# Quantitative Proteomics Uncovers Novel Factors Involved in Developmental Differentiation of *Trypanosoma brucei*

**DOI:** 10.1371/journal.ppat.1005439

**Published:** 2016-02-24

**Authors:** Mario Dejung, Ines Subota, Ferdinand Bucerius, Gülcin Dindar, Anja Freiwald, Markus Engstler, Michael Boshart, Falk Butter, Christian J. Janzen

**Affiliations:** 1 Institute of Molecular Biology (IMB), Ackermannweg 4, Mainz, Germany; 2 Department of Cell and Developmental Biology, Biocenter, University Wuerzburg, Am Hubland, Wuerzburg, Germany; 3 Department Biology I, Genetics, University of Munich (LMU), Großhaderner, Martinsried, Germany; The University of Maryland, UNITED STATES

## Abstract

Developmental differentiation is a universal biological process that allows cells to adapt to different environments to perform specific functions. African trypanosomes progress through a tightly regulated life cycle in order to survive in different host environments when they shuttle between an insect vector and a vertebrate host. Transcriptomics has been useful to gain insight into RNA changes during stage transitions; however, RNA levels are only a moderate proxy for protein abundance in trypanosomes. We quantified 4270 protein groups during stage differentiation from the mammalian-infective to the insect form and provide classification for their expression profiles during development. Our label-free quantitative proteomics study revealed previously unknown components of the differentiation machinery that are involved in essential biological processes such as signaling, posttranslational protein modifications, trafficking and nuclear transport. Furthermore, guided by our proteomic survey, we identified the cause of the previously observed differentiation impairment in the histone methyltransferase DOT1B knock-out strain as it is required for accurate karyokinesis in the first cell division during differentiation. This epigenetic regulator is likely involved in essential chromatin restructuring during developmental differentiation, which might also be important for differentiation in higher eukaryotic cells. Our proteome dataset will serve as a resource for detailed investigations of cell differentiation to shed more light on the molecular mechanisms of this process in trypanosomes and other eukaryotes.

## Author Summary

*Trypanosoma brucei* is a member of a large group of flagellated protozoan parasites that threatens the lives and husbandry of millions of people worldwide. This group includes parasites that cause devastating diseases such as leishmaniasis (caused by different species of *Leishmania*) and Chagas disease in South America (caused by *Trypanosoma cruzi*). One common feature of these parasites is a sophisticated life cycle, which requires transmission between a mammalian host and an insect vector. Over the course of this life cycle, the parasites follow a sequence of distinct developmental forms, which are perfectly adapted to the different host environments. We employed quantitative mass spectrometry techniques to unravel the molecular mechanisms that drive developmental differentiation of *Trypanosoma brucei*, the causative agent of African sleeping sickness. We followed the changes of expression of 4270 proteins during the development of the human-infective to the insect form of trypanosomes. The insights gained should help us to better understand not only how these dangerous parasites are able to survive in different environments, but also how they evade the defense mechanisms of their hosts. Unraveling the requirements needed for adaptation to human hosts might also be useful for targeted drug development to fight the devastating neglected tropical diseases caused by these parasites.

## Introduction

*Trypanosoma brucei* is a protozoan pathogen, which threatens thousands of people and kills millions of farm animals in sub-Saharan Africa [1]. In order to shuttle between different hosts, this parasite had to develop a complex life cycle, which includes two very different host environments: the vascular system and tissue fluids in the mammalian host and the intestinal tract and salivary glands of the vector, the tsetse fly. Many basic biological processes like motility, energy metabolism and morphology have to be adapted during several developmental differentiation events in order to survive and proliferate in these different environments (reviewed in [2,3]). Trypanosomes differentiate from the bloodstream form (BSF) in the mammalian host to the procyclic form (PF), which is adapted to live in the insect vector. Developmental differentiation in the mammalian host can be divided into two steps. First, proliferating bloodstream forms (called long slender, LS) differentiate into cell cycle-arrested bloodstream forms (called short stumpy, SS). Only the SS form is capable of differentiating efficiently to the PF that can resume proliferation in the fly. Interestingly, a form of quorum sensing pathway controls differentiation to the SS form. As parasitemia increases during the proliferation of slender LS, a parasite derived factor, so-called stumpy-induction factor (SIF), accumulates and promotes formation of SS forms, which arrest in G1/G0 phase of the cell cycle [4]. Although in general cell cycle arrest appears to be a prerequisite for differentiation to procyclic form, the link between cell cycle control and the differentiation process remains elusive. For example overexpression of the variant surface glycoprotein (VSG) in the long slender form causes only a G1 dormancy but initiates the slender-to-stumpy pathway in a reversible way [5]. Several components that might be associated with differentiation have been identified recently including kinases, phosphatases and components of a cAMP-signaling pathway ([6–8] reviewed in [8]). However, the consequences of these signaling events are largely unknown. Every step of the differentiation process involves coordinated changes of the parasite's gene expression profiles to provide host specific surface proteins or to change metabolism, morphology and organelle activity. There has been substantial progress in understanding the differentiation process of trypanosomes after several groups analyzed changes in transcription profiles during this process ([9–10] reviewed in [11]). However, it has to be taken into account that trypanosomes regulate steady-state protein levels mainly by posttranscriptional mechanisms [12]. Recently, genome-wide comparative ribosome profiling confirmed the importance of translation efficiency to regulate protein abundance in two different life cycle stages of trypanosomes [13–14]. Because translation efficiency can vary up to 100-fold between individual genes, substantial differences in the level of ribosome-bound mRNAs for the same transcripts were detected in different life cycle stages. These experiments demonstrate that translational control regulates protein abundance to a similar extent as RNA stability. Hence, to understand the developmental differentiation of trypanosomes, it is necessary to analyze the proteome during differentiation. Comparison of steady-state proteomes of SS, LS and PF have already shed more light on the differences between these life cycle stages [15–18]. To fully understand the required dynamics of proteome remodeling during the differentiation process, we here used quantitative label-free proteomics to monitor changes in protein expression during transition from LS to SS form and subsequent synchronous differentiation to the PF form of the parasite. While our analysis suggests previously unknown components of the differentiation machinery, we were also able to clarify the involvement of the histone methyltransferase DOT1B (disrupter of telomeric silencing) in this process. DOT1B was already reported to be essential for the differentiation process [19]. However, the exact step of the differentiation process that was impaired remained enigmatic. Our proteomic survey detected upregulation of the DOT1B protein in the first S-phase during differentiation of short stumpy forms to PF. Careful dissection of the phenotype of DOT1B-depleted parasites suggests a function in chromatin remodeling during developmental differentiation.

## Results and Discussion

### Changes of the trypanosome proteome during developmental differentiation

**Fig 1:**
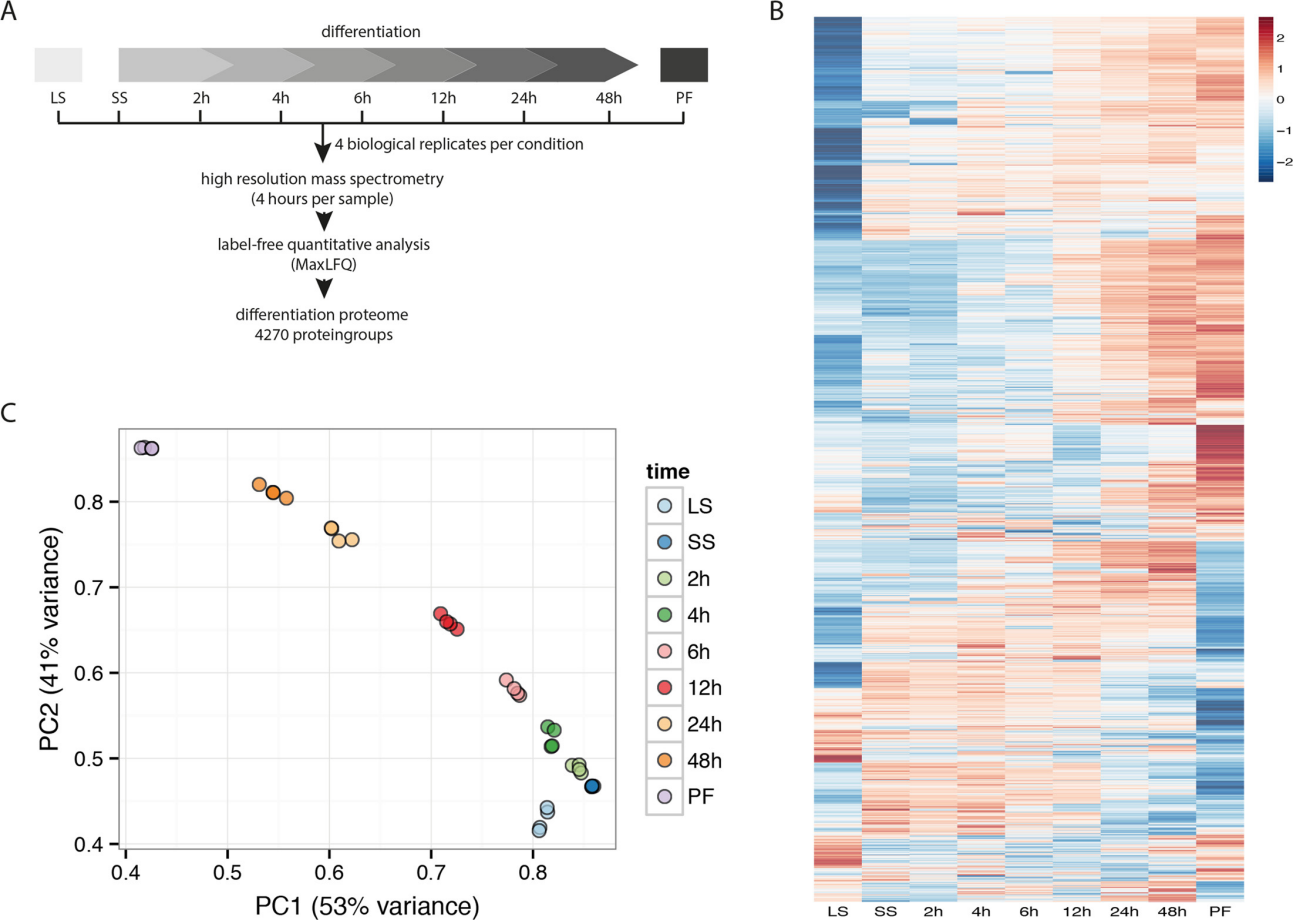
Experimental setup and differentiation proteome. (A) Schemata of the differentiation process and the experimental time points. Long slender bloodstream forms (LS) were differentiated to short stumpy forms (SS) followed by differentiation to insect form trypanosomes (PF). Parasites were harvested and analyzed by label-free quantitative mass spectrometry at different time points during the differentiation process as indicated. (B) The heat map encompassing 4270 z-scored LFQ quantified protein groups illustrates the major proteome remodeling. Significant expression differences can be compared between individual time points during the differentiation process. (C) Principle component analysis shows high reproducibility of replicates (r = 0.92–0.99) and consecutive progression of the differentiation process that can be described by the first two components PC1 and PC2.

To study the differentiation process, we treated short stumpy (SS) trypanosomes with citrate/cis-aconitate to initiate the transformation. We measured the proteome at 0 h (SS), 2 h, 4 h, 6 h, 12 h, 24 h and 48 h after the treatment and additionally included the long slender (LS) form and the established procyclic (PF) stage in our analysis (Fig 1A). To account for biological variability of the differentiation process, we measured 4 replicates each treated independently with citrate/cis-aconitate. In contrast to our previous experiment comparing LS and PF by mass spectrometry using stable isotope labeling [15], we here used label-free quantitative mass spectrometry empowered by the LFQ algorithm of MaxQuant [20–21]. We identified 4814 protein groups (each protein group may contain multiple proteins that are indistinguishable by mass spectrometry as they share all the identified peptides) to be expressed during differentiation and quantified 4270 of them (Fig 1B) covering differences in expression levels over 3 orders of magnitude. All expression profiles were compiled in a searchable interactive PDF file (S1 Fig). The differentiation process can be represented by principle component analysis with the first two components PC1 and PC2 (Fig 1C). In this representation the differentiation process follows a consecutive progression from LS (lower right) to PF (upper left). This consecutive change is detectable in each of the 4 replicates arguing for a strongly regulated effect involving major proteome remodeling. Replicates of the same time points cluster closely together with a correlation of r = 0.92–0.99 demonstrating minimal experimental variation during differentiation.

Differentiation has already been studied in trypanosomes by following transcriptome changes [9,10]. However, we have previously shown for LS and PF comparison [15] that the transcriptome is only a moderate proxy of proteome expression (r = 0.57). This also applies to the differentiation as we find the highest correlation between the transcriptome and our proteome at r = 0.39 (S2 Fig). In general, the correlation between transcriptome and proteome between the studies is low. While this might reflect different modes of regulation, we cannot exclude any systematic artifact due to the employed technologies. The authors used microarrays for the transcriptome measurement and already noted in their study that while the direction of regulation was reproducible, the degree of regulation did not match well between microarray and qRT-PCR. As we here compare the levels of up- and downregulation, this will compromise any comparison. However, it also needs to be noted that there are different experimental conditions (mouse infections in the transcriptome study). Due to these limitations we are ultimately unable to make a conclusion about the degree of differential regulation of transcriptome and proteome during the differentiation process.

Previously, stable isotope labeling (SILAC) was used to analyze the difference between LS and PF stage [15–17]. All three previous studies from different laboratories show a high correlation with our current label-free dataset. When we compare the correlation of 1680 proteins common to all four datasets, we find a high similarity between LS and PF stages (r~0.7, S3 Fig). This is reassuring as it demonstrates that reproducible results can be obtained by independent studies using either SILAC or label-free quantitation. We thus conclude that we have measured a high quality proteome dataset to investigate the differentiation process and infer that the data will be widely useable.

### Assessment of the proteomic survey by marker proteins

We report individual expression profiles for 4270 protein groups quantified in our experiment in an interactive file (S1 Fig). On a global level, 2805 protein groups do not significantly change during pre-adaptation from LS to SS, 1308 protein groups are upregulated and 157 protein groups are downregulated during this step in differentiation. During the differentiation of SS into PF 1224 protein groups are upregulated and 658 are downregulated (S1 Table). The remaining 2388 protein groups show a constant expression profile during differentiation (1736 protein groups) or the same protein expression levels in SS and PF but different levels during the differentiation process (652 protein groups).

**Fig 2:**
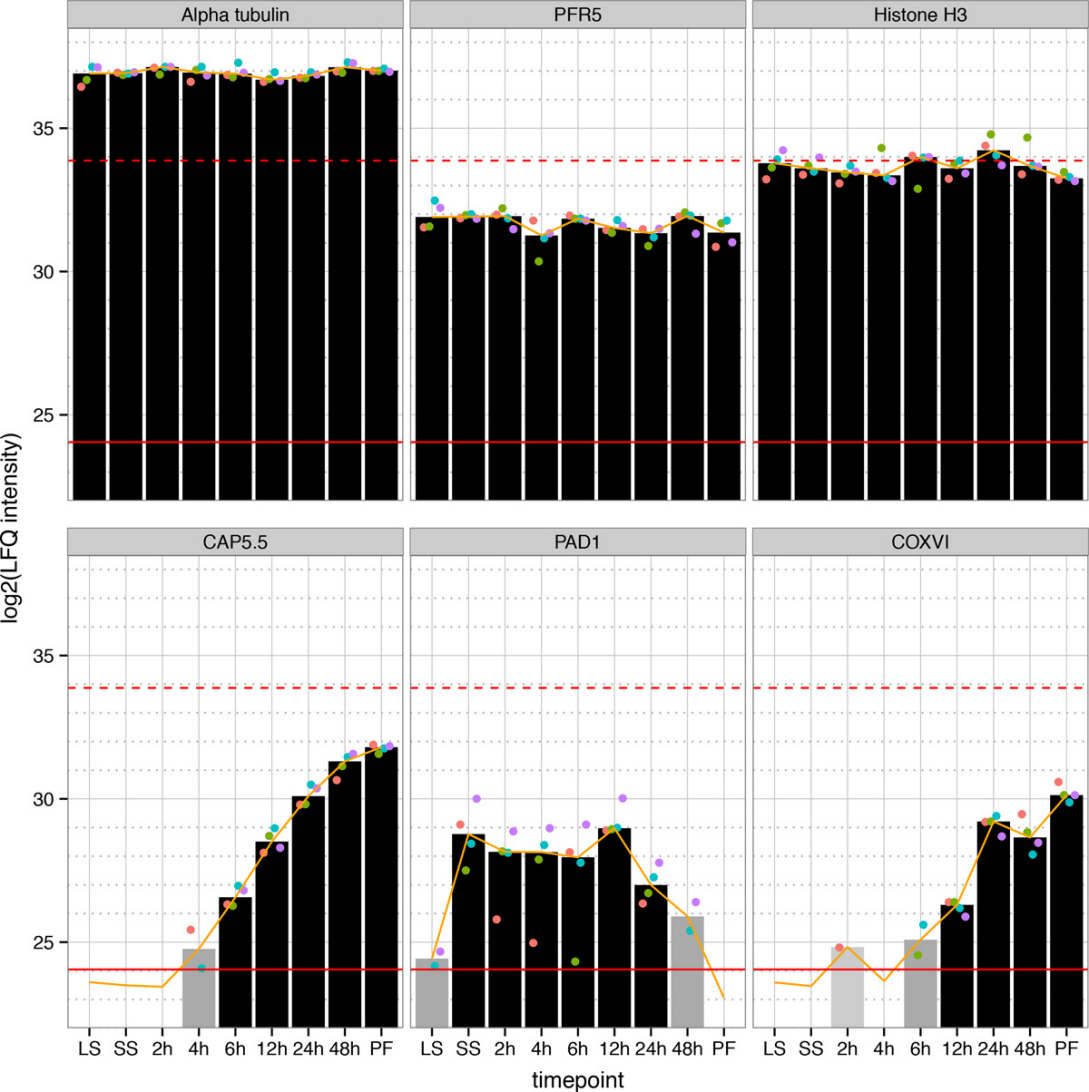
Individual protein expression profiles of selected proteins during differentiation. The expression profile for three structural proteins (upper panel) and proteins known to be regulated during differentiation (lower panel) are shown. The bars represent the calculated median LFQ intensities based on up to four measurements (ranging from light grey (quantified in 1 replicate) to black (quantified in 4 replicates). Individual replicates are indicated for each measurement (colored dots). The red lines represent the upper and lower quantile of measured LFQ intensities (99% and 1%, respectively). The orange line is the bioinformatically obtained expression profile for each protein.

To verify our dataset, we investigated profiles of selected individual proteins. First, we checked the expression profiles of highly abundant structural proteins such as tubulin (alpha tubulin, Tb927.1.2400), paraflagellar rod components (Pfr5, Tb927.2.4330) and histones (histone H3, Tb927.1.2550), (Fig 2 and S1 Fig). Neither the cell size nor the amount of chromatin seems to change dramatically during the differentiation process and thus substantial abundance changes of these proteins were not observed during differentiation [22]. Cytoskeleton associated protein 5.5 (CAP5.5, Tb927.4.3950) is upregulated at the mRNA level during differentiation [23,9]. Interestingly, CAP5.5 is already detectable at 4 hours post induction in our study. This underscores the sensitivity of mass spectrometry as a previous antibody based assay was only able to show expression in later stages of the differentiation process [24]. Additionally, as expected PAD1 (Tb927.7.5930) (Fig 2), another known marker of differentiation [25], is detectable in SS and during early differentiation but not measurable in PF.

Our dataset contains 727 quantified putative mitochondrial proteins [26]: 355 upregulated, 46 downregulated, 126 transiently regulated and 200 protein groups with constant expression levels. In agreement with major mitochondrial reorganization, cytochrome oxidase subunit 5 (COXV), which was demonstrated to be upregulated at the mRNA level during differentiation [23,9], and all seven nuclear-encoded cytochrome oxidases (COXIV–COXX, Tb927.1.4100, Tb927.9.3170, Tb927.10.280, Tb927.3.1410, Tb927.4.4620, Tb927.10.8320, Tb927.11.13140, respectively, S4 Fig) show a continuously upregulated profile in our analysis. The detection of proteins by mass spectrometry depends on their relative abundance in the cell. After 2 hours COXVI was quantified in one of 4 replicates, and evaded detection after 4 hours (Fig 2). After 6 hours we were able to quantify the protein in 2 replicates and at later stages of differentiation (12 hours and beyond) the expression was sufficiently abundant for quantitation in each of the 4 replicates. Between 2–6 hours, the expression of COXVI was close to the sensitivity limit of the measurement. In case we quantified a protein in less than 3 replicates, additional values had to be imputed (see methods section), making the quantitation for COXVI slightly less reliable at the early time points. In summary, assessment of candidates with known expression profiles confirms that our data very well recapitulates the described differentiation program of trypanosomes.

### Systematic global assessment of proteome remodeling during differentiation

**Fig 3:**
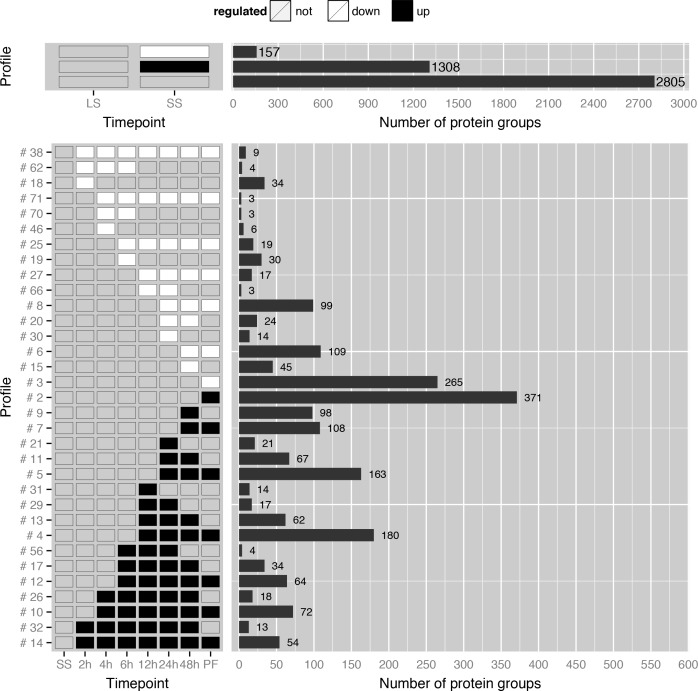
Categorization of protein groups with similar expression behavior into profile classes. Selection of expression profiles changes between LS and SS (top panel) as well as during the differentiation from SS to PF (bottom panel). In the upper panel the first line are proteins downregulated from LS to SS transition, the second line are upregulated and the third line are unchanged proteins. Numbers of protein groups within each class are indicated next to the bars. In the lower panel, all down-regulated (white) and up-regulated proteins (black) are shown compared to the SS stage. Proteins were accounted as regulated if fold change > 2 and p-value < 0.05. An overview of all 207 profile classes is available in the S1 Fig on page 2. (LS: long slender form, SS: short stumpy form, PF: procyclic form)

To systematically assess the proteome remodeling and to compare our data, we sorted individual protein groups with similar expression behavior into profile classes (S1 Fig, page 2 and Fig 3). 207 profile classes were obtained, 77 with more than 2 protein groups. For example, profile class #14 contains 54 protein groups upregulated at each measured time point starting 2h post induction of differentiation (Fig 3, bottom profile). This class includes a putative cystathionine-beta synthase (Tb11.02.5400), the metalloendopeptidase MSP-B (Tb927.8.1630), surface proteins such as PSSA-2 (Tb927.10.11220), metabolic proteins such as a glycosomal malate dehydrogenase (Tb927.10.15410), a serine/threonine kinase (Tb927.11.15010), three proteins with nucleic acid-binding activity (Tb927.6.1650, Tb927.6.2890, Tb927.7.2680), the well-characterized protein PTP1-interacting protein (Tb927.9.6090,[27]), 13 mitochondrial and 25 proteins of unknown function. Furthermore, profile class #10 includes CAP5.5 together with 71 other protein groups upregulated at 4 hours post induction. These profile classes (#14, #32, #10 and #26) could be of special interest to identify further early markers of differentiation.

### New insights into the differentiation process

Several proteins show a clear developmental regulation pattern. These proteins might shed more light on the function of several basic biological processes such as signaling, trafficking and remodeling of chromatin during the differentiation process of trypanosomes. Transient up- and downregulated expression profiles suggest the involvement of these proteins at specific periods of the differentiation process.

For example a putative Rab GTPase-regulating protein (Tb927.9.4370, S1 Fig) is upregulated during LS to SS transition but is not detectable anymore 48 h post induction of differentiation. Rab GTPases are a large heterogeneous family. However, most of their members are involved in vesicle trafficking or signaling and it is intriguing to observe that some Rab GTPase might have a function during early differentiation, but not in established LS and PF trypanosomes.

Interestingly, within this transient profile classes, we also identified enzymes for posttranslational modification of proteins, suggesting that ADP-ribosylation and palmitoylation play a role during differentiation. For example, a putative ADP-ribosylation factor-like protein (ARL1B, Tb927.9.7230, S1 Fig), which might function in vesicle trafficking is among the candidates, as well as a palmitoyl acyltransferase (TbPAT11, Tb927.7.3350, S1 Fig) recently described to putatively be involved in protein targeting to flagellar membranes [28]. RNAi-mediated depletion of TbPAT11 had no effect on growth in LS nor PF. Our data suggest TbPAT11 is transiently expressed only in stumpy forms and early during differentiation (2–6 h), giving an explanation for the lack of a phenotype after knock-down. In fact, also substrate proteins of palmitoylation, like a non-characterized conserved protein (Tb927.8.960, Fig 3, #4) shown to be palmitoylated [29] is upregulated late during differentiation (12 h) and in PF.

Cell differentiation is a carefully regulated process and several kinases that are involved in LS to SS formation have been identified [30–32]. Ground-breaking work recently unraveled many other components of a complex signaling cascade that ensures that the initiation of the LS to SS differentiation process occurs only after receiving the proper external stimuli [8]. However, little is known about signaling events during SS to PF differentiation. A MAP kinase (MKK1, Tb927.3.4860, S1 Fig) is necessary for transmission of trypanosomes by the tsetse vector [33] and only very recently, a kinome-wide RNAi screen identified the first two kinases (RDK1, Tb927.11.14070 and RDK2, Tb927.4.5310, S1 Fig), which regulate SS to PF differentiation [7]. Both proteins are present in our differentiation proteome. While RDK1 was only detectable in PF, the repressor of differentiation kinase 2 (RDK2) is very abundant and was quantified in all stages during differentiation. Interestingly, RDK2 is downregulated 24 hours after induction of differentiation (Fig 3, #8) suggesting that the inhibitory function of RDK2 is either not required after this time point or already established by its kinase activity. In our proteome analysis, we found additional kinases and phosphatases that might be involved in the differentiation process due to their expression profiles. For example, the protein level of a putative serine/threonine kinase (Tb927.11.5860, Fig 3, #8) is substantially upregulated in SS forms but decreases slowly 24h post induction of differentiation and is strongly downregulated in PF, which suggests a function early during differentiation. Another putative kinase (Tb927.8.6930, Fig 3, #8) shows a similar regulation pattern. Interestingly, this kinase was shown to play a role in developmental commitment recently [34]. The putative kinase Tb927.10.2040 (S1 Fig) is upregulated in SS but is also present throughout differentiation and in PF. This kinase seems to be important for the differentiation process according to a genome-wide RNAi screen [35]. Other putative kinases are transiently upregulated (Tb927.10.3230, Fig 3, #26) or are only reproducibly detectable during late time points of the differentiation process (Tb927.4.2680, Fig 3, #5). These proteins with previously unknown expression patterns might shed more light on the function of kinases during distinguishable periods of the differentiation process and could be the basis for molecular biology-based experiments to further unravel the signaling cascade that regulates differentiation progression.

It has been described already many years ago that chromatin composition and nuclear architecture is different in LS and PF [36–38]. However, the molecular machinery that mediates chromatin remodeling and changes in nuclear architecture during stage transition is completely unknown. The nuclear periphery and nuclear pore complexes are described to be involved in the spatial organization of chromatin in most eukaryotes (reviewed in [39]). We could quantify the expression of 20 nucleoporins constituting the nuclear pore complex (S4 Fig). Although most of them do not show a substantial difference in abundance during differentiation, Nup155 (Tb927.10.8170) and Nup59 (Tb927.11.2670) seem to be slightly upregulated after transition to SS (S4 Fig). Interestingly, nucleoporin Nup48 (Tb927.11.4540, S4 Fig) is not detectable in LS or SS but is upregulated 6h post induction of differentiation to PF, which suggests a stage-specific function in nuclear structure or transport. Notably, there is no *S*. *cerevisiae* homologue [40] arguing that this nucleoporin is not required in yeast, while the human tbNUP48 homologue, ALADIN, is involved in oxidative stress response and mutations cause a strong disease phenotype [41].

### The role of the epigenetic regulator DOT1B during differentiation

**Fig 4:**
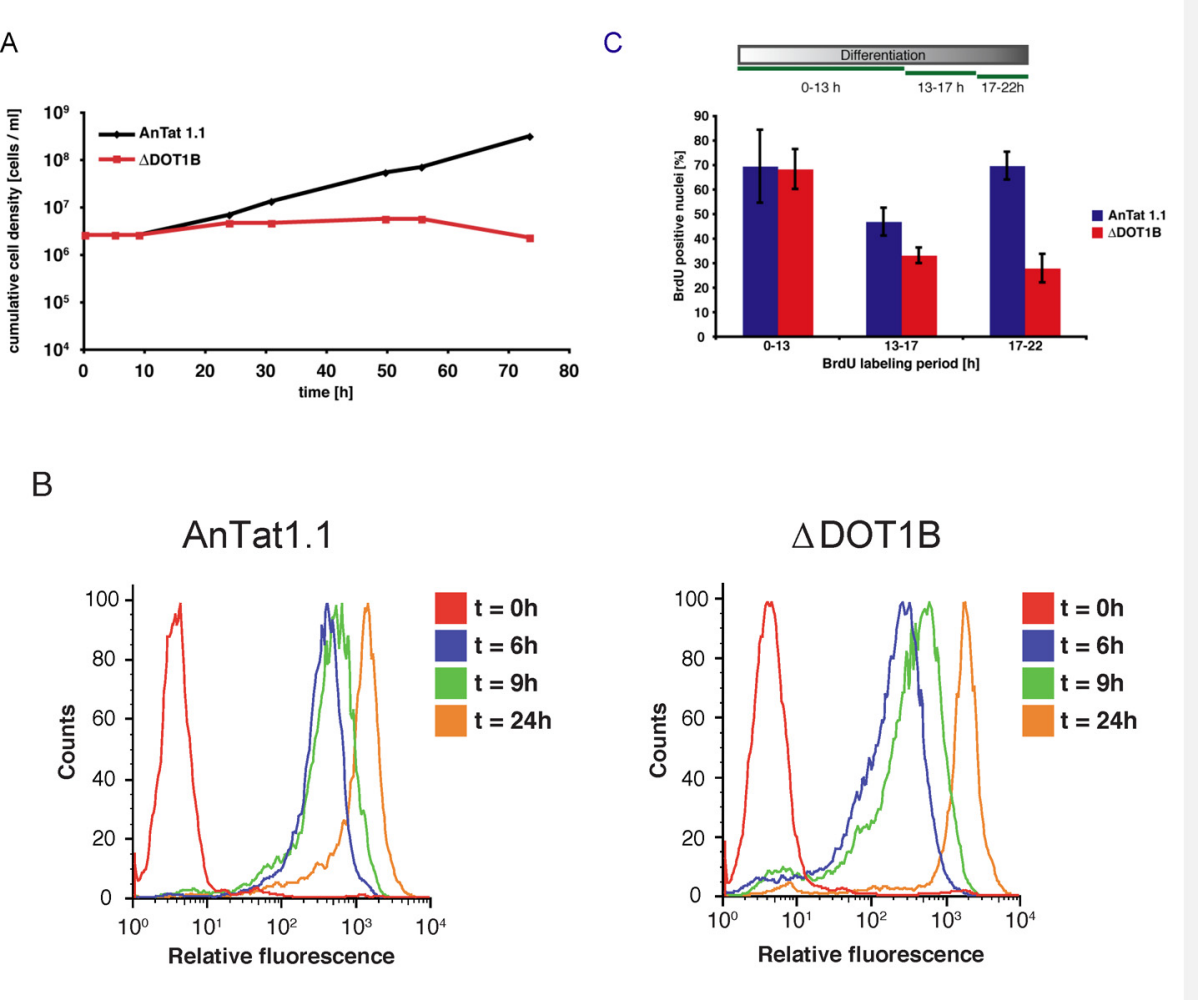
Analysis of early differentiation events from short stumpy to procyclic form. (A) Cumulative growth curve after induction of *in vitro* differentiation. After one population doubling ∆DOT1B trypanosomes undergo growth arrest and die after several days. (B) Detection of procyclin on the surface of differentiating trypanosomes by flow cytometry. Increasing relative fluorescence correlates with the amount of surface exposed procyclin during the course of differentiation. Procyclin expression is indistinguishable in wild-type and ∆DOT1B populations. (C) BrdU incorporation of differentiating trypanosomes. When differentiating cells re-enter the cell cycle both cell lines incorporate BrdU into freshly replicated DNA. Addition of BrdU for different time periods during differentiation demonstrates that the number of actively replicating cells decreases in DOT1B-deficient cells. The statistical analysis with an unpaired t-test shows significance between the data sets marked with bars and asterisks (* p-value <0.05, ** p-value <0.01).

**Fig 5:**
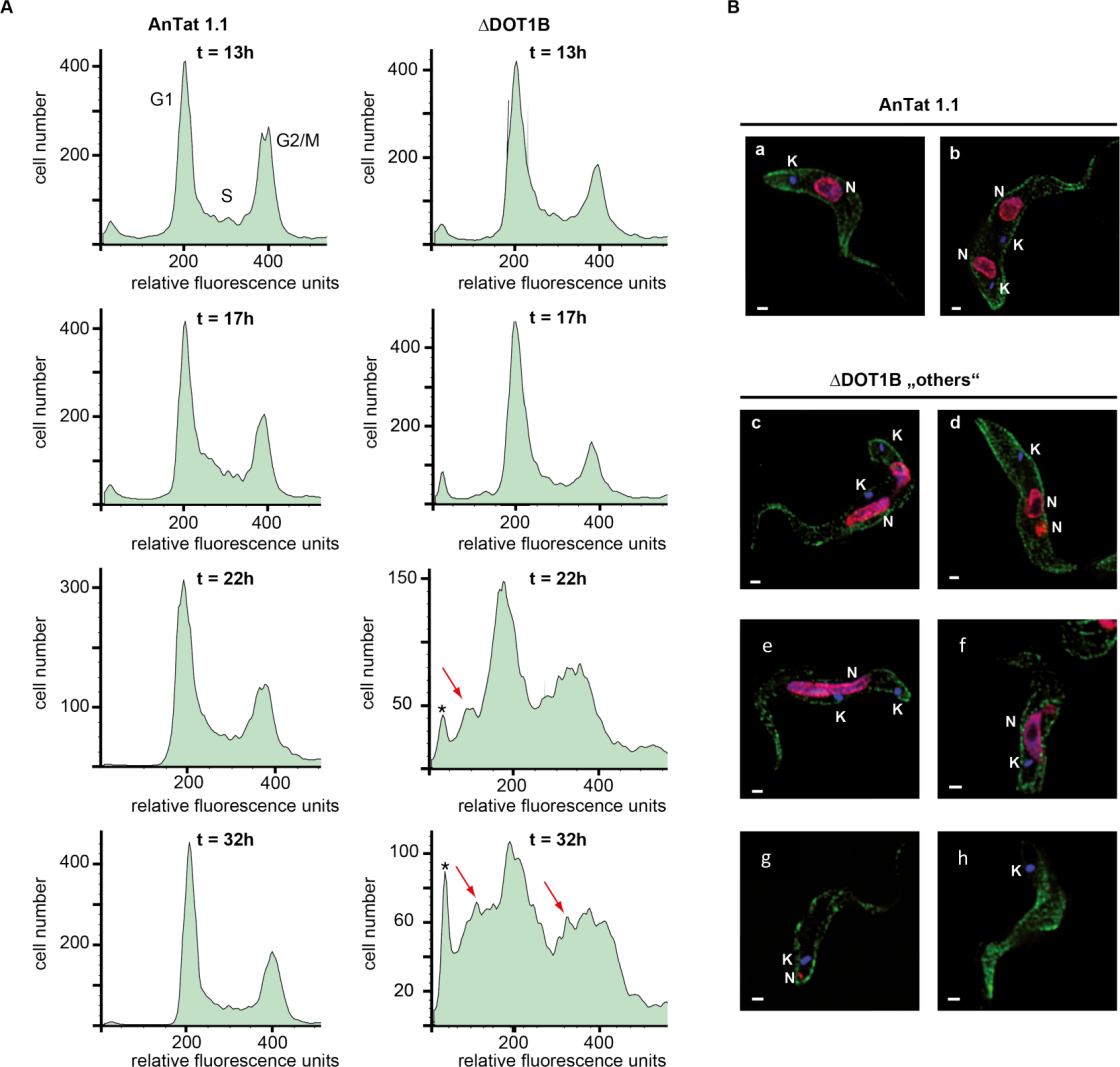
Differentiation defect of ∆DOT1B trypanosomes. (A) Cell cycle profiles of propidium iodide stained differentiating trypanosomes (flow cytometry). ∆DOT1B cells re-enter the cell cycle normally (t = 13h). Later in differentiation (t = 22h; t = 32h) the profiles show peak broadening indicating the appearance of cells with abnormal DNA content (red arrows) and enucleated “zoids” (asterisks). (B) Indirect immunofluorescence analysis of differentiating ∆DOT1B trypanosomes with karyokinesis defects (t = 22h). Wild-type cells shows normally shaped nuclei and kinetoplasts (a: 1N1K, b: 2N2K). During differentiation ∆DOT1B cells are unable to divide their nucleus properly resulting in cells with two kinetoplasts and one elongated or asymmetrically divided nucleus (c-f). Cytokinesis is not impaired demonstrated by the appearance of cells with asymmetrically divided nuclei and one kinetoplast (g) or anucleated “zoids” (h) (green: tubulin; red: histone 3; blue: DAPI; bars: 1 μm).

Next, we wanted to further exploit our data set to learn more about the function of dynamic changes in chromatin structure during developmental differentiation. To this end, we focused on nuclear proteins that are transiently upregulated during differentiation. We have previously shown that DOT1B (Tb927.1.570, Fig 3 #31), a histone methyltransferase responsible for methylation of histone H3 on lysine 76 [42] is essential for differentiation [19]. We could demonstrate in other studies that this histone methyltransferase is involved in transcriptional regulation of the major BSF variant surface glycoprotein (VSG) [43,5]. However, it remained unclear, which step of the differentiation process is impaired in DOT1B-depleted parasites. In our survey DOT1B shows a very peculiar expression pattern. We only detected its expression at 12h post induction of differentiation to PF. As trypanosomes synchronously re-enter the cell cycle and reach S-phase after 8–12 hours during differentiation [44], this suggested a function of DOT1B during or after the first S-phase of the differentiation process. To test this hypothesis, we generated pleomorphic ∆DOT1B trypanosomes (in strain AnTat1.1) using a previously described knock-out strategy [19]. DOT1B-depleted cells grow normally and were morphologically indistinguishable from wild-type cells, although the typical methylation pattern is absent (S5A–S5C Fig). As expected, the ability of ∆DOT1B trypanosomes to differentiate from LS to SS forms and to enter cell density-dependent growth arrest was indistinguishable between mutant and wild-type parasites (S5D Fig). Cell cycle arrest in G0/G1 was detectable by flow cytometry analysis of propidium iodide-stained cells in both cell lines as well as the expression of the stumpy marker PAD1 (S5E and S5F Fig). Thus, these events that pre-adapt the cells for differentiation to PF are not impaired in ∆DOT1B parasites suggesting that this first step of the differentiation process proceeds normally. We thus focused our further studies on the suggested cell cycle-dependent effect of DOT1B depletion. We therefore treated the parasites with citrate/cis-acconitate to induce the differentiation process to PF. Re-entry into cell cycle progression can simply be detected by resumed growth after 24 hours post induction. Although re-entry into the cell cycle was clearly detectable with both lines, ∆DOT1B cells did not grow (as reported previously) suggesting that the failure to differentiate happens during the first cell cycles as indicated by the detection of DOT1B protein at 12 h post induction (Fig 4A). We thus decided to follow the parasites through the first cell cycle and monitor changes of morphological markers, DNA replication and cell division in shorter intervals compared to the proteomic survey. One of the hallmarks of early differentiation is the exchange of surface proteins. The VSG coat is exchanged with a procyclic-specific surface protein called procyclin. The surface coat did not show differences during the first 24 hours of differentiation between wild type and DOT1B-deficient cells (Fig 4B) suggesting that at least some characteristic morphological changes are initiated. To test whether the progression through S-phase is compromised in DOT1B-deficient cells, we monitored BrdU-incorporation as a marker for DNA synthesis after the cells entered S phase. There was no difference in regards to incorporation rate (both close to 70%) between wild-type cells and ∆DOT1B mutants during the first S phase (Fig 4C, 0-13h). However, when BrdU-incorporation was monitored at later time points that reflected mainly replication in the second S phase the signals in the ∆DOT1B strain decreased dramatically suggesting that the differentiation defect is associated with G2 phase or mitosis of the first cell cycle or G1 and/or S phase of the second cell cycle. To determine what exactly could be impaired during G2 phase or mitosis progression, we monitored the cell cycle profiles after the time point we detected expression of DOT1B (13–32 hours post induction, Fig 5A). 13 hours post induction both cell lines re-entered the cell cycle synchronously, which results in a normal cell cycle profile. An accumulation of G2/ M phase cells suggests that cell cycle progression continued normally until the beginning of karyokinesis. However, the profiles began to change dramatically in ∆DOT1B mutants after the first division of the nuclei (Fig 5A, bottom panels ≥22h). Both, the G1 phase and the G2/M phase peak began to broaden due to the appearance of additional heterogeneous populations of cells with a decreased DNA content (Fig 5A, arrows). About 50% of the DOT1B-depleted cells developed an aberrant phenotype 22 h post induction of differentiation judging by unusual nuclei/kinetoplast configuration or obvious inaccurate nuclear division (S6 Fig).

With the help of our proteomic dataset, we could finally search for the differentiation defect more precisely and discovered a failure to complete accurate karyokinesis in the first cell division during differentiation in ∆DOT1B trypanosomes. To confirm this observation we performed indirect immunofluorescence analysis (IFA) with wild-type and DOT1B-deficient cells 22 hours post induction of differentiation that are in the middle of karyokinesis or cell division (Fig 5B). As expected, we could observe two accurately segregated nuclei and two kinetoplasts in an elongated parasite that is ready for cell division in wild-type cells (Fig 5B, panel b). However, a variety of unusual cells were detectable in ∆DOT1B mutants. Some cells failed to divide their nucleus properly and displayed extremely elongated or asymmetrically divided nuclei (Fig 5B, panels c-e). Surprisingly, cell division is not impaired demonstrated by parasites with one kinetoplast and two asymmetrical nuclei or nucleus fragments (Fig 5B, panels f-g). Infrequently, cells without a nucleus (so-called "zoids") were detectable (Fig 5B, panel h).

**Fig 6:**
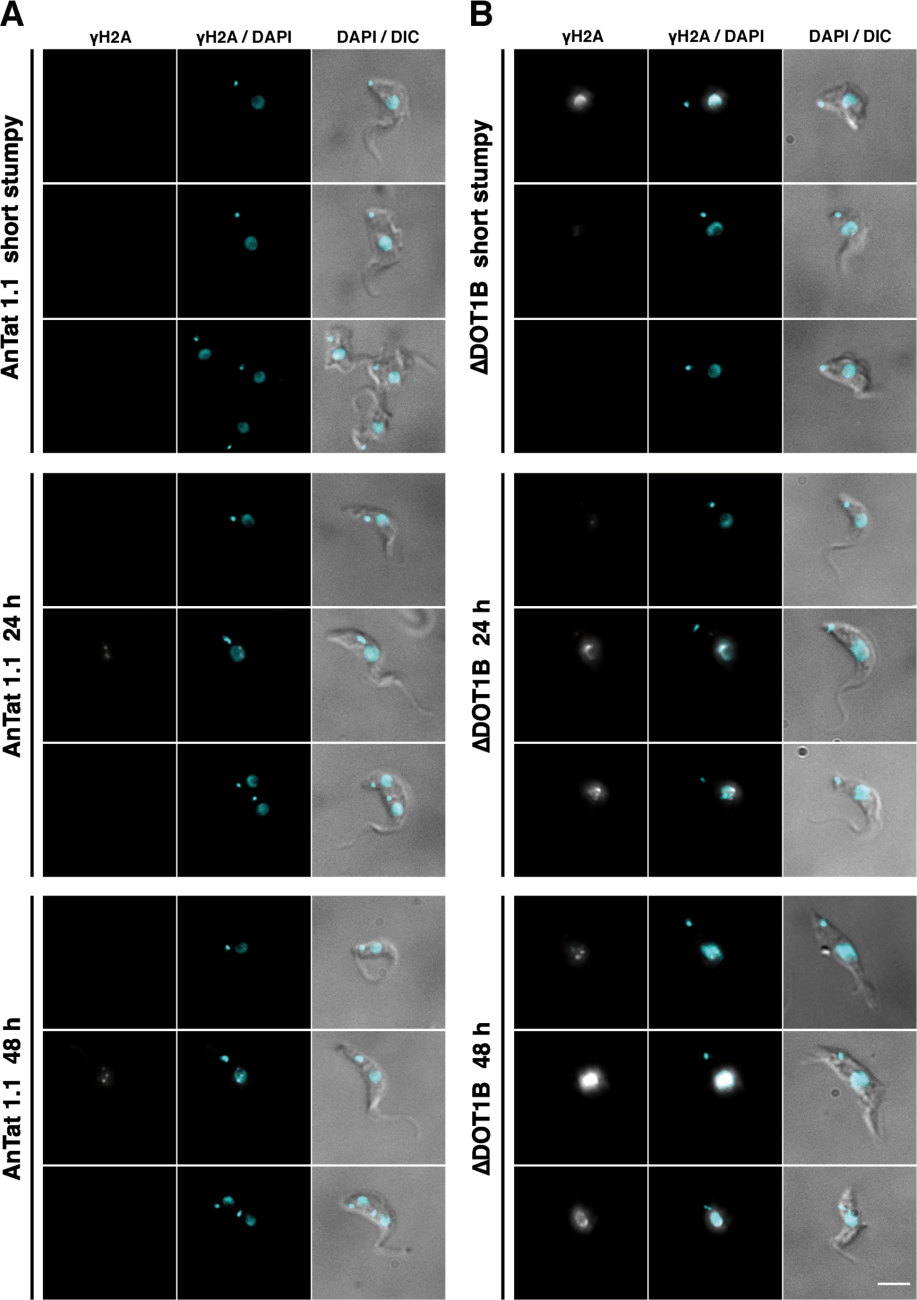
Accumulation of DNA damage in ∆DOT1B trypanosomes during differentiation. Indirect immunofluorescence analysis of differentiating wild-type (A) and ∆DOT1B trypanosomes (B) using an antibody specific for phosphorylated H2A (γH2A). Representative cells are shown before induction of differentiation (upper panels), 24h post induction (middle panels) and 48h post induction (bottom panel). DNA was stained with DAPI (blue). Cells were visualized with differential interference contrast (DIC) microscopy. Scale bars: 5 μm.

To further elucidate details of the differentiation defect, we performed IFA with an antibody specific for a threonine-phosphorylated form of histone H2A (γH2A), which accumulates at sites of DNA-damage [45]. In wild-type cells, only few distinguishable γH2A-foci can be detected during S-phase, which has been described before (Fig 6A, 24h and 48h, middle panels). In contrast, a continuous accumulation of γH2A-foci can be observed during the course of differentiation (Fig 6B, 24h and 48h and S7 Fig) suggesting that genome integrity is massively impaired in ∆DOT1B trypanosomes. Although we can detect a few SS trypanosomes with γH2A-foci (Fig 6B, top panel), there is a clear increase of DNA damage after induction of differentiation, which suggests that this phenomenon is differentiation dependent. This is in accordance with the fact that we do not observe a growth phenotype in LS trypanosomes (S4 Fig).

Guided by the proteomic survey, we could demonstrate that in ∆DOT1B trypanosomes the first karyokinesis after the parasites resume cell cycle progression after 8–12 hours post induction of differentiation is impaired. This is striking, considering that we could never observe this phenotype in a logarithmically growing LS or PF cell culture [19]. Hence, this appears to be a phenomenon specific for developmental differentiation. Interestingly, similar observations were reported during early development of mice. Germ line deletion of mouse DOT1L results in early embryonic lethality due to failures during organogenesis of the cardiovascular system [46]. These defects coincide with abnormal chromatin structure such as impaired heterochromatin development, telomere elongation and aneuploidy. As trypanosomes do not regulate transcription initiation of individual genes, it is unlikely that the differentiation phenotype of ∆DOT1B parasites is based on aberrant transcription regulation. Taking into account that chromatin structure and nuclear architecture was reported to be different in LS and PF parasites [36–38], we suggest that chromatin remodeling takes place during the first S-phase after initiation of differentiation and that DOT1B is essential for this process. The identification and characterization of the molecular machinery that is responsible for this process will be in the focus of future research. This example thus illustrates the usefulness of our dataset for further investigations of many aspects of developmental differentiation by the community to shed more light on the molecular mechanisms of this process in trypanosomes and other eukaryotes.

## Methods

### Differentiation

*Trypanosoma brucei* AnTat1.1 BSF were cultured in HMI-9 medium [47] complemented with 1.1% methylcellulose [30,48]. Cells were grown to a density of 4x10^6^ cells/ml and kept for additional 24h at 37°C/5% CO_2_ until efficient stumpy formation could be detected by flow cytometry analysis. The cell culture was diluted 1:5 in pre-warmed PBS at 37°C, filtered through MN 615^1^/_4_ filter papers (Macherey-Nagel), harvested by centrifugation (1400g, 10 min) and resuspended in DTM medium [49]. To induce differentiation, 6 mM citrate/cis-aconitate was added and trypanosomes were kept at 27°C with 5% CO_2_.

### MS sample preparation and measurement

Equal amounts of cells (2x10^7^) were harvested for each time point by centrifugation (1400g, 10 min), resuspended in NuPAGE Novex buffer (Thermo) and boiled for 10 min at 95°C. In gel digestion was performed essentially as described before [50,51]. Samples were separated by SDS-PAGE at 180V for 10 min, fixated with methanol and subsequently stained with colloidal Coomassie (Life Technologies). Gel lanes were minced, destained with 50% ethanol/50mM ammonium bicarbonate buffer and then reduced with 10mM DTT for 1 hour at 56°C, alkylated with 50mM iodoacetamide for 30 min at room temperature and digested with 1μg of trypsin (Promega). Peptides were eluted from the gel with acetonitrile, which was removed in a concentrator (Eppendorf). Each peptide fraction was desalted on a C18 StageTips (Empore) [52] and separated by nanoflow liquid chromatography on an EASY-nLC 1000 system (Thermo) coupled to a Q Exactive or Q Exactive Plus mass spectrometer (Thermo). Separation on the capillary was achieved by an immobilized C18-reversed phase (25 cm long, 75 μm inner diameter, packed in-house with ReproSil-Pur C18-AQ 1.9 μm resin (Dr. Maisch). The tip of the capillary was directed to the electrospray ion source with a spray voltage of 2.0–2.4 kV. A 240 min gradient from 2% to 95% acetonitrile in 0.5% formic acid at a flow of 200 nl/min was used. The Q Exactive type mass spectrometer measured in a positive mode performing data-dependent Top10 MS/MS (15,000 resolution) HCD spectra acquisition method per MS full scan (70,000 resolution), rejecting unassigned or +1 charges and excluding sequenced masses for 35 sec with a dynamic exclusion list. The normalized collision energy was set to 25. The mass spectrometry proteomics data have been deposited to the ProteomeXchange Consortium via the PRIDE partner repository with the dataset identifier PXD003319.

### MS data analysis

The raw measurement files were analysed with MaxQuant 1.5.2.8 [20] standard settings and searched against the *Trypanosoma brucei* TREU927 protein database (version 8.1, 11567 entries) available at tritrypDB (http://tritrypdb.org). The Andromeda search engine [53], which is part of the MaxQuant software suite, was used for peptide identification. Enzyme search specificity was set to Trypsin/P (Promega) with two miscleavages allowed per peptide. Carbamidomethylation on cysteines was set as fixed modification while methionine oxidation and protein N-acetylation were considered as variable modifications. False discovery rate was fixed at 1 percent on peptide and protein level. In the label-free quantification settings a minimum ratio count of 2 was set. Match between run option was activated with 0.7 minutes time window was allowed to match between runs. In the MaxQuant protein groups output table, peptides mapped to known contaminants, reverse hits and protein groups only identified by site were removed before further analysis. Furthermore, only protein groups identified with at least 2, one of them unique, peptides were used for downstream analysis. The identified proteins were further filtered for two measured LFQ intensities in at least one time point.

### Bioinformatics analysis

To substitute missing values, which cannot be measured because they are below the detection limit of the instrument or not expressed in the sample, we looked at each time point individually. For better comparison of up and down regulation we transferred all LFQ intensities into log2 values.

First we determined the LFQ intensity variance at each timepoint (distance from the protein group average) of protein groups with minimum of 3 replicates measured. Secondly we used this distribution to impute the missing values around the median of each protein group.

Thirdly, for protein groups for which none of the four replicates contained LFQ intensities, the same variance was applied but the average was set to 0.001 quantile of the measured LFQ intensities of the related timepoint. A Welch t-test was performed between SS or LS and all time points. If less than three measured LFQ intensities were available, the imputed values were included in the calculation.

The principal component analysis was subsequently performed using the psych R-package with standard settings. The heatmap was created with the NMF R-package, all other plots where produced with the ggplot2 R-package. For the comparison the following datasets were downloaded from tritrypDB (version 8.1): Gunasekera et al. (SILAC proteome), Urbaniak et al. (SILAC proteome), Queiroz et al. (microarray transcriptome) and Kabani et al. (microarray transcriptome) [9,10,16,17]. The data from Butter et al. [15] was used as provided as supplementary and was merged into a single value after restoring the reported tritrypDB data in some of the experiments by inverting values back to their original state, the other data sets were used as supplied. For the SILAC proteome, bloodstream forms and procyclic forms were compared using the cor function of the stats R-package reporting a Pearson correlation used on complete observations only. To create groups of same expression profiles, the fold change was calculated to a specific time point (e.g. SS). Any fold change larger than 2 with p<0.05 (Welch t-test) was reported as significantly up or downregulated. Proteins following the same expression pattern were grouped together producing 207 possible groups resulting in an interactive PDF with bookmarks for important proteins (S1 Fig). For a better overview 33 groups were manually selected representing possible differentiation marker (Fig 3). Gene names and GO annotations were also downloaded from tritrypDB (version 8.1) and mapped to the trypanosome identifiers. In protein groups with multiple tb identifiers annotation, each protein was considered.

### DNA cloning and plasmid construction

To generate the DOT1B gene deletion constructs, a phleomycin resistance gene was amplified from pLew100 with primers F9 (GACTACTAGTGGGCACAGCAAGGTCTTC) and F10 (GACTACTAGTACTATTTCAATCATGTCGACAC). The puromycin resistance gene was amplified using primers F11 (GACTGCTAGCGGGCACAGCAAGGTCTTC) and F12 (GACTGCTAGCACTATTTCAATCATGTCGACAC). The phleomycin PCR products and the target vector CJ44 were digested with *Spe*I, the puromycin PCR product and target vector CJ44 were digested with *Nhe*I prior to ligation. 10 μg of plasmid was digested with FspI and EcoNI prior to electroporation.

### Transfection of AnTat 1.1

*Trypanosoma brucei* bloodstream forms AnTat1.1 were cultured in HMI-9-methylcellulose medium at 37°C as described previously [30]. 2x10^7^ cells were harvested for each transfection. Methylcellulose was diluted 1:5 in pre-warmed PBS at 37°C, filtered through 185 mm filter papers (Macherey and Nagel). After filtration, trypanosomes were harvested by centrifugation (1400 g, 10 min, 37°C), washed in 10 ml PBS and resuspended in 100 μl of pre-warmed transfection buffer (90 mM Na_2_HPO_4_, 5 mM KCl, 0.15 mM CaCl_2_, 50 mM HEPES pH 7.3). Cells were transfected using AMAXA Nucleofector II (Lonza), transferred into 50 ml pre-warmed HMI-9 methylcellulose and distributed into two 6 well plates. Puromycin (0.2 μg/ml^-^ and phleomycin (2 μg/ml) were applied 12 h after transfection.

### Flow cytometry analysis

2x10^7^ cells were harvested from HMI-9 methylcellulose medium as described above. Trypanosomes were resuspended in 1 ml PBS and fixed with 2.5 ml ice cold ethanol for 30 min. Fixed cells were harvested by centrifugation (1400 g, 10 min, 37°C), washed once in 5 ml PBS and resuspended in 1 ml PBS/2 mM EDTA. 10 μg RNase A (Applichem) and 10 μg of propidium iodide (Sigma) were added, followed by 30 min incubation at 37°C. Trypanosomes were measured with a FACSCalibur (BD Biosciences) and analyzed with CellQuest 3.3 (BD Biosciences) or FlowJo 8.8.6 (TreeStar Inc.) software.

### SDS-PAGE, Western Blot and Immunofluorescence analysis (IFA)

Trypanosome cell extract from 1x10^6^ cells were separated on a 15% SDS-PAGE. Proteins were transferred onto a PVDF (Millipore) membrane using semi-dry apparatus (Bio-Rad). Membranes were incubated with 3% BSA/PBS for 1h at RT. Primary antibodies (mouse anti-PFR L13D6 (1:2000 in PBS/0,1%Tween): rabbit anti-PAD1 (kindly provided by Keith Matthews), rabbit anti-H3K76me3 (1:2000 in PBS/0,1%TWEEN) were incubated with PBS/3%BSA/0.1%Tween. Secondary antibody (IRDye 800CW Goat anti-Rabbit and IRDye 680LT Goat anti-Mouse (LI-COR Biosciences) signals were detected with an Odyssey infrared imaging system (LI-COR). For immunofluorescence analysis, 1x10^7^ cells were harvested and resuspended in 1ml HMI-9 medium. Cells were fixed in 2% formaldehyde for 5 min. Cells were permeabilized in 0.2% NP-40/PBS for 5 min at room temperature. Immobilized cells were blocked in 3% BSA/PBS for 30 min, followed by incubation with the primary antibody (anti-tubulin antibody, anti-H3 antibody) diluted in 0.1% BSA / PBS for 1 h at RT. Cellular DNA was stained with 1 μg/ml 4,6-diamidino-2-phenylindole (DAPI) in PBS for 5 min at RT. Cells were embedded in Vectashield antifade medium (Vector laboratories). For the IFA of phosphorylated H2A (γH2A), cells were fixed in 4% formaldehyde for 15 min at room temperature, then harvested by centrifugation (1000 g, 5 min, 4°C) and washed once with PBS. Cells were left to settle on poly-lysine slides, then permeabilised in 0.2% NP-40/PBS for 5 min at room temperature. Immobilised cells were blocked in 10% FCS/PBS for 1 h, followed by incubation with the primary antibody (1:200, gift from David Horn) diluted in 3% FCS/PBS for 1 h at RT. Cellular DNA was stained with 5 μg/ml 4,6-diamidino-2-phenylindole (DAPI) in PBS for 1 min at RT. Cells were embedded in Vectashield antifade medium (Vector laboratories). Images were acquired with a Leica DMI6000 B microscope at [1]100x magnification. Fields of view containing several cells, were acquired with the epifluorescence microscope Nikon Eclipse TS100 using the 100x magnification and a defined exposure time of 800 ms for the quantification of the yH2A signal. The processing was performed with the ImageJ software (National Institutes of Health). First, a region of interest (ROI) with identical dimensions was set around the area of kinetoplast and nucleus of >100 individual cells (per time point) in the DAPI images. Then the signal intensity was measured in the same ROIs of the corresponding captures with the yH2A signal to define the integrated fluorescence density (the product of area and mean grey value). Statistical analysis was performed with the GraphPad Prism software.

### BrdU labeling

For BrdU labeling, cells were supplemented with 100 μM 5-bromo-2’deoxyuridine (BrdU) (Sigma) and 50 μM of 2-deoxycytidine. 1x10^6^ cells were resuspended in 100 μl PBS, fixed in ice cold (-20°C) 75% ethanol for 1 min and transferred to a cover slip. DNA was denatured in 2M HCl for 30 min at RT. Cells were neutralized in 0.1 sodium borate for 5 min, followed by four washing steps with PBS. Incorporated BrdU was detected by a monoclonal α-BrdU mouse antibody (Caltag MedSystems) and visualized by the secondary antibody Alexa 488 α-mouse (Invitrogen).

## Supporting Information

S1 Fig Quantification of 4270 protein groups during developmental differentiation. **Page 1** Example page of individual expression profile. The bars in the bottom panel show the calculated median LFQ intensities with color indicating the amount of measured instances in 4 replicates ranging from light grey (1 value) to black (4 values). Colored dots represent individual replicates. The orange line and dots show the bioinformatically obtained expression profile for each protein group. Missing values are imputed (see methods section). The red line marks the lower range of 1% of measured values, the dashed red line represents 99% of all measured values. Stages and timepoints sampled during differentiation are indicated on the x-axis. The top panels display fold changes compared to long slender (left panel) or short stumpy forms (right panel). **Page 2**: Groups of proteins with similar regulation pattern. Click on the profiles to see the individual protein profile. Groups of proteins discussed in the manuscript are compiled in the table of content of this PDF file where they can be selected.
(PDF)

S2 Fig Proteome complexity and comparison of proteome expression profiles with the transcriptome during the differentiation process. (A) Protein groups count per timepoint. (B) The overall correlation ranges from r = 0.23 to r = 0.39. A global maximum is found when comparing the transcriptome 18 h post induction with the proteome 24 h post induction. The transcriptome and proteome of the LS stage shows the second highest correlation.
(PDF)

S3 Fig Comparison with available SILAC proteomes of PF and LS stages. (A) Venn diagram with the number of individual protein IDs comparing to previously published datasets. (B) Scatterplot of LS/PF enrichment of 1680 protein groups overlapping with all previous studies. Each plot compares to the corresponding study. For each case, we obtain a pearson coefficient of r~0.7.
(PDF)

S4 Fig Changes in LFQ intensity of cytochrome oxidase subunits and nuclear pore complex proteins. Log2 LFQ intensities of proteins are shown for long slender (LS) and short stumpy (SS) forms and consecutive time points during the differentiation as indicated.
(PDF)

S5 Fig Characterization of DOT1B-depleted pleomorphic trypanosomes. (A) Cumulative growth curve of wild-type AnTat 1.1 and ∆DOT1B trypanosomes. AnTat 1.1 and ∆DOT1B cells were cultivated in high viscosity HMI-9-methylcellulose medium and show same population doubling time (5.5h). (B) AnTat 1.1 and ∆DOT1B long slender forms are morphologically indistinguishable (representative phase contrast microscopy pictures; bars 10μm). (C) Western Blot analysis shows H3K76 tri-methylation in wild-type AnTat 1.1 and loss of the modification in ∆DOT1B trypanosomes. (D) Cell density-dependent entry into stationary phase is indistinguishable in wild-type and ∆DOT1B cells. (E) Western blot analysis of PAD1 expression. Stumpy formation marker PAD1 is detectable in SS populations of both cell lines (LS: long slender; SS: short stumpy). The structural protein PFR serves as a loading control. (F) Cell cycle profiles of propidium iodide stained logarithmically growing parasites (upper panel) and arrested stumpy cell analysed by flow cytometry. Stumpy populations of both cell lines accumulate in G0/G1 phase of the cell cycle.
(PDF)

S6 Fig Quantitative analysis of nuclei (N) and kinetoplast (K) configuration in differentiating wild-type (WT) and DOT1B-depleted (∆DOT1B) cells. “Others” include cells with unusual nuclei/kinetoplast configuration or obvious inaccurate nuclear division (n = 100). Samples were taken at different timepoints after differentiation initiation as indicated below.
(PDF)

S7 Fig Quantification of DNA damage in ∆DOT1B trypanosomes during differentiation. Signal intensity of indirect immunofluorescence analysis (anti-γH2A) of differentiating AnTat1.1 wild-type versus ∆DOT1B trypanosomes was quantified using the ImageJ software. More than 100 individual cells were analysed for each time point and each cell line during the differentiation process (before differentiation in stumpy cells, 24 h and 48 h after the onset of differentiation). The measured integrated fluorescence density defined for each cell is shown in a scatter dot plot. The red lines delimit the mean value and standard deviation. The statistical analysis with a one-way Anova and Tukey’s multiple comparison test shows significance between the data sets marked with bars and asterisks (*** p-value <0.001).
(PDF)

S1 Table Accession numbers of groups of proteins with similar regulation pattern. Compilation of protein groups with similar expression pattern during the differentiation of SS into PF as displayed on page 2 of supplementary S1 Fig.
(XLSX)
