# Supplementary material for: Quantitative Proteomics Uncovers Novel Factors Involved in Developmental Differentiation of Trypanosoma brucei
Source: PLoS Pathog. 2016 Feb 24;12(2):e1005439. doi: 10.1371/journal.ppat.1005439 (PMC4765897; doi:10.1371/journal.ppat.1005439)
Supplement: S2 Fig — (A) Protein groups count per timepoint. (B) The overall correlation ranges from r = 0.23 to r = 0.39. A global maximum is found when comparing the transcriptome 18 h post induction with the proteome 24 h post induction. The transcriptome and proteome of the LS stage shows the second highest correlation. (PDF) [file ppat.1005439.s002.pdf]

Supplementary Figure 2

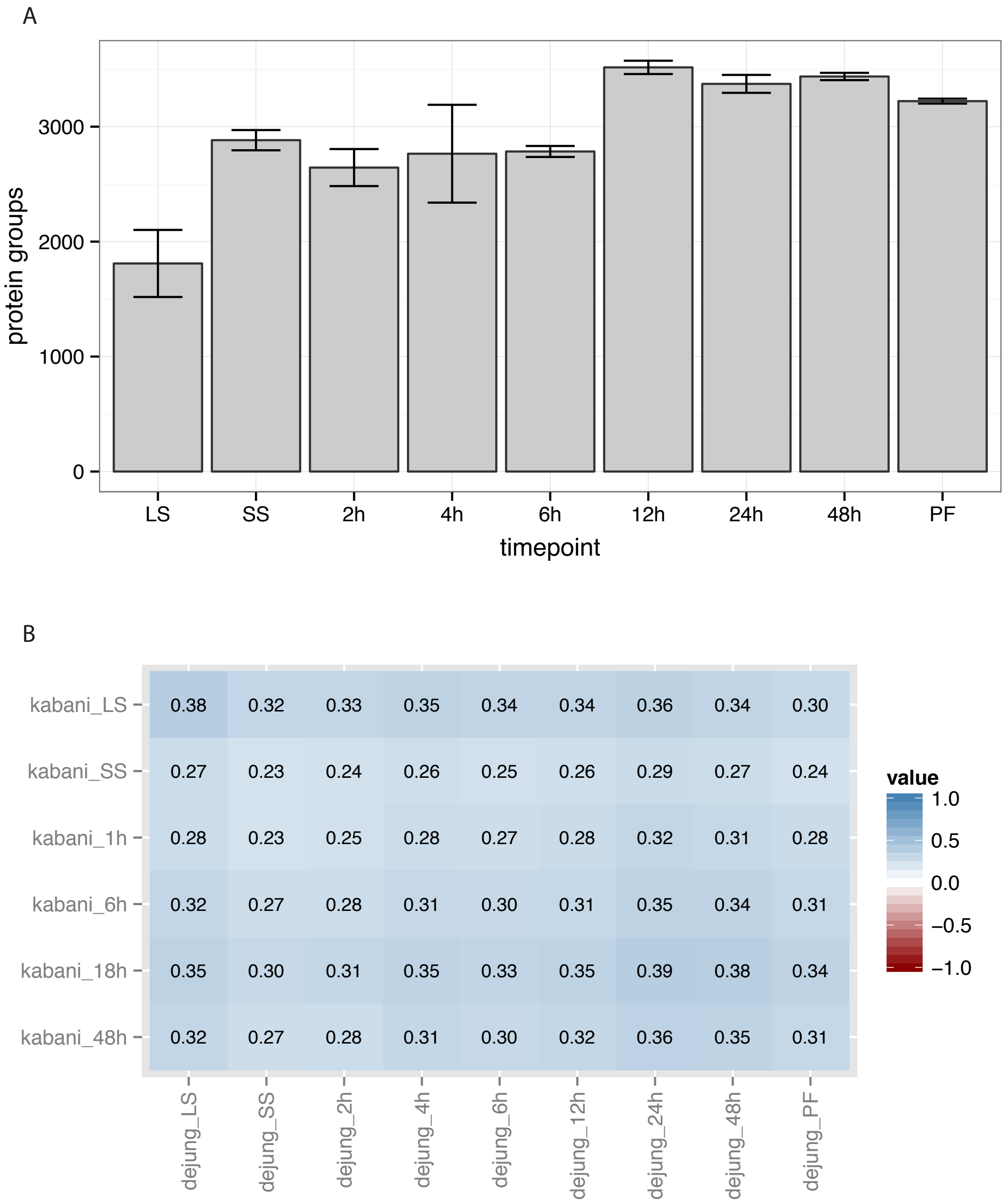

Fig S2: Proteome complexity and comparison of proteome expression profiles with the transcriptome during the differentiation process. (A) Protein groups count per timepoint. **(B)** The overall correlation ranges from r=0.23 to r=0.39. A global maximum is found when comparing the transcriptome 18 h post induction with the proteome 24 h post induction. The transcriptome and proteome of the LS stage shows the second highest correlation.
