# Supplementary material for: Quantitative Proteomics Uncovers Novel Factors Involved in Developmental Differentiation of Trypanosoma brucei
Source: PLoS Pathog. 2016 Feb 24;12(2):e1005439. doi: 10.1371/journal.ppat.1005439 (PMC4765897; doi:10.1371/journal.ppat.1005439)
Supplement: S3 Fig — (A) Venn diagram with the number of individual protein IDs comparing to previously published datasets. (B) Scatterplot of LS/PF enrichment of 1680 protein groups overlapping with all previous studies. Each plot compares to the corresponding study. For each case, we obtain a pearson coefficient of r~0.7. (PDF) [file ppat.1005439.s003.pdf]

Supplementary Figure 3

A

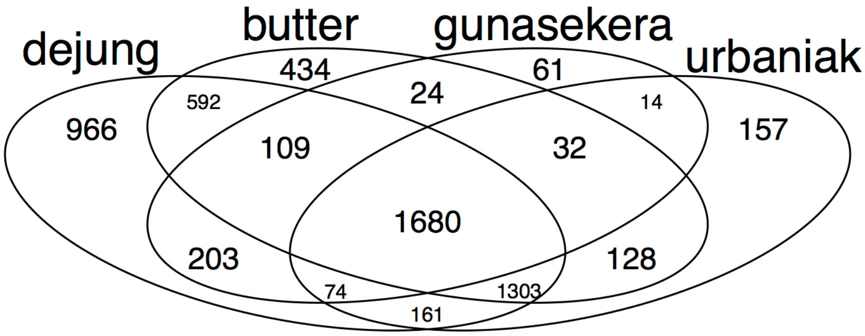

B

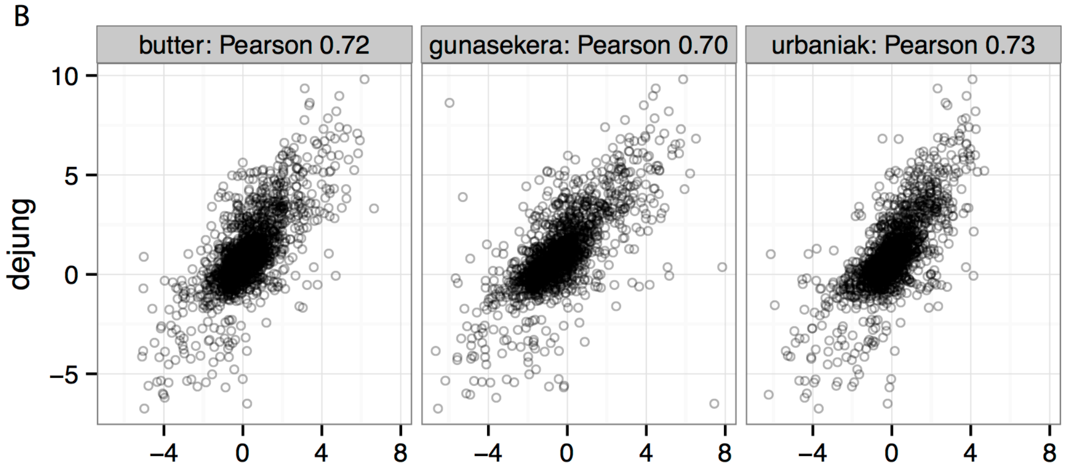

Fig S3: Comparison with available SILAC proteomes of PF and LS states. (A) Venn diagram with the number of individual protein IDs comparing to previously published datasets. **(B)** Scatterplot of LS/PF enrichment of 1680 protein groups overlapping with all previous studies. Each plot compares to the corresponding study. For each case, we obtain a pearson coefficient of r~0.7.
