# Supplementary material for: Quantitative Proteomics Uncovers Novel Factors Involved in Developmental Differentiation of Trypanosoma brucei
Source: PLoS Pathog. 2016 Feb 24;12(2):e1005439. doi: 10.1371/journal.ppat.1005439 (PMC4765897; doi:10.1371/journal.ppat.1005439)
Supplement: S4 Fig — Log2 LFQ intensities of proteins are shown for long slender (LS) and short stumpy (SS) forms and consecutive time points during the differentiation as indicated. (PDF) [file ppat.1005439.s004.pdf]

Supplementary Figure 4

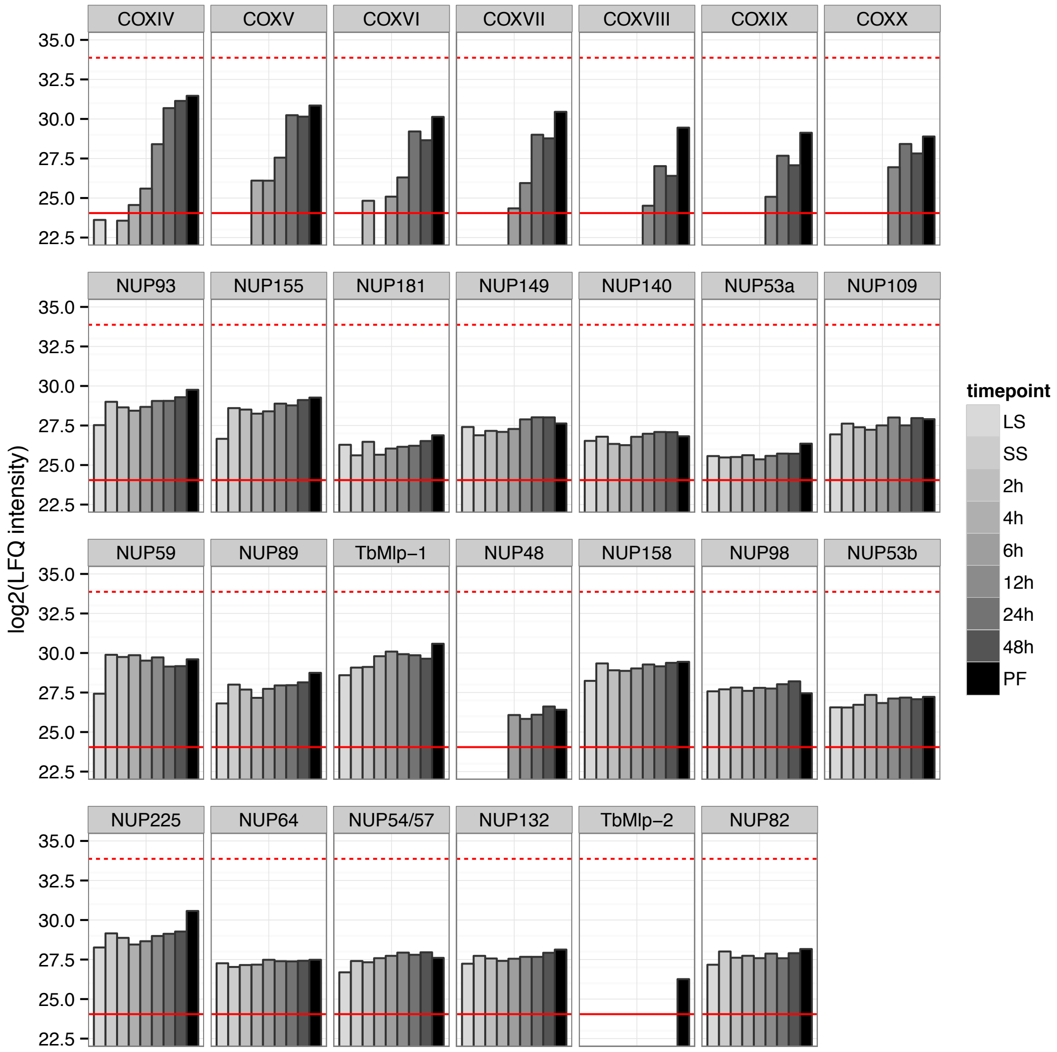

Fig S4: Changes in LFQ intensity of cytochrome oxidase subunits and nuclear pore complex proteins. Log2 LFQ intensities of proteins are shown for long slender (LS) and short stumpy (SS) forms and consecutive time points during the differentiation as indicated.
