# Supplementary material for: Quantitative Proteomics Uncovers Novel Factors Involved in Developmental Differentiation of Trypanosoma brucei
Source: PLoS Pathog. 2016 Feb 24;12(2):e1005439. doi: 10.1371/journal.ppat.1005439 (PMC4765897; doi:10.1371/journal.ppat.1005439)
Supplement: S5 Fig — (A) Cumulative growth curve of wild-type AnTat 1.1 and ∆DOT1B trypanosomes. AnTat 1.1 and ∆DOT1B cells were cultivated in high viscosity HMI-9-methylcellulose medium and show same population doubling time (5.5h). (B) AnTat 1.1 and ∆DOT1B long slender forms are morphologically indistinguishable (representative phase contrast microscopy pictures; bars 10μm). (C) Western Blot analysis shows H3K76 tri-methylation in wild-type AnTat 1.1 and loss of the modification in ∆DOT1B trypanosomes. (D) Cell density-dependent entry into stationary phase is indistinguishable in wild-type and ∆DOT1B cells. (E) Western blot analysis of PAD1 expression. Stumpy formation marker PAD1 is detectable in SS populations of both cell lines (LS: long slender; SS: short stumpy). The structural protein PFR serves as a loading control. (F) Cell cycle profiles of propidium iodide stained logarithmically growing parasites (upper panel) and arrested stumpy cell analysed by flow cytometry. Stumpy populations of both cell lines accumulate in G0/G1 phase of the cell cycle. (PDF) [file ppat.1005439.s005.pdf]

Supplementary Figure 5

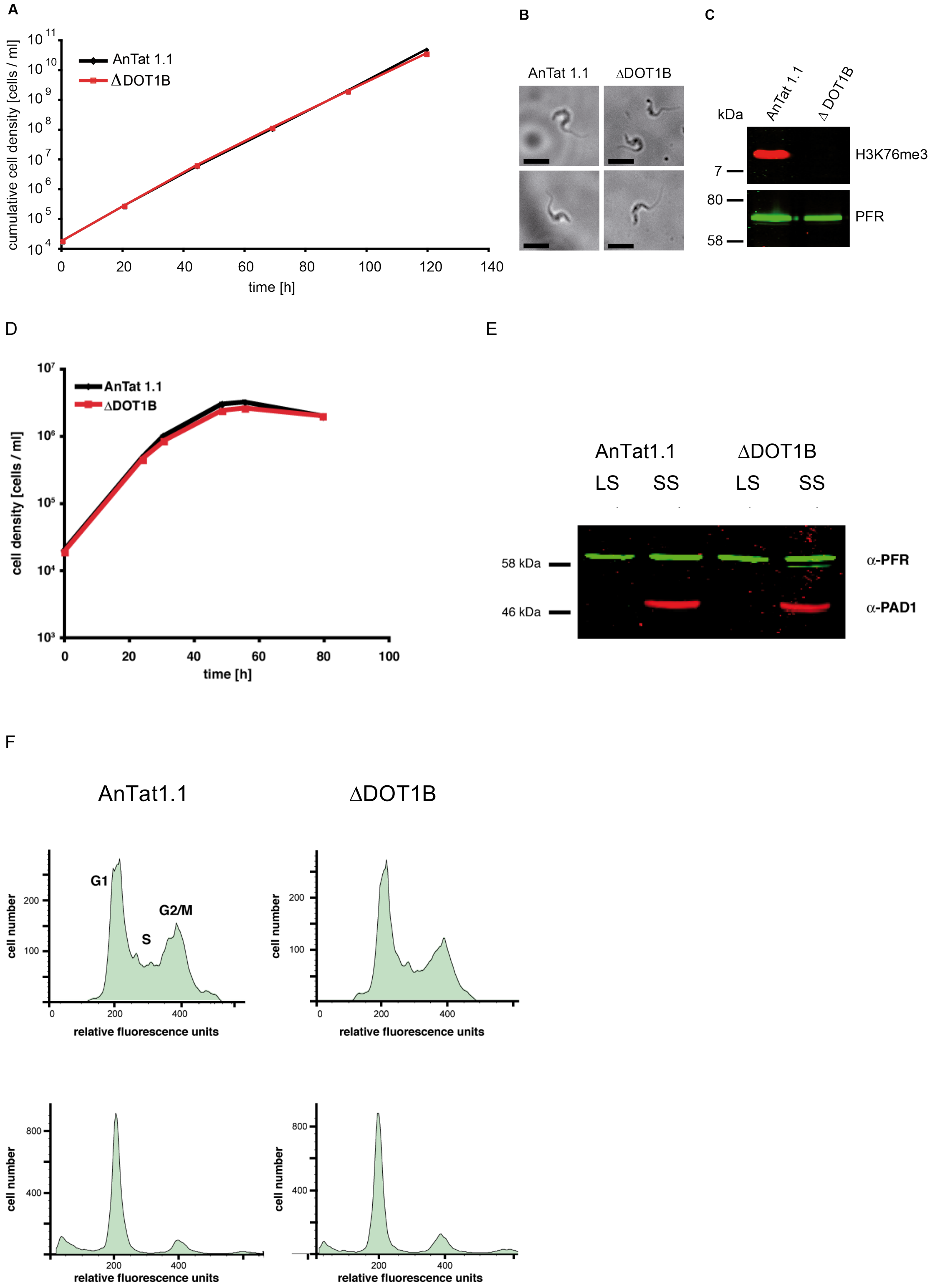

Fig S5: Characterization of DOT1B-depleted pleomorphic trypanosomes. (A) Cumulative growth curve of wild-type AnTat 1.1 and ΔDOT1B trypanosomes. AnTat 1.1 and ΔDOT1B cells were cultivated in high viscosity HMI-9-methylcellulose medium and show same population doubling time (5.5h). **(B)** AnTat 1.1 and ΔDOT1B long slender forms are morphologically indistinguishable (representative phase contrast microscopy pictures; bars 10µm). **(C)** Western Blot analysis shows H3K76 tri-methylation in wild-type AnTat 1.1 and loss of the modification in ΔDOT1B trypanosomes. **(D)** Cell density-dependent entry into stationary phase is indistinguishable in wild-type and ΔDOT1B cells. **(E)** Western blot analysis of PAD1 expression. Stumpy formation marker PAD1 is detectable in SS populations of both cell lines (LS: long slender; SS: short stumpy). The structural protein PFR serves as a loading control. **(F)** Cell cycle profiles of propidium iodide stained logarithmically growing parasites (upper panel) and arrested stumpy cell analysed by flow cytometry. Stumpy populations of both cell lines accumulate in G0/G1 phase of the cell cycle.
