# Supplementary material for: Quantitative Proteomics Uncovers Novel Factors Involved in Developmental Differentiation of Trypanosoma brucei
Source: PLoS Pathog. 2016 Feb 24;12(2):e1005439. doi: 10.1371/journal.ppat.1005439 (PMC4765897; doi:10.1371/journal.ppat.1005439)
Supplement: S6 Fig — “Others” include cells with unusual nuclei/kinetoplast configuration or obvious inaccurate nuclear division (n = 100). Samples were taken at different timepoints after differentiation initiation as indicated below. (PDF) [file ppat.1005439.s006.pdf]

Supplementary Figure 6

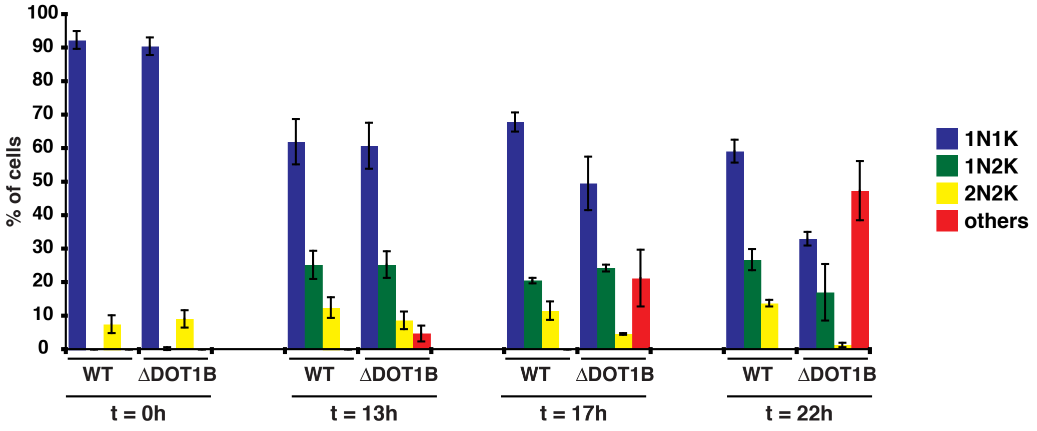

Fig S6: Quantitative analysis of nuclei (N) and kinetoplast (K) configuration in differentiating wild-type (WT) and DOT1B-depleted (ΔDOT1B) cells. "Others" include cells with unusual nuclei/kinetoplast configuration or obvious inaccurate nuclear division (n=100). Samples were taken at different timepoints after differentiation initiation as indicated below.
