# Supplementary material for: Quantitative Proteomics Uncovers Novel Factors Involved in Developmental Differentiation of Trypanosoma brucei
Source: PLoS Pathog. 2016 Feb 24;12(2):e1005439. doi: 10.1371/journal.ppat.1005439 (PMC4765897; doi:10.1371/journal.ppat.1005439)
Supplement: S7 Fig — Signal intensity of indirect immunofluorescence analysis (anti-γH2A) of differentiating AnTat1.1 wild-type versus ∆DOT1B trypanosomes was quantified using the ImageJ software. More than 100 individual cells were analysed for each time point and each cell line during the differentiation process (before differentiation in stumpy cells, 24 h and 48 h after the onset of differentiation). The measured integrated fluorescence density defined for each cell is shown in a scatter dot plot. The red lines delimit the mean value and standard deviation. The statistical analysis with a one-way Anova and Tukey’s multiple comparison test shows significance between the data sets marked with bars and asterisks (*** p-value <0.001). (PDF) [file ppat.1005439.s007.pdf]

Supplementary Figure 7

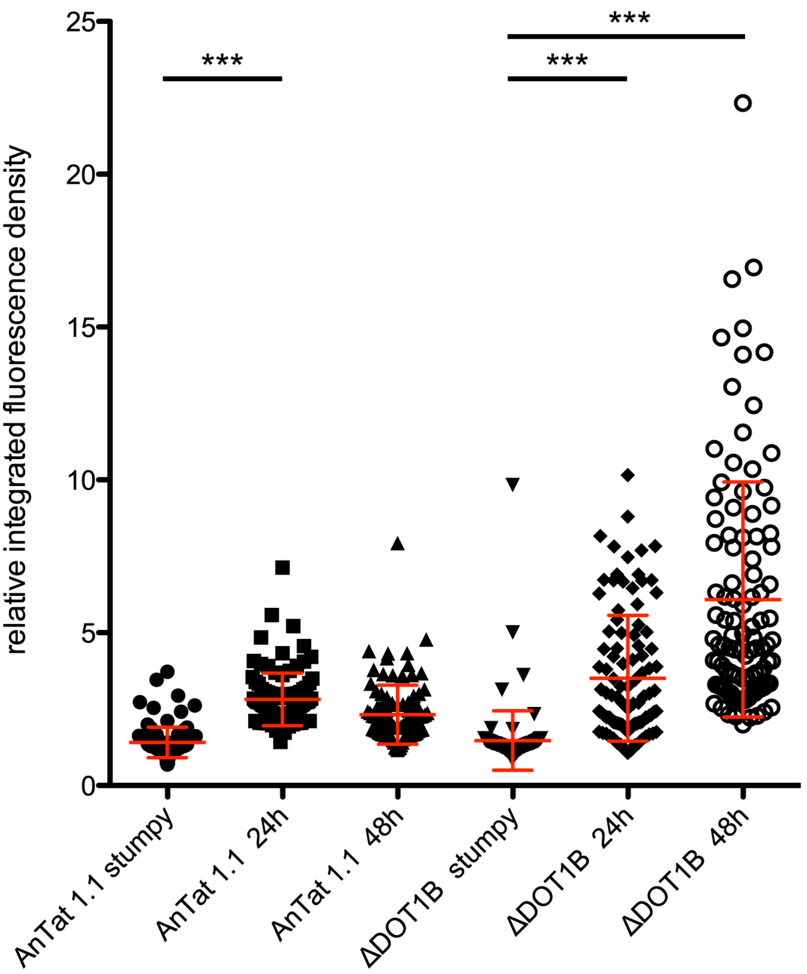

Fig S7 **Quantification of DNA damage in ΔDOT1B trypanosomes during differentiation**. Signal intensity of indirect immunofluorescence analysis (anti-γH2A) of differentiating AnTat1.1 wild-type versus ΔDOT1B trypanosomes was quantified using the ImageJ software. More than 100 individual cells were analysed for each time point and each cell line during the differentiation process (before differentiation in stumpy cells, 24 h and 48 h after the onset of differentiation). The measured integrated fluorescence density defined for each cell is shown in a scatter dot plot. The red lines delimit the mean value and standard deviation. The statistical analysis with a one-way Anova and Tukey's multiple comparison test shows significance between the data sets marked with bars and asterisks (*** p-value <0.001).
