# Supplementary material for: Quantitative Proteomics Uncovers Novel Factors Involved in Developmental Differentiation of Trypanosoma brucei
Source: PLoS Pathog. 2016 Feb 24;12(2):e1005439. doi: 10.1371/journal.ppat.1005439 (PMC4765897; doi:10.1371/journal.ppat.1005439)
Supplement: S1 Fig — Page 1 Example page of individual expression profile. The bars in the bottom panel show the calculated median LFQ intensities with color indicating the amount of measured instances in 4 replicates ranging from light grey (1 value) to black (4 values). Colored dots represent individual replicates. The orange line and dots show the bioinformatically obtained expression profile for each protein group. Missing values are imputed (see methods section). The red line marks the lower range of 1% of measured values, the dashed red line represents 99% of all measured values. Stages and timepoints sampled during differentiation are indicated on the x-axis. The top panels display fold changes compared to long slender (left panel) or short stumpy forms (right panel). Page 2: Groups of proteins with similar regulation pattern. Click on the profiles to see the individual protein profile. Groups of proteins discussed in the manuscript are compiled in the table of content of this PDF file where they can be selected. (PDF) [file ppat.1005439.s001.pdf]

Supplemental figure 1

Quantitative proteomics uncovers novel factors for developmental differentiation of *Trypanosoma brucei*

Mario Dejung, Ines Subota, Ferdinand Brucerius, Gülcin Dindar, Anja Freiwald, Markus Engstler, Michael Boshart, Falk Butter, Christian Janzen

Note: Please switch on table of contents.

Example page:

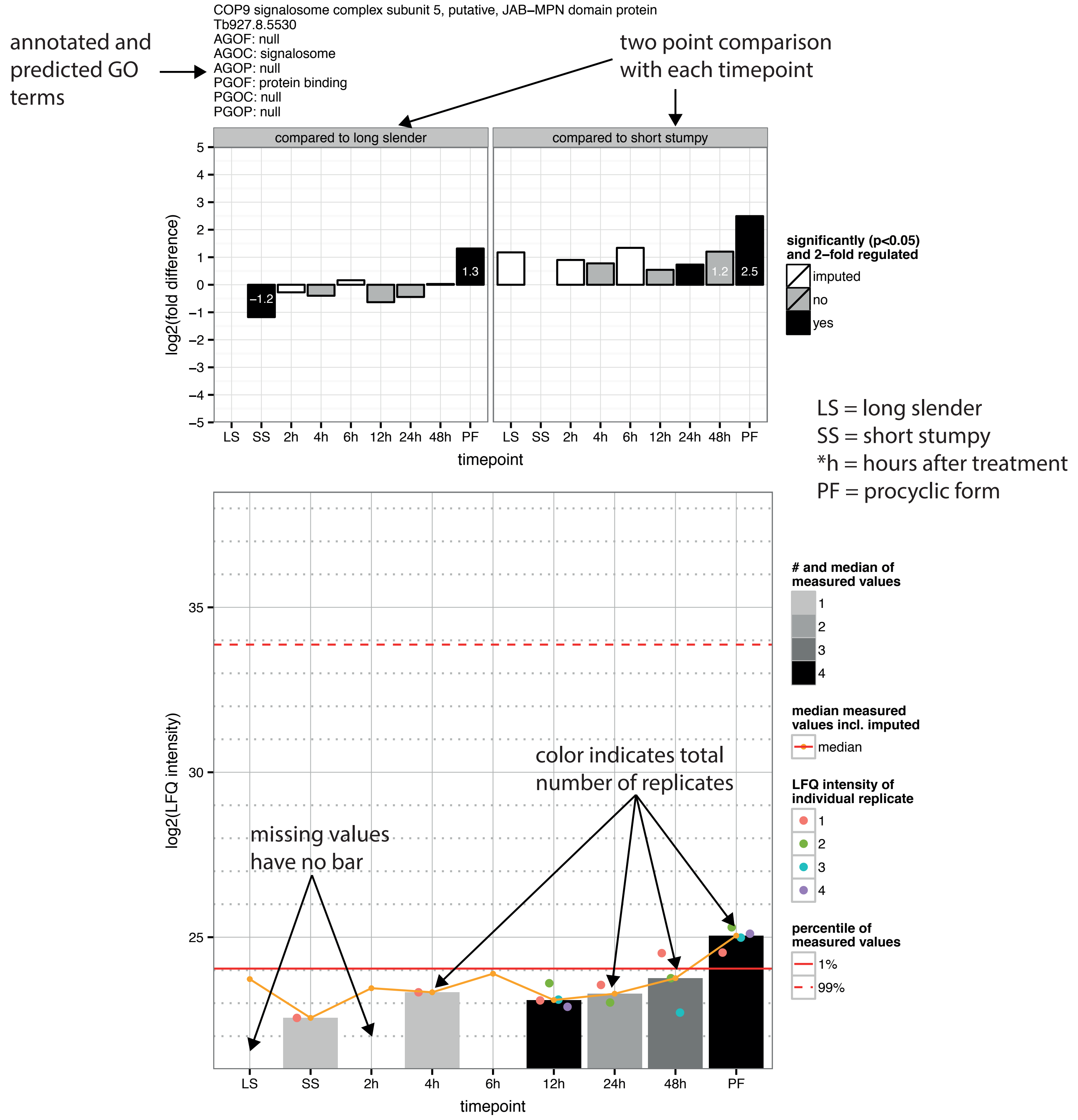

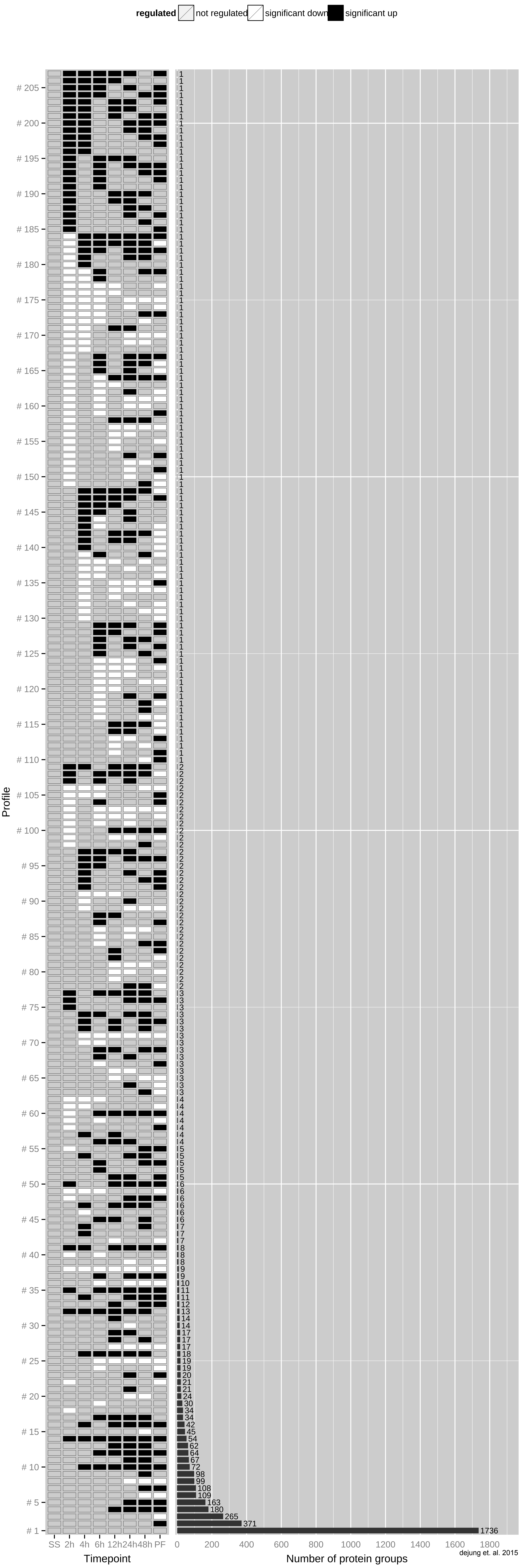
regulated
not regulated
significant down
significant up
Profile
Timepoint
SS 2h 4h 6h 12h 24h 48h PF
Number of protein groups
0 200 400 600 800 1000 1200 1400 1600 1800
dejung et. al. 2015

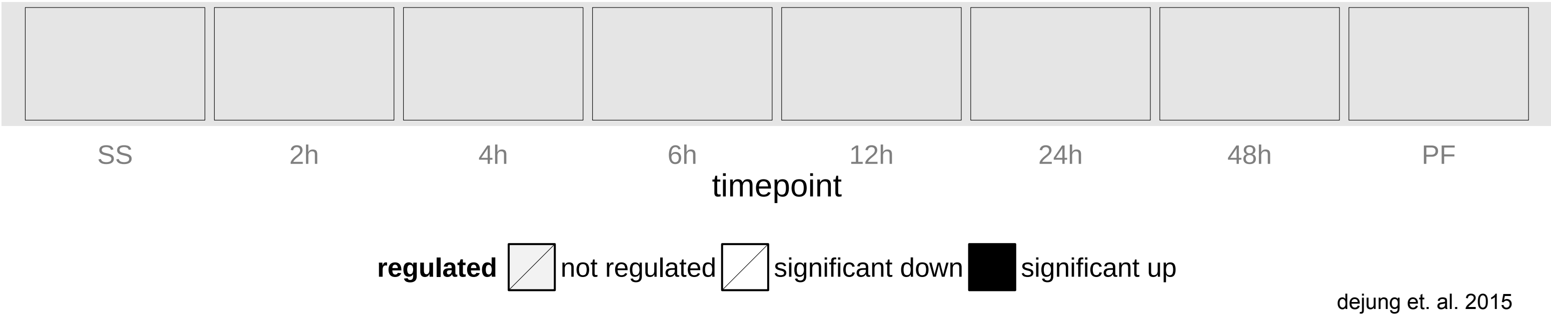

SS
2h
4h
6h
12h
24h
48h
PF
timepoint
regulated
not regulated
significant down
significant up
dejung et. al. 2015

major vault protein, putative, BAC from homologous region on chr5, putative (MVP)
Tb927.5.4460;Tb05.5K5.110;Tb11.v5.0866
AGOF: null
AGOC: null
AGOP: null
PGOF: null
PGOC: null
PGOP: null

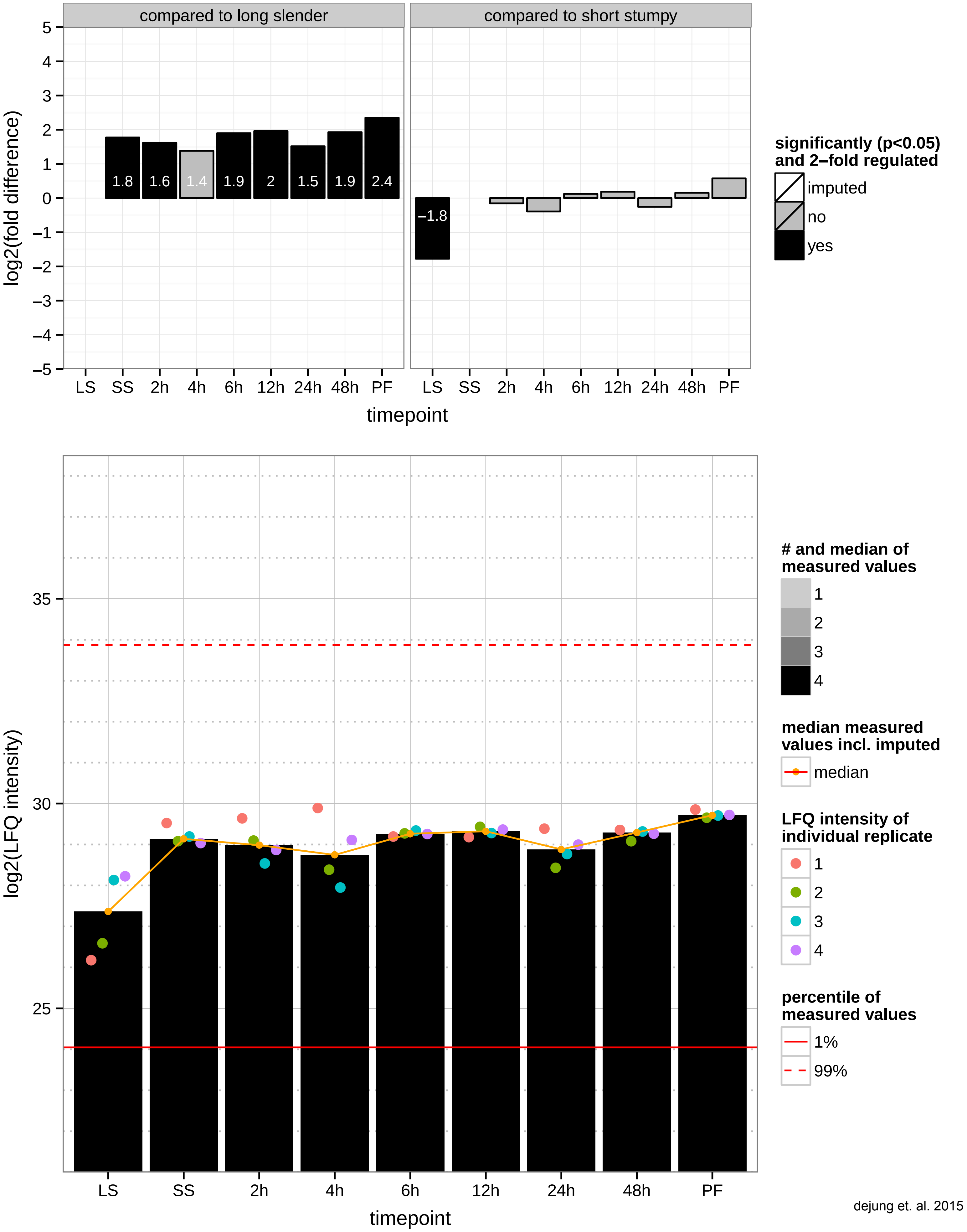

dejung et. al. 2015

paraflagellar rod component par4, putative, BAC from homologous region on chr5
Tb927.5.4480;Tb05.5K5.130
AGOF: null
AGOC: null
AGOP: null
PGOF: null
PGOC: null
PGOP: null

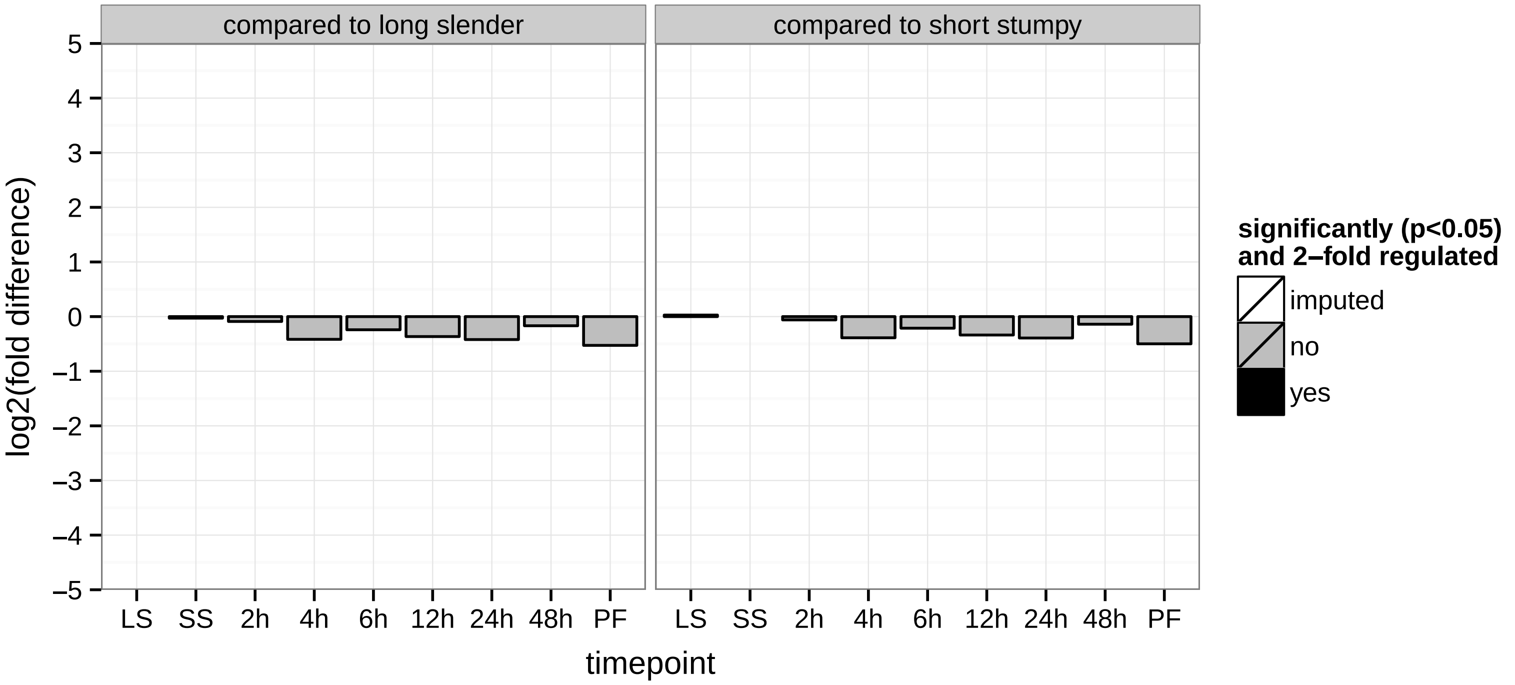

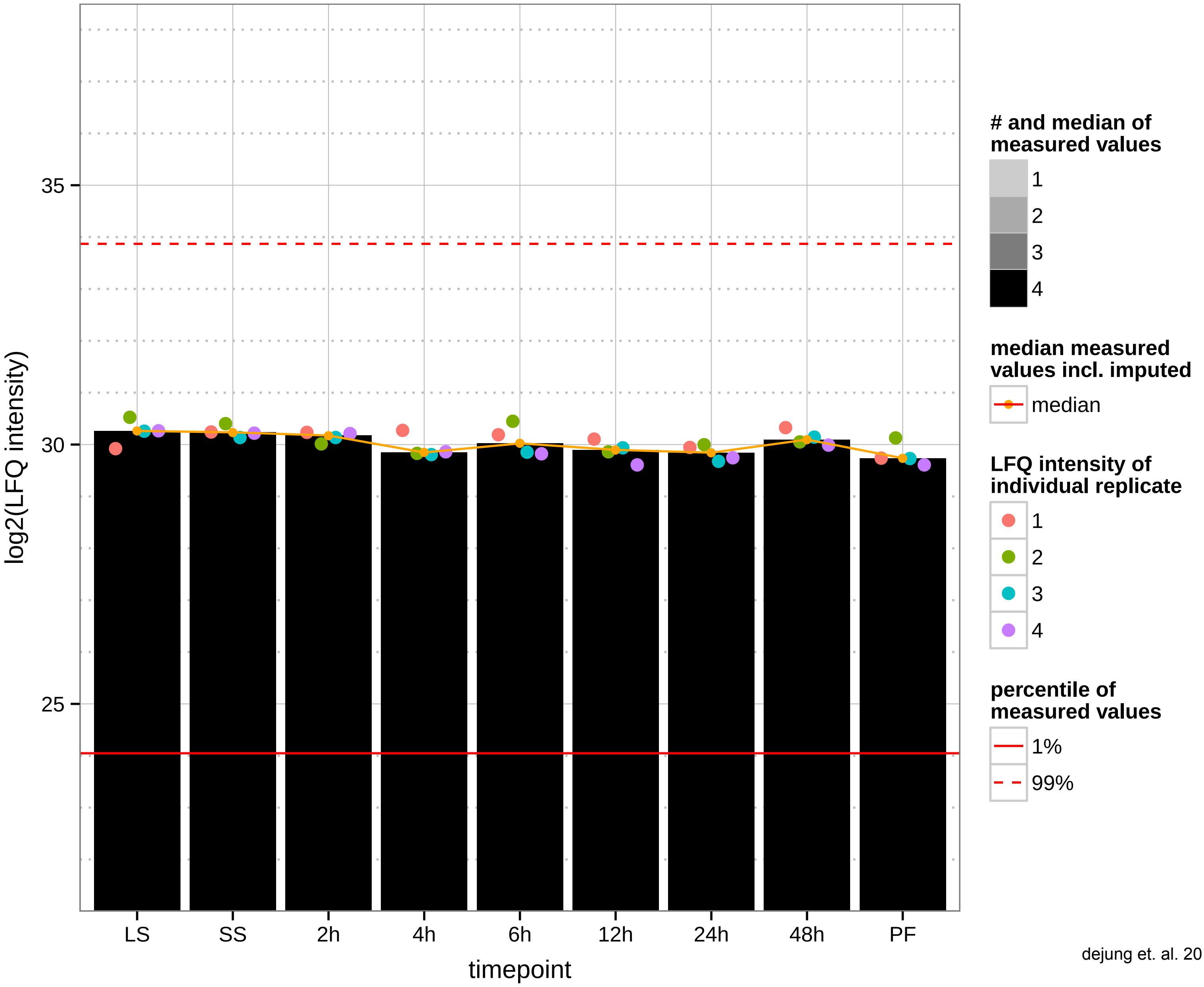

dejung et. al. 2015

guanine deaminase, putative, guanase, guanine aminase, guanine aminohydrolase, BAC from homologous region on chr5
Tb927.5.4560;Tb11.v5.0409;Tb05.5K5.200
AGOF: guanine deaminase activity, zinc ion binding, null
AGOC: null
AGOP: null
PGOF: guanine deaminase activity, hydrolase activity, acting on carbon–nitrogen (but not peptide) bonds, zinc ion binding
PGOC: null
PGOP: null

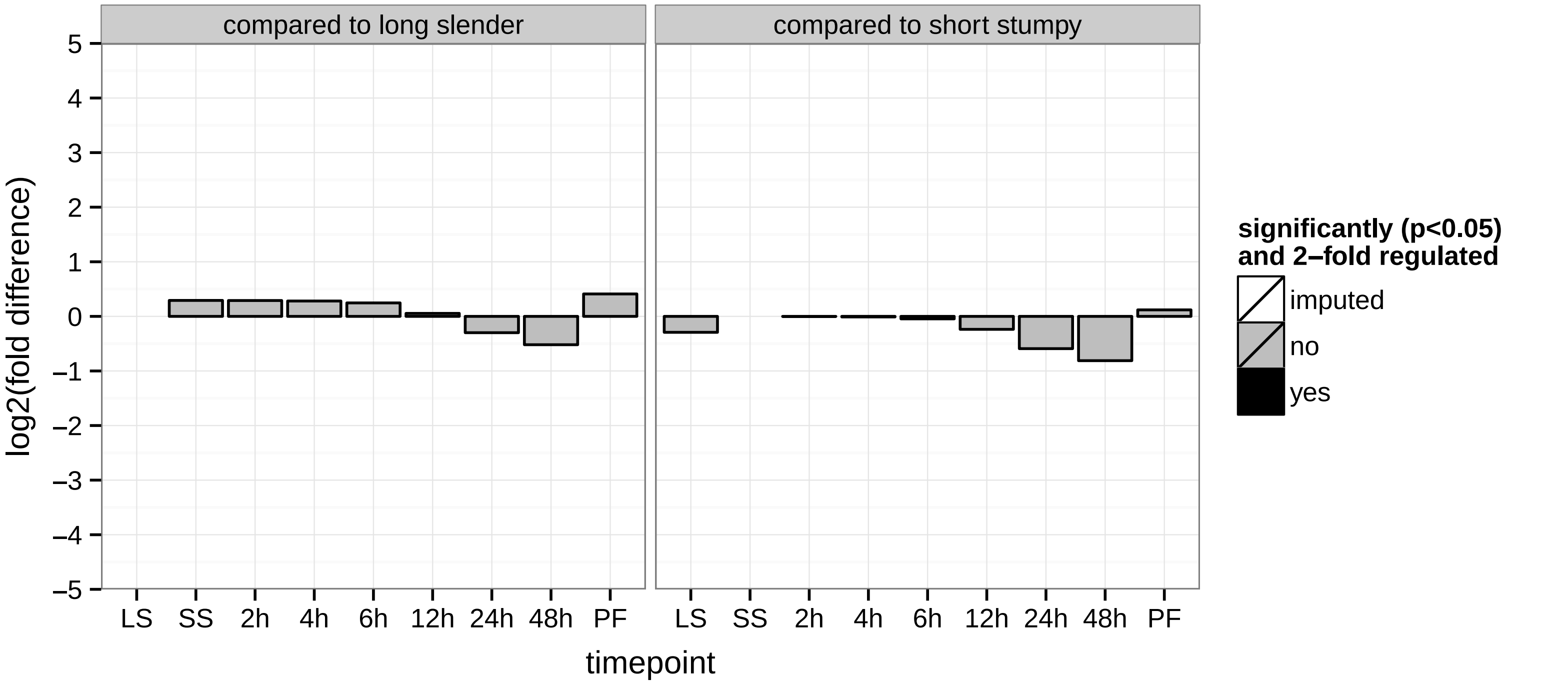

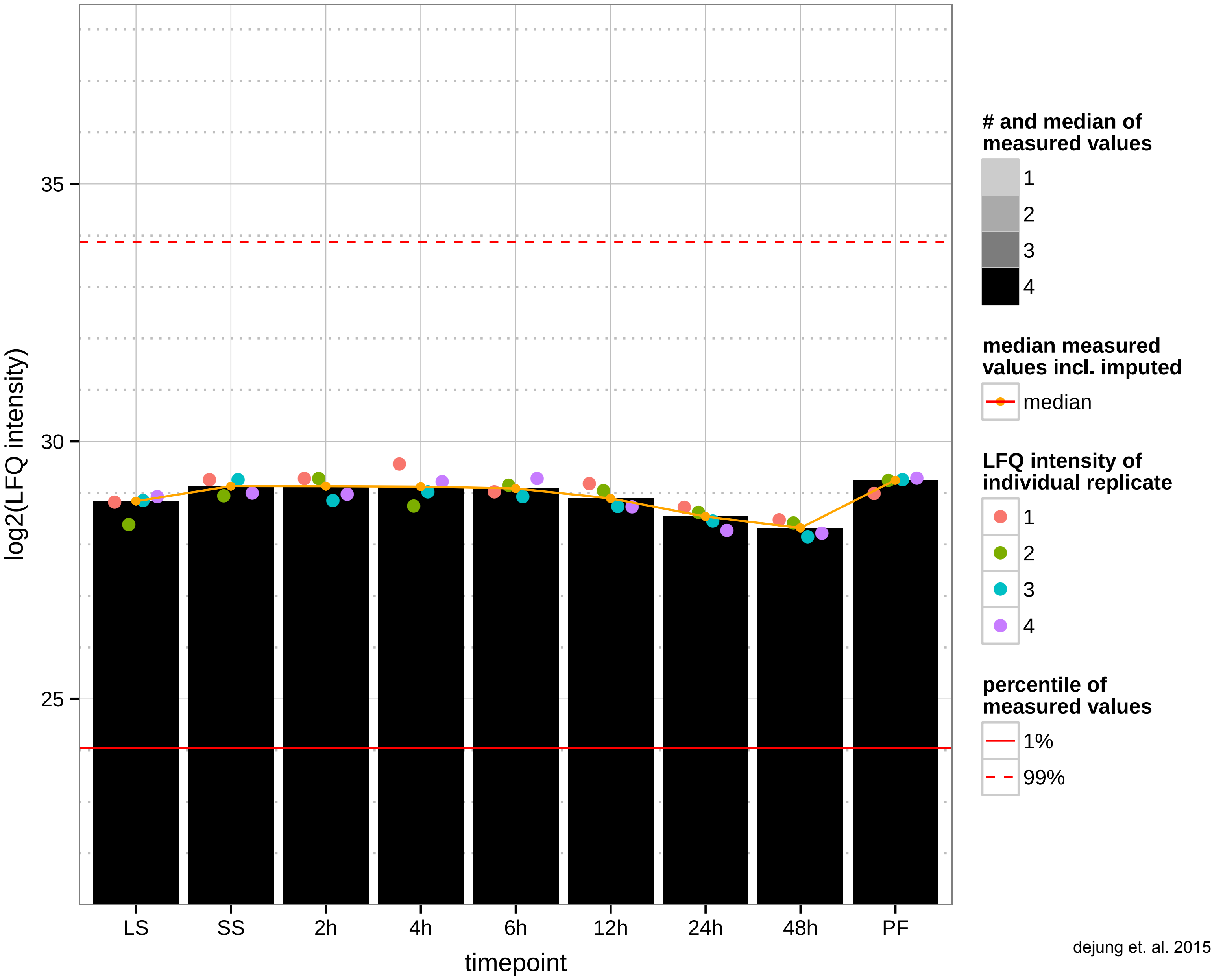

dejung et. al. 2015

hypothetical protein, conserved, BAC from homologous region on chr5
Tb927.5.4400;Tb05.5K5.50
AGOF: null
AGOC: null
AGOP: null
PGOF: null
PGOC: null
PGOP: null

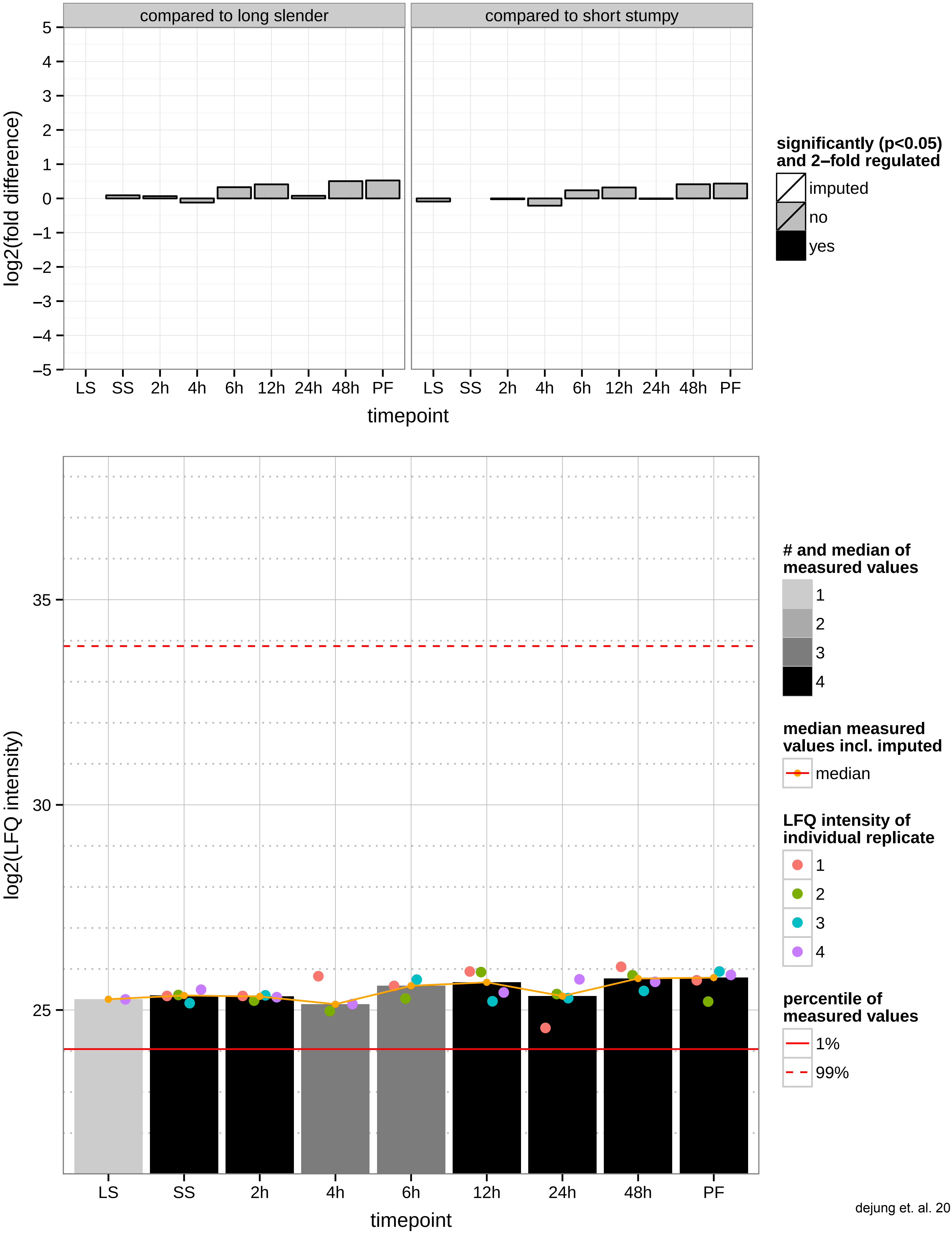

dejung et. al. 2015

hypothetical protein, conserved, BAC from homologous region on chr5
Tb927.5.4410;Tb05.5K5.60
AGOF: null
AGOC: null
AGOP: null
PGOF: null
PGOC: null
PGOP: null

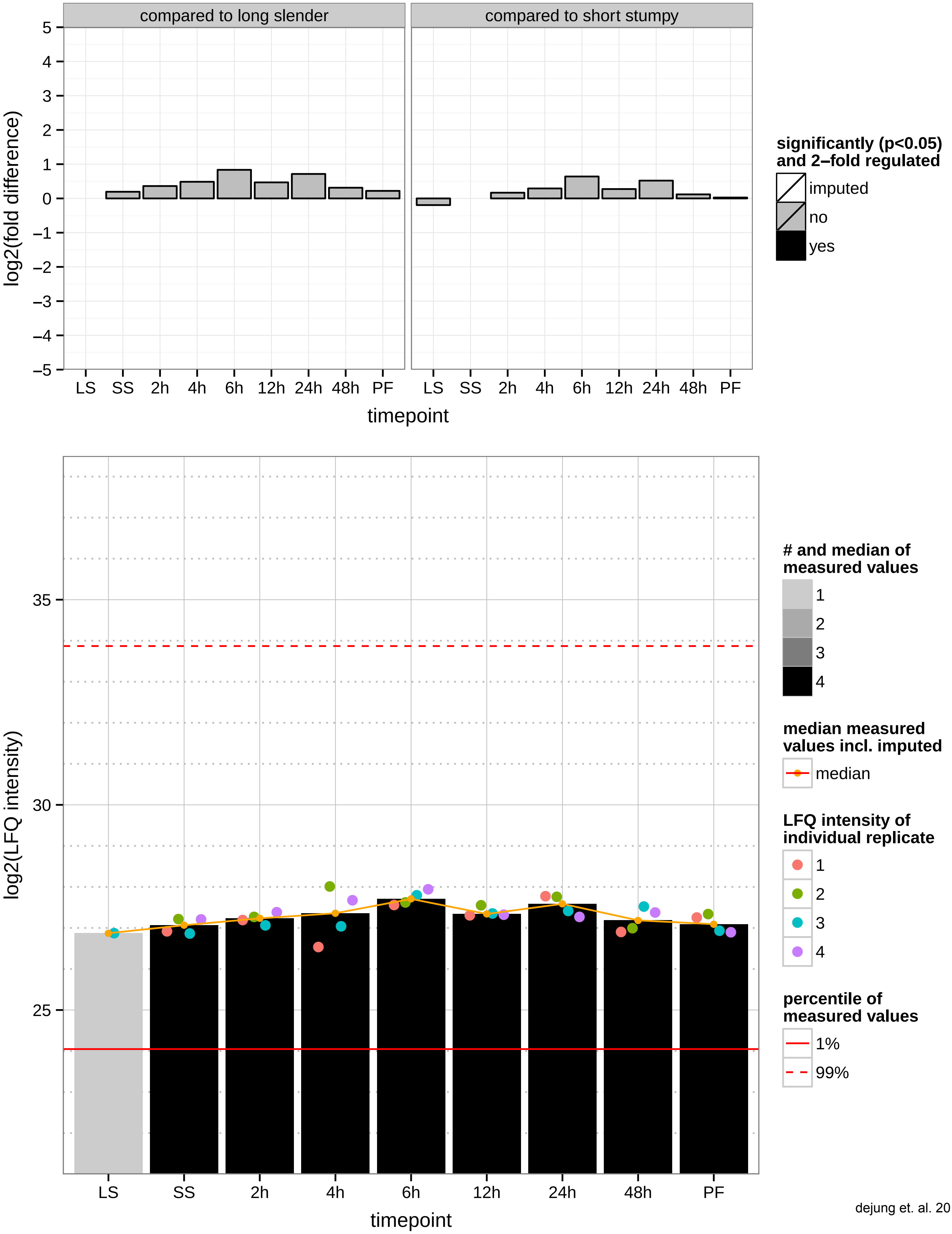

dejung et. al. 2015

dynein light chain, putative, BAC from homologous region on chr5
Tb927.5.4440;Tb05.5K5.90
AGOF: microtubule motor activity
AGOC: microtubule associated complex
AGOP: microtubule–based process
PGOF: null
PGOC: microtubule associated complex
PGOP: microtubule–based process

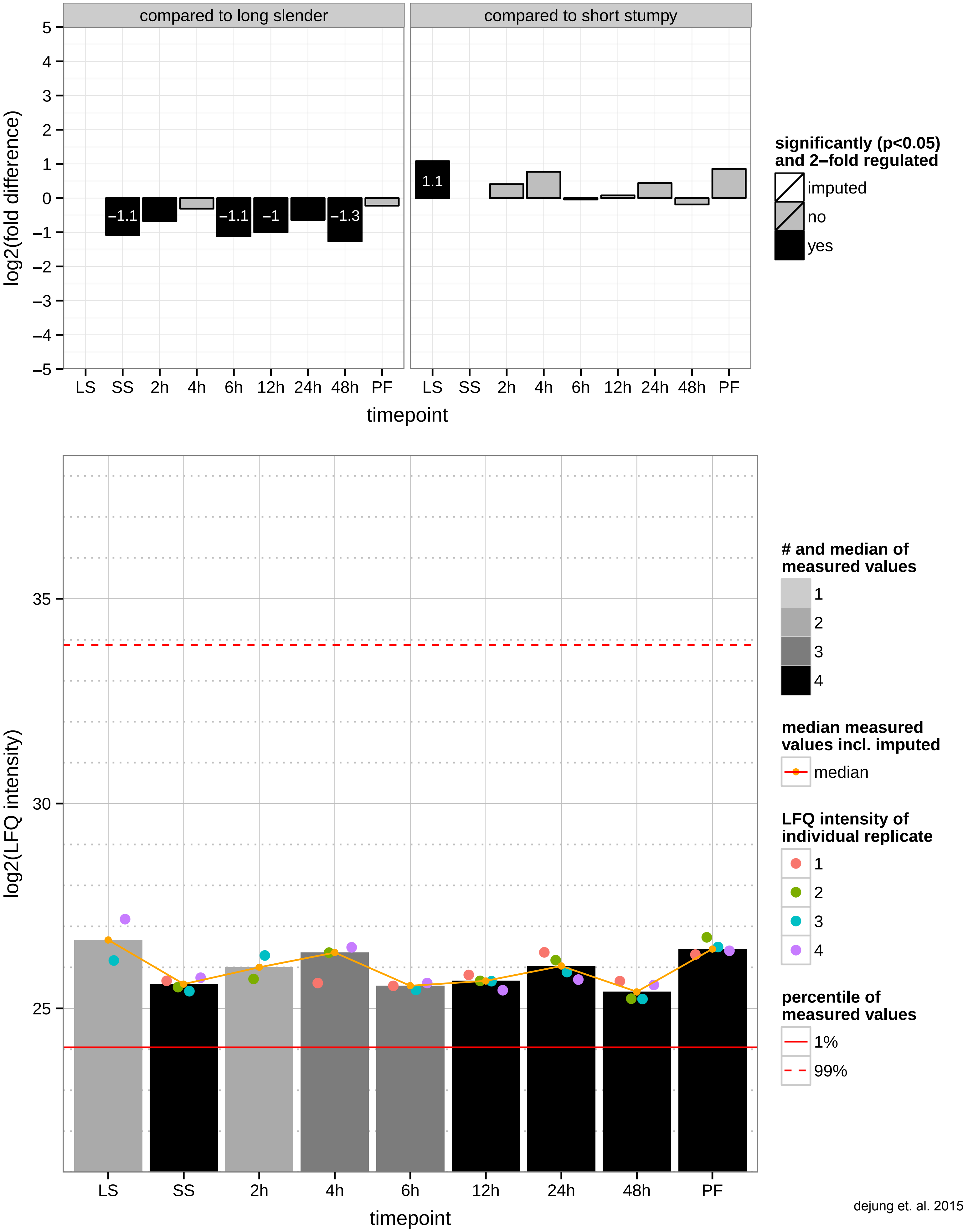

dejung et. al. 2015

stress–inducible protein STI1–like, putative, chrX additional, unordered contigs, variant surface glycoprotein, fragment
Tb927.10.4450;Tb10.v4.0035;Tb11.v5.0757
AGOF: null
AGOC: null
AGOP: null
PGOF: protein binding, null
PGOC: null
PGOP: null

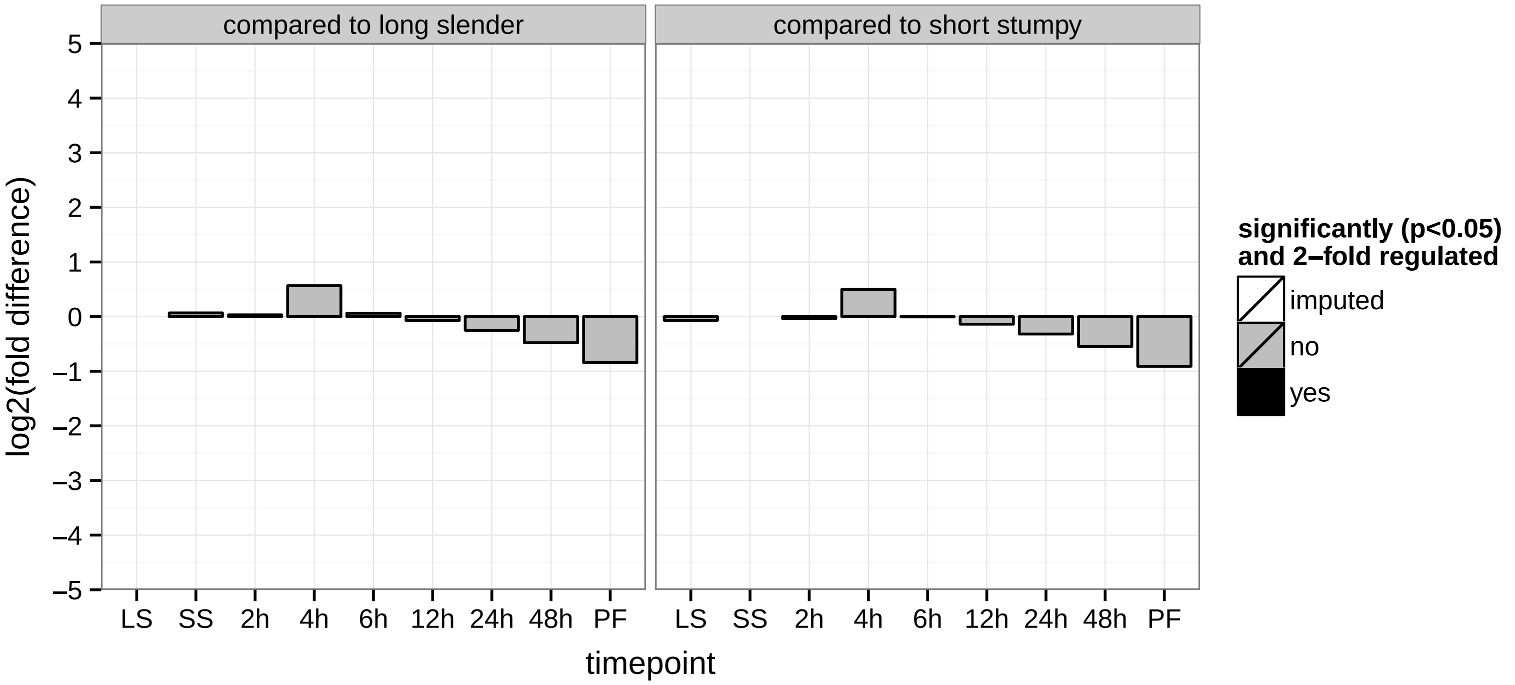

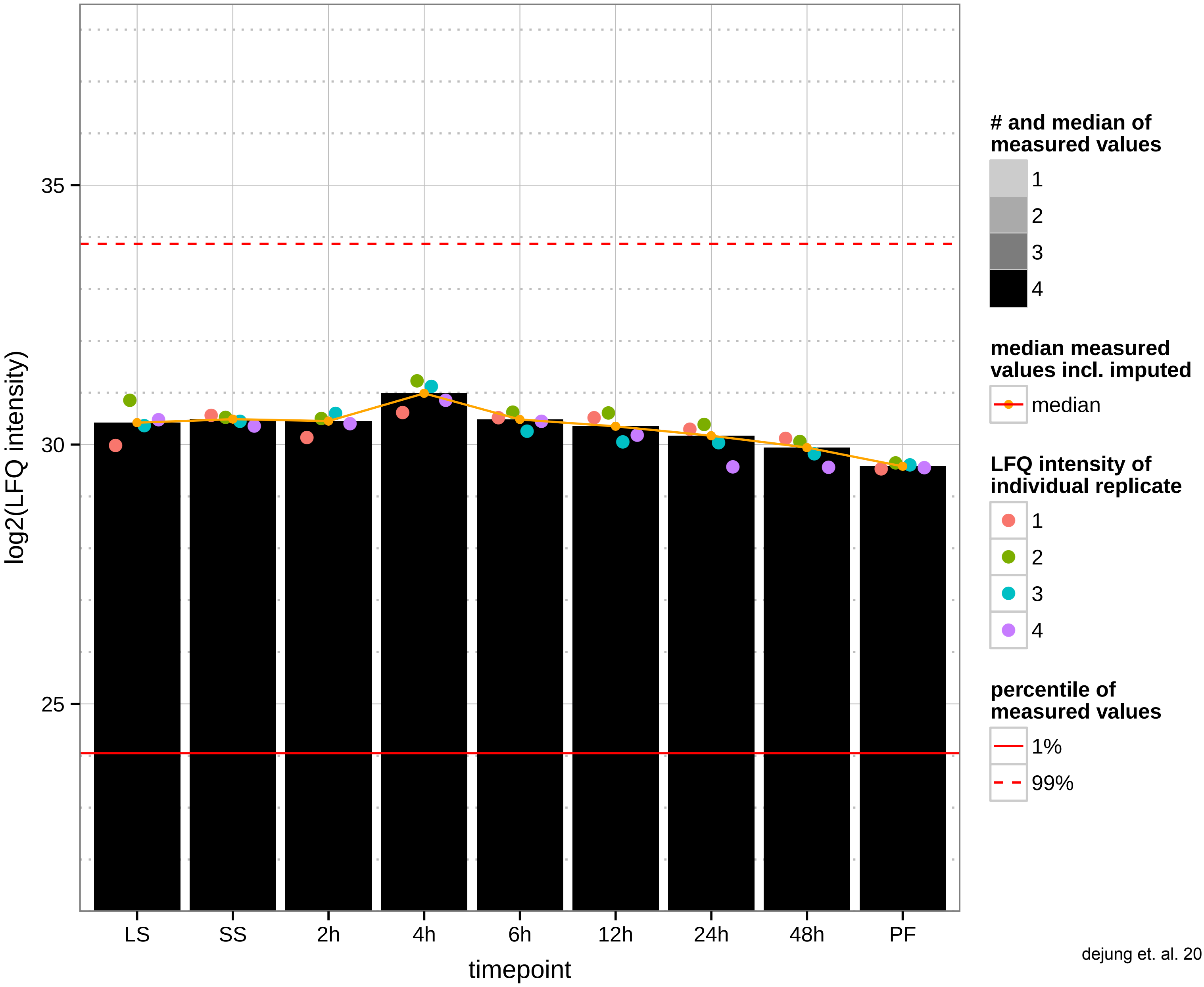

dejung et. al. 2015

leucine carboxyl methyltransferase, putative, chrX additional, unordered contigs
Tb927.10.4460;Tb10.v4.0036
AGOF: methyltransferase activity, protein C−terminal carboxyl O−methyltransferase activity
AGOC: null
AGOP: null, cellular protein modification process
PGOF: methyltransferase activity
PGOC: null
PGOP: null

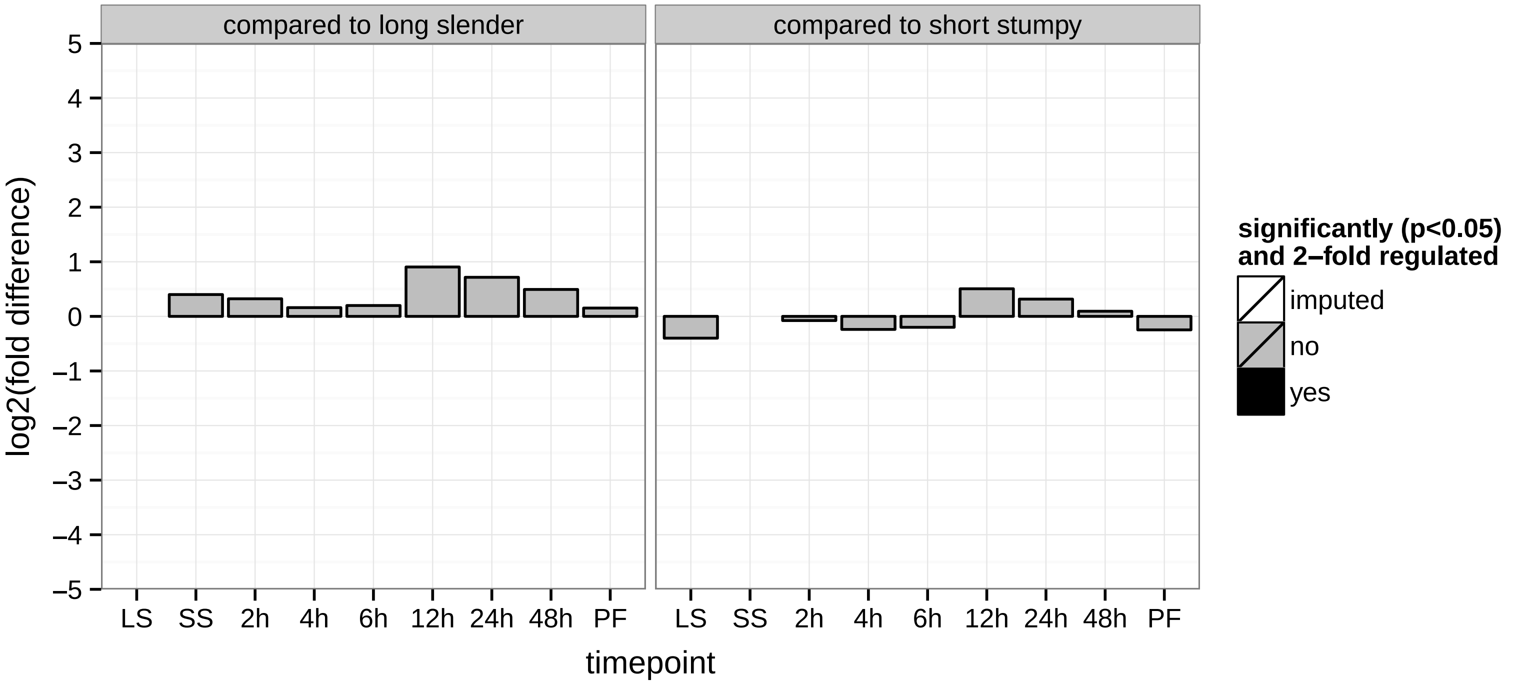

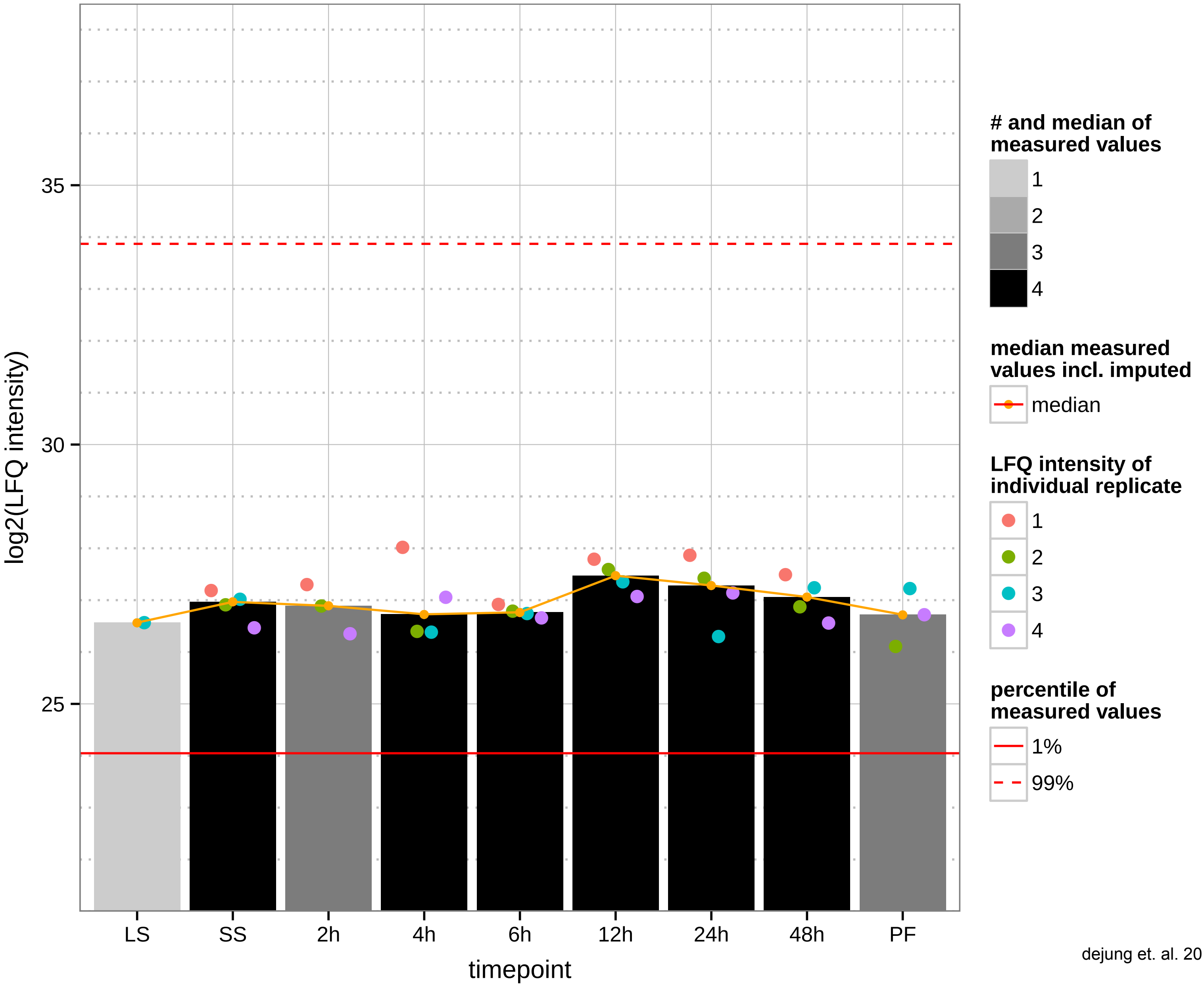

dejung et. al. 2015

mRNA capping methyltransferase, putative, chrX additional, unordered contigs
Tb927.10.4500;Tb10.v4.0040
AGOF: null, methyltransferase activity
AGOC: null
AGOP: 7−methylguanosine mRNA capping
PGOF: null
PGOC: null
PGOP: 7−methylguanosine mRNA capping

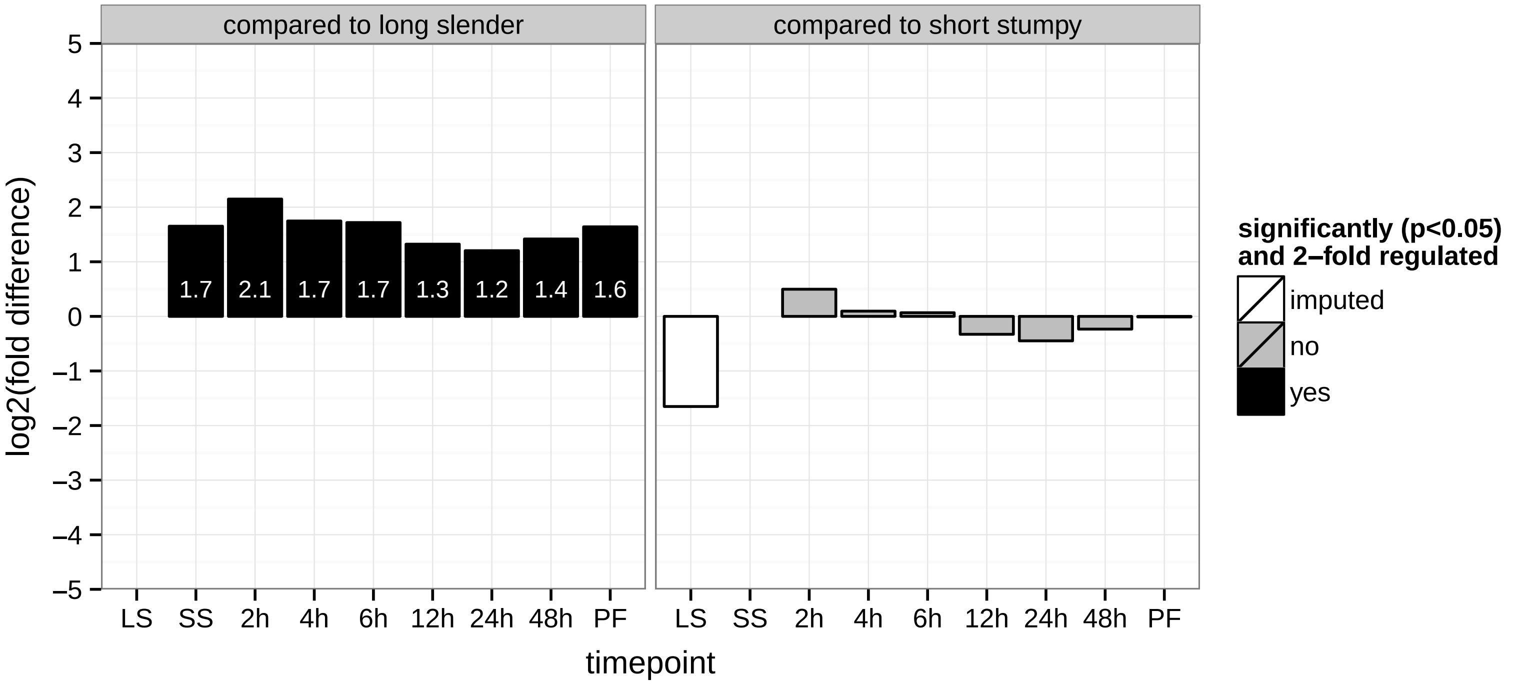

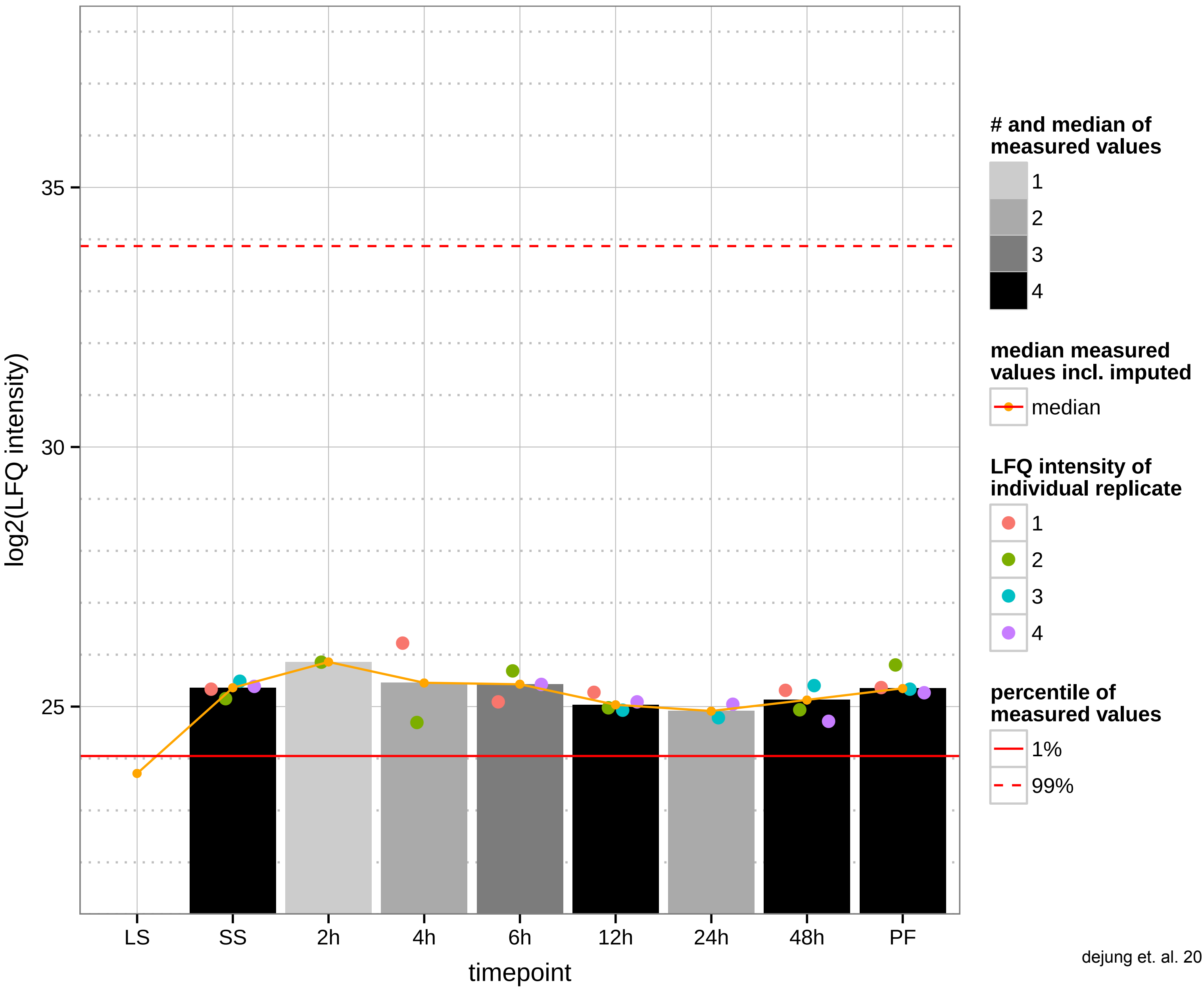

dejung et. al. 2015

mitochondrial edited mRNA stability factor 1 subunit, putative
Tb927.11.7218;Tb11.02.5120b
AGOF: RNA binding
AGOC: null
AGOP: RNA polyadenylation, mRNA stabilization
PGOF: null
PGOC: null
PGOP: null

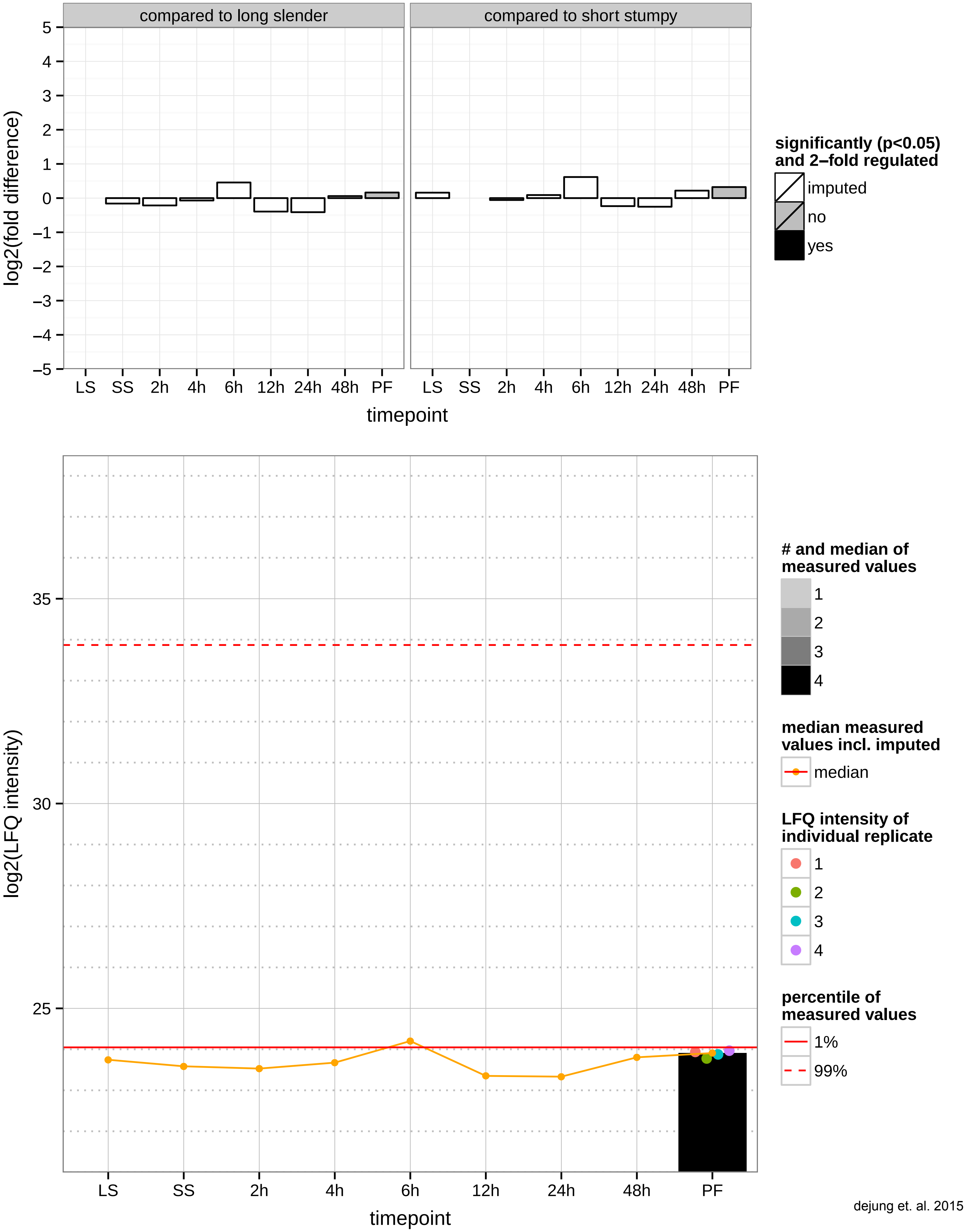

dejung et. al. 2015

NADPH–cytochrome p450 reductase, putative (CPR), putative
Tb11.02.5420b;Tb11.02.5420
AGOF: FMN binding, electron carrier activity, iron ion binding, oxidoreductase activity
AGOC: integral to membrane
AGOP: oxidation–reduction process
PGOF: FMN binding, oxidoreductase activity
PGOC: null
PGOP: oxidation–reduction process

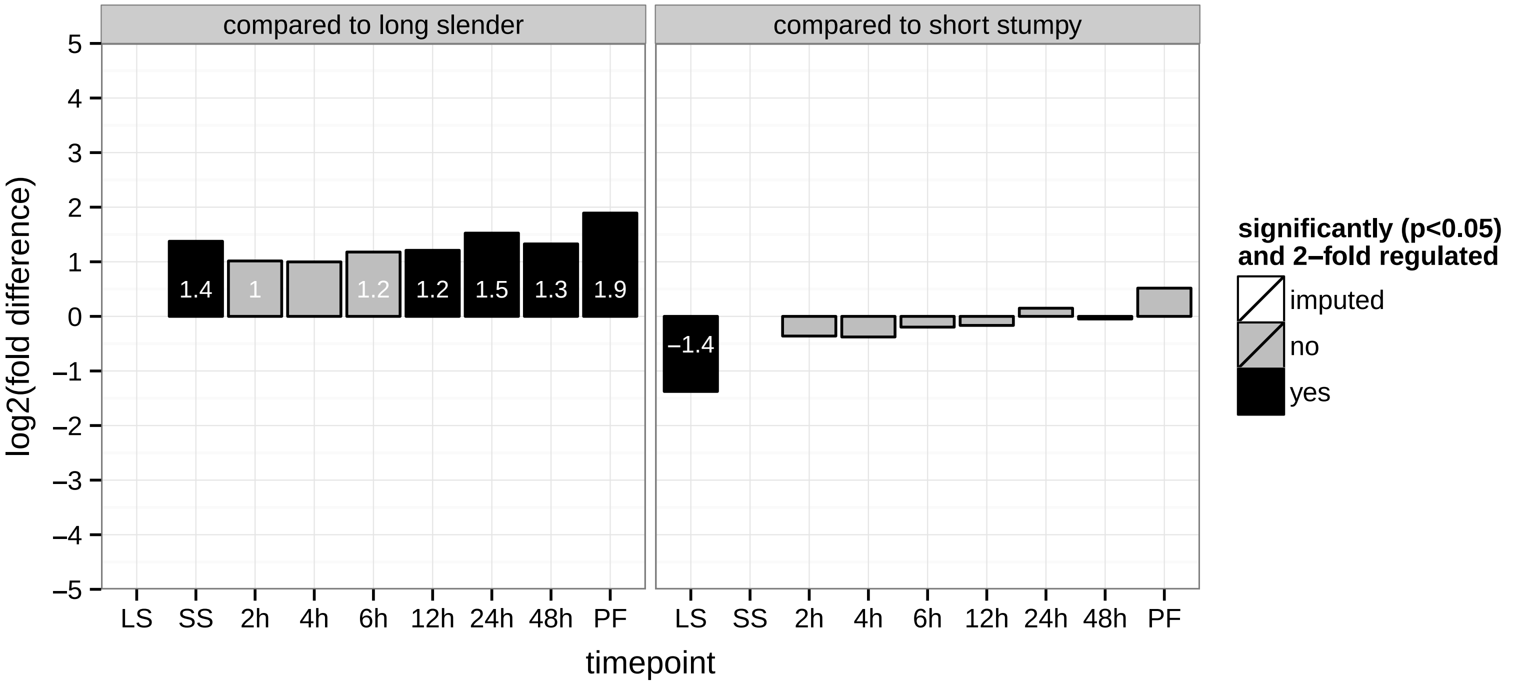

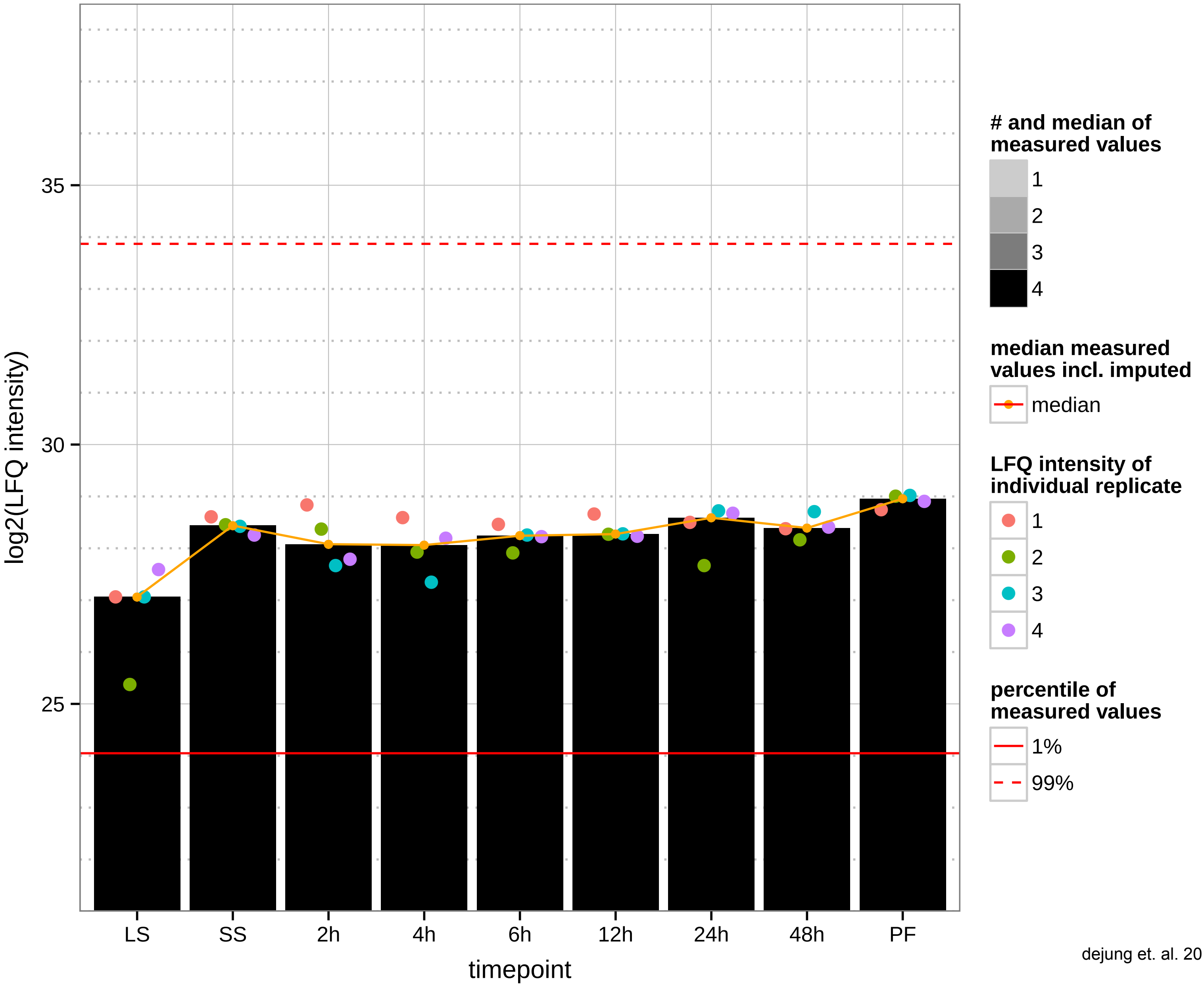

dejung et. al. 2015

40S ribosomal protein S14, putative, chrXI additional, unordered contigs (RPS14), 40S ribosomal protein S14 (RPS14)
Tb927.6.4980;Tb11.0390;Tb11.0290
AGOF: structural constituent of ribosome
AGOC: intracellular, ribosome, small ribosomal subunit
AGOP: translation
PGOF: structural constituent of ribosome
PGOC: intracellular, ribosome
PGOP: translation

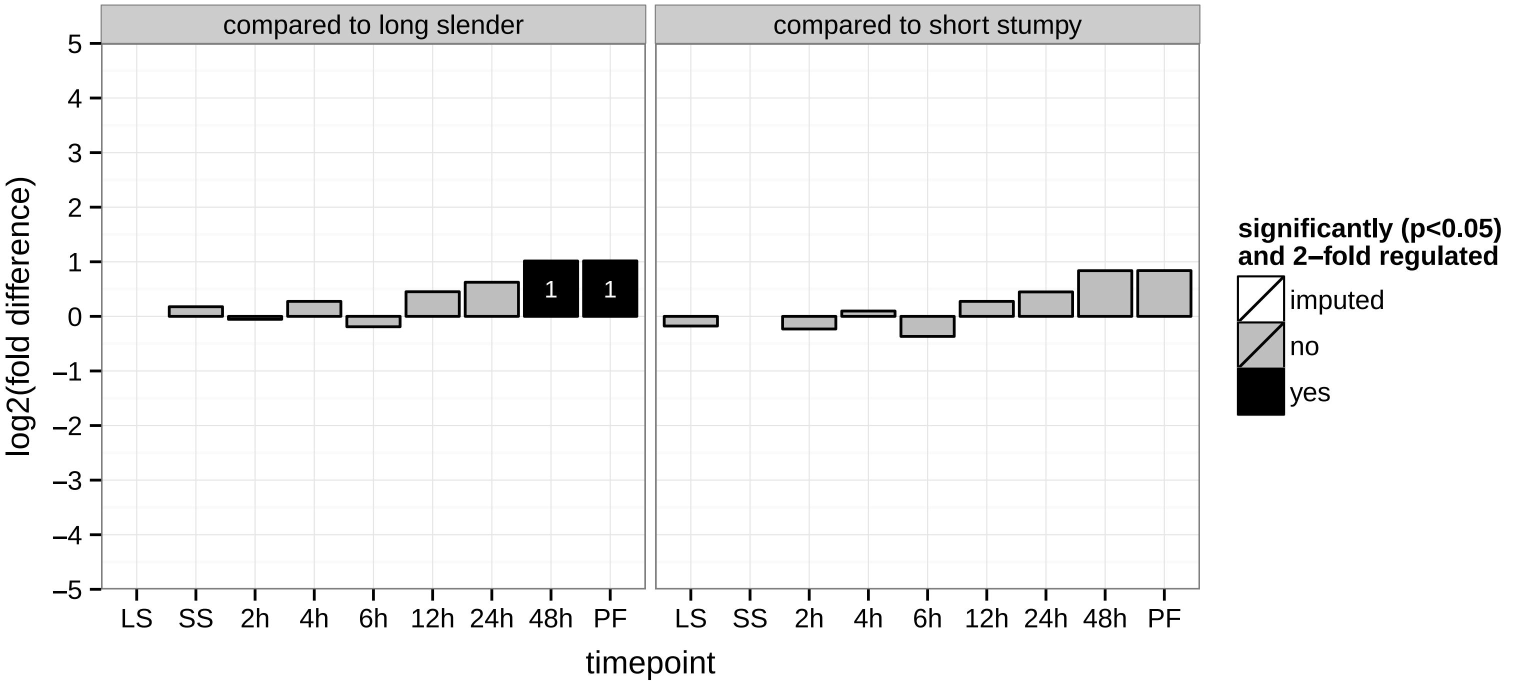

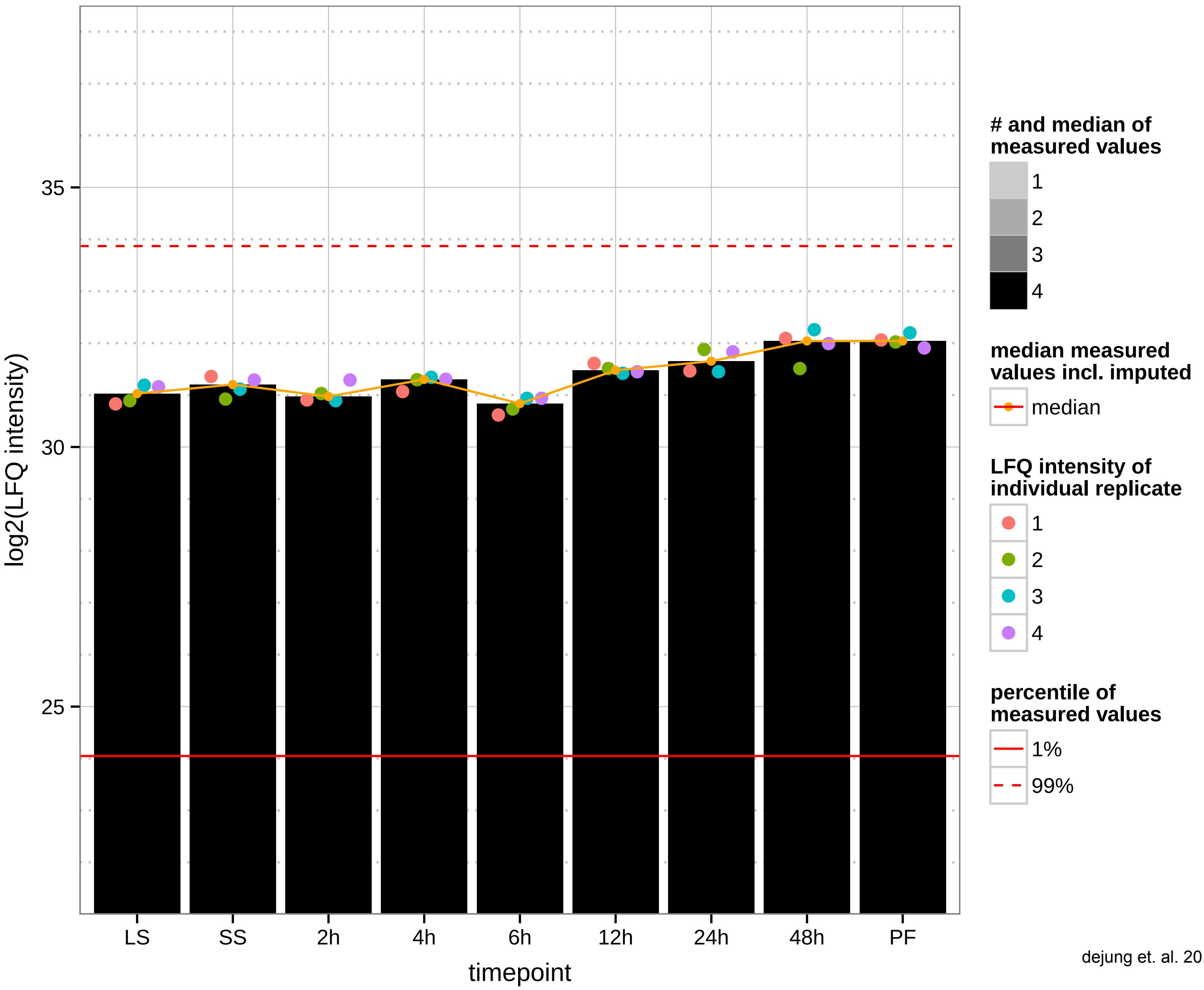

dejung et. al. 2015

hypothetical protein, chrXI additional, unordered contigs
Tb927.11.7670;Tb11.0330
AGOF: null
AGOC: null
AGOP: null
PGOF: null
PGOC: cytoplasm
PGOP: peptide cross–linking

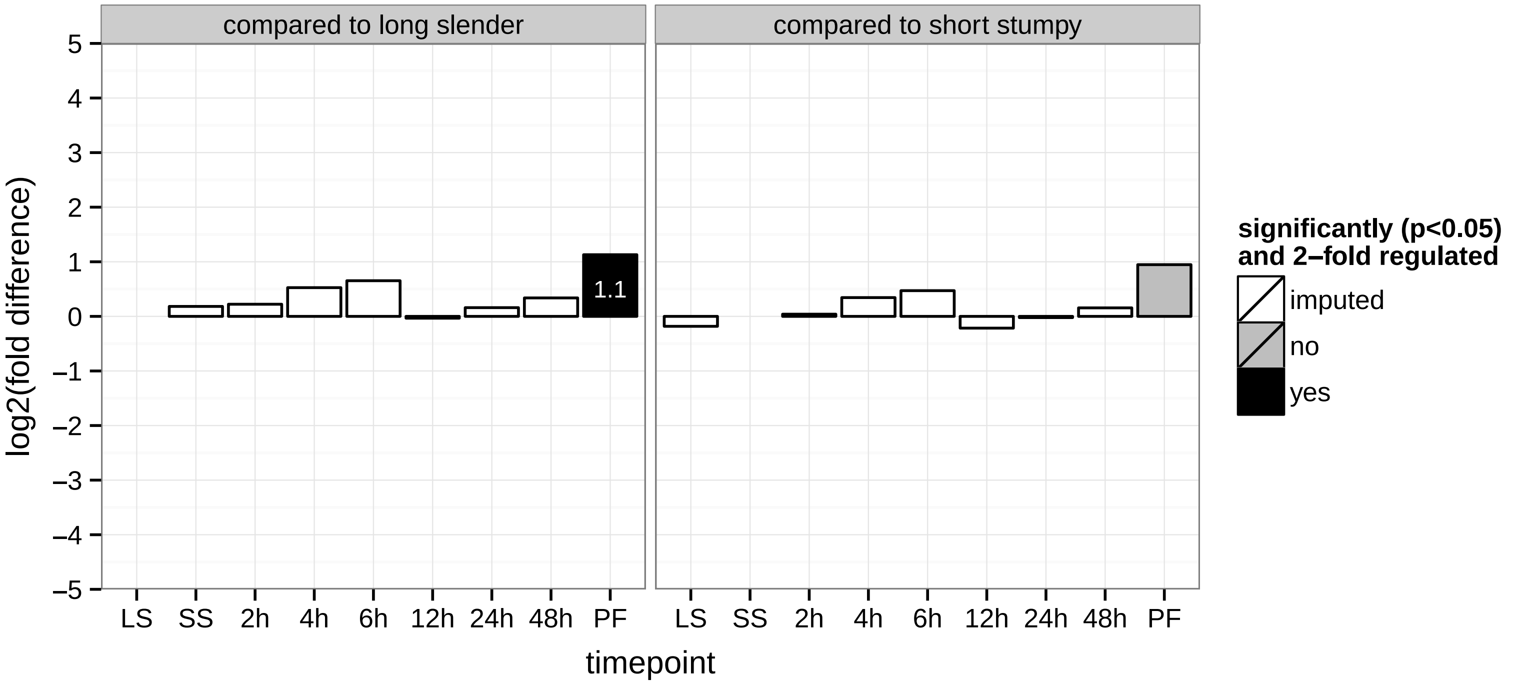

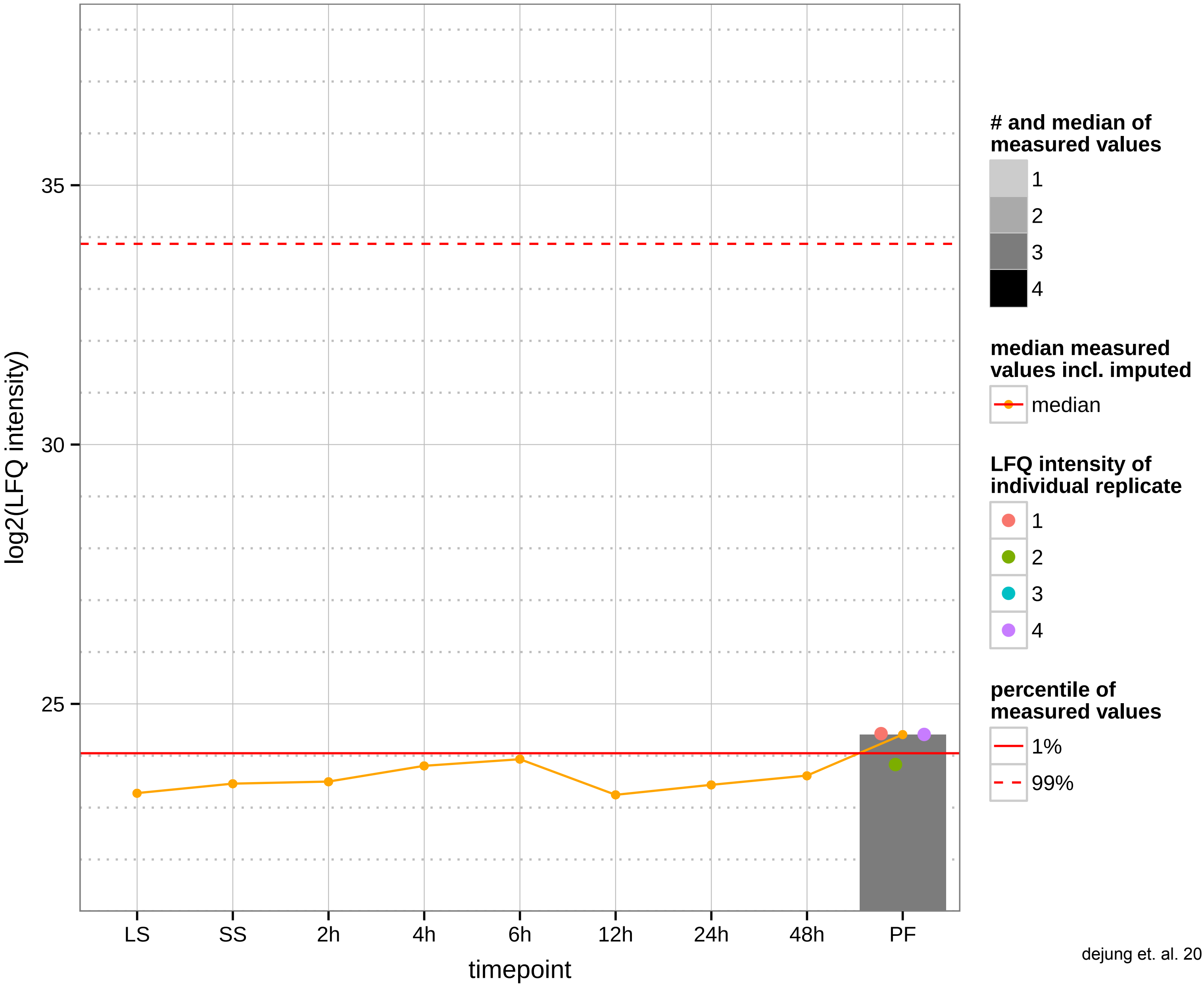

dejung et. al. 2015

hypothetical protein, chrXI additional, unordered contigs
Tb927.11.7650;Tb11.0350
AGOF: null
AGOC: null
AGOP: null
PGOF: null
PGOC: null
PGOP: null

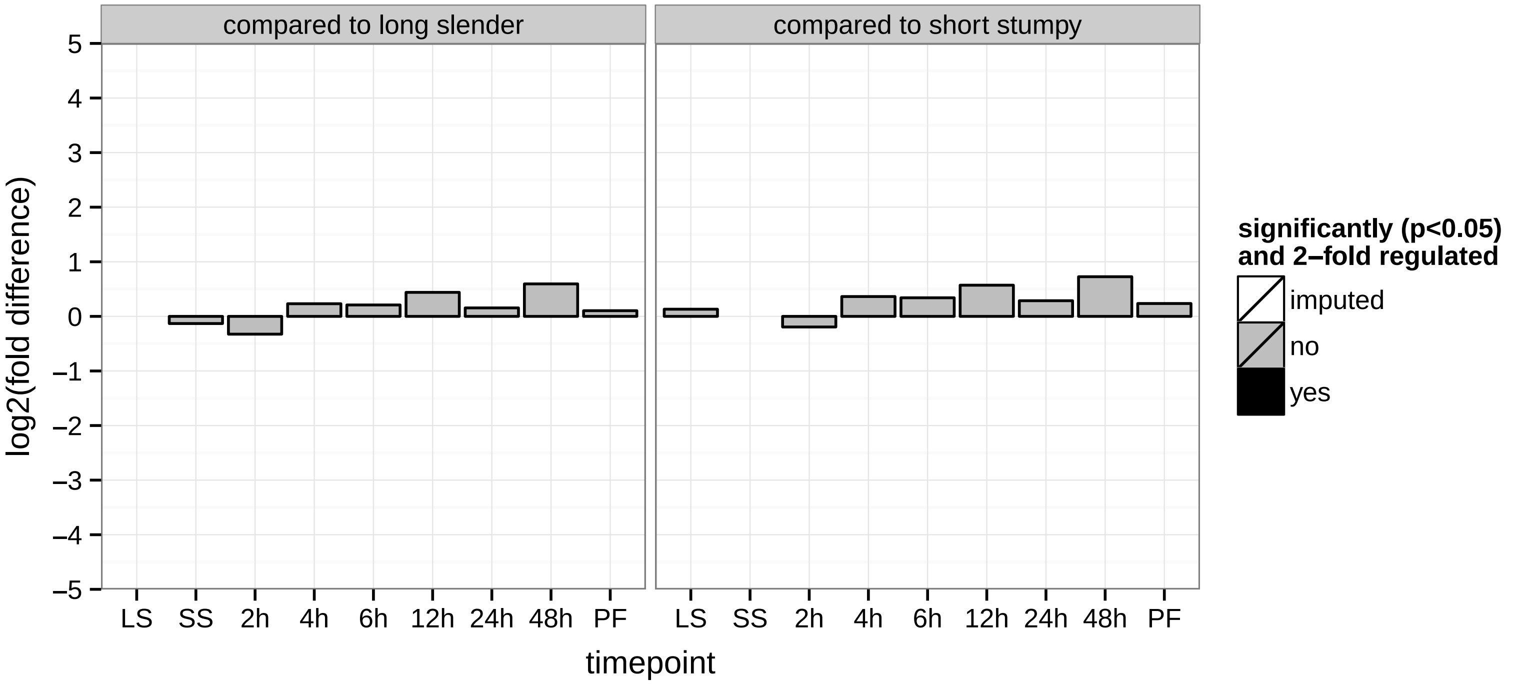

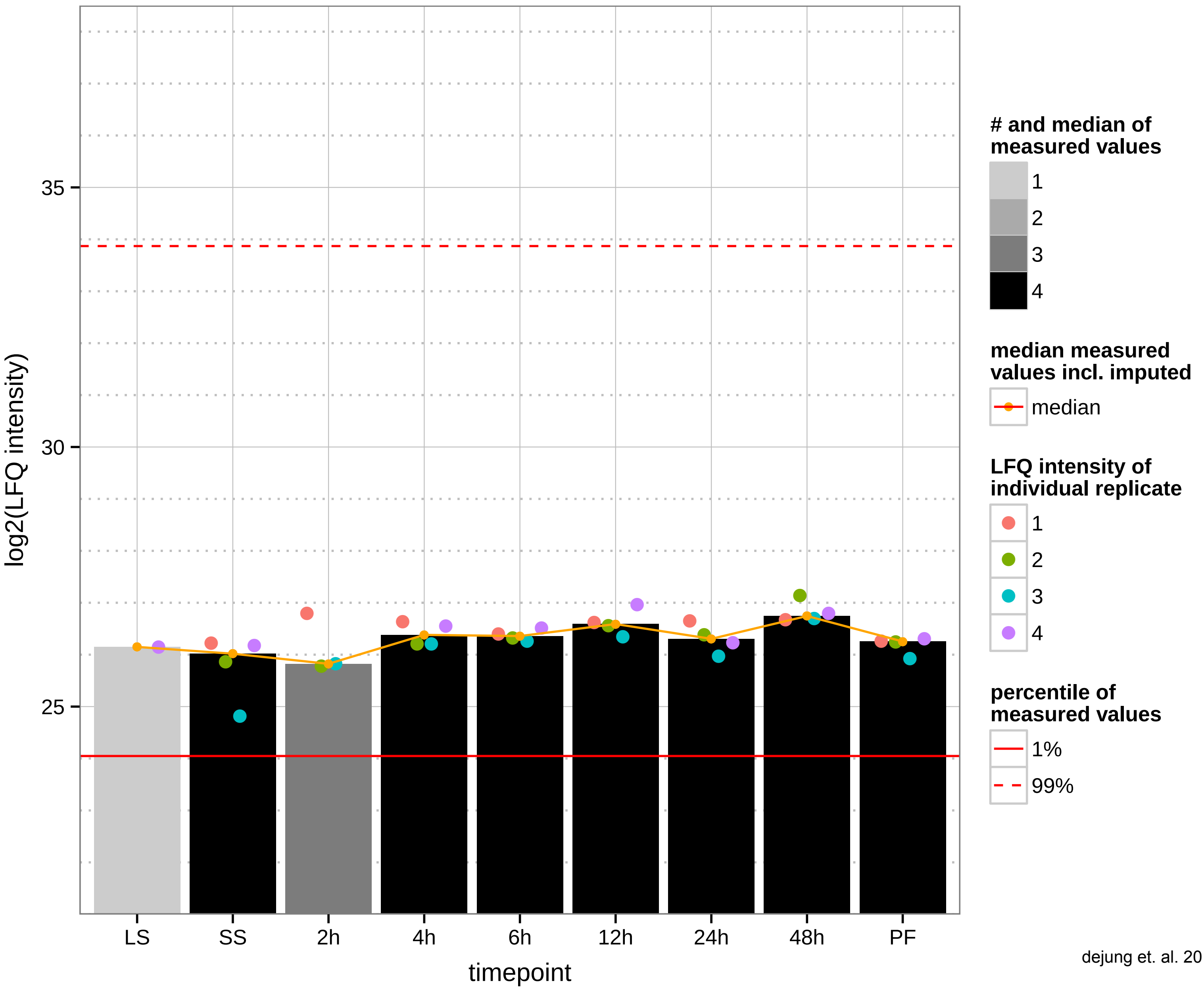

dejung et. al. 2015

chrX additional, unordered contigs, dynein light chain, putative, dynein arm light chain, axonemal
Tb927.10.1130;Tb11.0520
AGOF: null, microtubule motor activity
AGOC: null, axonemal dynein complex
AGOP: null, cellular component movement
PGOF: null
PGOC: null
PGOP: null

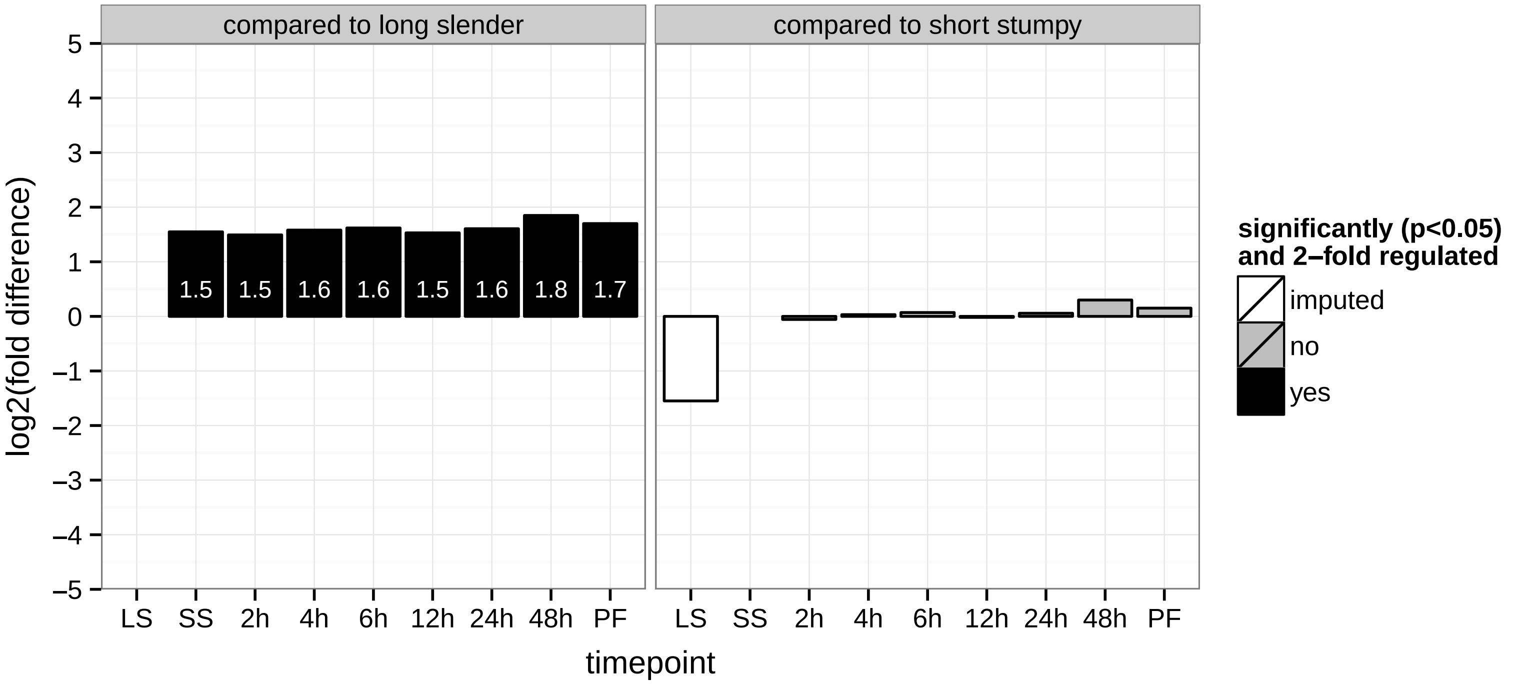

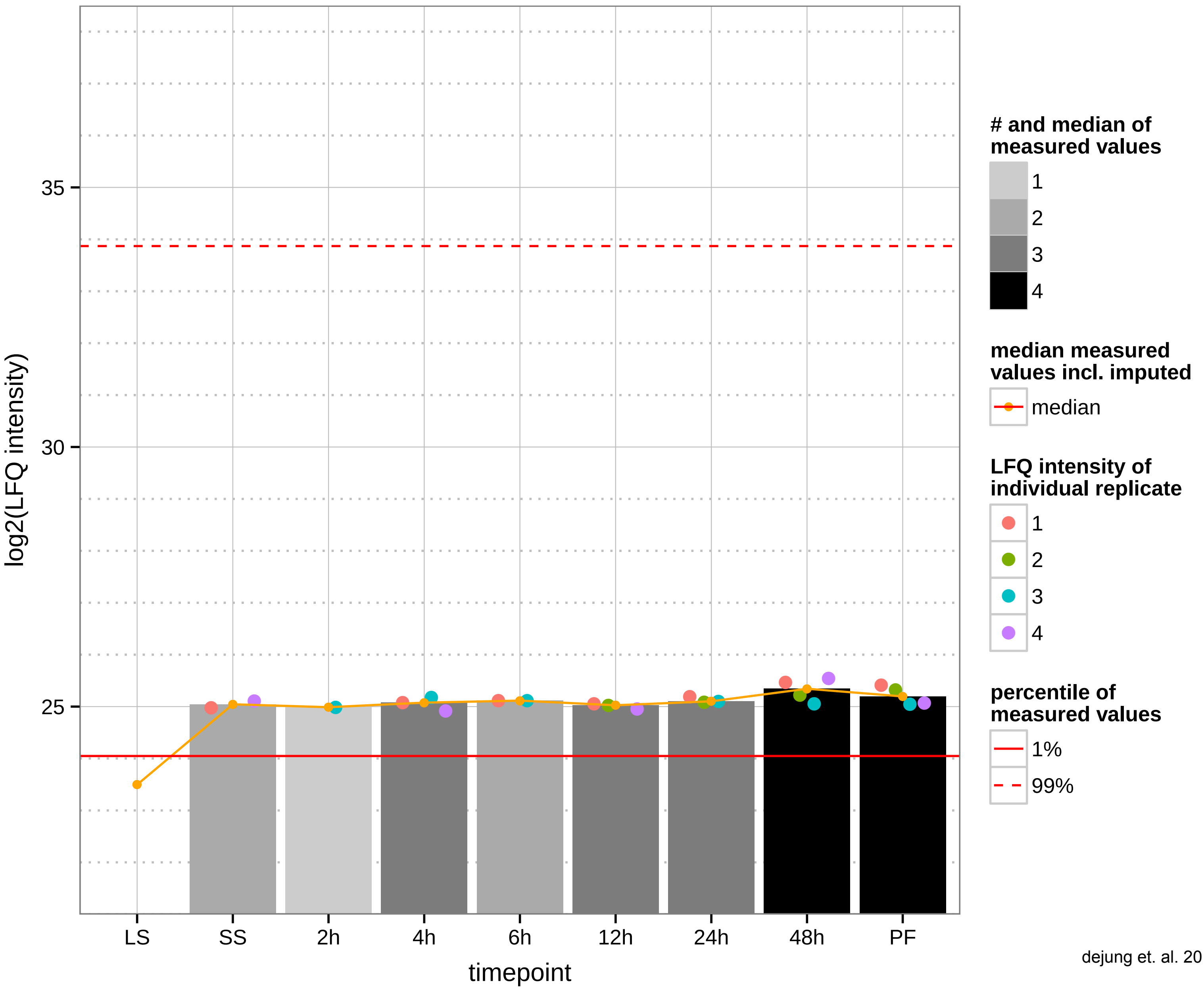

dejung et. al. 2015

hypothetical protein, chrXI additional, unordered contigs, conserved, paraflagellar rod component, putative (PFC15)
Tb11.v5.0996;Tb11.1220;Tb927.10.15250
AGOF: null
AGOC: null, mitochondrion
AGOP: null
PGOF: protein binding
PGOC: null
PGOP: null

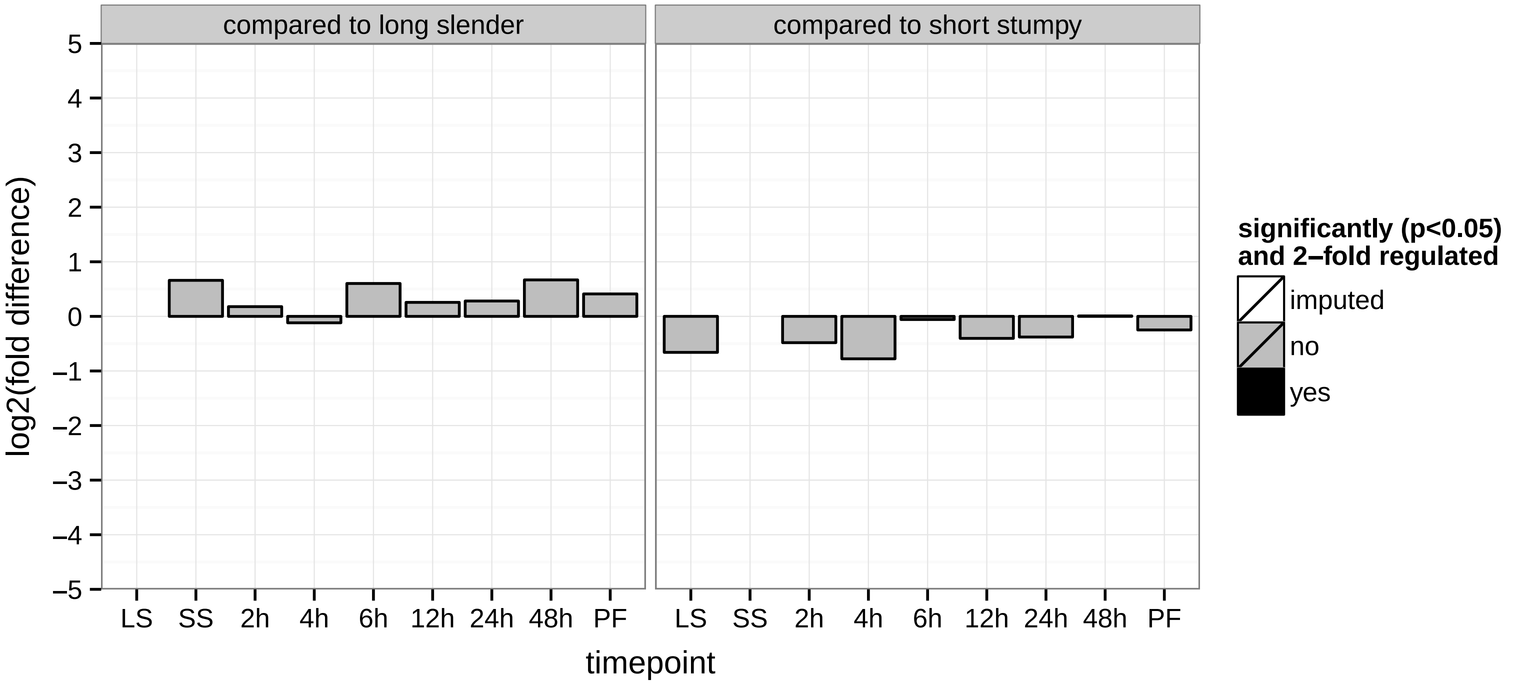

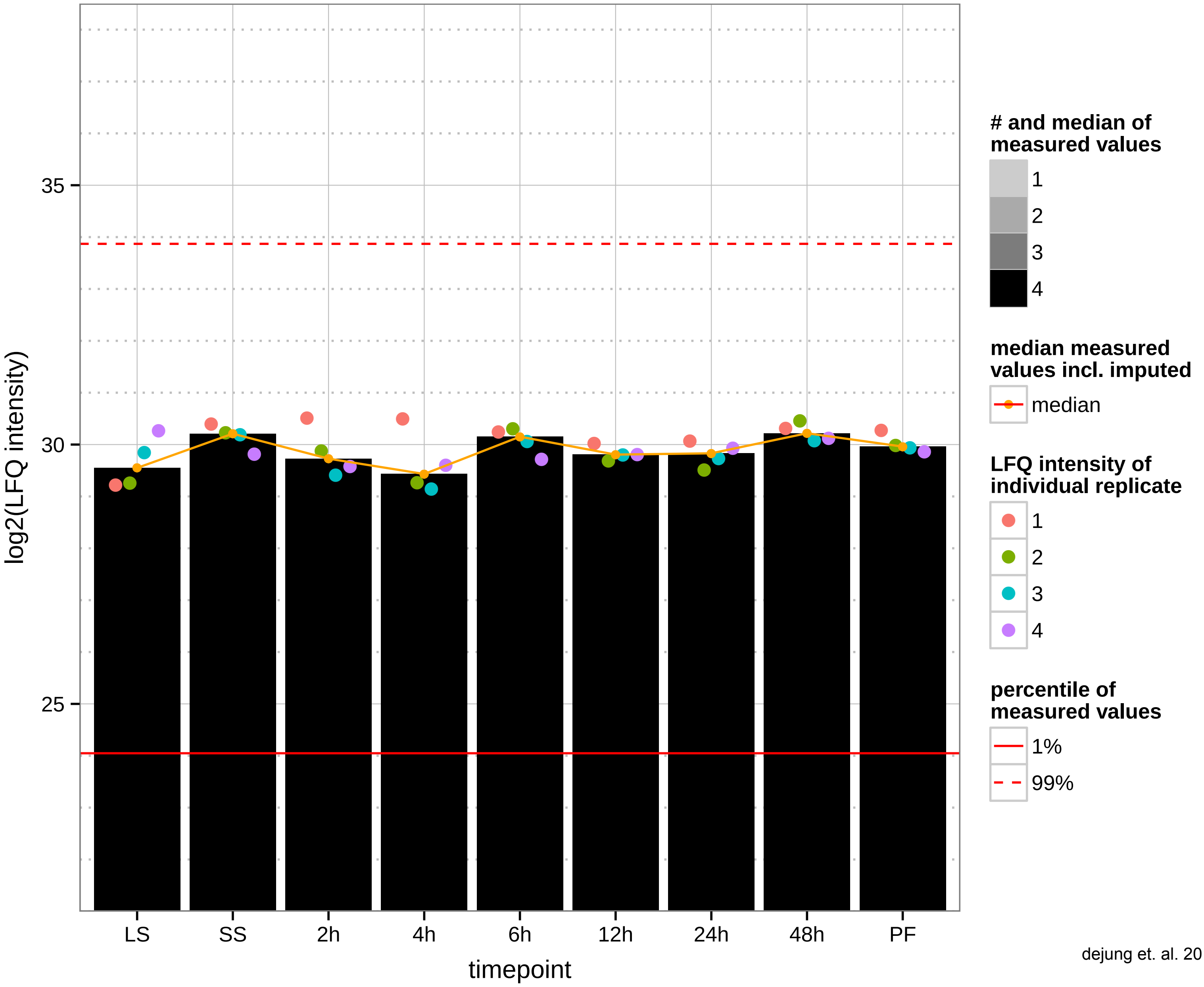

dejung et. al. 2015

hypothetical protein, conserved
Tb927.11.1840;Tb11.1380
AGOF: null
AGOC: null
AGOP: null
PGOF: null
PGOC: null
PGOP: null

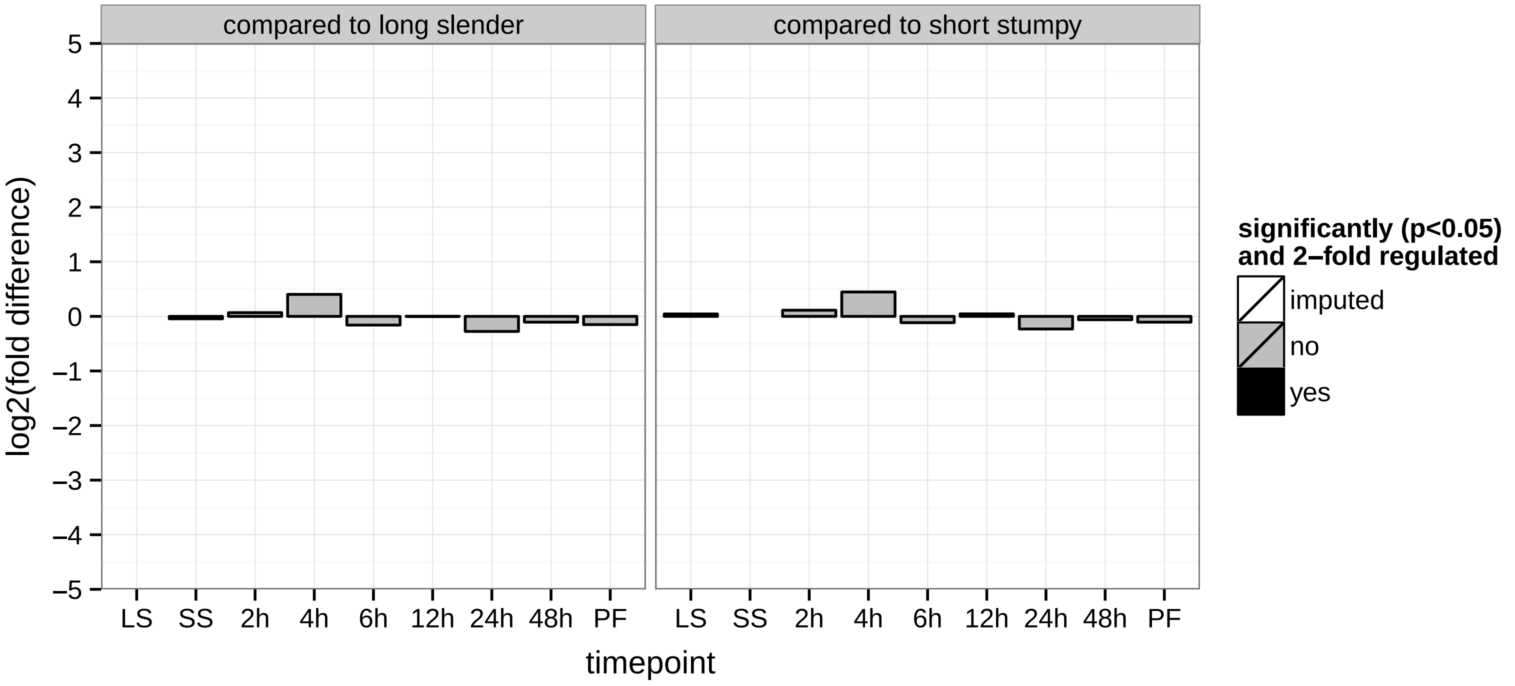

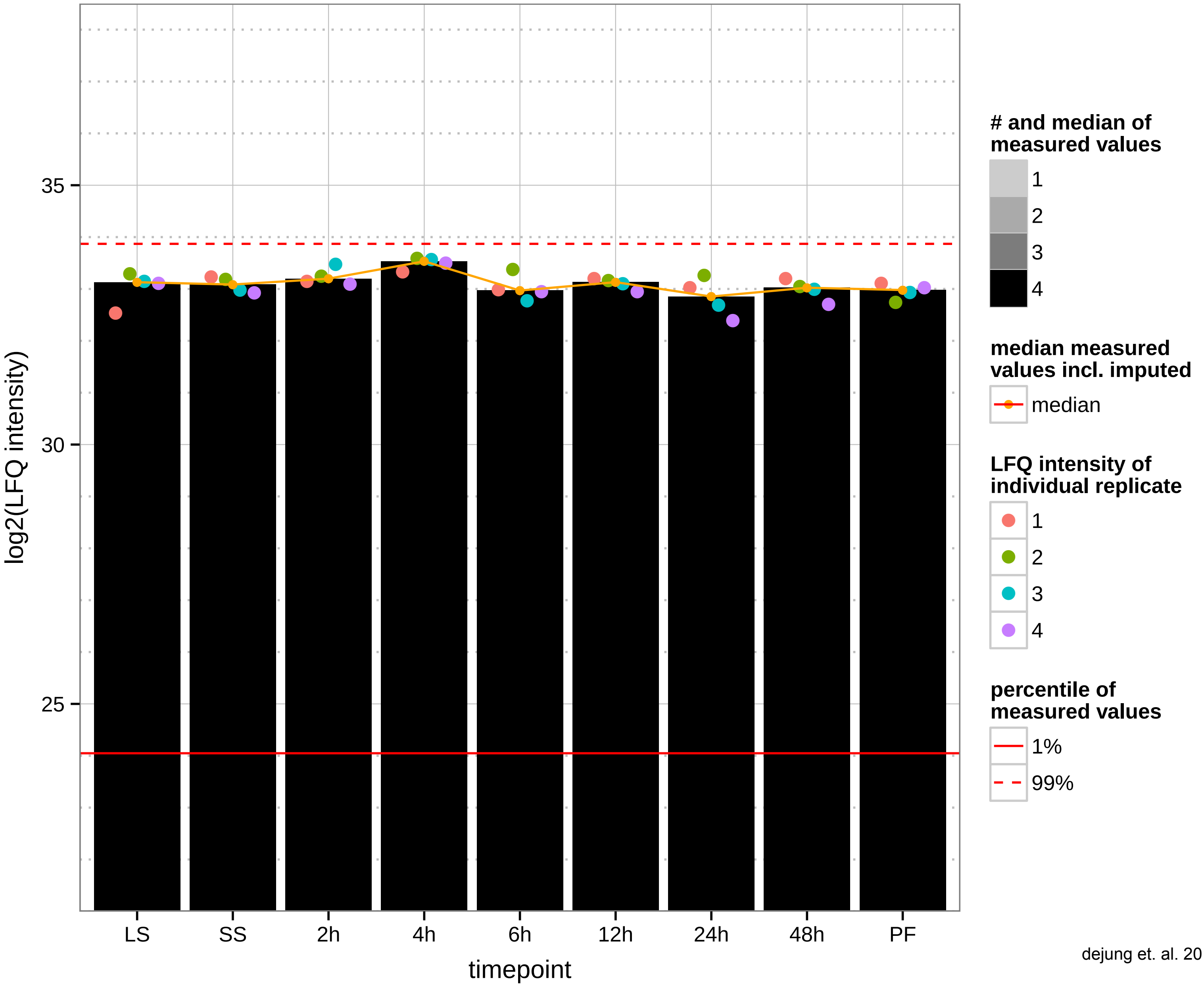

dejung et. al. 2015

hypothetical protein, conserved
Tb927.11.1830;Tb11.1390
AGOF: null
AGOC: null
AGOP: null
PGOF: null
PGOC: null
PGOP: null

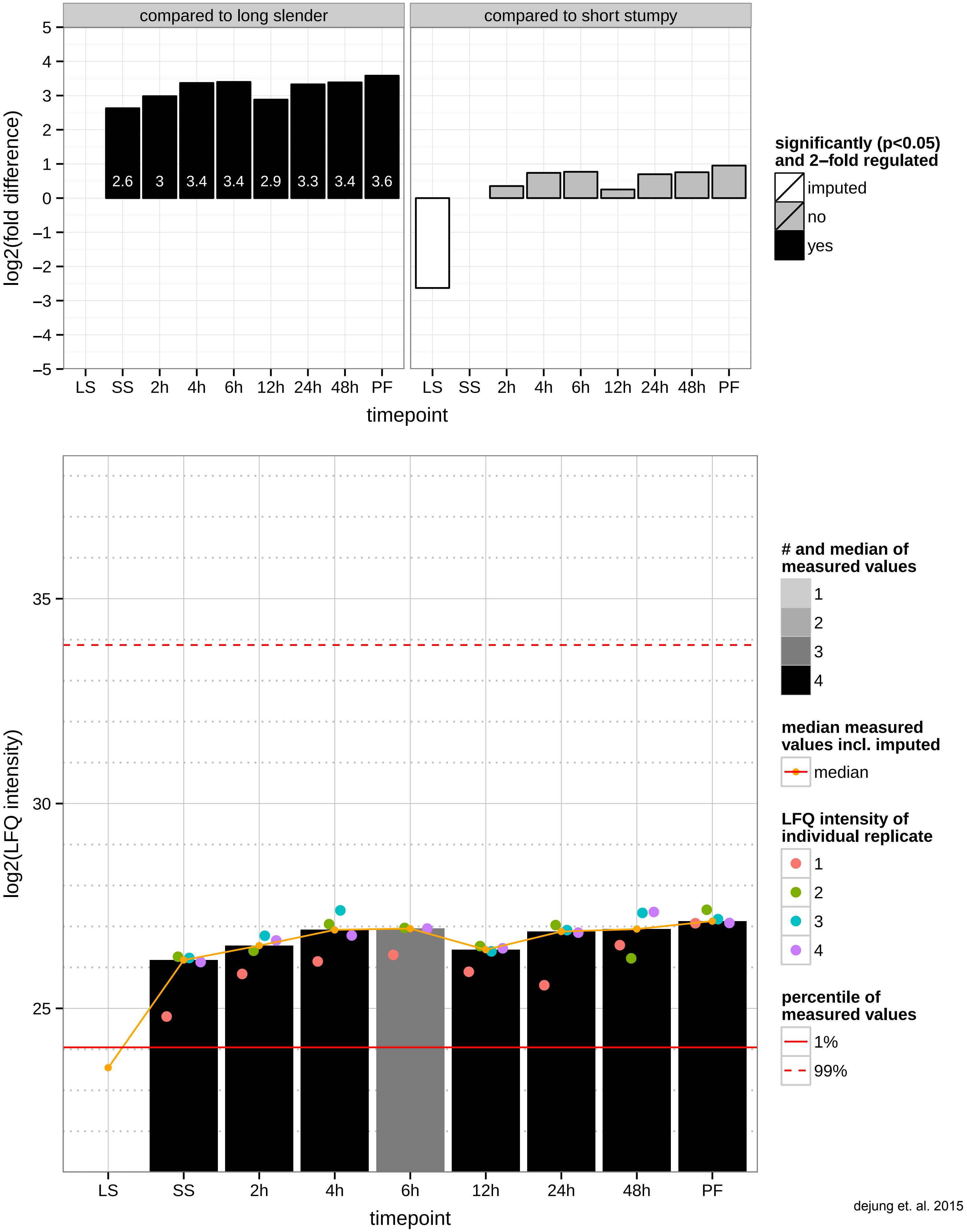

dejung et. al. 2015

retrotransposon hot spot protein 1 (RHS1, pseudogene), putative
Tb11.1780
AGOF: null
AGOC: null
AGOP: null
PGOF: null
PGOC: null
PGOP: null

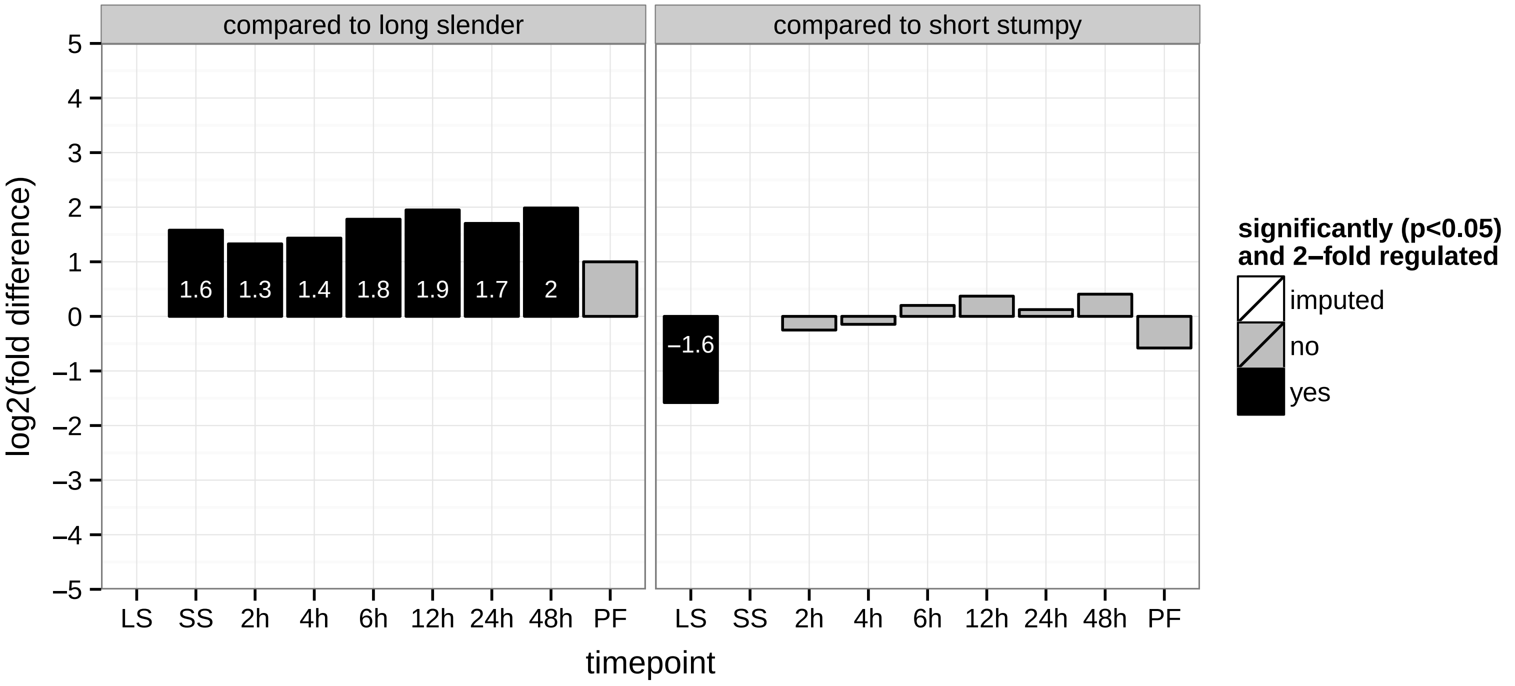

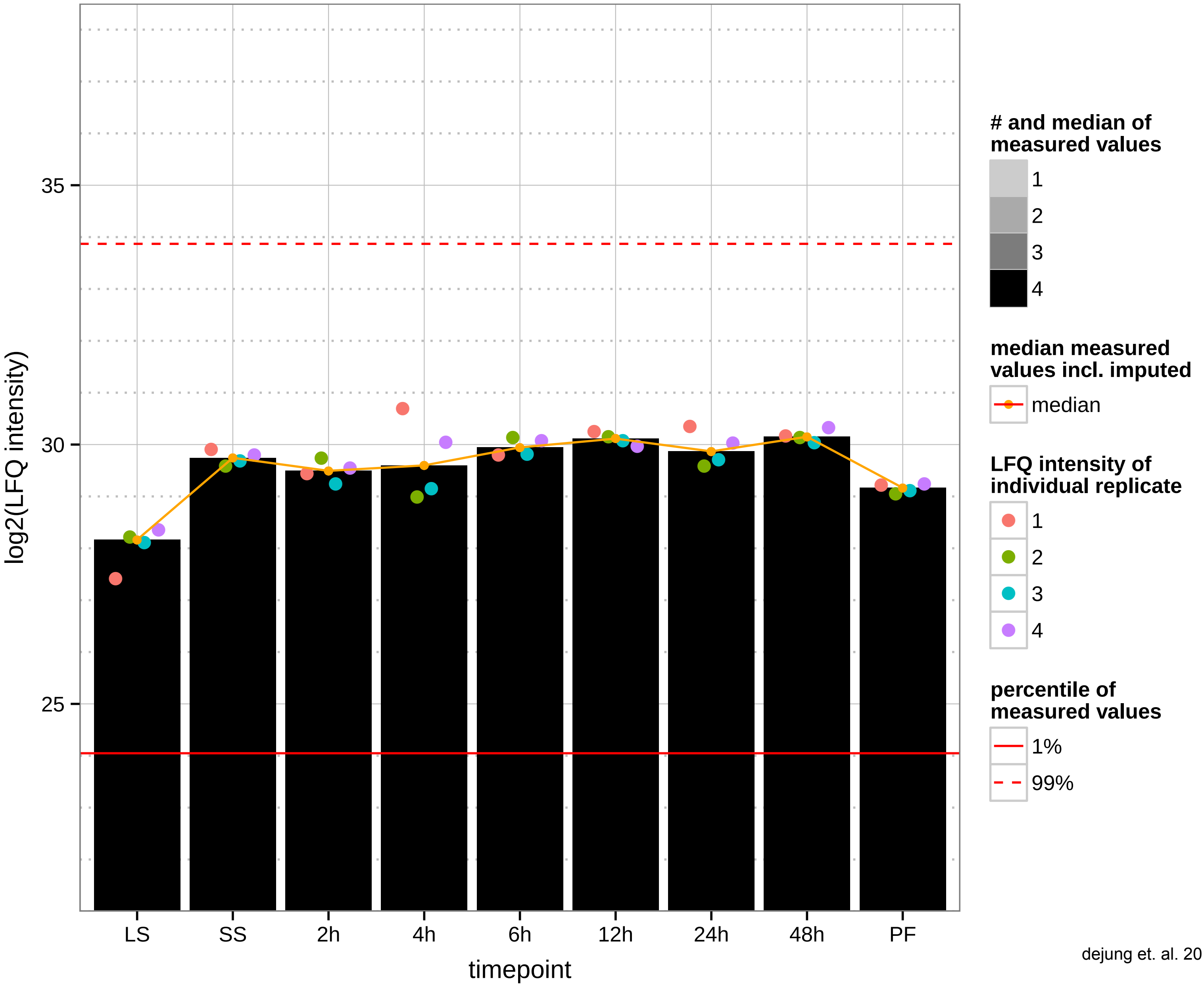

dejung et. al. 2015

retrotransposon hot spot protein (RHS, pseudogene), putative
Tb11.1790
AGOF: null
AGOC: null
AGOP: null
PGOF: null
PGOC: null
PGOP: null

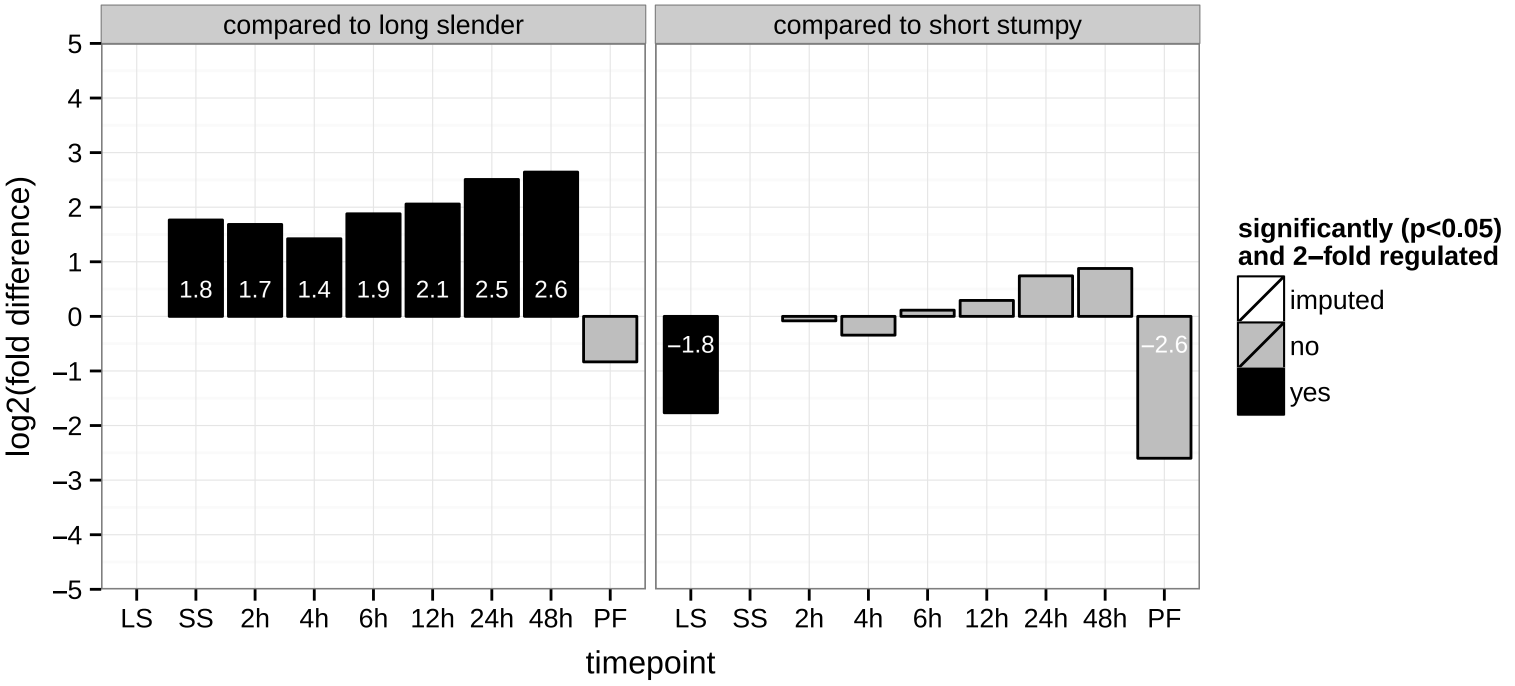

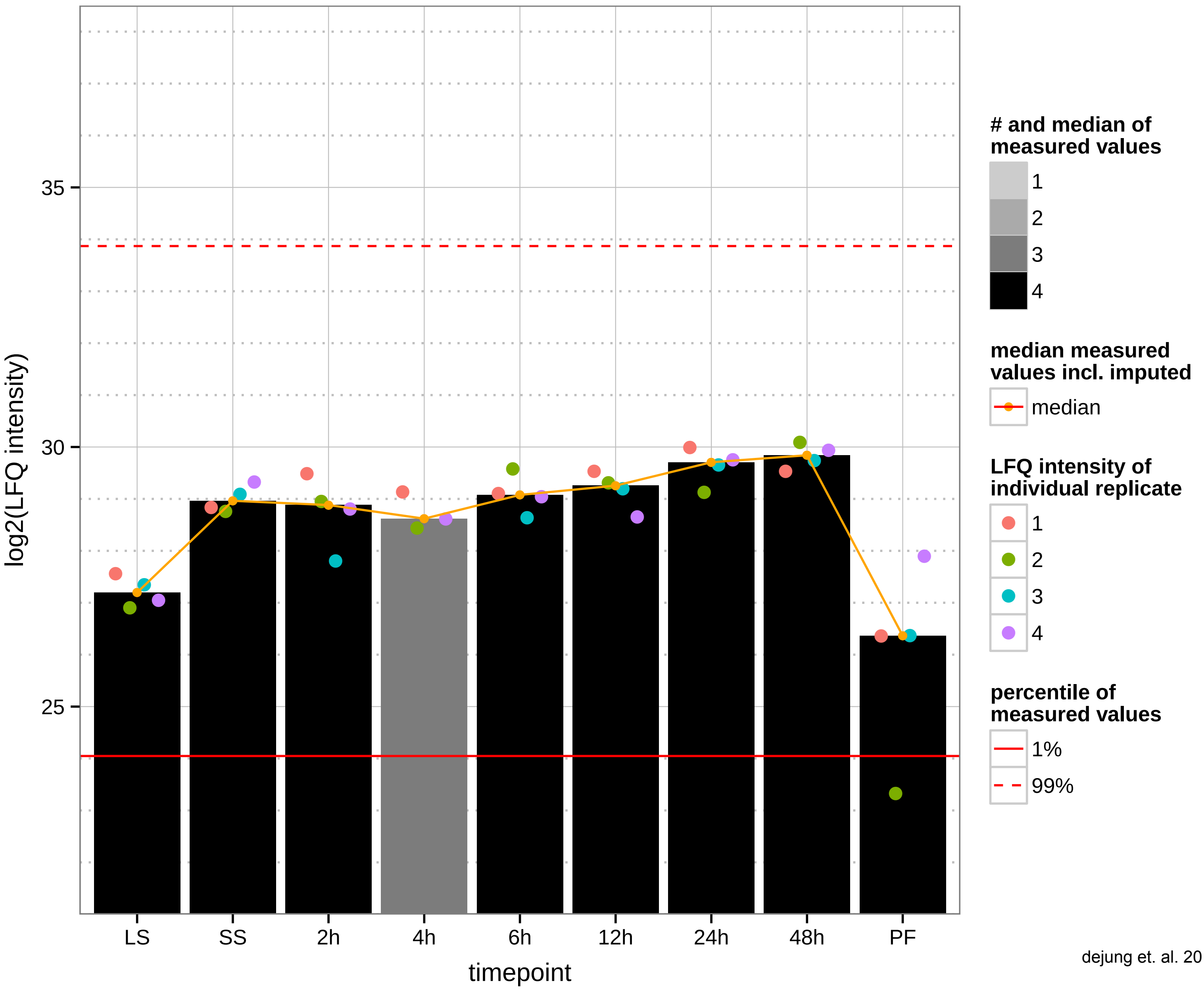

dejung et. al. 2015

retrotransposon hot spot (RHS) protein, (pseudogene), putative, retrotransposon hot spot protein (RHS, pseudogene)
Tb11.1810b;Tb11.1810
AGOF: null
AGOC: null
AGOP: null
PGOF: null
PGOC: null
PGOP: null

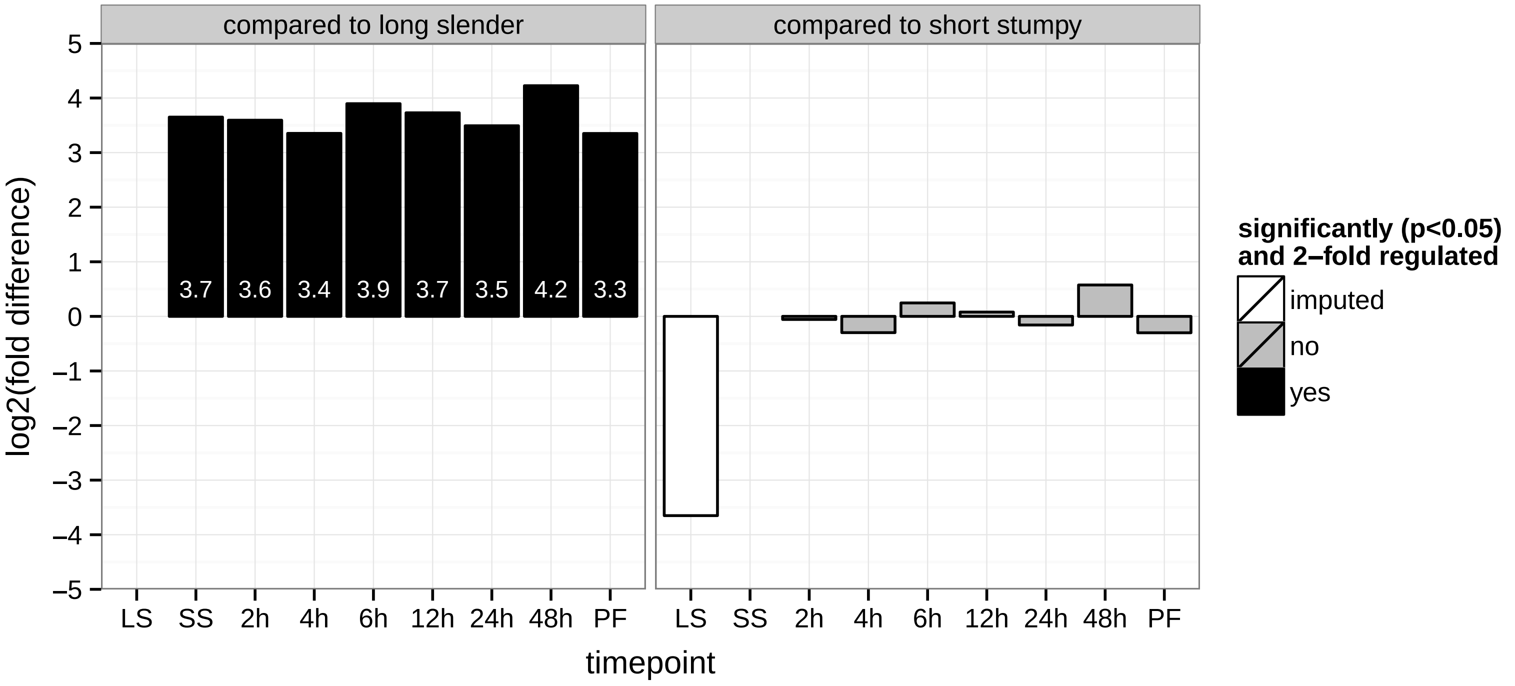

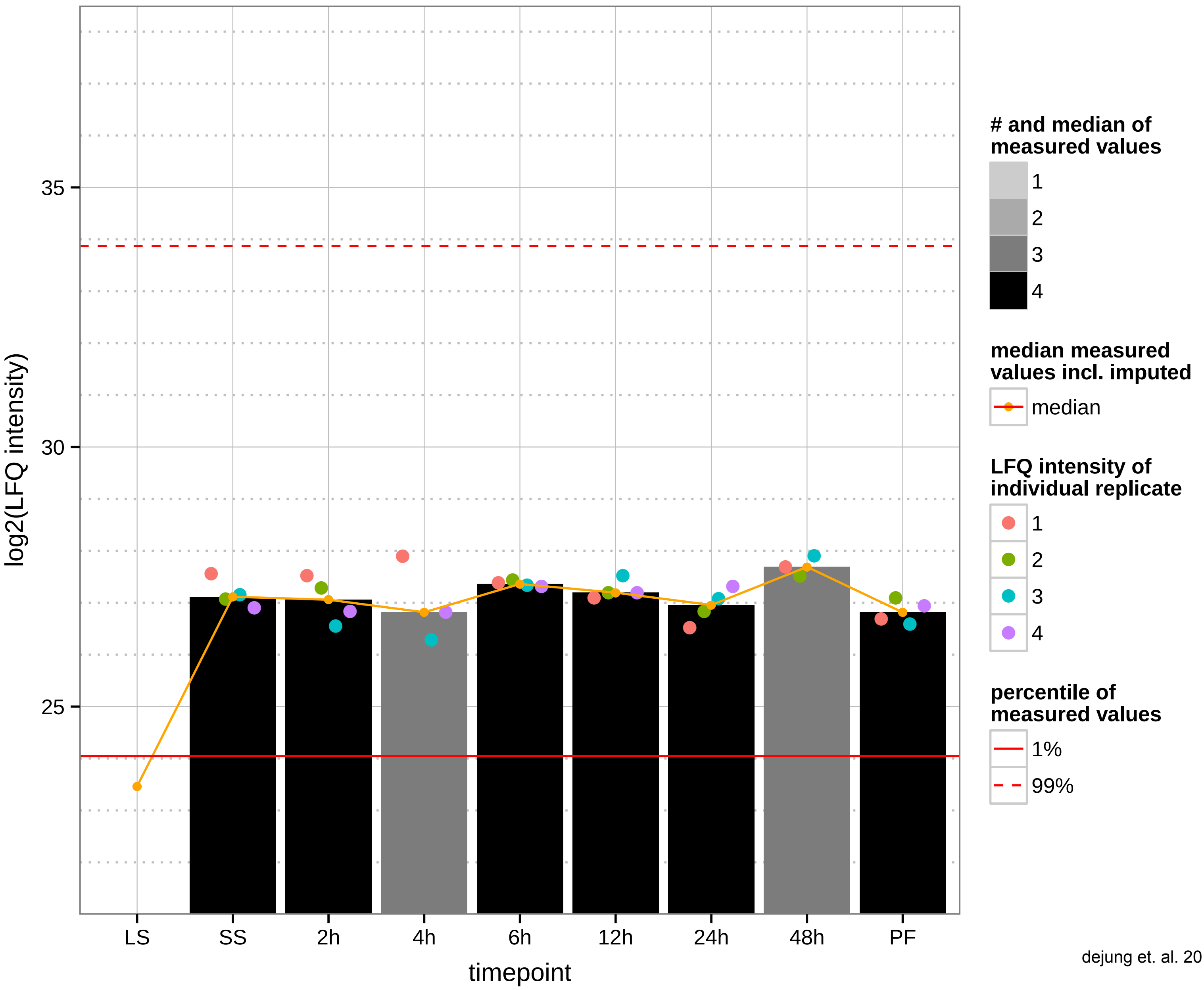

dejung et. al. 2015

ribosomal RNA processing protein 4, putative, exosome complex exonuclease (RRP4)
Tb927.7.4670;Tb11.v5.0152
AGOF: null, 3'−5'−exoribonuclease activity, RNA binding
AGOC: null, cytoplasmic exosome (RNase complex), exosome (RNase complex), nuclear exosome (RNase complex)
AGOP: null, cell growth, rRNA processing
PGOF: RNA binding
PGOC: null
PGOP: null

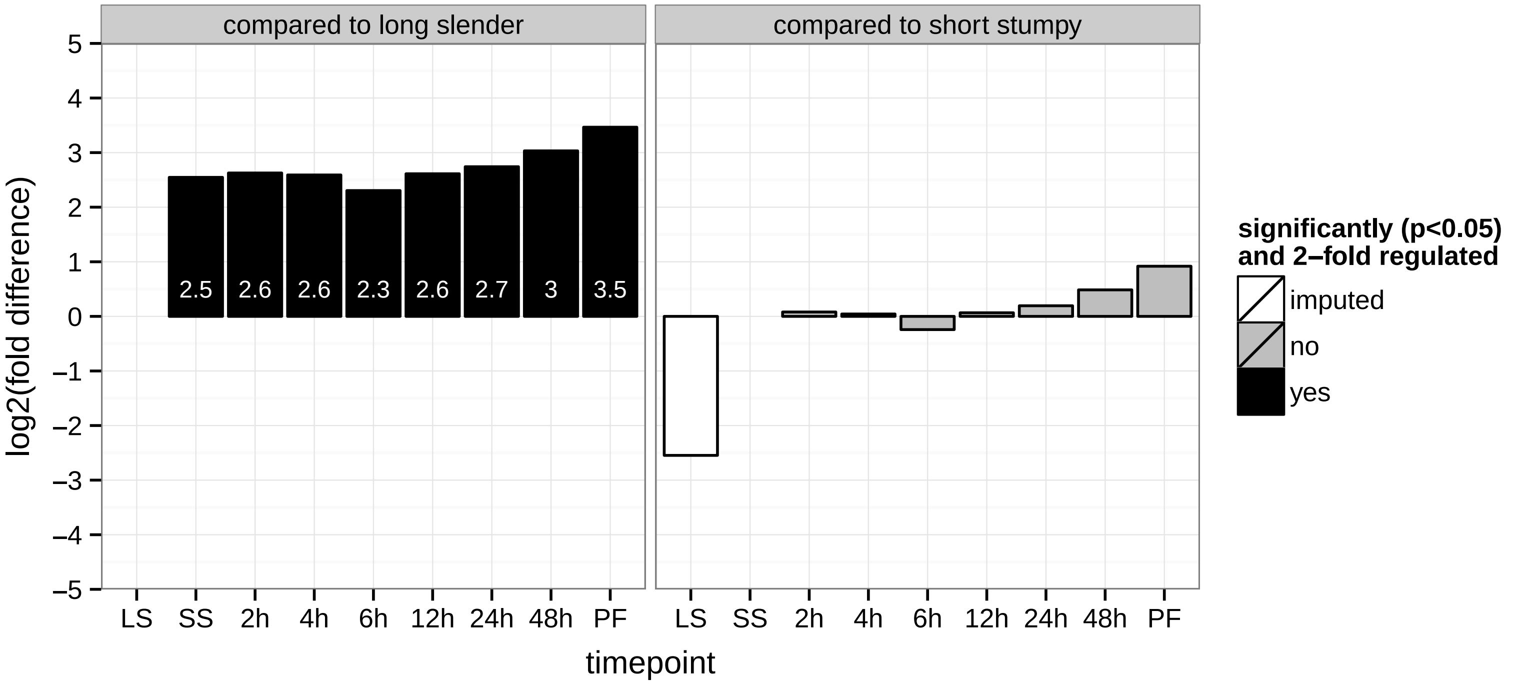

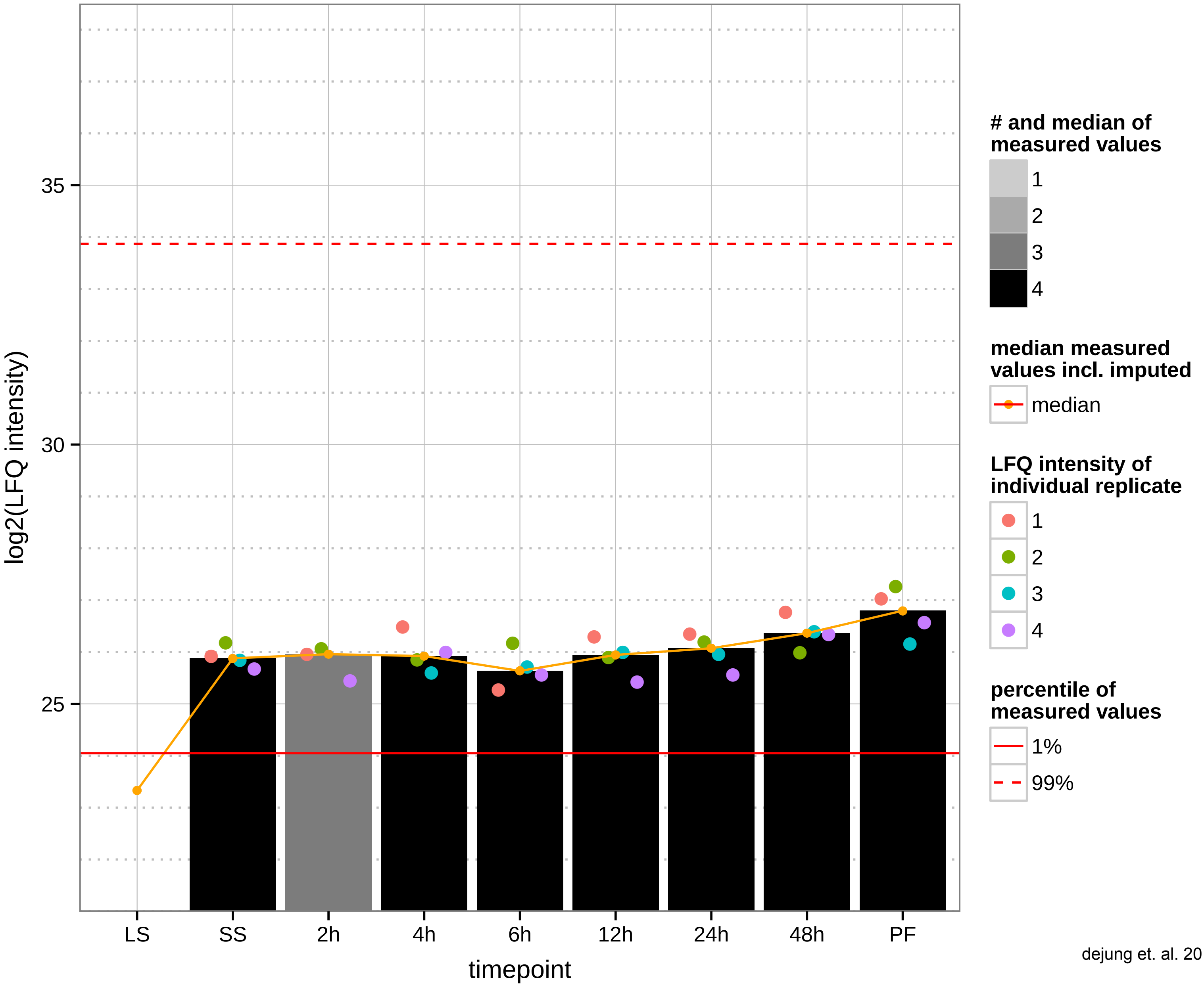

dejung et. al. 2015

hypothetical protein, conserved
Tb11.v5.0154
AGOF: null
AGOC: null
AGOP: null
PGOF: protein binding
PGOC: null
PGOP: null

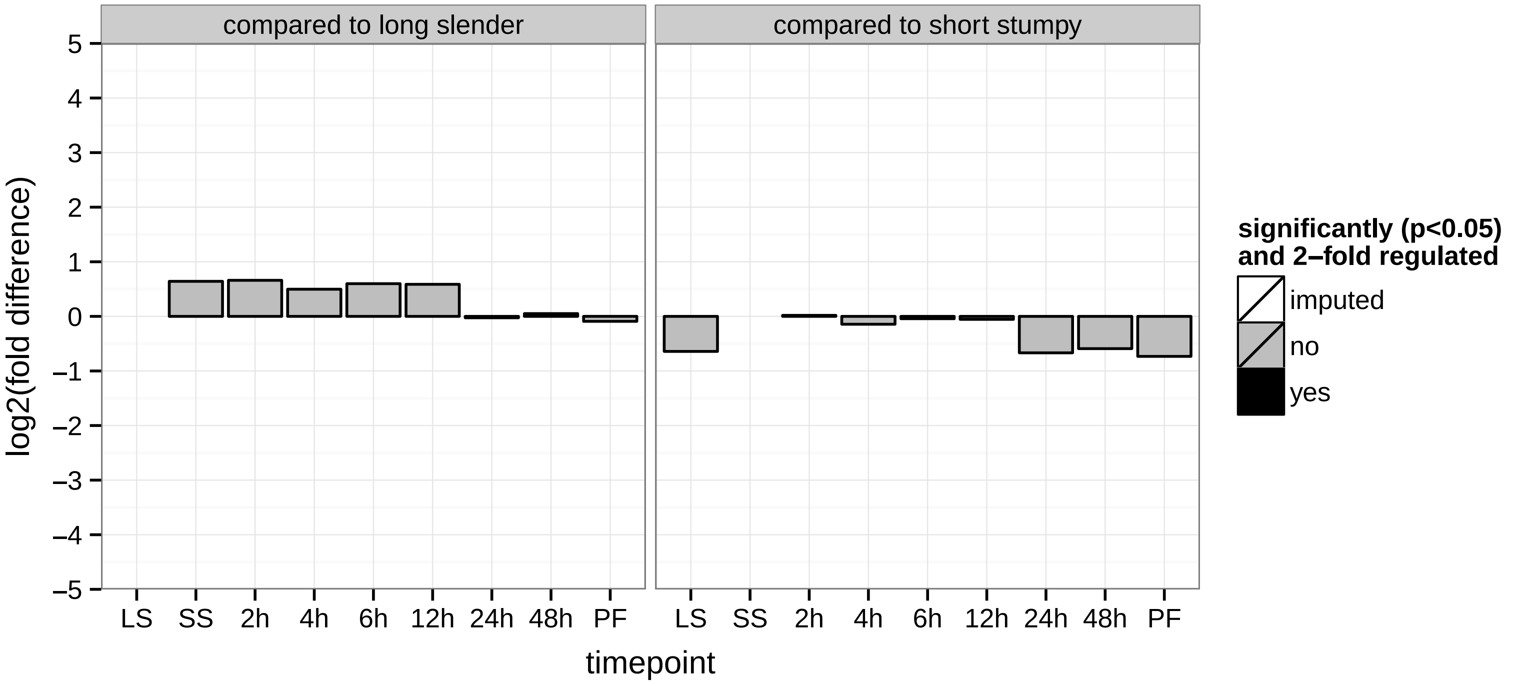

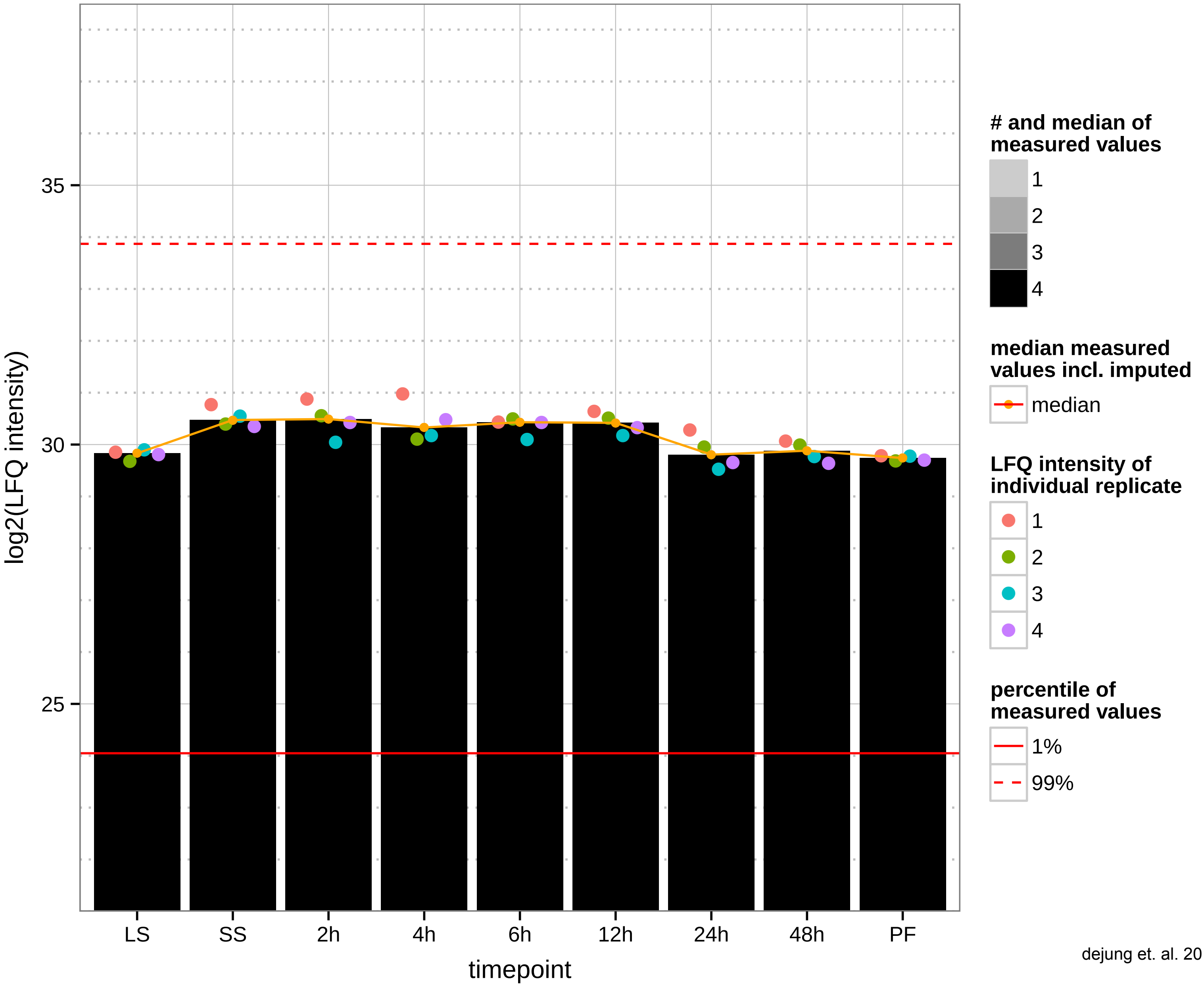

dejung et. al. 2015

cyclophilin–type peptidyl–prolyl cis–trans isomerase, putative, putative (PPIase)
Tb11.v5.0160;Tb927.7.4770
AGOF: null, peptidyl–prolyl cis–trans isomerase activity
AGOC: null
AGOP: null, protein folding
PGOF: peptidyl–prolyl cis–trans isomerase activity
PGOC: null
PGOP: protein folding

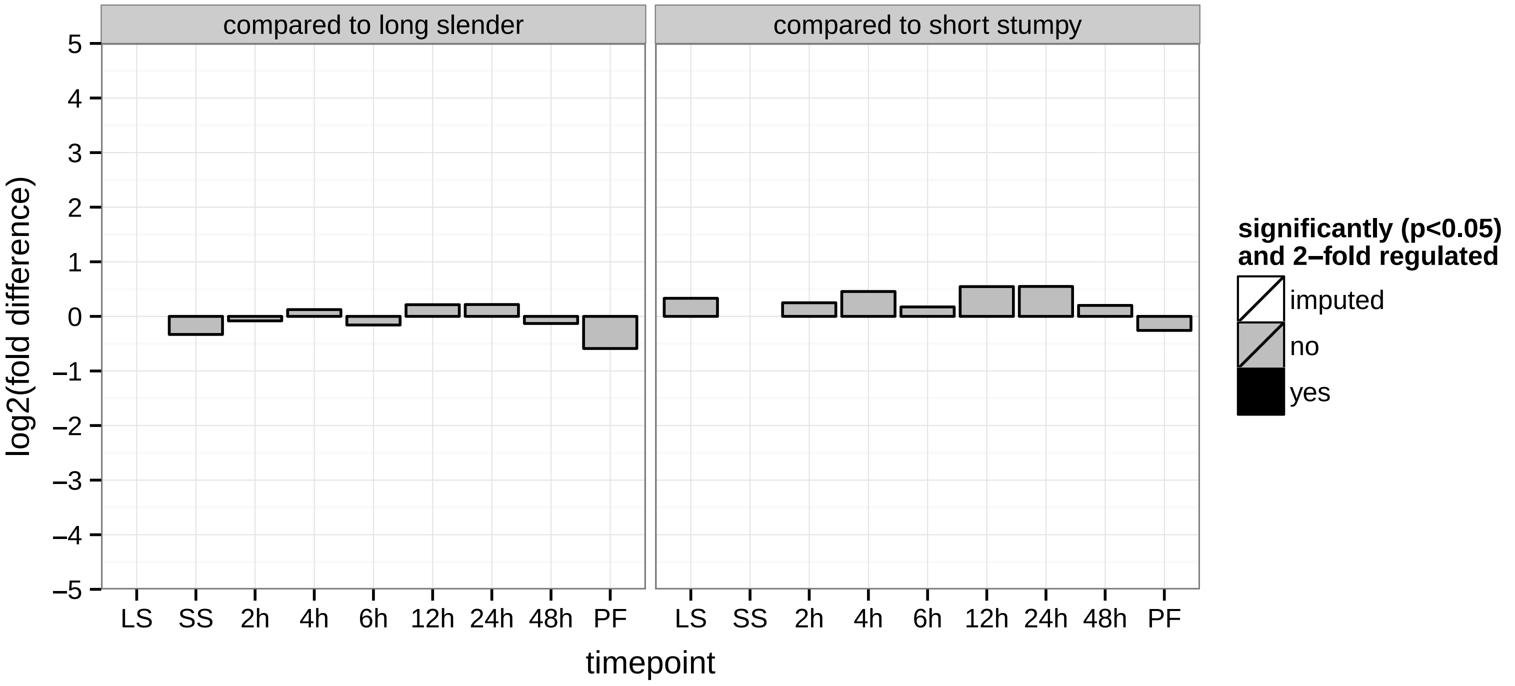

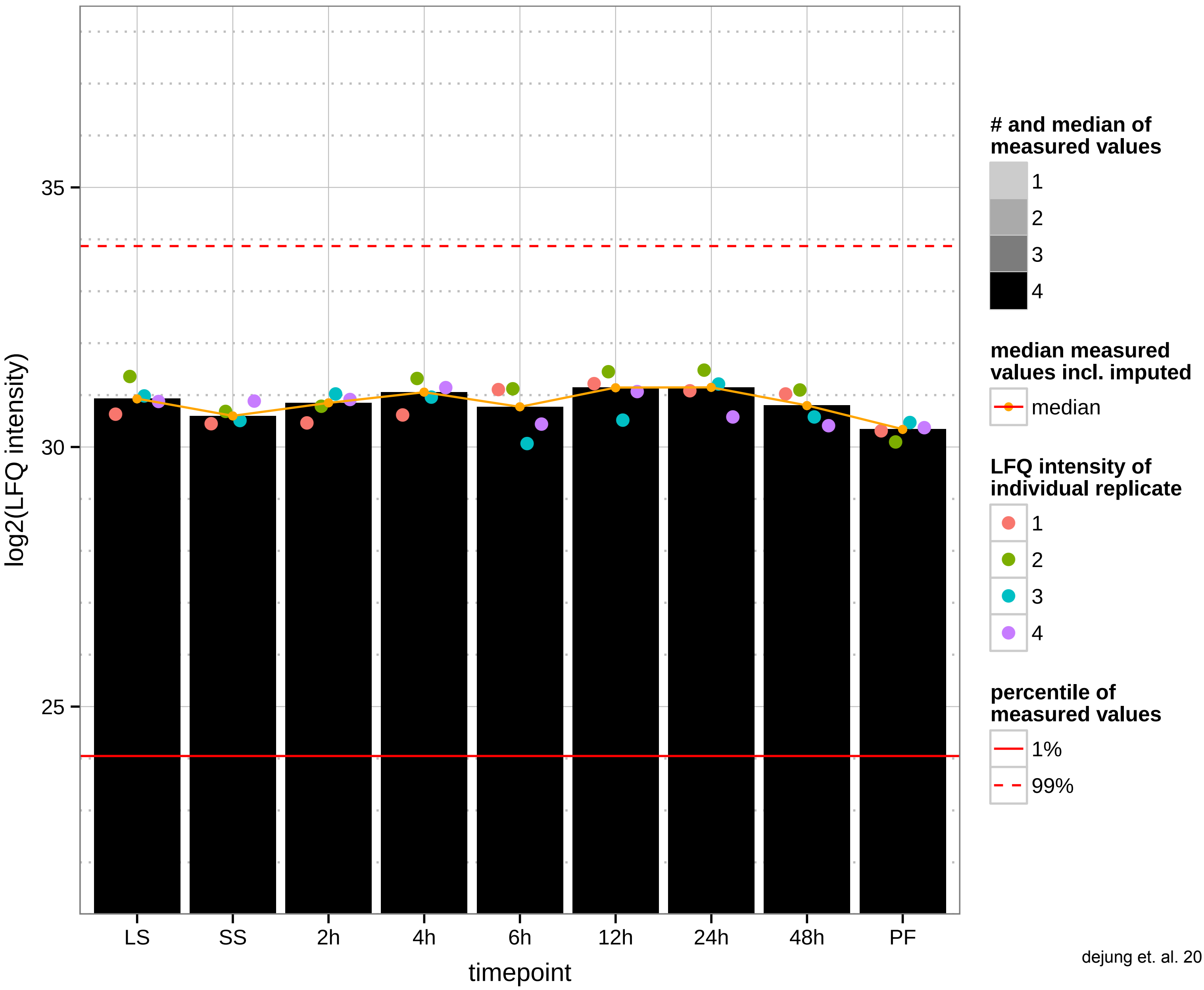

dejung et. al. 2015

proteasome beta 6 subunit, 20S proteasome beta 6 subunit, putative (BETA6)
Tb927.7.4790;Tb11.v5.0162
AGOF: null, endopeptidase activity, threonine−type endopeptidase activity
AGOC: null, proteasome core complex, proteasome core complex, beta−subunit complex
AGOP: null, ubiquitin−dependent protein catabolic process
PGOF: threonine−type endopeptidase activity
PGOC: proteasome core complex
PGOP: proteolysis involved in cellular protein catabolic process

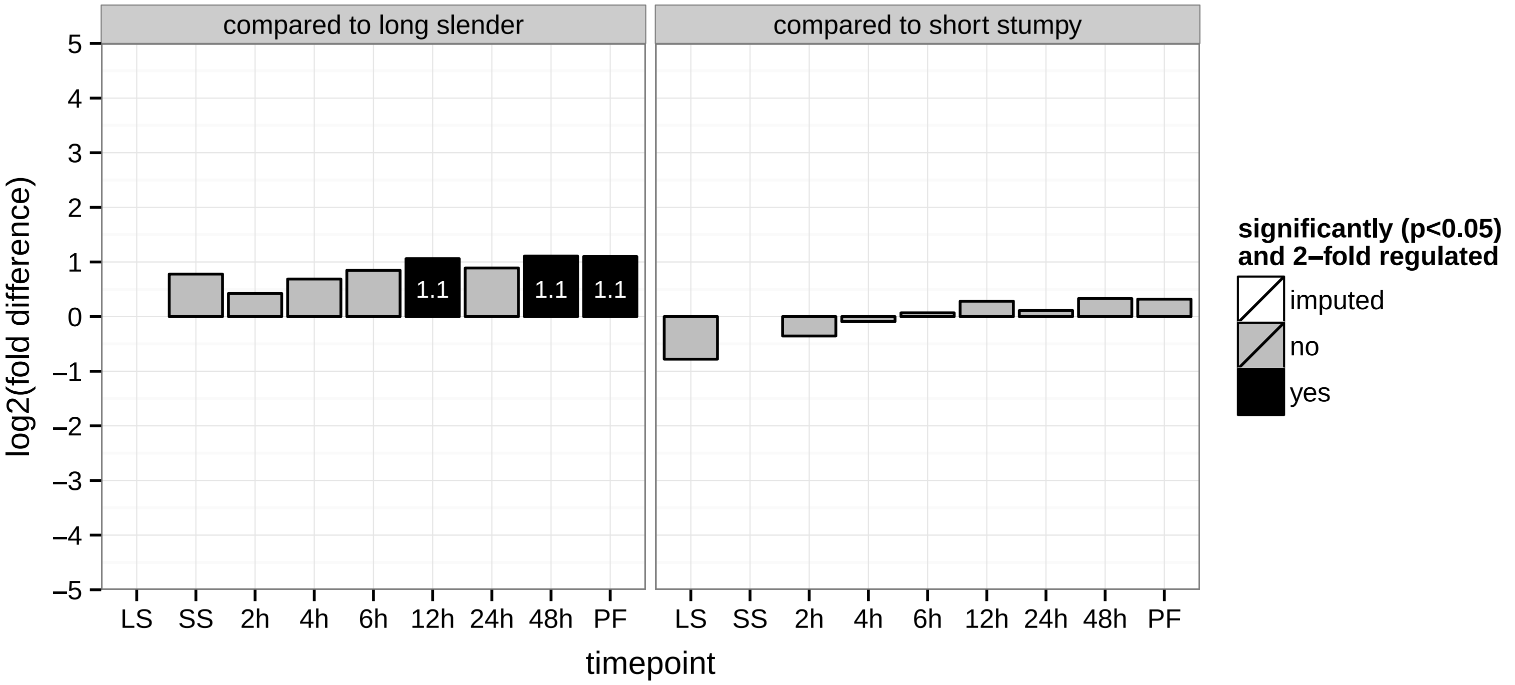

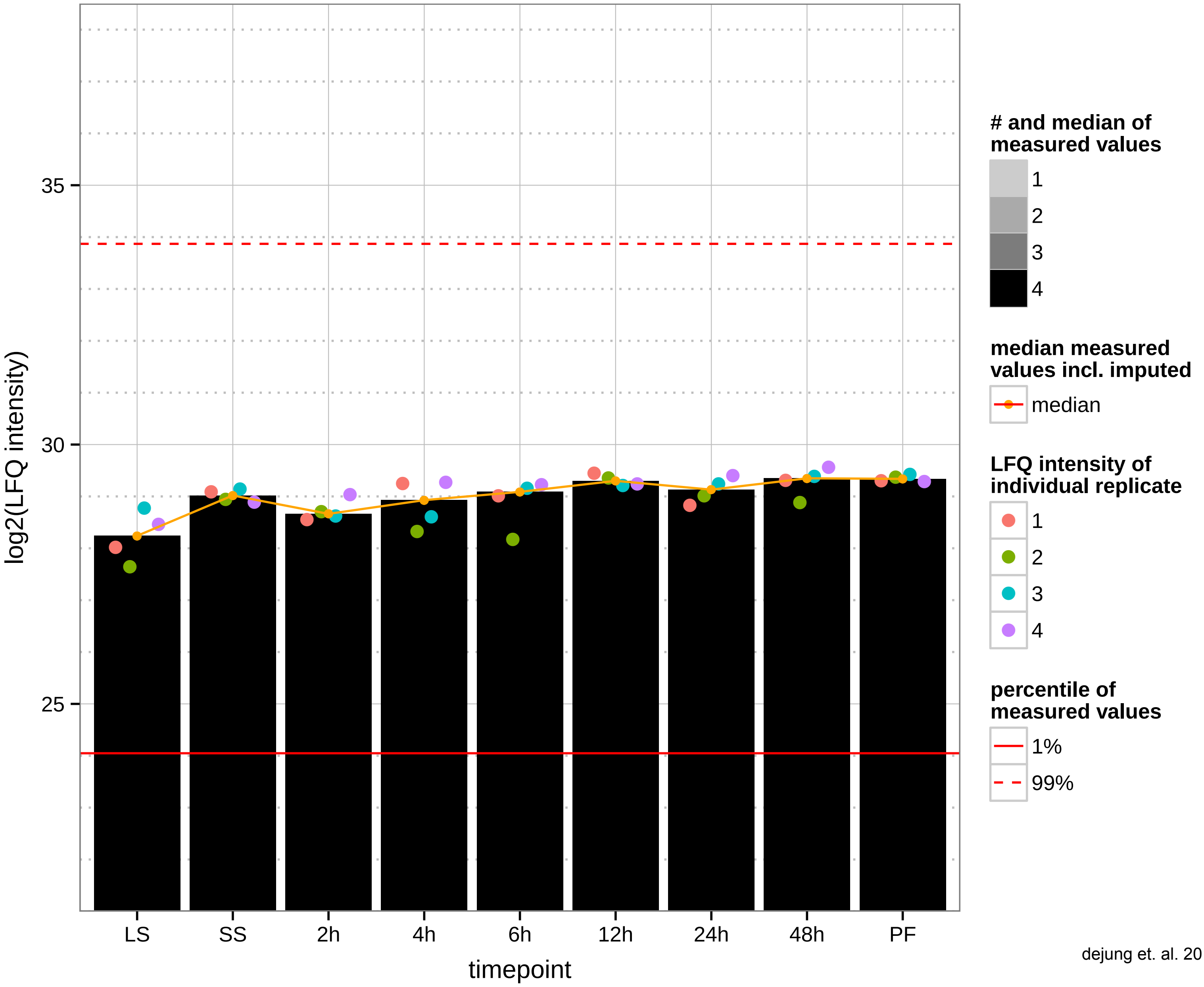

dejung et. al. 2015

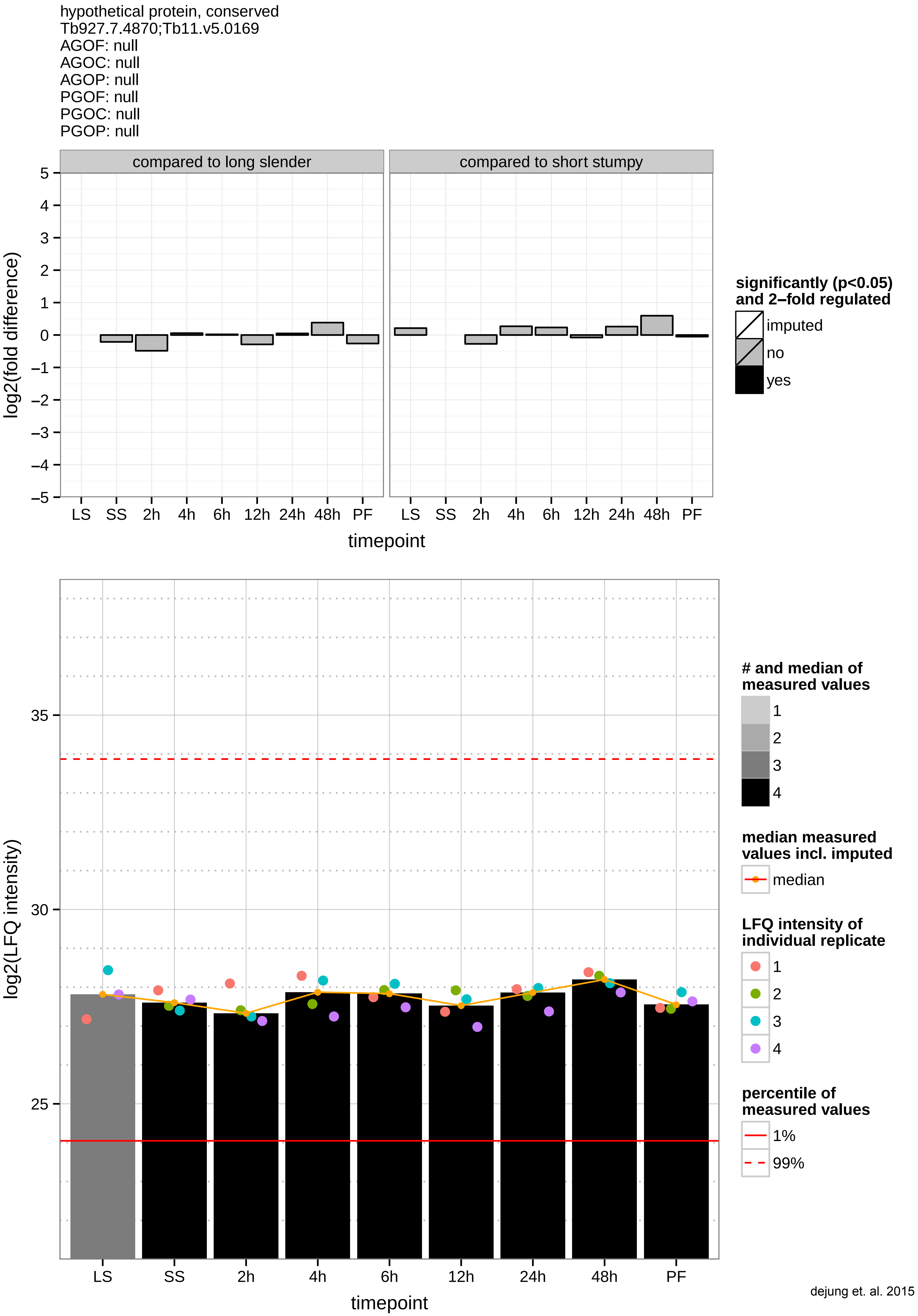
hypothetical protein, conserved
Tb927.7.4870;Tb11.v5.0169
AGOF: null
AGOC: null
AGOP: null
PGOF: null
PGOC: null
PGOP: null
compared to long slender
compared to short stumpy
log2(fold difference)
timepoint
LS SS 2h 4h 6h 12h 24h 48h PF
significantly (p<0.05) and 2–fold regulated
imputed
no
yes
log2(LFQ intensity)
# and median of measured values
1
2
3
4
median measured values incl. imputed
median
LFQ intensity of individual replicate
percentile of measured values
1%
99%
dejung et. al. 2015

5'–3' exonuclease XRNA, putative, exoribonuclease 1, putative (XRNA)
Tb927.7.4900;Tb11.v5.0171
AGOF: null, exonuclease activity, nucleic acid binding
AGOC: null, intracellular
AGOP: null
PGOF: exonuclease activity, nucleic acid binding
PGOC: intracellular
PGOP: null

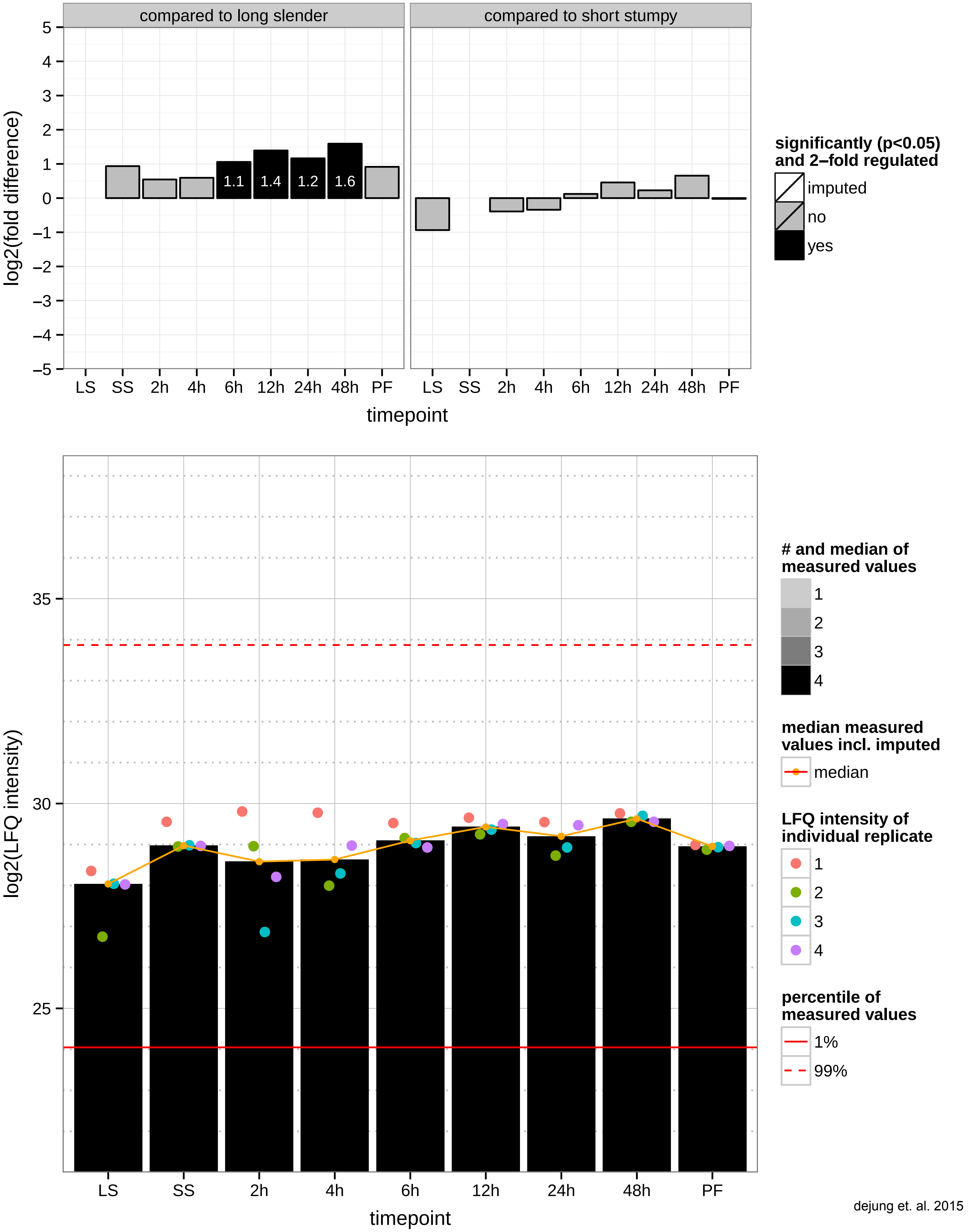

dejung et. al. 2015

hypothetical protein, conserved
Tb927.7.4910;Tb11.v5.0172
AGOF: null
AGOC: null
AGOP: null
PGOF: null
PGOC: null
PGOP: null

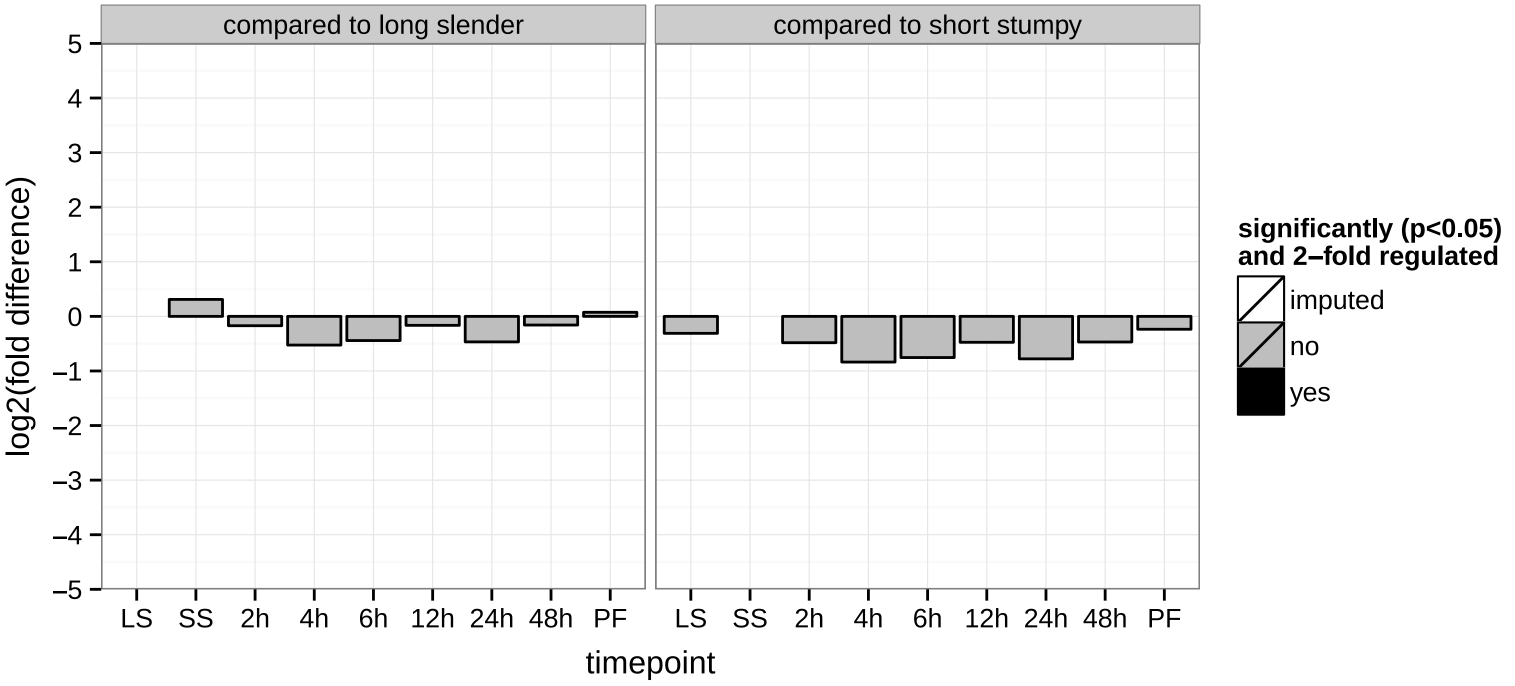

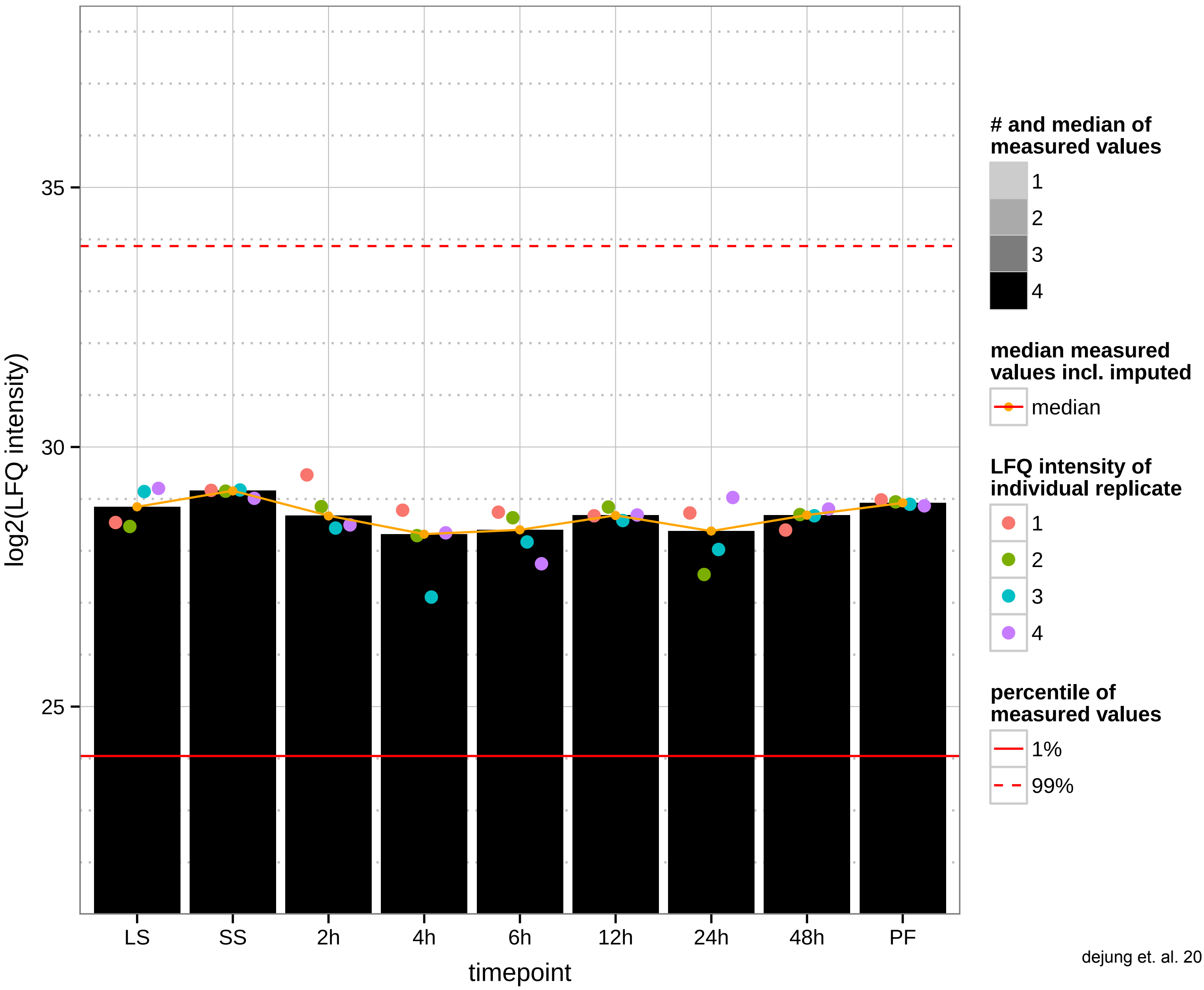

dejung et. al. 2015

hypothetical protein, conserved
Tb927.7.4960;Tb11.v5.0177
AGOF: null
AGOC: null
AGOP: null
PGOF: null
PGOC: null
PGOP: null

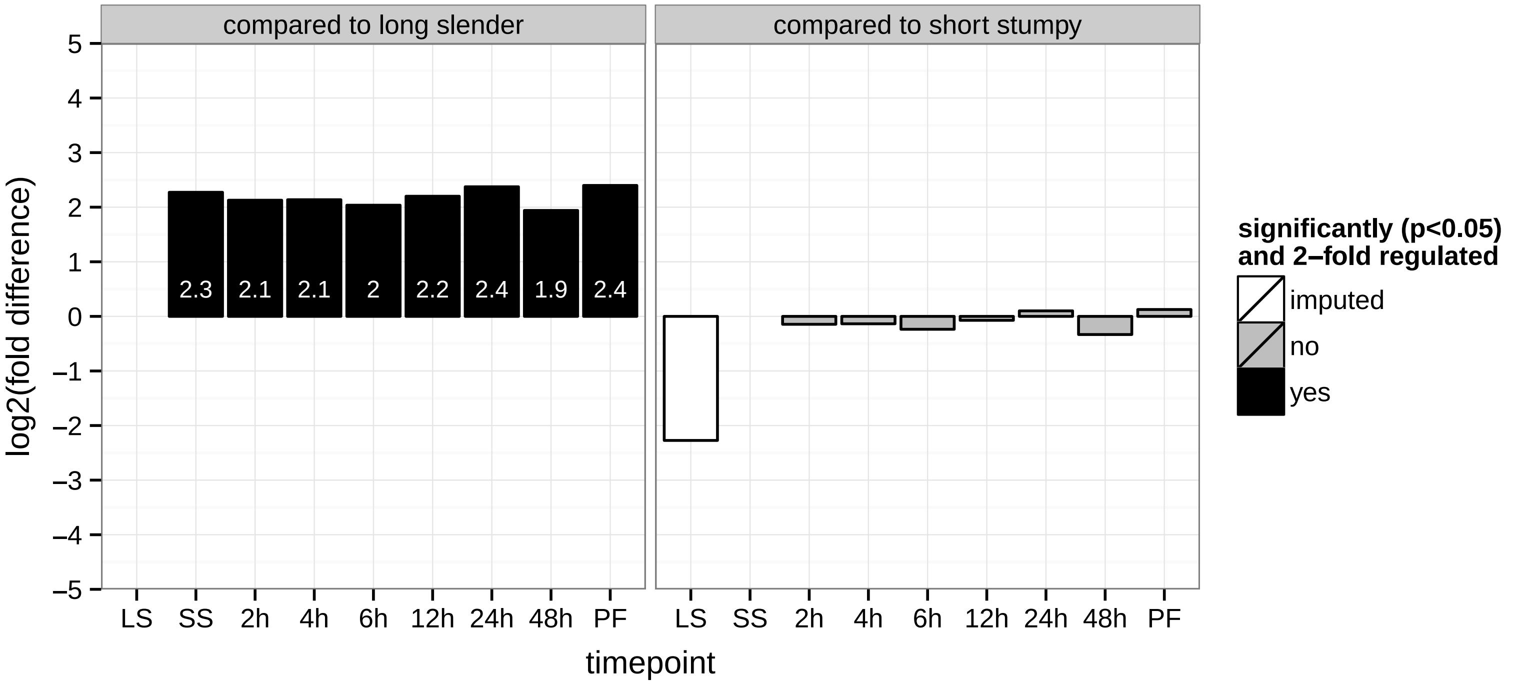

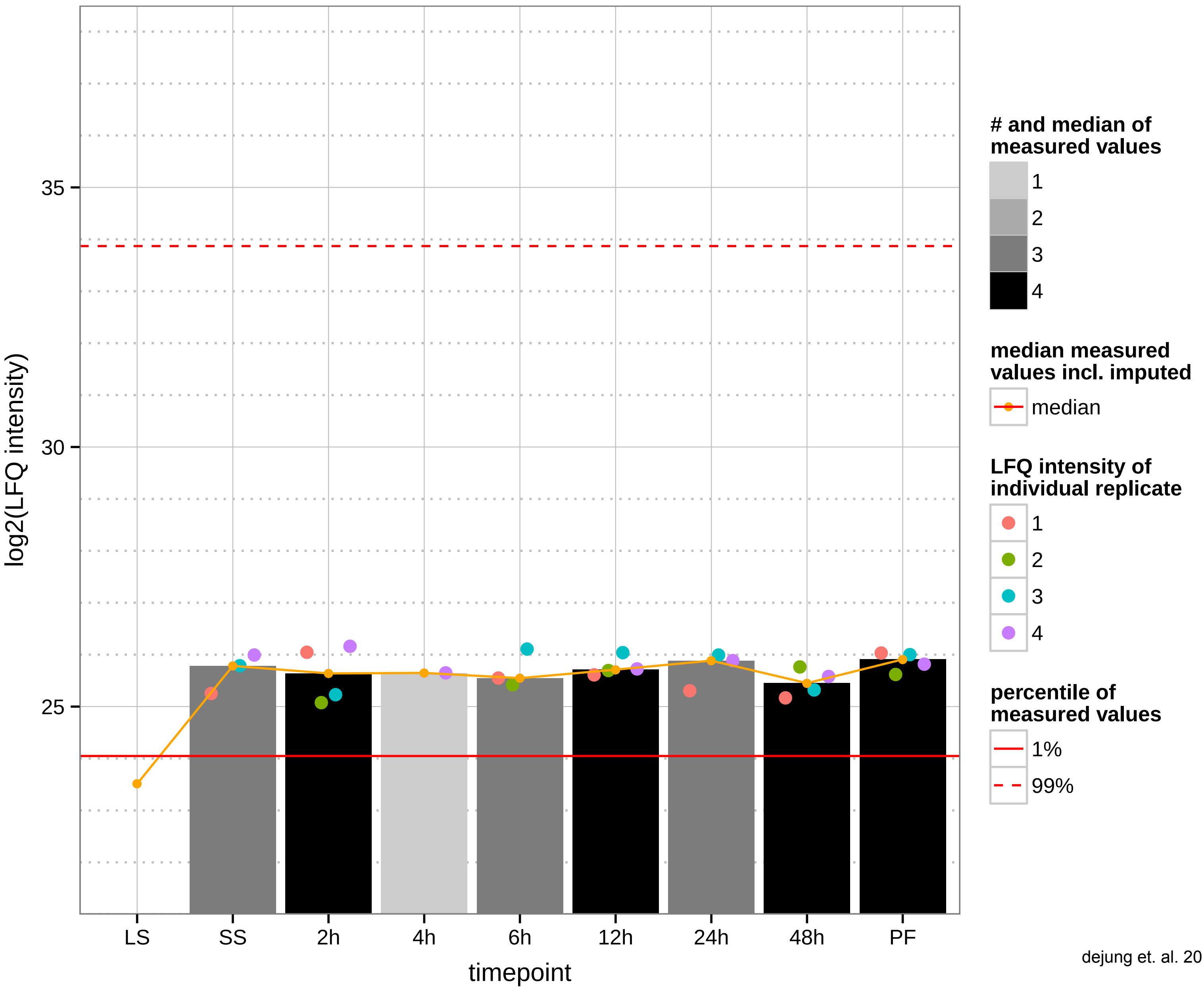

dejung et. al. 2015

hypothetical protein, conserved, zinc finger protein family member, putative (ZC3H23)
Tb927.7.4980;Tb11.v5.0179
AGOF: null, RNA binding, zinc ion binding
AGOC: null
AGOP: null
PGOF: nucleic acid binding, zinc ion binding
PGOC: null
PGOP: null

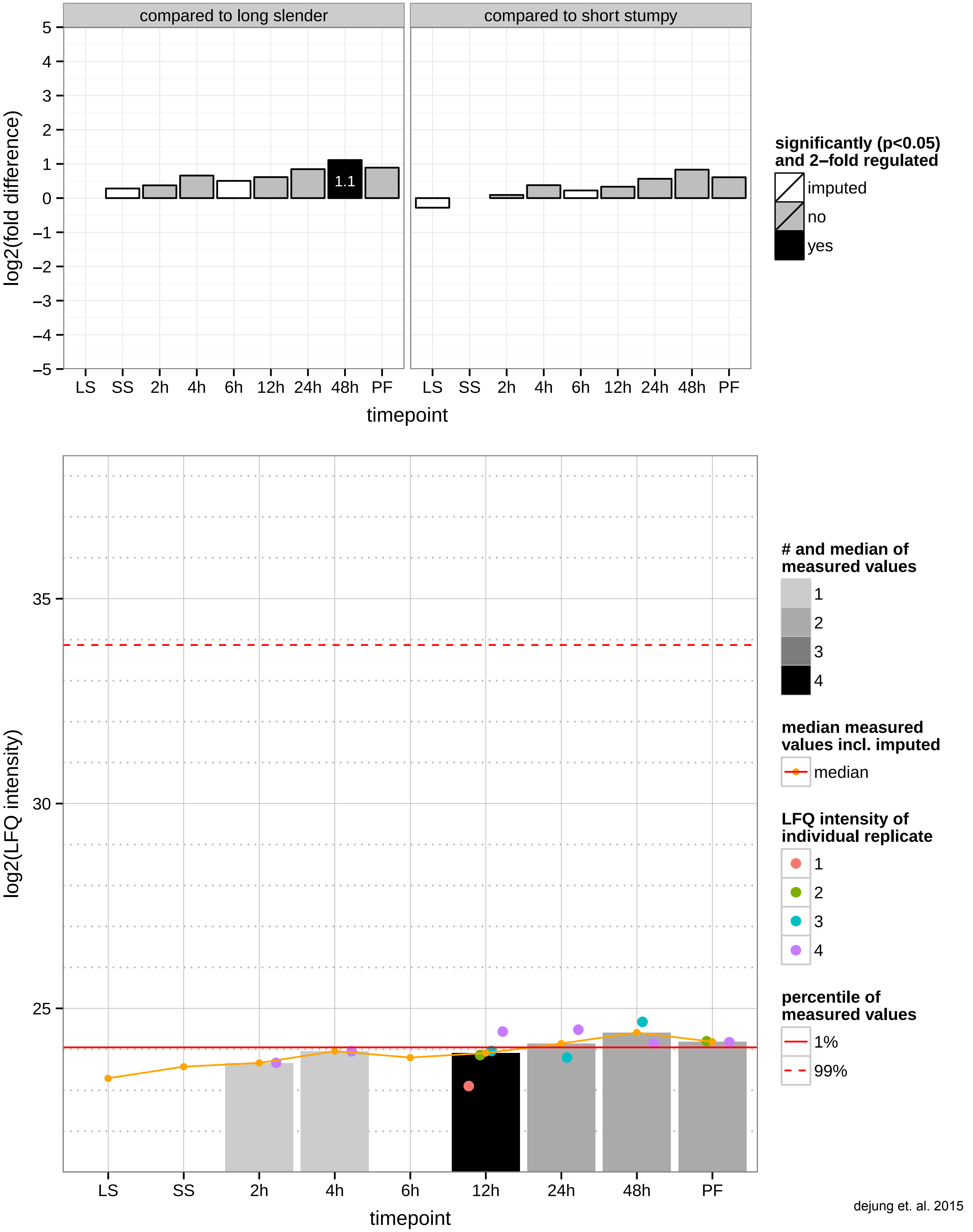

dejung et. al. 2015

hypothetical protein, conserved
Tb927.7.4990;Tb11.v5.0180
AGOF: null
AGOC: null
AGOP: null
PGOF: null
PGOC: null
PGOP: null

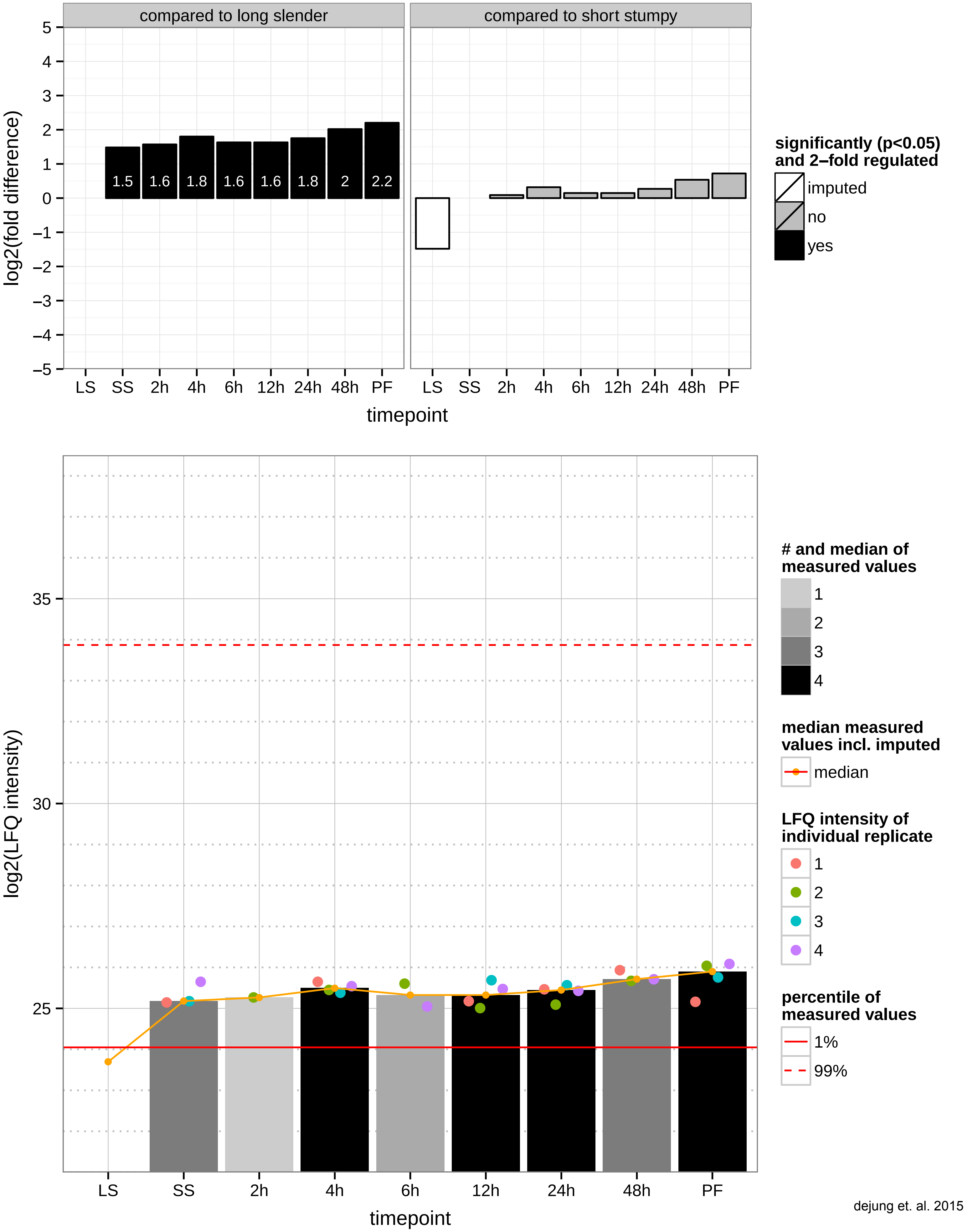

dejung et. al. 2015

hypothetical protein, conserved
Tb927.7.5030;Tb11.v5.0183
AGOF: null
AGOC: null
AGOP: null
PGOF: null
PGOC: null
PGOP: null

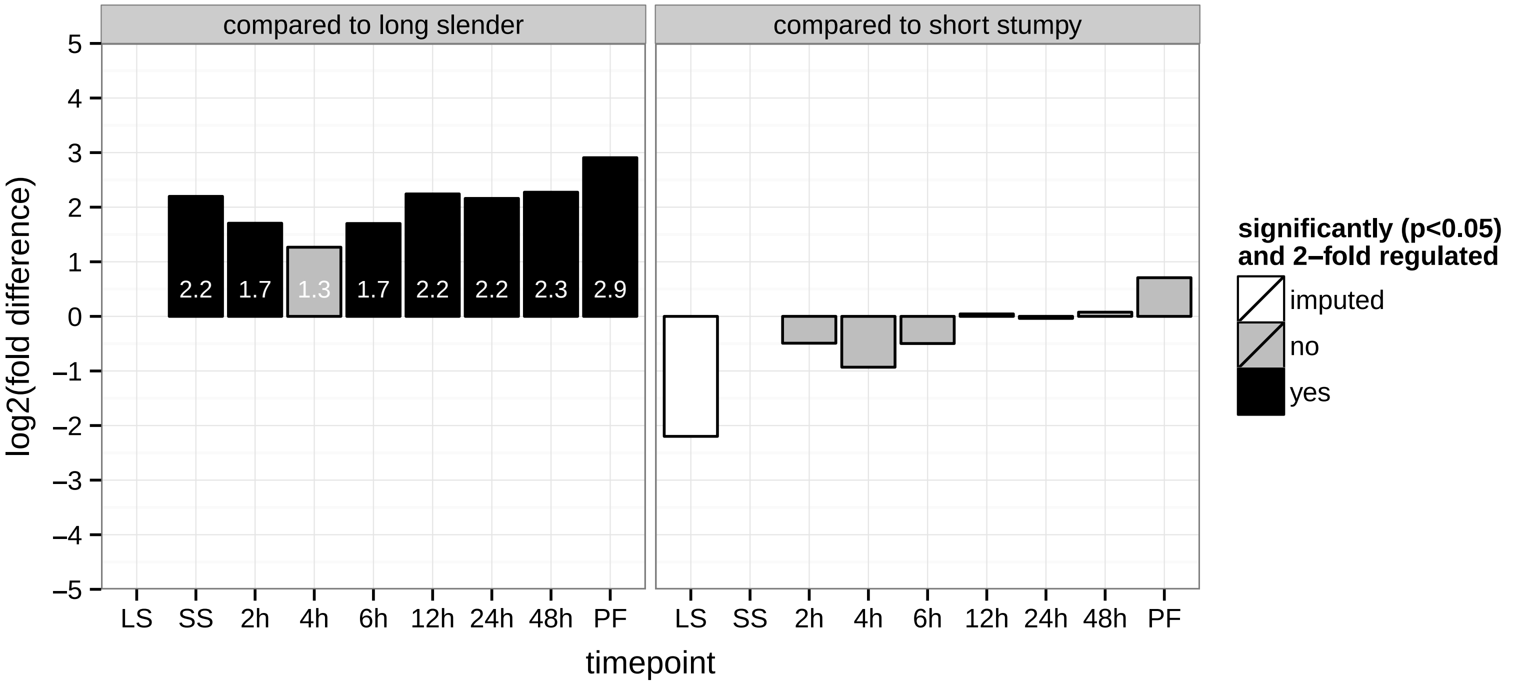

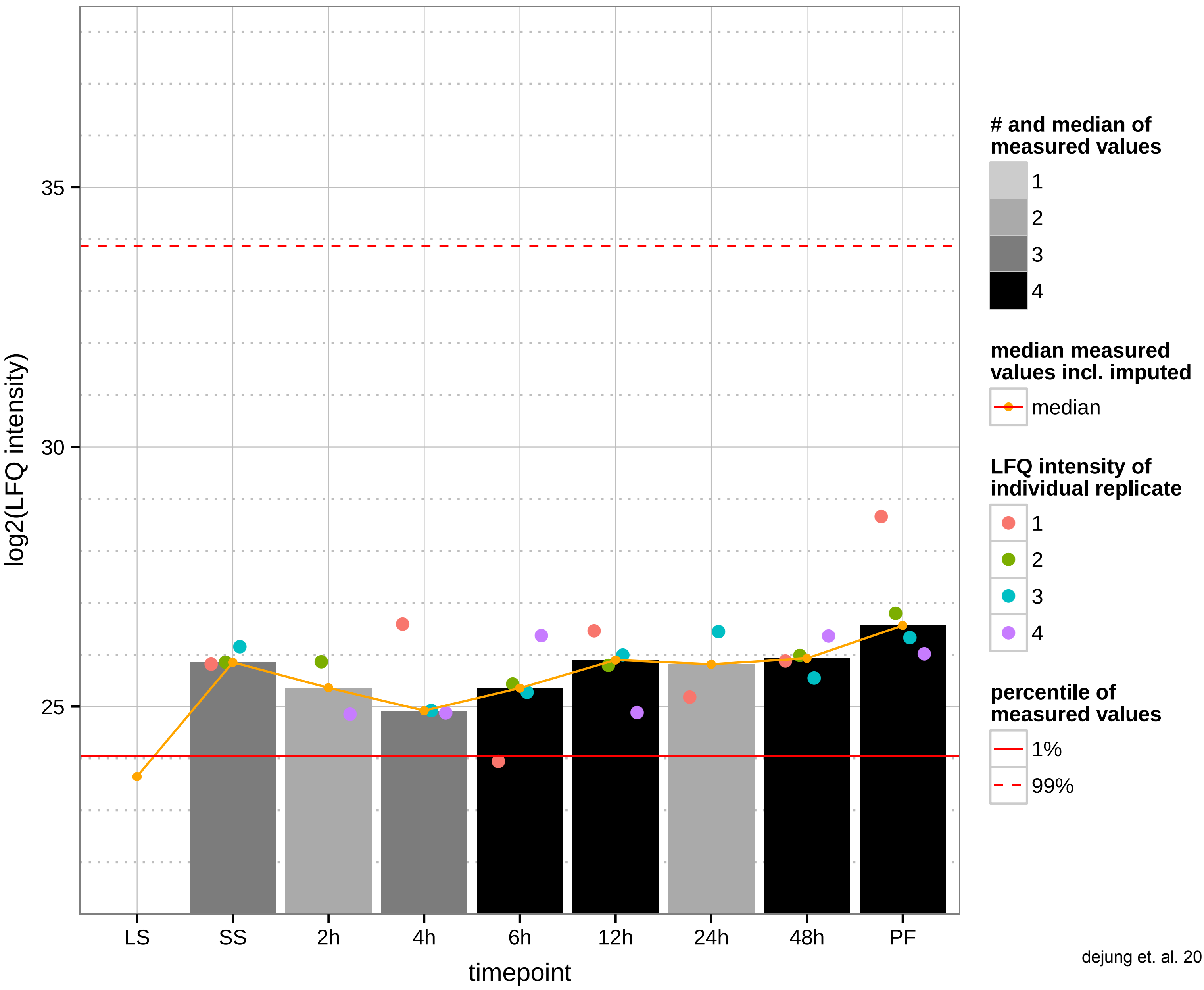

dejung et. al. 2015

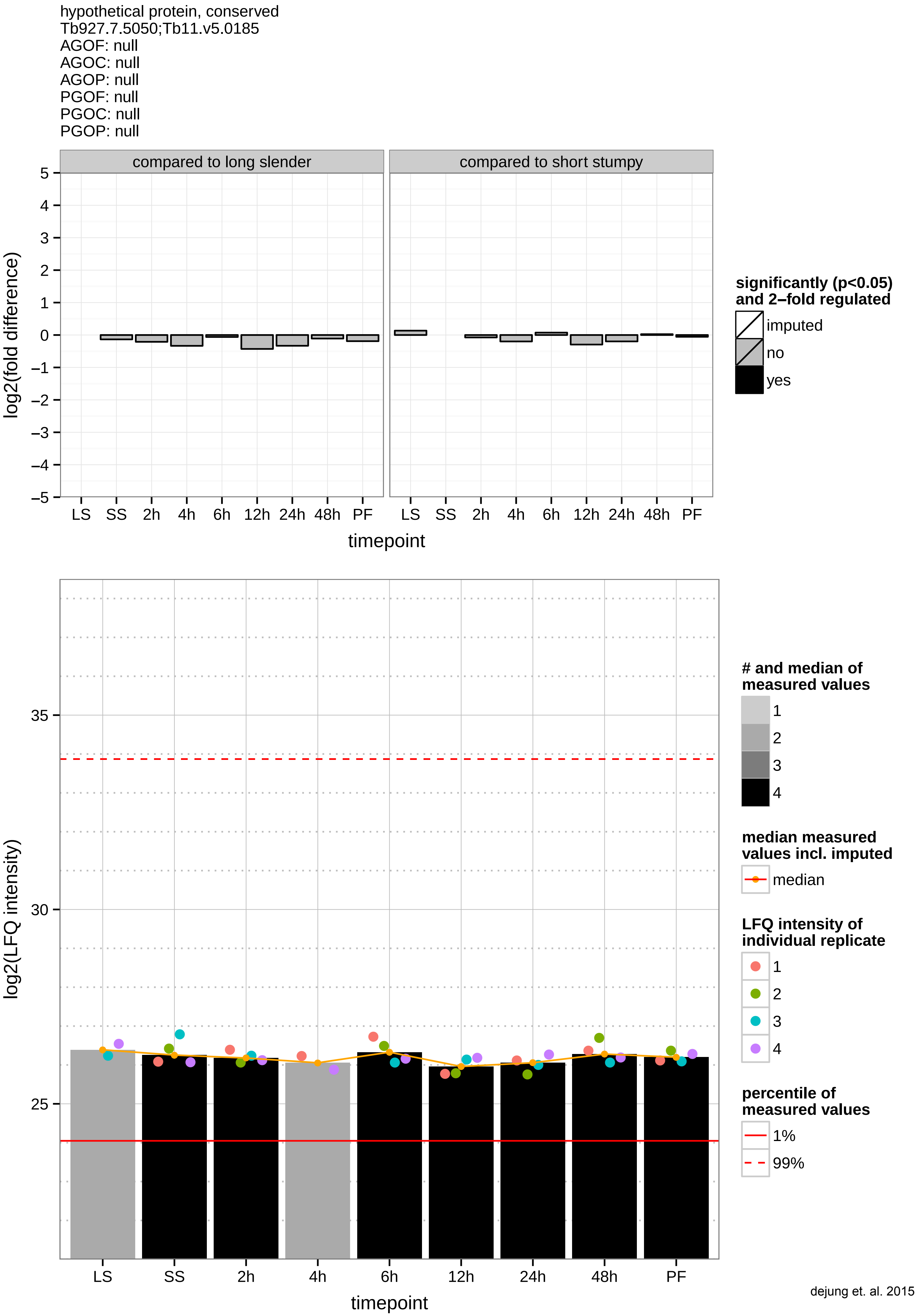

hypothetical protein, conserved
Tb927.7.5050;Tb11.v5.0185
AGOF: null
AGOC: null
AGOP: null
PGOF: null
PGOC: null
PGOP: null
compared to long slender
compared to short stumpy
log2(fold difference)
timepoint
LS SS 2h 4h 6h 12h 24h 48h PF
significantly (p<0.05) and 2–fold regulated
imputed
no
yes
log2(LFQ intensity)
# and median of measured values
1
2
3
4
median measured values incl. imputed
median
LFQ intensity of individual replicate
percentile of measured values
1%
99%
dejung et. al. 2015

protein kinase, putative, (OTHER) NEK family, HsNEK1–like
Tb927.10.5950;Tb11.v5.0190
AGOF: null, ATP binding, protein serine/threonine kinase activity, protein tyrosine kinase activity
AGOC: null
AGOP: null, modulation of development of symbiont involved in interaction with host, protein phosphorylation, quorum sensing
PGOF: ATP binding, phospholipid binding, protein binding, protein kinase activity, transferase activity, transferring phosphorus
PGOC: null
PGOP: protein phosphorylation

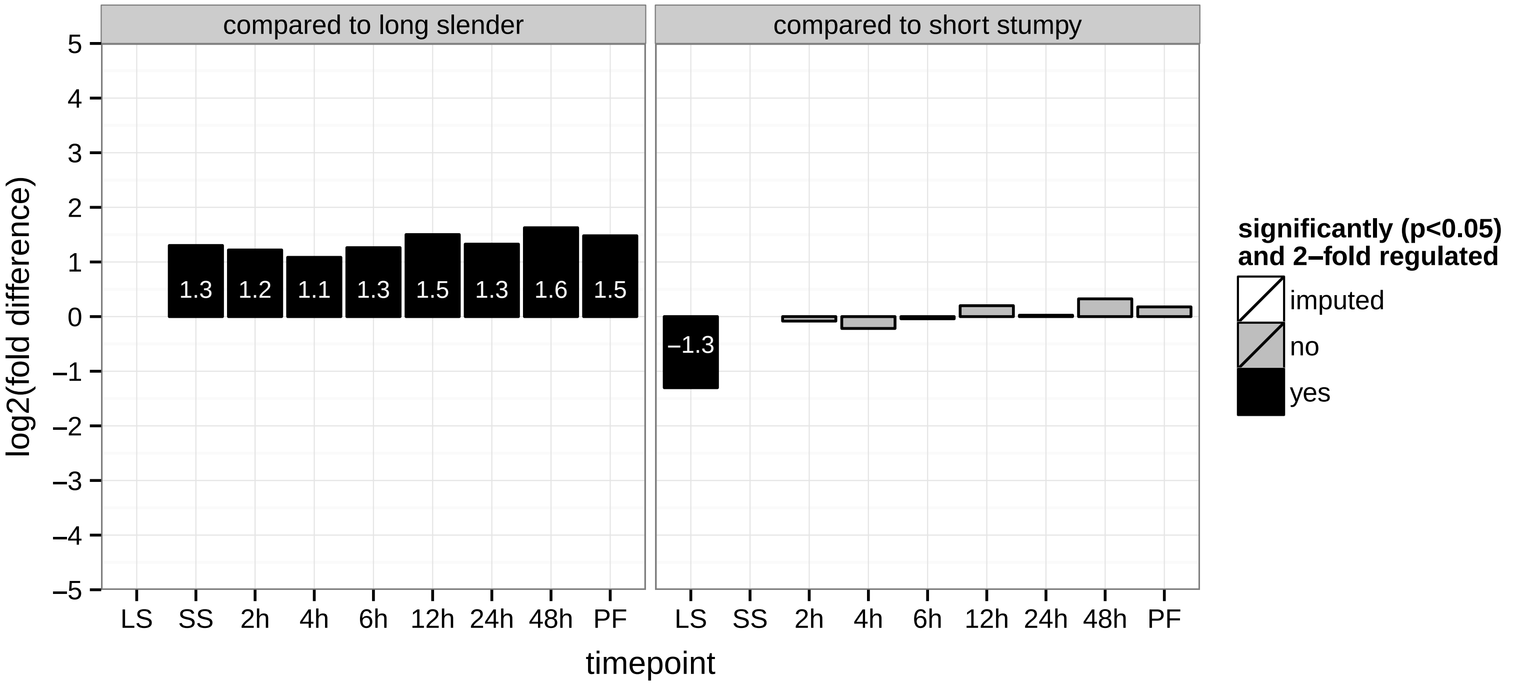

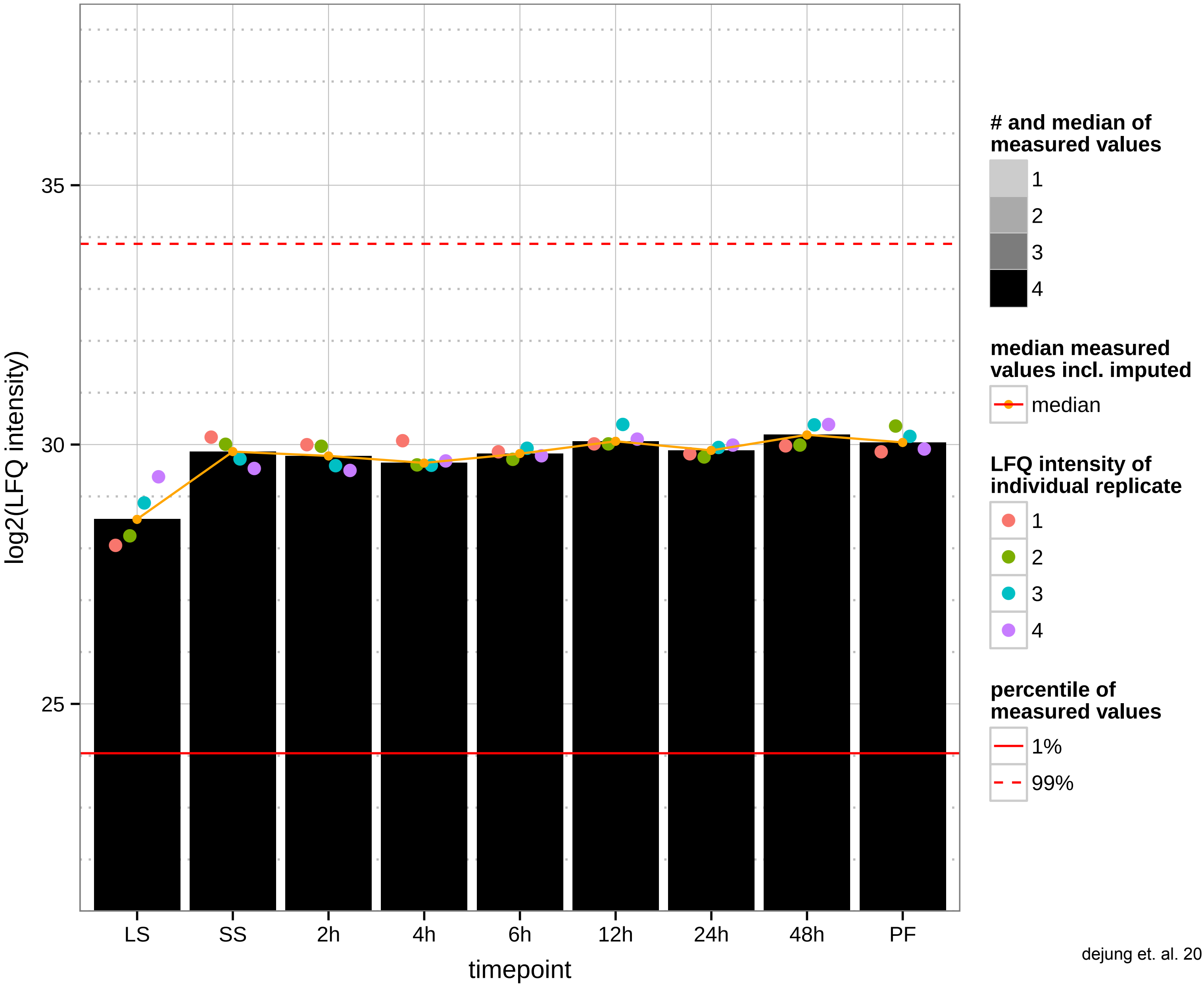

dejung et. al. 2015

hypothetical protein, conserved, proteasome alpha 1 subunit, putative, 20S proteasome subunit alpha–6, (putative) (TbPSA6)
Tb11.v5.0196;Tb927.10.6030
AGOF: null, endopeptidase activity, threonine–type endopeptidase activity
AGOC: null, mitochondrion, proteasome core complex
AGOP: null, ubiquitin–dependent protein catabolic process
PGOF: endopeptidase activity, threonine–type endopeptidase activity
PGOC: proteasome core complex, proteasome core complex, alpha–subunit complex
PGOP: proteolysis involved in cellular protein catabolic process, ubiquitin–dependent protein catabolic process

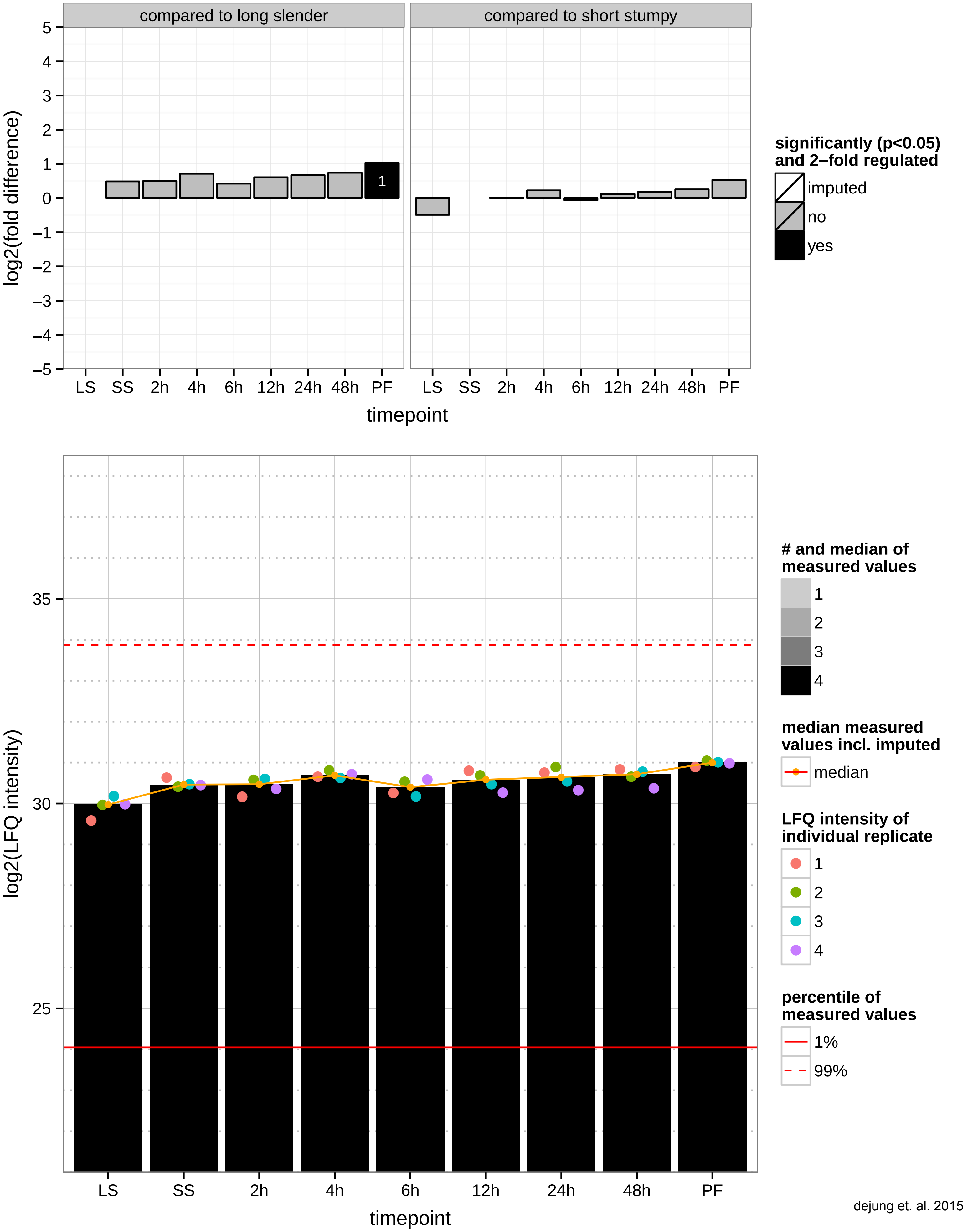

dejung et. al. 2015

proteasome beta 5 subunit, putative, proteasome beta 5 subunit (PRCE)
Tb927.10.6080;Tb11.v5.0200
AGOF: null, endopeptidase activity, threonine–type endopeptidase activity
AGOC: null, proteasome core complex, proteasome core complex, beta–subunit complex
AGOP: null, ubiquitin–dependent protein catabolic process
PGOF: threonine–type endopeptidase activity
PGOC: proteasome core complex
PGOP: proteolysis involved in cellular protein catabolic process

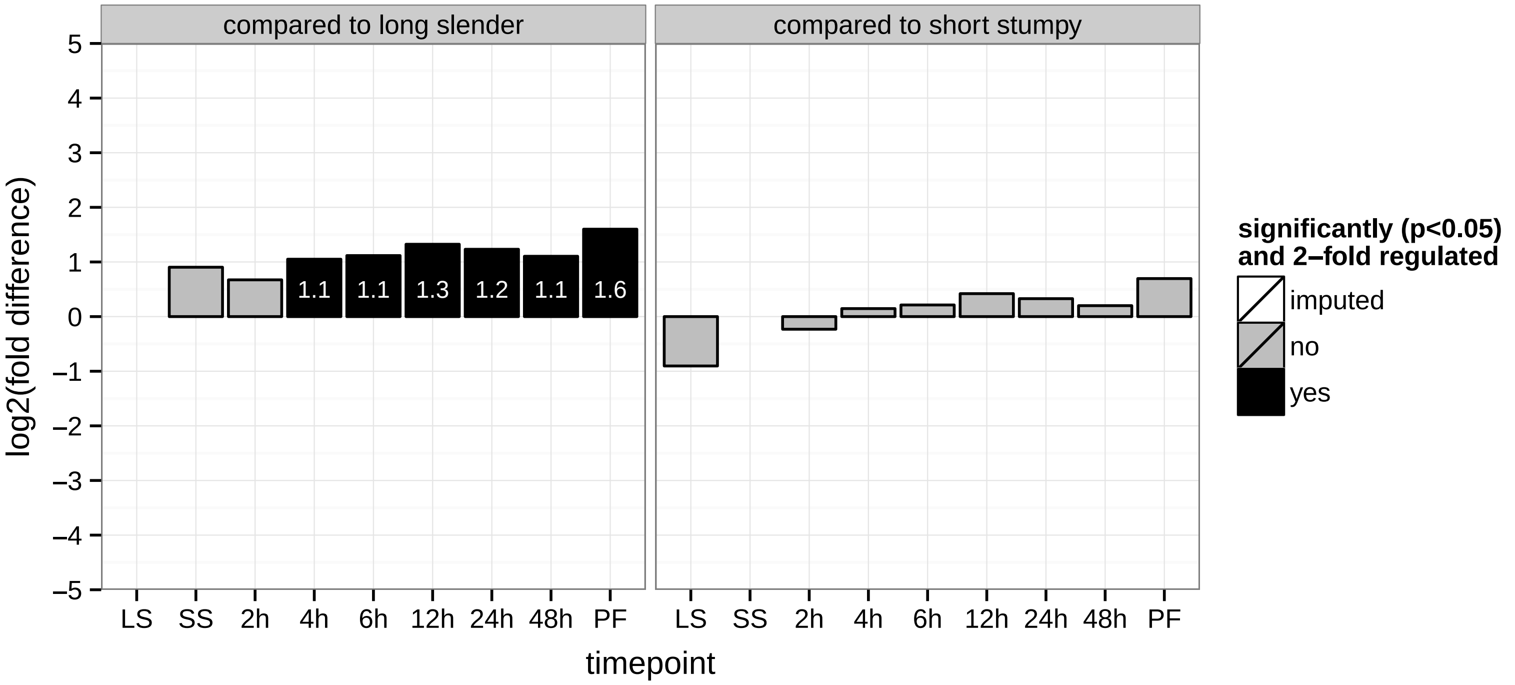

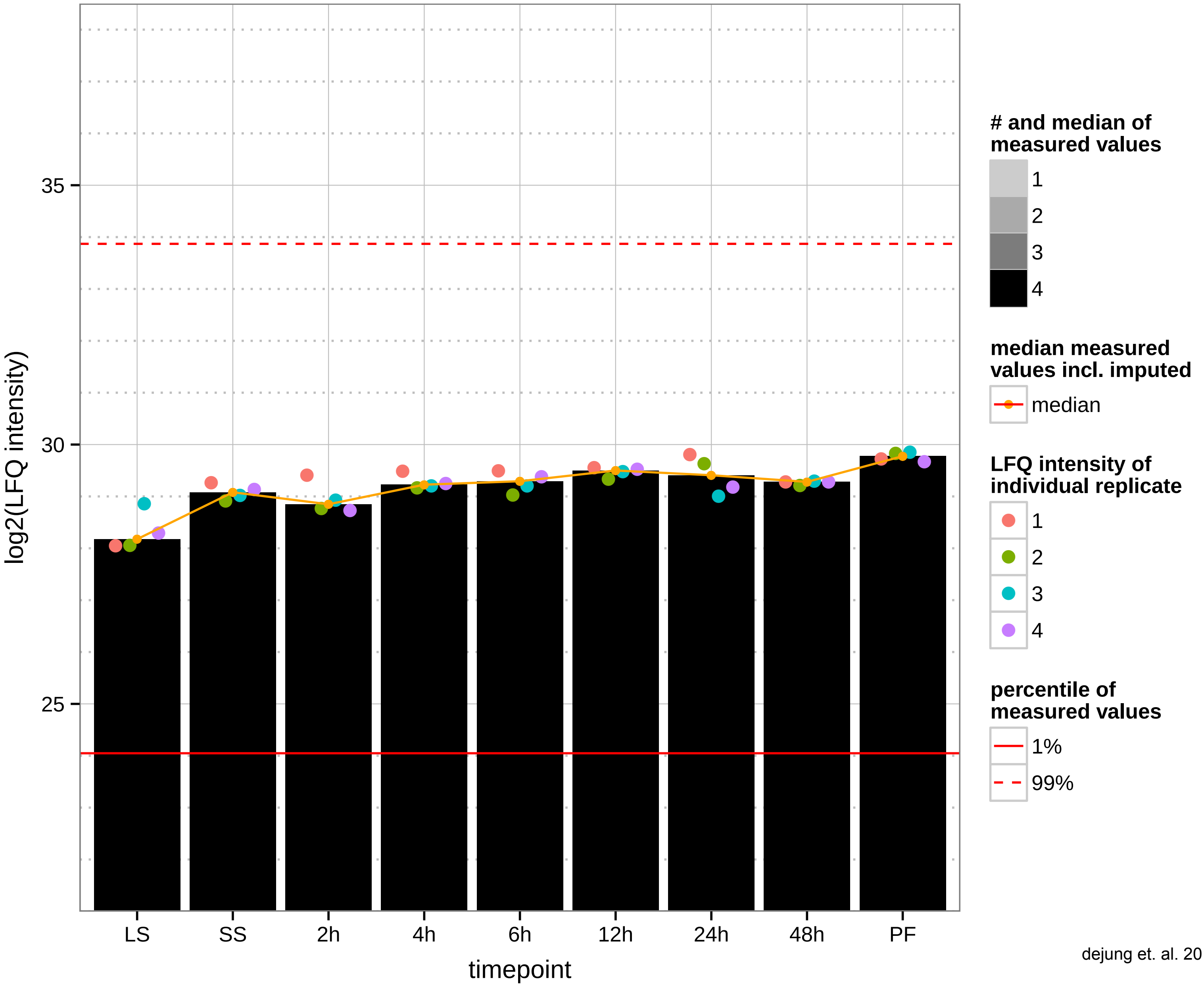

dejung et. al. 2015

tRNA pseudouridine synthase A, putative
Tb11.v5.0201;Tb927.10.6090
AGOF: null, RNA binding, pseudouridine synthase activity
AGOC: null
AGOP: null, pseudouridine synthesis
PGOF: RNA binding, pseudouridine synthase activity
PGOC: null
PGOP: RNA modification, pseudouridine synthesis

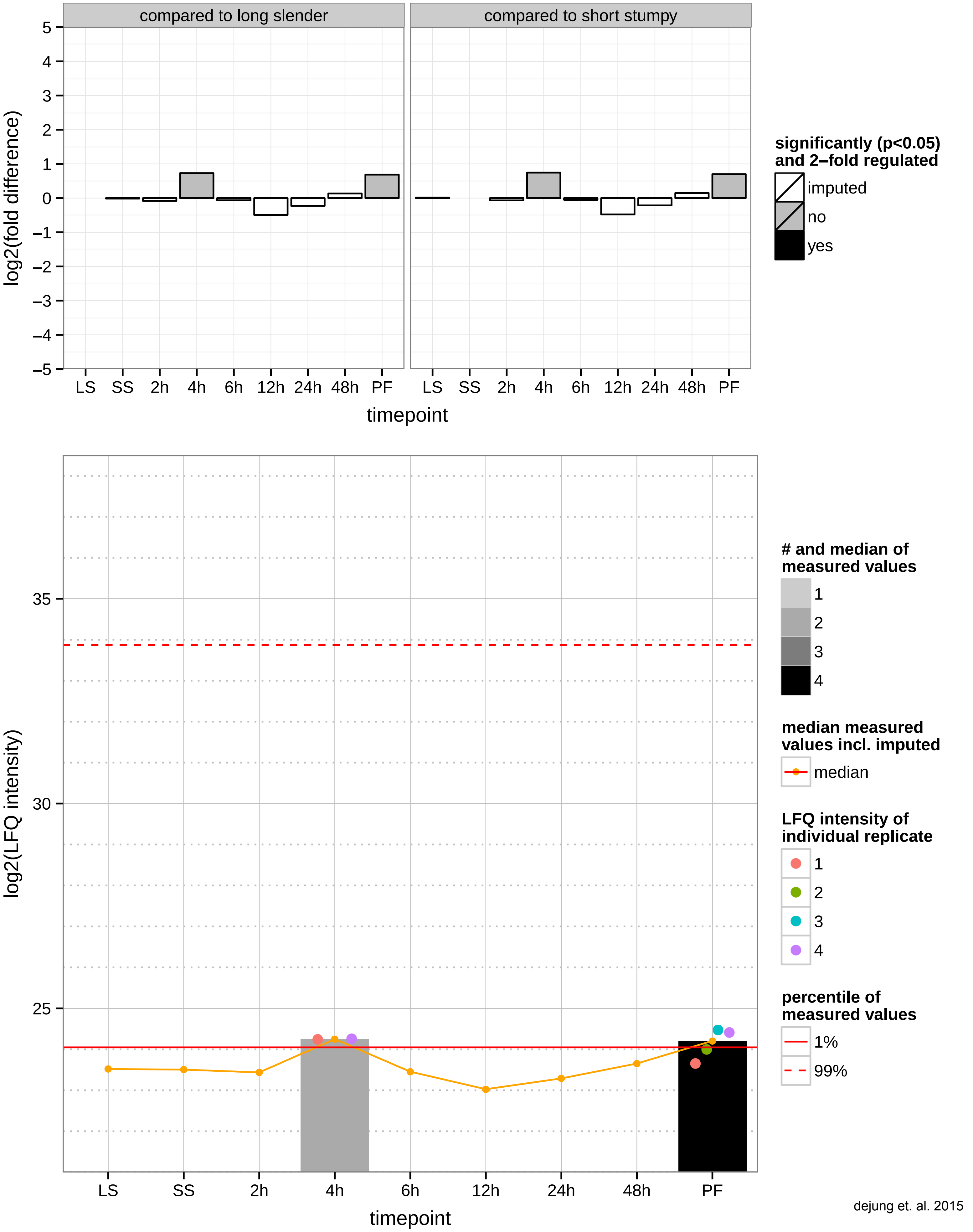

dejung et. al. 2015

iron/ascorbate oxidoreductase family protein, putative, thymine−7−hydroxylase, putative (TLP5), 2−oxoglutarate (2OG) and Fe
Tb927.5.300b;Tb927.5.300;Tb11.v5.0215
AGOF: null, ferrous iron binding, oxidoreductase activity, iron ion binding
AGOC: null
AGOP: null, metabolic process
PGOF: oxidoreductase activity, oxidoreductase activity, acting on paired donors, with incorporation or reduction of molecular o
PGOC: null
PGOP: oxidation−reduction process

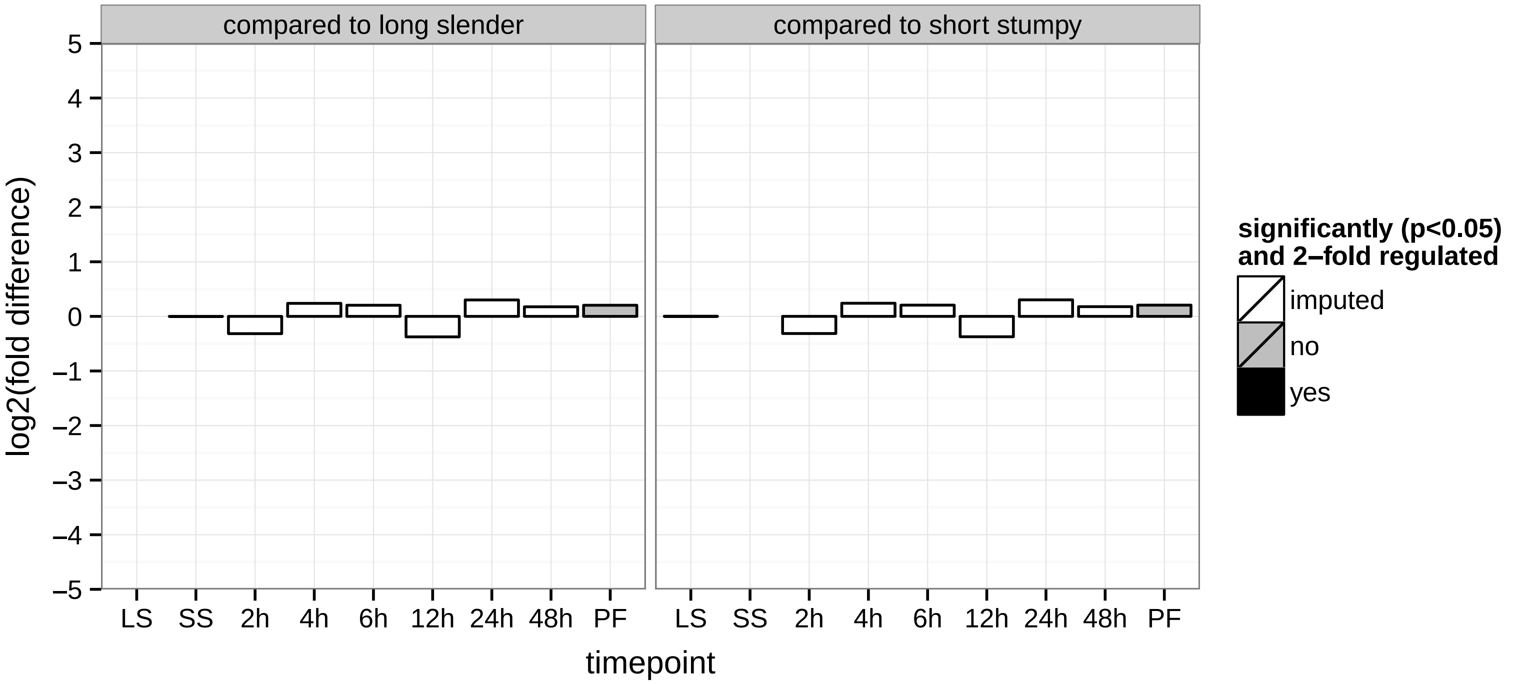

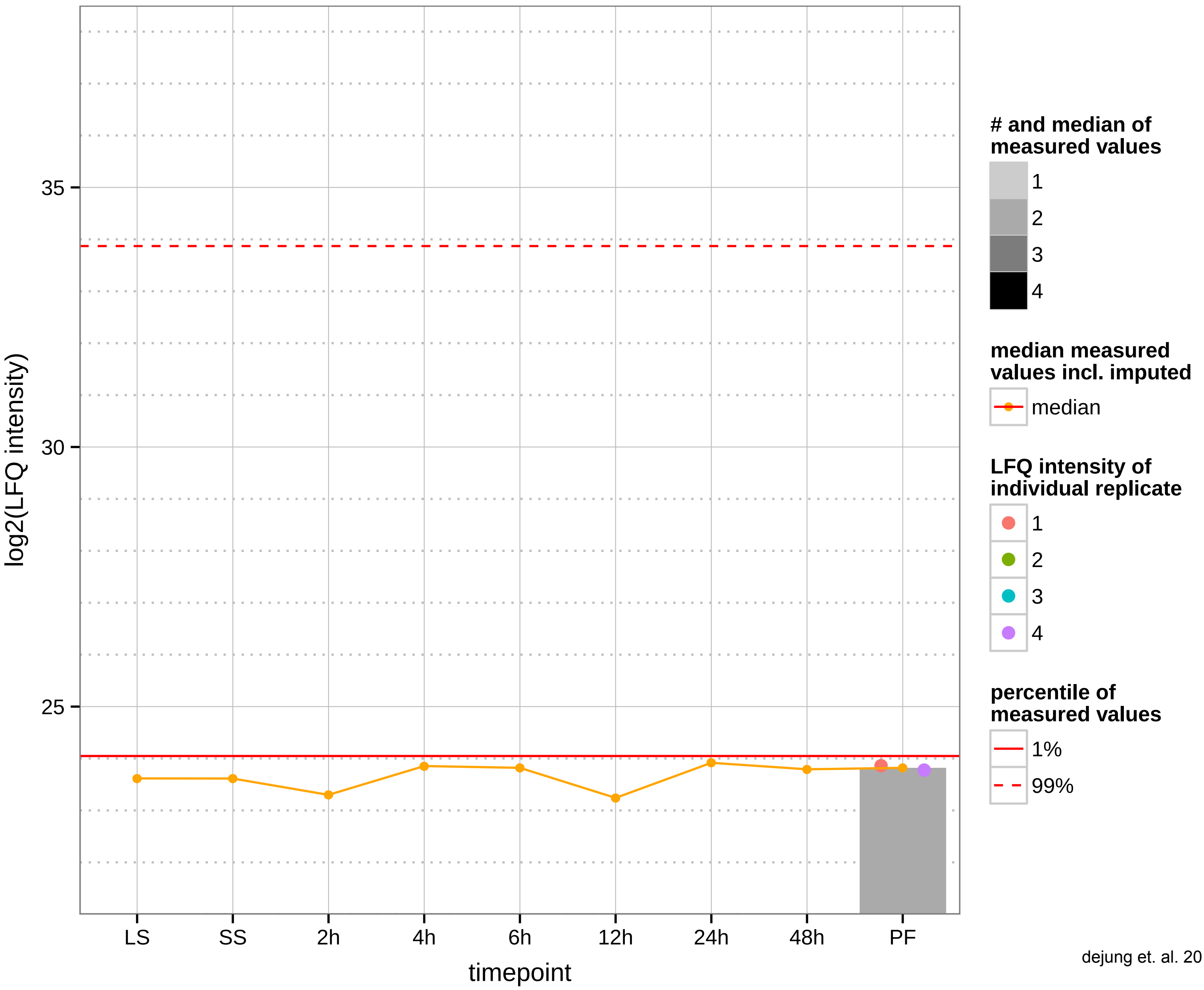

dejung et. al. 2015

hypothetical protein, conserved
Tb927.5.500;Tb11.v5.0218
AGOF: null
AGOC: null, mitochondrion
AGOP: null
PGOF: null
PGOC: null
PGOP: null

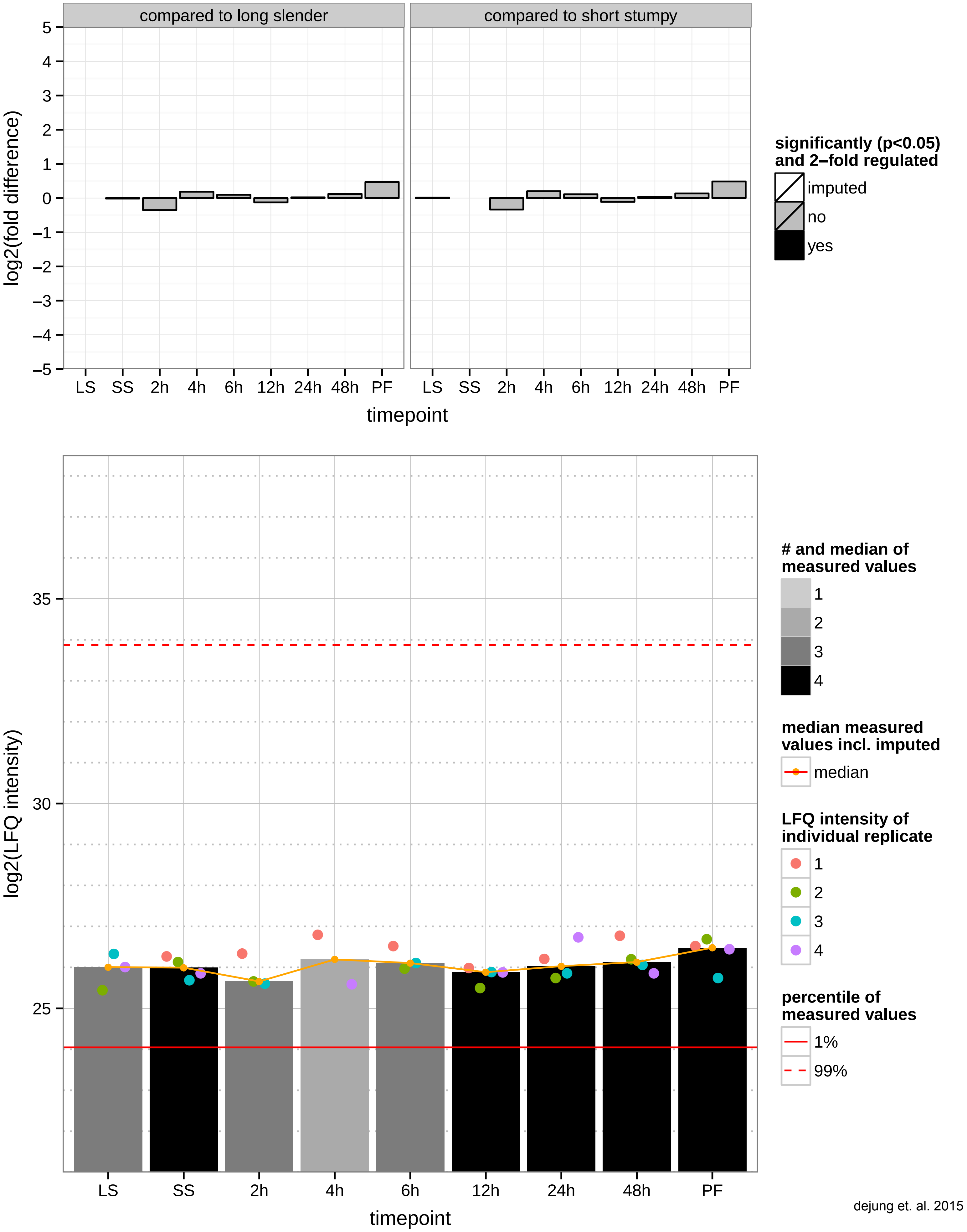

dejung et. al. 2015

75 kDa invariant surface glycoprotein, putative, putative (ISG75)
Tb927.5.400;Tb11.v5.0227;Tb927.5.350;Tb11.v5.0421
AGOF: null
AGOC: null, plasma membrane
AGOP: null
PGOF: null
PGOC: null
PGOP: null

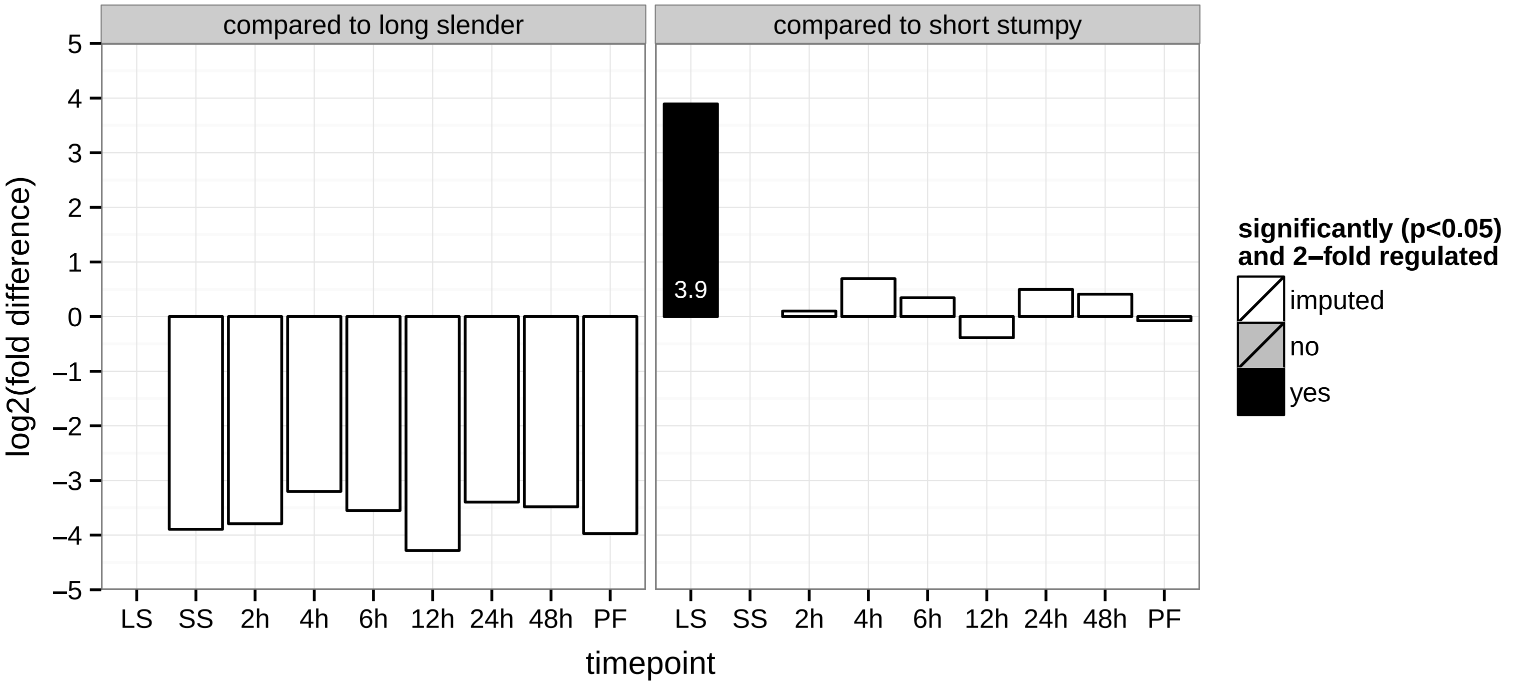

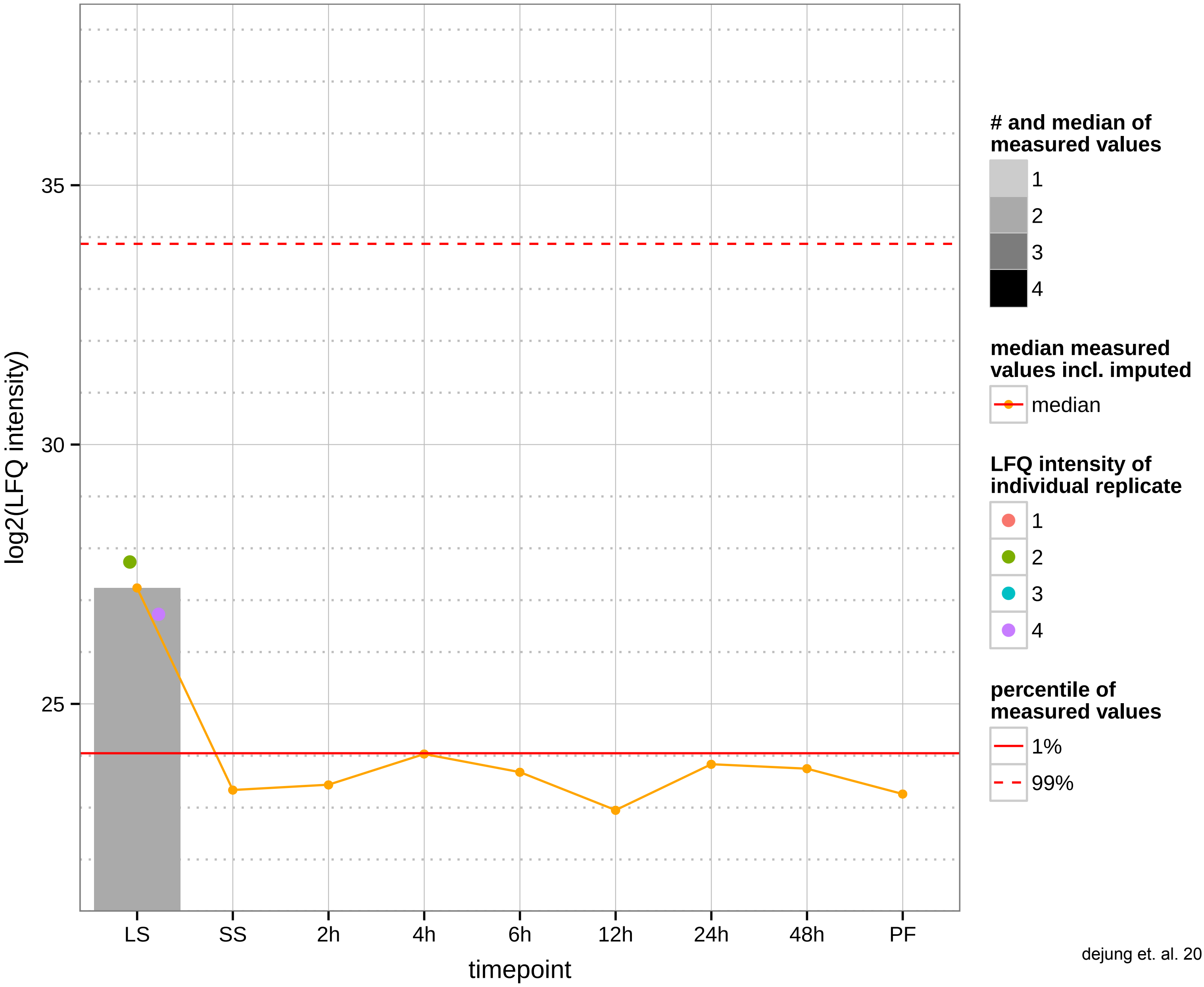

dejung et. al. 2015

protein kinase, putative, SNF1–related protein kinases
Tb927.10.5310;Tb11.v5.0245
AGOF: null, ATP binding, protein serine/threonine kinase activity, protein tyrosine kinase activity
AGOC: null
AGOP: null, protein phosphorylation
PGOF: ATP binding, protein kinase activity, protein serine/threonine kinase activity, transferase activity, transferring phosphoru
PGOC: null
PGOP: protein phosphorylation

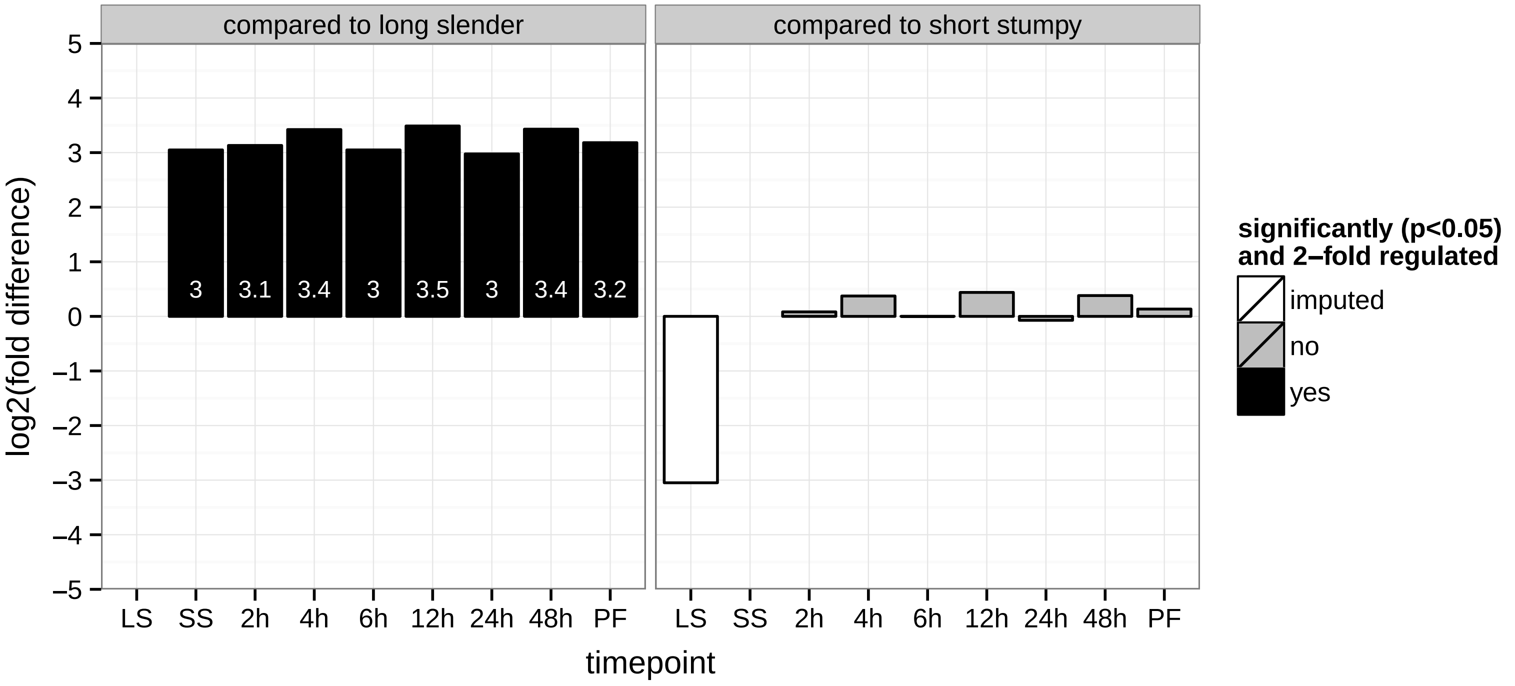

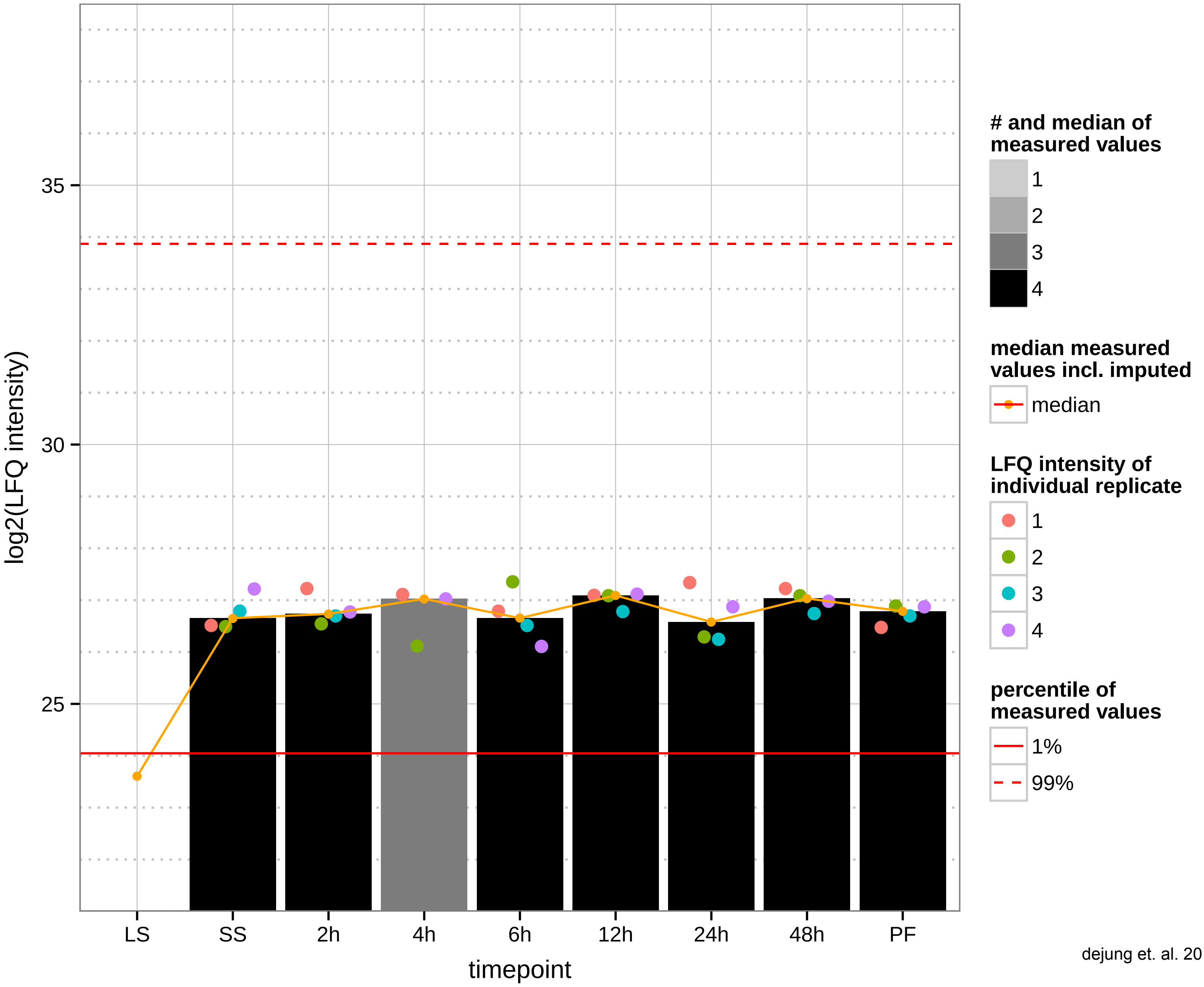

dejung et. al. 2015

eukaryotic translation initiation factor 6, putative, eukaryotic translation initiation factor 6 (eIF−6)
Tb927.10.5300;Tb11.v5.0246
AGOF: null, ribosome binding
AGOC: null
AGOP: null, mature ribosome assembly
PGOF: ribosome binding
PGOC: null
PGOP: mature ribosome assembly

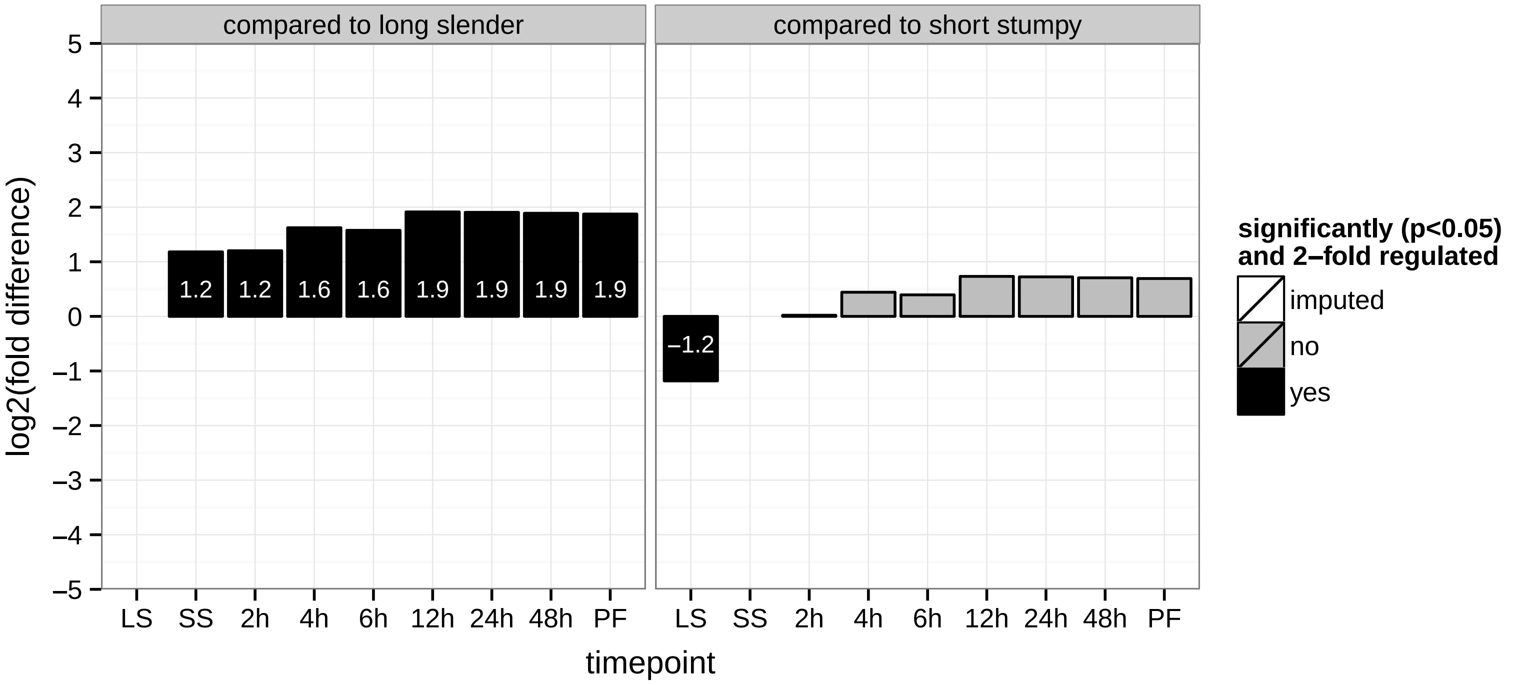

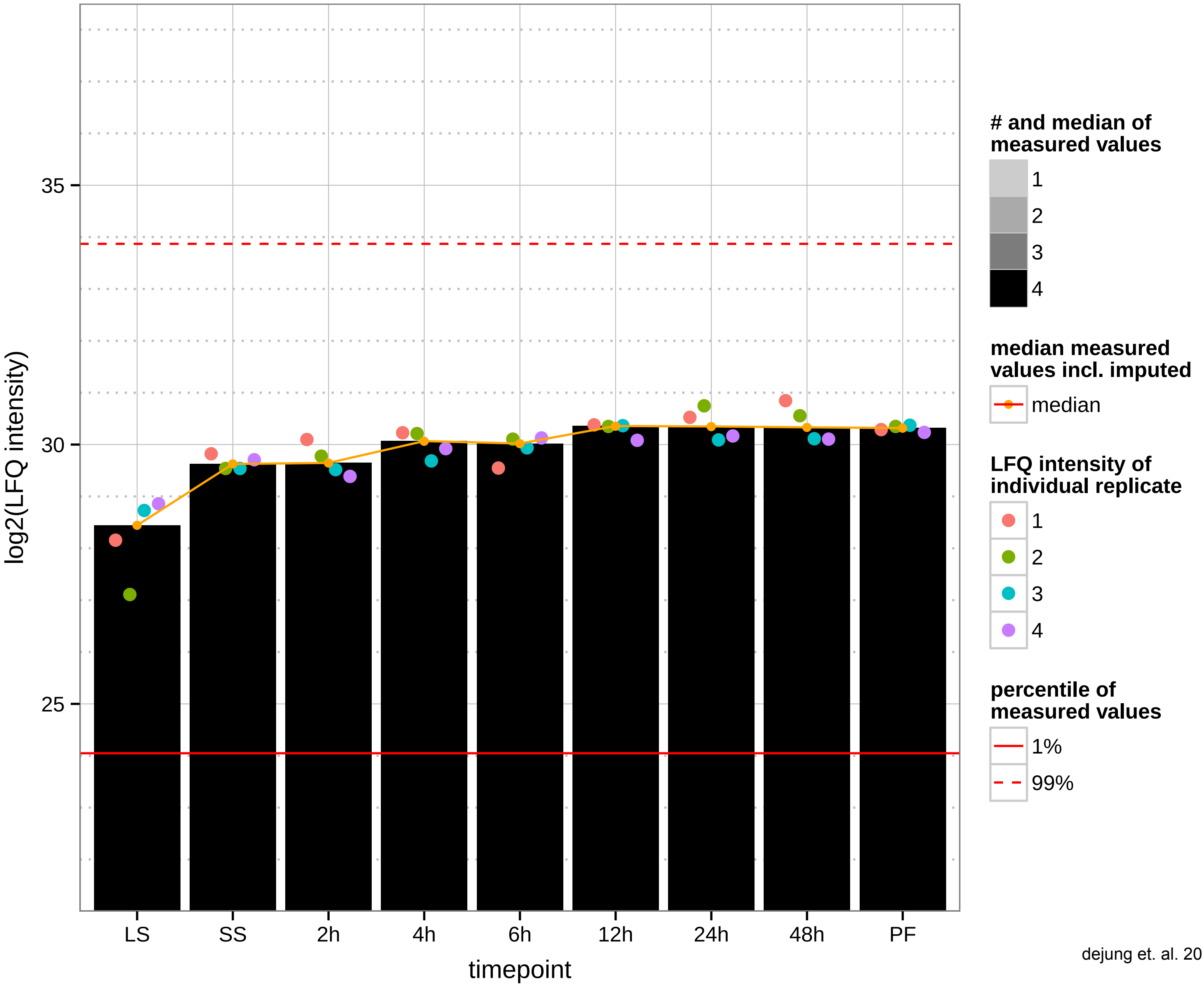

dejung et. al. 2015

RNA binding protein, putative
Tb927.11.7310;Tb11.v5.0252
AGOF: null, RNA binding
AGOC: null
AGOP: null
PGOF: nucleic acid binding
PGOC: null
PGOP: null

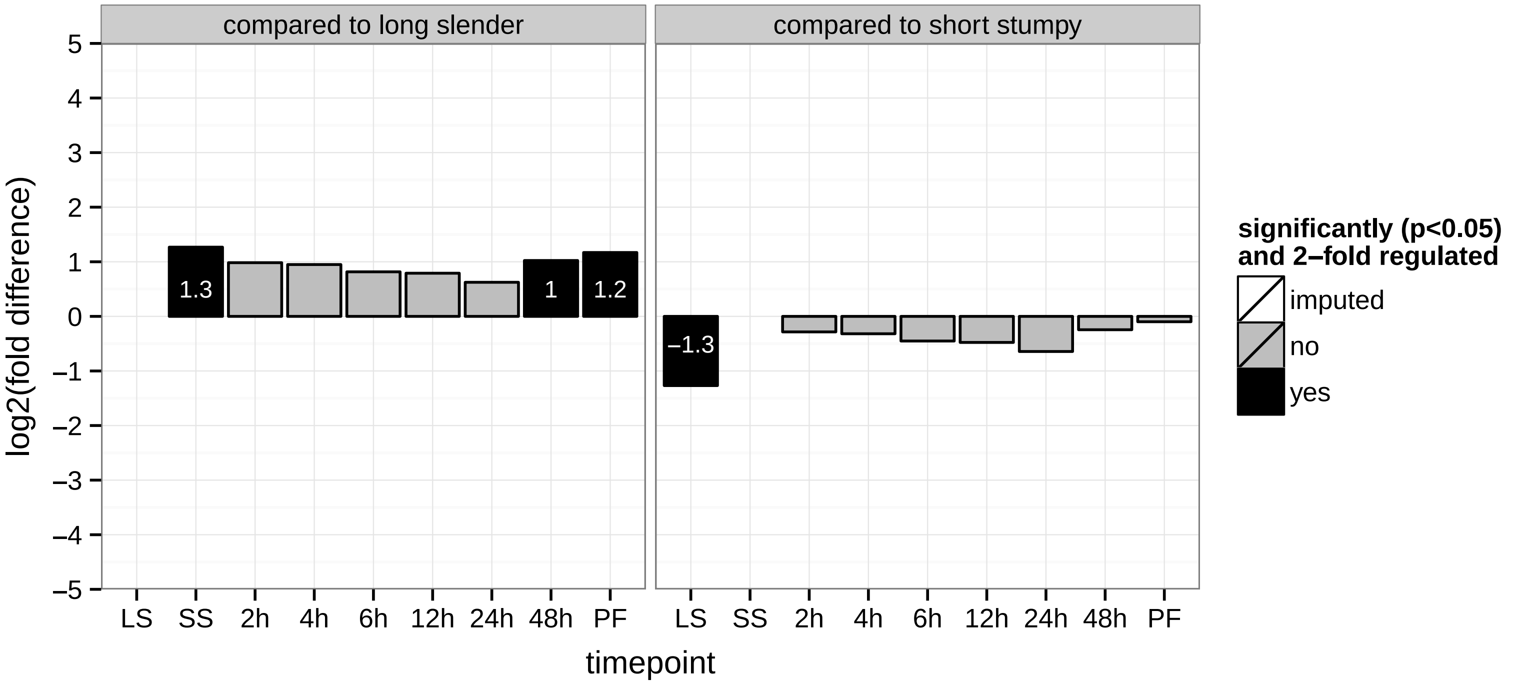

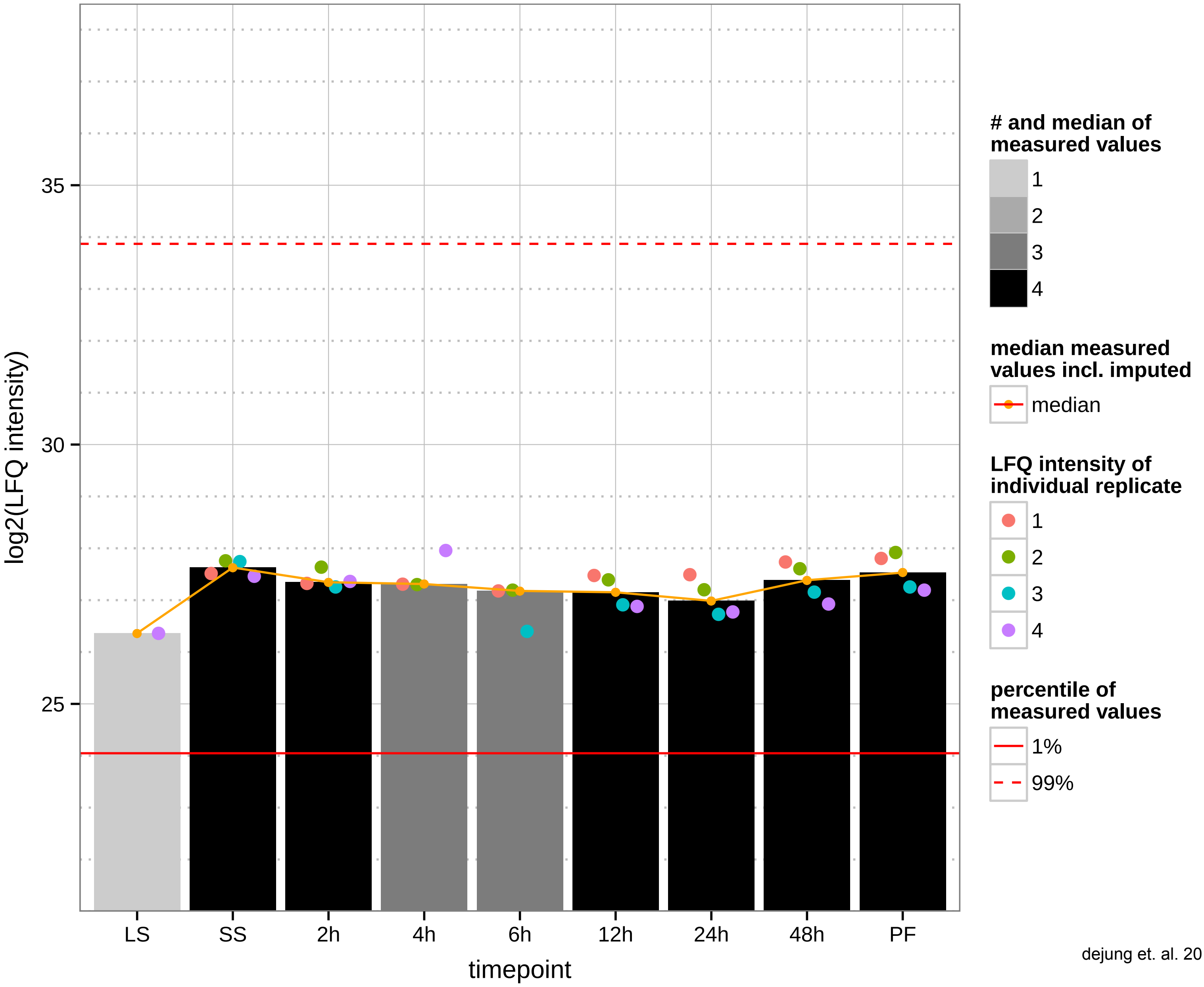

dejung et. al. 2015

proteasome beta 3 subunit, putative, putative (PSB3)
Tb927.11.7270;Tb11.v5.0256
AGOF: null, endopeptidase activity, threonine–type endopeptidase activity
AGOC: null, proteasome core complex, proteasome core complex, beta–subunit complex
AGOP: null, ubiquitin–dependent protein catabolic process
PGOF: threonine–type endopeptidase activity
PGOC: proteasome core complex
PGOP: proteolysis involved in cellular protein catabolic process

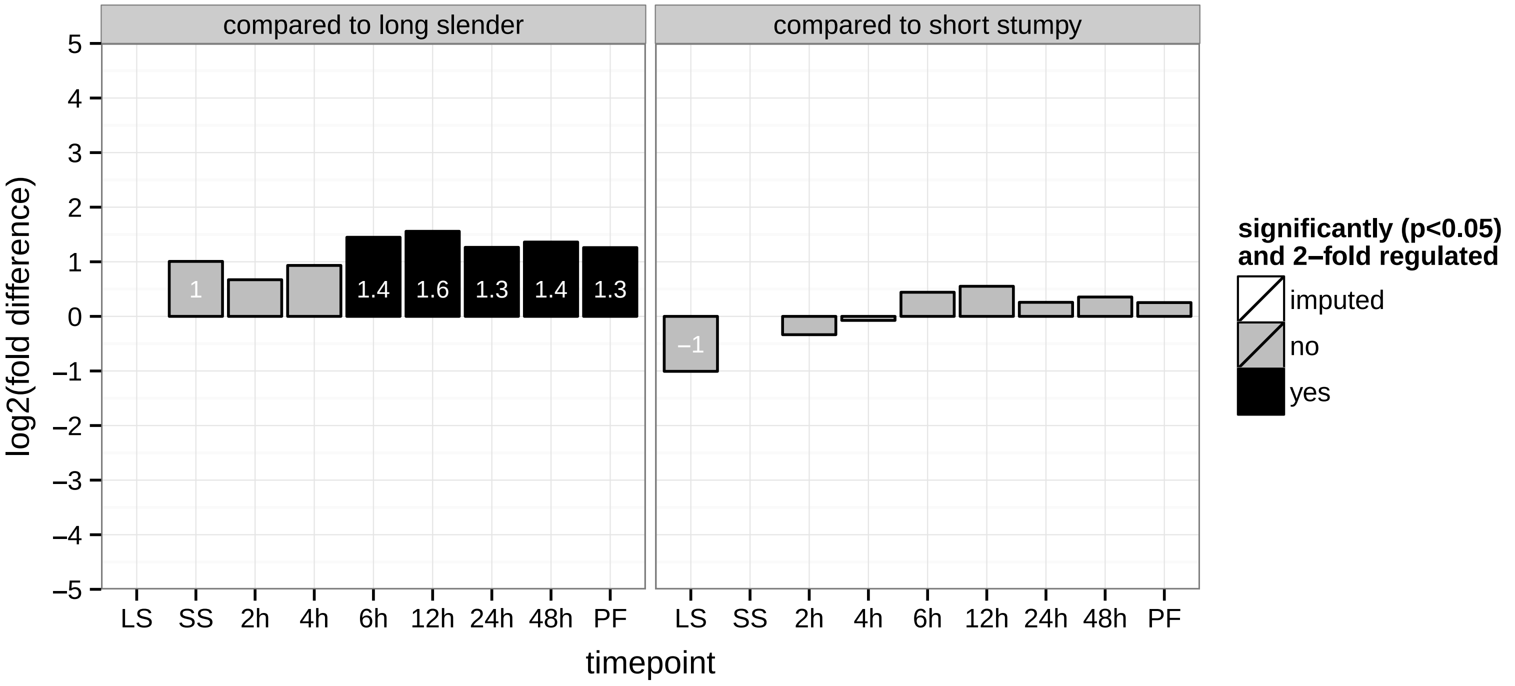

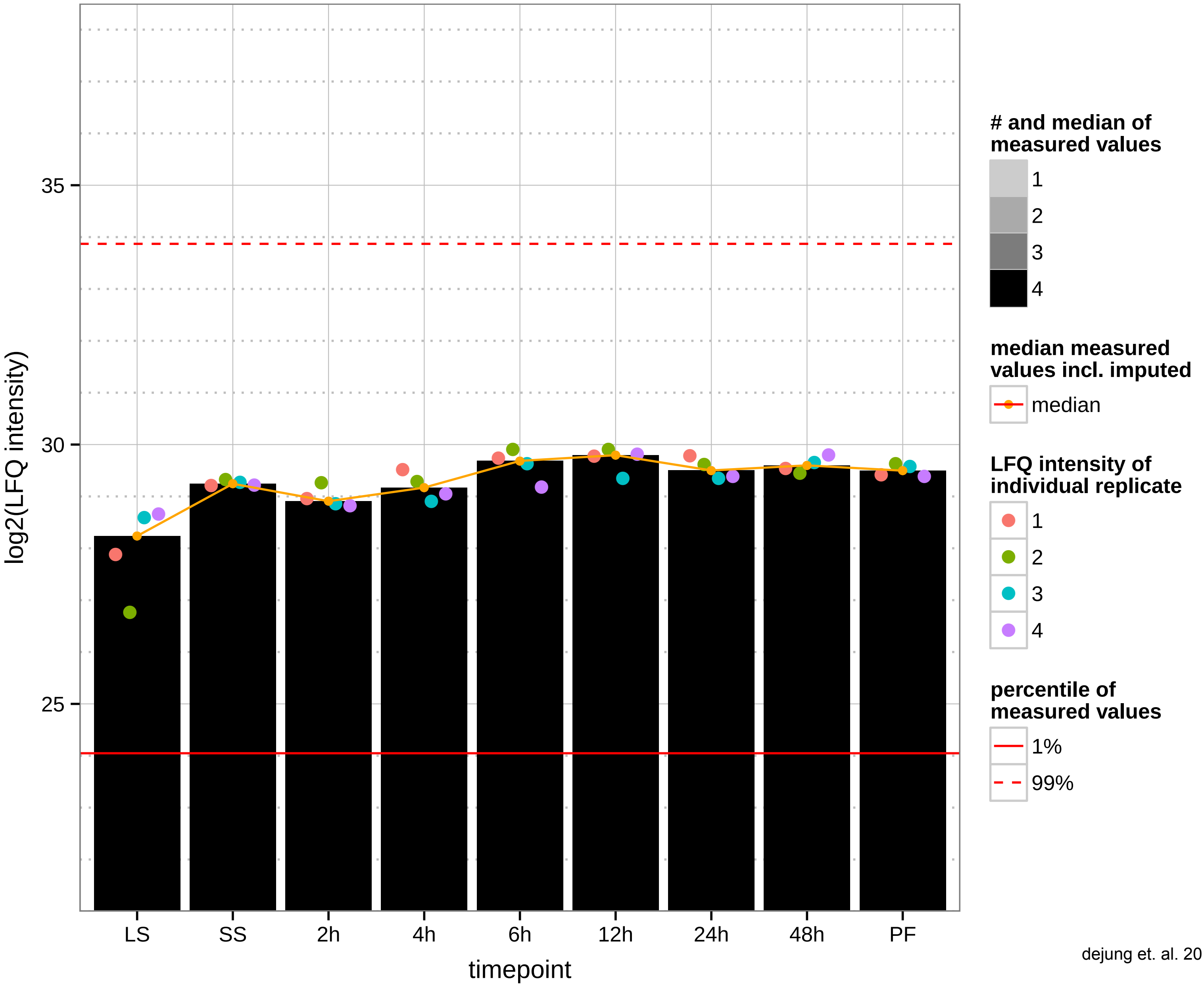

dejung et. al. 2015

hypothetical protein, conserved, dynein regulatory complex subunit (CMF70)
Tb927.11.7240;Tb11.v5.0258
AGOF: null
AGOC: null, axonemal dynein complex, cilium
AGOP: null, ciliary cell motility, ciliary or flagellar motility
PGOF: null
PGOC: null
PGOP: null

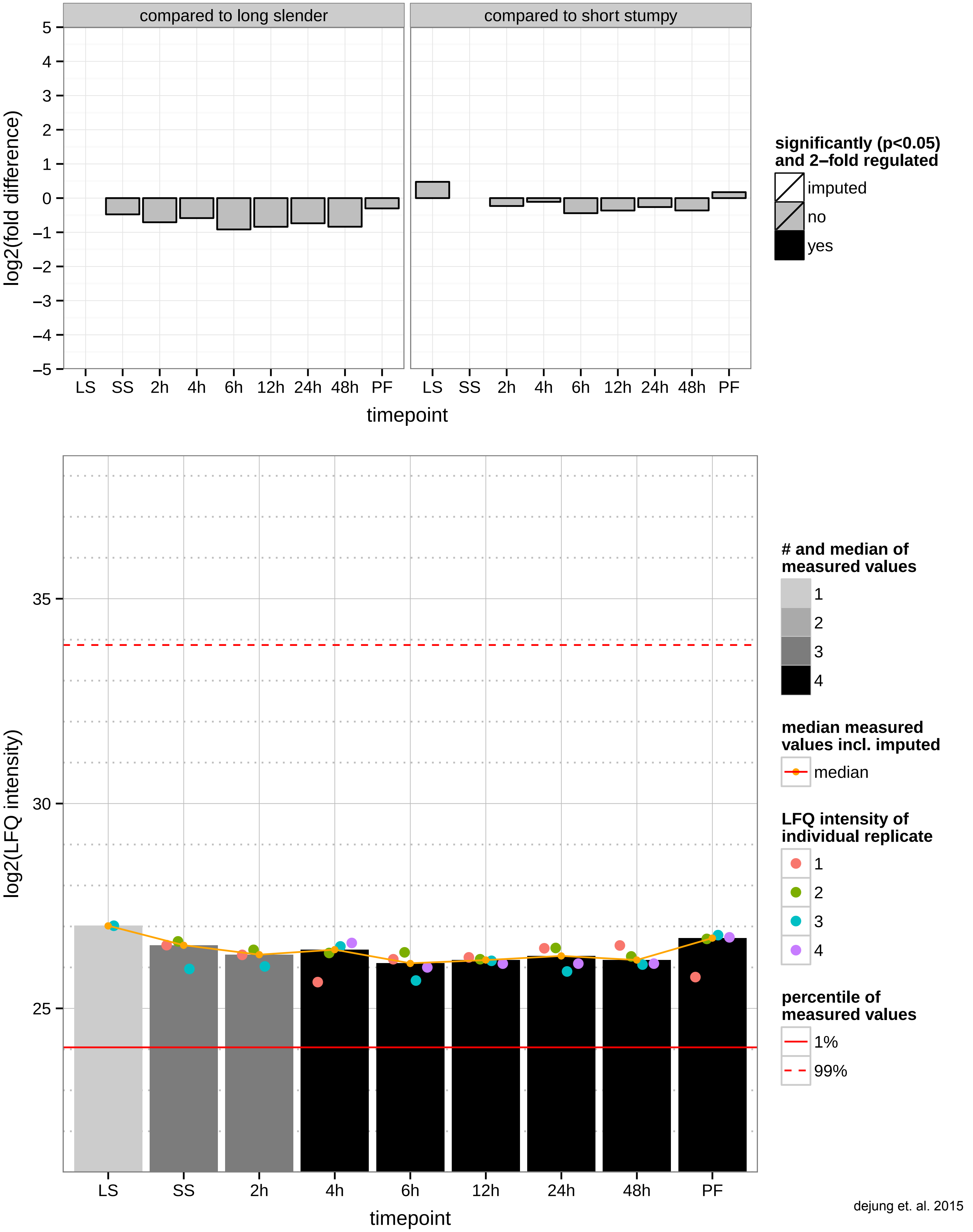

dejung et. al. 2015

adenosine 5'−monophosphoramidase, putative
Tb927.7.4480;Tb11.v5.0261
AGOF: null, catalytic activity
AGOC: null
AGOP: null
PGOF: catalytic activity
PGOC: null
PGOP: null

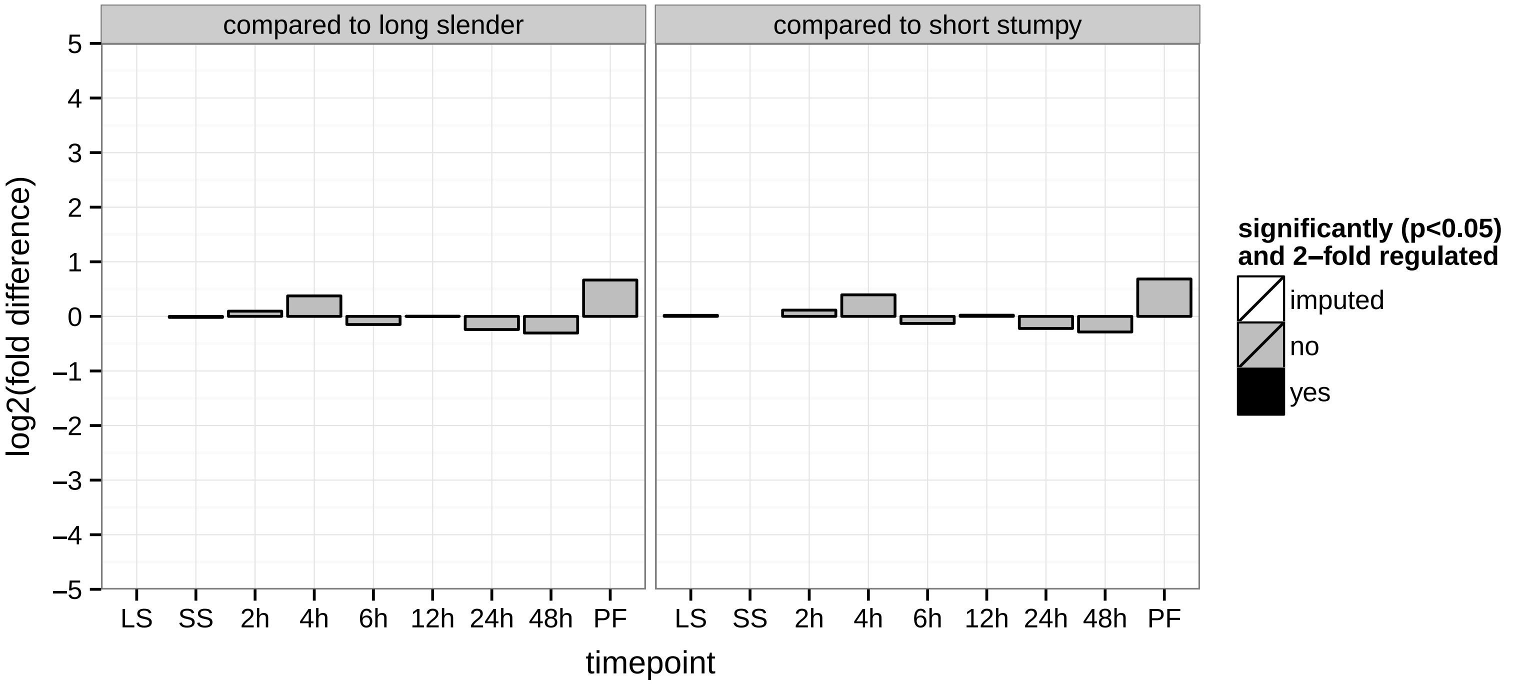

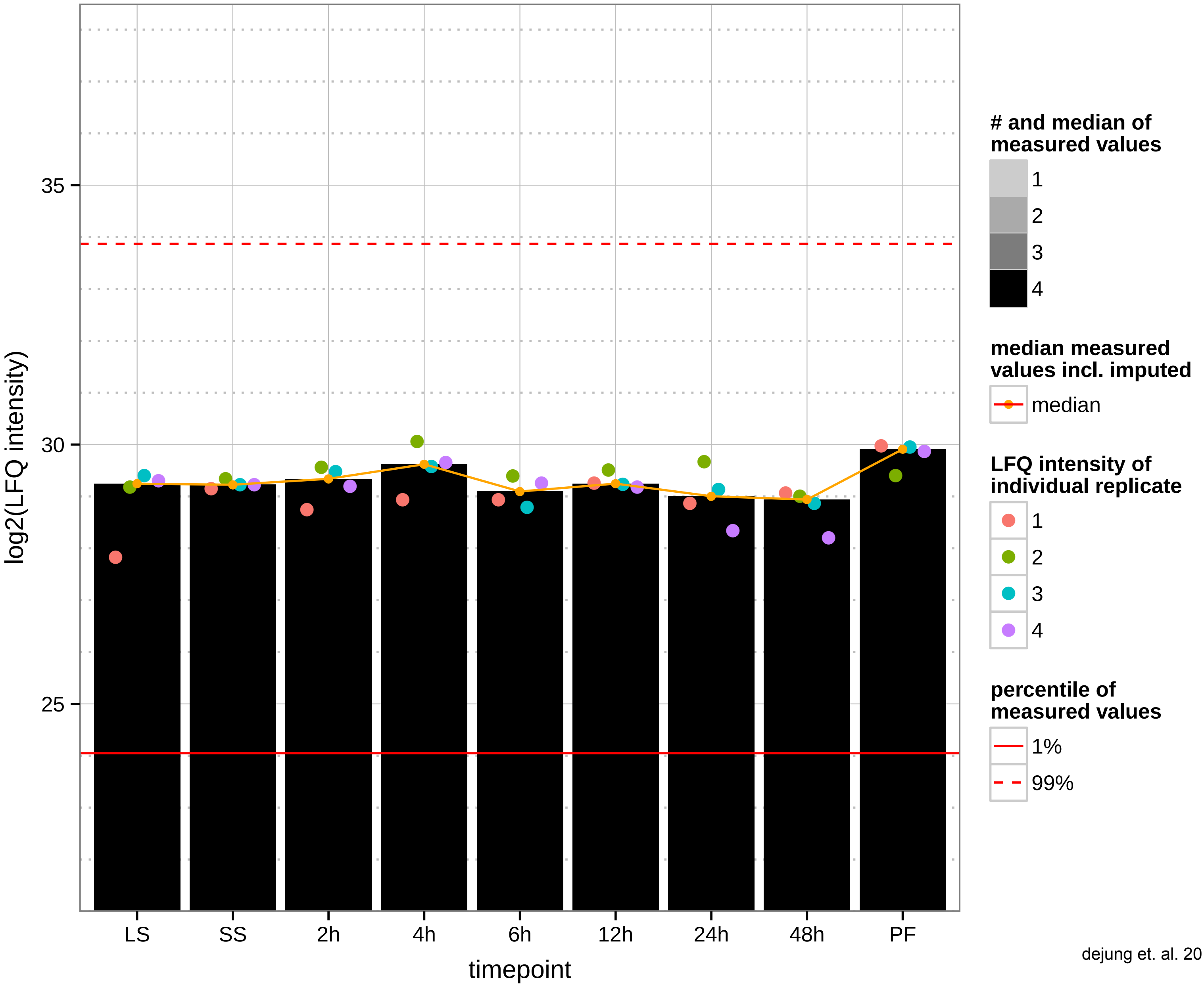

dejung et. al. 2015

hypothetical protein, conserved
Tb927.7.4520;Tb11.v5.0265
AGOF: null
AGOC: null
AGOP: null, cell redox homeostasis
PGOF: null
PGOC: null
PGOP: null

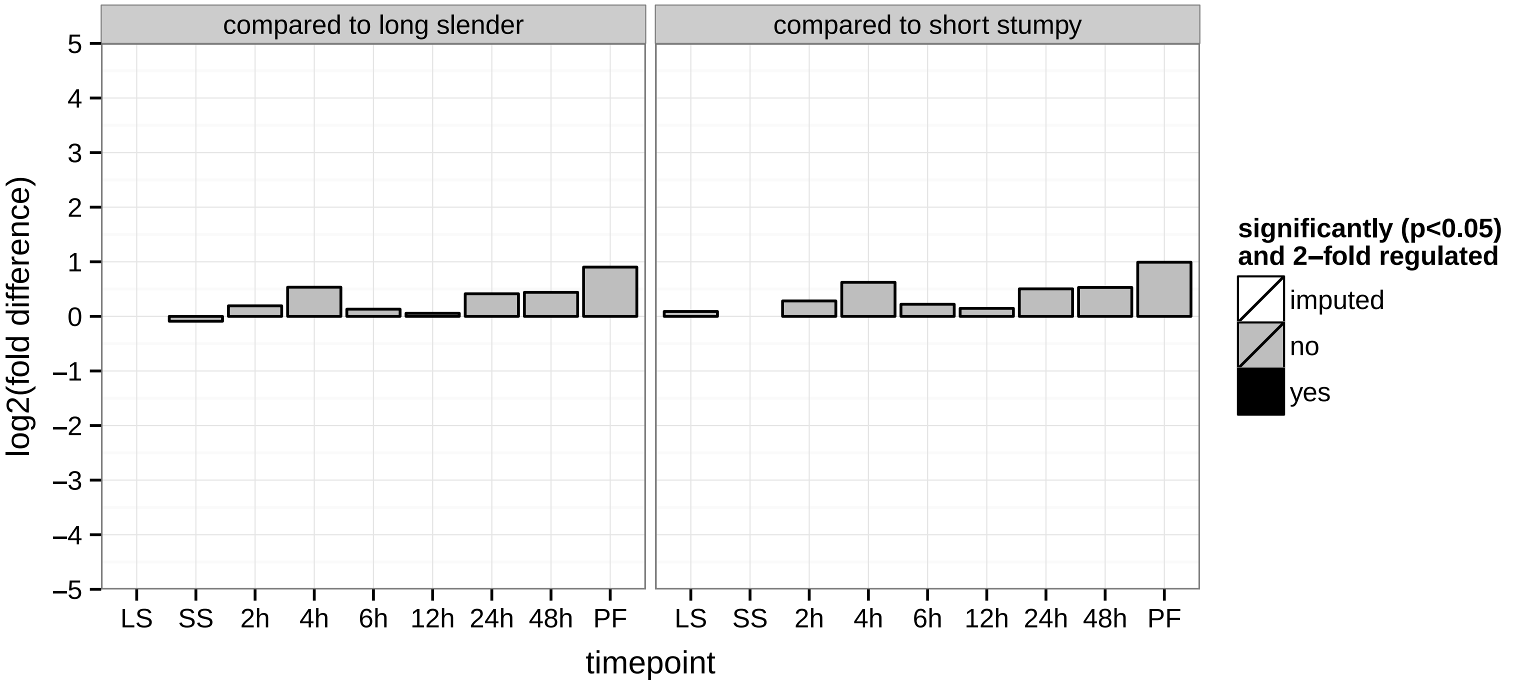

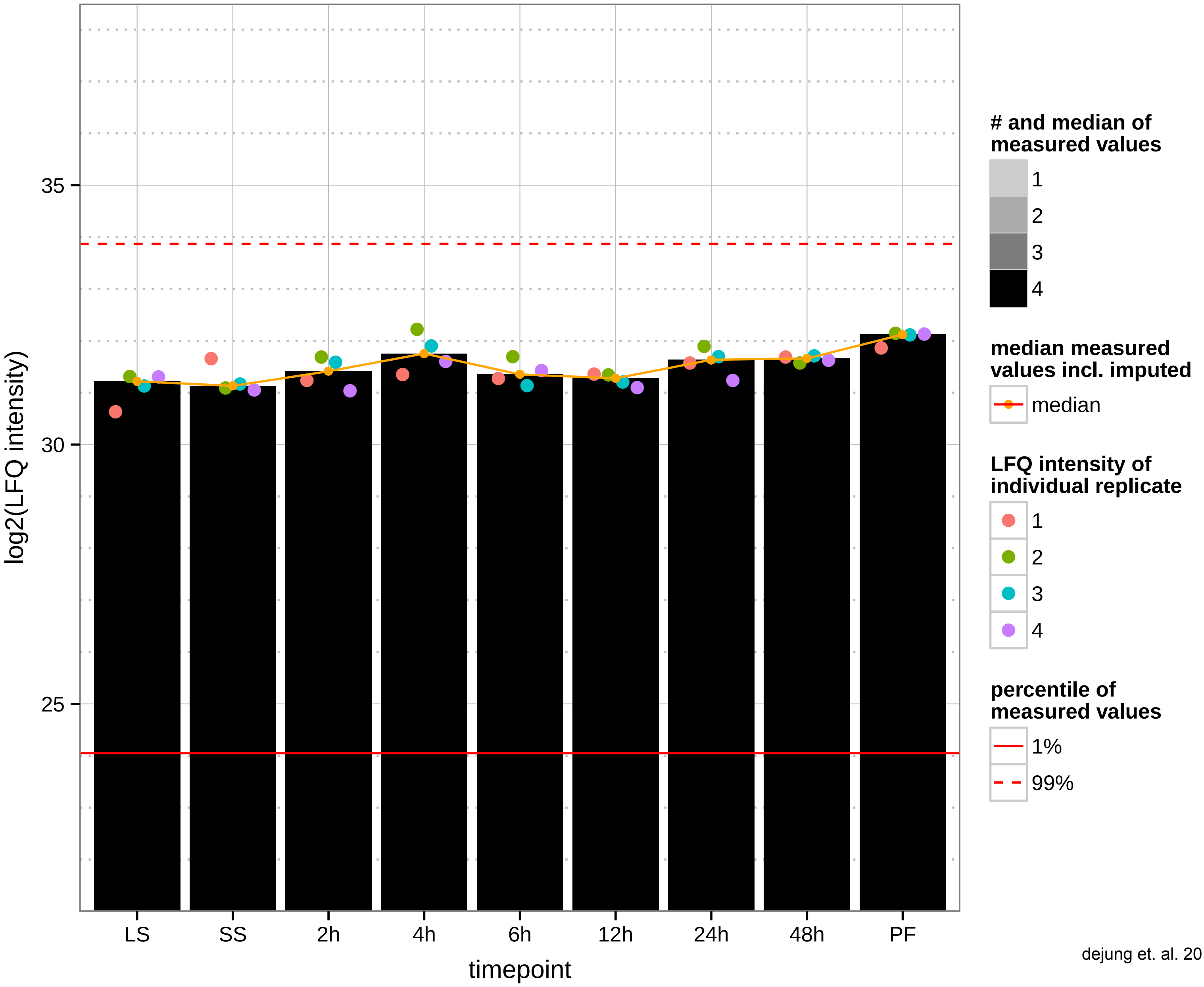

dejung et. al. 2015

nucleoside hydrolase, putative, inosine–guanine nucleoside hydrolase (IG–NH)
Tb927.7.4570;Tb11.v5.0270
AGOF: null, inosine nucleosidase activity
AGOC: null
AGOP: null, guanosine salvage, inosine salvage
PGOF: null
PGOC: null
PGOP: null

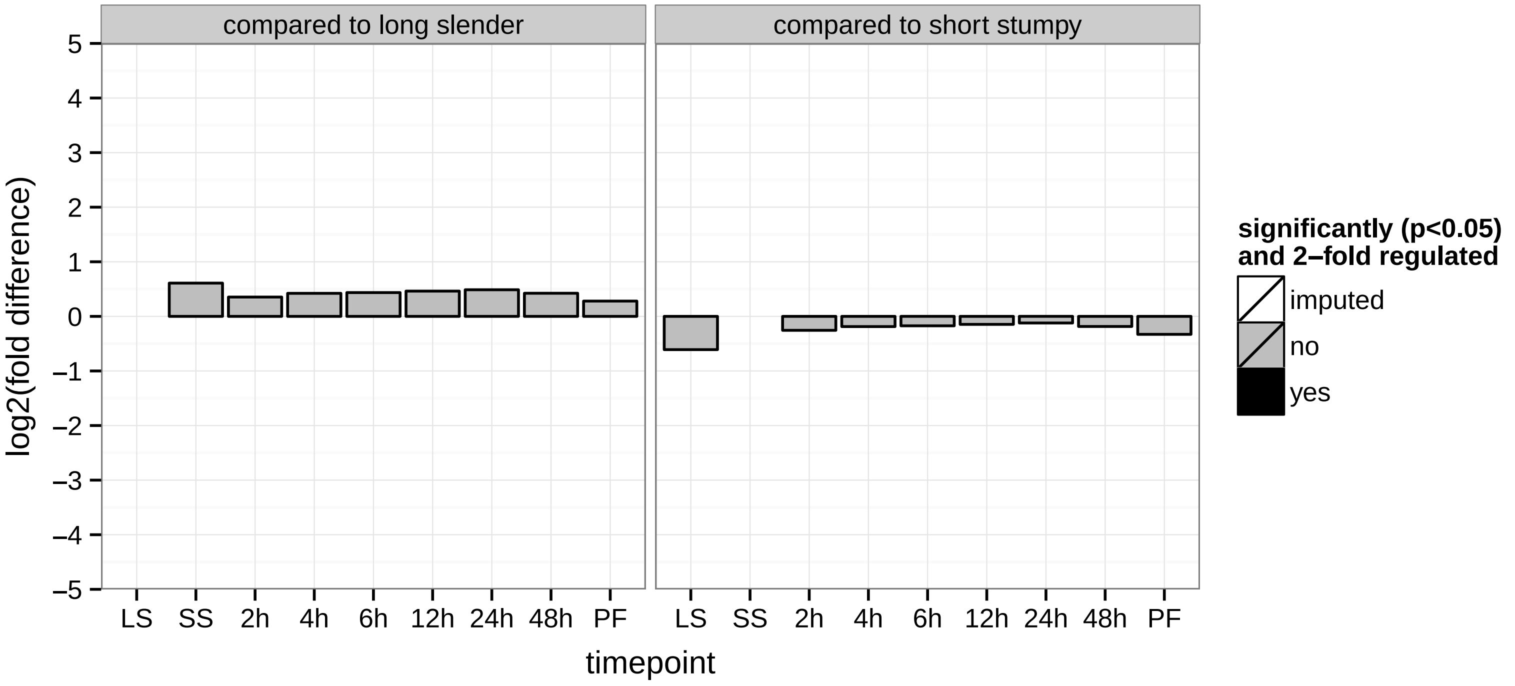

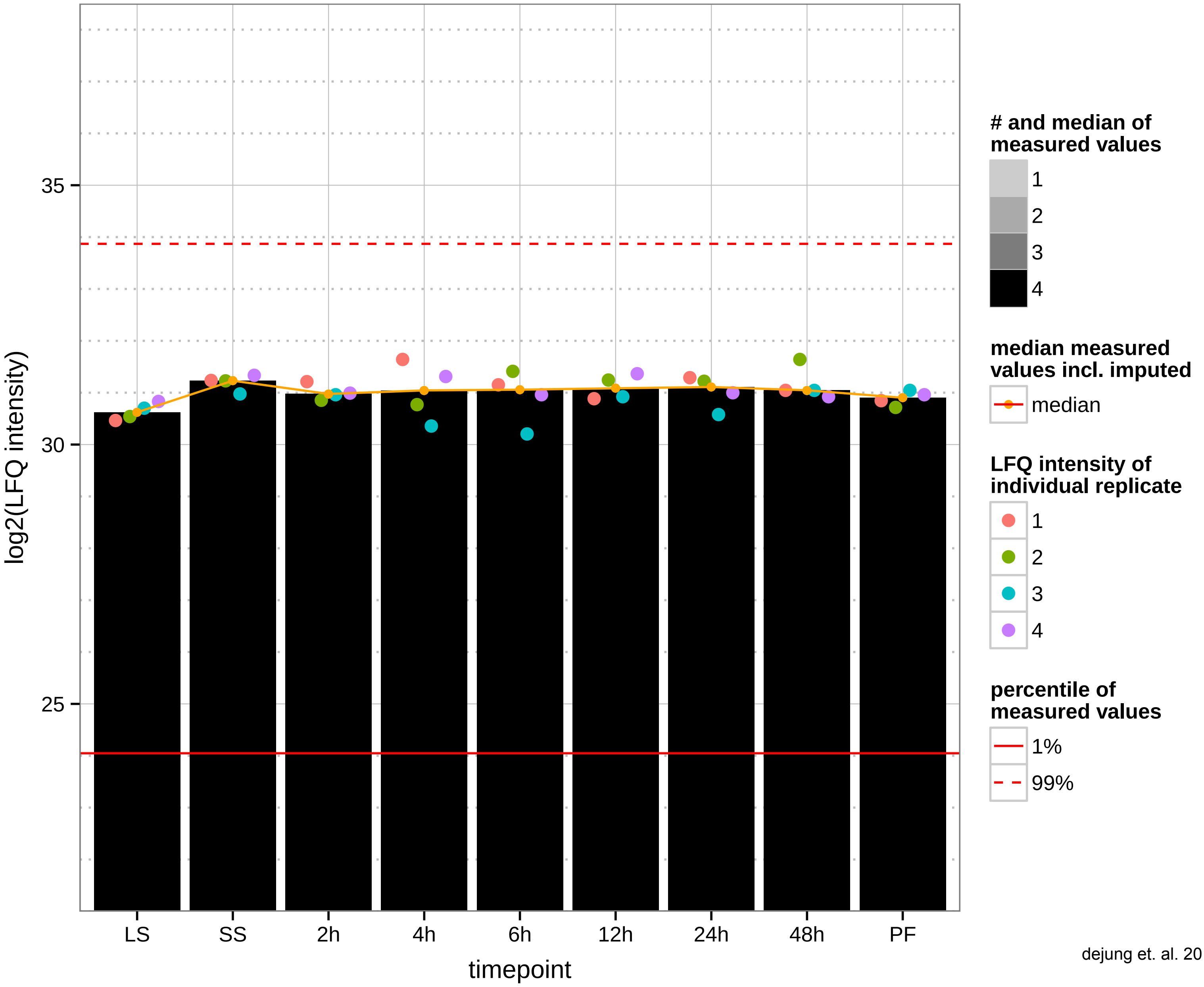

dejung et. al. 2015

trypanin, putative, trypanin (trypanin)
Tb927.10.6350;Tb11.v5.0271
AGOF: null
AGOC: null, cilium
AGOP: null, cellular component movement
PGOF: null
PGOC: microtubule–based flagellum
PGOP: cell motility

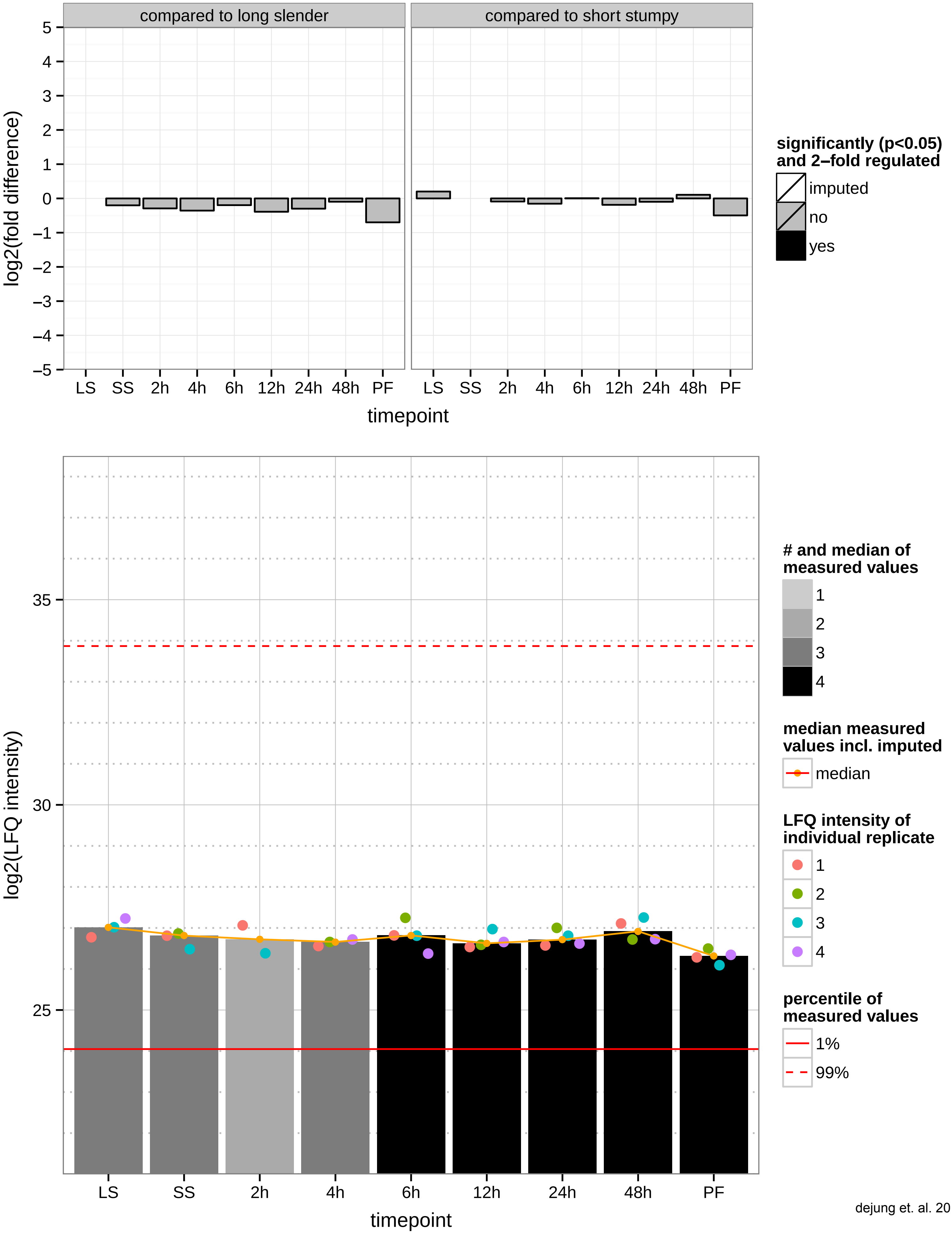

dejung et. al. 2015

hypothetical protein, conserved, kinetoplastid kinetochore protein 1 (kkt1)
Tb927.10.6330;Tb11.v5.0273
AGOF: null
AGOC: null, kinetochore
AGOP: null, chromosome segregation
PGOF: null
PGOC: null
PGOP: null

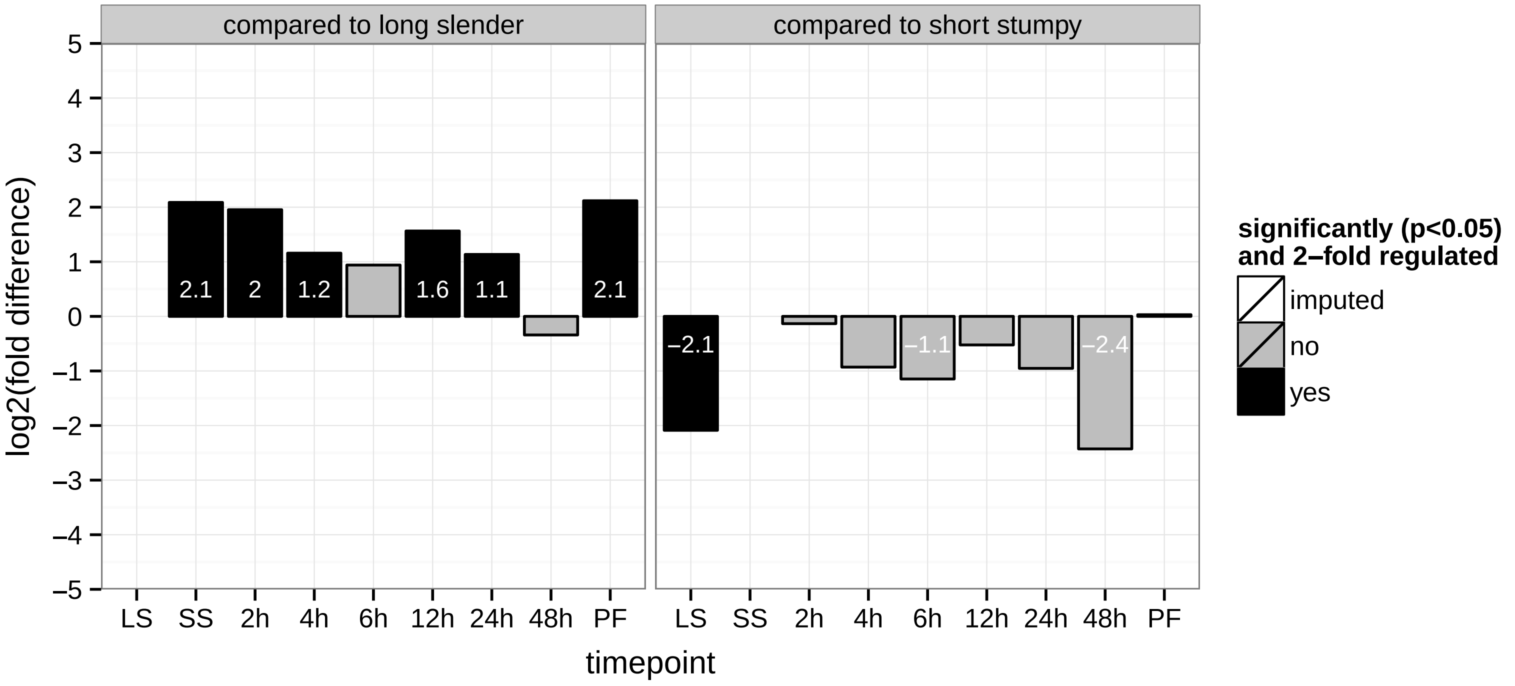

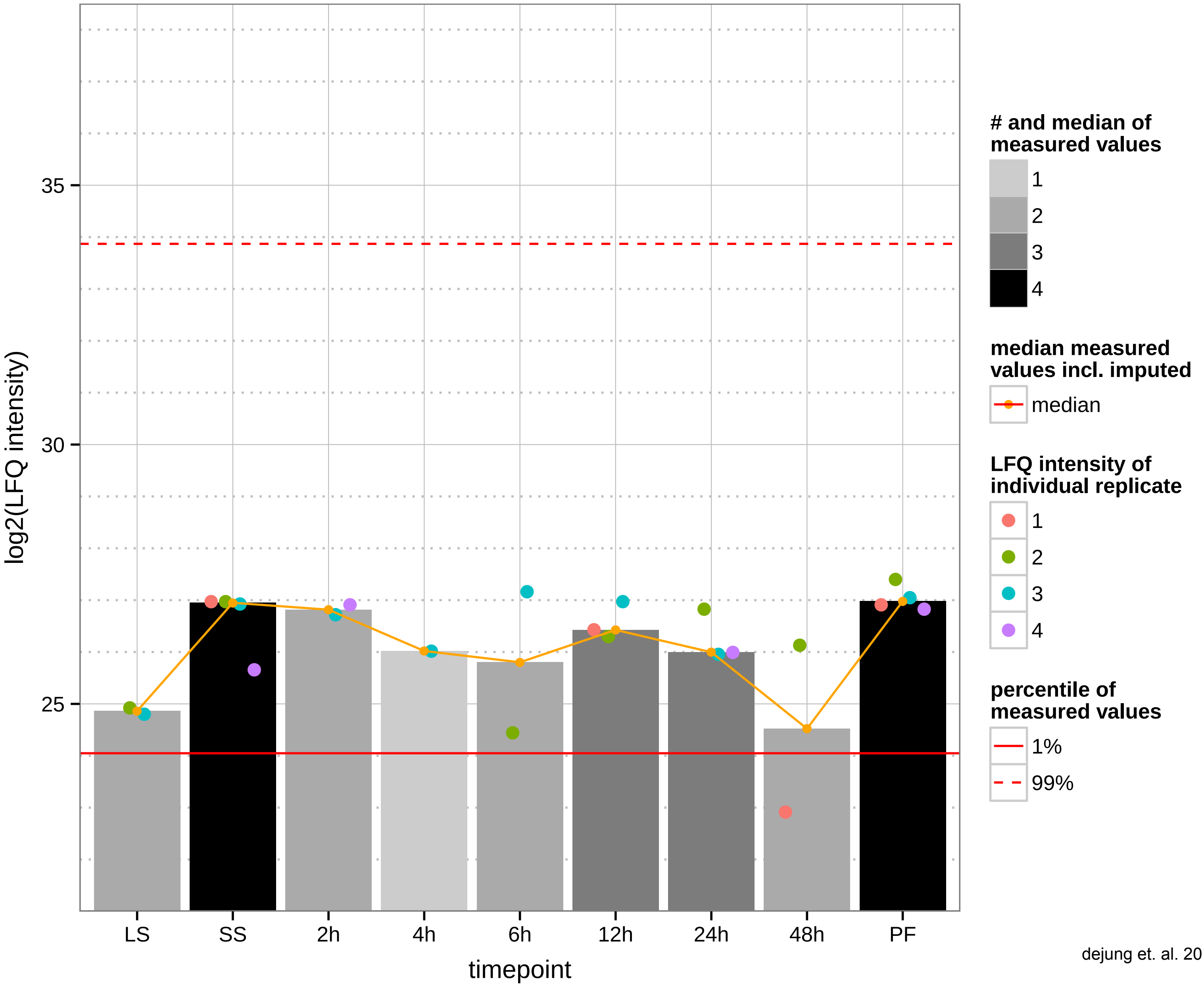

dejung et. al. 2015

major vault protein, putative, putative (MVP)
Tb927.10.6310;Tb11.v5.0275
AGOF: null
AGOC: null
AGOP: null, negative regulation of signaling
PGOF: null
PGOC: null
PGOP: null

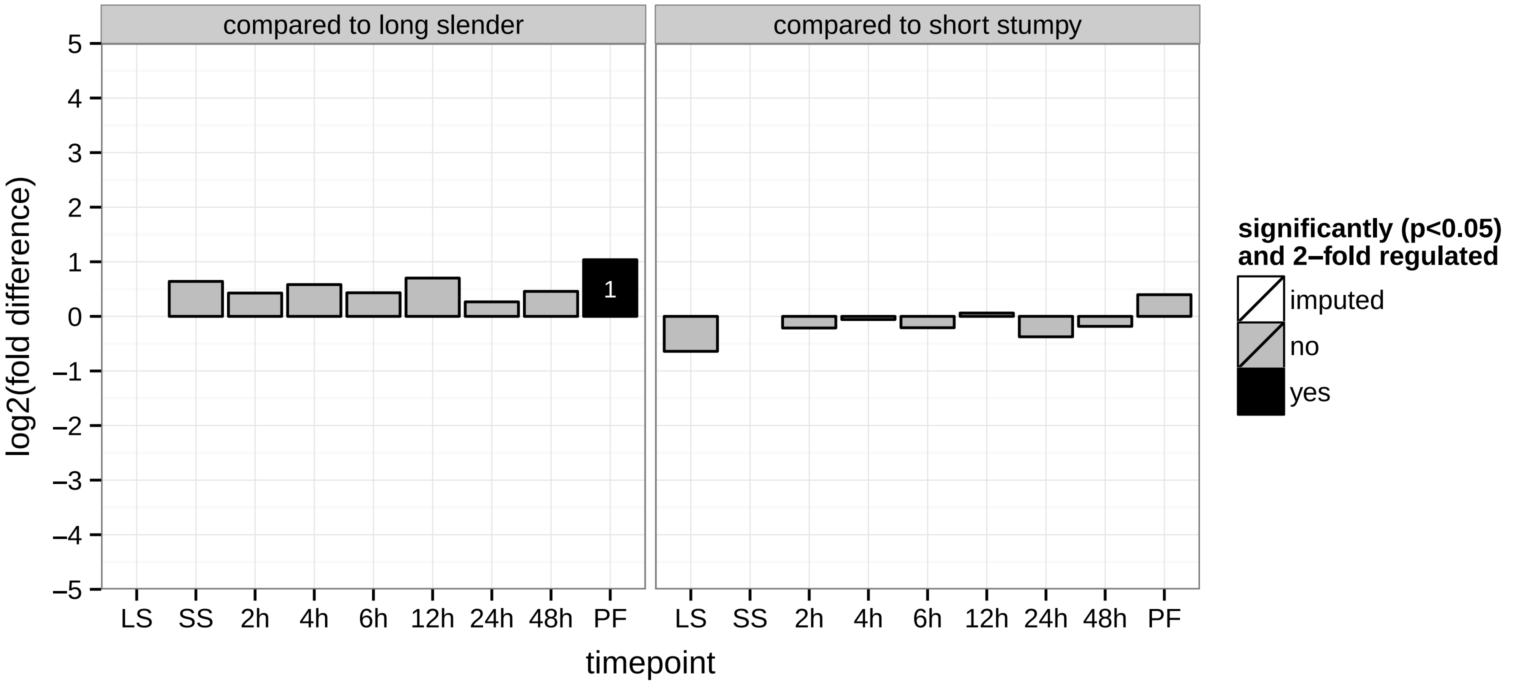

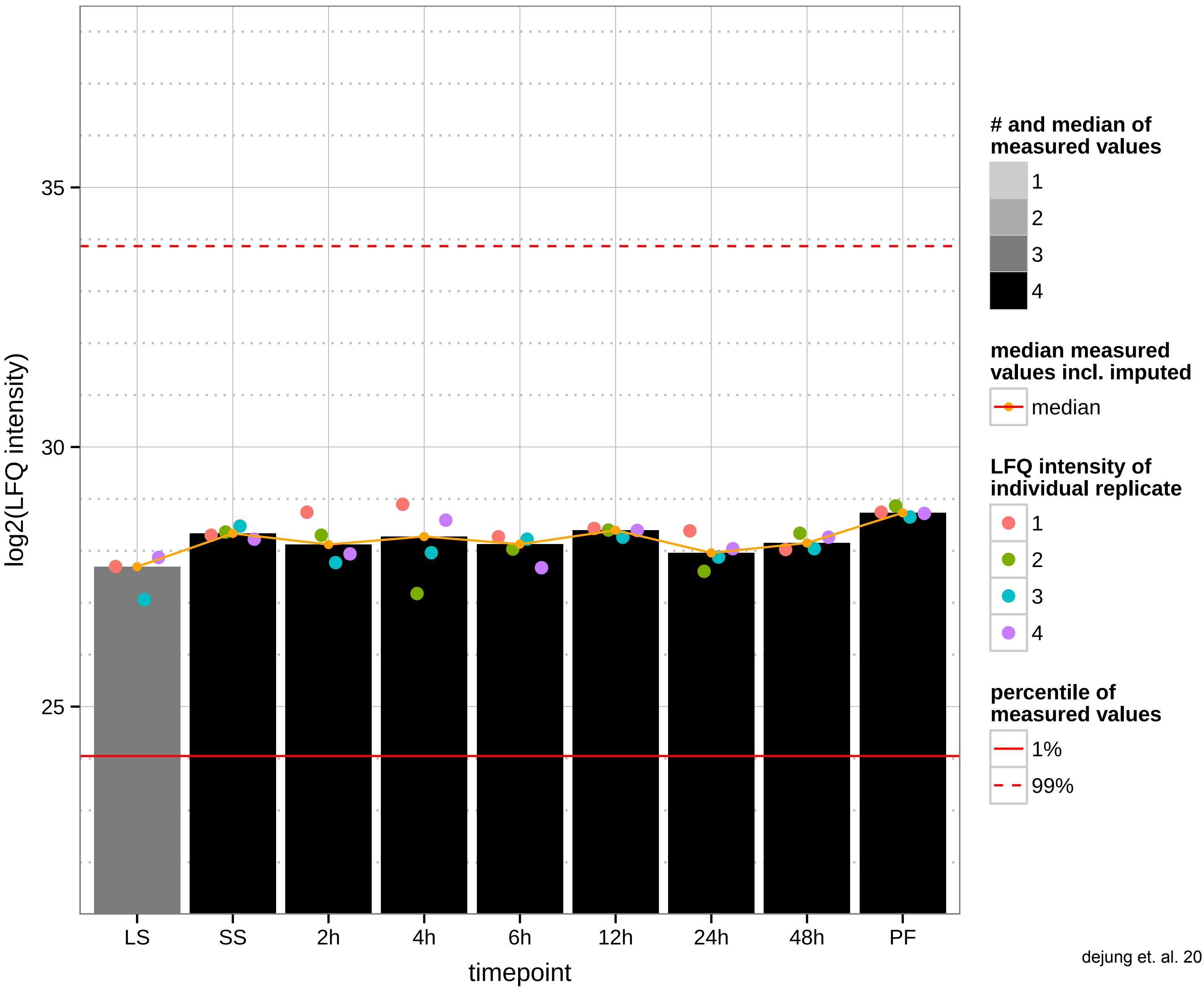

dejung et. al. 2015

ATP−dependent DEAD/H RNA helicase, putative
Tb927.10.6290;Tb11.v5.0277
AGOF: null, ATP binding, ATP−dependent helicase activity, helicase activity, nucleic acid binding
AGOC: null
AGOP: null, nucleobase−containing compound metabolic process
PGOF: ATP binding, ATP−dependent helicase activity, helicase activity, nucleic acid binding
PGOC: null
PGOP: null

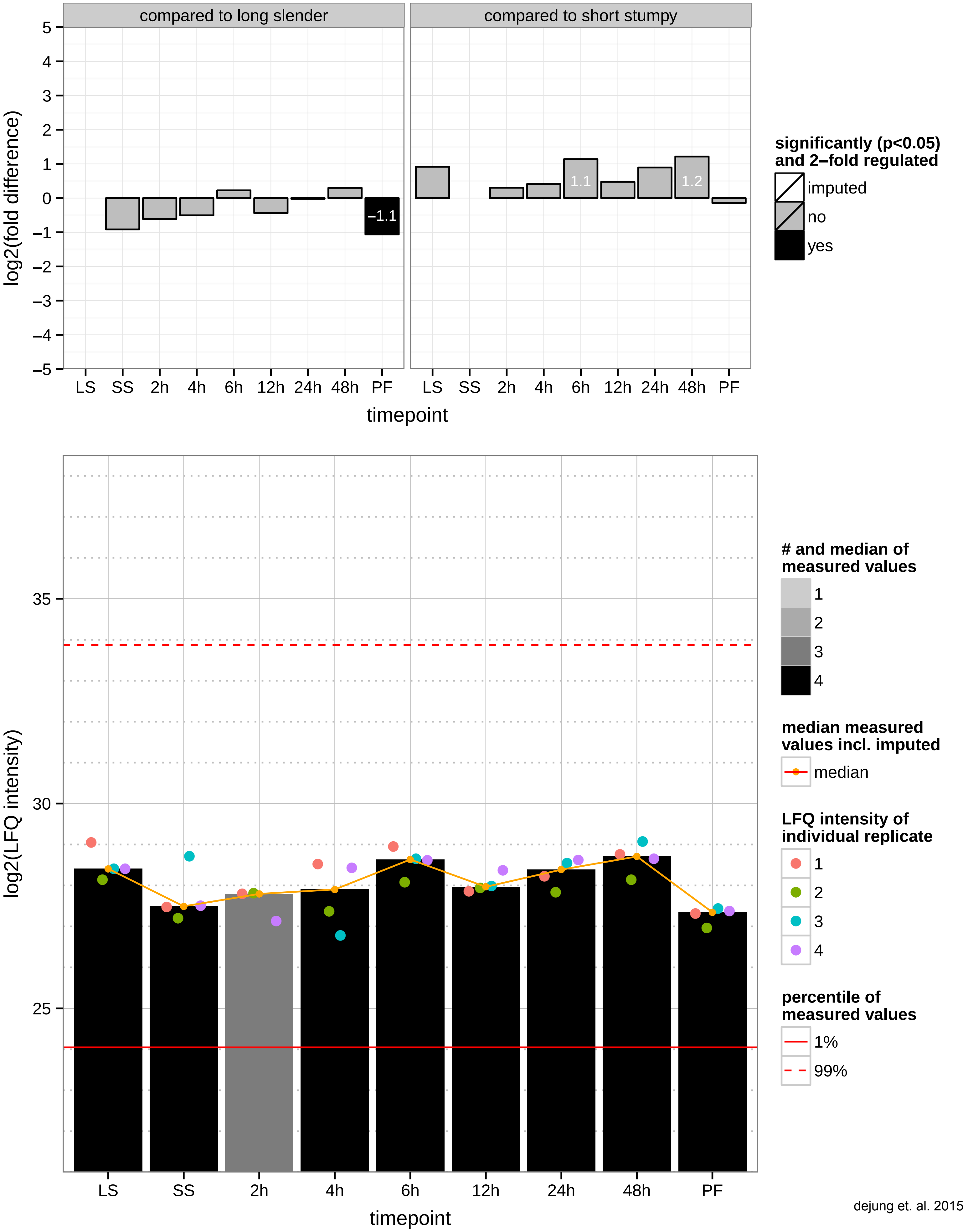

dejung et. al. 2015

cleavage and polyadenylation specificity factor 30 kDa subunit, putative, CPSF 30 kDa subunit, NS1 effector domain– binding
Tb927.11.12750;Tb11.v5.0291
AGOF: null, RNA binding, nucleic acid binding, zinc ion binding
AGOC: null, mRNA cleavage and polyadenylation specificity factor complex
AGOP: null, RNA processing
PGOF: nucleic acid binding, zinc ion binding
PGOC: null
PGOP: null

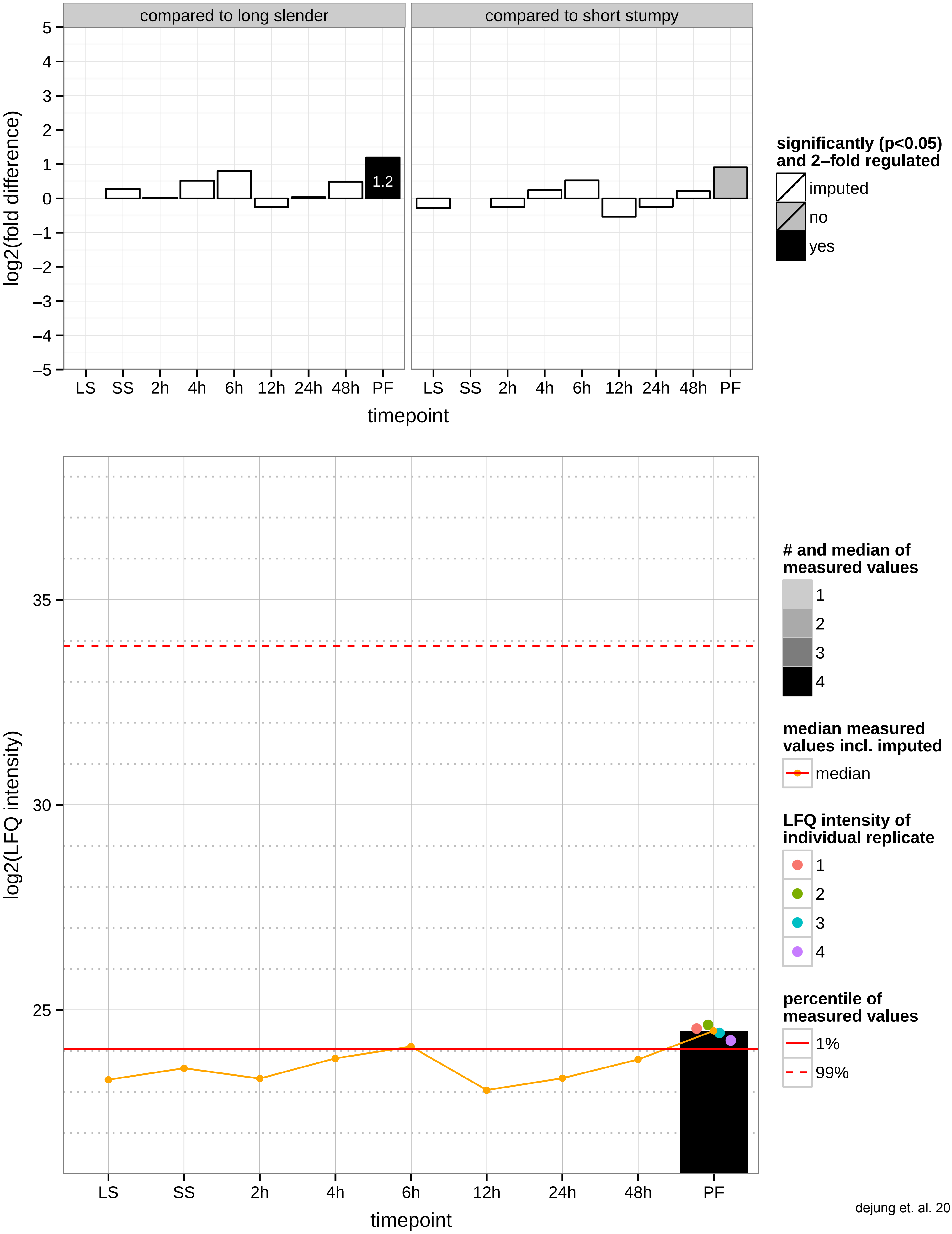

dejung et. al. 2015

TFIID–like protein, putative, putative (TRF4)
Tb927.10.15950;Tb11.v5.0305
AGOF: null, DNA binding
AGOC: null, nucleus, transcription factor TFIID complex
AGOP: null, regulation of transcription, DNA–dependent, transcription initiation from RNA polymerase II promoter
PGOF: DNA binding
PGOC: null
PGOP: regulation of transcription, DNA–dependent, transcription initiation from RNA polymerase II promoter

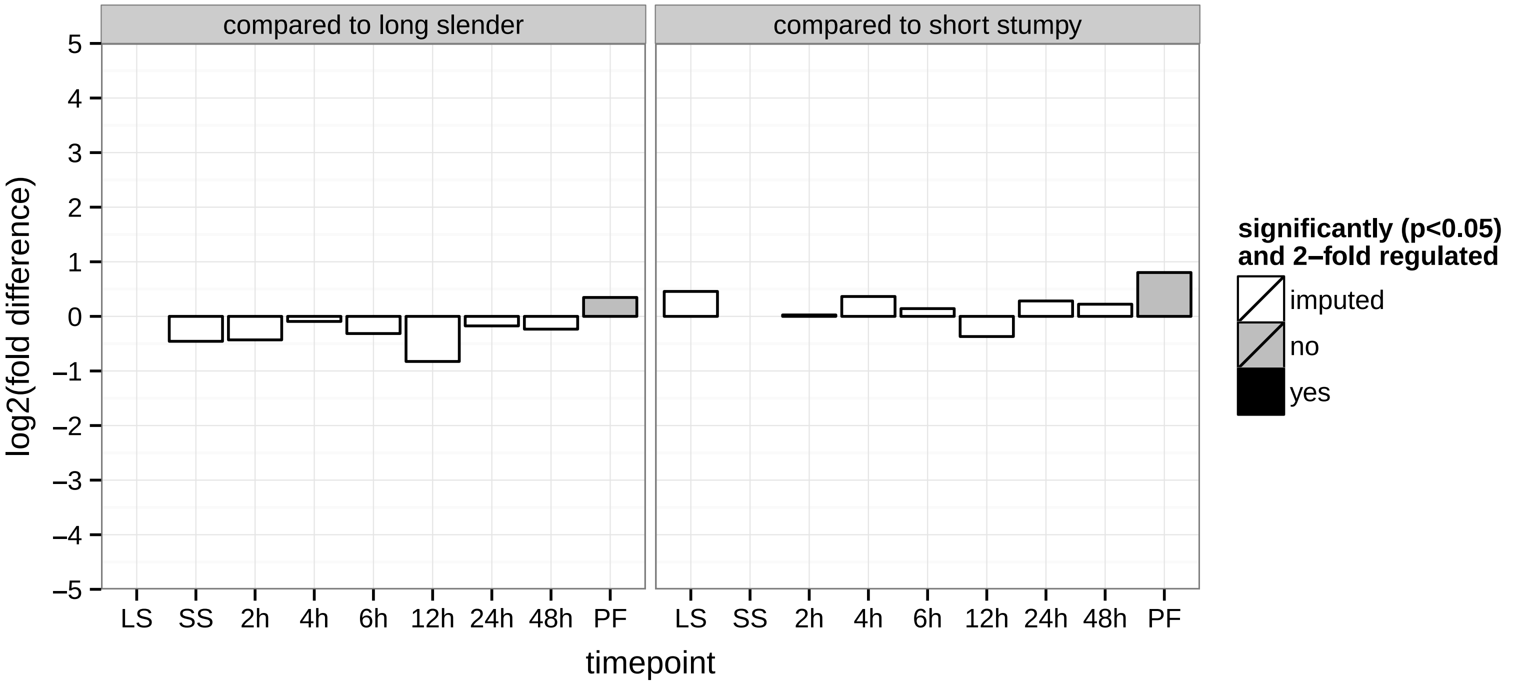

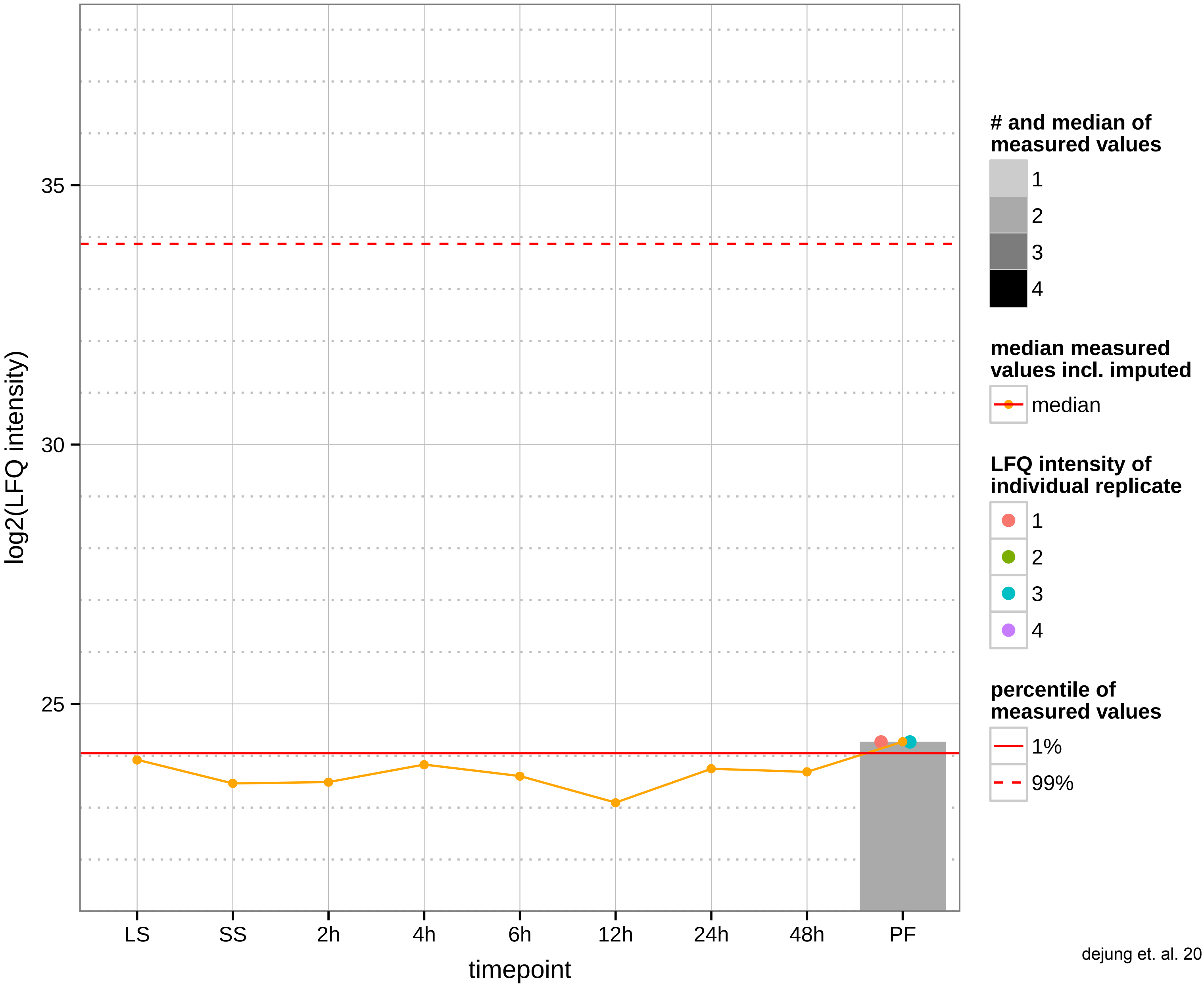

dejung et. al. 2015

retrotransposon hot spot (RHS) protein, putative
Tb11.v5.0325
AGOF: null
AGOC: null
AGOP: null
PGOF: null
PGOC: null
PGOP: null

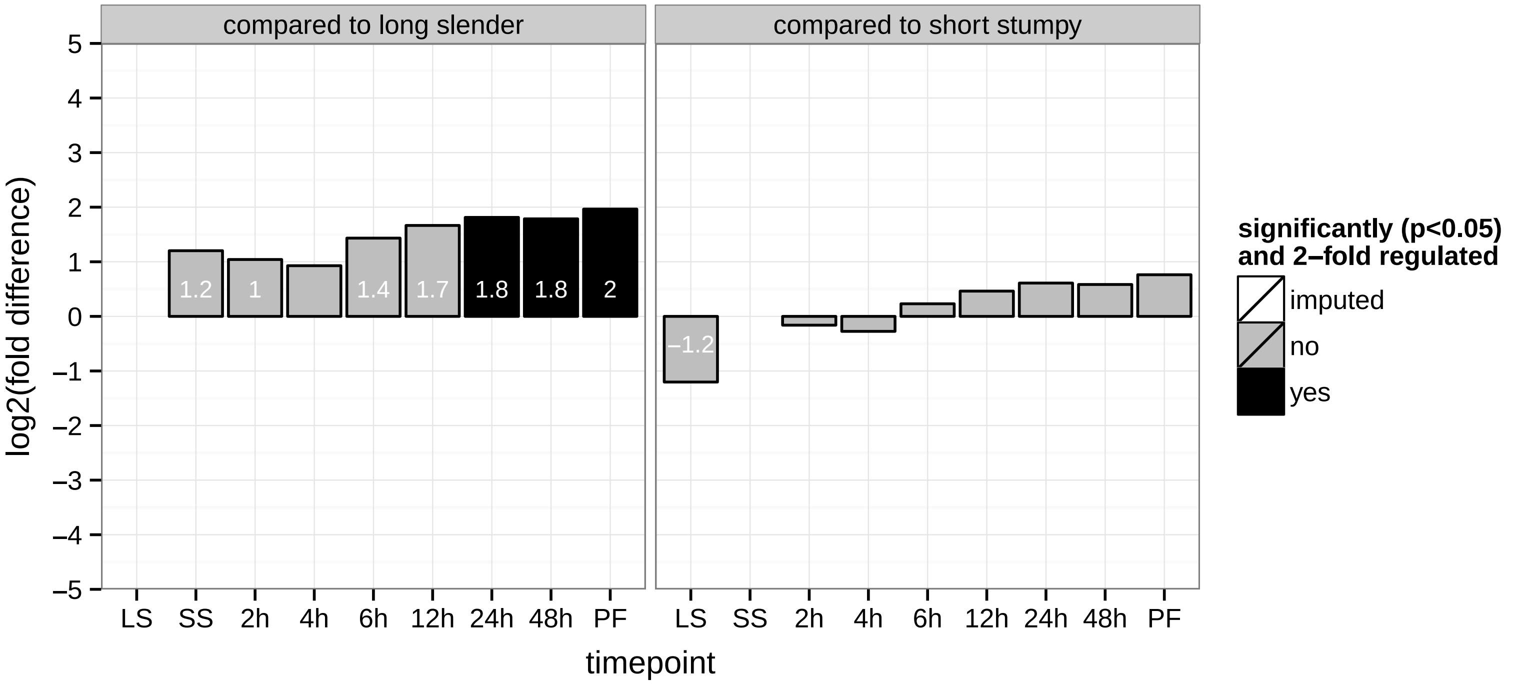

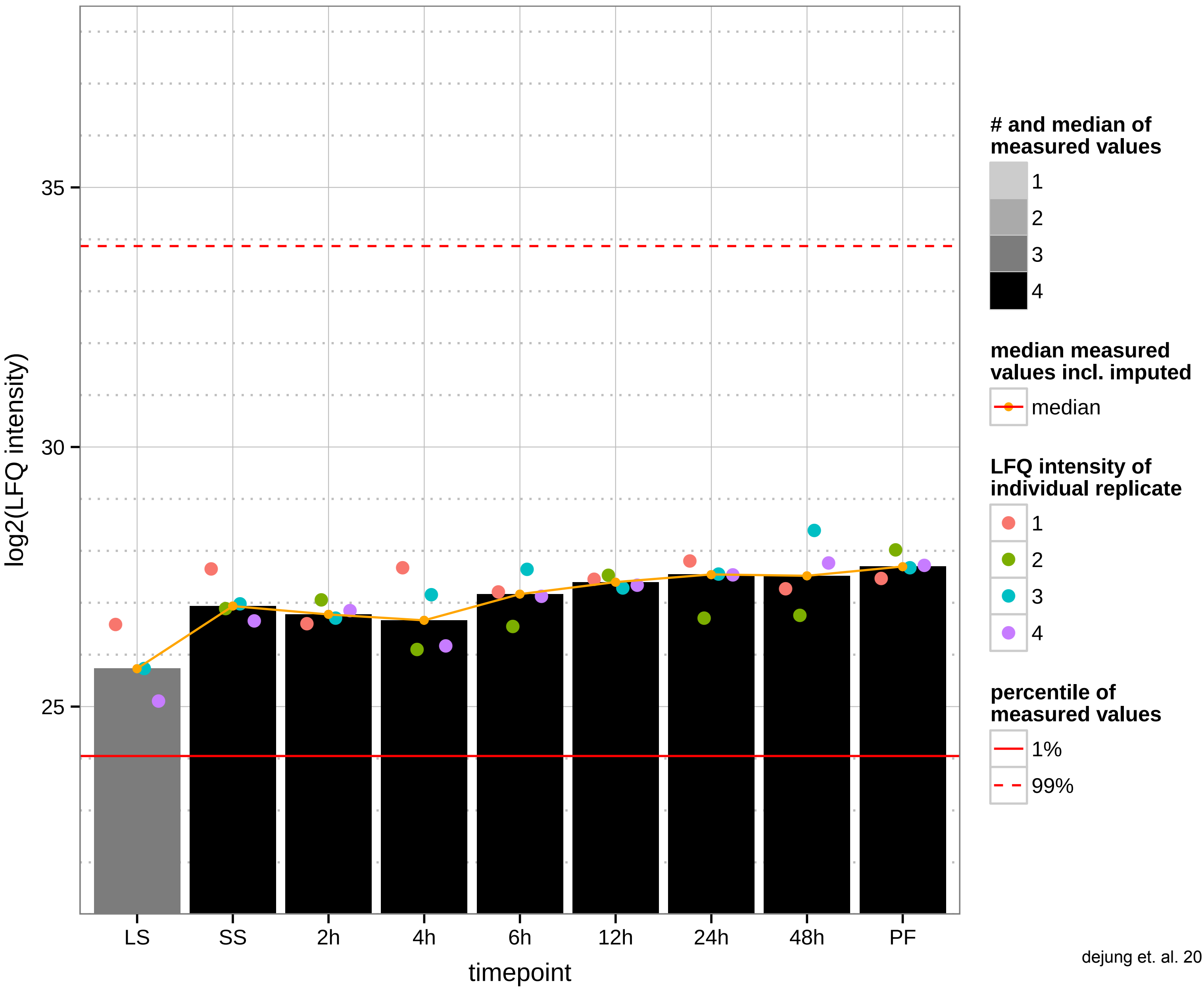

dejung et. al. 2015

hypothetical protein, conserved, ribonuclease H, putative
Tb927.10.5070;Tb11.v5.0340
AGOF: null, RNA binding, ribonuclease H activity
AGOC: null, nucleus
AGOP: null, RNA catabolic process
PGOF: nucleic acid binding, ribonuclease H activity
PGOC: null
PGOP: RNA metabolic process

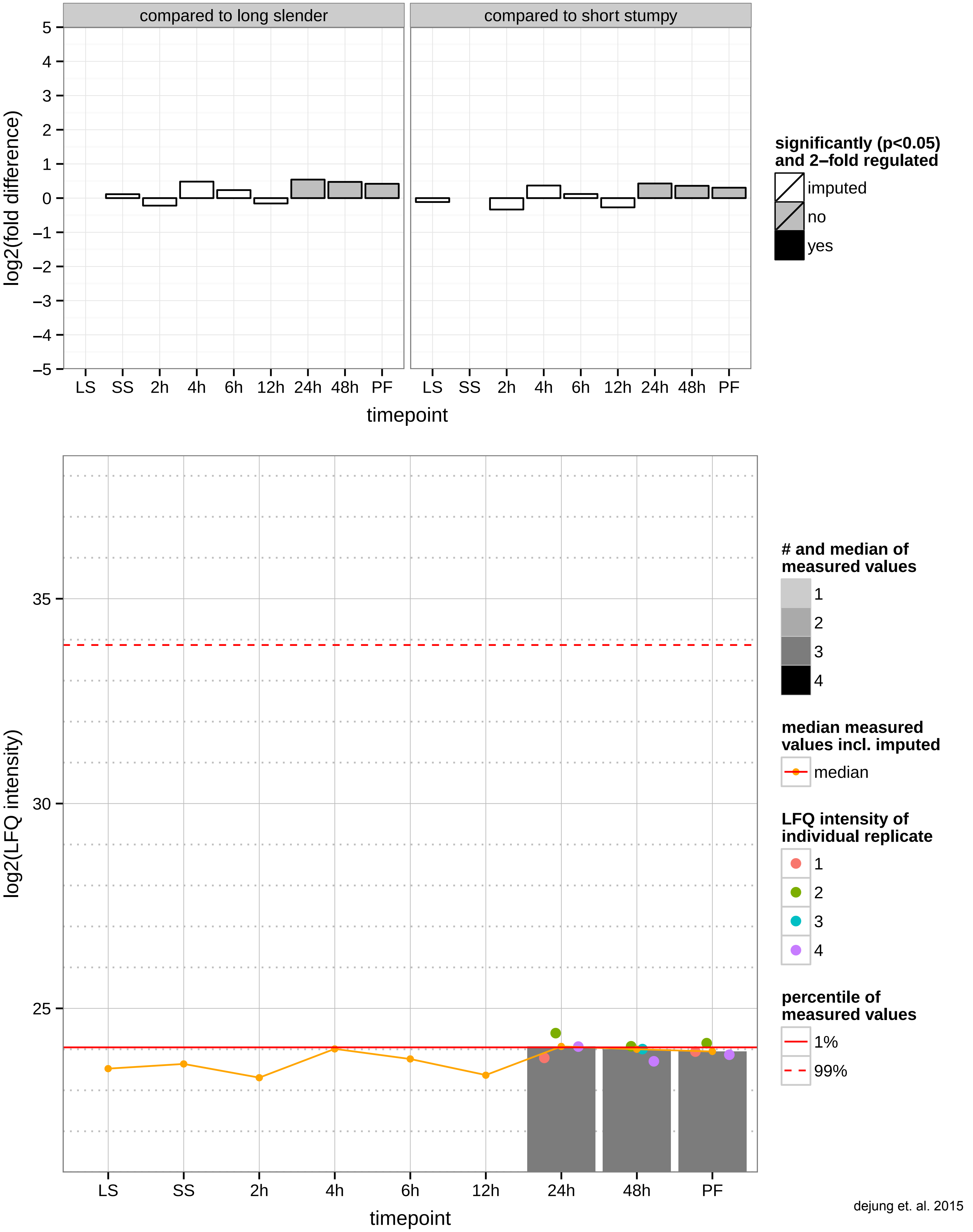

dejung et. al. 2015

chaperone protein DNAj, putative
Tb927.10.5040;Tb11.v5.0342
AGOF: null, heat shock protein binding, unfolded protein binding
AGOC: null
AGOP: null, protein folding
PGOF: heat shock protein binding
PGOC: null
PGOP: null

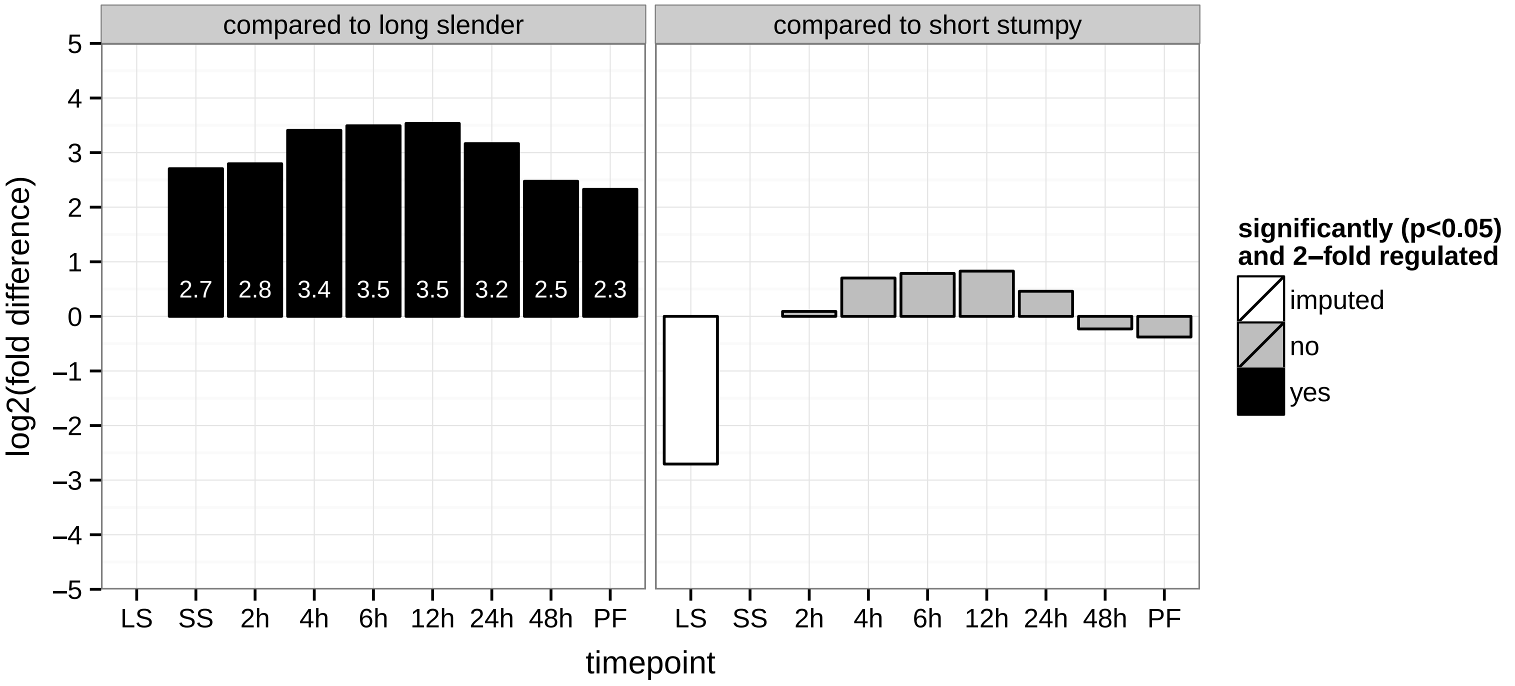

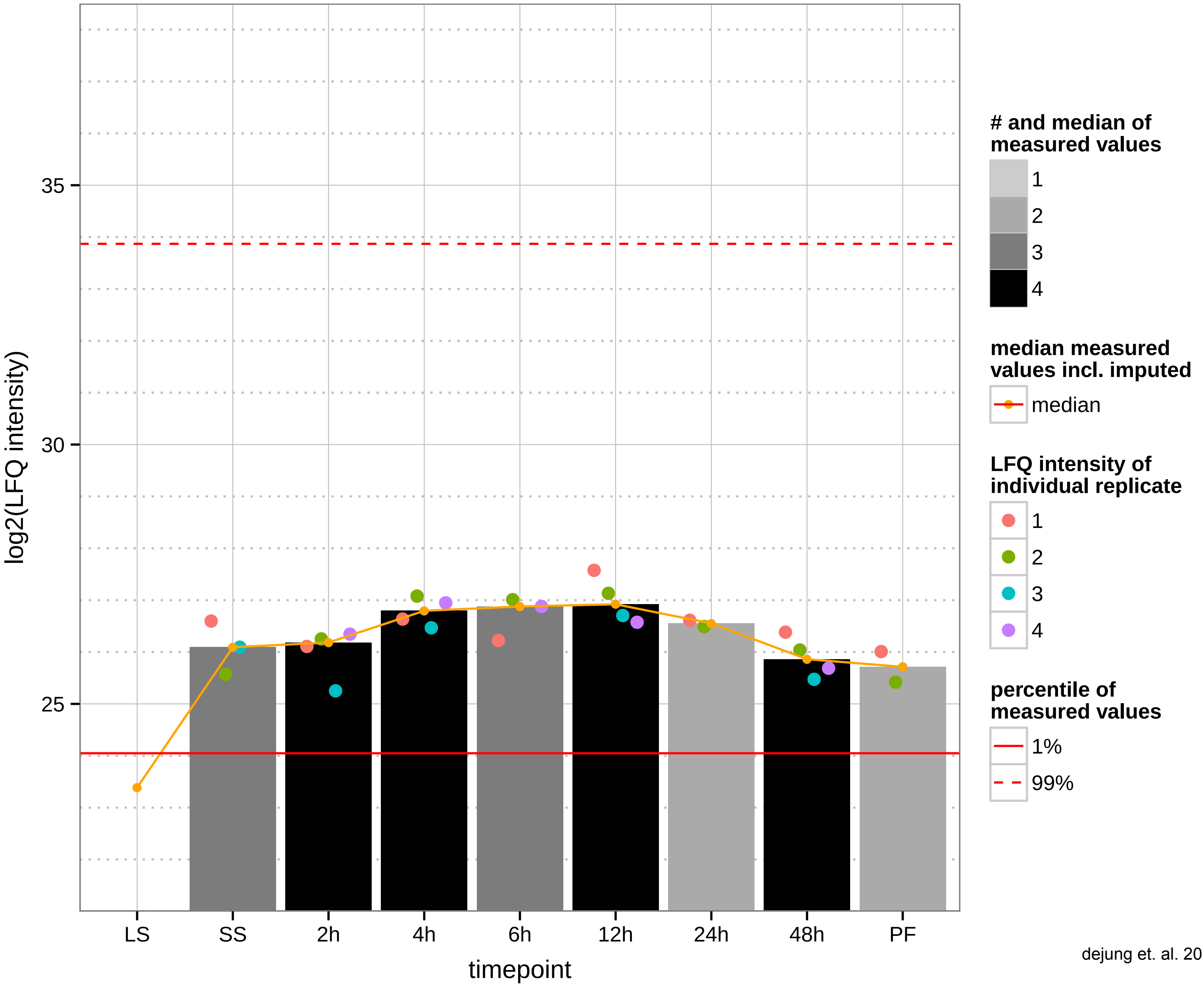

dejung et. al. 2015

cytidine triphosphate synthase, putative
Tb927.1.1240;Tb11.v5.0356
AGOF: null, CTP synthase activity
AGOC: null, cytosol
AGOP: null, CTP biosynthetic process
PGOF: CTP synthase activity
PGOC: null
PGOP: pyrimidine nucleotide biosynthetic process

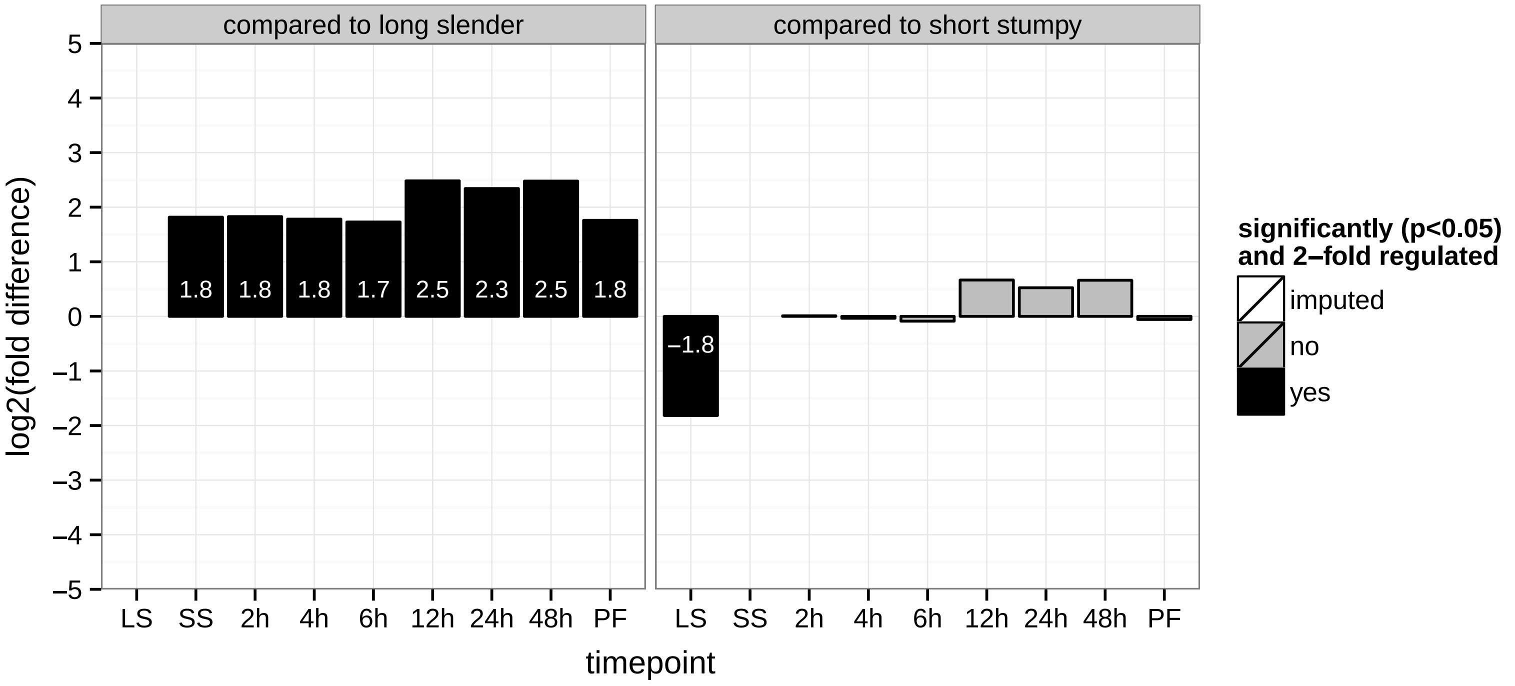

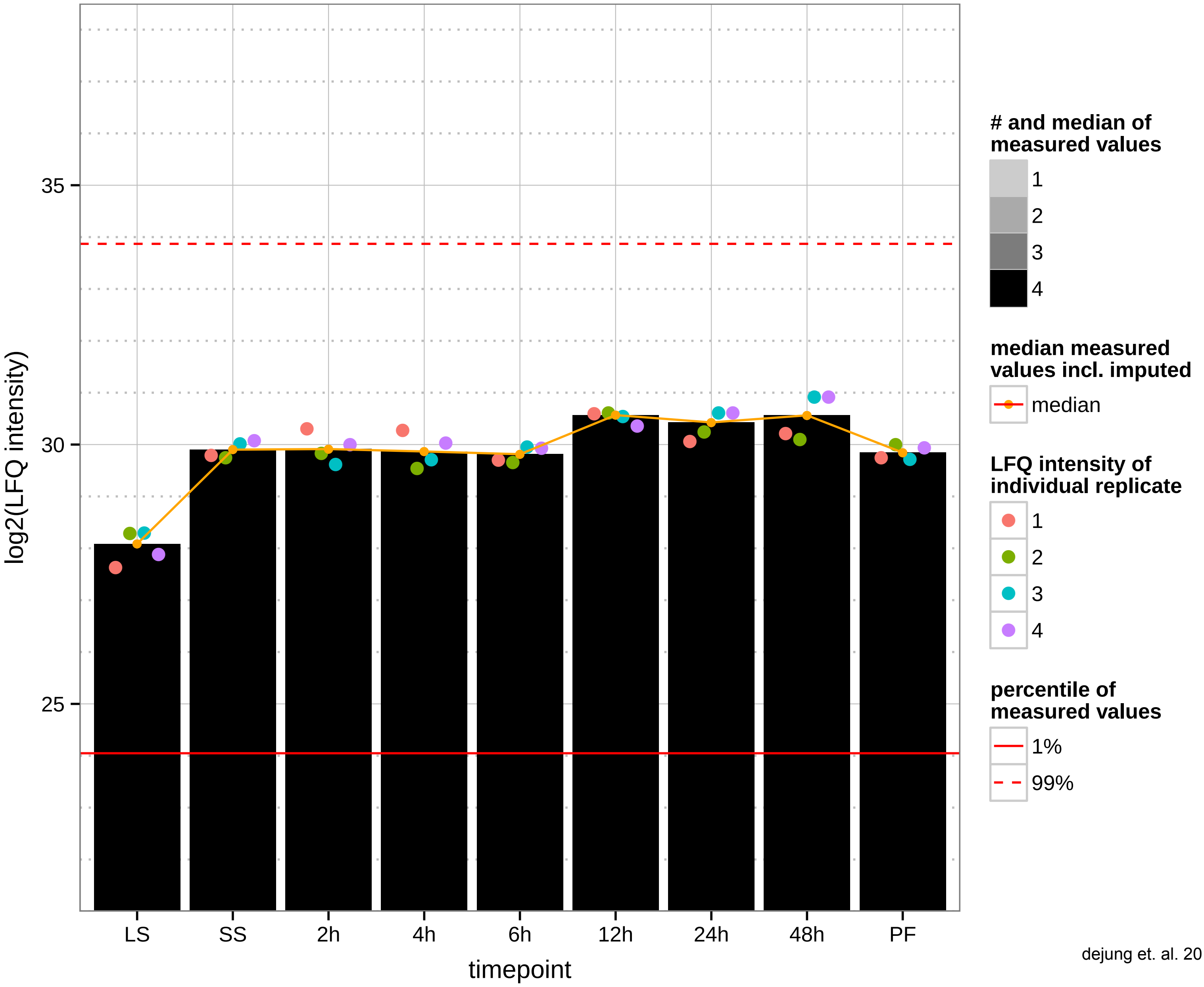

dejung et. al. 2015

hypothetical protein, conserved, conserved protein
Tb11.v5.0366;Tb927.11.12880
AGOF: null
AGOC: null
AGOP: null
PGOF: null
PGOC: null
PGOP: null

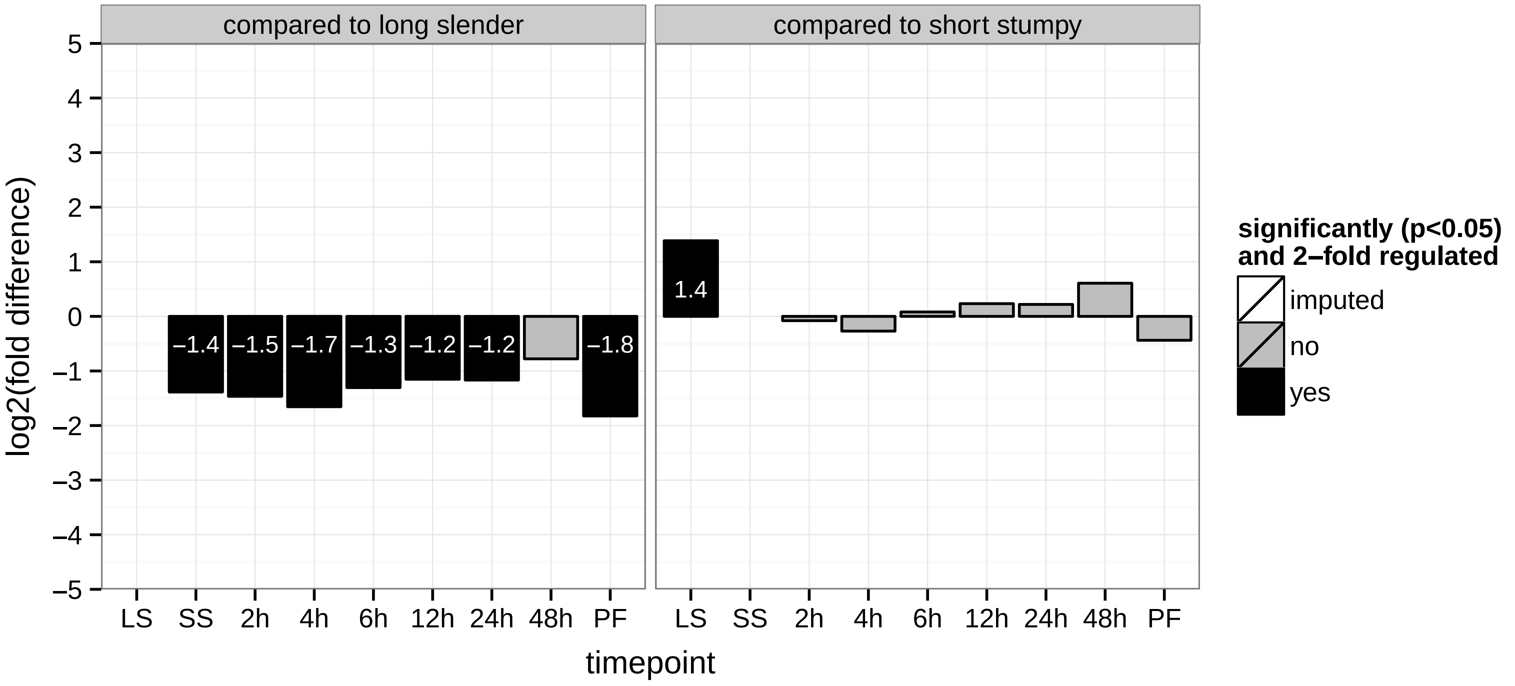

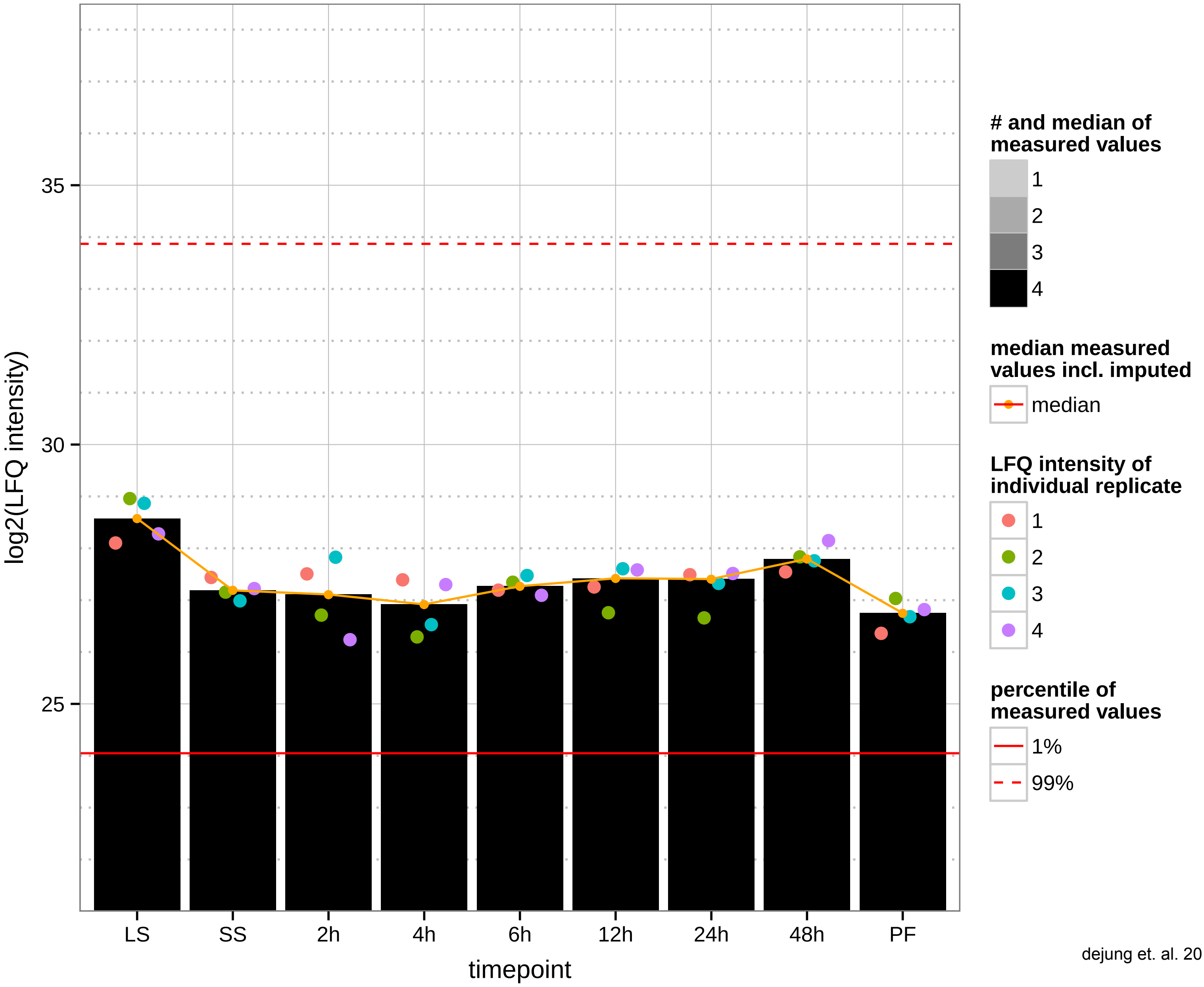

dejung et. al. 2015

rRNA biogenesis protein, putative
Tb927.1.1370;Tb11.v5.0391
AGOF: null, RNA binding
AGOC: null
AGOP: null, rRNA processing
PGOF: null
PGOC: null
PGOP: null

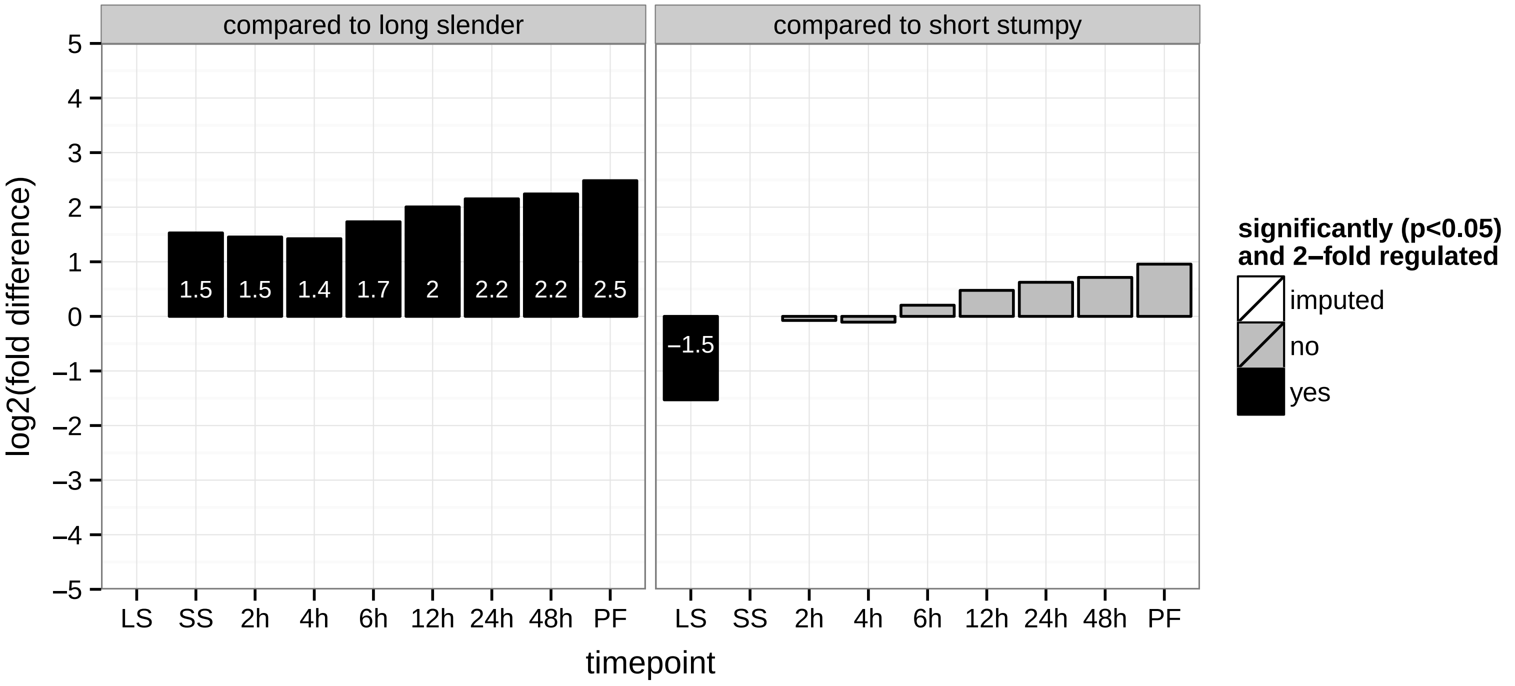

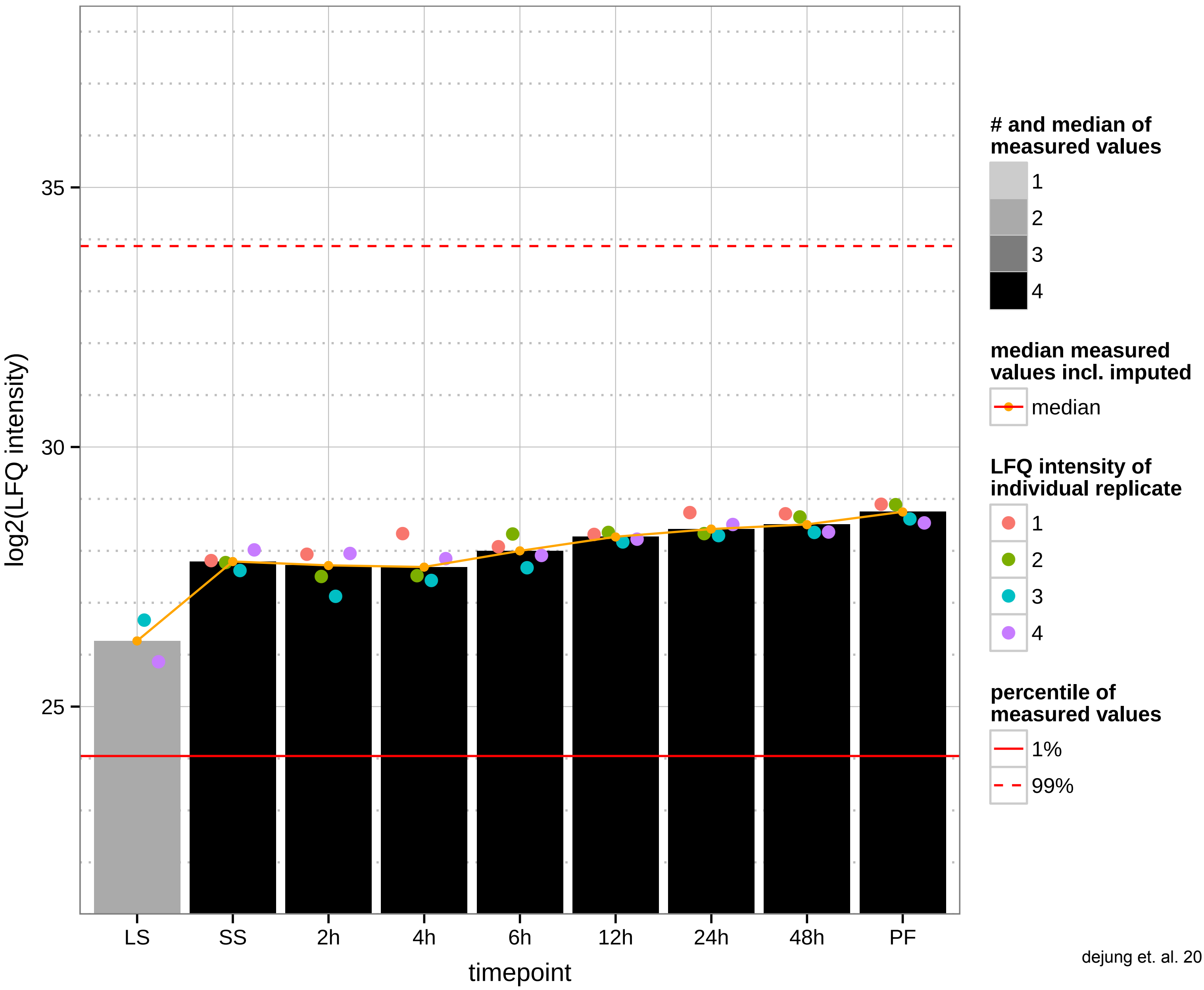

dejung et. al. 2015

hypothetical protein, conserved
Tb11.v5.0394;Tb927.10.15750
AGOF: null, zinc ion binding
AGOC: null, bacterial–type flagellum basal body
AGOP: null
PGOF: protein binding, zinc ion binding
PGOC: null
PGOP: null

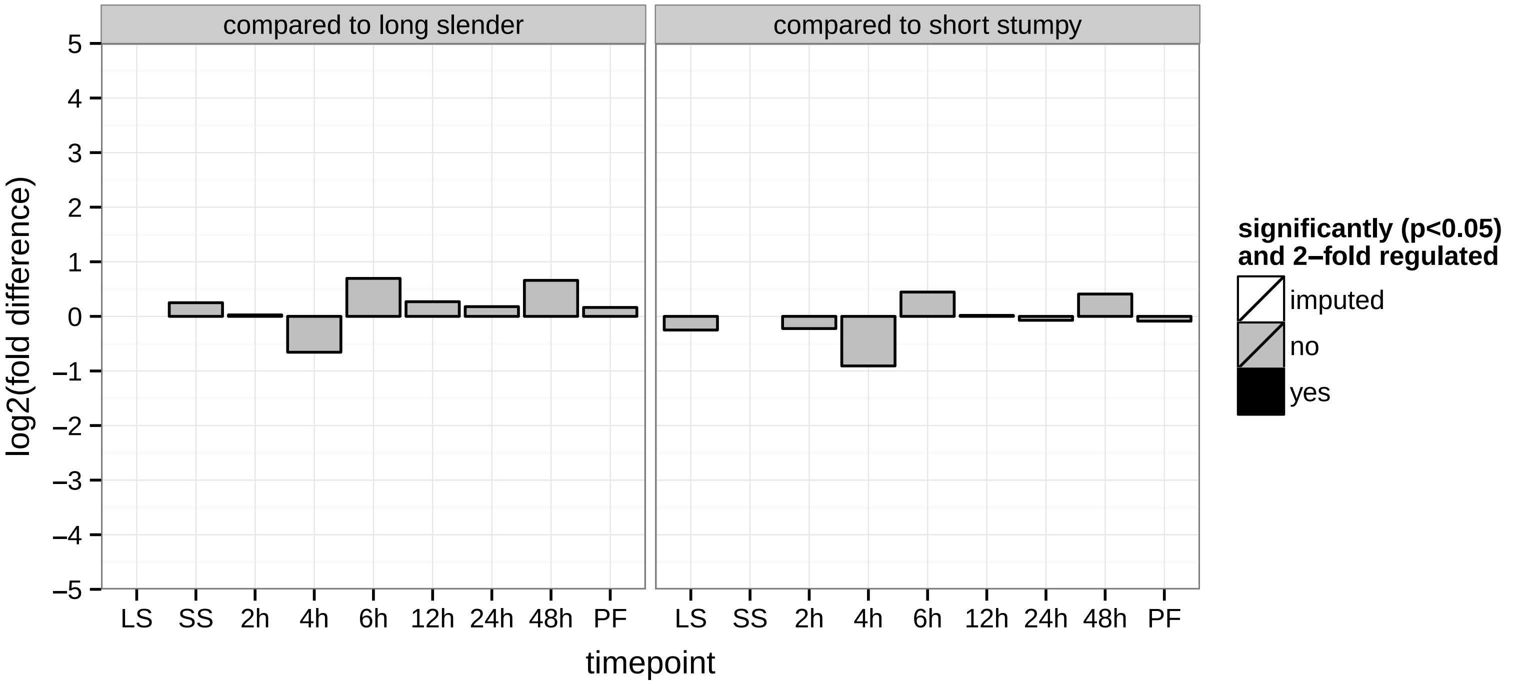

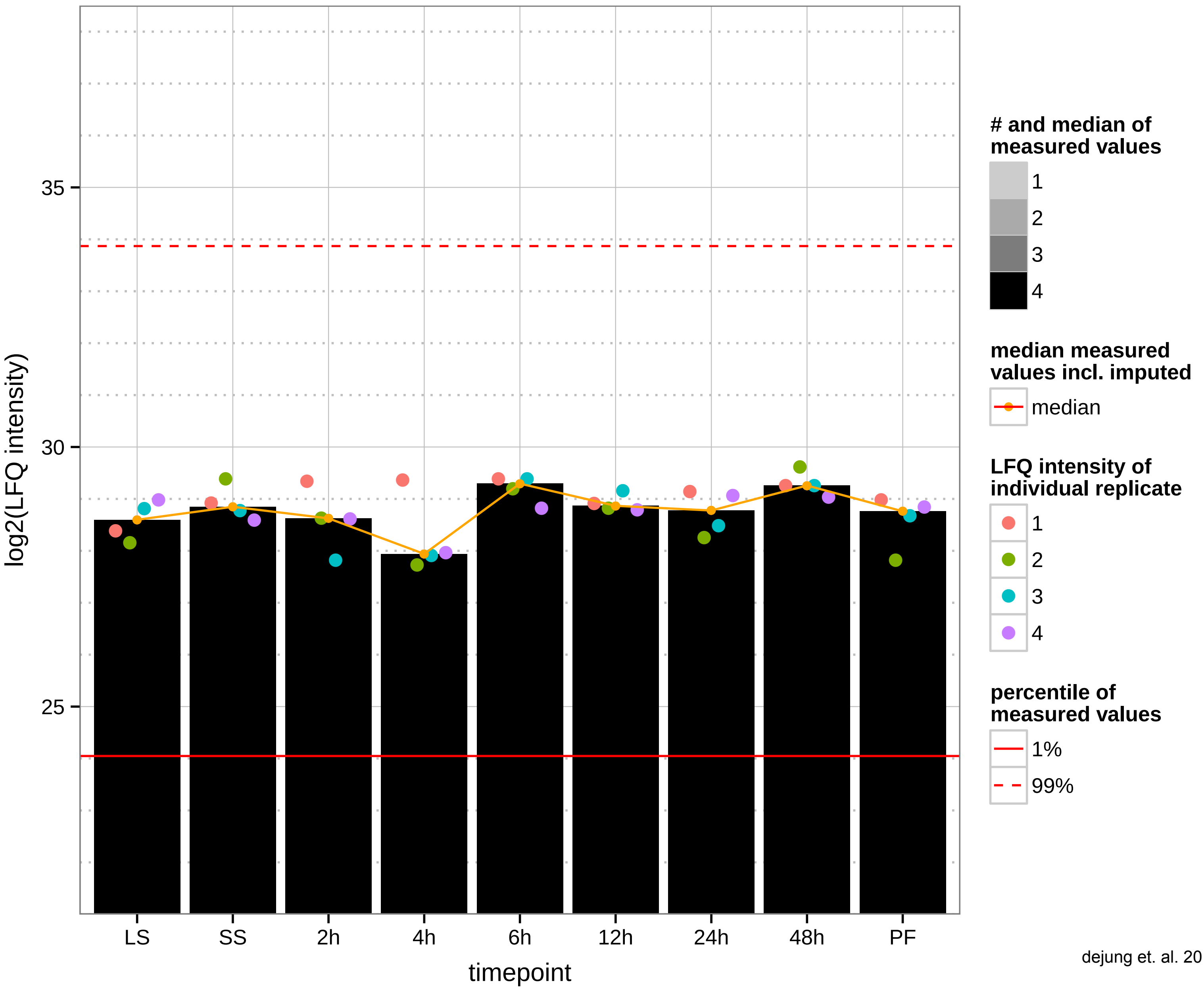

dejung et. al. 2015

potassium voltage–gated channel, putative
Tb927.10.16170;Tb11.v5.0396
AGOF: null, ATP–activated inward rectifier potassium channel activity, voltage–gated potassium channel activity
AGOC: null, membrane, mitochondrion, voltage–gated potassium channel complex
AGOP: null, potassium ion transport
PGOF: null
PGOC: null
PGOP: protein homooligomerization

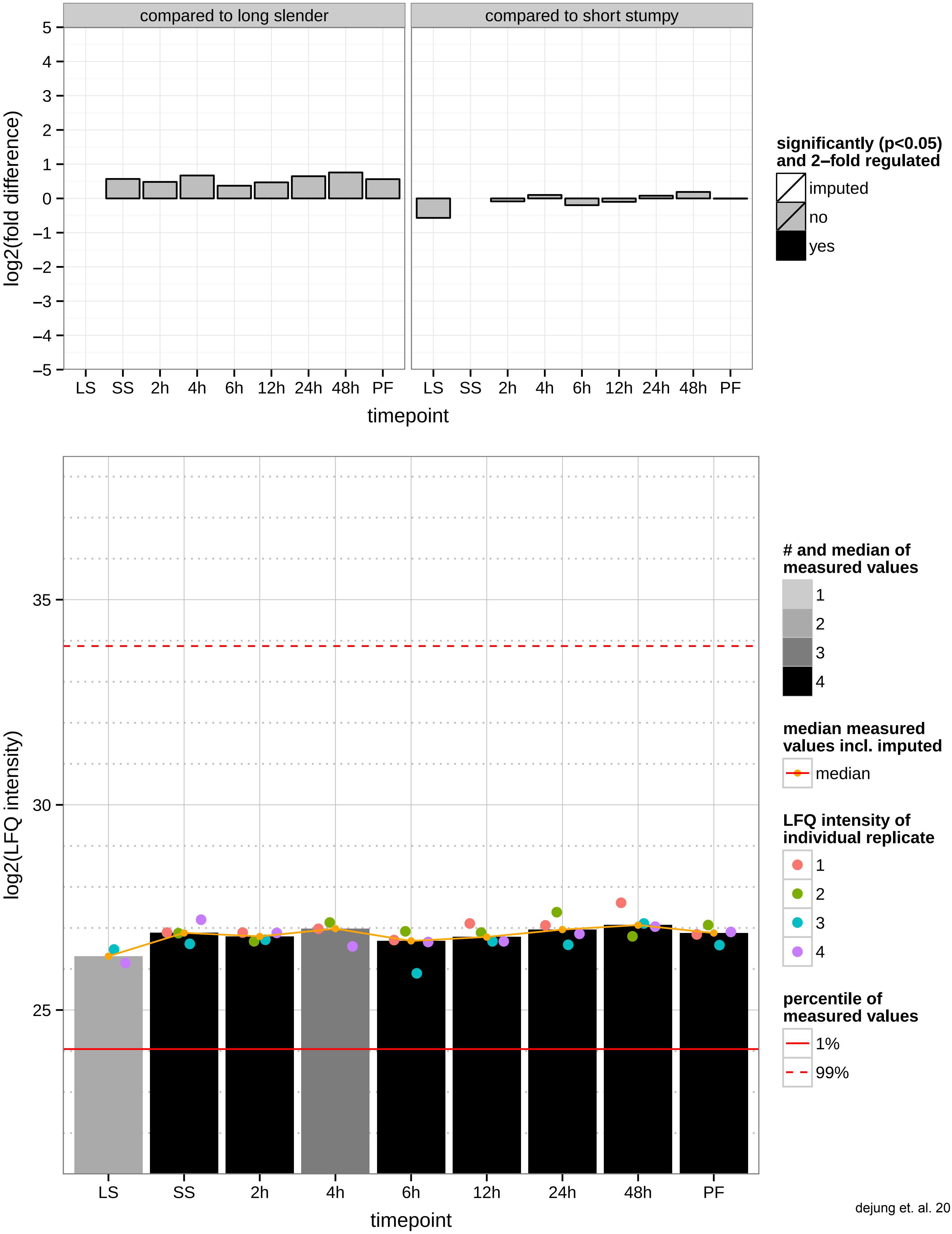

dejung et. al. 2015

ATP−dependent DEAD/H RNA helicase, putative, DEAD box RNA helicase
Tb927.10.540;Tb11.v5.0414
AGOF: null, ATP binding, ATP−dependent helicase activity, helicase activity, nucleic acid binding
AGOC: null
AGOP: null
PGOF: ATP binding, ATP−dependent helicase activity, helicase activity, nucleic acid binding
PGOC: null
PGOP: null

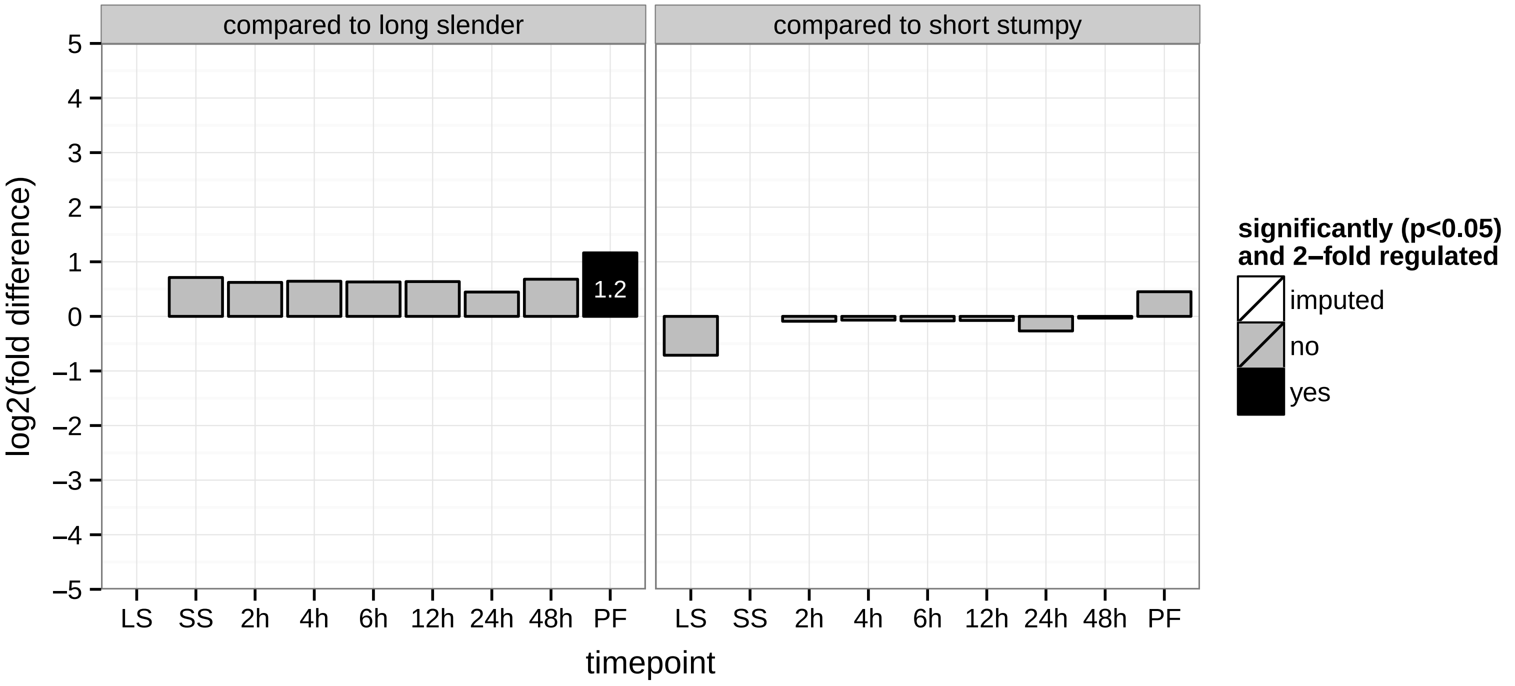

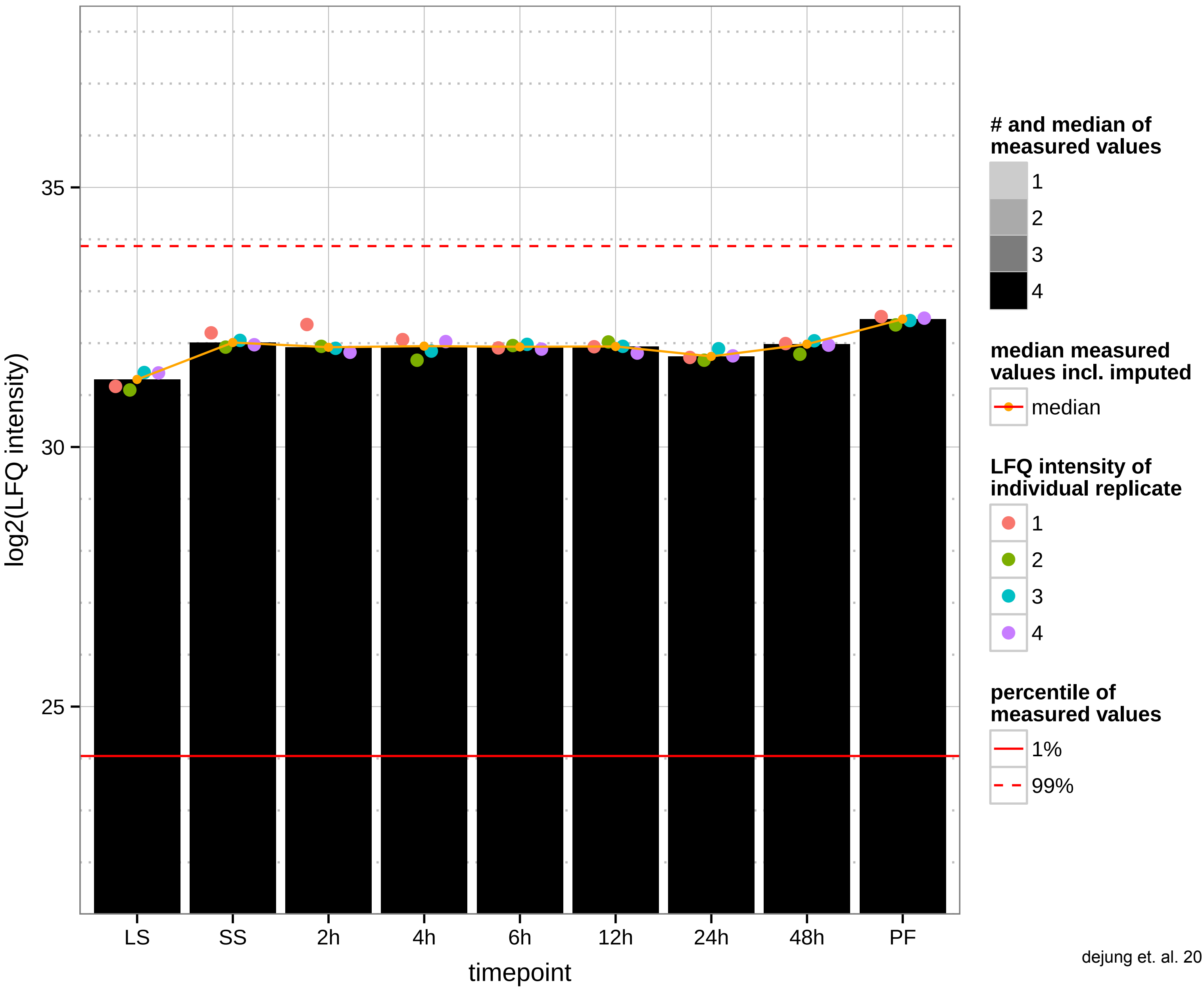

dejung et. al. 2015

hypothetical protein, conserved
Tb927.10.14490;Tb11.v5.0420
AGOF: null
AGOC: null
AGOP: null
PGOF: null
PGOC: null
PGOP: null

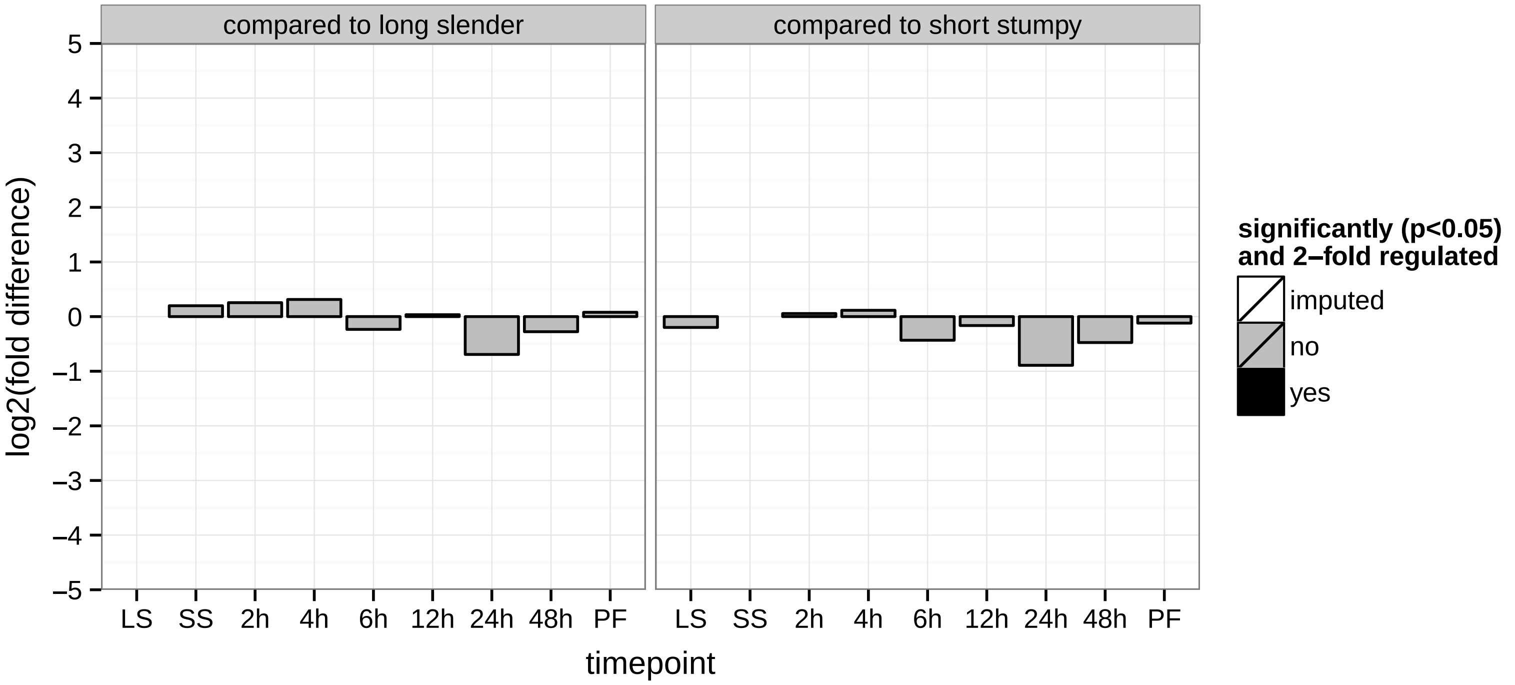

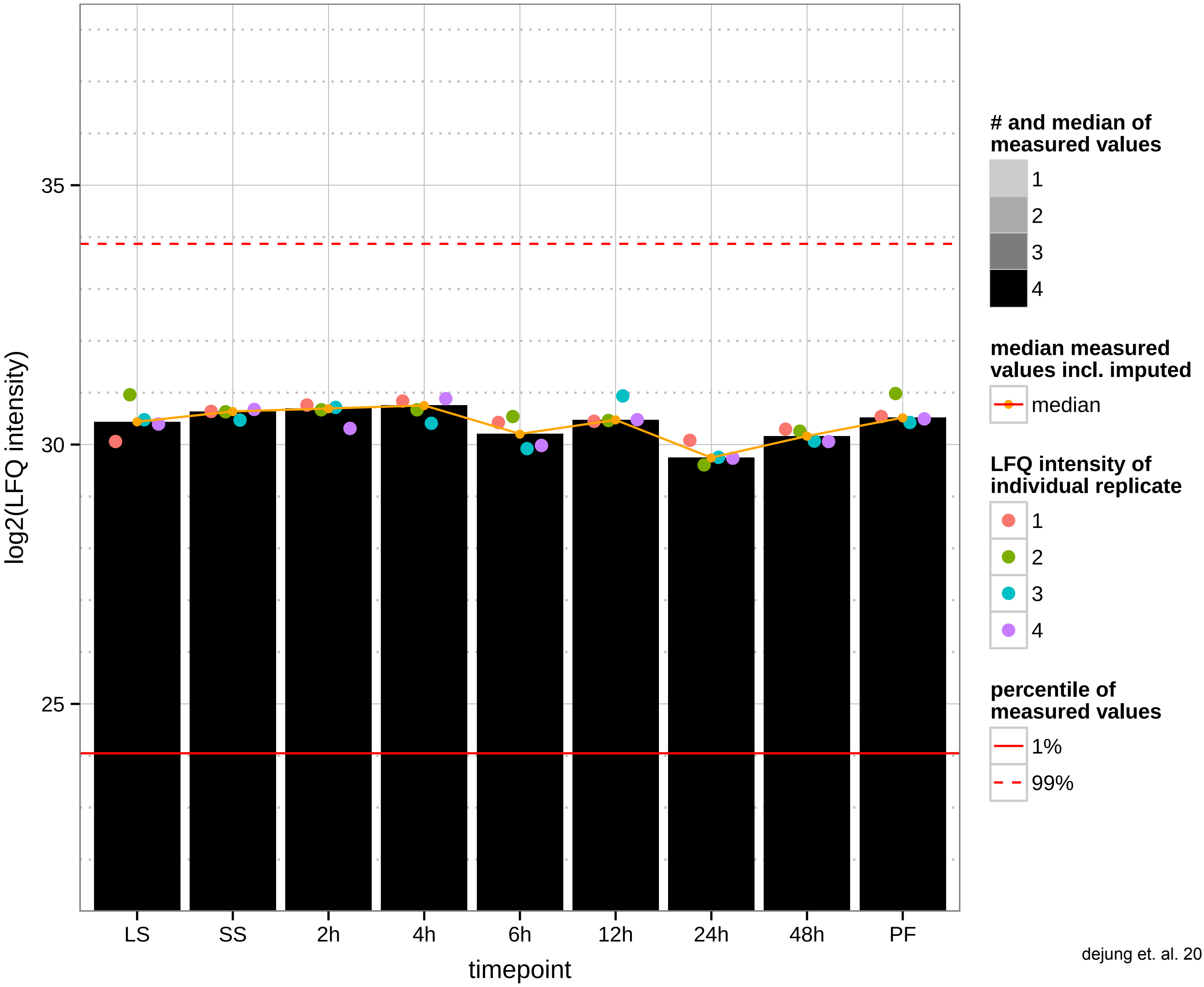

dejung et. al. 2015

peroxisome assembly protein, putative, peroxin 12, putative (PEX12)
Tb927.10.15850;Tb11.v5.0427
AGOF: null
AGOC: null, glycosome, glycosome membrane, integral to membrane, peroxisomal membrane
AGOP: null, peroxisome organization
PGOF: null
PGOC: null
PGOP: null

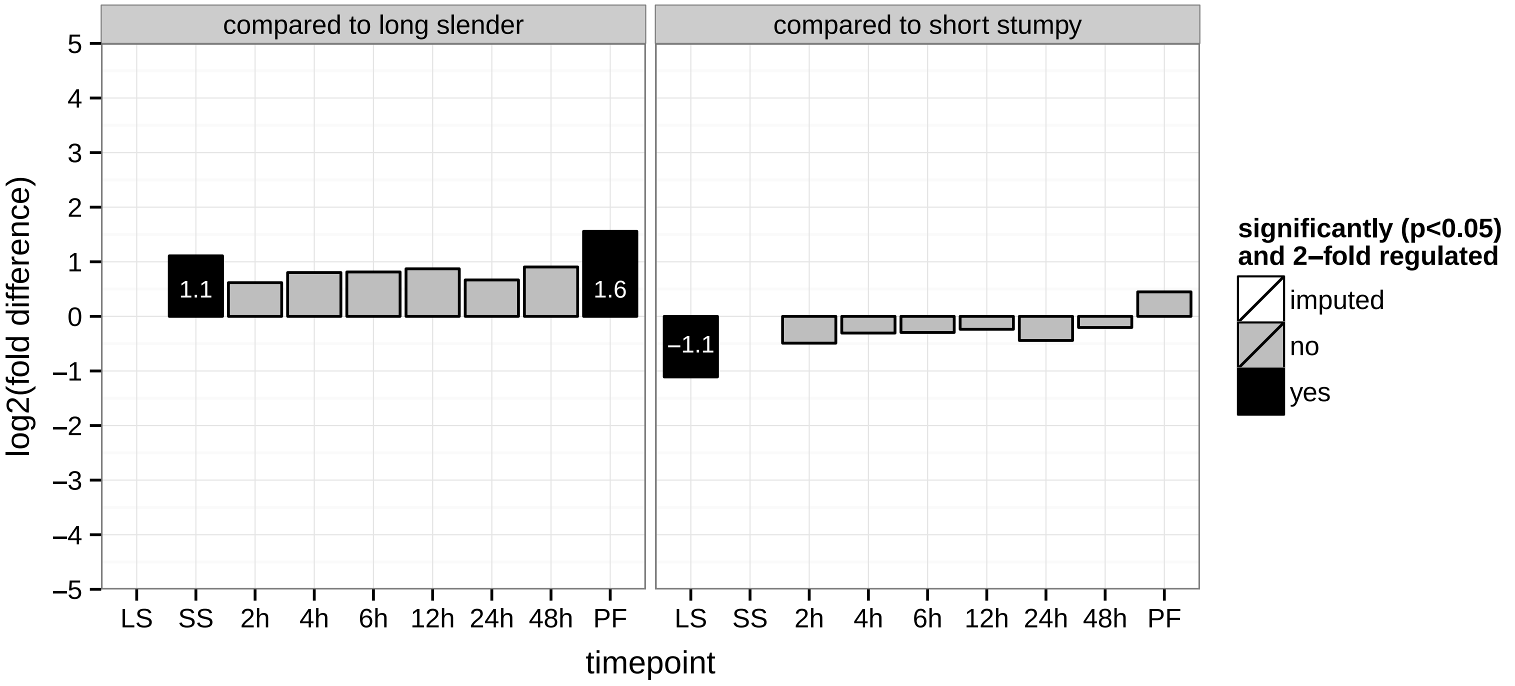

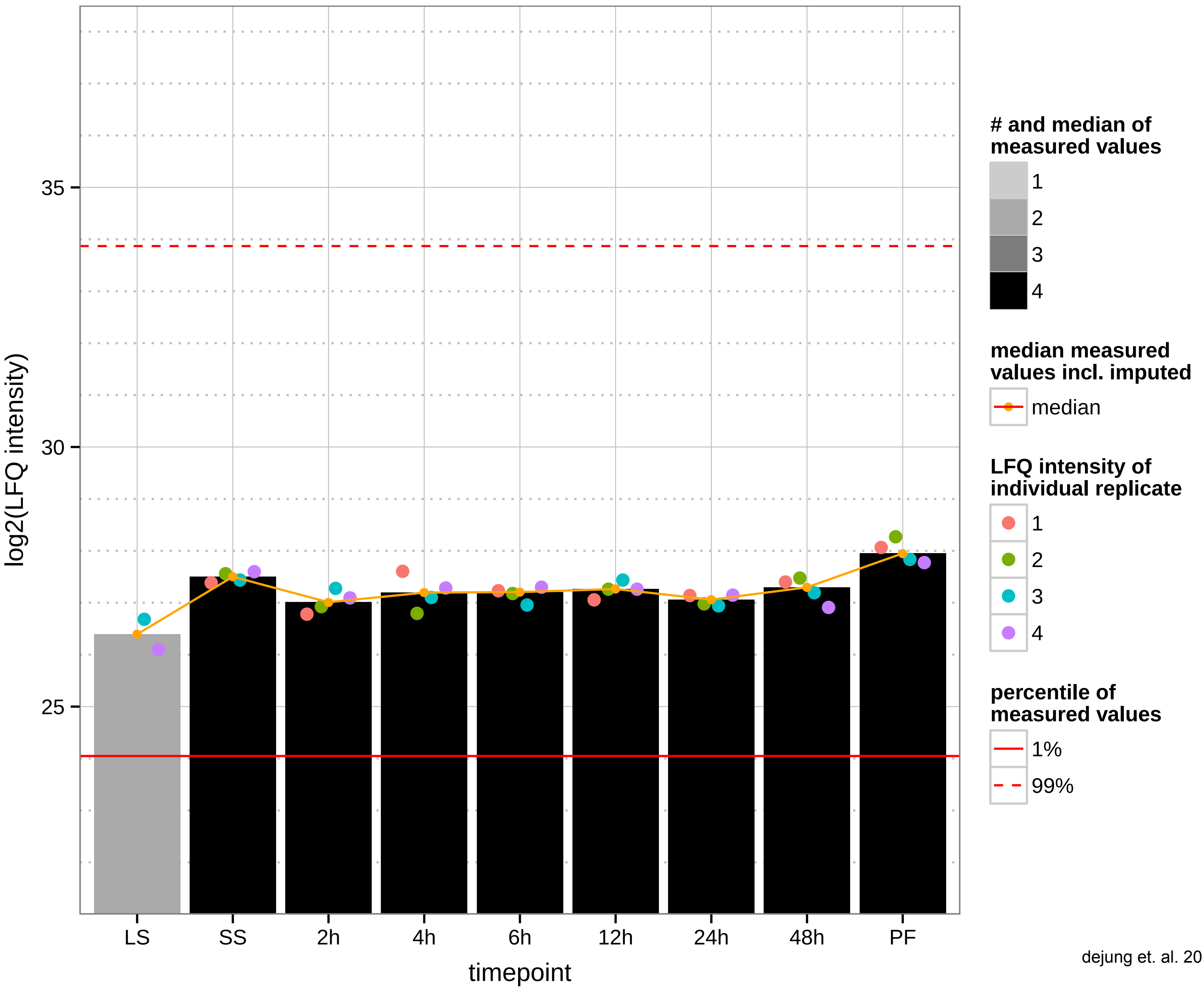

dejung et. al. 2015

hypothetical protein, conserved, leucine−rich repeat protein (LRRP), putative
Tb927.1.1030;Tb11.v5.0436
AGOF: null
AGOC: null
AGOP: null
PGOF: null
PGOC: null
PGOP: null

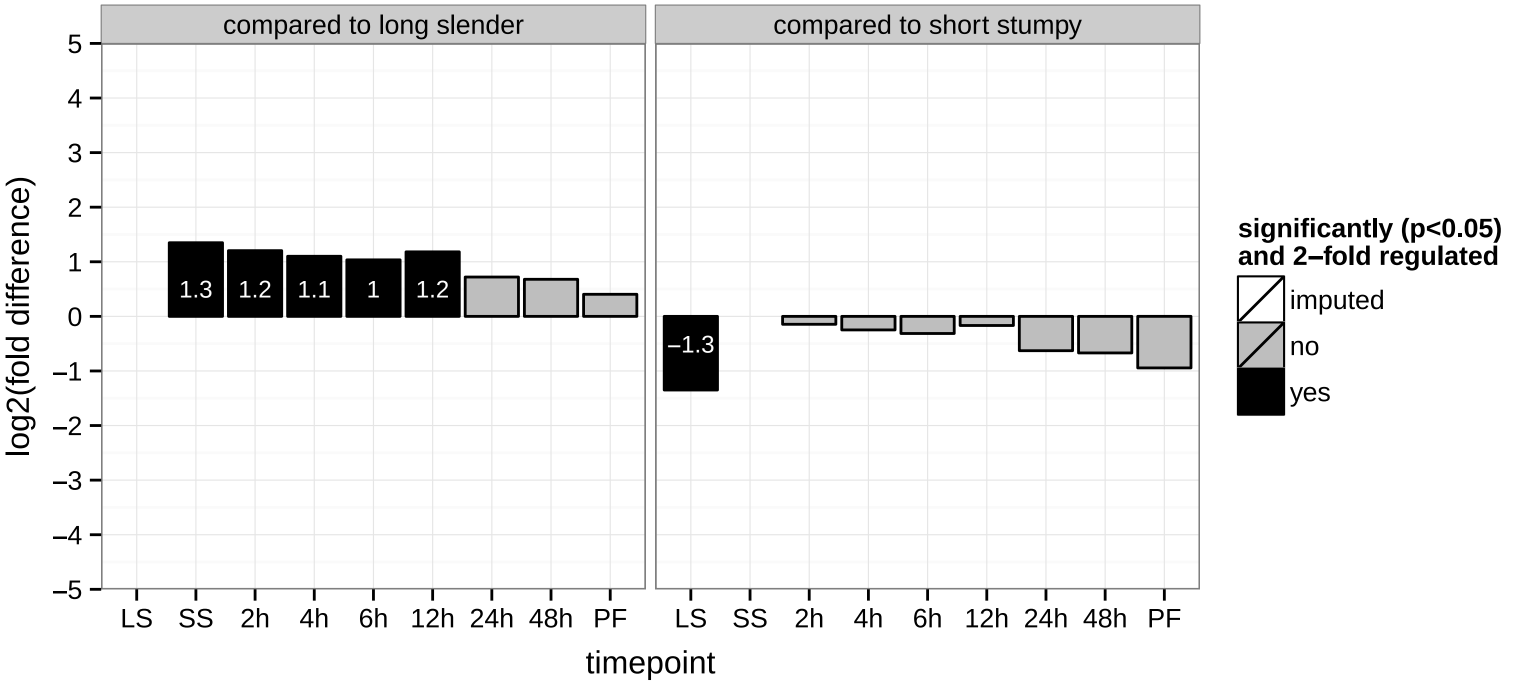

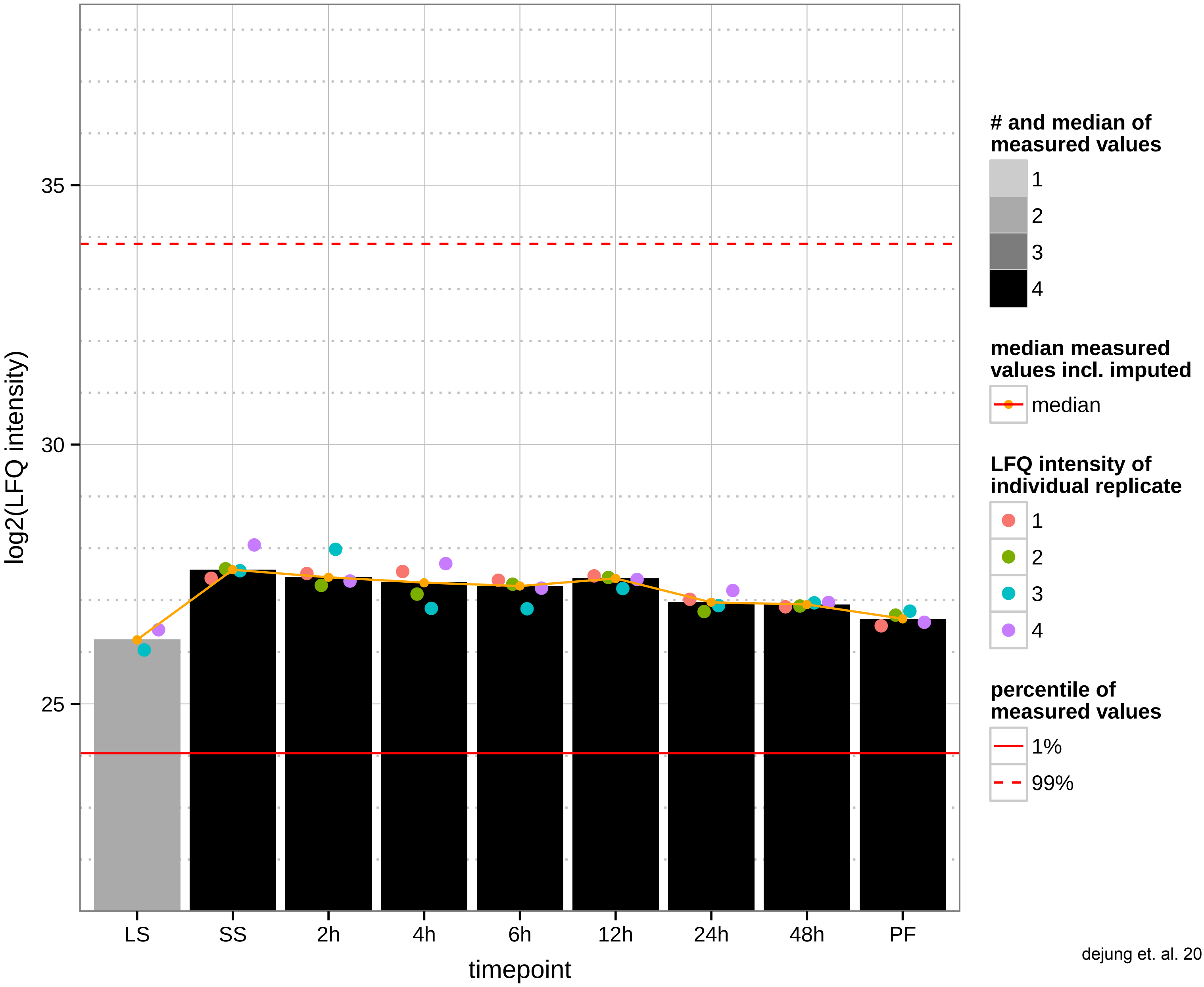

dejung et. al. 2015

alpha tubulin, putative
Tb11.v5.0468
AGOF: null
AGOC: null
AGOP: null
PGOF: GTP binding, GTPase activity
PGOC: protein complex
PGOP: GTP catabolic process, protein polymerization

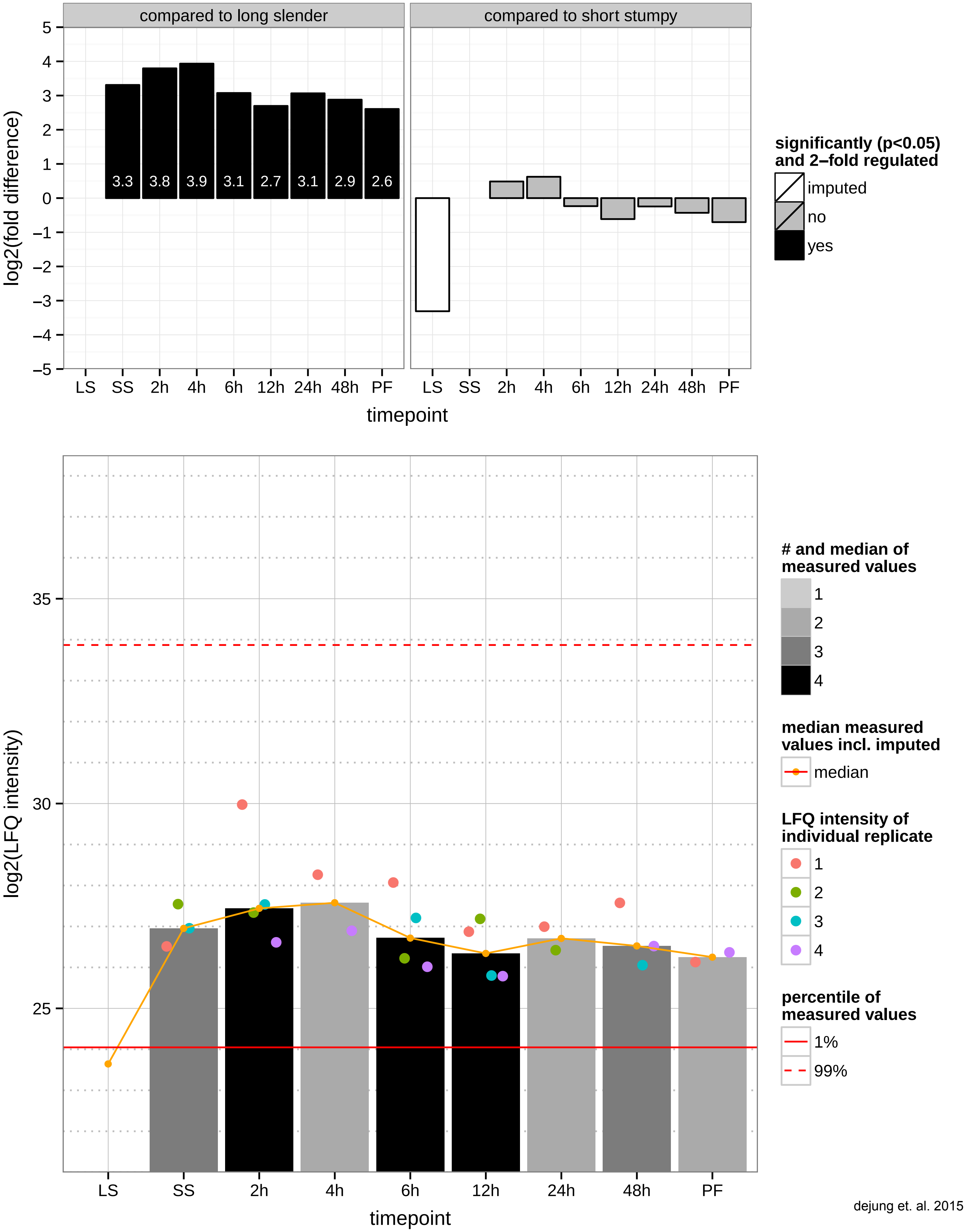

dejung et. al. 2015

beta tubulin, putative, pseudogene
Tb927.1.2390;Tb927.1.2370;Tb927.1.2350;Tb927.1.2330;Tb11.v5.0469;Tb927.1.2410
AGOF: null, GTP binding, GTPase activity, structural constituent of cytoskeleton, structural molecule activity
AGOC: null, microtubule, protein complex
AGOP: null, cellular component movement, microtubule cytoskeleton organization, microtubule–based movement, protein poly
PGOF: GTP binding, GTPase activity, null
PGOC: protein complex, null
PGOP: GTP catabolic process, protein polymerization, null

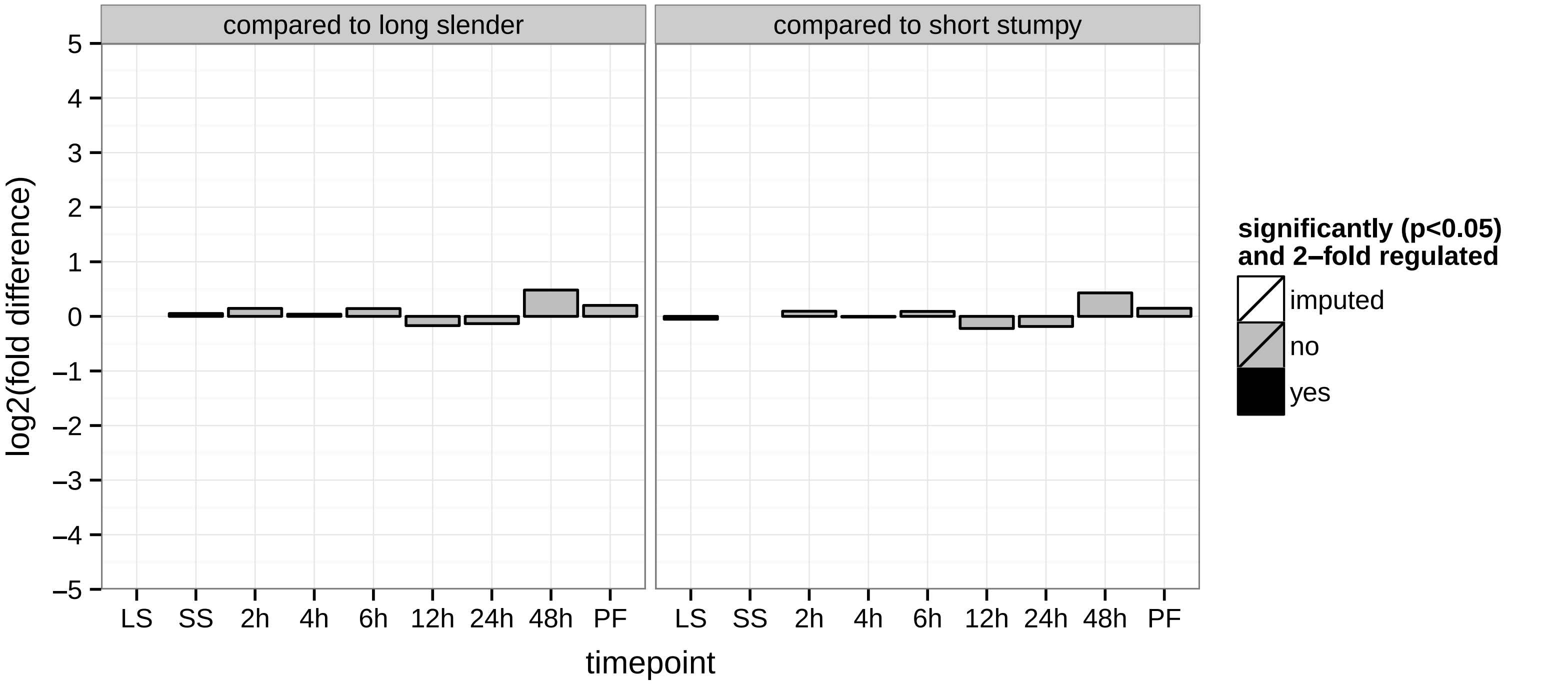

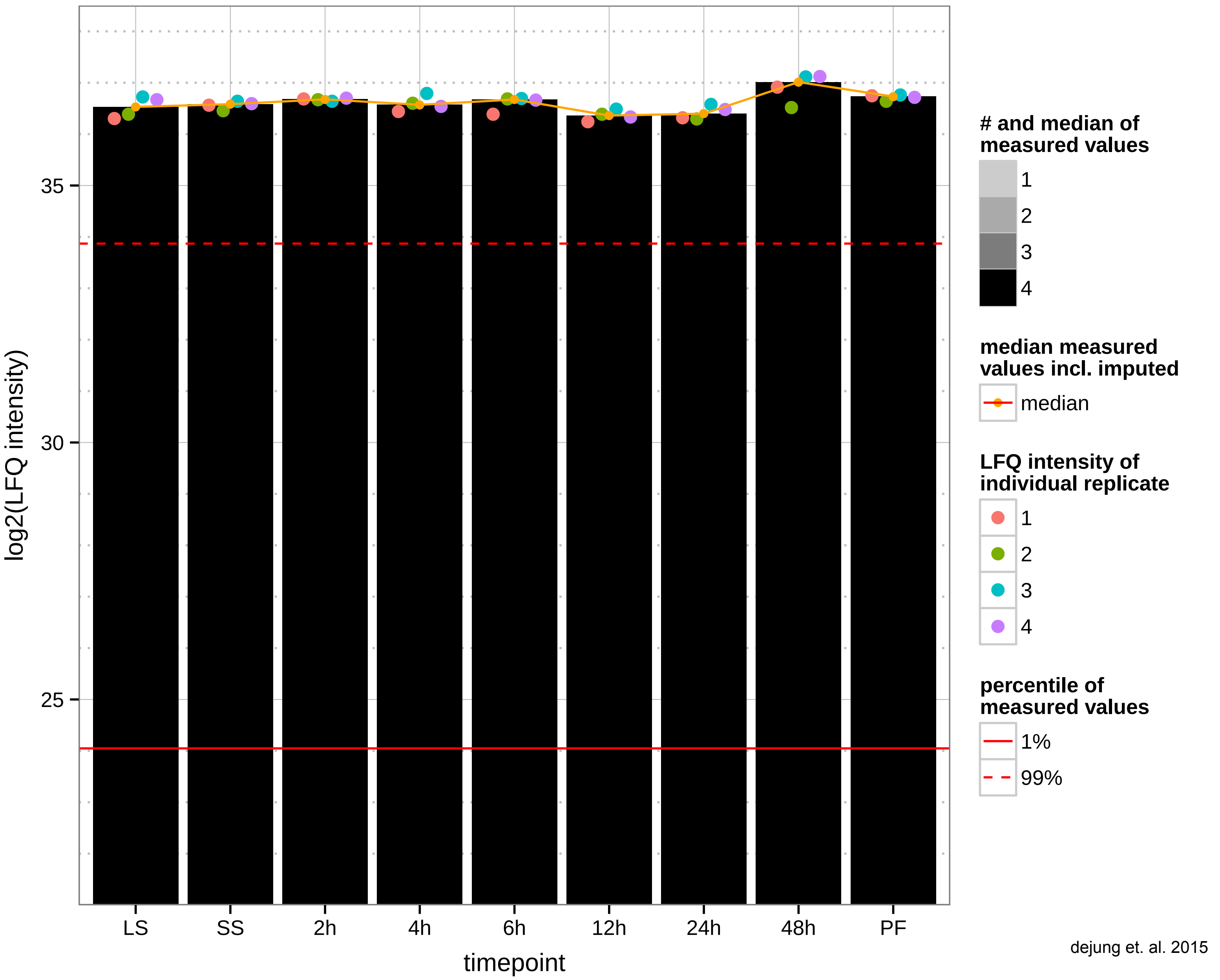

dejung et. al. 2015

DNA–directed RNA polymerase, alpha subunit, putative, DNA–directed RNA polymerases I and III subunit RPAC1, putative (F
Tb11.v5.0480;Tb927.10.15370
AGOF: null, DNA binding, DNA–directed RNA polymerase activity, protein dimerization activity
AGOC: null
AGOP: null, transcription, DNA–dependent
PGOF: DNA binding, DNA–directed RNA polymerase activity, protein dimerization activity
PGOC: null
PGOP: transcription, DNA–dependent

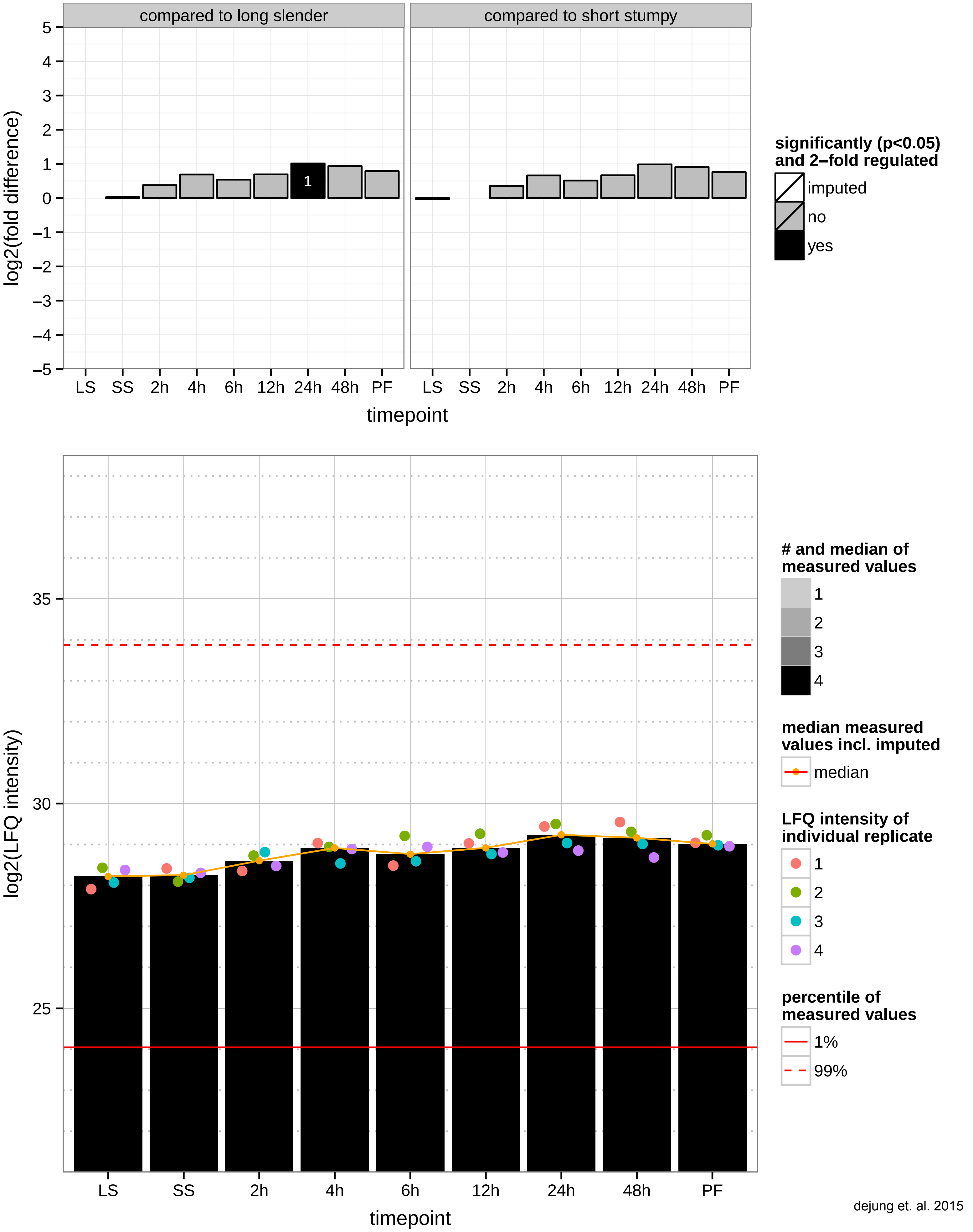

dejung et. al. 2015

U3 snoRNA–associated protein UTP11, putative
Tb927.10.15380;Tb11.v5.0481
AGOF: null
AGOC: null, ribonucleoprotein complex, small–subunit processome
AGOP: null, rRNA processing
PGOF: null
PGOC: small–subunit processome
PGOP: rRNA processing

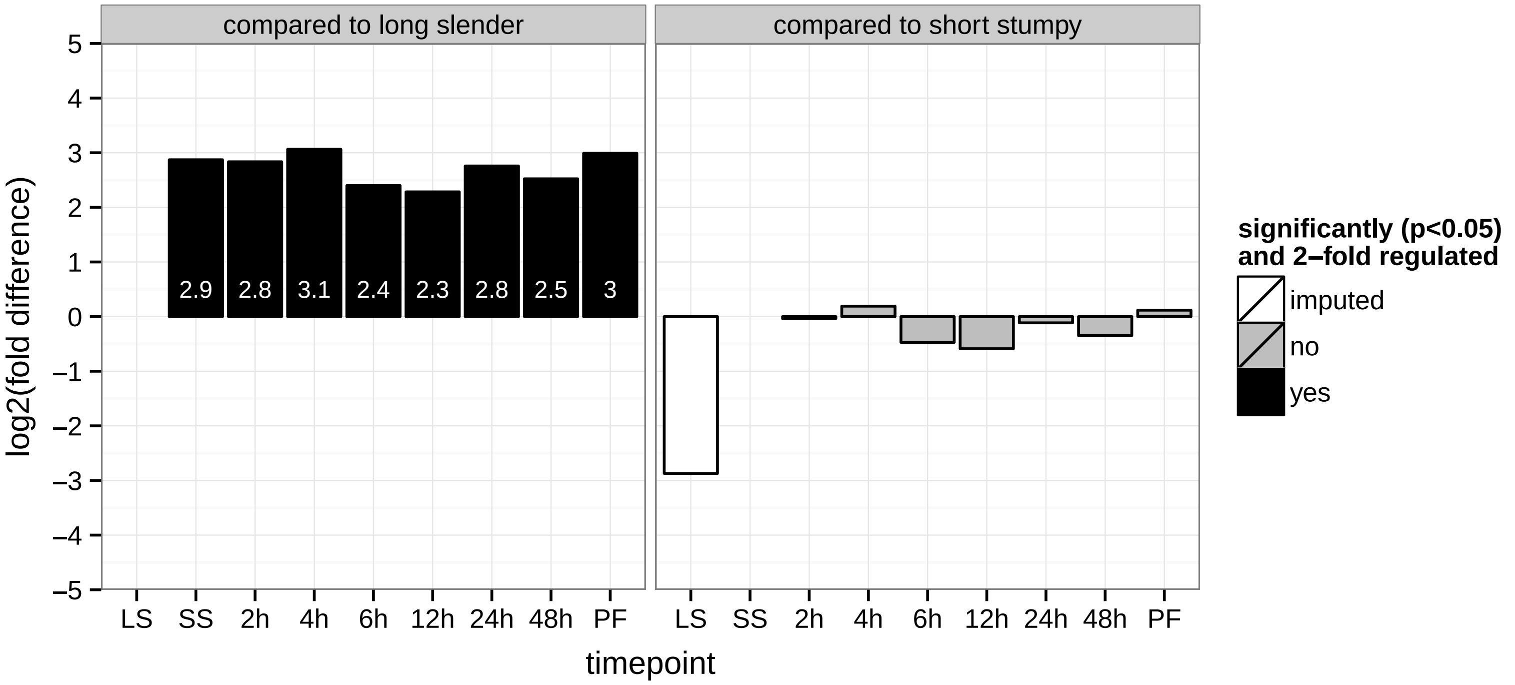

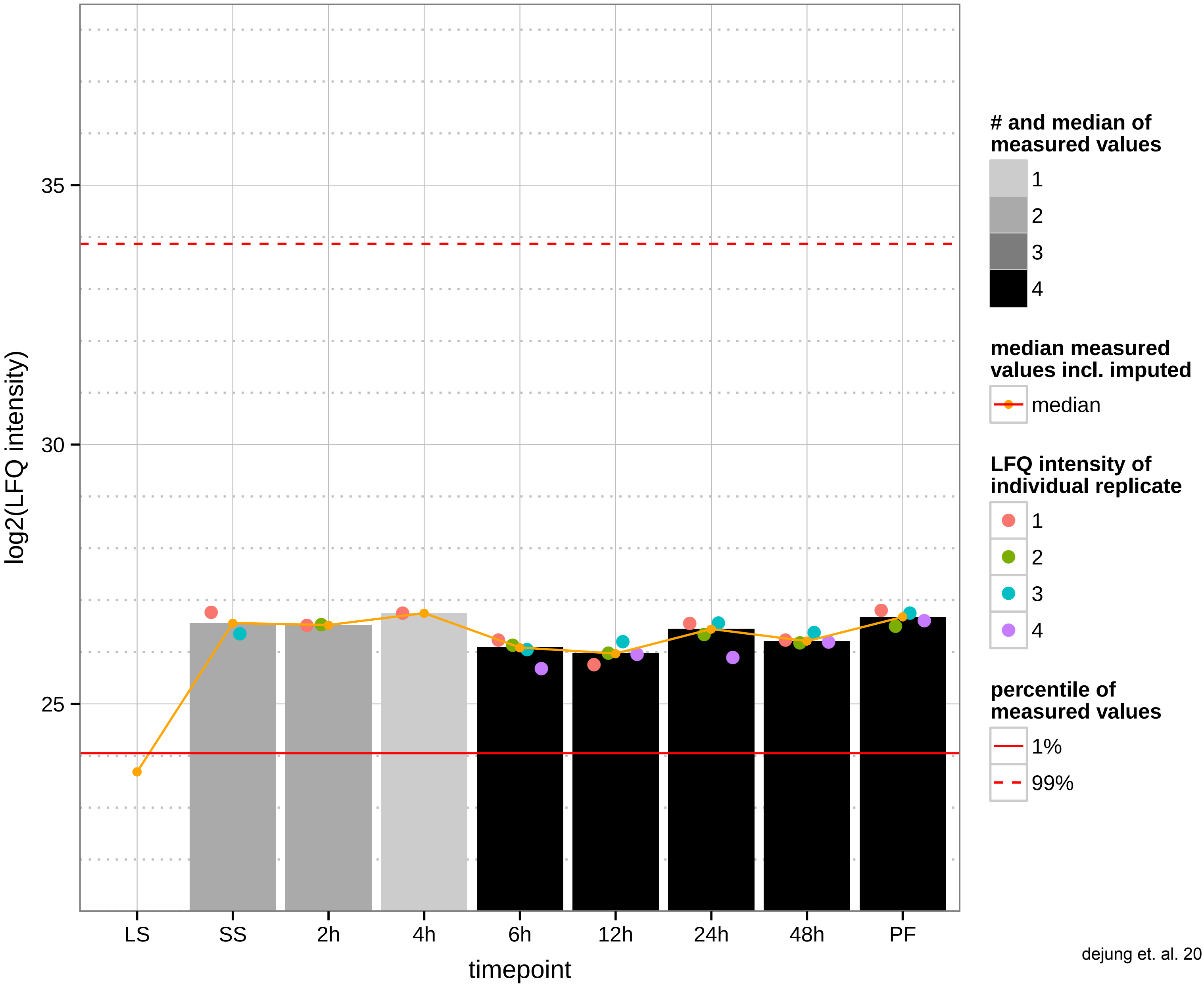

dejung et. al. 2015

retrotransposon hot spot (RHS) protein, putative
Tb11.v5.0489
AGOF: null
AGOC: null
AGOP: null
PGOF: null
PGOC: null
PGOP: null

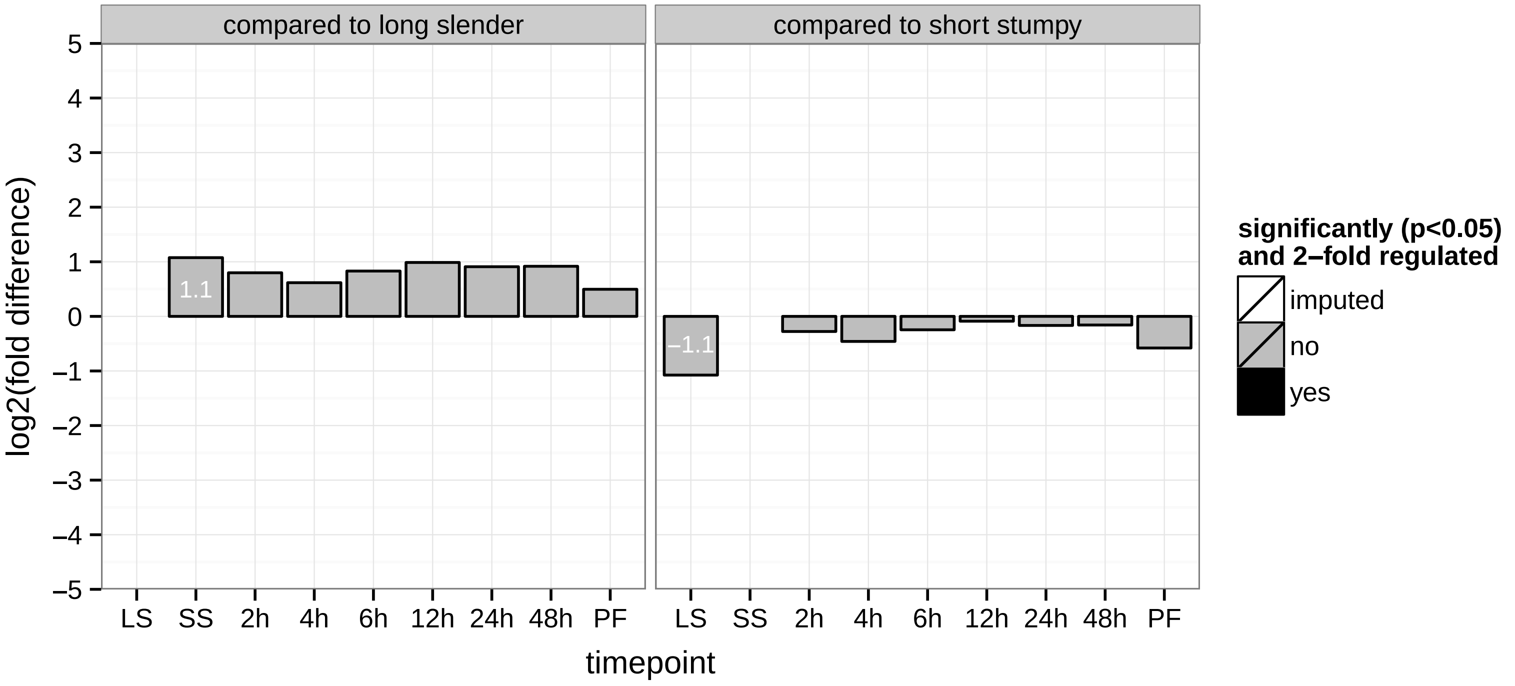

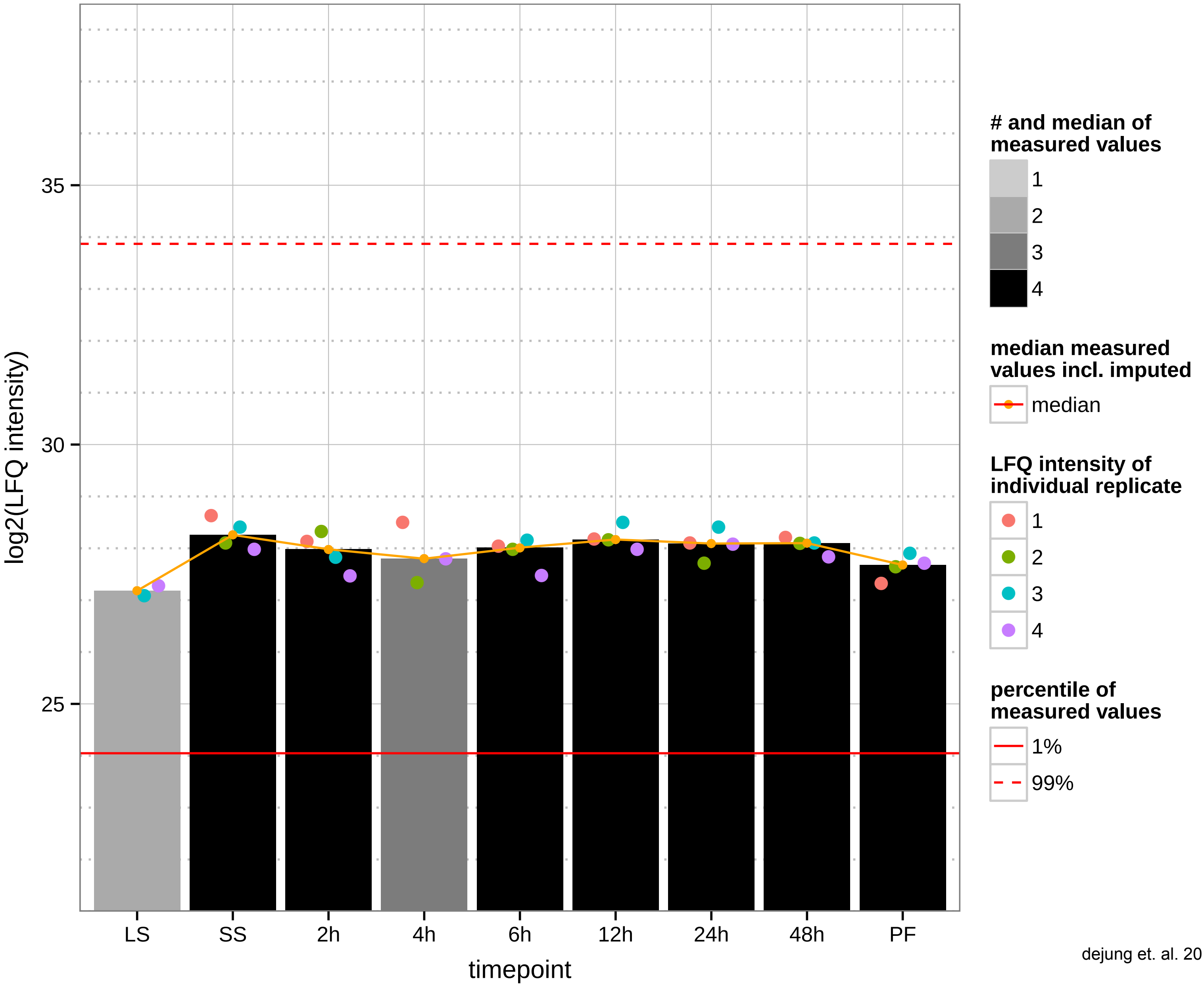

dejung et. al. 2015

40S ribosomal protein S3A, putative
Tb927.10.3940;Tb11.v5.0501;Tb927.10.3930
AGOF: null, structural constituent of ribosome
AGOC: null, intracellular, ribosome
AGOP: null, translation
PGOF: structural constituent of ribosome
PGOC: intracellular, ribosome
PGOP: translation

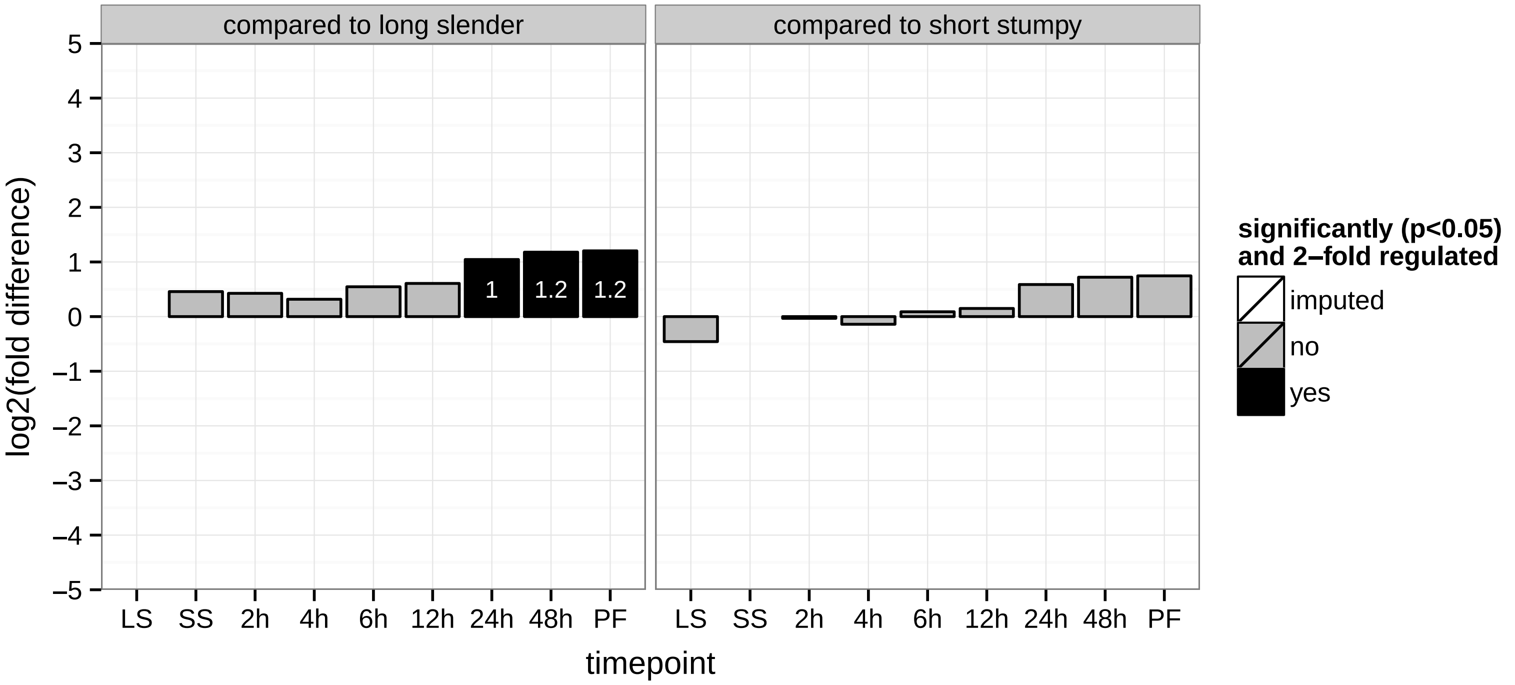

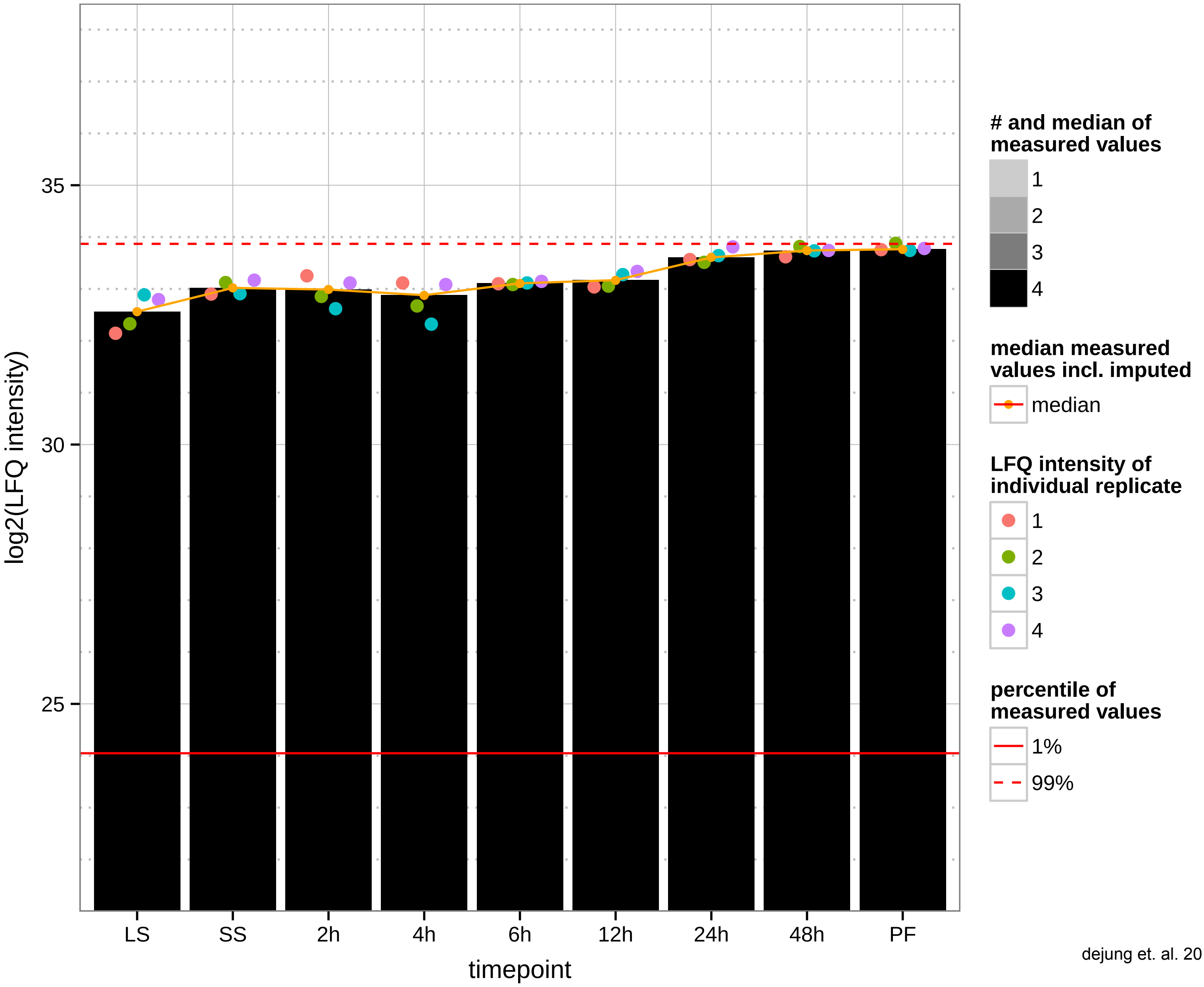

dejung et. al. 2015

hypothetical protein, conserved
Tb927.10.3920;Tb11.v5.0502
AGOF: null
AGOC: null
AGOP: null
PGOF: null
PGOC: null
PGOP: null

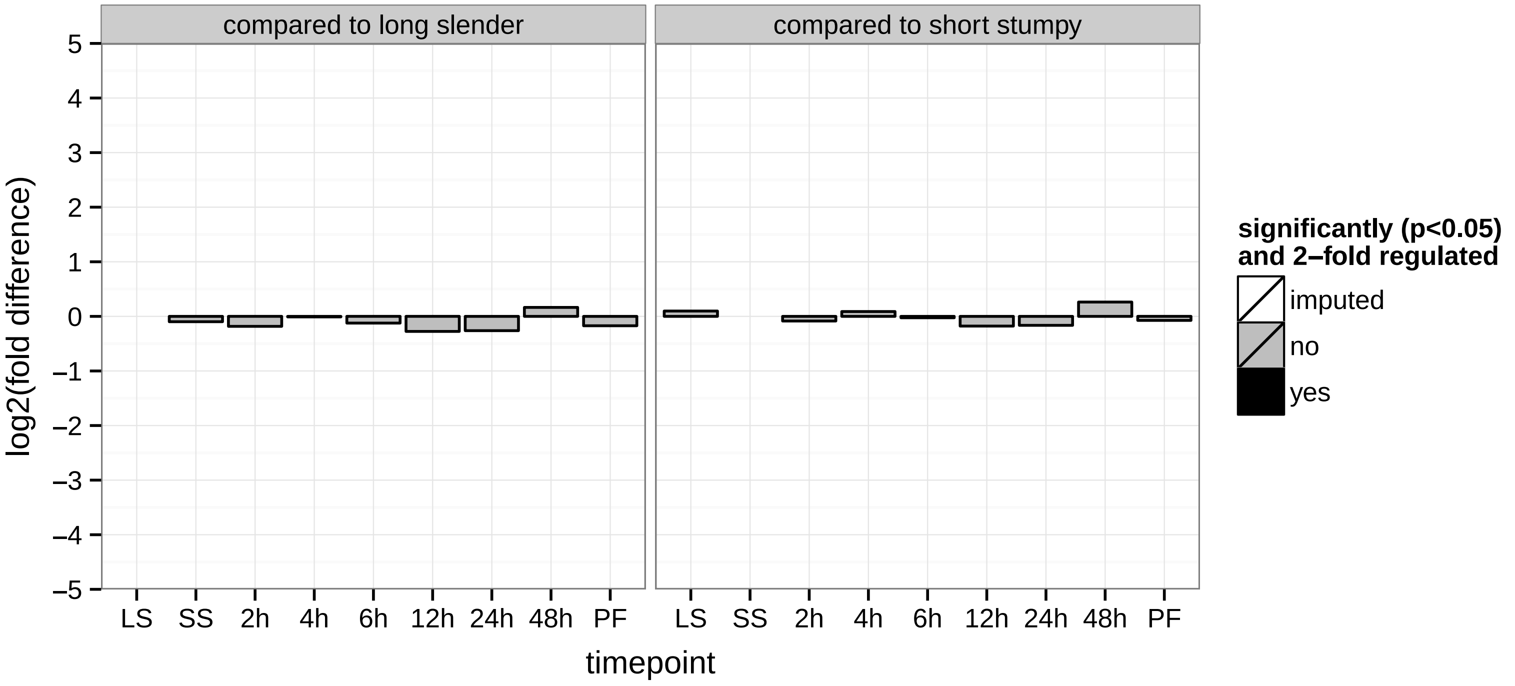

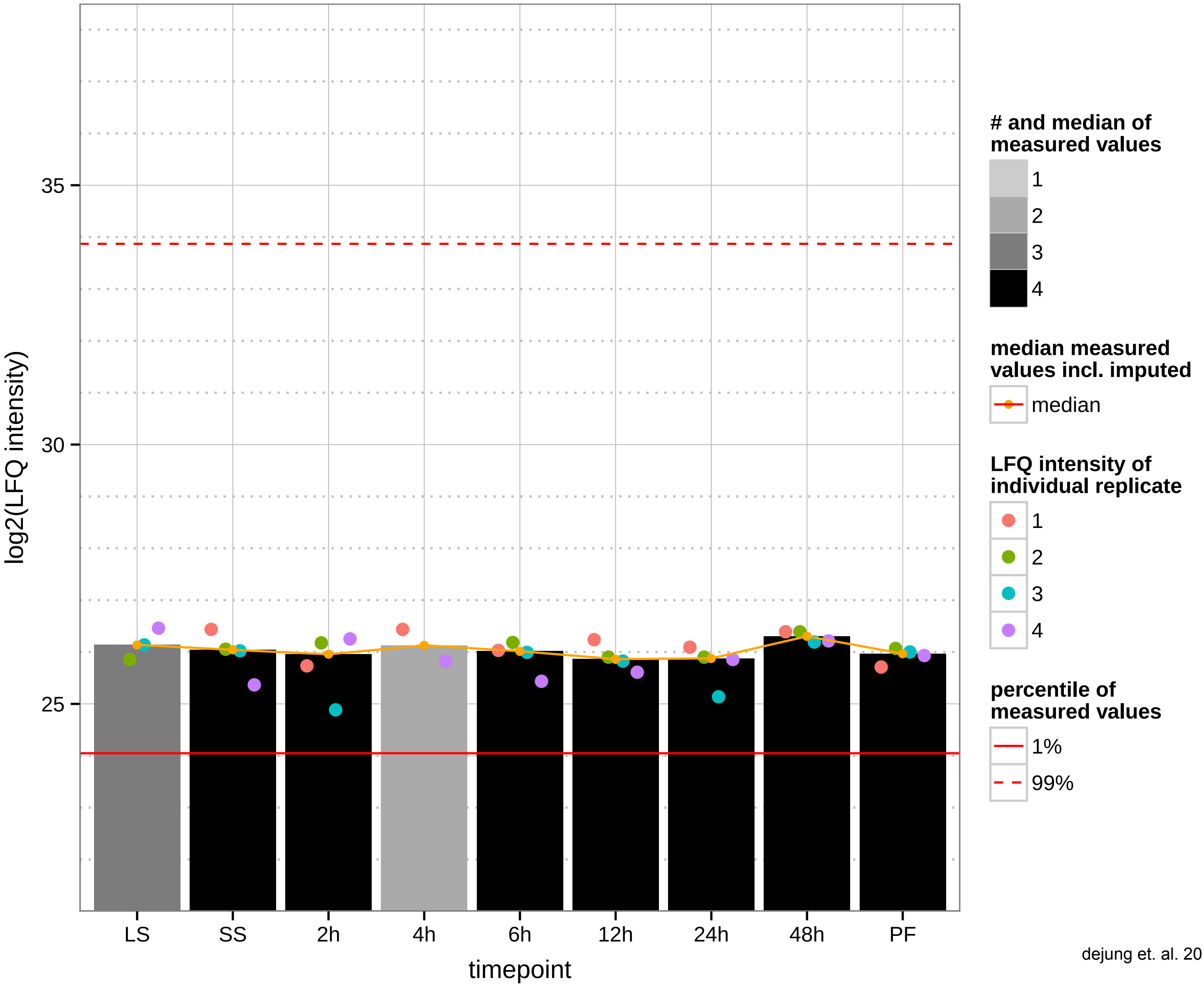

dejung et. al. 2015

hypothetical protein, conserved
Tb927.10.16110;Tb11.v5.0527
AGOF: null
AGOC: null
AGOP: null, ciliary or flagellar motility
PGOF: protein binding
PGOC: null
PGOP: null

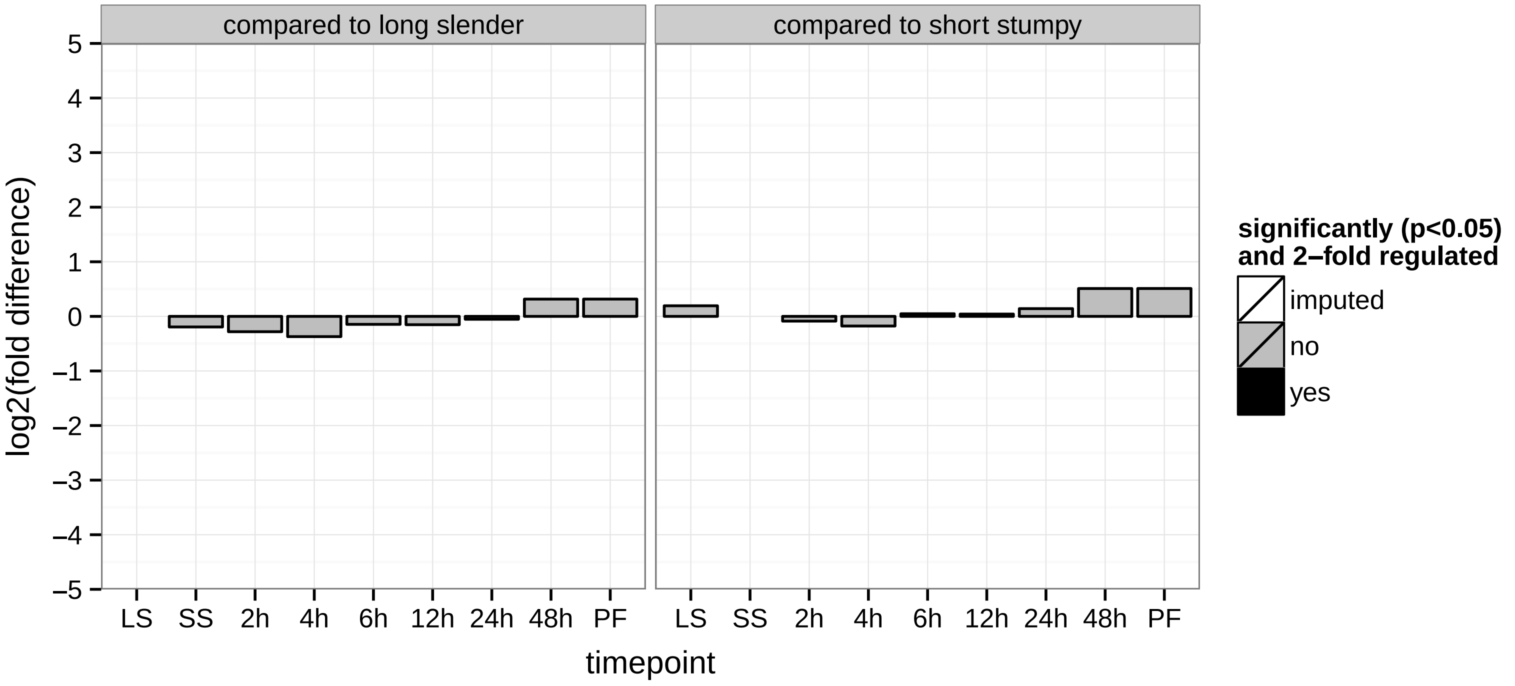

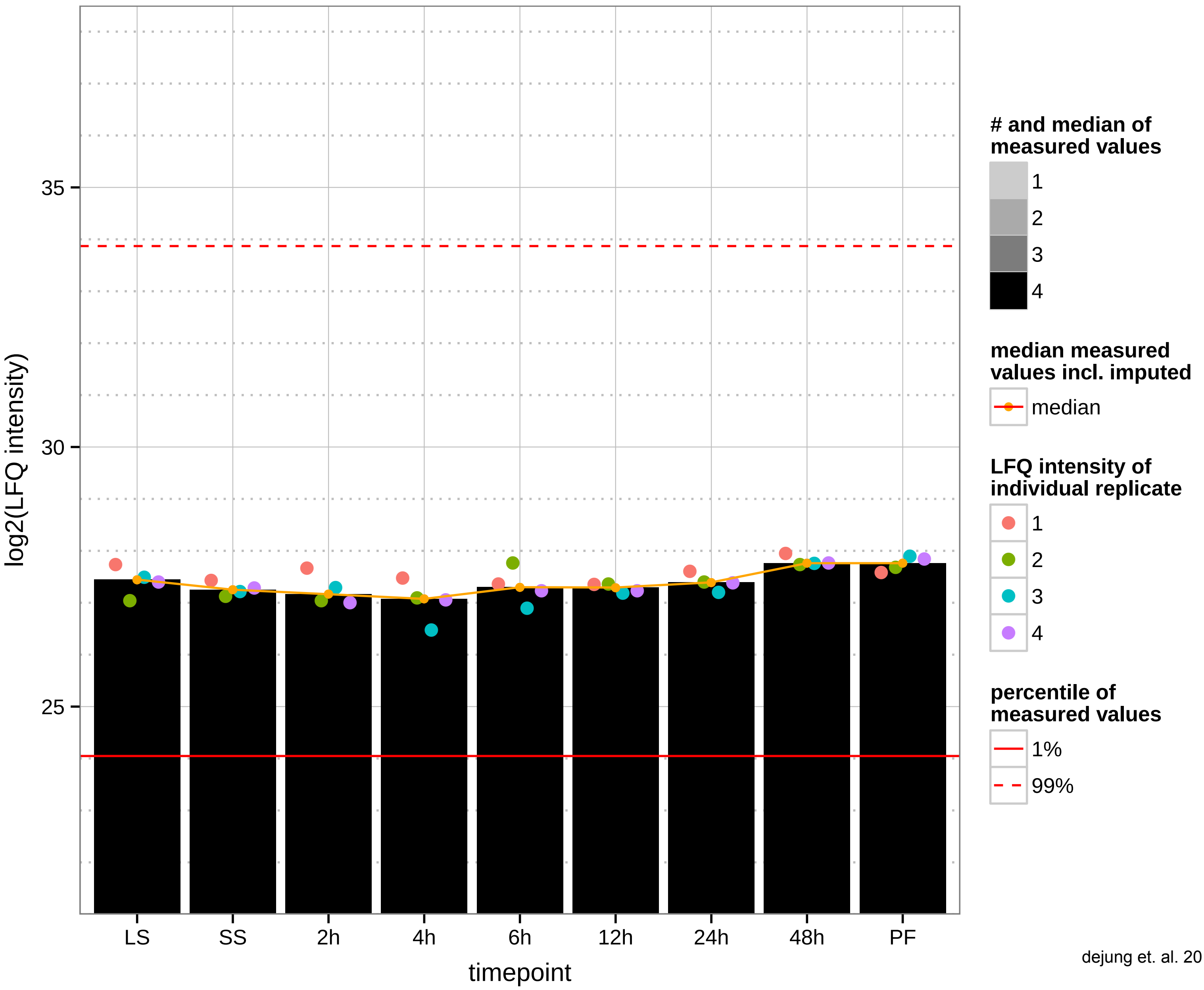

dejung et. al. 2015

69 kDa paraflagellar rod protein, putative, Paraflagellar rod protein 2, Paraflagellar rod protein A (PFR2)
Tb927.8.5010;Tb927.8.5000;Tb927.8.4990;Tb927.8.4980;Tb927.8.4970;Tb11.v5.0538
AGOF: null, calmodulin binding
AGOC: null, cilium, microtubule–based flagellum
AGOP: null, cellular component movement, forward locomotion
PGOF: calmodulin binding, null
PGOC: microtubule–based flagellum, null
PGOP: null

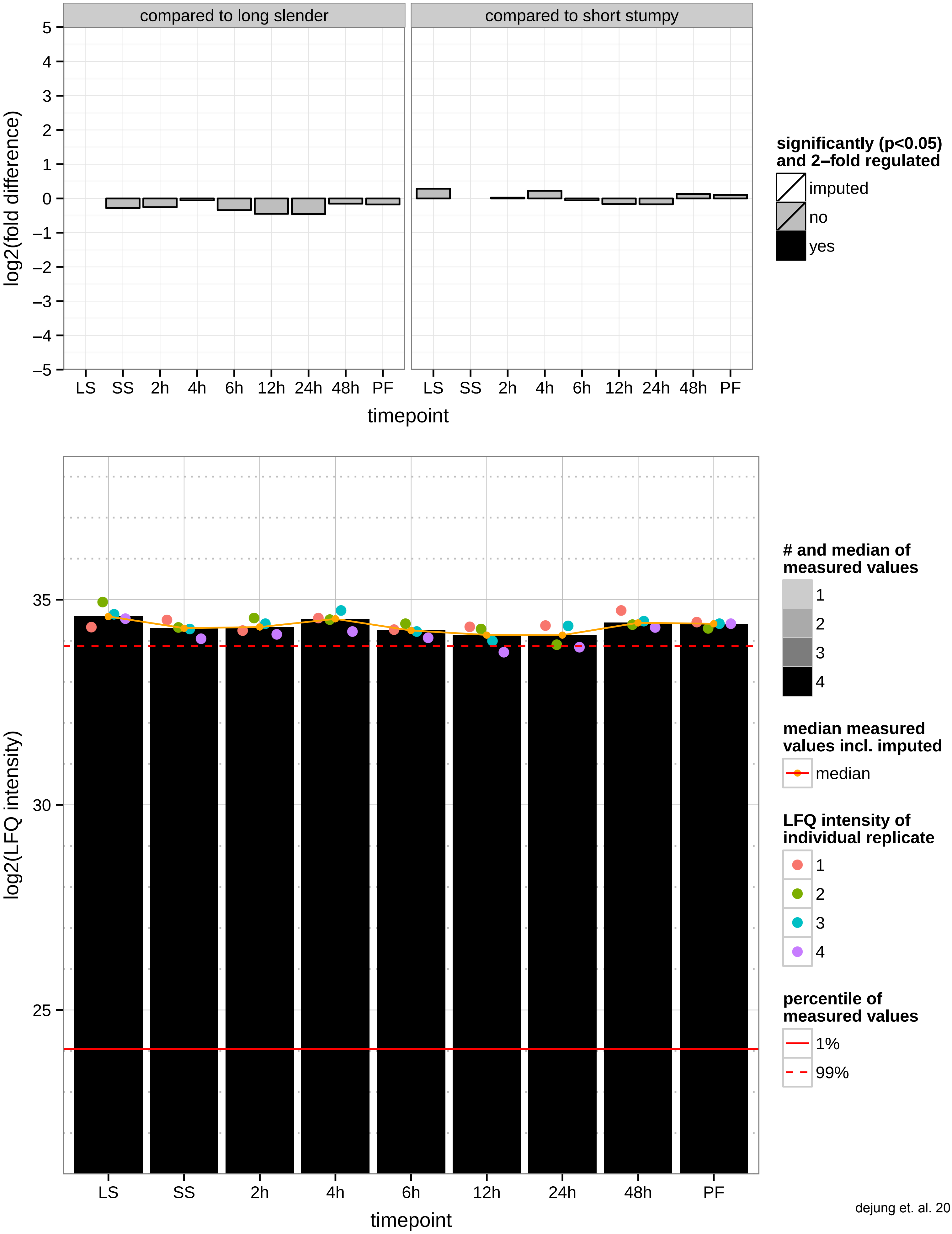

dejung et. al. 2015

Heat shock protein 83, putative, heat shock protein, heat shock protein 90
Tb927.10.10960;Tb927.10.10950;Tb927.10.10940;Tb927.10.10920;Tb927.10.10900;Tb927.10.10890;Tb11.v5.0543;Tb927.1
AGOF: null, ATP binding, unfolded protein binding, ATPase activity, coupled
AGOC: null, centrosome
AGOP: null, protein folding, response to stress, protein refolding, response to unfolded protein
PGOF: ATP binding, unfolded protein binding, null
PGOC: null
PGOP: protein folding, response to stress, null

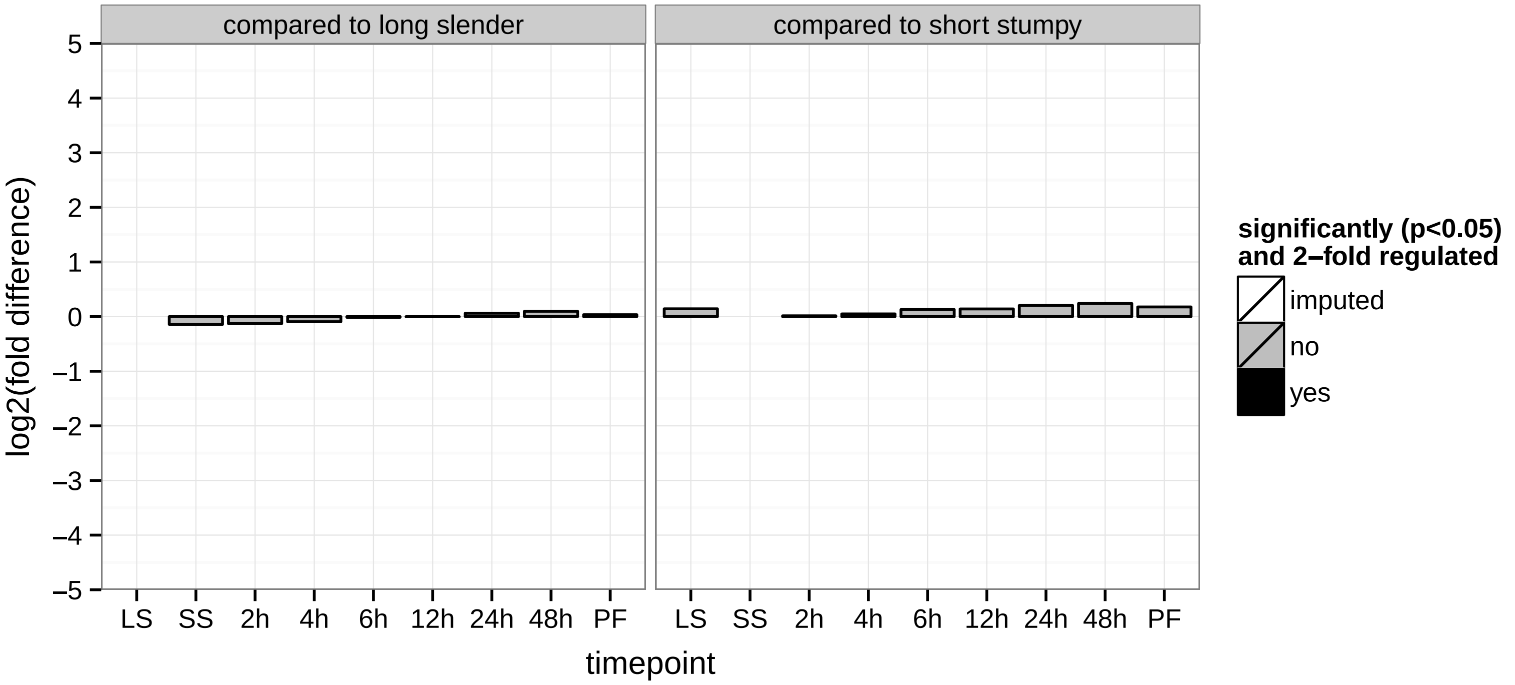

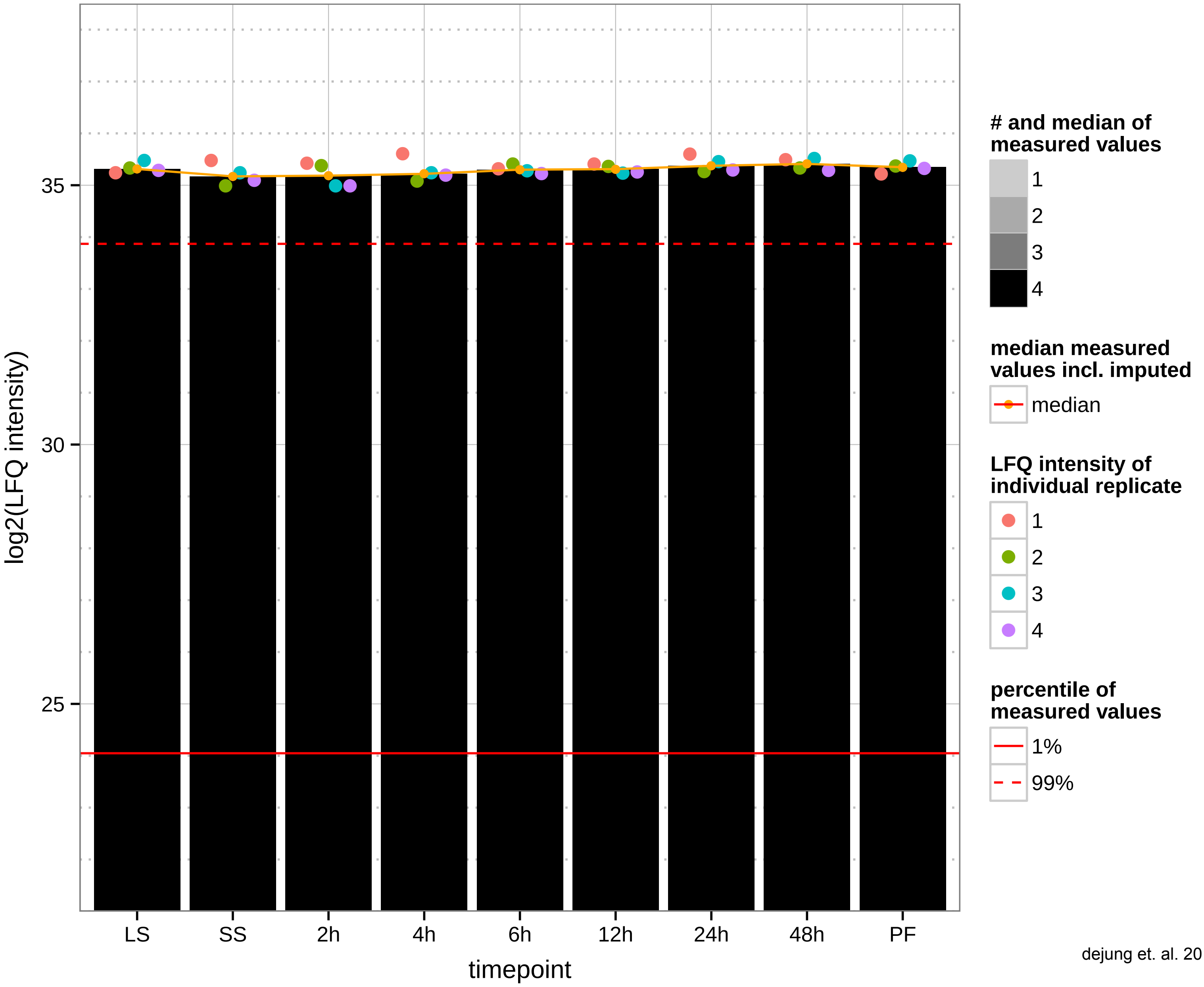

dejung et. al. 2015

protein kinase, putative
Tb927.10.350;Tb11.v5.0564
AGOF: null, ATP binding, protein kinase activity, protein serine/threonine kinase activity, protein tyrosine kinase activity
AGOC: null
AGOP: null, protein phosphorylation
PGOF: ATP binding, protein kinase activity, transferase activity, transferring phosphorus–containing groups
PGOC: null
PGOP: protein phosphorylation

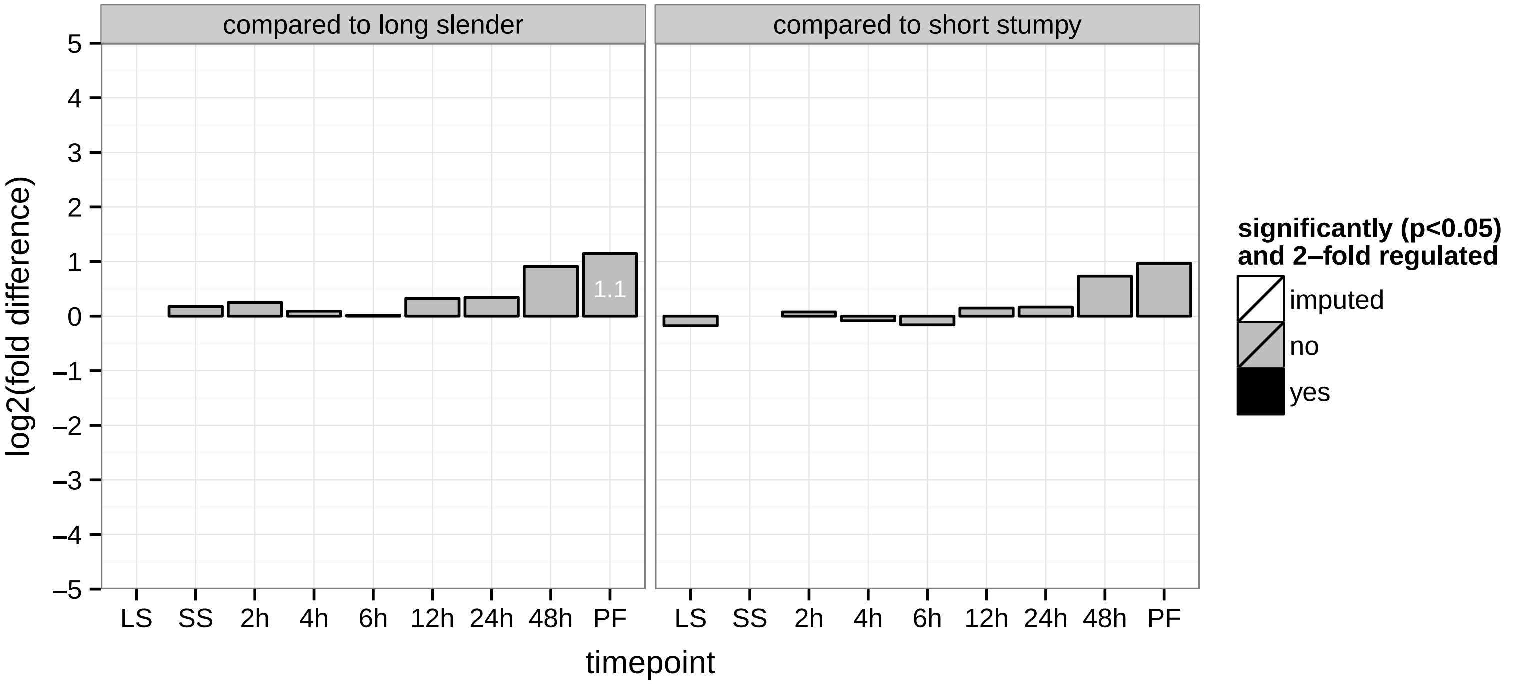

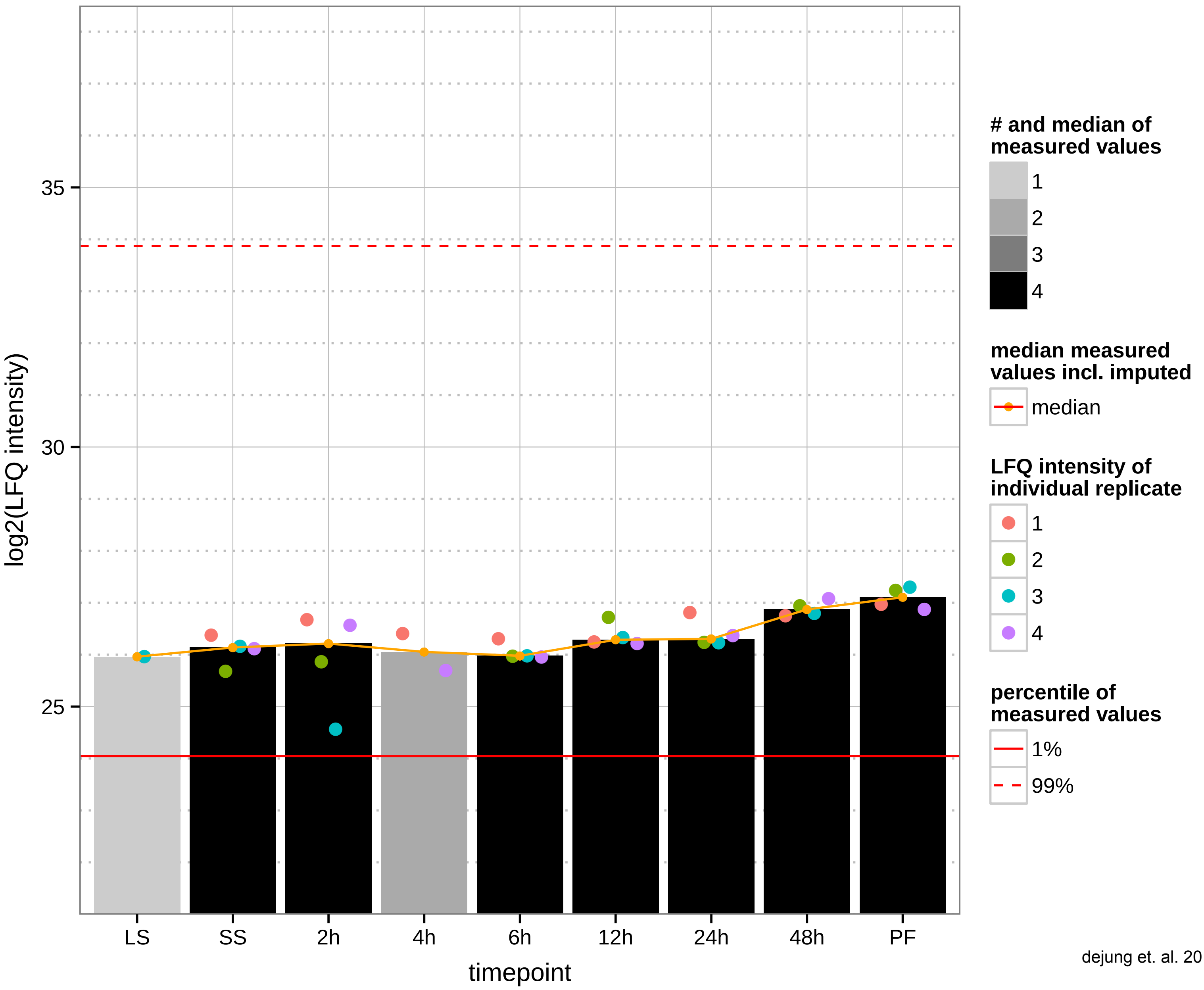

dejung et. al. 2015

hypothetical protein, conserved
Tb927.7.5620;Tb11.v5.0590
AGOF: null, zinc ion binding
AGOC: null
AGOP: null
PGOF: protein binding, zinc ion binding
PGOC: null
PGOP: null

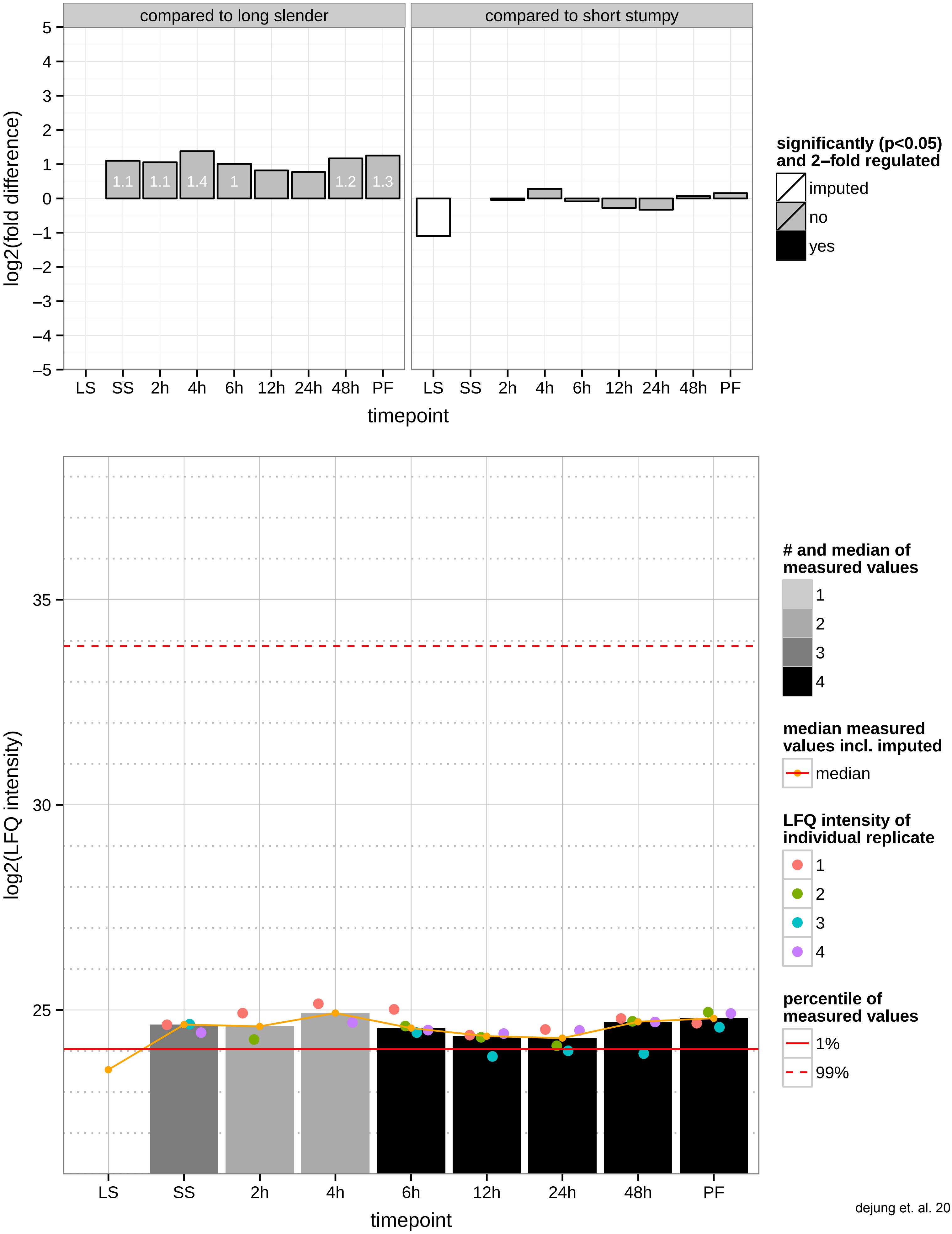

dejung et. al. 2015

hypothetical protein, conserved, guide RNA associated protein, GAP2, mitochondrial RNA binding protein 1
Tb927.7.2570;Tb11.v5.0611
AGOF: null, mRNA binding
AGOC: null, mitochondrion
AGOP: null, mitochondrial RNA processing
PGOF: null
PGOC: null
PGOP: null

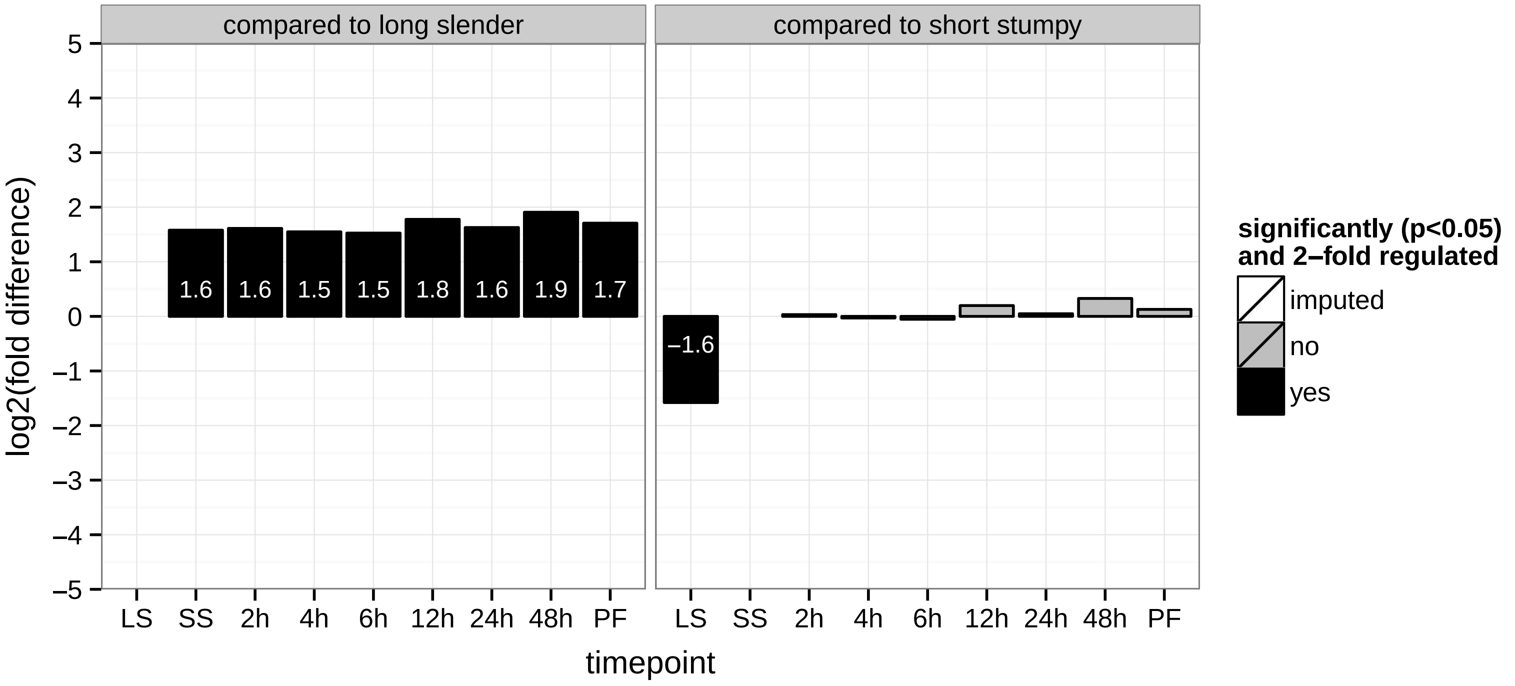

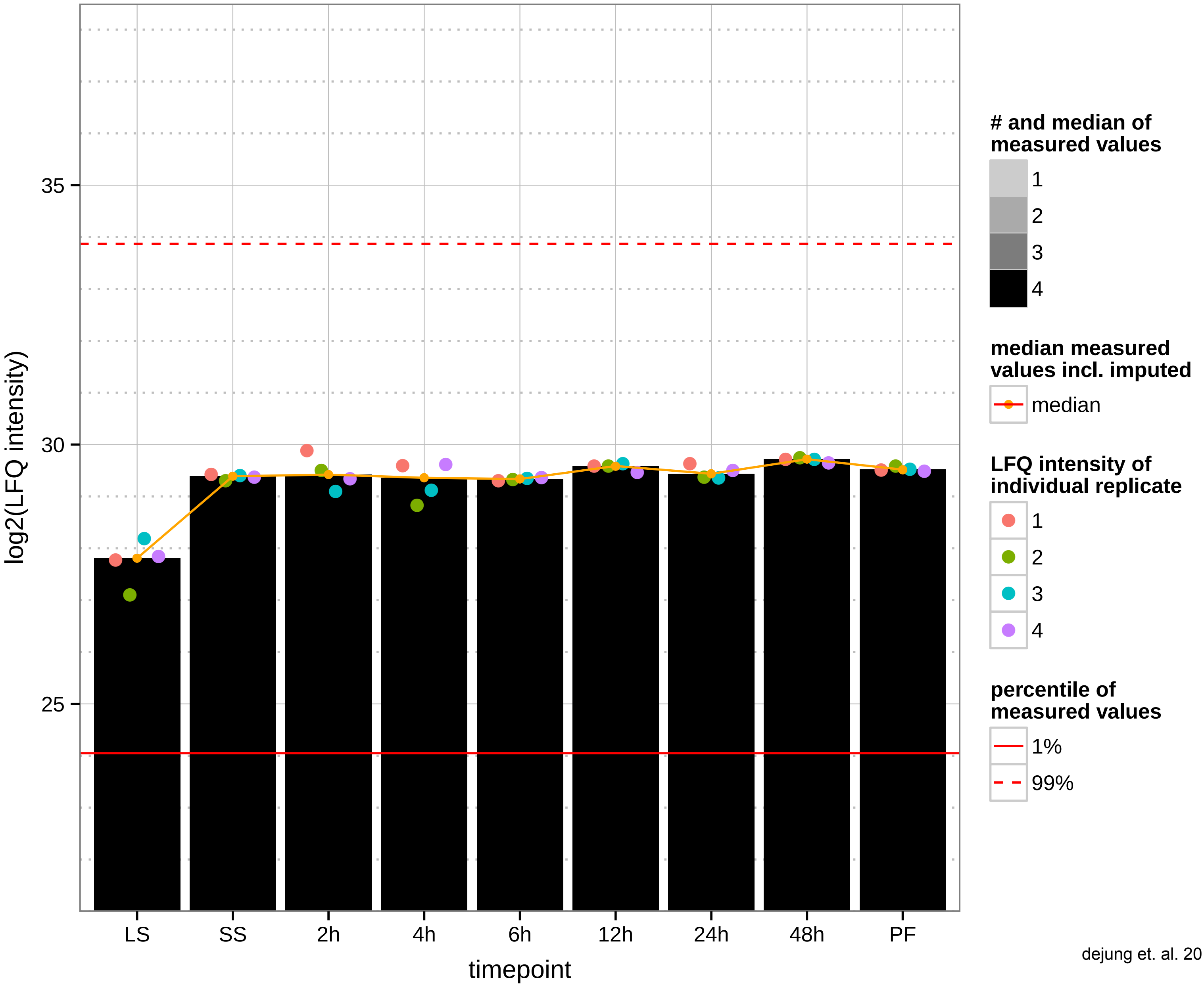

dejung et. al. 2015

hypothetical protein, conserved
Tb927.7.7070;Tb11.v5.0612
AGOF: null, zinc ion binding
AGOC: null
AGOP: null
PGOF: zinc ion binding
PGOC: null
PGOP: null

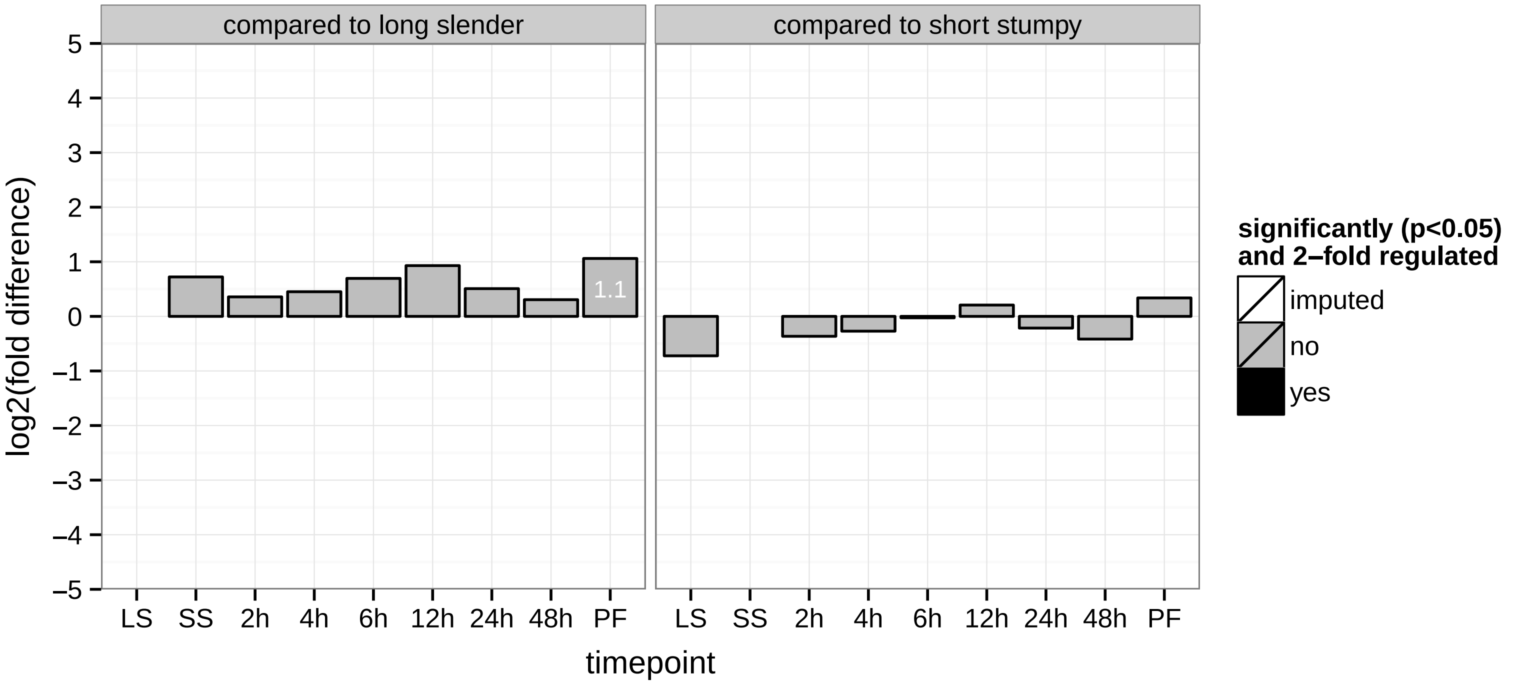

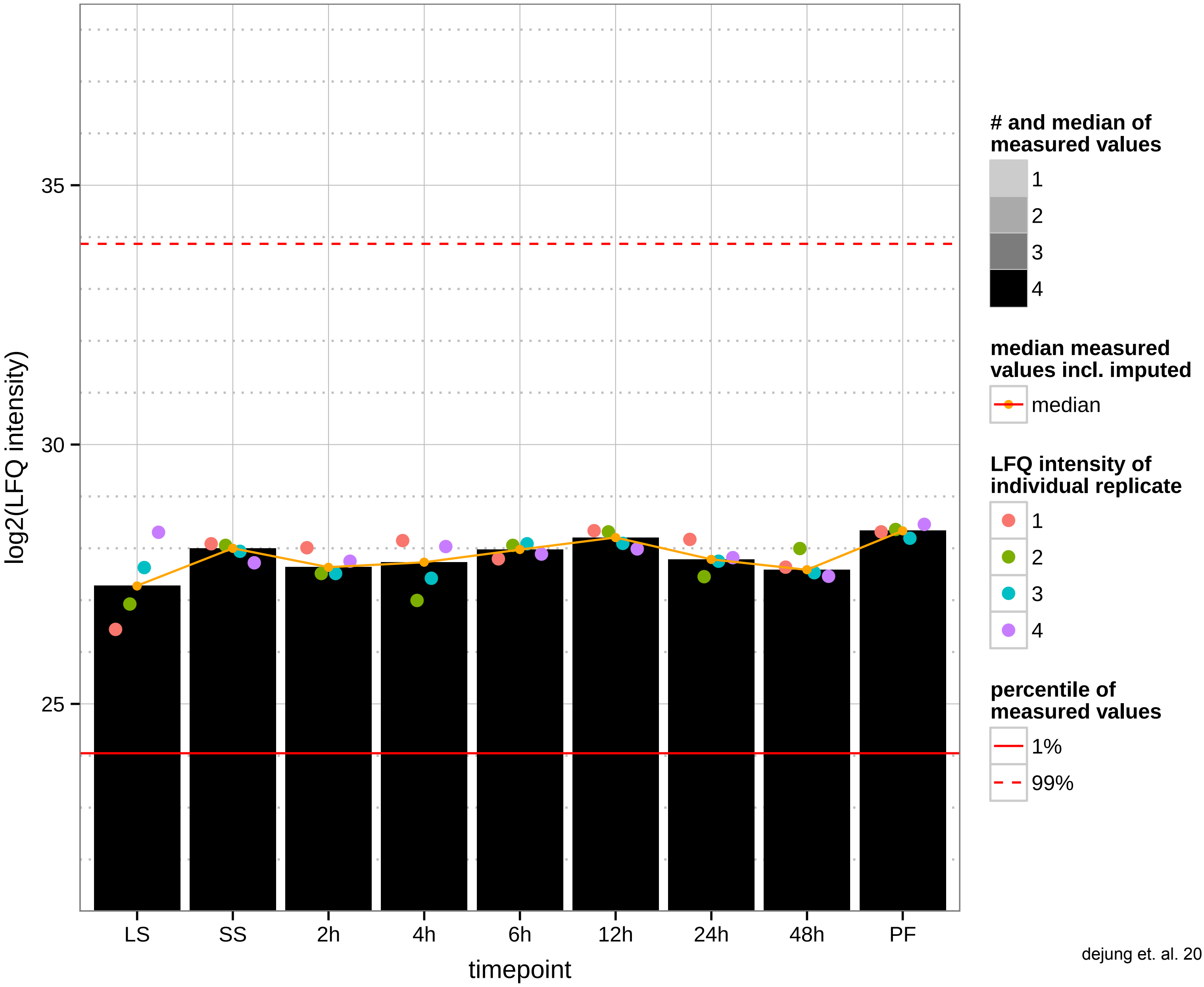

dejung et. al. 2015

paraflagellar rod protein, putative
Tb11.v5.0616
AGOF: null
AGOC: null
AGOP: null
PGOF: calmodulin binding
PGOC: microtubule–based flagellum
PGOP: null

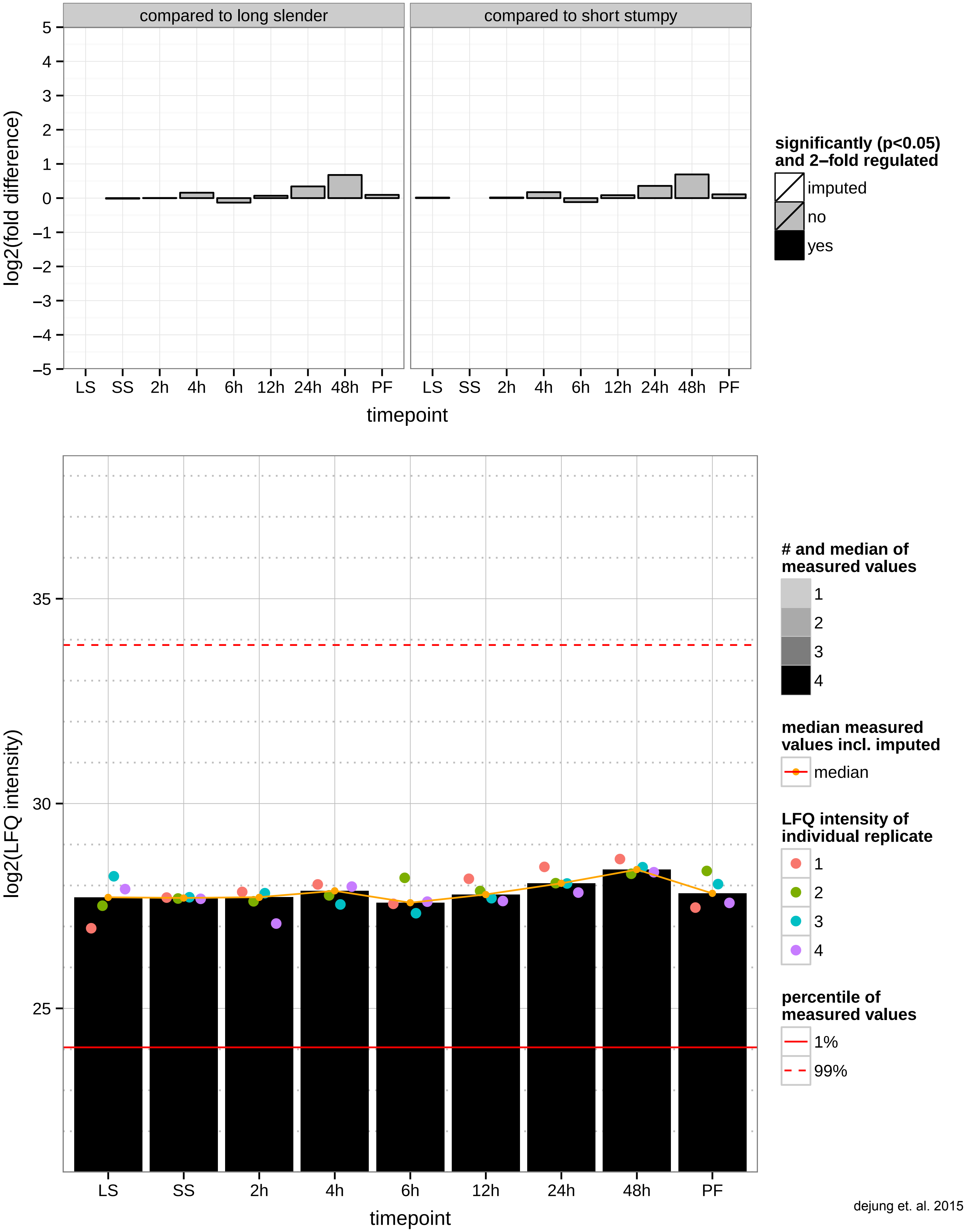

dejung et. al. 2015

retrotransposon hot spot protein (PHS), putative
Tb11.v5.0624
AGOF: null
AGOC: null
AGOP: null
PGOF: null
PGOC: null
PGOP: null

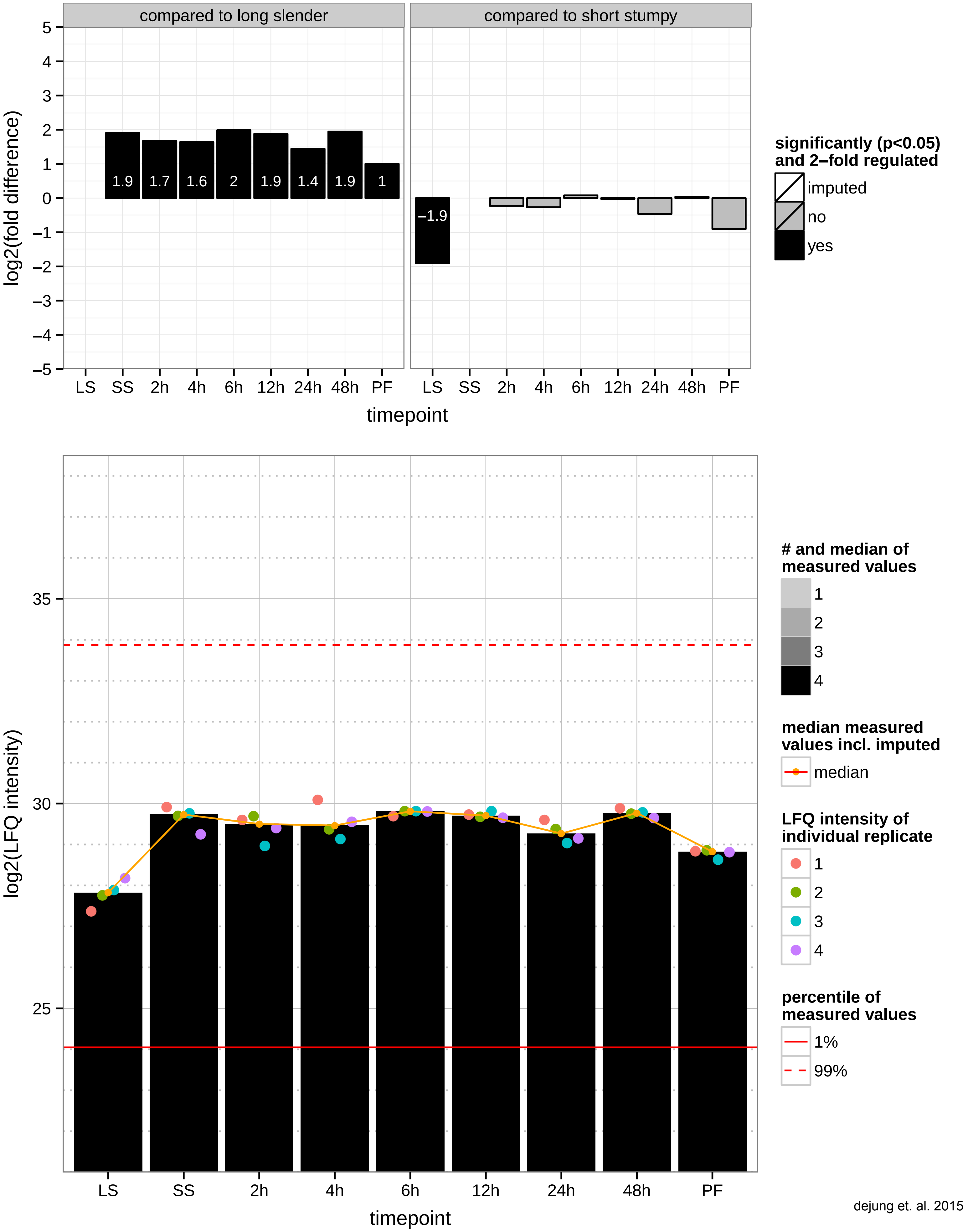

dejung et. al. 2015

hypothetical protein, conserved
Tb927.5.1230;Tb11.v5.0639
AGOF: null
AGOC: null
AGOP: null
PGOF: null
PGOC: null
PGOP: null

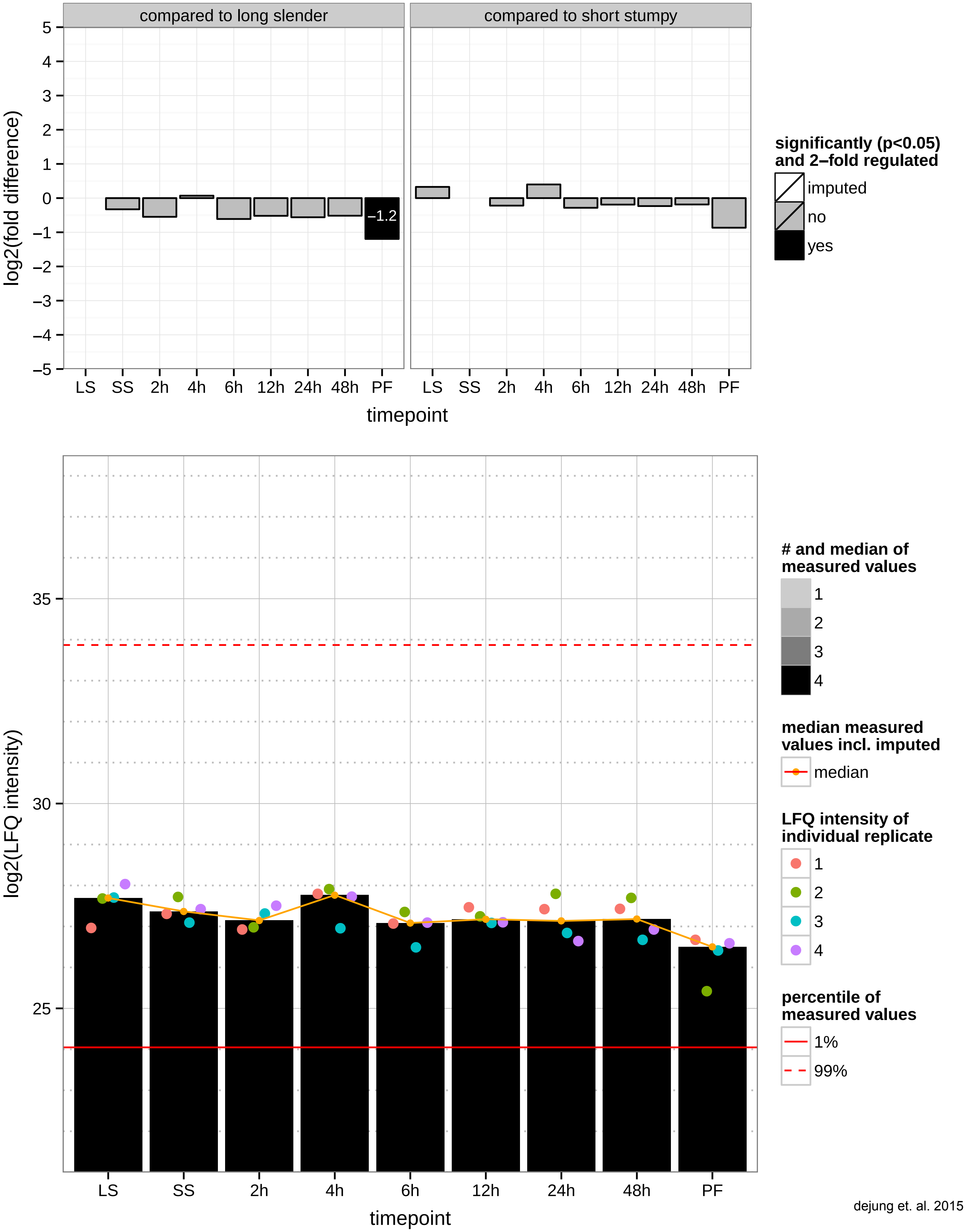

dejung et. al. 2015

enolase, putative
Tb927.10.2890;Tb11.v5.0650
AGOF: null, magnesium ion binding, phosphopyruvate hydratase activity
AGOC: null, phosphopyruvate hydratase complex
AGOP: null
PGOF: magnesium ion binding, phosphopyruvate hydratase activity
PGOC: phosphopyruvate hydratase complex
PGOP: glycolysis

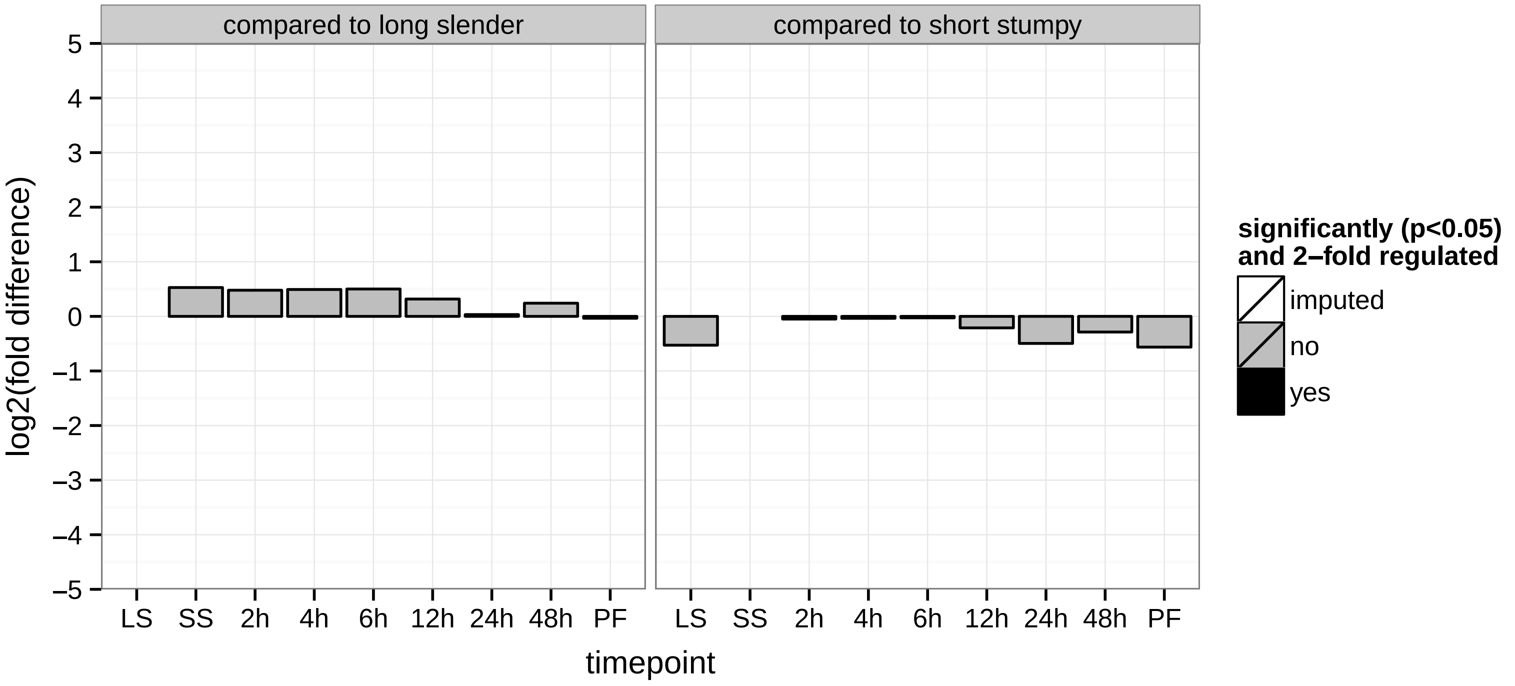

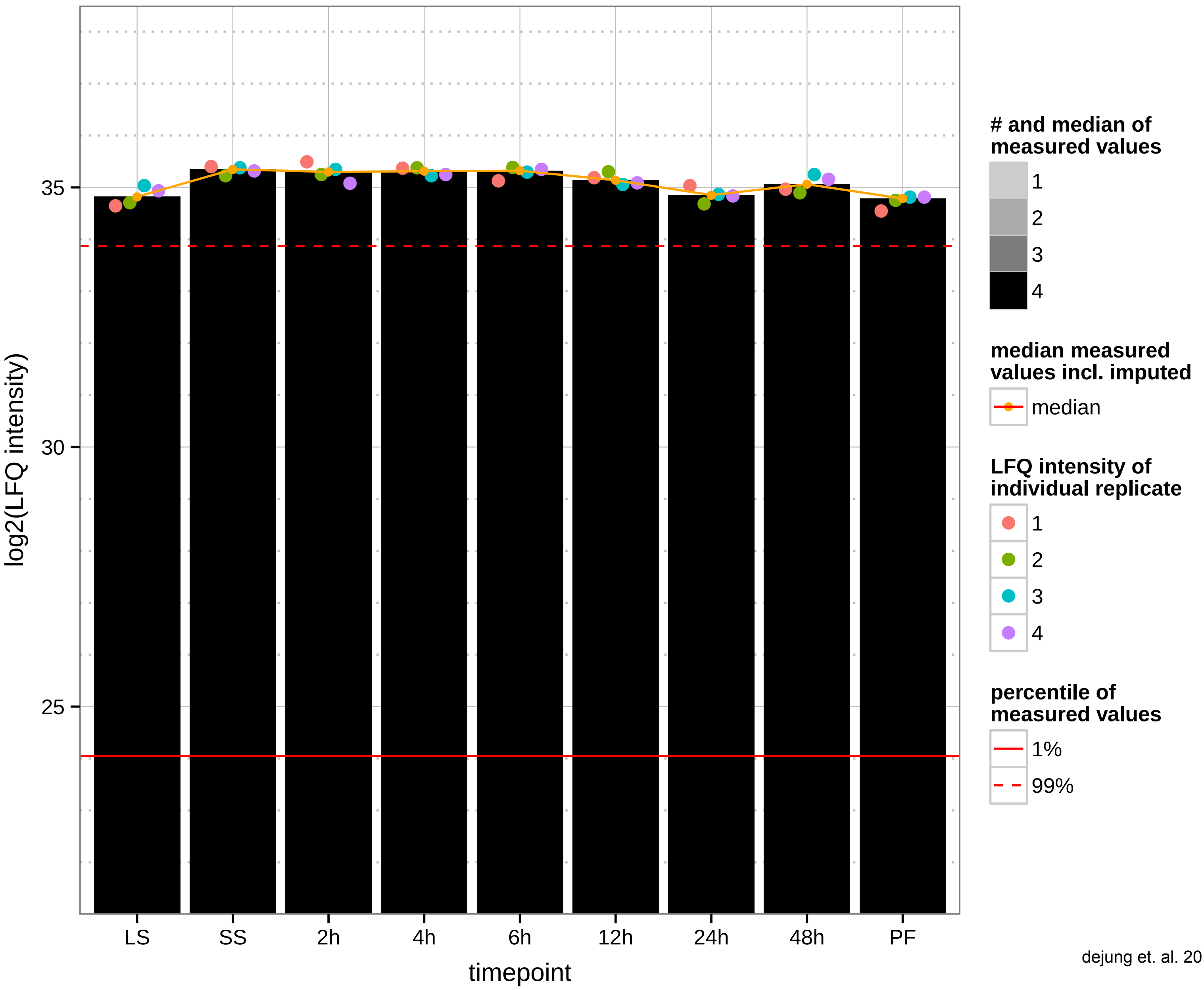

dejung et. al. 2015

hypothetical protein, conserved, retrotransposon hot spot protein (RHS, pseudogene), putative, retrotransposon hot spot prote
Tb927.2.830;Tb11.v5.0655
AGOF: null
AGOC: null
AGOP: null
PGOF: null
PGOC: null
PGOP: null

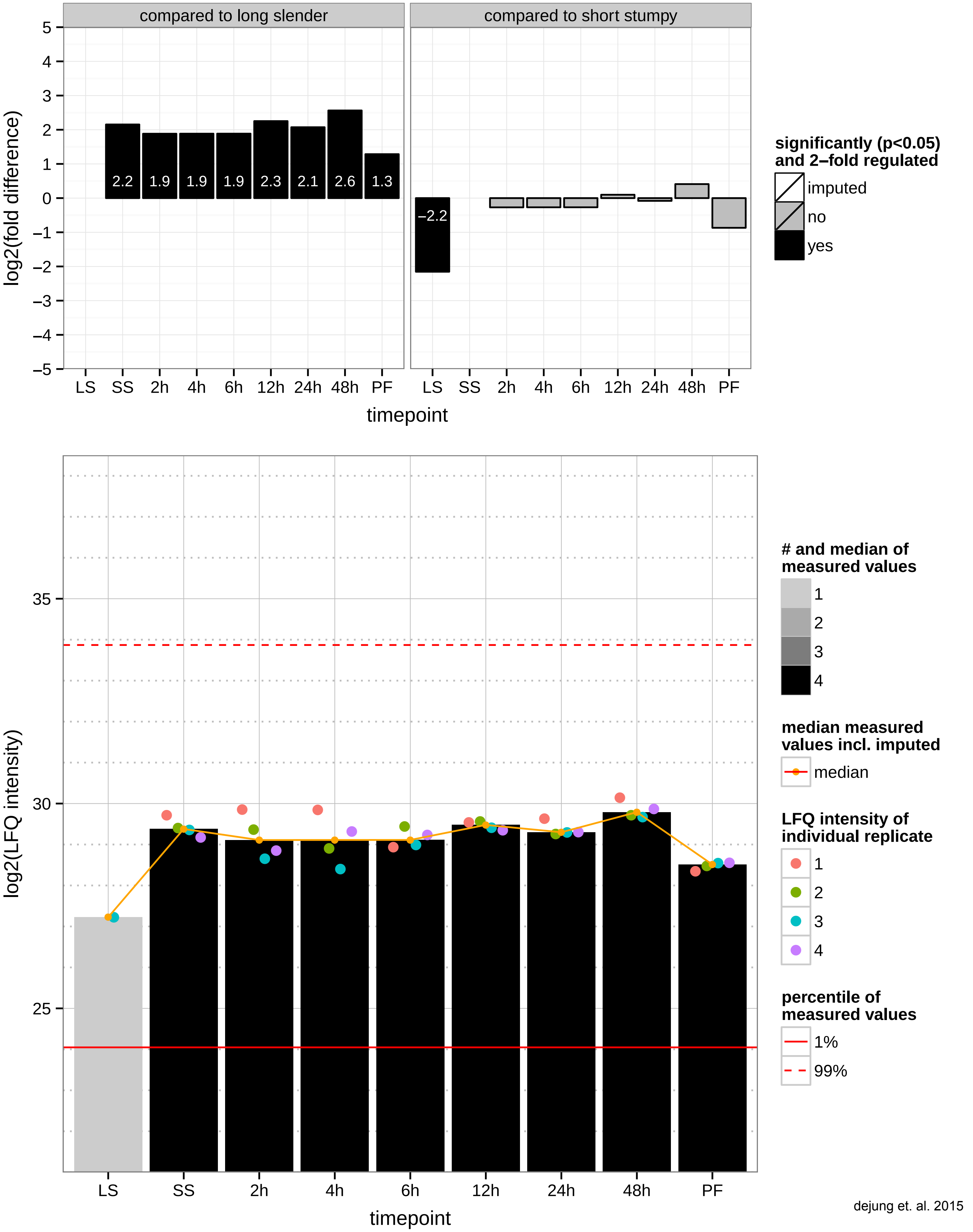

dejung et. al. 2015

histone deacetylase, putative, histone deacetylase 3 (HDAC3)
Tb11.v5.0660;Tb927.2.2190
AGOF: null, histone deacetylase activity, zinc ion binding
AGOC: null, nucleus
AGOP: null, negative regulation of chromatin silencing, regulation of transcription, DNA−dependent
PGOF: null
PGOC: null
PGOP: null

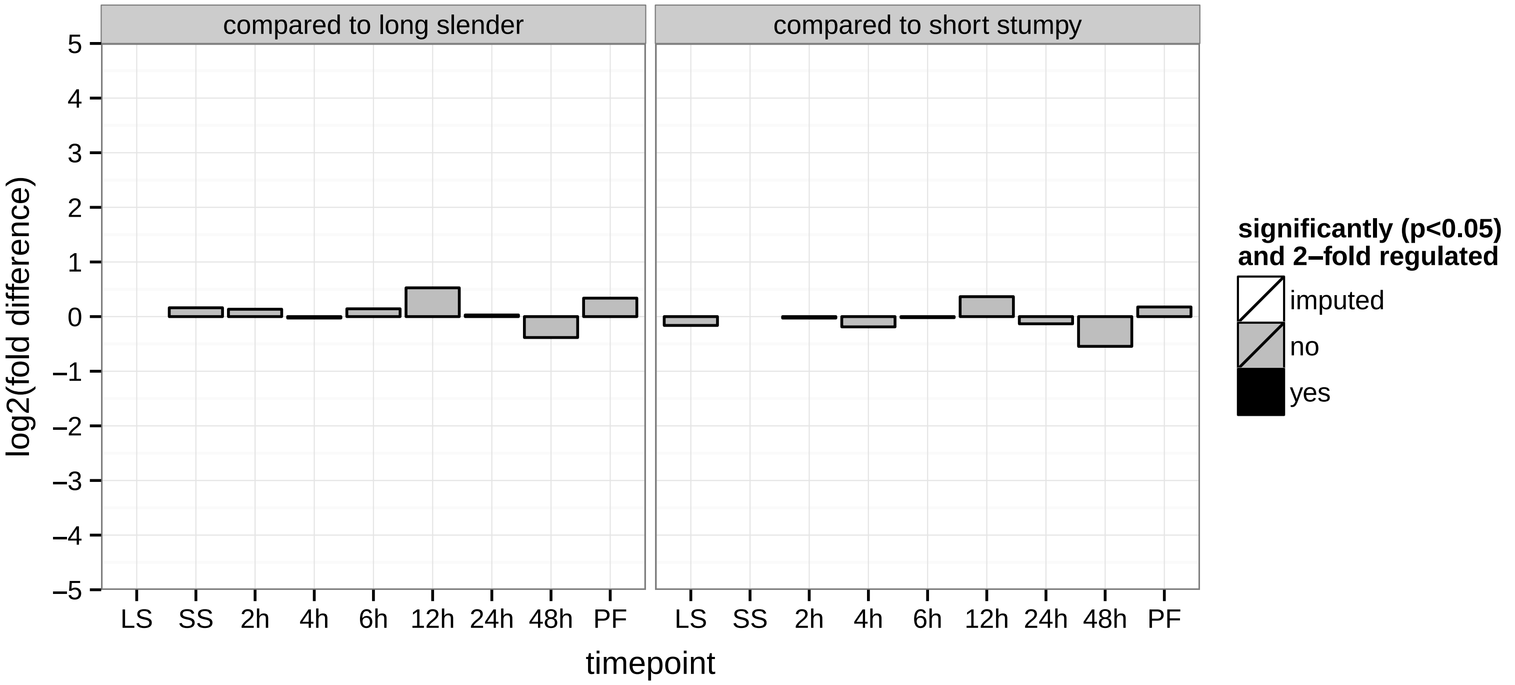

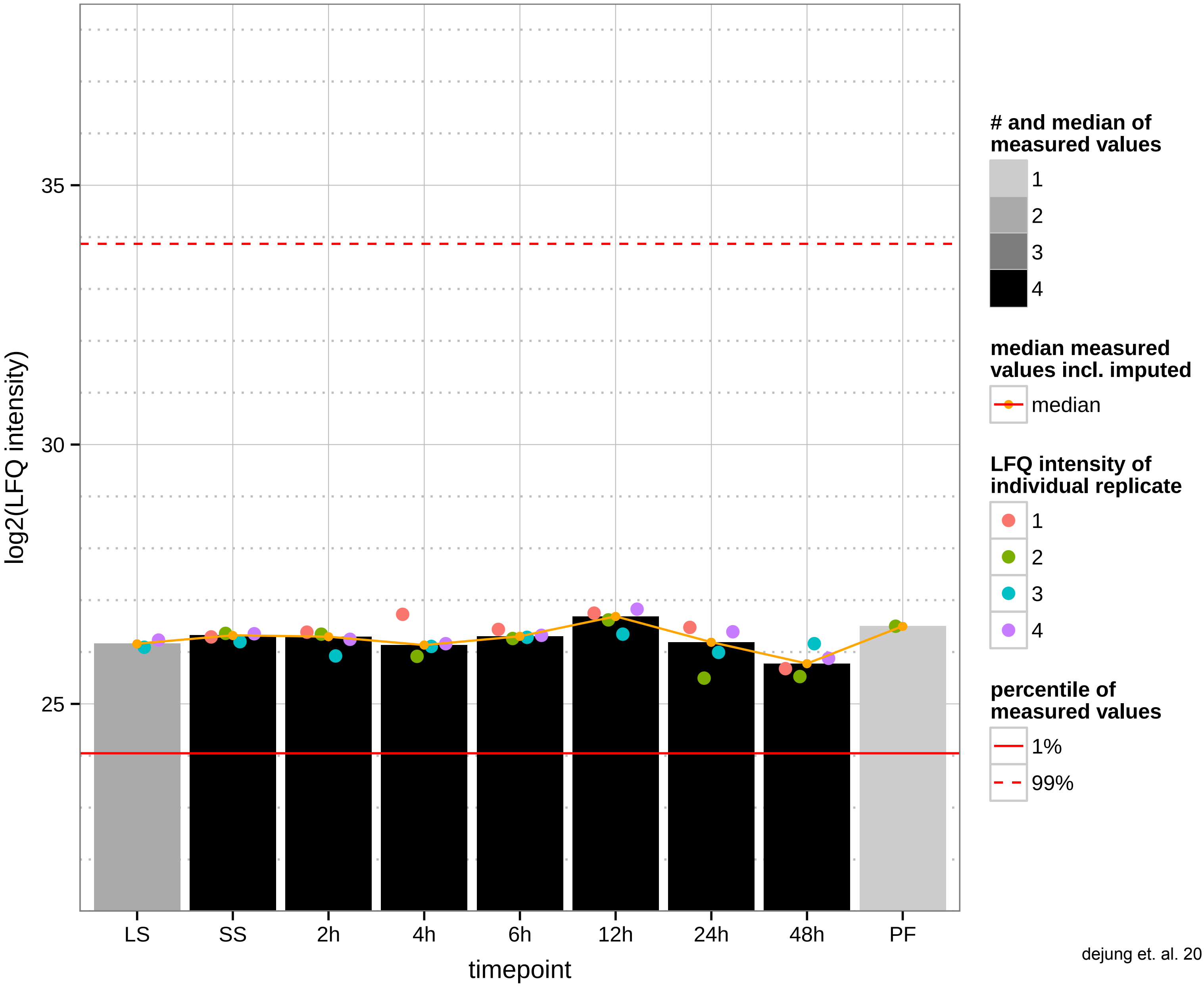

dejung et. al. 2015

retrotransposon hot spot (RHS) protein, putative
Tb11.v5.0670
AGOF: null
AGOC: null
AGOP: null
PGOF: null
PGOC: null
PGOP: null

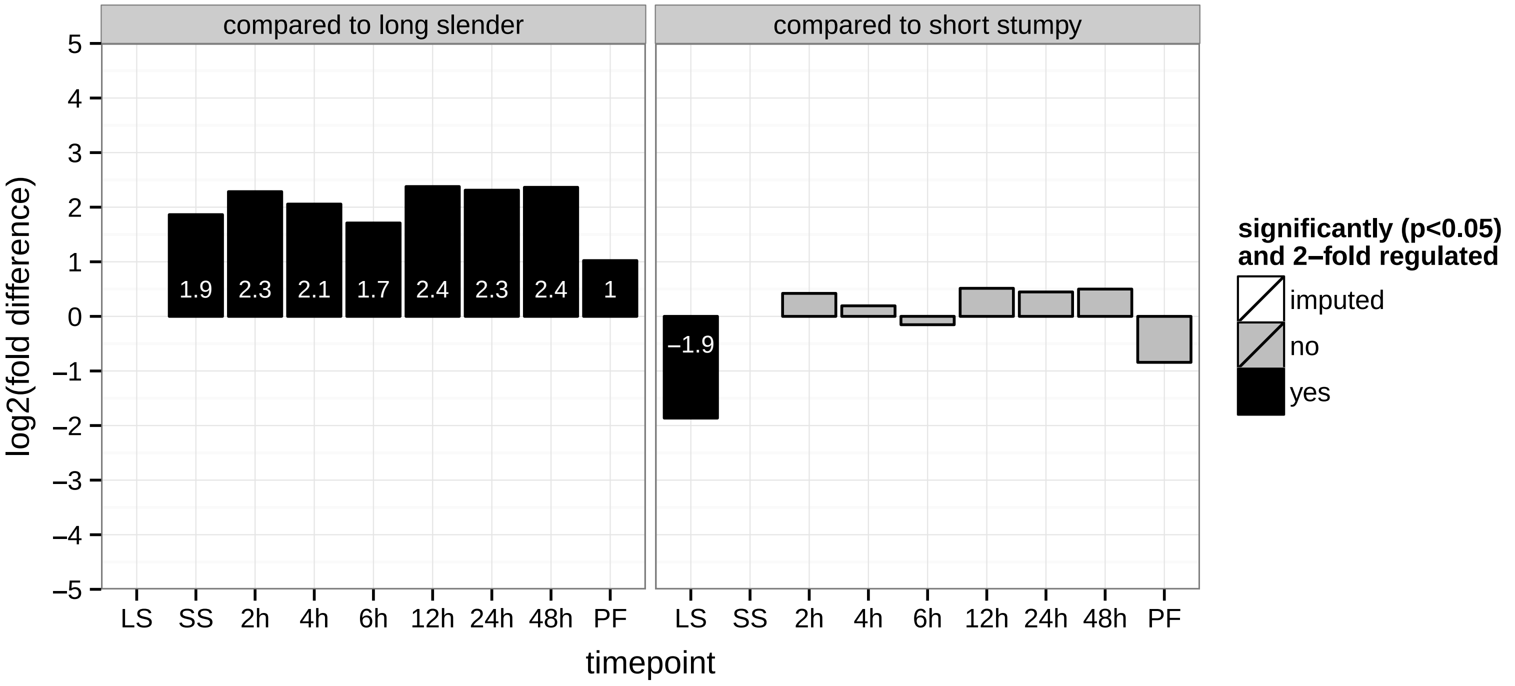

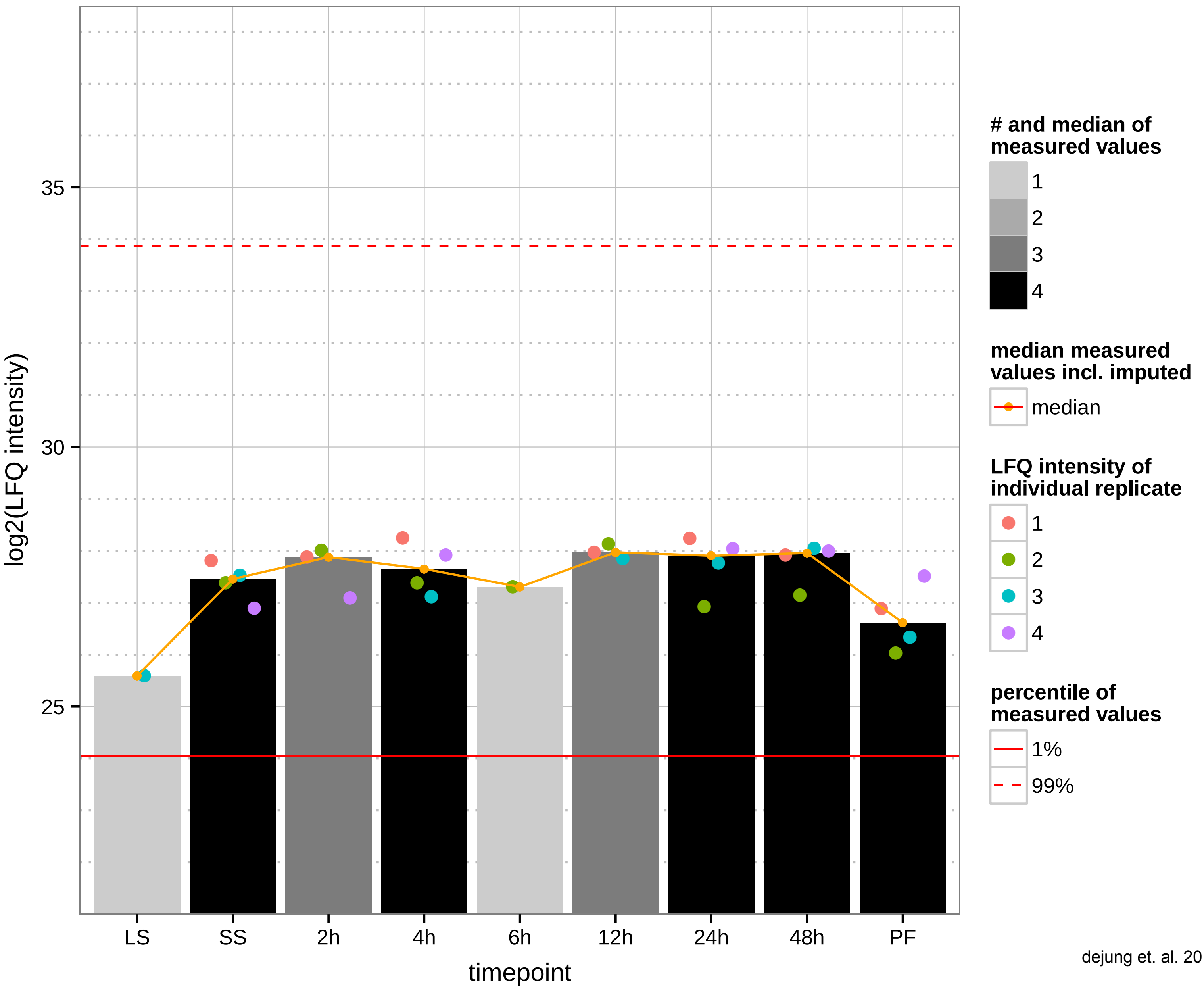

dejung et. al. 2015

hypothetical protein, conserved
Tb11.v5.0692;Tb927.5.3000
AGOF: null, thiopurine S–methyltransferase activity
AGOC: null, cytoplasm, mitochondrion
AGOP: null, metabolic process
PGOF: S–adenosylmethionine–dependent methyltransferase activity
PGOC: null
PGOP: null

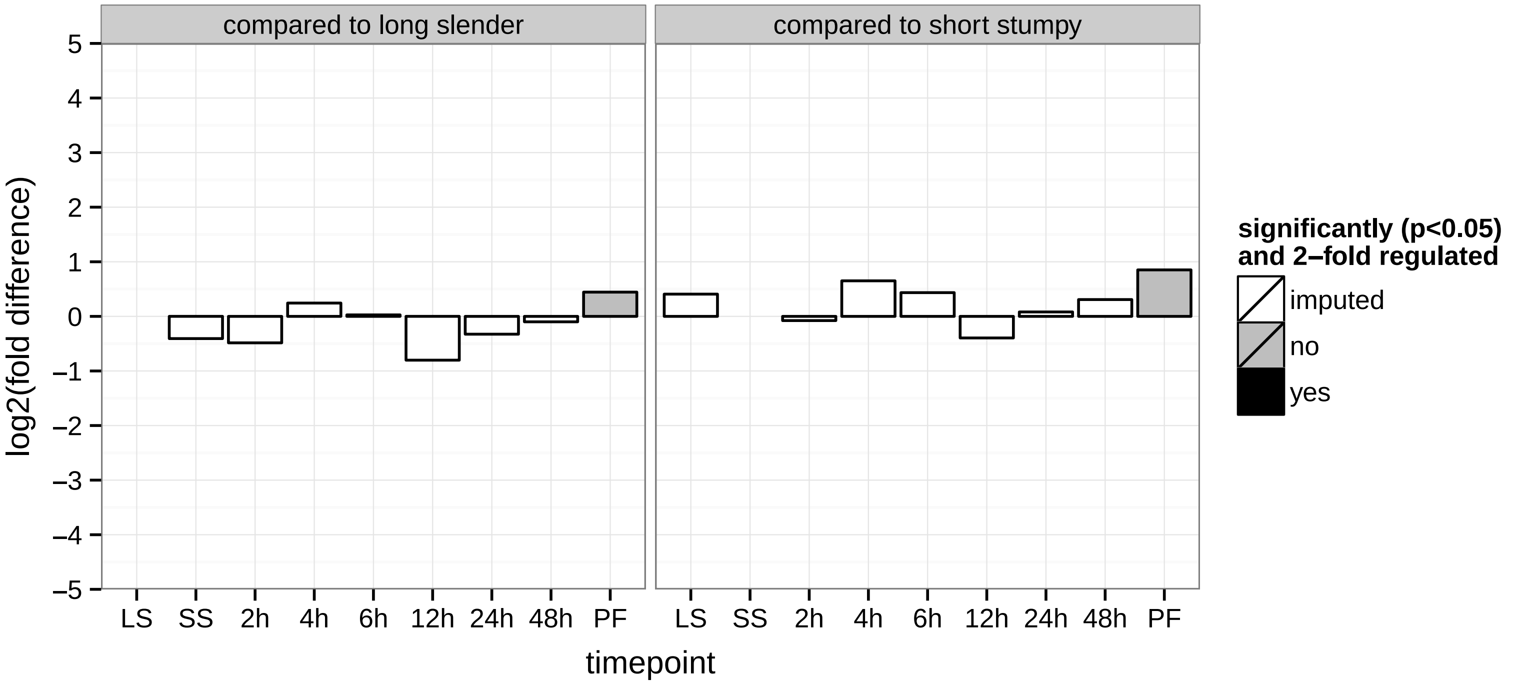

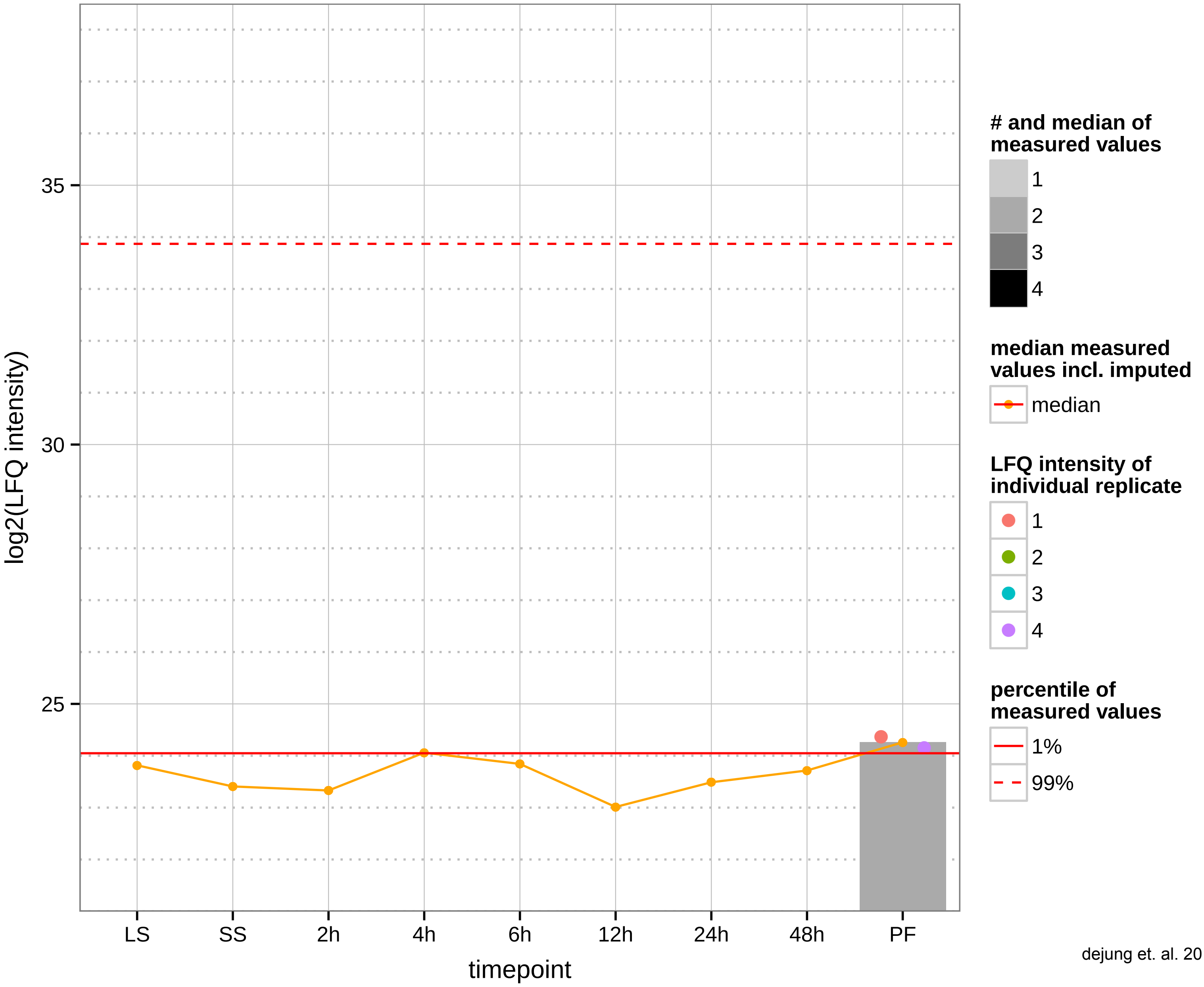

dejung et. al. 2015

hypothetical protein, conserved
Tb927.10.8650;Tb11.v5.0707
AGOF: null
AGOC: null
AGOP: null, intracellular transport
PGOF: null
PGOC: null
PGOP: intracellular transport

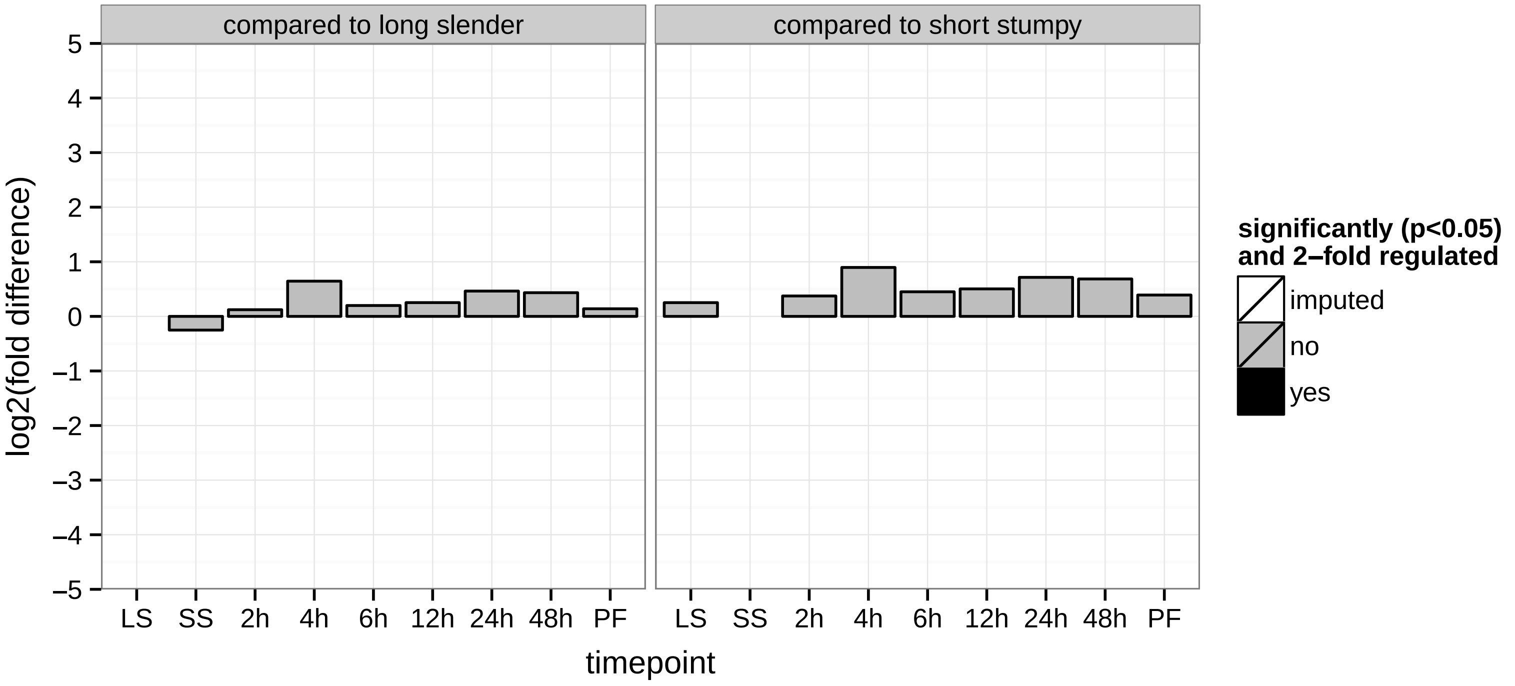

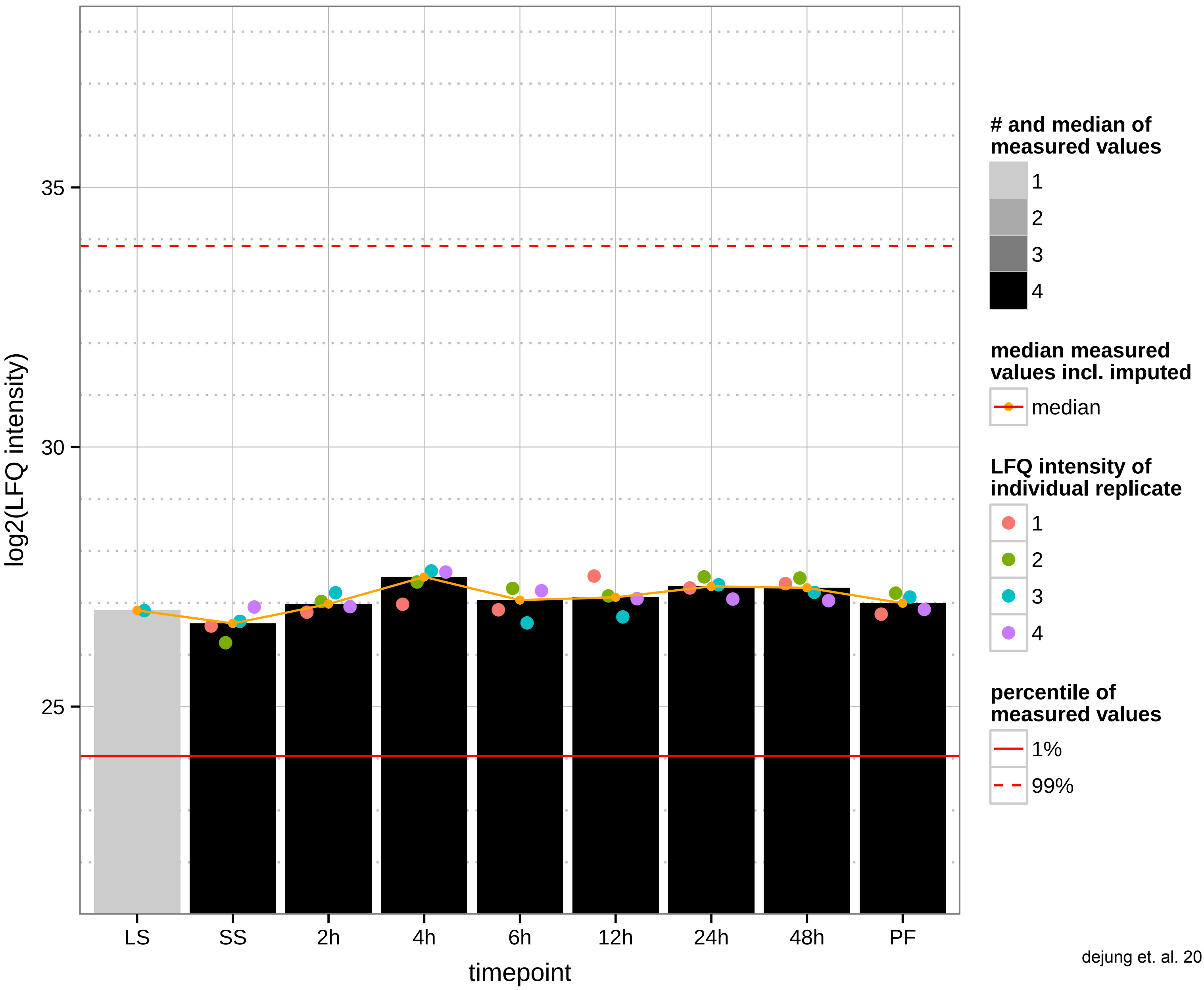

dejung et. al. 2015

protein kinase, putative, glycogen synthase kinase 3 (GSK3), glycogen synthase kinase−3 alpha
Tb927.10.13780;Tb11.v5.0718
AGOF: null, ATP binding, protein serine/threonine kinase activity, protein tyrosine kinase activity, protein kinase activity
AGOC: null
AGOP: null, growth, protein phosphorylation, protein phosphorylation
PGOF: ATP binding, protein kinase activity, transferase activity, transferring phosphorus−containing groups
PGOC: null
PGOP: protein phosphorylation

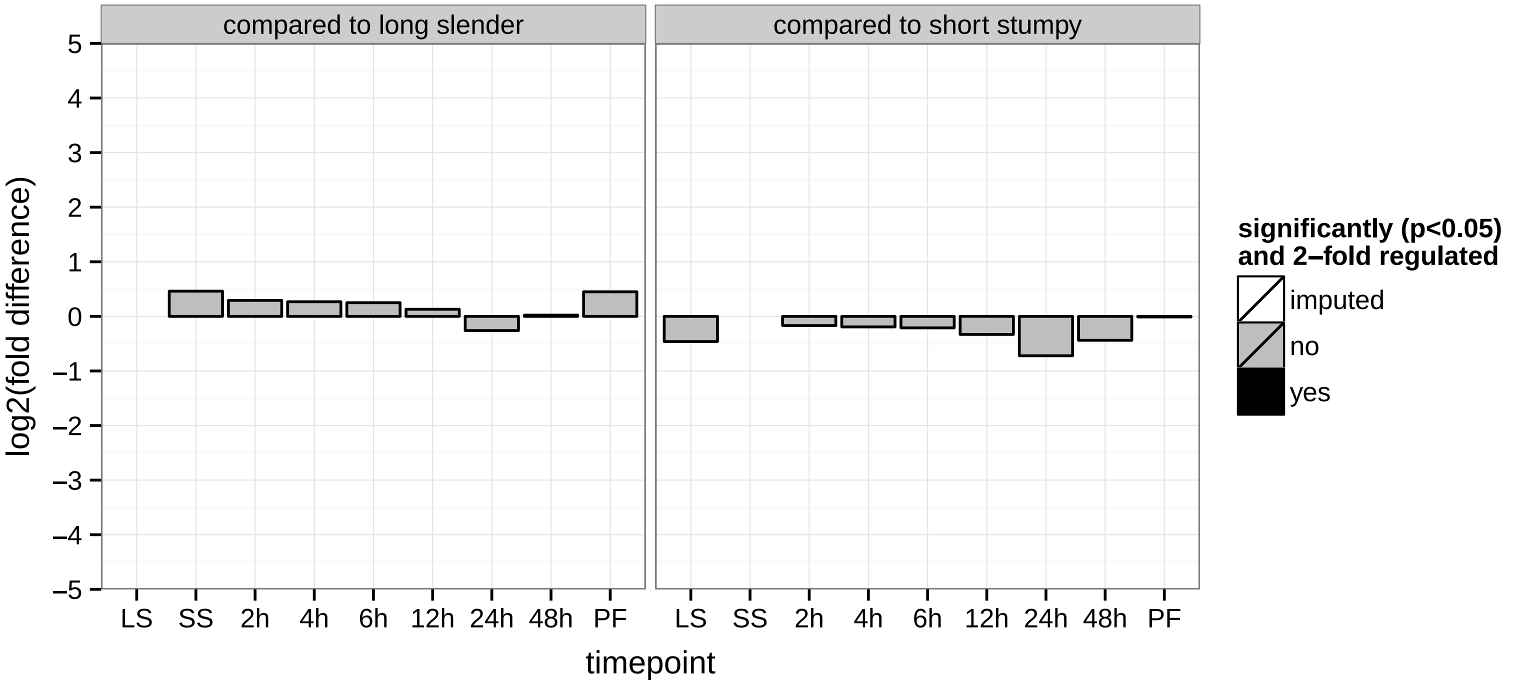

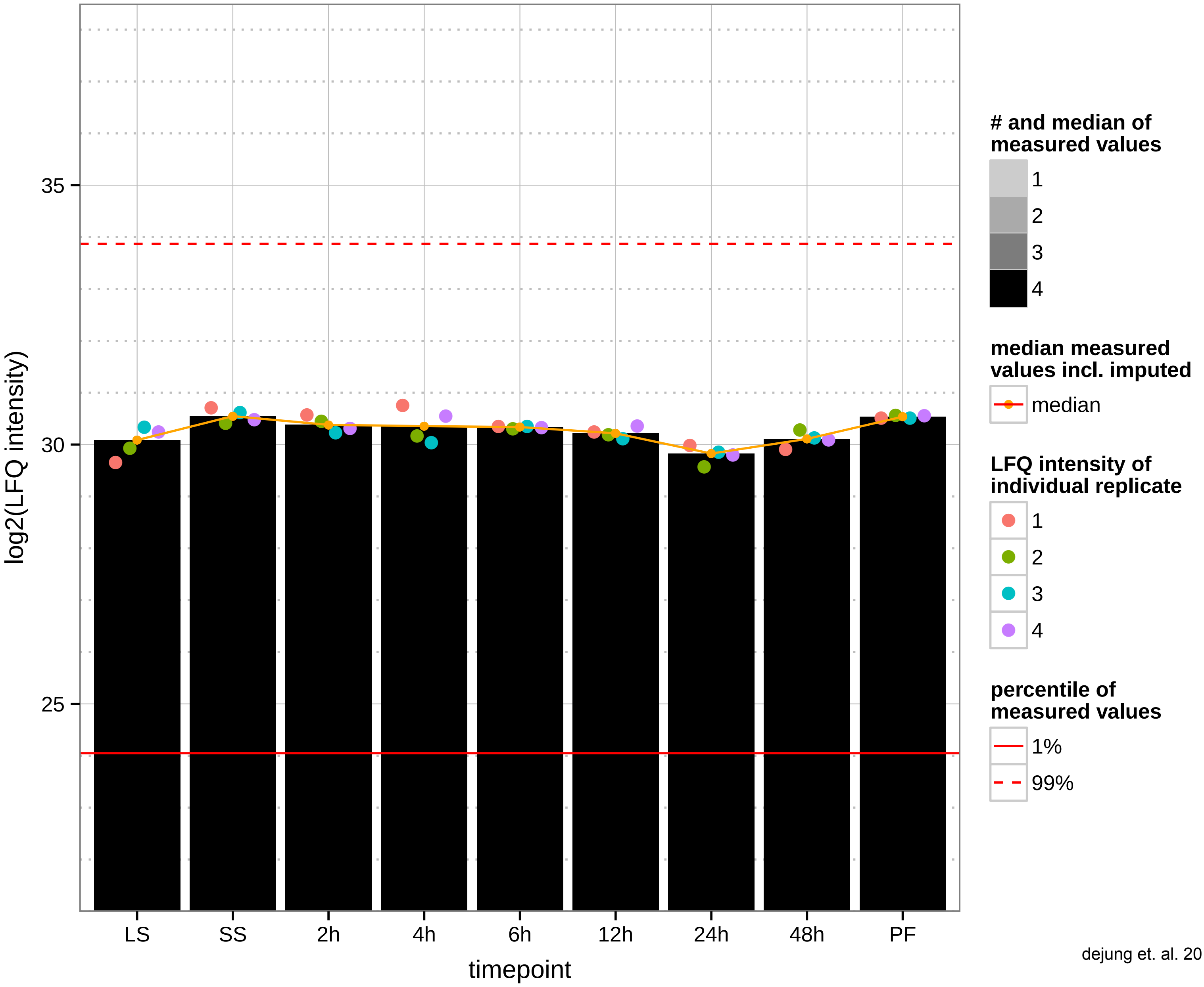

dejung et. al. 2015

hypothetical protein, conserved, paraflagellar rod component, putative (PFC6)
Tb927.3.3790;Tb927.3.3770;Tb11.v5.0741
AGOF: null
AGOC: null
AGOP: null
PGOF: null
PGOC: null
PGOP: null

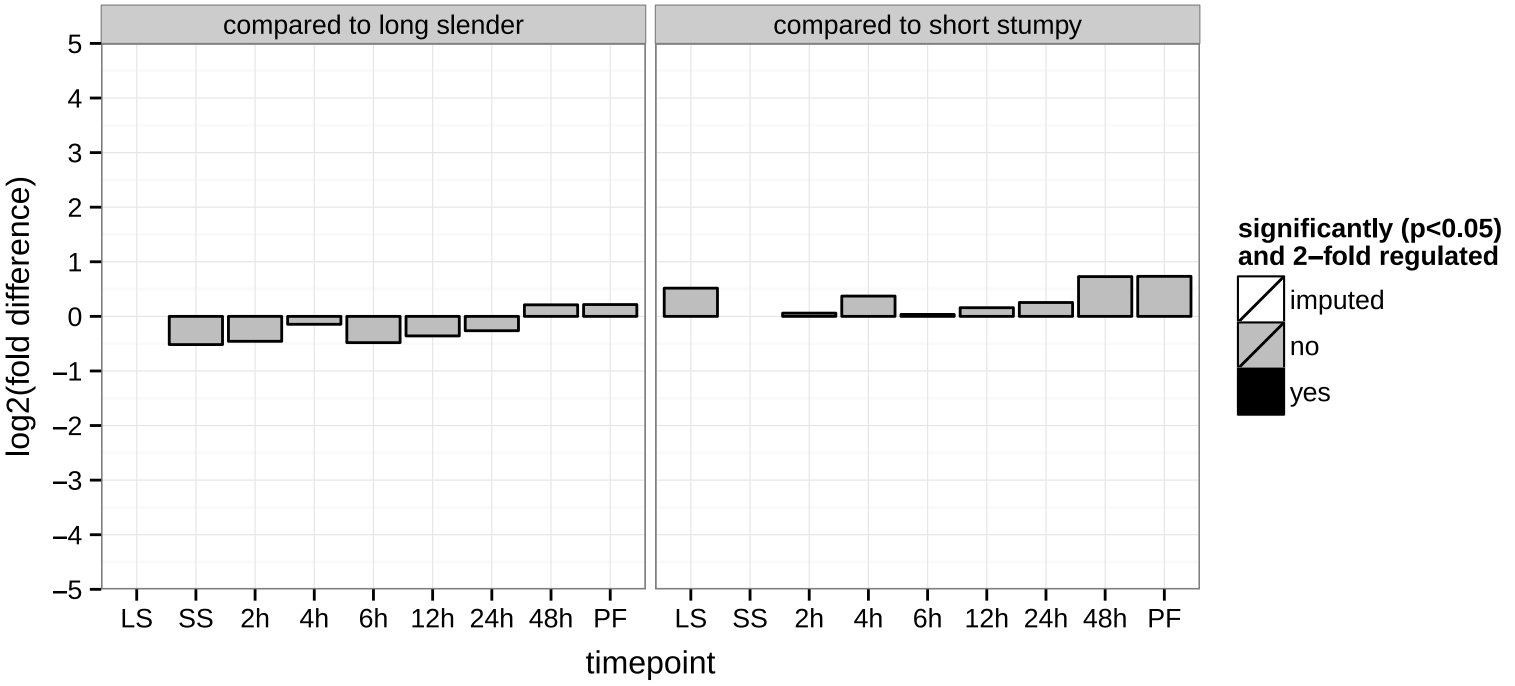

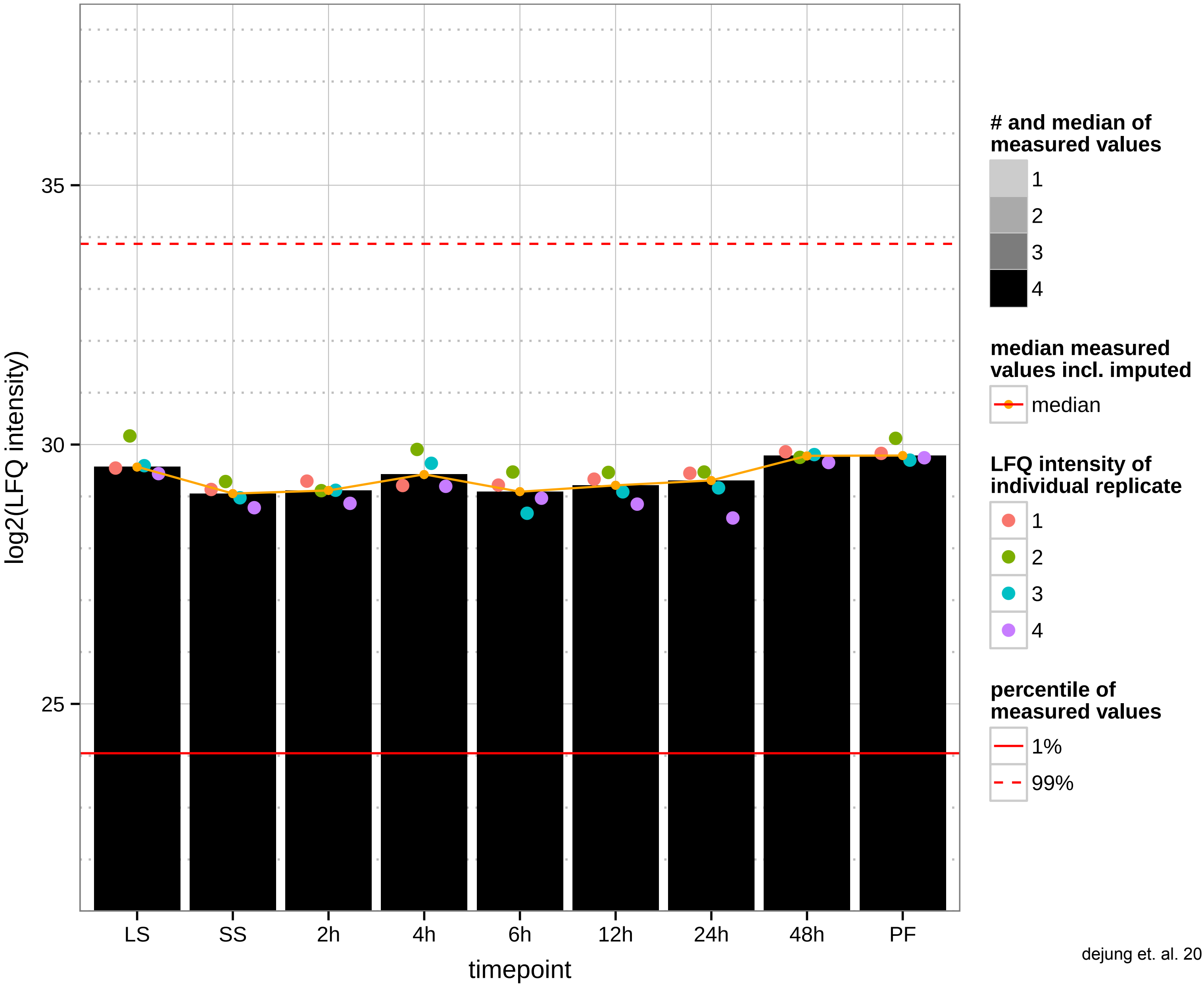

dejung et. al. 2015

proteasome beta−1 subunit, putative, putative (TbPSB1)
Tb927.6.1260;Tb11.v5.0742
AGOF: null, peptidase activity, acting on L−amino acid peptides, threonine−type endopeptidase activity
AGOC: null, proteasome core complex, proteasome core complex, beta−subunit complex
AGOP: null, protein metabolic process, proteolysis involved in cellular protein catabolic process
PGOF: threonine−type endopeptidase activity
PGOC: proteasome core complex
PGOP: proteolysis involved in cellular protein catabolic process

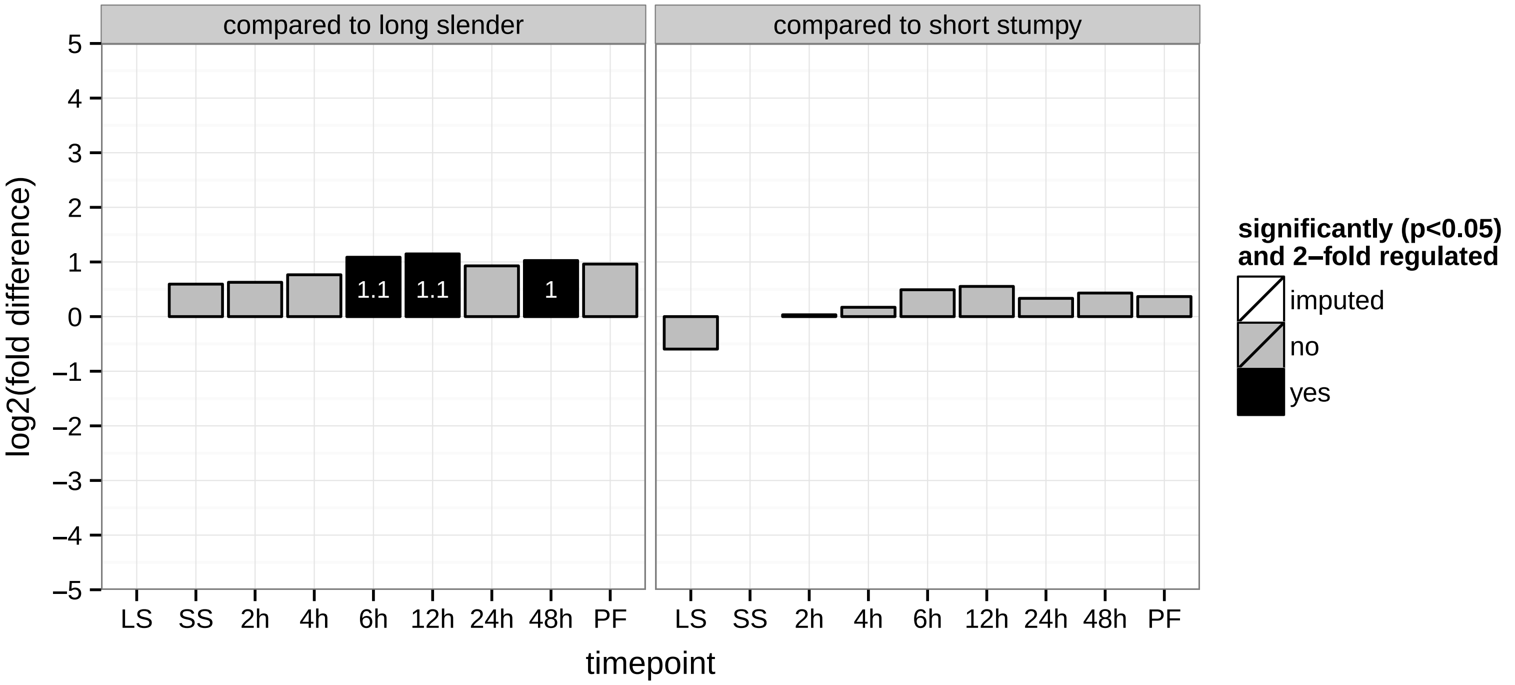

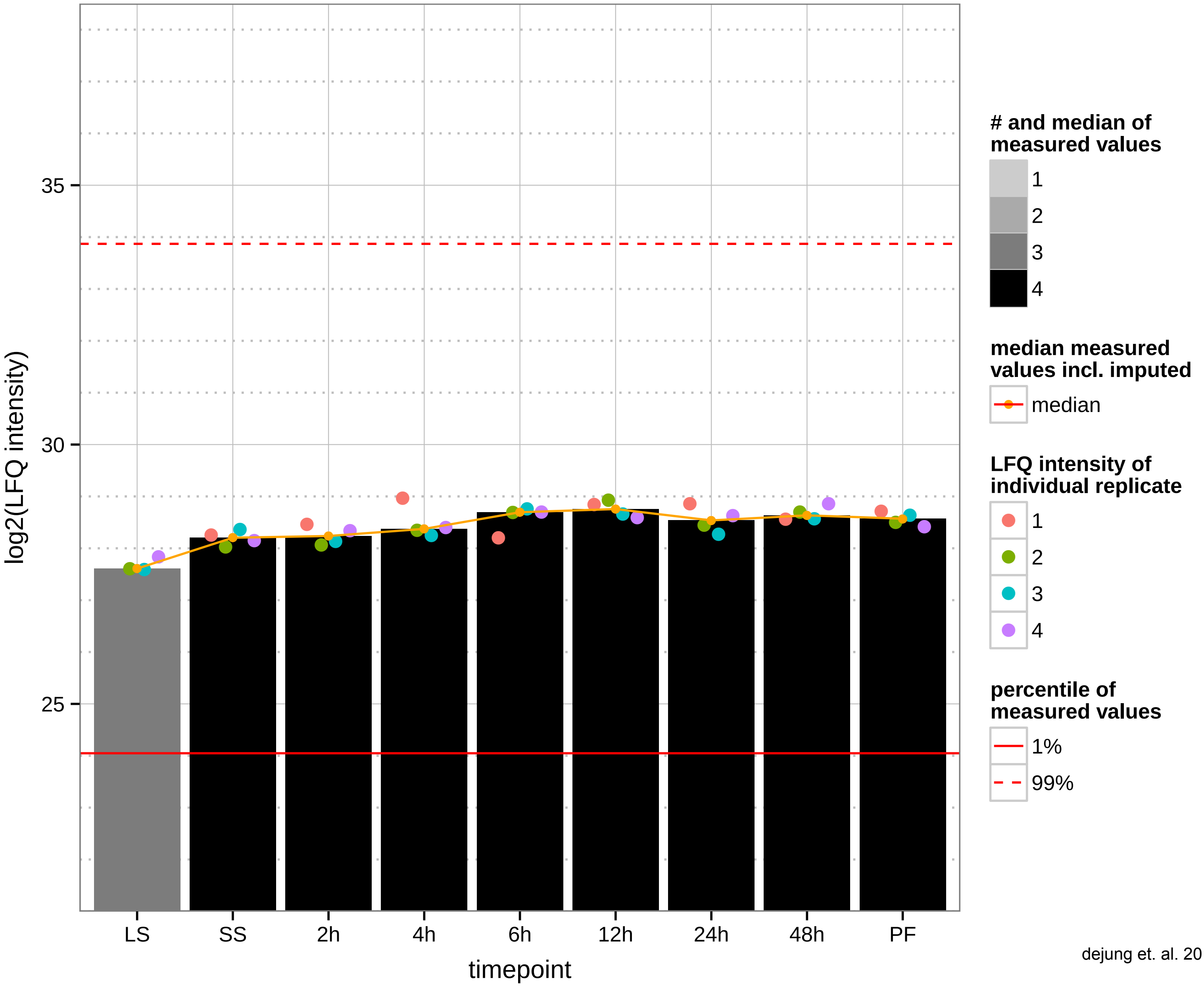

dejung et. al. 2015

ribonuclease, putative, Poly(A)–specific ribonuclease PARN–3 (parn3)
Tb11.v5.0807;Tb927.9.13510
AGOF: null, nucleic acid binding
AGOC: null, nucleus
AGOP: null
PGOF: nucleic acid binding
PGOC: nucleus
PGOP: null

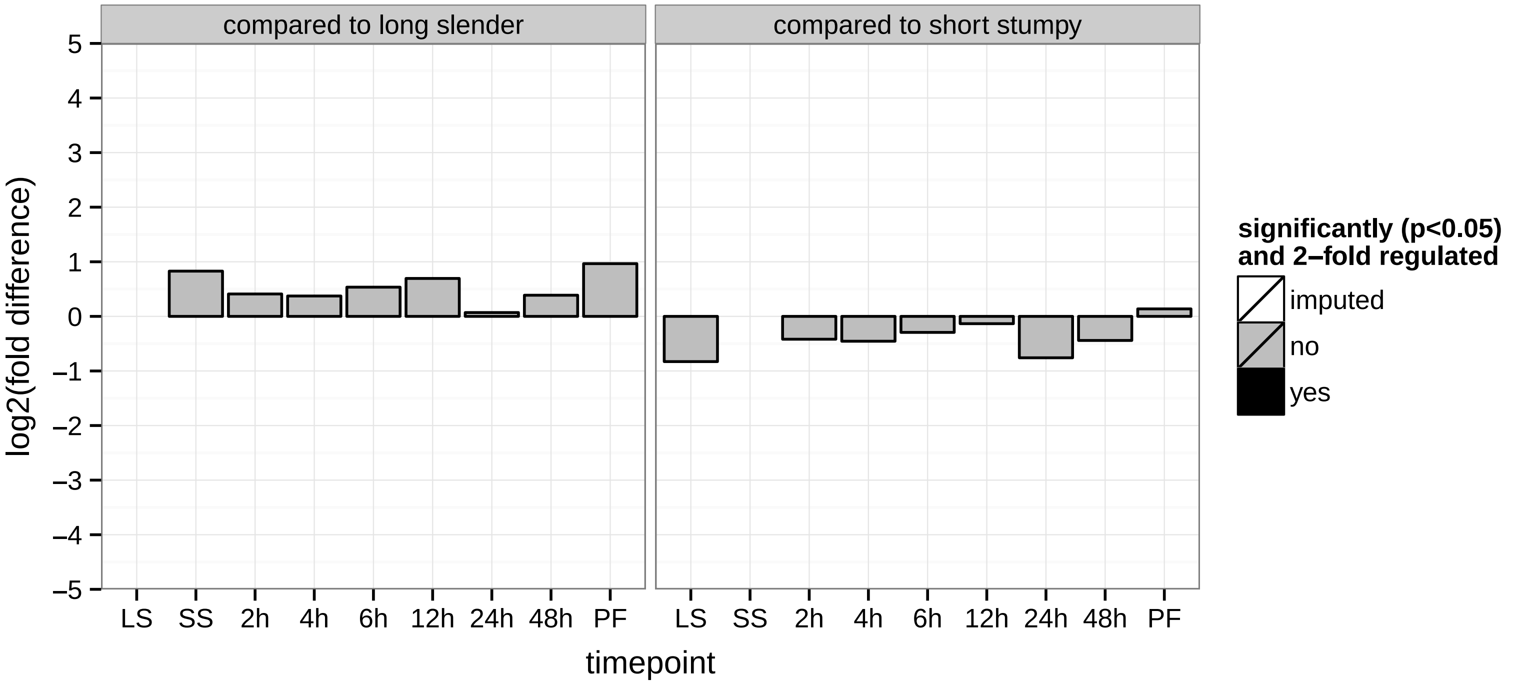

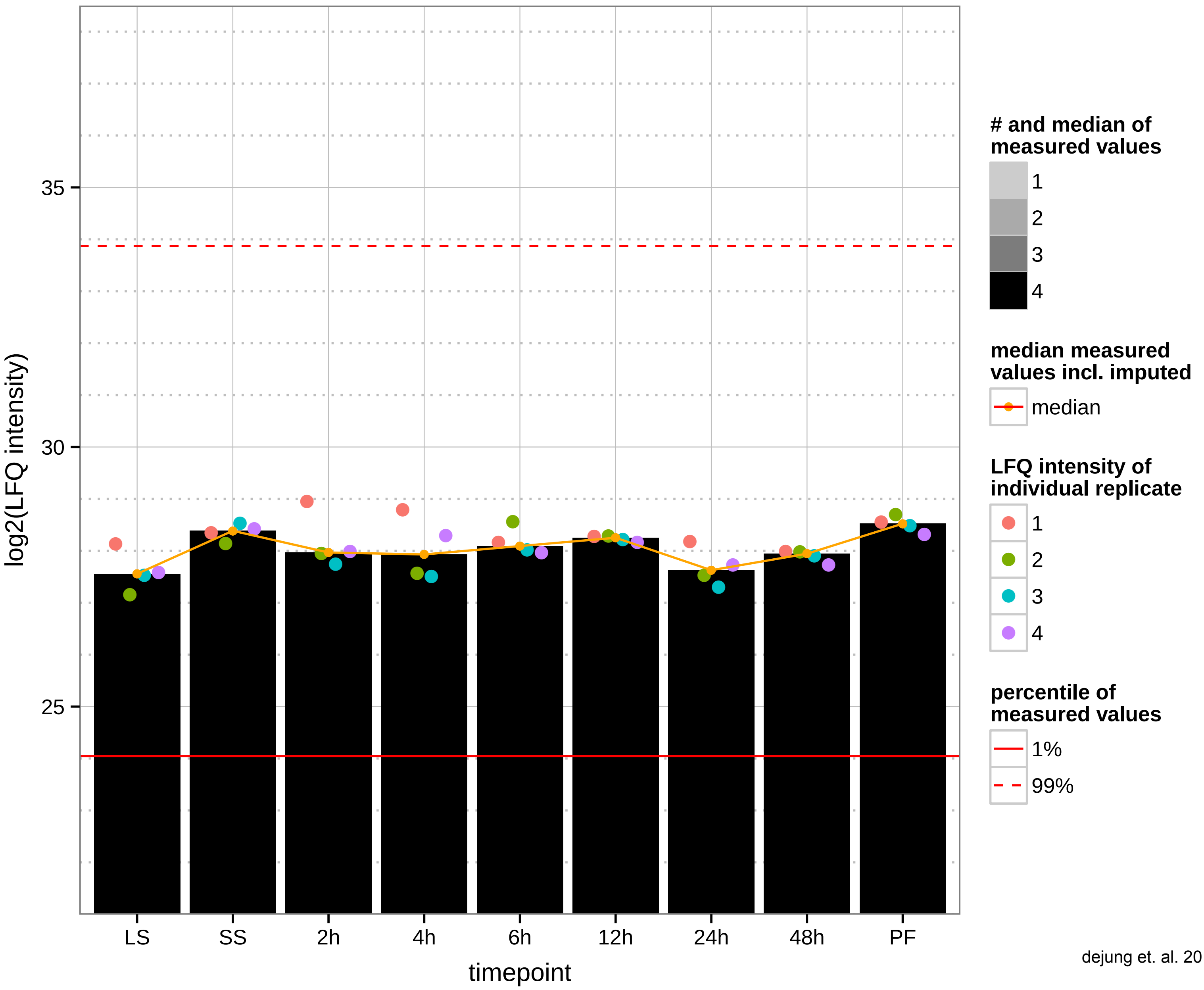

dejung et. al. 2015

peroxin–2, putative, glycosome import protein (gim1) (PEX2)
Tb927.3.2340;Tb11.v5.0818
AGOF: null, zinc ion binding
AGOC: null, glycosome membrane, peroxisomal membrane
AGOP: null, glycolysis, peroxisome membrane biogenesis, peroxisome organization, protein import
PGOF: protein binding, zinc ion binding
PGOC: null
PGOP: null

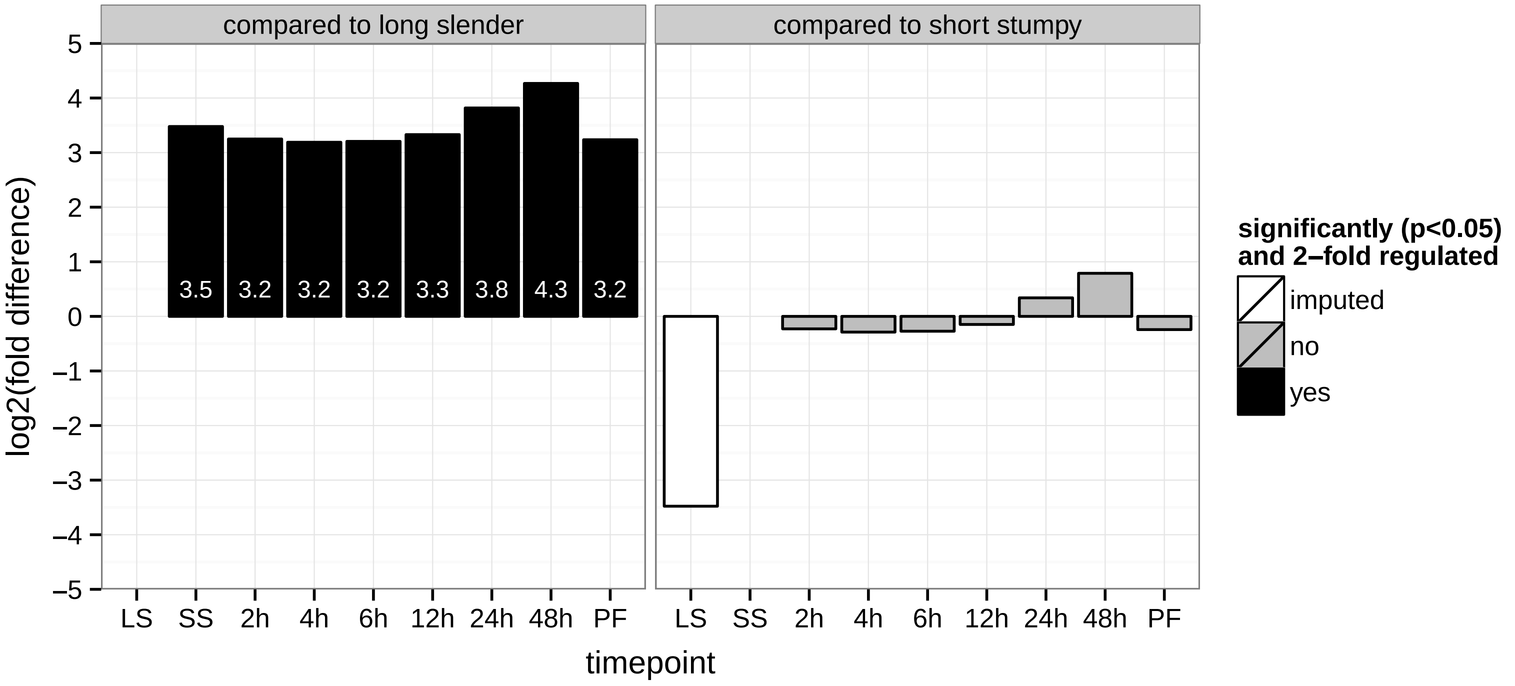

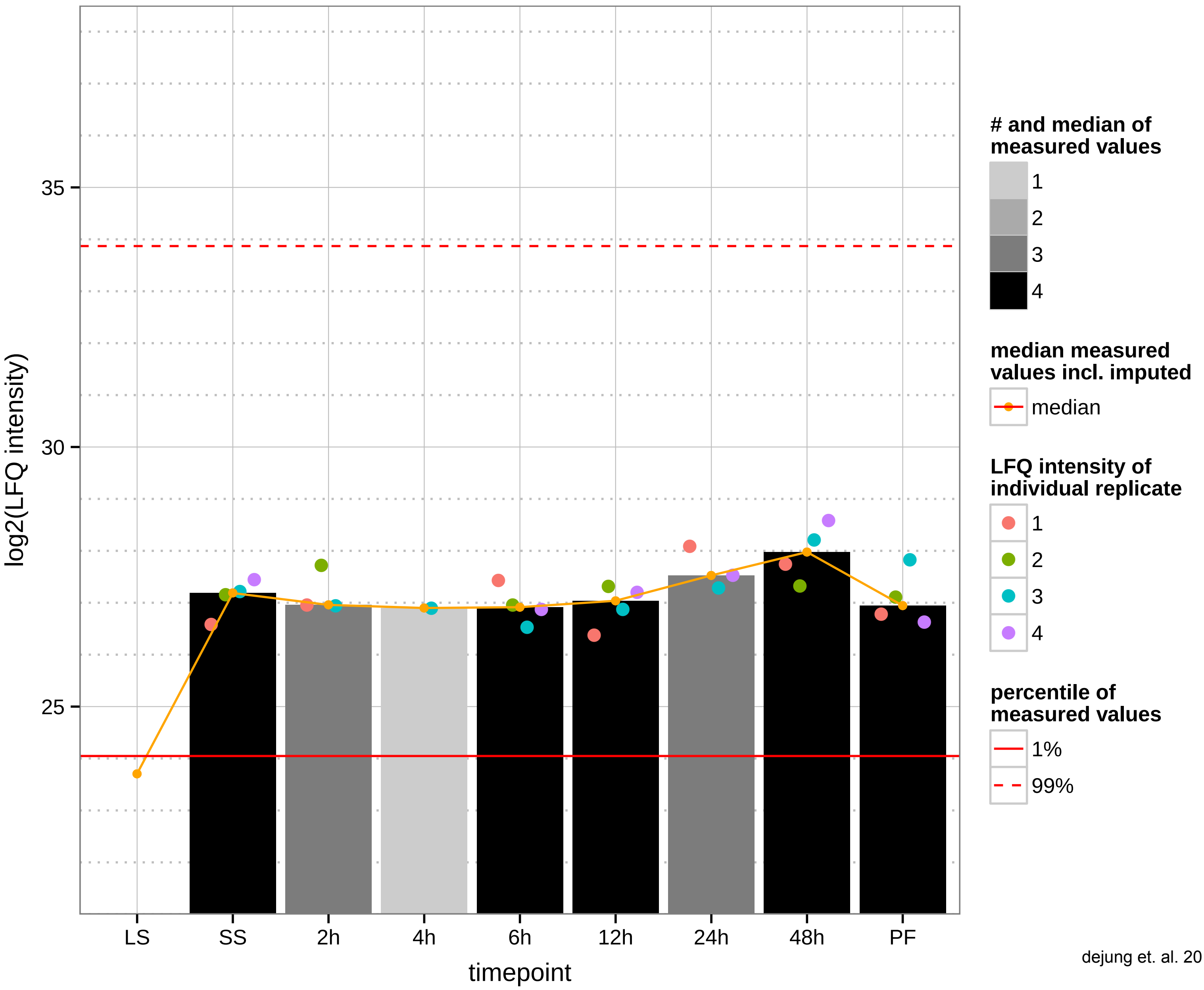

dejung et. al. 2015

procyclin−associated gene 2−like protein, putative
Tb927.11.14600;Tb11.v5.1026
AGOF: null
AGOC: null
AGOP: null, evasion or tolerance of host immune response
PGOF: null
PGOC: null
PGOP: evasion or tolerance of host immune response

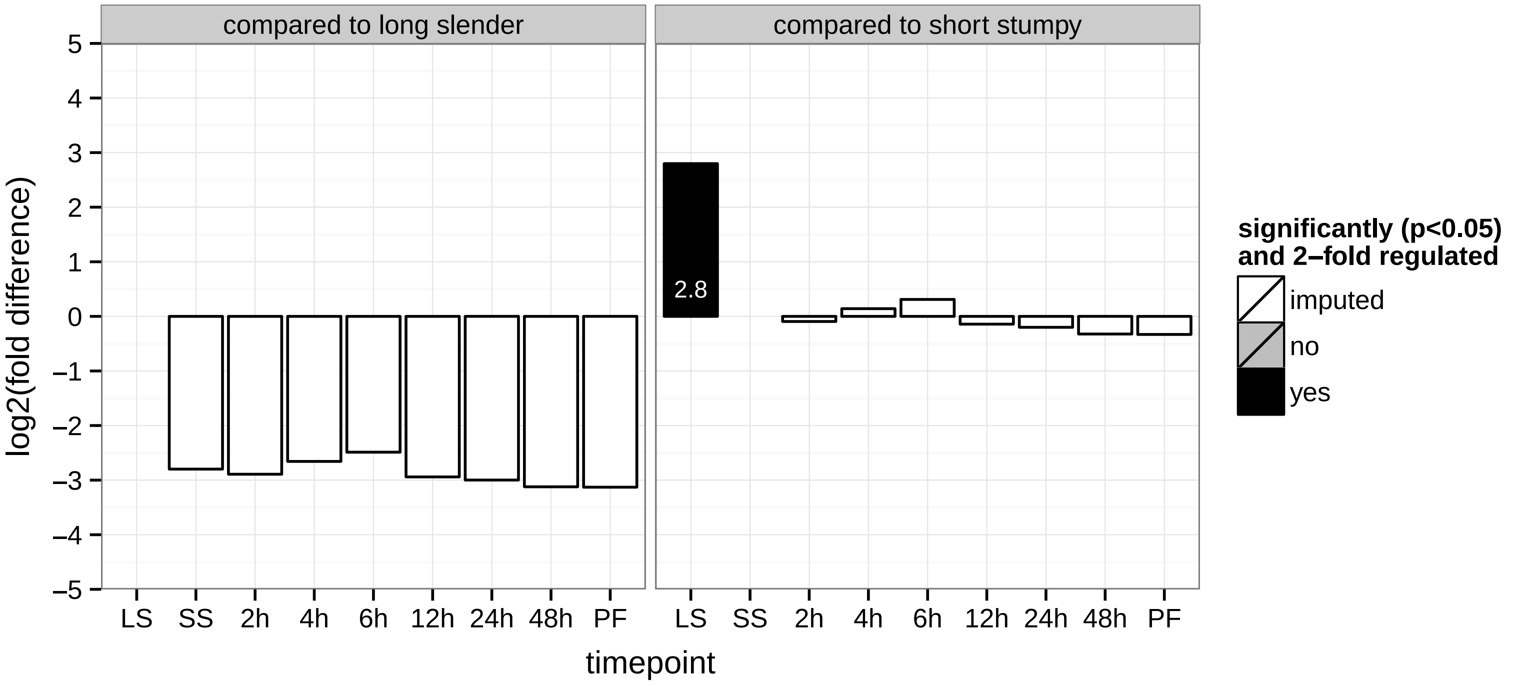

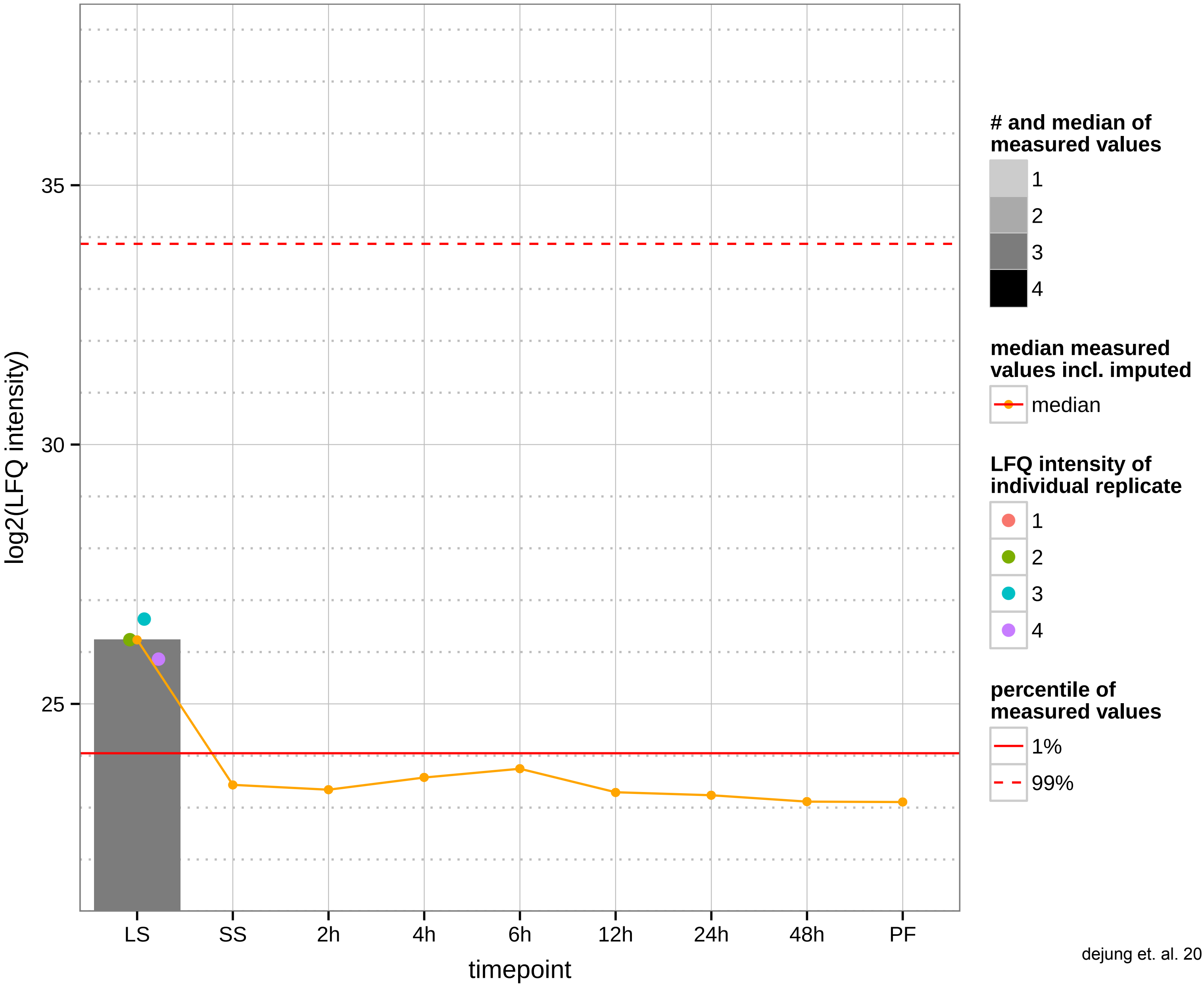

dejung et. al. 2015

elongation factor 1–alpha, putative, EF–1–alpha (TEF1), elongation factor 1–alpha (TEF1)
Tb927.10.2110;Tb927.10.2100;Tb11.v5.1046;Tb927.10.2090
AGOF: null, GTP binding, GTPase activity, calmodulin binding, translation elongation factor activity
AGOC: null, cytoplasm
AGOP: null, calcium–mediated signaling, microtubule–based process, translational elongation
PGOF: GTP binding, GTPase activity, translation elongation factor activity
PGOC: cytoplasm
PGOP: translational elongation

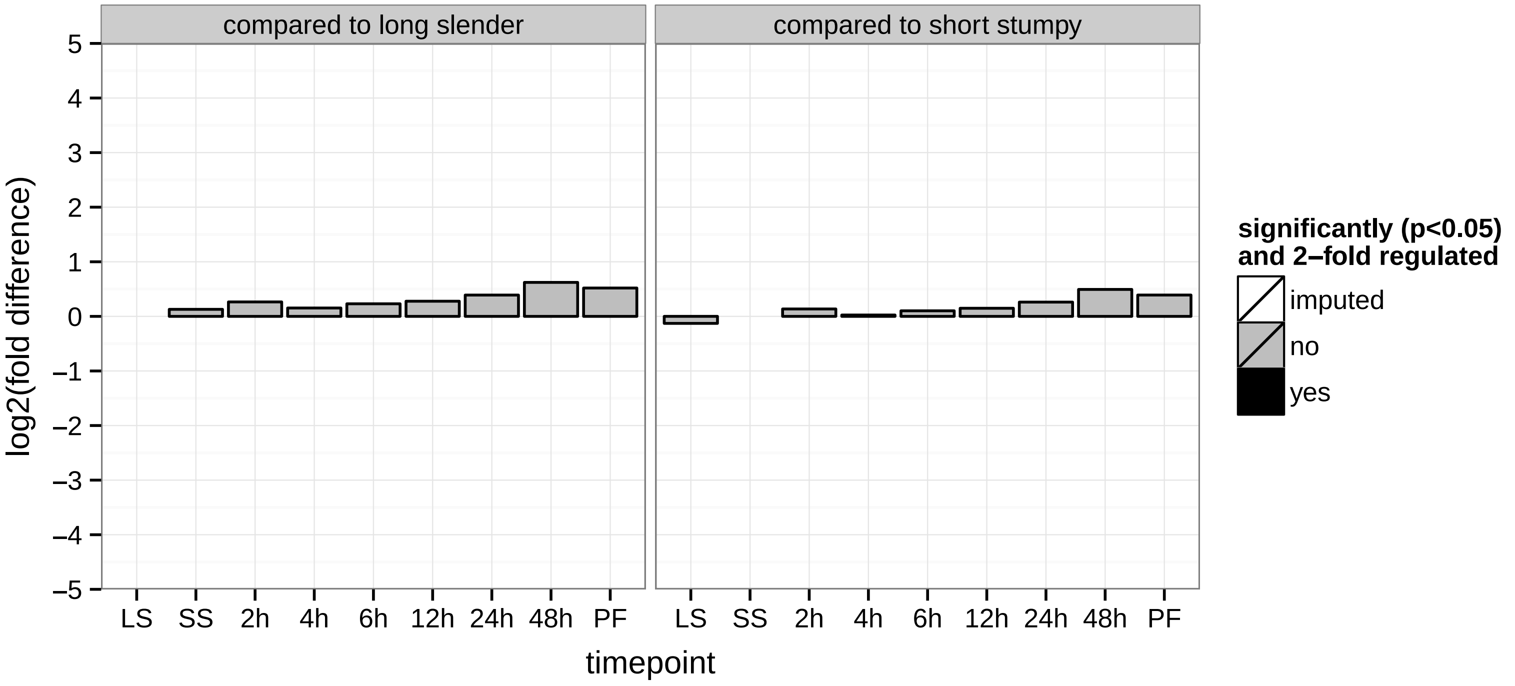

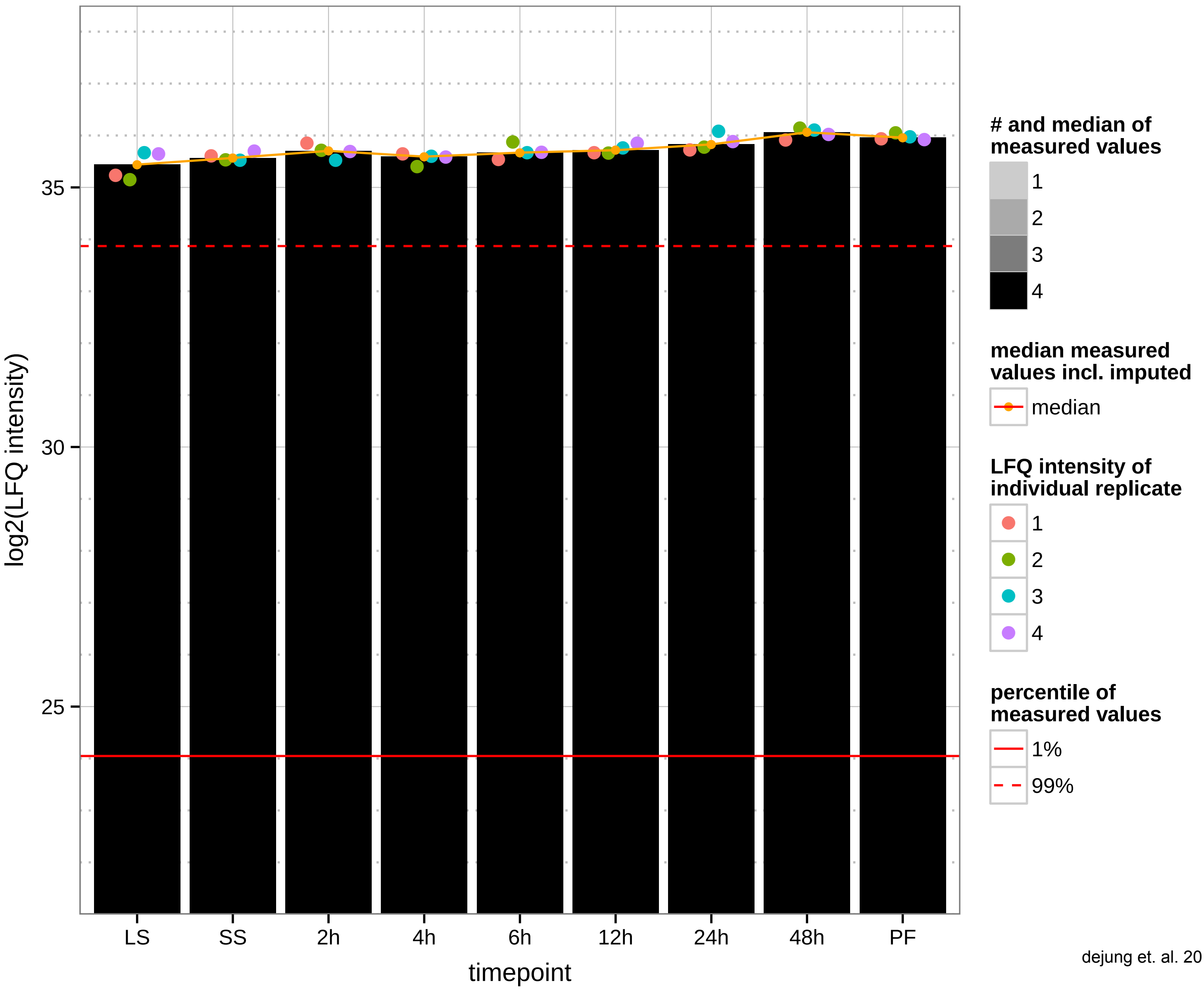

dejung et. al. 2015

73 kDa paraflagellar rod protein, putative, PFR1 (PFR1)
Tb927.3.4330;Tb927.3.4320;Tb927.3.4310;Tb927.3.4300;Tb927.3.4290;Tb11.v5.1055
AGOF: null, calmodulin binding, molecular_function
AGOC: null, cilium, microtubule–based flagellum
AGOP: null, cellular component movement, forward locomotion
PGOF: calmodulin binding, null
PGOC: microtubule–based flagellum, null
PGOP: null

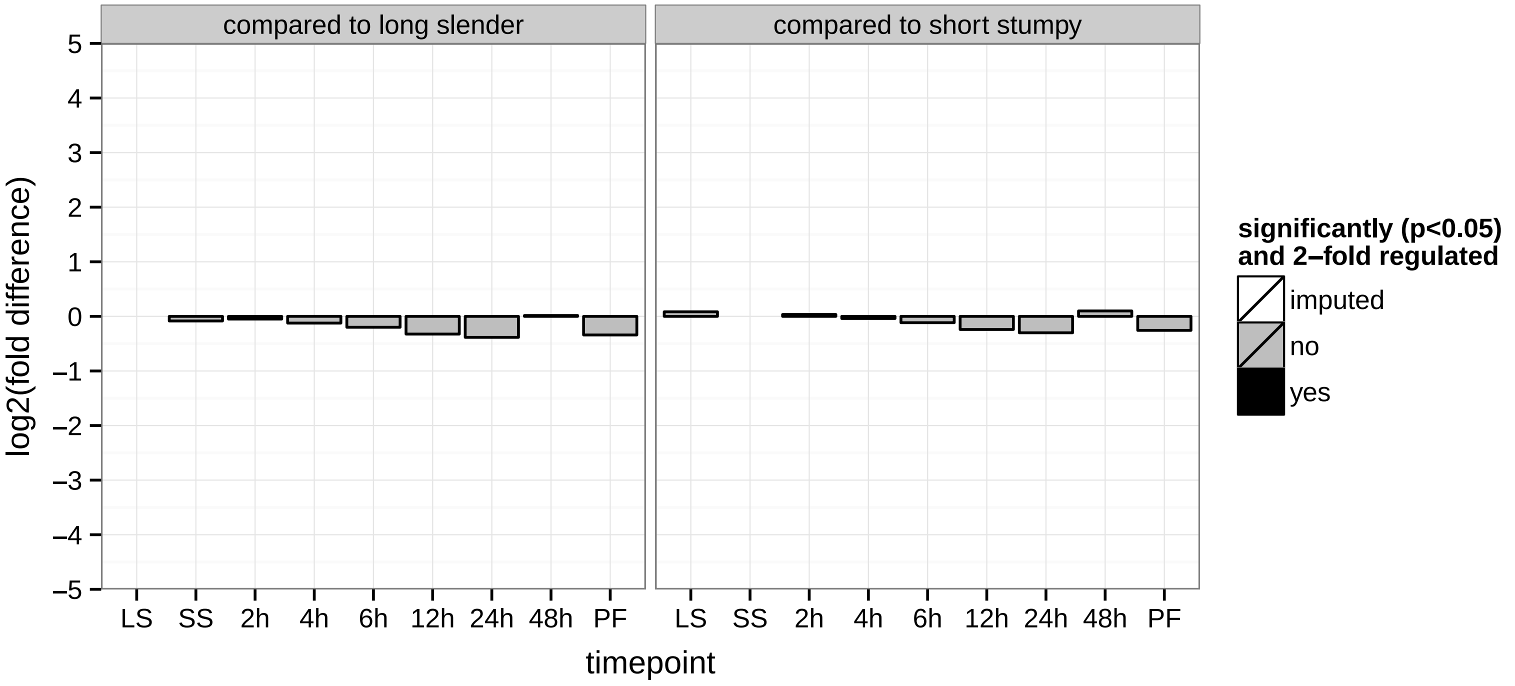

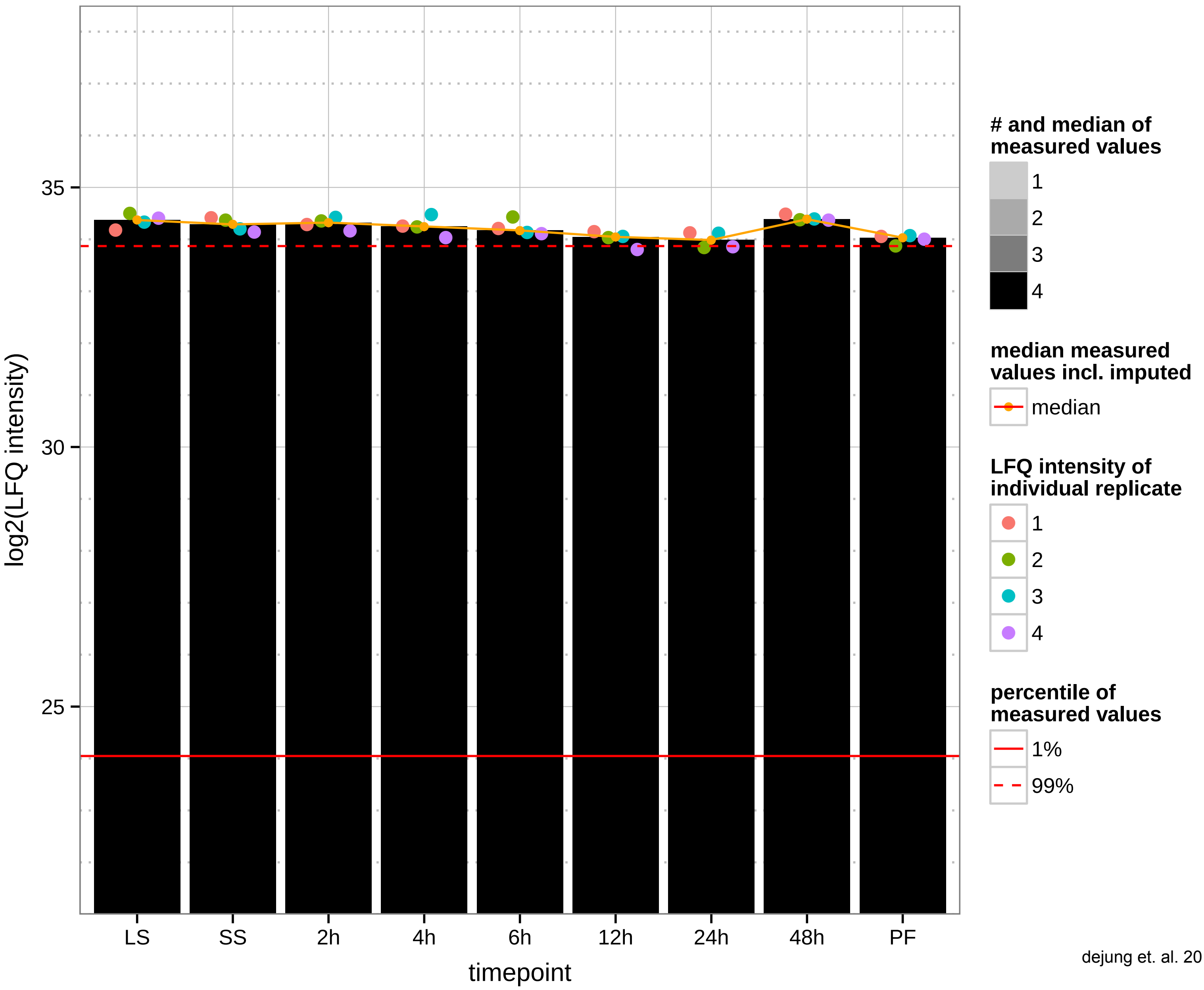

dejung et. al. 2015

protein kinase, putative, mitogen–activated protein kinase 11
Tb927.10.12040;Tb11.v5.1057
AGOF: null, ATP binding, protein kinase activity, protein serine/threonine kinase activity, protein tyrosine kinase activity
AGOC: null
AGOP: null, protein phosphorylation
PGOF: ATP binding, protein kinase activity, transferase activity, transferring phosphorus–containing groups
PGOC: null
PGOP: protein phosphorylation

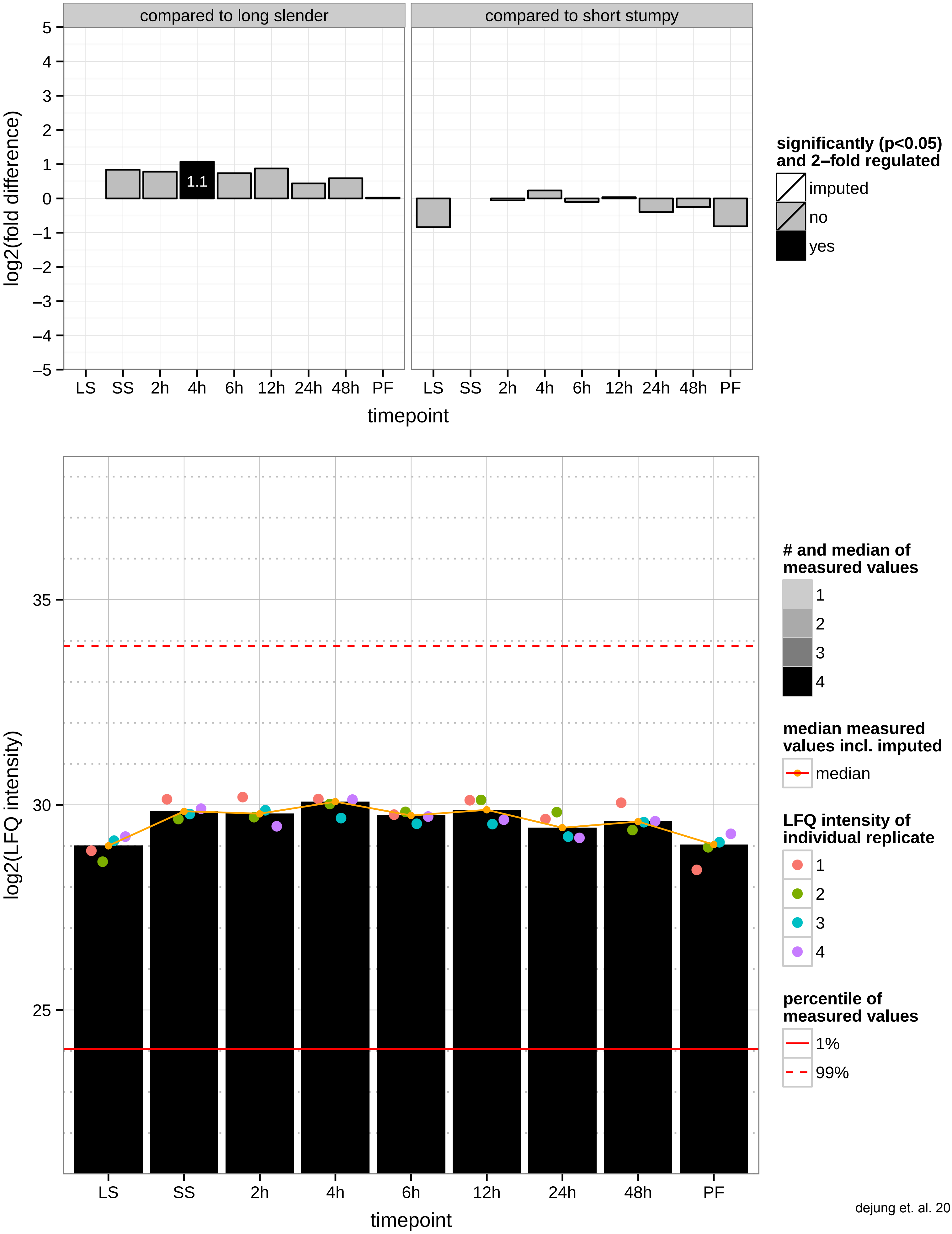

dejung et. al. 2015

developmentally regulated phosphoprotein
Tb927.1.1000
AGOF: ATP binding
AGOC: mitochondrion
AGOP: null
PGOF: ATP binding
PGOC: null
PGOP: null

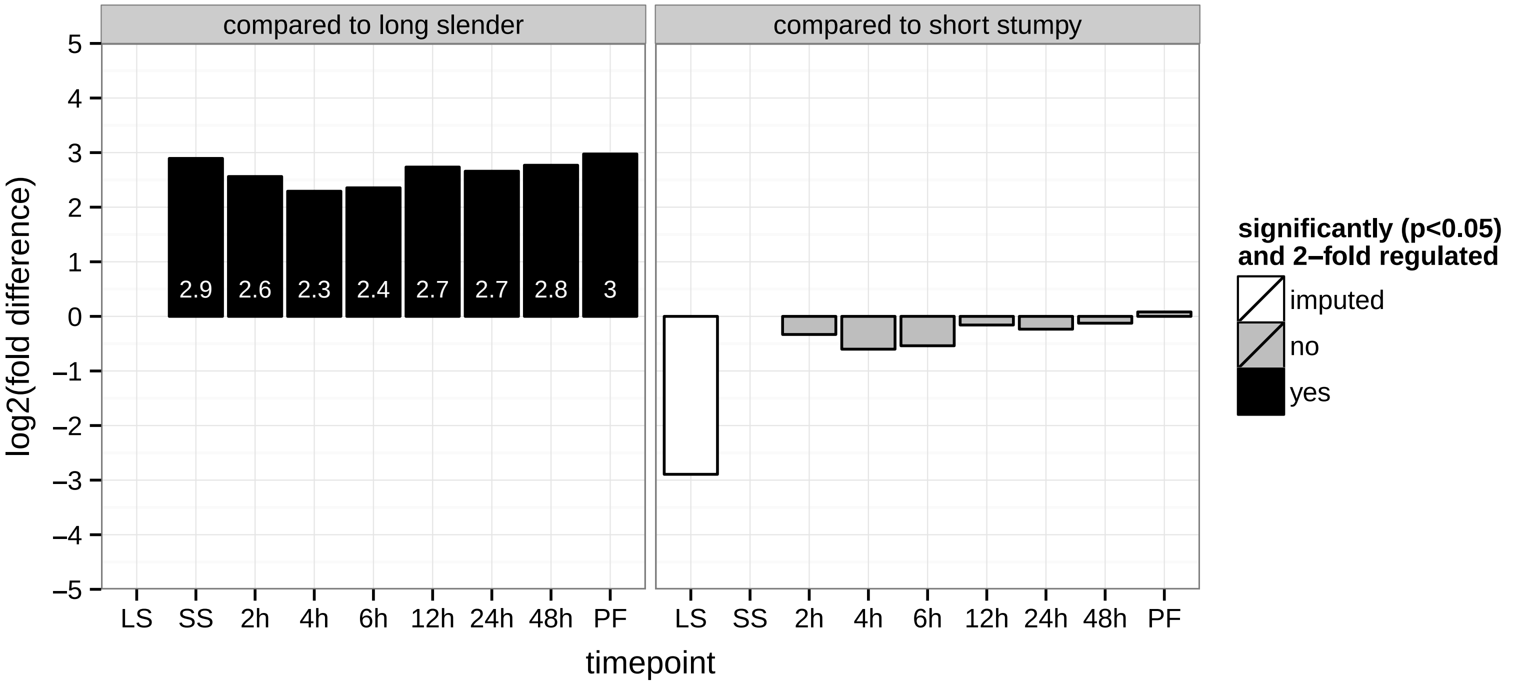

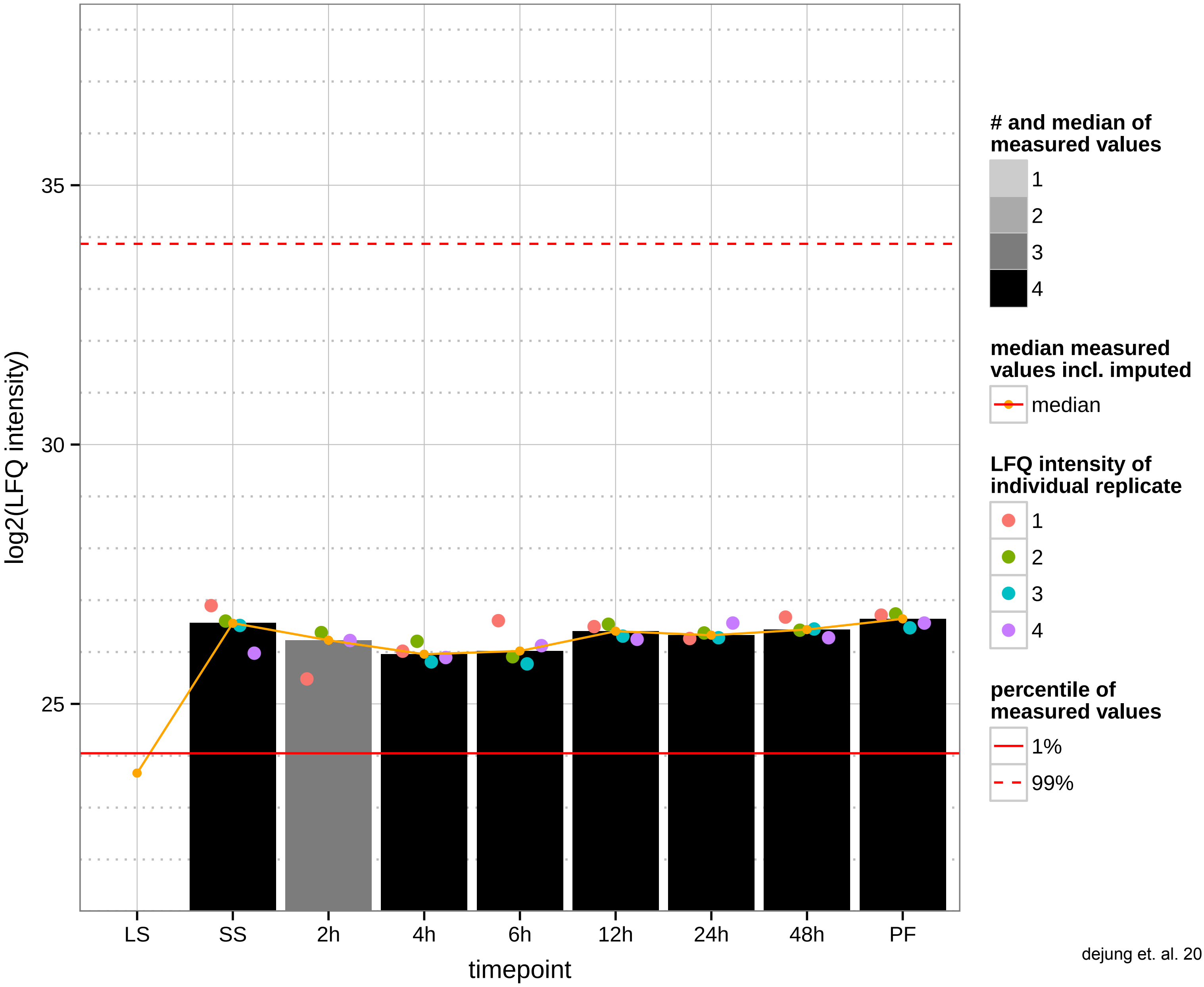

dejung et. al. 2015

retrotransposon hot spot protein 4 (RHS4), putative
Tb927.1.120
AGOF: null
AGOC: nucleus
AGOP: null
PGOF: null
PGOC: null
PGOP: null

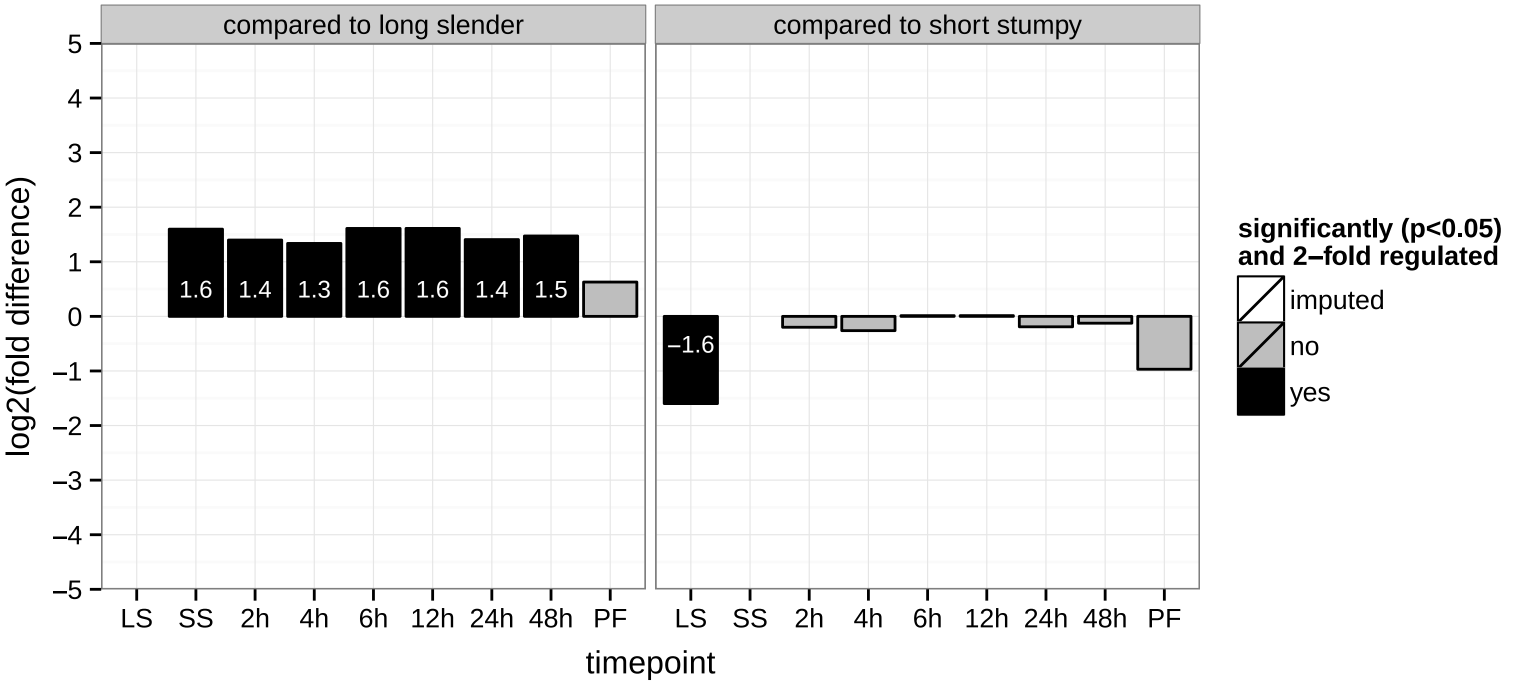

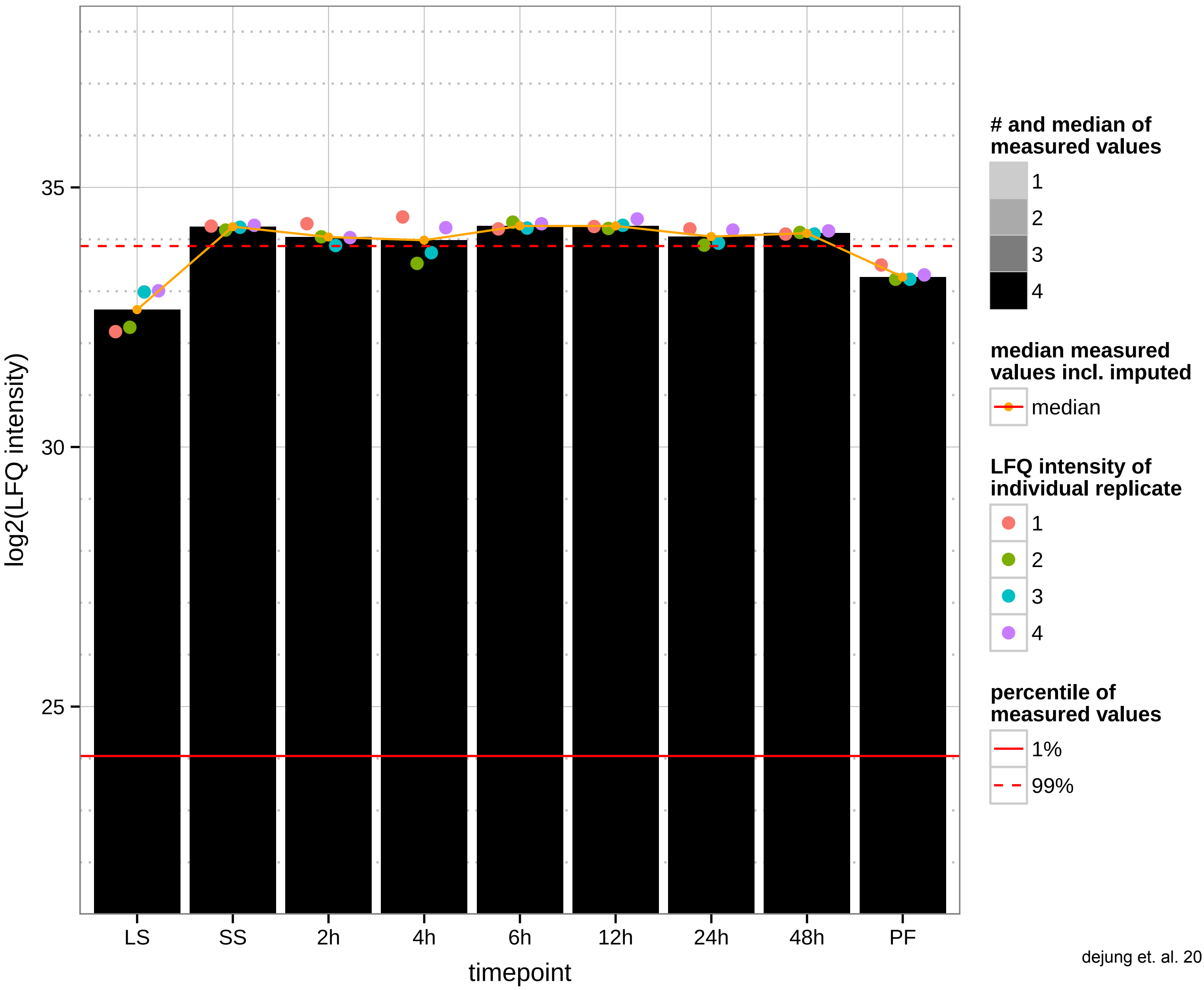

dejung et. al. 2015

serine/threonine protein phosphatase 2A regulatory subunit, putative
Tb927.1.1380;Tb11.v5.0390
AGOF: null
AGOC: null, protein serine/threonine phosphatase complex
AGOP: null, protein dephosphorylation
PGOF: binding
PGOC: null
PGOP: null

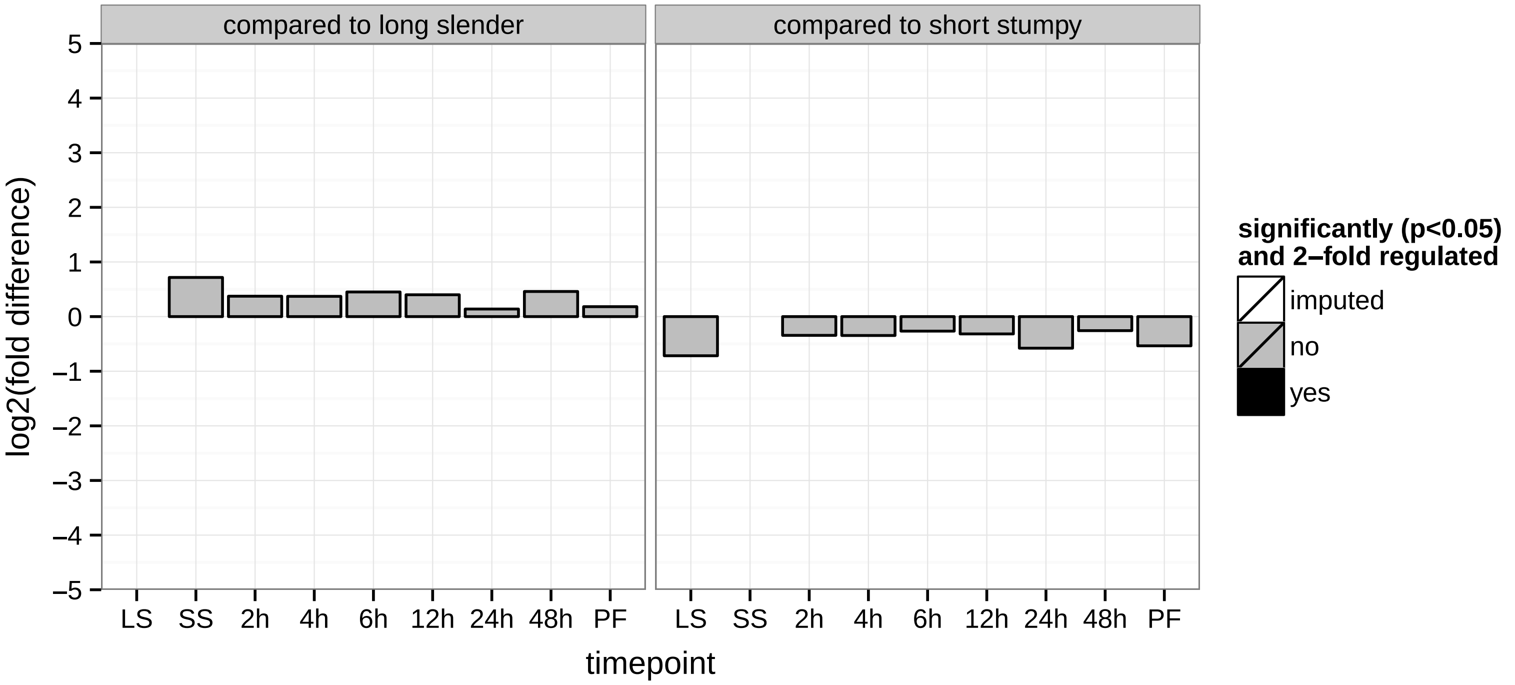

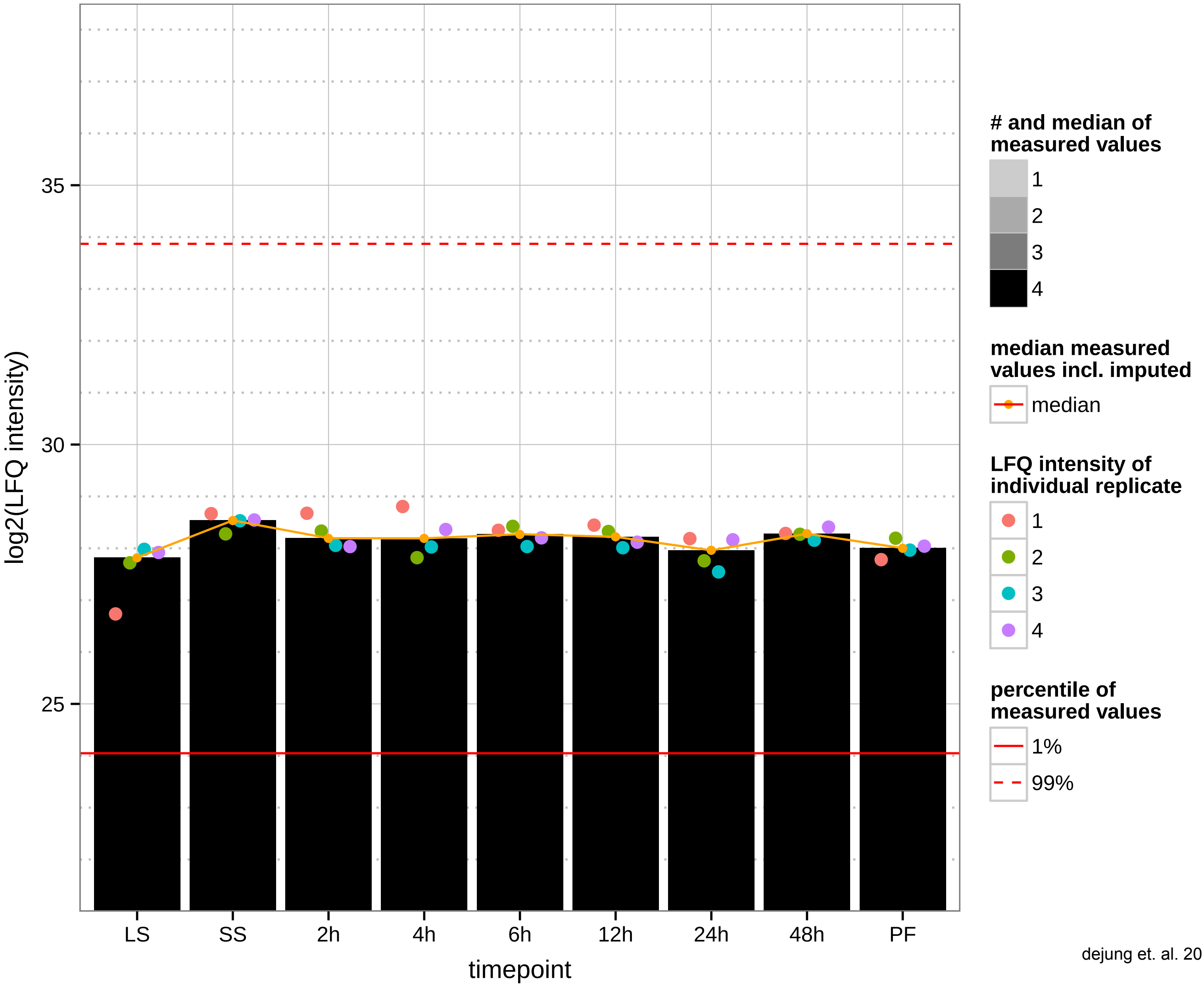

dejung et. al. 2015

ATP−dependent DEAD/H RNA helicase, putative
Tb927.1.1620
AGOF: null, ATP binding, ATP−dependent RNA helicase activity, nucleic acid binding
AGOC: null
AGOP: null, RNA metabolic process
PGOF: ATP binding, ATP−dependent helicase activity, nucleic acid binding, helicase activity
PGOC: null
PGOP: null

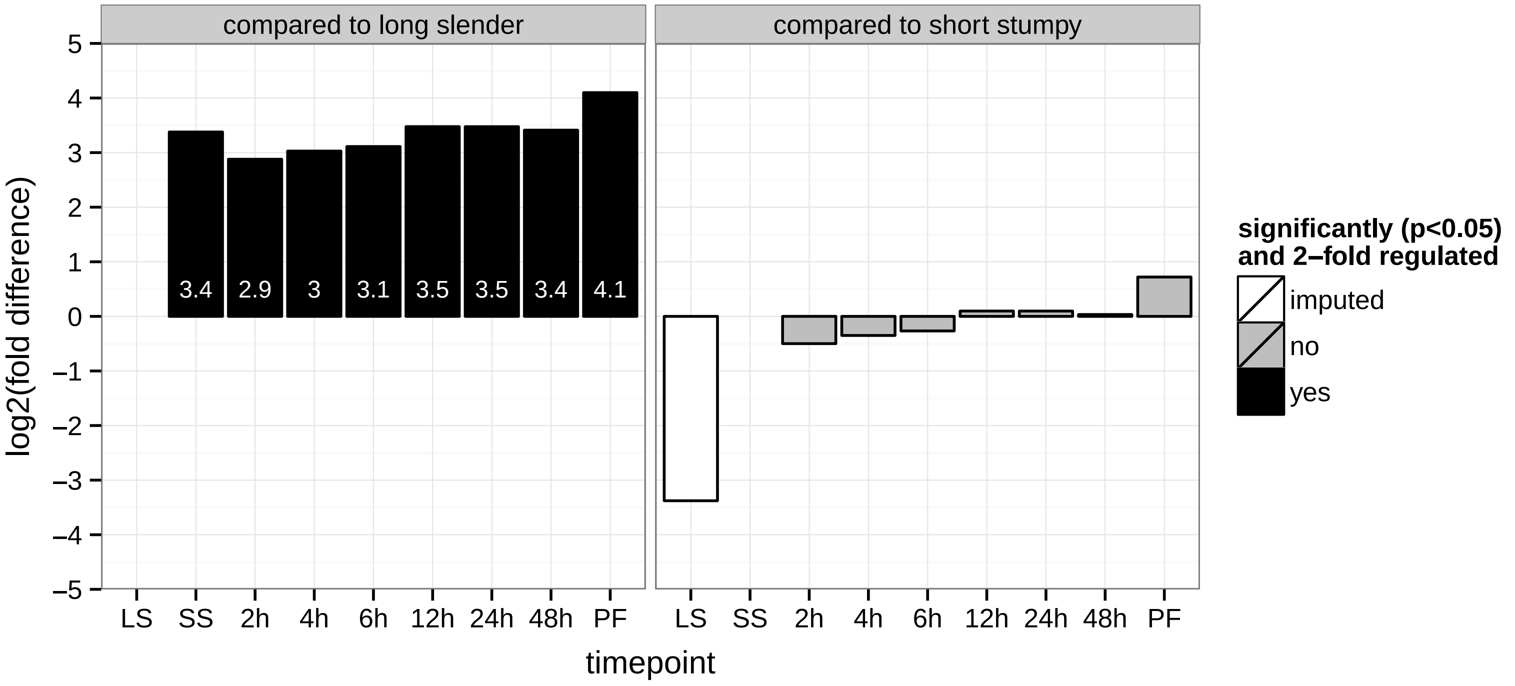

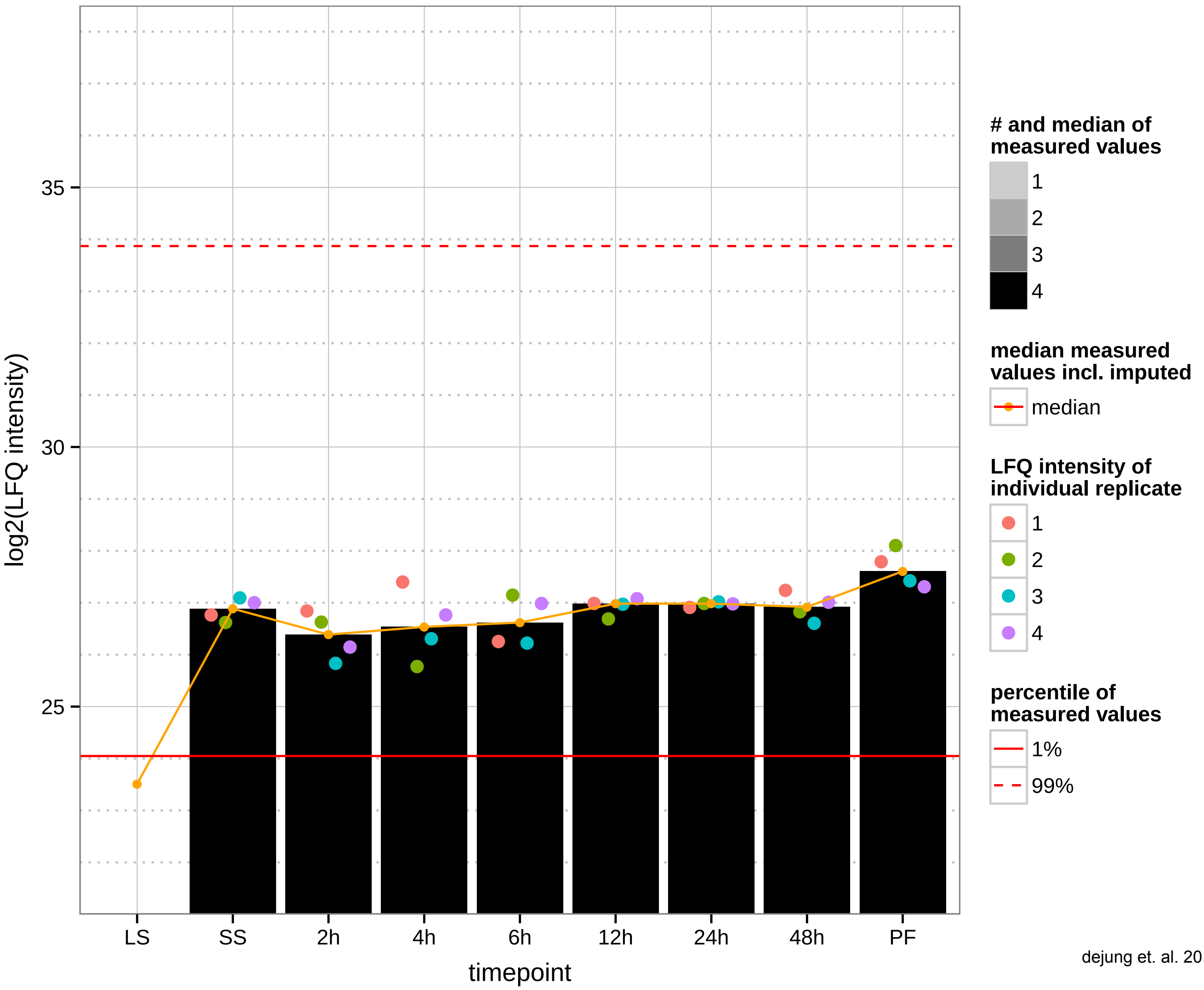

dejung et. al. 2015

hypothetical protein, conserved, ARM–like helical domain–containing protein
Tb927.1.1670
AGOF: null
AGOC: null
AGOP: null
PGOF: binding
PGOC: null
PGOP: null

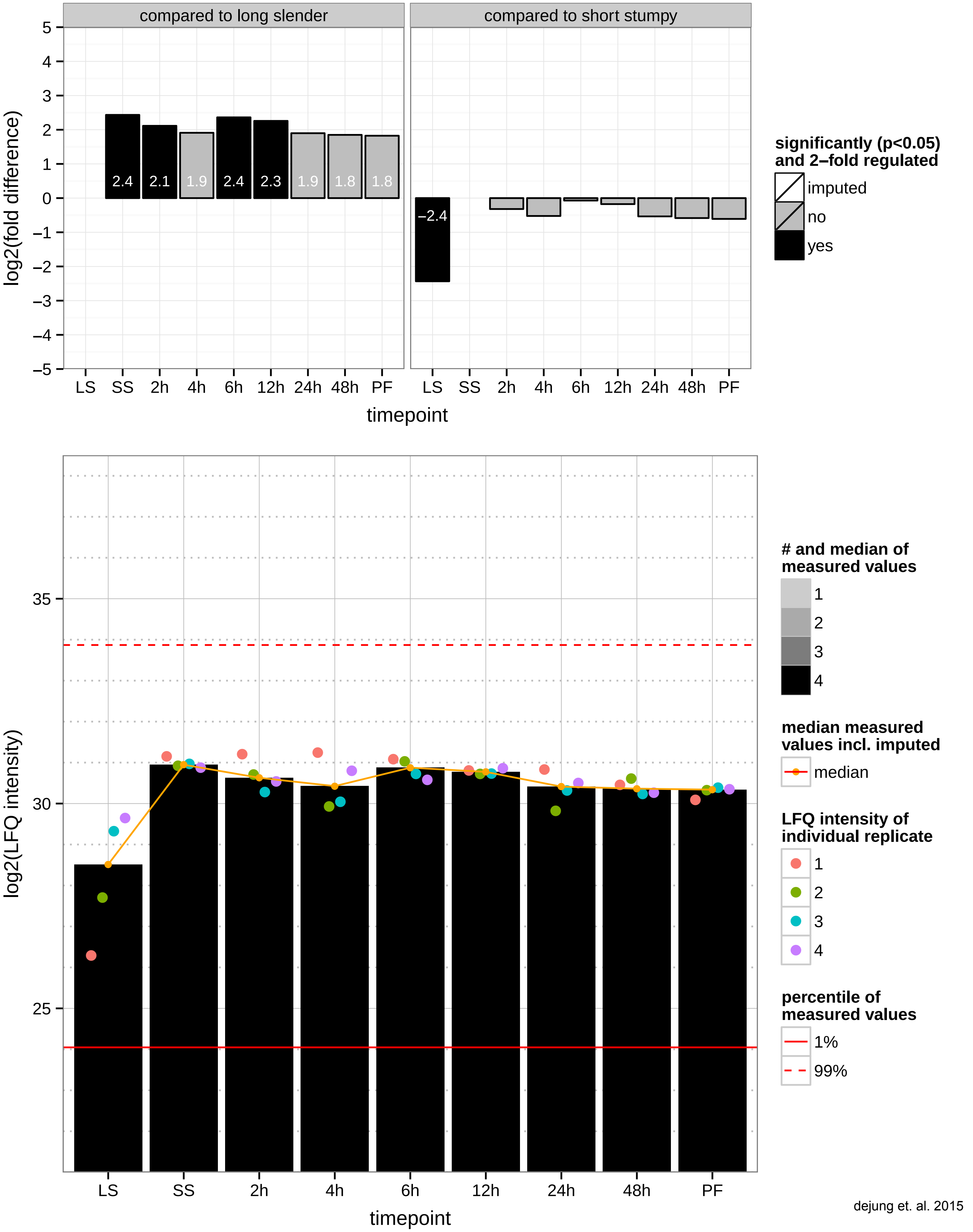

dejung et. al. 2015

AATF protein, putative
Tb927.1.1700
AGOF: null
AGOC: null
AGOP: null
PGOF: null
PGOC: null
PGOP: null

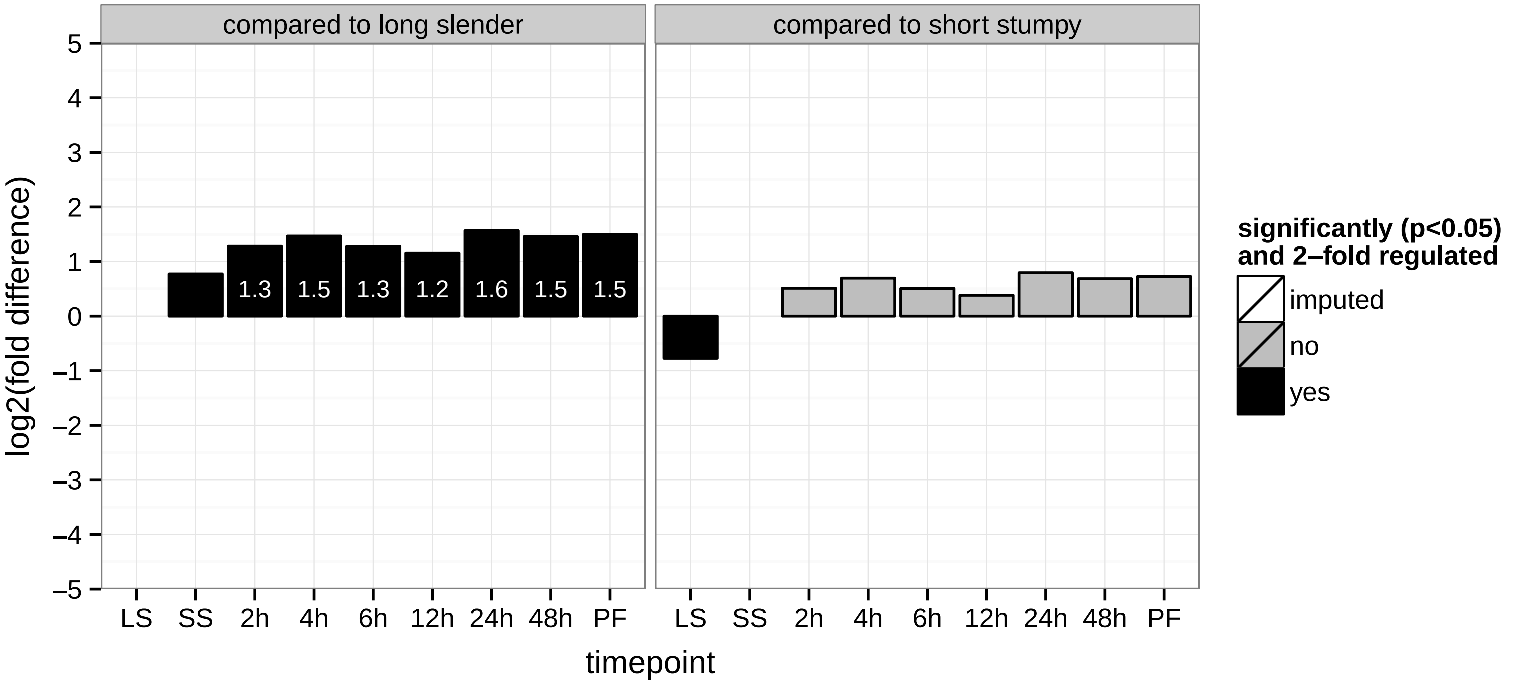

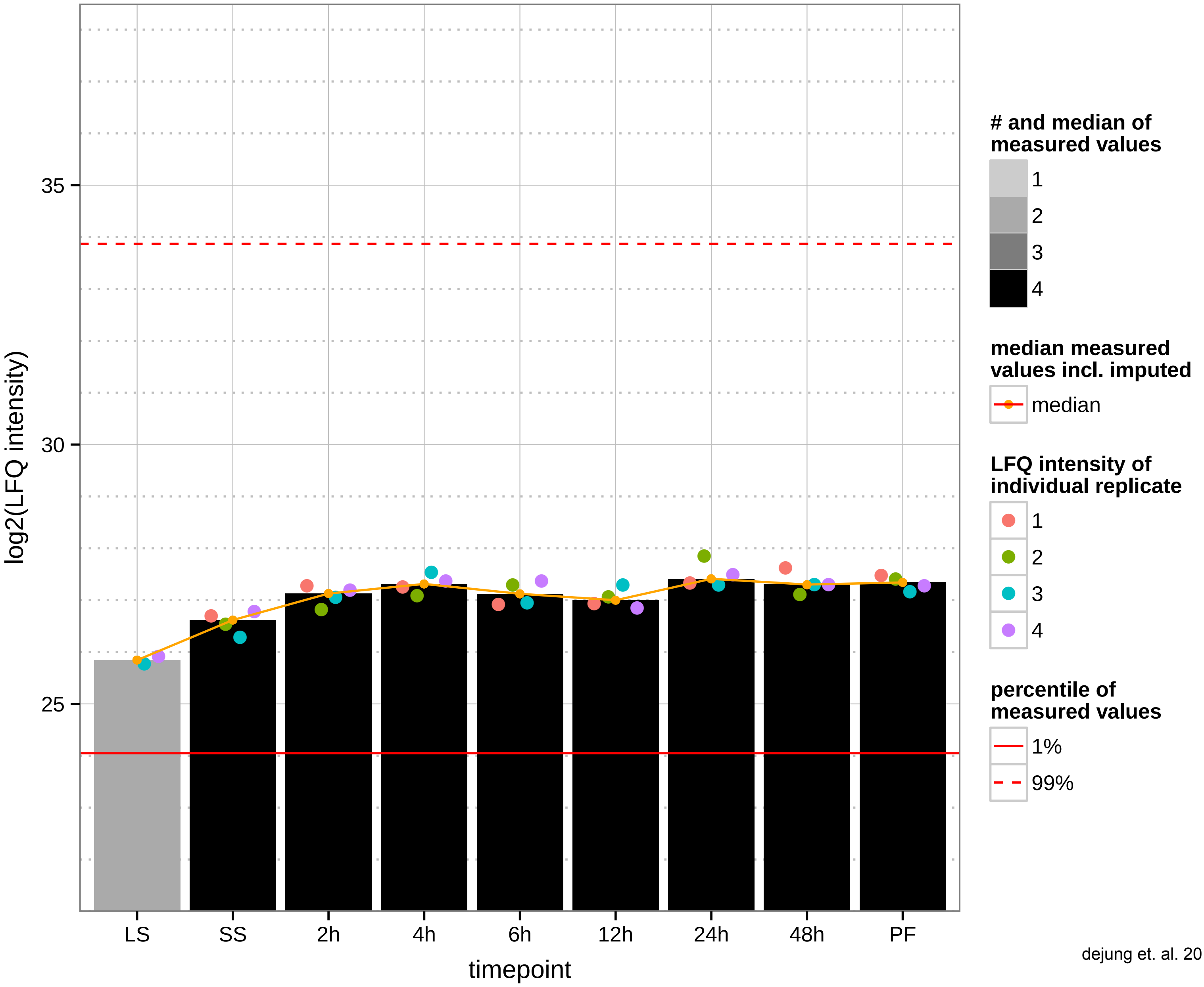

dejung et. al. 2015

transmembrane protein, conserved
Tb927.1.1710
AGOF: null
AGOC: null
AGOP: null
PGOF: null
PGOC: null
PGOP: null

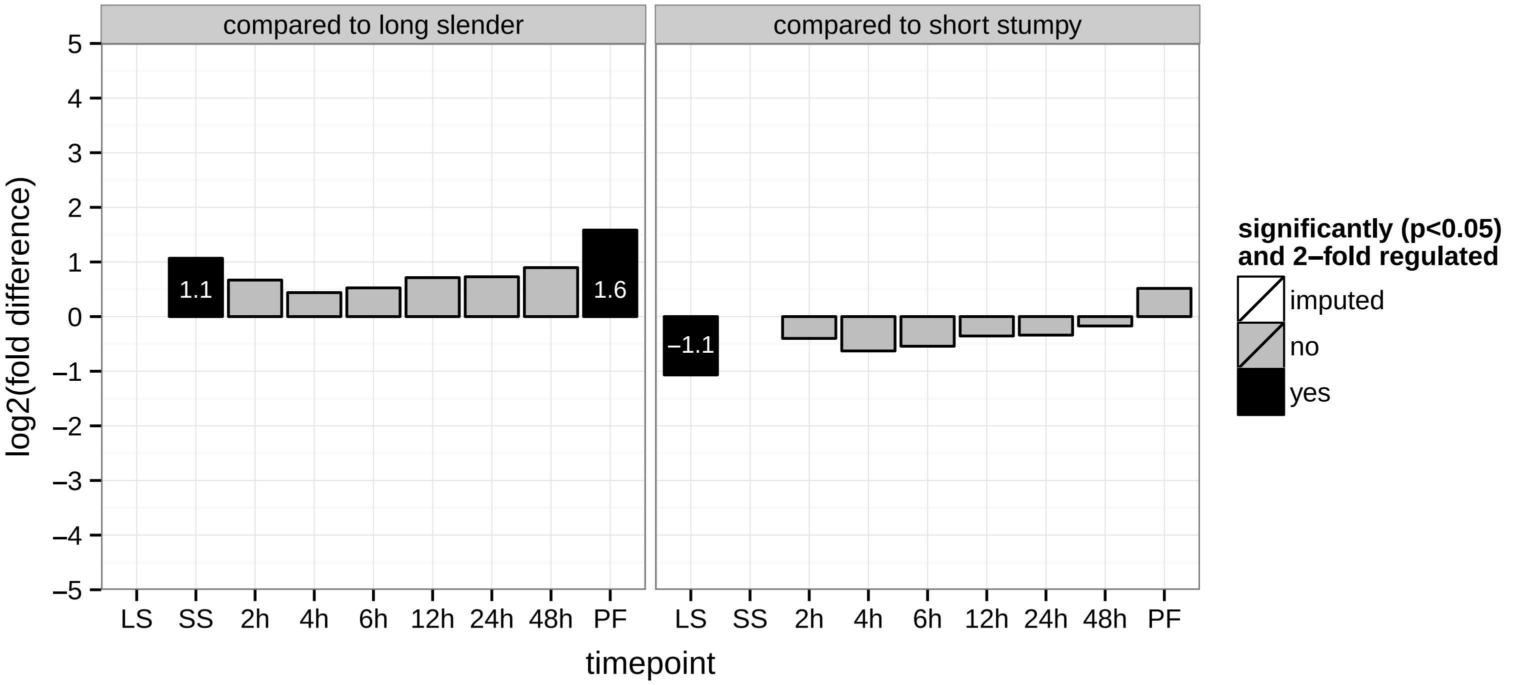

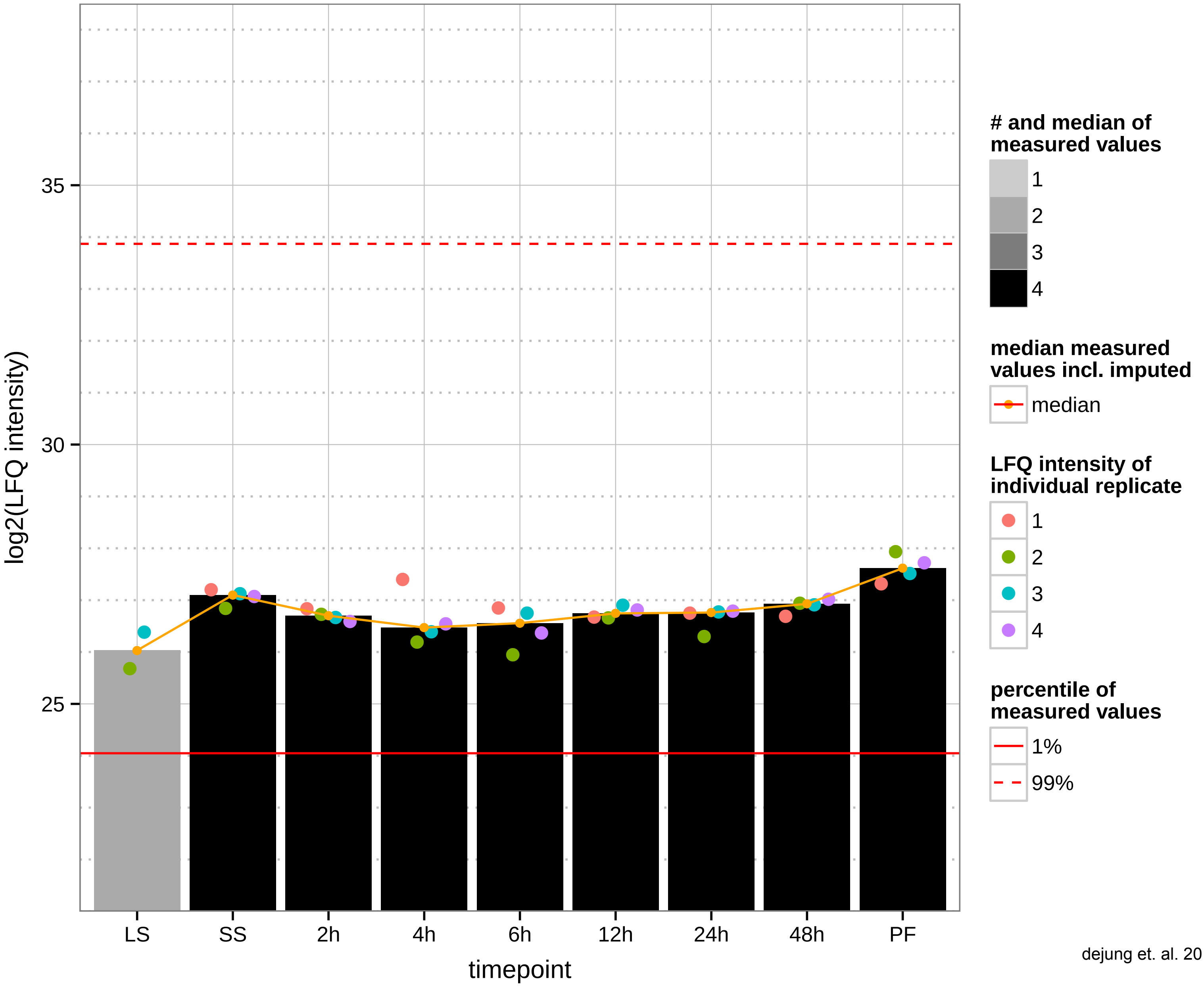

dejung et. al. 2015

cyclophilin 15, putative
Tb927.1.1720
AGOF: peptidyl–prolyl cis–trans isomerase activity
AGOC: null
AGOP: null
PGOF: peptidyl–prolyl cis–trans isomerase activity
PGOC: null
PGOP: protein folding

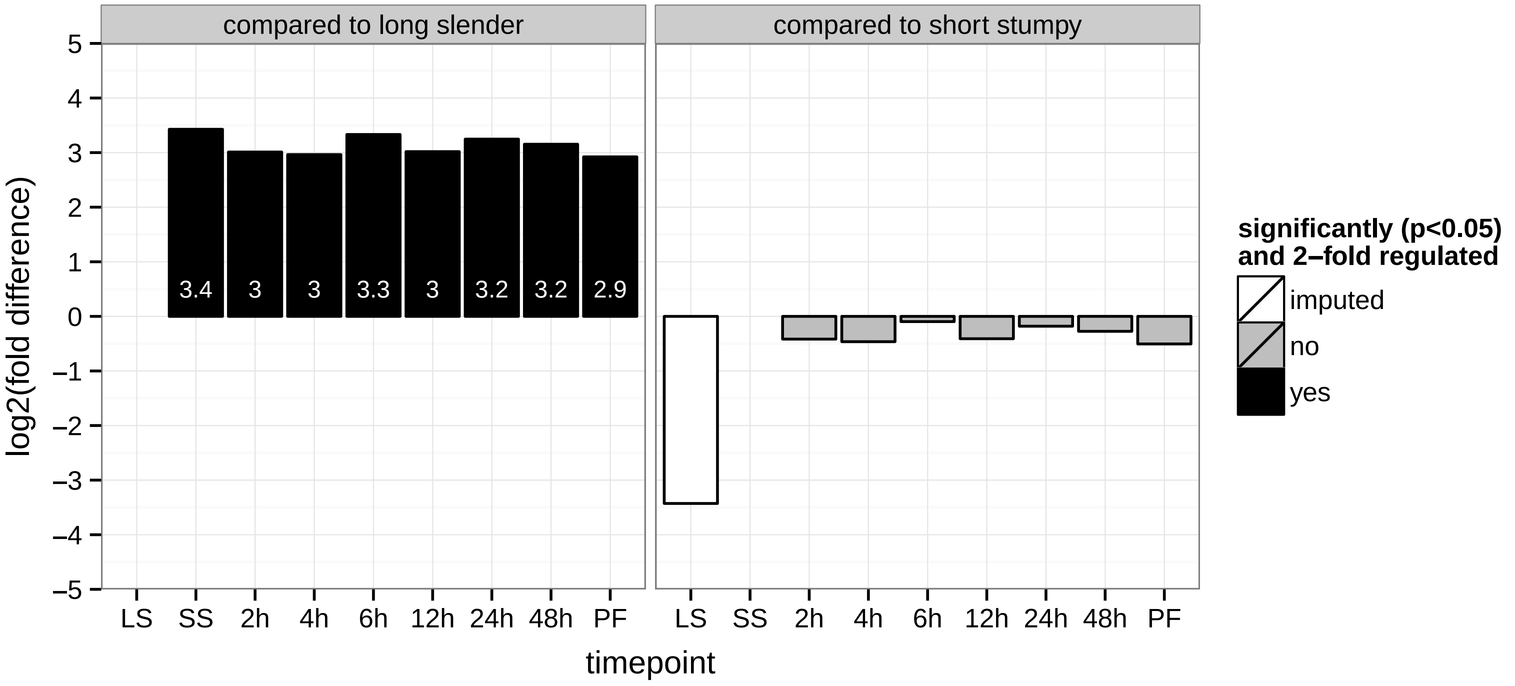

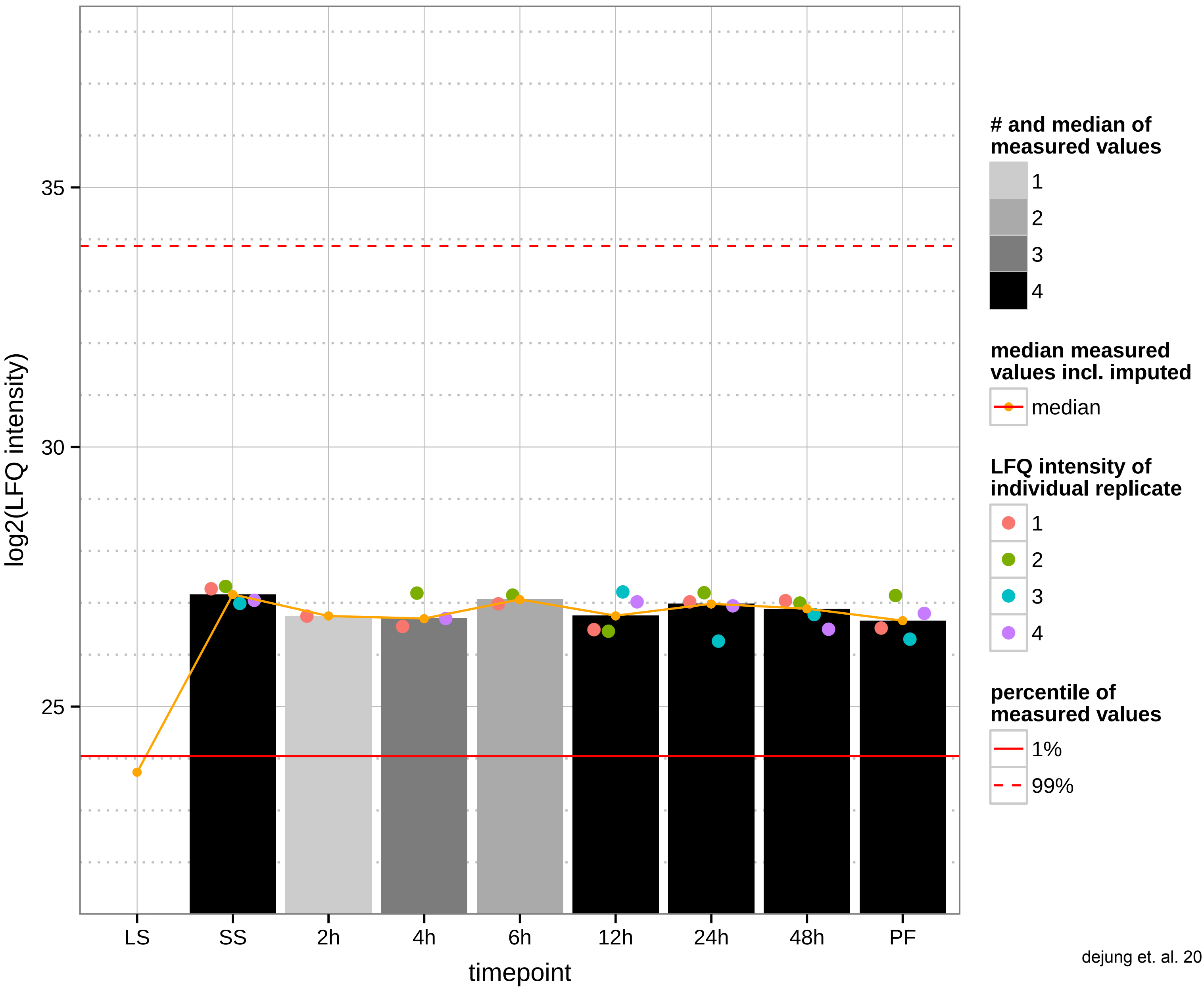

dejung et. al. 2015

conserved protein, unknown function
Tb927.1.1730
AGOF: null
AGOC: mitochondrion
AGOP: null
PGOF: null
PGOC: null
PGOP: null

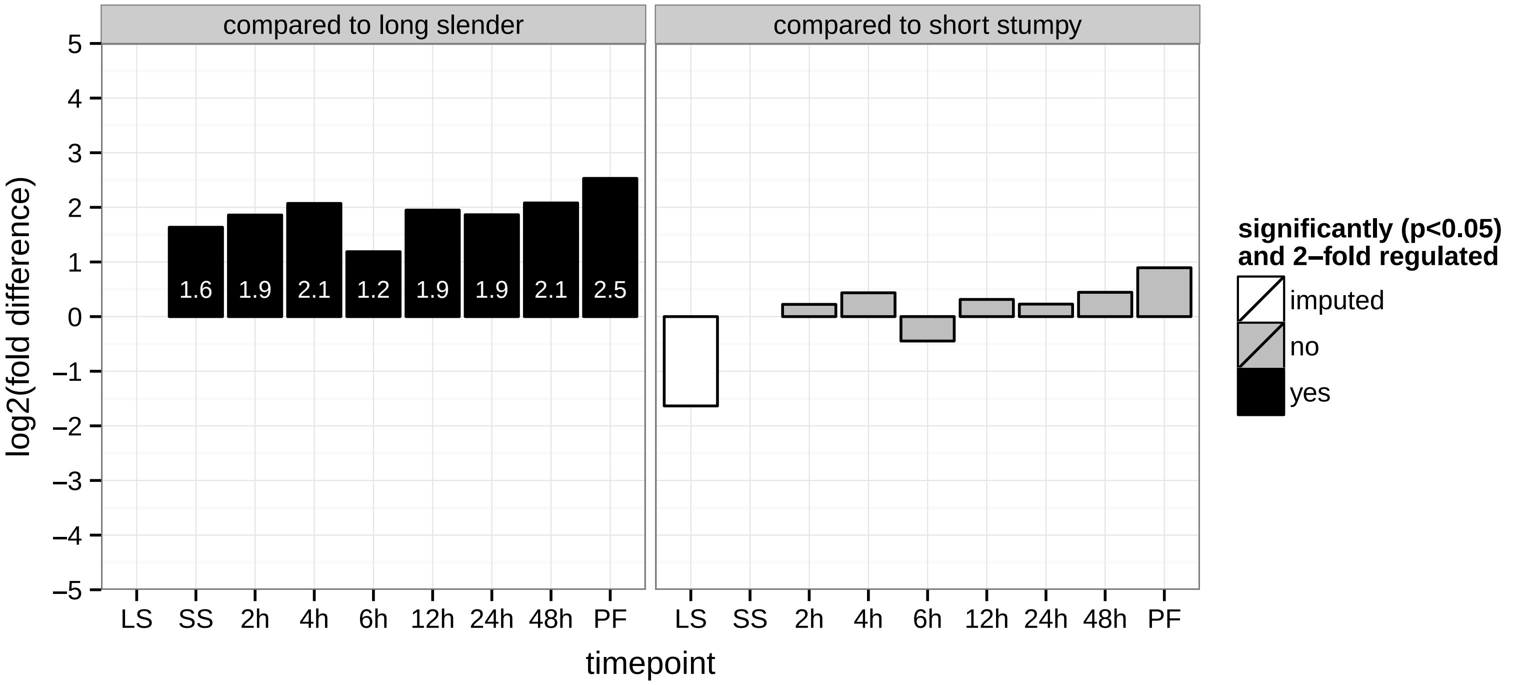

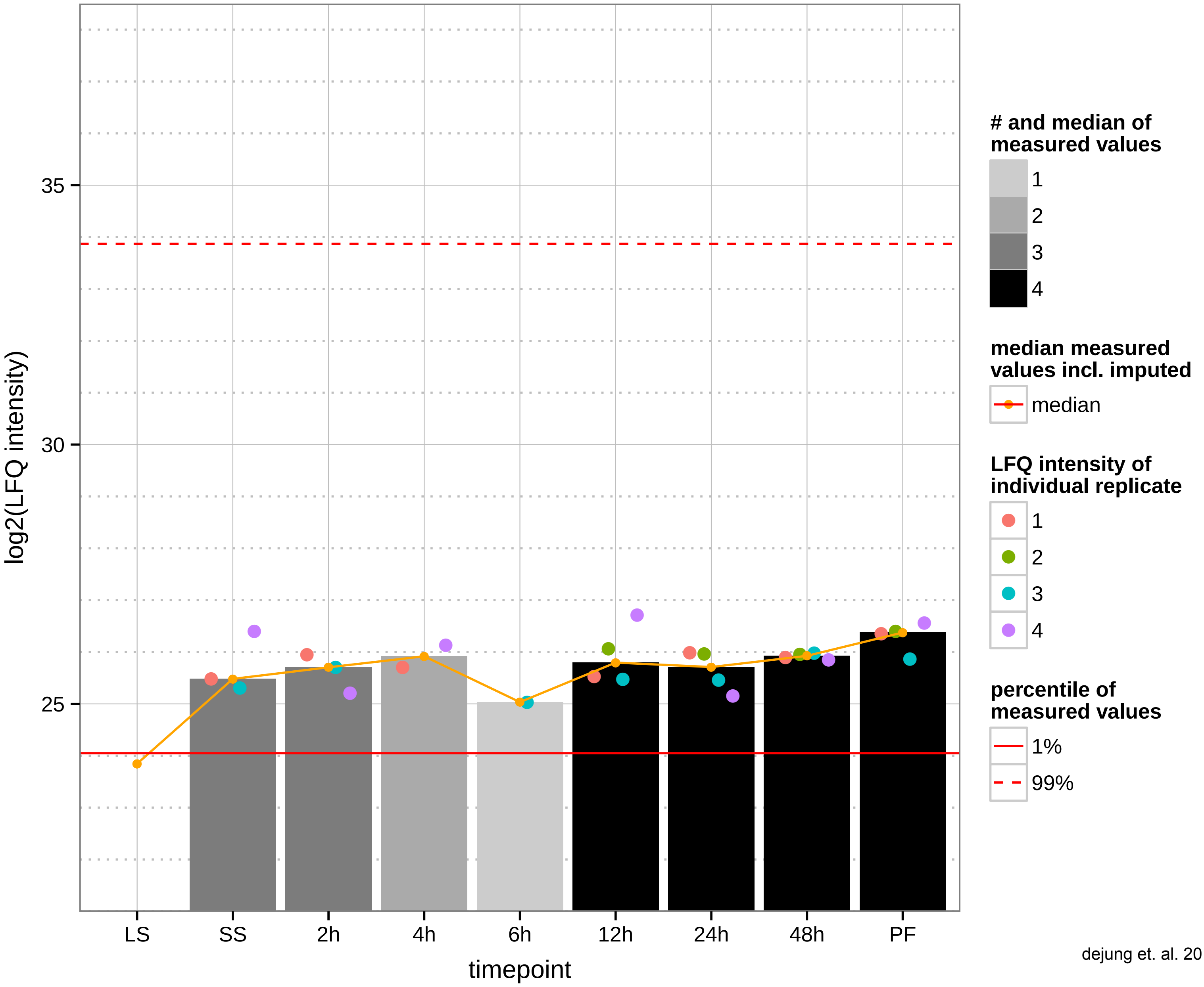

dejung et. al. 2015

retrotransposon hot spot protein 1 (RHS1), putative
Tb927.1.180
AGOF: null
AGOC: nucleus
AGOP: null
PGOF: null
PGOC: null
PGOP: null

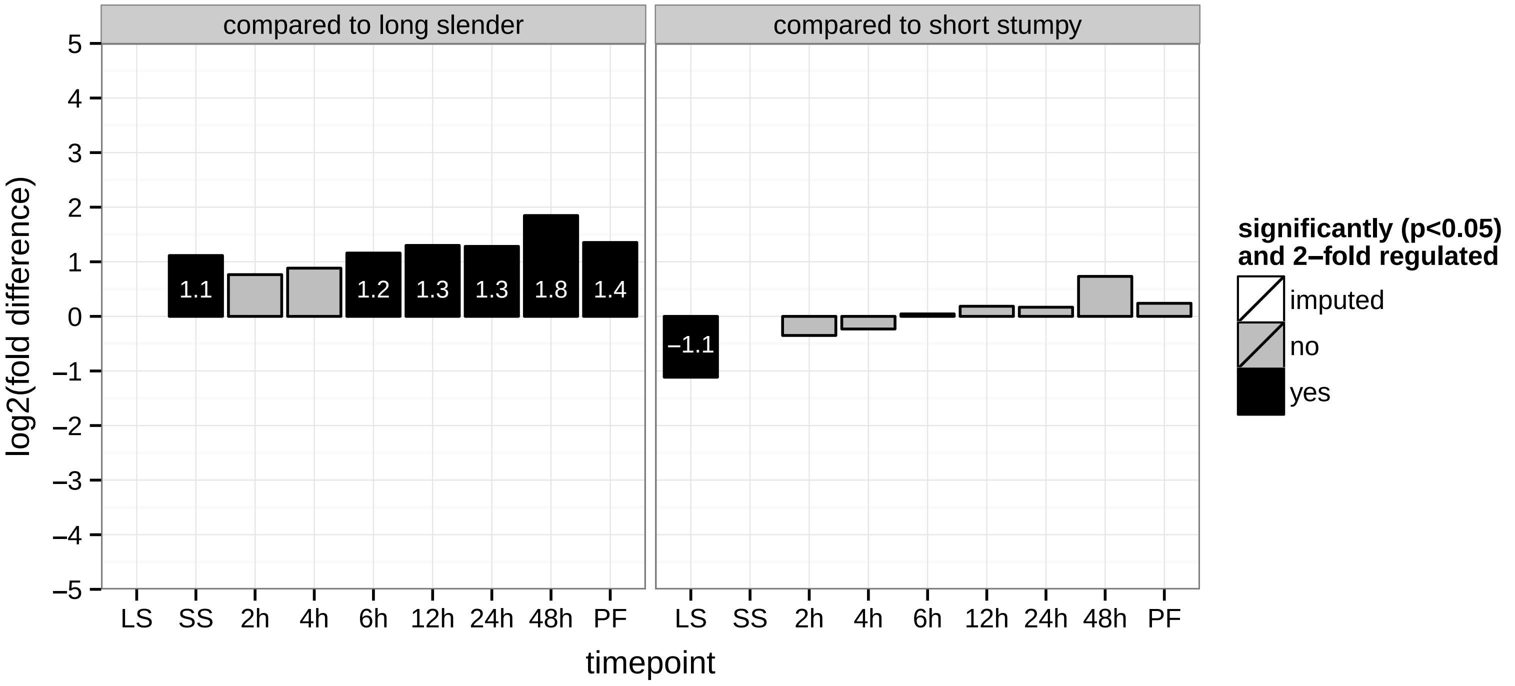

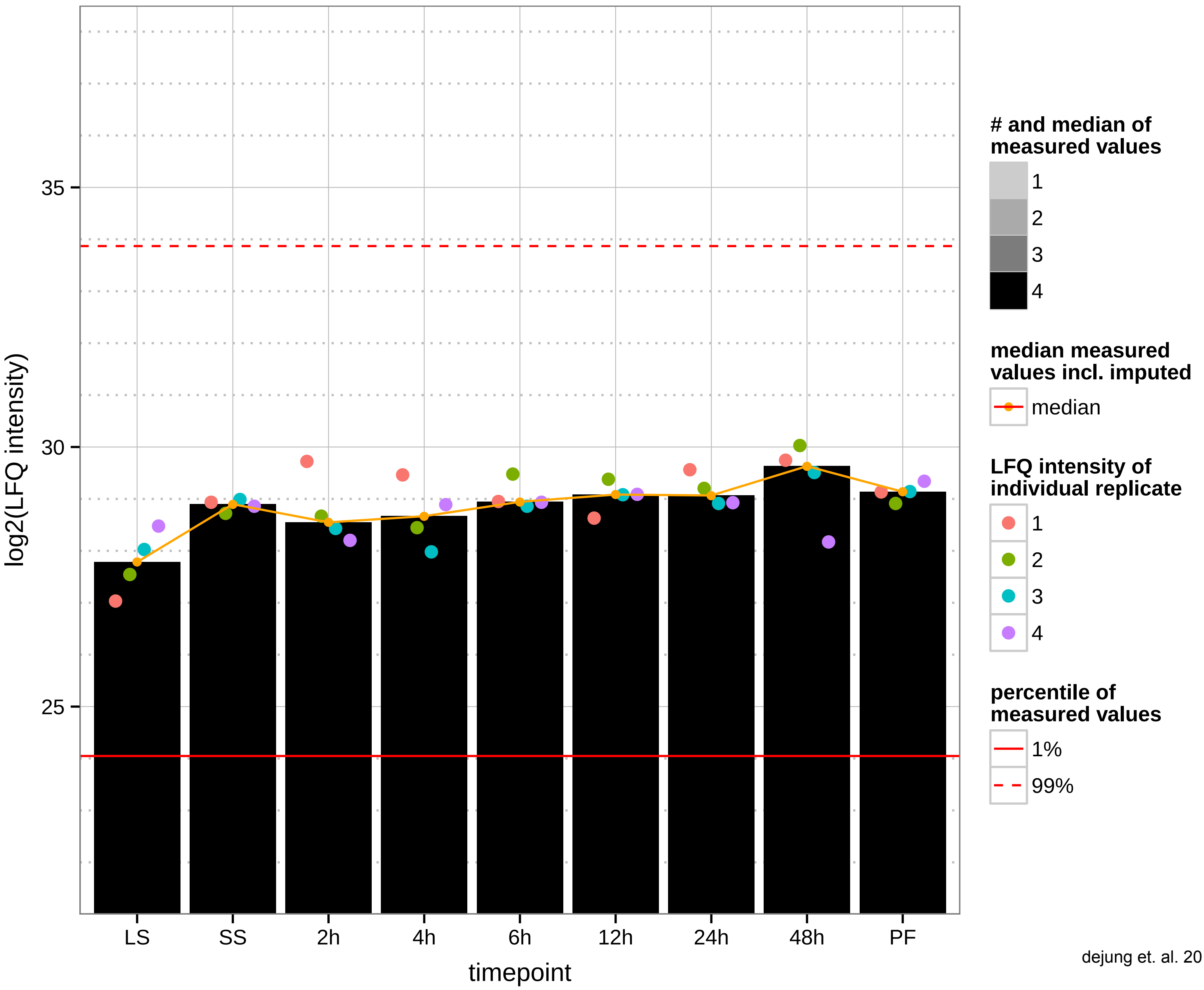

dejung et. al. 2015

retrotransposon hot spot protein (RHS, pseudogene), putative
Tb927.1.190
AGOF: null
AGOC: null
AGOP: null
PGOF: null
PGOC: null
PGOP: null

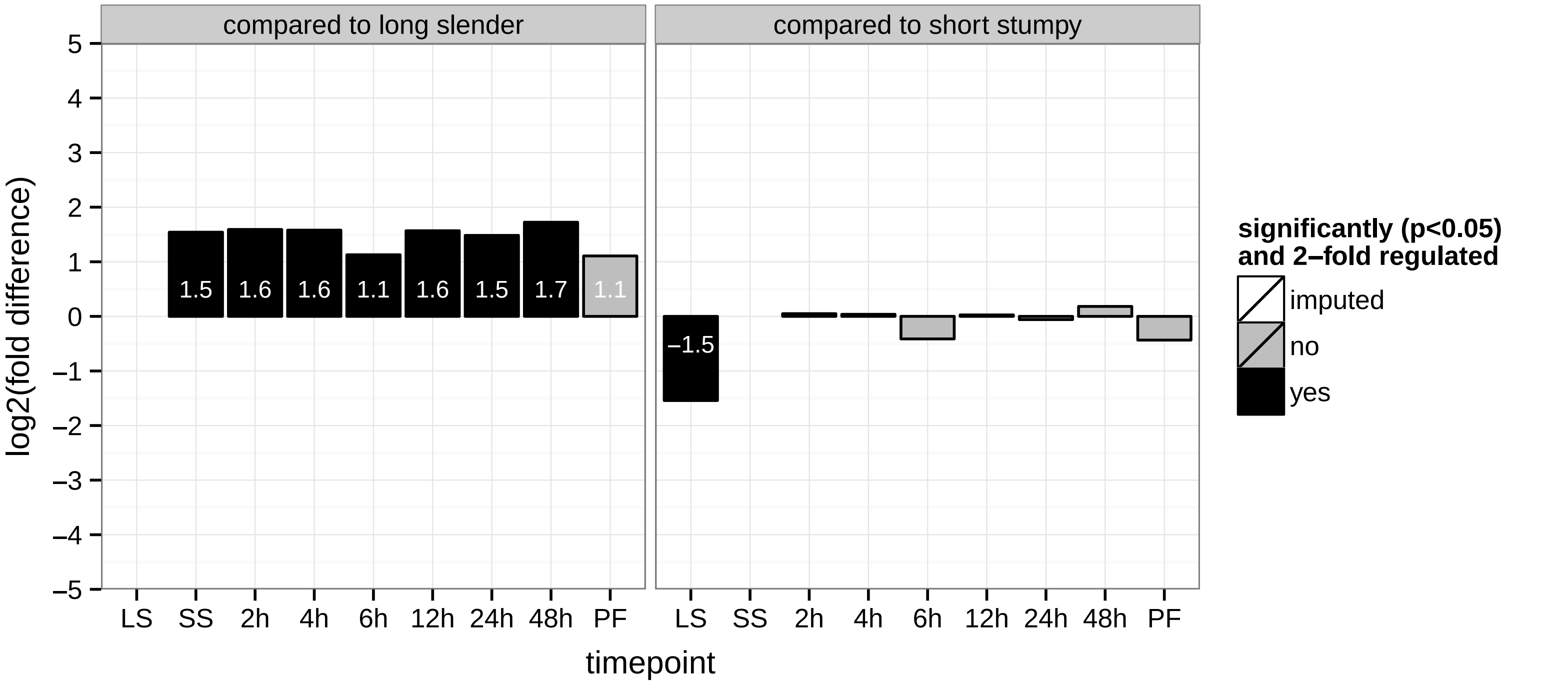

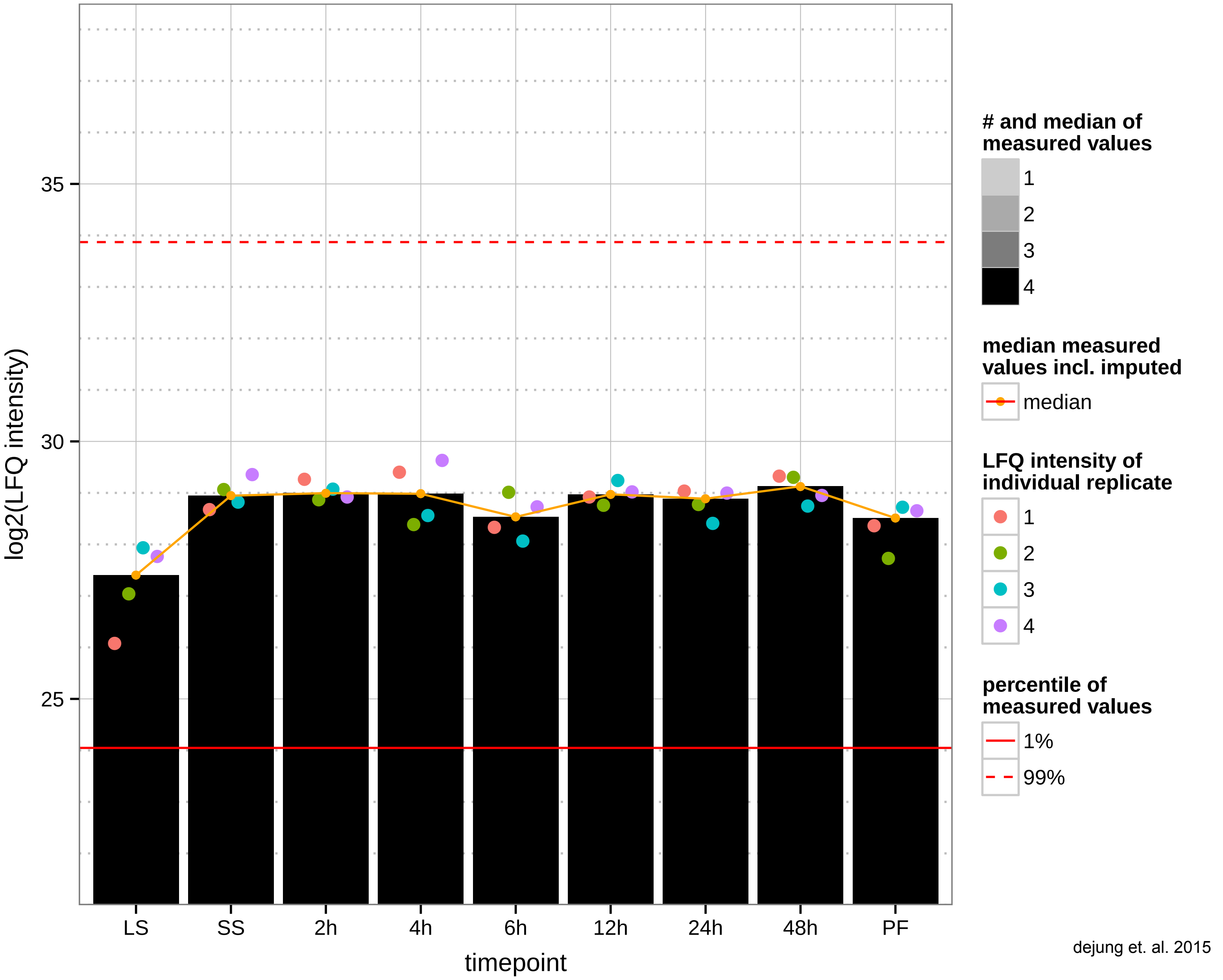

dejung et. al. 2015

chaperone protein DNAj, putative
Tb927.1.1960
AGOF: DNA binding, heat shock protein binding, unfolded protein binding
AGOC: null
AGOP: protein folding
PGOF: DNA binding, heat shock protein binding
PGOC: null
PGOP: null

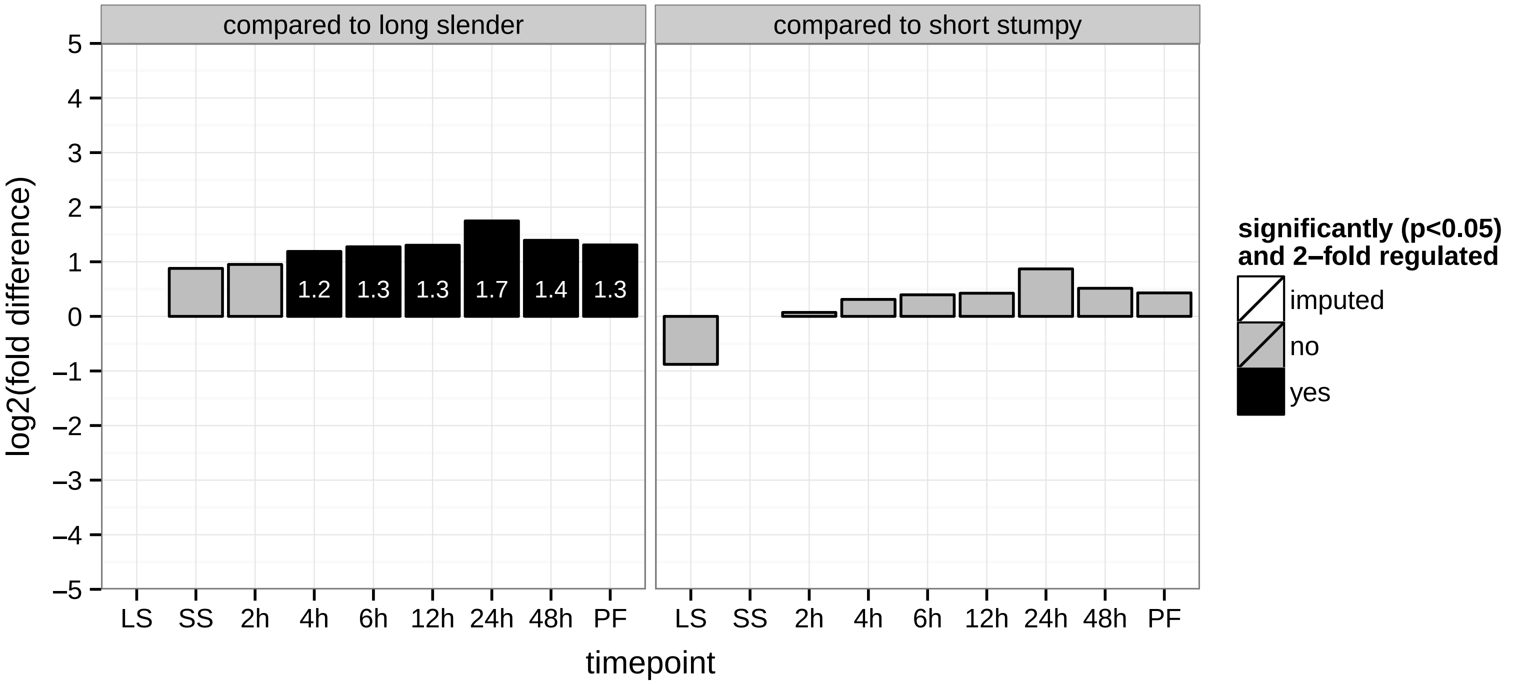

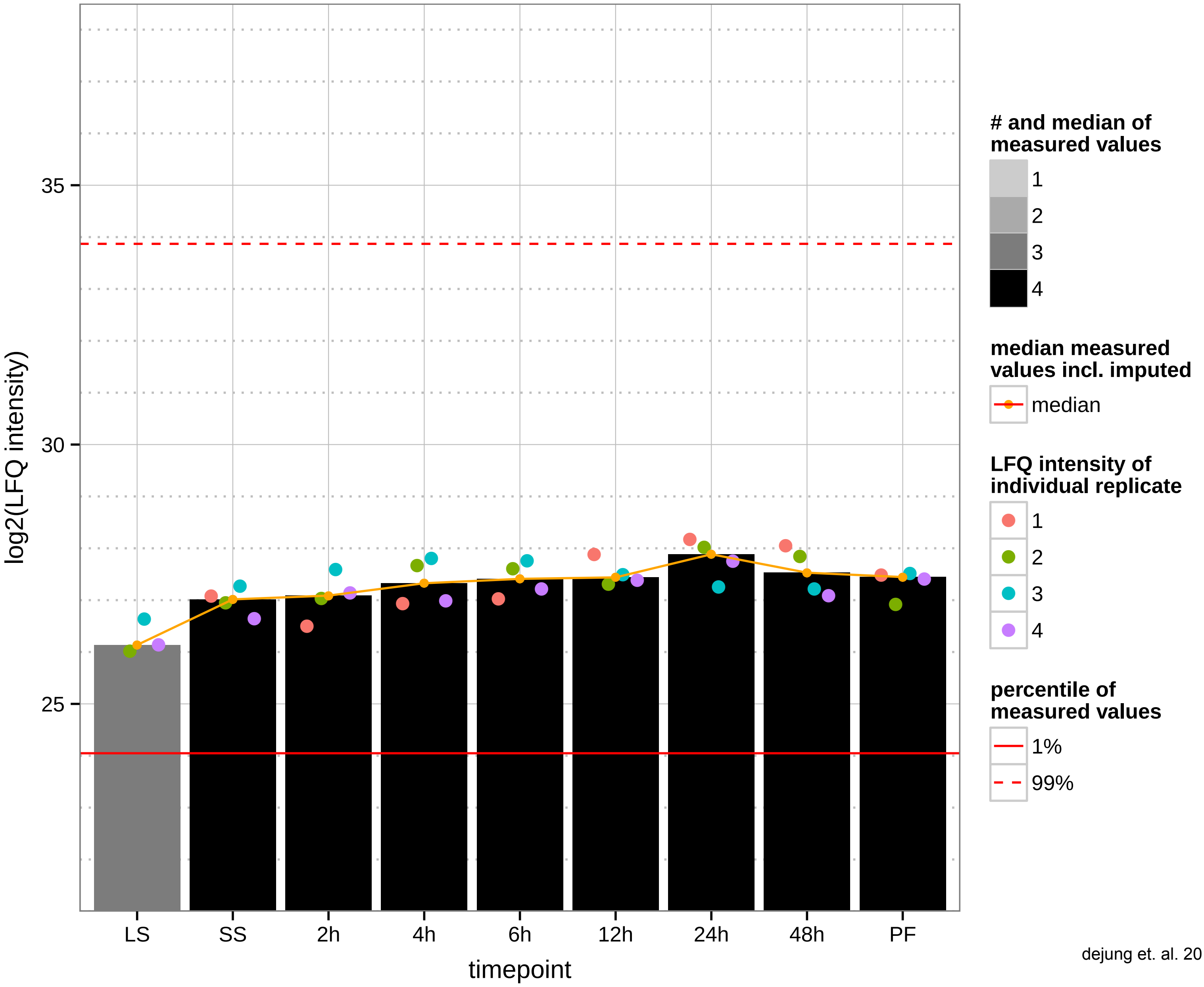

dejung et. al. 2015

cysteine peptidase, Clan CA, family C2, putative, calpain−like cysteine peptidase
Tb927.1.2100
AGOF: calcium−dependent cysteine−type endopeptidase activity
AGOC: intracellular
AGOP: proteolysis
PGOF: calcium−dependent cysteine−type endopeptidase activity
PGOC: intracellular
PGOP: proteolysis

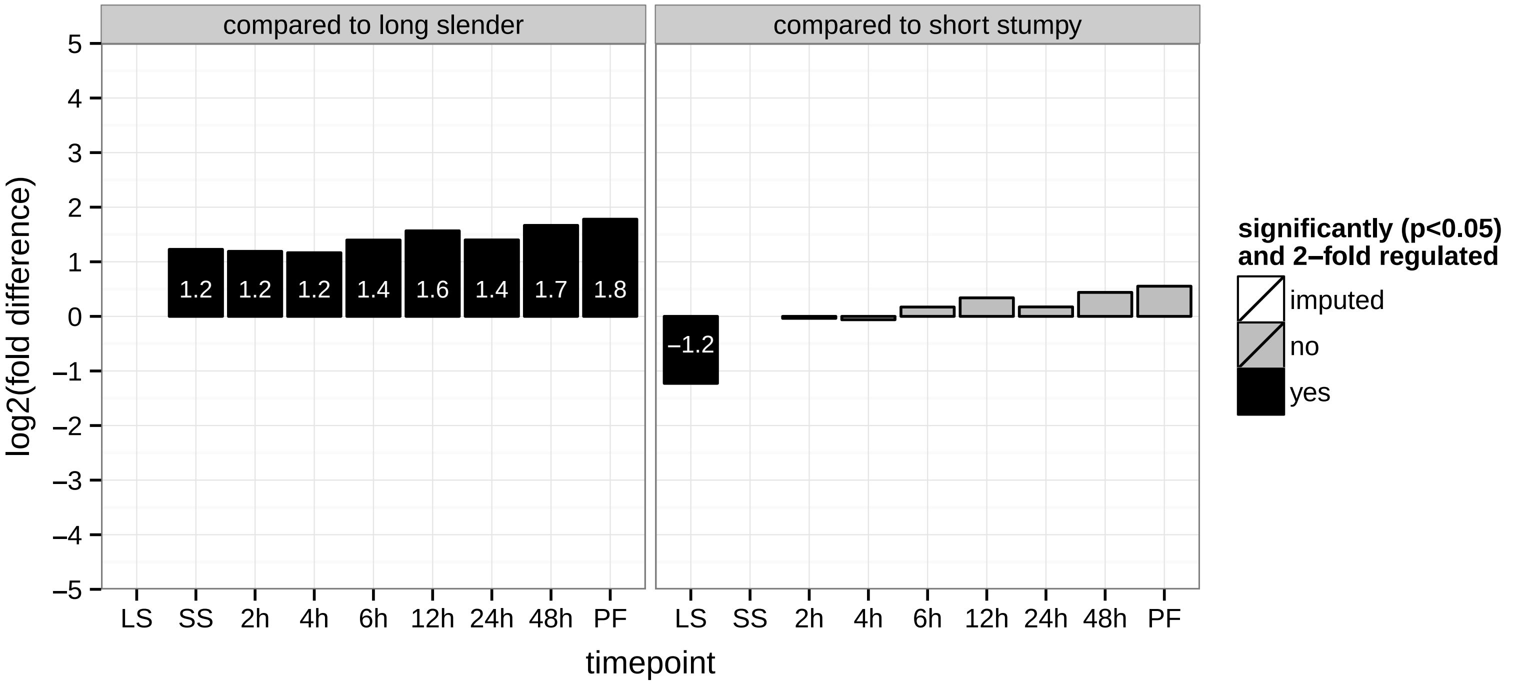

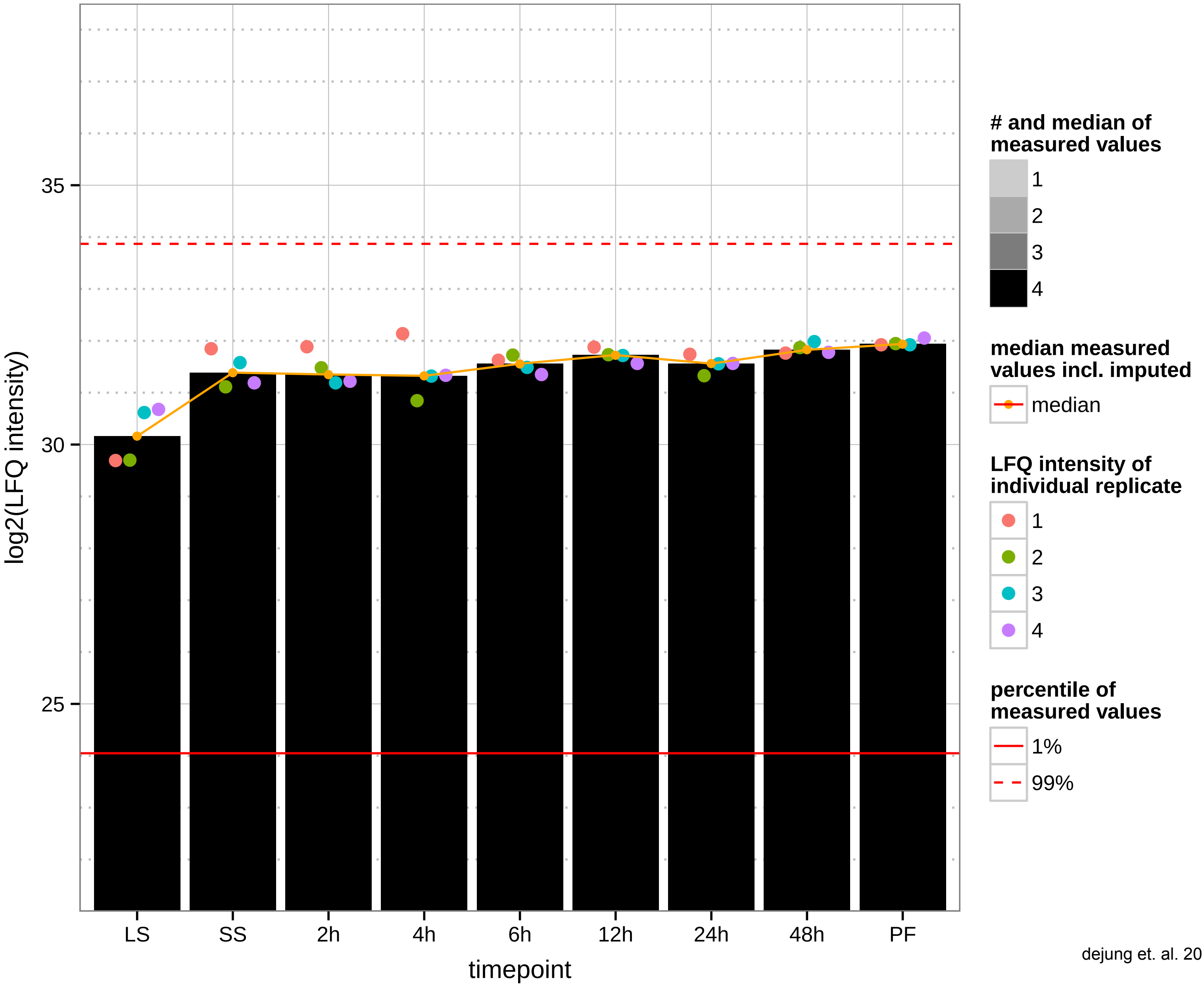

dejung et. al. 2015

calpain, putative, cysteine peptidase, Clan CA, family C2
Tb927.1.2120
AGOF: calcium−dependent cysteine−type endopeptidase activity
AGOC: intracellular
AGOP: proteolysis
PGOF: calcium−dependent cysteine−type endopeptidase activity
PGOC: intracellular
PGOP: proteolysis

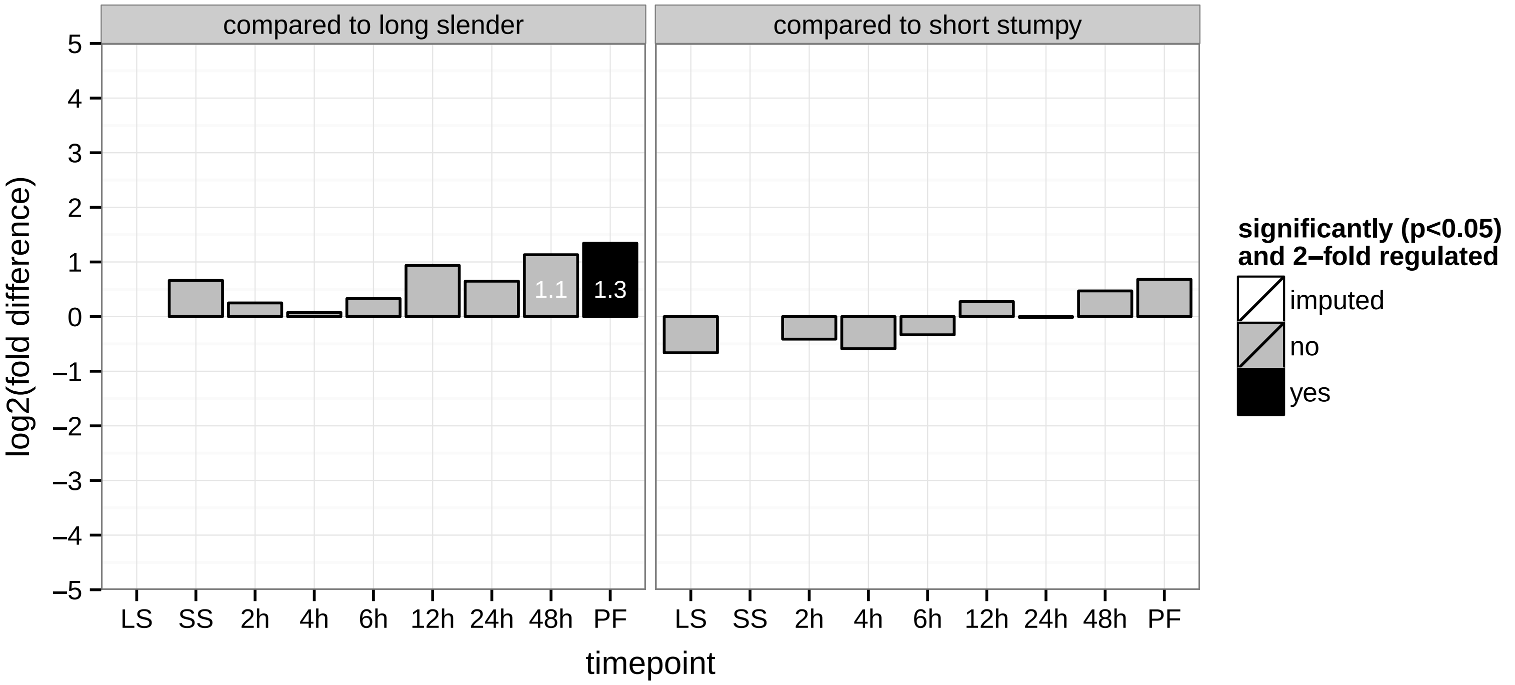

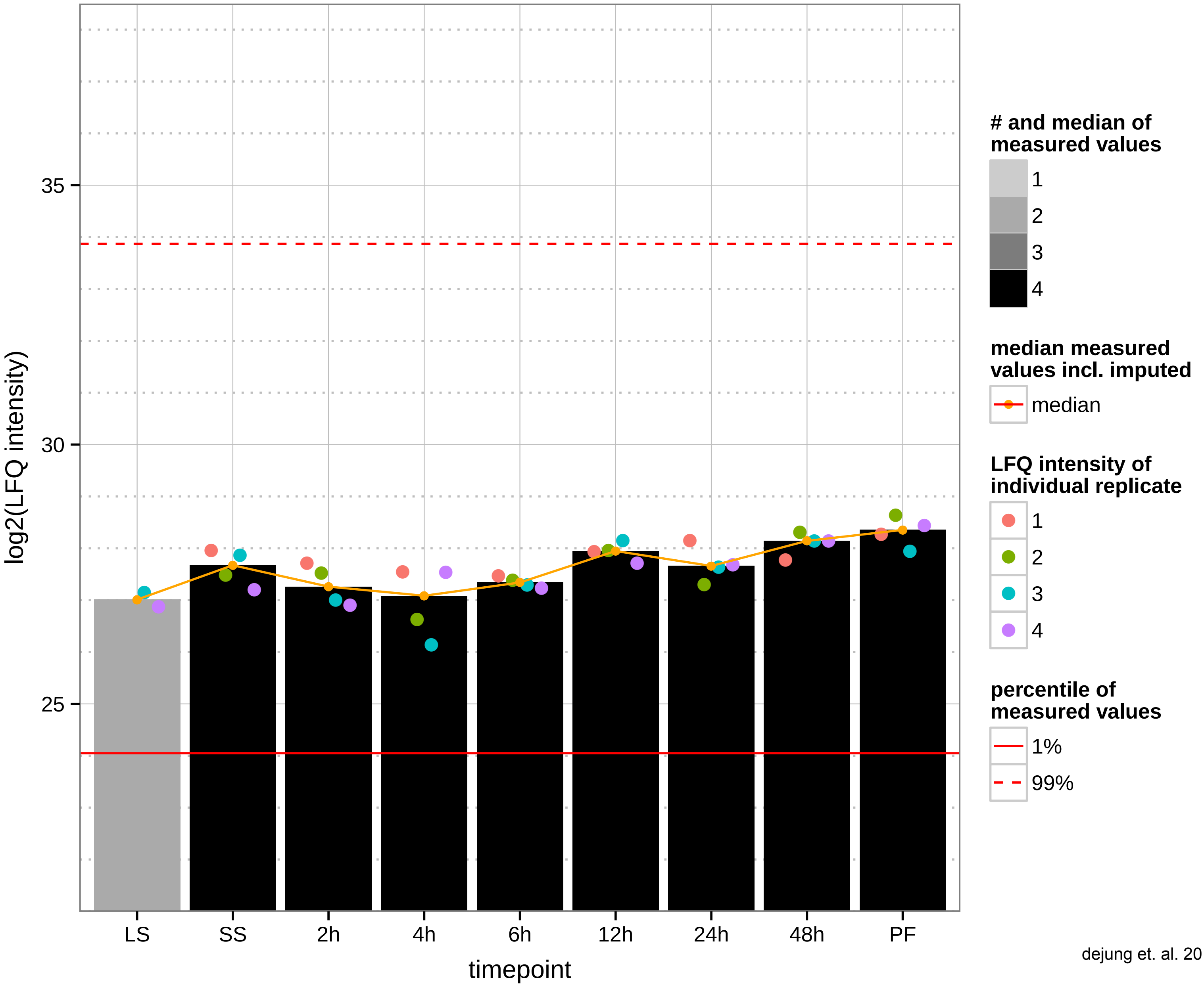

dejung et. al. 2015

alpha tubulin
Tb927.1.2400;Tb927.1.2380;Tb927.1.2360;Tb927.1.2340
AGOF: GTP binding, GTPase activity, structural constituent of cytoskeleton
AGOC: microtubule, protein complex
AGOP: cellular component movement, microtubule cytoskeleton organization, microtubule−based movement, protein polymer
PGOF: GTP binding, GTPase activity, null
PGOC: protein complex, null
PGOP: GTP catabolic process, protein polymerization, null

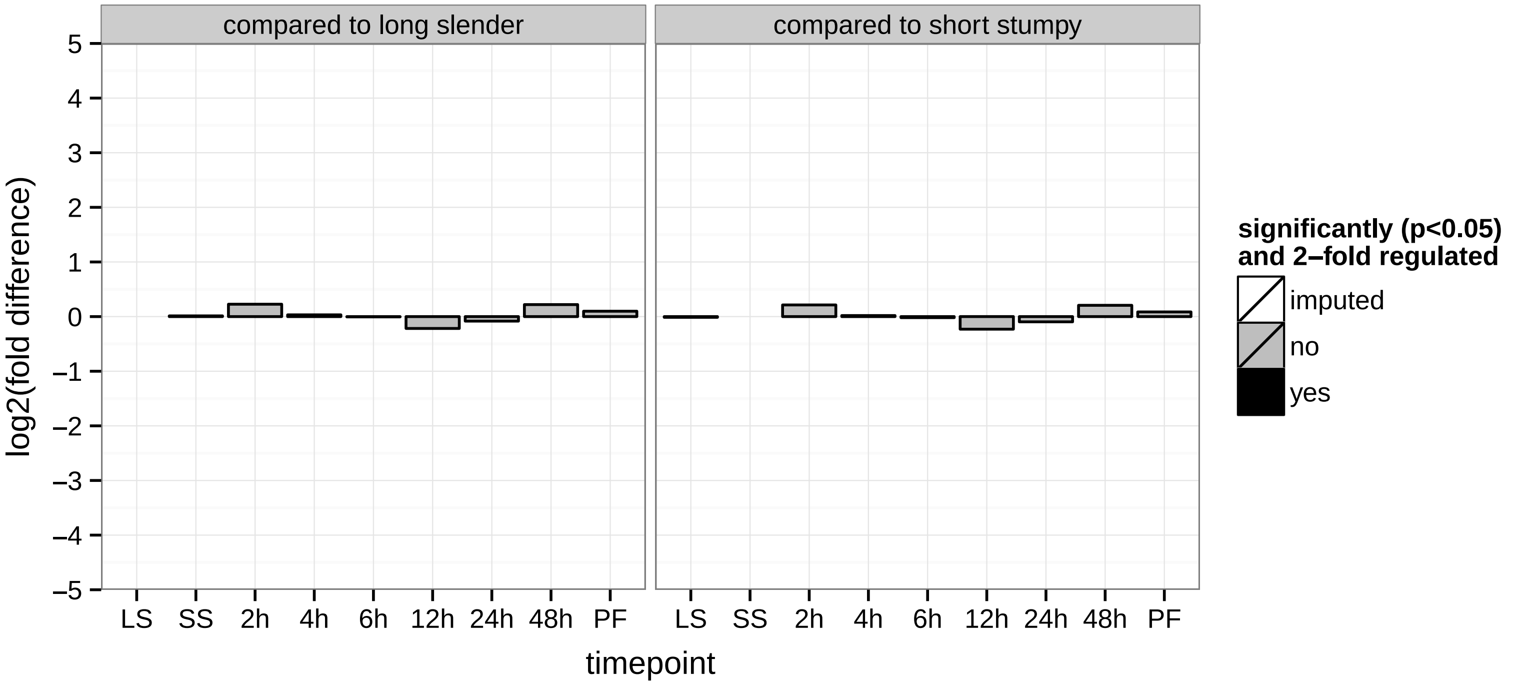

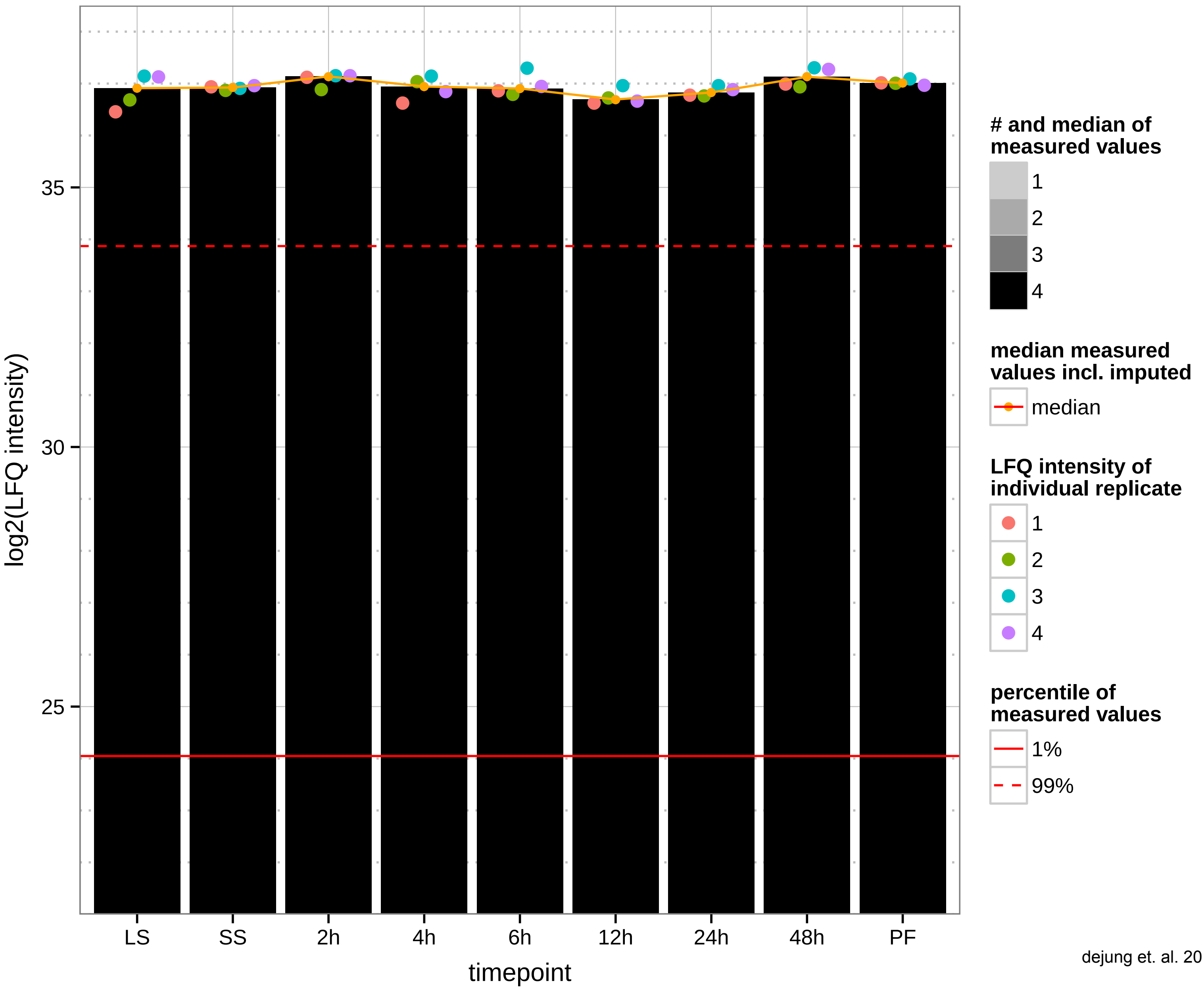

dejung et. al. 2015

retrotransposon hot spot protein (RHS, pseudogene), putative
Tb927.1.240
AGOF: RNA binding, RNA−directed DNA polymerase activity, ribonuclease H activity, zinc ion binding
AGOC: intracellular
AGOP: RNA−dependent DNA replication
PGOF: RNA binding, RNA−directed DNA polymerase activity, nucleic acid binding, ribonuclease H activity, zinc ion binding
PGOC: intracellular
PGOP: RNA−dependent DNA replication

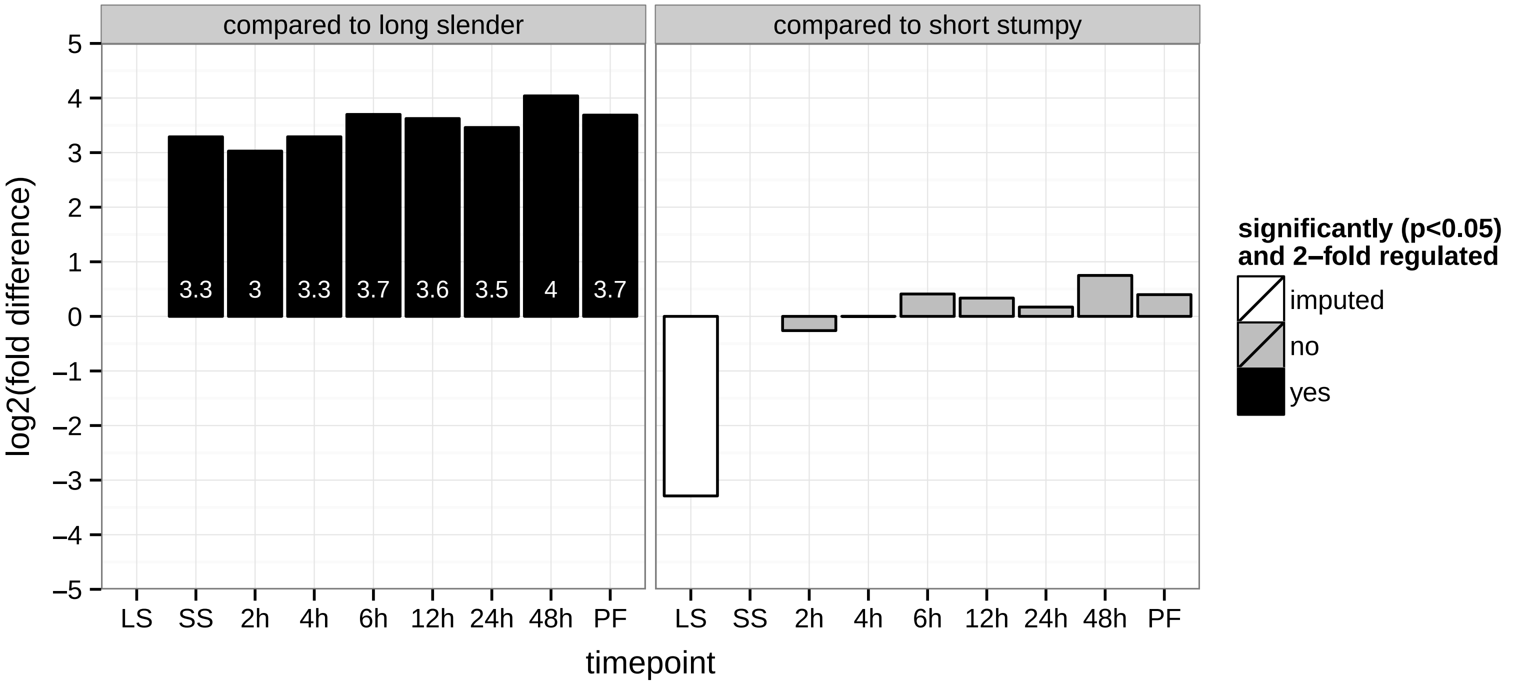

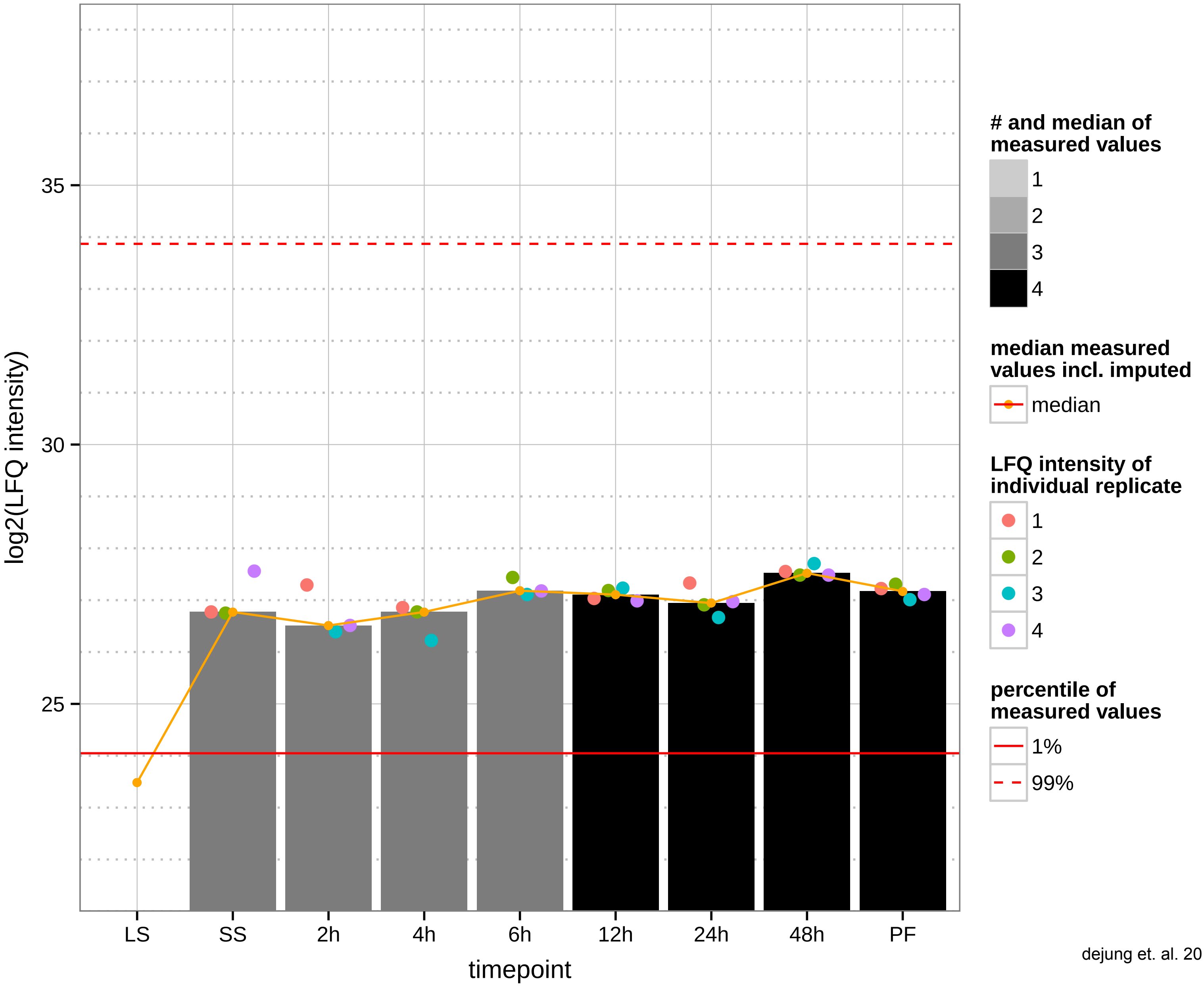

dejung et. al. 2015

histone H3, putative
Tb927.1.2550;Tb927.1.2530;Tb927.1.2510;Tb927.1.2490;Tb927.1.2470;Tb927.1.2450;Tb927.1.2430
AGOF: DNA binding
AGOC: nucleosome, nucleus
AGOP: nucleosome assembly
PGOF: null, DNA binding
PGOC: null, nucleosome
PGOP: null, nucleosome assembly

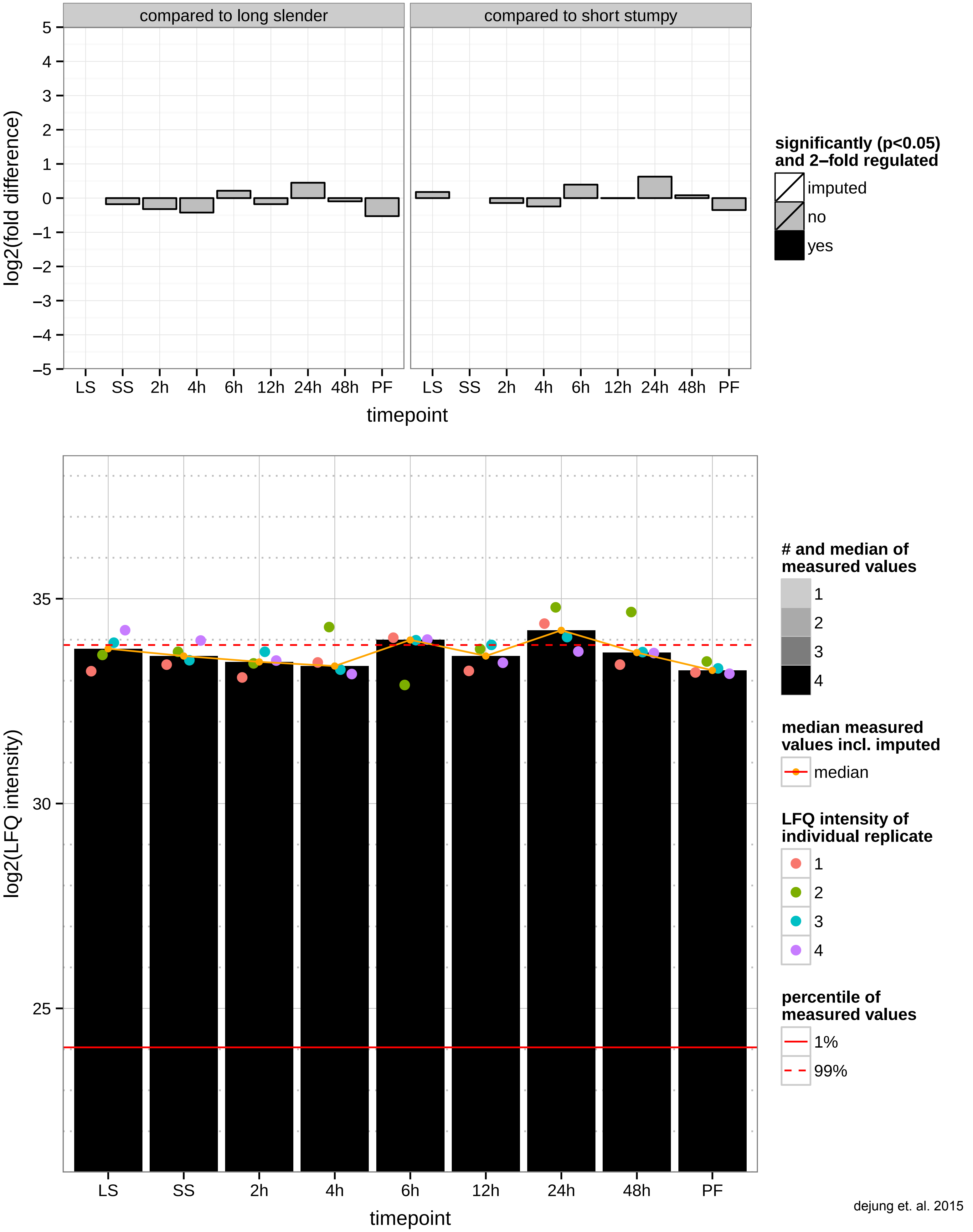

dejung et. al. 2015

coatomer beta subunit (beta–coP)
Tb927.1.2570
AGOF: structural molecule activity
AGOC: membrane coat
AGOP: intracellular protein transport, vesicle–mediated transport
PGOF: binding, structural molecule activity
PGOC: COPI vesicle coat, cytoplasm, membrane coat
PGOP: intracellular protein transport, vesicle–mediated transport

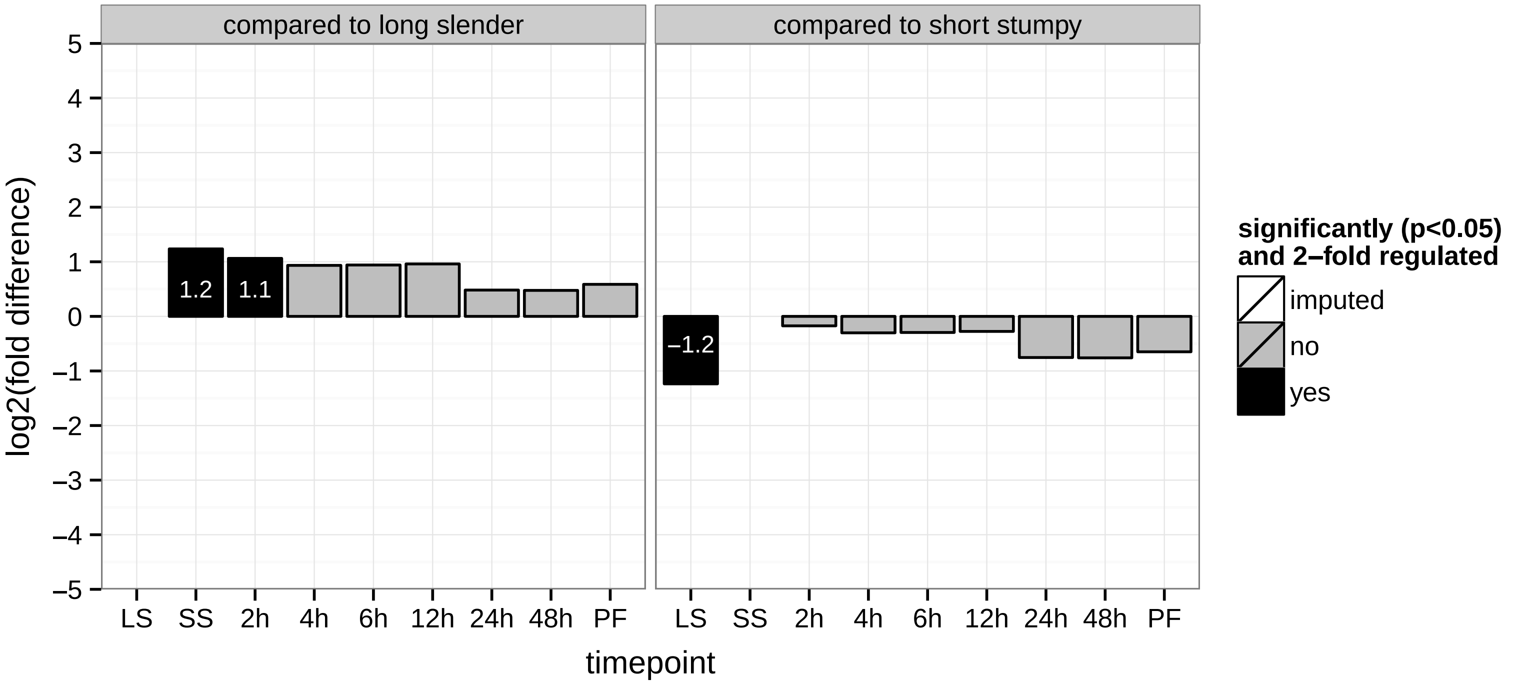

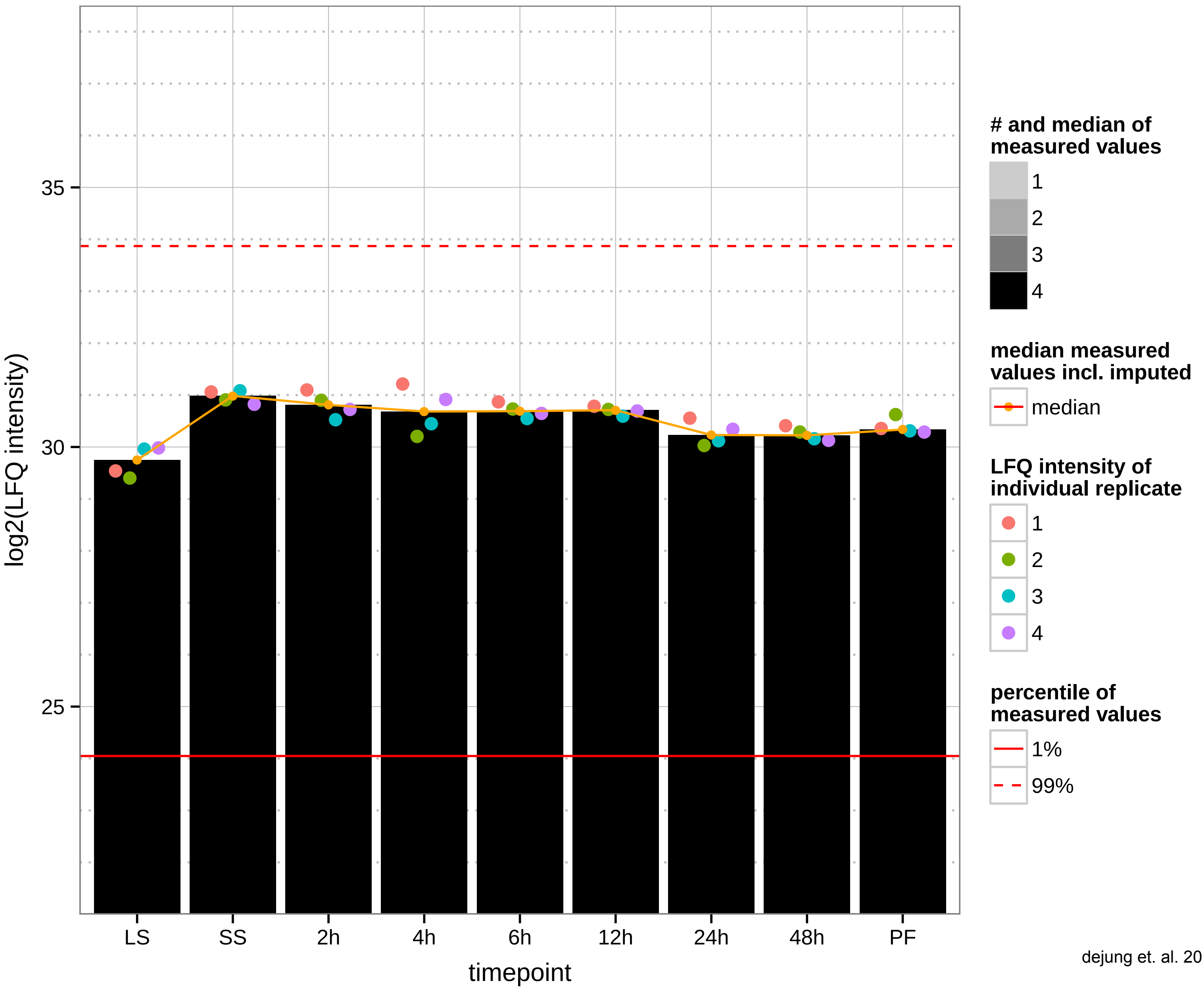

dejung et. al. 2015

flagellar protein PF16, axoneme central apparatus protein
Tb927.1.2670
AGOF: null
AGOC: axoneme
AGOP: axoneme assembly, ciliary or flagellar motility
PGOF: binding, protein binding
PGOC: null
PGOP: null

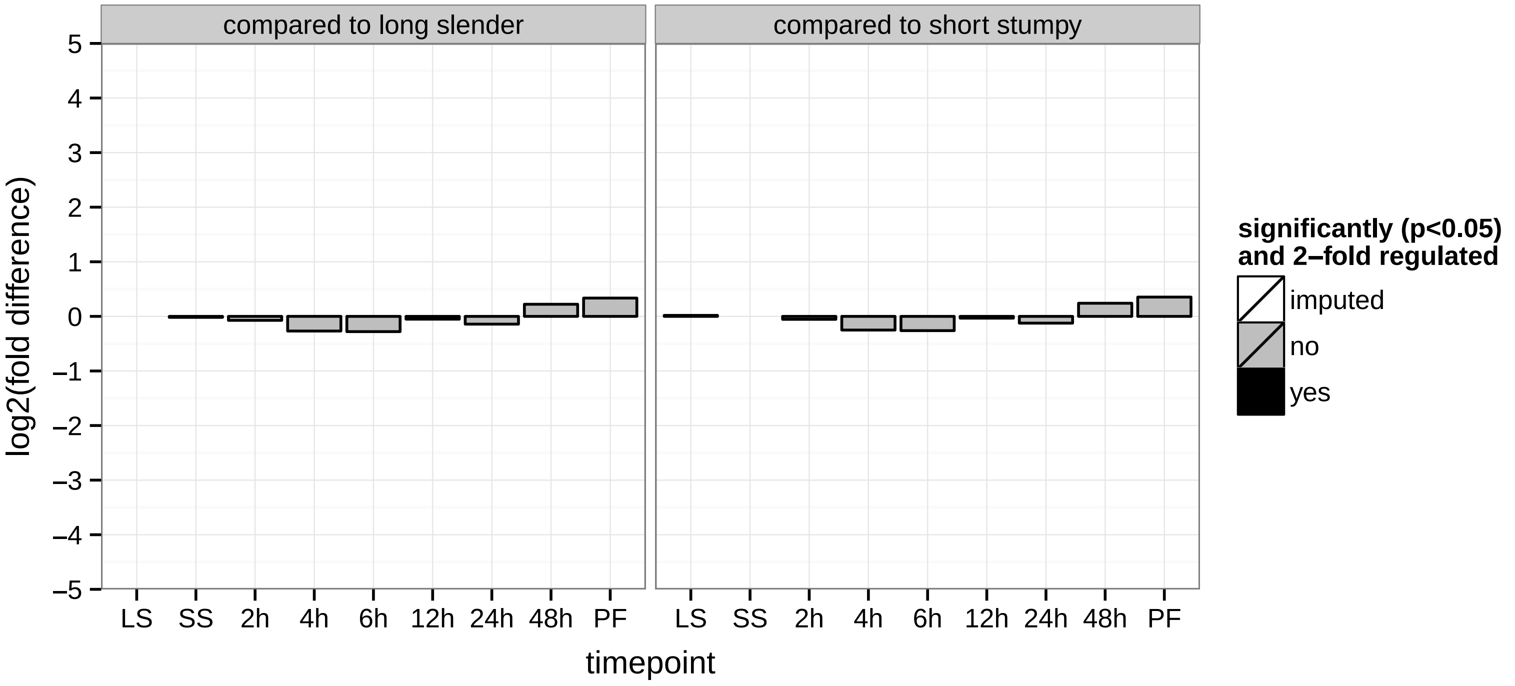

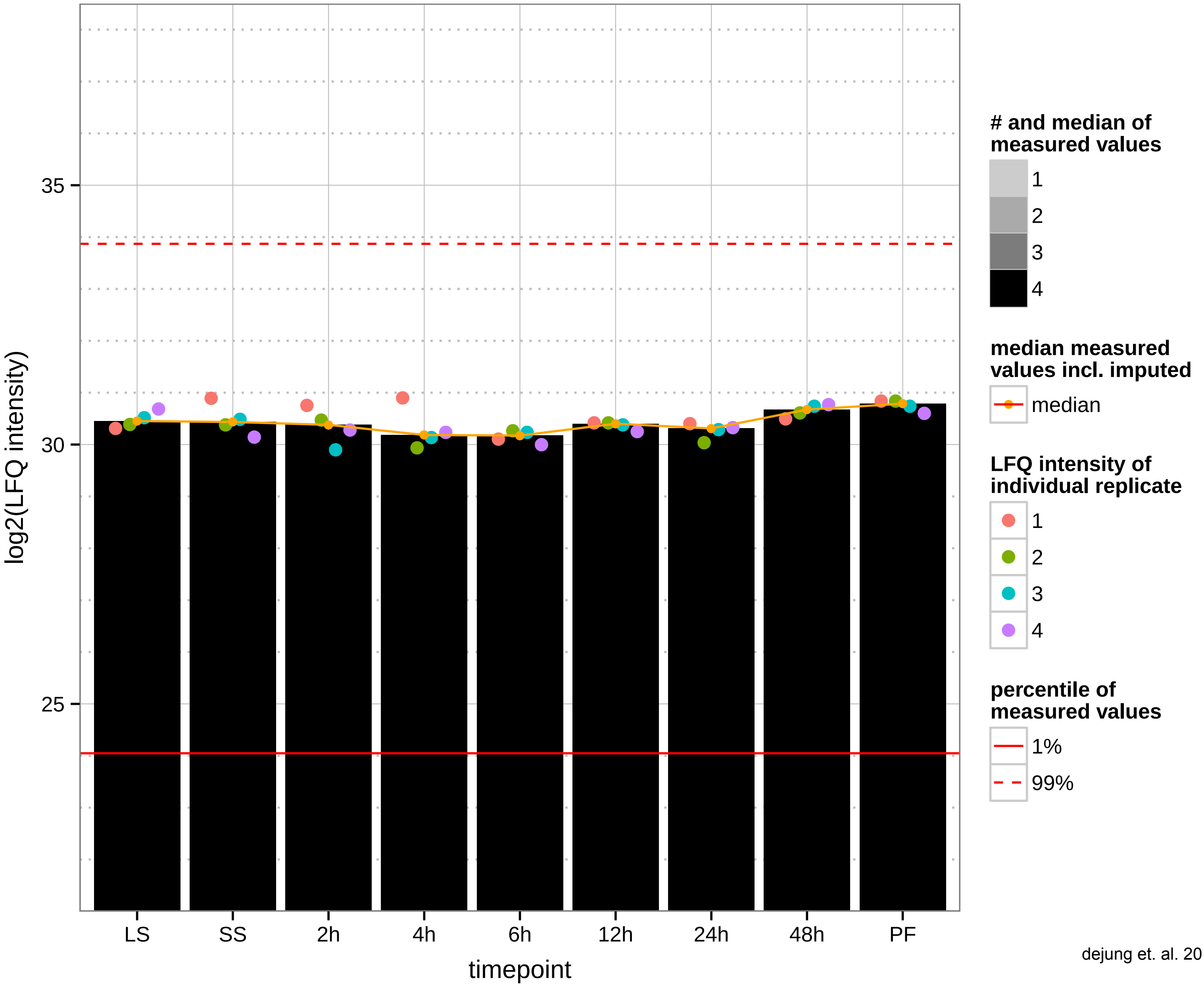

dejung et. al. 2015

KREL2, RNA–editing complex protein, RNA editing ligase (KREL2)
Tb927.1.3030
AGOF: ATP binding, RNA ligase (ATP) activity
AGOC: mRNA editing complex, mitochondrial mRNA editing complex, mitochondrion
AGOP: mRNA modification
PGOF: ATP binding, RNA ligase (ATP) activity, ligase activity
PGOC: null
PGOP: null

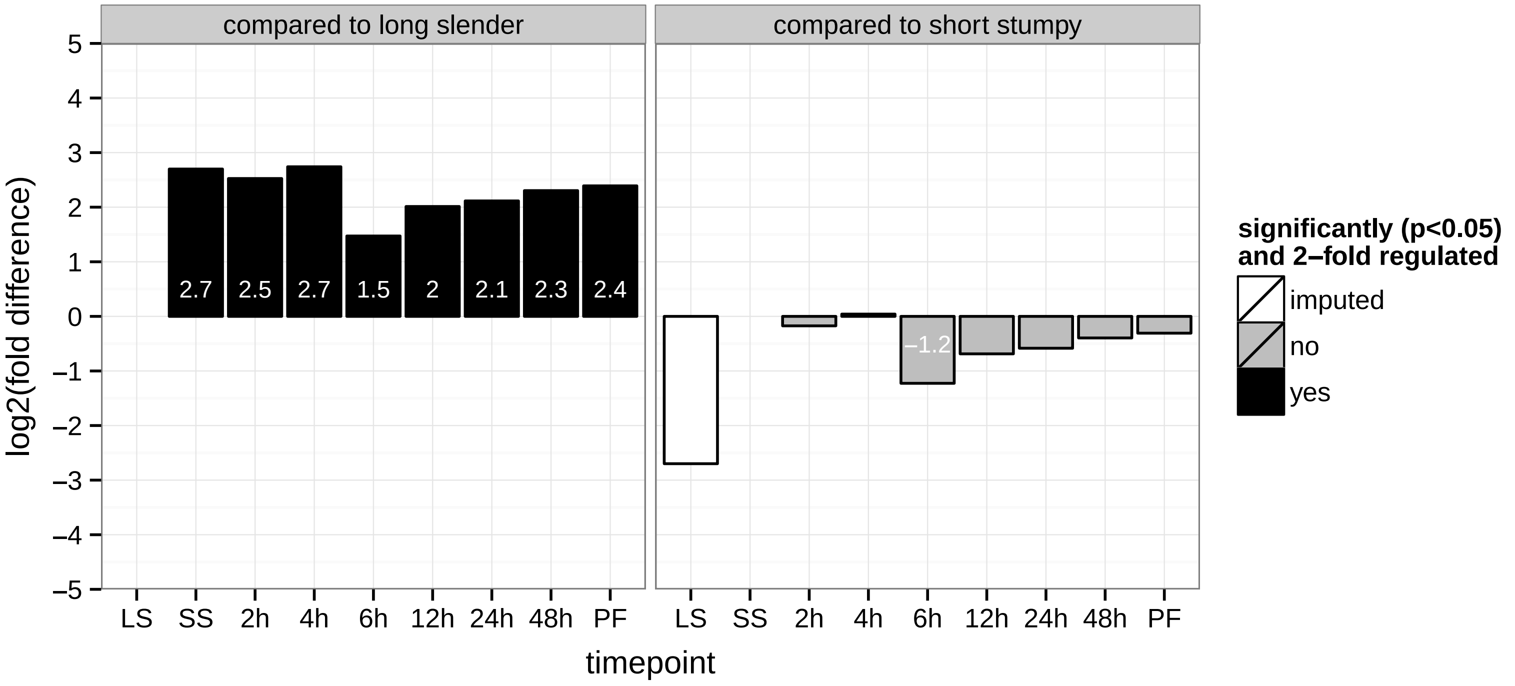

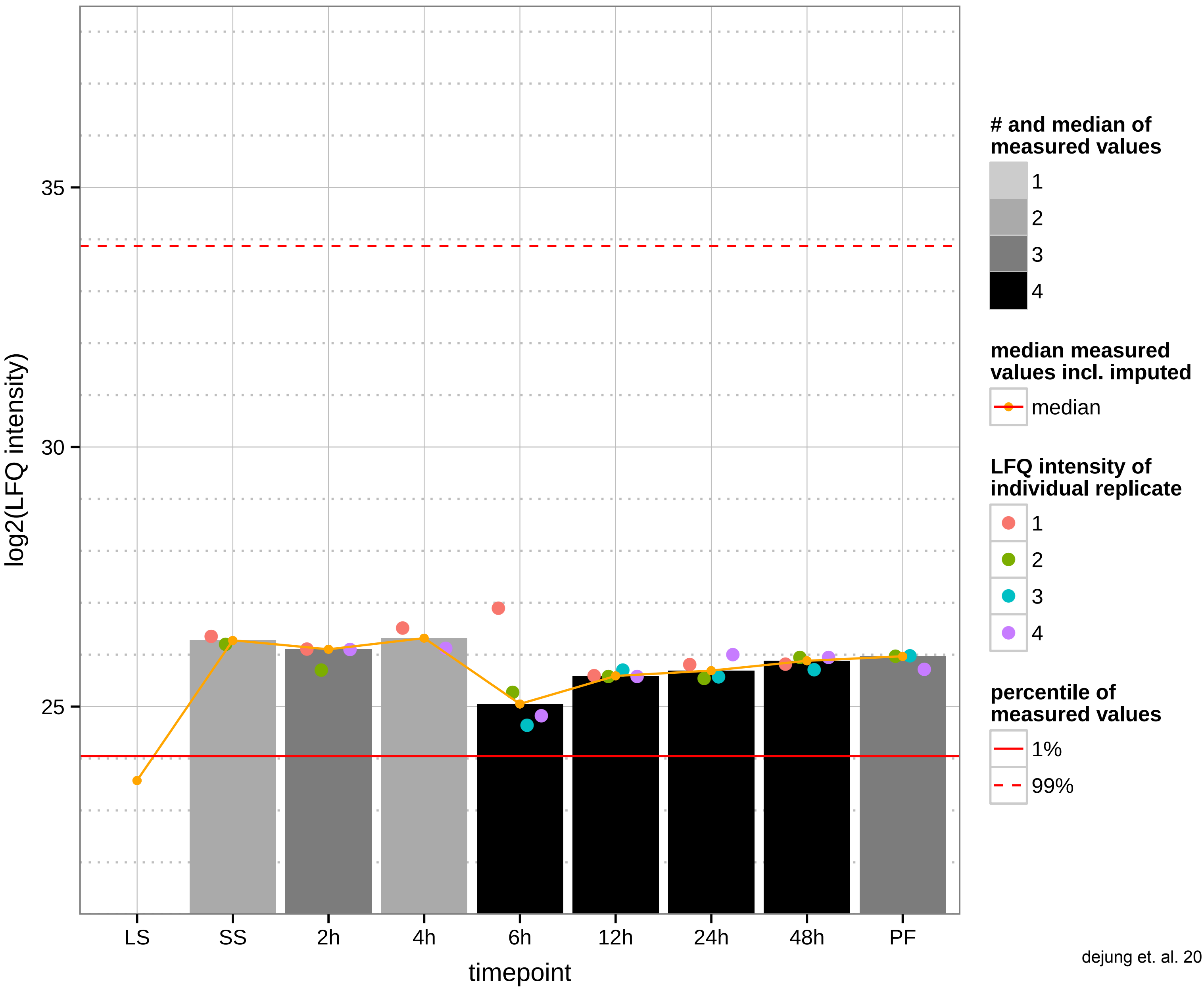

dejung et. al. 2015

soluble N–ethylmaleimide sensitive factor (NSF) attachment protein, putative
Tb927.1.3110
AGOF: soluble NSF attachment protein activity
AGOC: Golgi apparatus, endoplasmic reticulum
AGOP: intracellular protein transport
PGOF: null
PGOC: null
PGOP: null

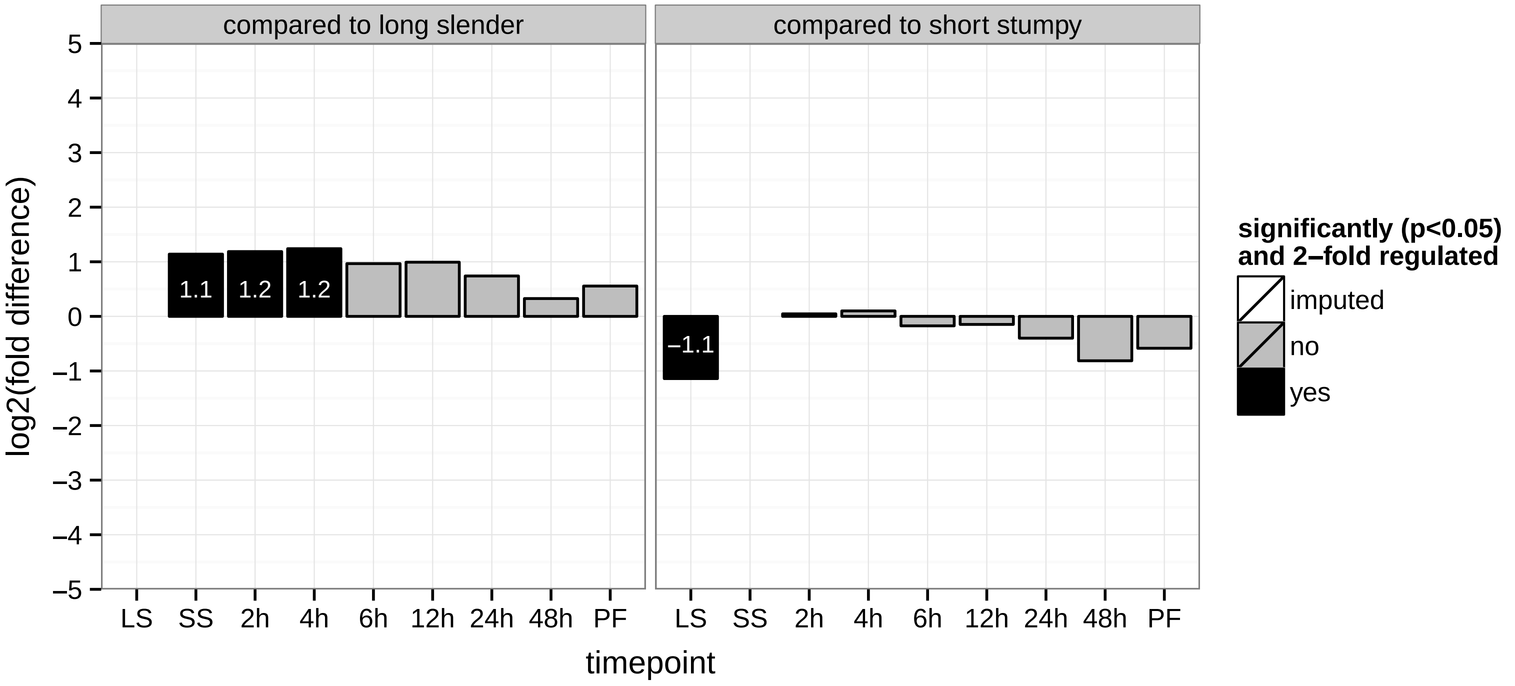

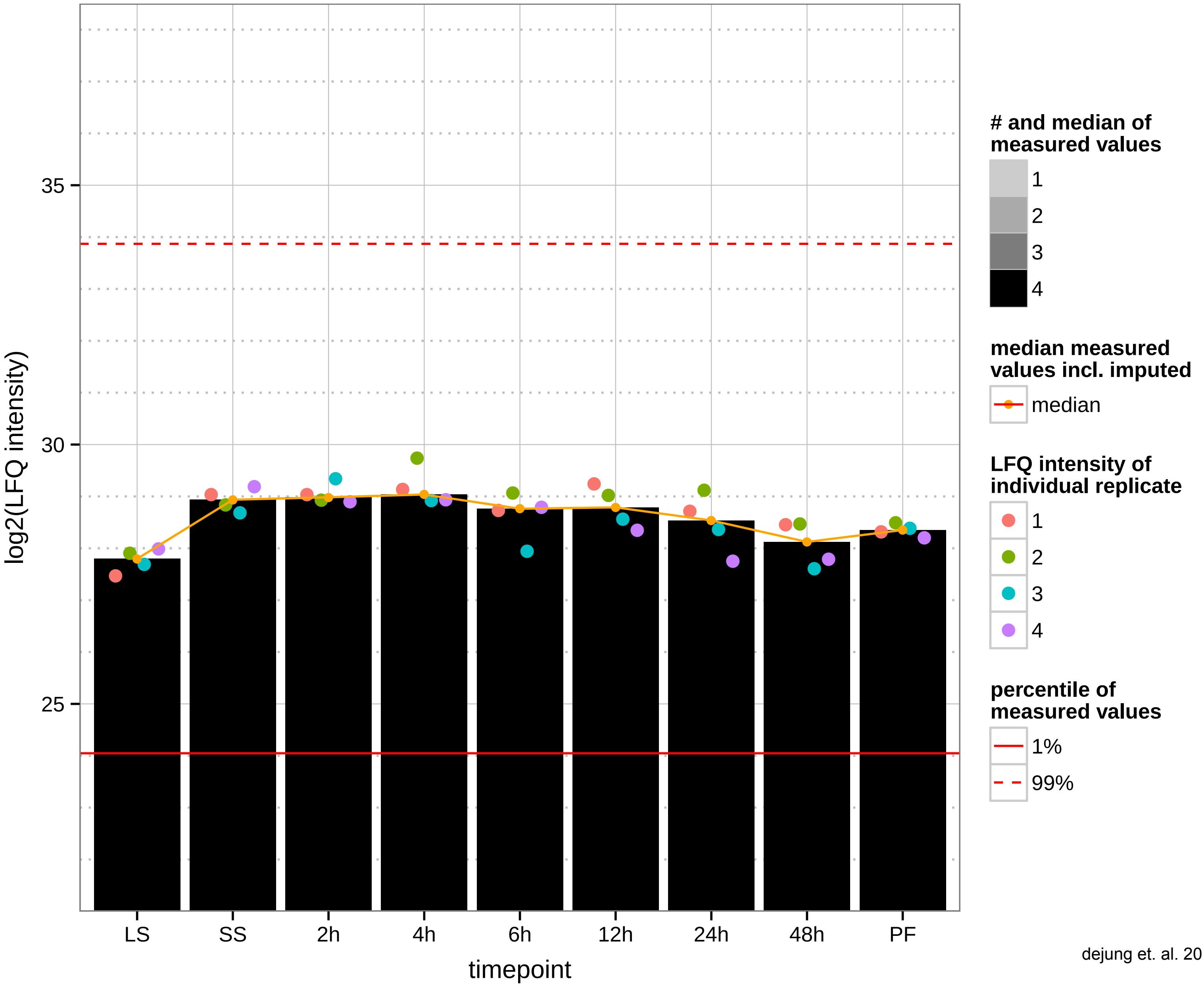

dejung et. al. 2015

Suppressor of G2 allele of SKP1, putative
Tb927.1.3200
AGOF: null
AGOC: ubiquitin ligase complex
AGOP: null
PGOF: null
PGOC: null
PGOP: null

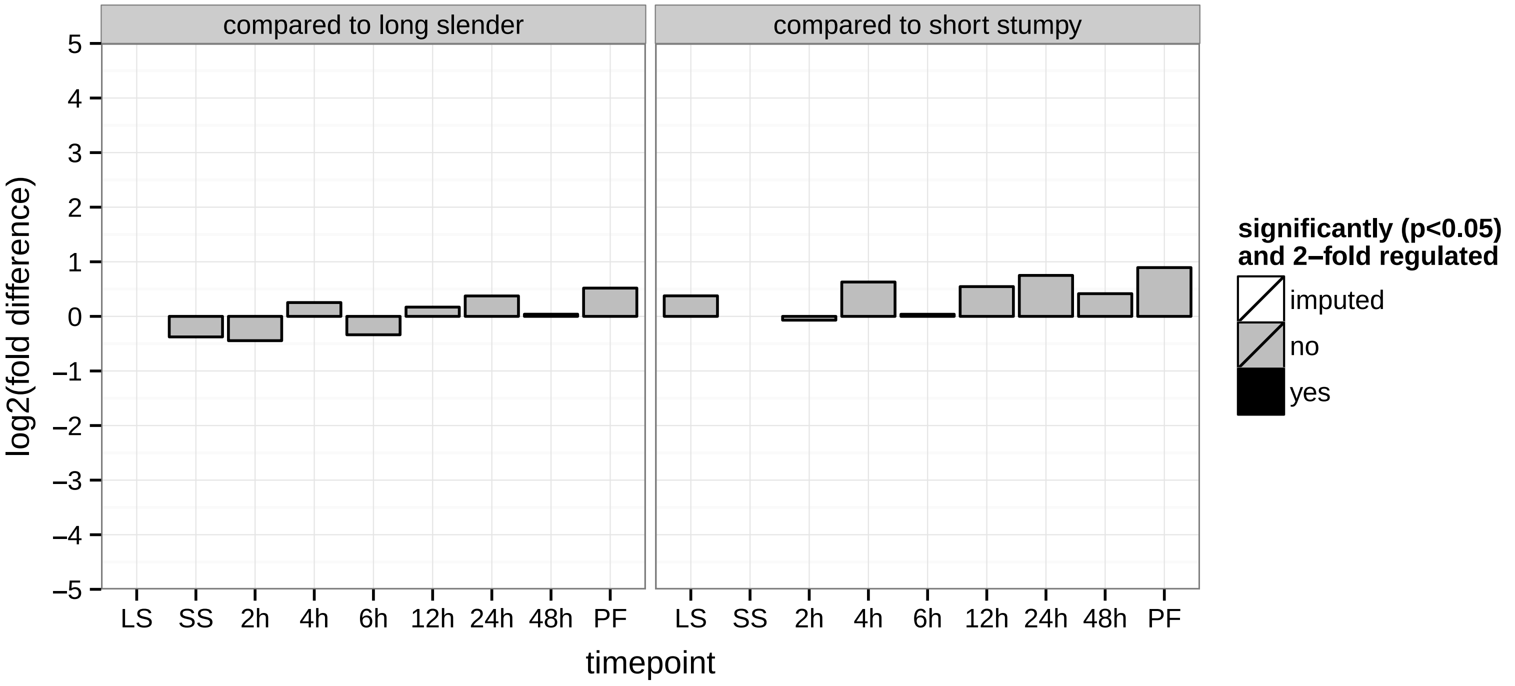

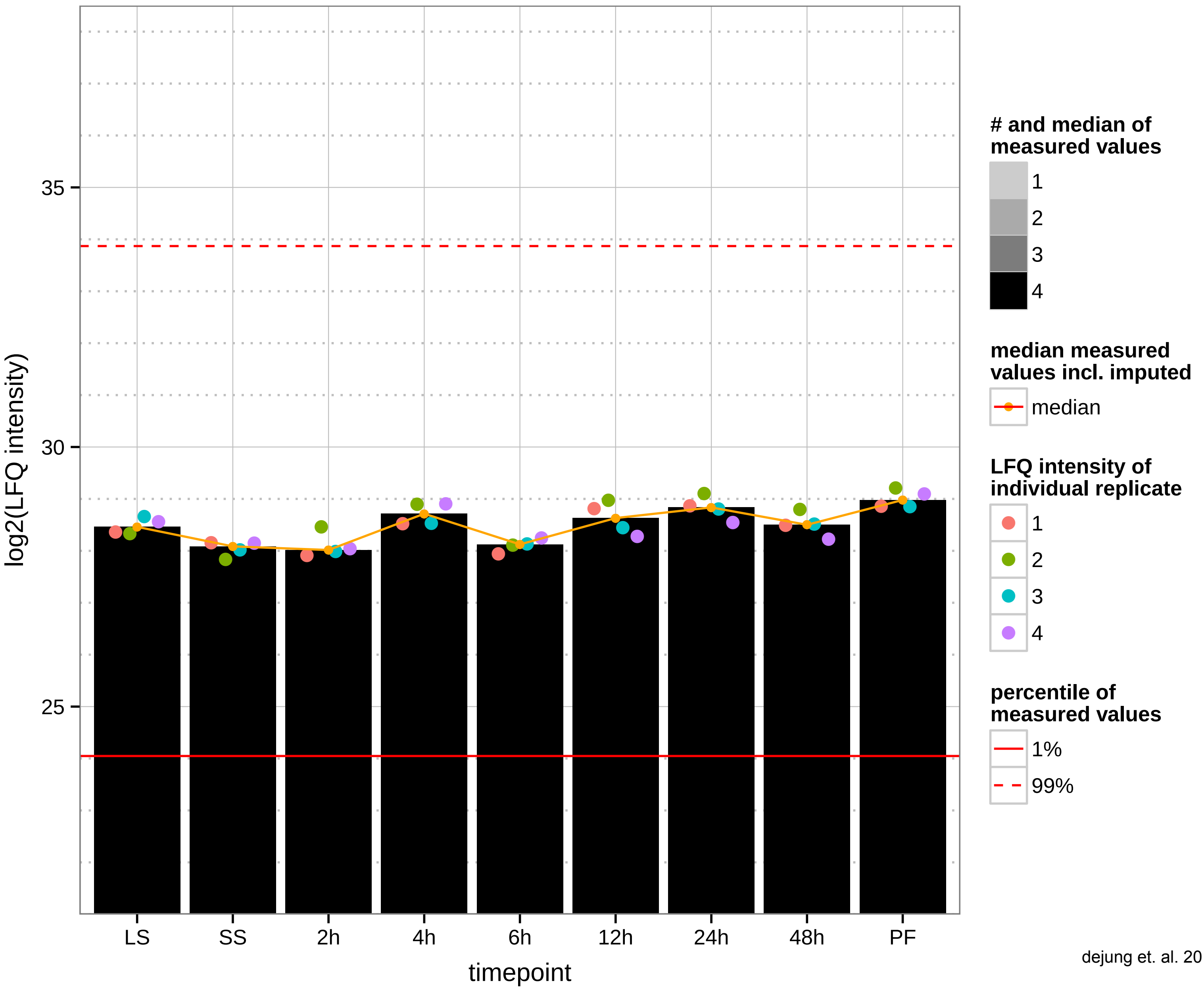

dejung et. al. 2015

TBC1 domain family member 20/GTPase, putative
Tb927.1.3220
AGOF: Rab GTPase activator activity
AGOC: intracellular
AGOP: regulation of Rab GTPase activity
PGOF: Rab GTPase activator activity
PGOC: intracellular
PGOP: regulation of Rab GTPase activity

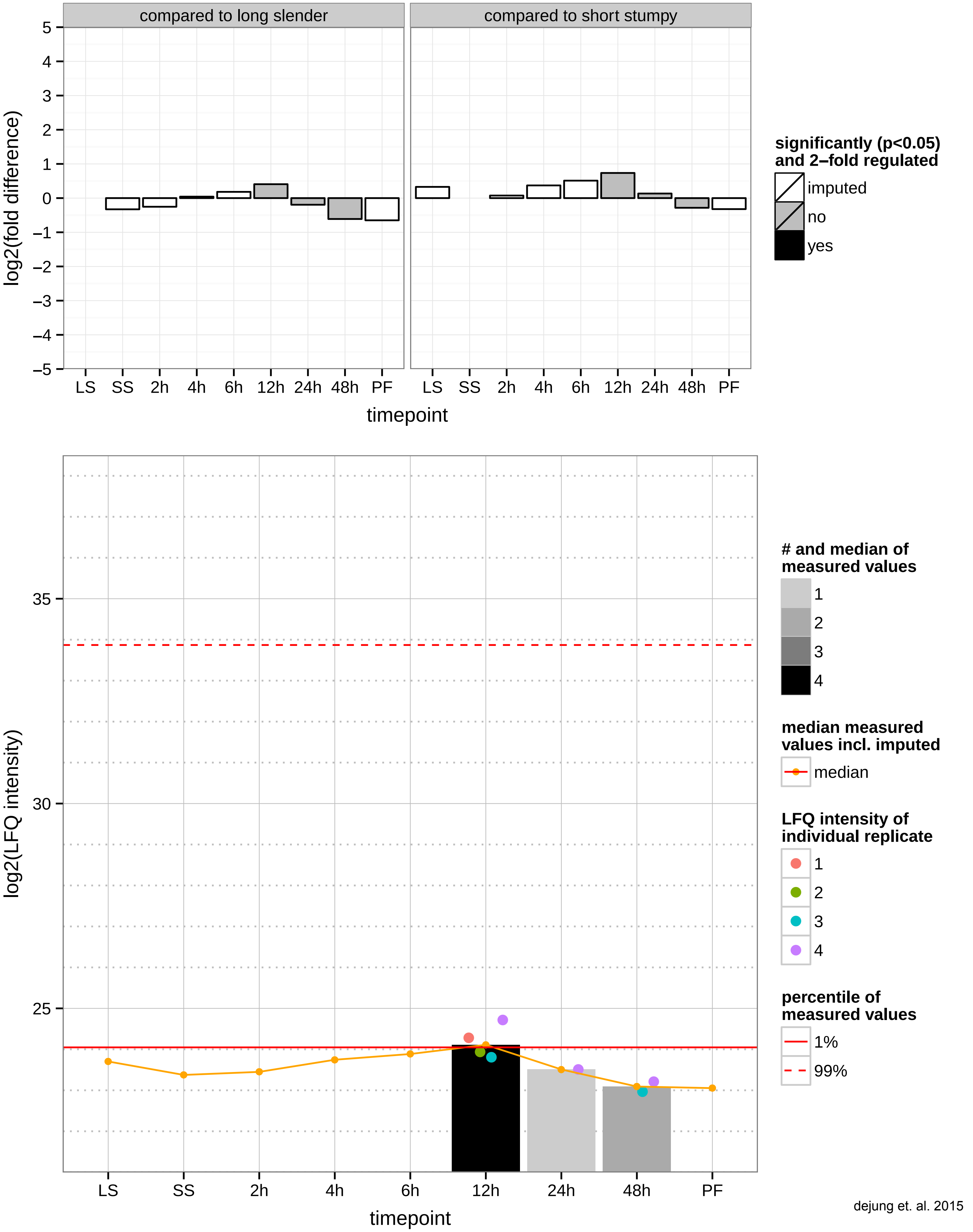

dejung et. al. 2015

cell division cycle protein, putative
Tb927.1.3230
AGOF: ATP binding, nucleoside–triphosphatase activity
AGOC: null
AGOP: null
PGOF: ATP binding, nucleoside–triphosphatase activity, nucleotide binding
PGOC: null
PGOP: null

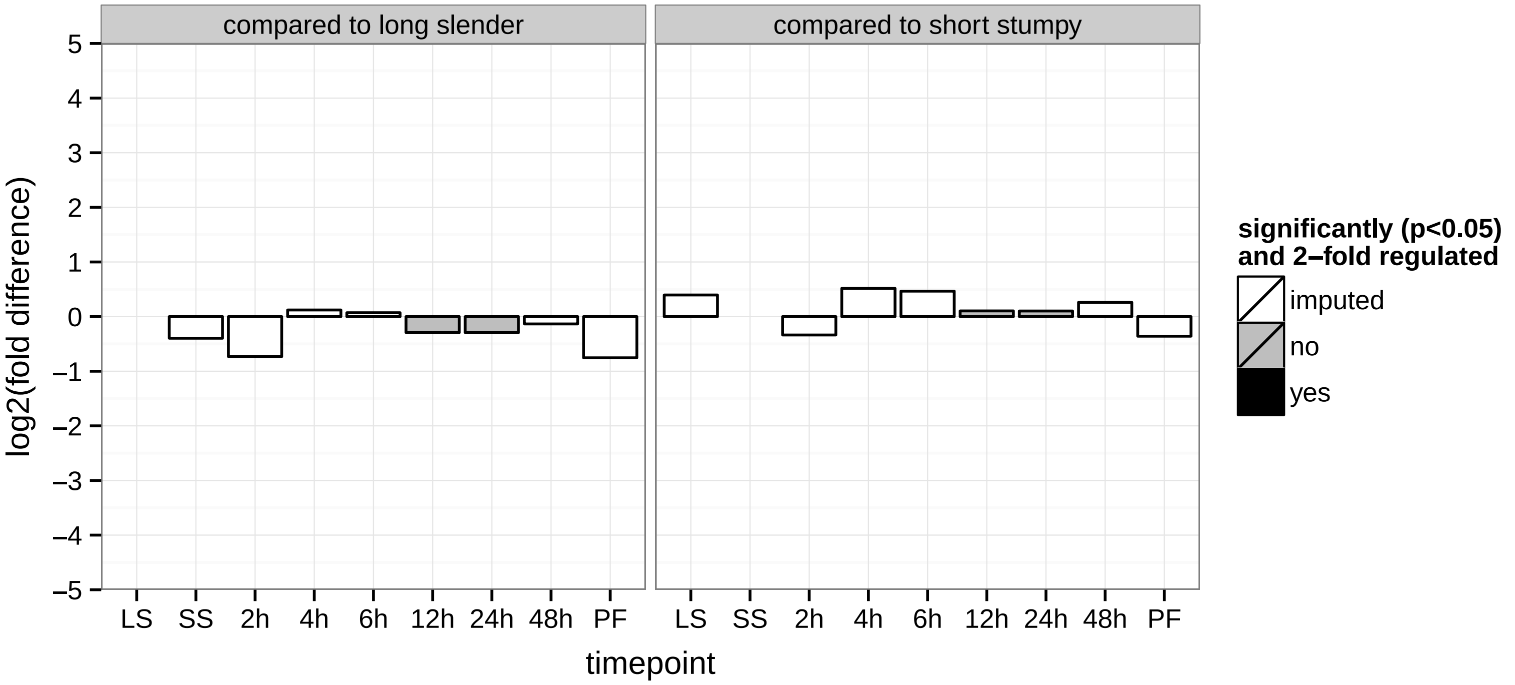

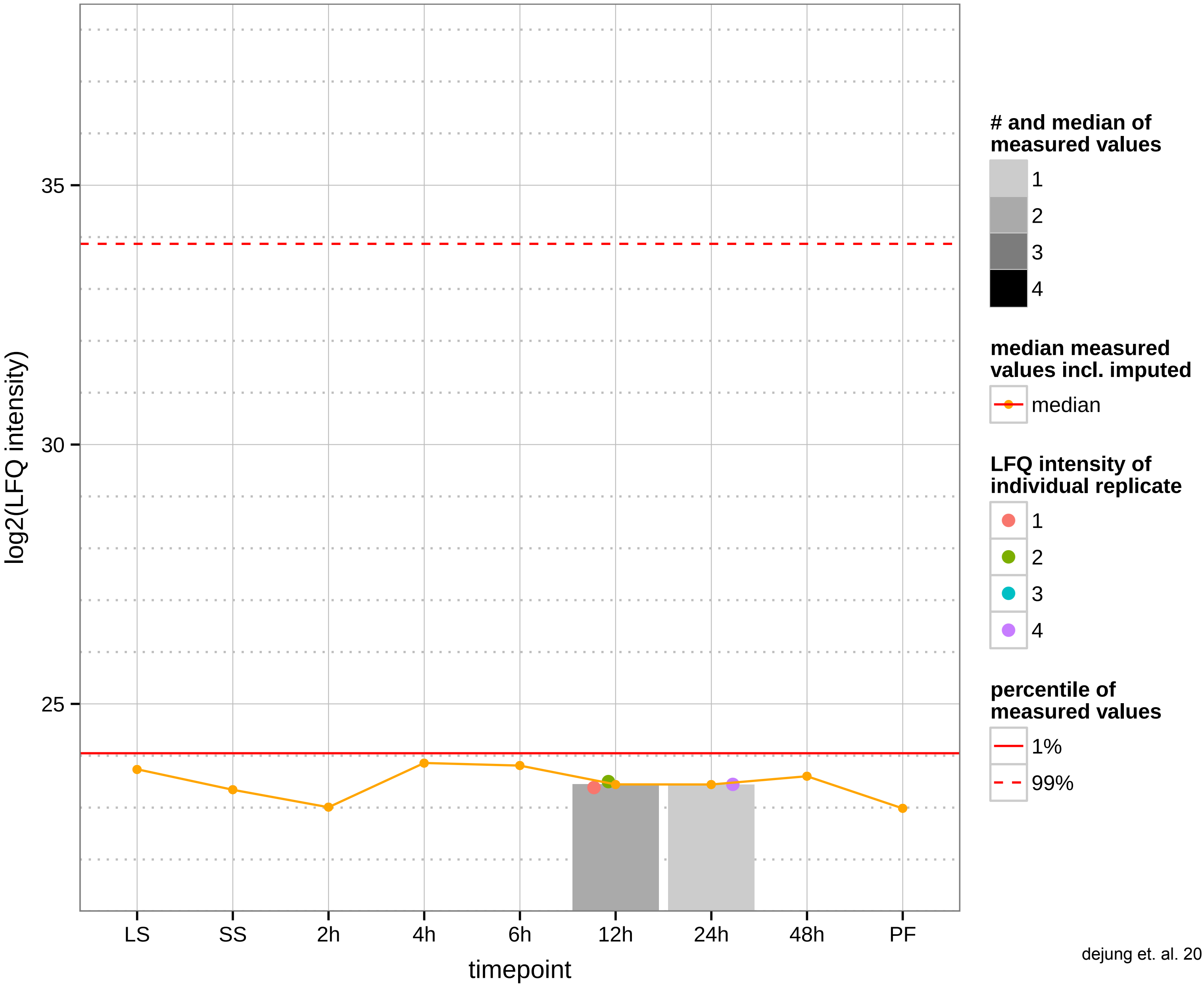

dejung et. al. 2015

myotubularin, putative, zinc–binding phosphatase
Tb927.1.3300
AGOF: phosphatase activity
AGOC: null
AGOP: dephosphorylation
PGOF: metal ion binding, phosphatase activity
PGOC: null
PGOP: dephosphorylation

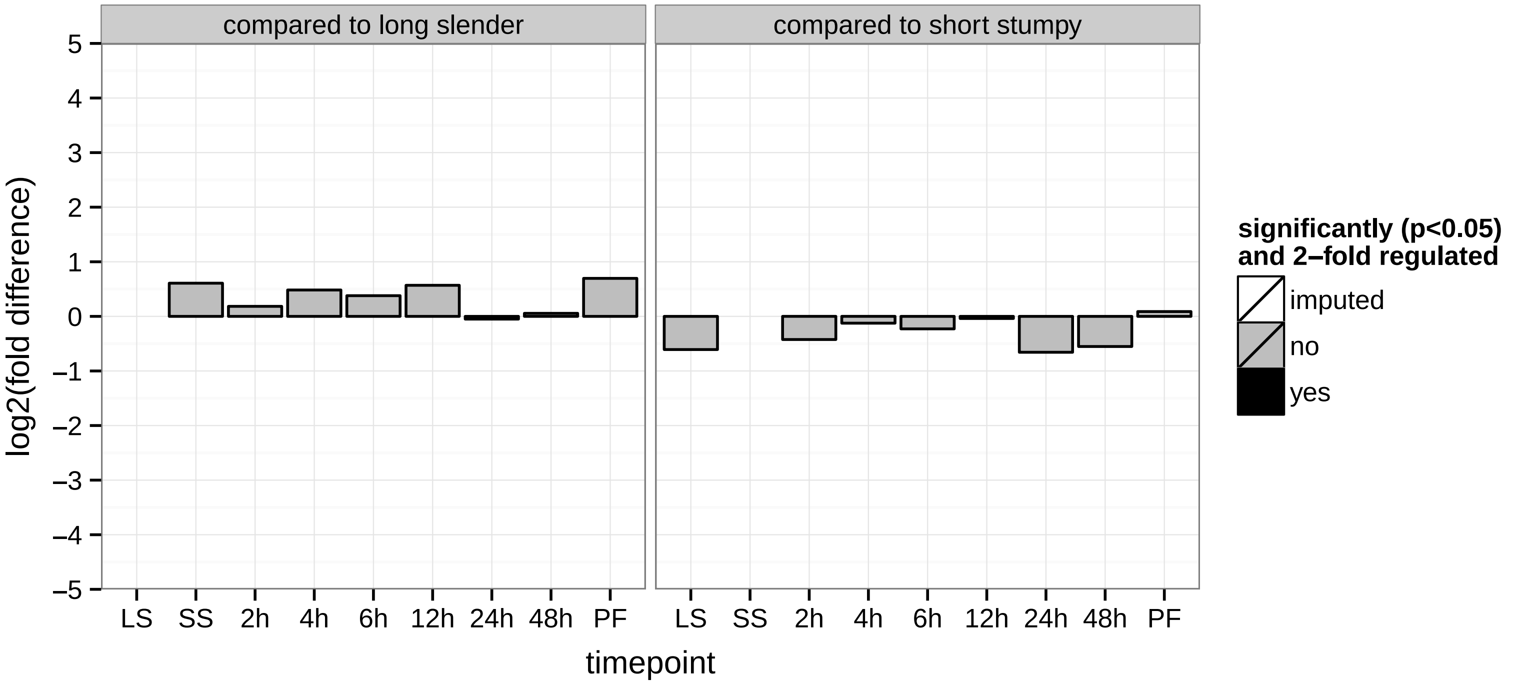

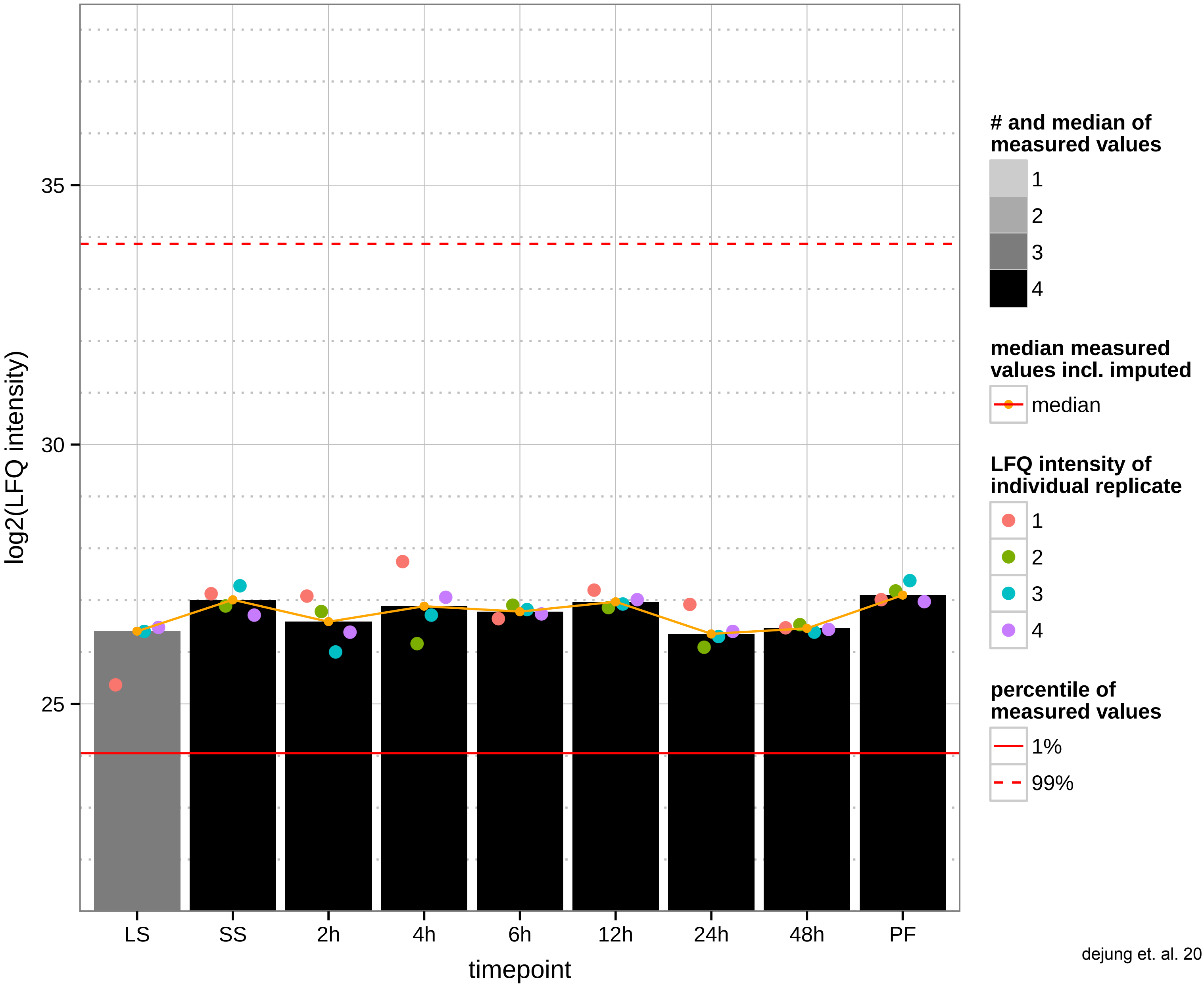

dejung et. al. 2015

hypothetical protein, conserved
Tb927.1.3450;Tb11.v5.0619
AGOF: null
AGOC: null
AGOP: null
PGOF: null
PGOC: null
PGOP: null

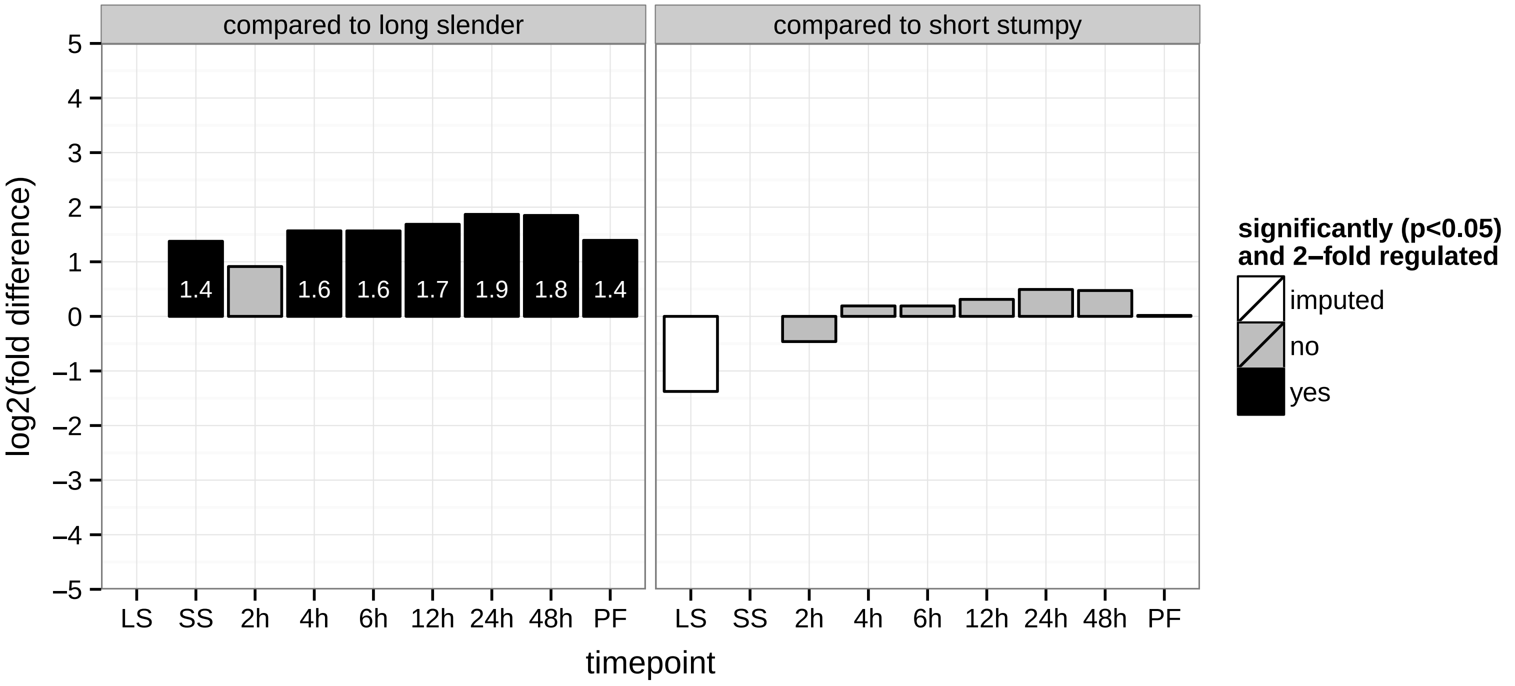

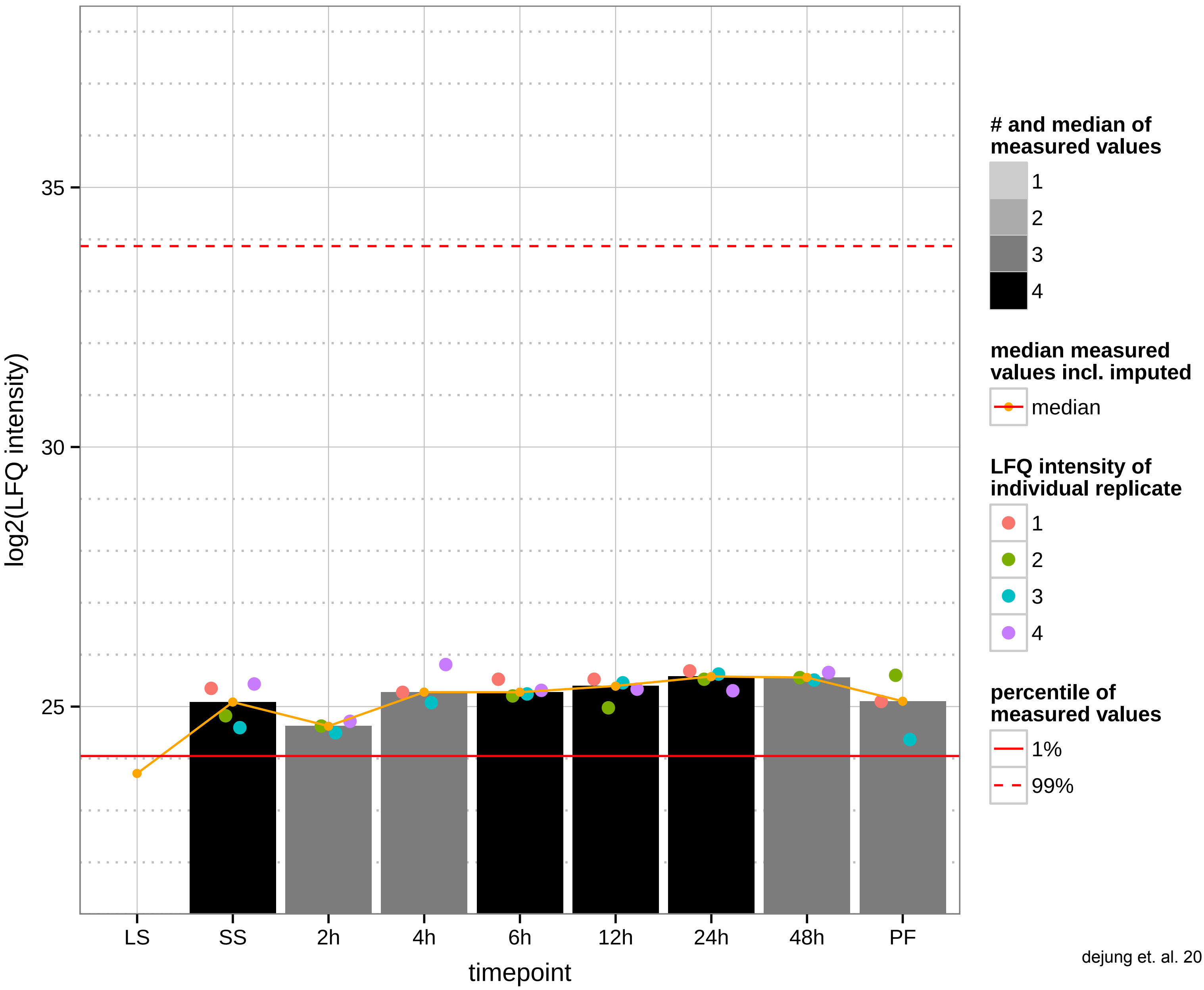

dejung et. al. 2015

protein phosphatase with EF−Hand domains (PPEF), ser/thr protein phosphatase, putative
Tb927.1.4050
AGOF: protein serine/threonine phosphatase activity
AGOC: protein serine/threonine phosphatase complex
AGOP: protein dephosphorylation
PGOF: hydrolase activity
PGOC: null
PGOP: null

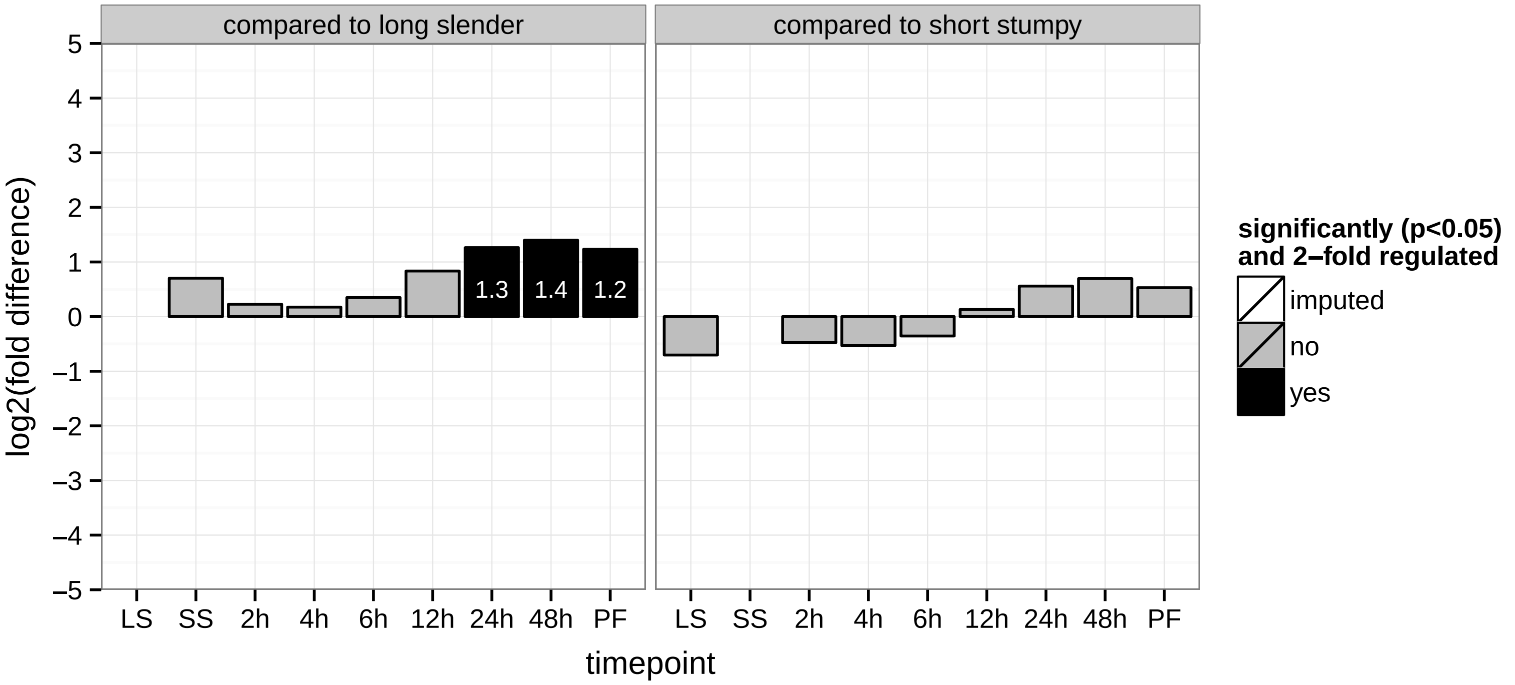

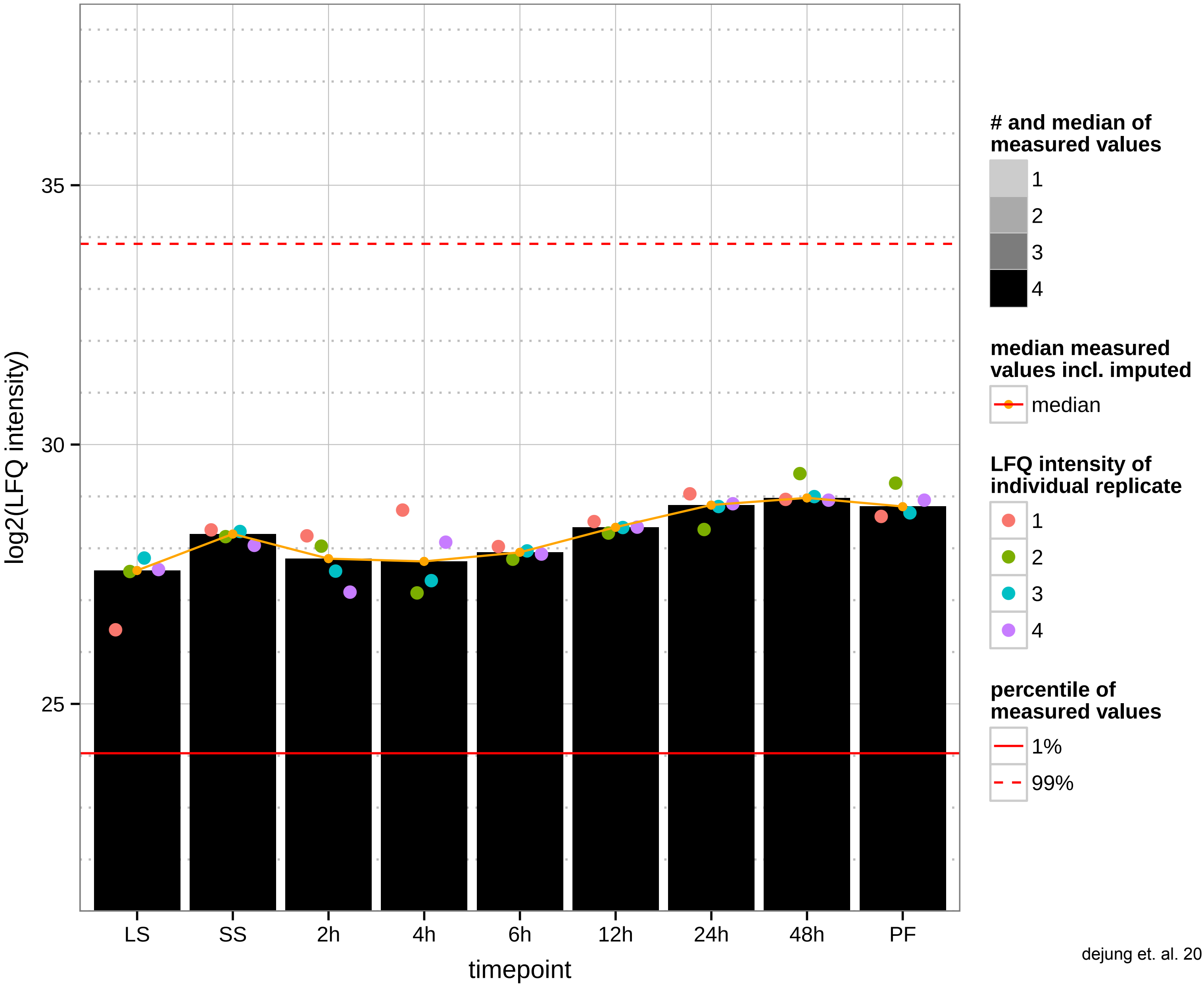

dejung et. al. 2015

hypothetical protein, conserved
Tb927.1.4250
AGOF: null
AGOC: null
AGOP: null
PGOF: null
PGOC: null
PGOP: null

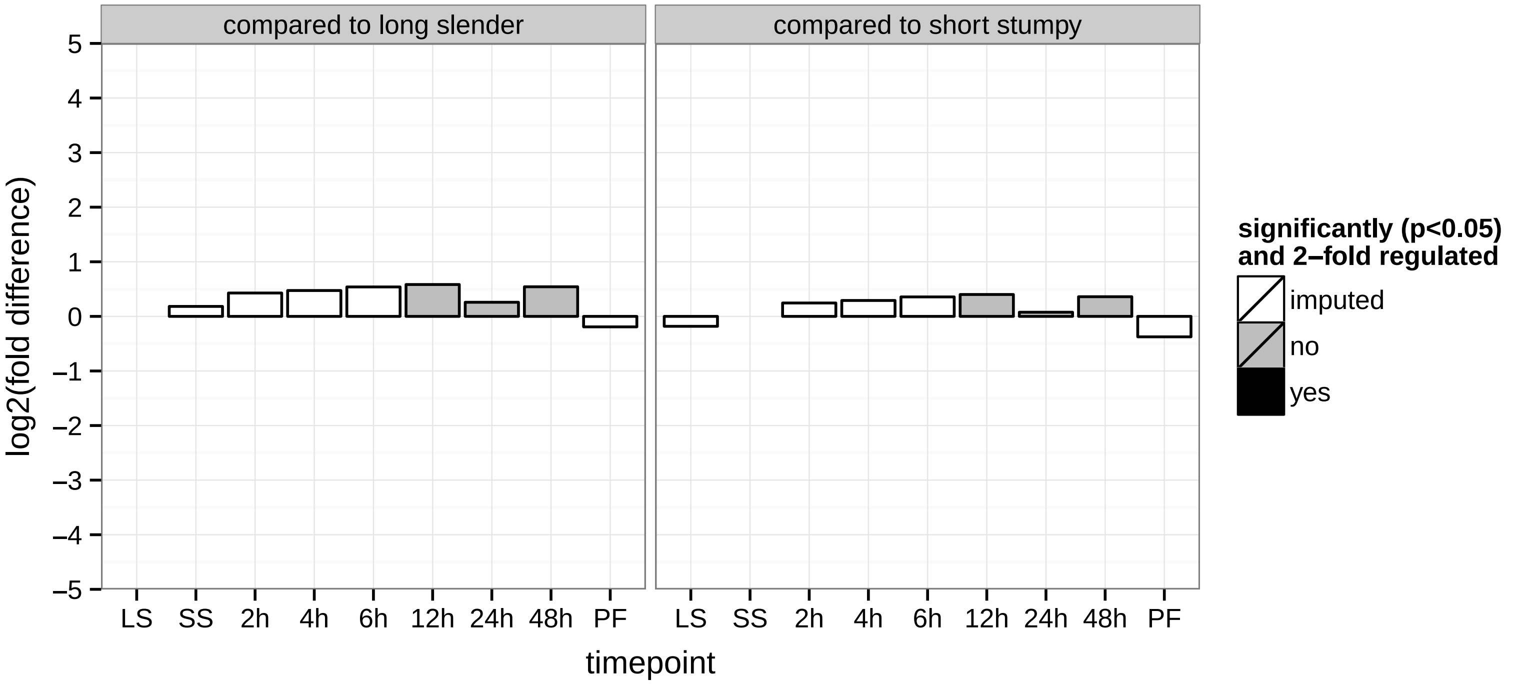

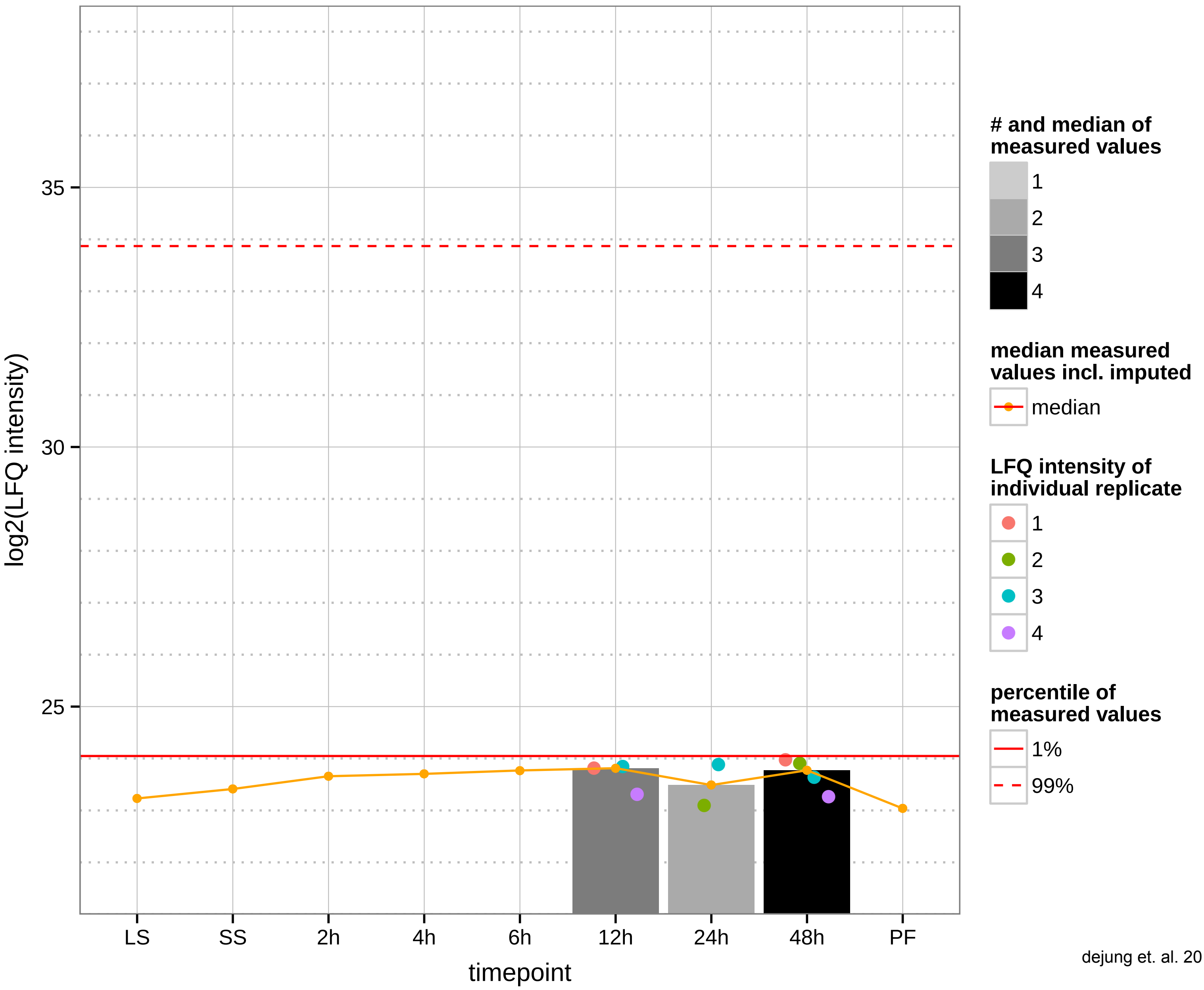

dejung et. al. 2015

hypothetical protein, conserved
Tb927.1.4280
AGOF: null
AGOC: null
AGOP: null
PGOF: protein binding
PGOC: null
PGOP: null

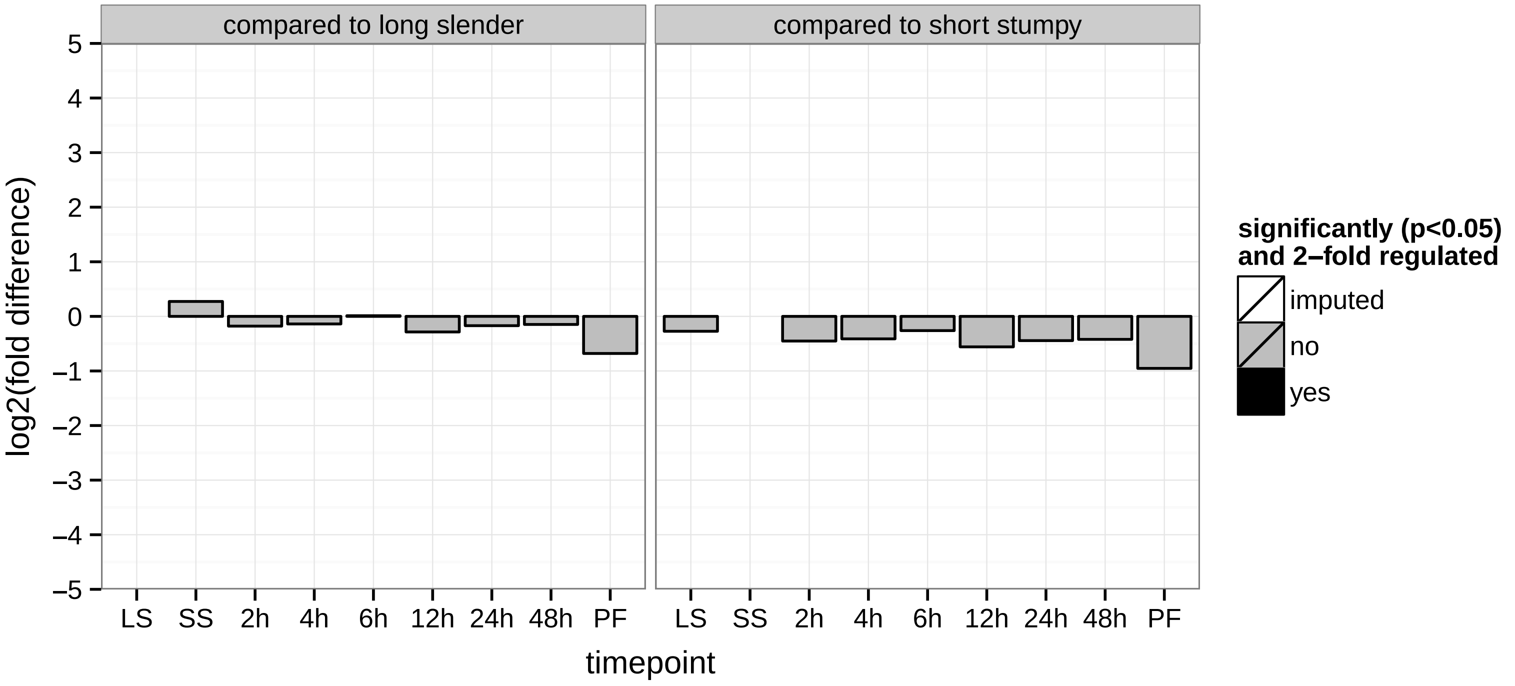

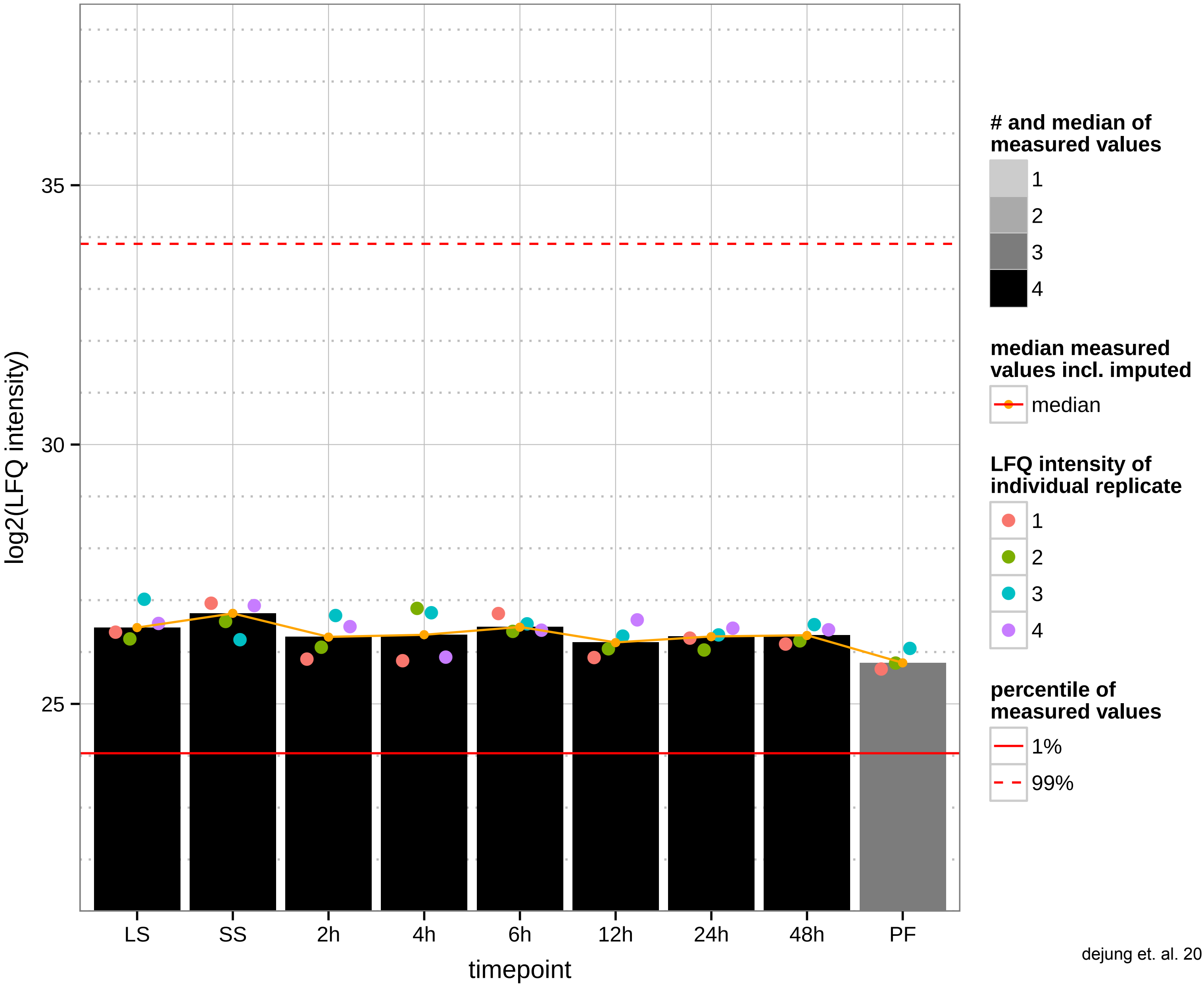

dejung et. al. 2015

hypothetical protein, conserved
Tb927.1.4310
AGOF: null
AGOC: null
AGOP: null
PGOF: null
PGOC: null
PGOP: null

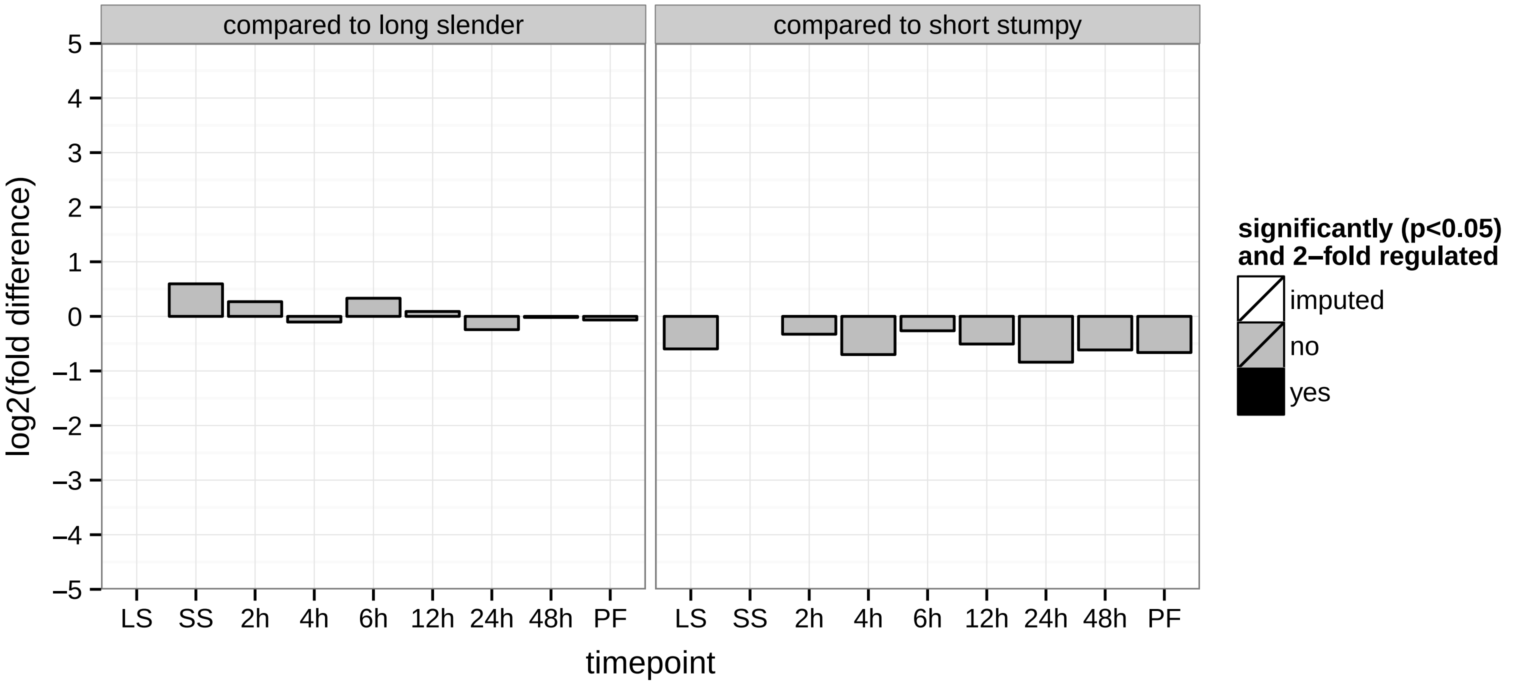

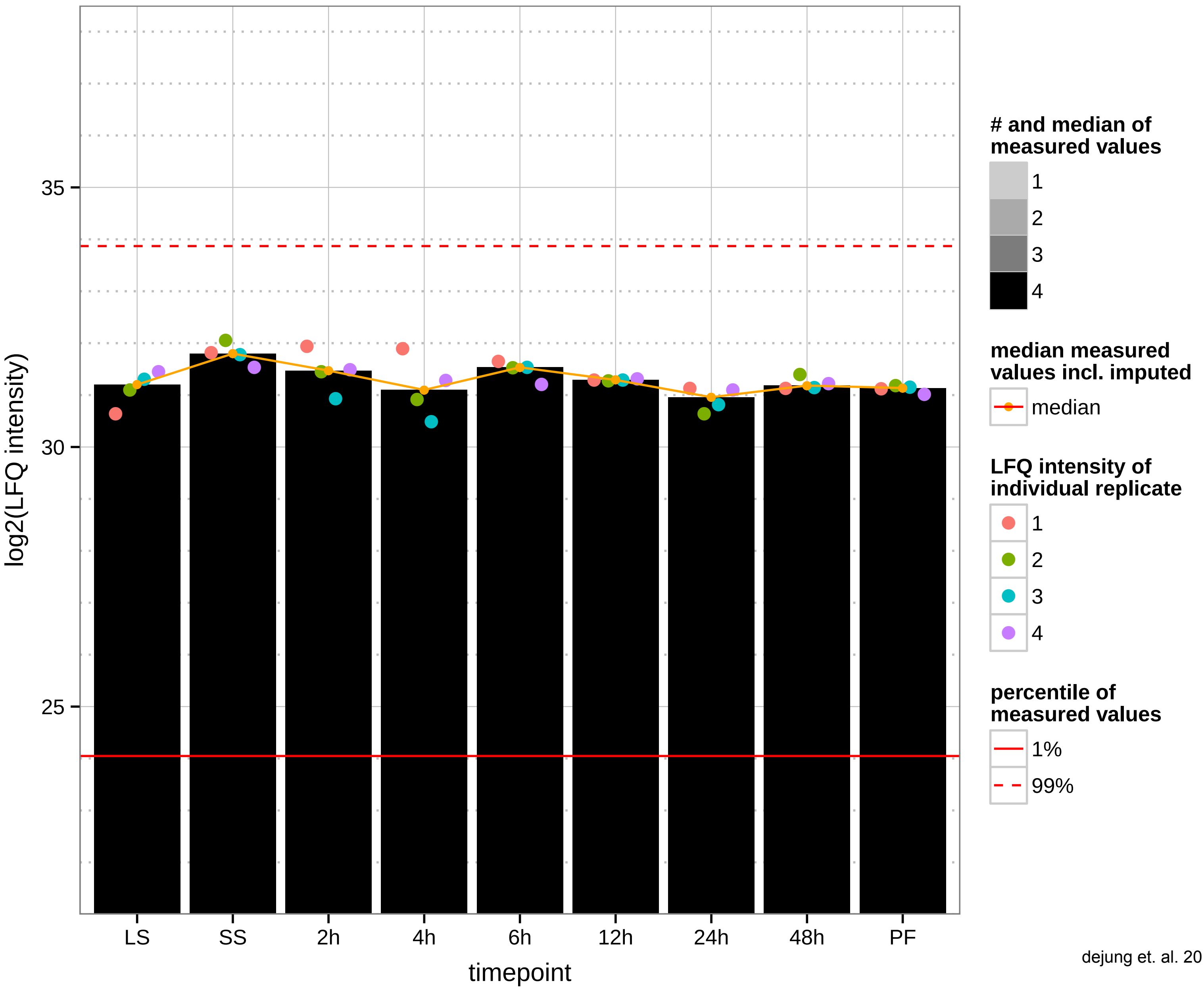

dejung et. al. 2015

hypothetical protein, conserved
Tb927.1.4340
AGOF: null
AGOC: null
AGOP: null
PGOF: null
PGOC: null
PGOP: null

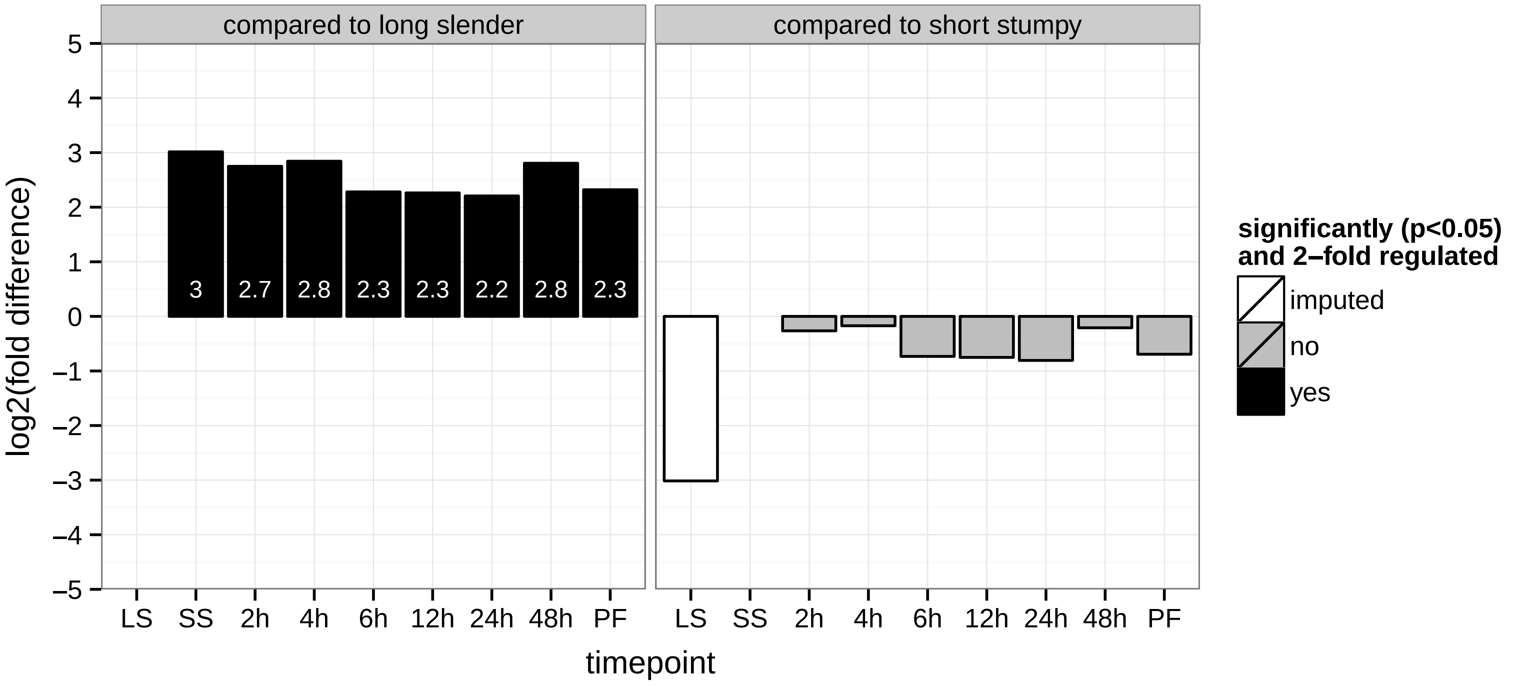

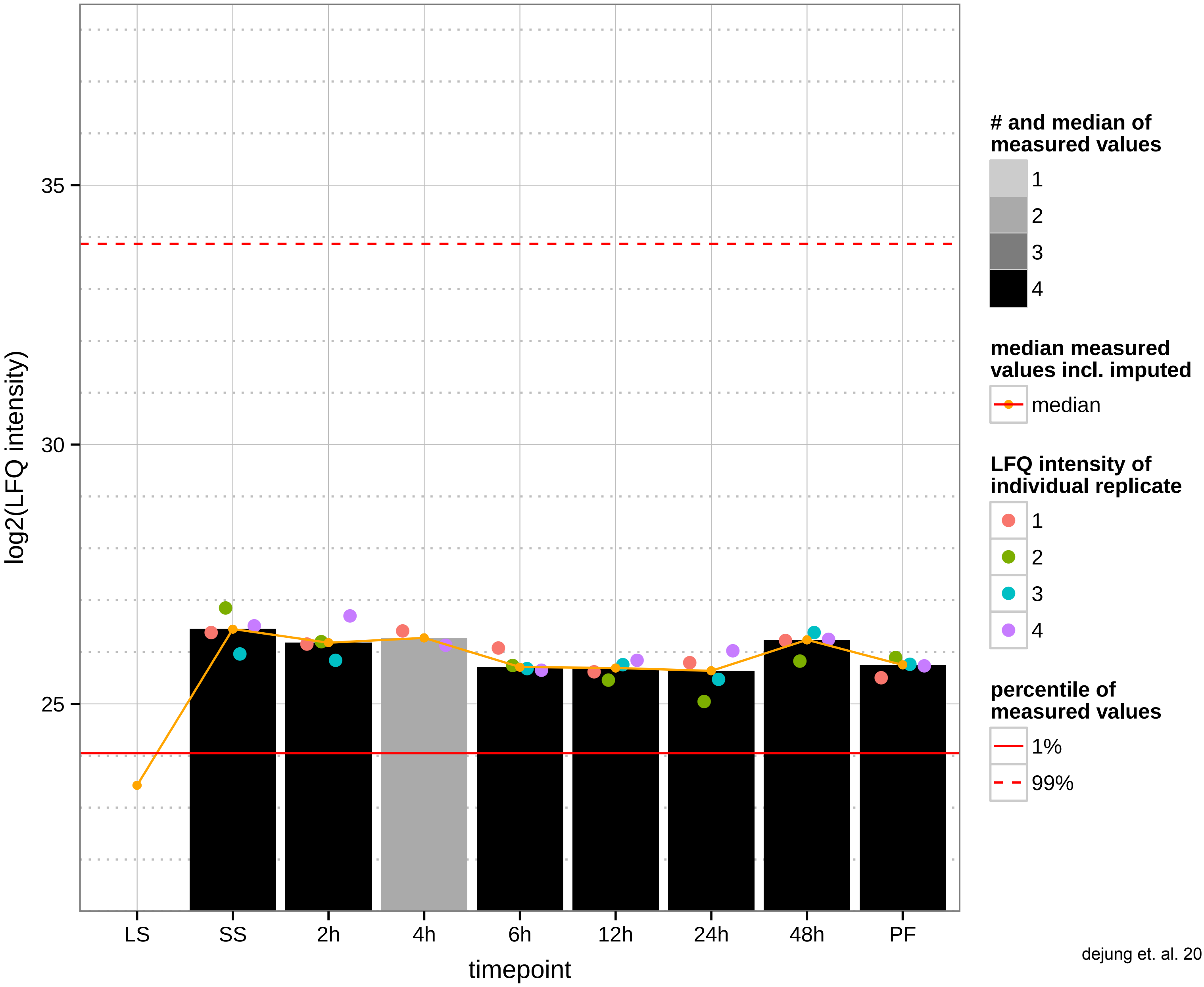

dejung et. al. 2015

hypothetical protein, conserved
Tb927.1.4410
AGOF: null
AGOC: null
AGOP: null
PGOF: null
PGOC: null
PGOP: null

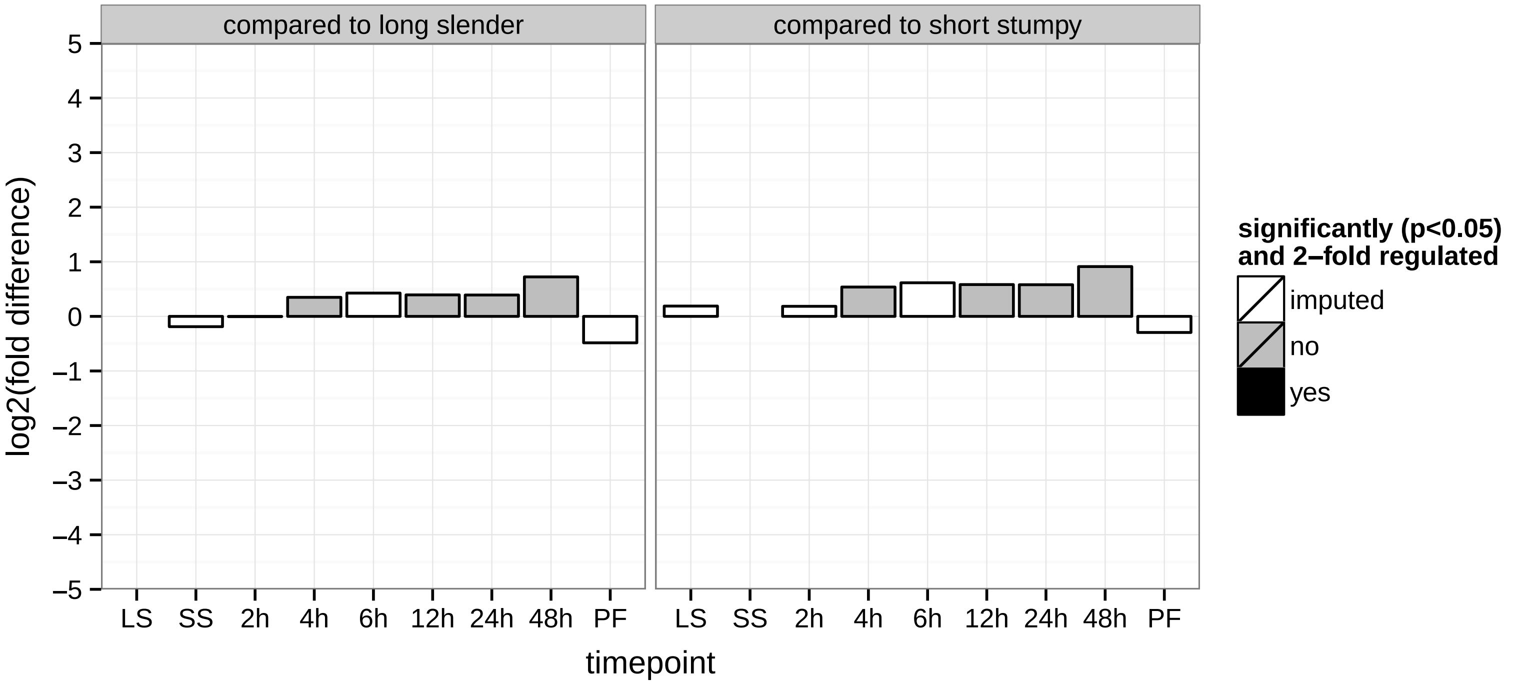

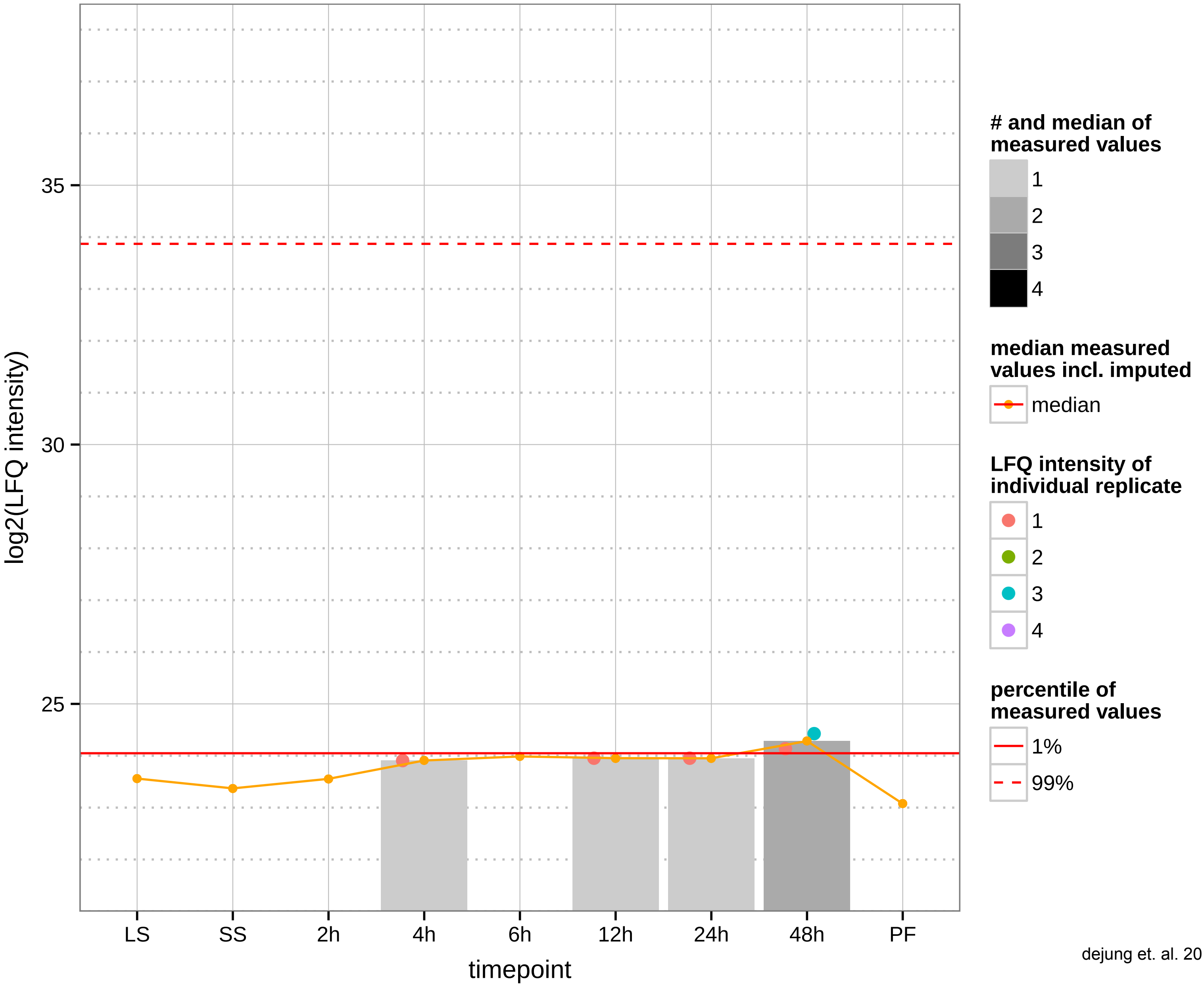

dejung et. al. 2015

retrotransposon hot spot protein (RHS, pseudogene), putative
Tb927.1.450
AGOF: null
AGOC: null
AGOP: null
PGOF: null
PGOC: null
PGOP: null

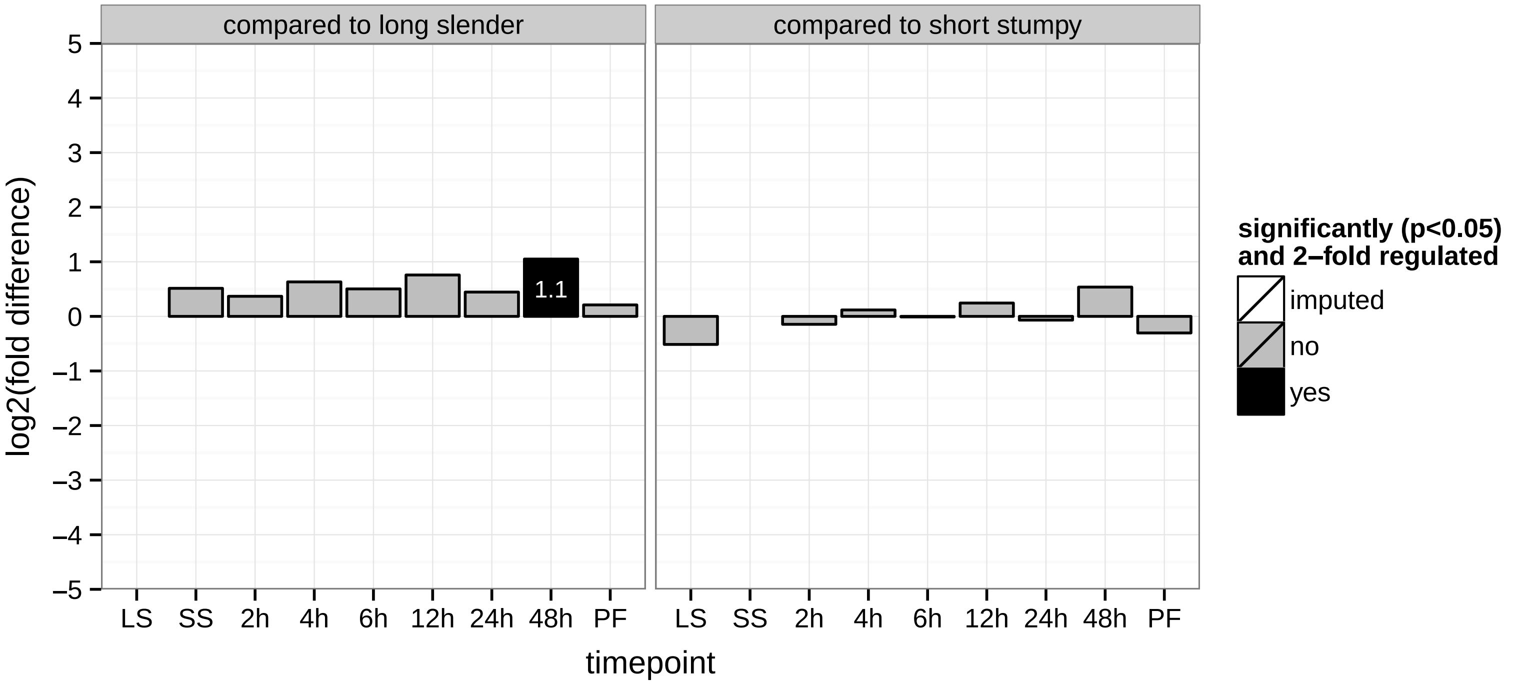

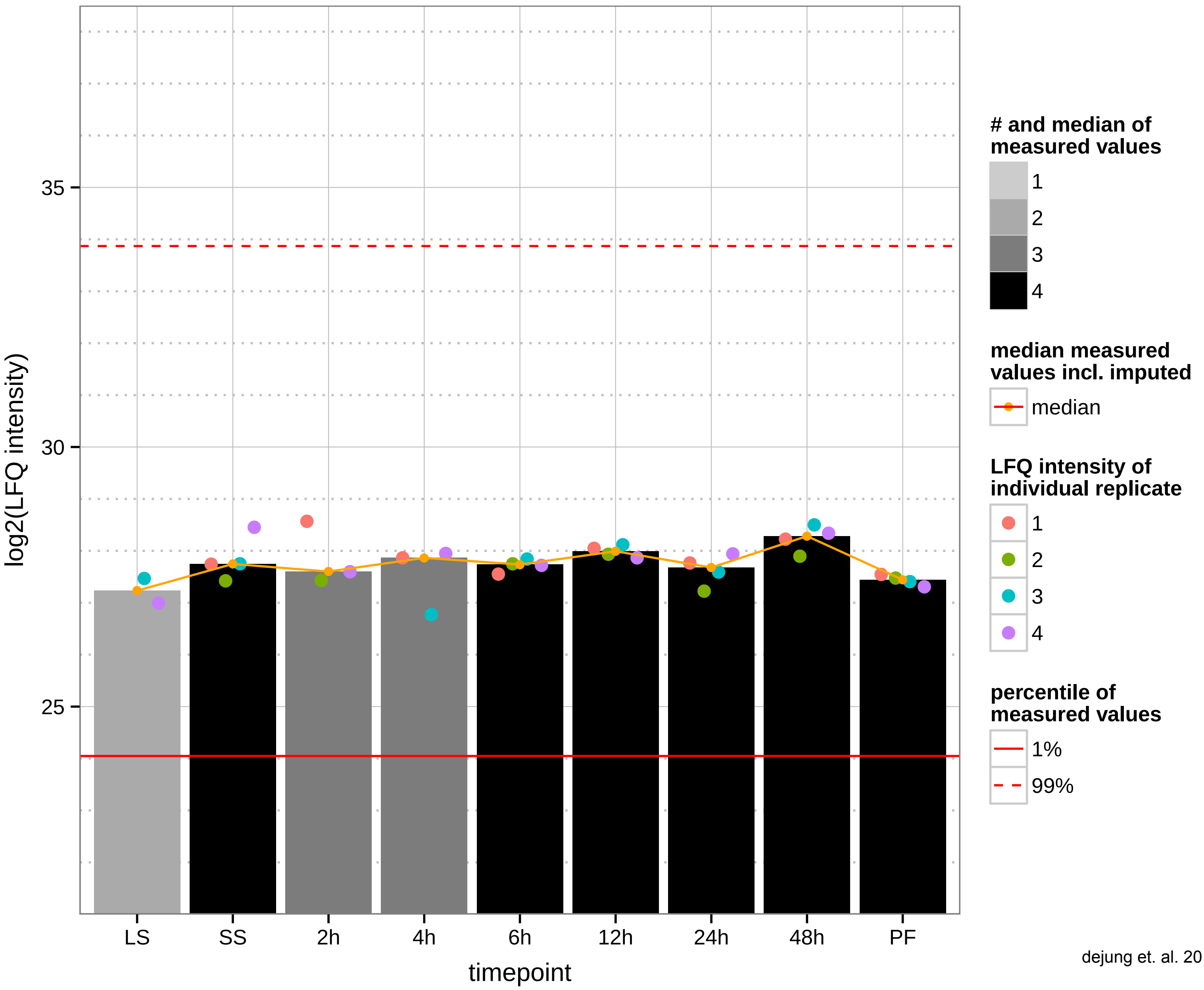

dejung et. al. 2015

hypothetical protein, conserved
Tb927.1.4680
AGOF: null
AGOC: null
AGOP: null
PGOF: null
PGOC: null
PGOP: null

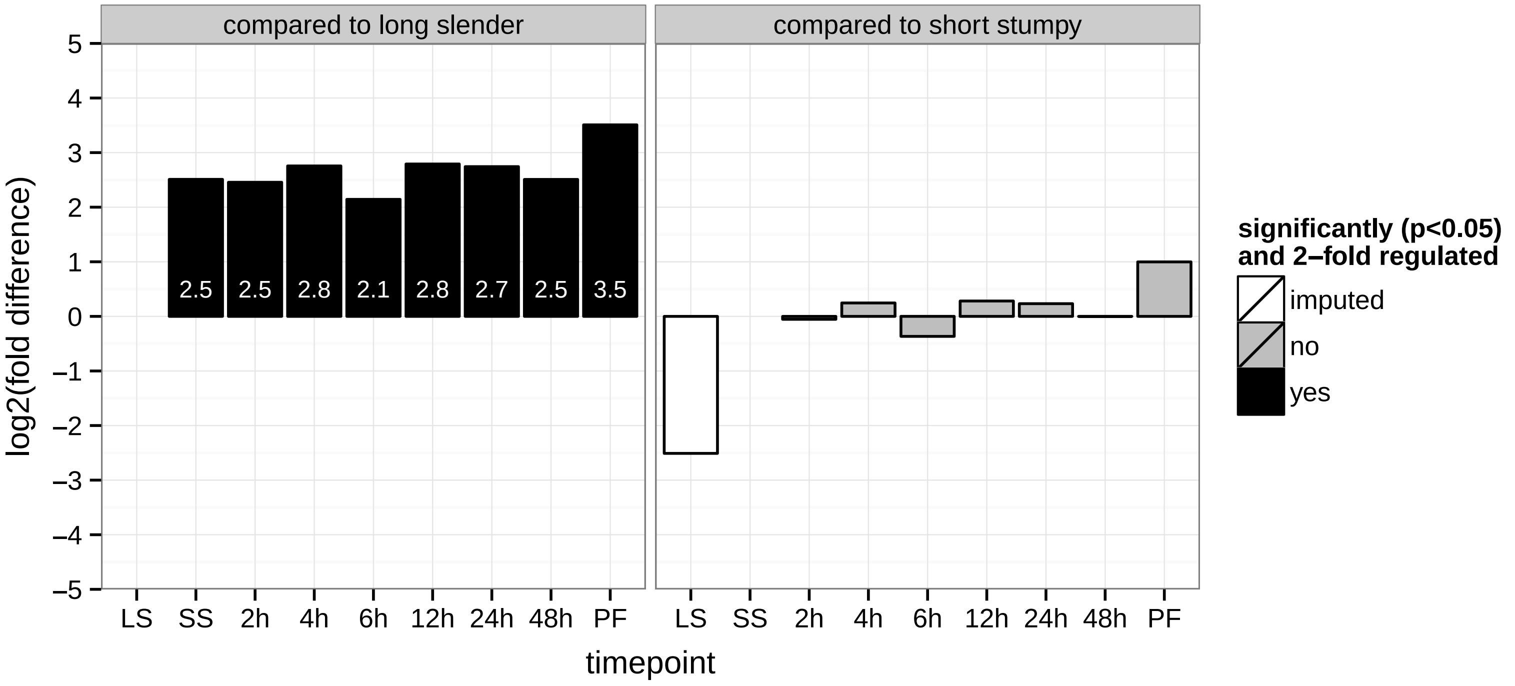

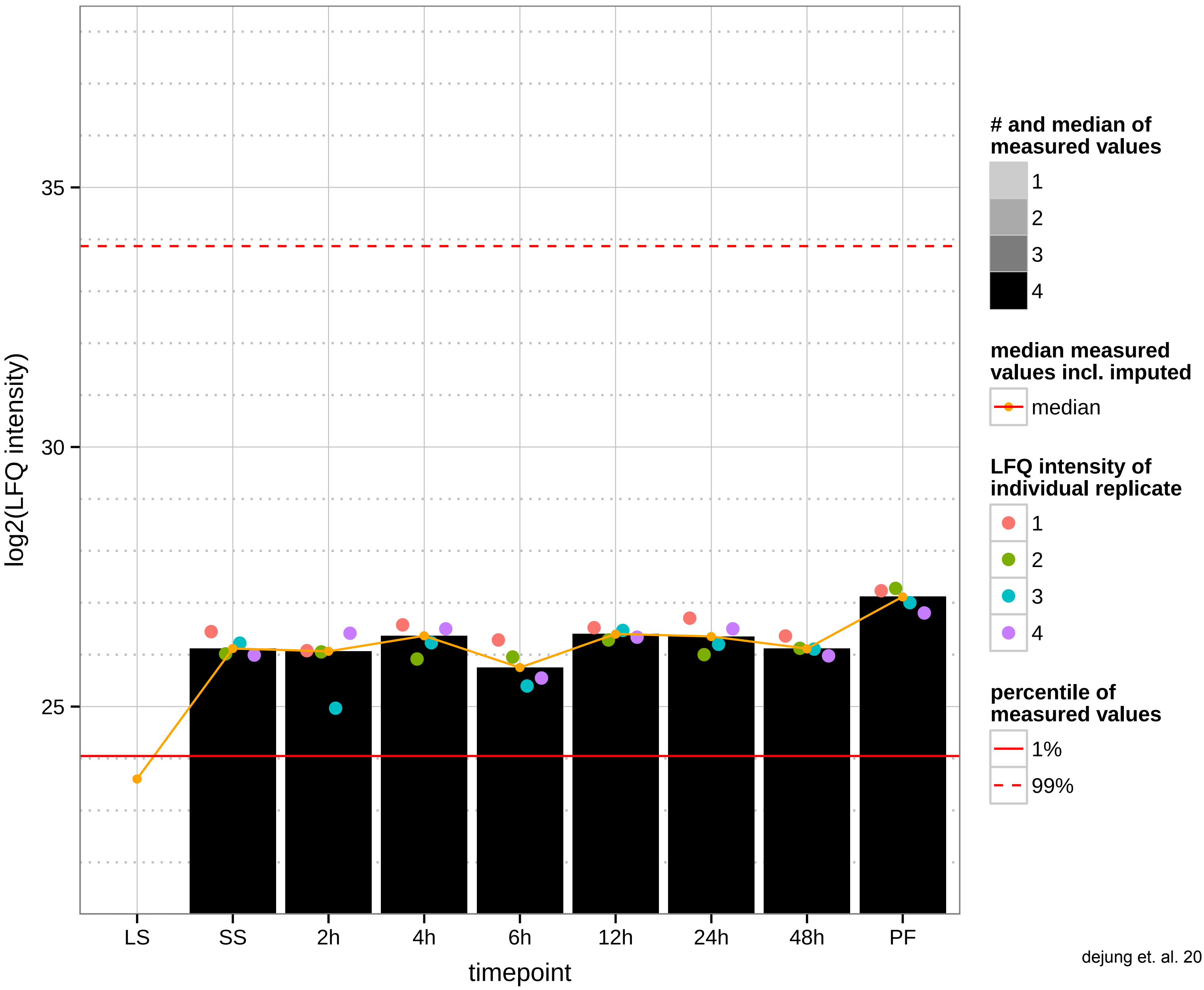

dejung et. al. 2015

hypothetical protein, conserved
Tb927.1.4720
AGOF: null
AGOC: null
AGOP: null
PGOF: protein binding
PGOC: null
PGOP: null

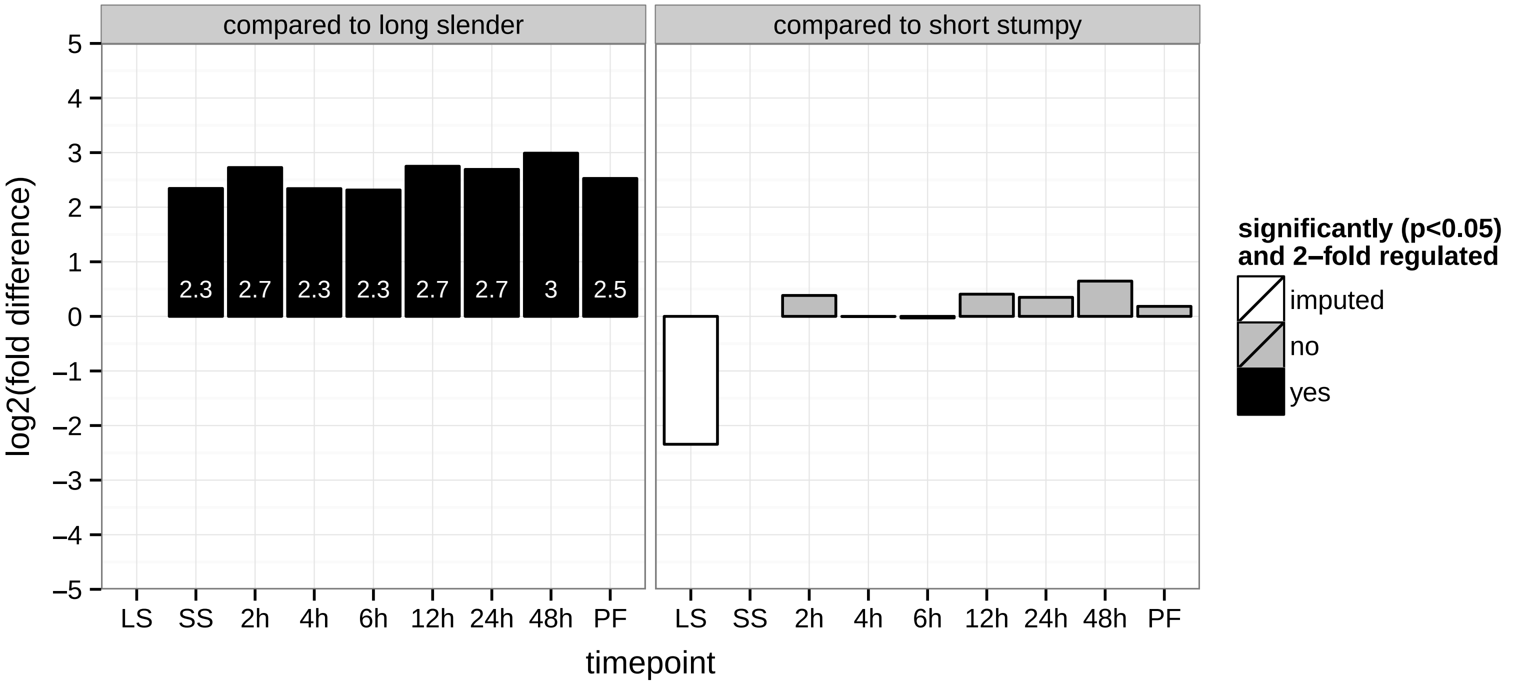

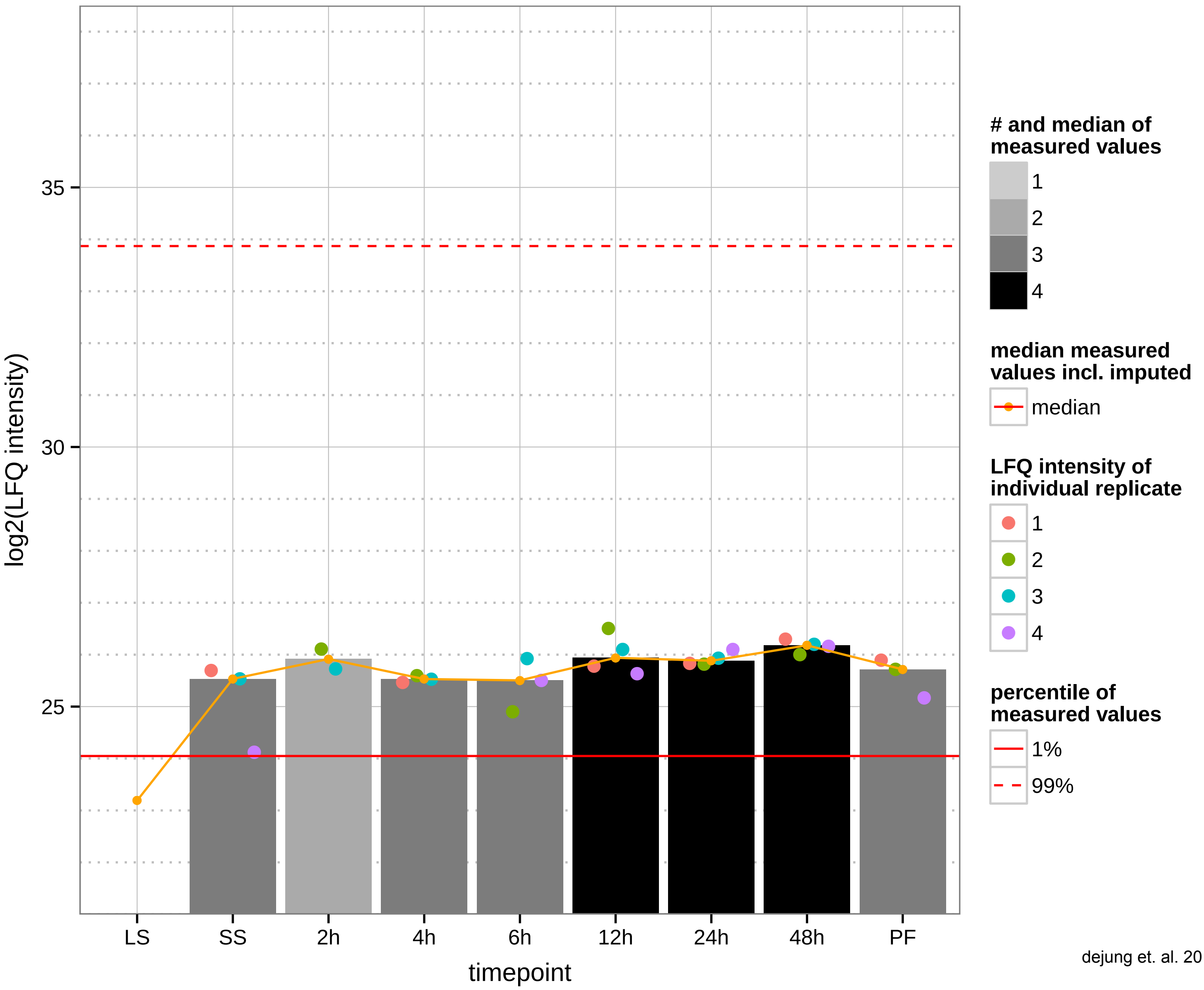

dejung et. al. 2015

hypothetical protein, conserved
Tb927.1.4740
AGOF: null
AGOC: null
AGOP: null
PGOF: null
PGOC: null
PGOP: null

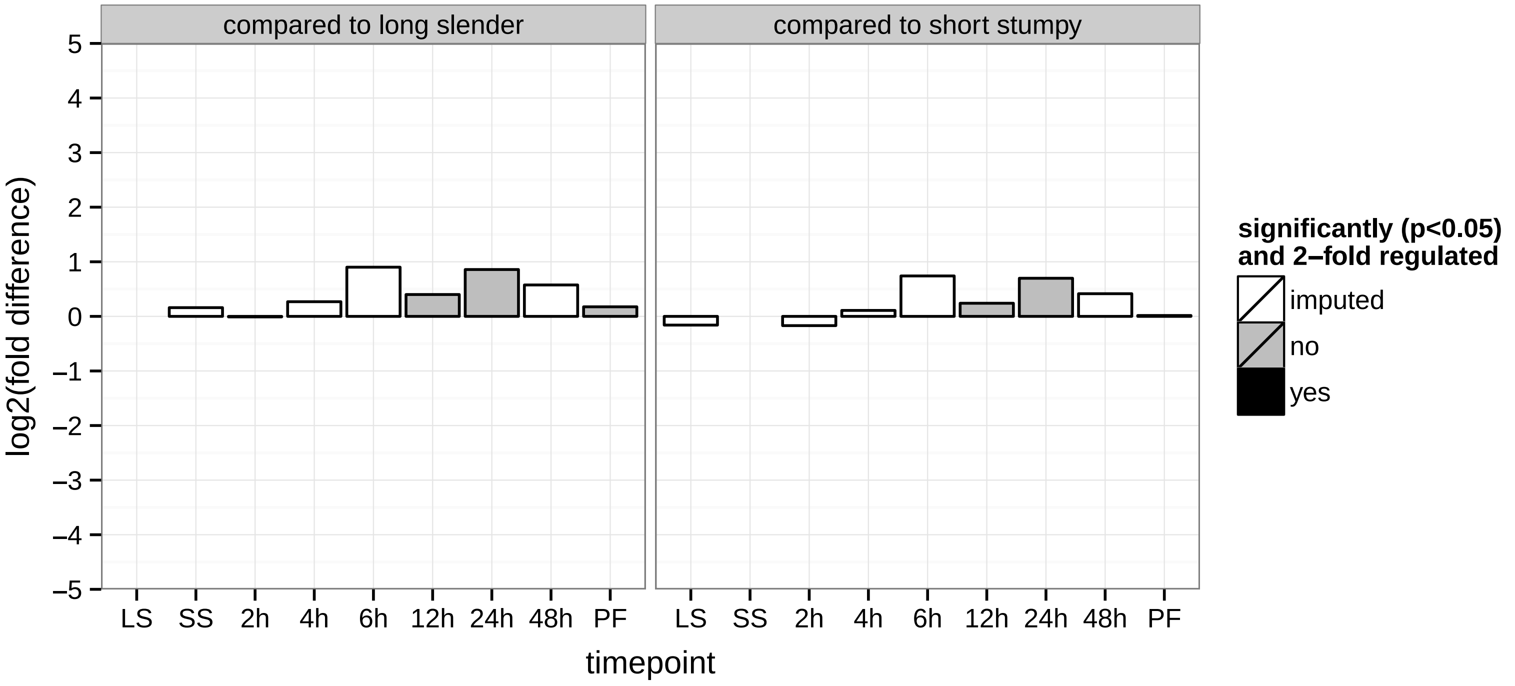

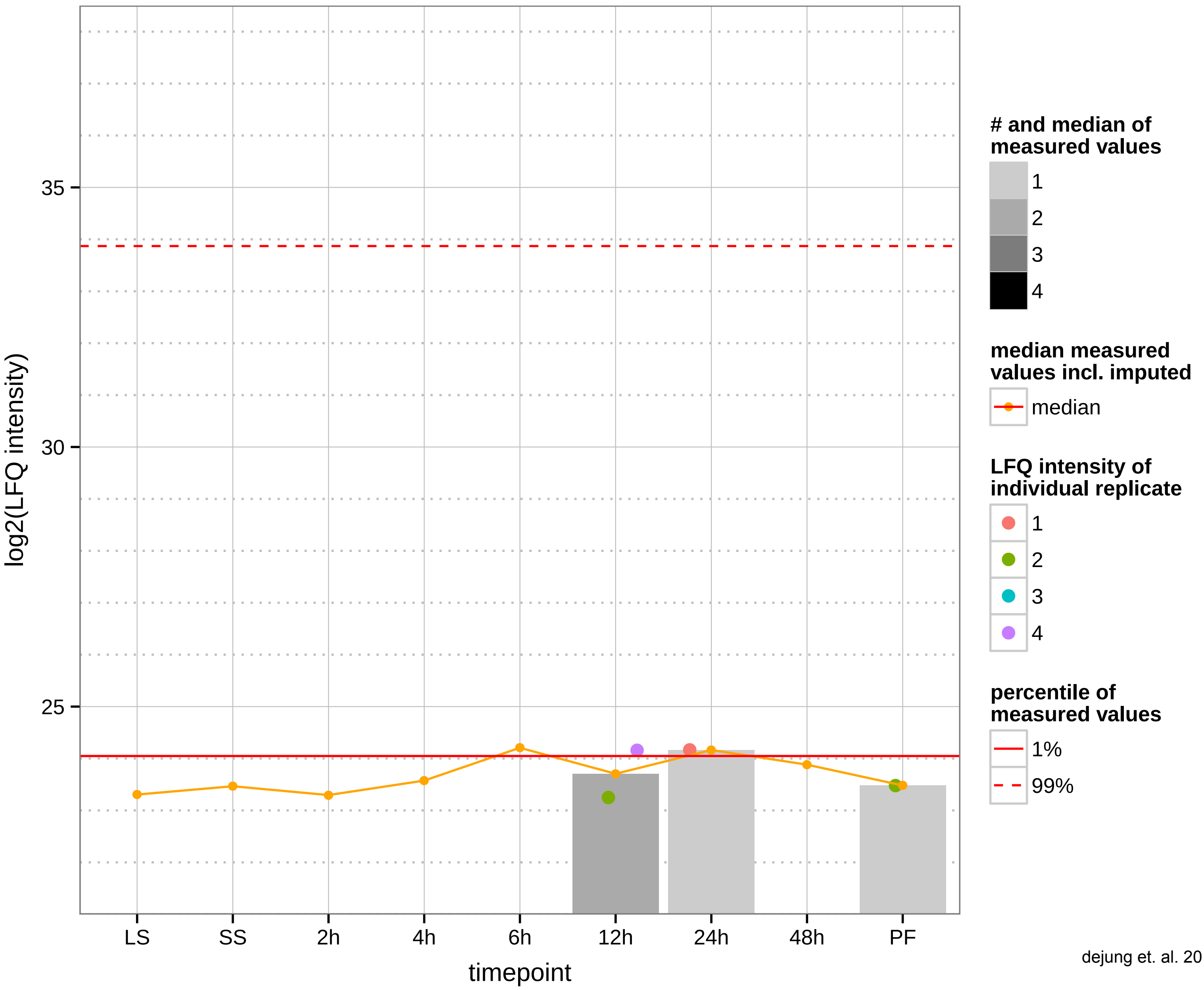

dejung et. al. 2015

leucine–rich repeat protein (LRRP), putative
Tb927.1.5030
AGOF: null
AGOC: null
AGOP: null
PGOF: protein binding
PGOC: null
PGOP: null

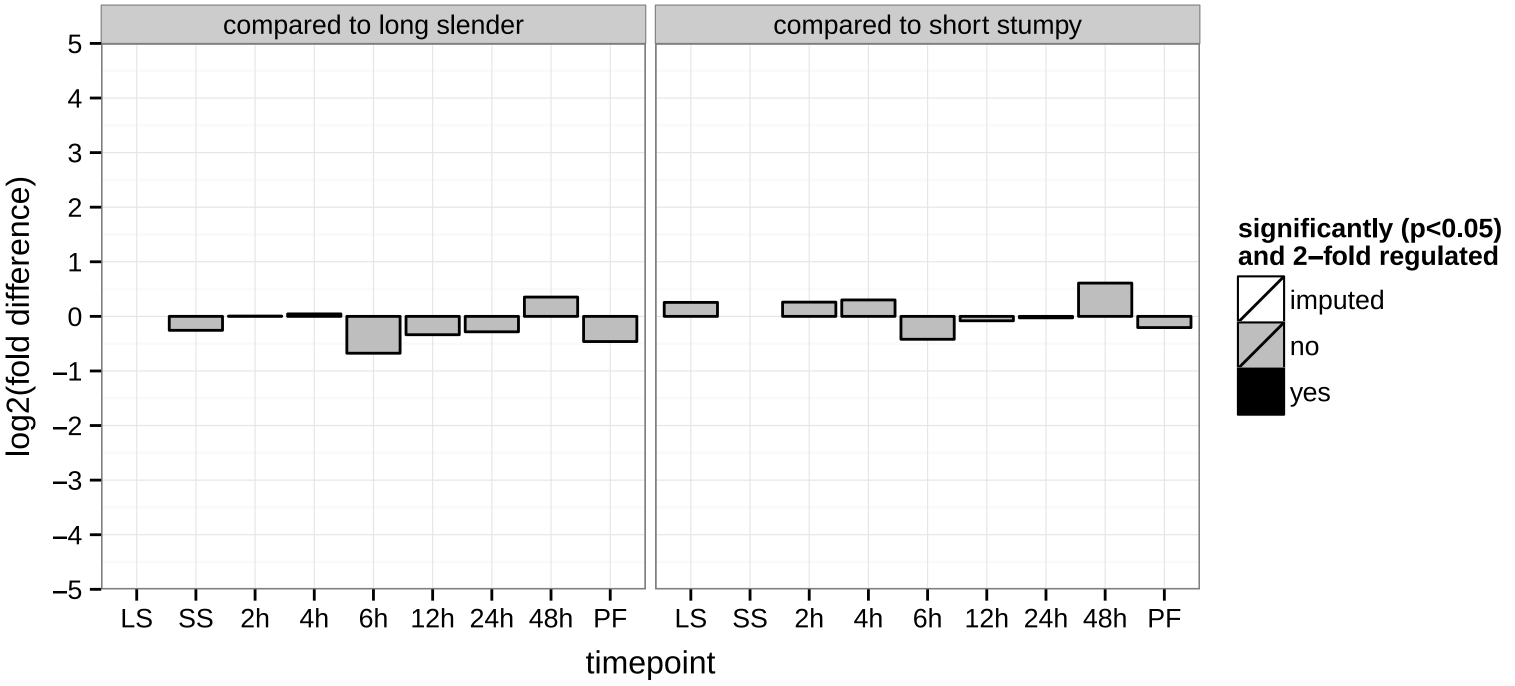

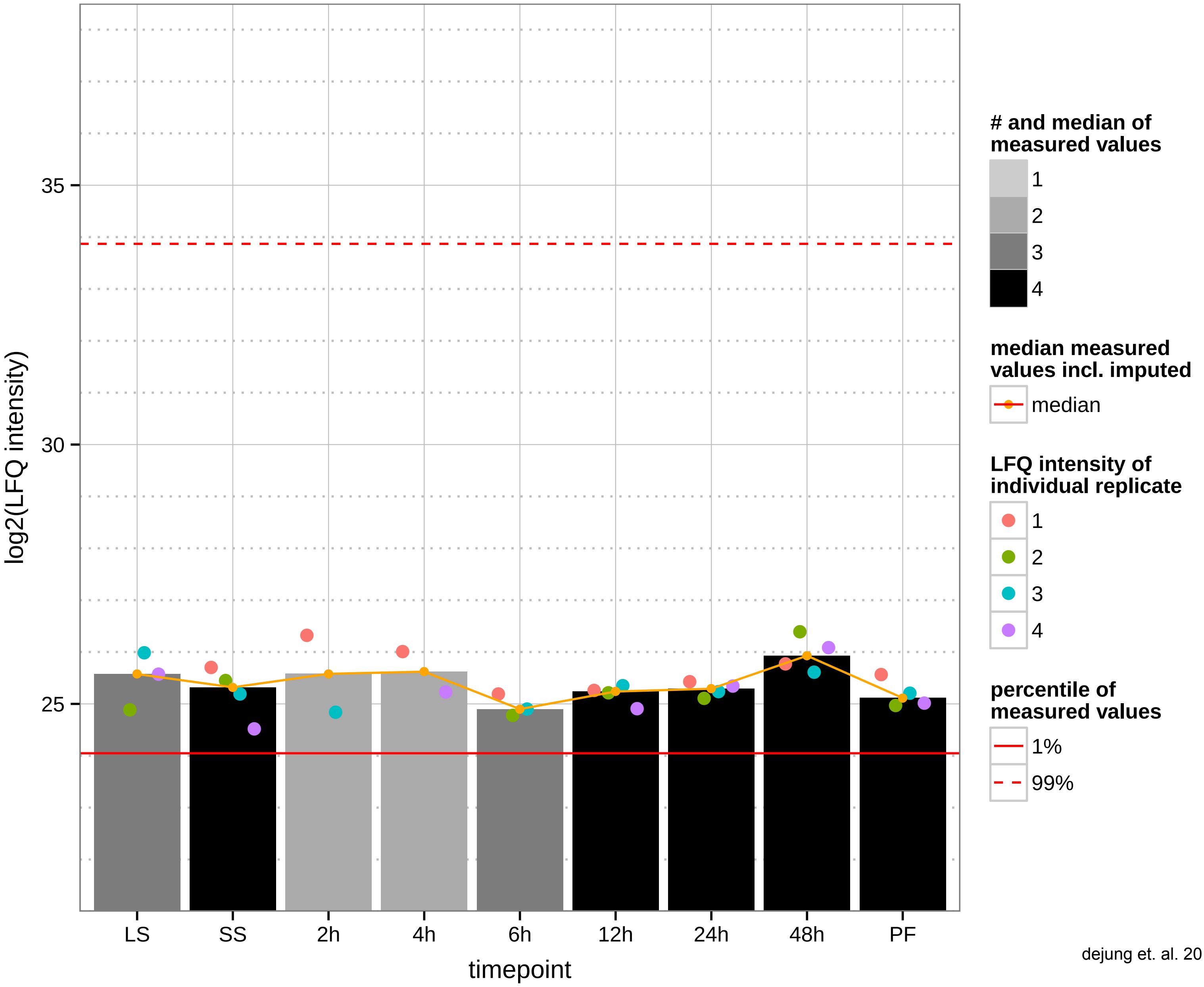

dejung et. al. 2015

variant surface glycoprotein (VSG)–related, putative
Tb927.1.5170
AGOF: host cell surface receptor binding
AGOC: integral to membrane
AGOP: antigenic variation, symbiosis, encompassing mutualism through parasitism
PGOF: null
PGOC: null
PGOP: null

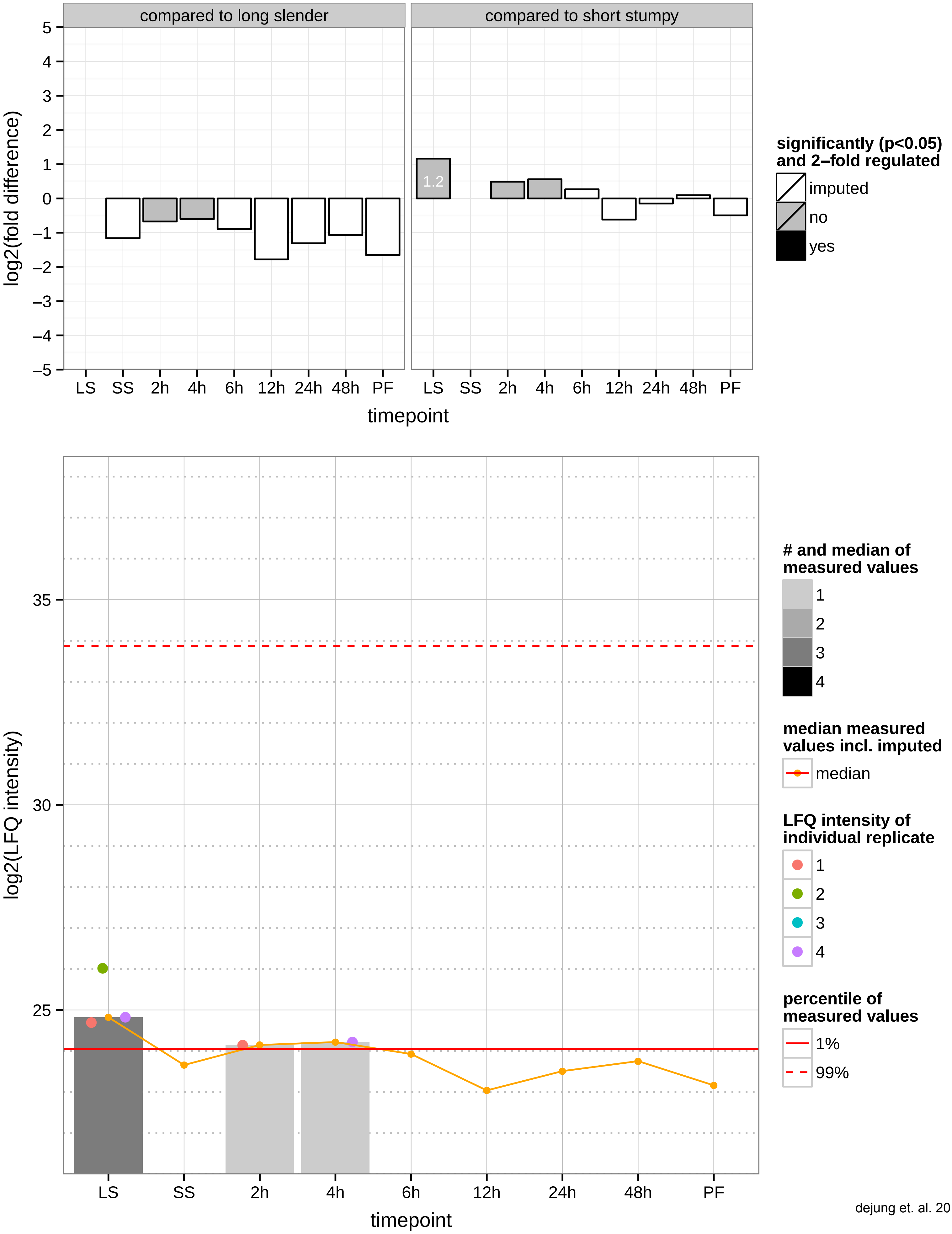

dejung et. al. 2015

DNA–directed RNA polymerase III, putative, RNA polymerase subunit, putative (RPC128), RNA polymerase (pseudogene), D
Tb927.1.540
AGOF: DNA binding, DNA–directed RNA polymerase activity, ribonucleoside binding
AGOC: DNA–directed RNA polymerase III complex, null
AGOP: transcription from RNA polymerase III promoter, transcription, DNA–dependent
PGOF: DNA binding, DNA–directed RNA polymerase activity
PGOC: null
PGOP: transcription, DNA–dependent

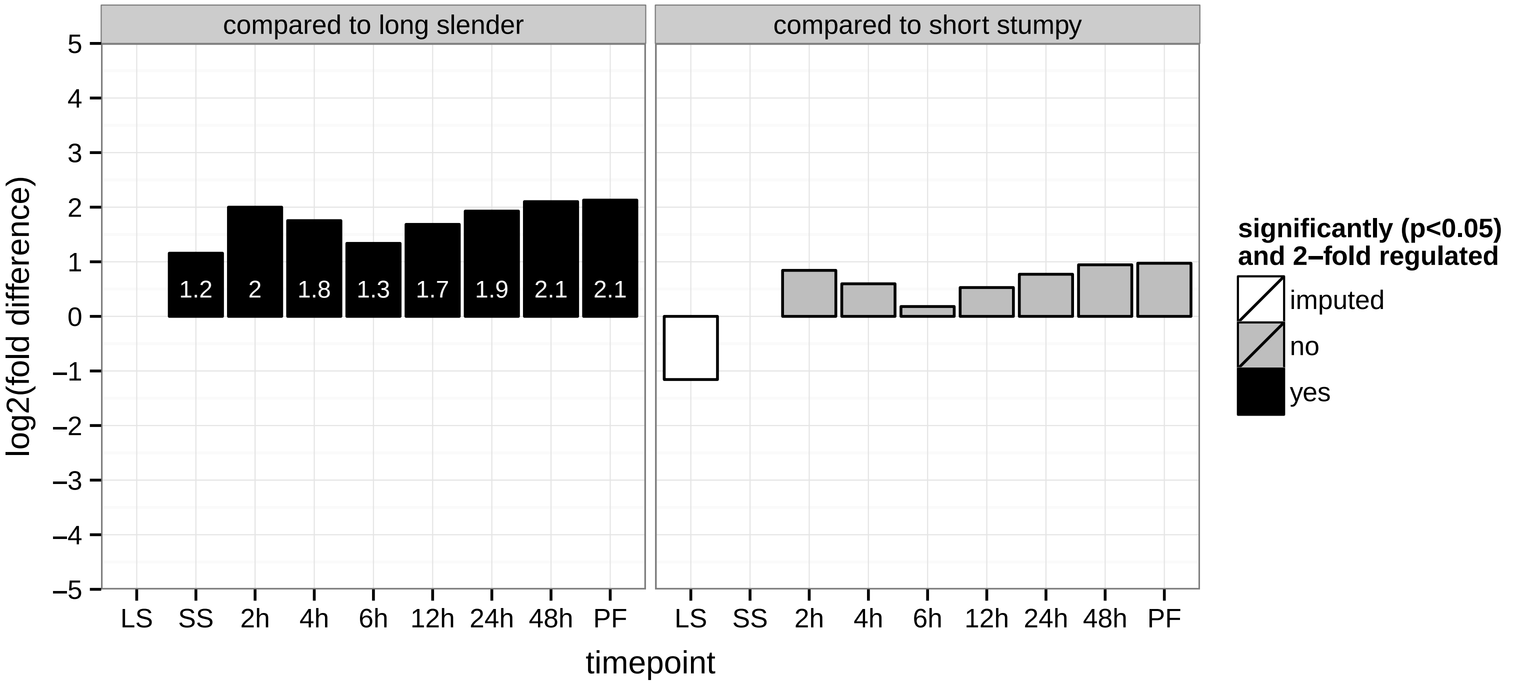

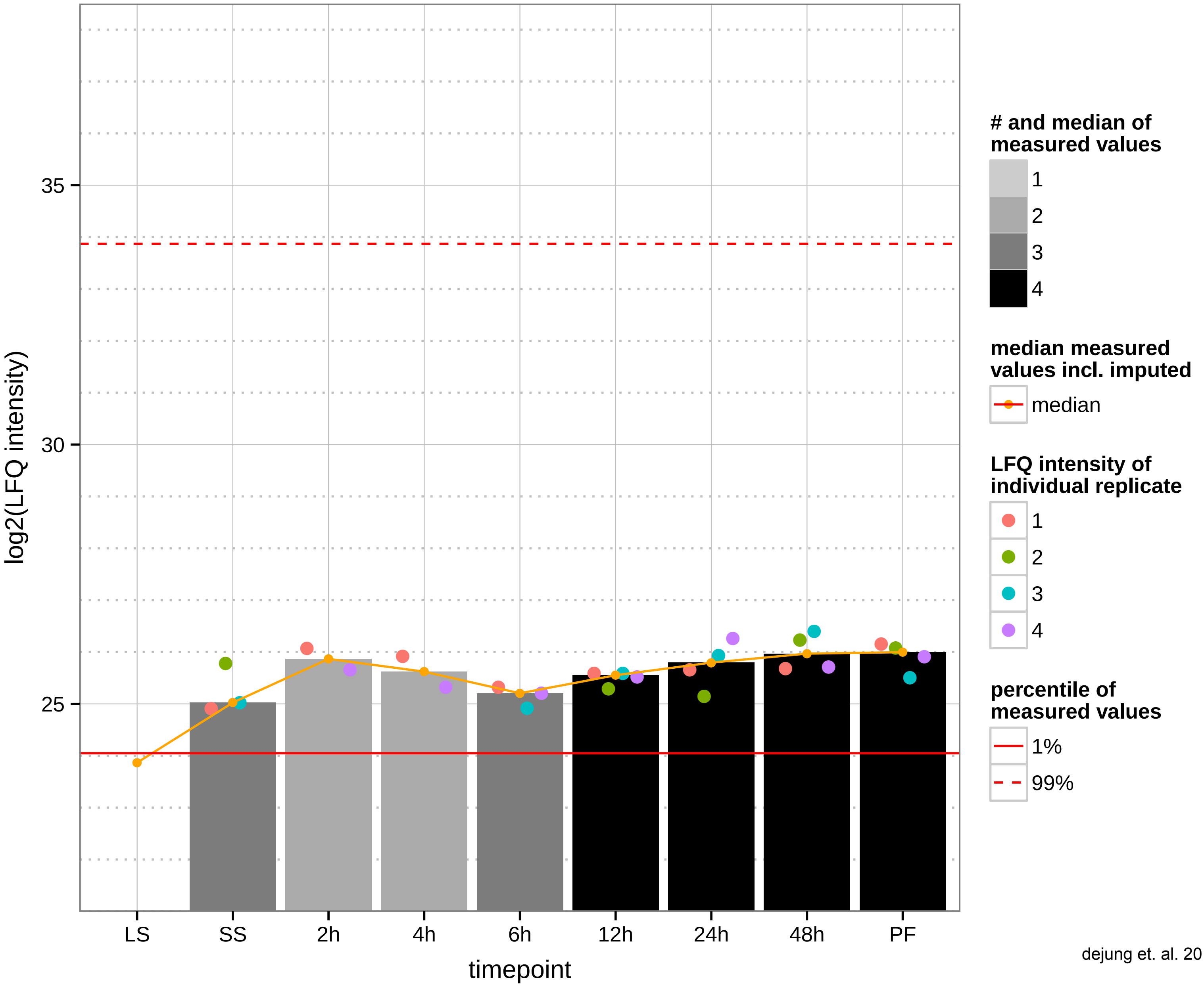

dejung et. al. 2015

BRCA2
Tb927.1.640
AGOF: damaged DNA binding
AGOC: nucleus
AGOP: antigenic variation
PGOF: null
PGOC: null
PGOP: double−strand break repair via homologous recombination

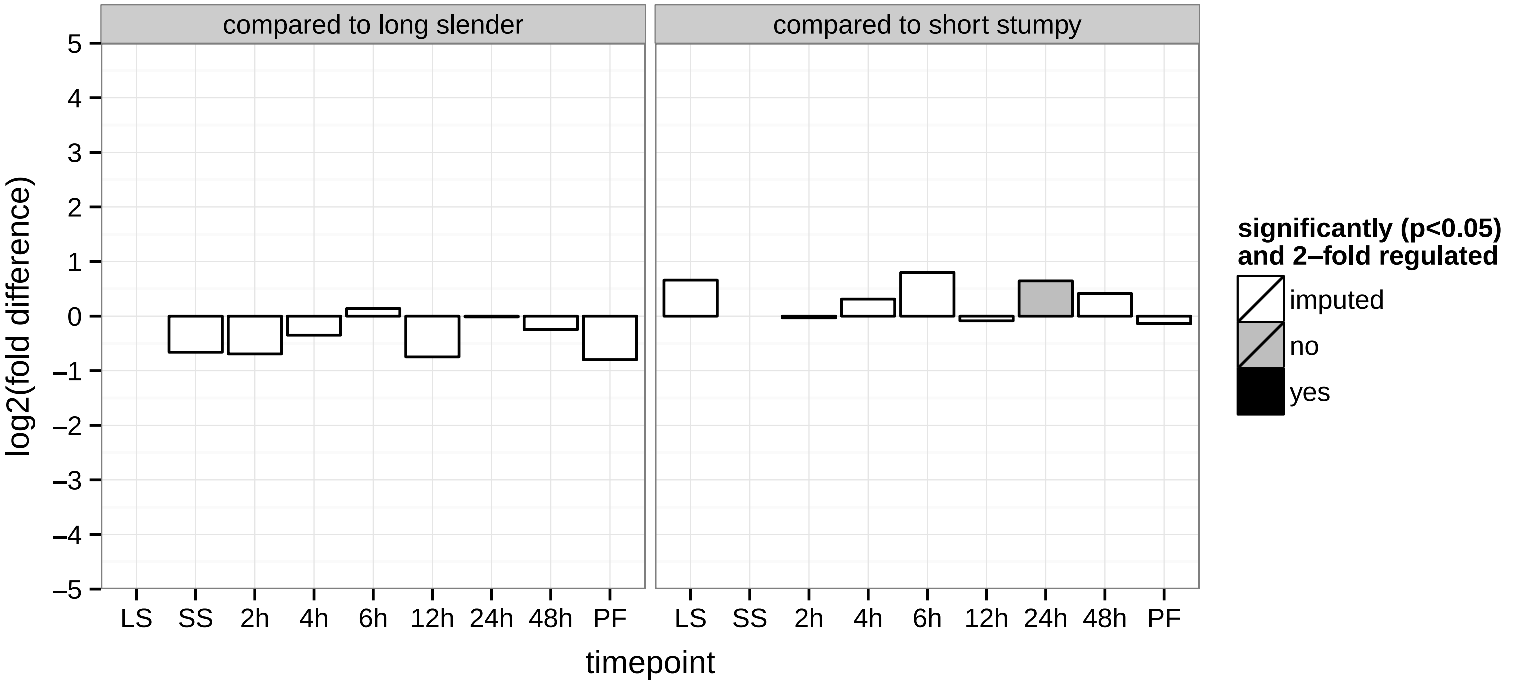

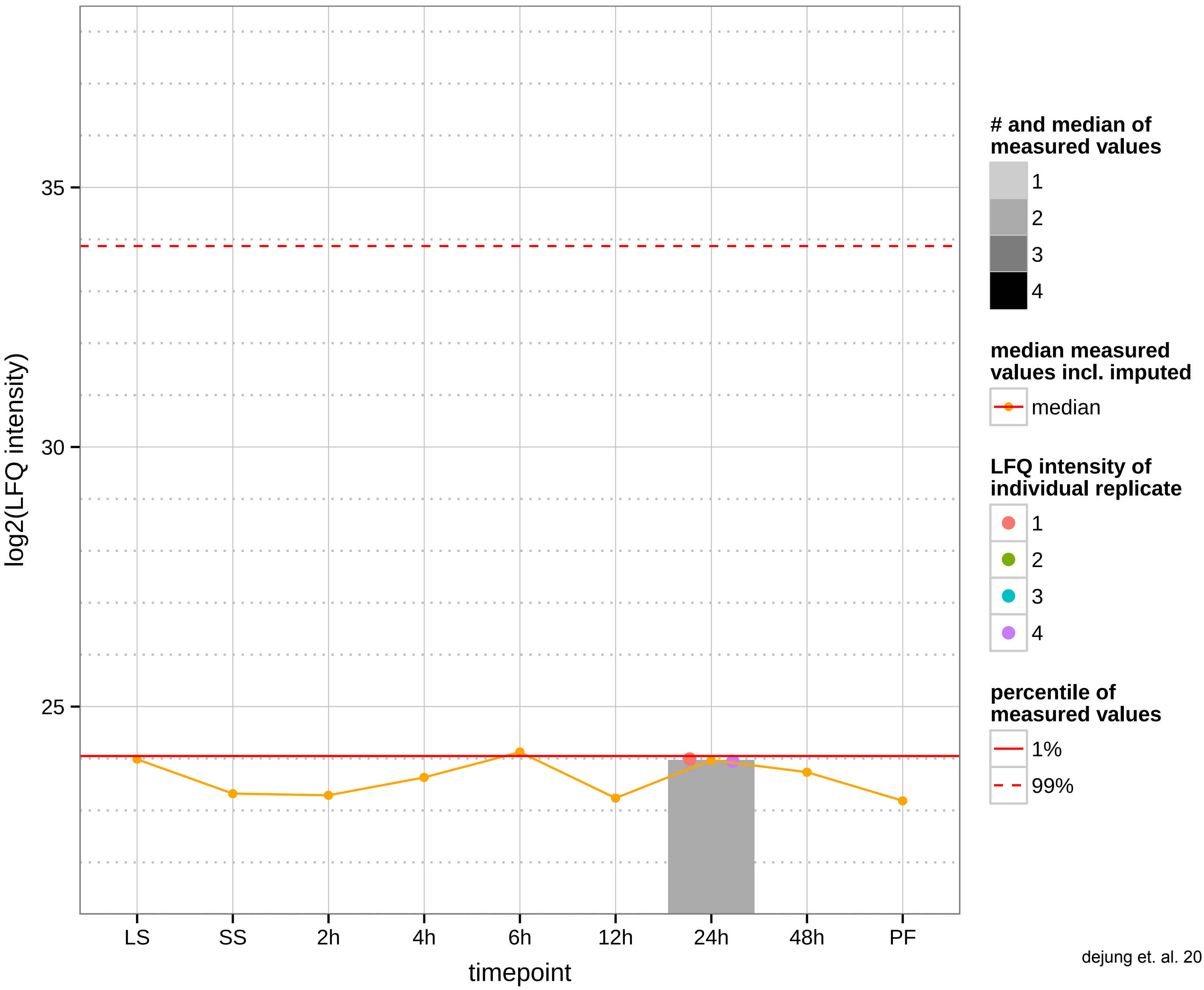

dejung et. al. 2015

conserved protein, unknown function
Tb927.1.670
AGOF: null
AGOC: null
AGOP: null
PGOF: null
PGOC: null
PGOP: null

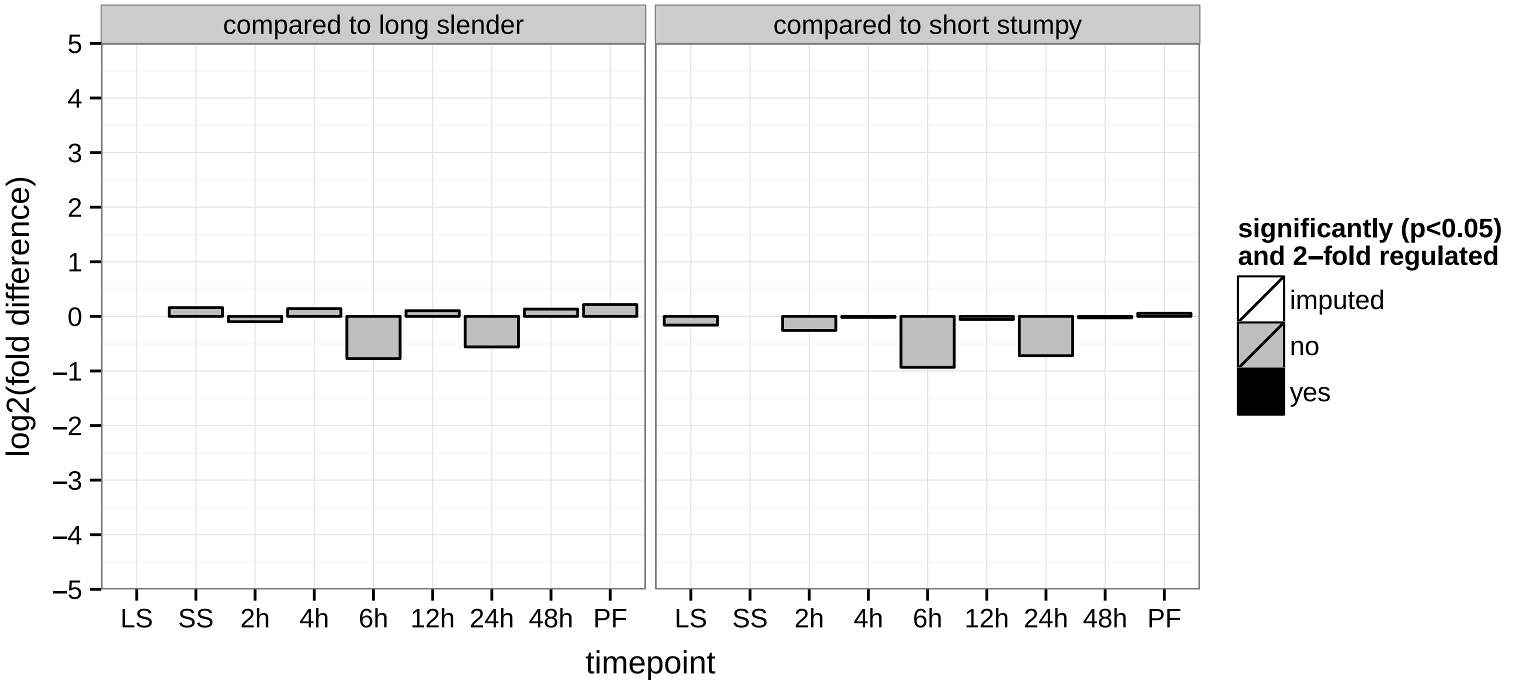

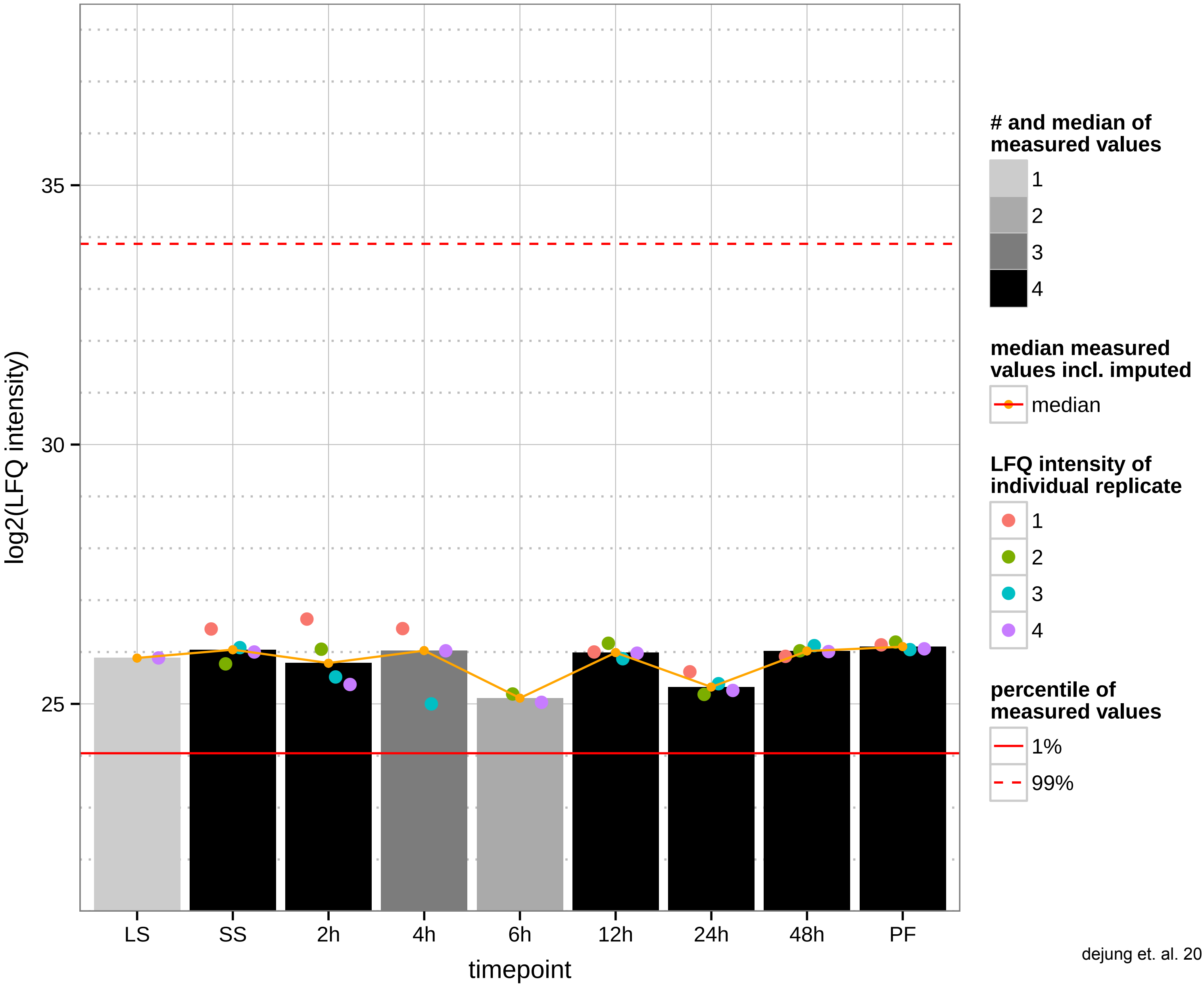

dejung et. al. 2015

phosphoglycerate kinase (PGKC)
Tb927.1.700
AGOF: phosphoglycerate kinase activity
AGOC: glycosome
AGOP: glycolysis
PGOF: phosphoglycerate kinase activity
PGOC: null
PGOP: glycolysis

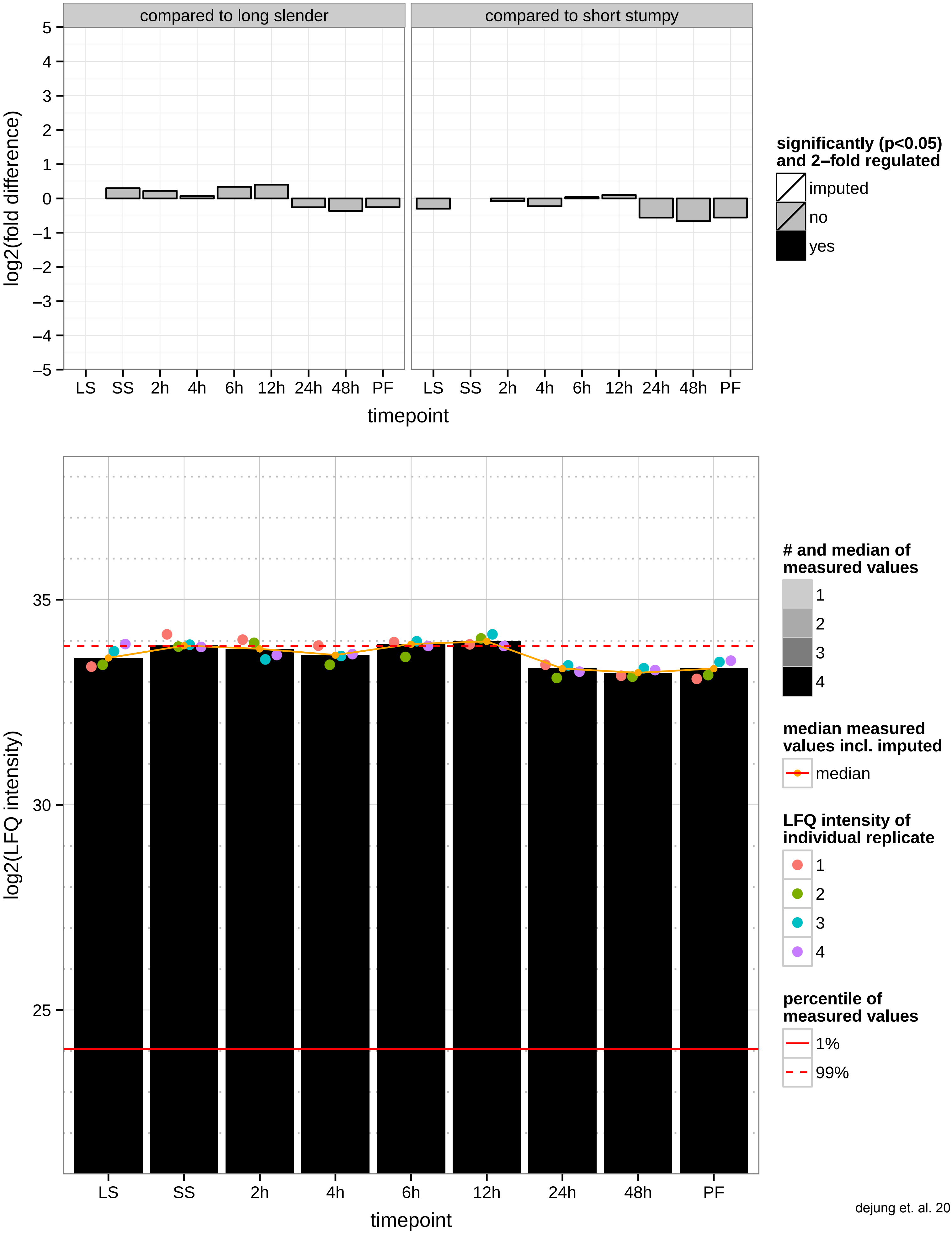

dejung et. al. 2015

deoxyhypusine synthase, putative
Tb927.1.870
AGOF: null
AGOC: null
AGOP: deoxyhypusine biosynthetic process from spermidine, peptidyl–lysine modification to hypusine
PGOF: null
PGOC: null
PGOP: peptidyl–lysine modification to hypusine

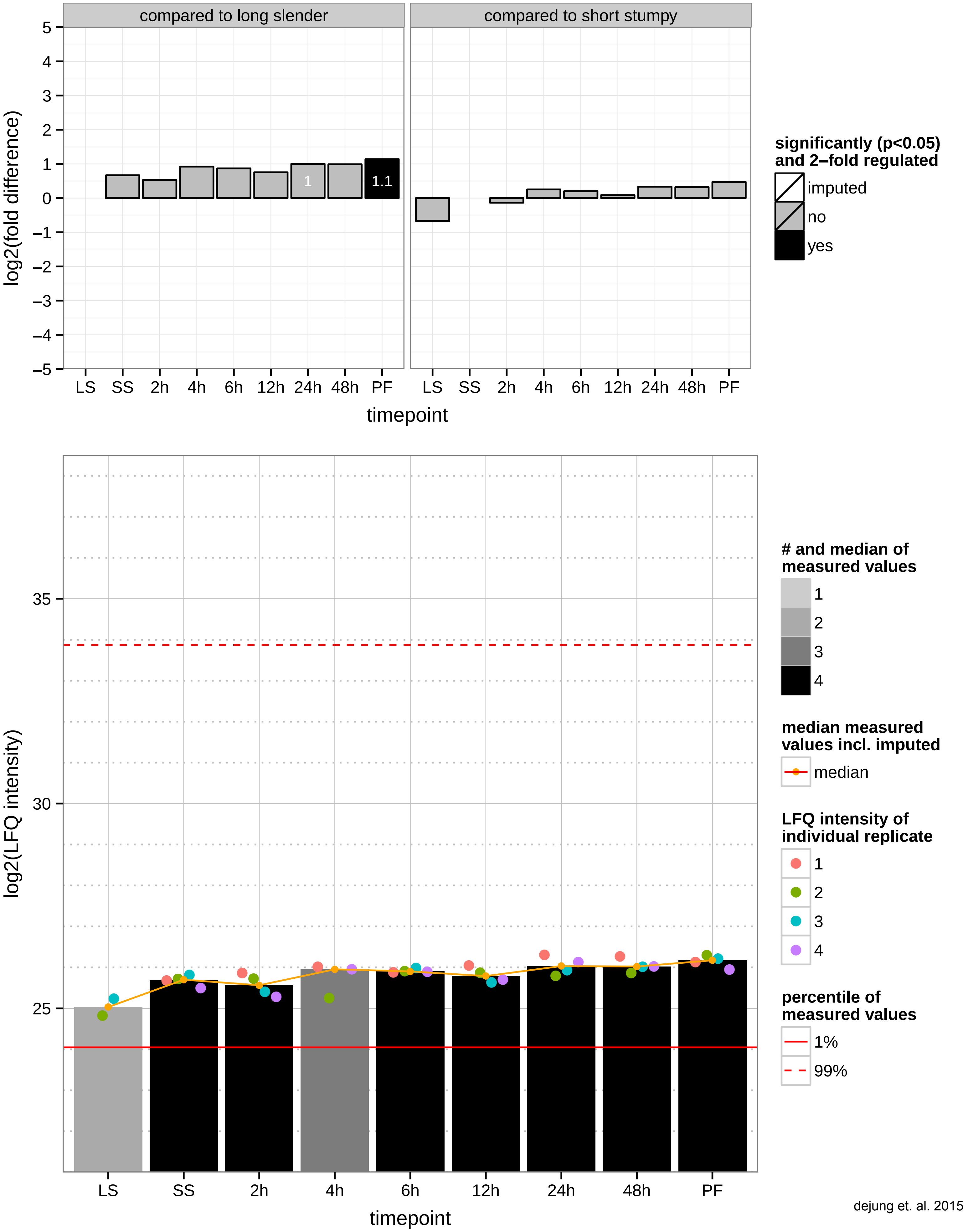

dejung et. al. 2015

hypothetical protein, conserved, Midasin, putative (MDN1)
Tb927.1.880
AGOF: null, ATP binding, ATPase activity
AGOC: null, nucleus
AGOP: null, regulation of protein complex assembly
PGOF: ATP binding, ATPase activity, nucleoside−triphosphatase activity, nucleotide binding, protein binding
PGOC: null, nucleus
PGOP: null, protein complex assembly

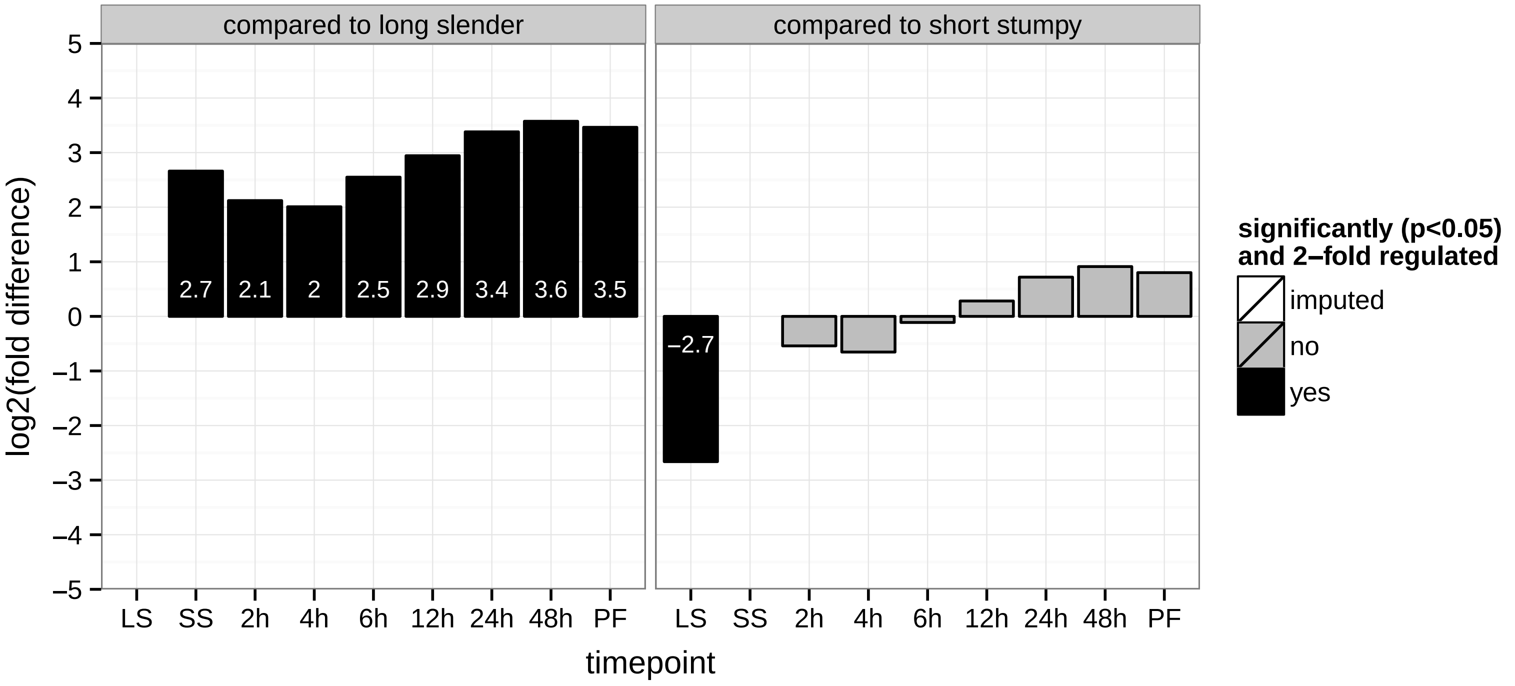

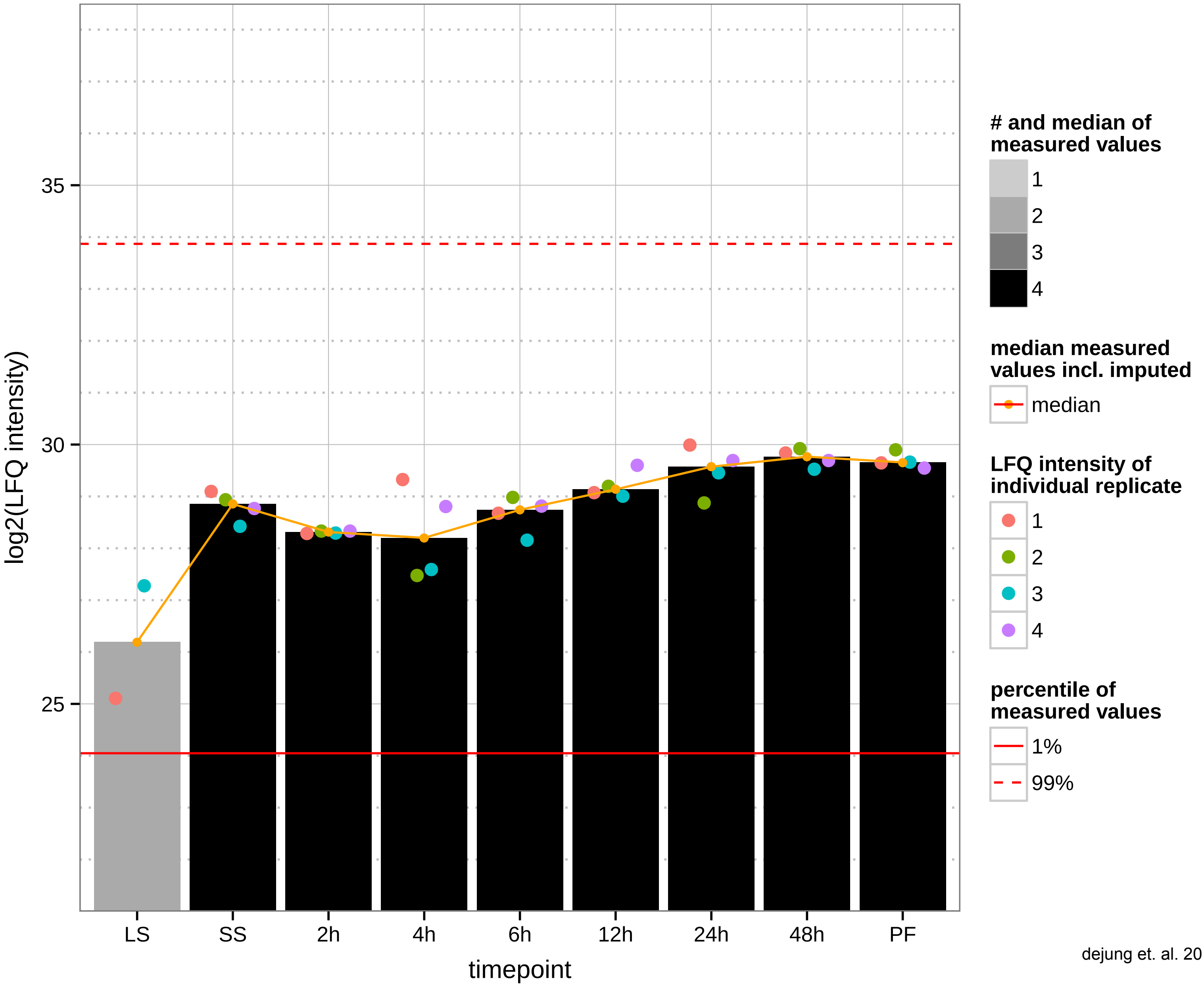

dejung et. al. 2015

Enkuring domain−containig protein
Tb927.1.990
AGOF: calmodulin binding
AGOC: null
AGOP: null
PGOF: null
PGOC: null
PGOP: null

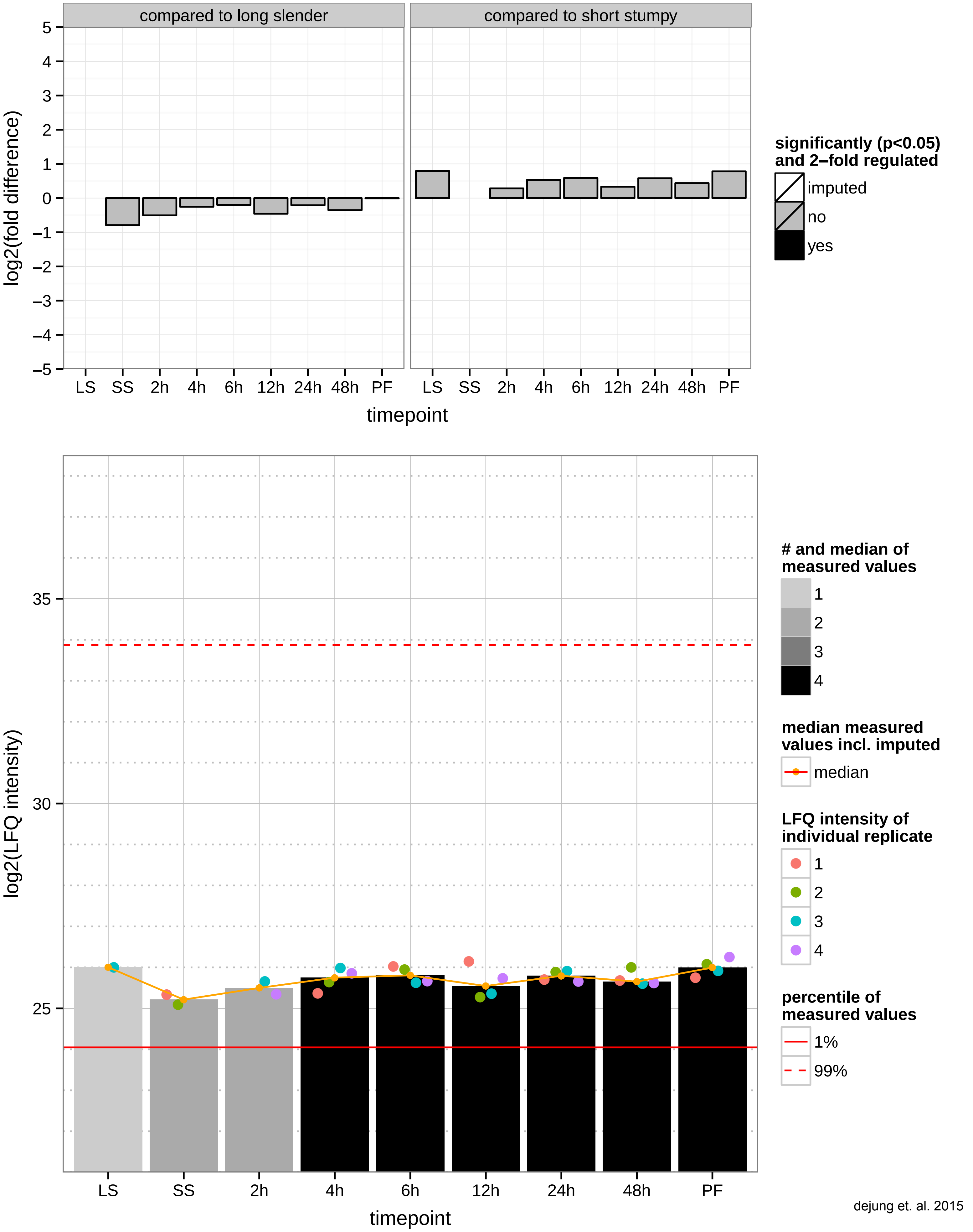

dejung et. al. 2015

60S acidic ribosomal protein, putative
Tb927.10.10010
AGOF: structural constituent of ribosome
AGOC: intracellular, ribosome
AGOP: translation
PGOF: null
PGOC: intracellular
PGOP: ribosome biogenesis

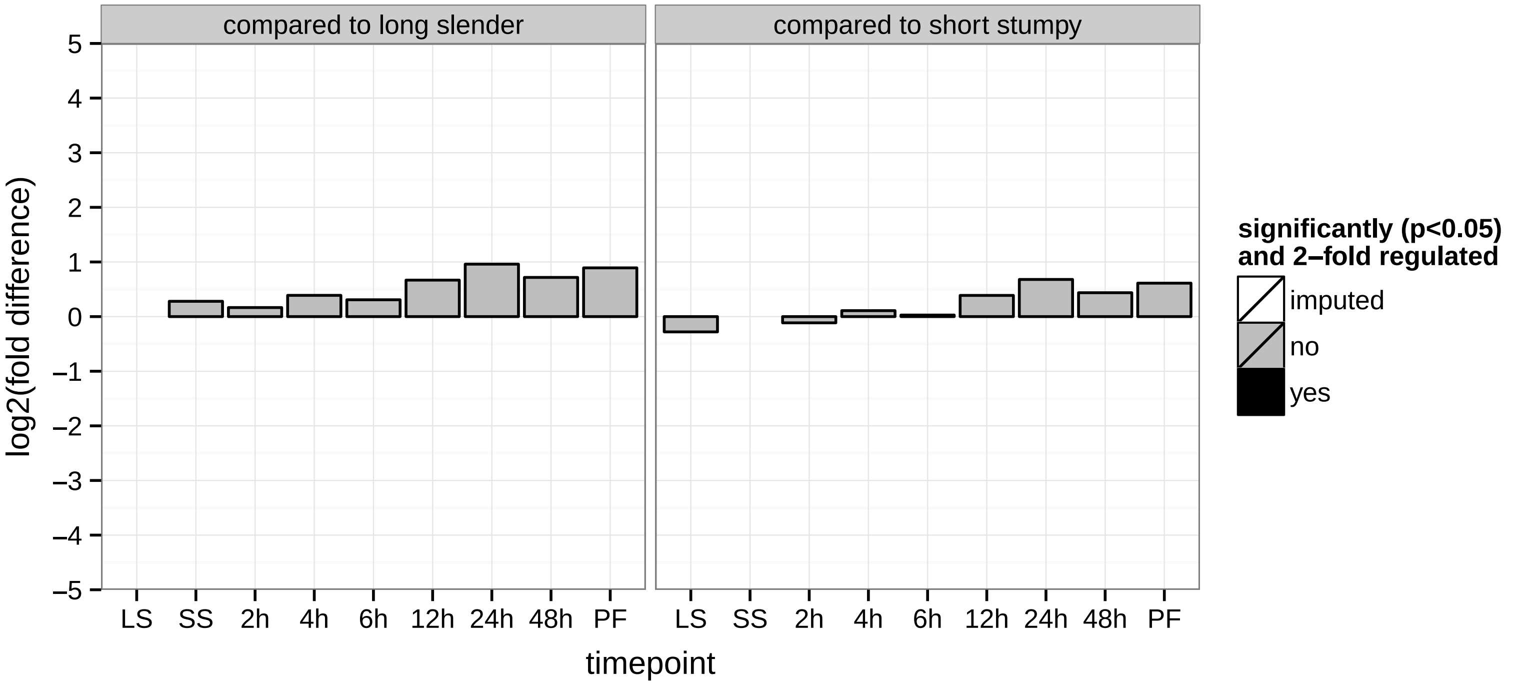

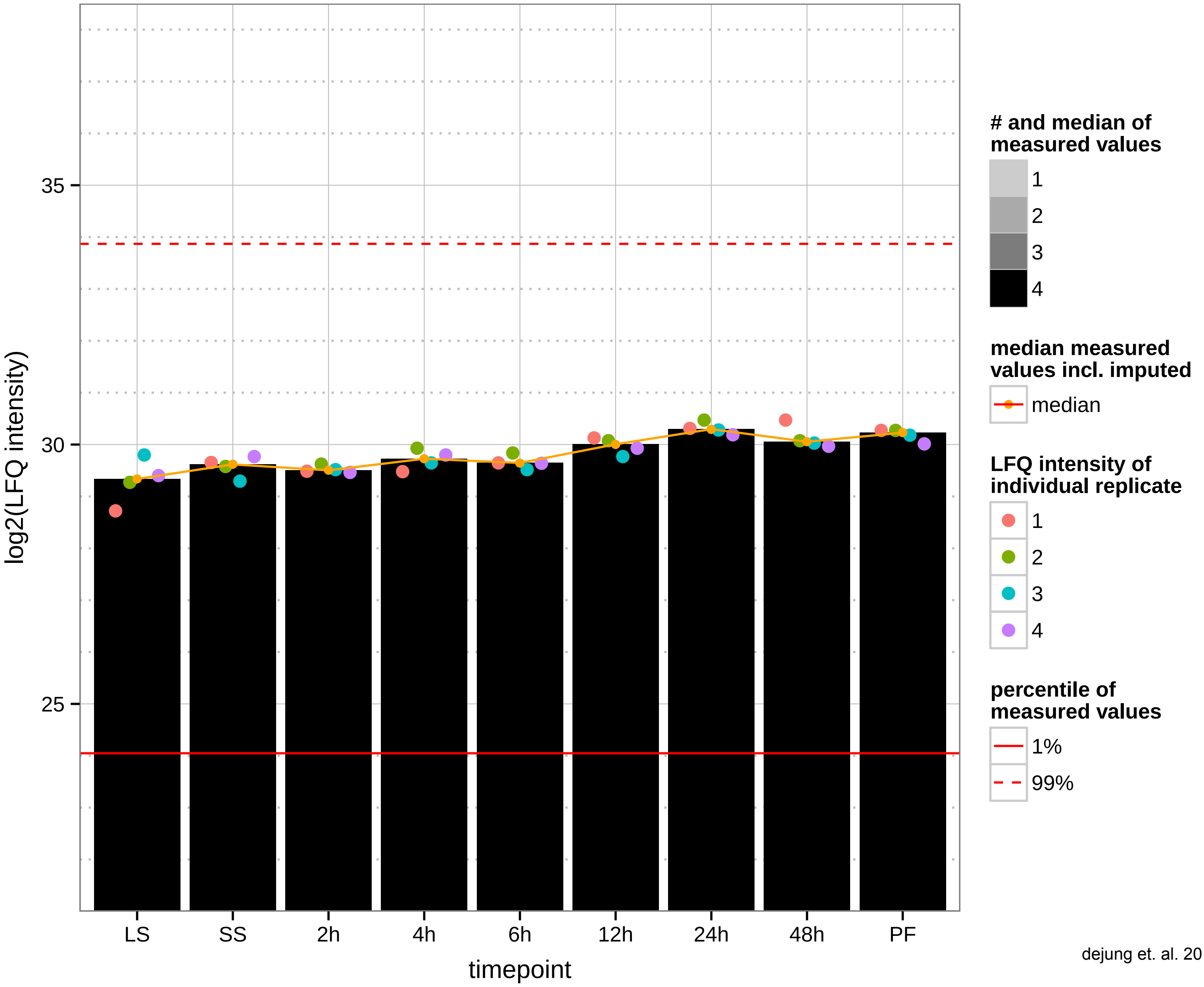

dejung et. al. 2015

hypothetical protein, conserved
Tb927.10.10030
AGOF: null
AGOC: null
AGOP: null
PGOF: null
PGOC: null
PGOP: null

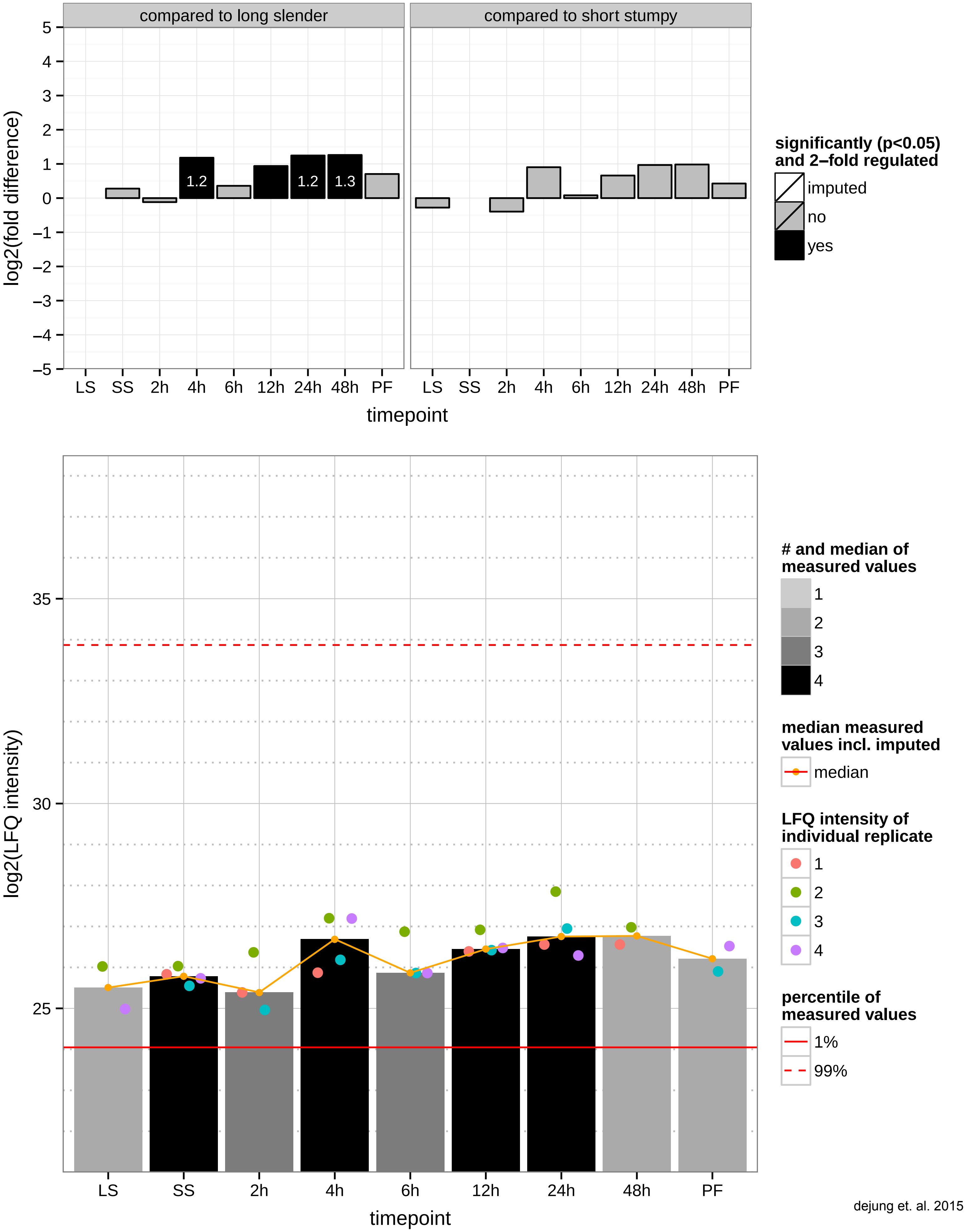

dejung et. al. 2015

mitochondrial RNA binding complex 1 subunit (MRB10130)
Tb927.10.10130
AGOF: RNA binding
AGOC: mitochondrion
AGOP: cytidine to uridine editing, mRNA stabilization
PGOF: binding
PGOC: null
PGOP: null

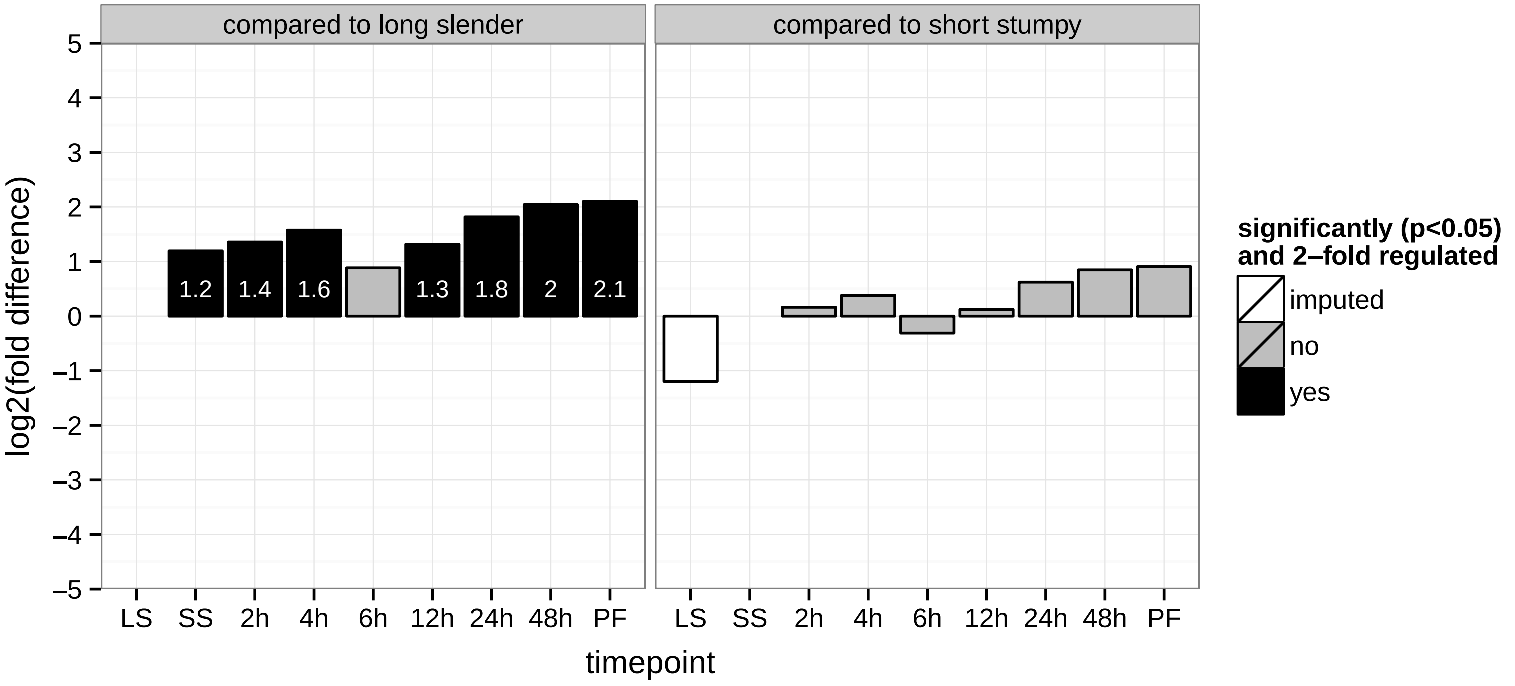

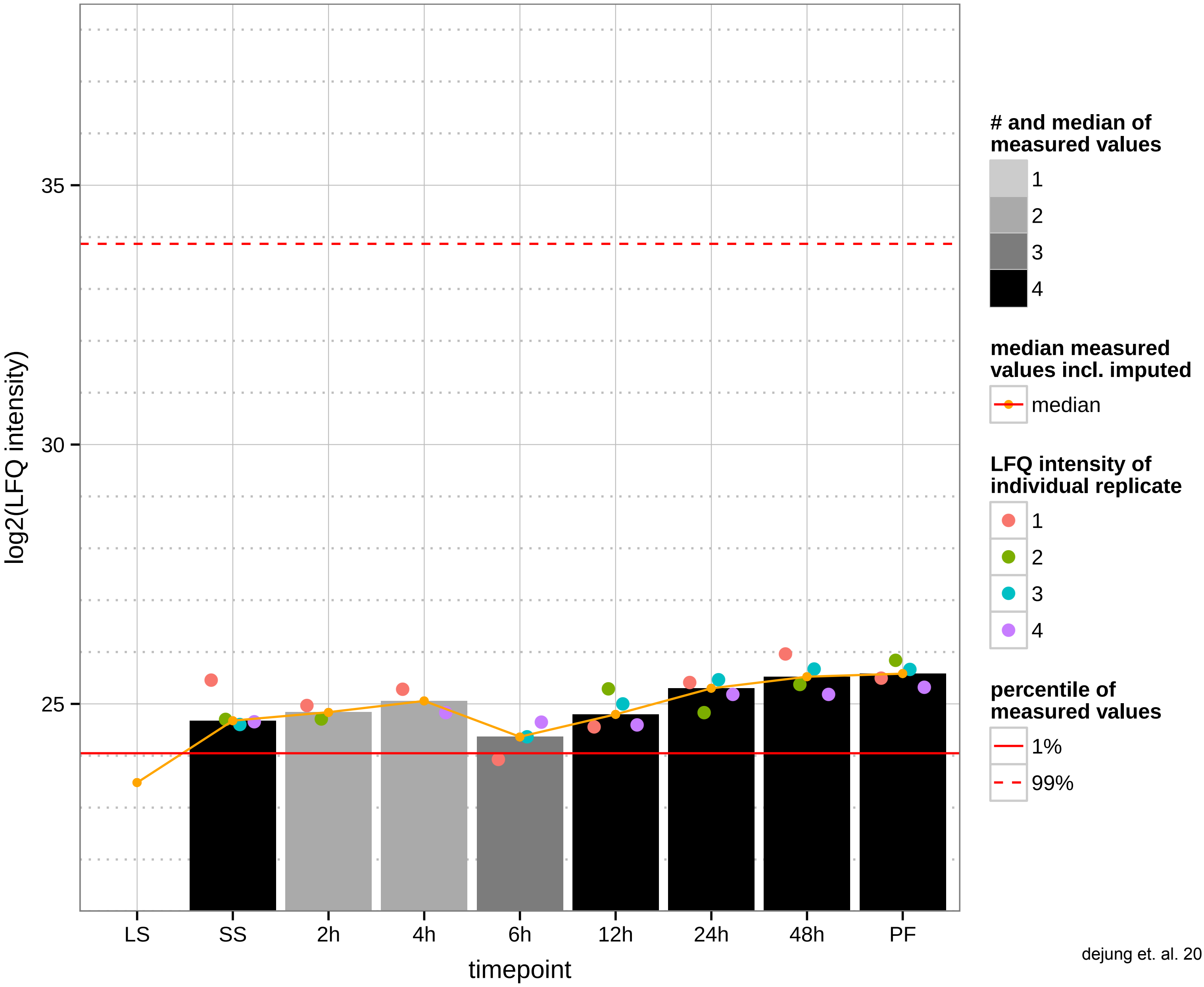

dejung et. al. 2015

paraflagellar rod component, putative (PFC19)
Tb927.10.10140
AGOF: null
AGOC: null
AGOP: null
PGOF: null
PGOC: null
PGOP: null

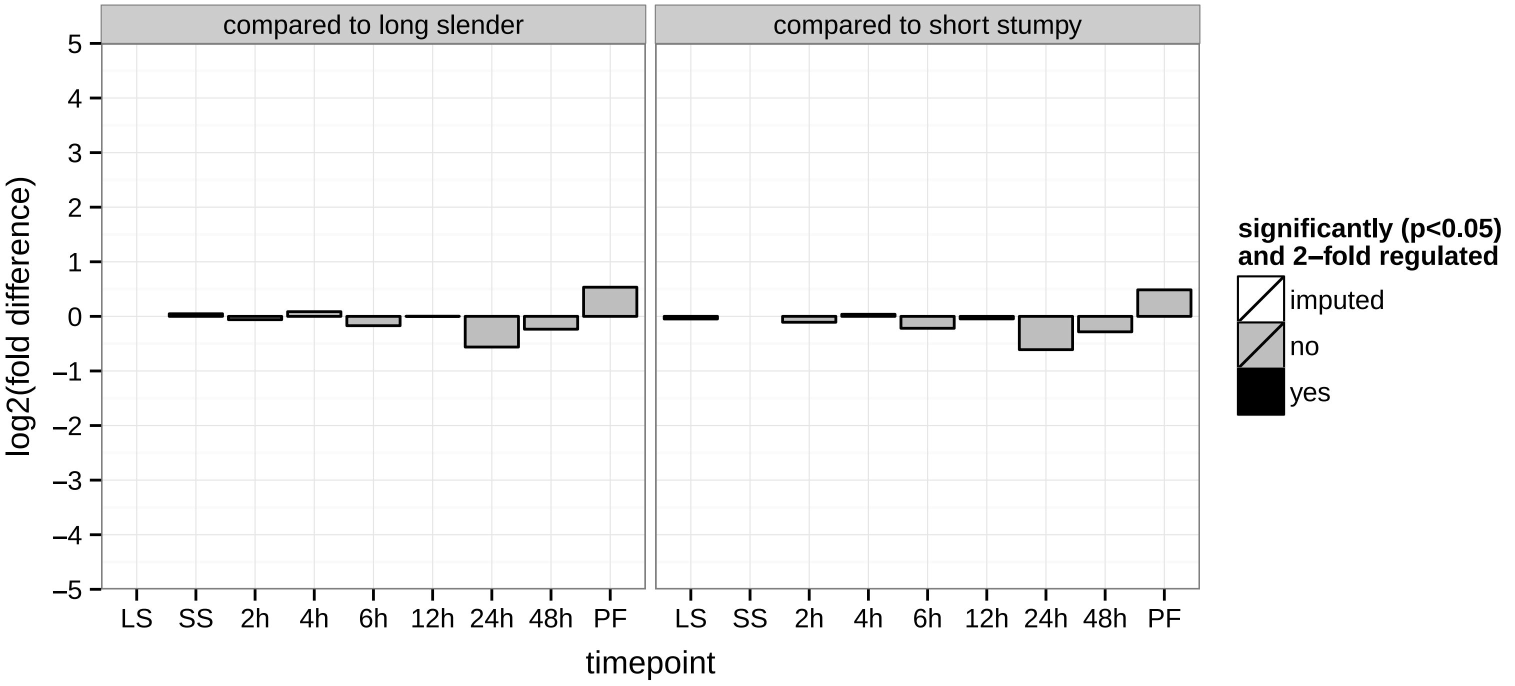

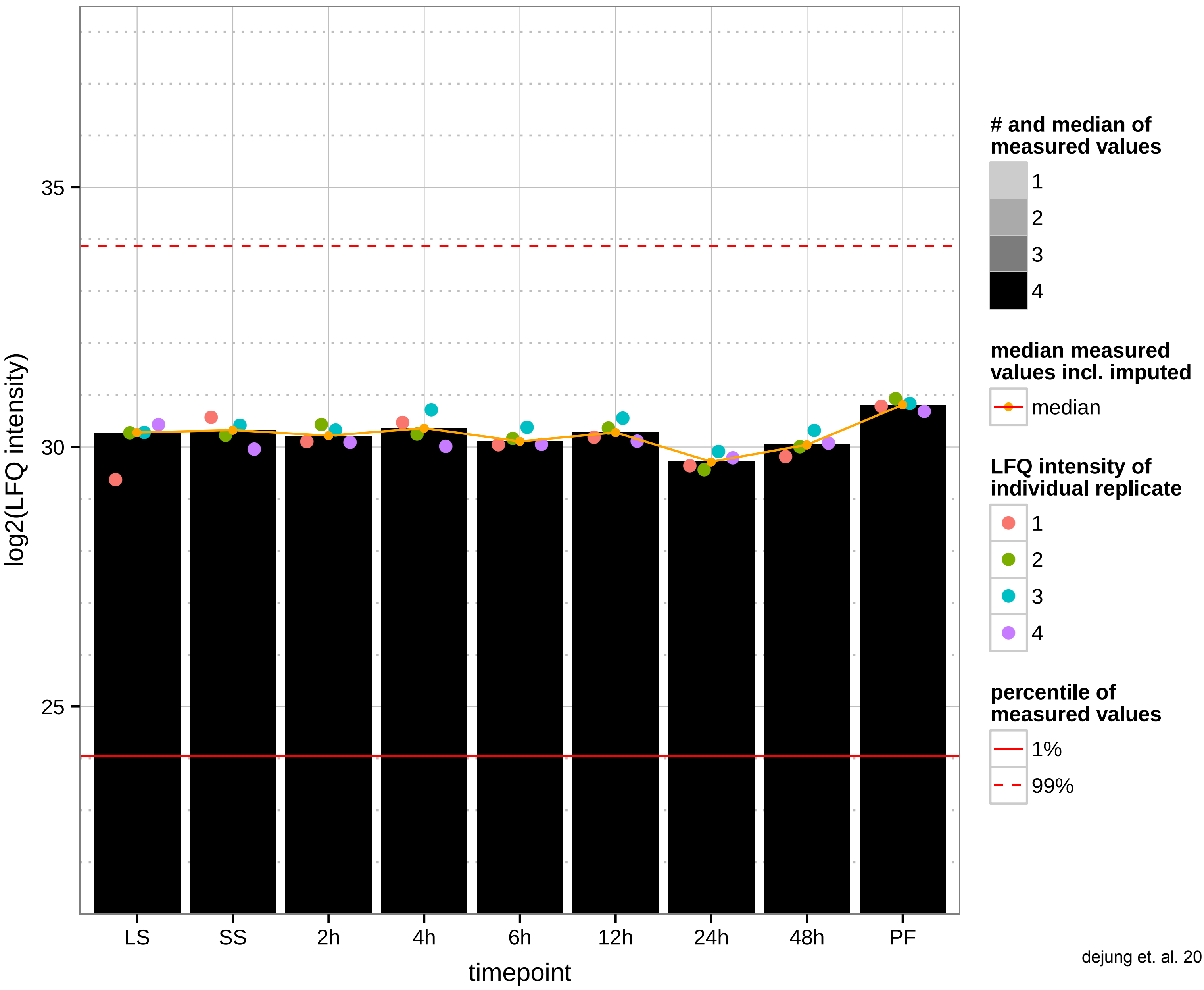

dejung et. al. 2015

hypothetical protein, conserved
Tb927.10.10160
AGOF: null
AGOC: mitochondrion
AGOP: null
PGOF: null
PGOC: null
PGOP: null

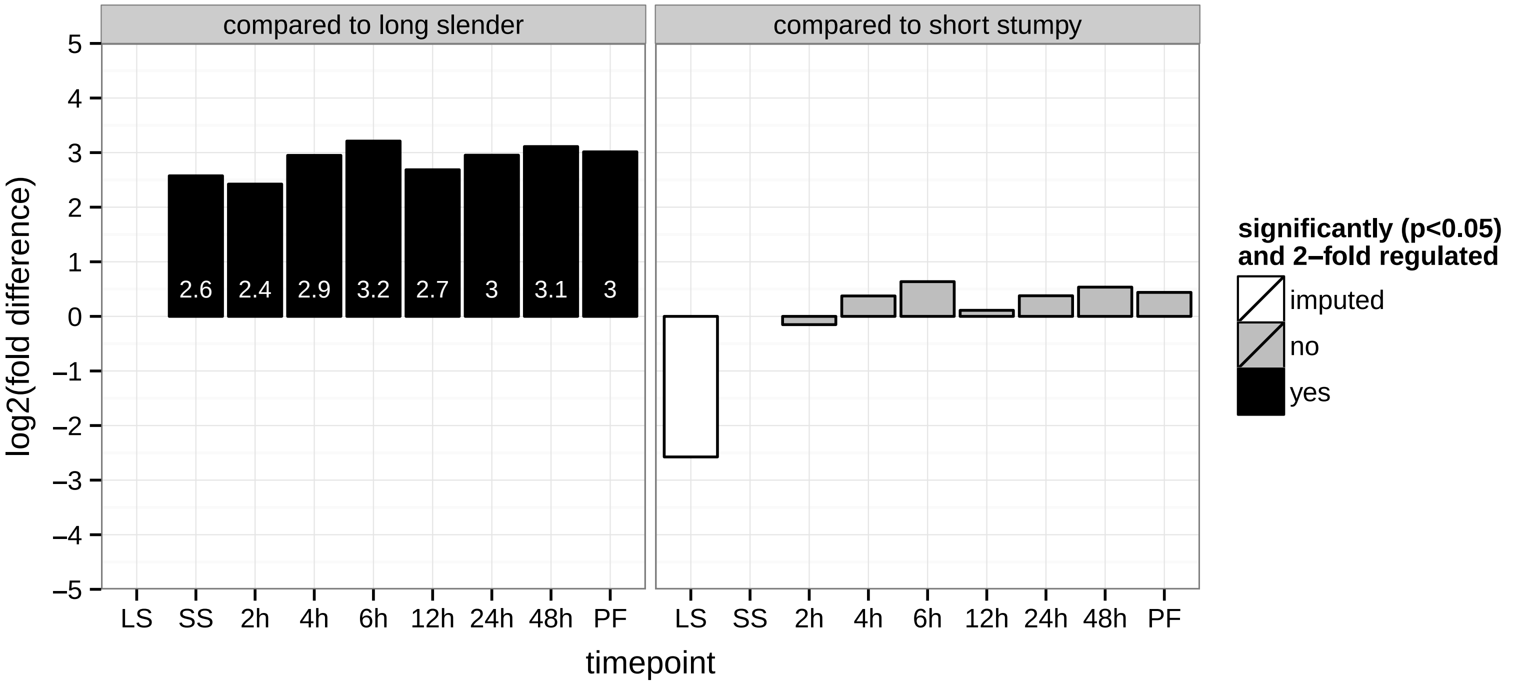

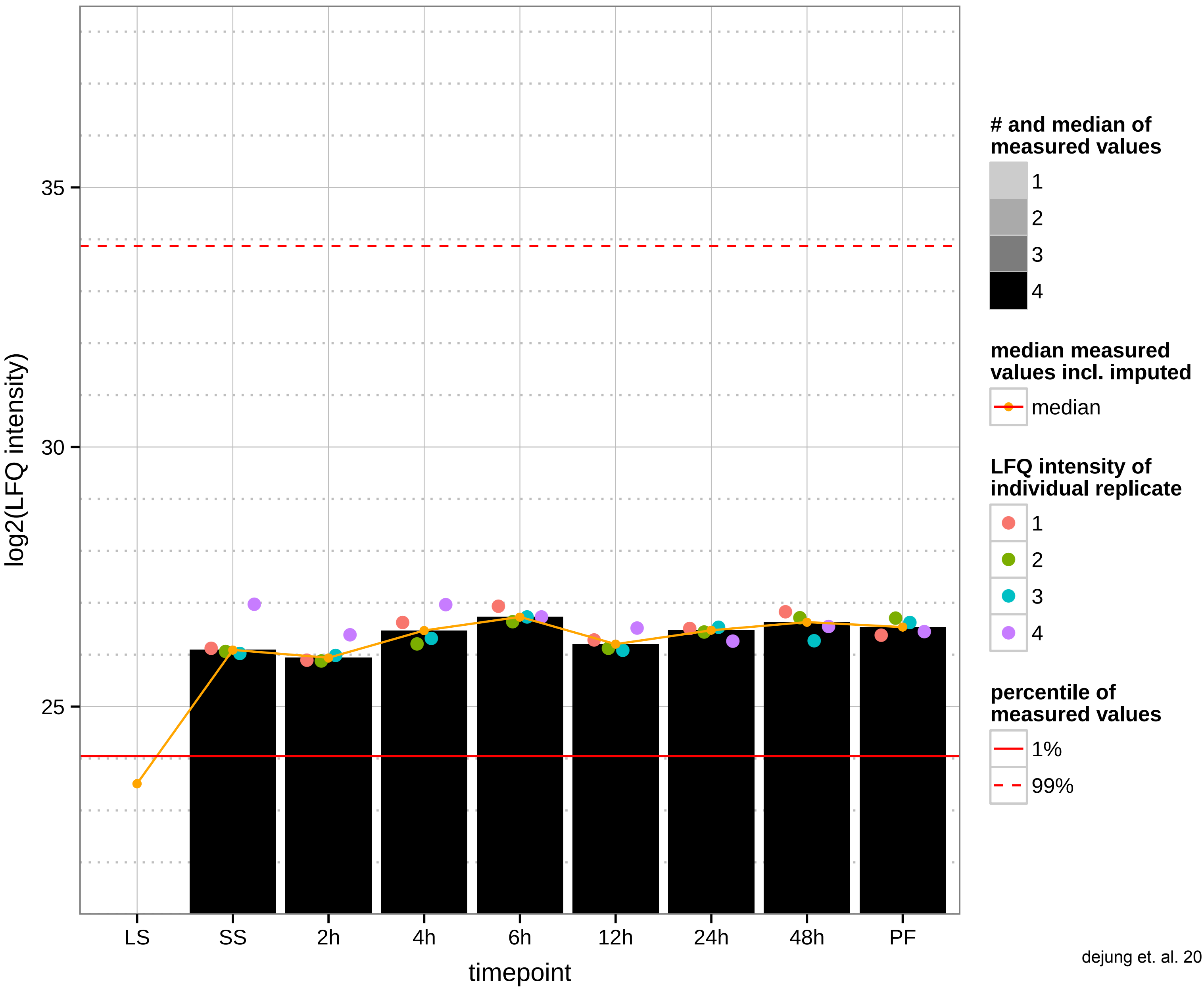

dejung et. al. 2015

hypothetical protein, conserved
Tb927.10.10330
AGOF: null
AGOC: null
AGOP: null
PGOF: protein binding
PGOC: null
PGOP: null

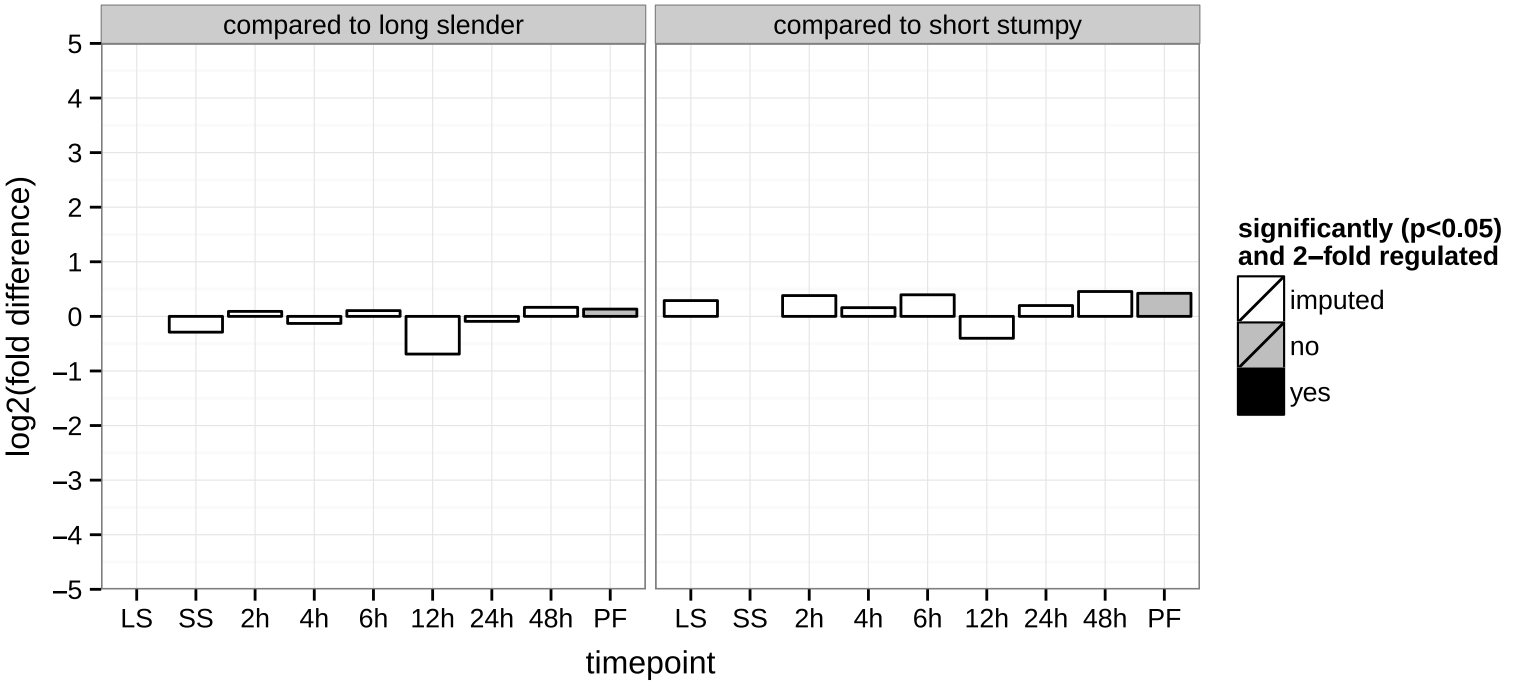

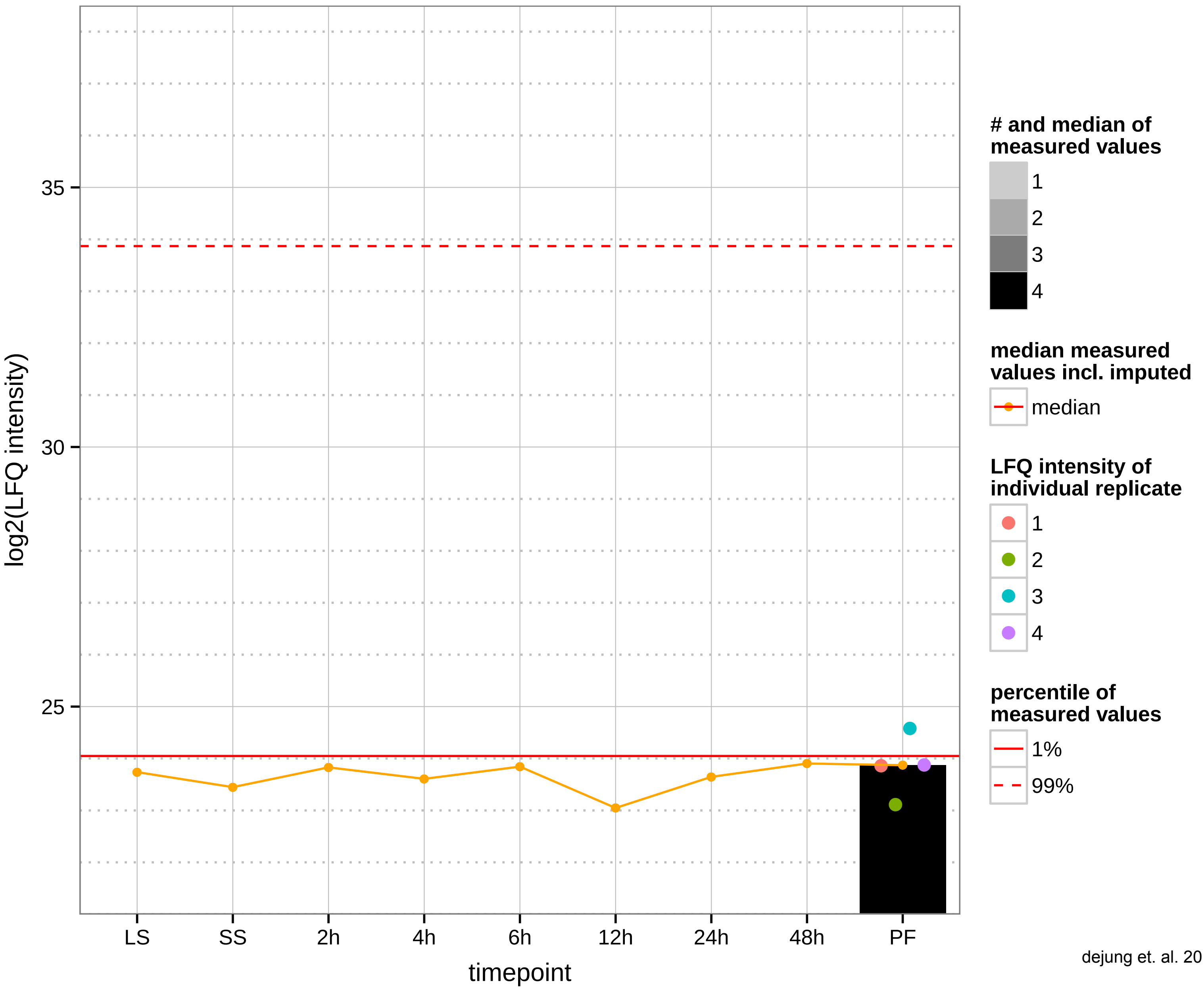

dejung et. al. 2015

protein kinase, putative
Tb927.10.10350
AGOF: ATP binding, protein serine/threonine kinase activity, protein tyrosine kinase activity
AGOC: null
AGOP: protein phosphorylation
PGOF: ATP binding, protein kinase activity, transferase activity, transferring phosphorus–containing groups
PGOC: null
PGOP: protein phosphorylation

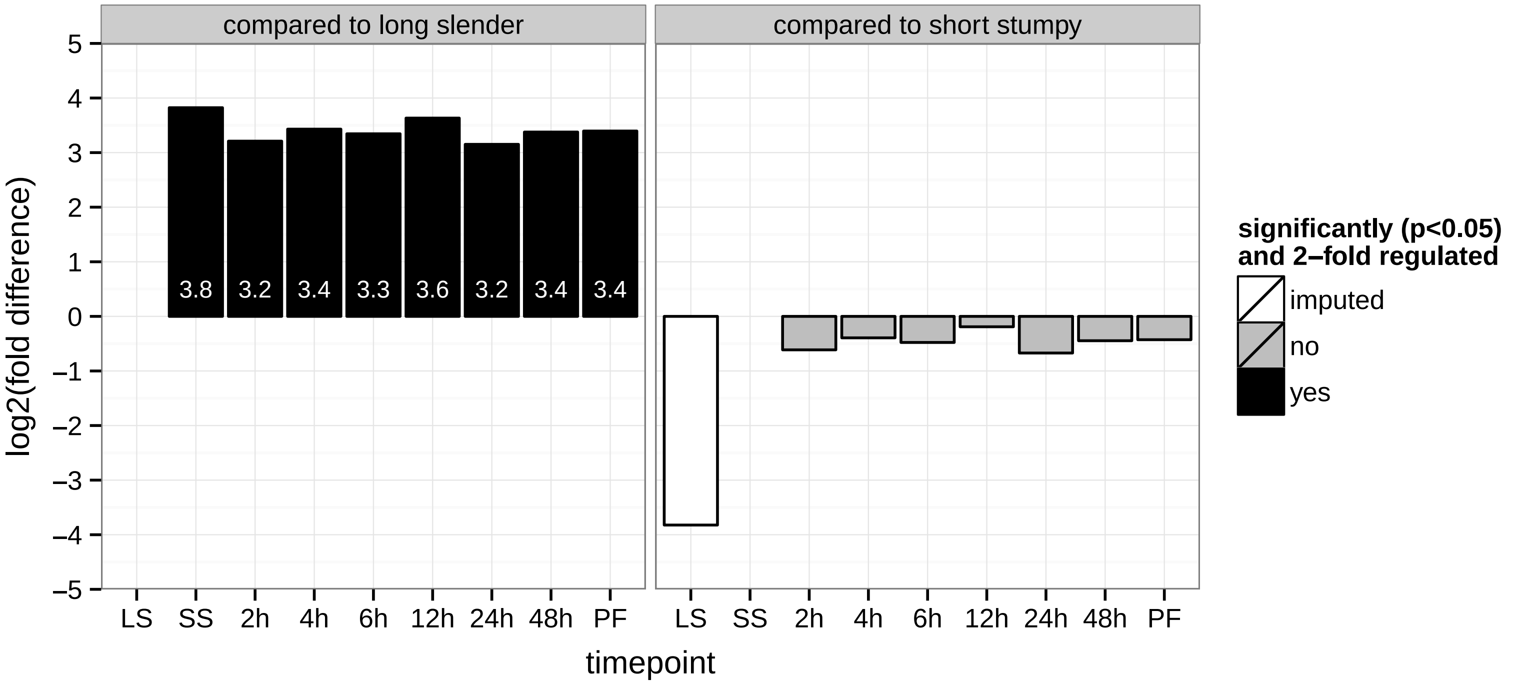

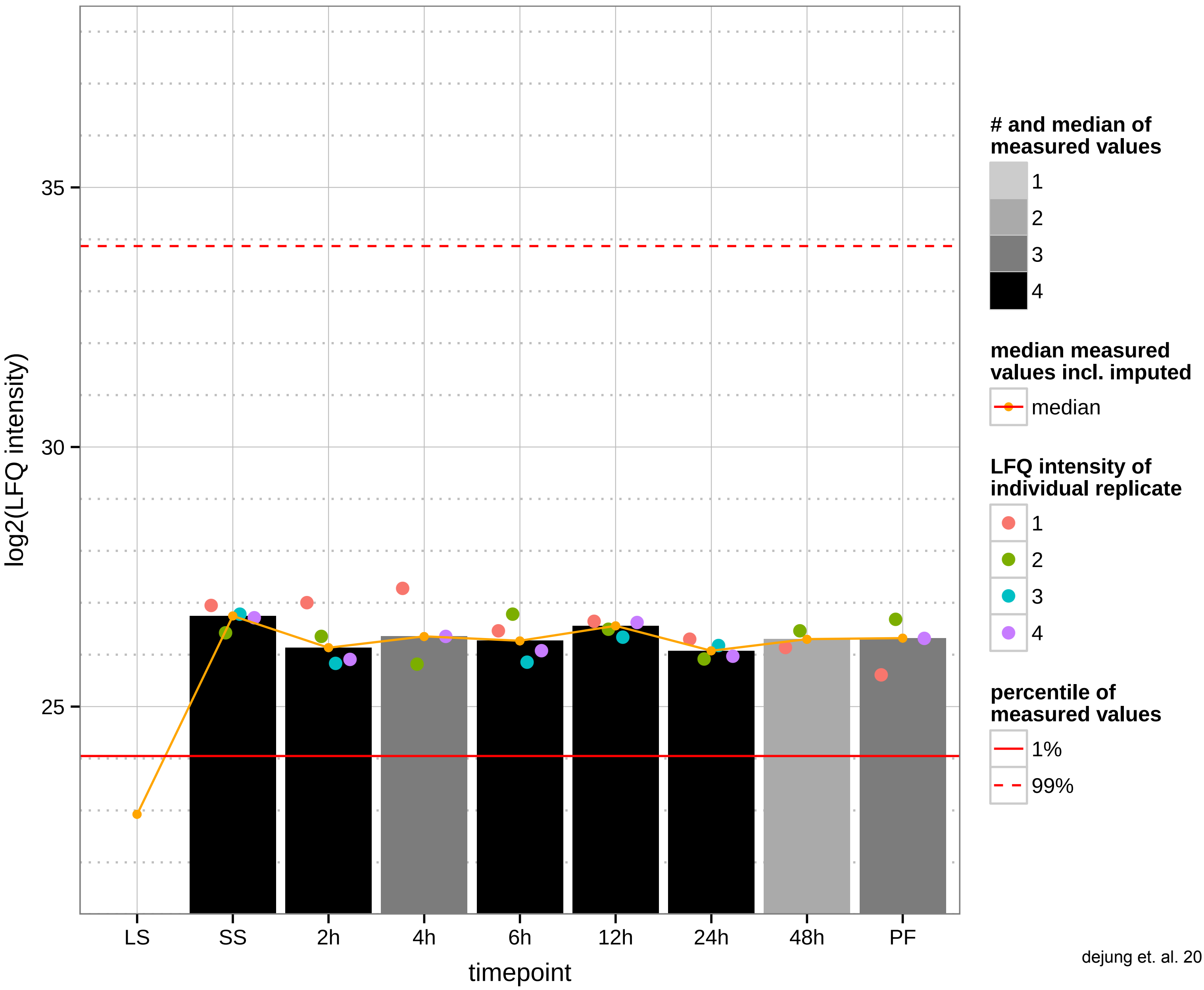

dejung et. al. 2015

hypothetical protein, conserved
Tb927.10.10370;Tb927.10.10290
AGOF: null
AGOC: null
AGOP: null
PGOF: null
PGOC: null
PGOP: null

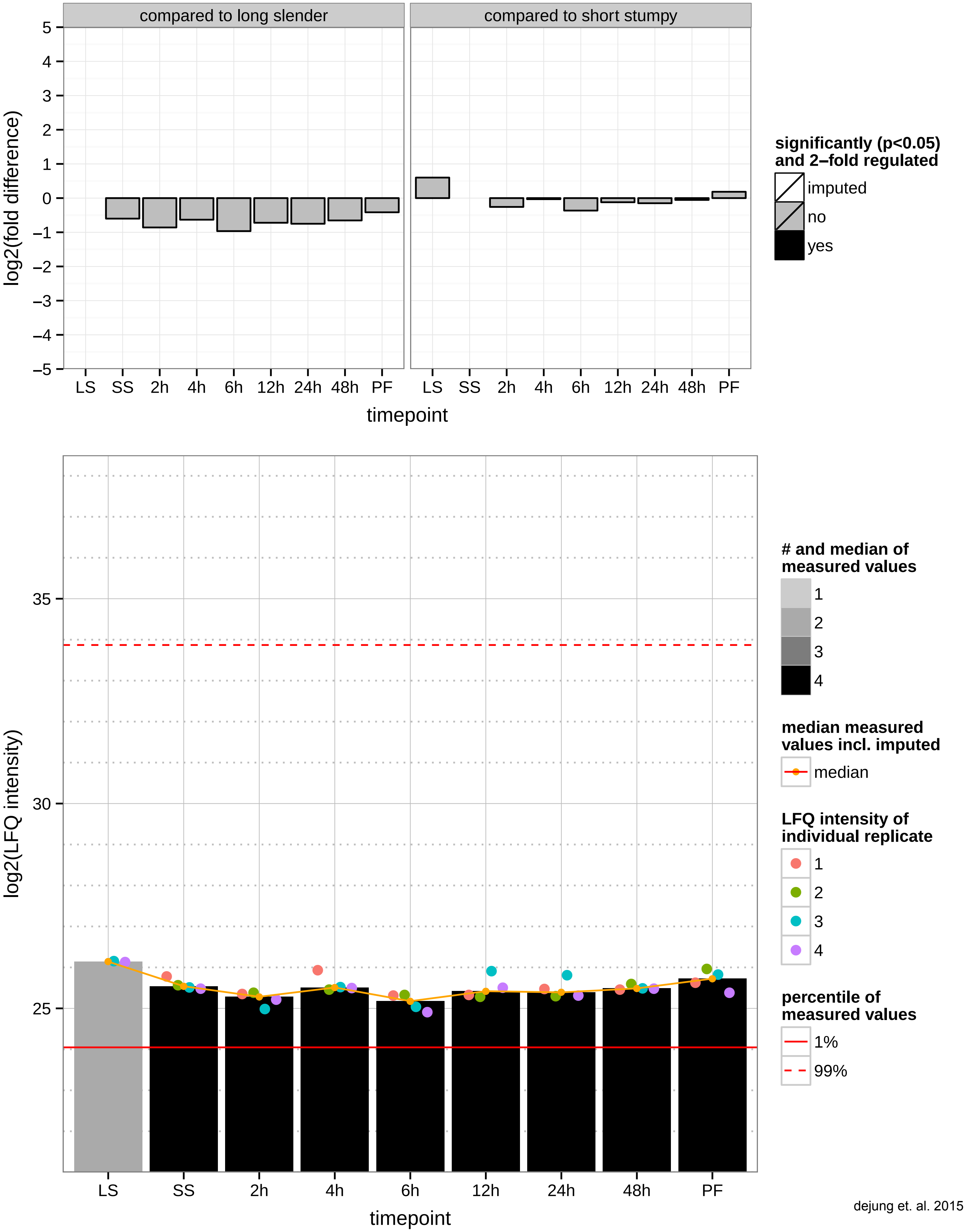

dejung et. al. 2015

ATP−dependent DEAD/H RNA helicase, putative
Tb927.10.10380
AGOF: ATP binding, ATP−dependent helicase activity, helicase activity, nucleic acid binding
AGOC: null
AGOP: nucleobase−containing compound metabolic process
PGOF: ATP binding, ATP−dependent helicase activity, helicase activity, nucleic acid binding
PGOC: null
PGOP: null

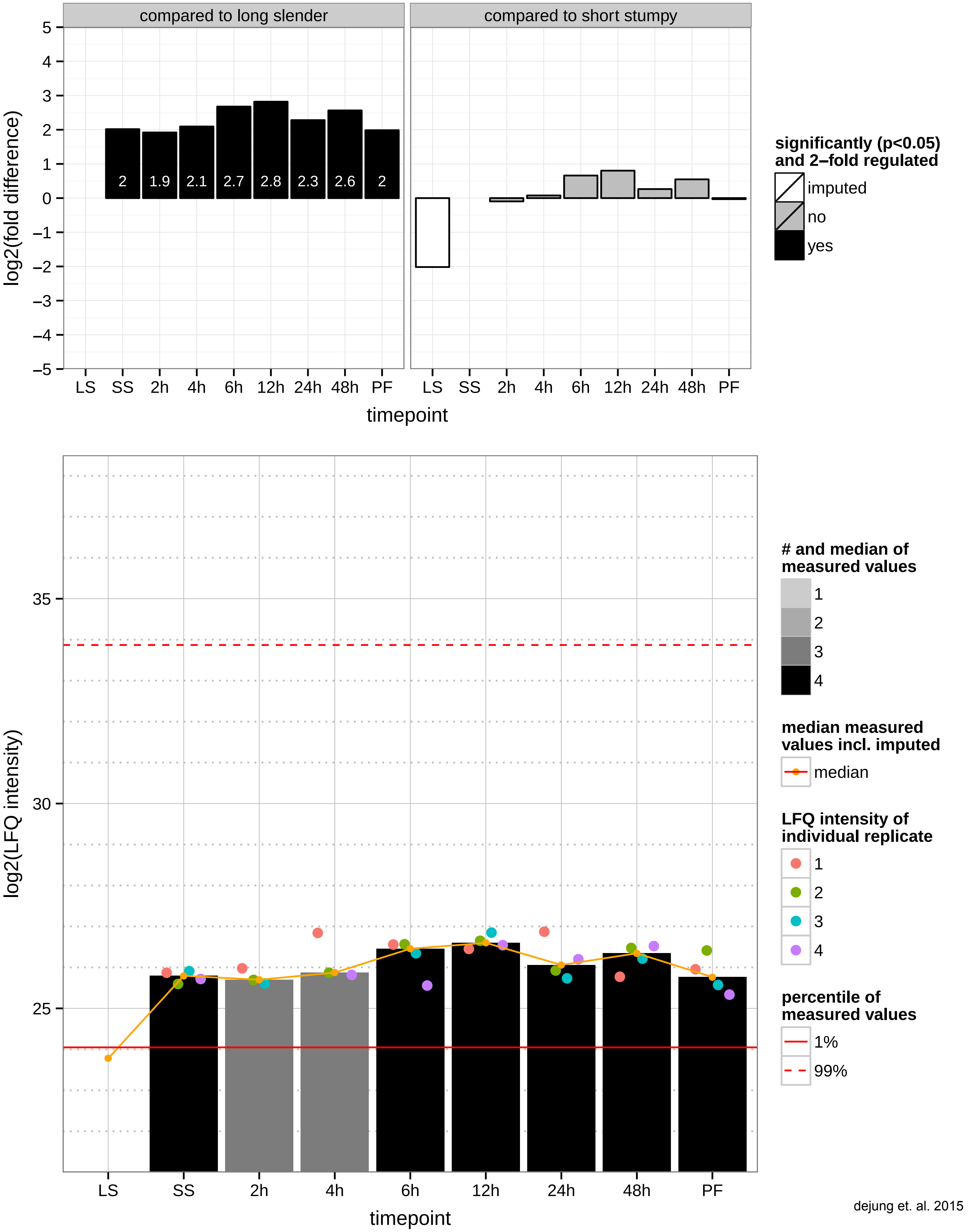

dejung et. al. 2015

trypanothione reductase
Tb927.10.10390
AGOF: disulfide oxidoreductase activity, flavin adenine dinucleotide binding, trypanothione–disulfide reductase activity
AGOC: cytoplasm
AGOP: cell redox homeostasis, oxidation–reduction process
PGOF: disulfide oxidoreductase activity, flavin adenine dinucleotide binding, oxidoreductase activity
PGOC: cytoplasm
PGOP: cell redox homeostasis, oxidation–reduction process

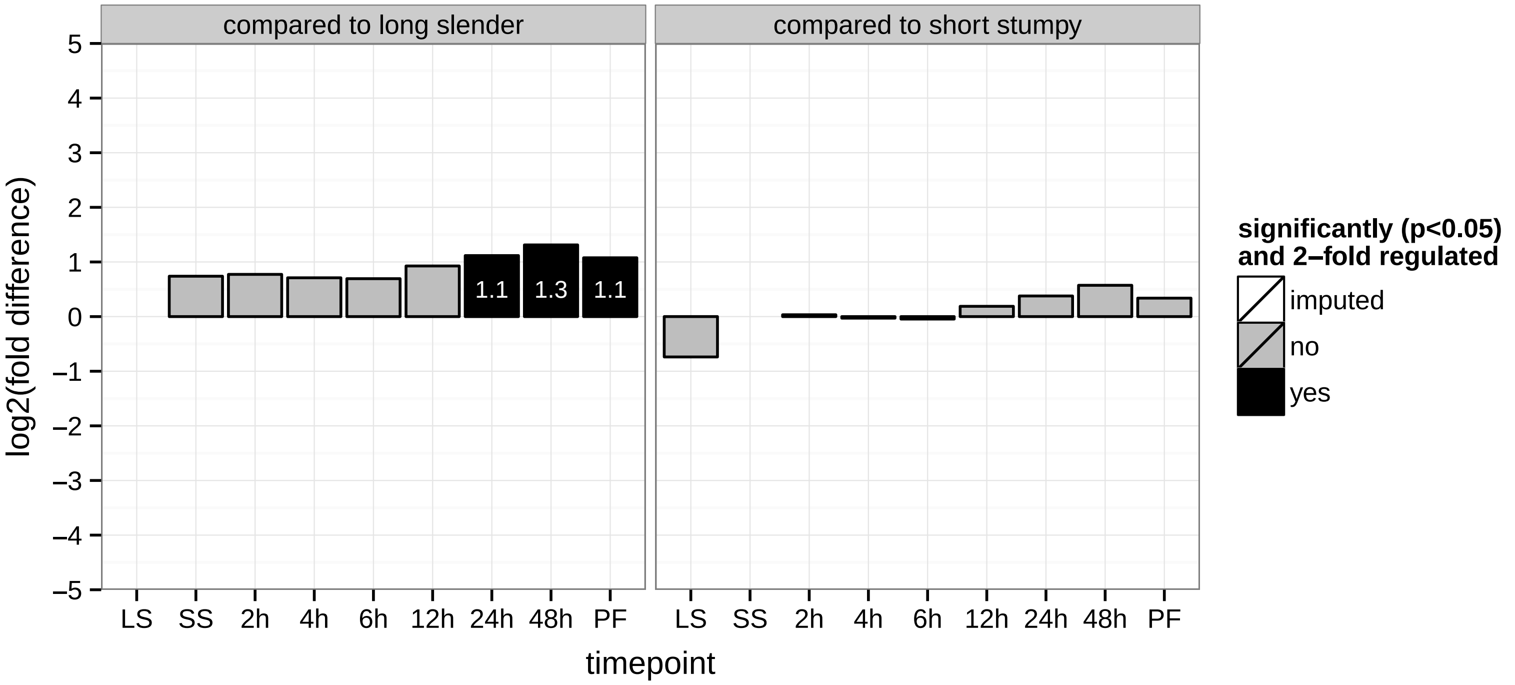

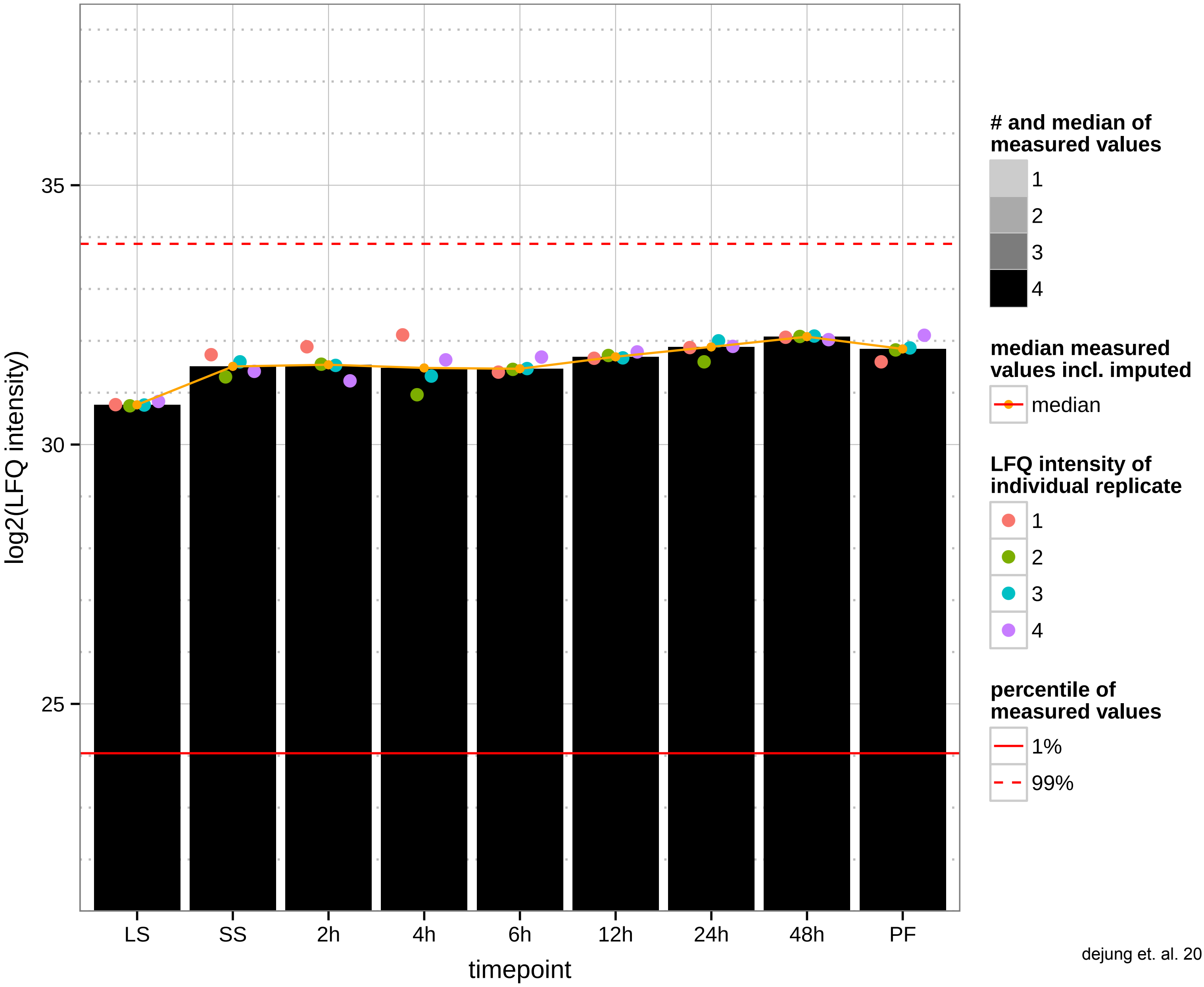

dejung et. al. 2015

histone H2B, putative
Tb927.10.10590;Tb927.10.10580;Tb927.10.10570;Tb927.10.10560;Tb927.10.10550;Tb927.10.10540;Tb927.10.10530;Tb92
AGOF: DNA binding
AGOC: nucleosome, nucleus
AGOP: chromosome organization, nucleosome assembly
PGOF: null, DNA binding
PGOC: null, nucleosome, nucleus
PGOP: null, nucleosome assembly

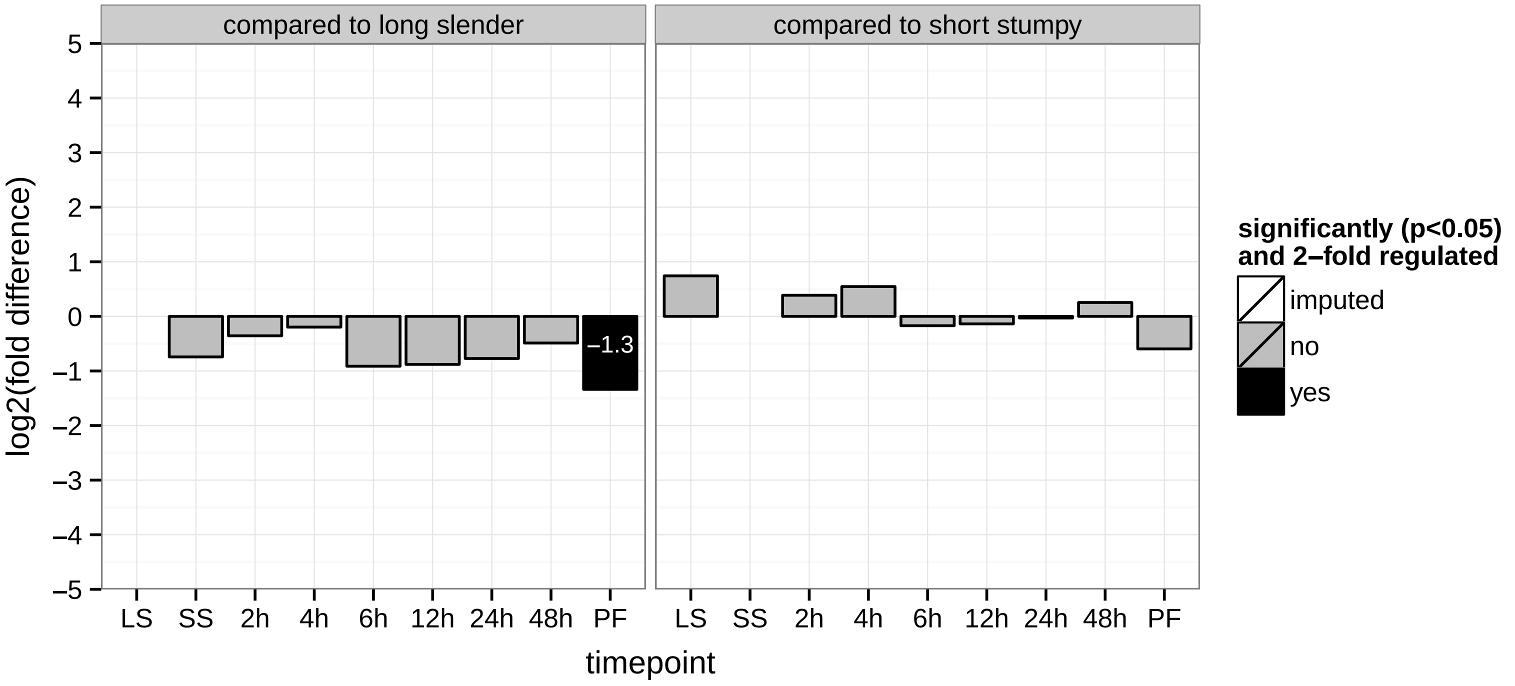

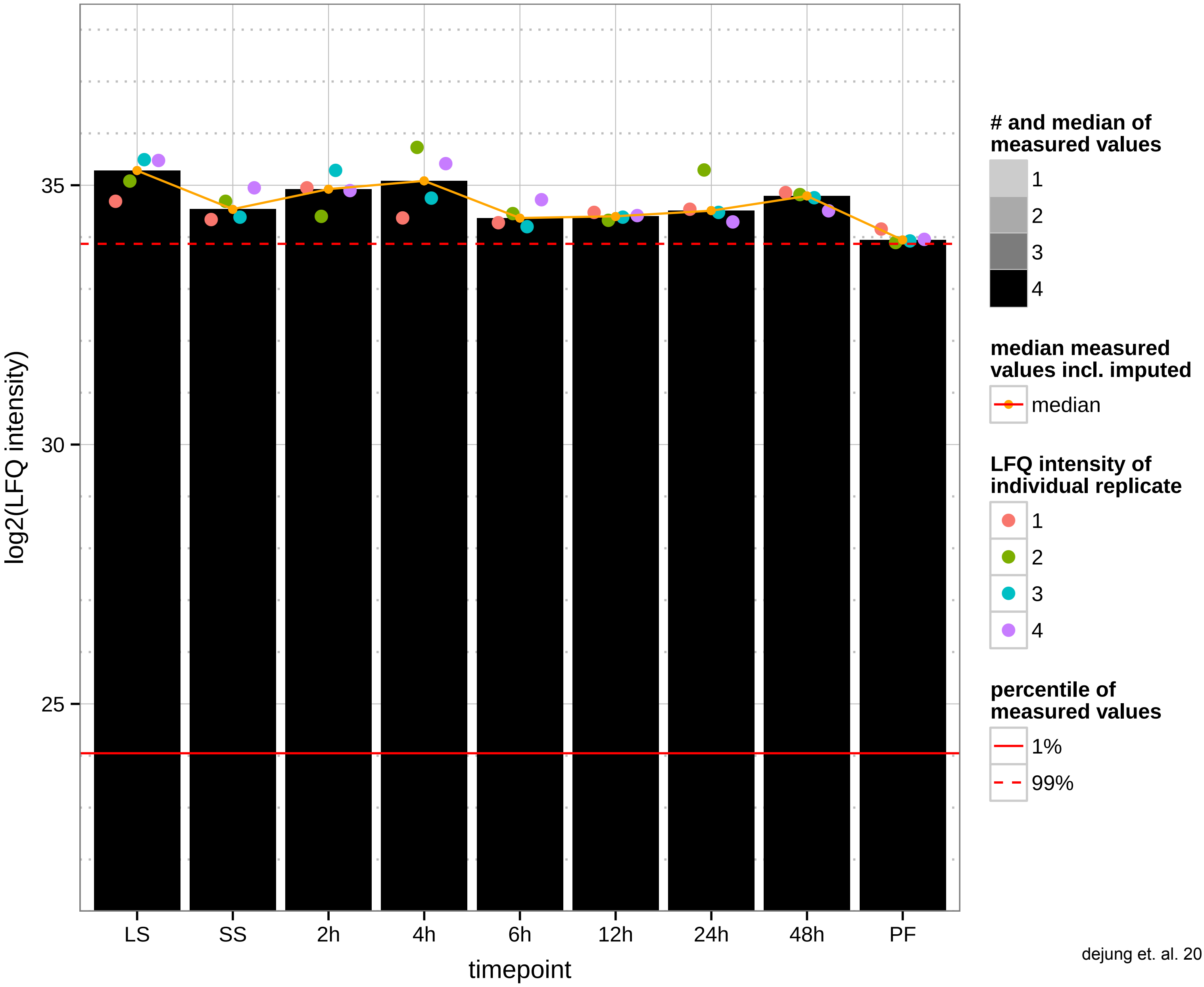

dejung et. al. 2015

hypothetical protein, conserved
Tb927.10.10620
AGOF: null
AGOC: null
AGOP: null
PGOF: null
PGOC: null
PGOP: null

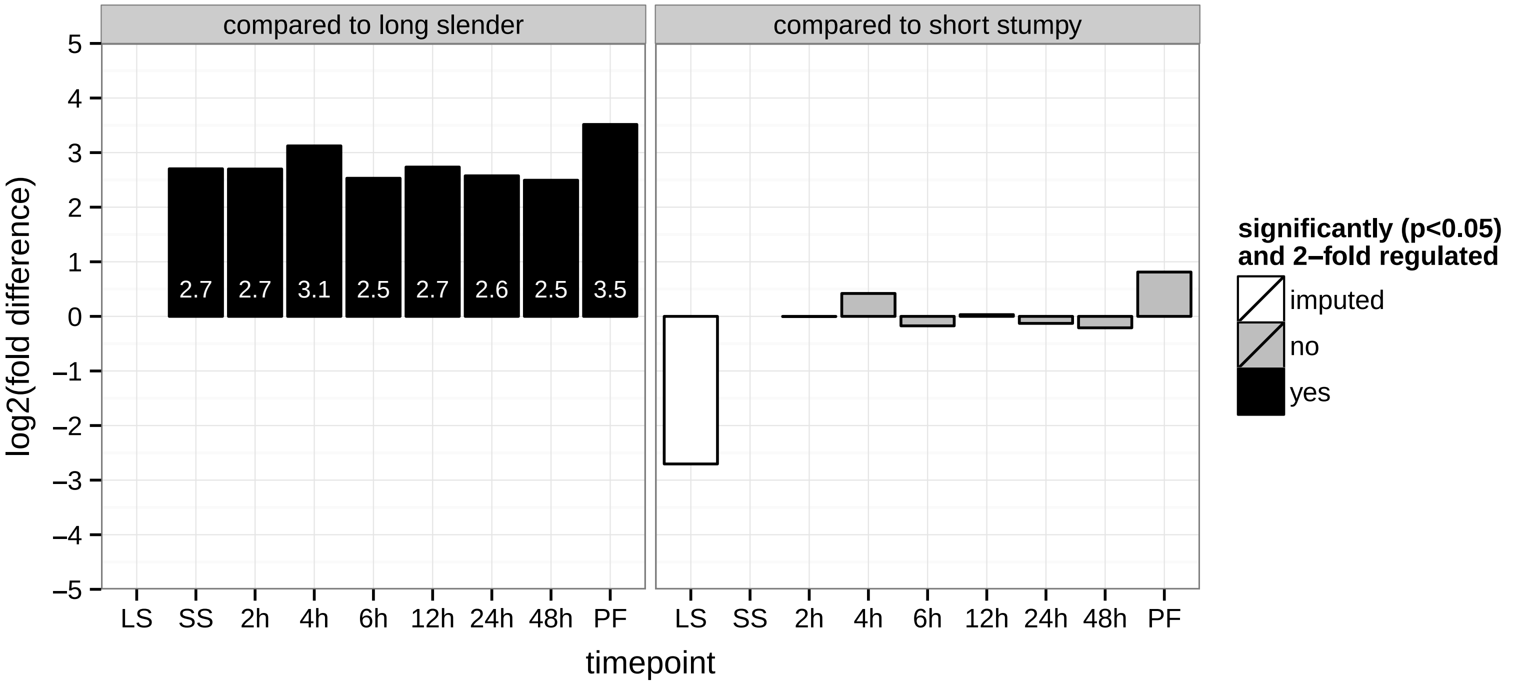

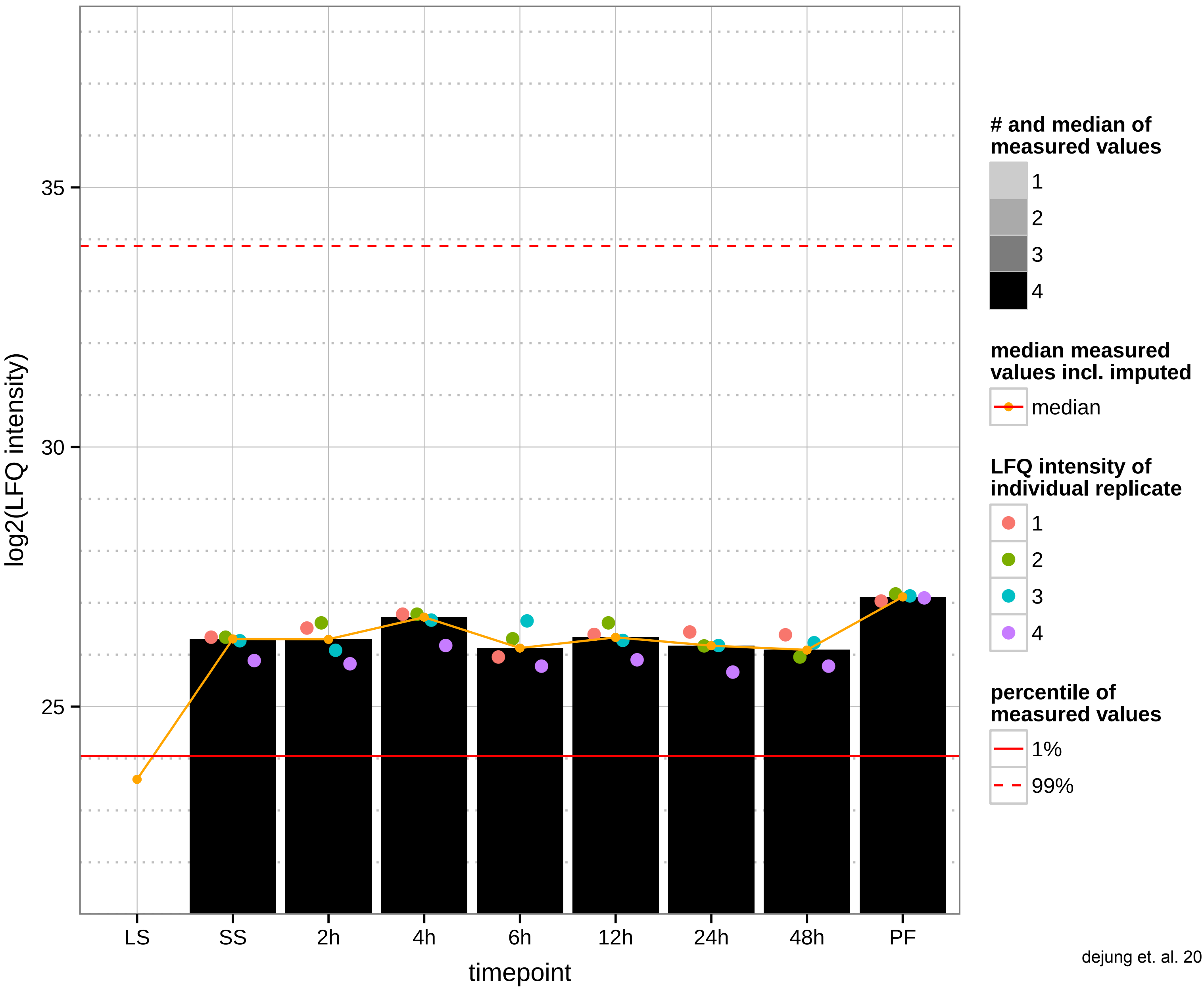

dejung et. al. 2015

hypothetical protein, conserved
Tb927.10.10630
AGOF: null
AGOC: null
AGOP: null
PGOF: null
PGOC: null
PGOP: null

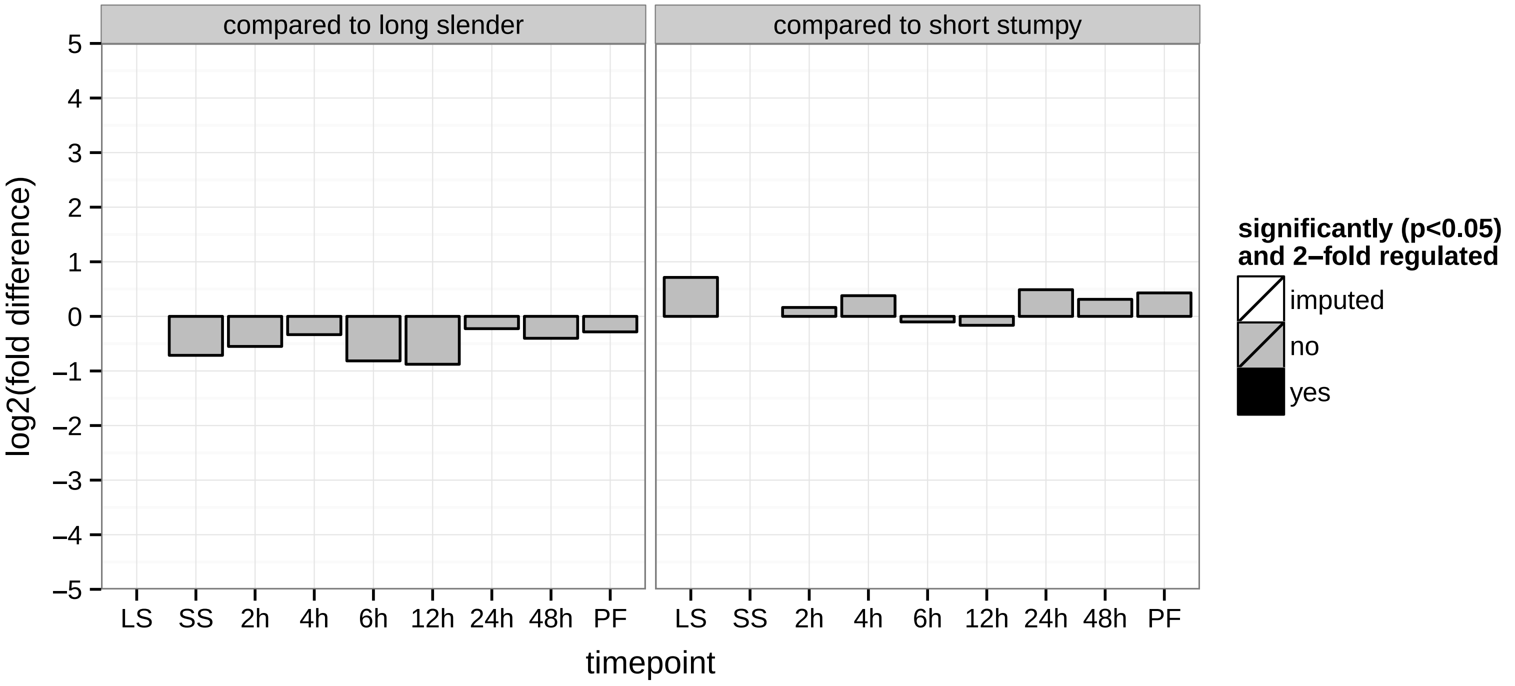

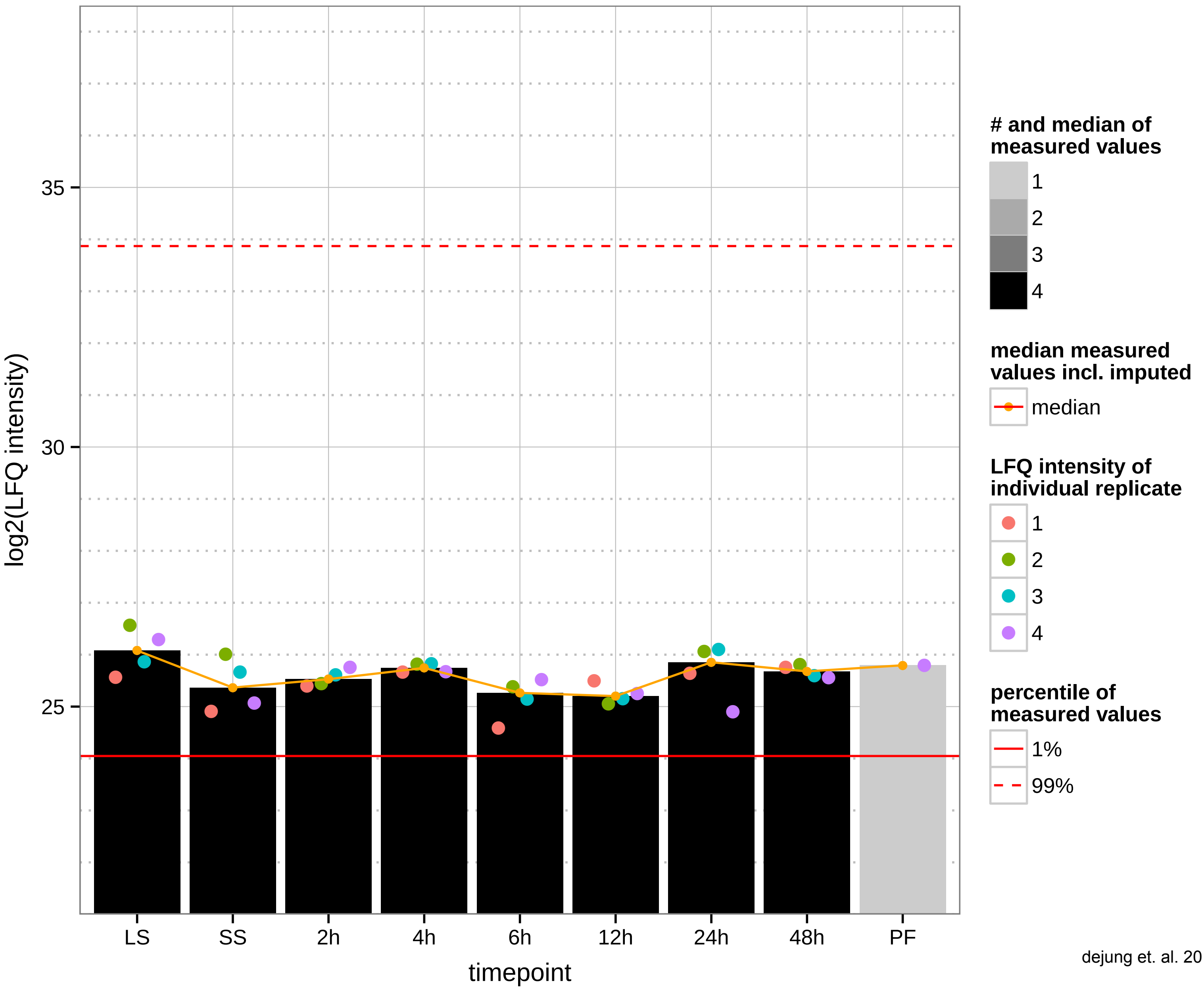

dejung et. al. 2015

cdc2–related kinase 1, putative, cell division protein kinase 2 homolog 1 (CRK1)
Tb927.10.1070
AGOF: ATP binding, protein serine/threonine kinase activity, protein tyrosine kinase activity
AGOC: cytosol
AGOP: cell cycle, growth, protein phosphorylation
PGOF: ATP binding, protein kinase activity, transferase activity, transferring phosphorus–containing groups
PGOC: null
PGOP: protein phosphorylation

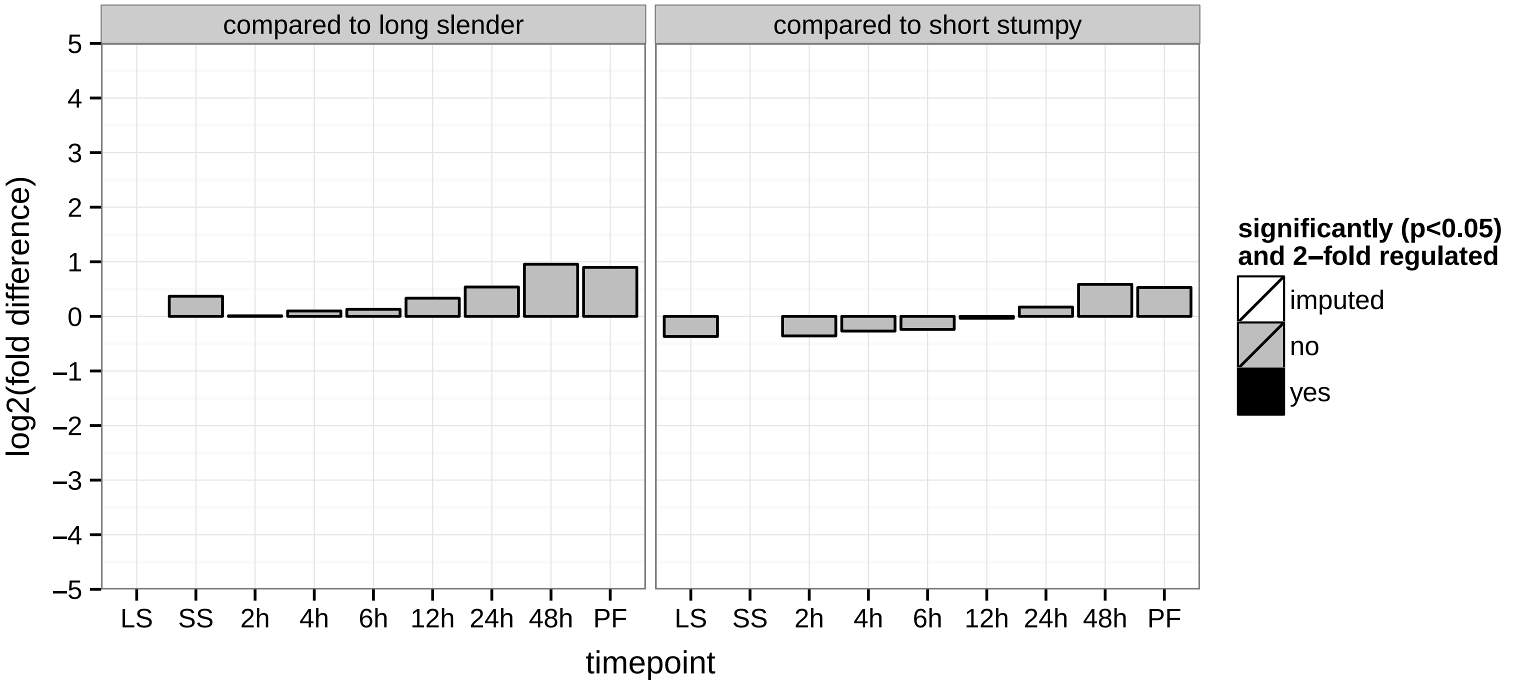

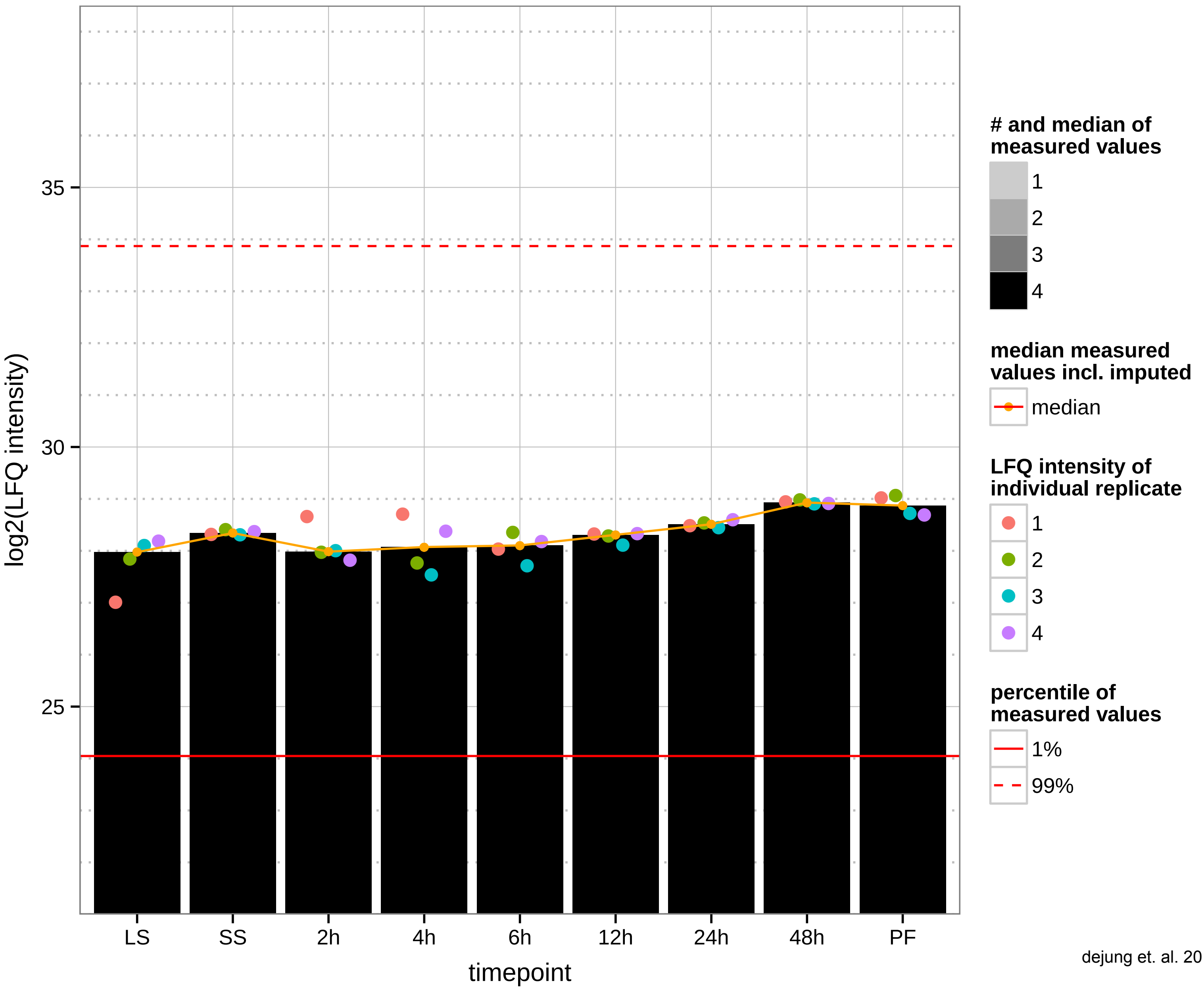

dejung et. al. 2015

hypothetical protein, conserved
Tb927.10.10720
AGOF: null
AGOC: null
AGOP: null
PGOF: null
PGOC: integral to membrane
PGOP: null

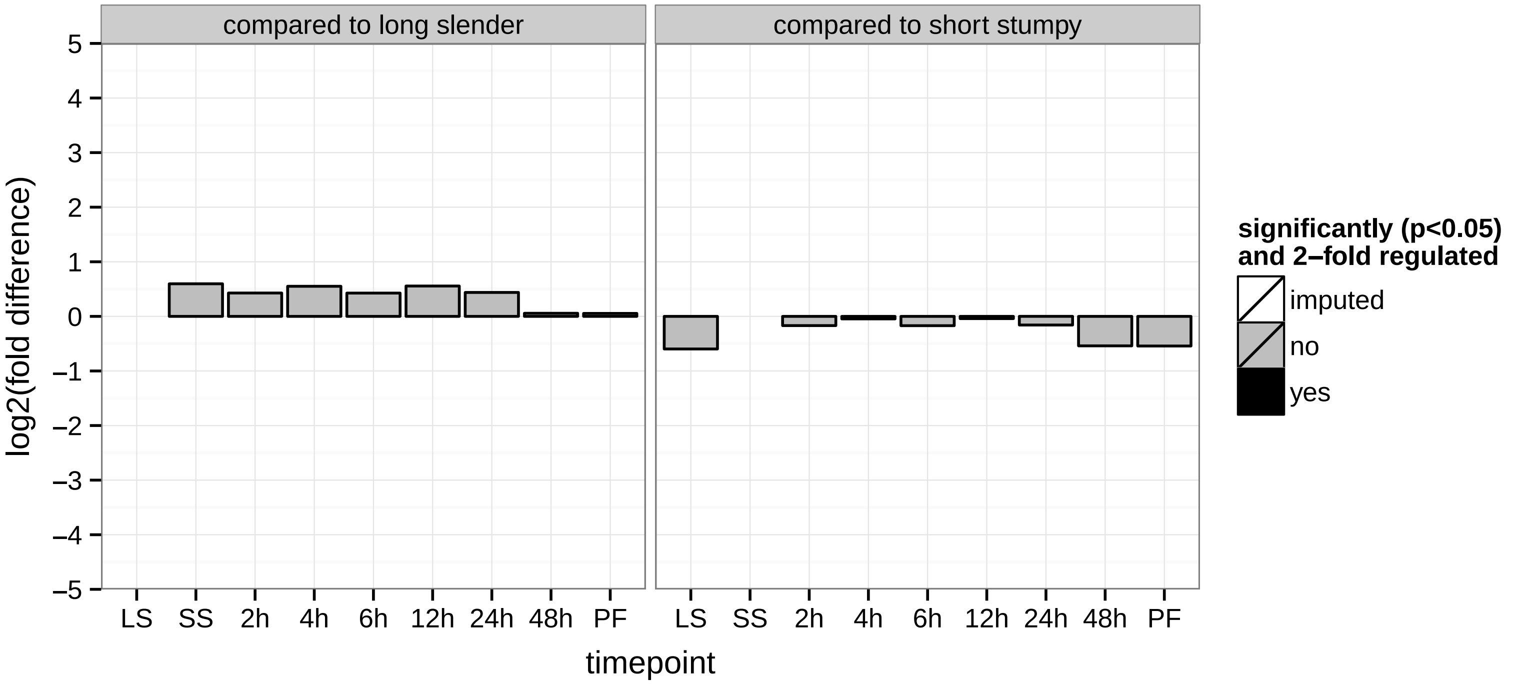

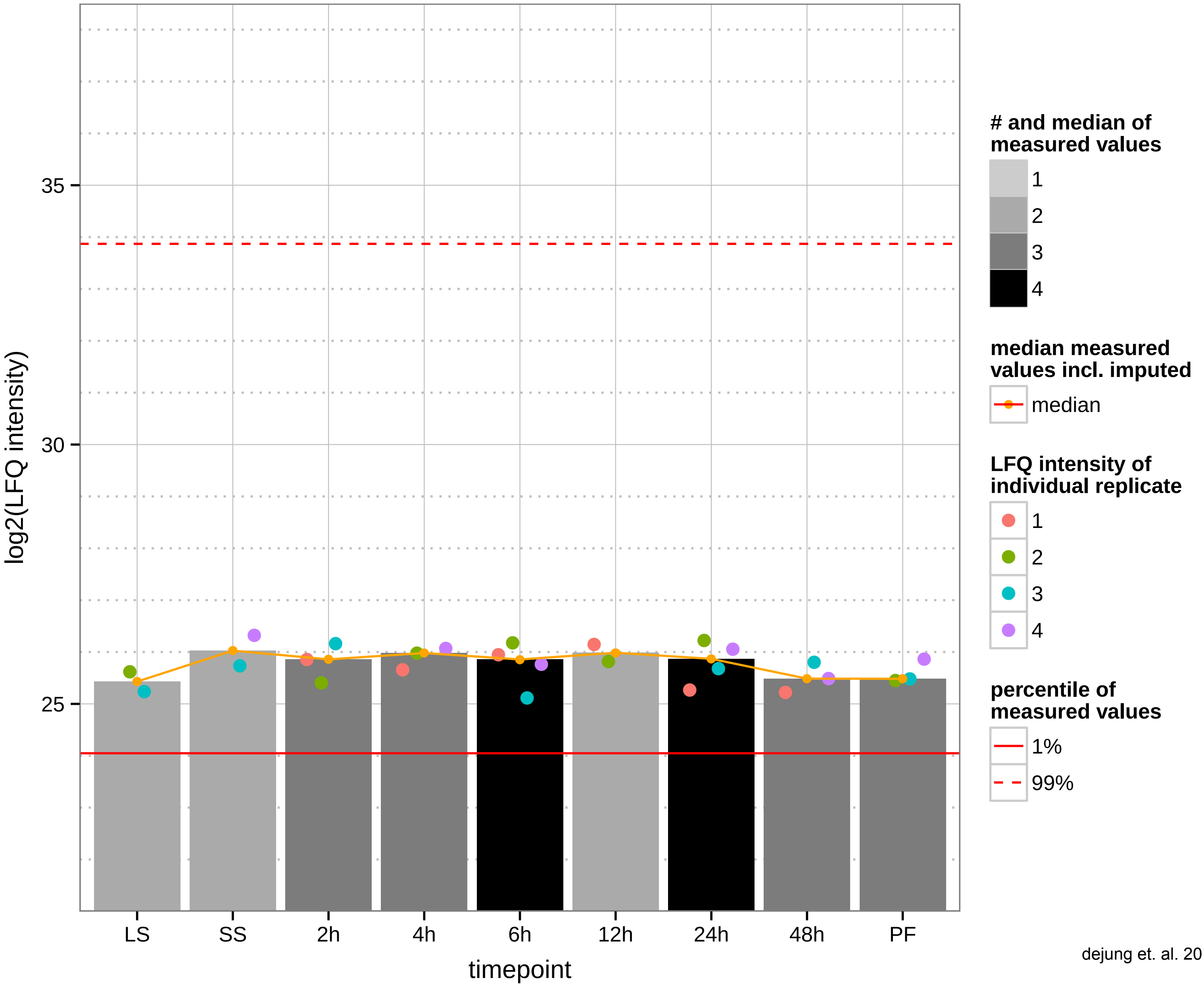

dejung et. al. 2015

hypothetical protein, conserved
Tb927.10.10740
AGOF: null
AGOC: mitochondrion
AGOP: null
PGOF: null
PGOC: null
PGOP: null

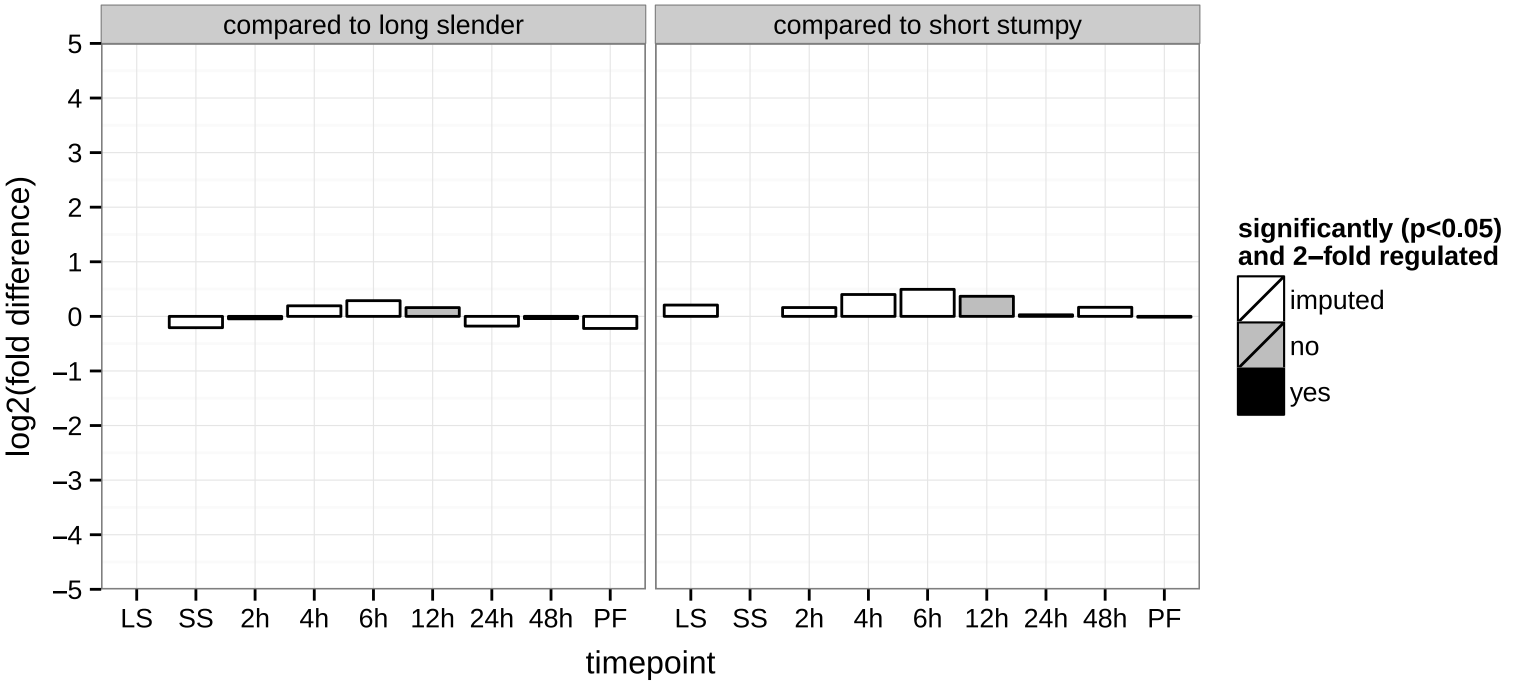

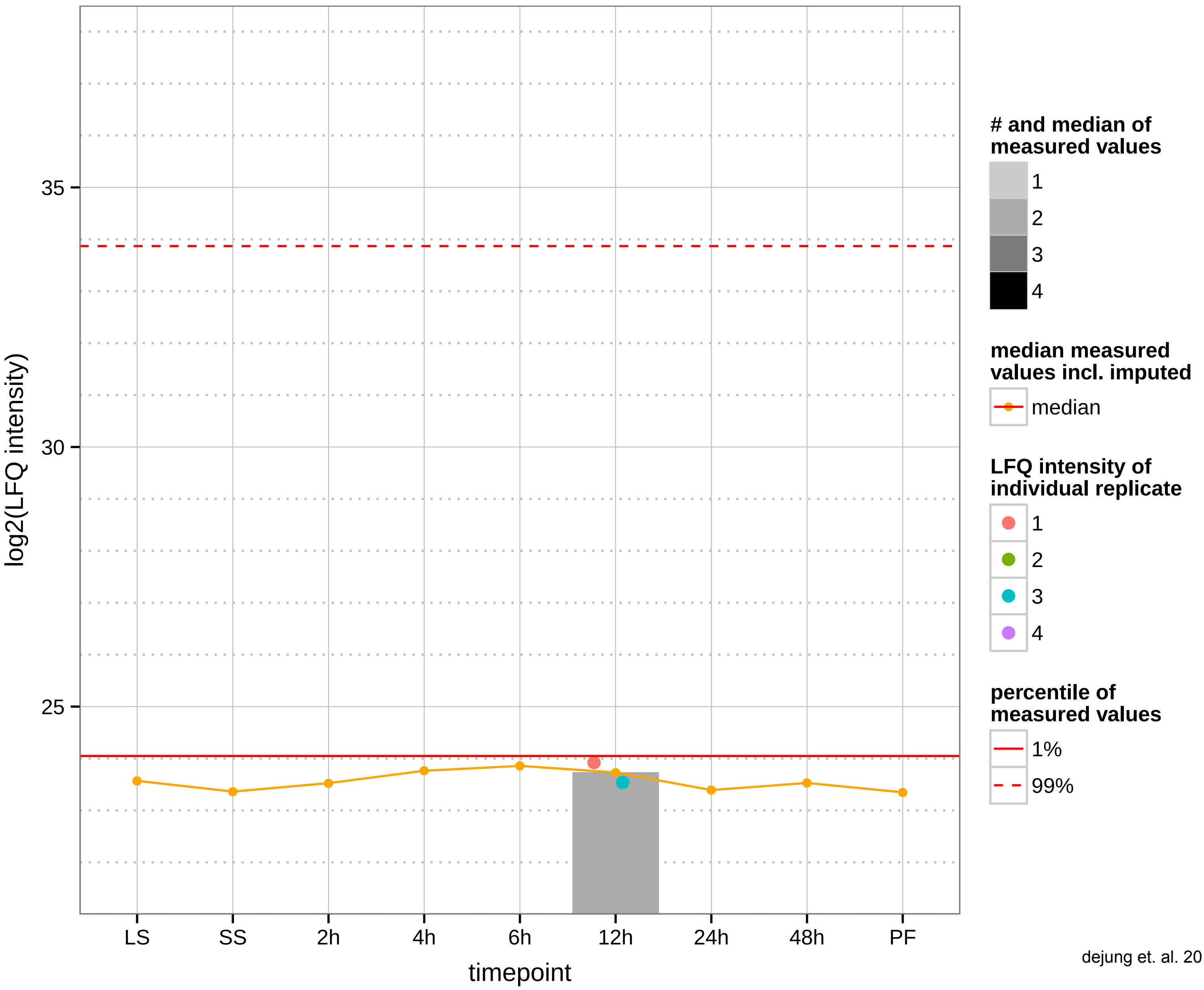

dejung et. al. 2015

40S ribosomal protein S23, putative (RPS23)
Tb927.10.1090;Tb927.10.1080
AGOF: RNA binding, structural constituent of ribosome
AGOC: intracellular, ribosome, small ribosomal subunit
AGOP: translation
PGOF: structural constituent of ribosome, null
PGOC: intracellular, ribosome, small ribosomal subunit, null
PGOP: translation, null

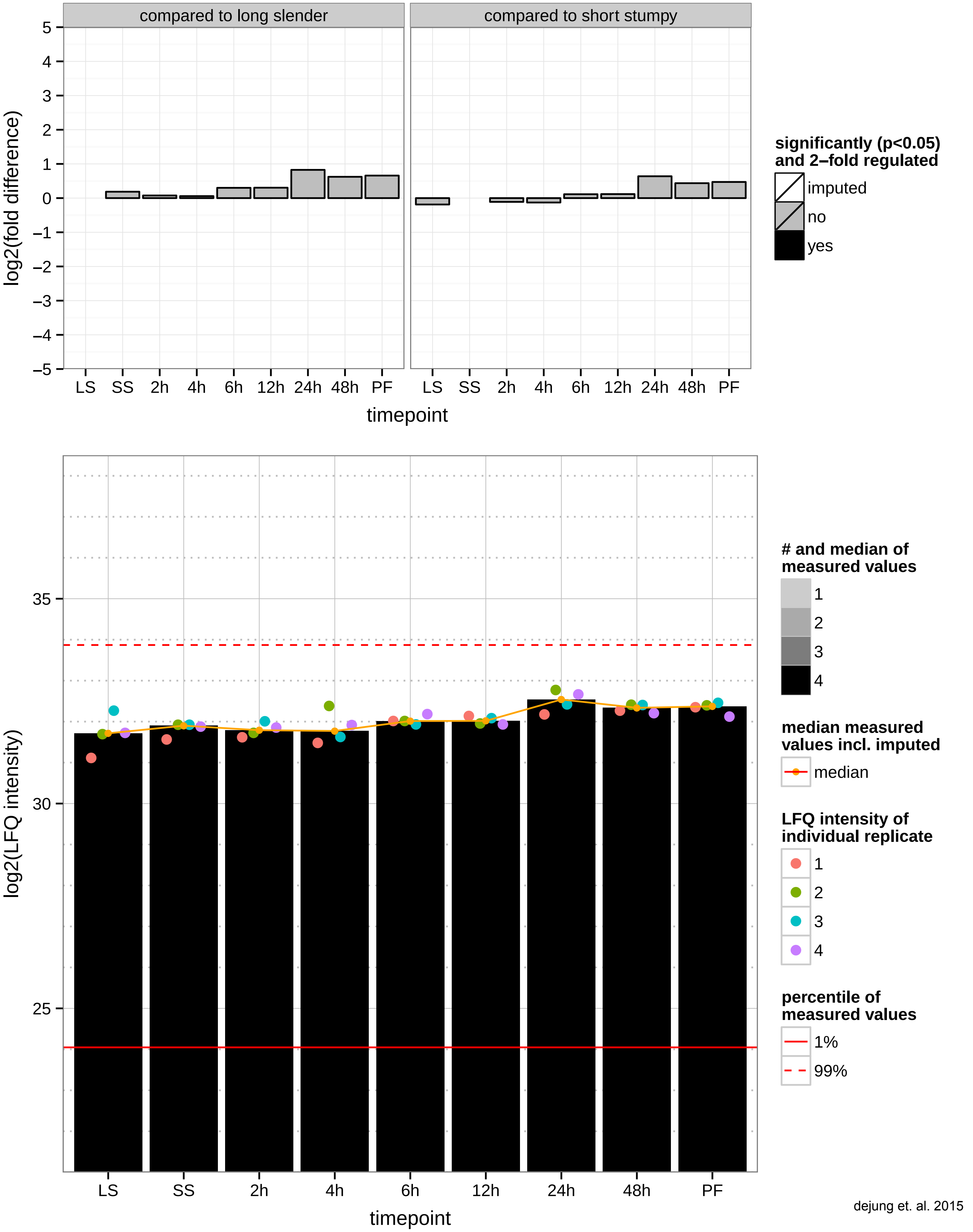

dejung et. al. 2015

argonaute–like protein (AGO1)
Tb927.10.10850
AGOF: ribosome binding
AGOC: cytoplasm
AGOP: RNA interference, production of siRNA involved in RNA interference
PGOF: nucleic acid binding, protein binding
PGOC: null
PGOP: null

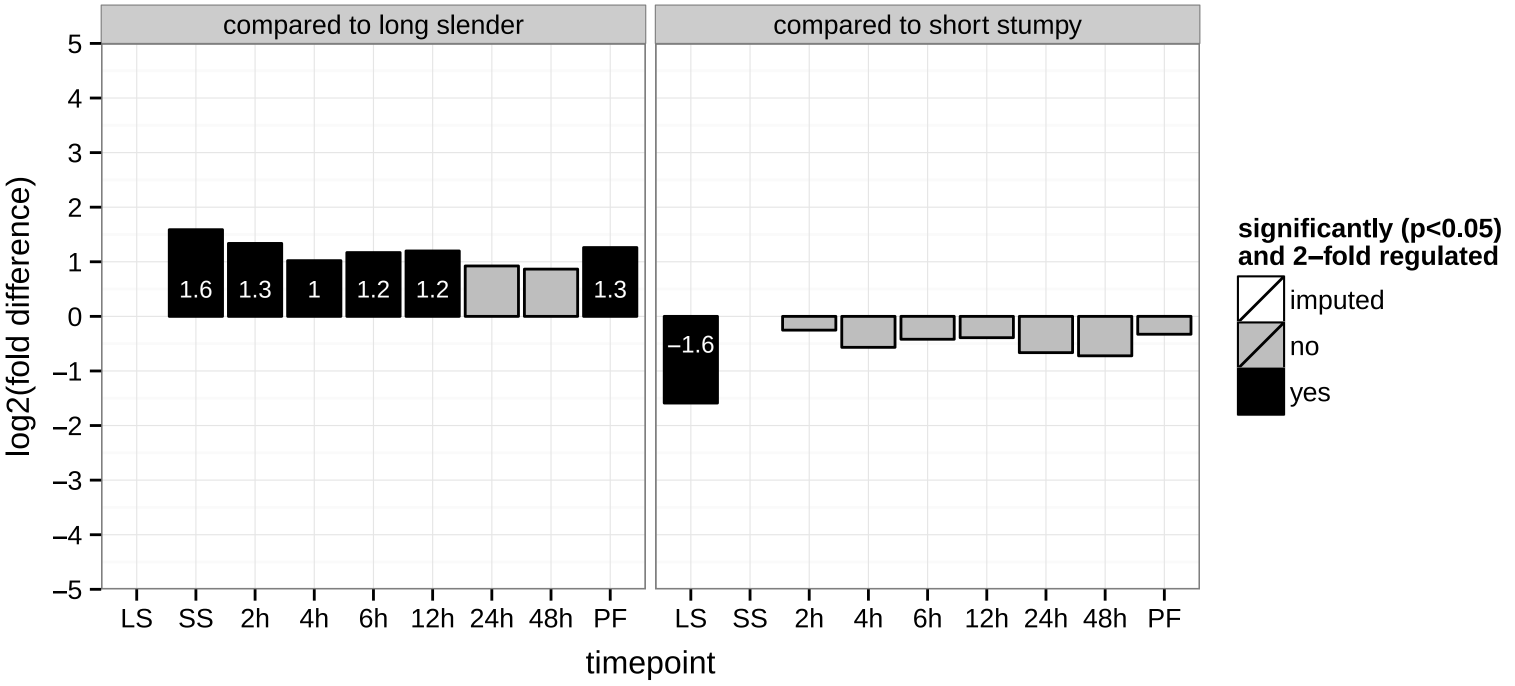

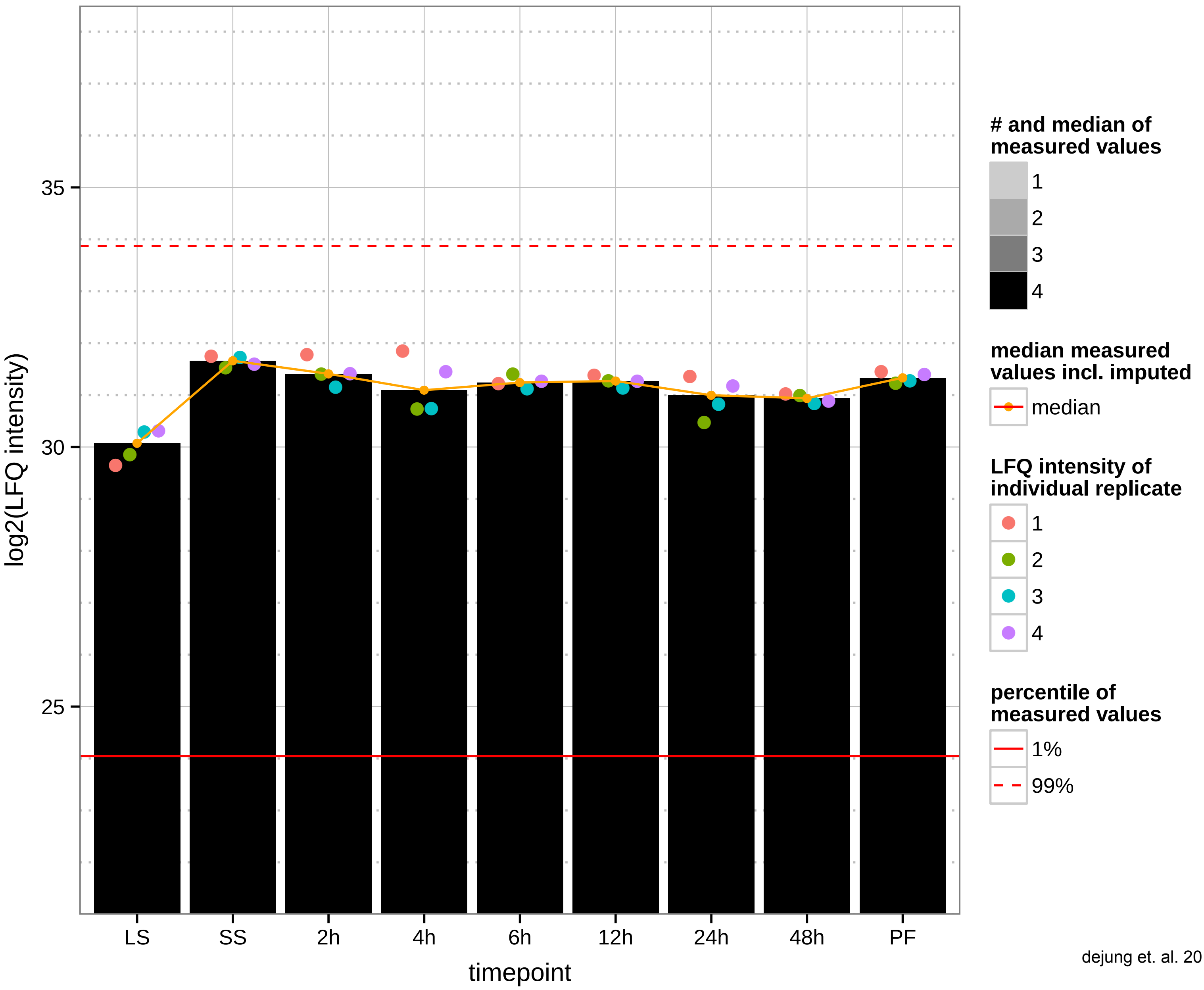

dejung et. al. 2015

60S ribosomal protein L9, putative
Tb927.10.1100;Tb927.6.4690
AGOF: rRNA binding, structural constituent of ribosome
AGOC: ribosome
AGOP: ribosome biogenesis, translation
PGOF: rRNA binding, structural constituent of ribosome
PGOC: ribosome
PGOP: translation

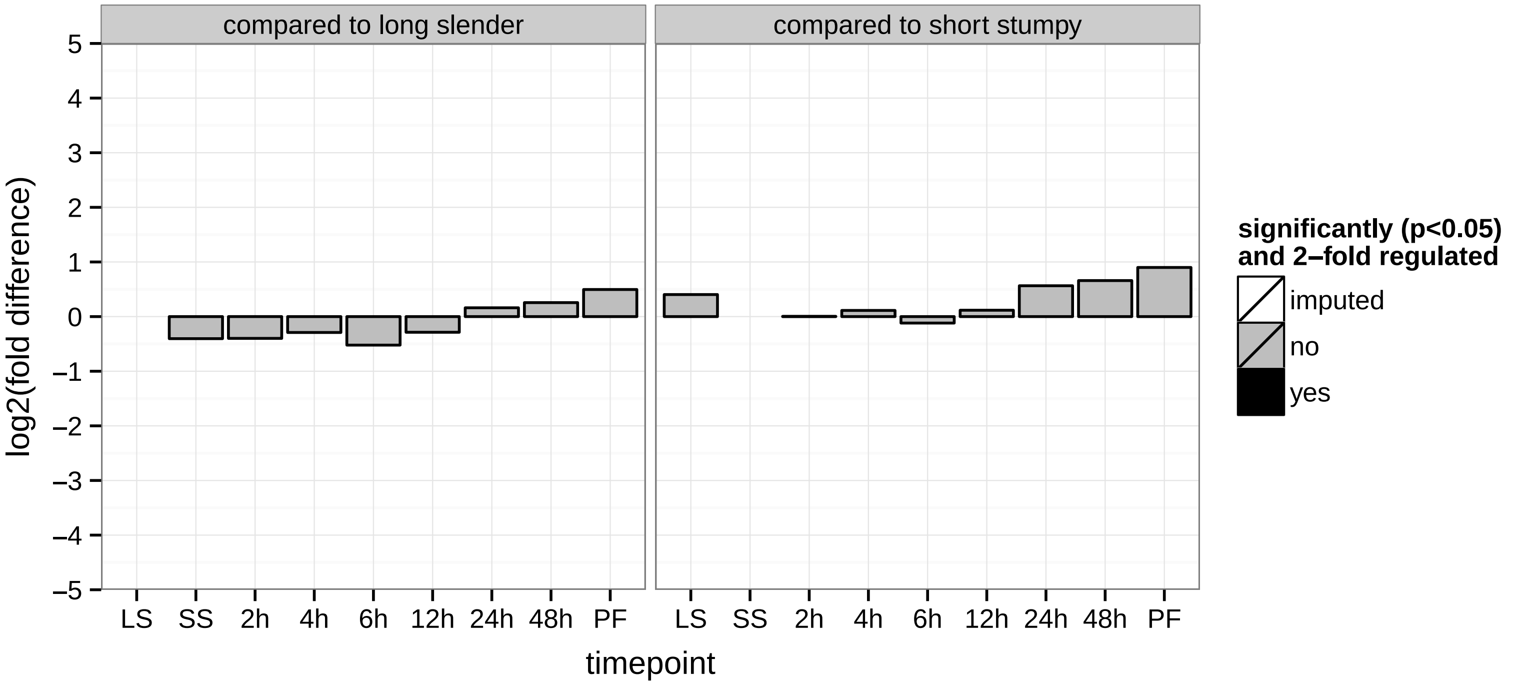

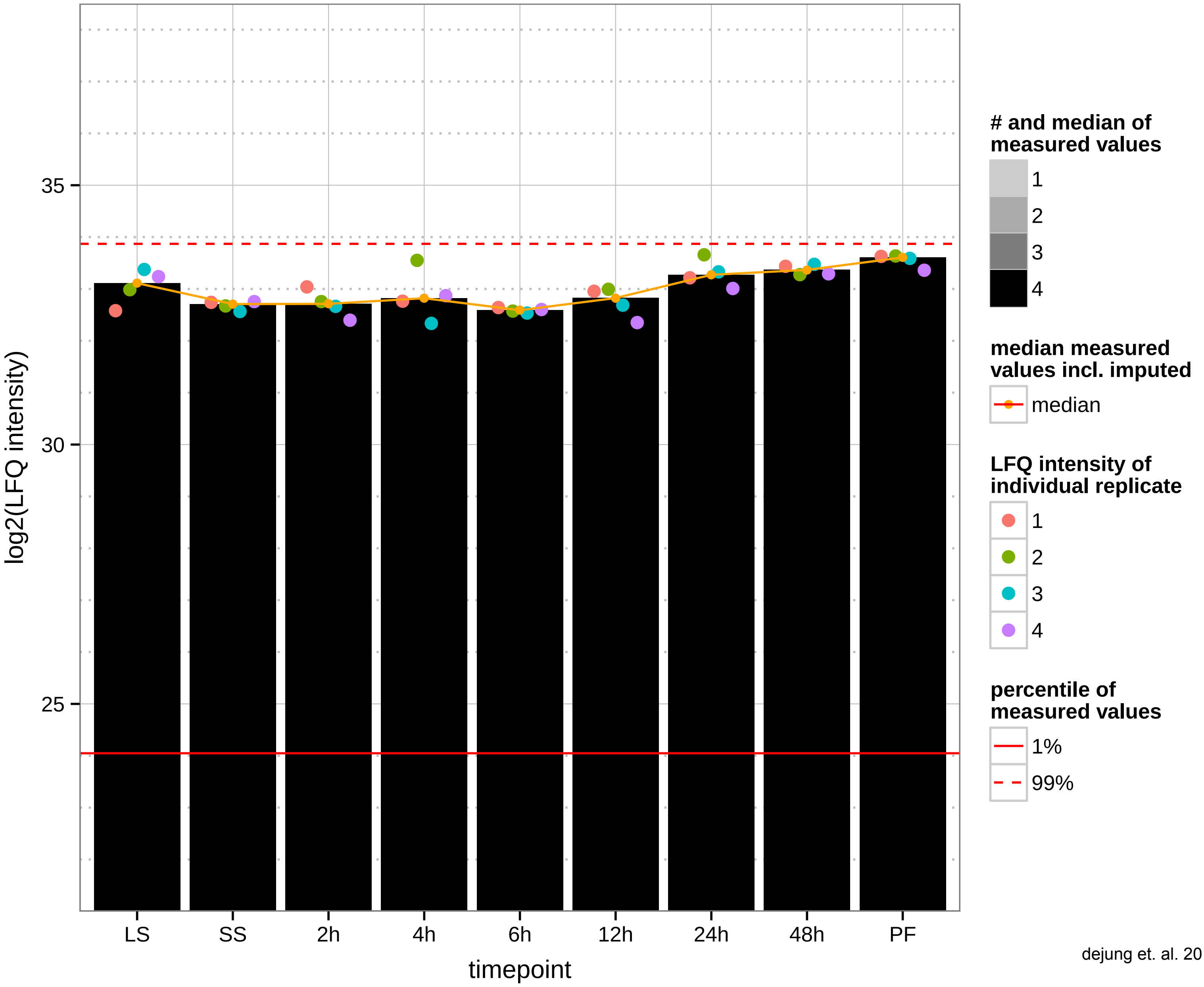

dejung et. al. 2015

paraflagellar rod protein
Tb927.10.11080
AGOF: null
AGOC: null
AGOP: null
PGOF: null
PGOC: null
PGOP: null

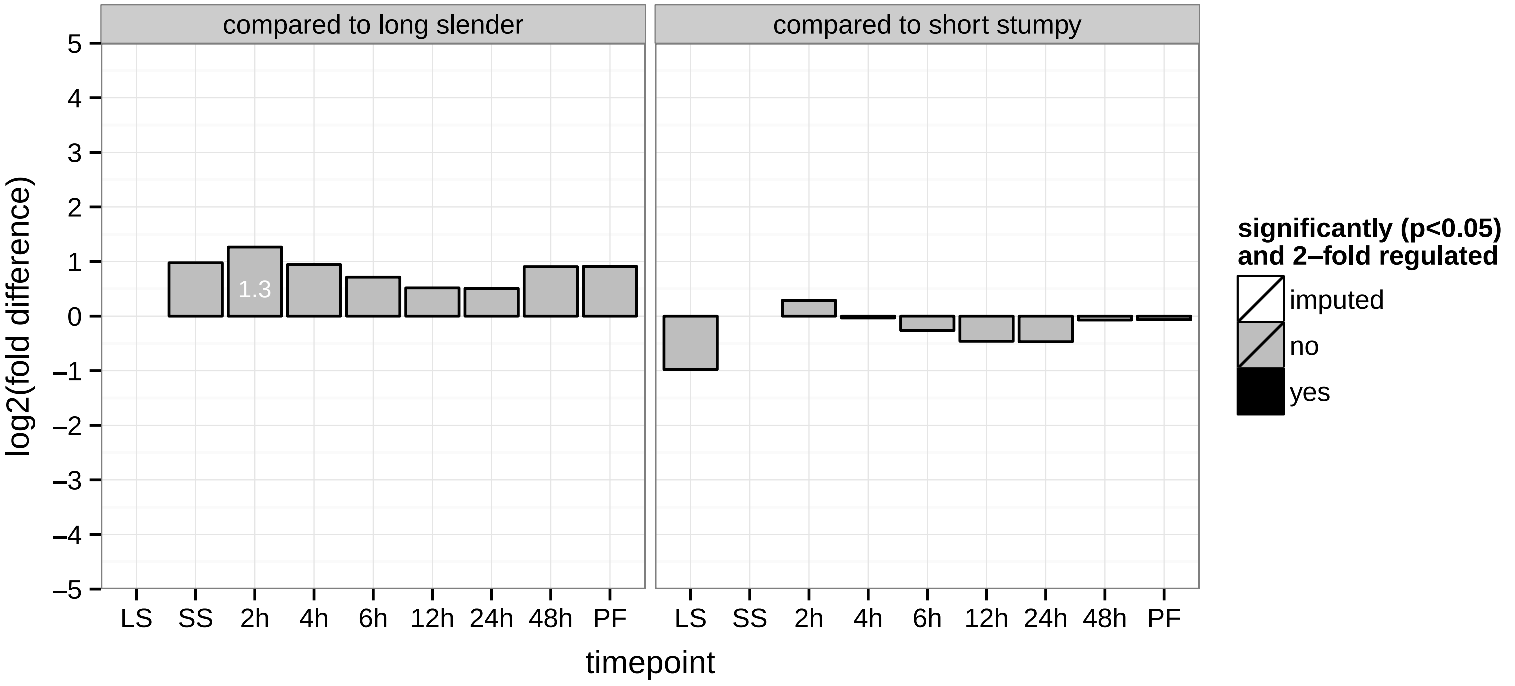

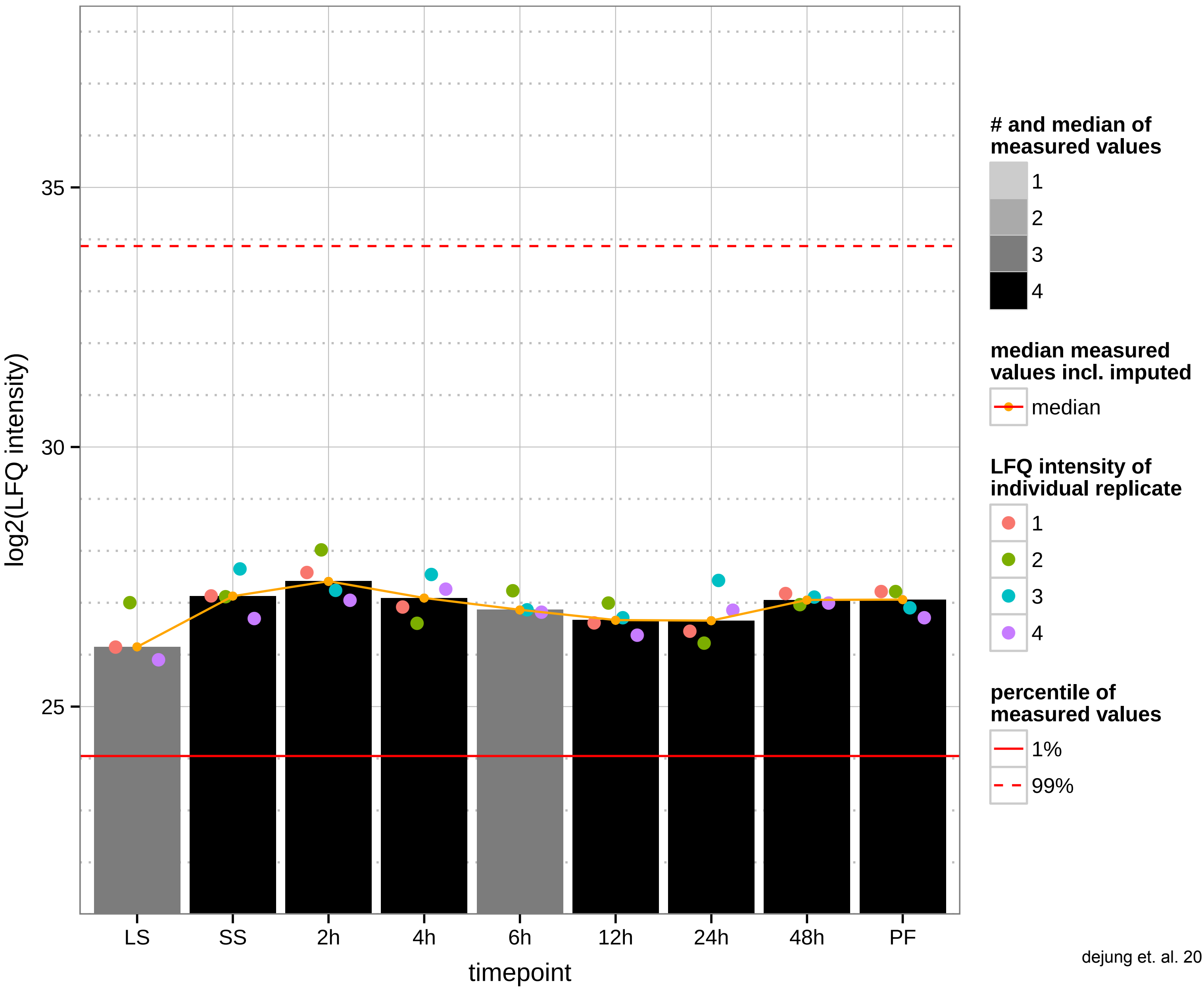

dejung et. al. 2015

hypothetical protein, conserved
Tb927.10.11150
AGOF: null
AGOC: null
AGOP: null
PGOF: null
PGOC: null
PGOP: null

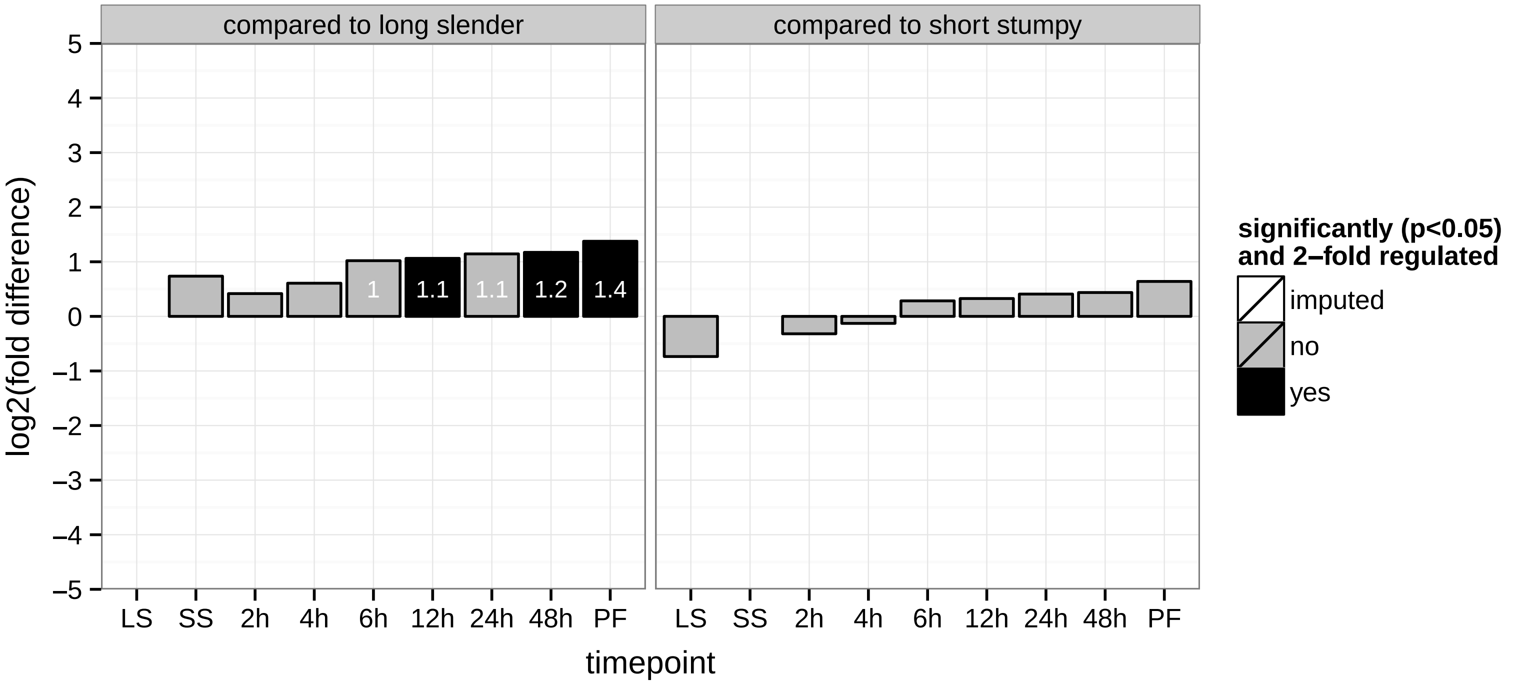

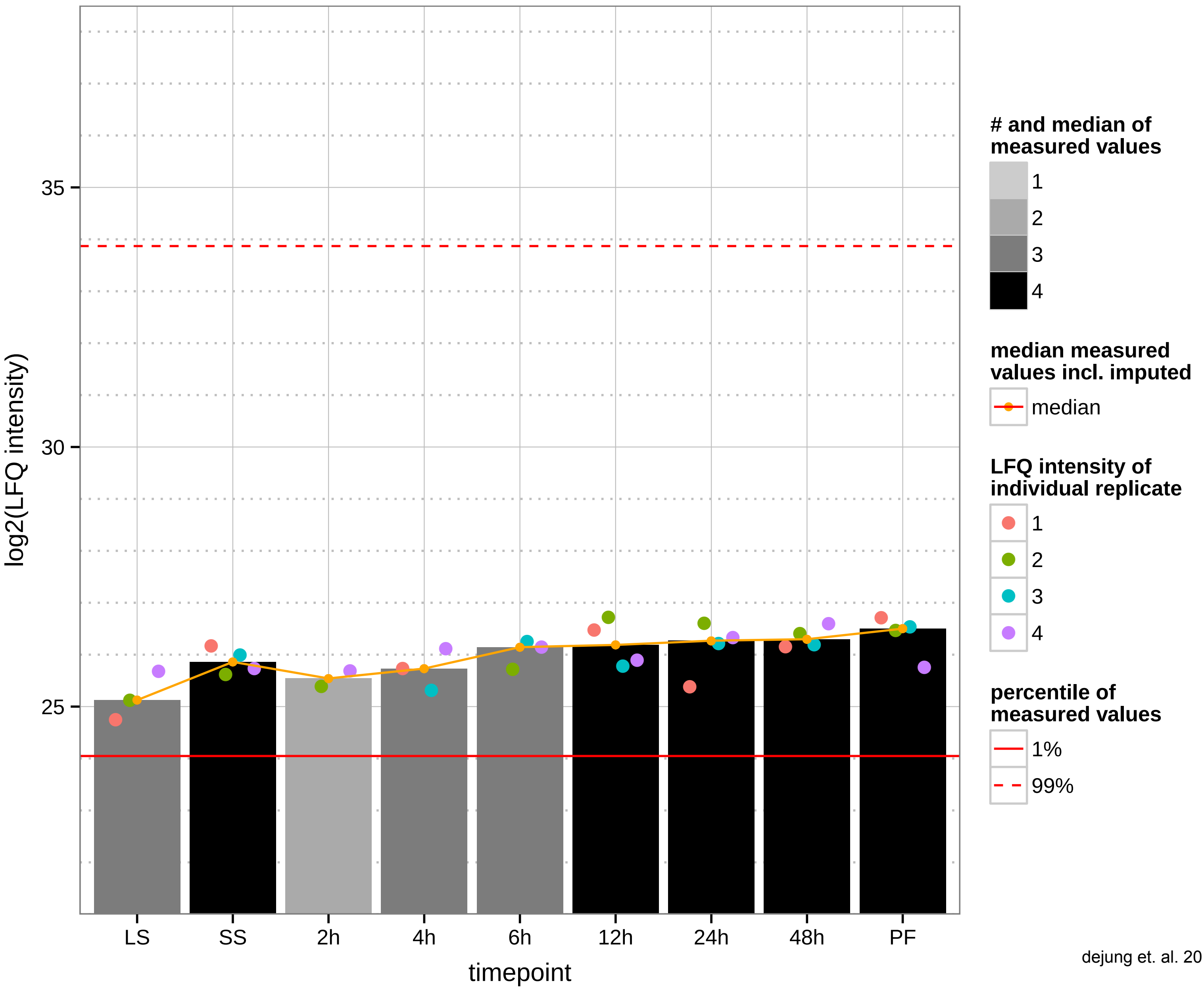

dejung et. al. 2015

hypothetical protein, conserved
Tb927.10.11210
AGOF: null
AGOC: null
AGOP: null
PGOF: protein binding
PGOC: null
PGOP: null

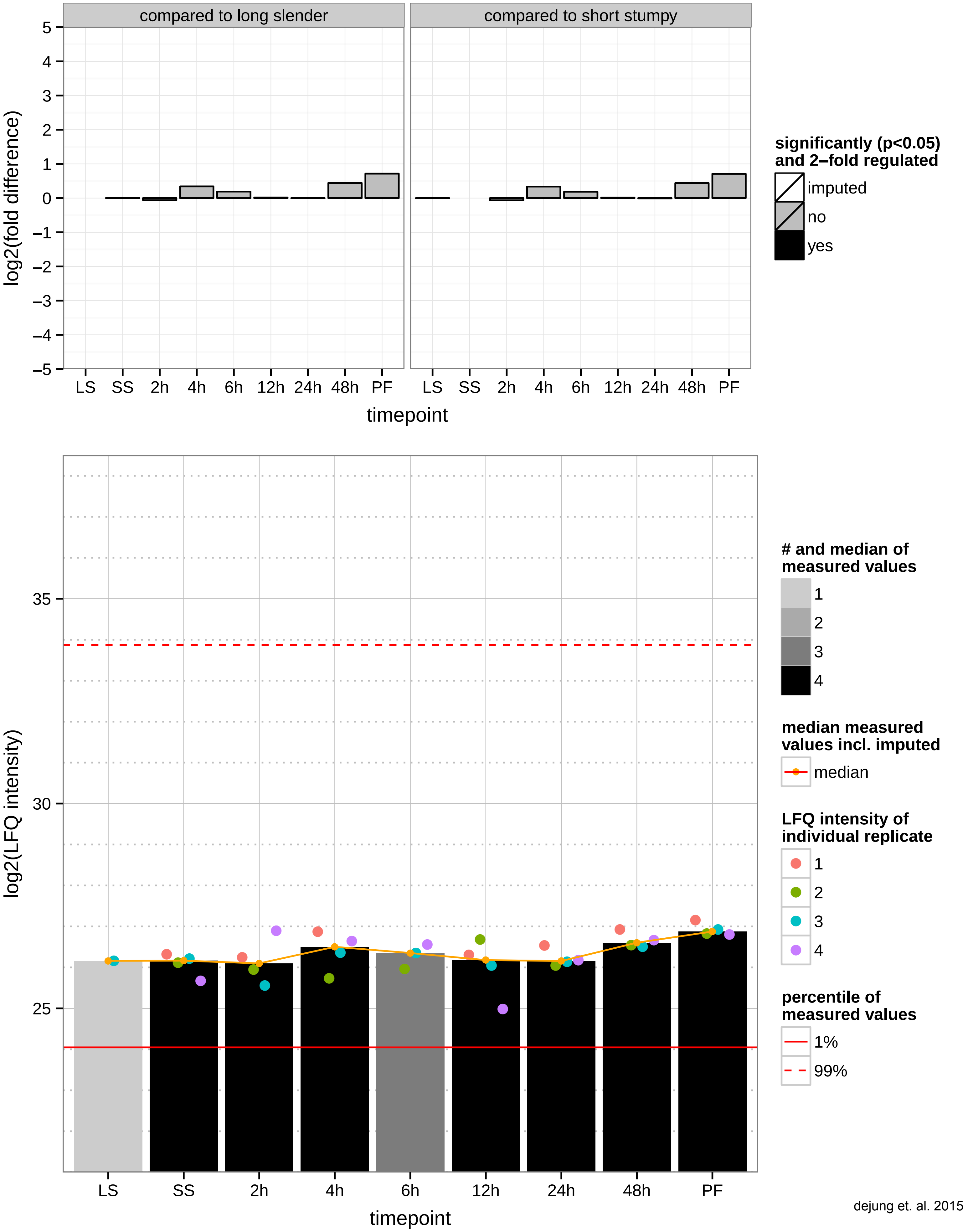

dejung et. al. 2015

hypothetical protein, conserved
Tb927.10.11250;Tb11.v5.0575
AGOF: null
AGOC: null
AGOP: null
PGOF: null
PGOC: null
PGOP: null

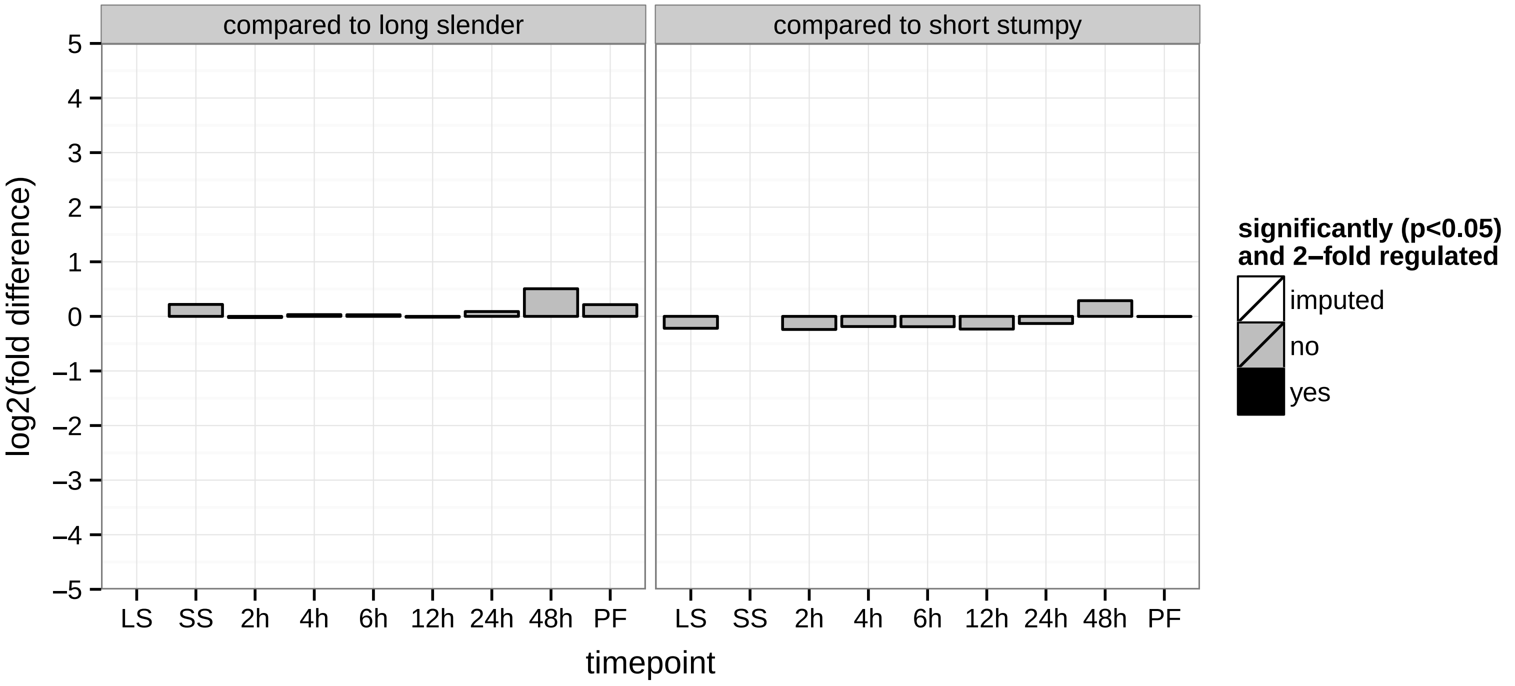

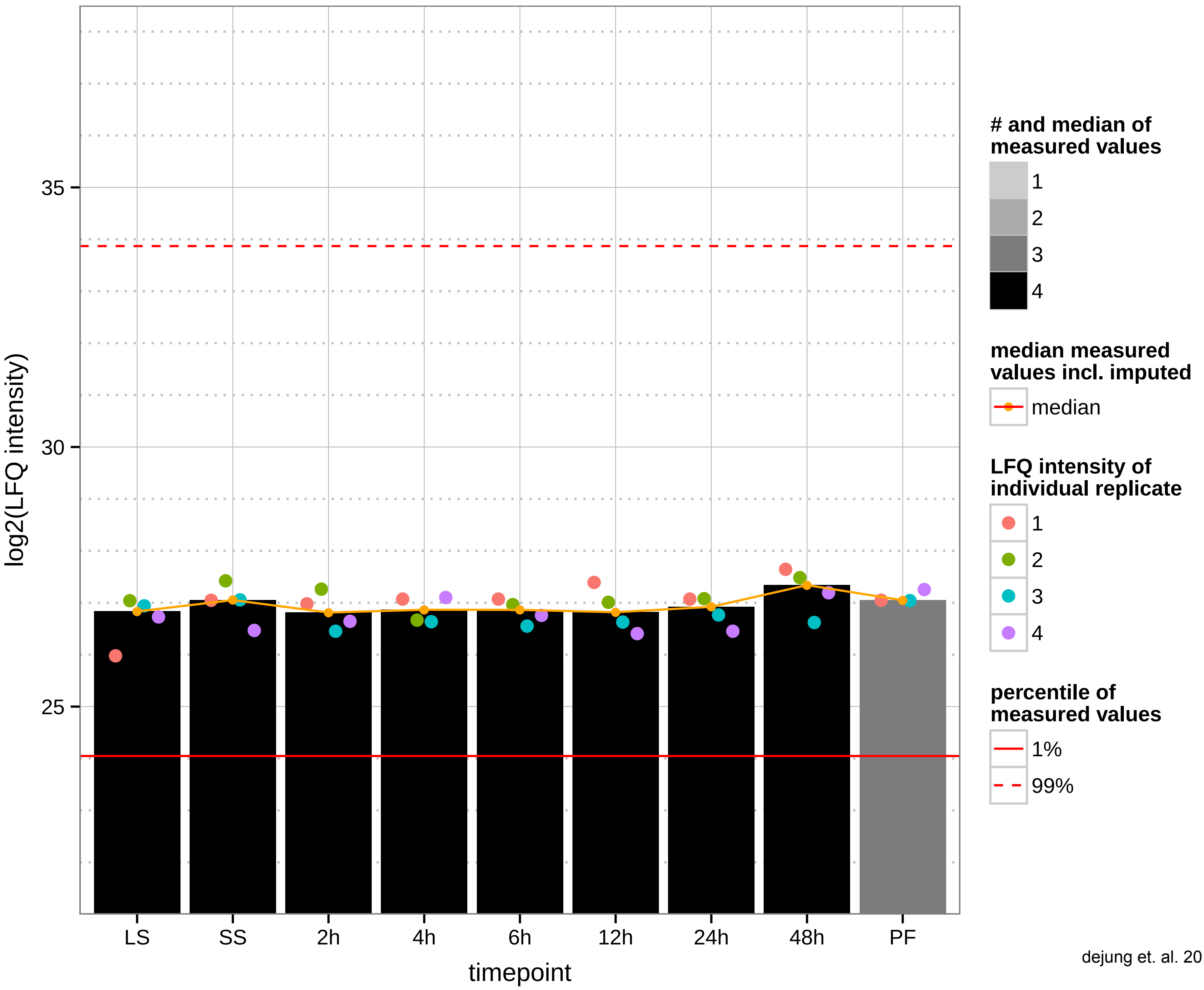

dejung et. al. 2015

hypothetical protein, conserved
Tb927.10.11260
AGOF: null
AGOC: mitochondrion
AGOP: null
PGOF: null
PGOC: null
PGOP: null

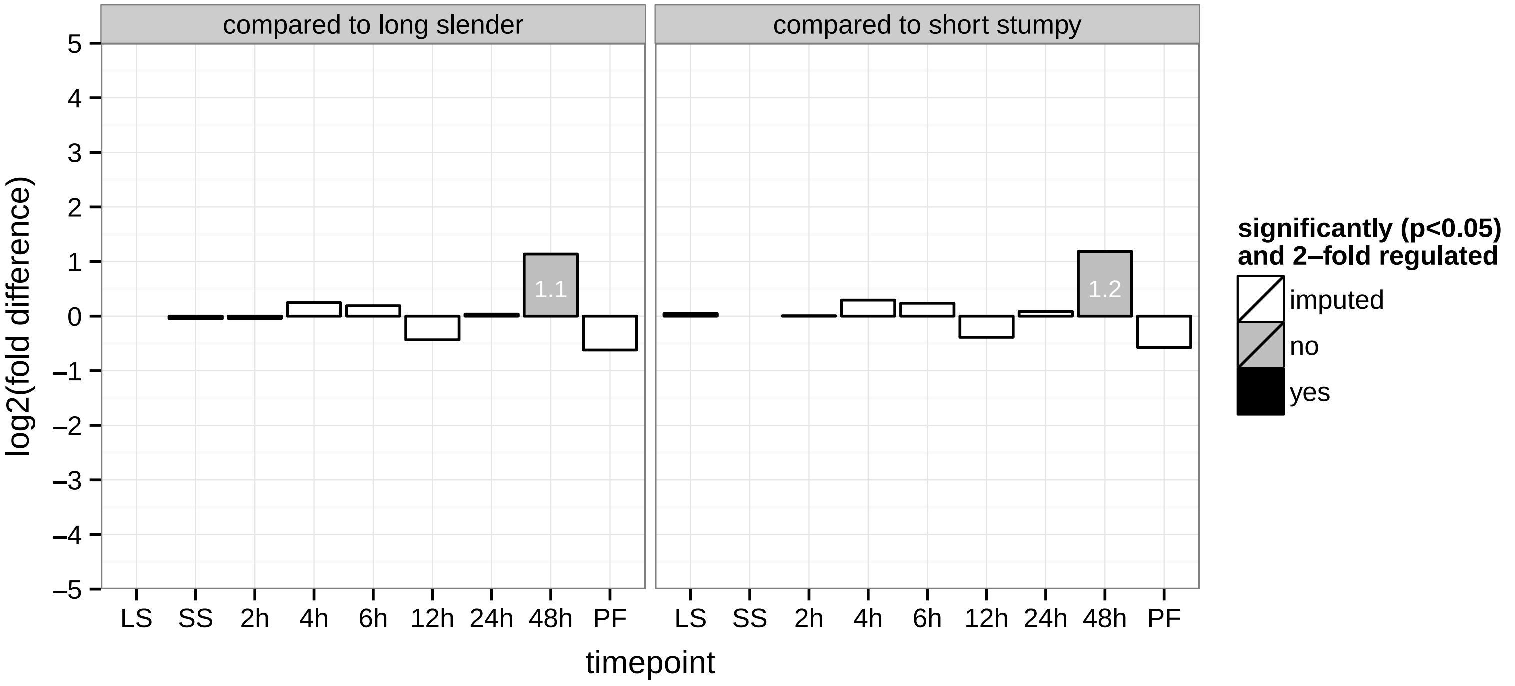

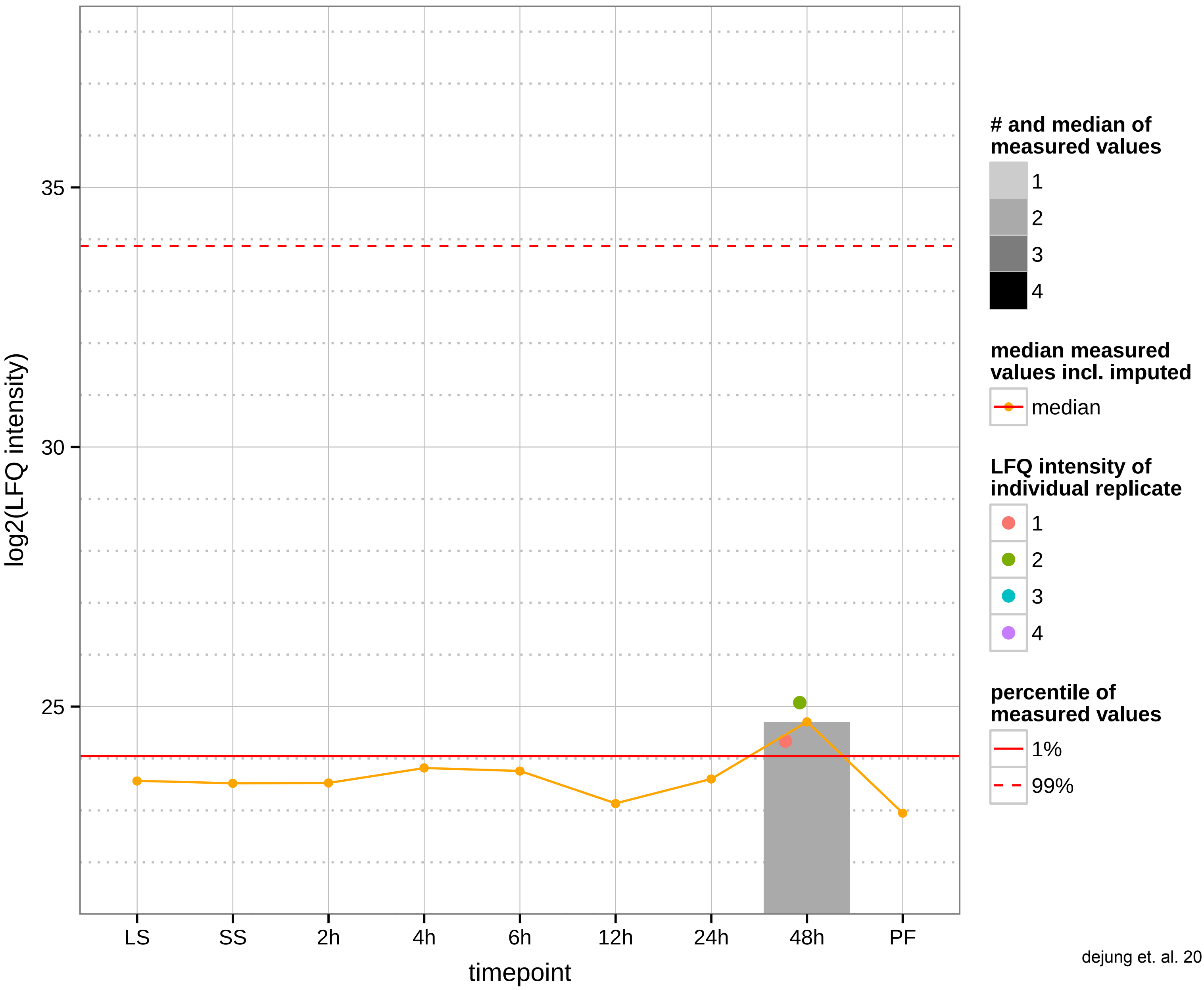

dejung et. al. 2015

paraflagellar rod component, putative (PFC16)
Tb927.10.11300
AGOF: null
AGOC: eukaryotic translation initiation factor 4F complex
AGOP: null
PGOF: null
PGOC: null
PGOP: null

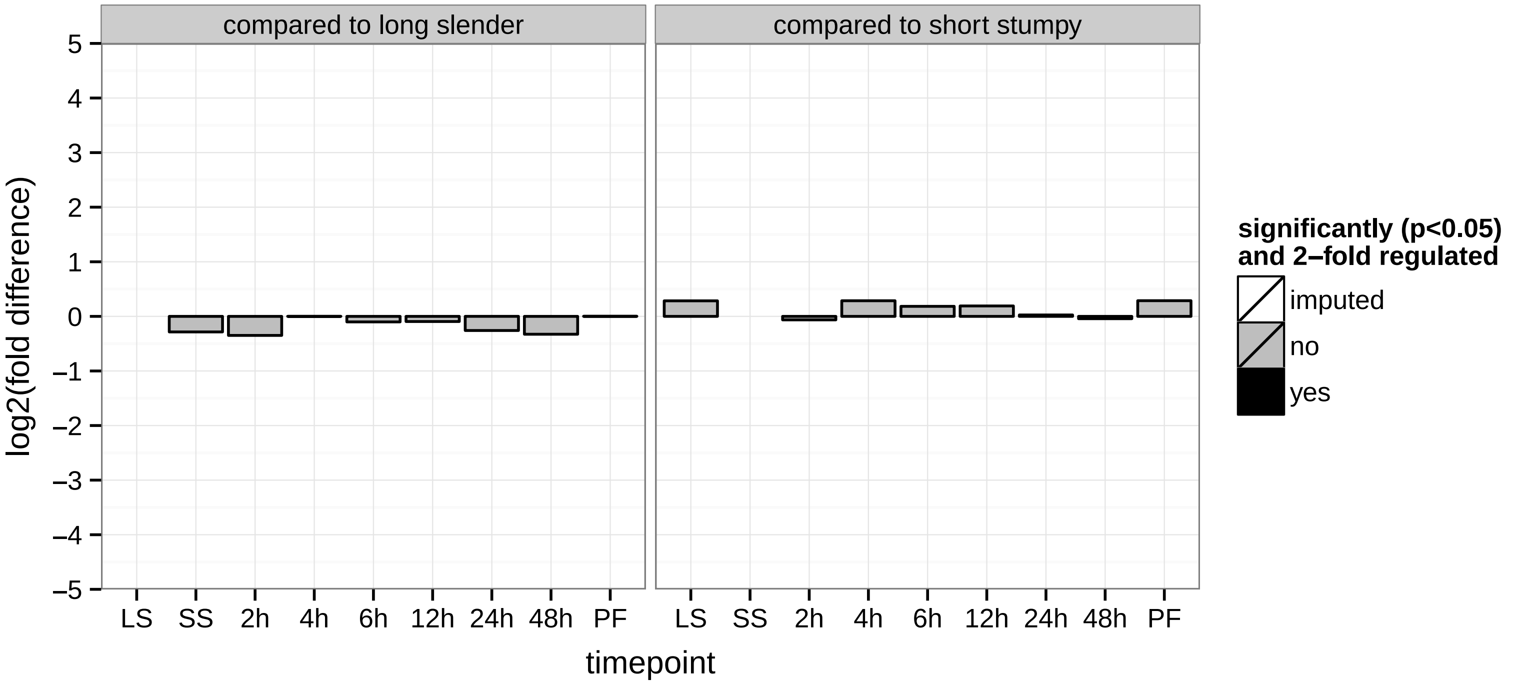

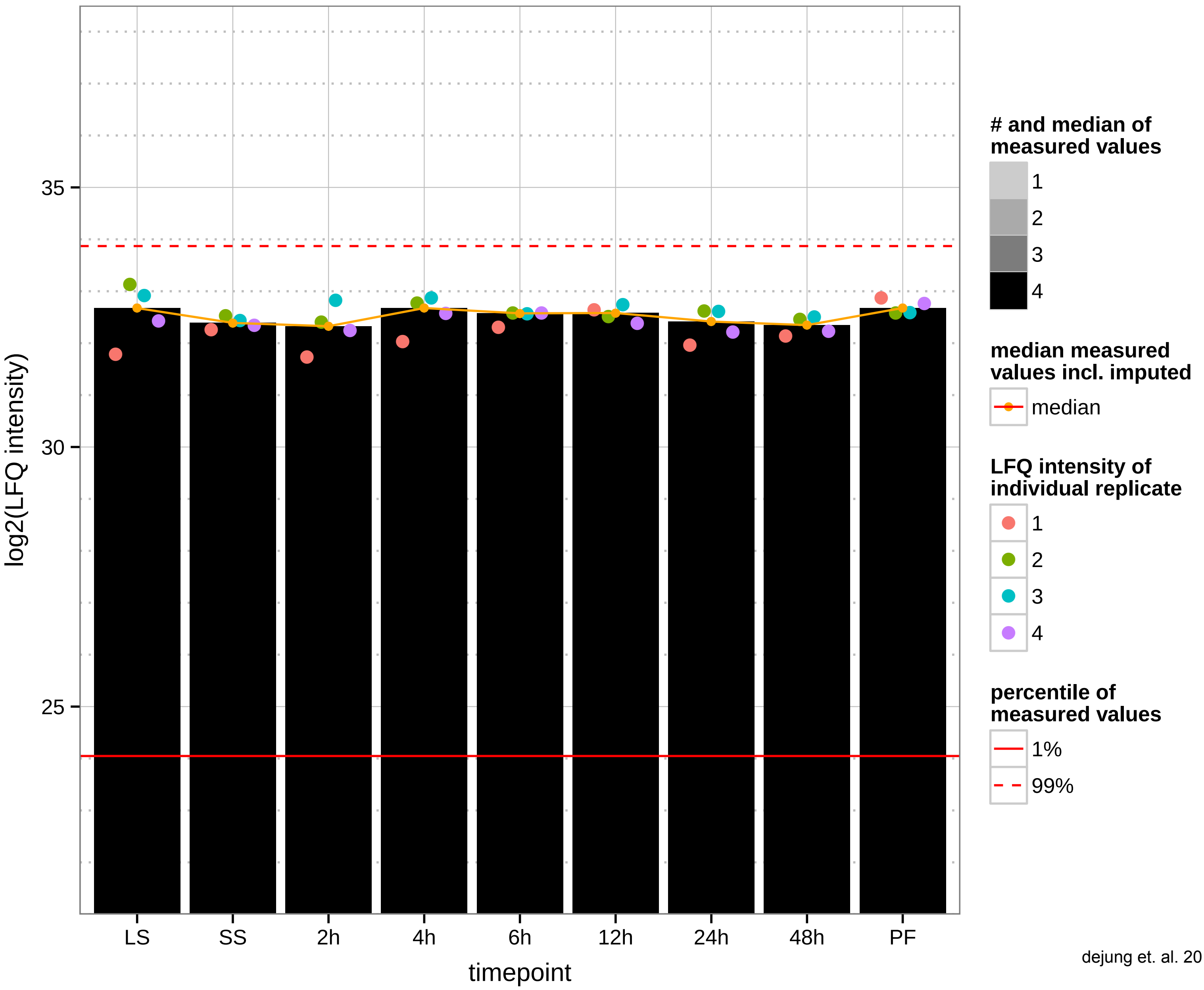

dejung et. al. 2015

intraflagellar transport protein IFT55/IFT57, putative (IFT57/55)
Tb927.10.11310
AGOF: null
AGOC: null
AGOP: null
PGOF: null
PGOC: null
PGOP: null

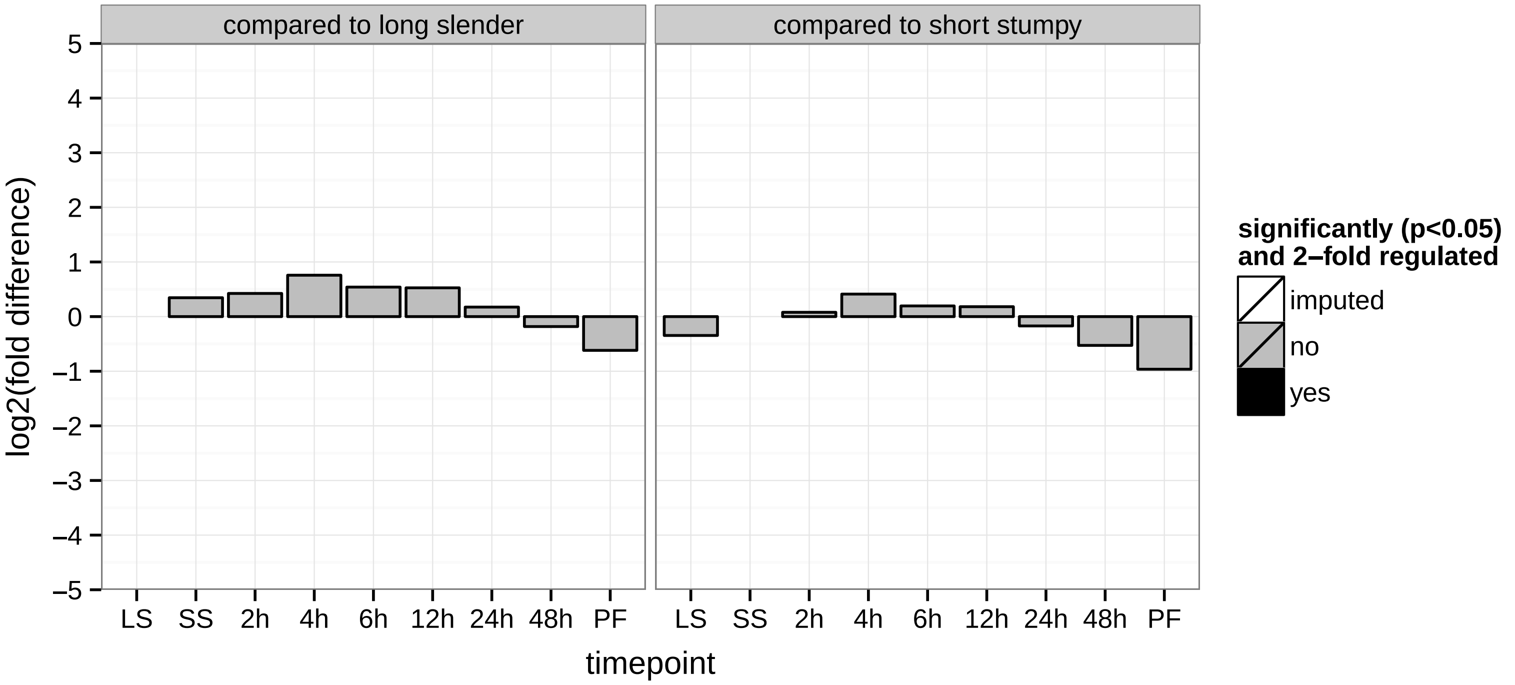

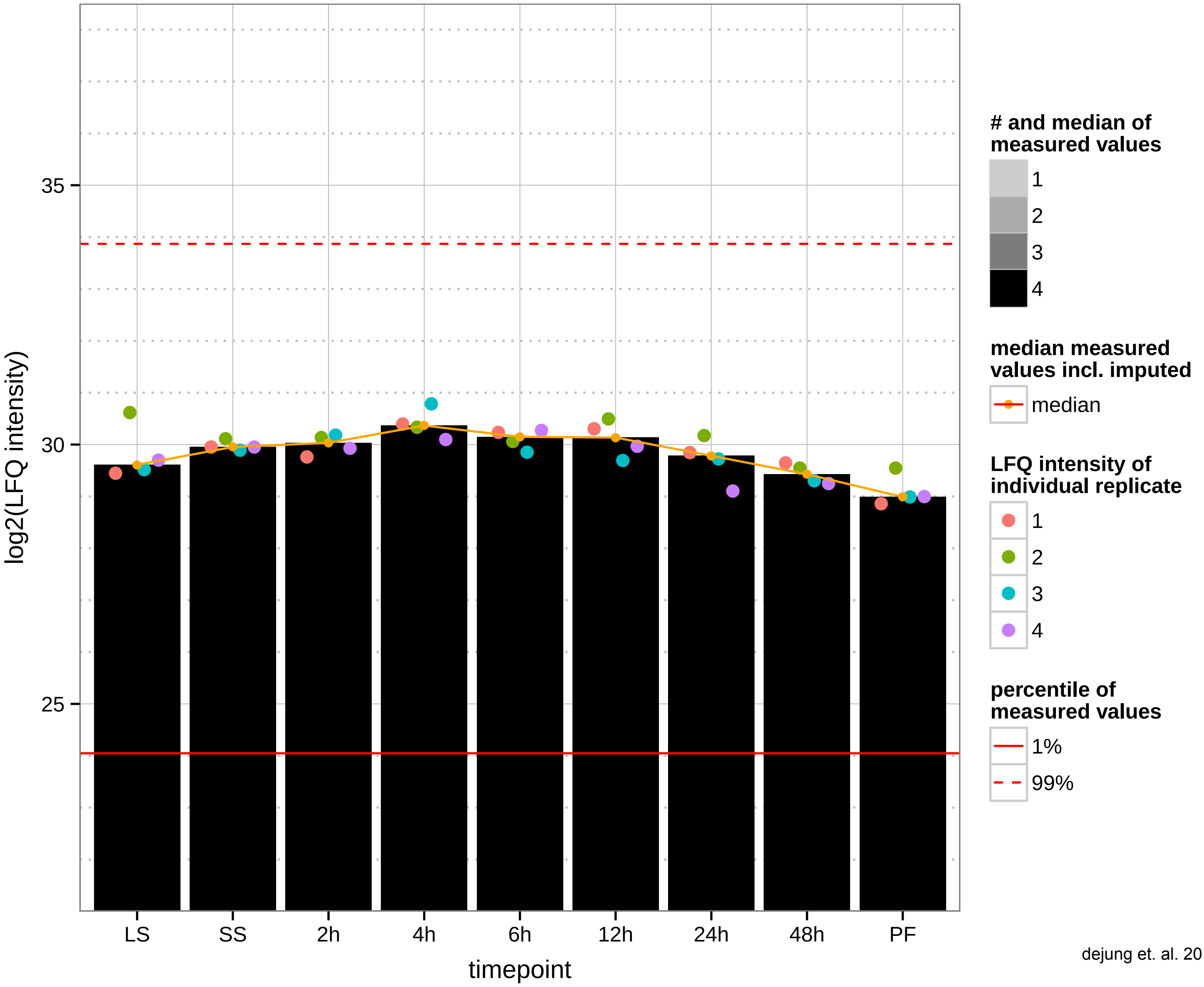

dejung et. al. 2015

hypothetical protein, conserved
Tb927.10.11510
AGOF: null
AGOC: null
AGOP: null
PGOF: null
PGOC: null
PGOP: null

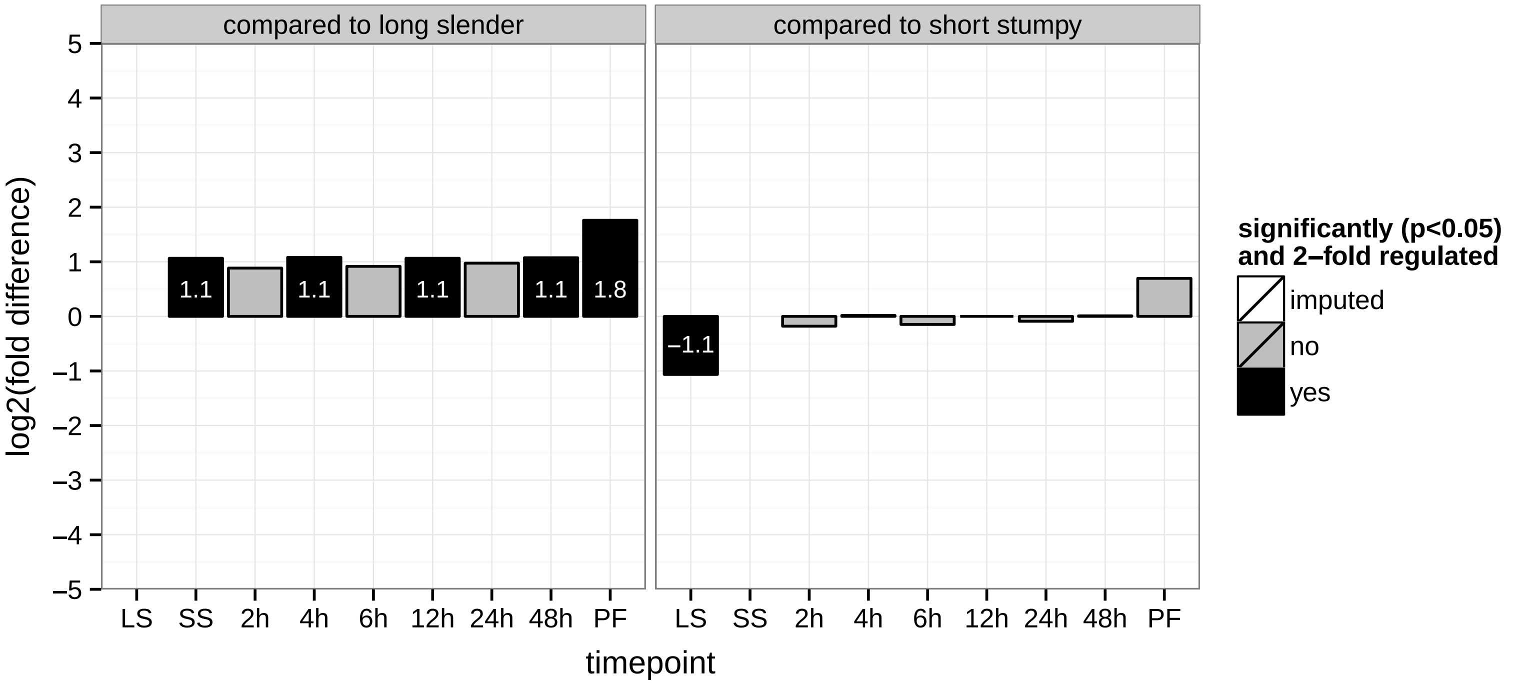

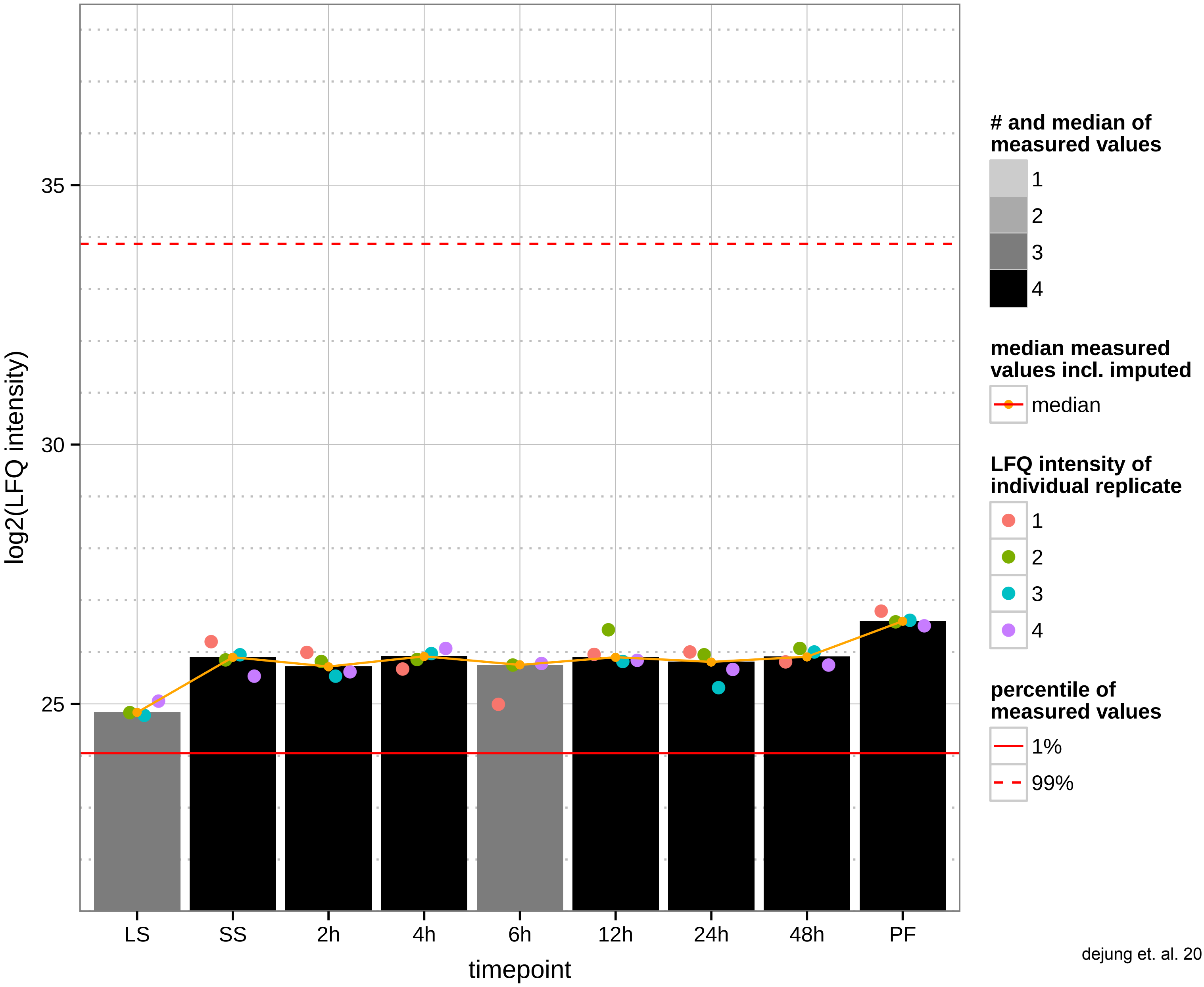

dejung et. al. 2015

hypothetical protein, conserved
Tb927.10.11530
AGOF: null
AGOC: null
AGOP: null
PGOF: null
PGOC: null
PGOP: null

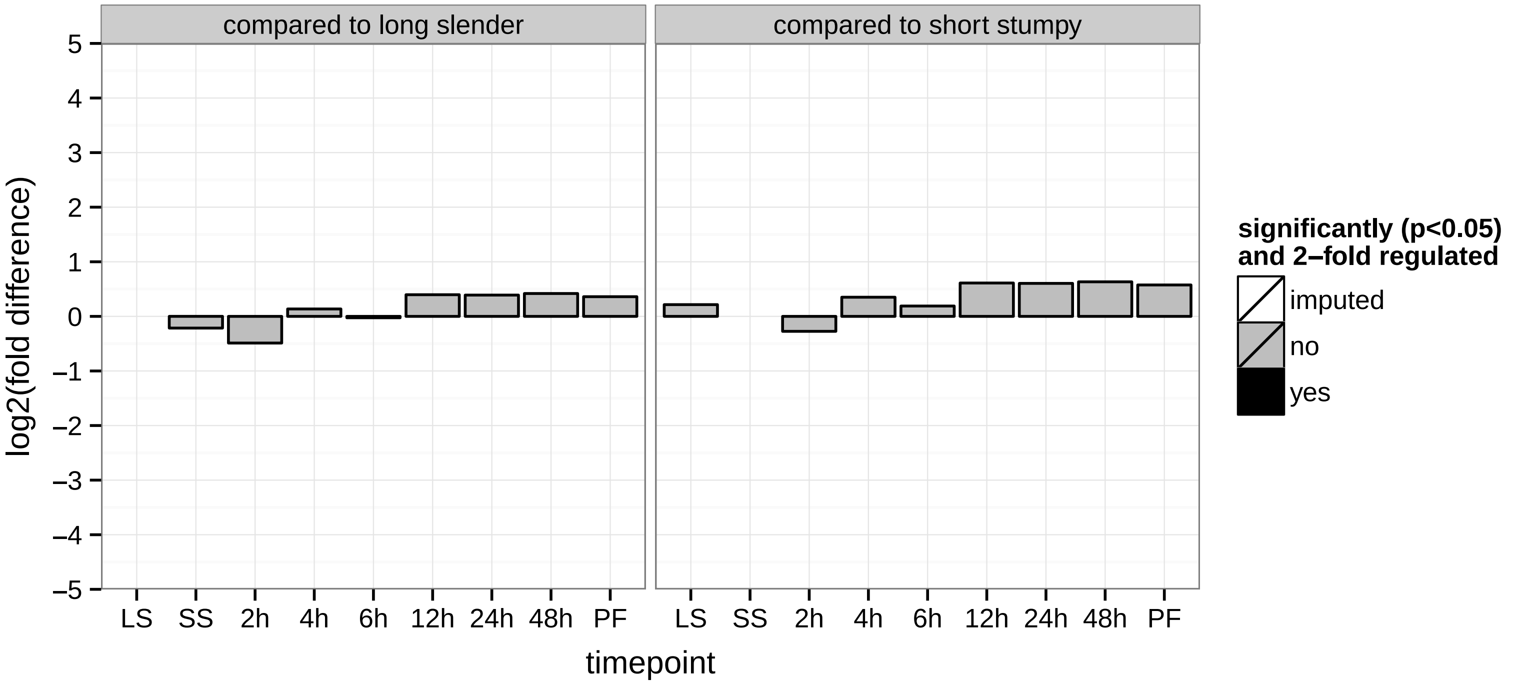

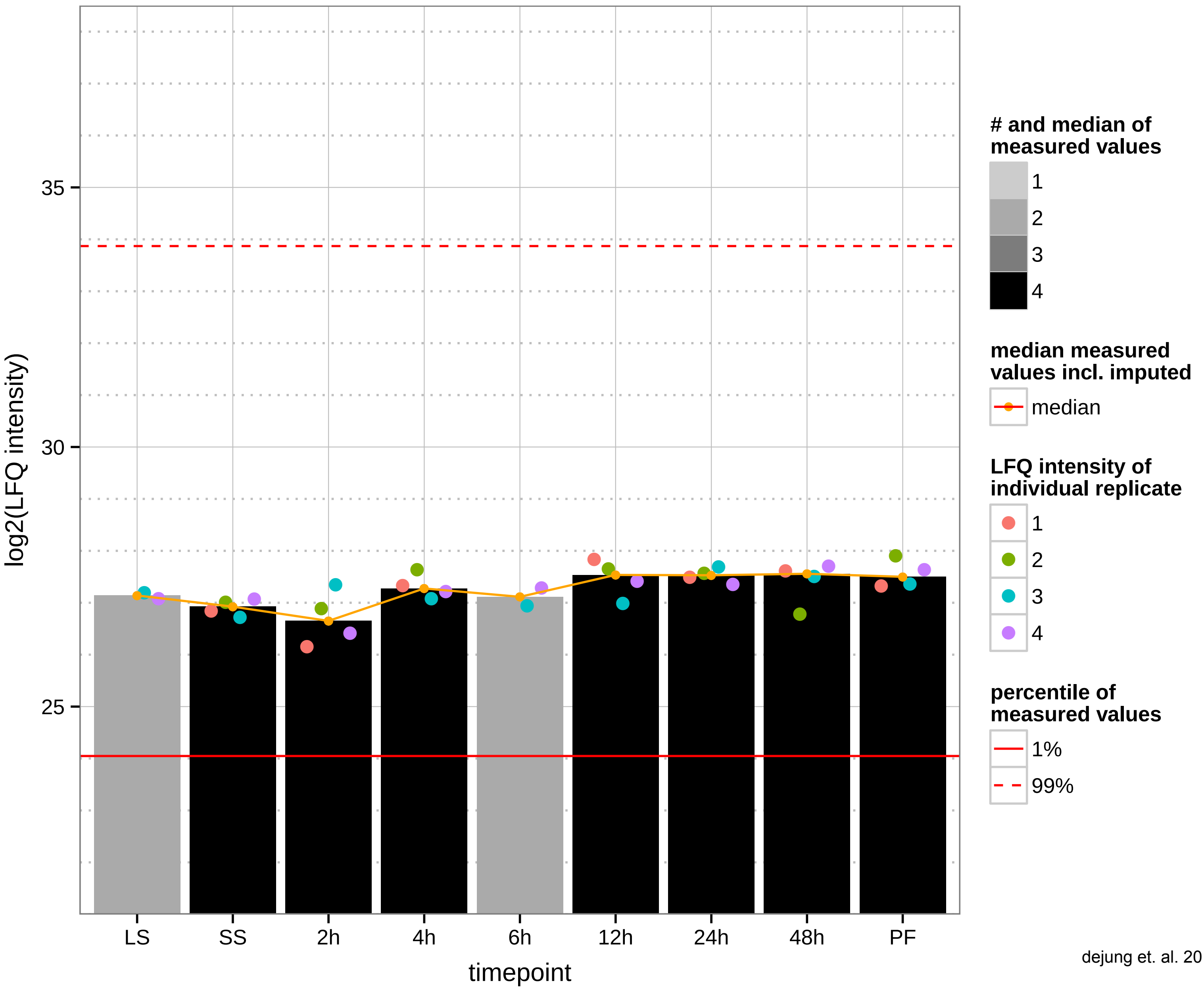

dejung et. al. 2015

40S ribosomal protein S3, putative (RPS3)
Tb927.9.6070;Tb927.10.11540
AGOF: RNA binding, nucleic acid binding, structural constituent of ribosome
AGOC: intracellular, ribosome, small ribosomal subunit
AGOP: ribosome biogenesis, translation
PGOF: RNA binding, structural constituent of ribosome
PGOC: intracellular, ribosome, small ribosomal subunit
PGOP: translation

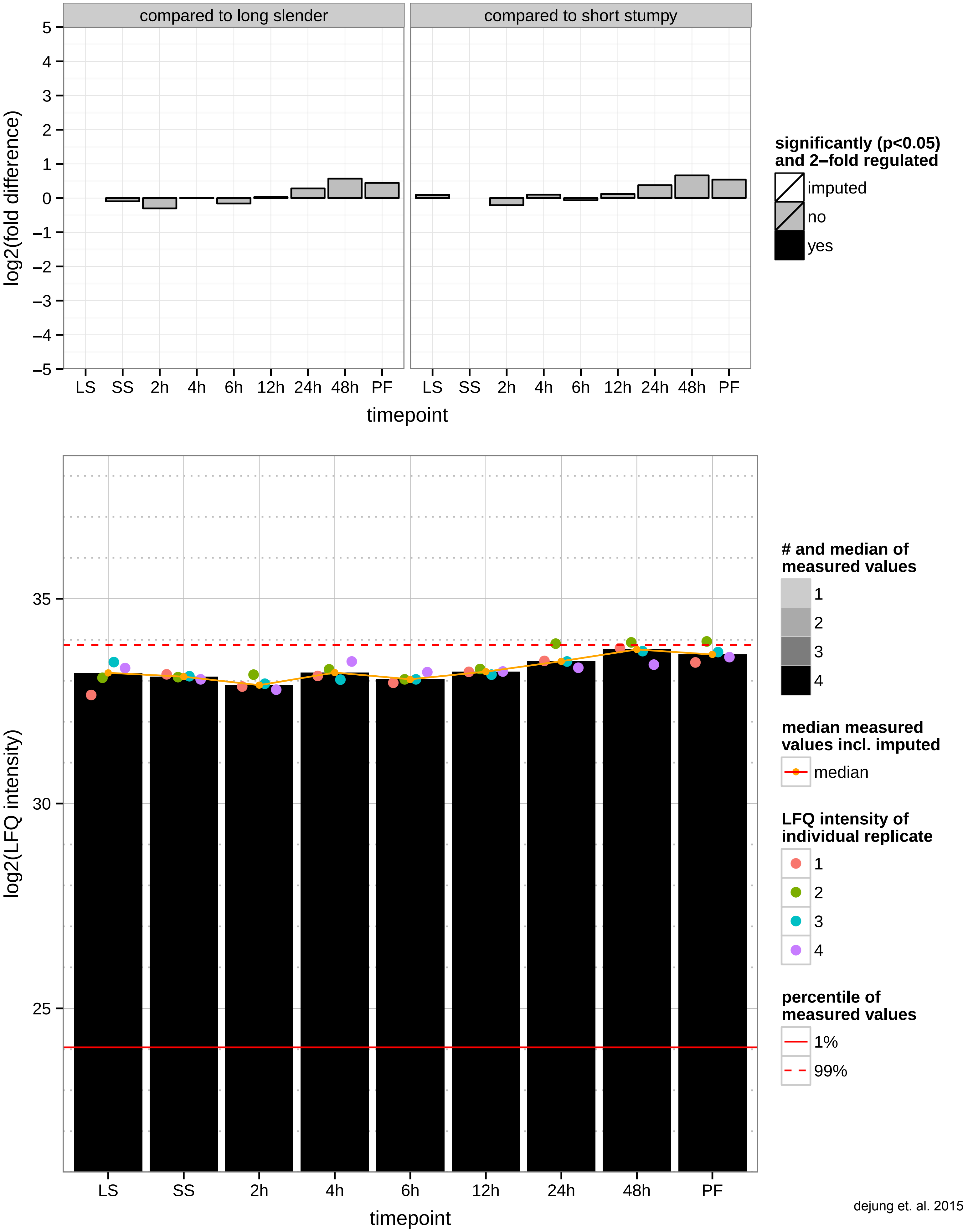

dejung et. al. 2015

hypothetical protein, conserved
Tb927.10.11660
AGOF: null
AGOC: null
AGOP: null
PGOF: null
PGOC: null
PGOP: null

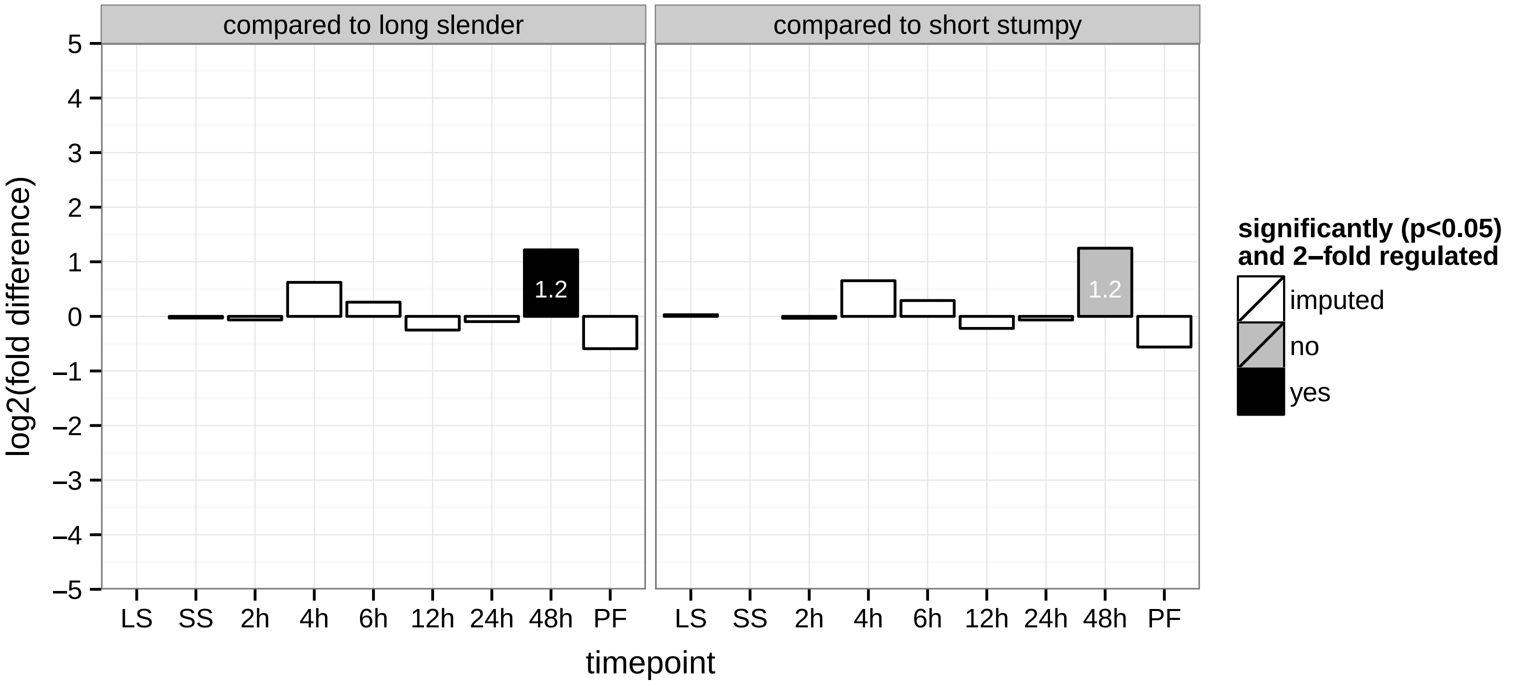

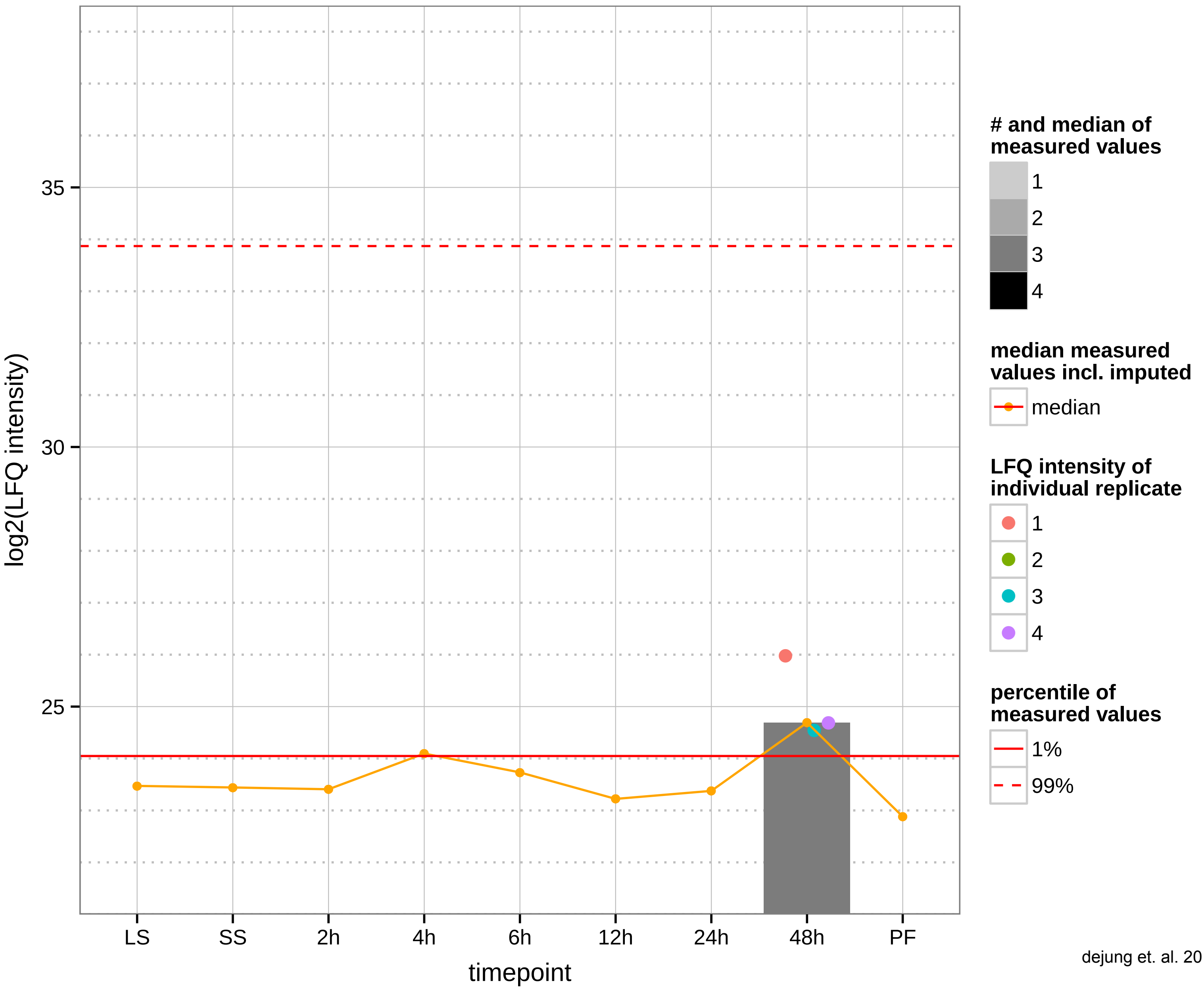

dejung et. al. 2015

guanylate kinase, putative
Tb927.10.11700
AGOF: guanylate kinase activity
AGOC: null
AGOP: nucleobase−containing small molecule interconversion
PGOF: protein binding
PGOC: null
PGOP: null

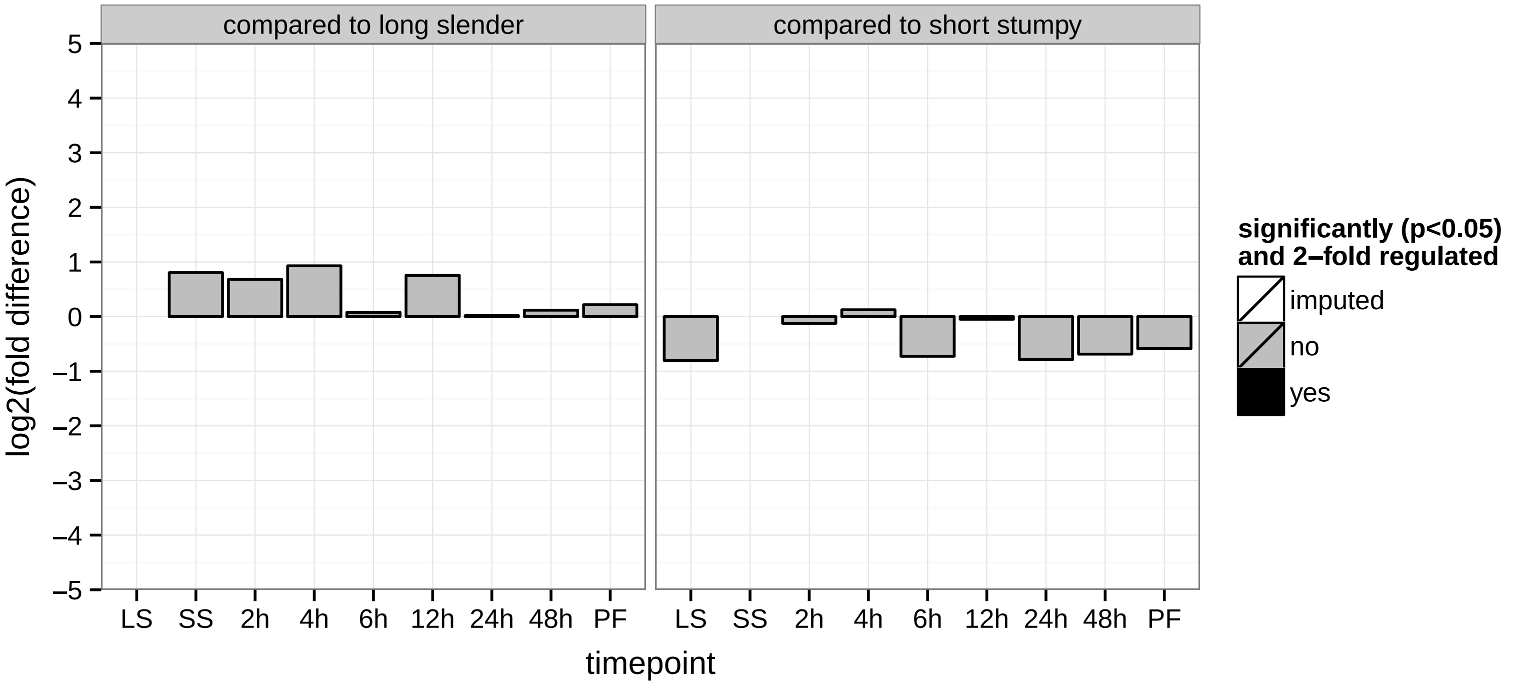

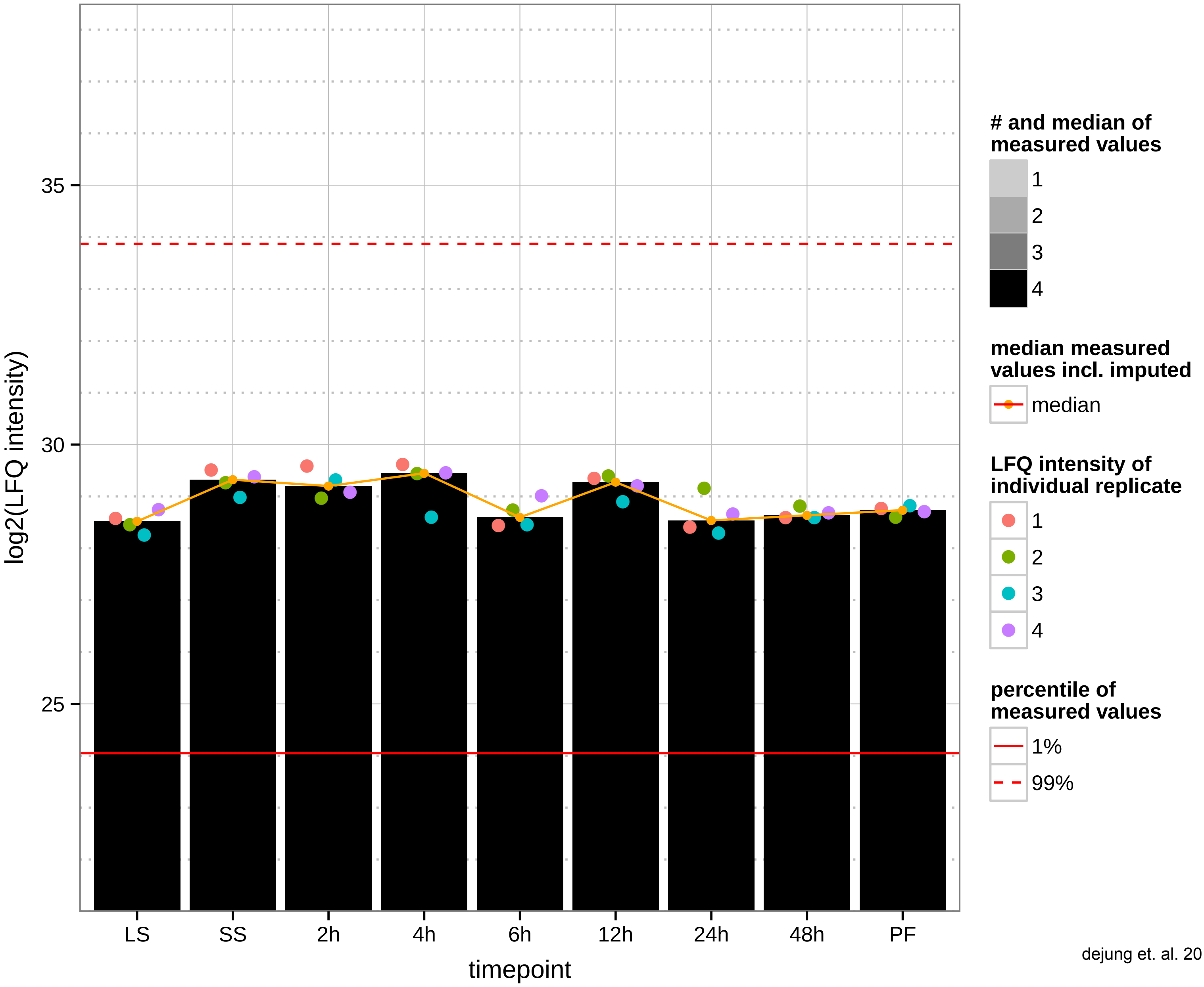

dejung et. al. 2015

hypothetical protein, conserved
Tb927.10.11780
AGOF: null
AGOC: null
AGOP: null
PGOF: null
PGOC: null
PGOP: null

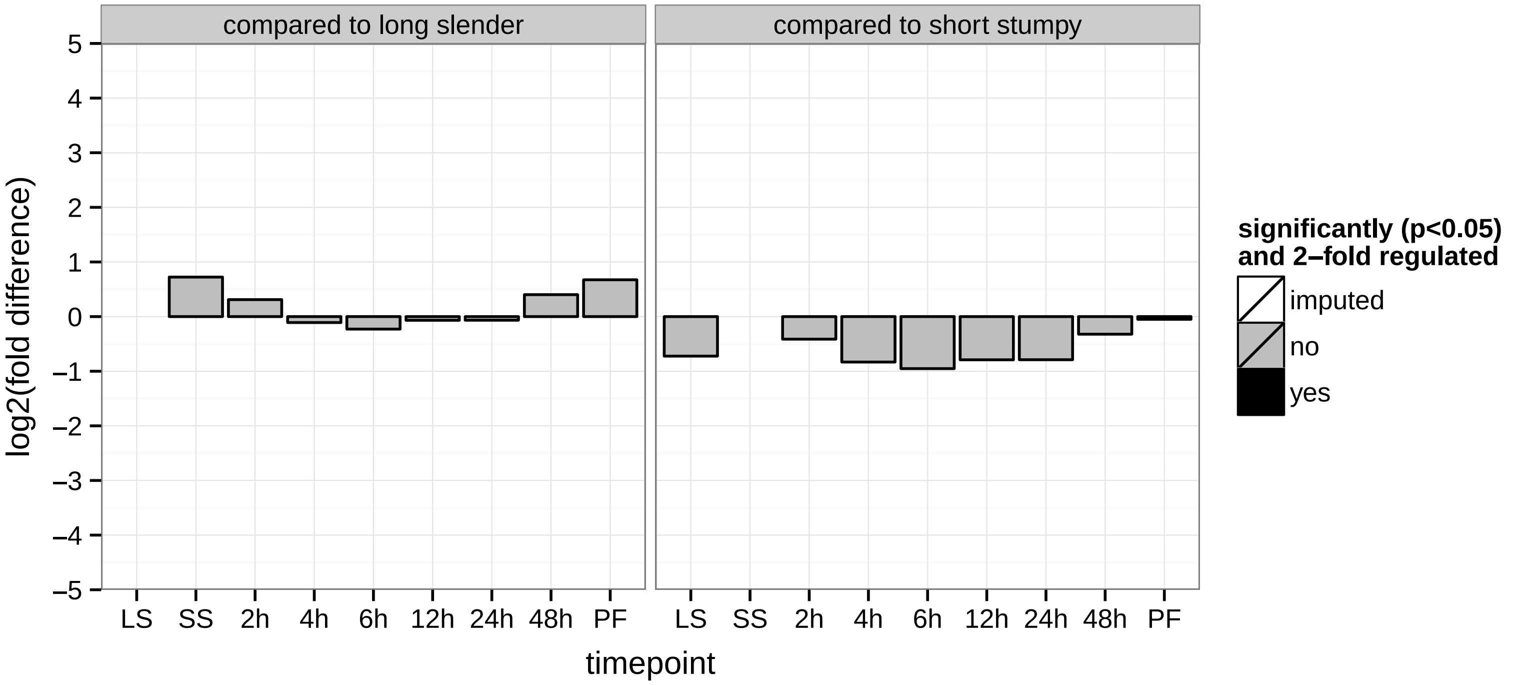

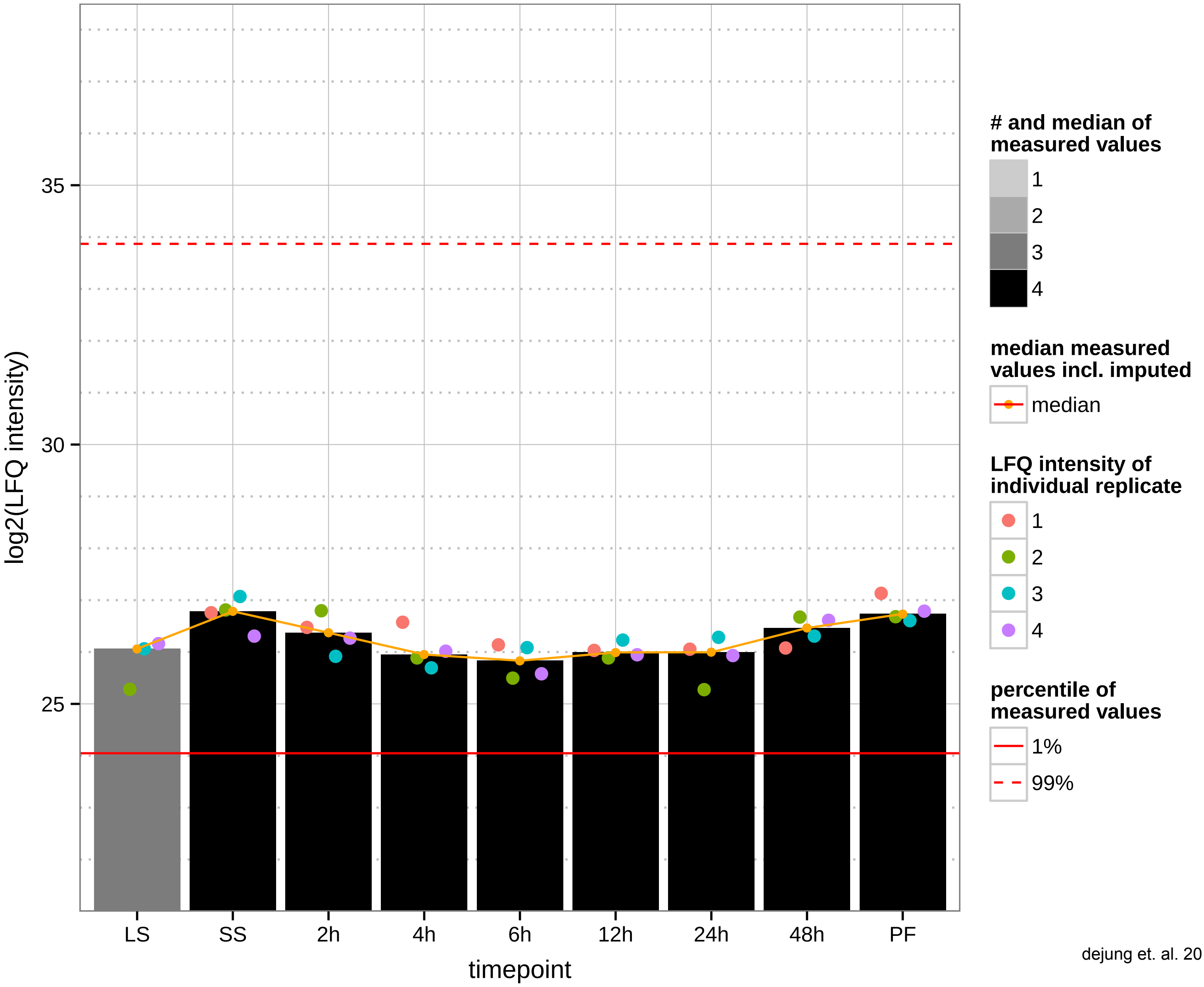

dejung et. al. 2015

Axonemal inner arm dynein light chain, putative
Tb927.10.11800
AGOF: microtubule motor activity
AGOC: null
AGOP: microtubule−based movement
PGOF: null
PGOC: null
PGOP: null

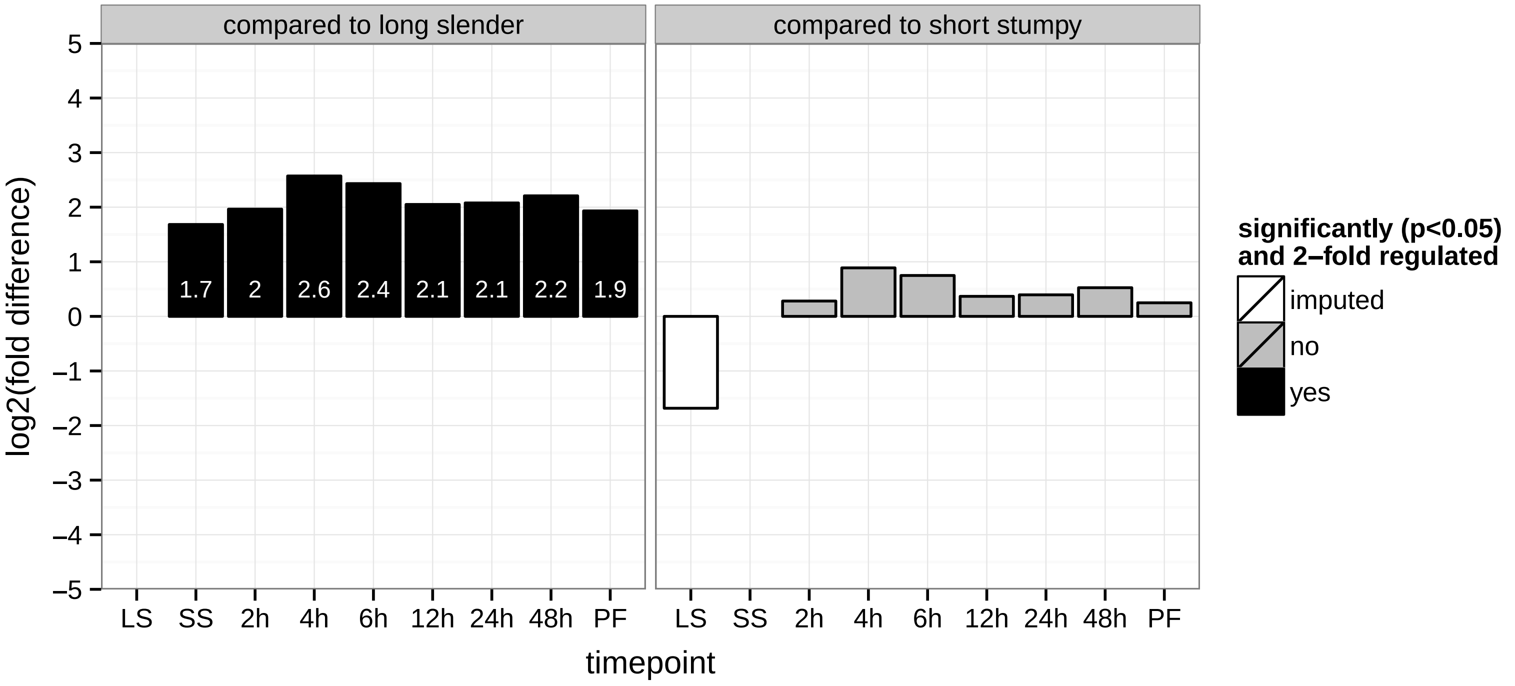

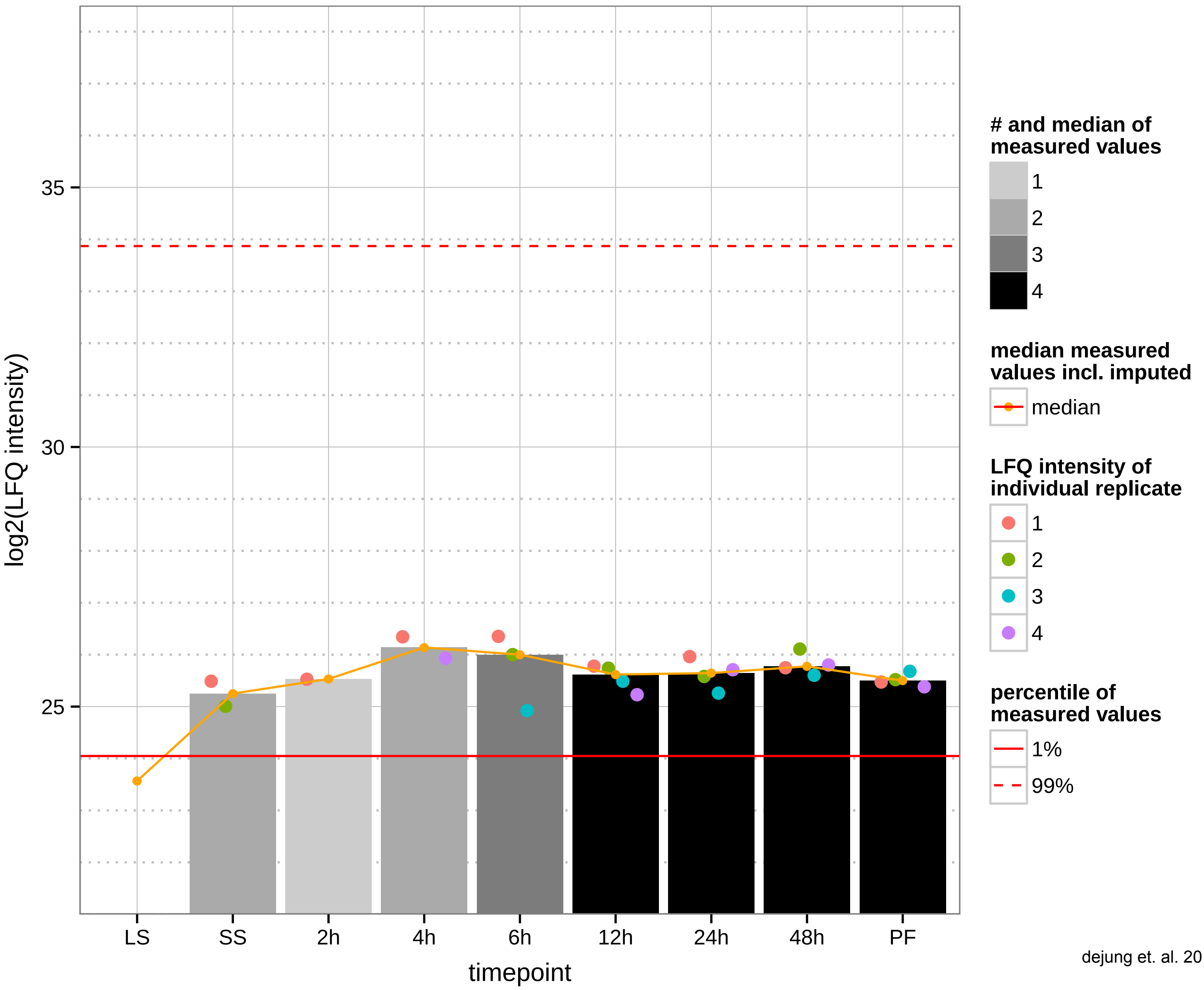

dejung et. al. 2015

mitochondrial RNA binding protein (MRB11870)
Tb927.10.11870
AGOF: RNA binding
AGOC: mitochondrion
AGOP: RNA metabolic process
PGOF: null
PGOC: null
PGOP: null

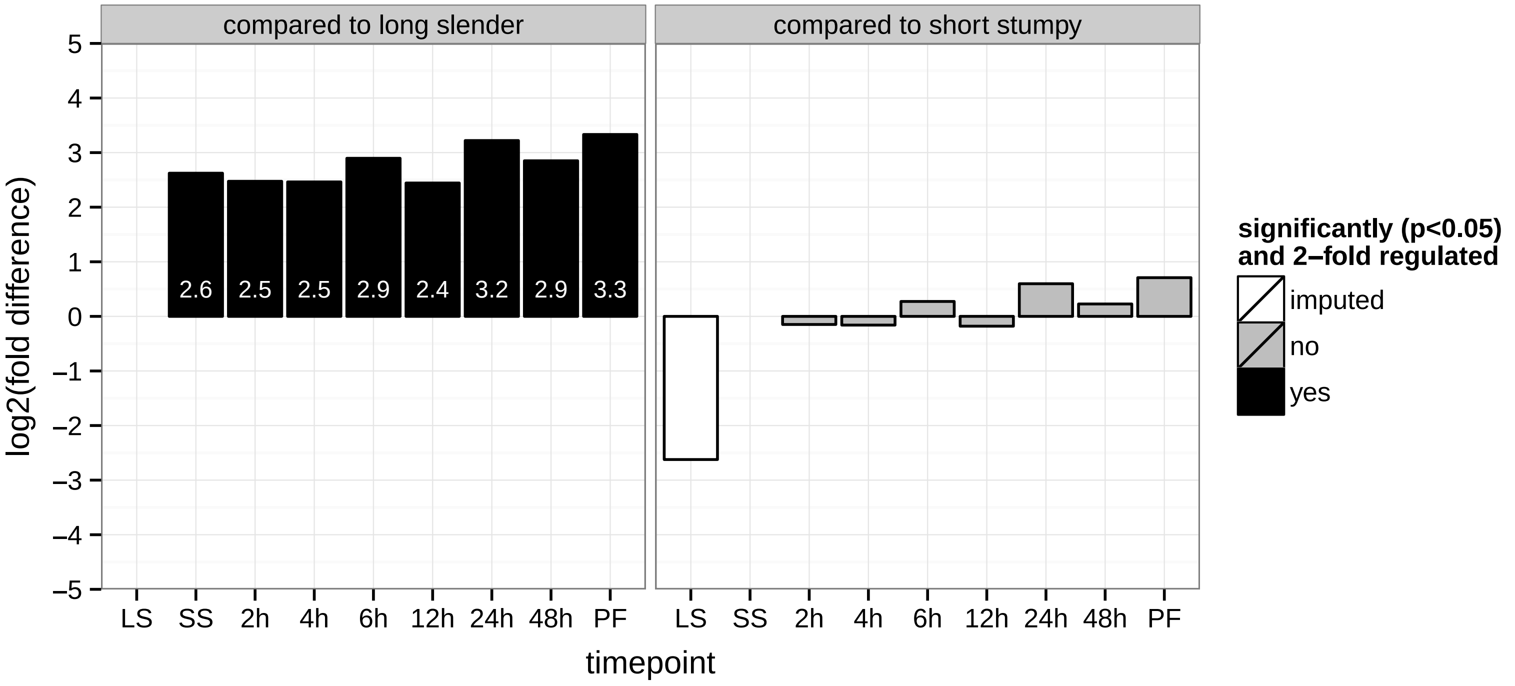

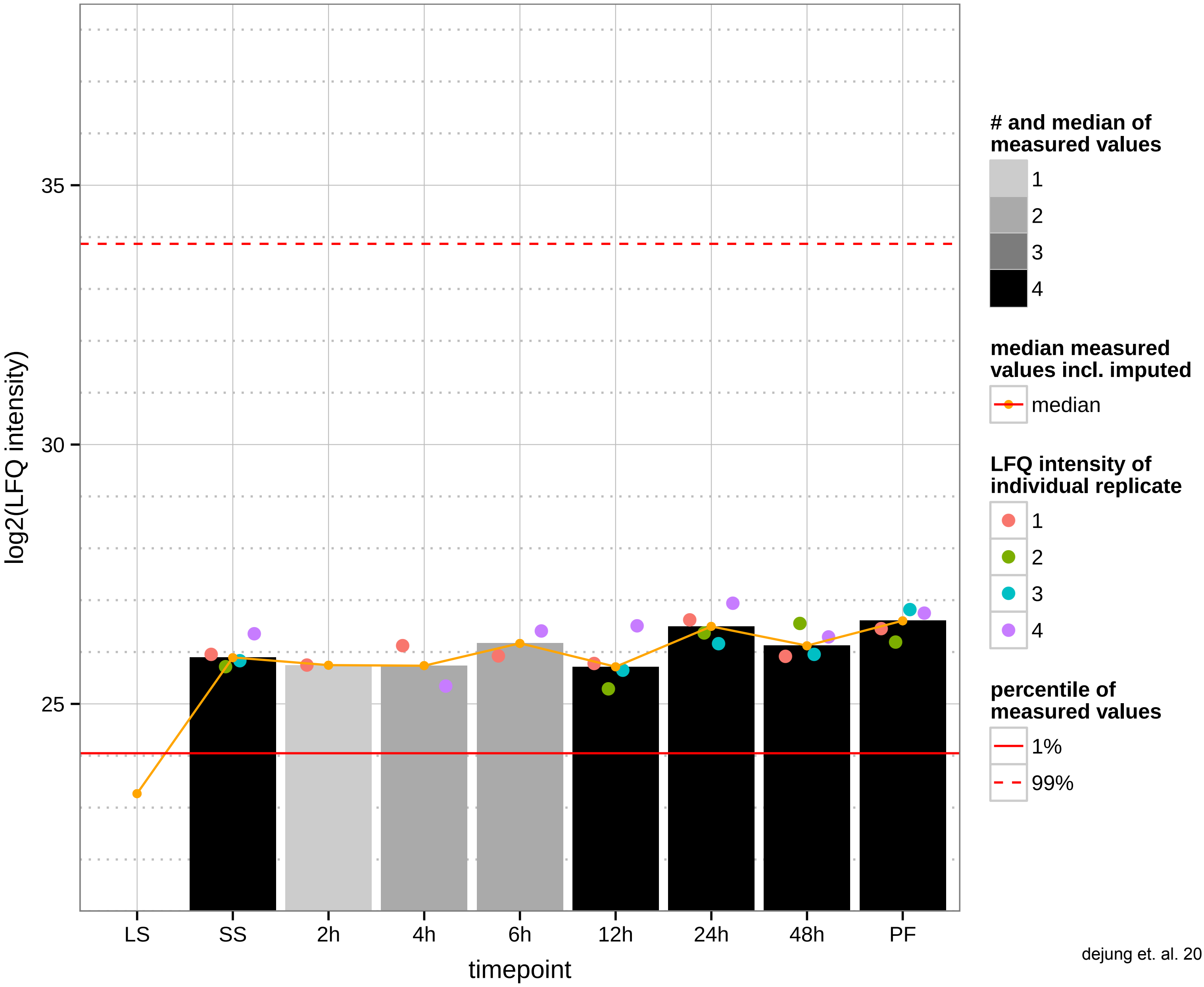

dejung et. al. 2015

glutamine aminotransferase (GlnAT) (GlnAT)
Tb927.10.11970
AGOF: 1–aminocyclopropane–1–carboxylate synthase activity, L–cysteine:2–oxoglutarate aminotransferase activity, L–glutam
AGOC: cytosol
AGOP: biosynthetic process
PGOF: pyridoxal phosphate binding, transferase activity
PGOC: null
PGOP: biosynthetic process

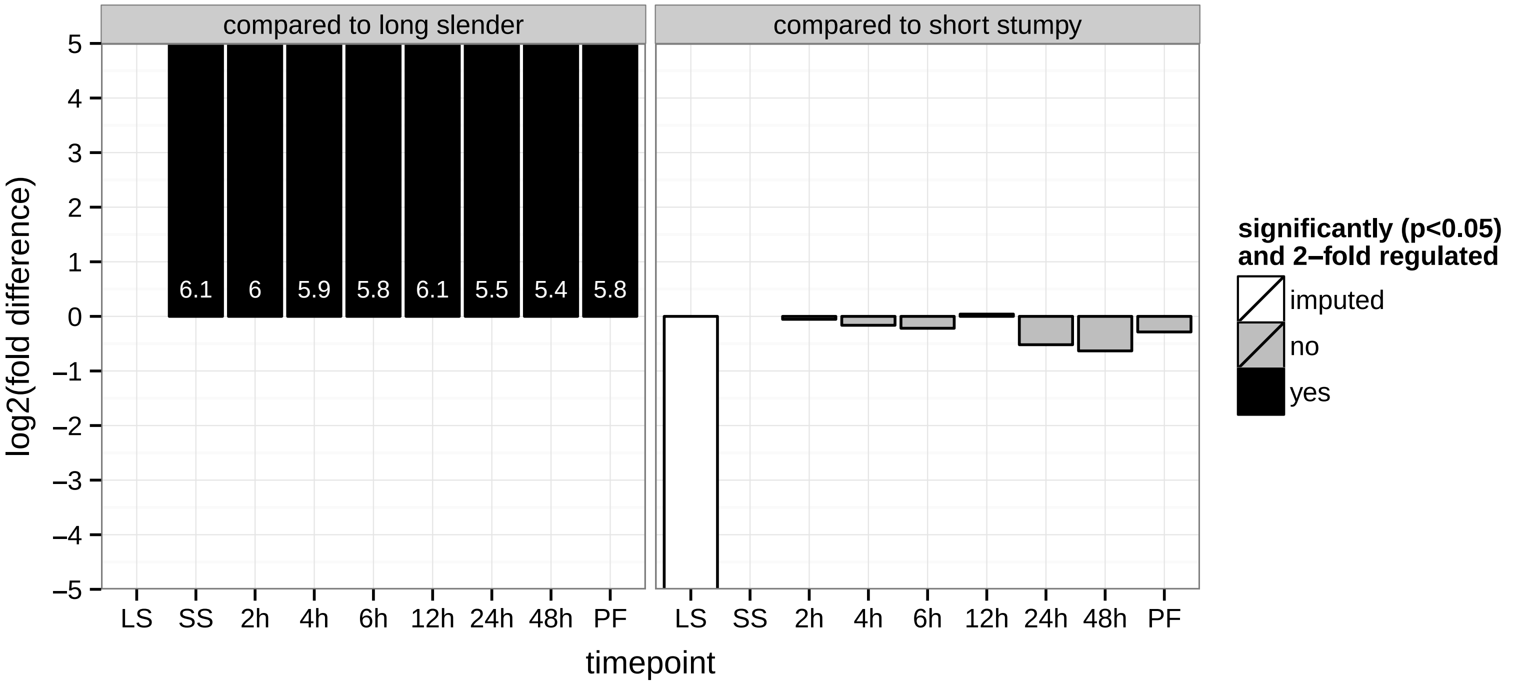

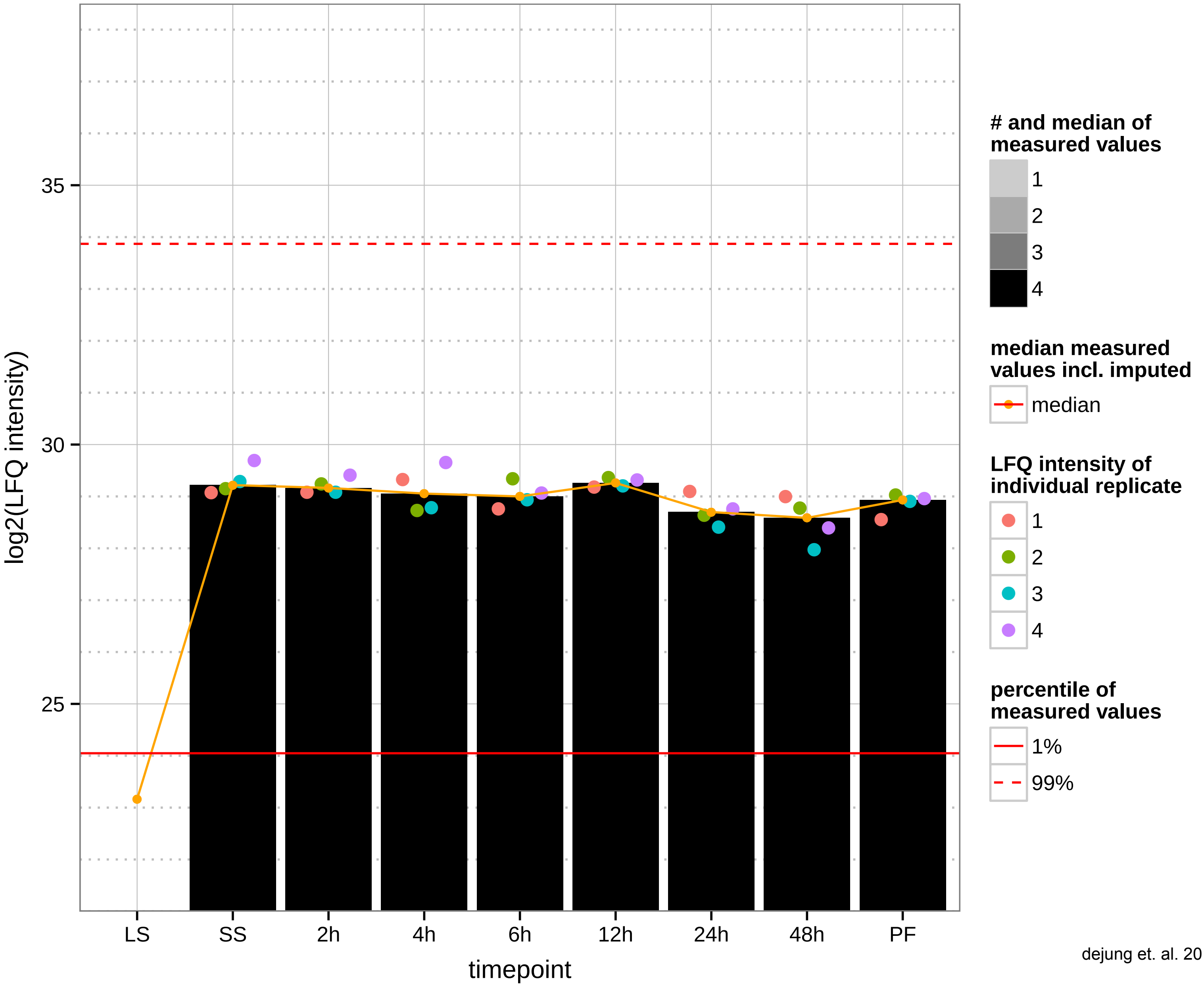

dejung et. al. 2015

RNA–binding protein
Tb927.10.11990
AGOF: RNA binding
AGOC: null
AGOP: null
PGOF: RNA binding
PGOC: nucleus
PGOP: ribosome assembly

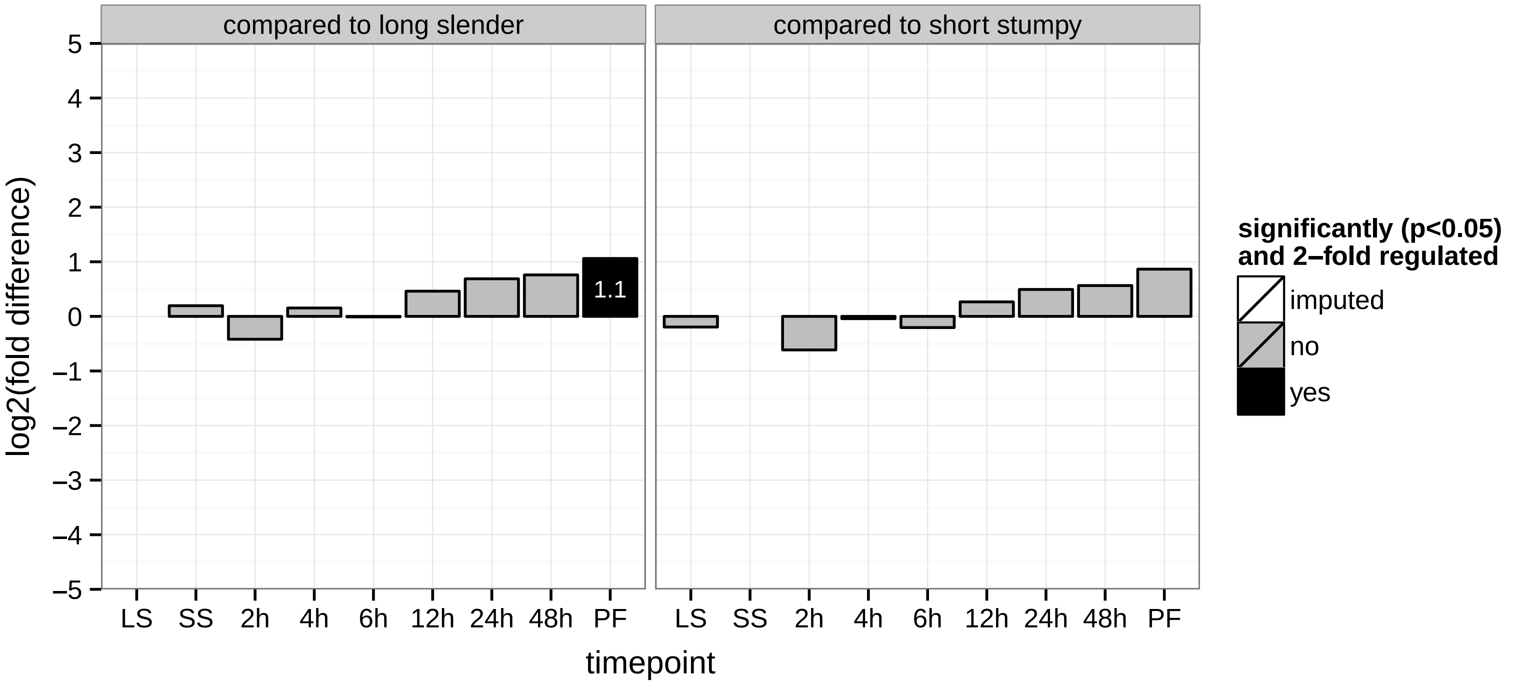

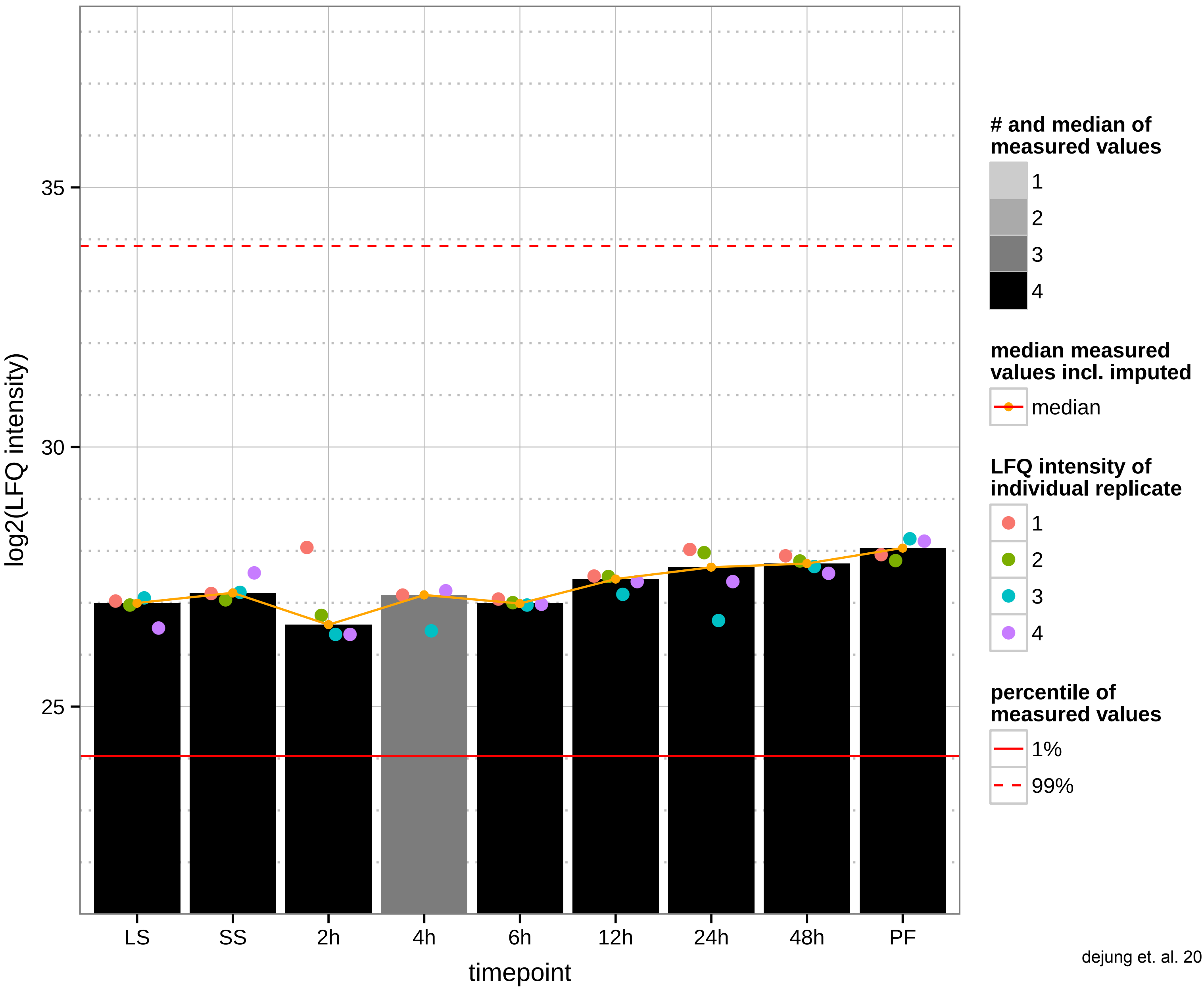

dejung et. al. 2015

katanin, putative
Tb927.10.1210
AGOF: null, ATP binding, ATPase activity
AGOC: null, katanin complex
AGOP: null, endosomal transport
PGOF: ATP binding, nucleoside–triphosphatase activity, nucleotide binding
PGOC: null
PGOP: null

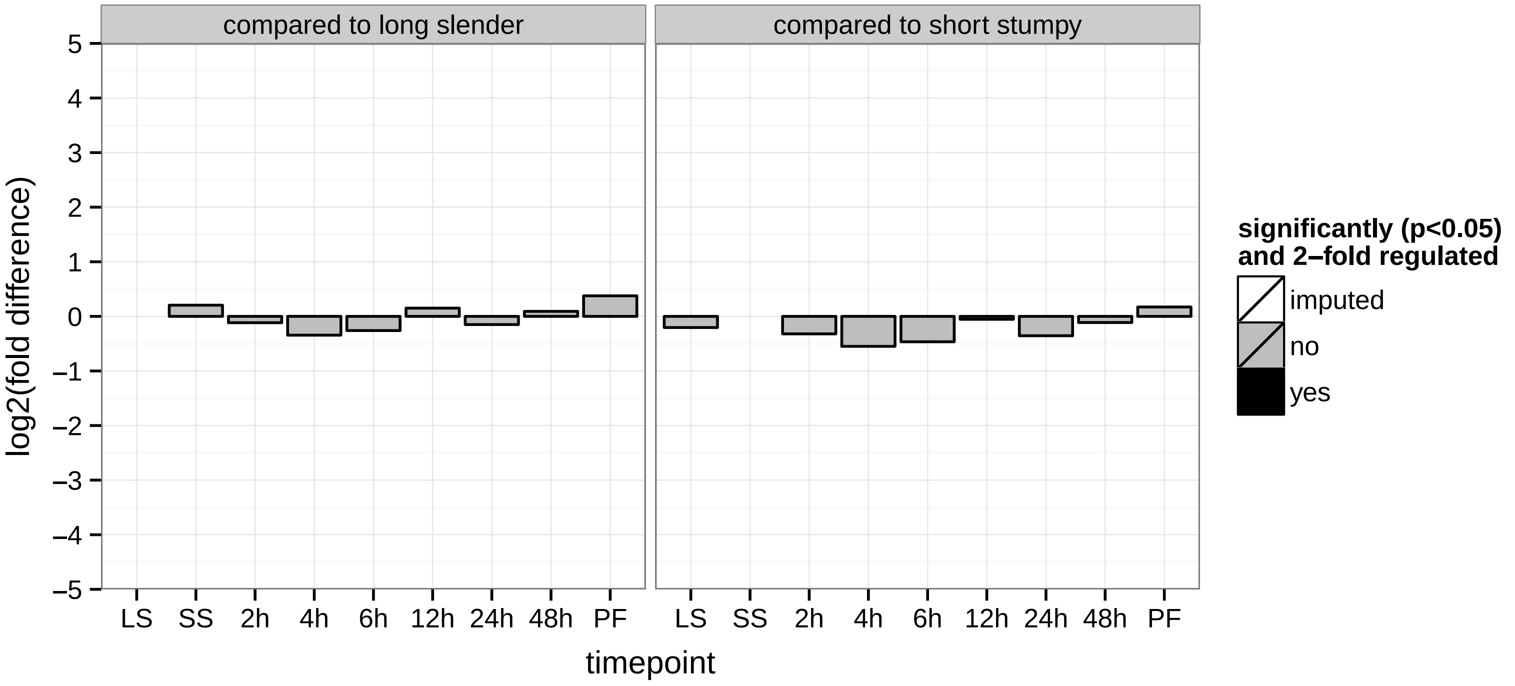

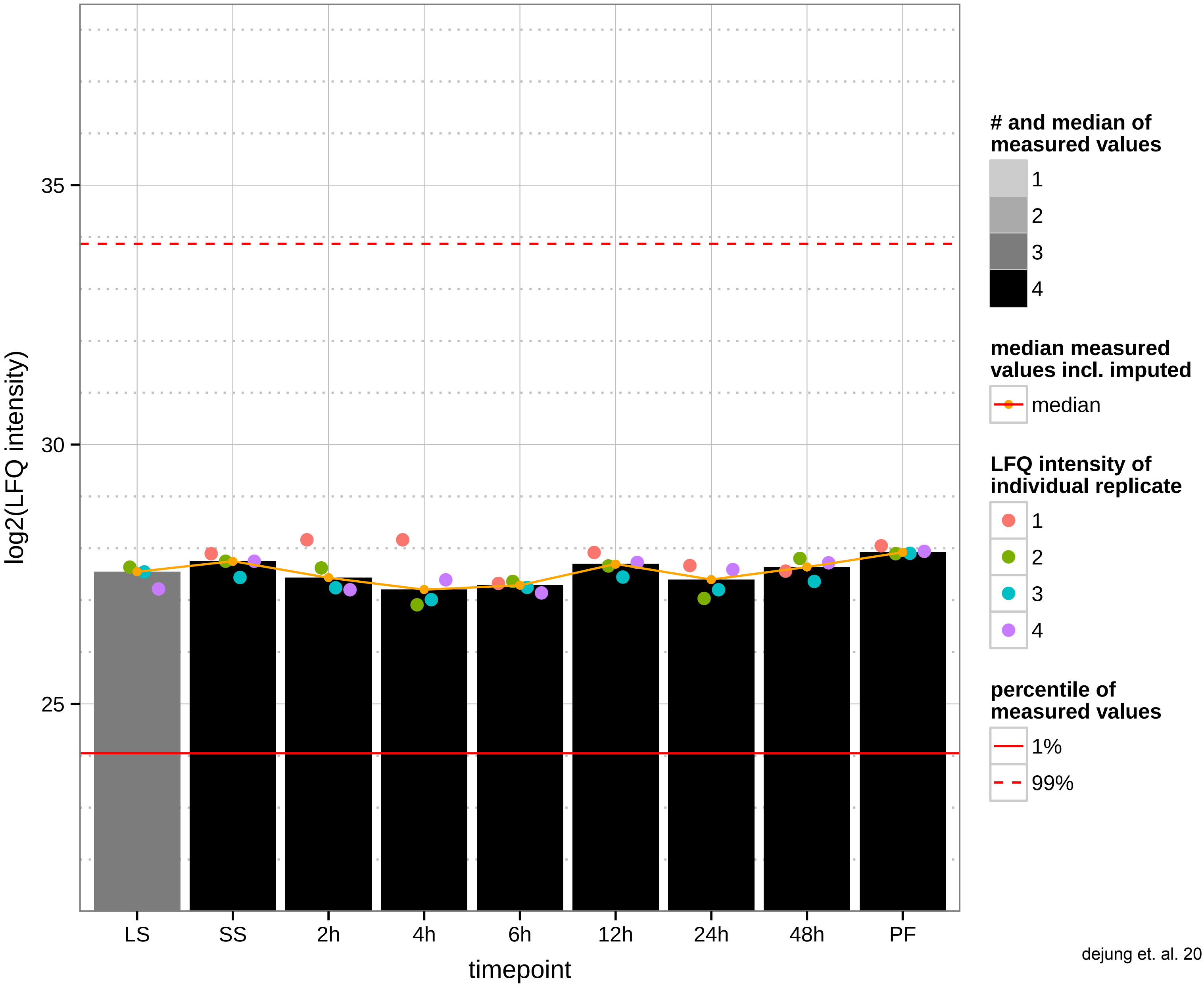

dejung et. al. 2015

hypothetical protein, conserved
Tb927.10.12110
AGOF: null
AGOC: null
AGOP: null
PGOF: null
PGOC: null
PGOP: null

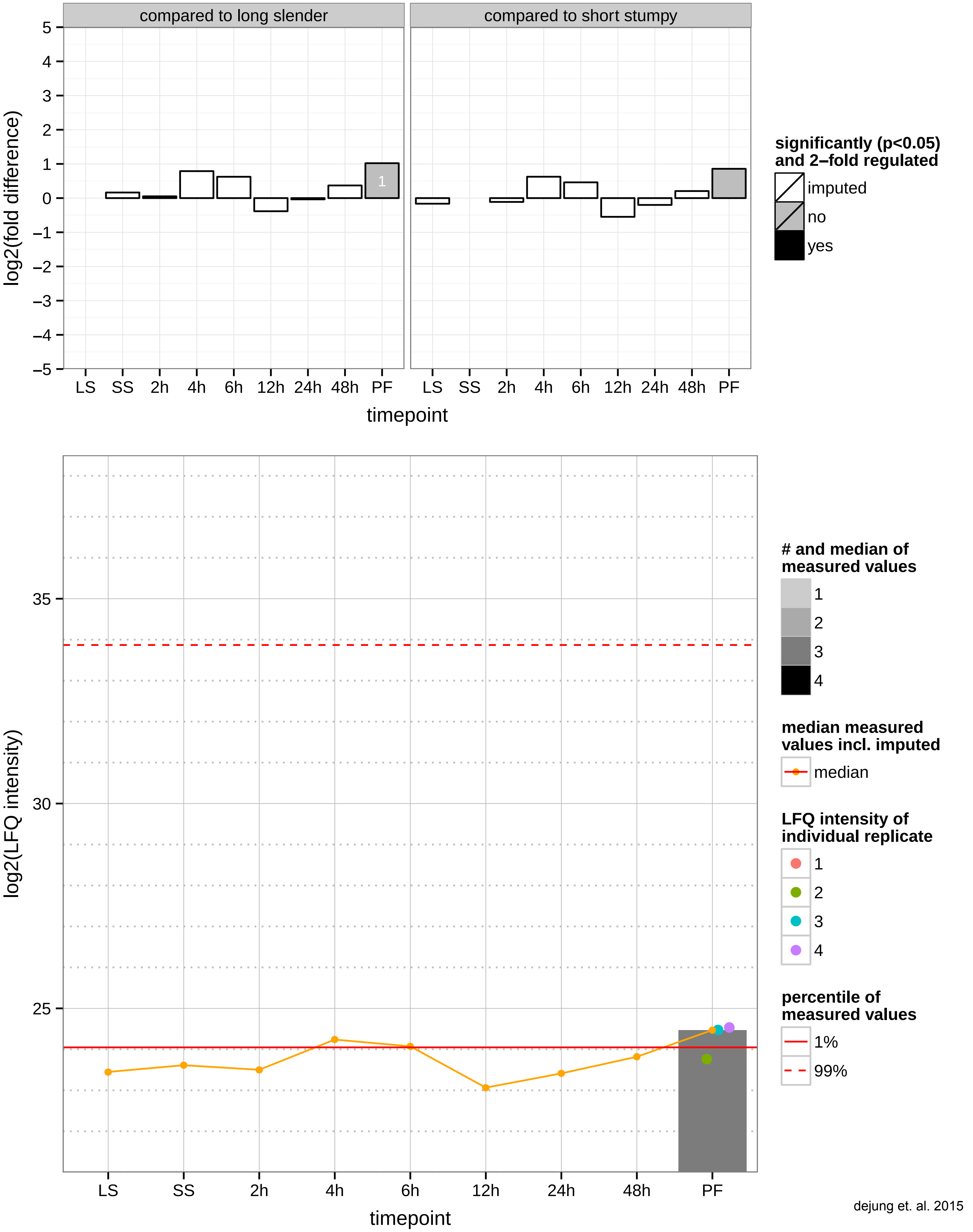

dejung et. al. 2015

RAB–interacting protein, putative
Tb927.10.12190
AGOF: Rab GTPase binding
AGOC: null
AGOP: null
PGOF: null
PGOC: null
PGOP: null

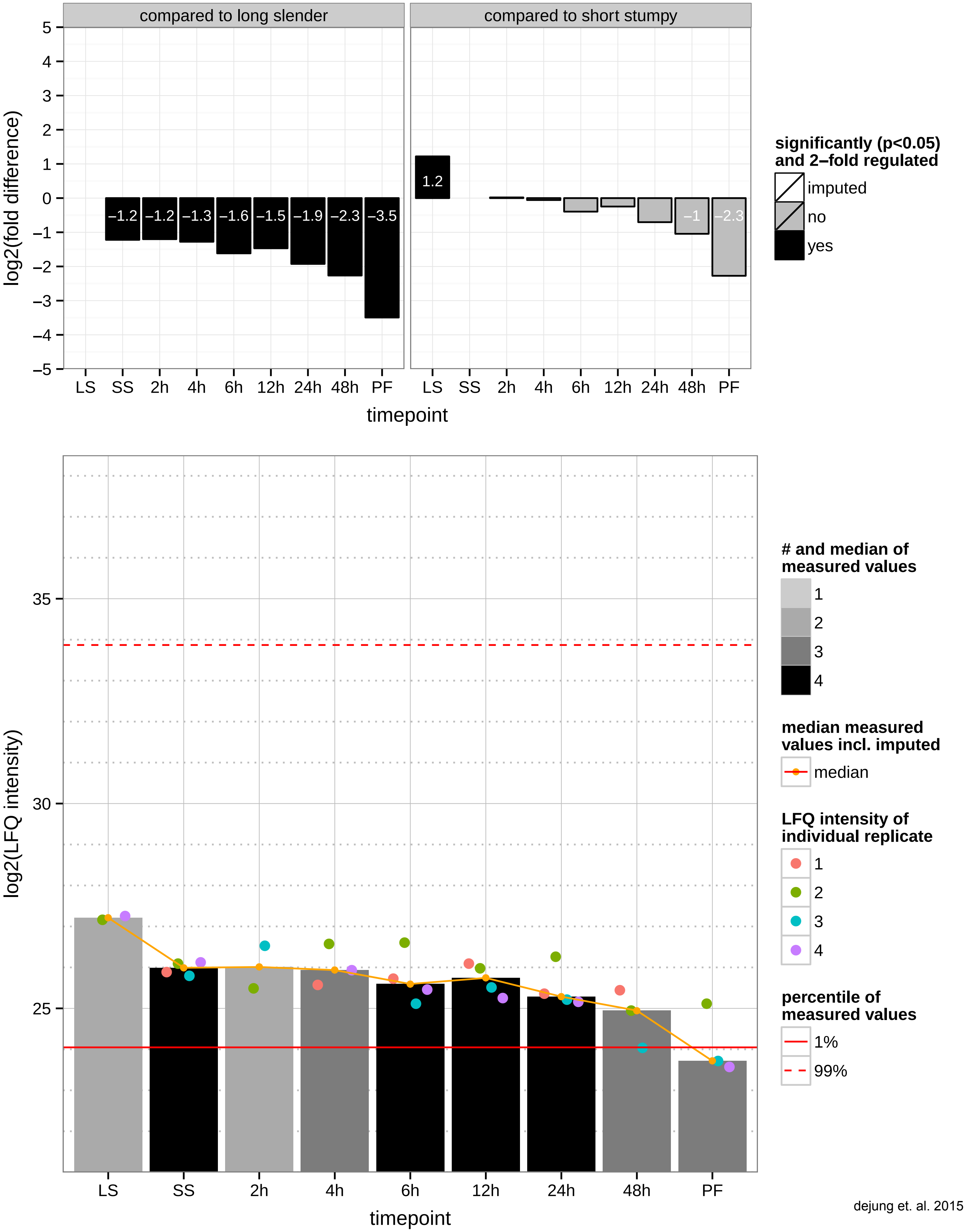

dejung et. al. 2015

hypothetical protein, conserved
Tb927.10.12360
AGOF: null
AGOC: null
AGOP: null
PGOF: null
PGOC: null
PGOP: null

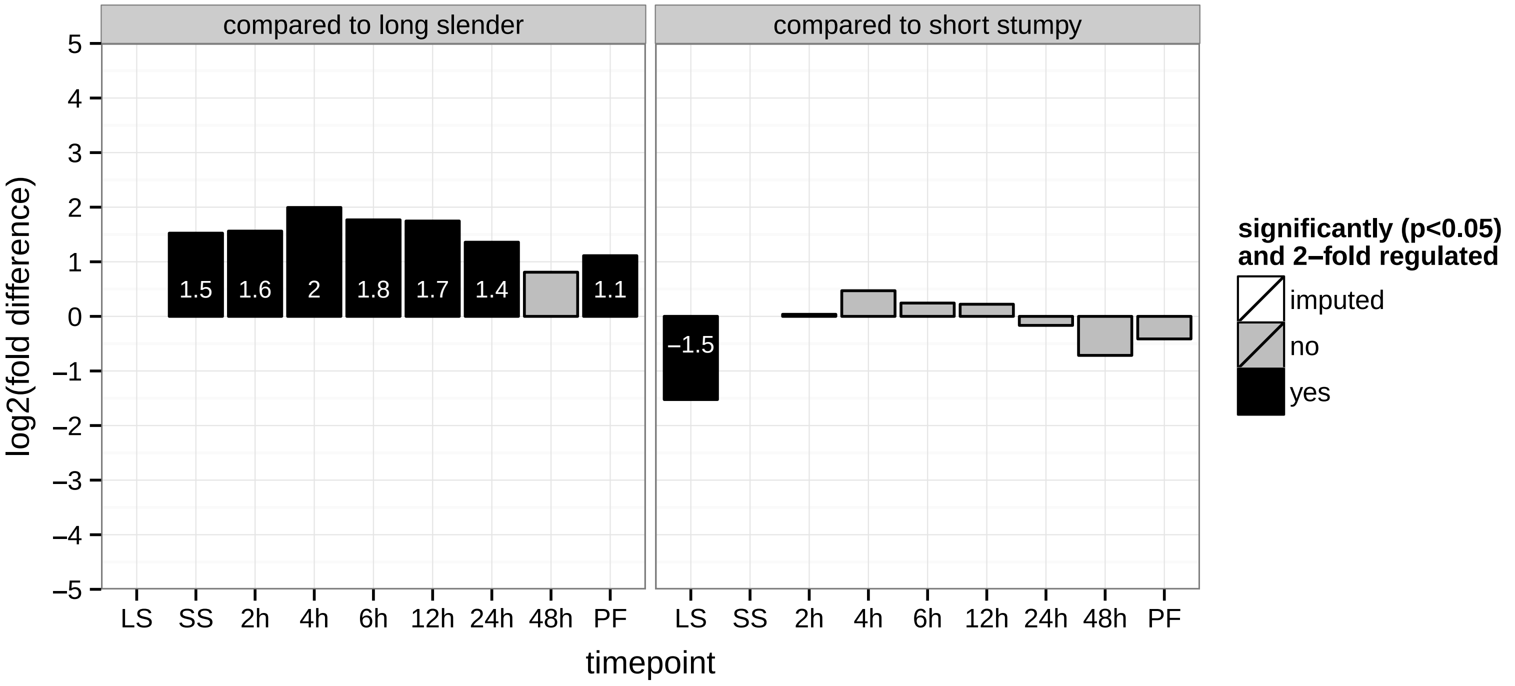

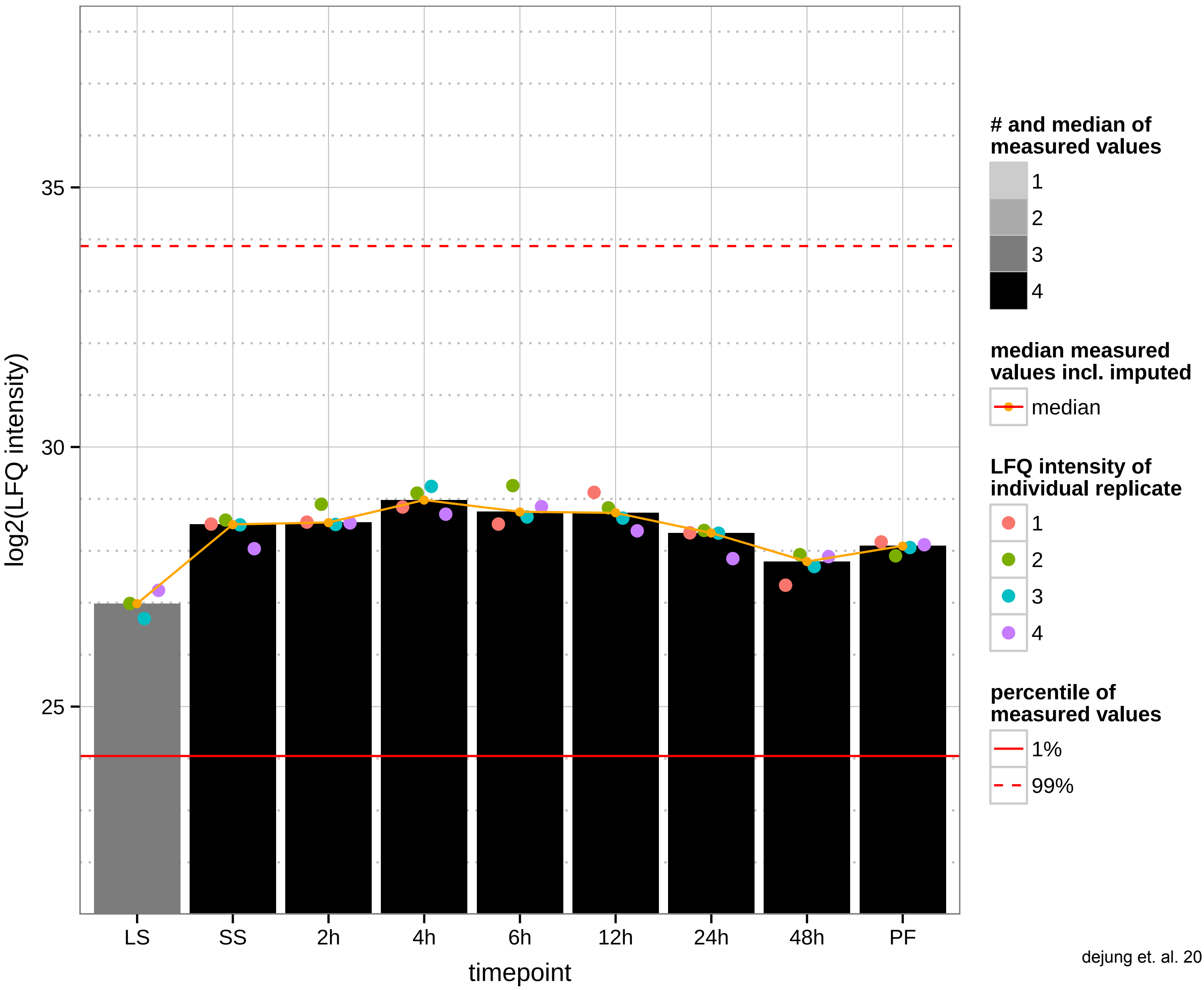

dejung et. al. 2015

hypothetical protein, conserved
Tb927.10.12410
AGOF: null
AGOC: null
AGOP: null
PGOF: null
PGOC: null
PGOP: null

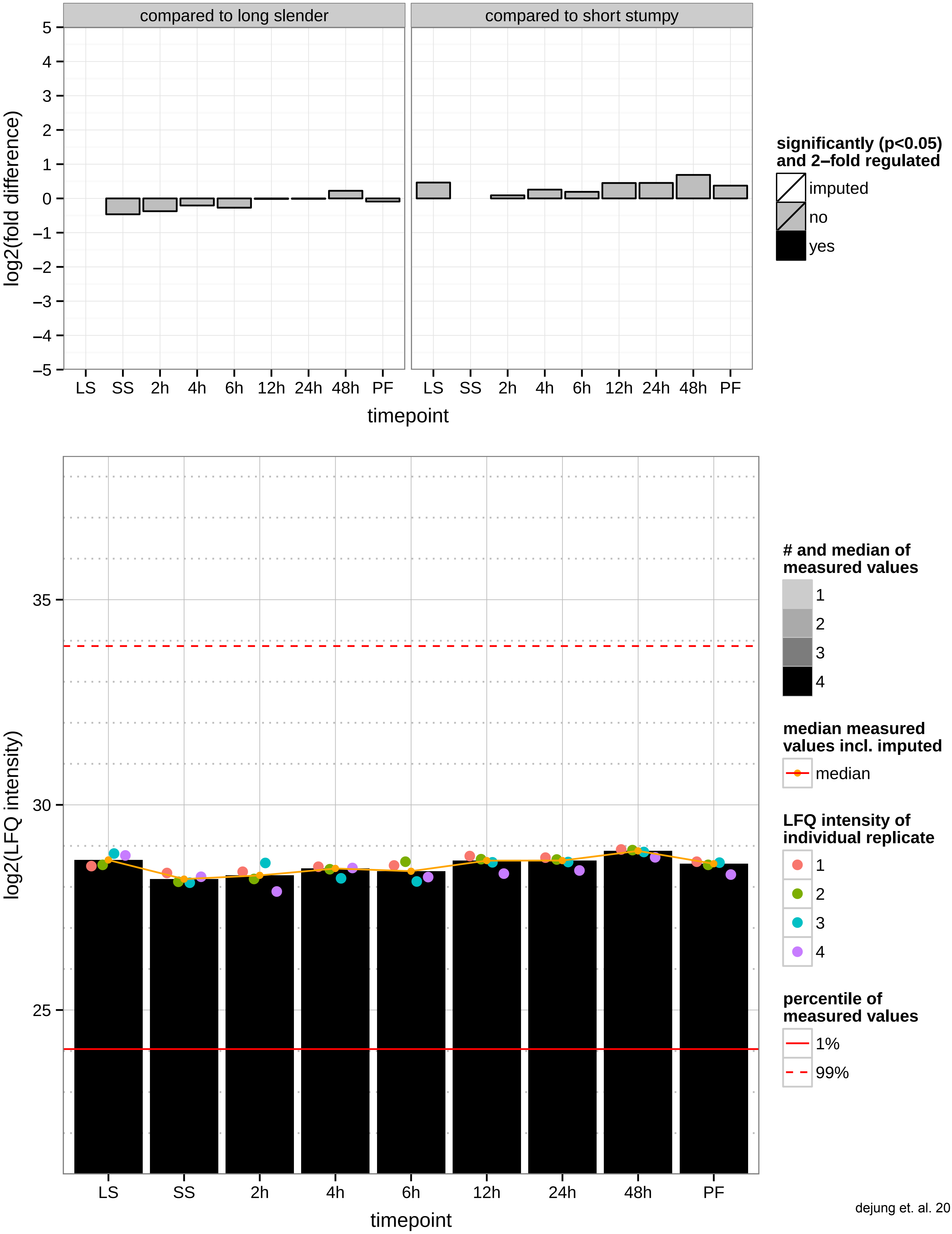

dejung et. al. 2015

predicted S. cerevisiae Got1 homologue
Tb927.10.12420
AGOF: null
AGOC: null
AGOP: vesicle–mediated transport
PGOF: null
PGOC: null
PGOP: vesicle–mediated transport

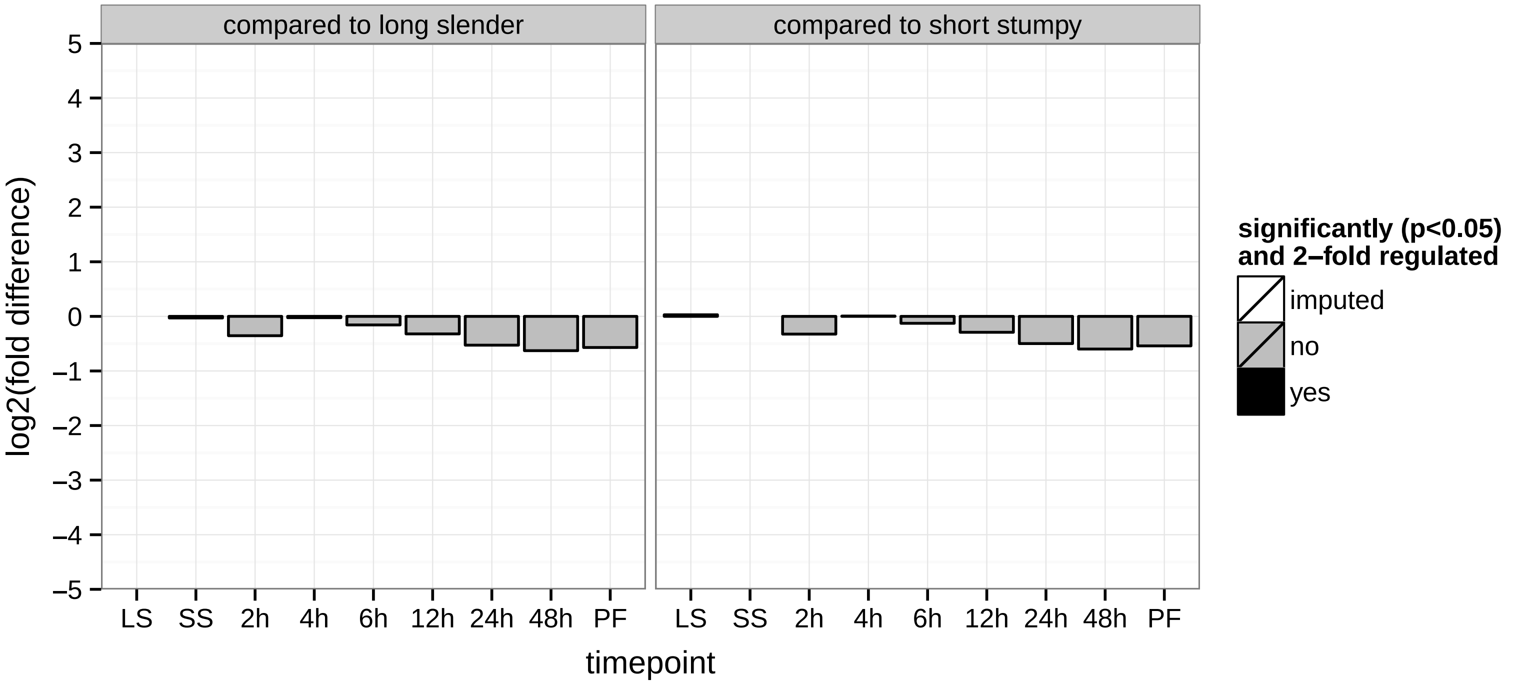

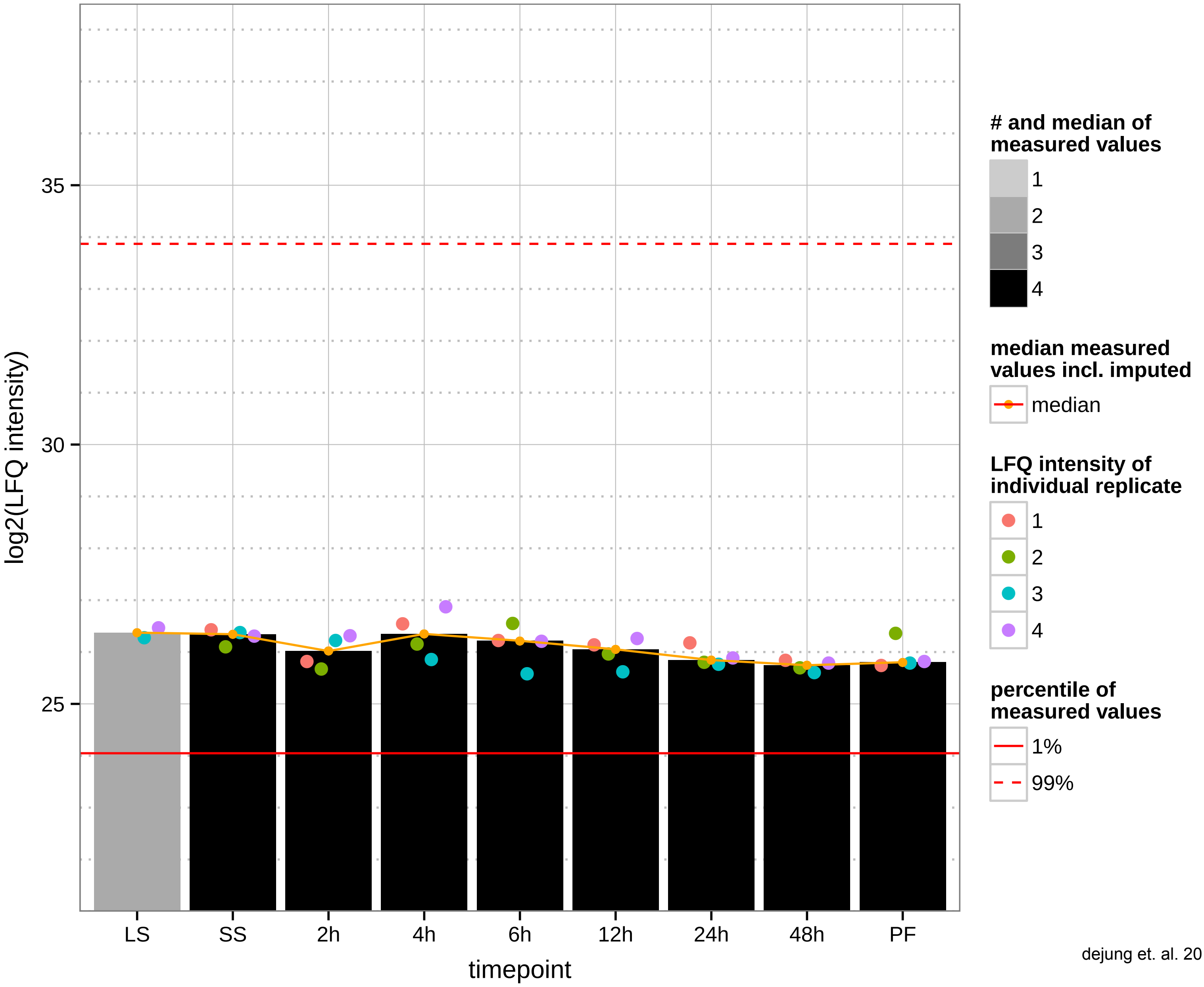

dejung et. al. 2015

hypothetical protein, conserved
Tb927.10.12430
AGOF: null
AGOC: null
AGOP: null
PGOF: binding
PGOC: null
PGOP: null

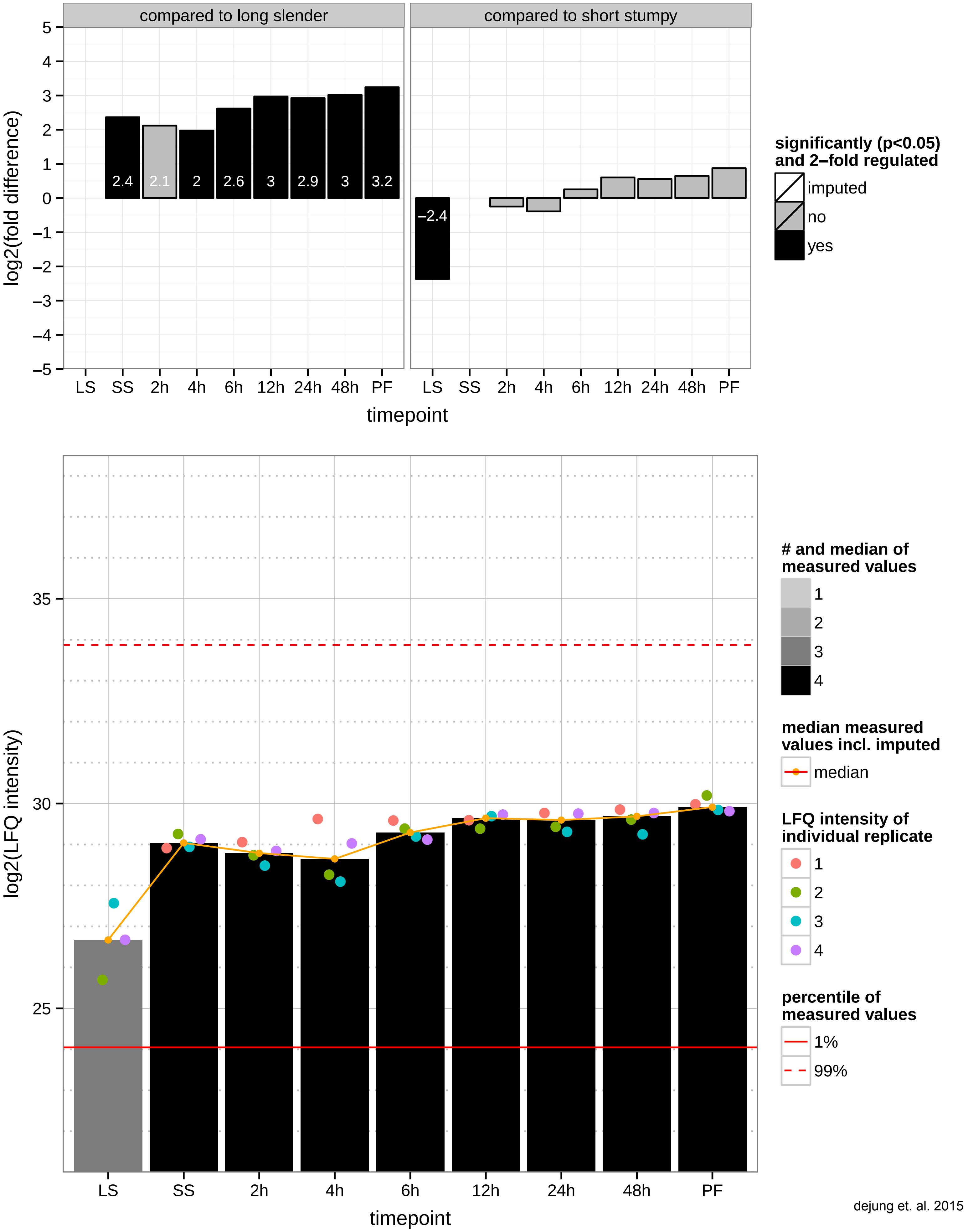

dejung et. al. 2015

P–type H+–ATPase, putative
Tb927.10.12500
AGOF: ATP binding, ATPase activity, coupled to transmembrane movement of ions, phosphorylative mechanism
AGOC: integral to membrane, plasma membrane
AGOP: ATP biosynthetic process, metabolic process, proton transport
PGOF: ATP binding, ATPase activity, coupled to transmembrane movement of ions, phosphorylative mechanism, catalytic acti
PGOC: integral to membrane, membrane
PGOP: ATP biosynthetic process, ATP catabolic process, cation transport, metabolic process

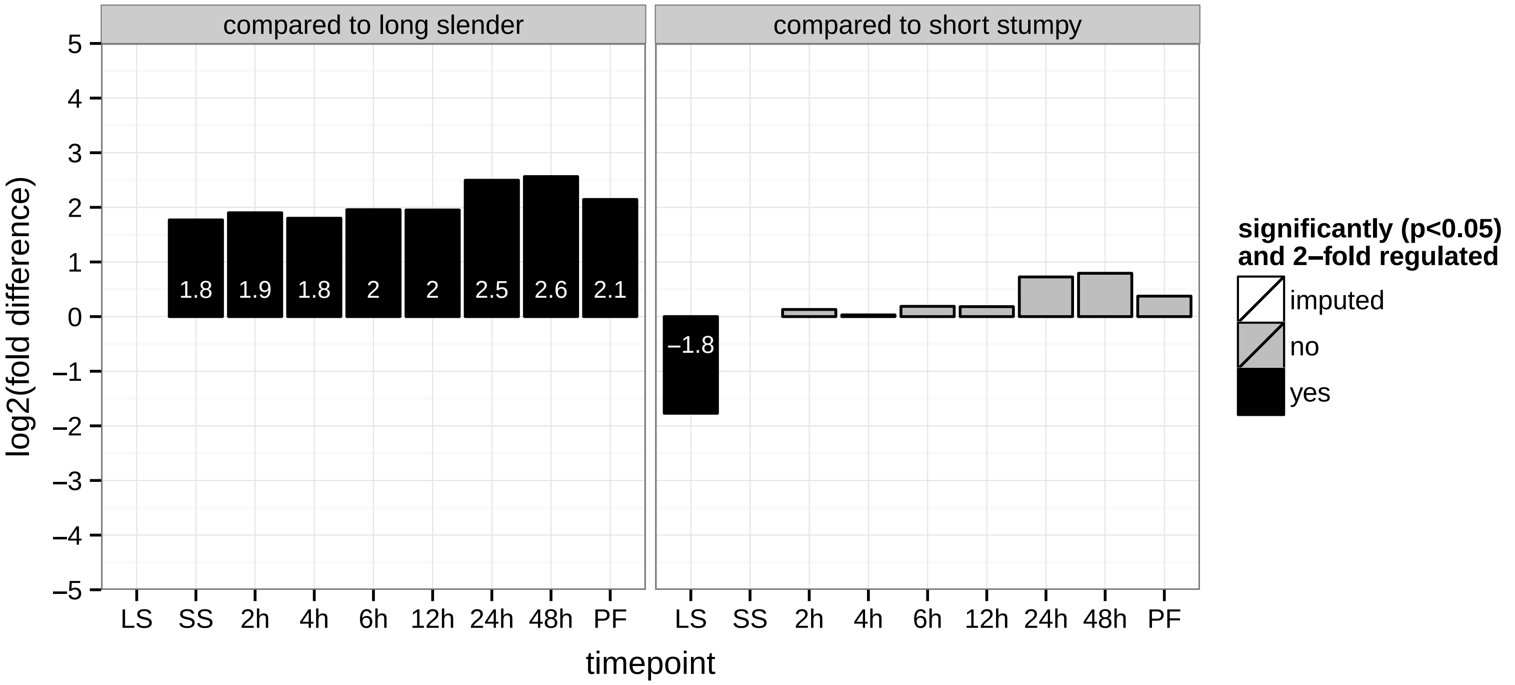

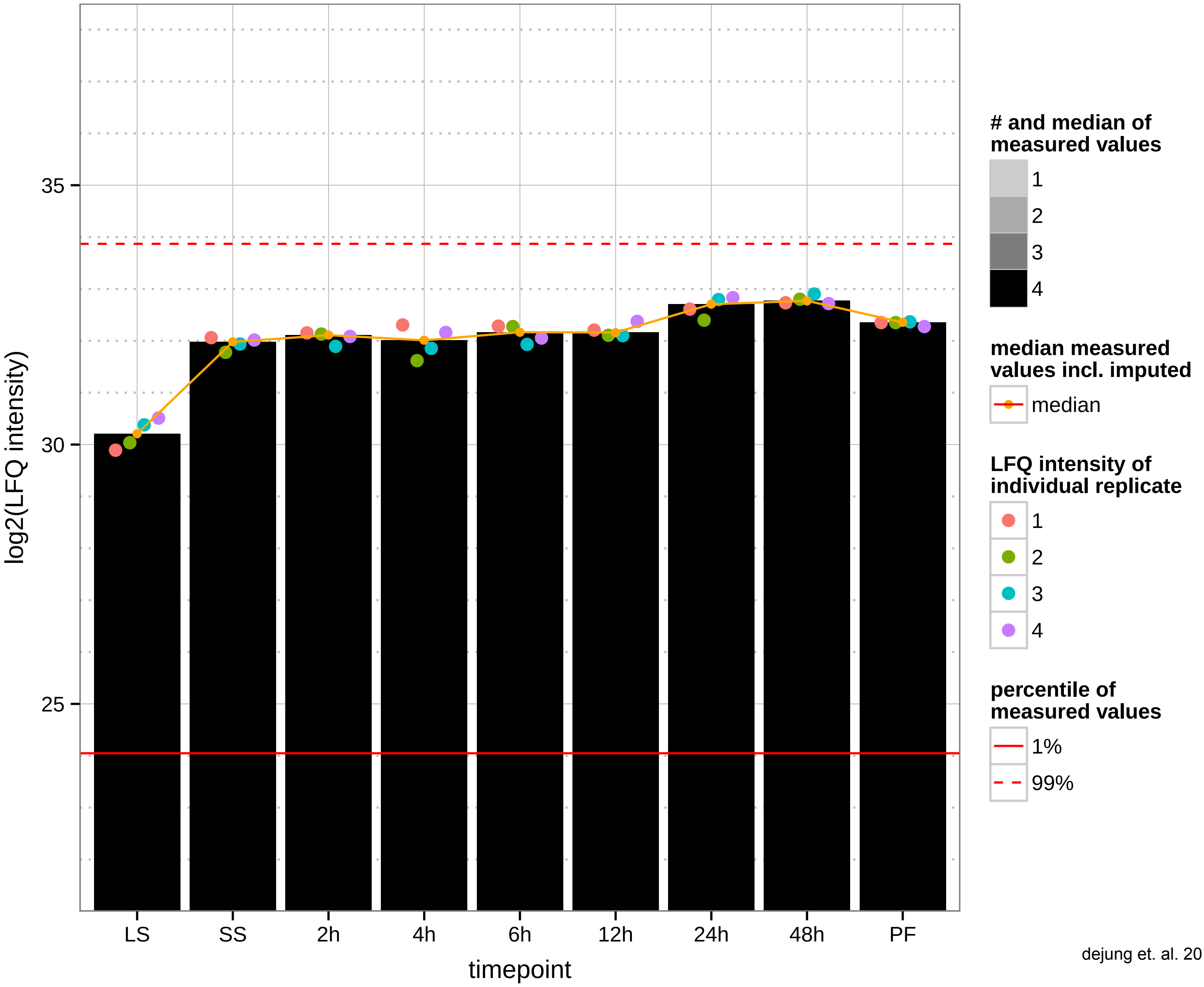

dejung et. al. 2015

hypothetical protein, conserved
Tb927.10.12630
AGOF: calcium ion binding
AGOC: null
AGOP: null
PGOF: calcium ion binding
PGOC: null
PGOP: null

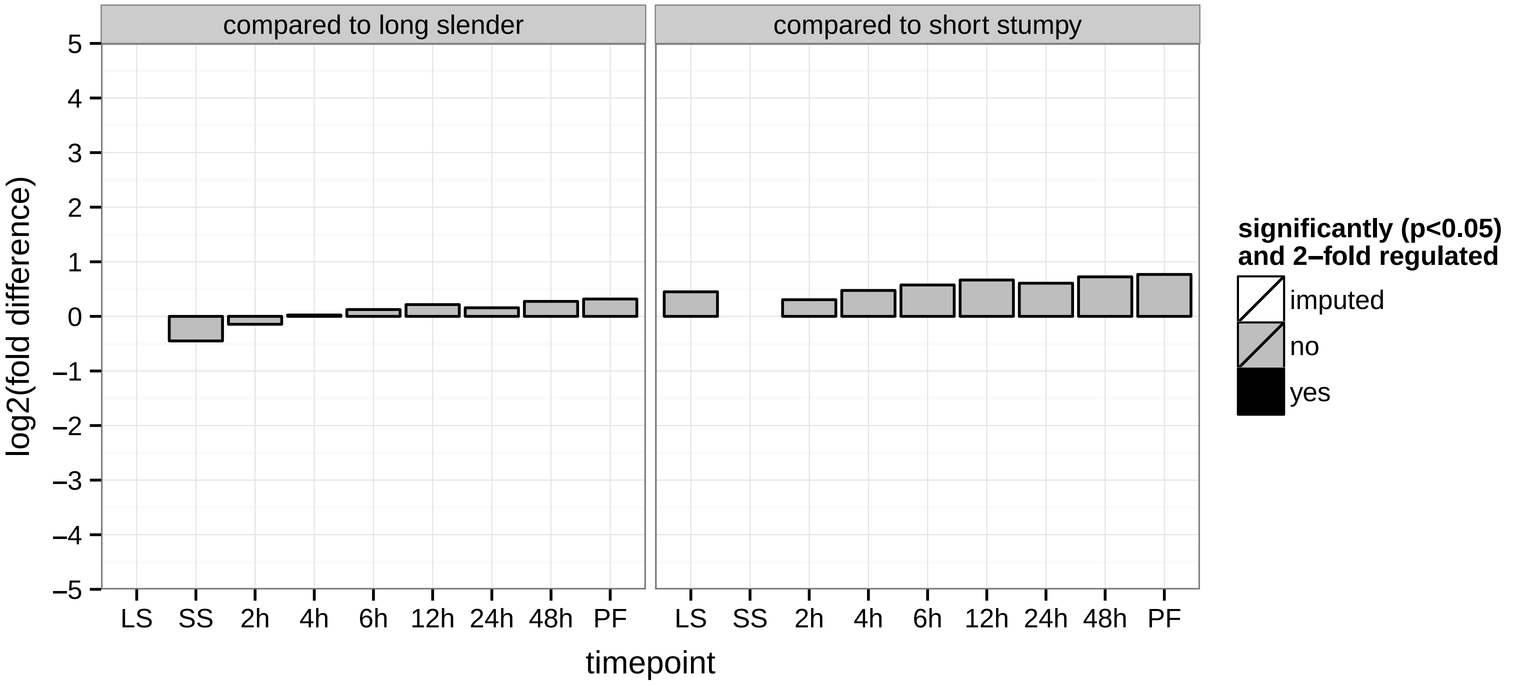

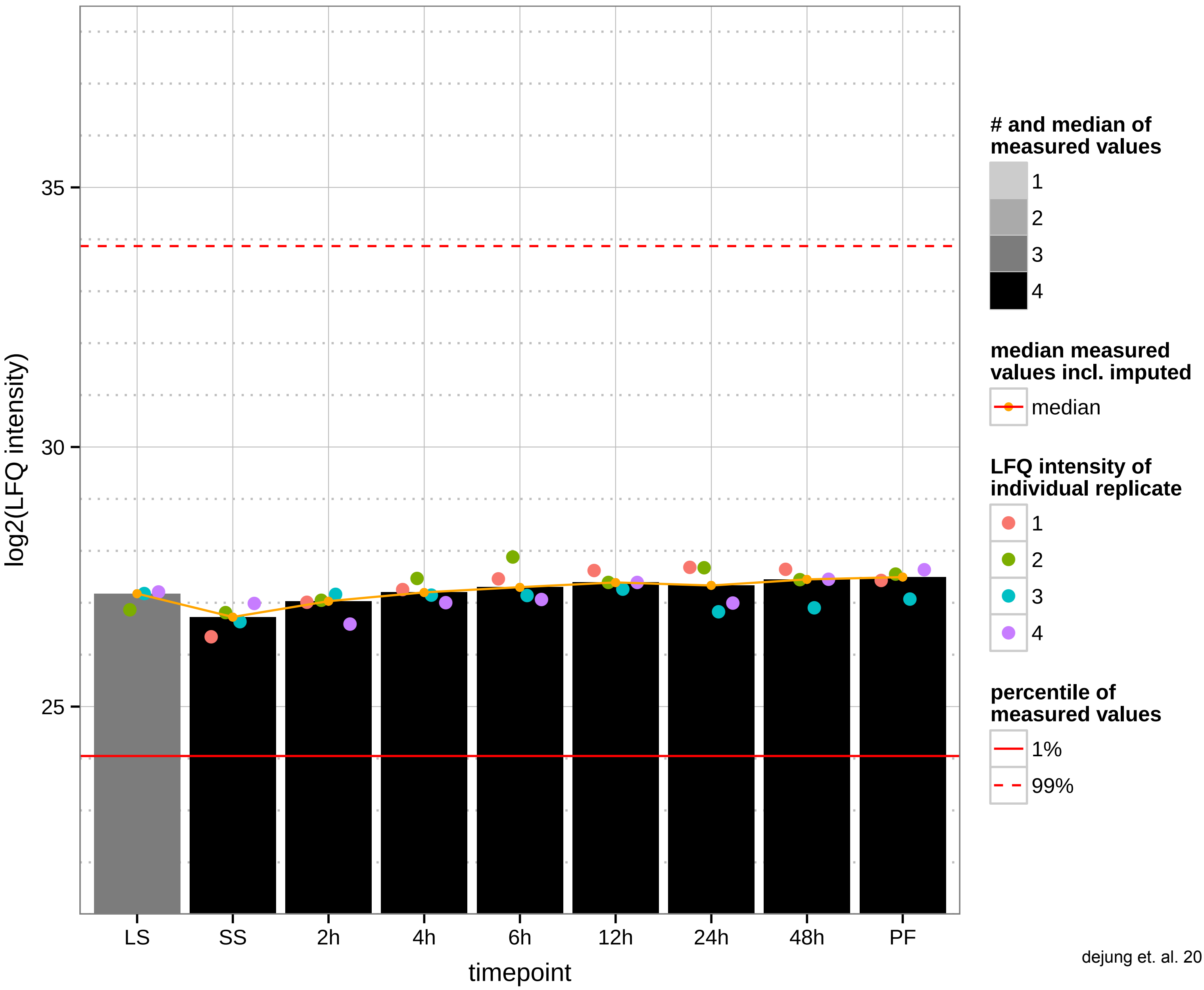

dejung et. al. 2015

60S ribosomal protein L34, putative
Tb927.11.9730;Tb927.10.12680
AGOF: structural constituent of ribosome
AGOC: intracellular, ribosome
AGOP: translation
PGOF: structural constituent of ribosome
PGOC: intracellular, ribosome
PGOP: translation

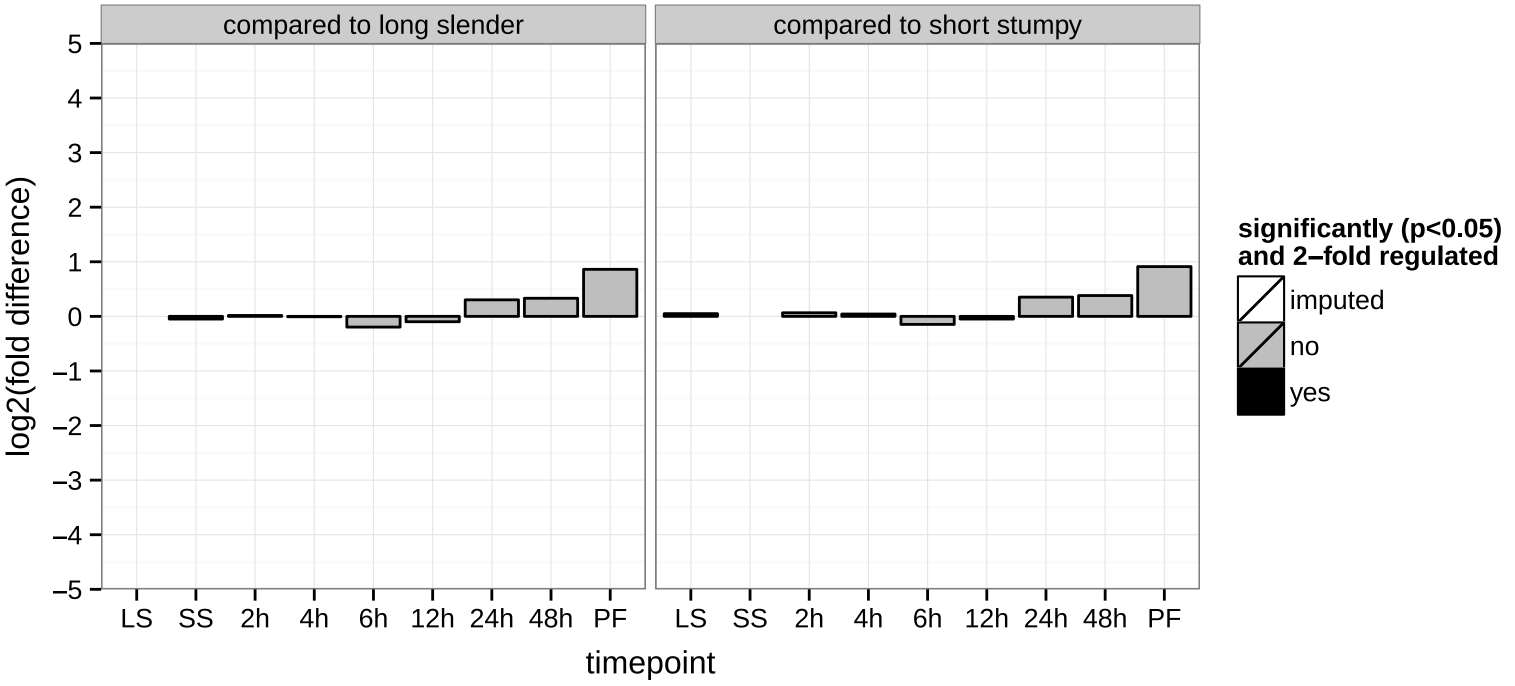

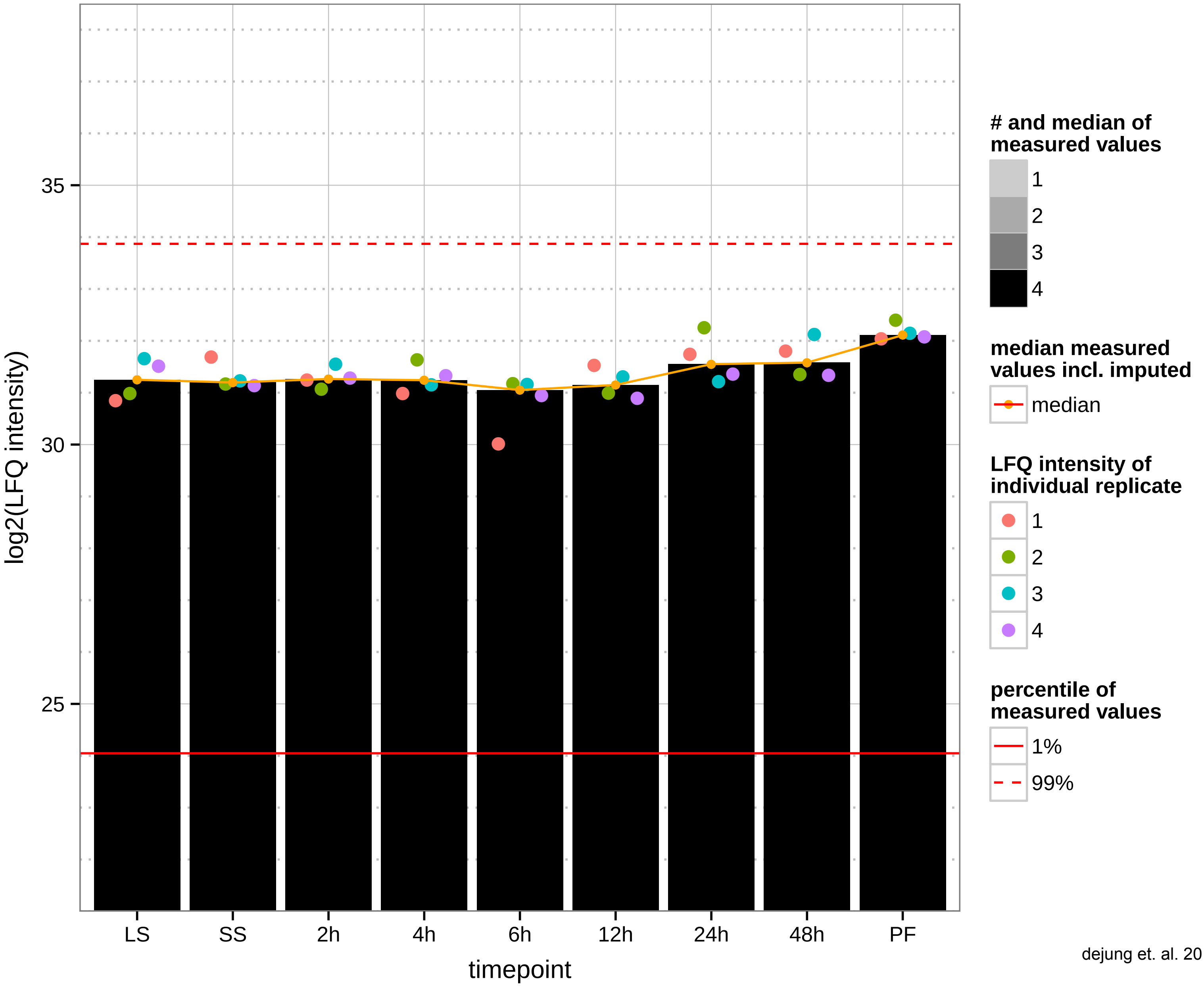

dejung et. al. 2015

pyruvate dehydrogenase E1 alpha subunit, putative
Tb927.10.12700
AGOF: oxidoreductase activity, acting on the aldehyde or oxo group of donors, disulfide as acceptor
AGOC: mitochondrion
AGOP: null
PGOF: oxidoreductase activity, acting on the aldehyde or oxo group of donors, disulfide as acceptor
PGOC: null
PGOP: metabolic process

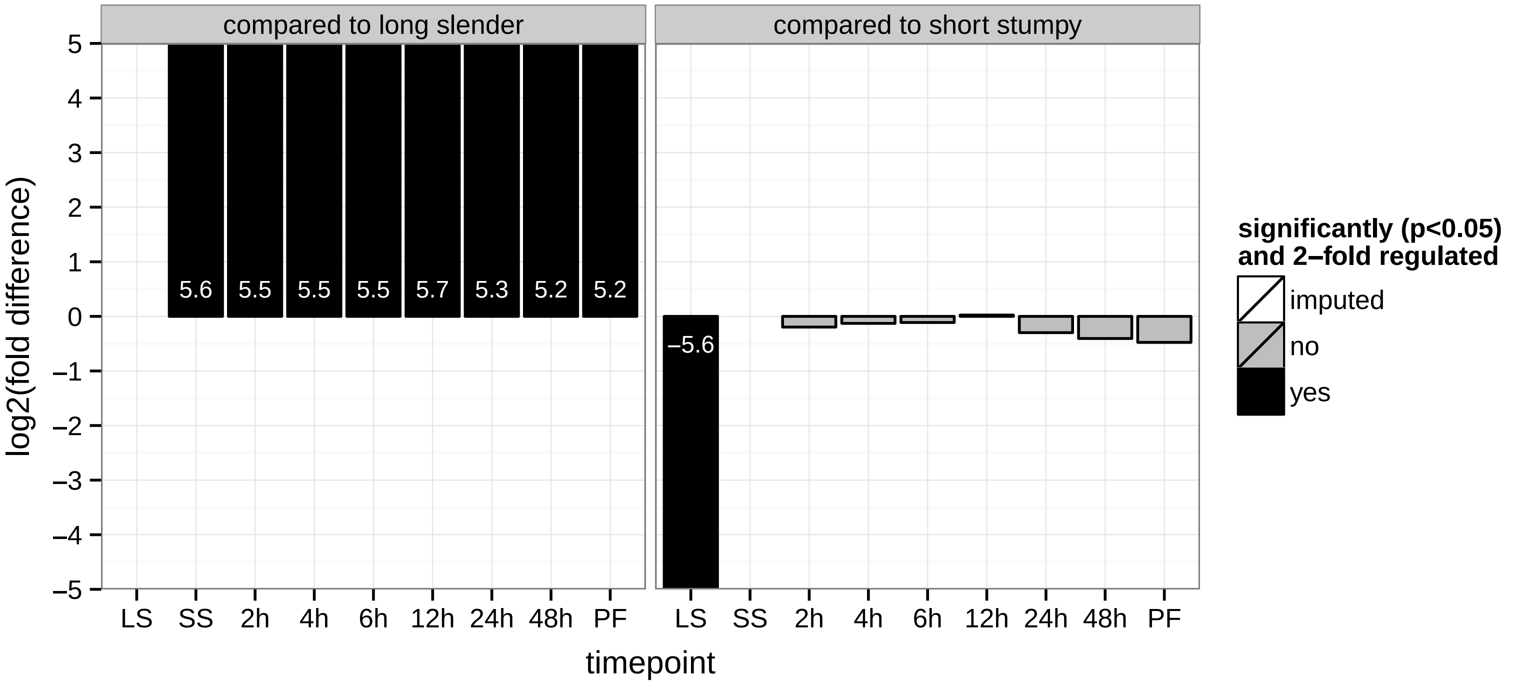

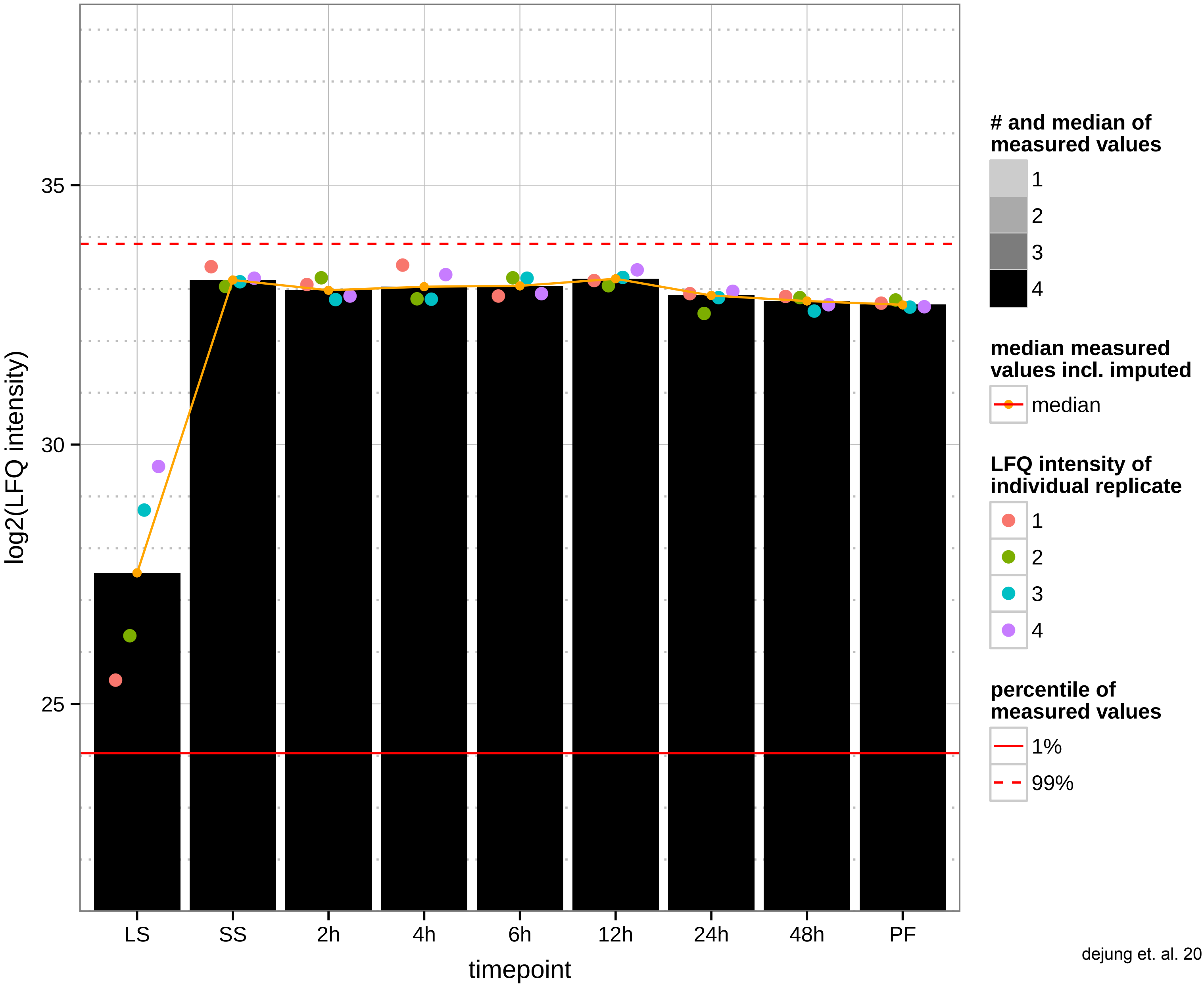

dejung et. al. 2015

heat shock protein 110, putative
Tb927.10.12710
AGOF: ATP binding
AGOC: null
AGOP: null
PGOF: null
PGOC: null
PGOP: null

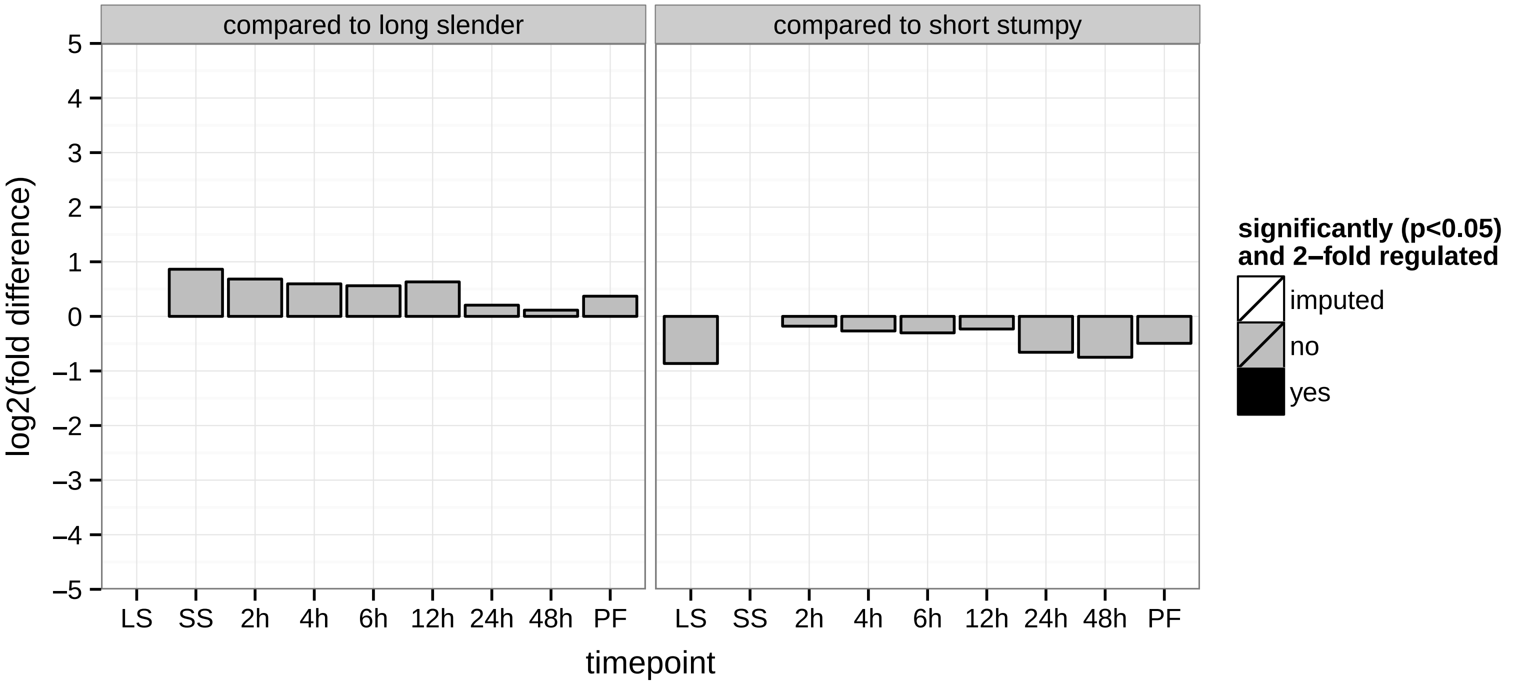

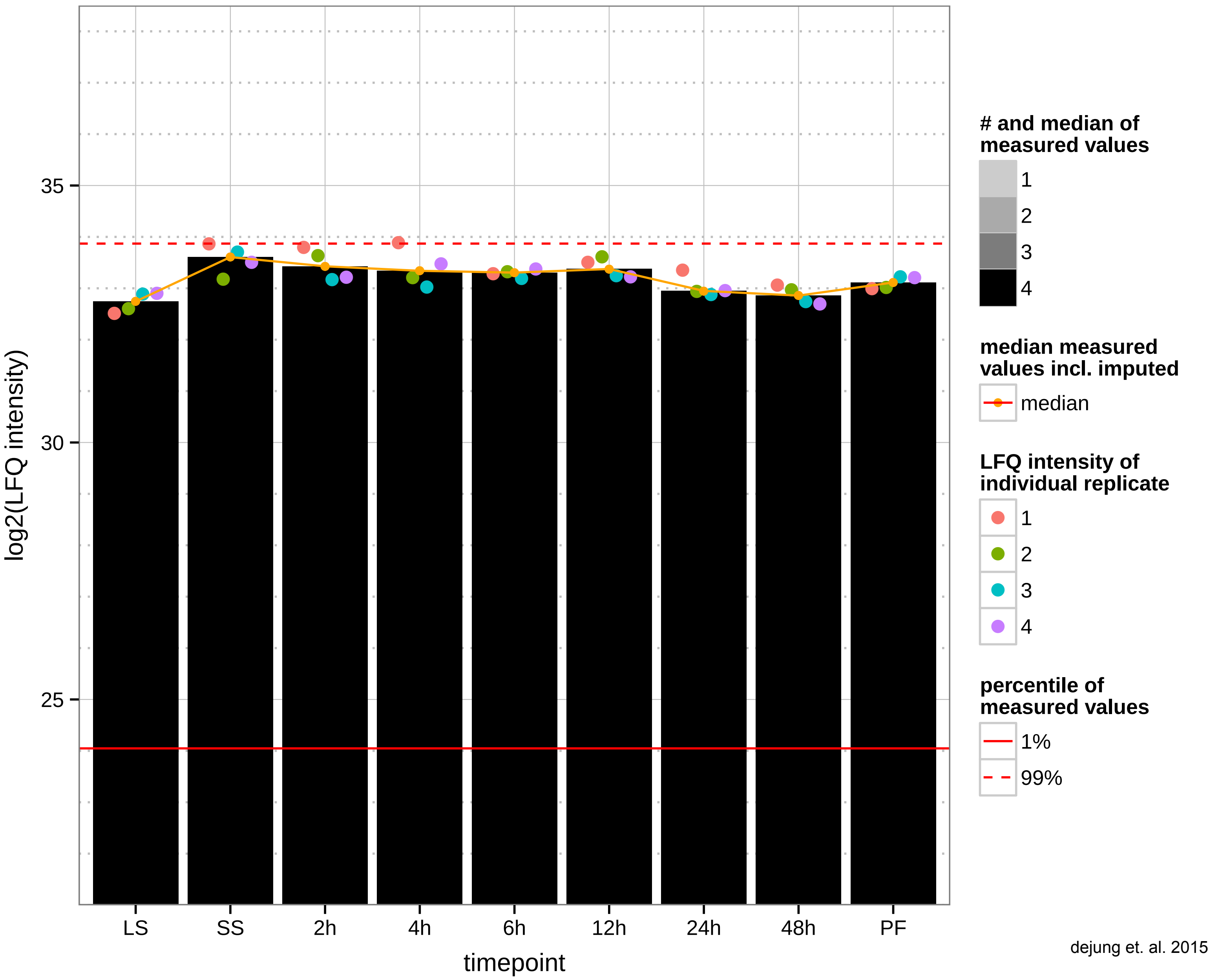

dejung et. al. 2015

mitochondrial carrier protein (MCP12)
Tb927.10.12840
AGOF: null
AGOC: membrane, mitochondrial inner membrane, mitochondrial part
AGOP: transport
PGOF: null
PGOC: null
PGOP: null

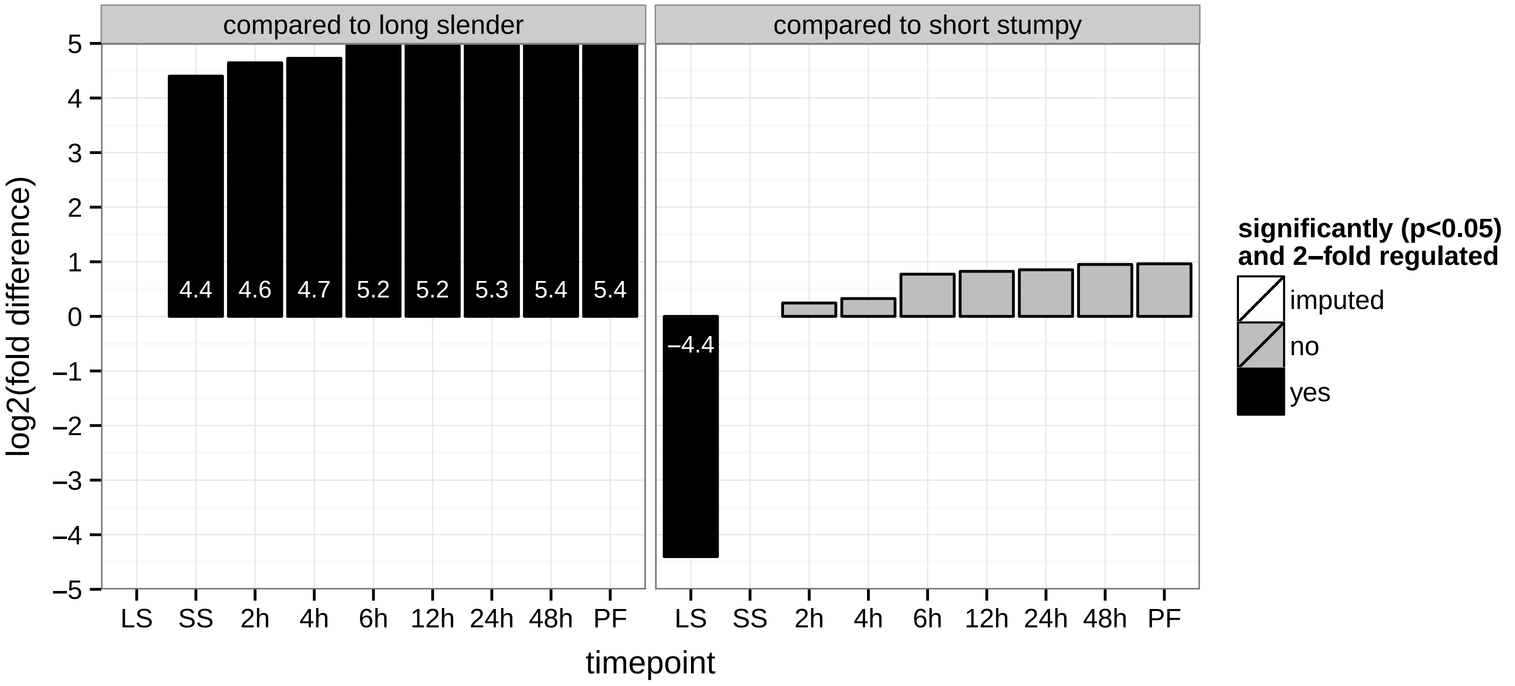

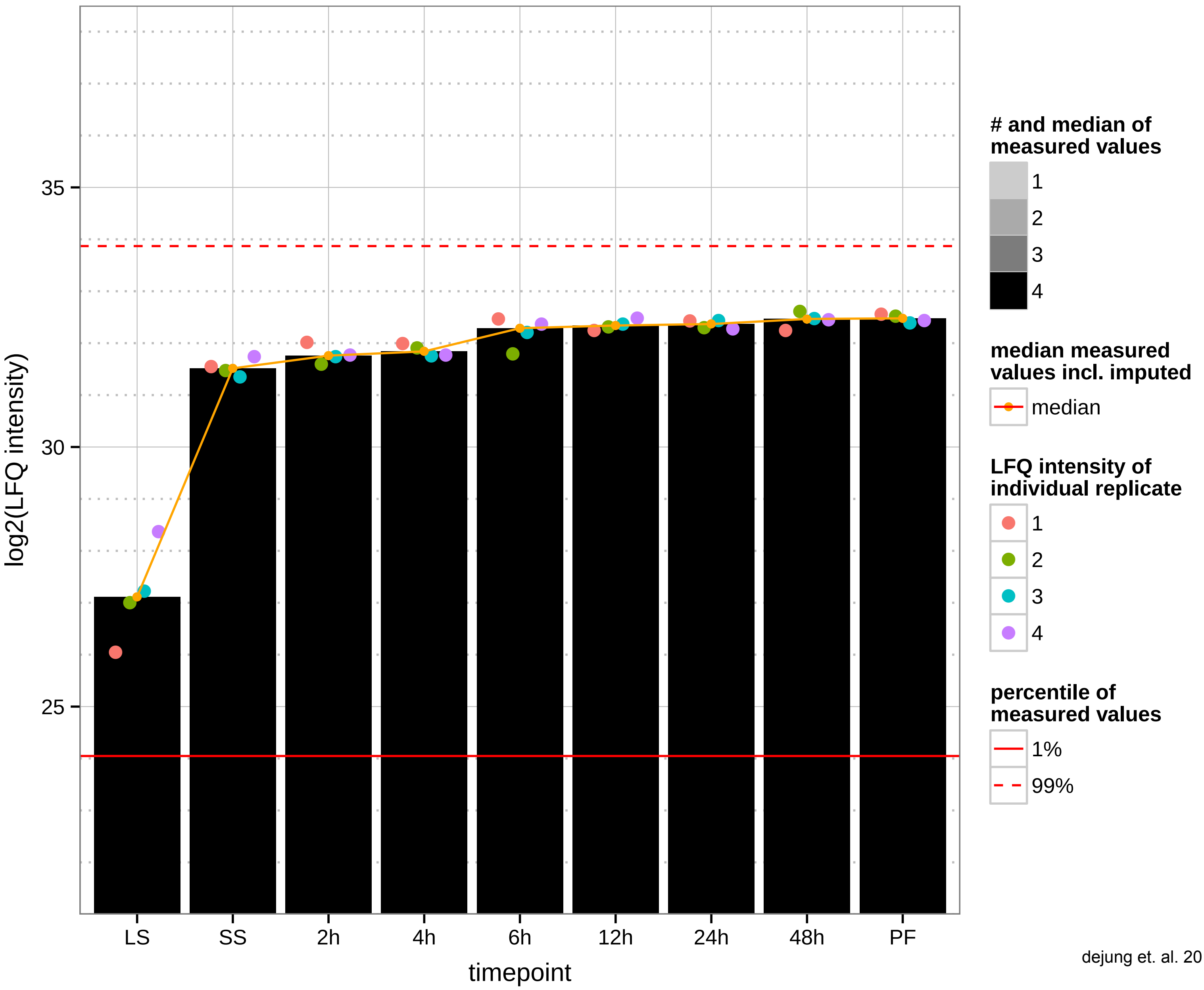

dejung et. al. 2015

ttaggg binding factor
Tb927.10.12850
AGOF: null
AGOC: null
AGOP: null
PGOF: DNA binding
PGOC: null
PGOP: null

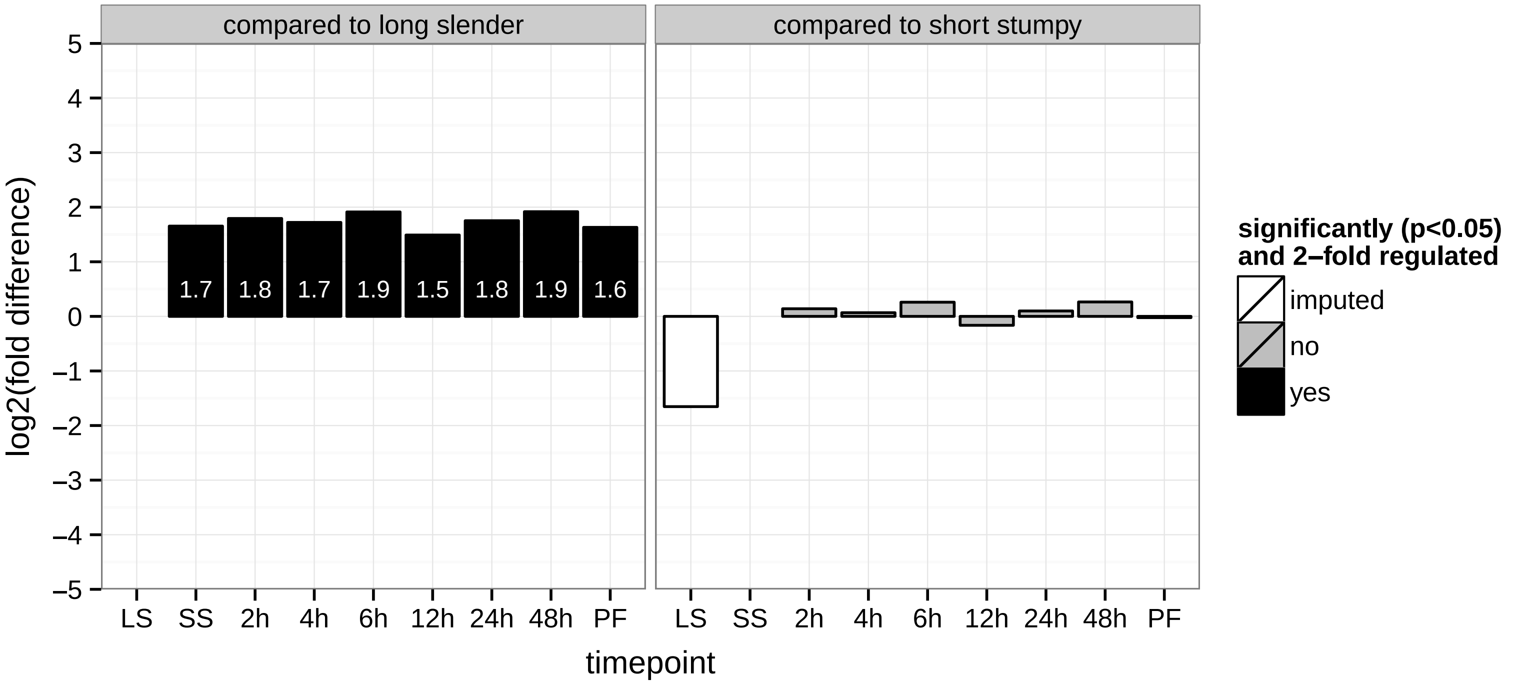

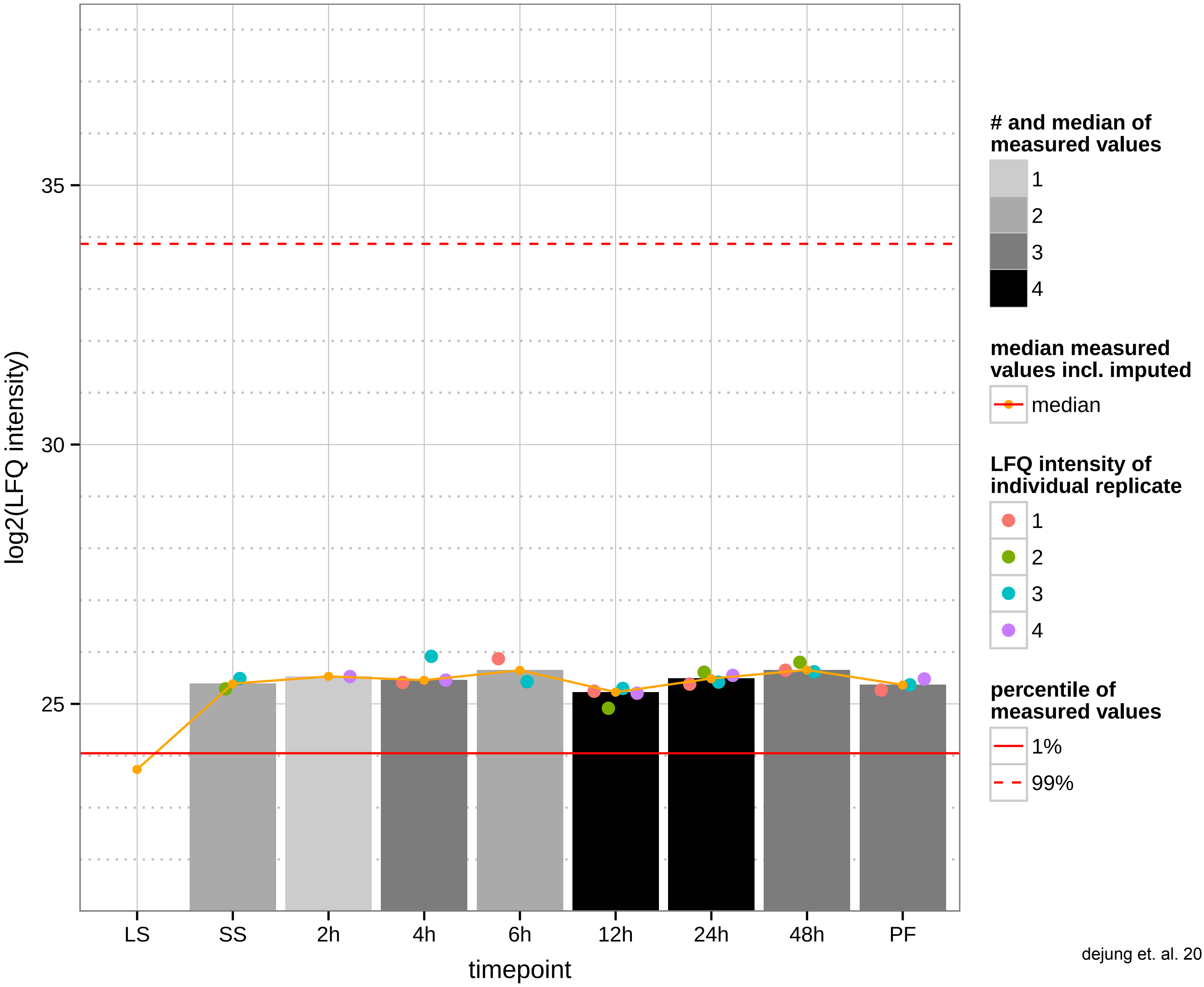

dejung et. al. 2015

bifunctional aminoacyl–tRNA synthetase, putative
Tb927.10.12890
AGOF: ATP binding, proline–tRNA ligase activity
AGOC: cytoplasm, mitochondrion
AGOP: prolyl–tRNA aminoacylation, translation
PGOF: ATP binding, aminoacyl–tRNA ligase activity, nucleotide binding, proline–tRNA ligase activity
PGOC: cytoplasm
PGOP: prolyl–tRNA aminoacylation, tRNA aminoacylation for protein translation

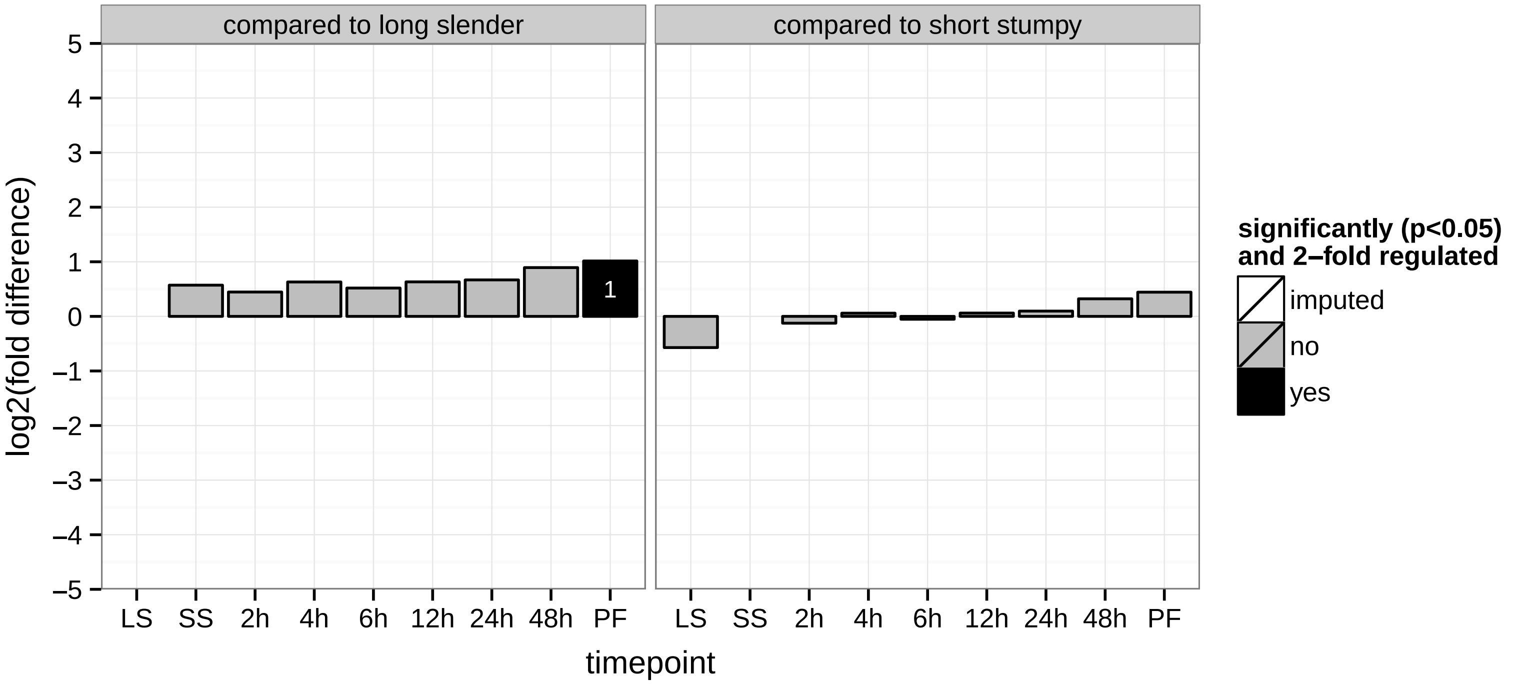

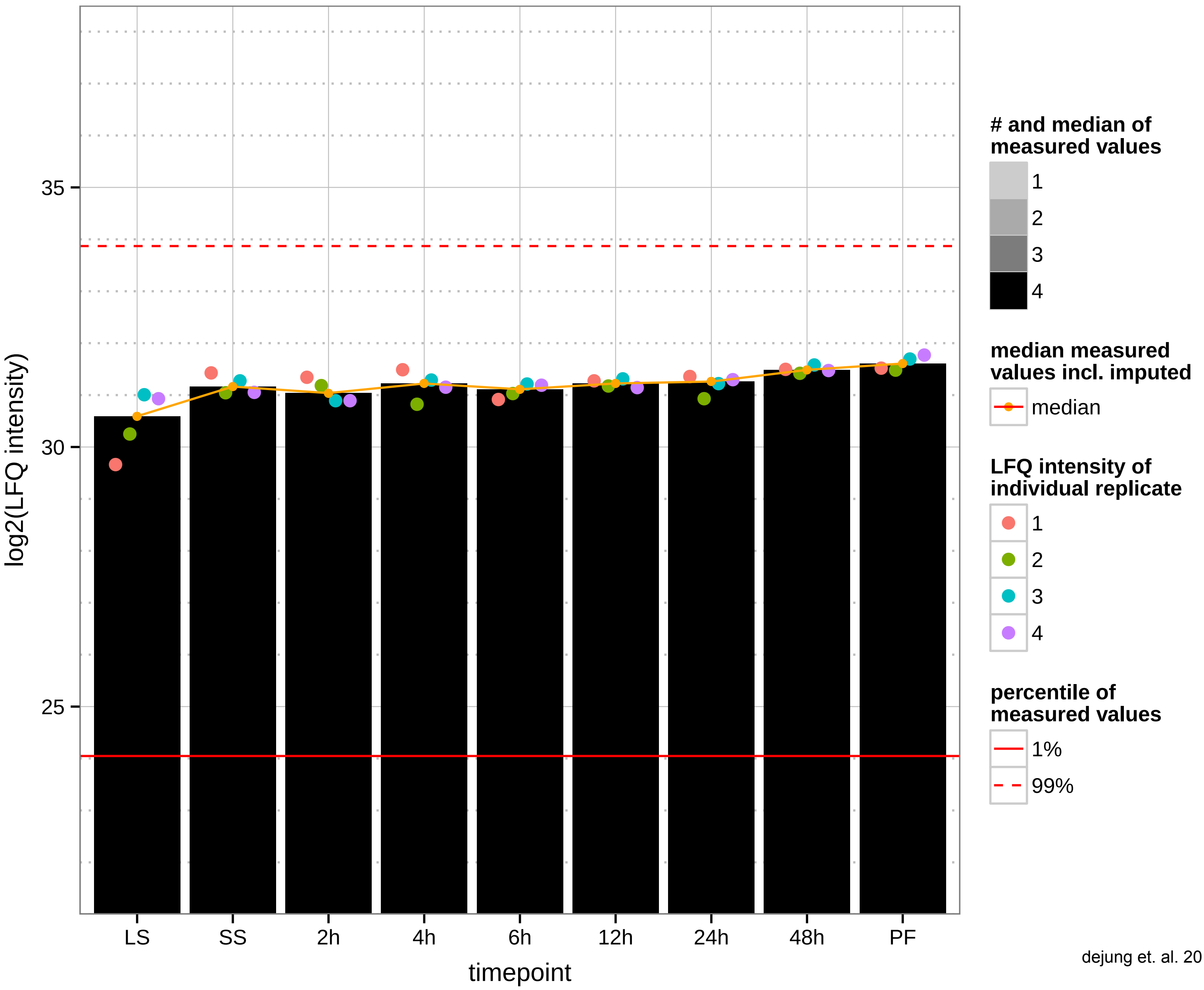

dejung et. al. 2015

Multisite−specific tRNA:(cytosine−C(5))−methyltransferase, putative, NCL1(Yeast)−related NOL1/NOP2/FMU(SUN) domain−c
Tb927.10.12980
AGOF: null
AGOC: mitochondrion
AGOP: tRNA methylation
PGOF: null
PGOC: null
PGOP: null

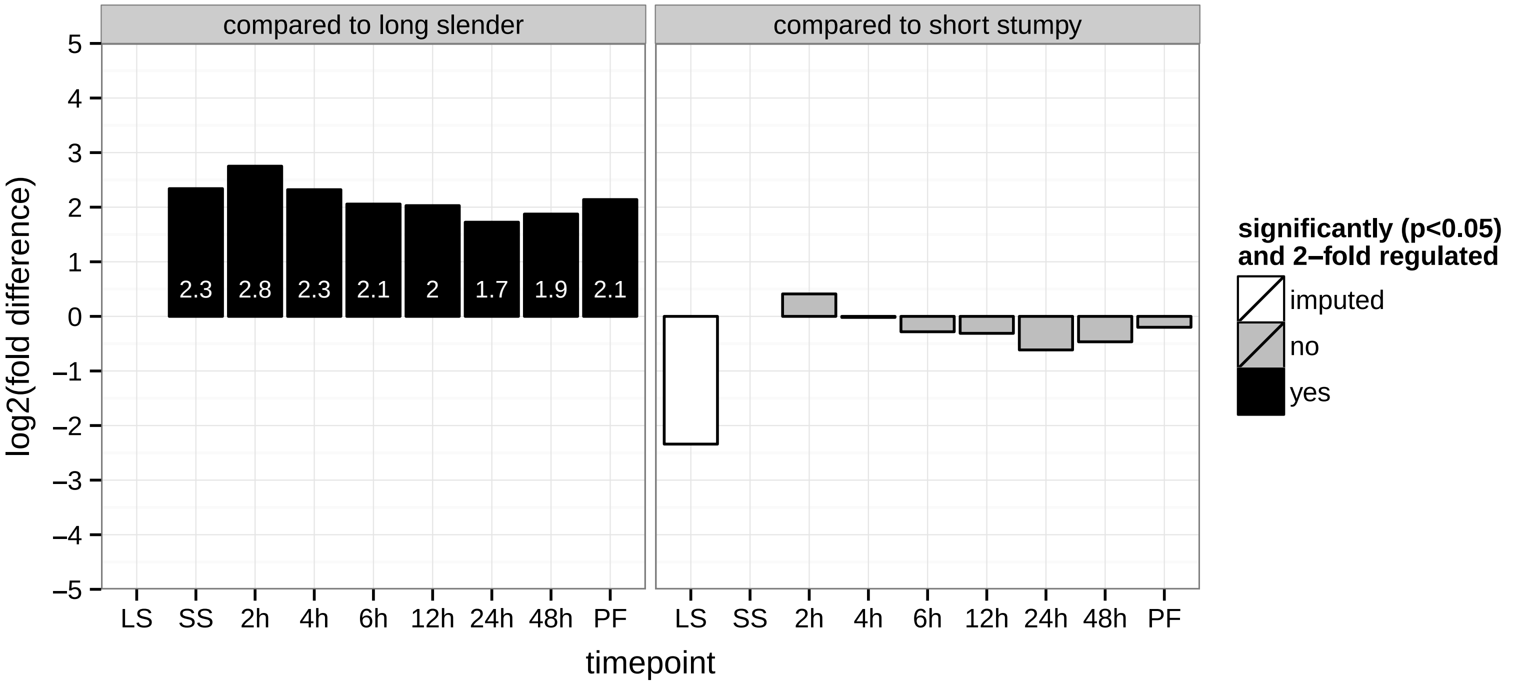

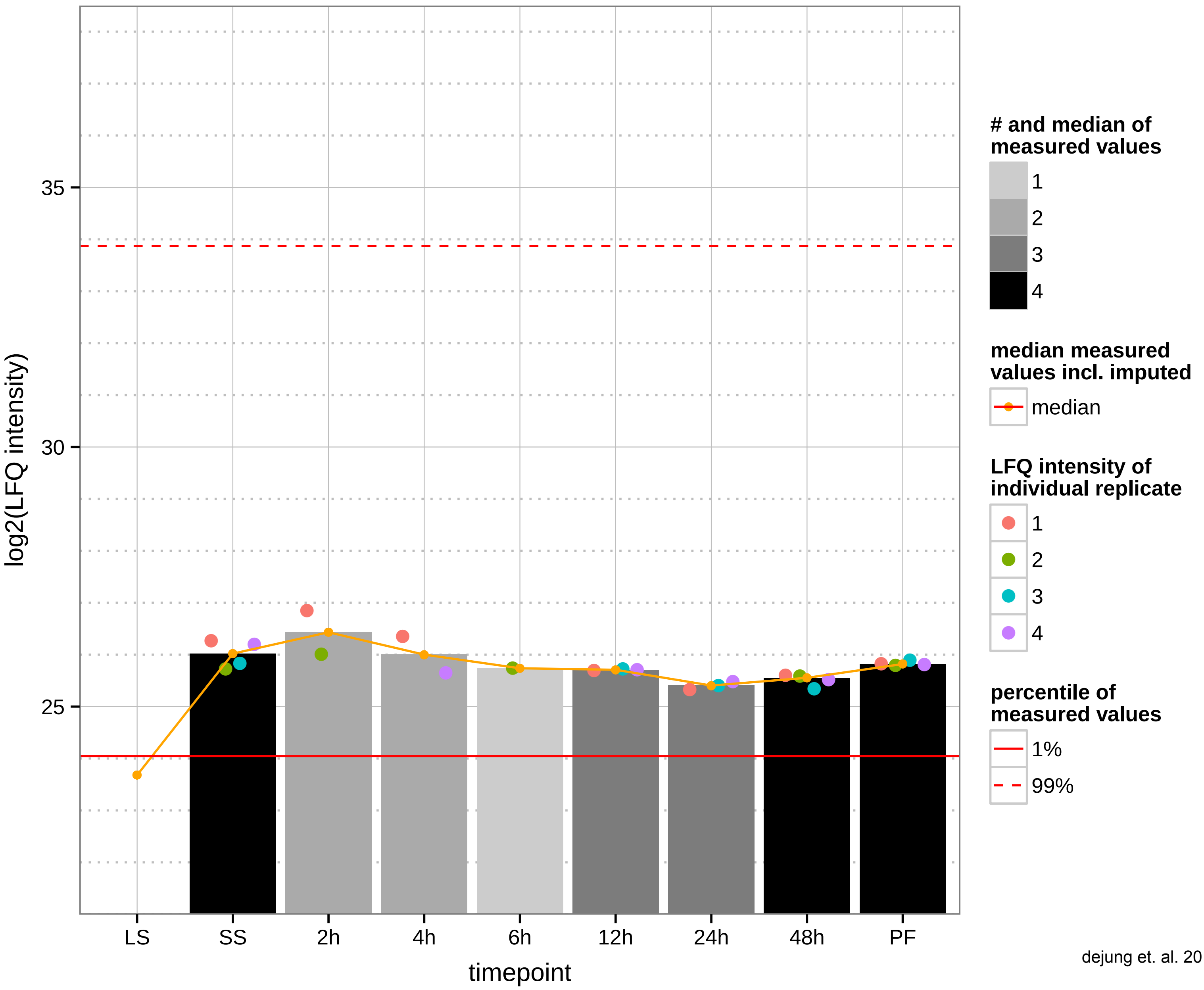

dejung et. al. 2015

predicted ankyrin repeat family protein
Tb927.10.12990
AGOF: null
AGOC: null
AGOP: null
PGOF: protein binding
PGOC: null
PGOP: null

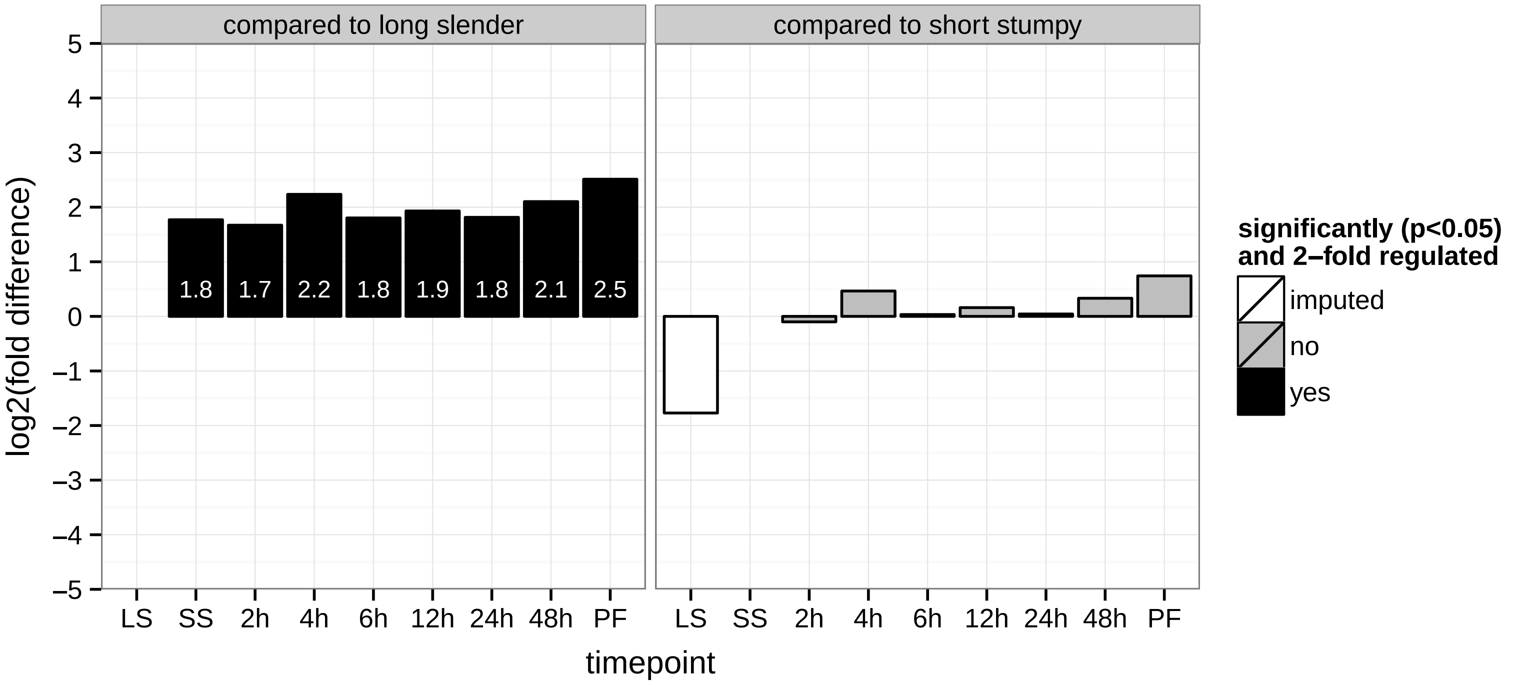

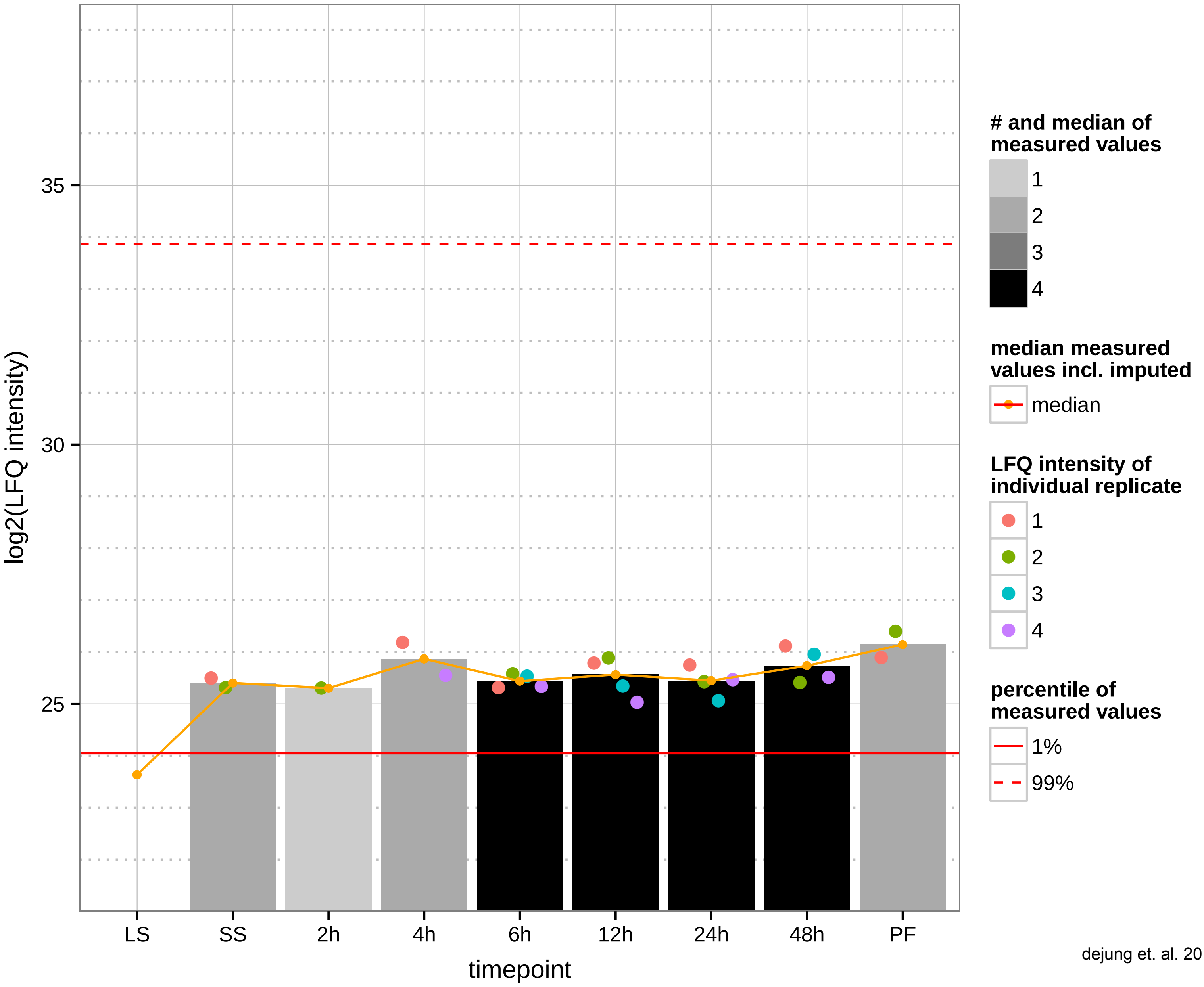

dejung et. al. 2015

receptor–type adenylate cyclase GRESAG 4, putative, gene related to ESAG4, putative (GRESAG4)
Tb927.10.13040
AGOF: adenylate cyclase activity
AGOC: null
AGOP: cyclic nucleotide biosynthetic process, intracellular signal transduction
PGOF: phosphorus–oxygen lyase activity
PGOC: null
PGOP: cyclic nucleotide biosynthetic process, intracellular signal transduction

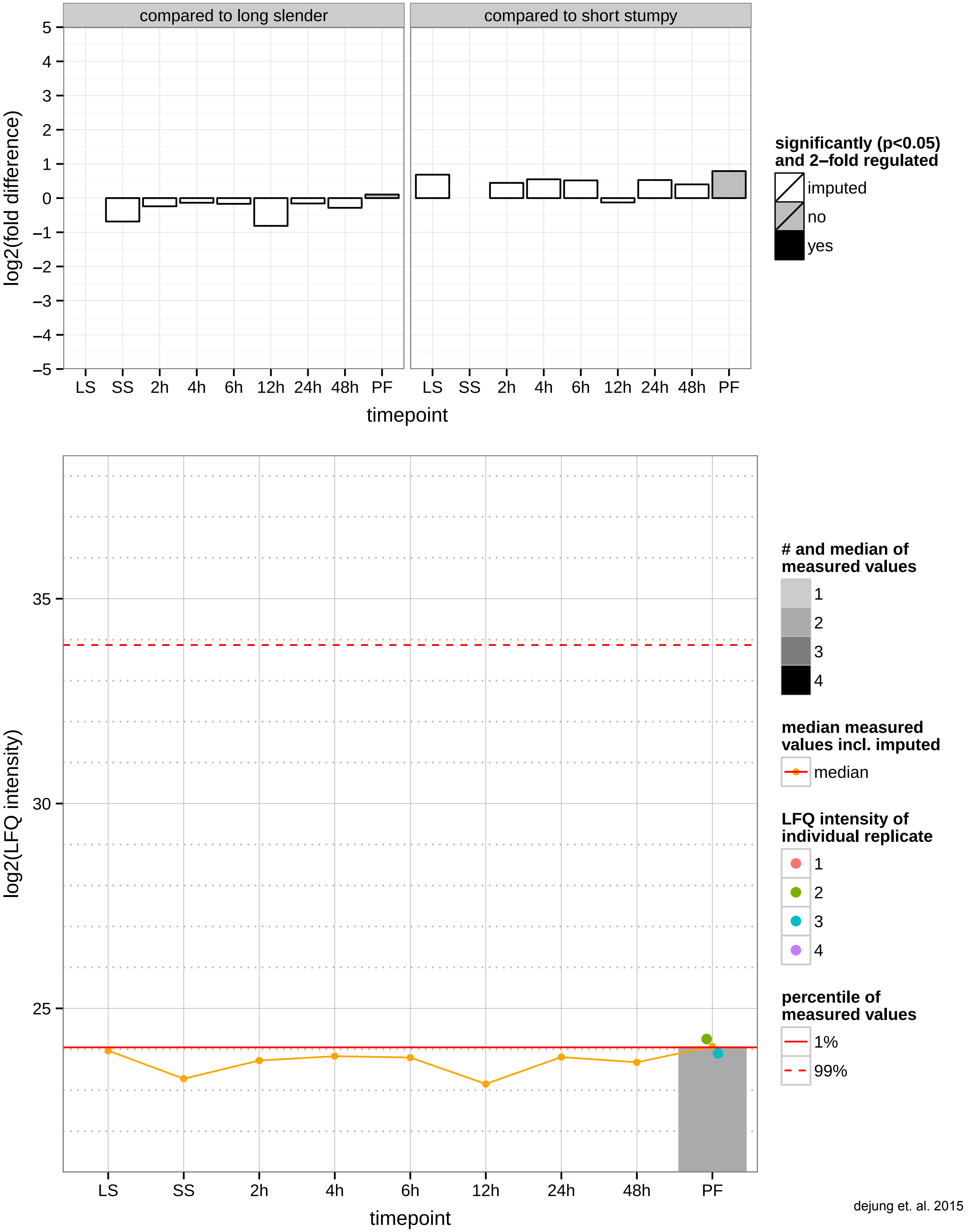

dejung et. al. 2015

Dynein light chain roadblock–type protein, putative
Tb927.10.13110
AGOF: microtubule motor activity
AGOC: cytoplasmic dynein complex
AGOP: microtubule–based movement
PGOF: null
PGOC: null
PGOP: null

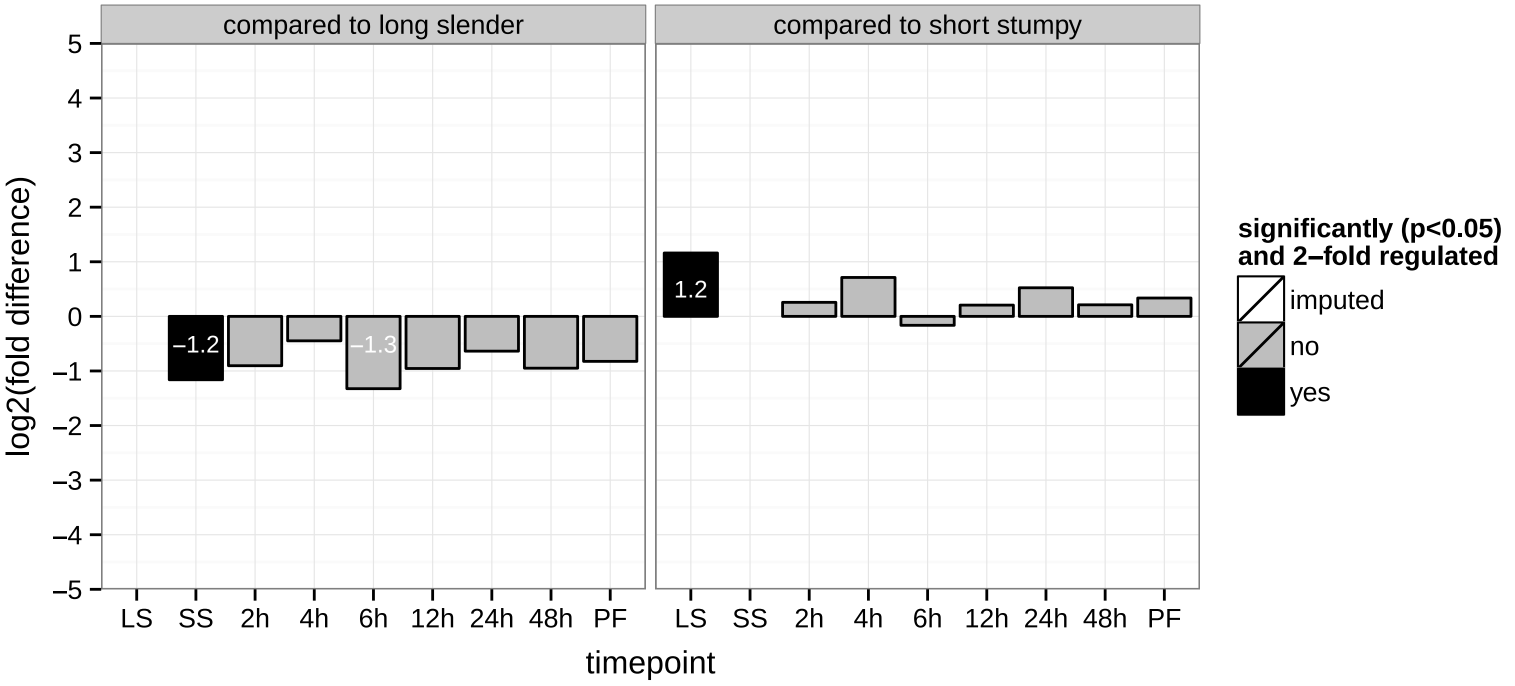

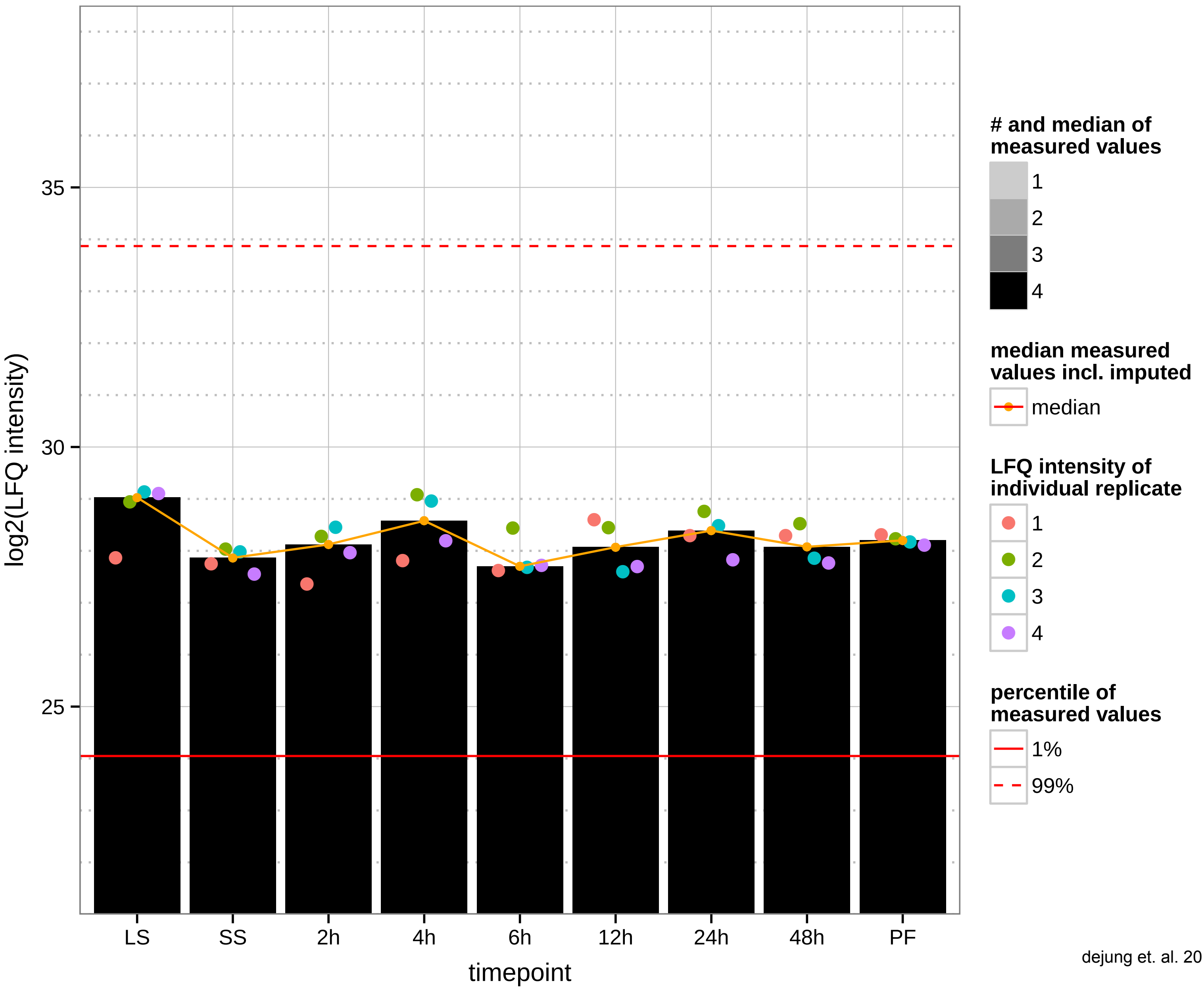

dejung et. al. 2015

UTP−glucose−1−phosphate uridylyltransferase
Tb927.10.13130
AGOF: UTP:glucose−1−phosphate uridylyltransferase activity, metal ion binding, nucleotidyltransferase activity
AGOC: glycosome
AGOP: UDP−glucose metabolic process
PGOF: nucleotidyltransferase activity
PGOC: null
PGOP: metabolic process

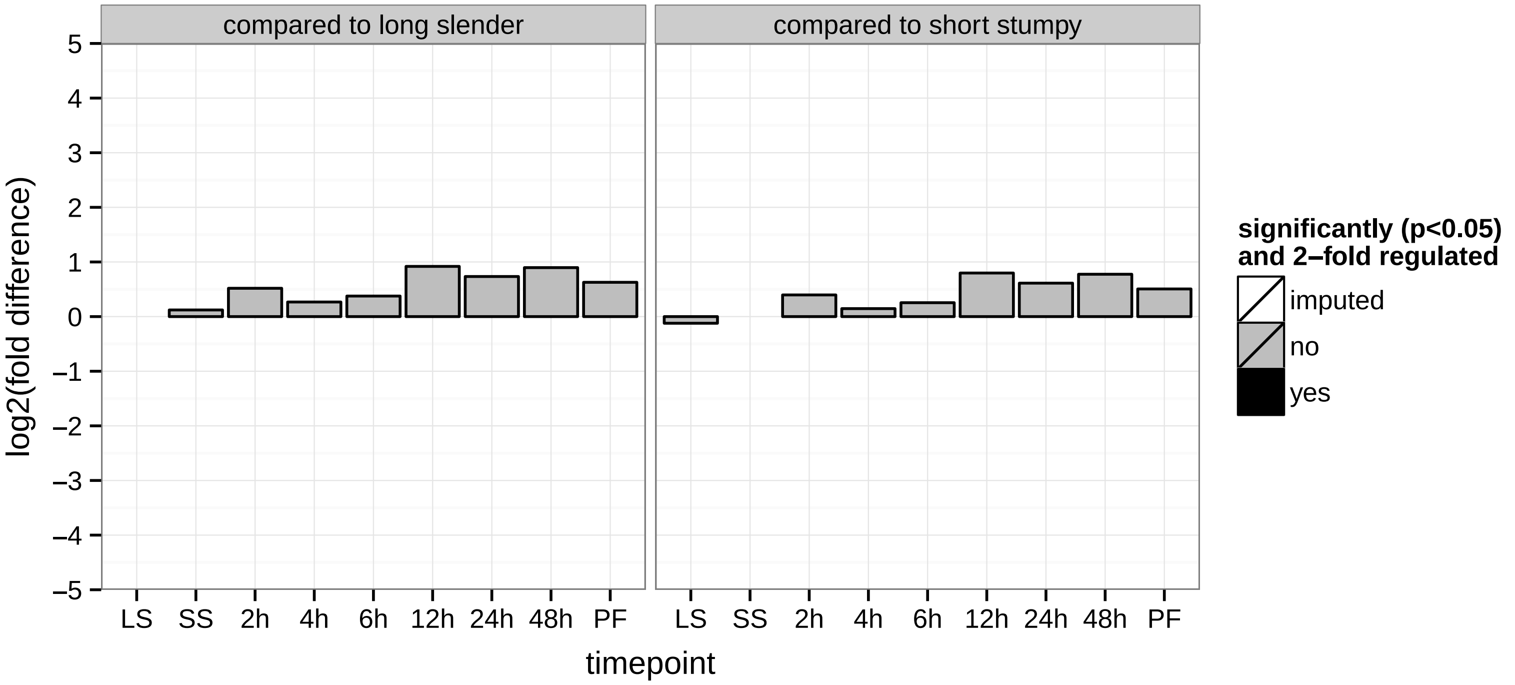

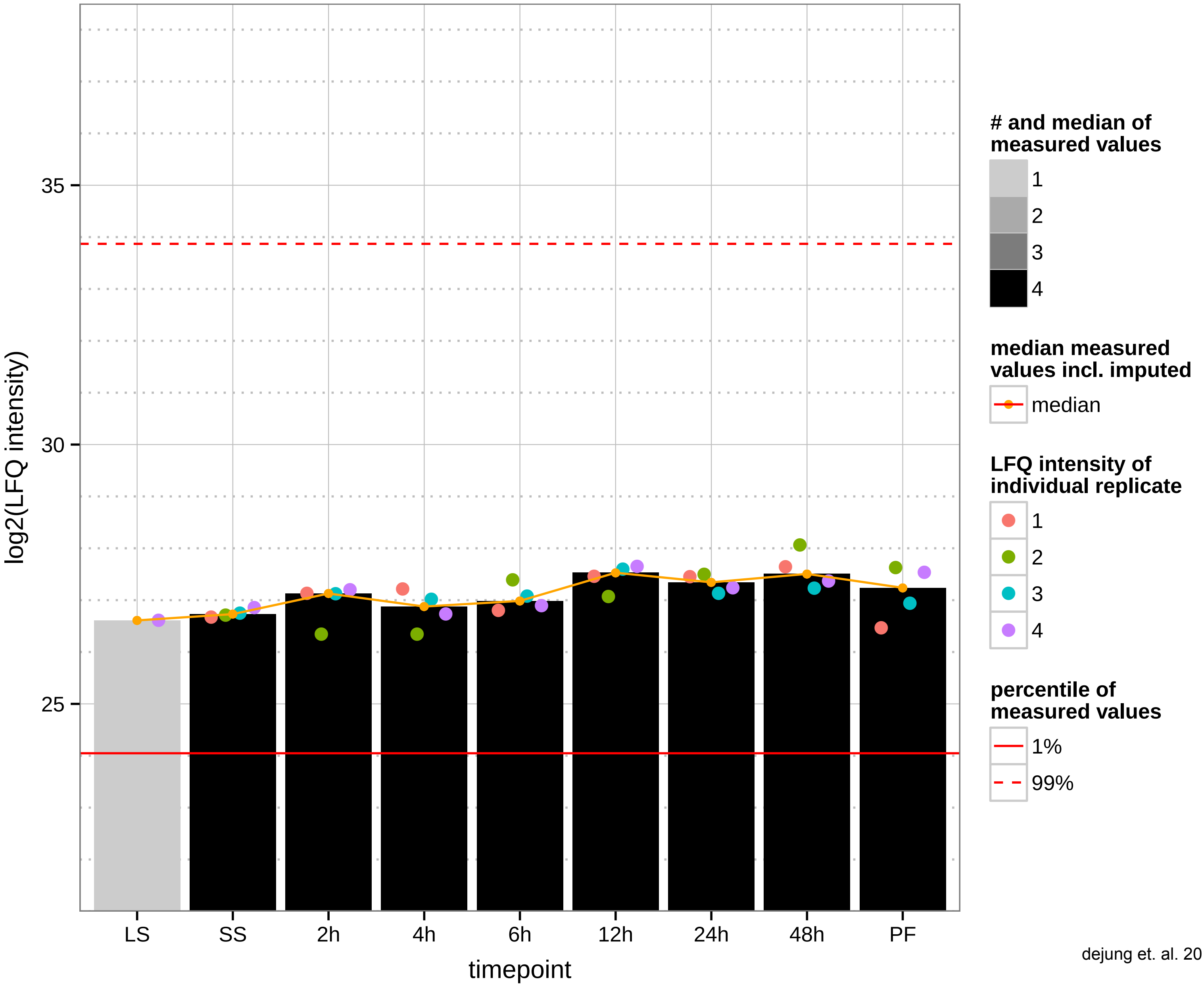

dejung et. al. 2015

hypothetical protein, conserved
Tb927.10.1320
AGOF: ATP binding, protein kinase activity
AGOC: null
AGOP: protein phosphorylation
PGOF: ATP binding, binding, protein kinase activity, transferase activity, transferring phosphorus–containing groups
PGOC: null
PGOP: protein phosphorylation

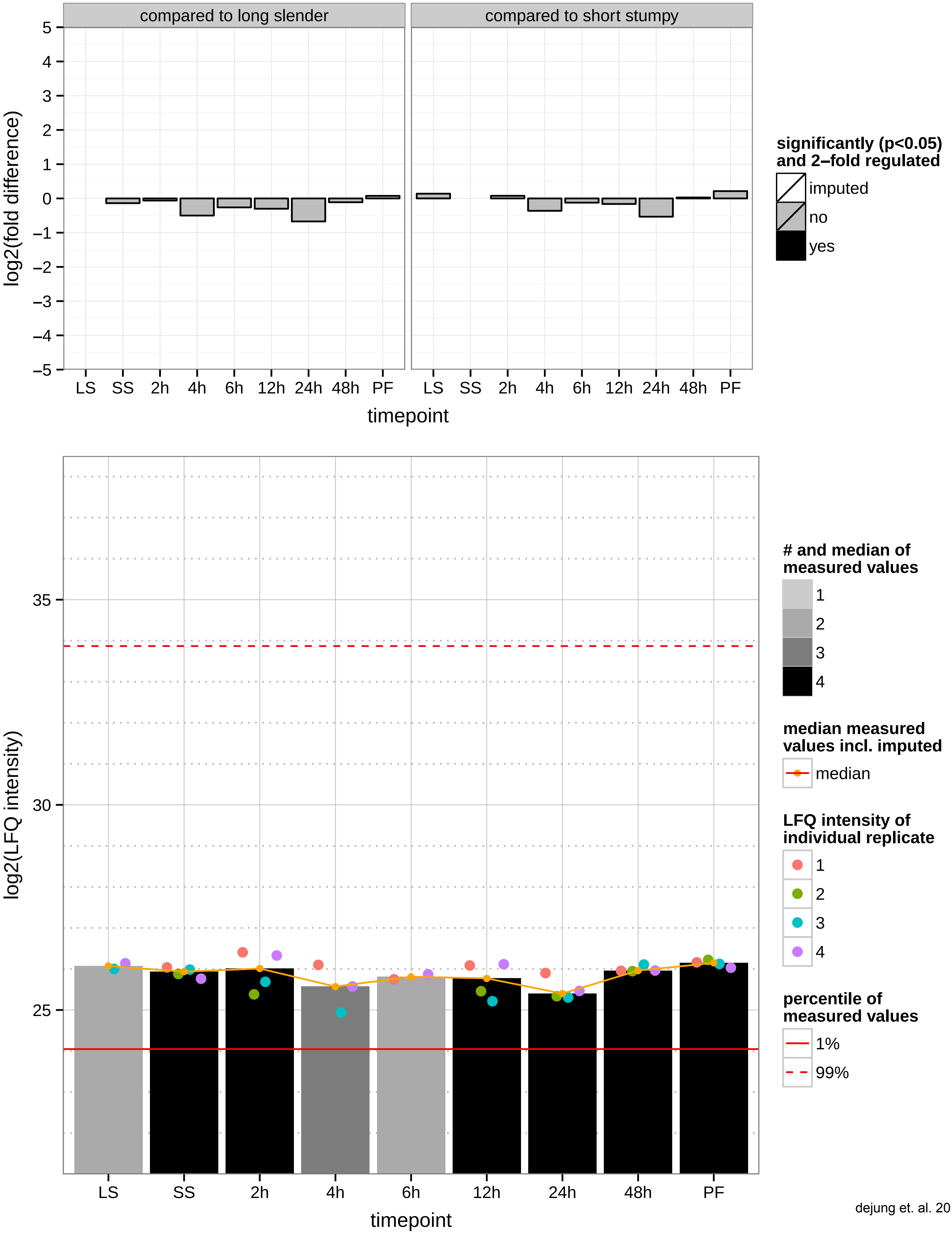

dejung et. al. 2015

hypothetical protein, conserved
Tb927.10.13250
AGOF: null
AGOC: null
AGOP: null
PGOF: protein binding
PGOC: null
PGOP: null

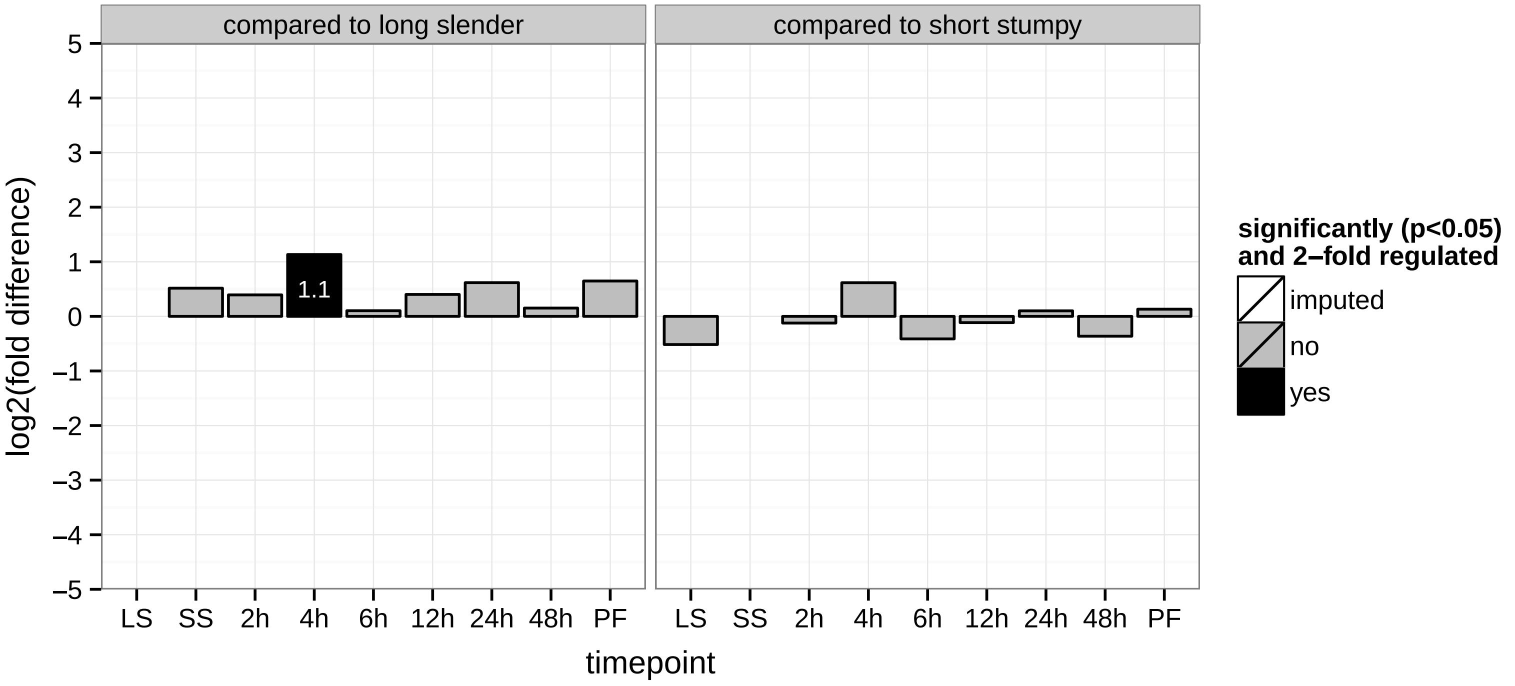

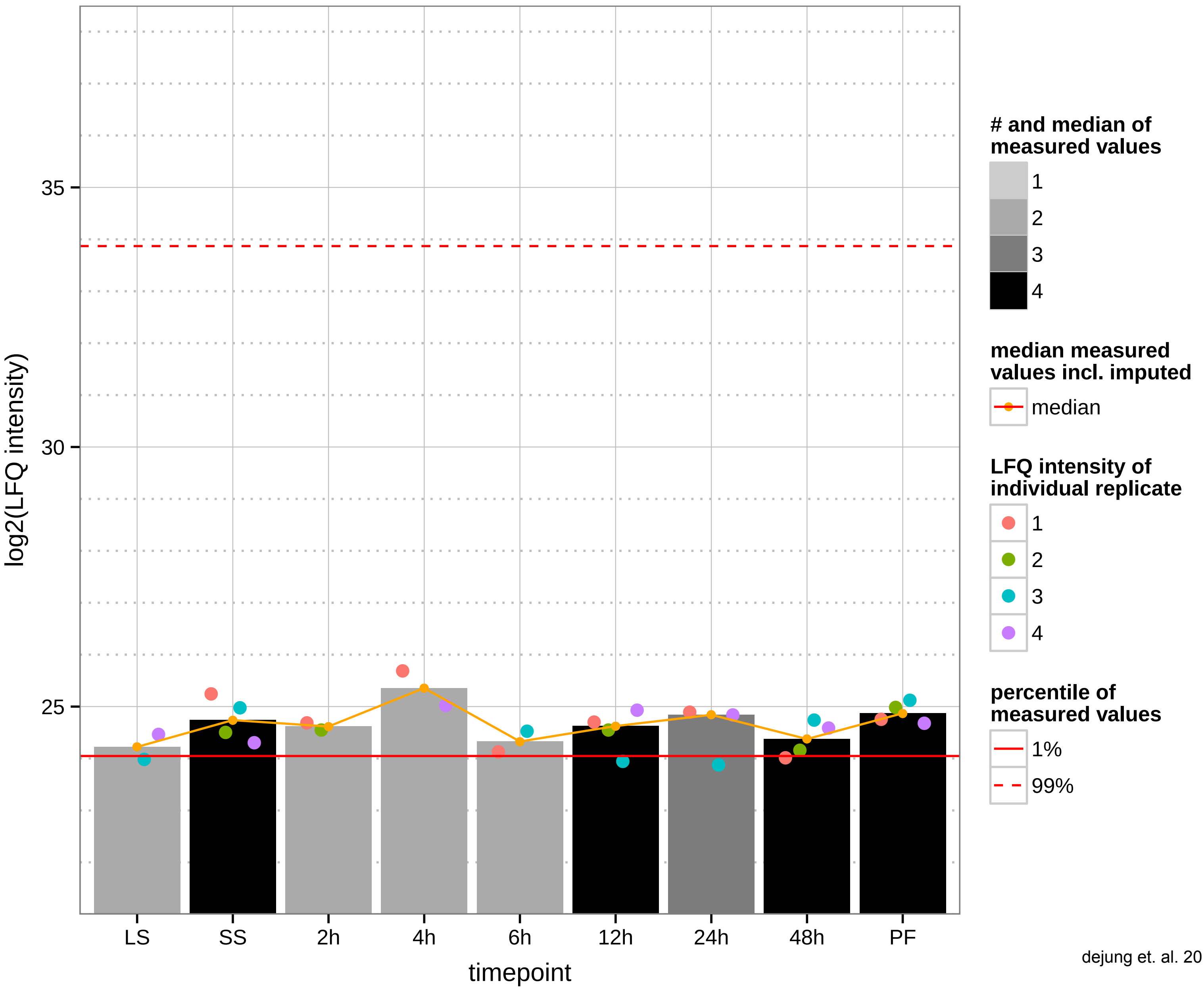

dejung et. al. 2015

DNA–directed RNA polymerase II/III subunit, putative (RPB5)
Tb927.10.13320
AGOF: DNA binding, DNA–directed RNA polymerase activity
AGOC: nucleus
AGOP: transcription, DNA–dependent
PGOF: DNA binding, DNA–directed RNA polymerase activity
PGOC: nucleus
PGOP: transcription, DNA–dependent

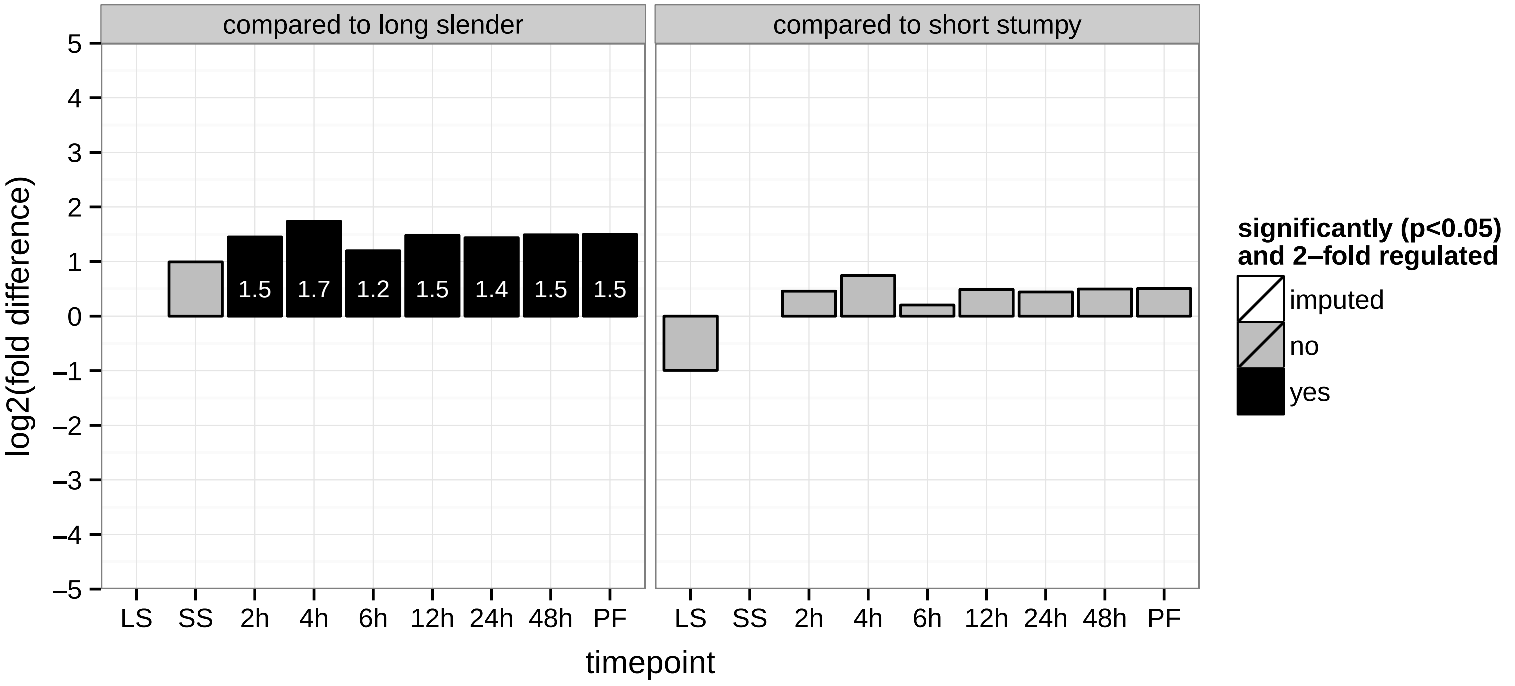

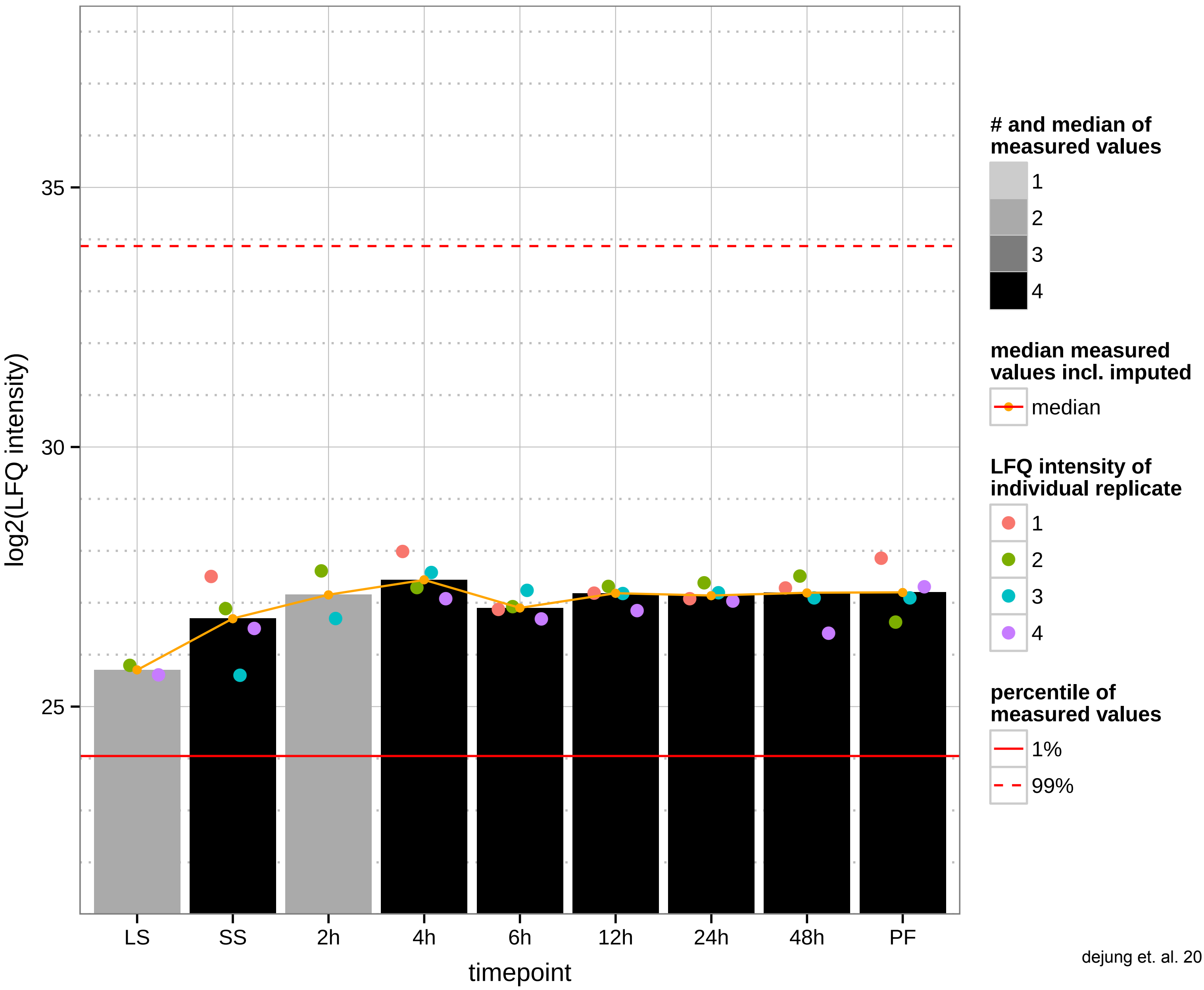

dejung et. al. 2015

citrate synthase, putative
Tb927.10.13430
AGOF: transferase activity, transferring acyl groups, acyl groups converted into alkyl on transfer
AGOC: mitochondrion
AGOP: cellular carbohydrate metabolic process
PGOF: transferase activity, transferring acyl groups, acyl groups converted into alkyl on transfer
PGOC: null
PGOP: cellular carbohydrate metabolic process

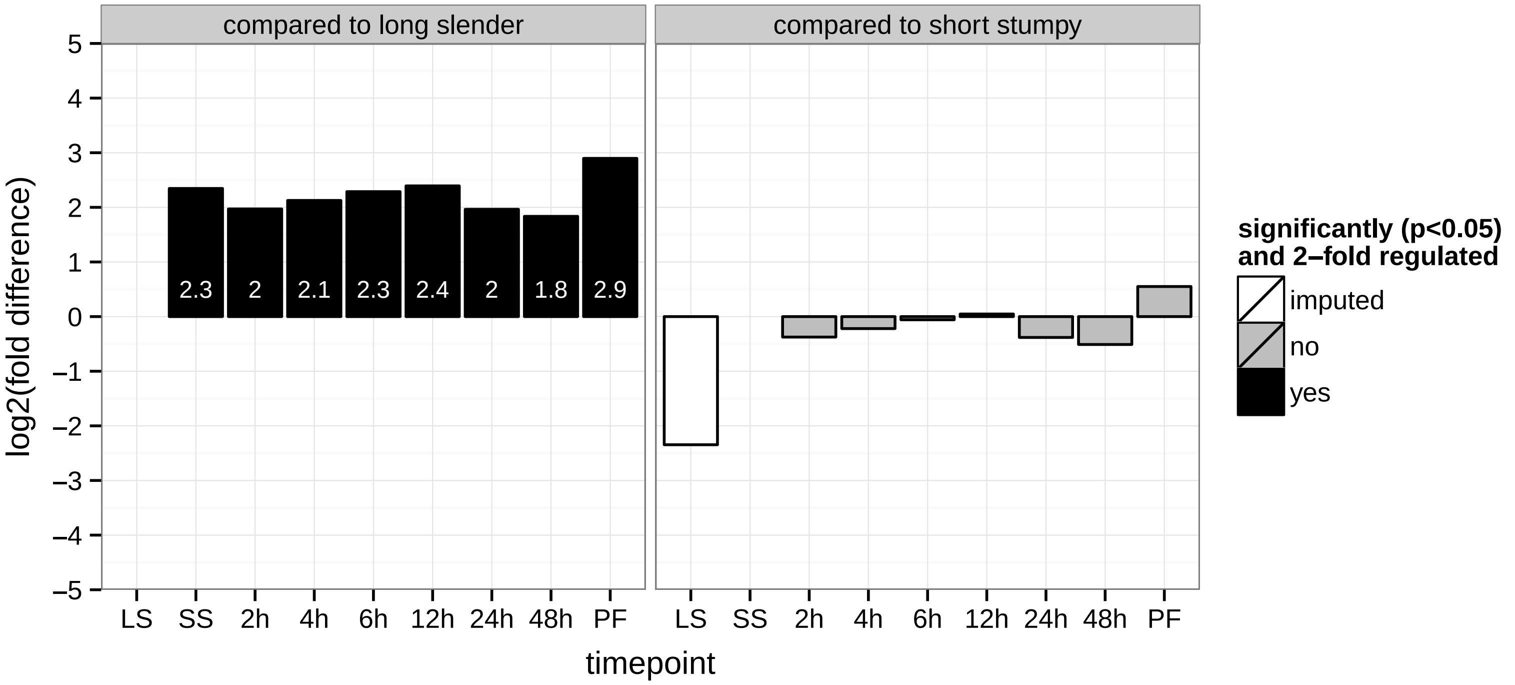

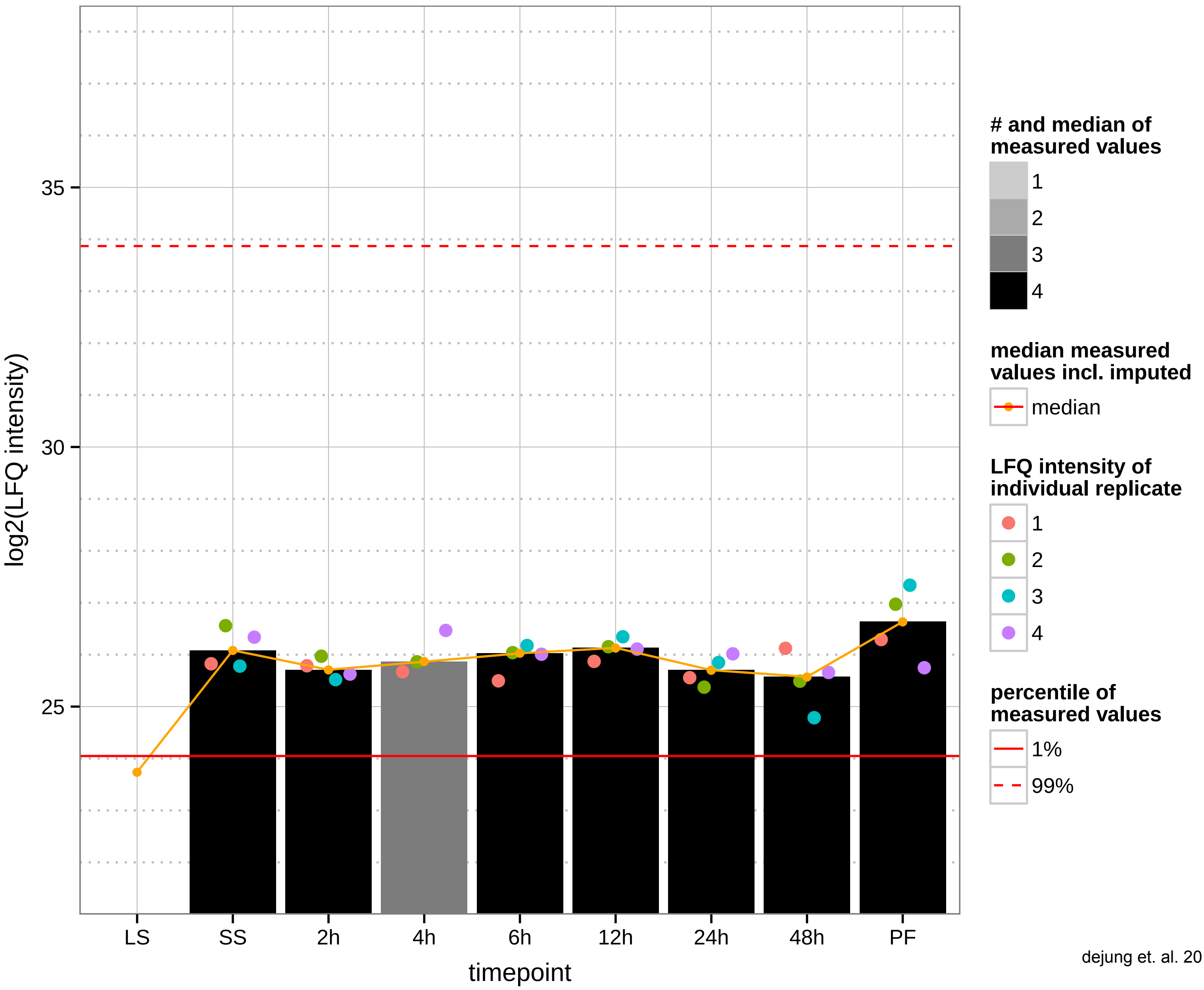

dejung et. al. 2015

hypothetical protein, conserved
Tb927.10.13450
AGOF: null
AGOC: null
AGOP: null
PGOF: binding
PGOC: null
PGOP: null

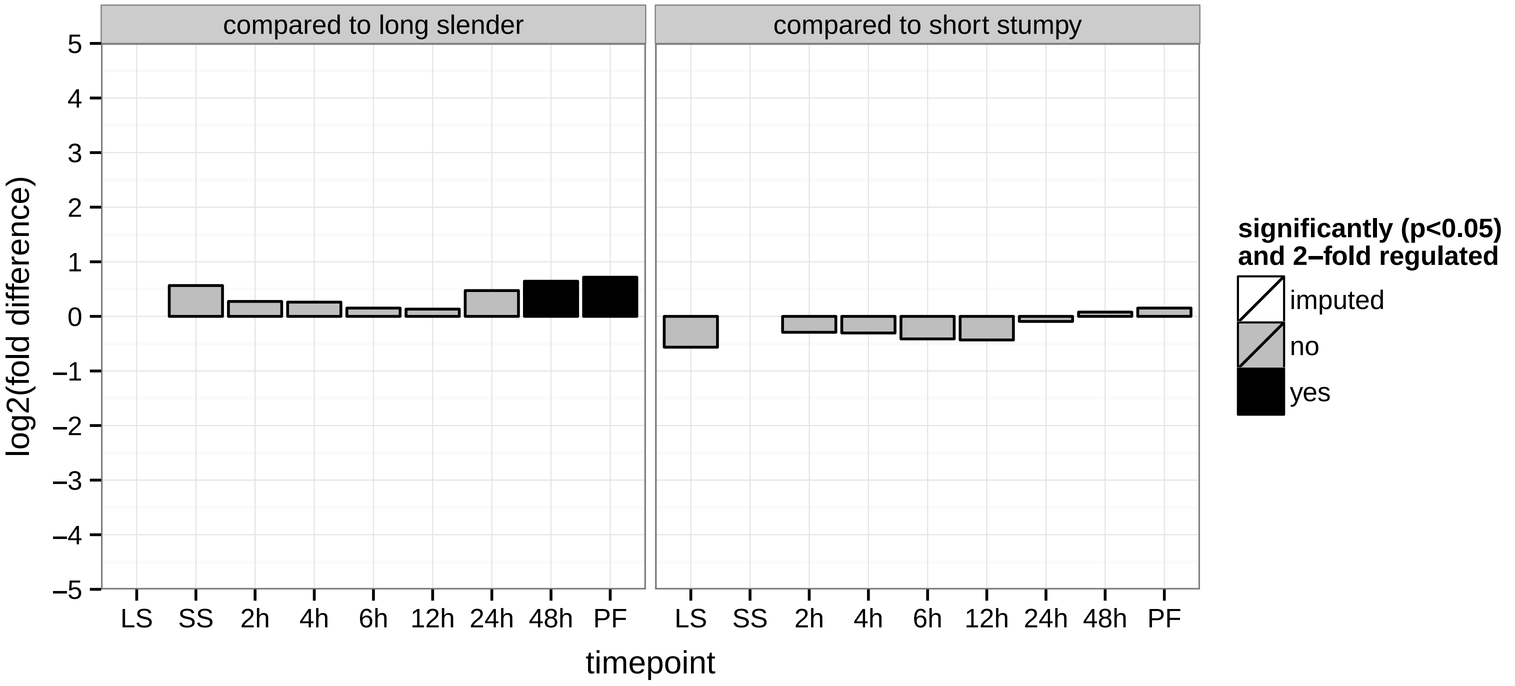

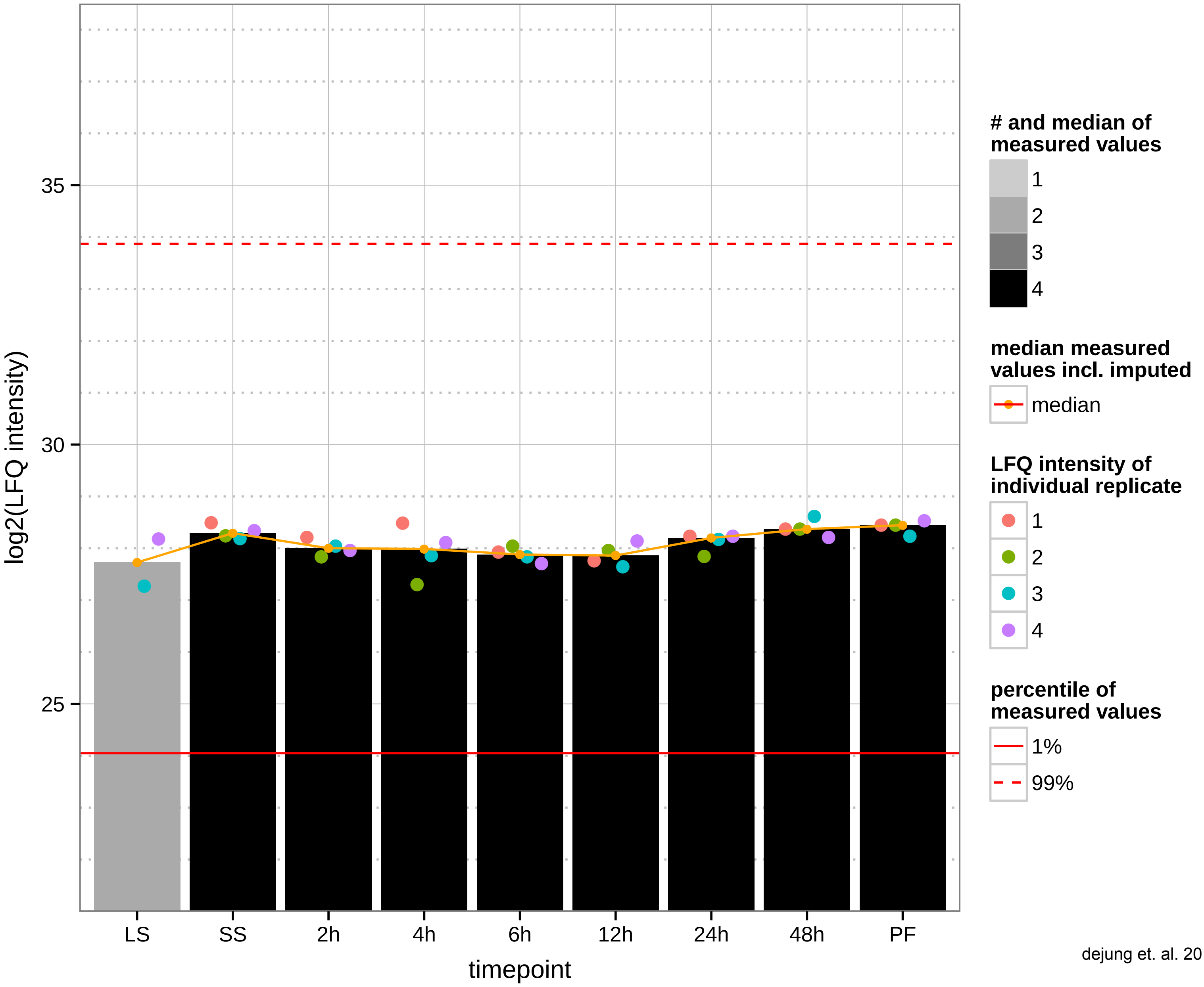

dejung et. al. 2015

protein kinase, putative
Tb927.10.13480
AGOF: ATP binding, protein serine/threonine kinase activity, protein tyrosine kinase activity
AGOC: mitochondrion
AGOP: protein phosphorylation
PGOF: ATP binding, protein kinase activity, transferase activity, transferring phosphorus–containing groups
PGOC: null
PGOP: protein phosphorylation

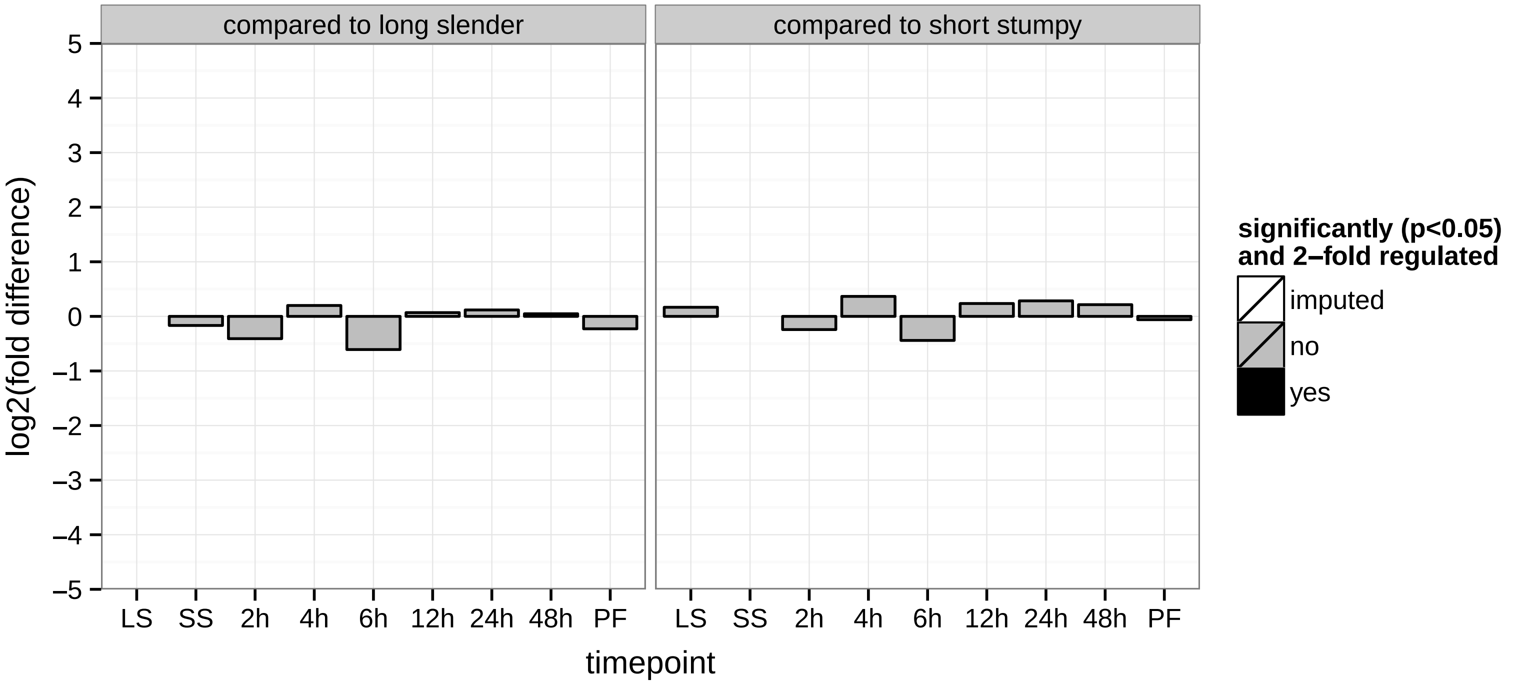

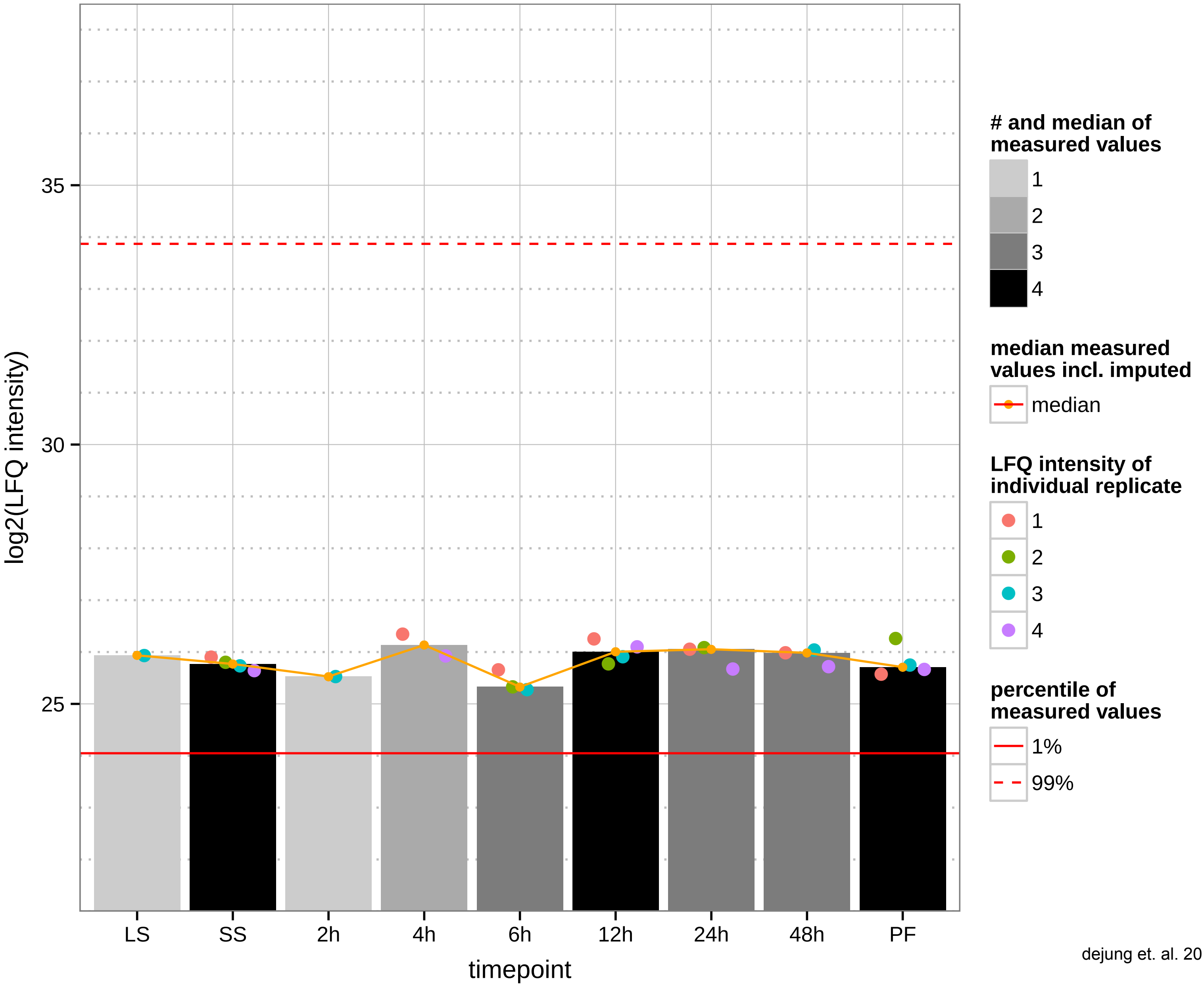

dejung et. al. 2015

zinc metallopeptidase, putative
Tb927.10.13510
AGOF: ATP binding, metalloendopeptidase activity, nucleoside−triphosphatase activity
AGOC: null
AGOP: proteolysis
PGOF: ATP binding, metalloendopeptidase activity, nucleoside−triphosphatase activity, nucleotide binding
PGOC: null
PGOP: proteolysis

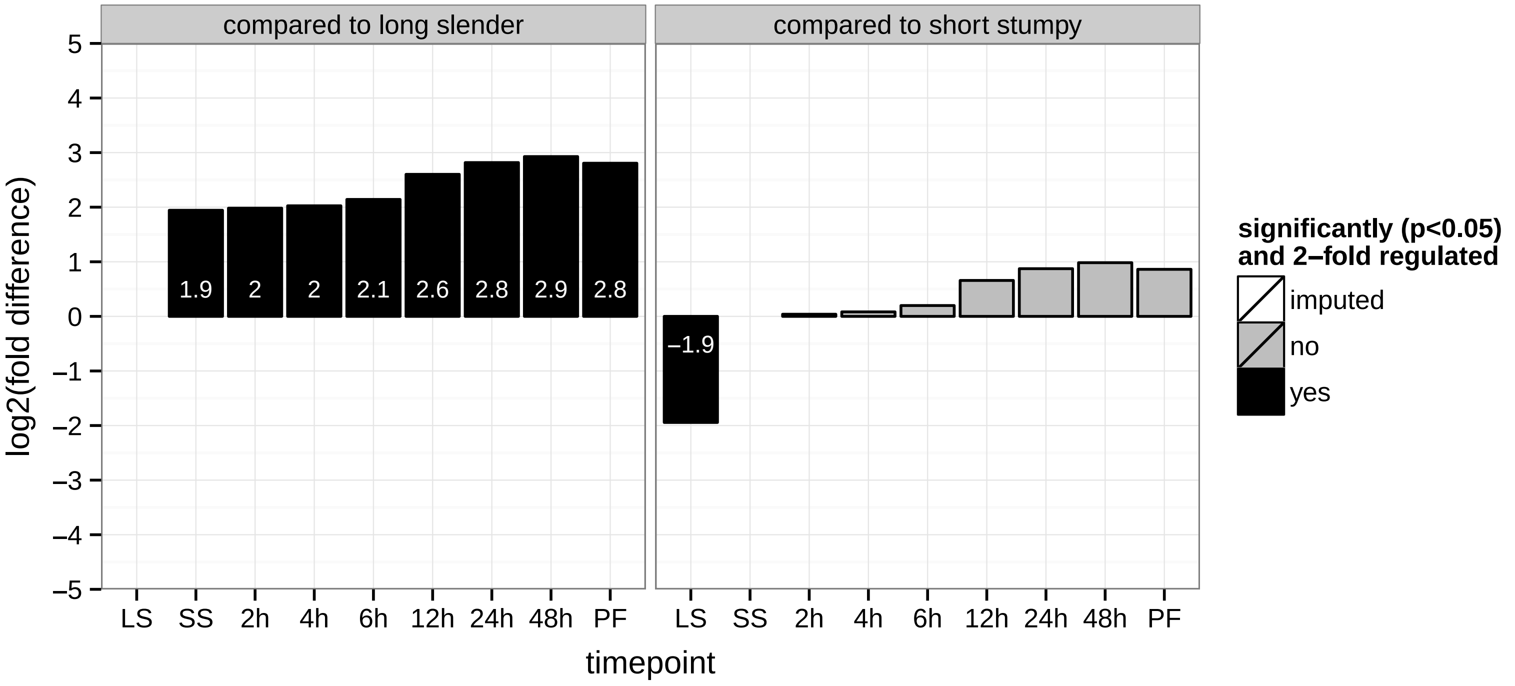

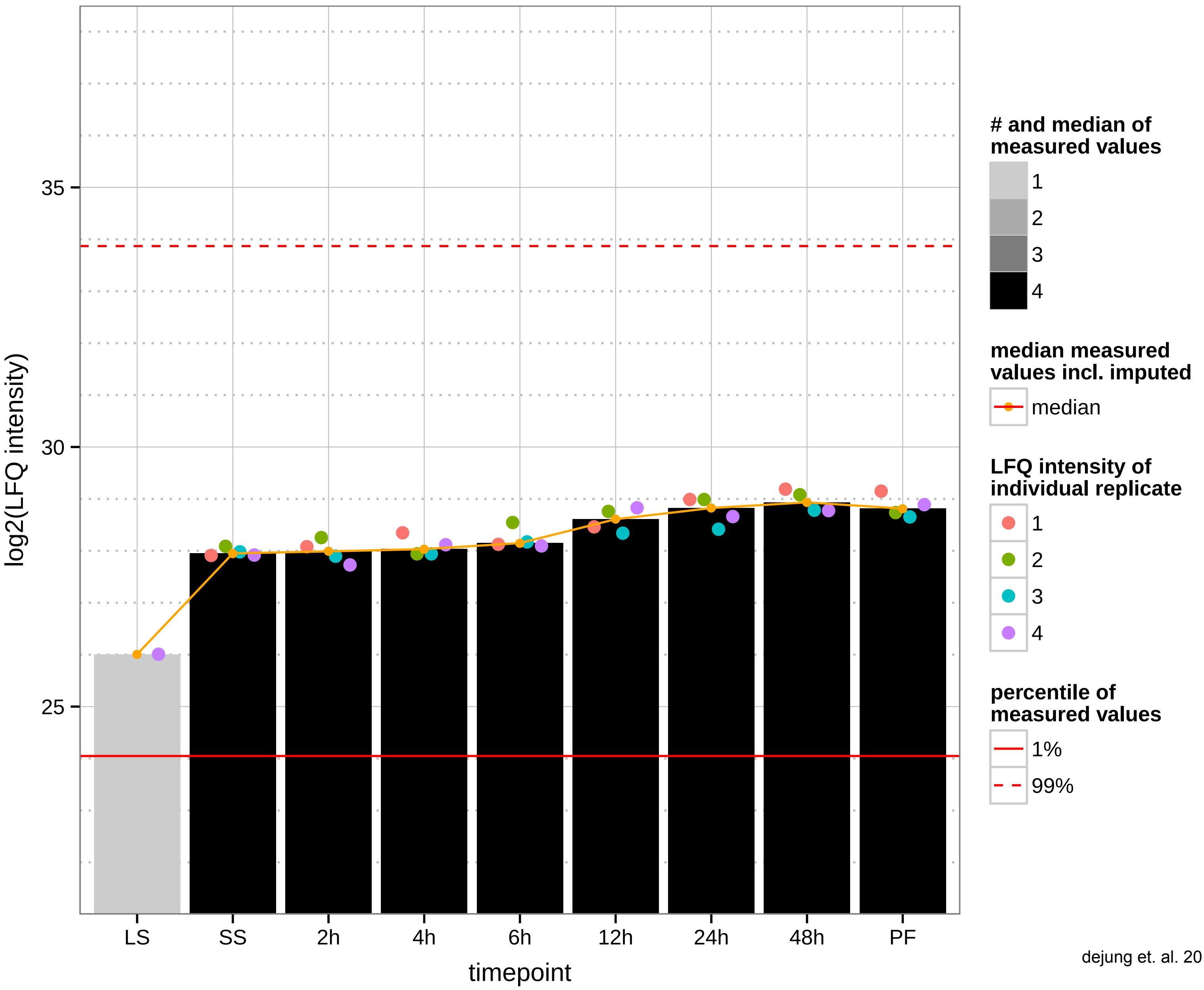

dejung et. al. 2015

mevalonate–diphosphate decarboxylase, mevalonate diphosphate decarboxylase
Tb927.10.13560;Tb927.10.14070
AGOF: ATP binding, diphosphomevalonate decarboxylase activity, kinase activity
AGOC: null
AGOP: isoprenoid biosynthetic process, phosphorylation
PGOF: ATP binding, diphosphomevalonate decarboxylase activity
PGOC: null
PGOP: isoprenoid biosynthetic process

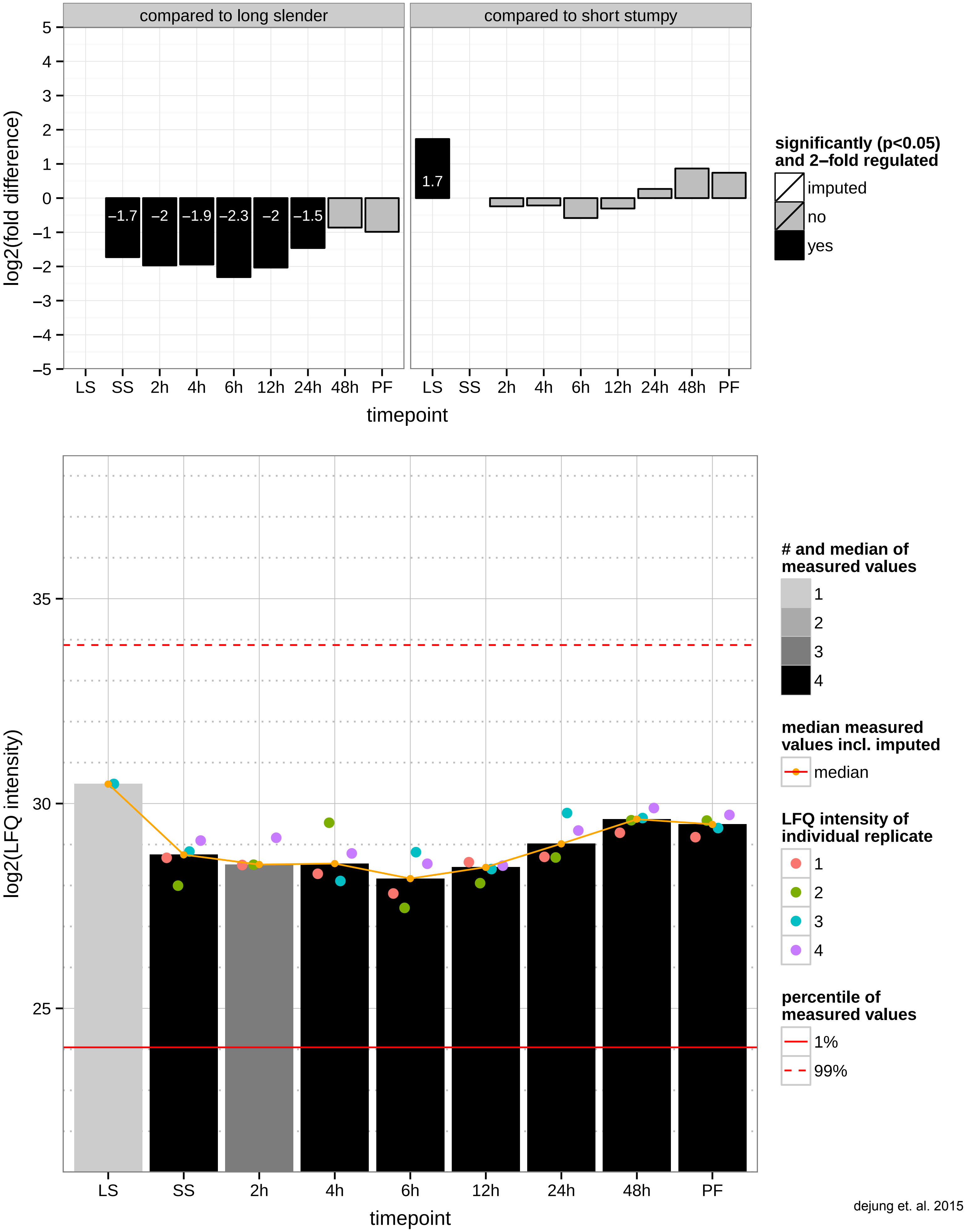

dejung et. al. 2015

glucosidase, putative
Tb927.10.13630
AGOF: hydrolase activity, hydrolyzing O−glycosyl compounds
AGOC: null
AGOP: carbohydrate metabolic process
PGOF: carbohydrate binding, catalytic activity, hydrolase activity, hydrolyzing O−glycosyl compounds
PGOC: null
PGOP: carbohydrate metabolic process

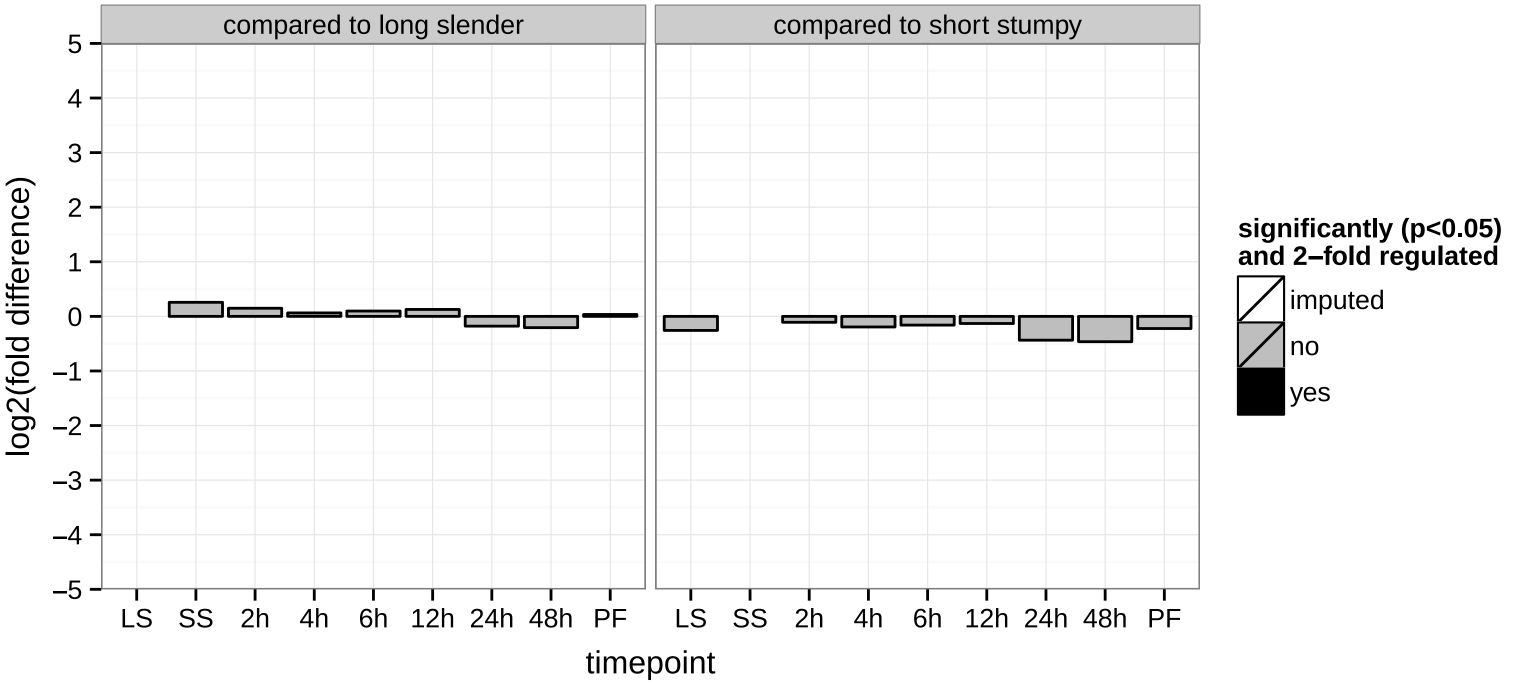

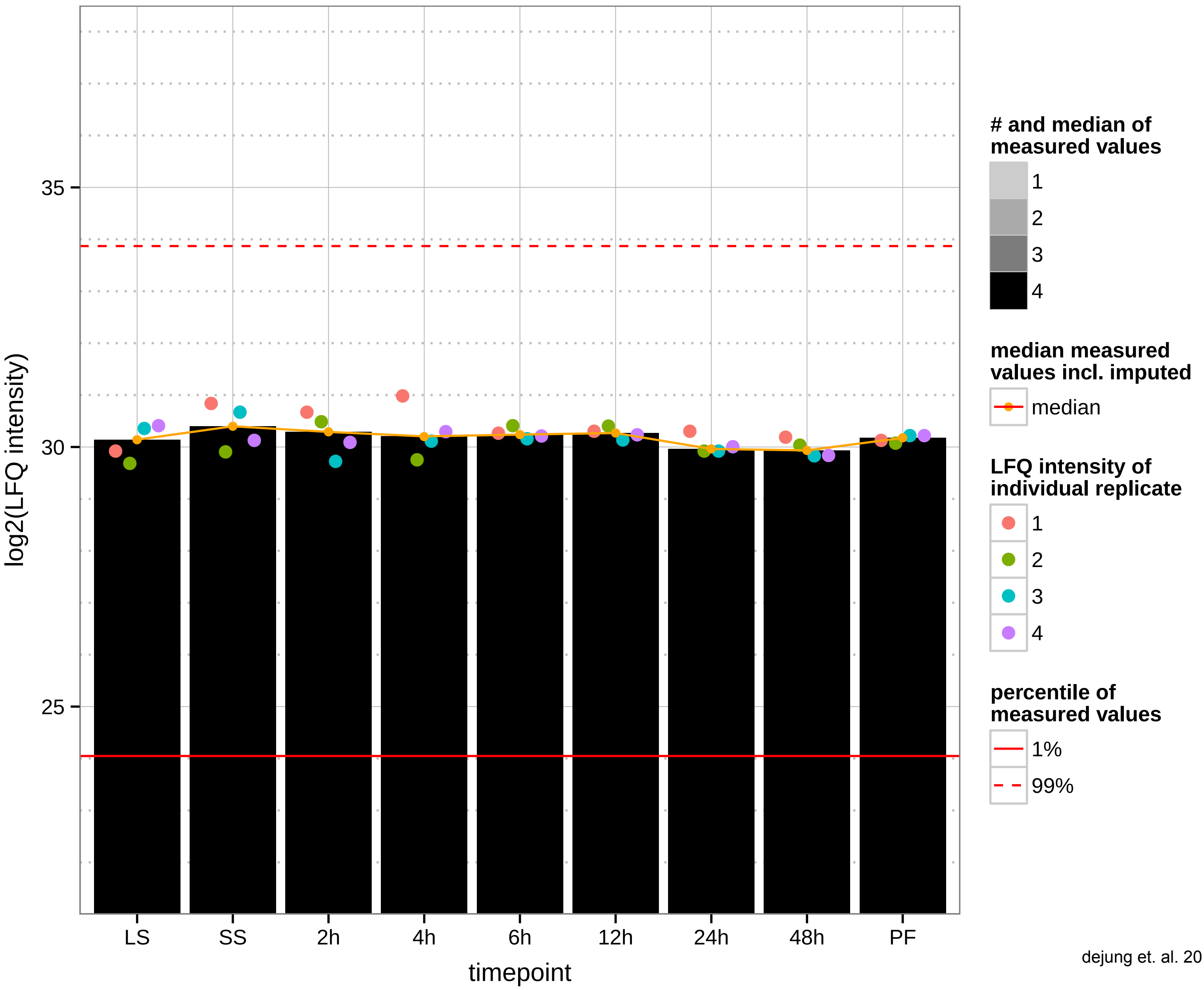

dejung et. al. 2015

ARF–like 2–binding protein, putative, BART
Tb927.10.13650
AGOF: null
AGOC: null
AGOP: null
PGOF: null
PGOC: null
PGOP: null

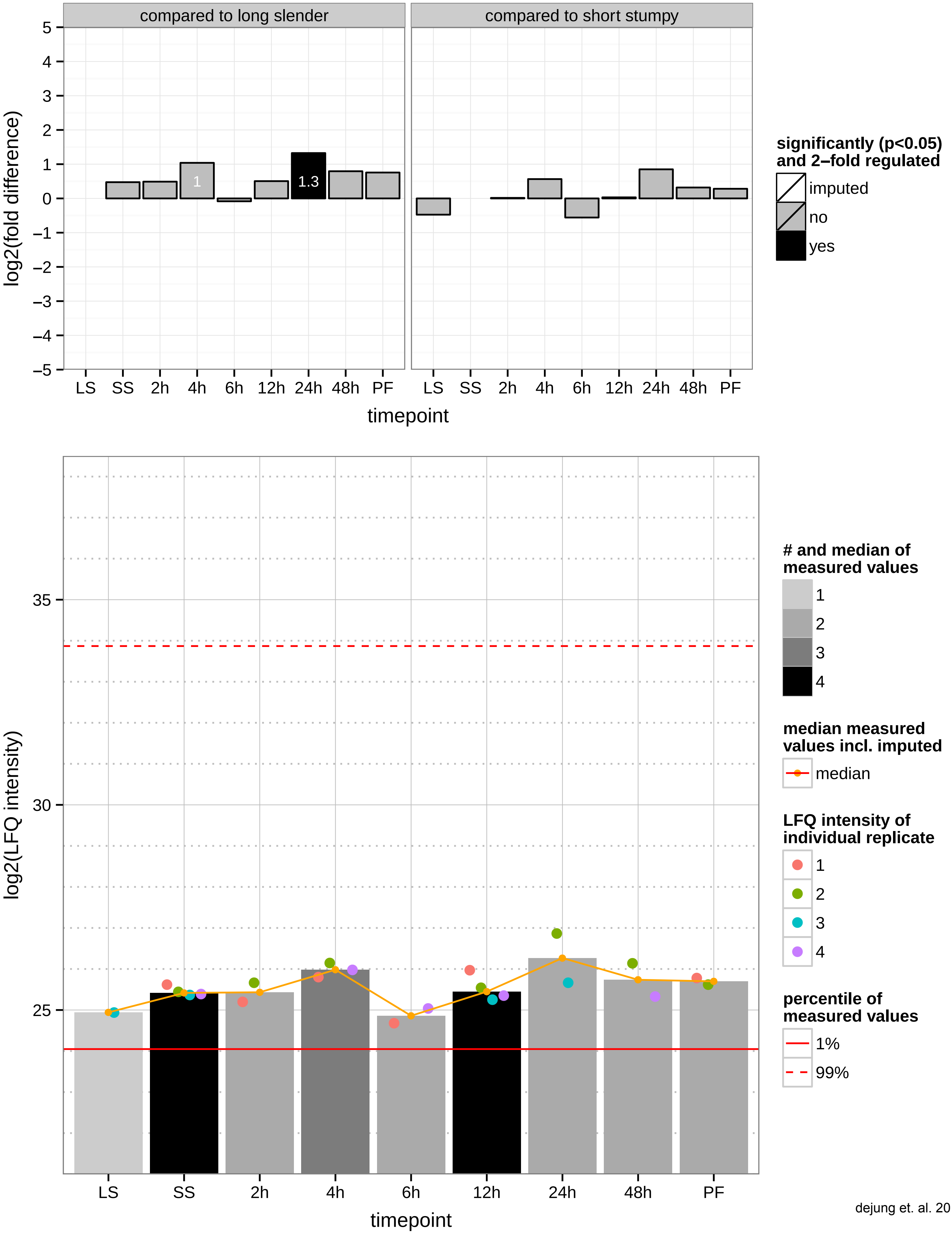

dejung et. al. 2015

serine/threonine protein phosphatase 5
Tb927.10.13670
AGOF: metal ion binding, protein serine/threonine phosphatase activity
AGOC: cytoplasm, nucleus
AGOP: protein dephosphorylation
PGOF: hydrolase activity, phosphoprotein phosphatase activity, protein binding
PGOC: cytoplasm, nucleus
PGOP: protein dephosphorylation

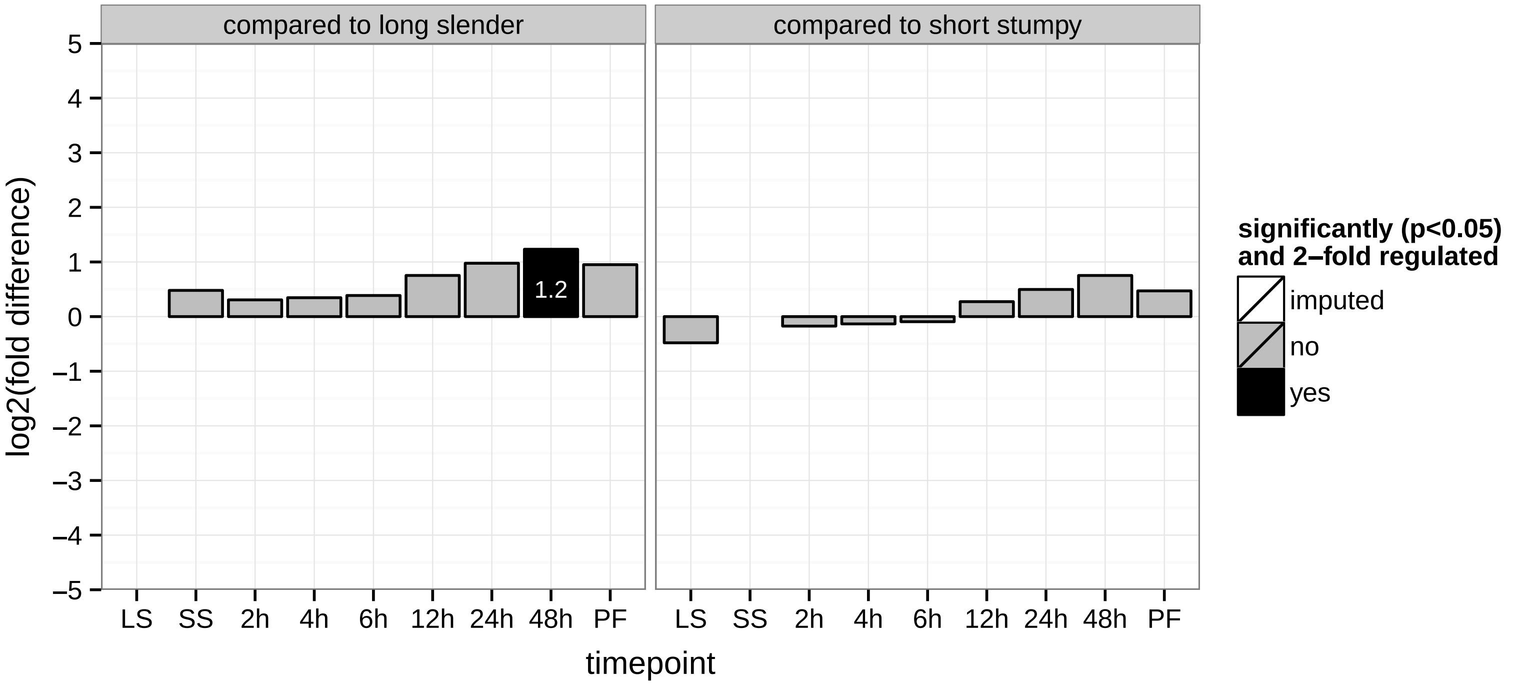

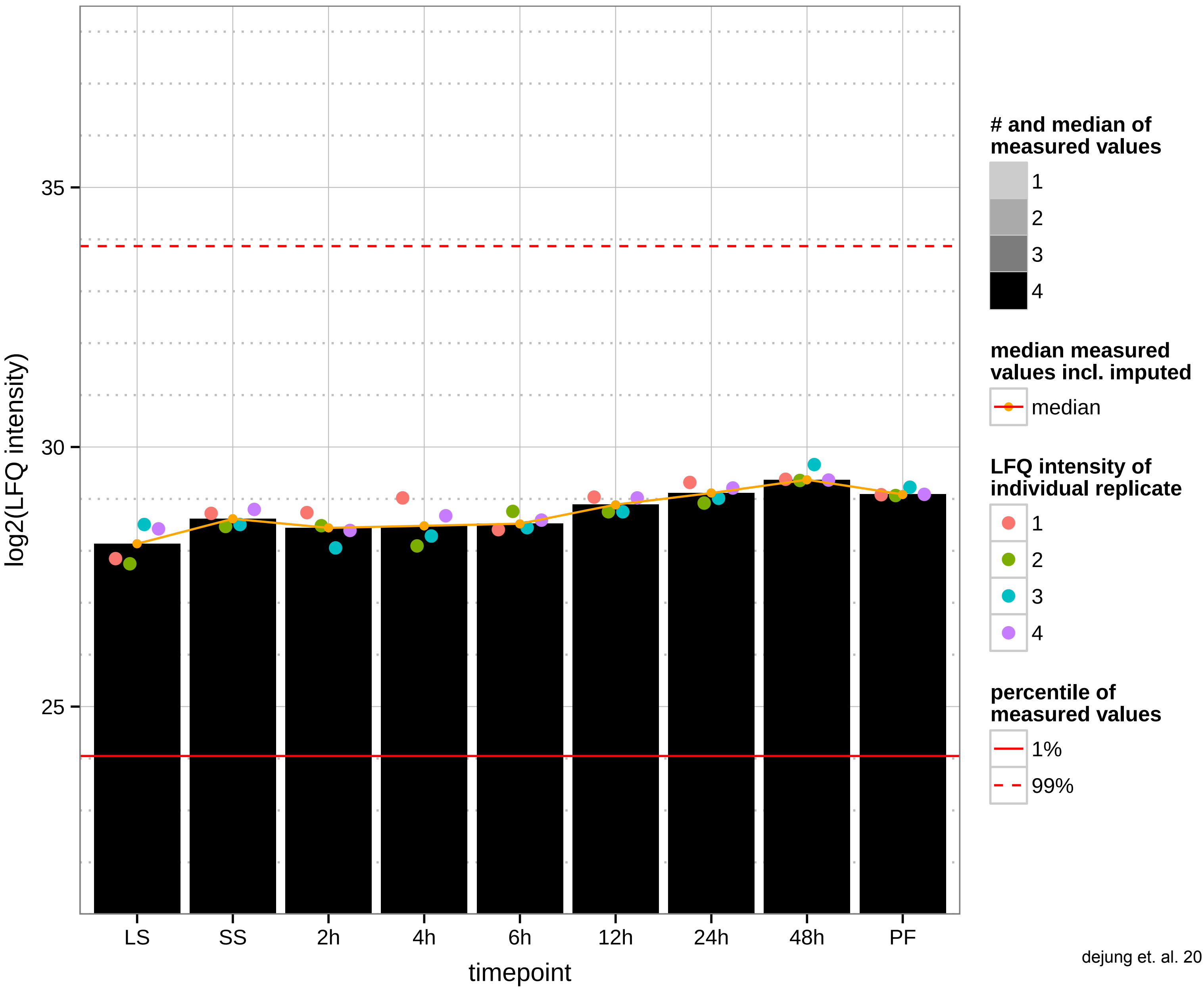

dejung et. al. 2015

hypothetical protein, conserved
Tb927.10.13710
AGOF: ATPase activator activity, chaperone binding
AGOC: cytoplasm
AGOP: response to stress
PGOF: ATPase activator activity, chaperone binding
PGOC: null
PGOP: response to stress

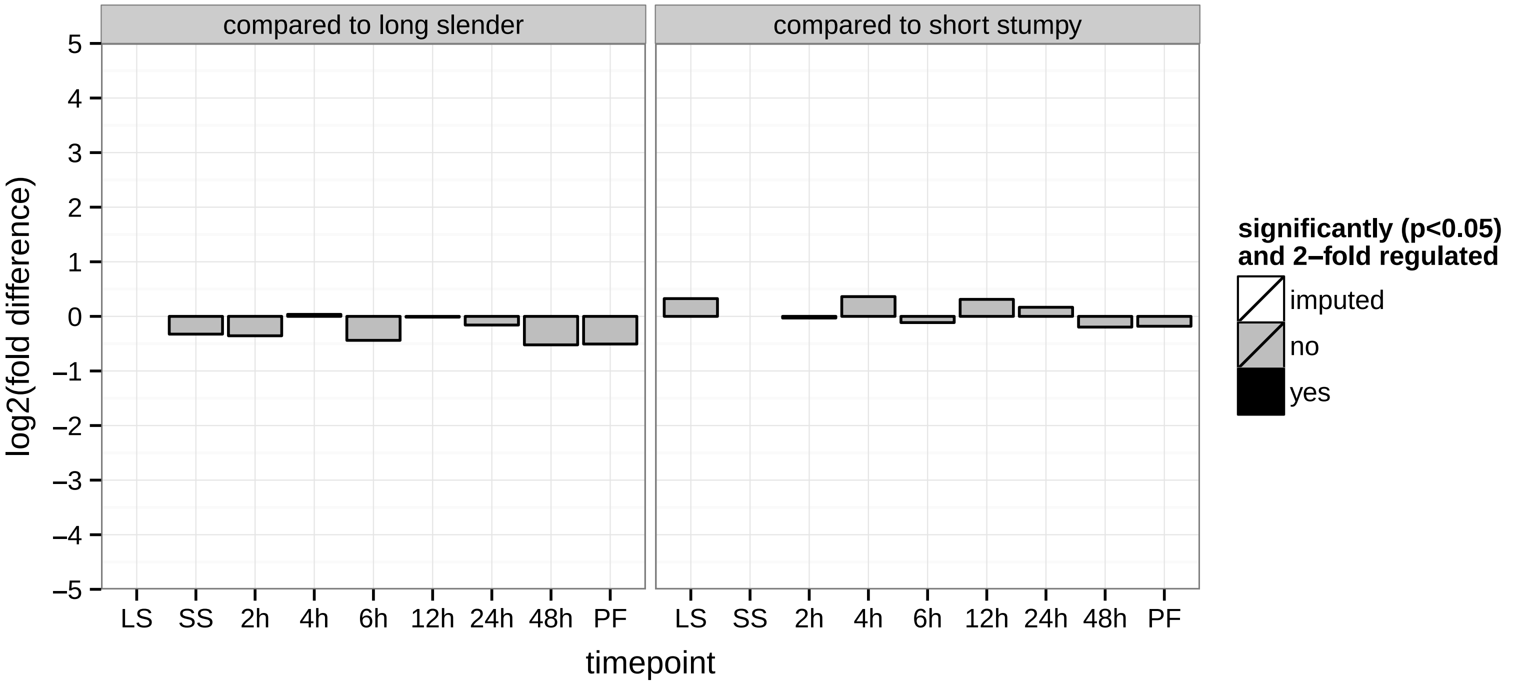

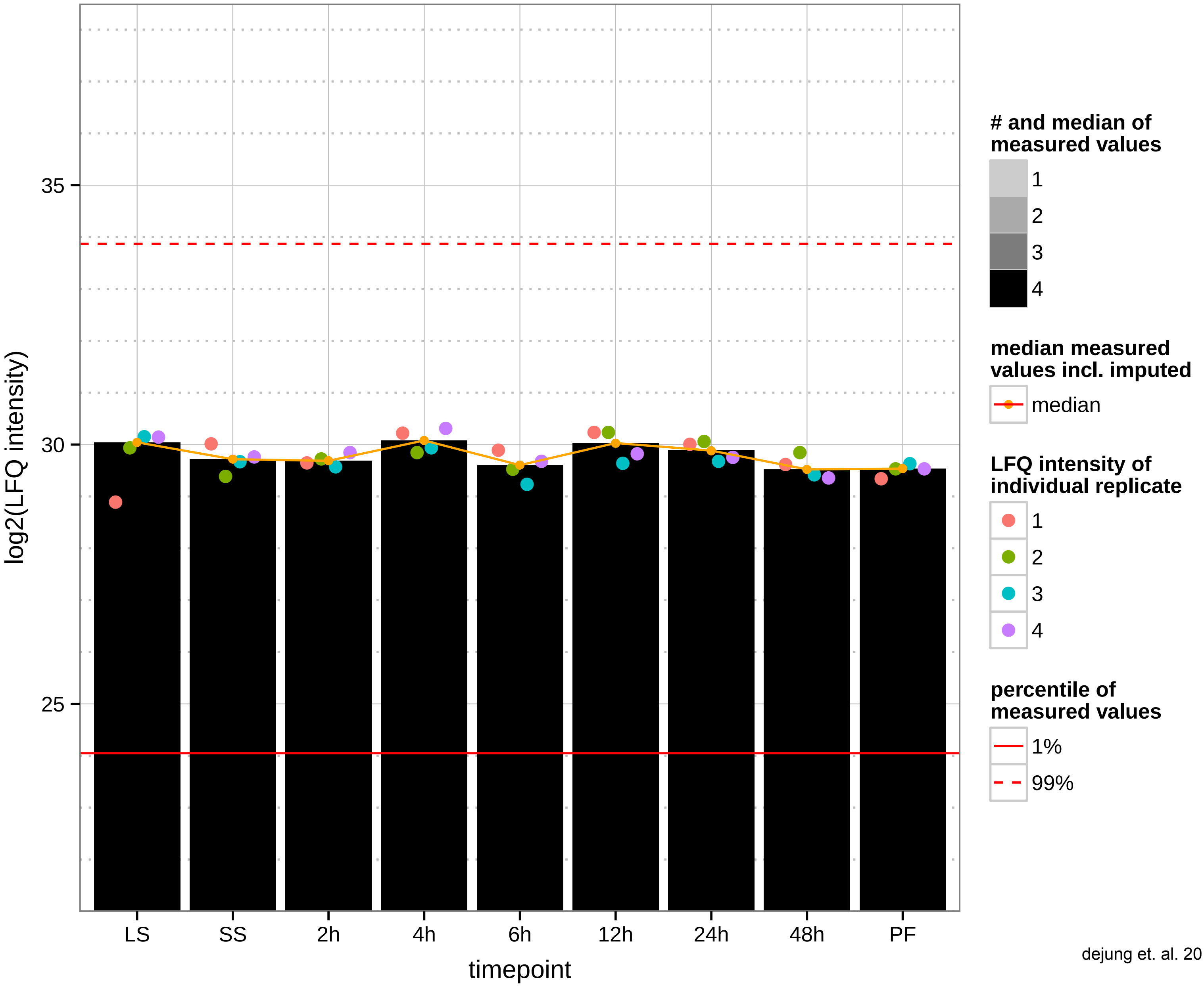

dejung et. al. 2015

RNA–binding protein 29, putative (RBP29)
Tb927.10.13720
AGOF: RNA binding
AGOC: nucleus
AGOP: RNA processing
PGOF: nucleic acid binding
PGOC: null
PGOP: null

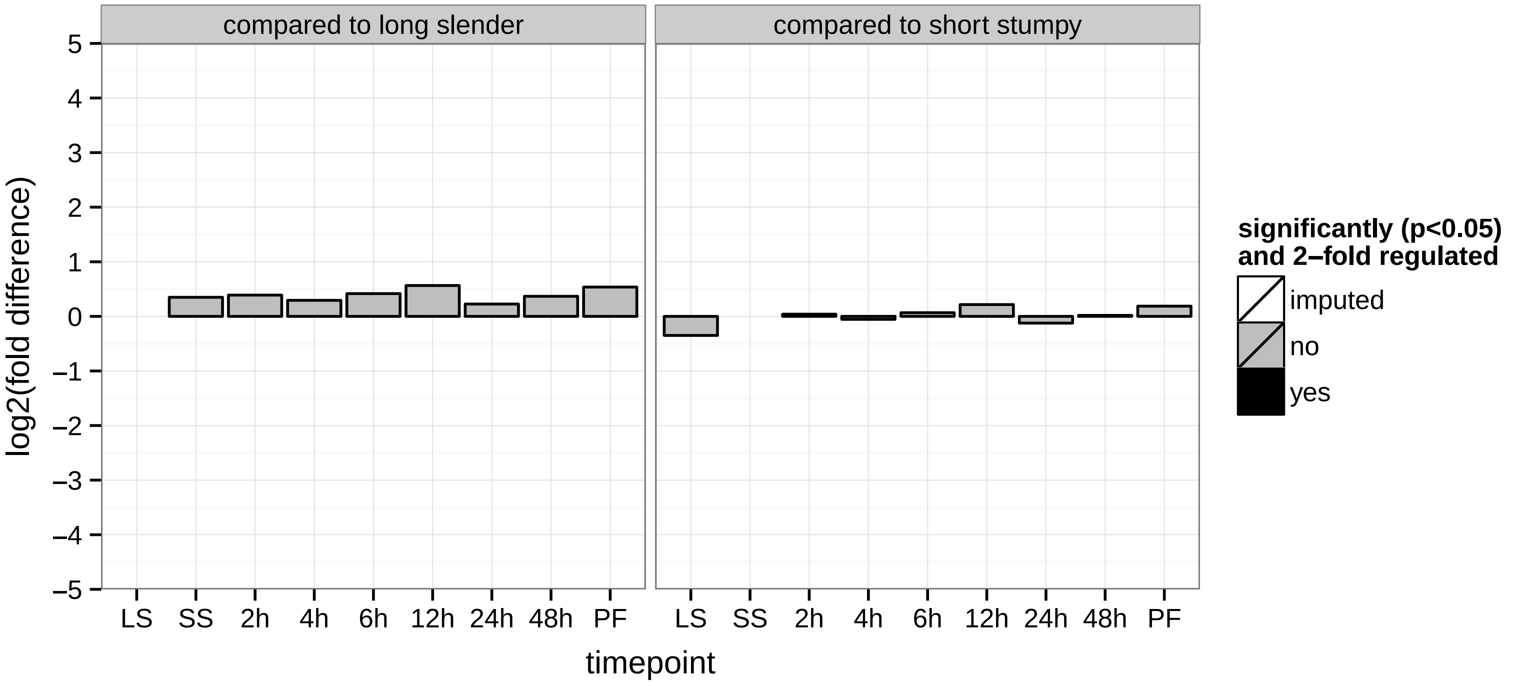

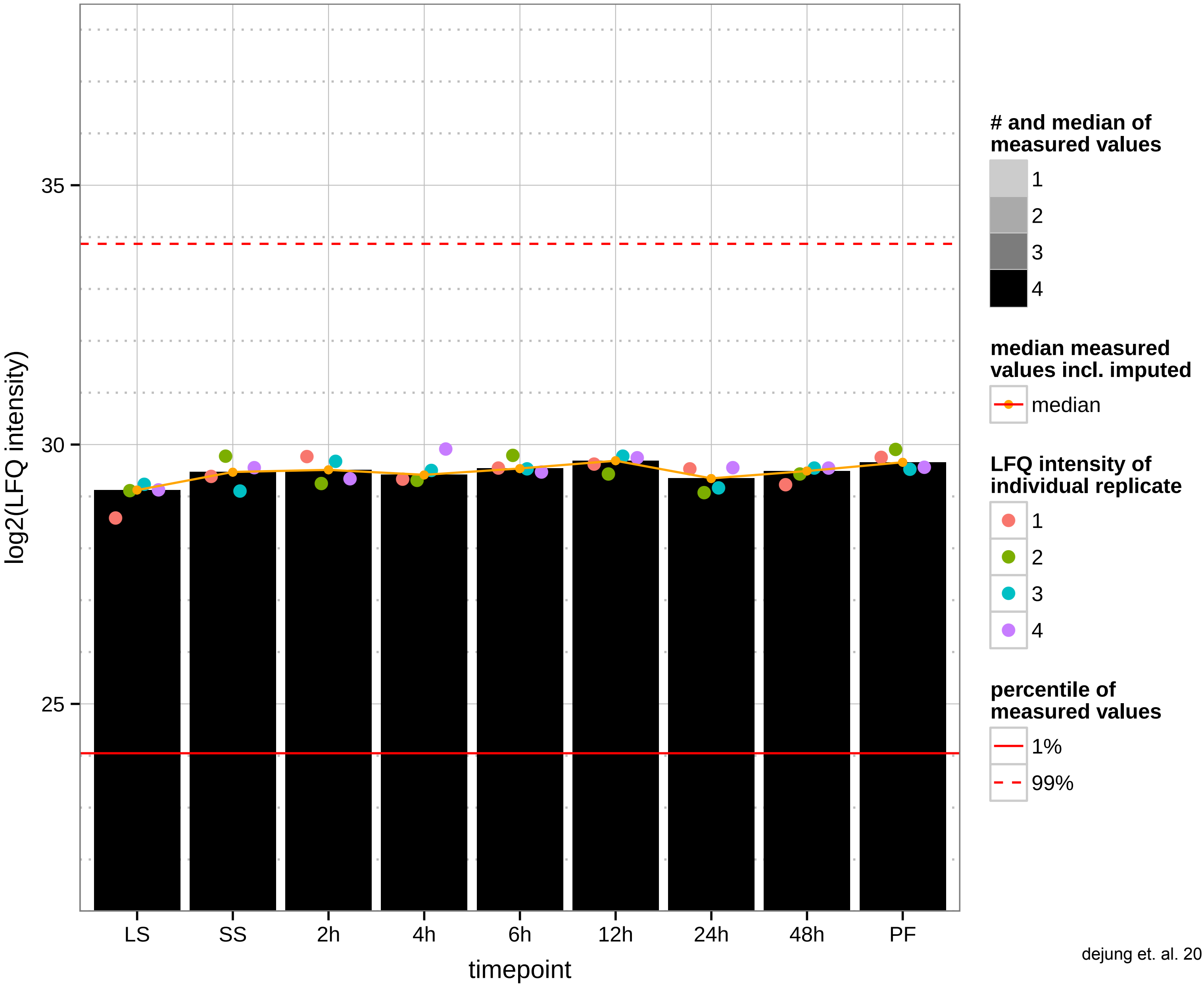

dejung et. al. 2015

synaptotagmin, putative, calcium−dependent lipid binding protein
Tb927.10.13740
AGOF: null
AGOC: null
AGOP: null
PGOF: protein binding
PGOC: null
PGOP: null

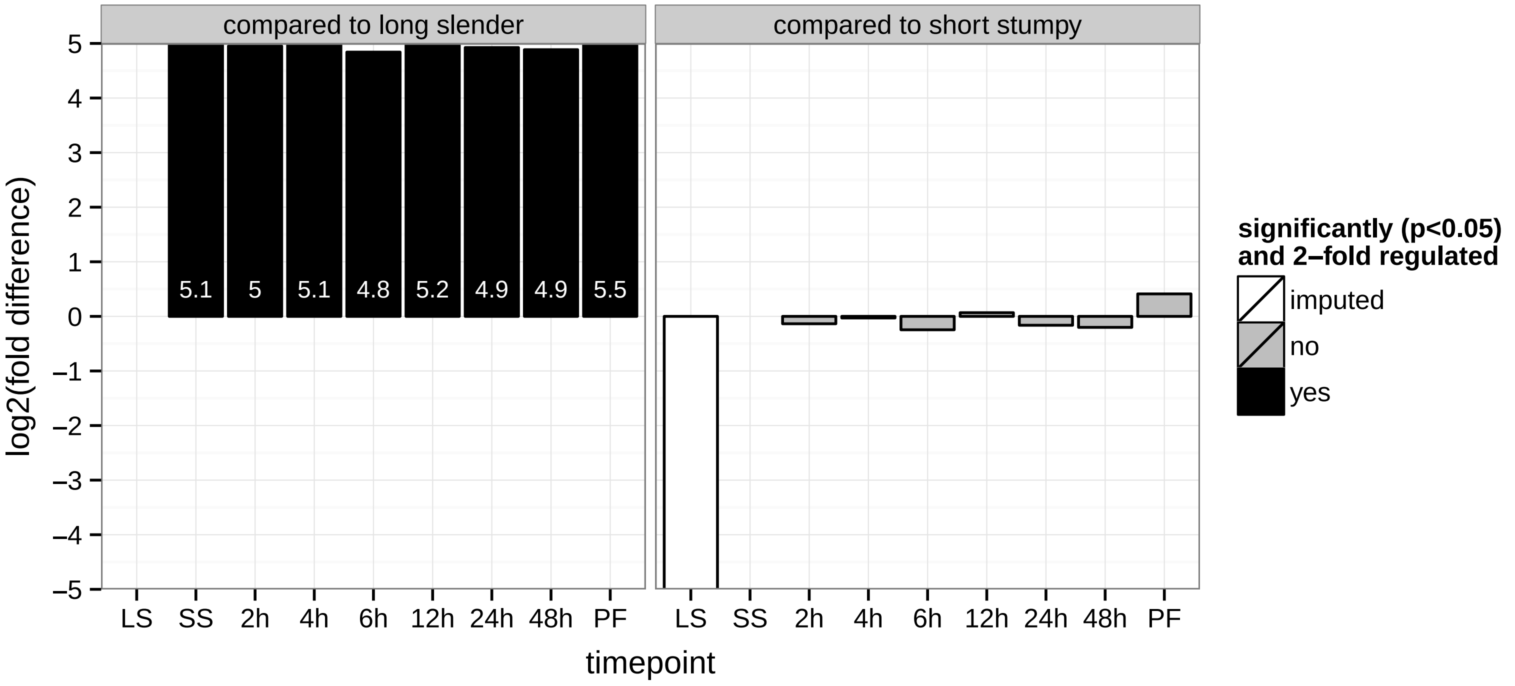

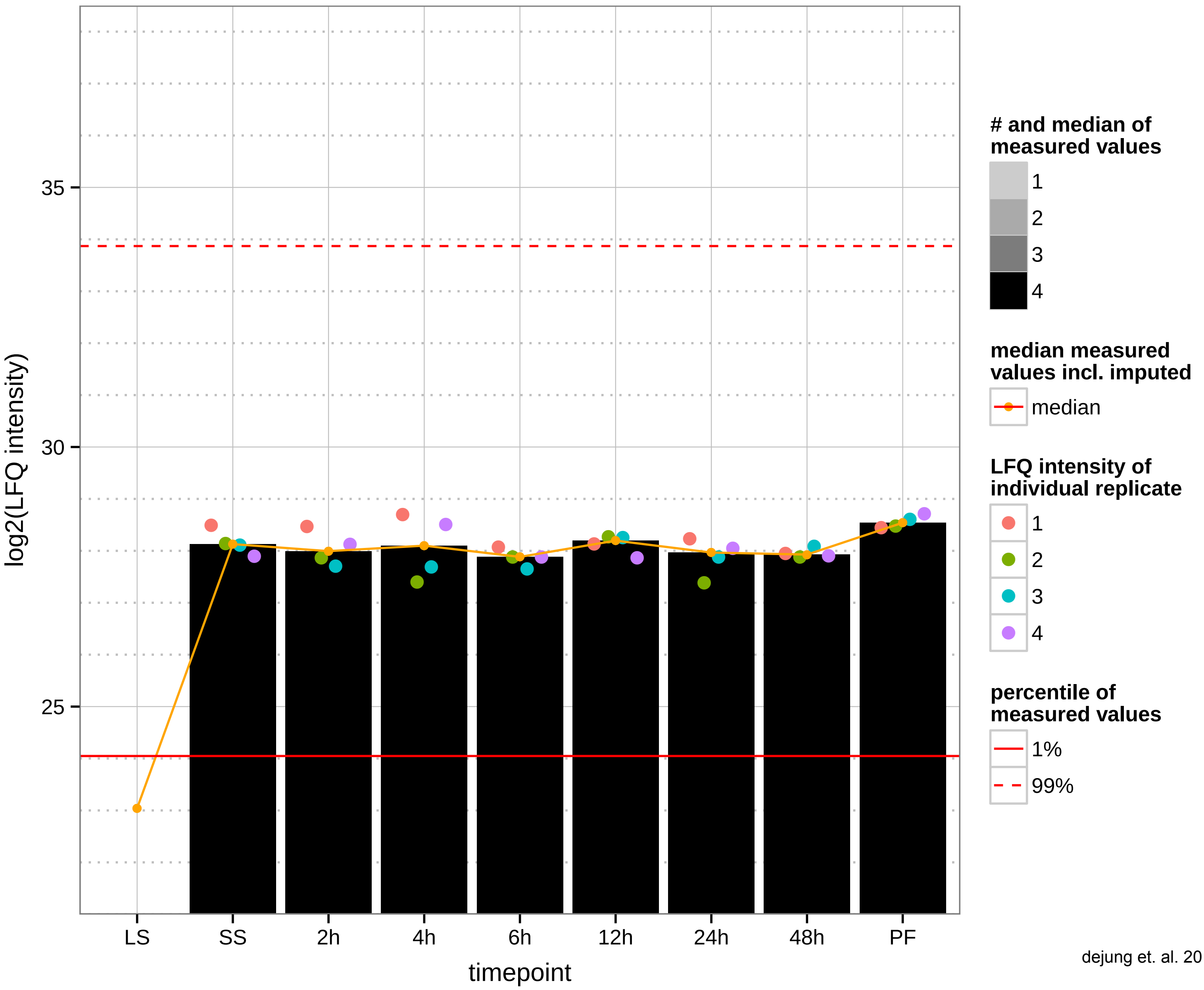

dejung et. al. 2015

GPI−anchor transamidase subunit 8 (GPI8), cysteine peptidase, Clan CD, family C13
Tb927.10.13860
AGOF: cysteine−type endopeptidase activity
AGOC: null
AGOP: proteolysis
PGOF: cysteine−type endopeptidase activity
PGOC: null
PGOP: proteolysis

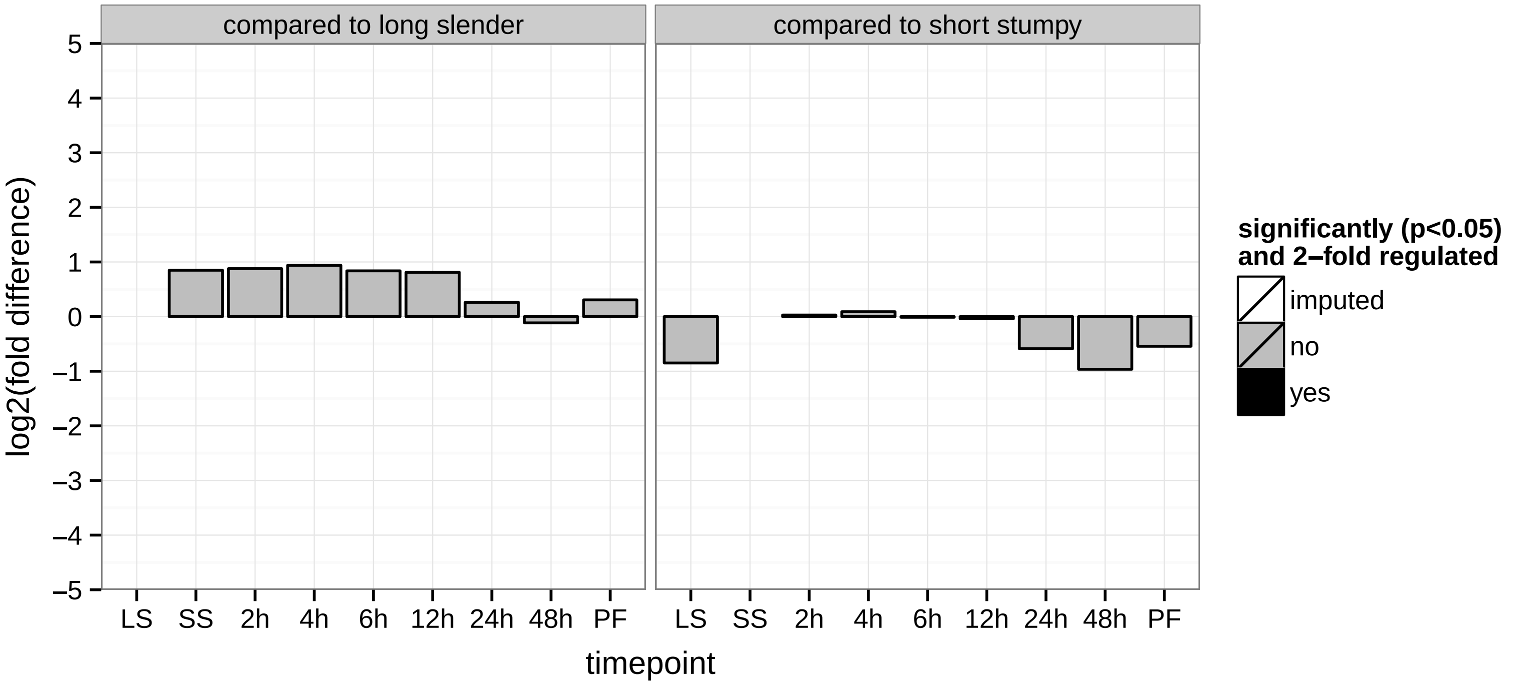

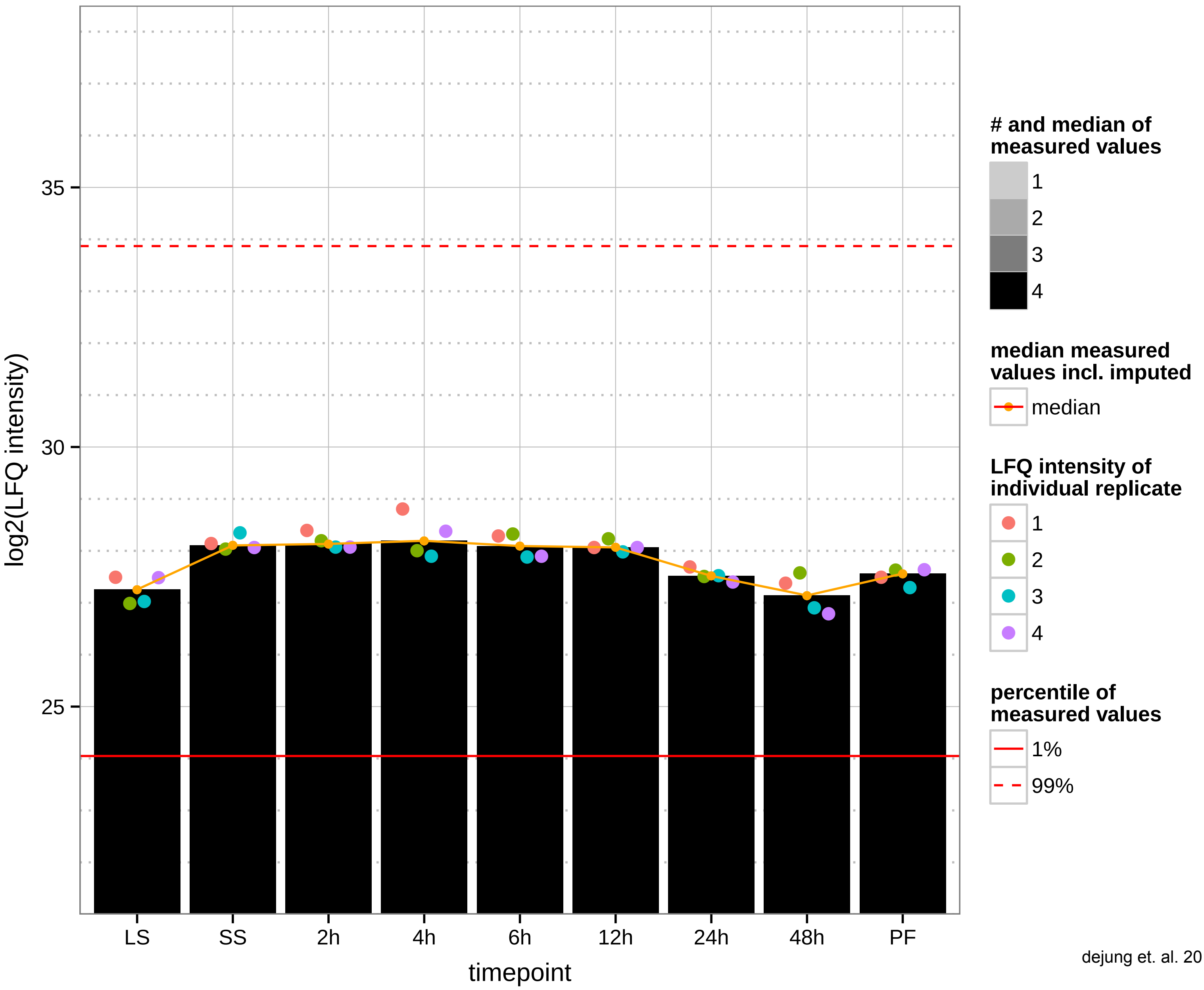

dejung et. al. 2015

hypothetical protein, conserved
Tb927.10.13880
AGOF: null
AGOC: null
AGOP: null
PGOF: binding
PGOC: null
PGOP: null

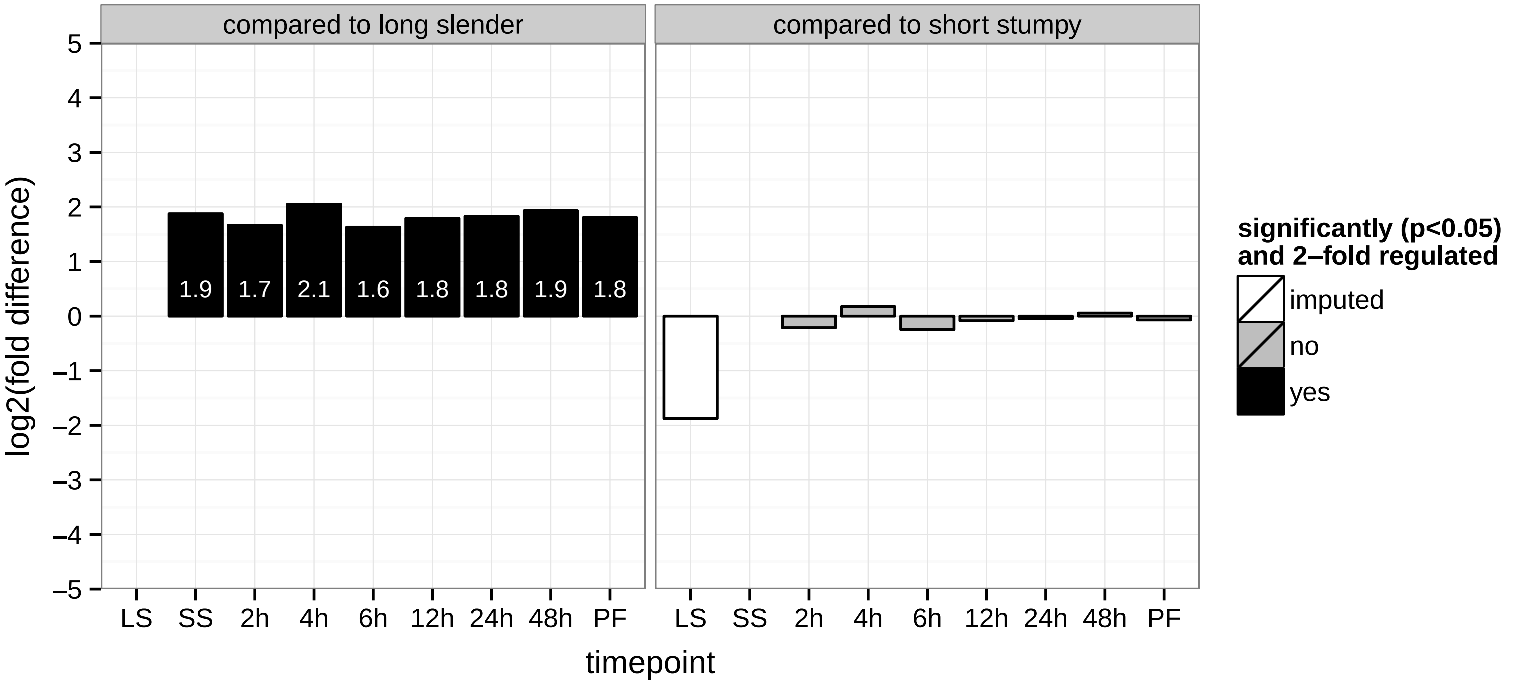

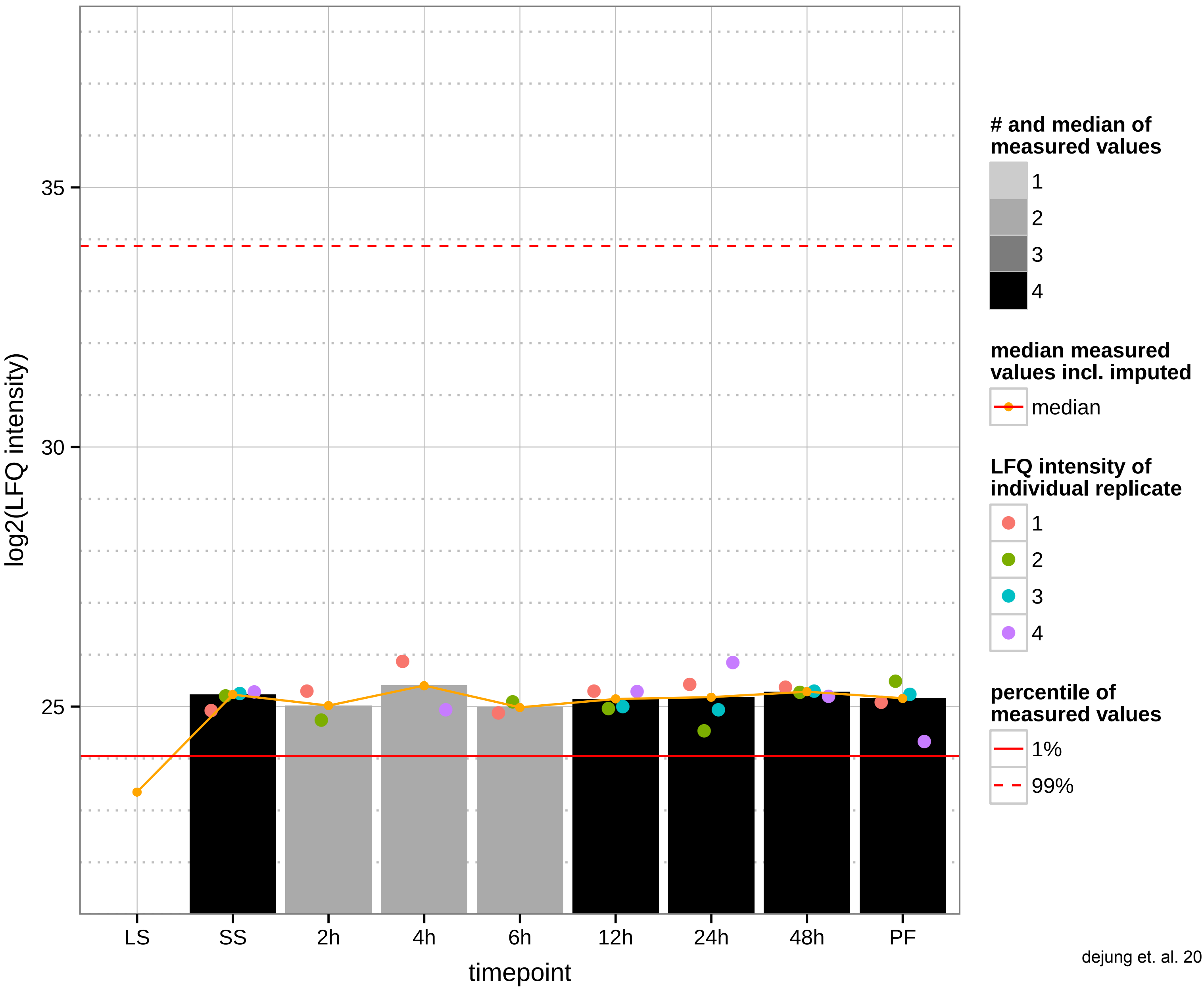

dejung et. al. 2015

hypoxanthine–guanine phosphoribosyltransferase (HGPRT)
Tb927.10.1400;Tb927.10.1470
AGOF: hypoxanthine phosphoribosyltransferase activity
AGOC: cytoplasm
AGOP: purine ribonucleoside salvage
PGOF: hypoxanthine phosphoribosyltransferase activity
PGOC: cytoplasm
PGOP: nucleoside metabolic process, purine ribonucleoside salvage

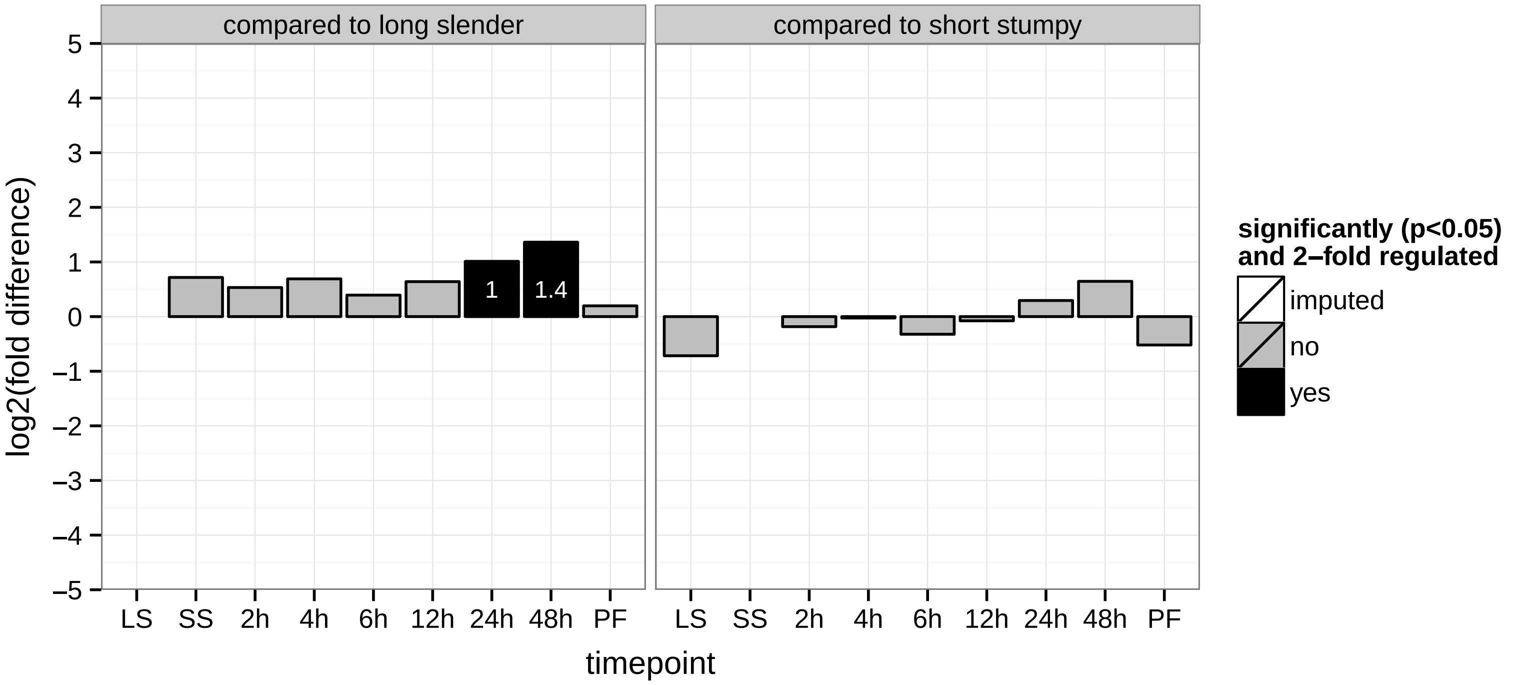

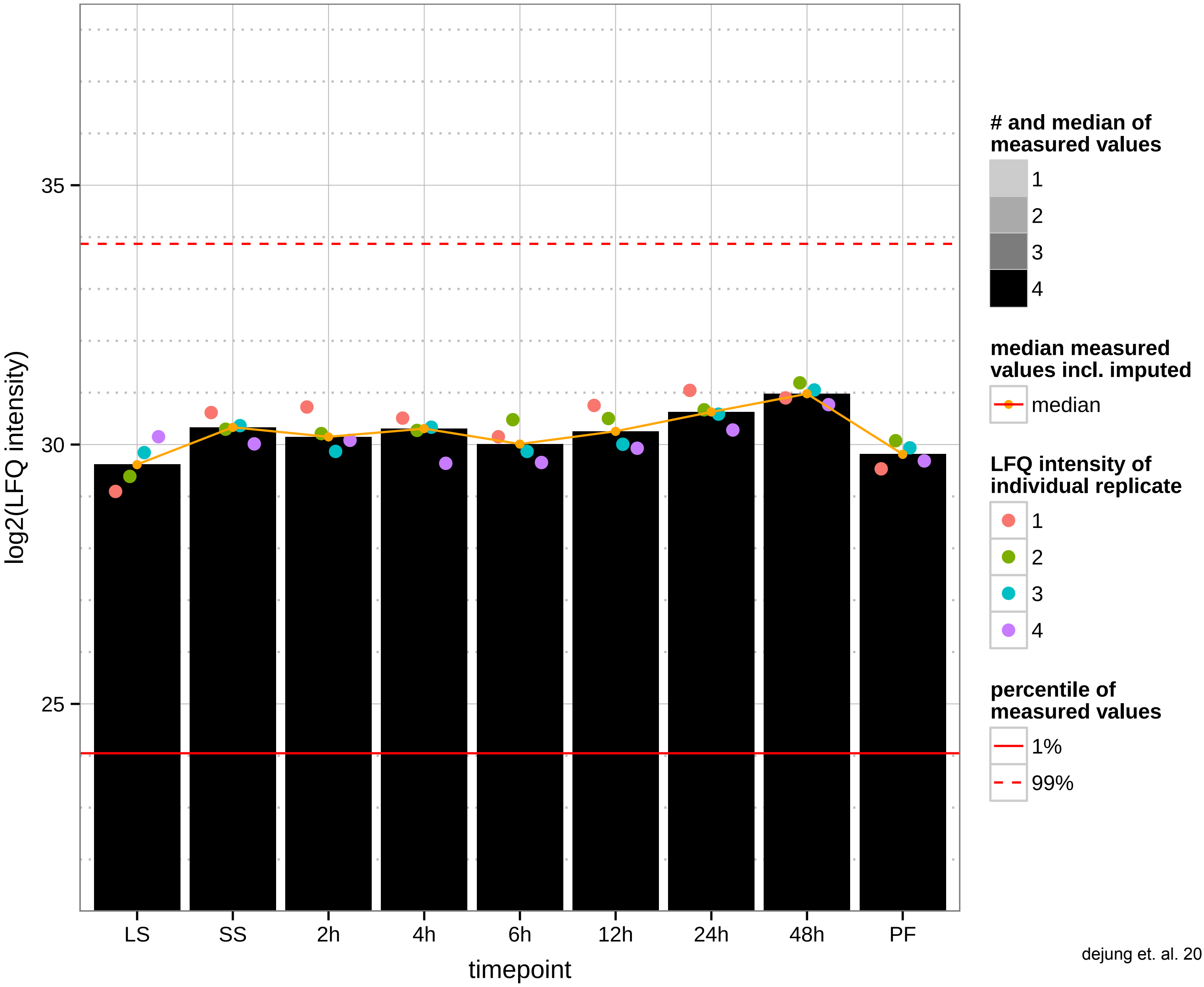

dejung et. al. 2015

tubulin cofactor C domain–containing protein RP2 (rp2)
Tb927.10.14010
AGOF: GTPase activator activity
AGOC: cilium basal body
AGOP: cell morphogenesis, motile cilium assembly, spindle assembly
PGOF: protein binding
PGOC: microtubule organizing center
PGOP: microtubule anchoring

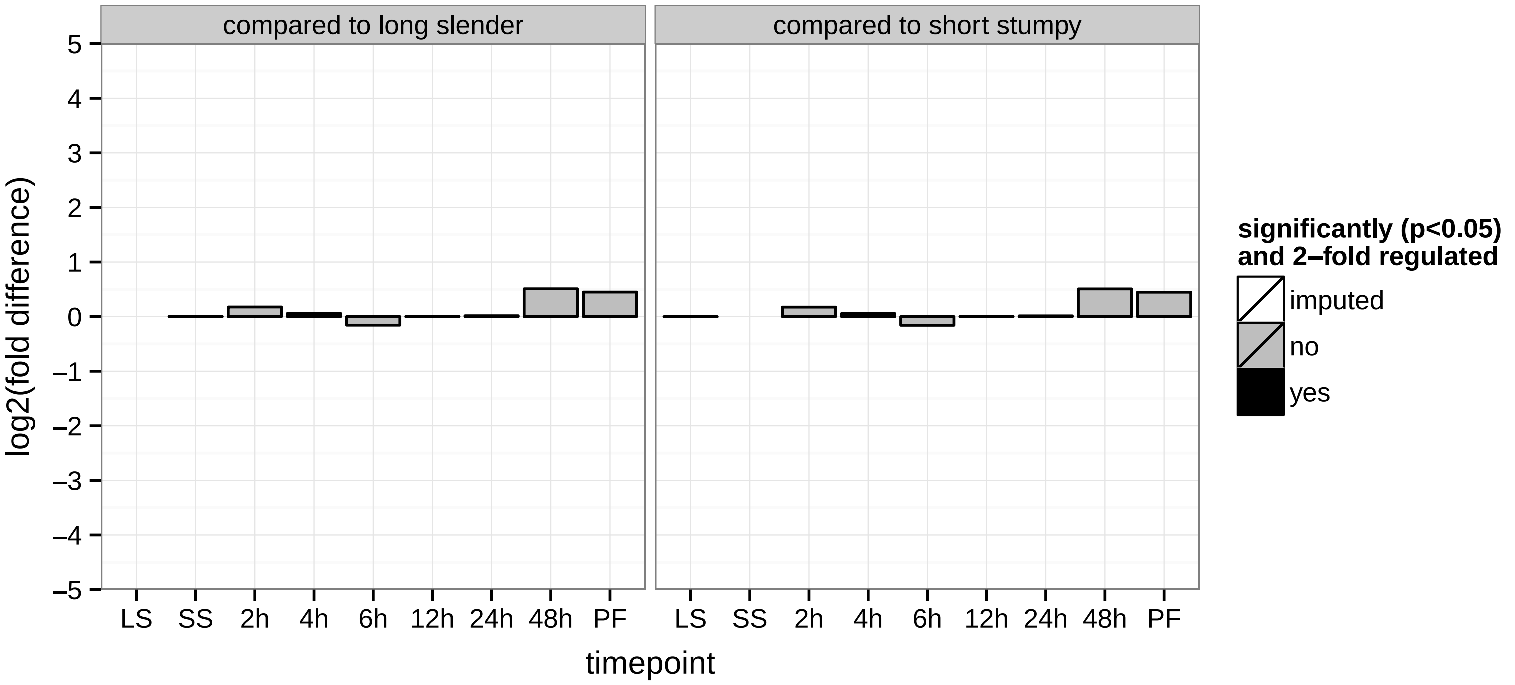

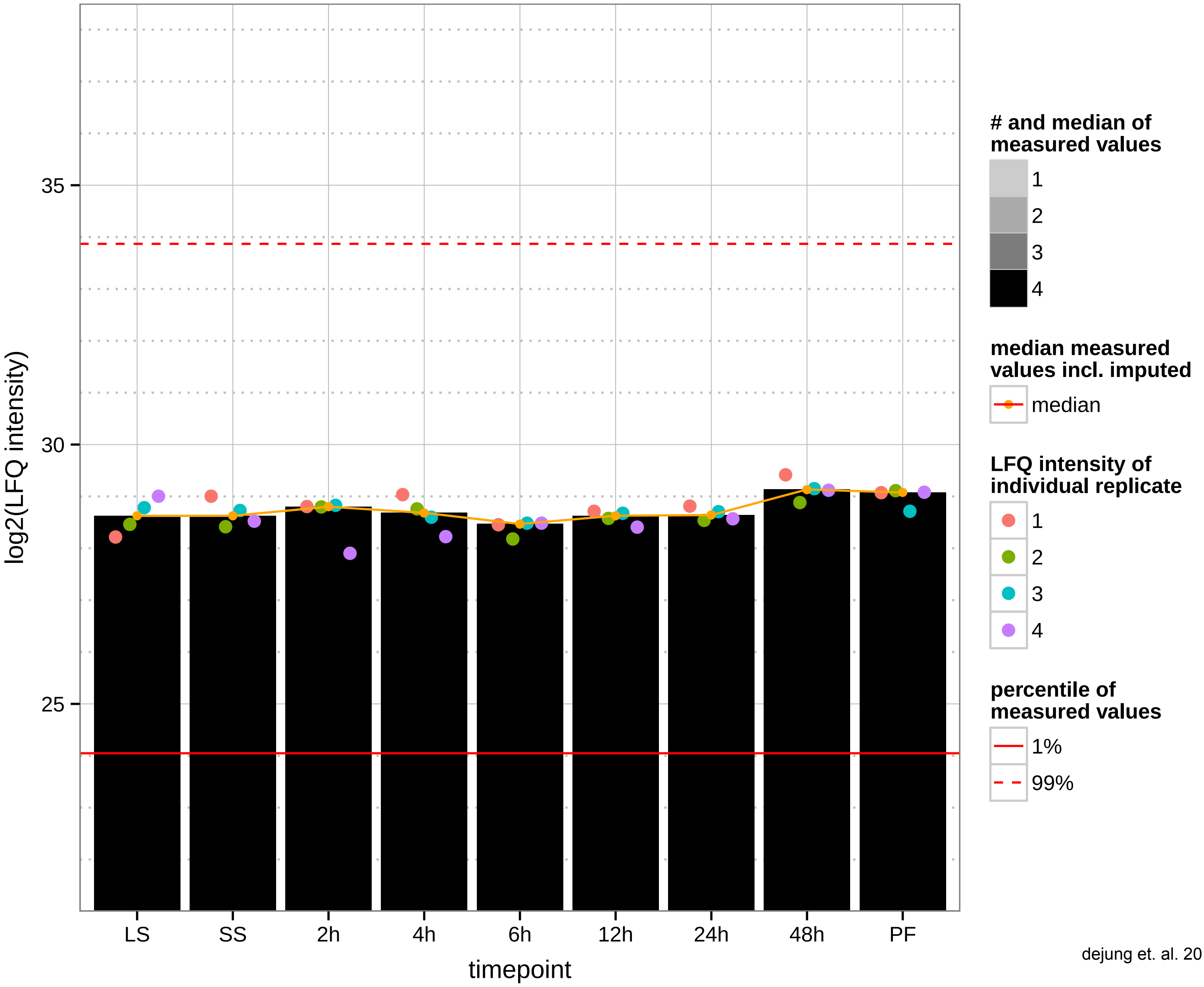

dejung et. al. 2015

vacuolar ATP synthase subunit c, putative
Tb927.10.14040
AGOF: hydrolase activity, acting on acid anhydrides, catalyzing transmembrane movement of substances
AGOC: proton−transporting V−type ATPase, V1 domain, proton−transporting two−sector ATPase complex
AGOP: ATP synthesis coupled proton transport
PGOF: hydrolase activity, acting on acid anhydrides, catalyzing transmembrane movement of substances
PGOC: proton−transporting V−type ATPase, V1 domain
PGOP: ATP hydrolysis coupled proton transport

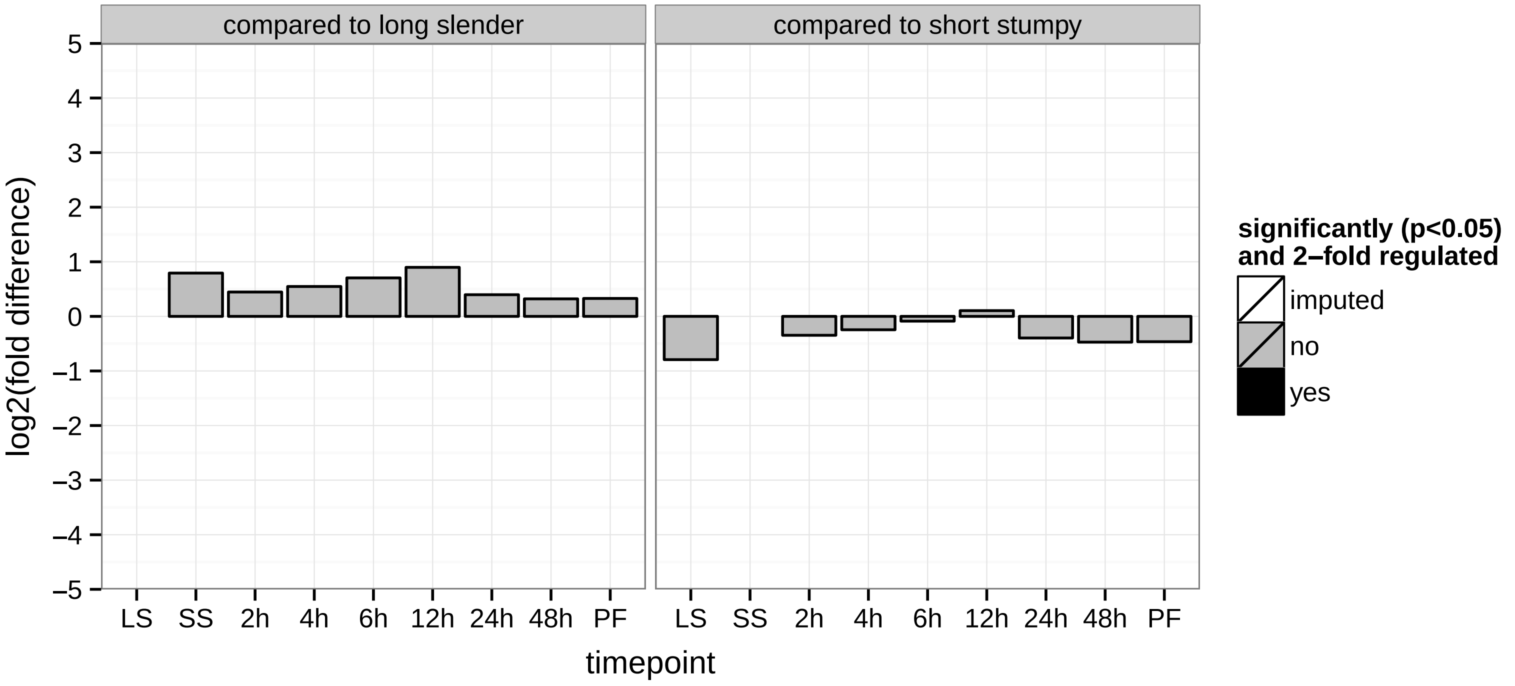

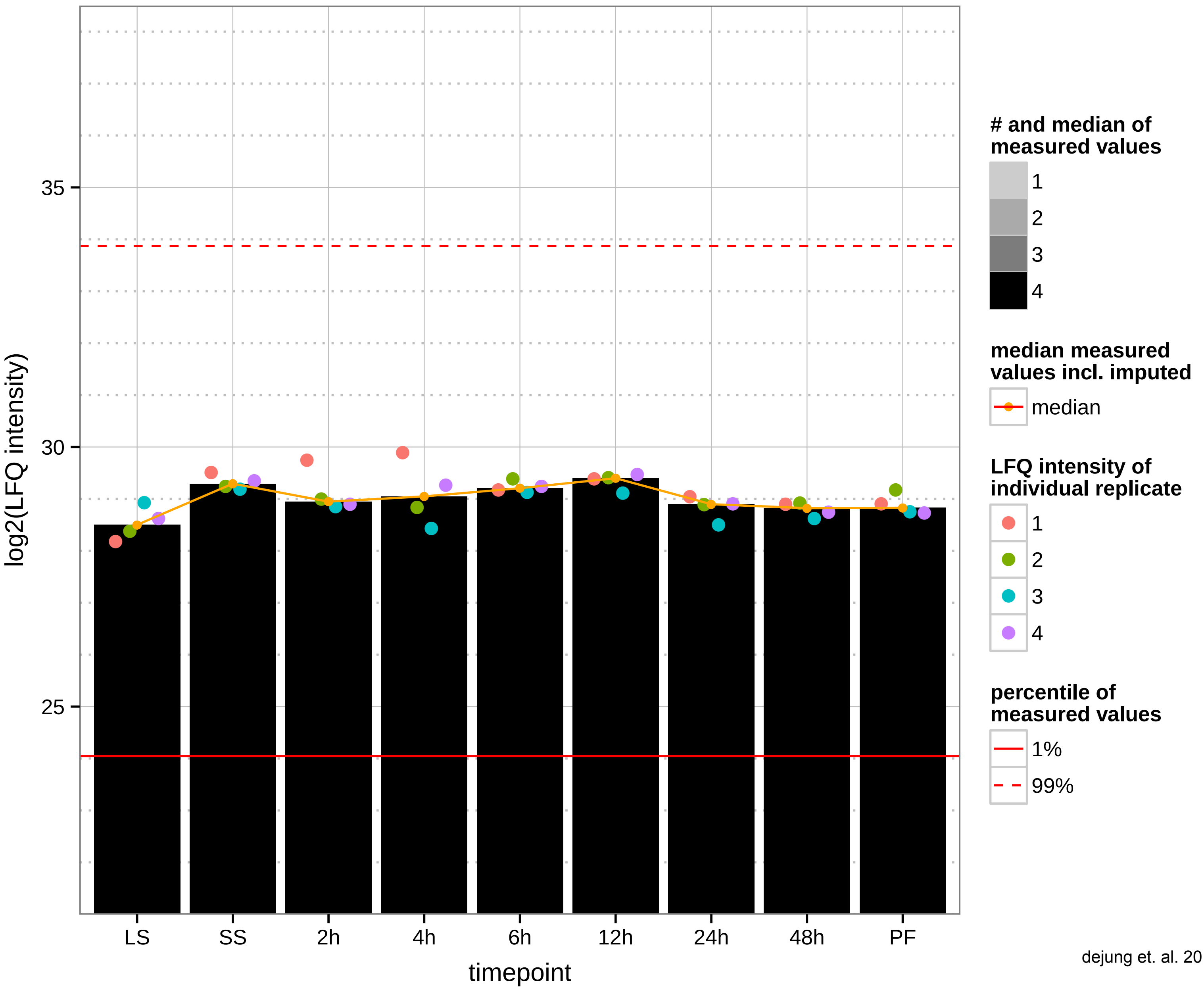

dejung et. al. 2015

protein transport protein SEC13, putative
Tb927.10.14180
AGOF: null
AGOC: null
AGOP: intracellular protein transport, membrane budding
PGOF: protein binding
PGOC: null
PGOP: null

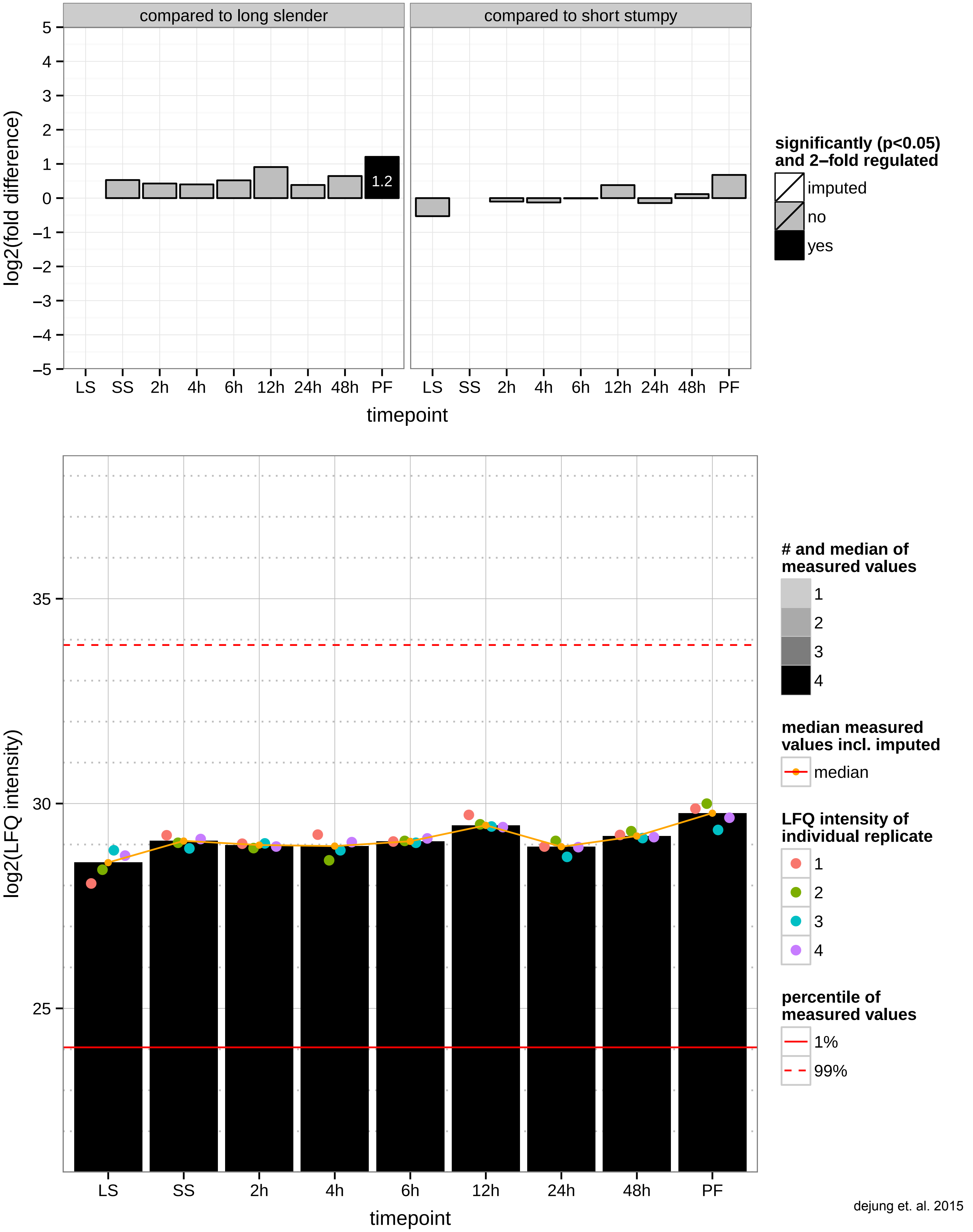

dejung et. al. 2015

methionine aminopeptidase 2, putative, metallo–peptidase, Clan MG, Family M24, putative (MetAP2)
Tb927.10.1420
AGOF: null, aminopeptidase activity, metallopeptidase activity
AGOC: null
AGOP: null, cellular process, proteolysis
PGOF: null, aminopeptidase activity, metalloexopeptidase activity
PGOC: null
PGOP: cellular process, proteolysis

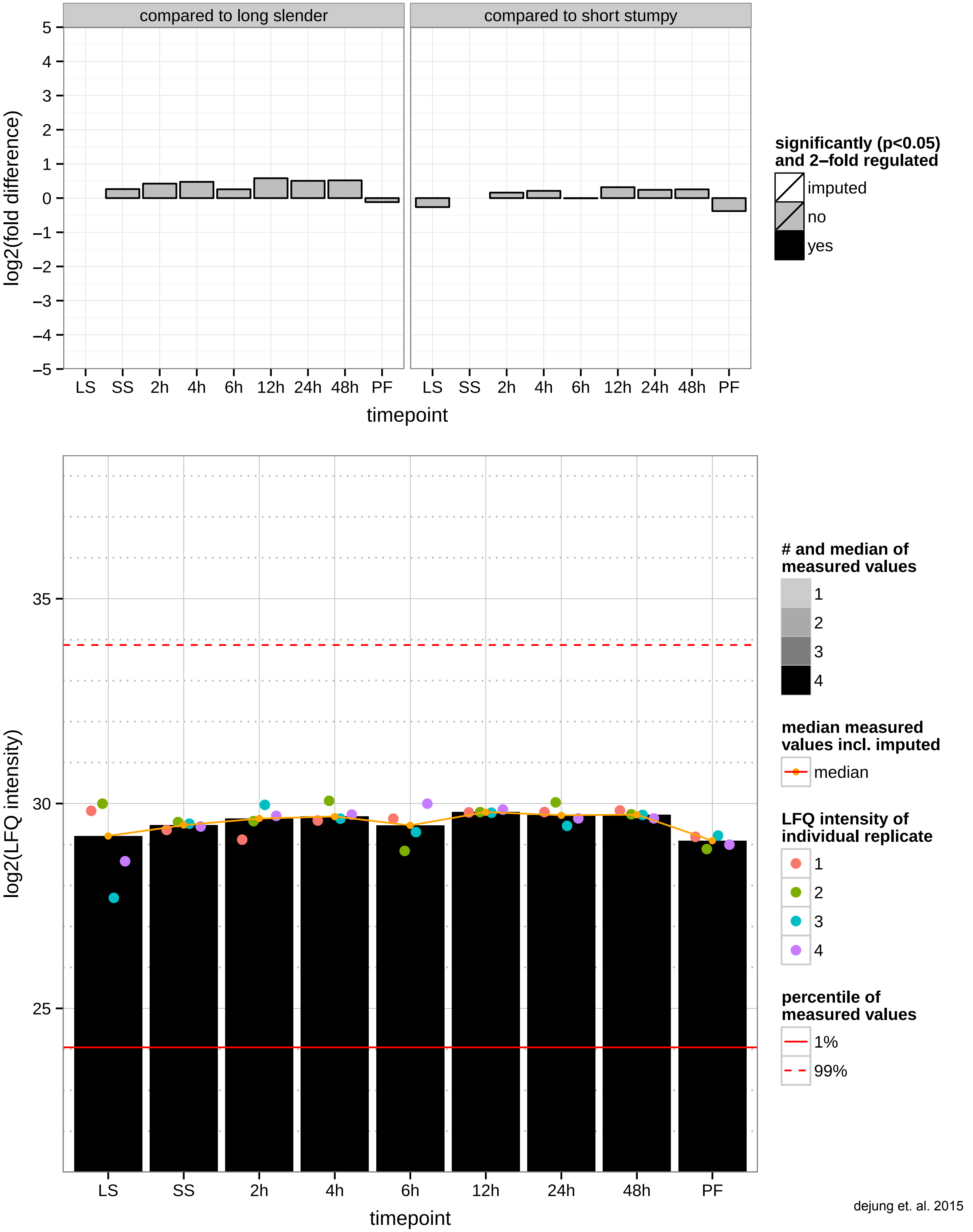

dejung et. al. 2015

syntaxin 5
Tb927.10.14200
AGOF: SNAP receptor activity
AGOC: membrane
AGOP: membrane fusion, vesicle–mediated transport
PGOF: protein binding
PGOC: membrane
PGOP: vesicle–mediated transport

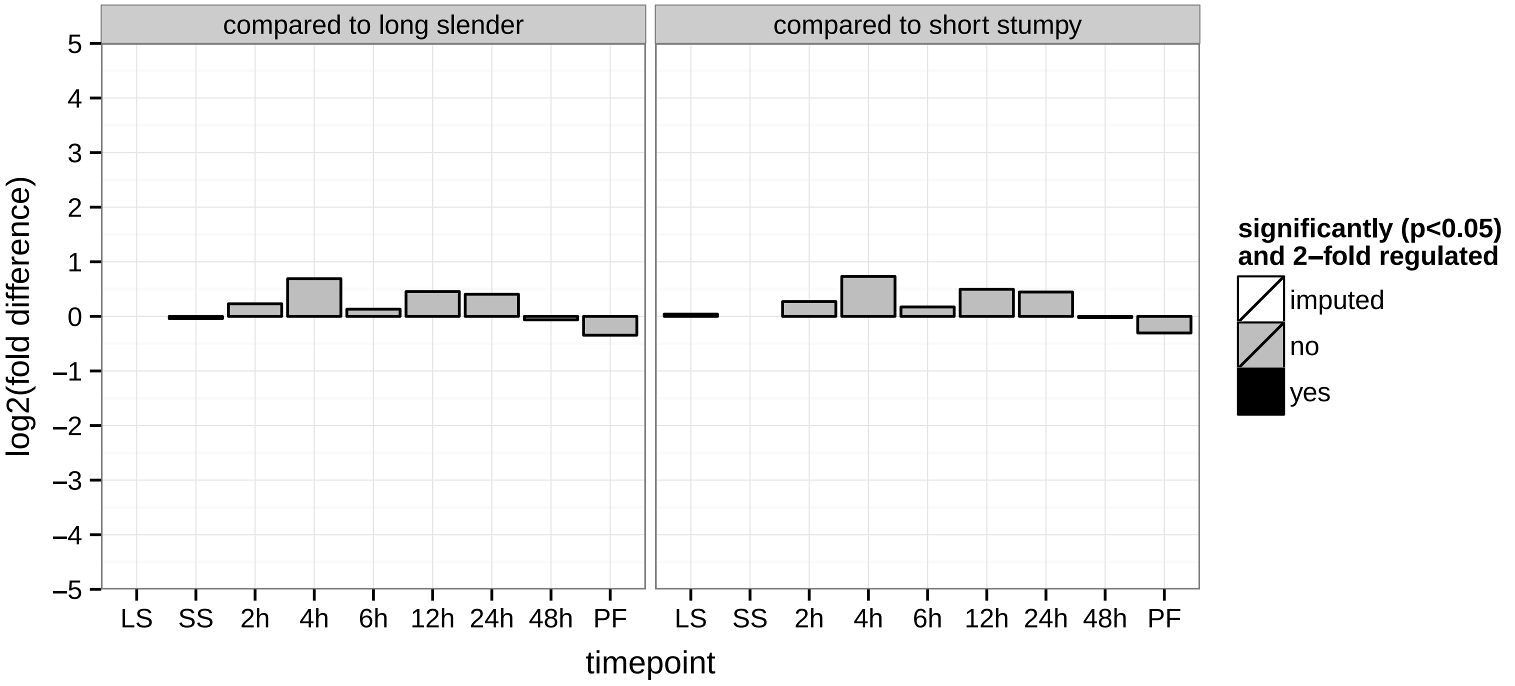

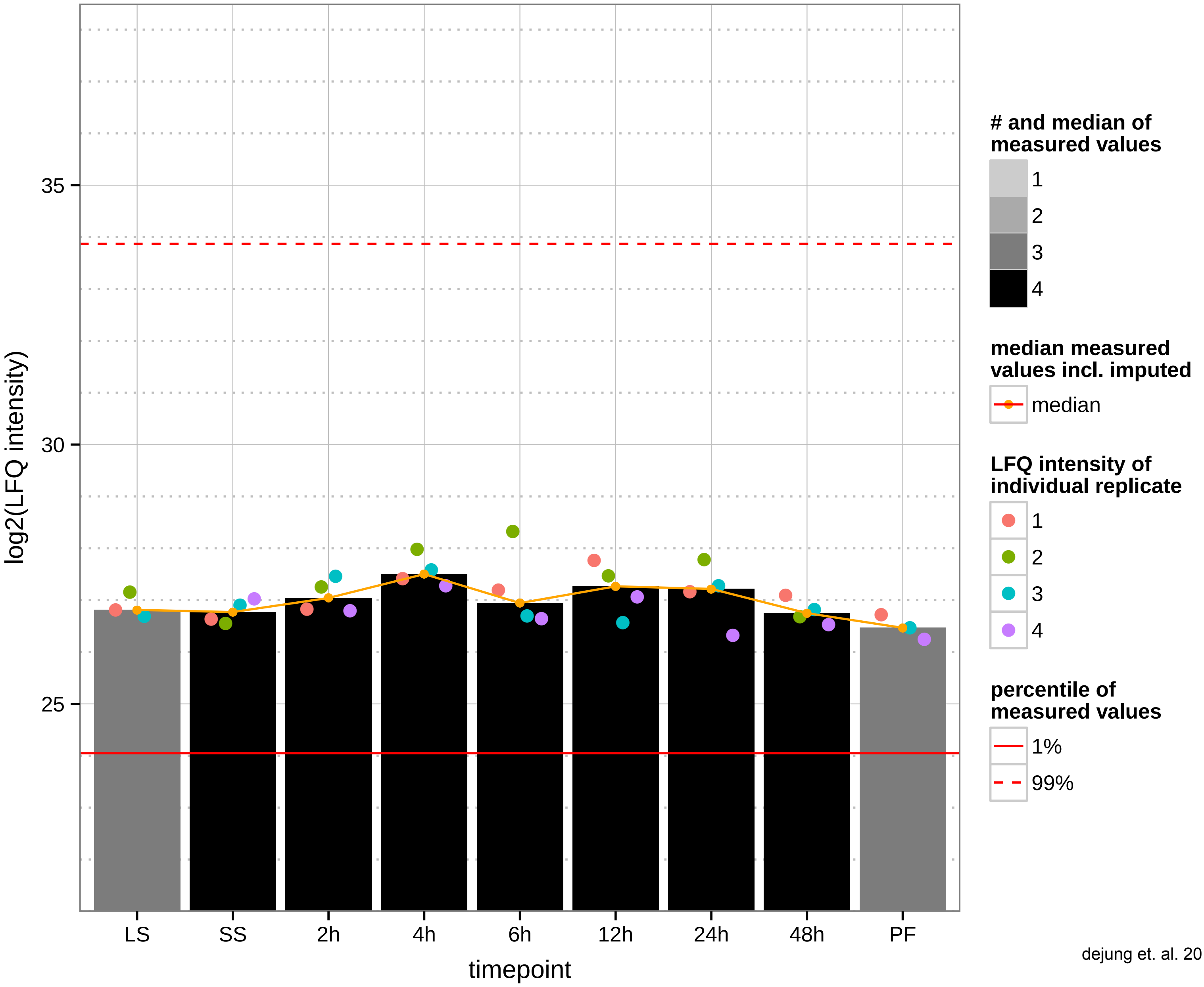

dejung et. al. 2015

N–myristoyl transferase, putative, N–myristoyltransferase (NMT)
Tb927.10.14240;Tb11.v5.0859
AGOF: null, glycylpeptide N–tetradecanoyltransferase activity, myristoyltransferase activity
AGOC: null
AGOP: null, N–terminal protein myristoylation
PGOF: glycylpeptide N–tetradecanoyltransferase activity
PGOC: null
PGOP: N–terminal protein myristoylation

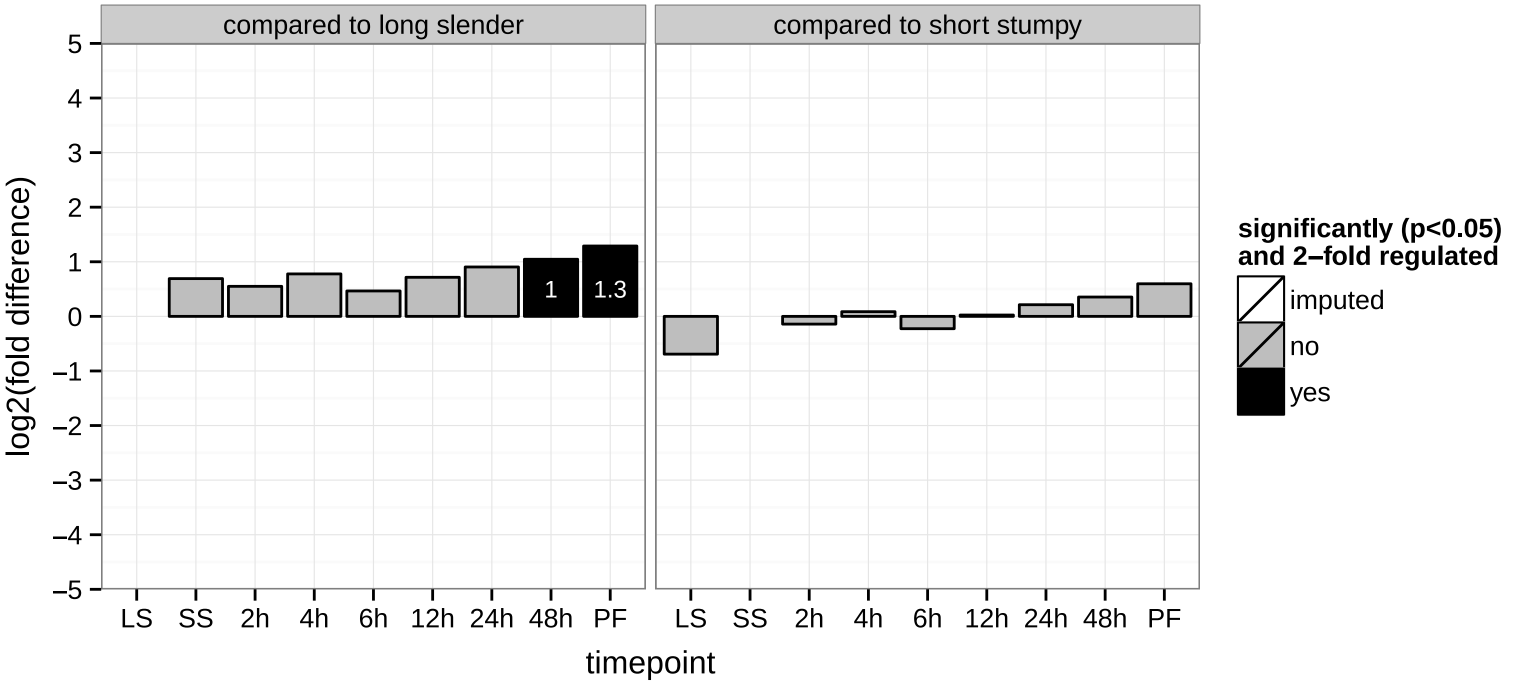

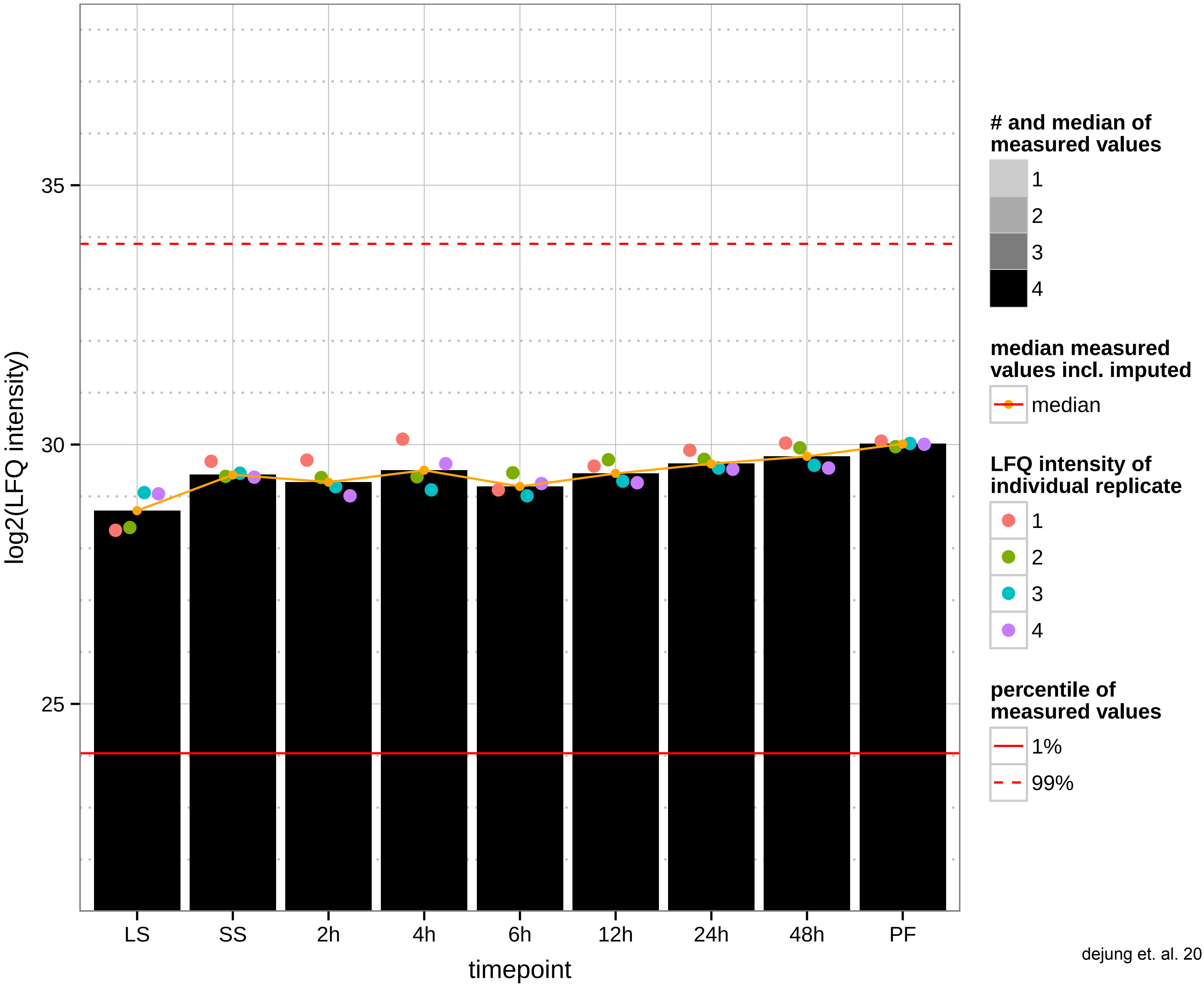

dejung et. al. 2015

U2 small nuclear ribonucleoprotein 40K (U2_40K)
Tb927.10.14360
AGOF: mRNA binding
AGOC: U2 snRNP
AGOP: nuclear mRNA cis splicing, via spliceosome
PGOF: null
PGOC: null
PGOP: null

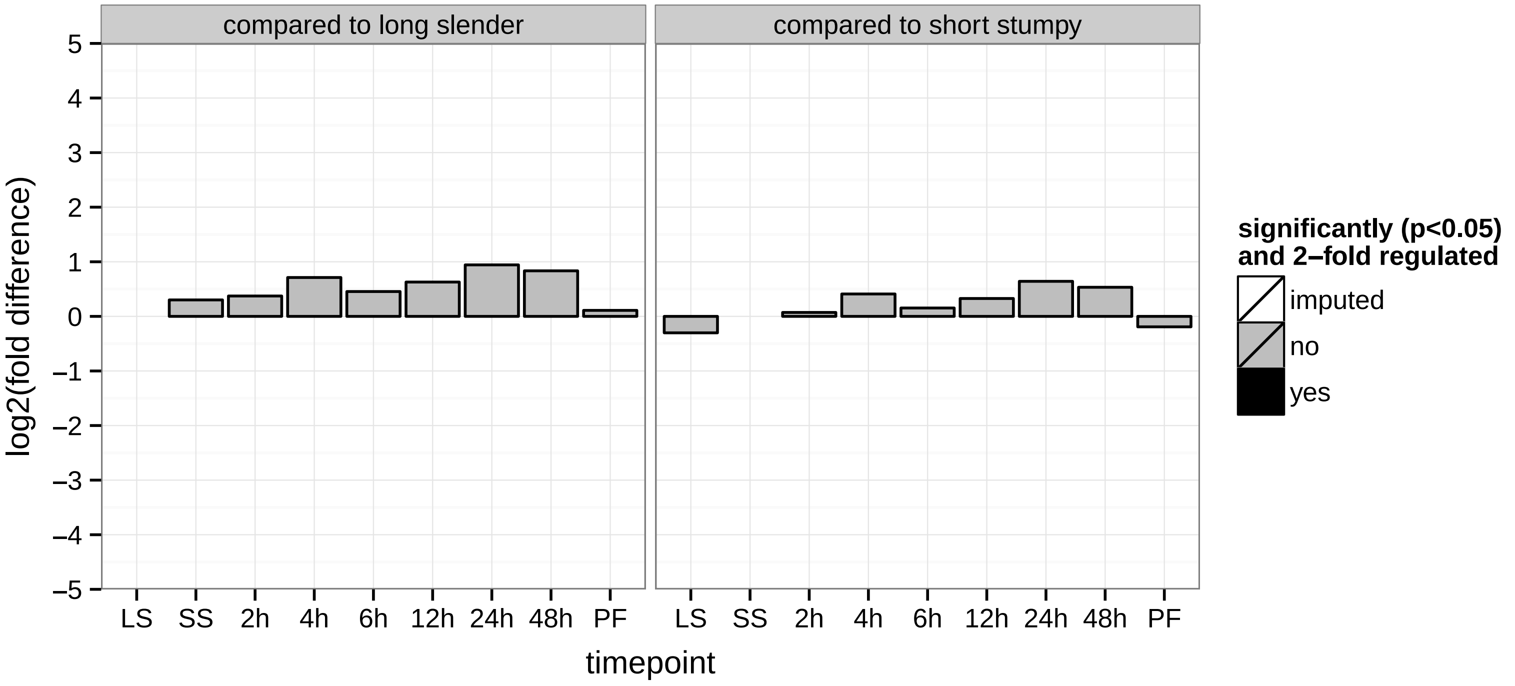

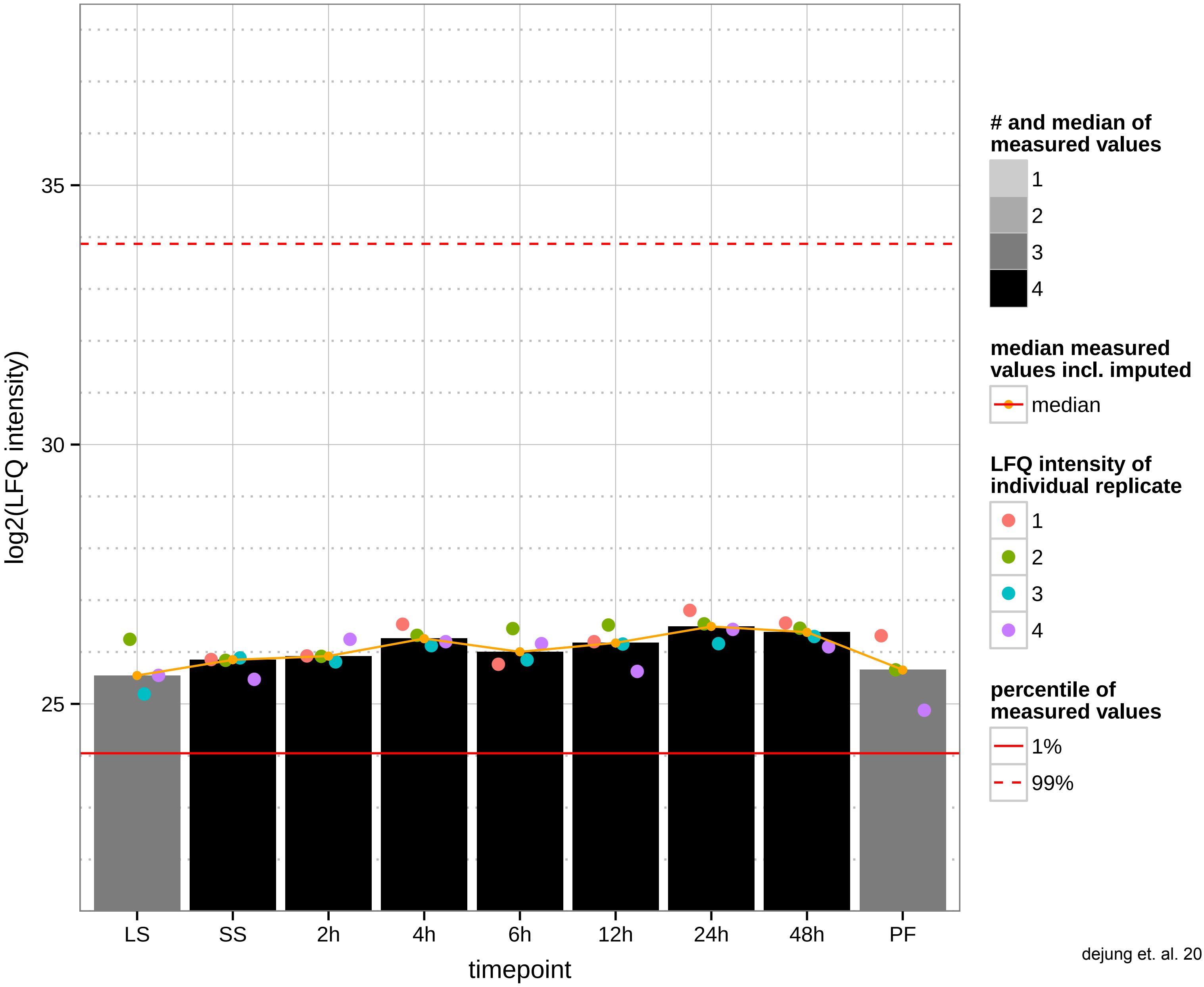

dejung et. al. 2015

hypothetical protein, conserved
Tb927.10.14390
AGOF: null
AGOC: null
AGOP: null
PGOF: binding
PGOC: null
PGOP: null

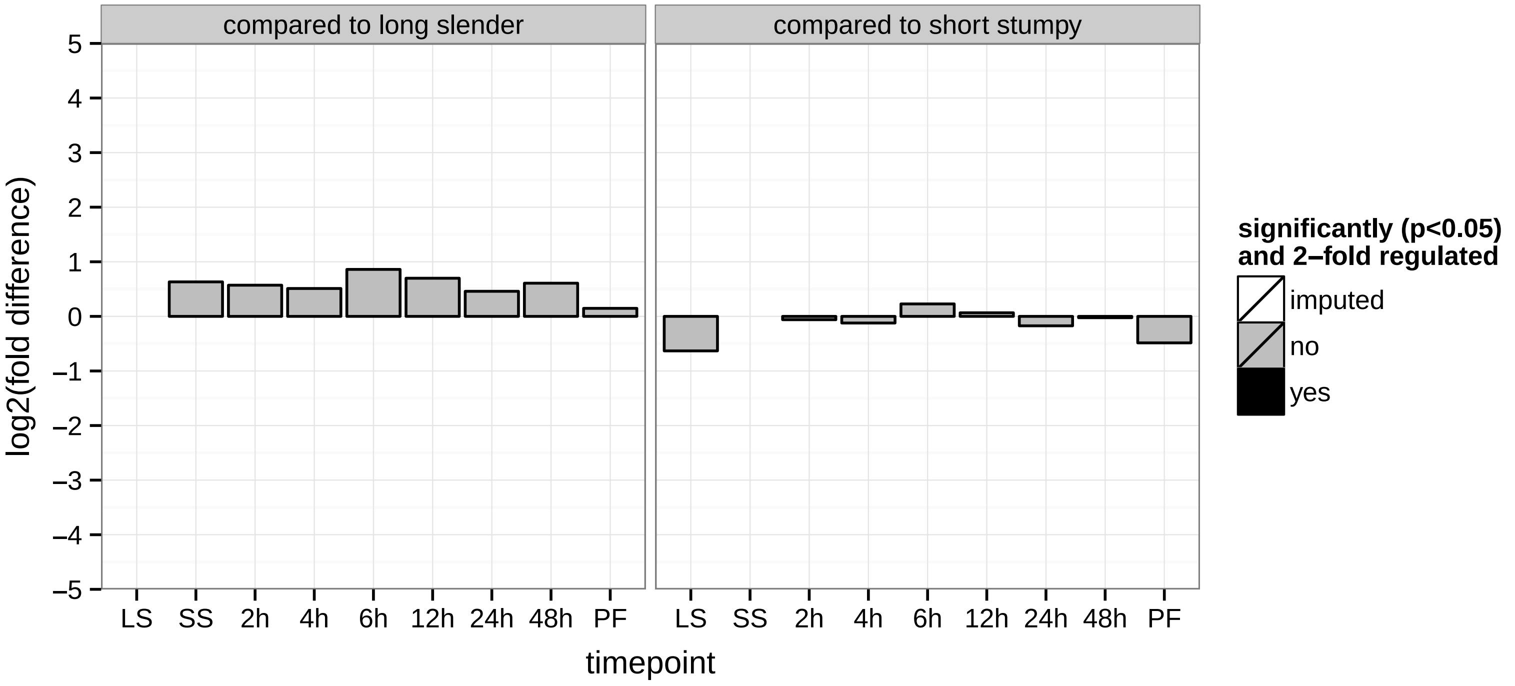

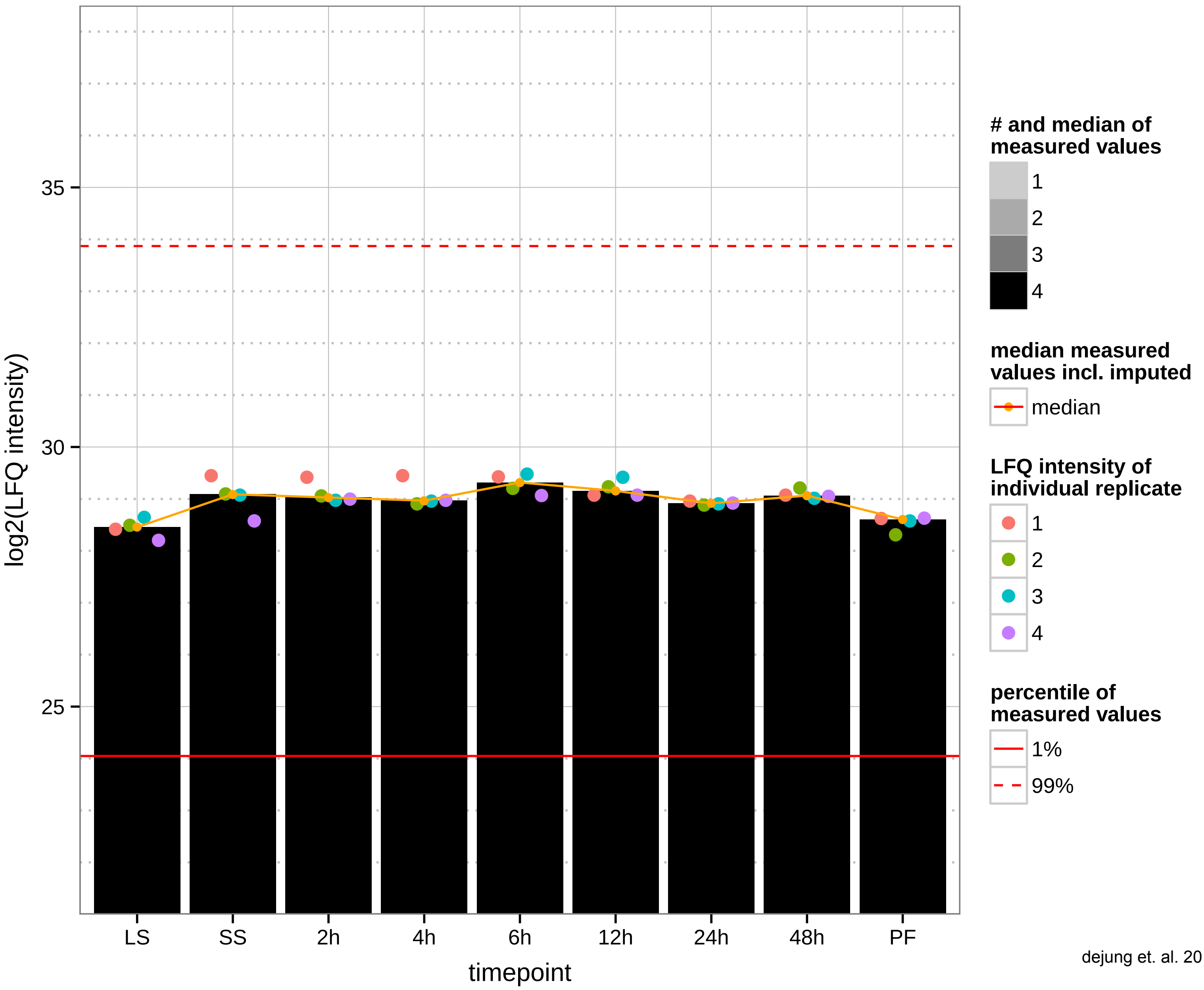

dejung et. al. 2015

intraflagellar transport protein IFT140, putative (IFT140)
Tb927.10.14470
AGOF: null
AGOC: null
AGOP: null
PGOF: protein binding
PGOC: null
PGOP: null

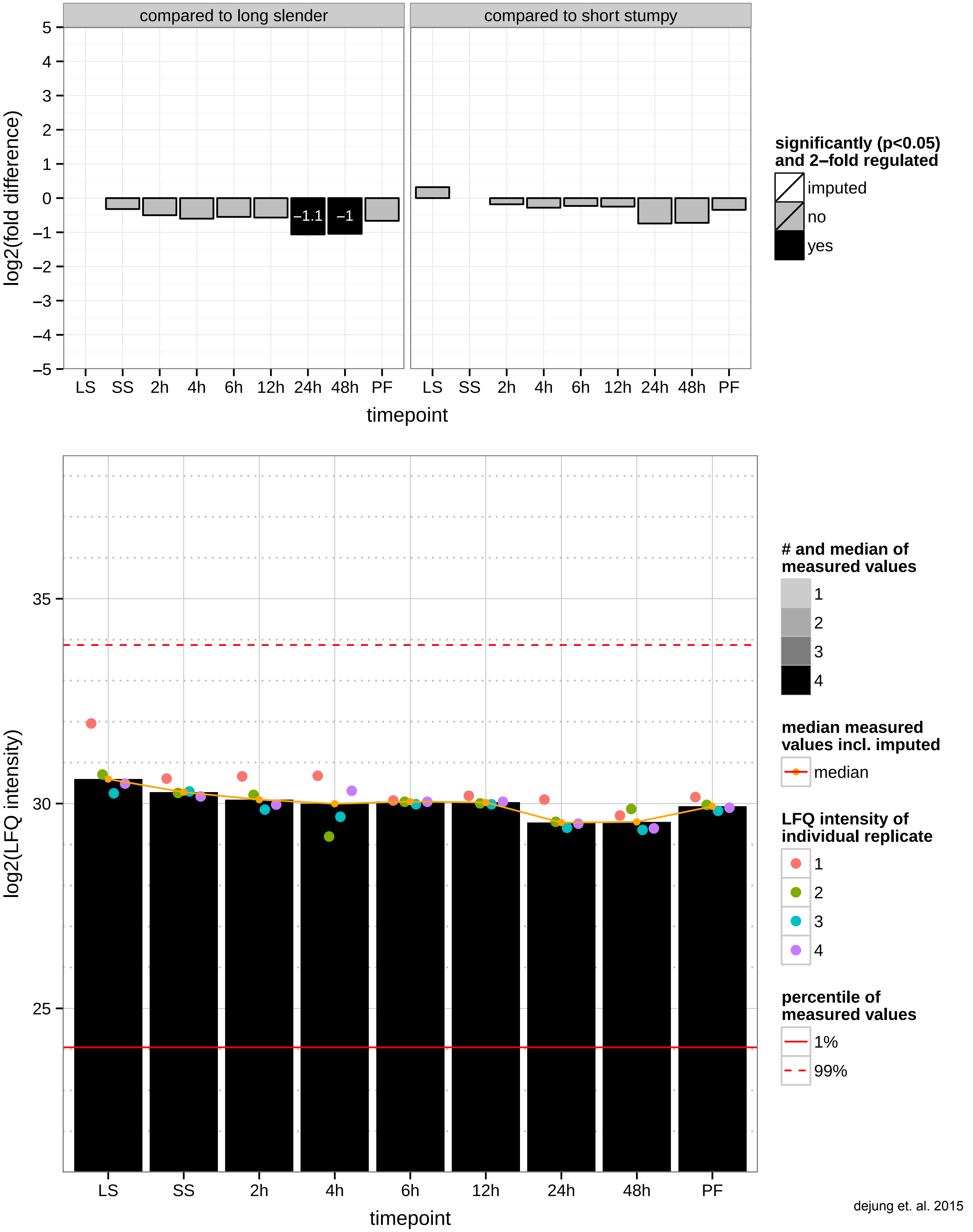

dejung et. al. 2015

hypothetical protein, conserved
Tb927.10.14480
AGOF: null
AGOC: null
AGOP: null
PGOF: null
PGOC: null
PGOP: null

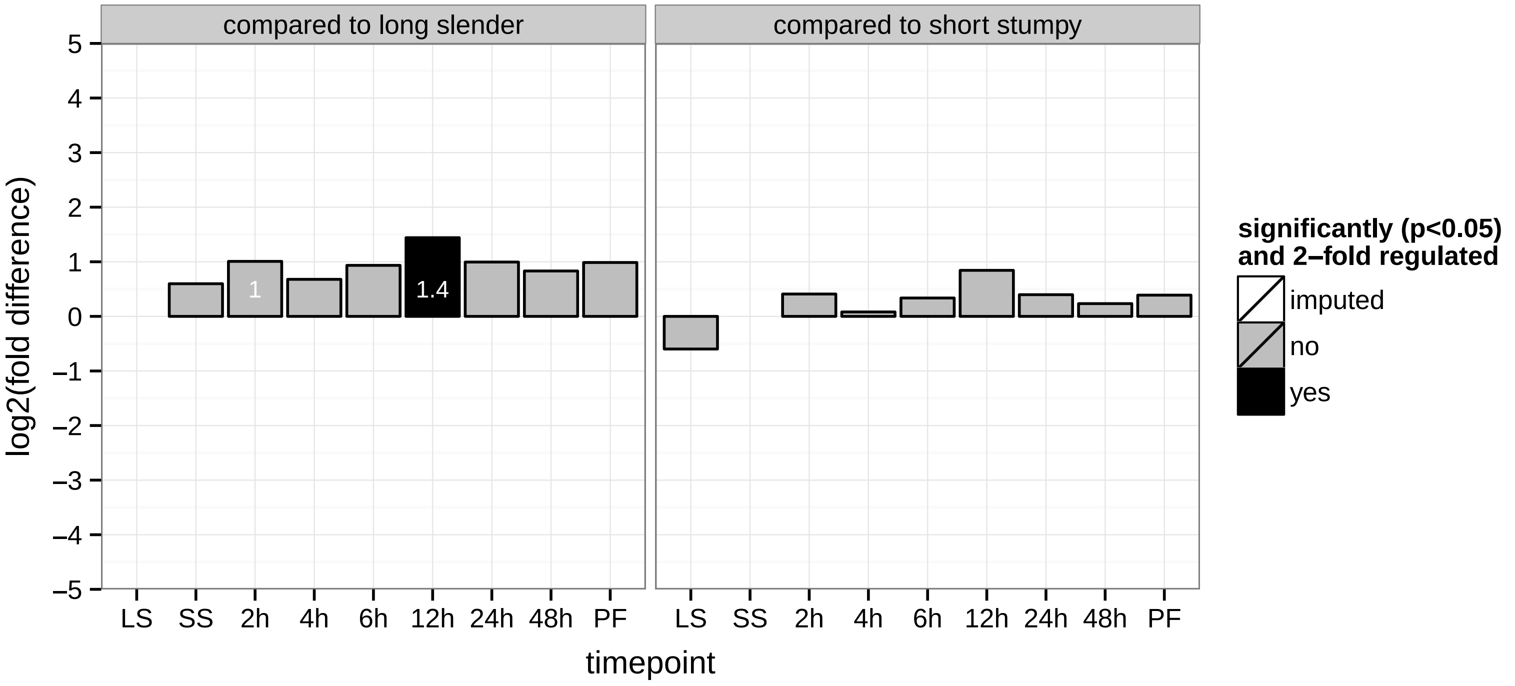

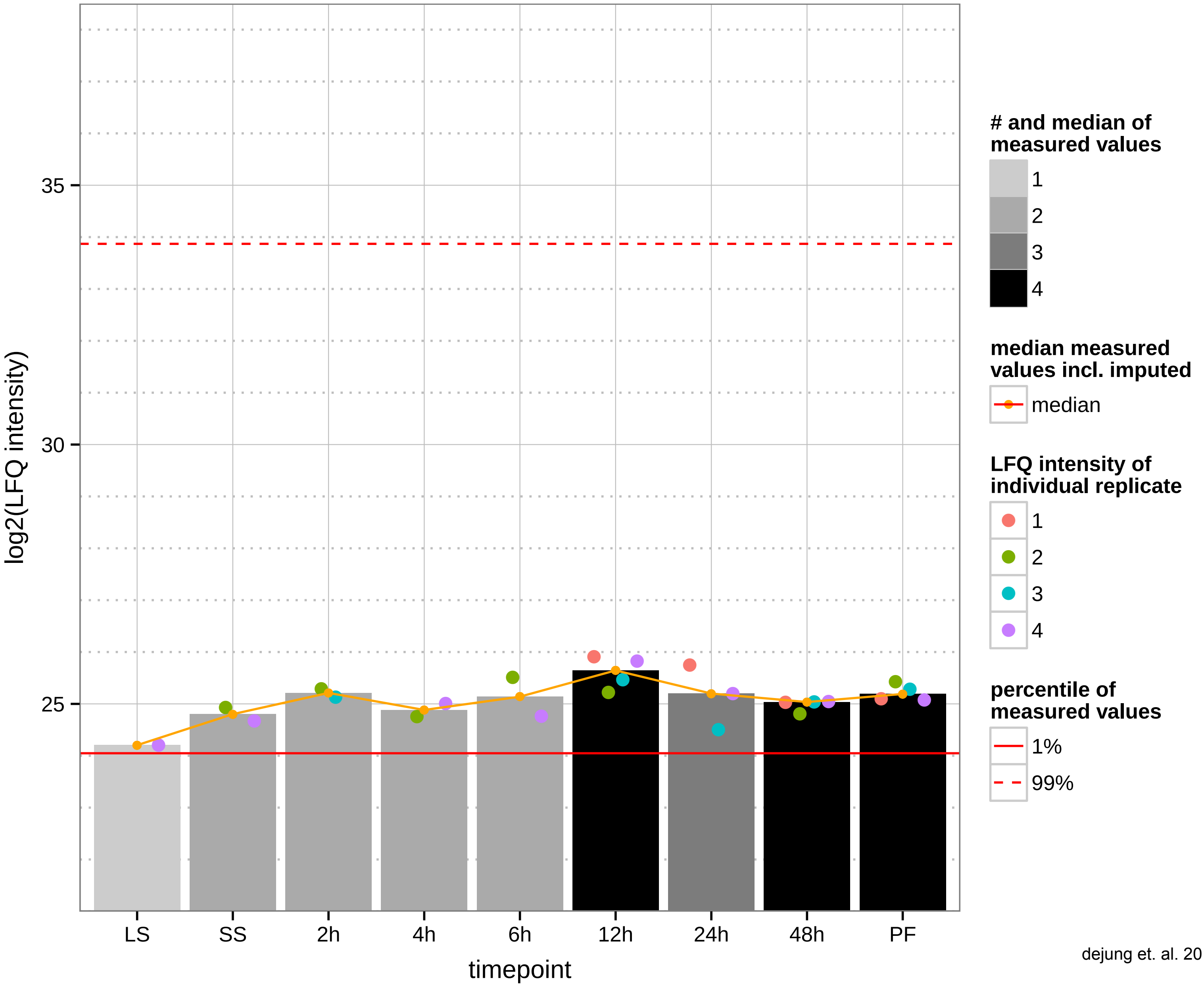

dejung et. al. 2015

hypothetical protein, conserved
Tb927.10.1450
AGOF: null
AGOC: null
AGOP: null
PGOF: null
PGOC: null
PGOP: null

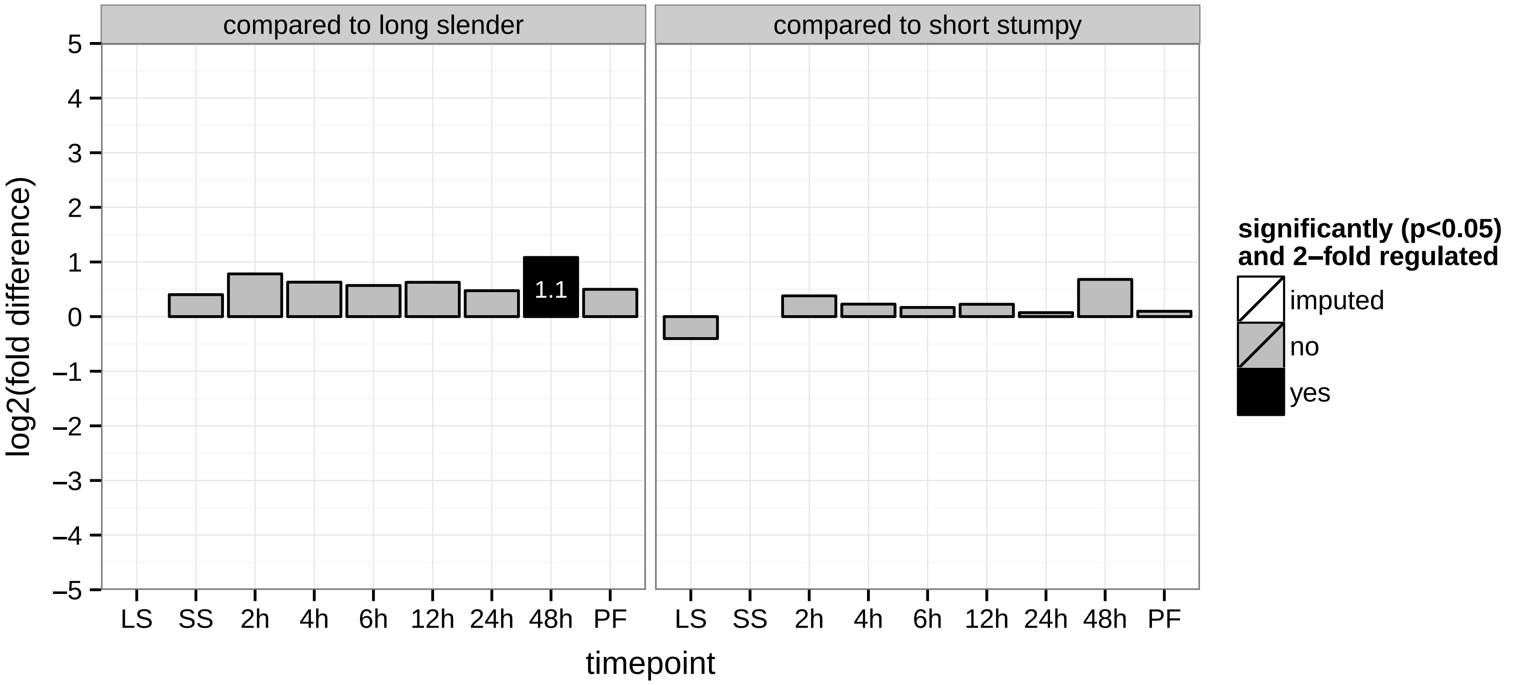

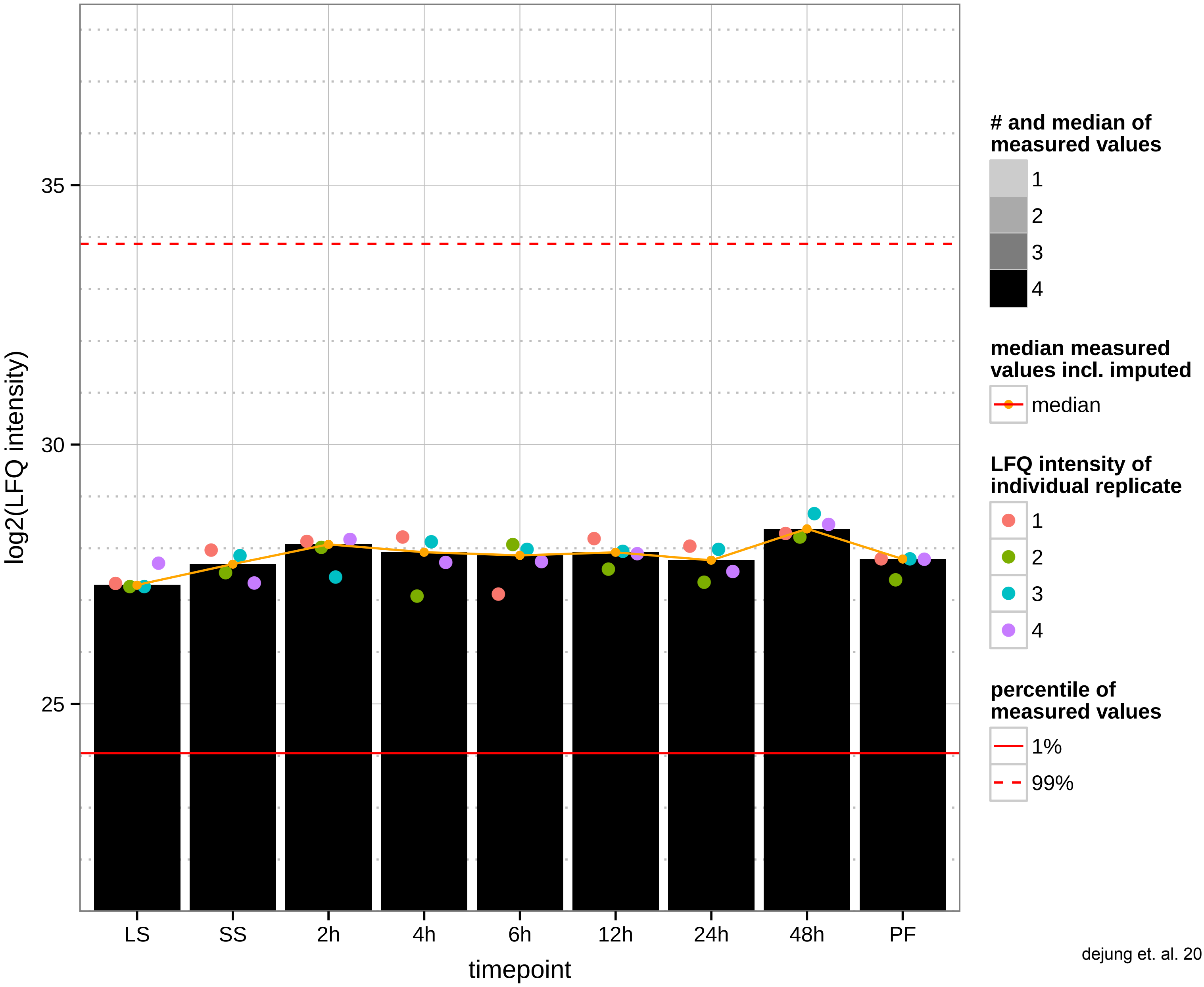

dejung et. al. 2015

hypothetical protein, conserved
Tb927.10.14500
AGOF: null
AGOC: null
AGOP: null
PGOF: null
PGOC: null
PGOP: null

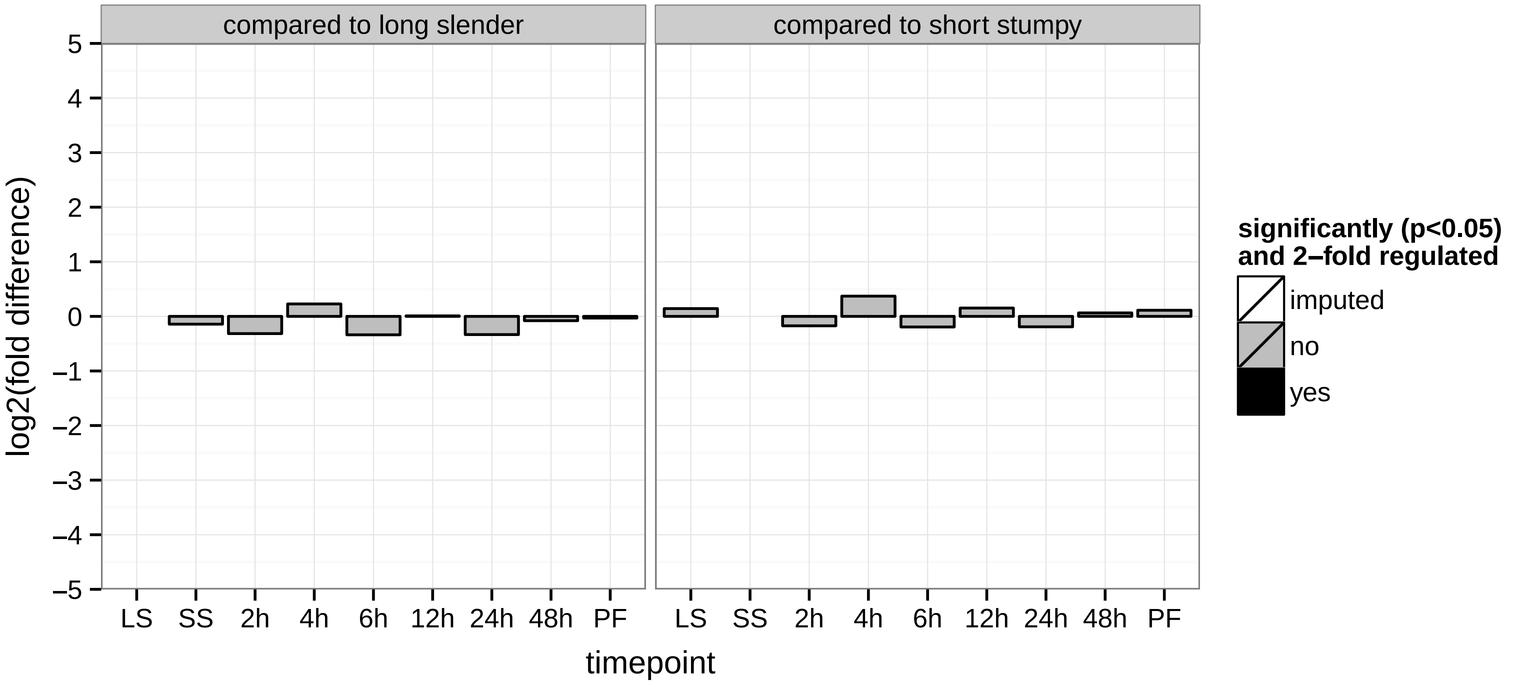

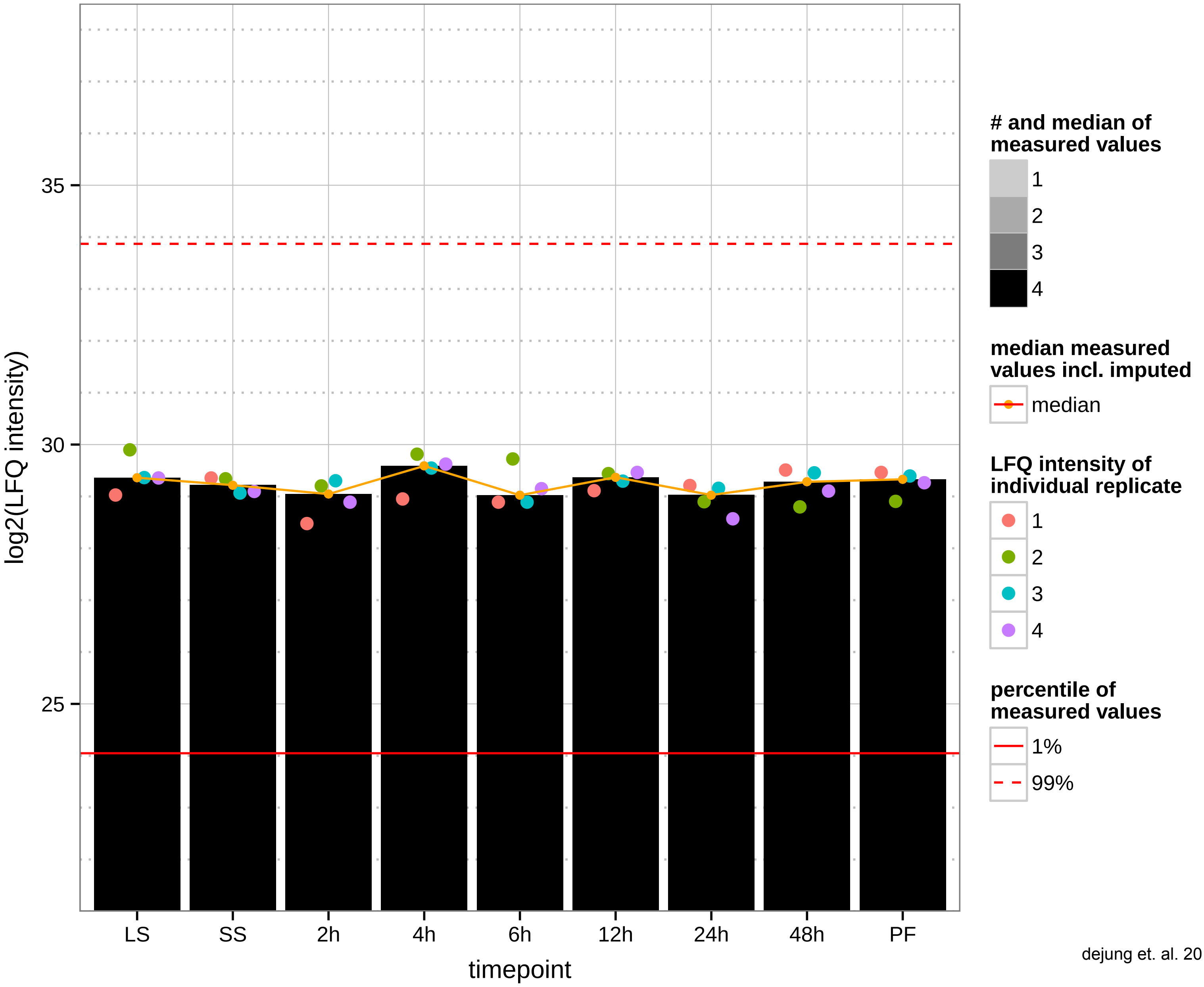

dejung et. al. 2015

hypothetical protein, conserved
Tb927.10.14510
AGOF: null
AGOC: null
AGOP: null
PGOF: hydrolase activity, acting on acid anhydrides
PGOC: null
PGOP: null

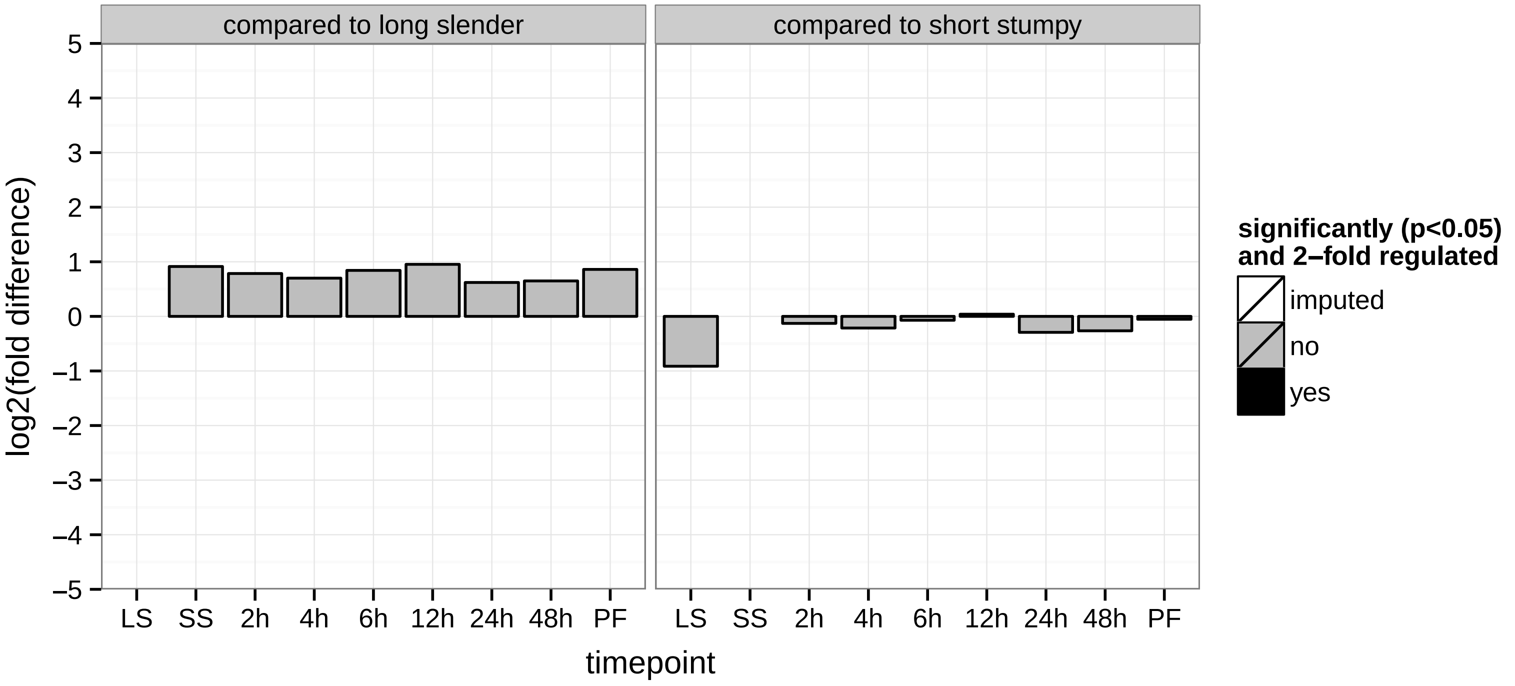

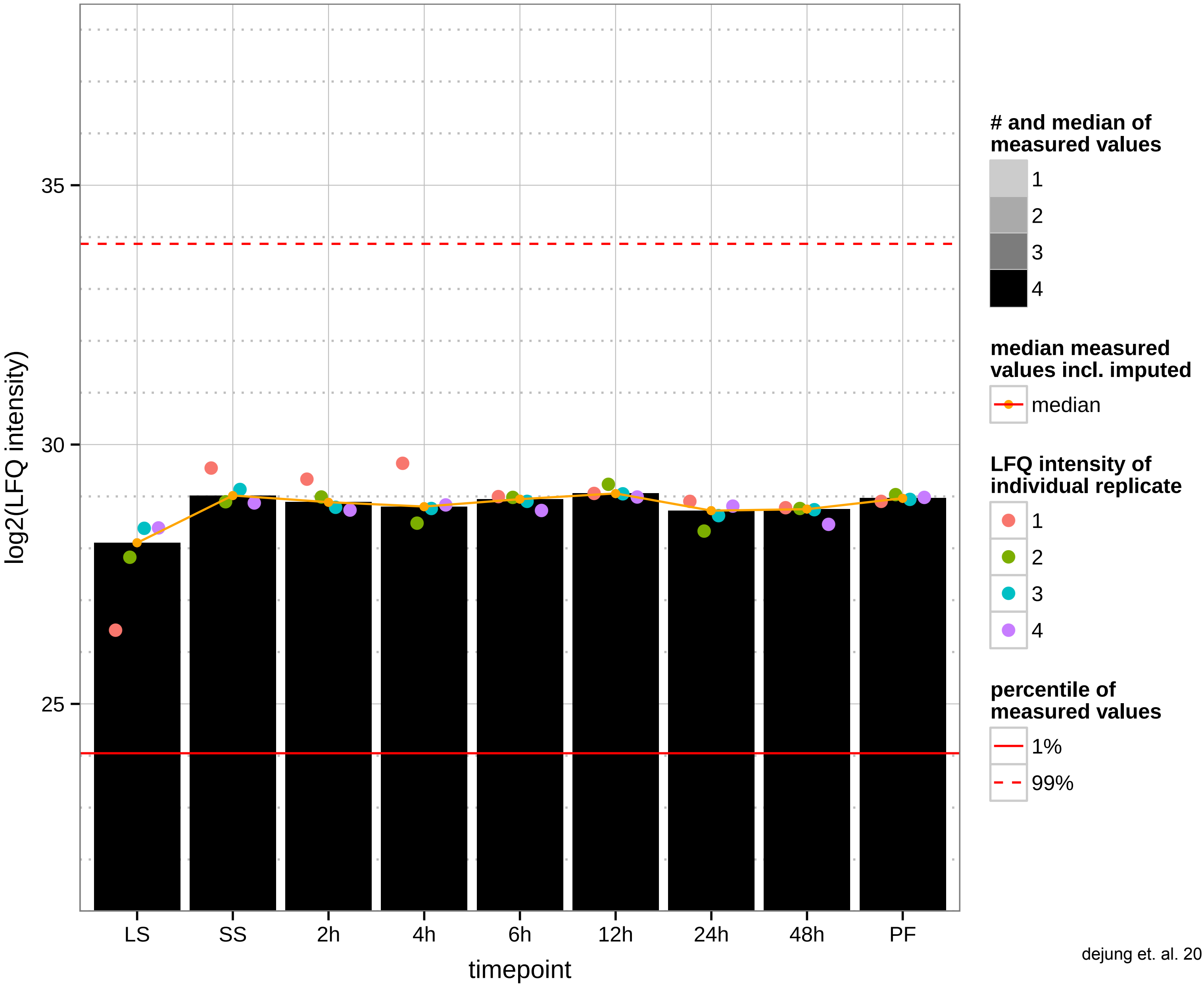

dejung et. al. 2015

hypothetical protein, conserved
Tb927.10.14520
AGOF: null
AGOC: bacterial–type flagellum basal body
AGOP: null
PGOF: null
PGOC: null
PGOP: null

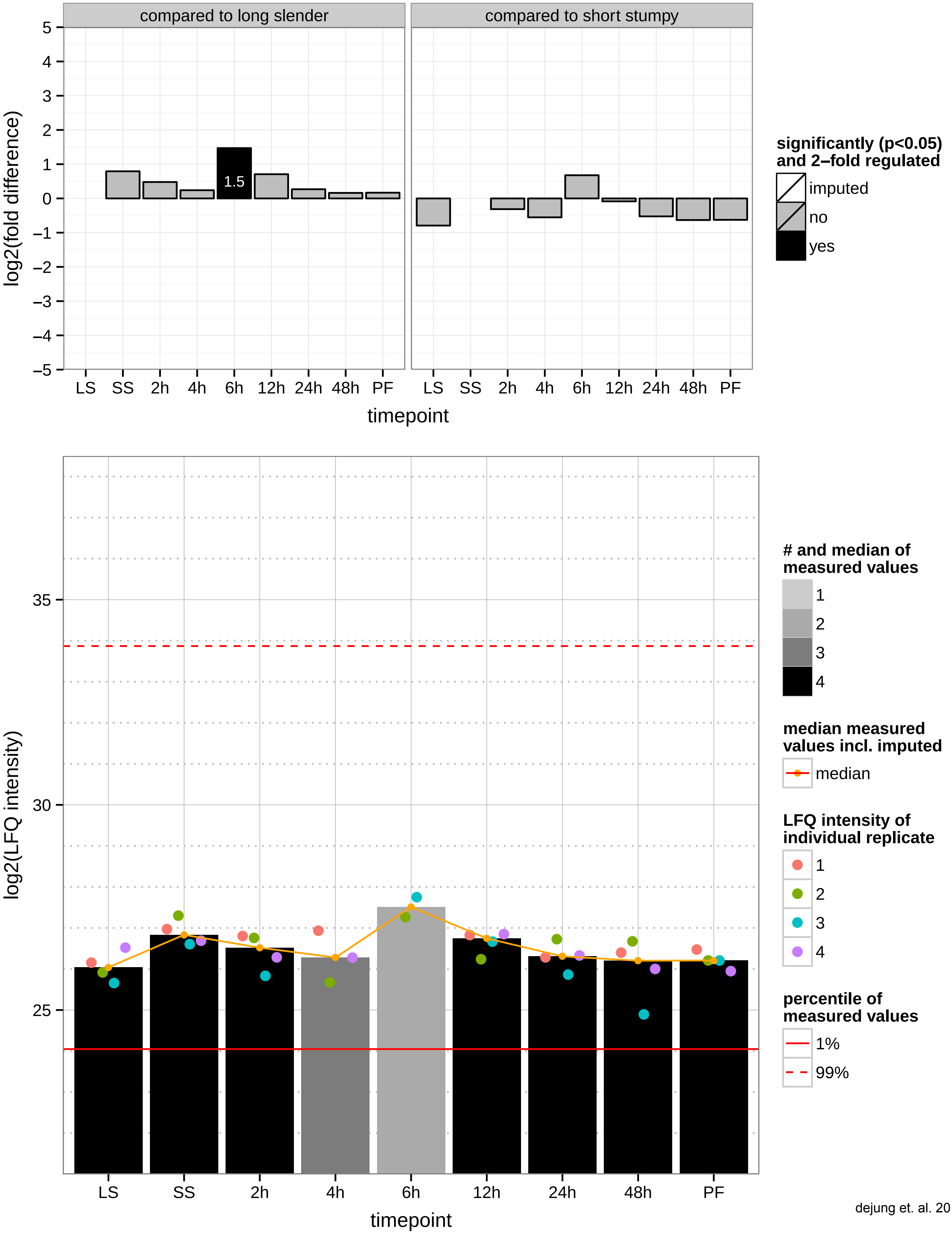

dejung et. al. 2015

19S proteasome non–atpase subunit 8, 26s proteasome non–atpase regulatory subunit 8 (Rpn8)
Tb927.10.14530
AGOF: endopeptidase activity
AGOC: proteasome regulatory particle
AGOP: cell growth, ubiquitin–dependent protein catabolic process
PGOF: protein binding
PGOC: null
PGOP: null

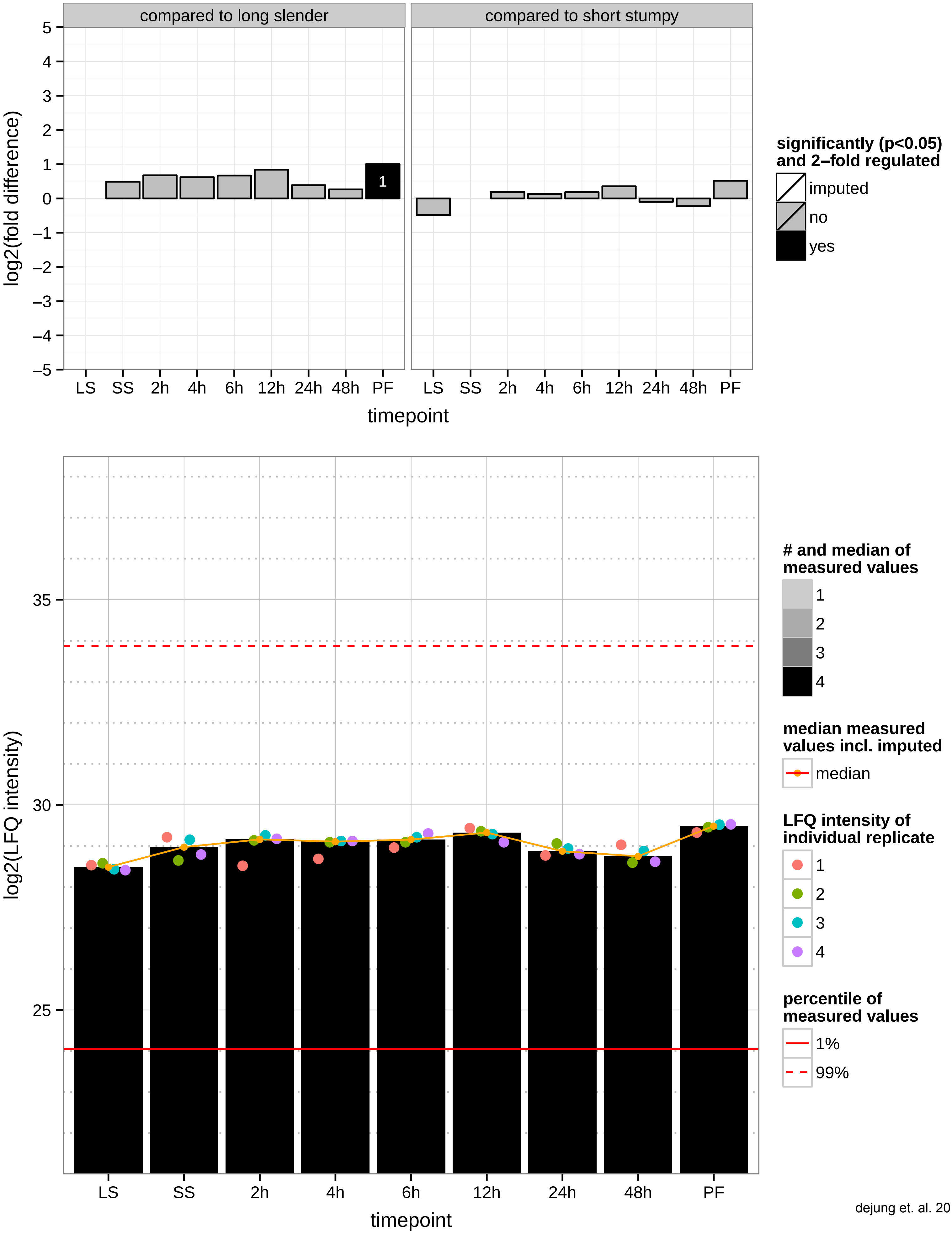

dejung et. al. 2015

hypothetical protein, conserved
Tb927.10.14570
AGOF: ATP binding, microtubule motor activity
AGOC: null
AGOP: microtubule–based movement
PGOF: ATP binding, microtubule motor activity
PGOC: null
PGOP: microtubule–based movement

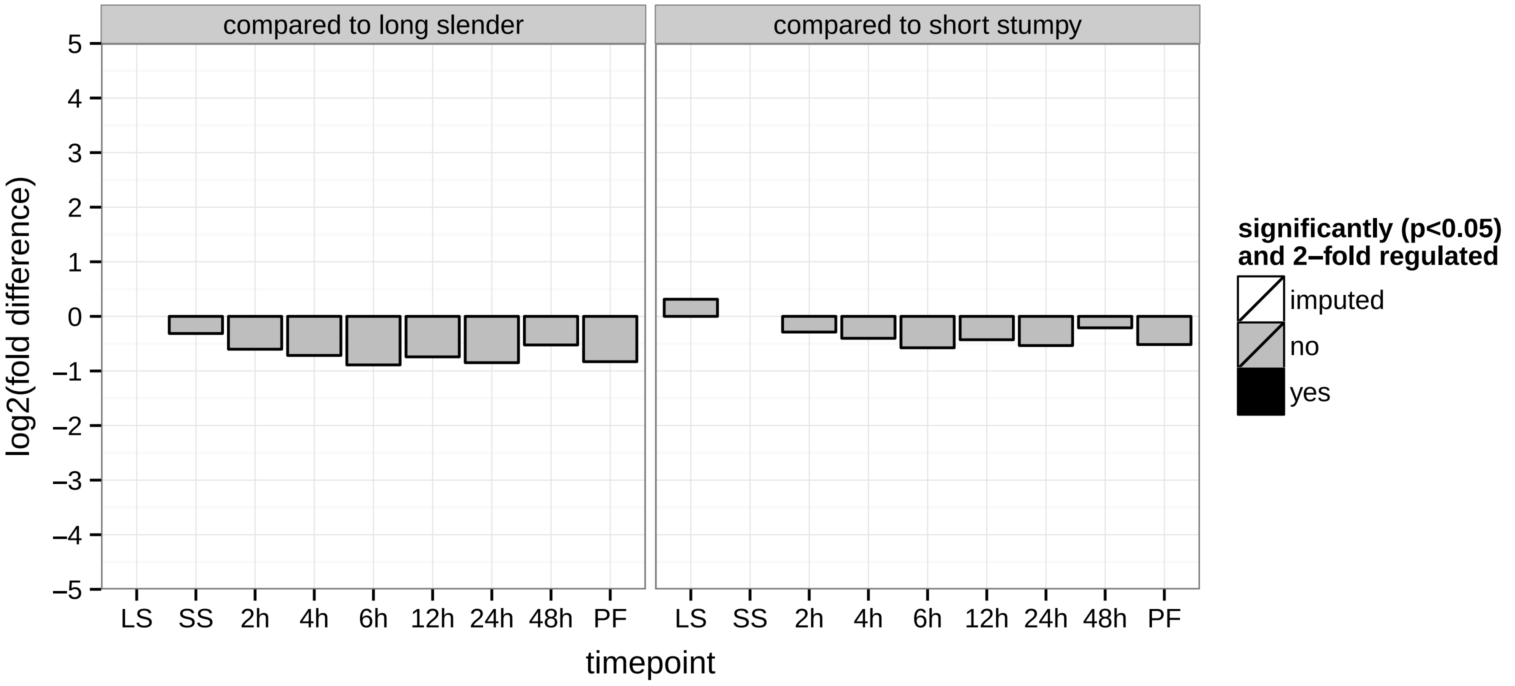

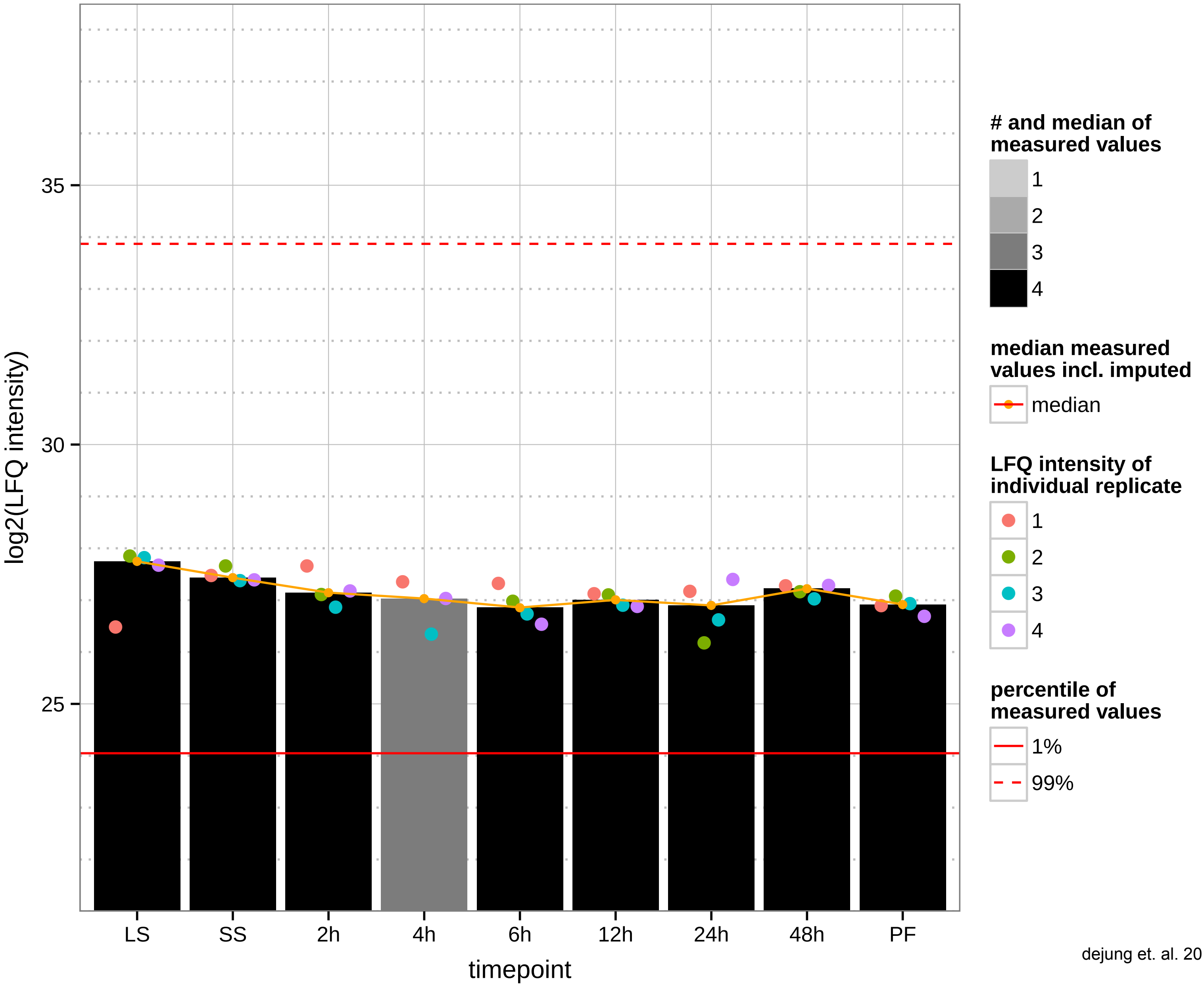

dejung et. al. 2015

40S ribosomal protein S2, putative (RPS2)
Tb927.10.14710;Tb927.10.14600
AGOF: RNA binding, structural constituent of ribosome
AGOC: intracellular, ribosome, small ribosomal subunit
AGOP: translation
PGOF: RNA binding, structural constituent of ribosome
PGOC: intracellular, ribosome, small ribosomal subunit
PGOP: translation

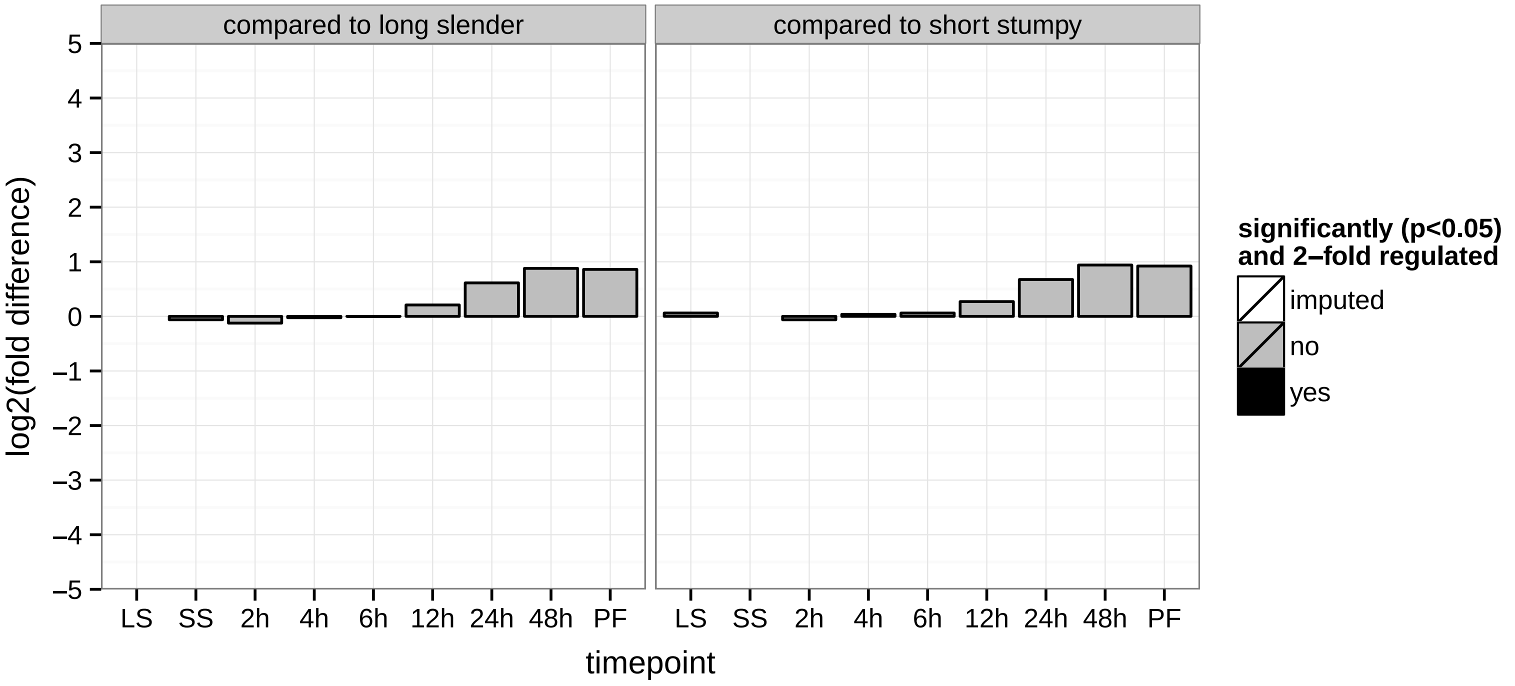

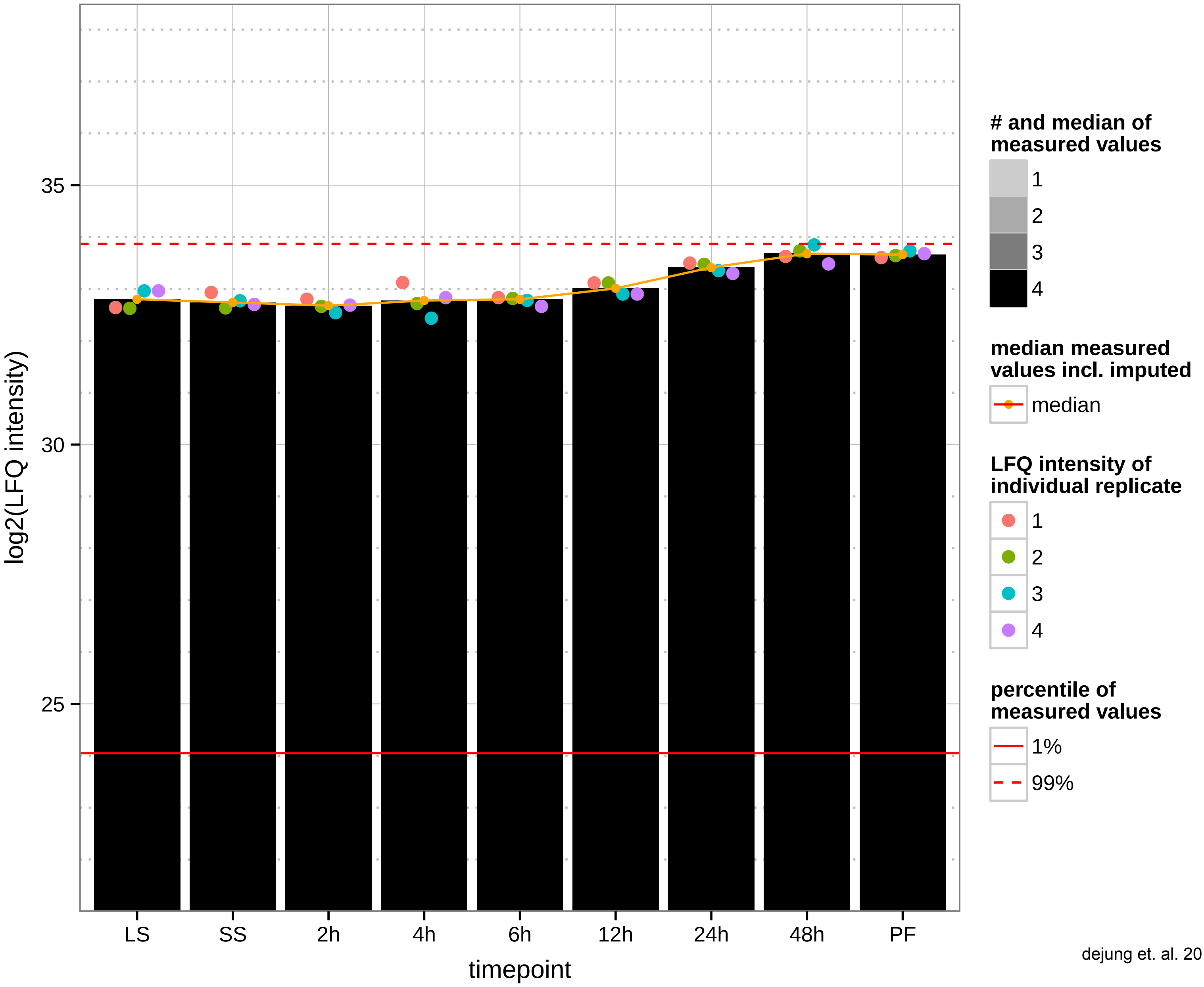

dejung et. al. 2015

leucine–rich repeat protein (LRRP), putative
Tb927.10.14610
AGOF: null
AGOC: null
AGOP: null
PGOF: null
PGOC: null
PGOP: null

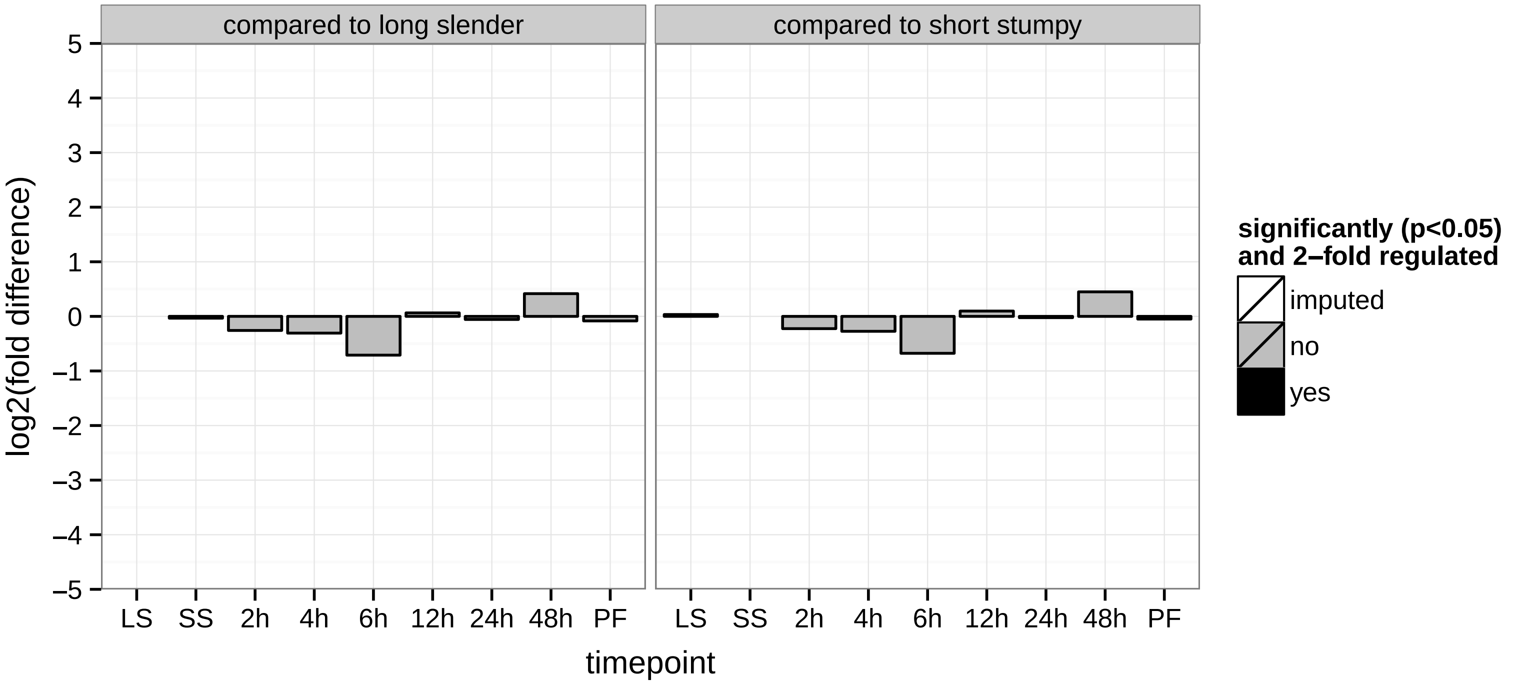

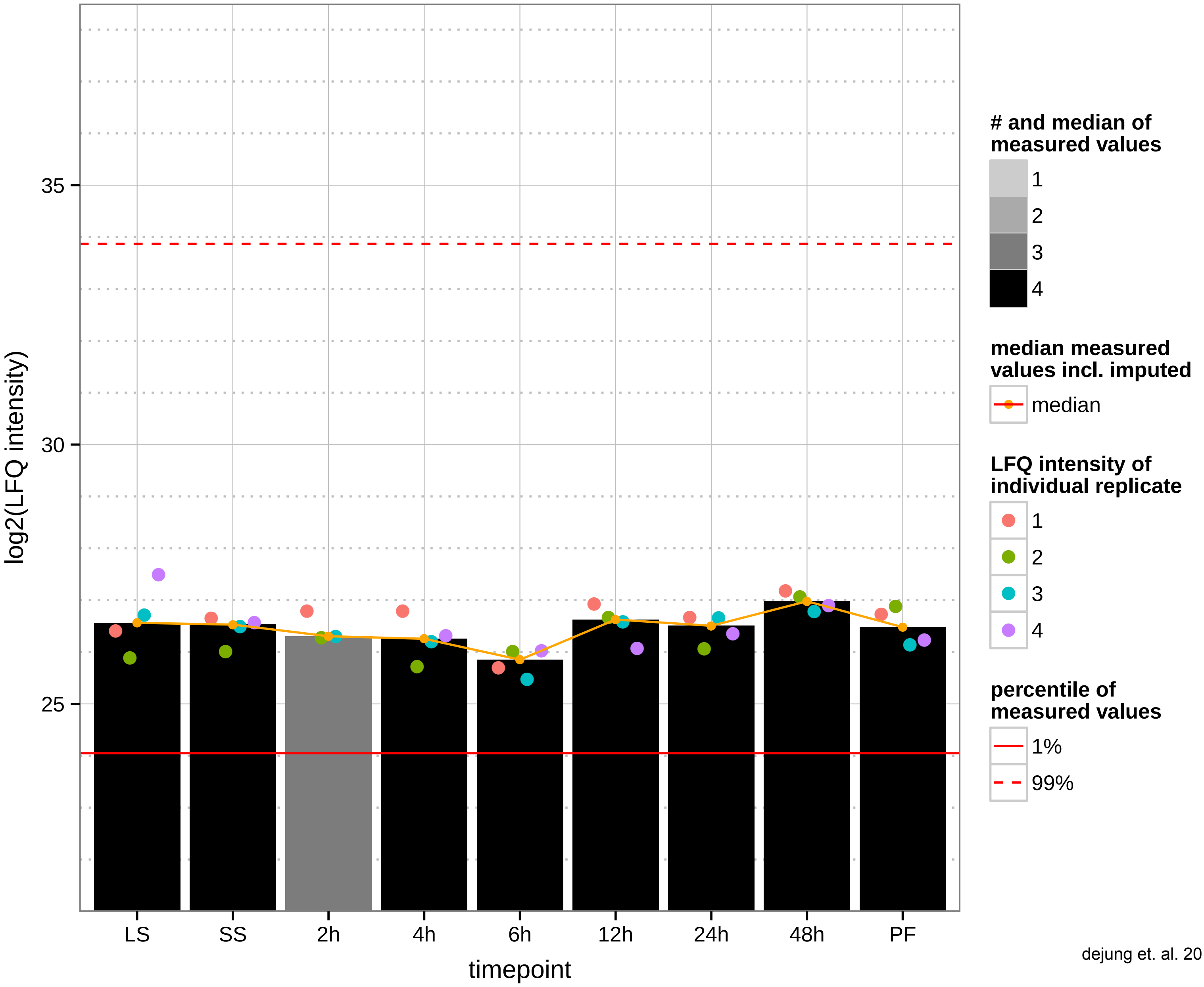

dejung et. al. 2015

ribosome biogenesis protein, putative
Tb927.10.14680
AGOF: rRNA binding
AGOC: nucleolus
AGOP: ribosomal large subunit assembly
PGOF: null
PGOC: null
PGOP: null

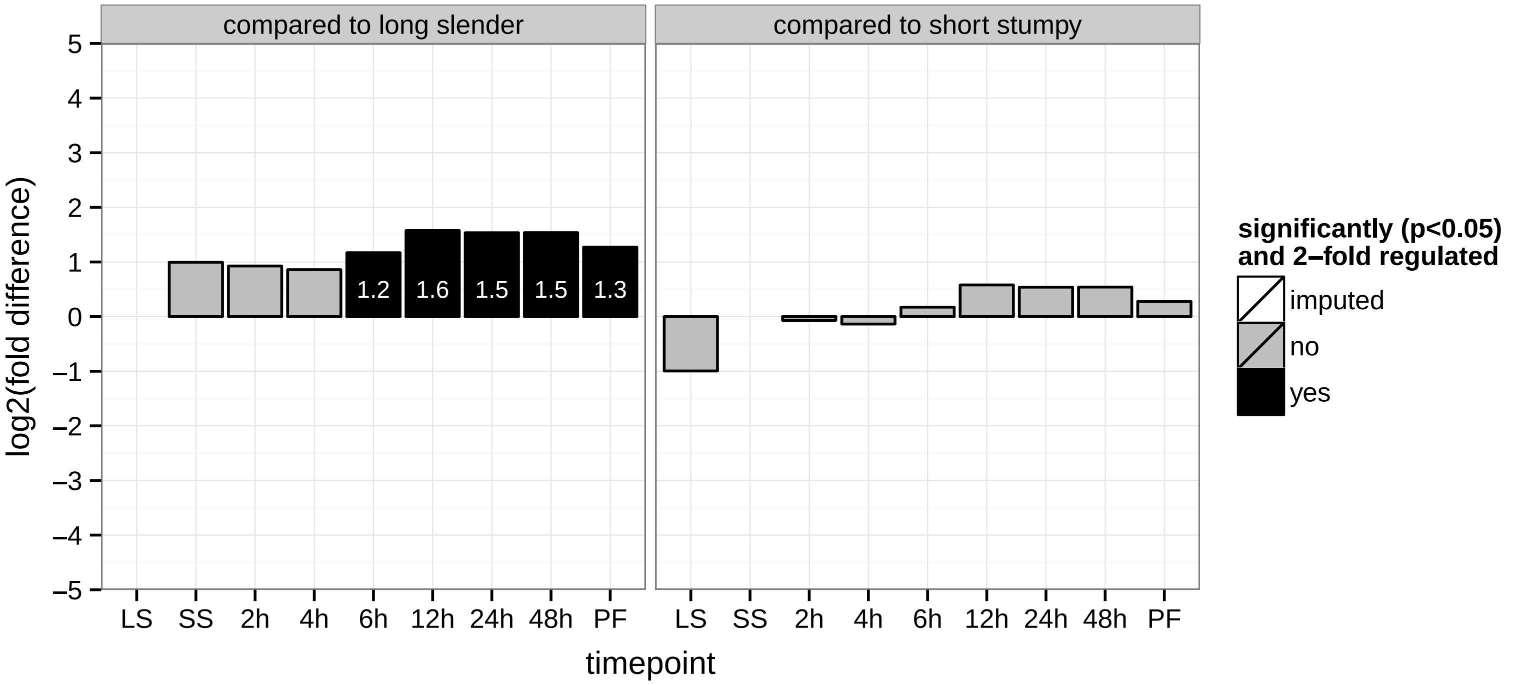

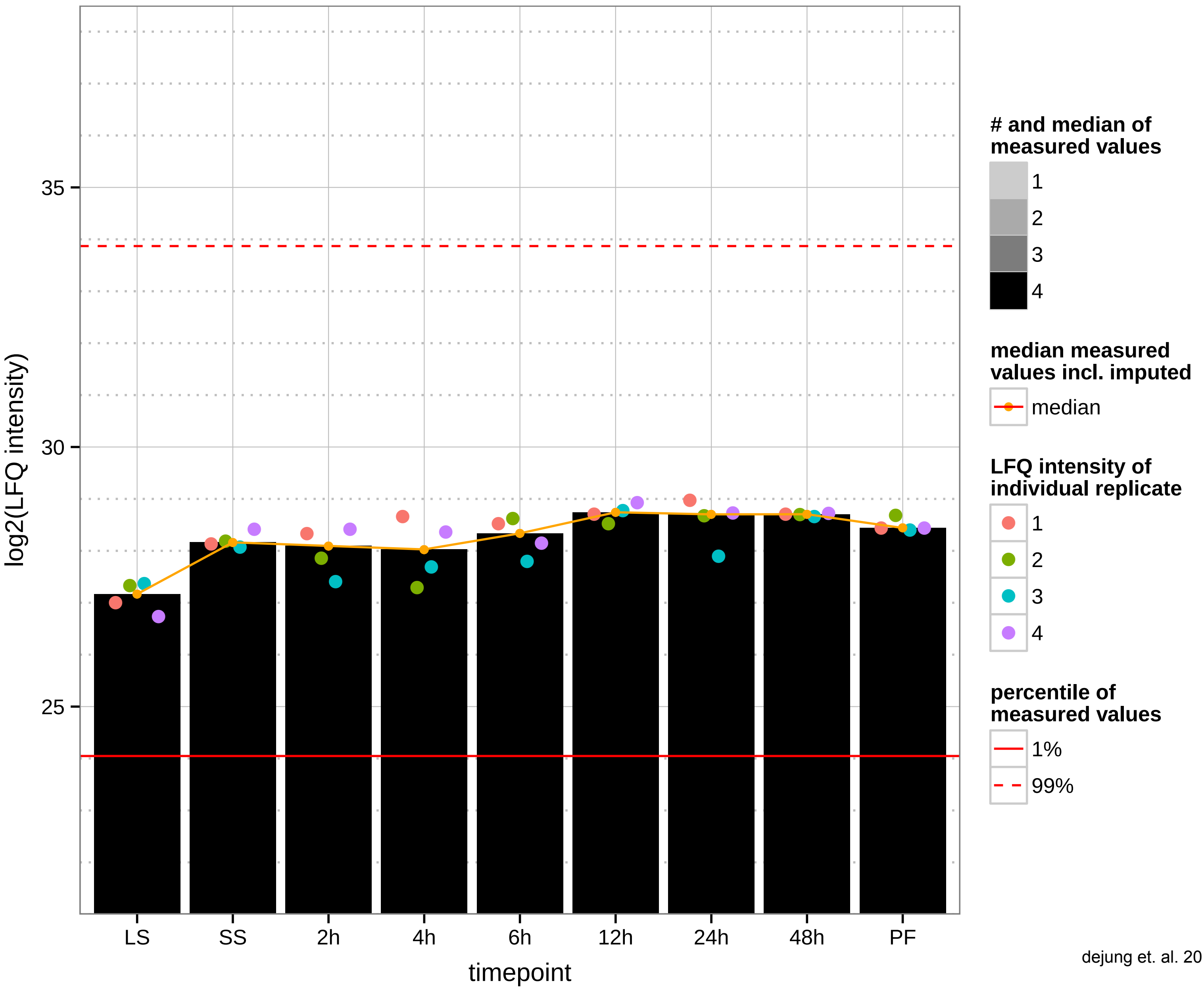

dejung et. al. 2015

fibrillarin, putative
Tb927.10.14750;Tb927.10.14630
AGOF: RNA binding
AGOC: nucleus
AGOP: rRNA processing
PGOF: RNA binding, methyltransferase activity
PGOC: null
PGOP: rRNA processing, tRNA processing

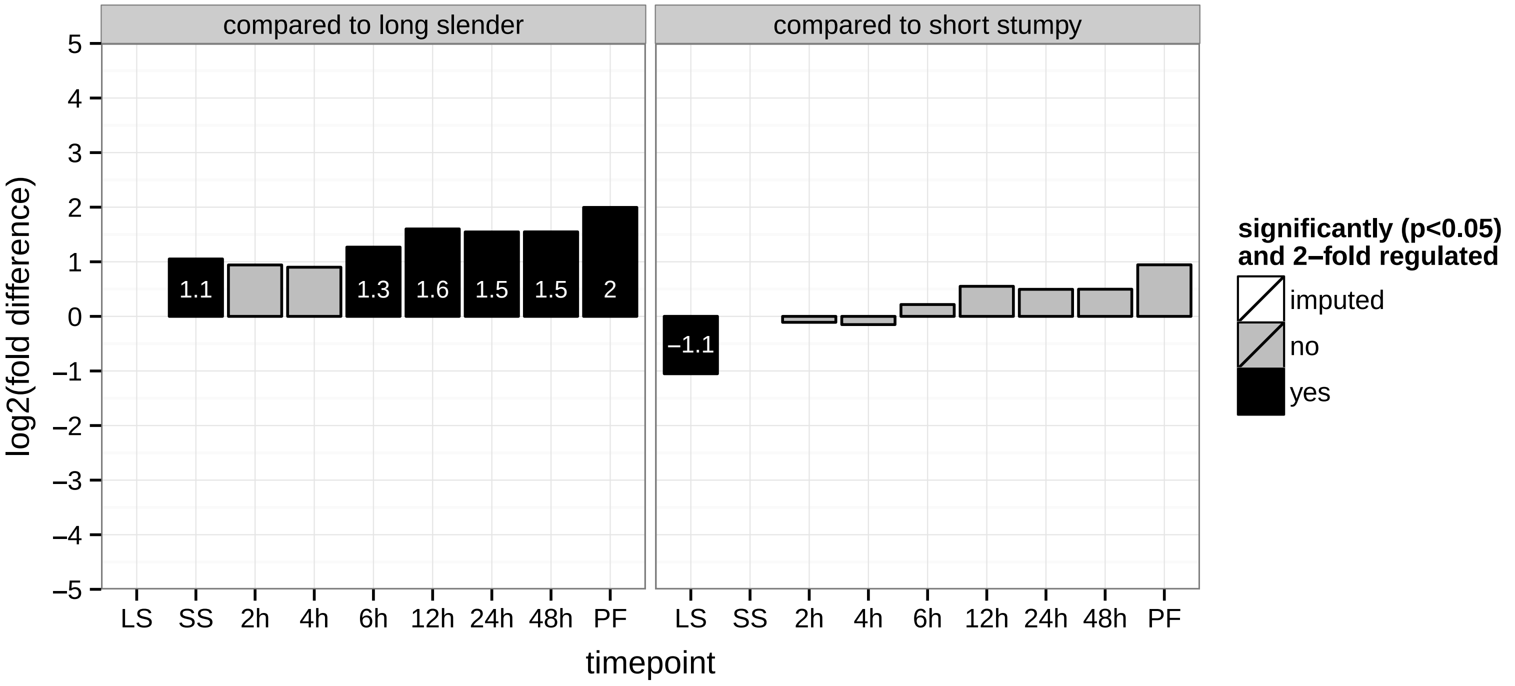

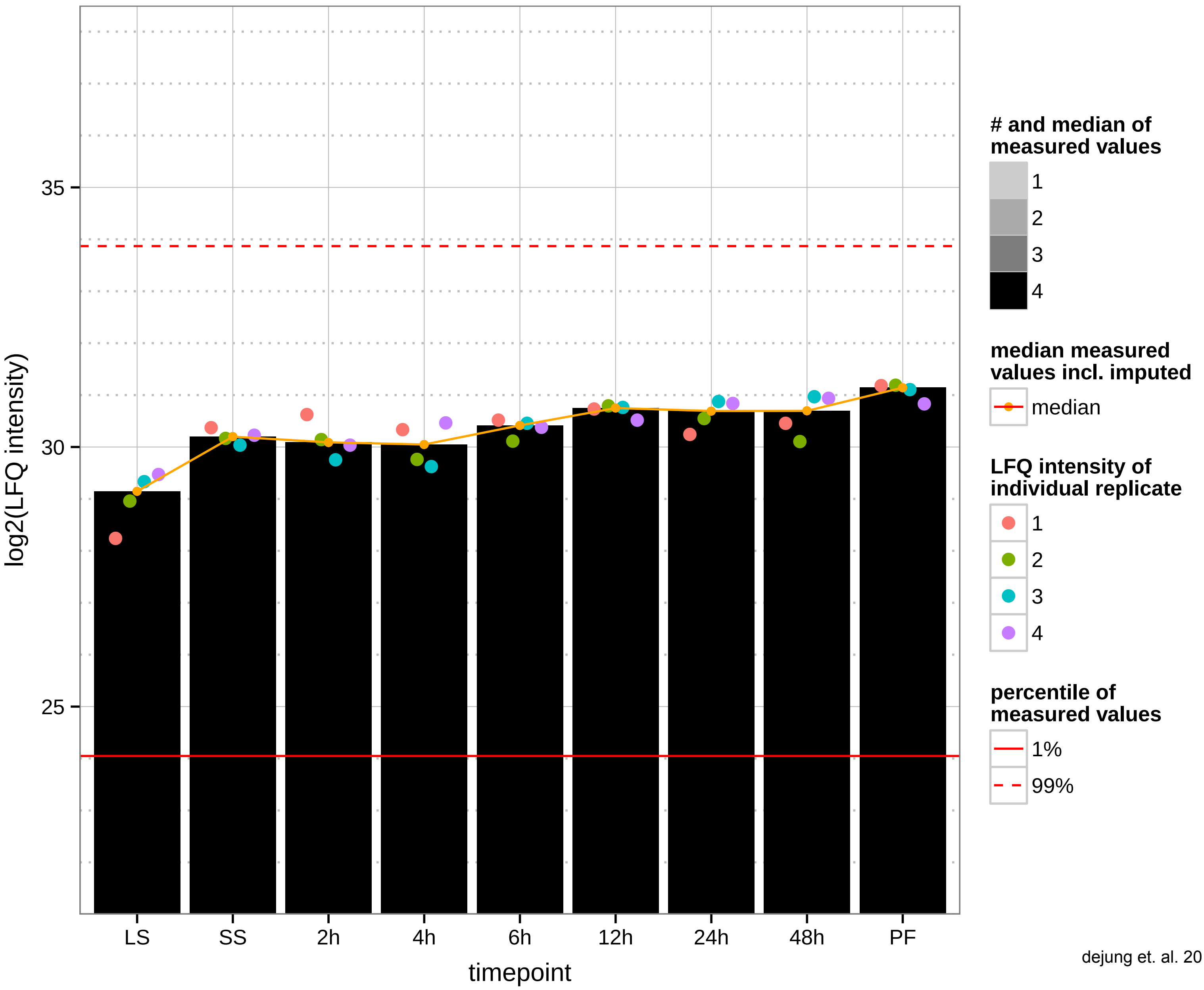

dejung et. al. 2015

protein kinase, putative
Tb927.10.14770
AGOF: ATP binding, protein serine/threonine kinase activity, protein tyrosine kinase activity
AGOC: null
AGOP: protein phosphorylation
PGOF: ATP binding, protein kinase activity, transferase activity, transferring phosphorus–containing groups
PGOC: null
PGOP: protein phosphorylation

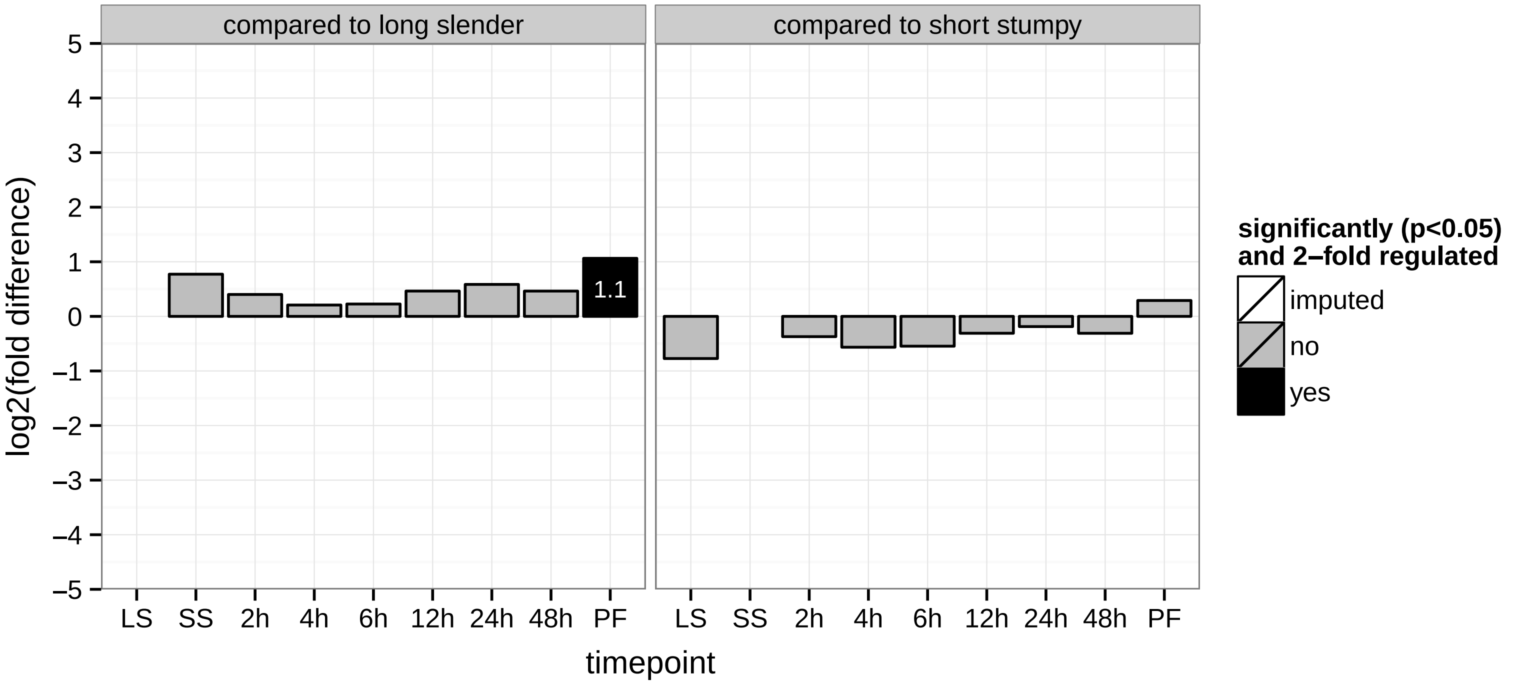

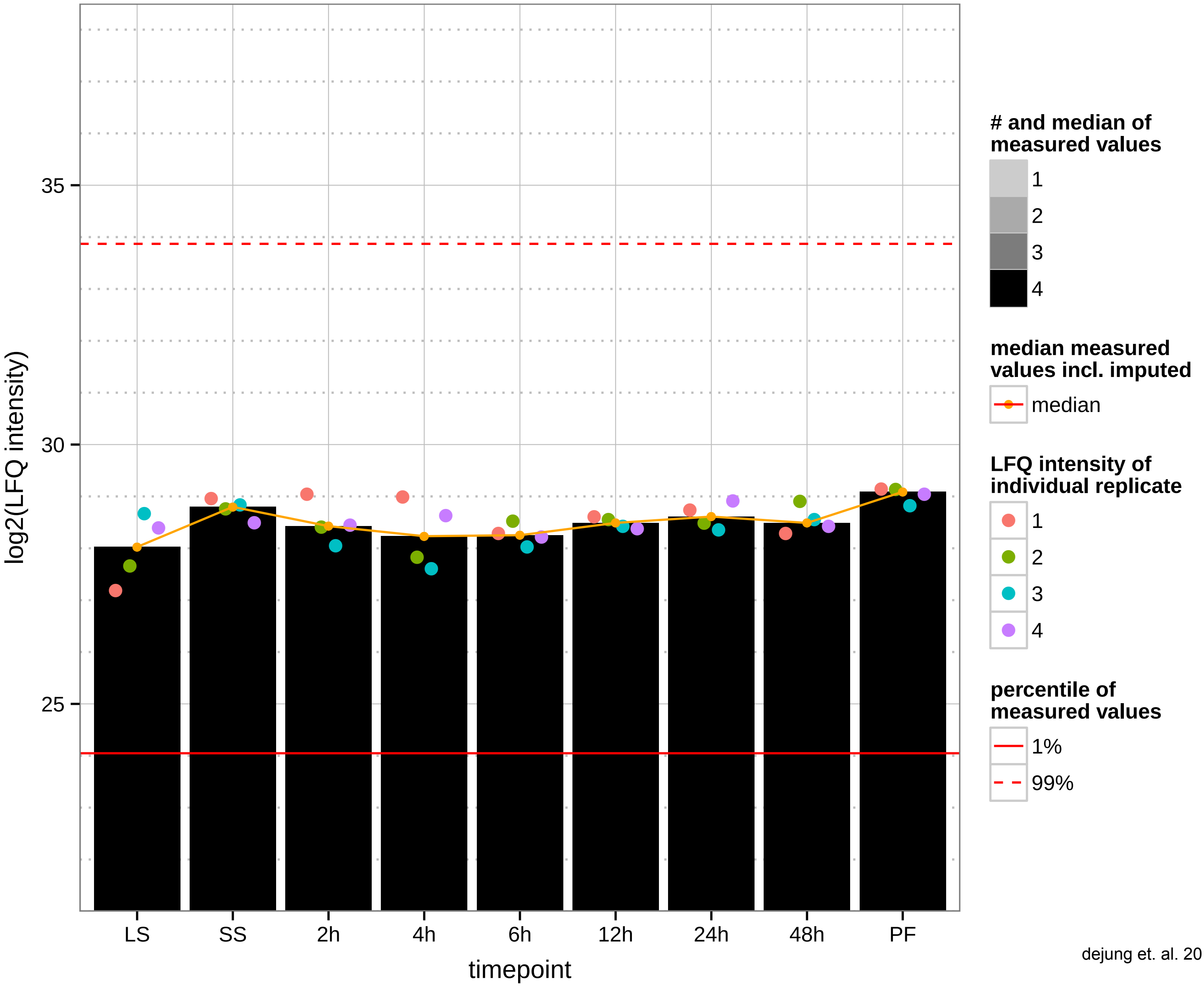

dejung et. al. 2015

mitogen–activated protein kinase kinase kinase, putative, canertinib–binding protein kinase–1 (CBPK1)
Tb927.10.14780
AGOF: ATP binding, protein serine/threonine kinase activity, protein tyrosine kinase activity
AGOC: null
AGOP: protein phosphorylation
PGOF: ATP binding, protein kinase activity, transferase activity, transferring phosphorus–containing groups
PGOC: null
PGOP: protein phosphorylation

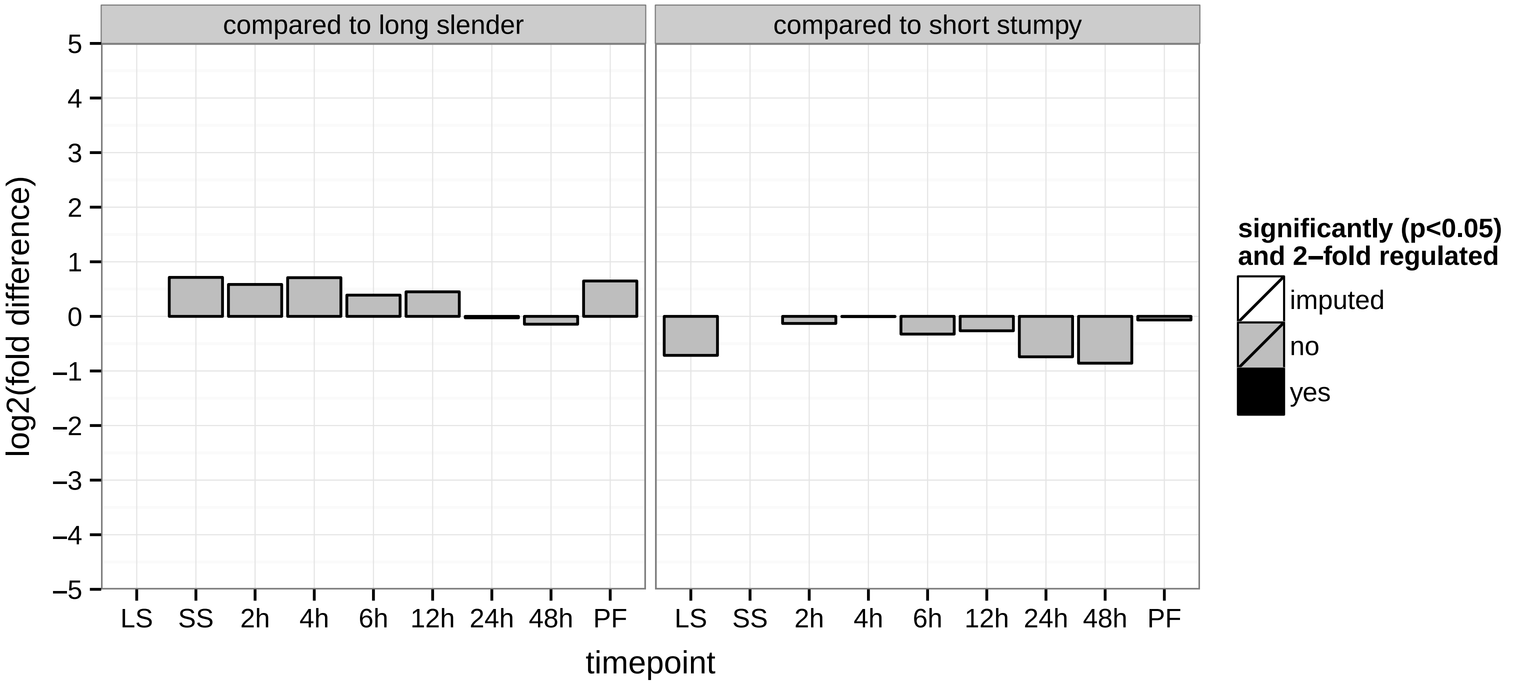

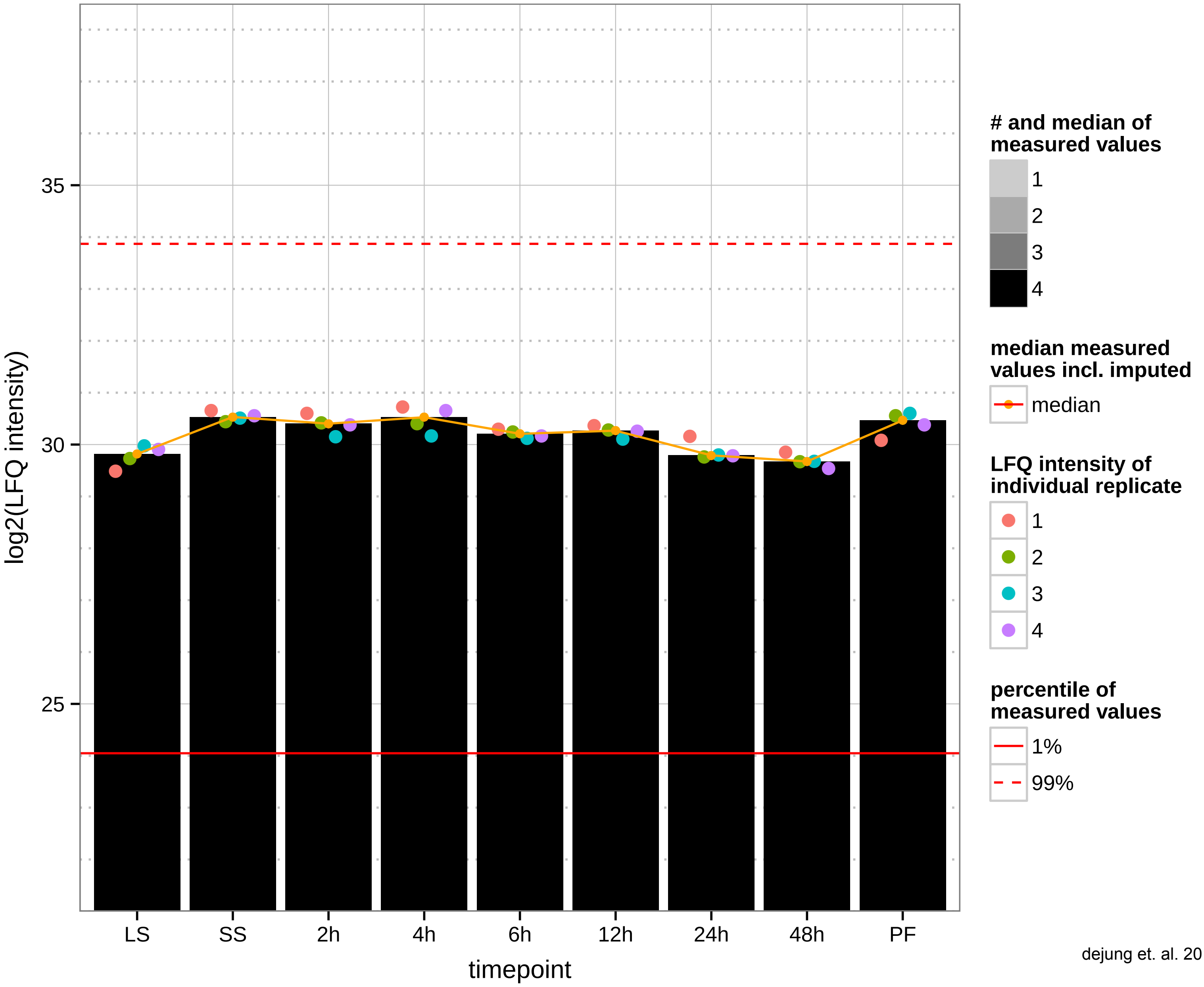

dejung et. al. 2015

hypothetical protein, conserved
Tb927.10.1490
AGOF: null
AGOC: null
AGOP: null
PGOF: null
PGOC: null
PGOP: null

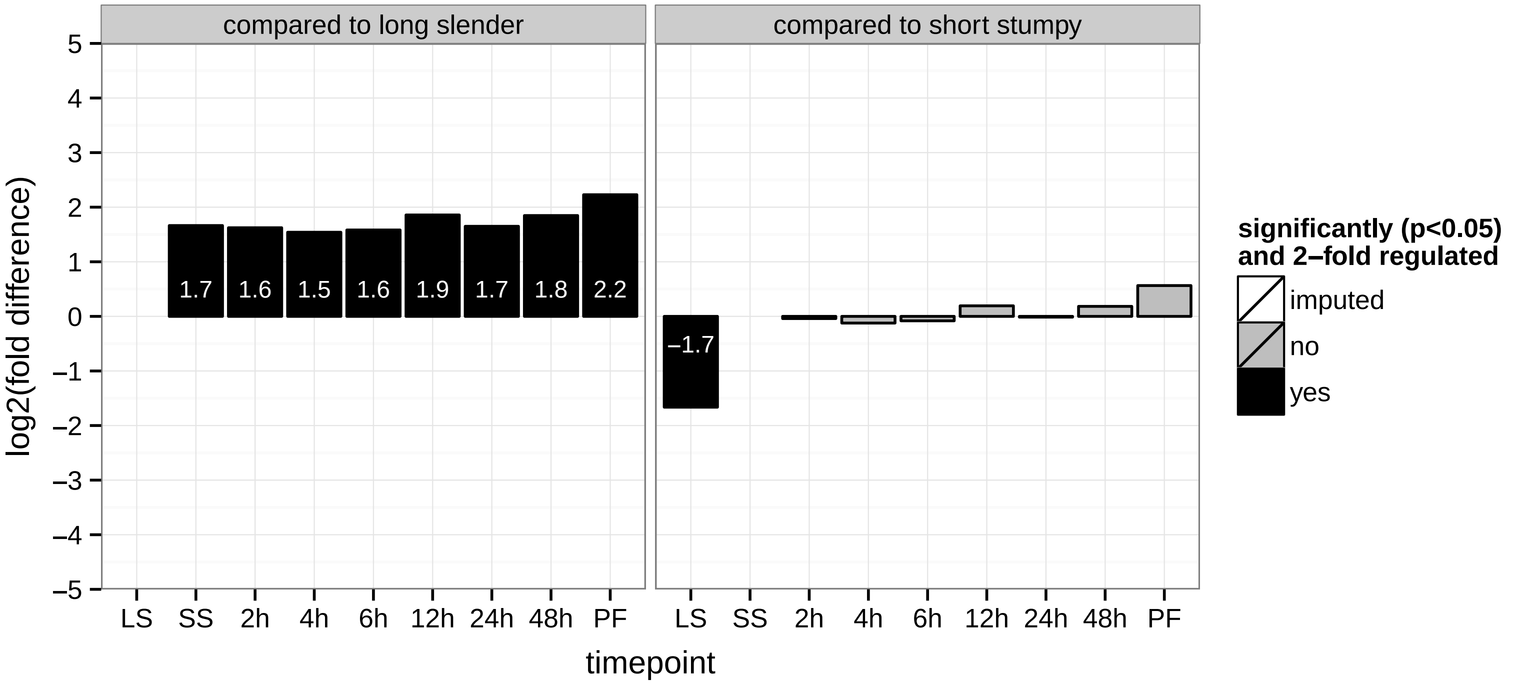

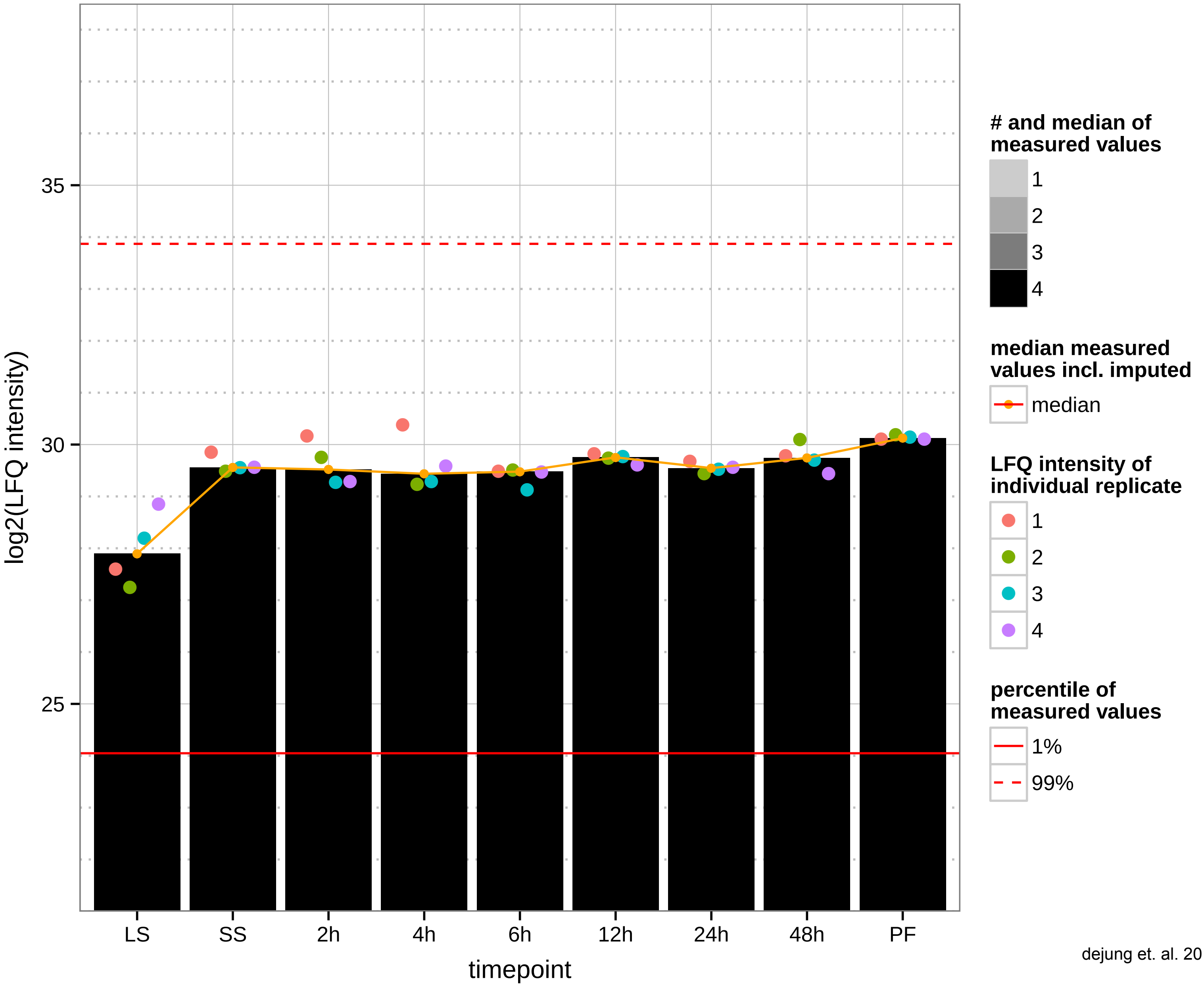

dejung et. al. 2015

intraflagellar transport protein IFT52, putative (IFT52)
Tb927.10.14980
AGOF: null
AGOC: cilium
AGOP: intraflagellar transport
PGOF: null
PGOC: null
PGOP: null

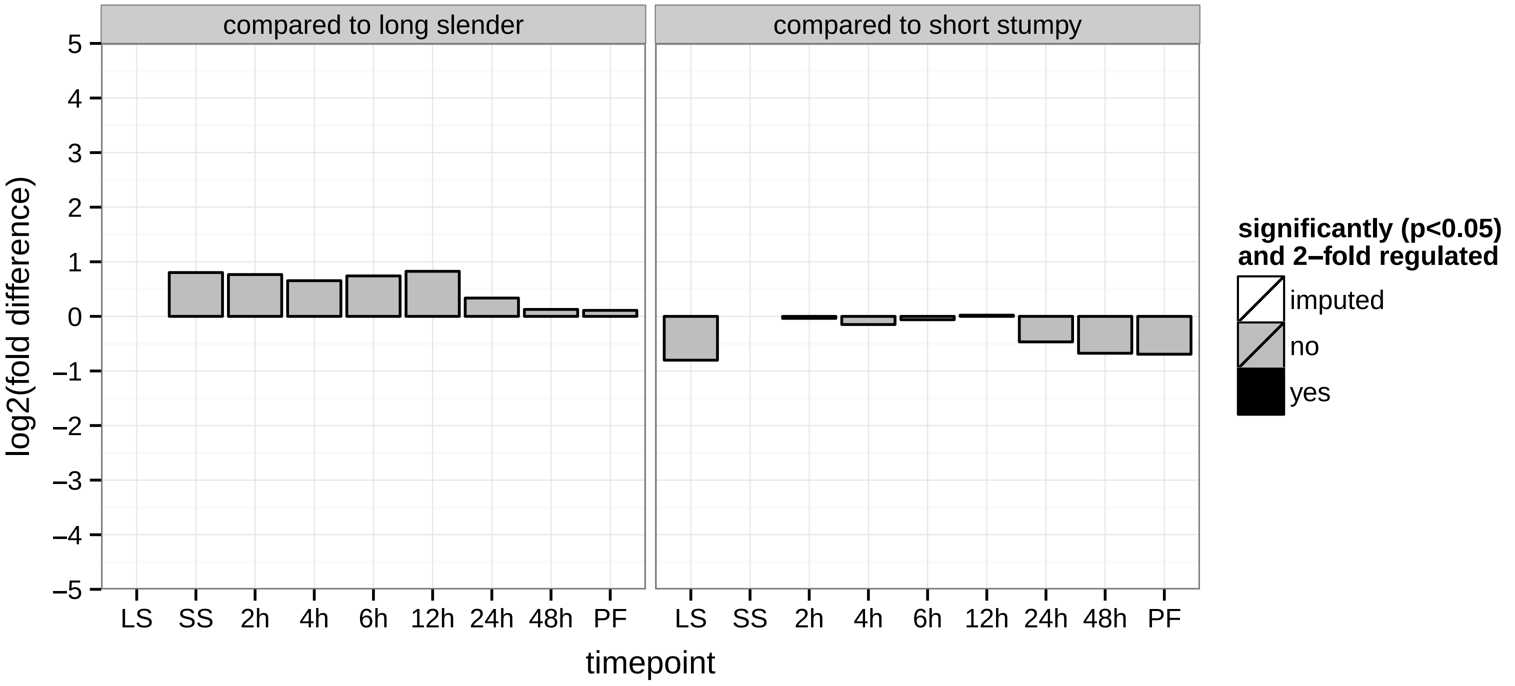

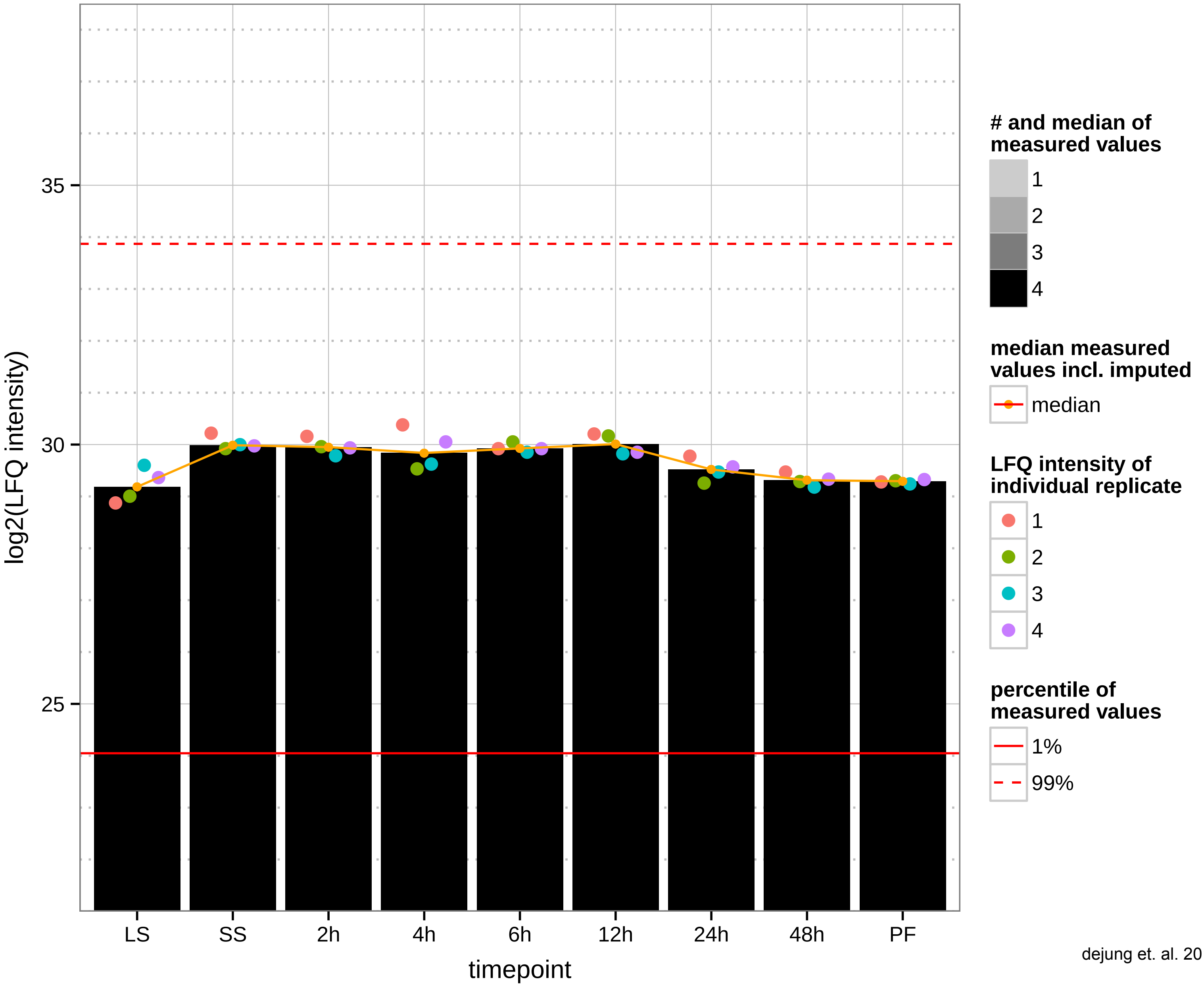

dejung et. al. 2015

hypothetical protein, conserved
Tb927.10.15000
AGOF: null
AGOC: null
AGOP: null
PGOF: null
PGOC: nucleus
PGOP: null

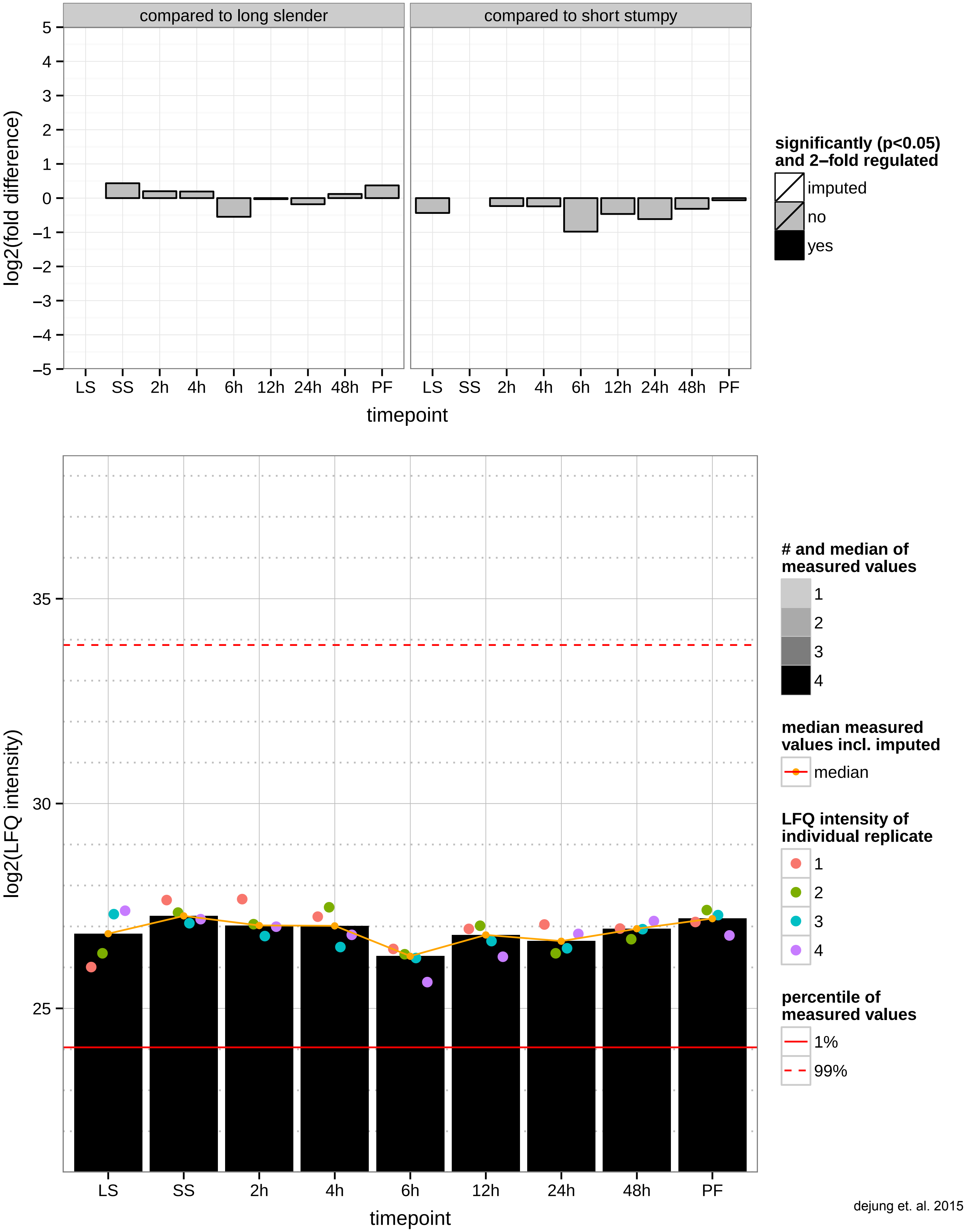

dejung et. al. 2015

lipoic acid synthetase, mitochondrial precursor, putative
Tb927.10.15010
AGOF: 4 iron, 4 sulfur cluster binding, iron ion binding, lipoate synthase activity
AGOC: mitochondrion
AGOP: lipoate biosynthetic process
PGOF: 4 iron, 4 sulfur cluster binding, catalytic activity, iron–sulfur cluster binding, lipoate synthase activity
PGOC: null
PGOP: lipoate biosynthetic process

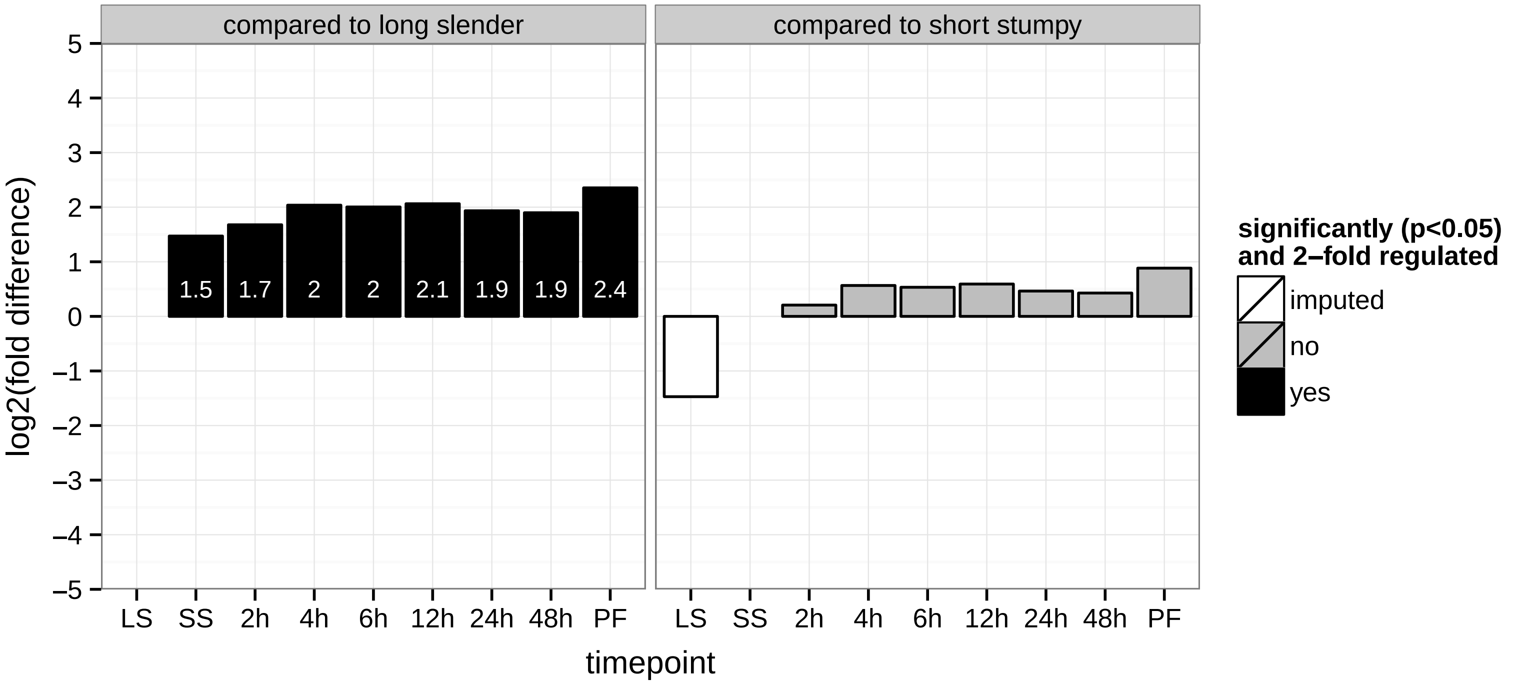

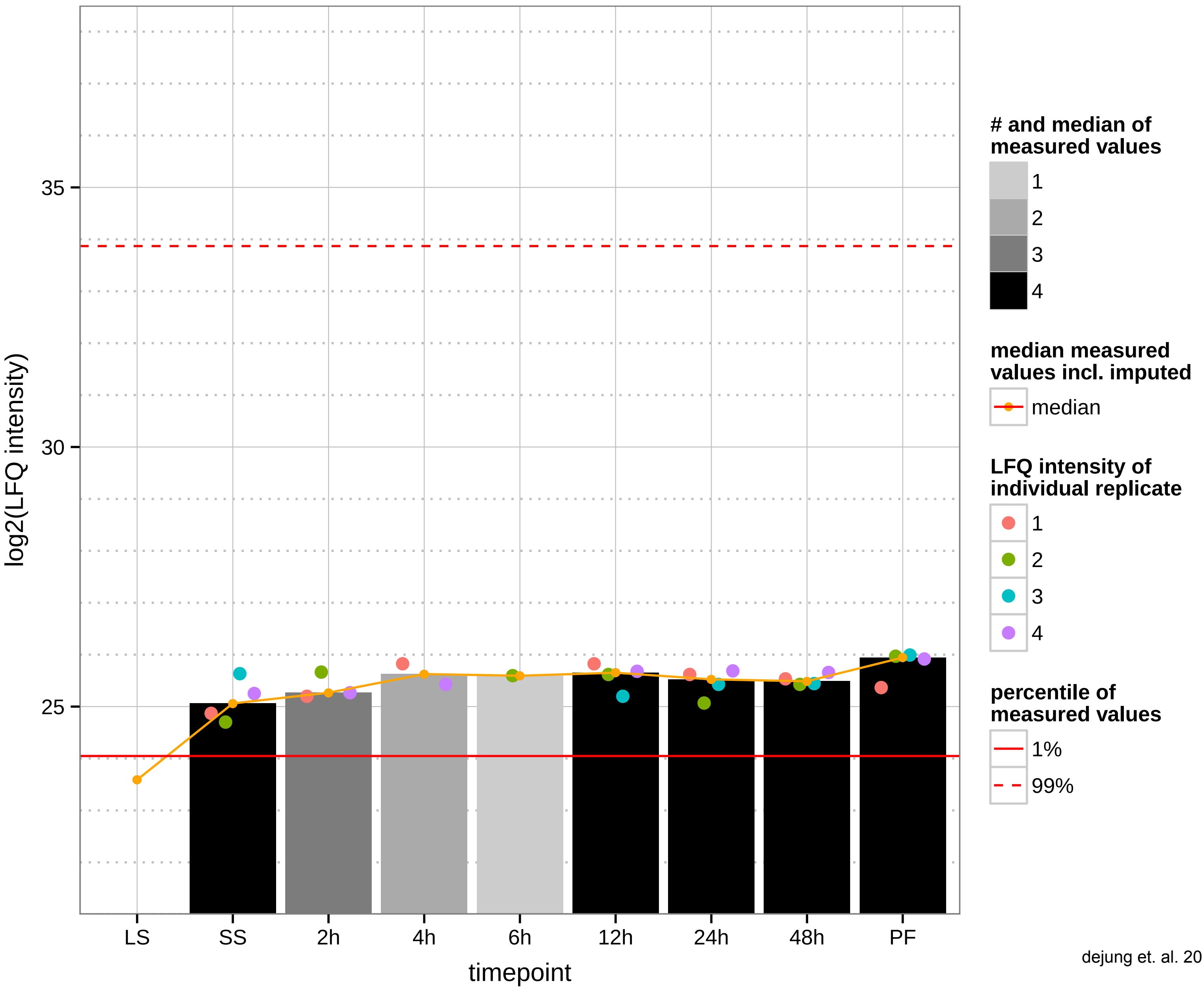

dejung et. al. 2015

NOT1 (NOT1)
Tb927.10.1510
AGOF: null
AGOC: null
AGOP: null
PGOF: null
PGOC: null
PGOP: null

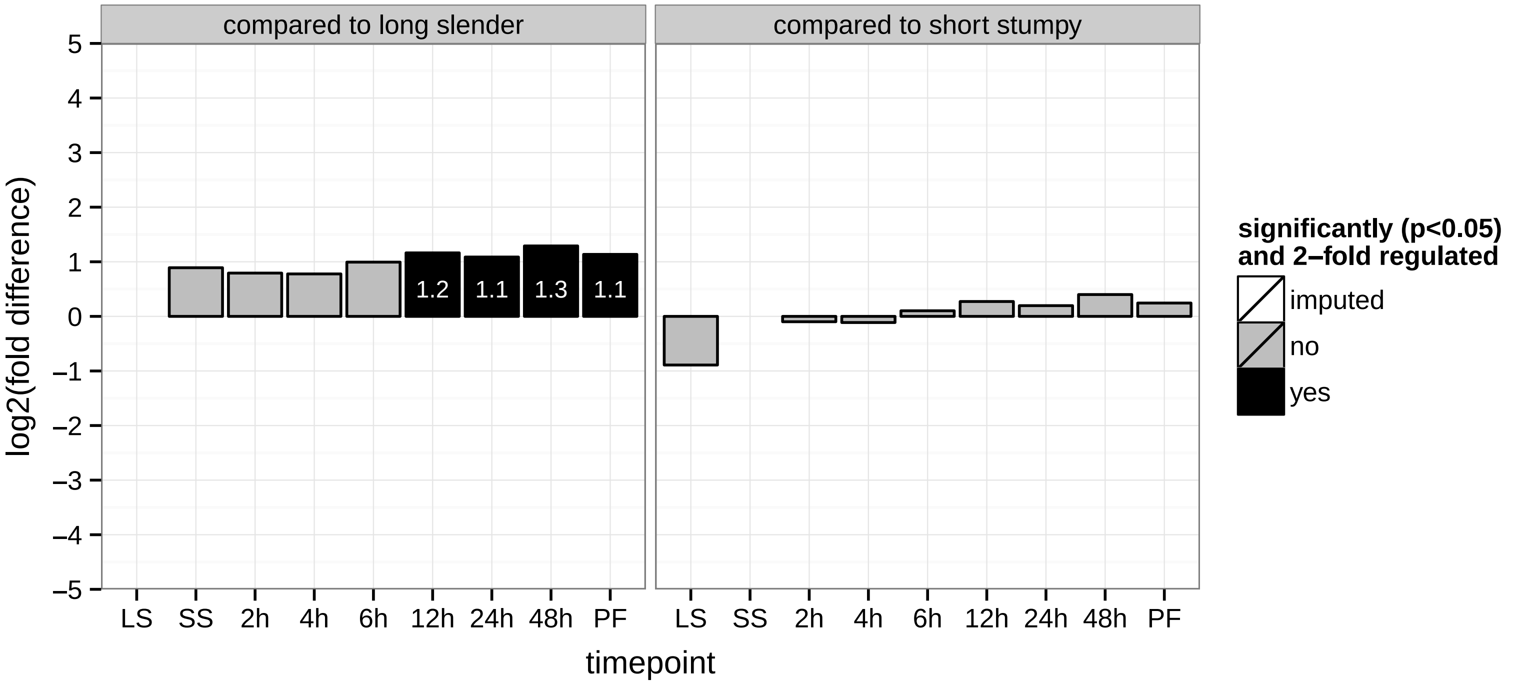

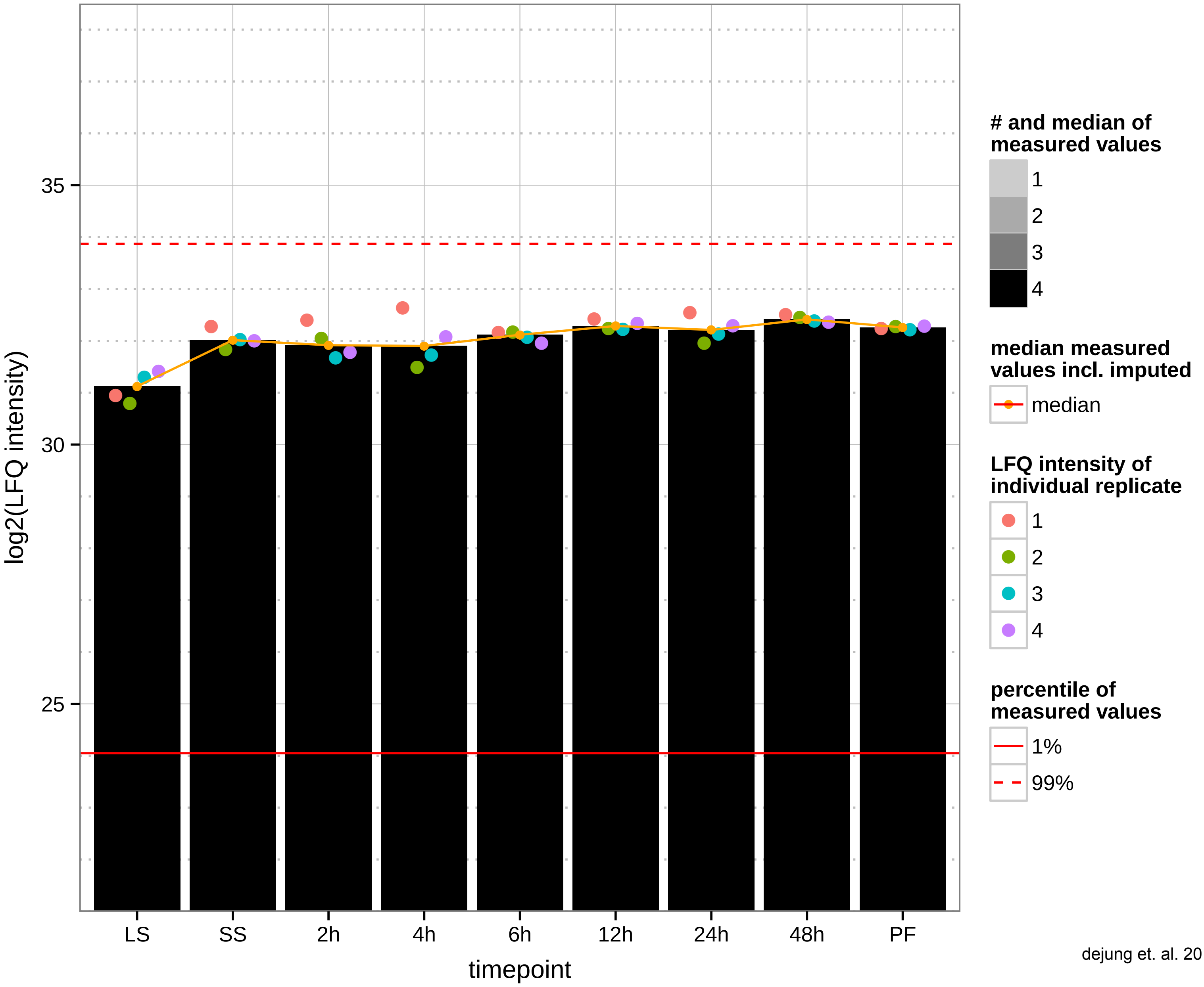

dejung et. al. 2015

40S ribosomal protein S13, putative
Tb927.2.5910;Tb927.10.15120
AGOF: structural constituent of ribosome
AGOC: intracellular, ribosome
AGOP: translation
PGOF: structural constituent of ribosome
PGOC: intracellular, ribosome
PGOP: translation

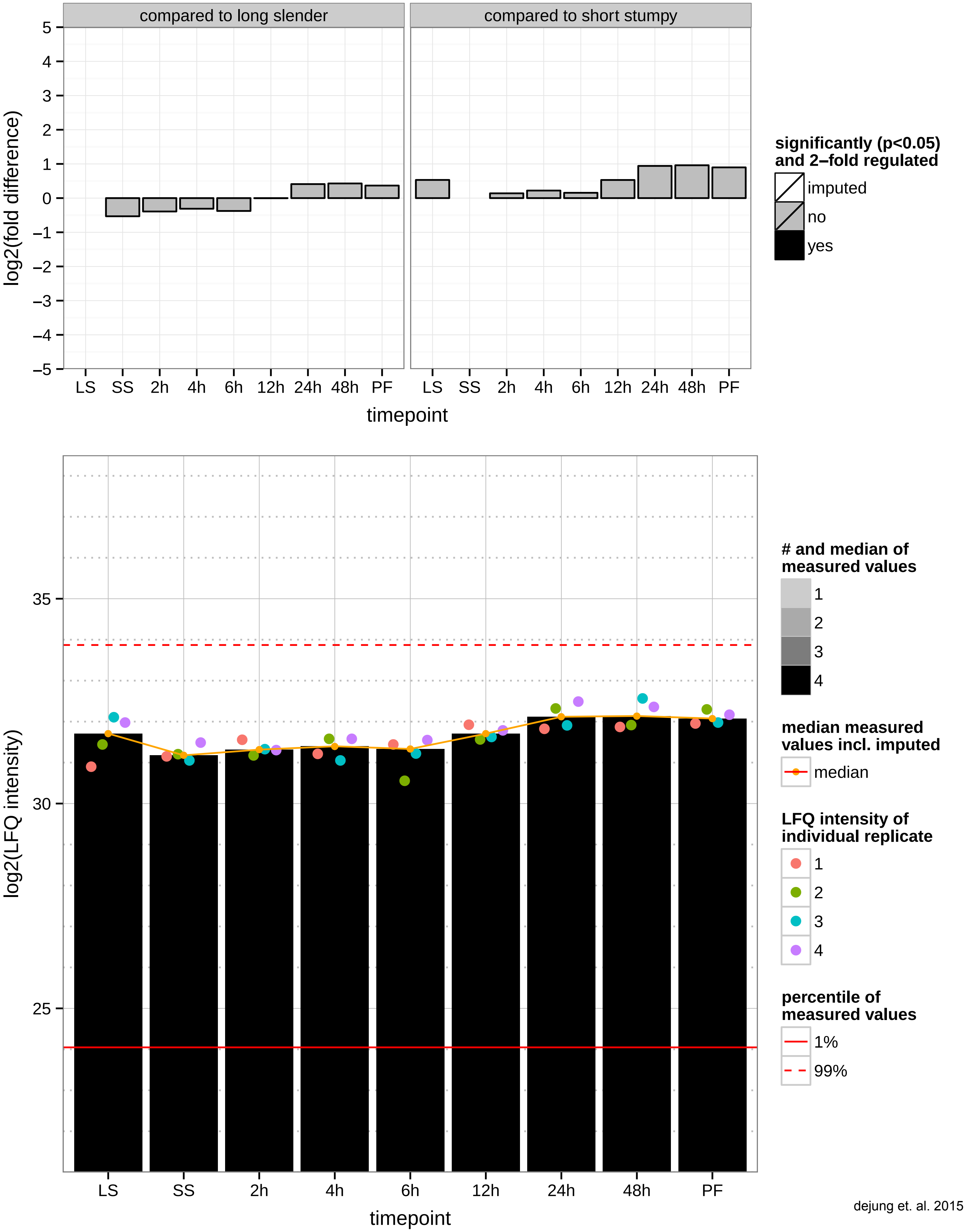

dejung et. al. 2015

Vesicle–trafficking protein SEC22, putative, putative (SEC22)
Tb927.10.15130
AGOF: SNAP receptor activity
AGOC: integral to membrane
AGOP: vesicle targeting, vesicle–mediated transport
PGOF: null
PGOC: integral to membrane
PGOP: transport, vesicle–mediated transport

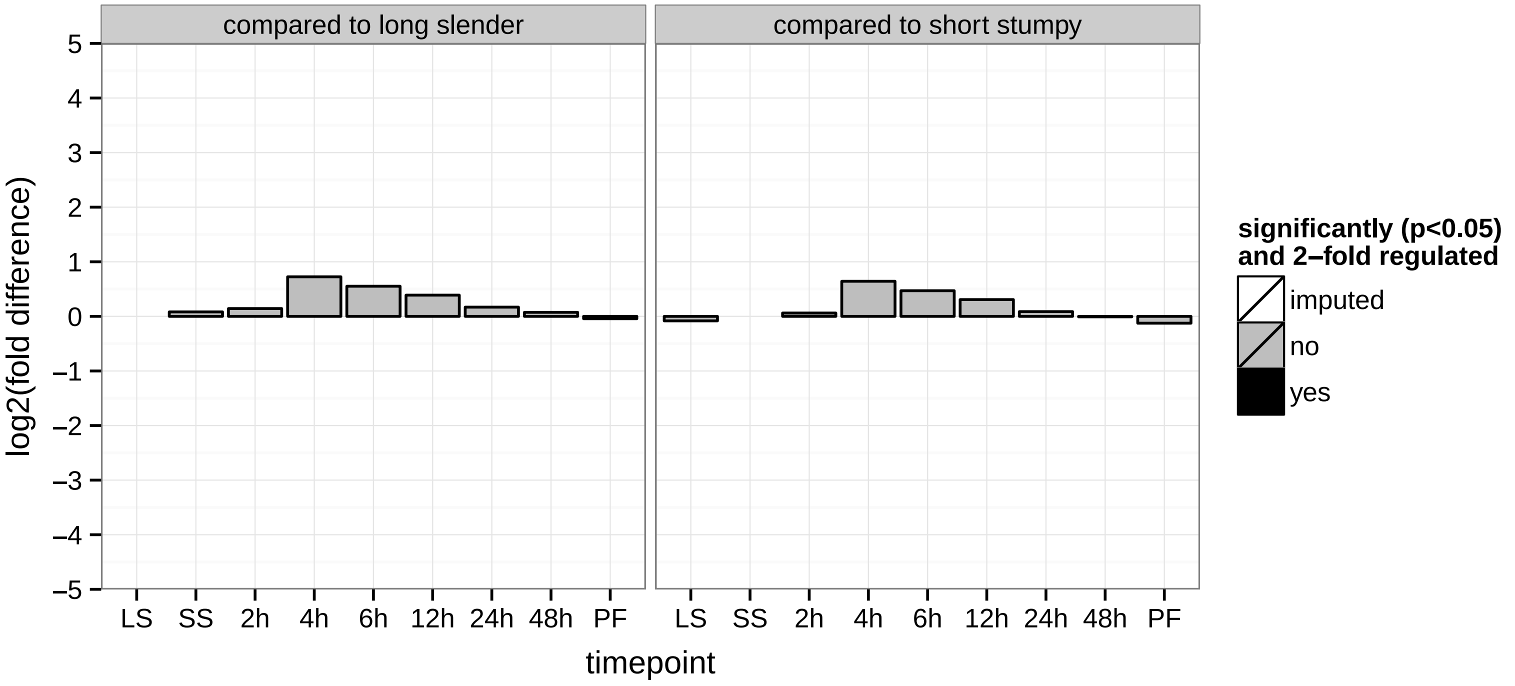

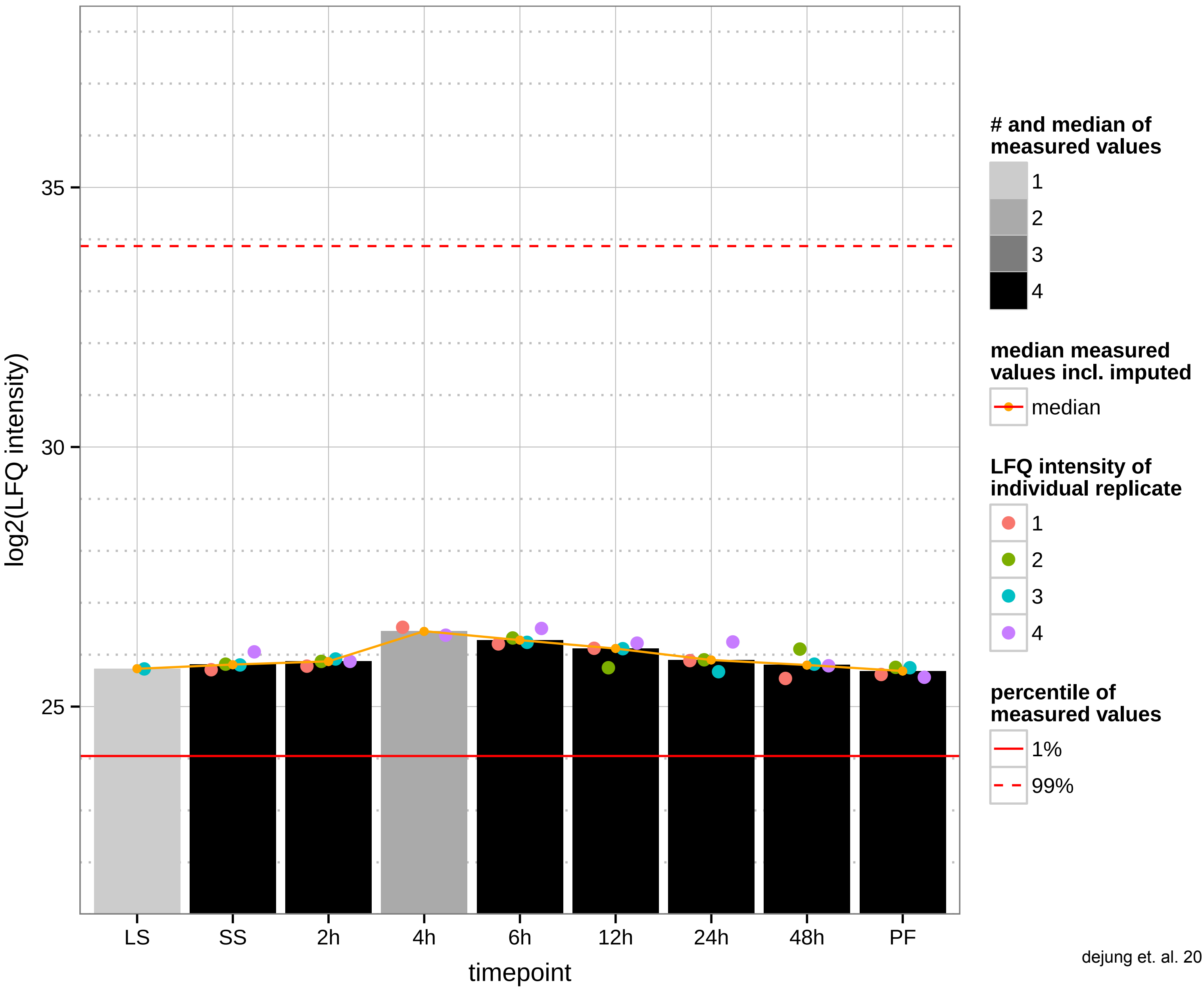

dejung et. al. 2015

nuclear cap binding complex subunit CBP30 (CBP30)
Tb927.10.15210
AGOF: null
AGOC: mitochondrion, nuclear cap binding complex, nucleus
AGOP: null
PGOF: null
PGOC: null
PGOP: null

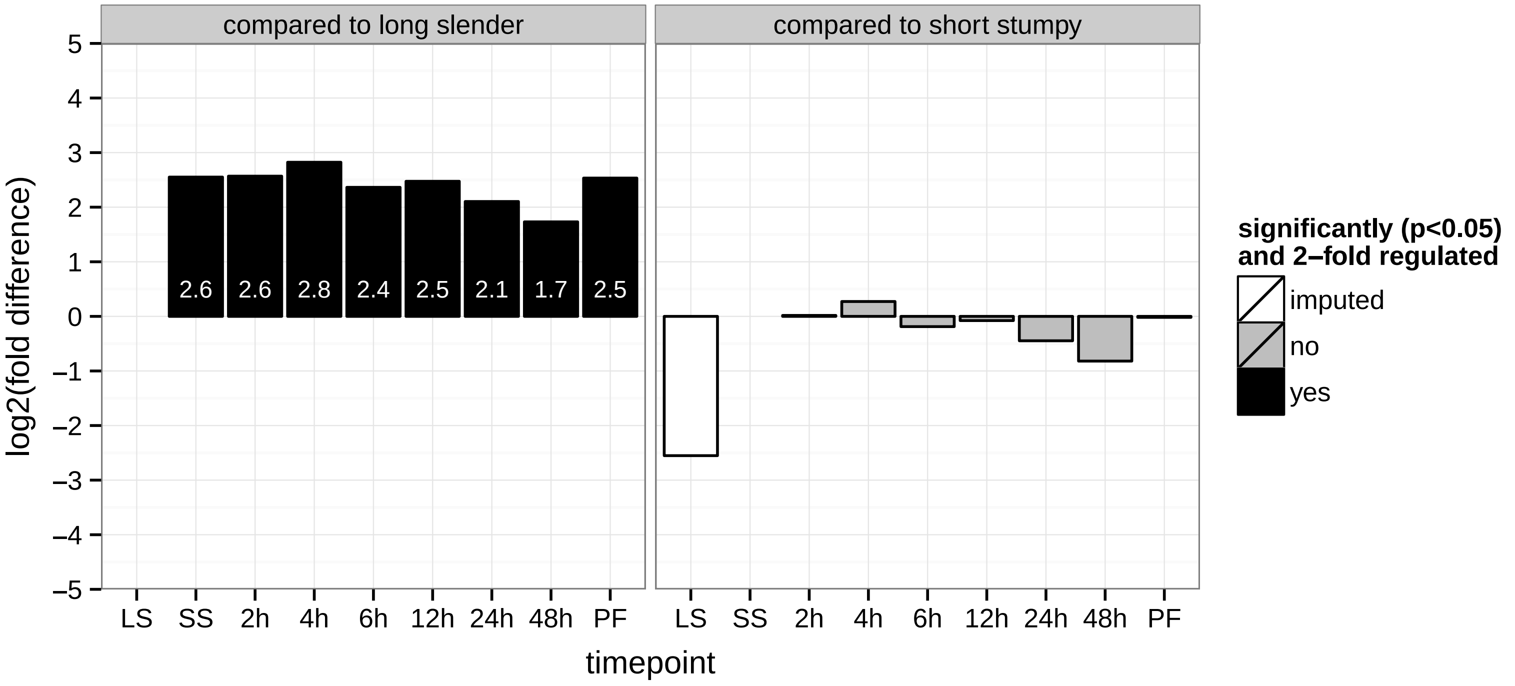

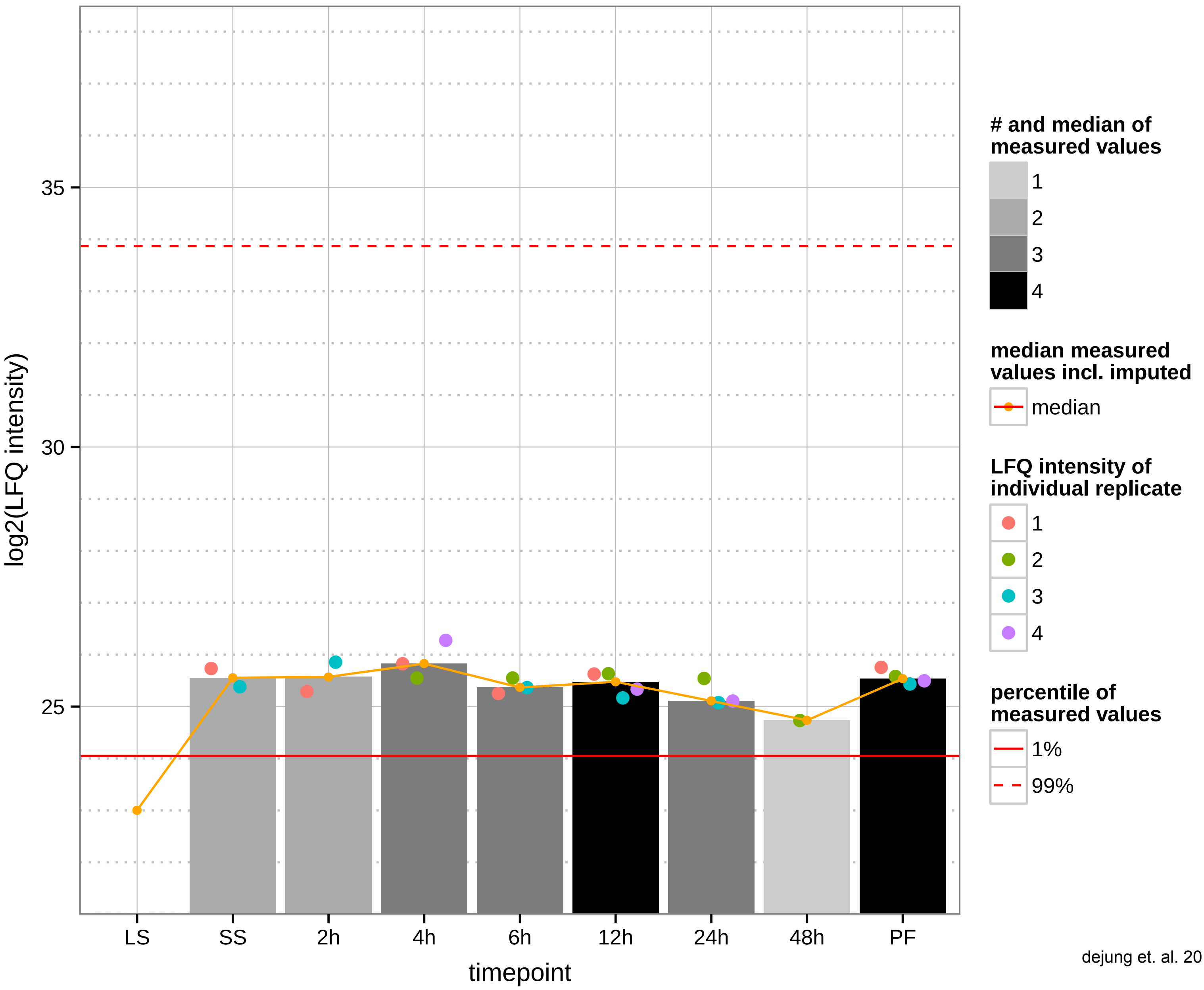

dejung et. al. 2015

hypothetical protein, conserved
Tb927.10.15290
AGOF: null, ATP binding, protein kinase activity
AGOC: null
AGOP: null, protein phosphorylation
PGOF: null, ATP binding, protein kinase activity, transferase activity, transferring phosphorus–containing groups
PGOC: null
PGOP: null, protein phosphorylation

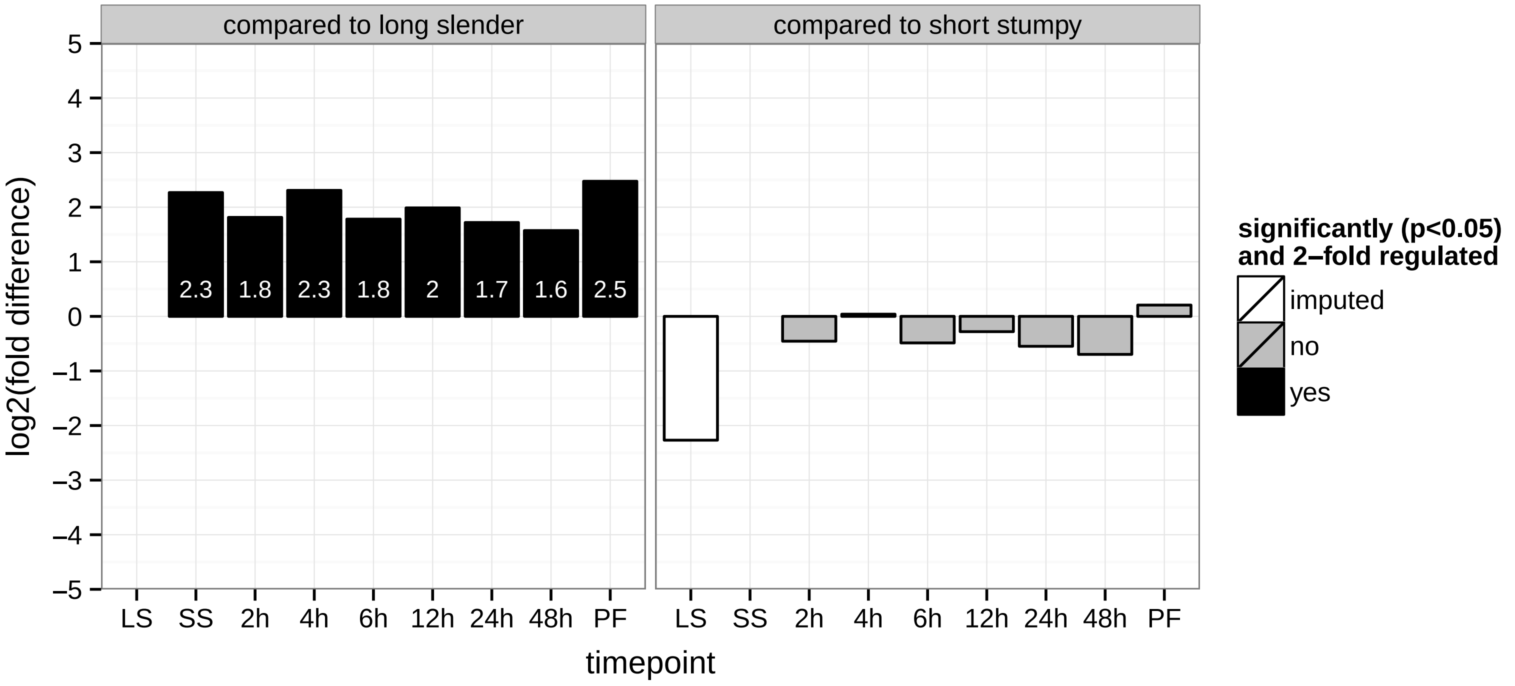

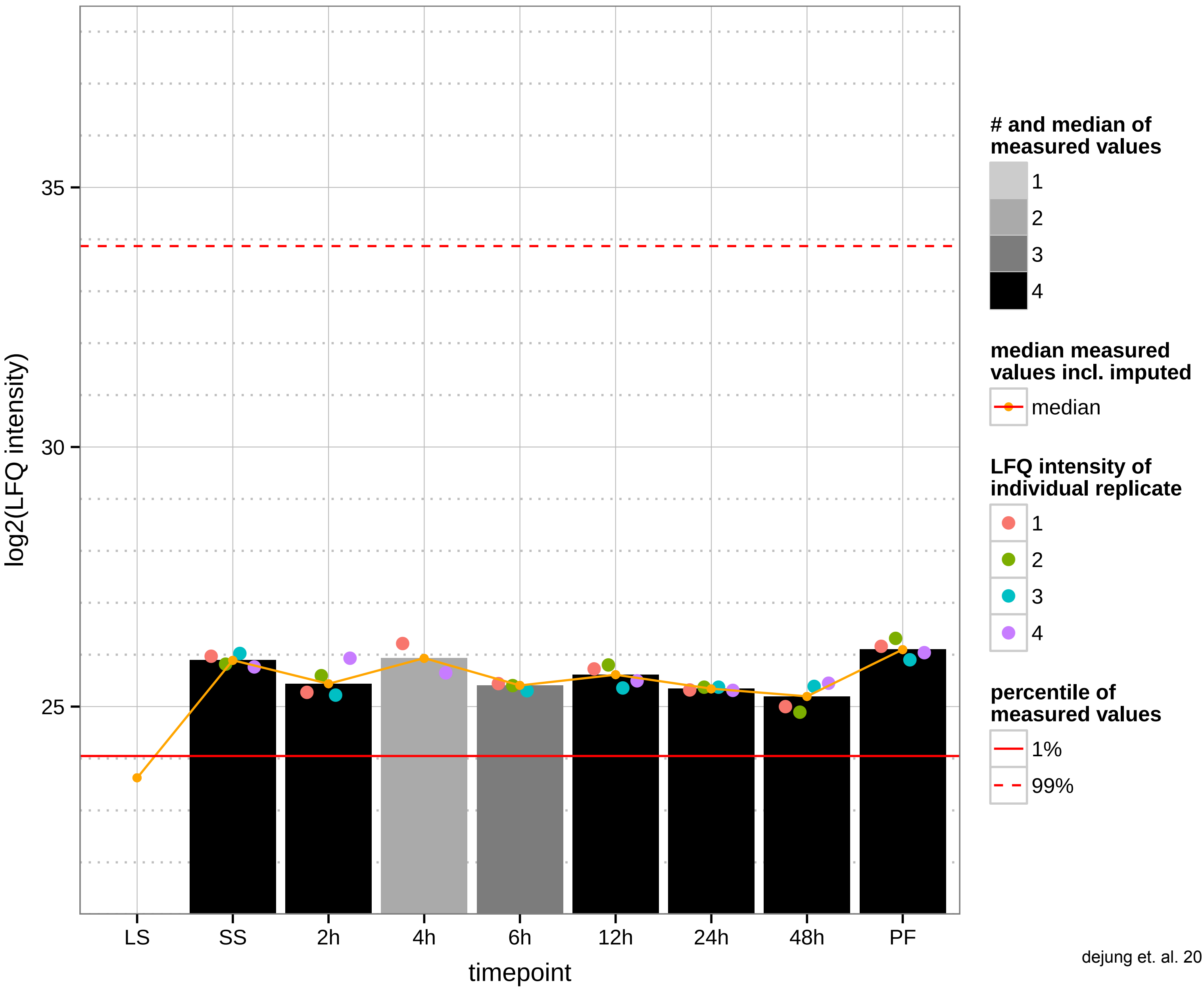

dejung et. al. 2015

hypothetical protein, conserved
Tb927.10.15310
AGOF: null
AGOC: null
AGOP: null
PGOF: null
PGOC: null
PGOP: null

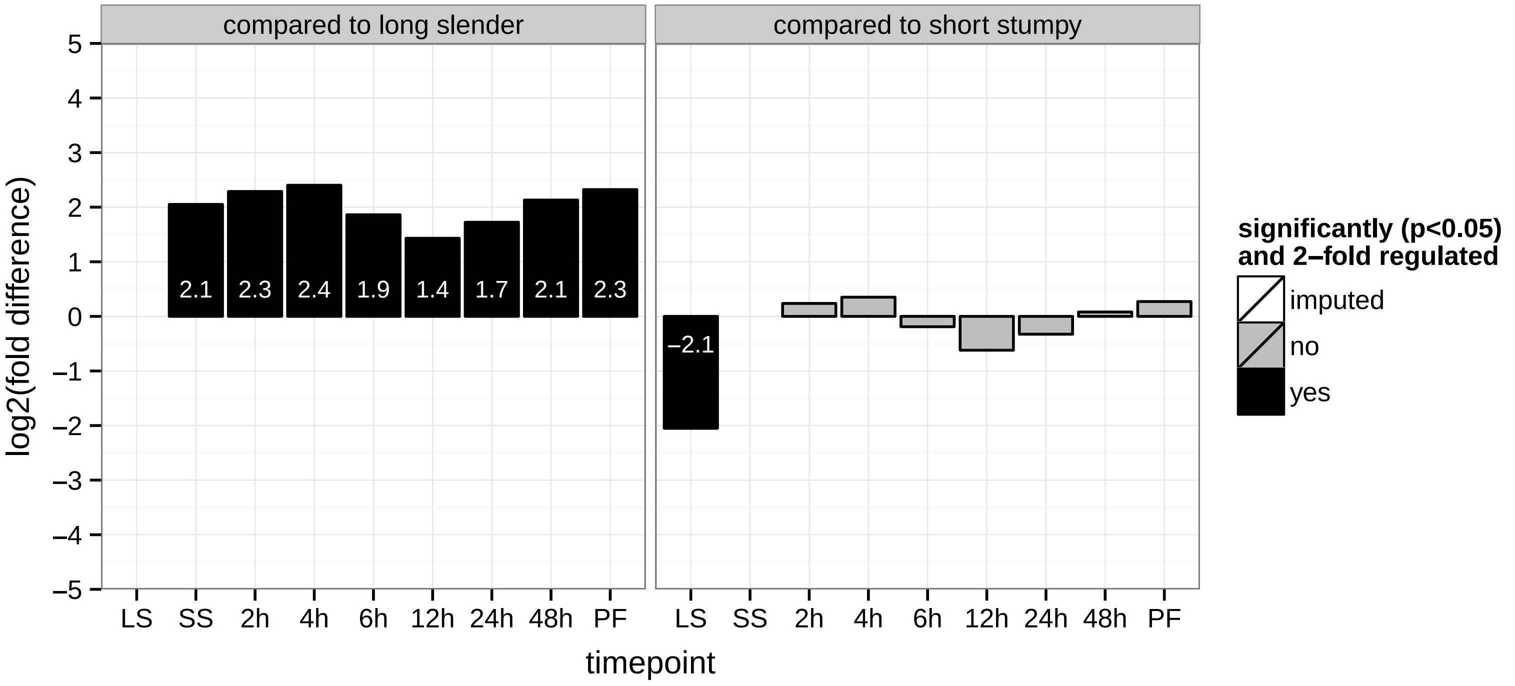

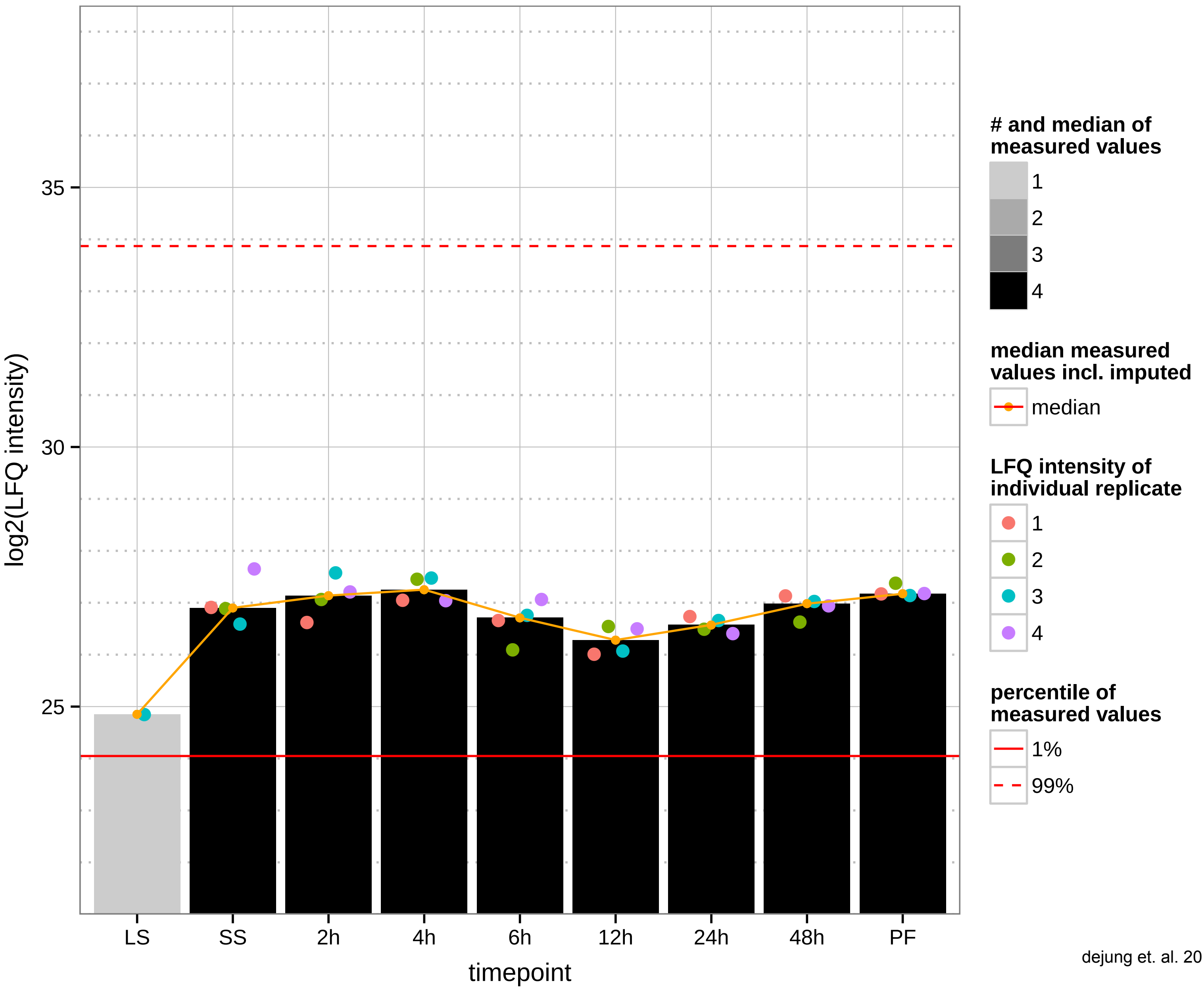

dejung et. al. 2015

hypothetical protein, conserved
Tb927.10.15330;Tb11.v5.0348
AGOF: null
AGOC: null
AGOP: null
PGOF: null
PGOC: null
PGOP: null, phosphate−containing compound metabolic process

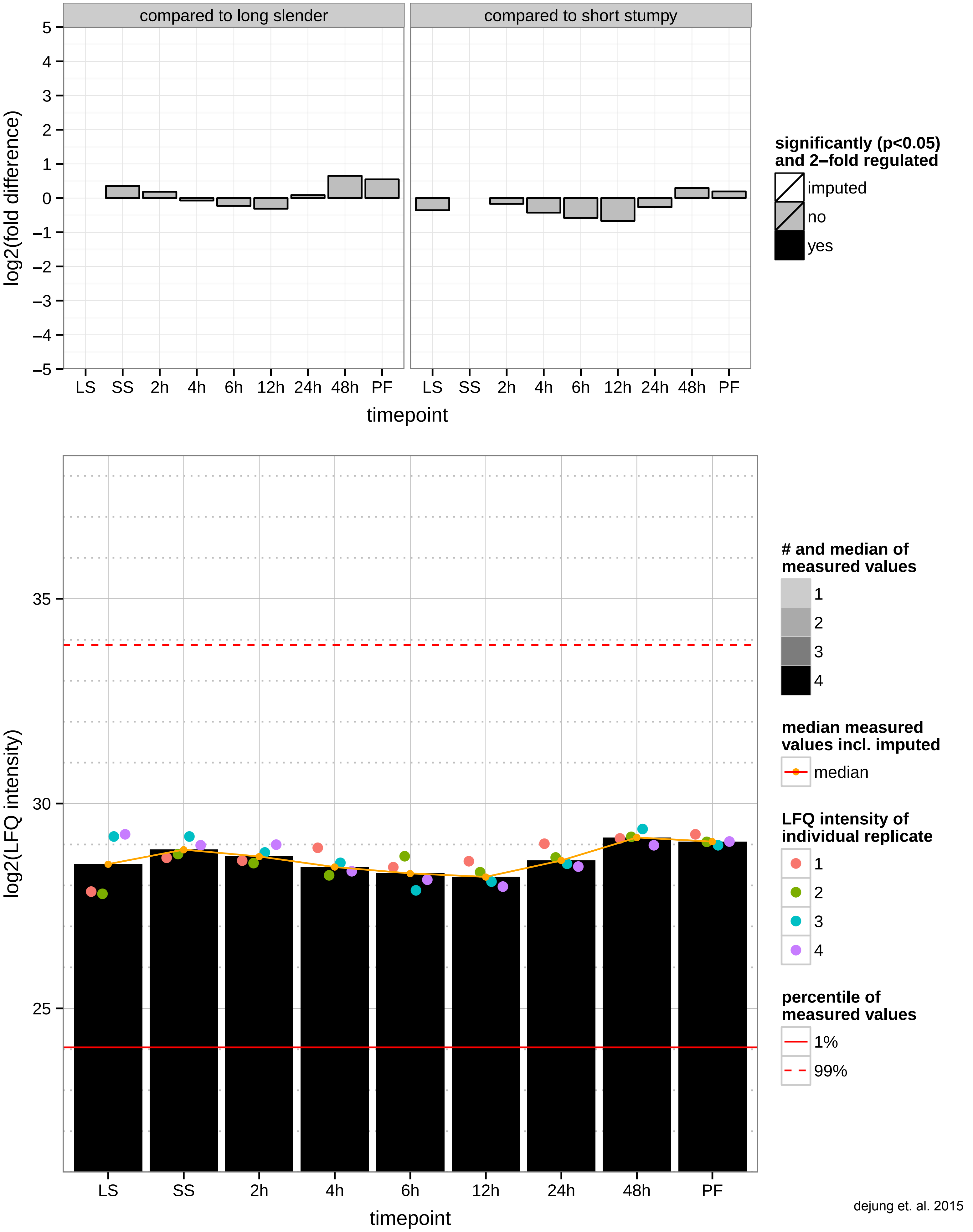

dejung et. al. 2015

histone H3 variant, H3V (h3vaR)
Tb927.10.15350
AGOF: DNA binding
AGOC: chromosome, telomeric region, nucleosome, nucleus
AGOP: chromosome organization, nucleosome assembly, termination of RNA polymerase II transcription
PGOF: DNA binding
PGOC: nucleosome
PGOP: nucleosome assembly

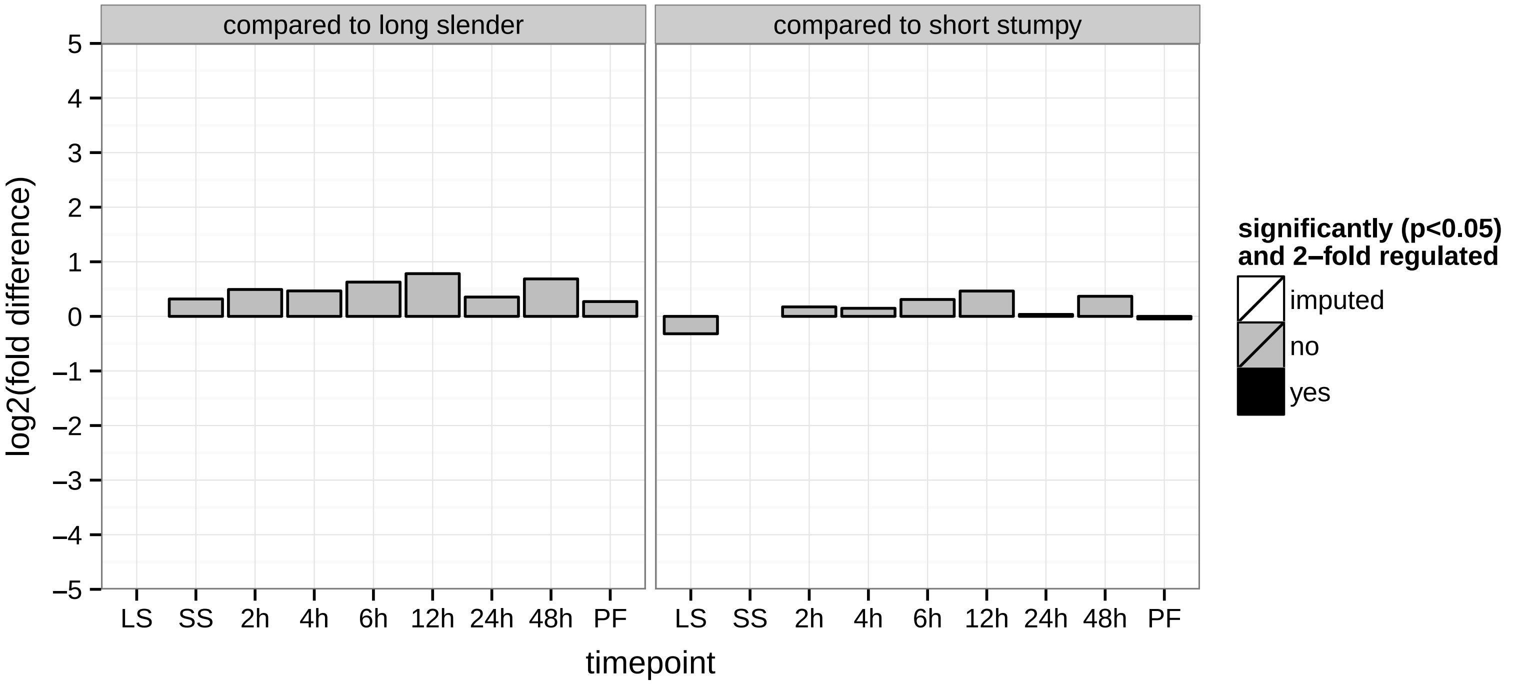

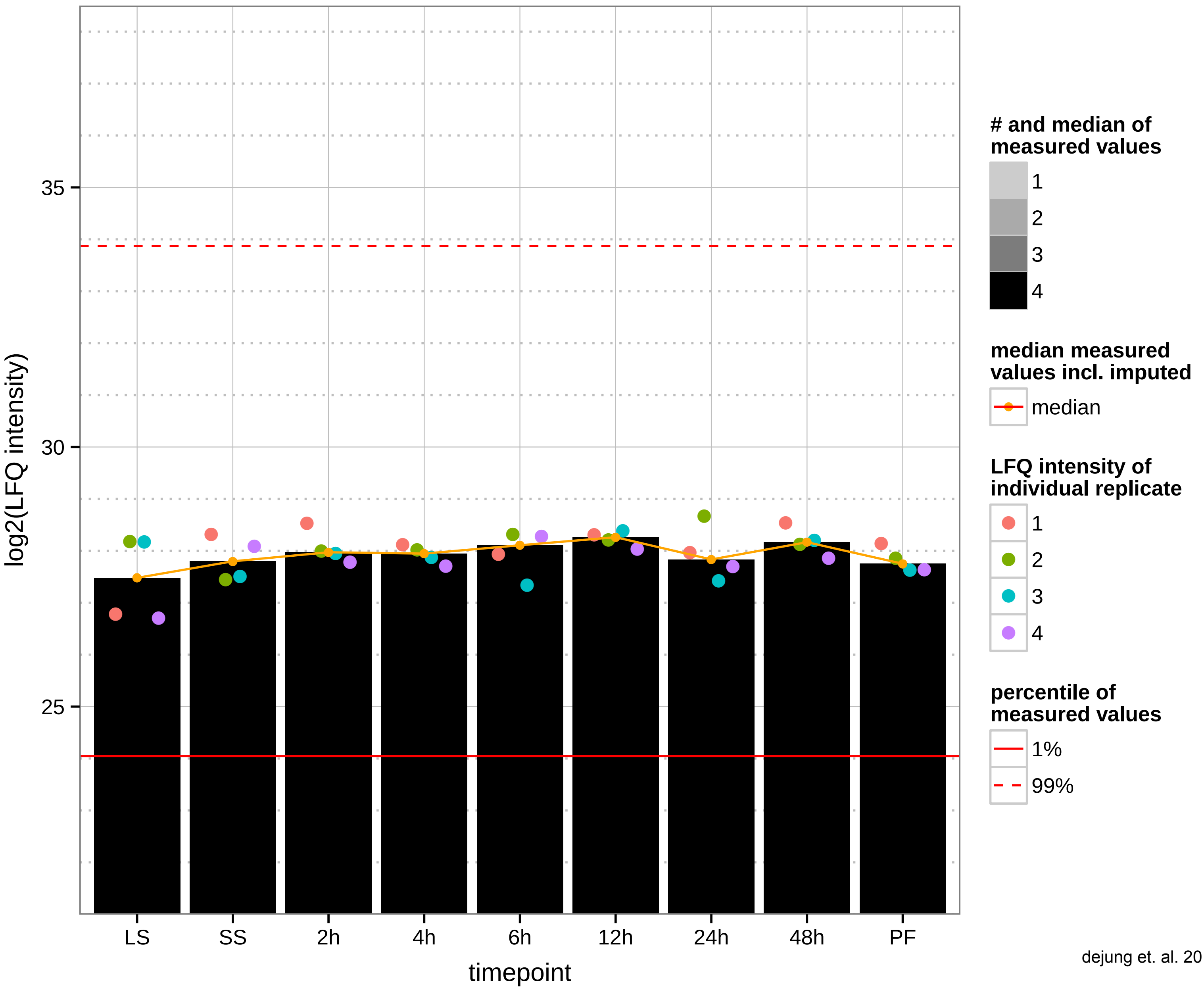

dejung et. al. 2015

hypothetical protein, conserved
Tb927.10.15360
AGOF: RNA binding
AGOC: null
AGOP: RNA processing
PGOF: ATP binding, ATP–dependent helicase activity, RNA binding, nucleic acid binding
PGOC: null
PGOP: RNA processing

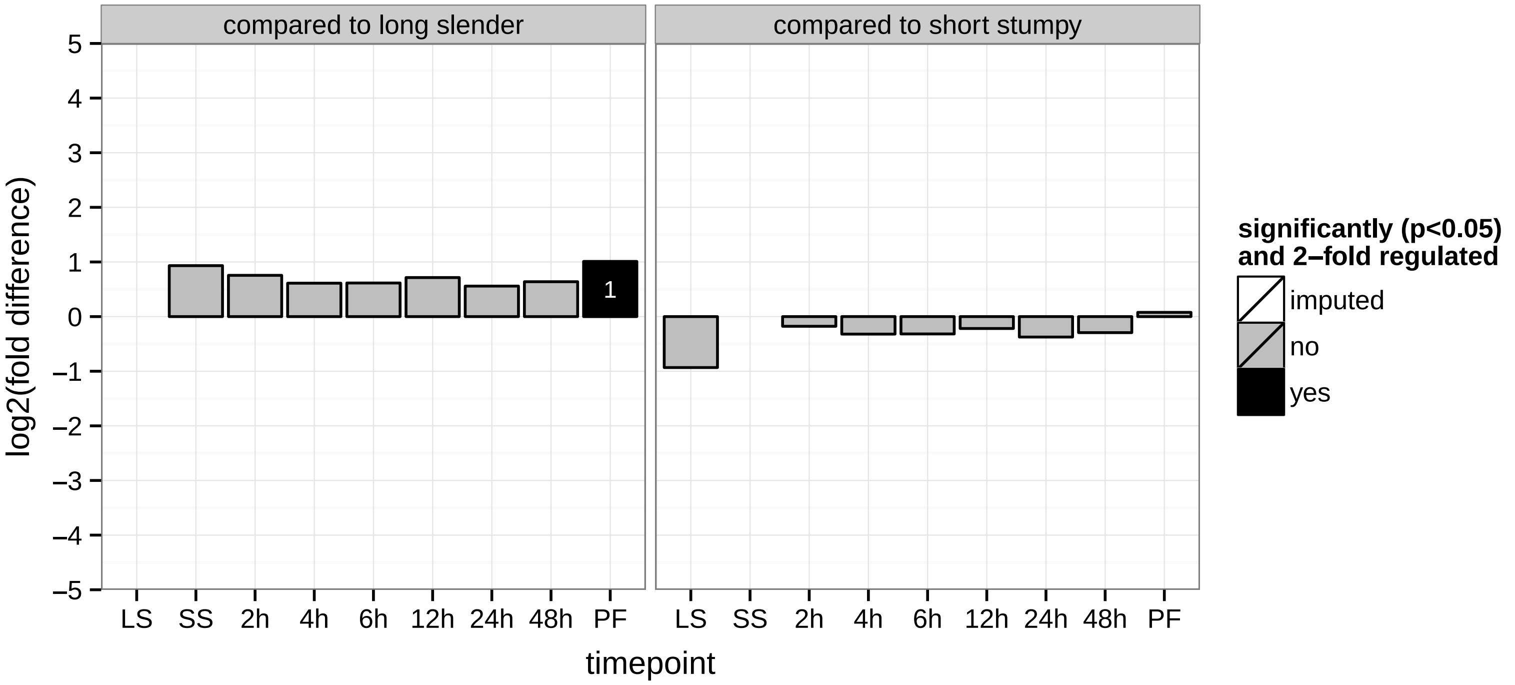

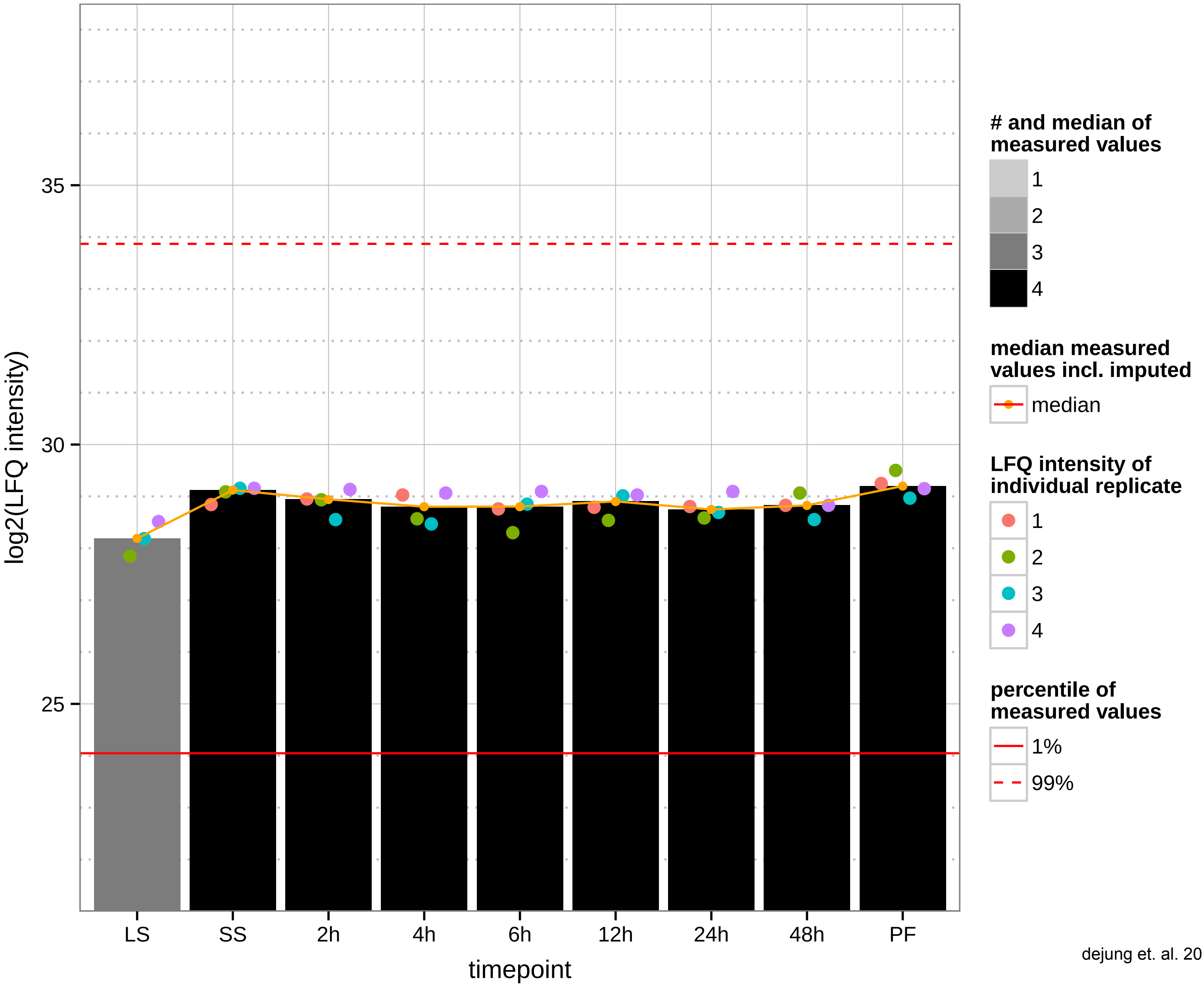

dejung et. al. 2015

kinesin, putative, hypothetical protein, conserved
Tb927.10.15400;Tb11.v5.0849
AGOF: null, ATP binding, microtubule motor activity, motor activity
AGOC: null, kinesin complex, microtubule associated complex
AGOP: null, microtubule–based movement
PGOF: null, ATP binding, microtubule motor activity
PGOC: null
PGOP: null, microtubule–based movement

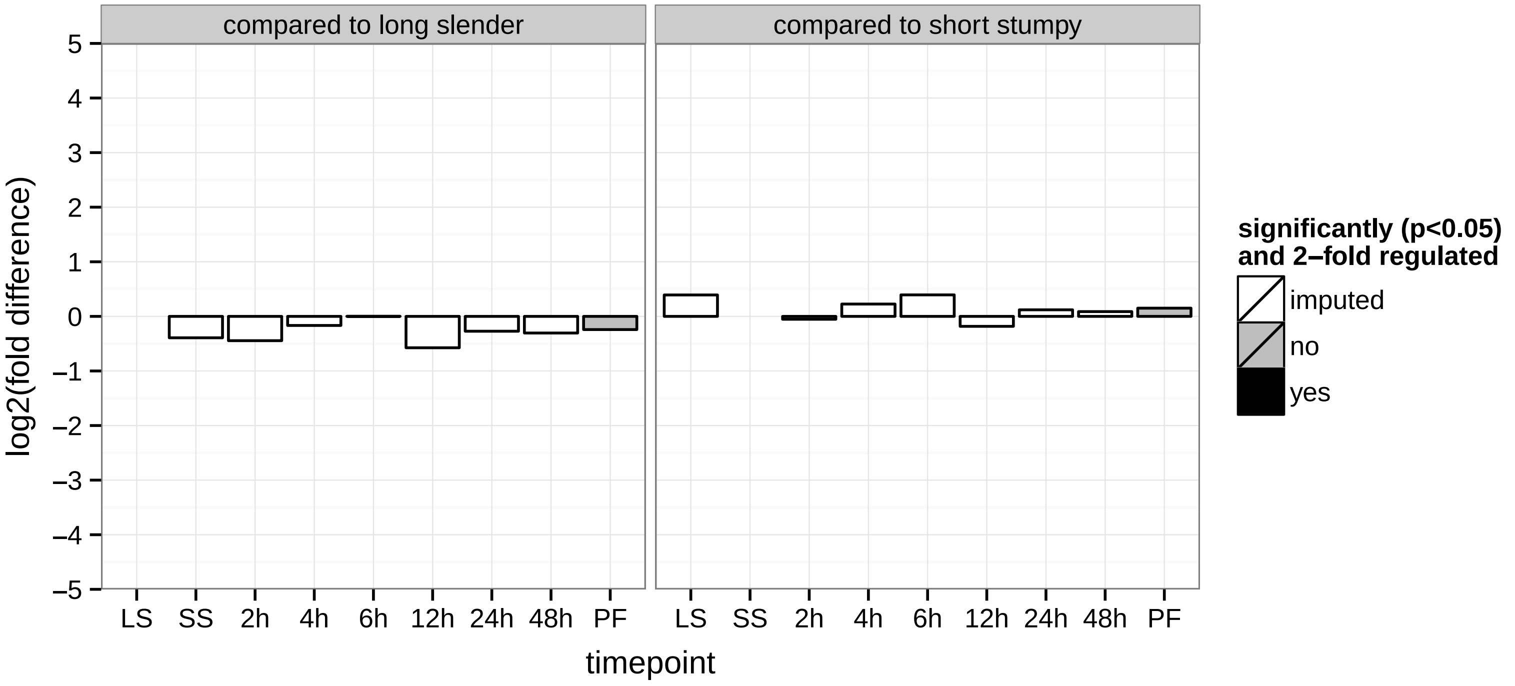

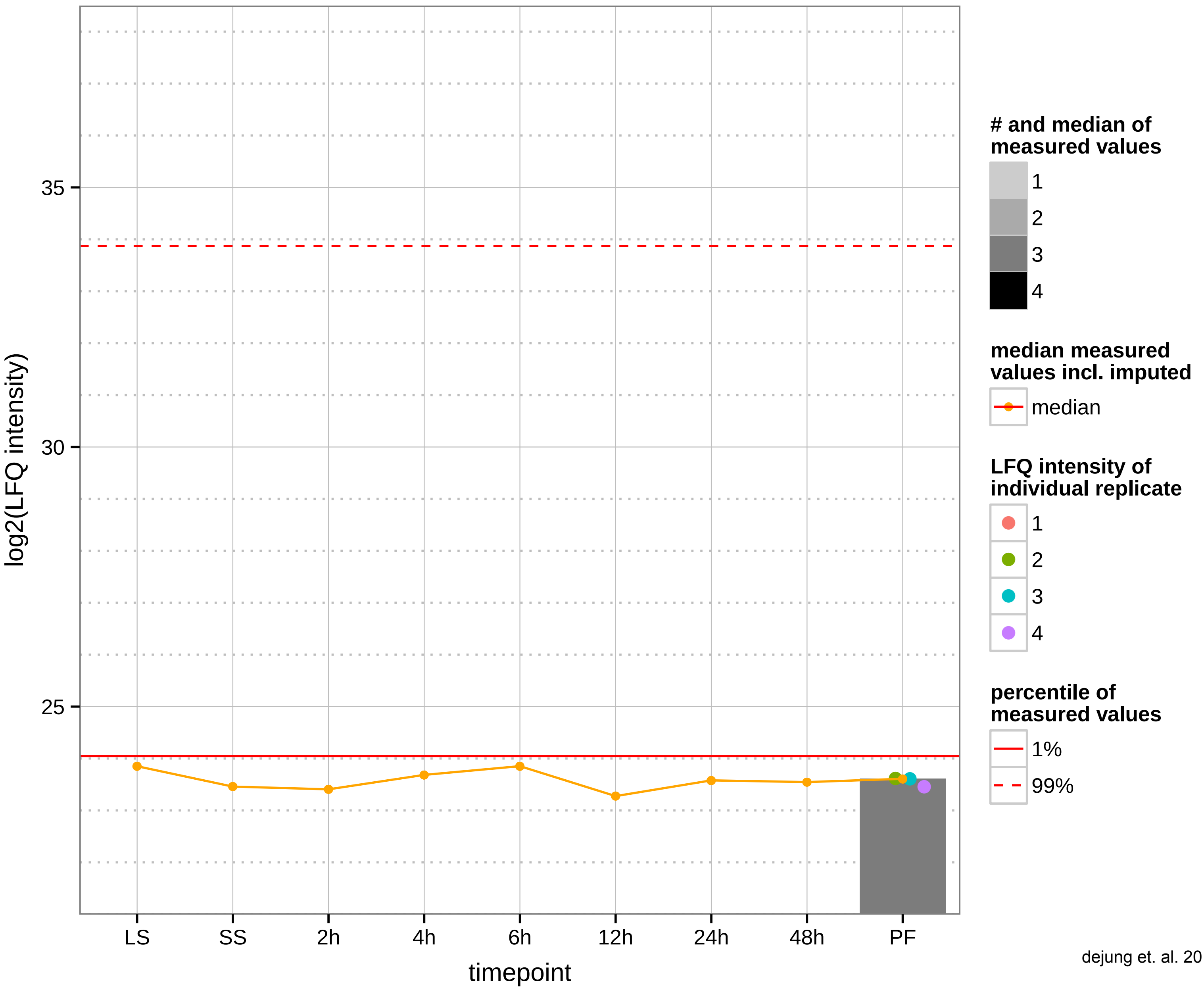

dejung et. al. 2015

hypothetical protein, conserved
Tb927.10.15470
AGOF: null
AGOC: null
AGOP: null
PGOF: null
PGOC: null
PGOP: null

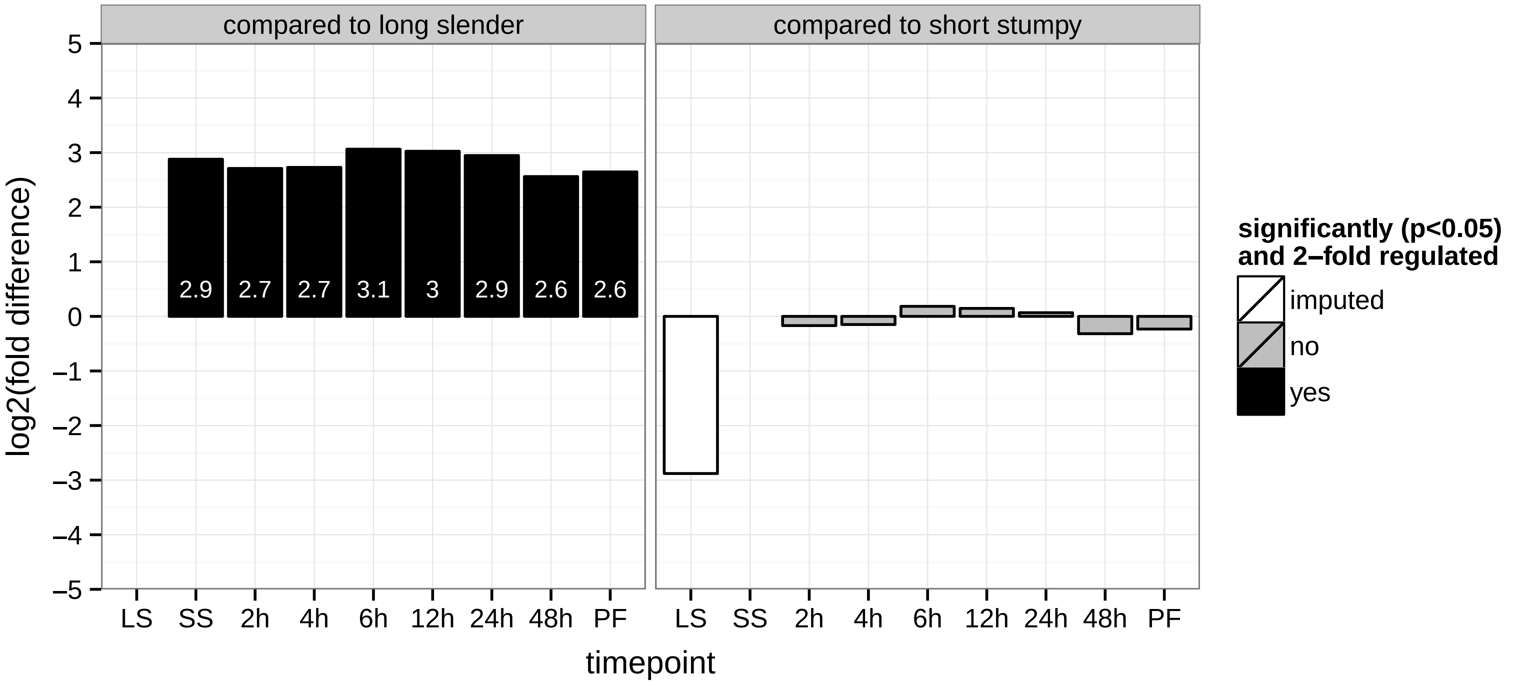

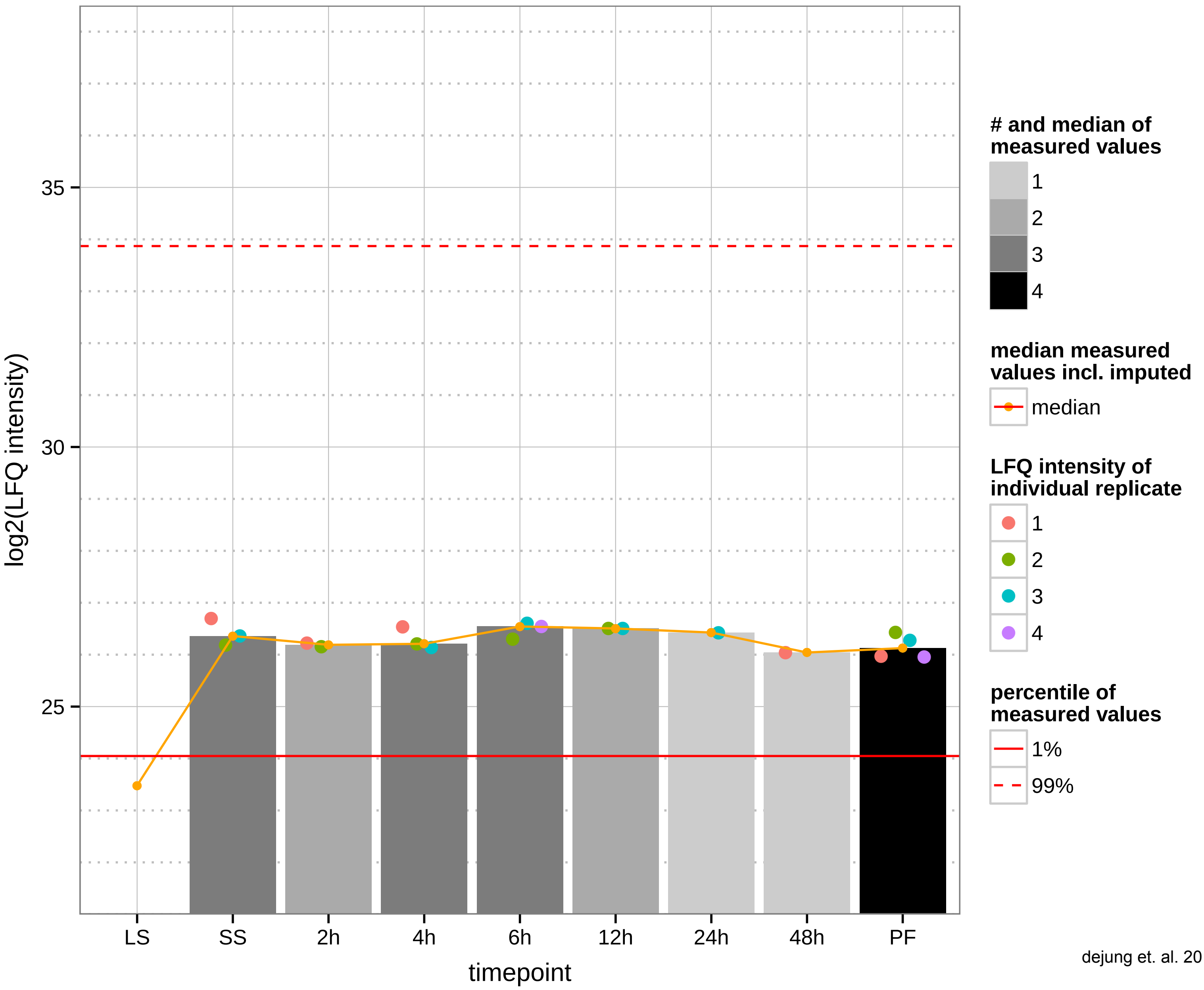

dejung et. al. 2015

proteasome regulatory non–ATP–ase subunit 5, 19S proteasome regulatory subunit 5 (RPN5)
Tb927.10.1550
AGOF: endopeptidase activity
AGOC: proteasome regulatory particle
AGOP: ubiquitin–dependent protein catabolic process
PGOF: protein binding
PGOC: null
PGOP: null

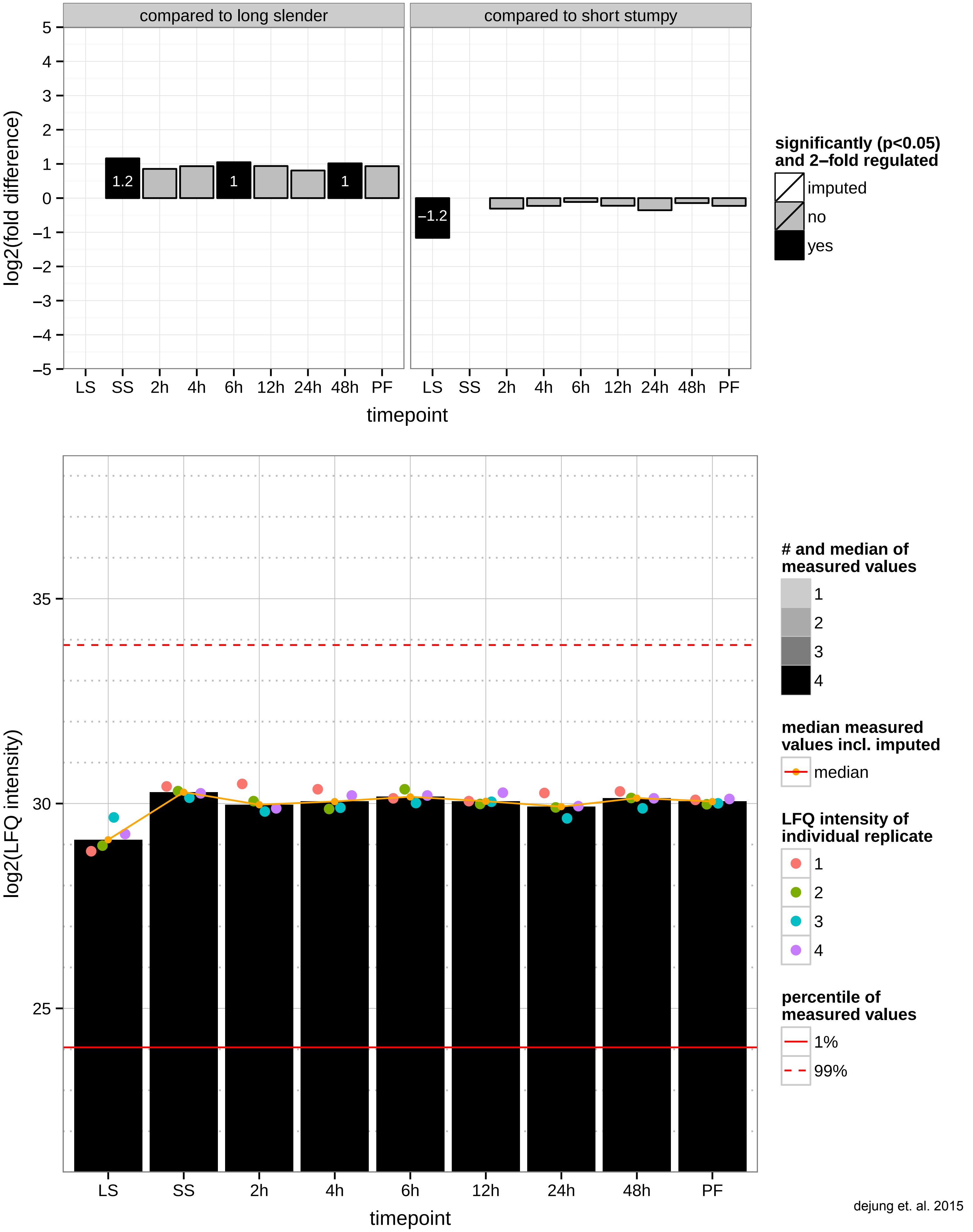

dejung et. al. 2015

signal recognition particle protein, putative
Tb927.10.15520
AGOF: 7S RNA binding
AGOC: signal recognition particle
AGOP: SRP−dependent cotranslational protein targeting to membrane
PGOF: 7S RNA binding
PGOC: signal recognition particle
PGOP: SRP−dependent cotranslational protein targeting to membrane

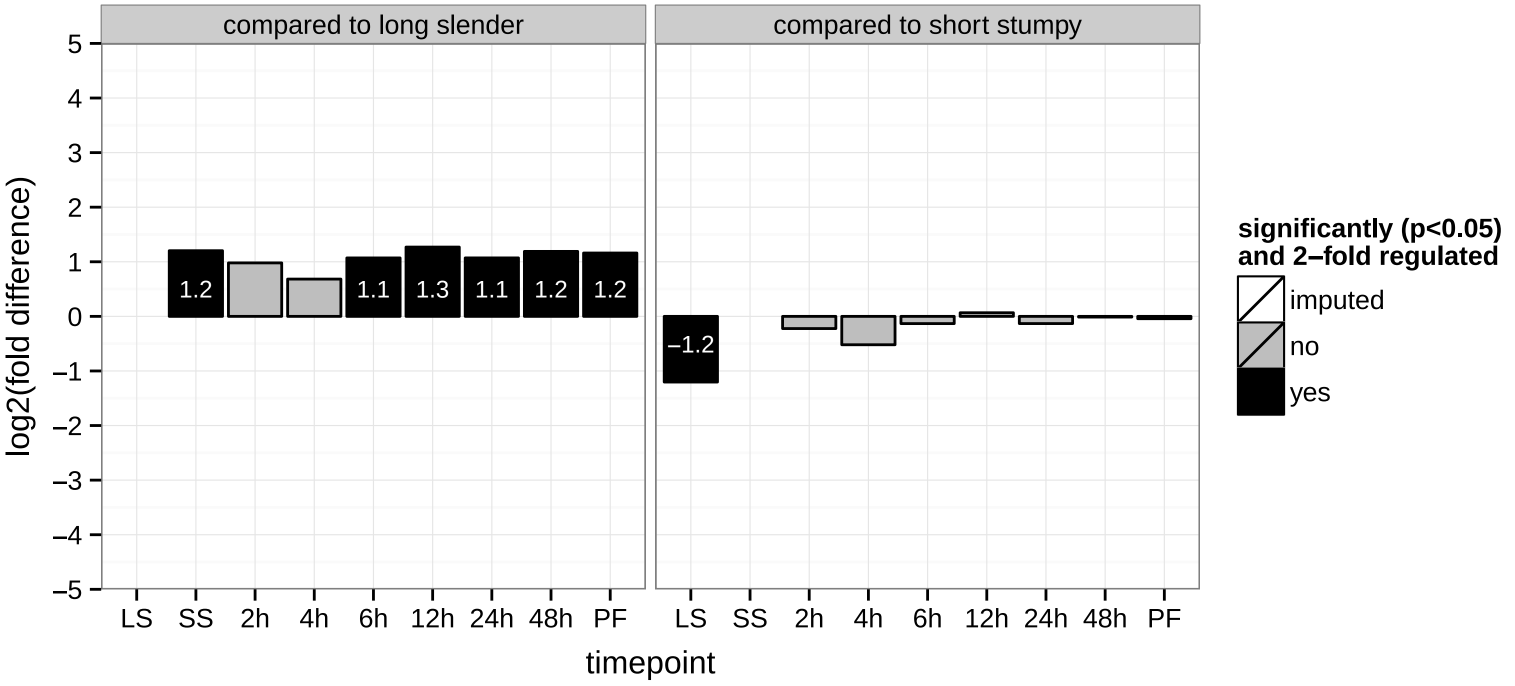

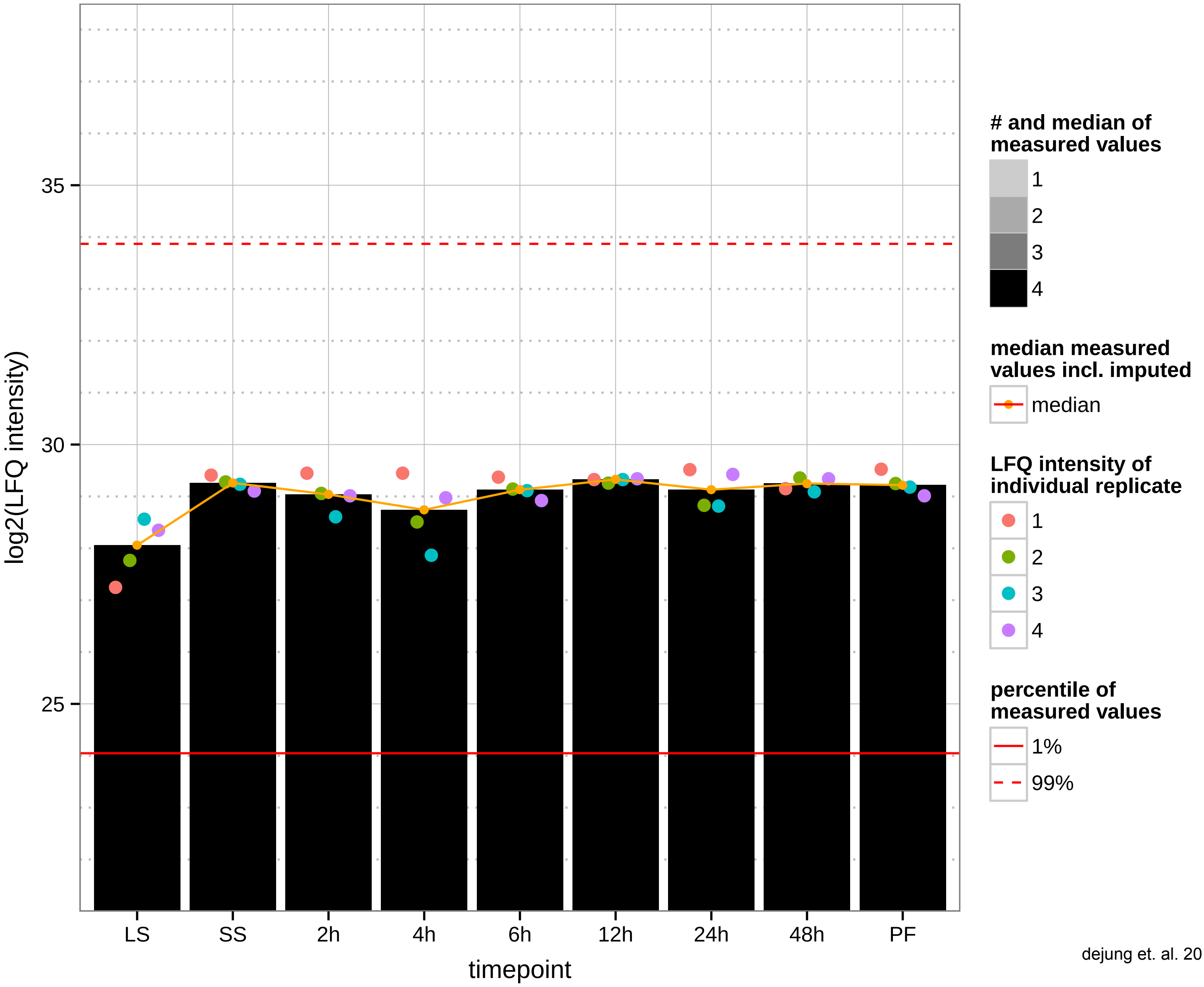

dejung et. al. 2015

hypothetical protein, conserved
Tb927.10.1560
AGOF: null
AGOC: mitochondrion
AGOP: null
PGOF: null
PGOC: null
PGOP: null

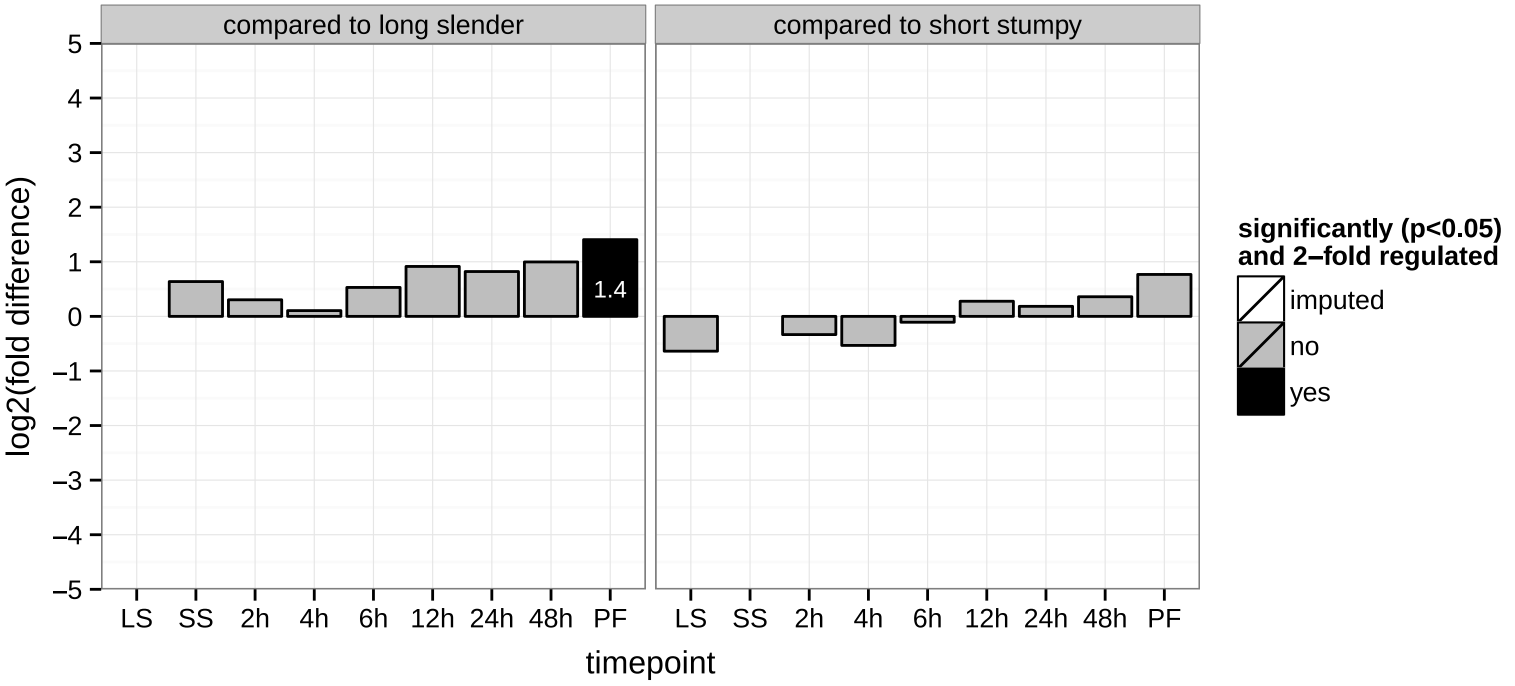

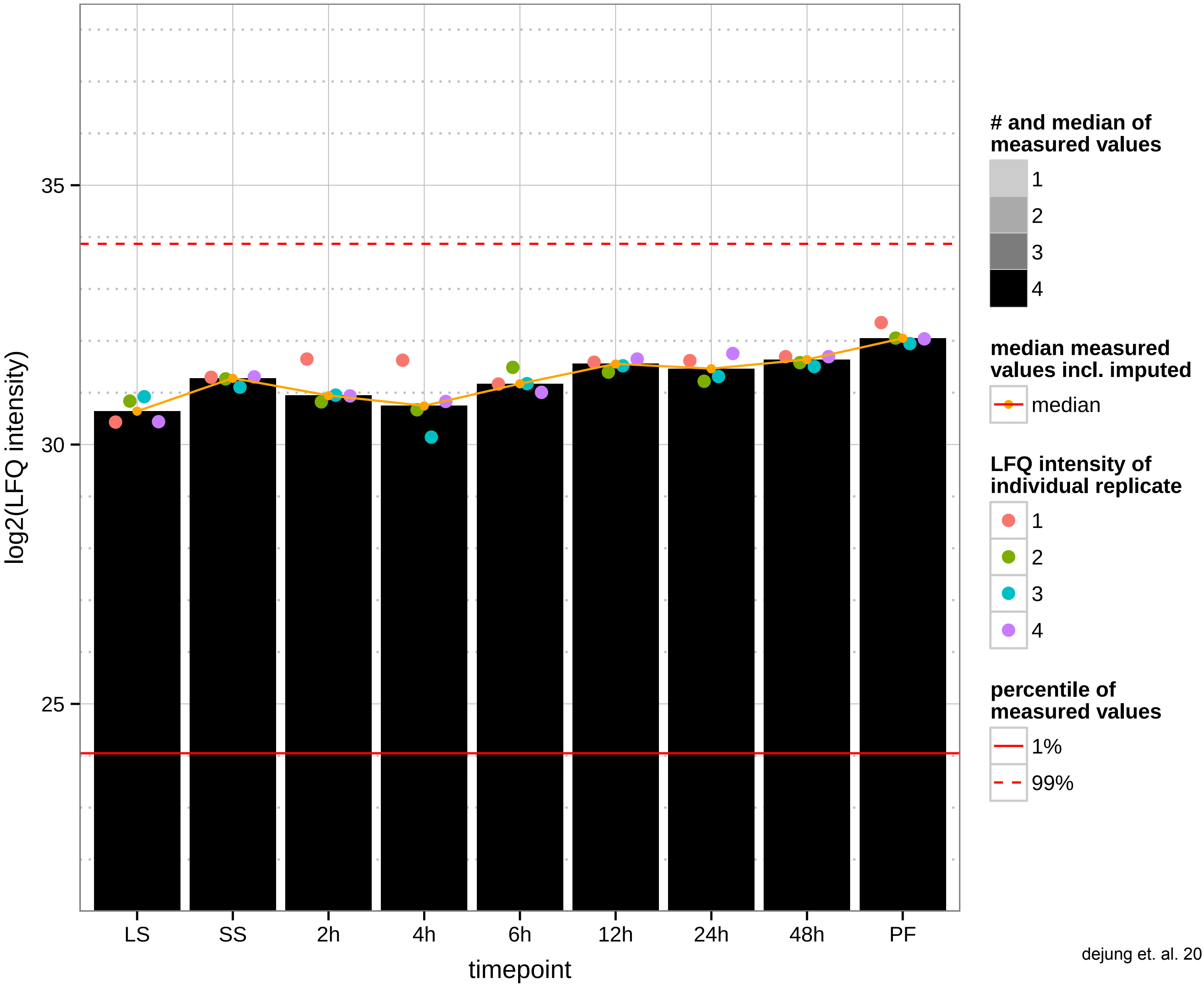

dejung et. al. 2015

hypothetical protein, conserved
Tb927.10.15660
AGOF: null
AGOC: null
AGOP: null
PGOF: null
PGOC: null
PGOP: null

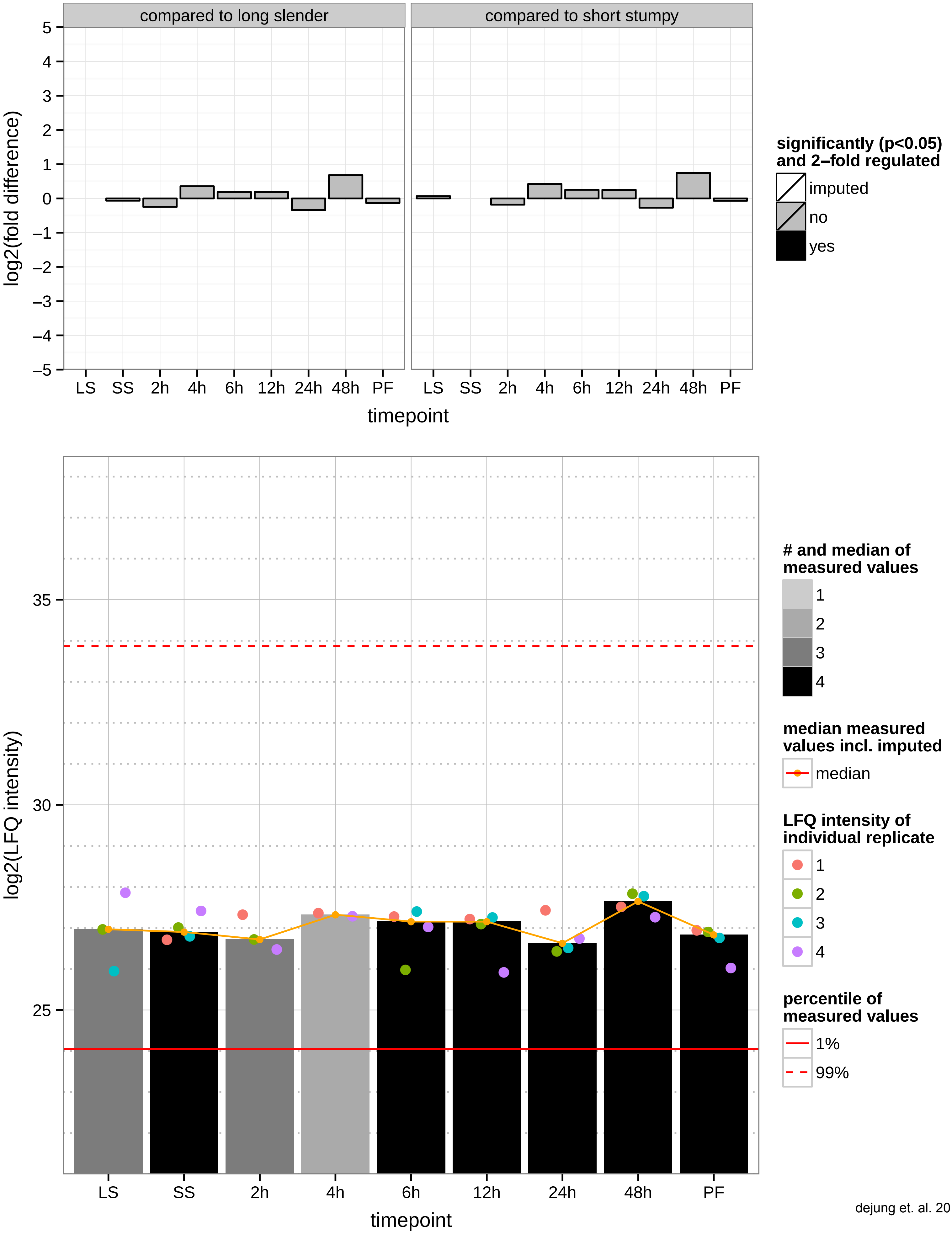

dejung et. al. 2015

mitochondrial carrier protein (MCP7)
Tb927.10.15710
AGOF: null
AGOC: mitochondrial inner membrane, mitochondrial part
AGOP: transport
PGOF: null
PGOC: null
PGOP: null

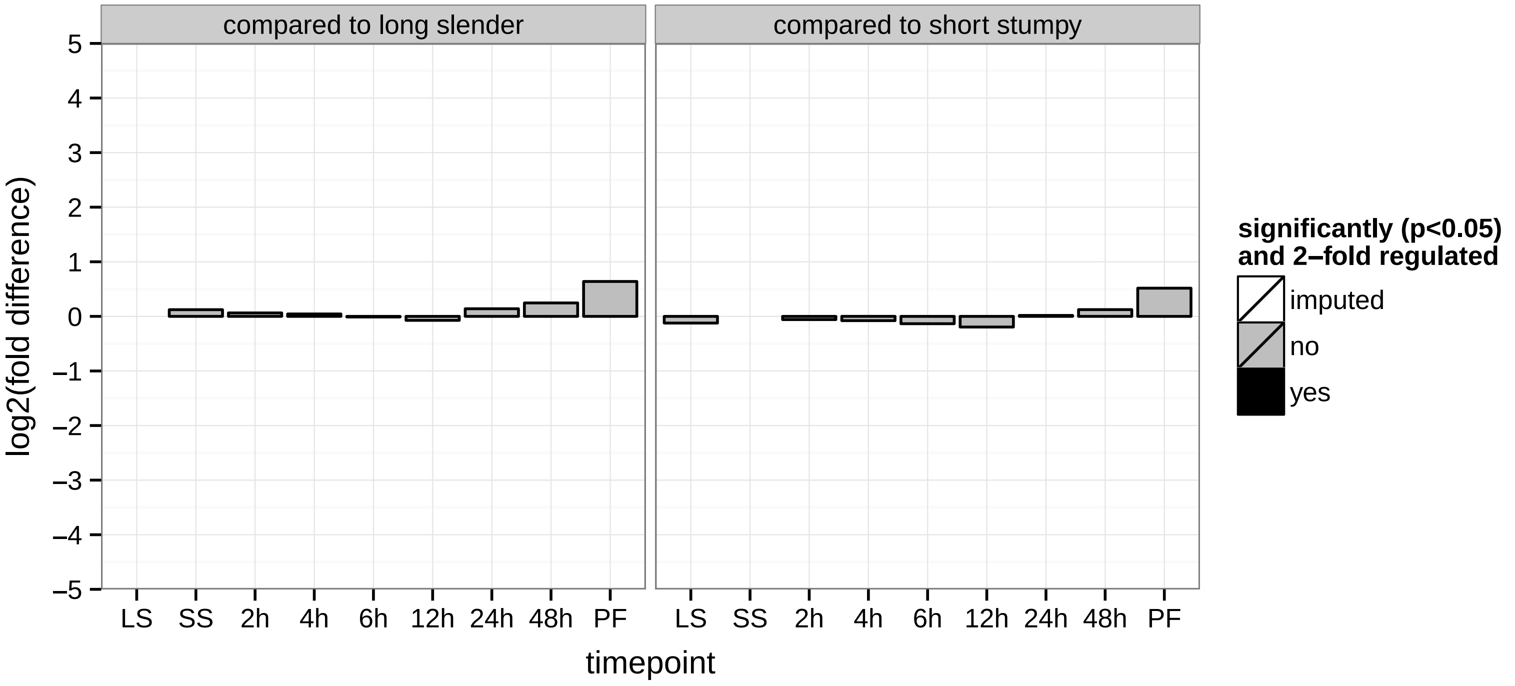

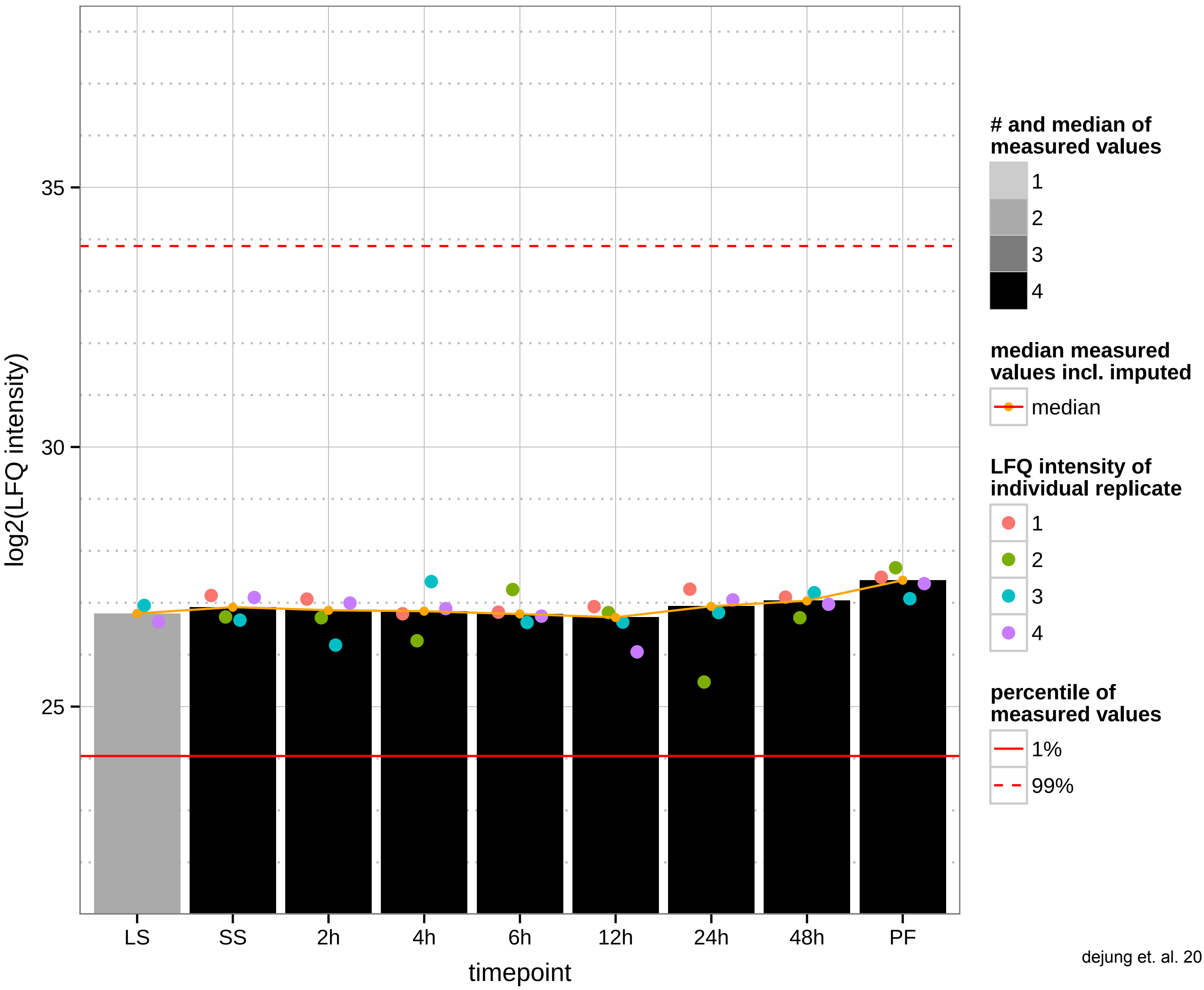

dejung et. al. 2015

proteasome regulatory non–ATP–ase subunit 9, 19S proteasome regulatory subunit 9 (RPN9)
Tb927.10.15720
AGOF: endopeptidase activity
AGOC: proteasome regulatory particle
AGOP: ubiquitin–dependent protein catabolic process
PGOF: protein binding
PGOC: null
PGOP: null

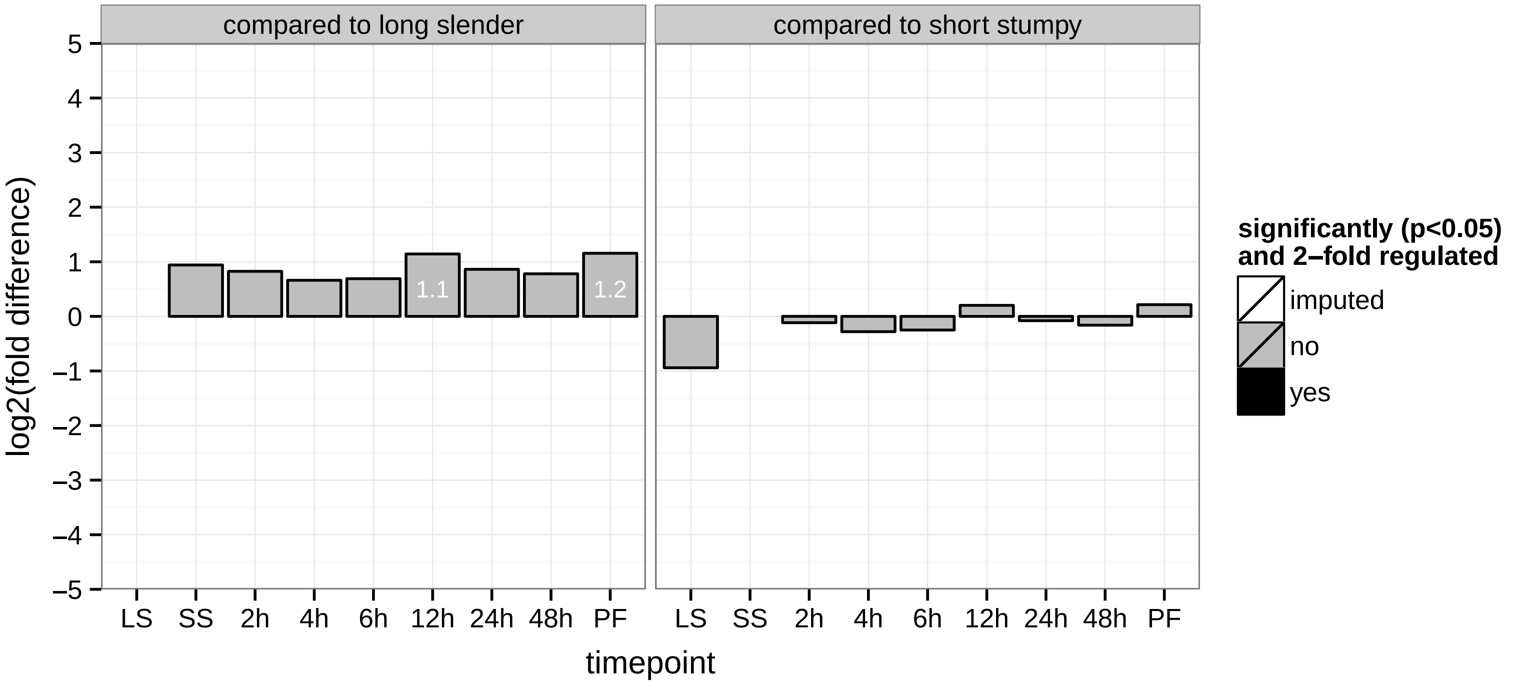

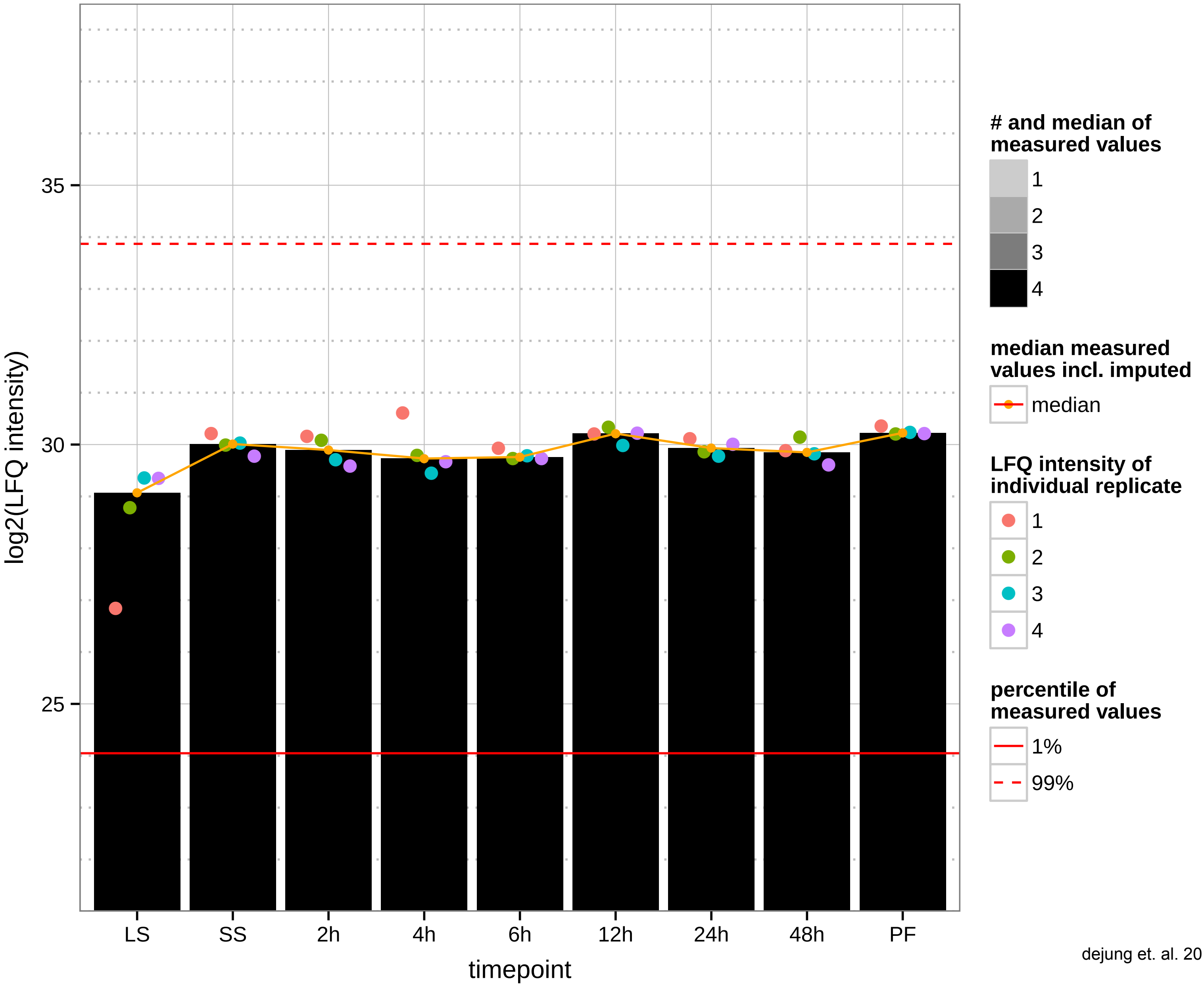

dejung et. al. 2015

hypothetical protein, conserved
Tb927.10.15740
AGOF: null
AGOC: null
AGOP: null
PGOF: protein binding
PGOC: null
PGOP: null

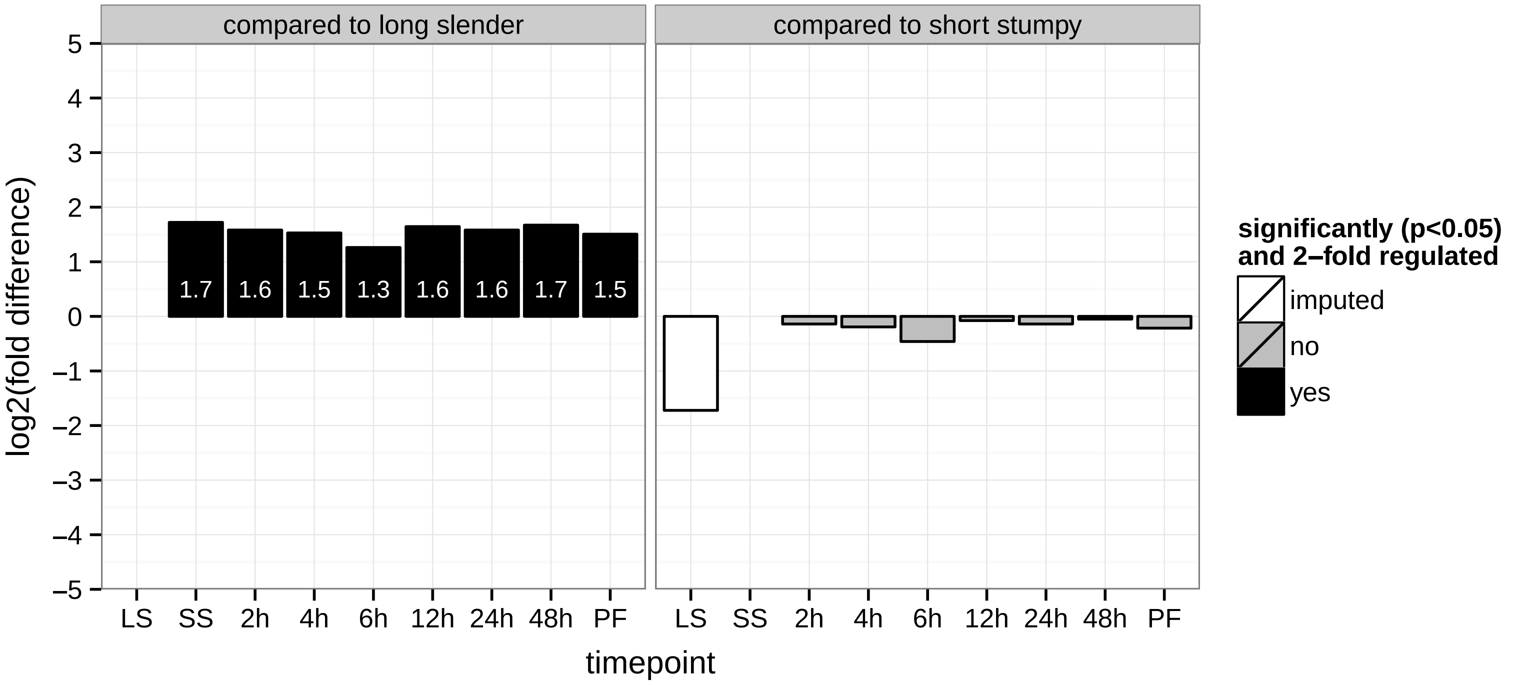

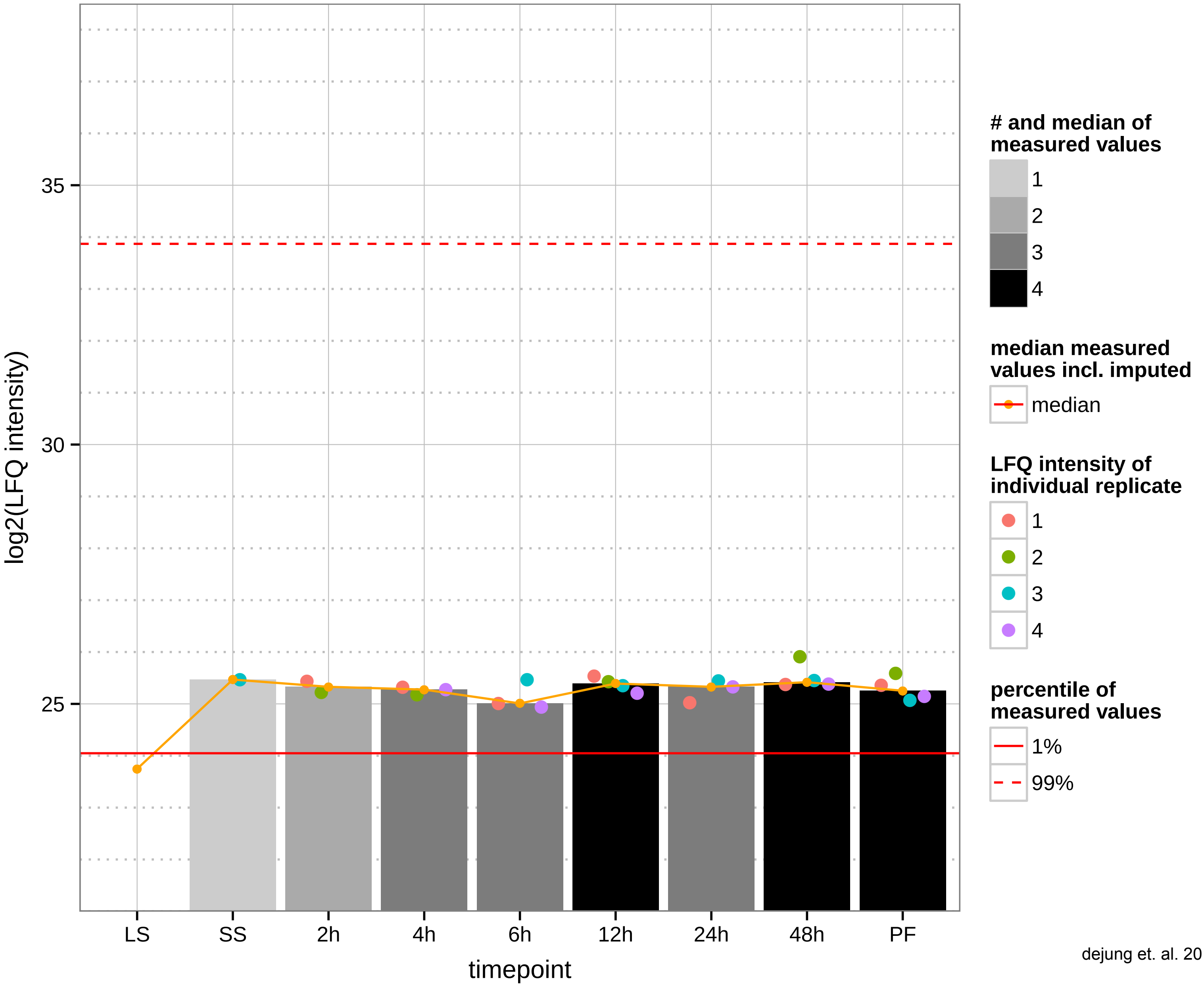

dejung et. al. 2015

hypothetical protein, conserved
Tb927.10.15760
AGOF: null
AGOC: null
AGOP: null
PGOF: null
PGOC: null
PGOP: null

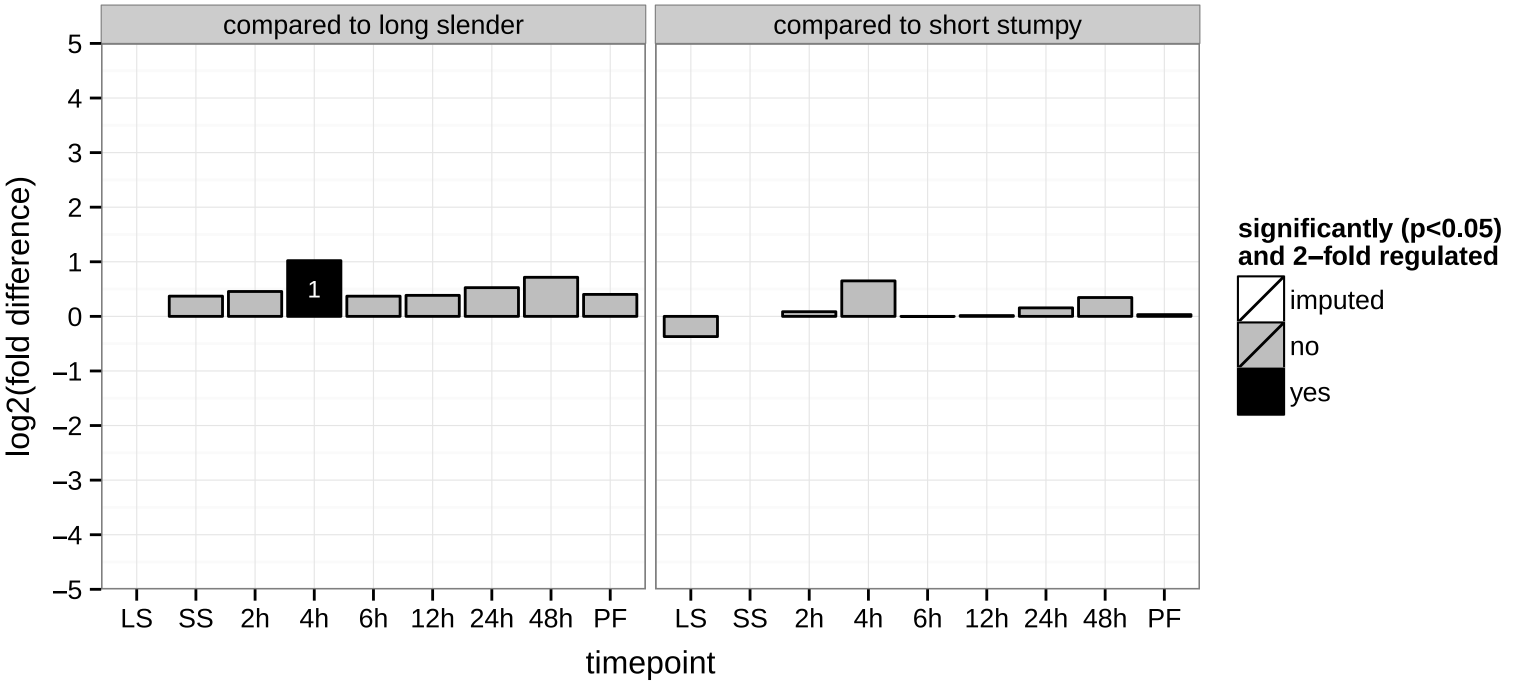

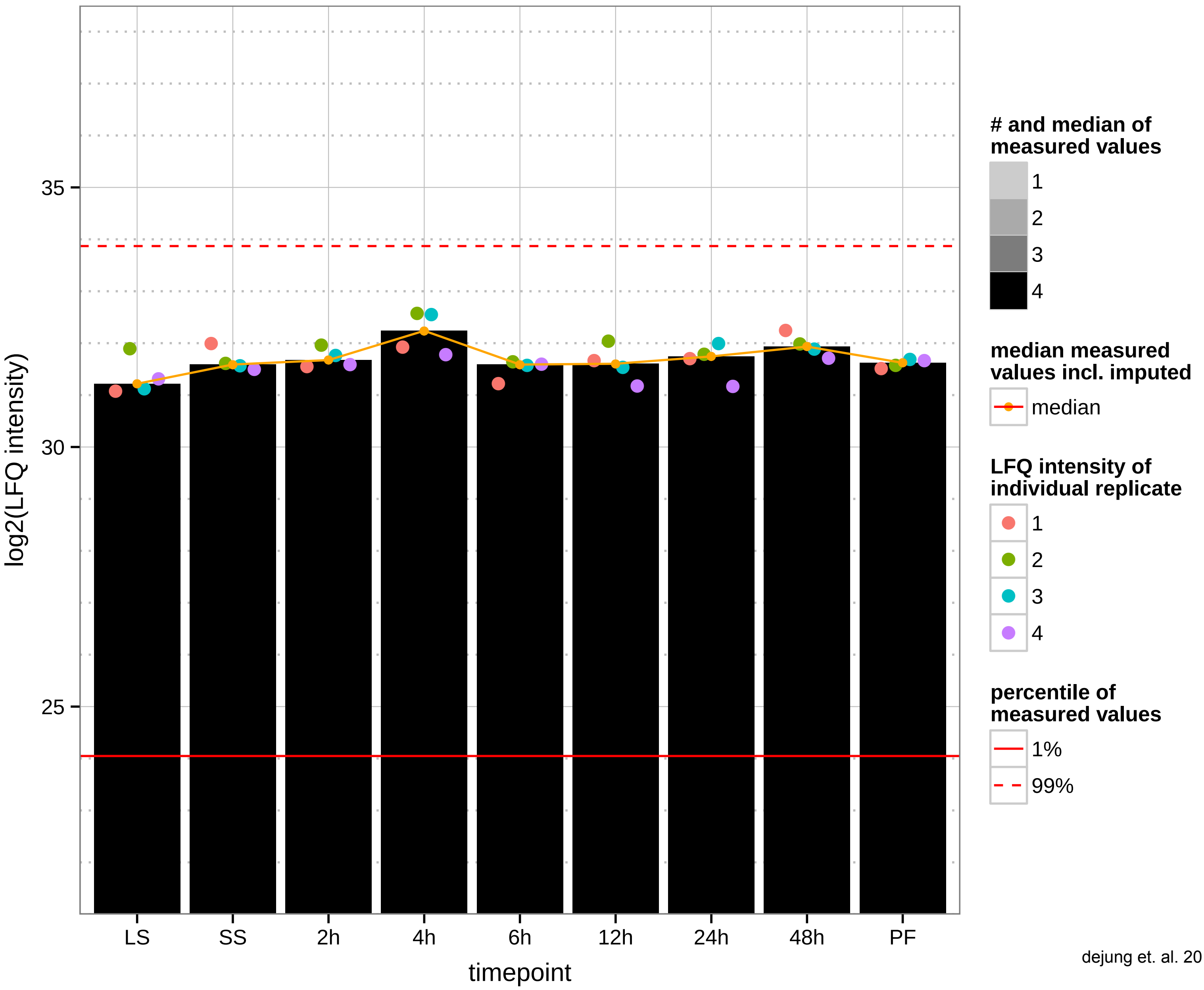

dejung et. al. 2015

hypothetical protein, conserved
Tb927.10.1580
AGOF: null
AGOC: null
AGOP: null
PGOF: null
PGOC: null
PGOP: null

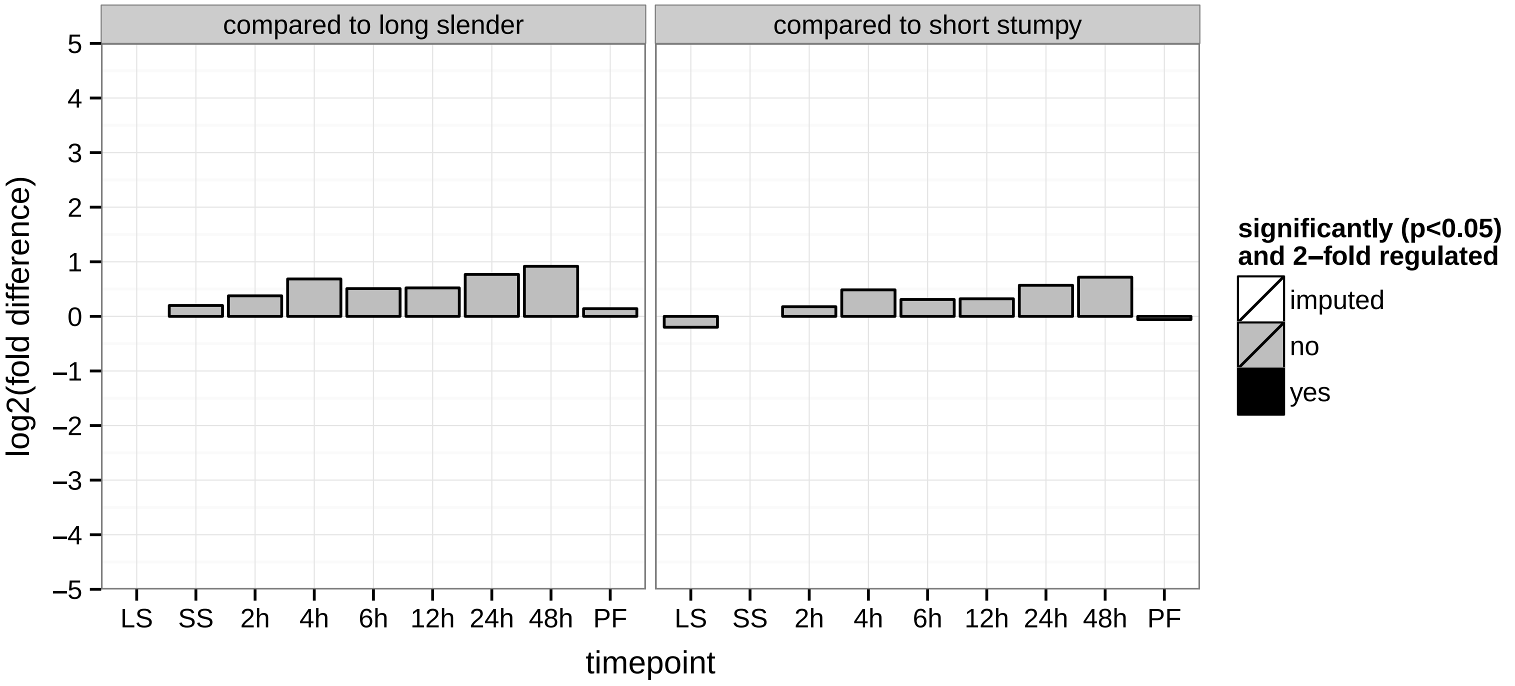

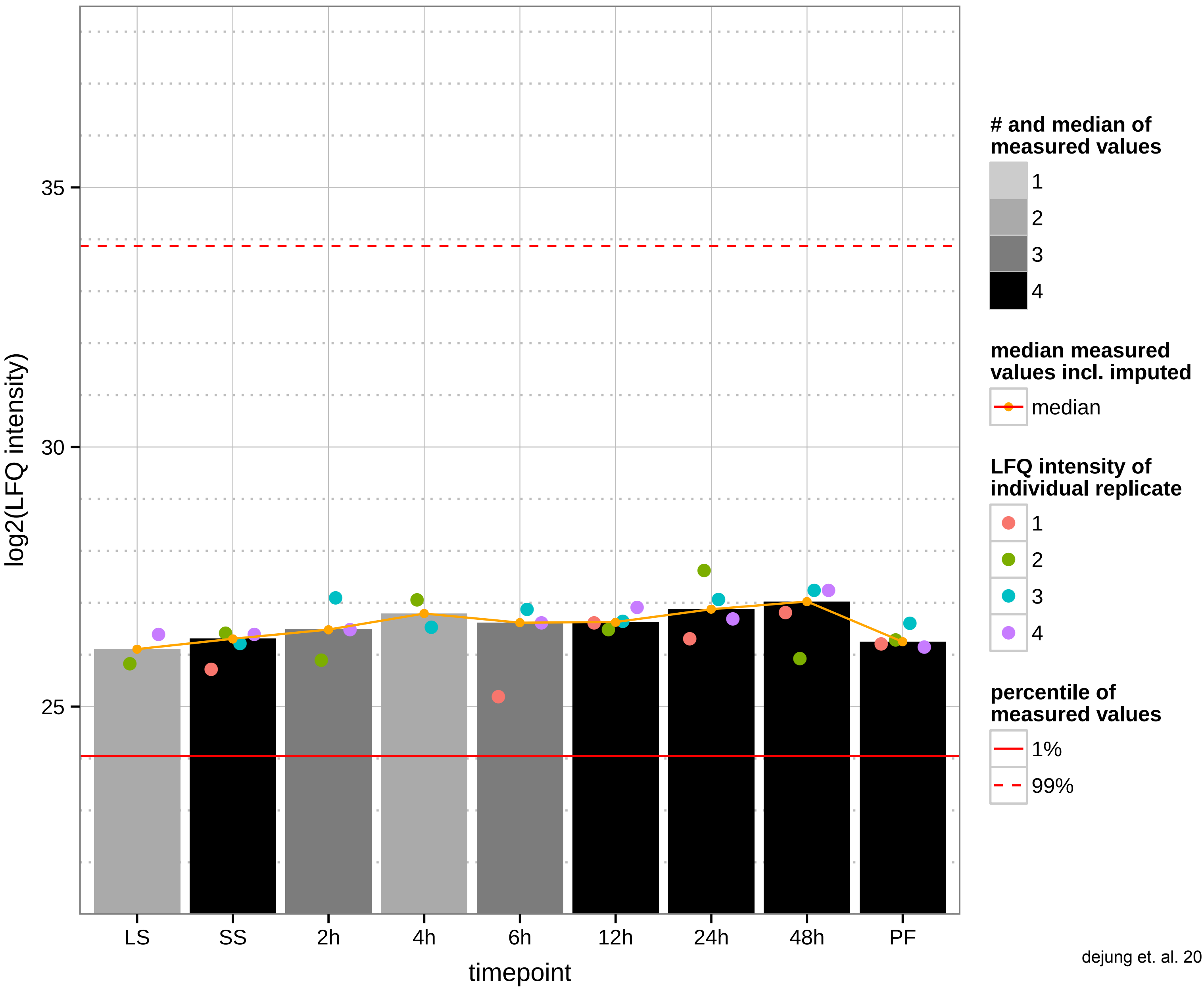

dejung et. al. 2015

RNA binding protein, putative
Tb927.10.15870
AGOF: RNA binding
AGOC: null
AGOP: RNA processing
PGOF: RNA binding
PGOC: null
PGOP: RNA processing

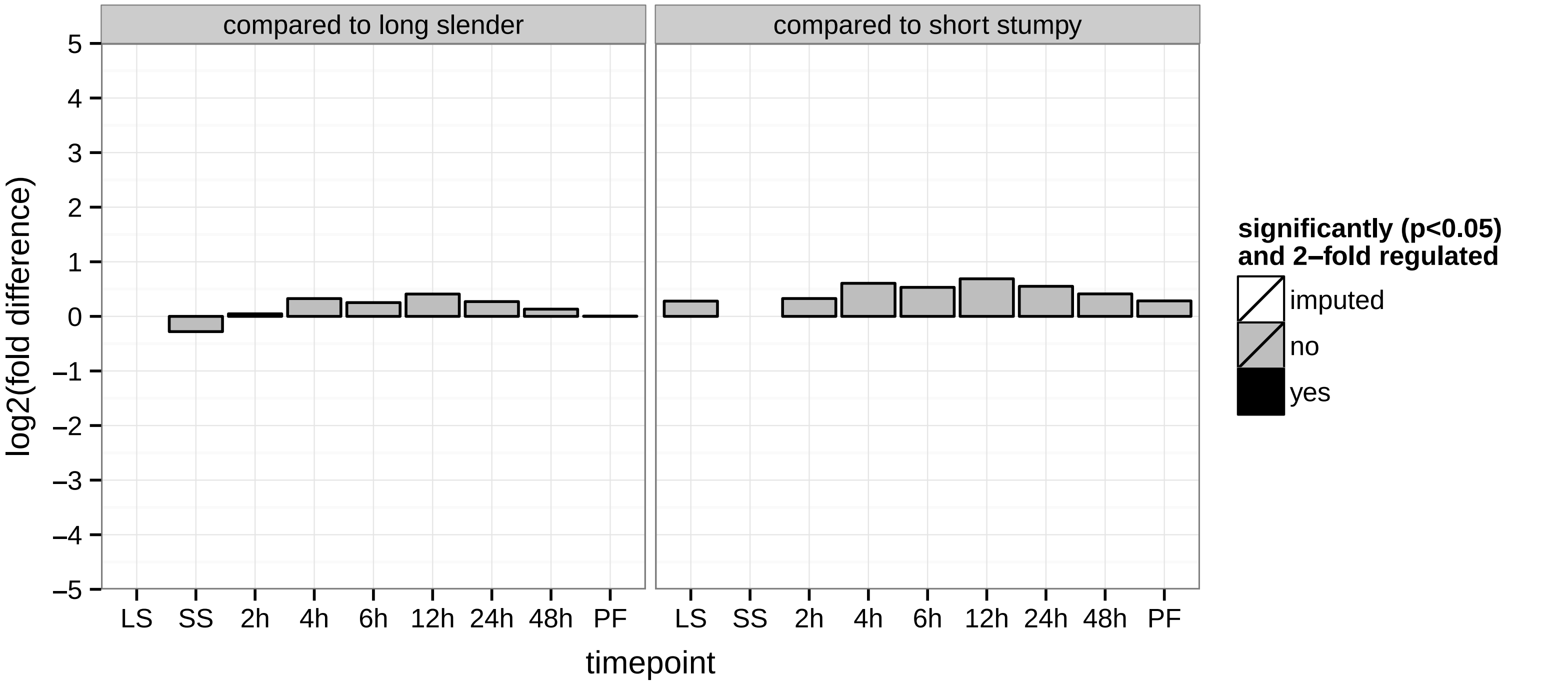

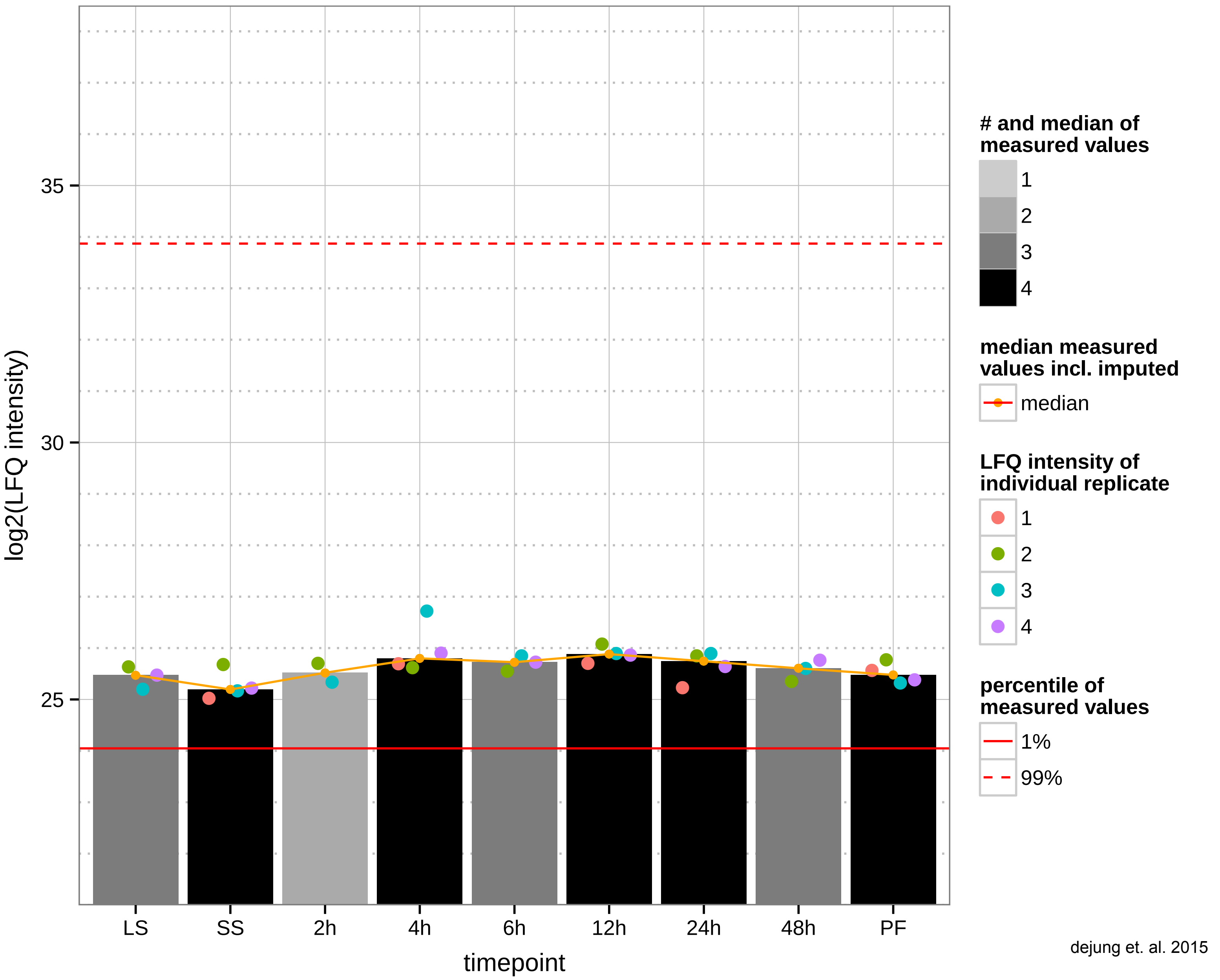

dejung et. al. 2015

ribosomal protein L36, putative
Tb927.9.15210;Tb927.10.1590
AGOF: structural constituent of ribosome
AGOC: intracellular, ribosome
AGOP: translation
PGOF: structural constituent of ribosome
PGOC: intracellular, ribosome
PGOP: translation

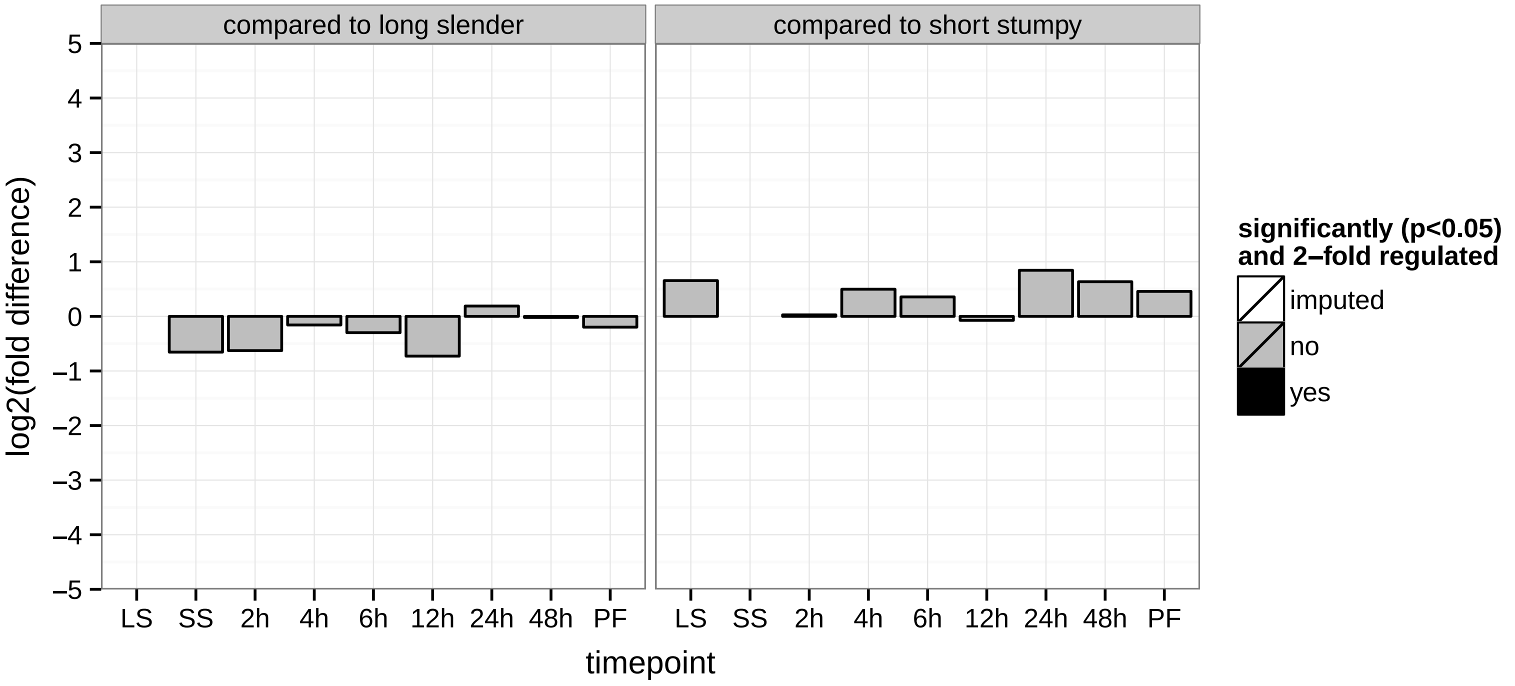

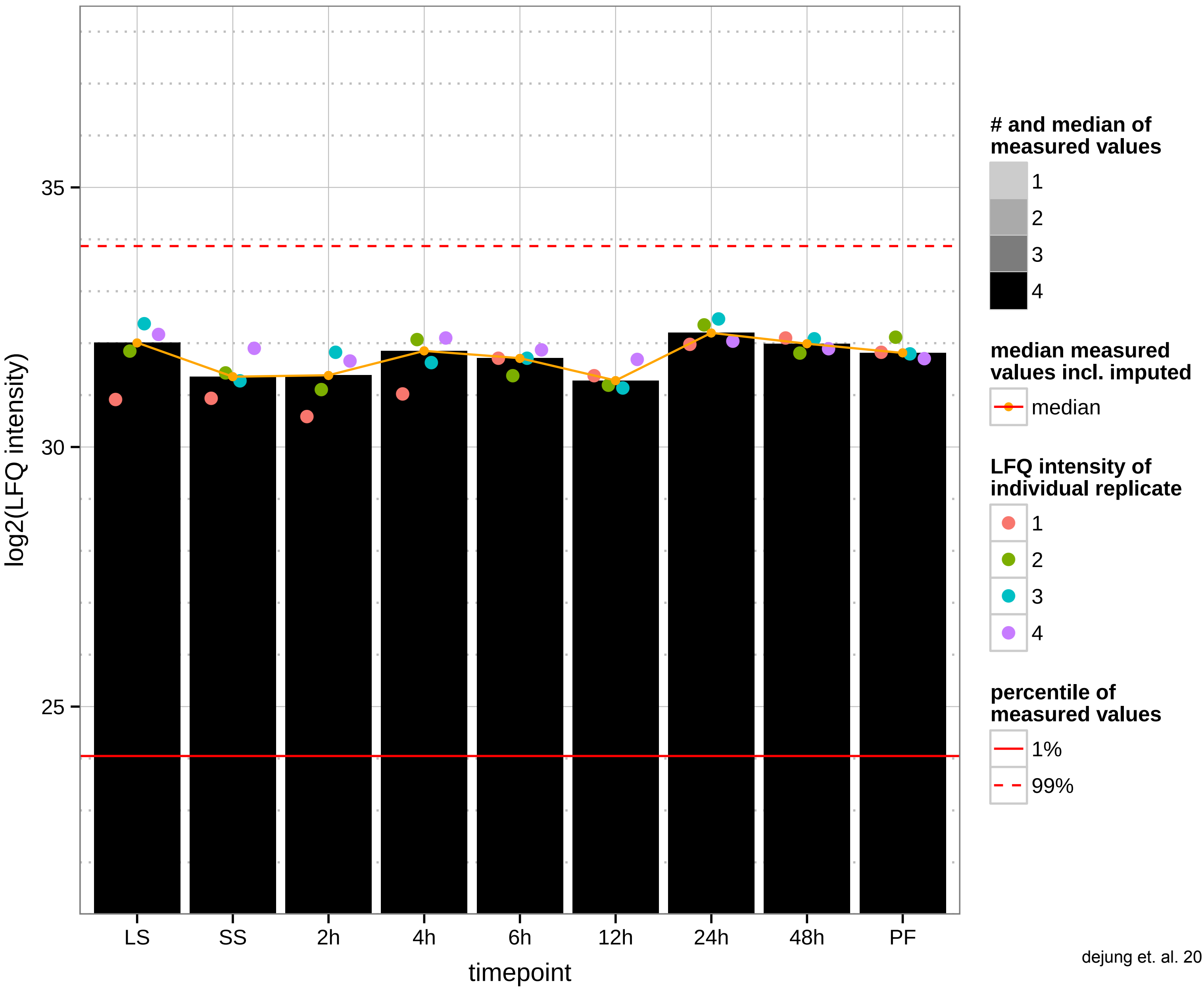

dejung et. al. 2015

Triglyceride lipase, putative, class 3 lipase
Tb927.10.15970;Tb11.v5.0306
AGOF: triglyceride lipase activity, zinc ion binding
AGOC: null
AGOP: lipid metabolic process
PGOF: triglyceride lipase activity
PGOC: null
PGOP: lipid metabolic process

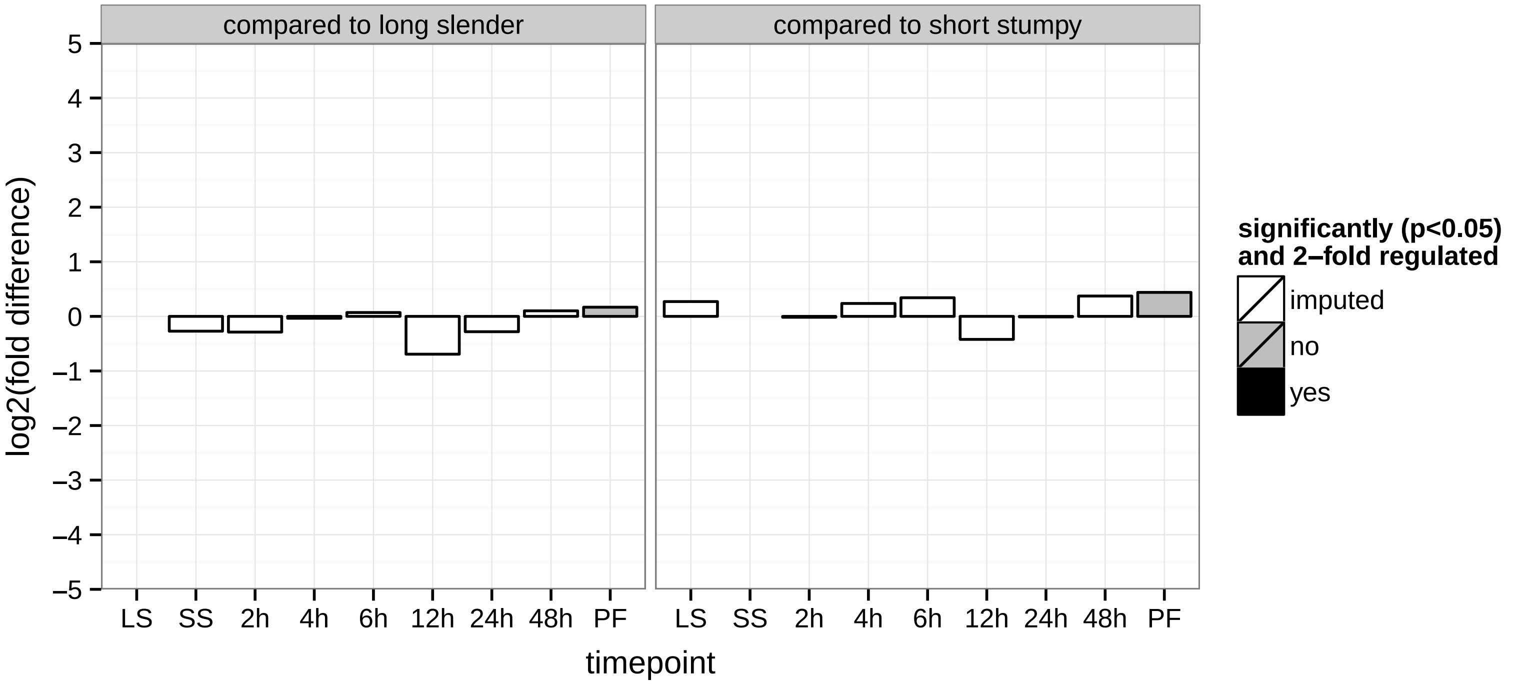

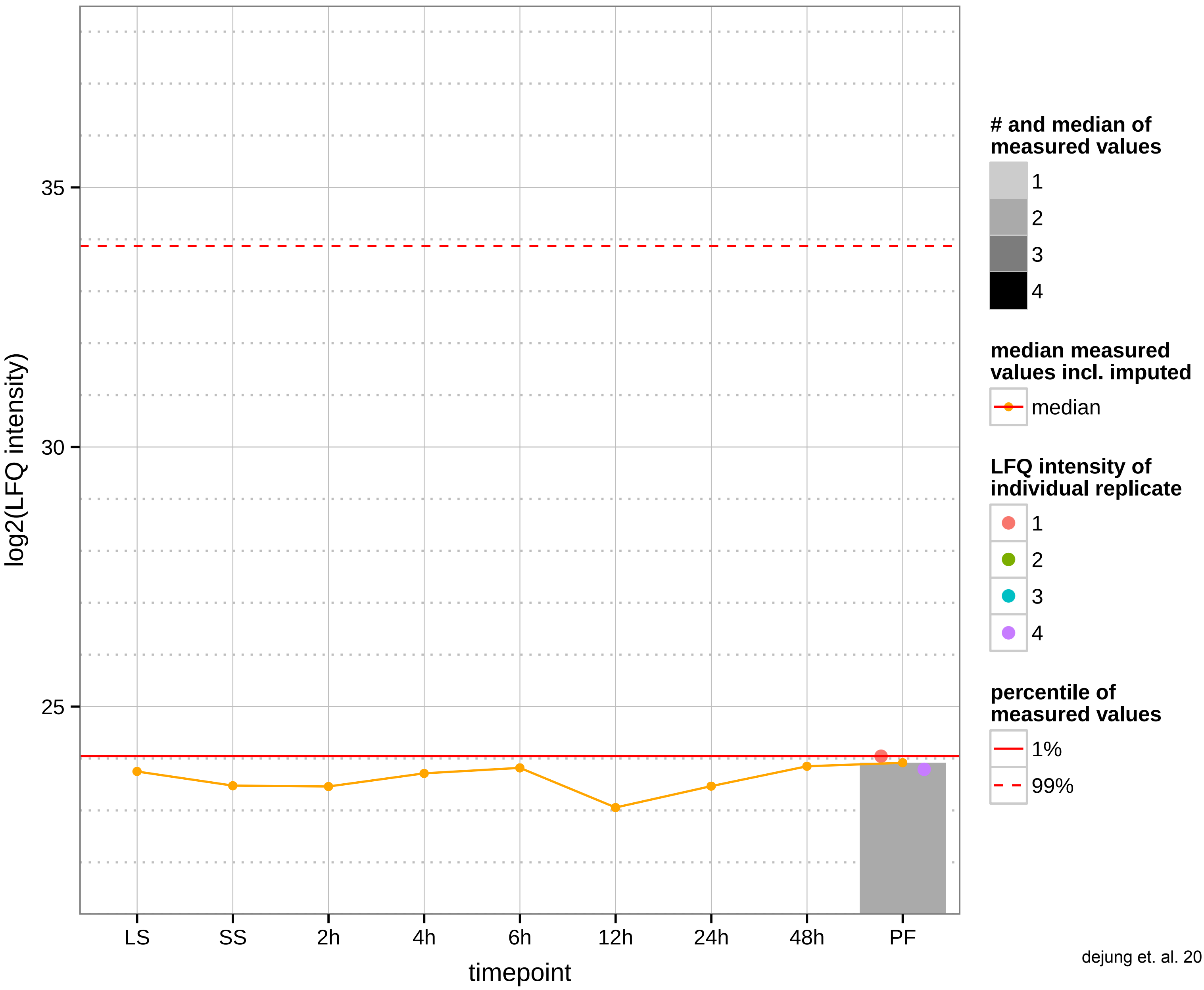

dejung et. al. 2015

inosine–5'–monophosphate dehydrogenase, IMP dehydrogenase
Tb927.10.16120
AGOF: IMP dehydrogenase activity
AGOC: null
AGOP: oxidation–reduction process, purine ribonucleotide biosynthetic process
PGOF: IMP dehydrogenase activity, catalytic activity, protein binding
PGOC: null
PGOP: oxidation–reduction process

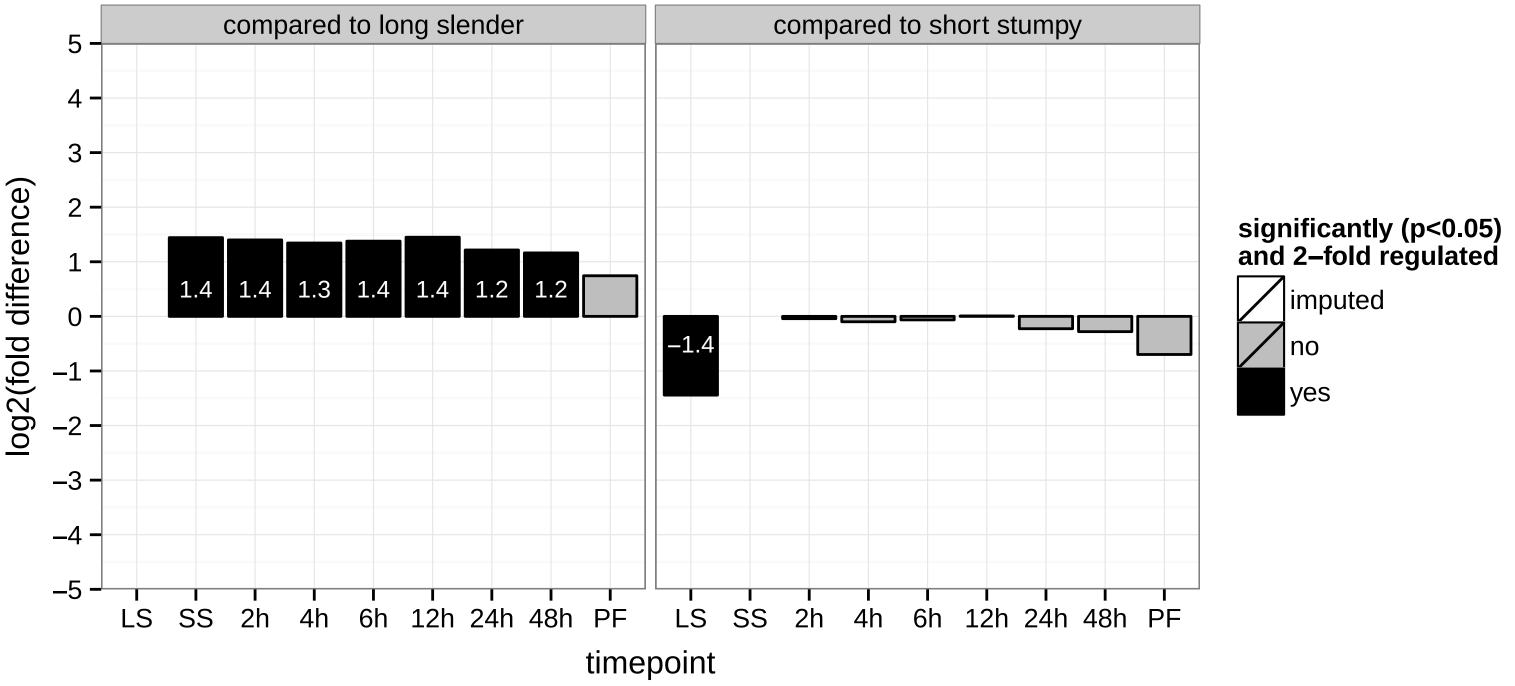

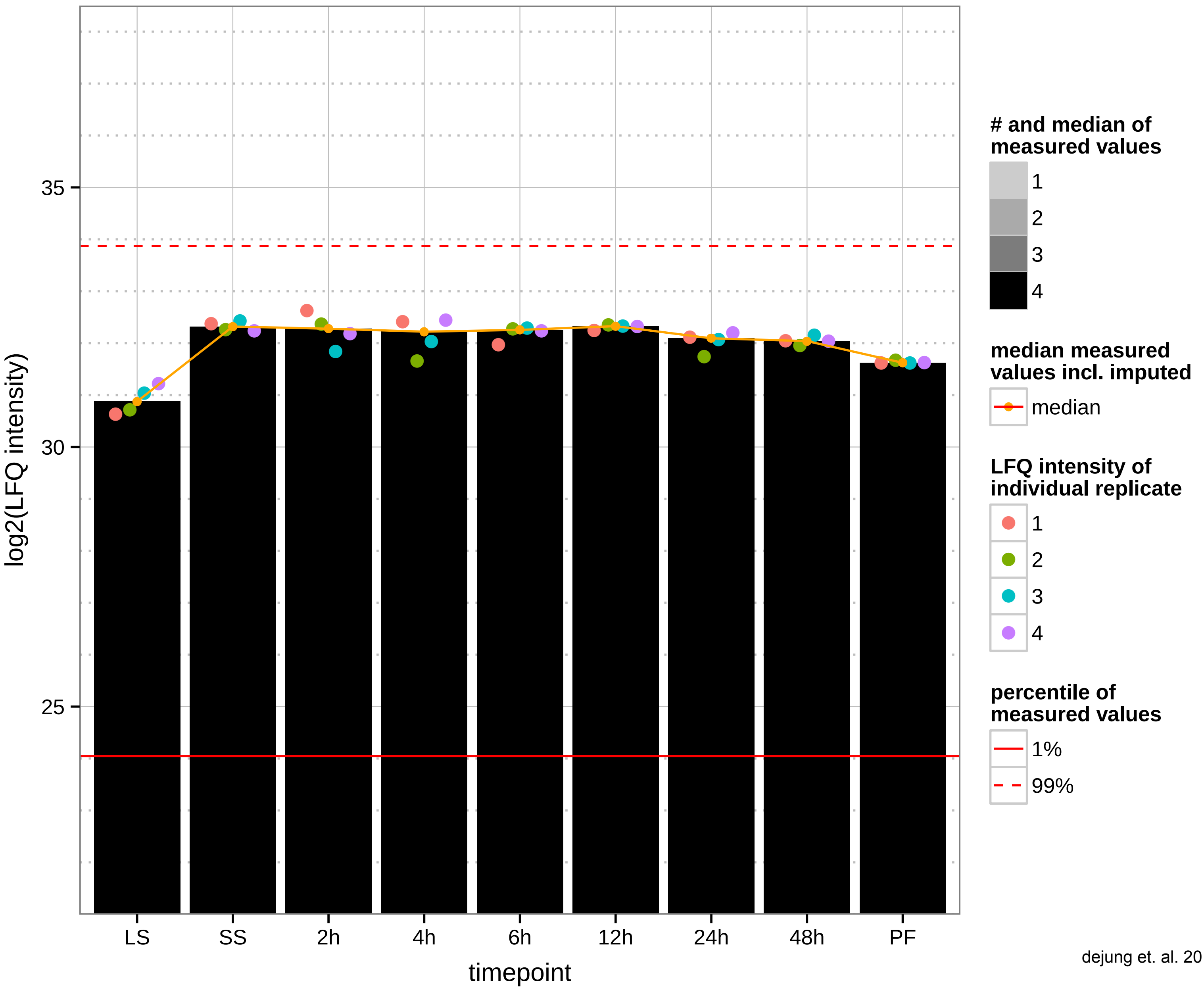

dejung et. al. 2015

atp–binding cassette sub–family e member 1 (ABCE1)
Tb927.10.1630
AGOF: ATP binding, ATPase activity, electron carrier activity, iron–sulfur cluster binding, ribonuclease activity
AGOC: cytoplasm
AGOP: nuclear–transcribed mRNA catabolic process, exonucleolytic
PGOF: ATP binding, ATPase activity, electron carrier activity, iron–sulfur cluster binding, nucleoside–triphosphatase activity, nu
PGOC: null
PGOP: null

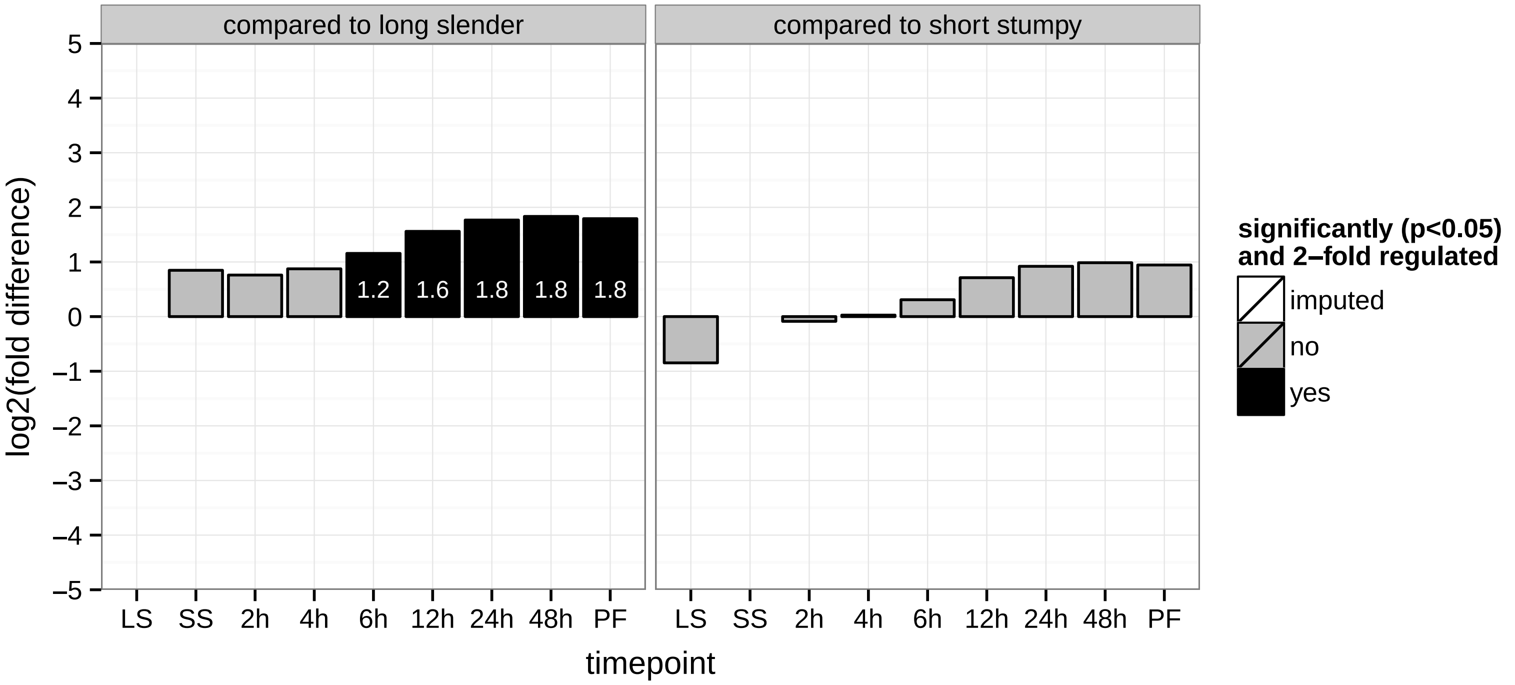

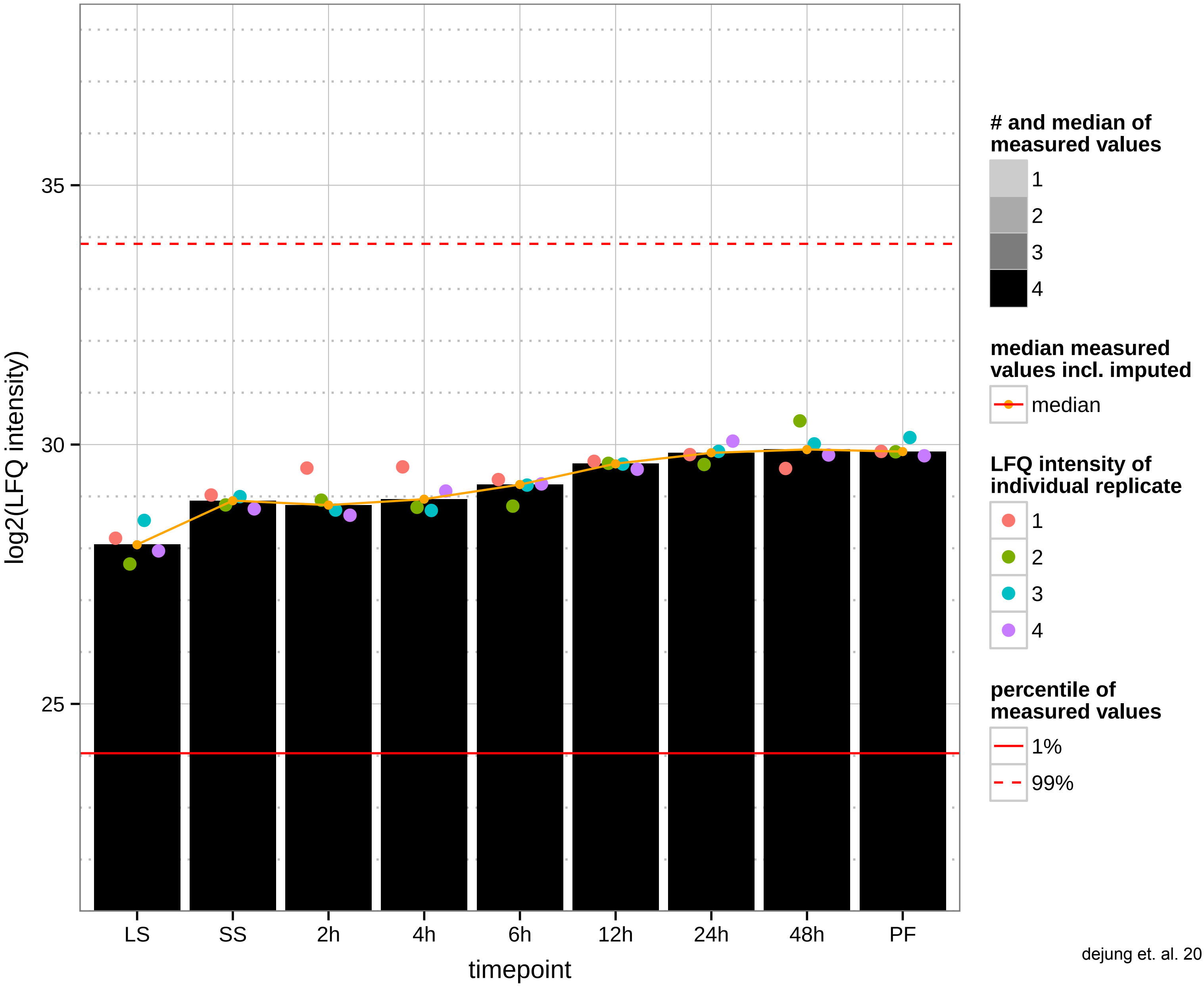

dejung et. al. 2015

nucleotide binding protein, putative (NBP 1)
Tb927.10.1690
AGOF: nucleotide binding
AGOC: cytosol
AGOP: null
PGOF: null
PGOC: null
PGOP: null

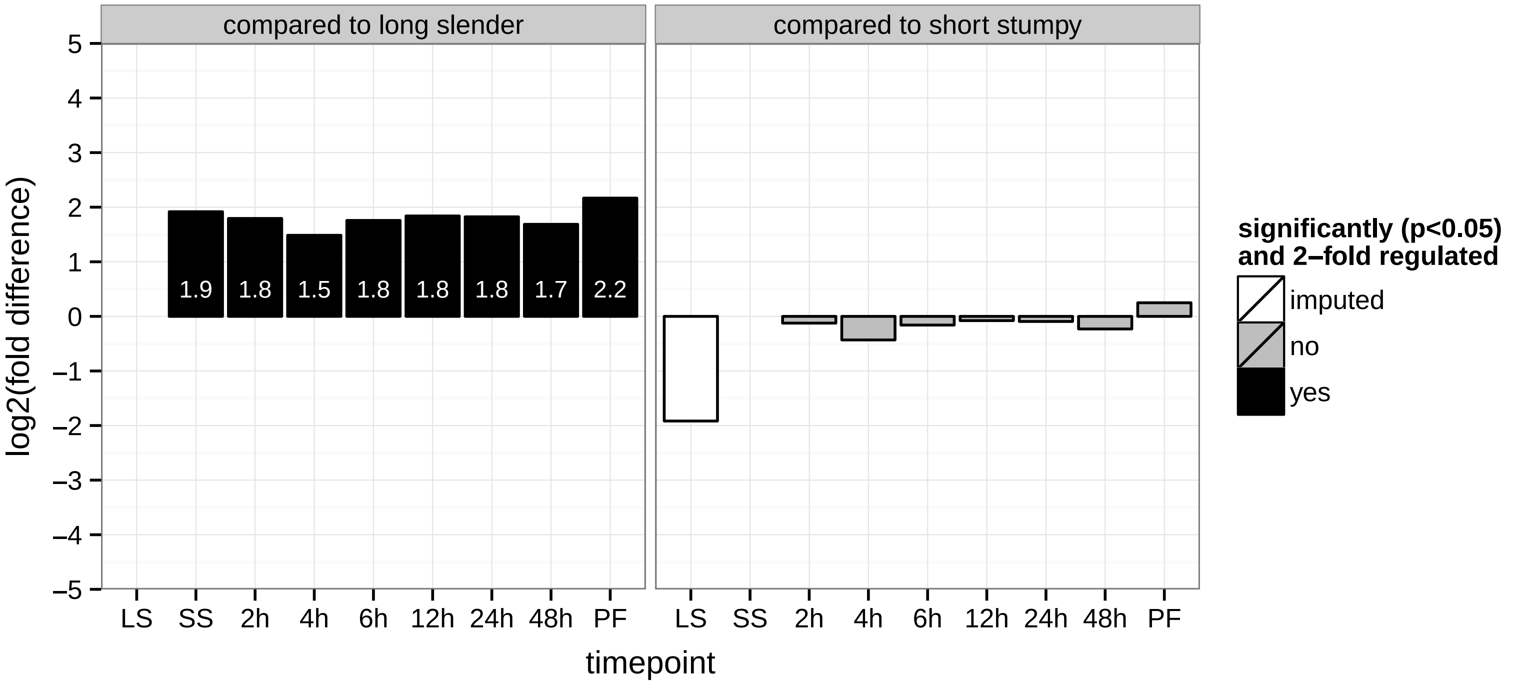

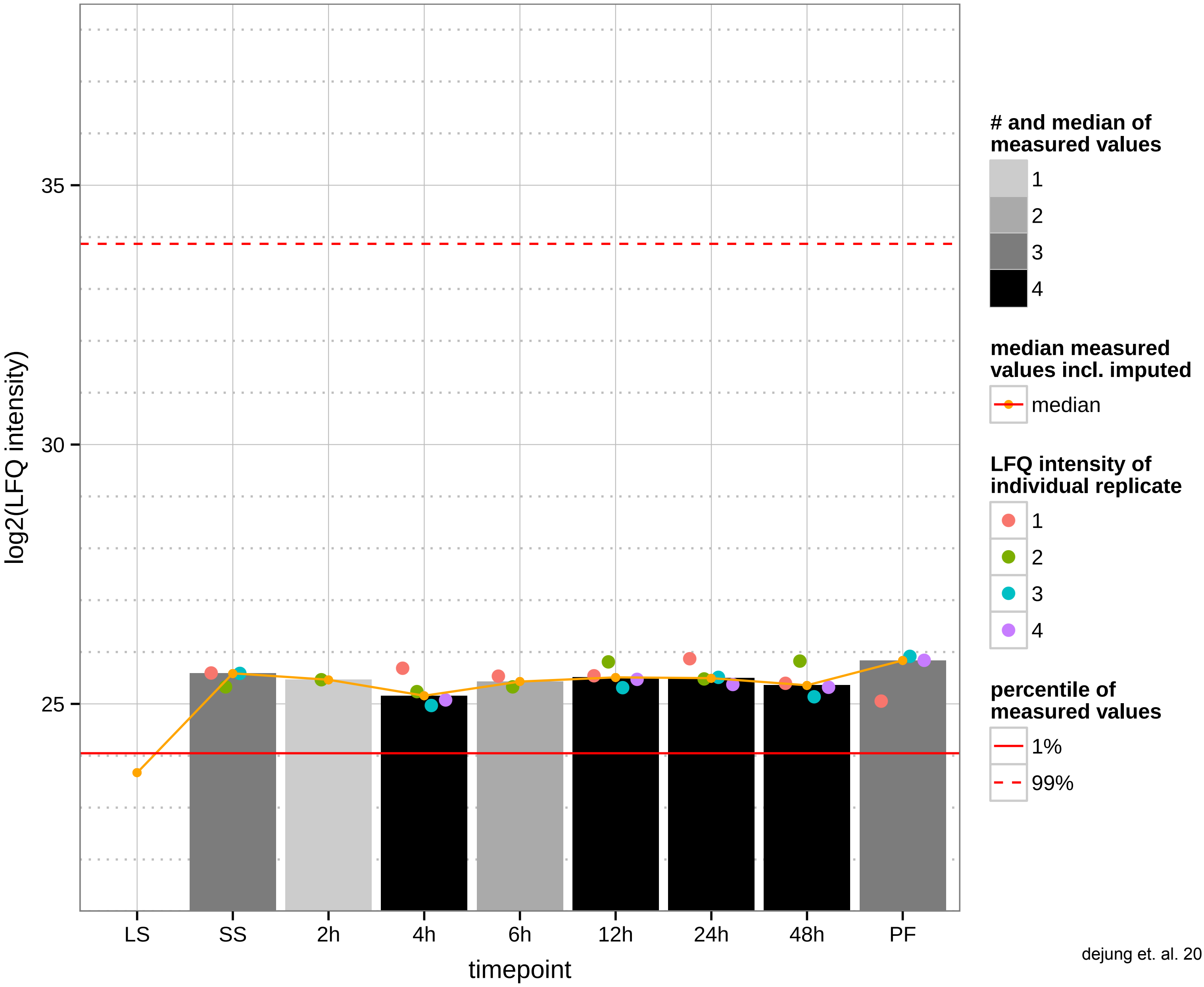

dejung et. al. 2015

pseudouridine synthase, Cbf5p
Tb927.10.170
AGOF: RNA binding, pseudouridine synthase activity, pseudouridylate synthase activity
AGOC: nucleus
AGOP: RNA processing, pseudouridine synthesis, rRNA modification, tRNA modification
PGOF: RNA binding, pseudouridine synthase activity
PGOC: null
PGOP: RNA modification, RNA processing, pseudouridine synthesis

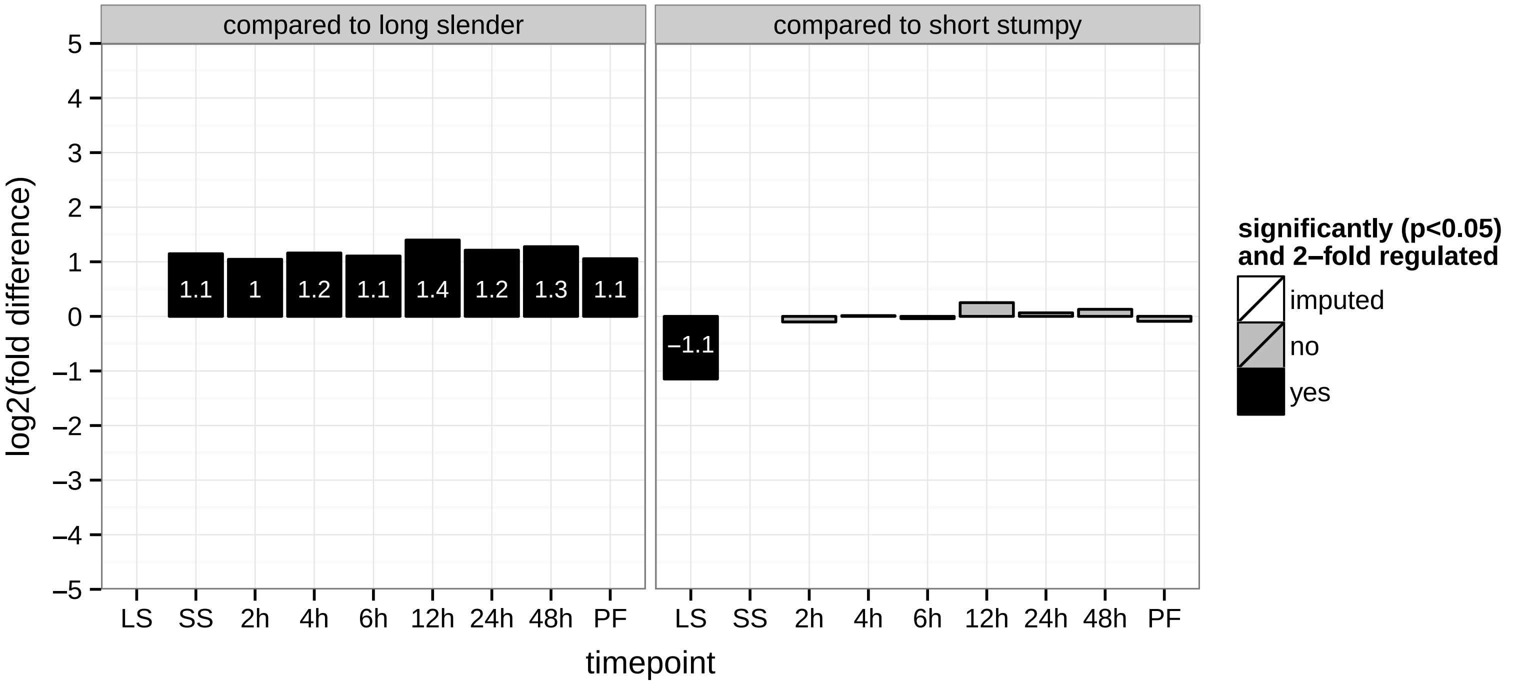

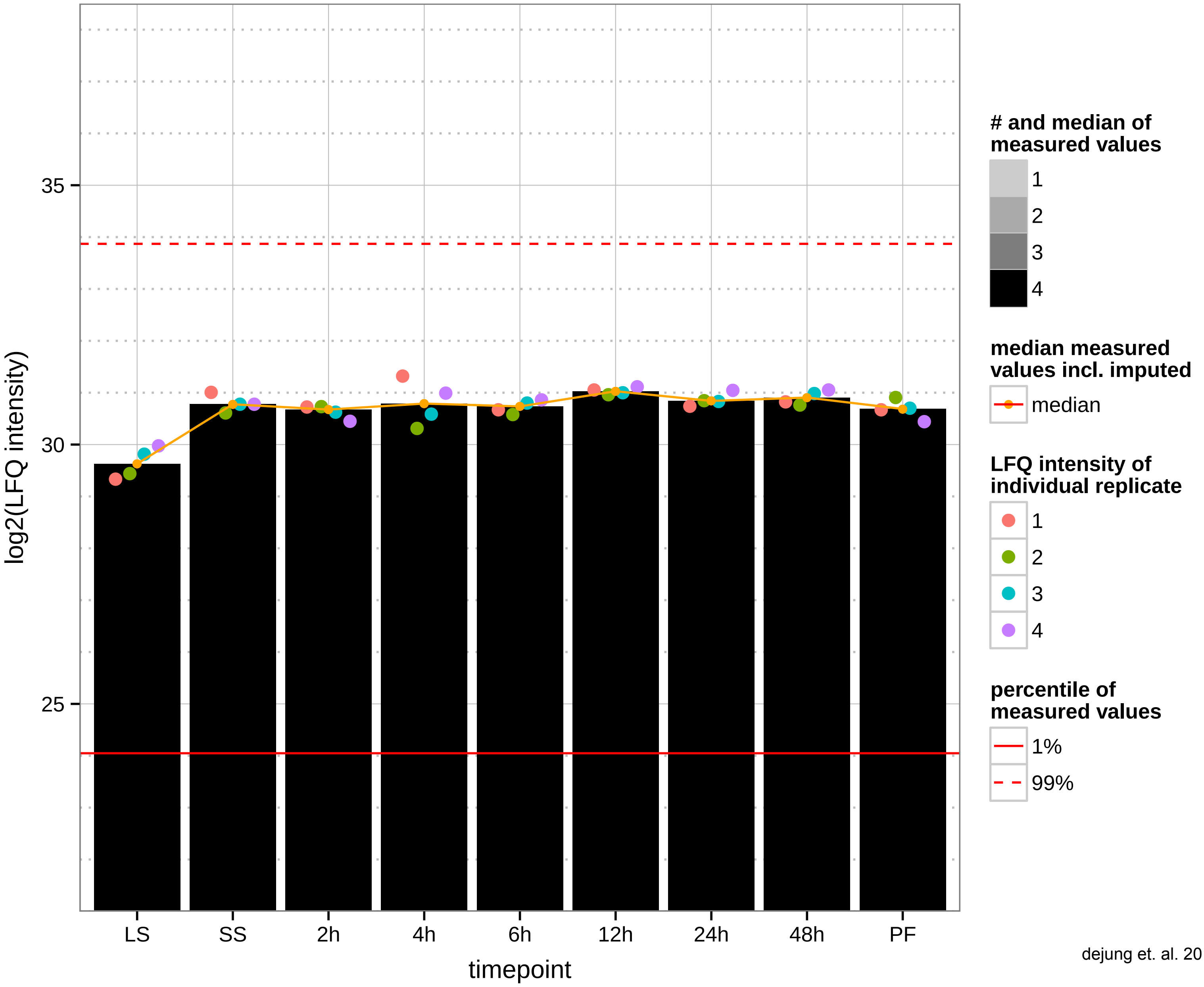

dejung et. al. 2015

hypothetical protein, conserved
Tb927.10.1860
AGOF: null
AGOC: null
AGOP: null
PGOF: null
PGOC: null
PGOP: null

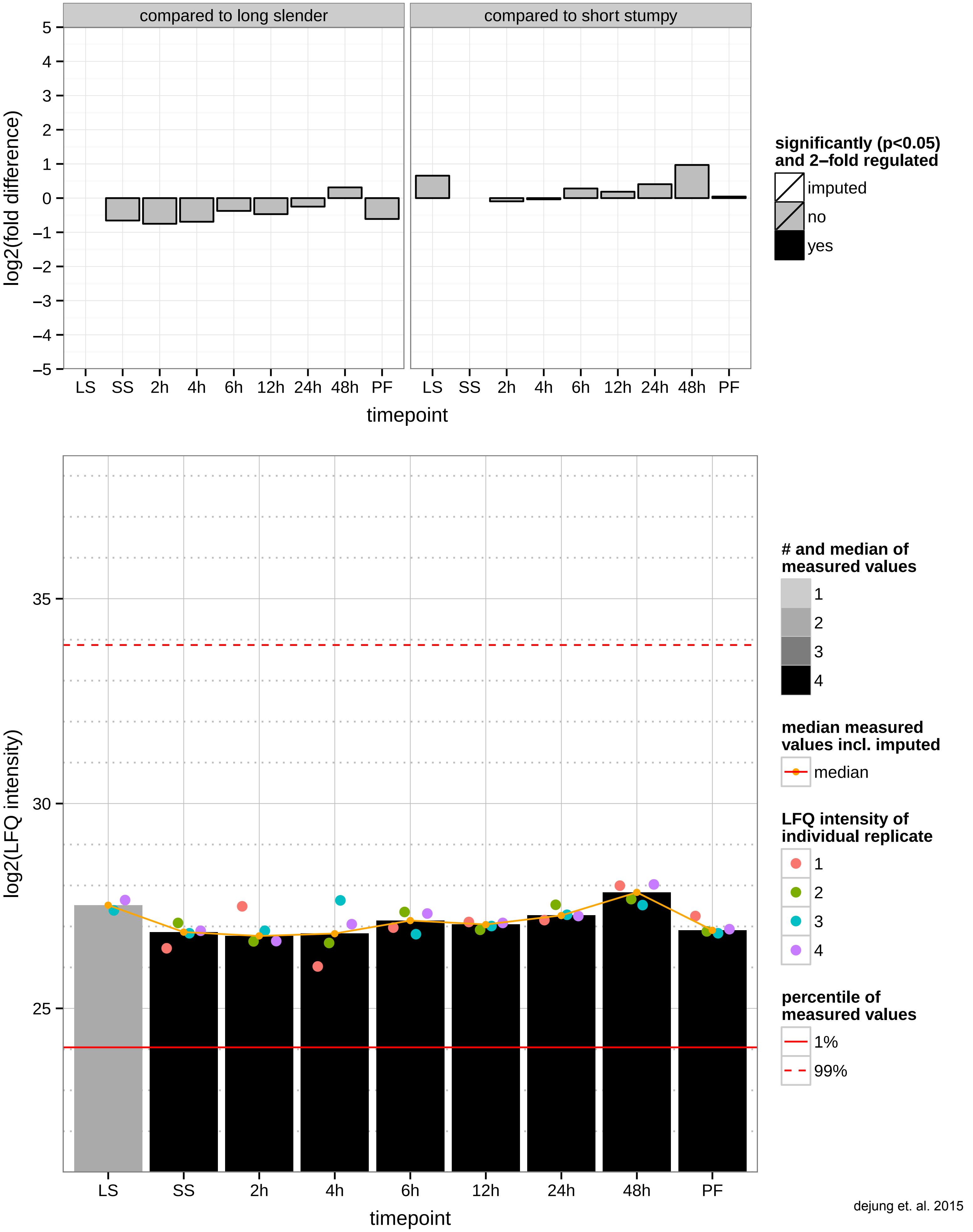

dejung et. al. 2015

cysteine peptidase, Clan CA, family C2, putative, calpain
Tb927.10.1890
AGOF: calcium−dependent cysteine−type endopeptidase activity
AGOC: intracellular
AGOP: proteolysis
PGOF: calcium−dependent cysteine−type endopeptidase activity
PGOC: intracellular
PGOP: proteolysis

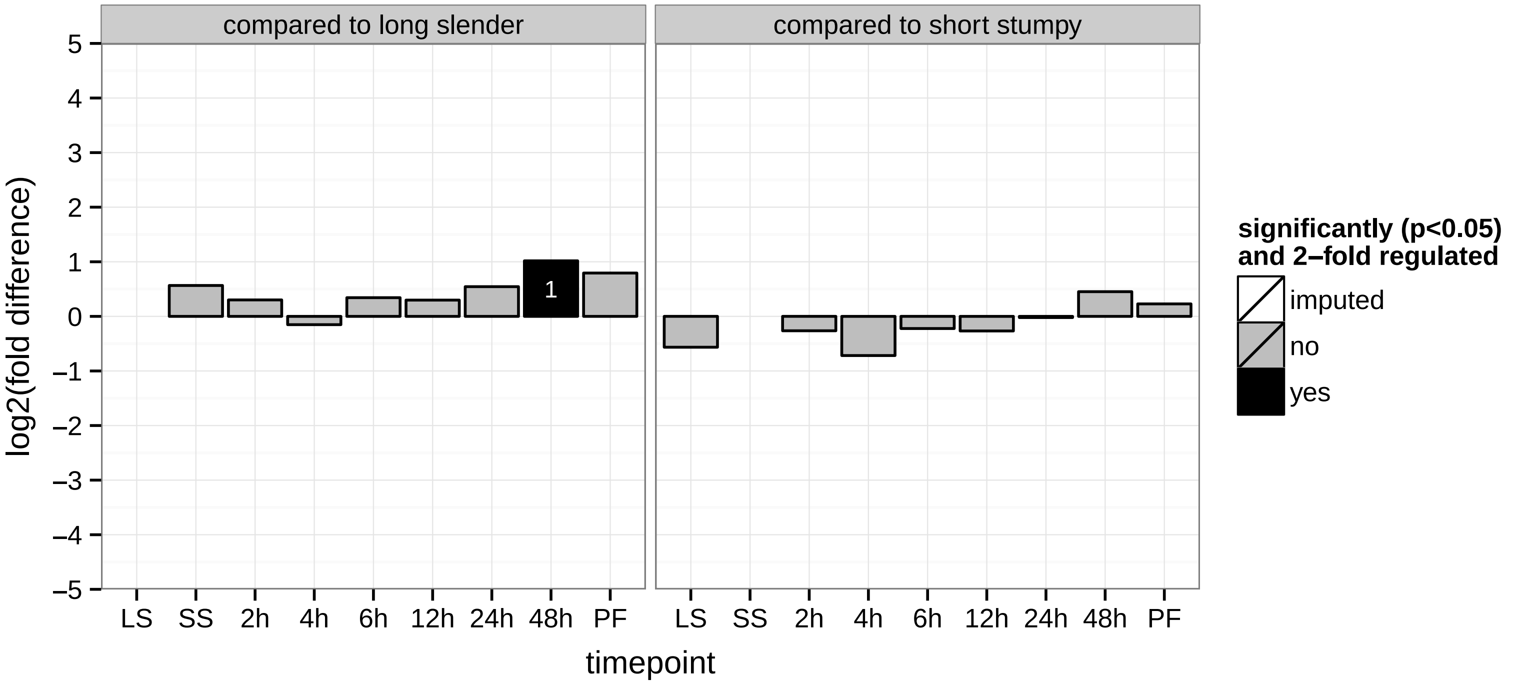

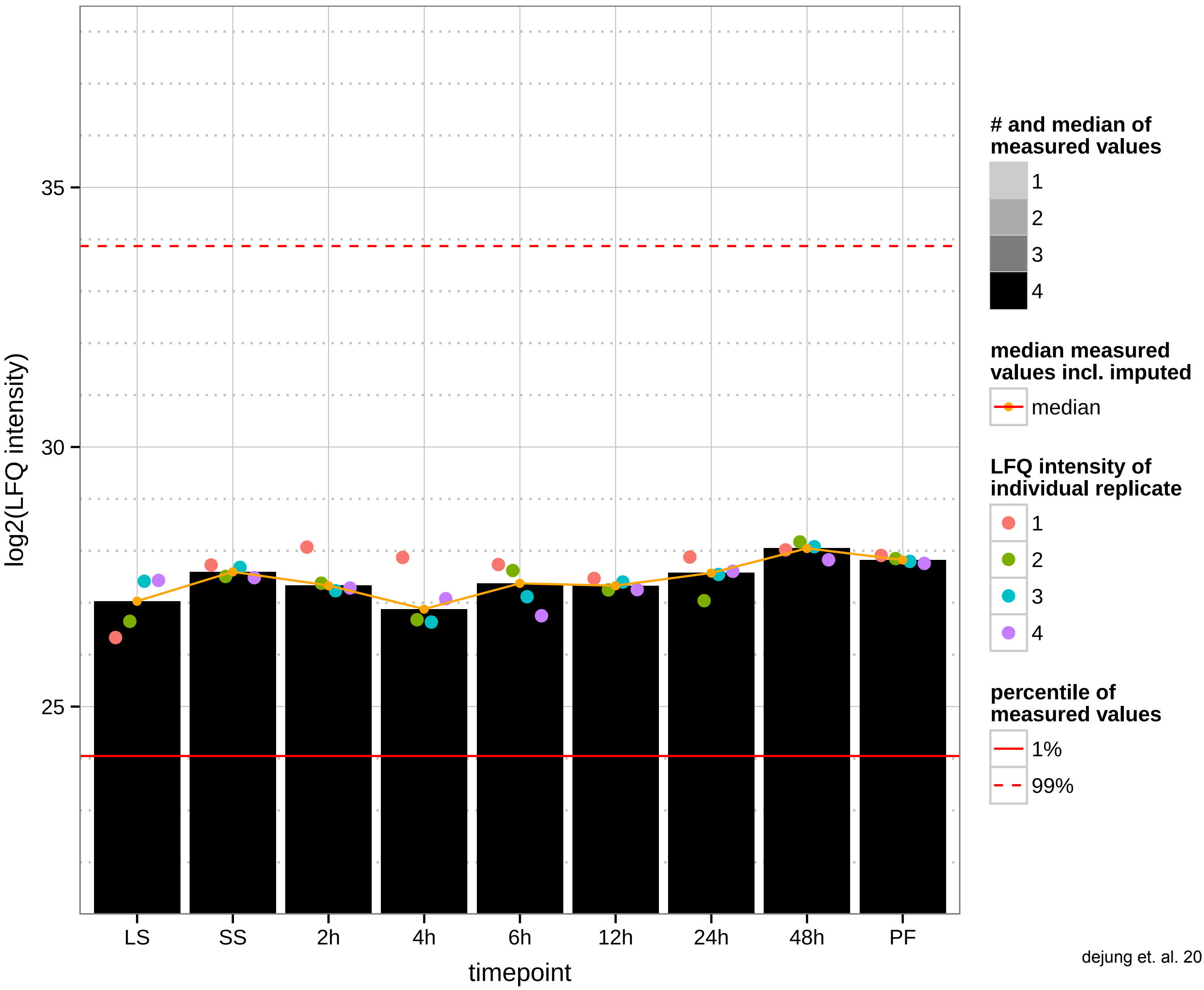

dejung et. al. 2015

hypothetical protein, conserved
Tb927.10.1970
AGOF: null
AGOC: null
AGOP: null
PGOF: null
PGOC: null
PGOP: null

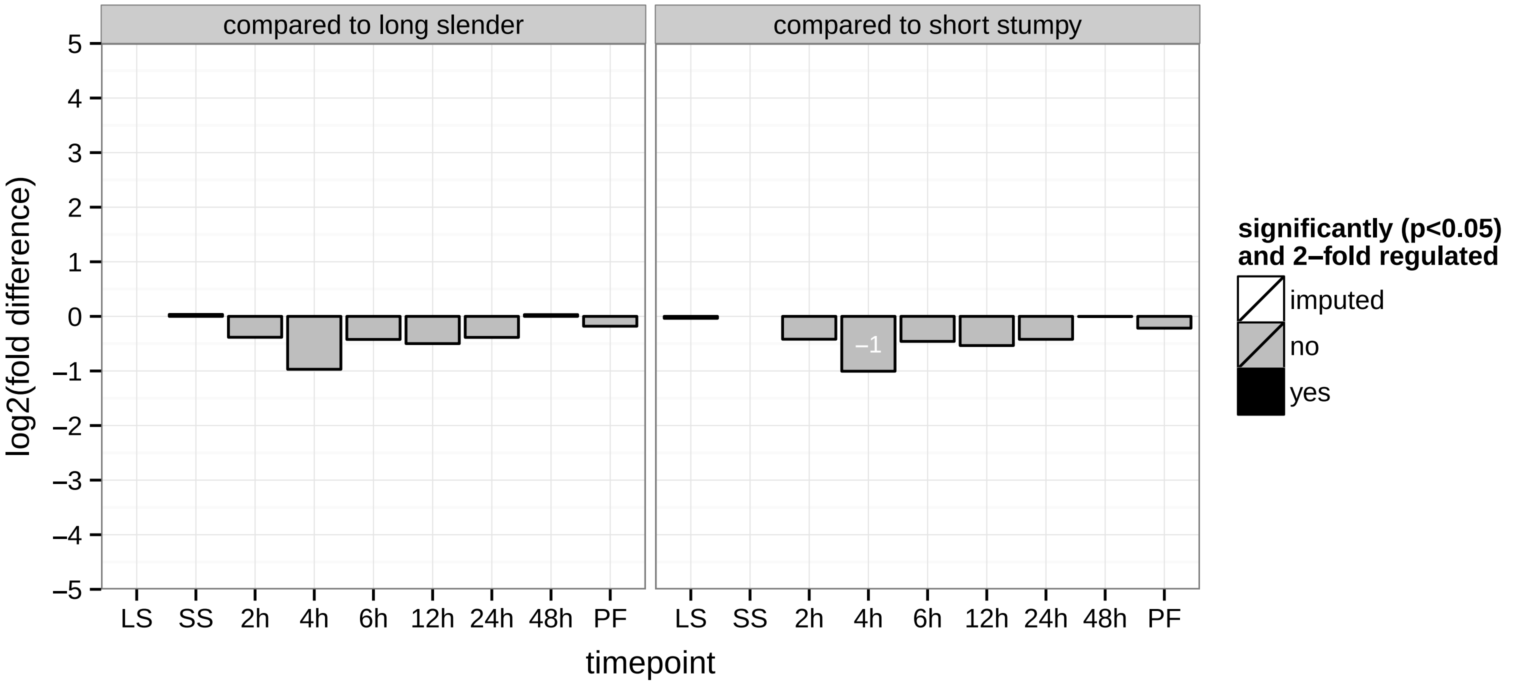

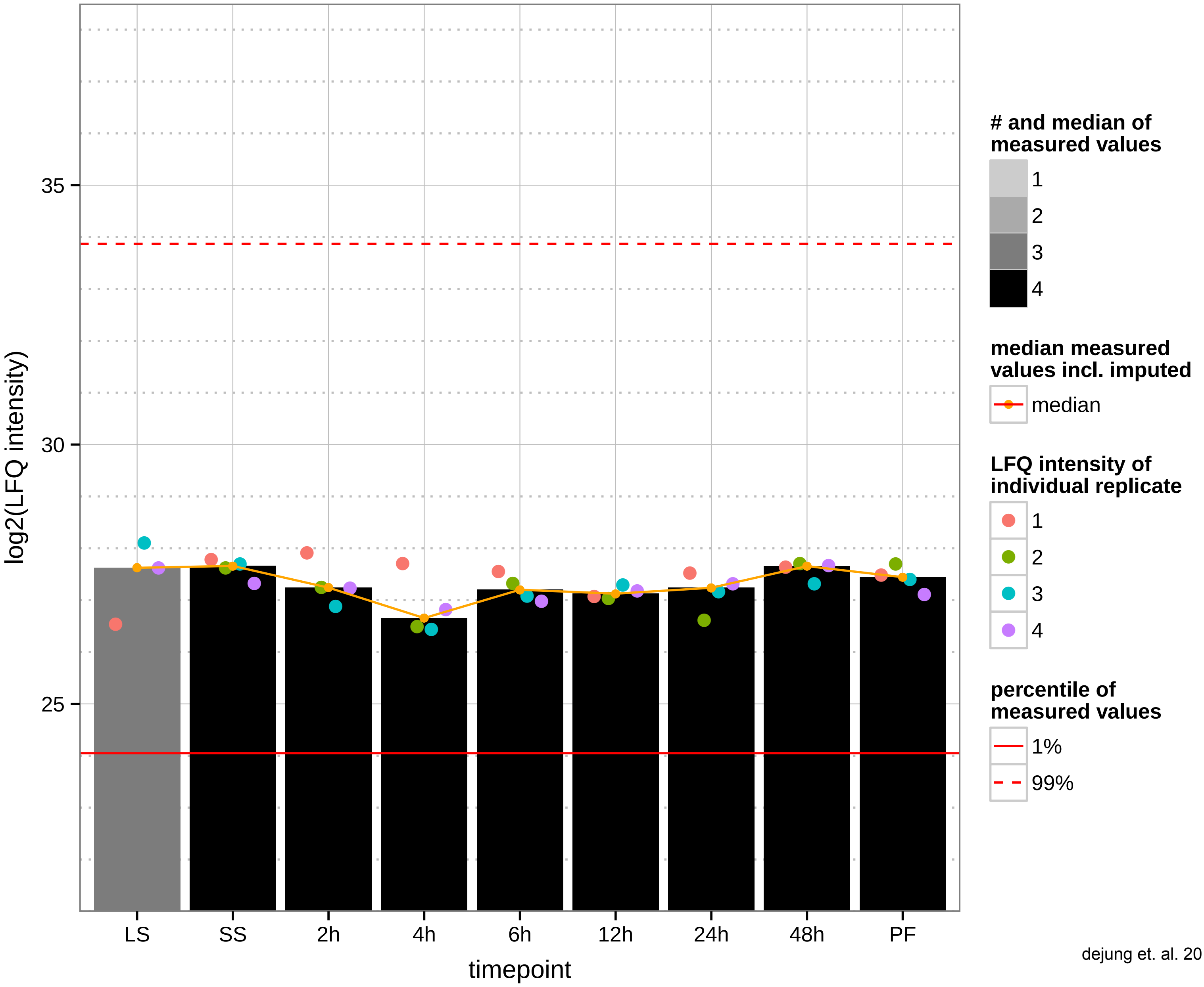

dejung et. al. 2015

major vault protein, putative, putative (MVP)
Tb927.10.1990
AGOF: null
AGOC: null
AGOP: null
PGOF: null
PGOC: null
PGOP: null

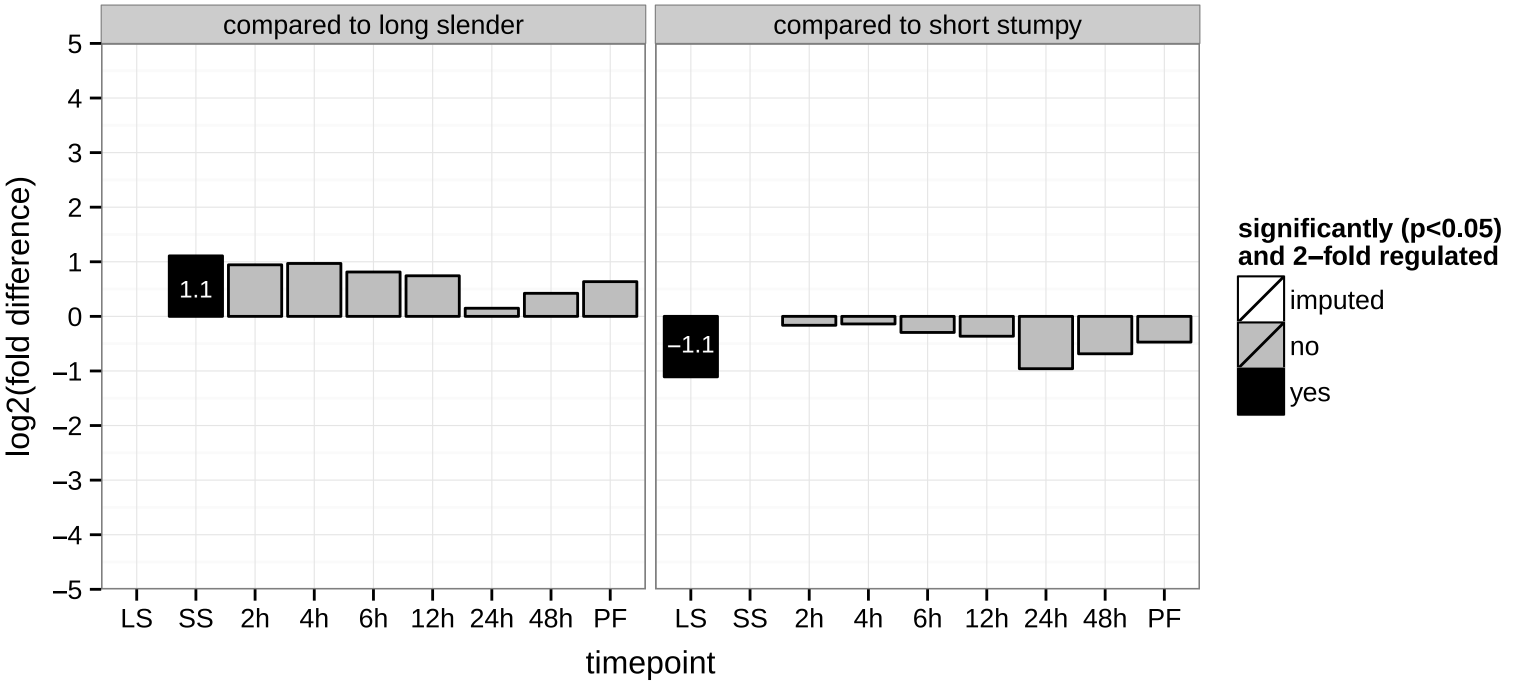

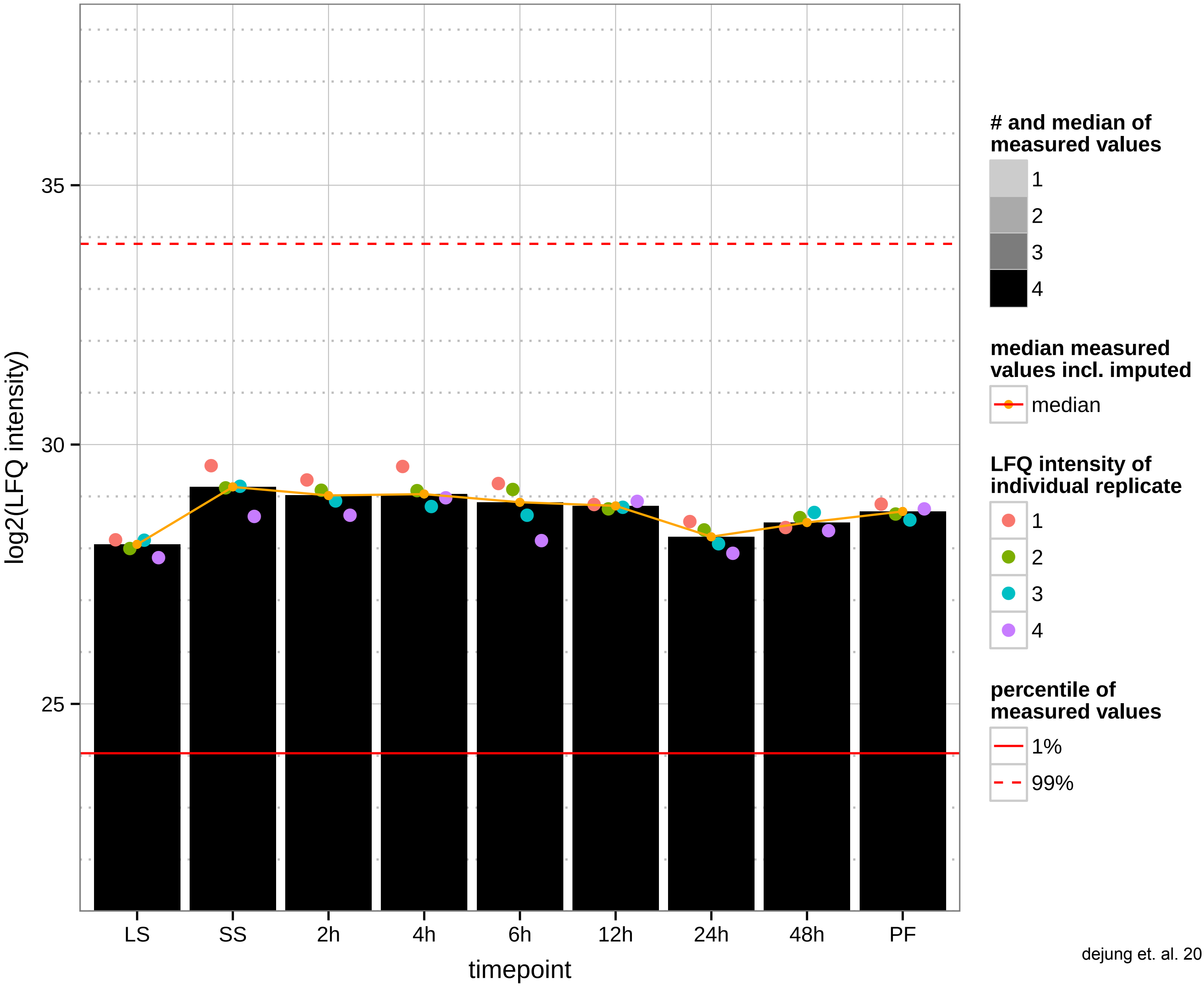

dejung et. al. 2015

protein kinase, putative
Tb927.10.2040
AGOF: ATP binding, protein serine/threonine kinase activity, protein tyrosine kinase activity
AGOC: null
AGOP: protein phosphorylation
PGOF: ATP binding, protein kinase activity, transferase activity, transferring phosphorus–containing groups
PGOC: null
PGOP: protein phosphorylation

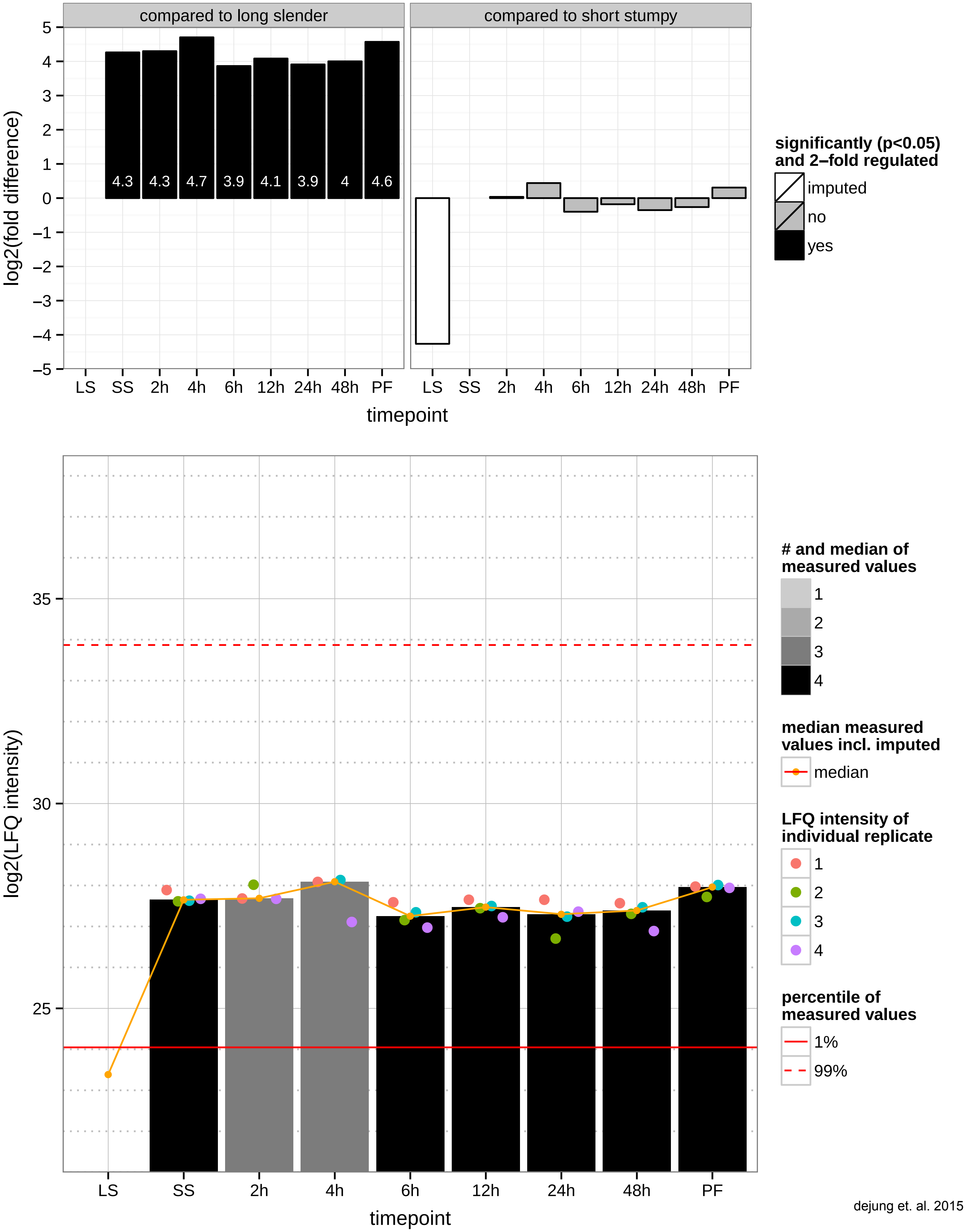

dejung et. al. 2015

hypothetical protein, conserved
Tb927.10.2190
AGOF: null
AGOC: null
AGOP: null
PGOF: null
PGOC: null
PGOP: null

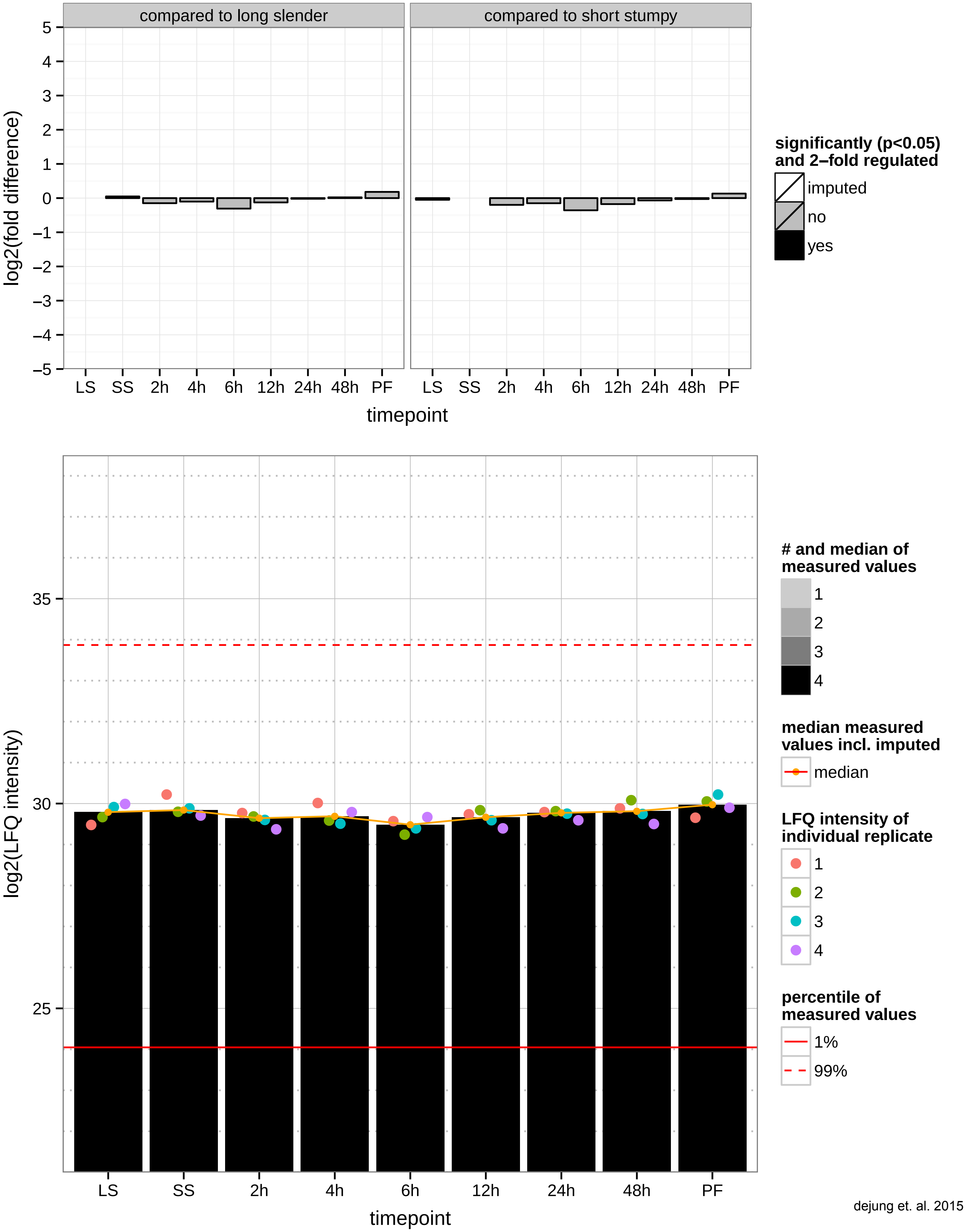

dejung et. al. 2015

60S ribosomal protein L37a, putative, 60S ribosomal proteins L37
Tb927.10.6370;Tb927.10.220
AGOF: structural constituent of ribosome, zinc ion binding
AGOC: intracellular, ribosome
AGOP: translation
PGOF: structural constituent of ribosome
PGOC: intracellular, ribosome
PGOP: translation

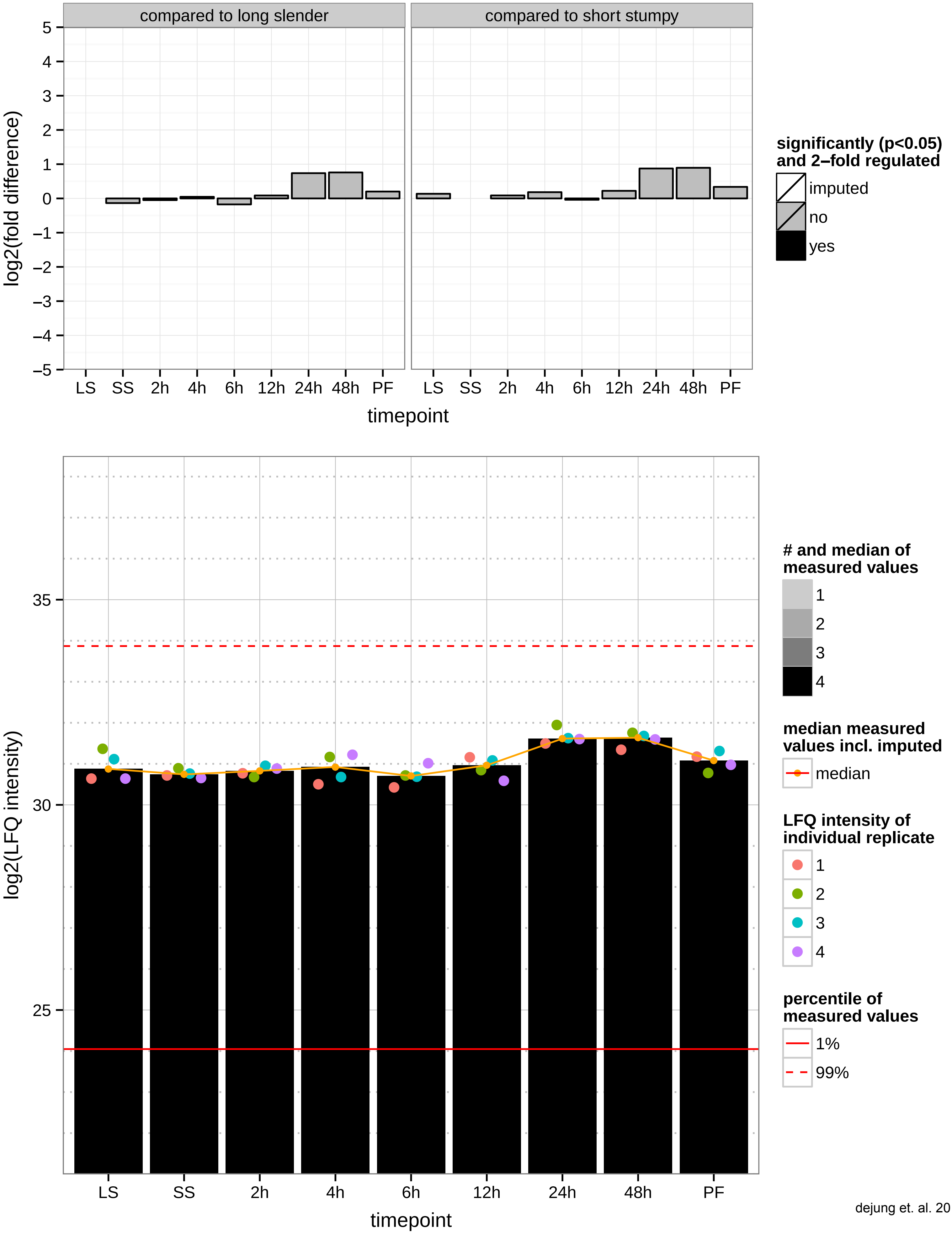

dejung et. al. 2015

vacuolar protein sorting–associated protein 35, putative
Tb927.10.2270
AGOF: null
AGOC: cytoplasm, membrane
AGOP: null
PGOF: binding
PGOC: null
PGOP: null

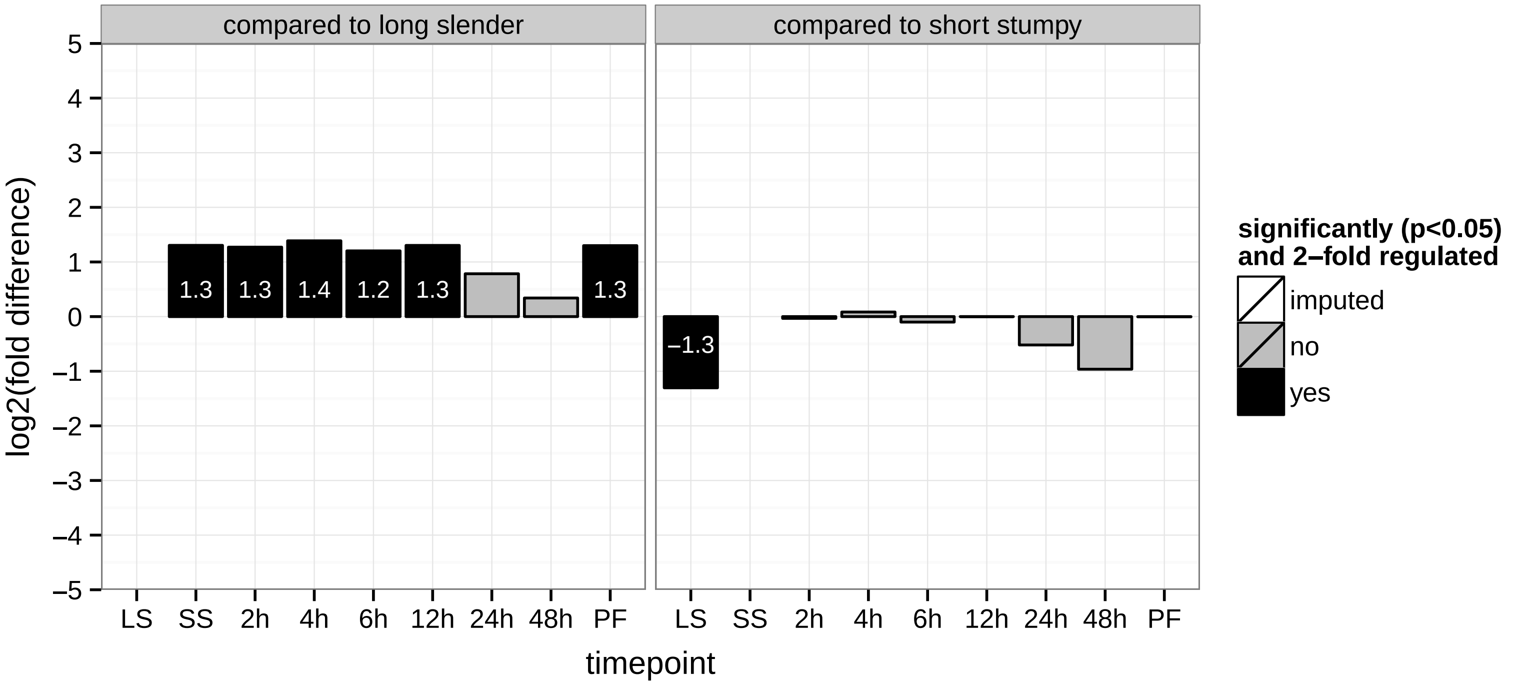

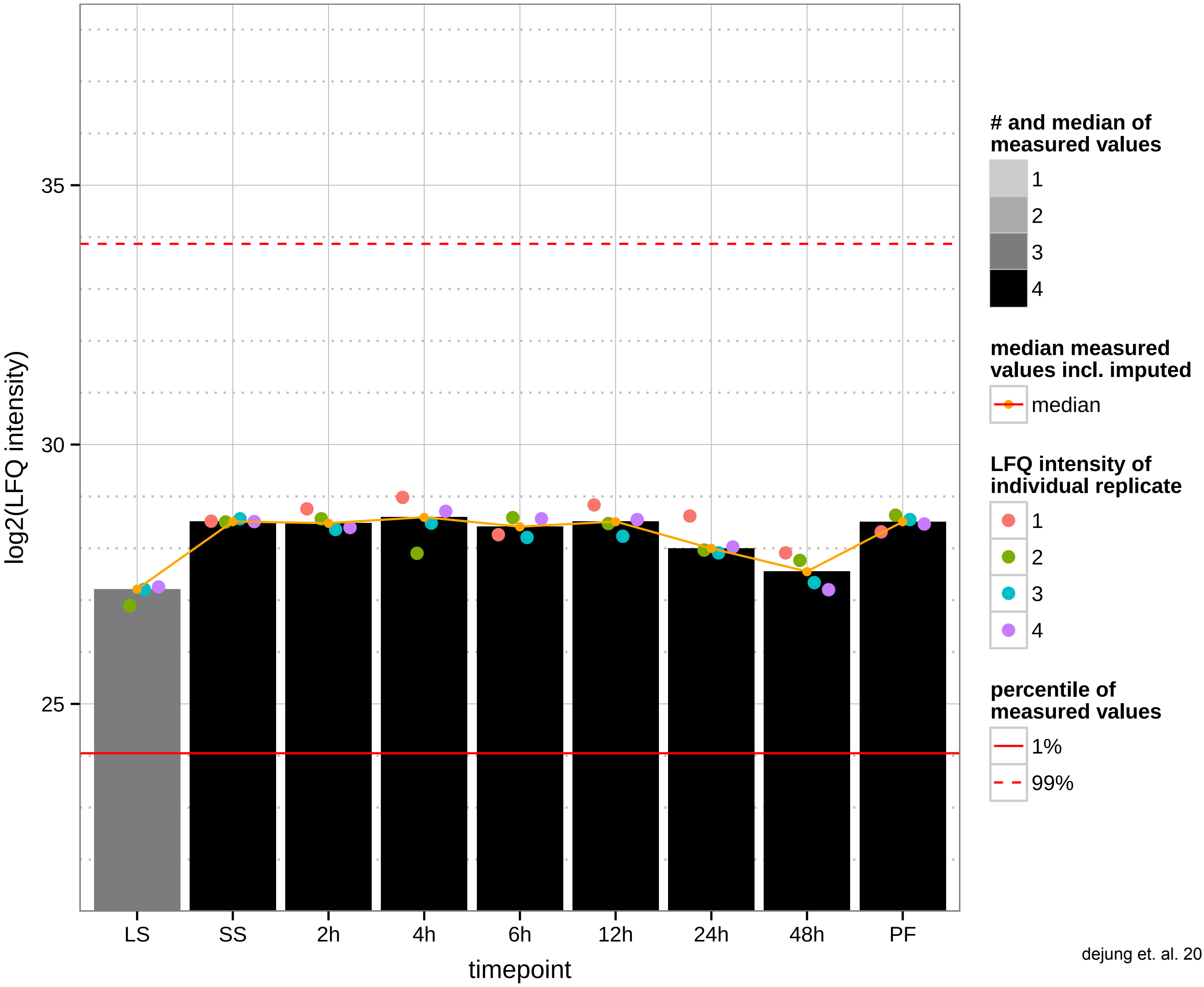

dejung et. al. 2015

proteasome subunit alpha type−5, putative, 20S proteasome subunit alpha 5
Tb927.10.230
AGOF: threonine−type endopeptidase activity
AGOC: cytosol, nucleus, proteasome core complex
AGOP: ubiquitin−dependent protein catabolic process
PGOF: endopeptidase activity, threonine−type endopeptidase activity
PGOC: proteasome core complex, proteasome core complex, alpha−subunit complex
PGOP: proteolysis involved in cellular protein catabolic process, ubiquitin−dependent protein catabolic process

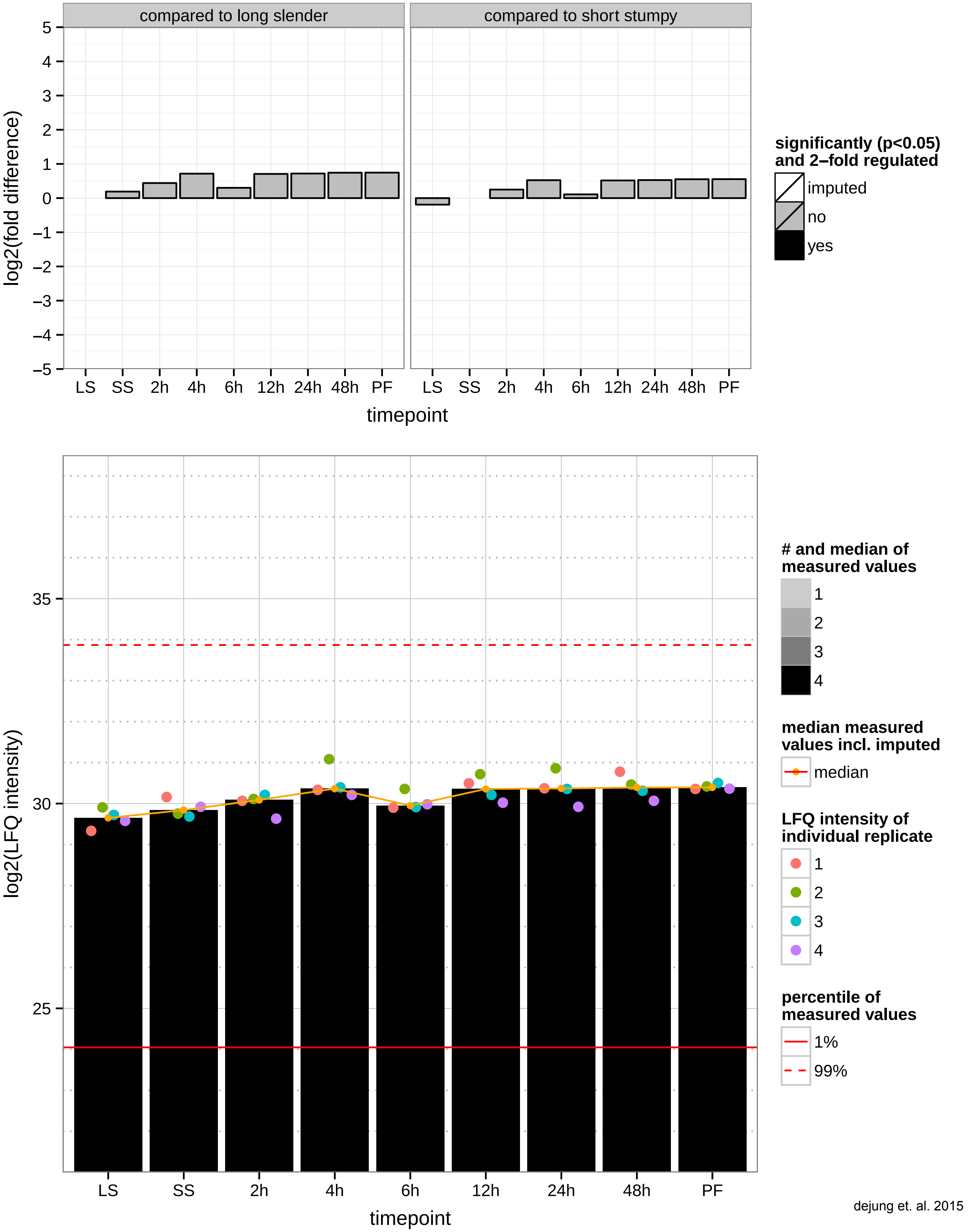

dejung et. al. 2015

pyruvate dehydrogenase complex E3 binding protein, putative
Tb927.10.2350
AGOF: biotin binding, dihydrolipoyllysine–residue acetyltransferase activity
AGOC: mitochondrion, pyruvate dehydrogenase complex
AGOP: generation of precursor metabolites and energy
PGOF: null
PGOC: null
PGOP: null

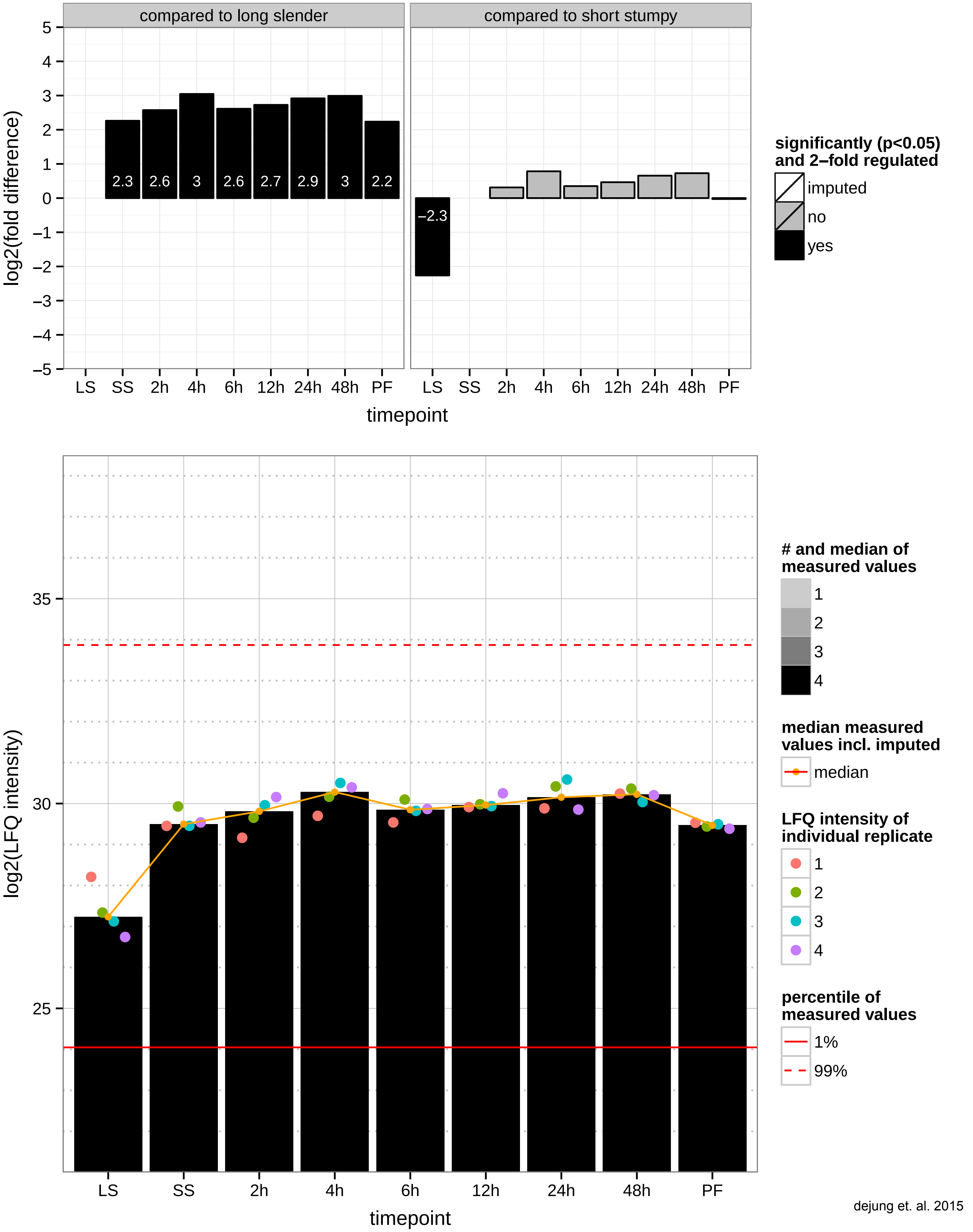

dejung et. al. 2015

RNA–binding protein, putative (LA)
Tb927.10.2370
AGOF: RNA binding
AGOC: nucleus, ribonucleoprotein complex
AGOP: RNA processing
PGOF: nucleic acid binding
PGOC: null
PGOP: null

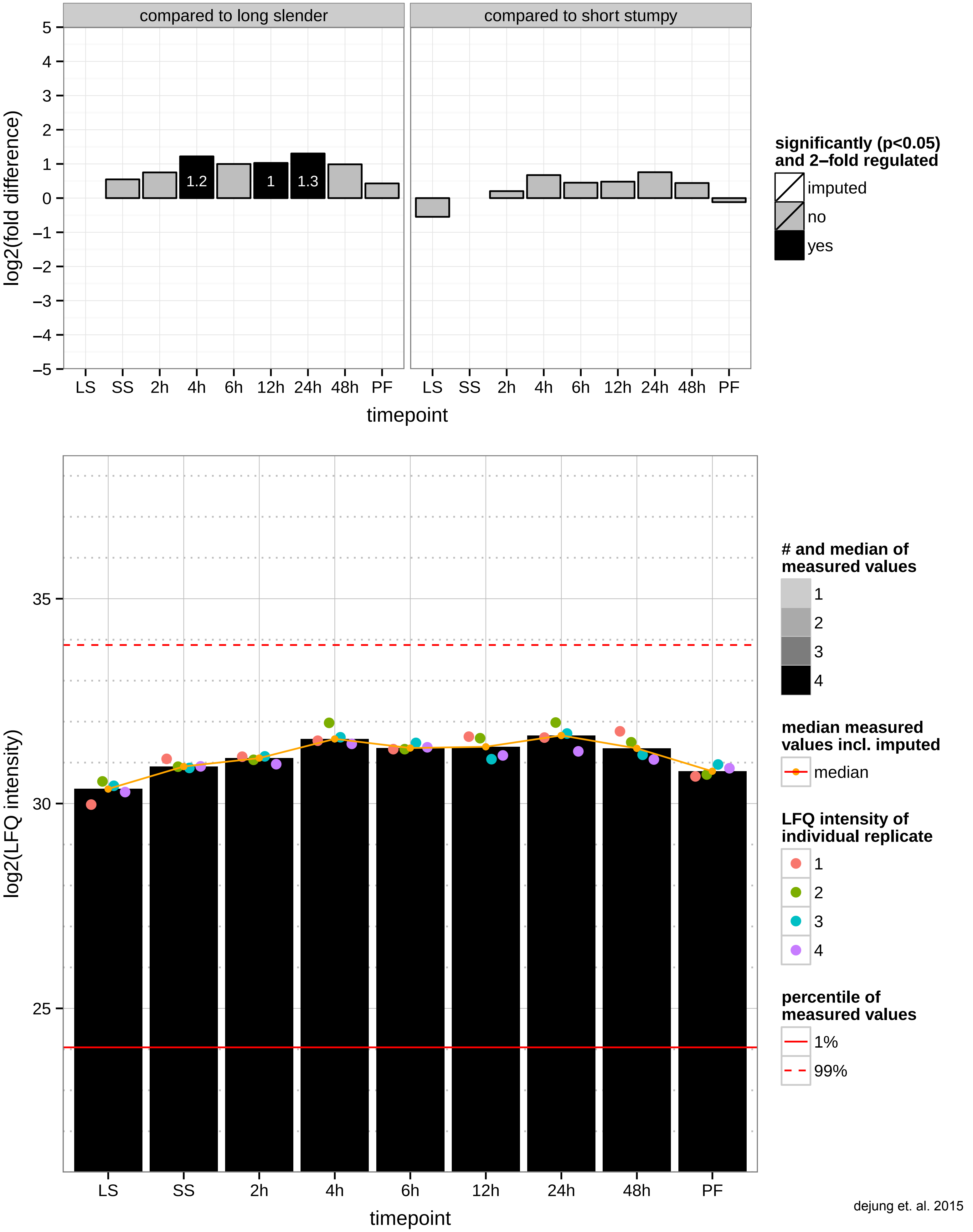

dejung et. al. 2015

hypothetical protein, conserved
Tb927.10.2380
AGOF: null
AGOC: null
AGOP: null
PGOF: protein binding
PGOC: null
PGOP: null

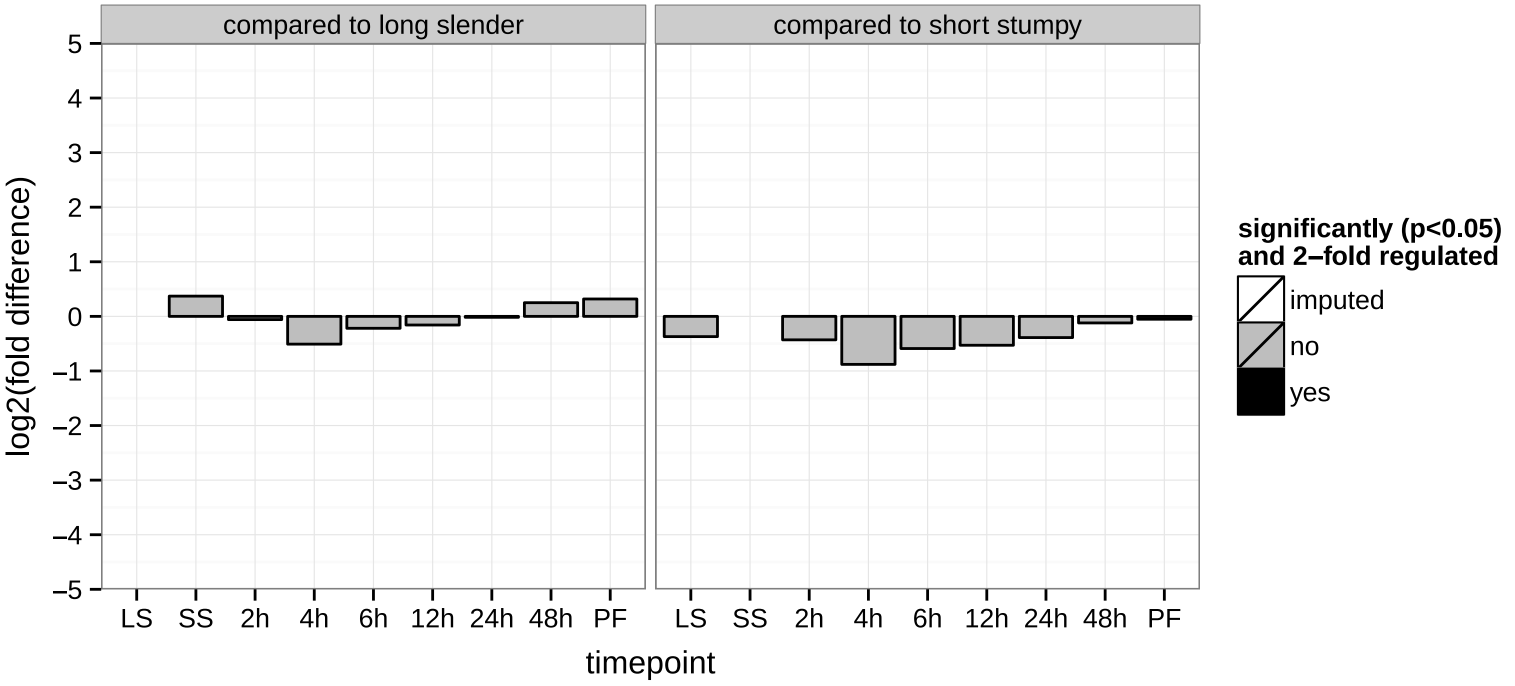

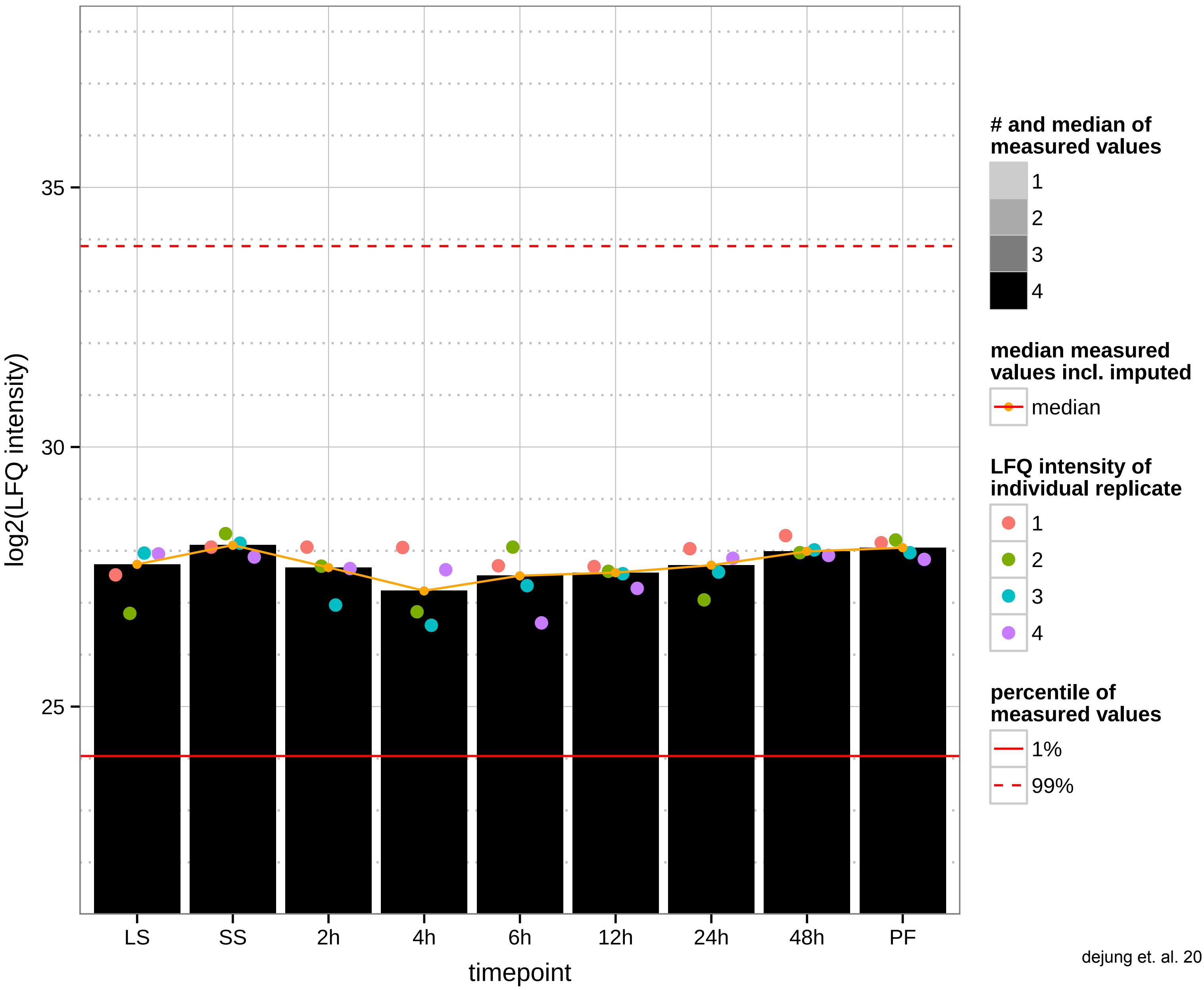

dejung et. al. 2015

hypothetical protein, conserved
Tb927.10.2470
AGOF: null
AGOC: null
AGOP: null
PGOF: null
PGOC: null
PGOP: null

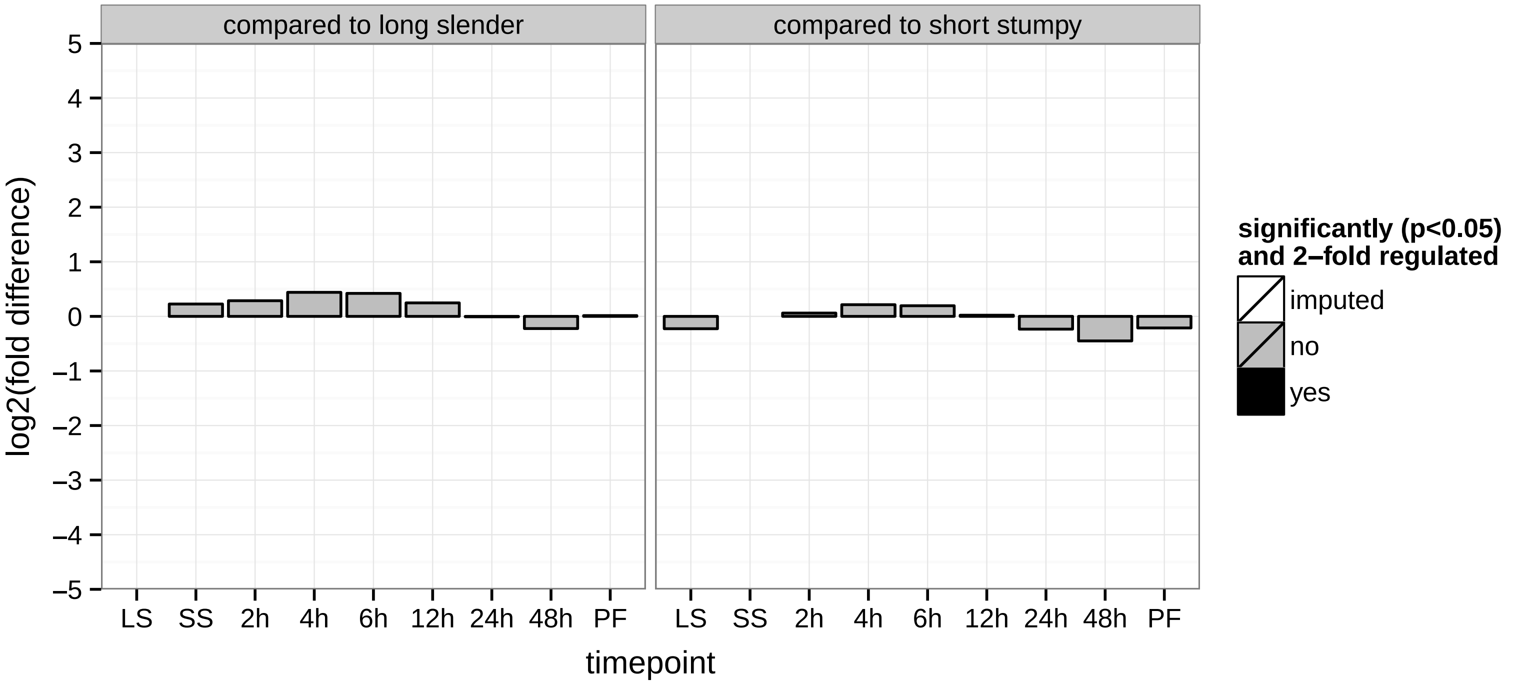

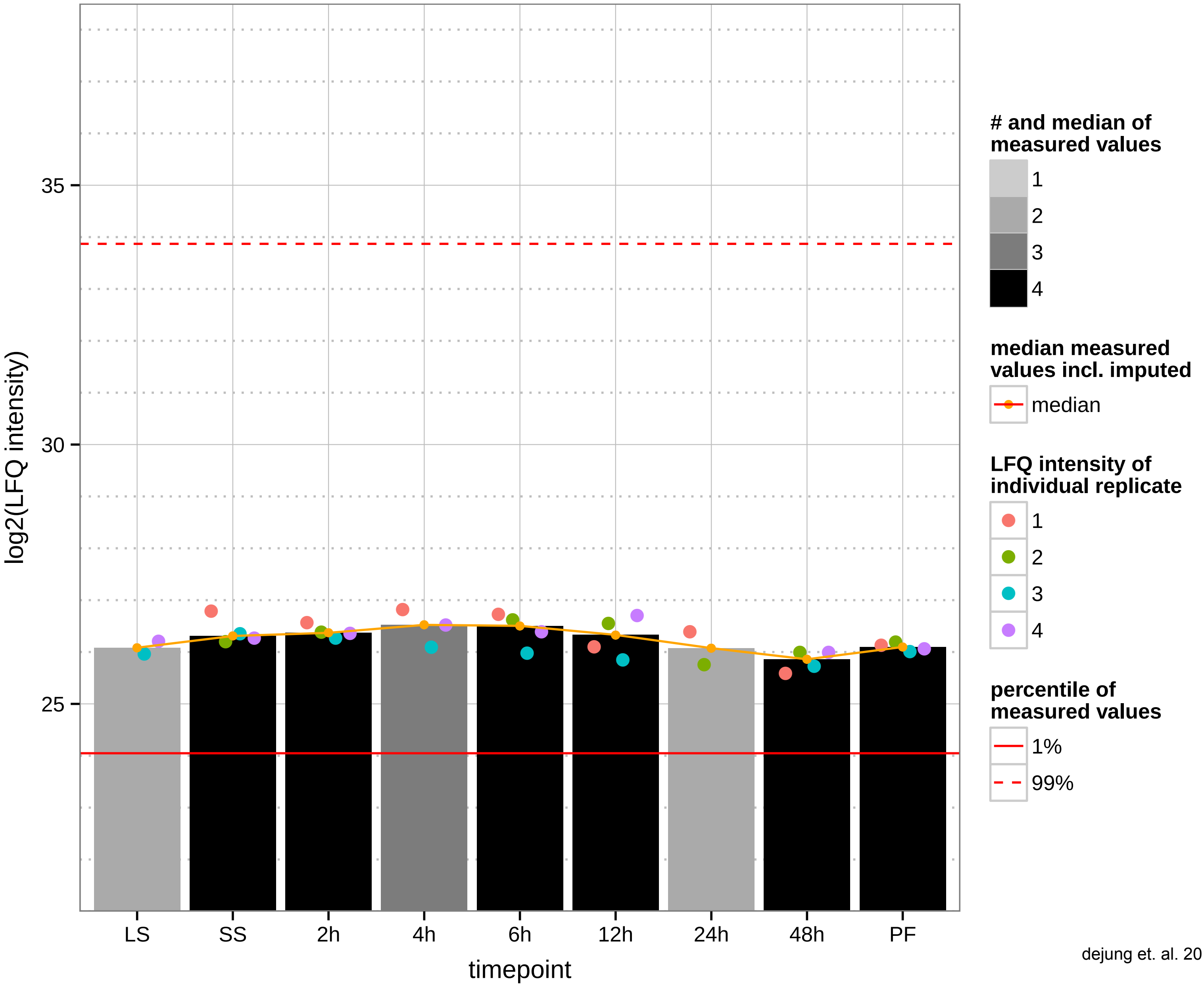

dejung et. al. 2015

adenylate kinase, putative
Tb927.10.2530
AGOF: ATP binding, adenylate kinase activity
AGOC: null
AGOP: nucleobase–containing compound metabolic process, purine ribonucleotide biosynthetic process
PGOF: ATP binding, adenylate kinase activity, nucleobase–containing compound kinase activity, nucleotide kinase activity, ph
PGOC: null
PGOP: nucleobase–containing compound metabolic process, nucleotide phosphorylation

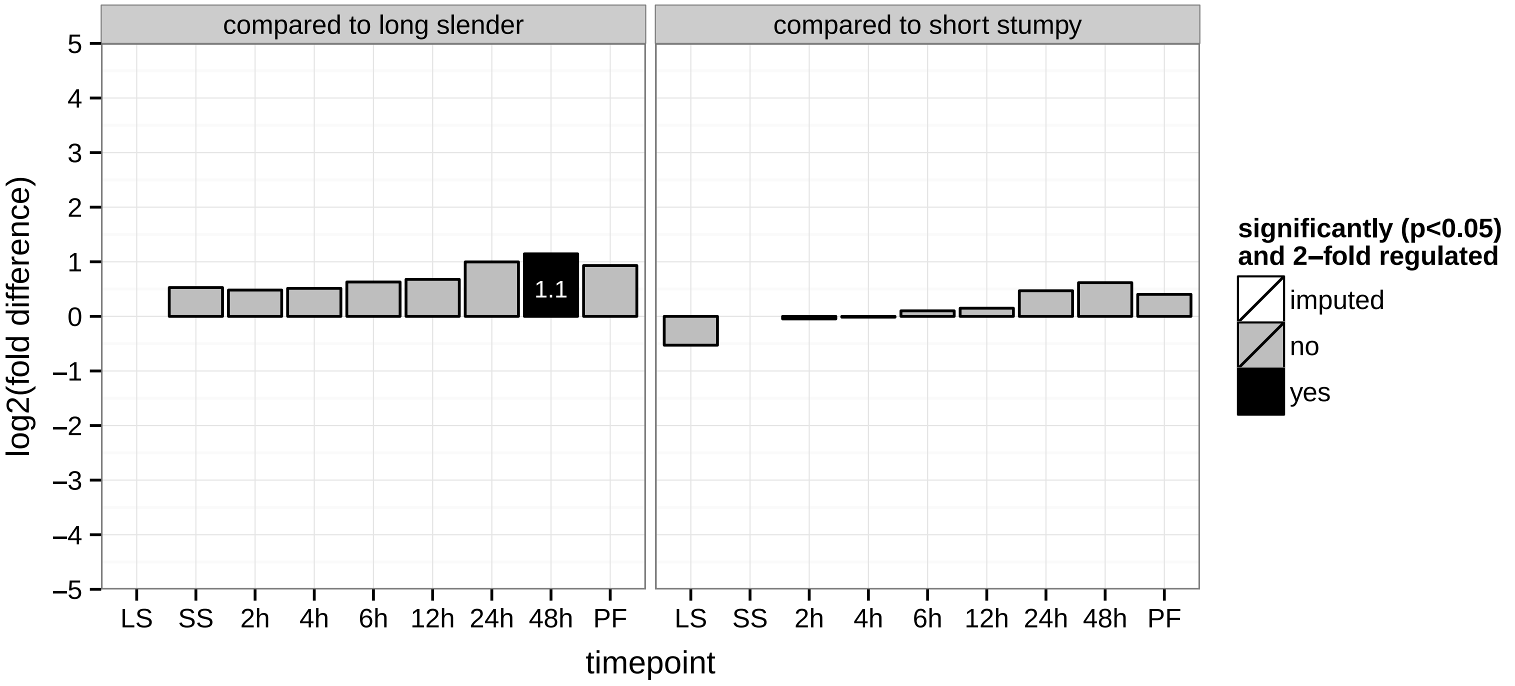

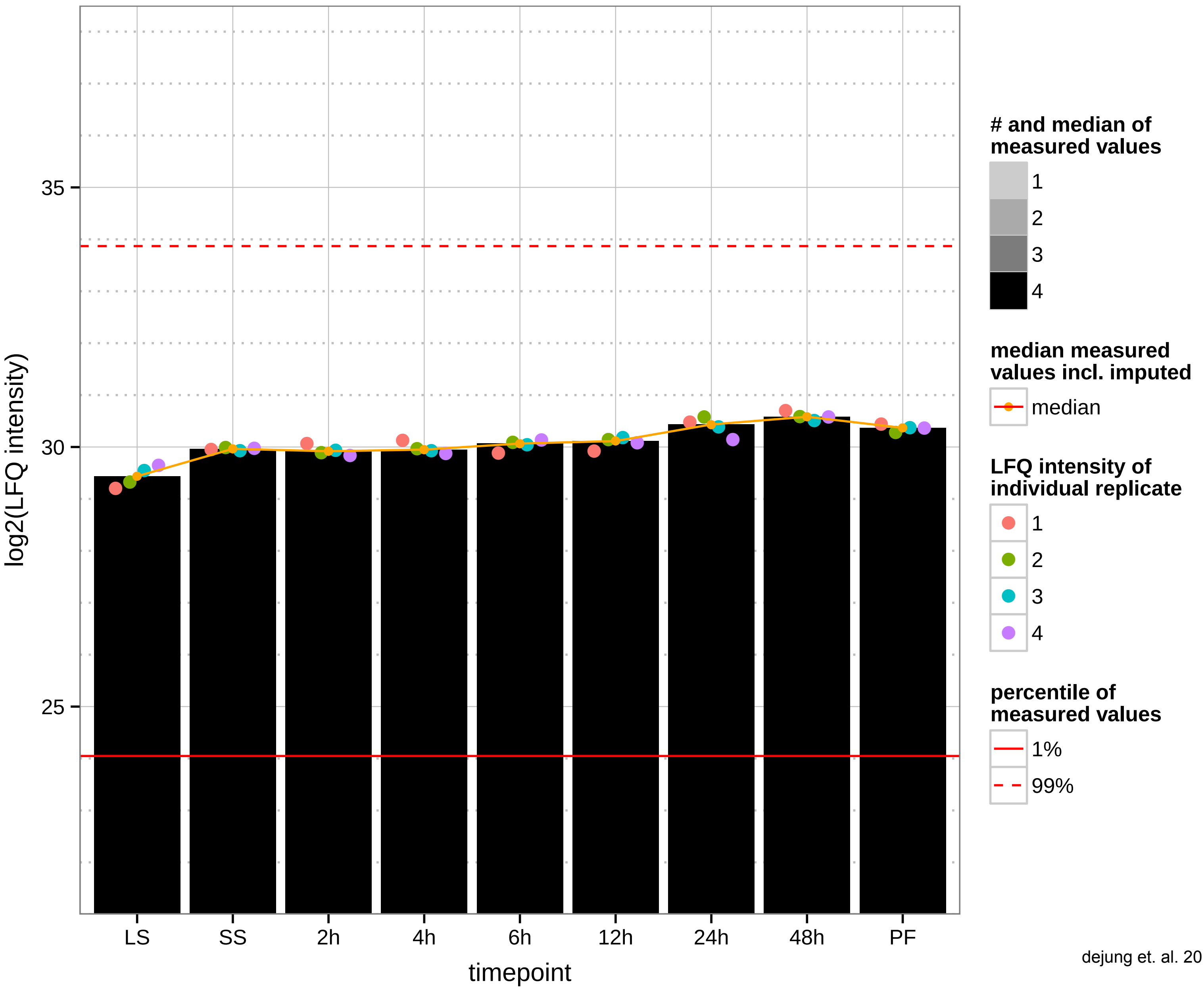

dejung et. al. 2015

adenylate kinase, putative
Tb927.10.2540
AGOF: ATP binding, adenylate kinase activity
AGOC: cytosol, glycosome, mitochondrion
AGOP: nucleobase–containing compound metabolic process
PGOF: ATP binding, adenylate kinase activity, nucleobase–containing compound kinase activity
PGOC: null
PGOP: nucleobase–containing compound metabolic process

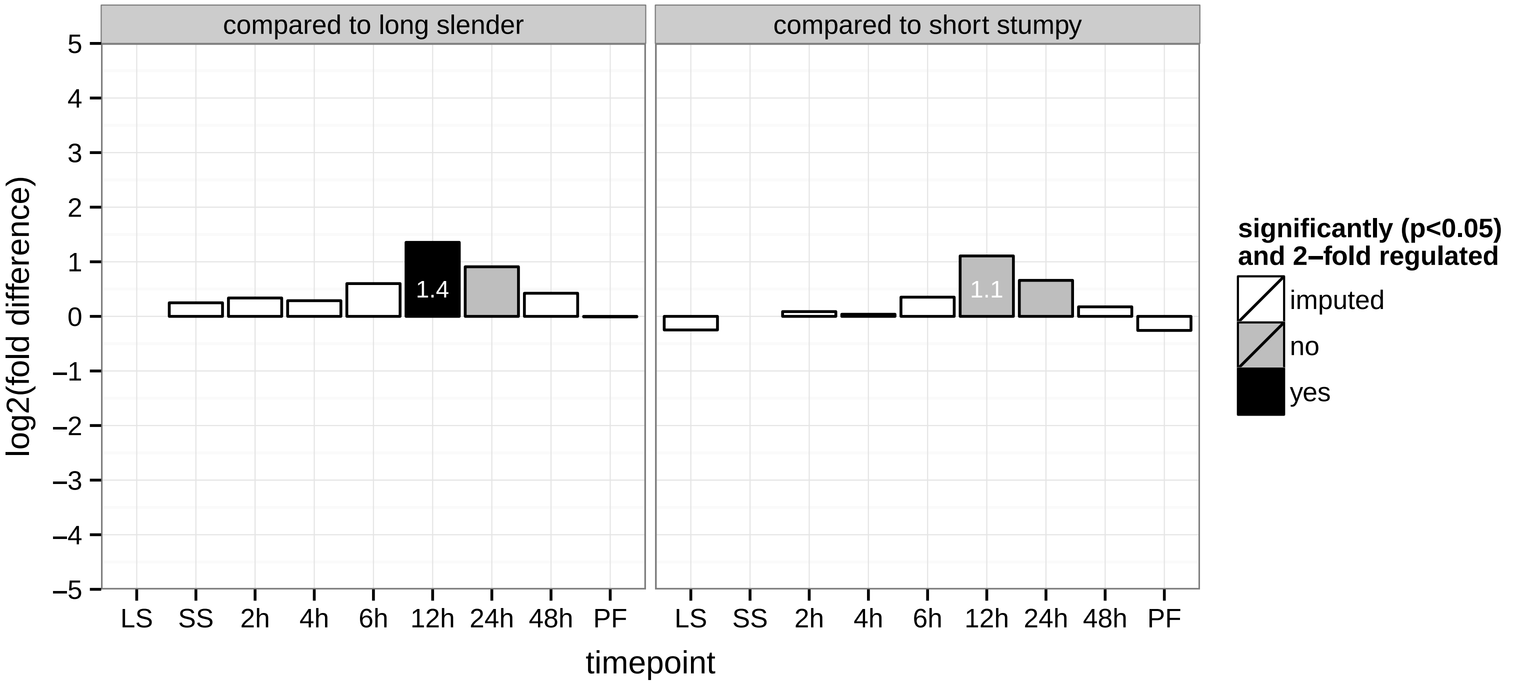

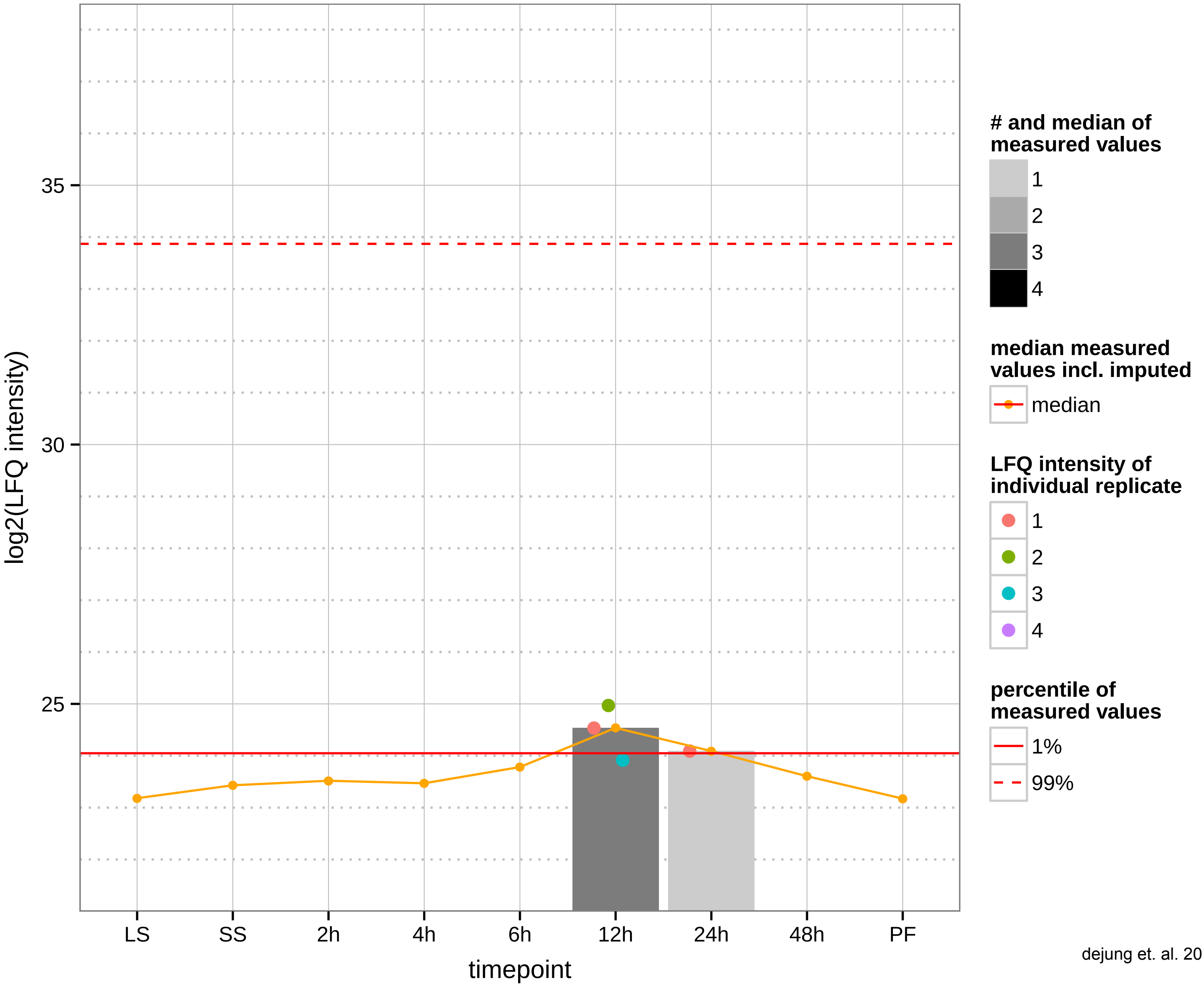

dejung et. al. 2015

hypothetical protein, conserved
Tb927.10.2630
AGOF: null
AGOC: null
AGOP: null
PGOF: null
PGOC: null
PGOP: null

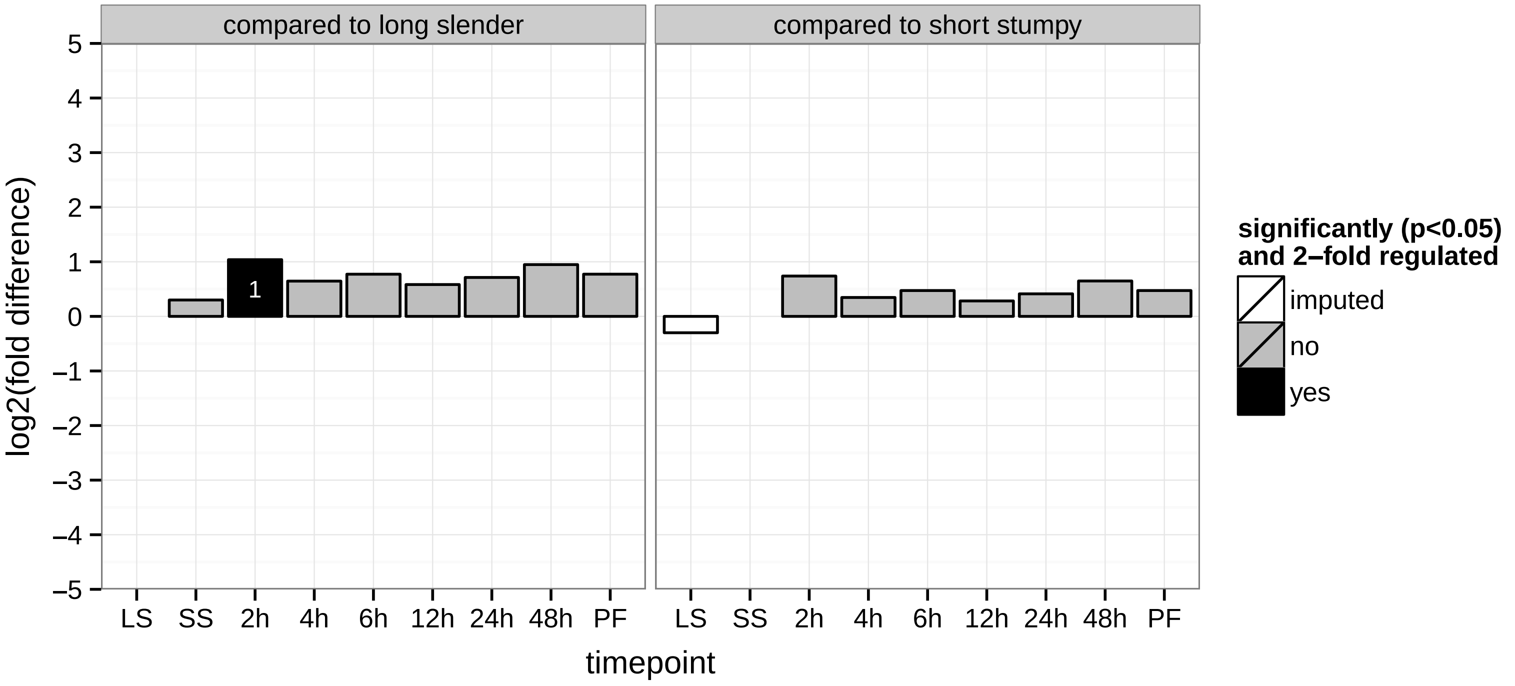

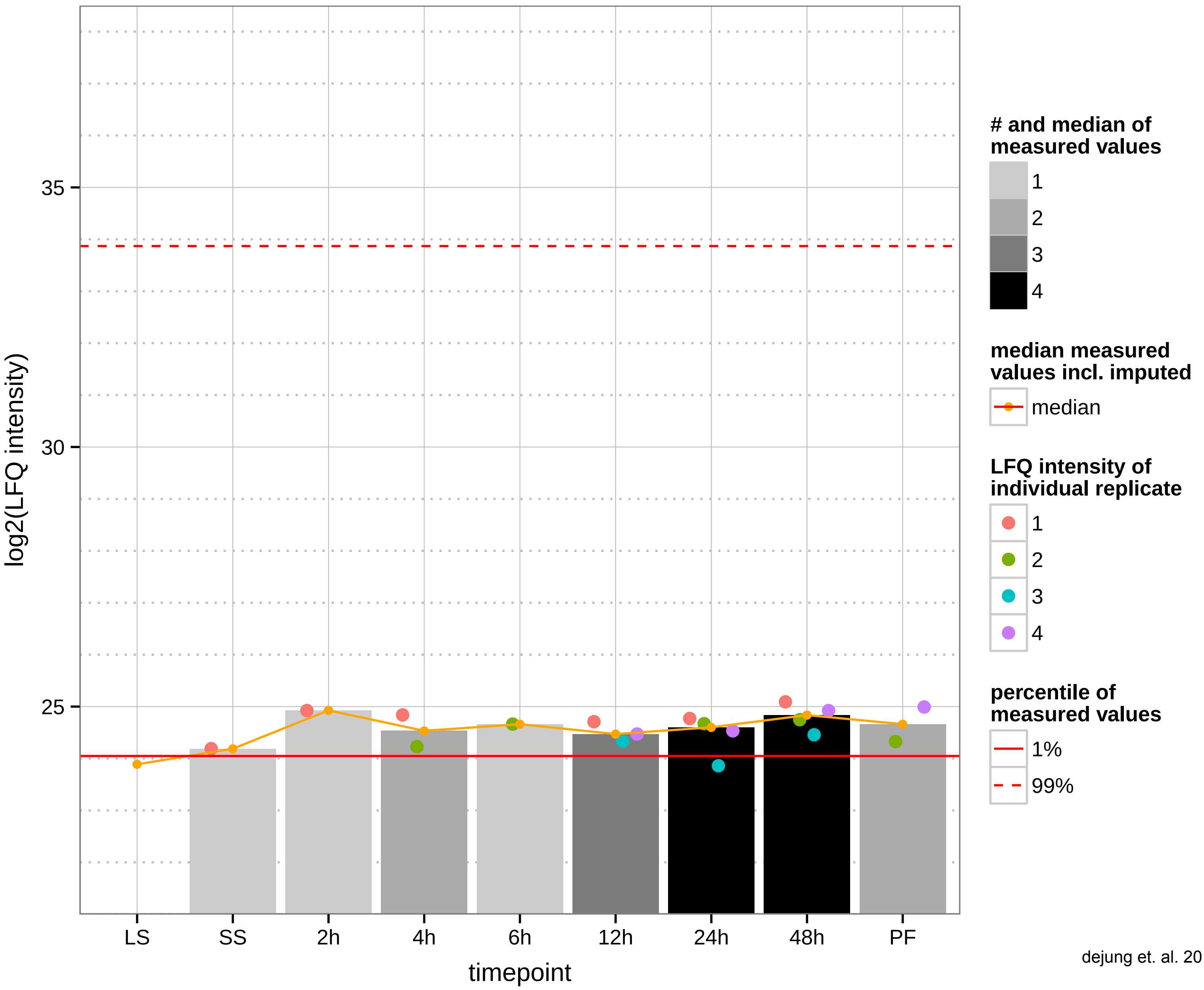

dejung et. al. 2015

hypothetical protein, conserved, intraflagellar transport protein IFT81, putative
Tb927.10.2640;Tb11.v5.0525
AGOF: null
AGOC: null
AGOP: null
PGOF: null
PGOC: null
PGOP: null

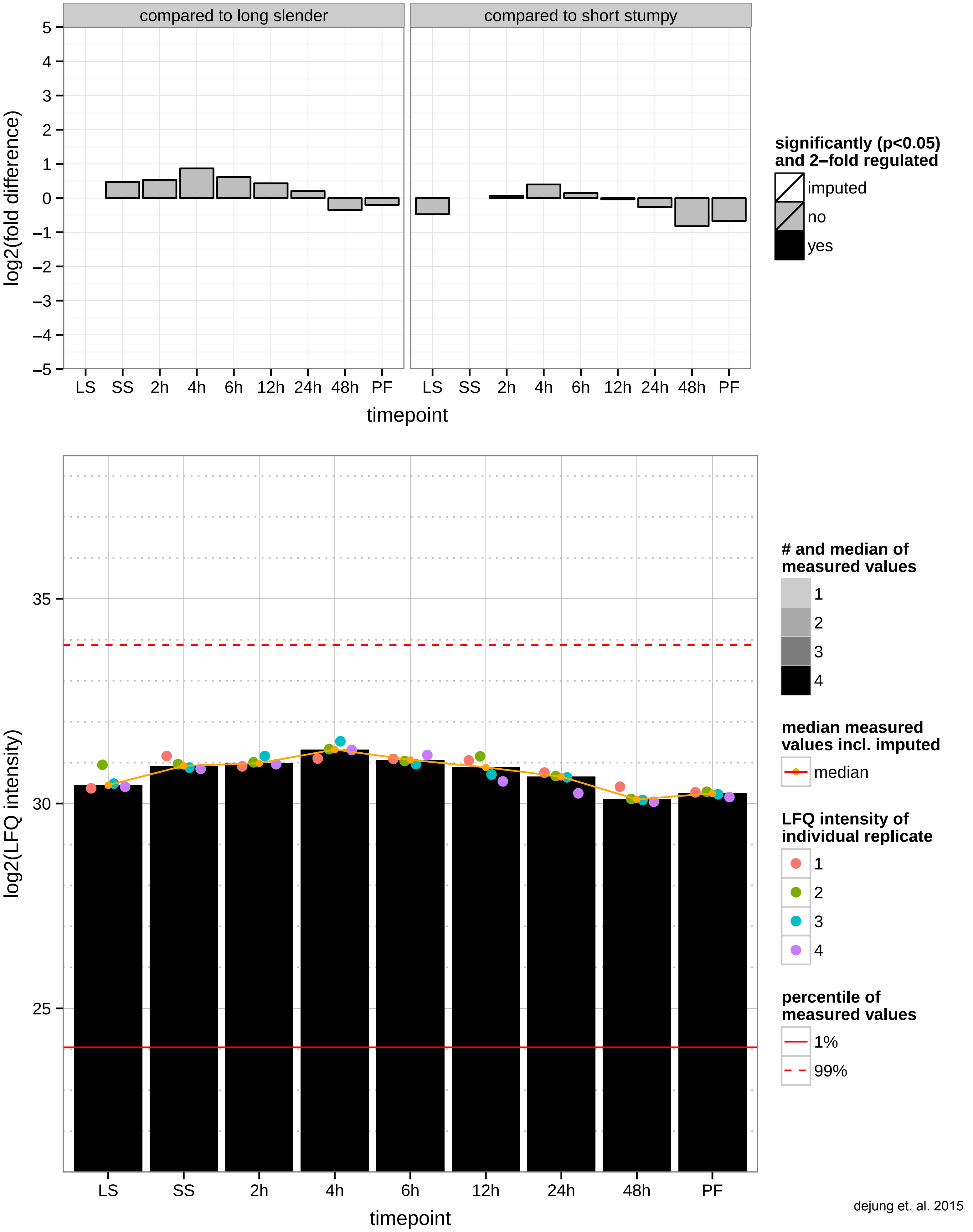

dejung et. al. 2015

hypothetical protein, conserved
Tb927.10.2650
AGOF: null
AGOC: null
AGOP: null
PGOF: null
PGOC: null
PGOP: null

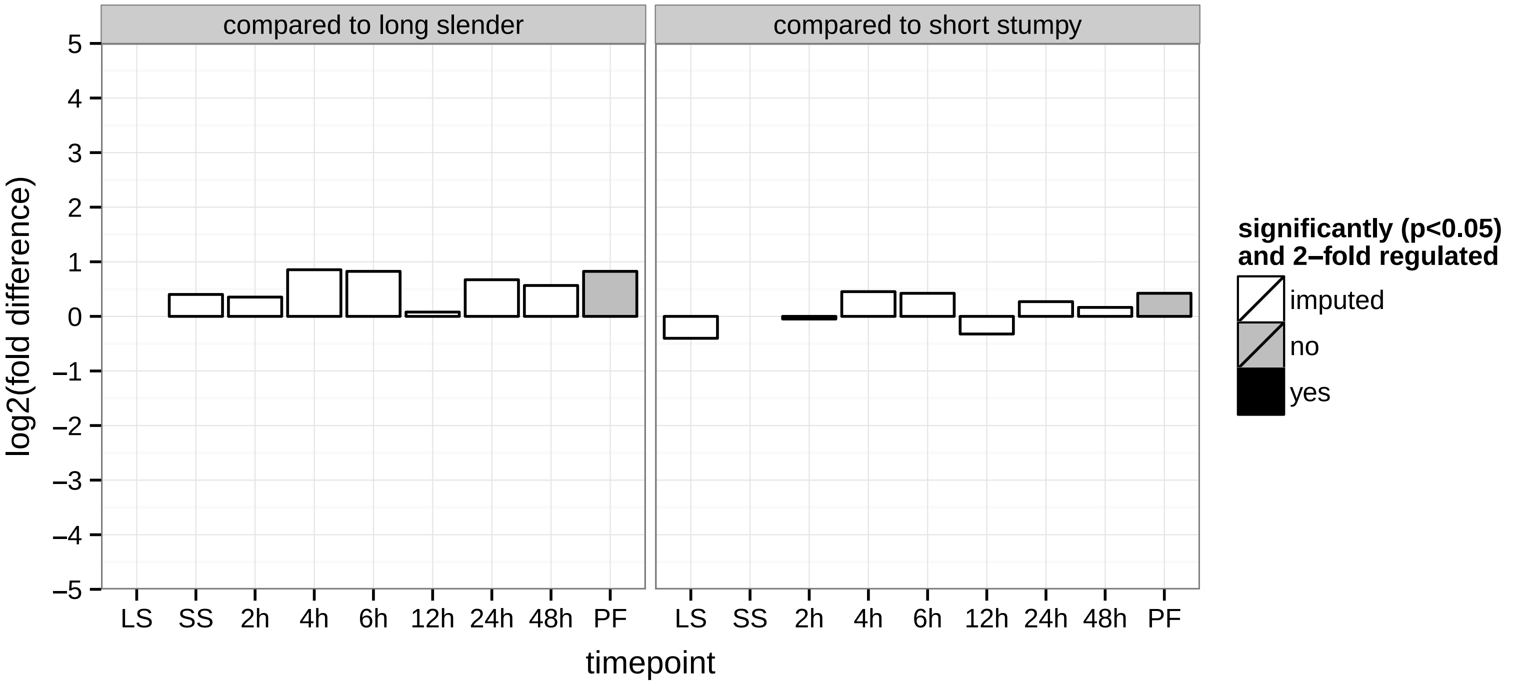

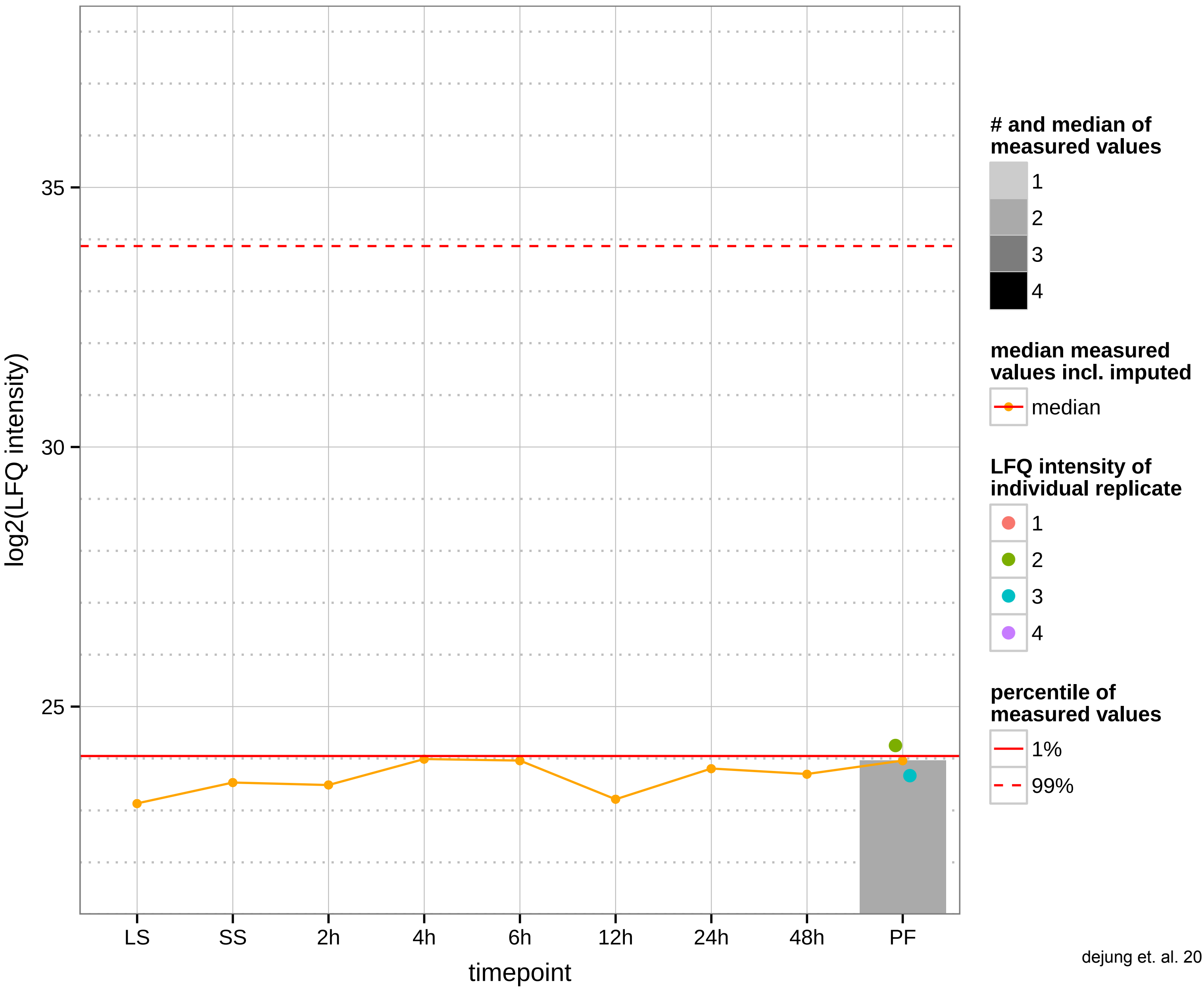

dejung et. al. 2015

predicted WD40 repeat protein
Tb927.10.2700
AGOF: null
AGOC: null
AGOP: null
PGOF: protein binding
PGOC: null
PGOP: null

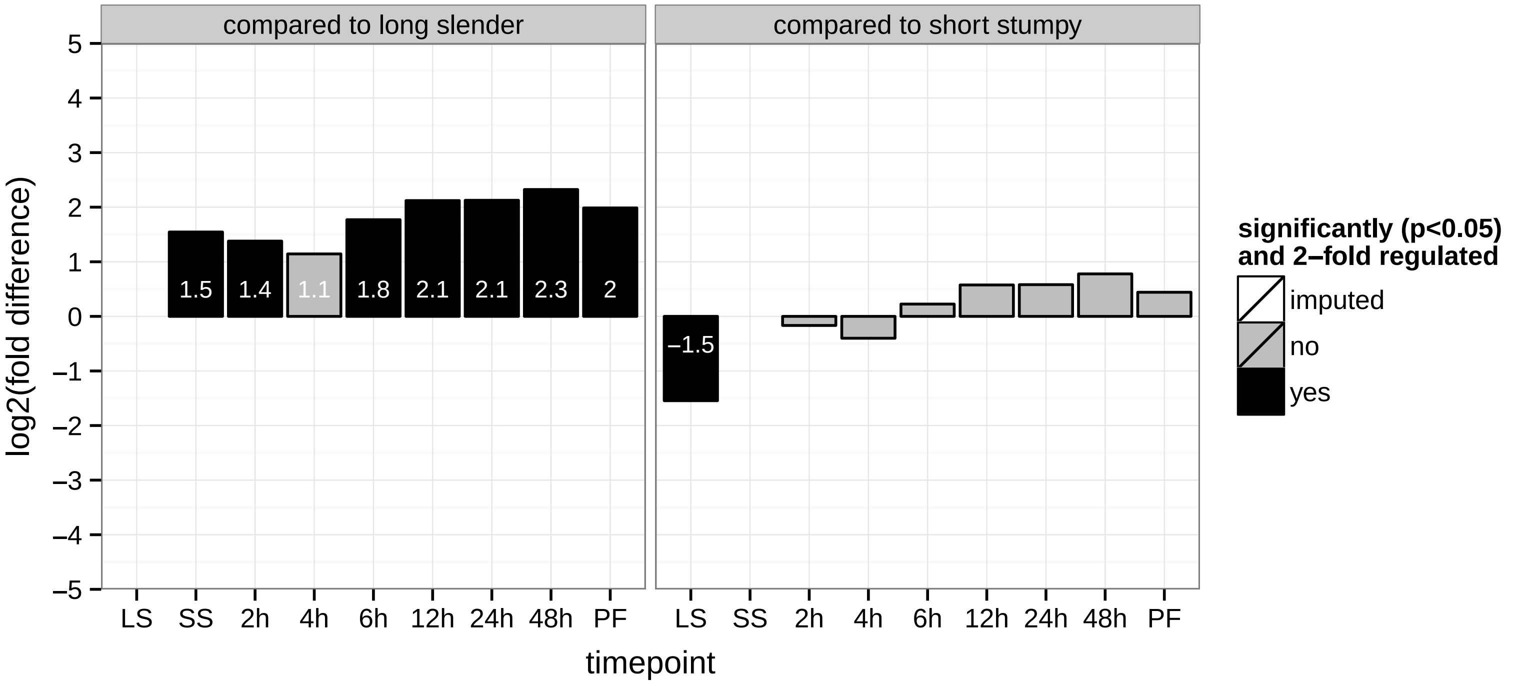

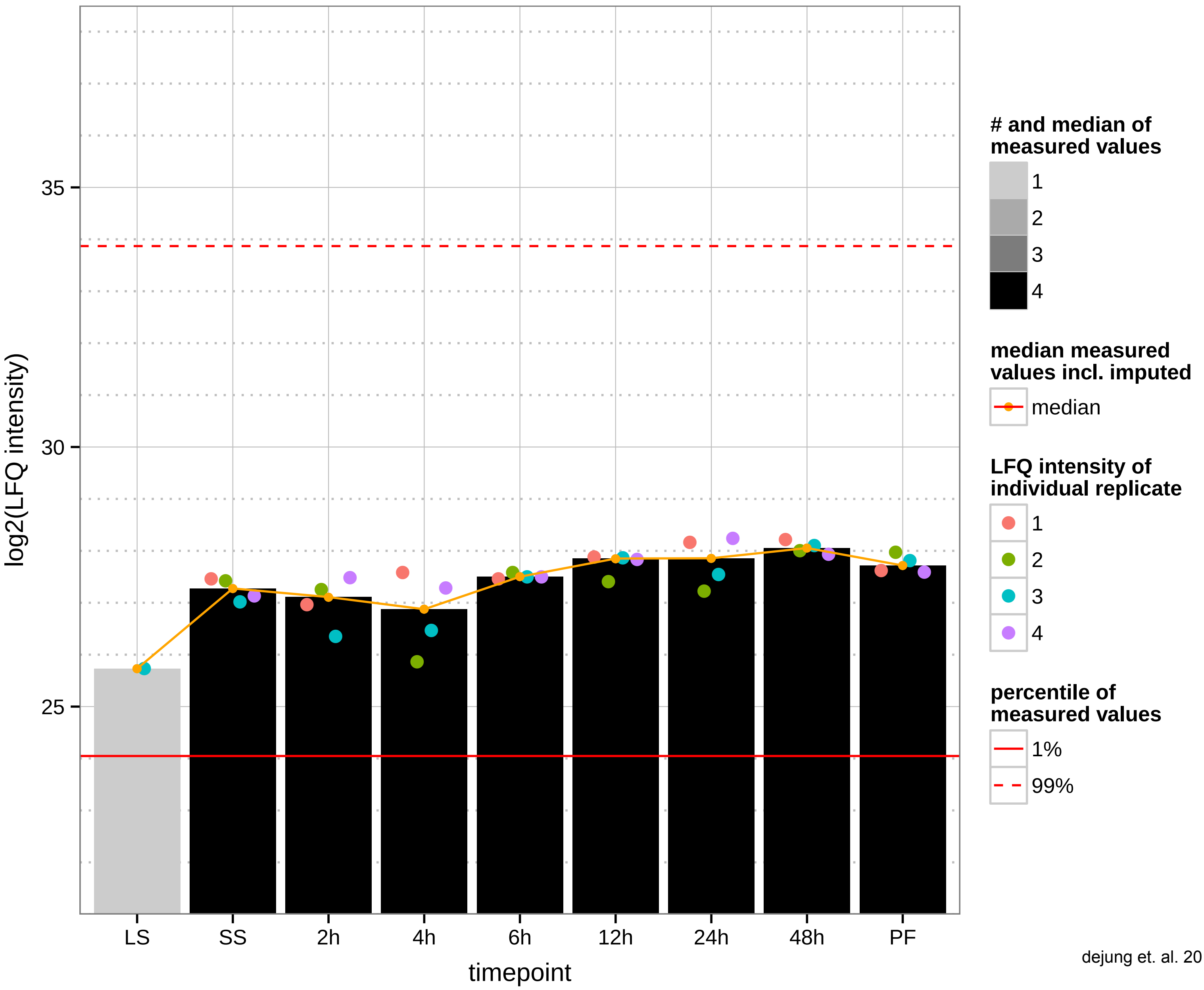

dejung et. al. 2015

hypothetical protein, conserved
Tb927.10.2720
AGOF: RNA binding, pseudouridine synthase activity
AGOC: null
AGOP: pseudouridine synthesis
PGOF: RNA binding, pseudouridine synthase activity
PGOC: null
PGOP: RNA modification, pseudouridine synthesis

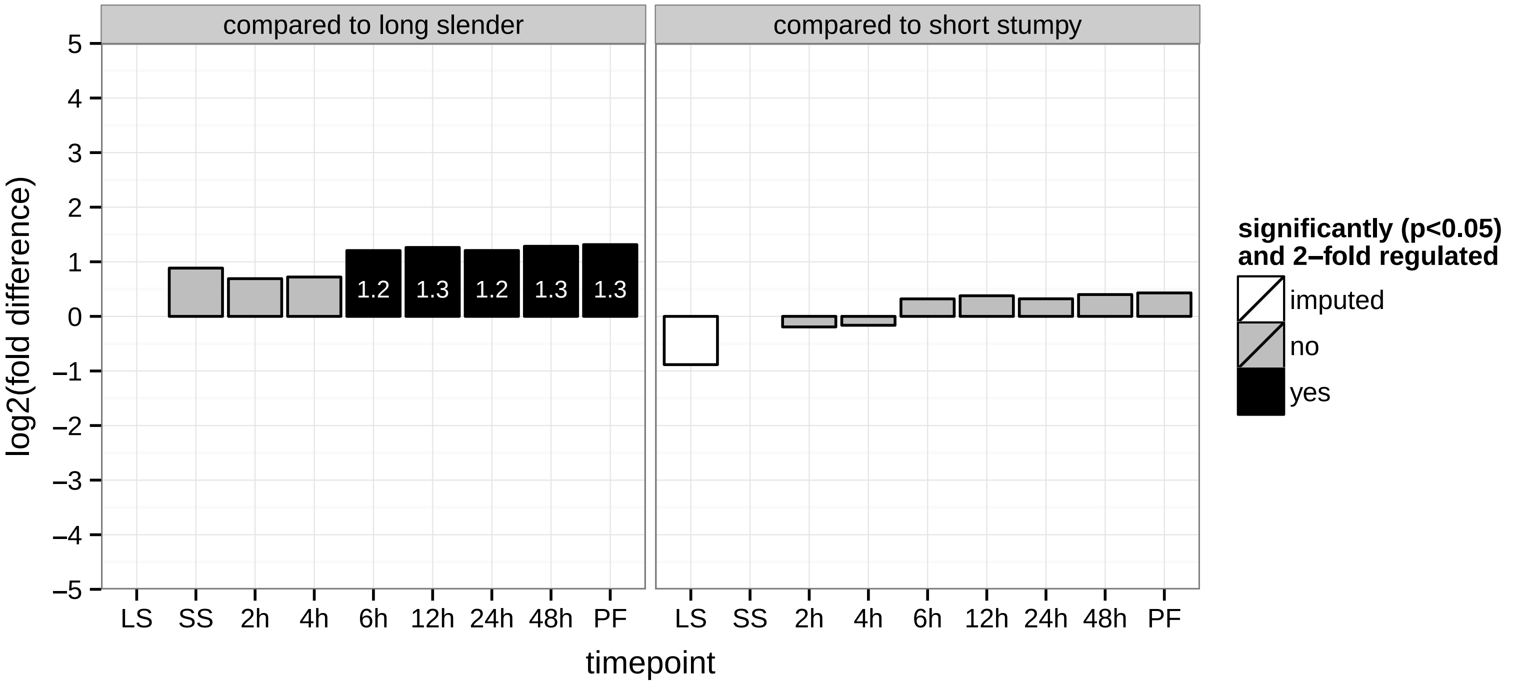

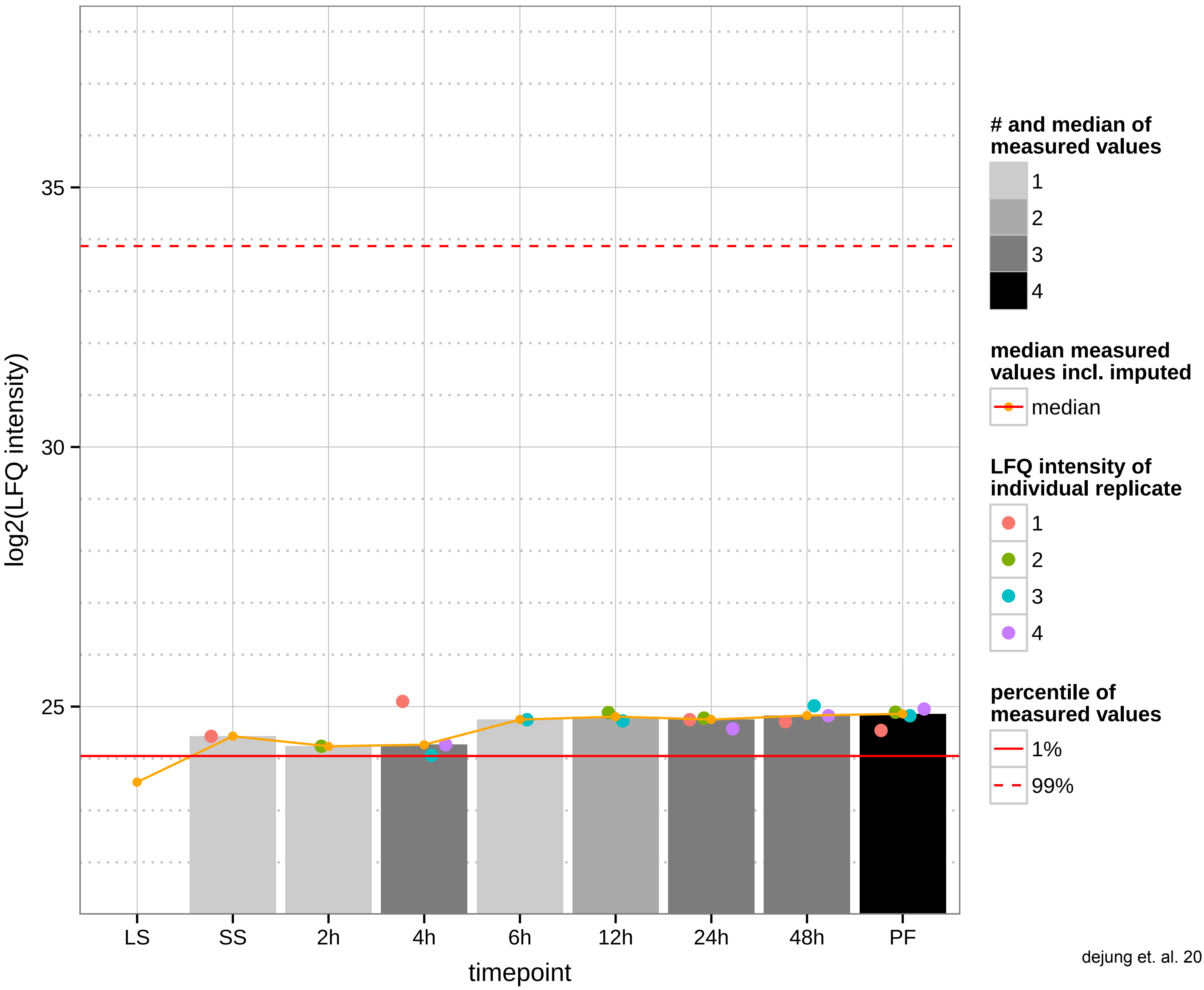

dejung et. al. 2015

hypothetical protein, conserved
Tb927.10.2730
AGOF: null
AGOC: null
AGOP: null
PGOF: null
PGOC: null
PGOP: null

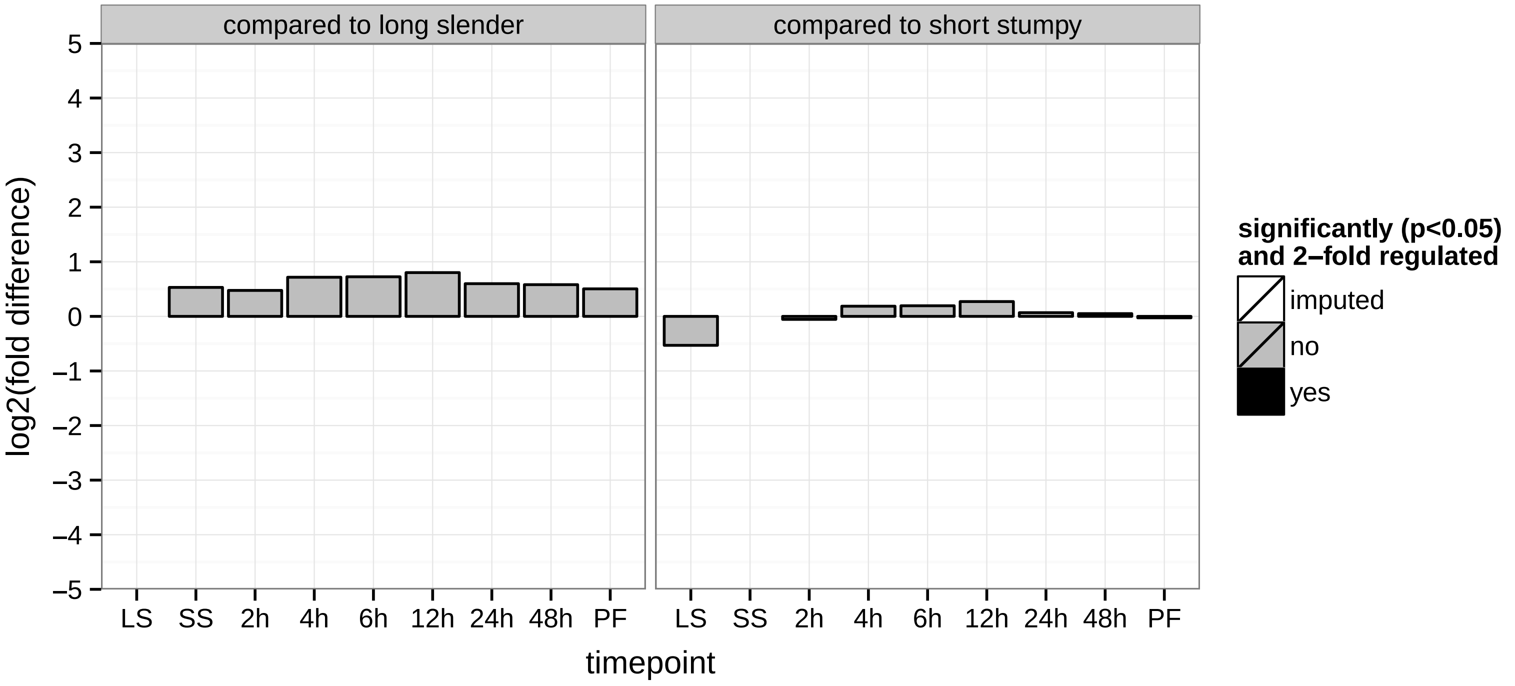

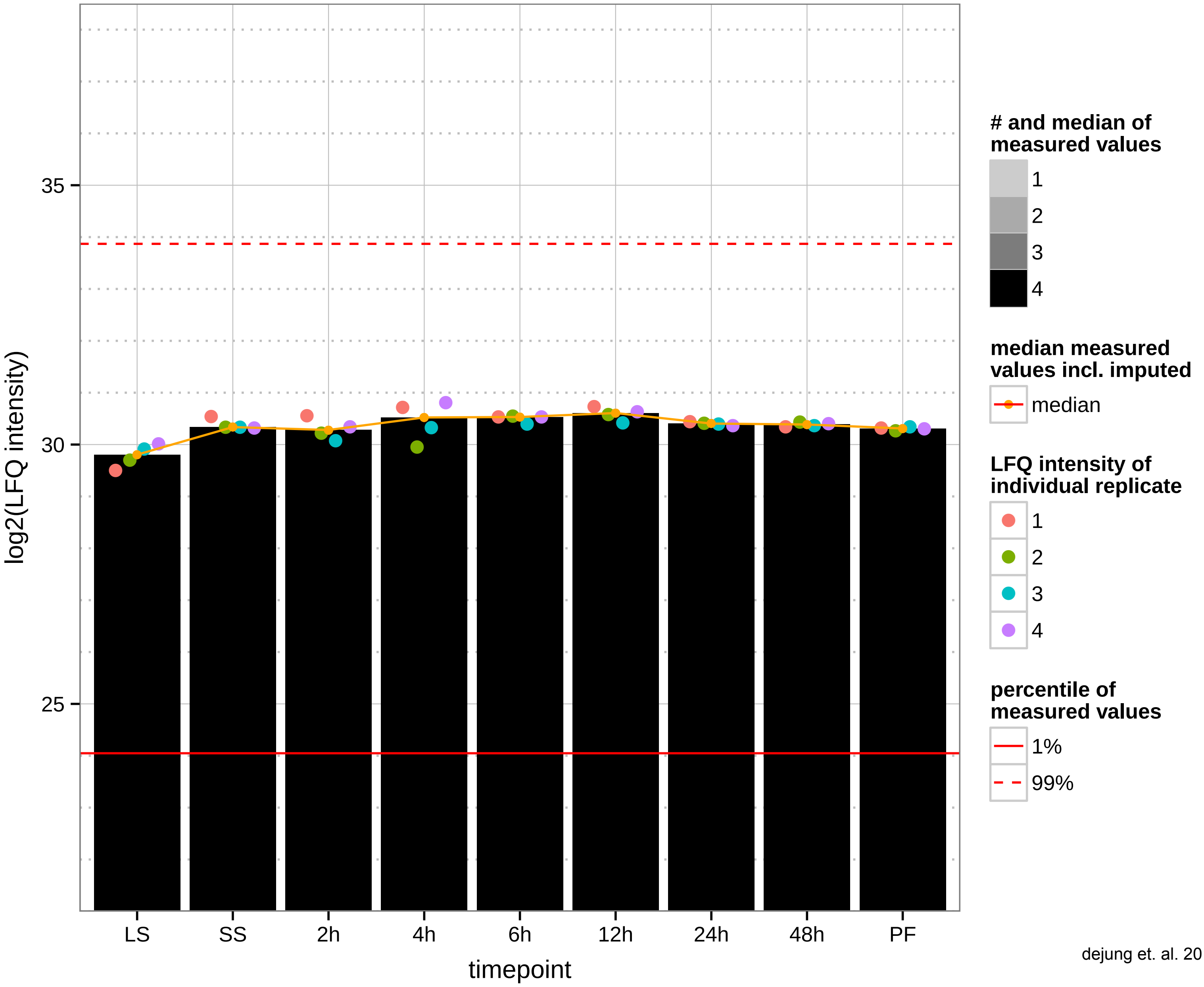

dejung et. al. 2015

DNA−directed RNA polymerase III largest subunit, putative, DNA−directed RNA polymerase III subunit C1, DNA−directed RN
Tb927.10.2780
AGOF: null, DNA binding, DNA−directed RNA polymerase activity, ribonucleoside binding, zinc ion binding
AGOC: null, nucleus
AGOP: null, transcription, DNA−dependent
PGOF: DNA binding, DNA−directed RNA polymerase activity
PGOC: null
PGOP: transcription, DNA−dependent

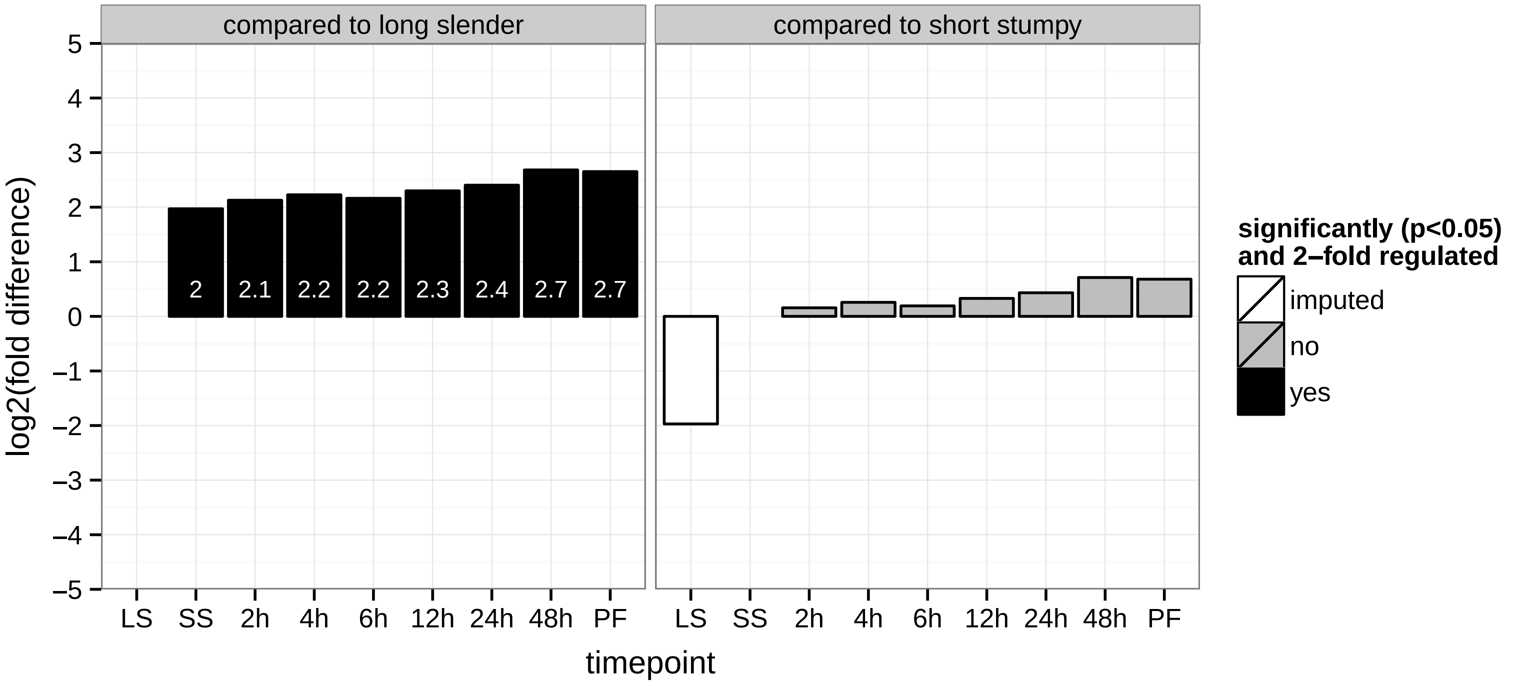

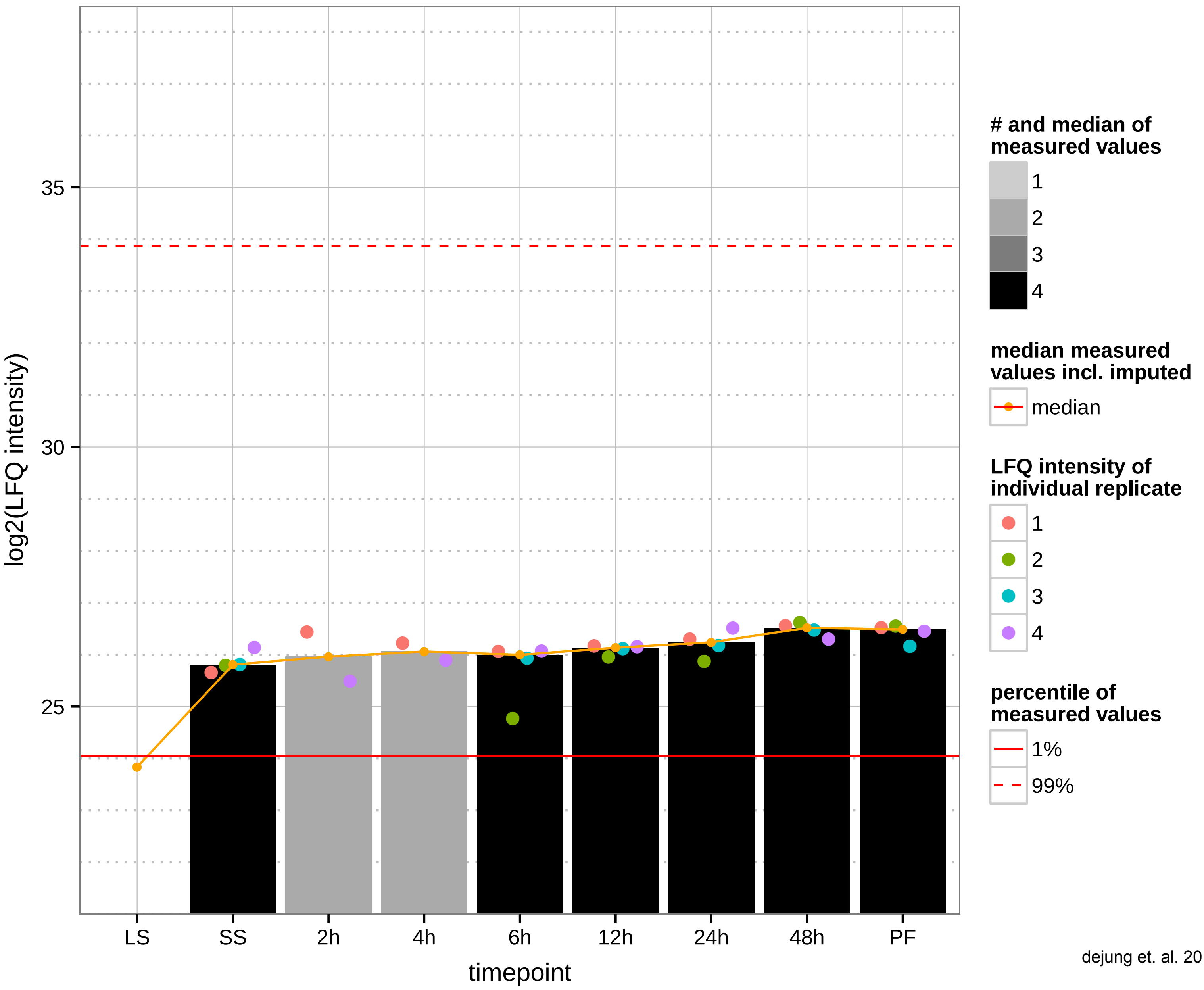

dejung et. al. 2015

hypothetical protein, conserved
Tb927.10.2810
AGOF: null
AGOC: null
AGOP: null
PGOF: null
PGOC: null
PGOP: null

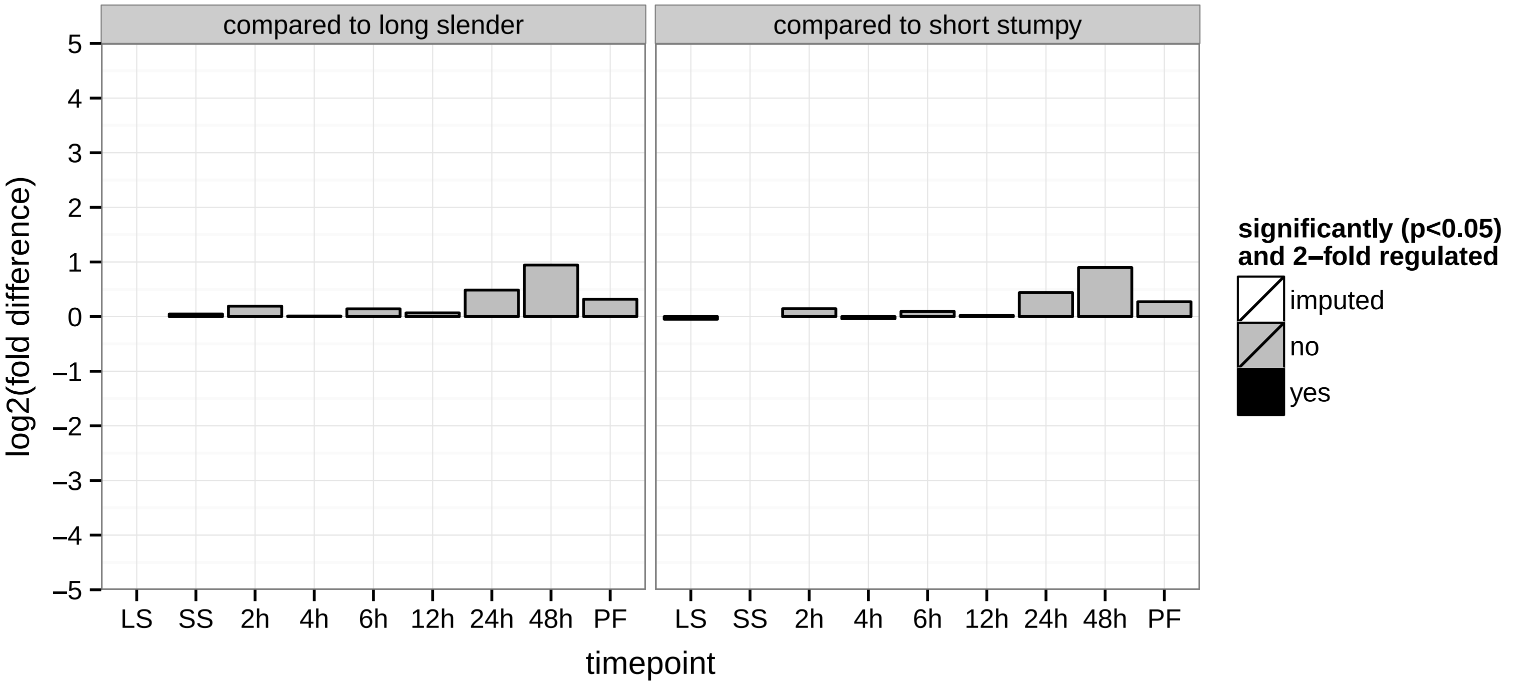

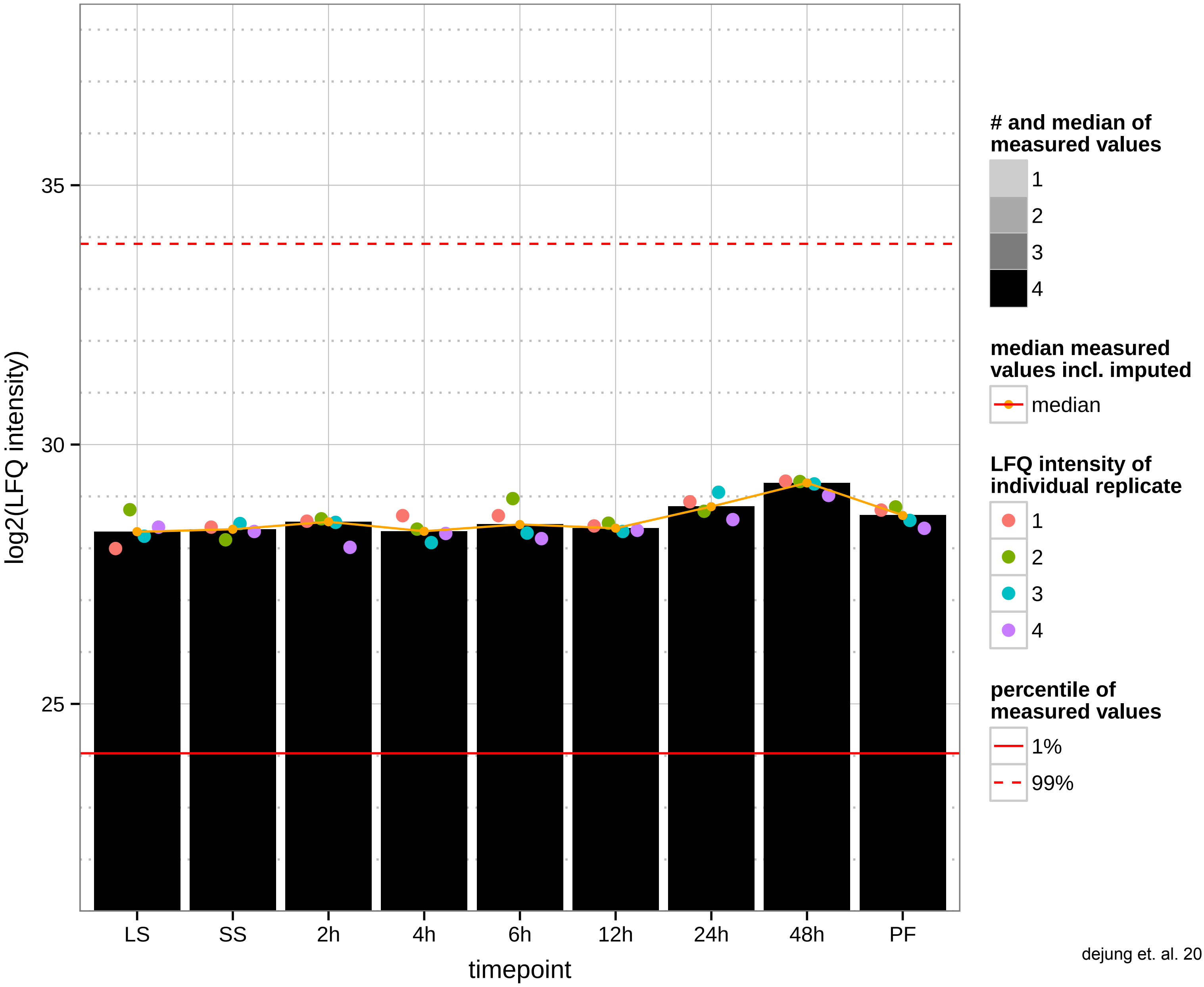

dejung et. al. 2015

ribosomal protein S25, putative, 40S ribosomal protein S25
Tb927.3.1370;Tb927.10.2840
AGOF: structural constituent of ribosome, null
AGOC: ribosome, null
AGOP: translation, null
PGOF: null
PGOC: null
PGOP: null

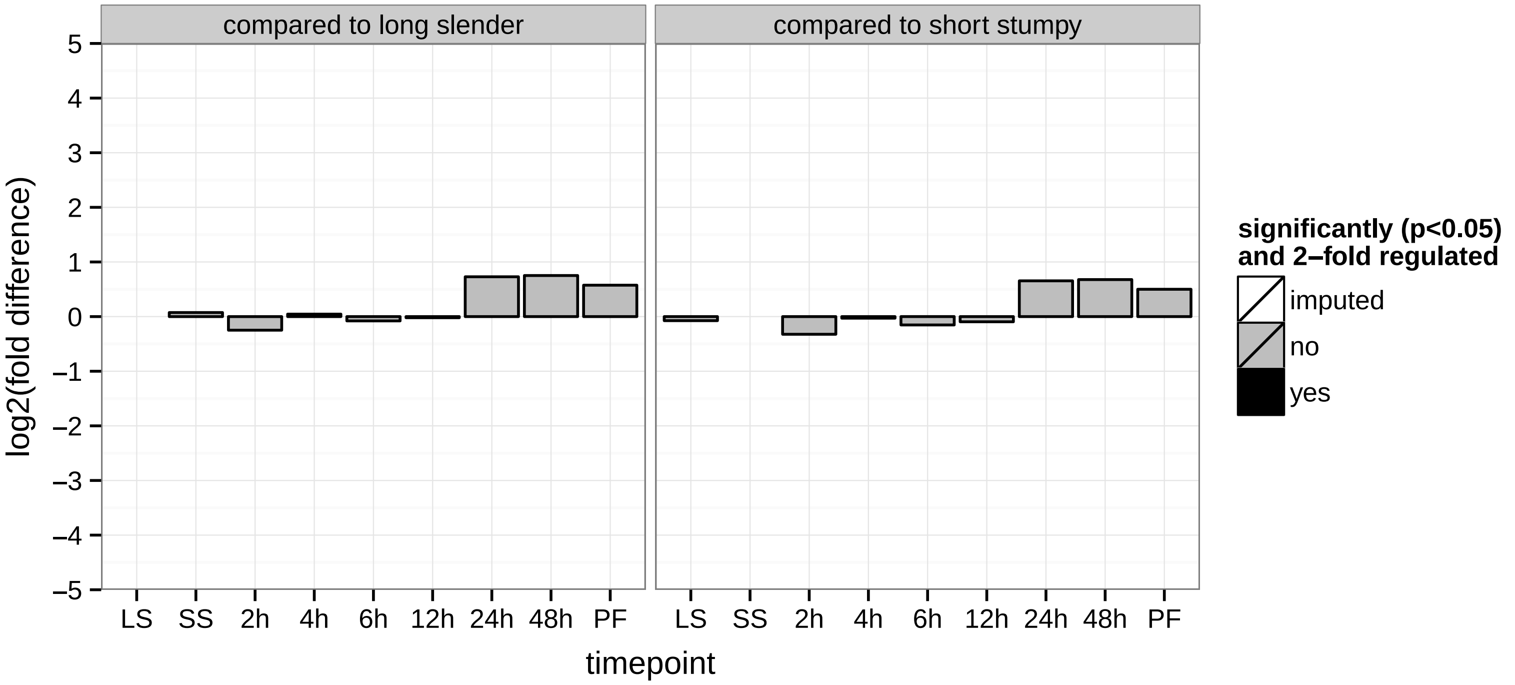

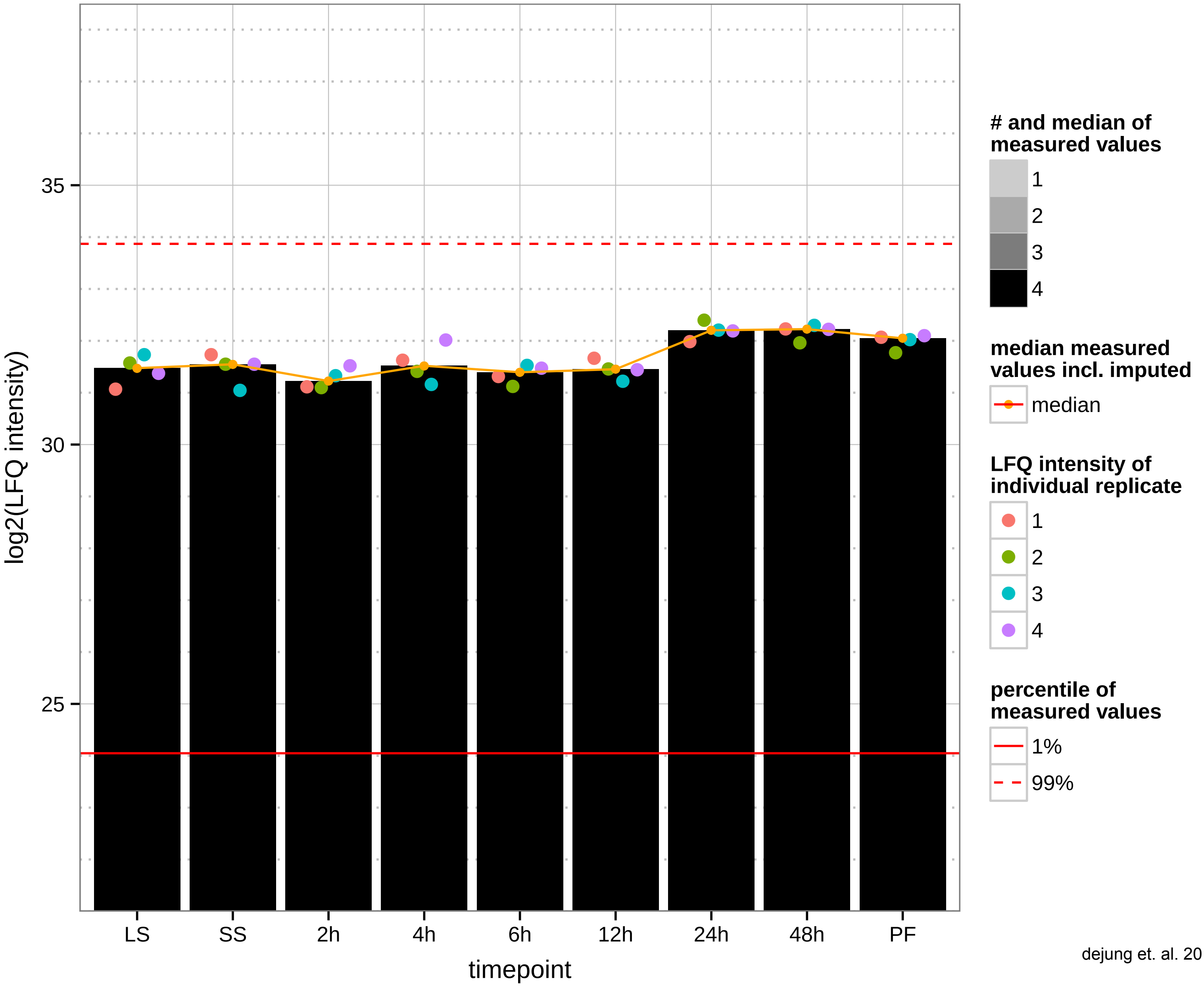

dejung et. al. 2015

hypothetical protein, conserved
Tb927.10.2860
AGOF: null
AGOC: bacterial–type flagellum basal body
AGOP: null
PGOF: protein binding
PGOC: null
PGOP: null

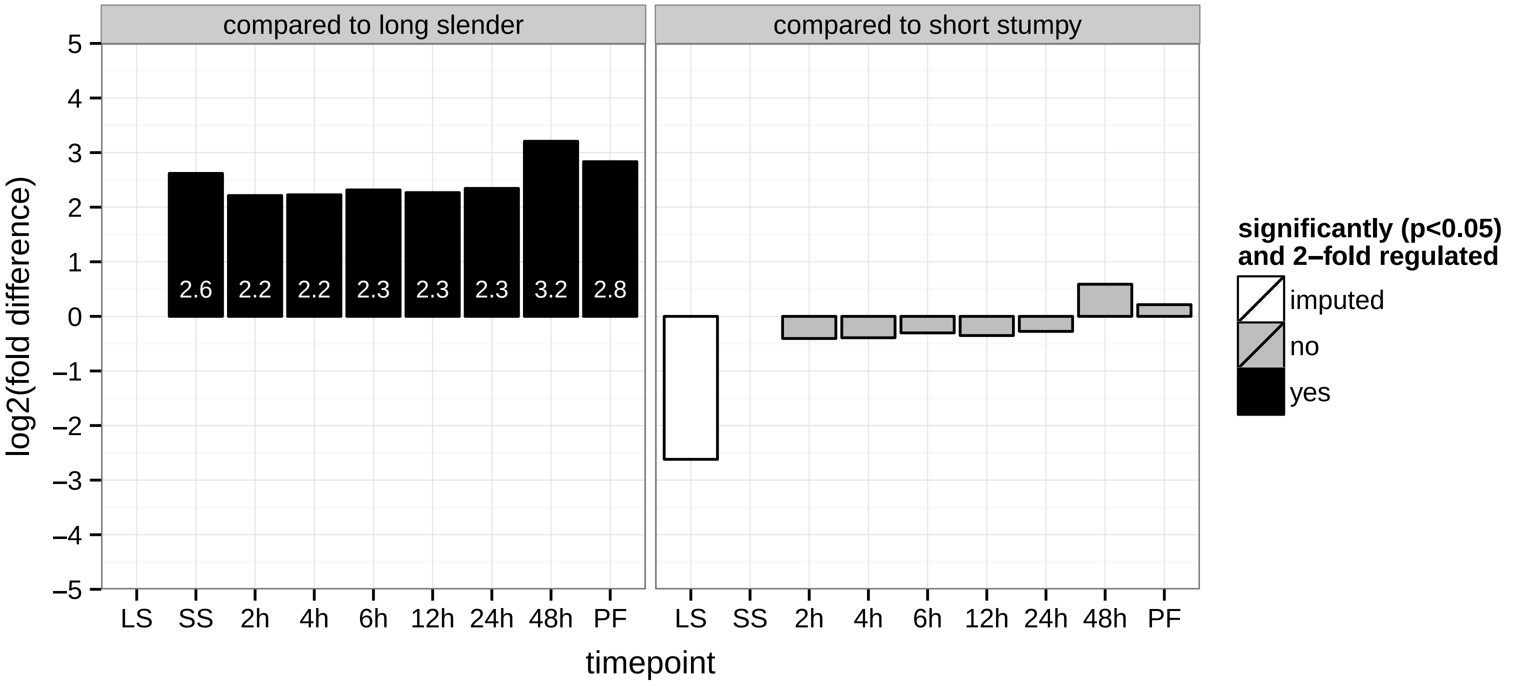

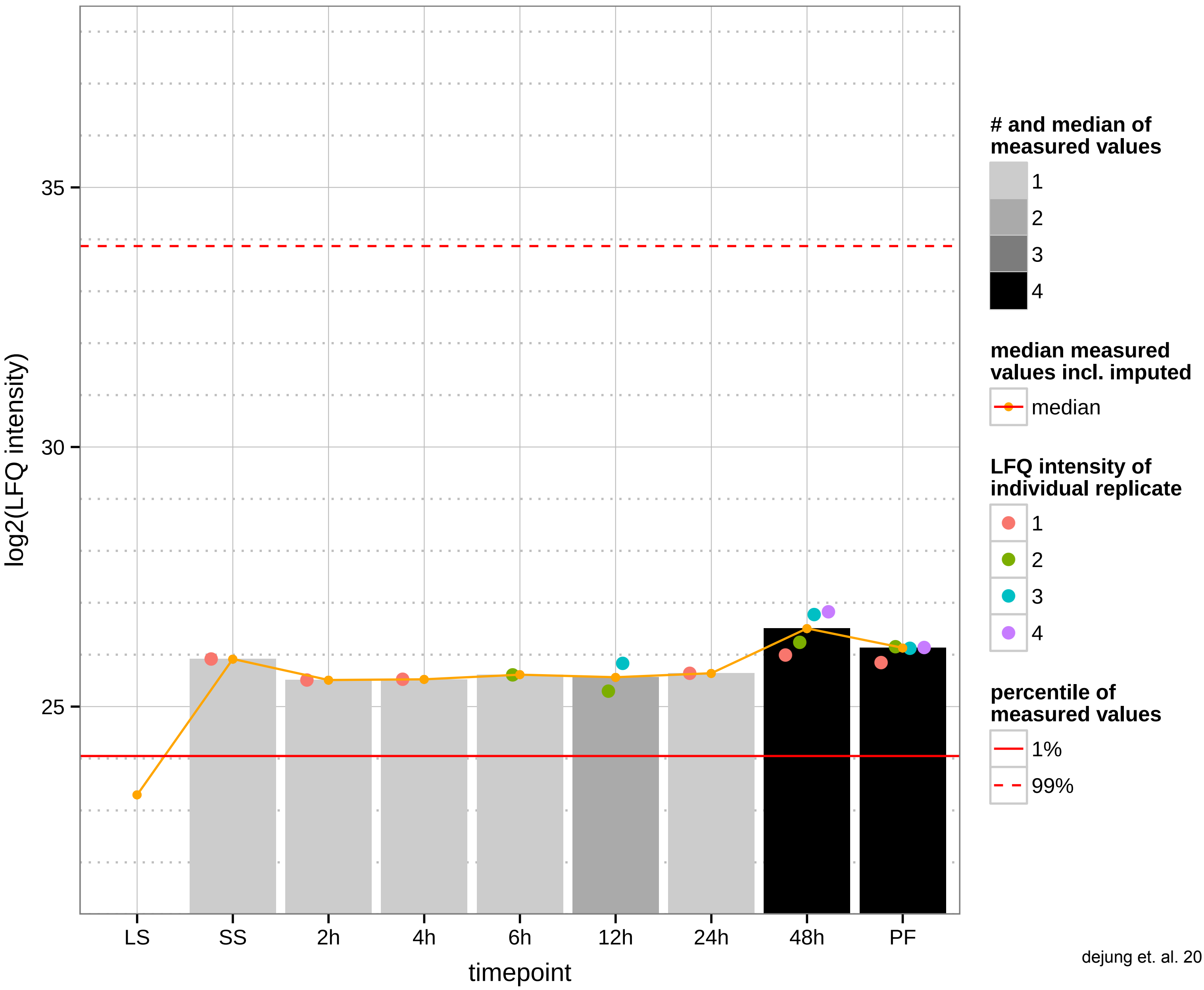

dejung et. al. 2015

calcium channel protein, putative
Tb927.10.2880
AGOF: ion channel activity
AGOC: null
AGOP: ion transport
PGOF: ion channel activity
PGOC: membrane
PGOP: ion transport, transmembrane transport

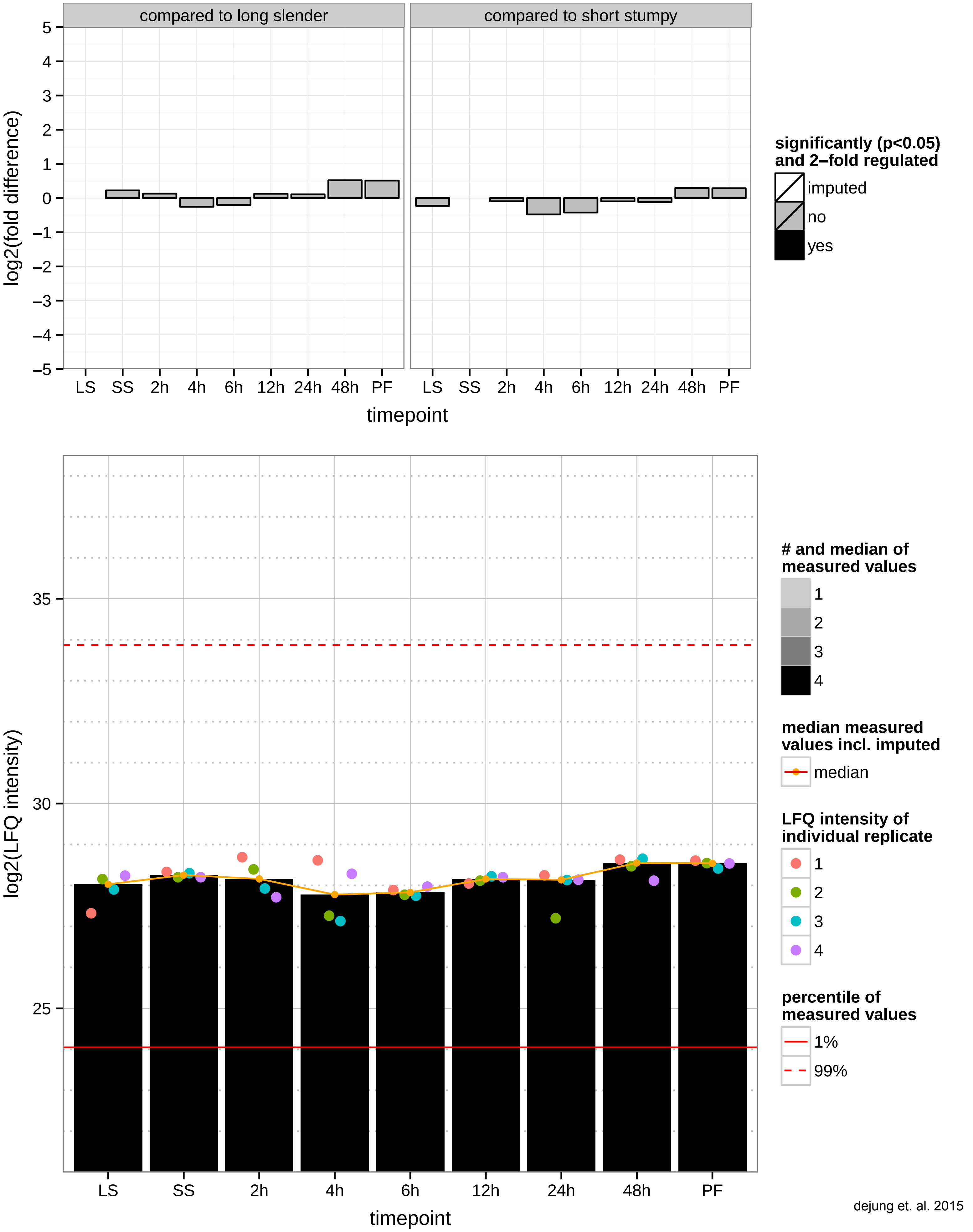

dejung et. al. 2015

proteasome alpha 2 subunit, putative
Tb927.10.290
AGOF: endopeptidase activity, threonine–type endopeptidase activity
AGOC: proteasome core complex
AGOP: ubiquitin–dependent protein catabolic process
PGOF: endopeptidase activity, threonine–type endopeptidase activity
PGOC: proteasome core complex, proteasome core complex, alpha–subunit complex
PGOP: proteolysis involved in cellular protein catabolic process, ubiquitin–dependent protein catabolic process

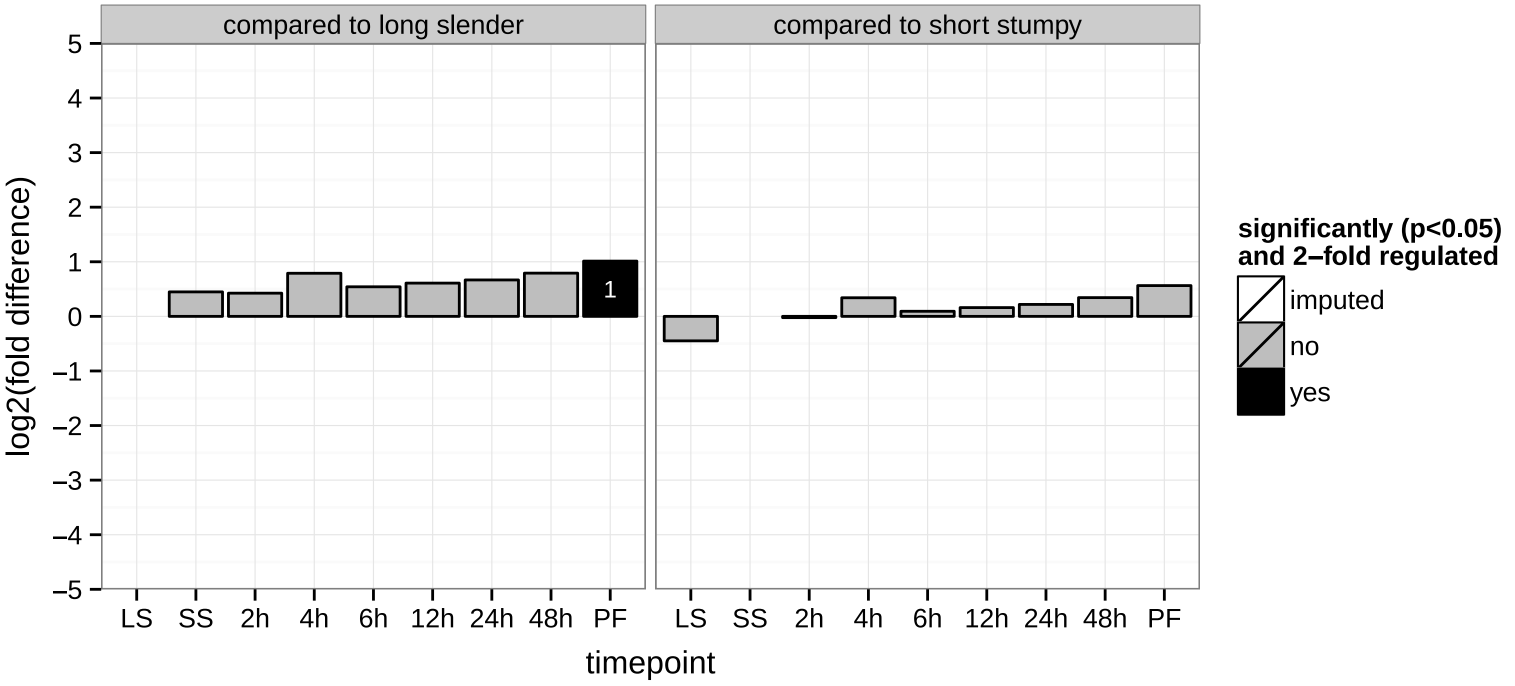

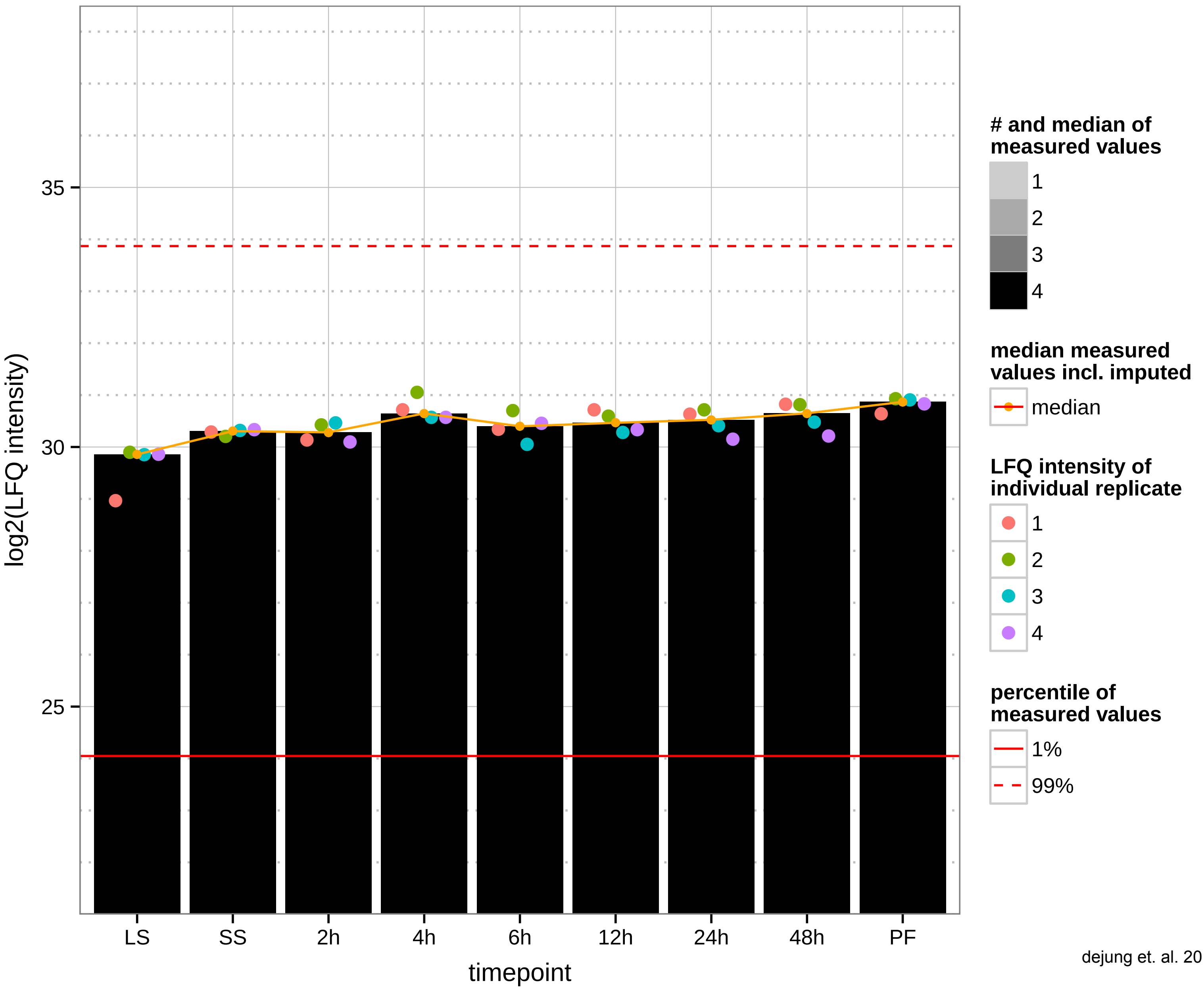

dejung et. al. 2015

importin beta–1 subunit, putative
Tb927.10.2900;Tb11.v5.0837
AGOF: null, protein transporter activity
AGOC: null, cytoplasm, nuclear pore, nucleus
AGOP: null, intracellular protein transport, nuclear import, protein import into nucleus, docking
PGOF: binding, protein transporter activity
PGOC: null
PGOP: intracellular protein transport

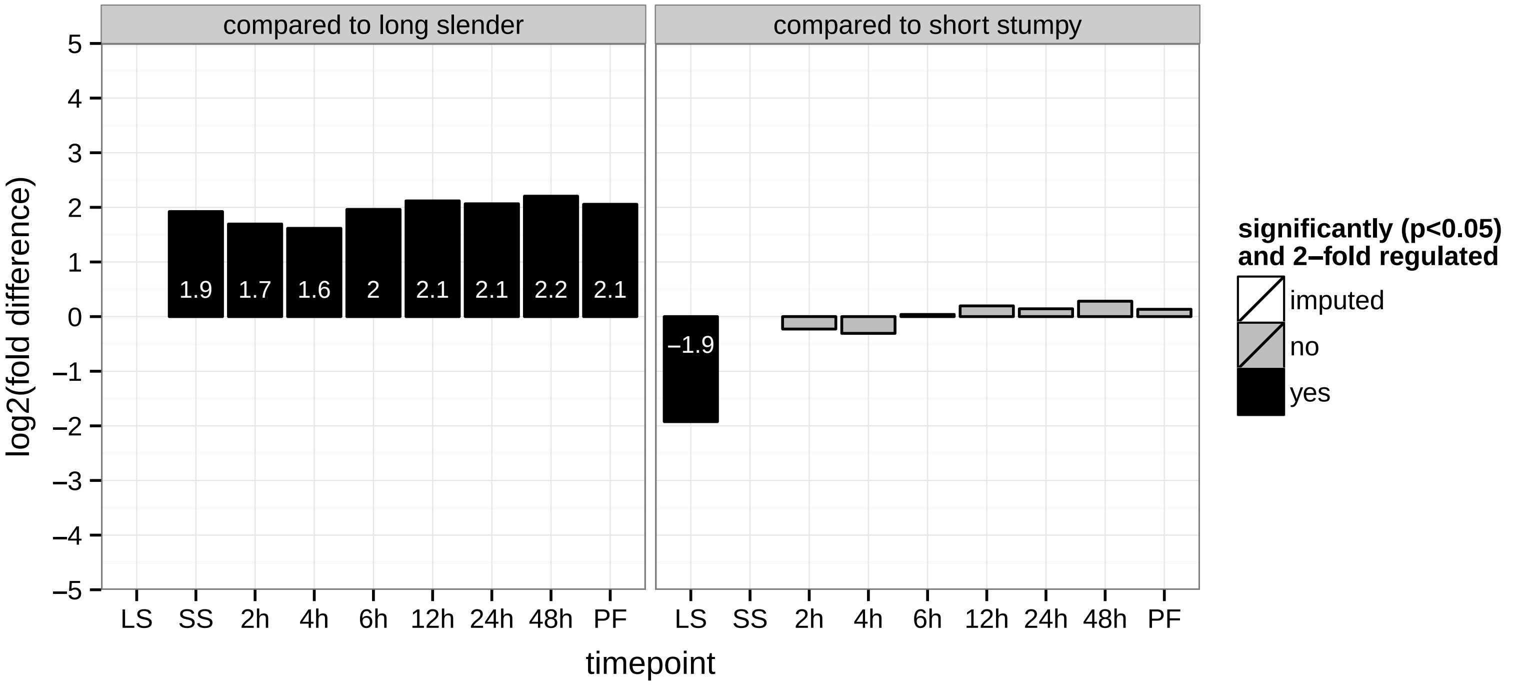

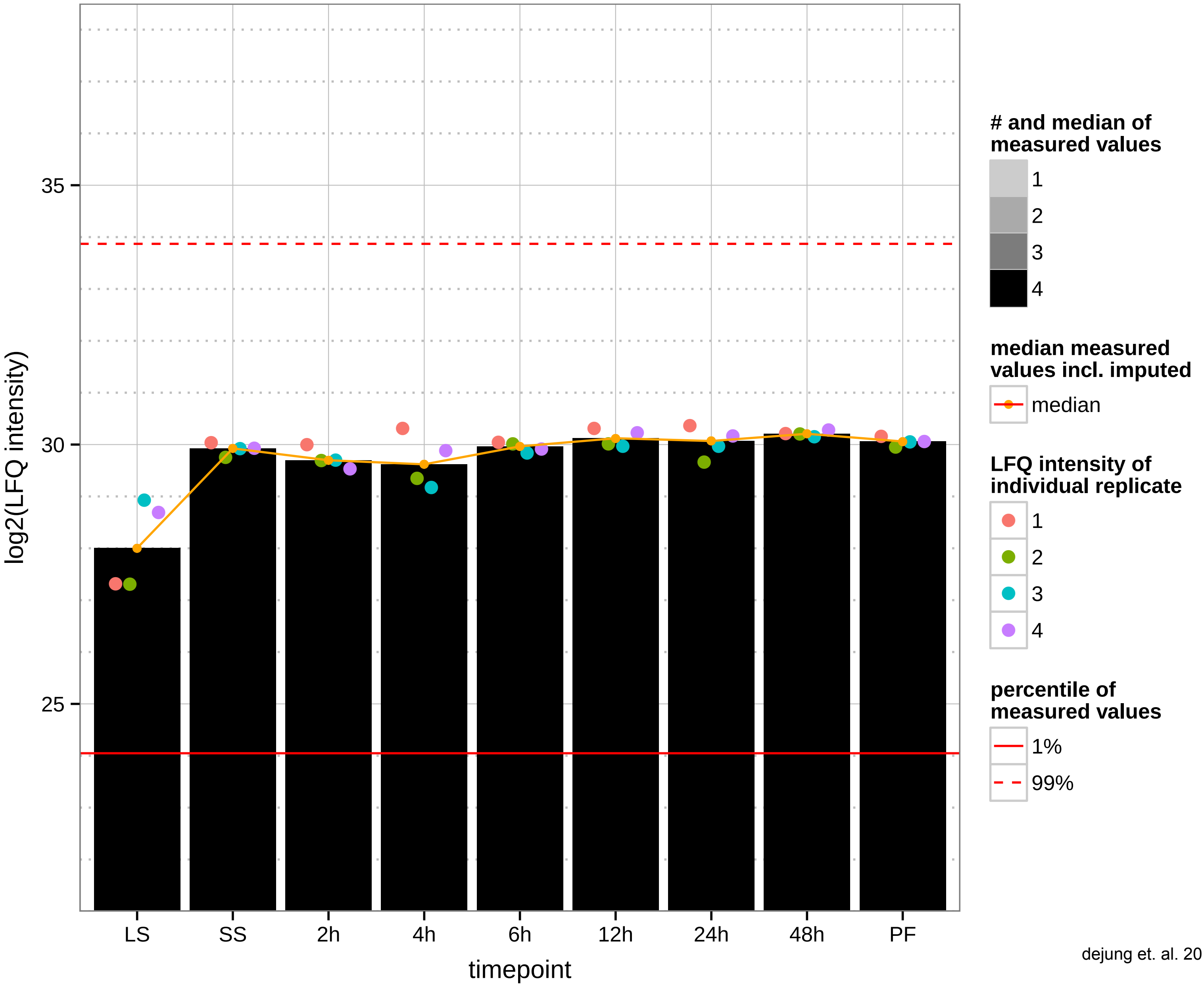

dejung et. al. 2015

splicing factor PTSR1 interacting protein (TSR1IP)
Tb927.10.2910
AGOF: RNA binding
AGOC: nucleus
AGOP: nuclear mRNA splicing, via spliceosome
PGOF: null
PGOC: null
PGOP: null

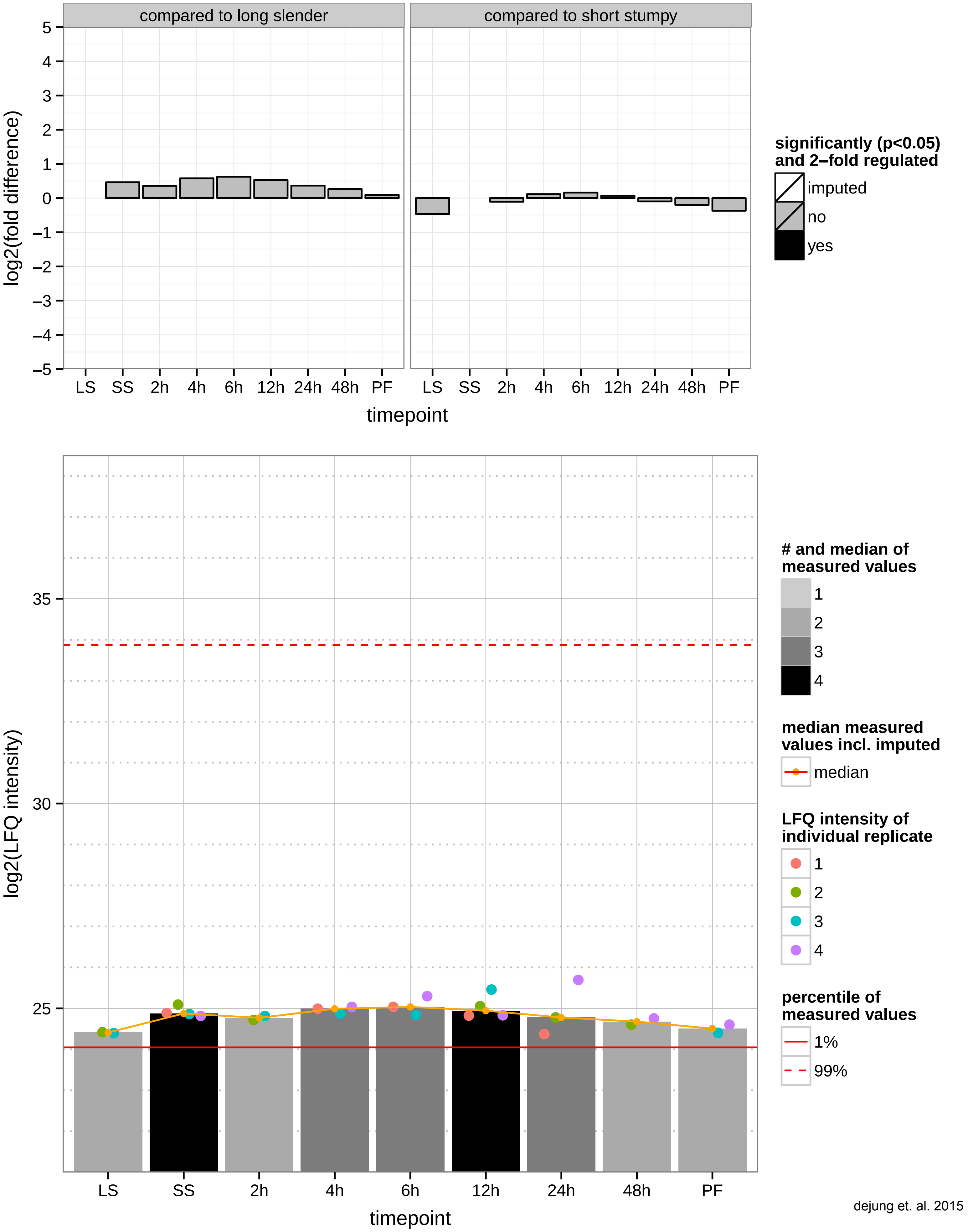

dejung et. al. 2015

hypothetical protein, conserved
Tb927.10.2950
AGOF: null
AGOC: null
AGOP: null
PGOF: null
PGOC: null
PGOP: null

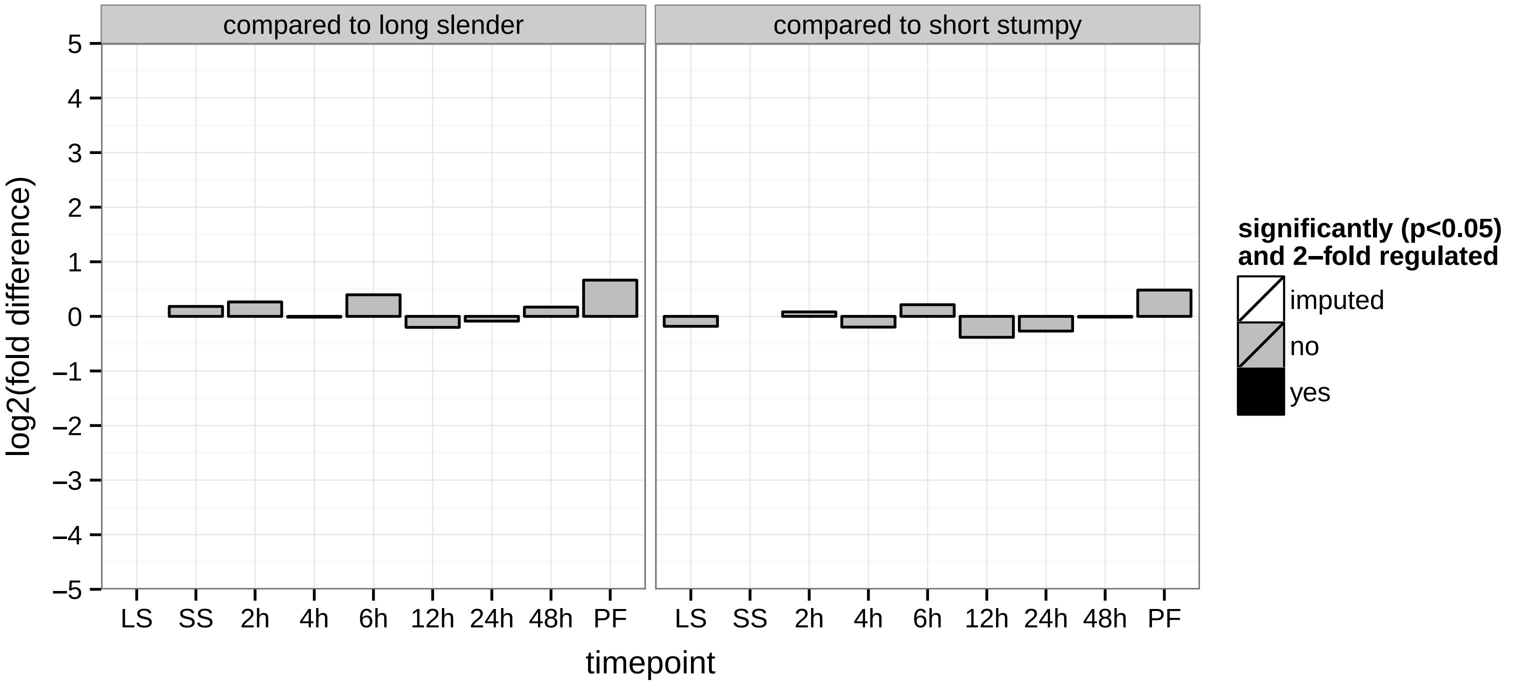

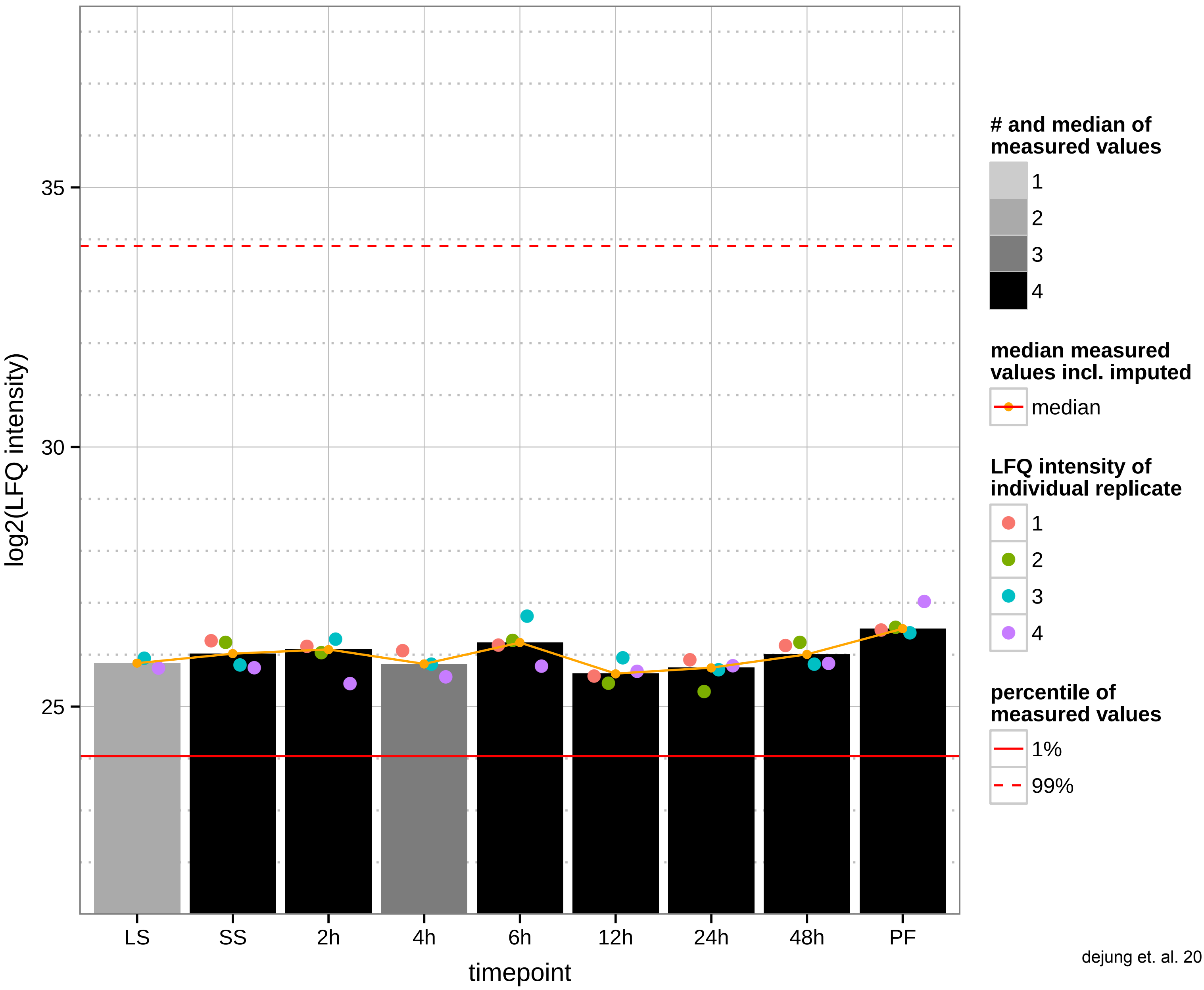

dejung et. al. 2015

proteasome regulatory non–ATPase subunit 11, Metallo–peptidase, Clan MP, Family M67, 19S proteasome regulatory subuni
Tb927.10.3030;Tb927.10.2980
AGOF: null, endopeptidase activity
AGOC: proteasome regulatory particle
AGOP: cell growth, ubiquitin–dependent protein catabolic process
PGOF: protein binding
PGOC: null
PGOP: null

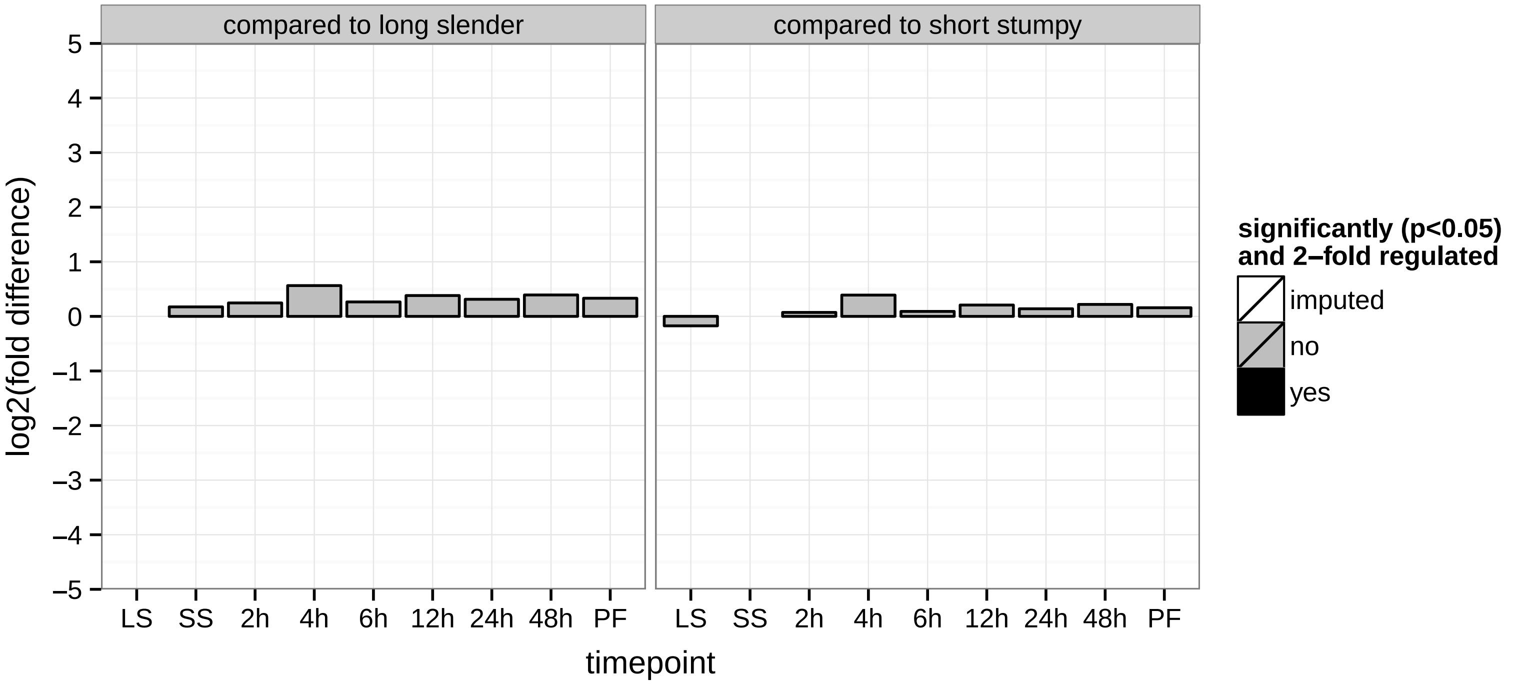

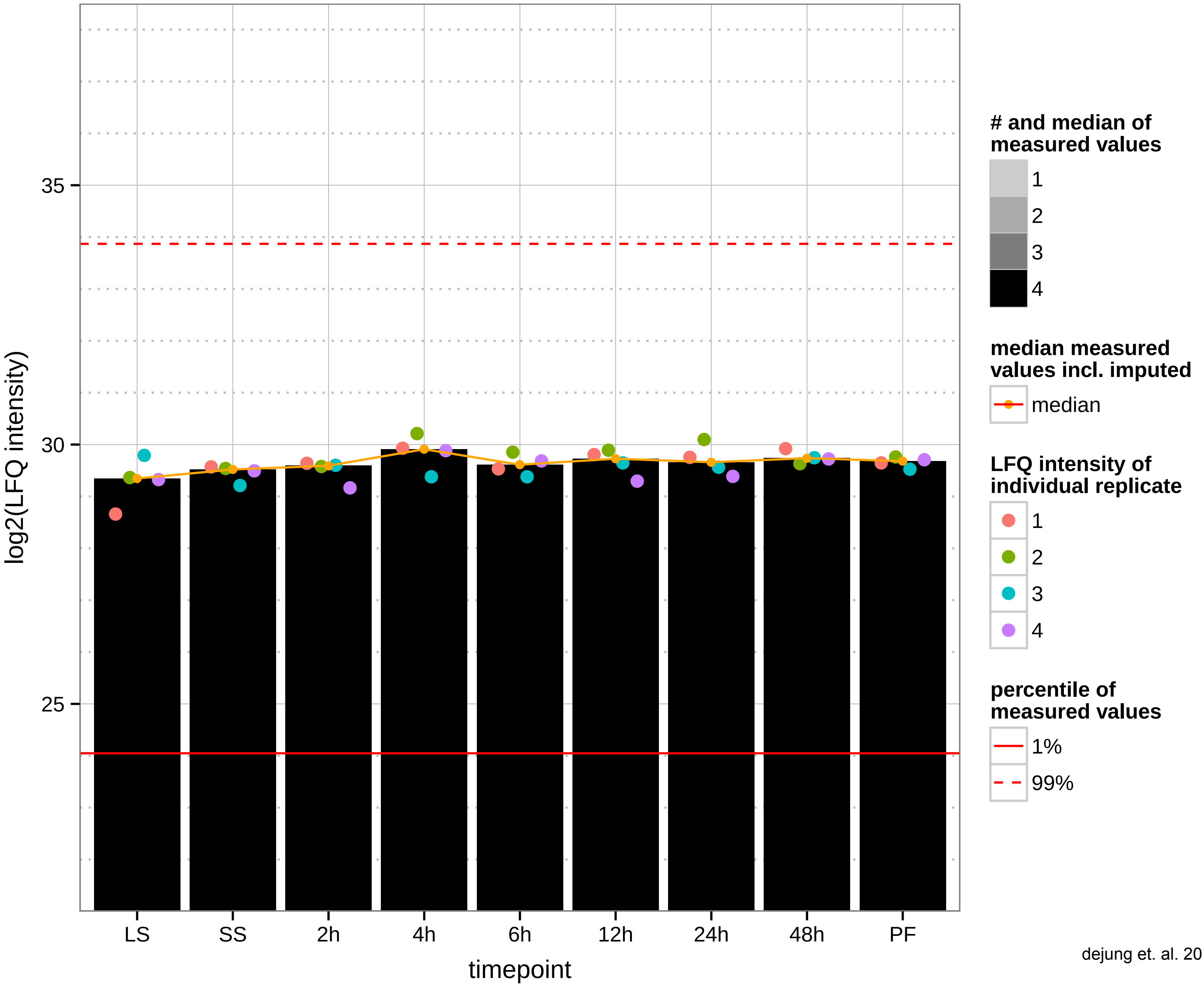

dejung et. al. 2015

hypothetical protein, conserved
Tb927.10.3010
AGOF: null
AGOC: null
AGOP: null
PGOF: null
PGOC: null
PGOP: null

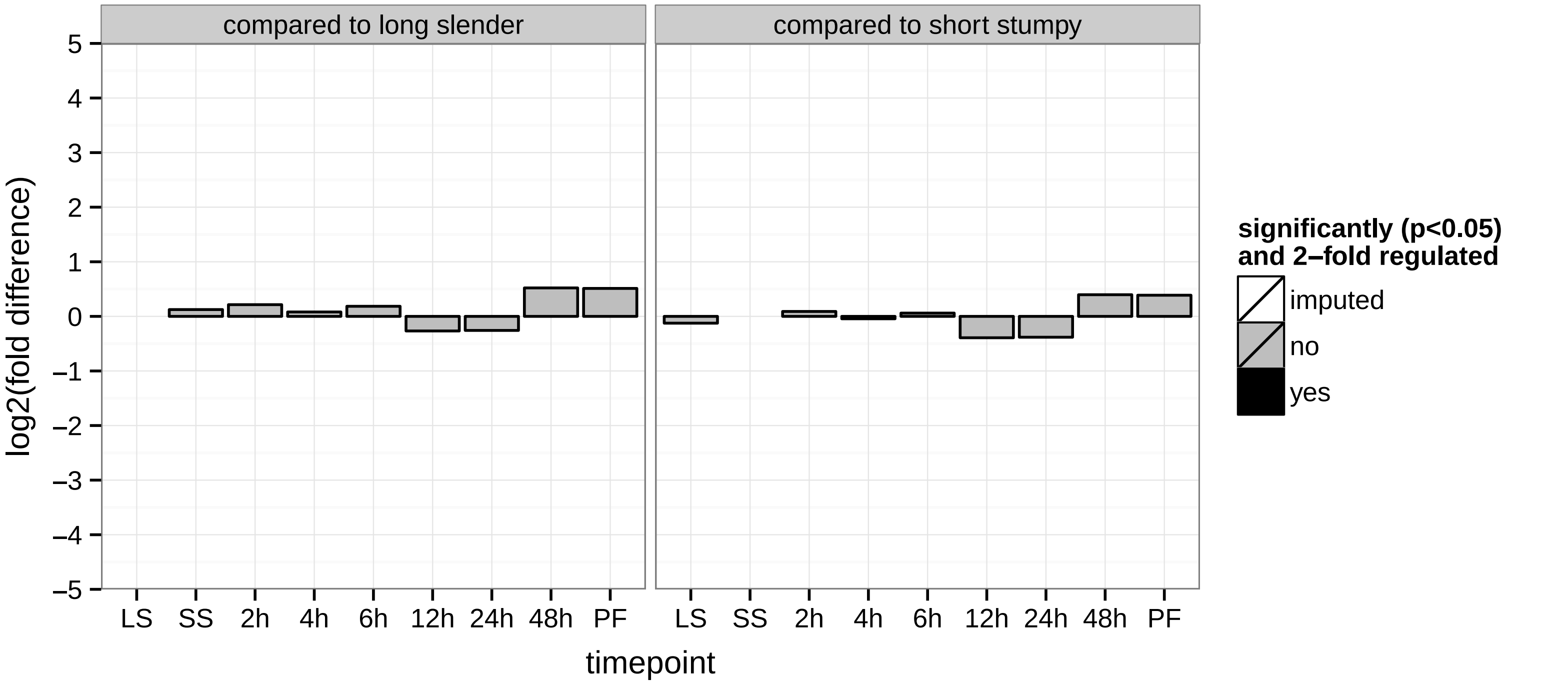

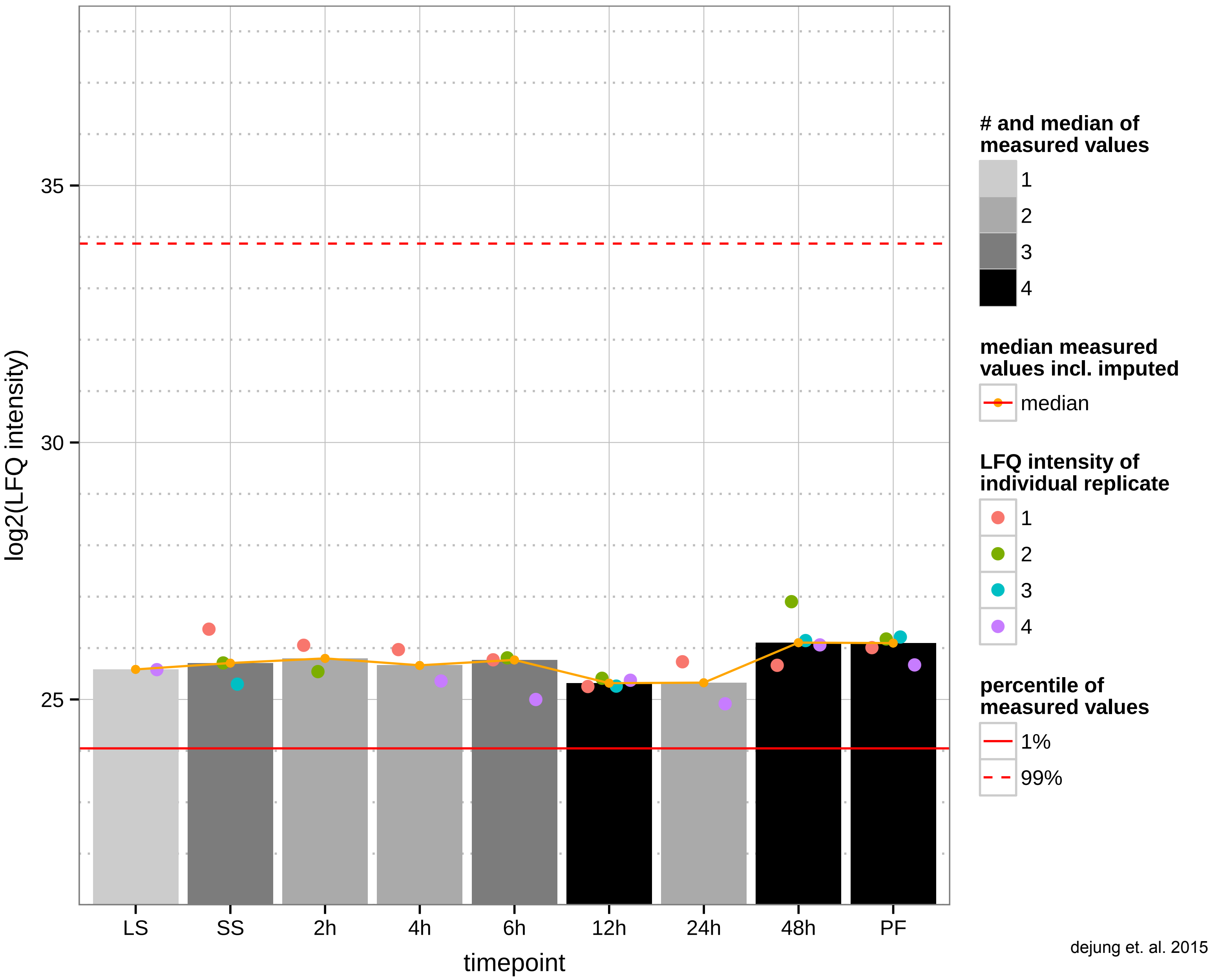

dejung et. al. 2015

hypothetical protein, conserved
Tb927.10.3070
AGOF: null
AGOC: null
AGOP: null
PGOF: null
PGOC: null
PGOP: null

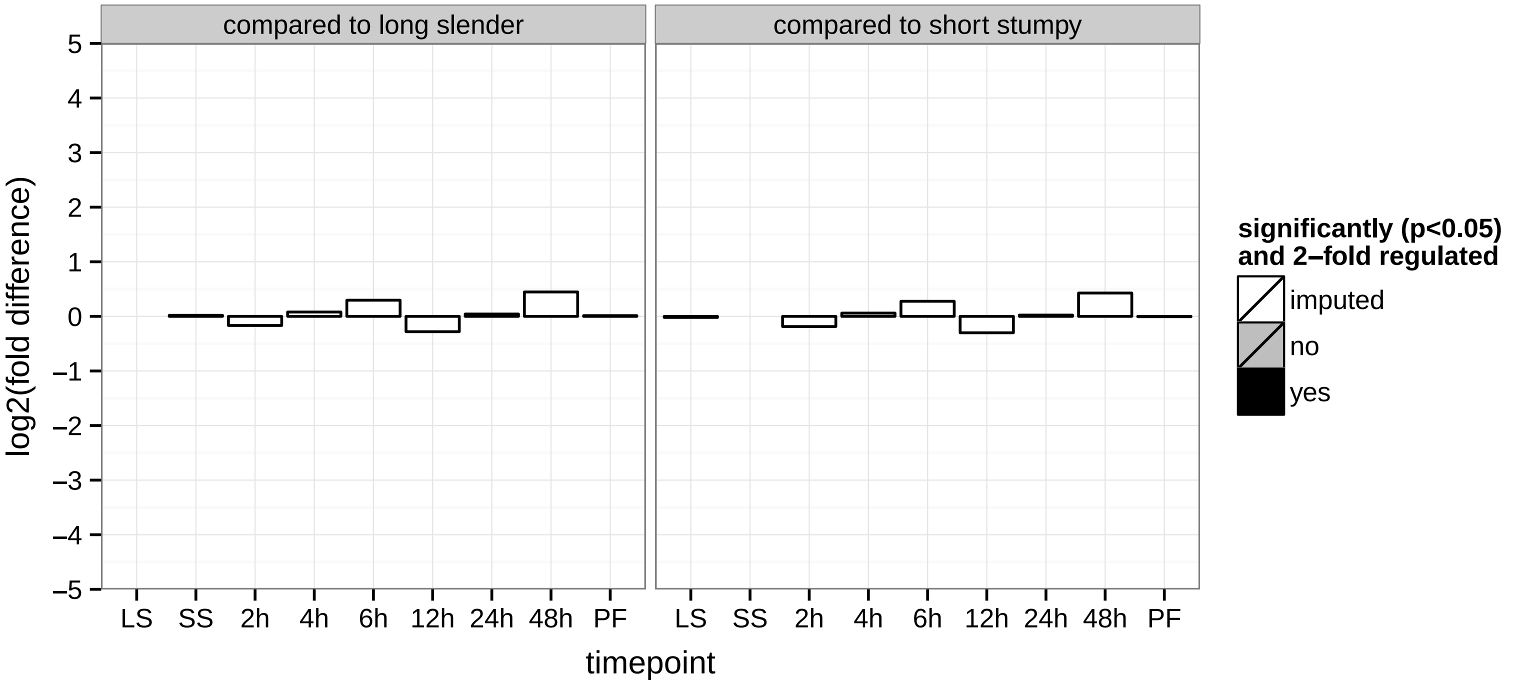

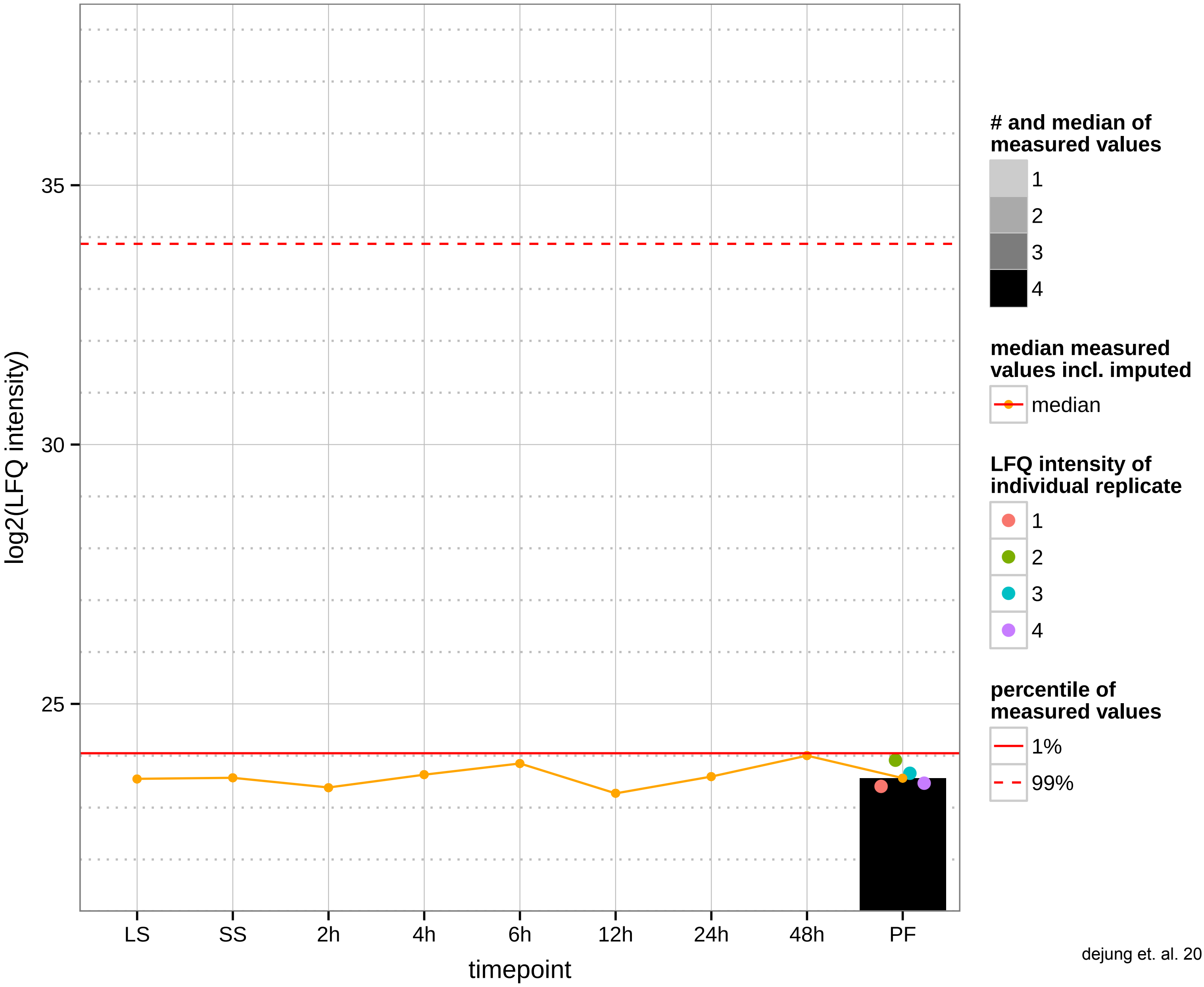

dejung et. al. 2015

methionine biosynthetic protein, putative
Tb927.10.3080
AGOF: null
AGOC: mitochondrial inner membrane
AGOP: null
PGOF: transferase activity
PGOC: null
PGOP: null

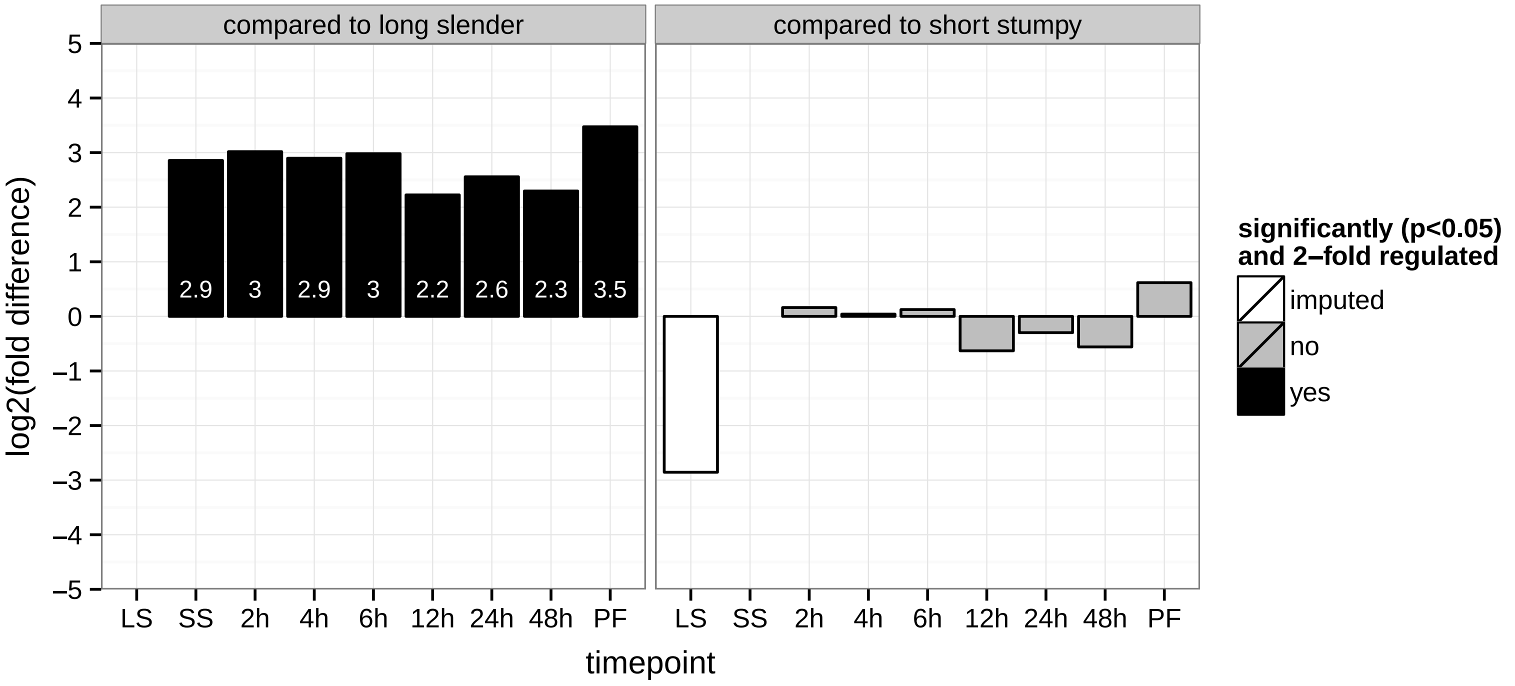

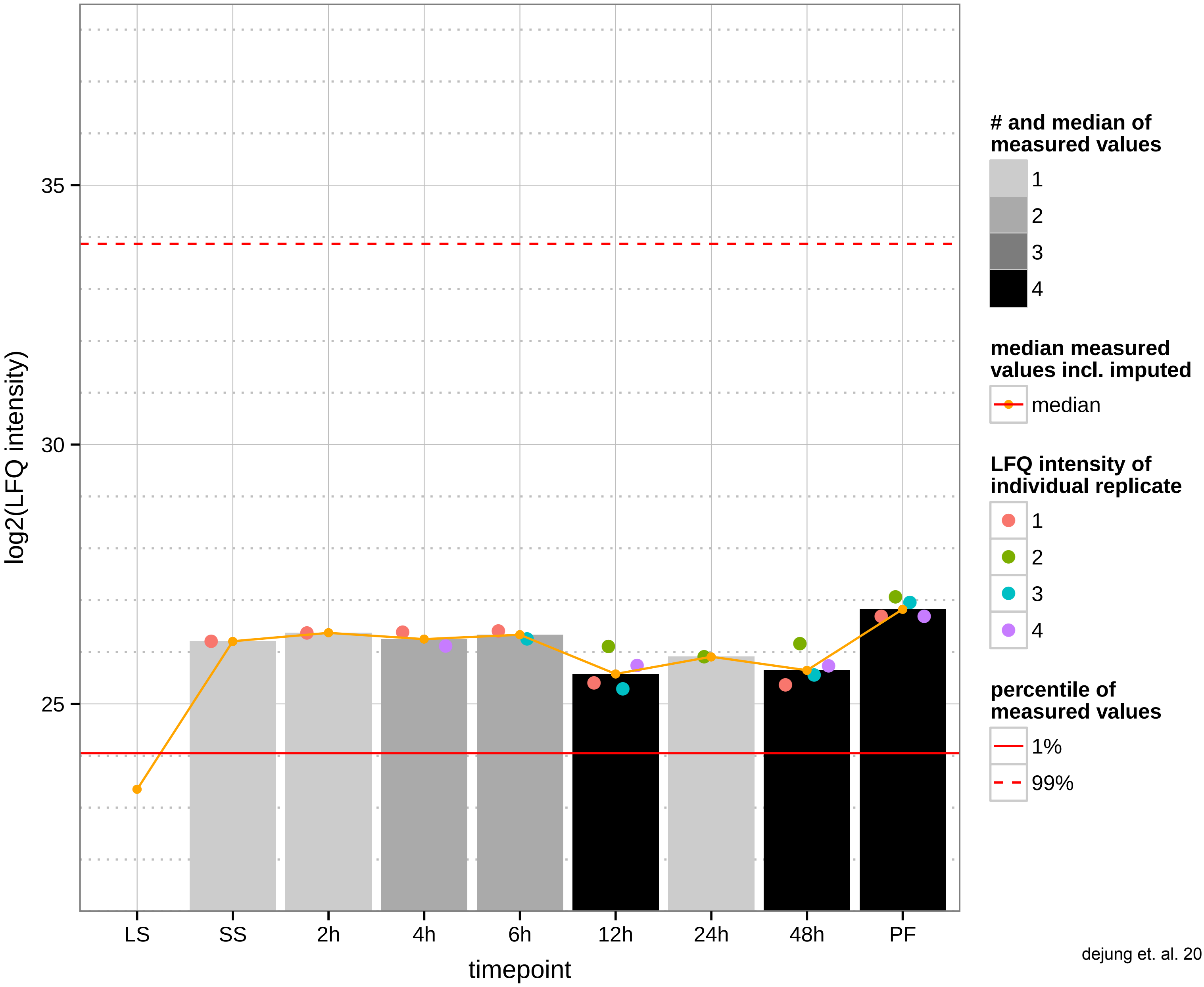

dejung et. al. 2015

hypothetical protein, conserved
Tb927.10.3090
AGOF: null
AGOC: null
AGOP: null
PGOF: null
PGOC: null
PGOP: null

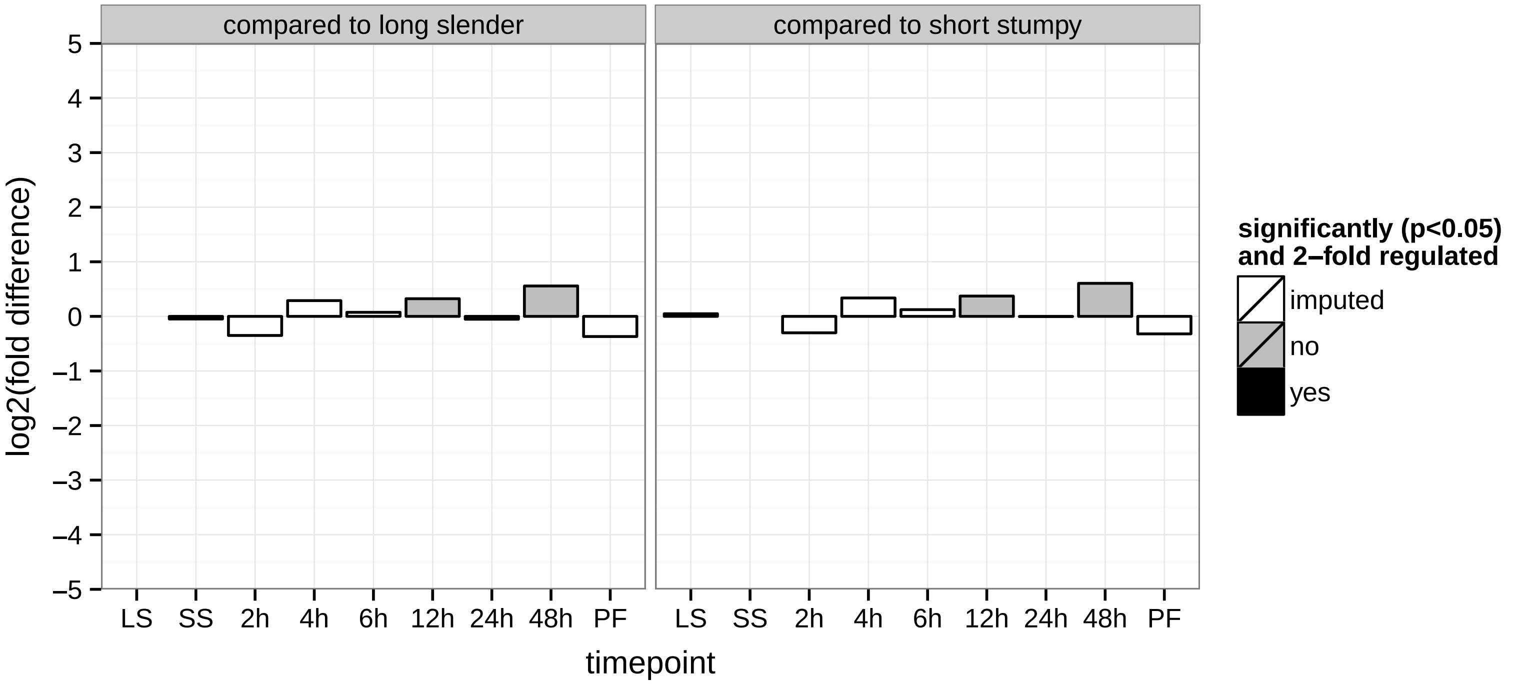

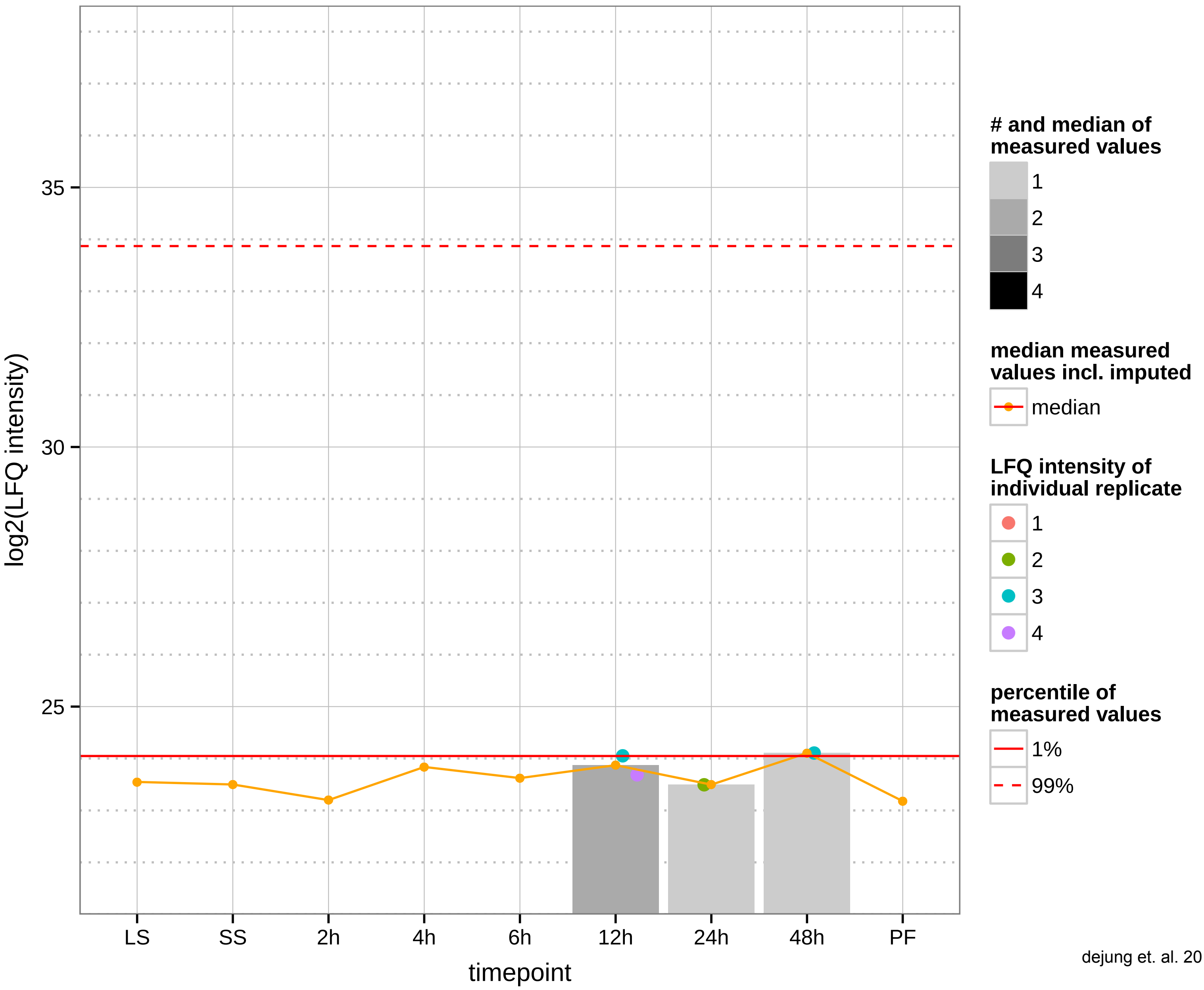

dejung et. al. 2015

N–acetyltransferase, putative
Tb927.10.3150
AGOF: N–acetyltransferase activity
AGOC: null
AGOP: metabolic process
PGOF: N–acetyltransferase activity
PGOC: null
PGOP: null

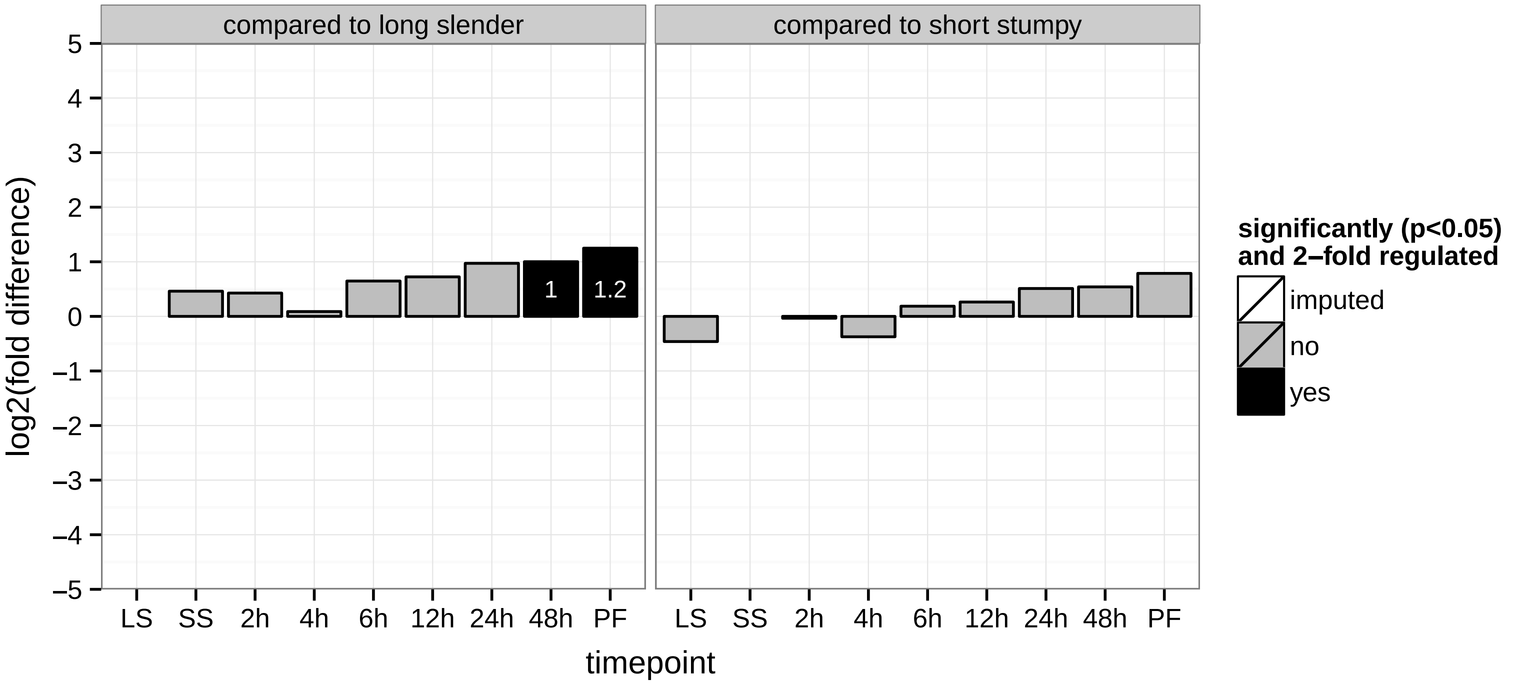

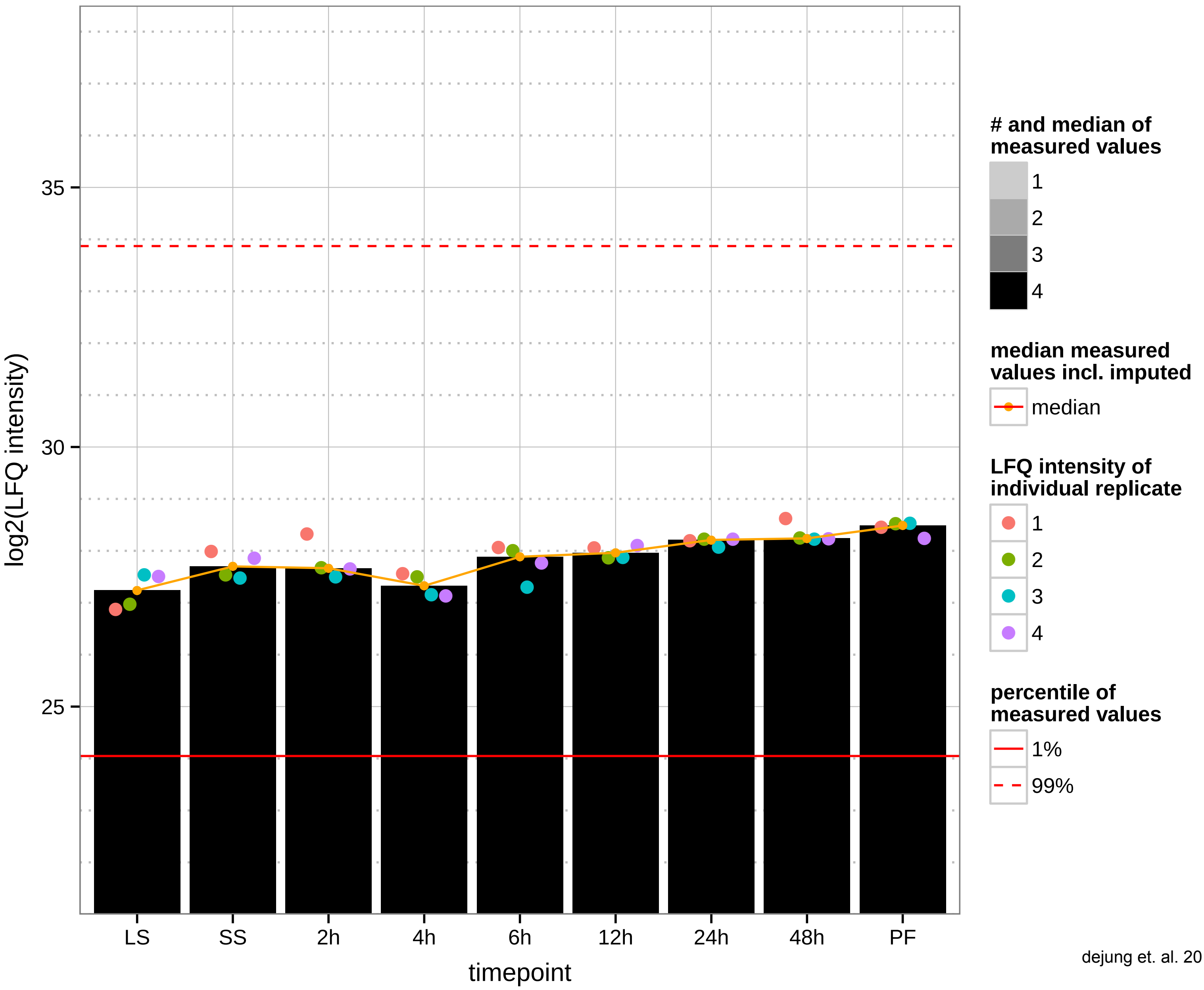

dejung et. al. 2015

U2 splicing auxiliary factor, putative (U2AF35)
Tb927.10.3200
AGOF: RNA binding, zinc ion binding
AGOC: nuclear speck, nucleus
AGOP: RNA splicing, RNA splicing, via transesterification reactions, mRNA processing
PGOF: nucleic acid binding, zinc ion binding
PGOC: null
PGOP: null

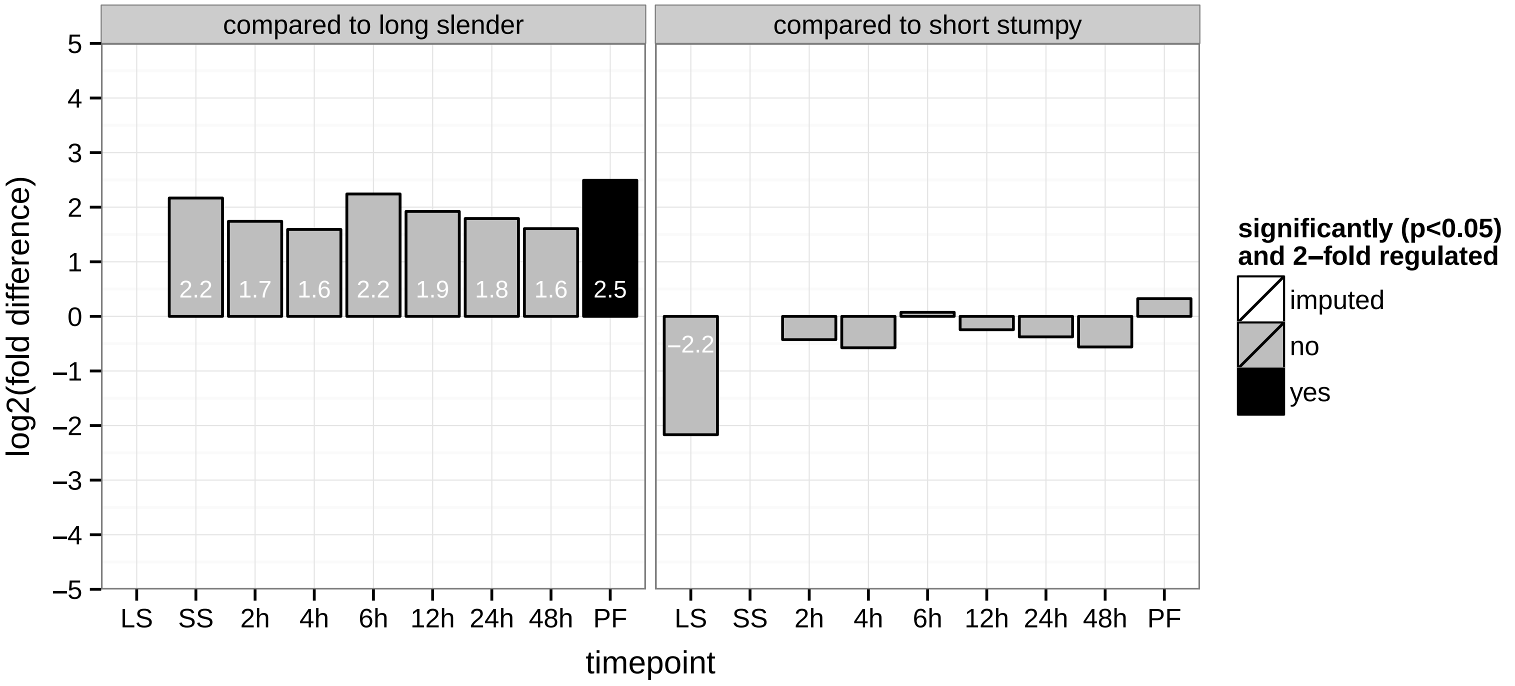

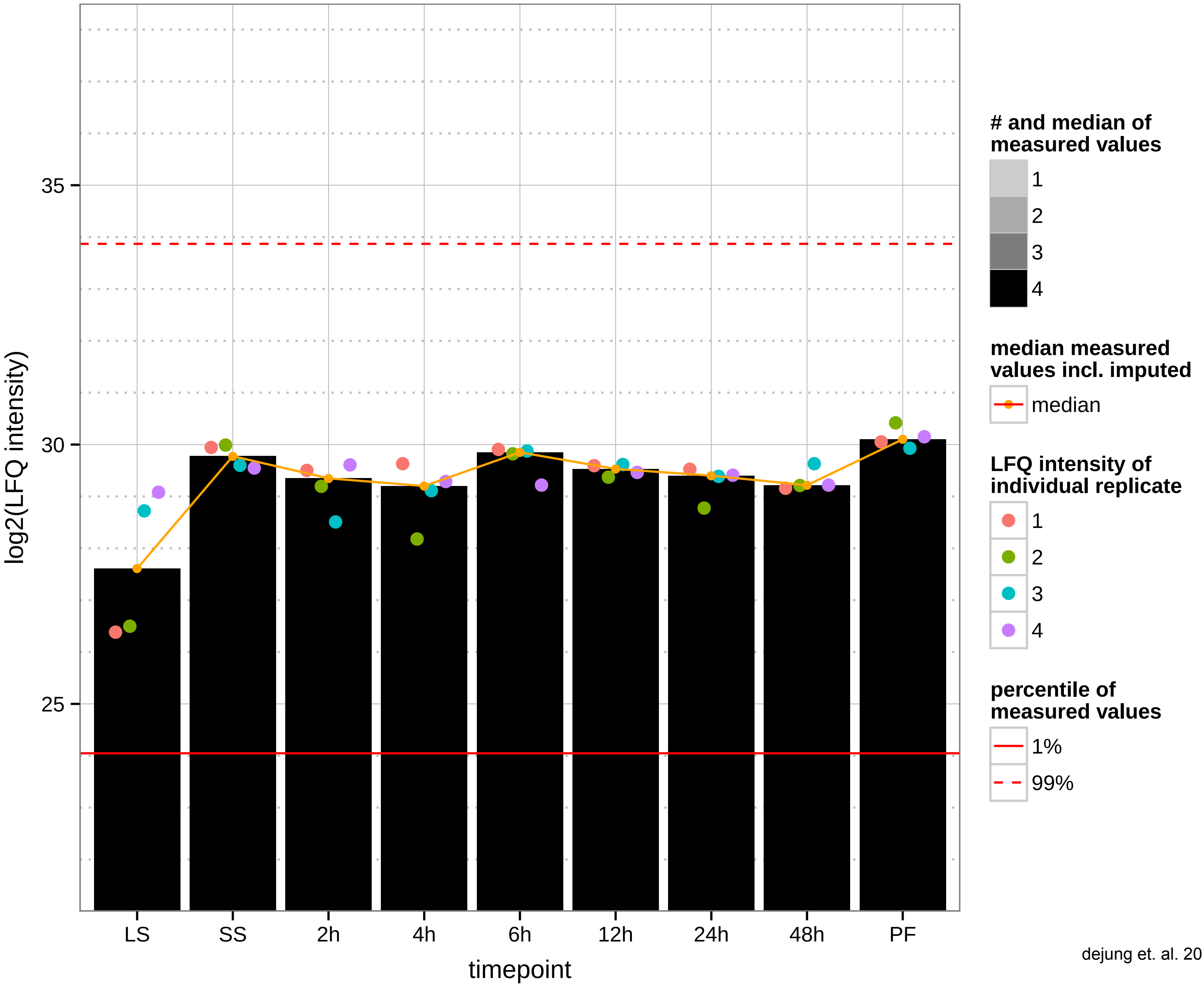

dejung et. al. 2015

delta–1–pyrroline–5–carboxylate dehydrogenase, putative
Tb927.10.3210
AGOF: 1–pyrroline–5–carboxylate dehydrogenase activity, 3–chloroallyl aldehyde dehydrogenase activity
AGOC: mitochondrial matrix, mitochondrion
AGOP: oxidation–reduction process, proline biosynthetic process, proline catabolic process
PGOF: 1–pyrroline–5–carboxylate dehydrogenase activity, oxidoreductase activity
PGOC: mitochondrial matrix
PGOP: metabolic process, oxidation–reduction process, proline biosynthetic process

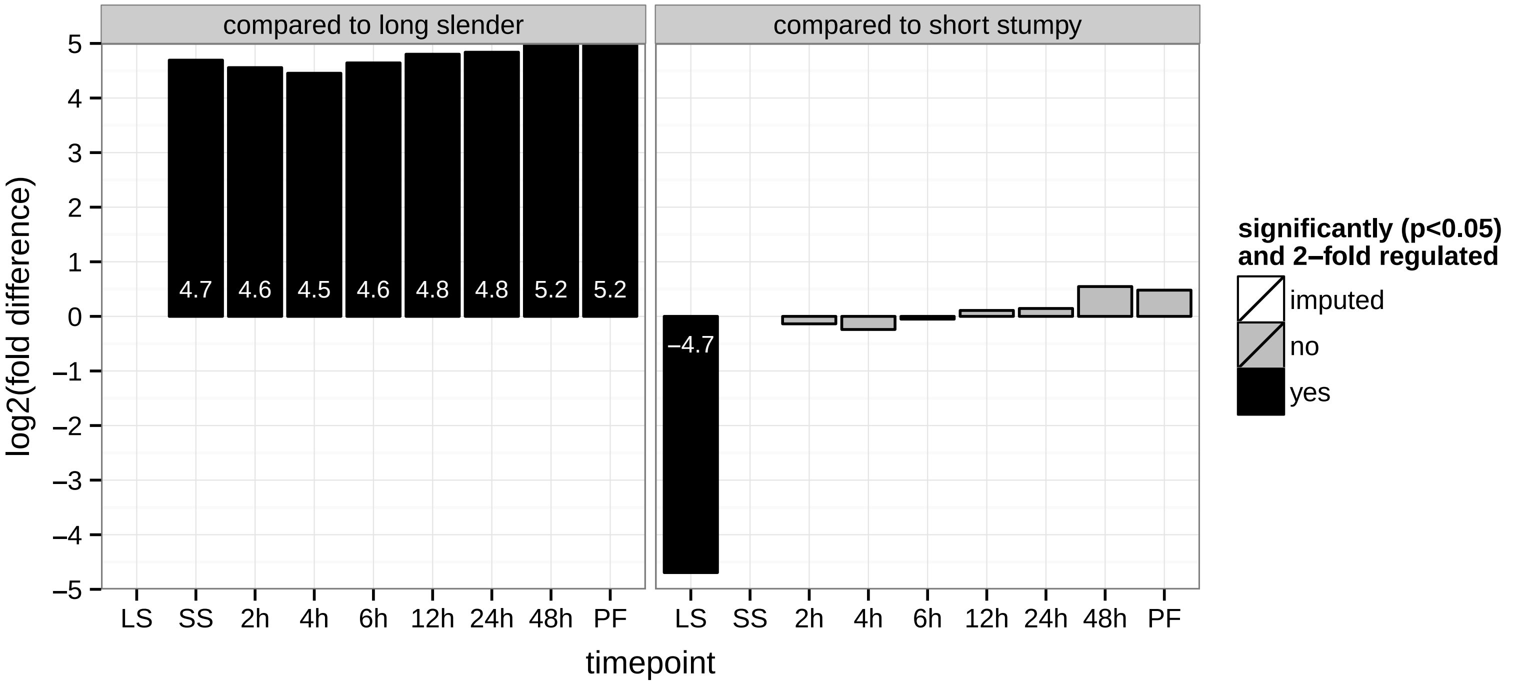

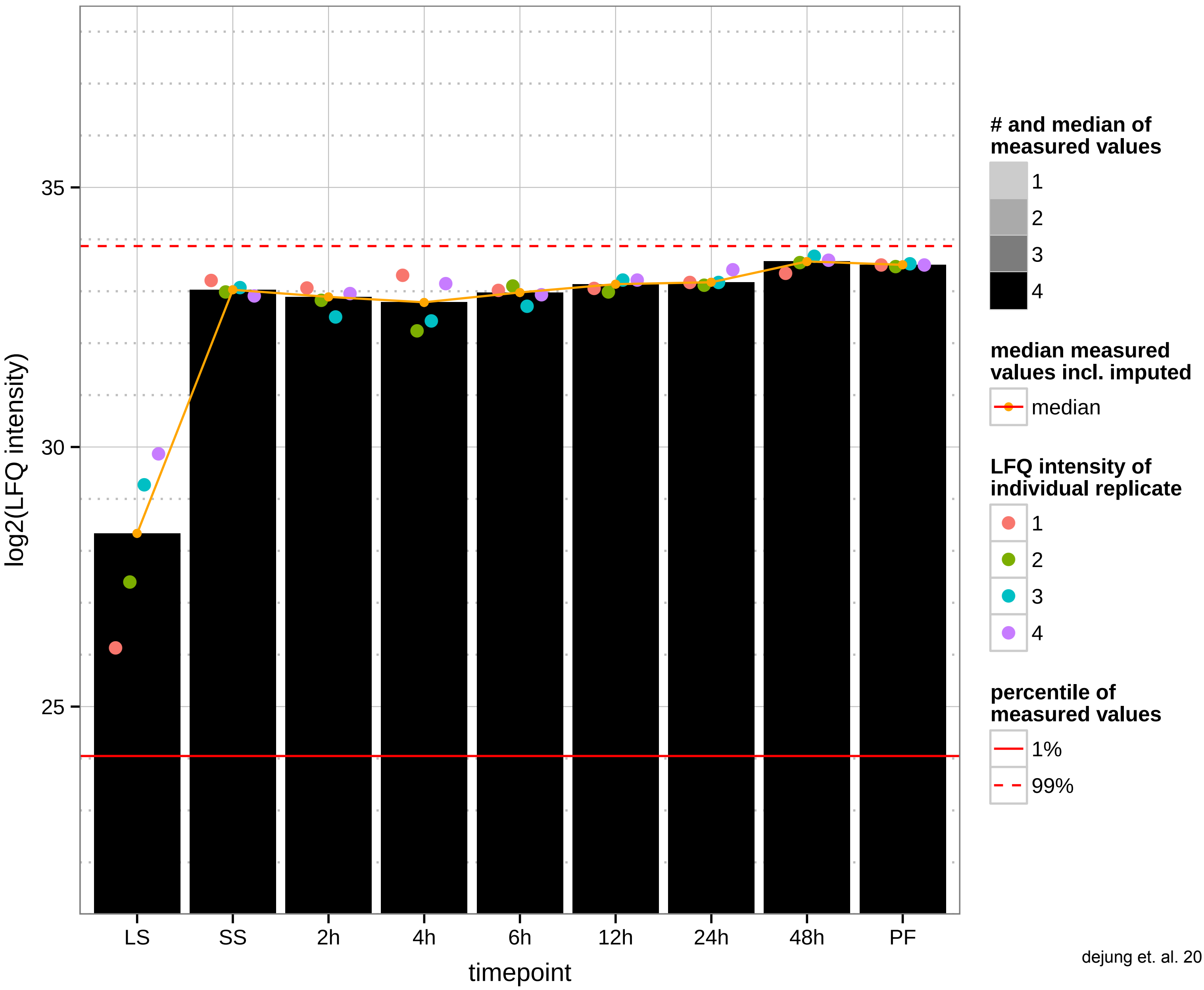

dejung et. al. 2015

hypothetical protein, conserved
Tb927.10.330
AGOF: null
AGOC: null
AGOP: null
PGOF: null
PGOC: null
PGOP: null

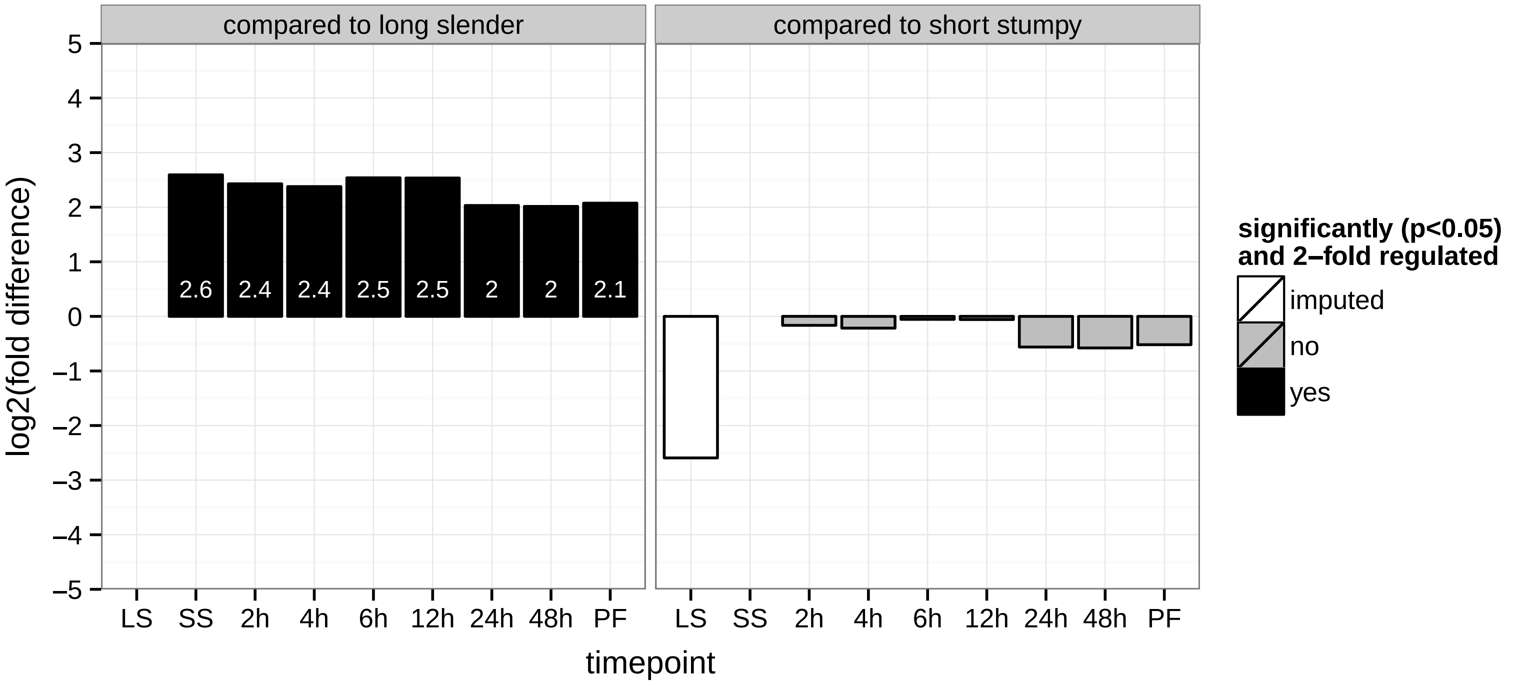

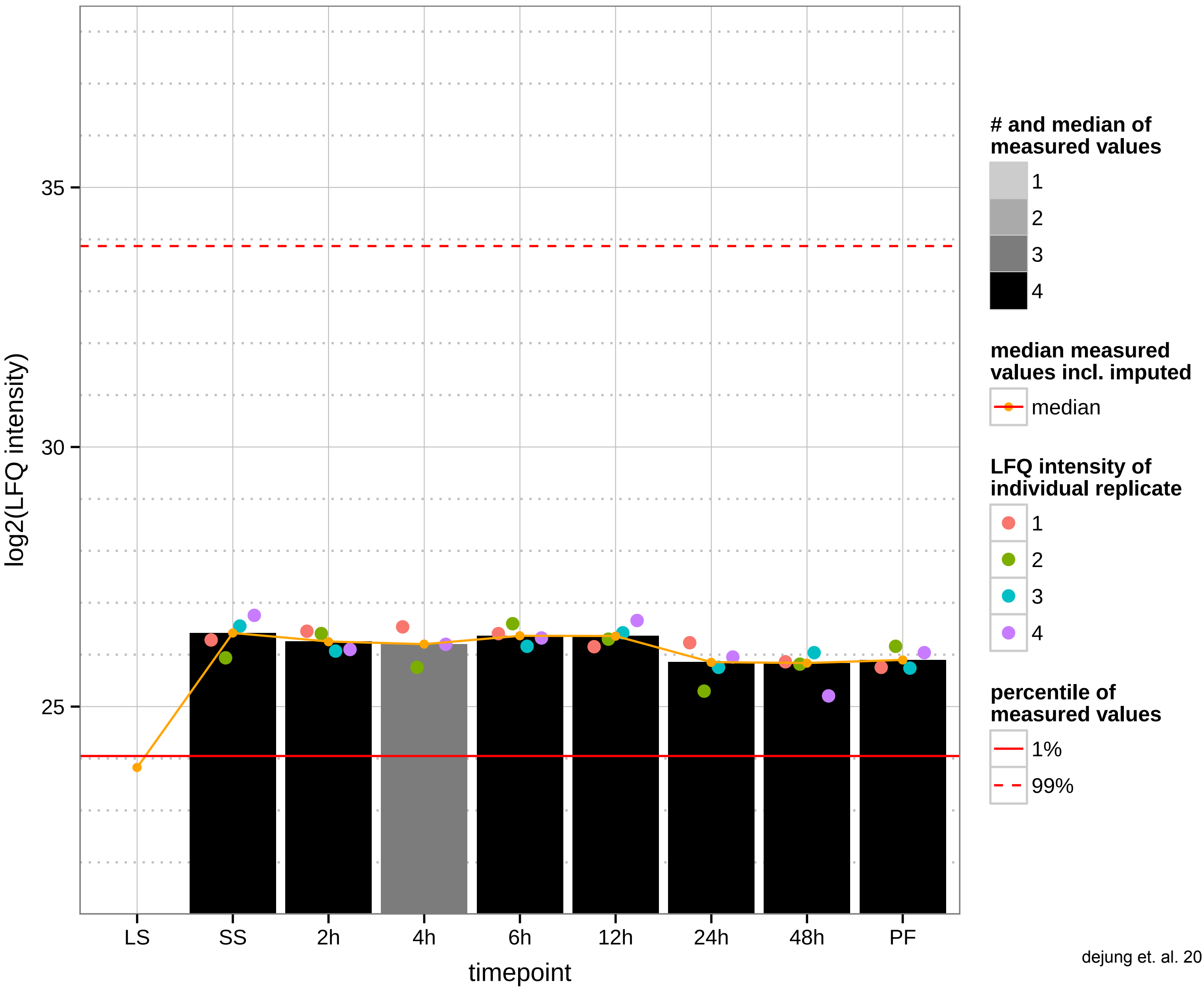

dejung et. al. 2015

hypothetical protein, conserved, predicted TLD domain protein
Tb927.10.3390;Tb11.v5.0724
AGOF: null
AGOC: null
AGOP: null
PGOF: null
PGOC: null
PGOP: null

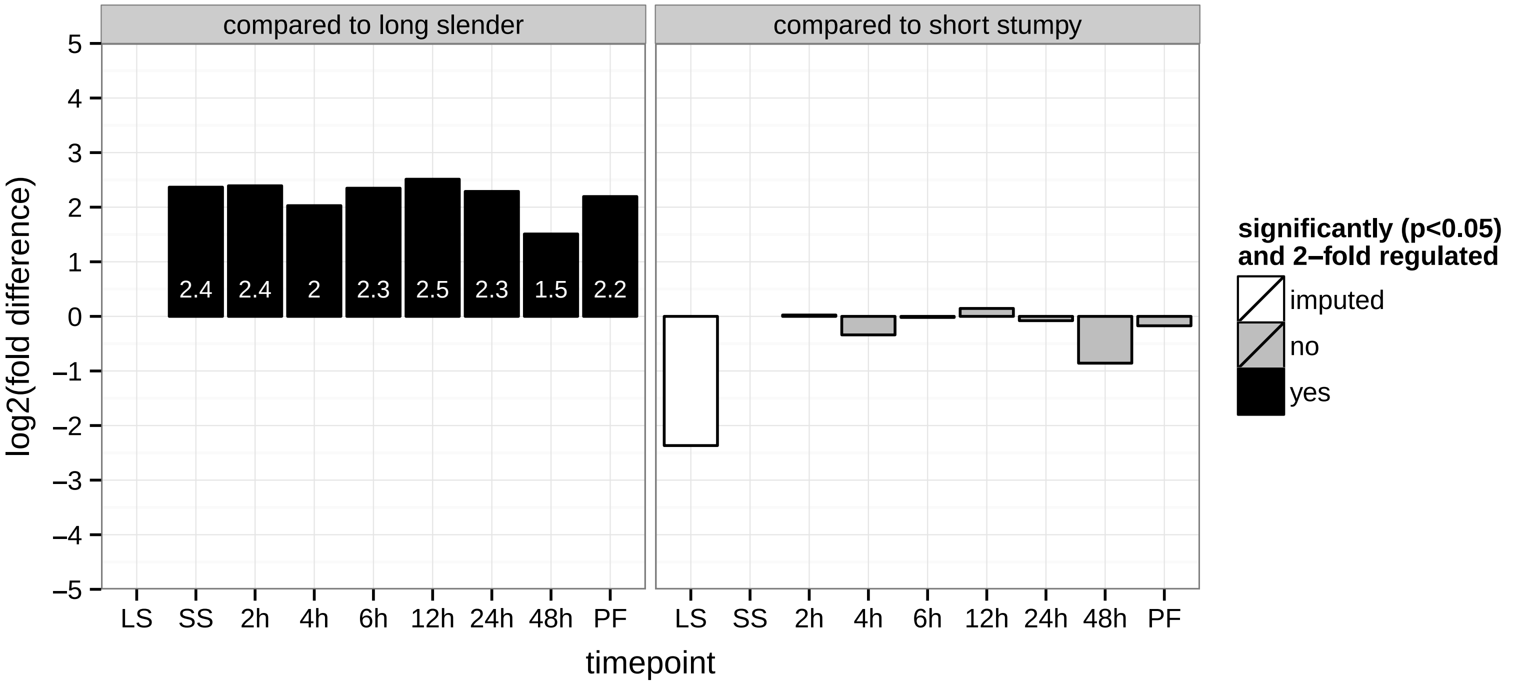

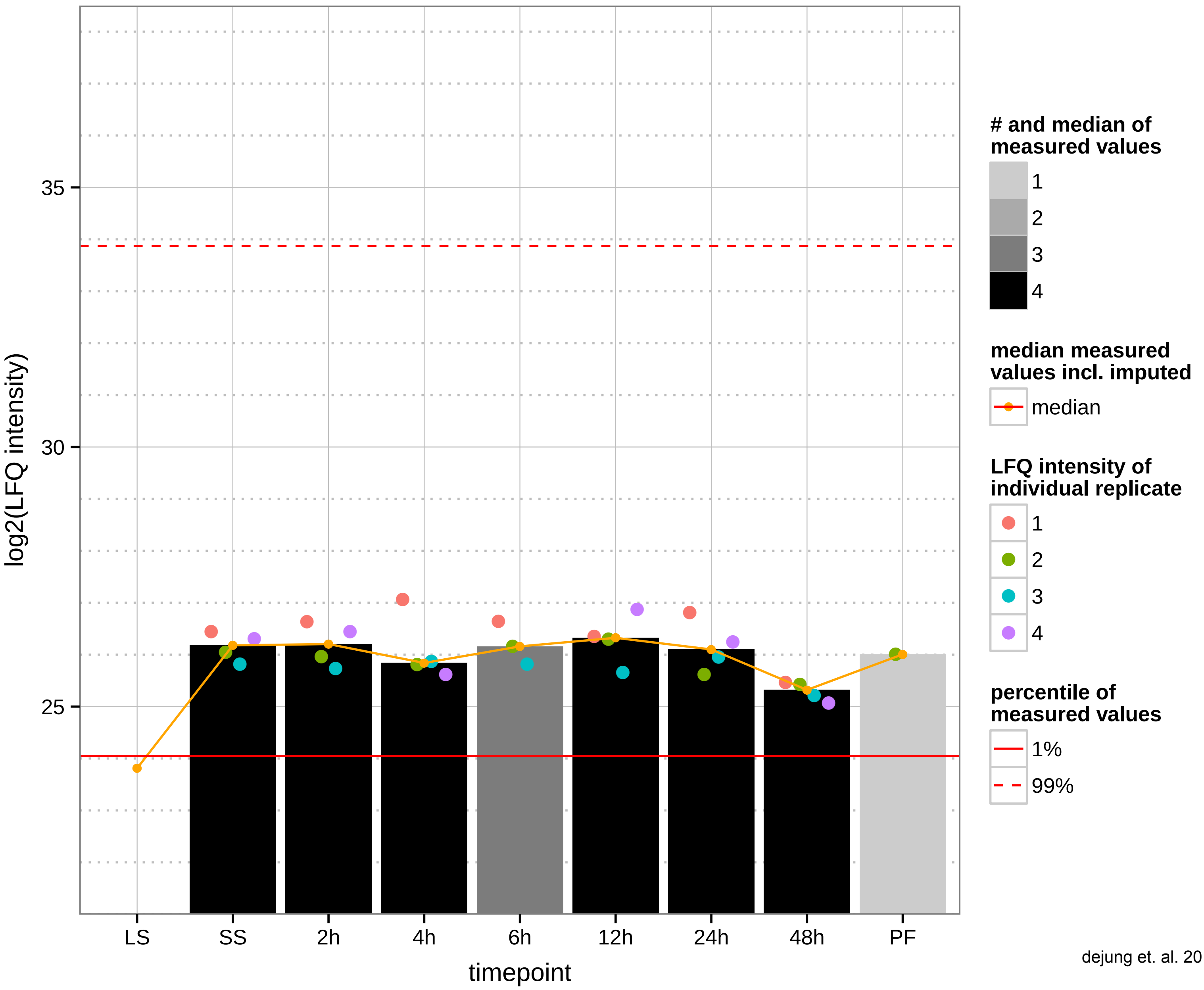

dejung et. al. 2015

RNA–binding protein, putative (RBSR4)
Tb927.10.3500
AGOF: RNA binding
AGOC: null
AGOP: RNA processing
PGOF: nucleic acid binding
PGOC: null
PGOP: null

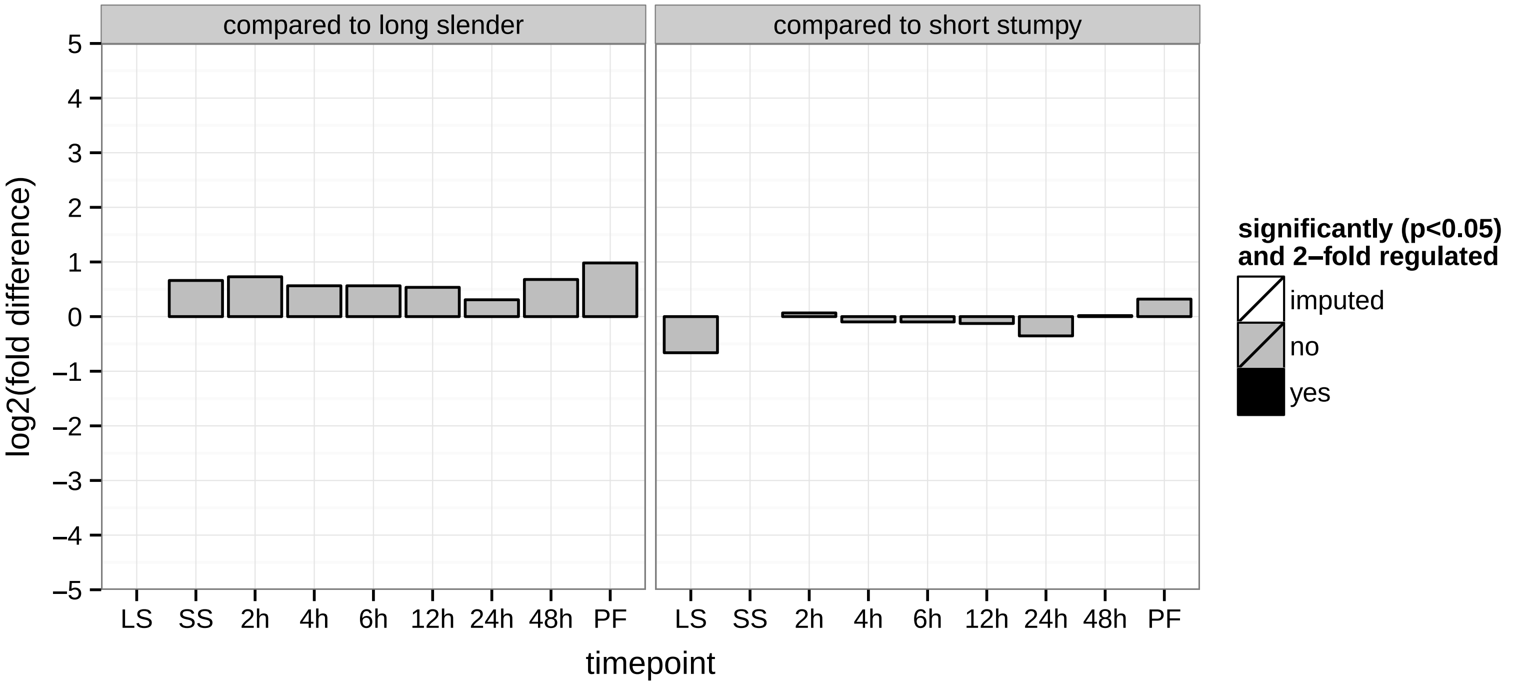

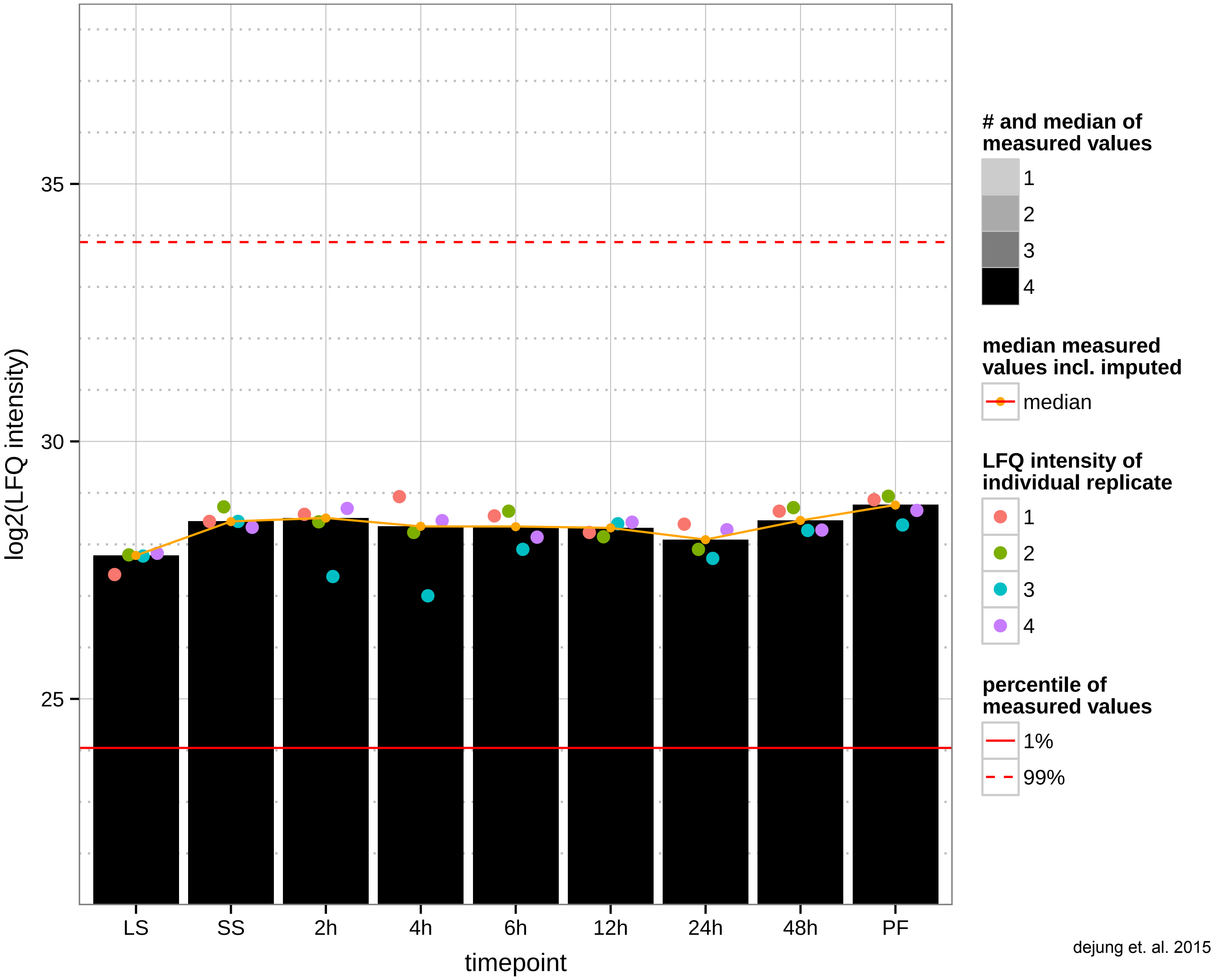

dejung et. al. 2015

protease regulatory ATPase subunit 4 (RPT4)
Tb927.10.3520
AGOF: ATP binding, ATPase activity
AGOC: cytoplasm, nucleus, proteasome regulatory particle
AGOP: ubiquitin–dependent protein catabolic process
PGOF: ATP binding, hydrolase activity, nucleoside–triphosphatase activity, nucleotide binding
PGOC: cytoplasm
PGOP: protein catabolic process

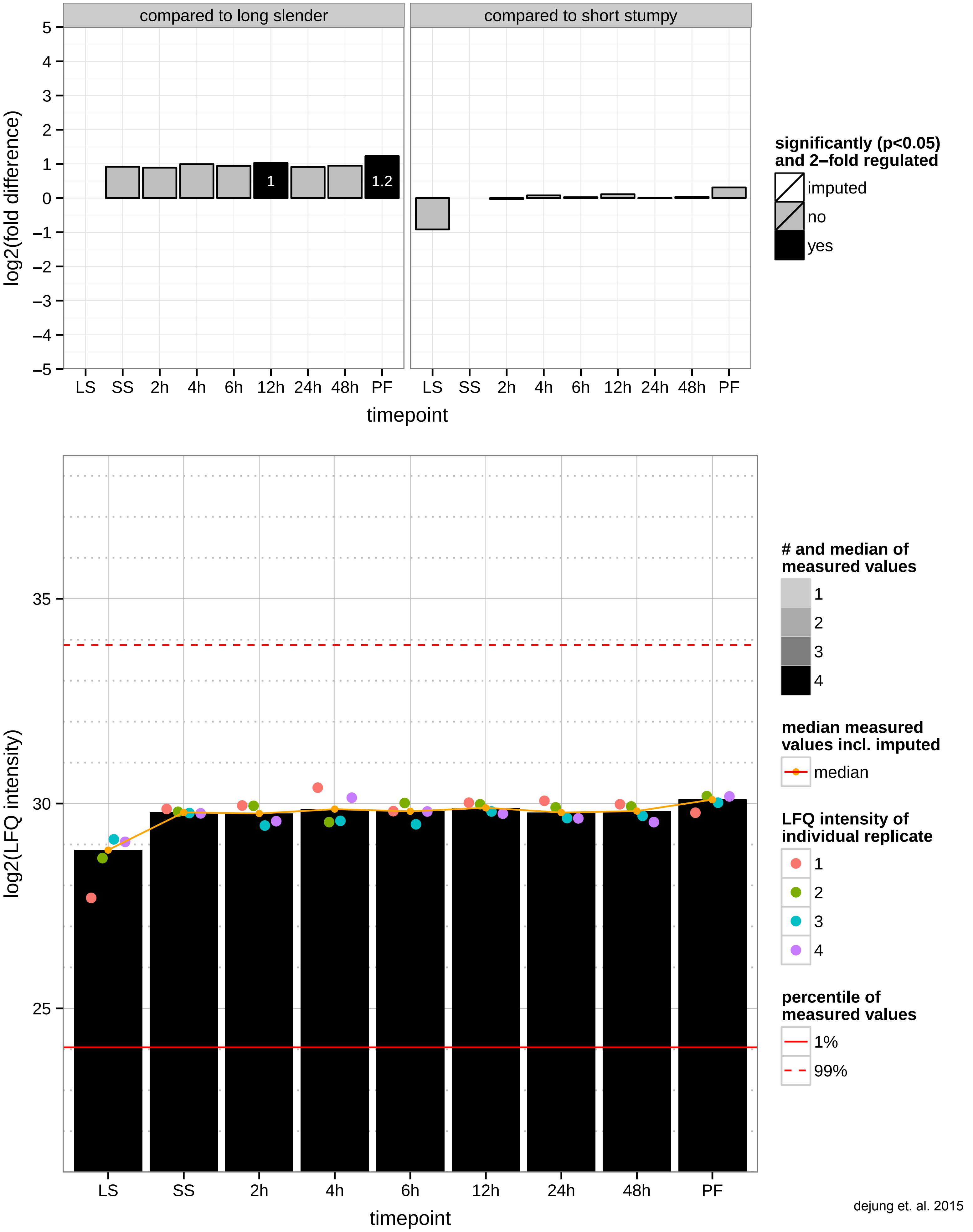

dejung et. al. 2015

hypothetical protein, conserved
Tb927.10.3540
AGOF: null
AGOC: null
AGOP: null
PGOF: null
PGOC: null
PGOP: null

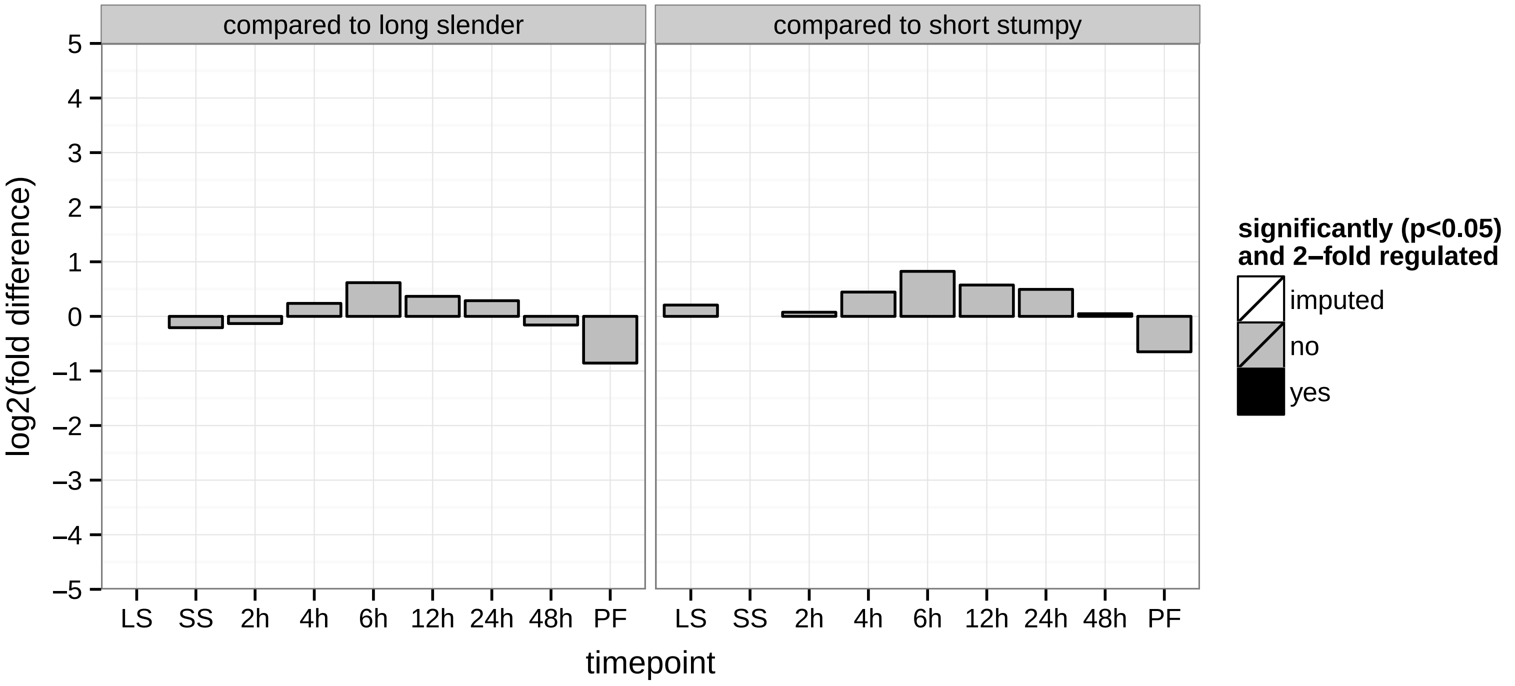

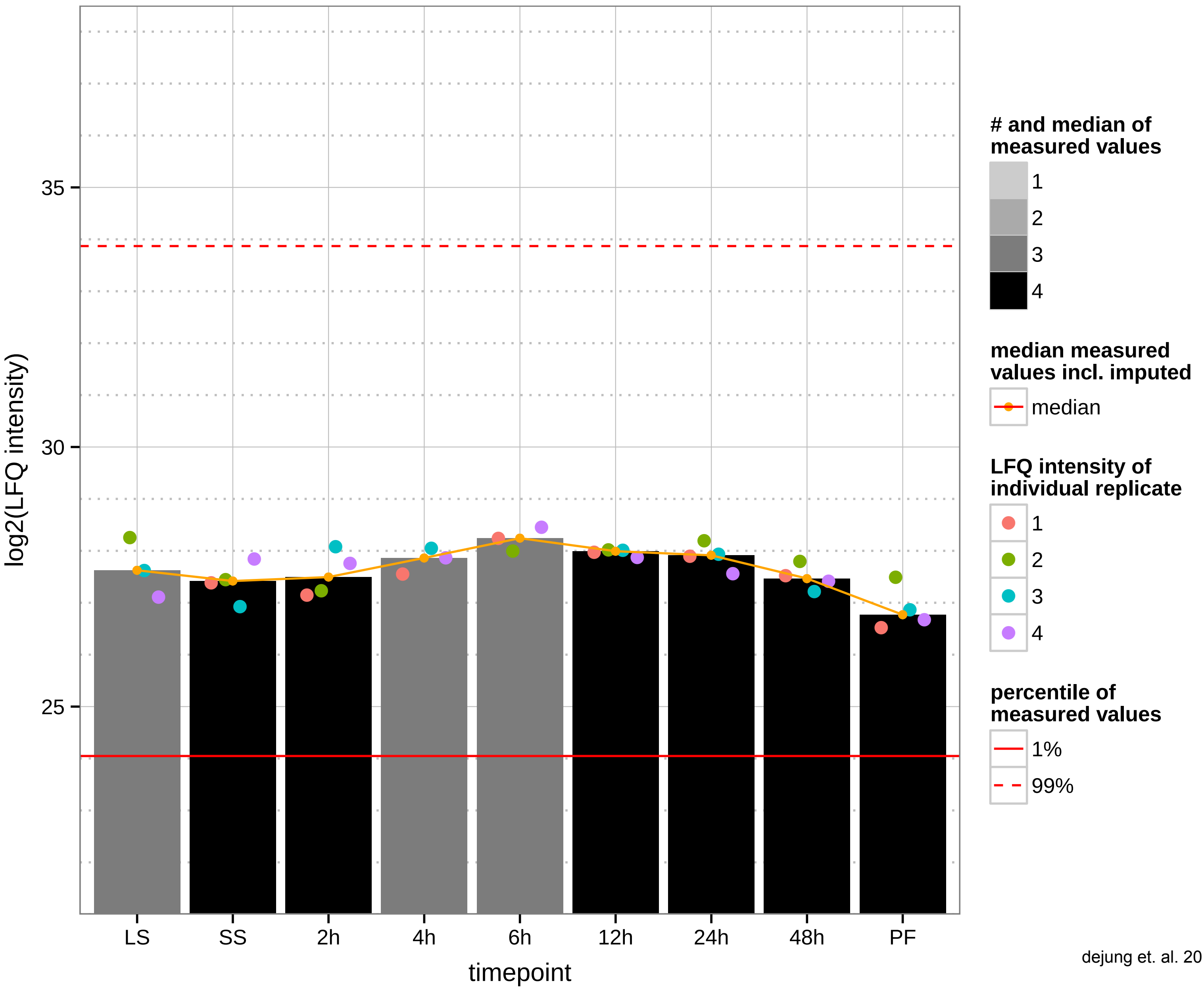

dejung et. al. 2015

arginine N–methyltransferase, putative
Tb927.10.3560
AGOF: histone–arginine N–methyltransferase activity
AGOC: histone methyltransferase complex
AGOP: histone methylation
PGOF: null
PGOC: null
PGOP: null

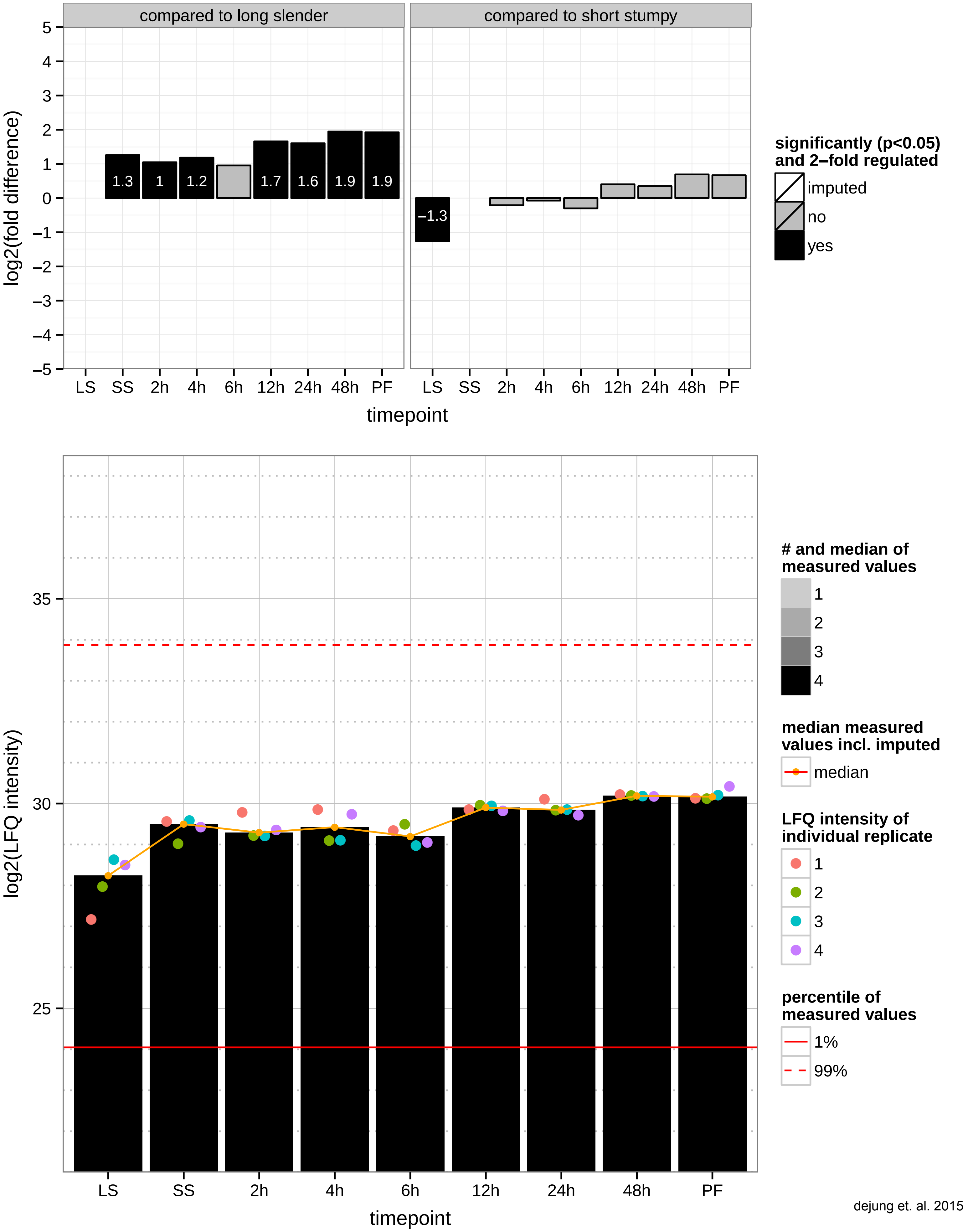

dejung et. al. 2015

RNA–editing complex protein, putative, RNA editing exonuclease 2, RNA editing 3' exouridylylase, RNA–editing complex prote
Tb927.10.3570
AGOF: null
AGOC: null, mitochondrial mRNA editing complex, mitochondrion
AGOP: null, RNA modification
PGOF: null
PGOC: null
PGOP: null

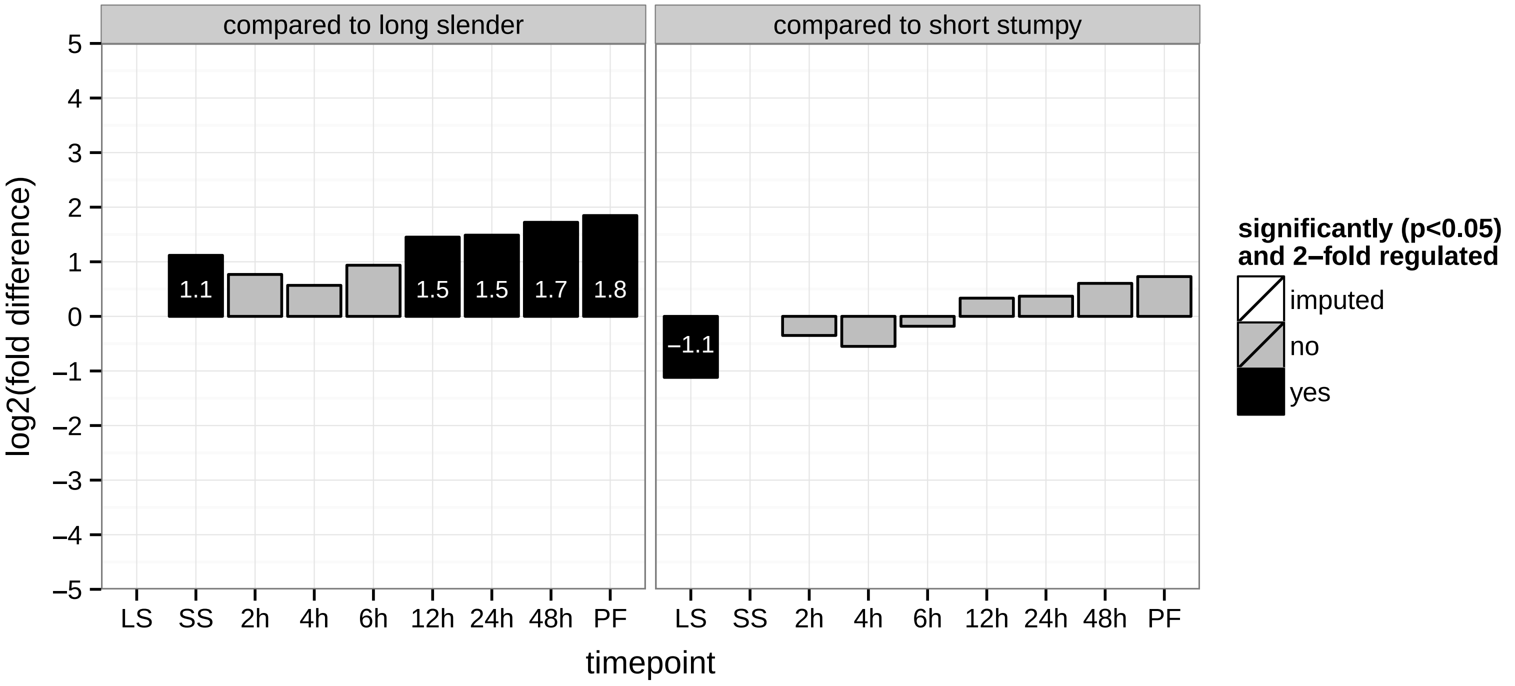

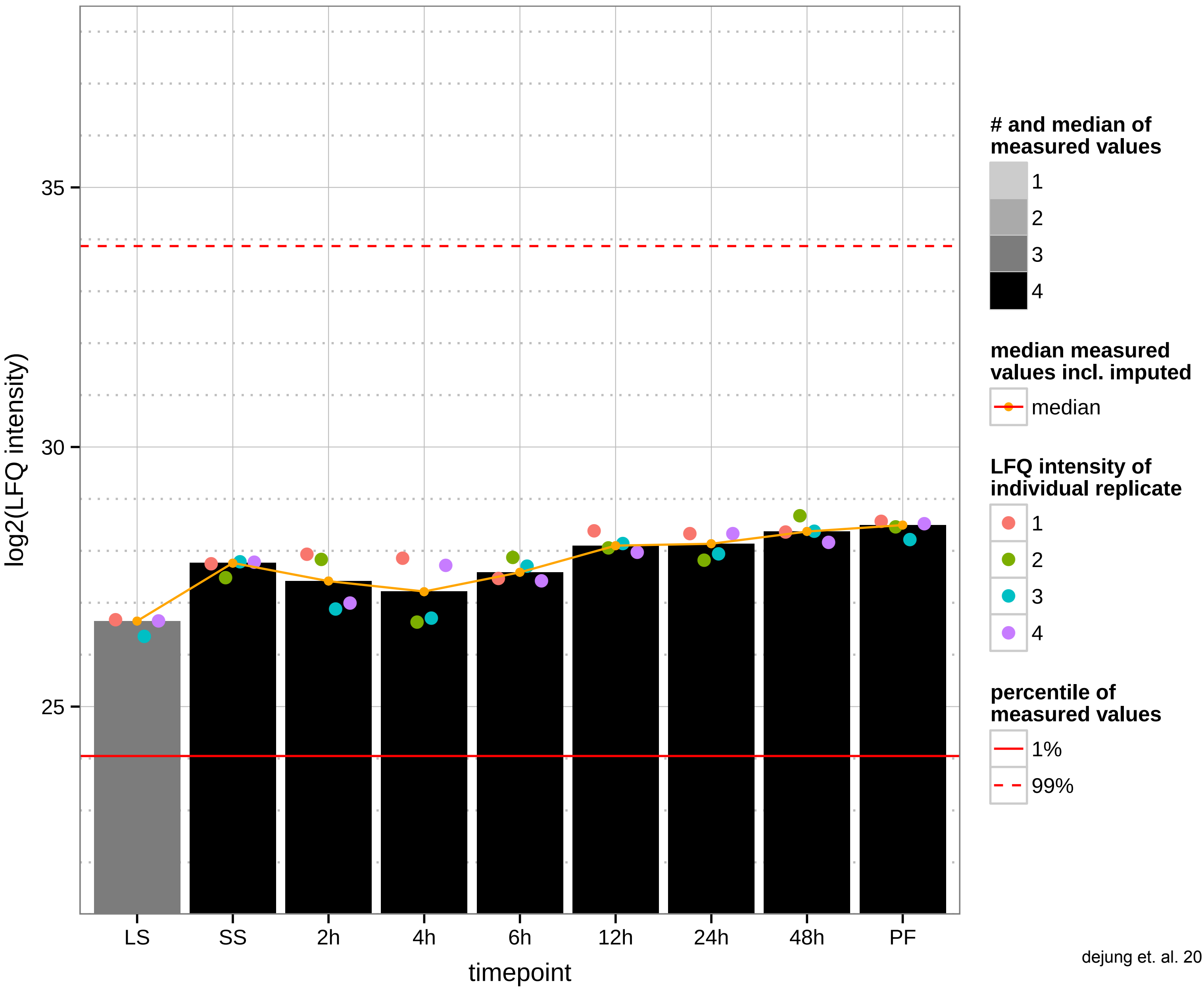

dejung et. al. 2015

hypothetical protein, conserved
Tb927.10.3590
AGOF: null
AGOC: mitochondrion
AGOP: null
PGOF: null
PGOC: null
PGOP: null

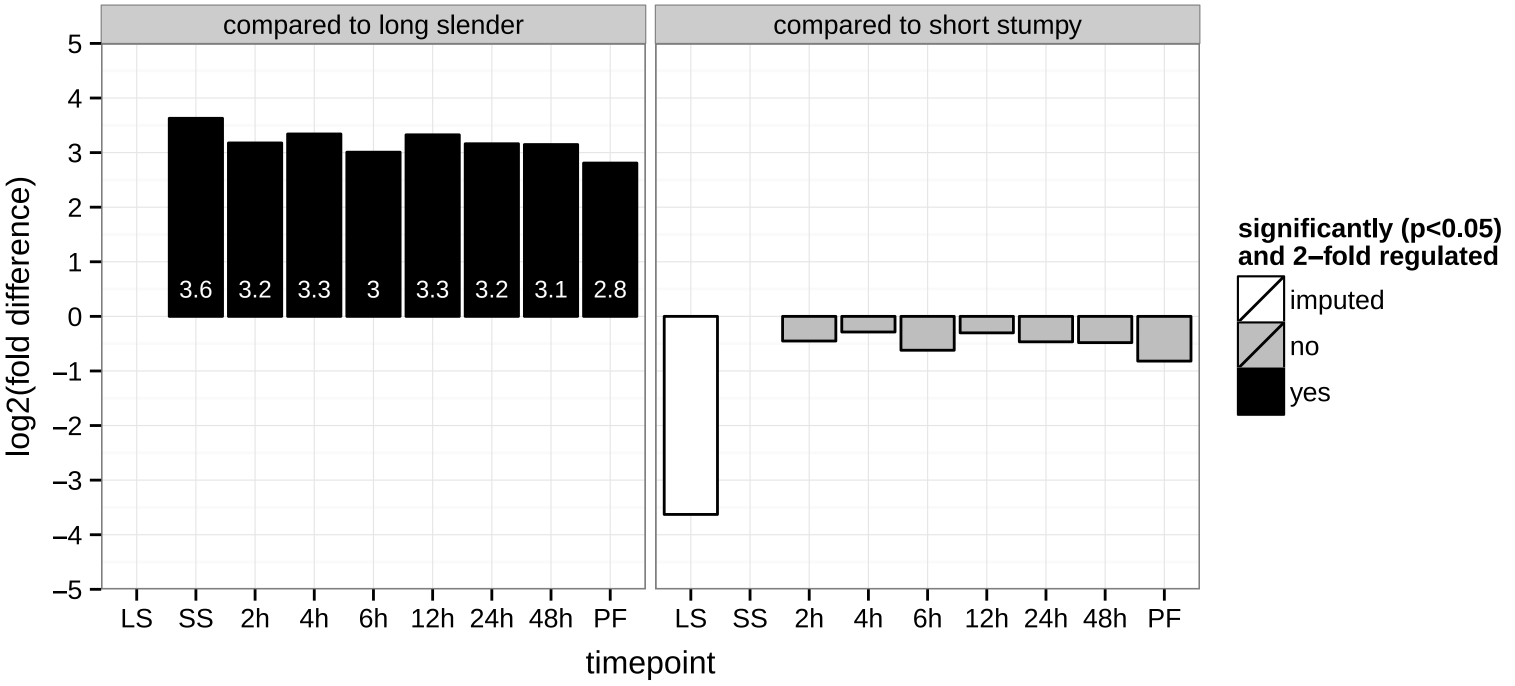

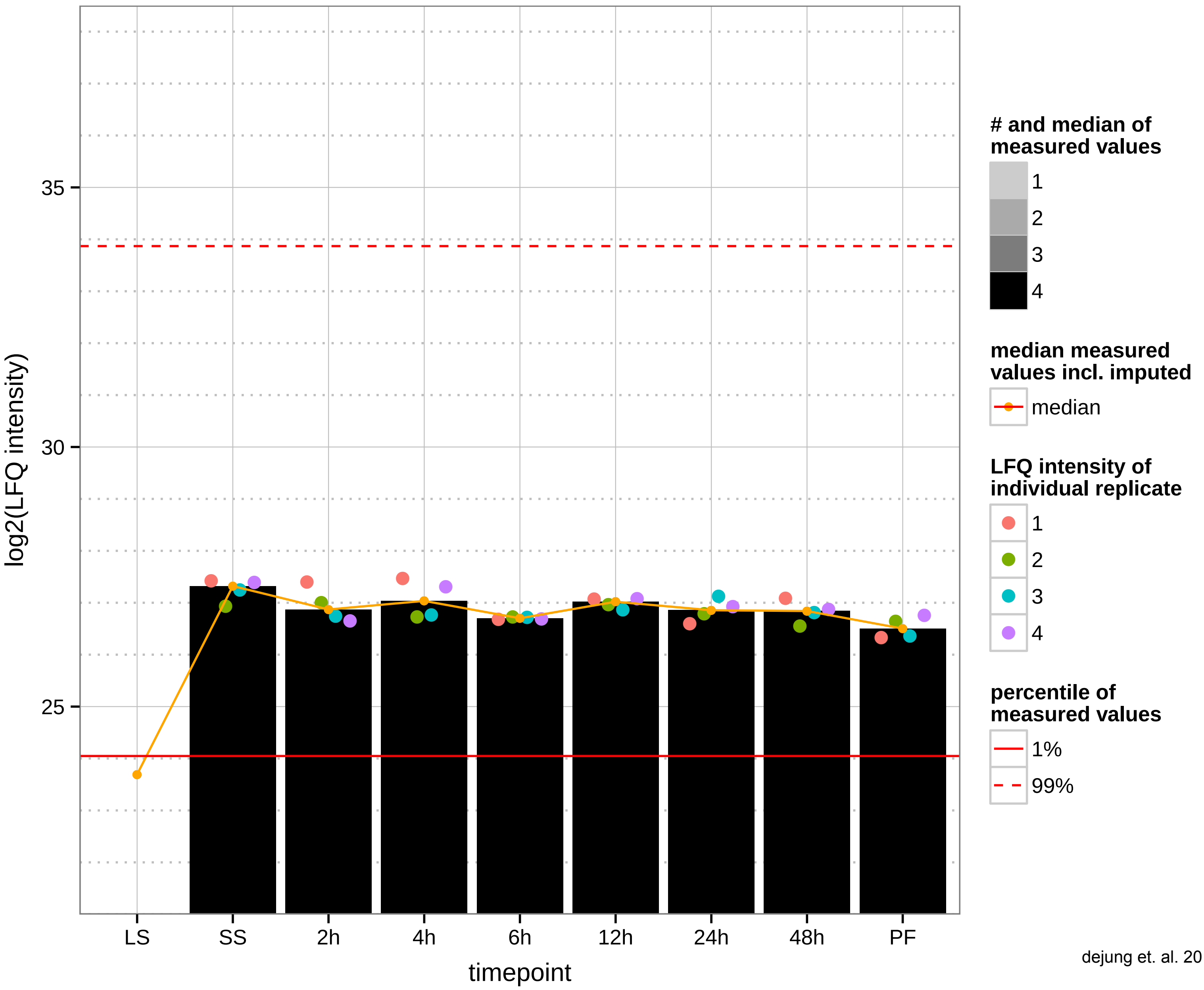

dejung et. al. 2015

hypothetical protein, conserved
Tb927.10.3640
AGOF: null
AGOC: null
AGOP: null
PGOF: null
PGOC: null
PGOP: null

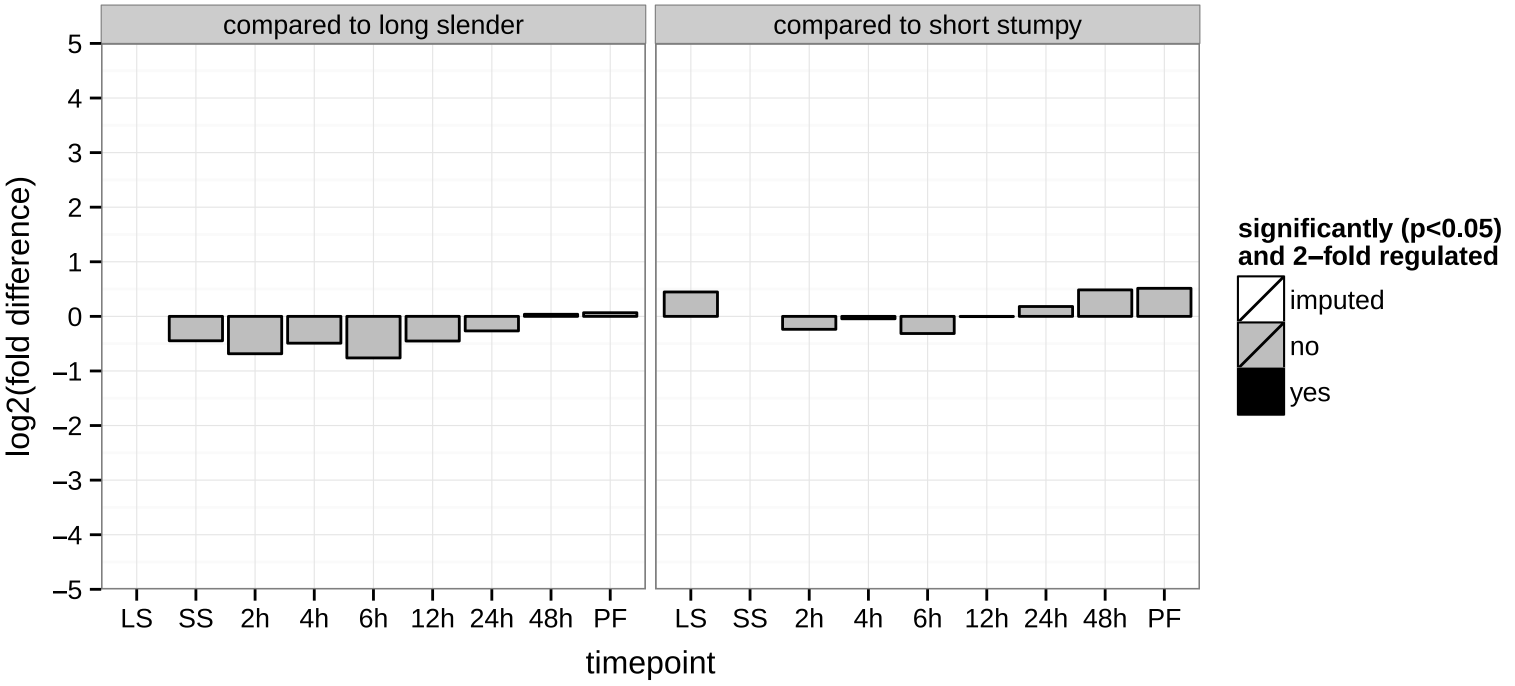

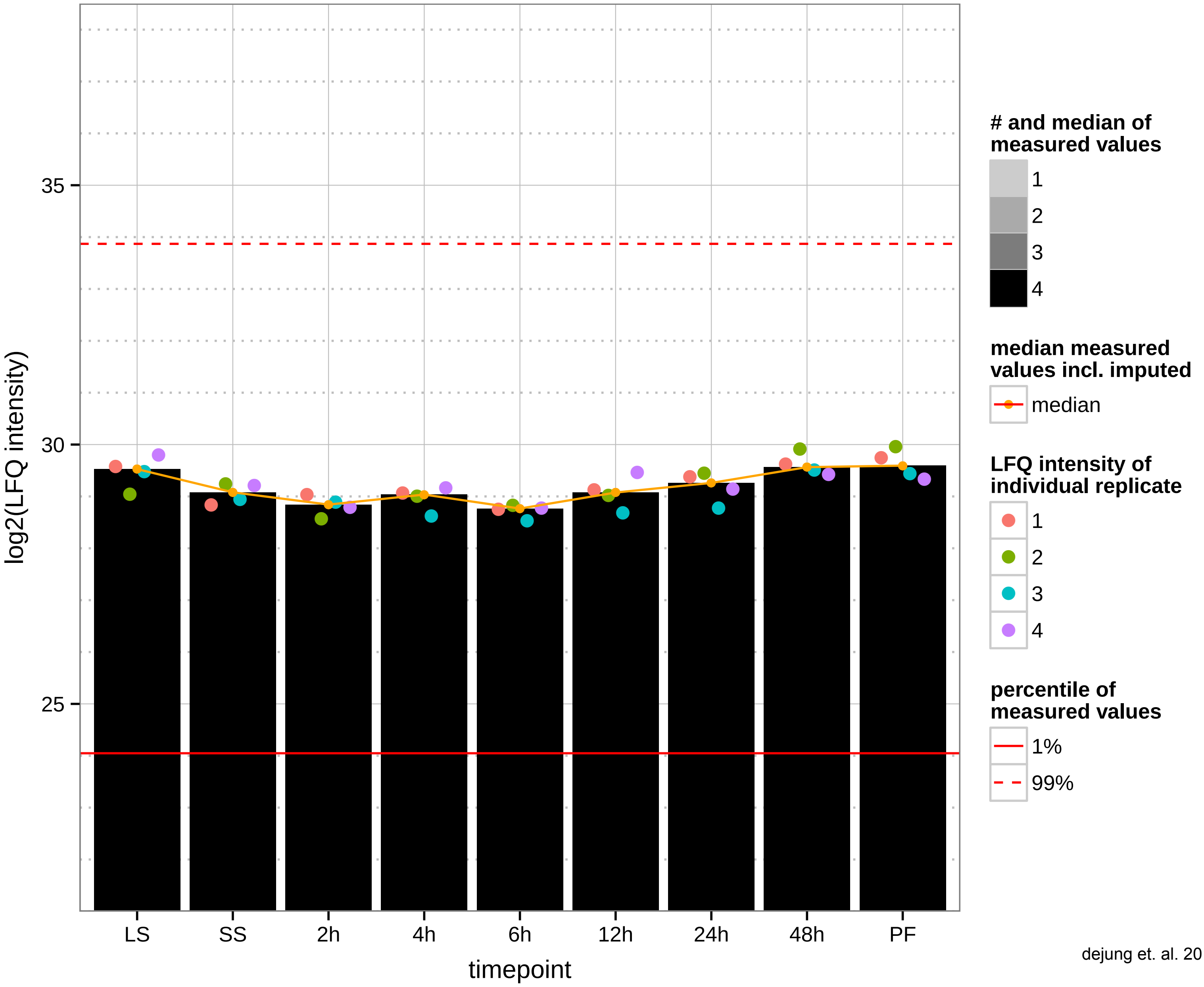

dejung et. al. 2015

predicted CBS domain protein
Tb927.10.3700
AGOF: null
AGOC: null
AGOP: null
PGOF: protein binding
PGOC: null
PGOP: null

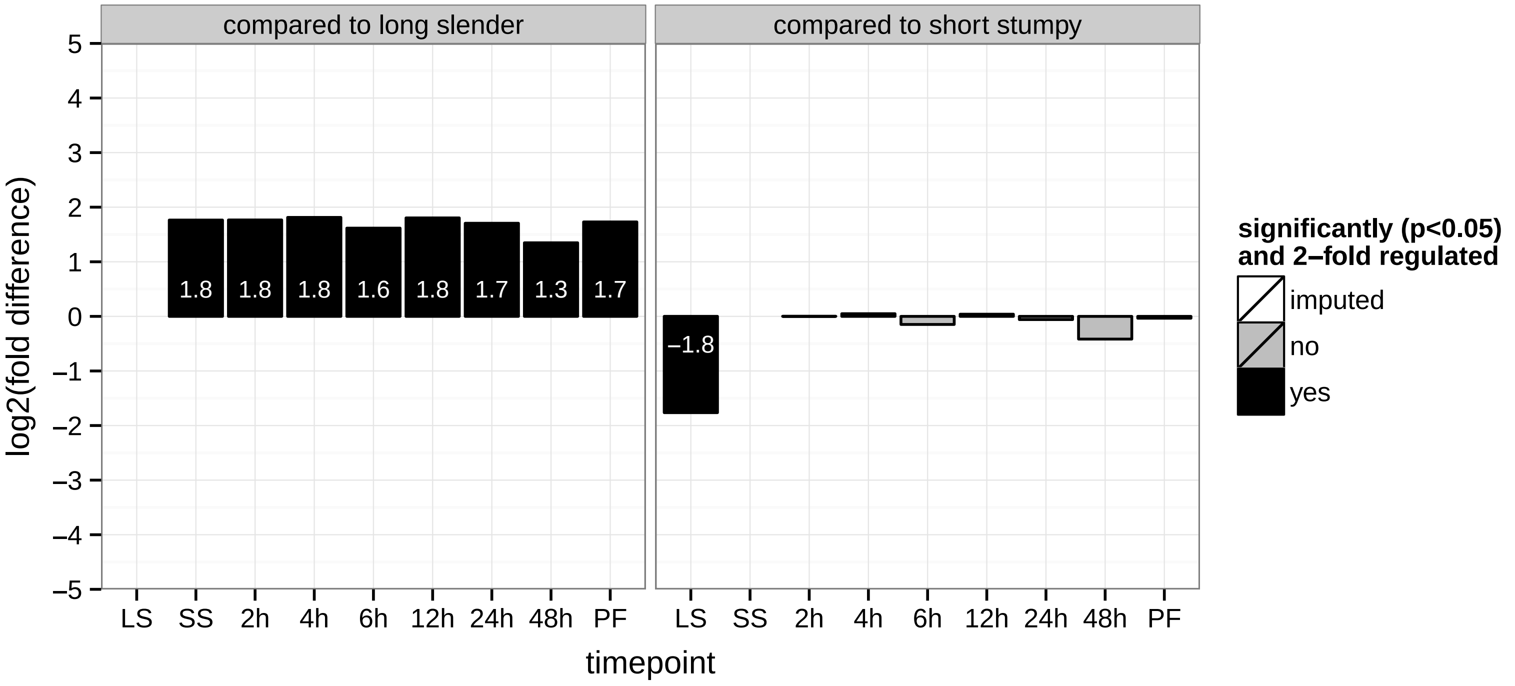

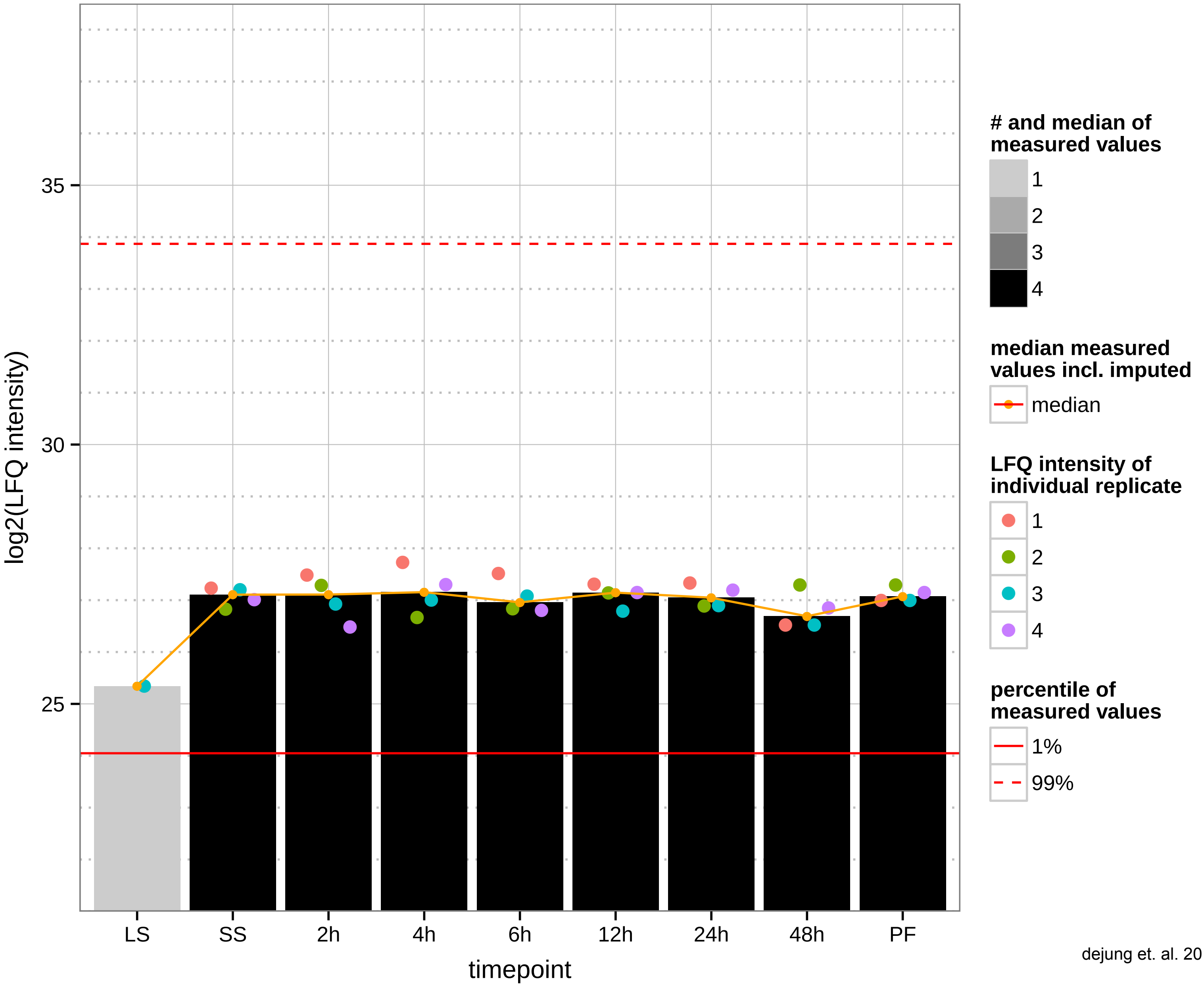

dejung et. al. 2015

proteasome activator protein pa26, putative, proteasome activator protein PA26 (pa26)
Tb927.10.3710;Tb11.v5.0761
AGOF: null
AGOC: null, proteasome activator complex
AGOP: null, proteolysis, regulation of proteasomal protein catabolic process
PGOF: null
PGOC: proteasome activator complex
PGOP: null

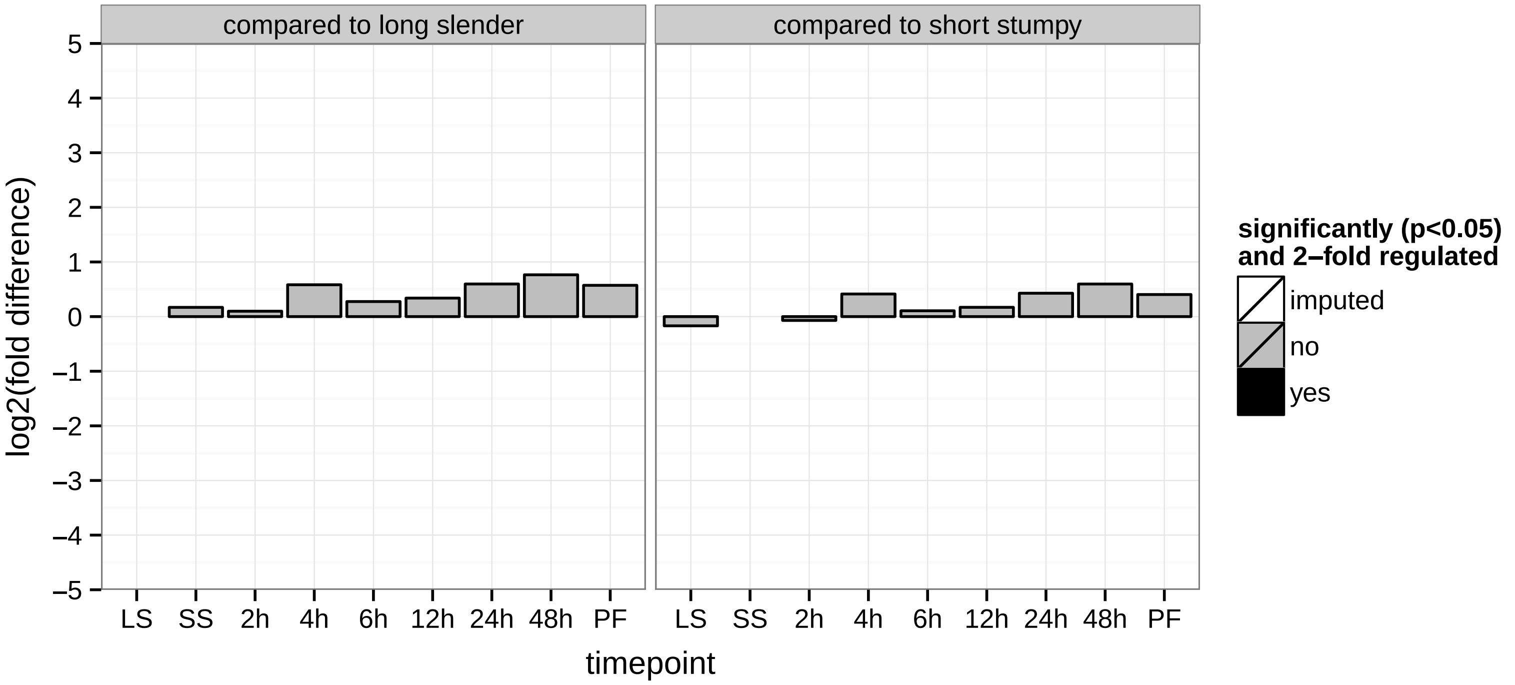

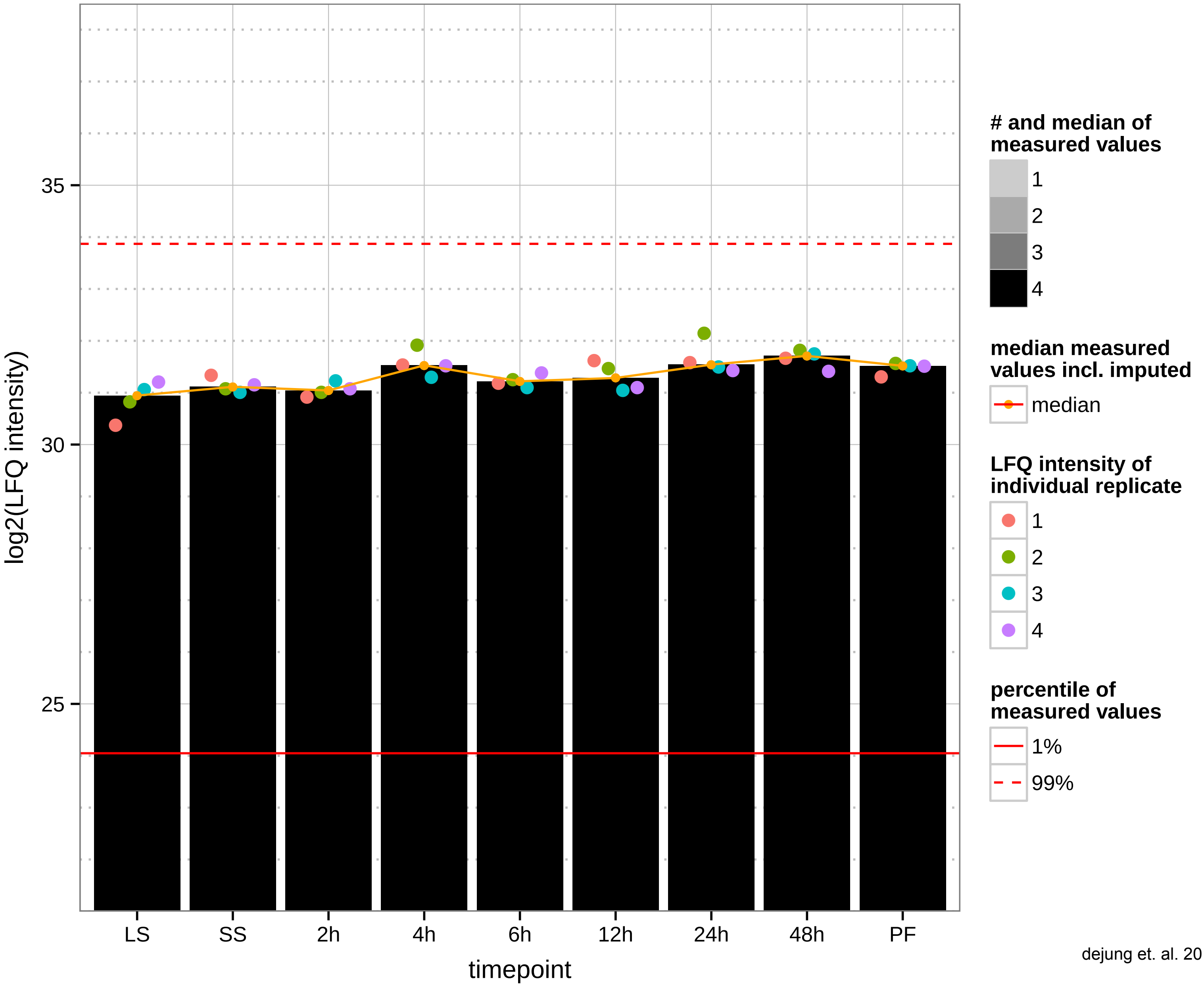

dejung et. al. 2015

vacuolar ATP synthase subunit d, putative
Tb927.10.3760
AGOF: hydrogen–exporting ATPase activity, phosphorylative mechanism, proton–transporting ATPase activity, rotational mech
AGOC: proton–transporting two–sector ATPase complex, catalytic domain, vacuolar proton–transporting V–type ATPase com
AGOP: ATP hydrolysis coupled proton transport, ATP synthesis coupled proton transport
PGOF: ATPase activity, coupled to transmembrane movement of substances
PGOC: null
PGOP: null

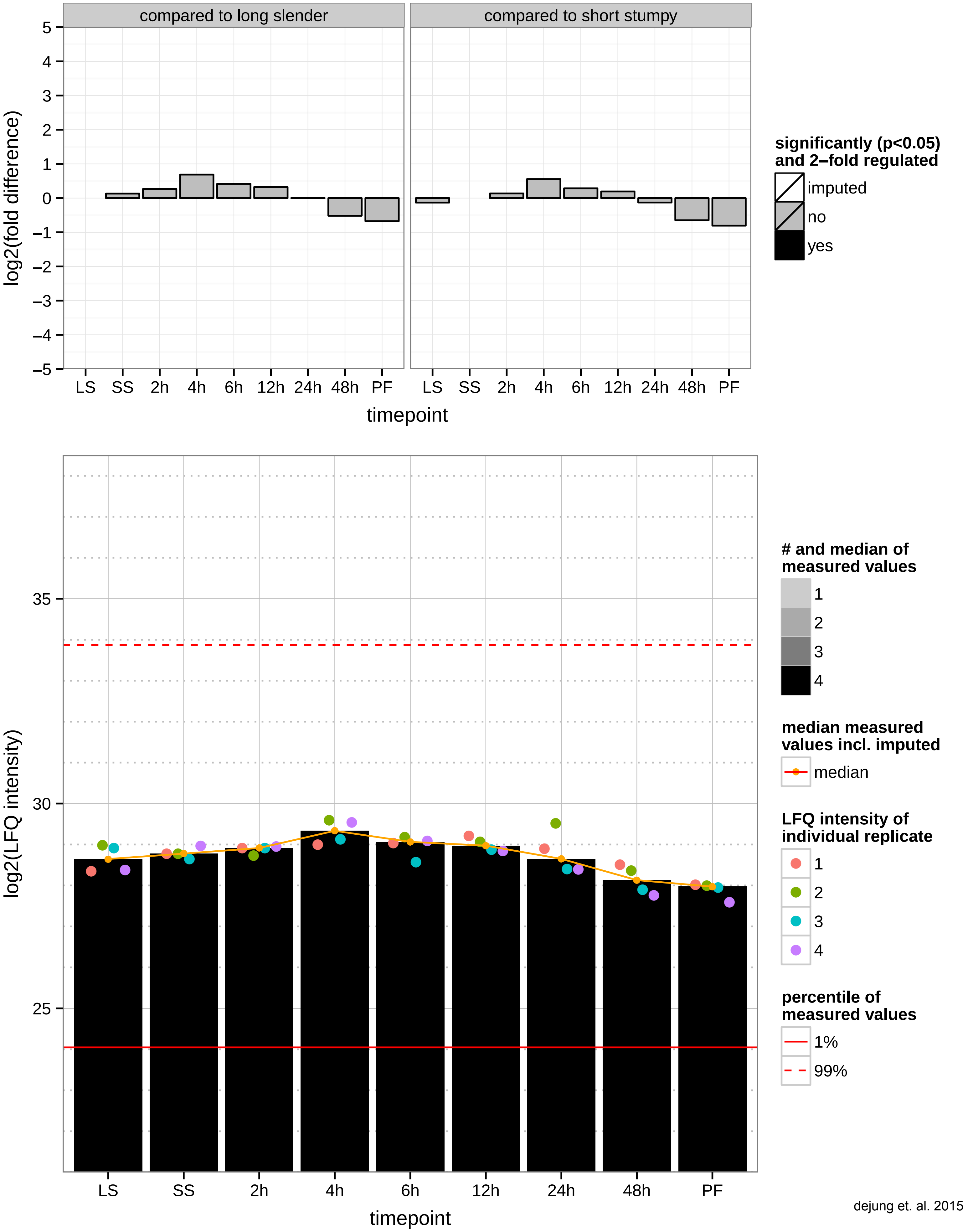

dejung et. al. 2015

predicted TPR repeat protein
Tb927.10.3770
AGOF: null
AGOC: mitochondrion
AGOP: null
PGOF: protein binding
PGOC: null
PGOP: null

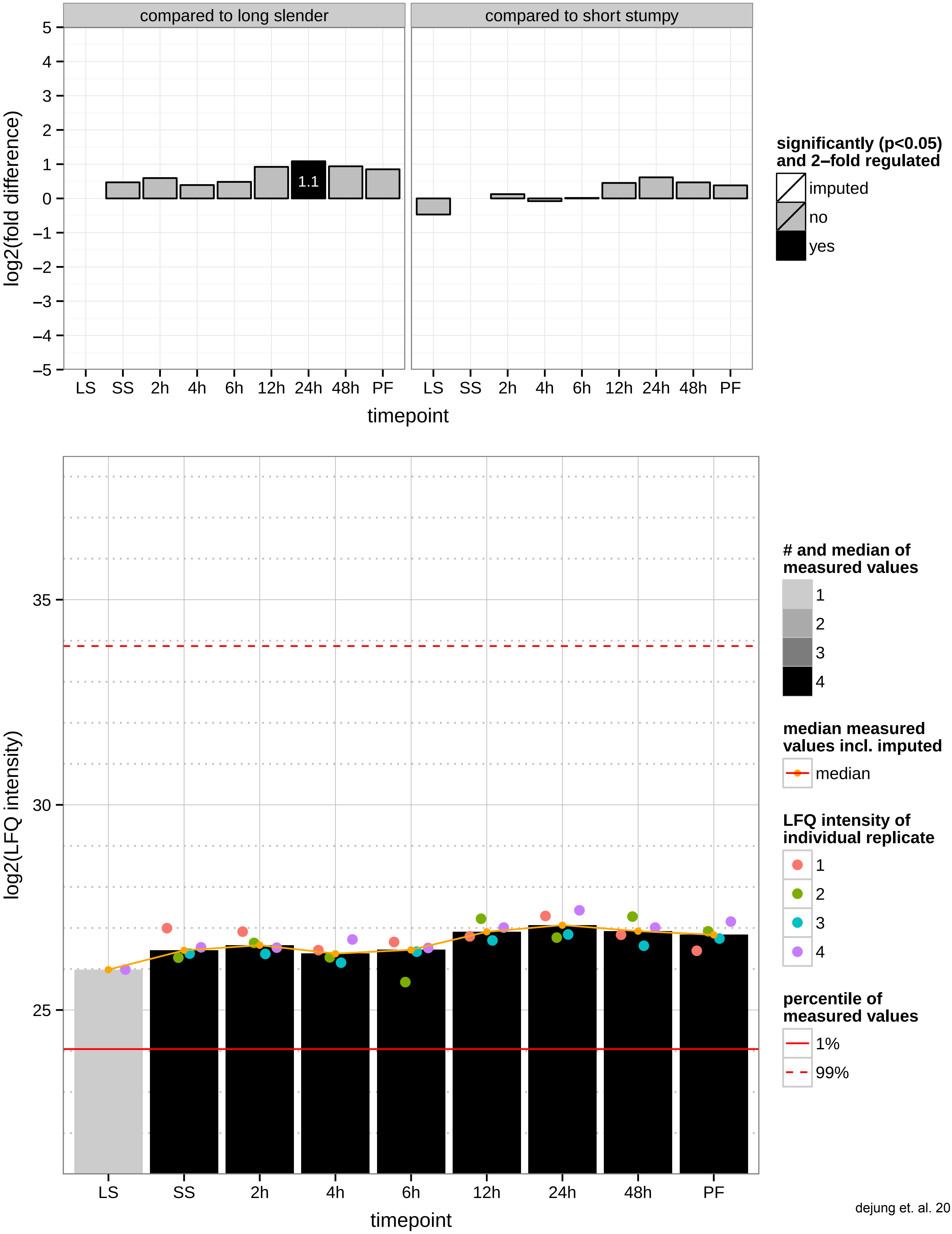

dejung et. al. 2015

hypothetical protein, conserved
Tb927.10.3820
AGOF: null
AGOC: null
AGOP: null
PGOF: null
PGOC: null
PGOP: null

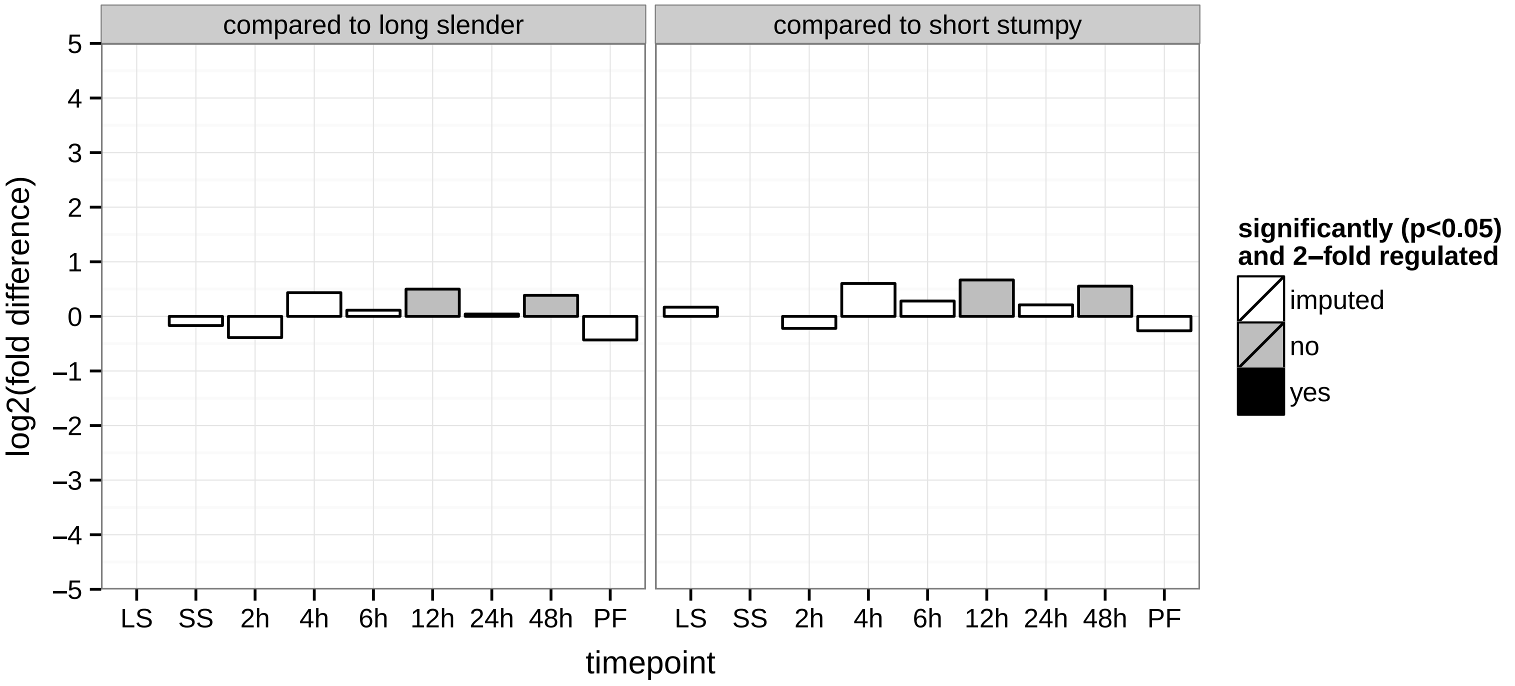

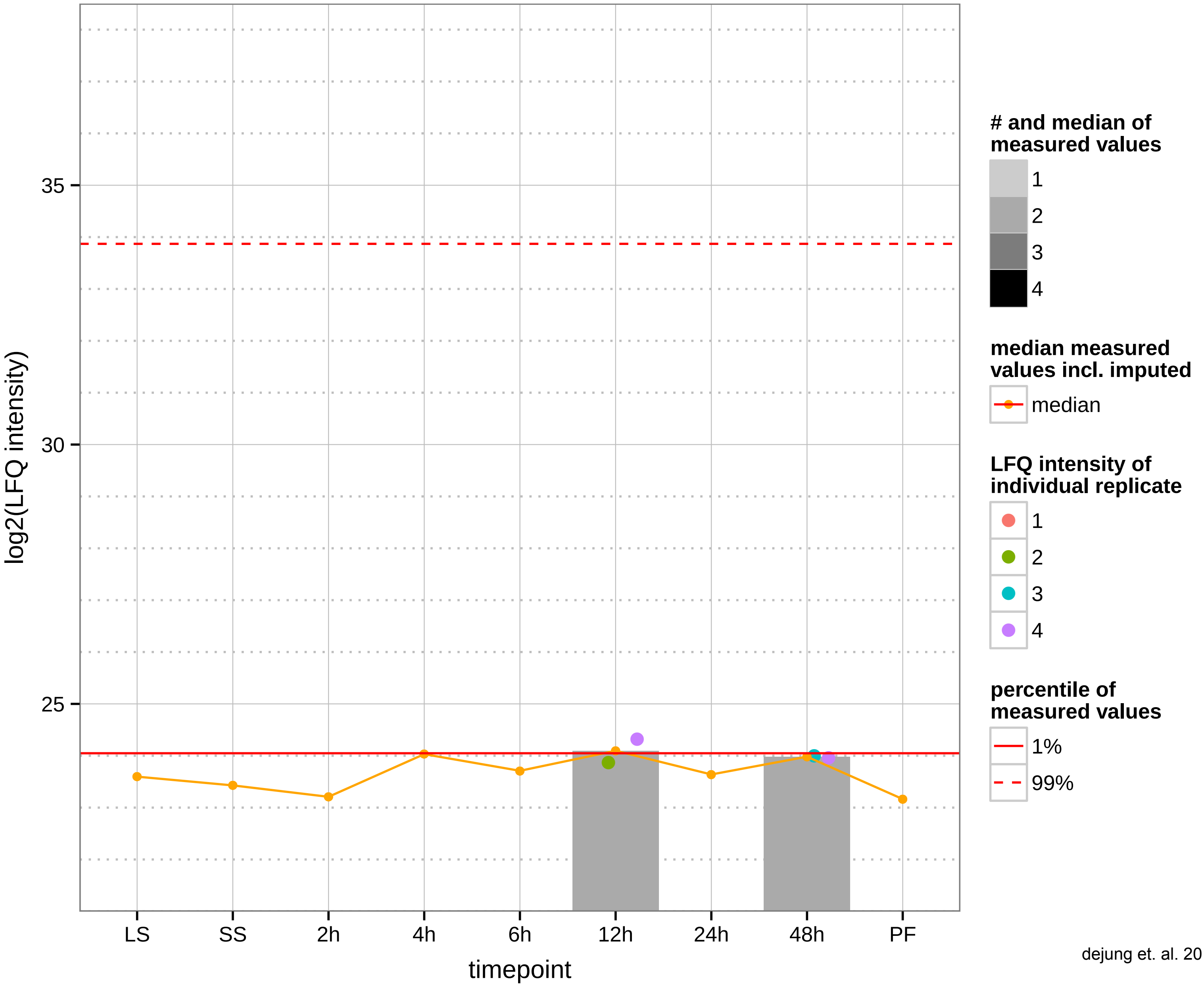

dejung et. al. 2015

60S ribosomal protein L18a, putative, ribosomal protein L18
Tb927.11.14130;Tb927.10.3840
AGOF: structural constituent of ribosome
AGOC: intracellular, ribosome
AGOP: translation
PGOF: structural constituent of ribosome
PGOC: ribosome
PGOP: translation

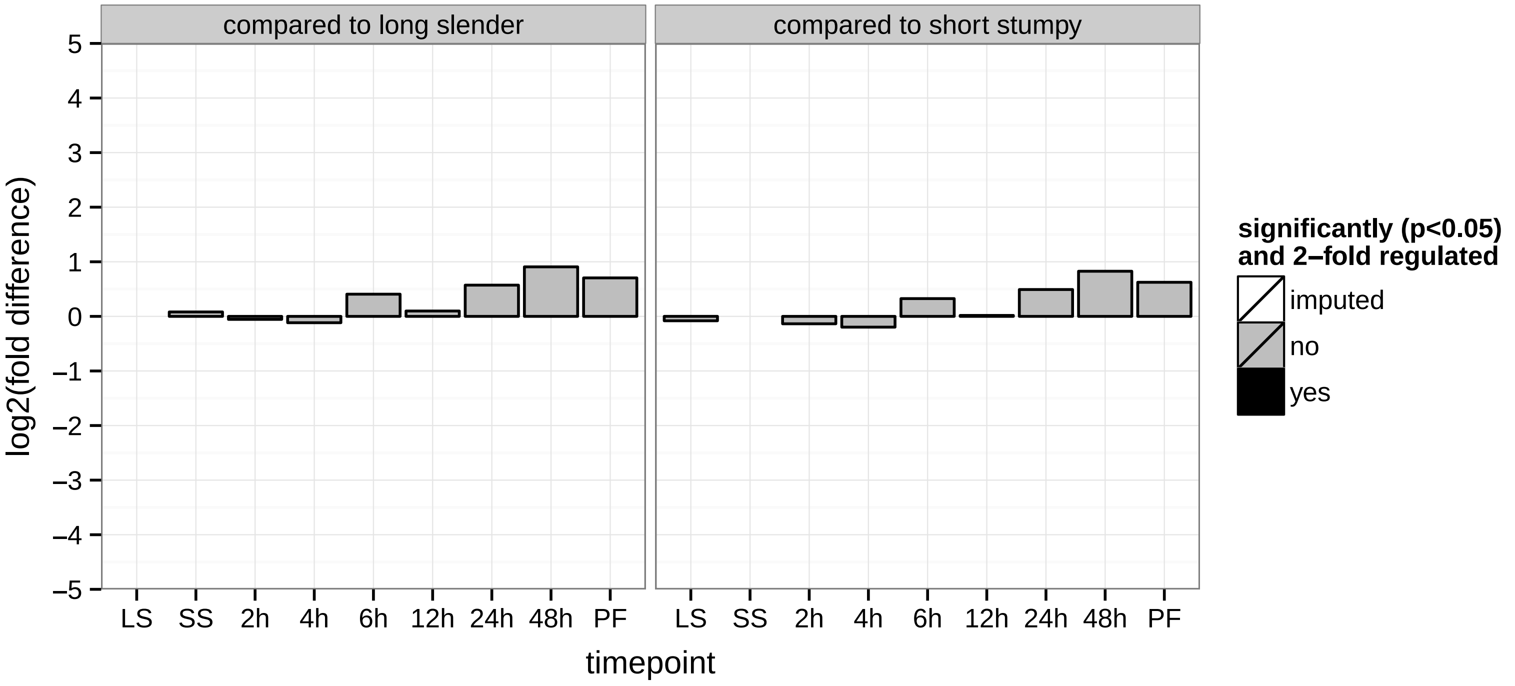

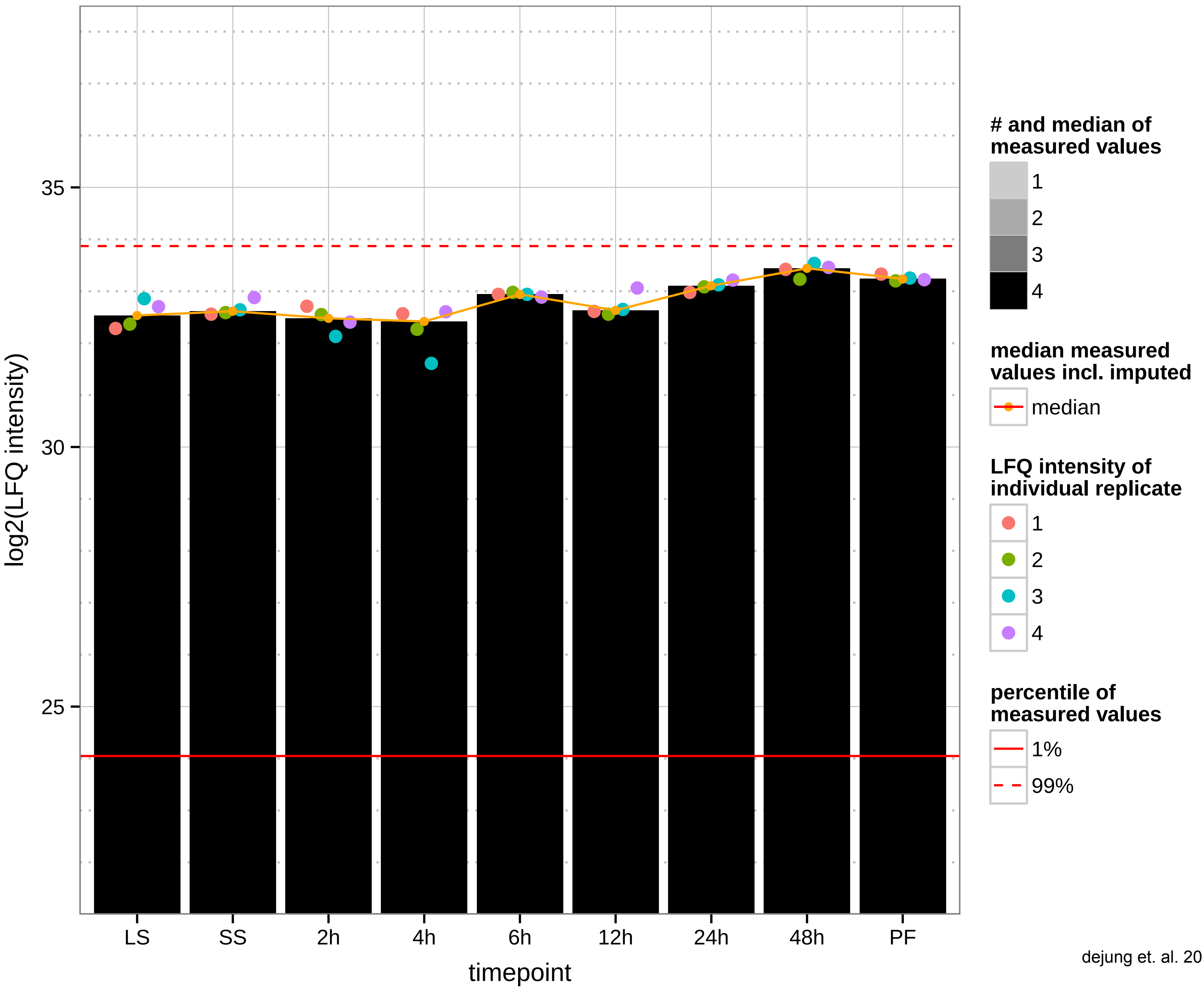

dejung et. al. 2015

hypothetical protein, conserved
Tb927.10.3950
AGOF: null
AGOC: null
AGOP: null
PGOF: null
PGOC: null
PGOP: null

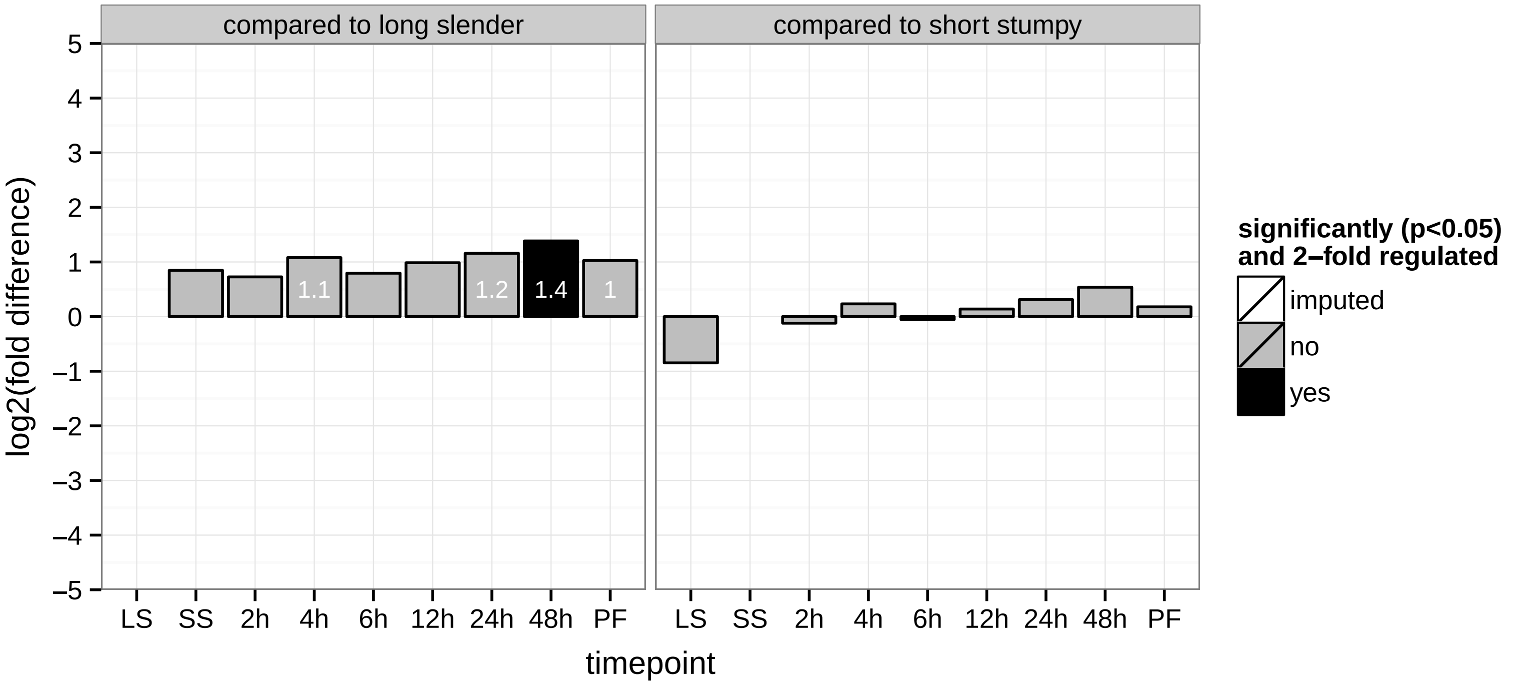

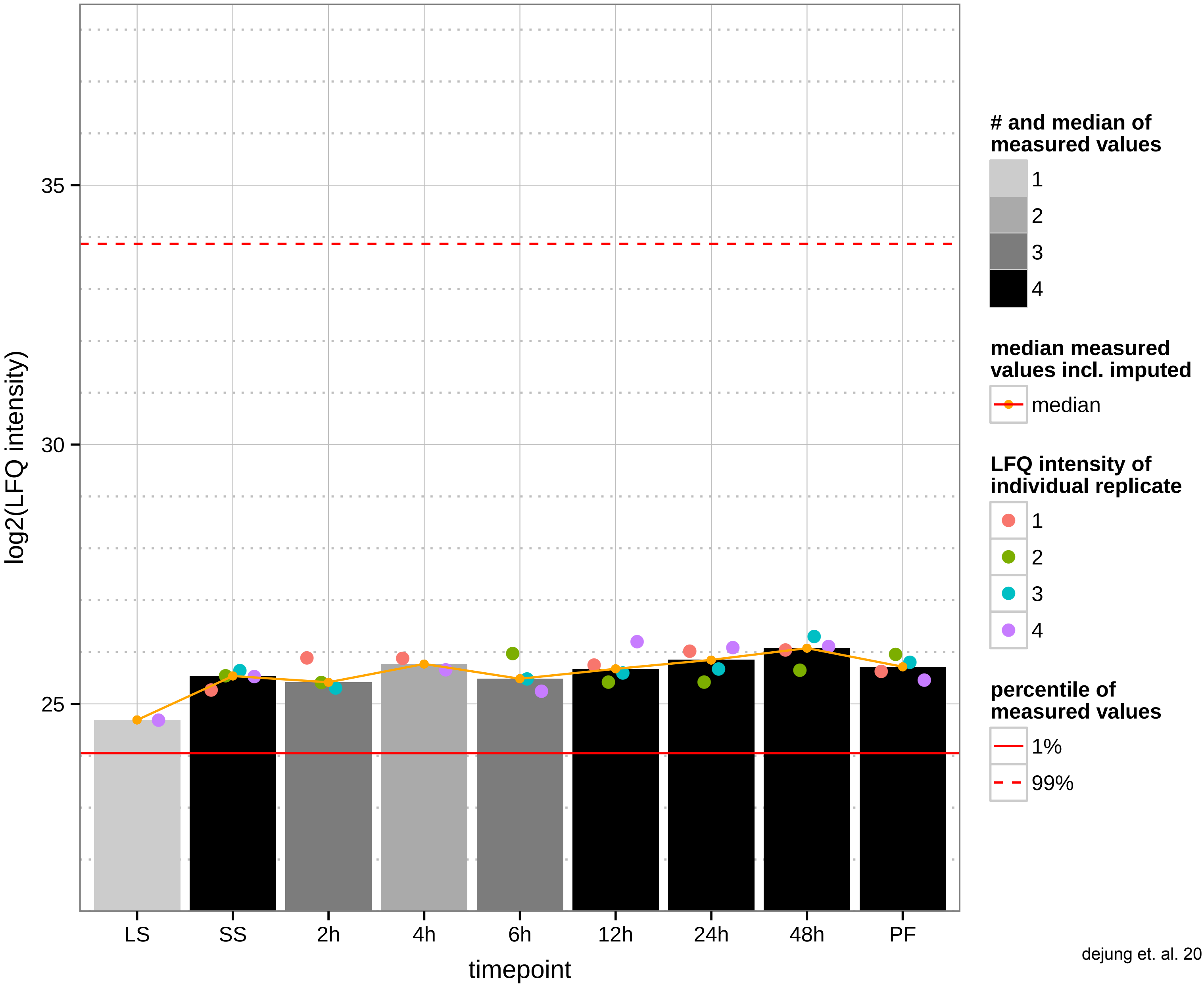

dejung et. al. 2015

DHH1 (DHH1)
Tb927.10.3990
AGOF: ATP binding, ATP–dependent helicase activity, helicase activity, nucleic acid binding
AGOC: null
AGOP: nucleobase–containing compound metabolic process
PGOF: ATP binding, ATP–dependent helicase activity, helicase activity, nucleic acid binding
PGOC: null
PGOP: null

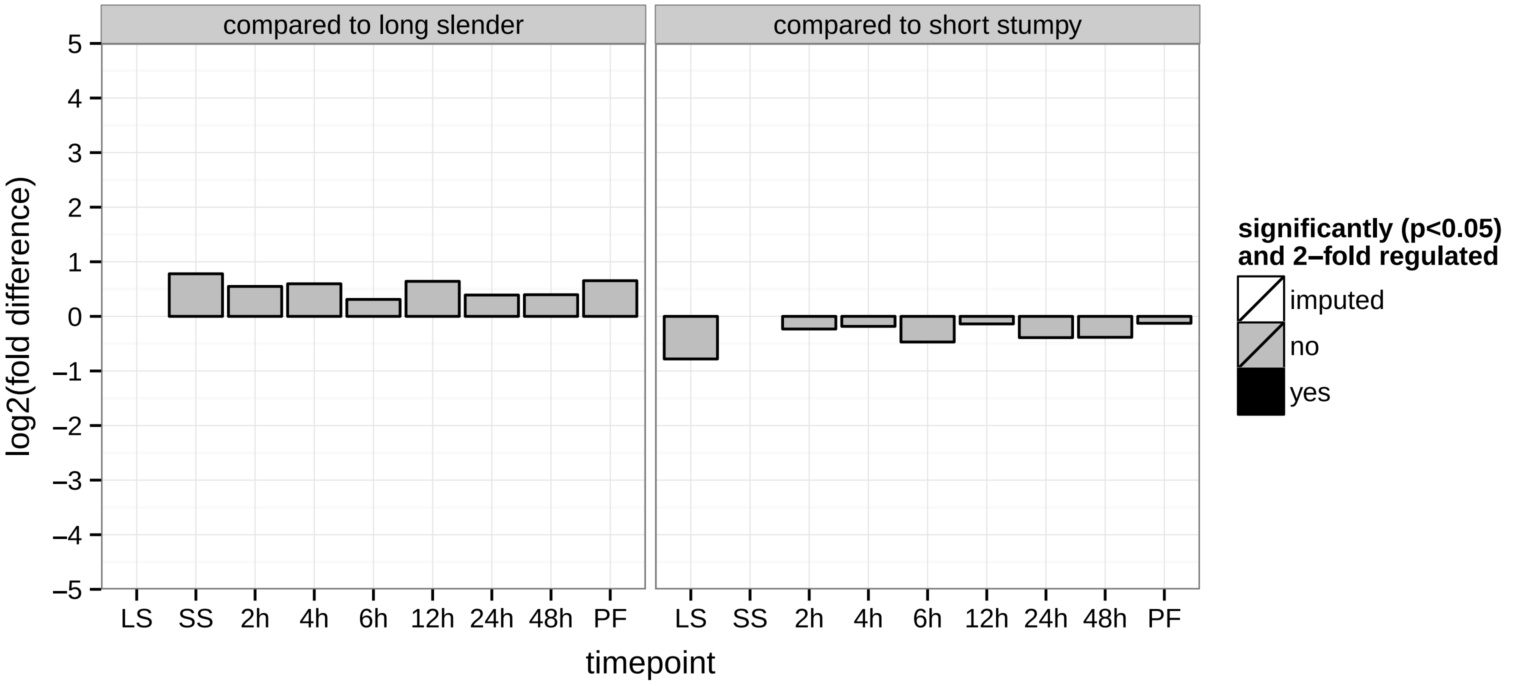

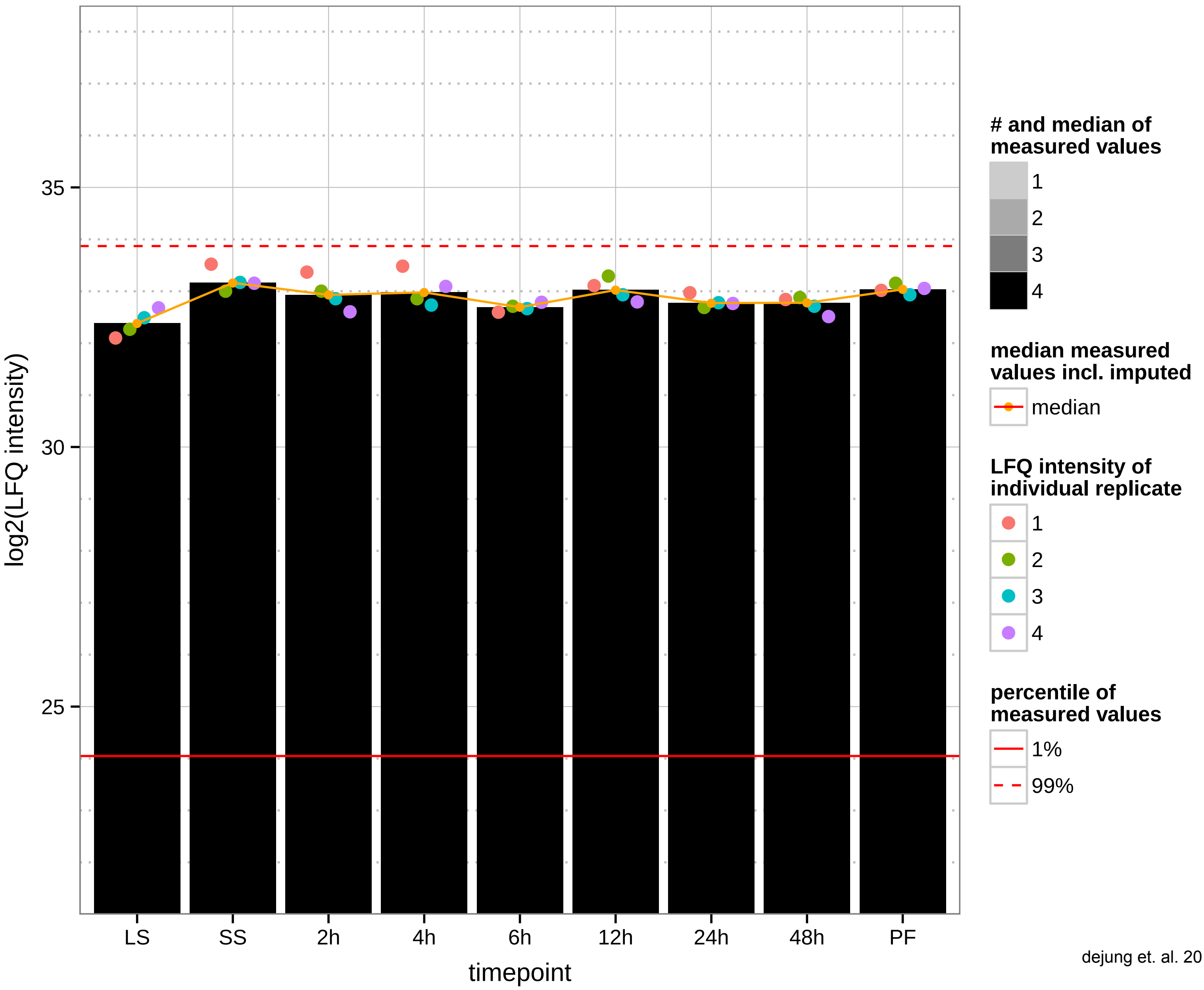

dejung et. al. 2015

methylglutaconyl−CoA hydratase, mitochondrial precursor, putative
Tb927.10.4000
AGOF: catalytic activity
AGOC: mitochondrion
AGOP: metabolic process
PGOF: catalytic activity
PGOC: null
PGOP: metabolic process

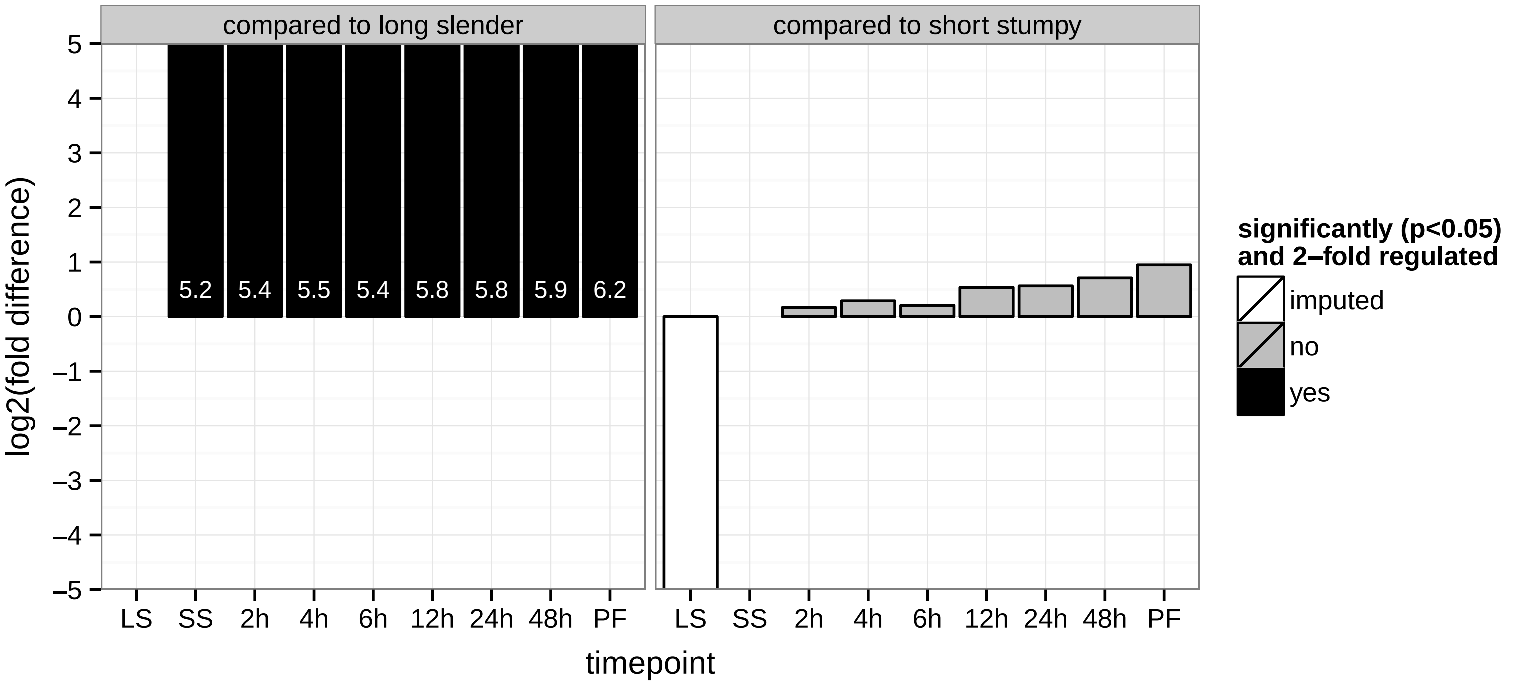

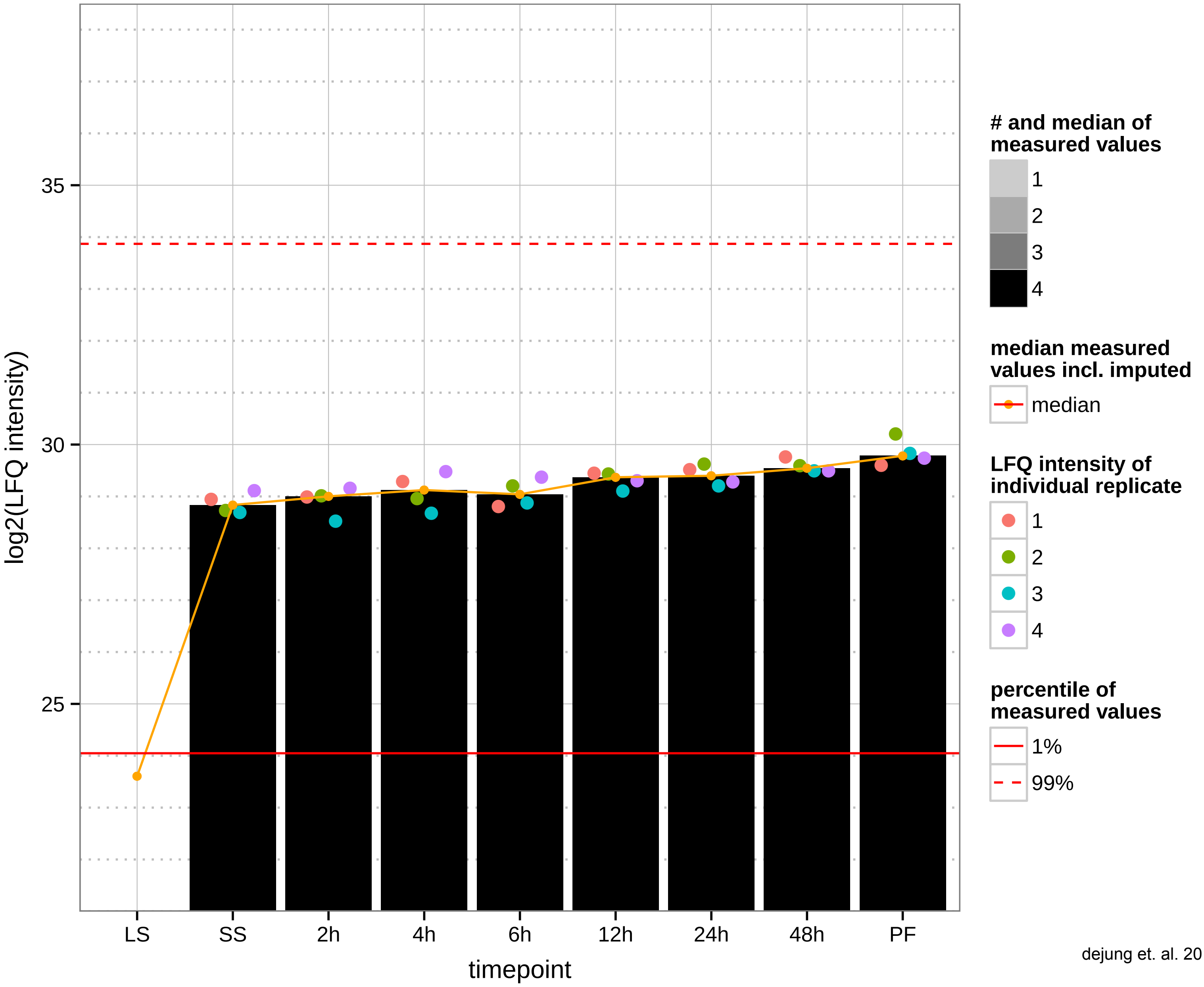

dejung et. al. 2015

hypothetical protein, conserved
Tb927.10.4010
AGOF: null
AGOC: null
AGOP: null
PGOF: null
PGOC: null
PGOP: null

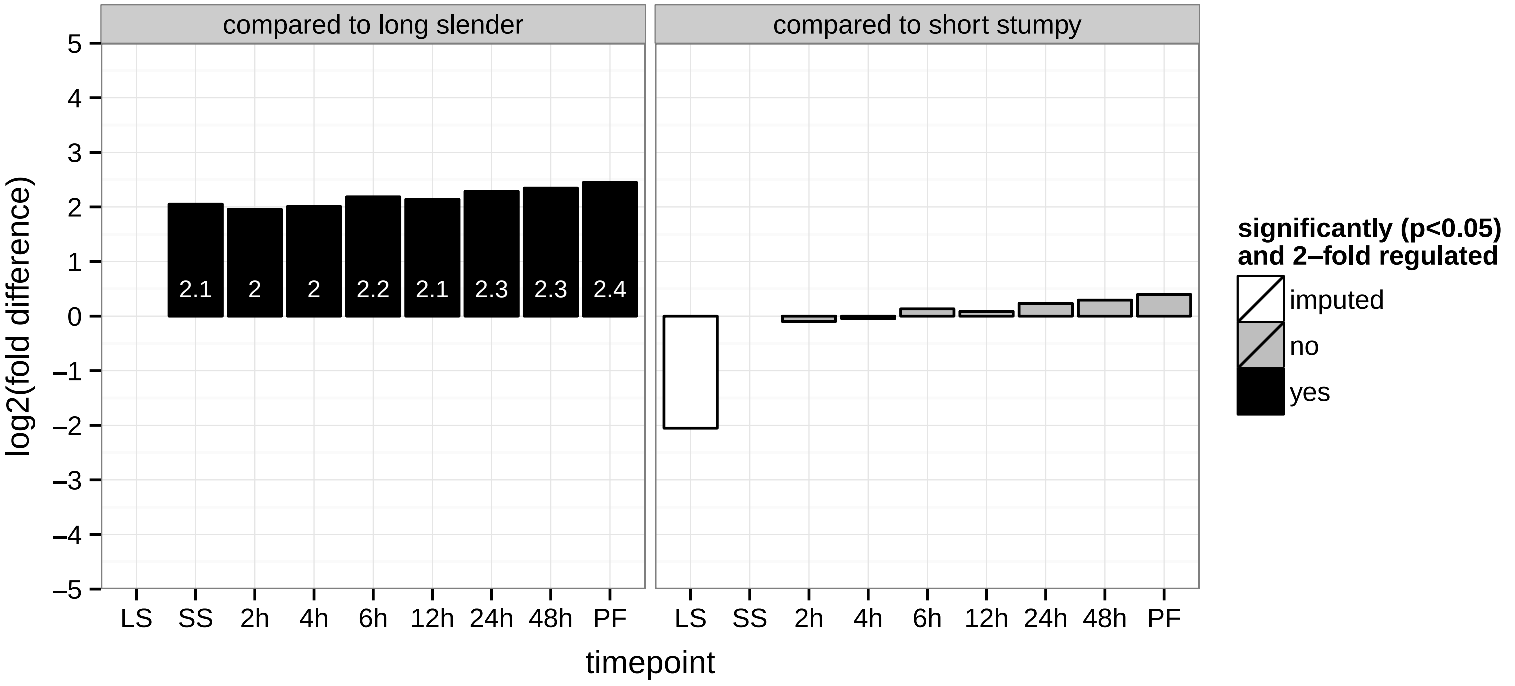

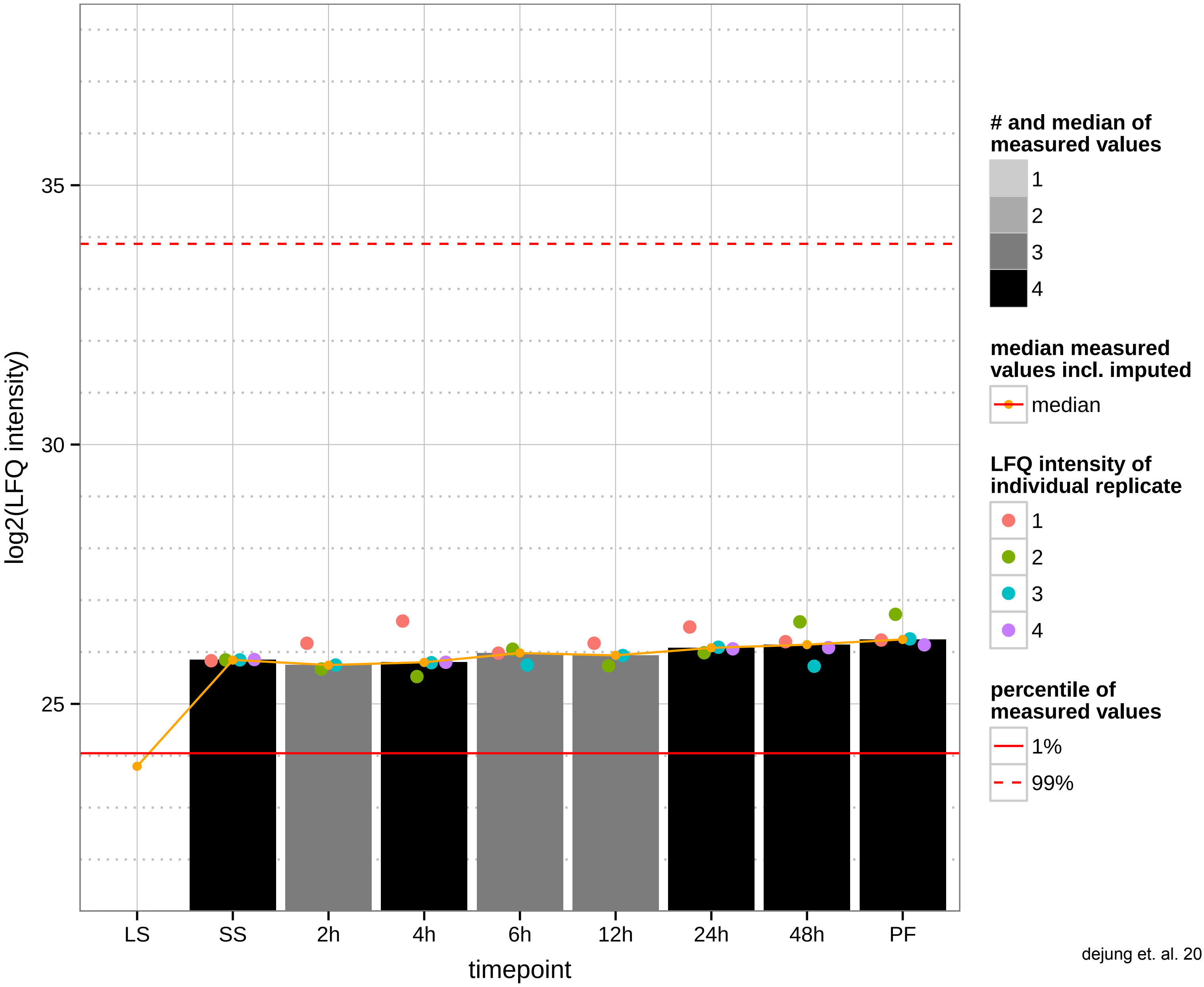

dejung et. al. 2015

3–keto–dihydrosphingosine reductase
Tb927.10.4040
AGOF: 3–dehydrosphinganine reductase activity
AGOC: endoplasmic reticulum membrane
AGOP: fatty acid biosynthetic process
PGOF: oxidoreductase activity
PGOC: null
PGOP: metabolic process

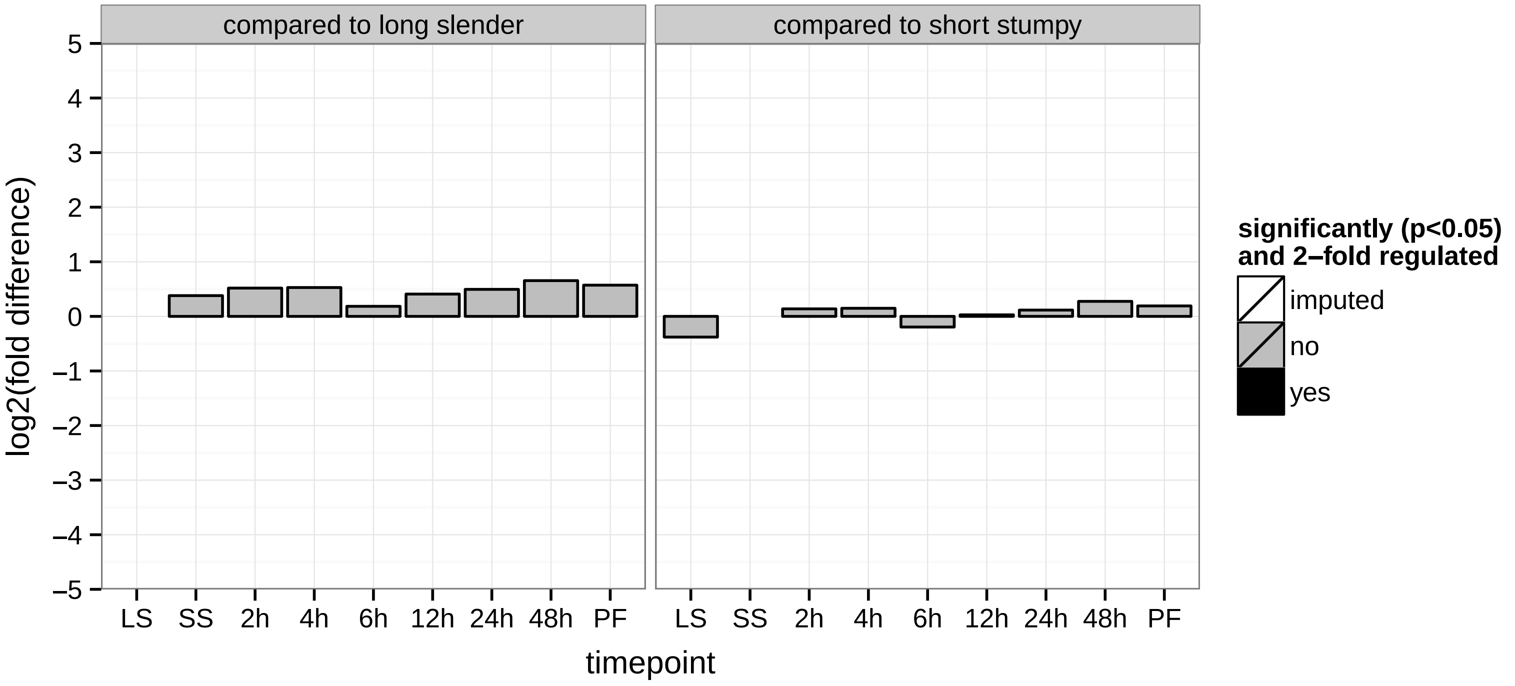

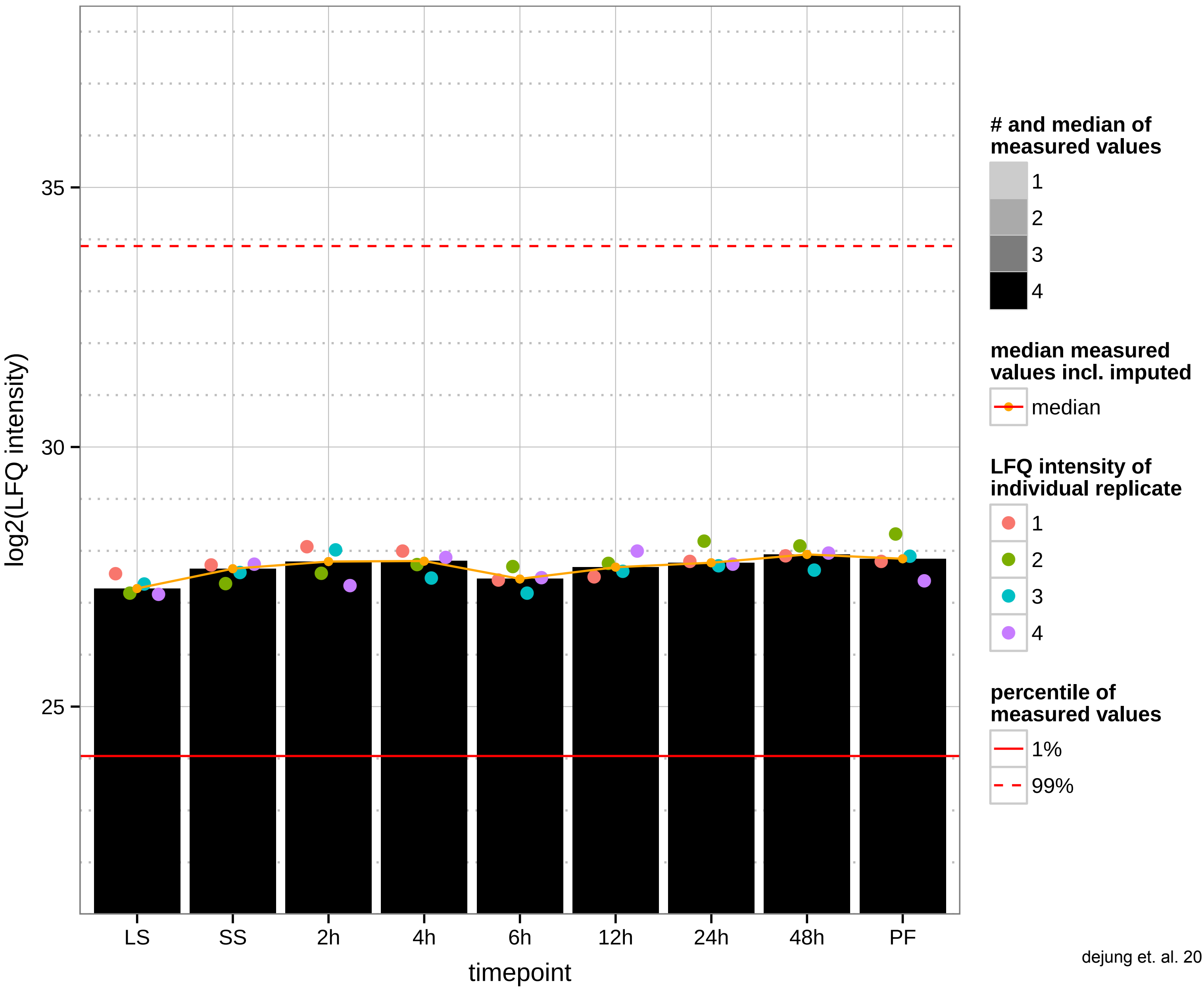

dejung et. al. 2015

hypothetical protein, conserved
Tb927.10.4060
AGOF: null
AGOC: null
AGOP: null
PGOF: protein binding
PGOC: null
PGOP: null

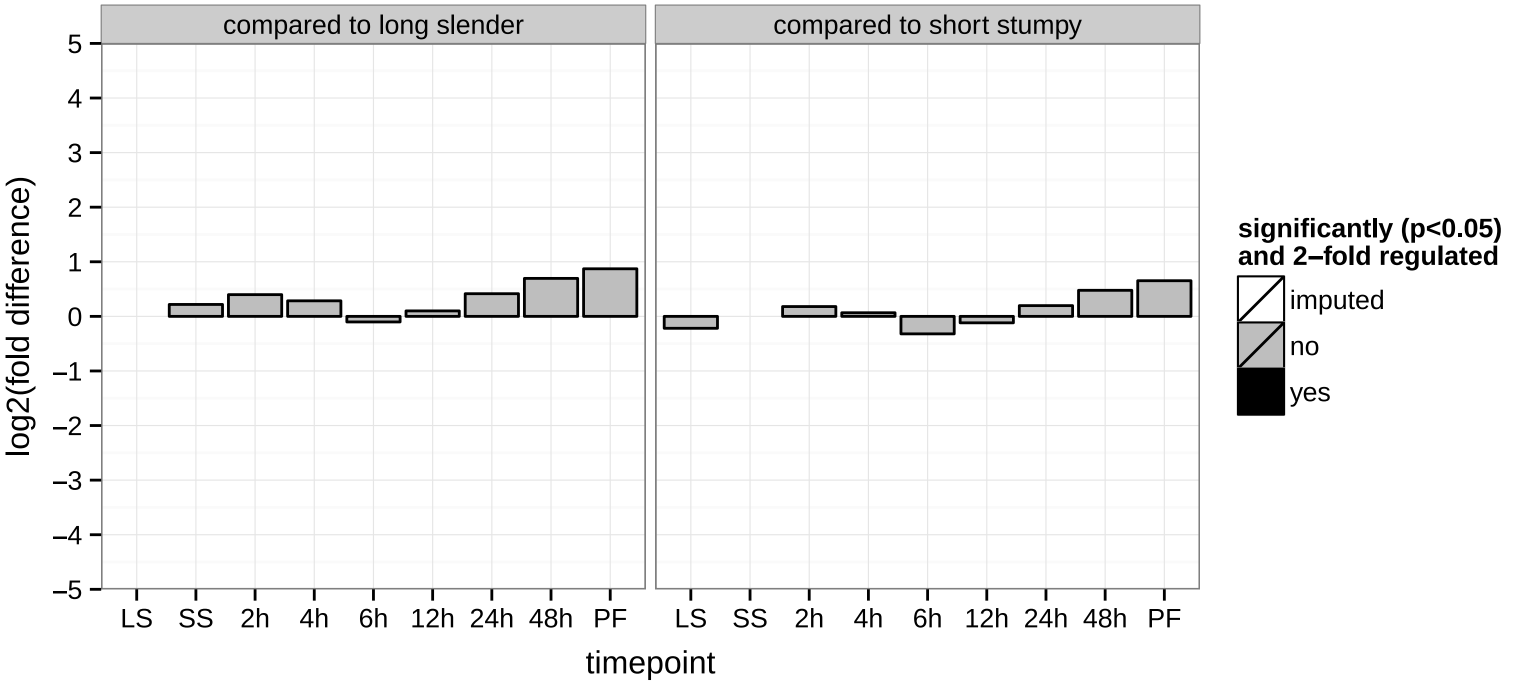

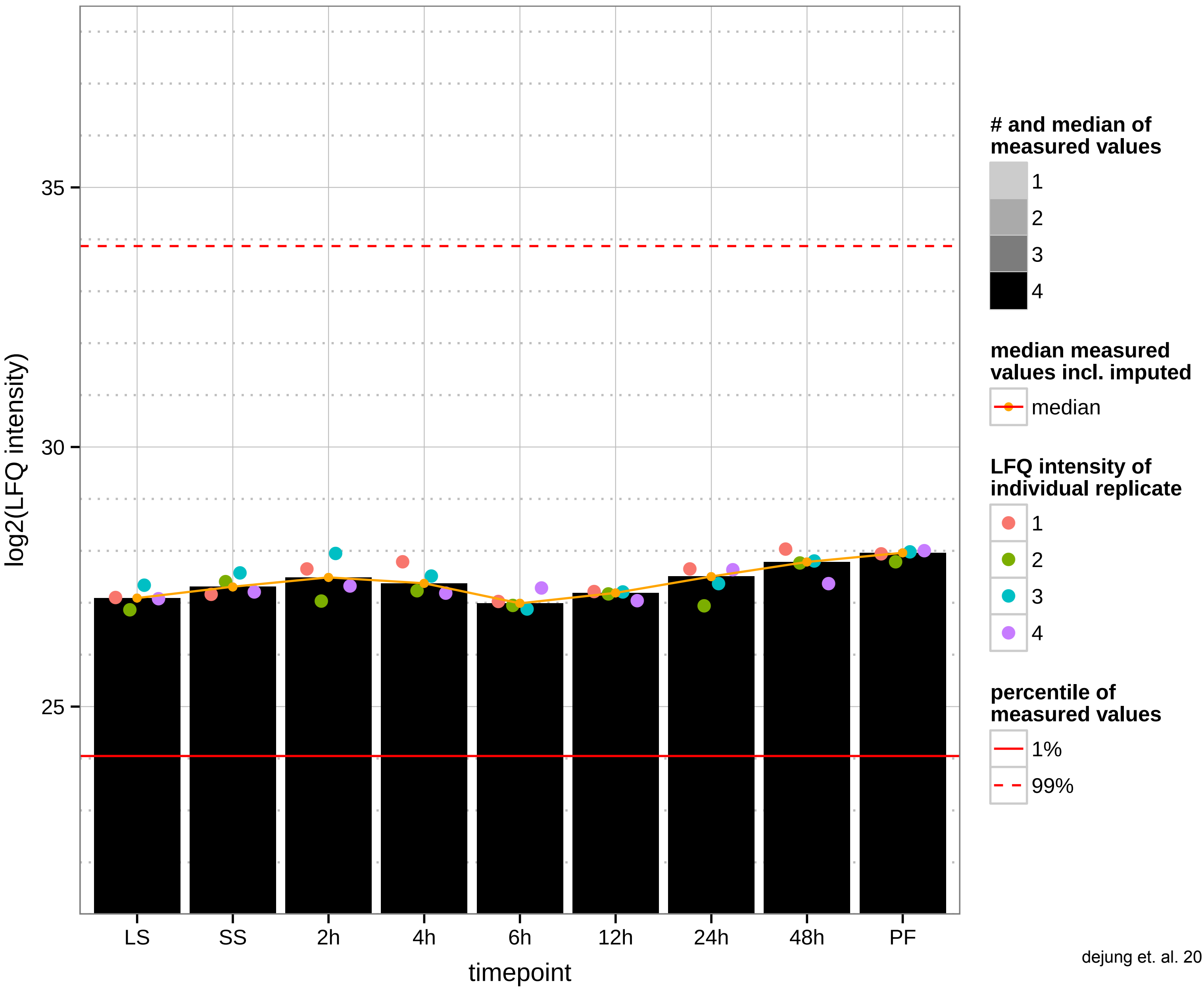

dejung et. al. 2015

60S ribosomal protein L30 (RPL30)
Tb927.10.4120;Tb927.10.4110
AGOF: structural constituent of ribosome
AGOC: ribosome, intracellular, ribosome
AGOP: translation
PGOF: null
PGOC: null
PGOP: null

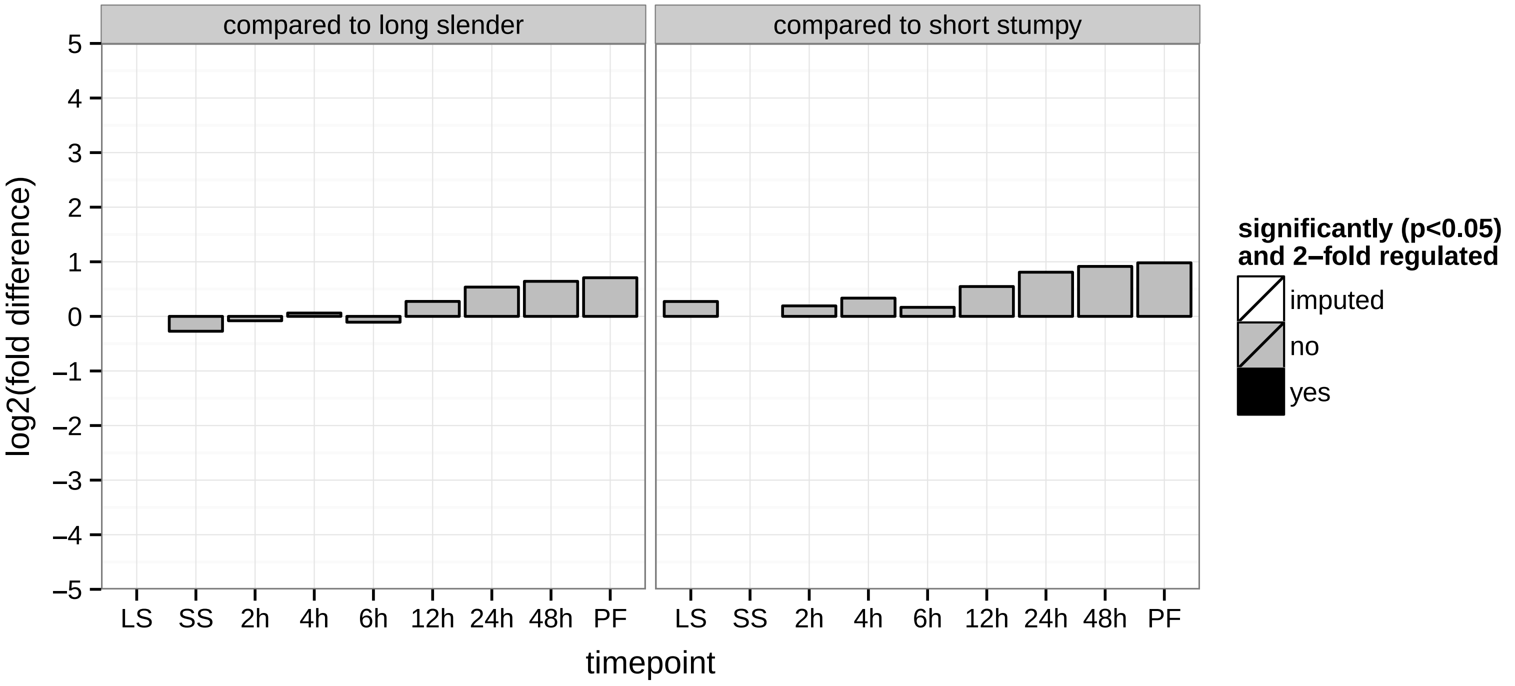

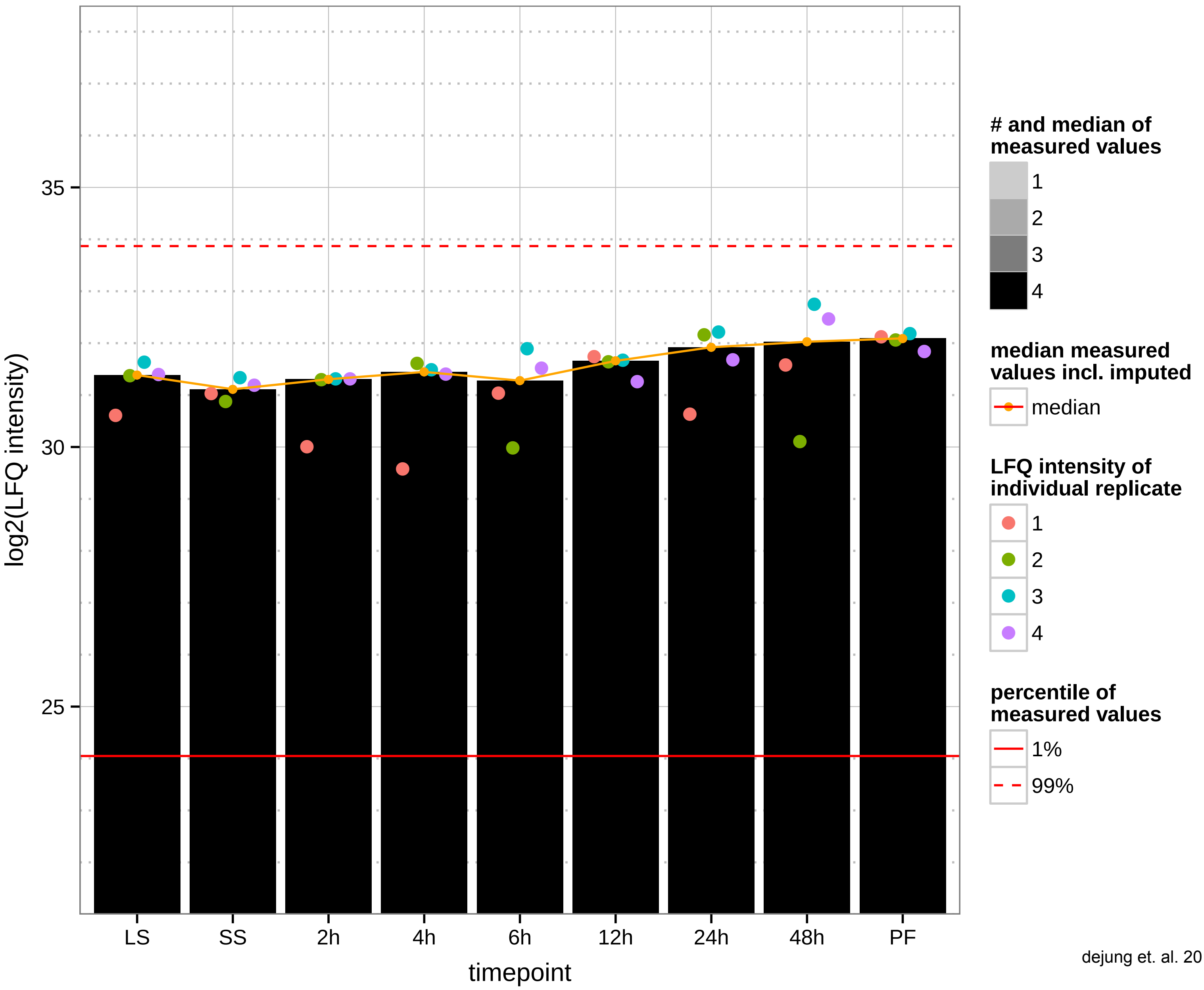

dejung et. al. 2015

ADP−ribosylation factor−like 2, arl2 (ARL2)
Tb927.10.4250
AGOF: null
AGOC: cytoplasm, intracellular
AGOP: cytokinesis, intracellular protein transport, small GTPase mediated signal transduction
PGOF: GTP binding
PGOC: intracellular
PGOP: intracellular protein transport, small GTPase mediated signal transduction

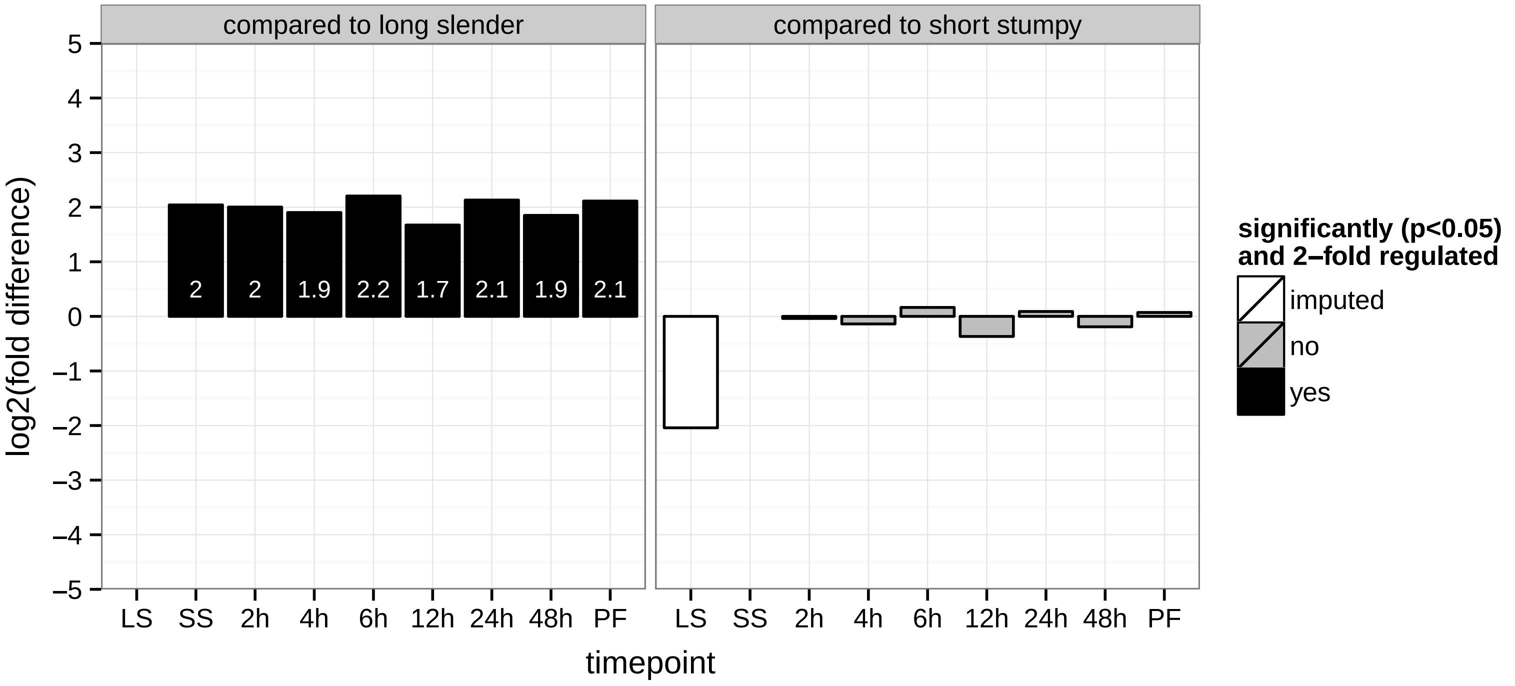

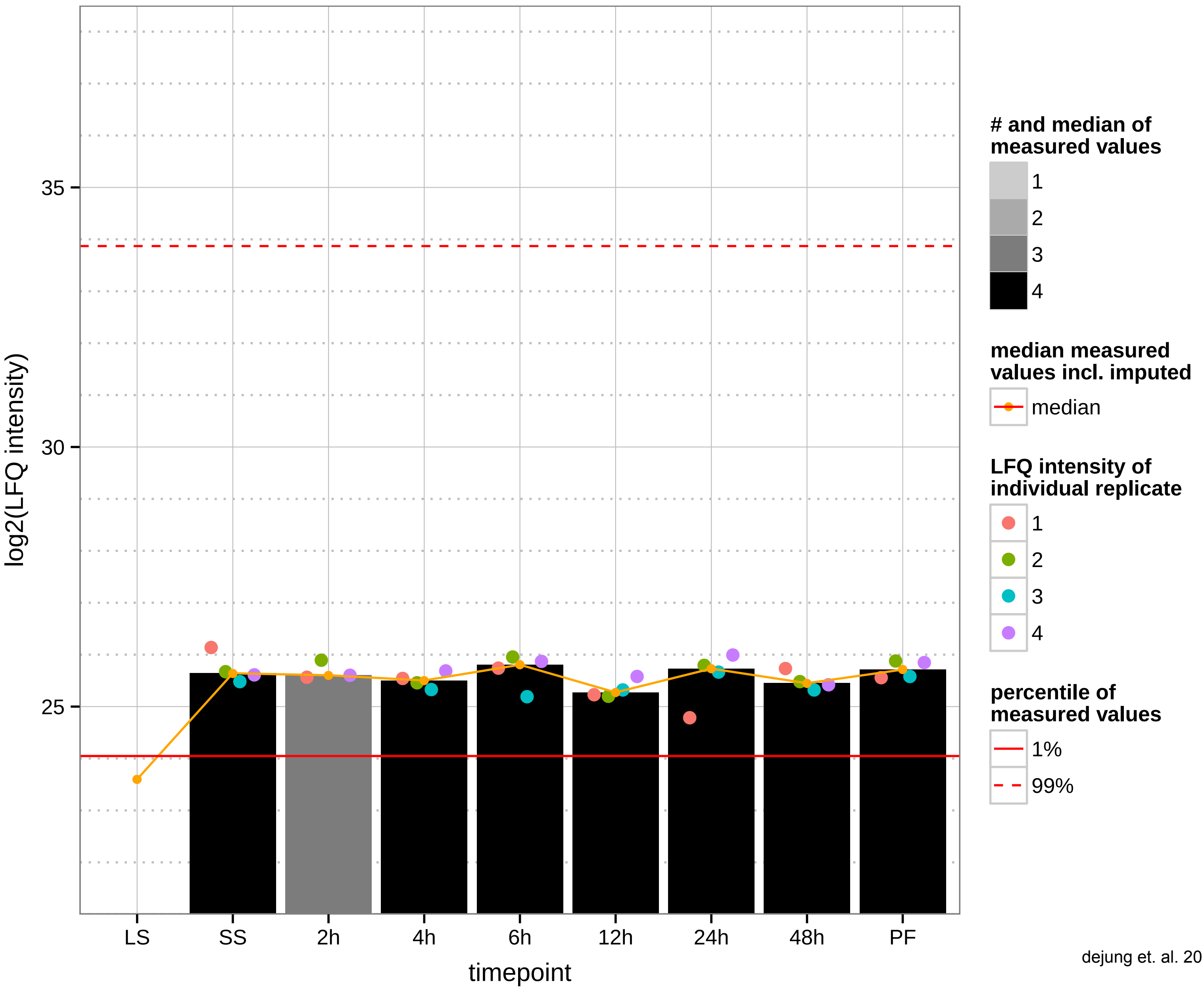

dejung et. al. 2015

dolicholphosphate−mannose synthase, putative (DPMS)
Tb927.10.4610
AGOF: dolichyl−phosphate beta−D−mannosyltransferase activity
AGOC: null
AGOP: GPI anchor biosynthetic process
PGOF: null
PGOC: null
PGOP: null

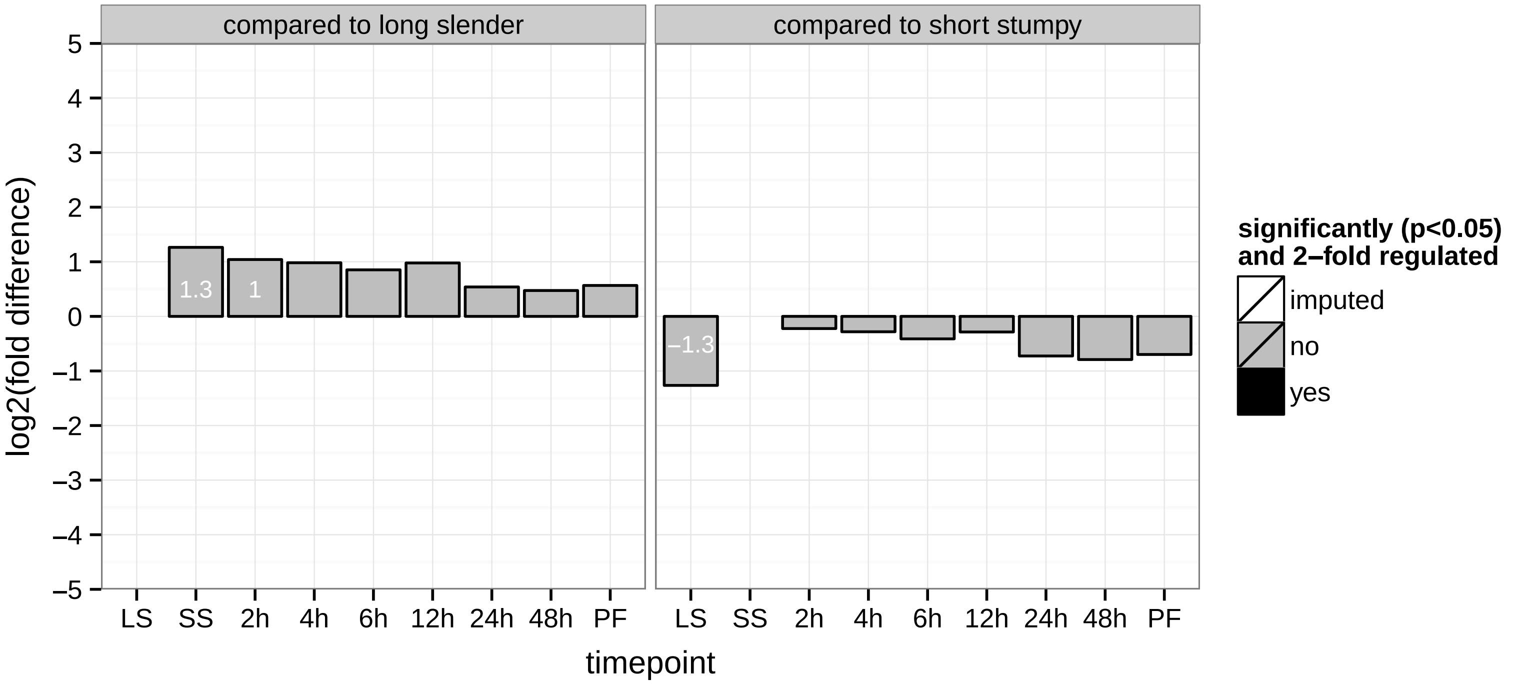

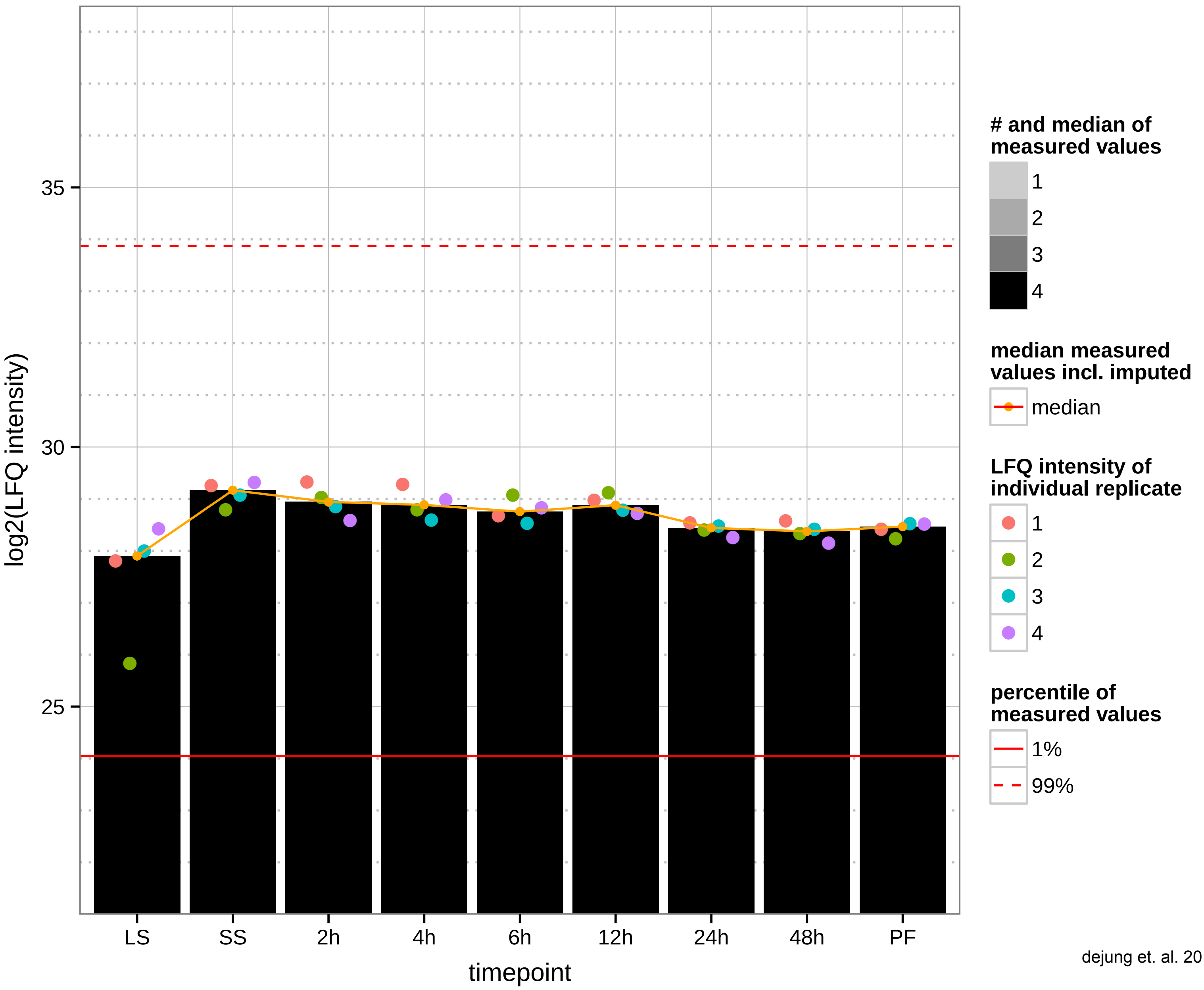

dejung et. al. 2015

eukaryotic translation initiation factor 3 subunit L, putative (EIF3L)
Tb927.10.4640
AGOF: translation initiation factor activity
AGOC: eukaryotic translation initiation factor 3 complex
AGOP: translational initiation
PGOF: null
PGOC: null
PGOP: null

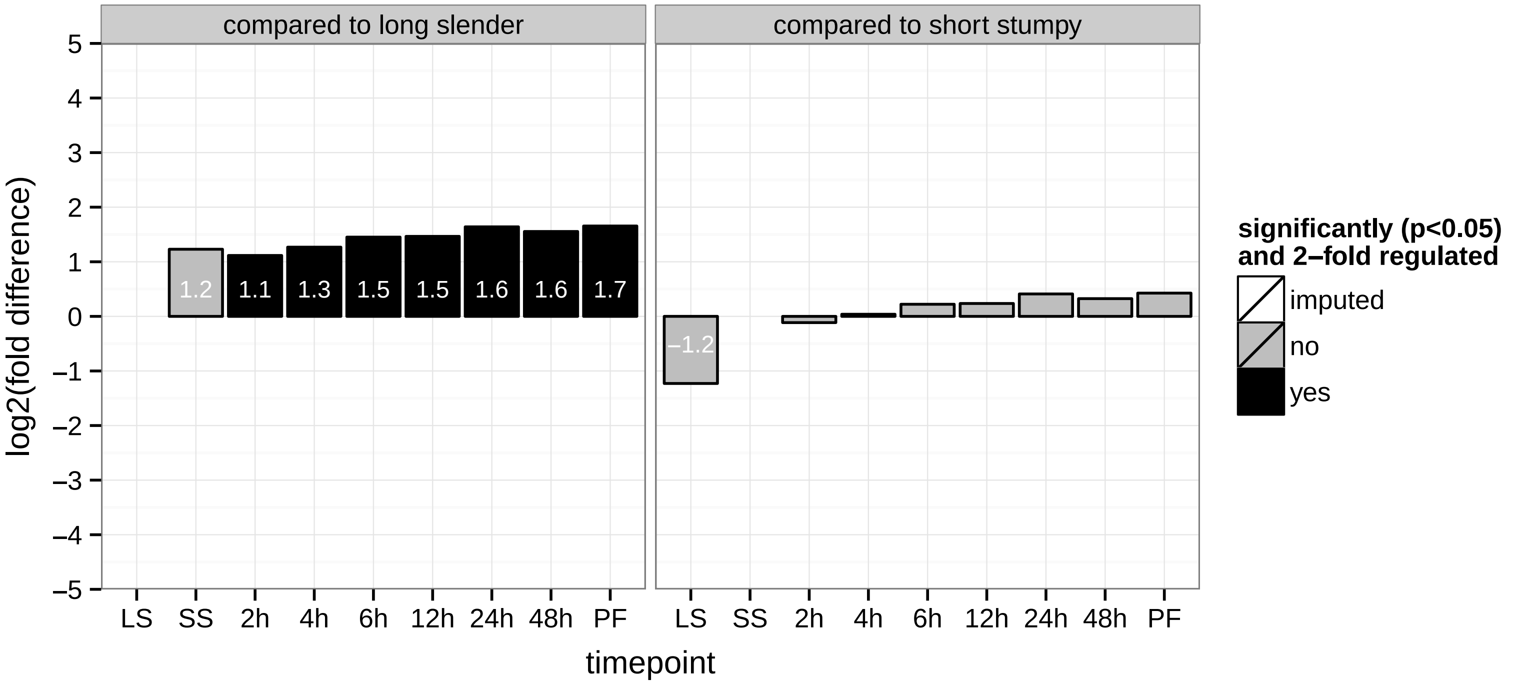

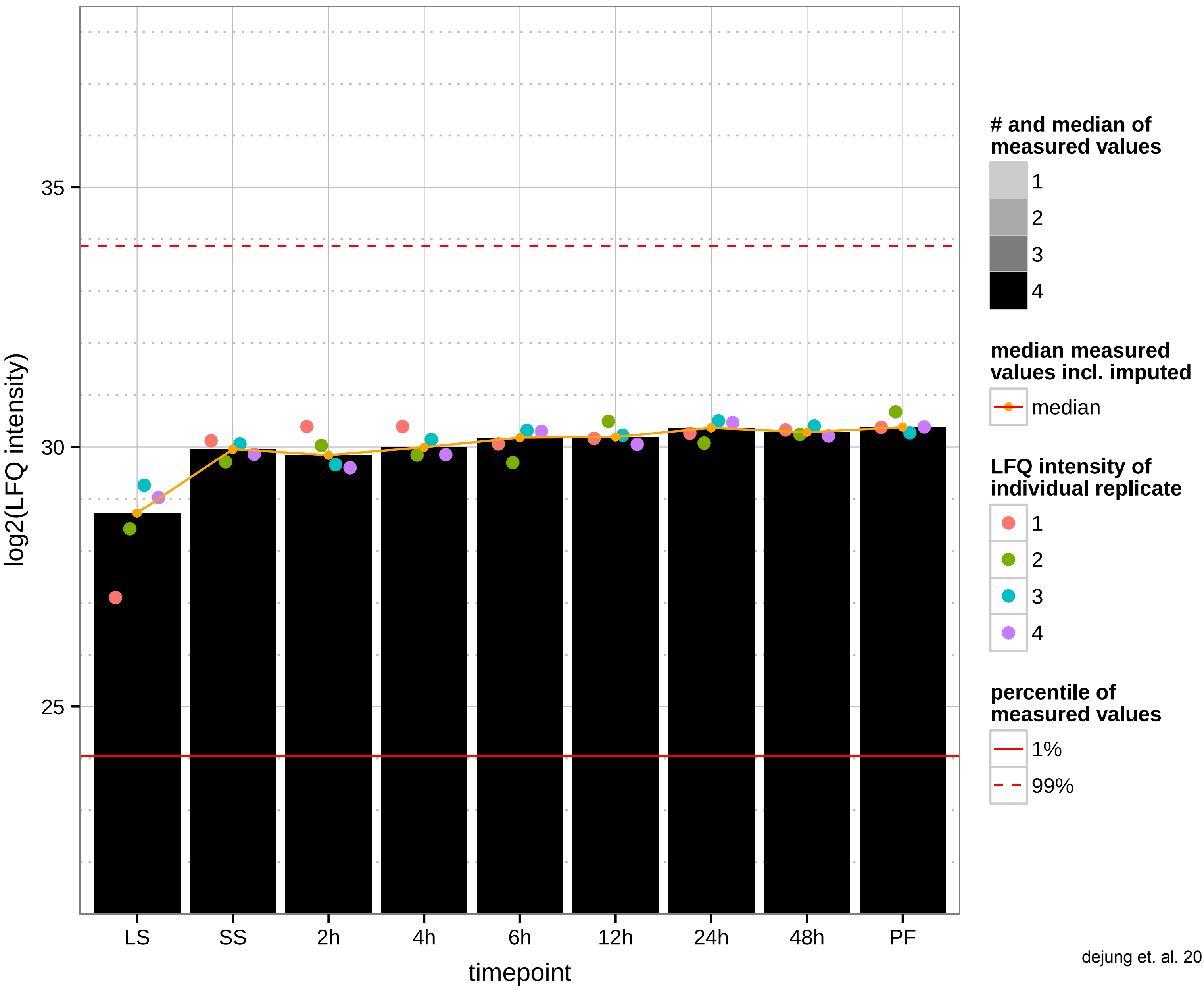

dejung et. al. 2015

hypothetical protein, conserved
Tb927.10.4660
AGOF: null
AGOC: null
AGOP: transcription, DNA–dependent
PGOF: null
PGOC: null
PGOP: transcription, DNA–dependent

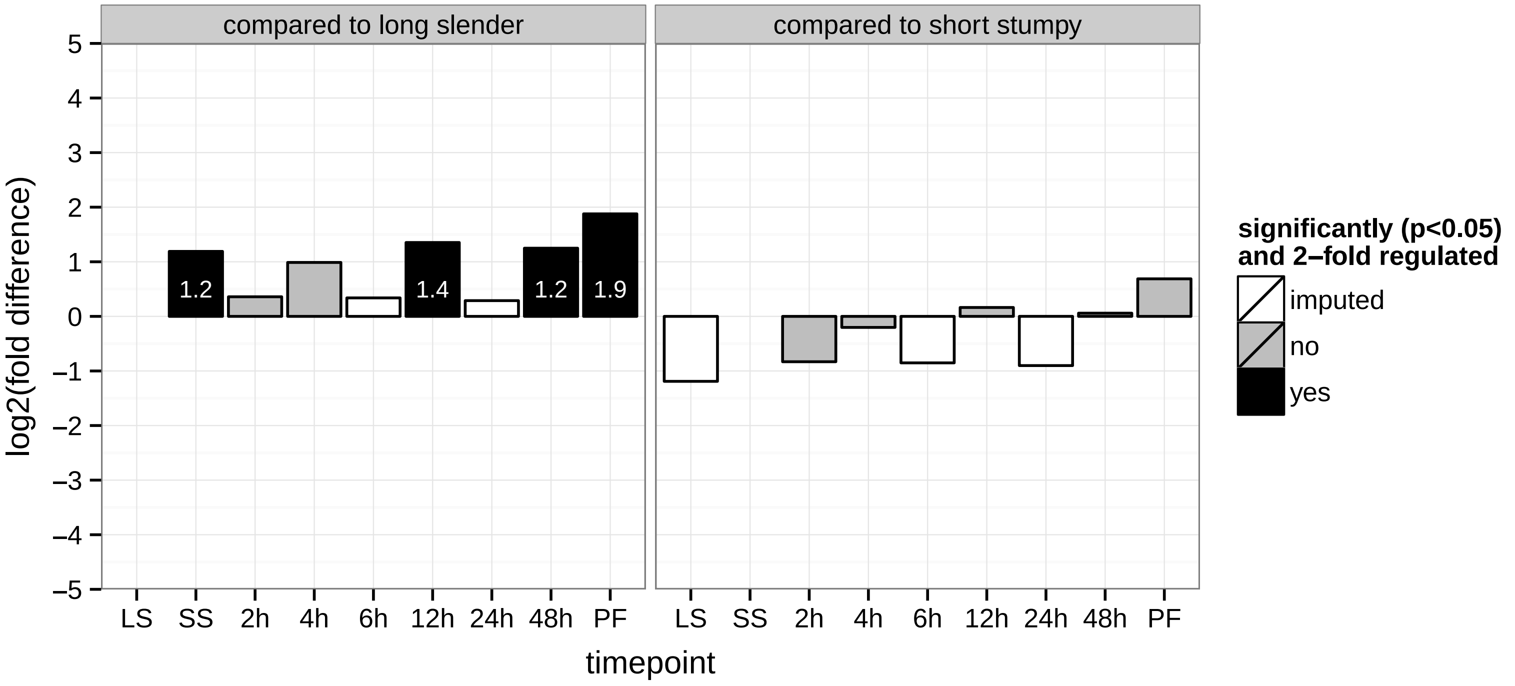

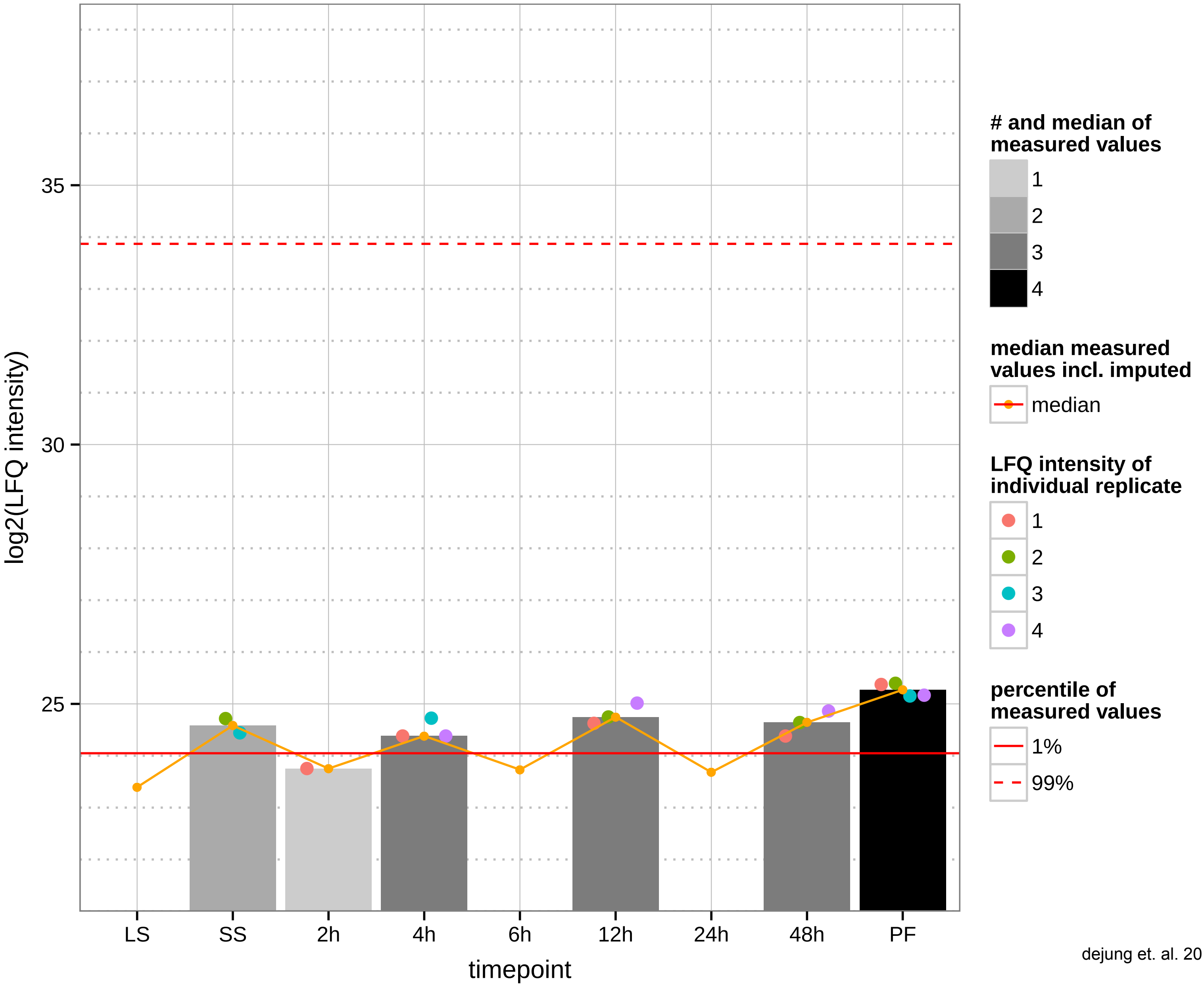

dejung et. al. 2015

dynein light chain, putative
Tb927.10.4690
AGOF: microtubule motor activity
AGOC: microtubule associated complex
AGOP: microtubule–based process
PGOF: null
PGOC: microtubule associated complex
PGOP: microtubule–based process

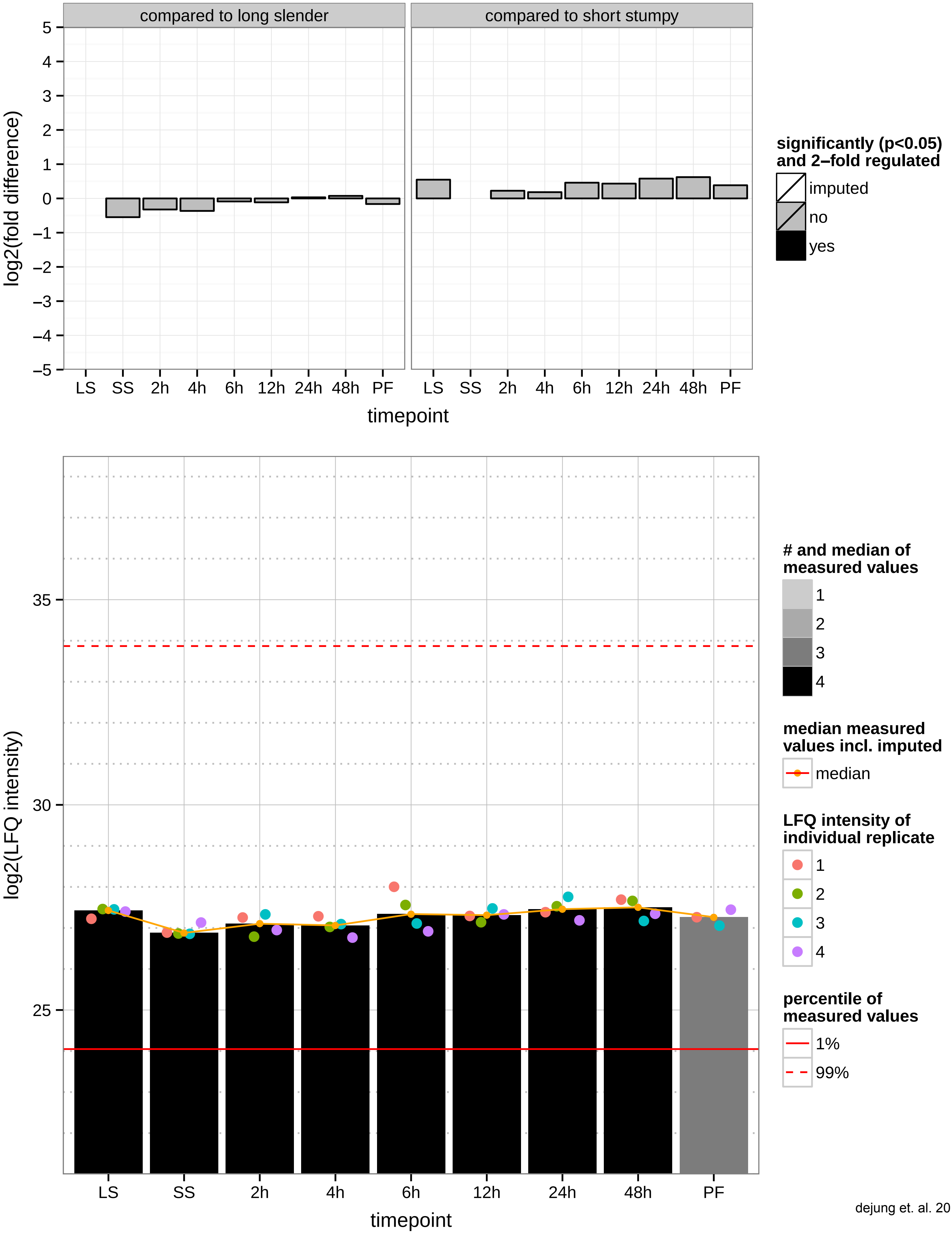

dejung et. al. 2015

dolicholphosphate–mannose synthase, putative
Tb927.10.4700
AGOF: dolichyl–phosphate beta–D–mannosyltransferase activity
AGOC: endoplasmic reticulum membrane
AGOP: dolichol–linked oligosaccharide biosynthetic process
PGOF: null
PGOC: null
PGOP: null

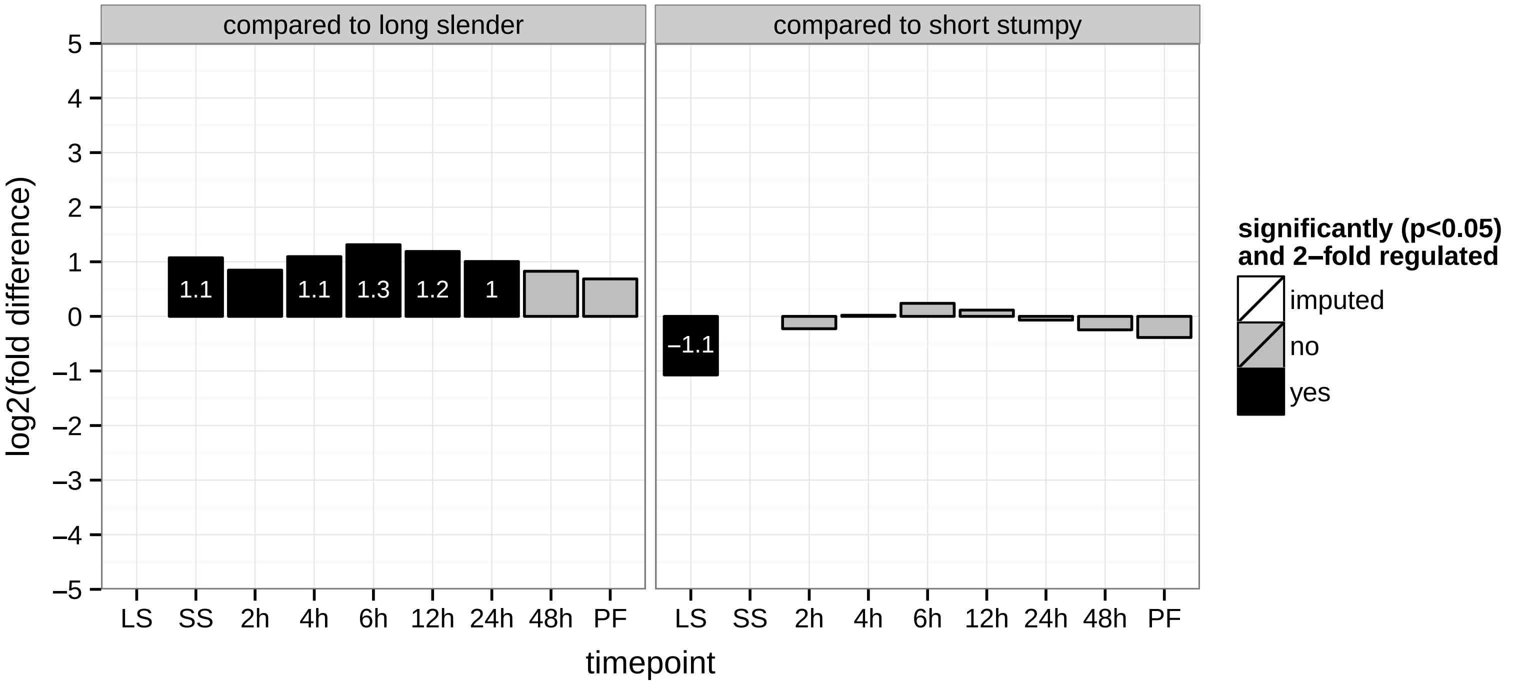

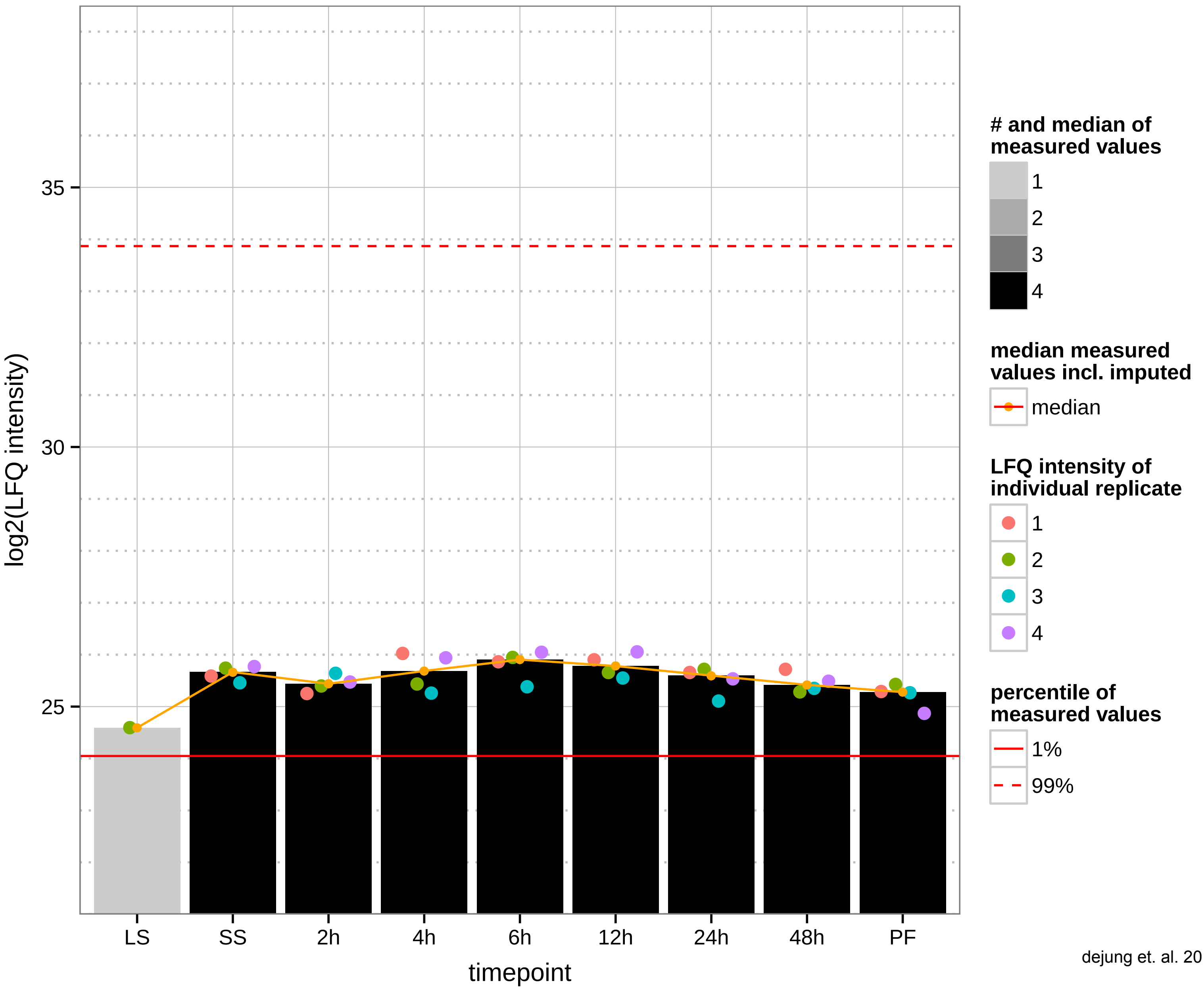

dejung et. al. 2015

proteasome beta 2 subunit, putative, 20S proteasome subunit
Tb927.10.4710
AGOF: endopeptidase activity, threonine−type endopeptidase activity
AGOC: proteasome core complex, proteasome core complex, beta−subunit complex
AGOP: ubiquitin−dependent protein catabolic process
PGOF: threonine−type endopeptidase activity
PGOC: proteasome core complex
PGOP: proteolysis involved in cellular protein catabolic process

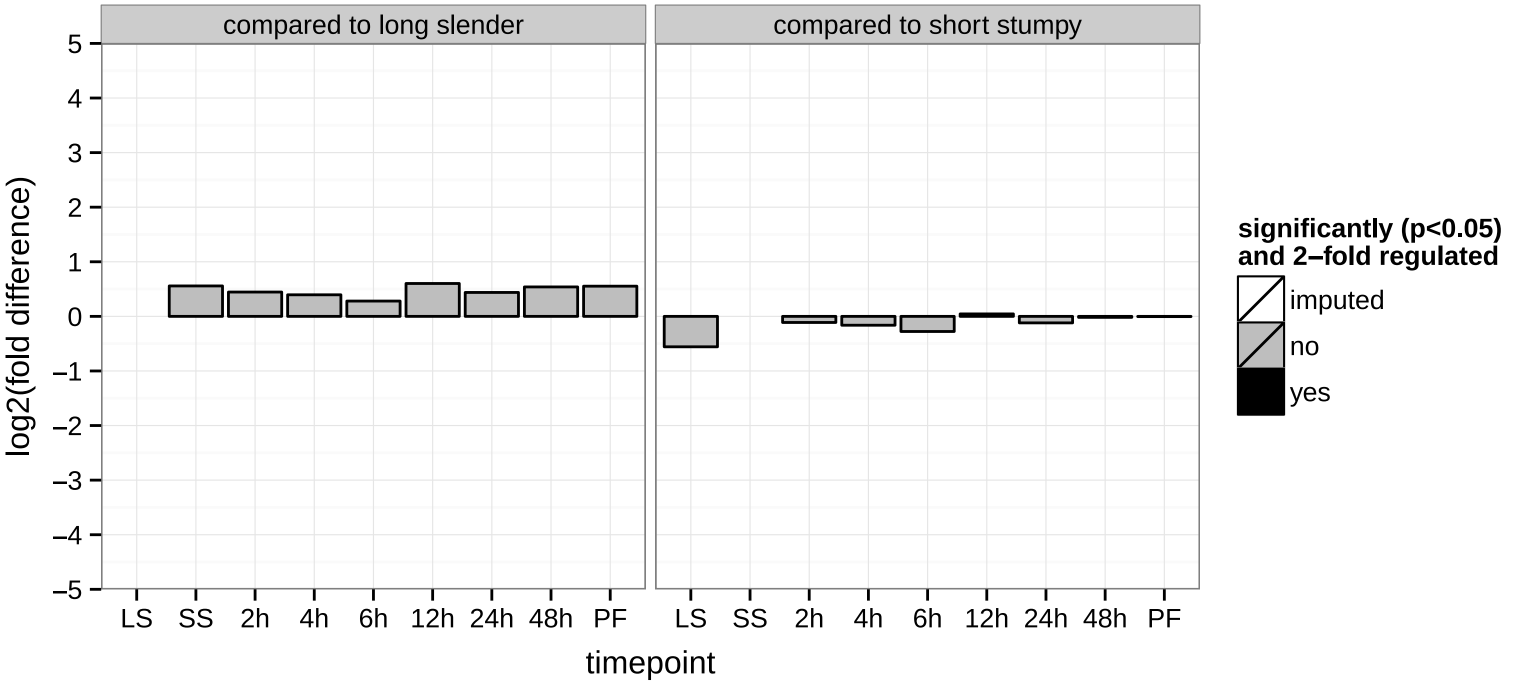

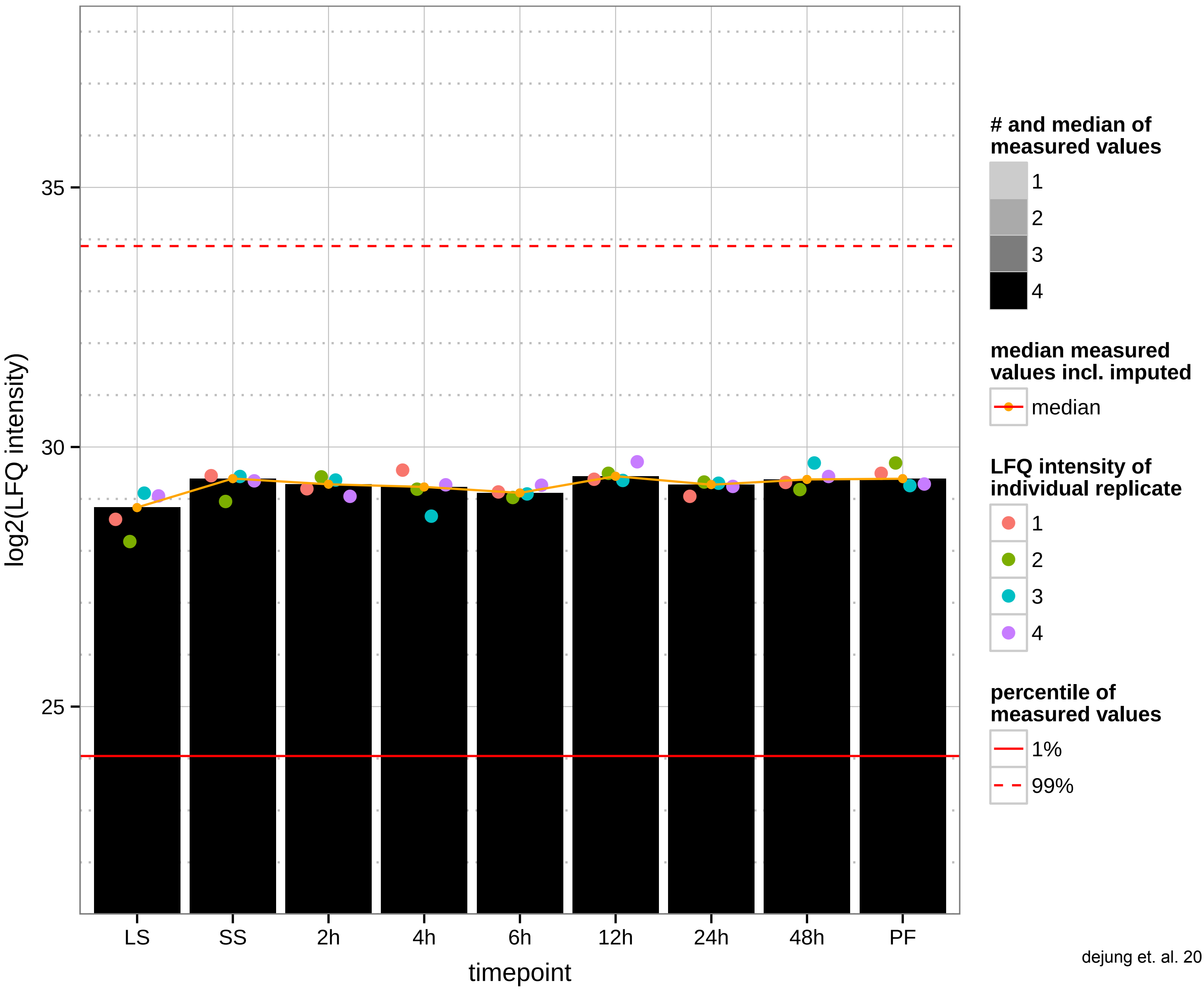

dejung et. al. 2015

hypothetical protein, conserved
Tb927.10.4850
AGOF: null
AGOC: mitochondrion
AGOP: null
PGOF: null
PGOC: null
PGOP: null

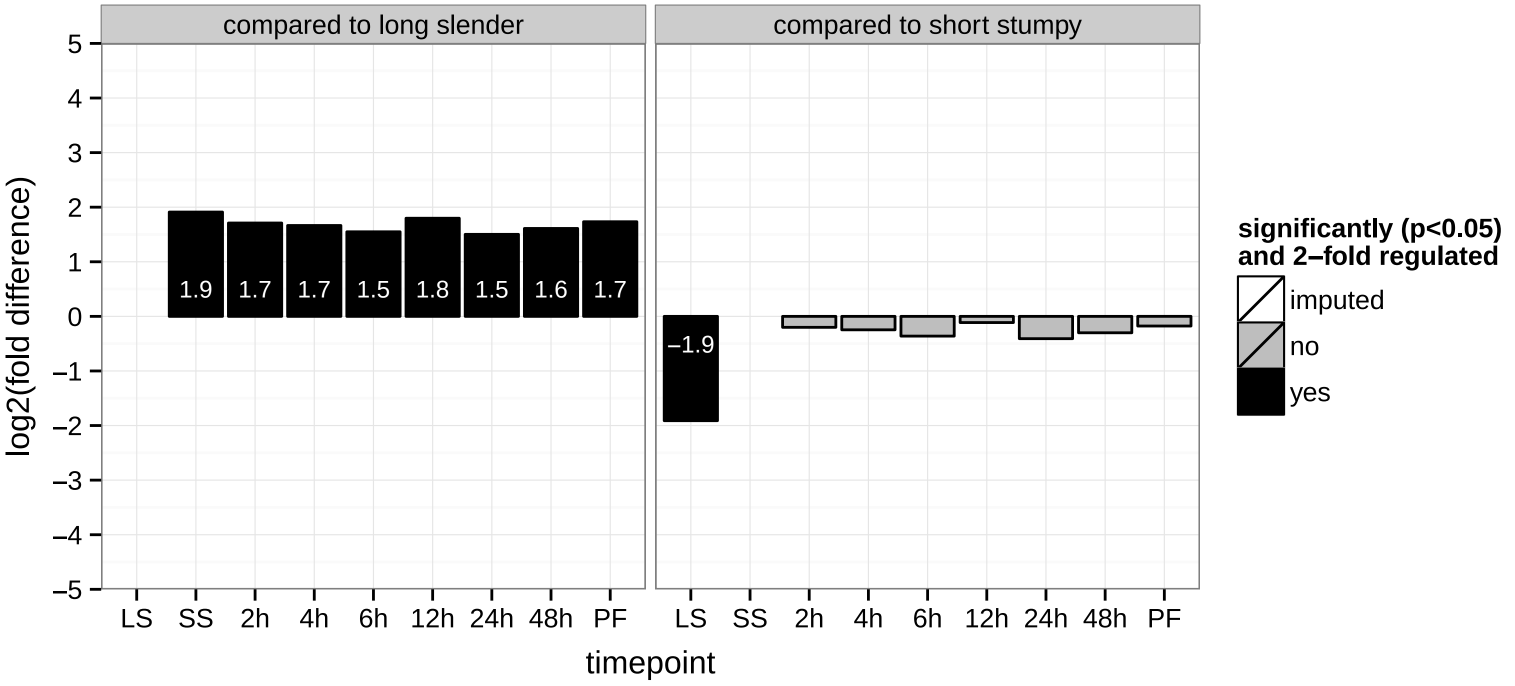

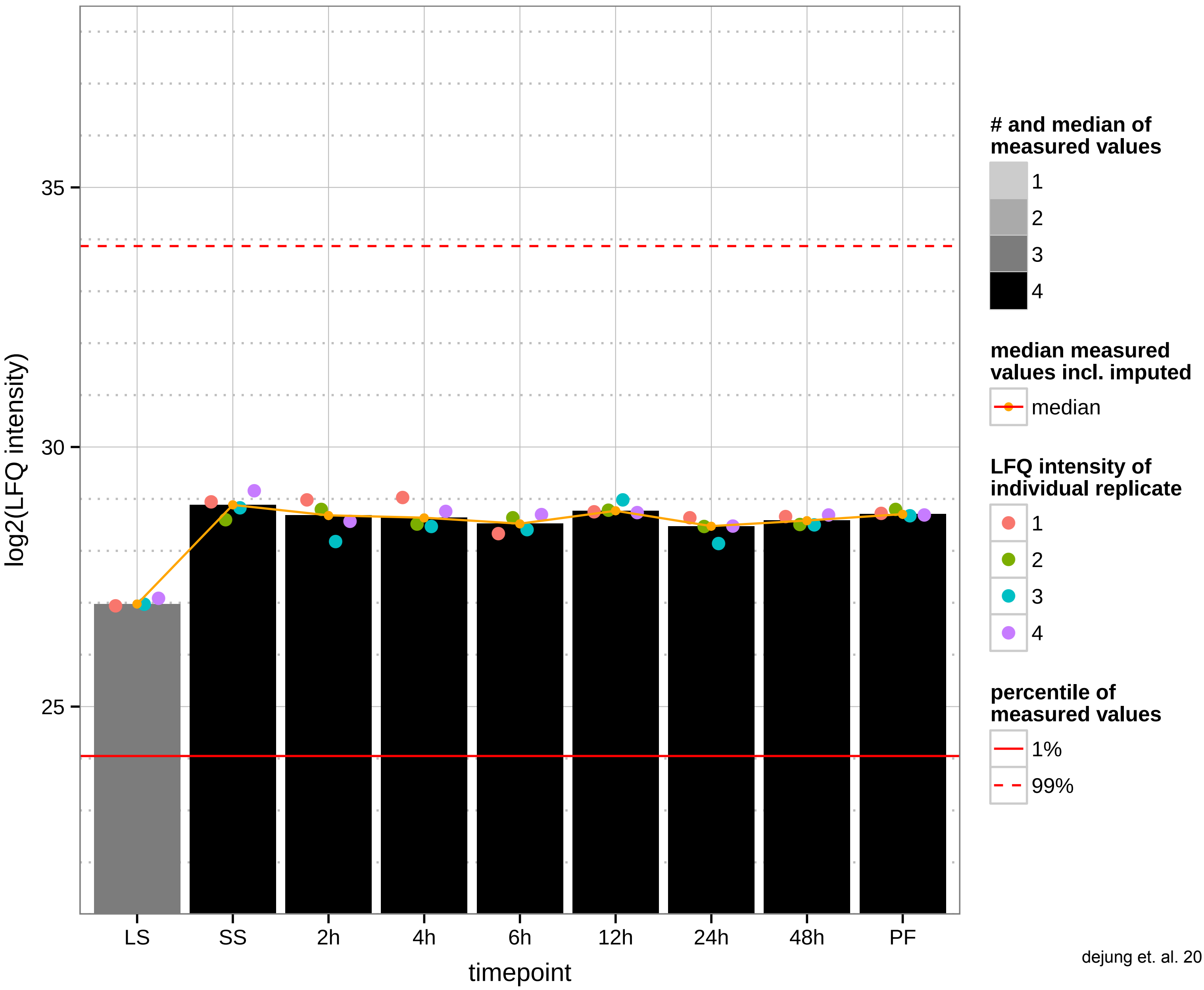

dejung et. al. 2015

protein phosphatase 2C, putative
Tb927.10.4930
AGOF: phosphatase activity
AGOC: protein serine/threonine phosphatase complex
AGOP: dephosphorylation
PGOF: catalytic activity
PGOC: null
PGOP: null

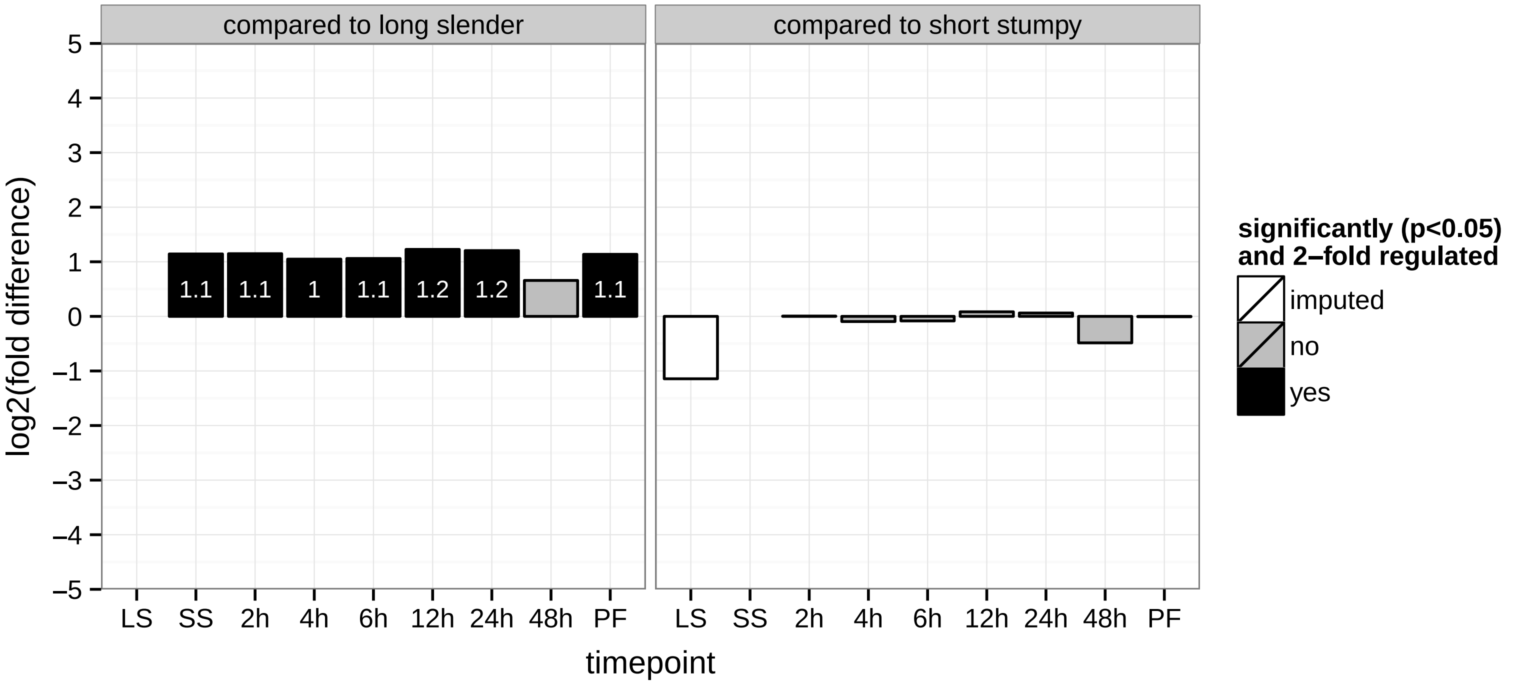

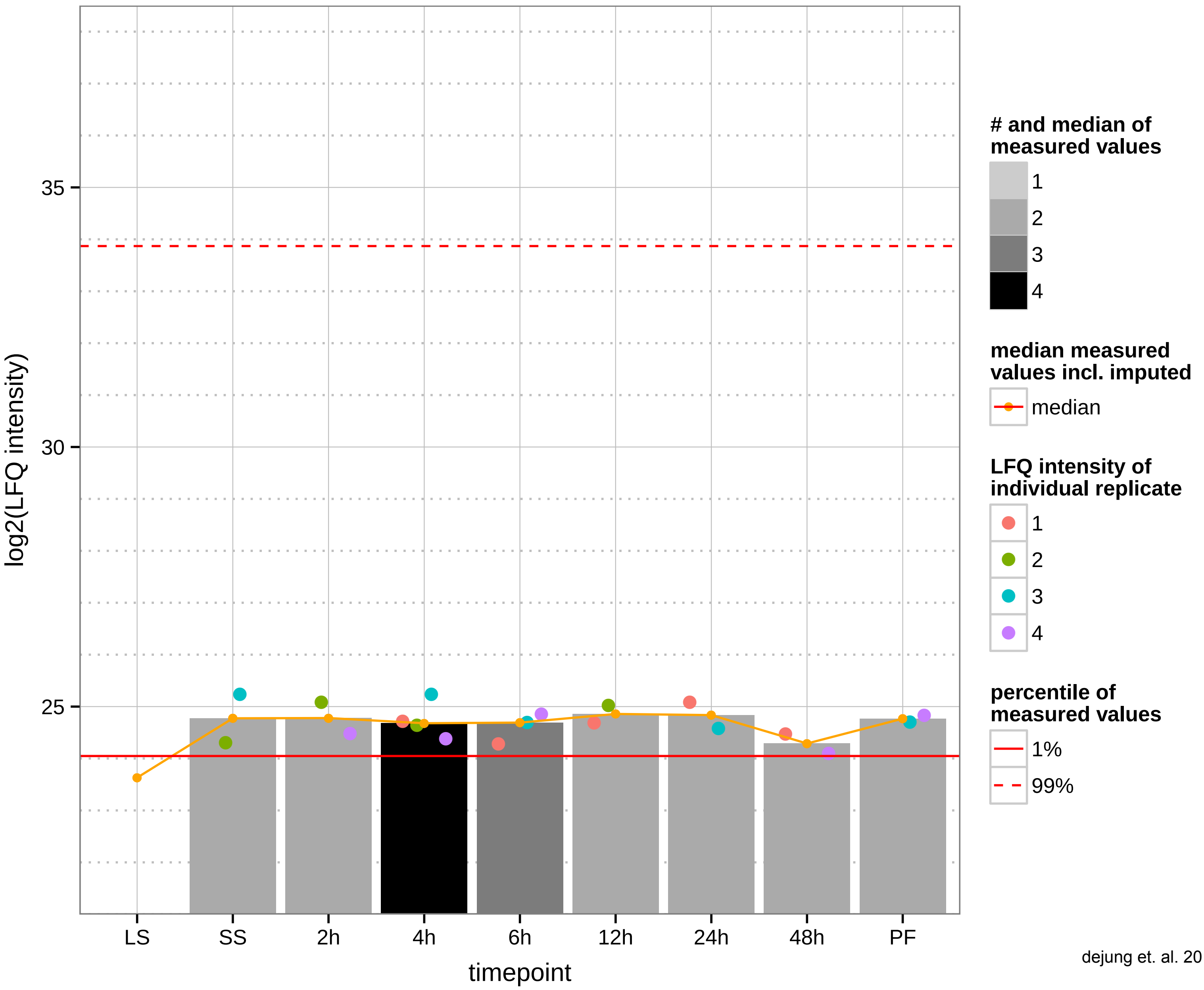

dejung et. al. 2015

serine/threonine protein kinase, putative, Nuclear Dbf2–related kinase (PK50)
Tb927.10.4940;Tb11.v5.0596
AGOF: null, ATP binding, protein kinase activity, protein tyrosine kinase activity
AGOC: null, cytosol, kinetoplast
AGOP: null, cytokinesis, growth, protein phosphorylation, regulation of cell cycle
PGOF: ATP binding, protein kinase activity, transferase activity, transferring phosphorus–containing groups
PGOC: null
PGOP: protein phosphorylation

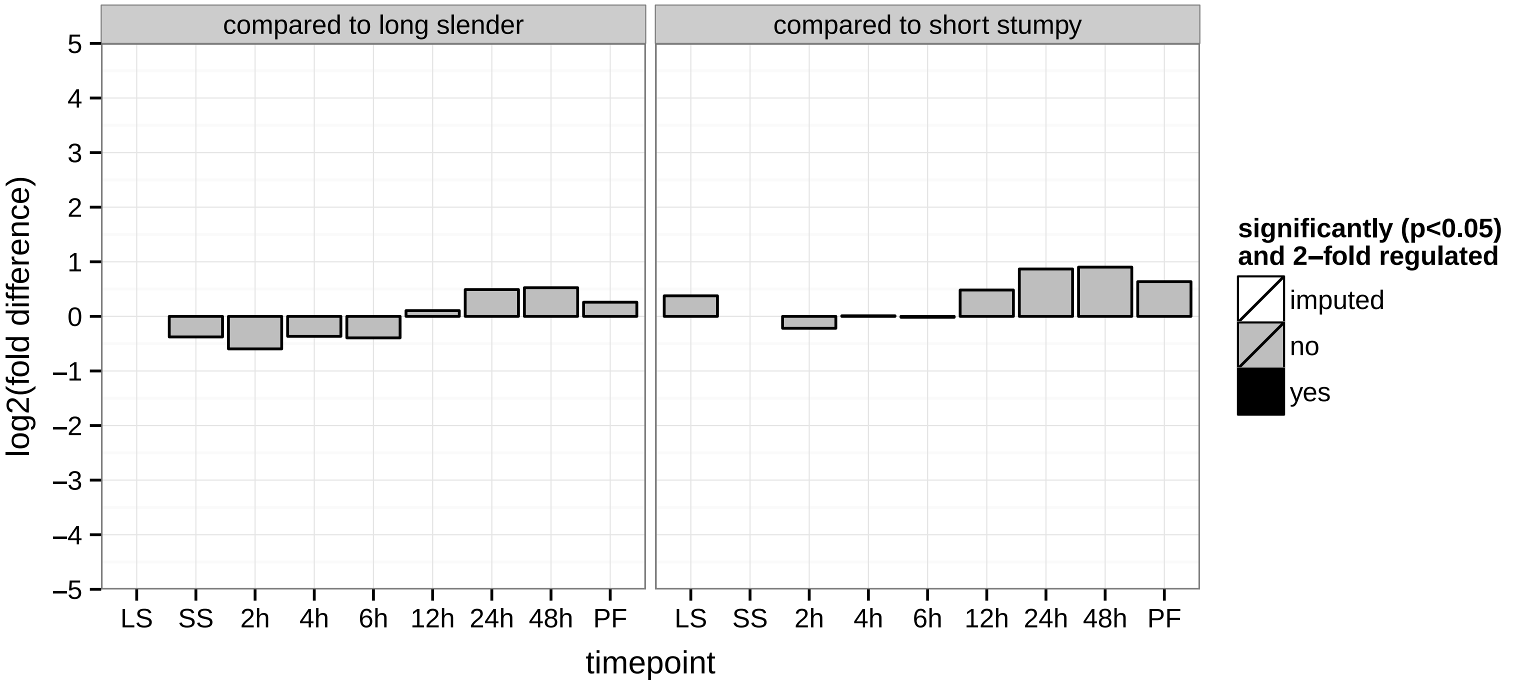

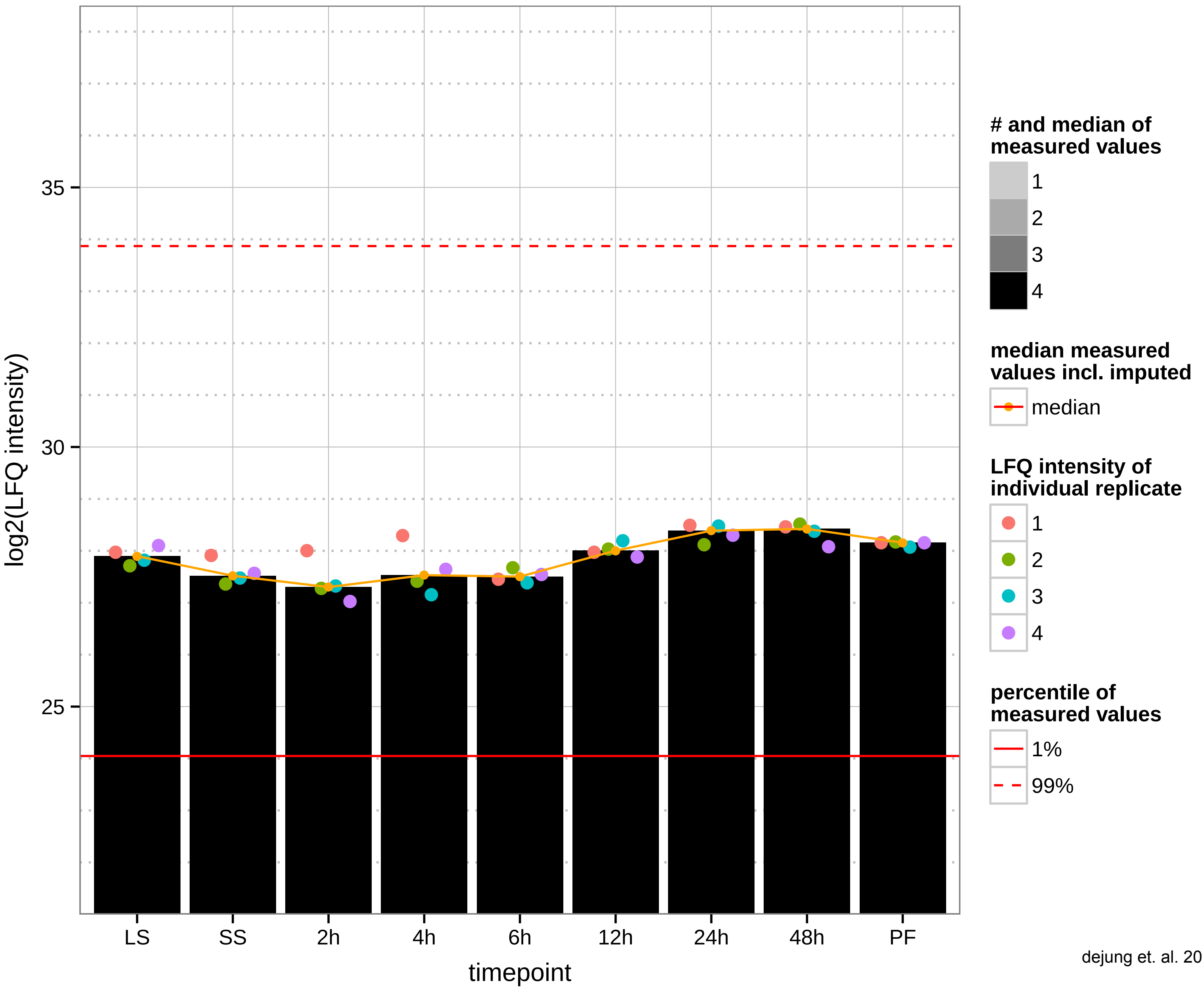

dejung et. al. 2015

ubiquitin–like protein DSK2, putative (DSK2)
Tb927.10.4980
AGOF: protein binding, bridging
AGOC: null
AGOP: DNA replication, chromosome organization, chromosome segregation
PGOF: protein binding
PGOC: null
PGOP: null

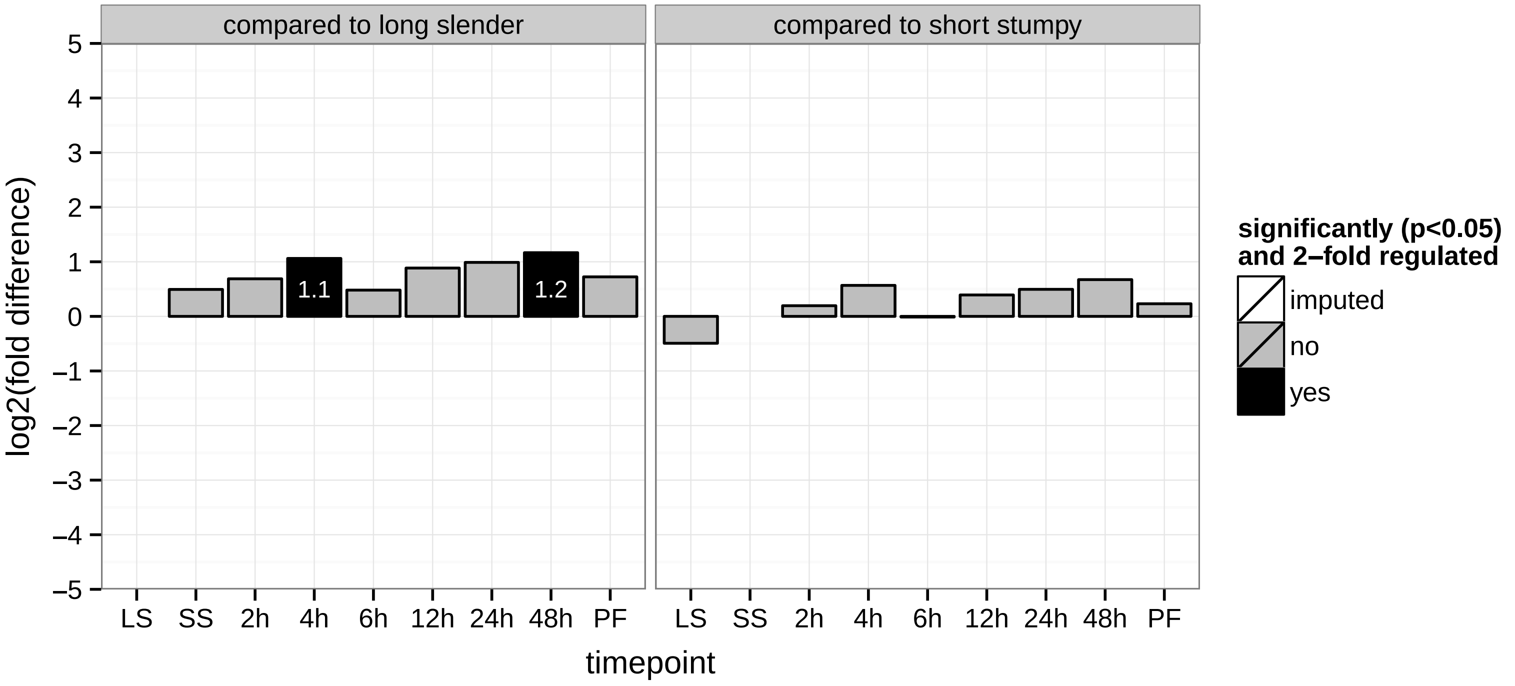

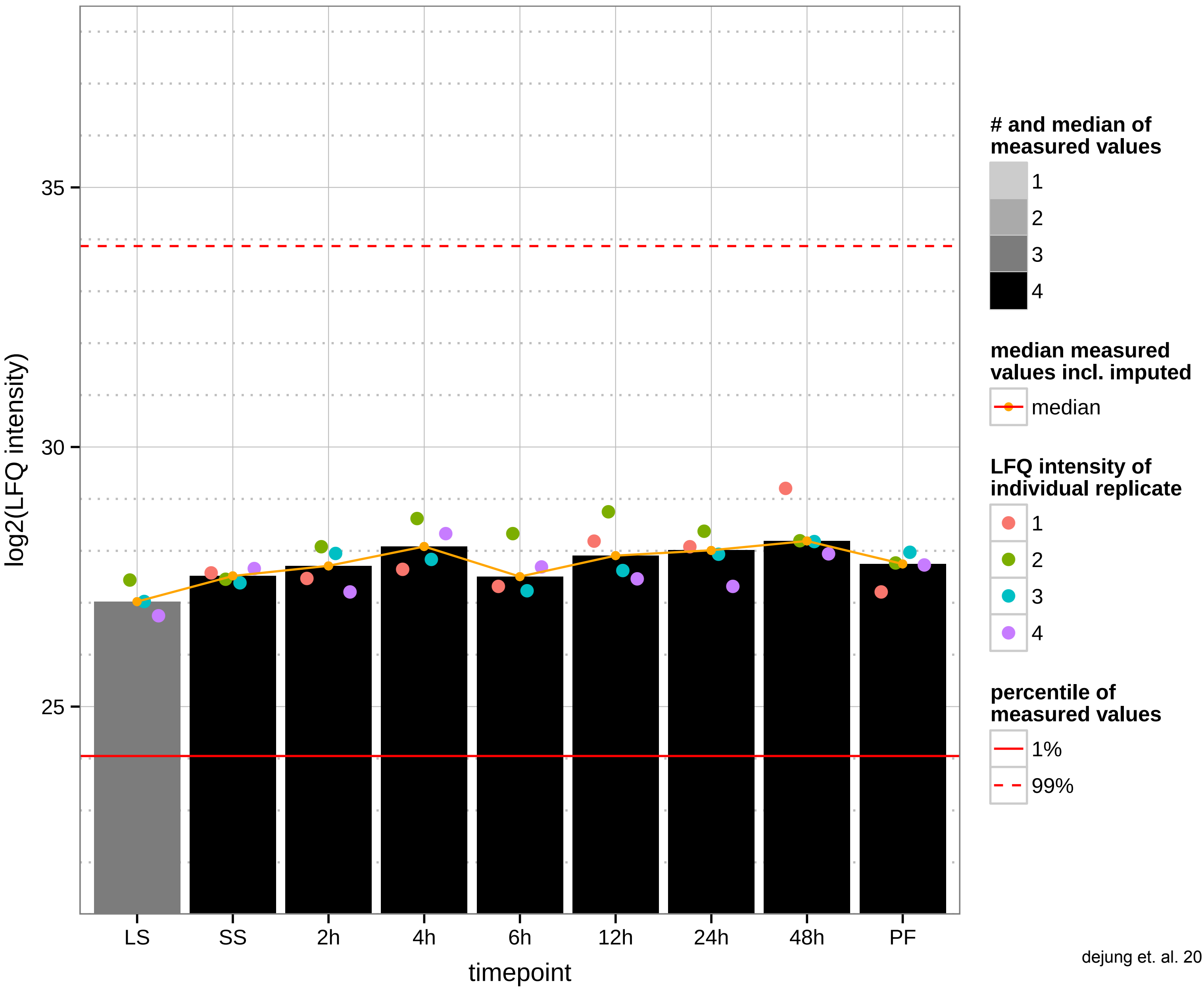

dejung et. al. 2015

ADP−ribosylation factor−like protein, putative, ADP−ribosylation factor
Tb927.10.5230
AGOF: null, GTP binding
AGOC: null, intracellular
AGOP: null, intracellular protein transport, protein transport, small GTPase mediated signal transduction
PGOF: GTP binding, cAMP−dependent protein kinase regulator activity
PGOC: intracellular
PGOP: small GTPase mediated signal transduction, intracellular protein transport, signal transduction, small GTPase mediate

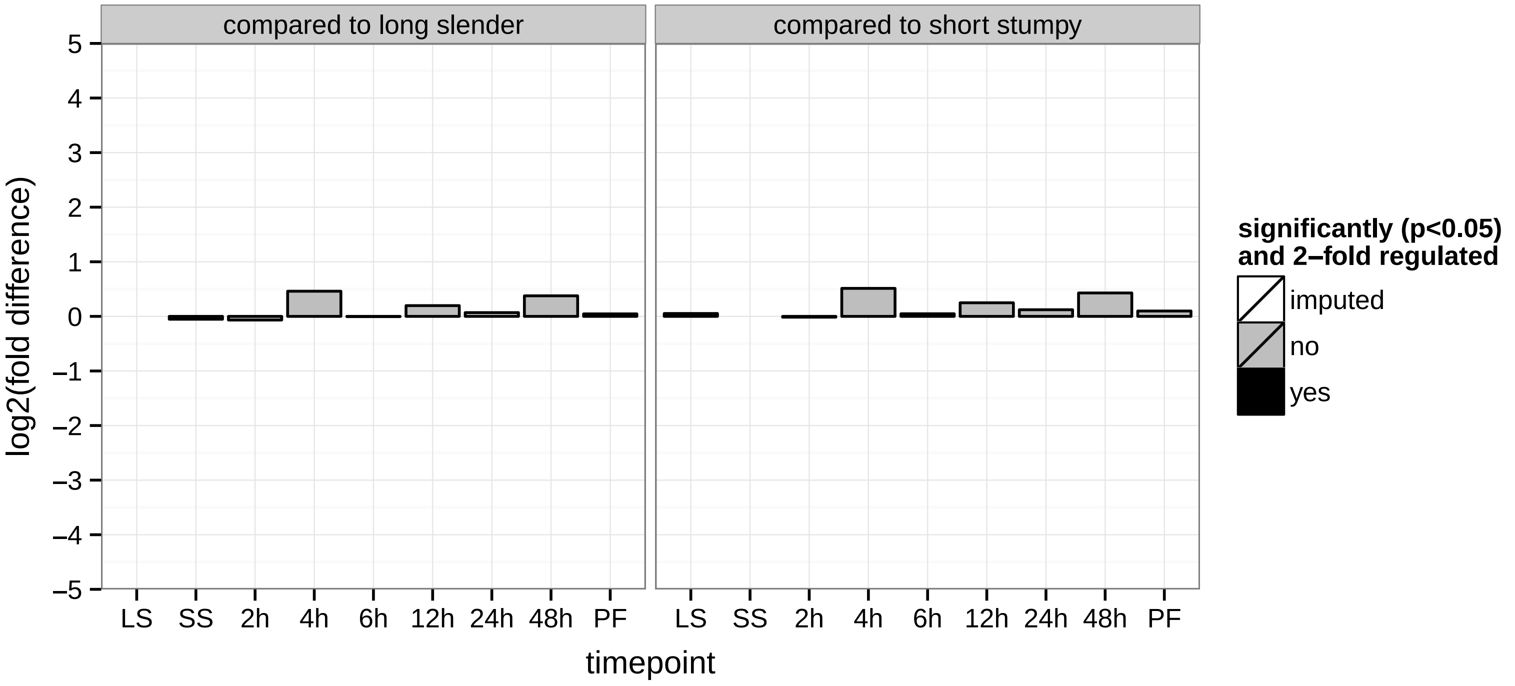

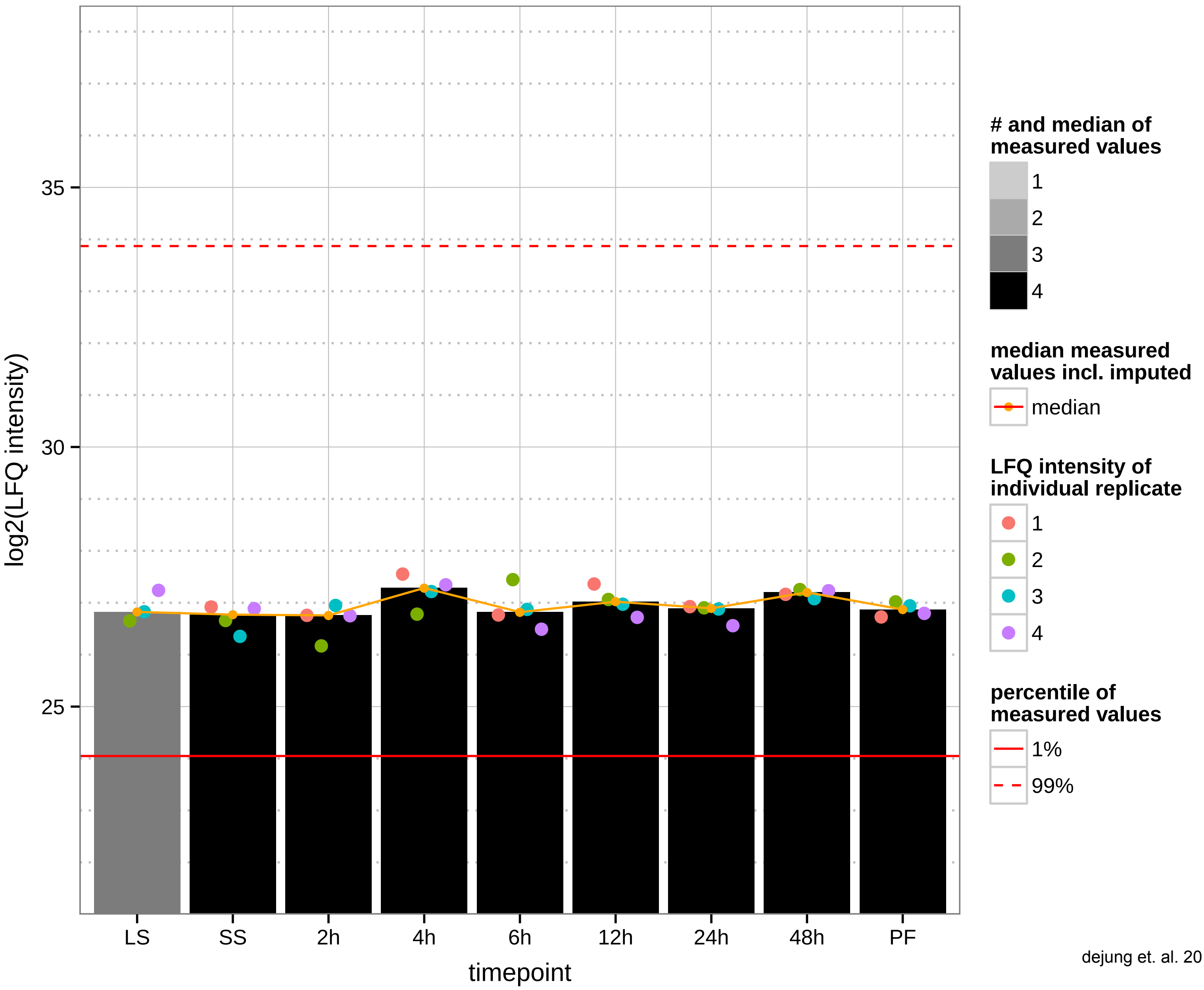

dejung et. al. 2015

dynein heavy chain, putative
Tb927.10.5350;Tb11.v5.0242
AGOF: null, ATP binding, ATPase activity, microtubule motor activity
AGOC: null, dynein complex
AGOP: null, microtubule–based movement
PGOF: microtubule motor activity, null
PGOC: dynein complex, null
PGOP: microtubule–based movement, null

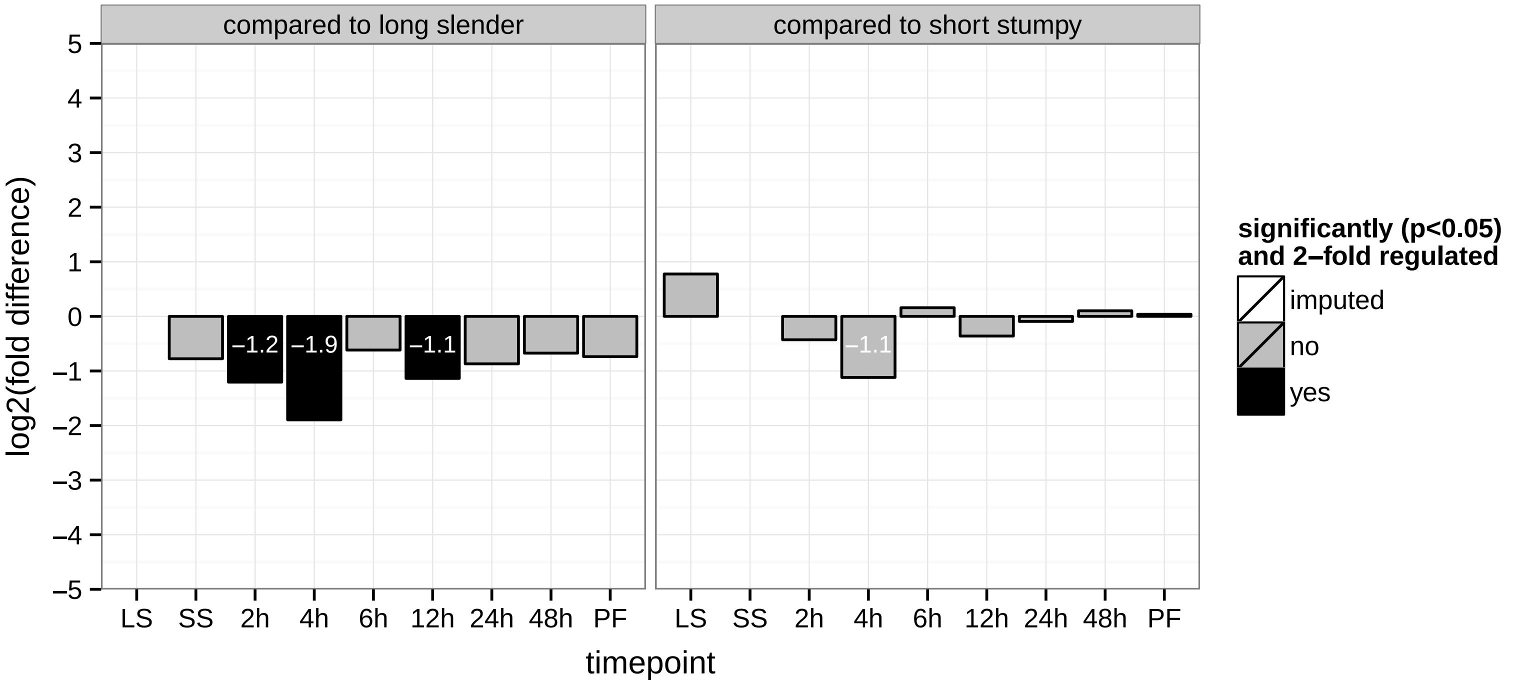

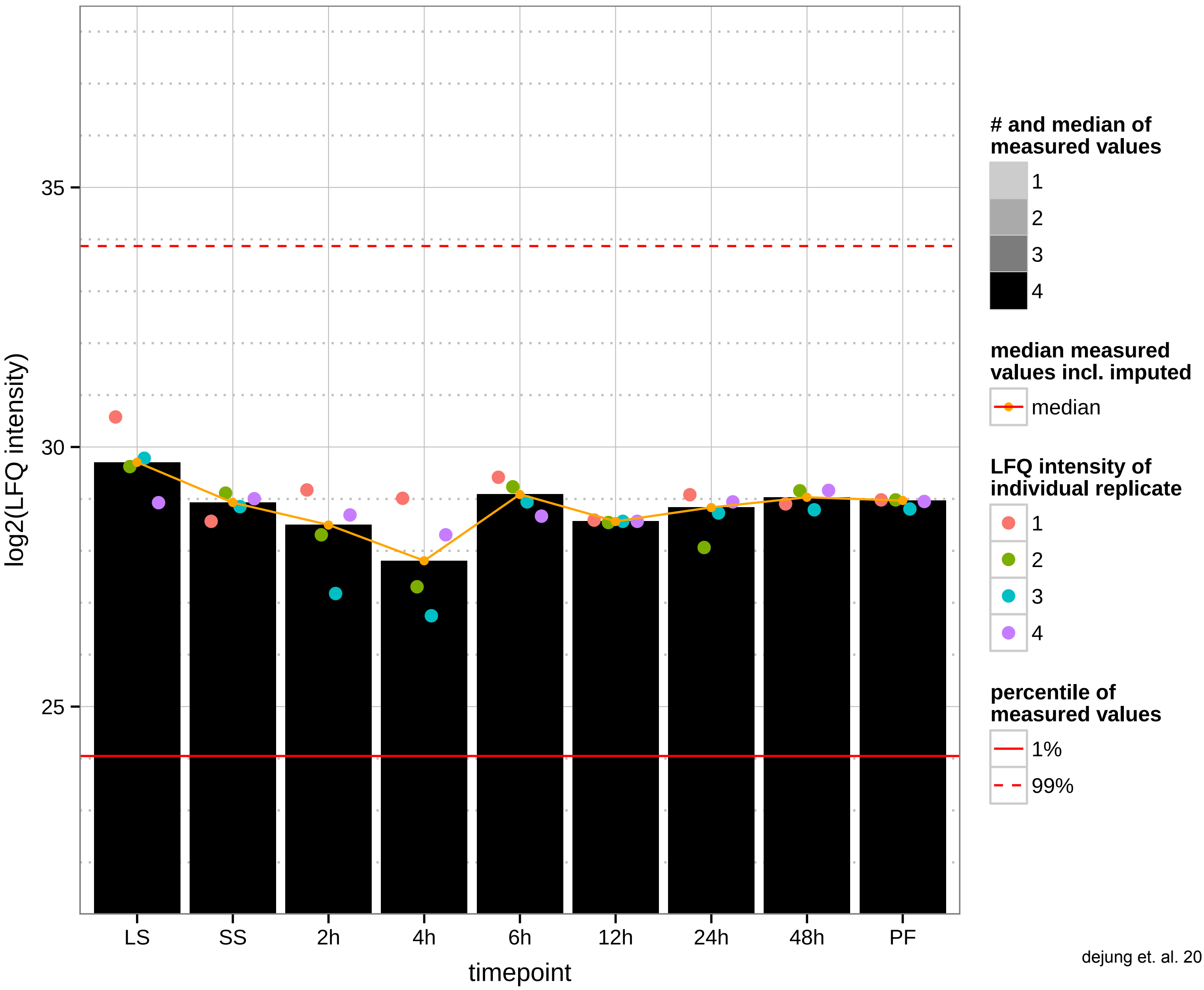

dejung et. al. 2015

hypothetical protein, conserved, intraflagellar transport protein IFT122, putative
Tb927.10.5380;Tb11.v5.0337
AGOF: null
AGOC: null
AGOP: null
PGOF: protein binding
PGOC: null
PGOP: null

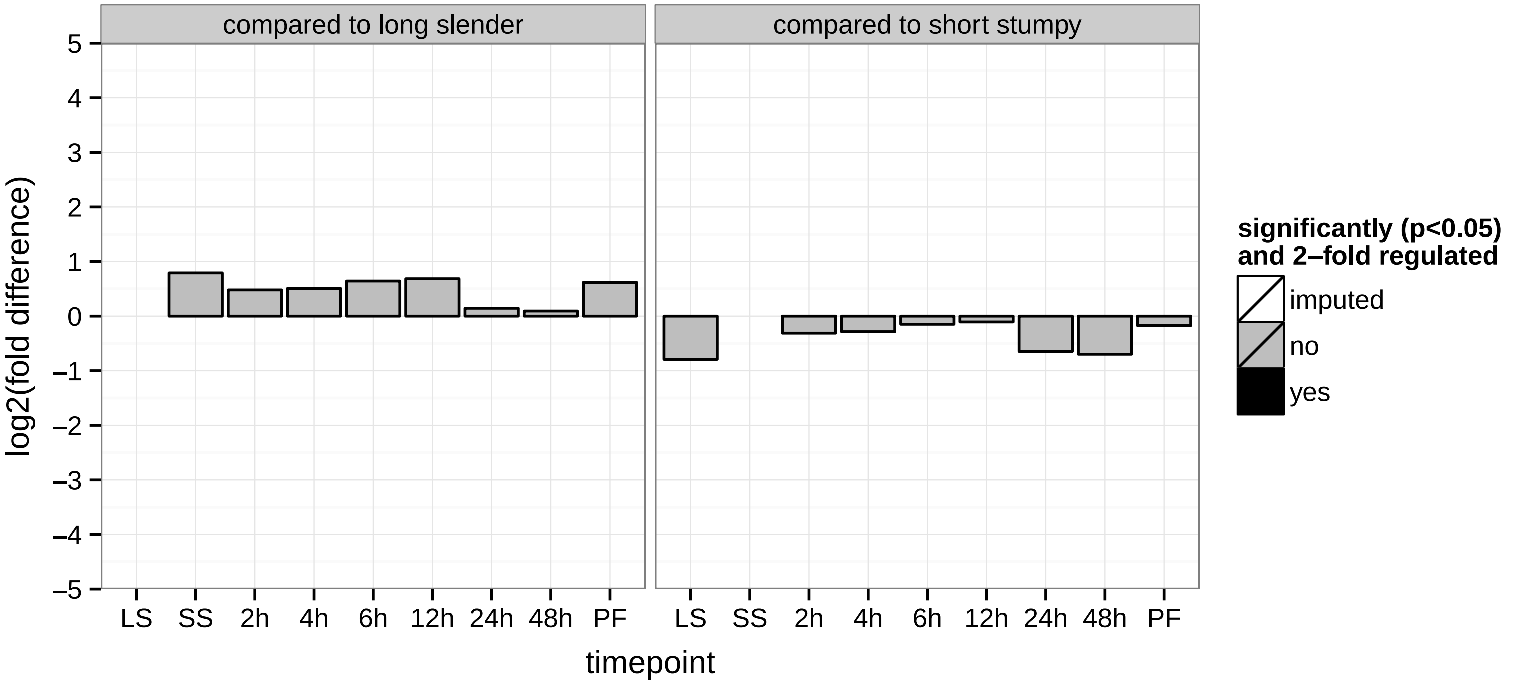

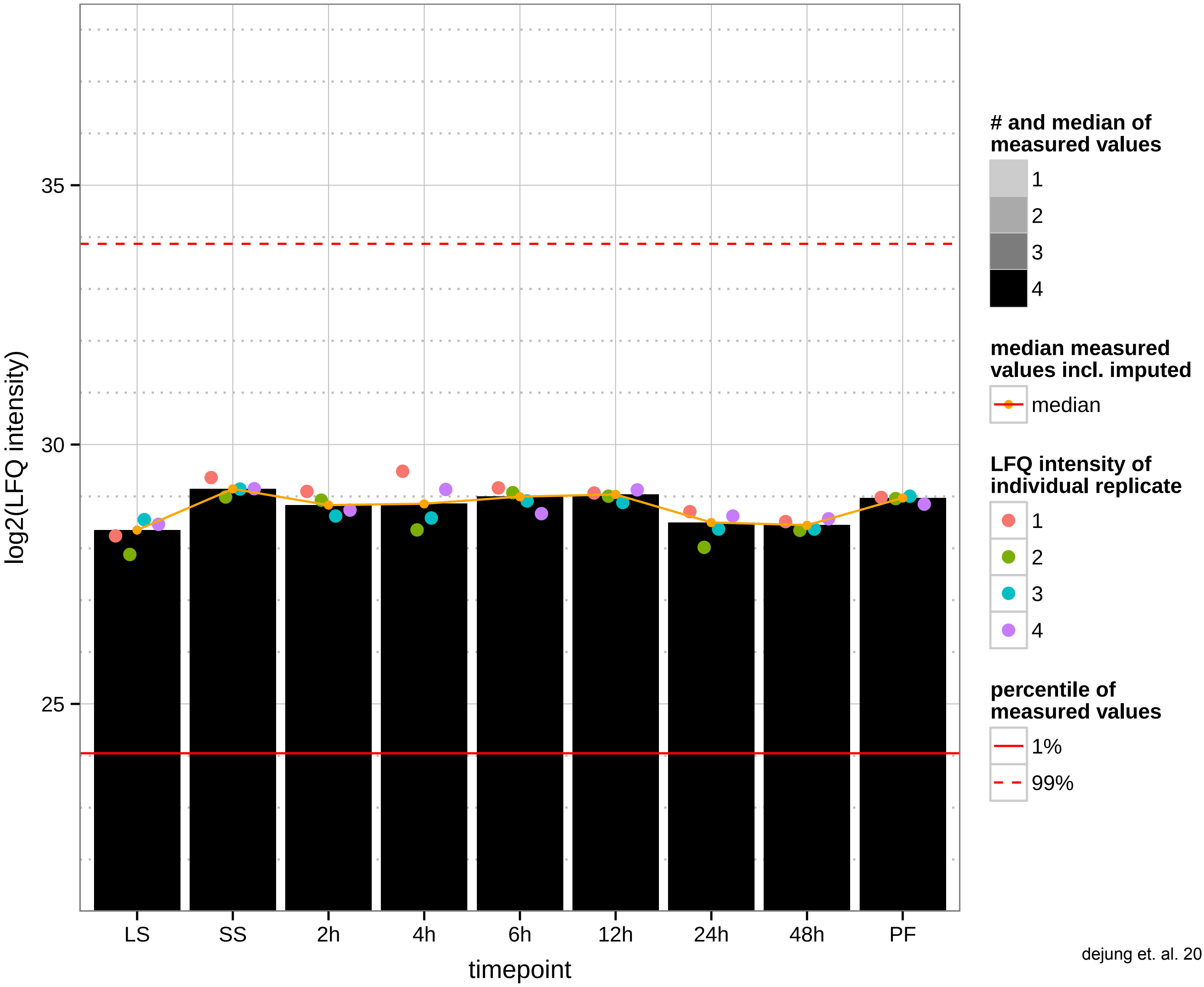

dejung et. al. 2015

hypothetical protein, conserved
Tb927.10.5430
AGOF: null
AGOC: null
AGOP: null
PGOF: null
PGOC: null
PGOP: null

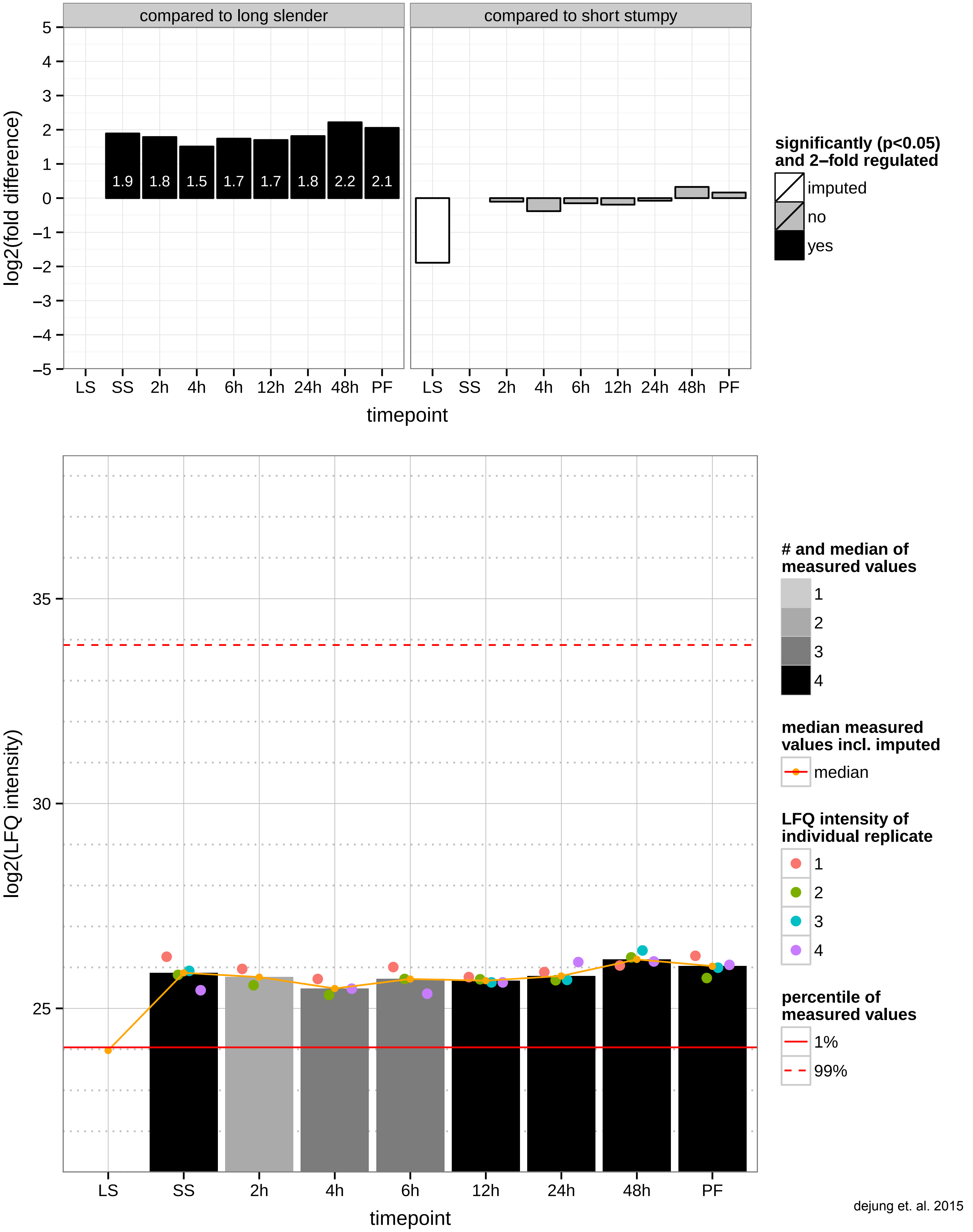

dejung et. al. 2015

nucleoplasmin–like protein (NLP) (nlp)
Tb927.10.5450
AGOF: DNA binding
AGOC: nucleus
AGOP: chromatin remodeling, negative regulation of transcription, DNA–dependent, positive regulation of transcription, regula
PGOF: binding
PGOC: null
PGOP: null

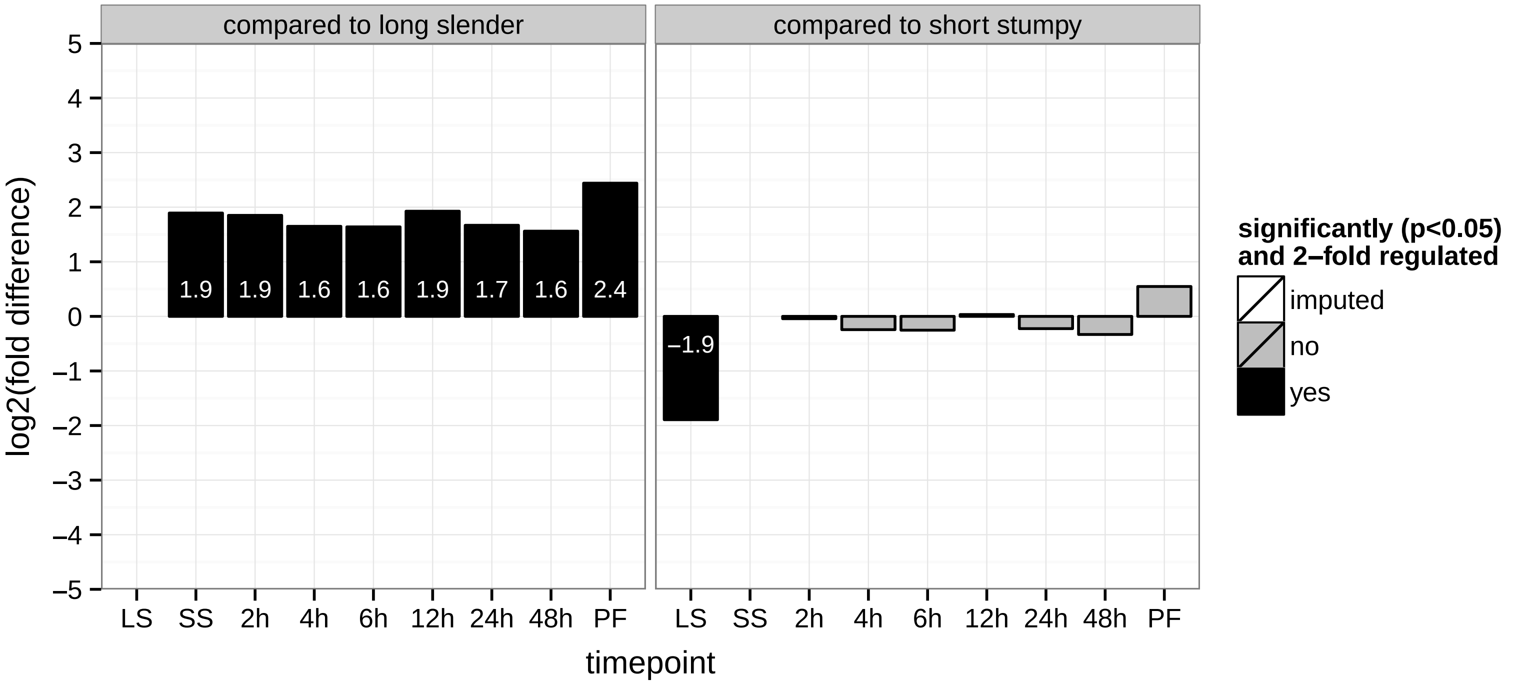

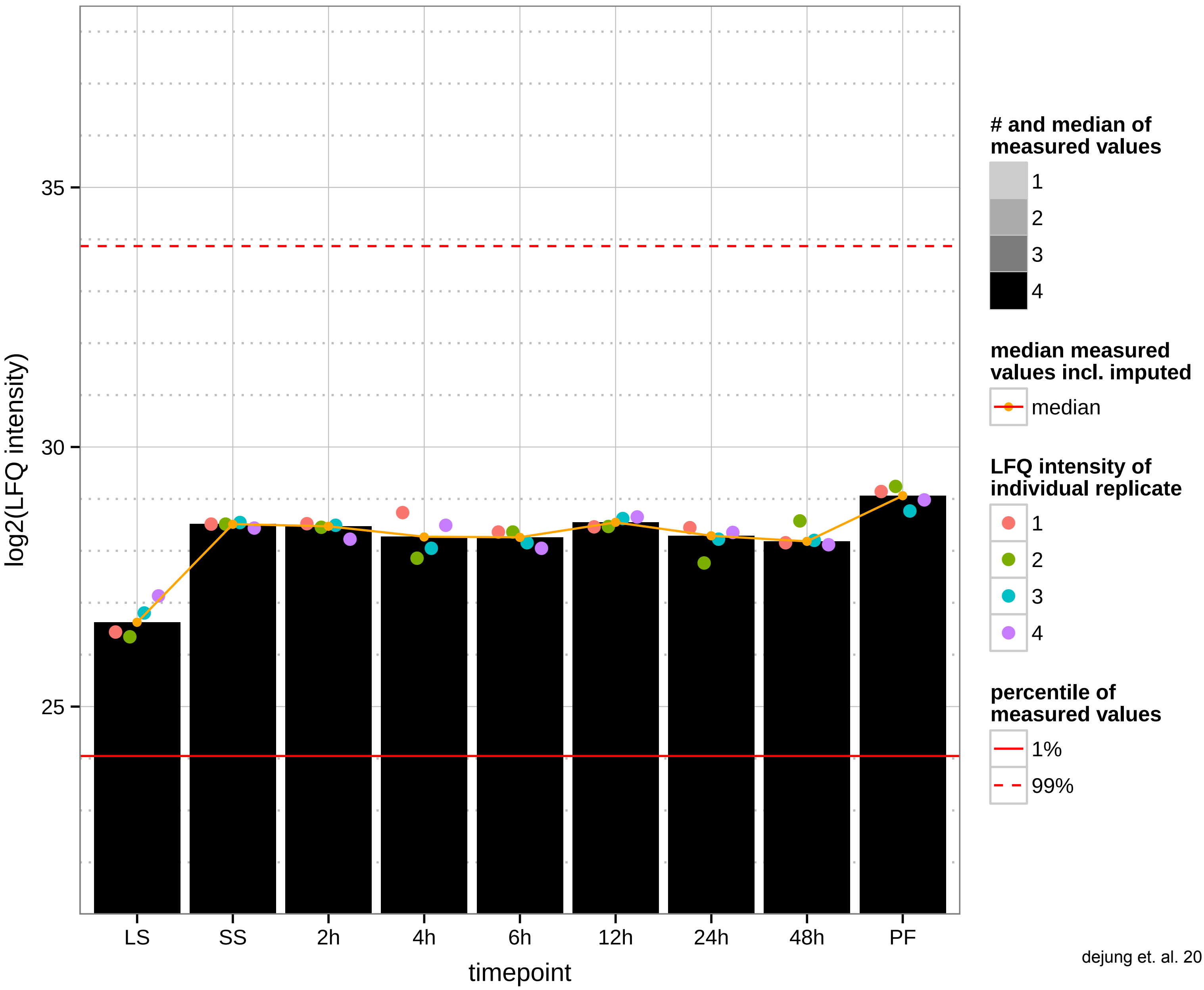

dejung et. al. 2015

hypothetical protein, conserved
Tb927.10.5520
AGOF: null
AGOC: null
AGOP: null
PGOF: null
PGOC: null
PGOP: null

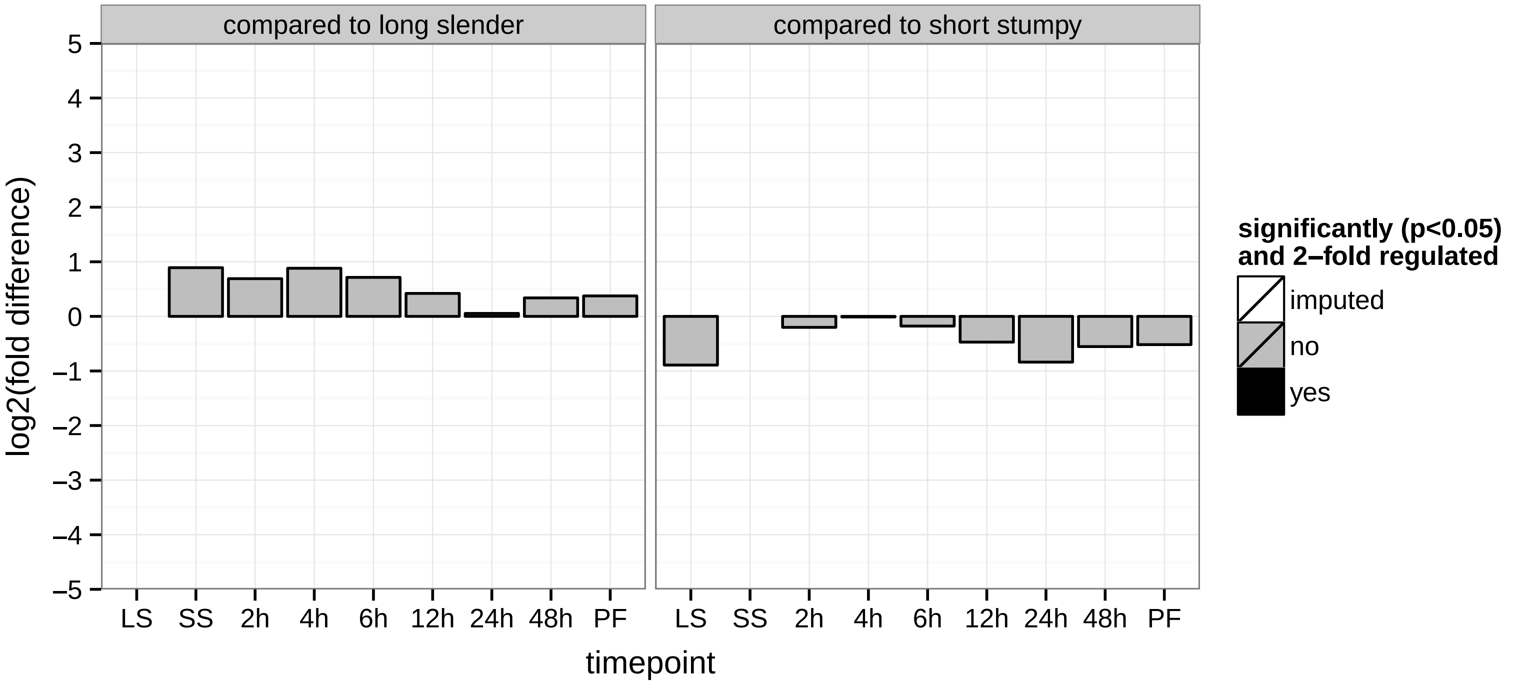

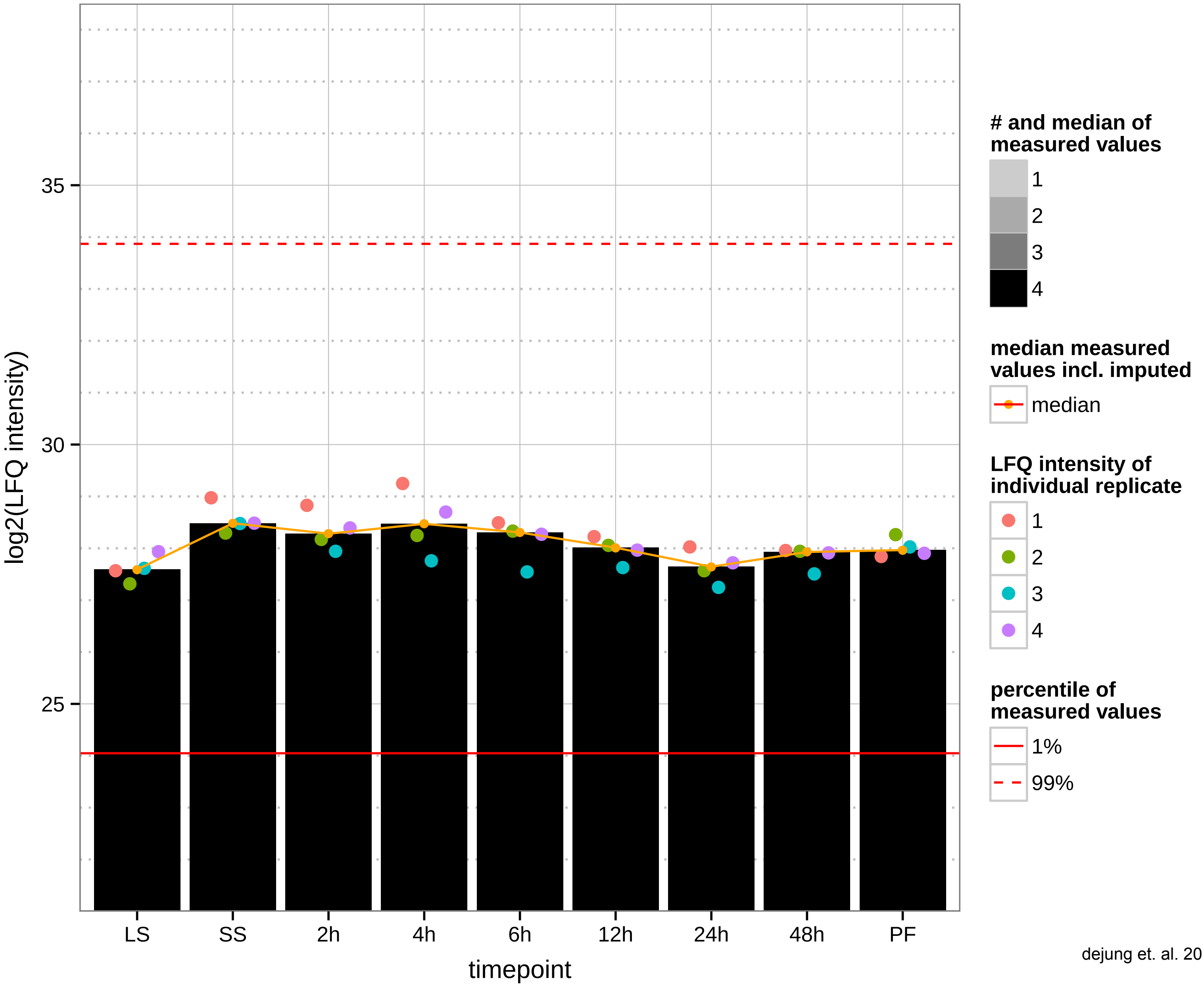

dejung et. al. 2015

hypothetical protein, conserved
Tb927.10.5540
AGOF: null
AGOC: mitochondrion
AGOP: null
PGOF: null
PGOC: null
PGOP: null

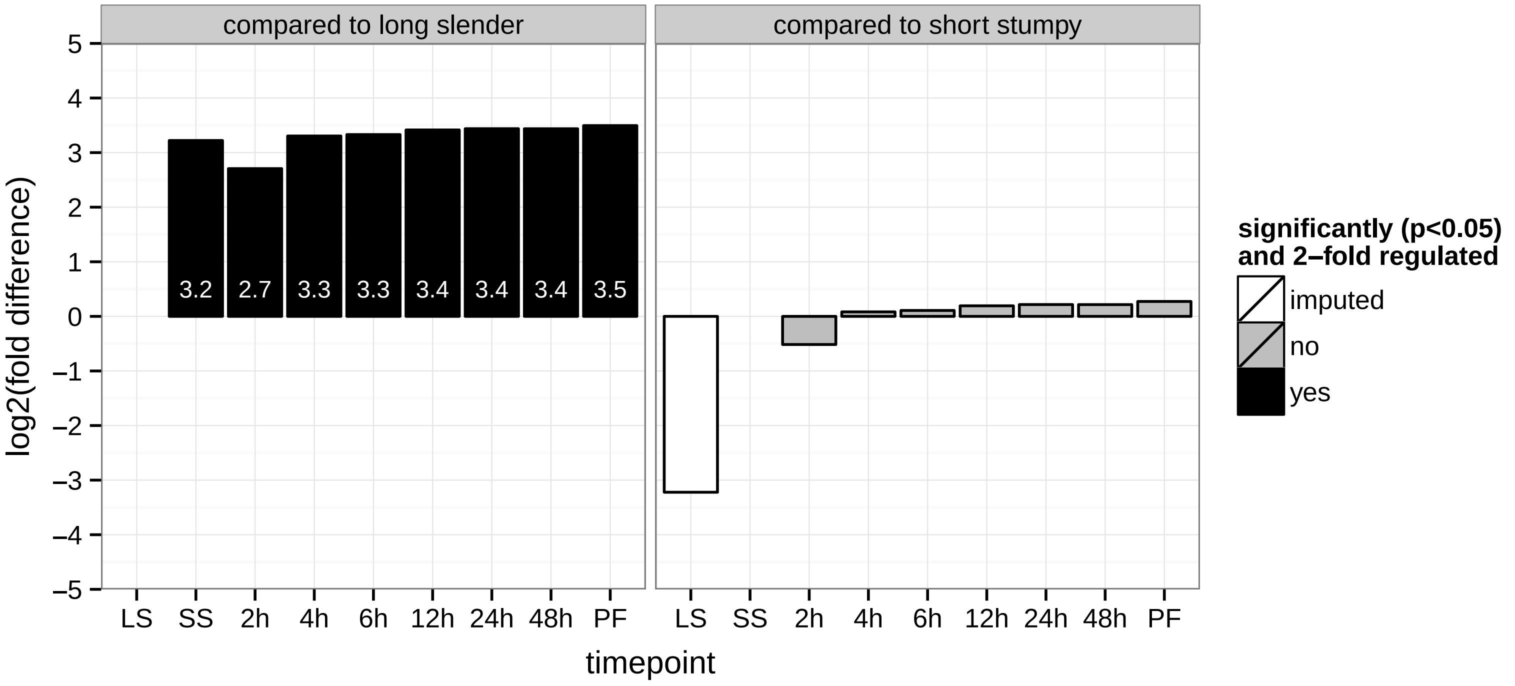

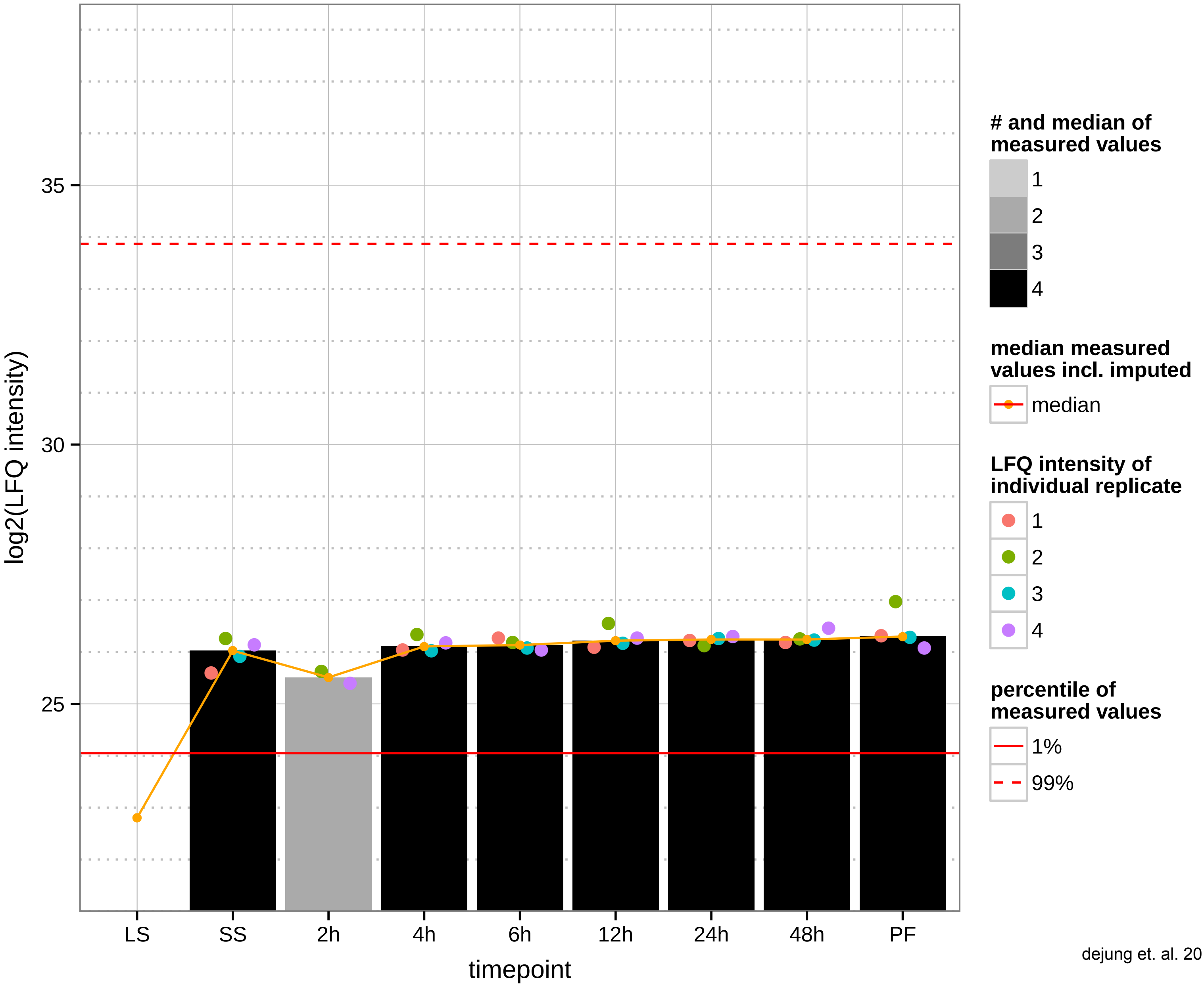

dejung et. al. 2015

hypothetical protein, conserved
Tb927.10.5630
AGOF: null
AGOC: null
AGOP: null
PGOF: null
PGOC: null
PGOP: null

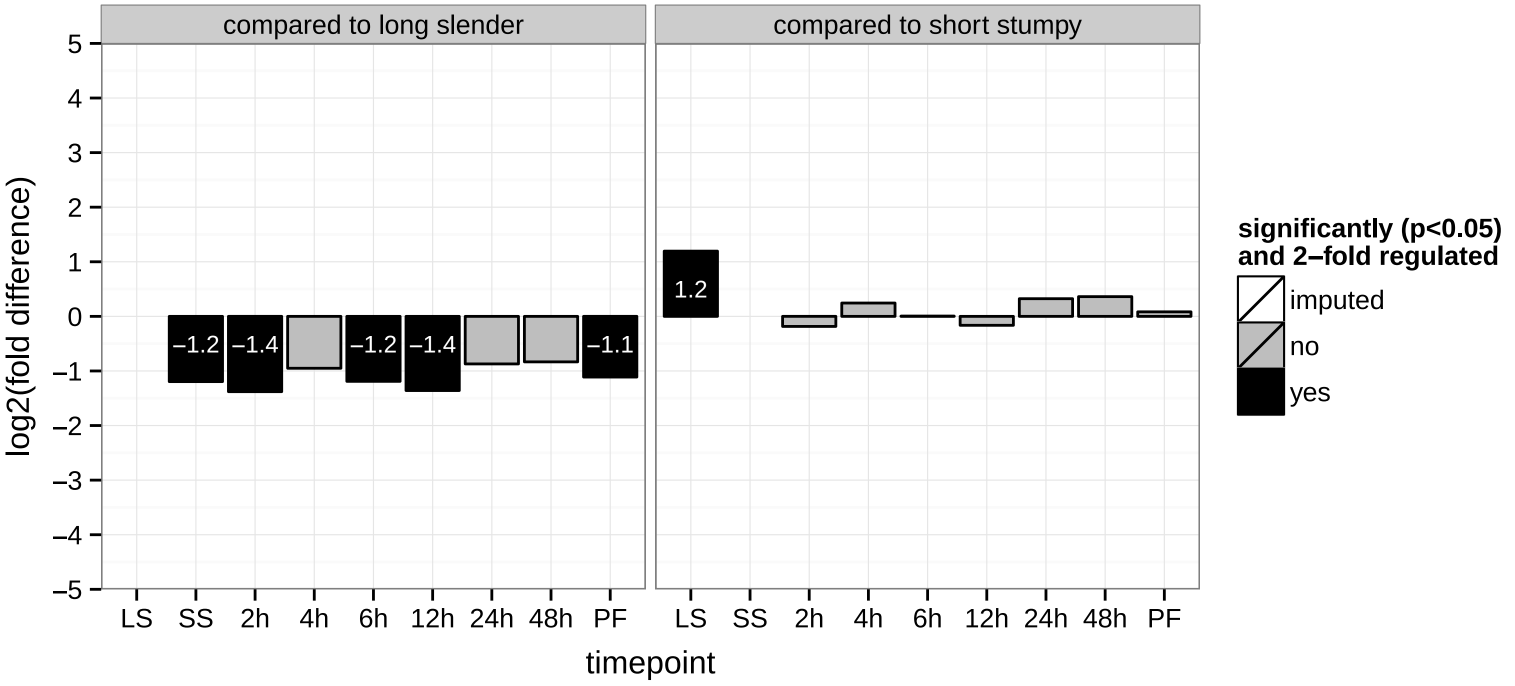

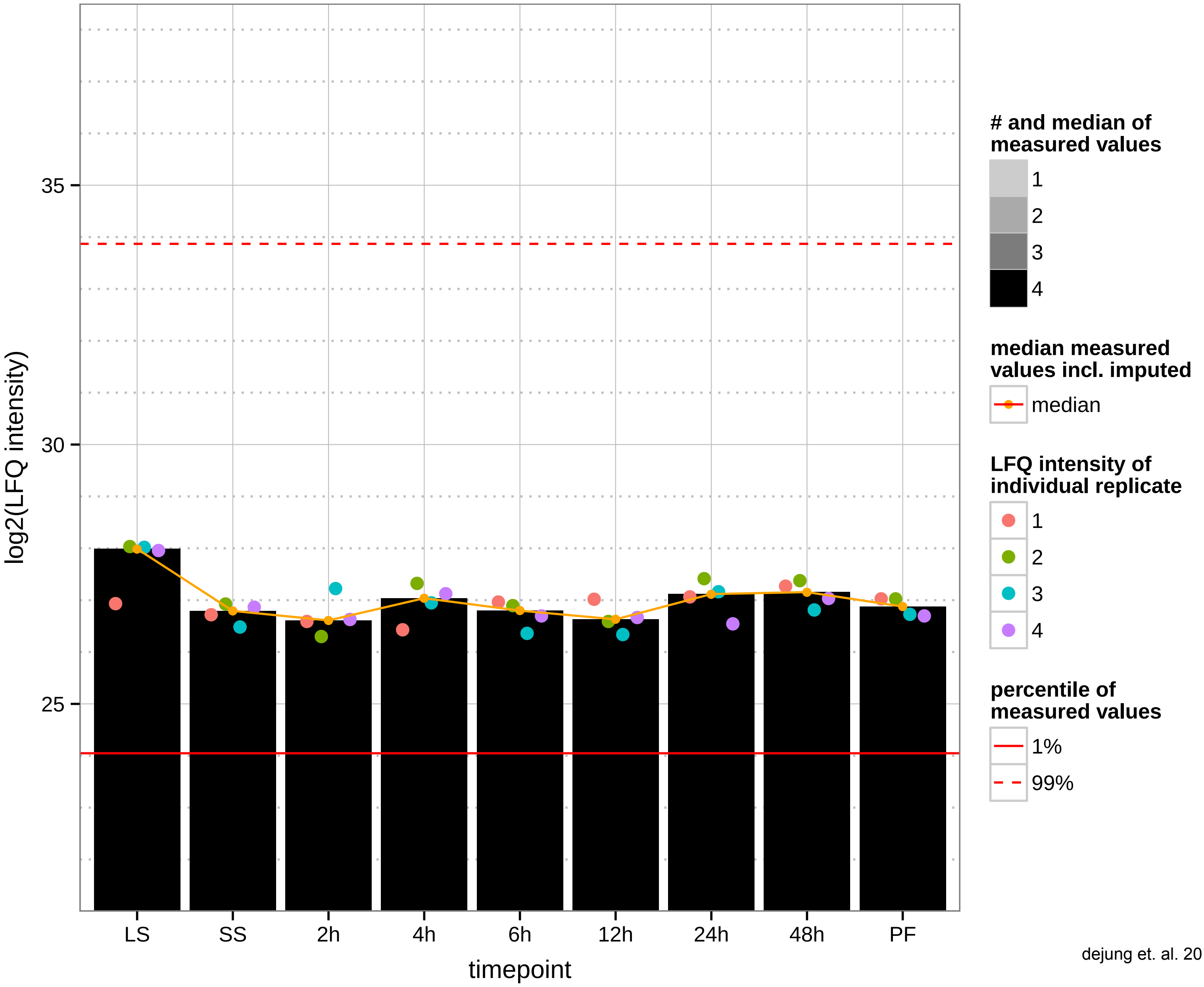

dejung et. al. 2015

hypothetical protein, conserved
Tb927.10.5640
AGOF: null
AGOC: null
AGOP: null
PGOF: null
PGOC: null
PGOP: null

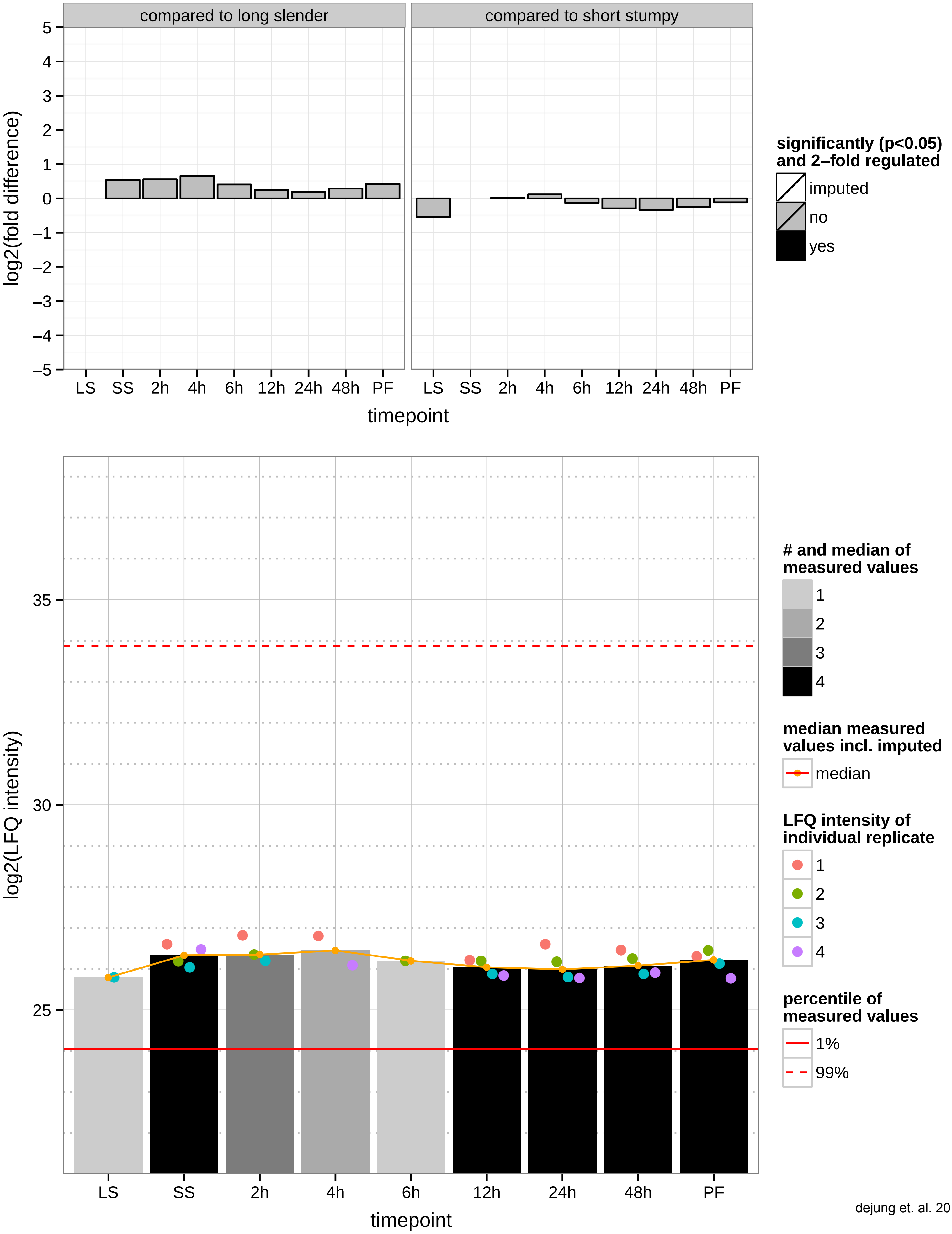

dejung et. al. 2015

procyclin–associated gene 1 (PAG1) protein, putative
Tb927.10.5680
AGOF: null
AGOC: null
AGOP: evasion or tolerance of host immune response
PGOF: null
PGOC: null
PGOP: evasion or tolerance of host immune response

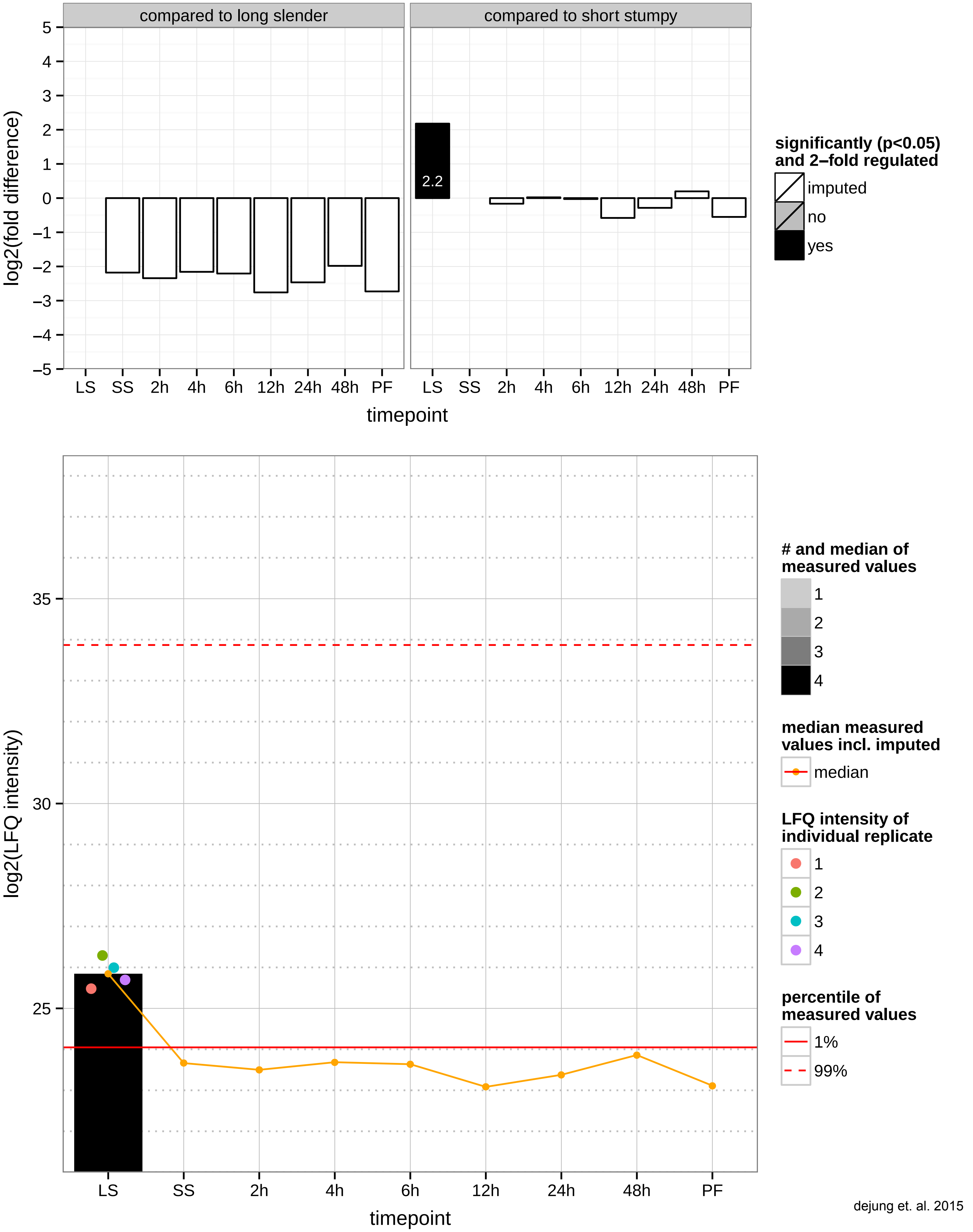

dejung et. al. 2015

valosin–containing protein homolog, Transitional endoplasmic reticulum ATPase, putative (VCP)
Tb927.10.5770
AGOF: ATP binding, ATPase activity
AGOC: cytoplasm, membrane
AGOP: biological_process
PGOF: ATP binding, hydrolase activity, nucleoside–triphosphatase activity, nucleotide binding
PGOC: null
PGOP: null

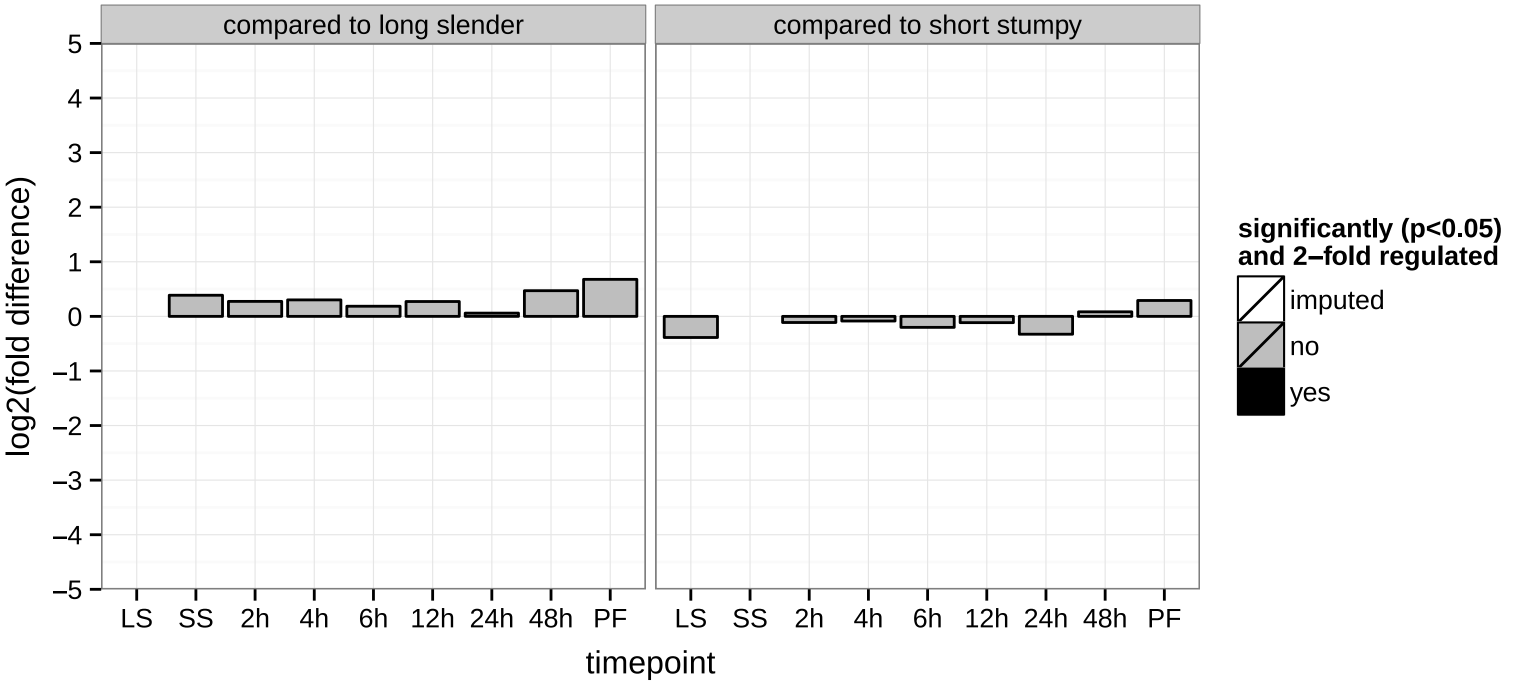

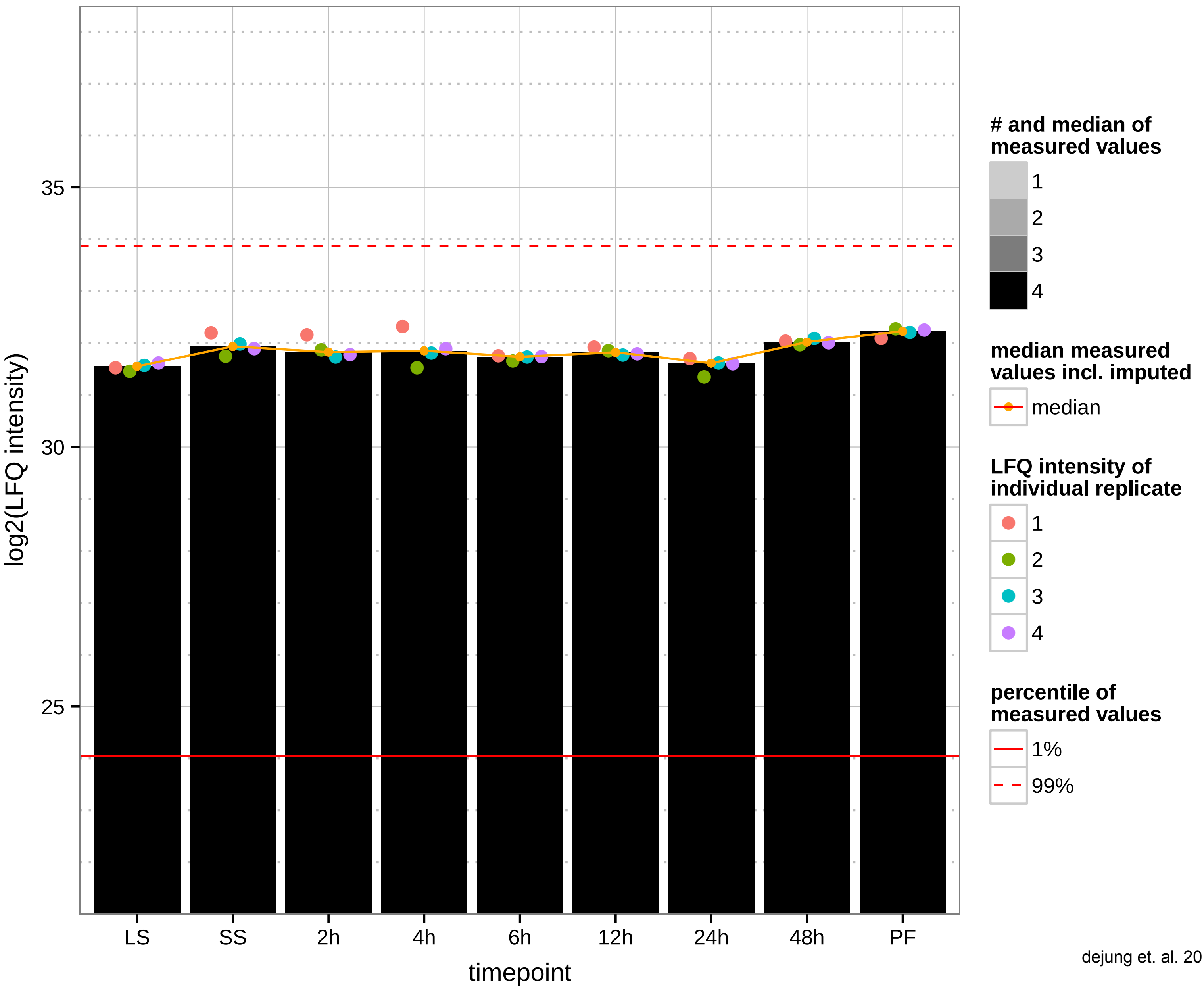

dejung et. al. 2015

hypothetical protein, conserved
Tb927.10.5790
AGOF: catalytic activity
AGOC: null
AGOP: null
PGOF: binding, catalytic activity, protein binding
PGOC: null
PGOP: null

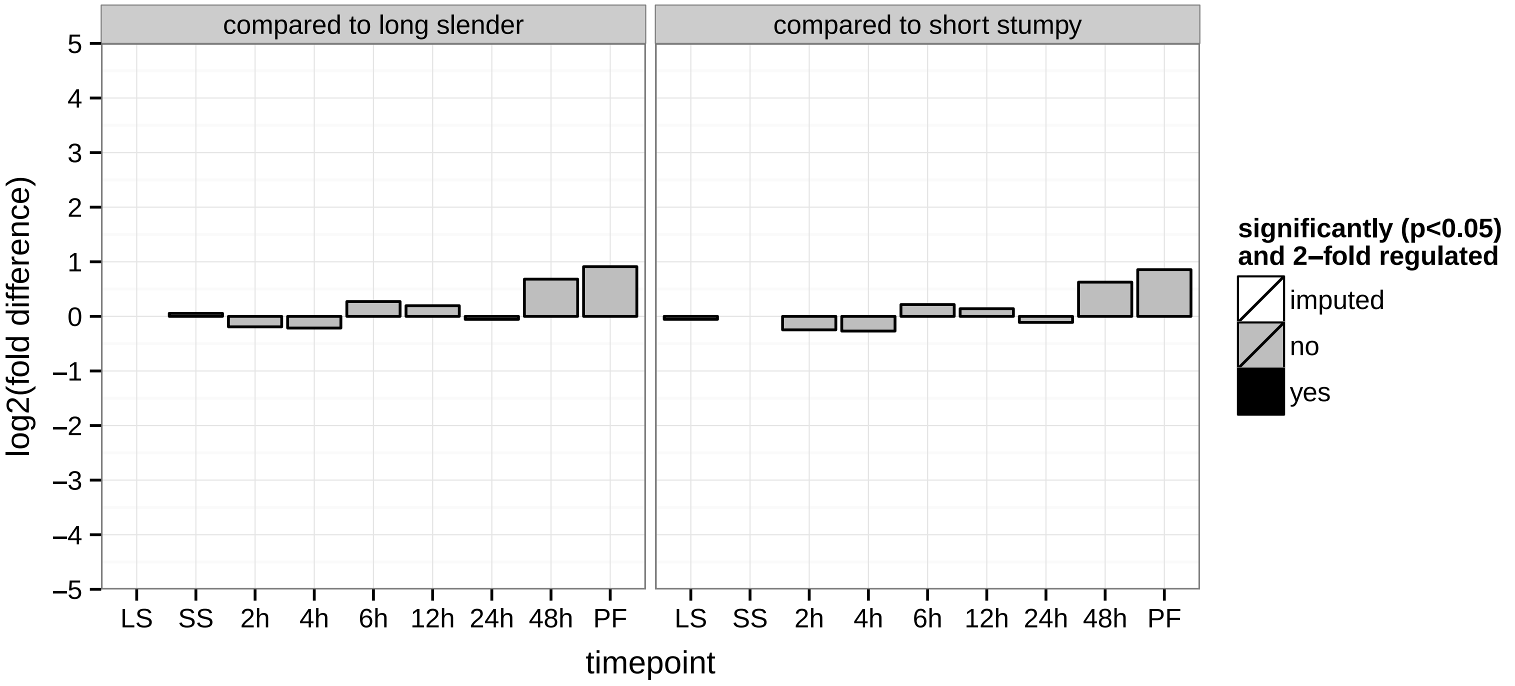

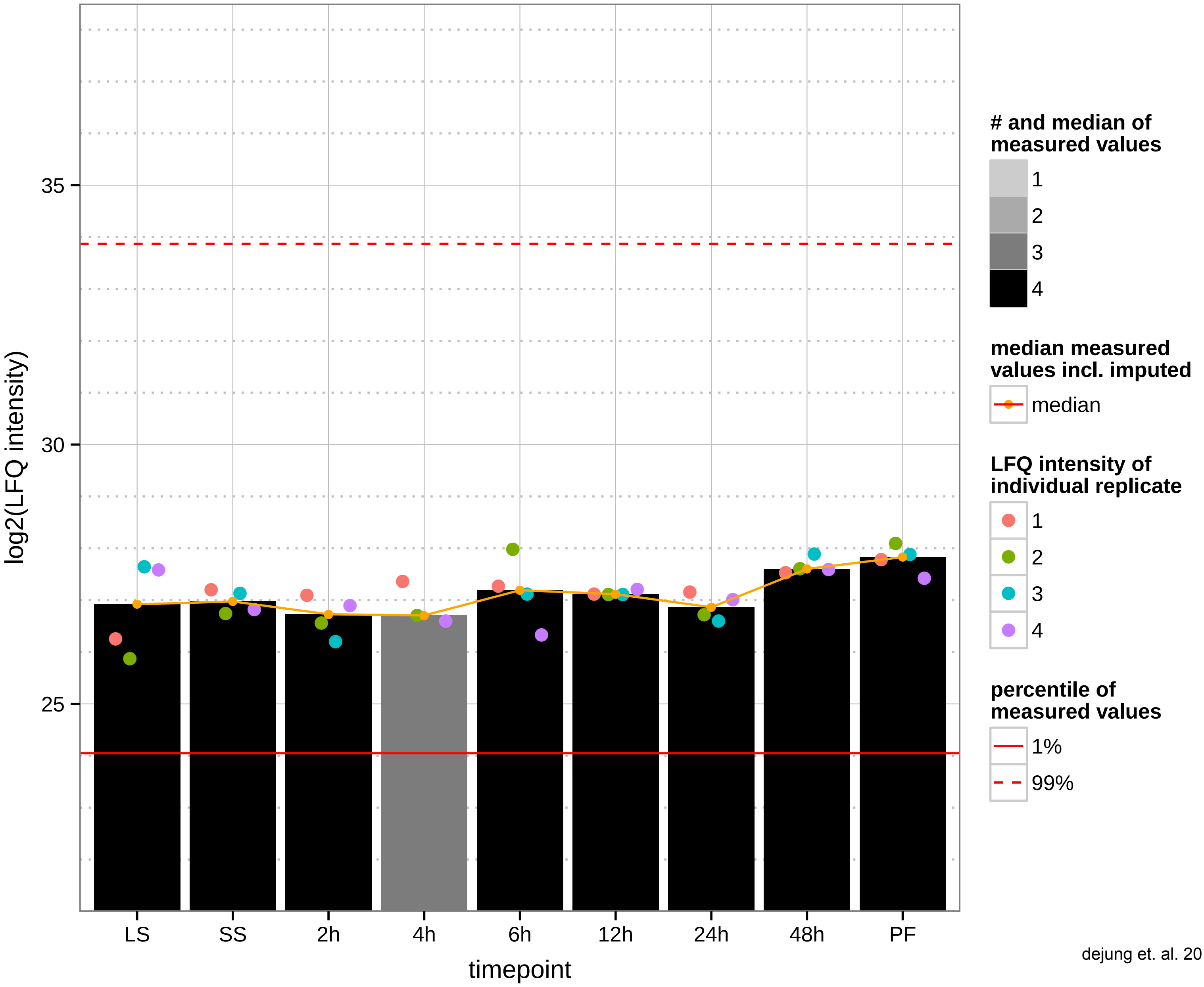

dejung et. al. 2015

hypothetical protein, conserved
Tb927.10.5830
AGOF: zinc ion binding
AGOC: intracellular
AGOP: null
PGOF: zinc ion binding
PGOC: intracellular
PGOP: null

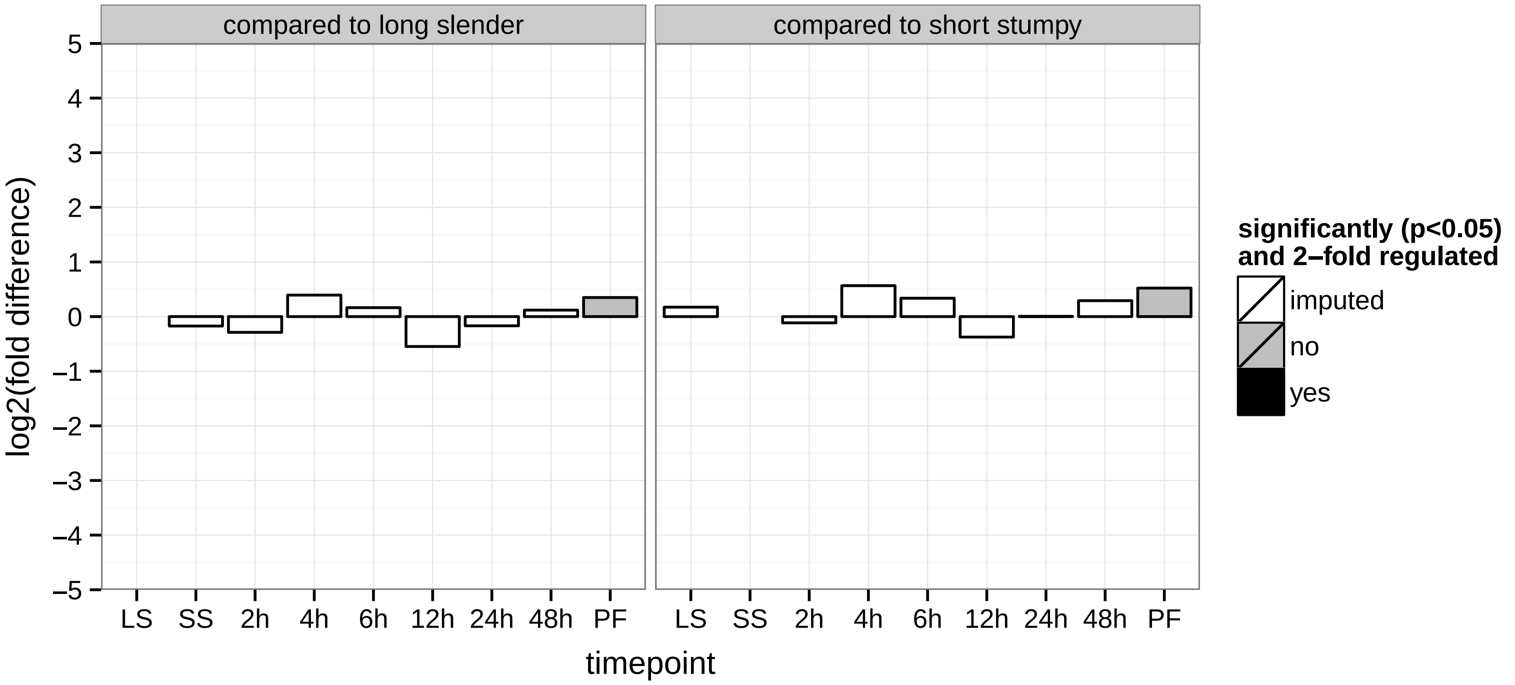

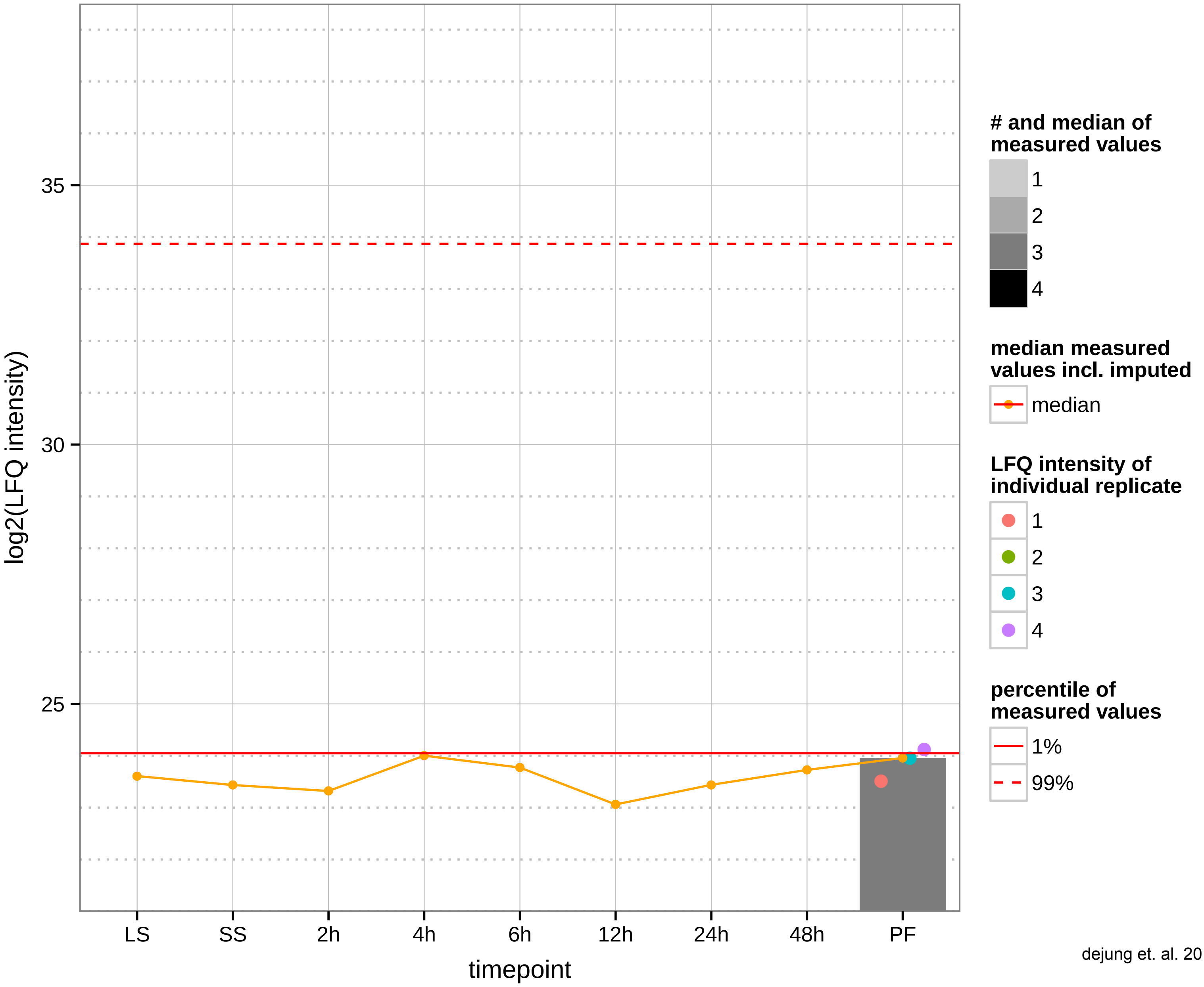

dejung et. al. 2015

hypothetical protein, conserved
Tb927.10.5900
AGOF: null
AGOC: mitochondrion
AGOP: null
PGOF: null
PGOC: null
PGOP: null

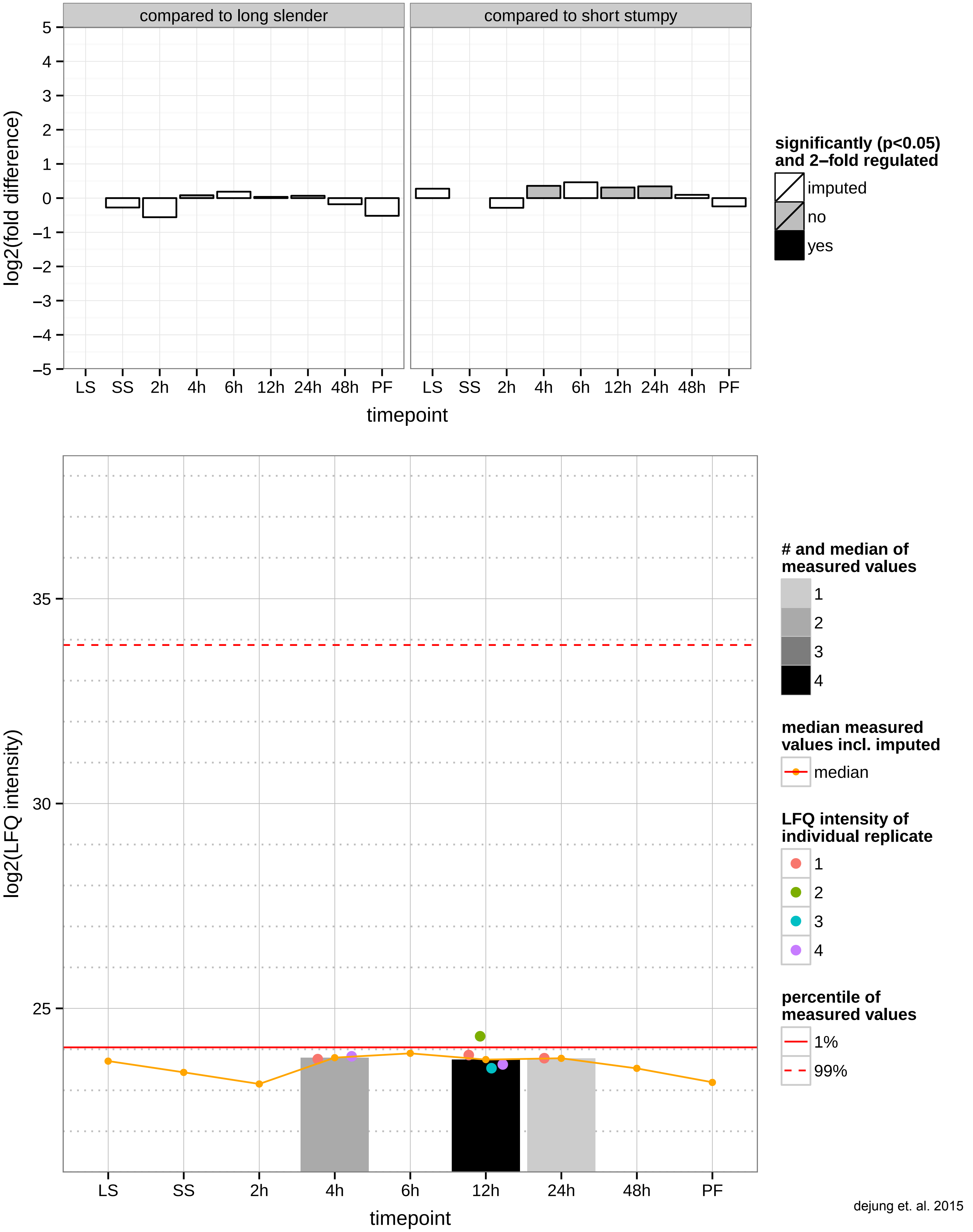

dejung et. al. 2015

leucine–rich repeat protein (LRRP), putative
Tb927.10.5980
AGOF: null
AGOC: null
AGOP: null
PGOF: protein binding
PGOC: null
PGOP: null

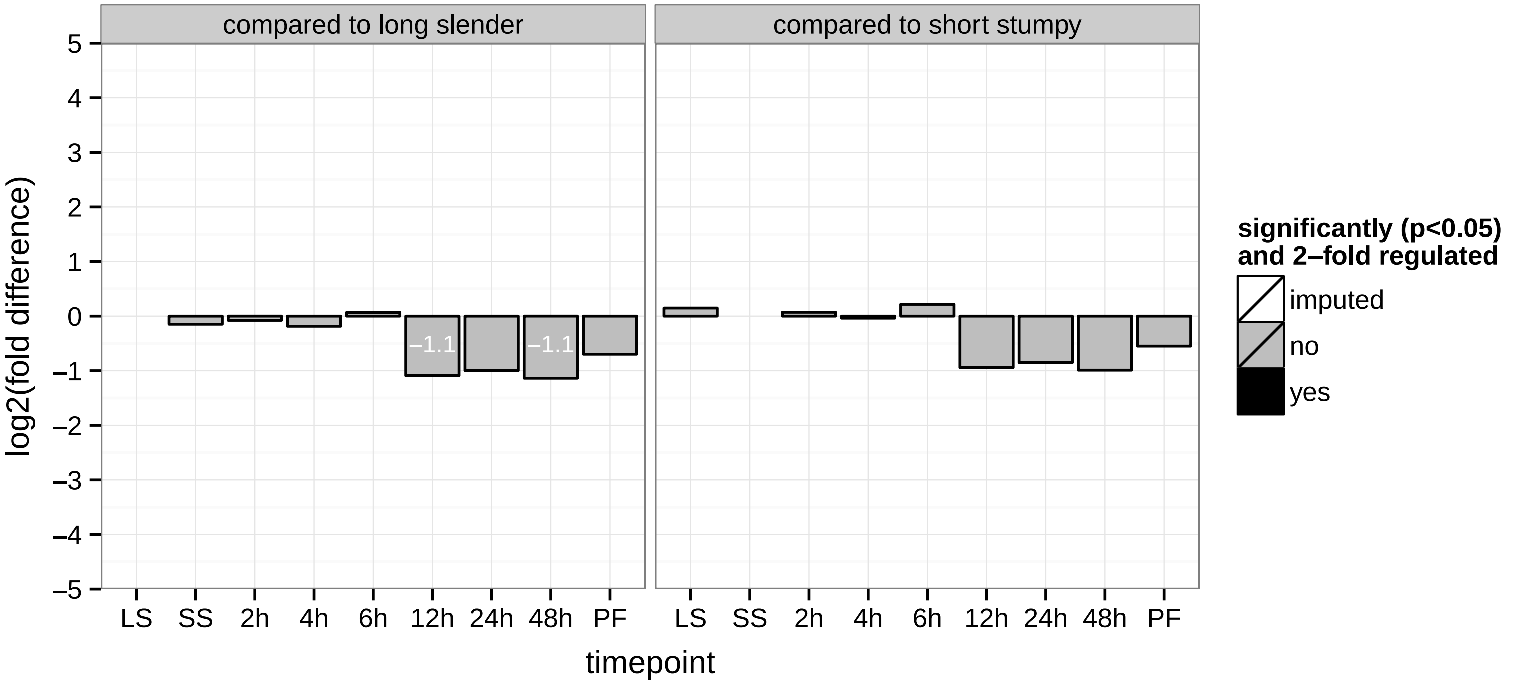

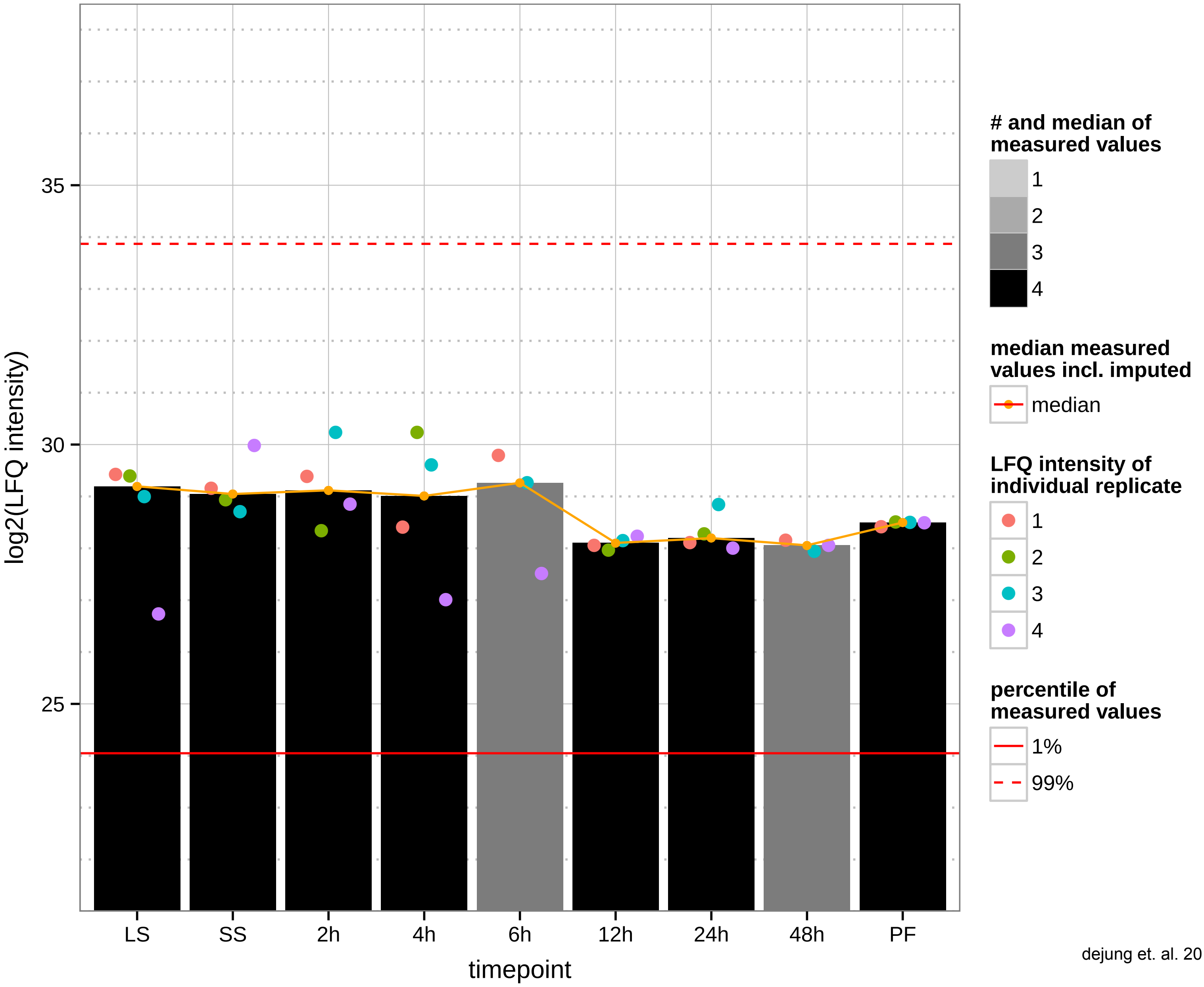

dejung et. al. 2015

hypothetical protein, conserved
Tb927.10.6010
AGOF: null
AGOC: null
AGOP: null
PGOF: binding
PGOC: null
PGOP: null

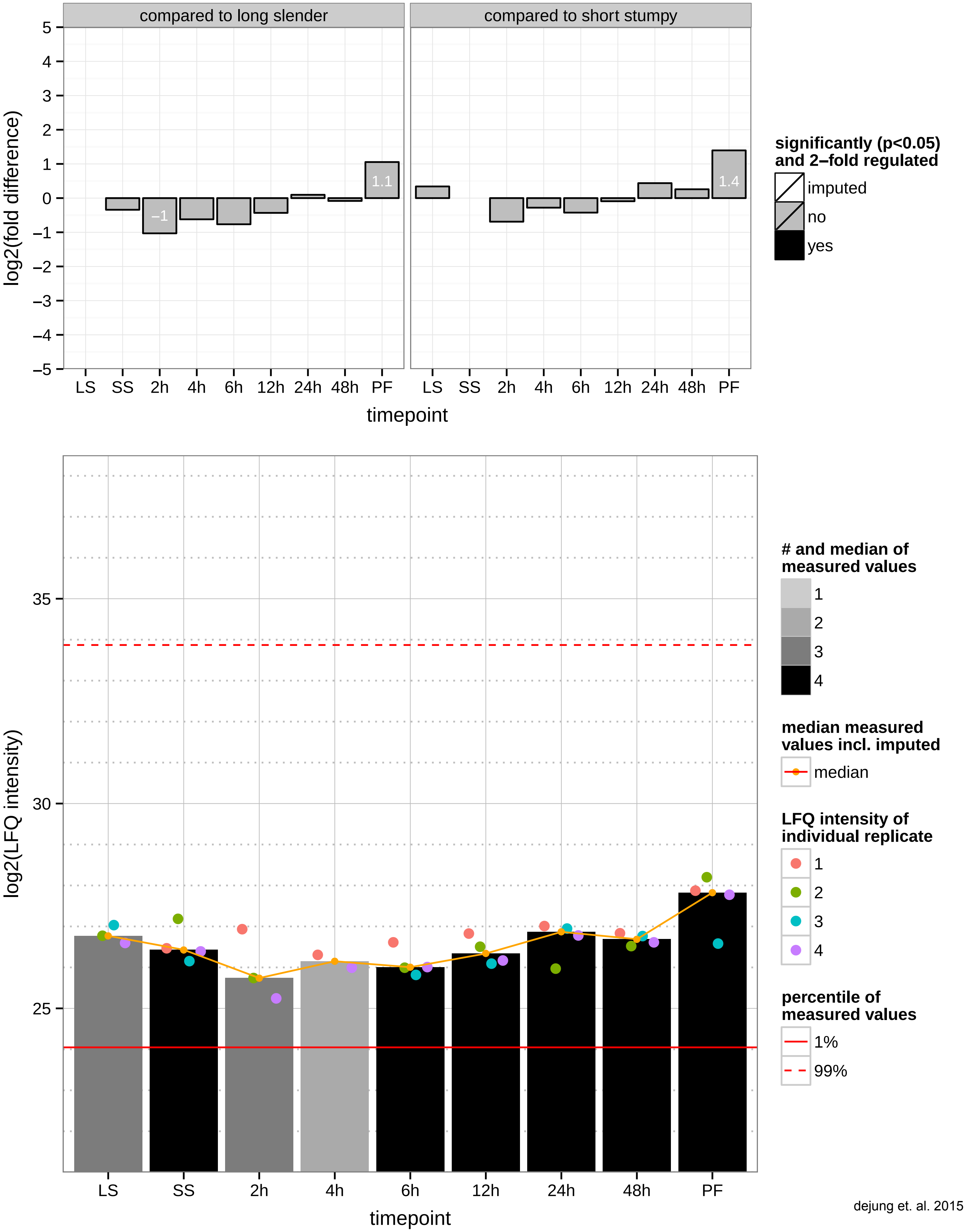

hypothetical protein, conserved
Tb927.10.6160
AGOF: null
AGOC: cis–Golgi network, membrane
AGOP: intracellular protein transport
PGOF: null
PGOC: cis–Golgi network, membrane
PGOP: intracellular protein transport

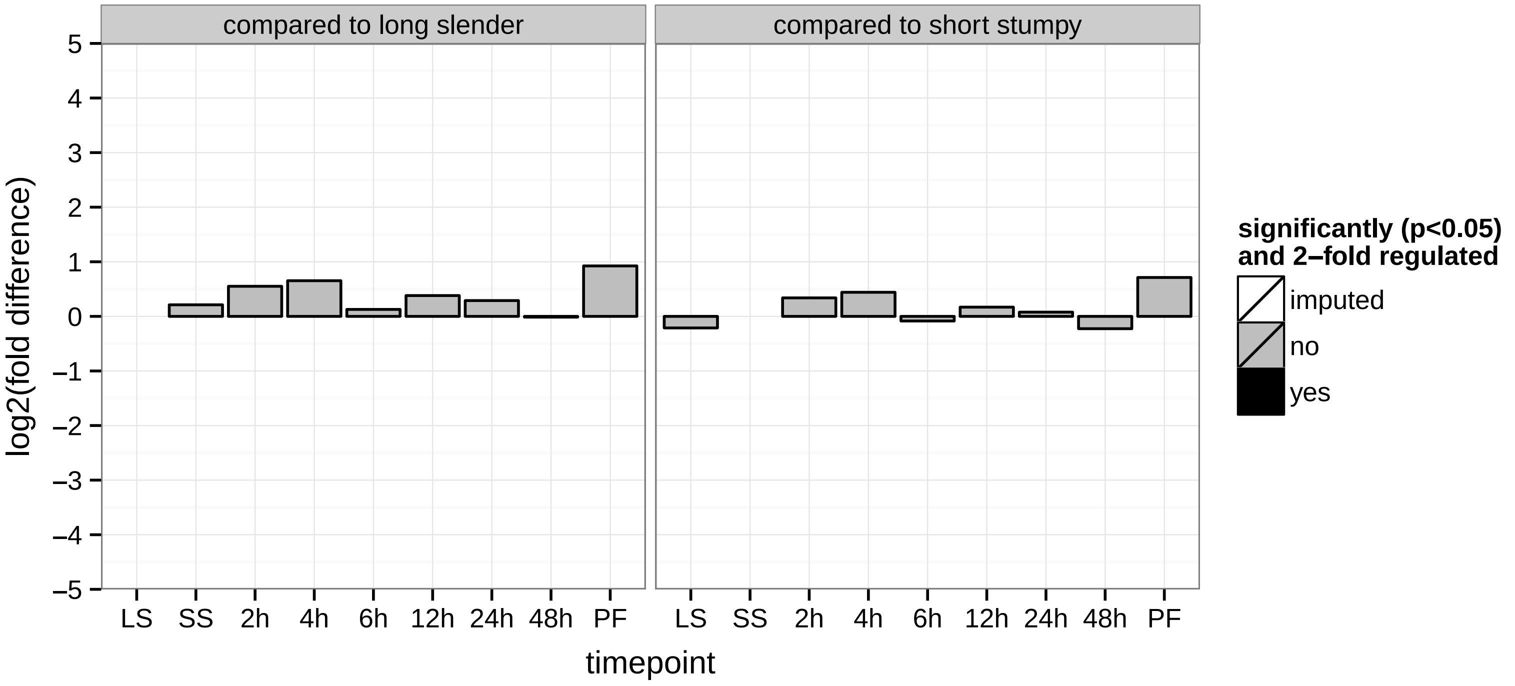

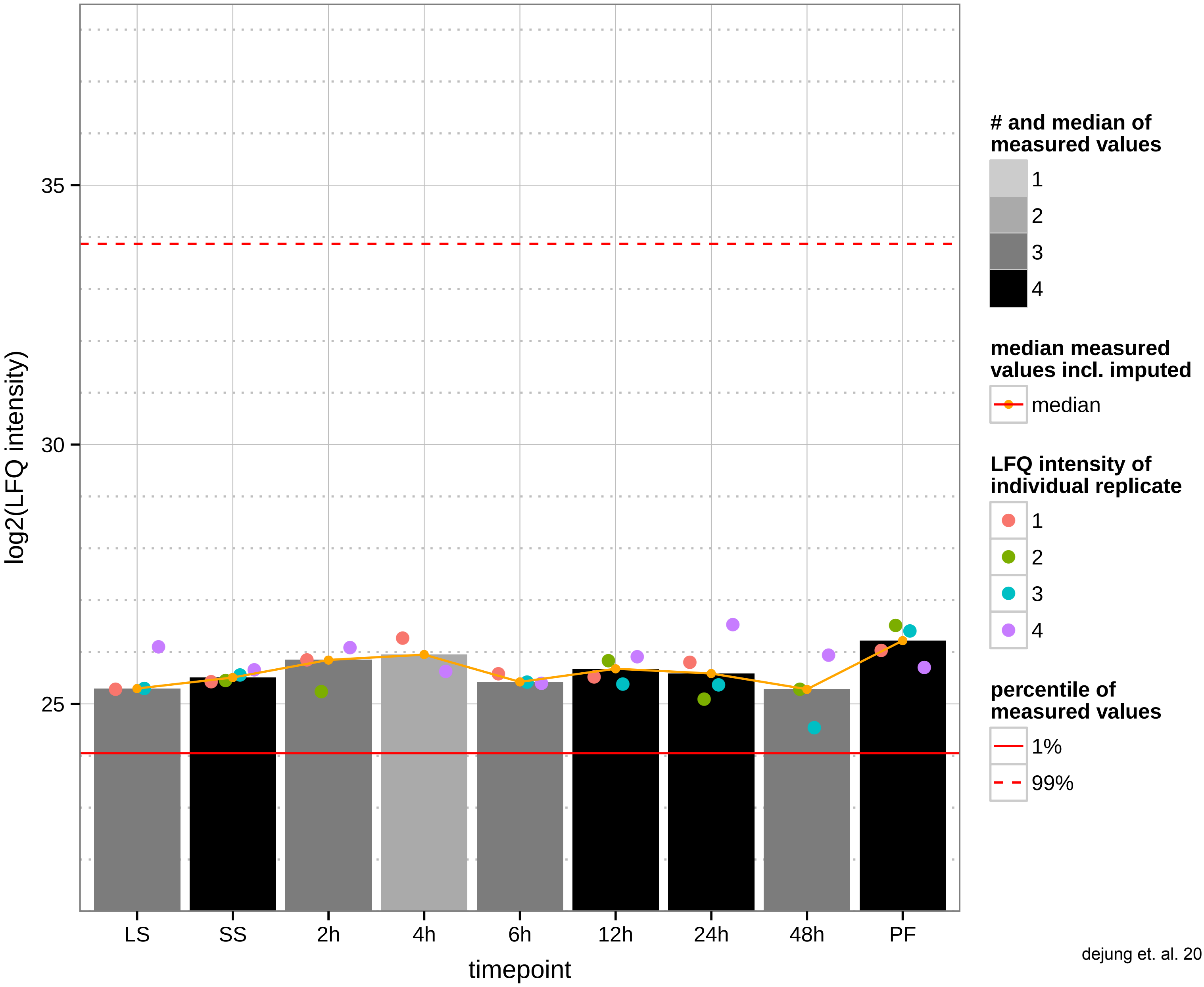

dejung et. al. 2015

hypothetical protein, conserved
Tb927.10.6170
AGOF: null
AGOC: mitochondrion
AGOP: null
PGOF: binding
PGOC: null
PGOP: null

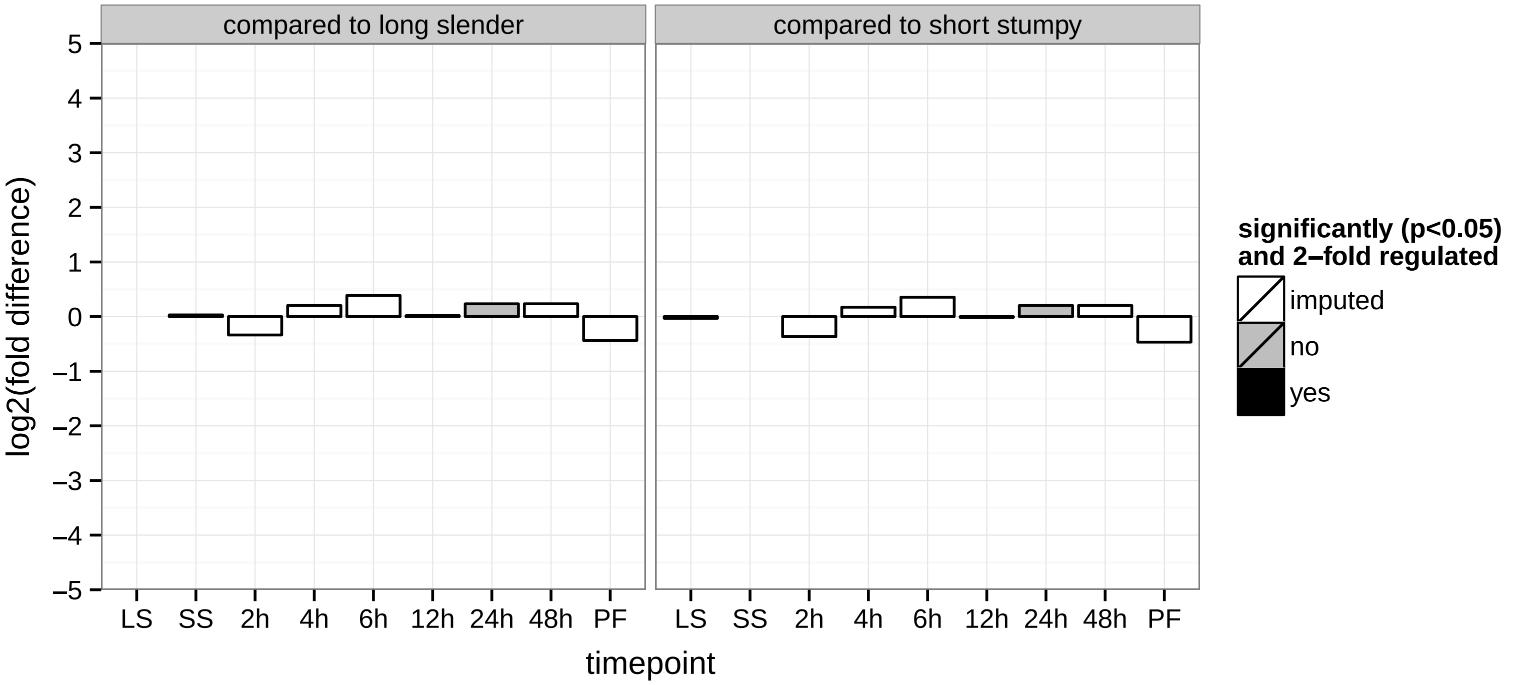

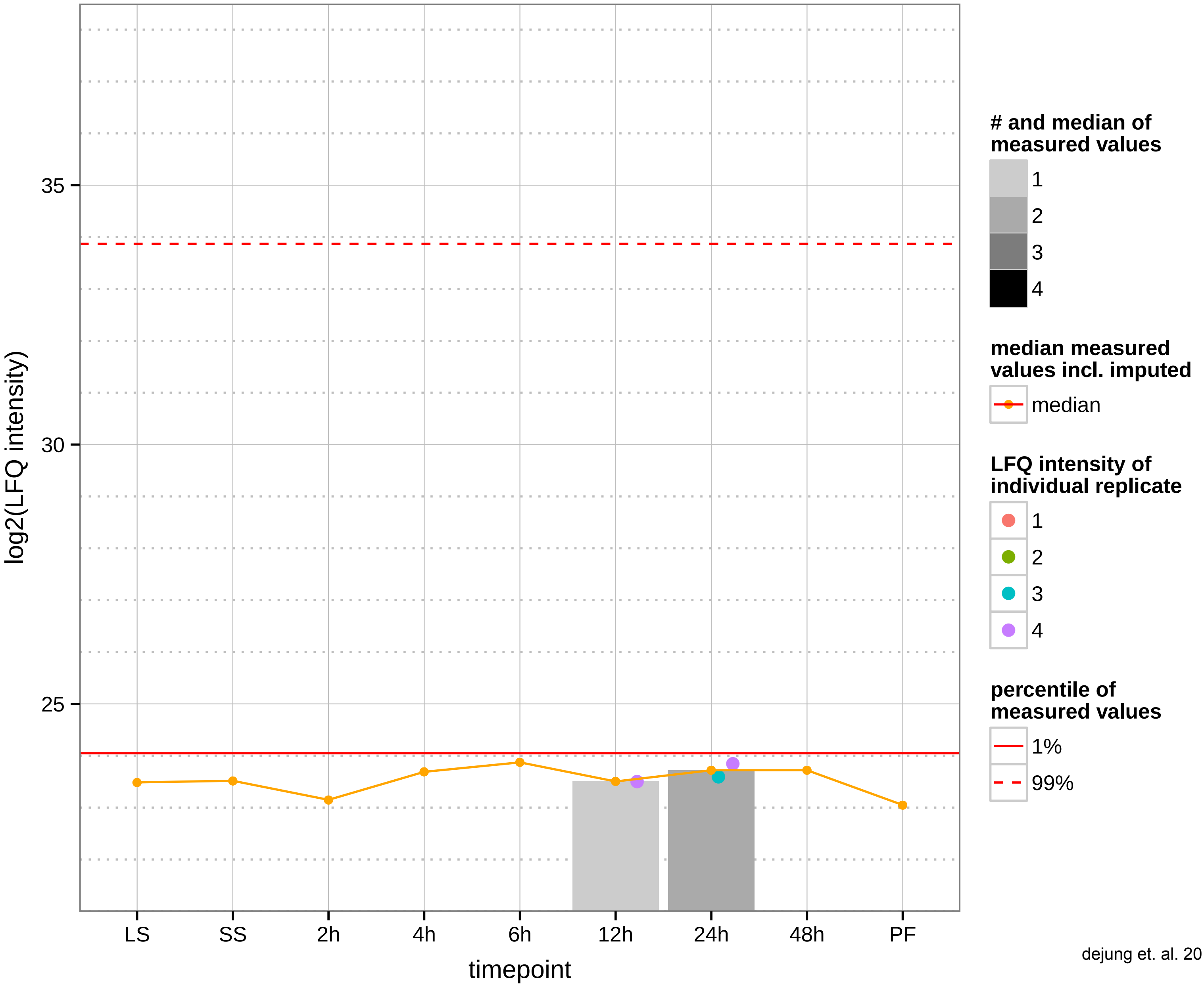

dejung et. al. 2015

5'–3' exoribonuclease 2, putative (XRND)
Tb927.10.6220
AGOF: exonuclease activity, nucleic acid binding
AGOC: intracellular, nucleus
AGOP: RNA processing
PGOF: exonuclease activity, nucleic acid binding
PGOC: intracellular
PGOP: null

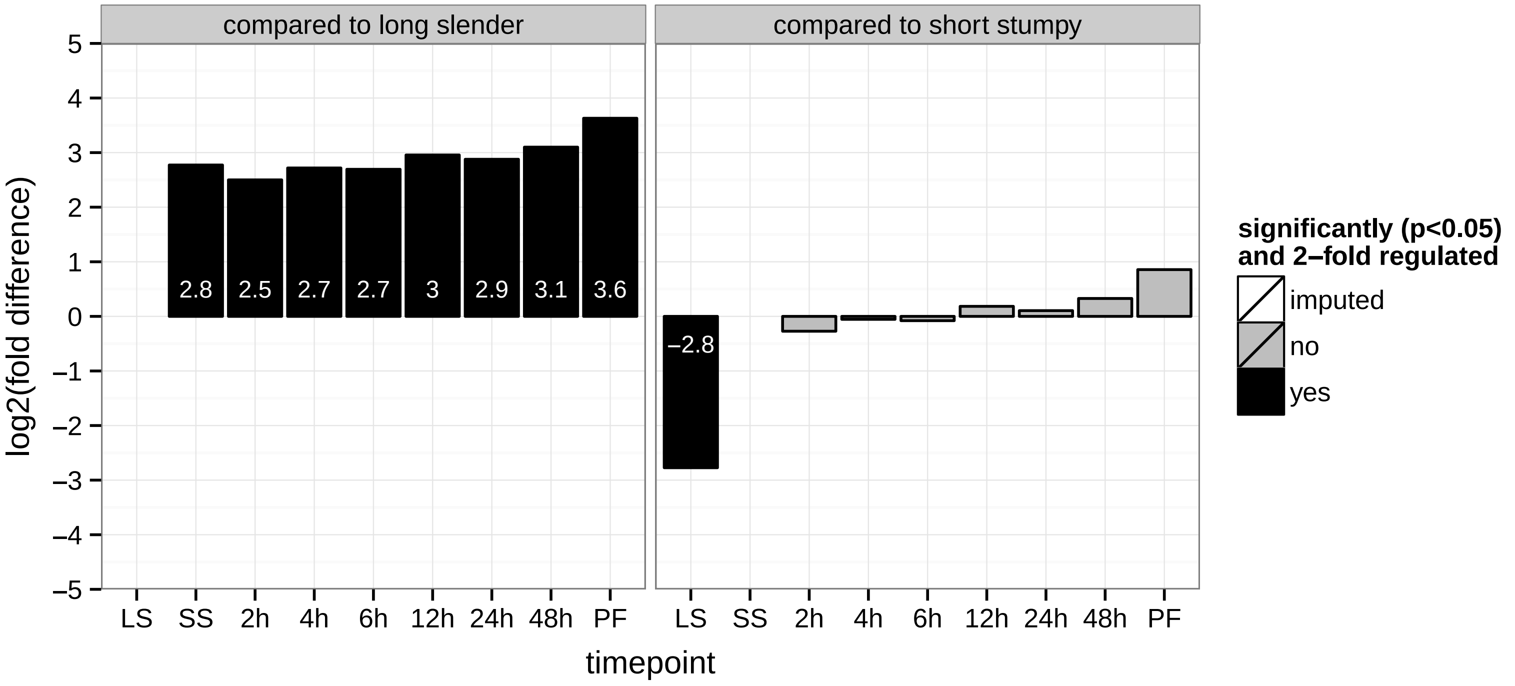

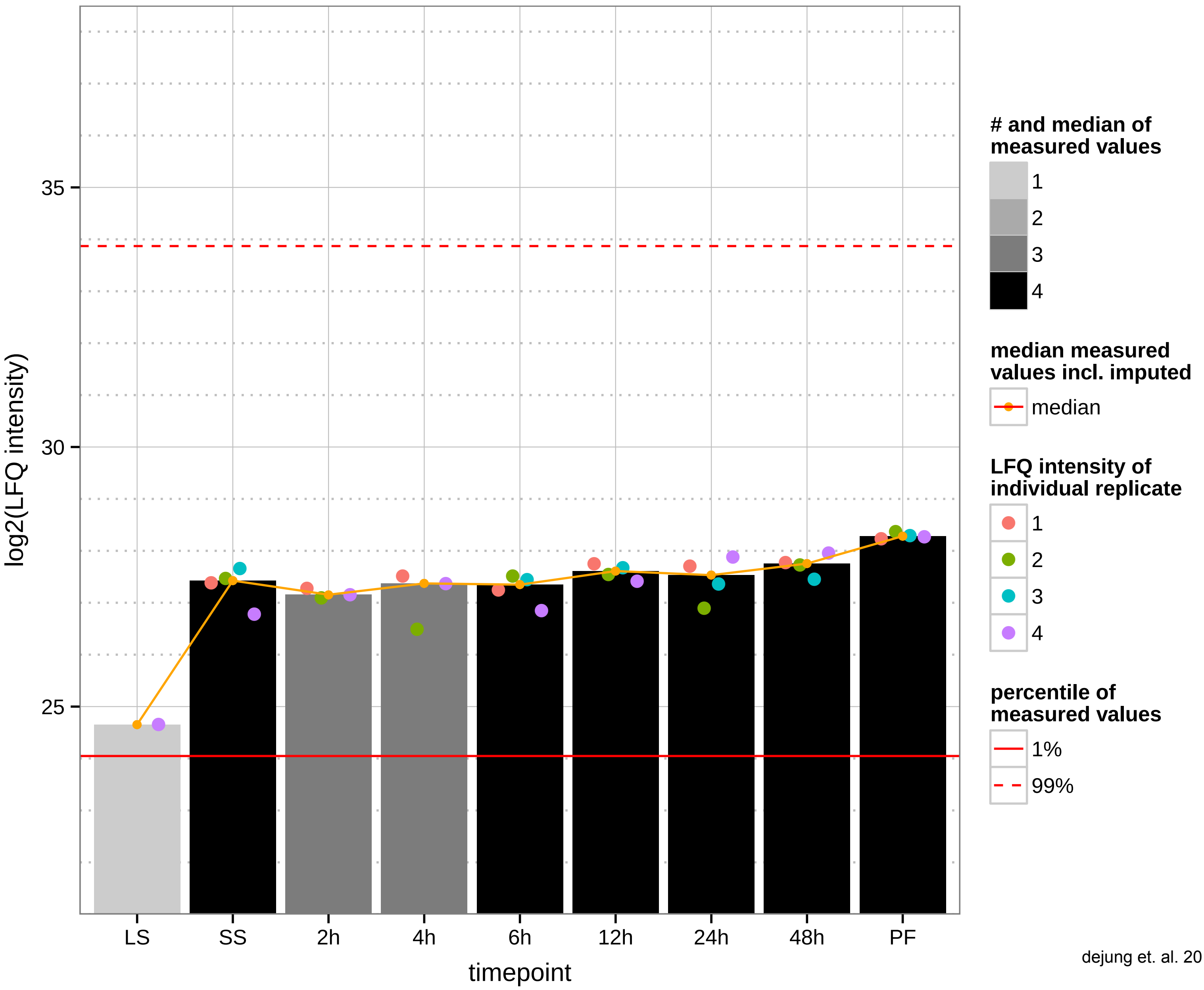

dejung et. al. 2015

ATP–dependent DEAD/H RNA helicase, putative
Tb927.10.6260;Tb11.v5.0279
AGOF: null, ATP binding, ATP–dependent helicase activity, helicase activity, nucleic acid binding
AGOC: null
AGOP: null, nucleobase–containing compound metabolic process
PGOF: ATP binding, ATP–dependent helicase activity, helicase activity, nucleic acid binding
PGOC: null
PGOP: null

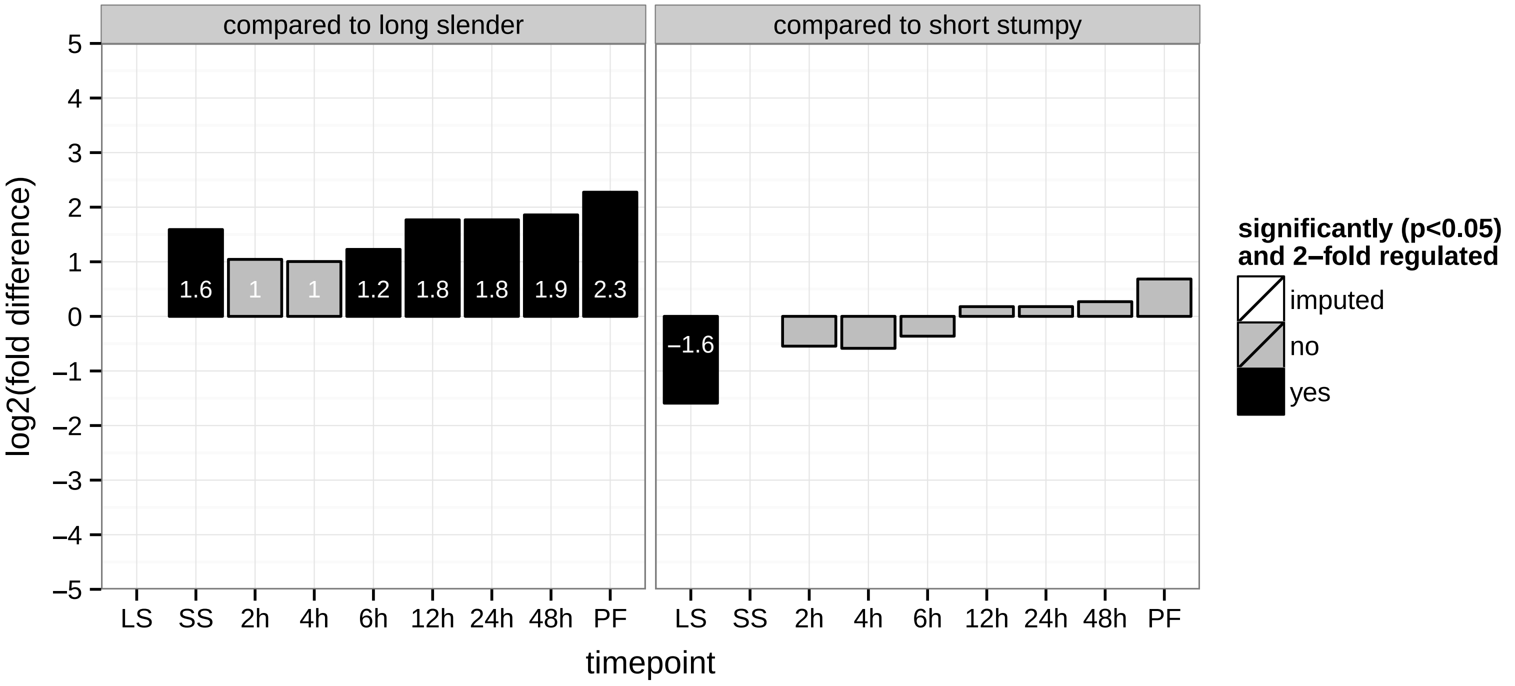

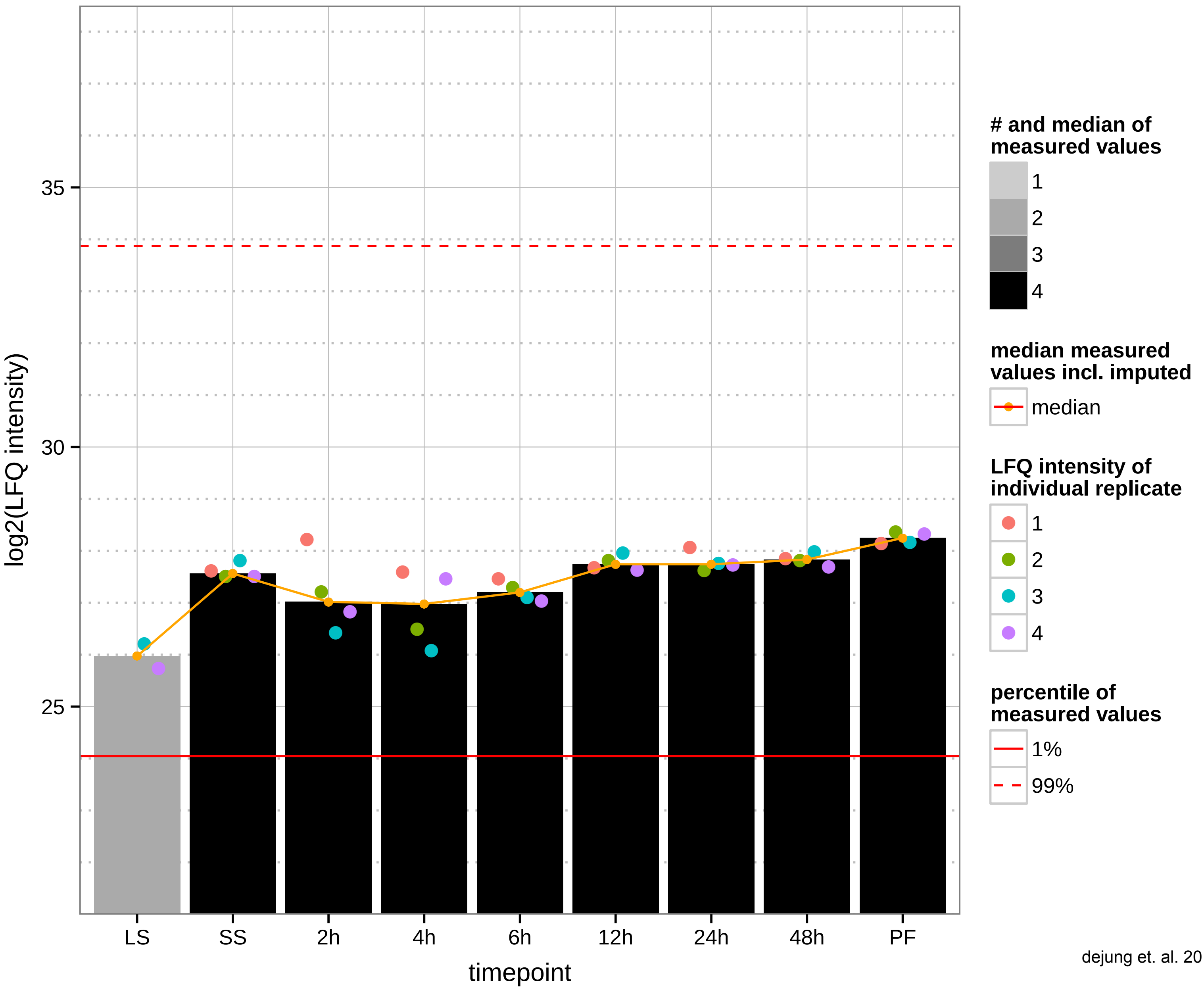

dejung et. al. 2015

chaperonin HSP60, mitochondrial precursor (HSP60)
Tb927.10.6510;Tb927.10.6400
AGOF: ATP binding, unfolded protein binding
AGOC: mitochondrion
AGOP: protein complex assembly, protein folding, protein import into mitochondrial matrix
PGOF: ATP binding
PGOC: cytoplasm
PGOP: cellular protein metabolic process, protein refolding

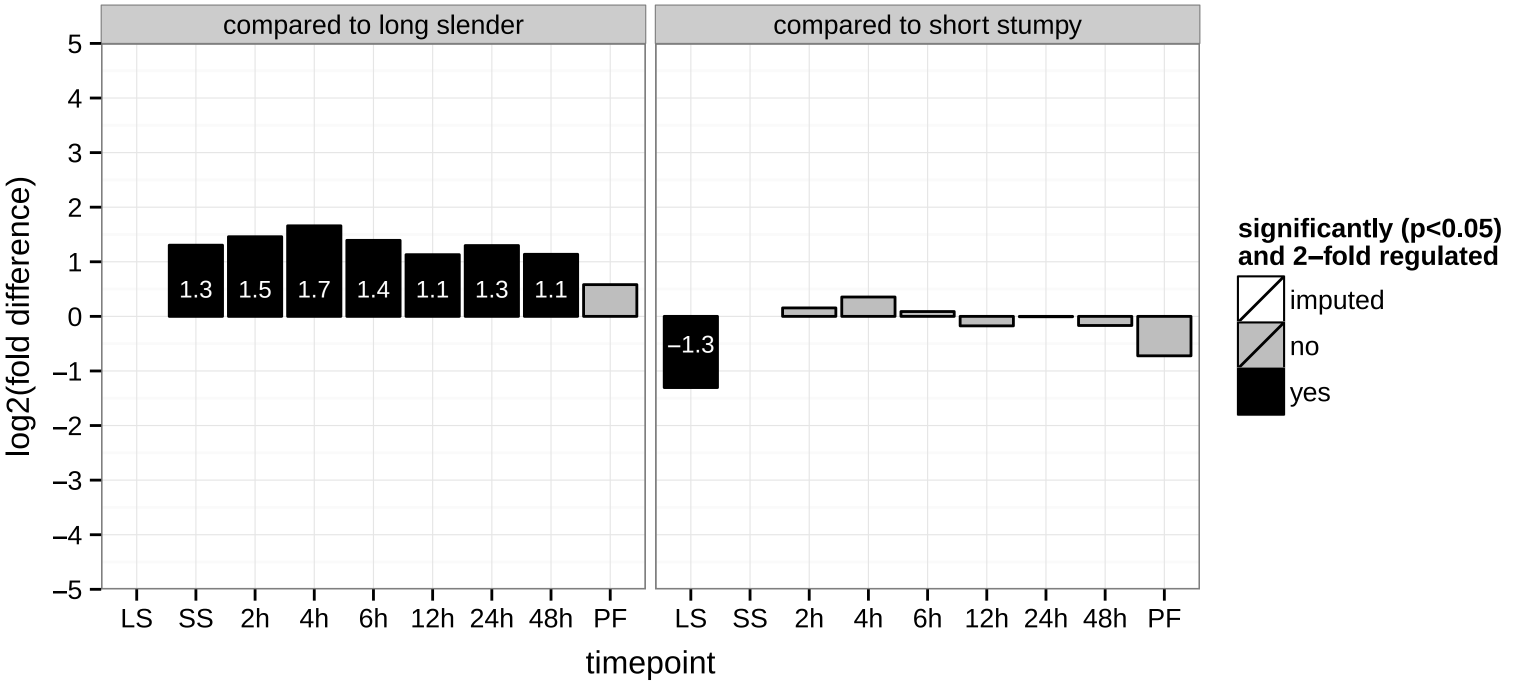

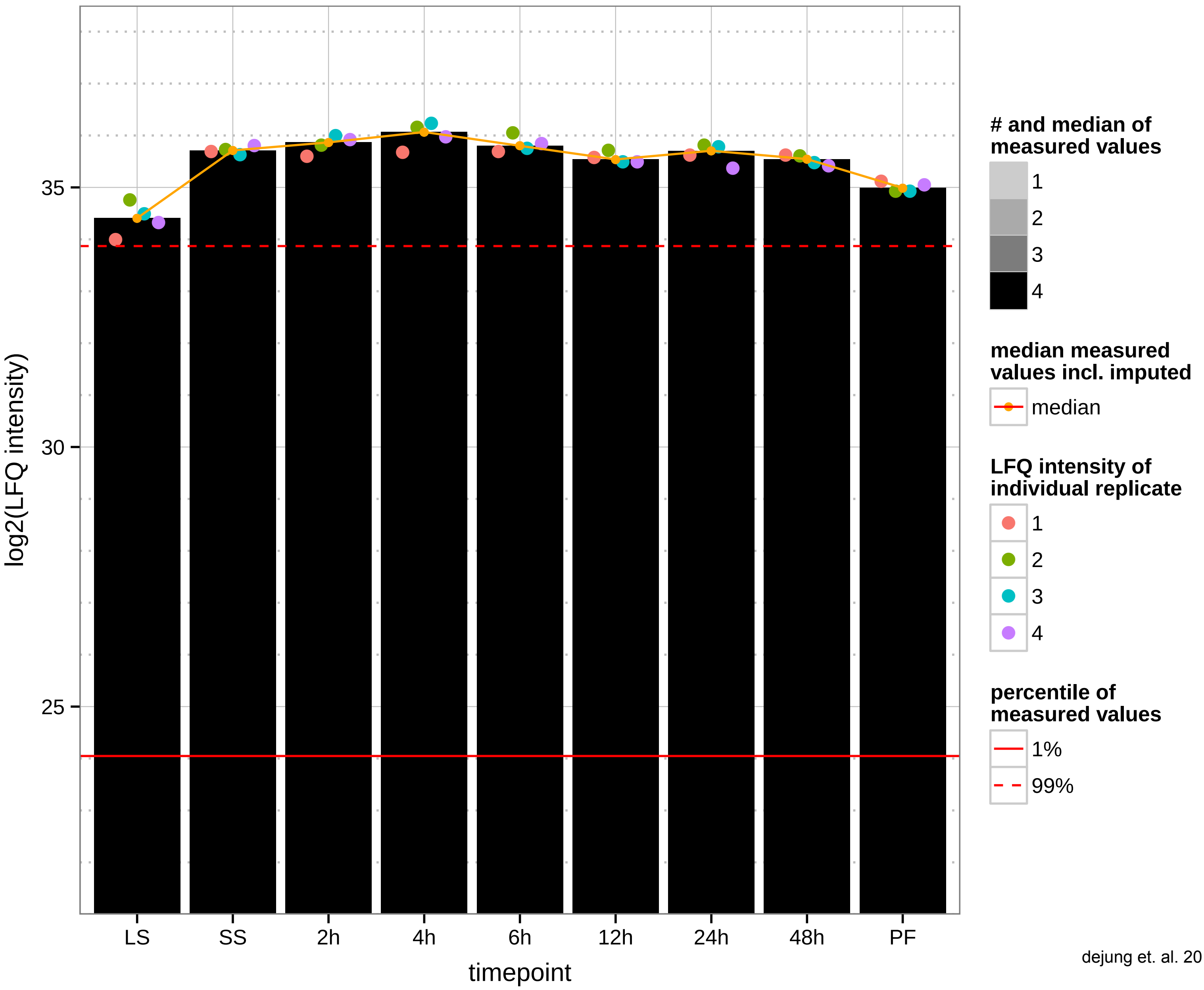

dejung et. al. 2015

phosphomannomutase, putative
Tb927.10.6440
AGOF: phosphomannomutase activity
AGOC: cytoplasm
AGOP: mannose biosynthetic process
PGOF: catalytic activity, phosphomannomutase activity
PGOC: cytoplasm
PGOP: mannose biosynthetic process, metabolic process

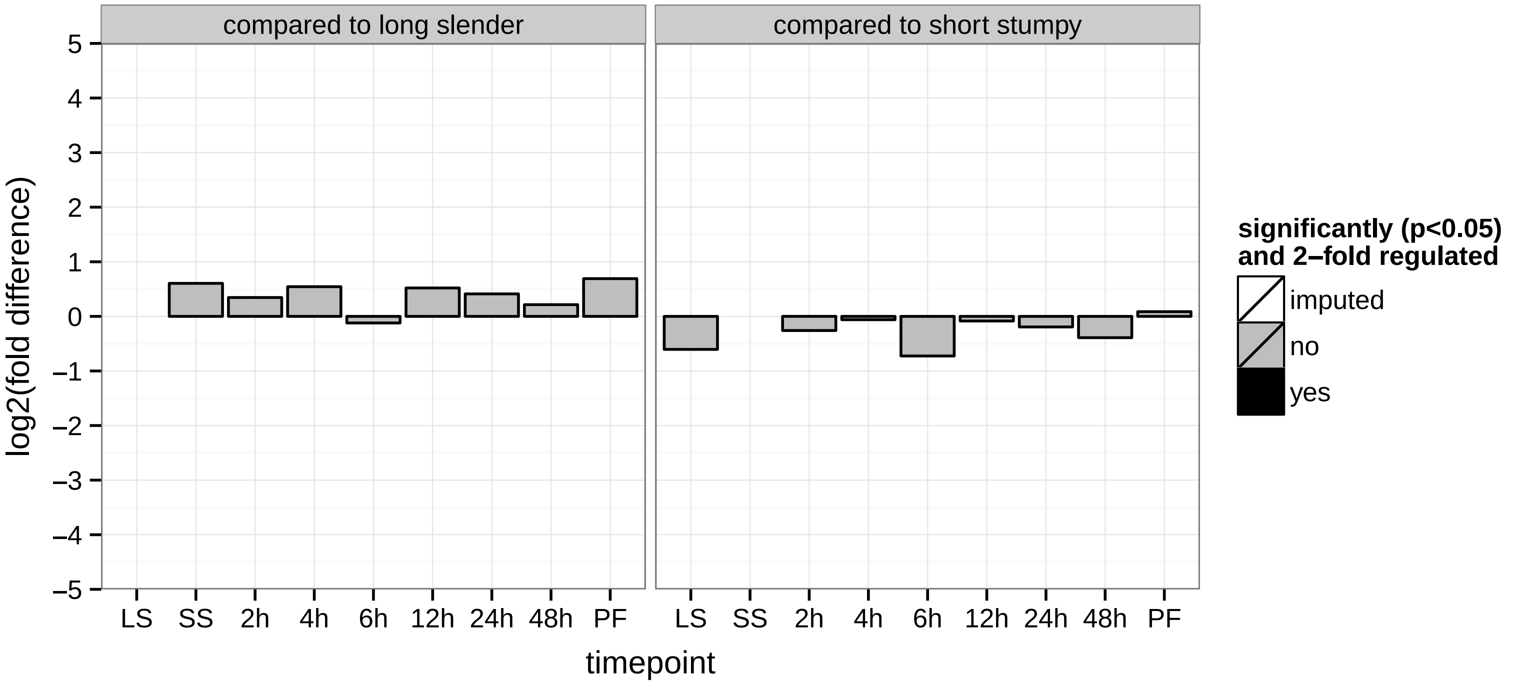

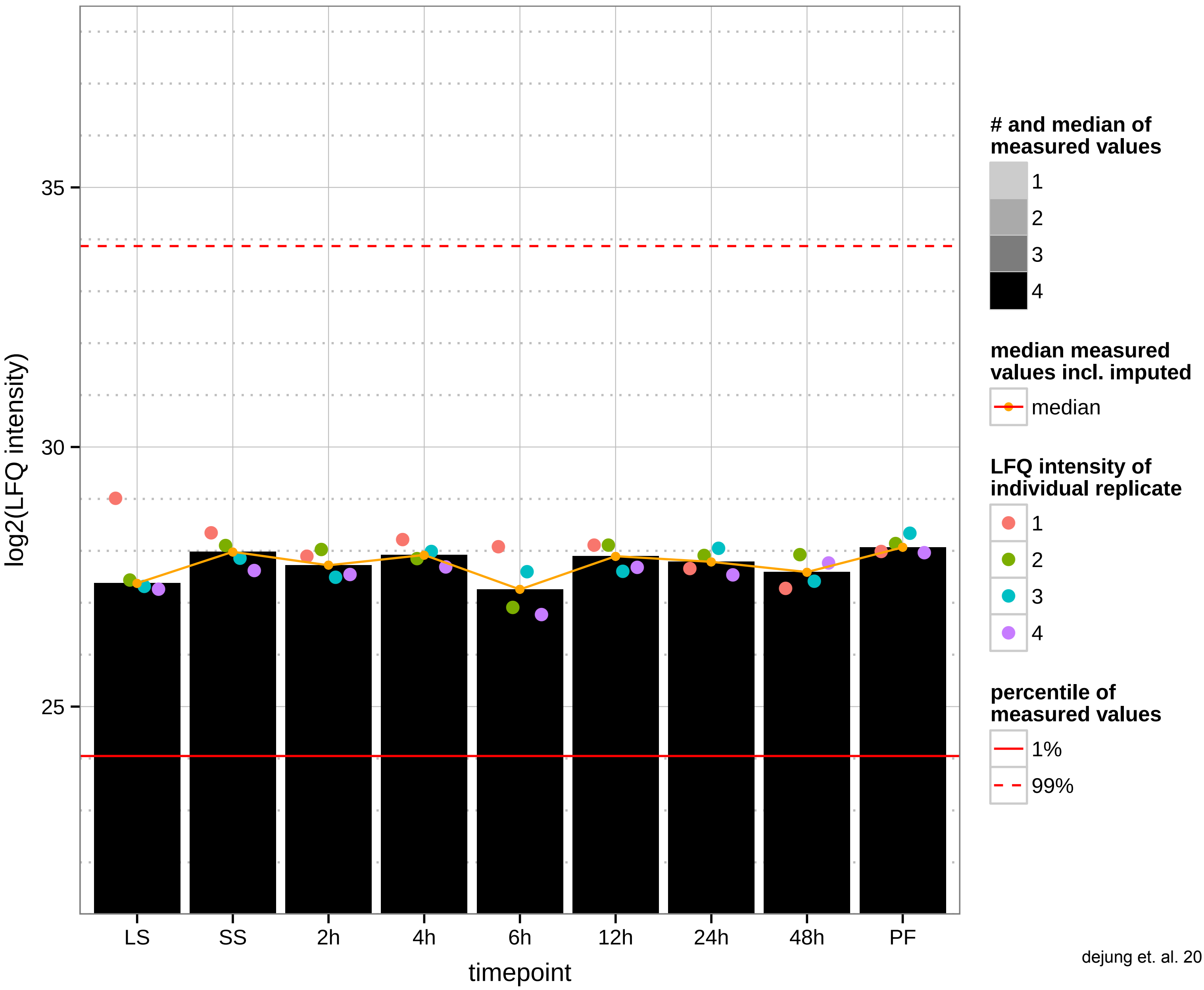

dejung et. al. 2015

hypothetical protein, conserved
Tb927.10.6490
AGOF: null
AGOC: null
AGOP: null
PGOF: null
PGOC: null
PGOP: null

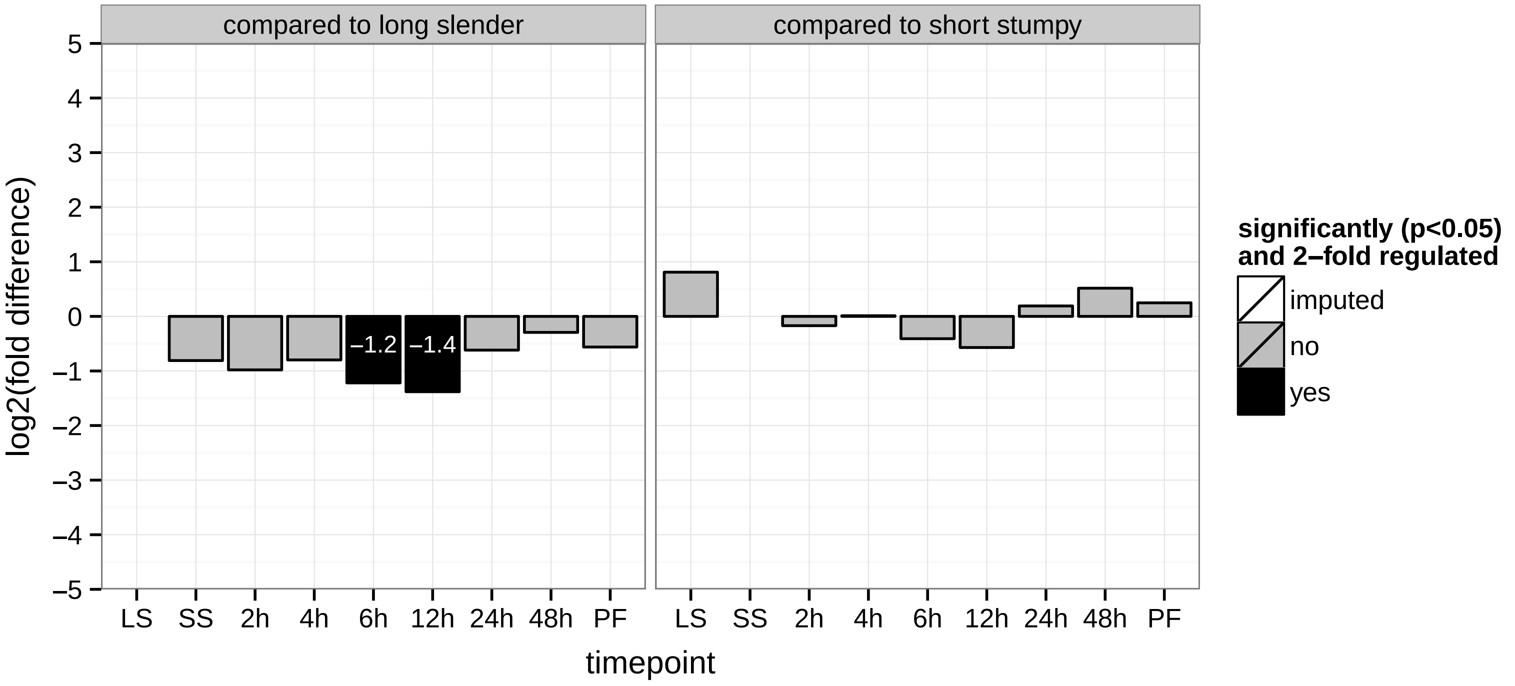

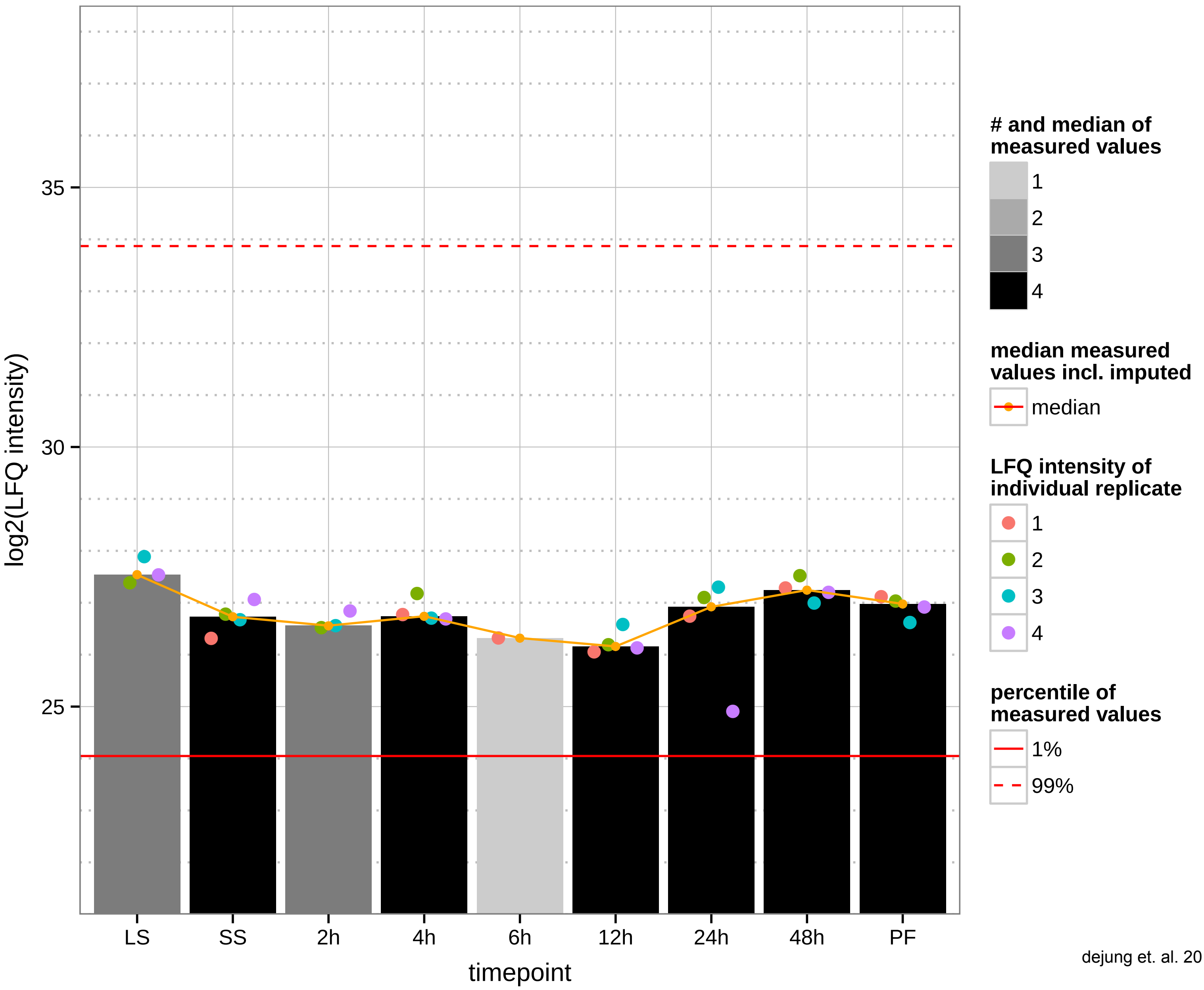

dejung et. al. 2015

TAX−2
Tb927.10.650
AGOF: heme binding
AGOC: cilium part
AGOP: ciliary or flagellar motility
PGOF: heme binding
PGOC: null
PGOP: null

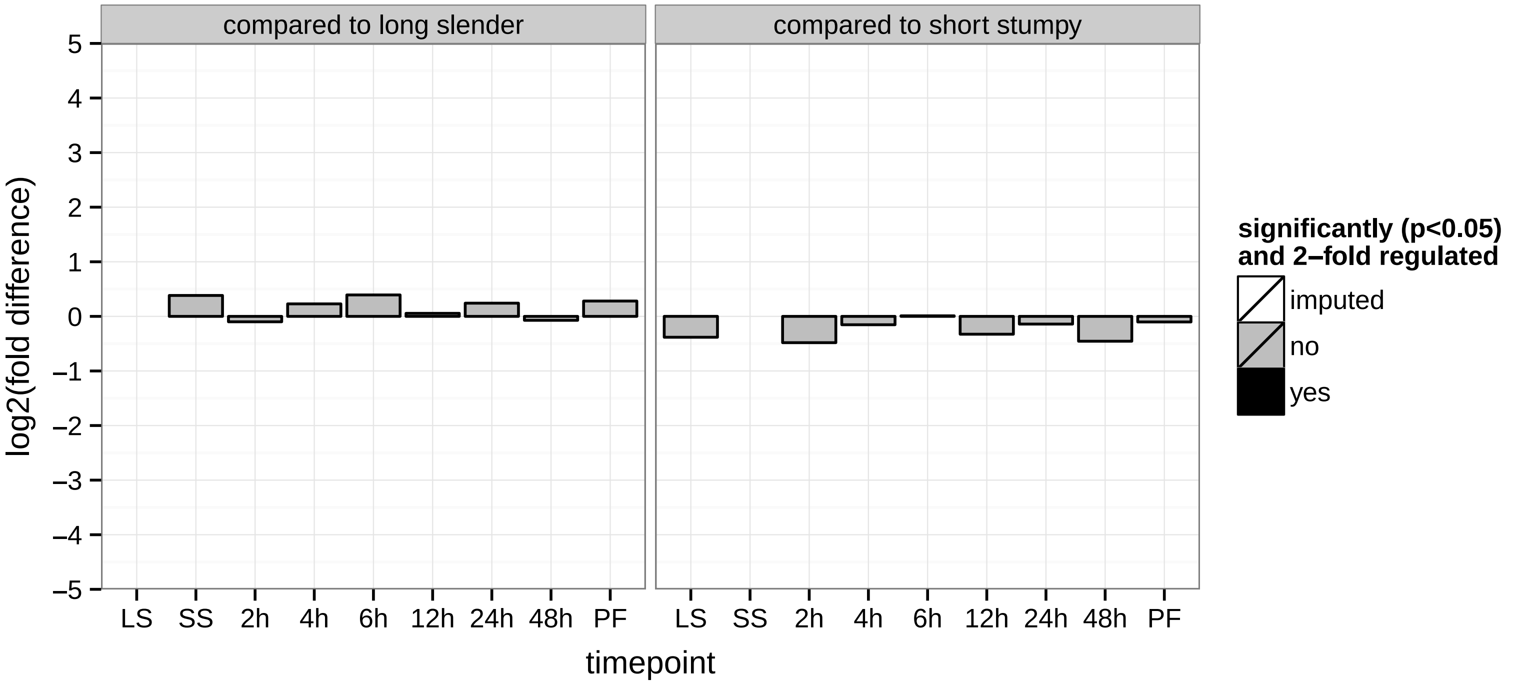

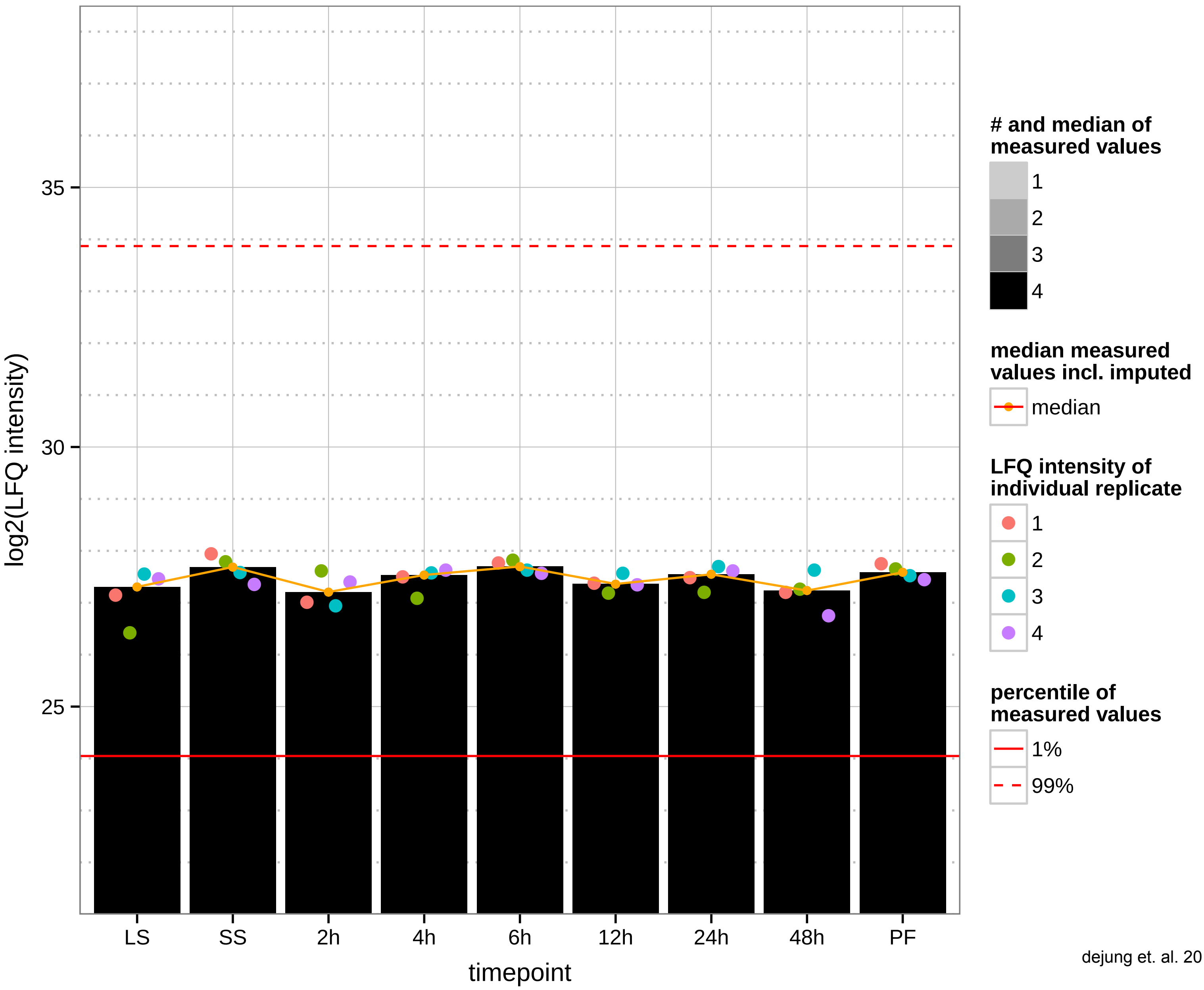

dejung et. al. 2015

chaperone protein DNAj, putative
Tb927.10.6610
AGOF: heat shock protein binding, unfolded protein binding
AGOC: null
AGOP: protein folding
PGOF: heat shock protein binding
PGOC: null
PGOP: null

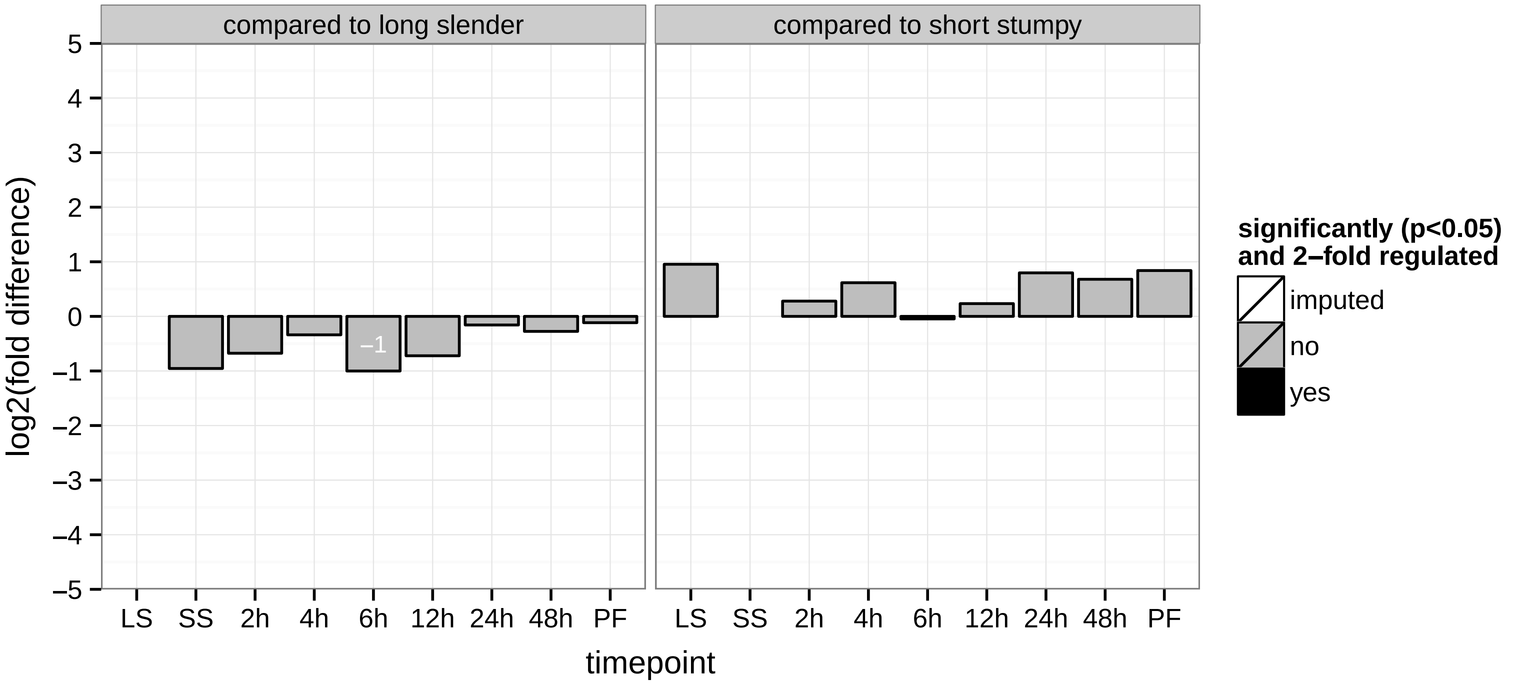

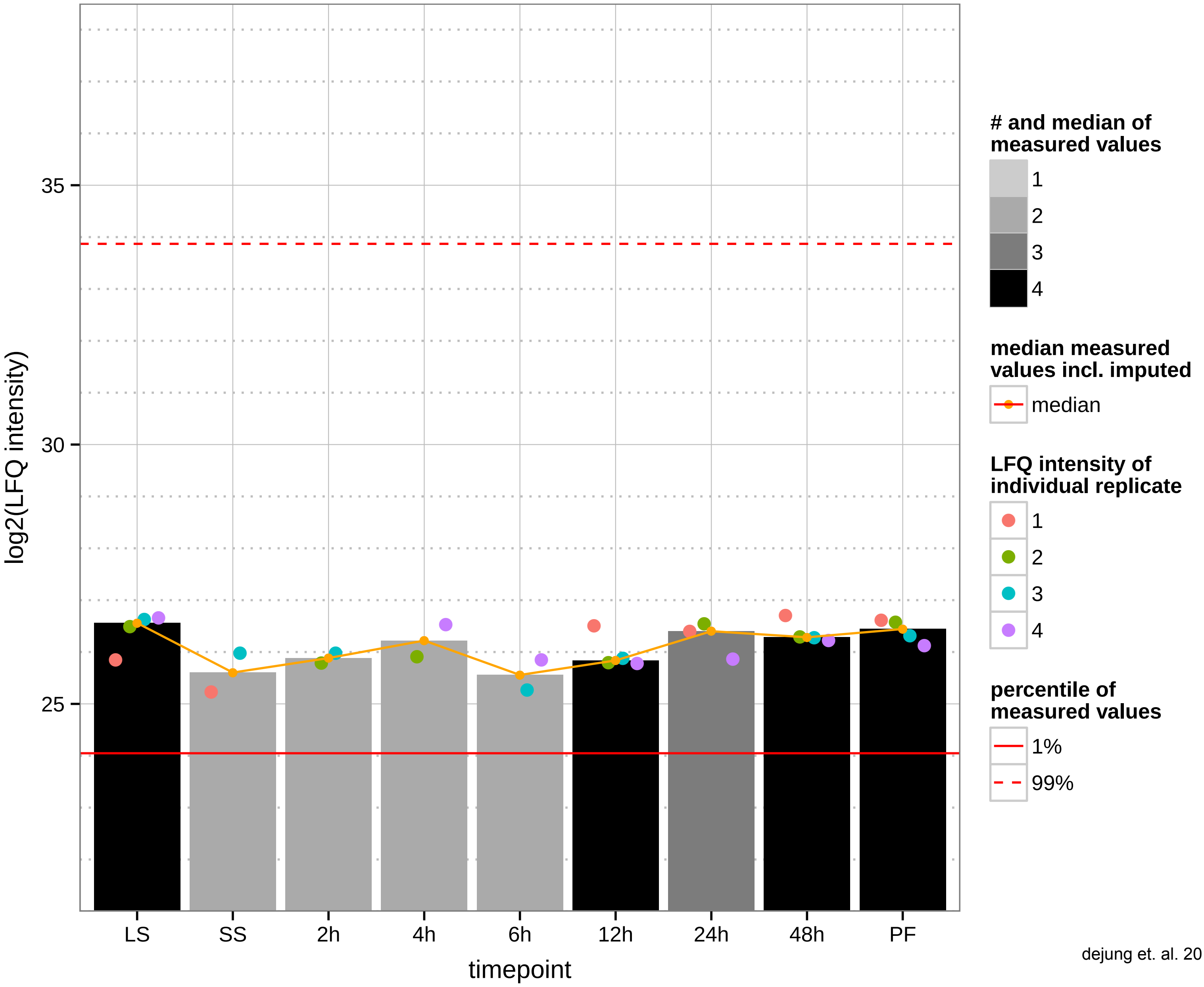

dejung et. al. 2015

COP–coated vesicle membrane protein erv25 precursor, putative, ER––golgi transport protein erv25 precursor
Tb927.10.6640
AGOF: null
AGOC: integral to membrane
AGOP: transport
PGOF: null
PGOC: integral to membrane
PGOP: transport

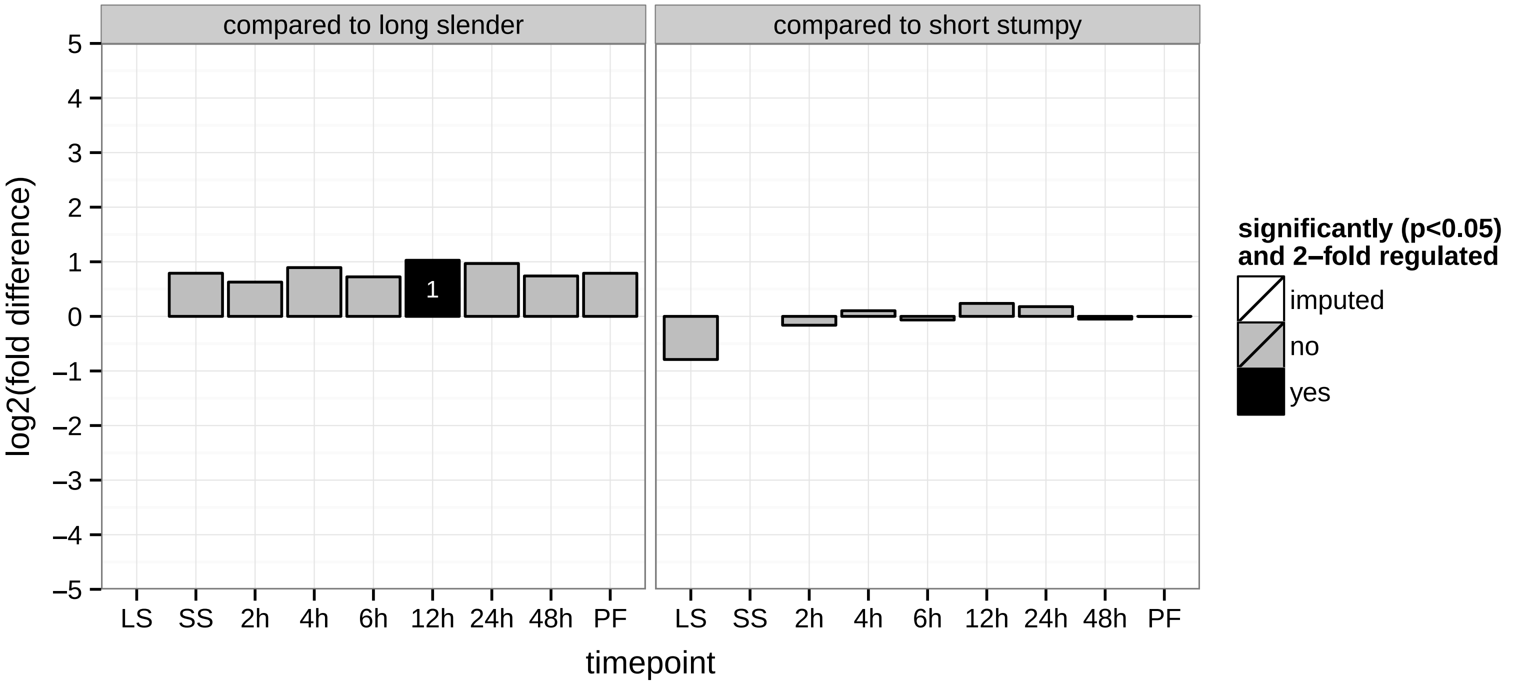

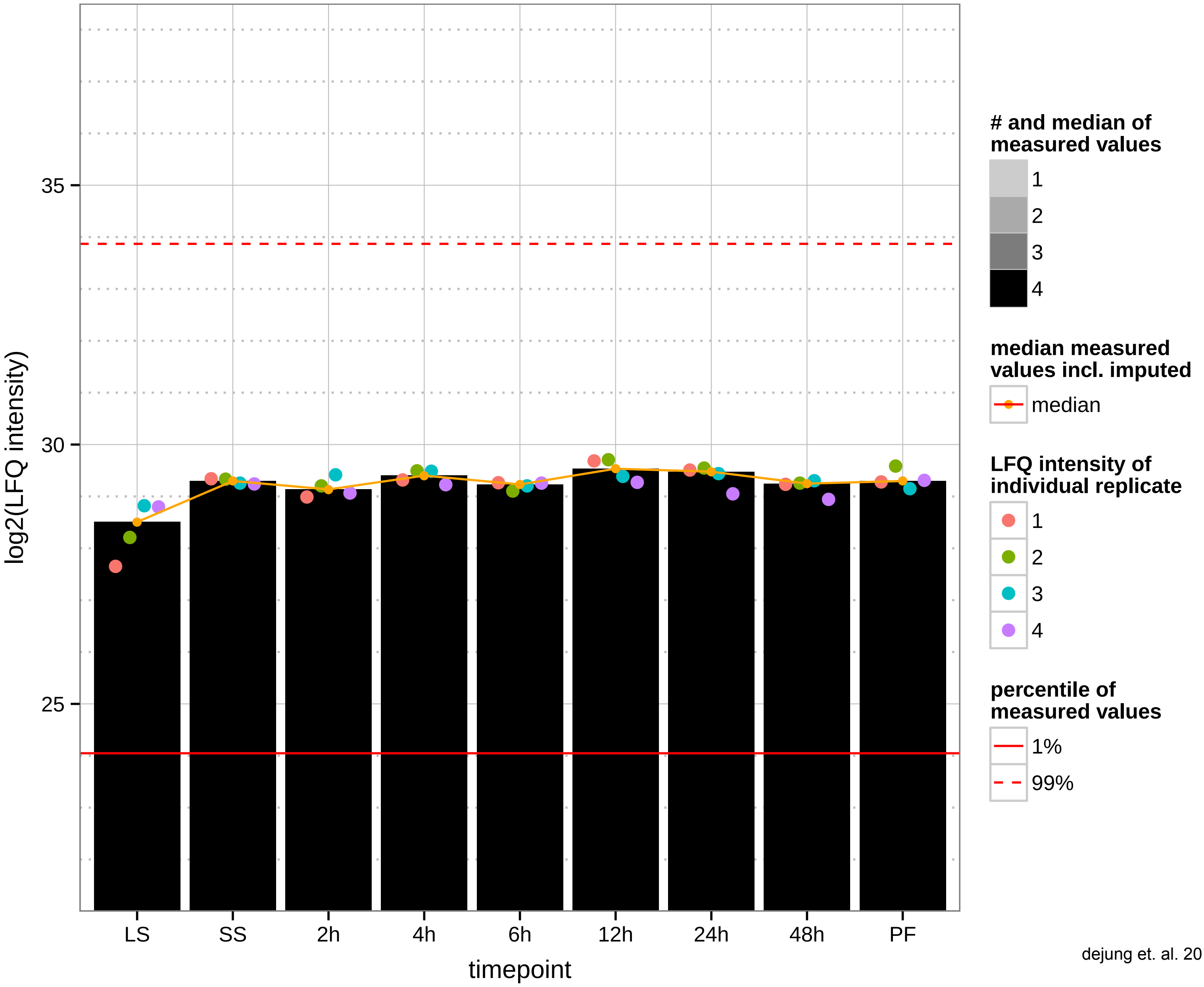

dejung et. al. 2015

dynein light chain, putative
Tb927.10.6670
AGOF: microtubule motor activity
AGOC: microtubule associated complex
AGOP: microtubule–based process
PGOF: null
PGOC: microtubule associated complex
PGOP: microtubule–based process

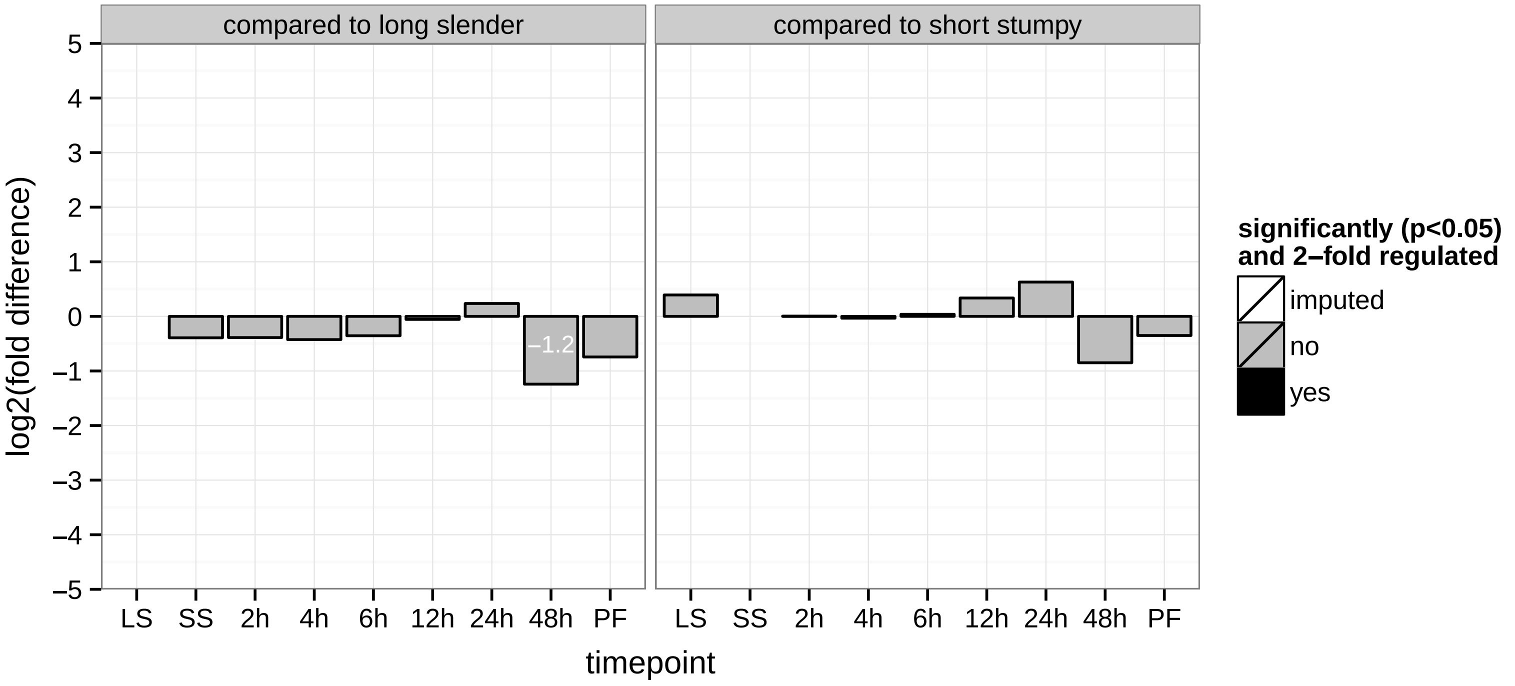

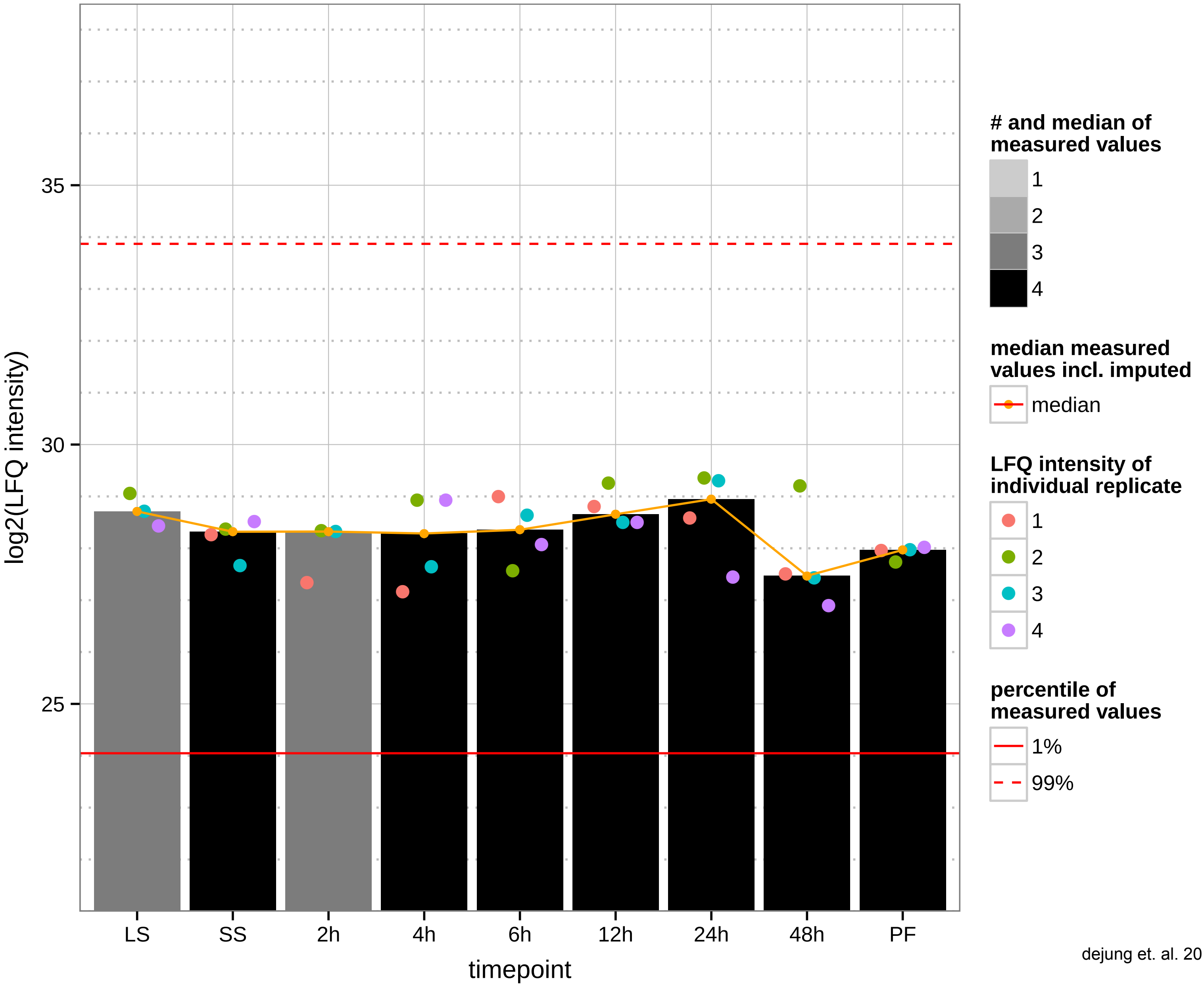

dejung et. al. 2015

developmentally regulated GTP−binding protein, putative
Tb927.10.6800
AGOF: GTP binding
AGOC: cytoplasm, intracellular
AGOP: cellular process
PGOF: GTP binding
PGOC: null
PGOP: null

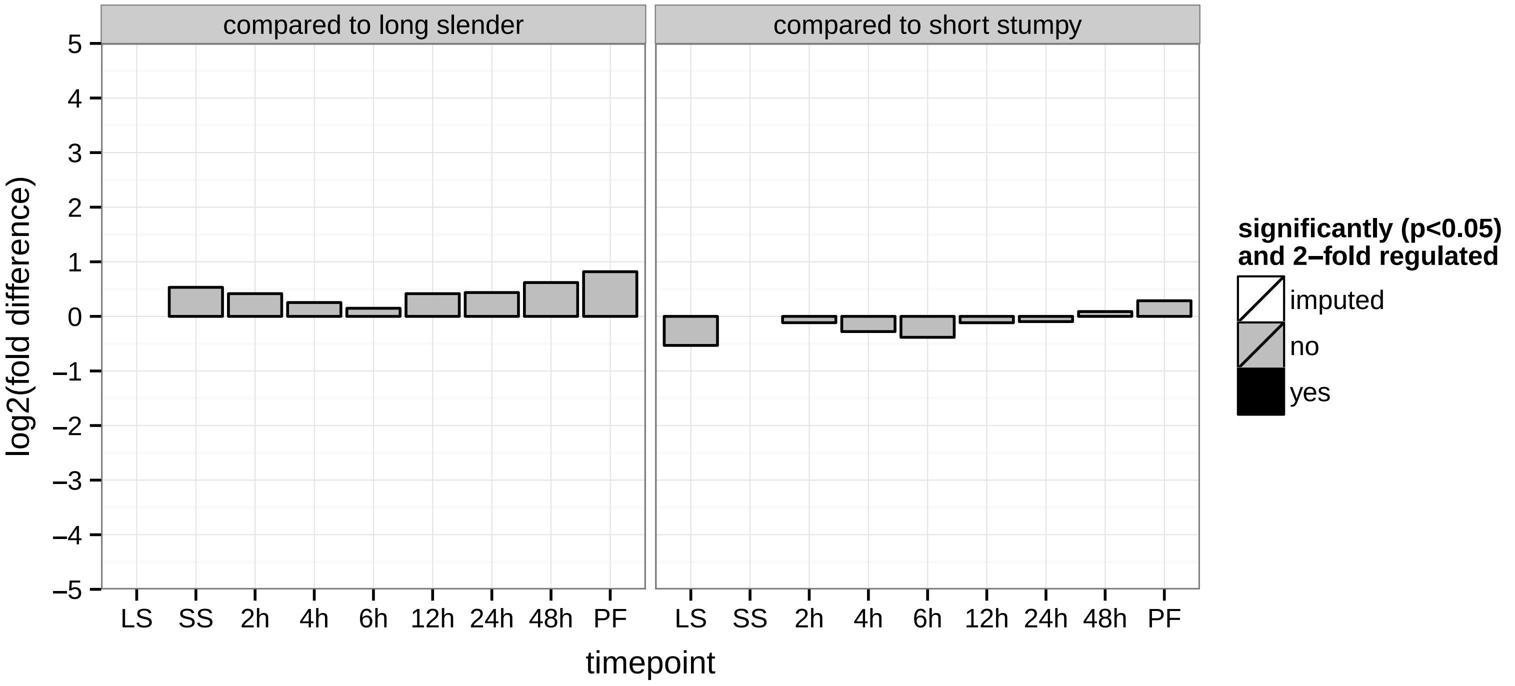

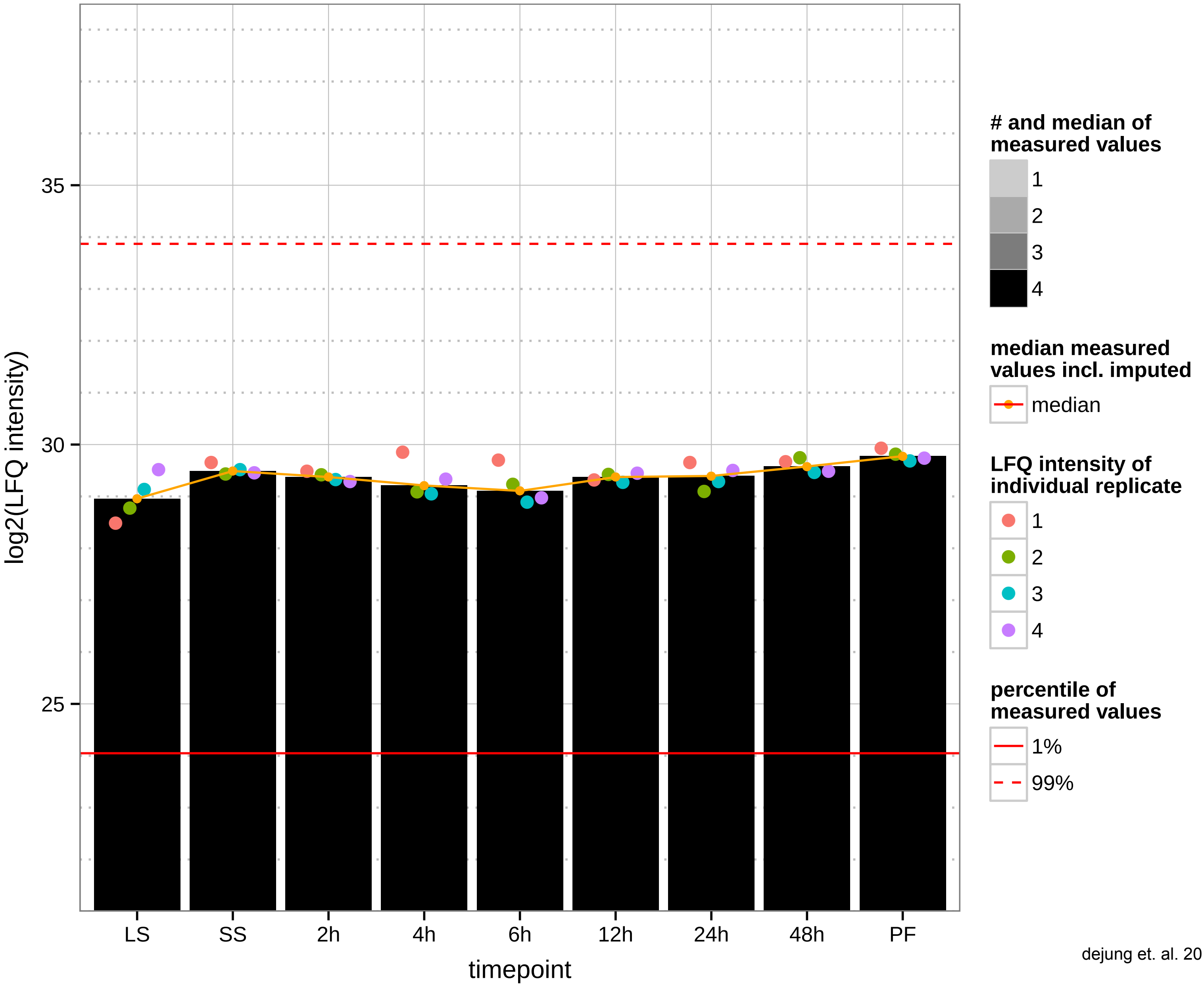

dejung et. al. 2015

guanylate kinase, putative
Tb927.10.6810
AGOF: null
AGOC: null
AGOP: nucleobase−containing small molecule interconversion
PGOF: protein binding
PGOC: null
PGOP: null

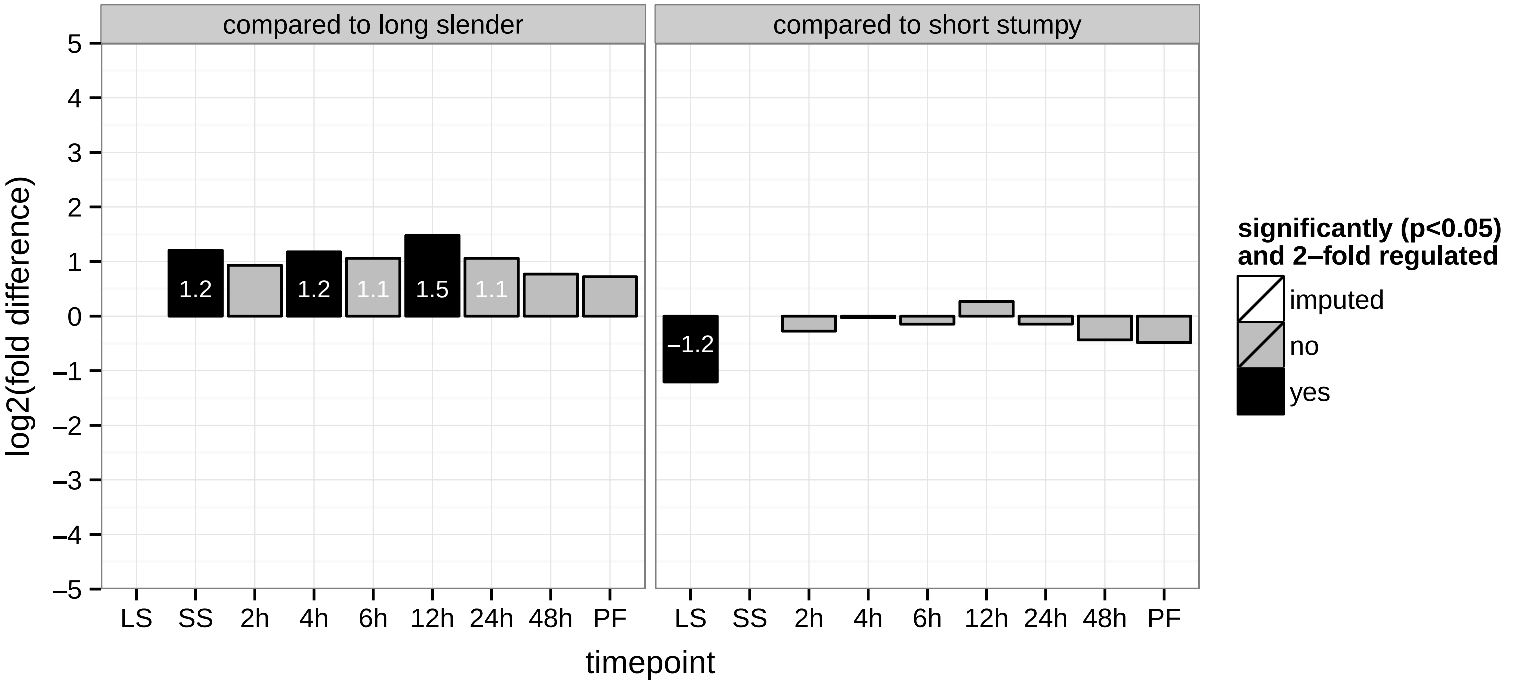

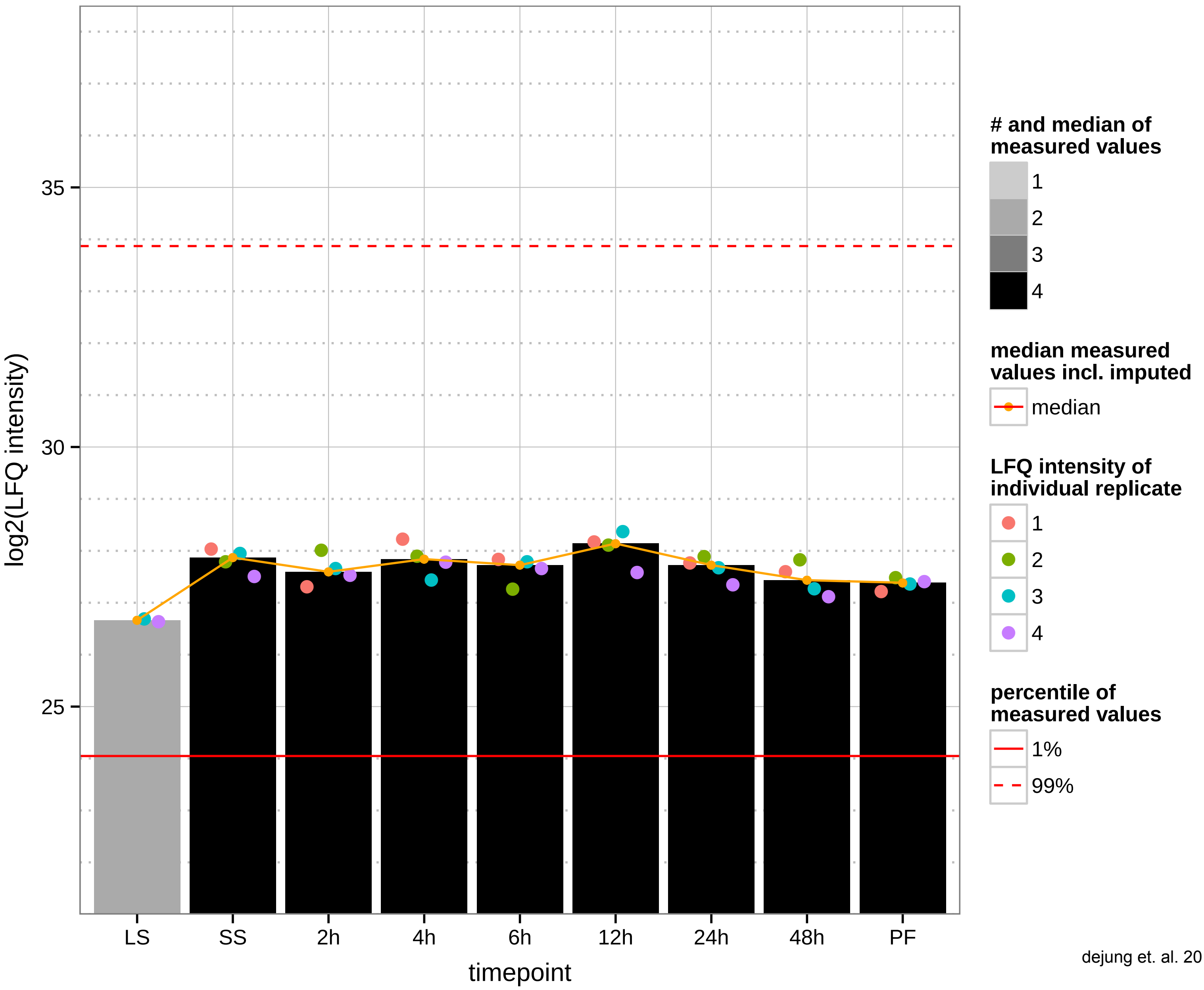

dejung et. al. 2015

hypothetical protein, conserved
Tb927.10.6820
AGOF: null
AGOC: null
AGOP: null
PGOF: null
PGOC: null
PGOP: null

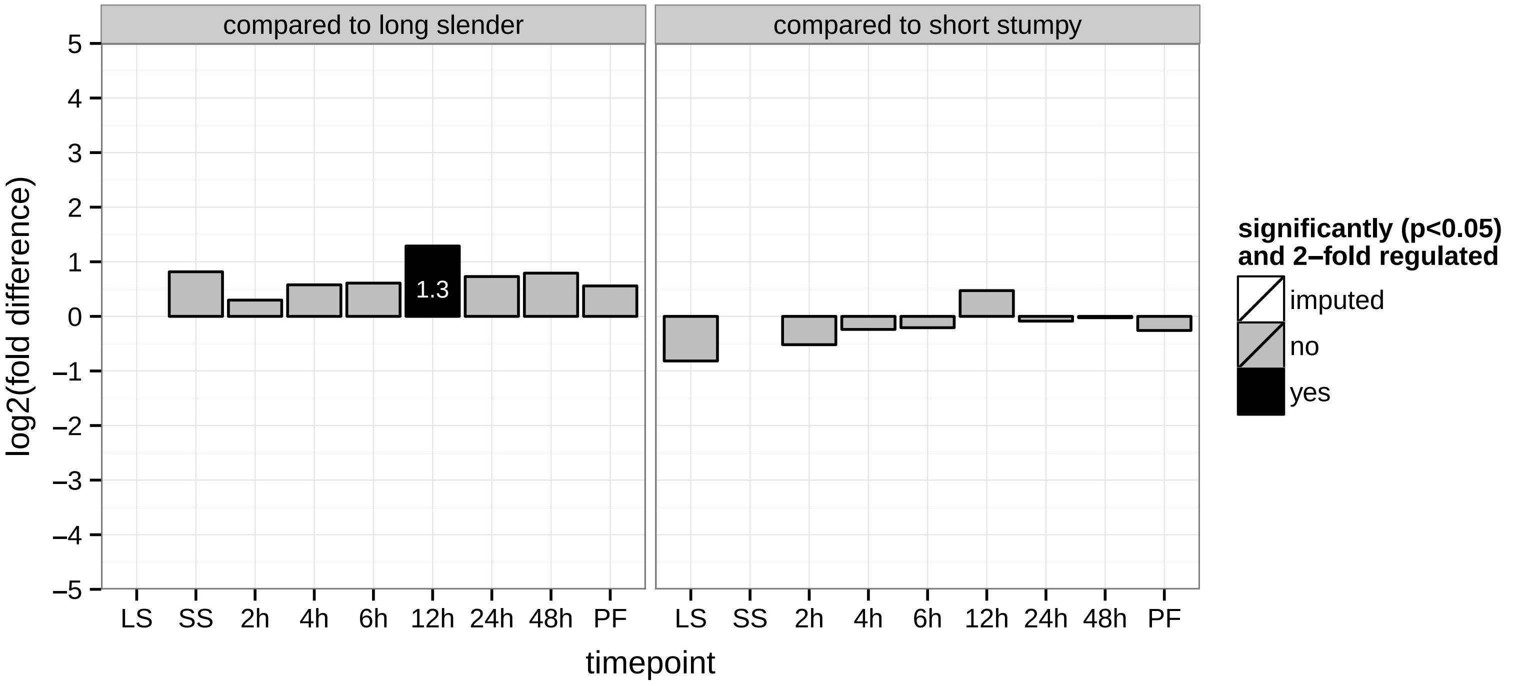

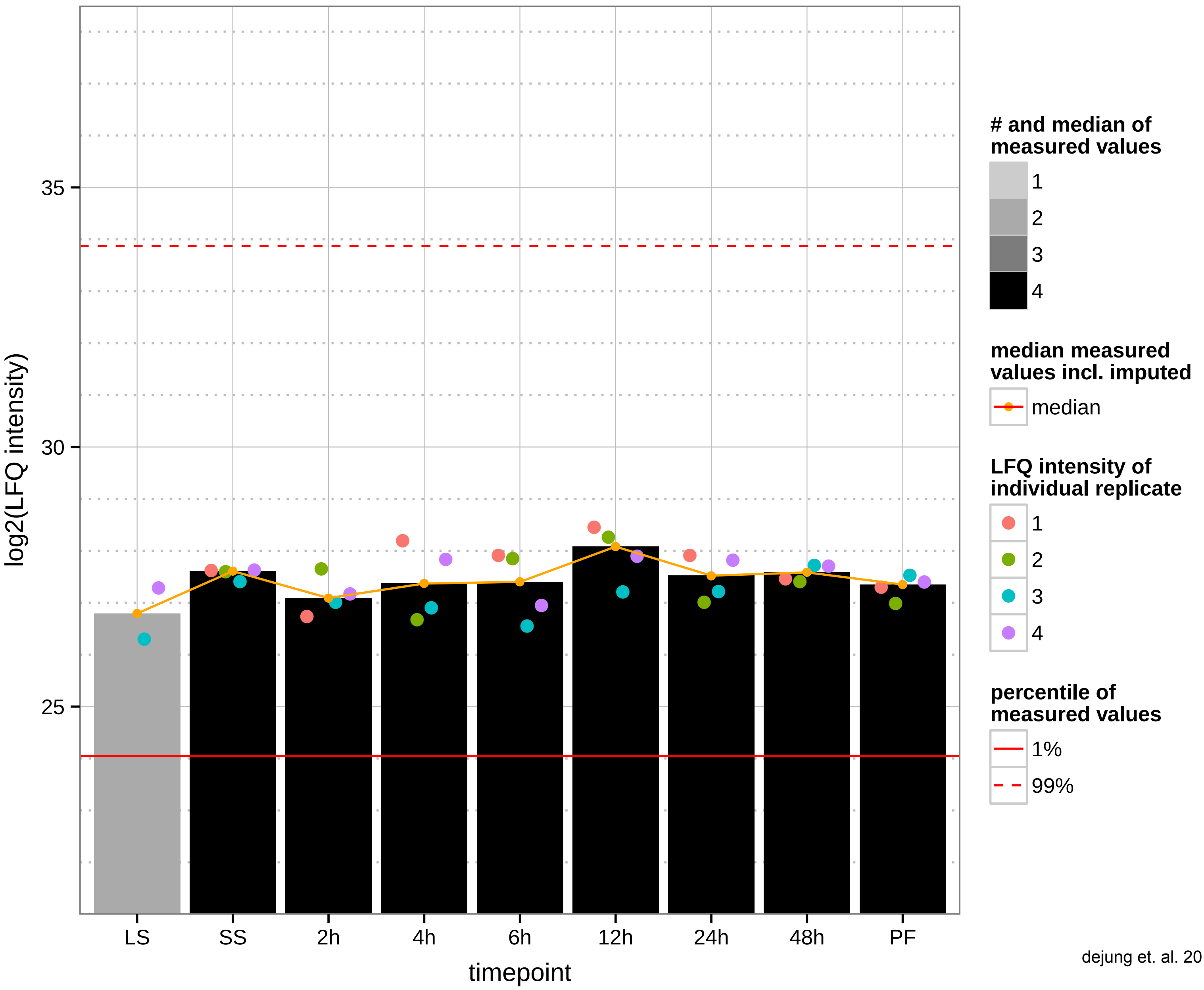

dejung et. al. 2015

hypothetical protein, conserved
Tb927.10.6840
AGOF: null
AGOC: null
AGOP: null
PGOF: null
PGOC: null
PGOP: null

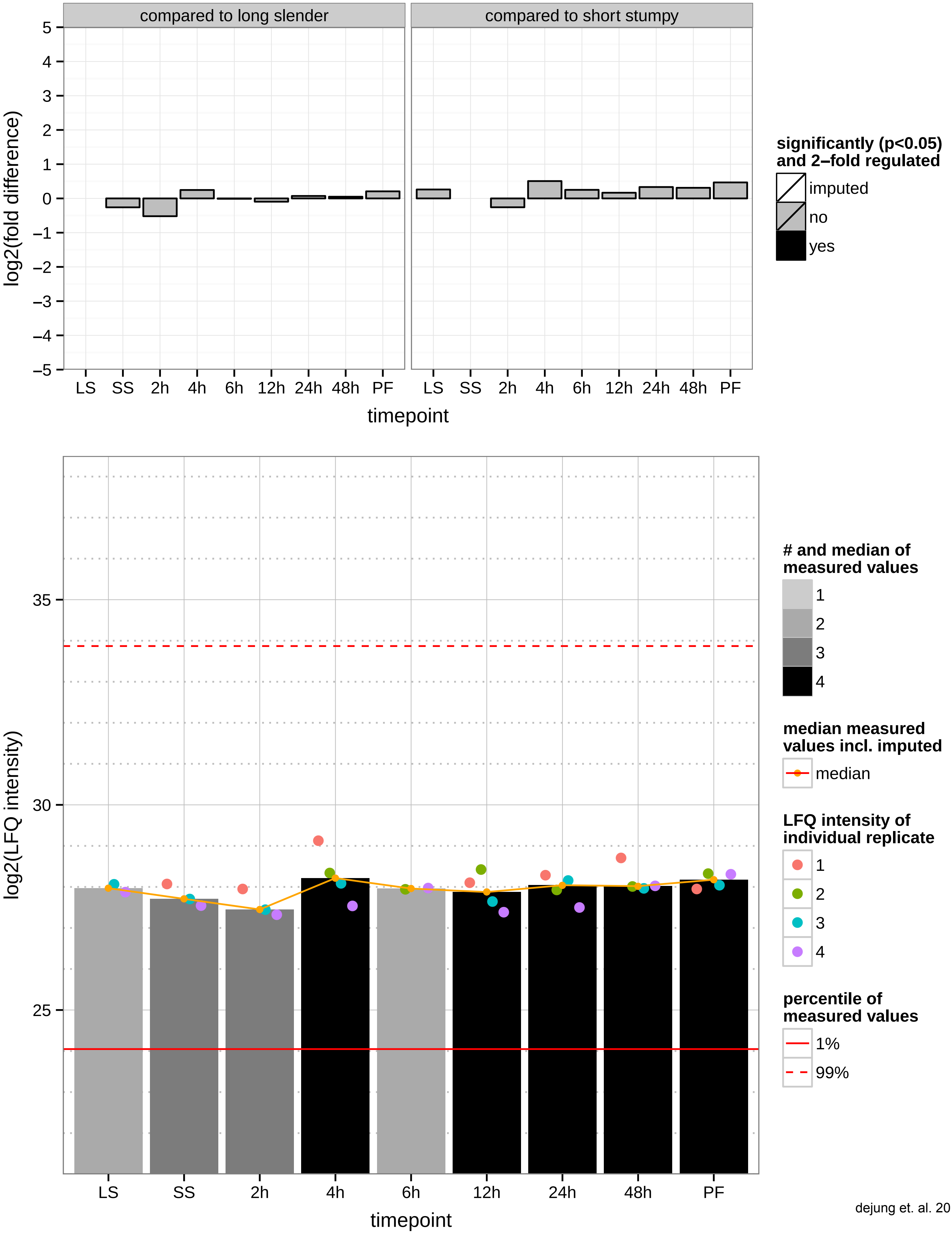

dejung et. al. 2015

Dipeptidyl–peptidase 8–like, putative, dipeptidyl–peptidase 8–like serine peptidase, serine peptidase, Clan SC, Family S9B
Tb927.10.6970;Tb927.10.6940
AGOF: serine–type peptidase activity
AGOC: membrane
AGOP: proteolysis
PGOF: serine–type peptidase activity
PGOC: membrane
PGOP: proteolysis

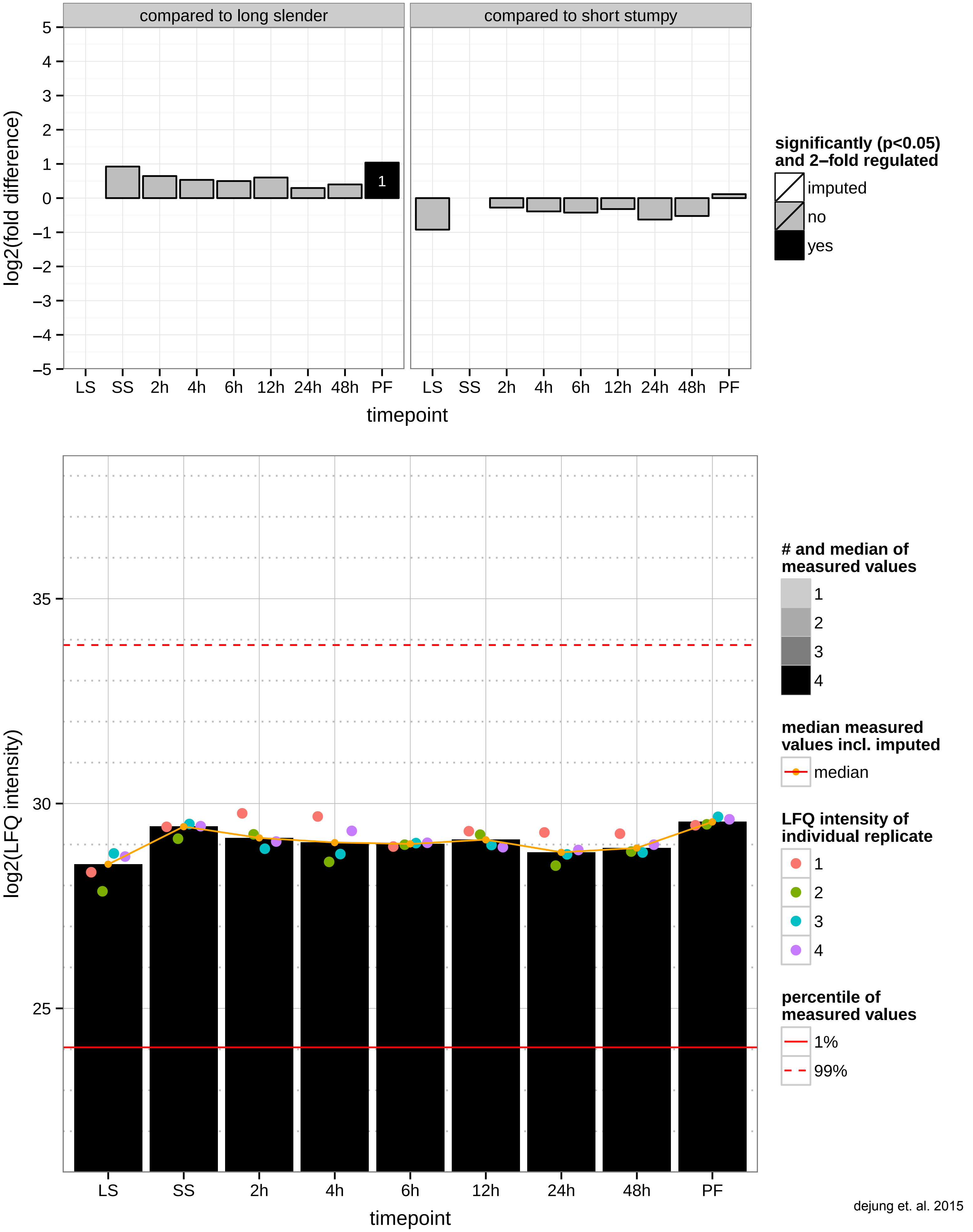

dejung et. al. 2015

hypothetical protein, conserved
Tb927.10.6960
AGOF: null
AGOC: null
AGOP: null
PGOF: null
PGOC: null
PGOP: null

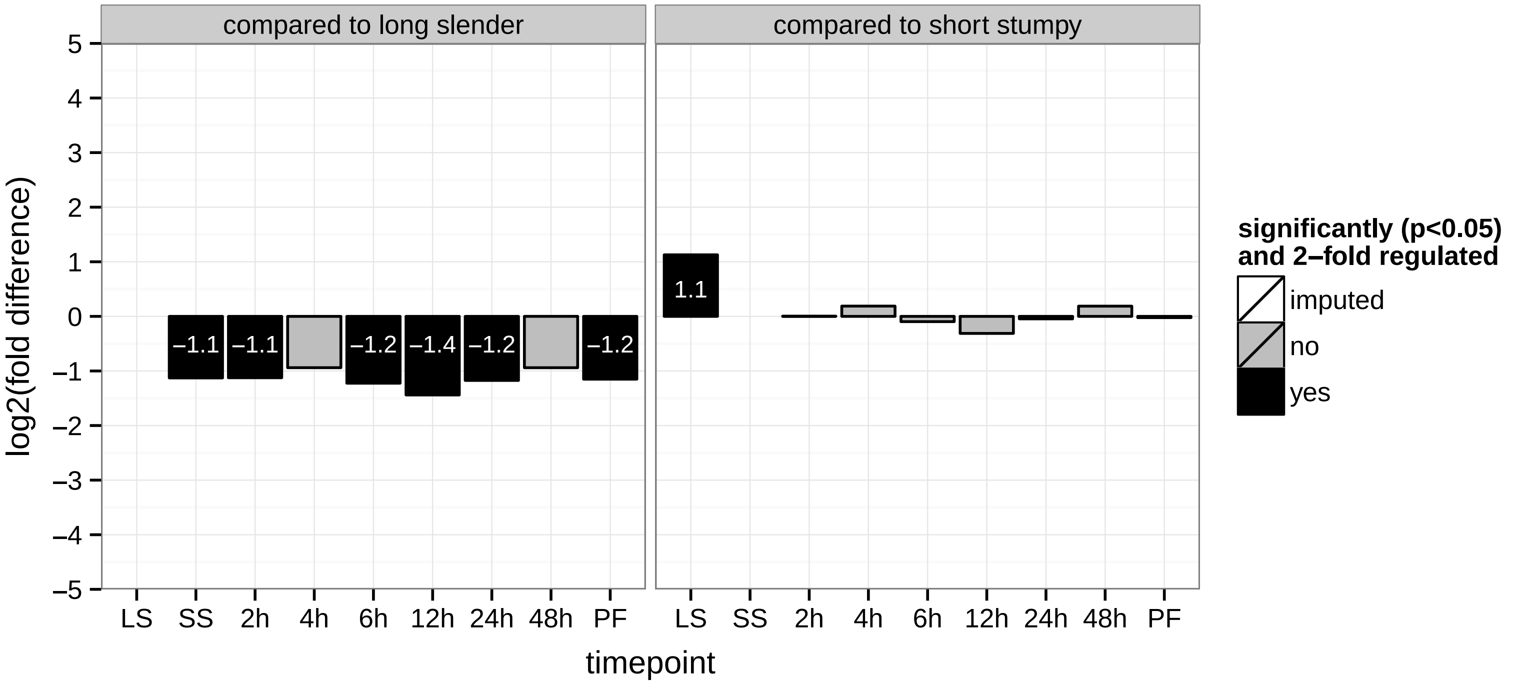

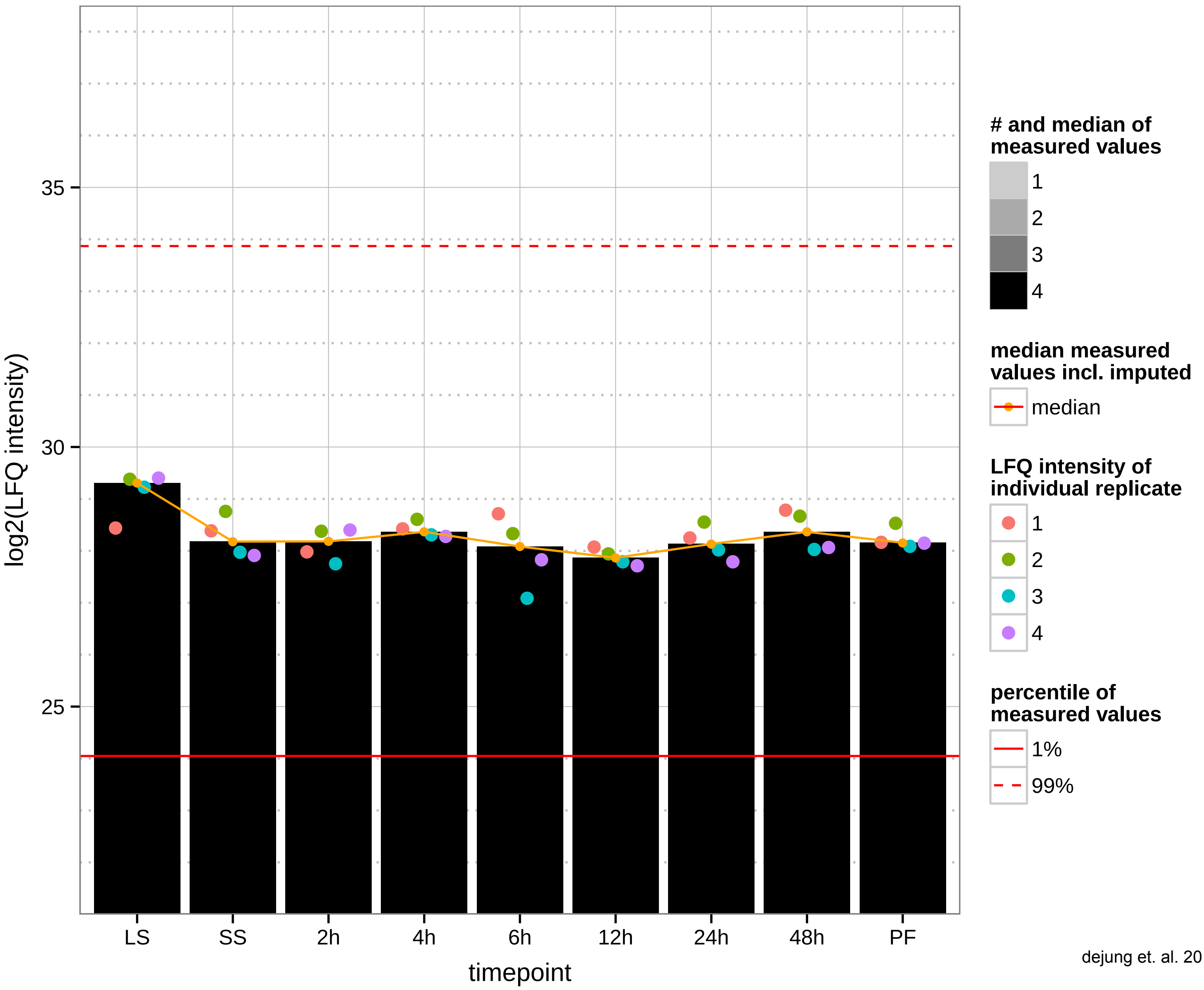

dejung et. al. 2015

hypothetical protein, conserved
Tb927.10.700
AGOF: null
AGOC: null
AGOP: null
PGOF: null
PGOC: null
PGOP: null

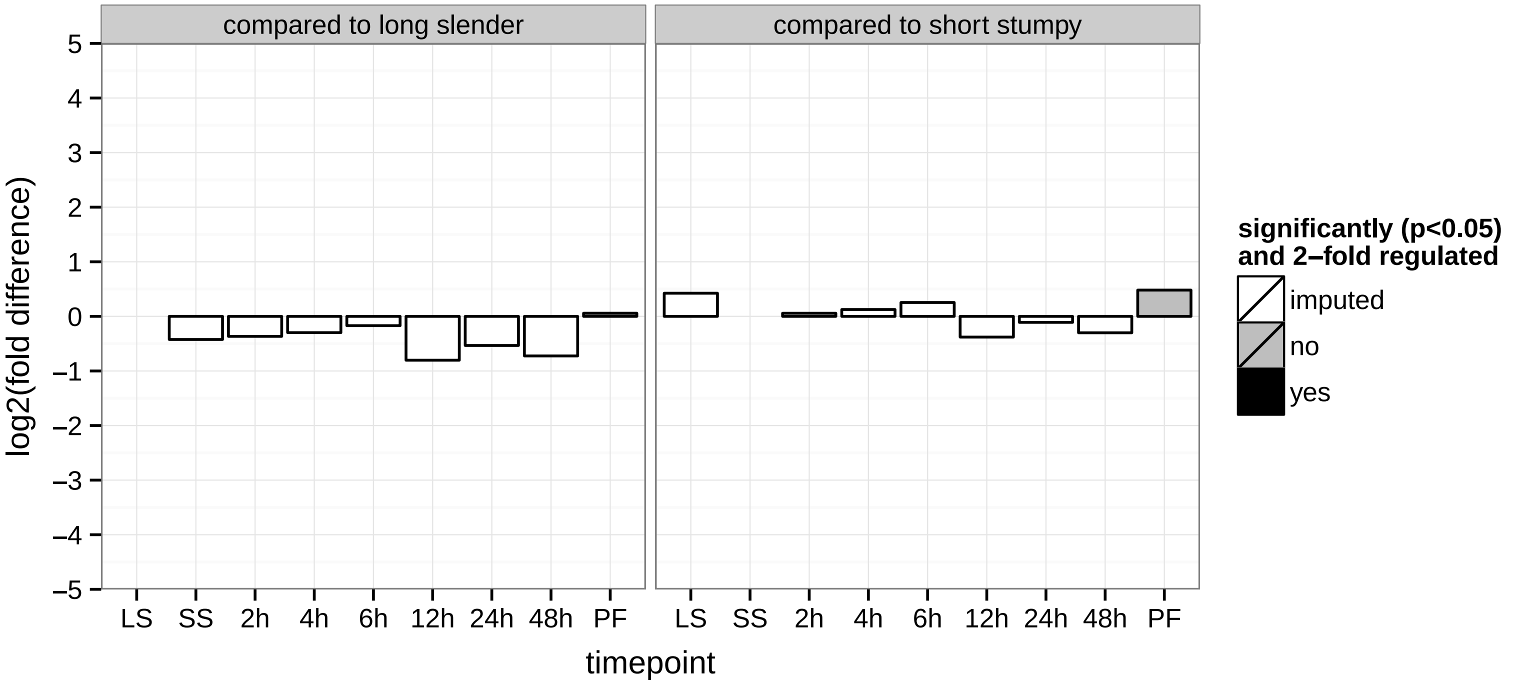

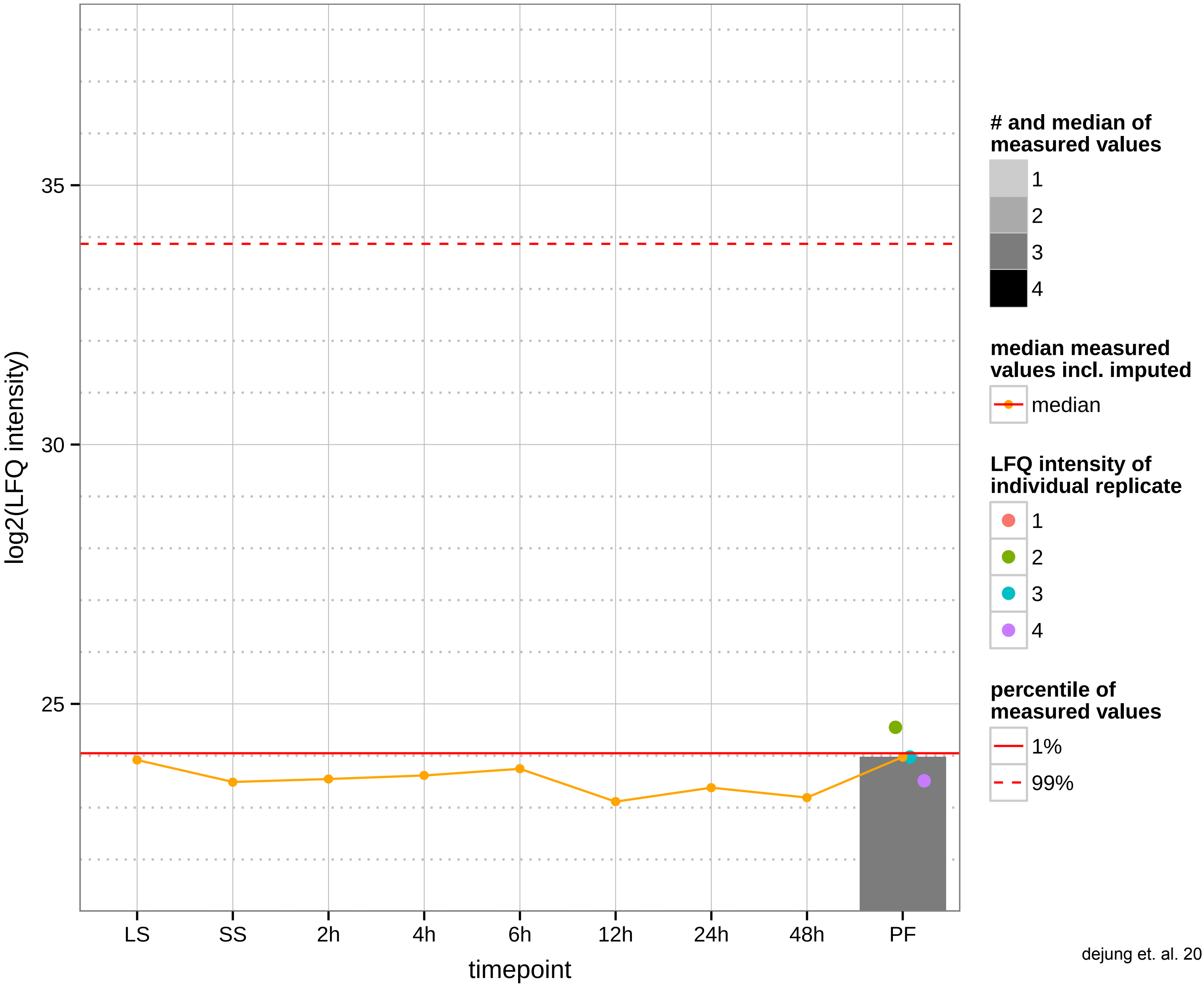

dejung et. al. 2015

acid phosphatase, putative
Tb927.10.7020
AGOF: acid phosphatase activity
AGOC: null
AGOP: null
PGOF: acid phosphatase activity
PGOC: null
PGOP: null

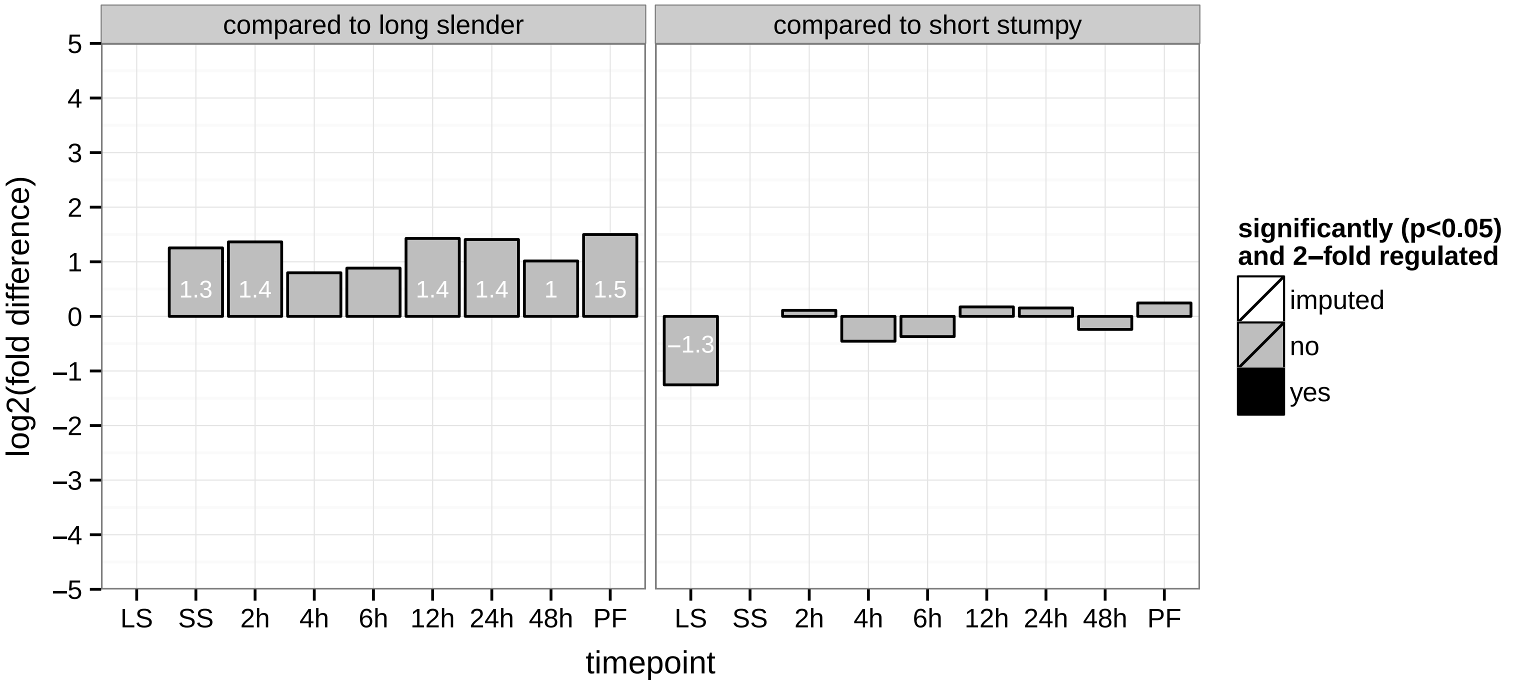

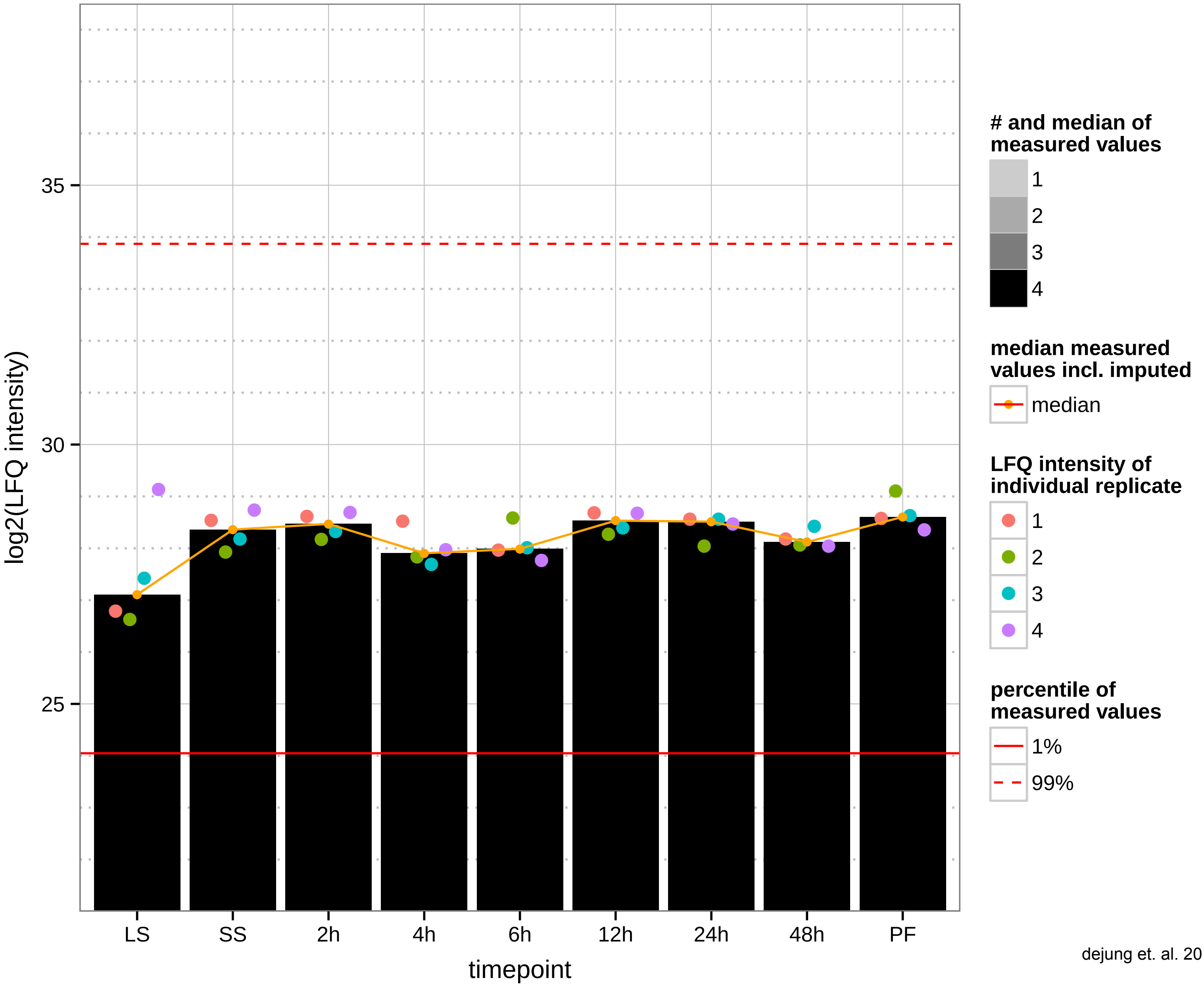

dejung et. al. 2015

nucleoporin interacting component (NUP93), putative
Tb927.10.7060
AGOF: null
AGOC: nuclear pore
AGOP: transport
PGOF: null
PGOC: nuclear pore
PGOP: transport

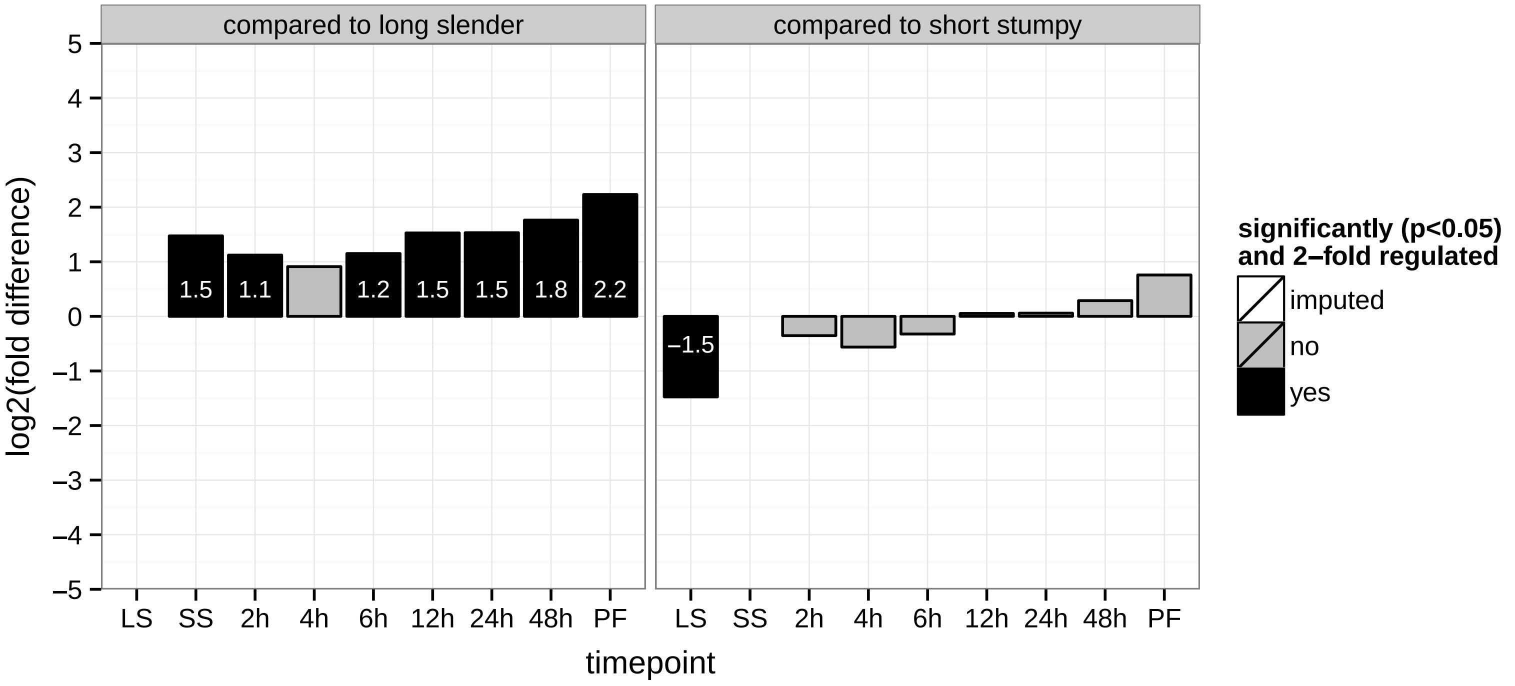

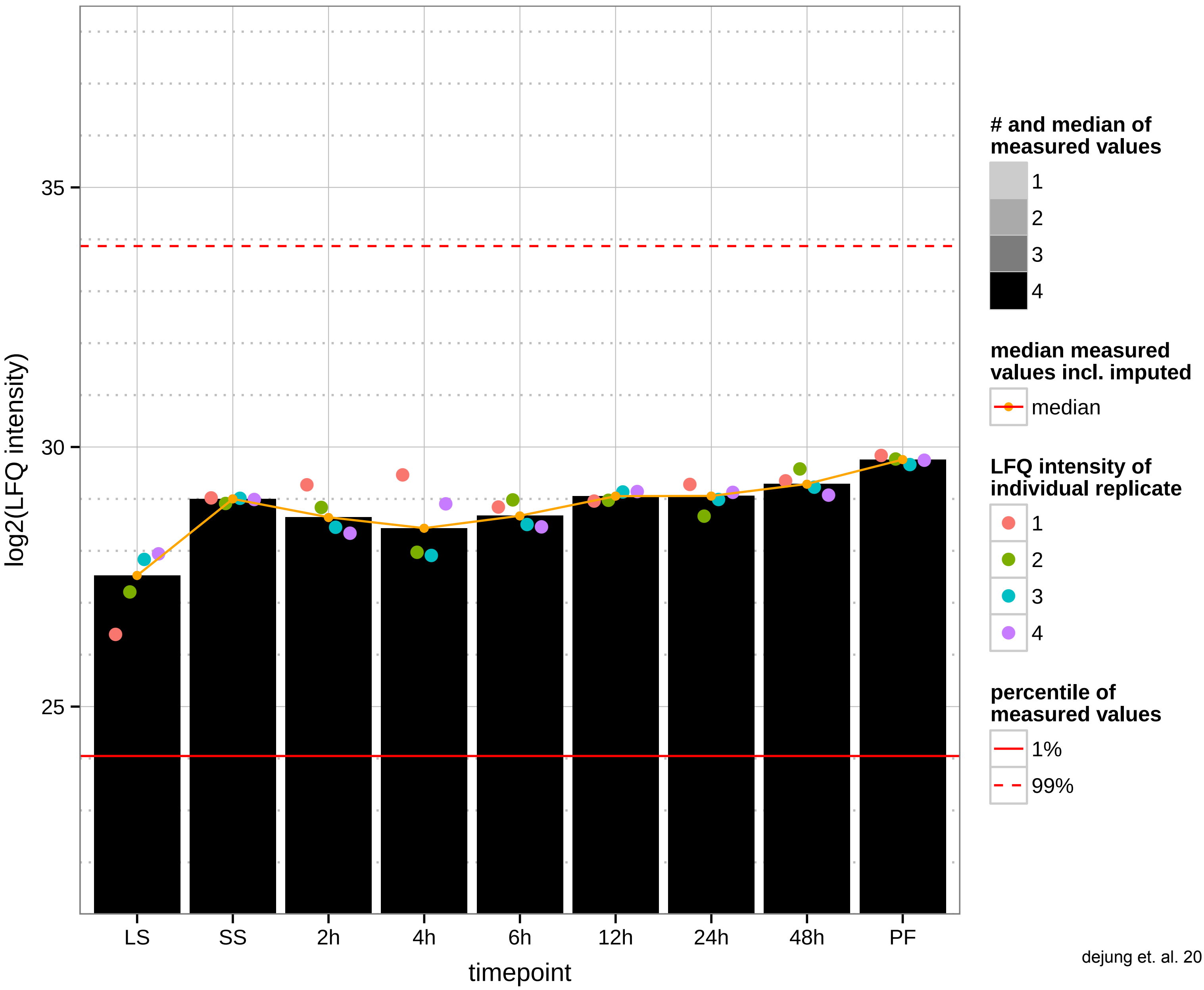

dejung et. al. 2015

ATP−dependent DEAD/H RNA helicase, putative, RNA helicase
Tb927.10.7080
AGOF: ATP binding, ATP−dependent helicase activity, helicase activity, nucleic acid binding
AGOC: null
AGOP: nucleobase−containing compound metabolic process
PGOF: ATP binding, ATP−dependent helicase activity, helicase activity, nucleic acid binding
PGOC: null
PGOP: null

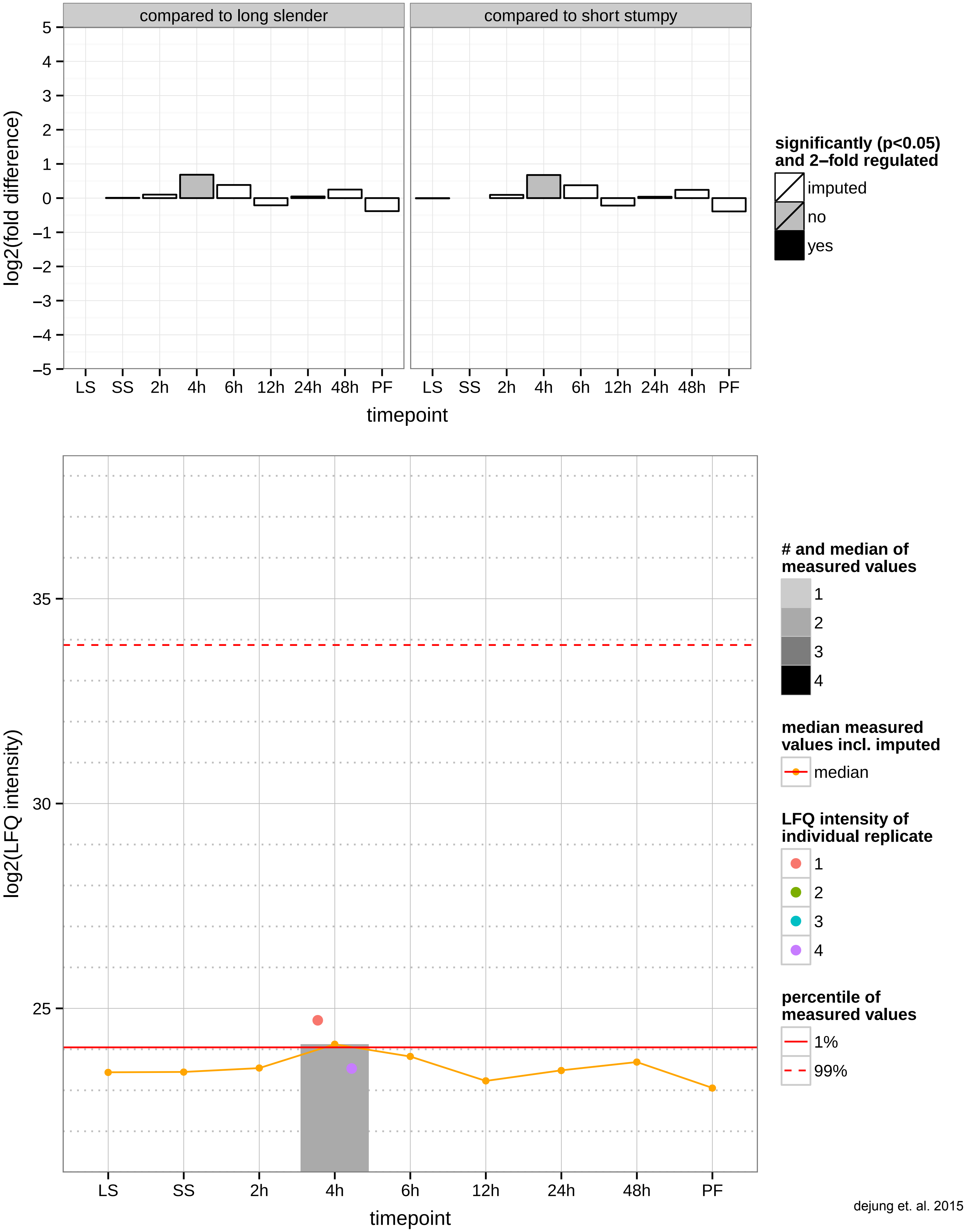

dejung et. al. 2015

hypothetical protein, conserved
Tb927.10.7120
AGOF: null
AGOC: null
AGOP: null
PGOF: null
PGOC: null
PGOP: null

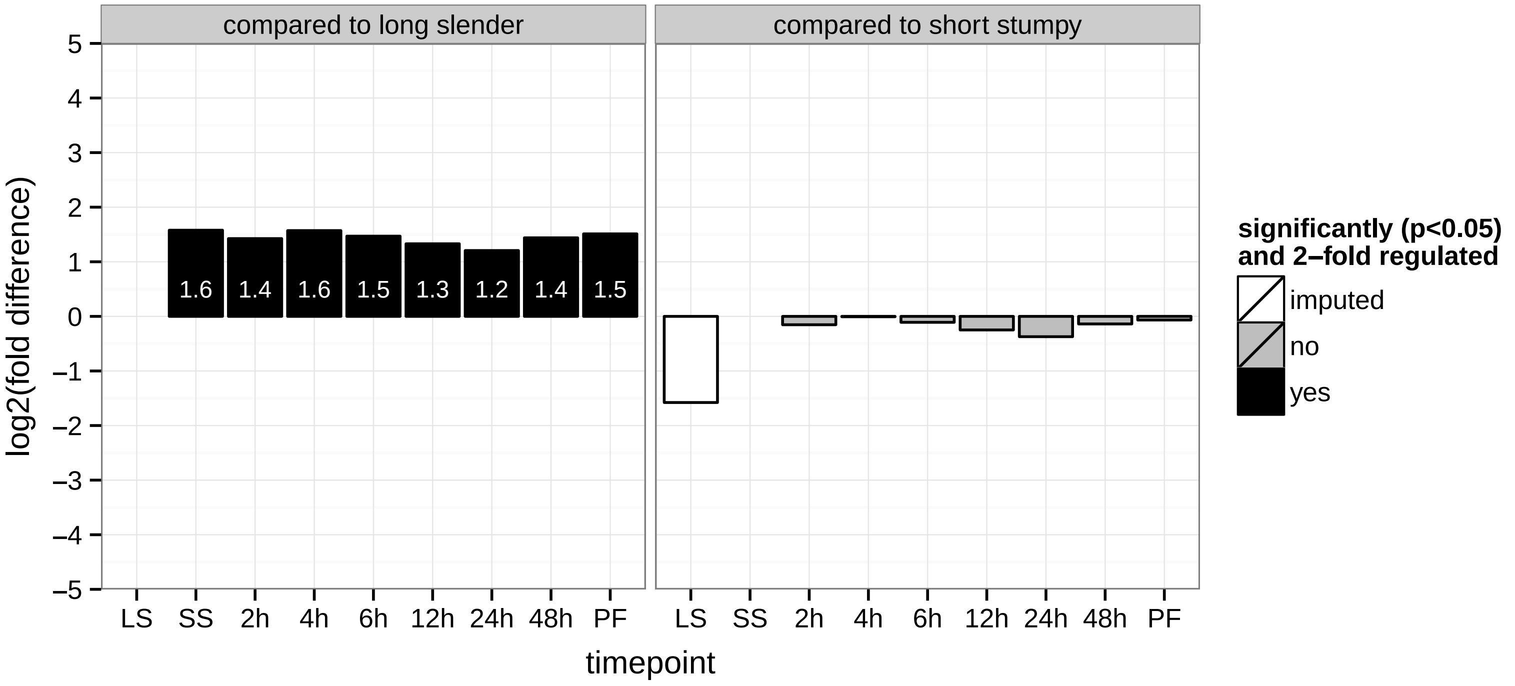

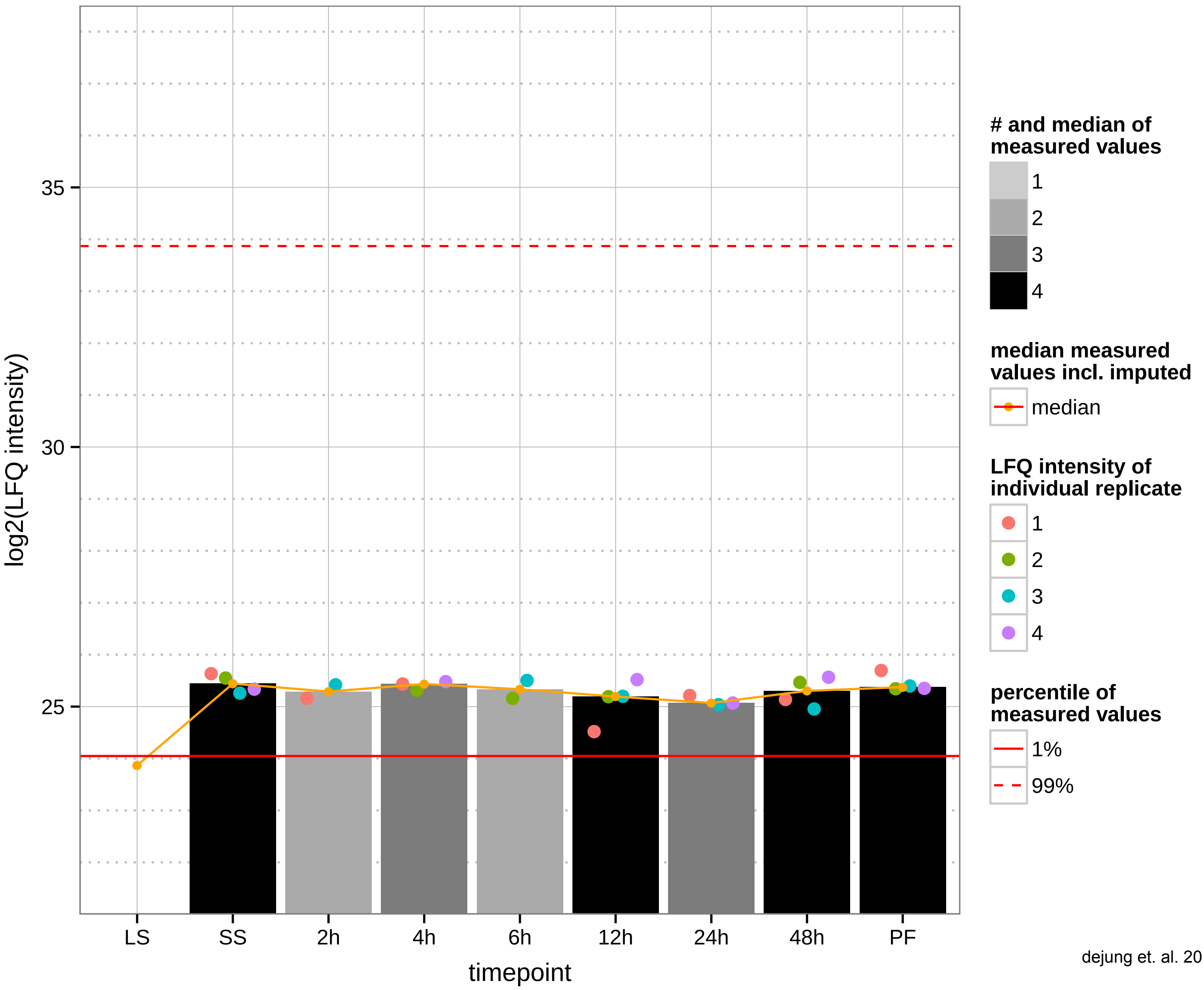

dejung et. al. 2015

hypothetical protein, conserved
Tb927.10.7130
AGOF: null
AGOC: null
AGOP: null
PGOF: binding
PGOC: null
PGOP: null

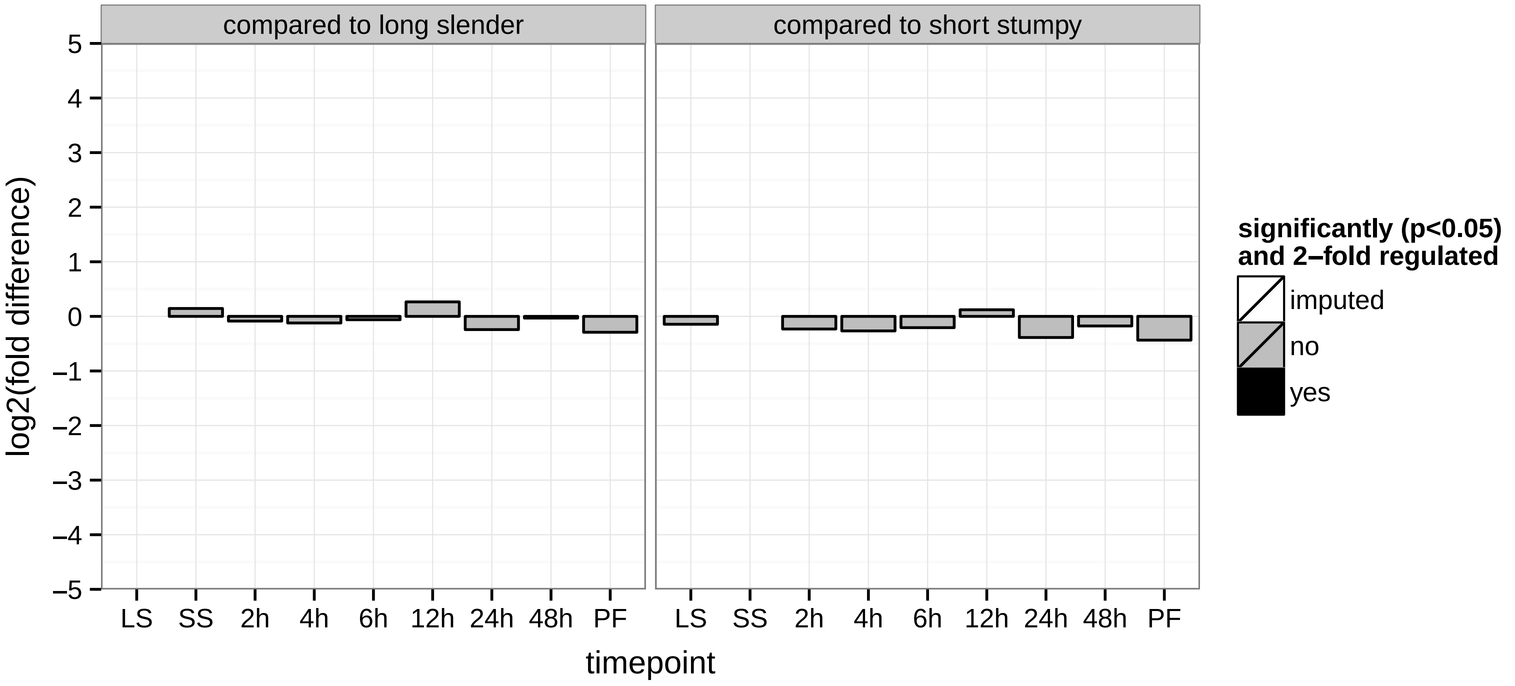

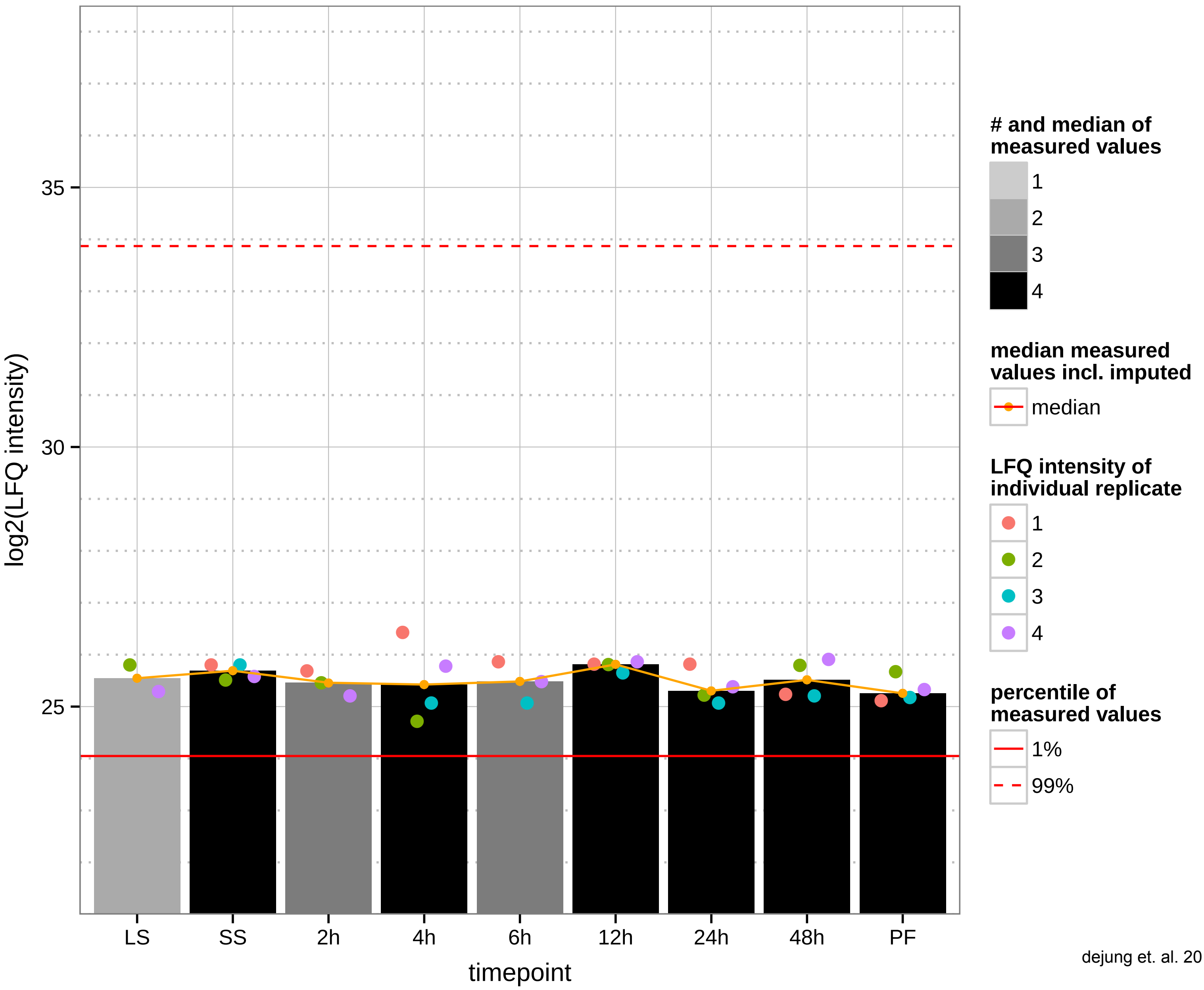

dejung et. al. 2015

hypothetical protein, conserved
Tb927.10.7200
AGOF: null
AGOC: null
AGOP: null
PGOF: protein binding
PGOC: null
PGOP: null

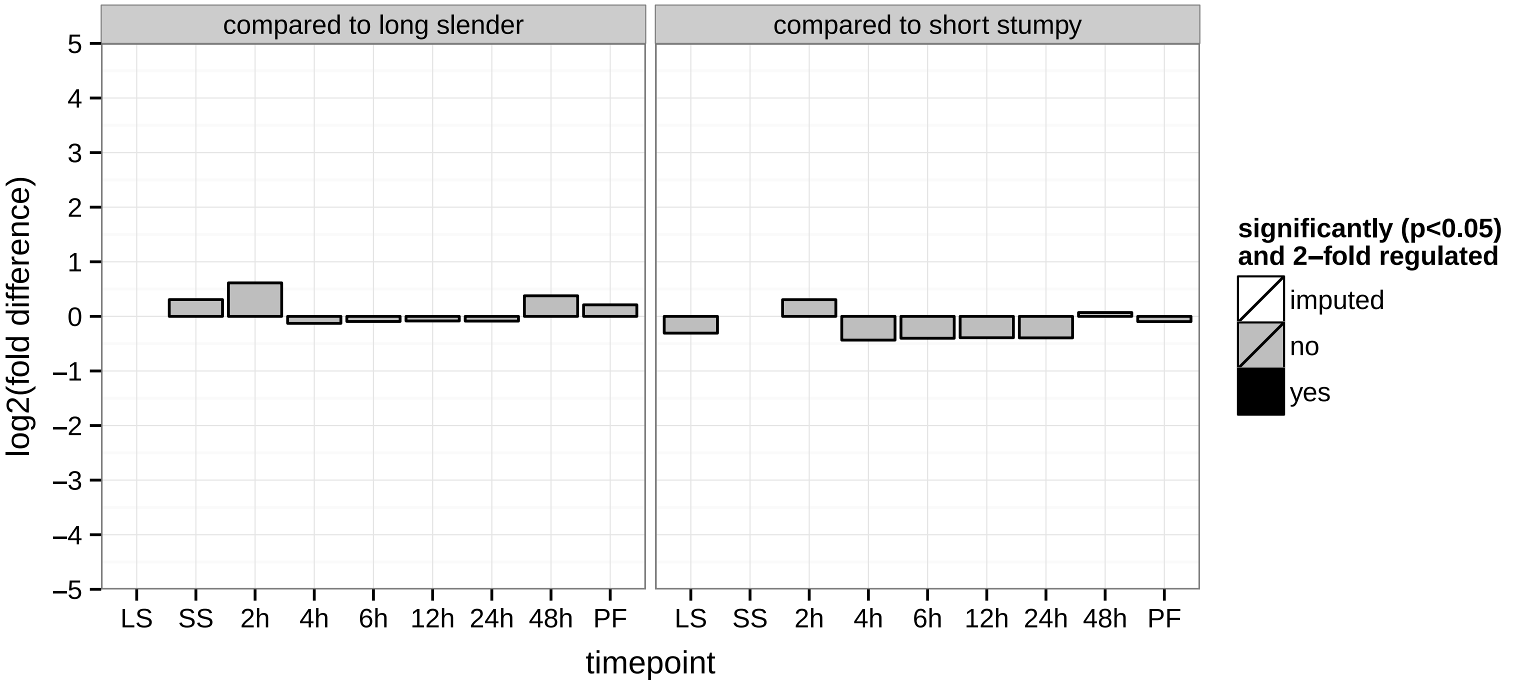

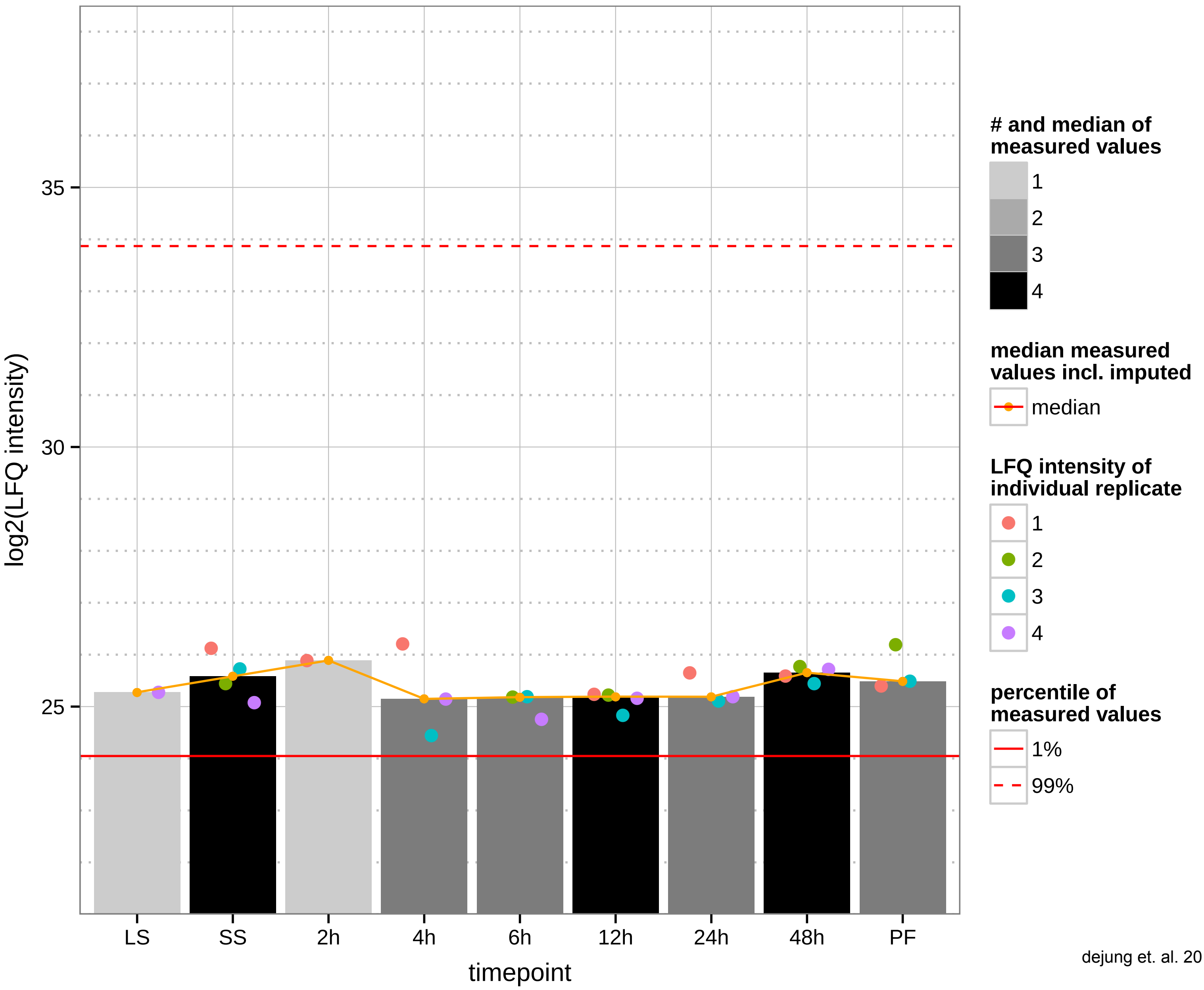

dejung et. al. 2015

hypothetical protein, conserved
Tb927.10.7230
AGOF: null, ATP binding, nucleoside diphosphate kinase activity
AGOC: null
AGOP: null, CTP biosynthetic process, GTP biosynthetic process, UTP biosynthetic process, oxidation–reduction process
PGOF: ATP binding, nucleoside diphosphate kinase activity
PGOC: null
PGOP: CTP biosynthetic process, GTP biosynthetic process, UTP biosynthetic process, nucleoside diphosphate phosphoryla

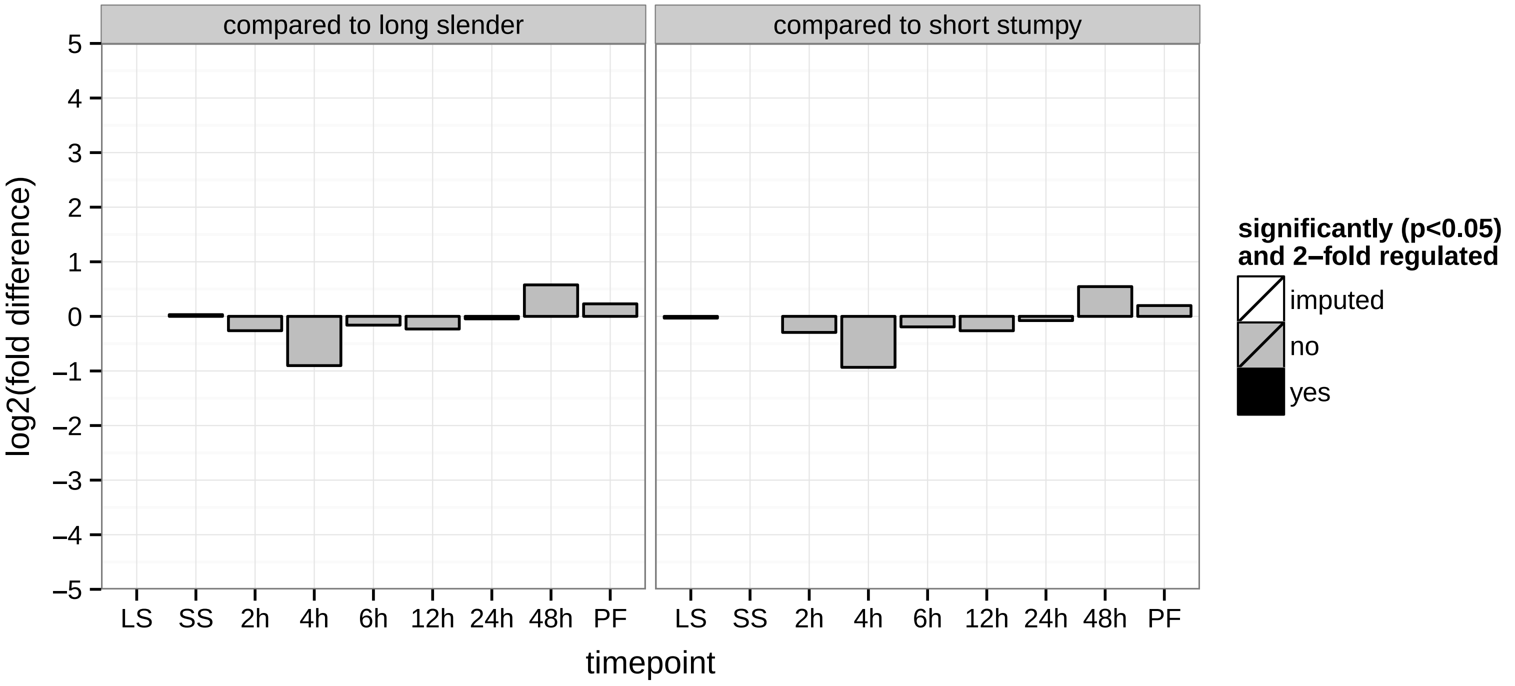

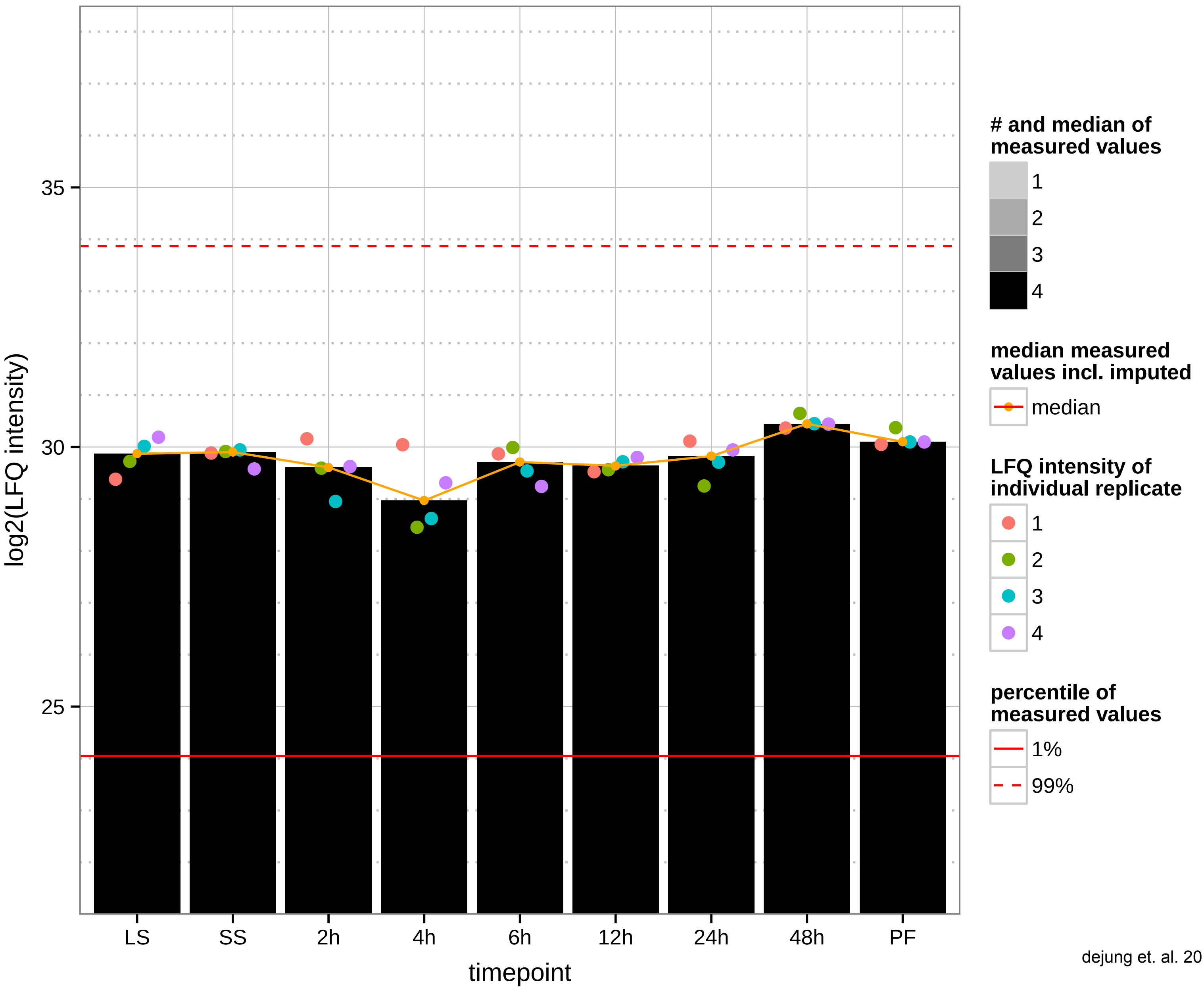

dejung et. al. 2015

pre–mRNA splicing factor ATP–dependent RNA helicase, putative, RNA helicase
Tb927.10.7280
AGOF: ATP binding, ATP–dependent RNA helicase activity, nucleic acid binding
AGOC: spliceosomal complex
AGOP: RNA splicing, RNA splicing, via transesterification reactions
PGOF: ATP binding, ATP–dependent helicase activity, helicase activity, nucleic acid binding
PGOC: null
PGOP: null

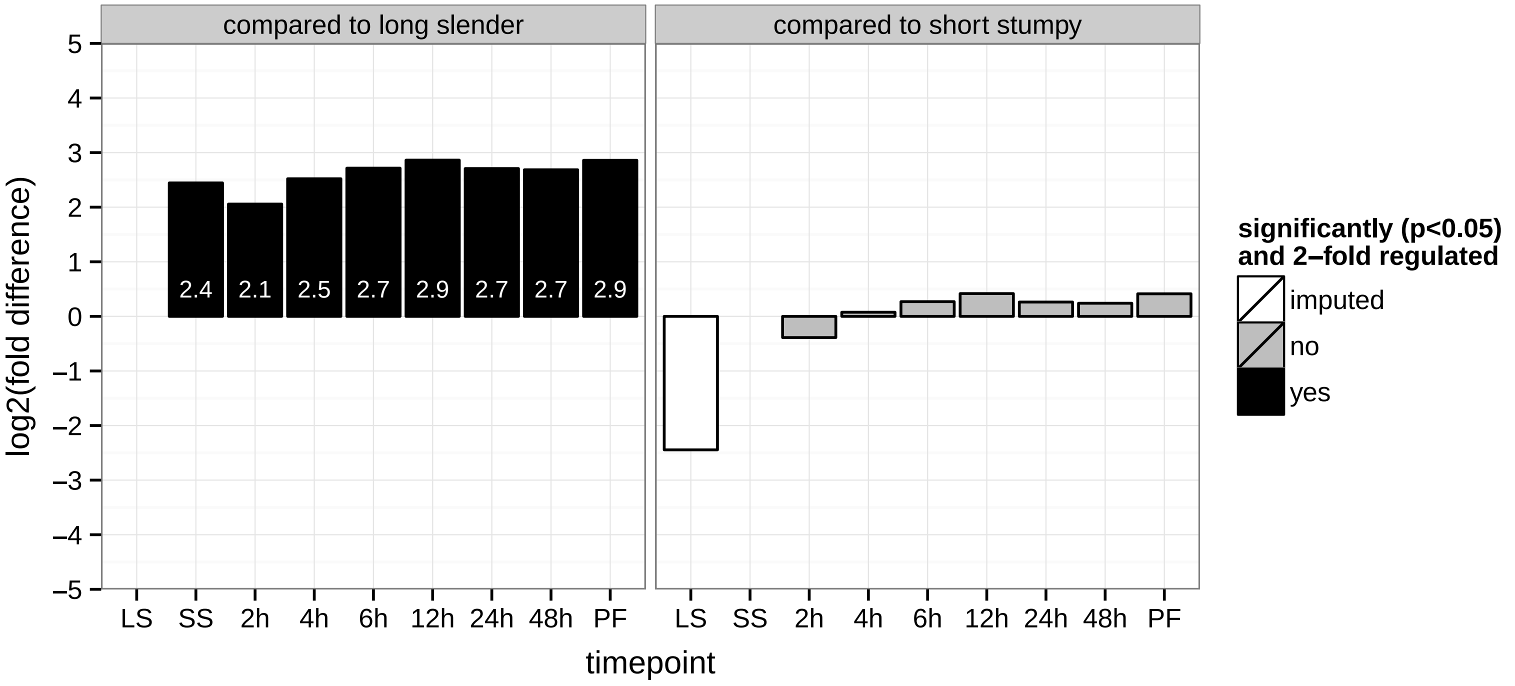

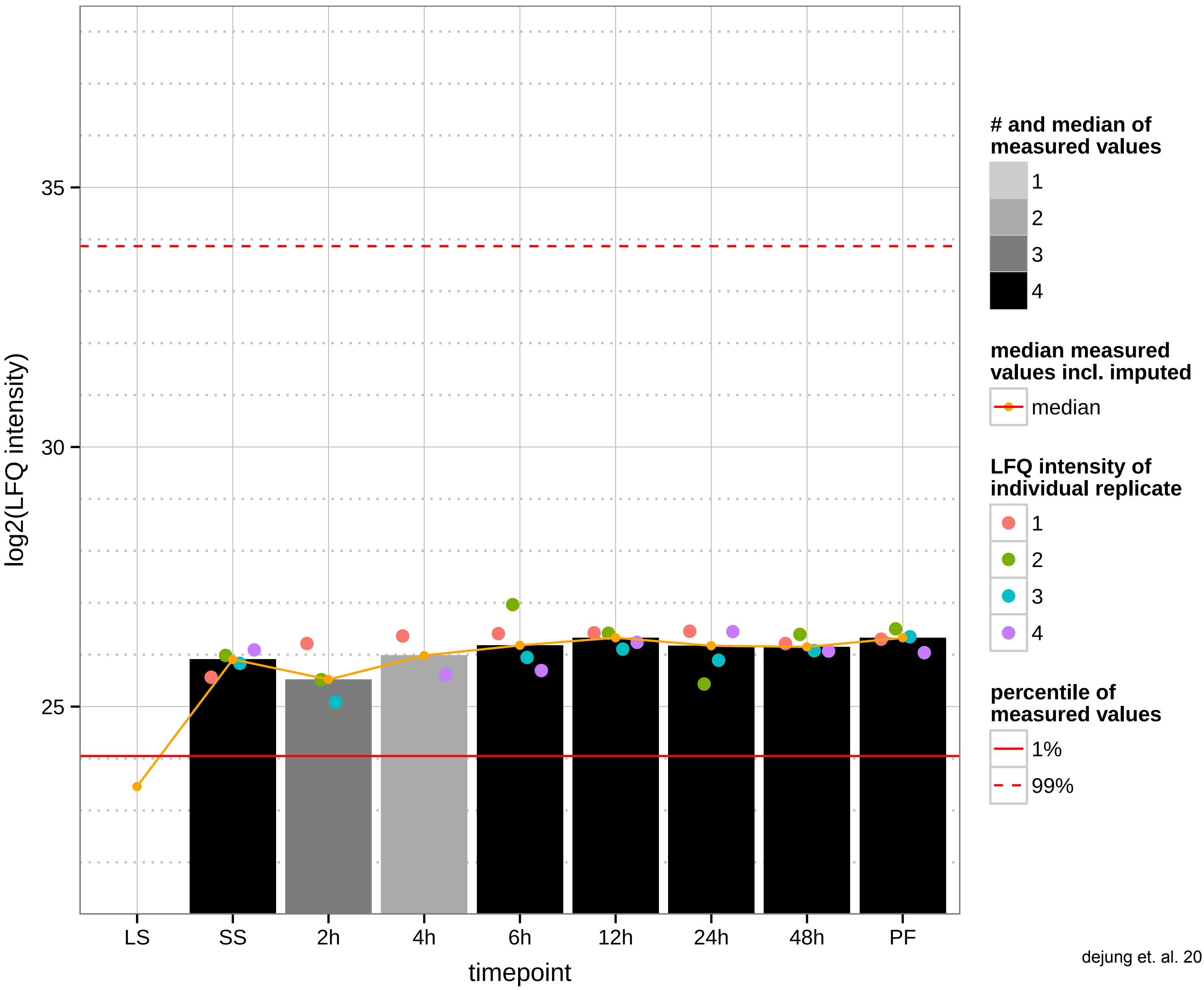

dejung et. al. 2015

hypothetical protein, conserved
Tb927.10.7350
AGOF: null
AGOC: null
AGOP: null
PGOF: null
PGOC: null
PGOP: null

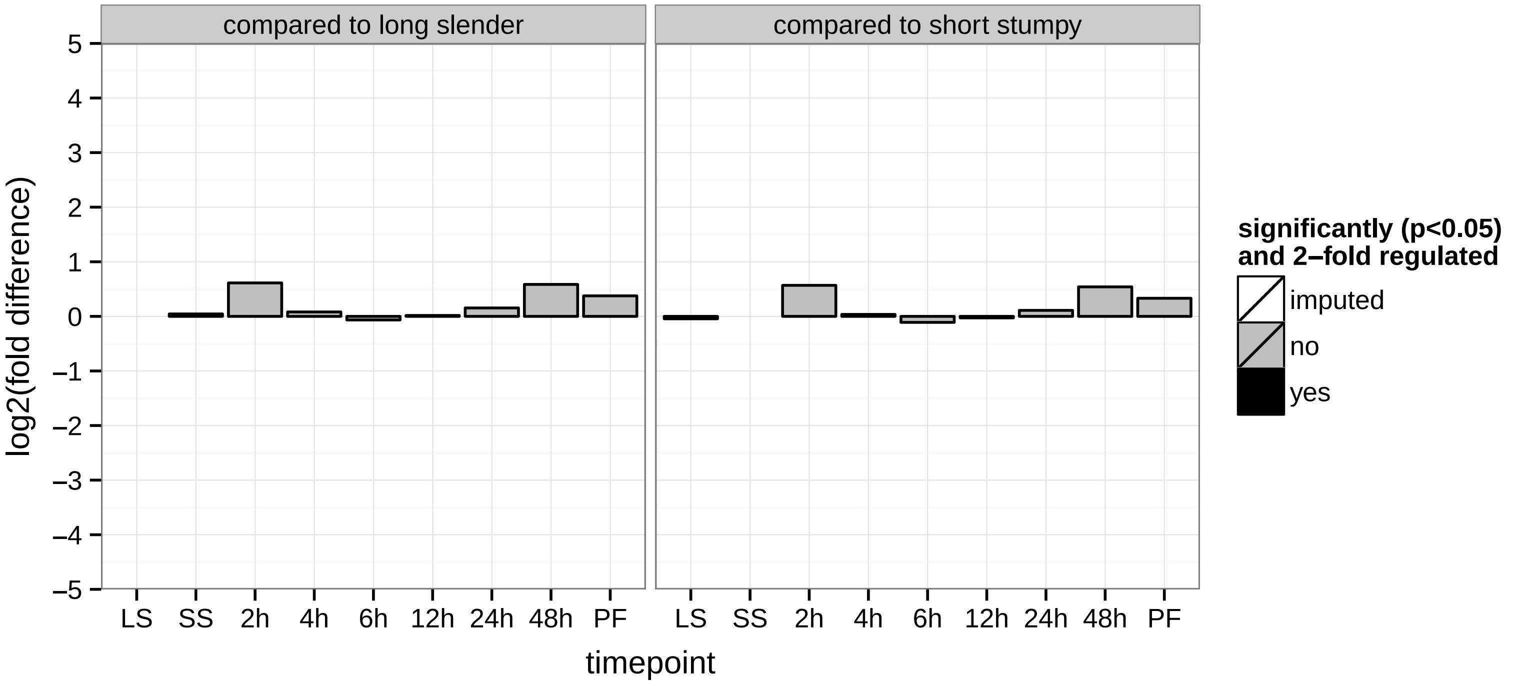

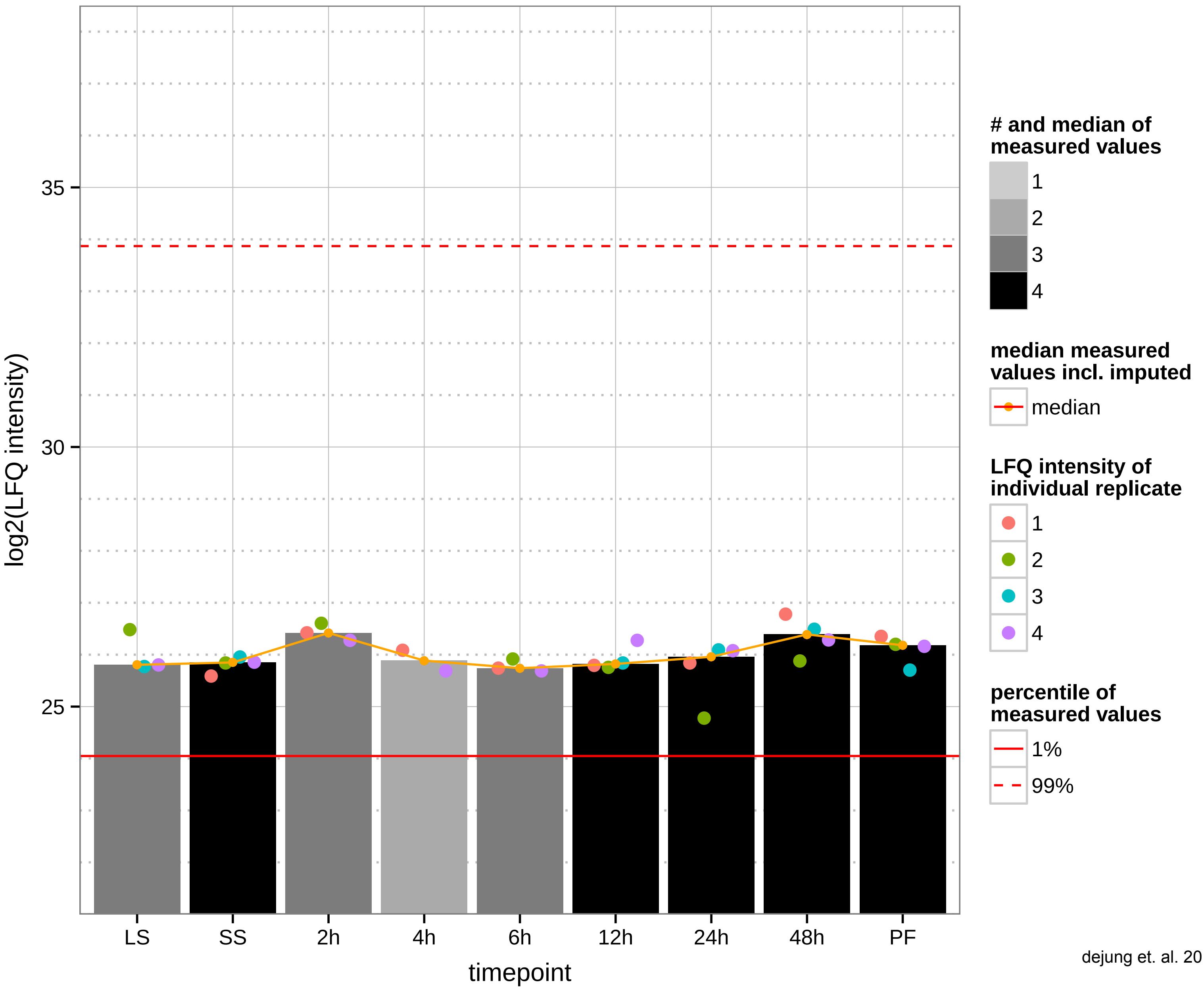

dejung et. al. 2015

succinyl–CoA ligase [GDP–forming] beta–chain, putative
Tb927.10.7410
AGOF: catalytic activity
AGOC: mitochondrion
AGOP: metabolic process
PGOF: catalytic activity
PGOC: null
PGOP: metabolic process

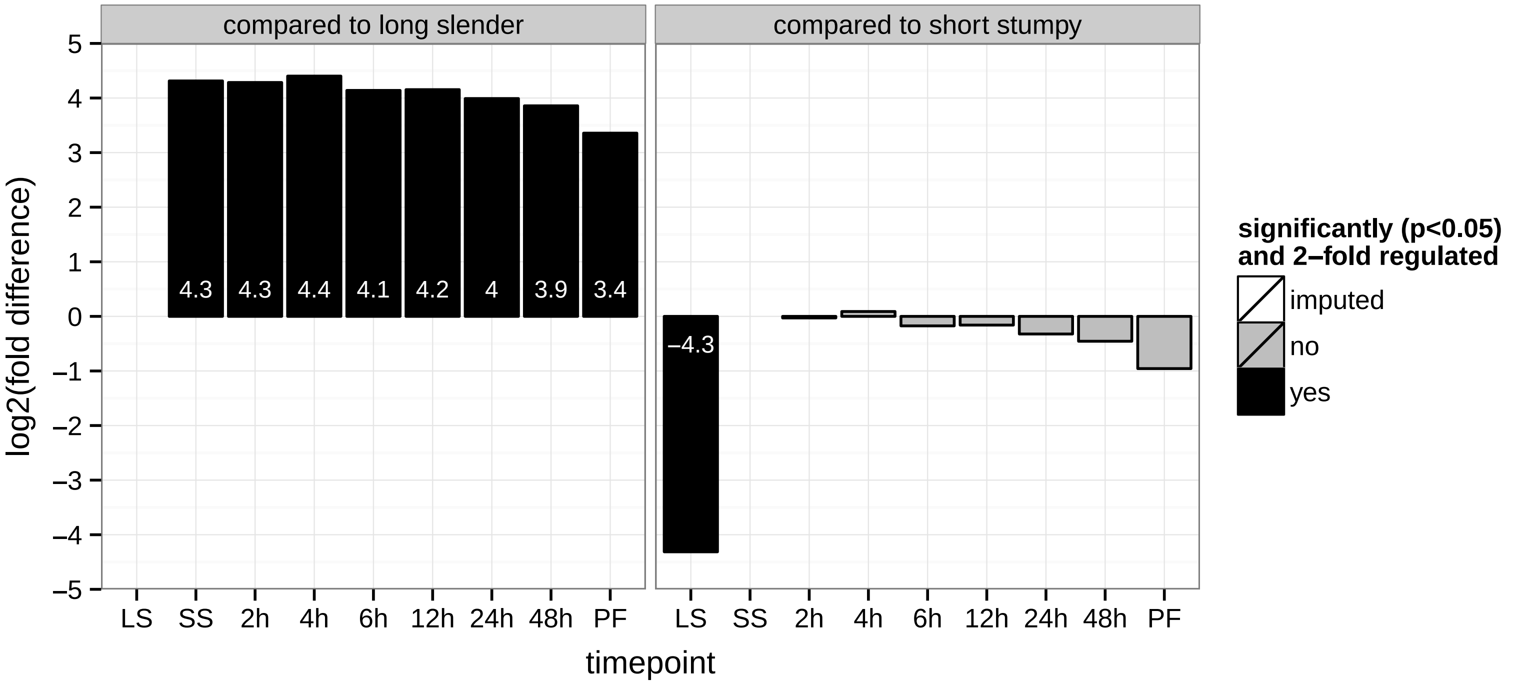

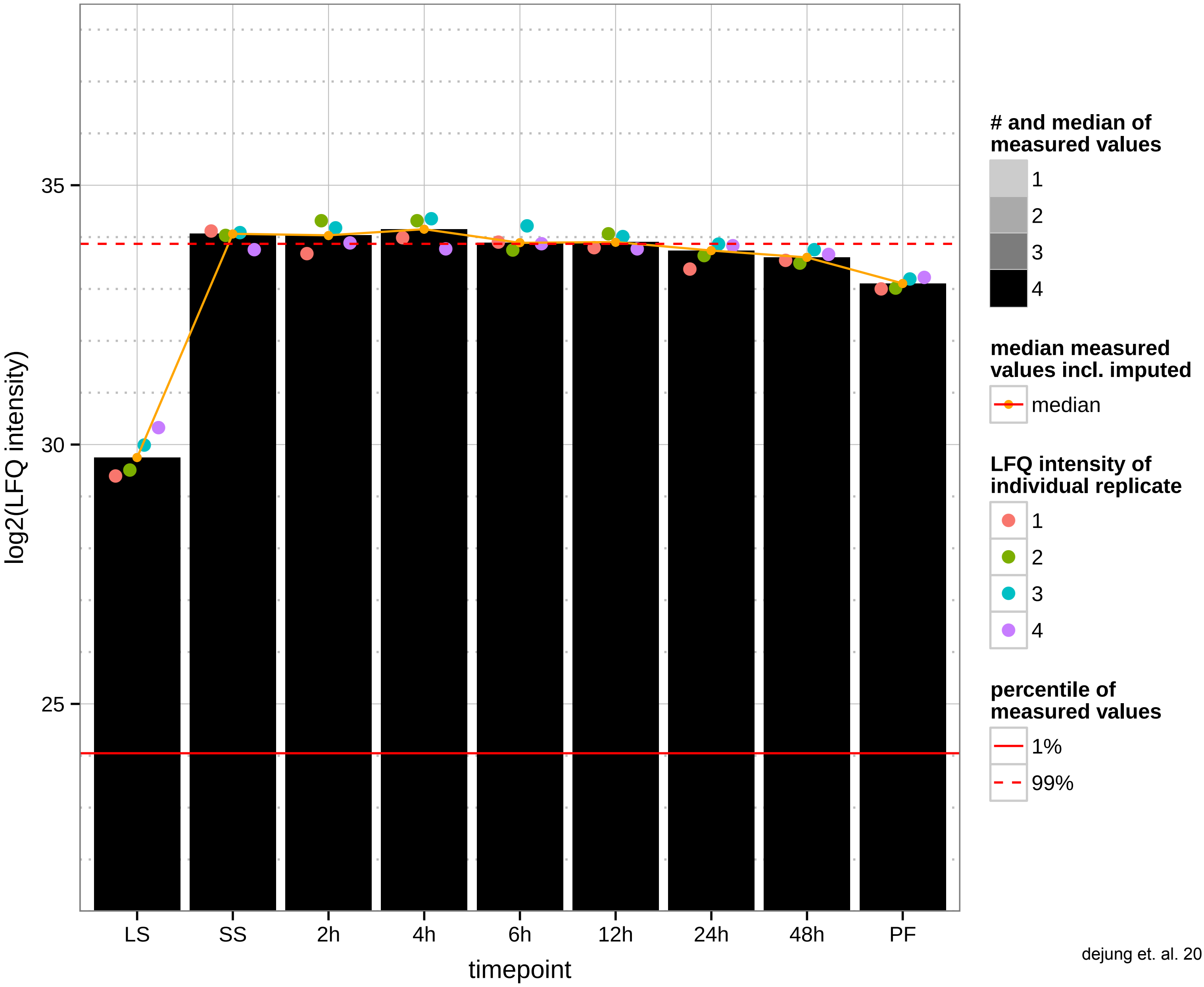

dejung et. al. 2015

bromodomain factor 2 protein, putative
Tb927.10.7420
AGOF: histone binding
AGOC: nucleus
AGOP: chromatin remodeling, histone modification
PGOF: protein binding
PGOC: null
PGOP: null

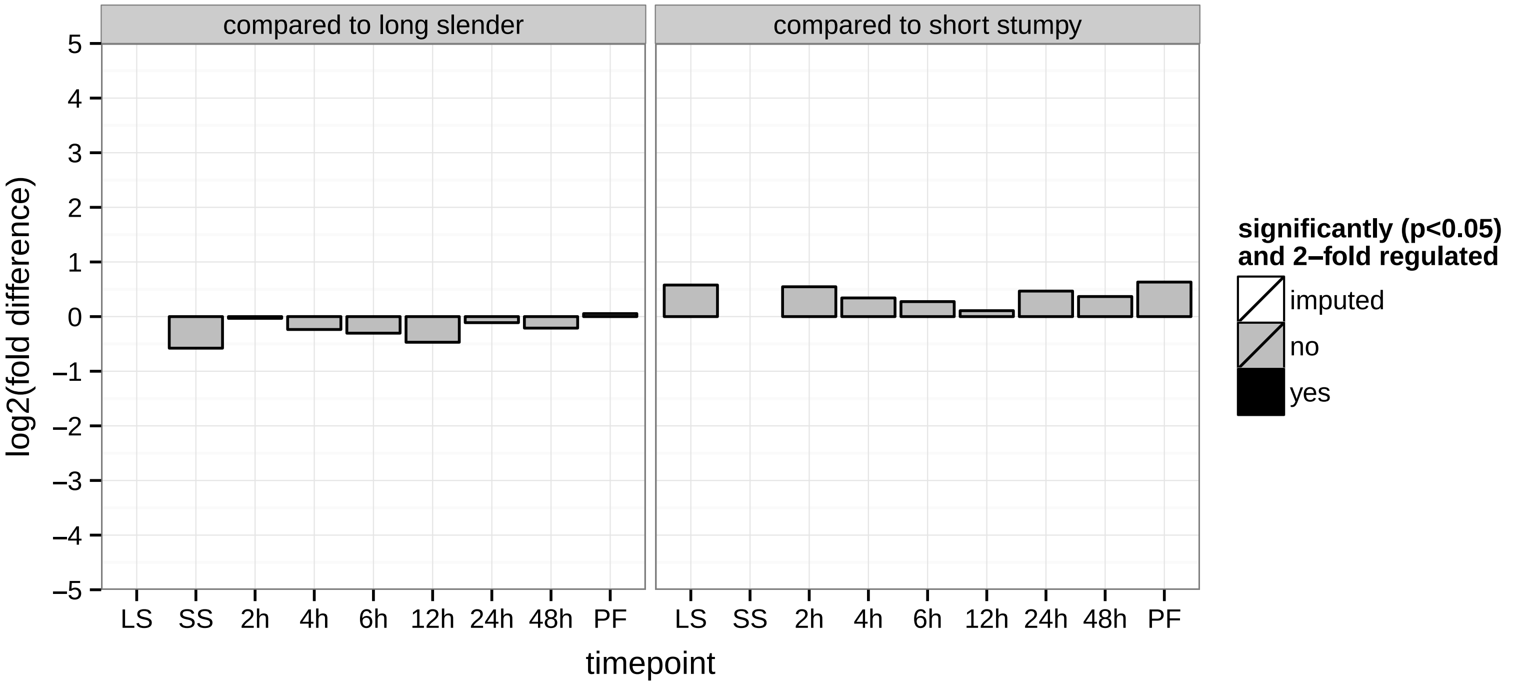

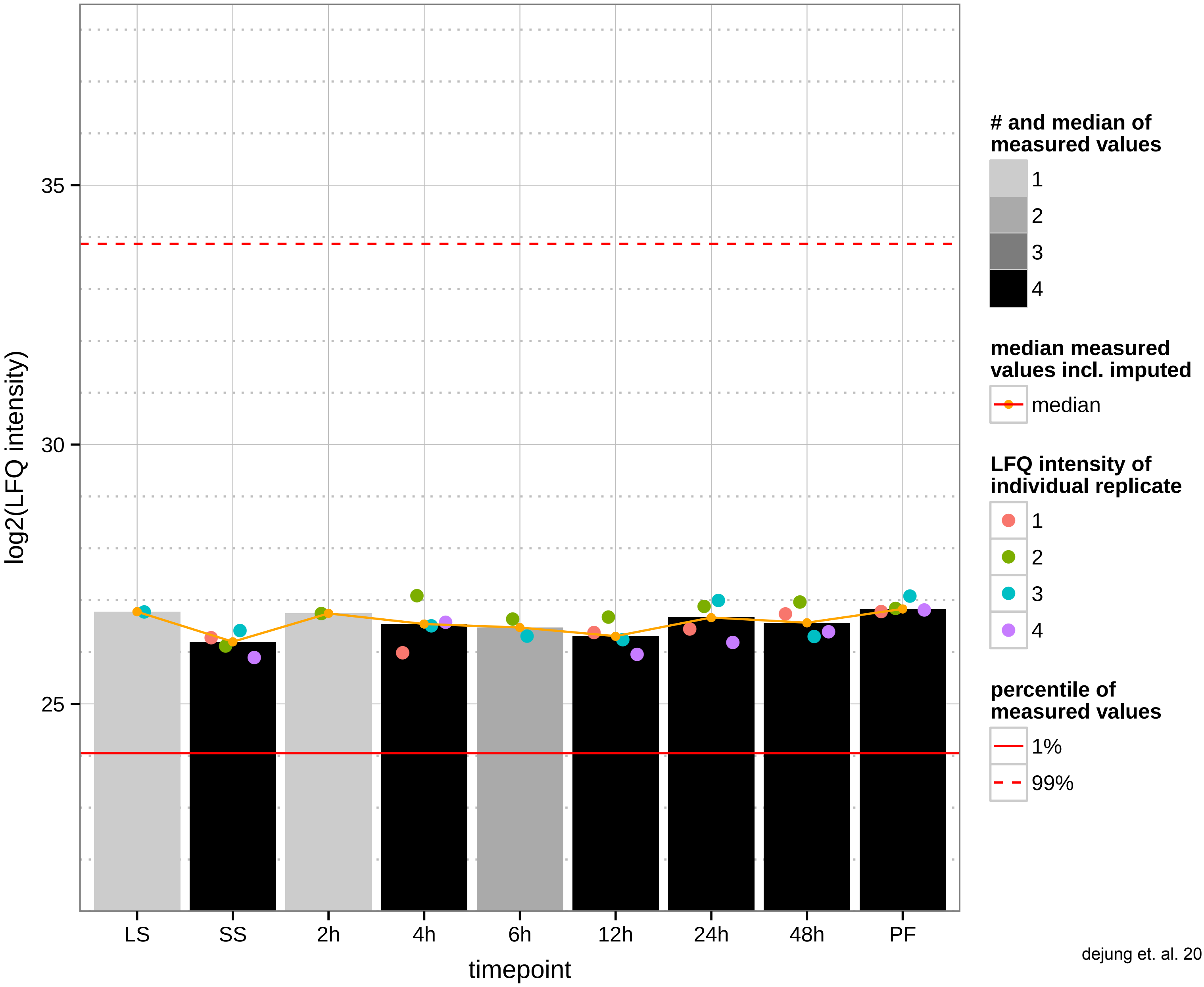

dejung et. al. 2015

MTR4, ATP–dependent DEAD/H RNA helicase (MTR4)
Tb927.10.7440
AGOF: ATP binding, ATP–dependent helicase activity, helicase activity, nucleic acid binding
AGOC: nucleus
AGOP: rRNA processing
PGOF: ATP binding, ATP–dependent helicase activity, helicase activity, hydrolase activity, acting on acid anhydrides, in phosp
PGOC: null
PGOP: null

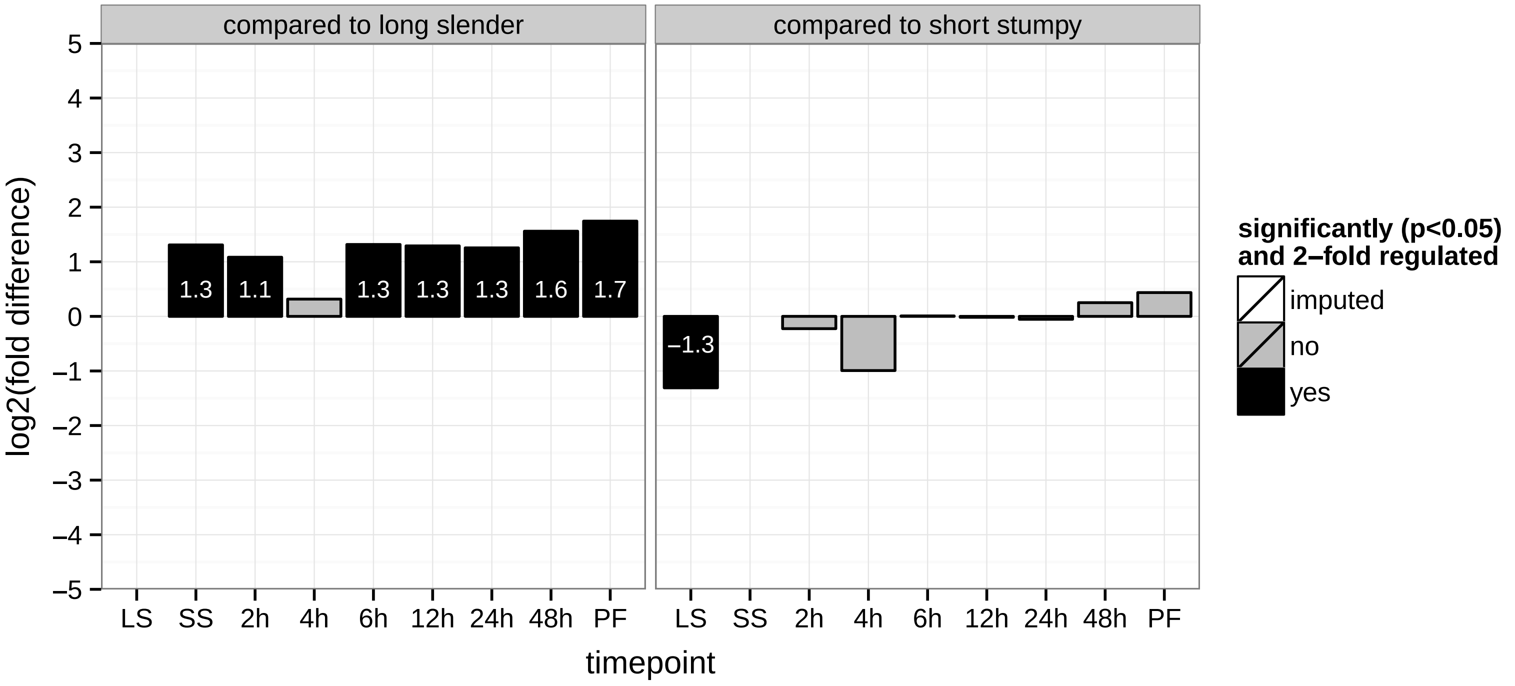

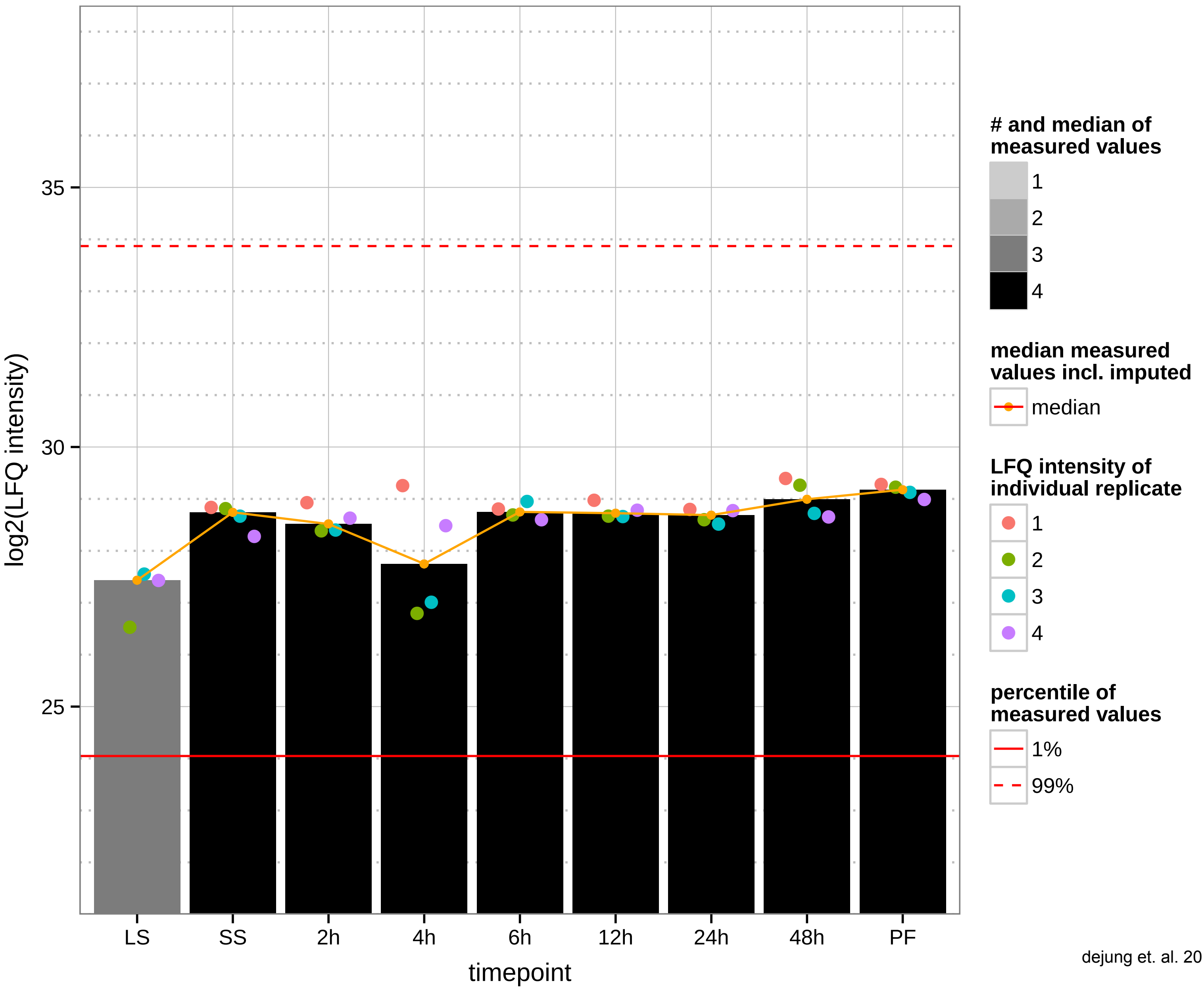

dejung et. al. 2015

exosome complex exonuclease RRP41A, Rrp41p homologue (RRP41A)
Tb927.10.7450
AGOF: 3'−5'−exoribonuclease activity, RNA binding
AGOC: null
AGOP: RNA processing, rRNA processing
PGOF: null
PGOC: null
PGOP: null

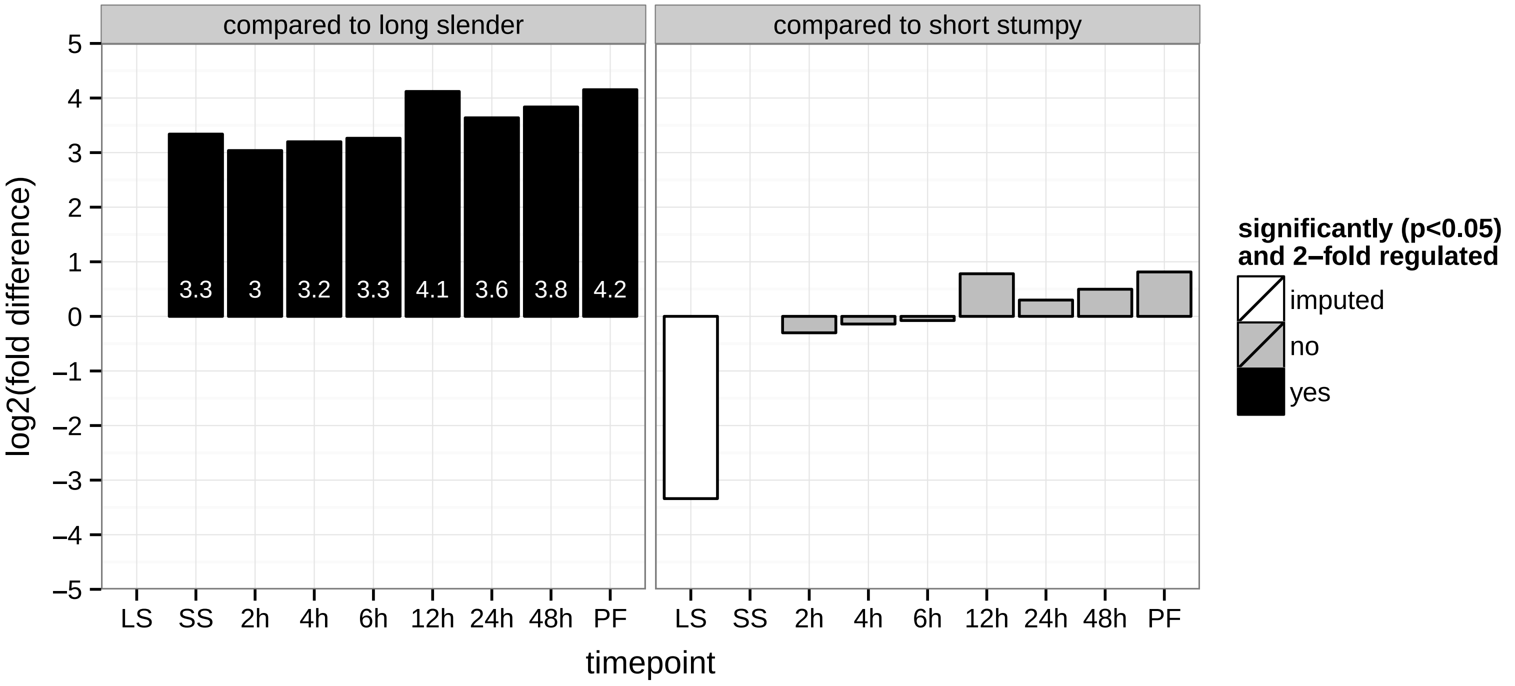

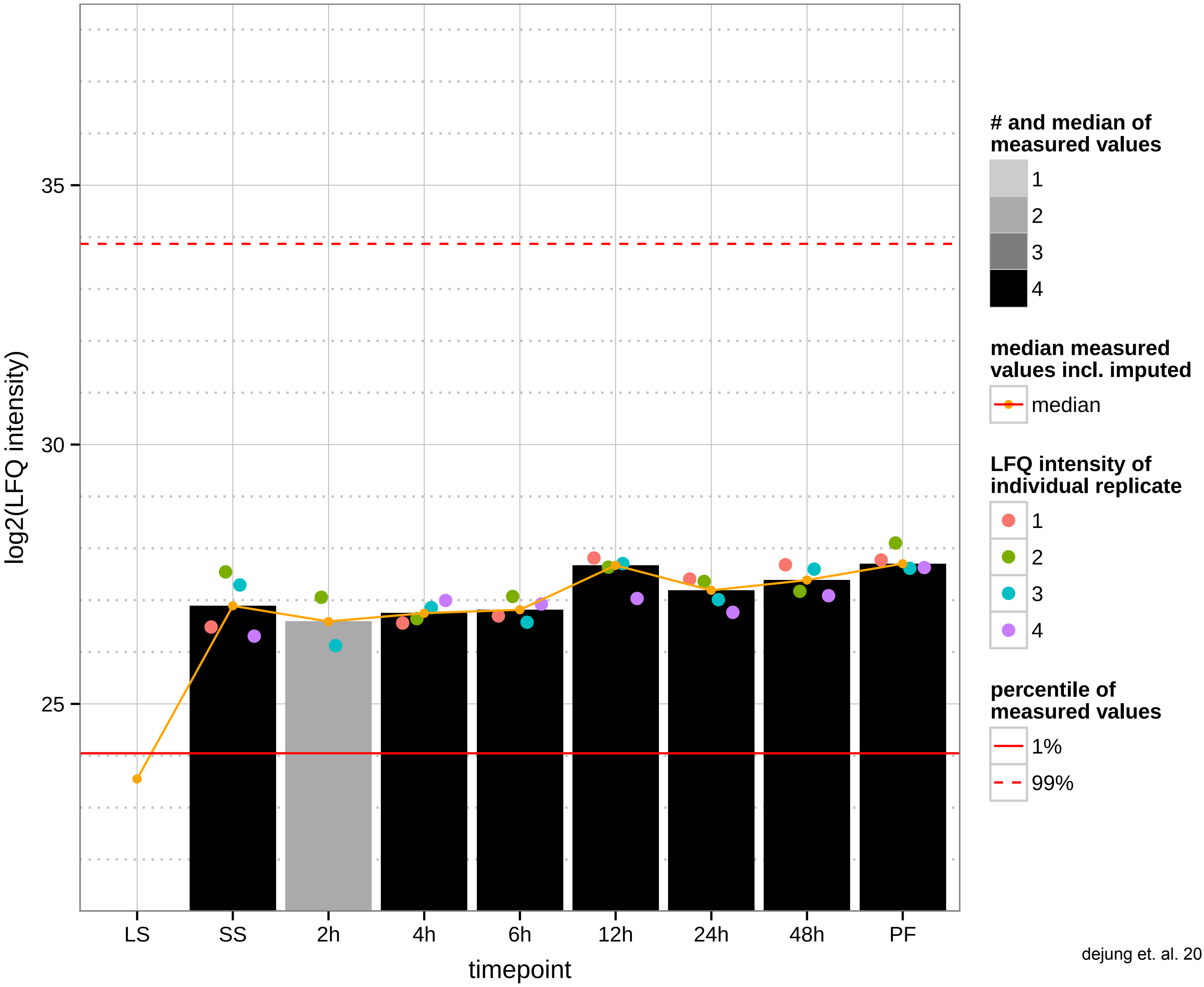

dejung et. al. 2015

fibrillarin (NOP1)
Tb927.10.7500
AGOF: RNA binding, rRNA 2'–O–ribose methylation guide activity
AGOC: box C/D snoRNP complex, nucleus
AGOP: rRNA processing
PGOF: RNA binding, methyltransferase activity
PGOC: null
PGOP: rRNA processing, tRNA processing

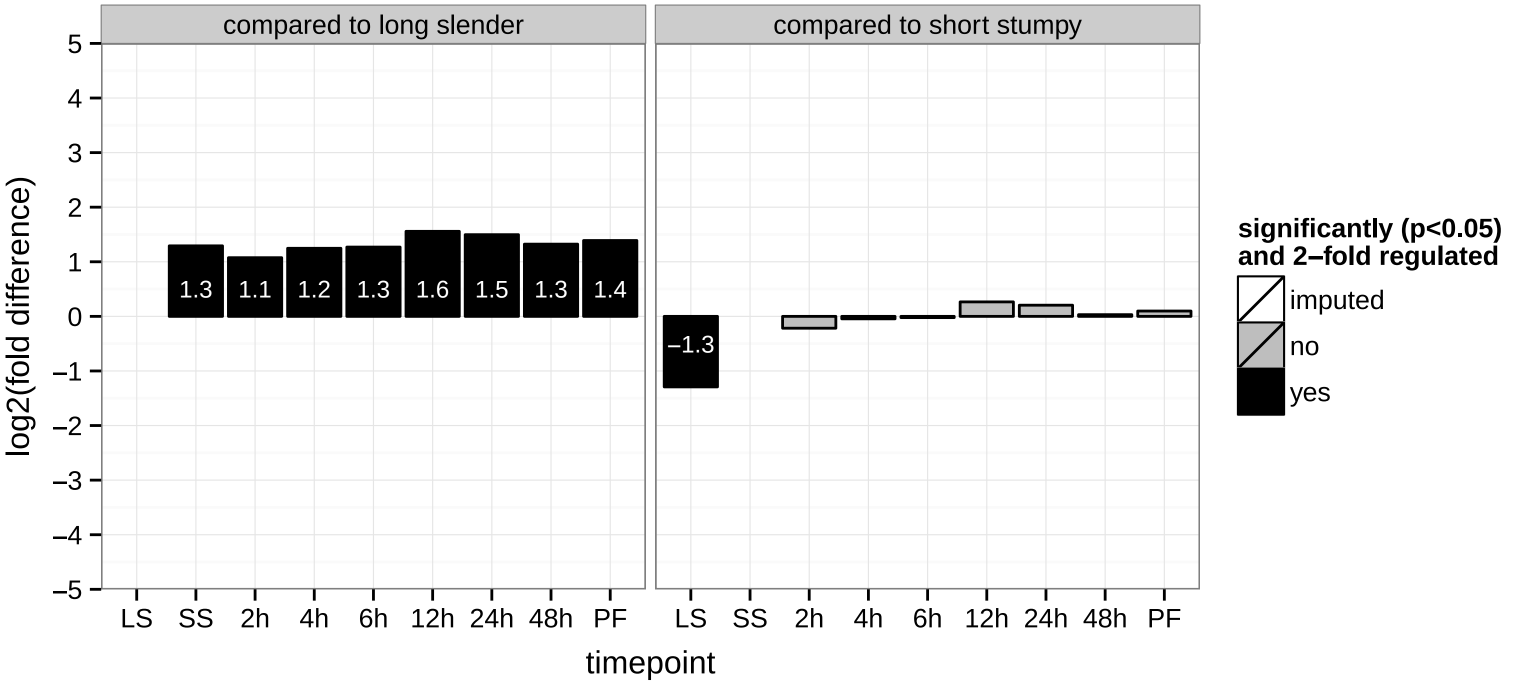

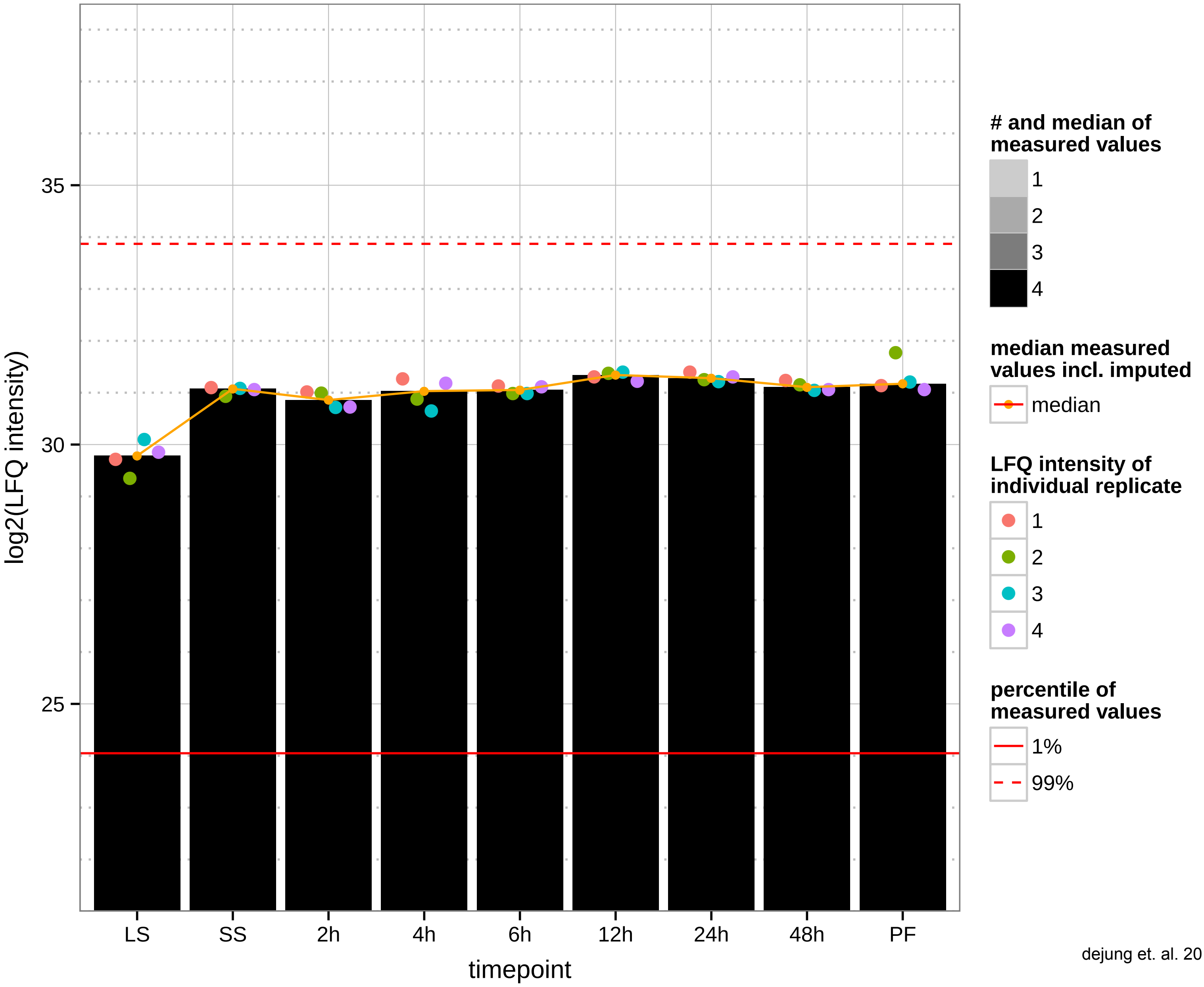

dejung et. al. 2015

chromatin binding protein, putative
Tb927.10.7510
AGOF: Ran GTPase binding, chromatin binding
AGOC: null
AGOP: chromosome organization
PGOF: null
PGOC: null
PGOP: null

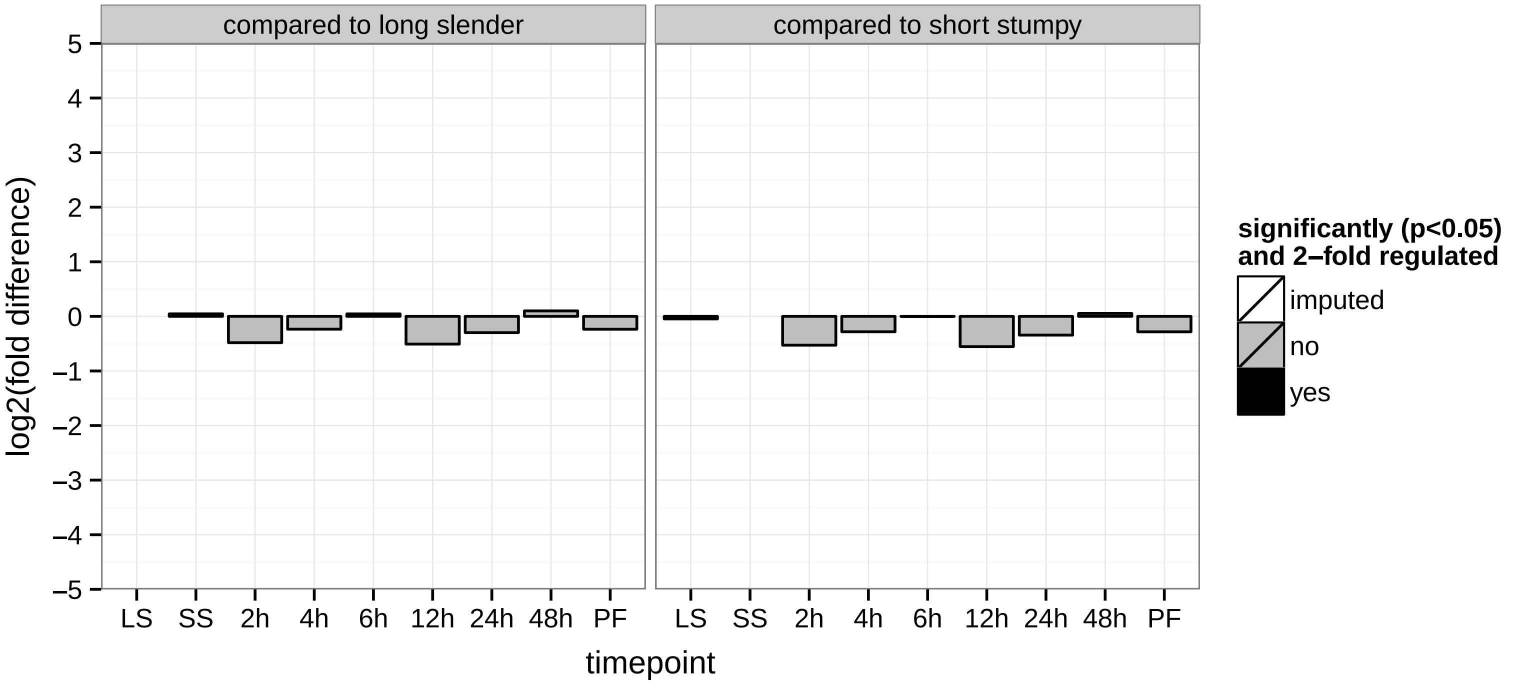

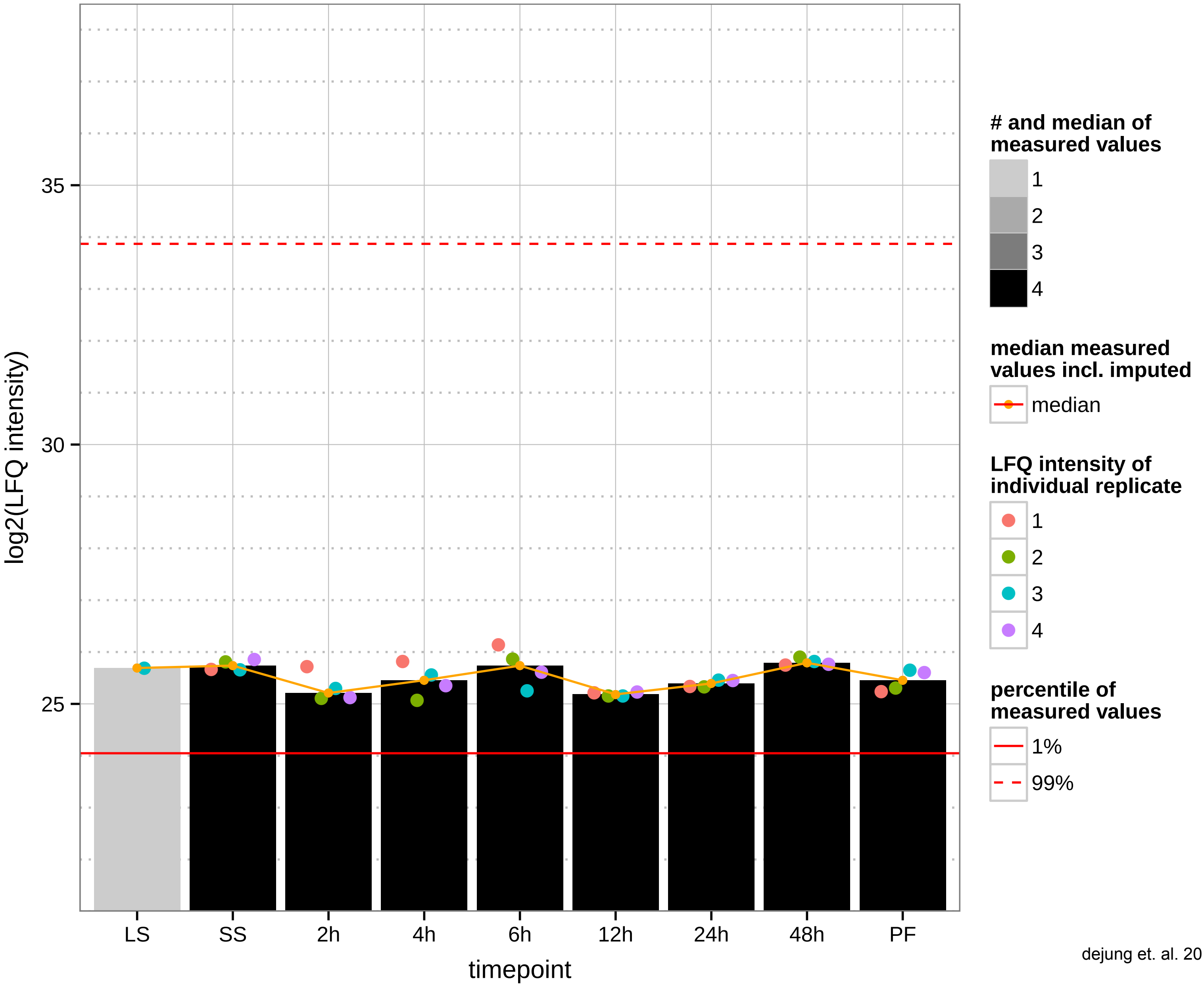

dejung et. al. 2015

hypothetical protein, conserved
Tb927.10.7550
AGOF: null
AGOC: null
AGOP: null
PGOF: null
PGOC: null
PGOP: null

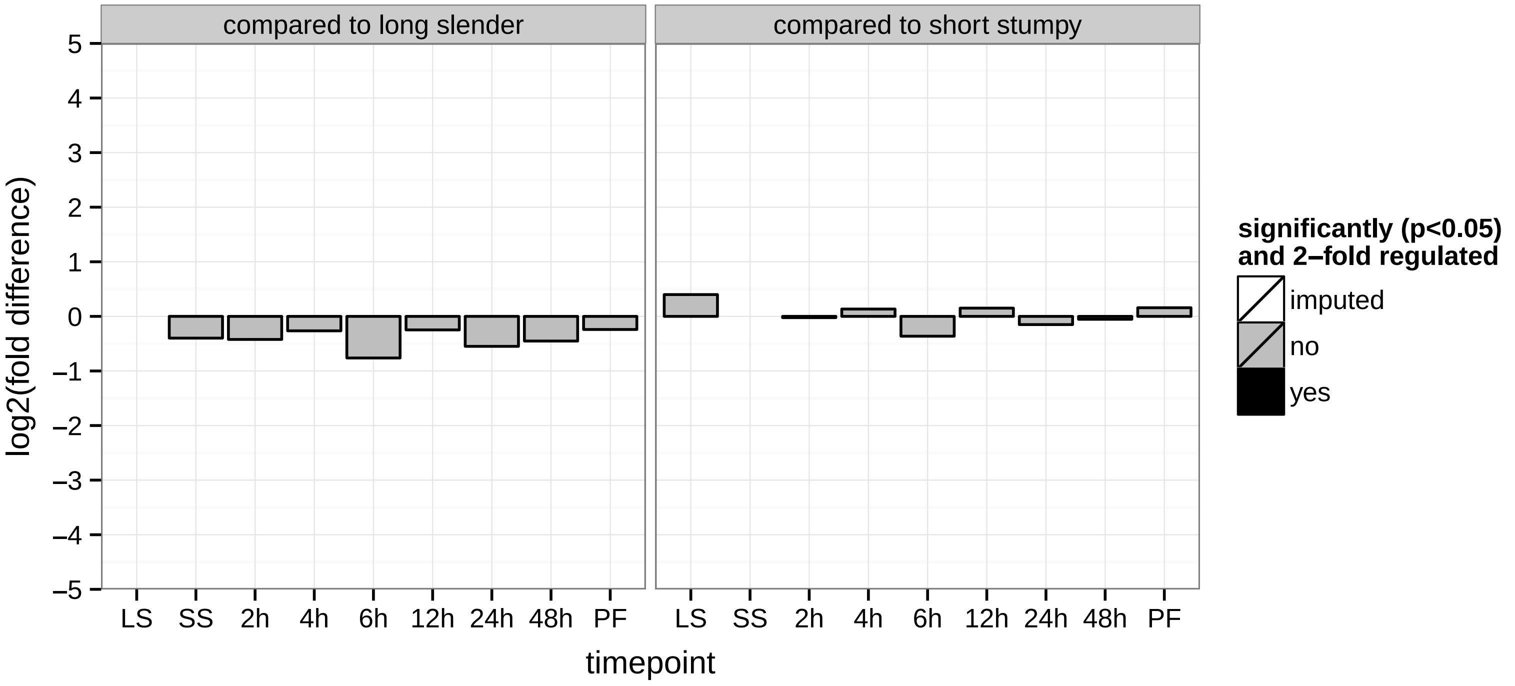

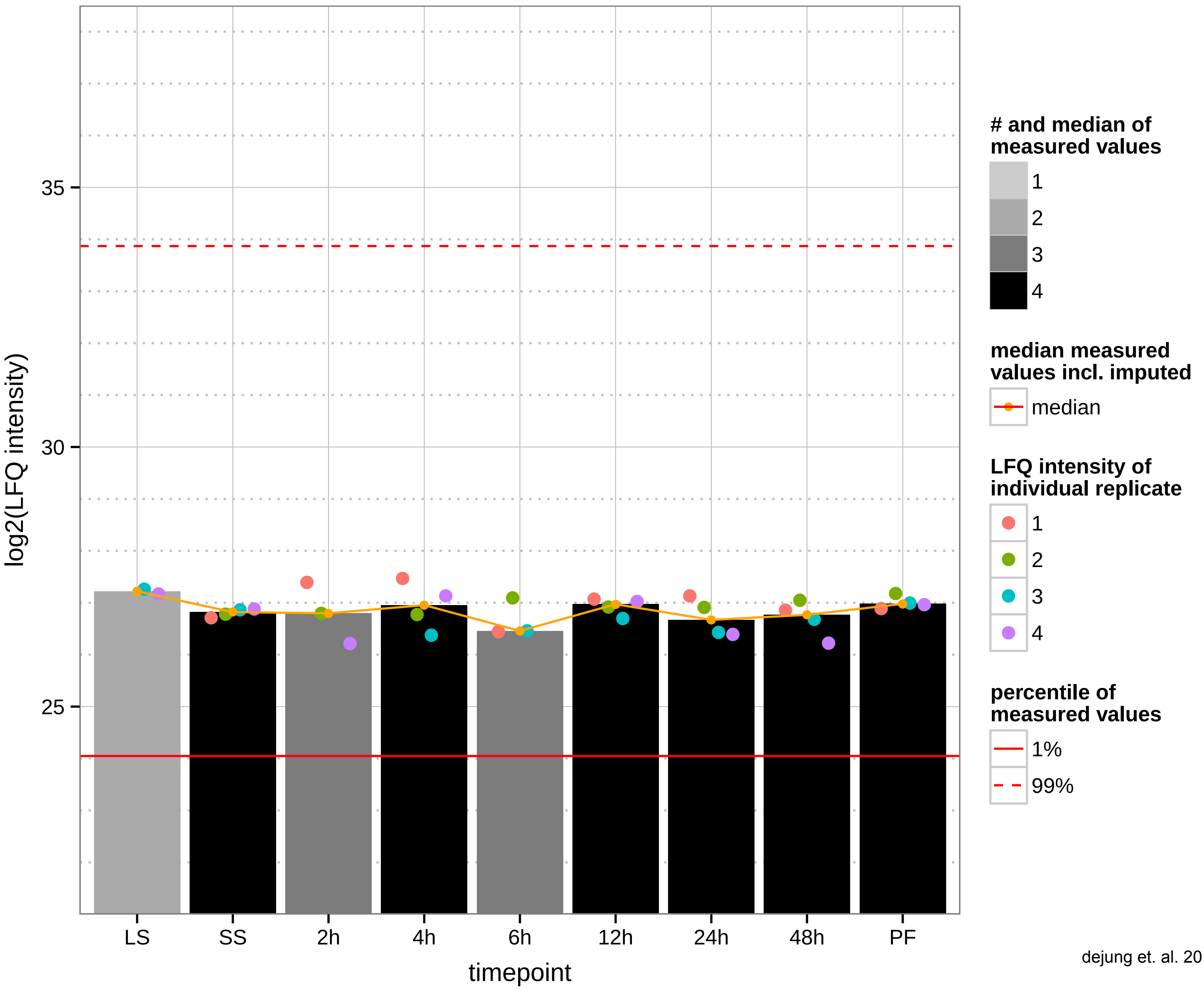

dejung et. al. 2015

dihydrolipoamide acetyltransferase precursor, putative, dihydrolipoamide acetyltransferase E2 subunit
Tb927.10.7570
AGOF: null, dihydrolipoyllysine−residue acetyltransferase activity, pyruvate dehydrogenase activity
AGOC: null, mitochondrial pyruvate dehydrogenase complex, mitochondrion
AGOP: null, acetyl−CoA biosynthetic process from pyruvate
PGOF: transferase activity, transferring acyl groups
PGOC: null
PGOP: metabolic process

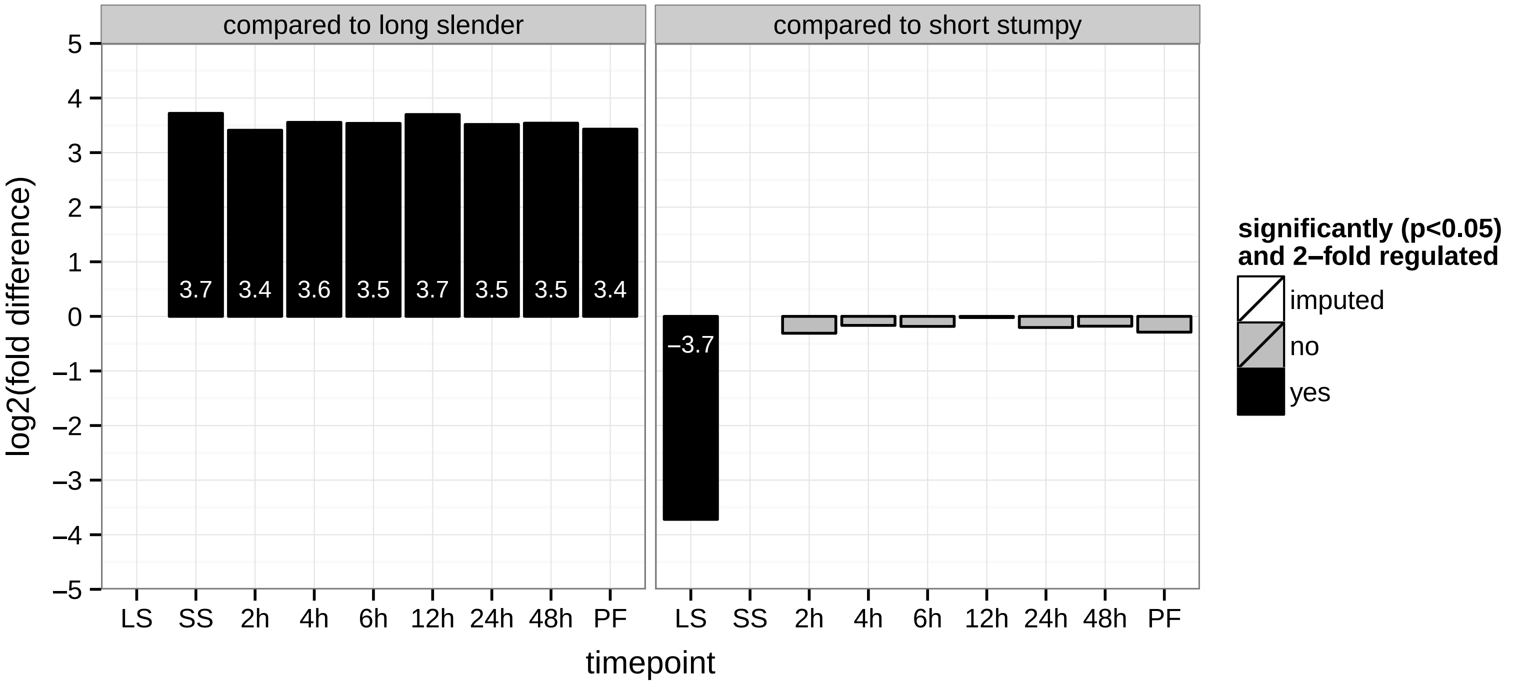

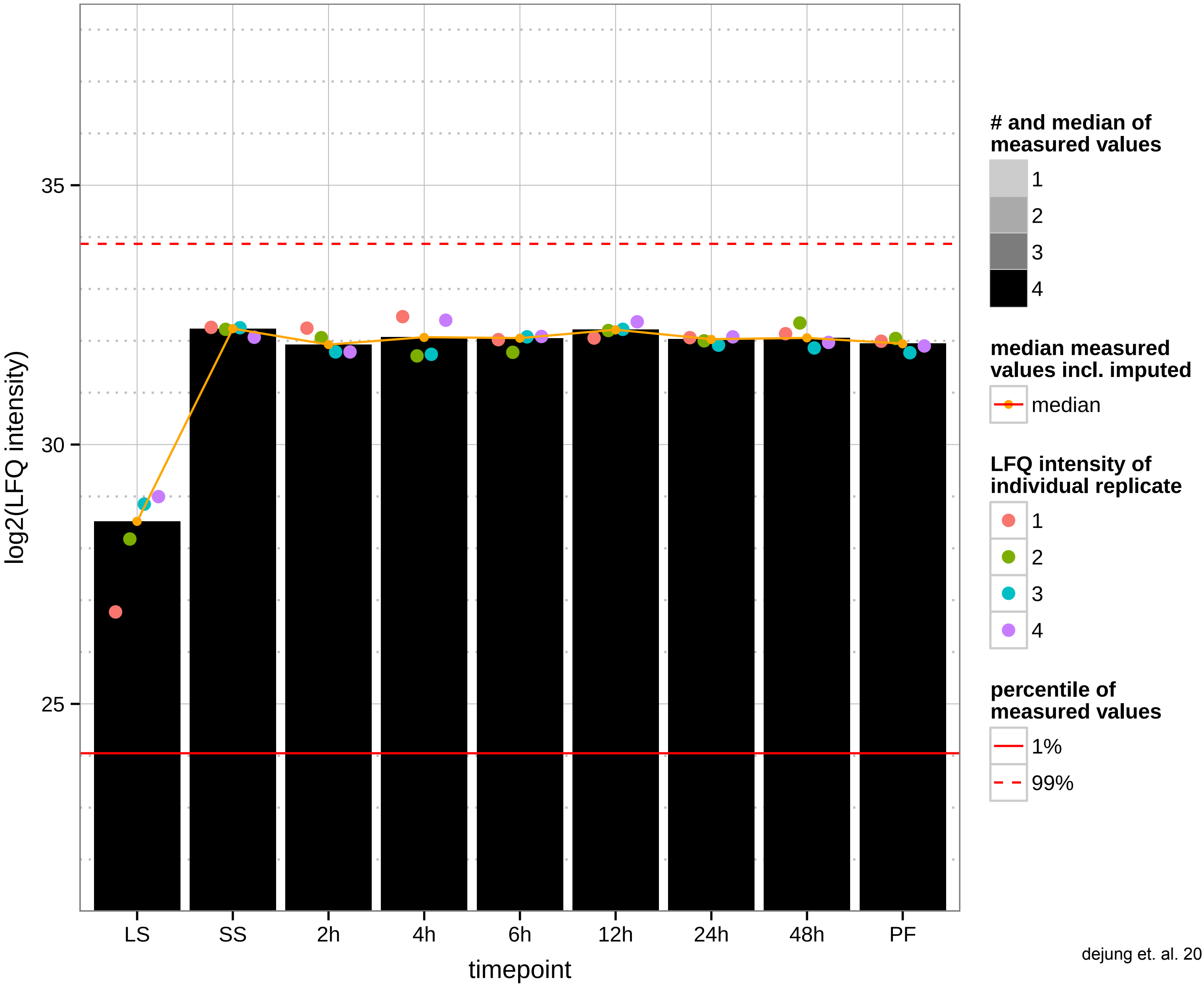

dejung et. al. 2015

hypothetical protein, conserved
Tb927.10.7580
AGOF: null
AGOC: null
AGOP: null
PGOF: binding
PGOC: null
PGOP: null

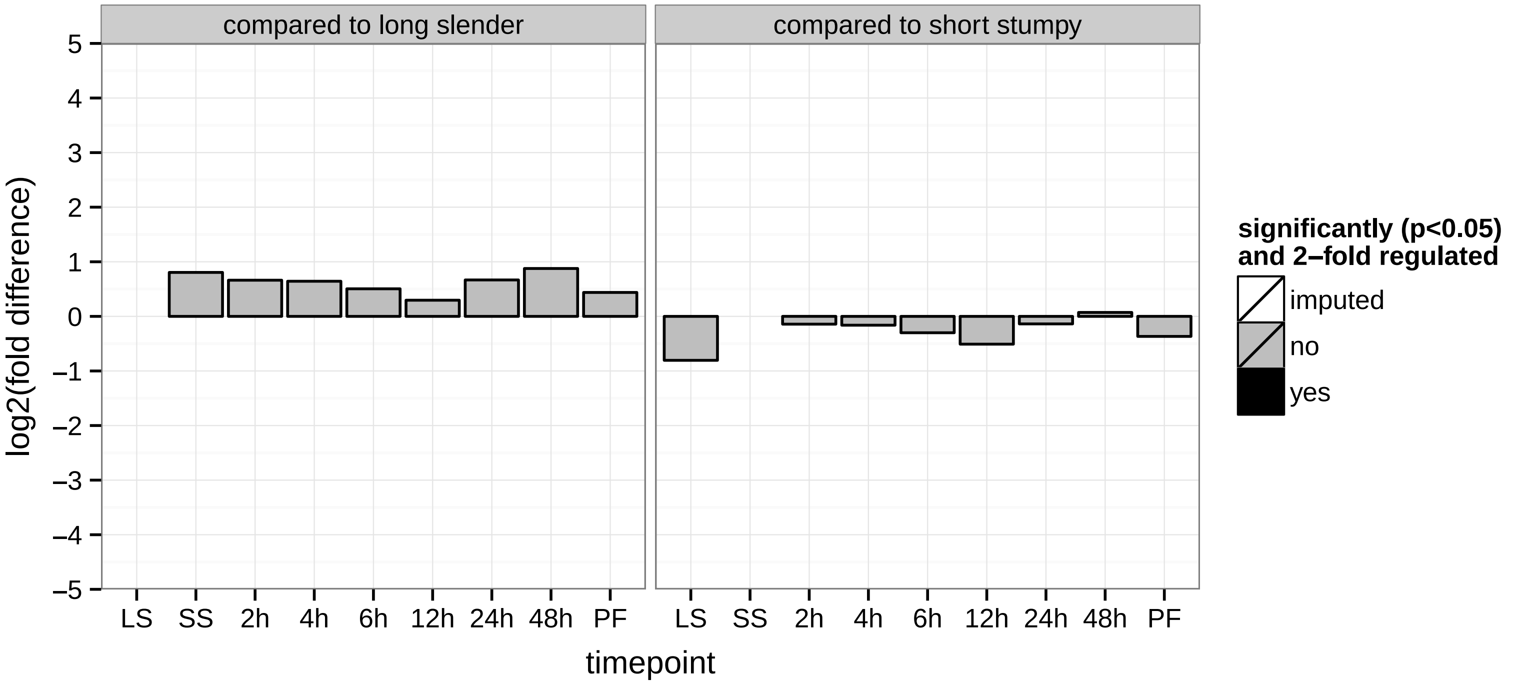

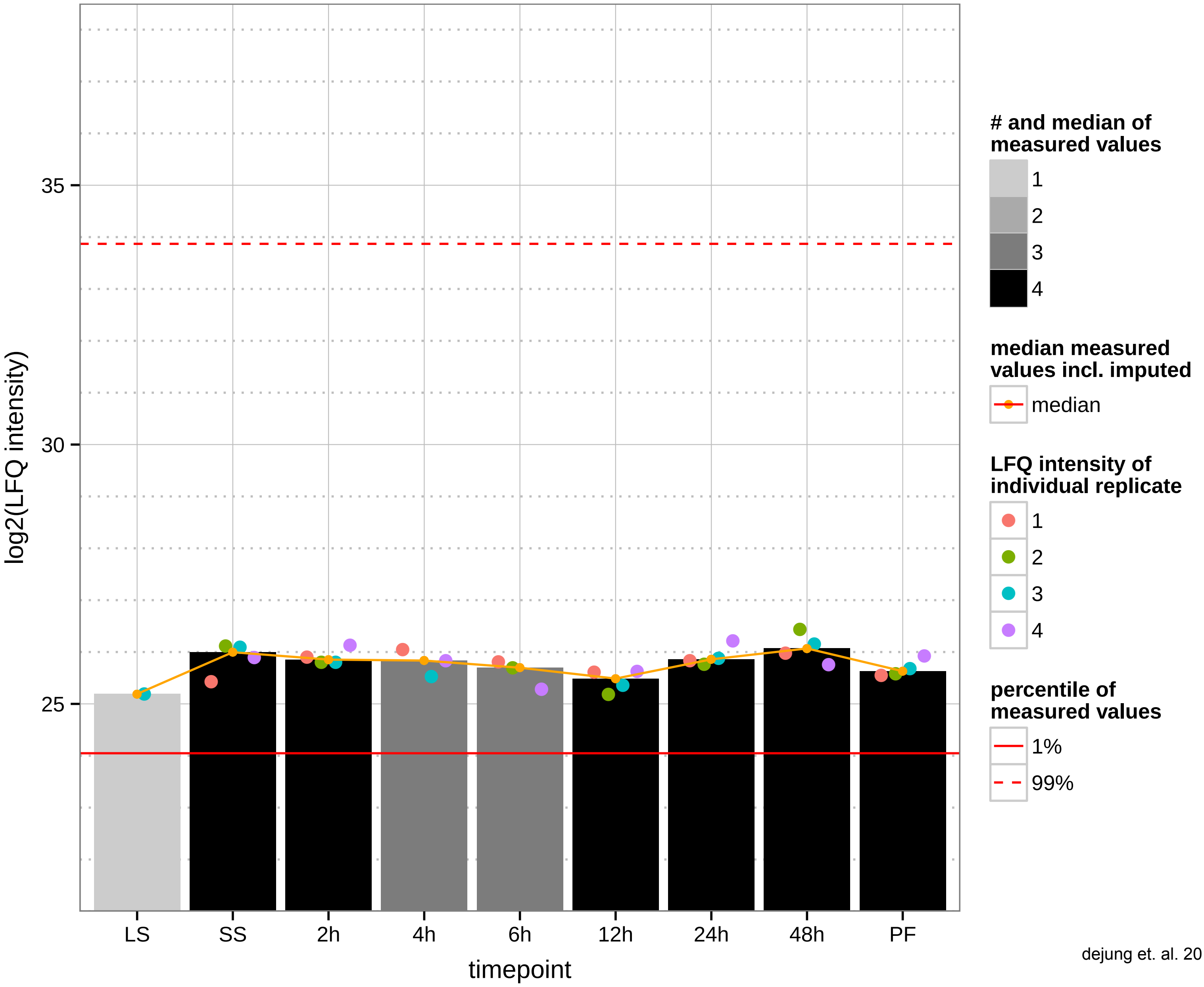

dejung et. al. 2015

hypothetical protein, conserved
Tb927.10.7610
AGOF: null
AGOC: mitochondrion
AGOP: null
PGOF: null
PGOC: null
PGOP: null

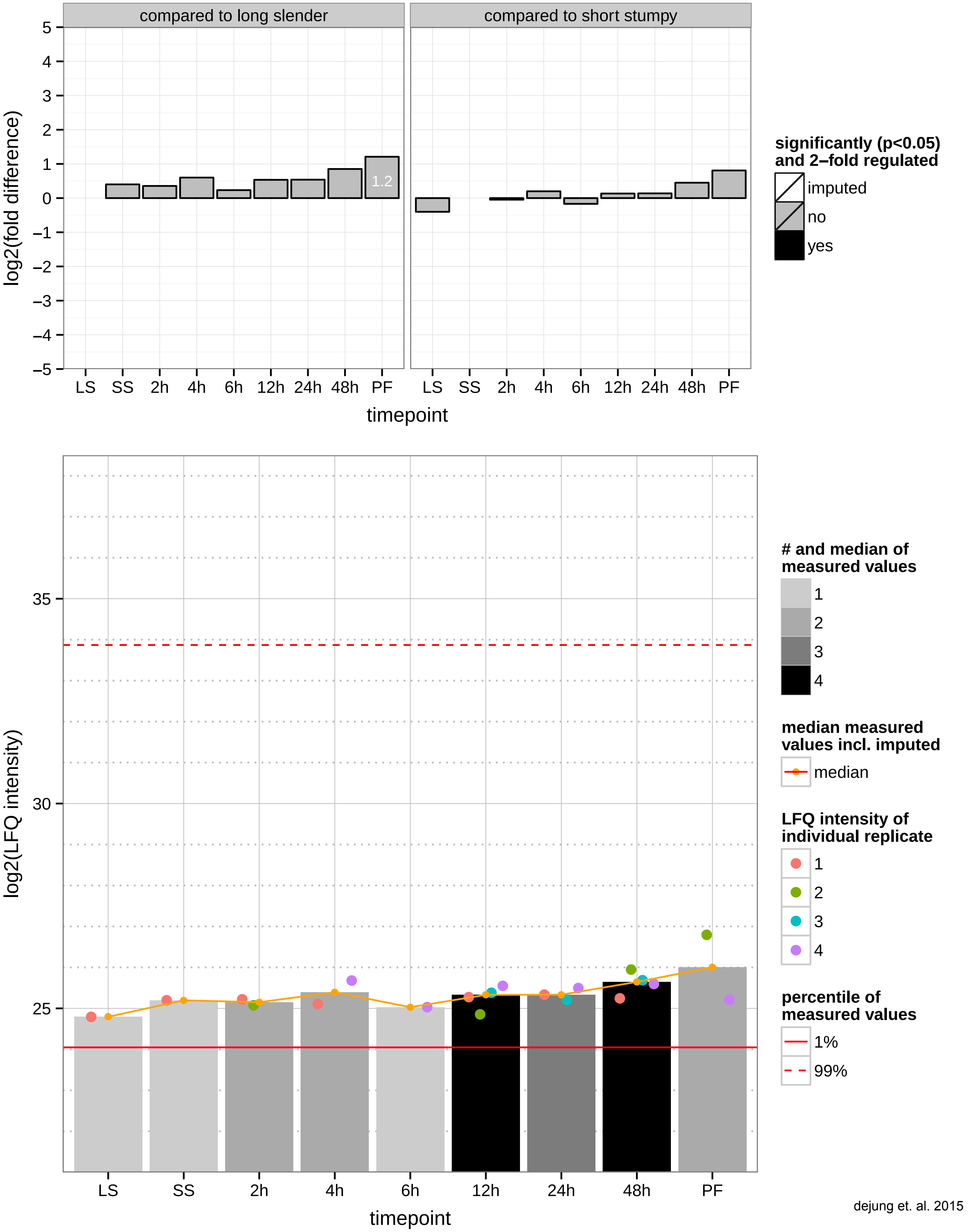

dejung et. al. 2015

transportin2– like protein
Tb927.10.7630
AGOF: null
AGOC: null
AGOP: null
PGOF: binding
PGOC: null
PGOP: null

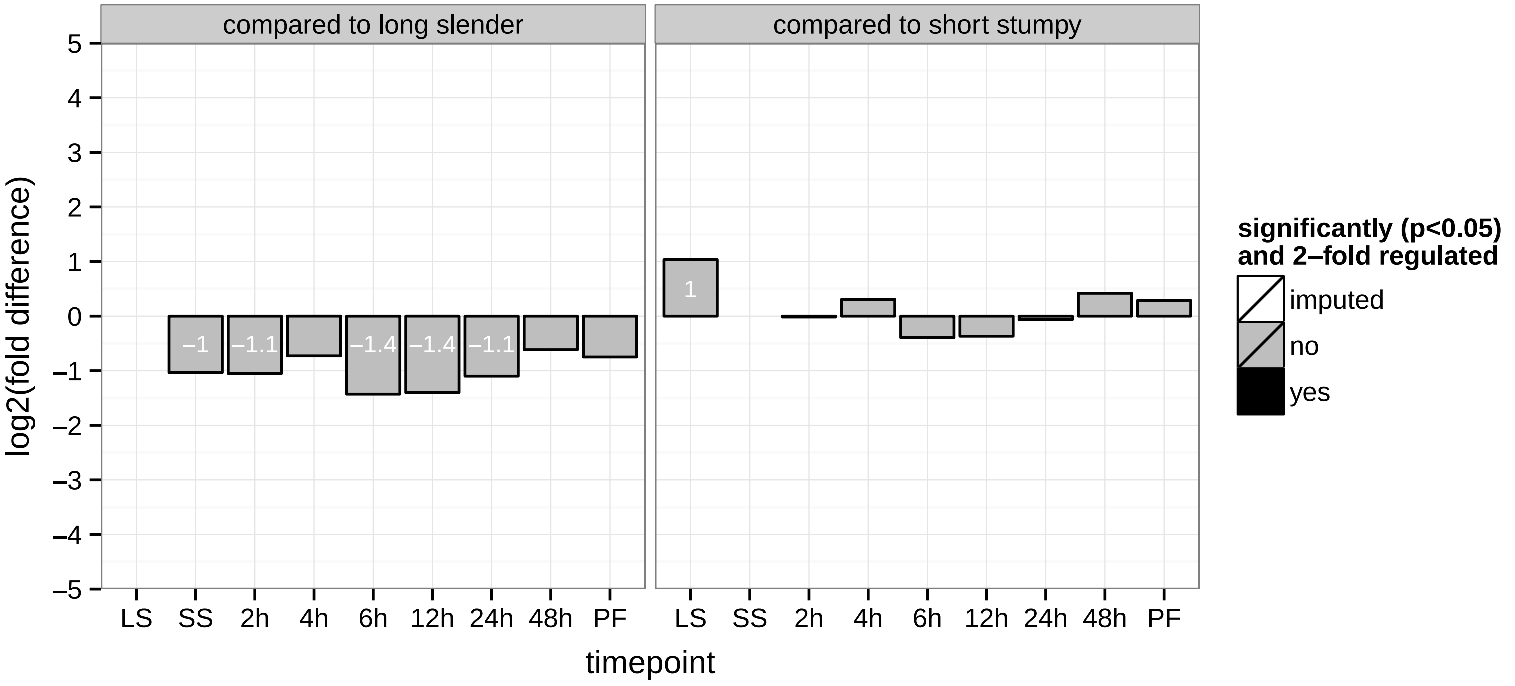

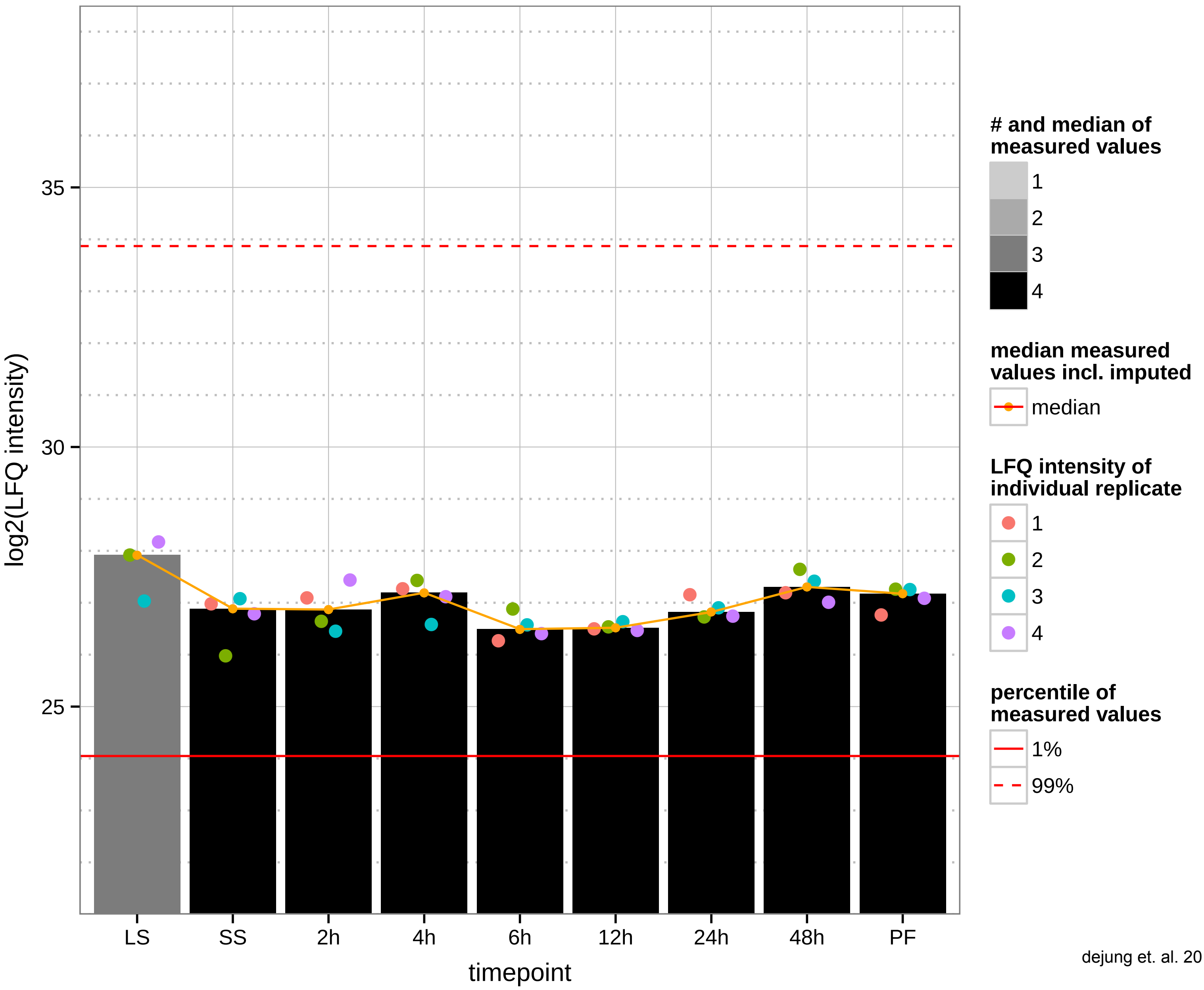

dejung et. al. 2015

rib72 protein– like protein
Tb927.10.7690
AGOF: null
AGOC: null
AGOP: ciliary or flagellar motility
PGOF: null
PGOC: null
PGOP: null

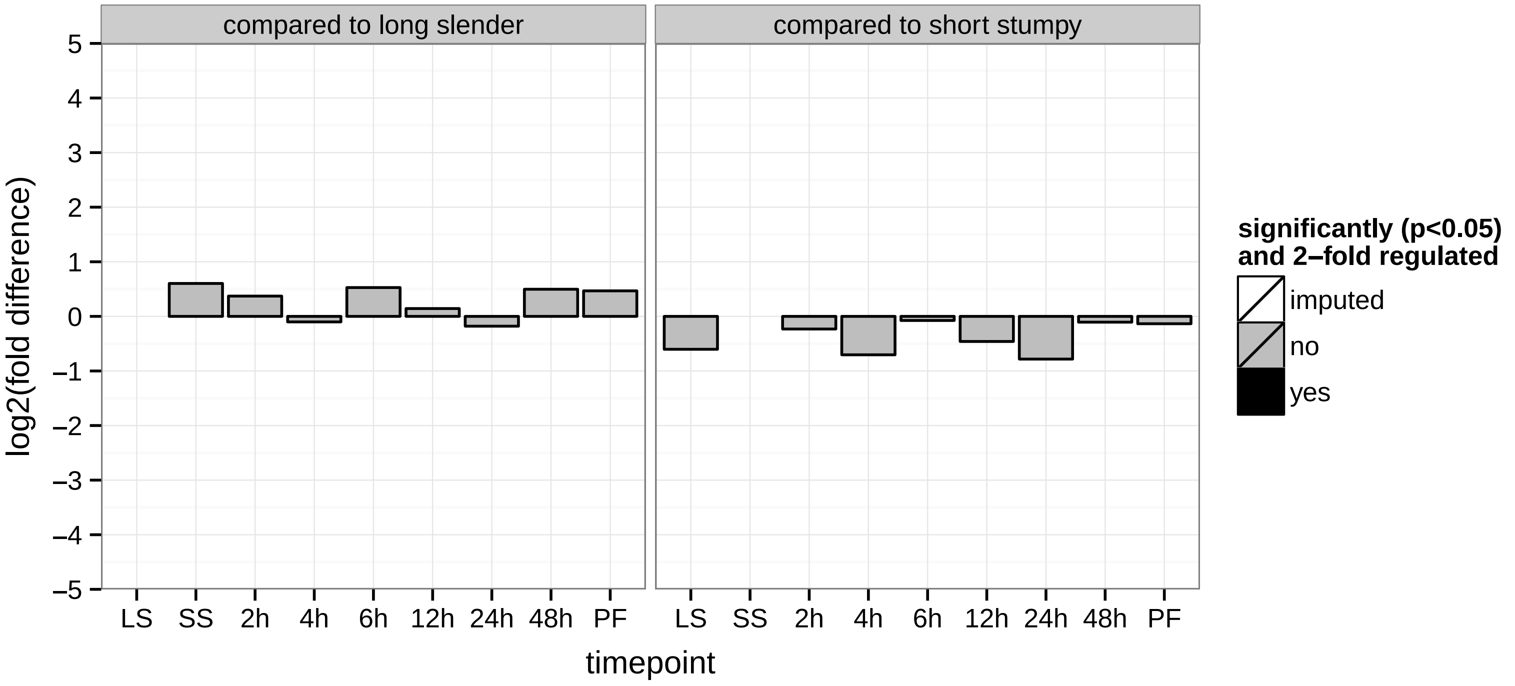

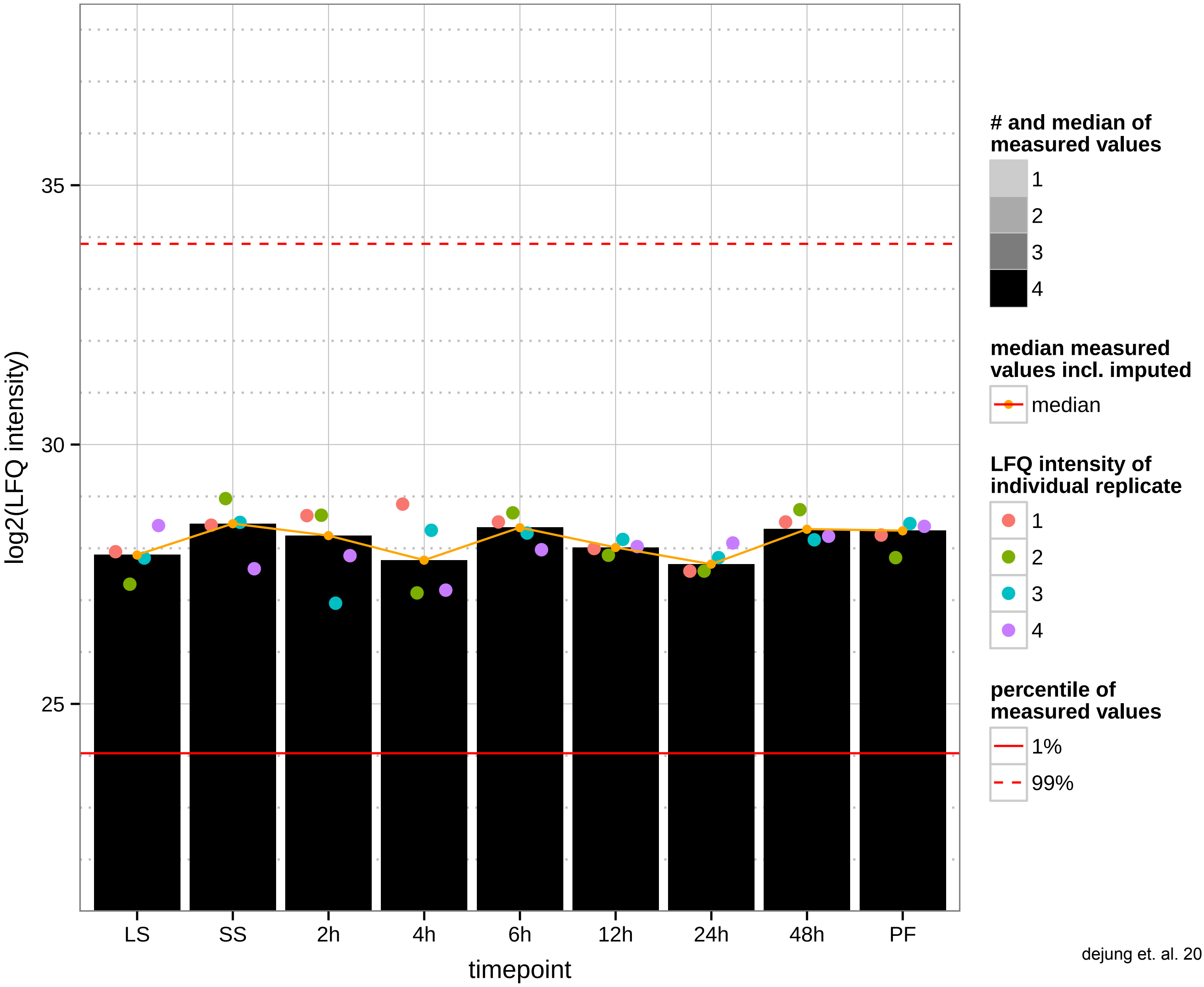

dejung et. al. 2015

protein transport protein SEC23 (SEC23.2)
Tb927.10.7740
AGOF: protein binding, zinc ion binding
AGOC: COPII vesicle coat
AGOP: ER to Golgi vesicle–mediated transport, intracellular protein transport
PGOF: zinc ion binding
PGOC: COPII vesicle coat
PGOP: ER to Golgi vesicle–mediated transport, intracellular protein transport

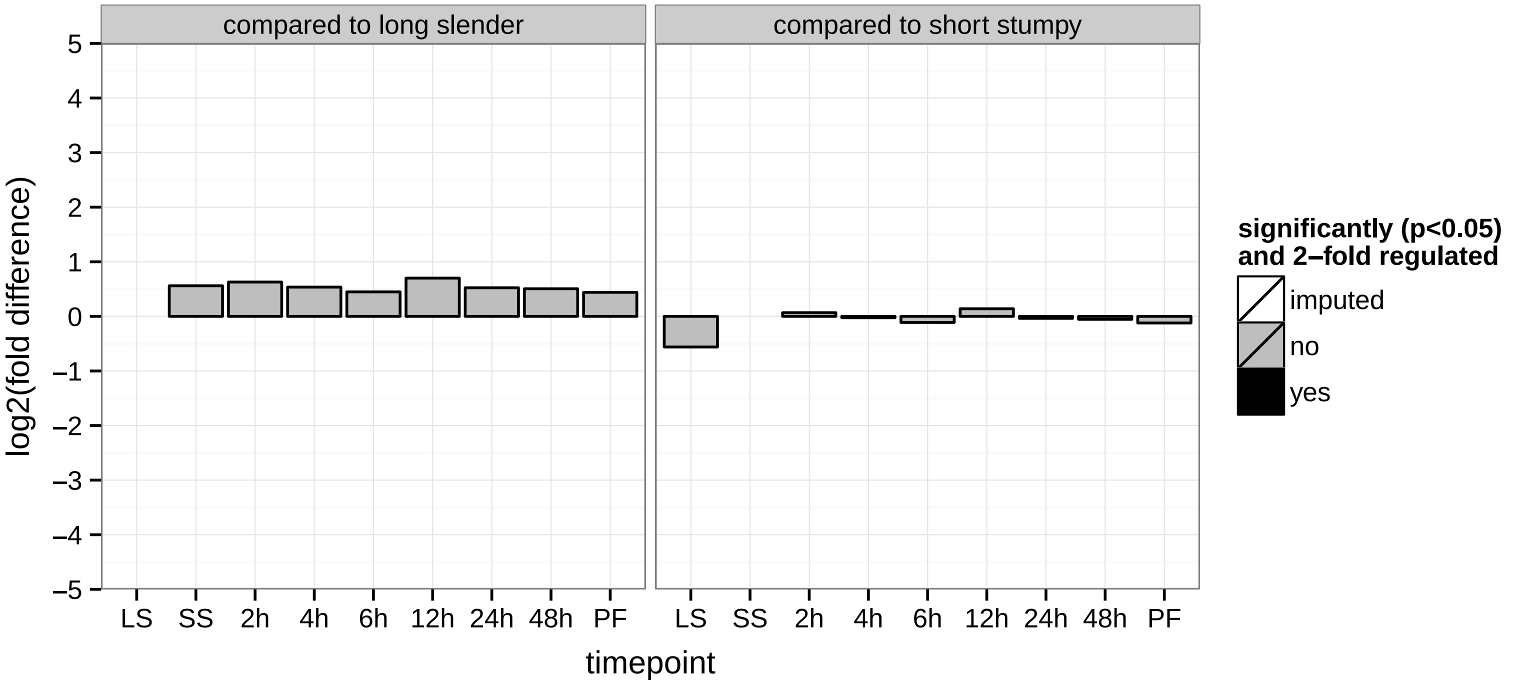

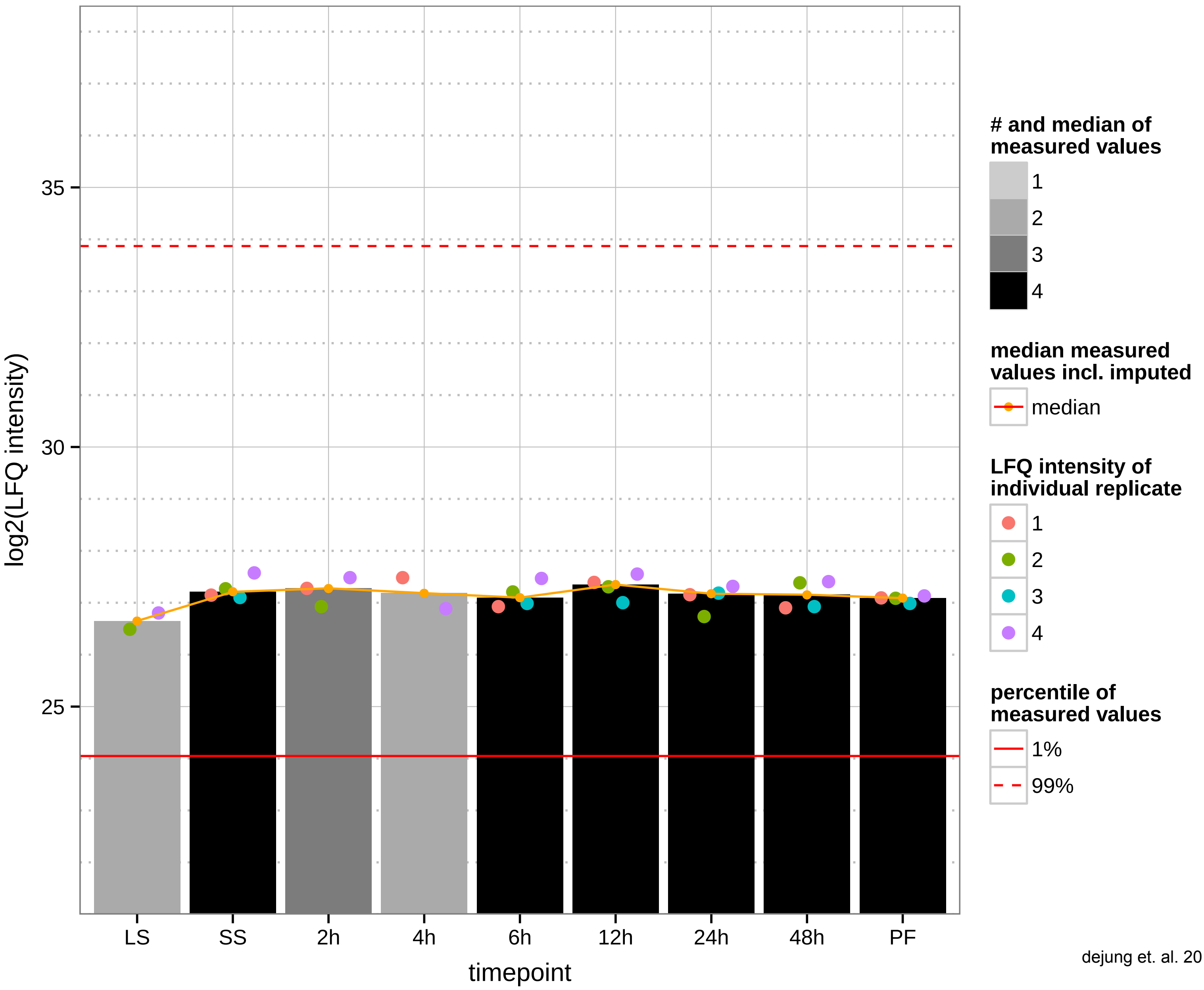

dejung et. al. 2015

ubiquitin fusion degradation protein, putative
Tb927.10.7790
AGOF: null
AGOC: null
AGOP: ubiquitin–dependent protein catabolic process
PGOF: null
PGOC: null
PGOP: ubiquitin–dependent protein catabolic process

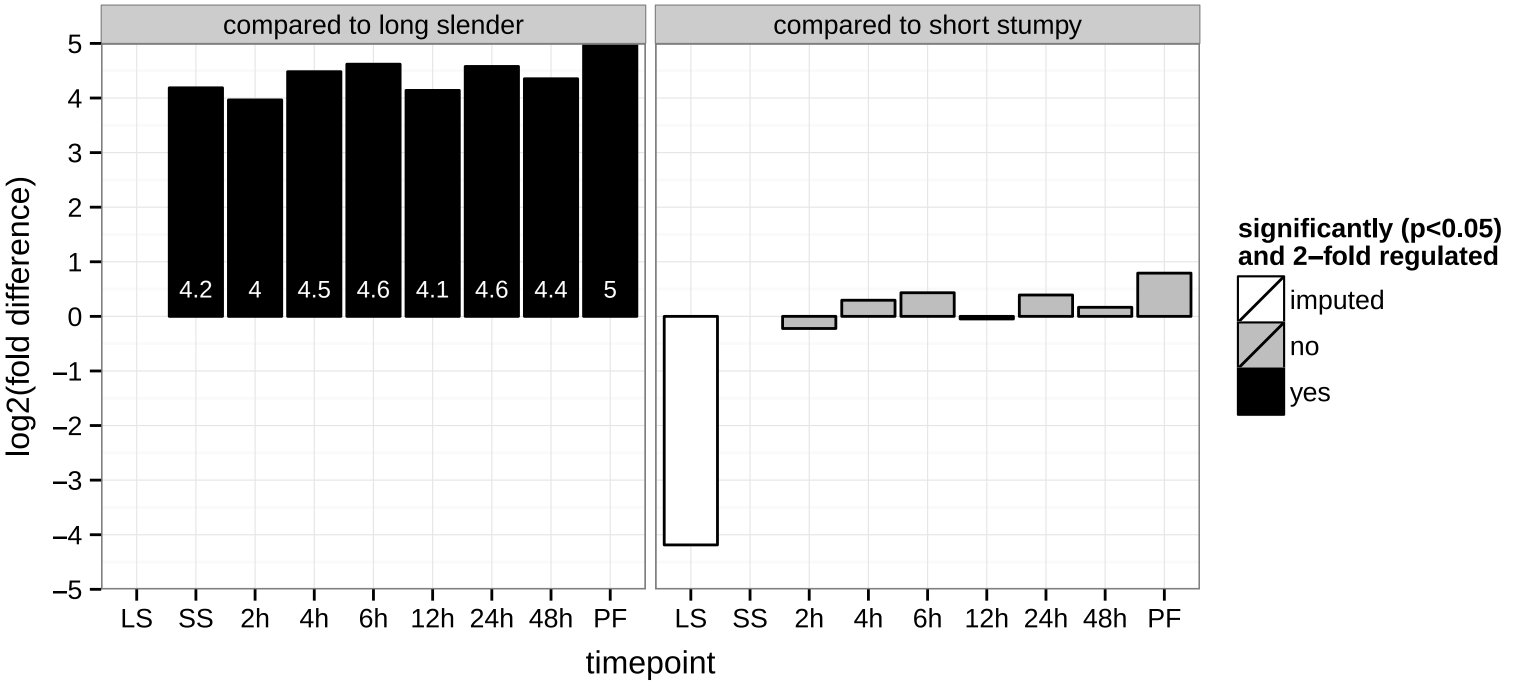

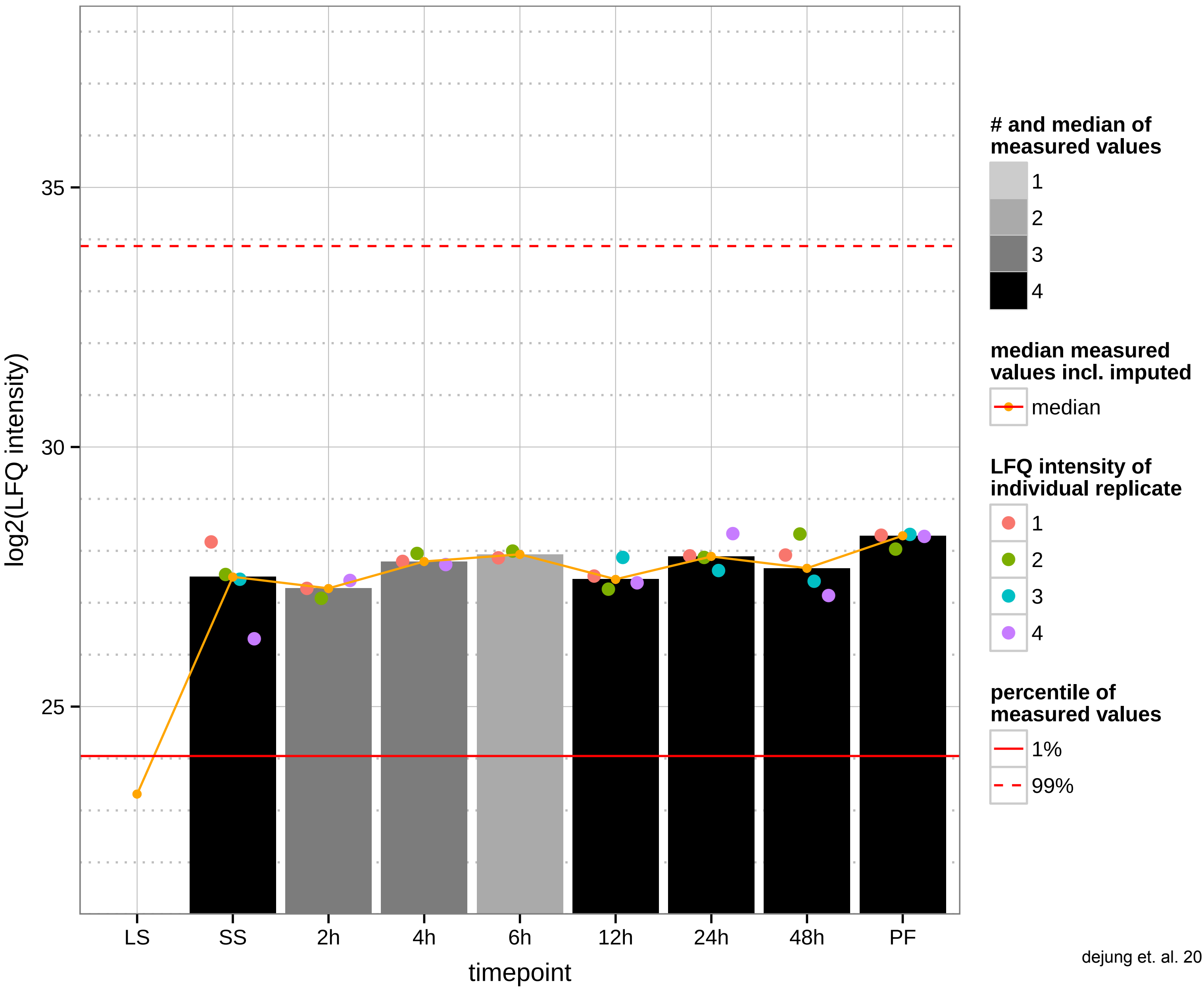

dejung et. al. 2015

hypothetical protein, conserved
Tb927.10.7810
AGOF: null
AGOC: null
AGOP: null
PGOF: null
PGOC: null
PGOP: null

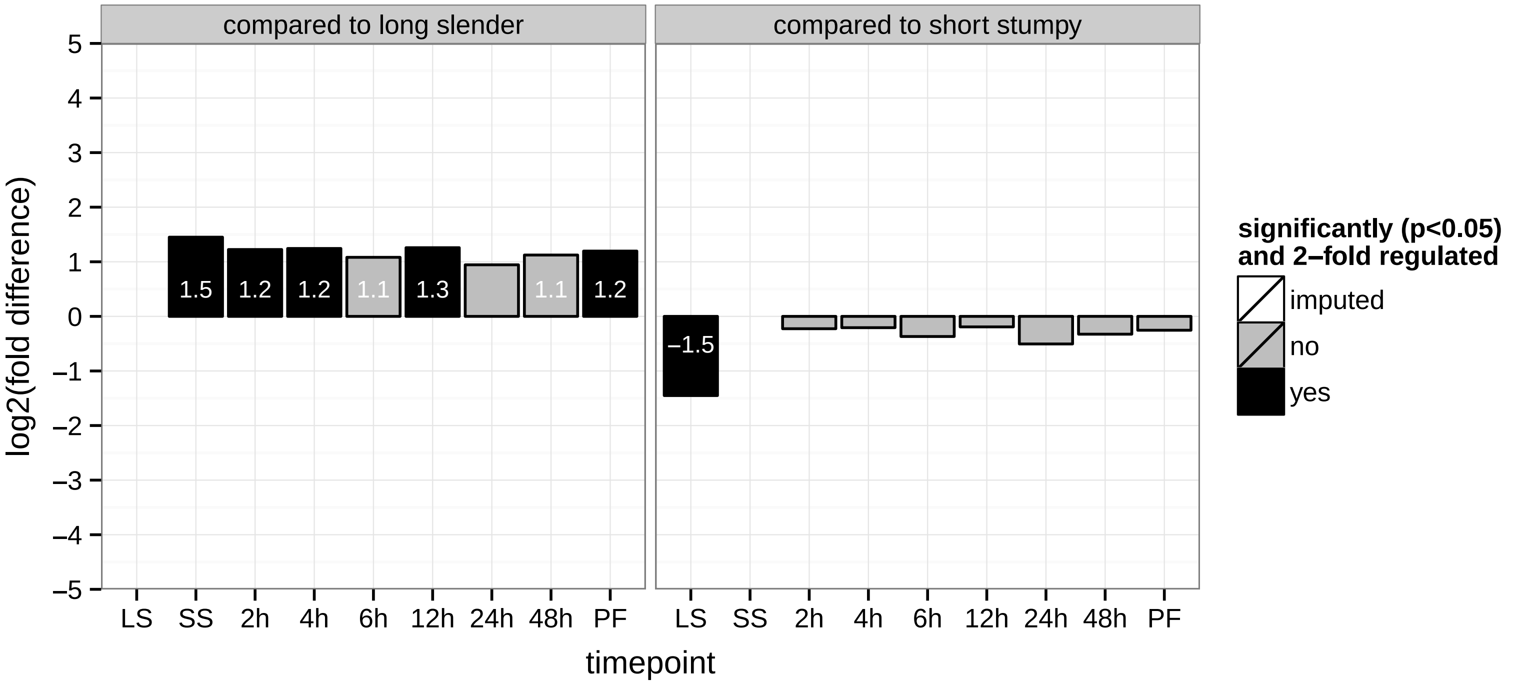

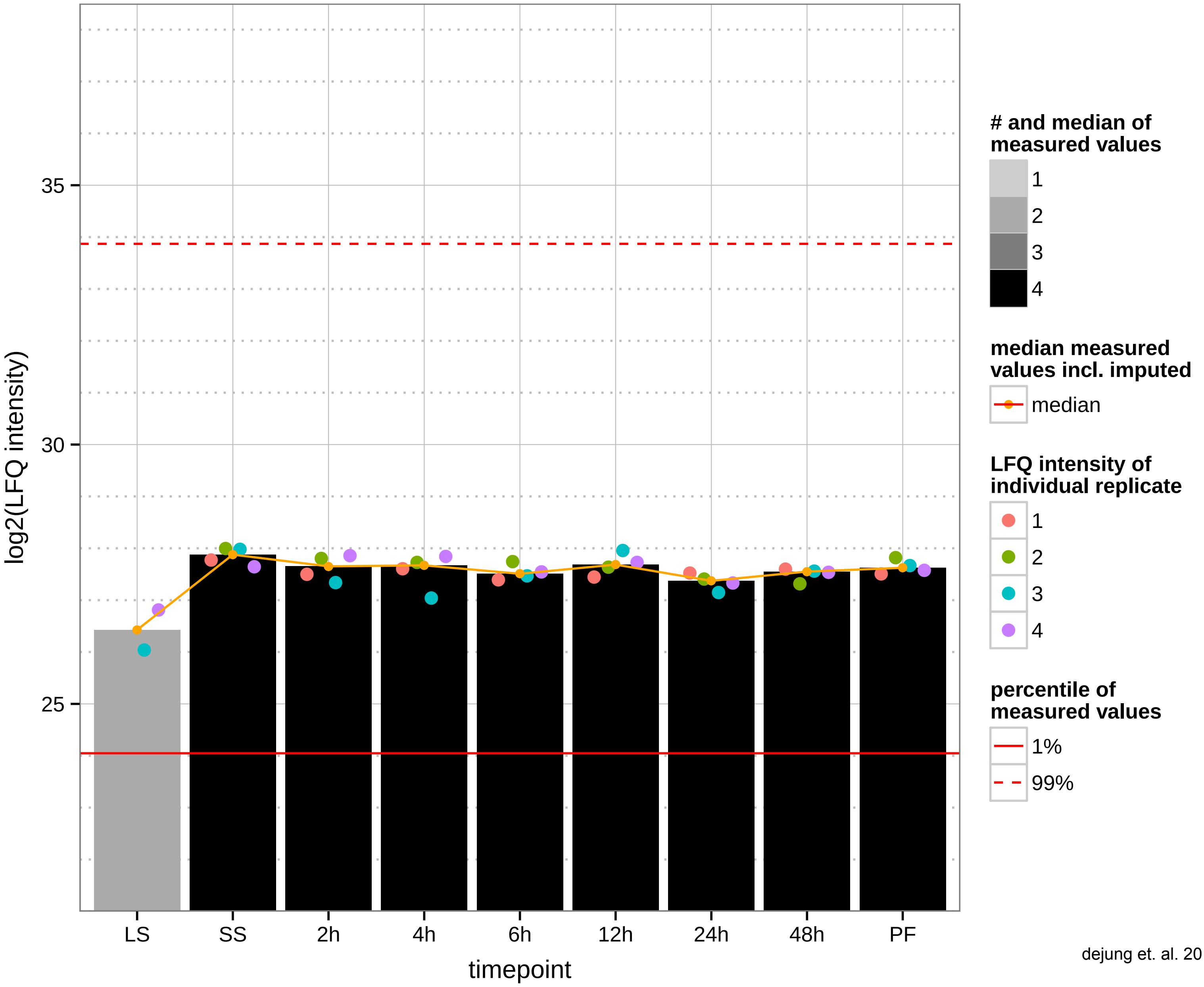

dejung et. al. 2015

hypothetical protein, conserved
Tb927.10.7870
AGOF: null
AGOC: null
AGOP: null
PGOF: null
PGOC: null
PGOP: null

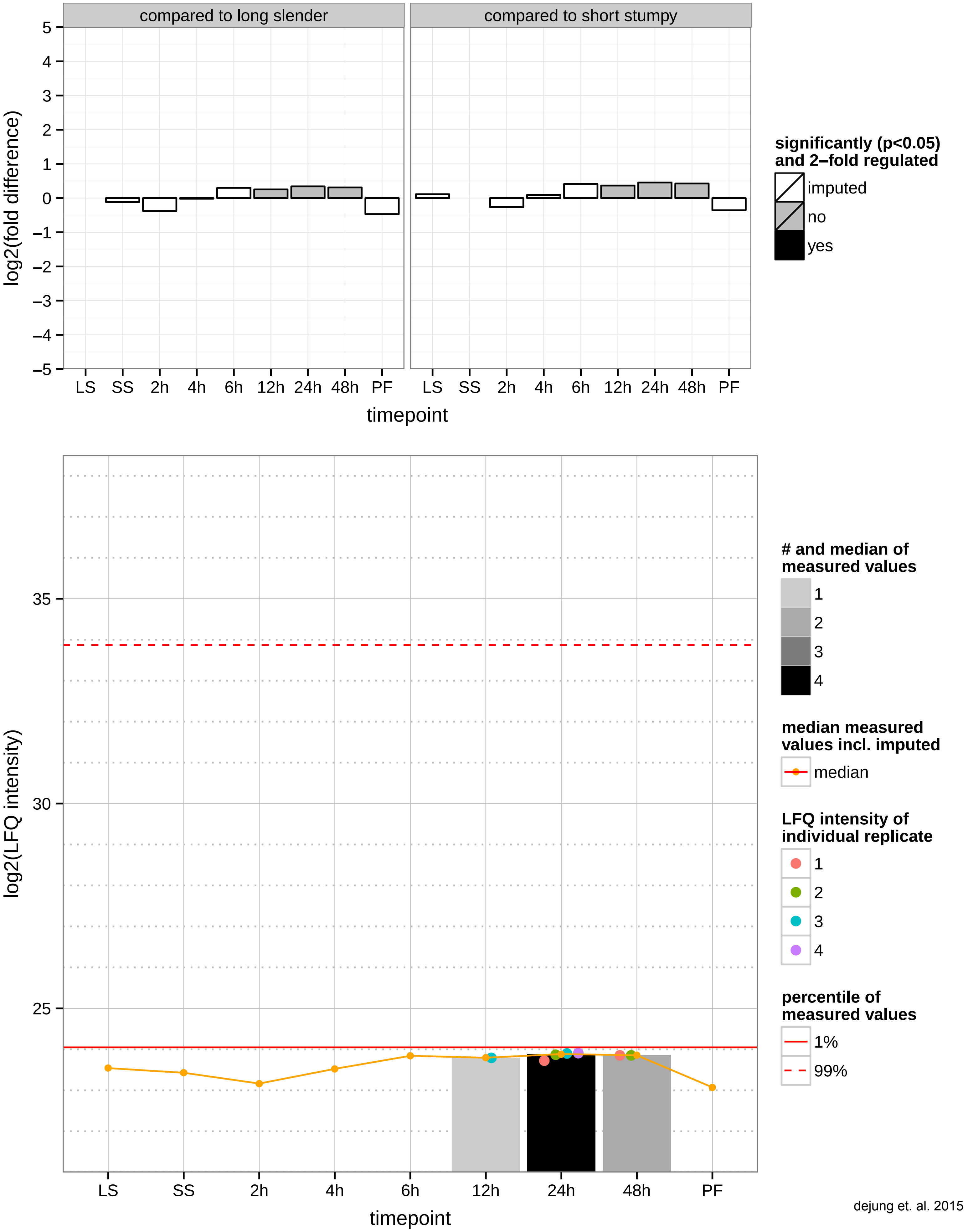

dejung et. al. 2015

hypothetical protein, conserved
Tb927.10.7880
AGOF: null
AGOC: null
AGOP: null
PGOF: null
PGOC: null
PGOP: null

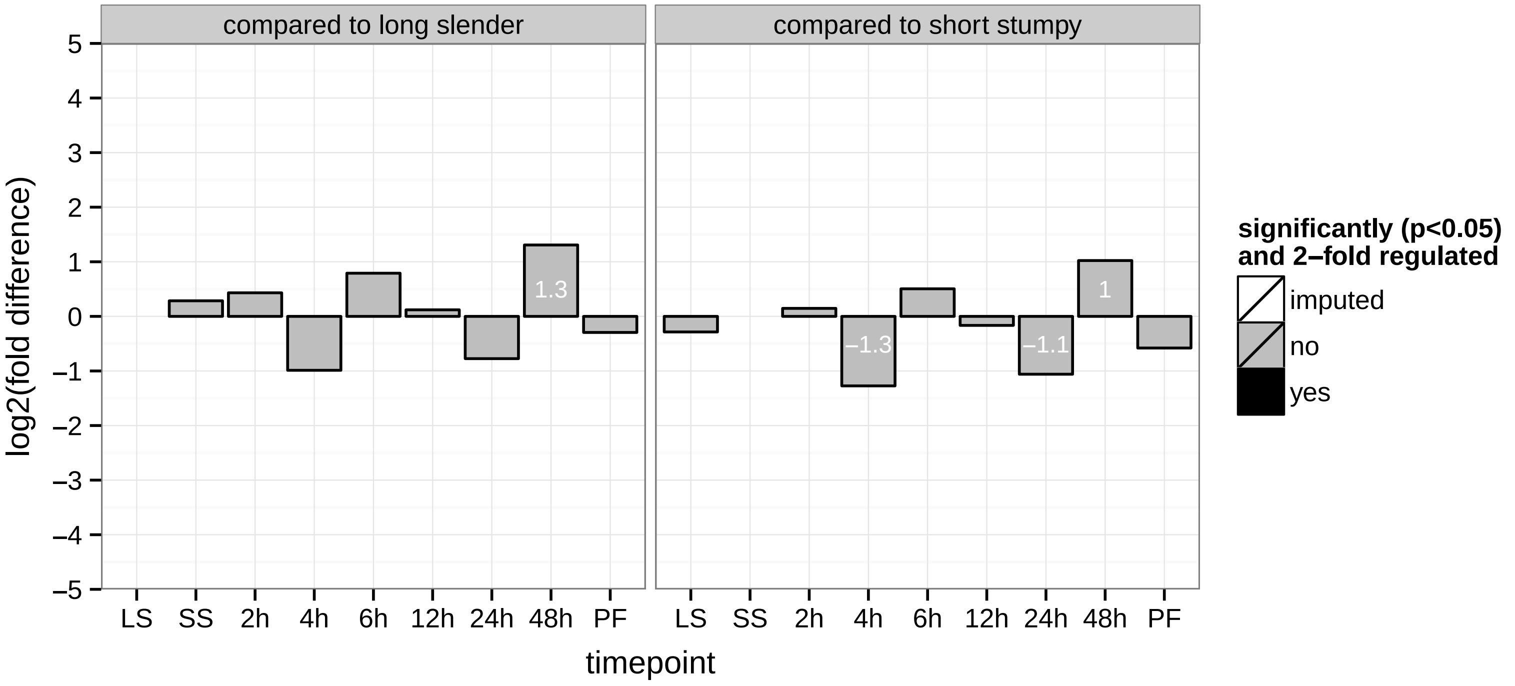

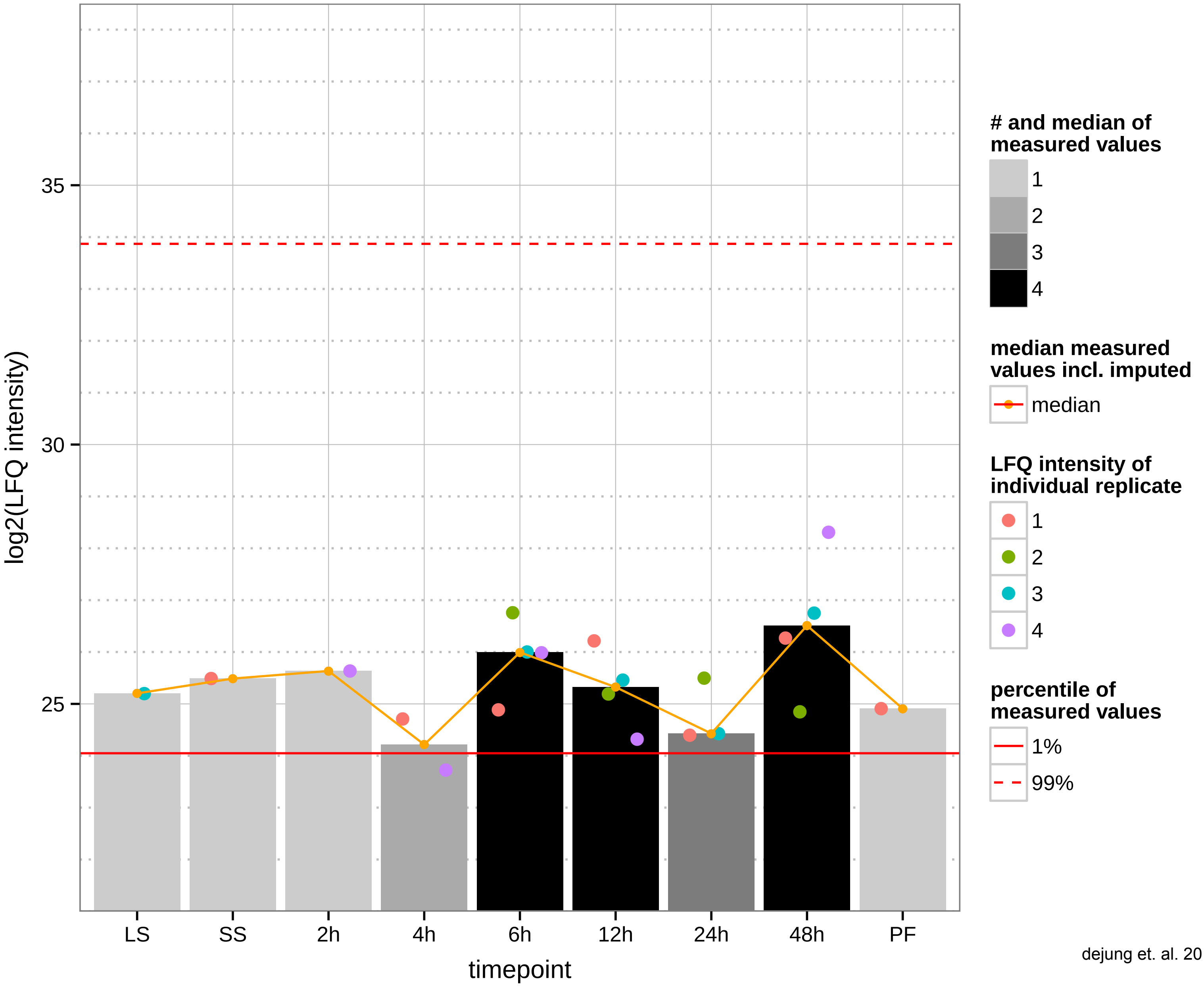

dejung et. al. 2015

hypothetical protein, conserved
Tb927.10.7920
AGOF: null
AGOC: null
AGOP: null
PGOF: null
PGOC: null
PGOP: null

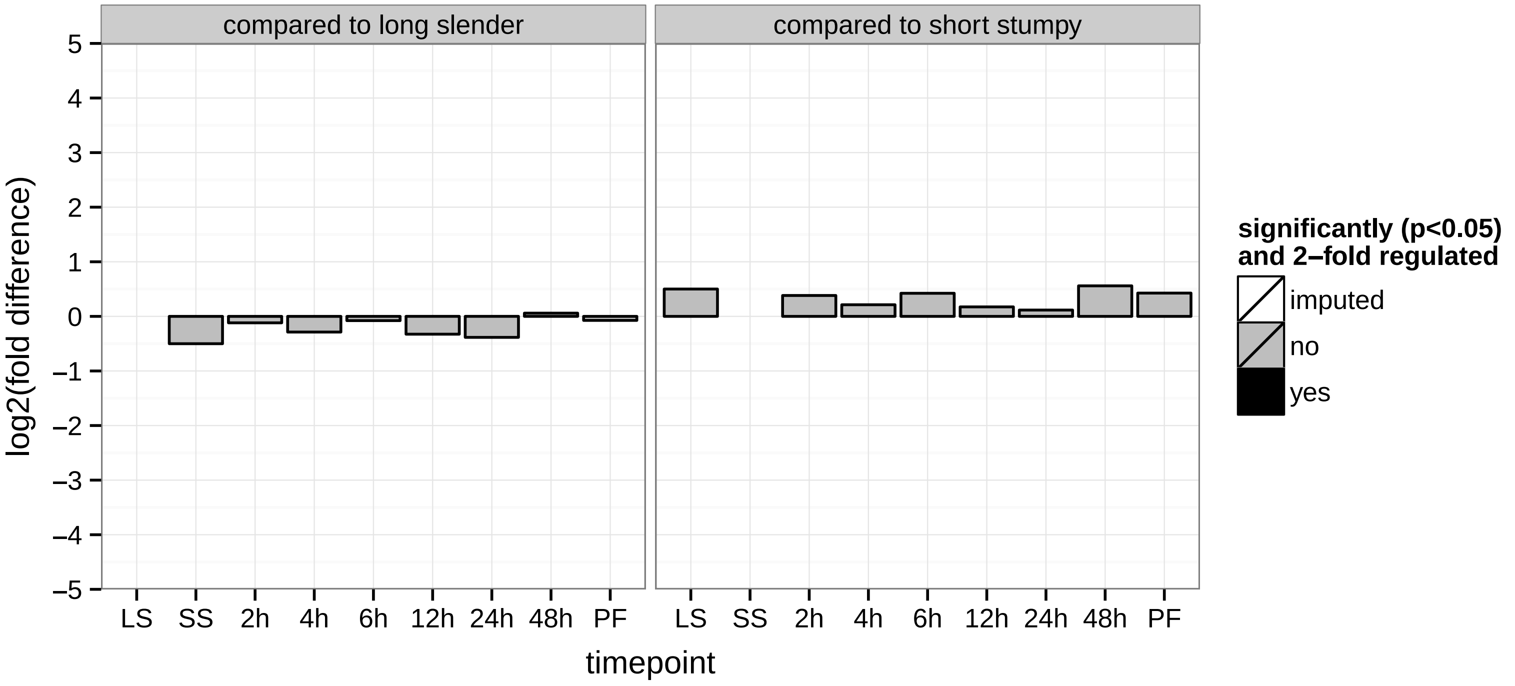

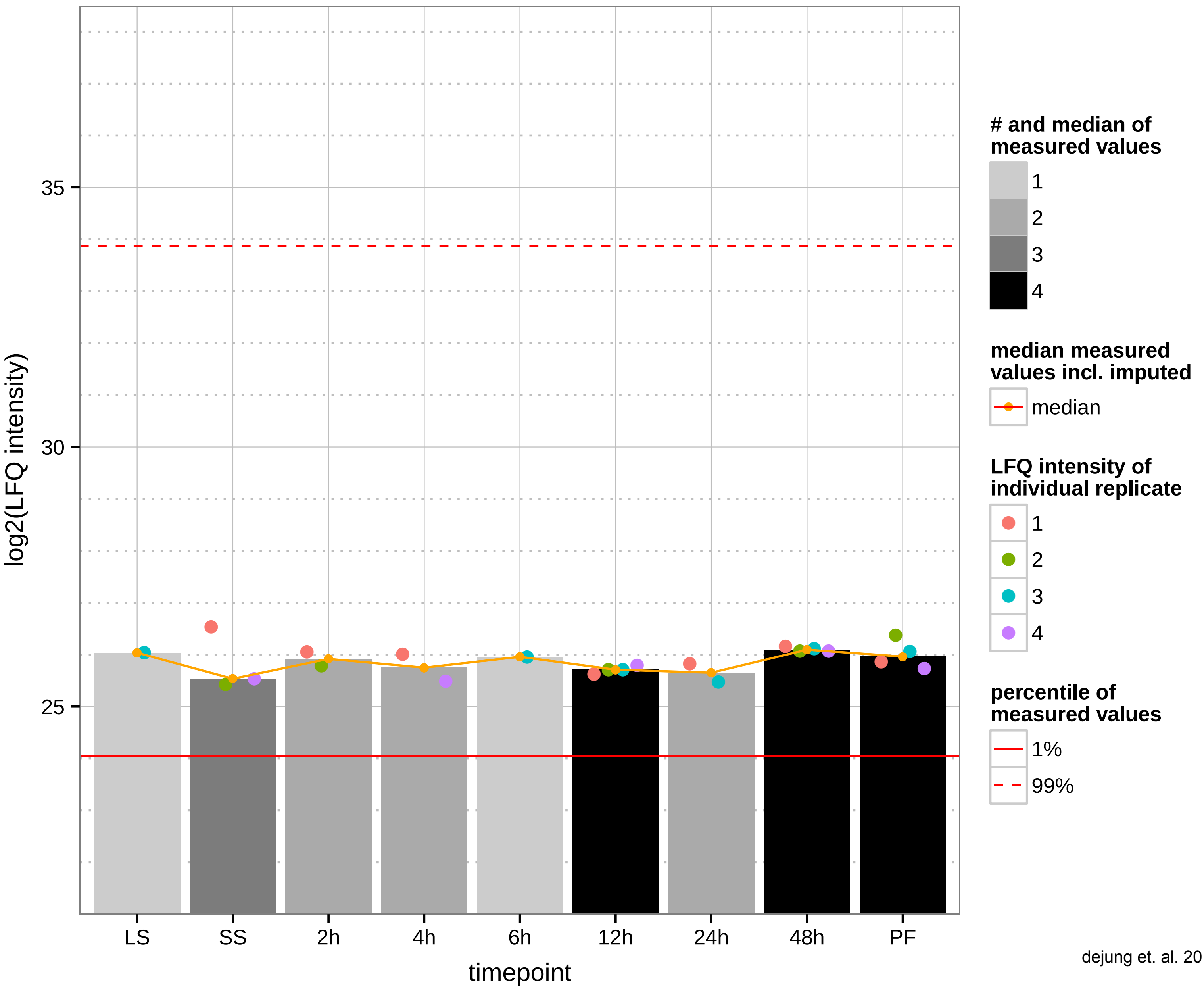

dejung et. al. 2015

2, 3–bisphosphoglycerate–independent phosphoglycerate mutase (PGAM)
Tb927.10.7930
AGOF: 2, 3–bisphosphoglycerate–independent phosphoglycerate mutase activity, manganese ion binding
AGOC: cytoplasm, cytosol
AGOP: gluconeogenesis, glycolysis
PGOF: catalytic activity, manganese ion binding, metal ion binding, phosphoglycerate mutase activity
PGOC: cytoplasm
PGOP: glucose catabolic process, metabolic process

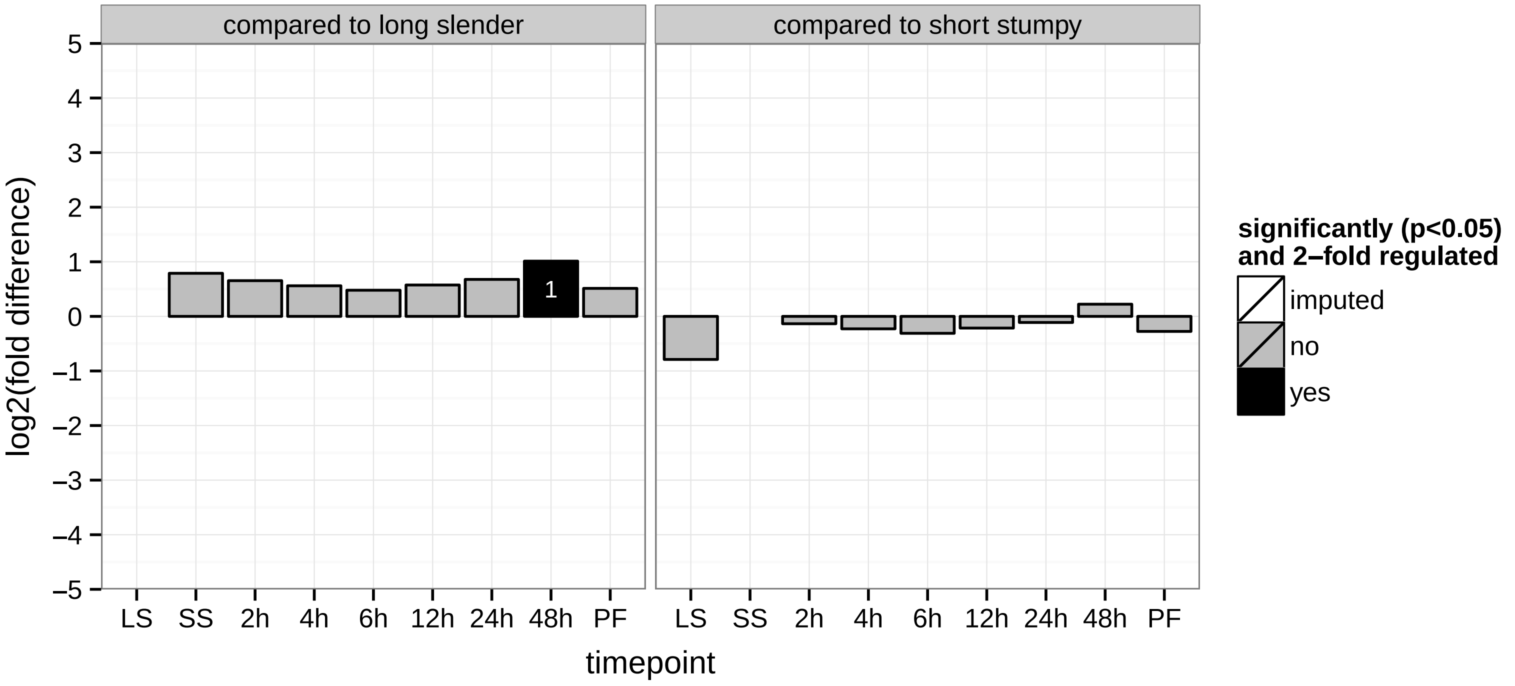

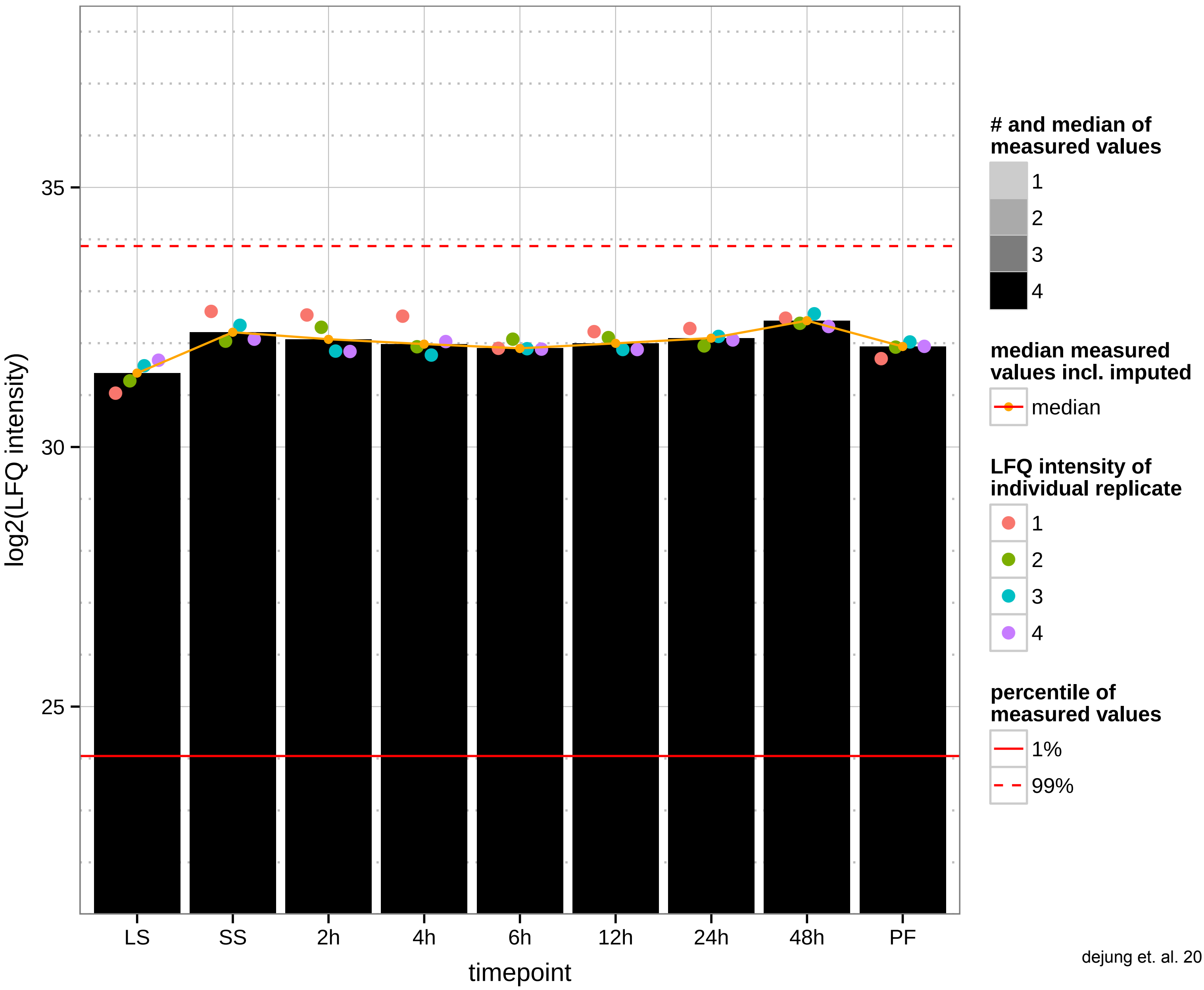

dejung et. al. 2015

methyltransferase, putative
Tb927.10.7940
AGOF: nucleic acid binding, rRNA methyltransferase activity
AGOC: null
AGOP: RNA methylation
PGOF: methyltransferase activity, nucleic acid binding
PGOC: null
PGOP: methylation

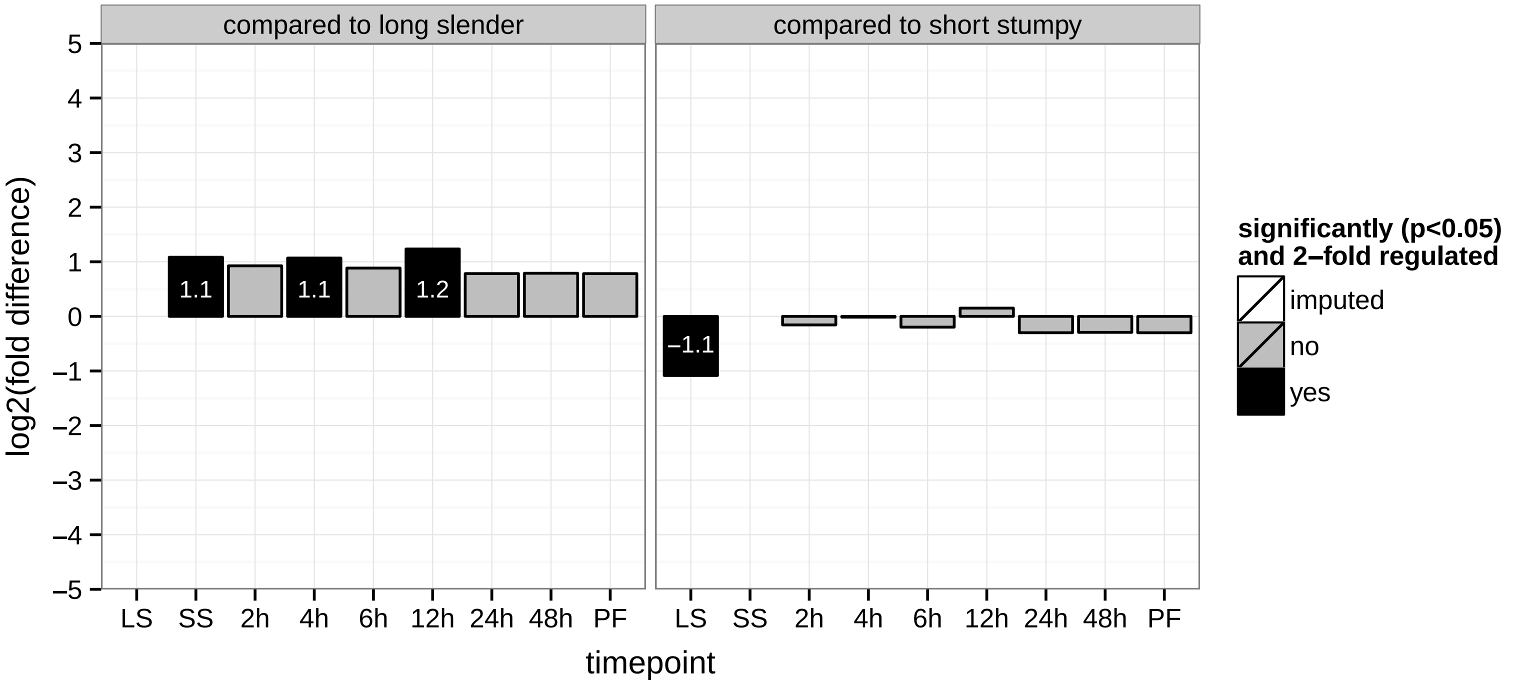

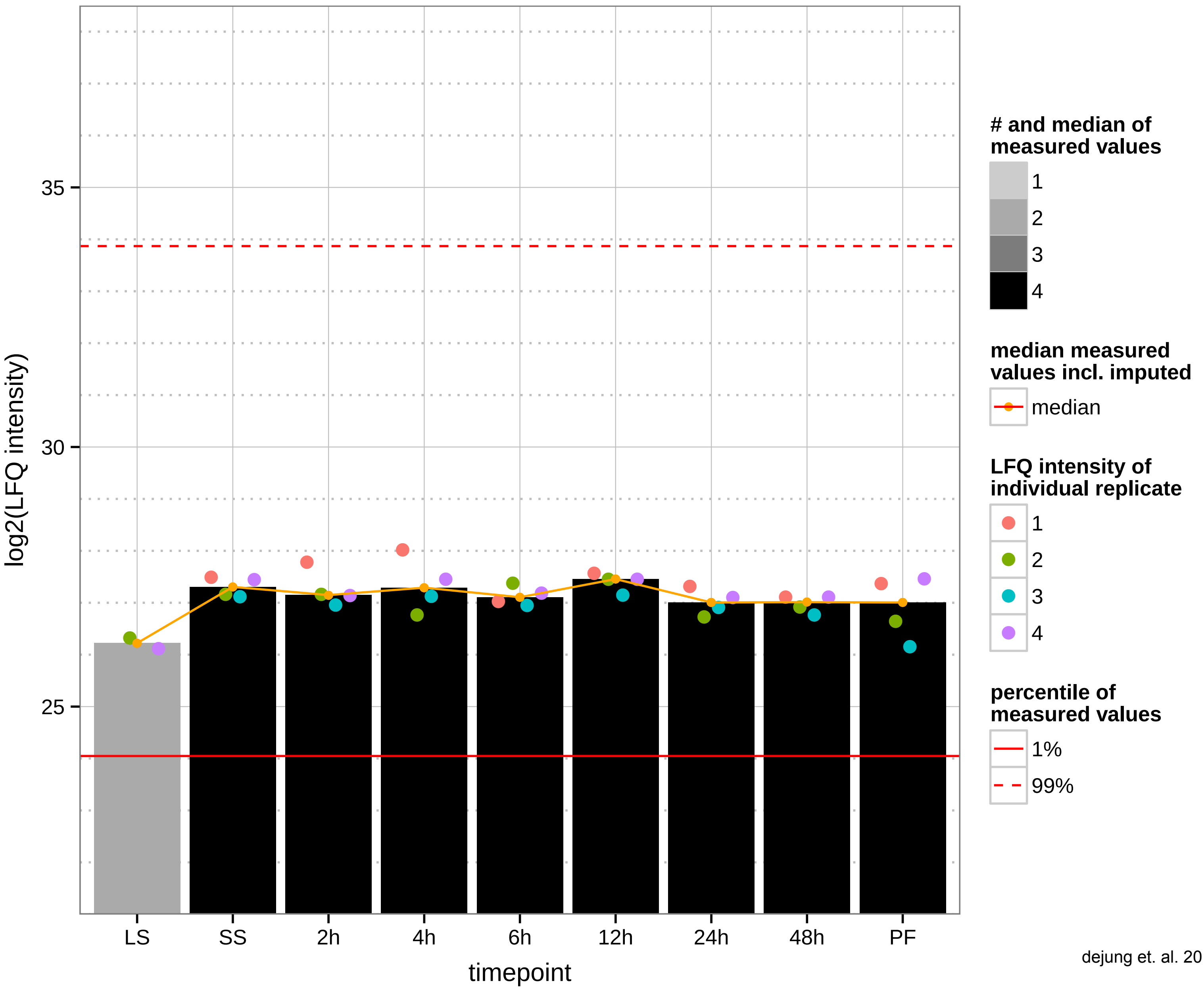

dejung et. al. 2015

replication factor C, subunit 5, putative, ATPase
Tb927.10.7990
AGOF: DNA binding, nucleoside–triphosphatase activity, nucleotide binding
AGOC: nucleus
AGOP: DNA replication
PGOF: DNA binding, nucleoside–triphosphatase activity, nucleotide binding
PGOC: null
PGOP: DNA replication

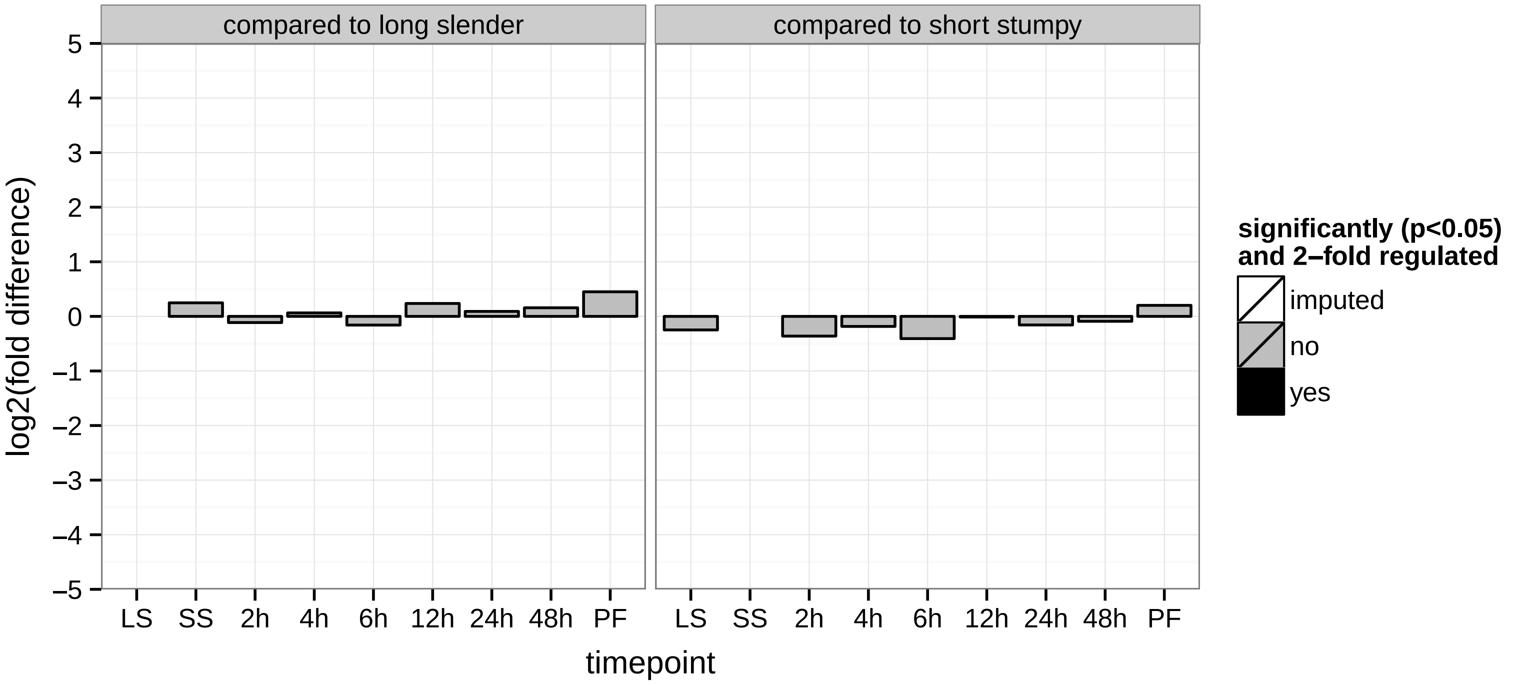

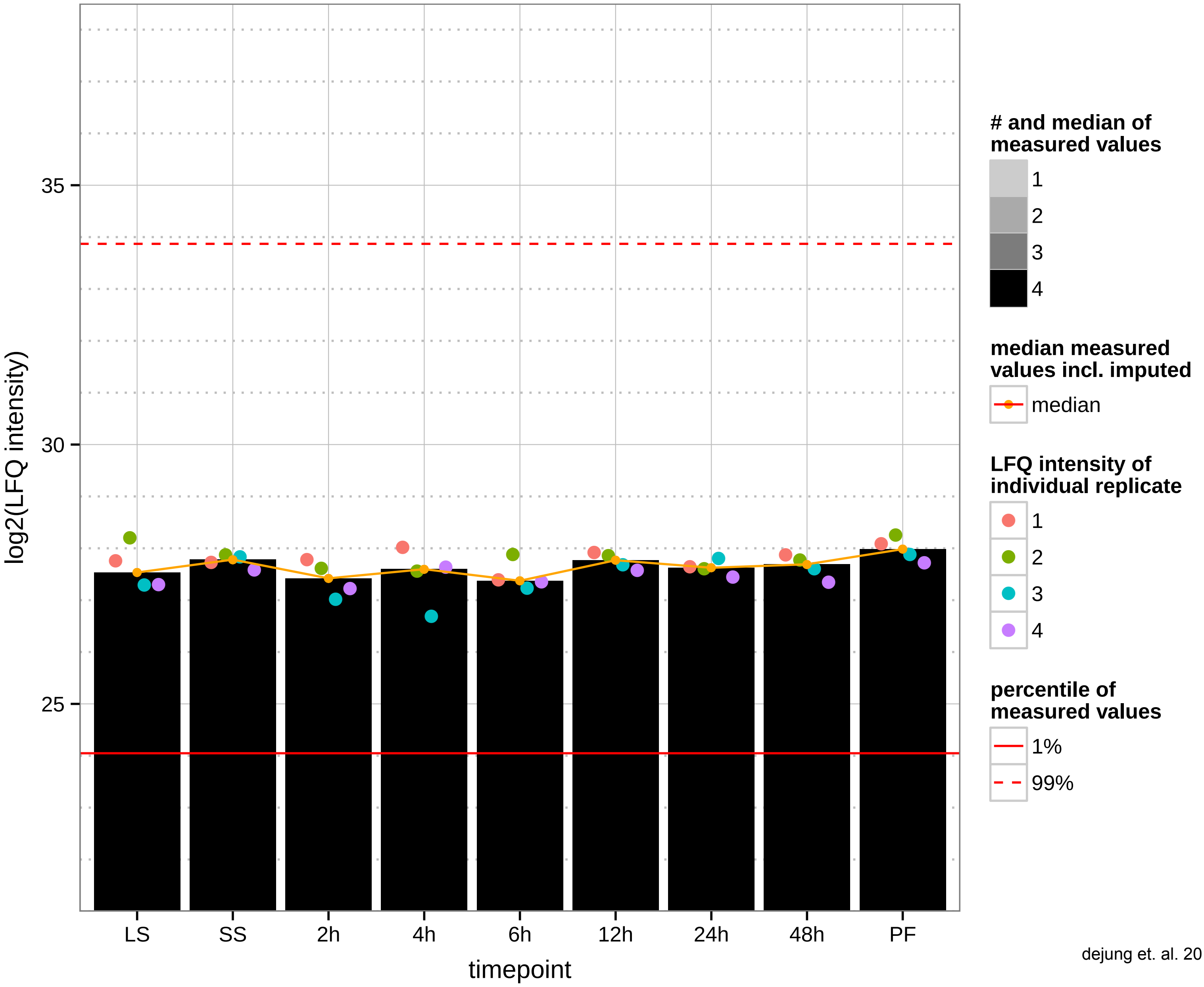

dejung et. al. 2015

hypothetical protein, conserved
Tb927.10.8000
AGOF: null
AGOC: null
AGOP: null
PGOF: adenylate kinase activity
PGOC: null
PGOP: null

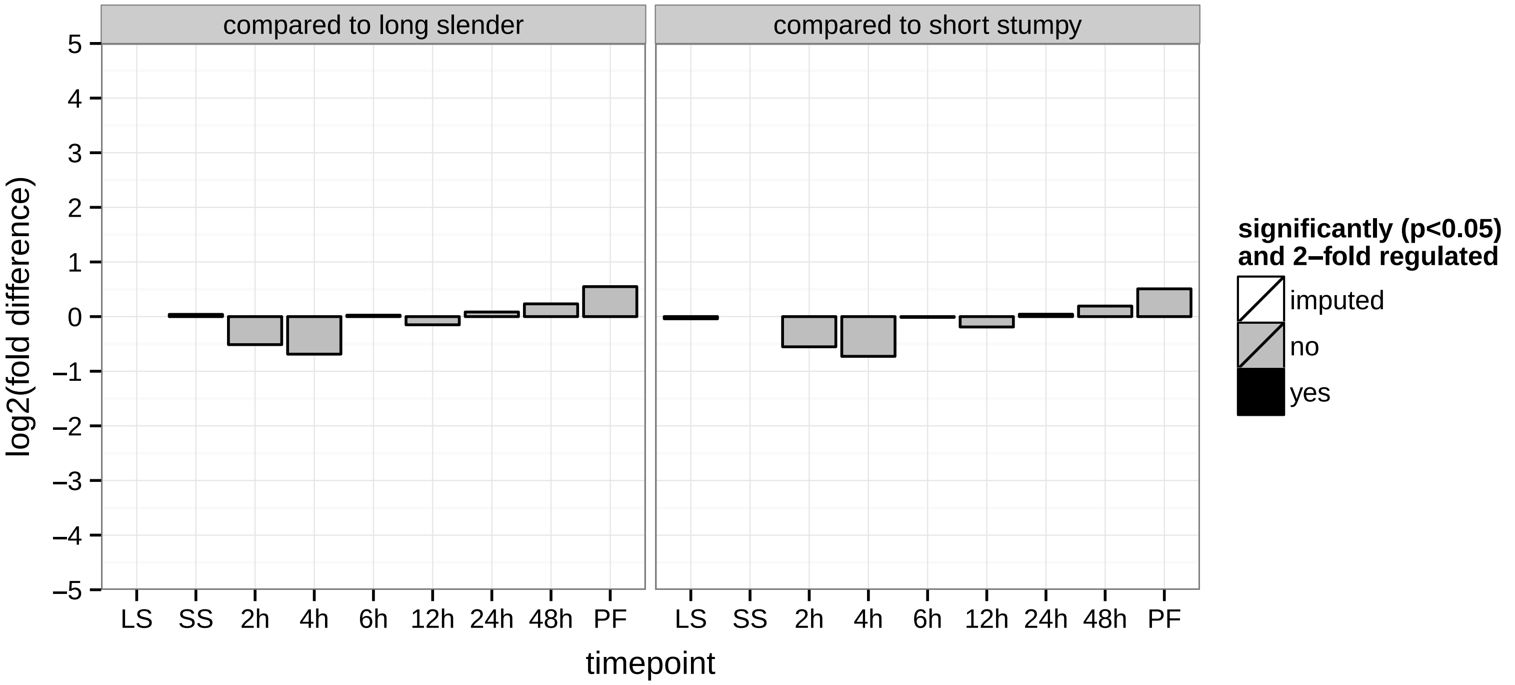

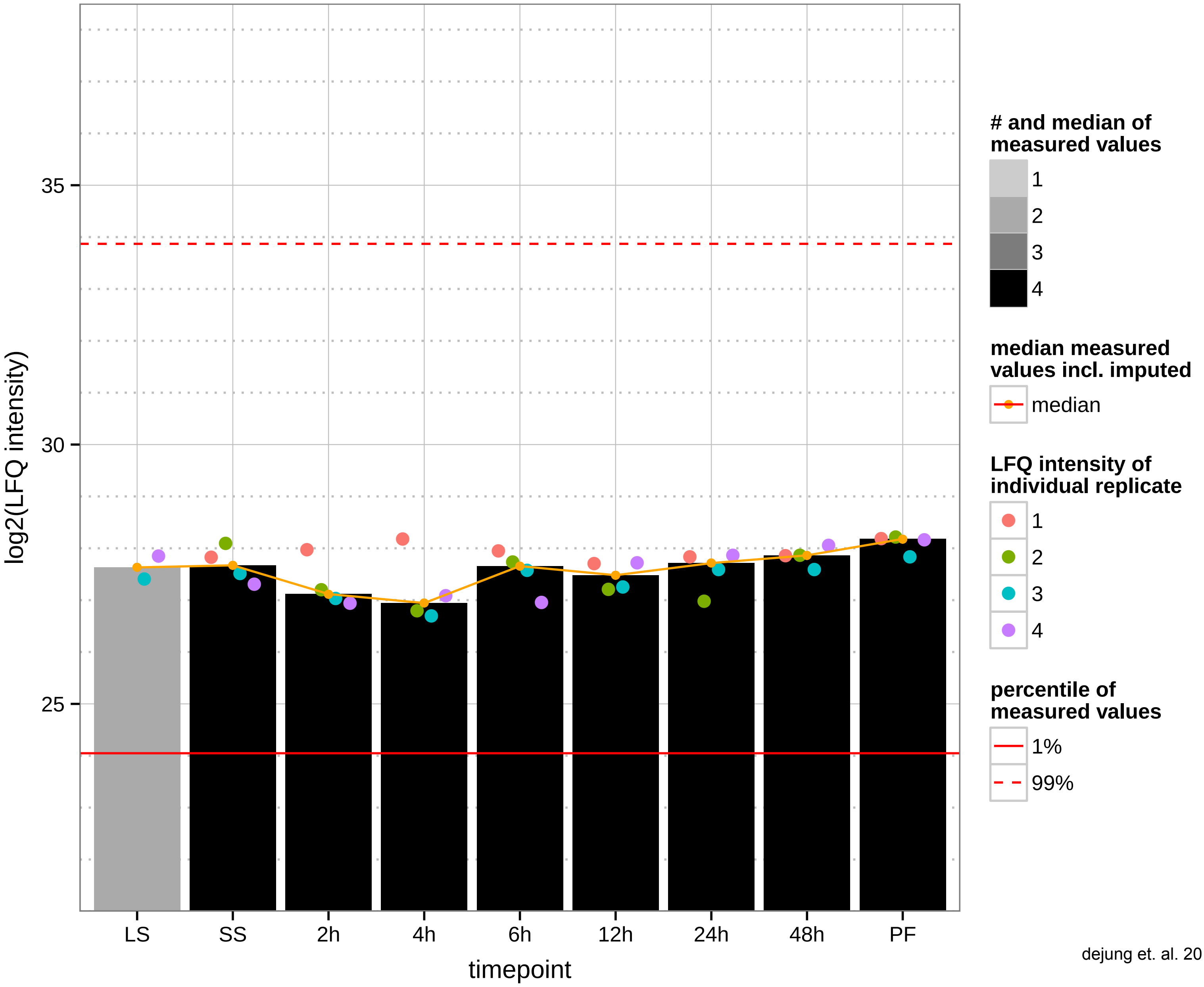

dejung et. al. 2015

hypothetical protein, conserved
Tb927.10.8010
AGOF: null
AGOC: mitochondrion
AGOP: null
PGOF: null
PGOC: null
PGOP: null

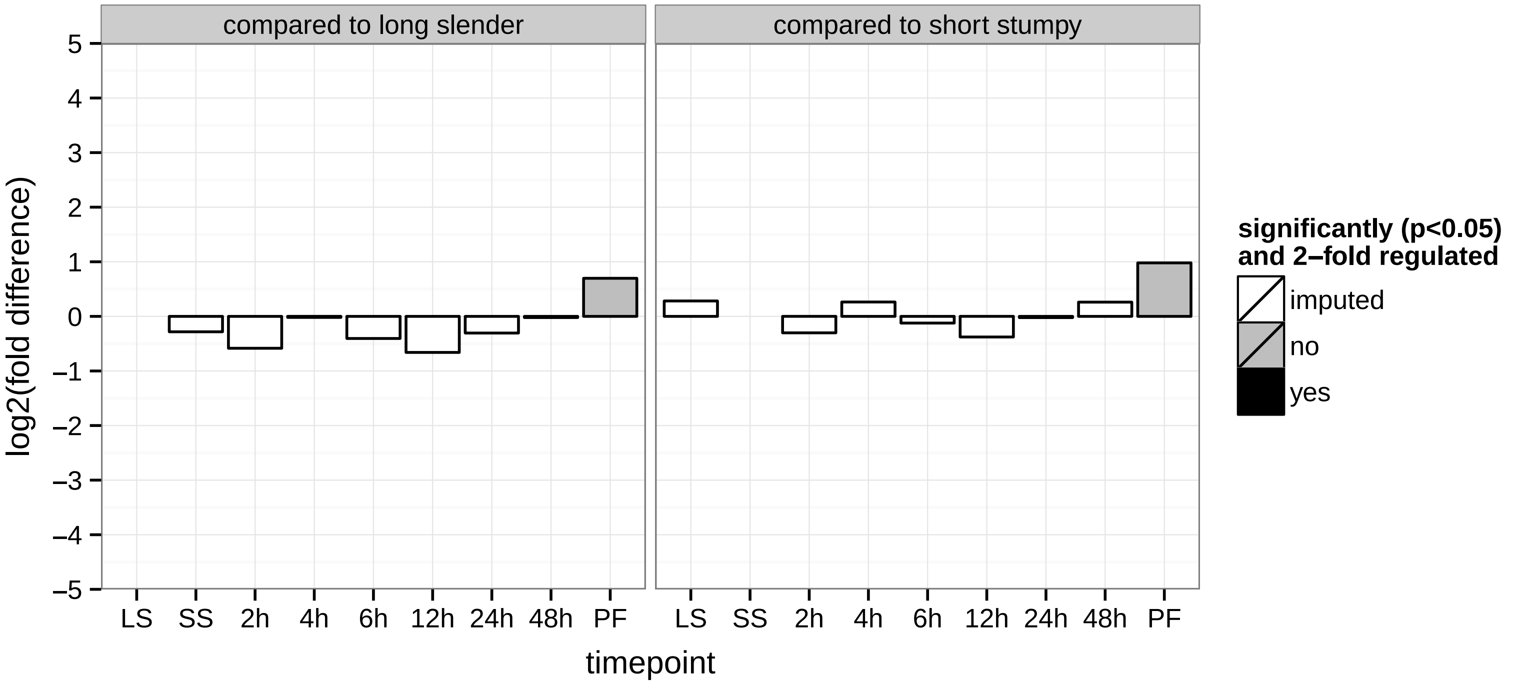

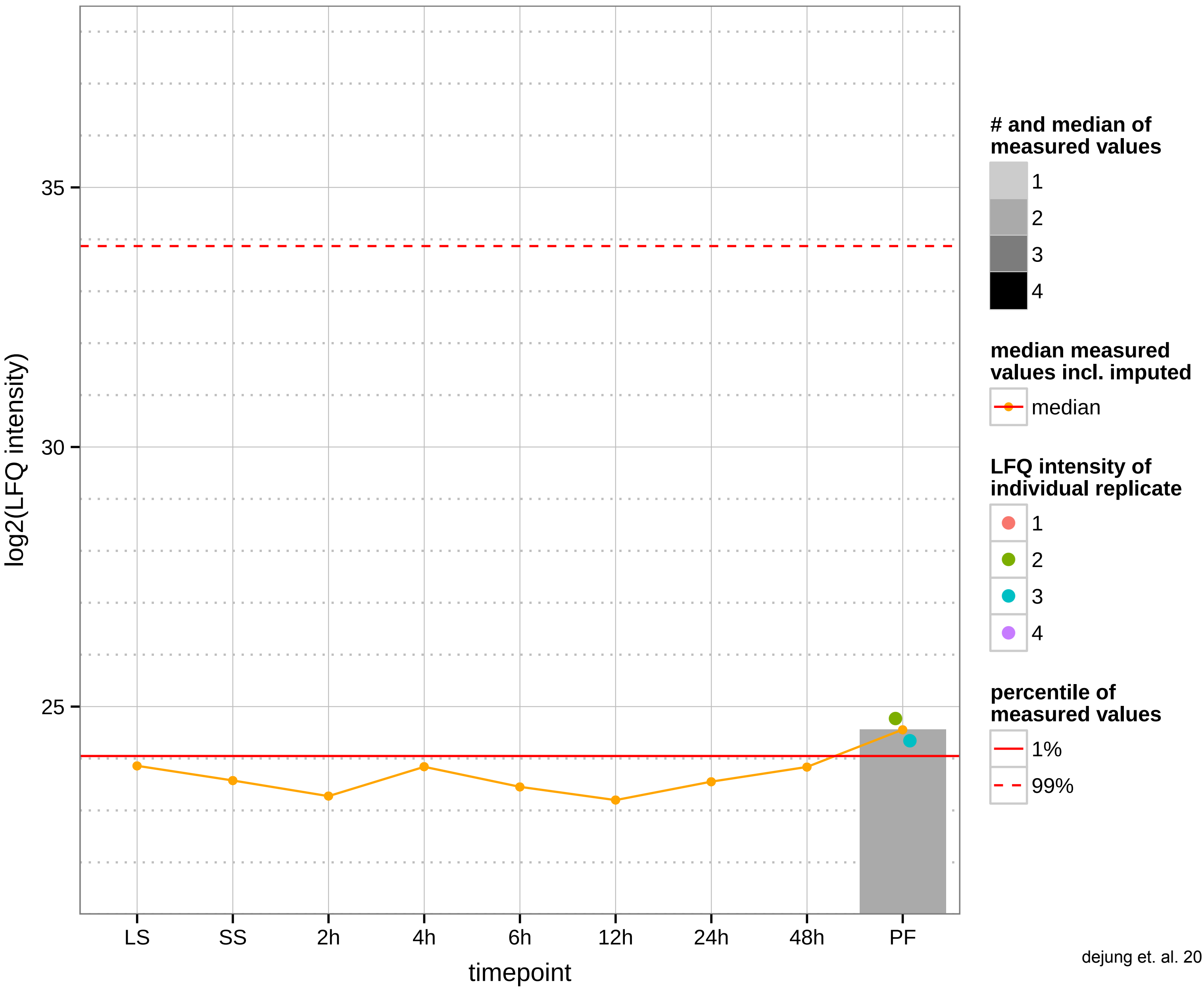

dejung et. al. 2015

adaptin complex 1 subunit, putative, beta–adaptin, fragment (BAD1)
Tb927.10.8040
AGOF: protein transporter activity
AGOC: Golgi apparatus, clathrin coat of trans–Golgi network vesicle, coated pit, trans–Golgi network
AGOP: intracellular protein transport, vesicle–mediated transport
PGOF: binding, protein transporter activity
PGOC: membrane coat
PGOP: intracellular protein transport, protein transport, vesicle–mediated transport

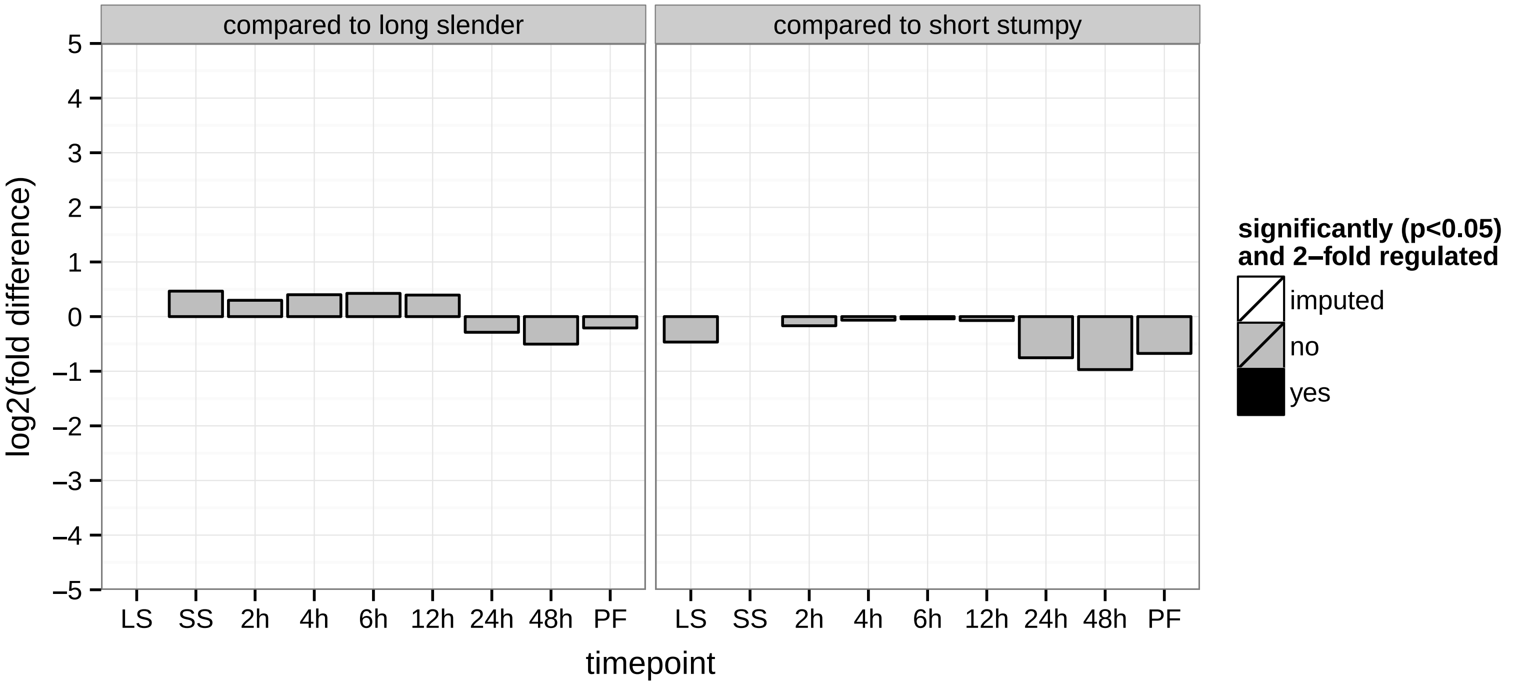

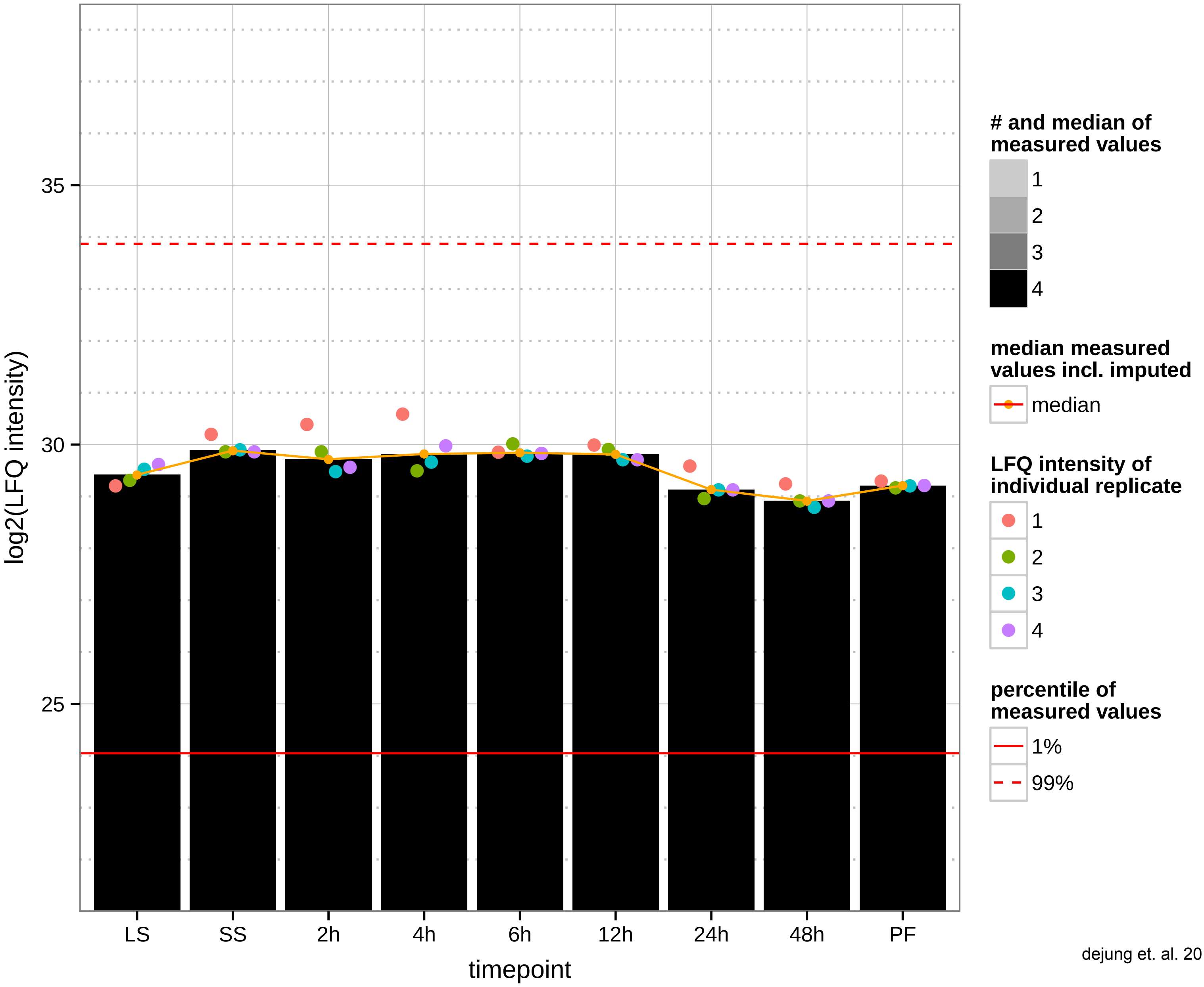

dejung et. al. 2015

hypothetical protein, conserved
Tb927.10.8060
AGOF: null
AGOC: null
AGOP: null
PGOF: protein binding
PGOC: null
PGOP: null

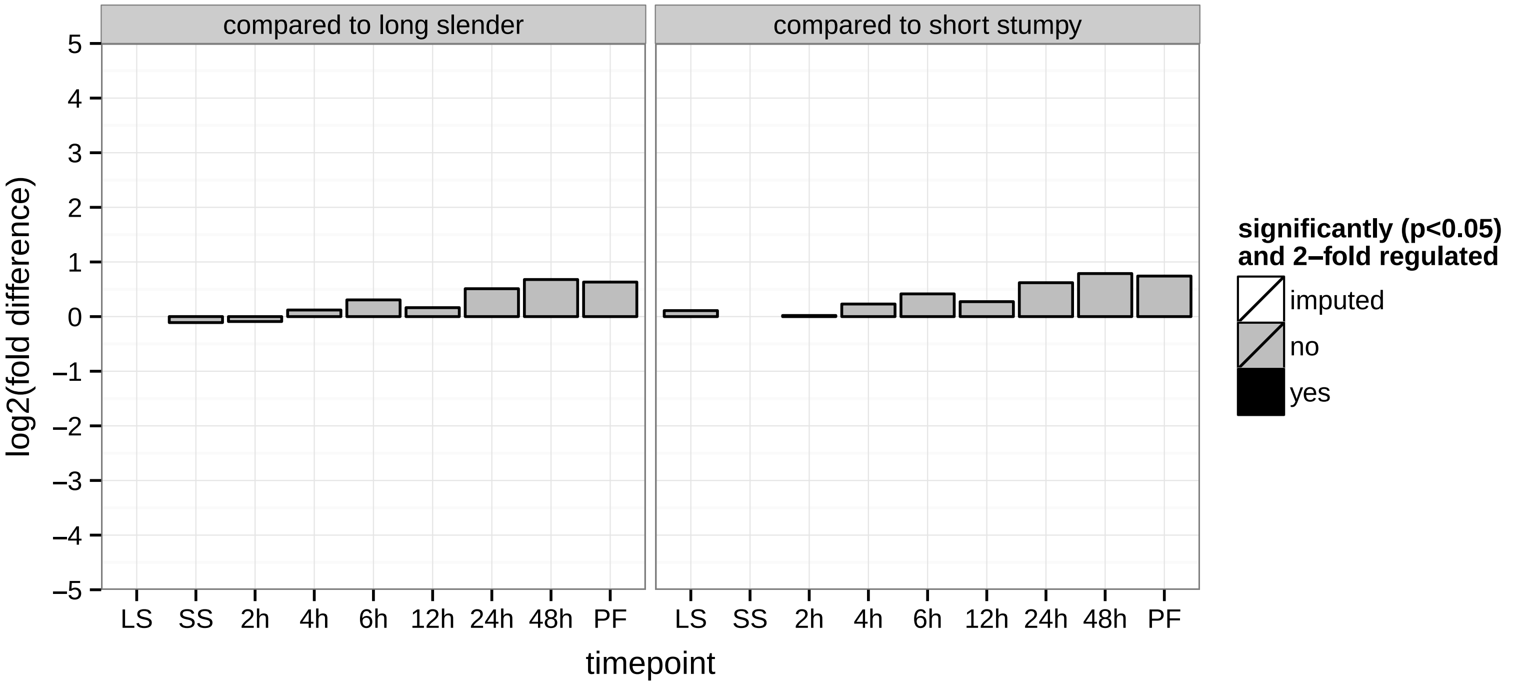

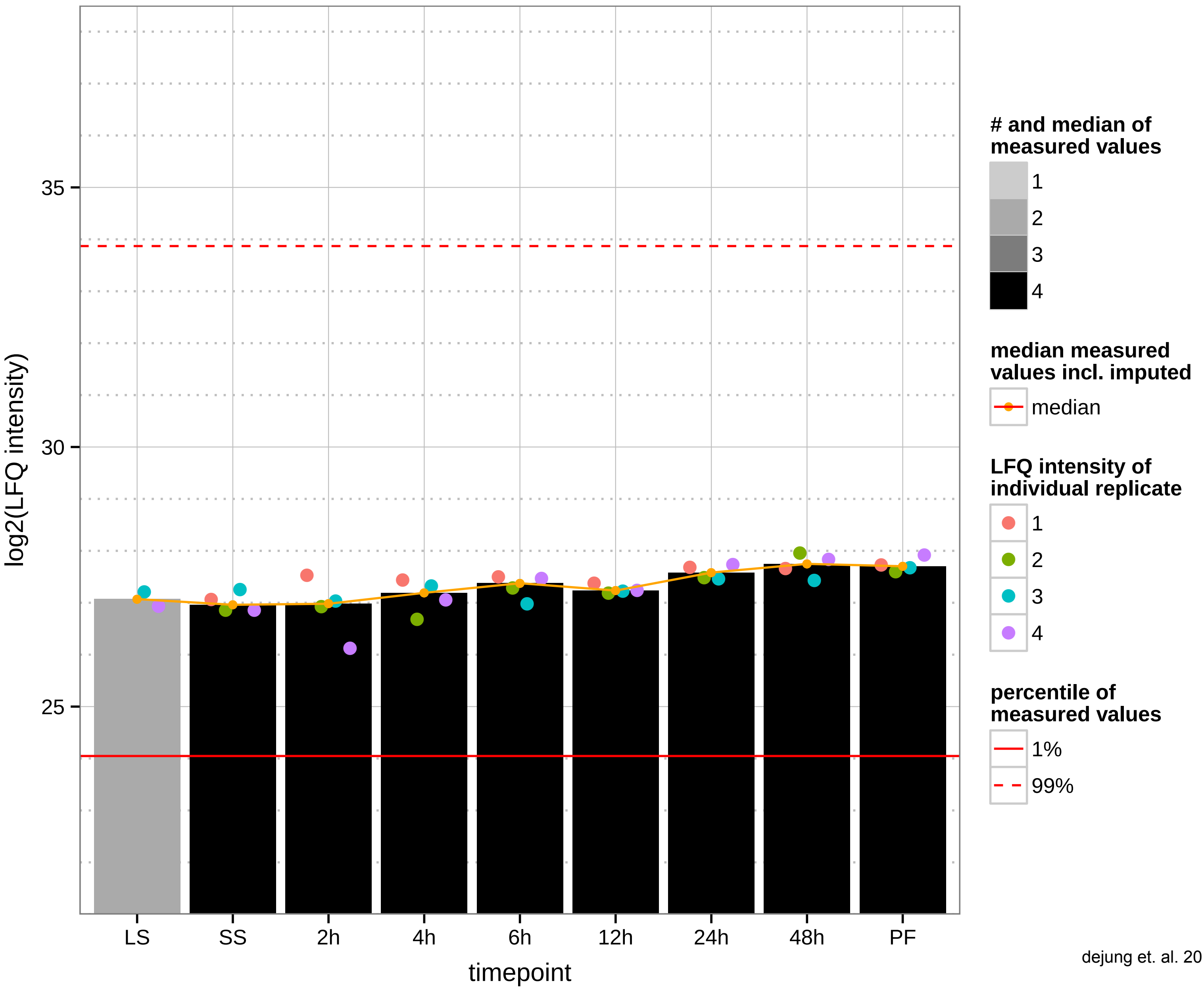

dejung et. al. 2015

hypothetical protein, conserved
Tb927.10.8080
AGOF: null
AGOC: null
AGOP: null
PGOF: null
PGOC: null
PGOP: null

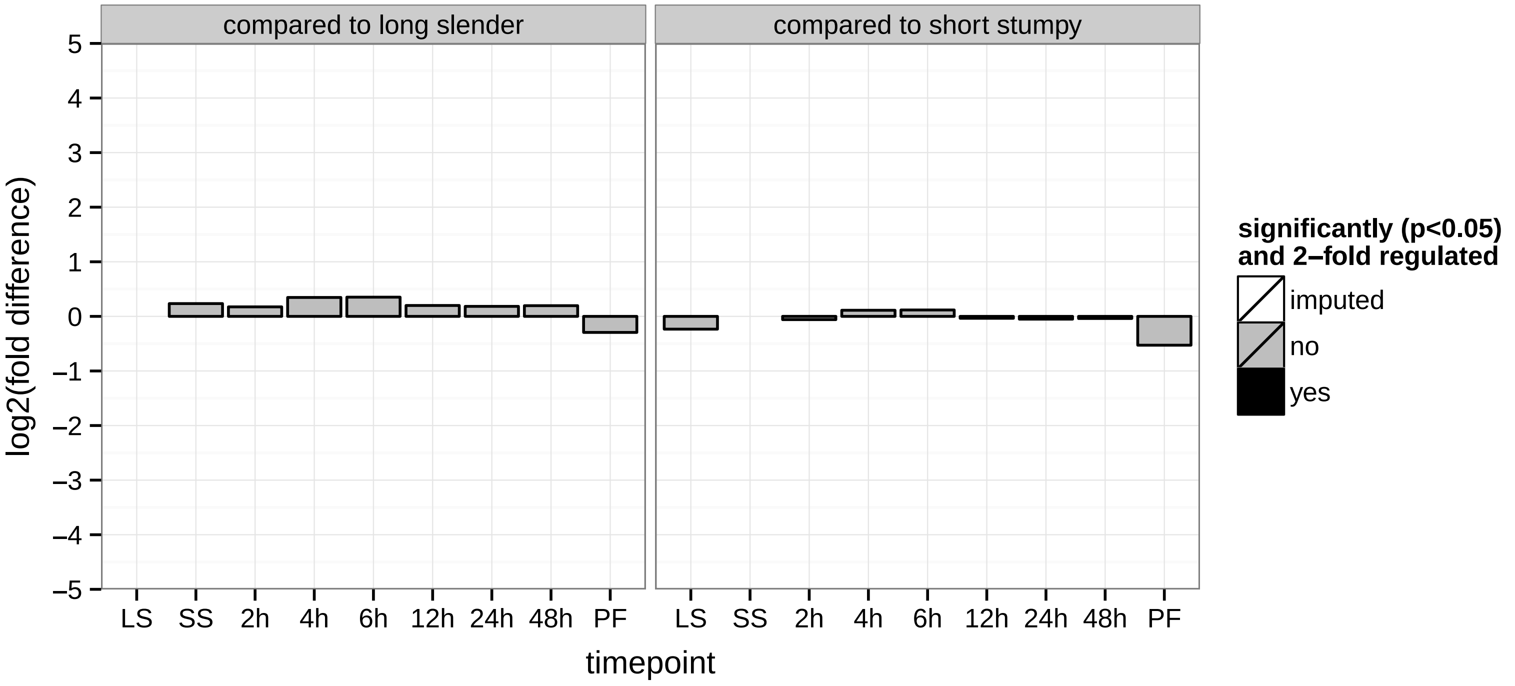

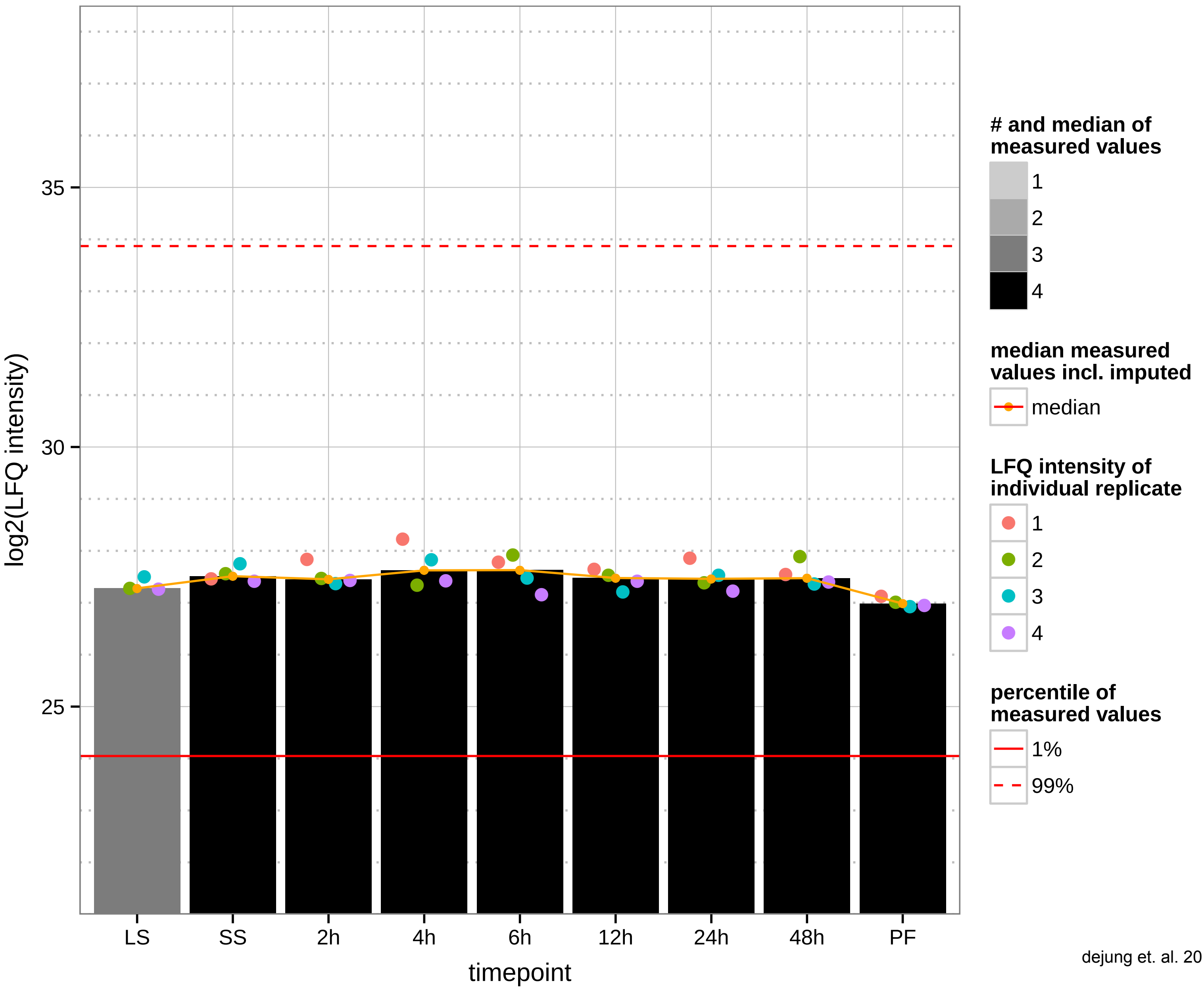

dejung et. al. 2015

hypothetical protein, conserved
Tb927.10.8090
AGOF: null
AGOC: null
AGOP: null
PGOF: null
PGOC: null
PGOP: null

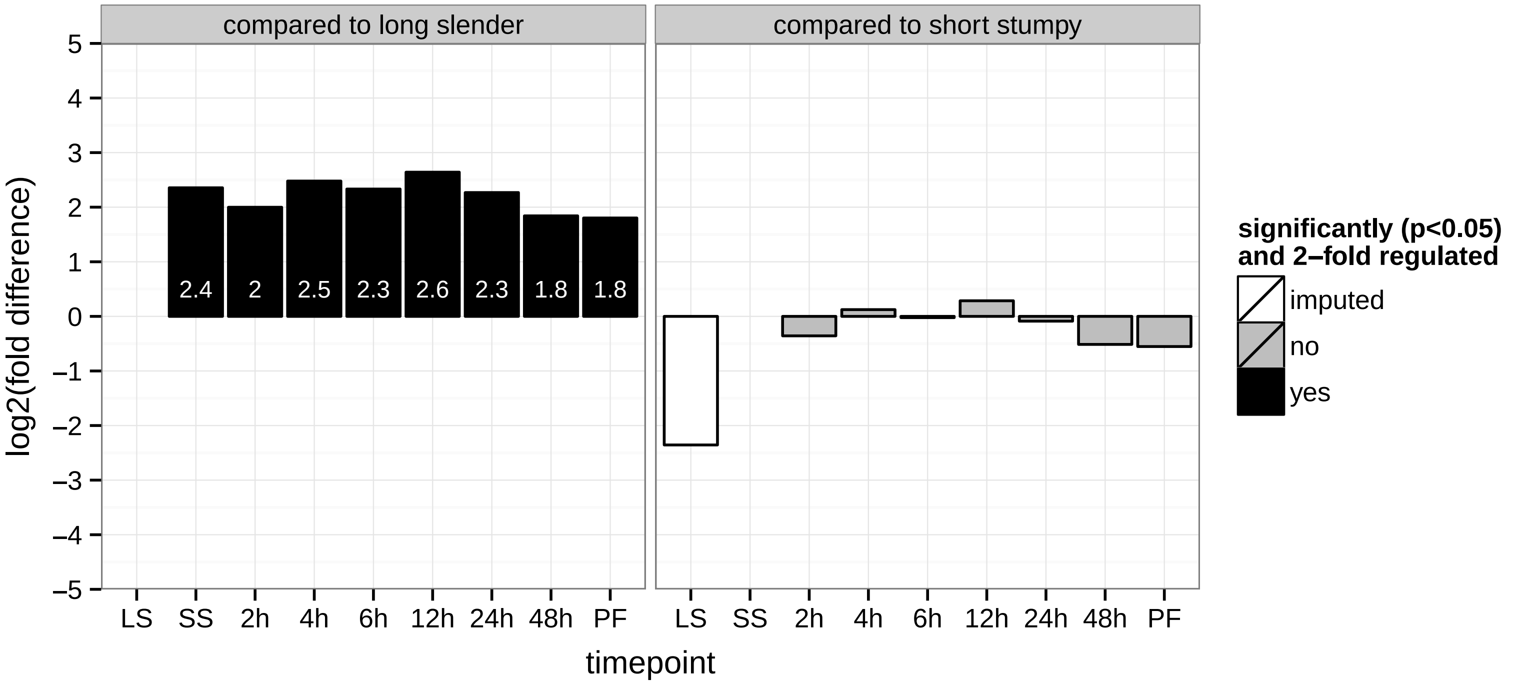

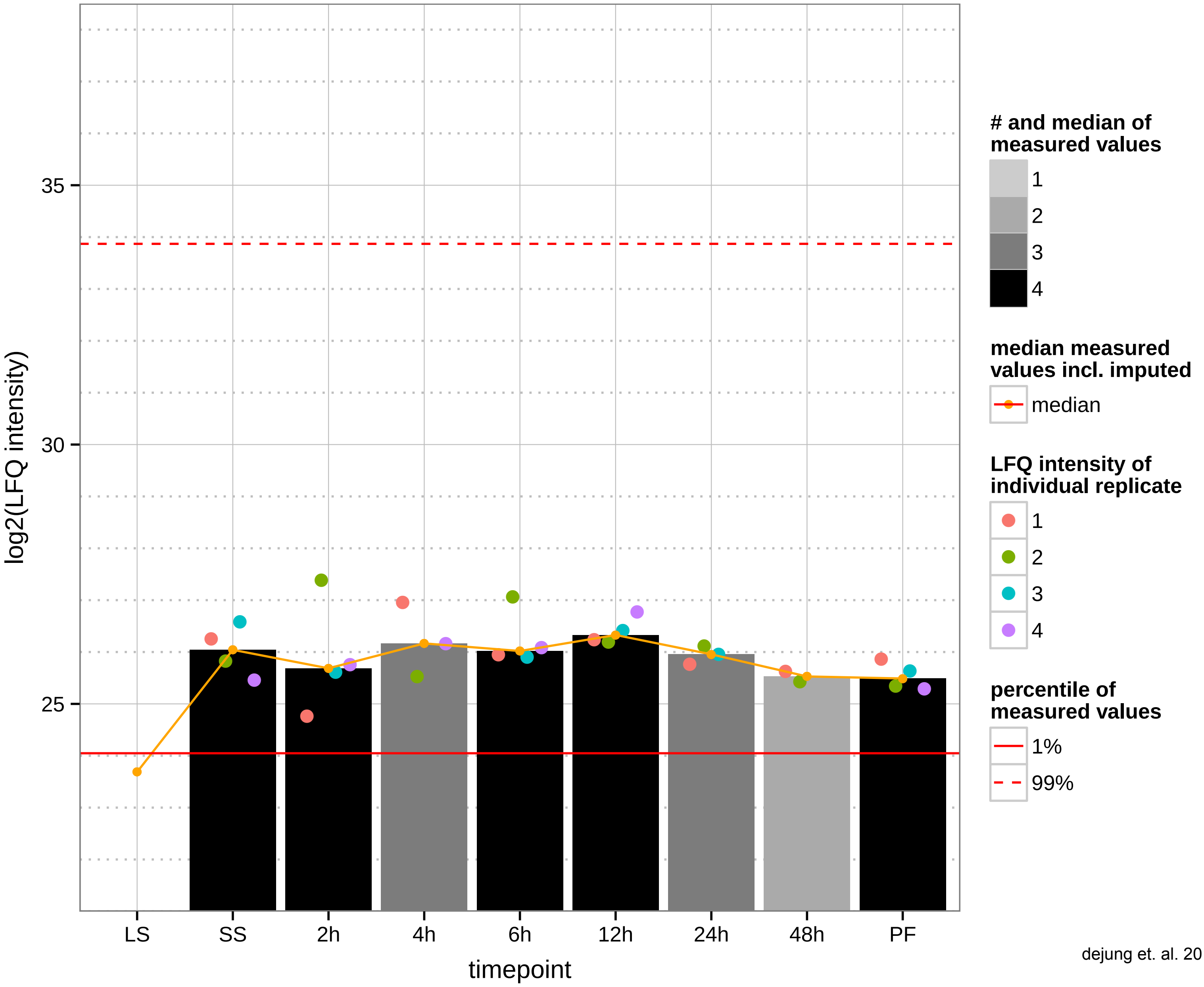

dejung et. al. 2015

hypothetical protein, conserved
Tb927.10.810
AGOF: null
AGOC: null
AGOP: null
PGOF: null
PGOC: null
PGOP: null

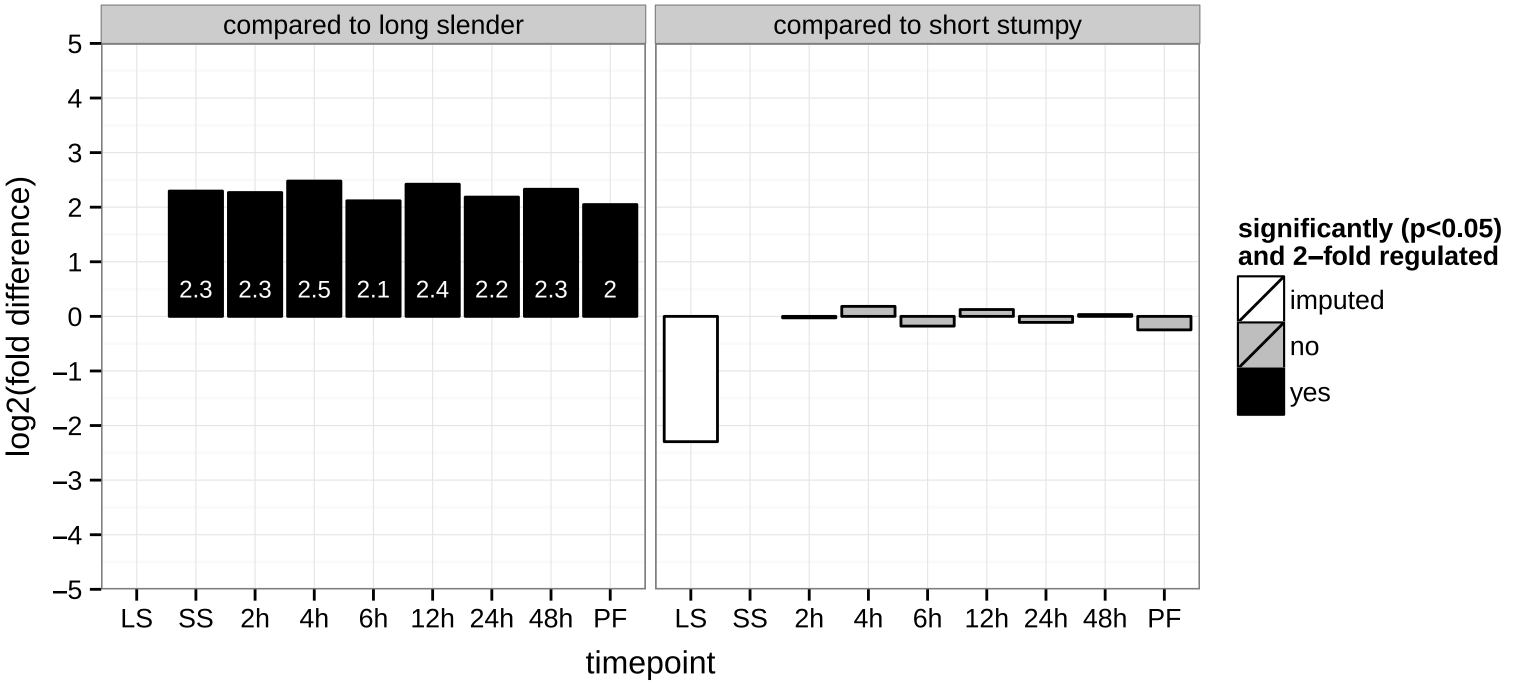

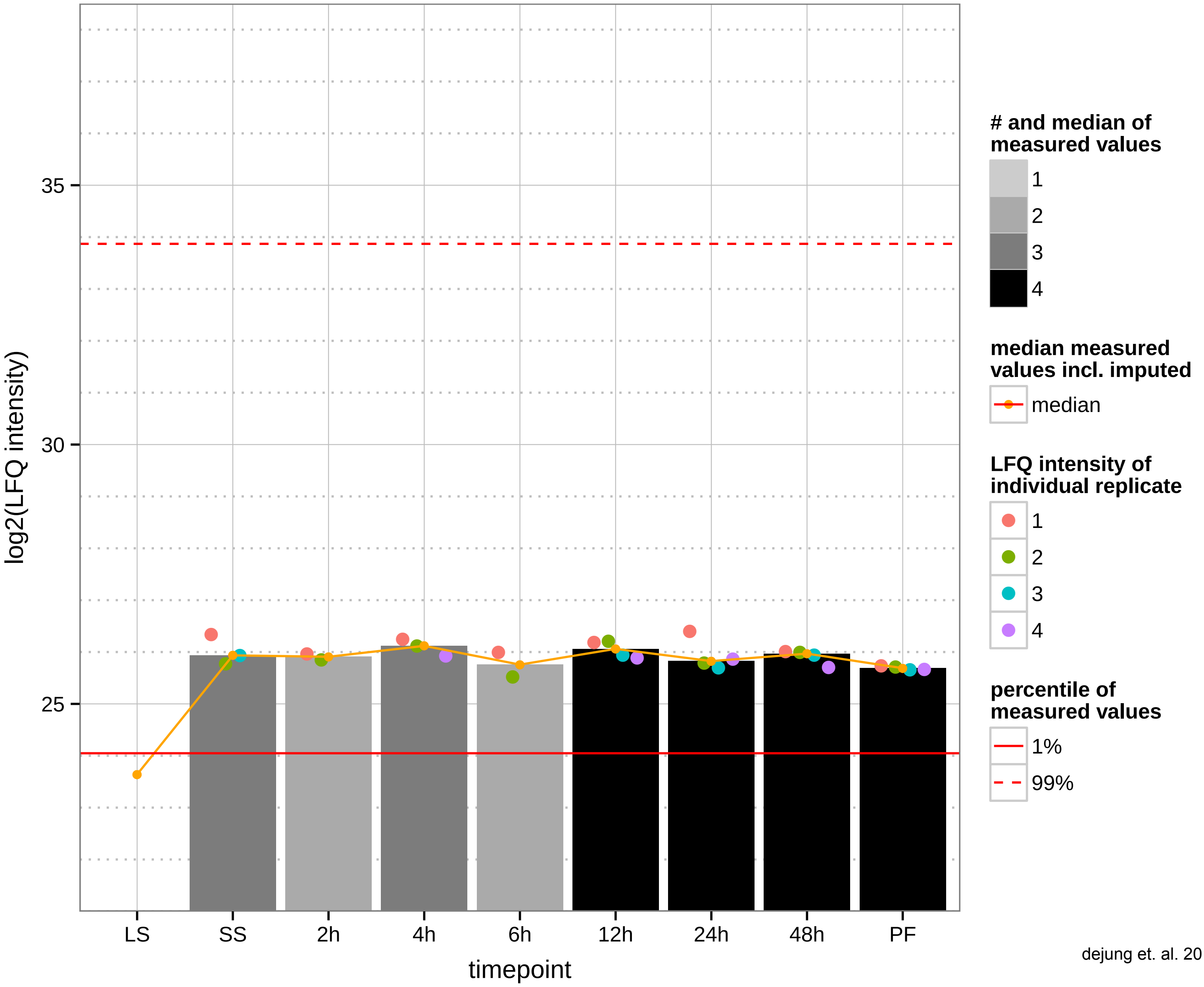

dejung et. al. 2015

hypothetical protein, conserved
Tb927.10.8110
AGOF: null
AGOC: mitochondrion
AGOP: null
PGOF: null
PGOC: null
PGOP: null

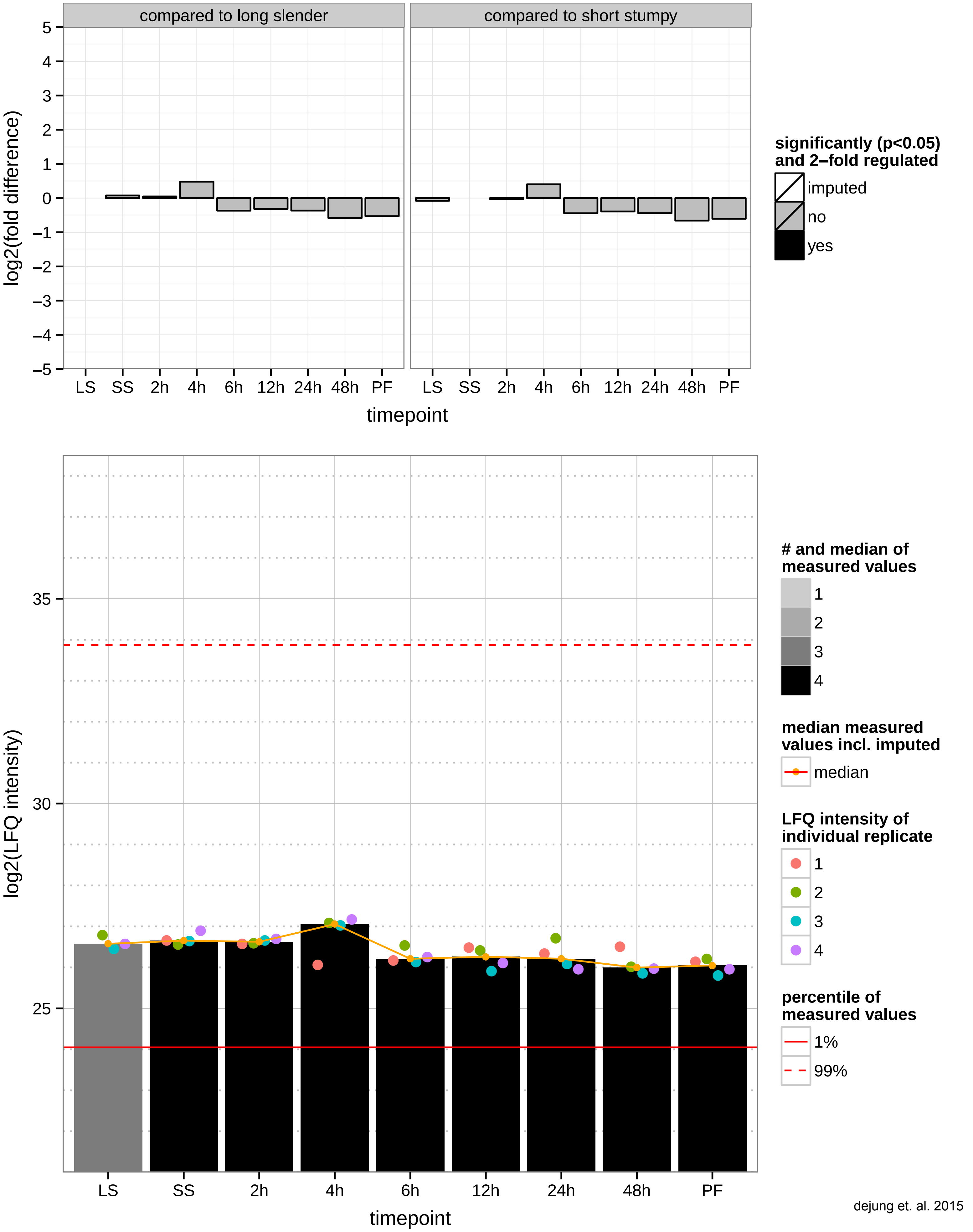

dejung et. al. 2015

nuclear pore complex protein (NUP155), putative, nucleoporin
Tb927.10.8170
AGOF: structural constituent of nuclear pore
AGOC: nuclear pore
AGOP: nucleocytoplasmic transport
PGOF: null
PGOC: null
PGOP: null

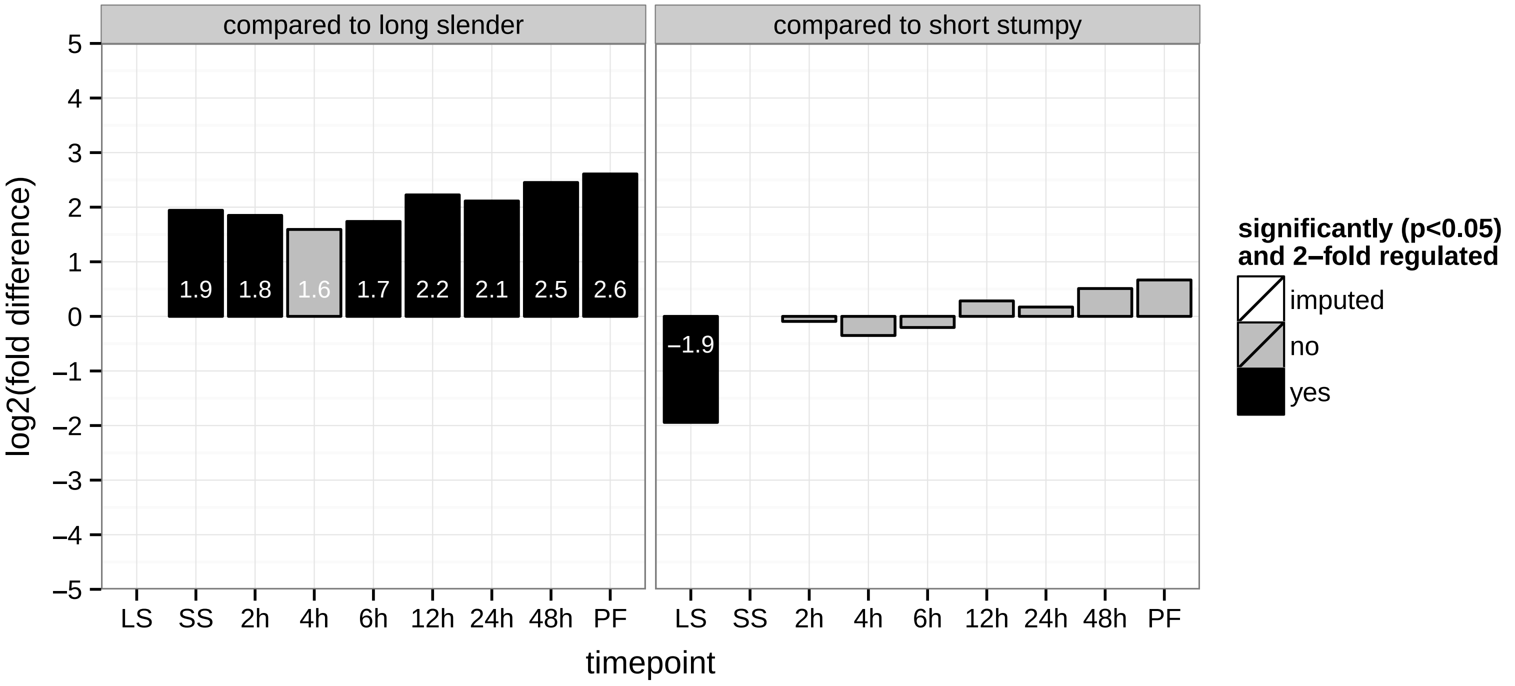

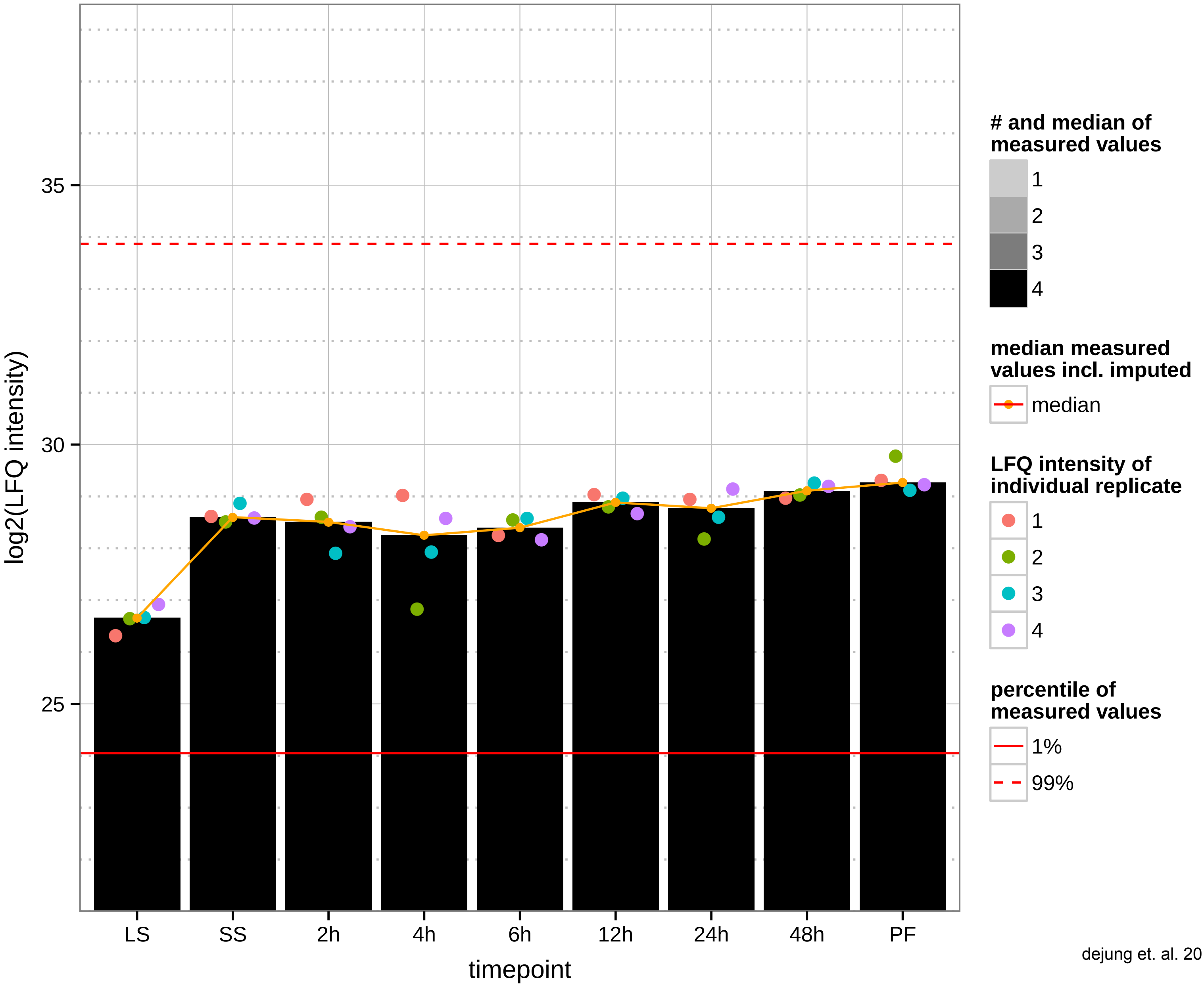

dejung et. al. 2015

KREPA2, RNA–editing complex protein (KREPA2)
Tb927.10.8210
AGOF: zinc ion binding
AGOC: intracellular, mRNA editing complex, mitochondrial mRNA editing complex, mitochondrion
AGOP: mRNA modification
PGOF: zinc ion binding
PGOC: intracellular
PGOP: null

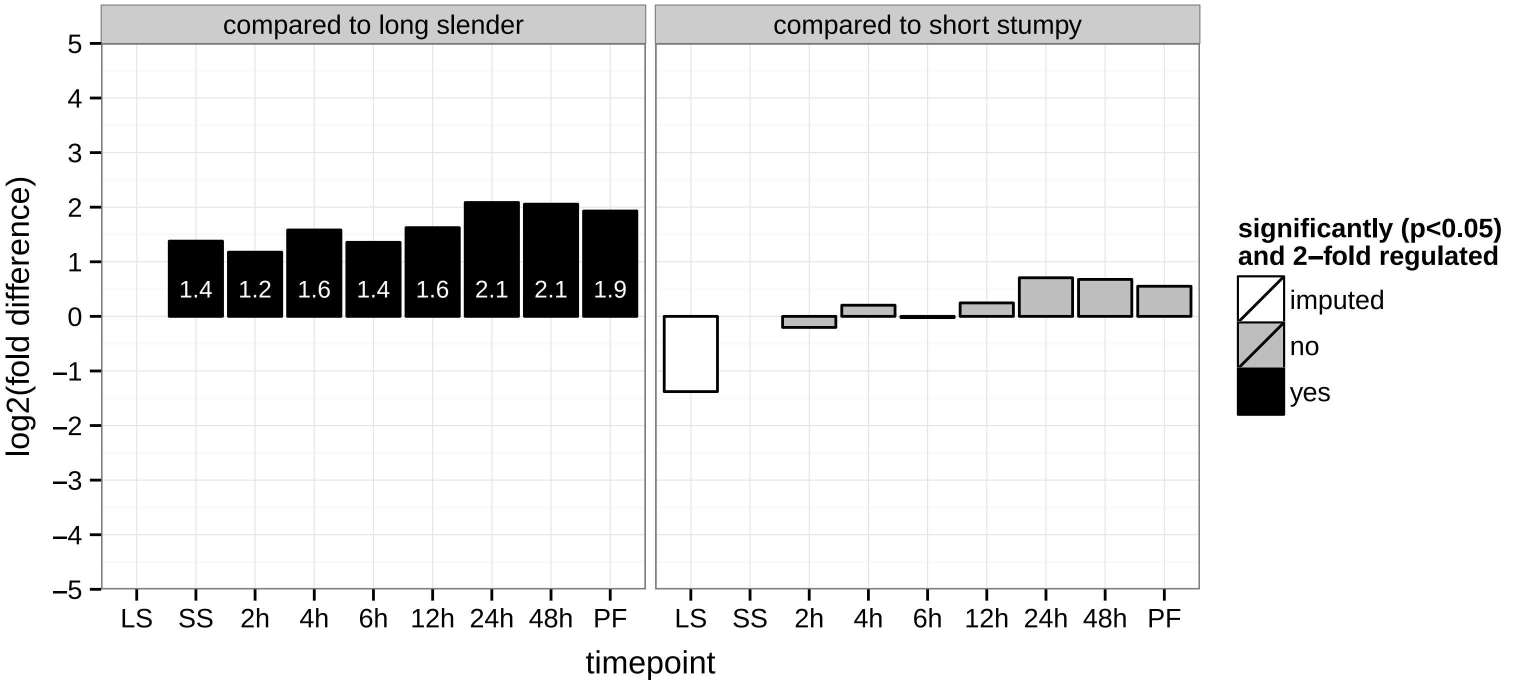

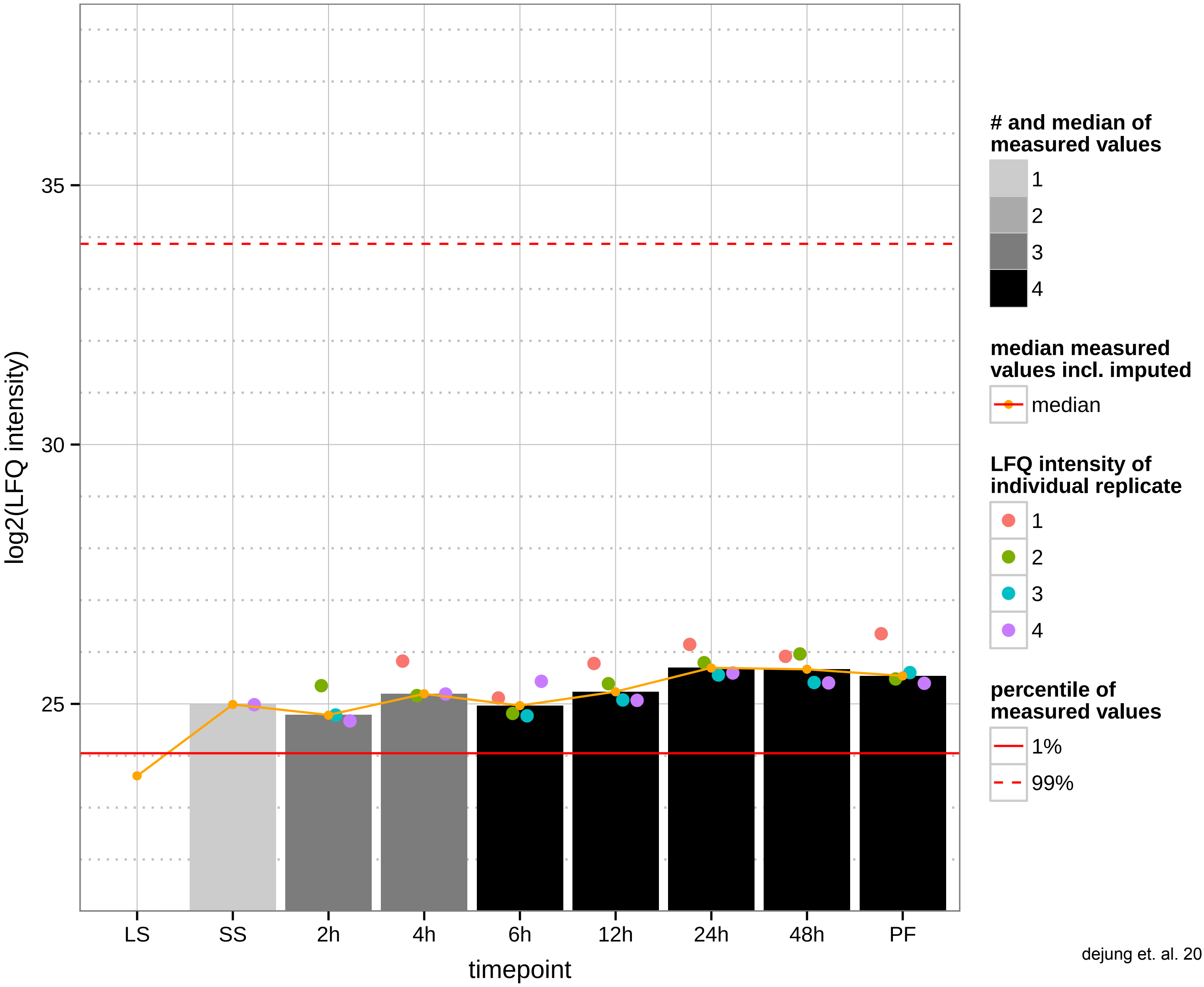

dejung et. al. 2015

eukaryotic translation initiation factor 3 subunit 8, putative
Tb927.10.8290;Tb927.10.8270;Tb11.v5.0877
AGOF: null, ribosomal small subunit binding, translation initiation factor activity
AGOC: null, eukaryotic translation initiation factor 3 complex
AGOP: null, translational initiation
PGOF: translation initiation factor activity
PGOC: eukaryotic translation initiation factor 3 complex
PGOP: translational initiation

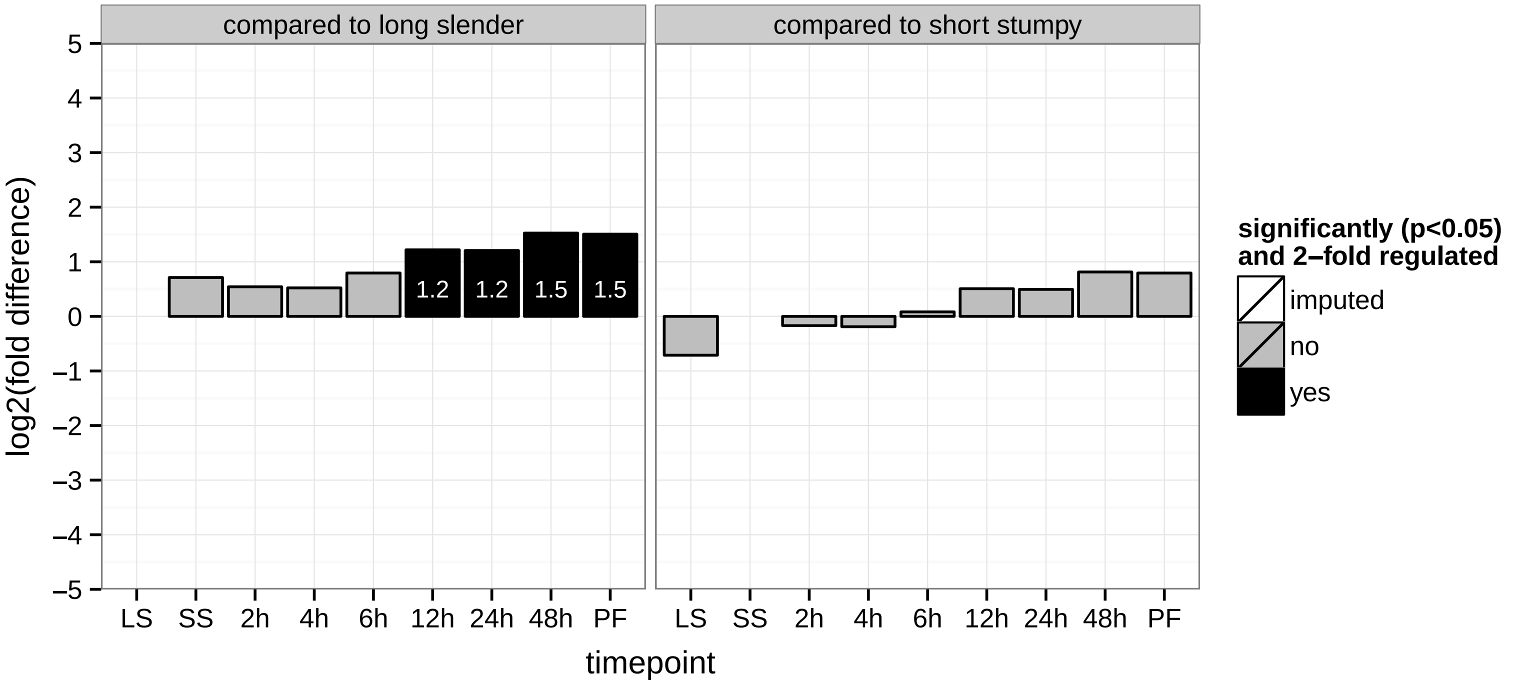

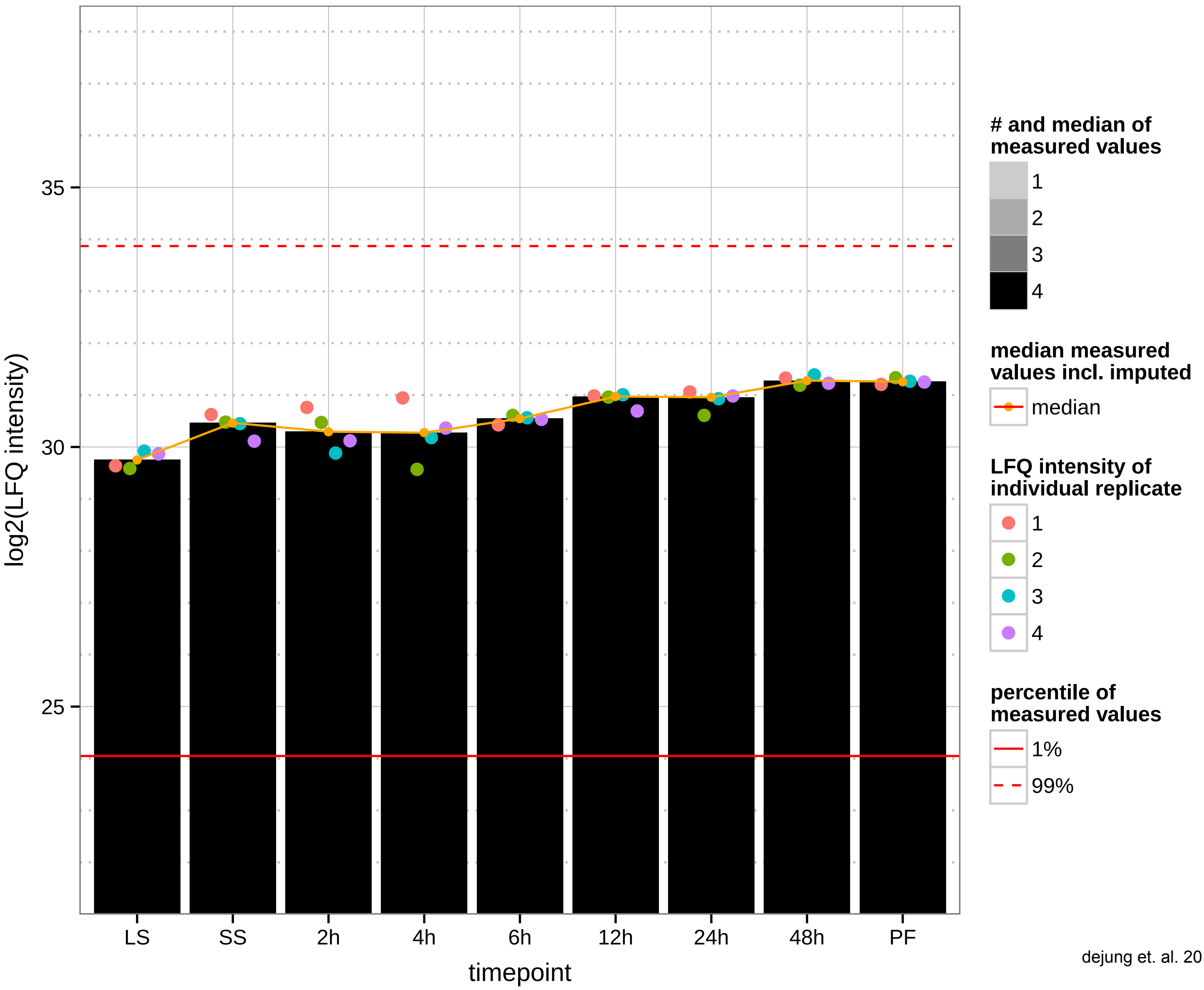

dejung et. al. 2015

adenylate kinase, putative
Tb927.10.830
AGOF: ATP binding, adenylate kinase activity
AGOC: cytosol, glycosome
AGOP: nucleobase−containing compound metabolic process
PGOF: ATP binding, adenylate kinase activity, cAMP−dependent protein kinase regulator activity, nucleobase−containing com
PGOC: null
PGOP: nucleobase−containing compound metabolic process, nucleotide phosphorylation, signal transduction

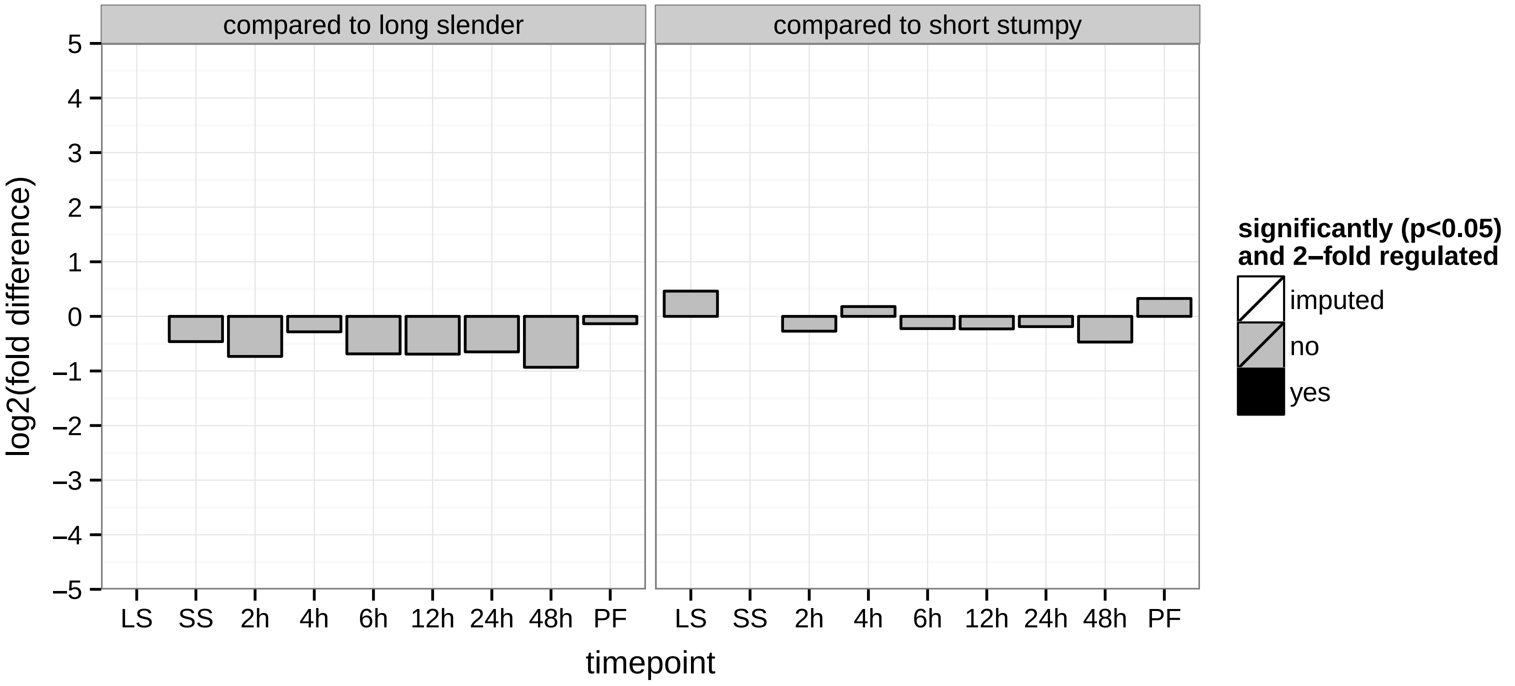

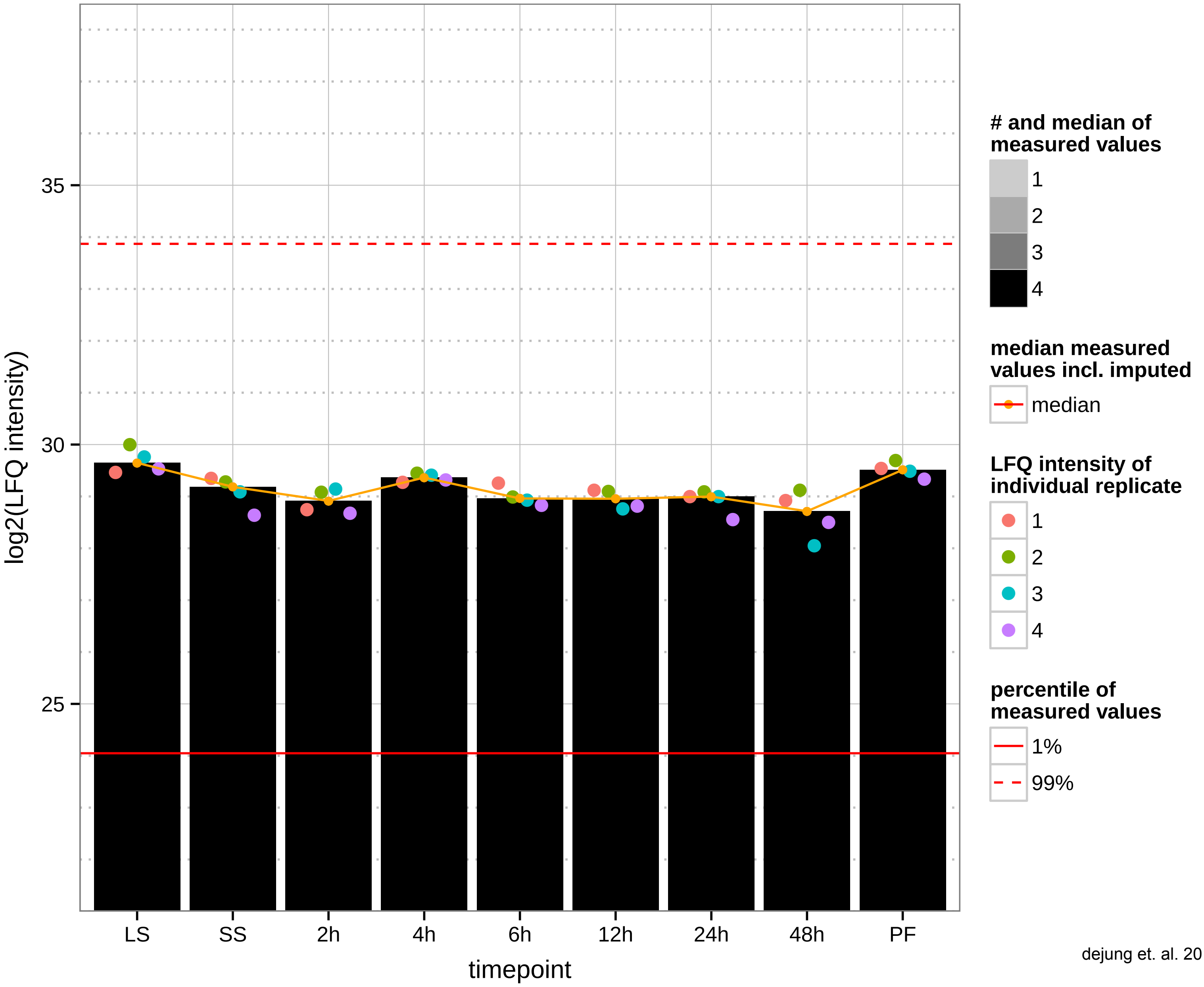

dejung et. al. 2015

hypothetical protein, conserved
Tb927.10.8410
AGOF: null
AGOC: null
AGOP: null
PGOF: null
PGOC: null
PGOP: null

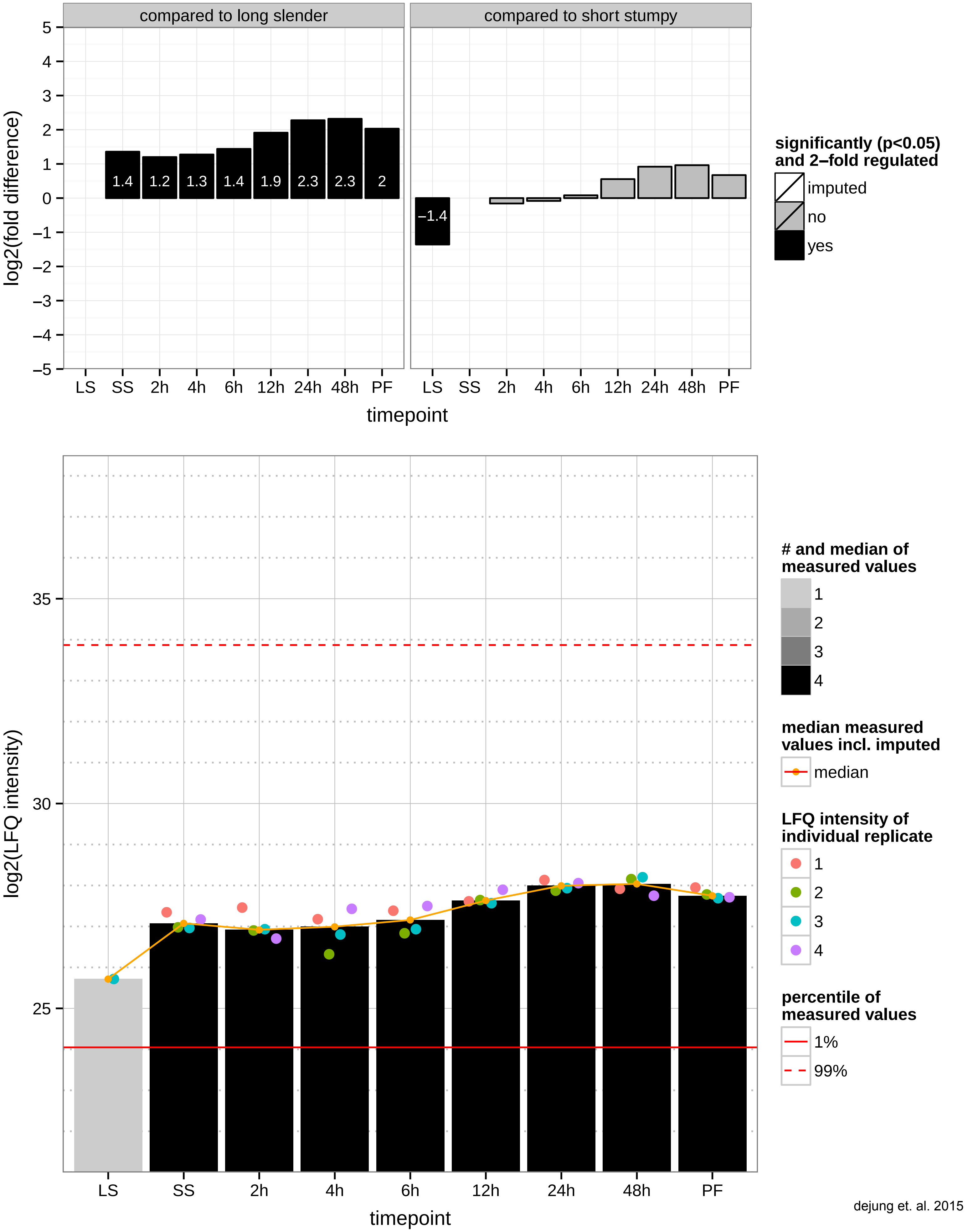

target of rapamycin (TOR) kinase 1 (TOR1)
Tb927.10.8420
AGOF: phosphotransferase activity, alcohol group as acceptor
AGOC: null
AGOP: growth, intracellular signal transduction
PGOF: binding, phosphotransferase activity, alcohol group as acceptor, protein binding, transferase activity, transferring phosp
PGOC: null
PGOP: null

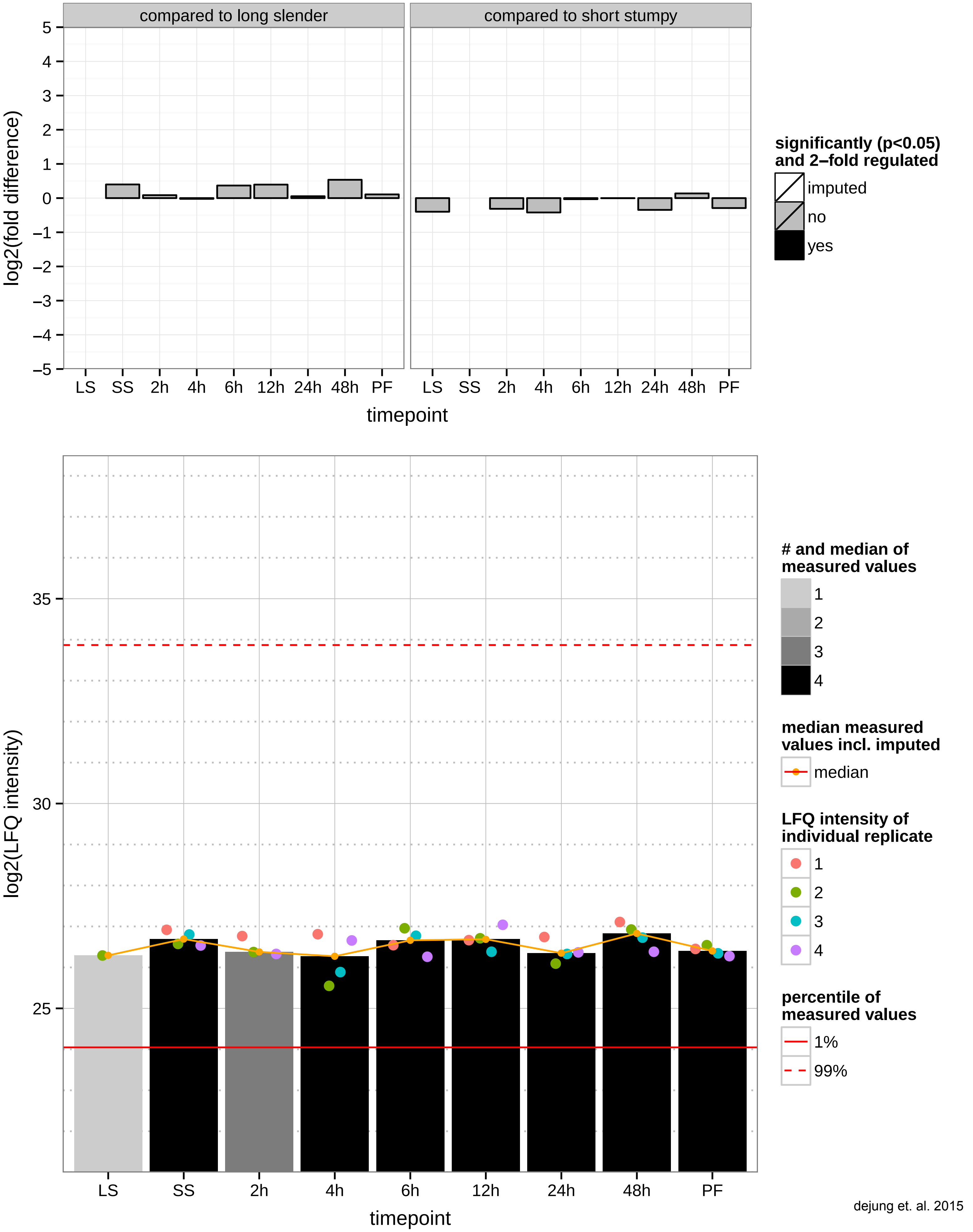

dejung et. al. 2015

hypothetical protein, conserved
Tb927.10.850
AGOF: null
AGOC: null
AGOP: null
PGOF: protein binding
PGOC: null
PGOP: null

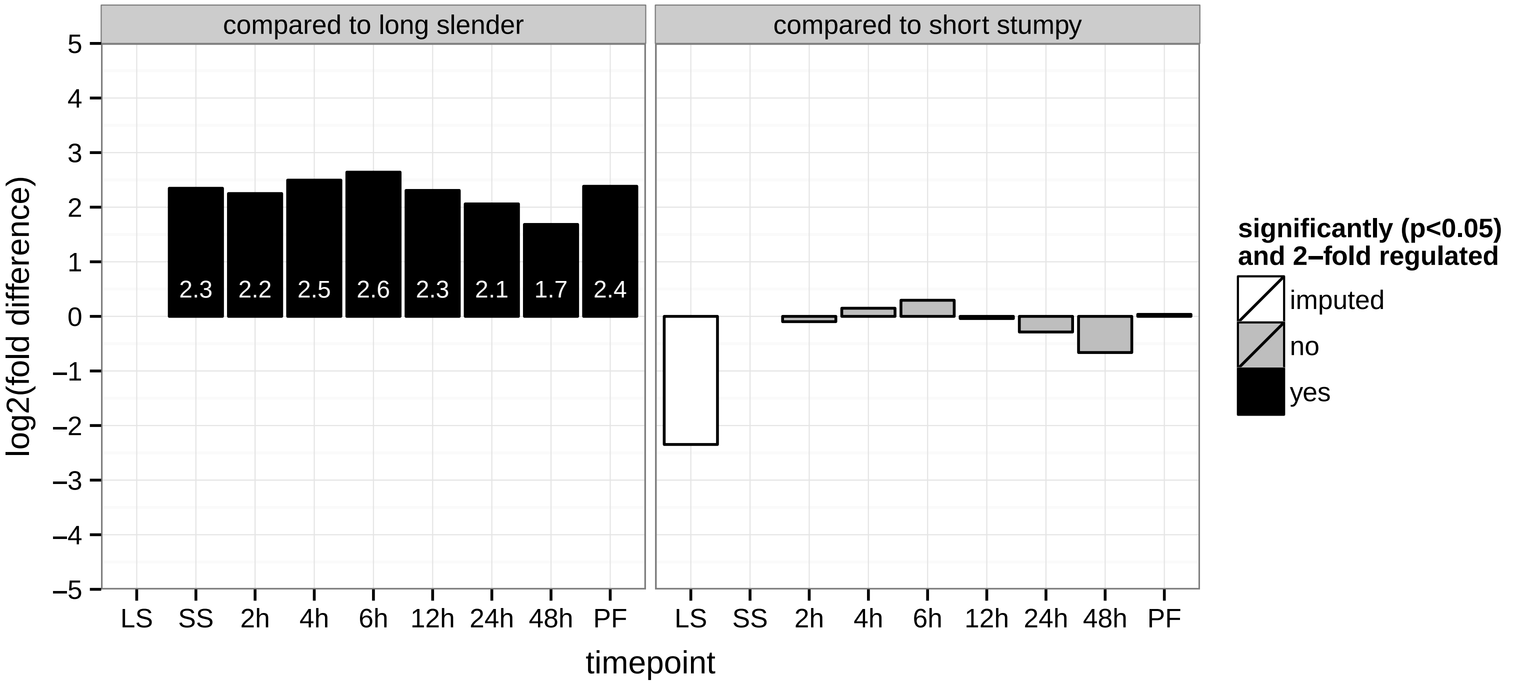

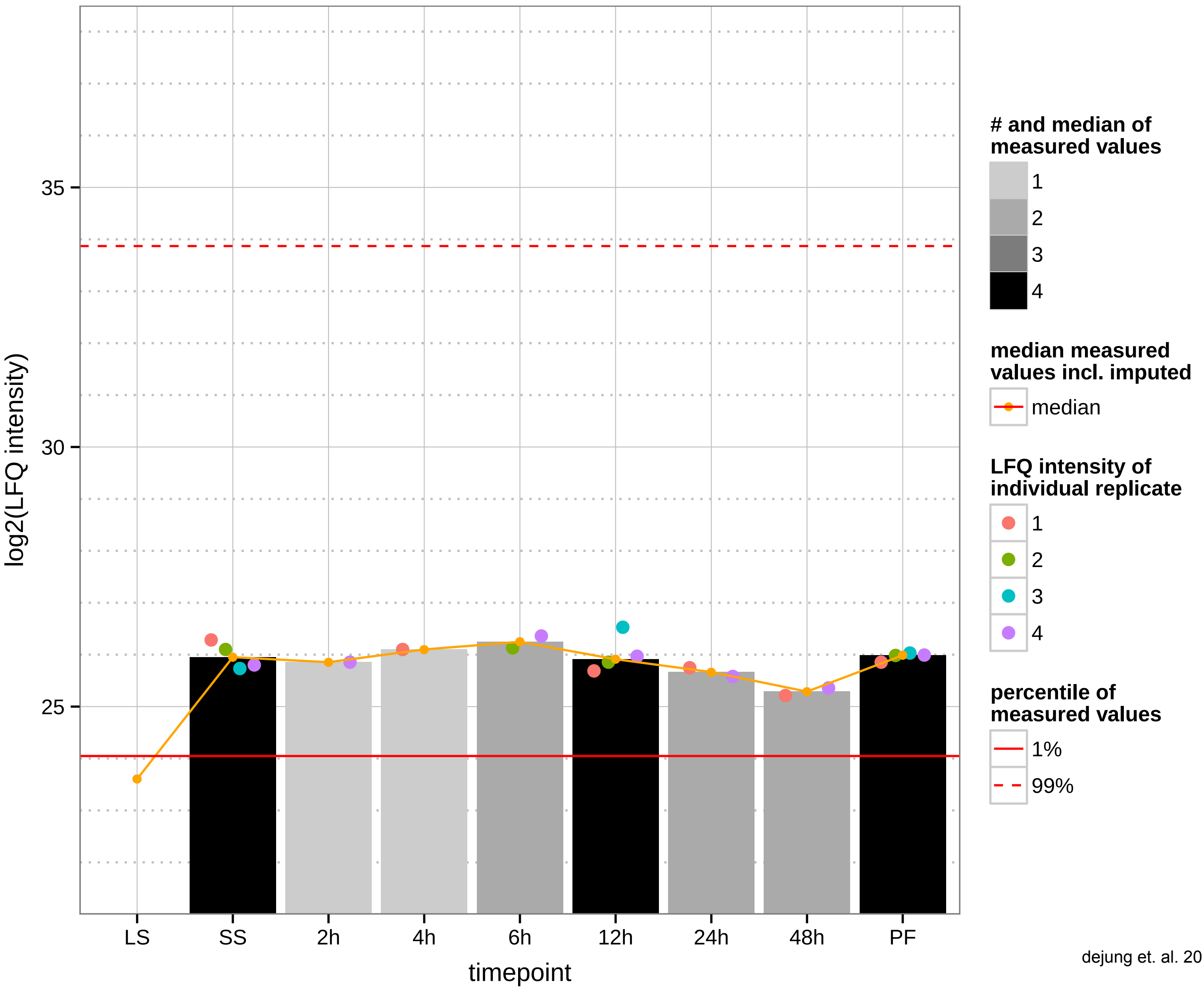

dejung et. al. 2015

flagellum transition zone component, putative, flagellum transition zone component (FTZC)
Tb927.10.8590
AGOF: null
AGOC: null, axoneme
AGOP: null
PGOF: null
PGOC: null
PGOP: null

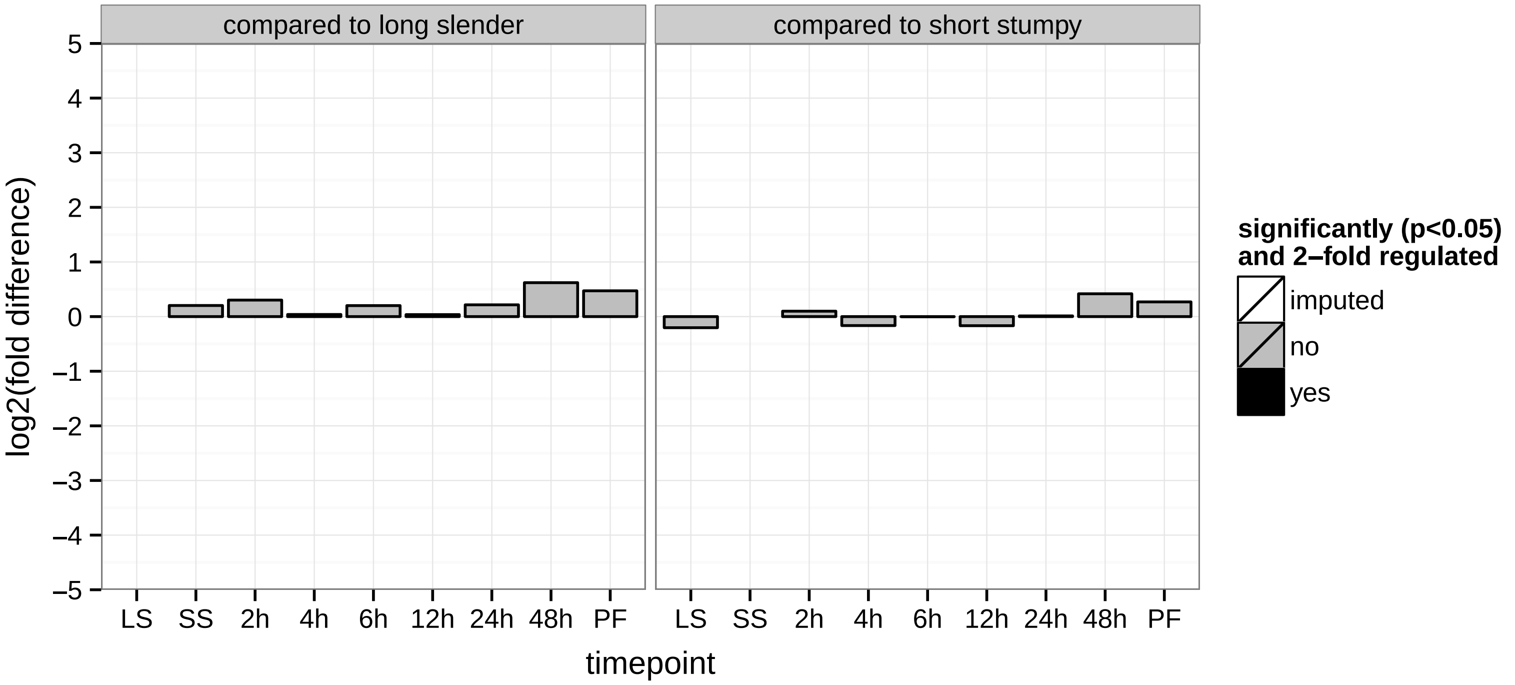

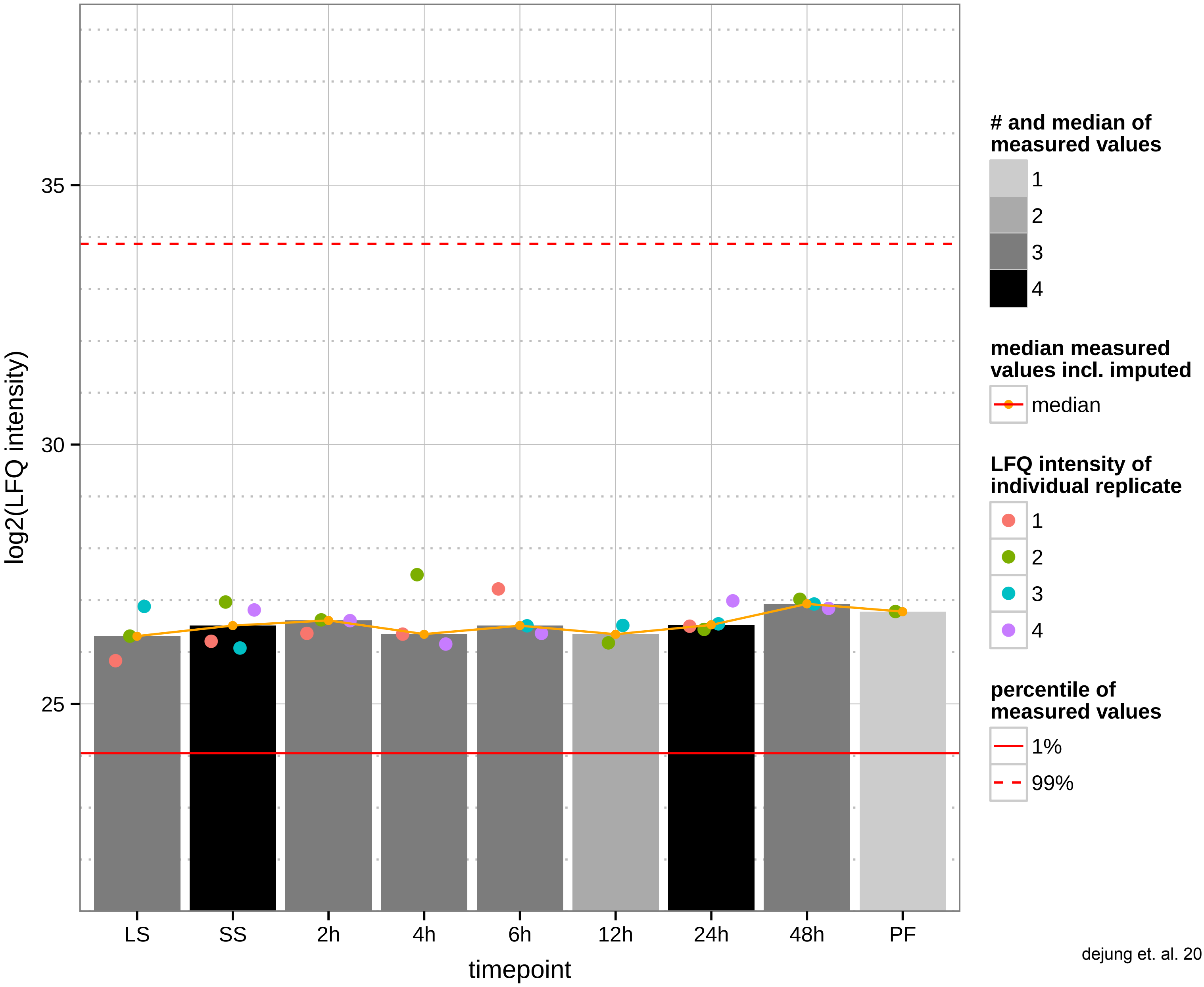

dejung et. al. 2015

hypothetical protein, conserved
Tb927.10.8640
AGOF: null
AGOC: null
AGOP: null
PGOF: null
PGOC: null
PGOP: null

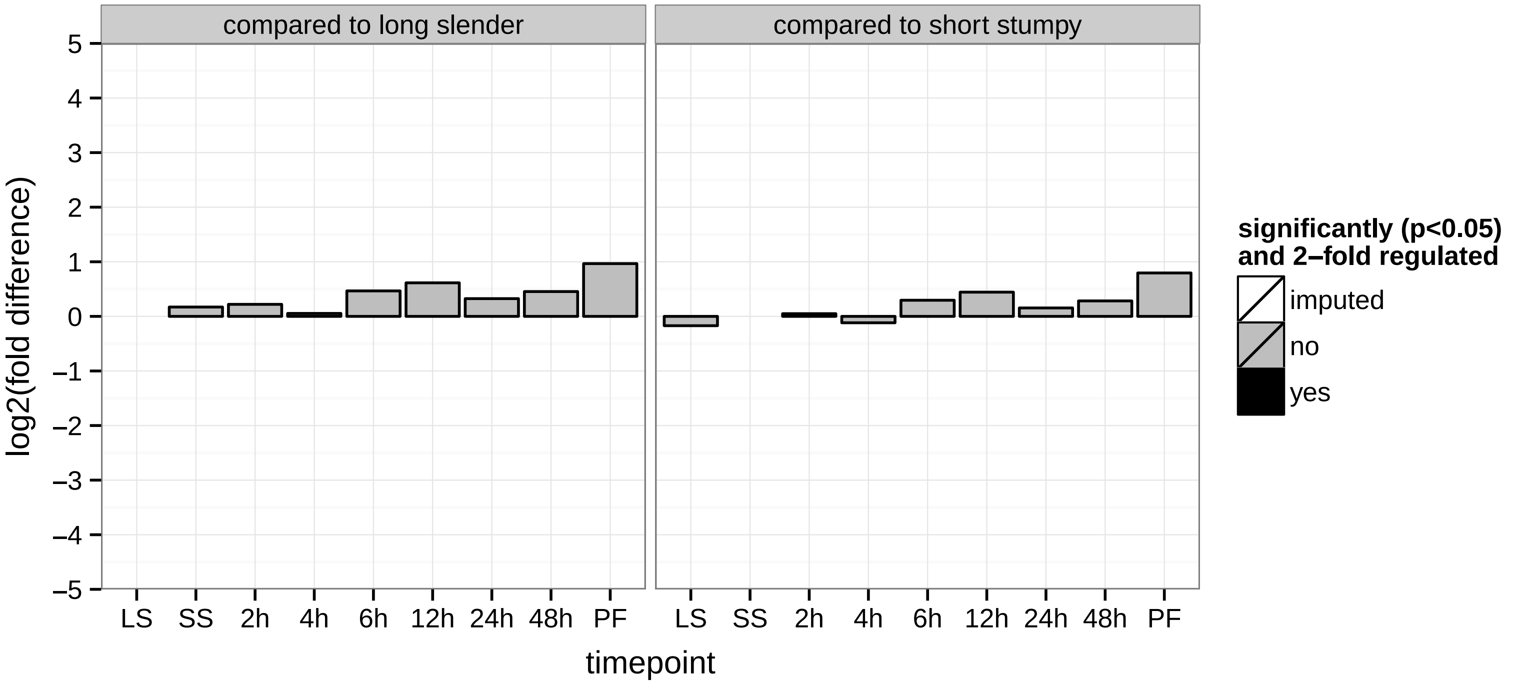

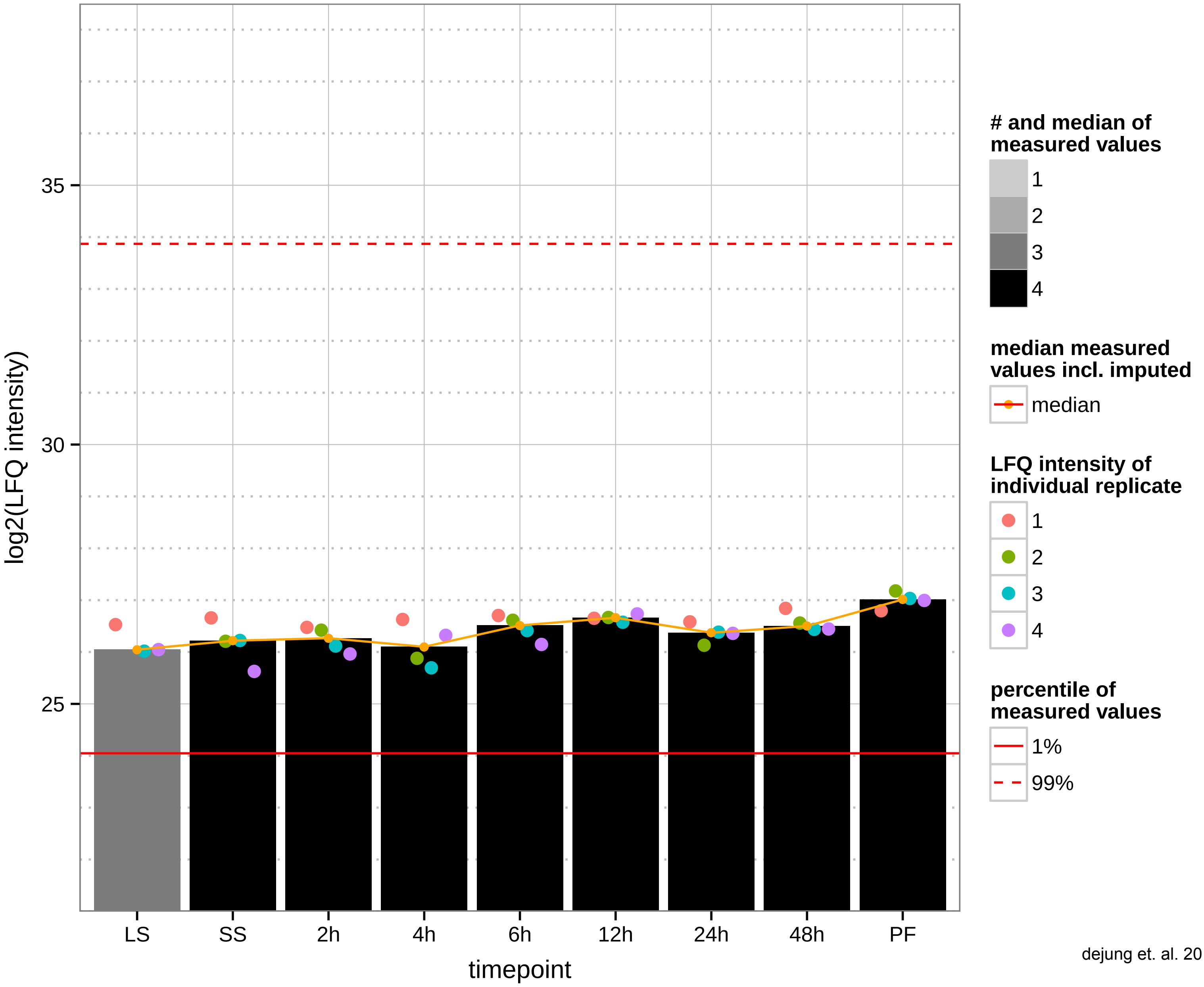

dejung et. al. 2015

hypothetical protein, conserved
Tb927.10.8660
AGOF: null
AGOC: null
AGOP: null
PGOF: protein binding
PGOC: null
PGOP: null

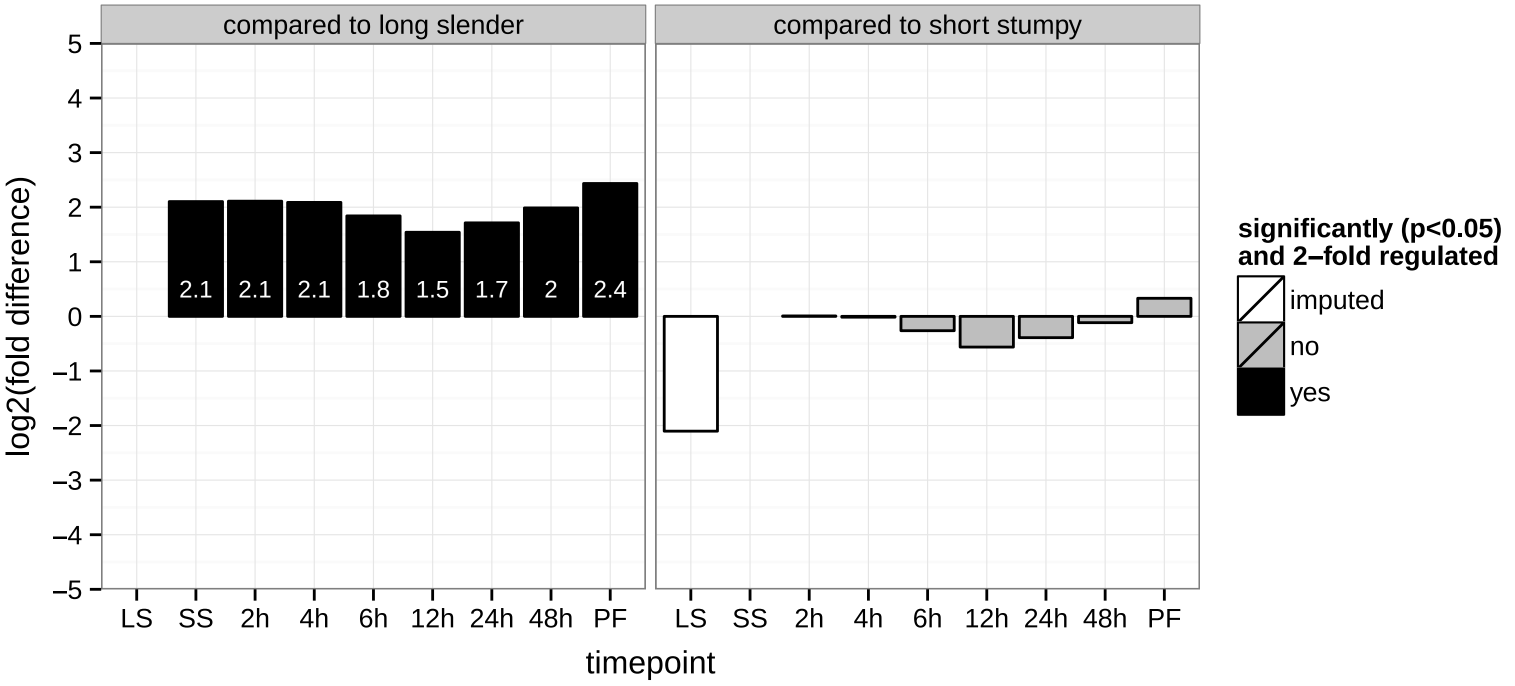

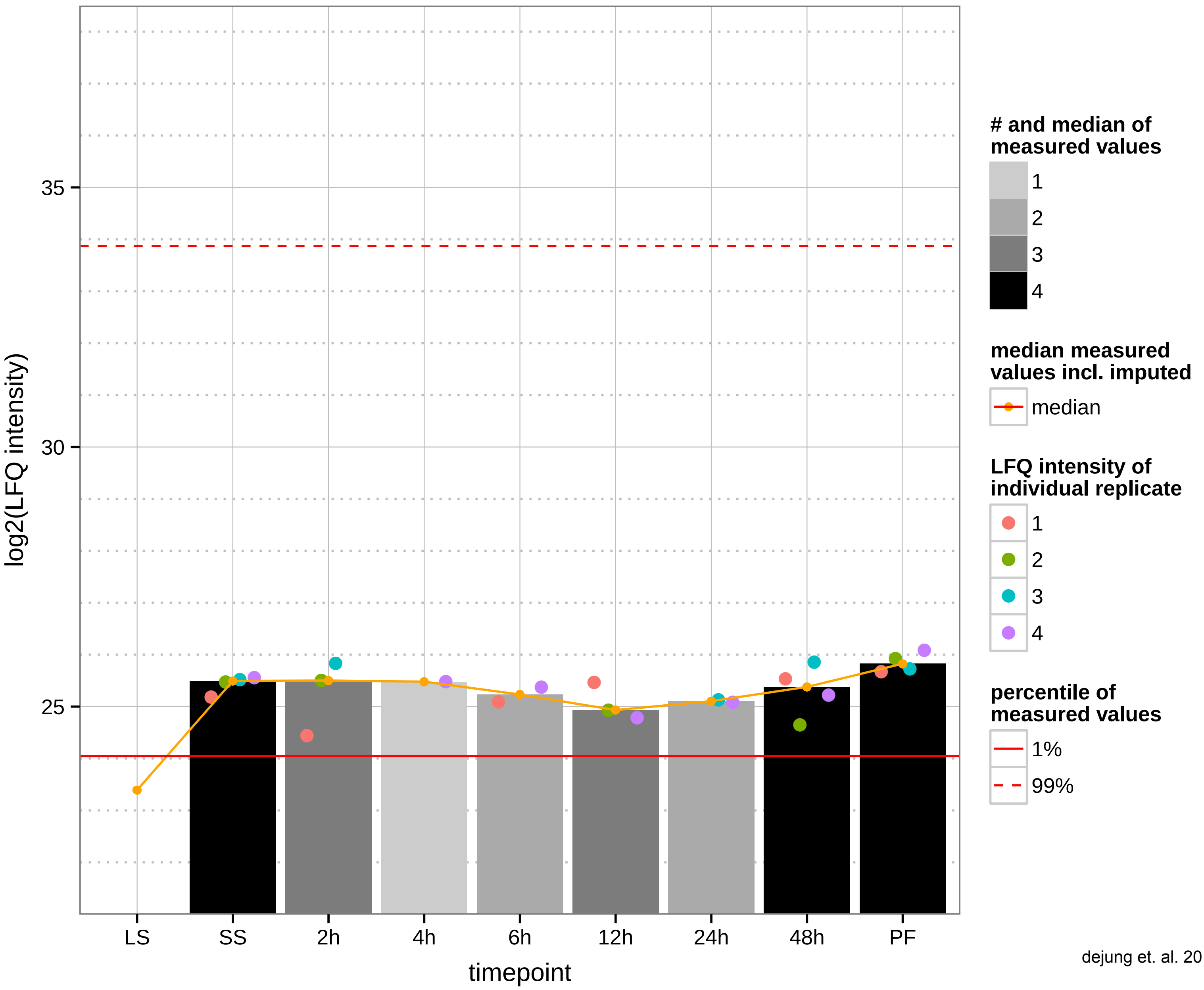

dejung et. al. 2015

CCR4–NOT transcription complex subunit 10, putative
Tb927.10.8720
AGOF: null
AGOC: cytoplasm
AGOP: null
PGOF: null
PGOC: null
PGOP: null

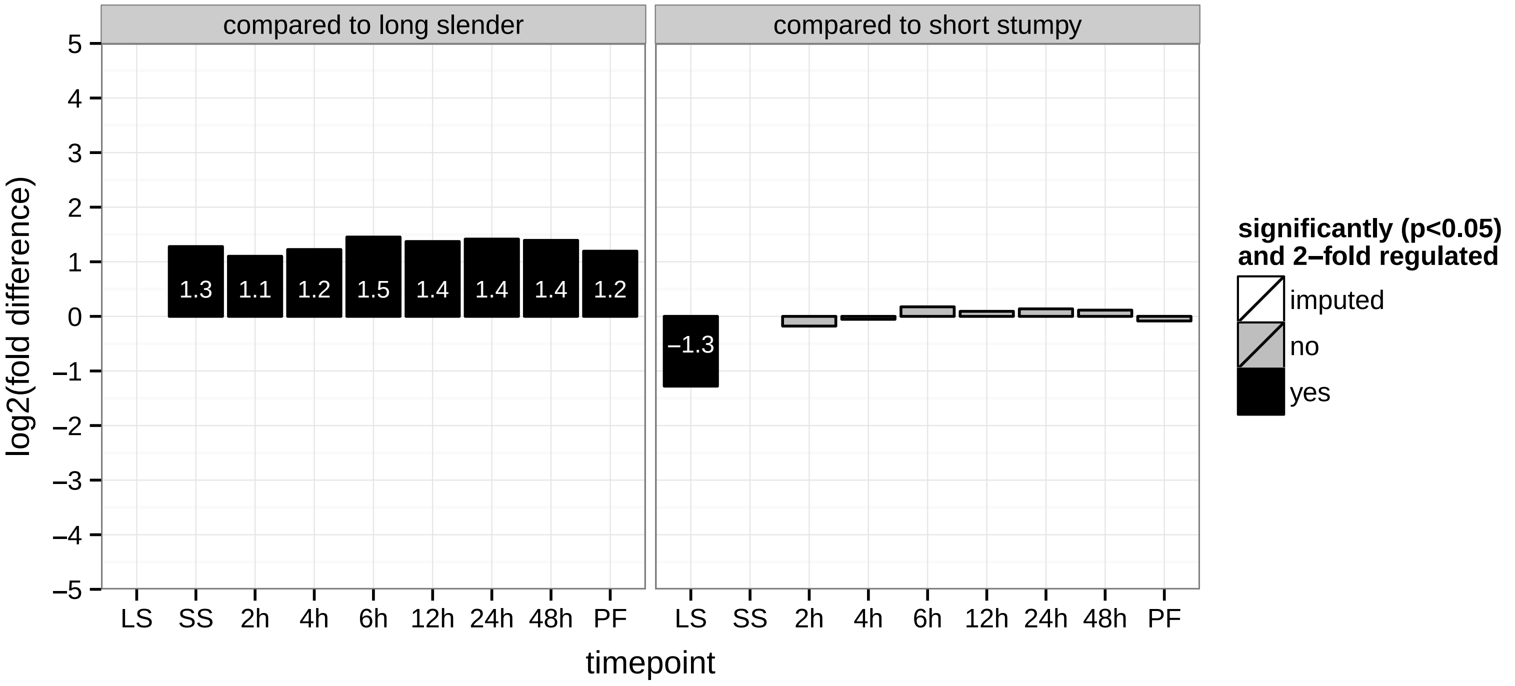

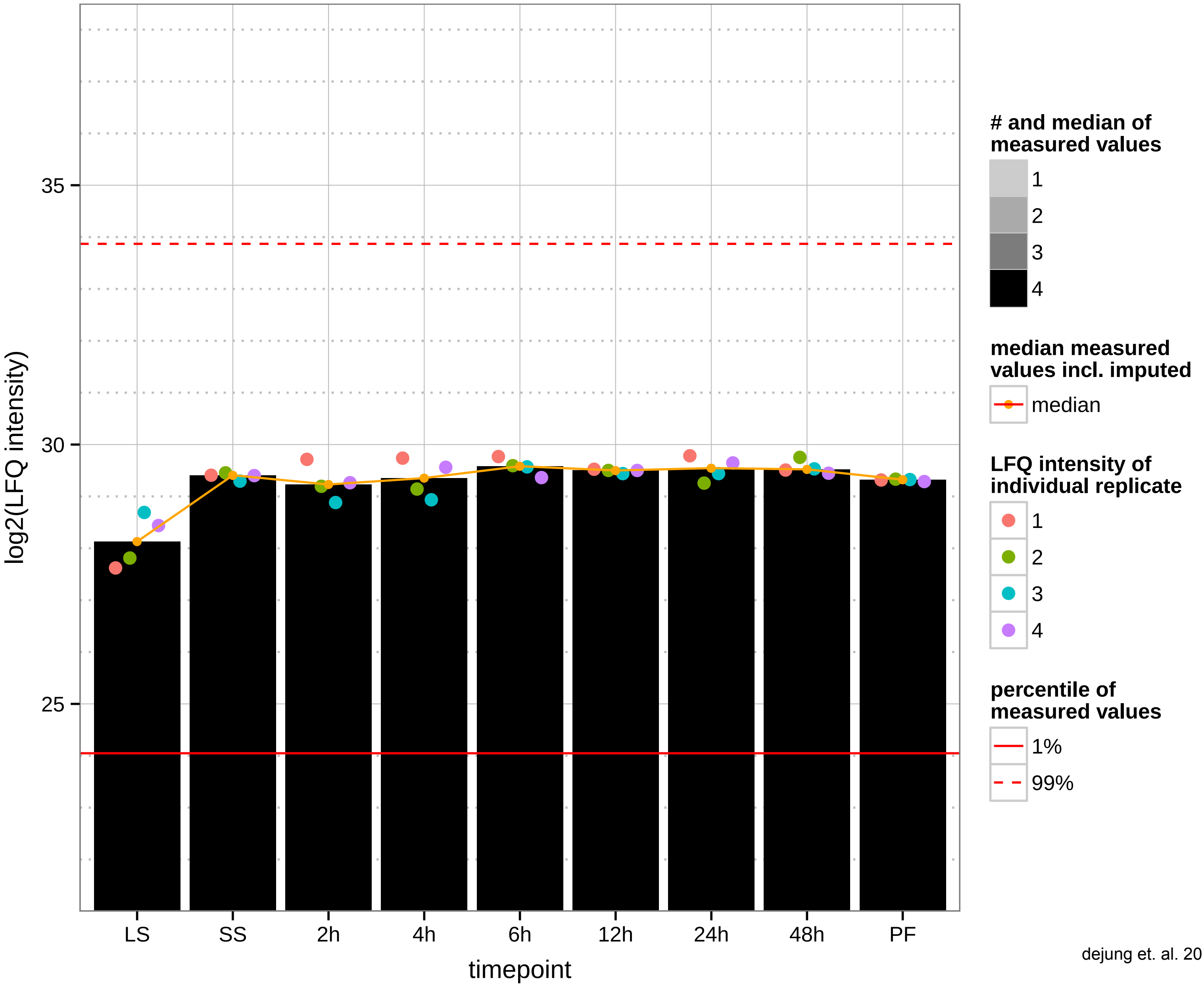

dejung et. al. 2015

hypothetical protein, conserved
Tb927.10.8780
AGOF: ATP binding, nucleobase–containing compound kinase activity
AGOC: null
AGOP: nucleobase–containing compound metabolic process
PGOF: null
PGOC: null
PGOP: null

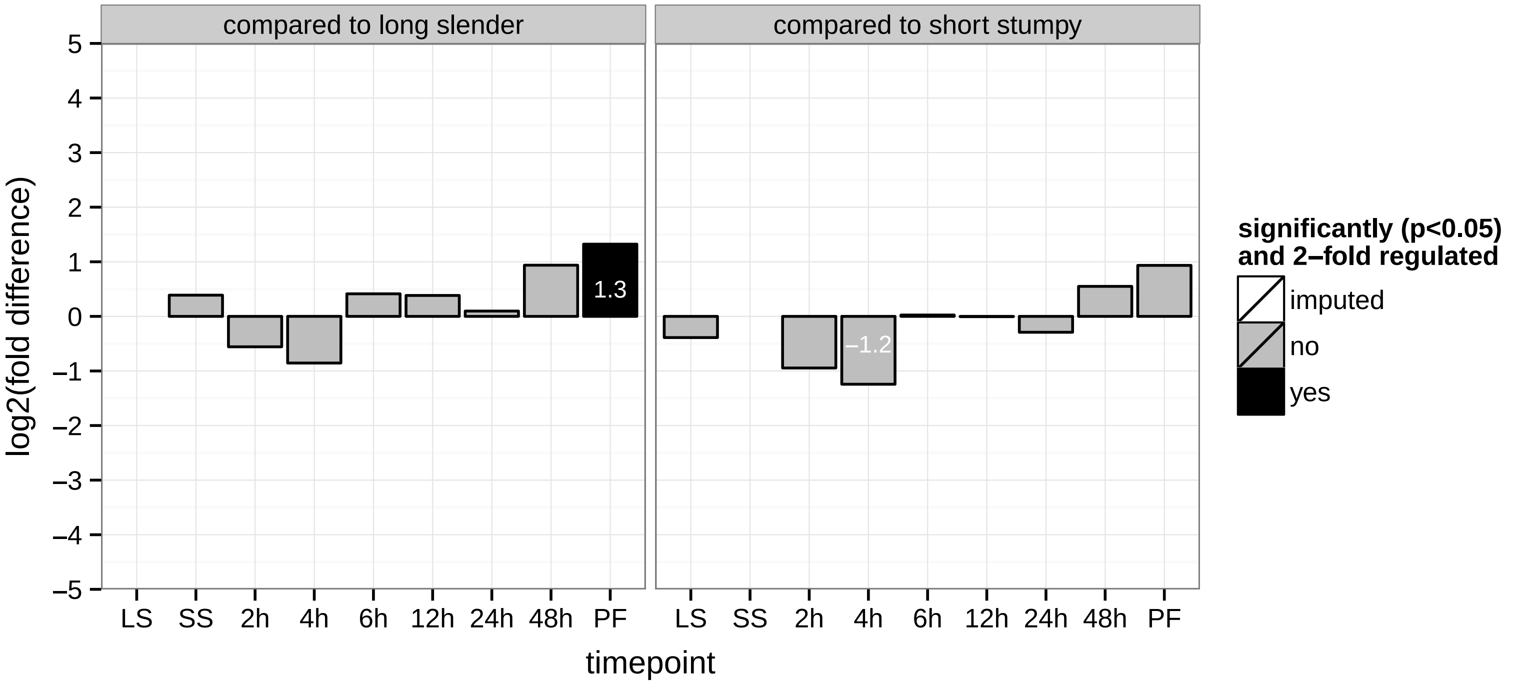

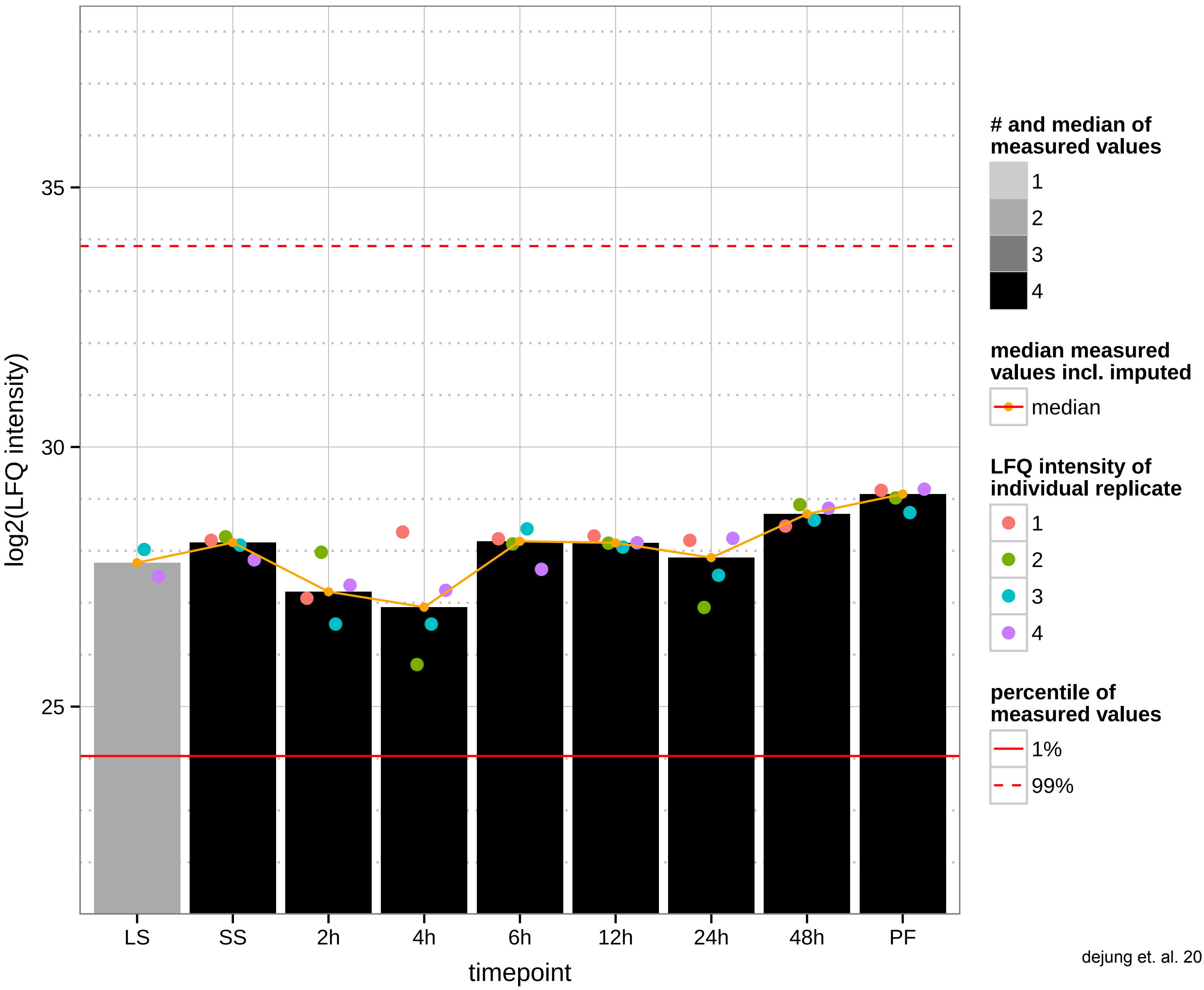

dejung et. al. 2015

hypothetical protein, conserved
Tb927.10.8790
AGOF: null
AGOC: null
AGOP: null
PGOF: null
PGOC: null
PGOP: null

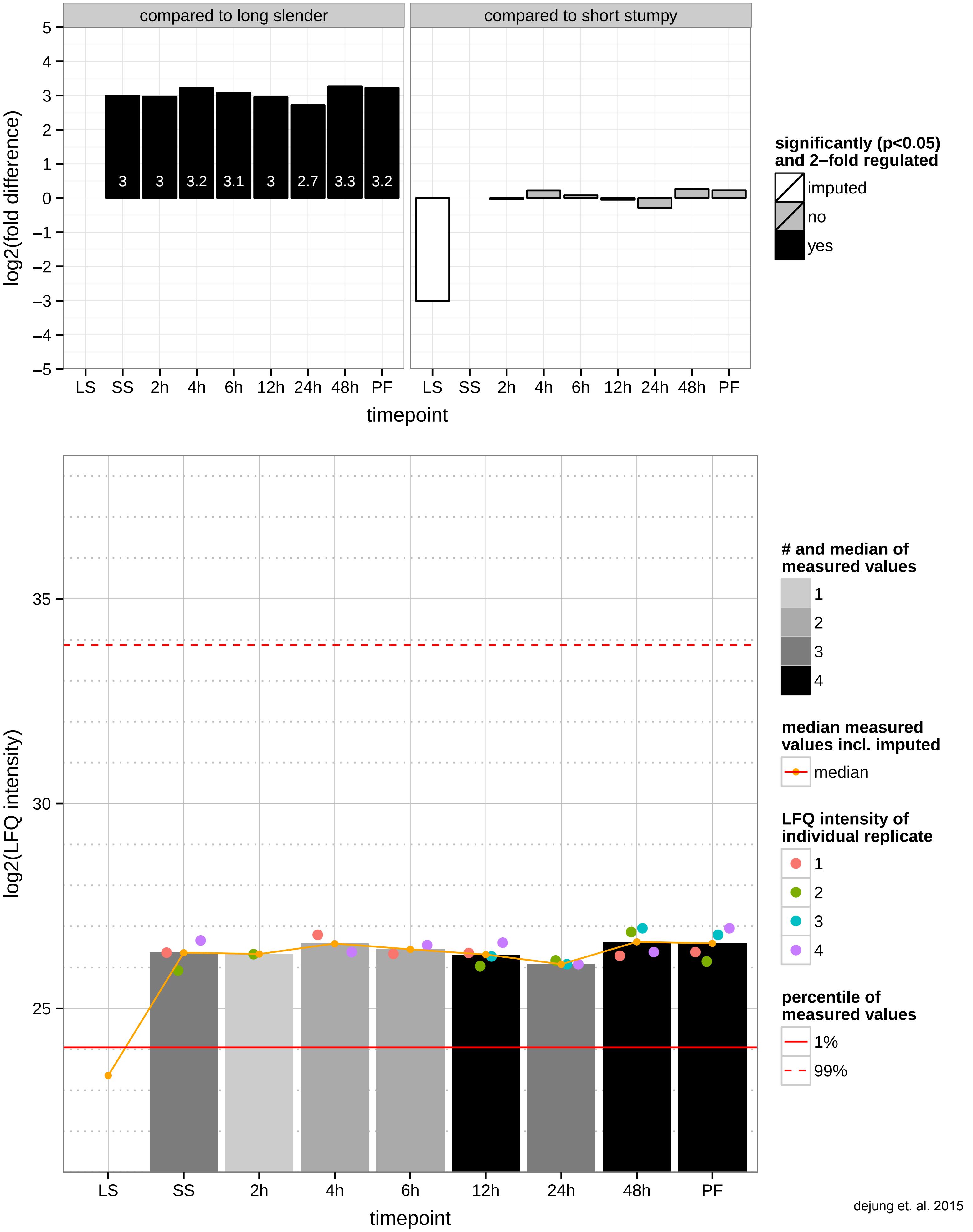

dejung et. al. 2015

hypothetical protein, conserved
Tb927.10.8820
AGOF: null
AGOC: null
AGOP: null
PGOF: null
PGOC: null
PGOP: null

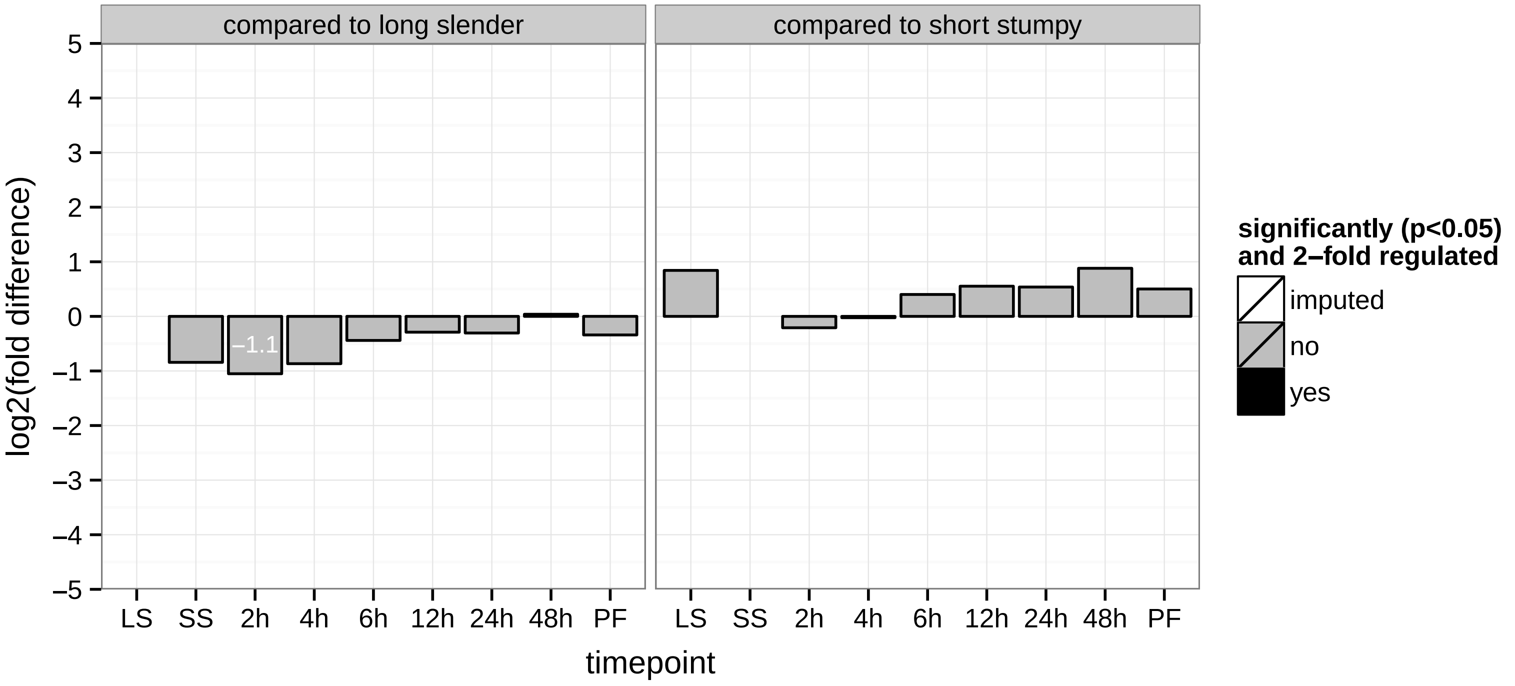

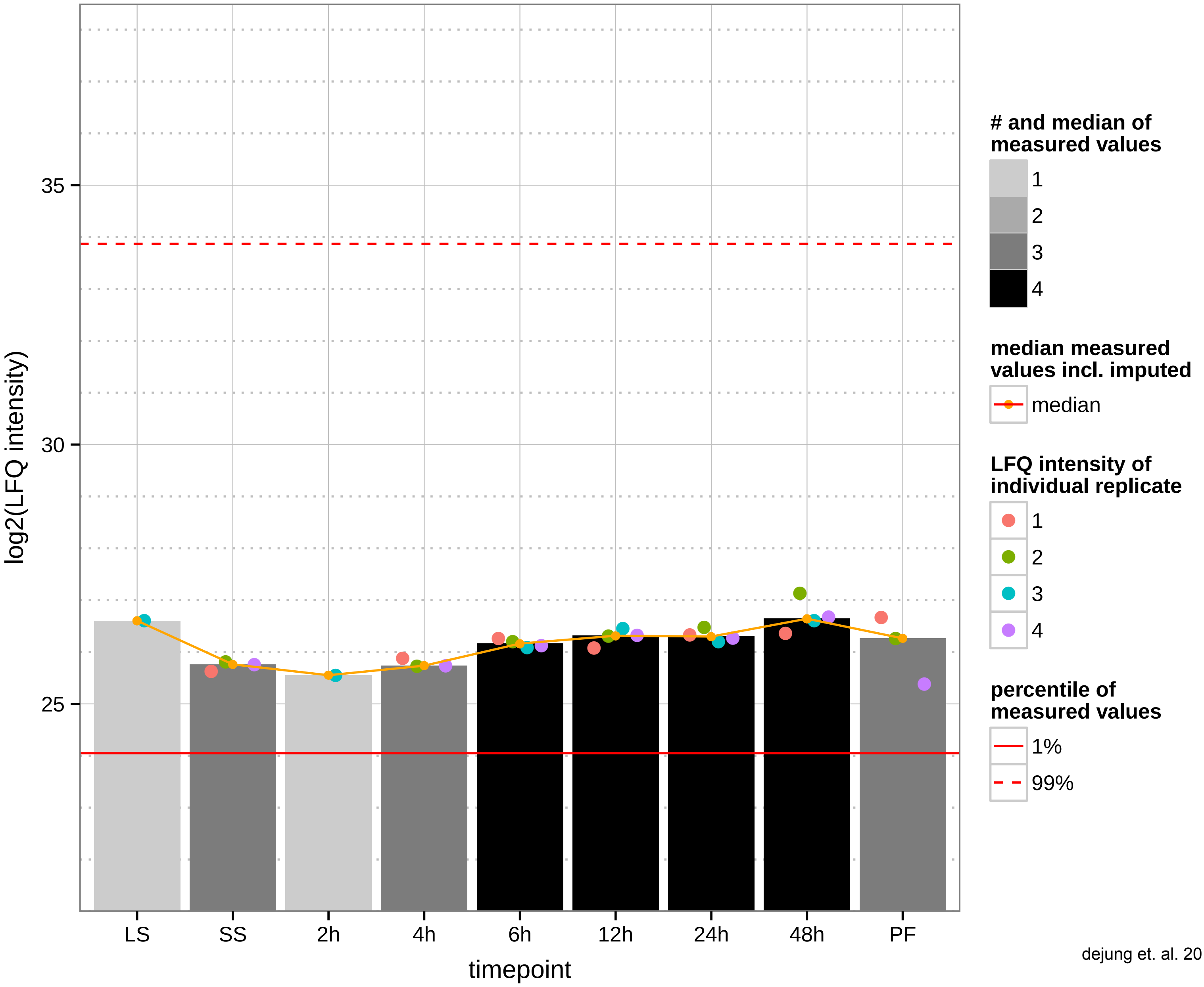

dejung et. al. 2015

hypothetical protein, conserved
Tb927.10.8830
AGOF: heat shock protein binding
AGOC: null
AGOP: null
PGOF: heat shock protein binding
PGOC: null
PGOP: null

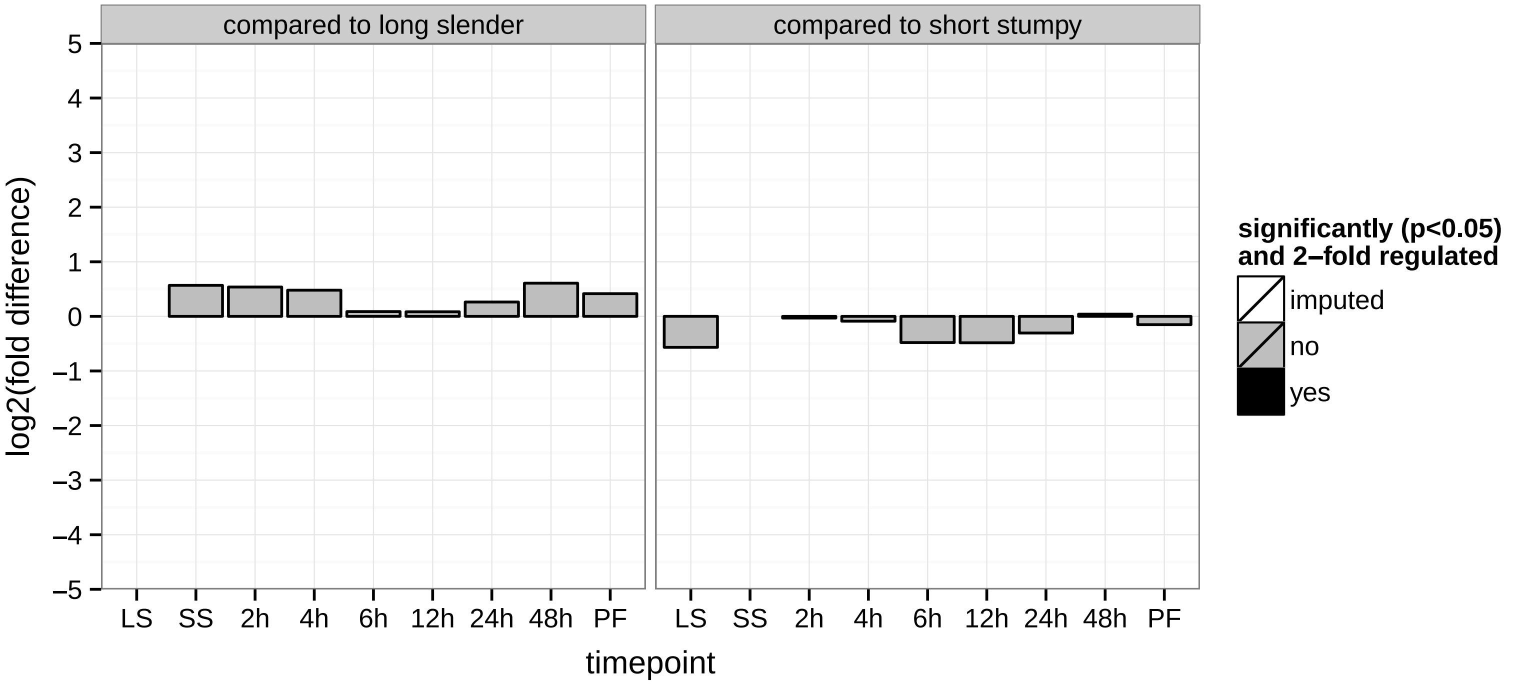

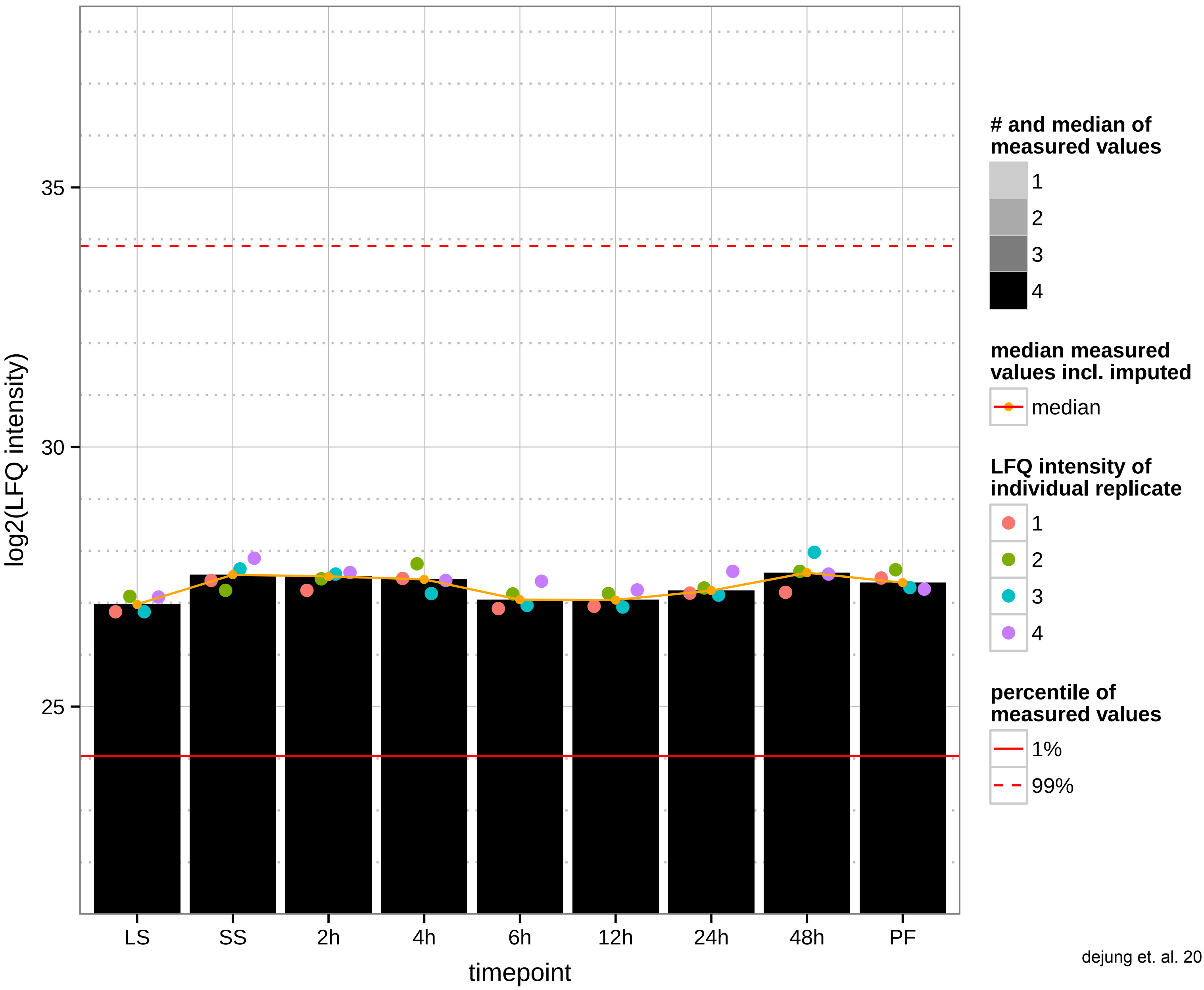

dejung et. al. 2015

kinetoplast DNA–associated protein, putative
Tb927.10.8890
AGOF: DNA binding
AGOC: kinetoplast, mitochondrion
AGOP: DNA packaging
PGOF: protein binding
PGOC: null
PGOP: null

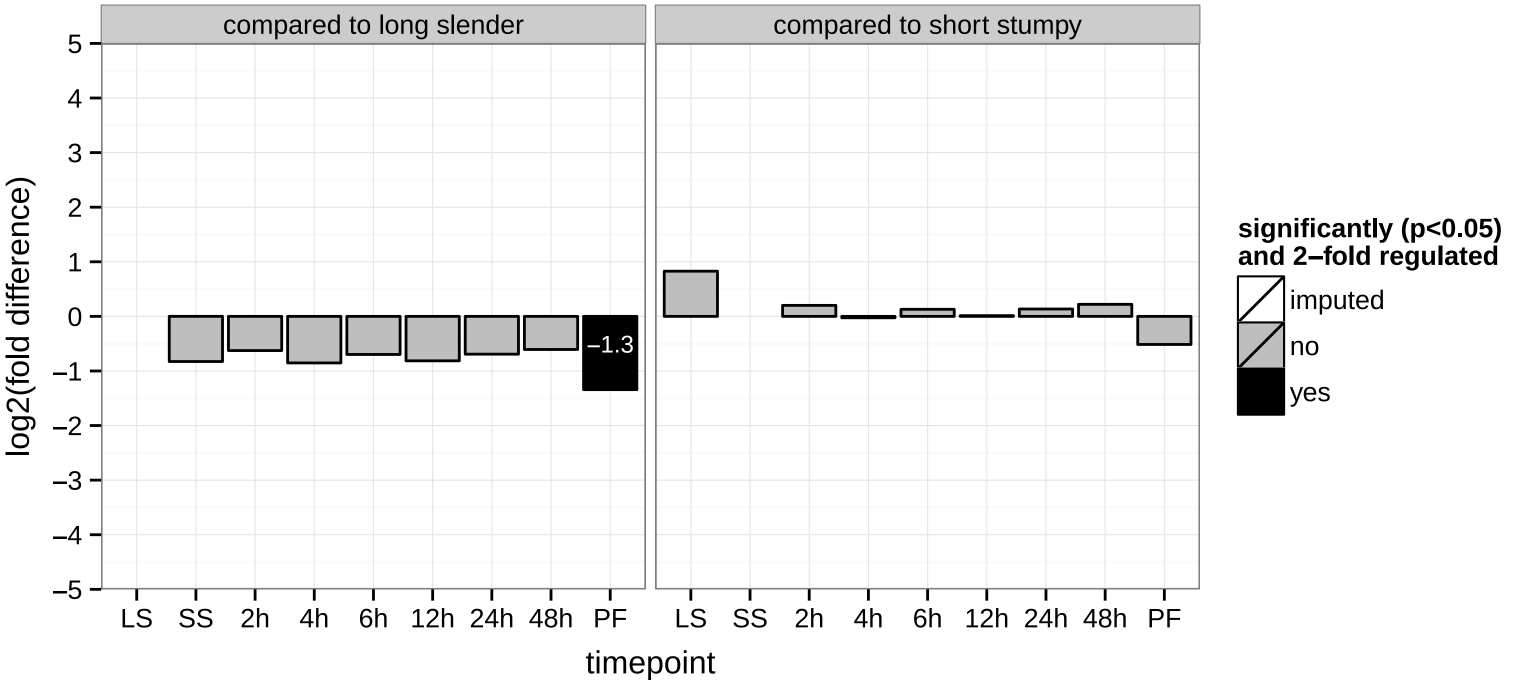

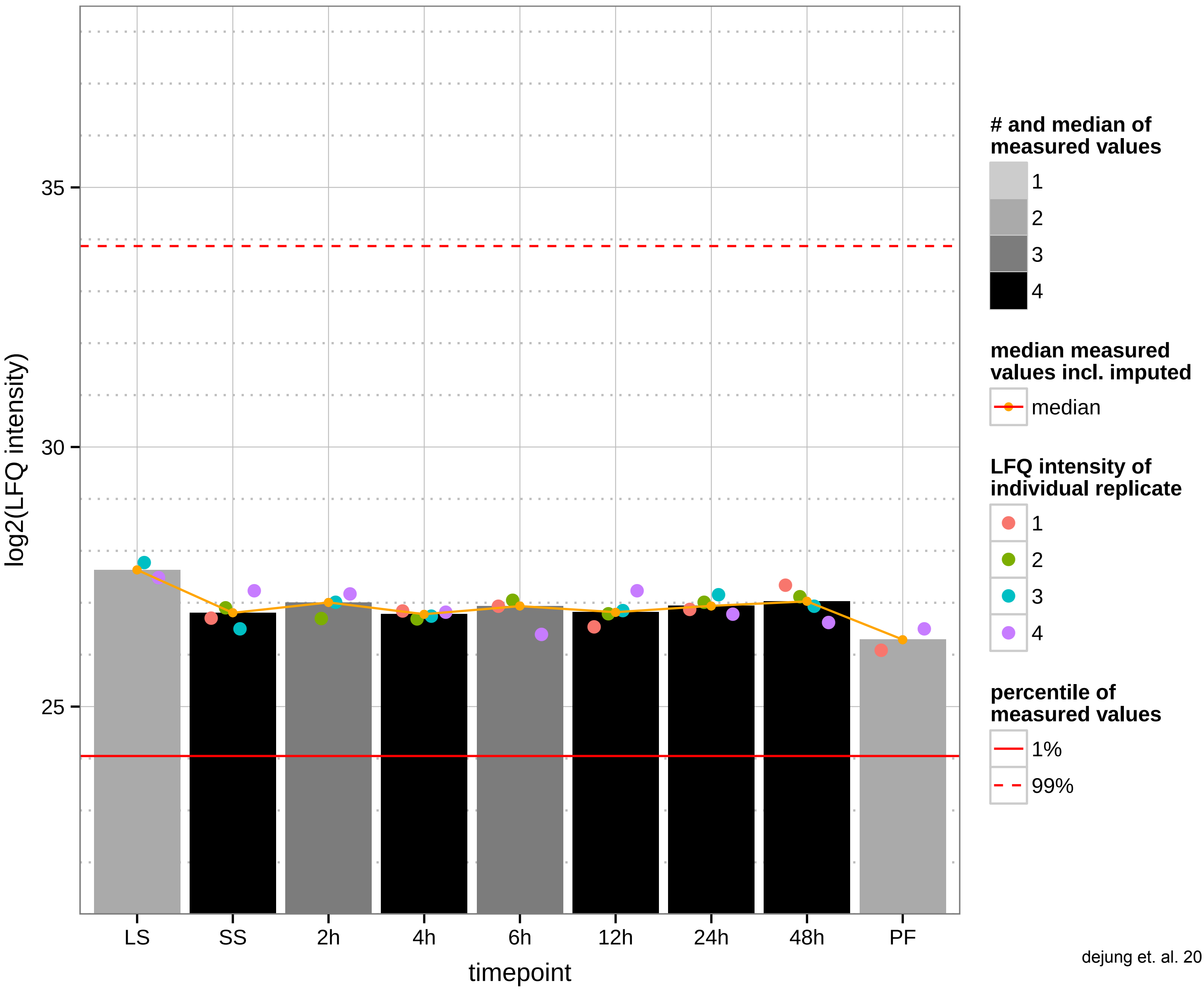

dejung et. al. 2015

paraflagellar rod component, putative (PFC18)
Tb927.10.8930
AGOF: null
AGOC: null
AGOP: null
PGOF: null
PGOC: null
PGOP: null

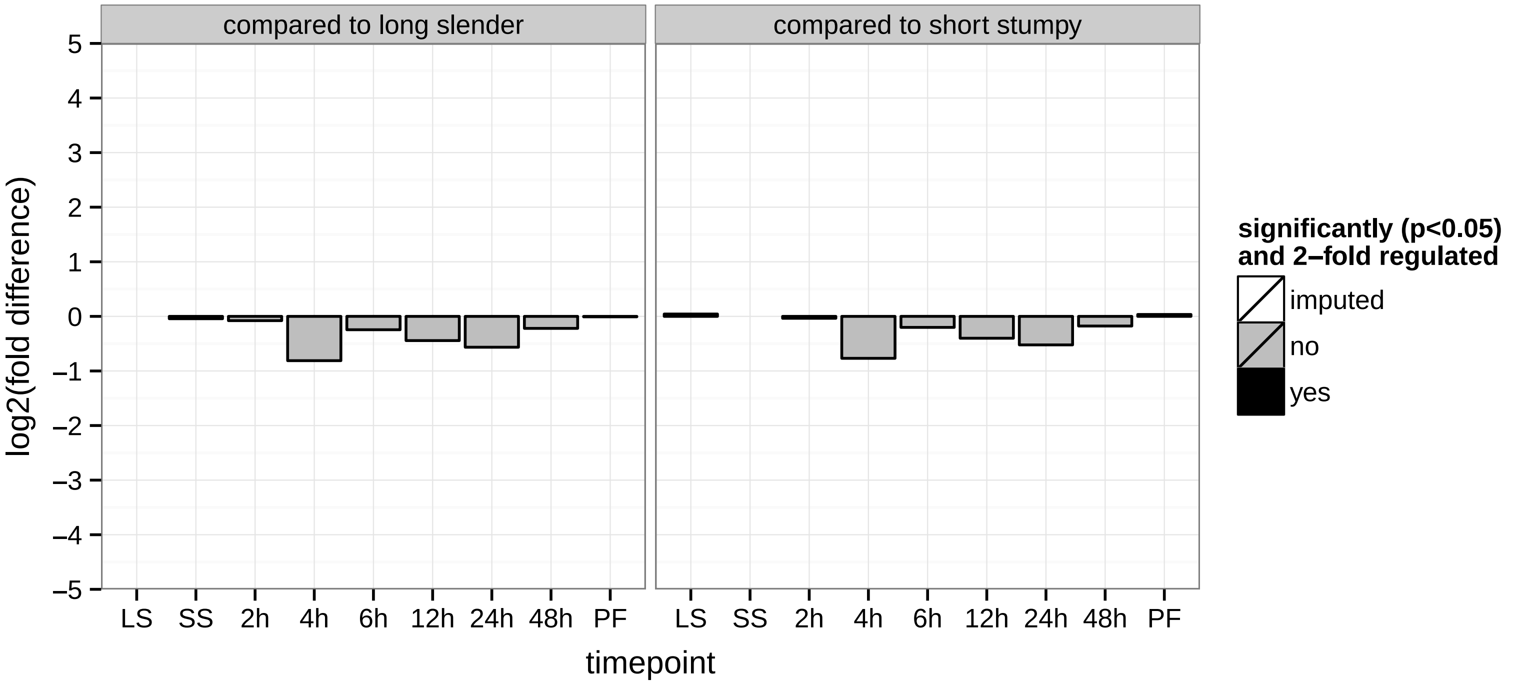

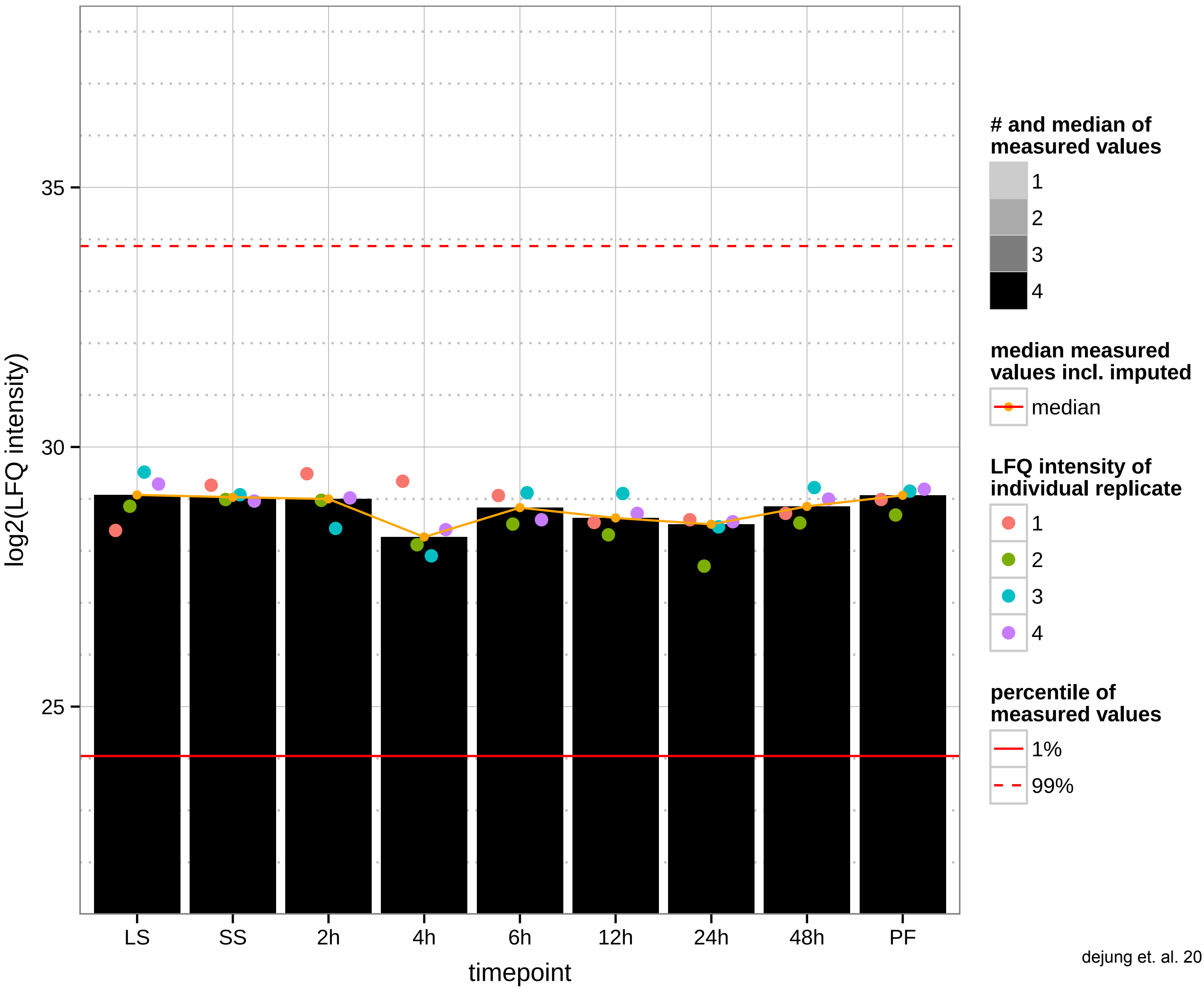

dejung et. al. 2015

flagellum targeting protein kharon1, putative (KH1)
Tb927.10.8940
AGOF: null
AGOC: flagellar pocket
AGOP: pathogenesis, protein targeting
PGOF: null
PGOC: null
PGOP: null

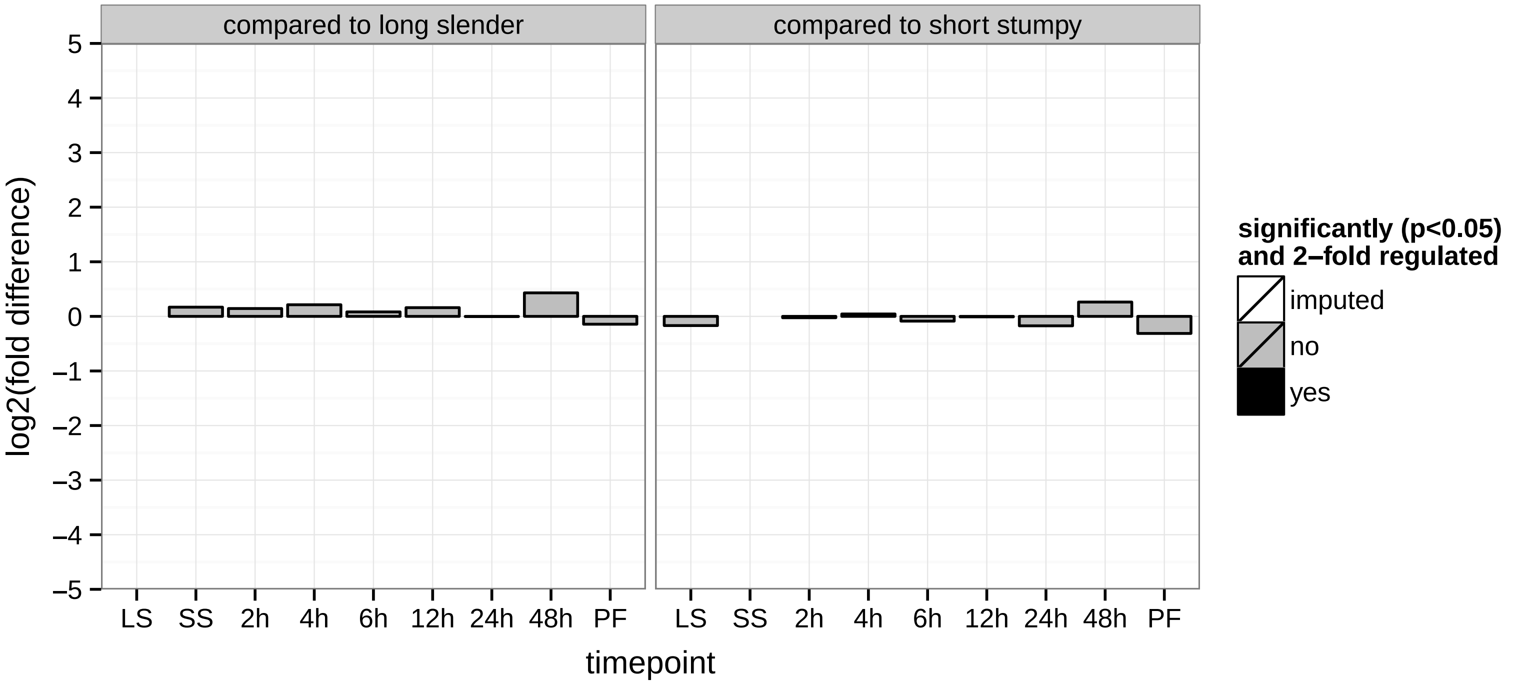

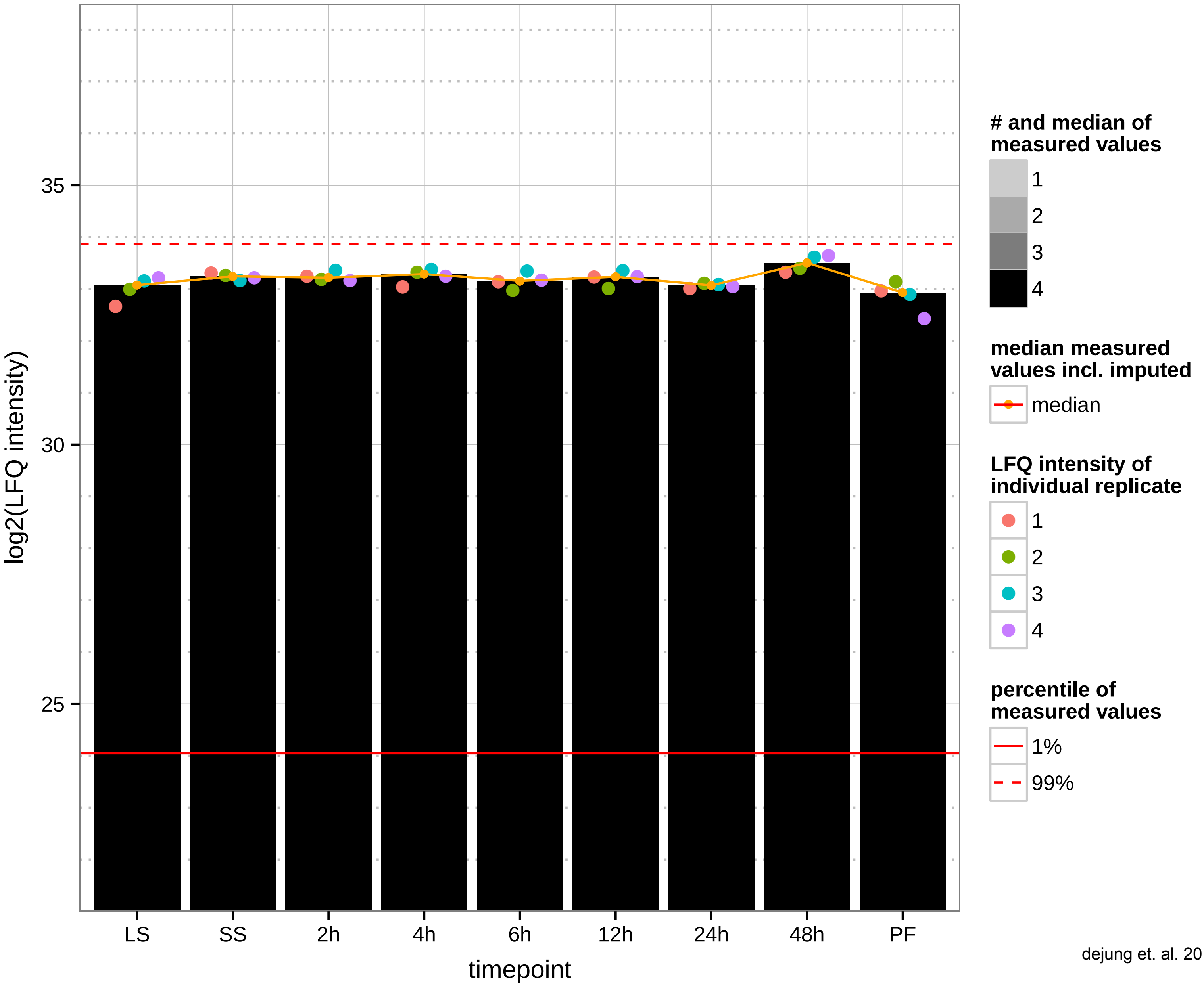

dejung et. al. 2015

kinetoplast DNA–associated protein, putative
Tb927.10.8950;Tb927.10.8970
AGOF: DNA binding
AGOC: kinetoplast, mitochondrion
AGOP: DNA packaging
PGOF: null
PGOC: null
PGOP: null

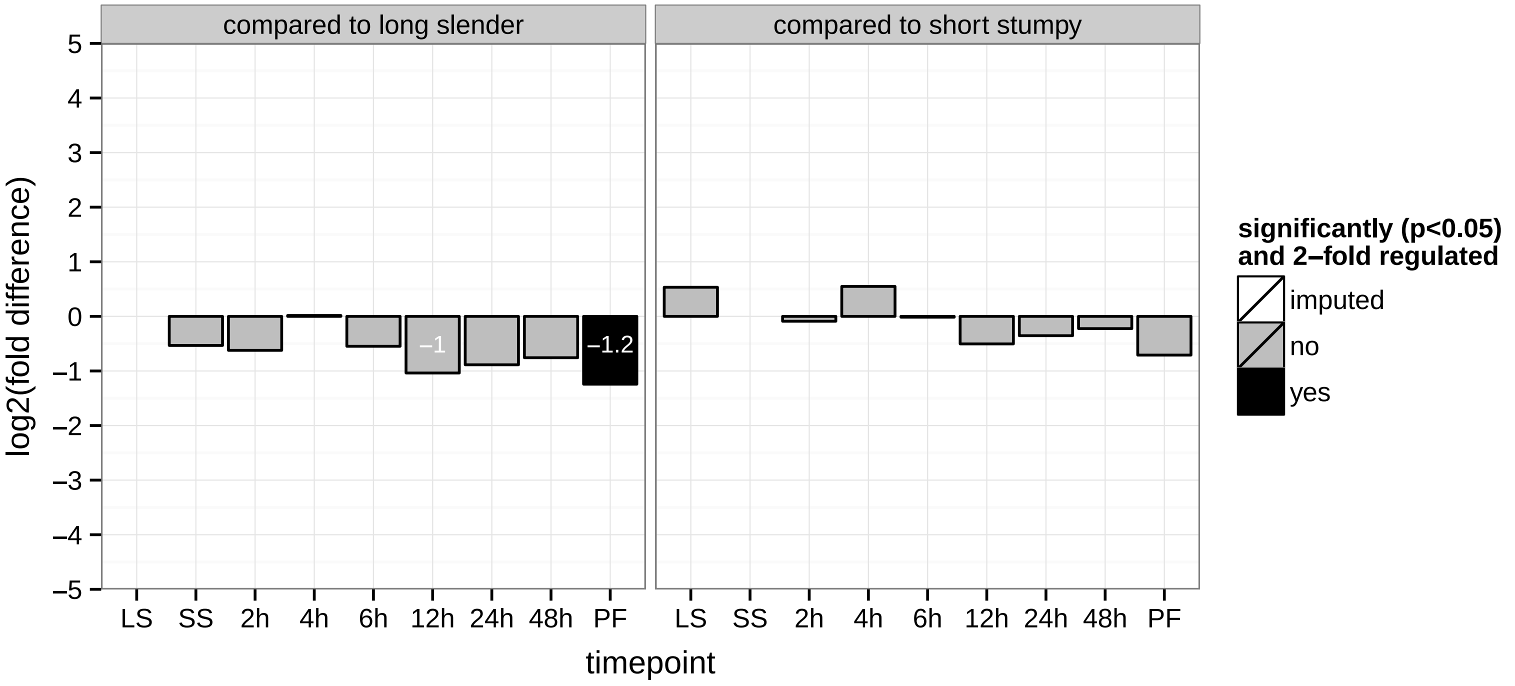

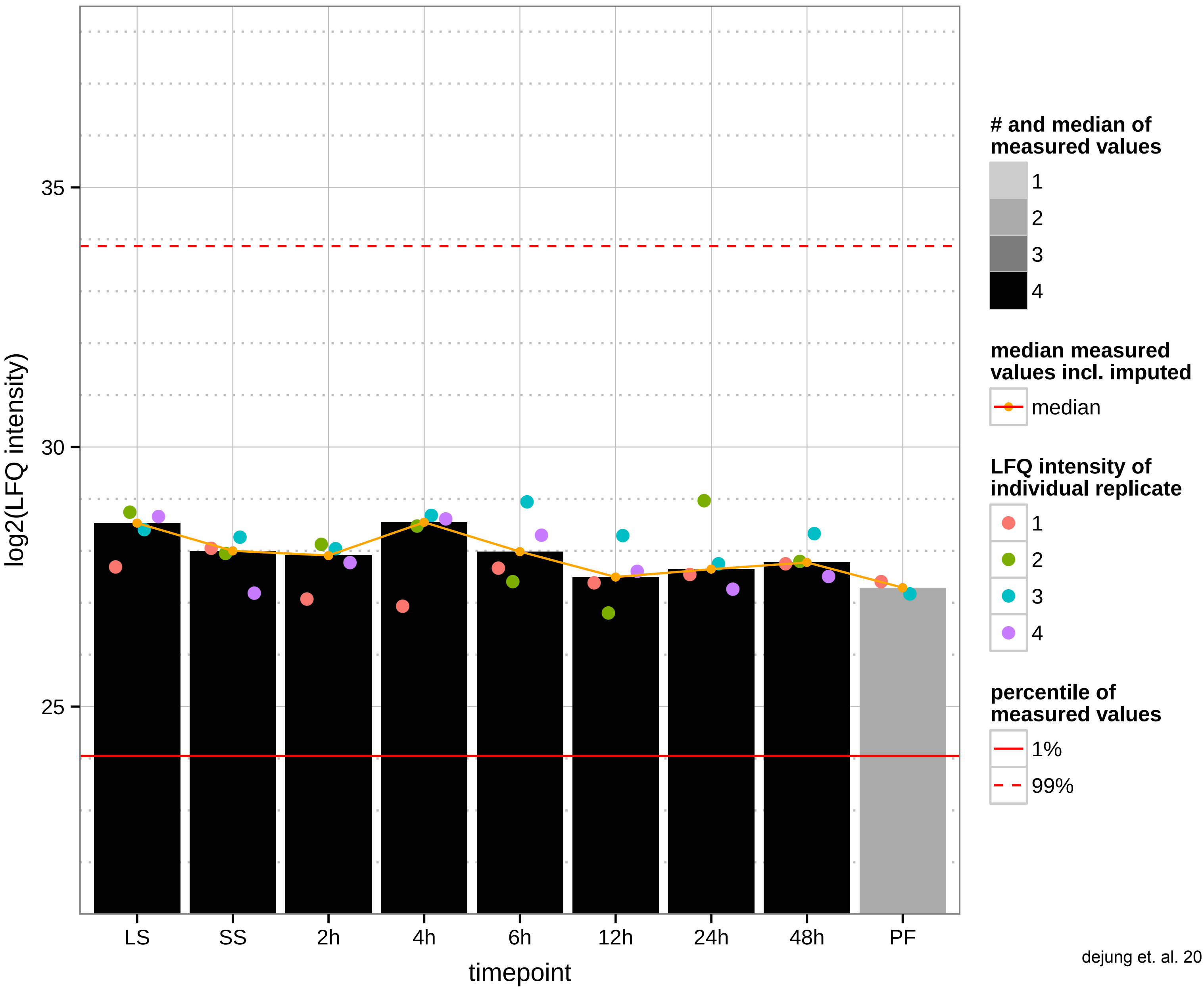

dejung et. al. 2015

hypothetical protein, conserved
Tb927.10.8960
AGOF: null
AGOC: null
AGOP: null
PGOF: protein binding
PGOC: null
PGOP: null

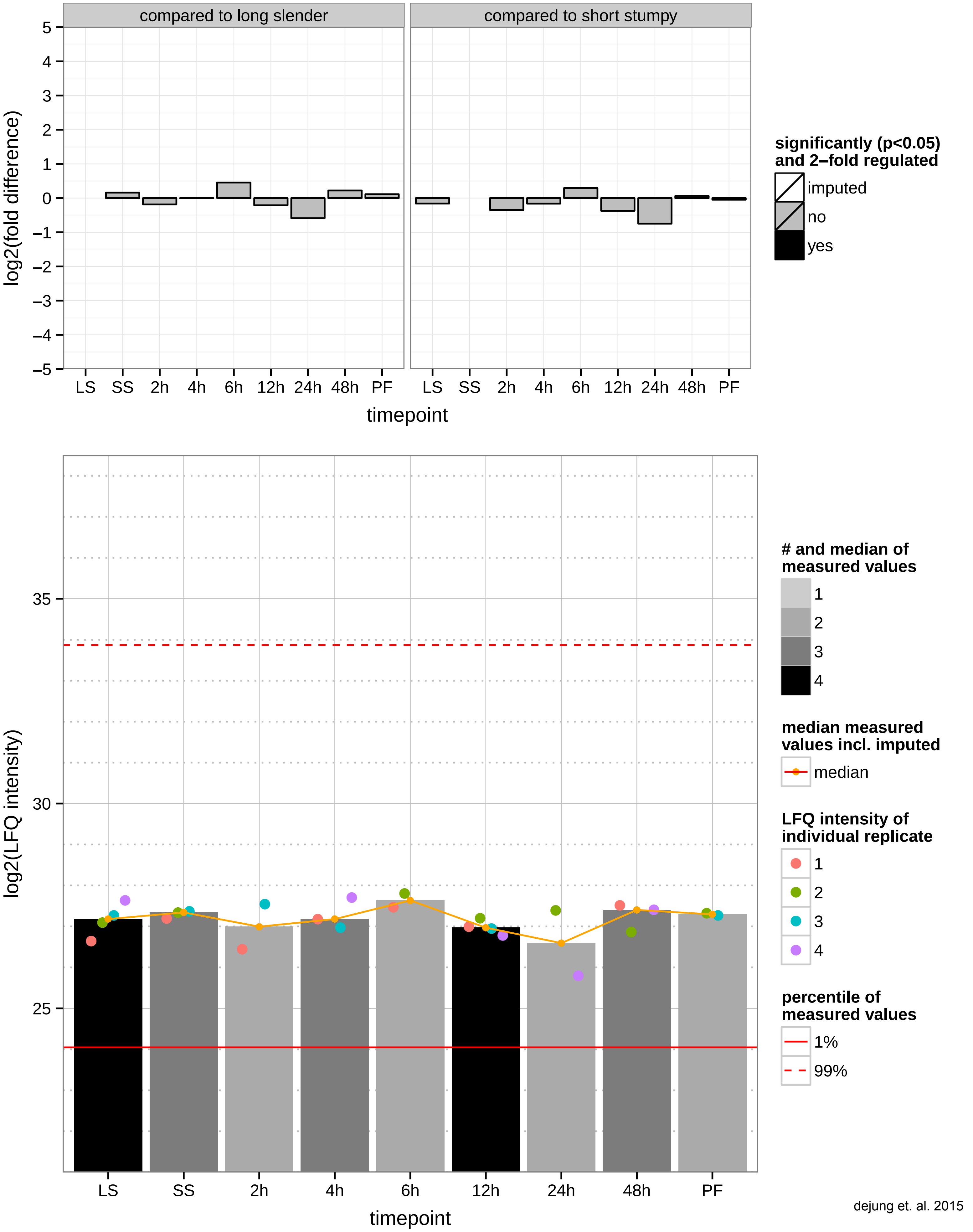

dejung et. al. 2015

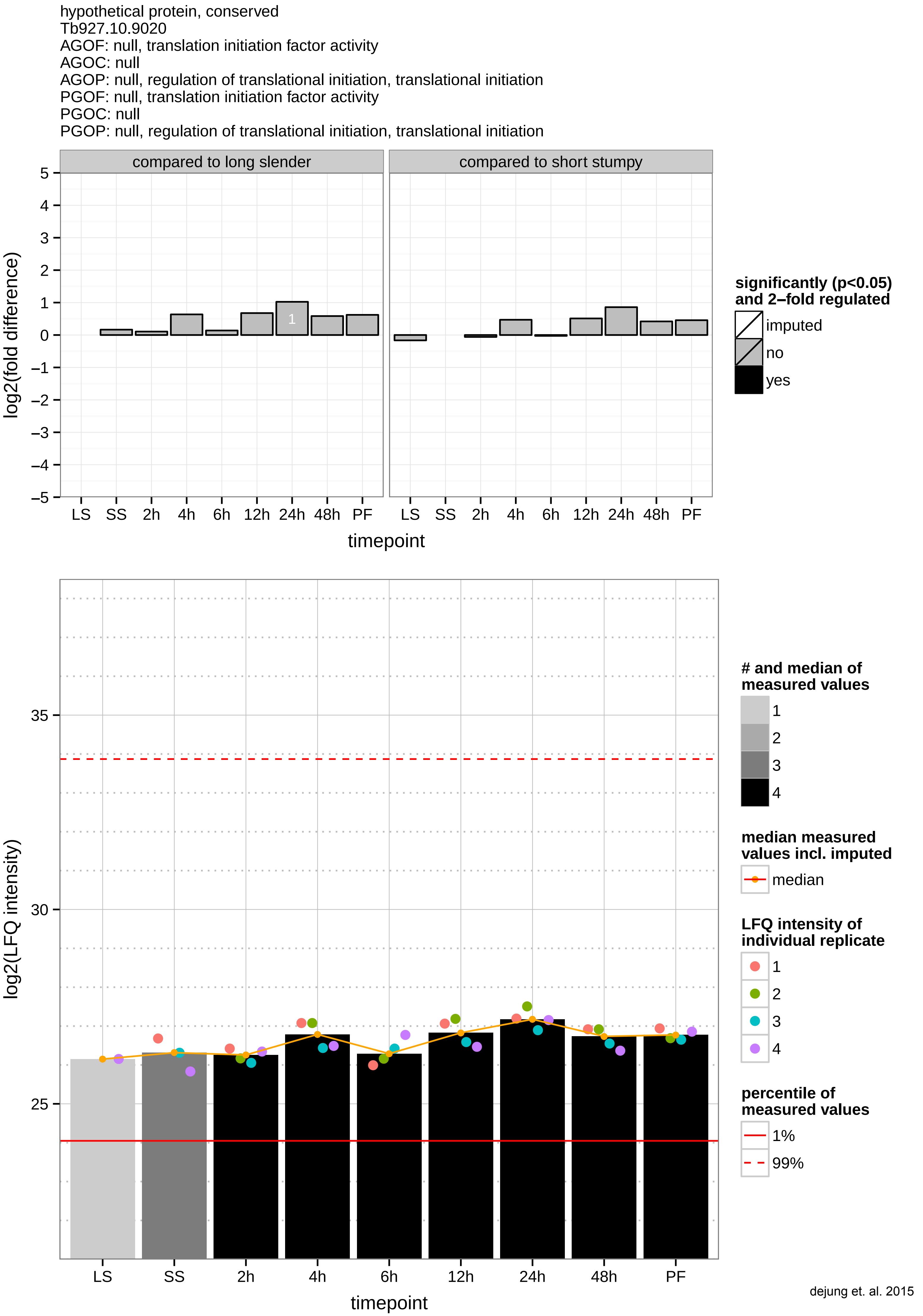

hypothetical protein, conserved
Tb927.10.9020
AGOF: null, translation initiation factor activity
AGOC: null
AGOP: null, regulation of translational initiation, translational initiation
PGOF: null, translation initiation factor activity
PGOC: null
PGOP: null, regulation of translational initiation, translational initiation
compared to long slender
compared to short stumpy
log2(fold difference)
timepoint
LS SS 2h 4h 6h 12h 24h 48h PF
significantly (p<0.05) and 2–fold regulated
imputed
no
yes
log2(LFQ intensity)
# and median of measured values
median measured values incl. imputed
median
LFQ intensity of individual replicate
percentile of measured values
1%
99%
dejung et. al. 2015

hypothetical protein, conserved
Tb927.10.9040
AGOF: null
AGOC: null
AGOP: null
PGOF: null
PGOC: null
PGOP: null

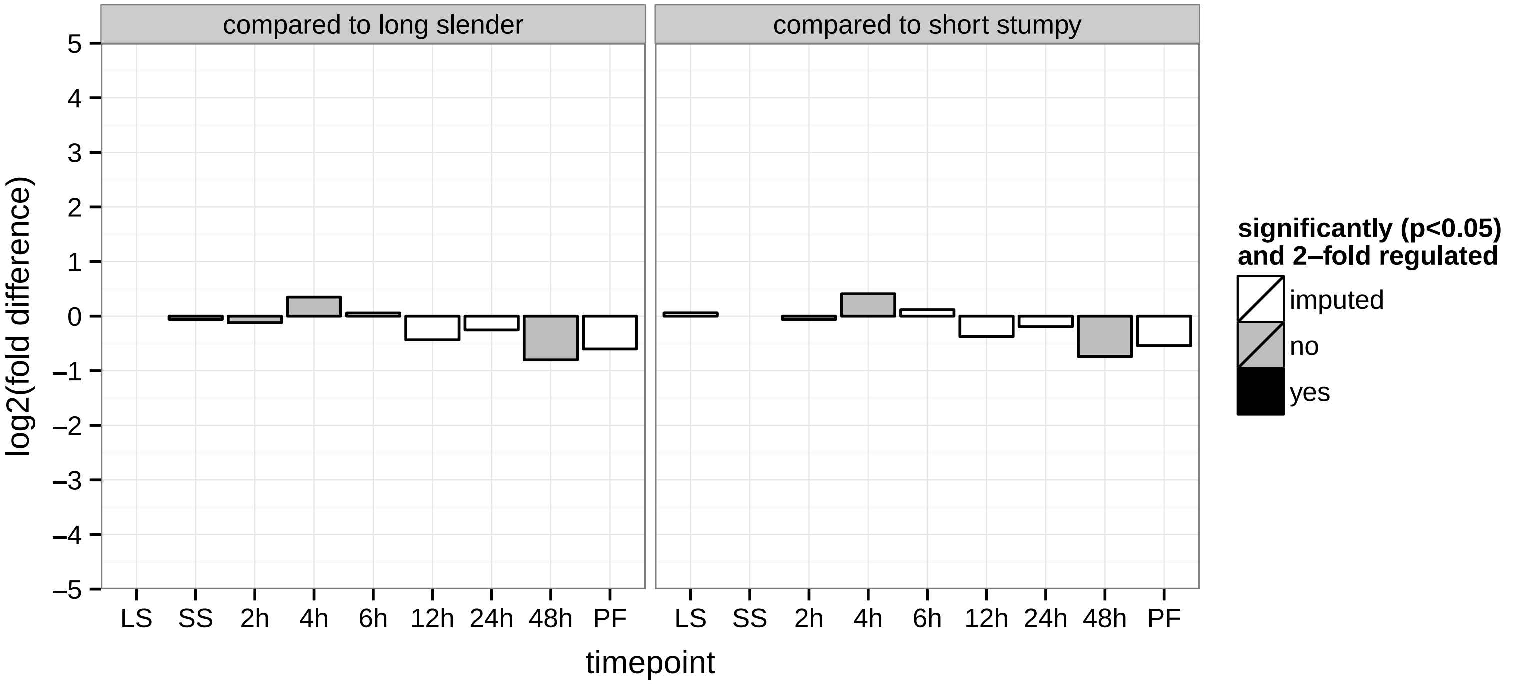

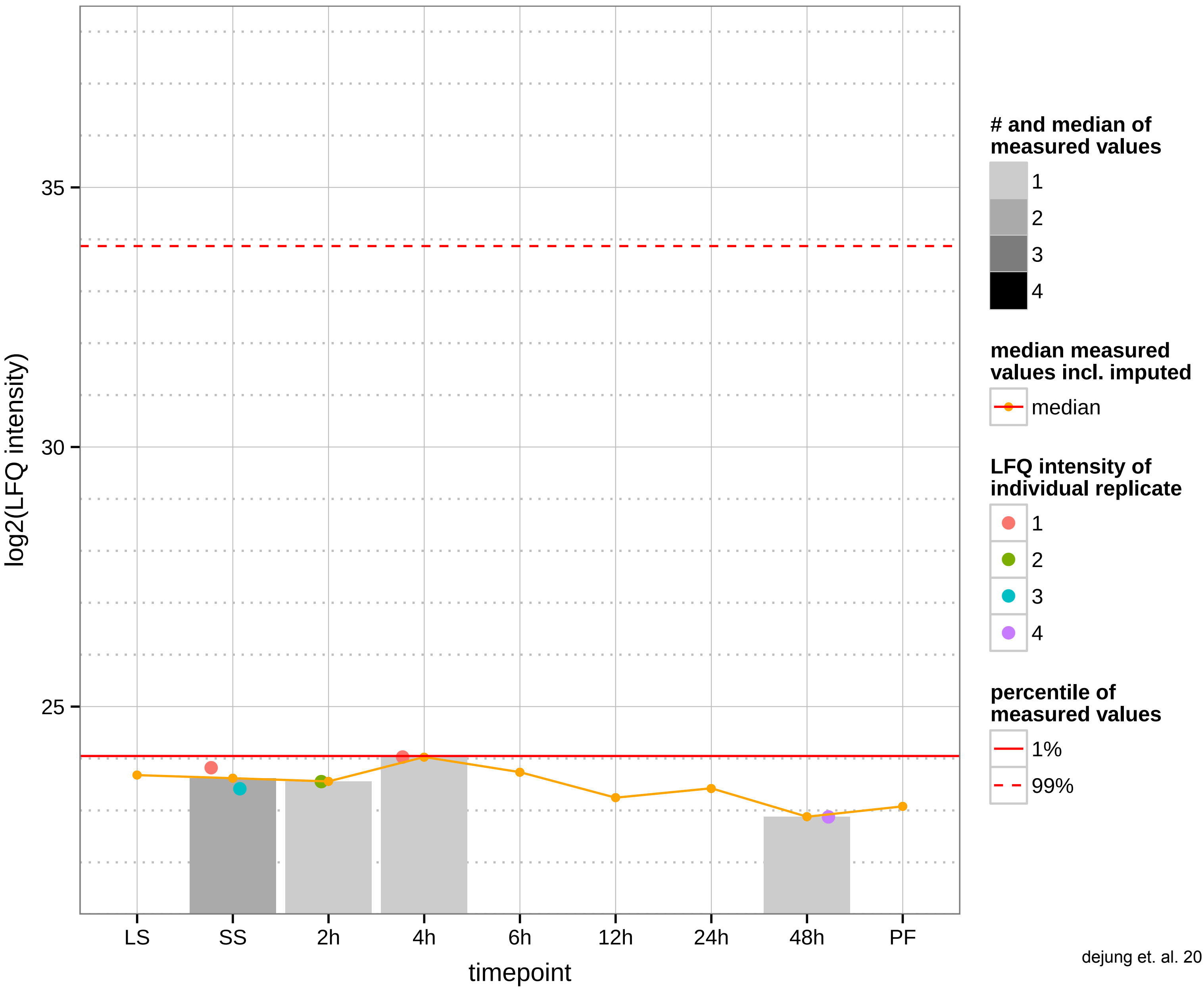

dejung et. al. 2015

hypothetical protein, conserved
Tb927.10.9090
AGOF: null
AGOC: null
AGOP: null
PGOF: null
PGOC: null
PGOP: null

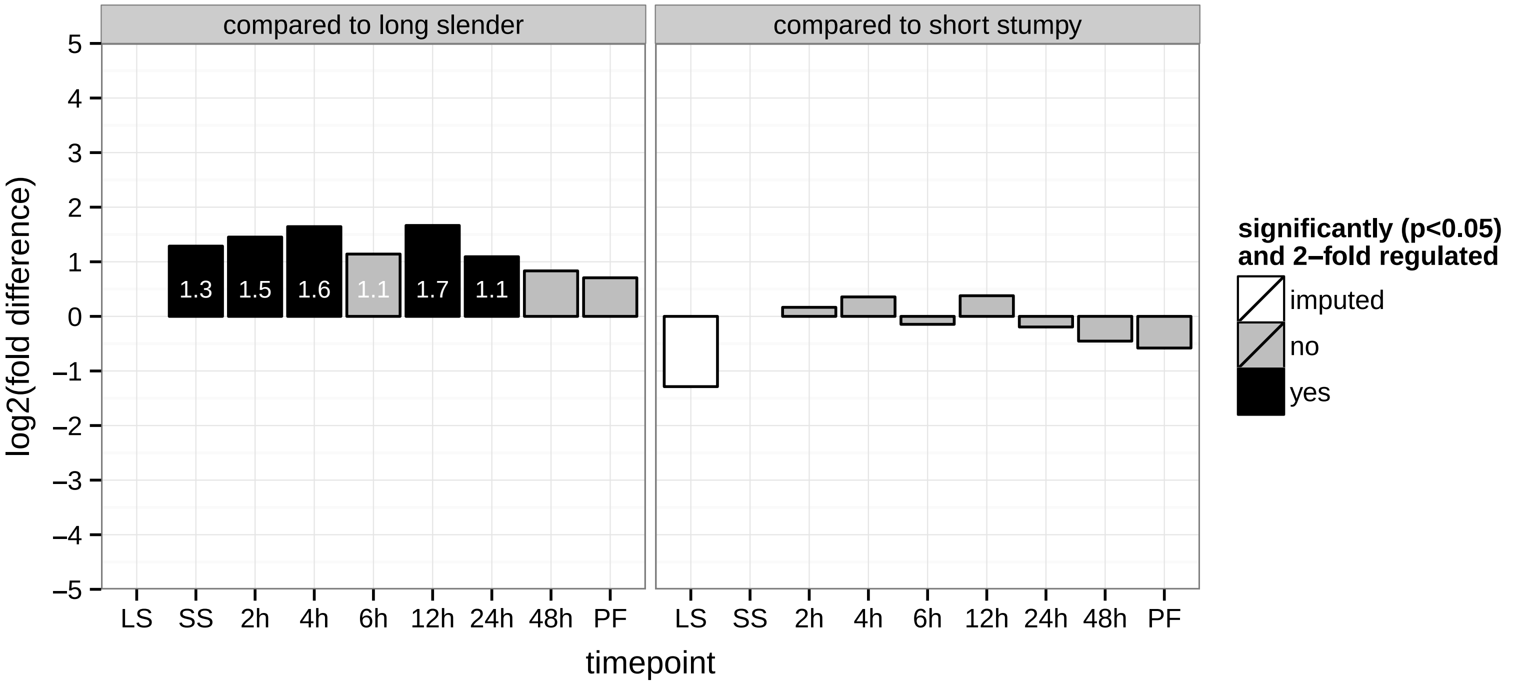

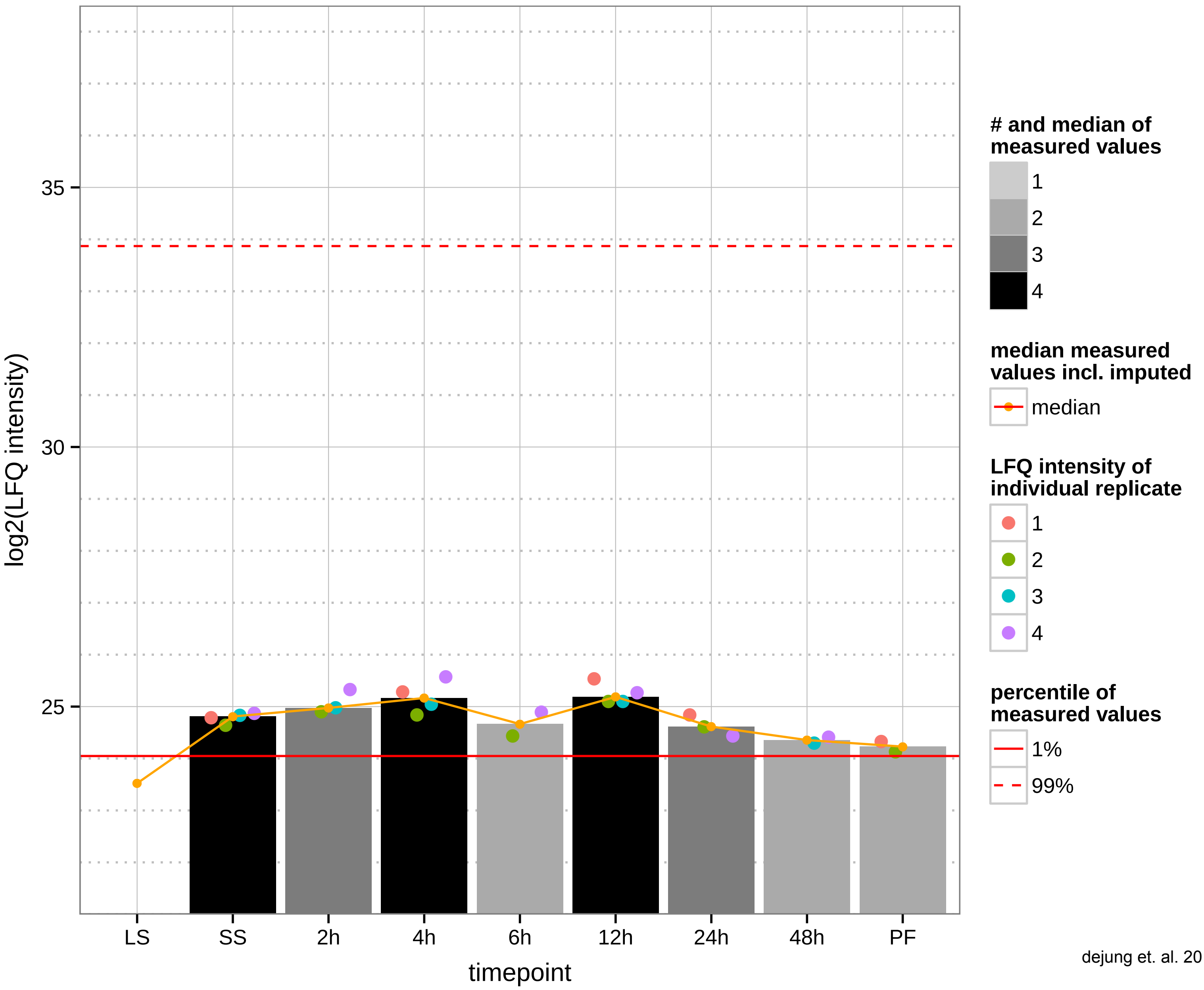

dejung et. al. 2015

pre−mRNA splicing factor ATP−dependent RNA helicase, putative
Tb927.10.9130
AGOF: ATP binding, ATP−dependent RNA helicase activity, nucleic acid binding
AGOC: spliceosomal complex
AGOP: RNA splicing, RNA splicing, via transesterification reactions
PGOF: ATP binding, ATP−dependent helicase activity, helicase activity, nucleic acid binding
PGOC: null
PGOP: null

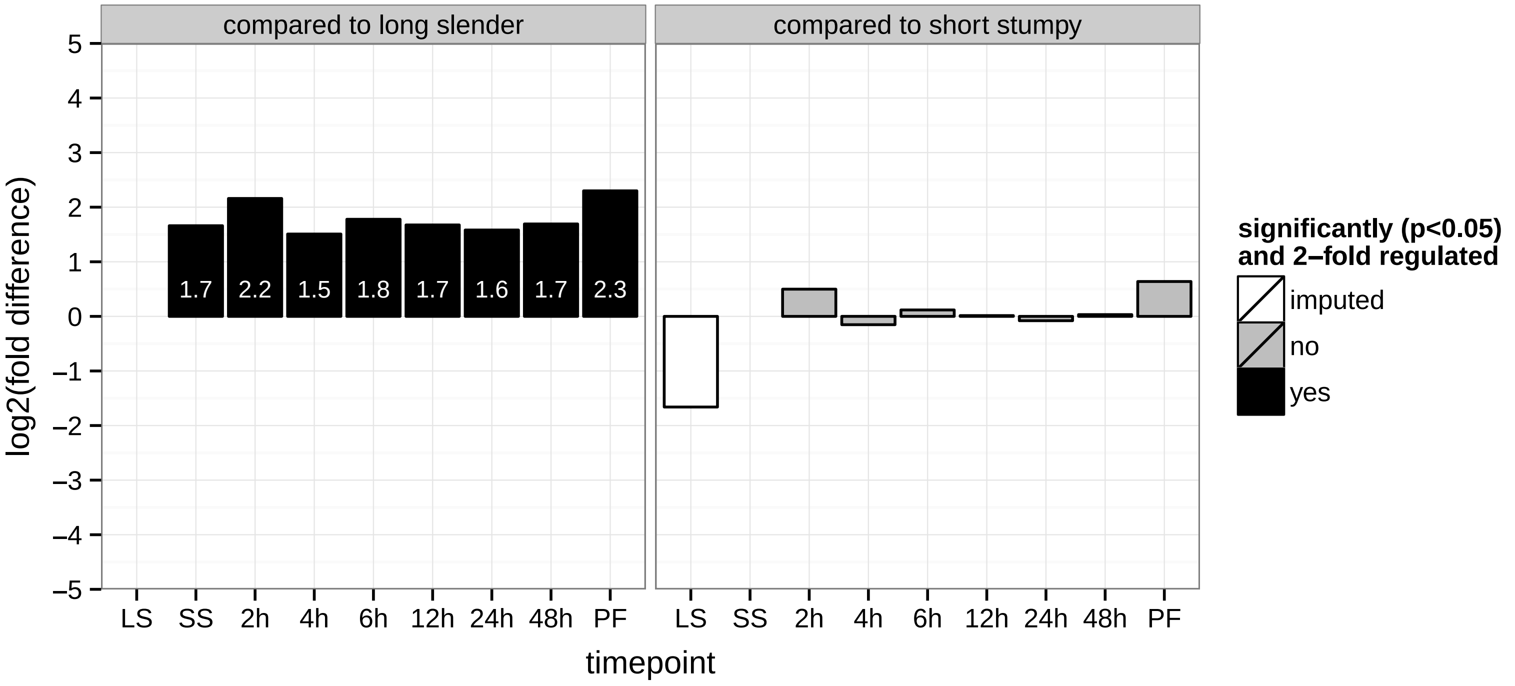

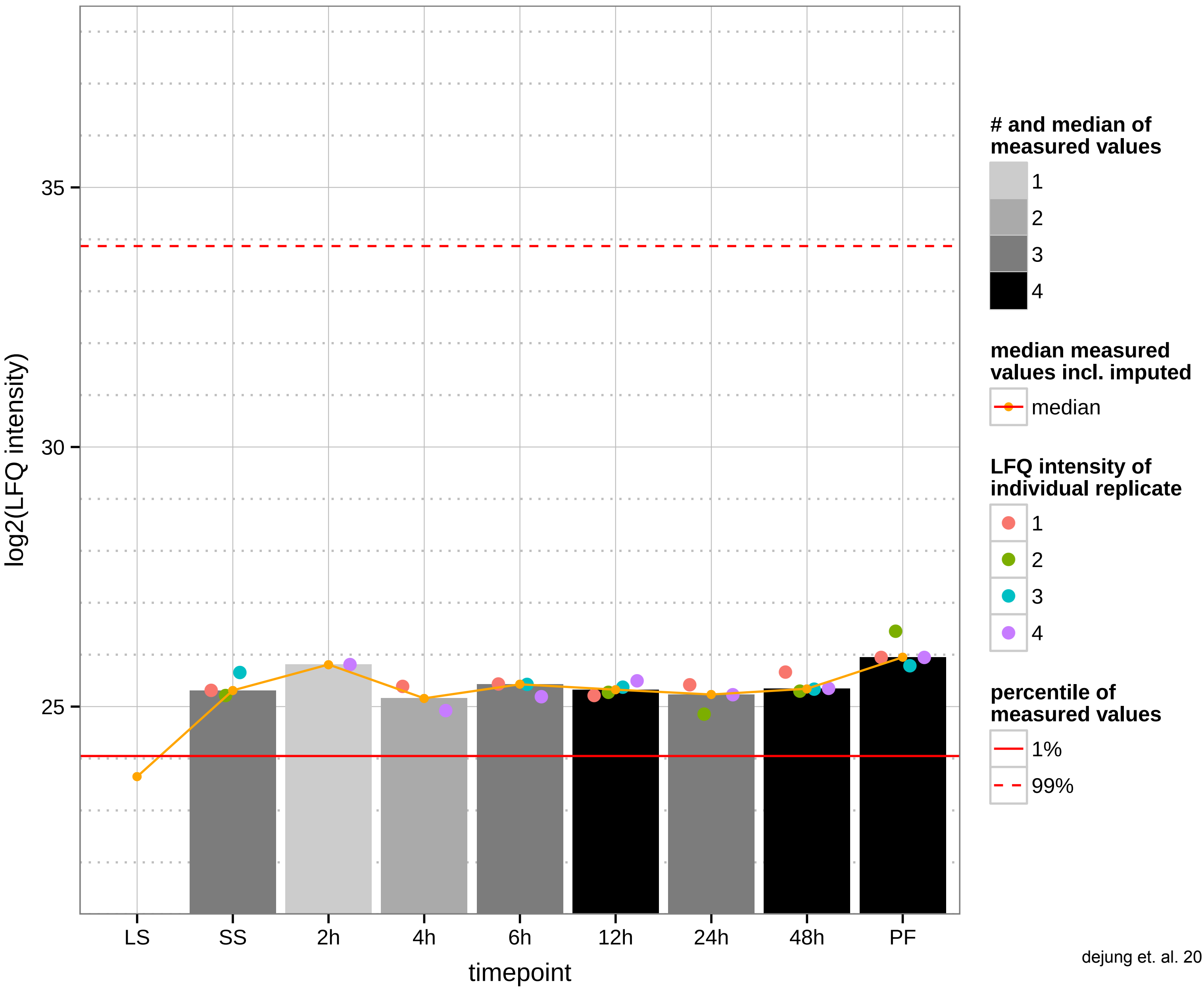

dejung et. al. 2015

hypothetical protein, conserved
Tb927.10.9140
AGOF: oxidoreductase activity
AGOC: mitochondrion
AGOP: null
PGOF: oxidoreductase activity
PGOC: null
PGOP: oxidation−reduction process

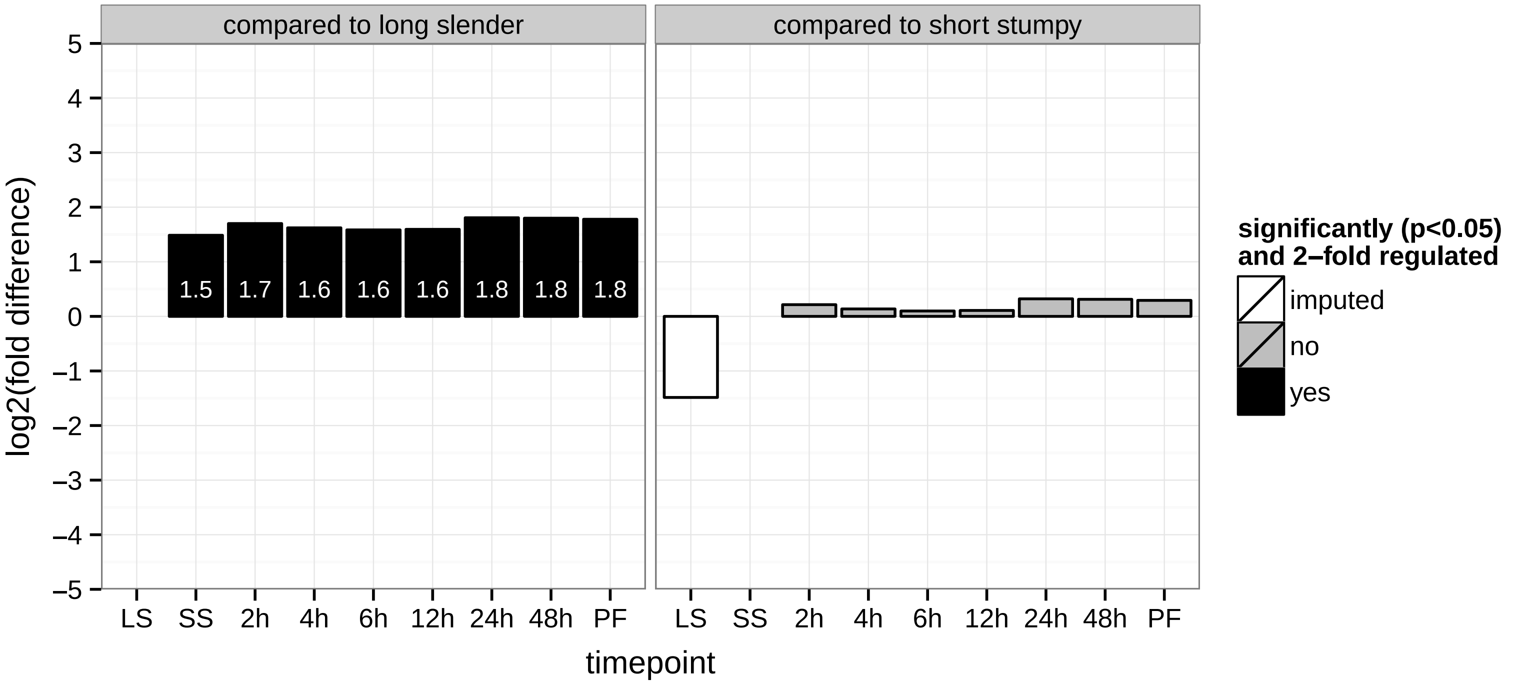

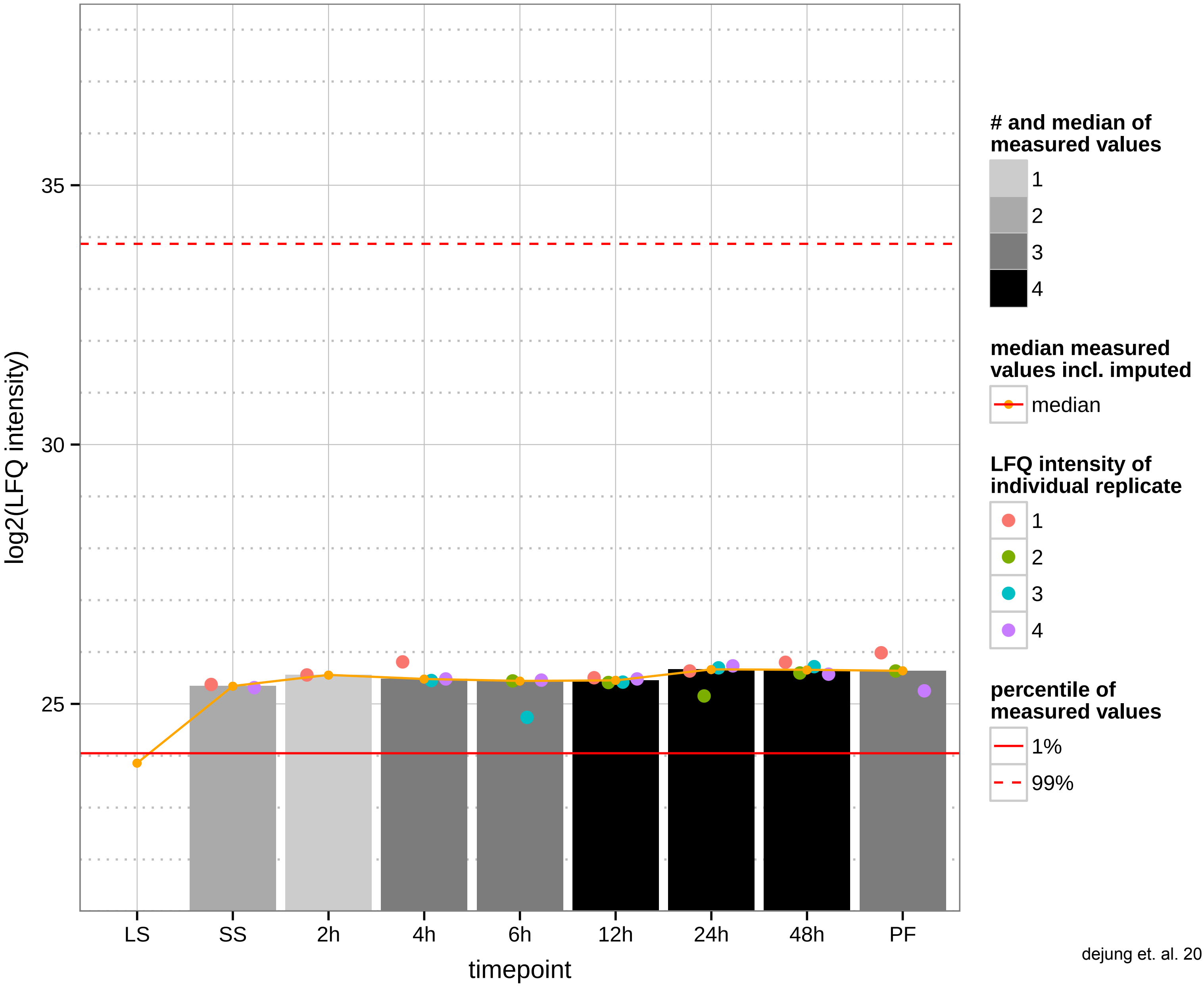

dejung et. al. 2015

isoleucyl–tRNA synthetase, putative (IleRS)
Tb927.10.9190
AGOF: ATP binding, isoleucine–tRNA ligase activity, zinc ion binding
AGOC: cytoplasm, mitochondrion
AGOP: isoleucyl–tRNA aminoacylation, translation
PGOF: ATP binding, aminoacyl–tRNA editing activity, aminoacyl–tRNA ligase activity, isoleucine–tRNA ligase activity, nucleoti
PGOC: cytoplasm
PGOP: isoleucyl–tRNA aminoacylation, tRNA aminoacylation for protein translation

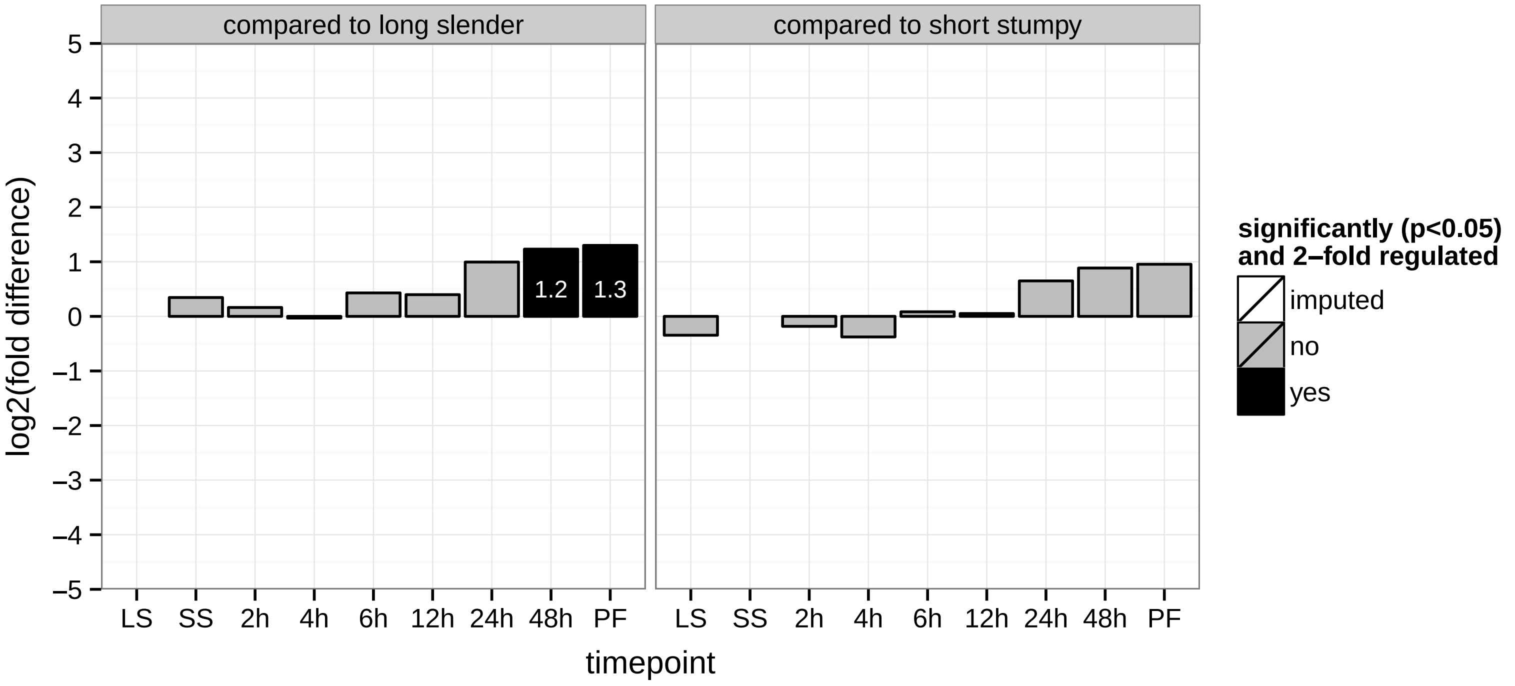

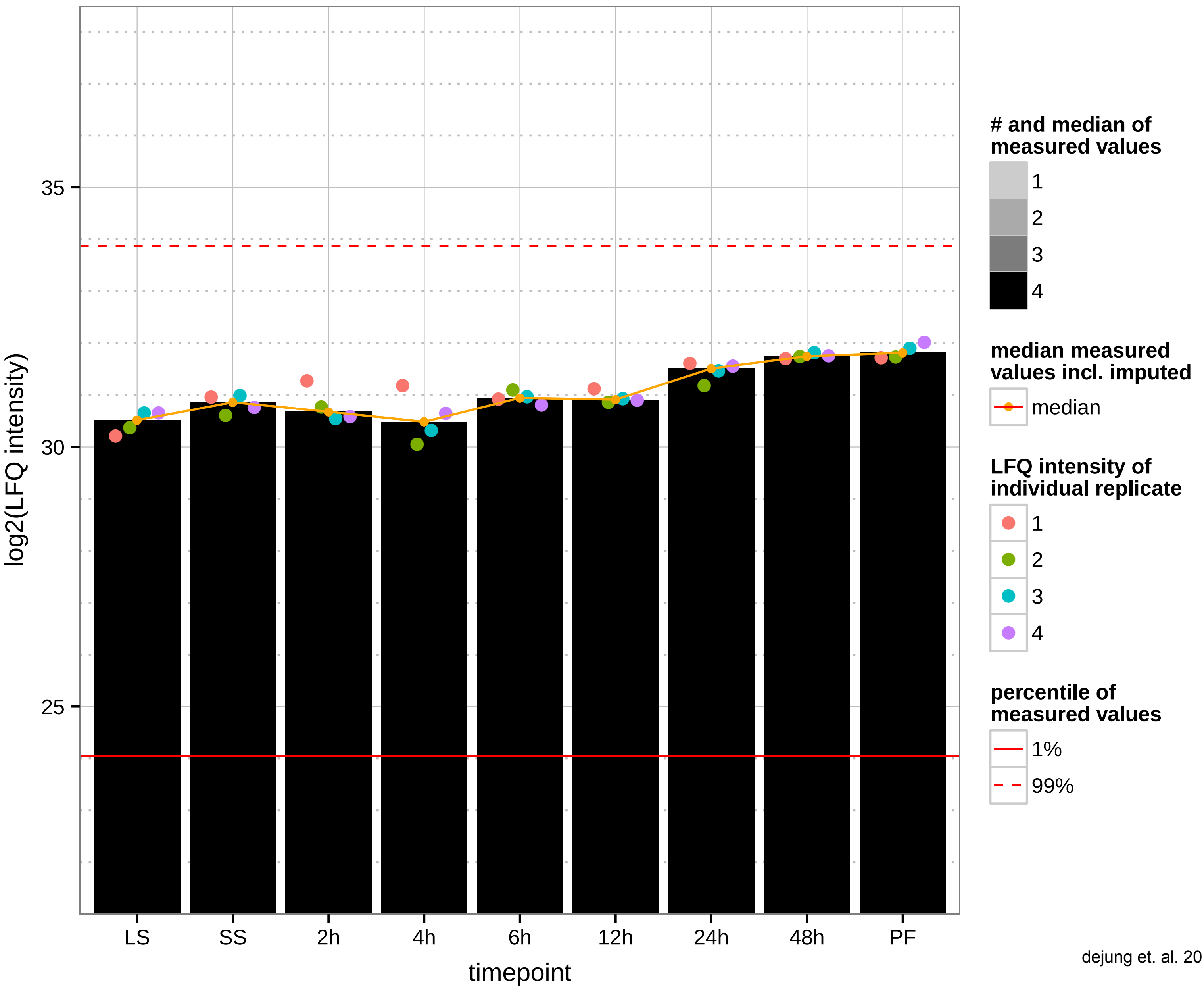

dejung et. al. 2015

hypothetical protein, conserved
Tb927.10.9240
AGOF: null
AGOC: cytoplasm
AGOP: pathogenesis, regulation of protein secretion
PGOF: null
PGOC: cytoplasm
PGOP: pathogenesis, regulation of protein secretion

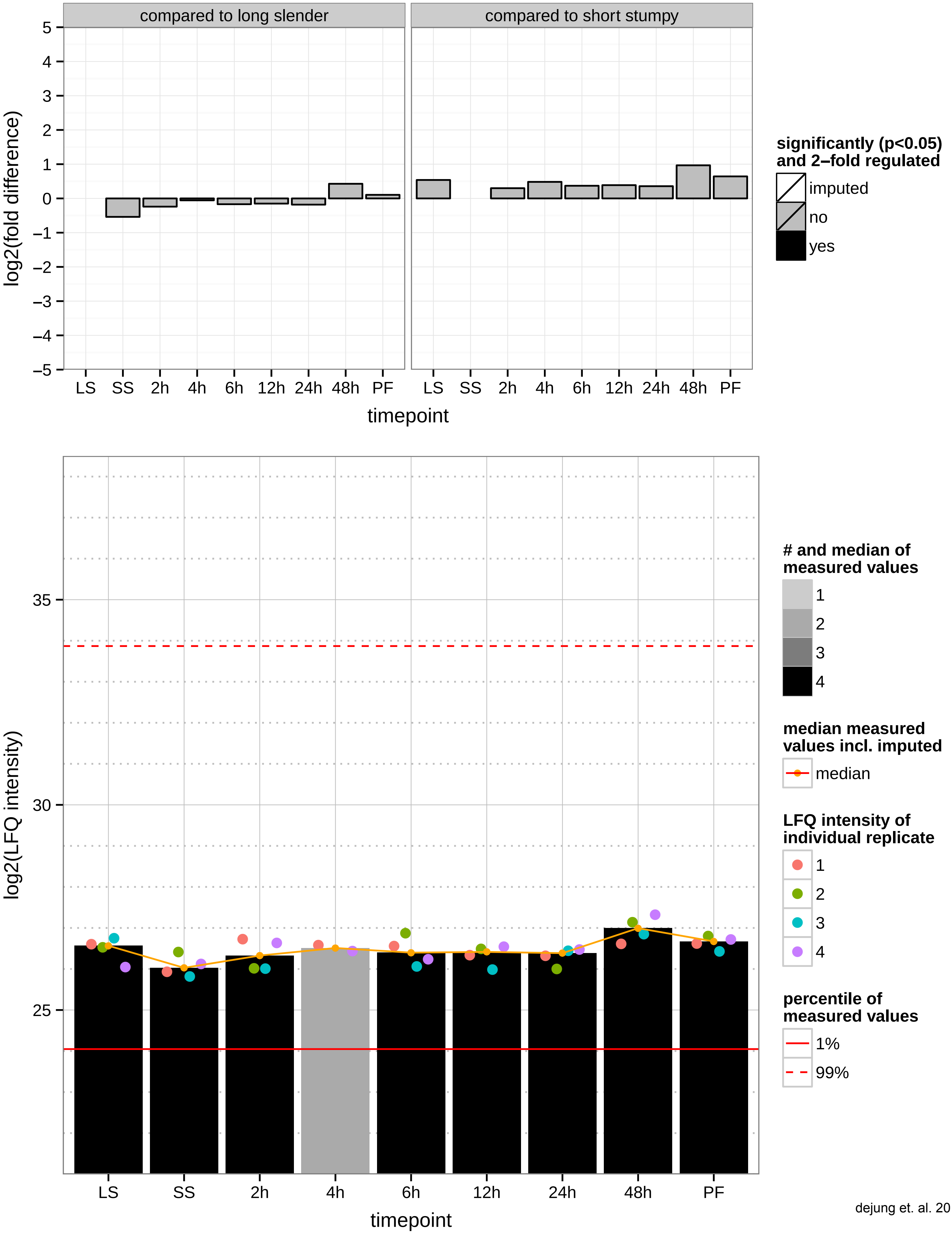

dejung et. al. 2015

G–actin binding protein, putative, CAP/Srv2p
Tb927.10.9250
AGOF: actin binding
AGOC: null
AGOP: activation of adenylate cyclase activity, cytoskeleton organization
PGOF: actin binding
PGOC: null
PGOP: cytoskeleton organization

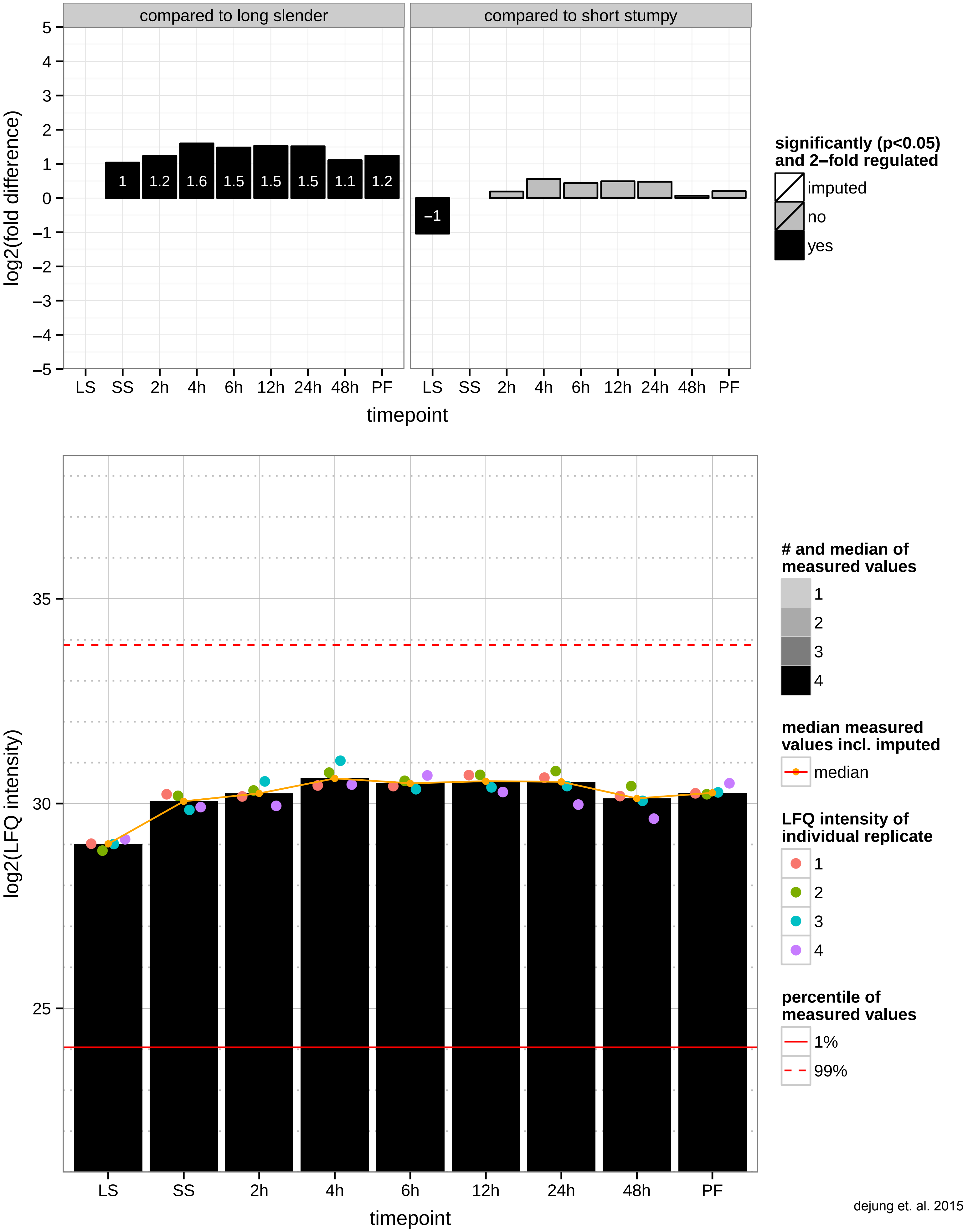

dejung et. al. 2015

hypothetical protein, conserved
Tb927.10.9260
AGOF: null
AGOC: null
AGOP: null
PGOF: binding
PGOC: null
PGOP: null

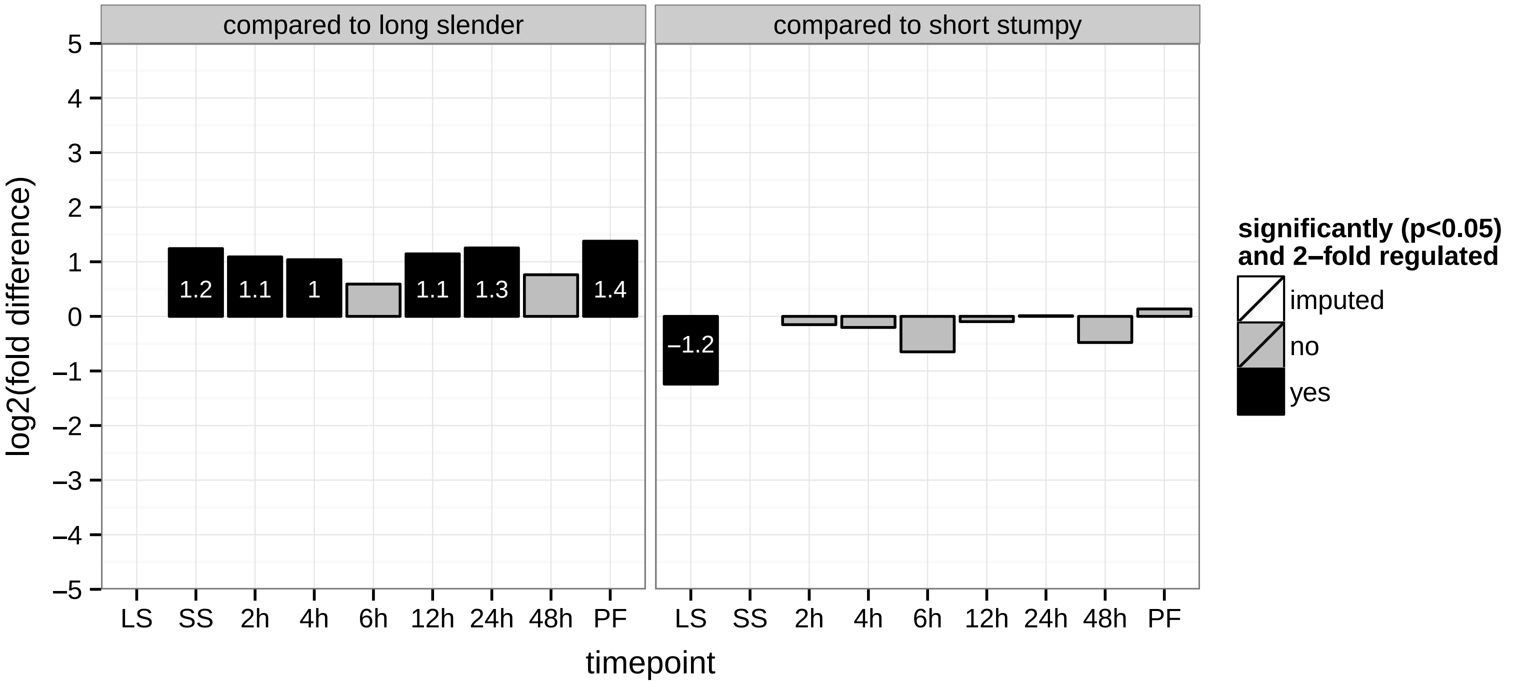

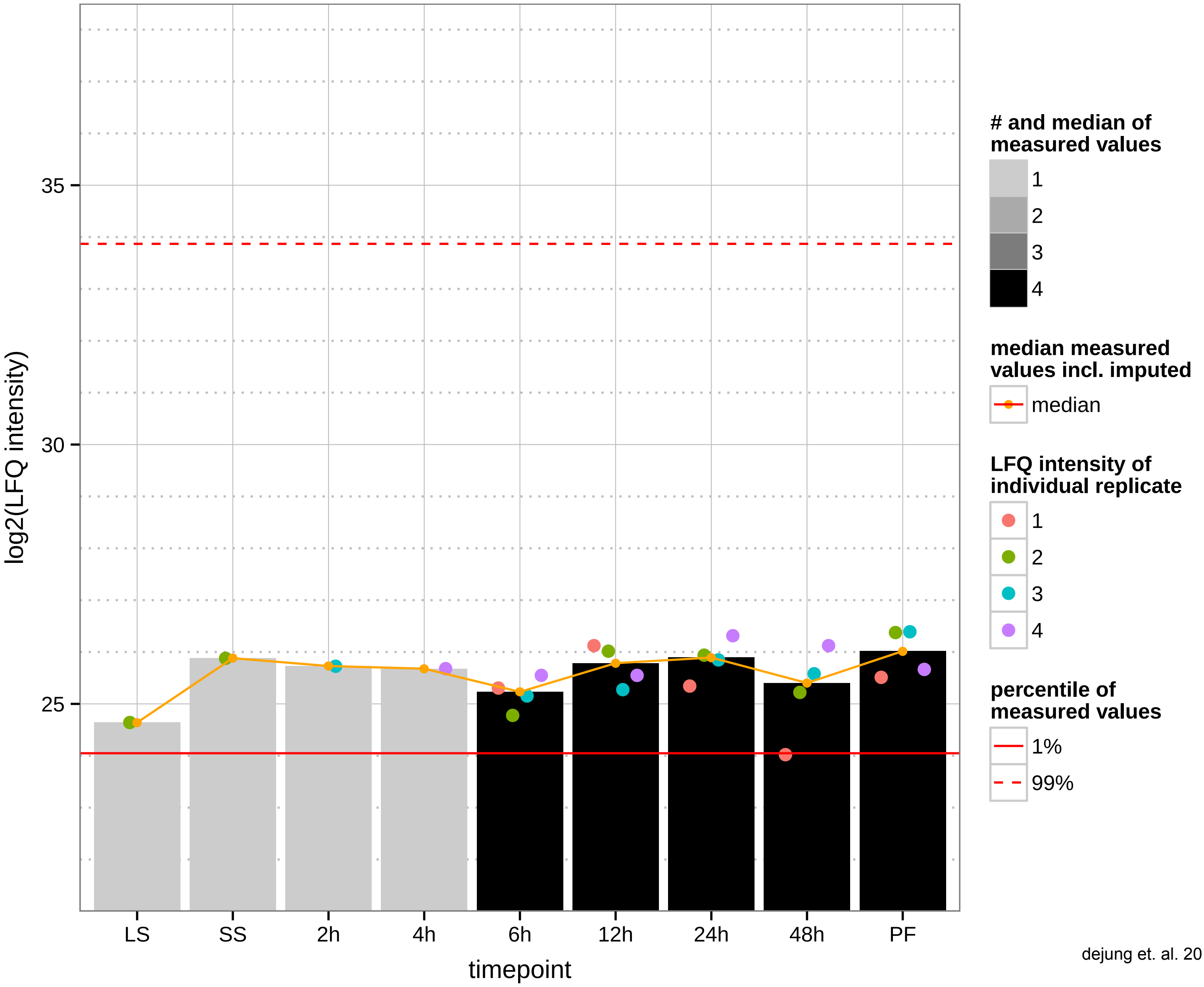

dejung et. al. 2015

hypothetical protein, conserved
Tb927.10.9390
AGOF: null
AGOC: null
AGOP: null
PGOF: null
PGOC: null
PGOP: null

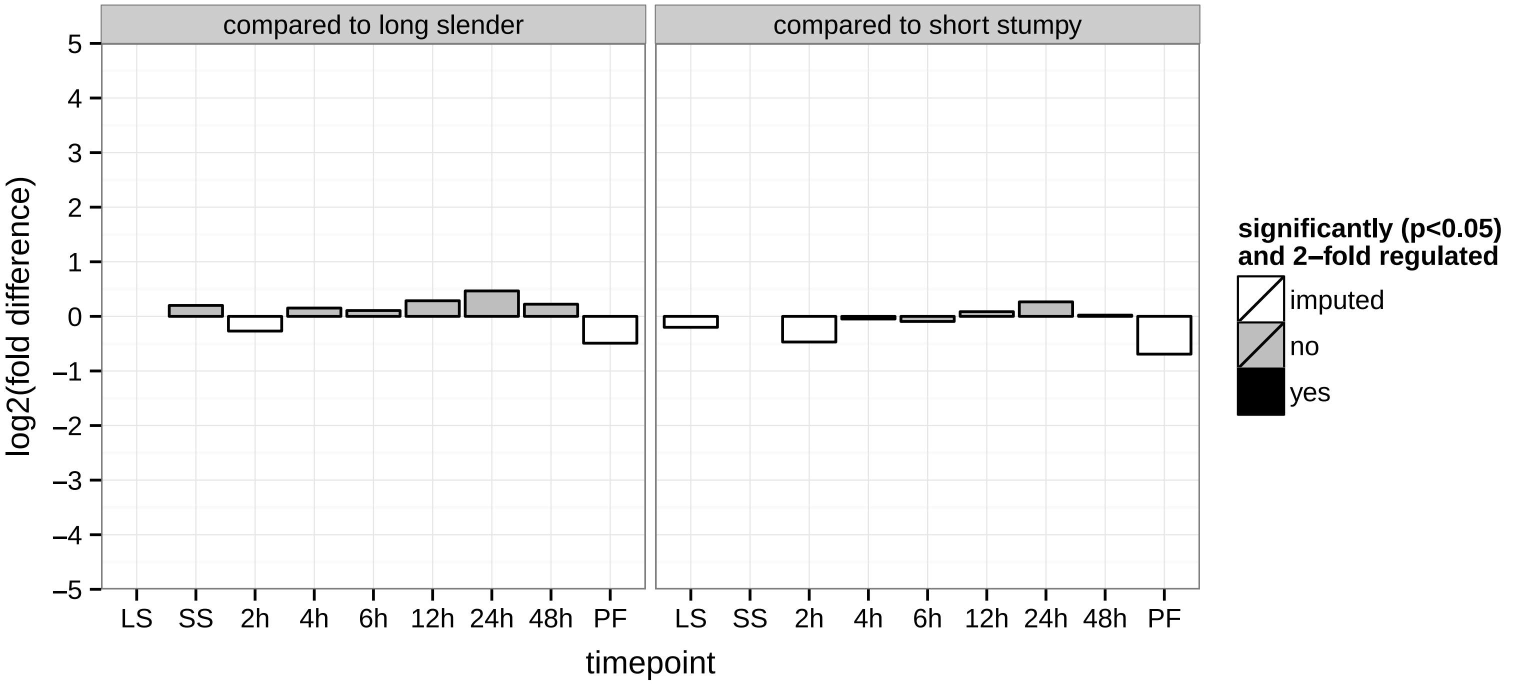

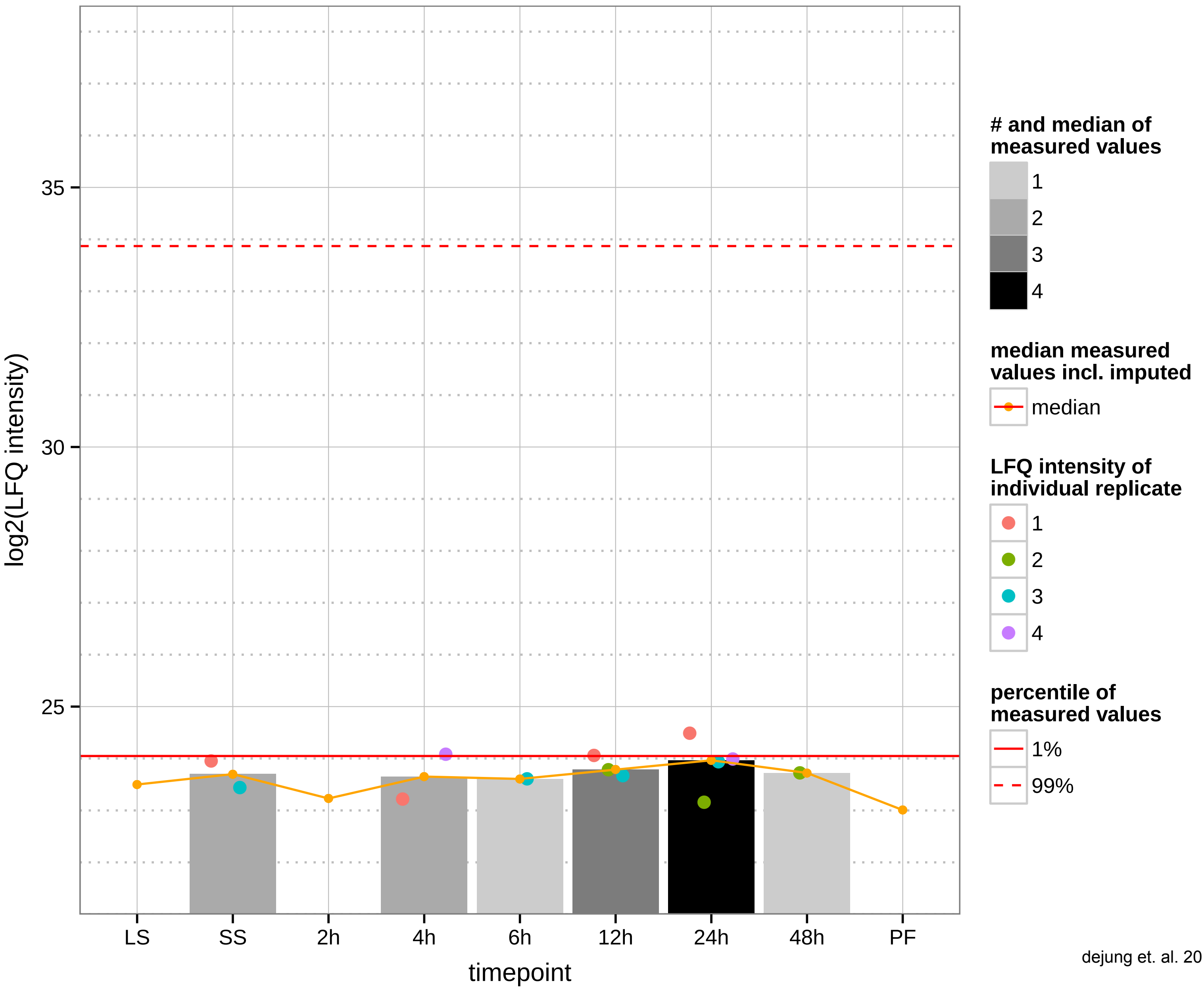

dejung et. al. 2015

branch point binding protein, putative
Tb927.10.9400
AGOF: transcription corepressor activity
AGOC: nucleus
AGOP: spliceosomal complex assembly
PGOF: null
PGOC: null
PGOP: null

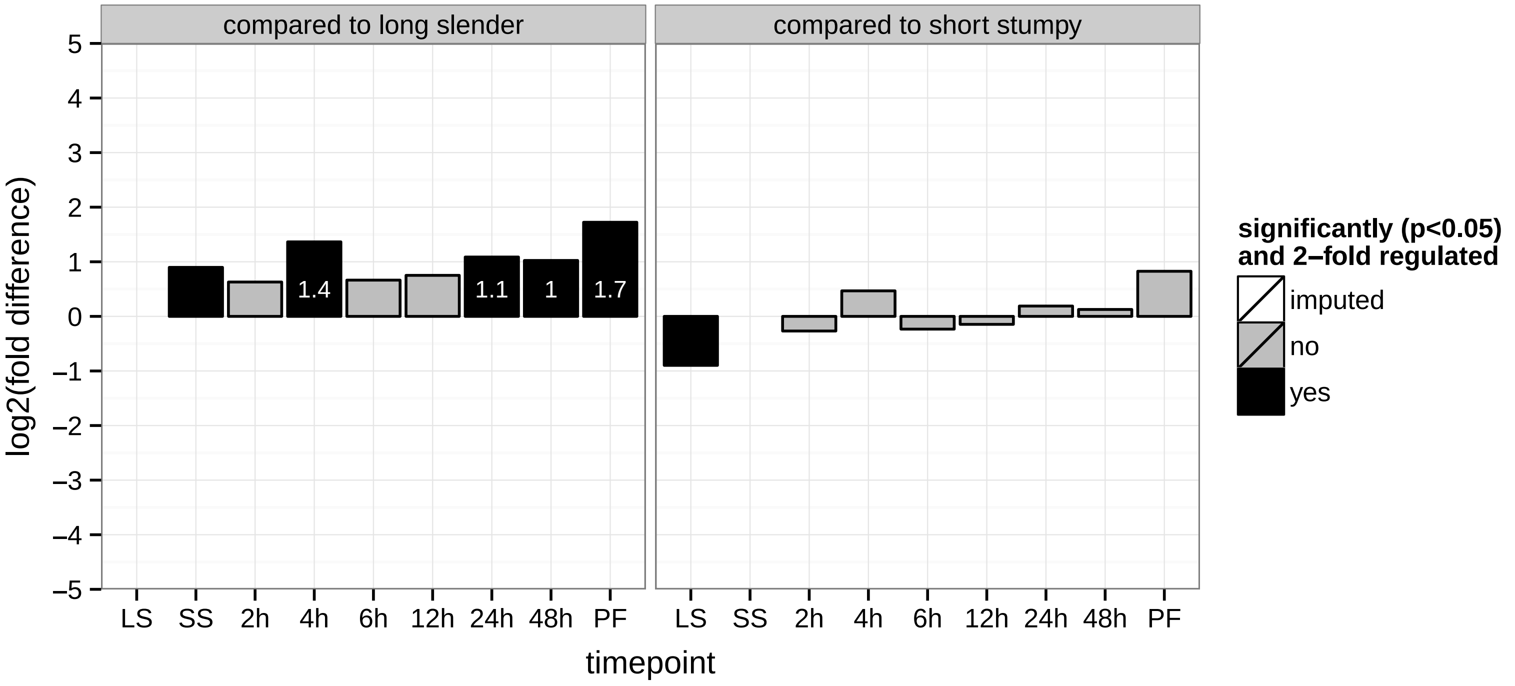

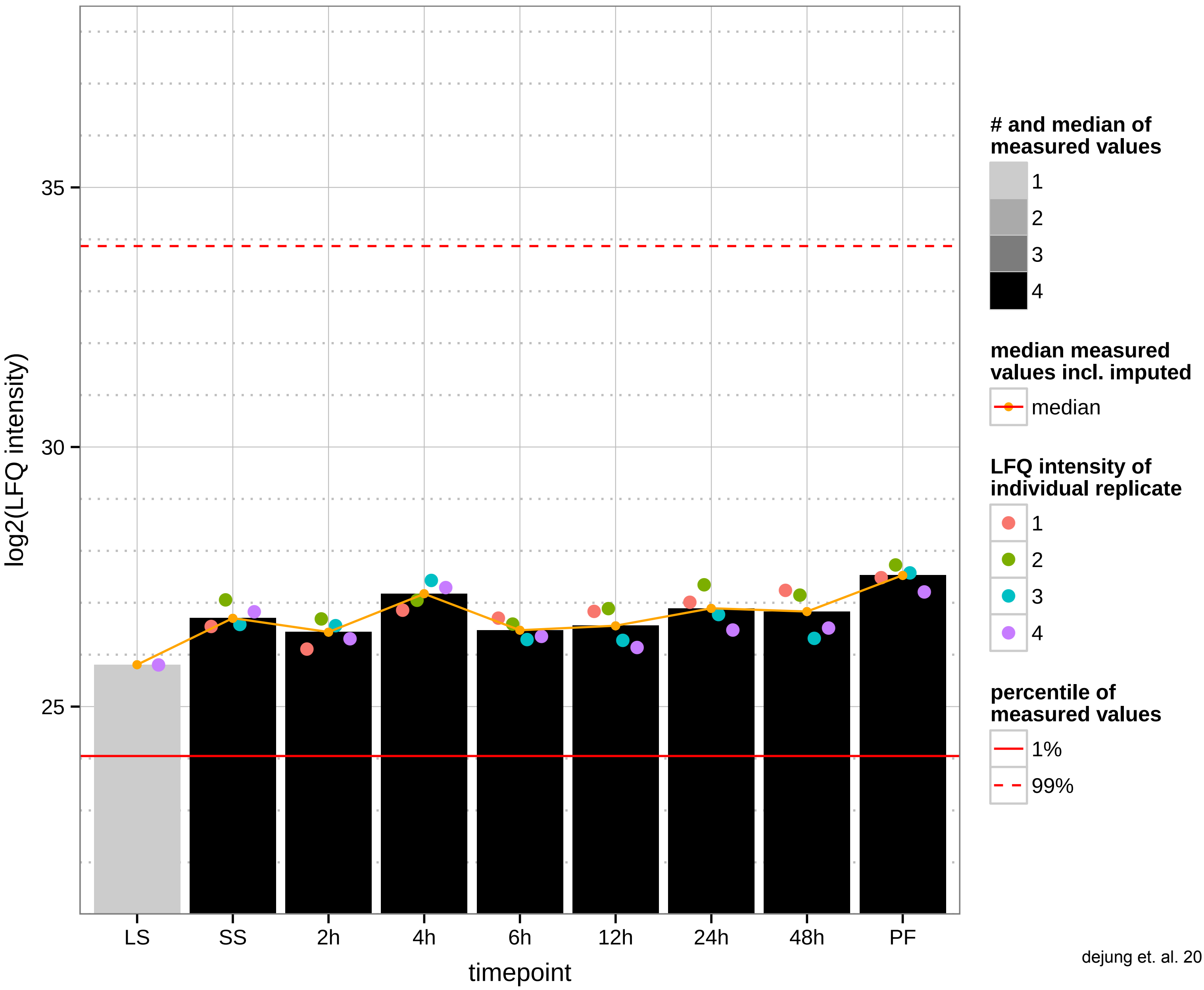

dejung et. al. 2015

selenophosphate synthetase, putative (SPS2)
Tb927.10.9410
AGOF: ATP binding, selenide, water dikinase activity
AGOC: null
AGOP: tRNA aminoacylation for protein translation
PGOF: ATP binding, catalytic activity, selenide, water dikinase activity
PGOC: null
PGOP: null

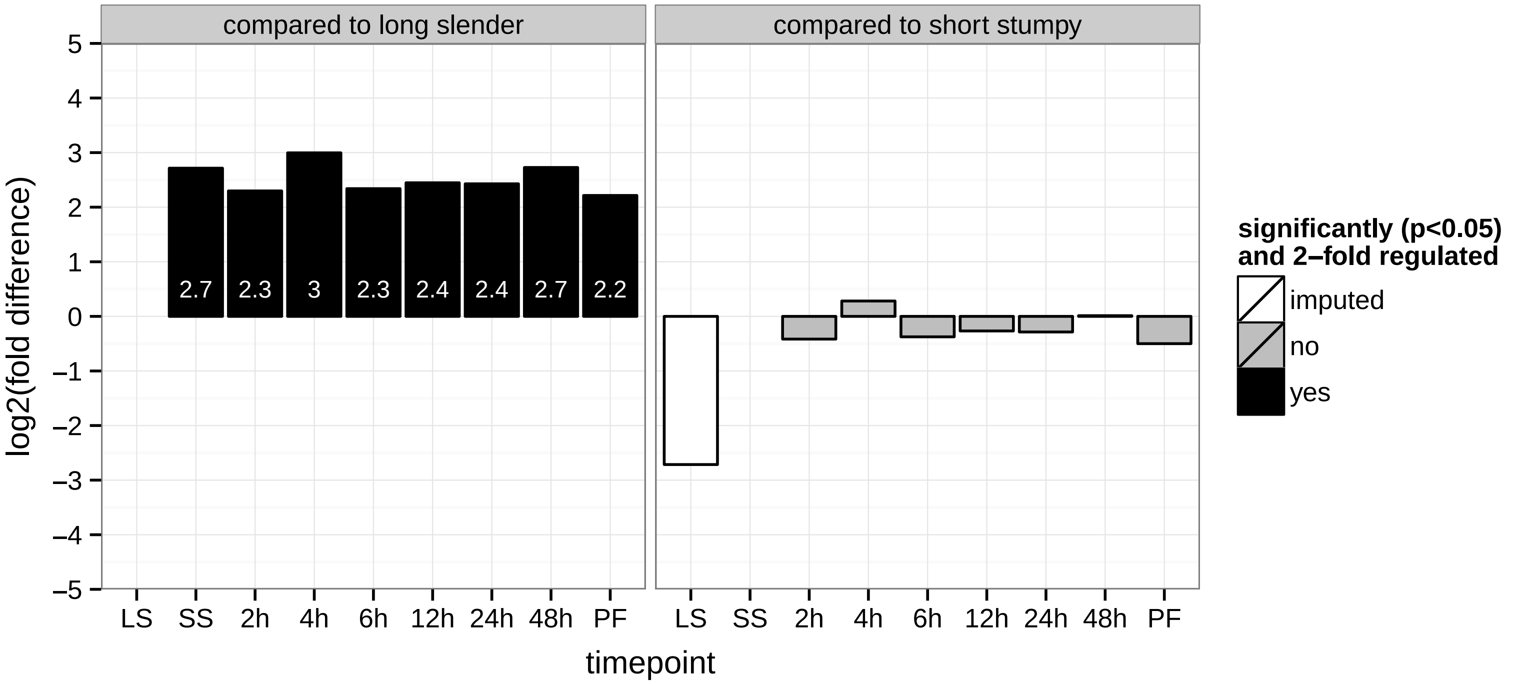

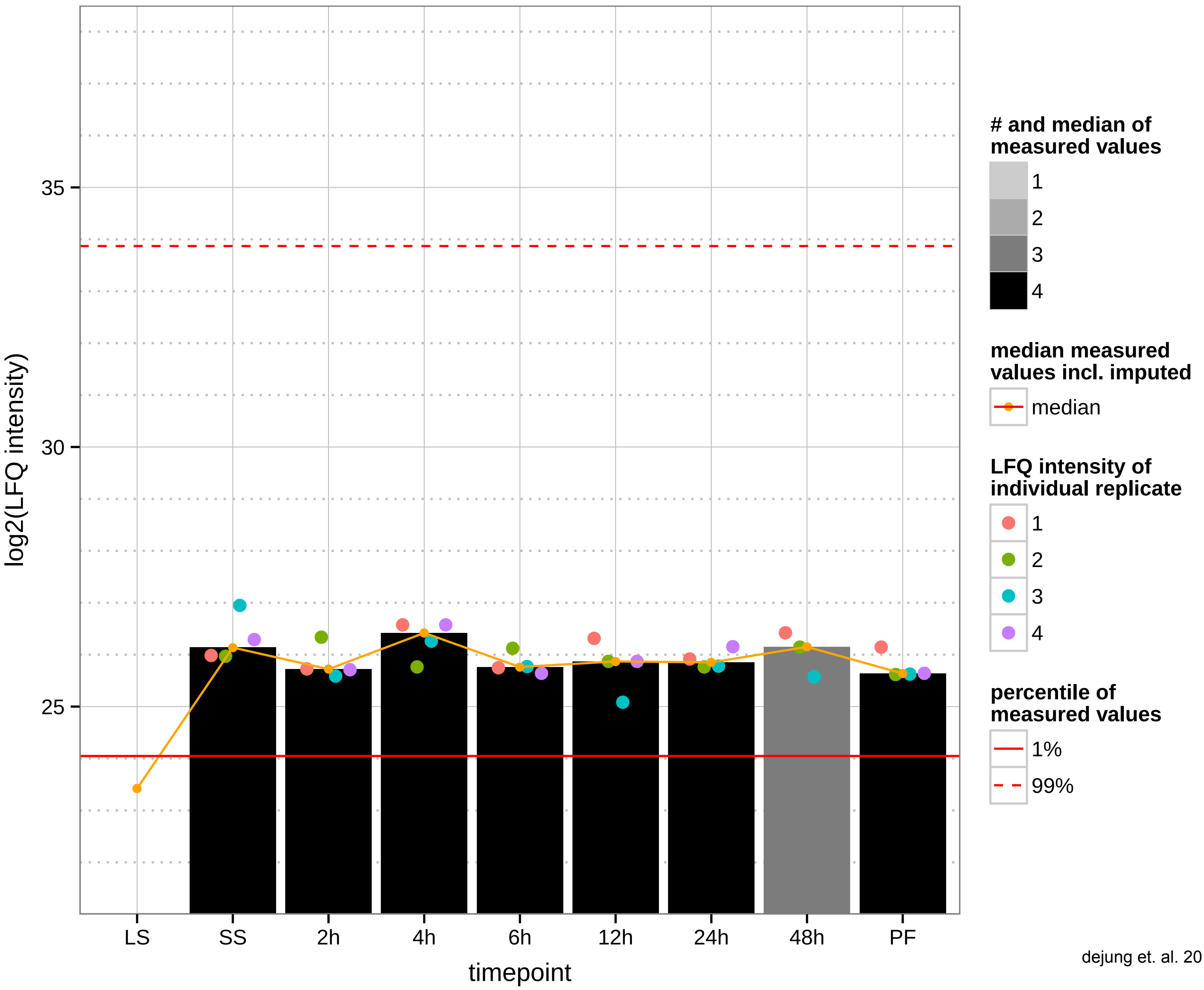

dejung et. al. 2015

phosphoribosylpyrophosphate synthetase, putative (PRS)
Tb927.10.9430
AGOF: ATP binding, magnesium ion binding, ribose phosphate diphosphokinase activity
AGOC: null
AGOP: nucleoside metabolic process, purine ribonucleotide biosynthetic process
PGOF: magnesium ion binding, ribose phosphate diphosphokinase activity
PGOC: null
PGOP: nucleoside metabolic process, nucleotide biosynthetic process

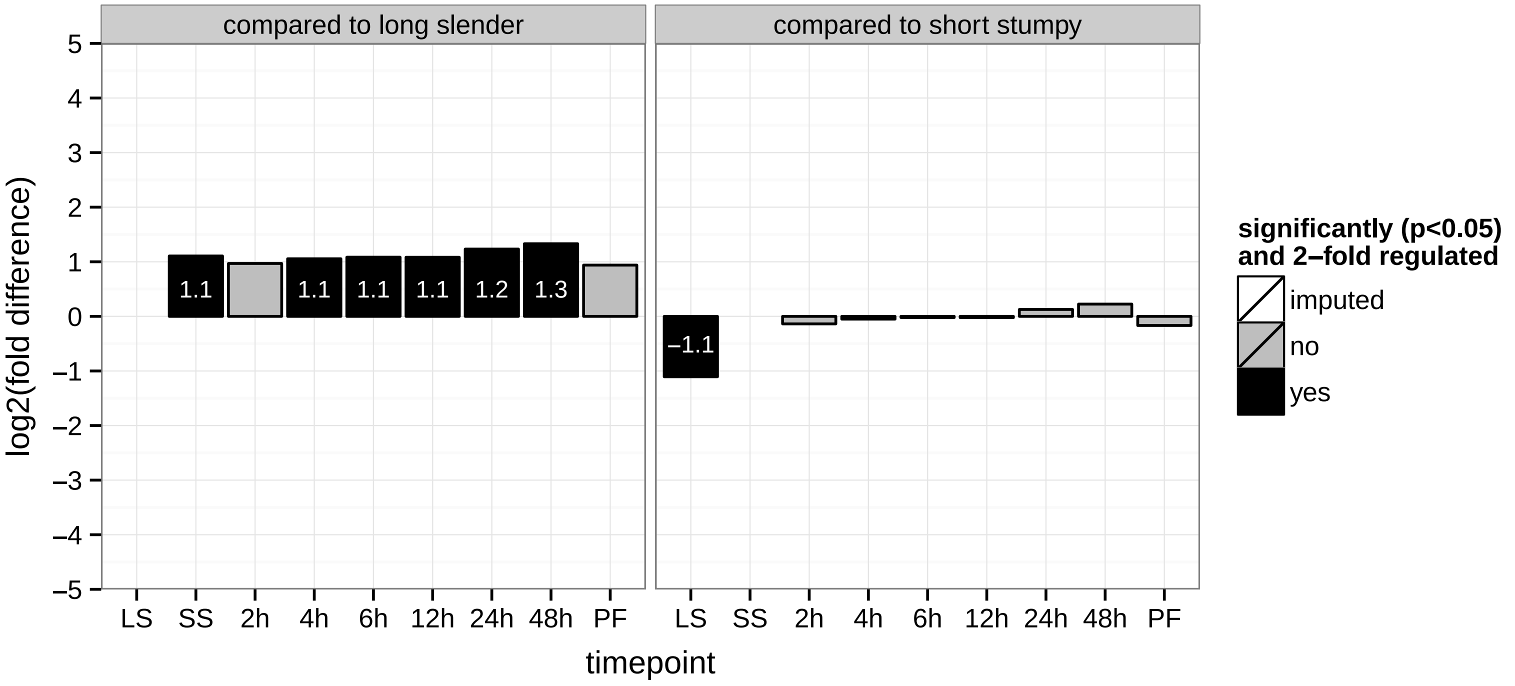

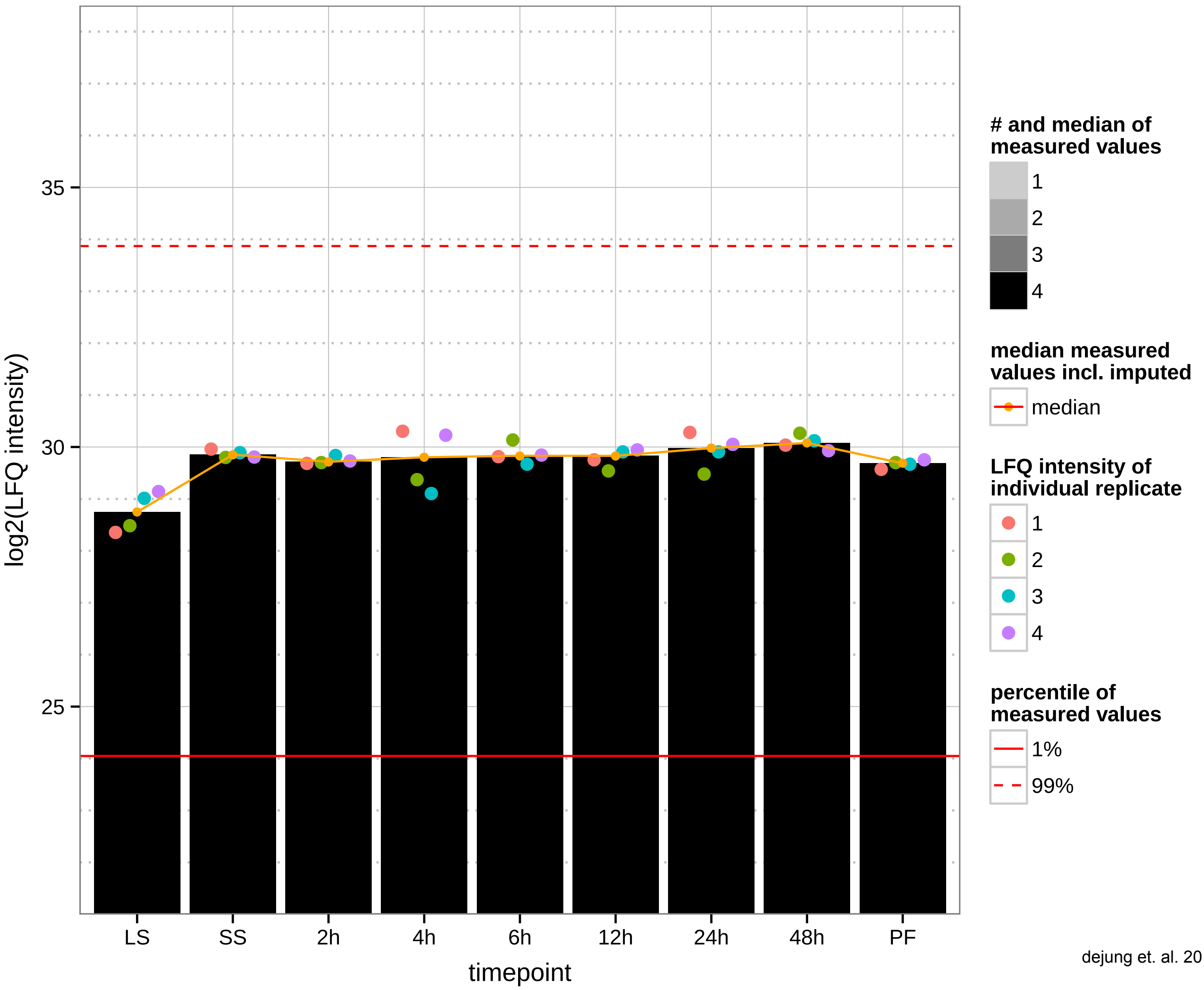

dejung et. al. 2015

NADH dehydrogenase (54 NDH2)
Tb927.10.9440
AGOF: NADH dehydrogenase (ubiquinone) activity
AGOC: mitochondrion
AGOP: mitochondrial electron transport, NADH to ubiquinone
PGOF: flavin adenine dinucleotide binding, oxidoreductase activity
PGOC: null
PGOP: oxidation–reduction process

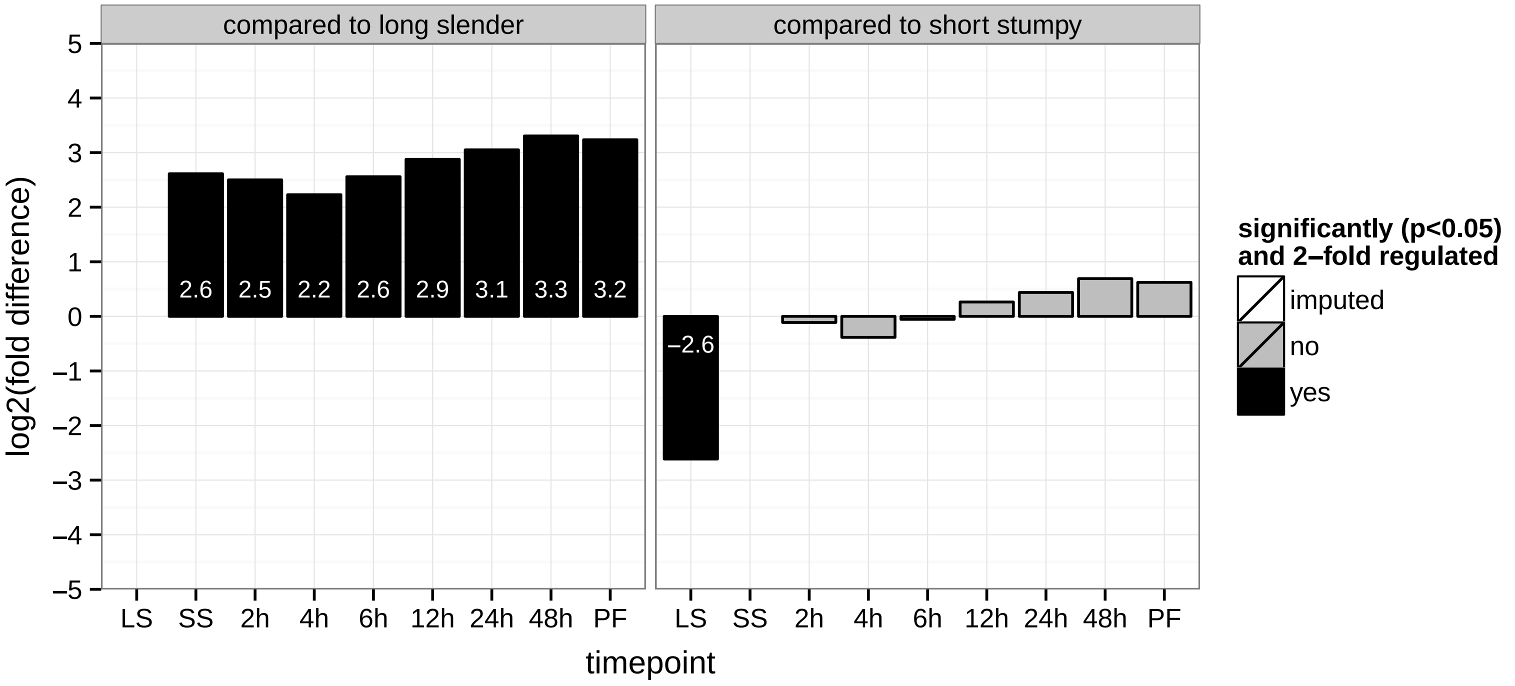

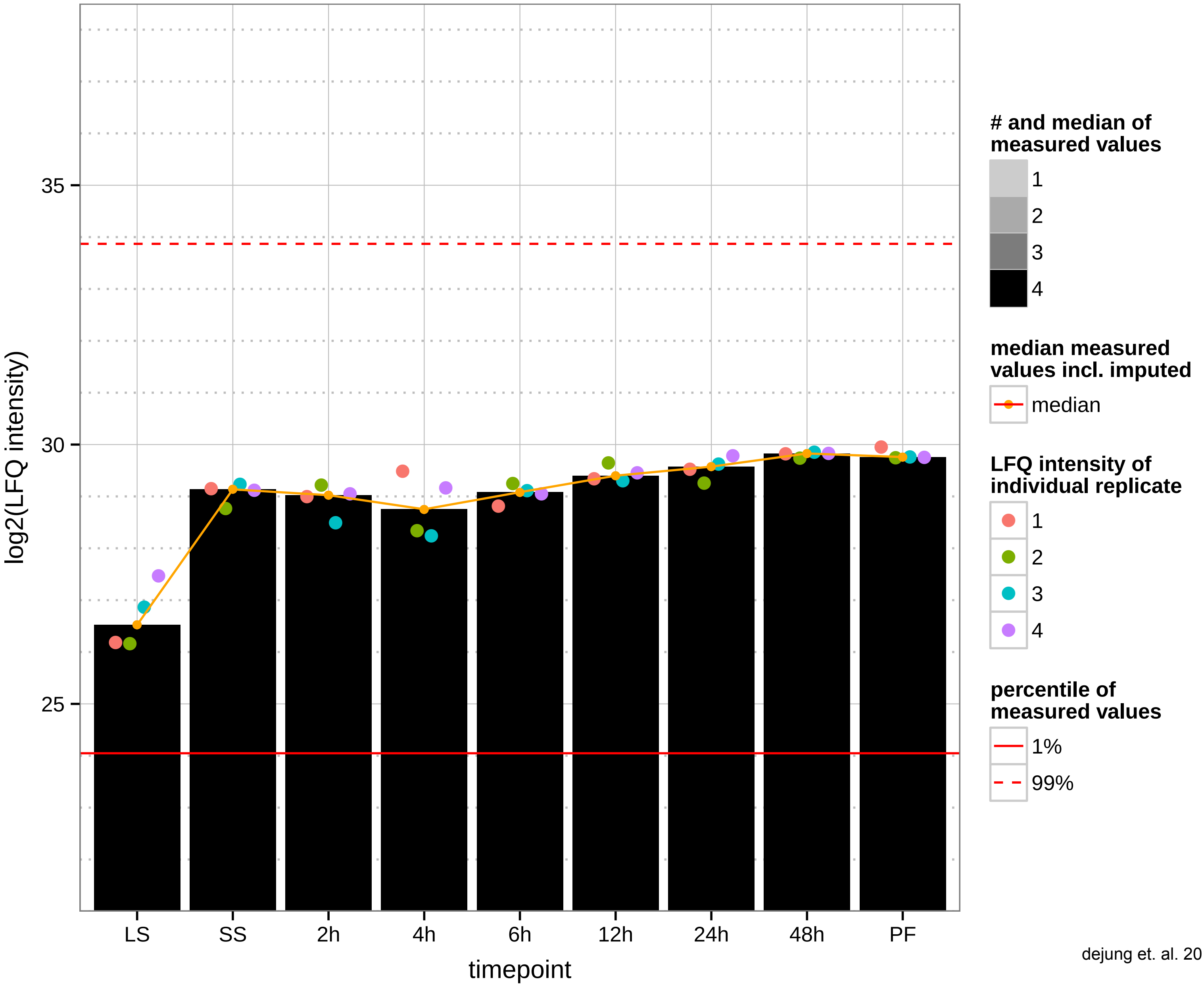

dejung et. al. 2015

oxidoreductase, putative
Tb927.10.9560
AGOF: alcohol dehydrogenase activity, zinc−dependent, oxidoreductase activity, zinc ion binding
AGOC: null
AGOP: metabolic process, oxidation−reduction process
PGOF: oxidoreductase activity, transferase activity, transferring acyl groups other than amino−acyl groups, zinc ion binding
PGOC: null
PGOP: oxidation−reduction process

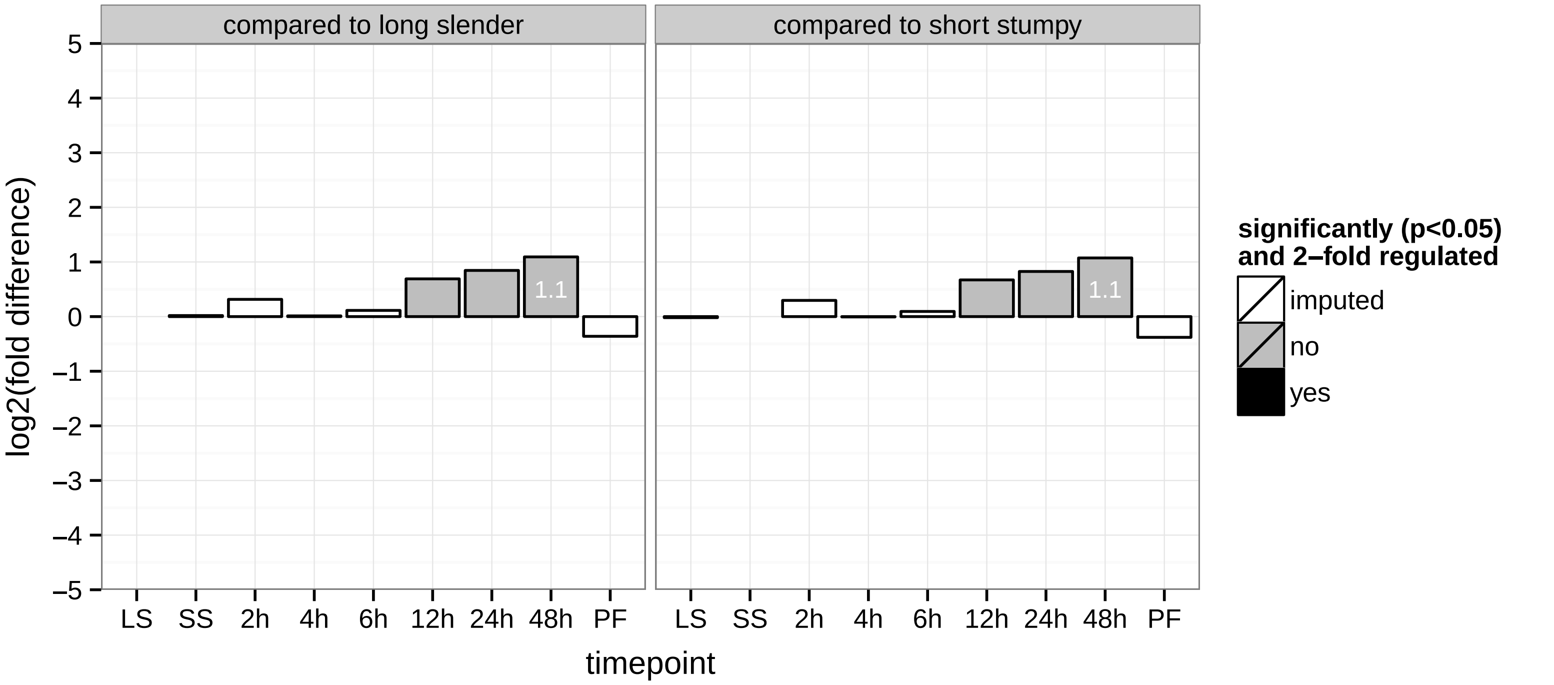

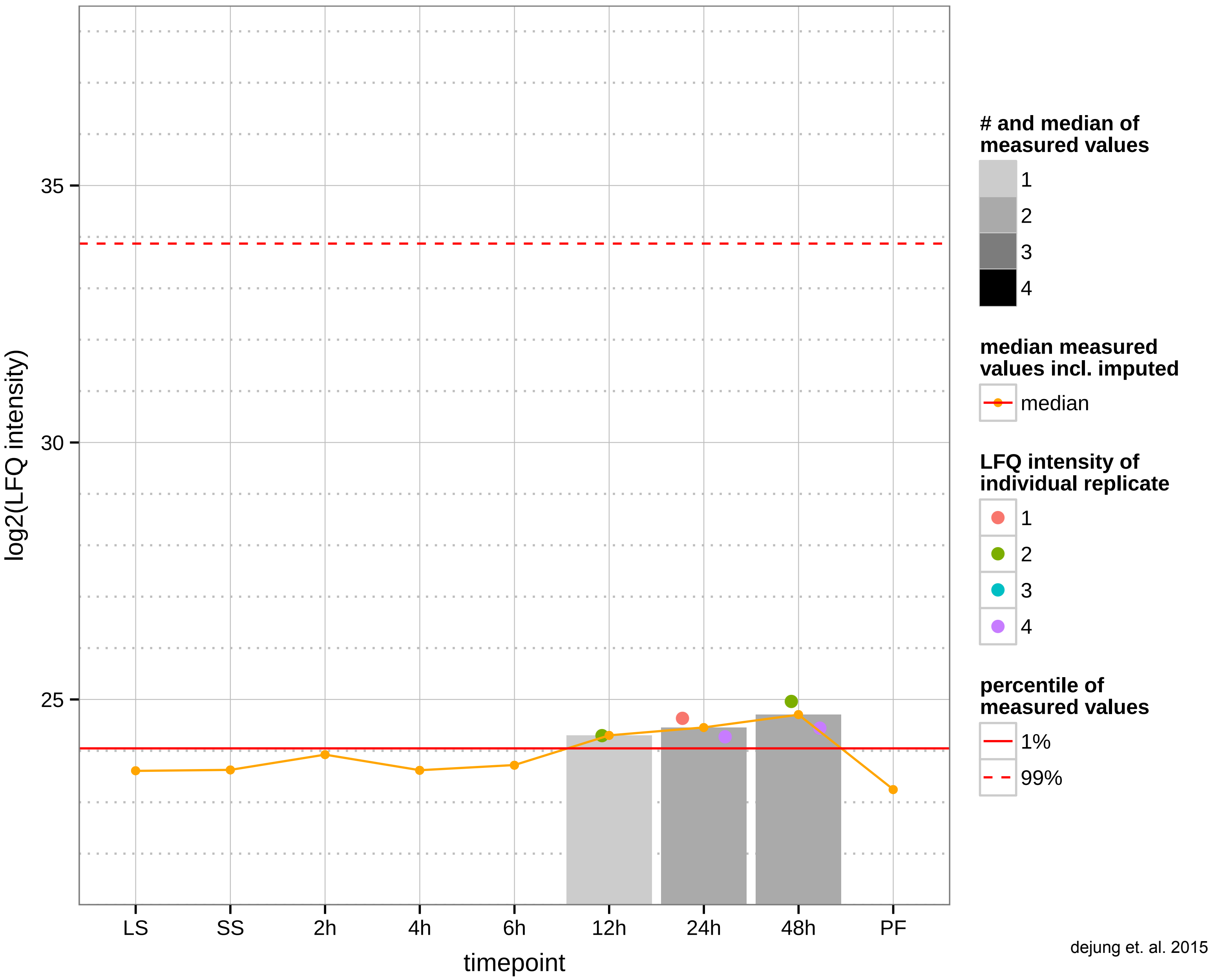

dejung et. al. 2015

hypothetical protein, conserved, paraflagellar rod component, putative (PFC14)
Tb927.10.9570
AGOF: null
AGOC: null
AGOP: null
PGOF: null, protein binding
PGOC: null
PGOP: null

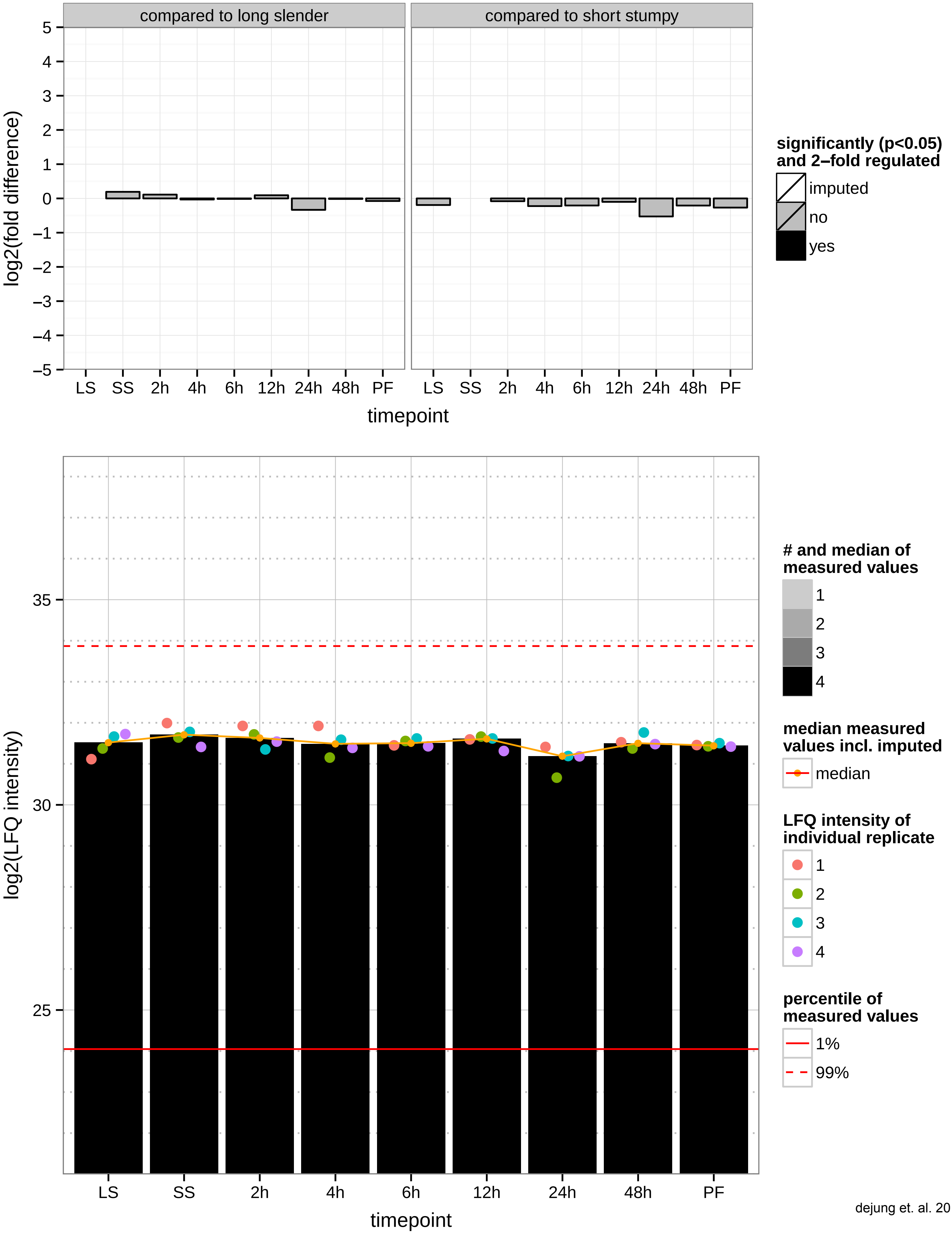

dejung et. al. 2015

hypothetical protein, conserved
Tb927.10.9640
AGOF: null
AGOC: null
AGOP: null
PGOF: null
PGOC: null
PGOP: null

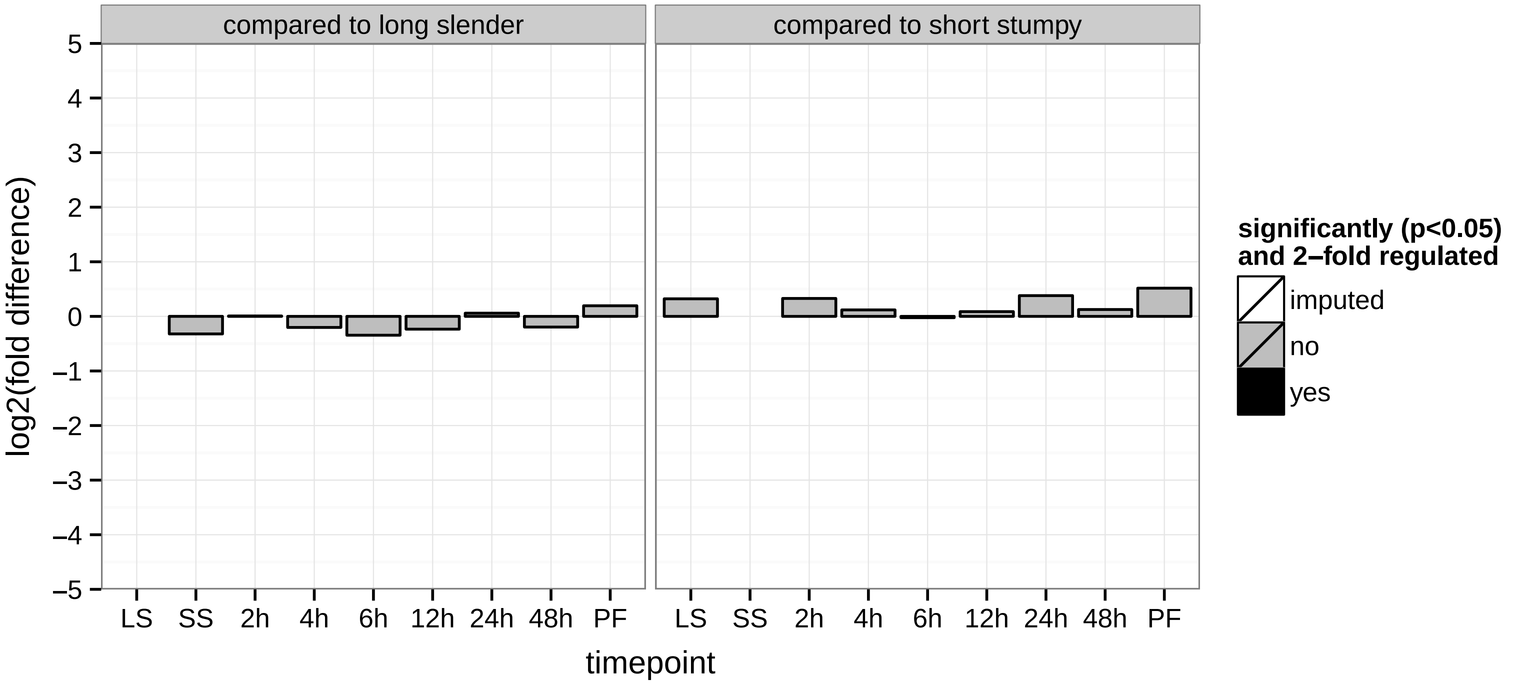

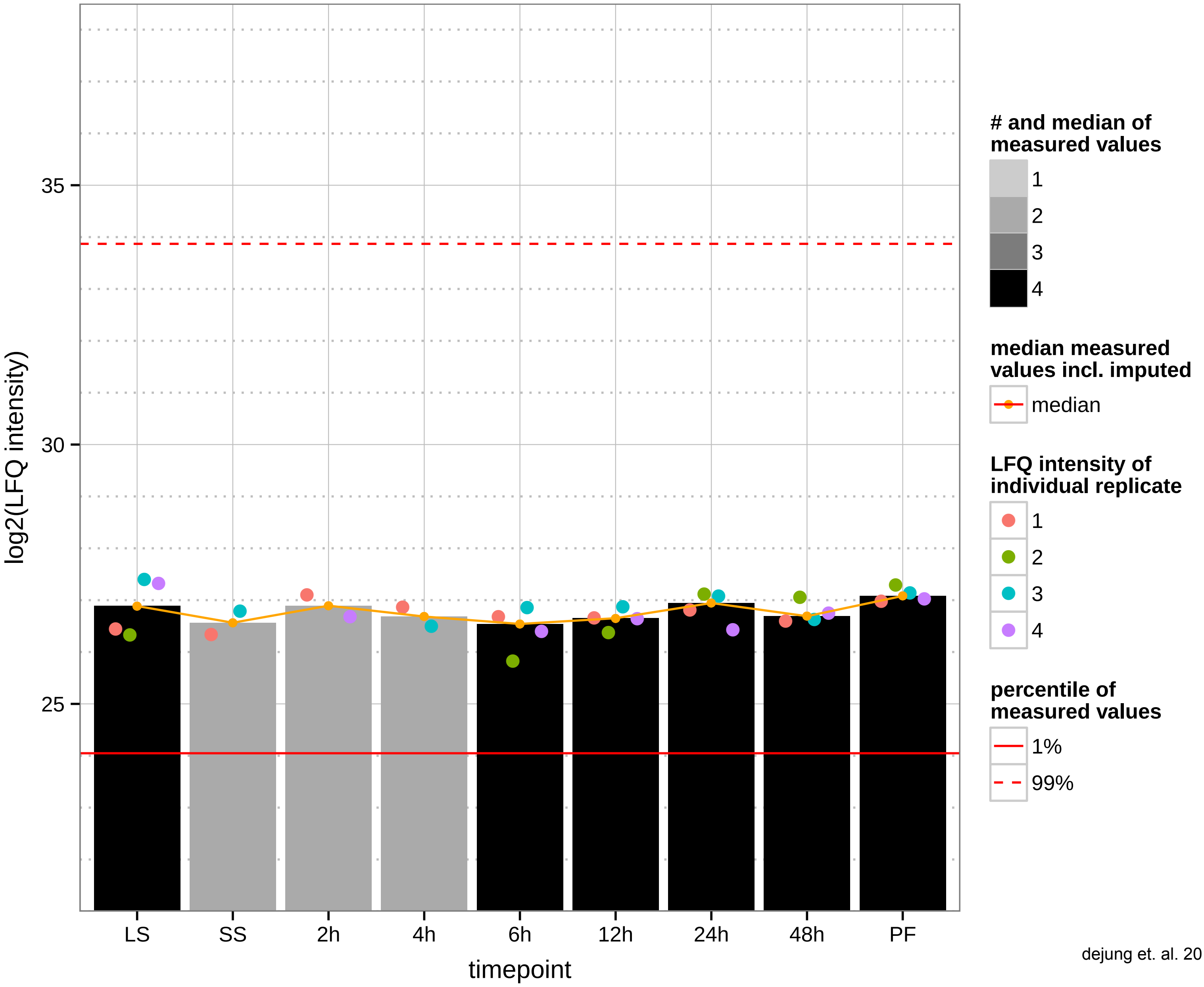

dejung et. al. 2015

hypothetical protein, conserved
Tb927.10.9650;Tb11.v5.0399
AGOF: null
AGOC: null
AGOP: null
PGOF: null
PGOC: null
PGOP: null

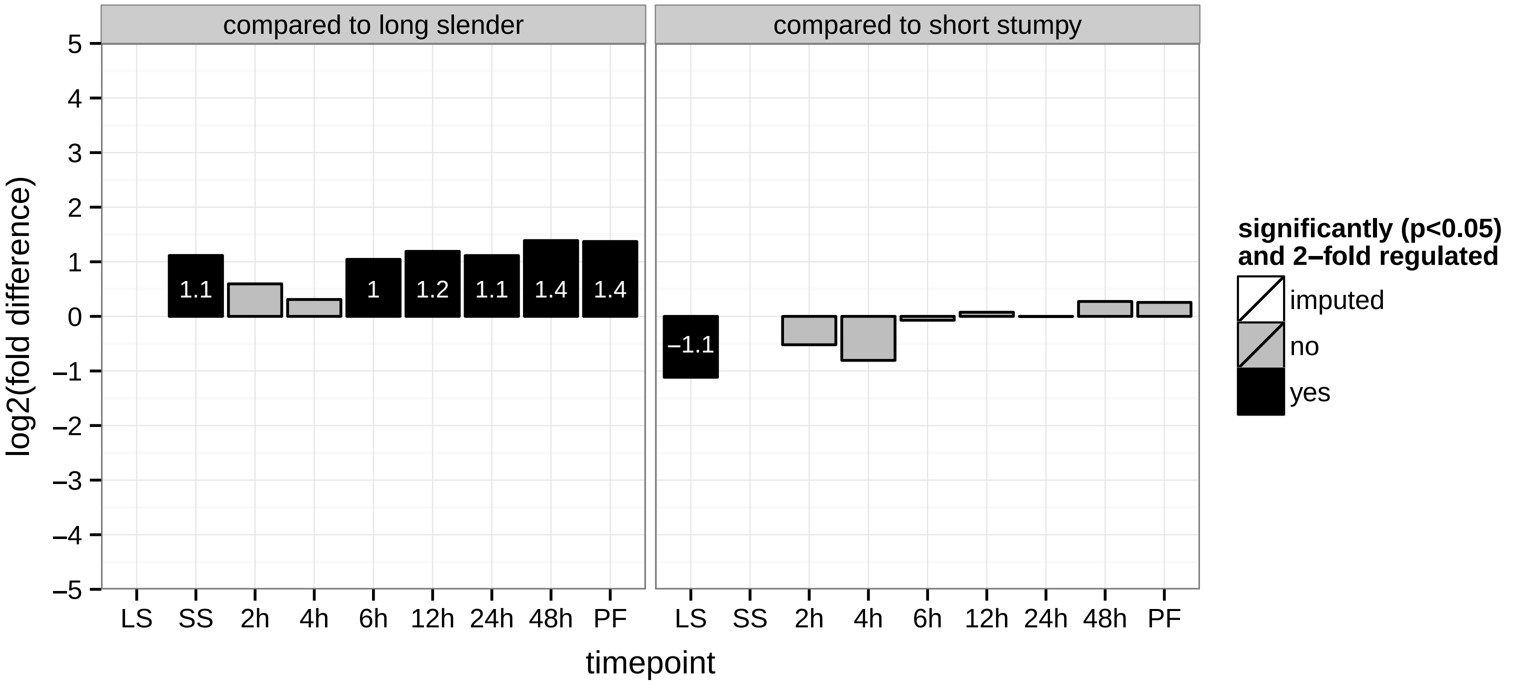

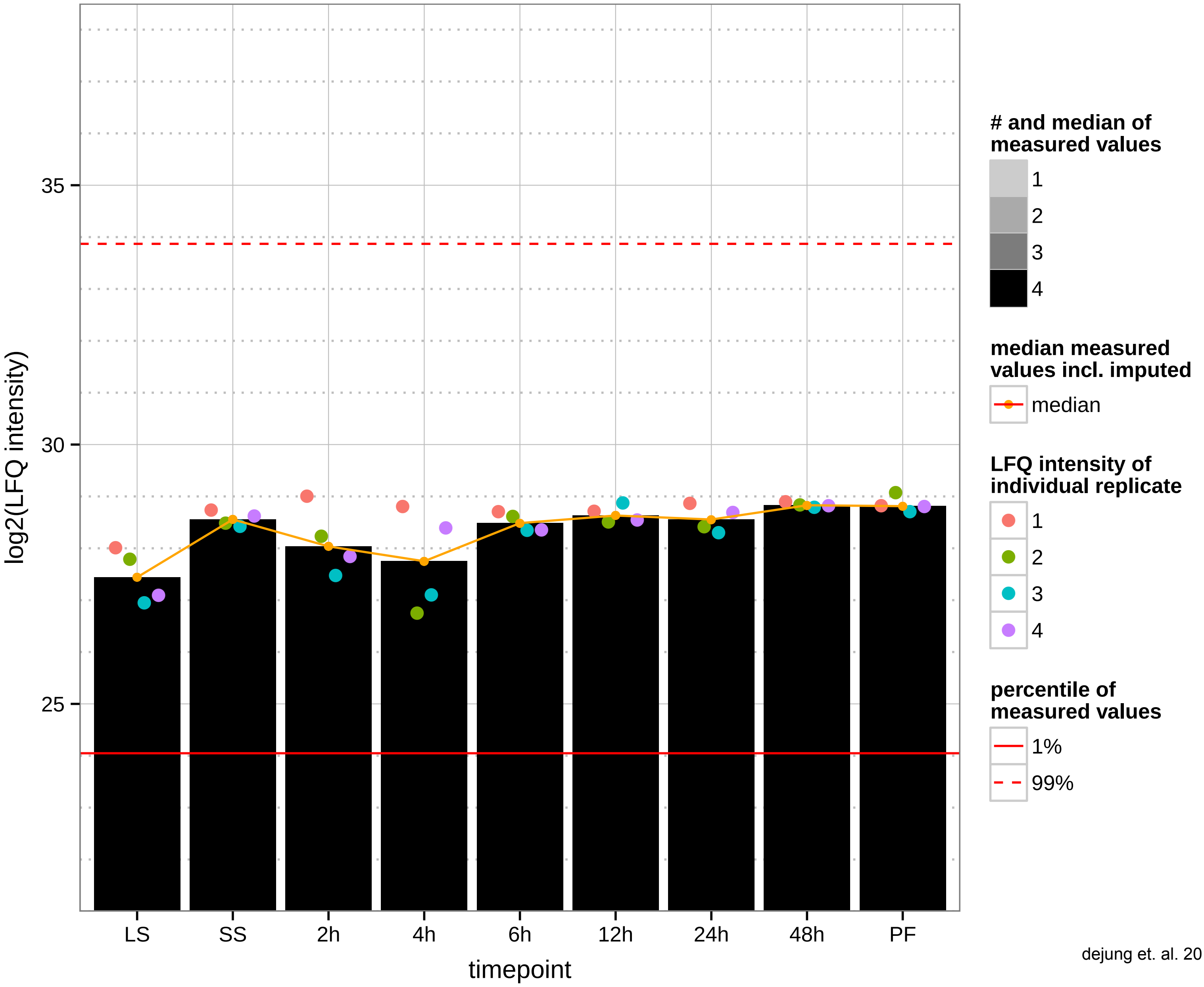

dejung et. al. 2015

hypothetical protein, conserved
Tb927.10.9660;Tb11.v5.0735
AGOF: null
AGOC: null, intracellular
AGOP: null, RNA processing
PGOF: null
PGOC: intracellular
PGOP: RNA processing

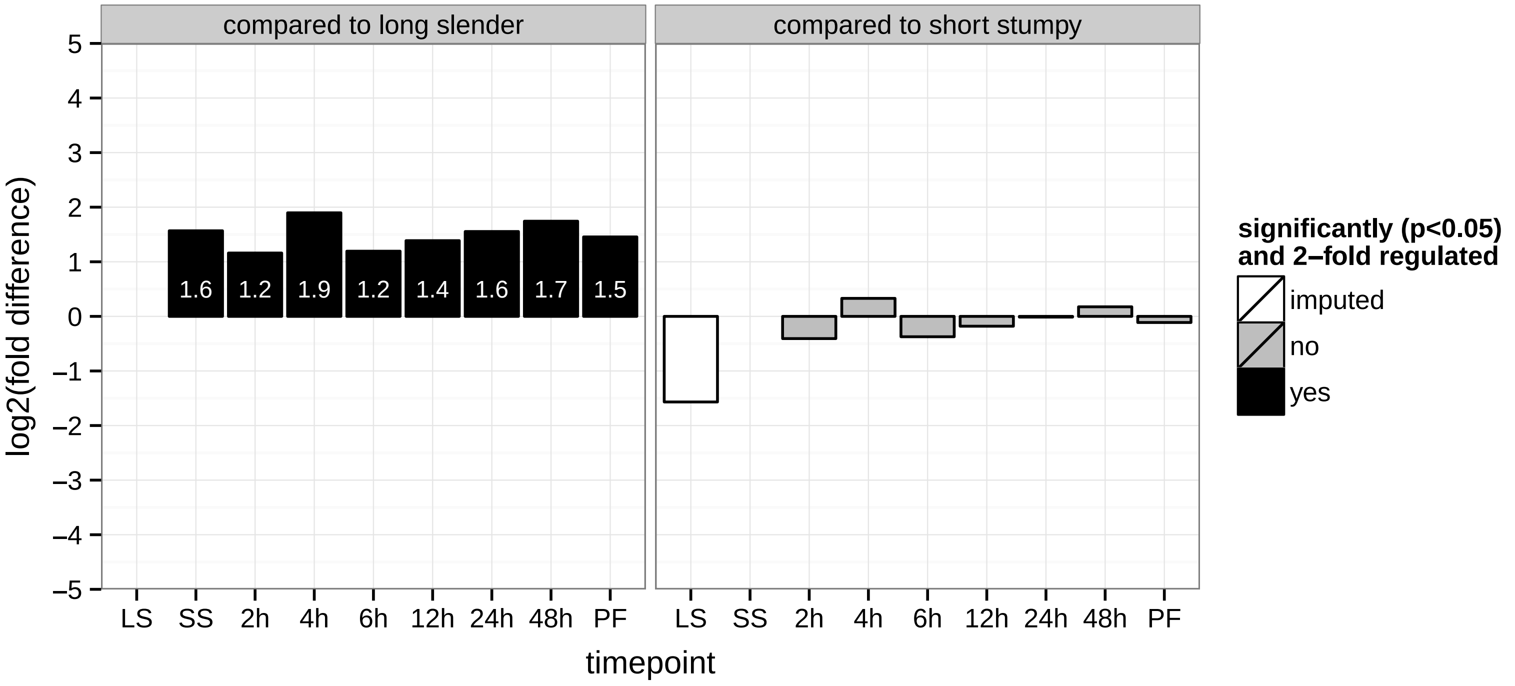

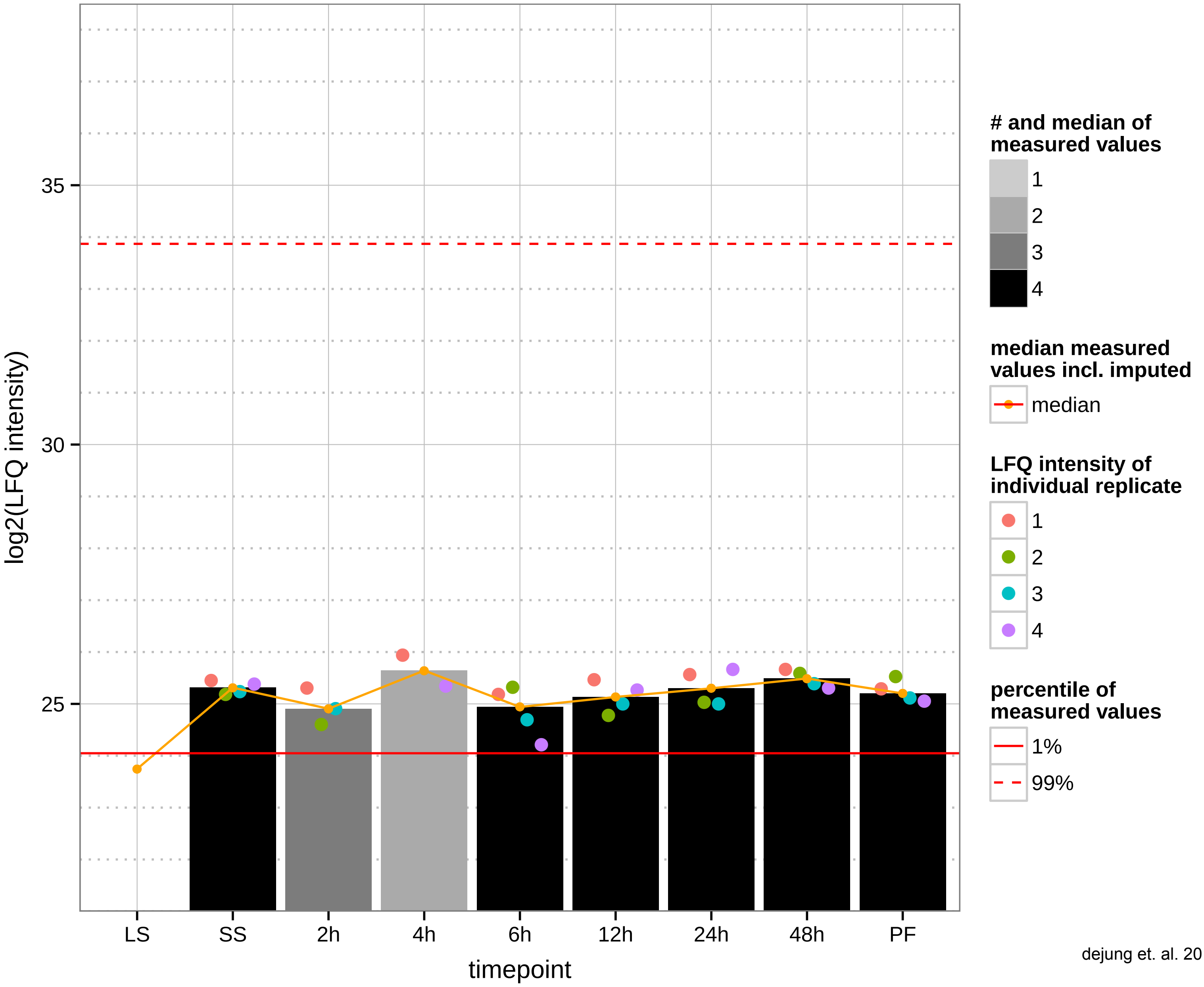

dejung et. al. 2015

hypothetical protein, conserved
Tb927.10.9680
AGOF: null
AGOC: null
AGOP: null
PGOF: protein binding
PGOC: null
PGOP: null

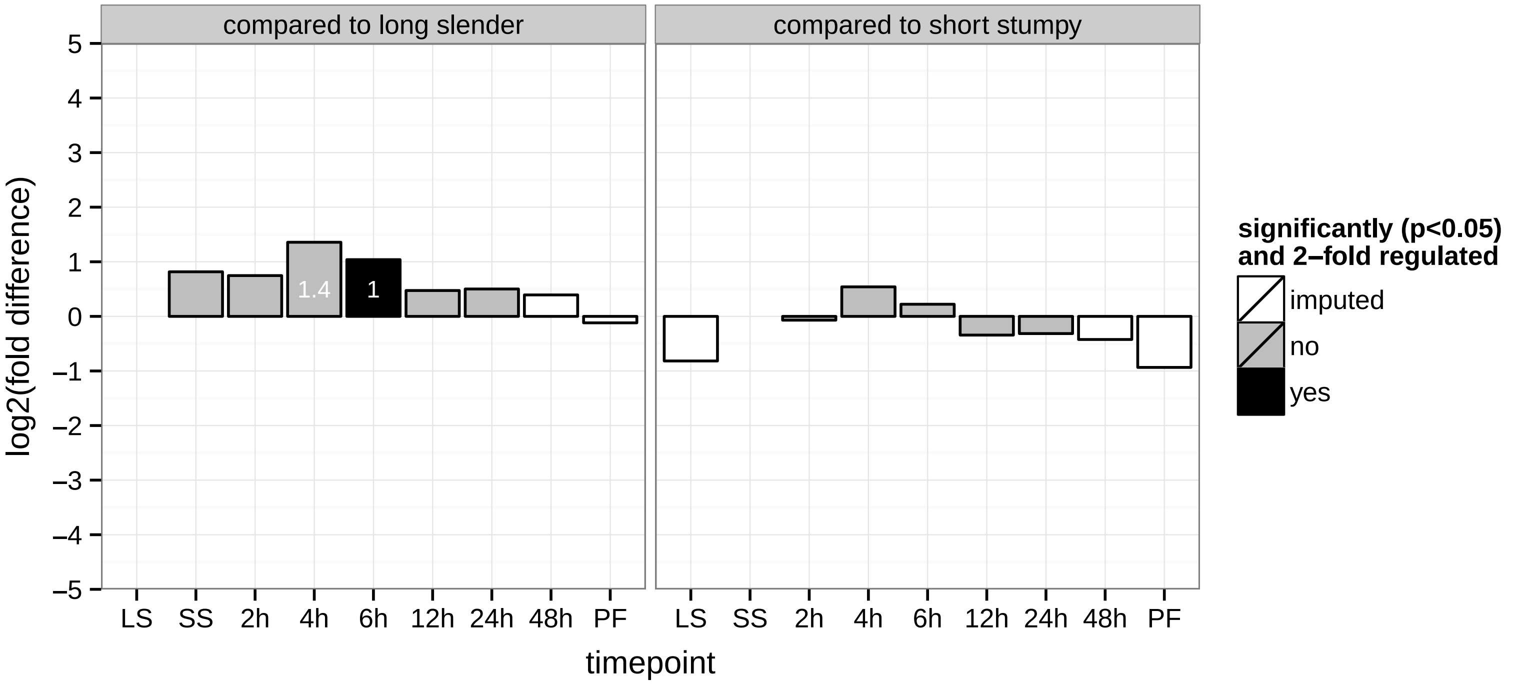

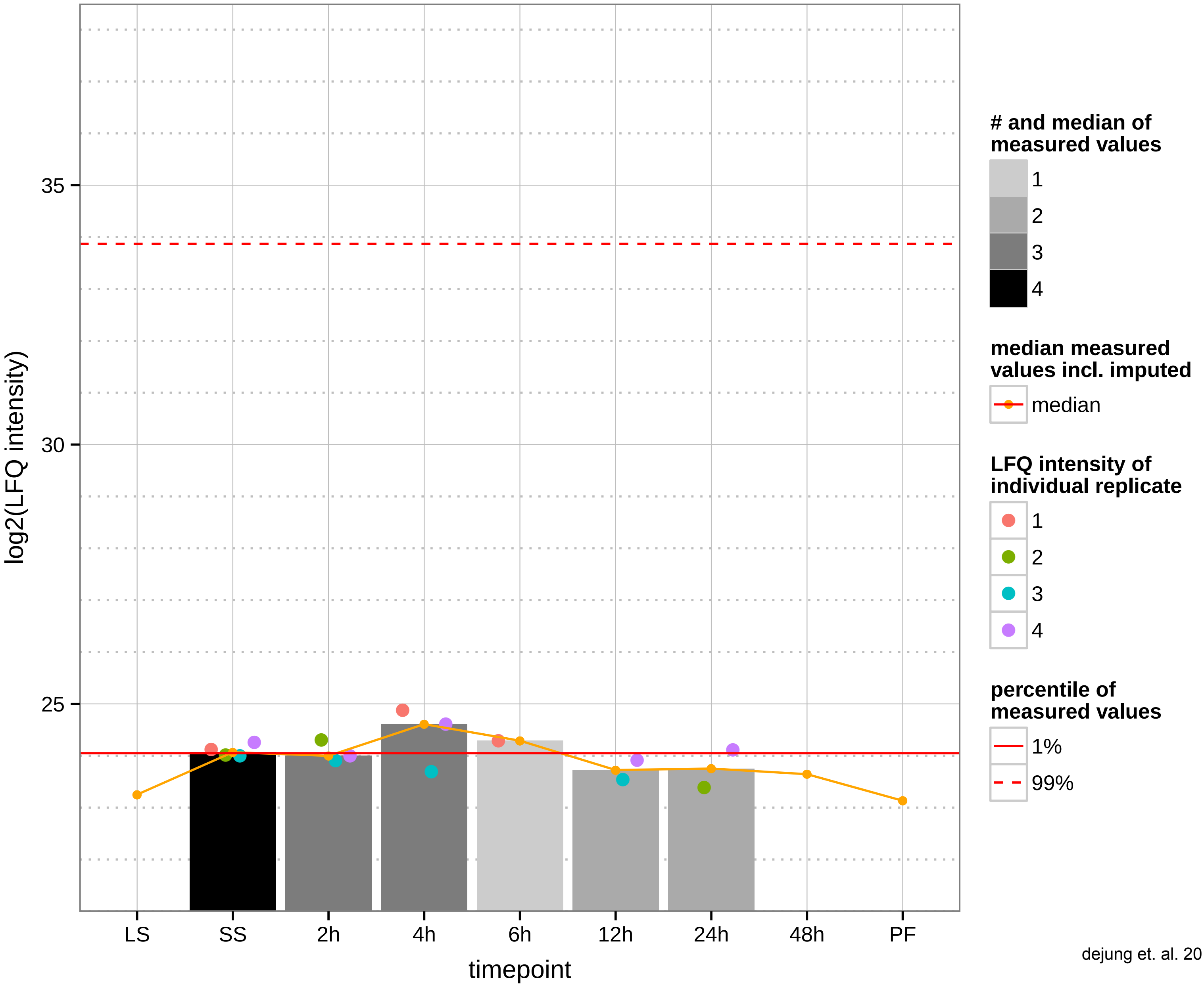

dejung et. al. 2015

hypothetical protein, conserved, predicted C2 domain protein
Tb927.10.9700
AGOF: null
AGOC: null
AGOP: null
PGOF: null, protein binding
PGOC: null
PGOP: null

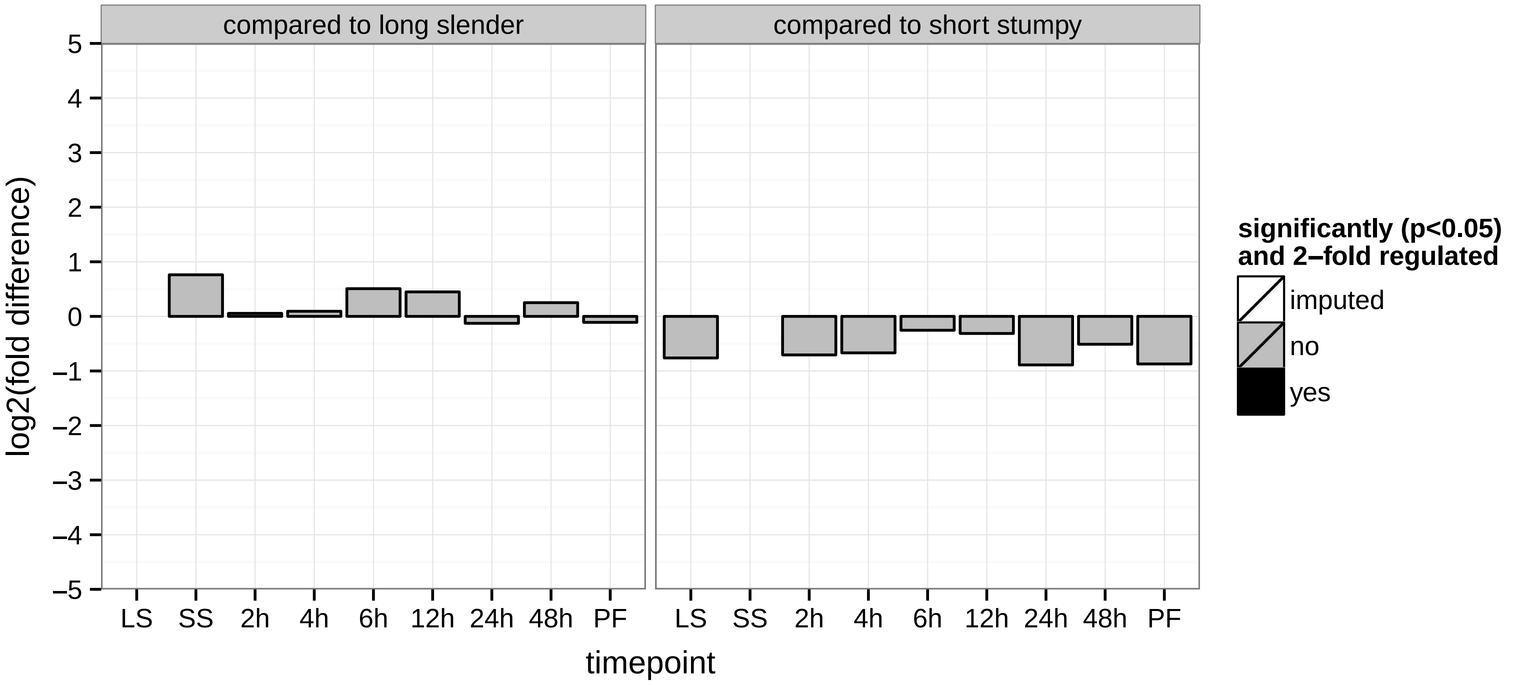

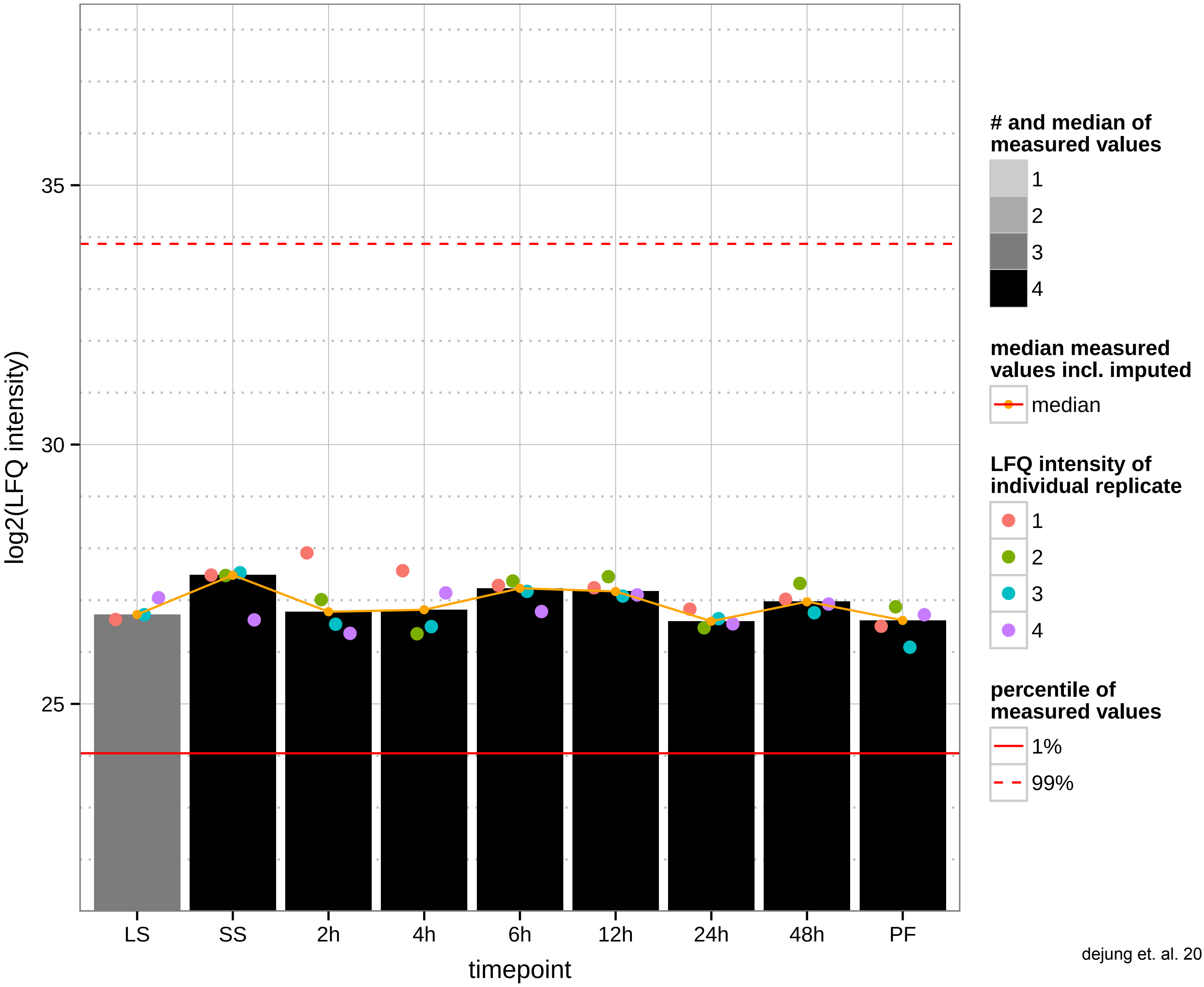

dejung et. al. 2015

proteasome regulatory ATPase subunit 6, 19S proteasome regulatory subunit (RPT6)
Tb927.10.9740
AGOF: ATP binding, nucleoside–triphosphatase activity
AGOC: cytoplasm, nucleus, proteasome regulatory particle
AGOP: ubiquitin–dependent protein catabolic process
PGOF: ATP binding, hydrolase activity, nucleoside–triphosphatase activity, nucleotide binding
PGOC: cytoplasm
PGOP: protein catabolic process

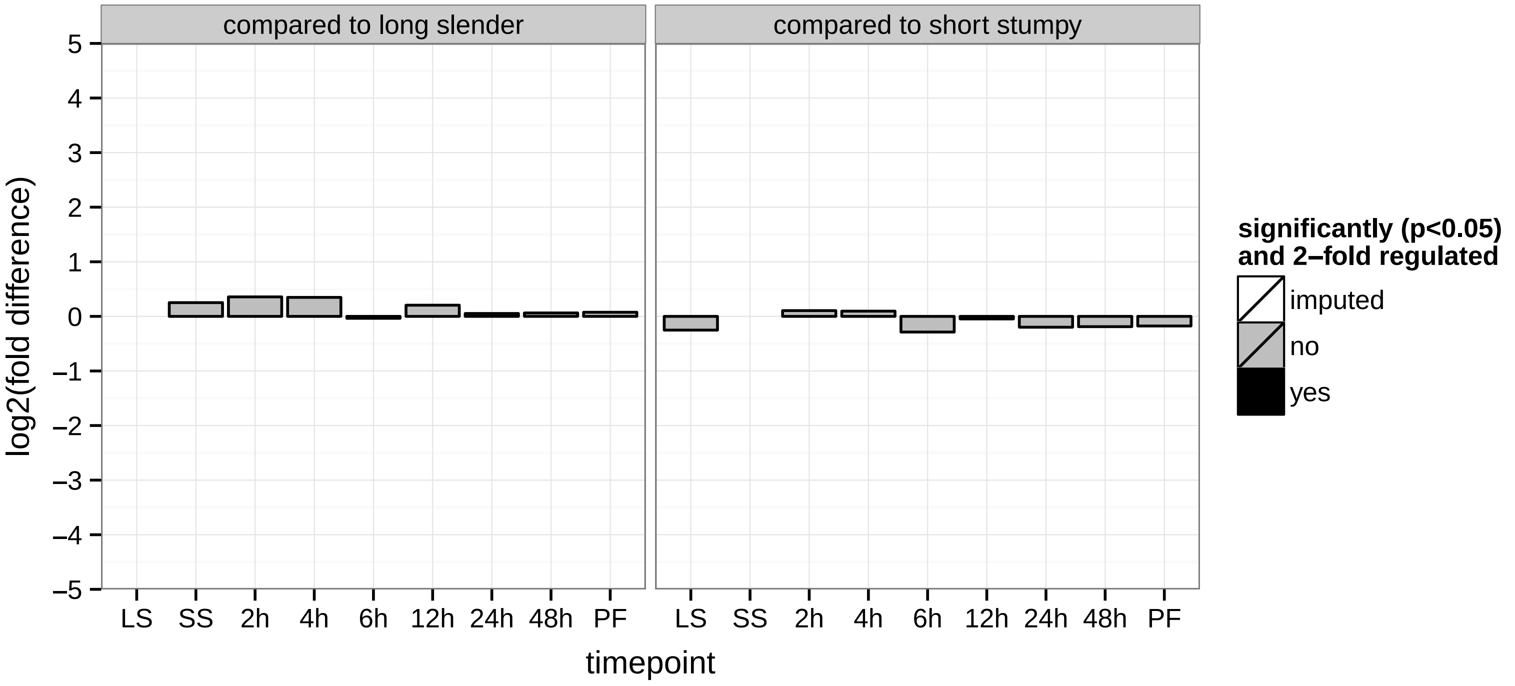

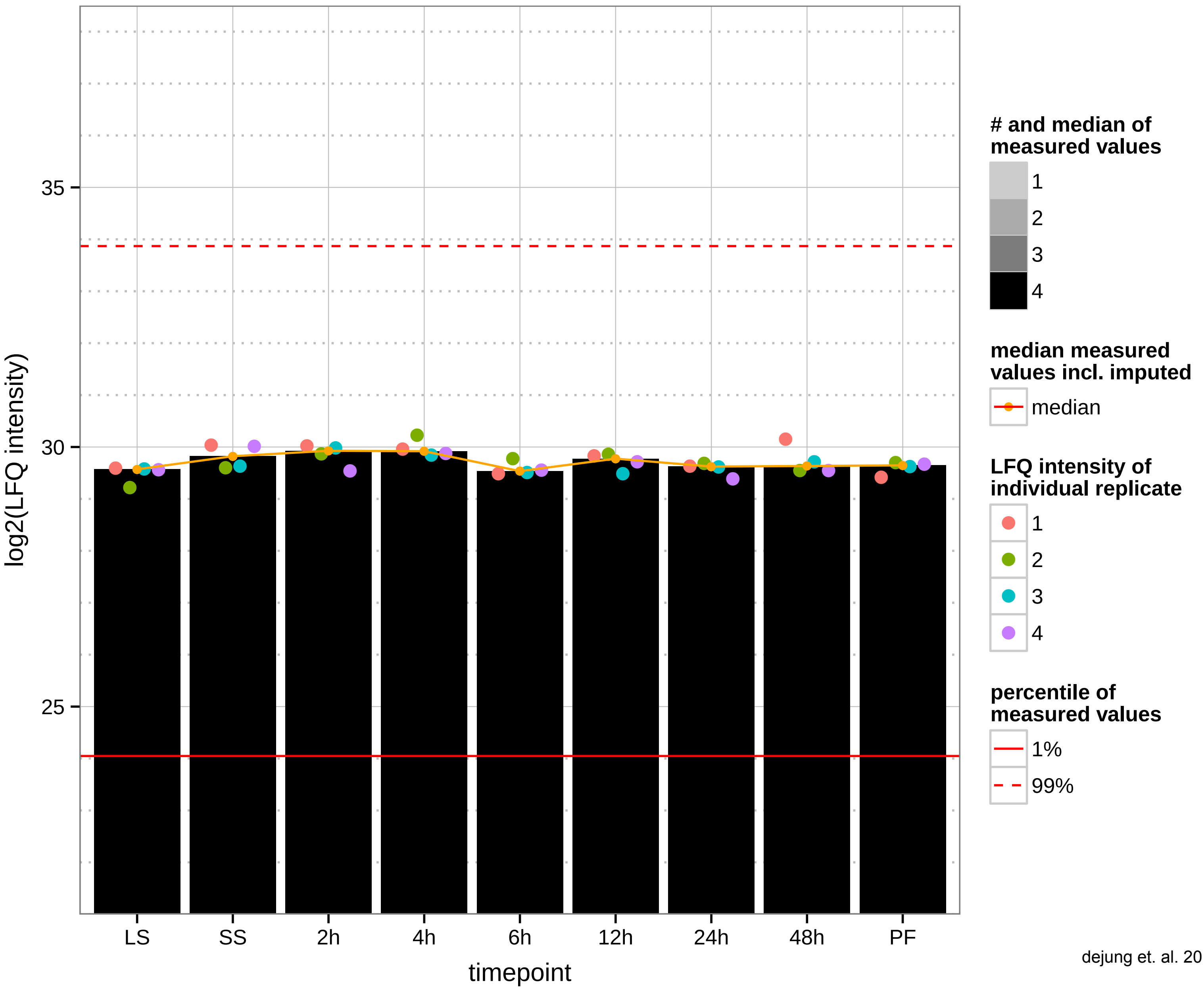

dejung et. al. 2015

alternative oxidase
Tb927.10.9760
AGOF: null
AGOC: mitochondrial envelope, mitochondrion
AGOP: respiratory gaseous exchange
PGOF: null
PGOC: mitochondrial envelope
PGOP: oxidation–reduction process, respiratory gaseous exchange

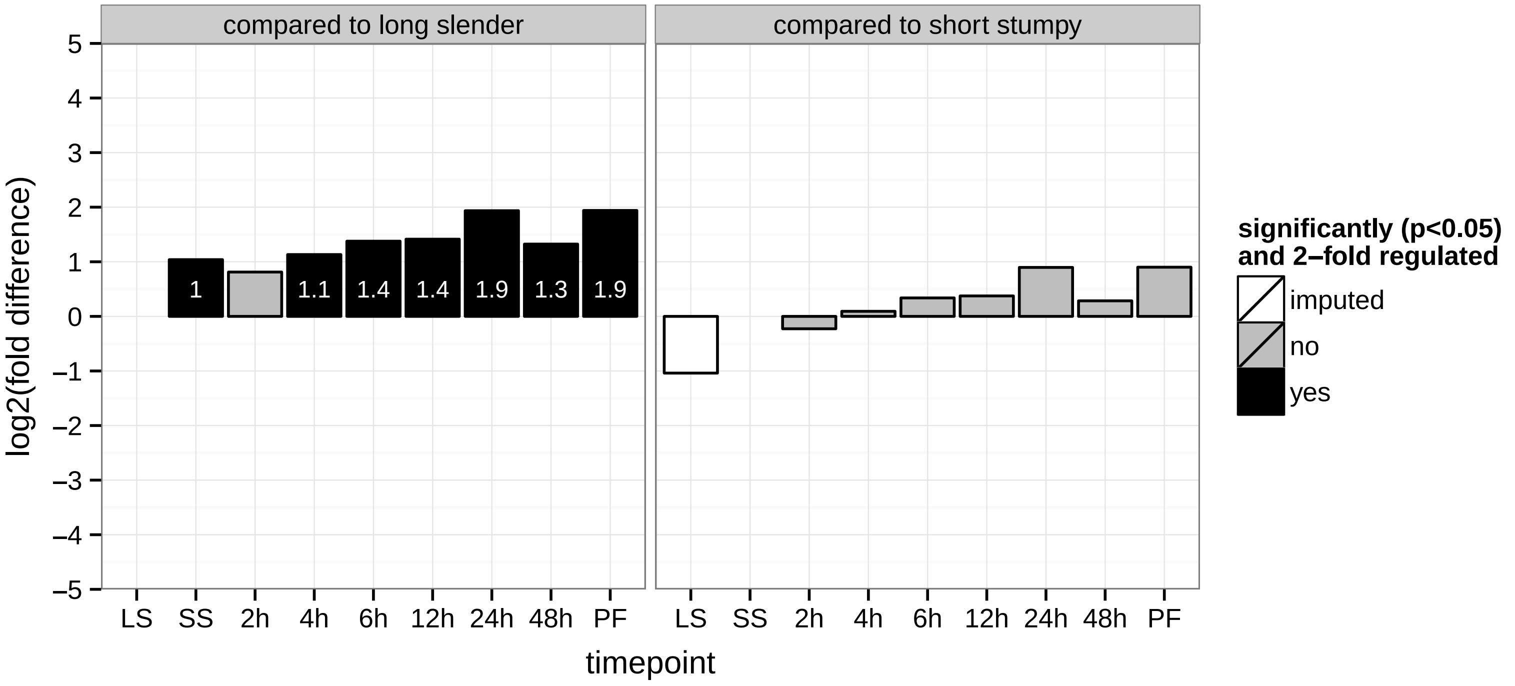

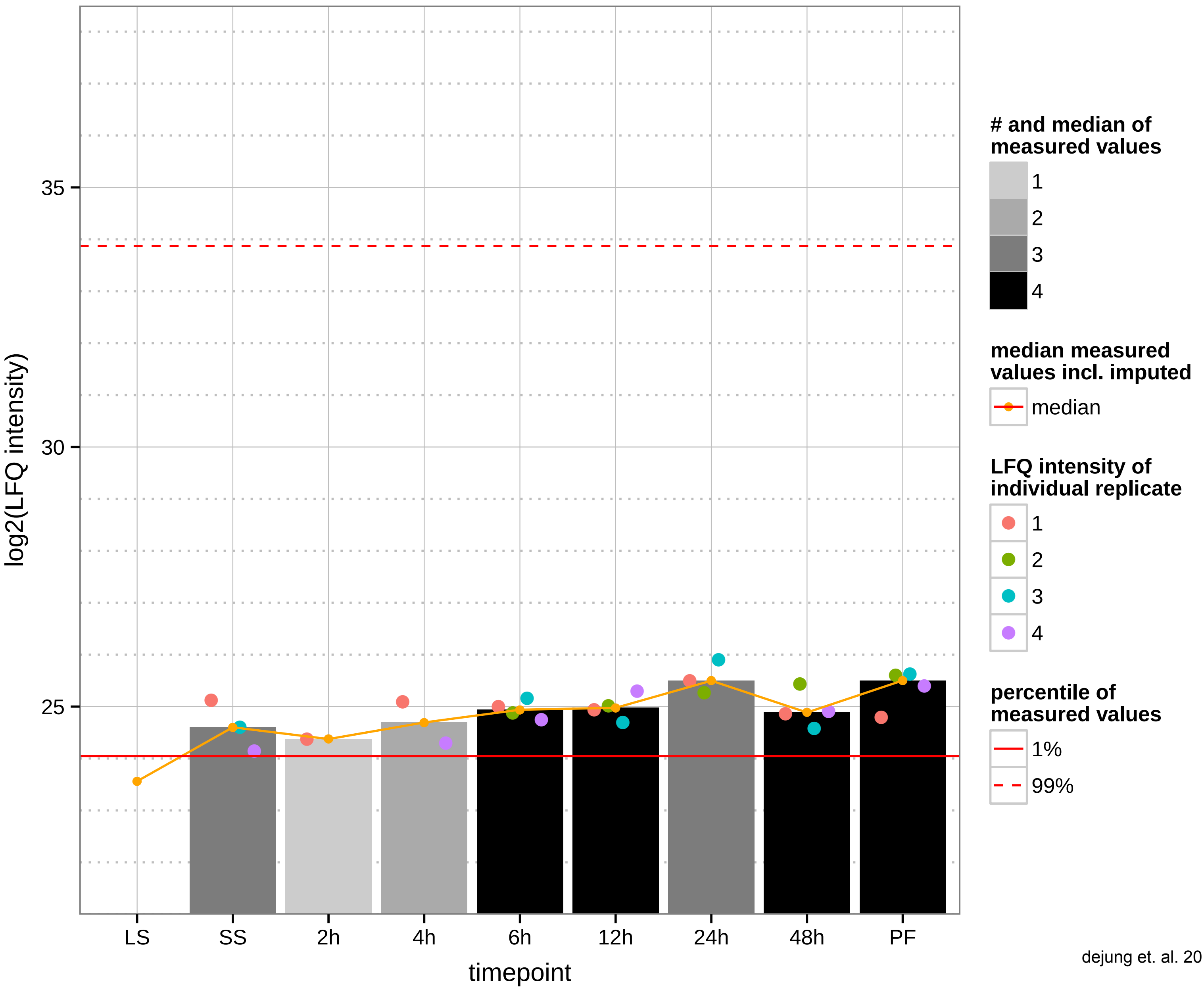

dejung et. al. 2015

60S ribosomal protein L22
Tb927.10.9800
AGOF: structural constituent of ribosome
AGOC: intracellular, ribosome
AGOP: translation
PGOF: structural constituent of ribosome
PGOC: intracellular, ribosome
PGOP: translation

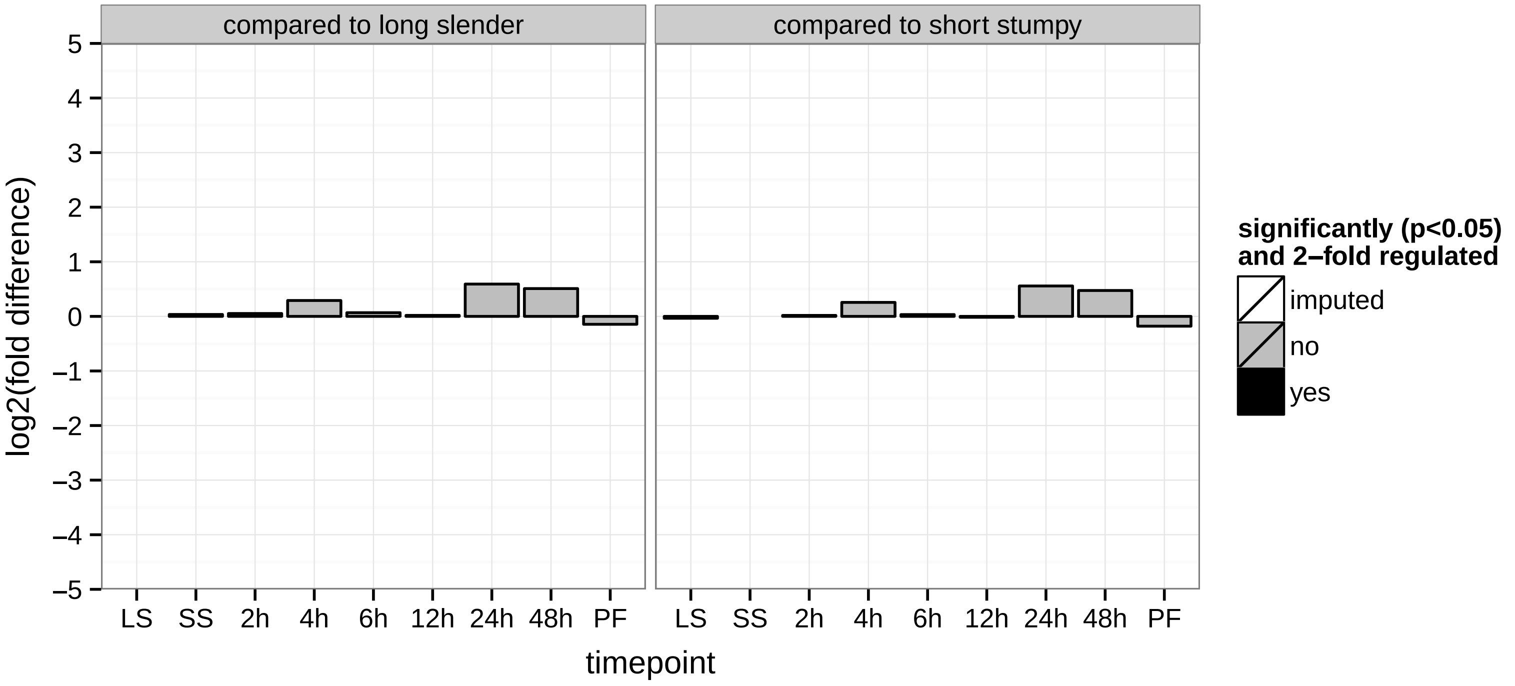

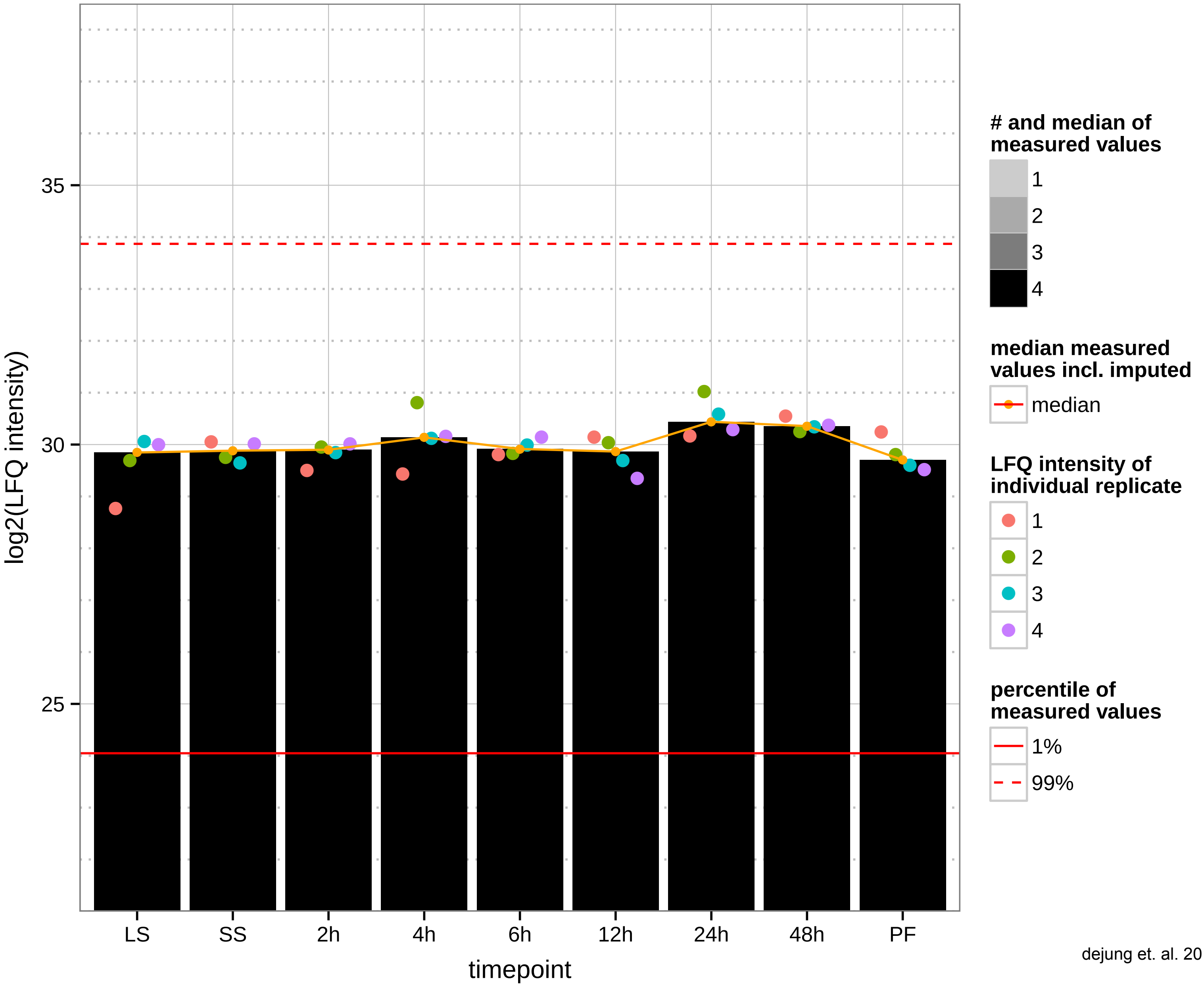

dejung et. al. 2015

chaperone protein DNAj, putative
Tb927.10.9840
AGOF: heat shock protein binding, unfolded protein binding
AGOC: null
AGOP: protein folding
PGOF: heat shock protein binding
PGOC: null
PGOP: null

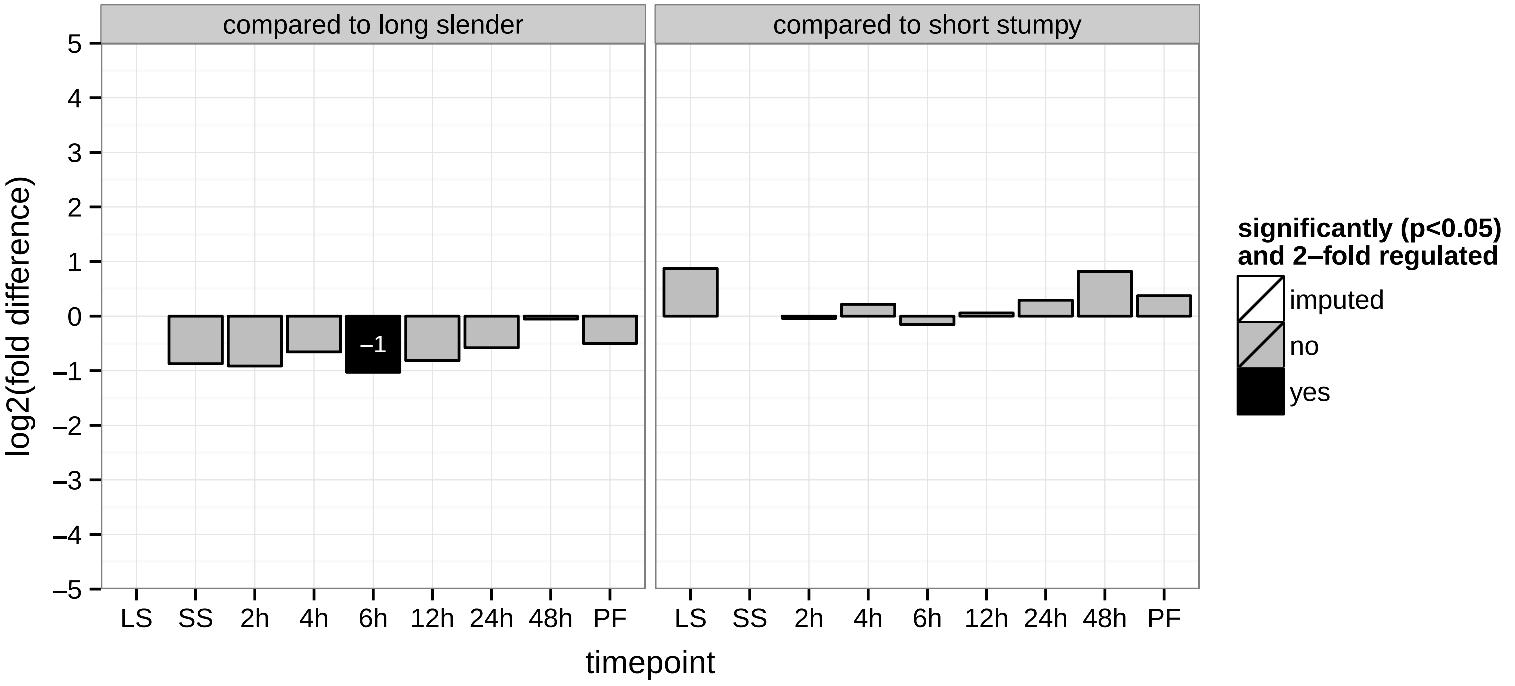

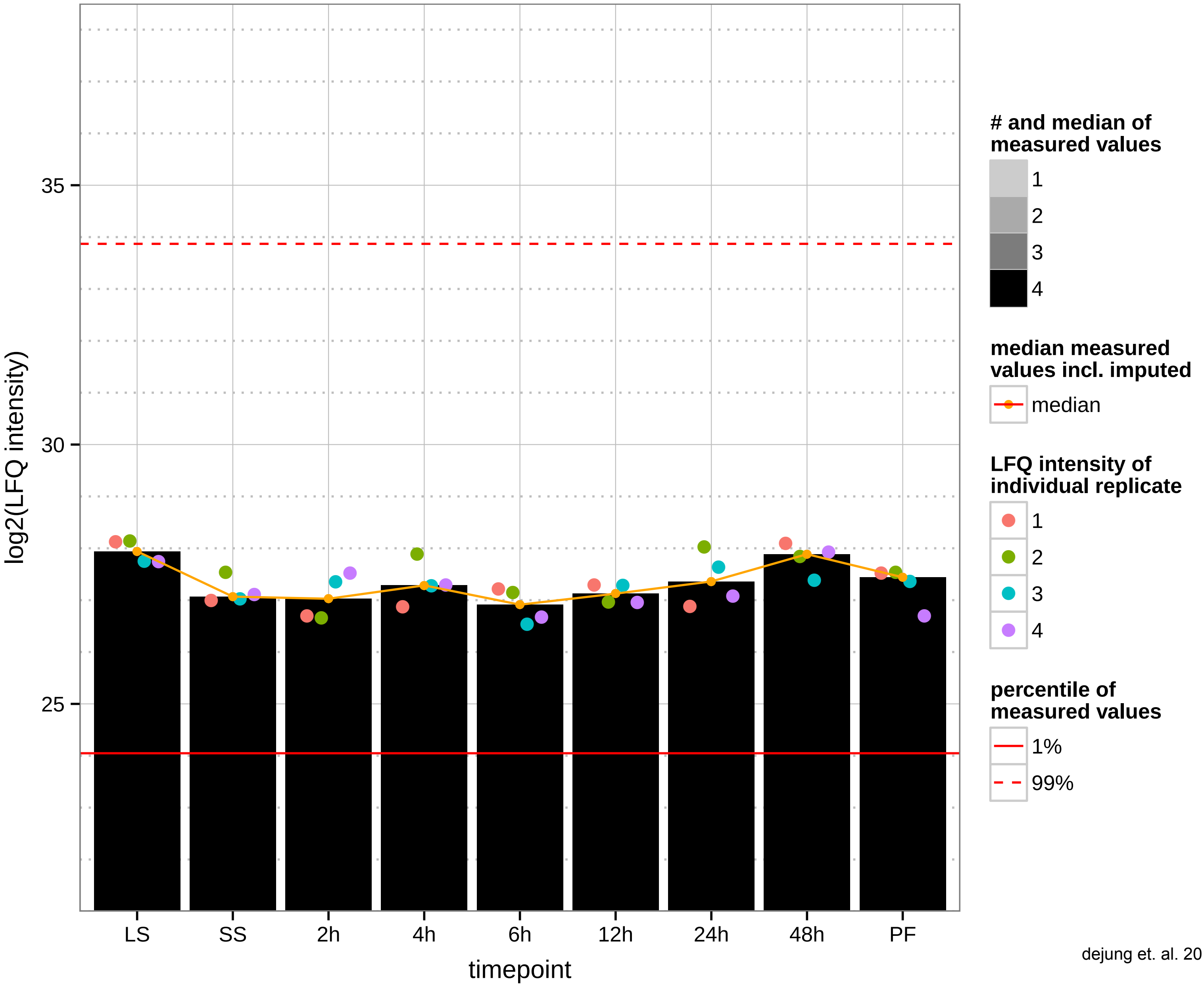

dejung et. al. 2015

Mitochondrial N(5)–glutamine methyltransferase MTQ1, putative
Tb927.10.9860
AGOF: S–adenosylmethionine–dependent methyltransferase activity, protein methyltransferase activity
AGOC: mitochondrion
AGOP: protein methylation, translational readthrough
PGOF: protein methyltransferase activity
PGOC: null
PGOP: protein methylation

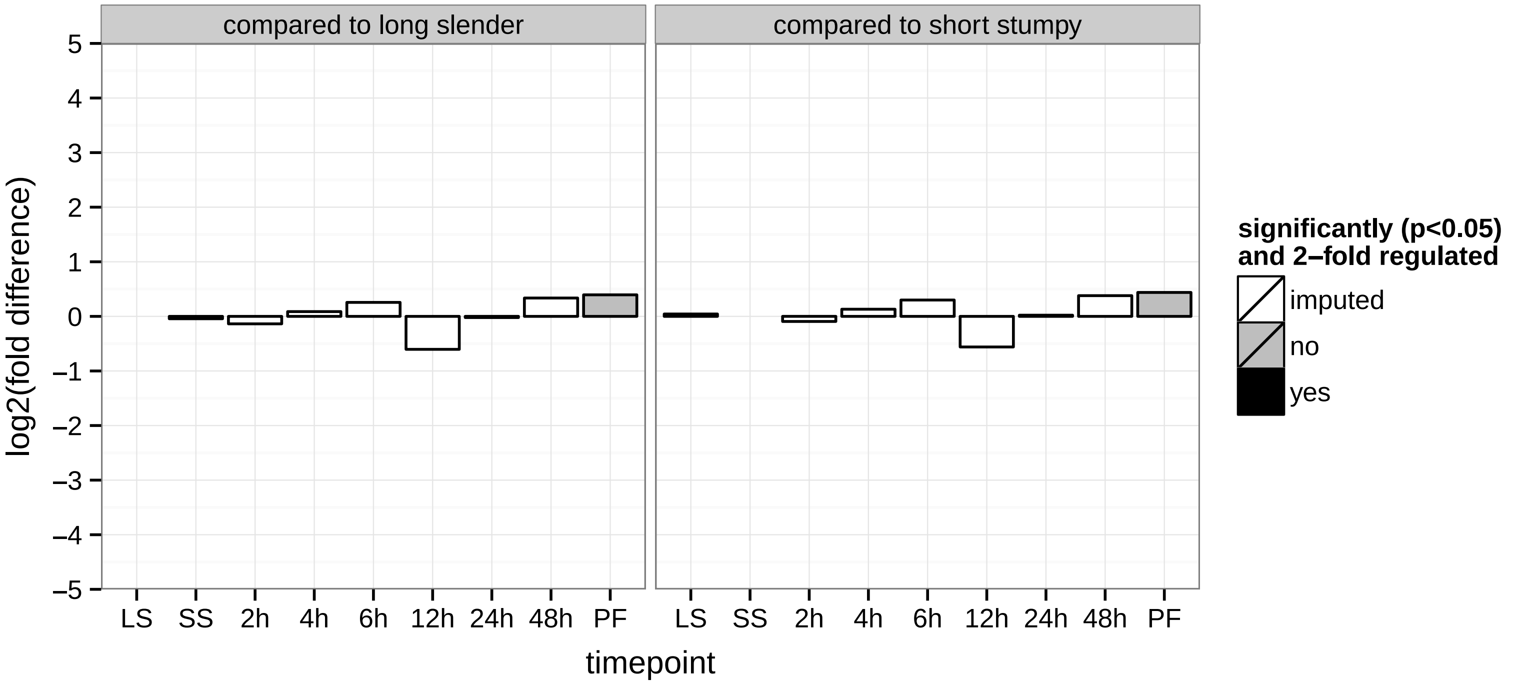

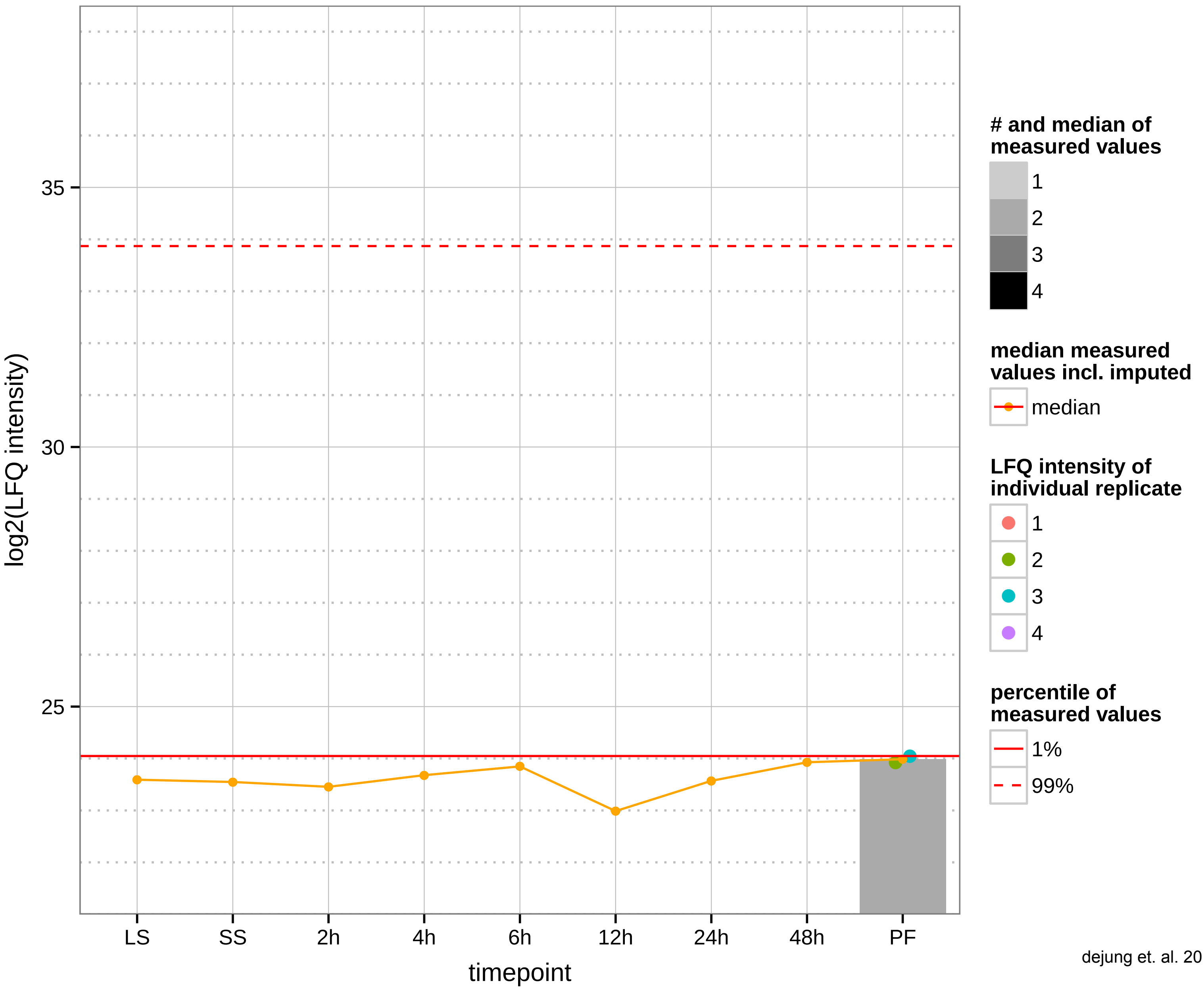

dejung et. al. 2015

hypothetical protein, conserved
Tb927.10.9890
AGOF: null
AGOC: null
AGOP: null
PGOF: null
PGOC: null
PGOP: null

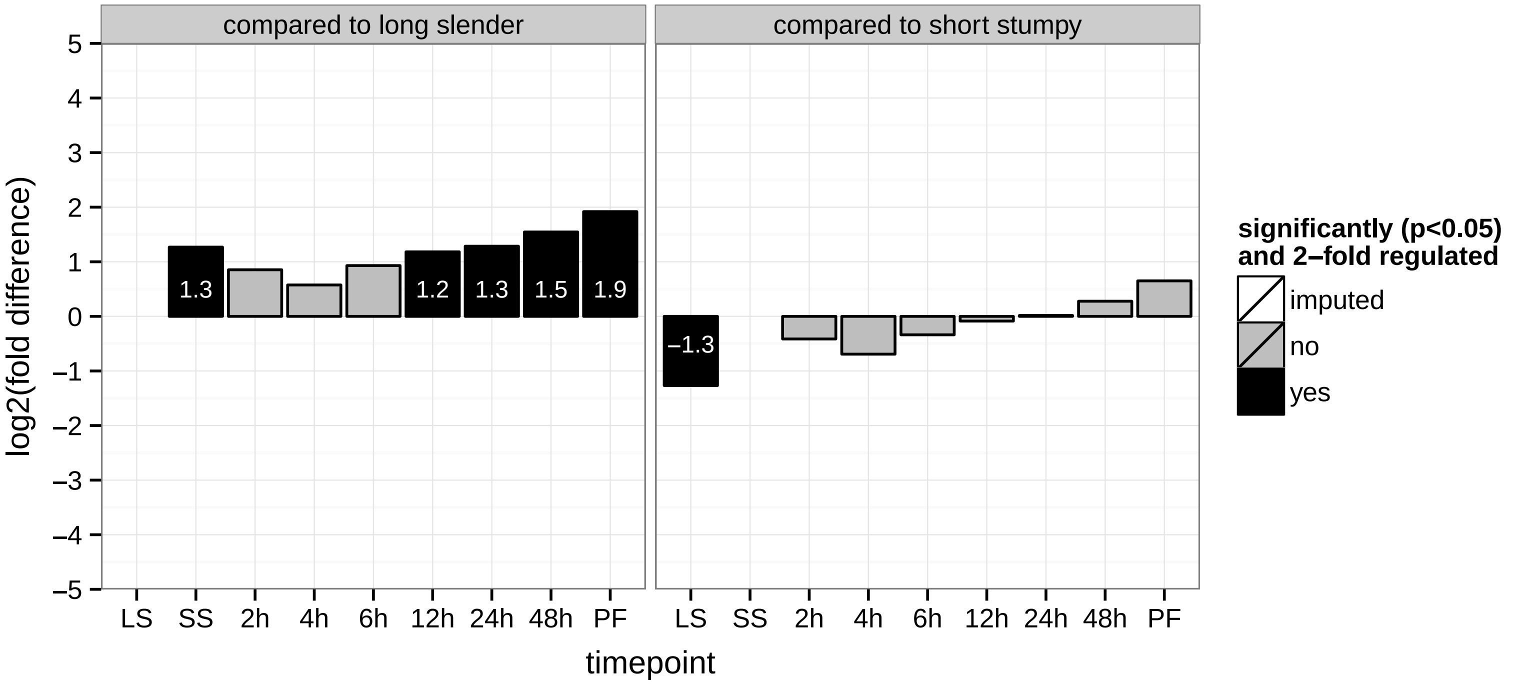

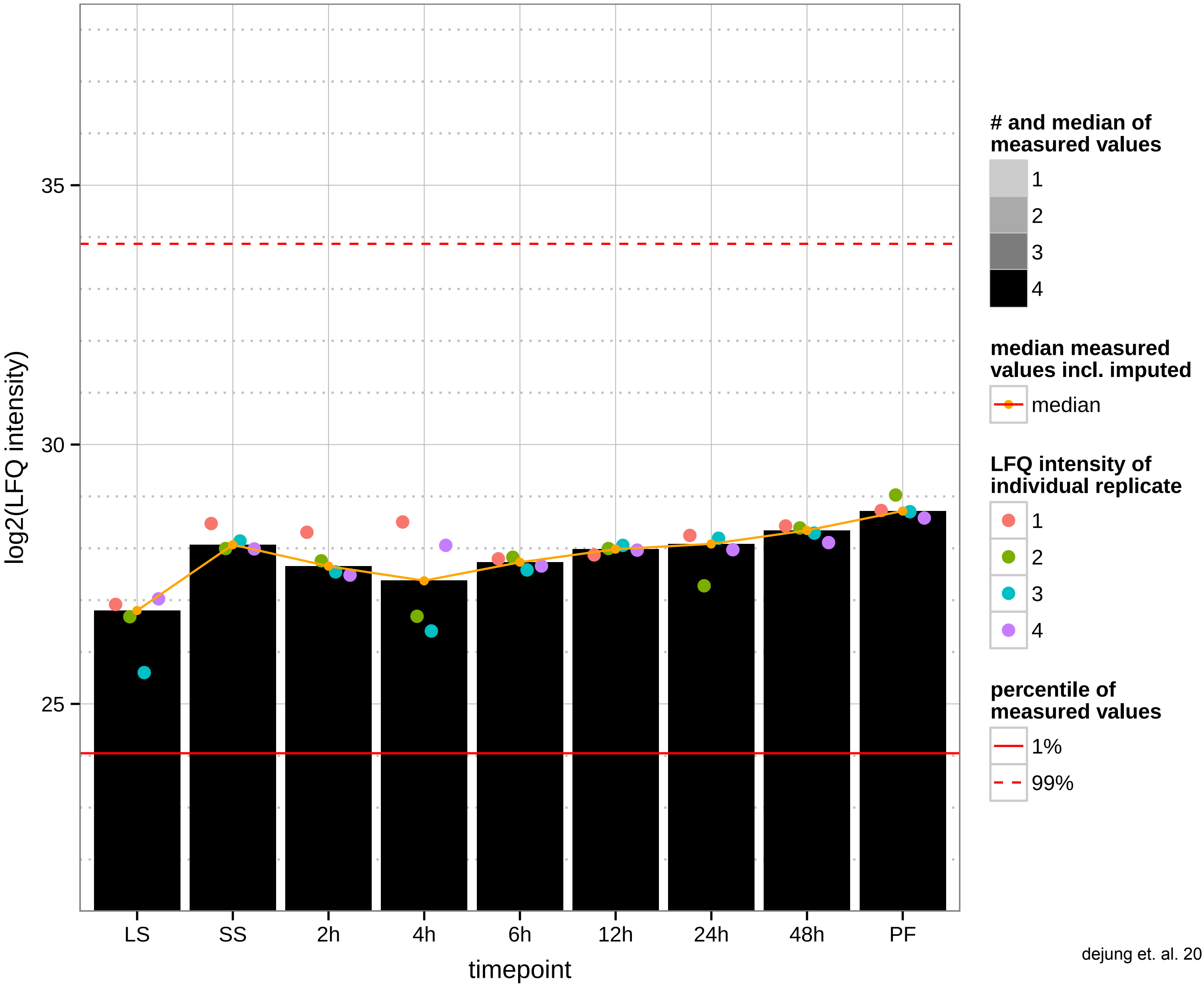

dejung et. al. 2015

ABC1 protein, putative
Tb927.10.9900
AGOF: protein kinase activity
AGOC: null
AGOP: null
PGOF: transferase activity, transferring phosphorus–containing groups
PGOC: null
PGOP: null

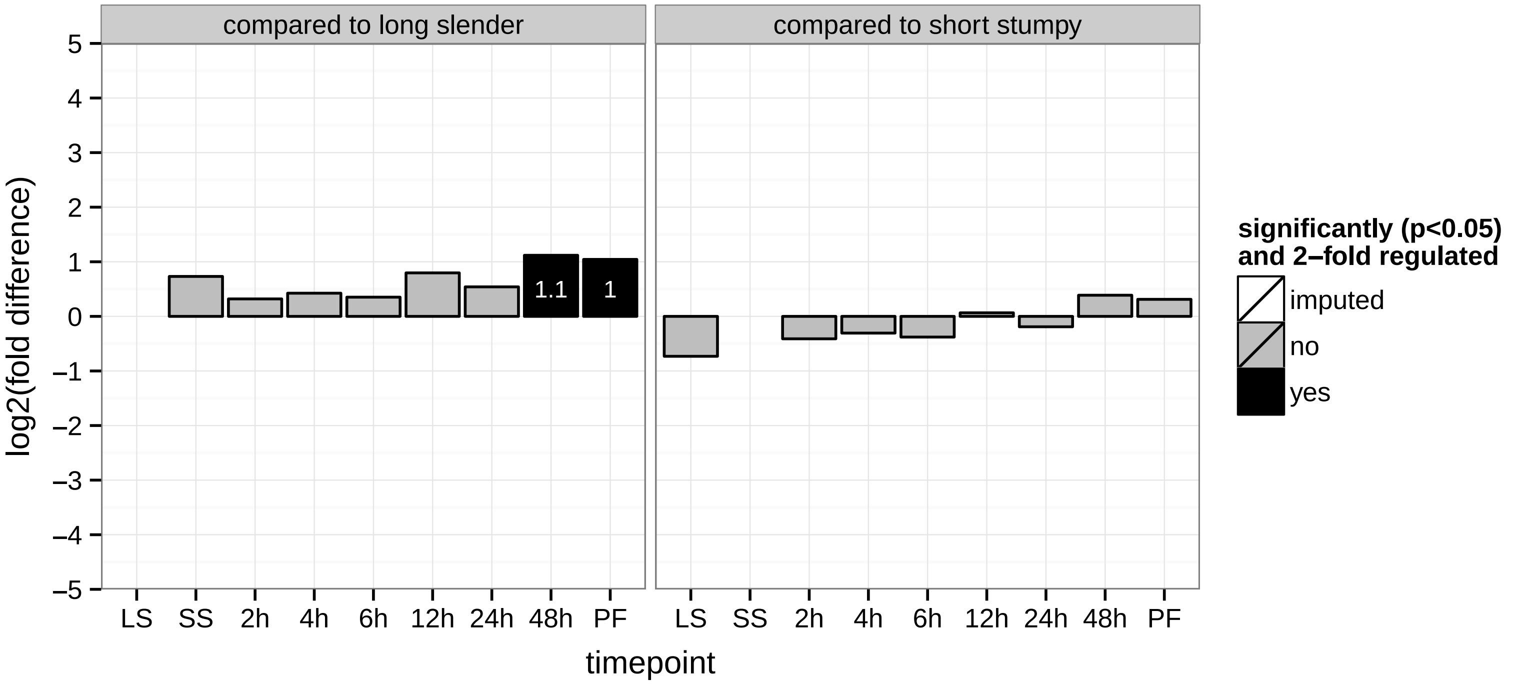

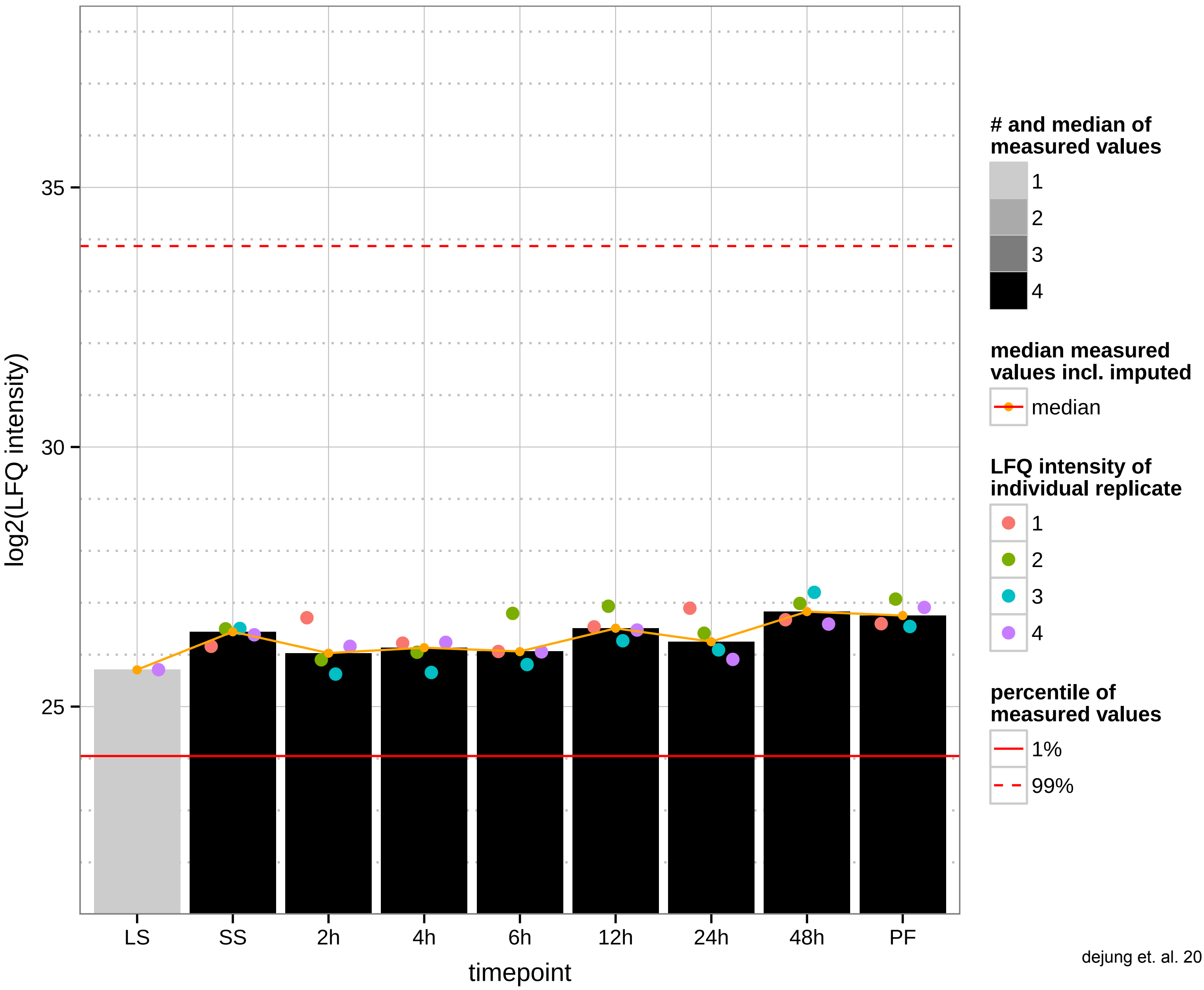

dejung et. al. 2015

hypothetical protein, conserved
Tb927.10.9910
AGOF: null
AGOC: null
AGOP: null
PGOF: null
PGOC: null
PGOP: null

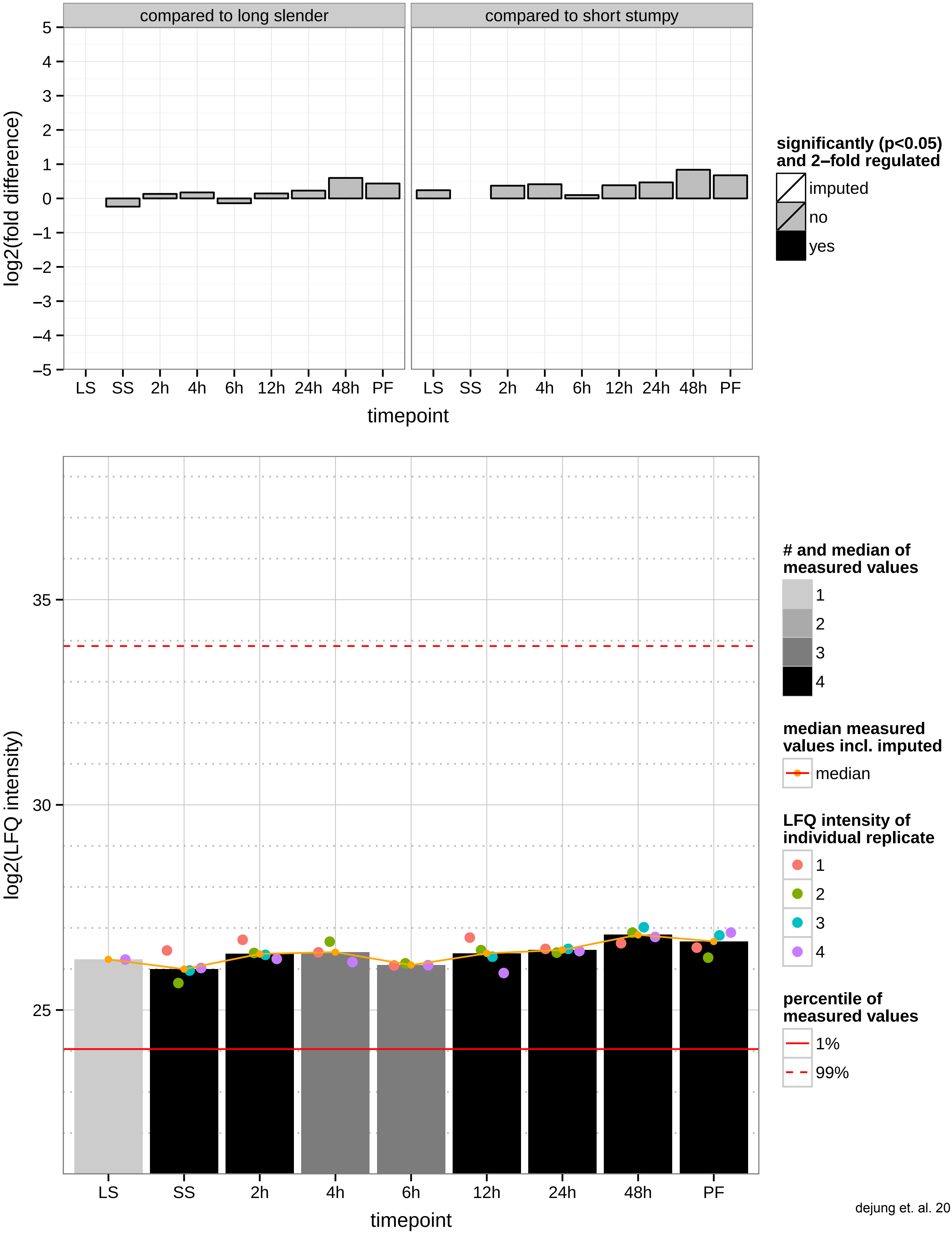

dejung et. al. 2015

hypothetical protein, conserved
Tb927.10.9970
AGOF: null
AGOC: mitochondrion
AGOP: null
PGOF: null
PGOC: null
PGOP: null

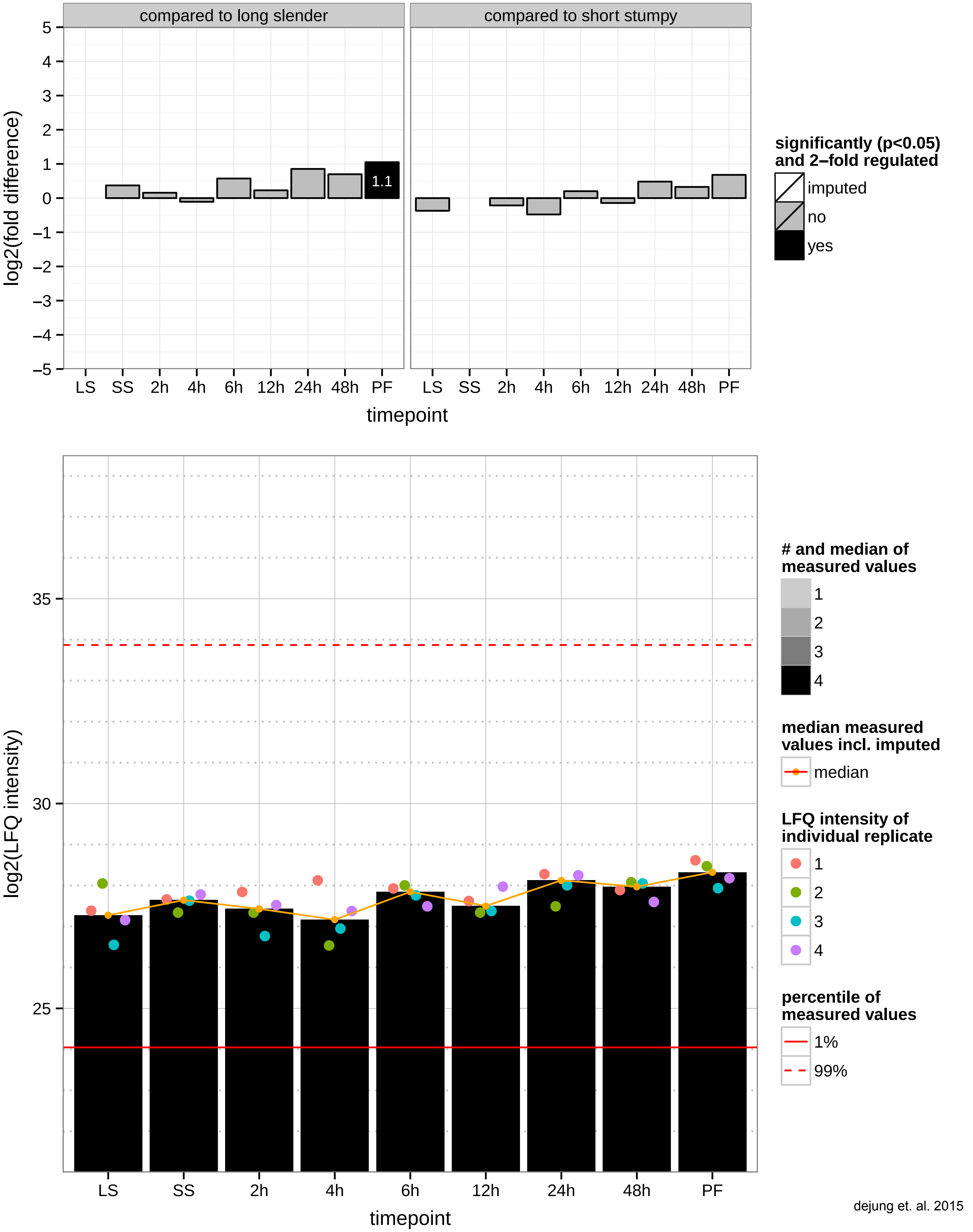

dejung et. al. 2015

short−chain dehydrogenase, putative
Tb927.11.10020
AGOF: oxidoreductase activity
AGOC: integral to membrane
AGOP: metabolic process
PGOF: oxidoreductase activity
PGOC: null
PGOP: metabolic process

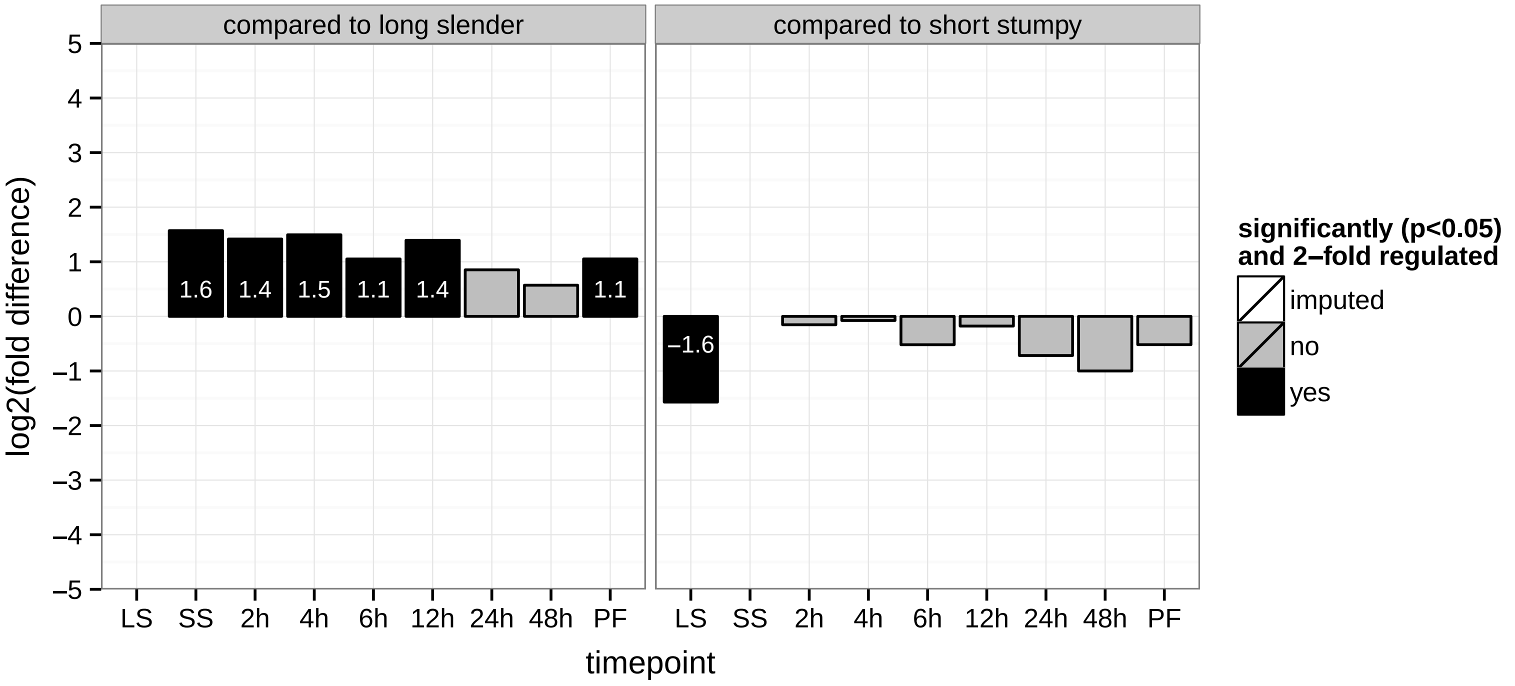

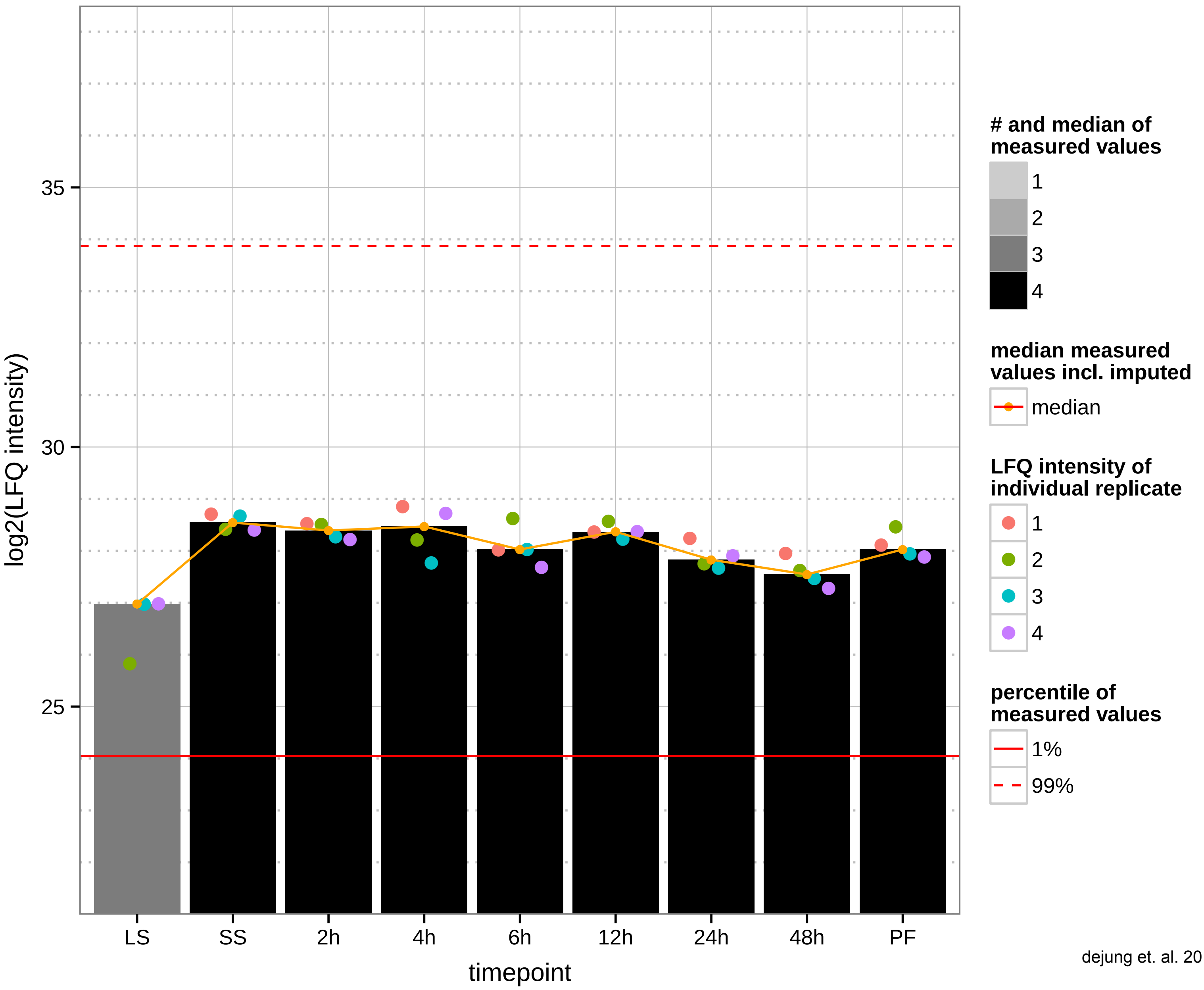

dejung et. al. 2015

Biotin−−acetyl−CoA−carboxylase ligase, putative, biotin−−protein ligase
Tb927.11.10060
AGOF: biotin−[acetyl−CoA−carboxylase] ligase activity
AGOC: null
AGOP: biotin biosynthetic process, cellular protein modification process
PGOF: biotin−[acetyl−CoA−carboxylase] ligase activity, catalytic activity
PGOC: null
PGOP: cellular protein modification process

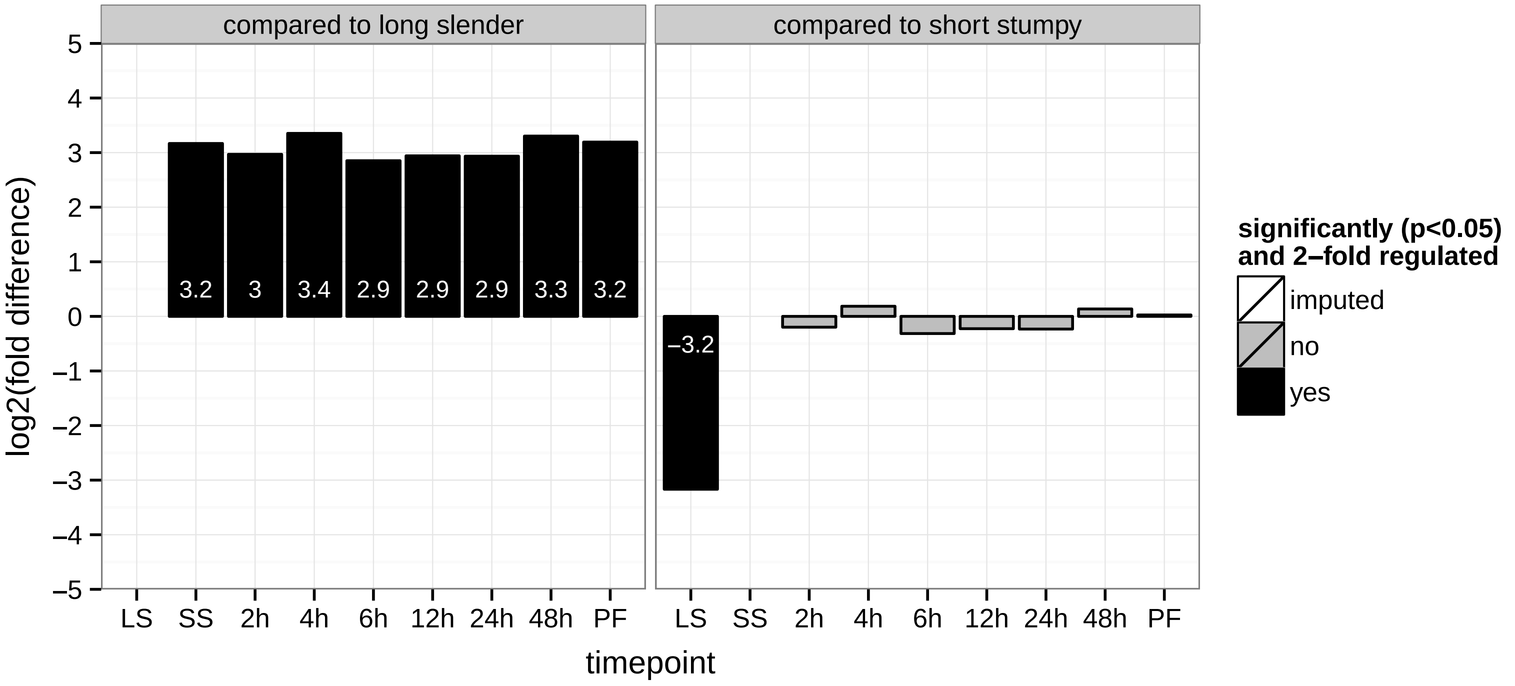

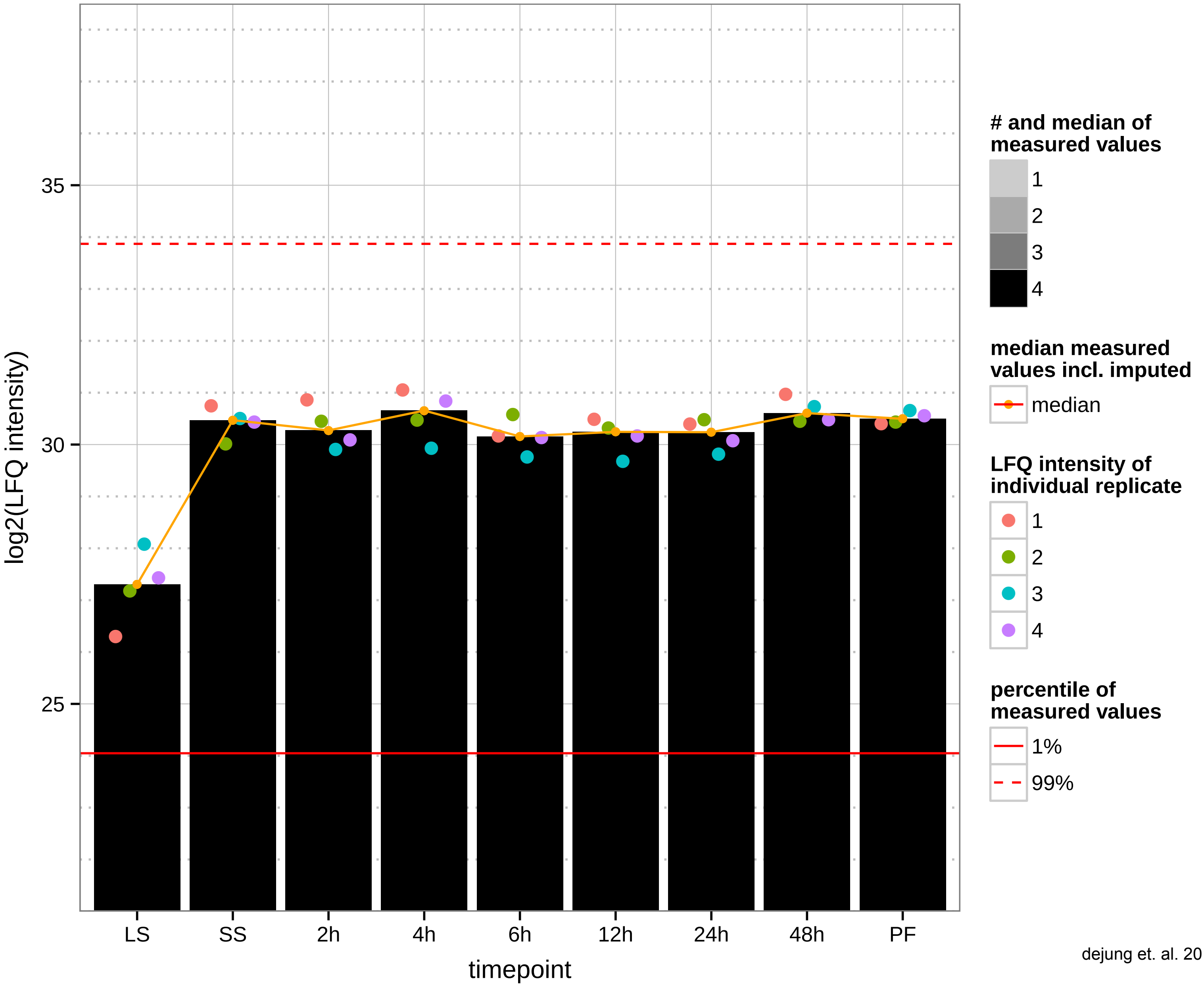

dejung et. al. 2015

hypothetical protein, conserved
Tb927.11.10070
AGOF: null
AGOC: null
AGOP: null
PGOF: protein binding
PGOC: null
PGOP: null

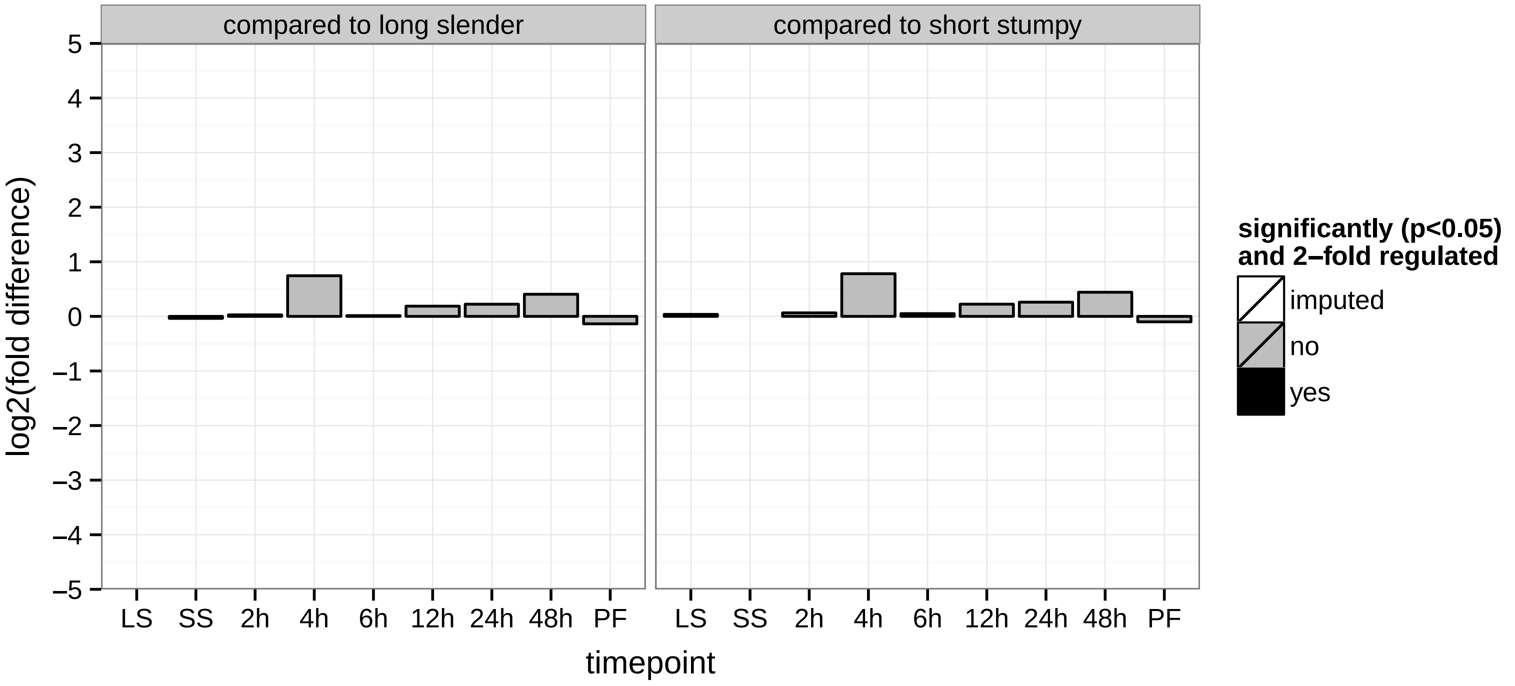

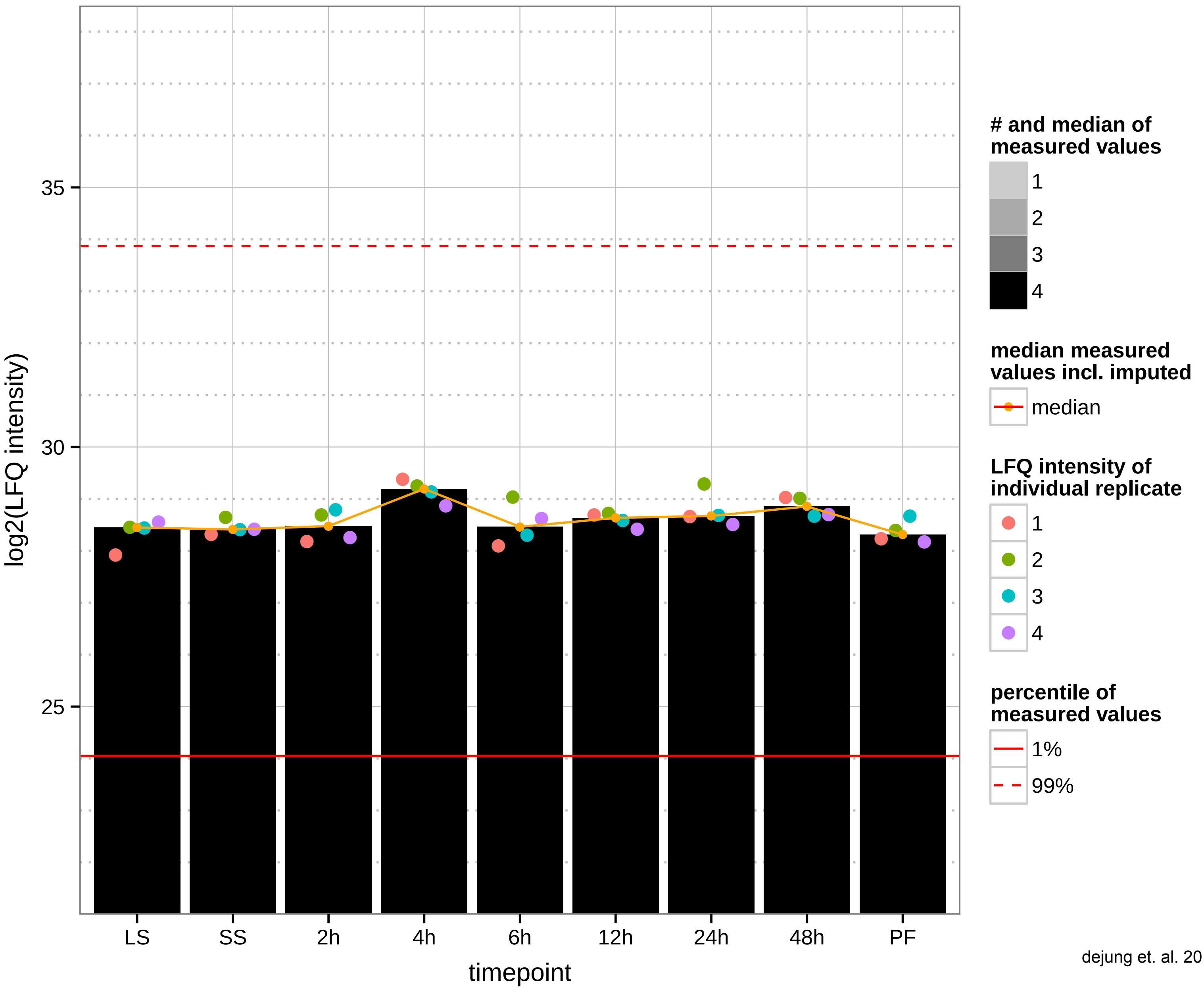

dejung et. al. 2015

hypothetical protein, conserved
Tb927.11.10090
AGOF: null
AGOC: null
AGOP: null
PGOF: null
PGOC: null
PGOP: null

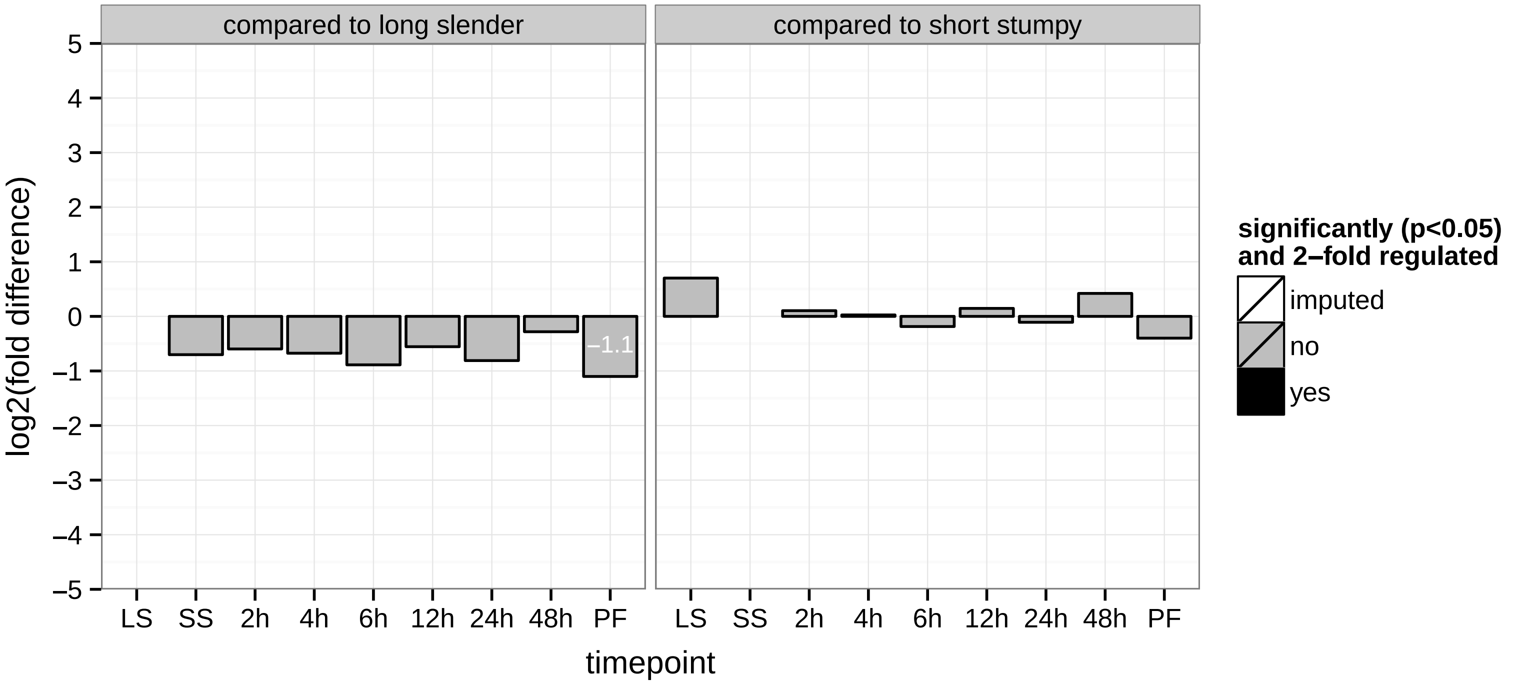

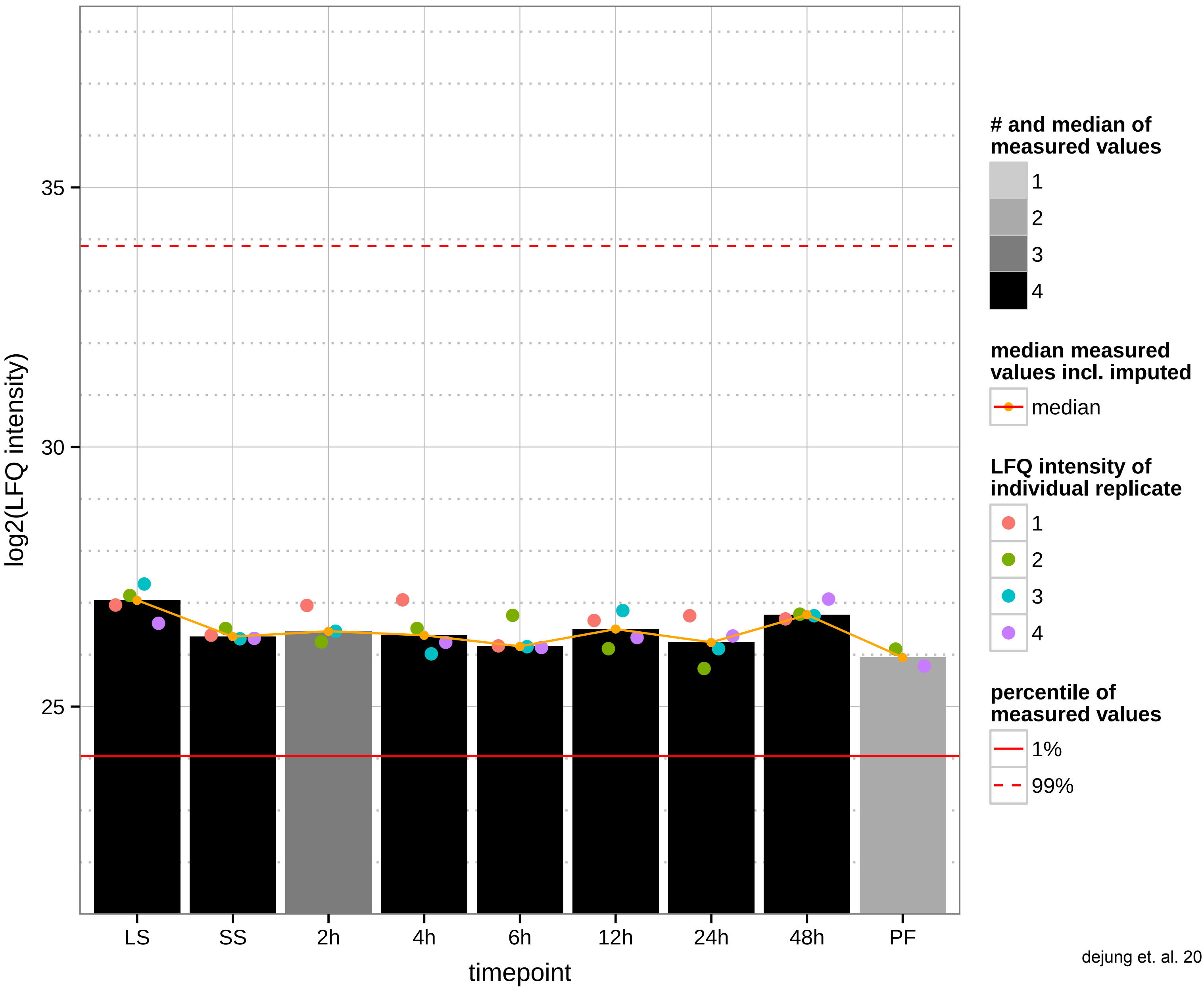

dejung et. al. 2015

chaperone protein DNAj, putative
Tb927.11.1010
AGOF: heat shock protein binding, unfolded protein binding
AGOC: integral to membrane, mitochondrial inner membrane, mitochondrion
AGOP: protein folding
PGOF: heat shock protein binding
PGOC: null
PGOP: null

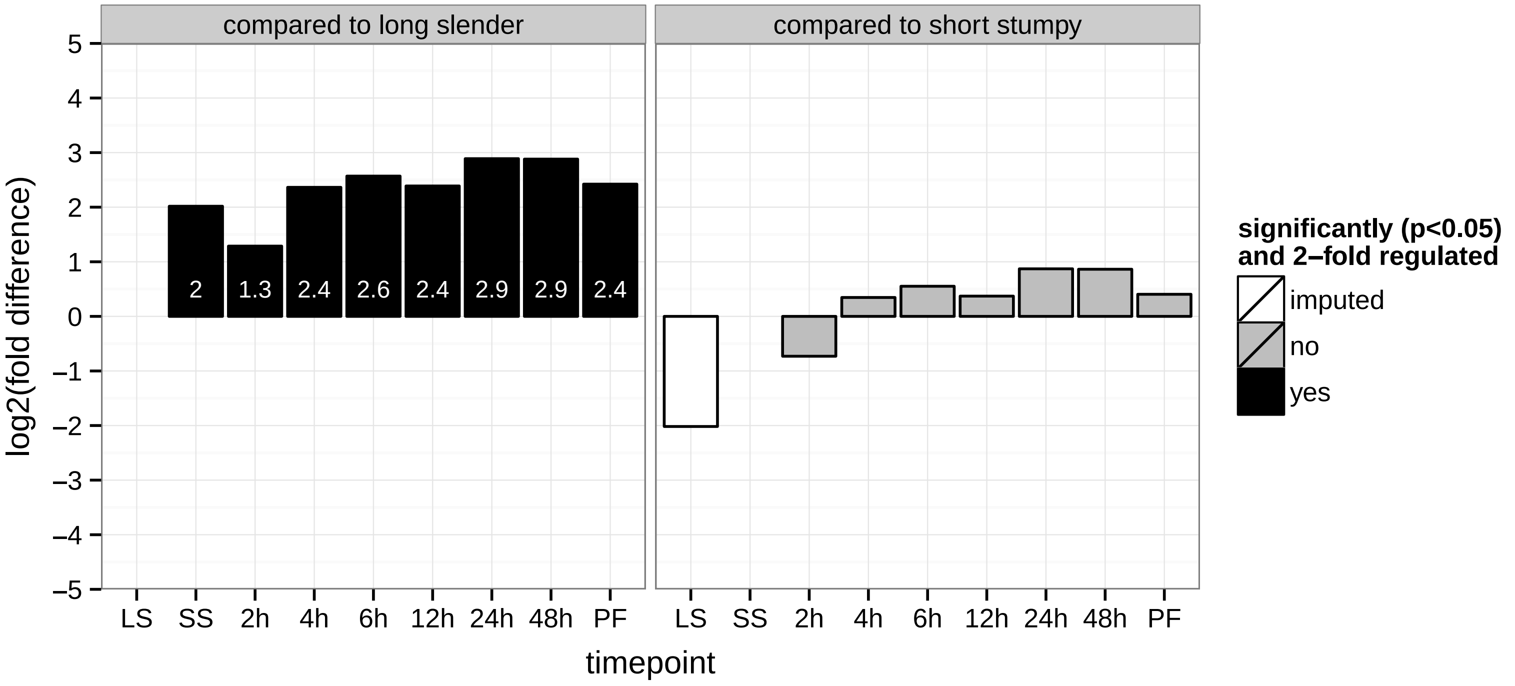

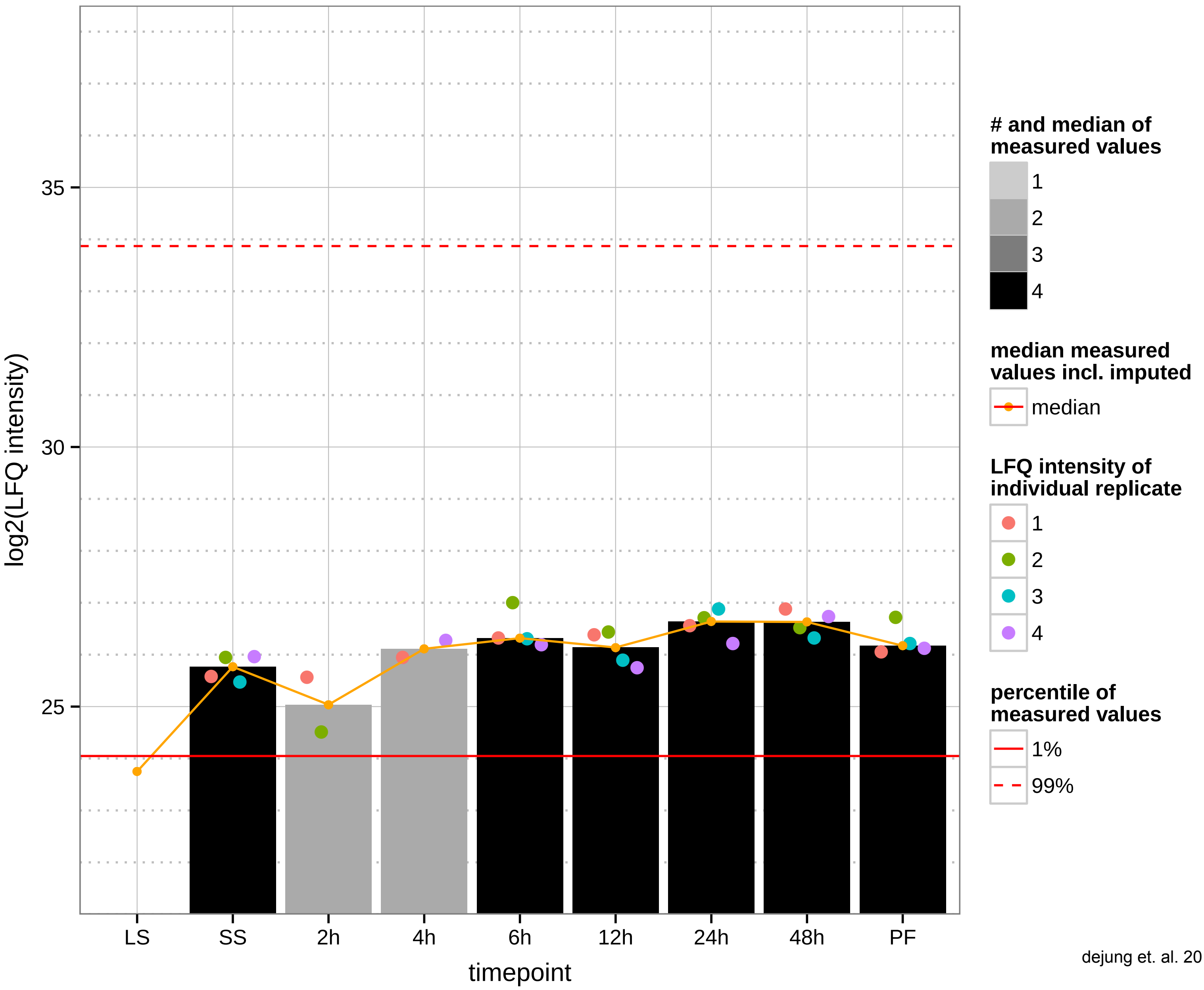

dejung et. al. 2015

hypothetical protein, conserved
Tb927.11.10130
AGOF: null
AGOC: null
AGOP: null
PGOF: null
PGOC: null
PGOP: null

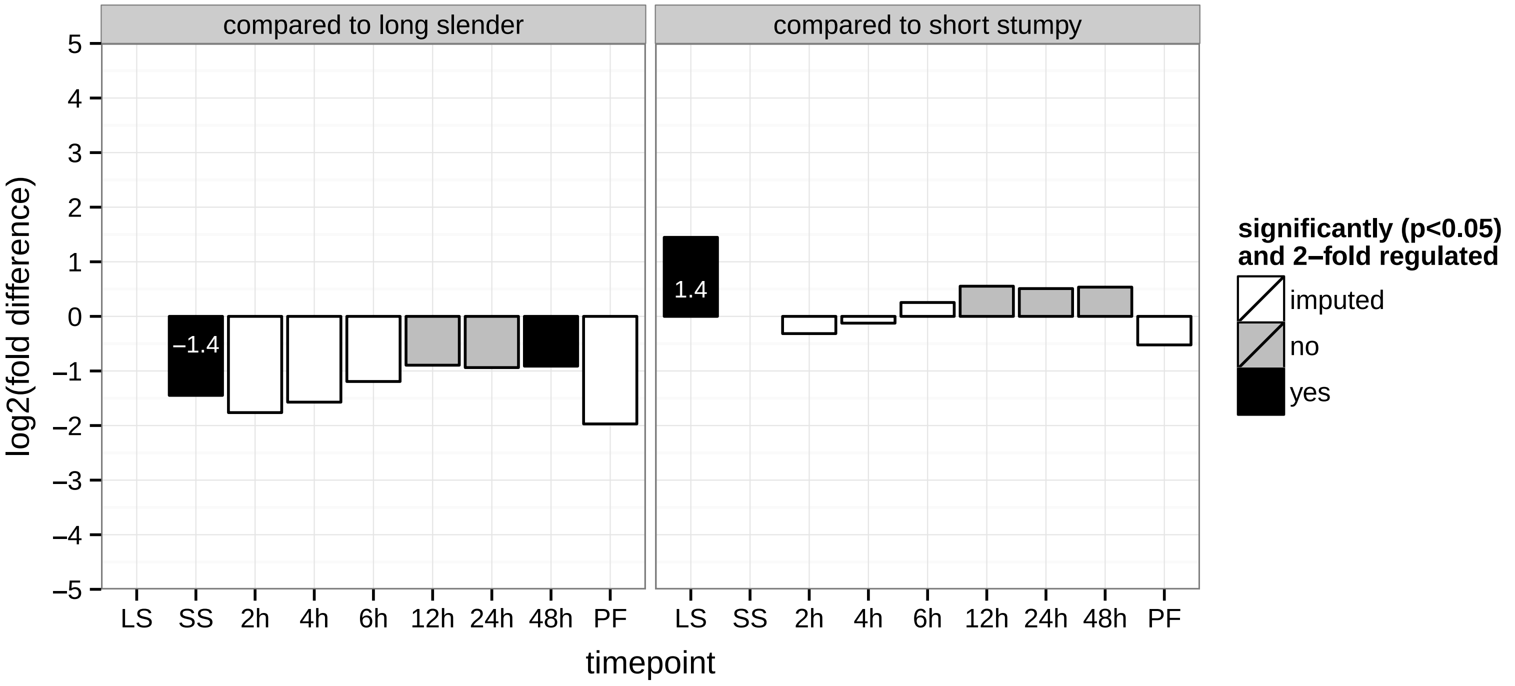

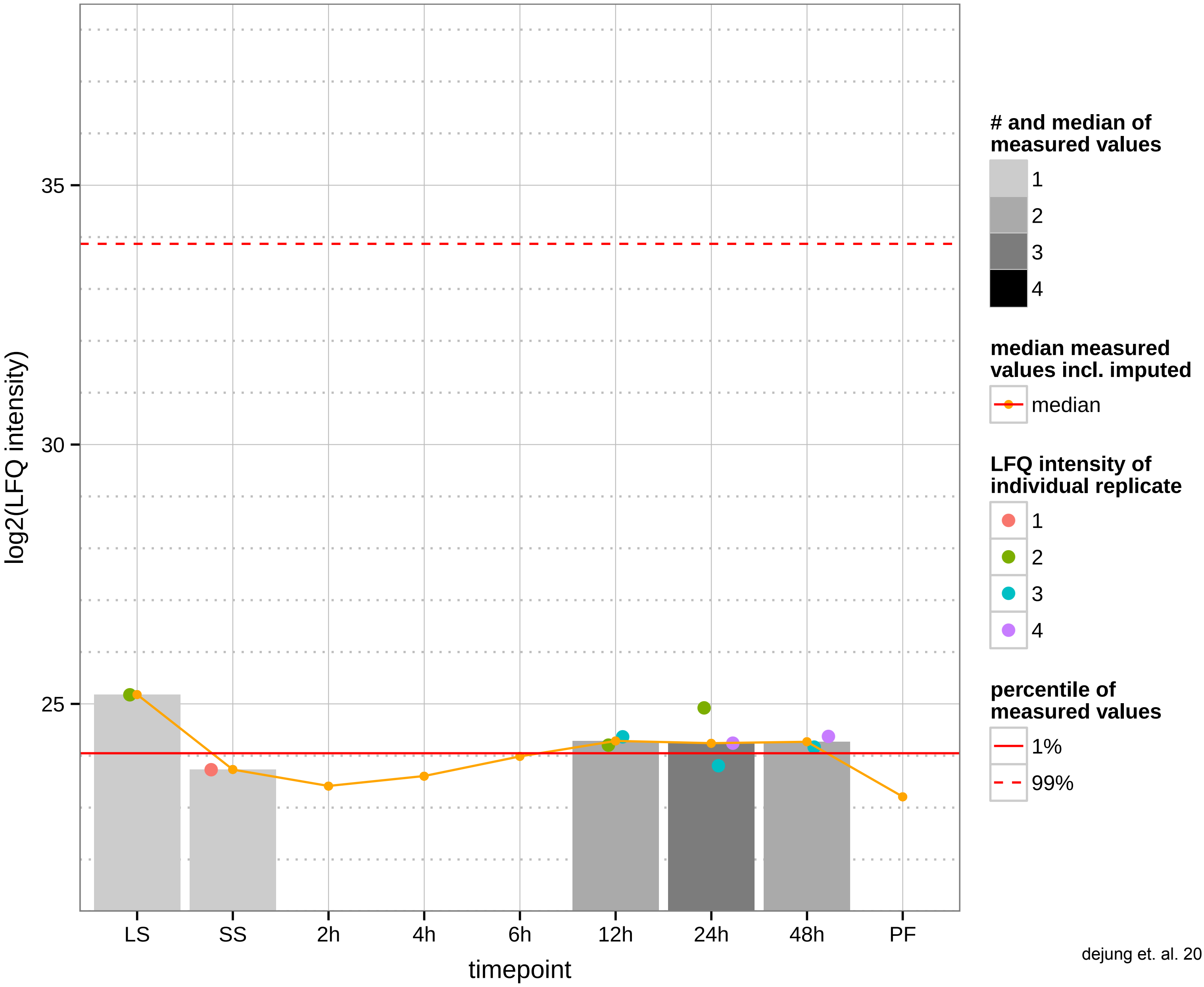

dejung et. al. 2015

ribokinase, putative
Tb927.11.1020
AGOF: ribokinase activity
AGOC: null
AGOP: D–ribose metabolic process
PGOF: null
PGOC: null
PGOP: null

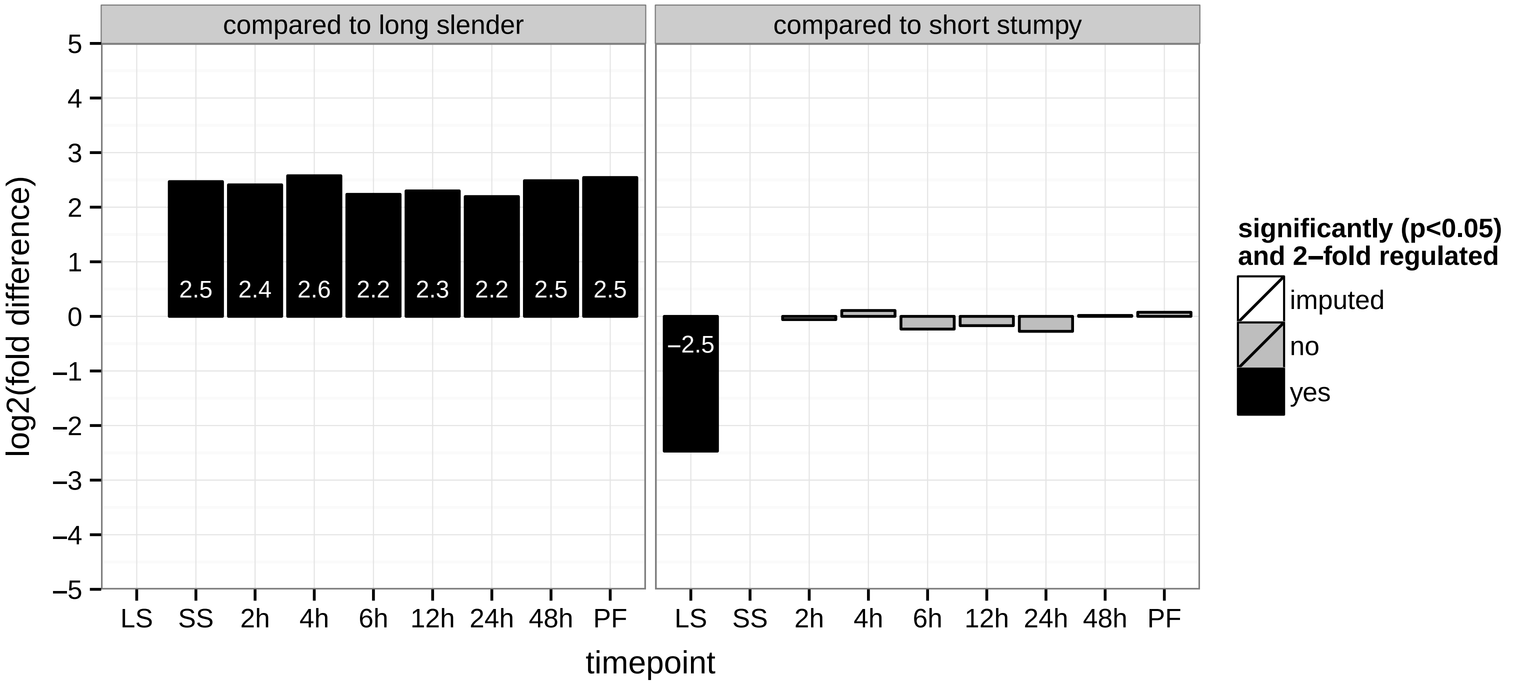

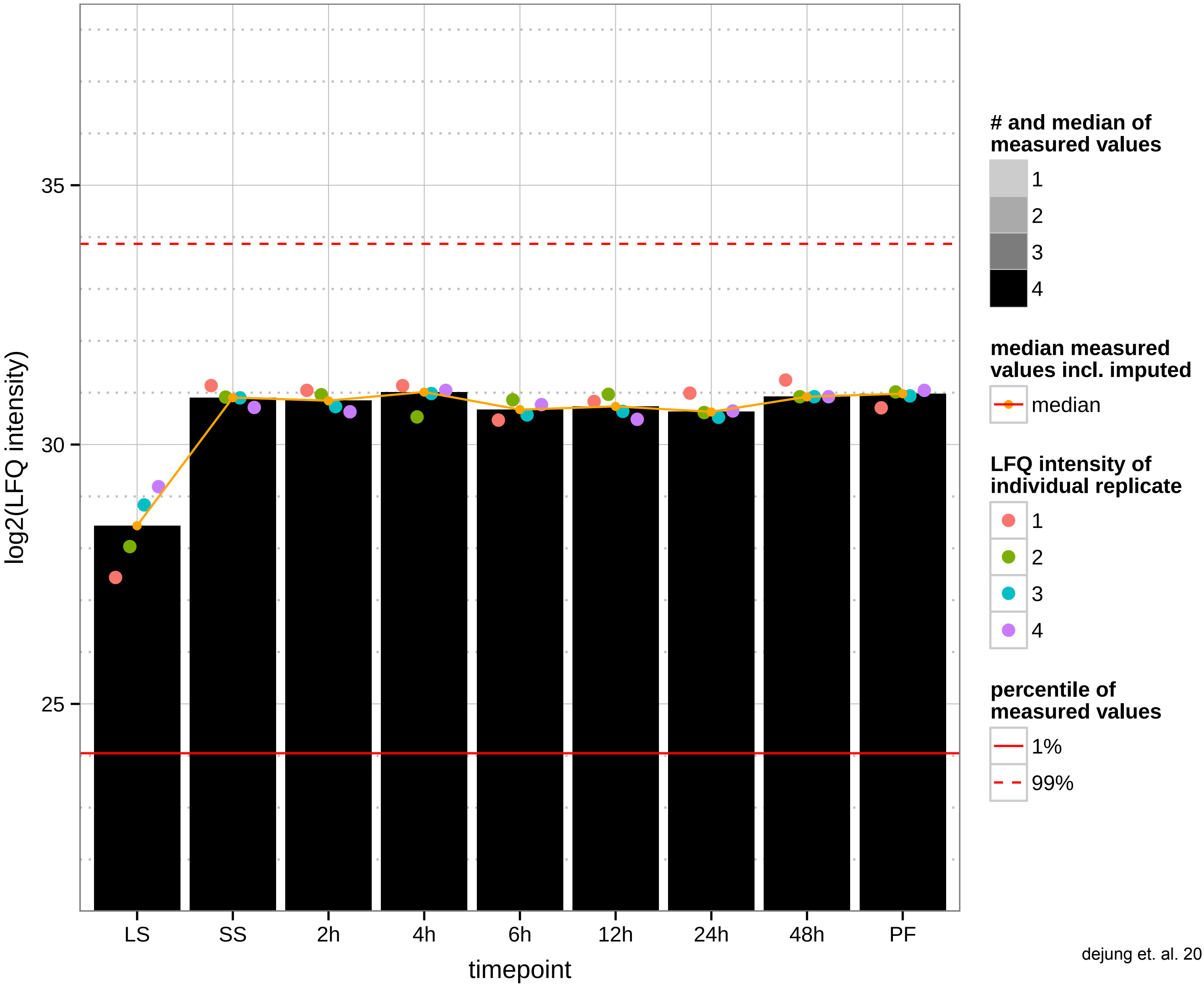

dejung et. al. 2015

hypothetical protein, conserved
Tb927.11.10200
AGOF: null
AGOC: null
AGOP: null
PGOF: null
PGOC: null
PGOP: null

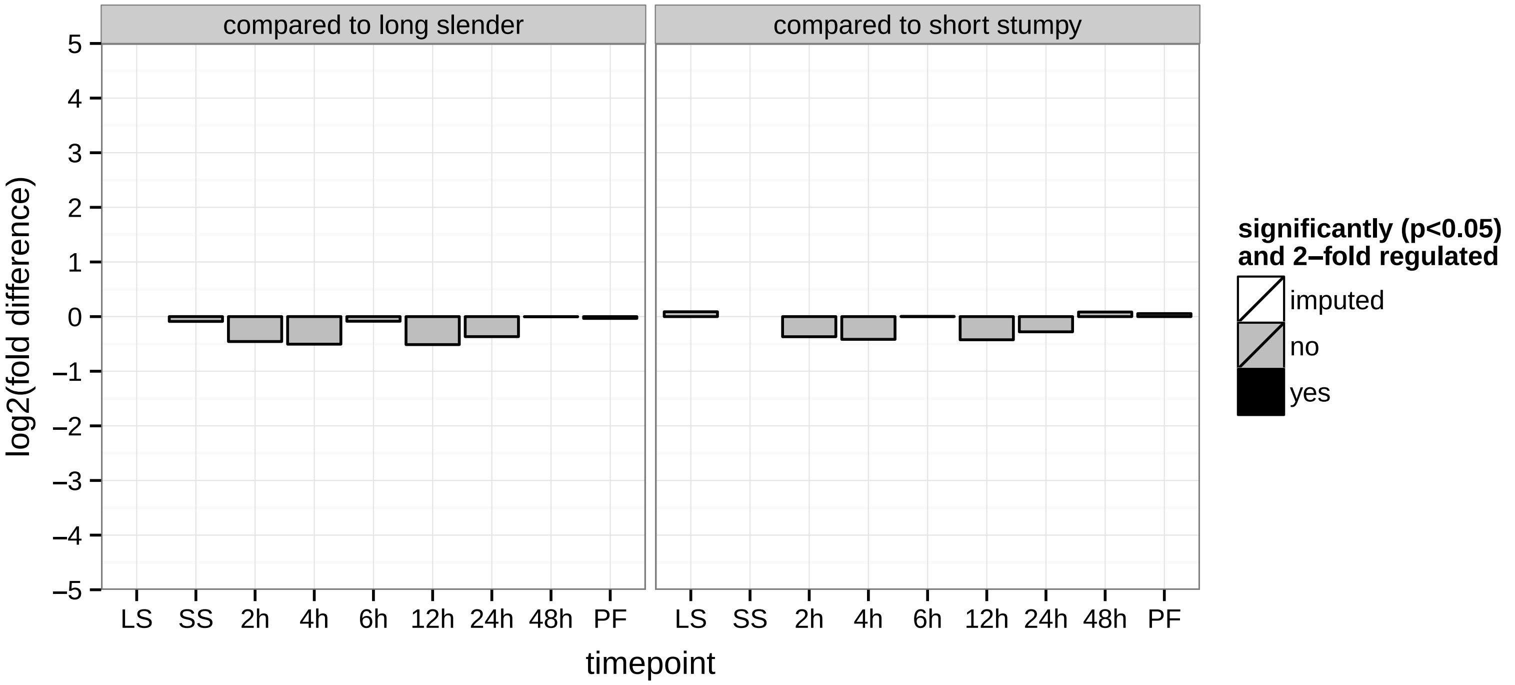

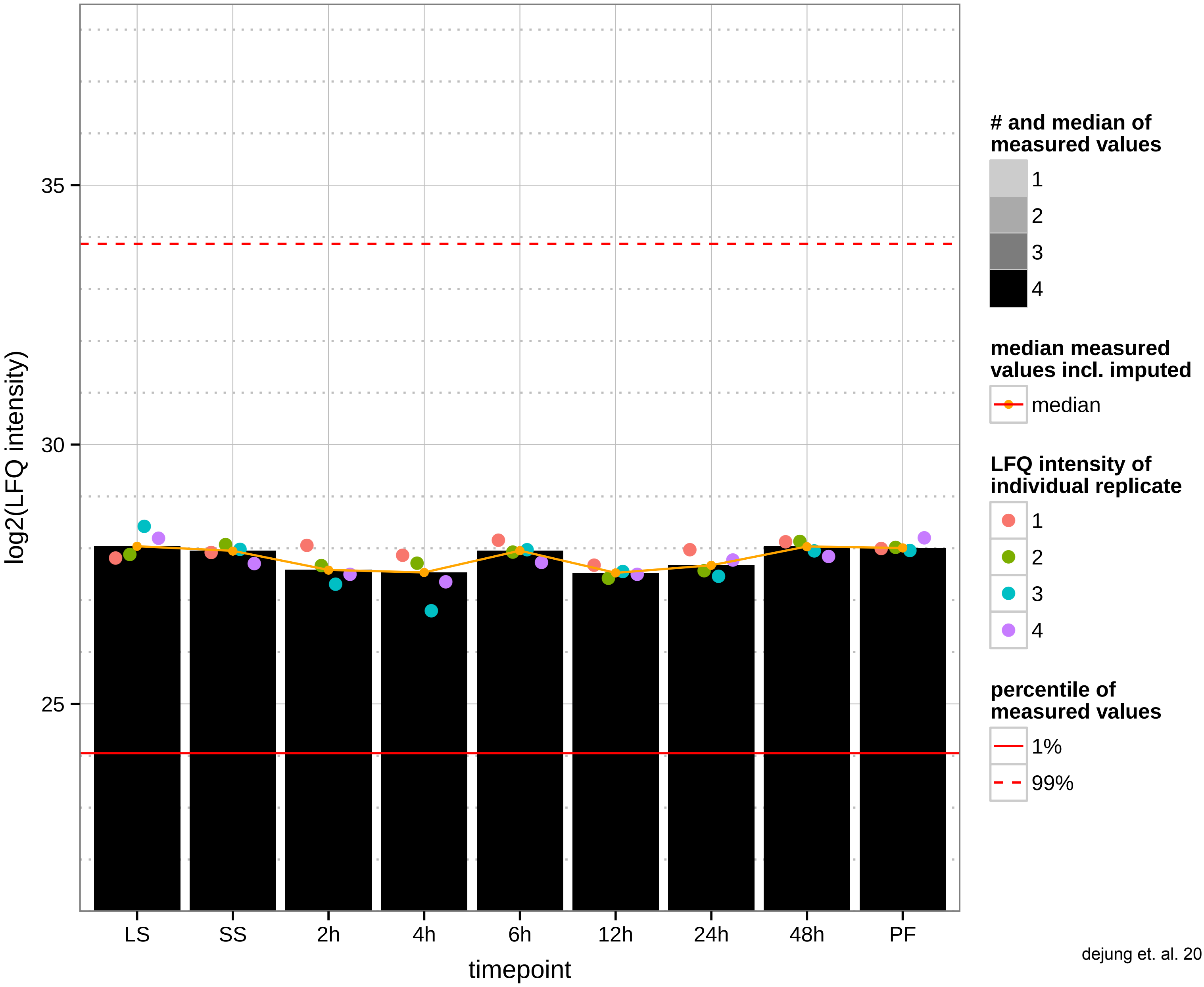

dejung et. al. 2015

kinetoplastid kinetochore protein 7 (kkt7)
Tb927.11.1030
AGOF: null
AGOC: kinetochore
AGOP: chromosome segregation
PGOF: null
PGOC: null
PGOP: null

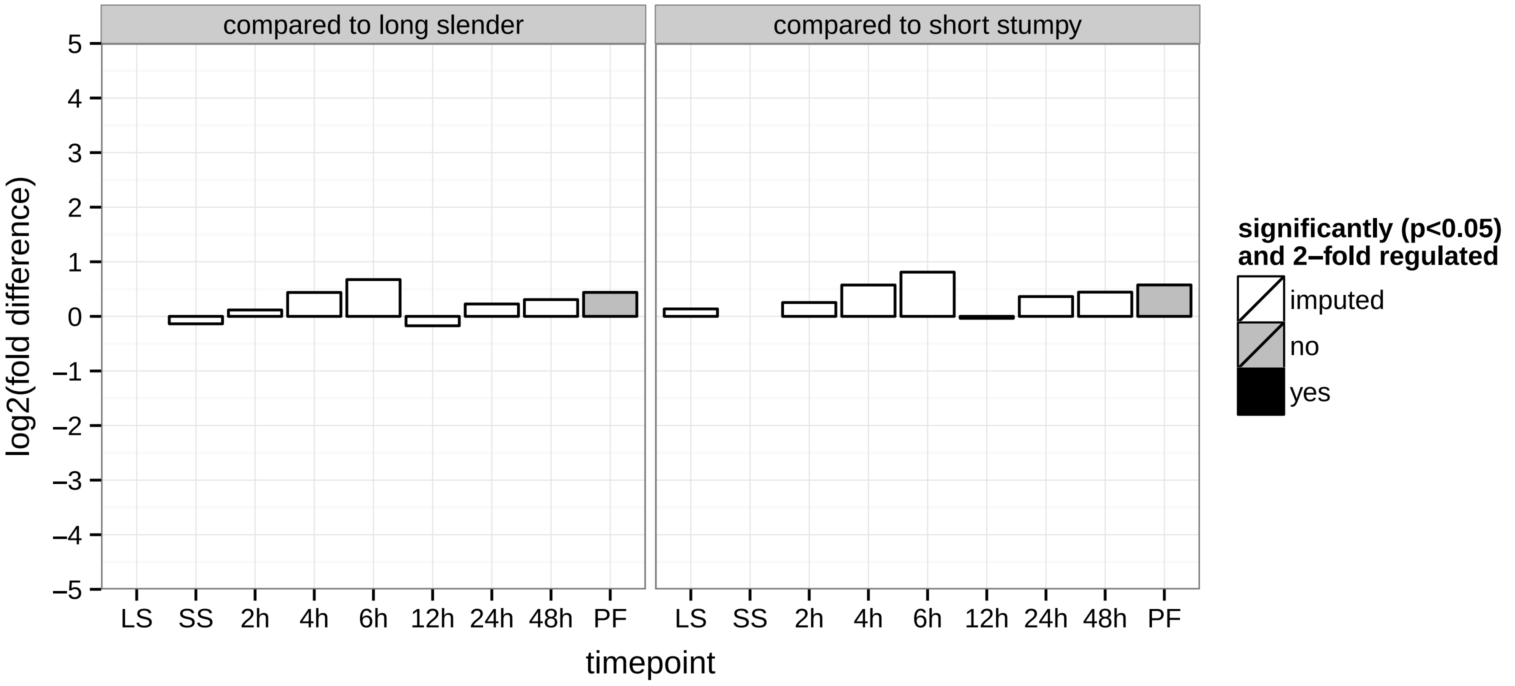

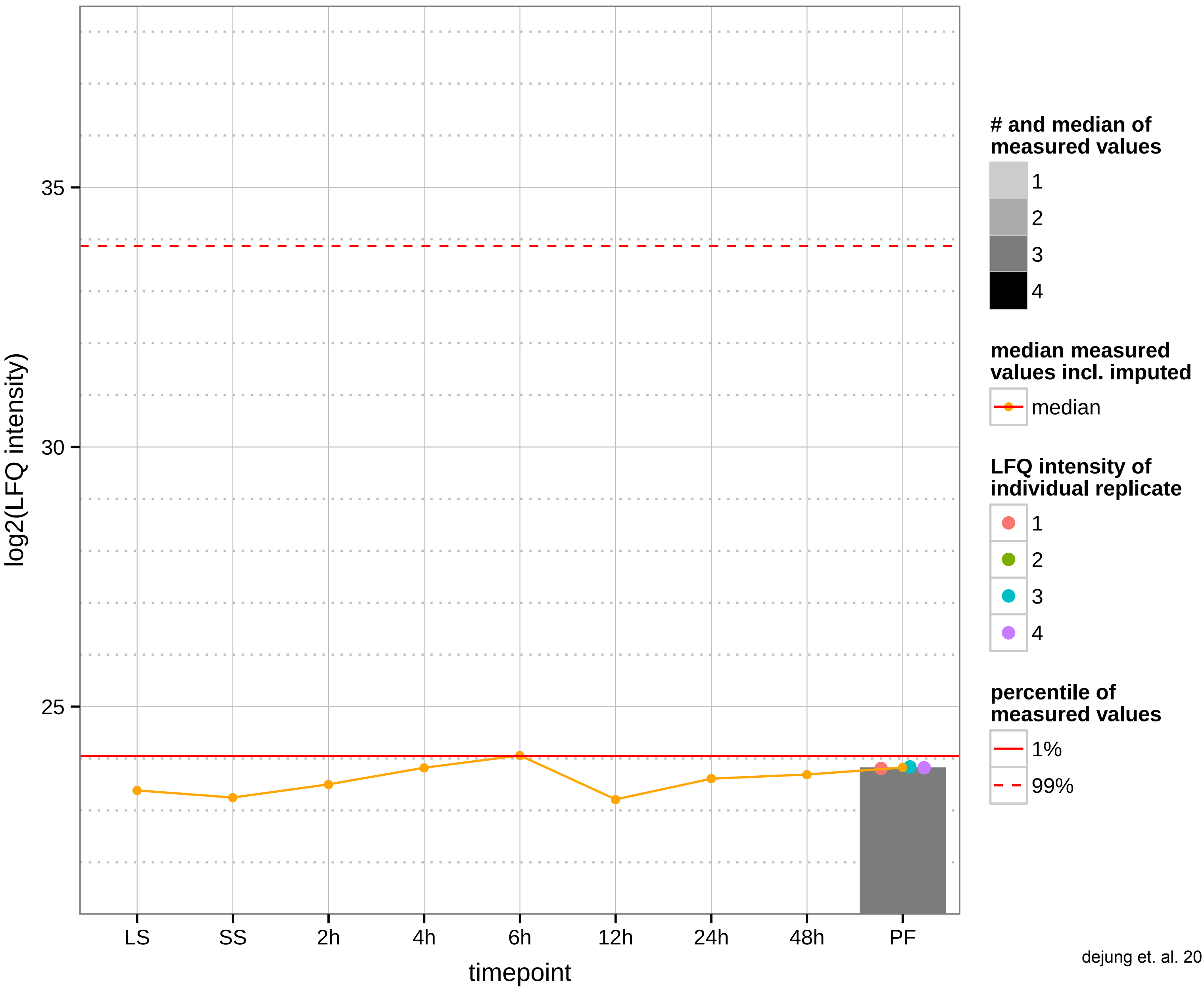

dejung et. al. 2015

hypothetical protein, conserved
Tb927.11.10330
AGOF: null
AGOC: null
AGOP: null
PGOF: null
PGOC: null
PGOP: null

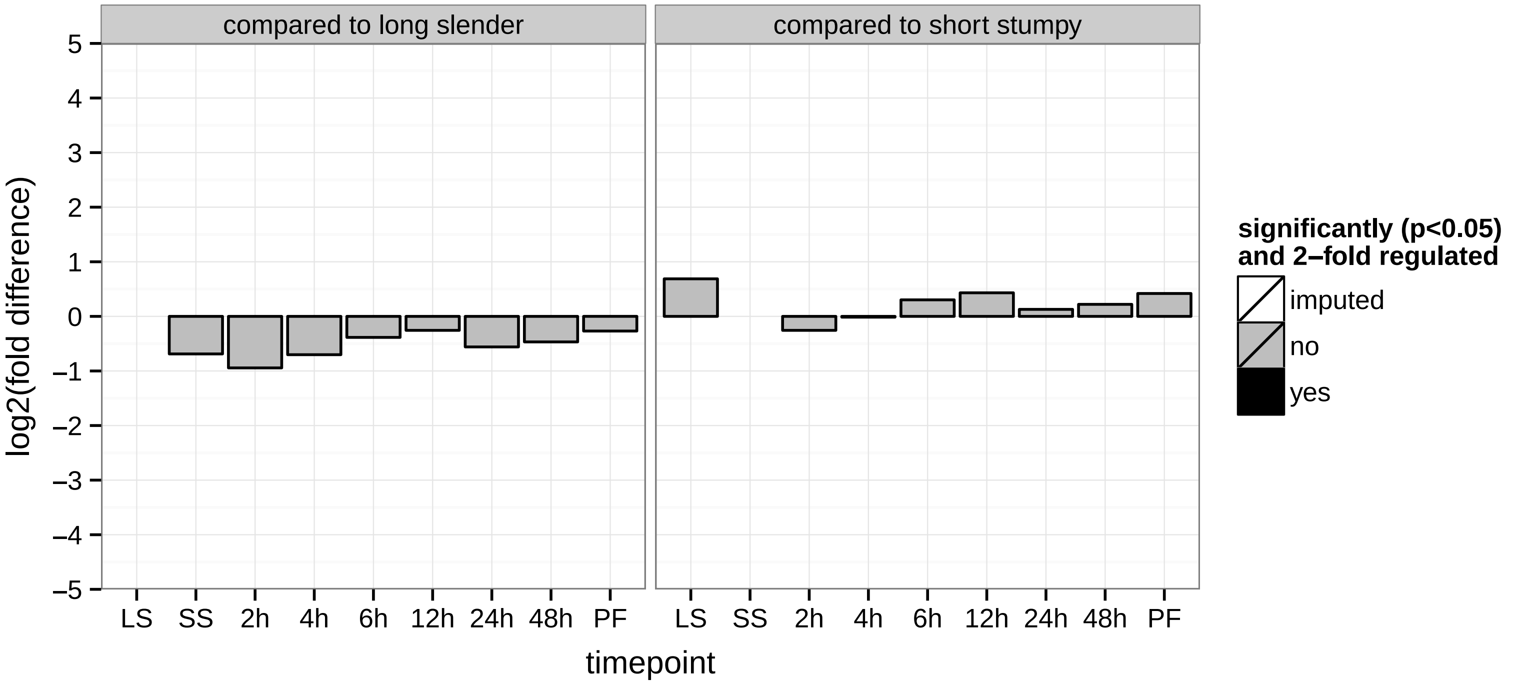

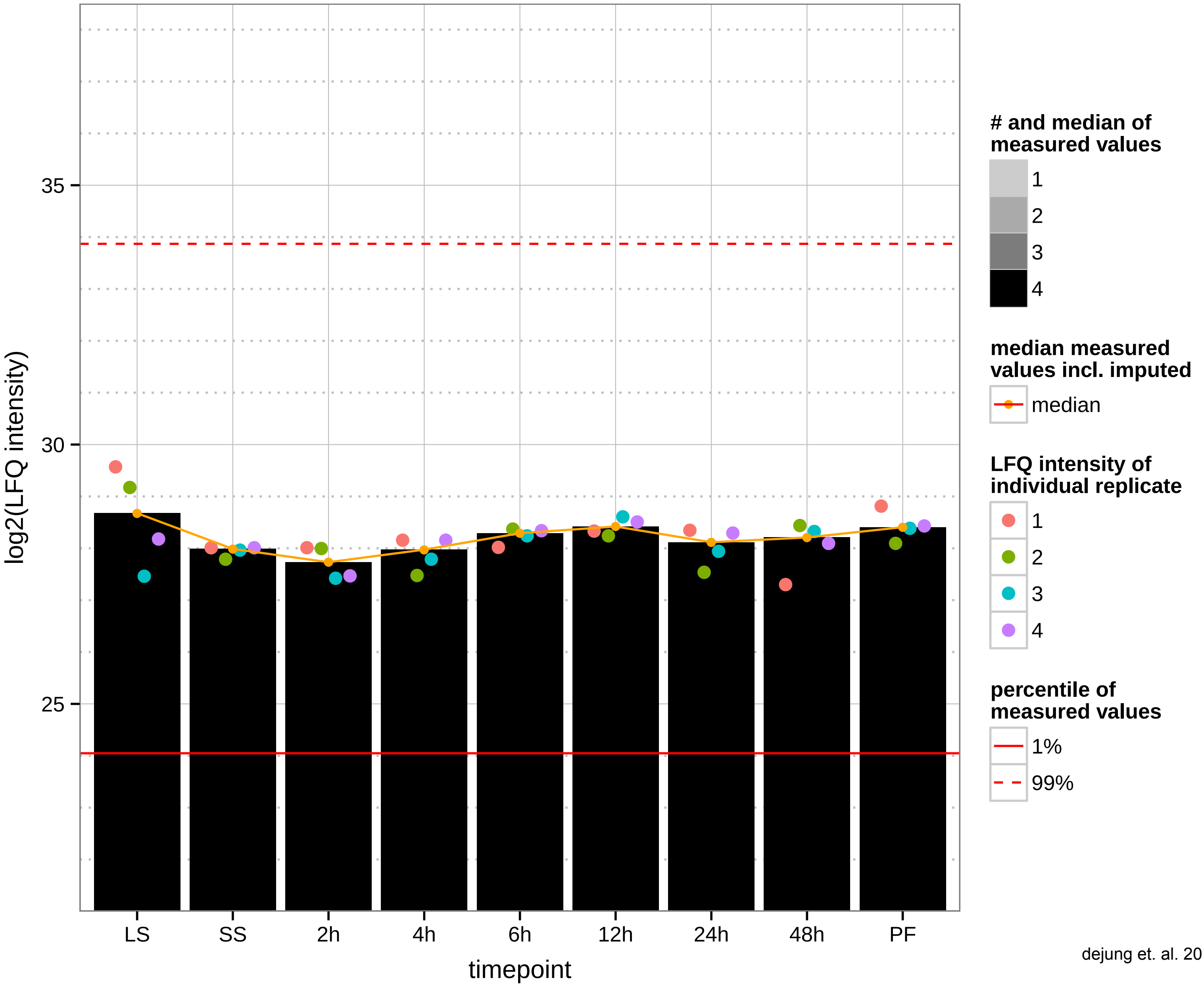

dejung et. al. 2015

Serine/threonine−protein phosphatase PGAM5, mitochondrial, putative, phosphoglycerate mutase family member 5, putative
Tb927.11.10340
AGOF: phosphoprotein phosphatase activity, protein serine/threonine kinase activator activity
AGOC: null
AGOP: regulation of mitochondrion organization
PGOF: null
PGOC: null
PGOP: null

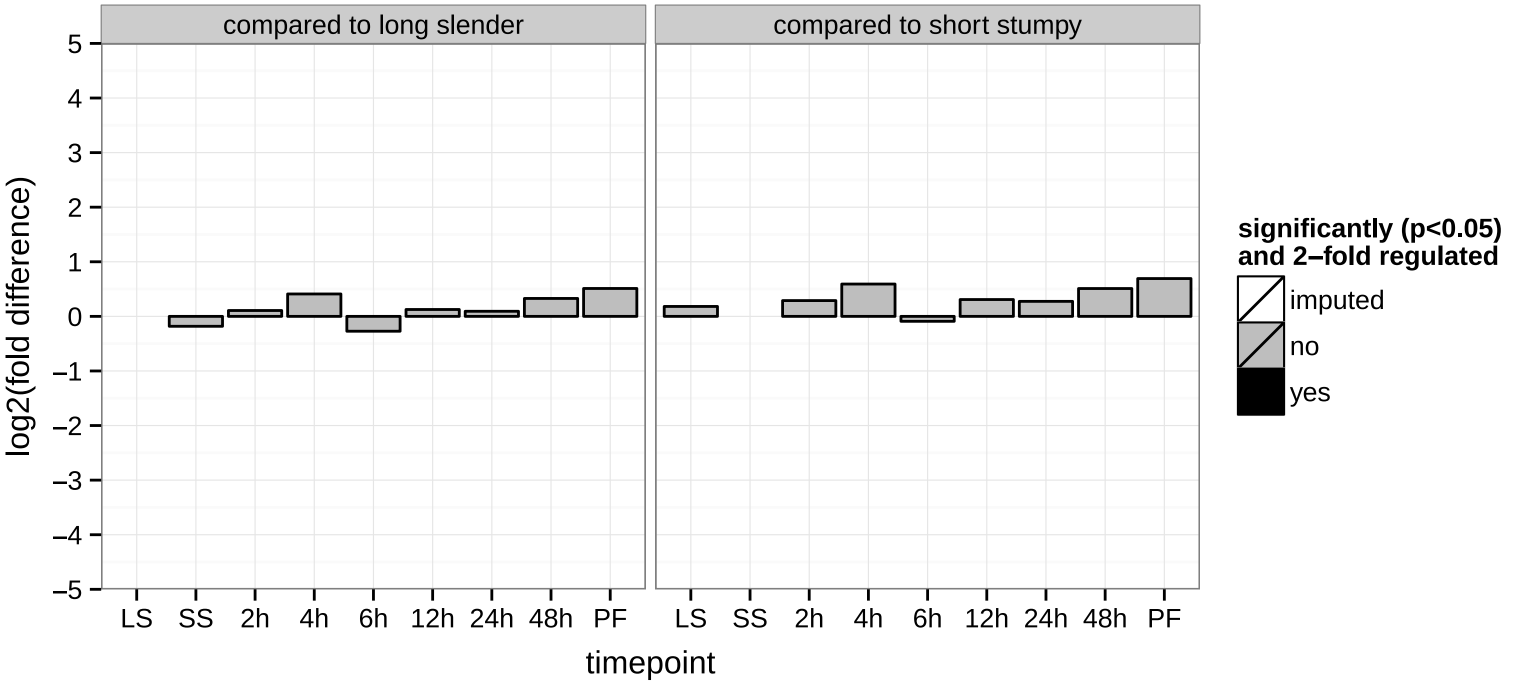

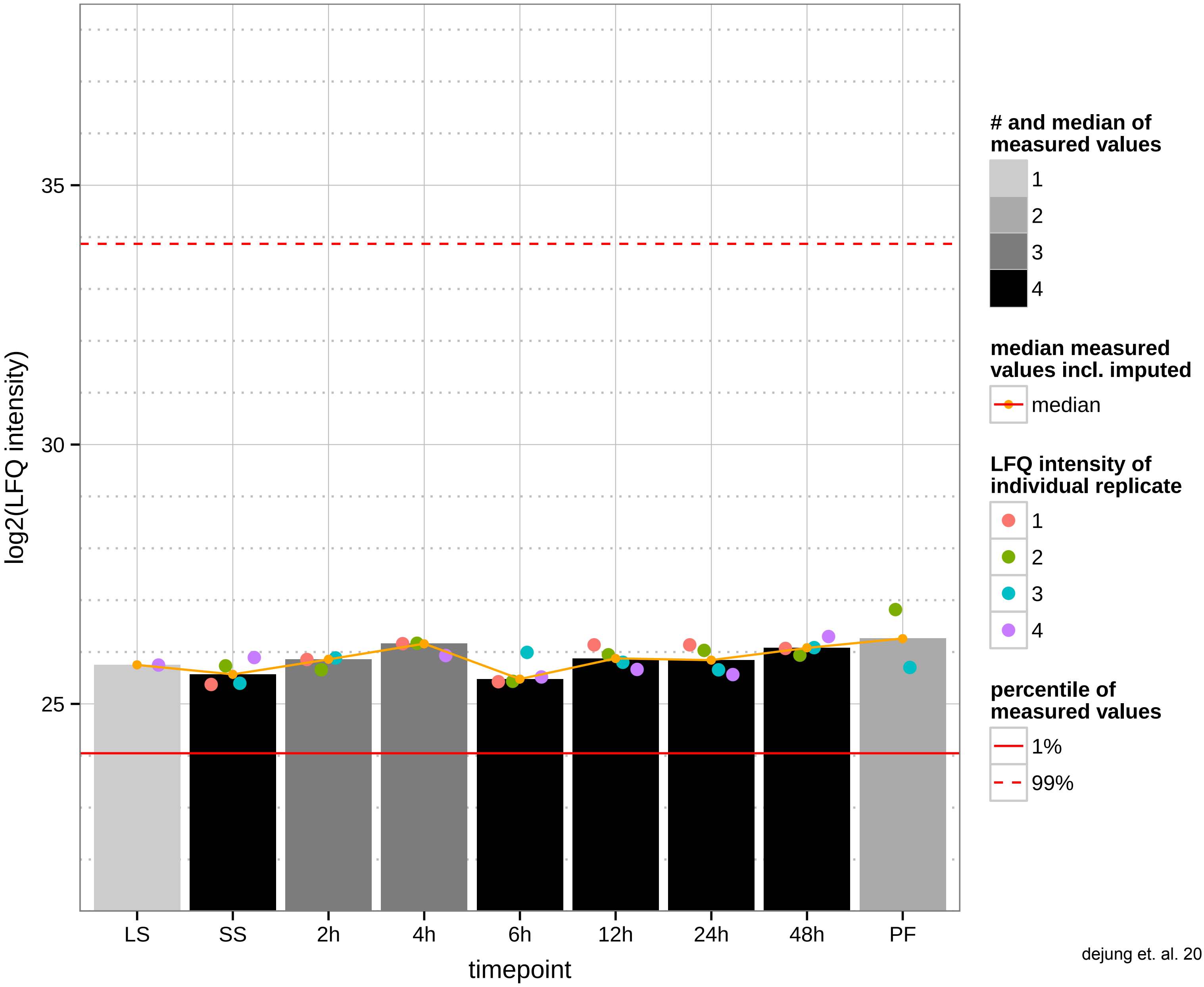

dejung et. al. 2015

hypothetical protein, conserved
Tb927.11.10380
AGOF: null
AGOC: null
AGOP: null
PGOF: null
PGOC: null
PGOP: null

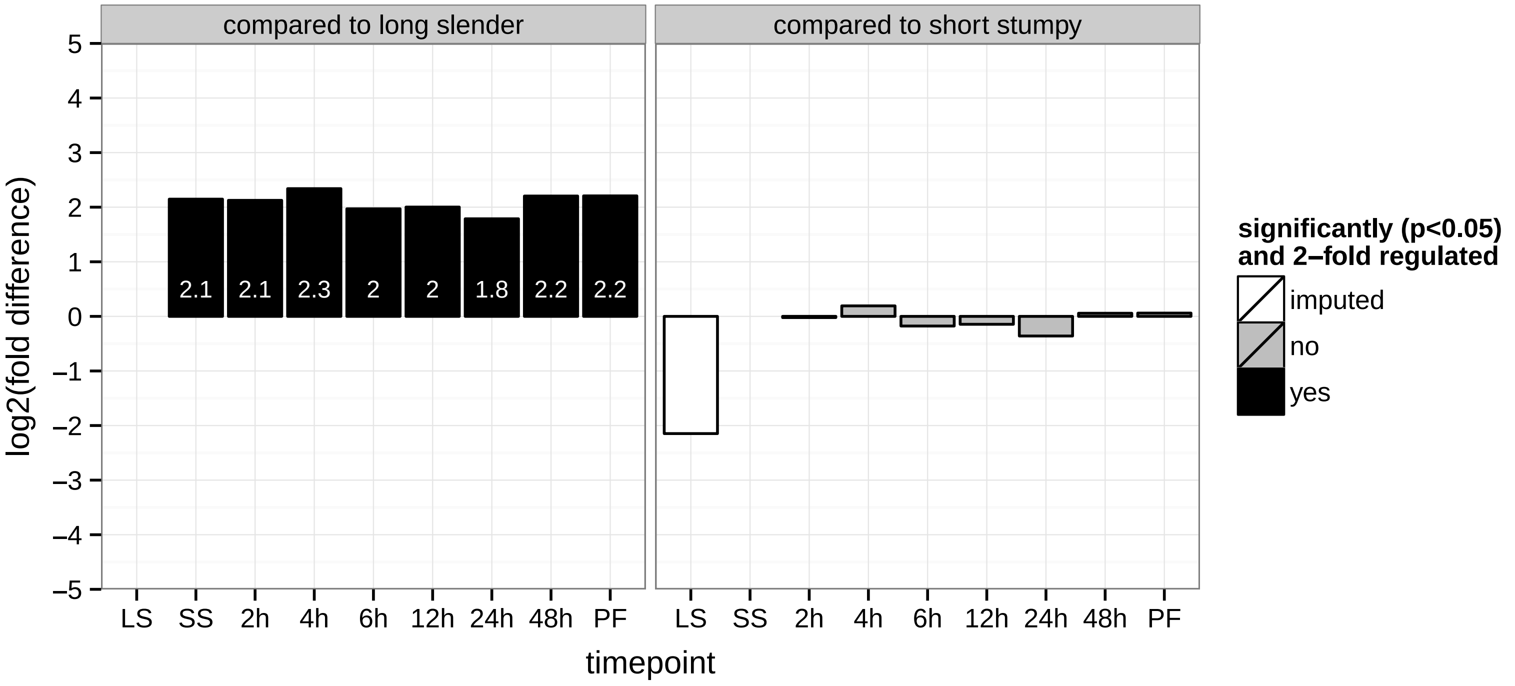

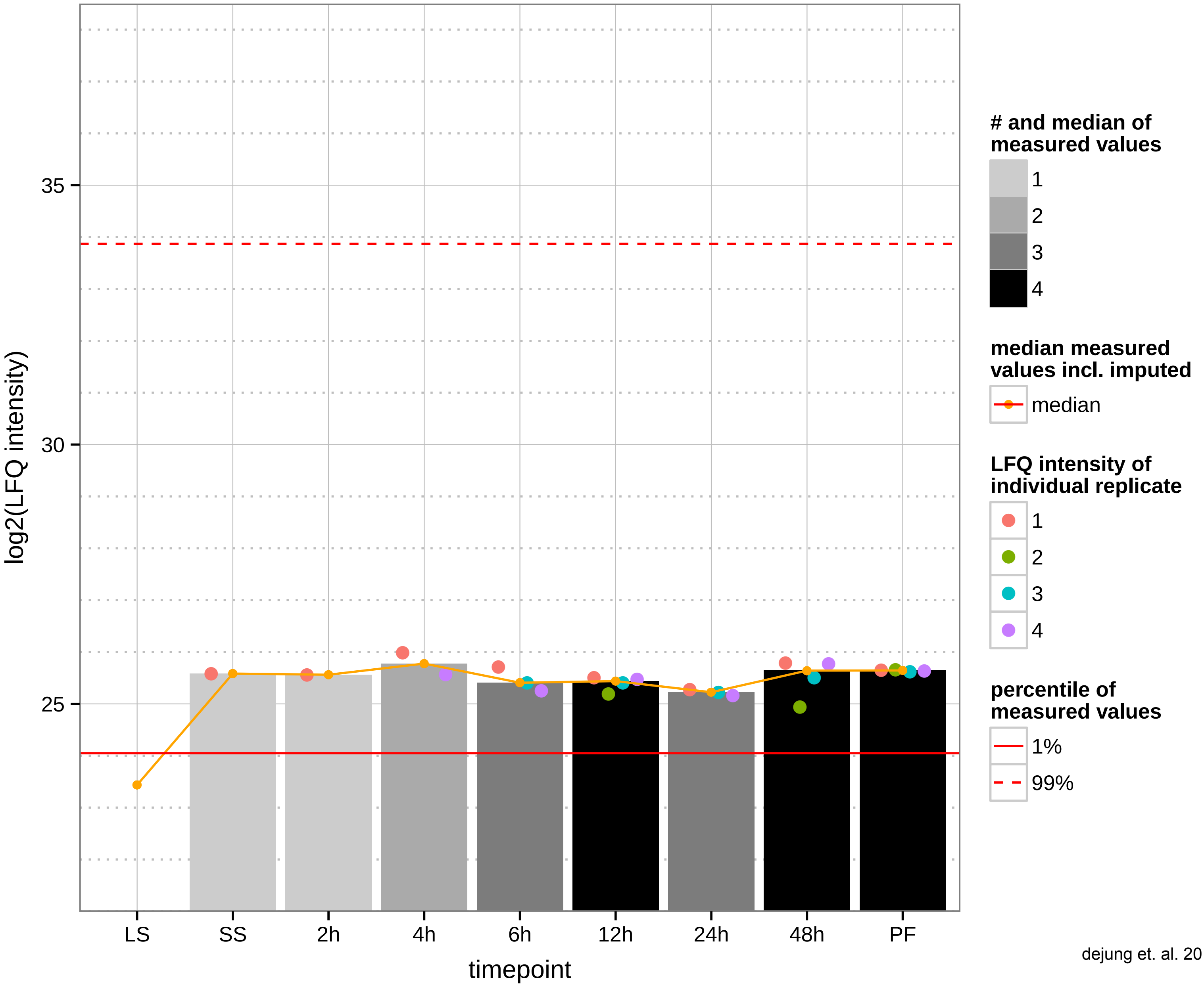

dejung et. al. 2015

hypothetical protein, conserved
Tb927.11.10430
AGOF: null
AGOC: null
AGOP: null
PGOF: protein binding
PGOC: null
PGOP: null

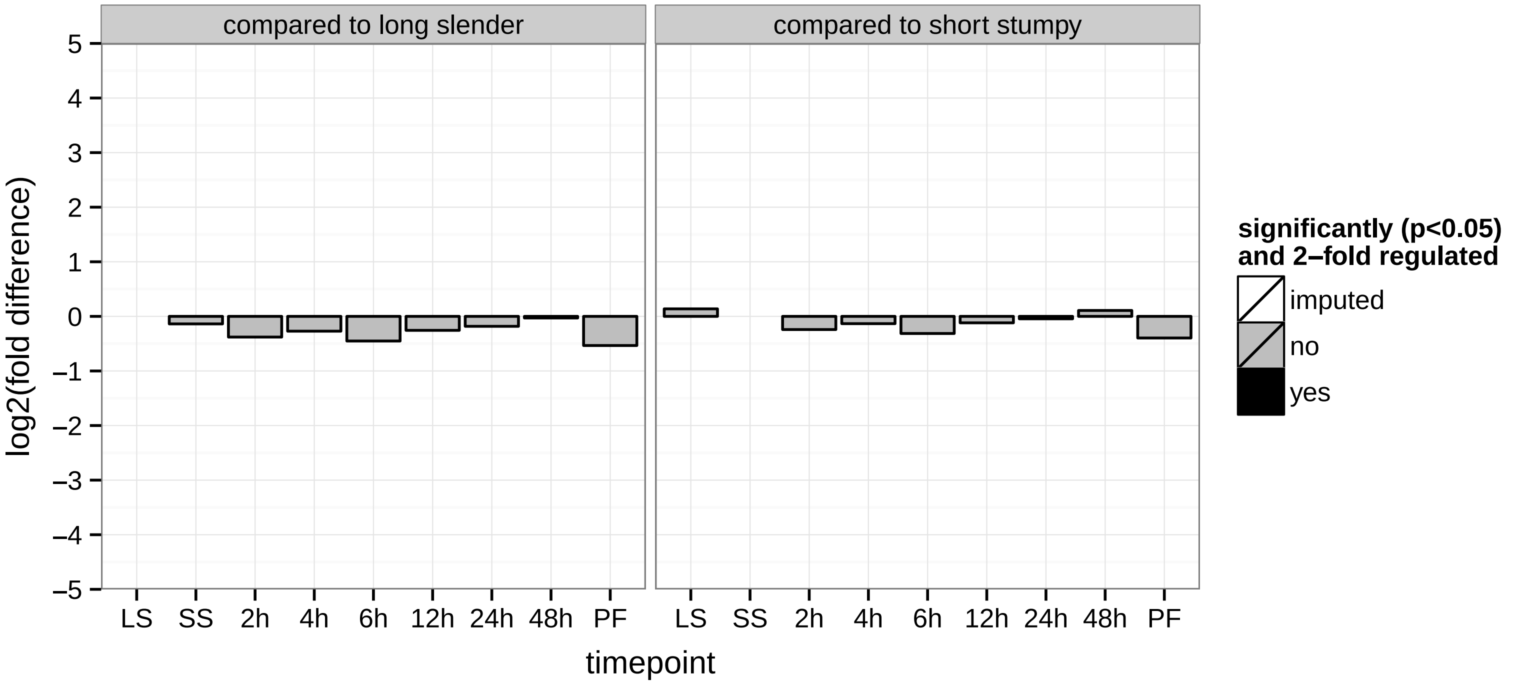

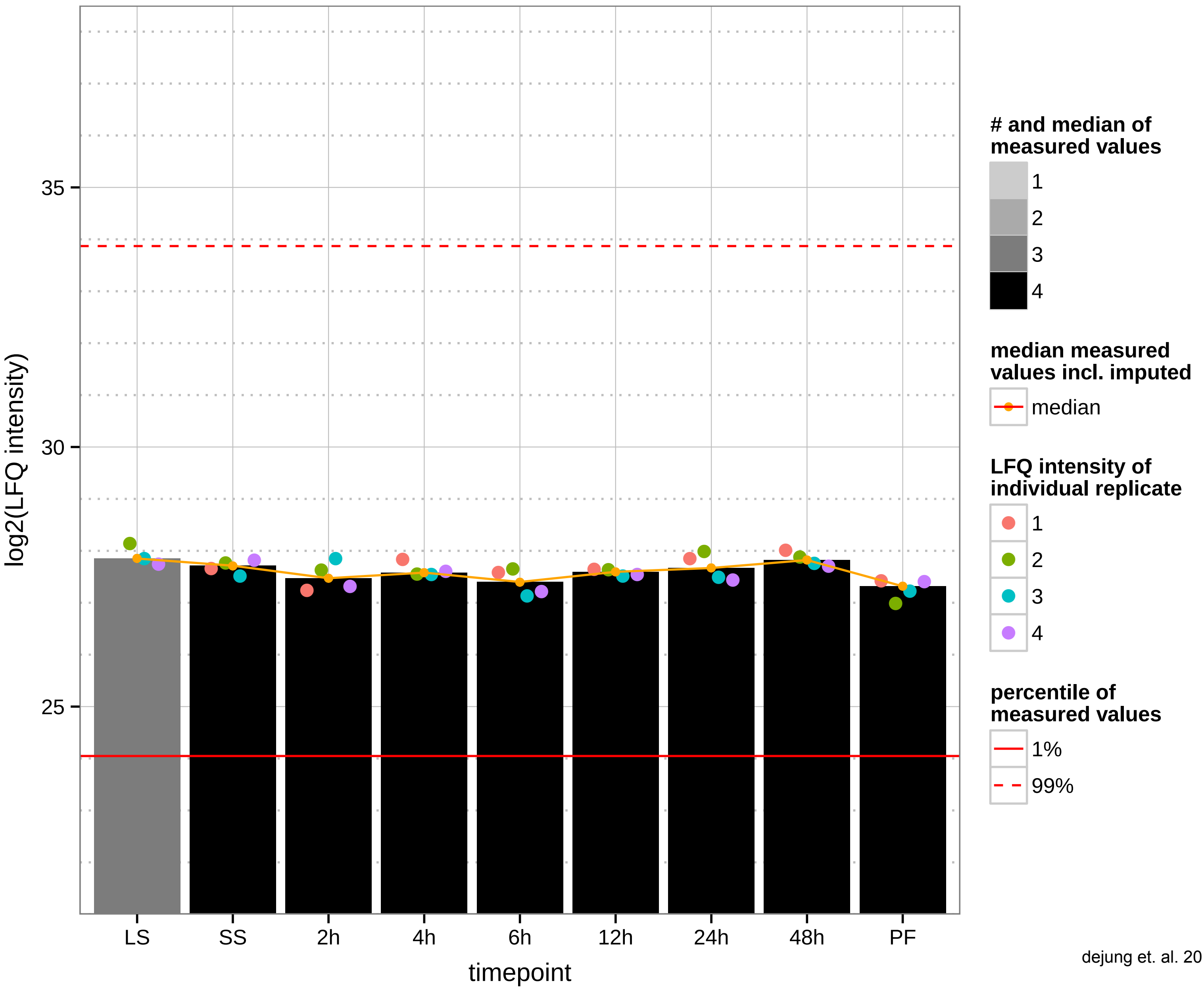

dejung et. al. 2015

UDP–N–acetylglucosamine–dolichyl–phosphate N–acetylglucosaminephosphotransferase, putative
Tb927.11.10450
AGOF: UDP–N–acetylglucosamine–dolichyl–phosphate N–acetylglucosaminephosphotransferase activity
AGOC: integral to membrane
AGOP: dolichol–linked oligosaccharide biosynthetic process
PGOF: phospho–N–acetylmuramoyl–pentapeptide–transferase activity
PGOC: integral to membrane
PGOP: null

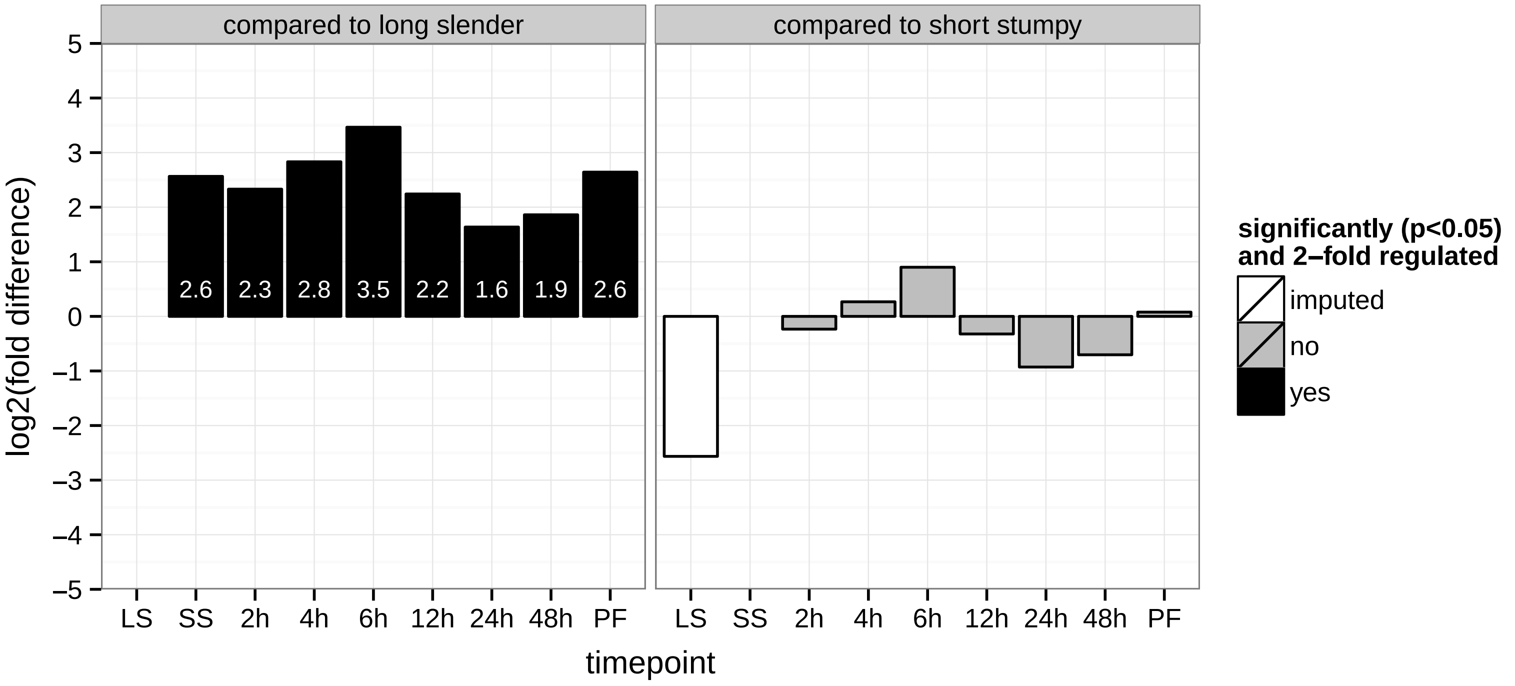

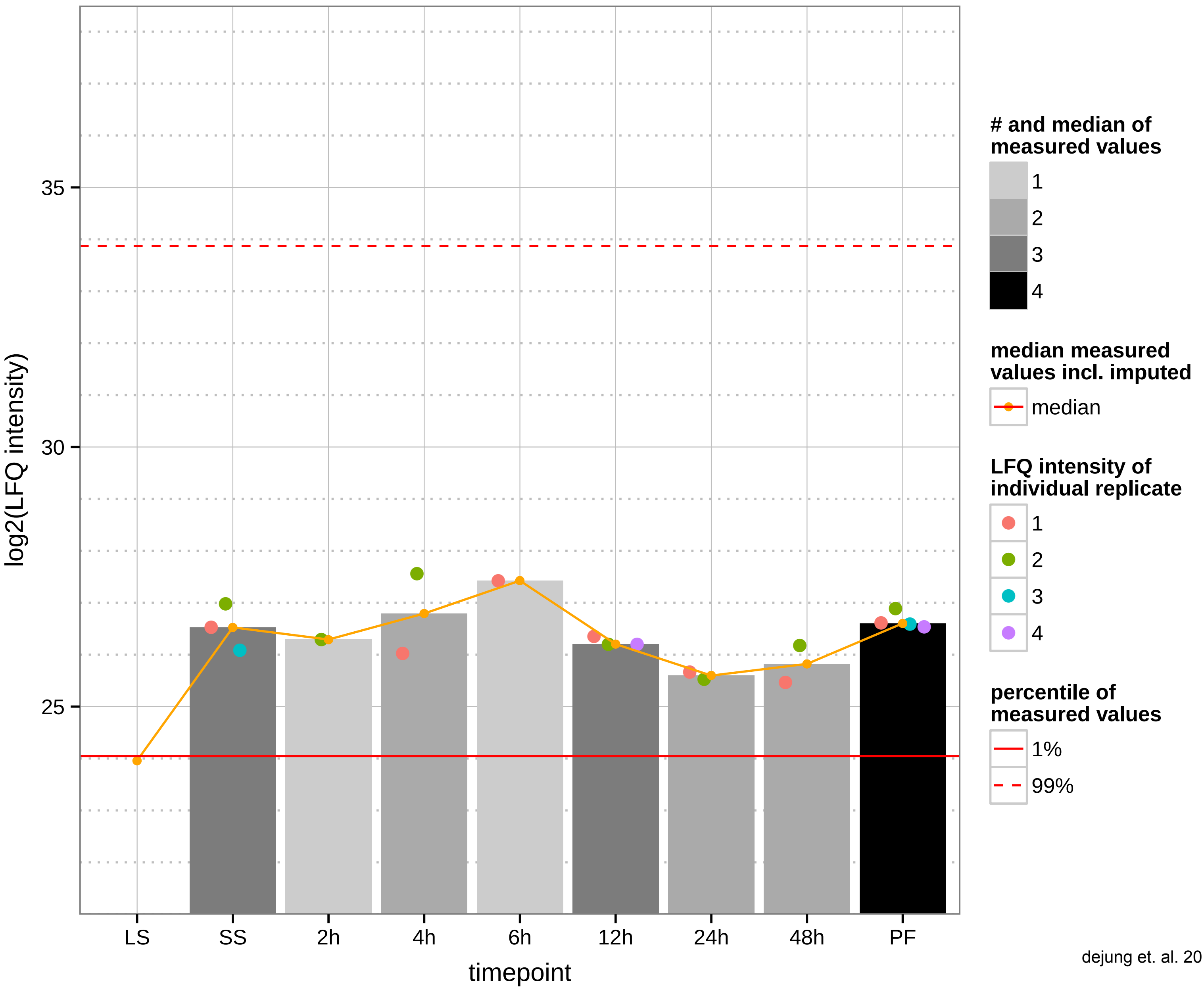

dejung et. al. 2015

hypothetical protein, conserved
Tb927.11.10480
AGOF: null
AGOC: null
AGOP: null
PGOF: protein binding
PGOC: small–subunit processome
PGOP: rRNA processing

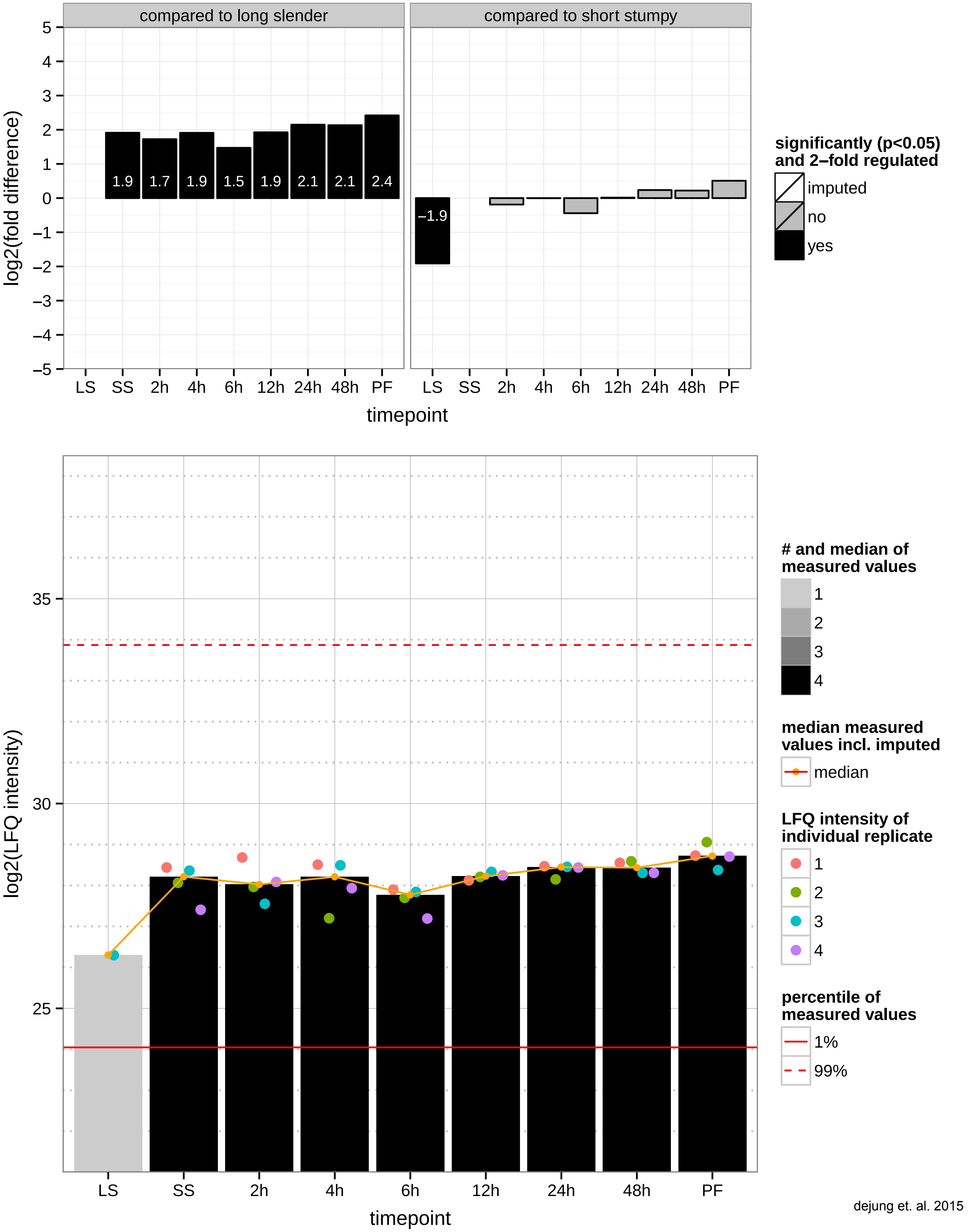

dejung et. al. 2015

ubiquinone biosynthesis methyltransferase, putative
Tb927.11.10510
AGOF: 2−polyprenyl−6−methoxy−1, 4−benzoquinone methyltransferase activity
AGOC: mitochondrion
AGOP: coenzyme biosynthetic process, ubiquinone metabolic process
PGOF: methyltransferase activity
PGOC: null
PGOP: null

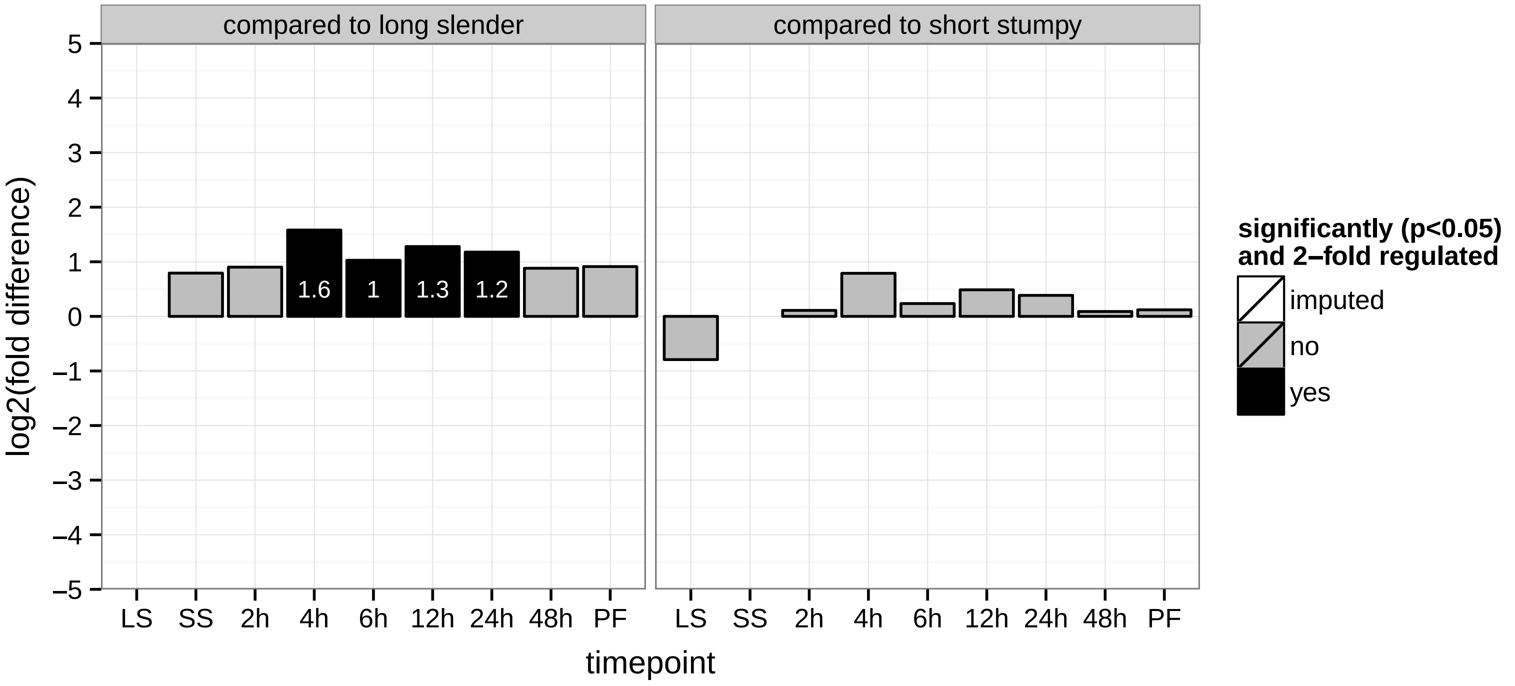

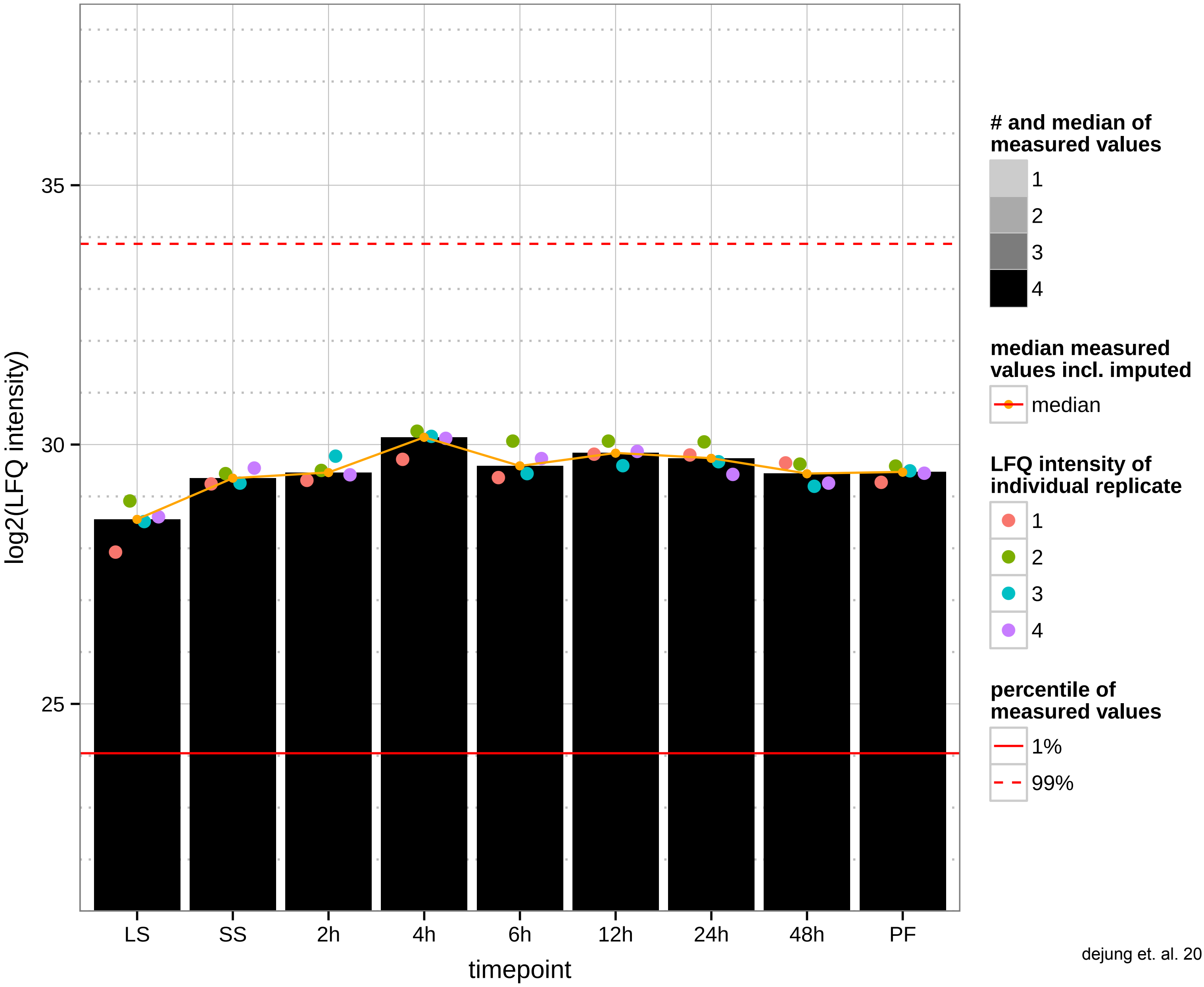

dejung et. al. 2015

hypothetical protein, conserved
Tb927.11.10540
AGOF: null
AGOC: null
AGOP: null
PGOF: null
PGOC: null
PGOP: null

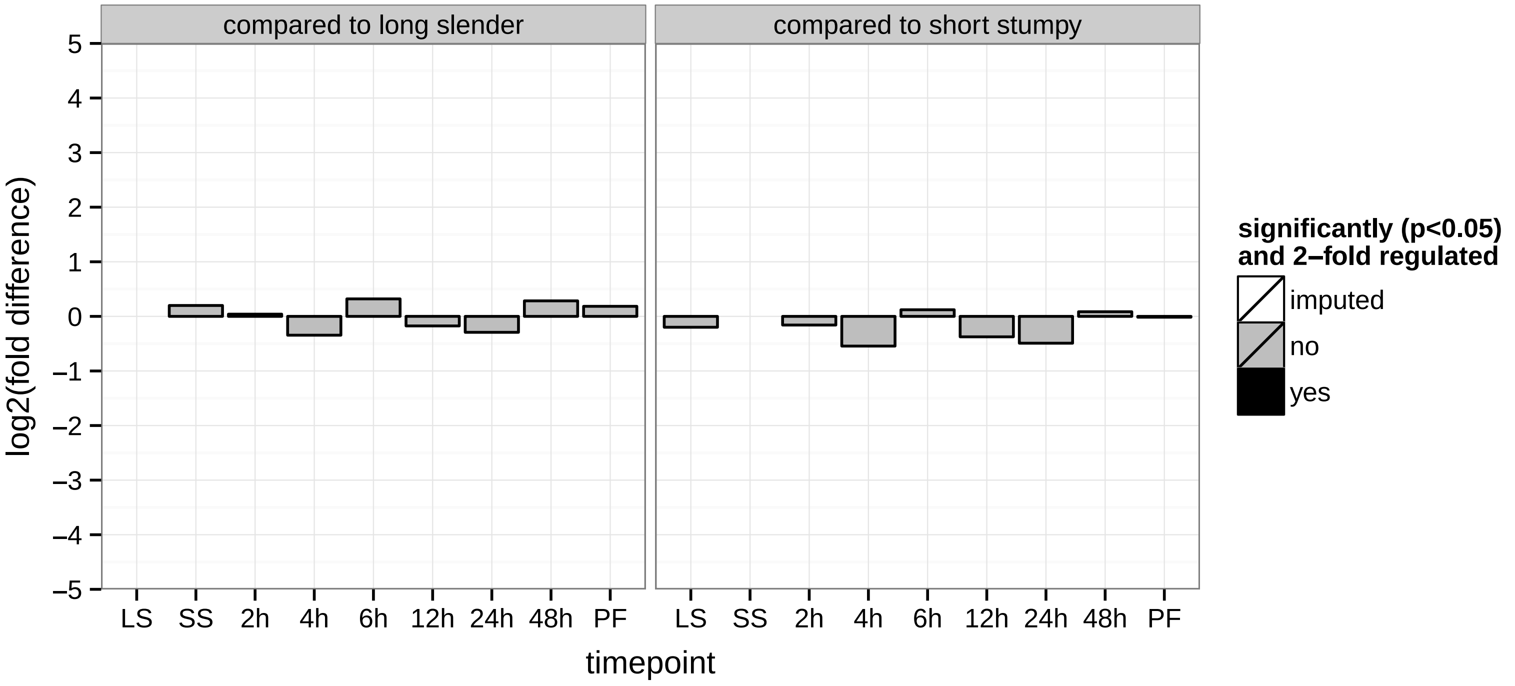

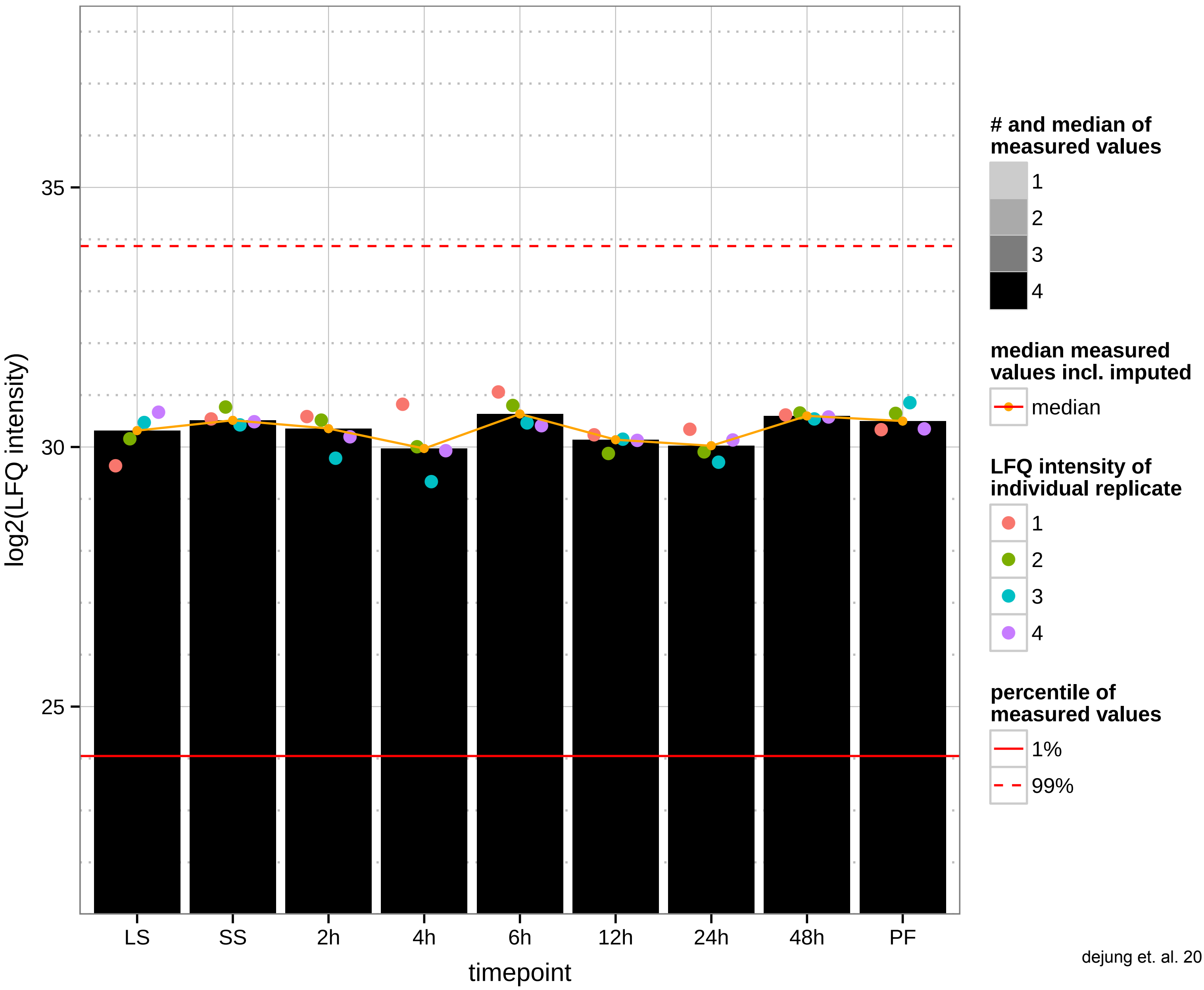

dejung et. al. 2015

hypothetical protein, conserved
Tb927.11.10580
AGOF: null
AGOC: membrane
AGOP: Golgi organization, protein transport
PGOF: null
PGOC: null
PGOP: null

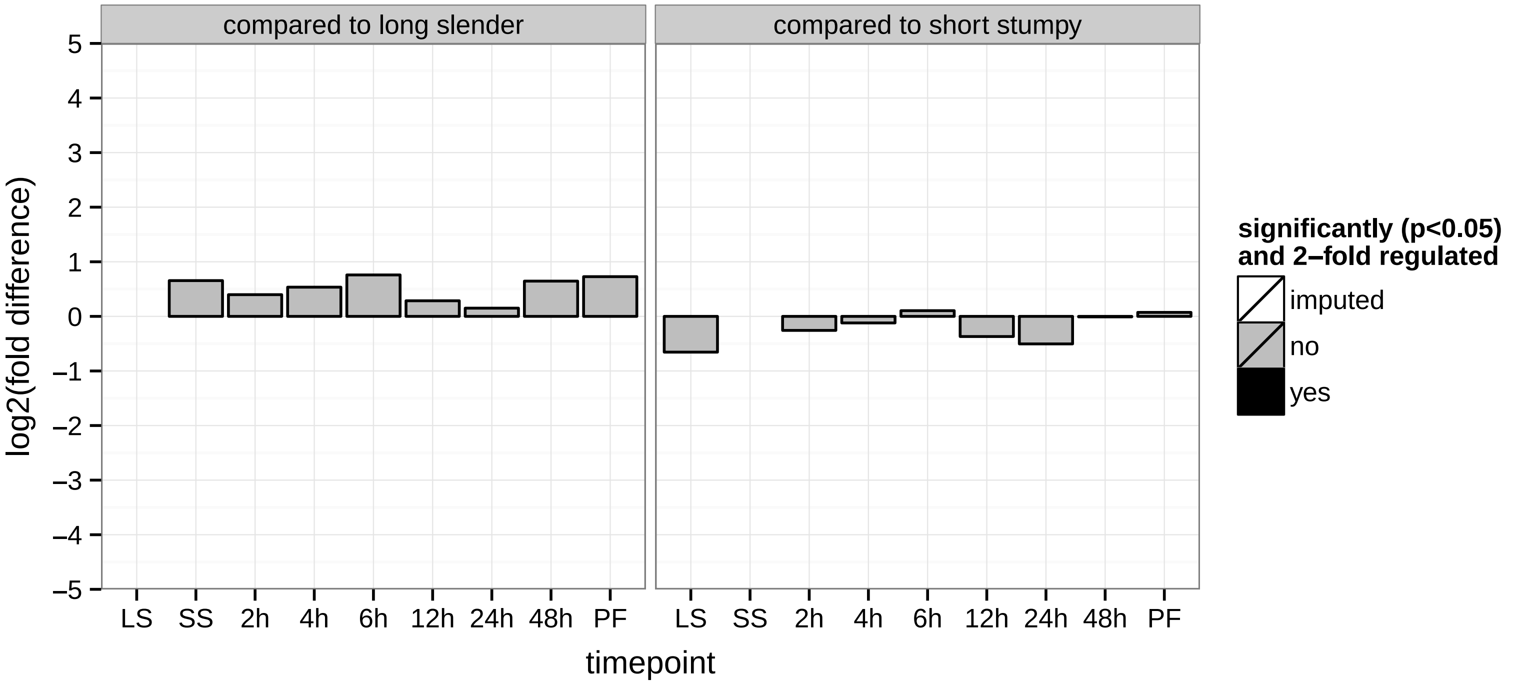

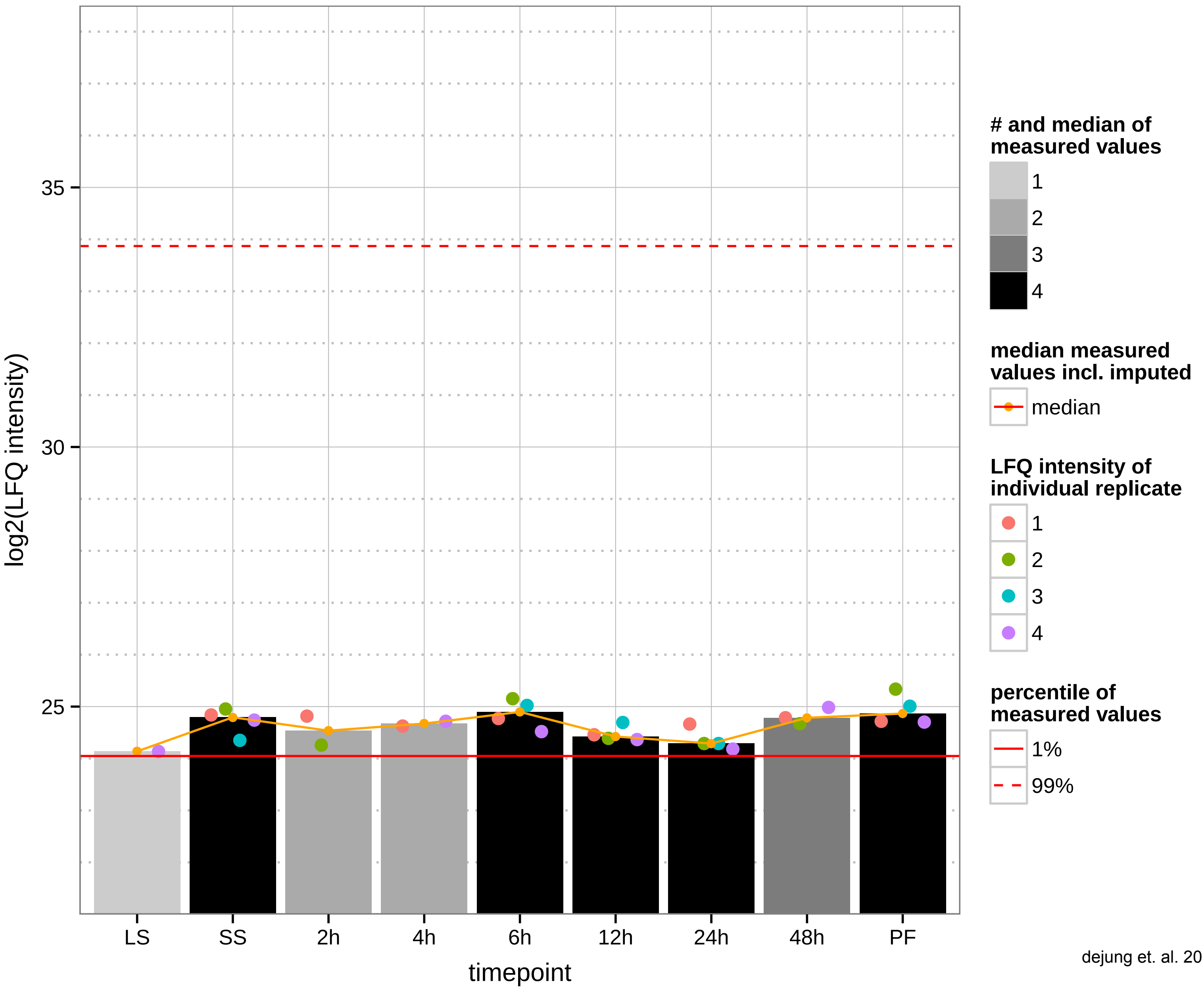

dejung et. al. 2015

hypothetical protein, conserved
Tb927.11.10720
AGOF: null
AGOC: mitochondrion
AGOP: null
PGOF: null
PGOC: null
PGOP: null

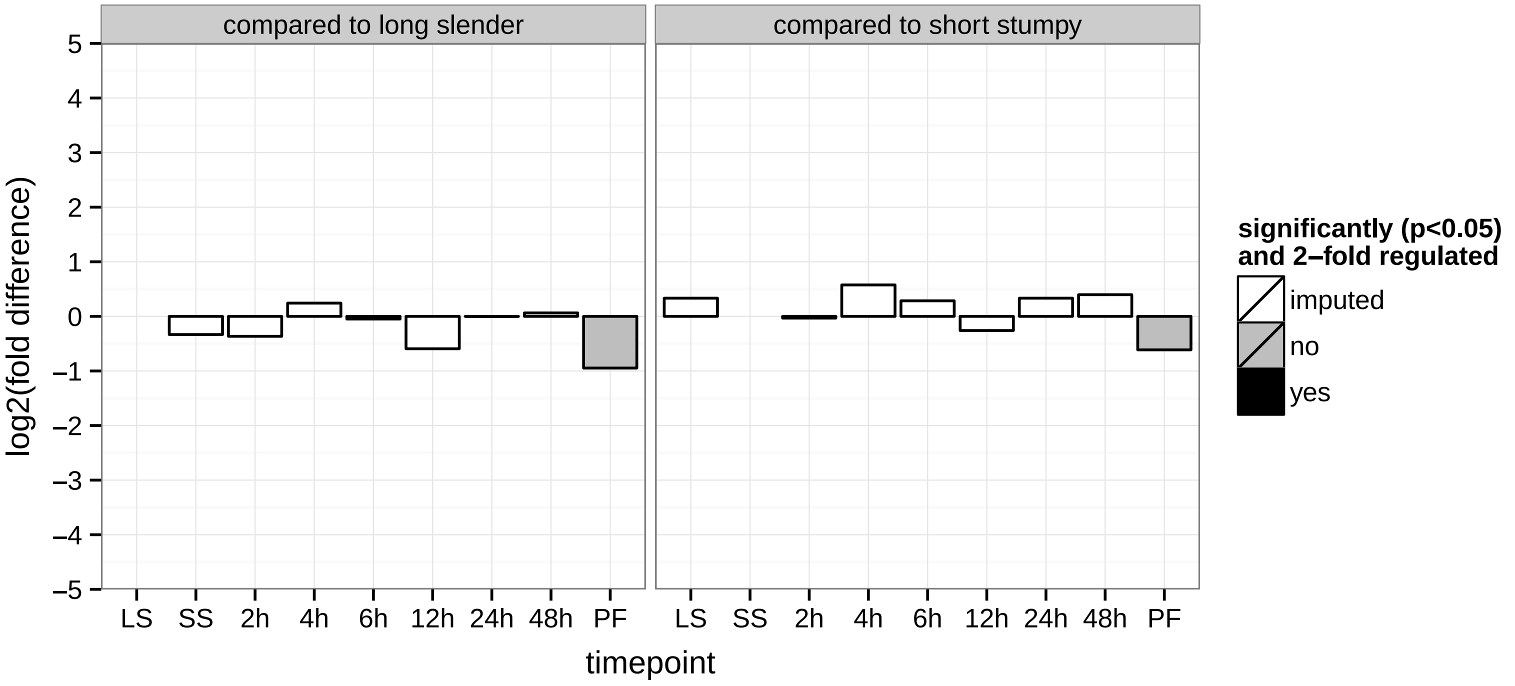

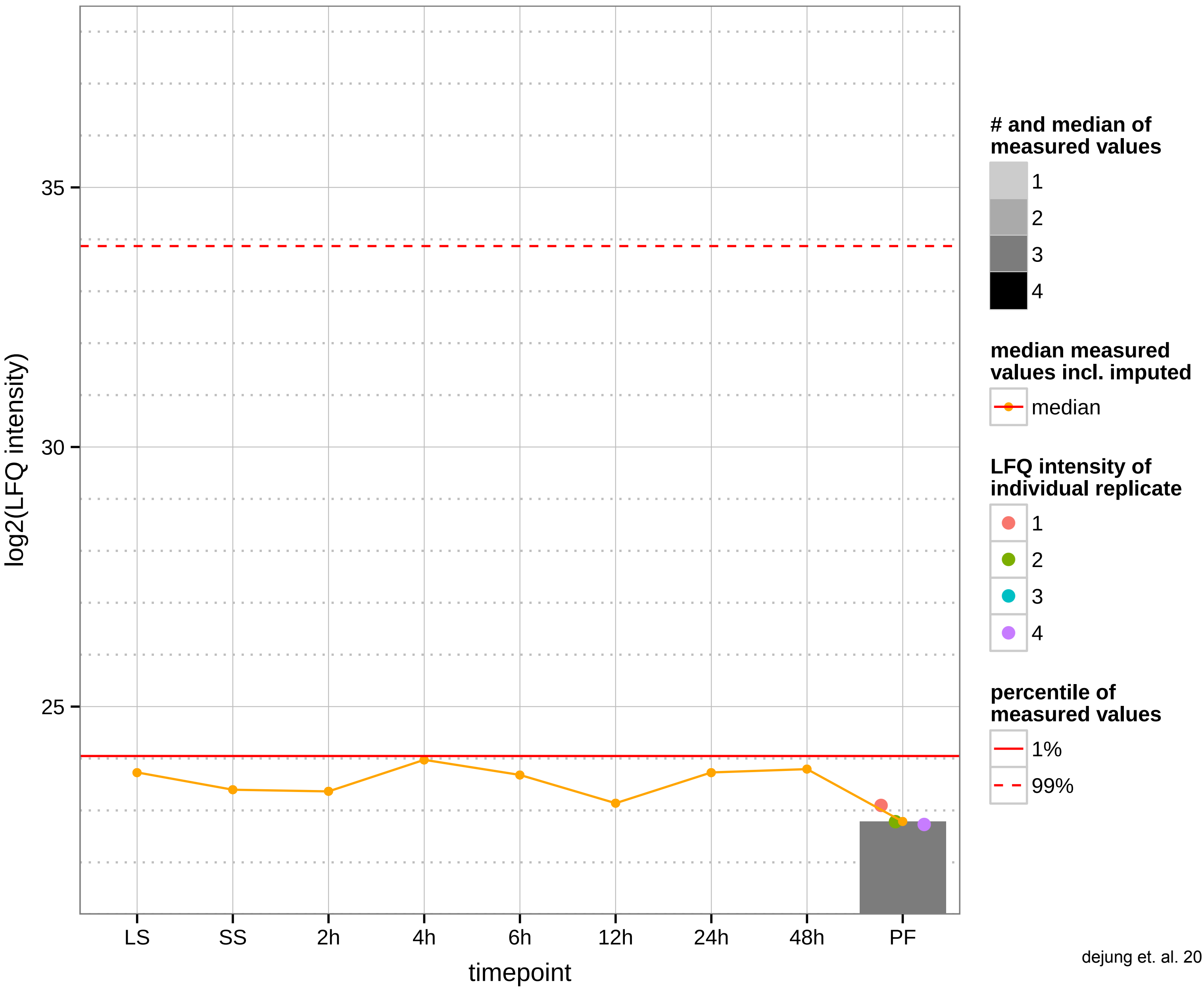

dejung et. al. 2015

vacuolar sorting–associated protein–like, putative
Tb927.11.10740
AGOF: null
AGOC: endosome
AGOP: protein targeting to vacuole
PGOF: null
PGOC: null
PGOP: null

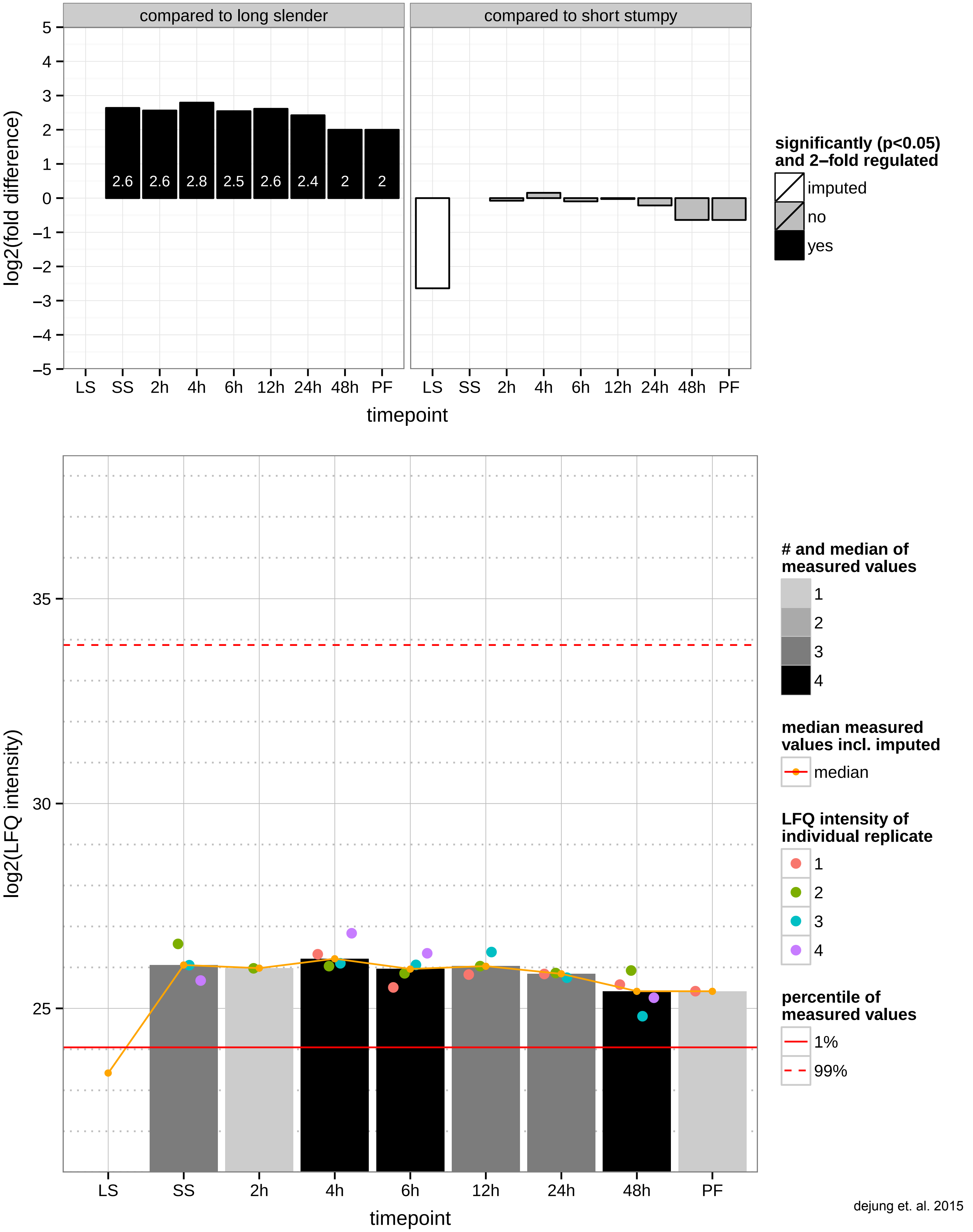

dejung et. al. 2015

hypothetical protein, conserved
Tb927.11.10780
AGOF: null
AGOC: null
AGOP: null
PGOF: null
PGOC: null
PGOP: null

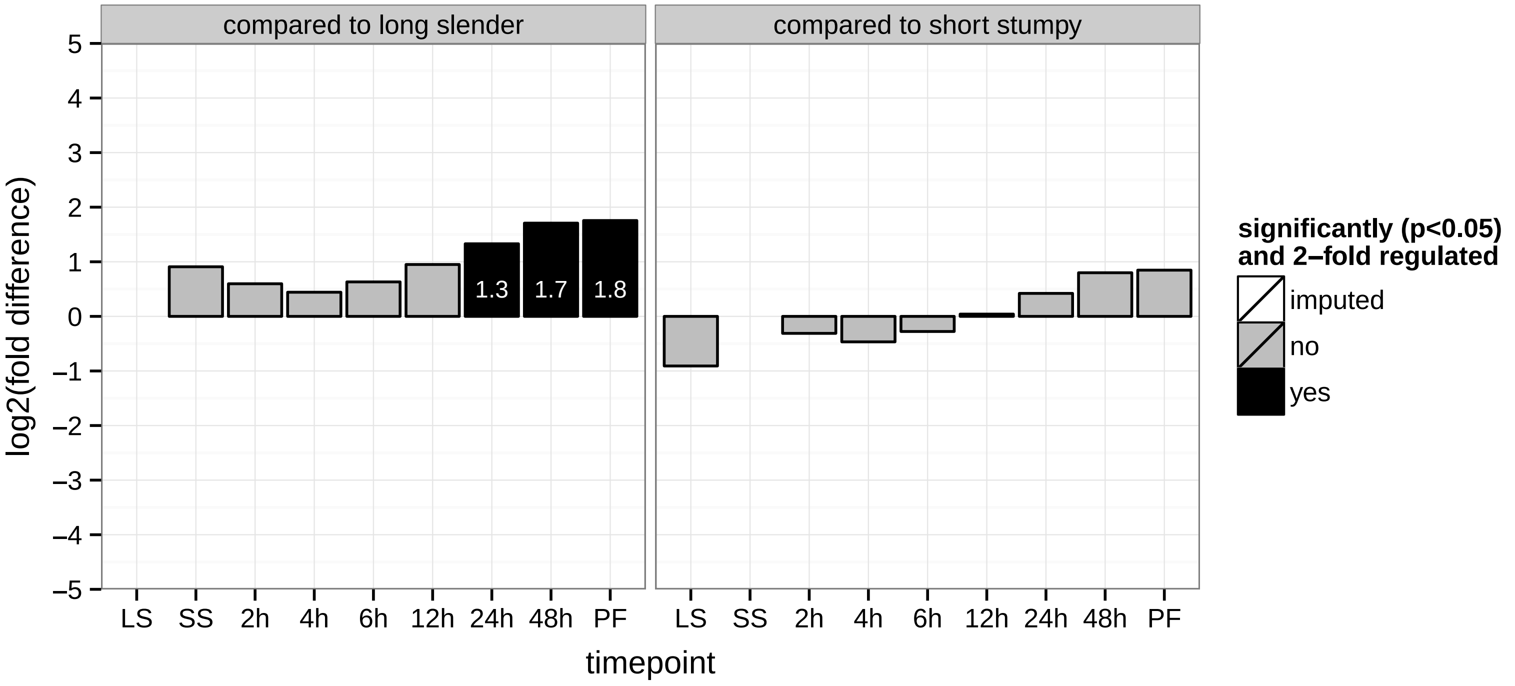

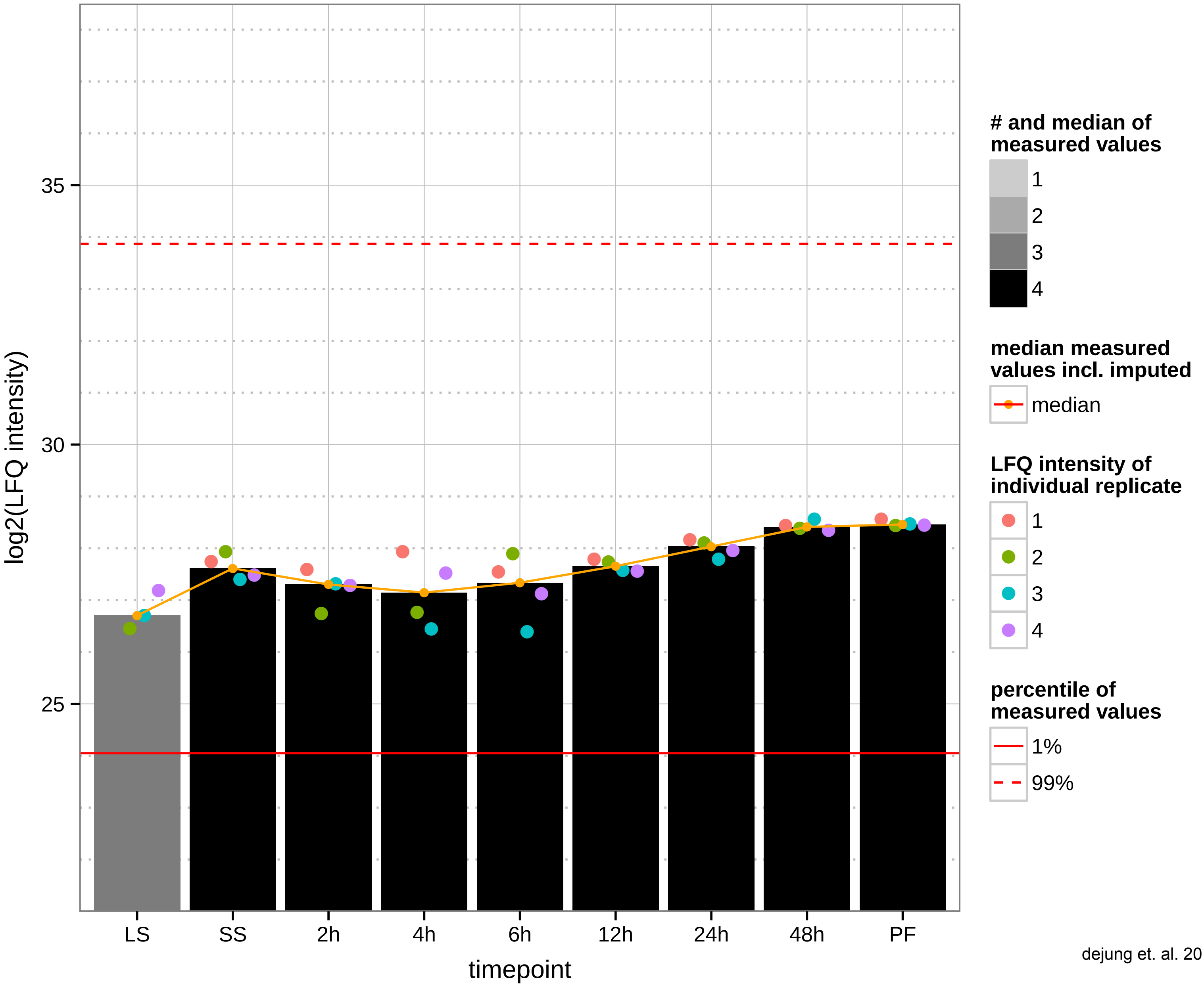

dejung et. al. 2015

40S ribosomal protein SA, putative
Tb927.11.10910;Tb927.11.10790
AGOF: structural constituent of ribosome
AGOC: intracellular, organellar small ribosomal subunit, ribosome
AGOP: translation
PGOF: structural constituent of ribosome
PGOC: intracellular, ribosome, small ribosomal subunit
PGOP: translation

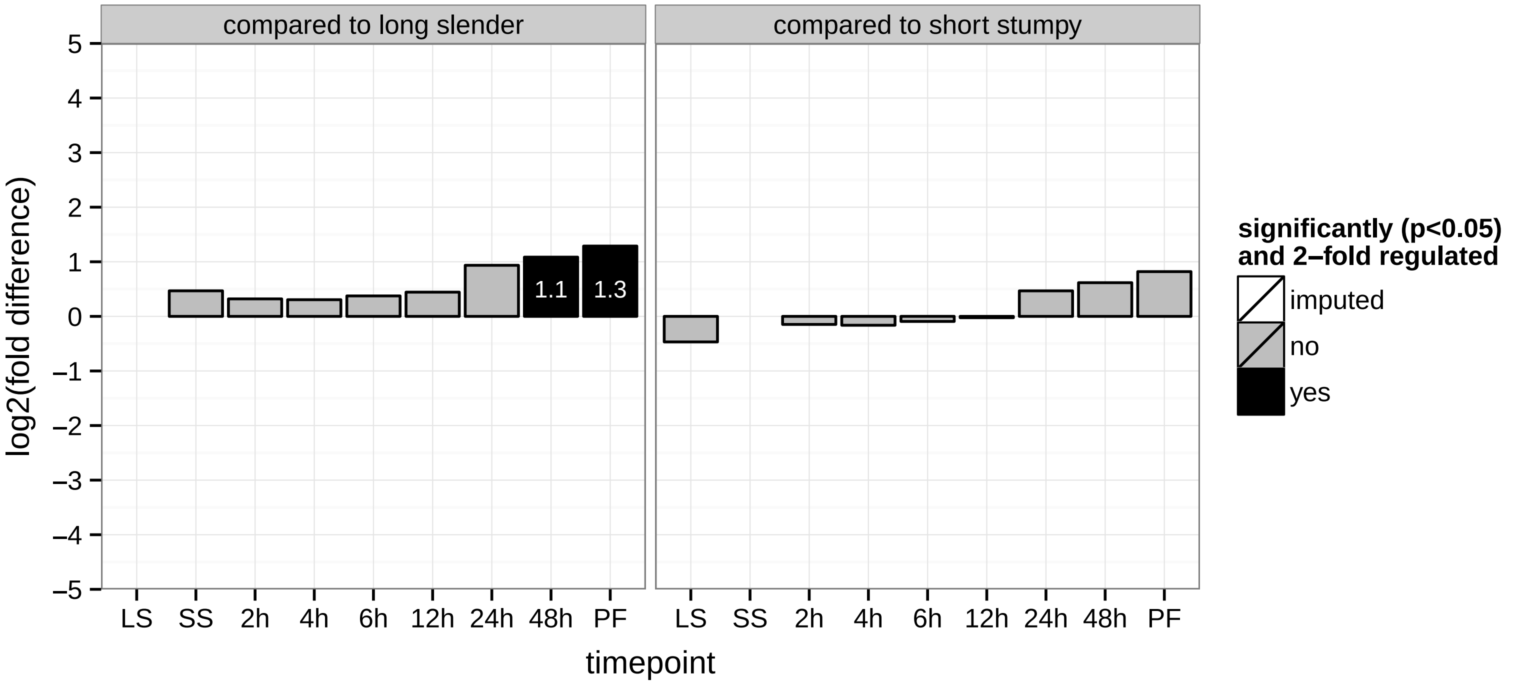

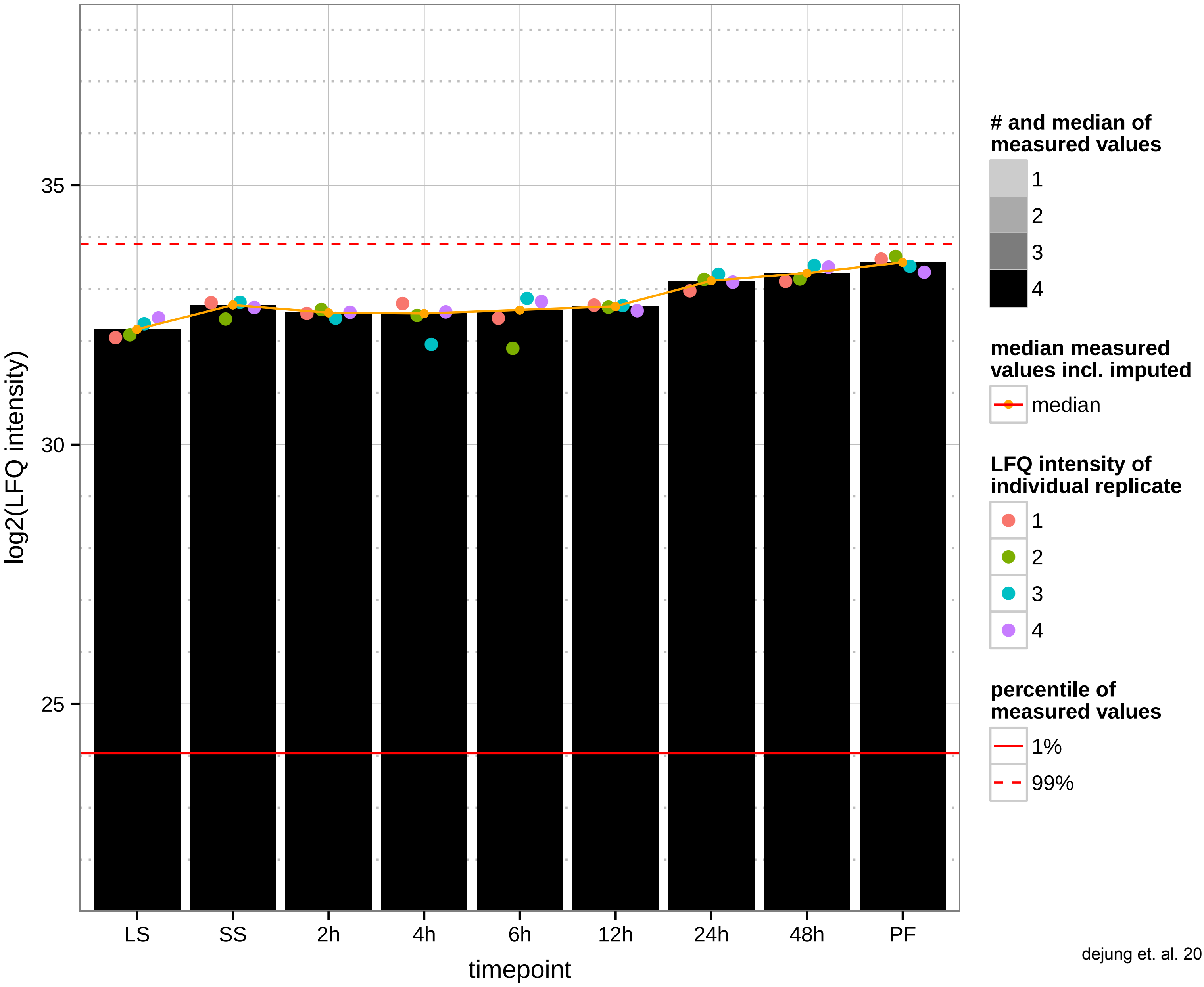

dejung et. al. 2015

hypothetical protein, conserved
Tb927.11.1080
AGOF: null
AGOC: null
AGOP: null
PGOF: null
PGOC: null
PGOP: null

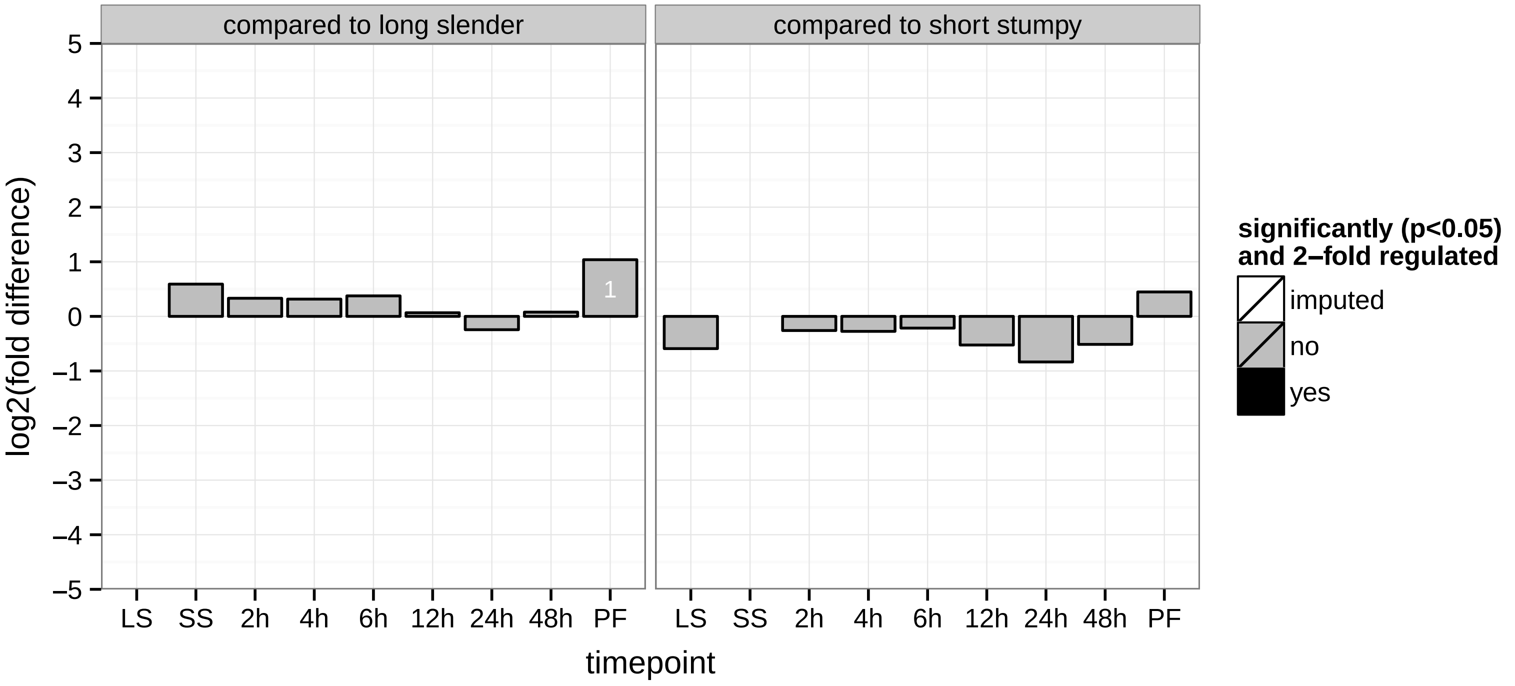

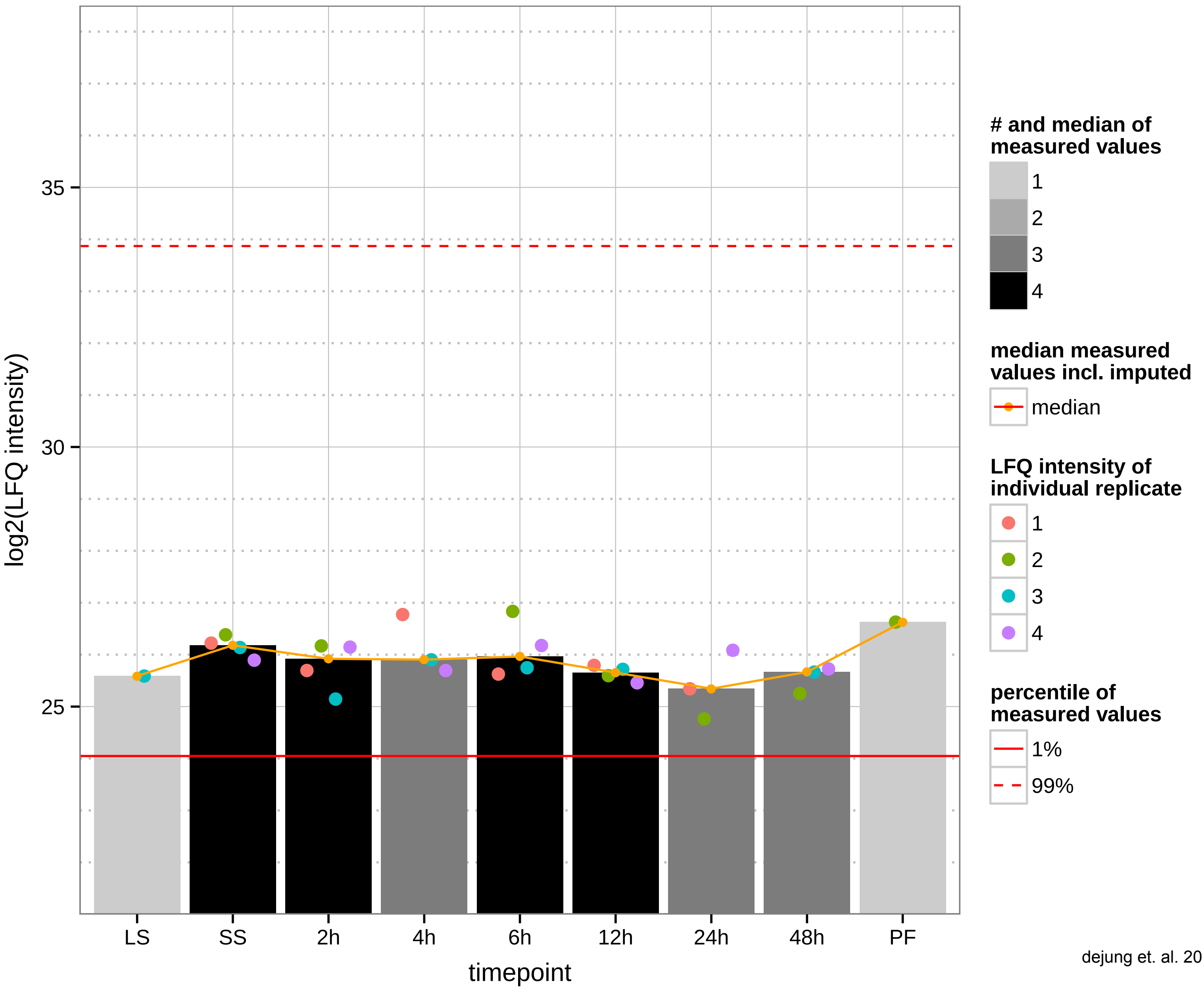

dejung et. al. 2015

hypothetical protein, conserved
Tb927.11.10800
AGOF: null
AGOC: null
AGOP: null
PGOF: null
PGOC: null
PGOP: null

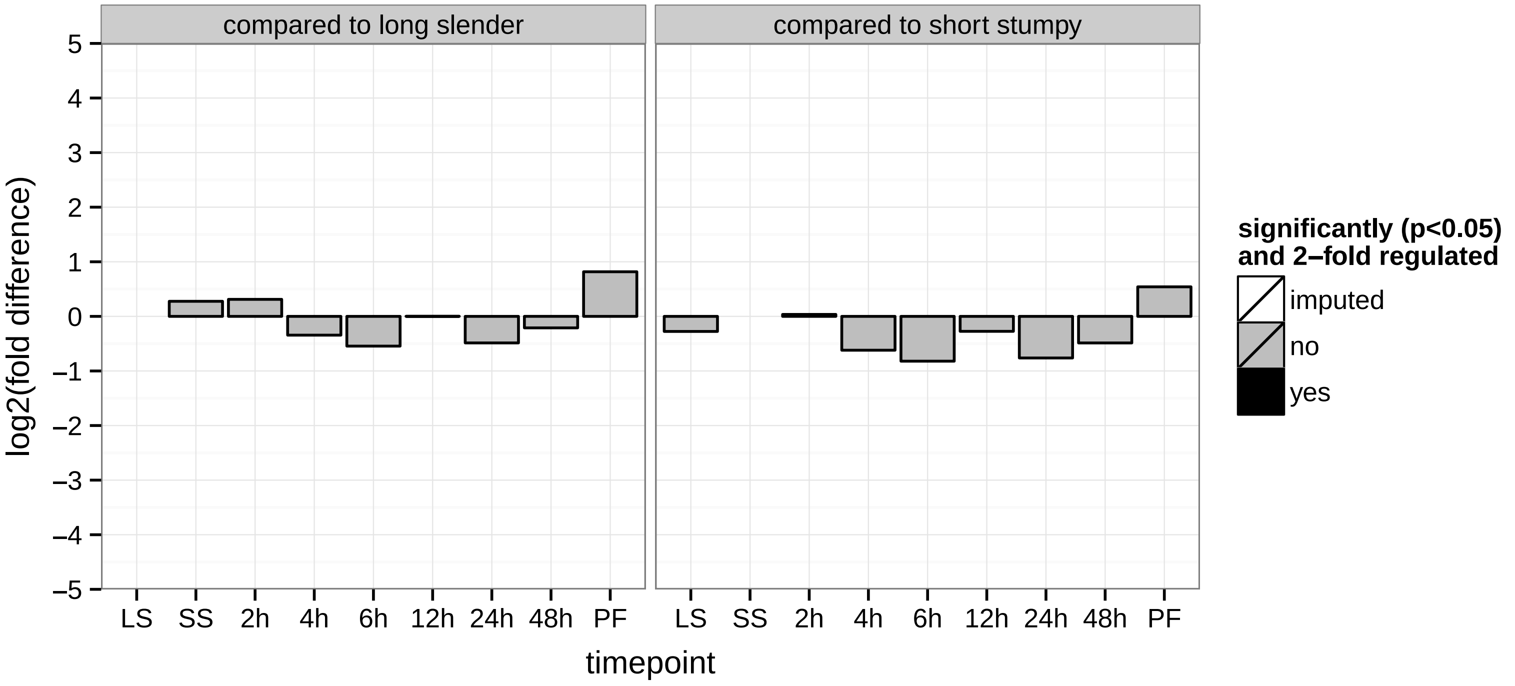

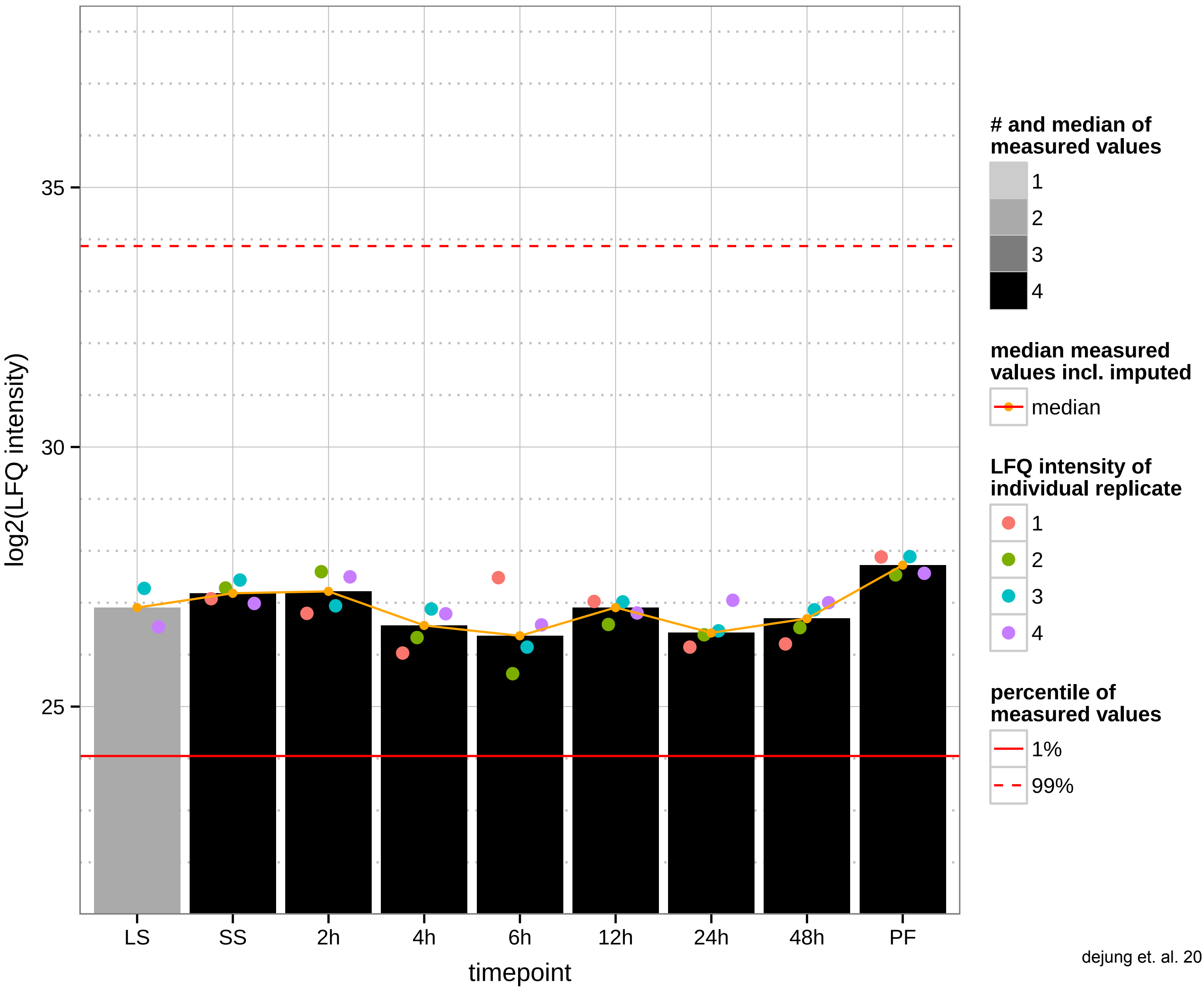

dejung et. al. 2015

protein kinase ck2 regulatory subunit, putative, casein kinase II beta chain
Tb927.11.10820
AGOF: protein kinase regulator activity
AGOC: protein kinase CK2 complex
AGOP: null
PGOF: protein kinase regulator activity
PGOC: protein kinase CK2 complex
PGOP: null

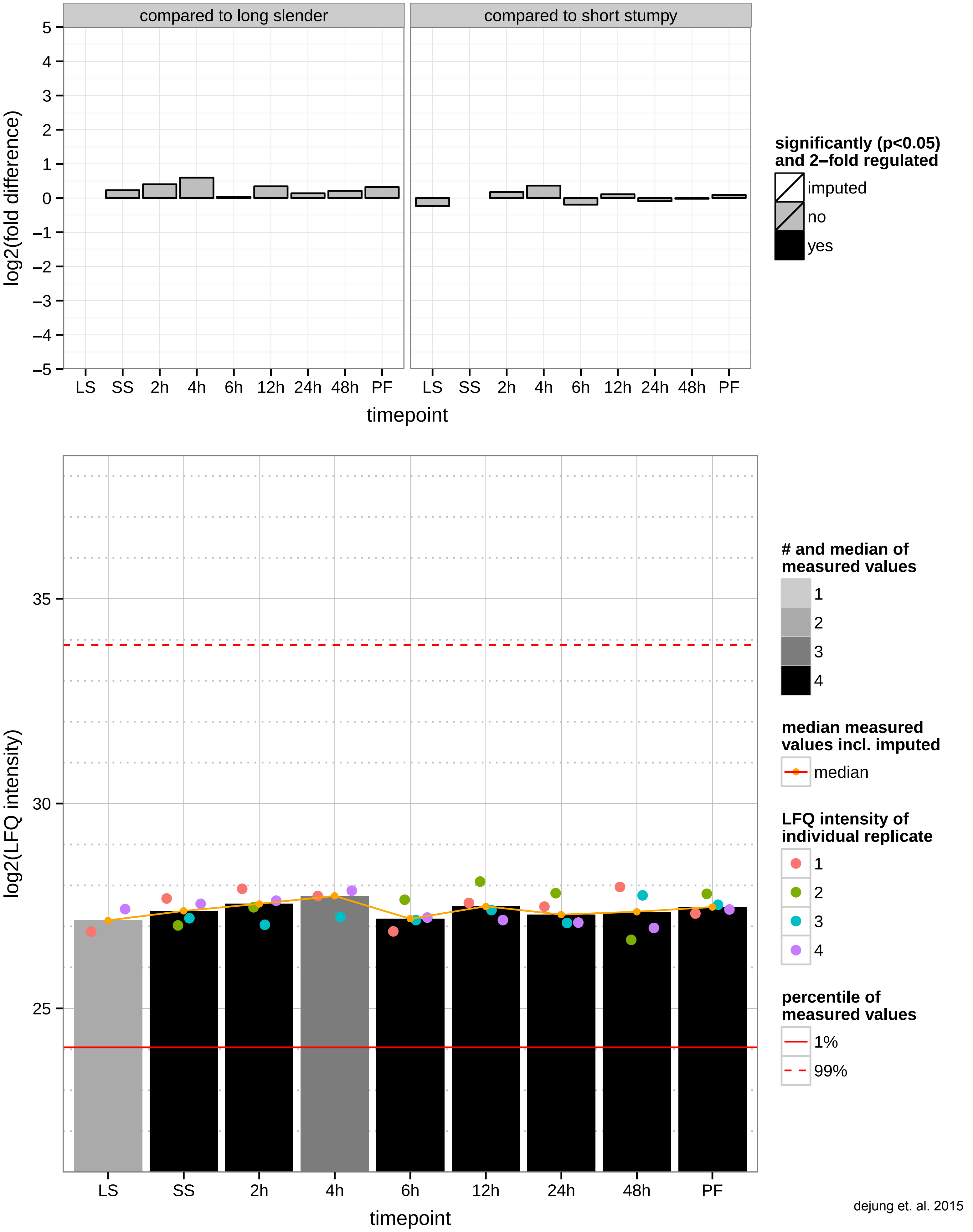

dejung et. al. 2015

hypothetical protein, conserved
Tb927.11.10840
AGOF: null
AGOC: null
AGOP: null
PGOF: null
PGOC: null
PGOP: null

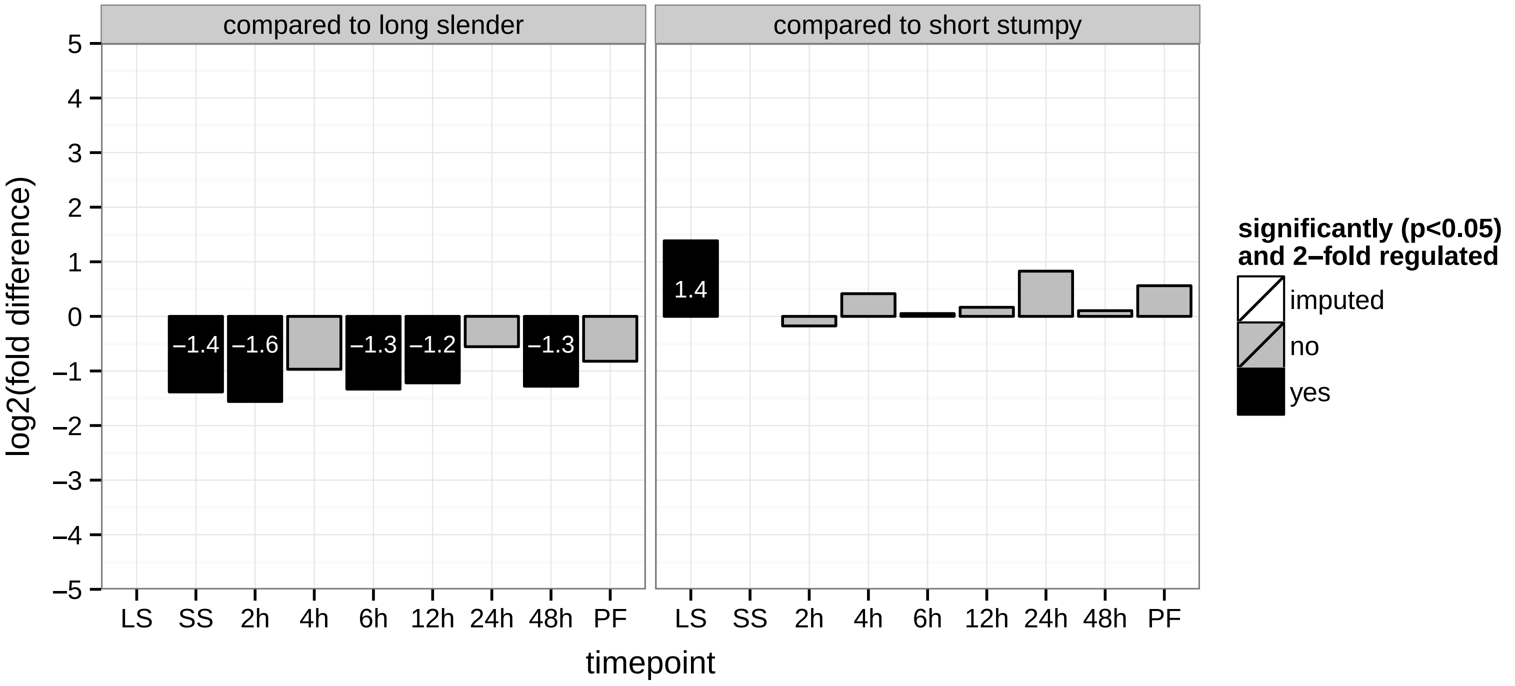

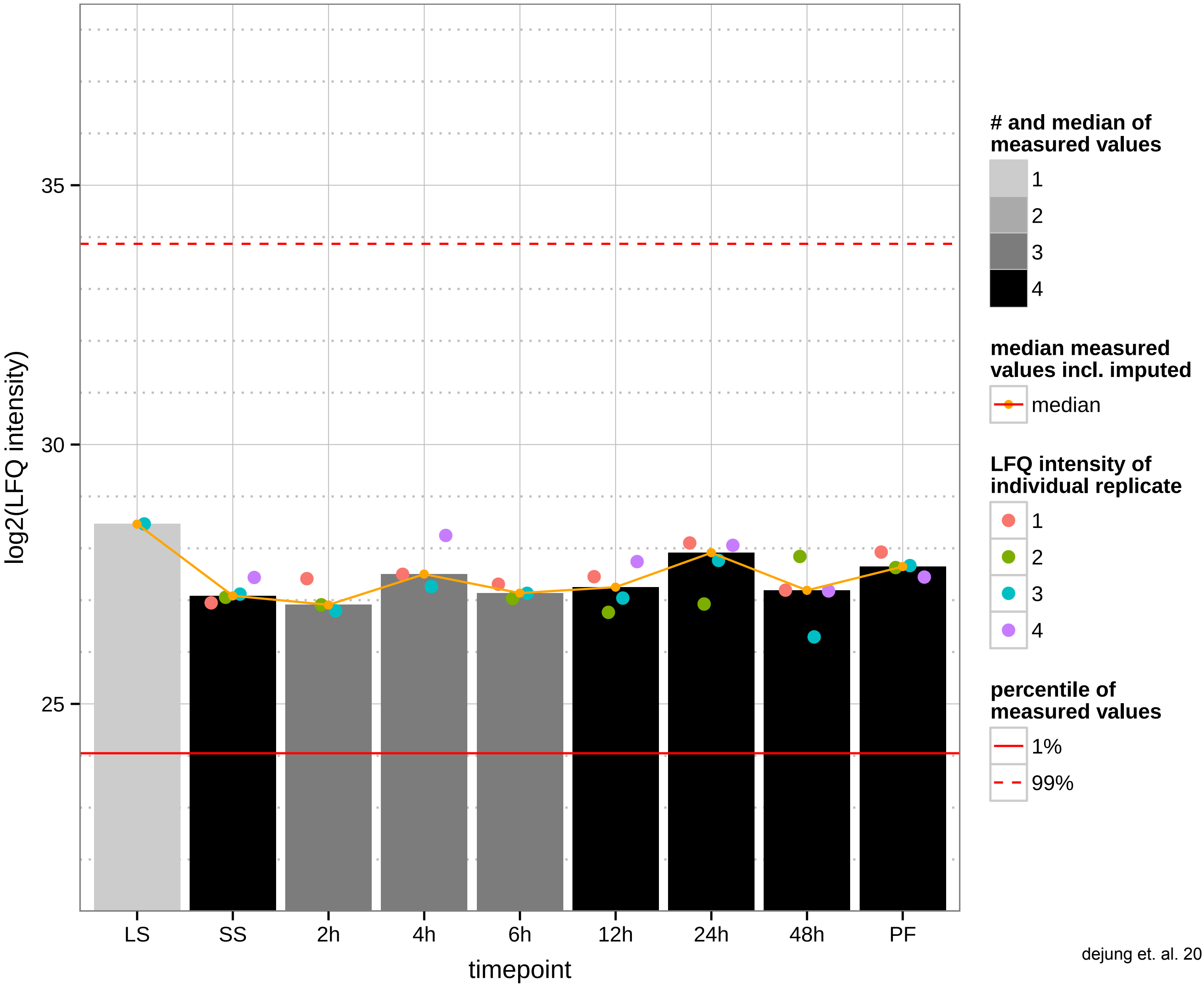

dejung et. al. 2015

32 kDa ER–associated protein (ERAP32)
Tb927.11.10870
AGOF: null
AGOC: endoplasmic reticulum
AGOP: null
PGOF: null
PGOC: null
PGOP: null

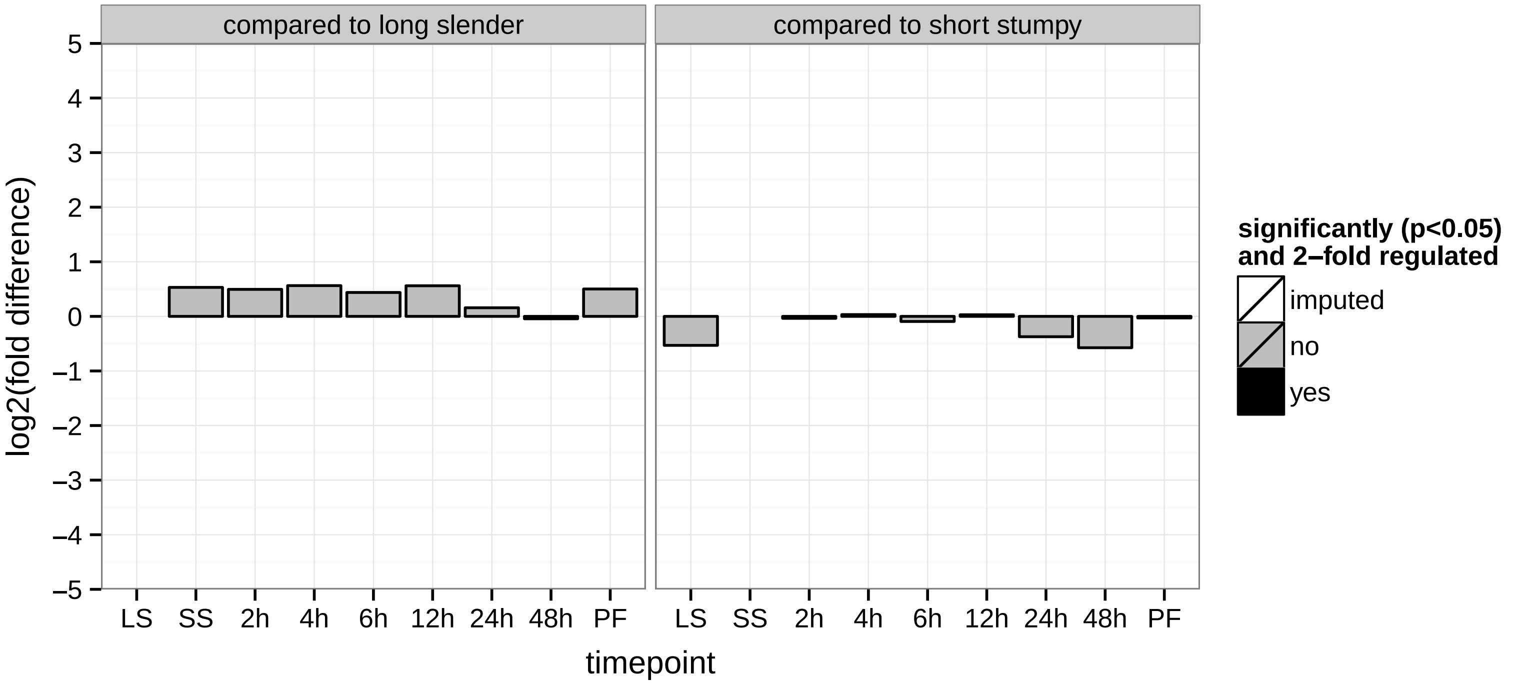

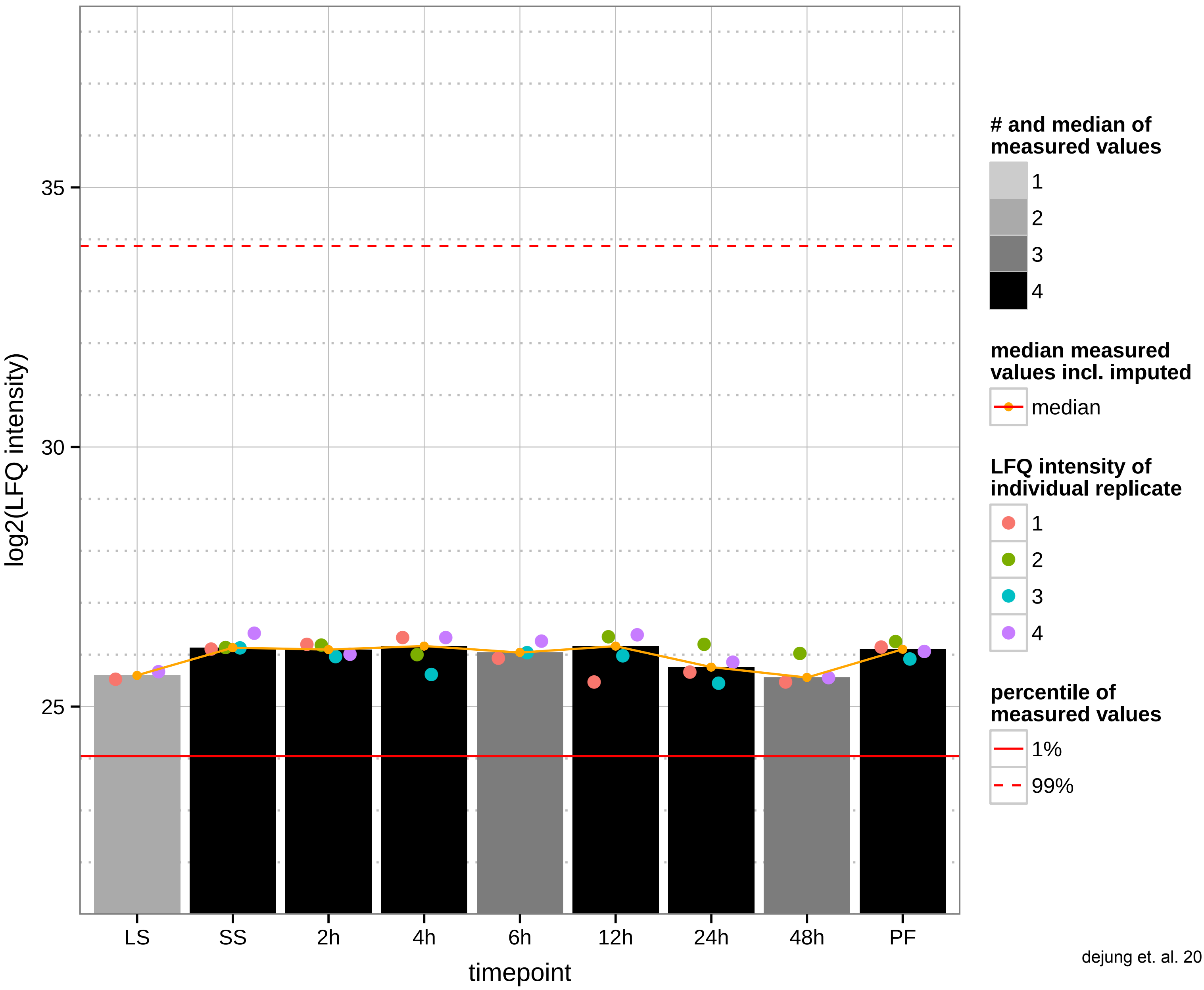

dejung et. al. 2015

calpain–like protein, putative, cytoskeleton assocociated protein, calpain, cysteine peptidase, Clan CA, family C2
Tb927.11.1090
AGOF: calcium–dependent cysteine–type endopeptidase activity
AGOC: intracellular, cytoplasm
AGOP: proteolysis
PGOF: calcium–dependent cysteine–type endopeptidase activity, null
PGOC: intracellular, null
PGOP: proteolysis, null

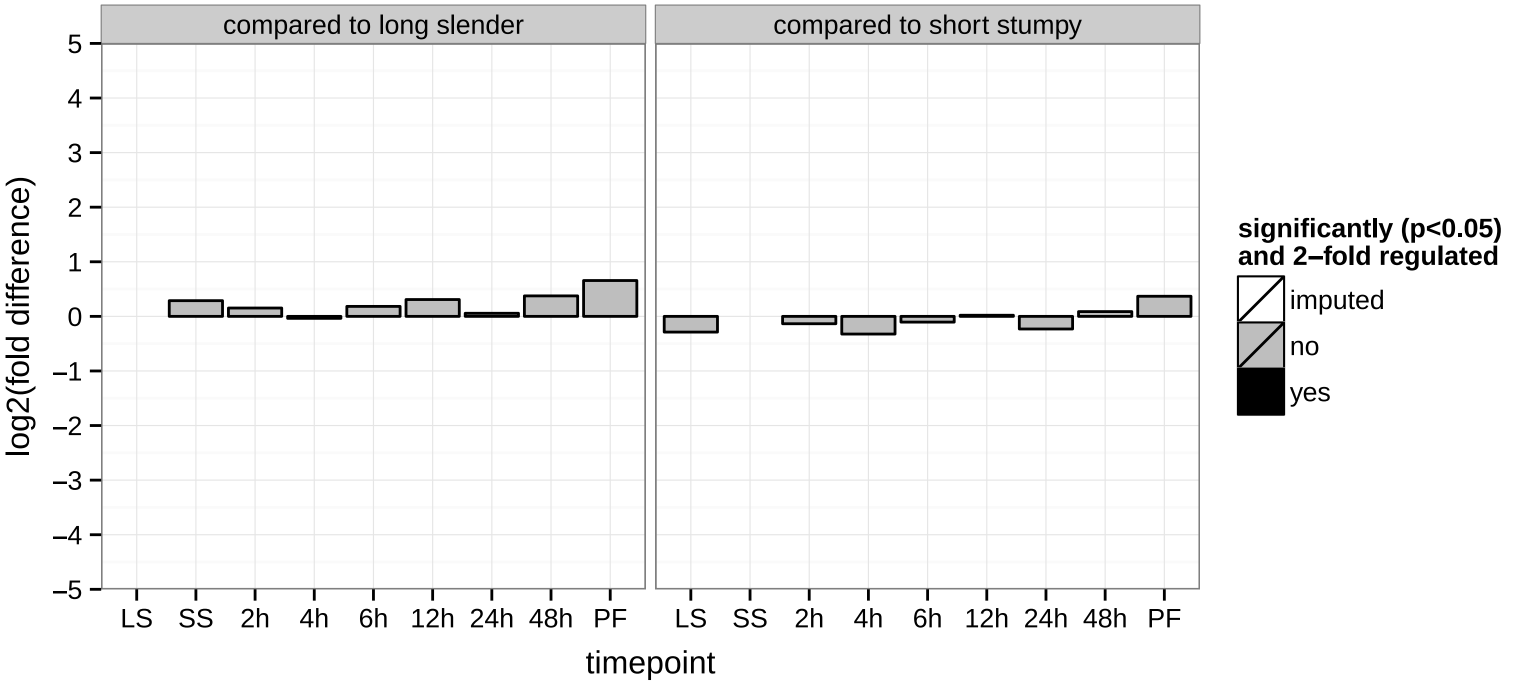

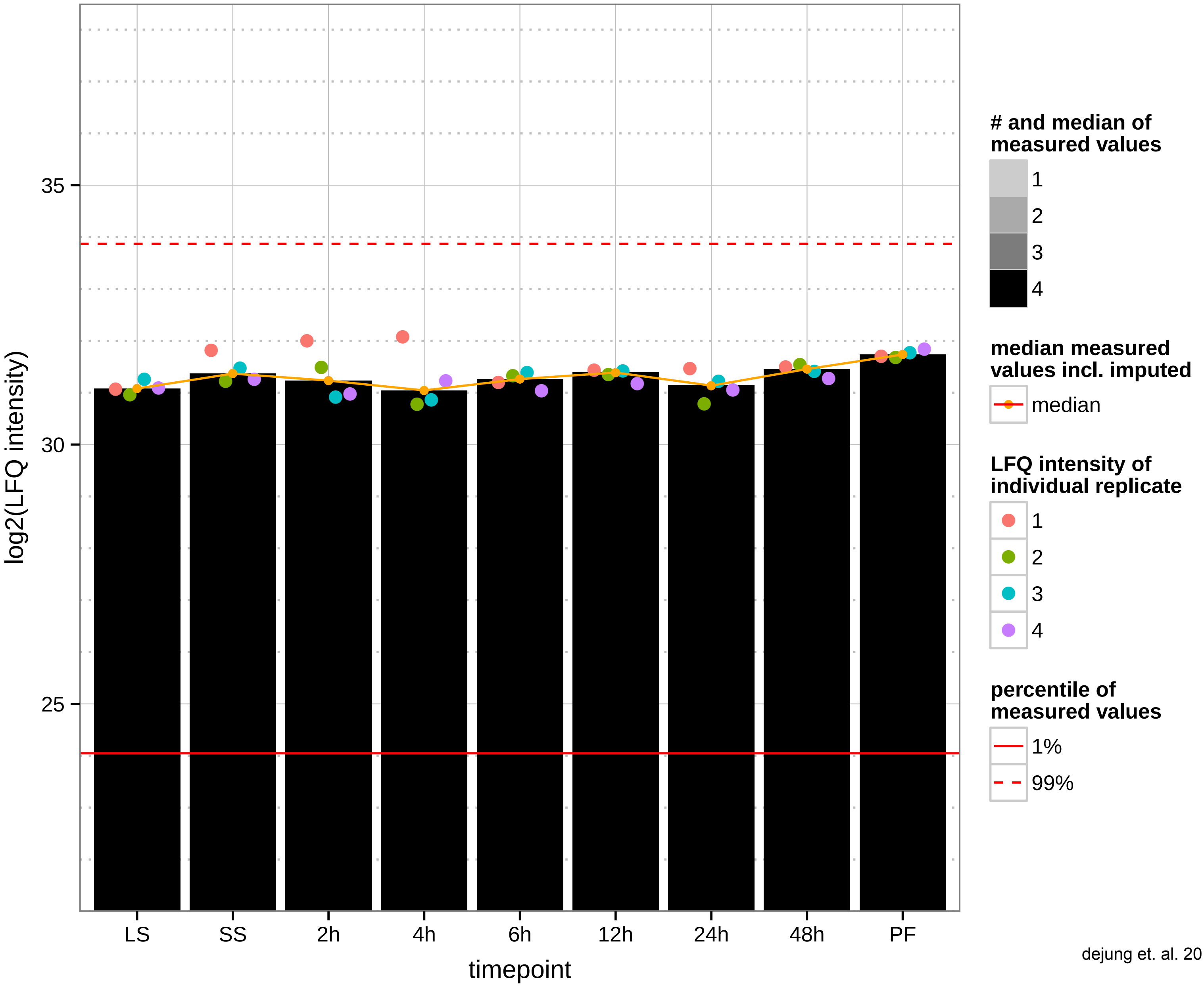

dejung et. al. 2015

delta tubulin, tubulin delta chain (TUBD1)
Tb927.11.10930
AGOF: GTP binding, structural molecule activity
AGOC: microtubule, protein complex
AGOP: cell growth, microtubule–based movement, protein polymerization
PGOF: null
PGOC: protein complex
PGOP: protein polymerization

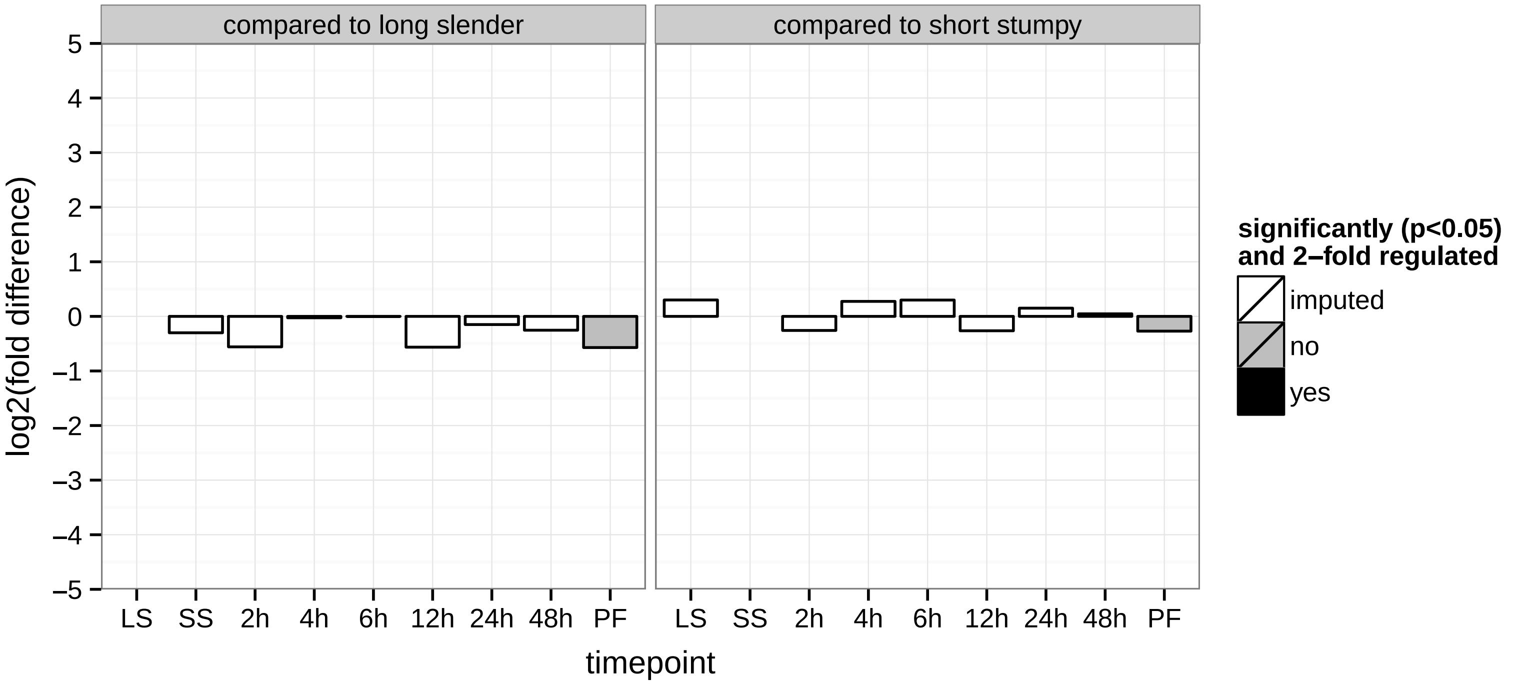

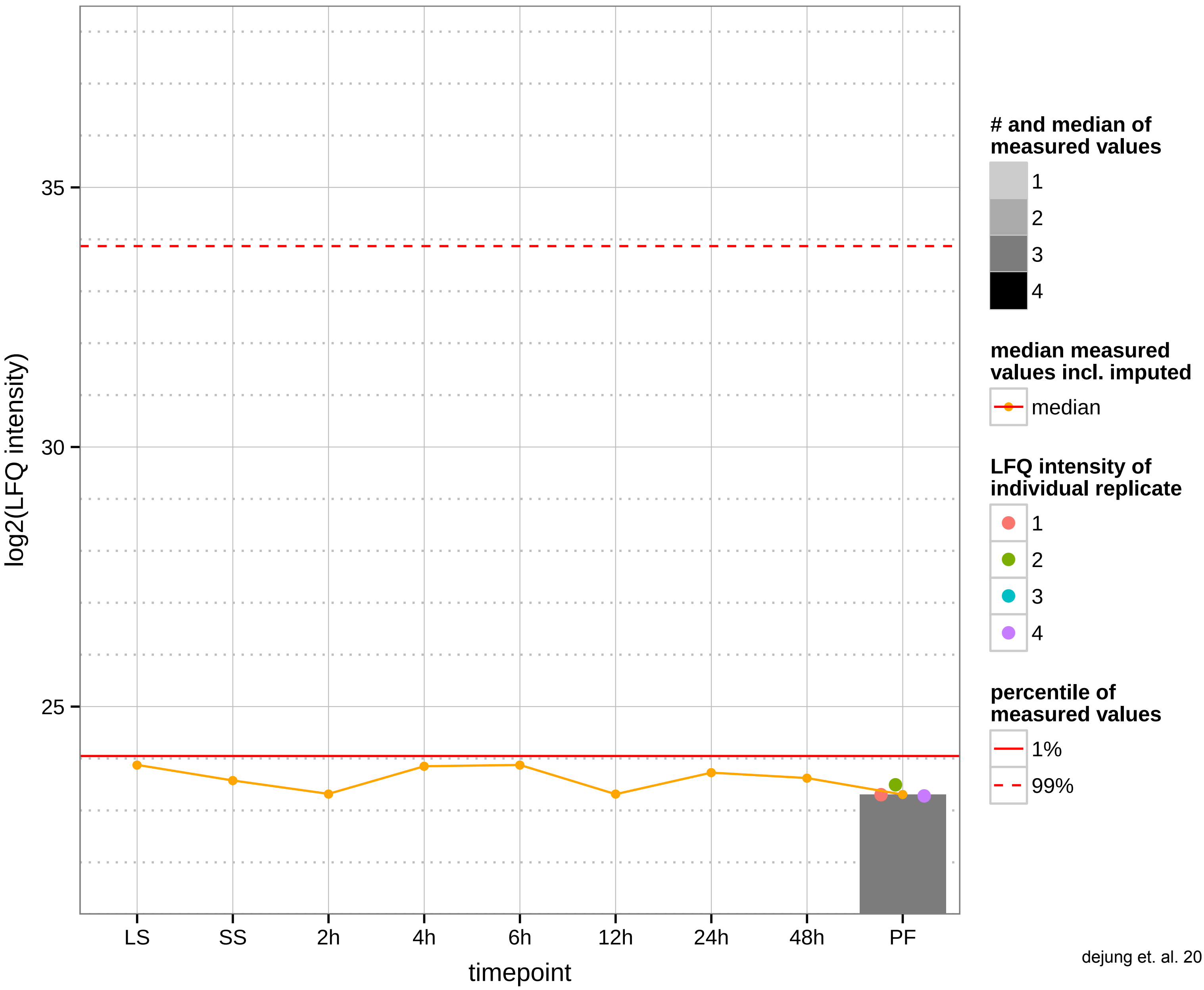

dejung et. al. 2015

translation initiation factor 2 subunit, putative
Tb927.11.10990
AGOF: GTP binding, translation initiation factor activity
AGOC: eukaryotic translation initiation factor 2B complex
AGOP: cellular biosynthetic process, translational initiation
PGOF: null
PGOC: null
PGOP: cellular biosynthetic process, cellular metabolic process

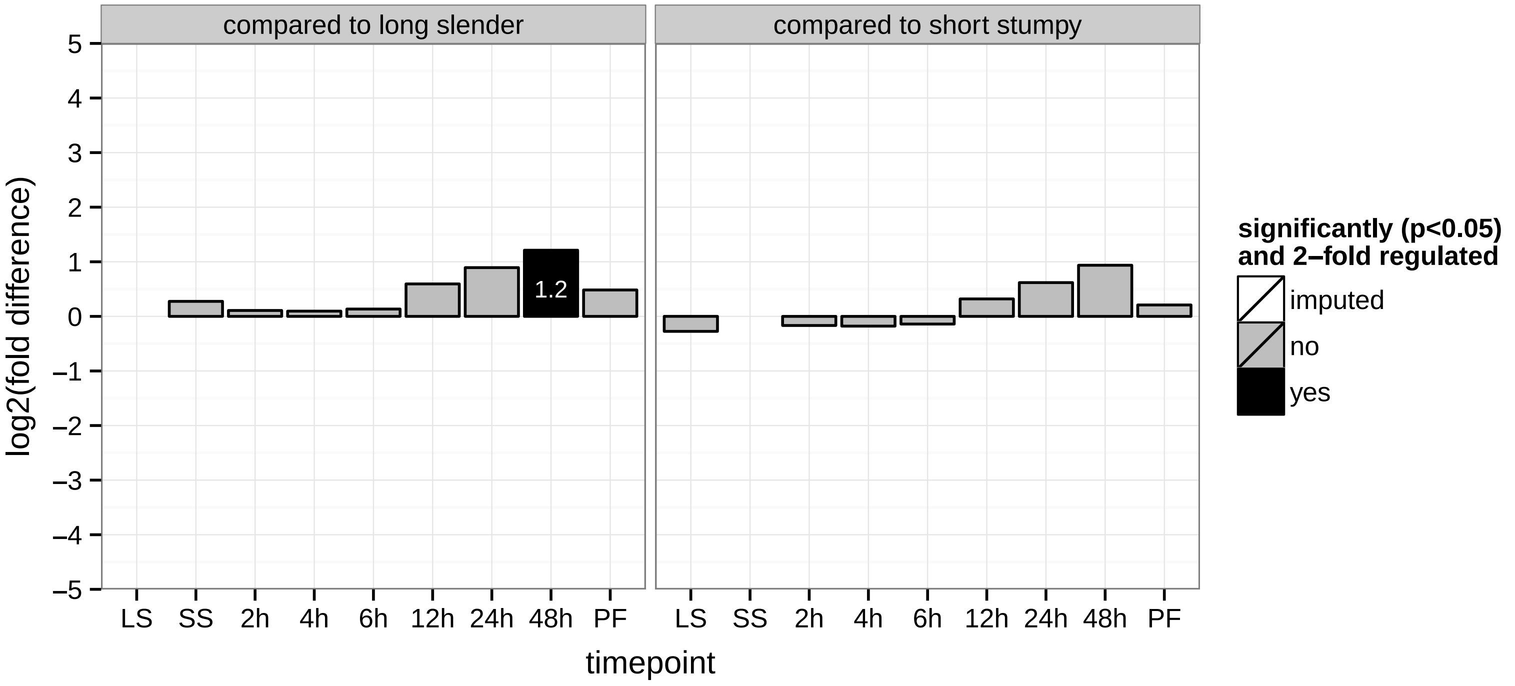

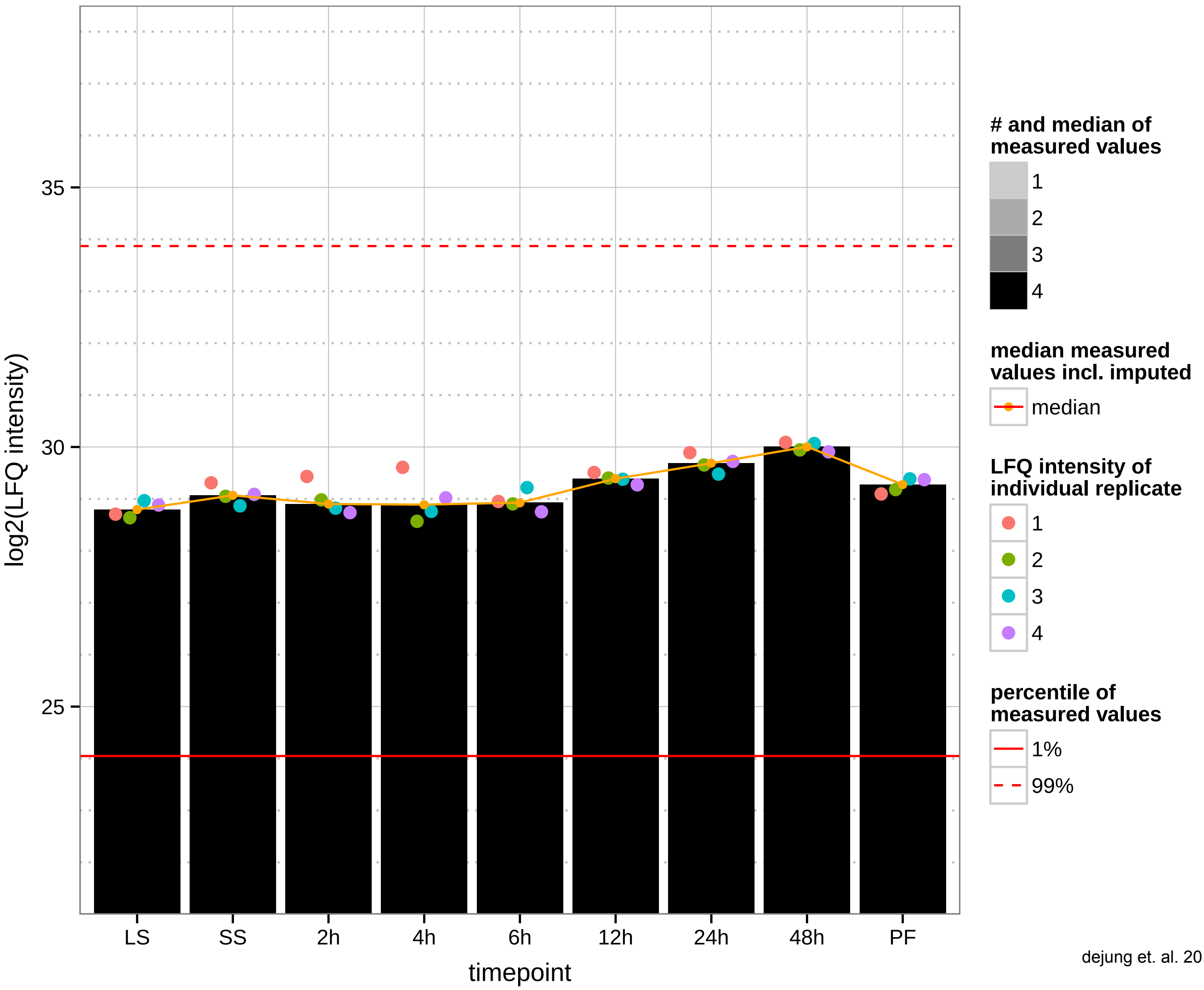

dejung et. al. 2015

hypothetical protein, conserved
Tb927.11.11000
AGOF: null
AGOC: null
AGOP: null
PGOF: protein binding
PGOC: null
PGOP: null

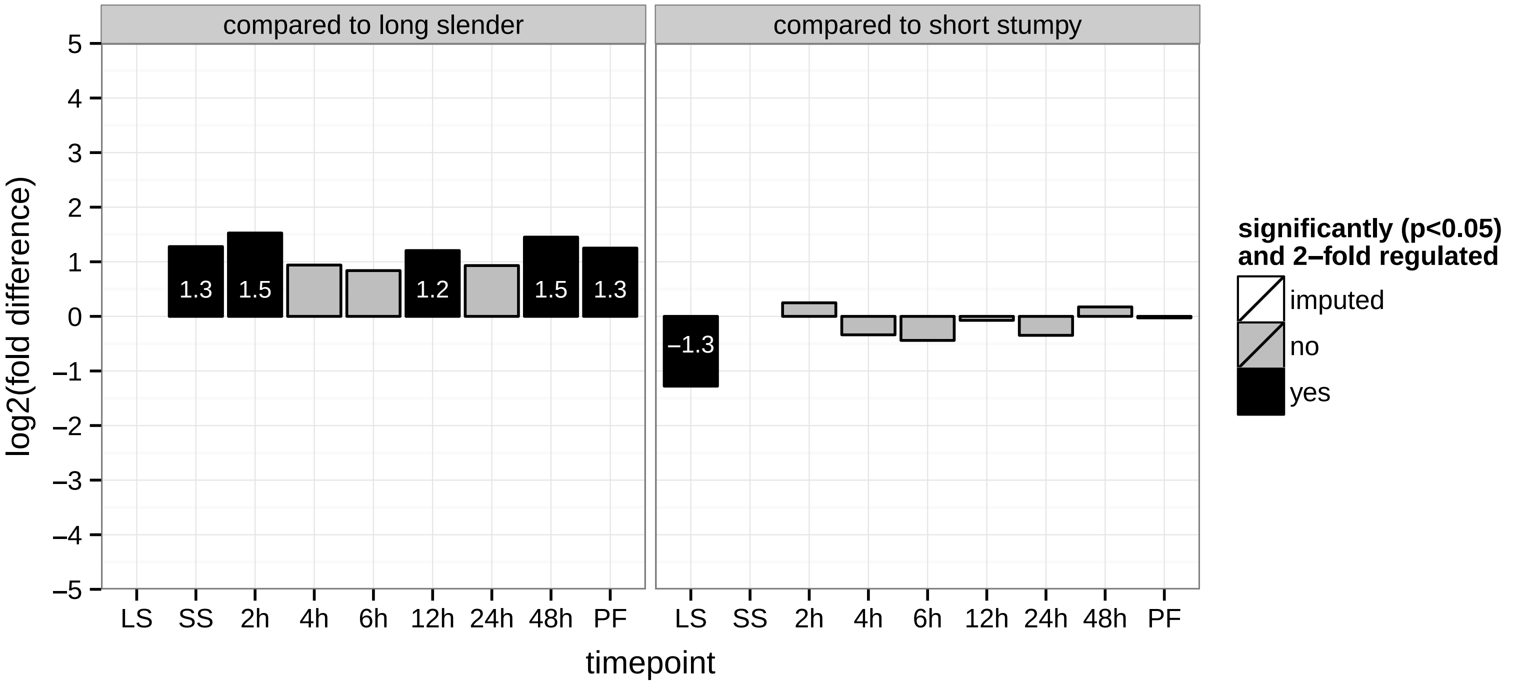

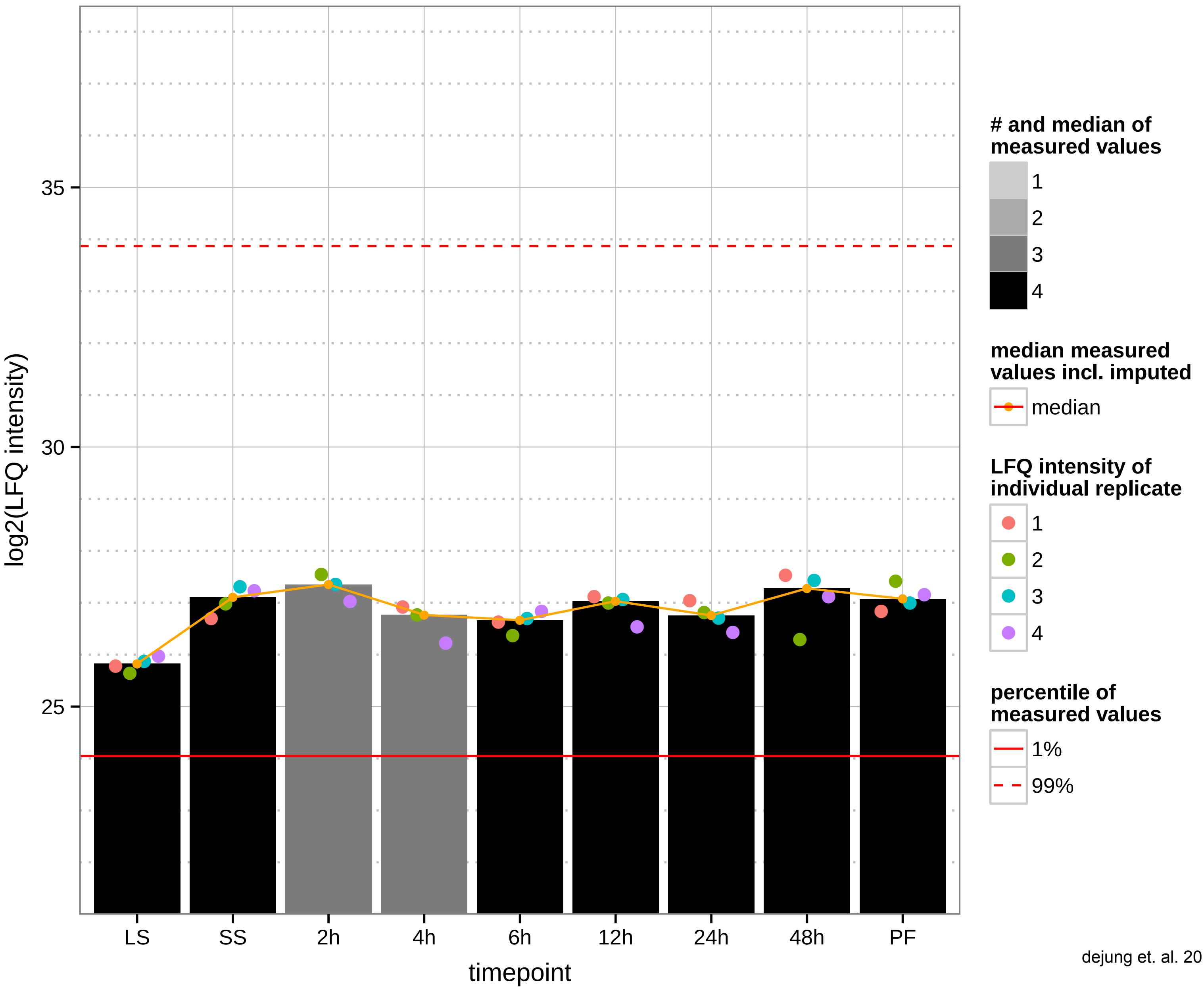

dejung et. al. 2015

hypothetical protein, conserved
Tb927.11.11010
AGOF: null
AGOC: null
AGOP: null
PGOF: null
PGOC: null
PGOP: null

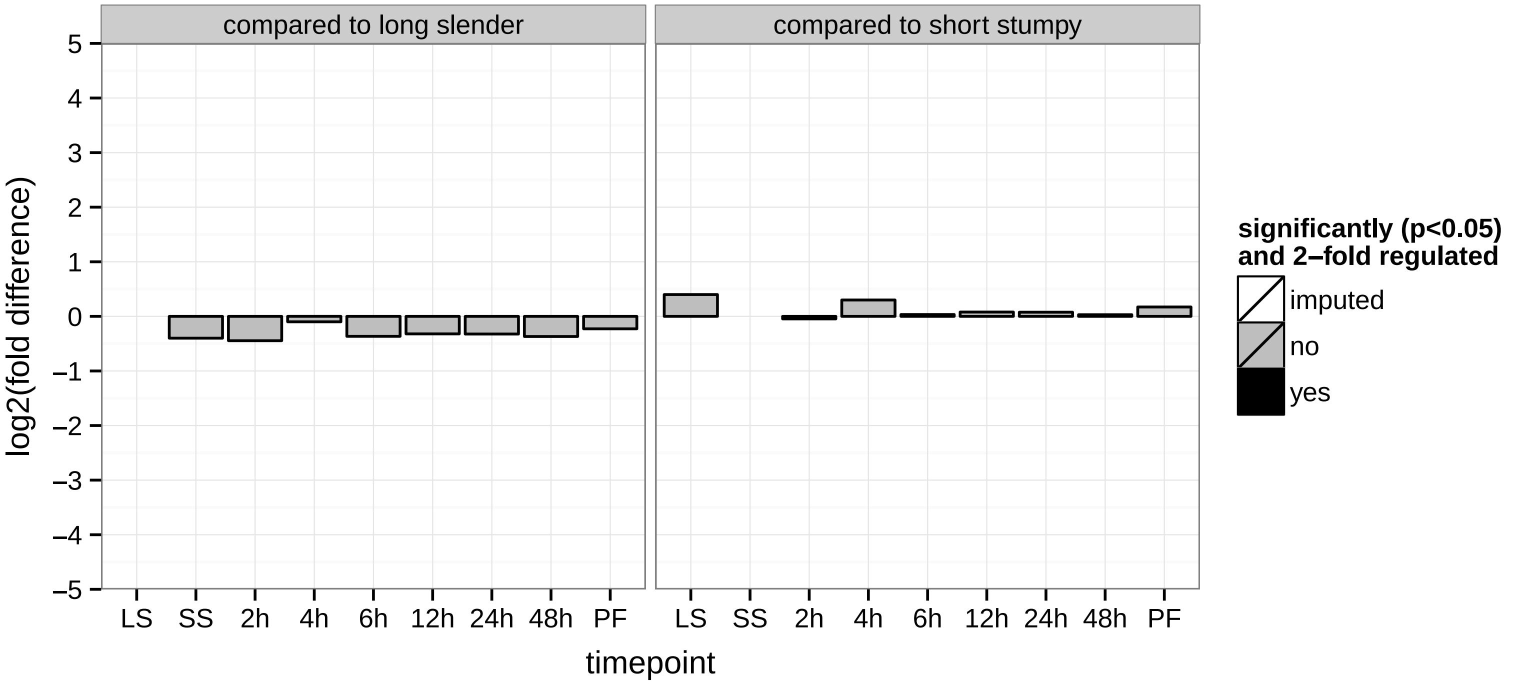

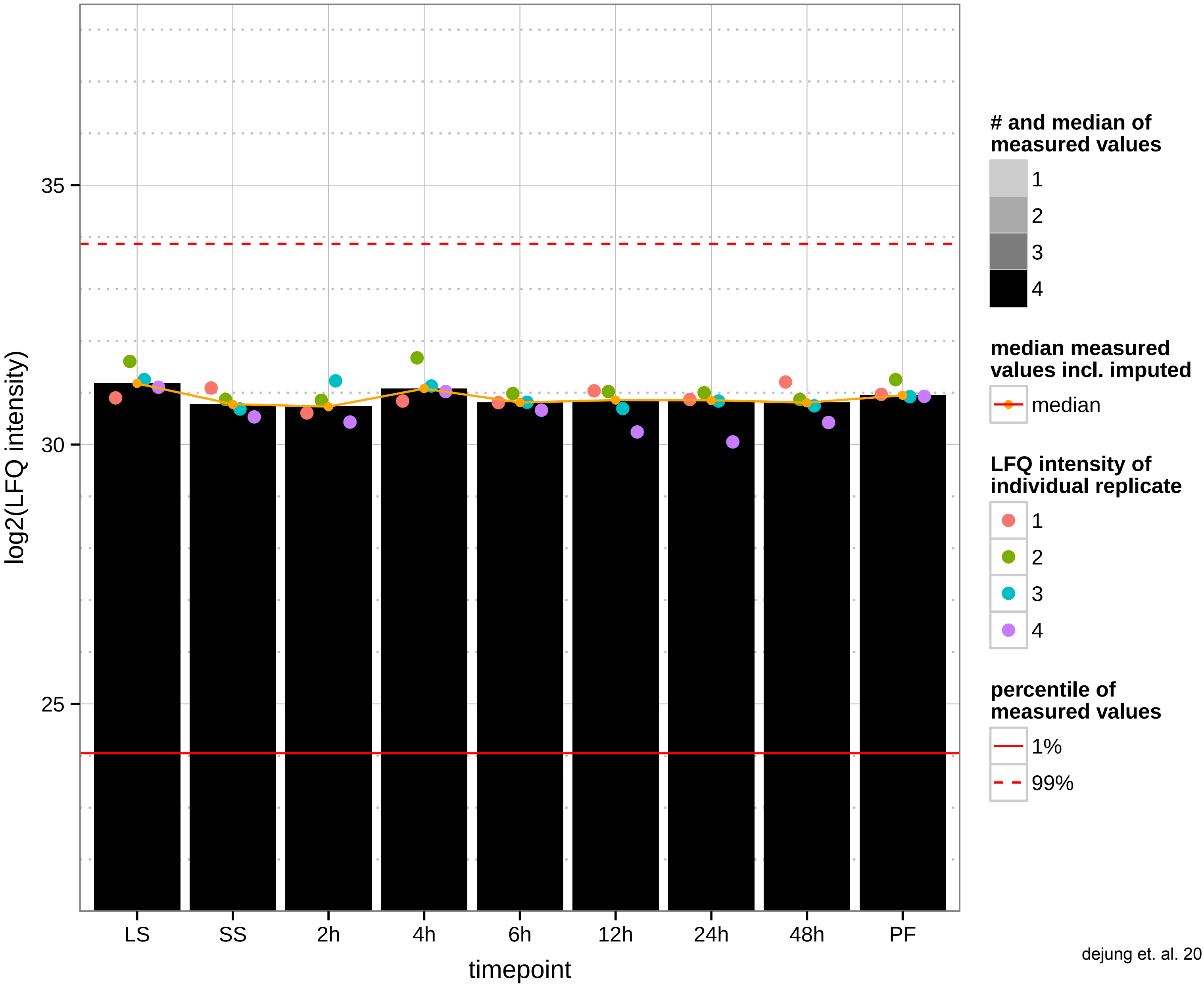

dejung et. al. 2015

Nucleoporin (TbNup140)
Tb927.11.11090
AGOF: null
AGOC: nuclear pore
AGOP: null
PGOF: protein binding
PGOC: null
PGOP: null

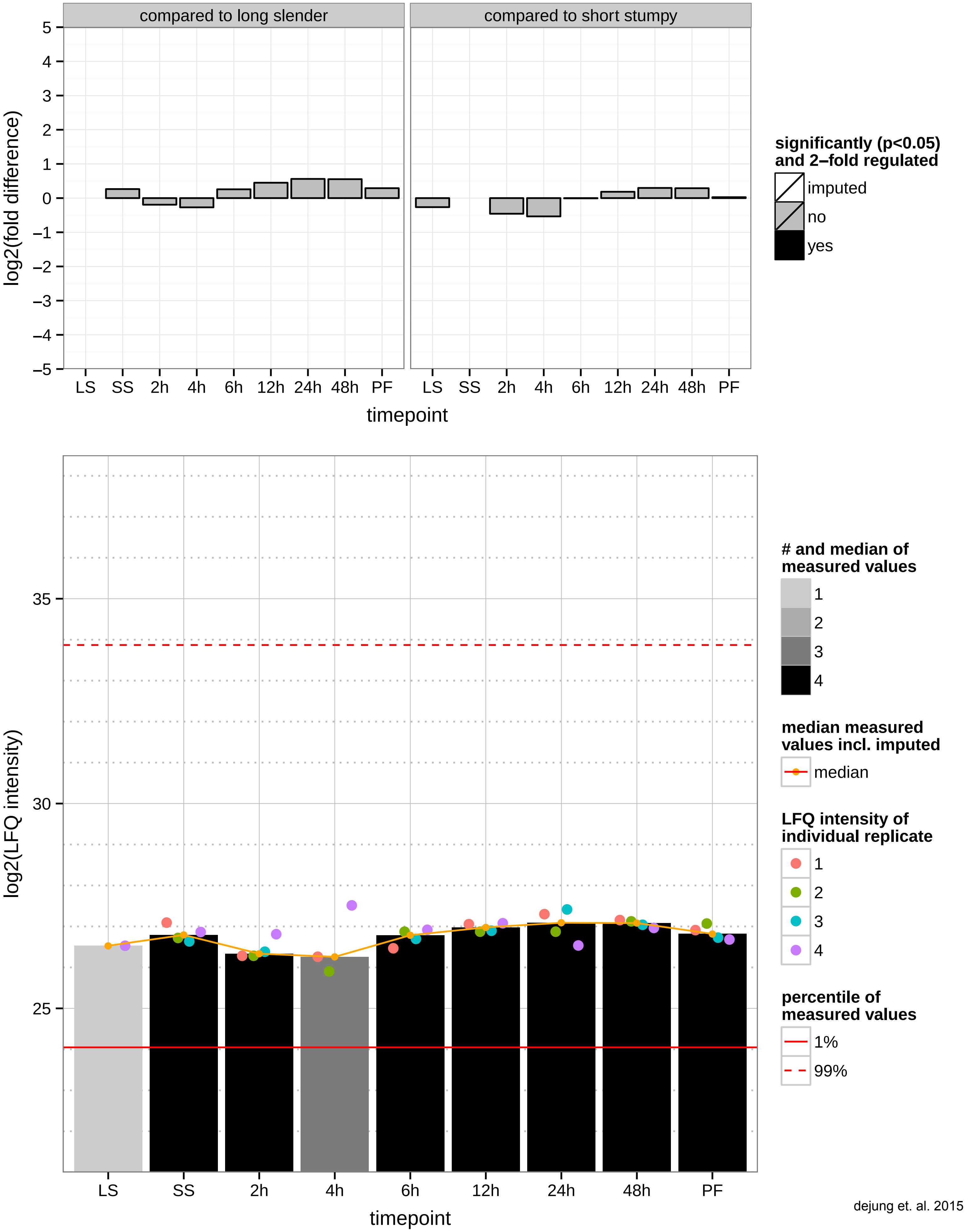

dejung et. al. 2015

calpain–like cysteine peptidase, putative, calpain
Tb927.11.1110
AGOF: null, calcium–dependent cysteine–type endopeptidase activity
AGOC: null, intracellular
AGOP: null, proteolysis
PGOF: null, calcium–dependent cysteine–type endopeptidase activity
PGOC: null, intracellular
PGOP: null, proteolysis

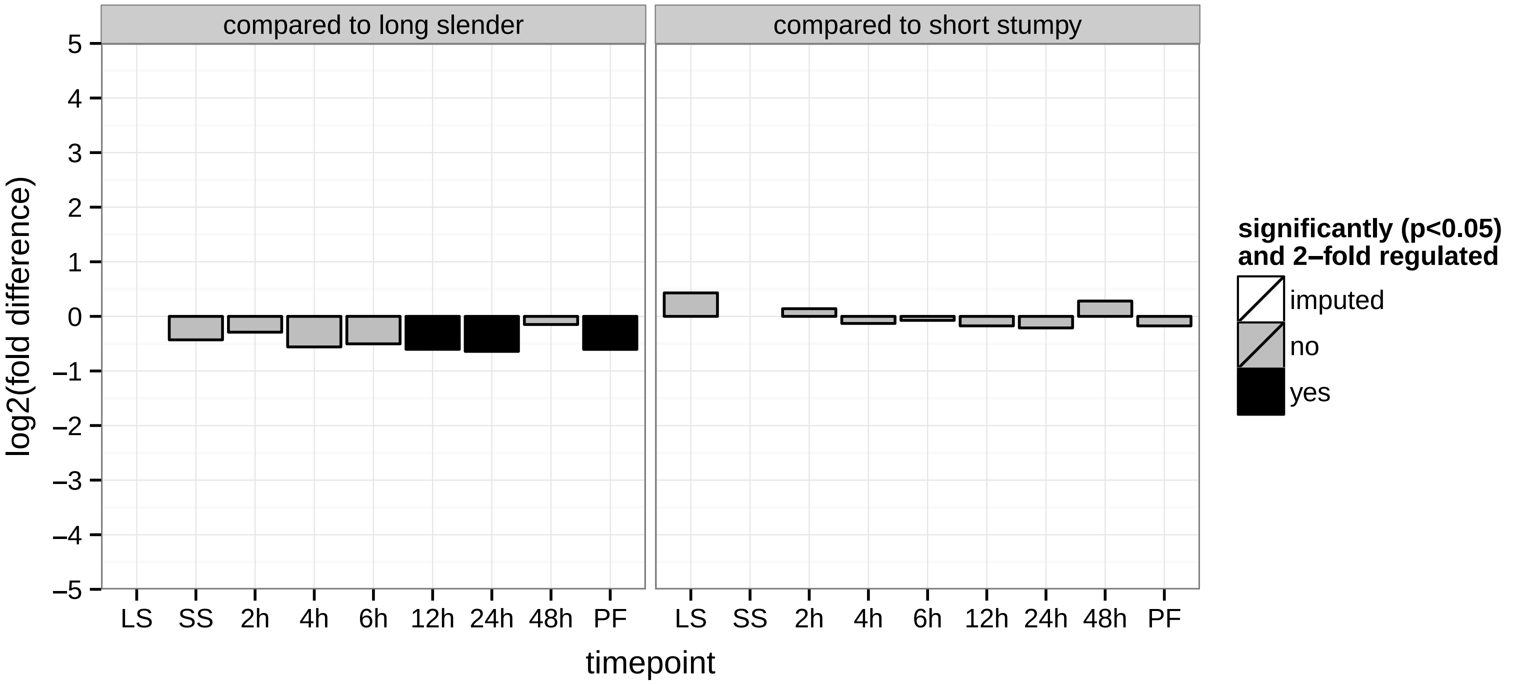

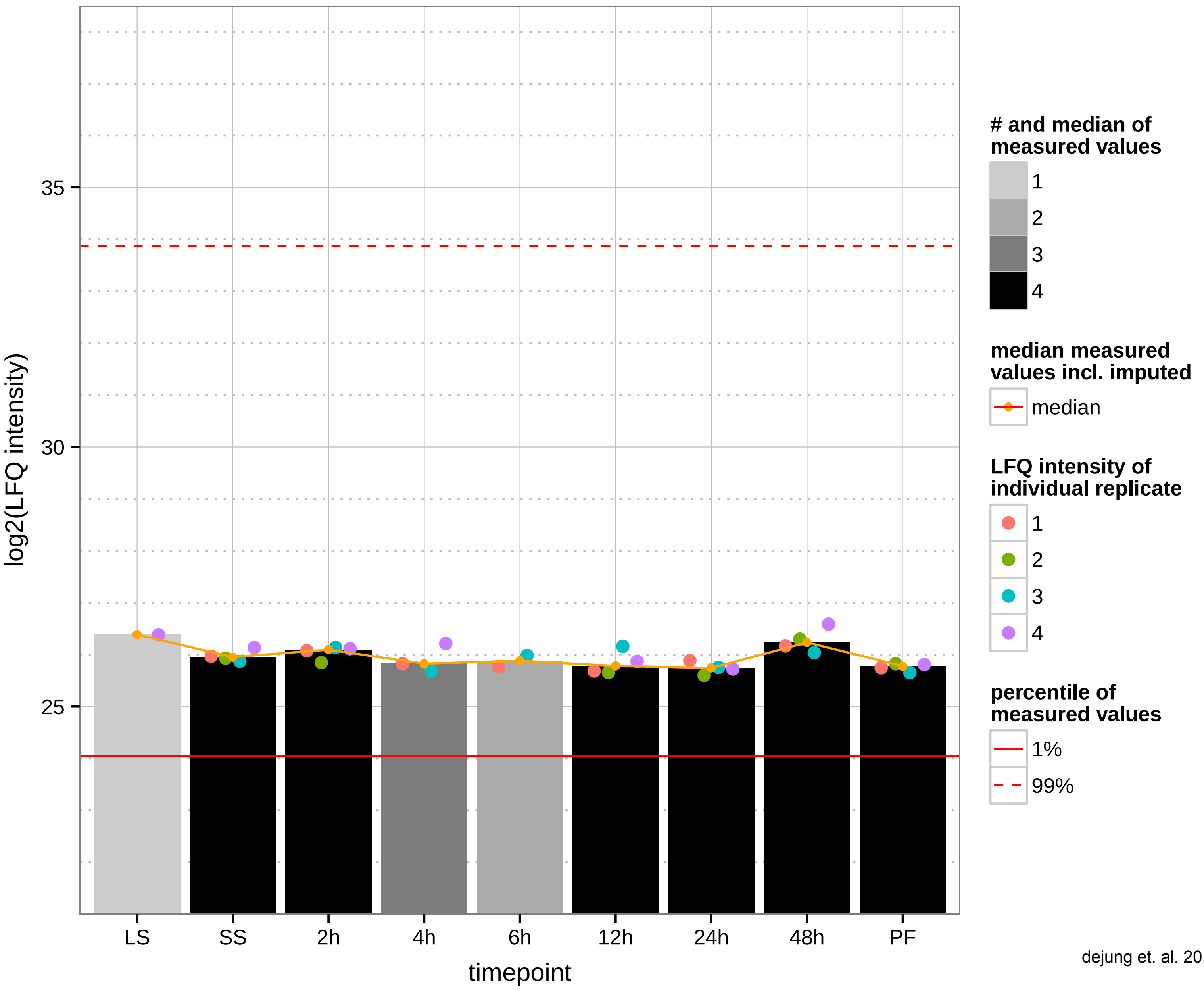

dejung et. al. 2015

U5 snRNP−specific 40 kDa protein
Tb927.11.11150
AGOF: pre−mRNA 5'−splice site binding
AGOC: cytosol, nucleus
AGOP: RNA splicing
PGOF: protein binding
PGOC: null
PGOP: null

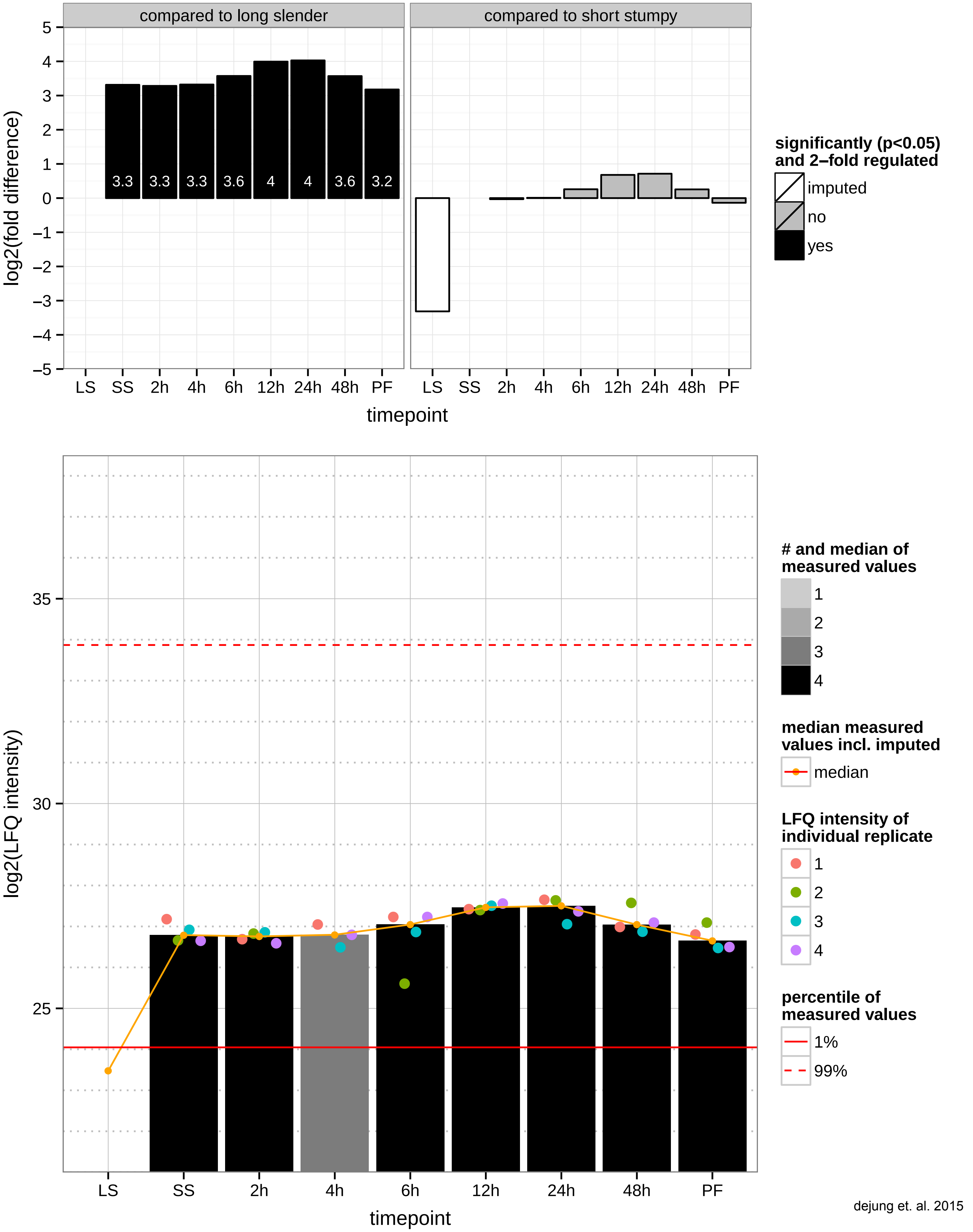

dejung et. al. 2015

phosphate transporter, putative
Tb927.11.11160
AGOF: citrate transmembrane transporter activity
AGOC: contractile vacuole, integral to membrane
AGOP: citrate transport
PGOF: transporter activity
PGOC: membrane
PGOP: sodium ion transport, transmembrane transport

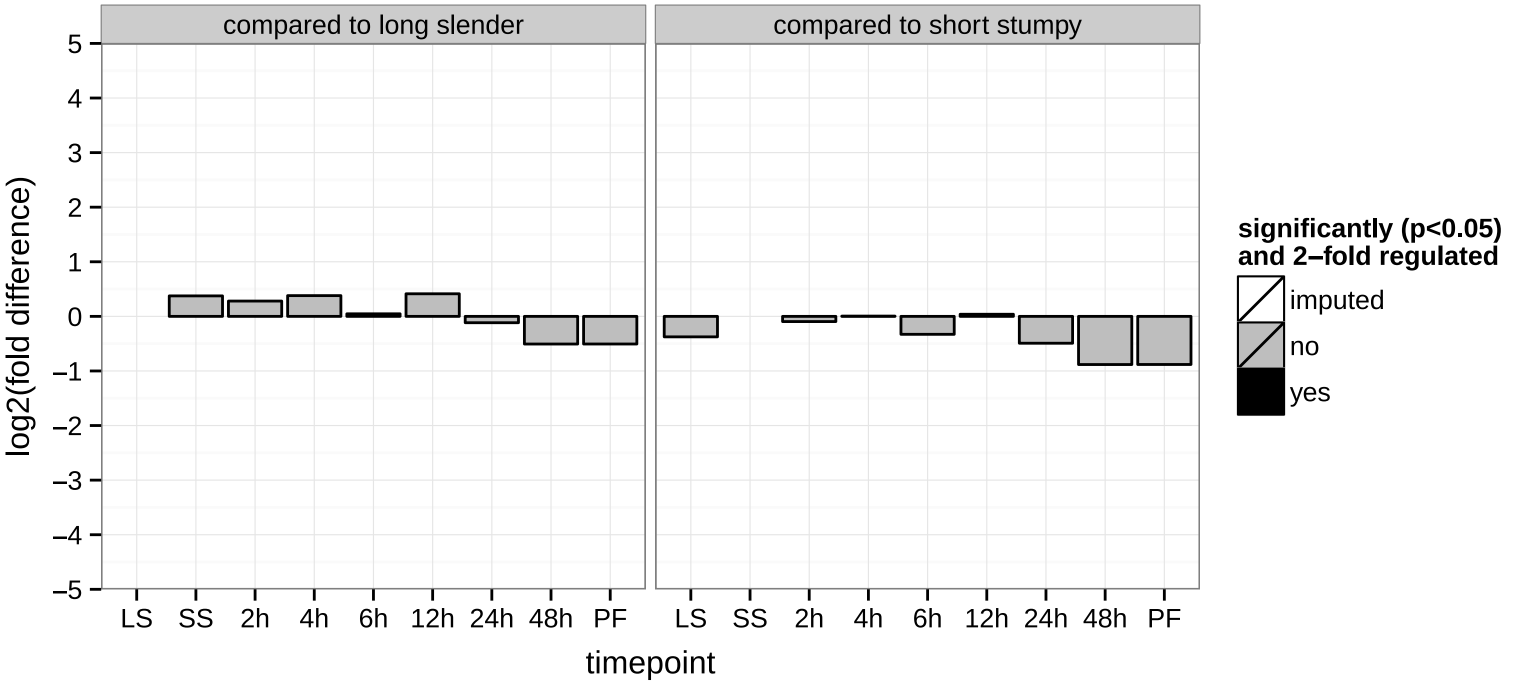

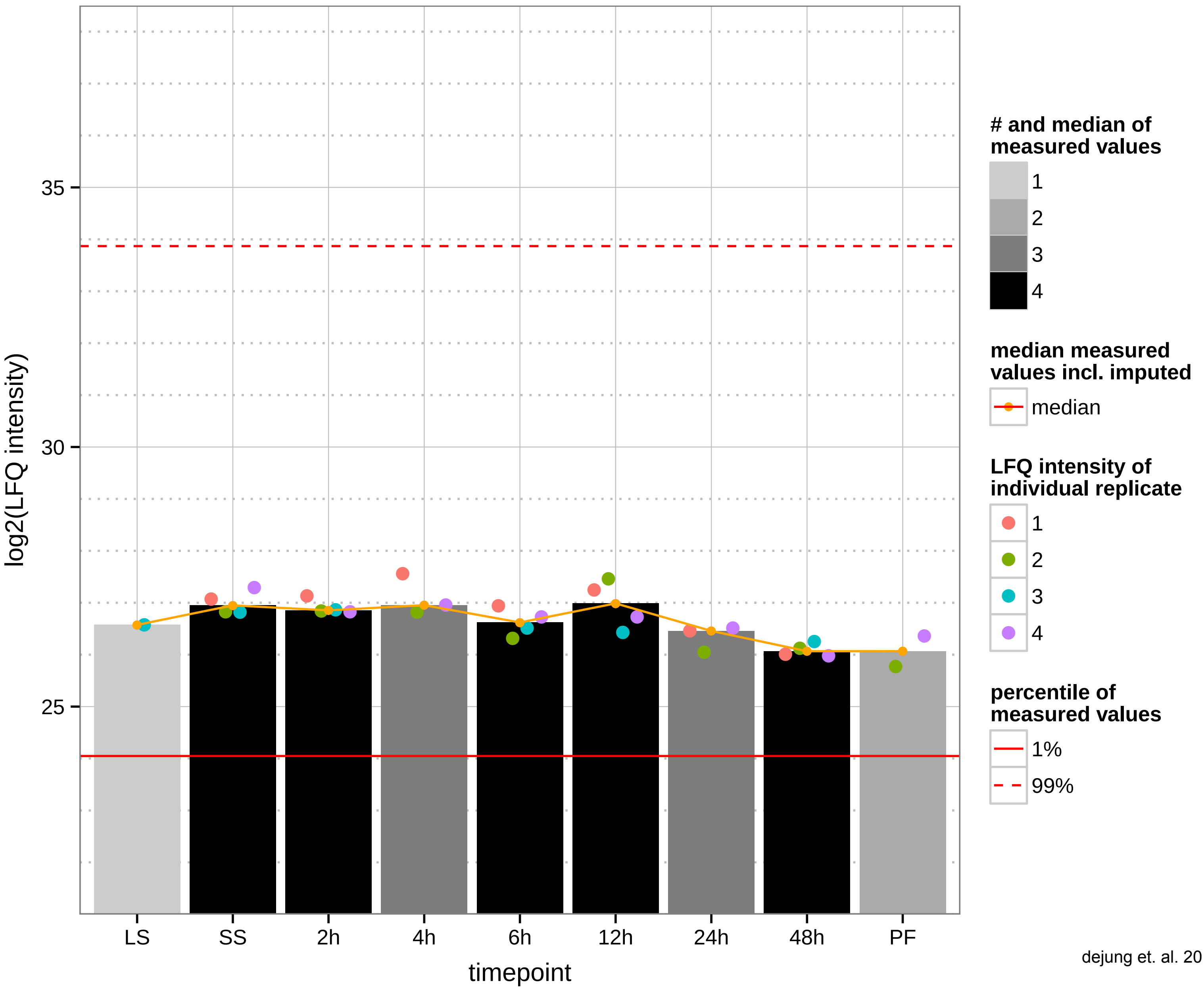

dejung et. al. 2015

dynein heavy chain, putative
Tb927.11.11220
AGOF: ATP binding, ATPase activity, microtubule motor activity
AGOC: dynein complex
AGOP: microtubule–based movement
PGOF: ATP binding, ATPase activity, microtubule motor activity, nucleoside–triphosphatase activity, nucleotide binding
PGOC: dynein complex
PGOP: microtubule–based movement

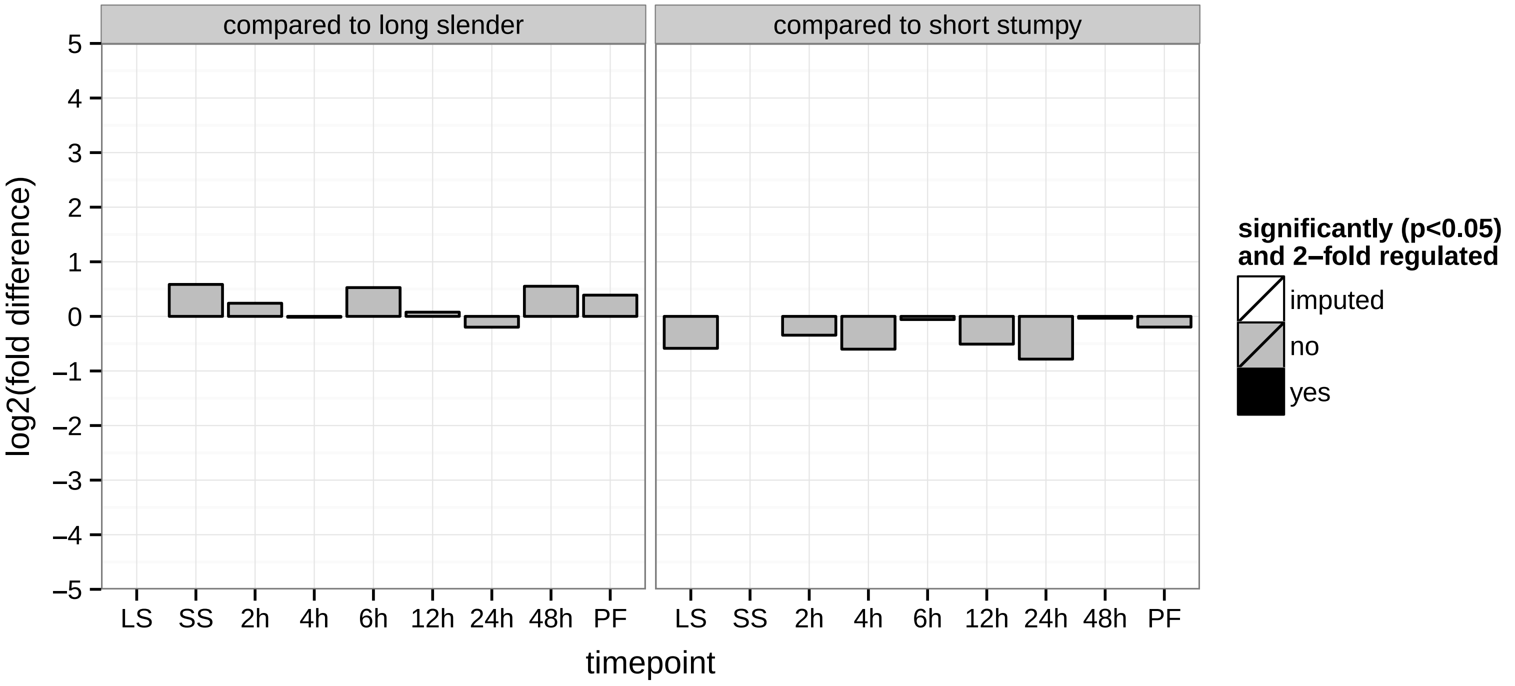

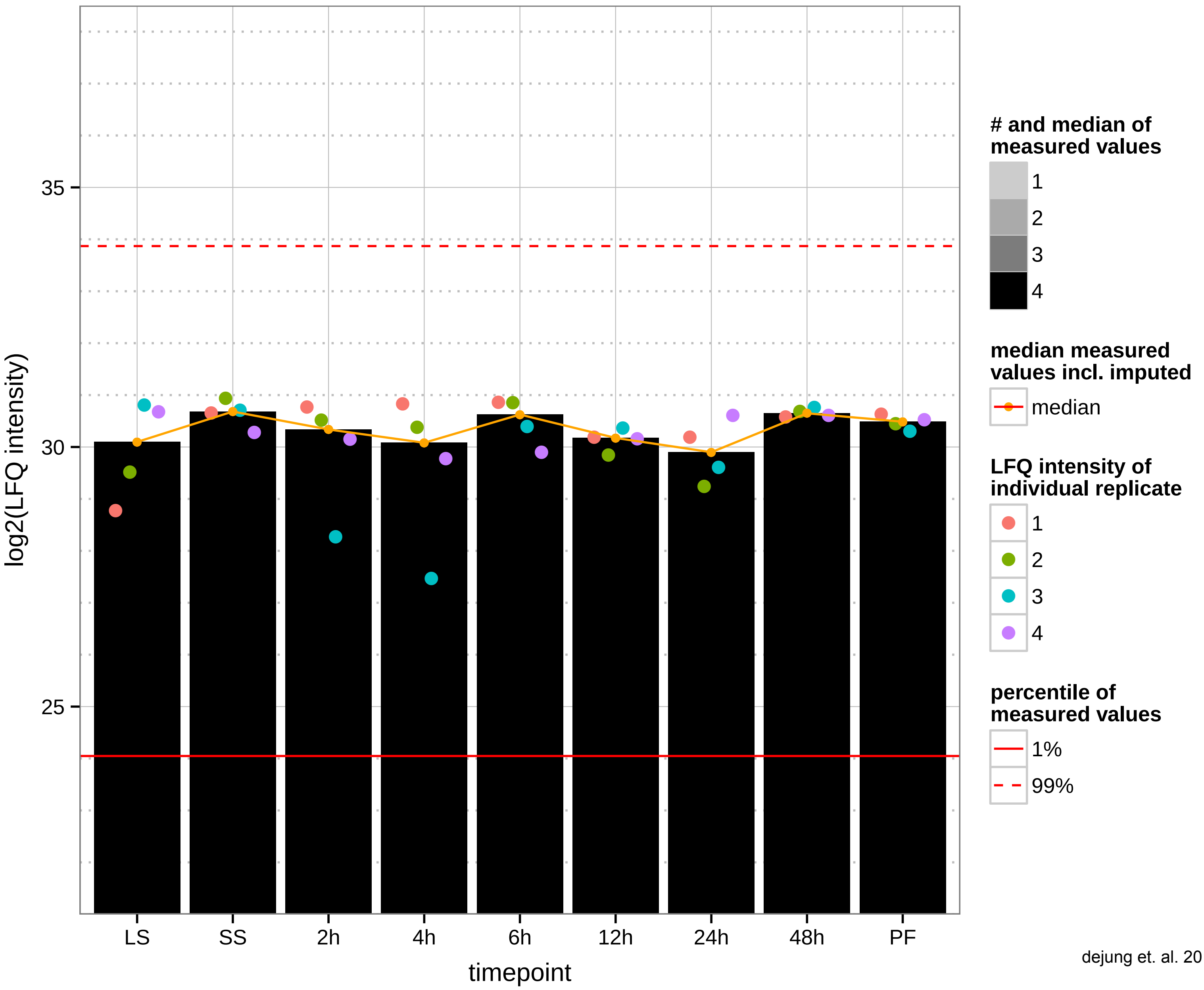

dejung et. al. 2015

cytosolic malate dehydrogenase (cMDH)
Tb927.11.11250
AGOF: L−malate dehydrogenase activity, malate dehydrogenase activity
AGOC: cytoplasm
AGOP: glycolysis, malate metabolic process, oxidation−reduction process
PGOF: L−malate dehydrogenase activity, catalytic activity, malate dehydrogenase activity, oxidoreductase activity, acting on th
PGOC: null
PGOP: carbohydrate metabolic process, cellular carbohydrate metabolic process, malate metabolic process, oxidation−reduct

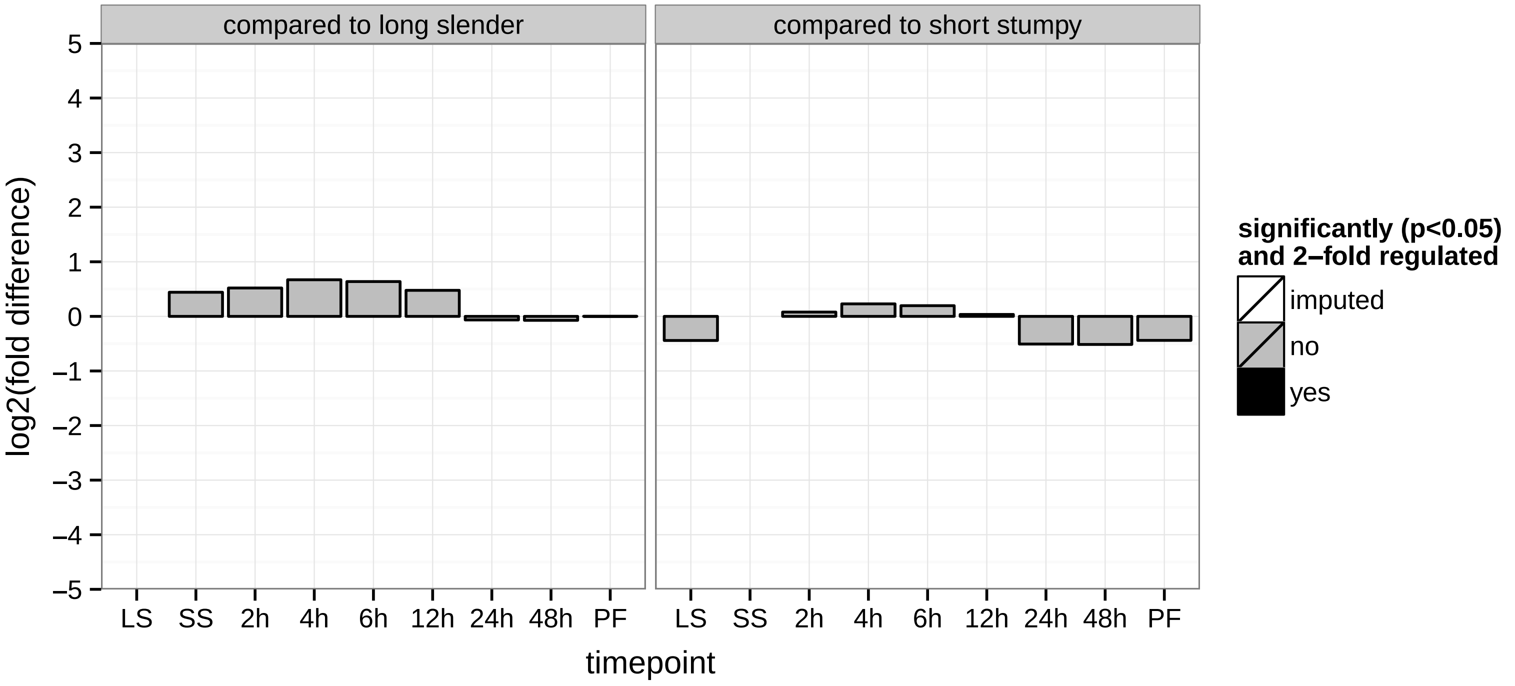

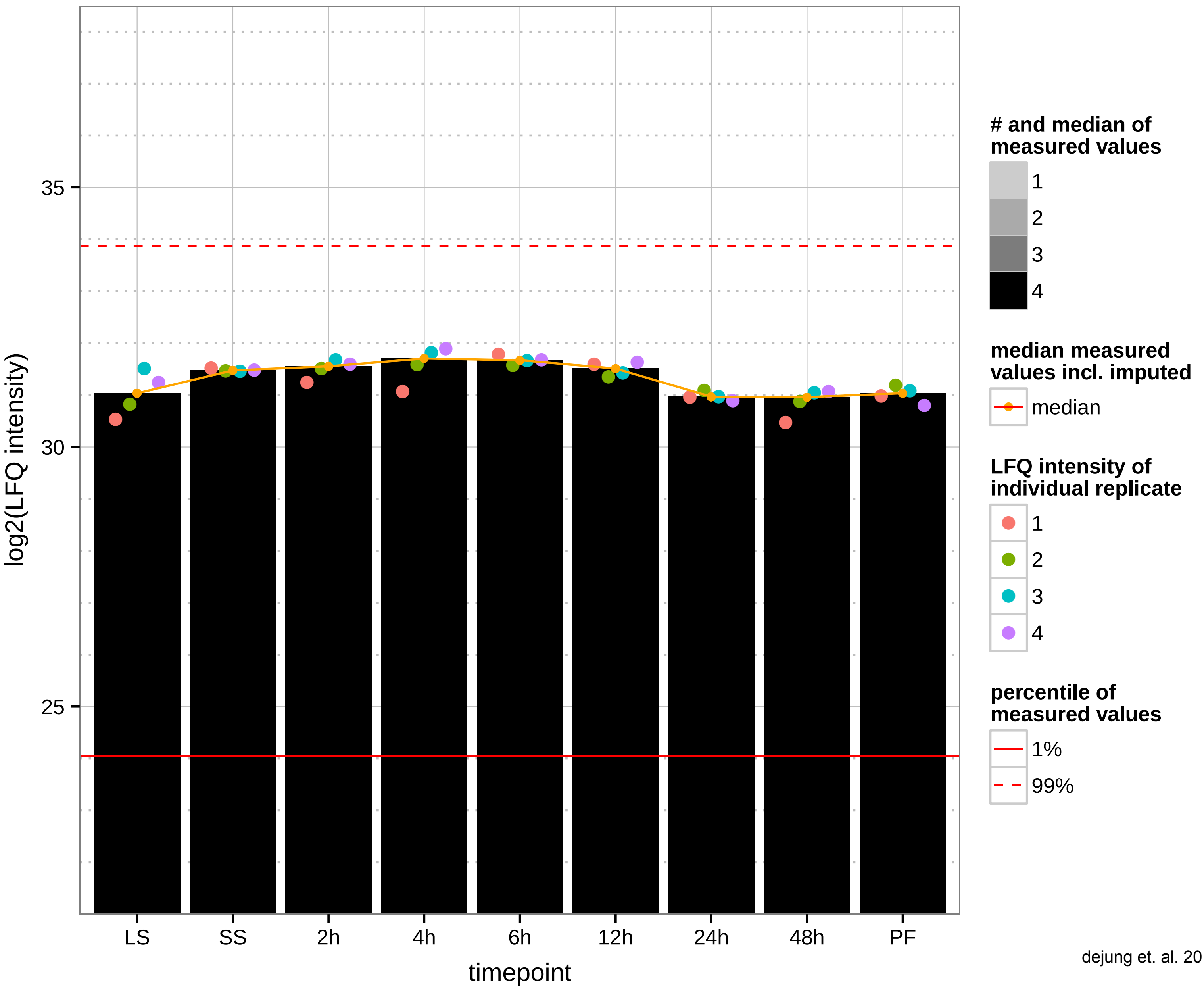

dejung et. al. 2015

hypothetical protein, conserved
Tb927.11.11280
AGOF: null
AGOC: null, mitochondrion
AGOP: null
PGOF: null
PGOC: null
PGOP: null

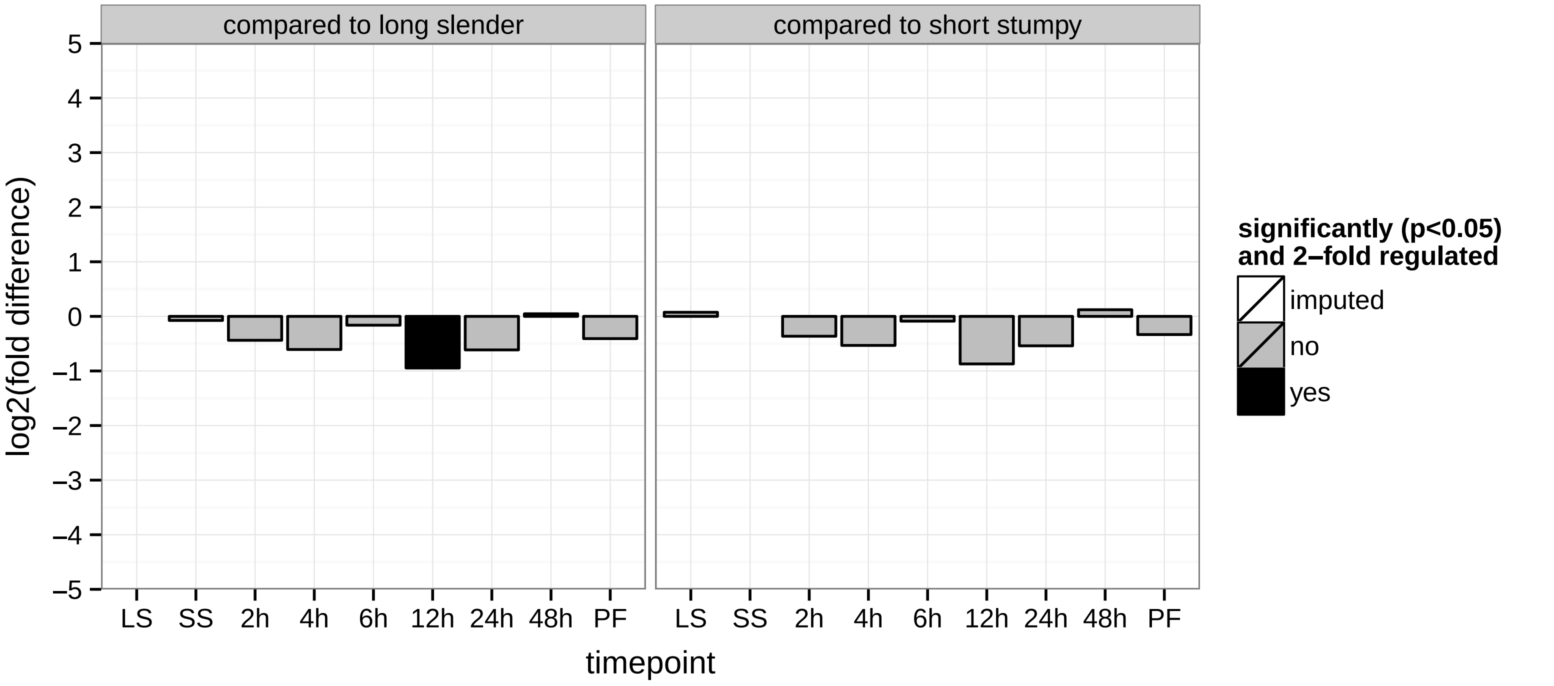

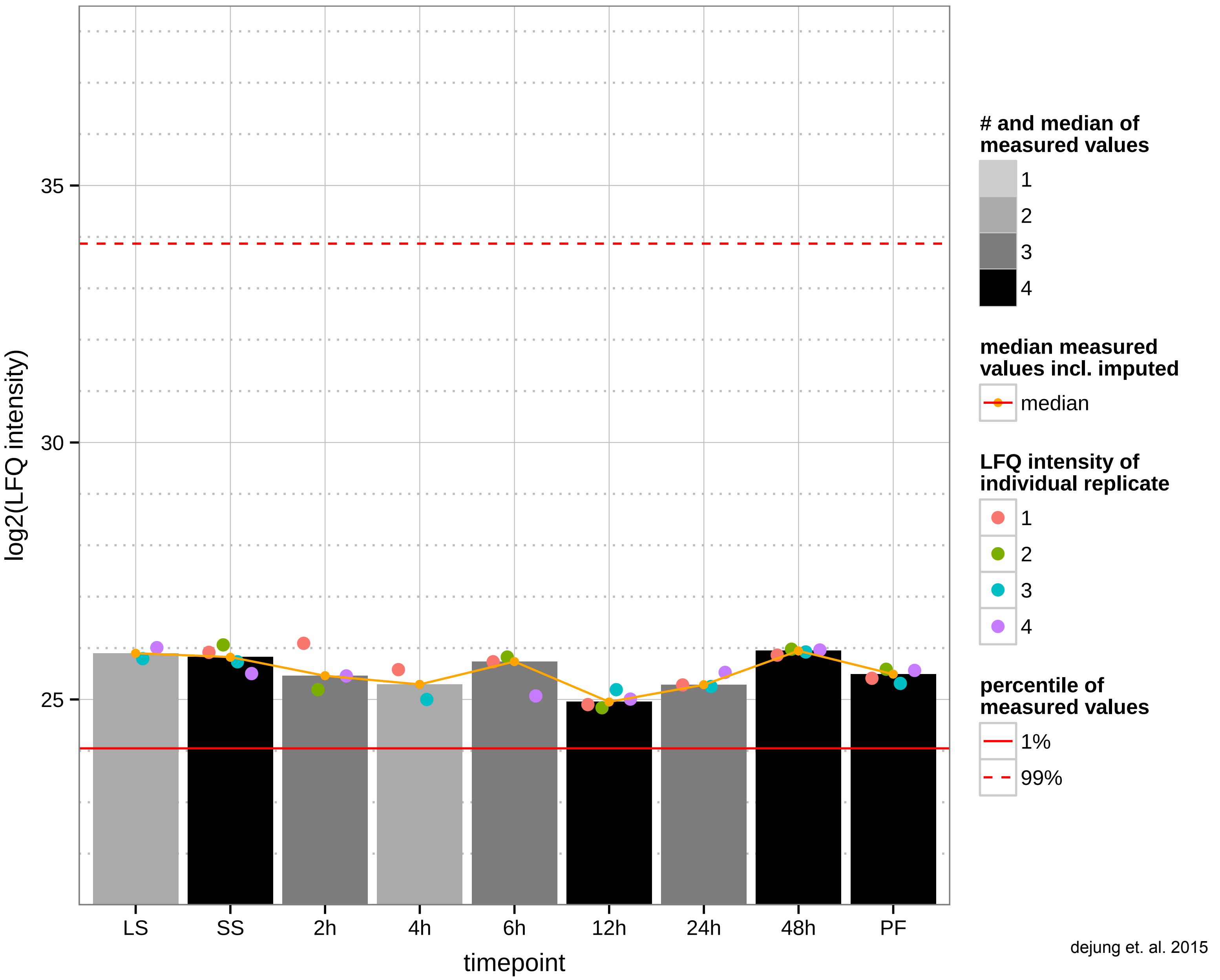

dejung et. al. 2015

heat shock protein 70, putative
Tb927.11.11290
AGOF: ATP binding
AGOC: null
AGOP: protein folding
PGOF: null
PGOC: null
PGOP: null

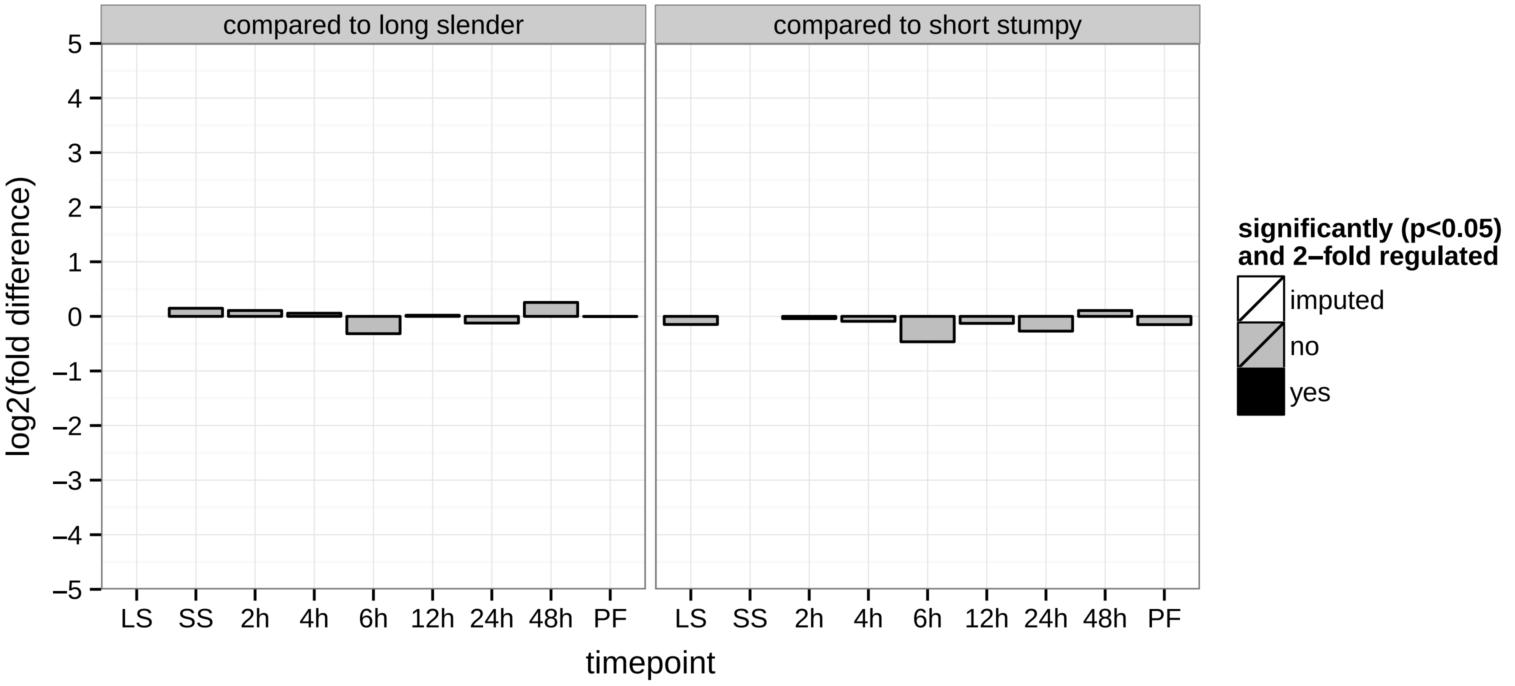

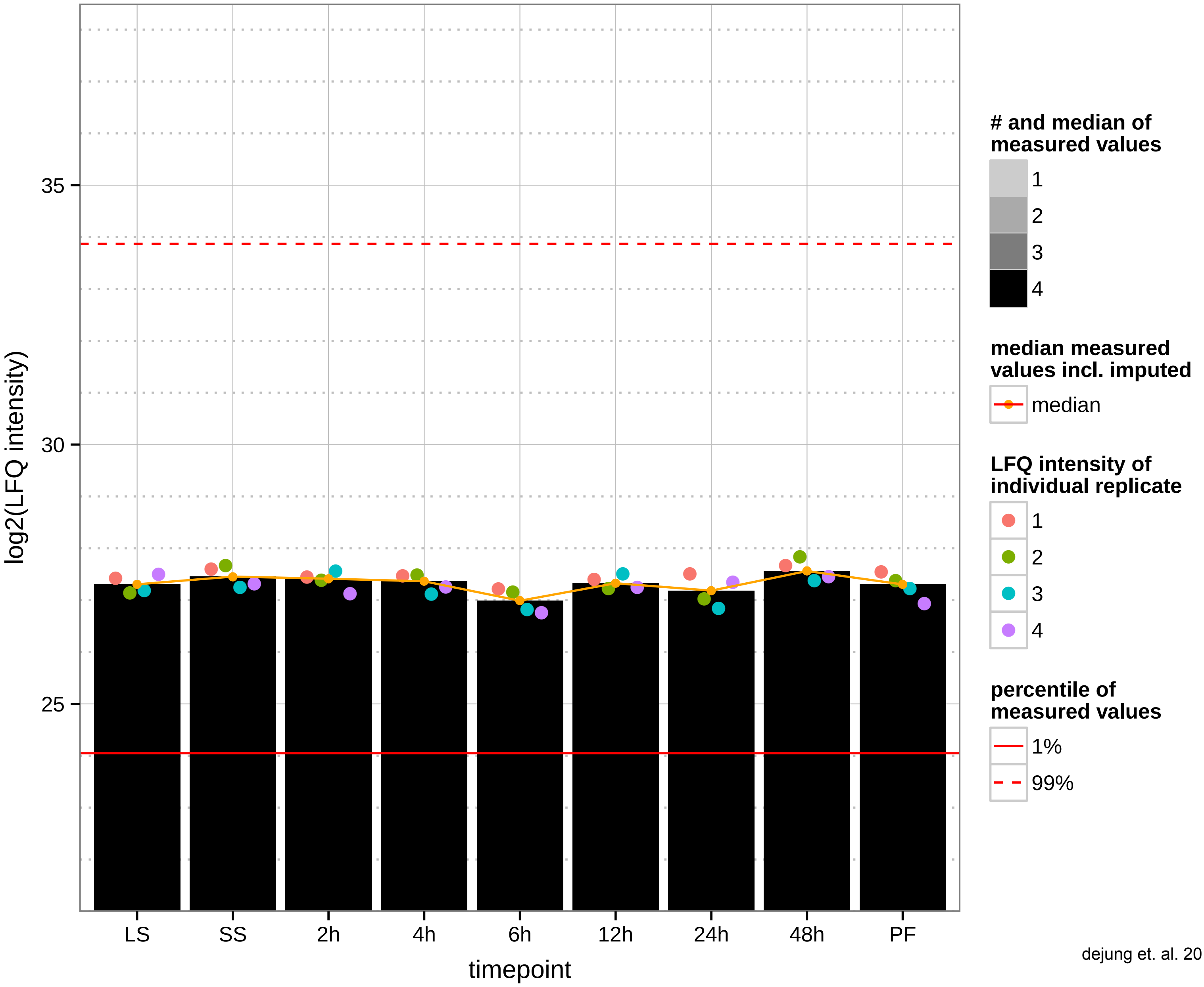

dejung et. al. 2015

heat shock protein 70 (hsp70), putative, heat shock protein 70
Tb927.11.11330;Tb11.v5.1035
AGOF: null, ATP binding
AGOC: null
AGOP: null, protein folding
PGOF: null
PGOC: null
PGOP: null

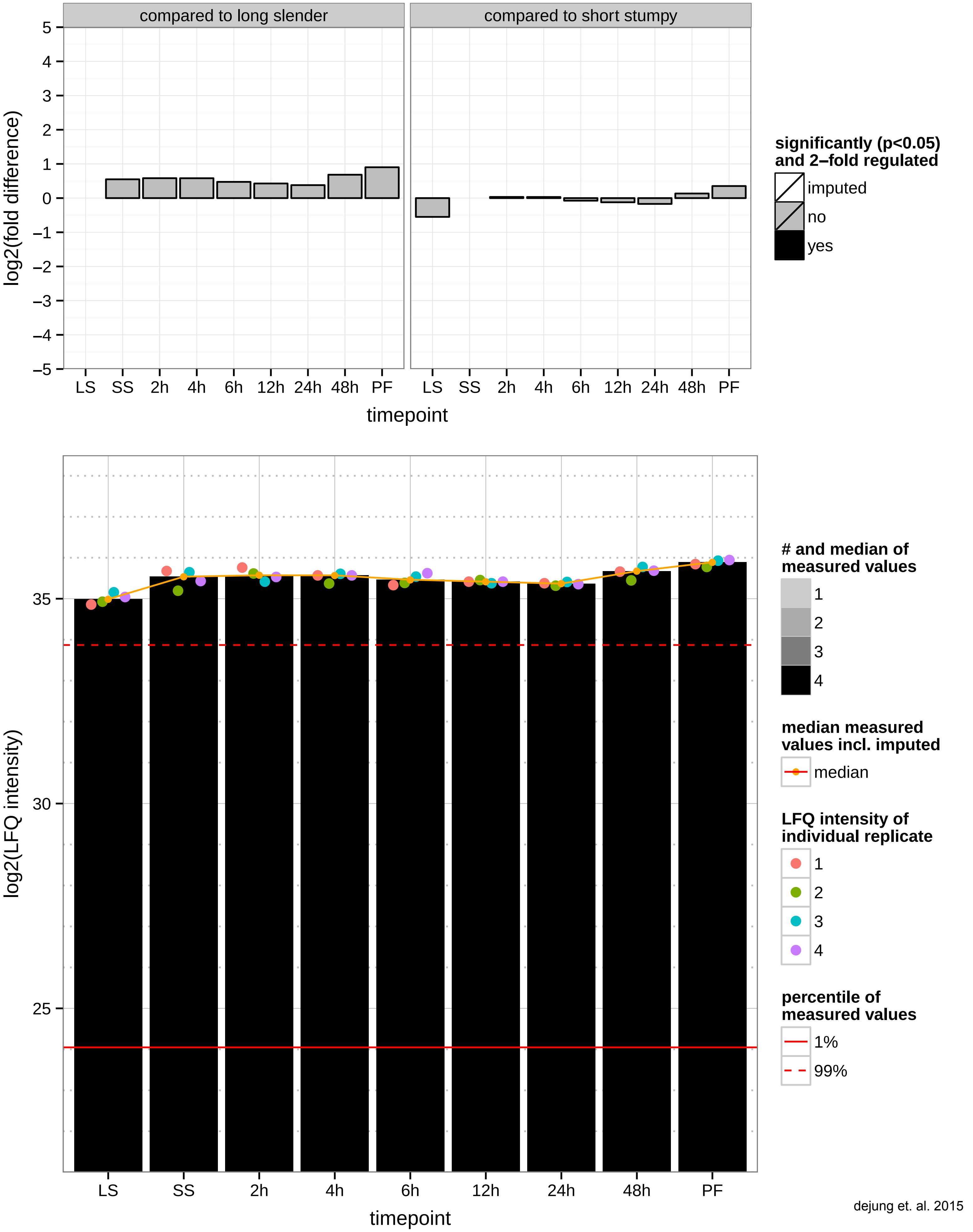

dejung et. al. 2015

gamma−tubulin complex subunit, putative, spindle pole body component alp6
Tb927.11.11340
AGOF: null
AGOC: null, integral to membrane, microtubule organizing center, spindle pole
AGOP: null, microtubule cytoskeleton organization
PGOF: null
PGOC: microtubule organizing center, spindle pole
PGOP: microtubule cytoskeleton organization

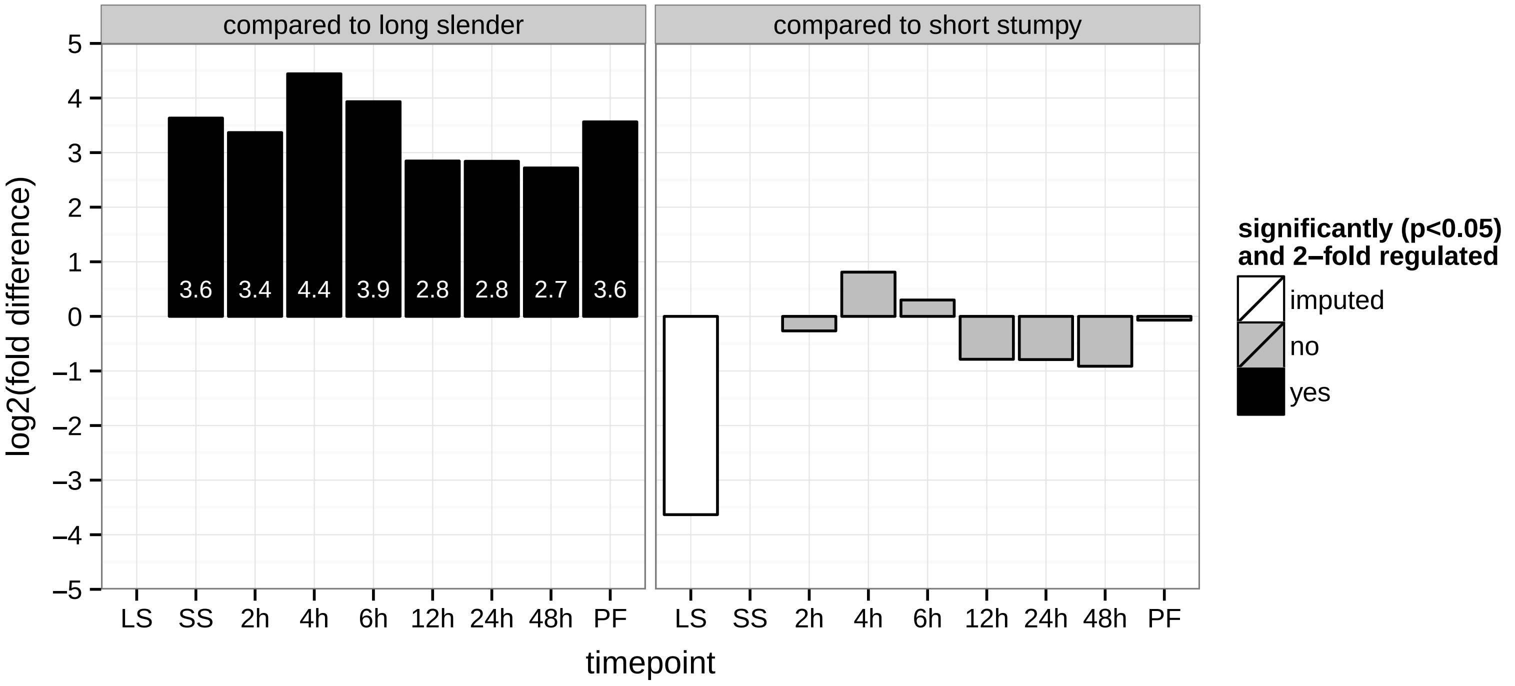

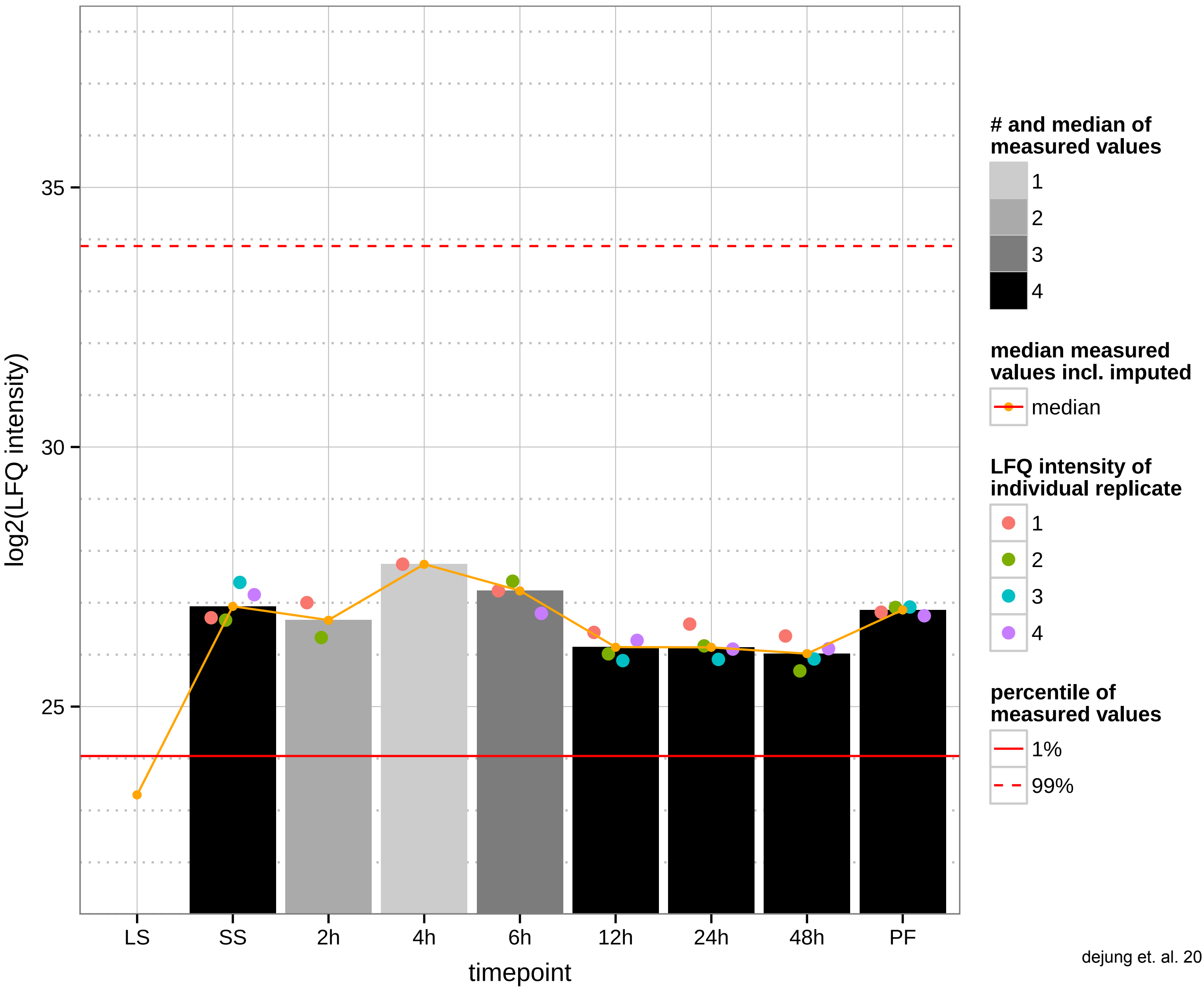

dejung et. al. 2015

hypothetical protein, conserved
Tb927.11.11380
AGOF: null
AGOC: mitochondrion
AGOP: null
PGOF: null
PGOC: null
PGOP: null

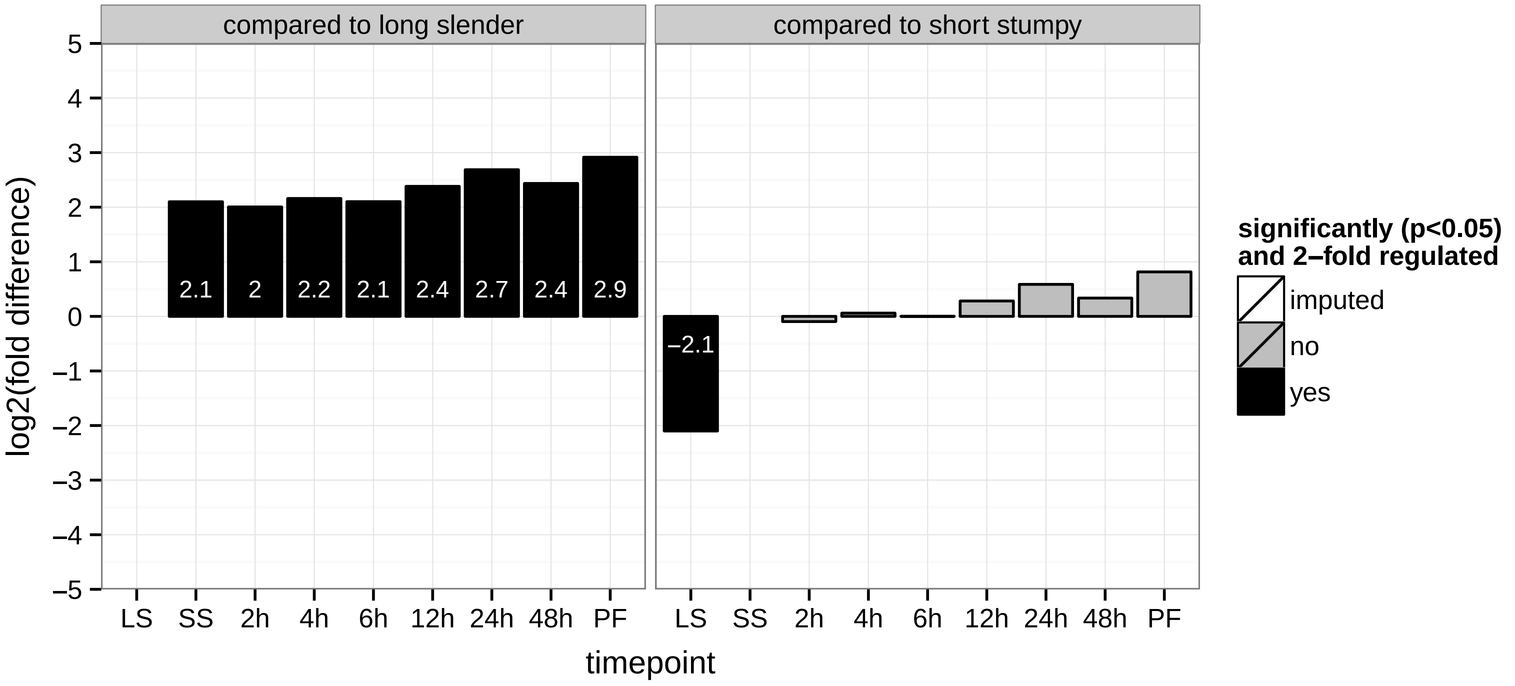

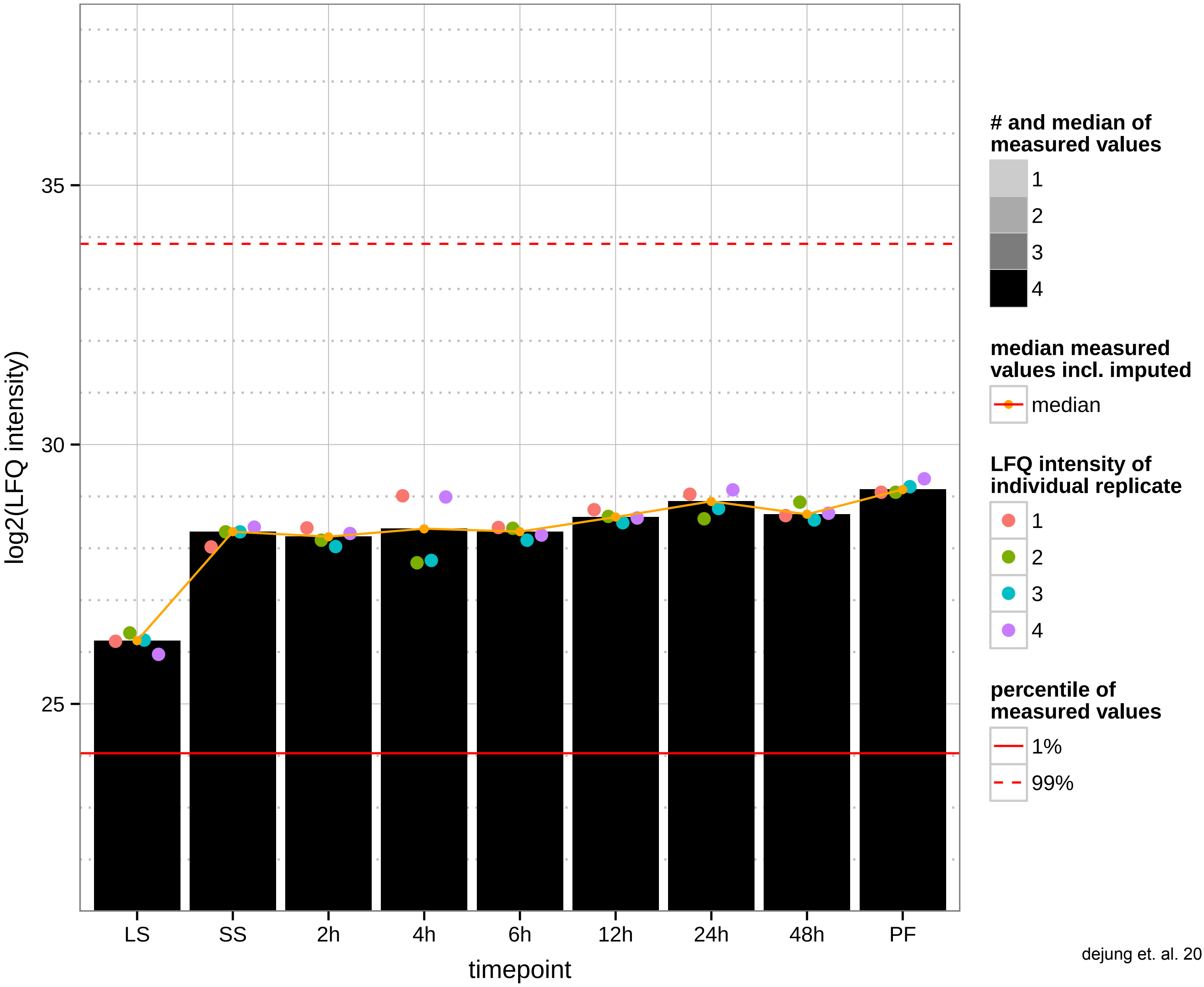

dejung et. al. 2015

cullin 1, putative
Tb927.11.11430
AGOF: ubiquitin protein ligase binding
AGOC: cullin–RING ubiquitin ligase complex
AGOP: cell proliferation, ubiquitin–dependent protein catabolic process
PGOF: ubiquitin protein ligase binding
PGOC: cullin–RING ubiquitin ligase complex
PGOP: ubiquitin–dependent protein catabolic process

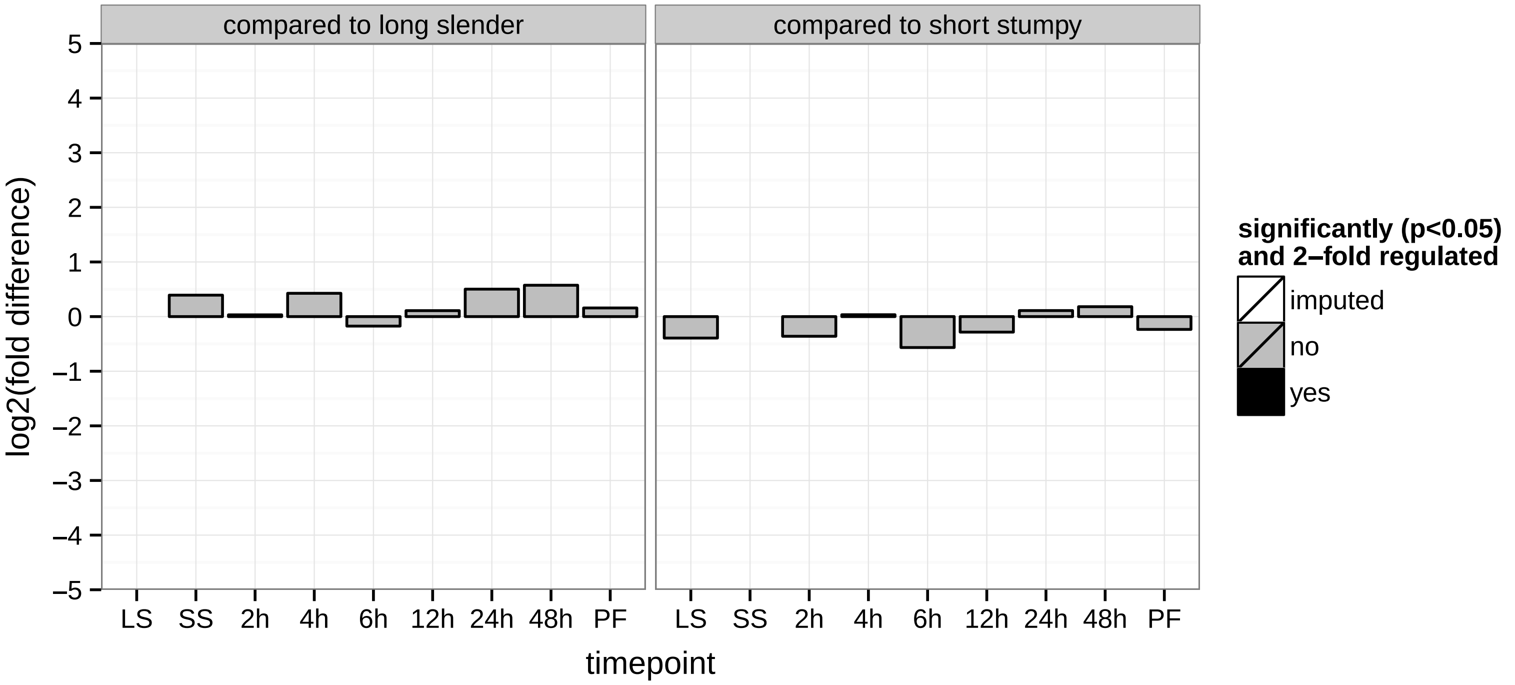

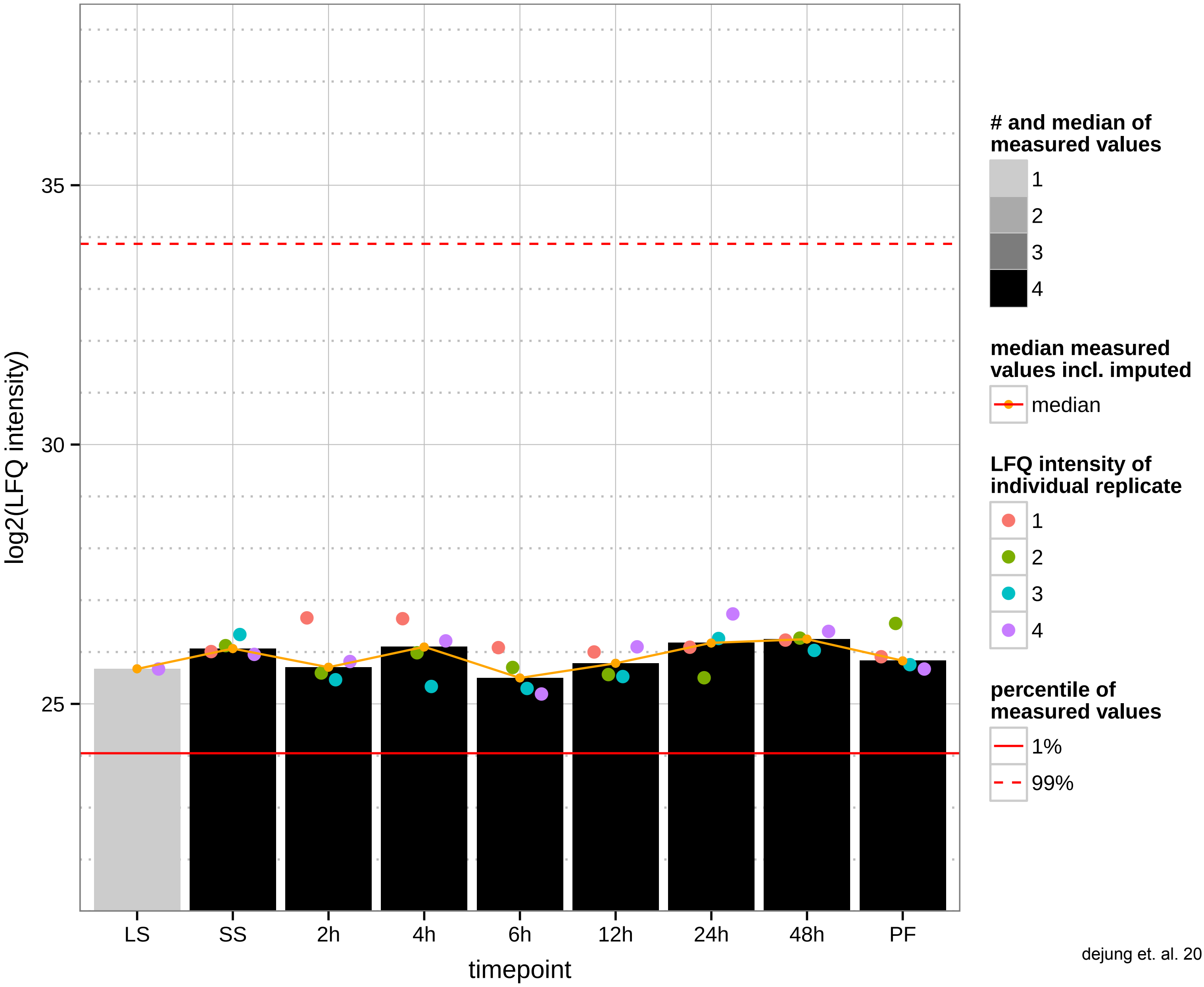

dejung et. al. 2015

radial spoke protein RSP3, putative
Tb927.11.1150
AGOF: null
AGOC: radial spoke
AGOP: ciliary or flagellar motility
PGOF: null
PGOC: null
PGOP: null

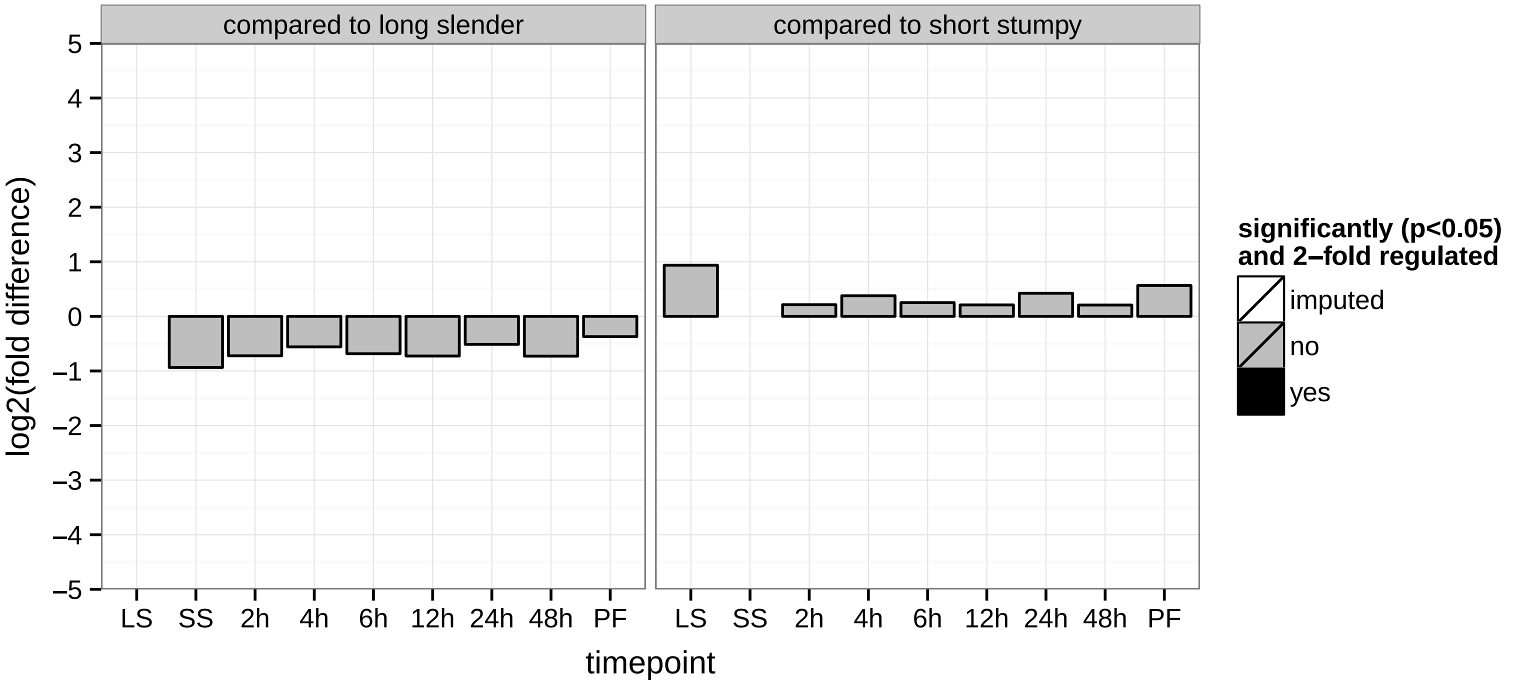

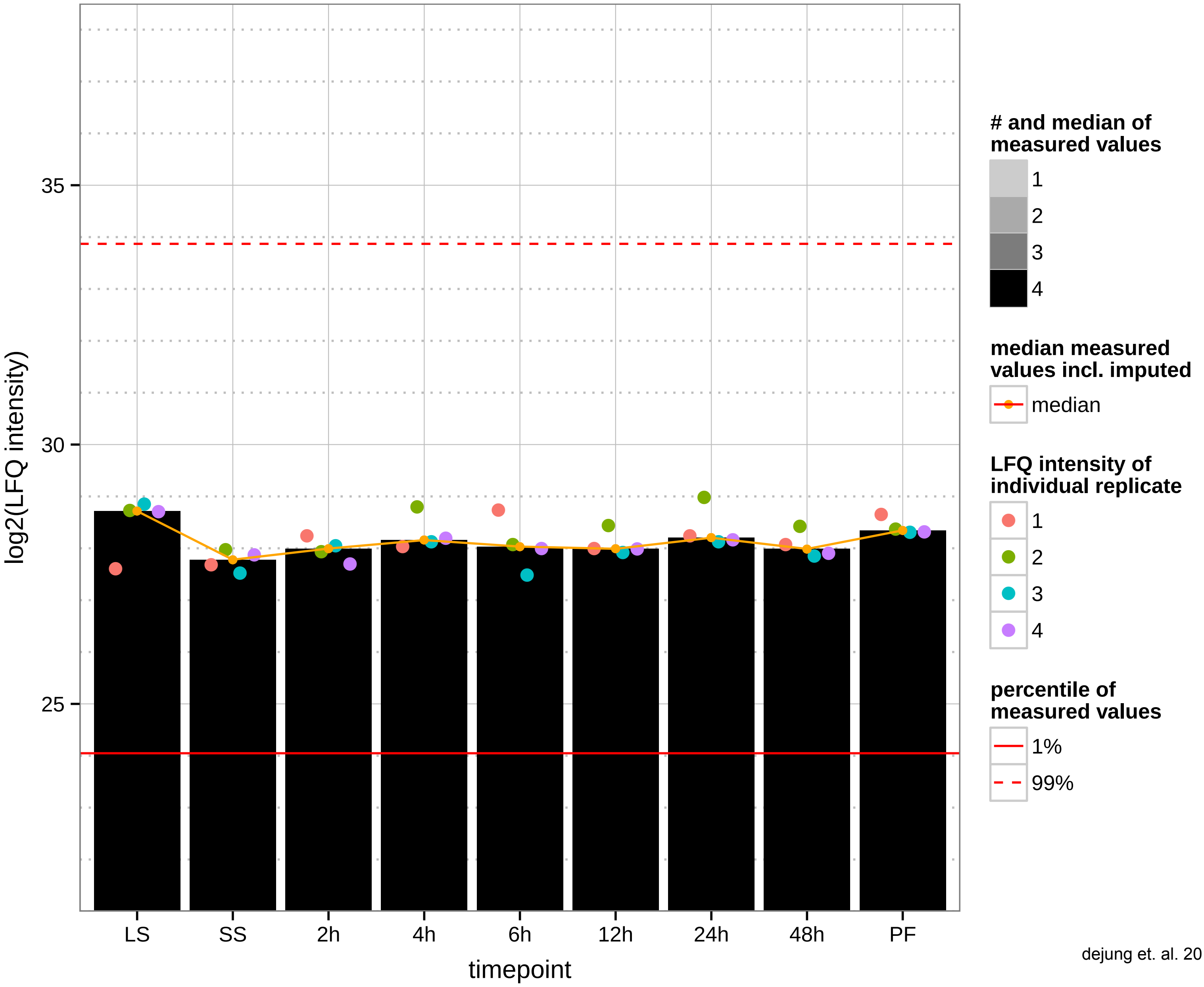

dejung et. al. 2015

glycosomal membrane protein (PEX11)
Tb927.11.11520
AGOF: null
AGOC: endoplasmic reticulum, glycosome membrane
AGOP: peroxisome fission
PGOF: null
PGOC: integral to peroxisomal membrane
PGOP: peroxisome fission

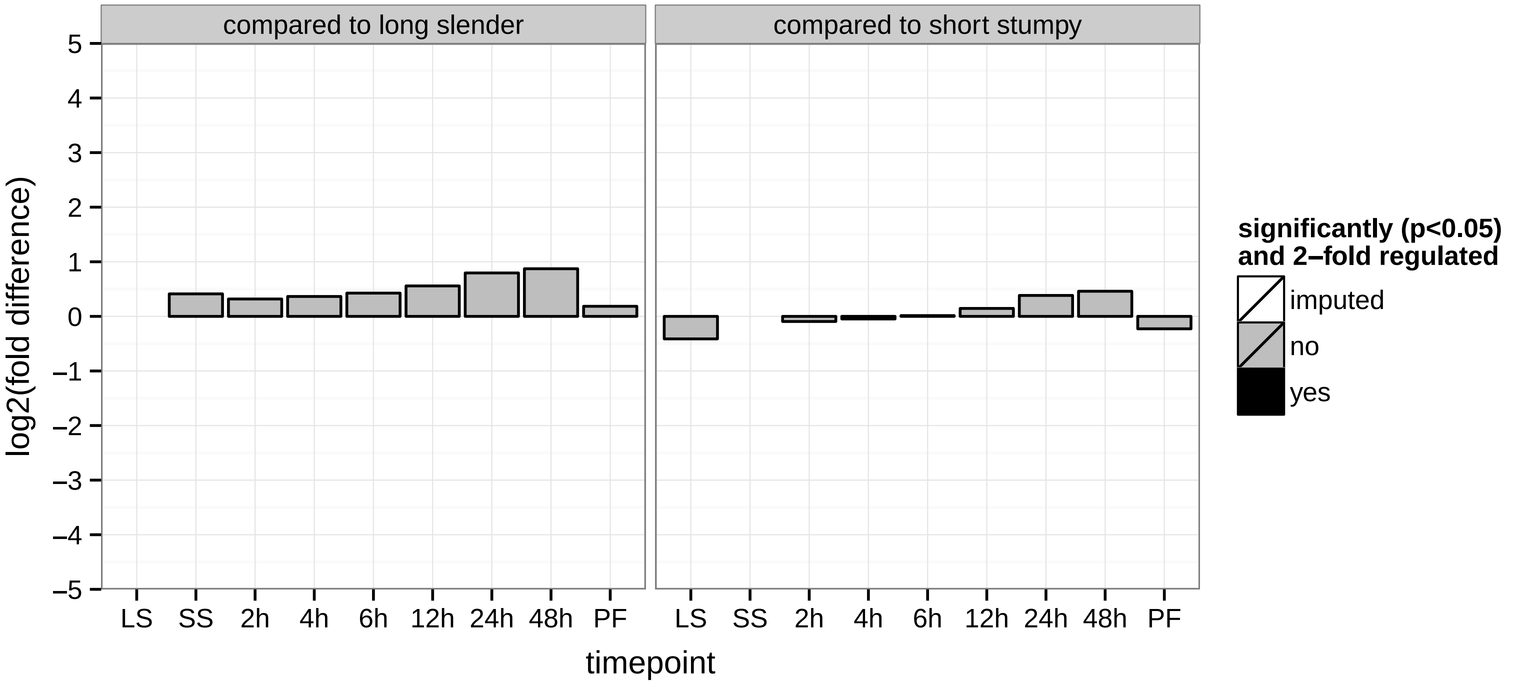

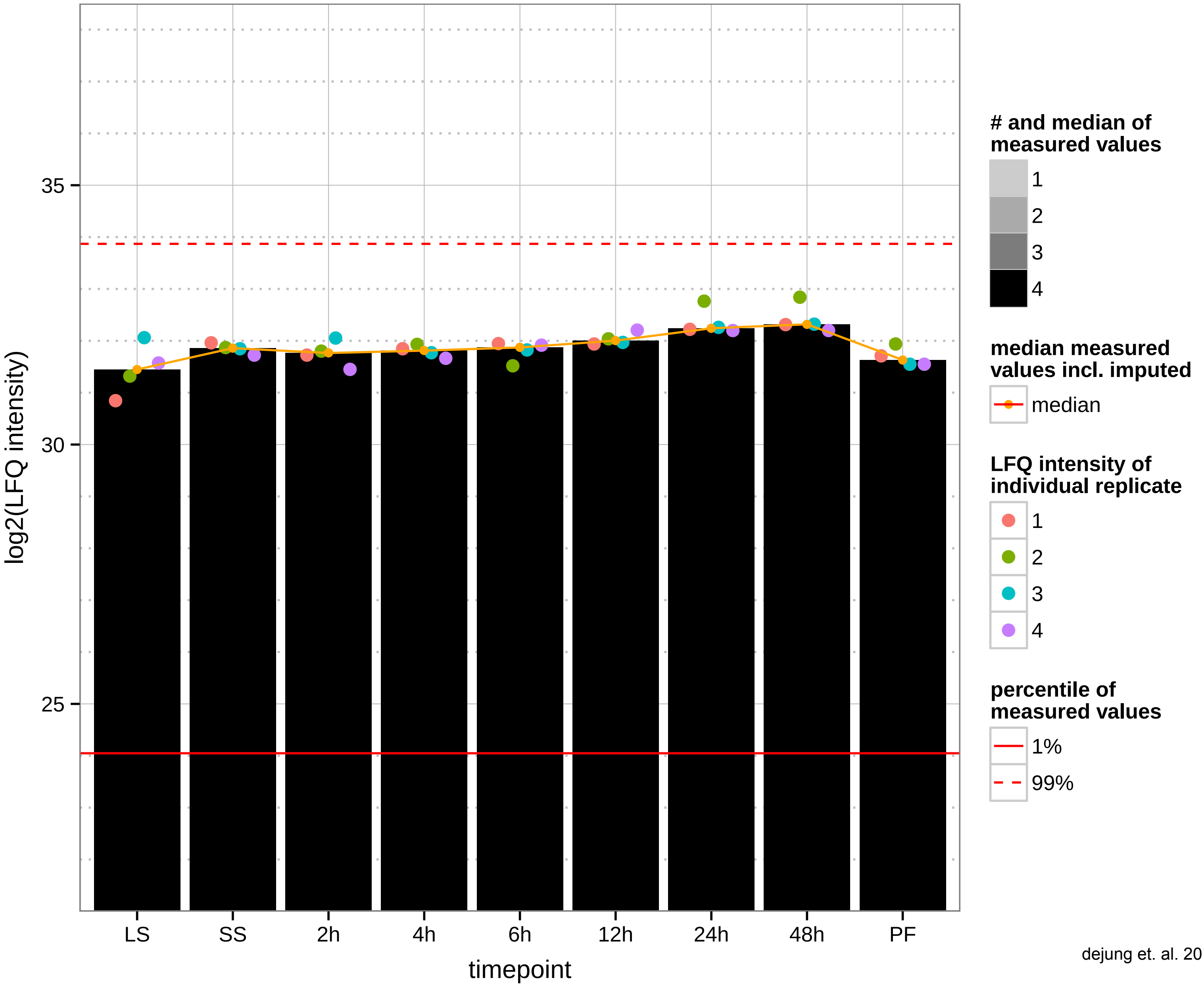

dejung et. al. 2015

unspecified product, DNA topoisomerase II, putative
Tb927.11.11550;Tb927.11.11540
AGOF: null, ATP binding, DNA topoisomerase (ATP−hydrolyzing) activity
AGOC: null, chromosome
AGOP: null, DNA topological change
PGOF: ATP binding, DNA binding, DNA topoisomerase (ATP−hydrolyzing) activity
PGOC: chromosome
PGOP: DNA topological change

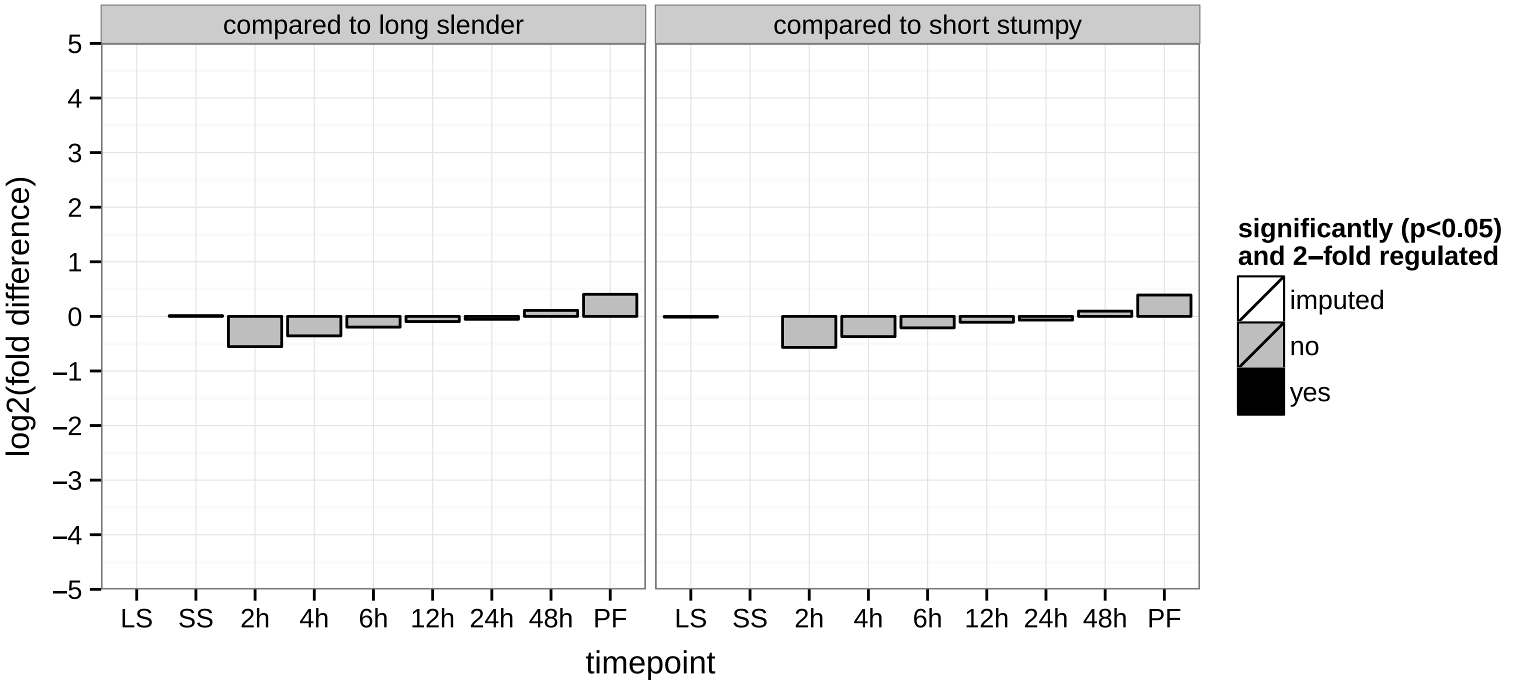

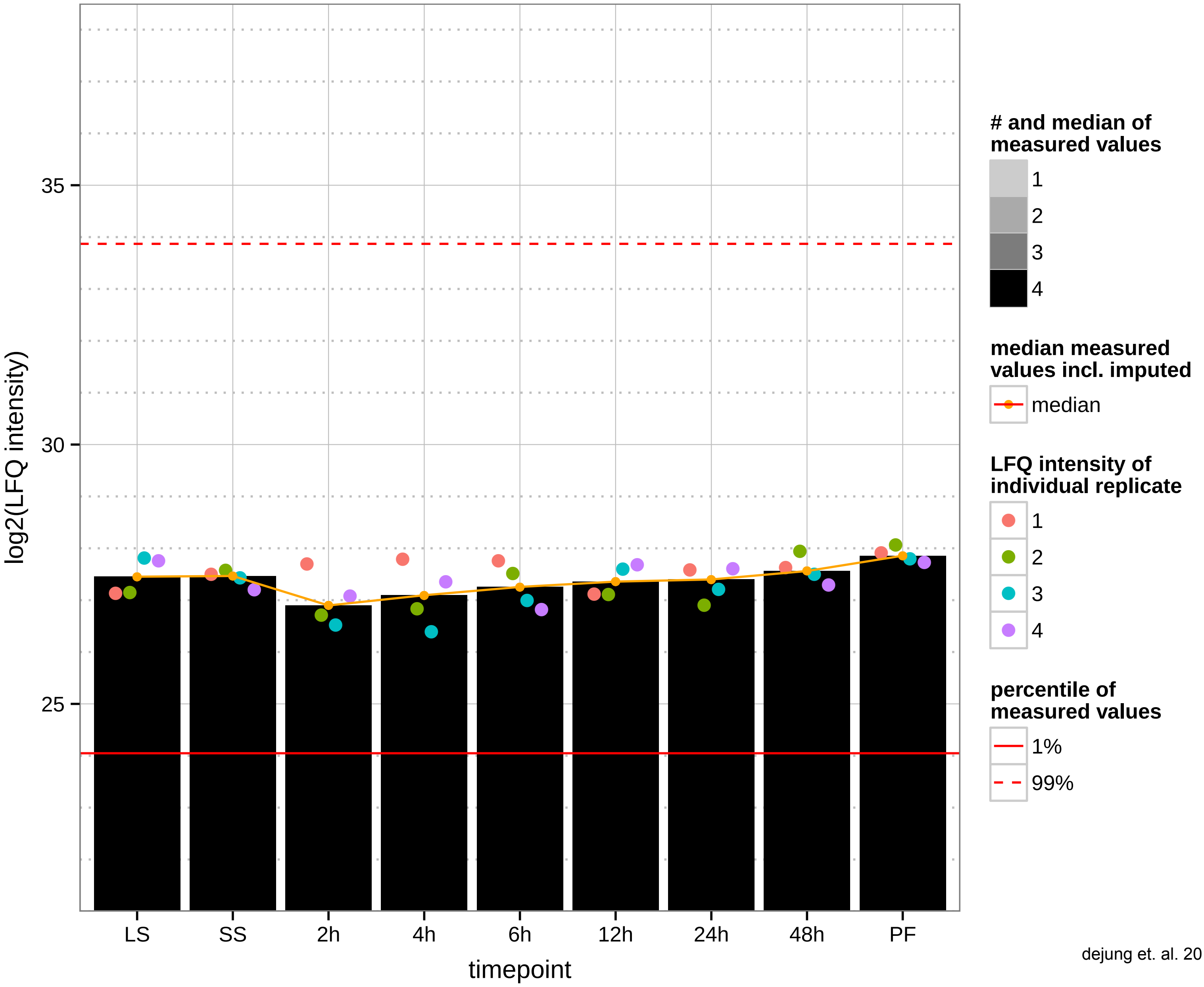

dejung et. al. 2015

Translation initiation factor eIF−2B subunit epsilon, putative, eIF−2B GDP−GTP exchange factor subunit epsilon (EIF2BE)
Tb927.11.11570
AGOF: null
AGOC: null
AGOP: null
PGOF: transferase activity
PGOC: null
PGOP: null

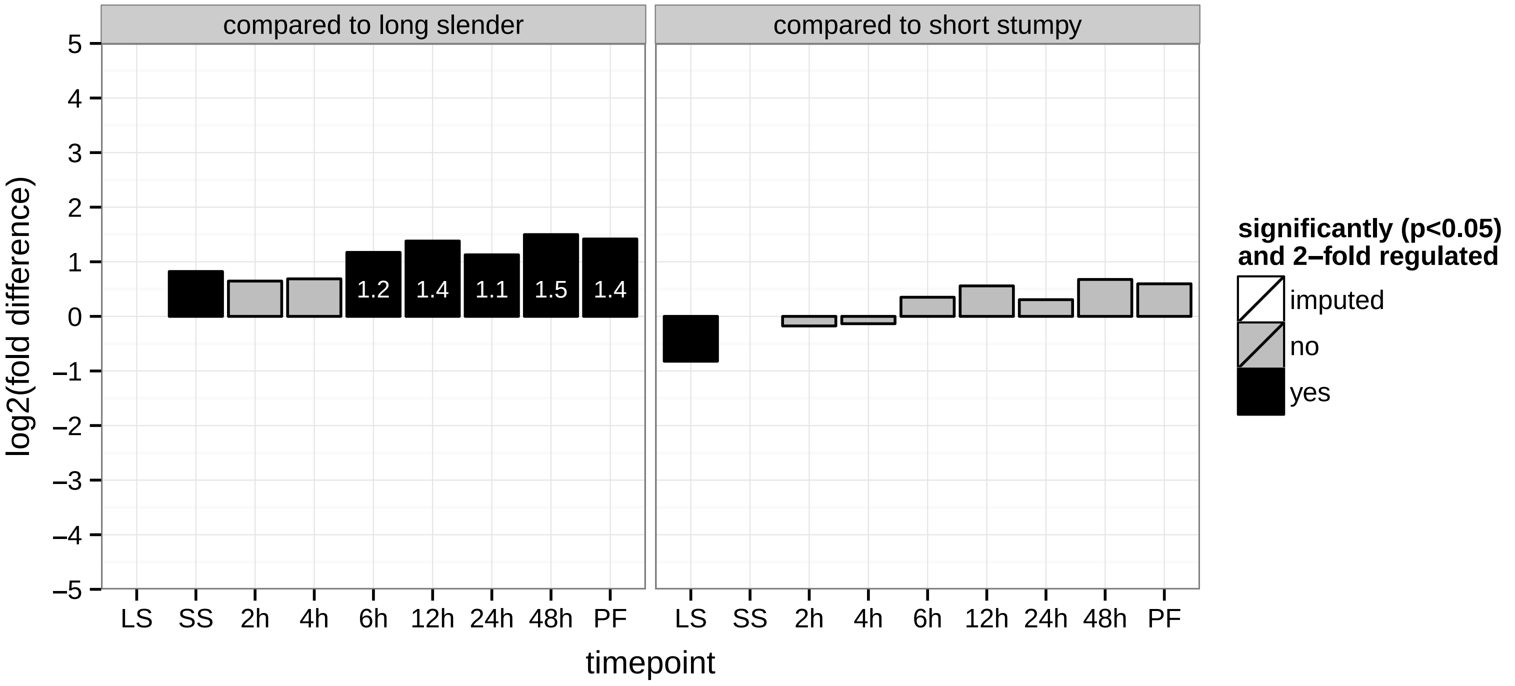

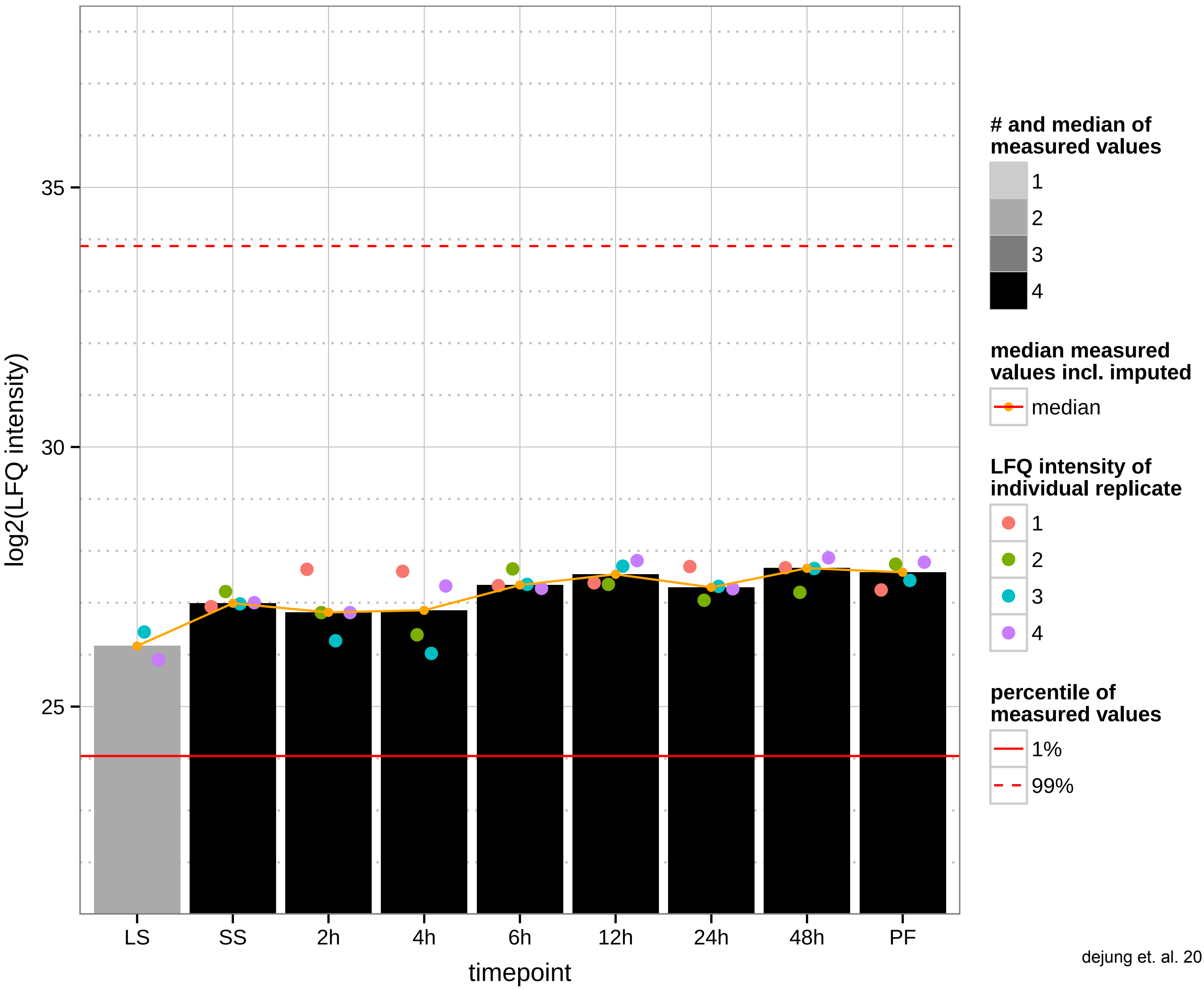

dejung et. al. 2015

eukaryotic translation initiation factor, putative
Tb927.11.11590
AGOF: translation initiation factor activity
AGOC: eukaryotic translation initiation factor 3 complex
AGOP: translational initiation
PGOF: protein binding
PGOC: null
PGOP: null

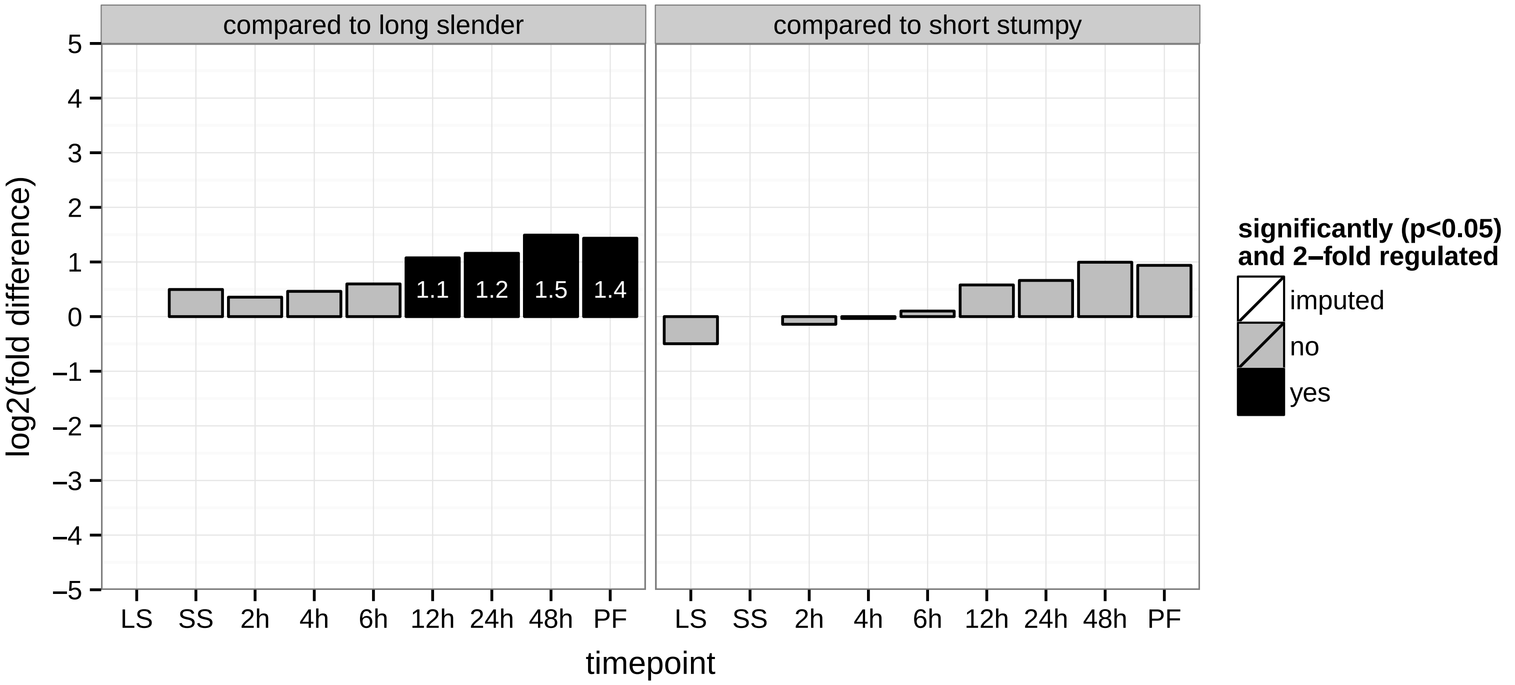

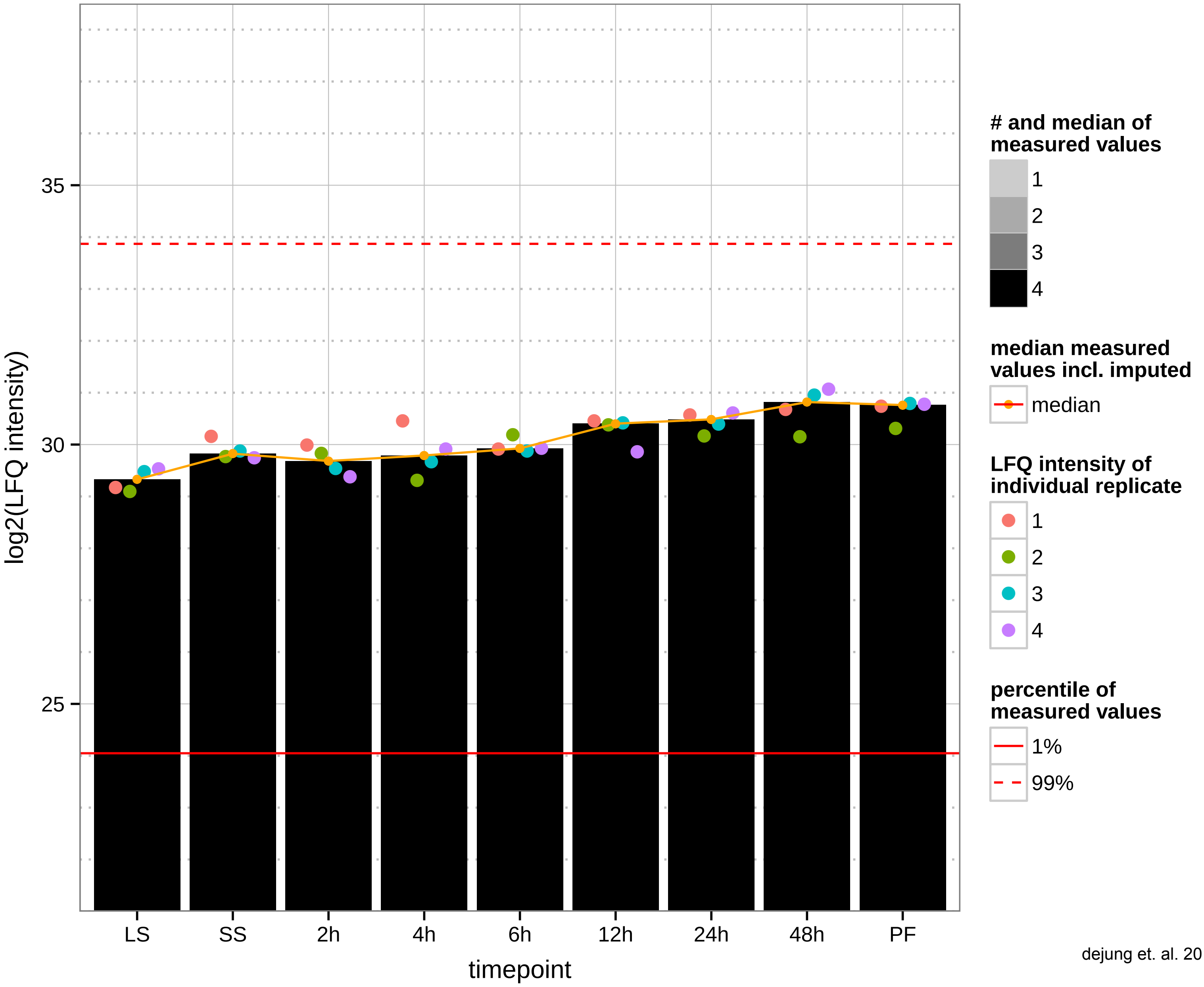

dejung et. al. 2015

hypothetical protein, conserved
Tb927.11.1160
AGOF: null
AGOC: null
AGOP: null
PGOF: null
PGOC: null
PGOP: intracellular protein transport, vesicle−mediated transport

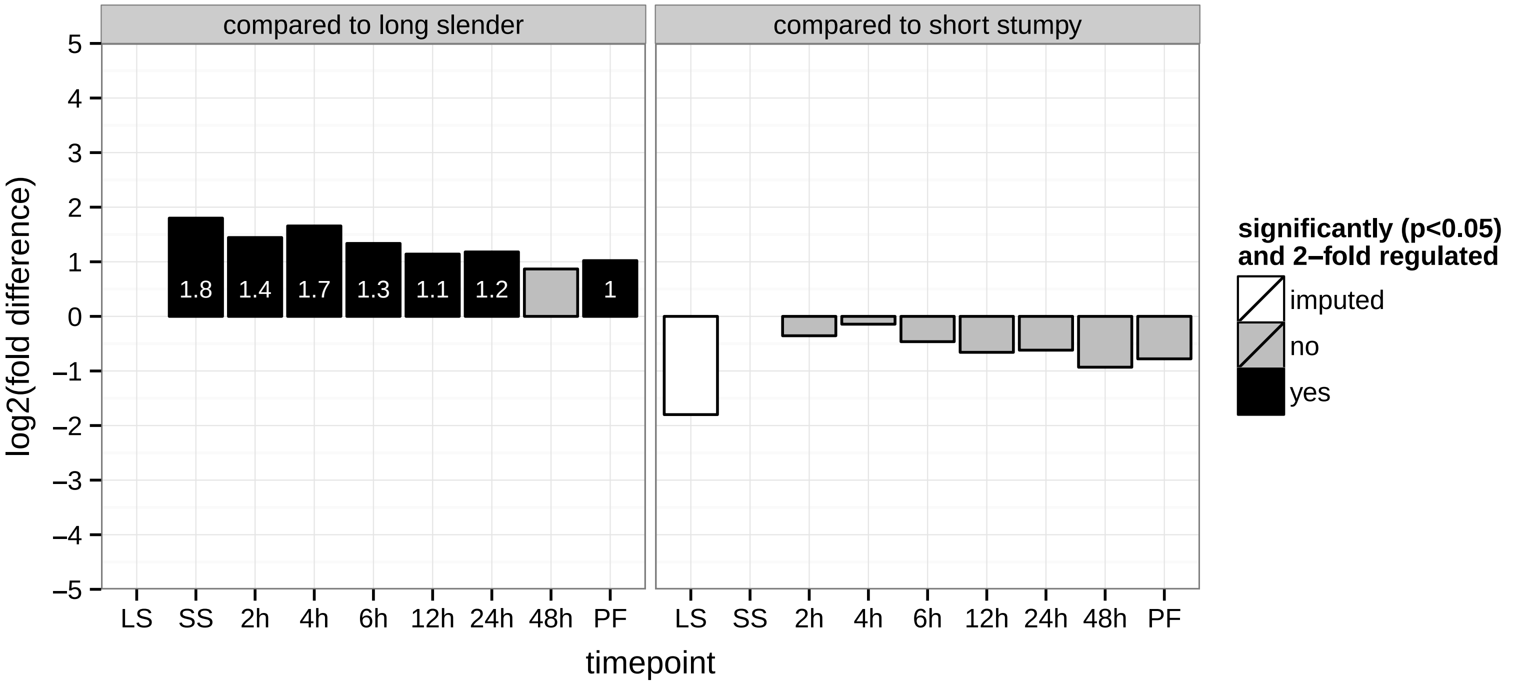

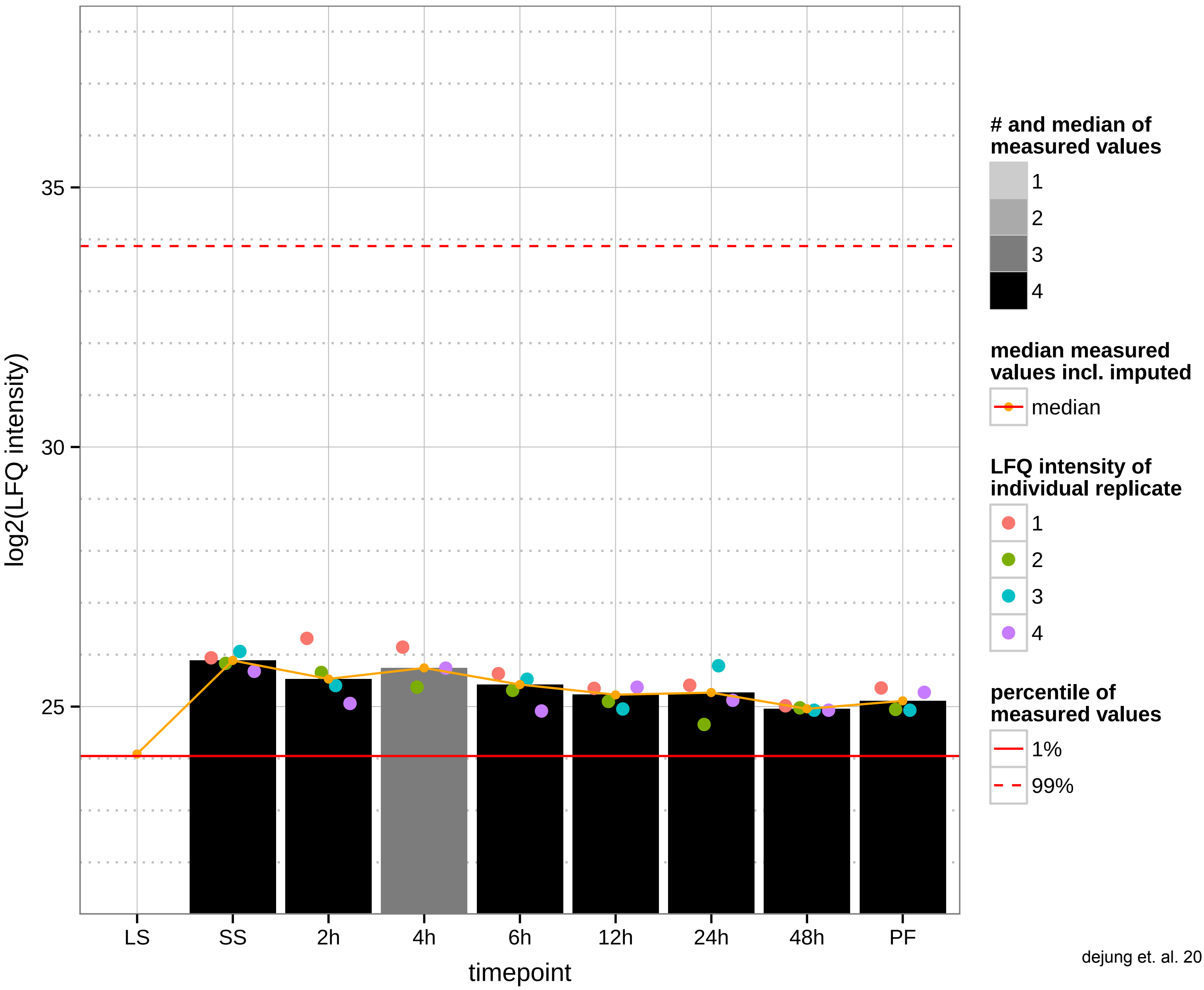

dejung et. al. 2015

conserved protein
Tb927.11.11660
AGOF: tRNA (adenine−N1−)−methyltransferase activity
AGOC: null
AGOP: tRNA methylation
PGOF: tRNA (adenine−N1−)−methyltransferase activity
PGOC: tRNA (m1A) methyltransferase complex
PGOP: tRNA methylation

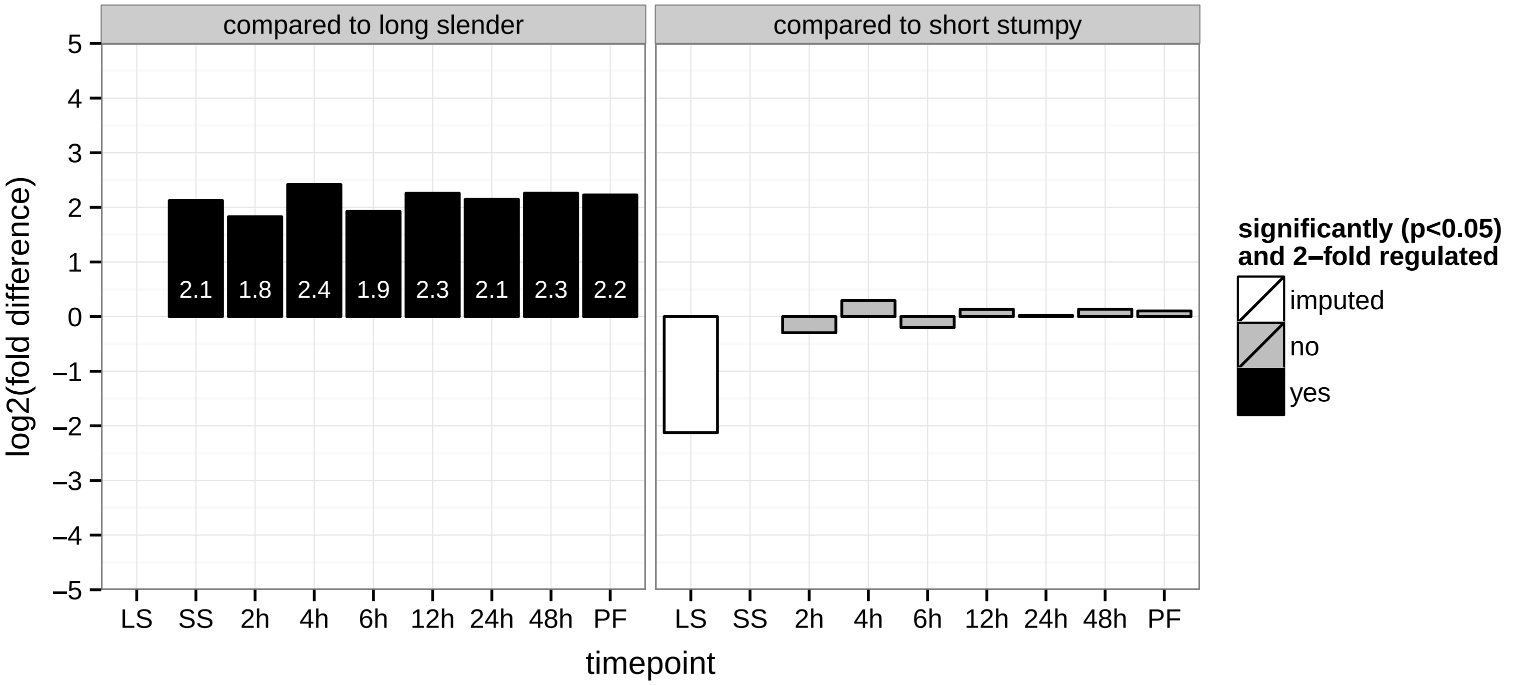

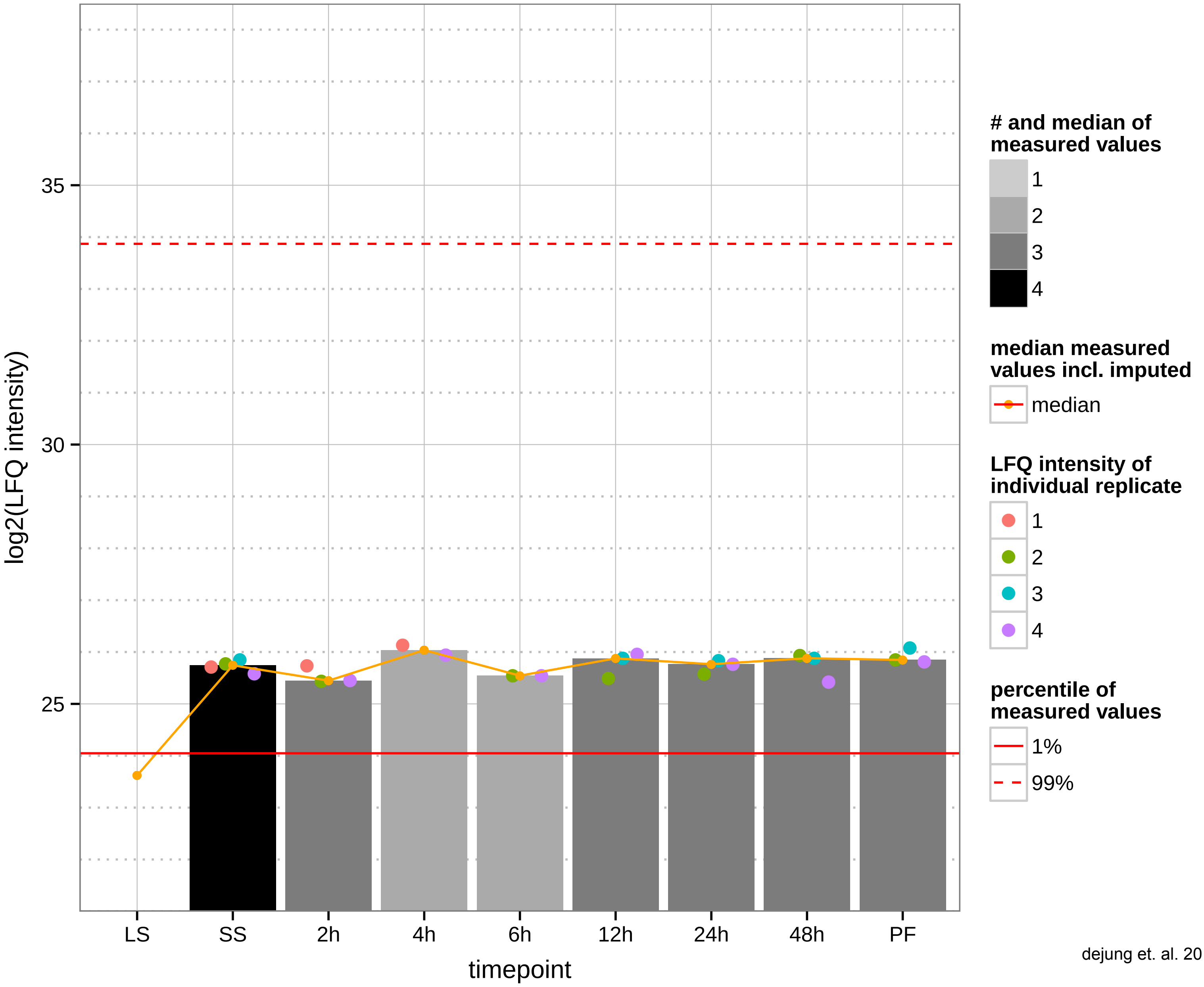

dejung et. al. 2015

2–oxoglutarate dehydrogenase E2 component, putative
Tb927.11.11680
AGOF: dihydrolipoyllysine–residue succinyltransferase activity
AGOC: mitochondrion, oxoglutarate dehydrogenase complex
AGOP: tricarboxylic acid cycle
PGOF: transferase activity, transferring acyl groups
PGOC: null
PGOP: metabolic process

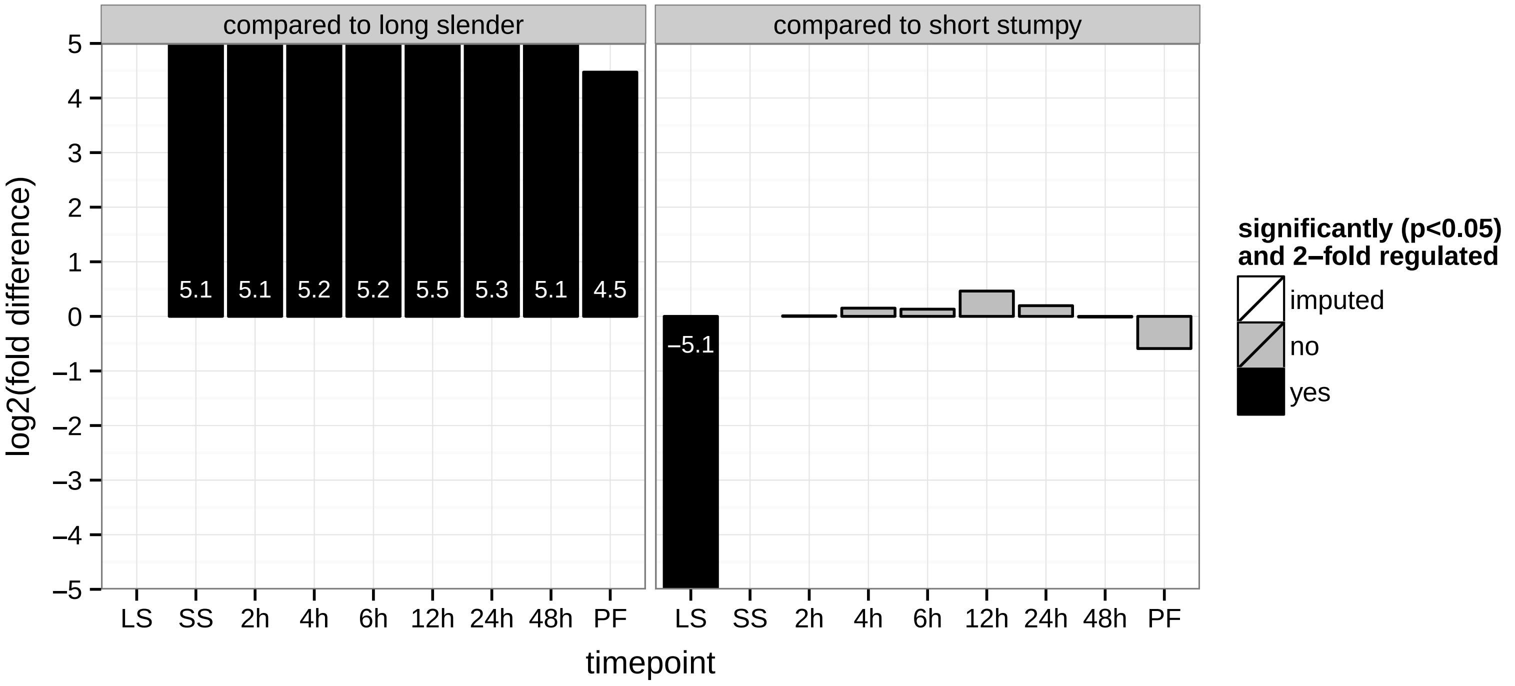

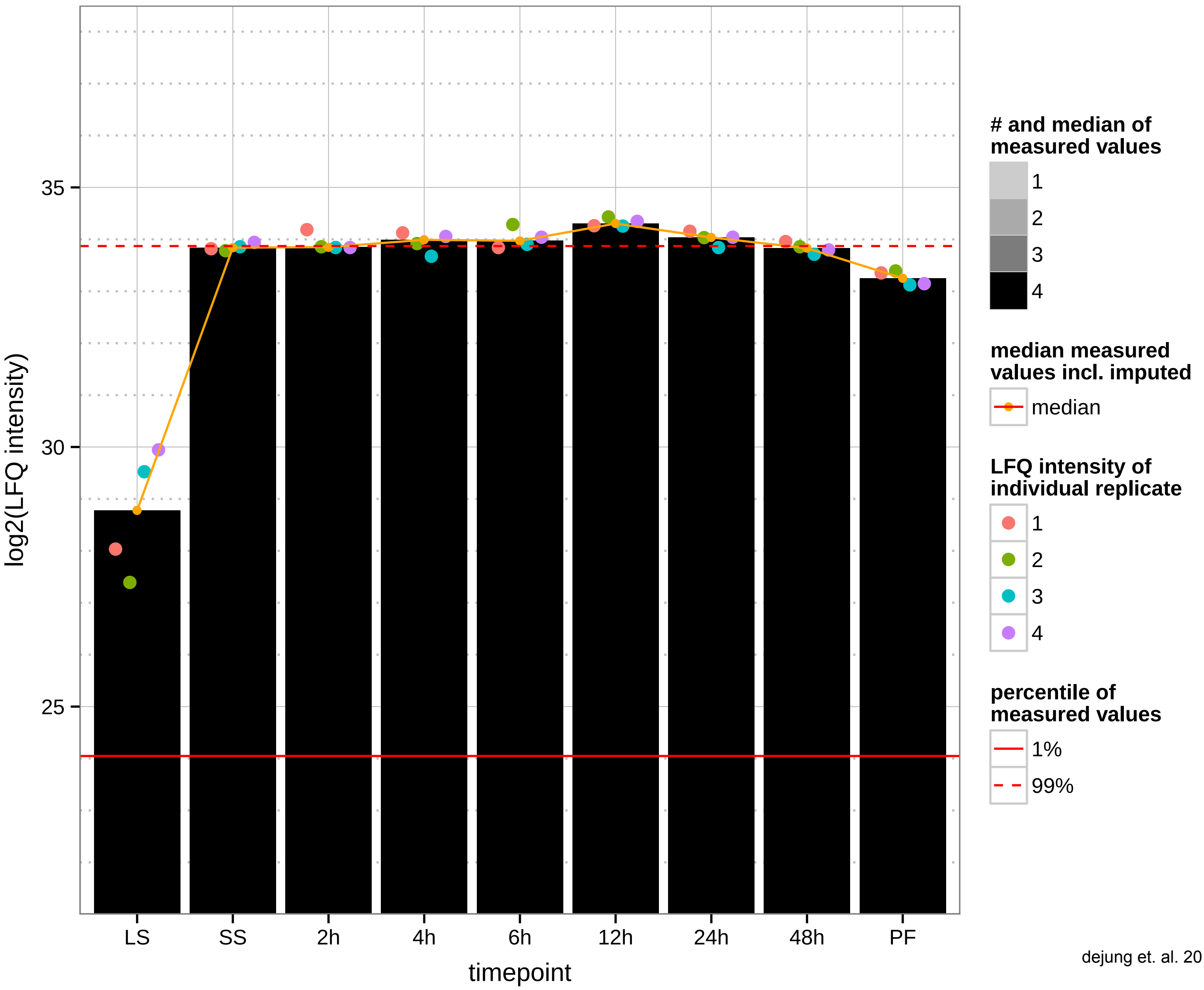

dejung et. al. 2015

hypothetical protein, conserved
Tb927.11.1170
AGOF: null
AGOC: null
AGOP: null
PGOF: null
PGOC: null
PGOP: null

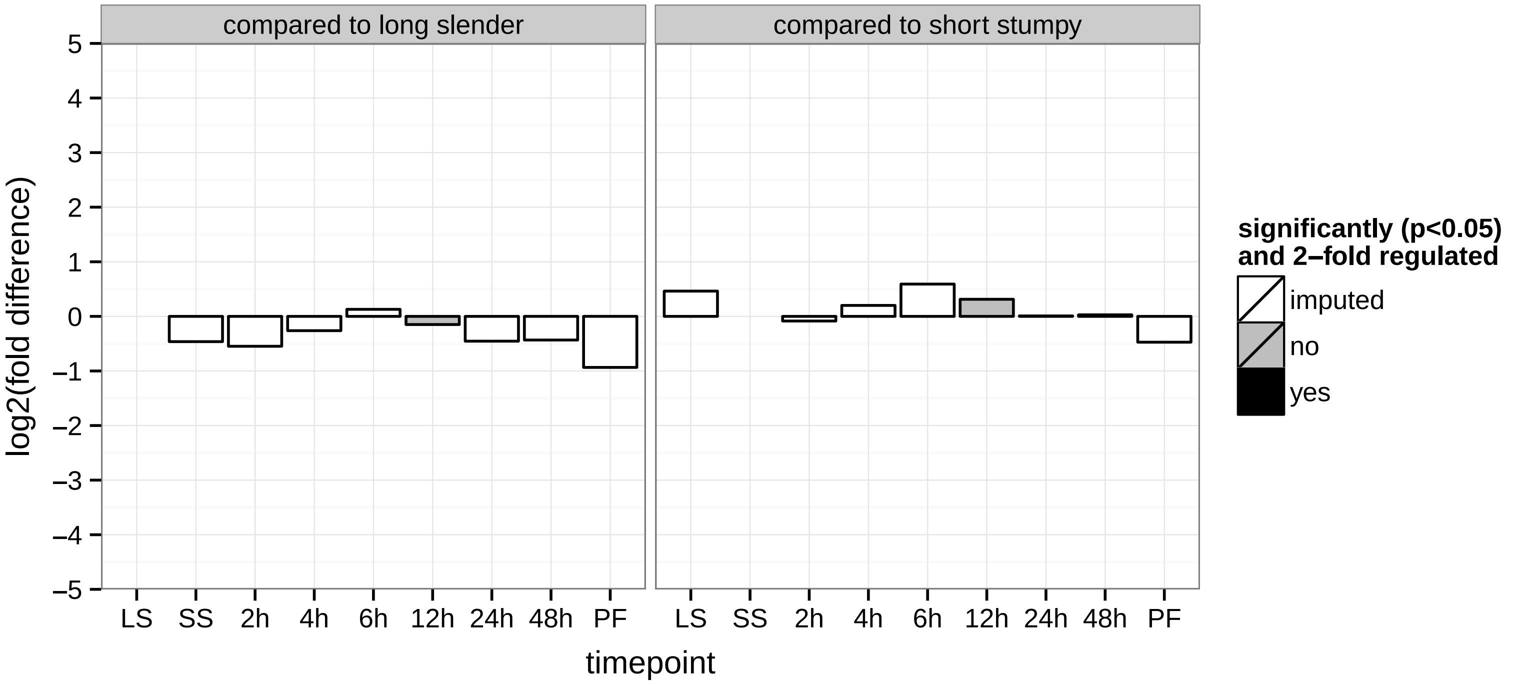

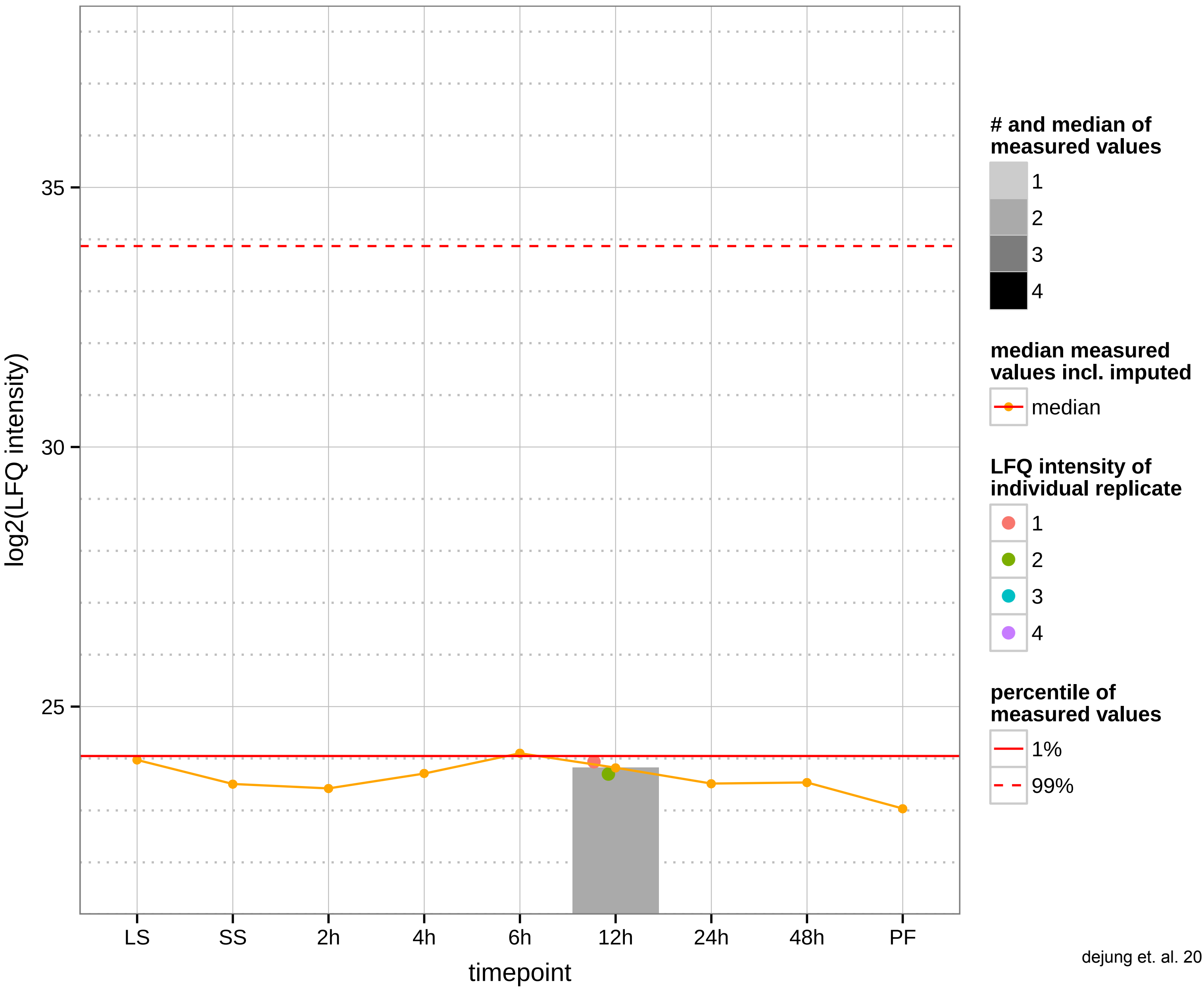

dejung et. al. 2015

hypothetical protein, conserved
Tb927.11.11700
AGOF: transferase activity
AGOC: null
AGOP: null
PGOF: nucleotidyltransferase activity, transferase activity
PGOC: null
PGOP: biosynthetic process

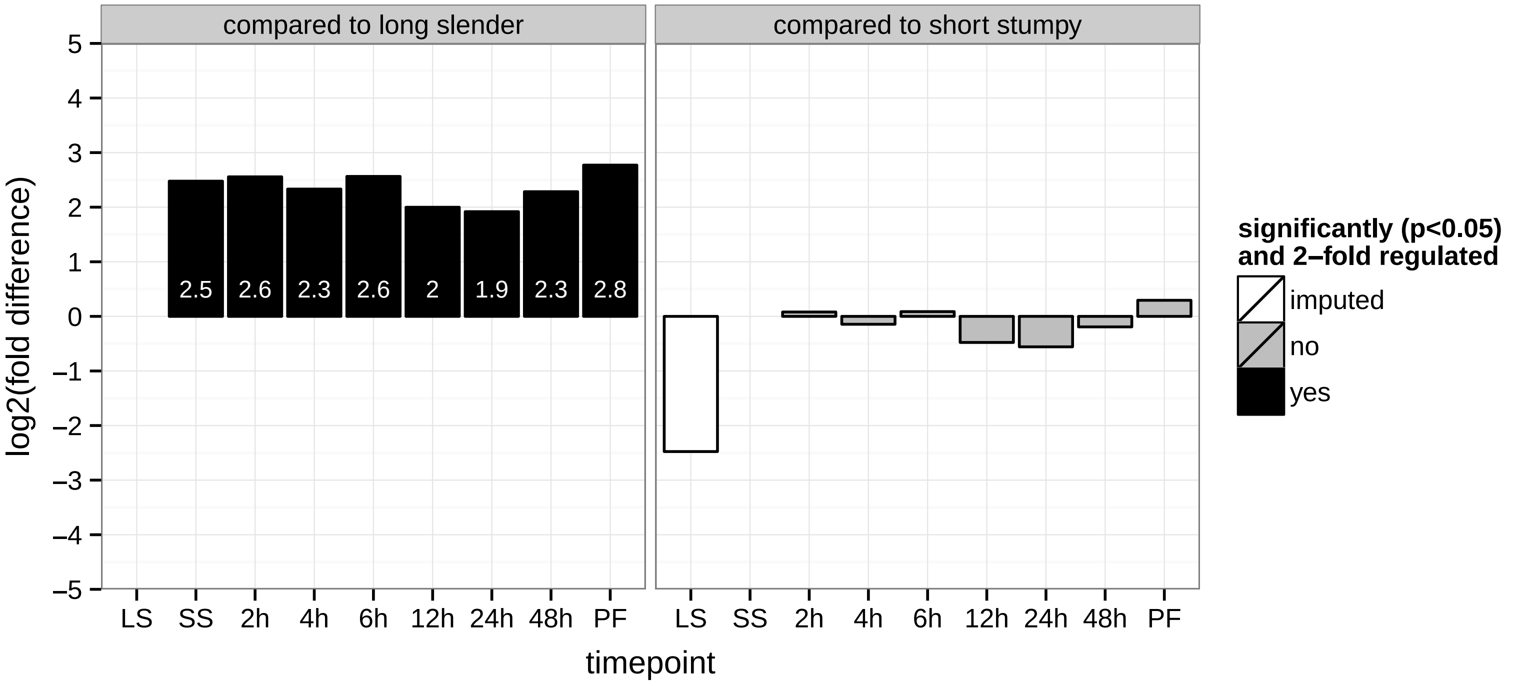

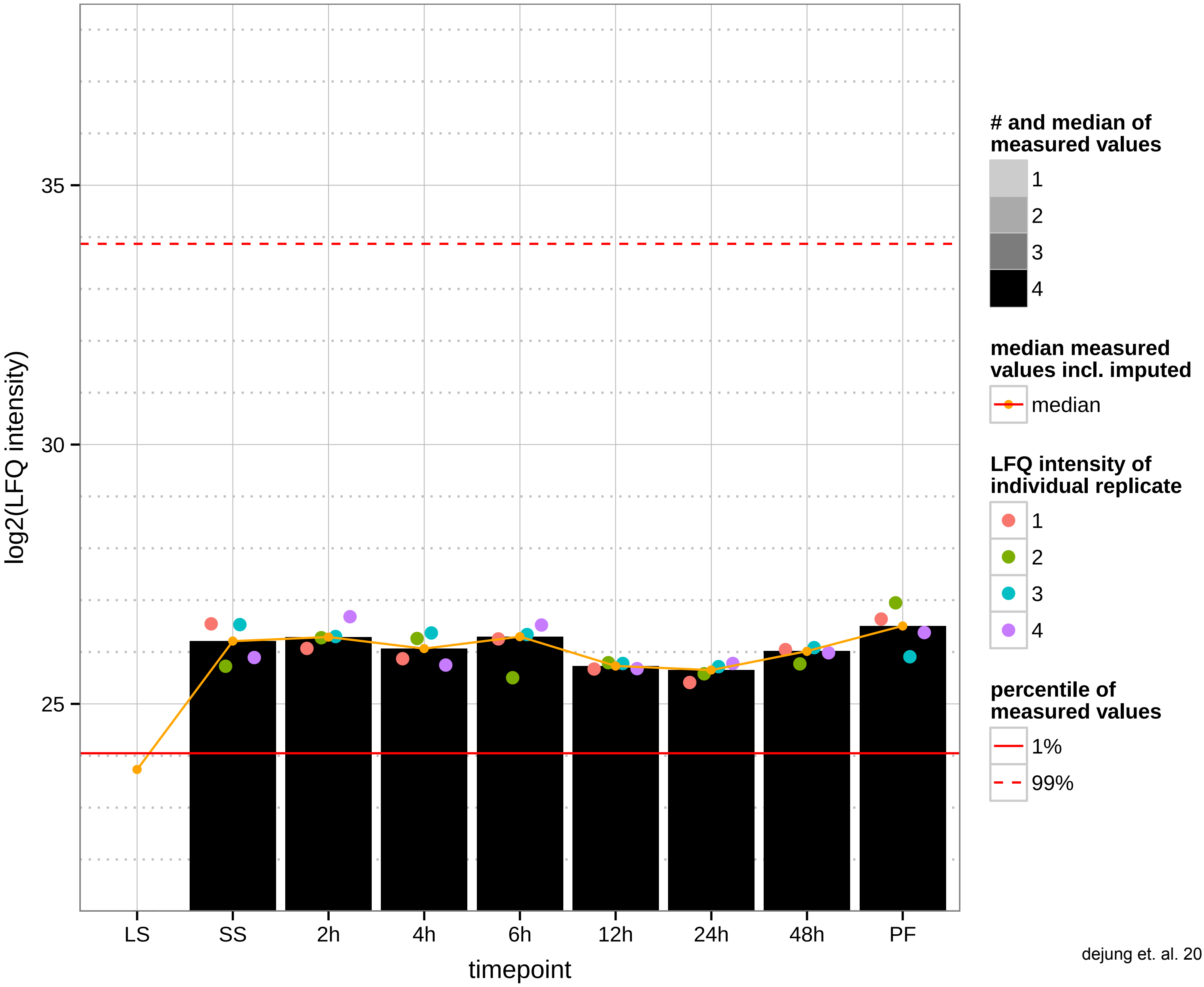

dejung et. al. 2015

FG−GAP repeat protein, putative, intergrin alpha chain protein
Tb927.11.11720
AGOF: null
AGOC: integral to membrane, integrin complex
AGOP: cell−matrix adhesion
PGOF: null
PGOC: null
PGOP: null

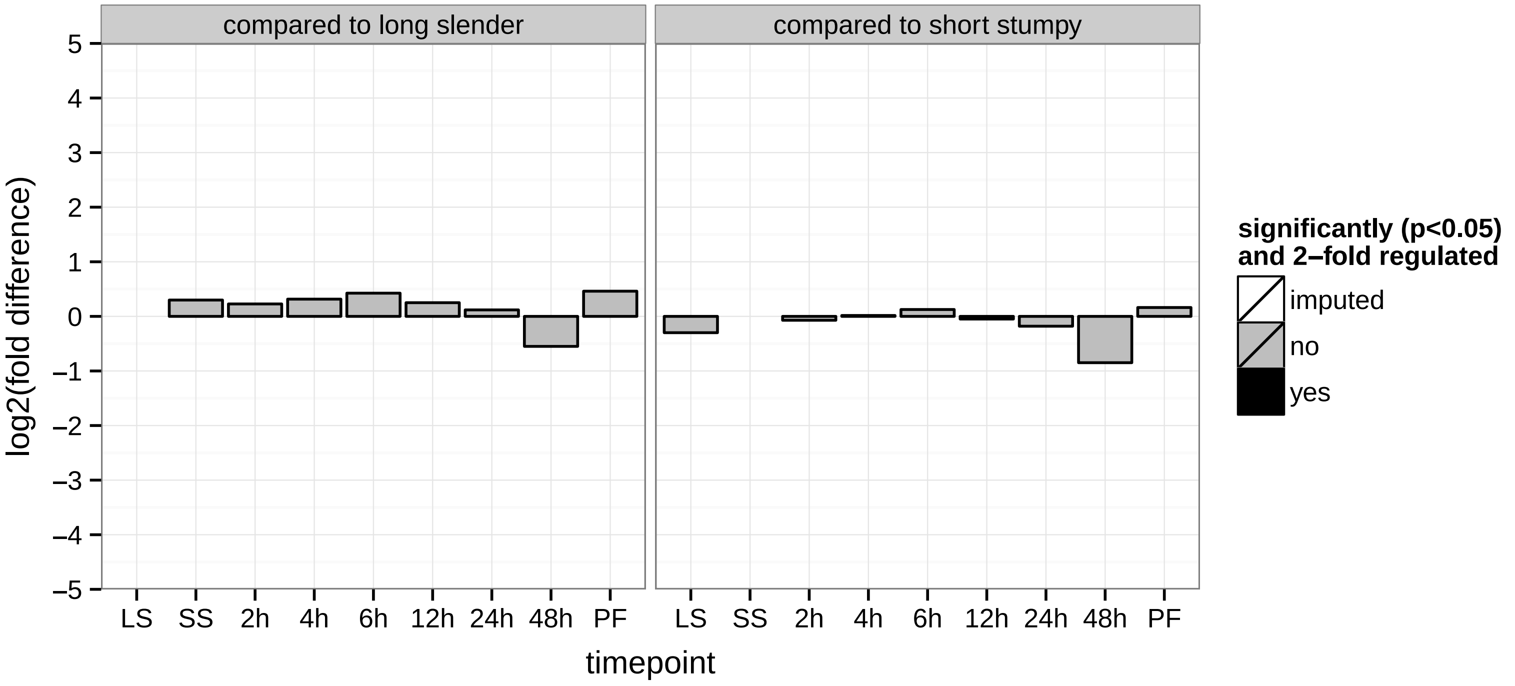

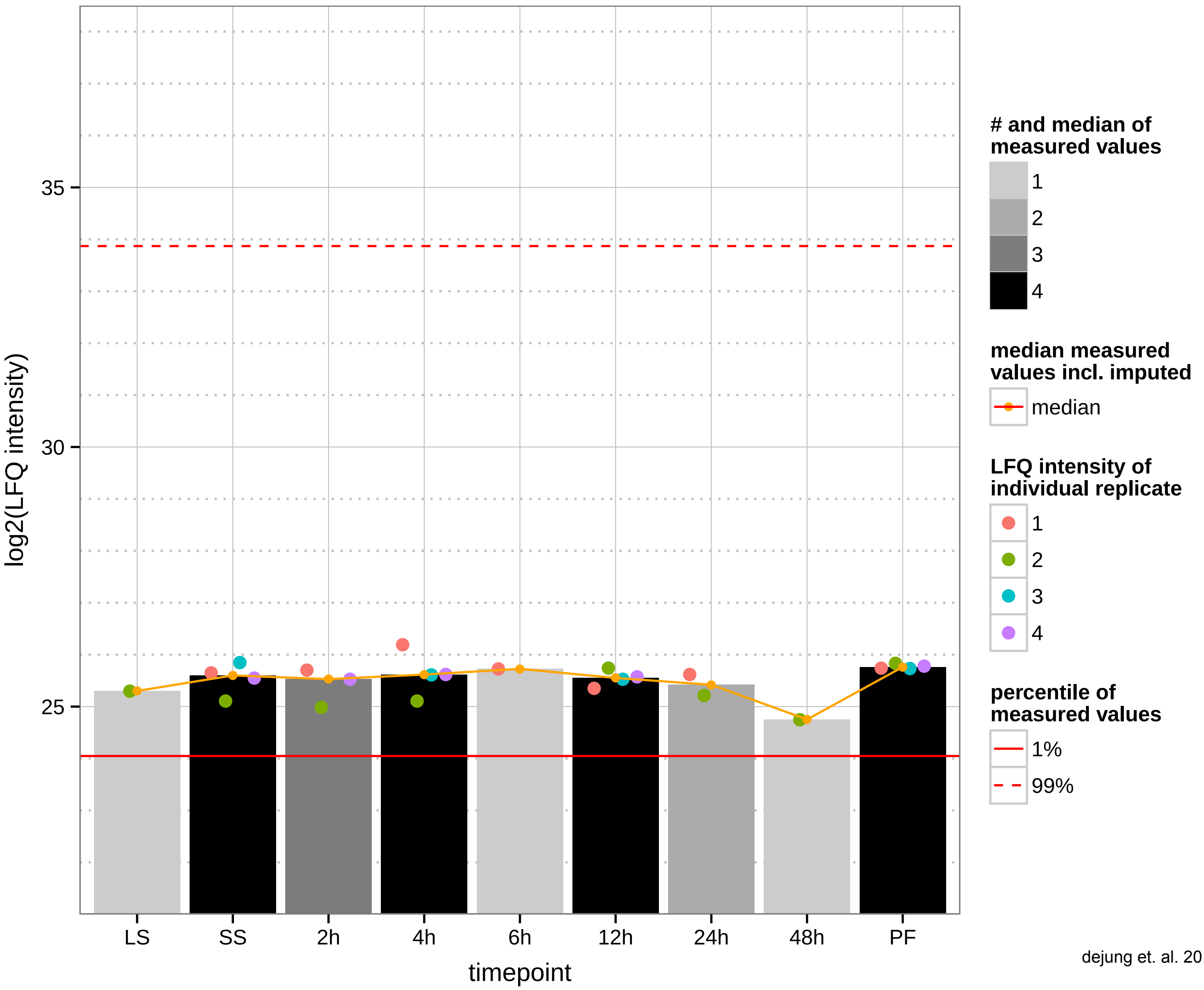

dejung et. al. 2015

MRP protein, putative
Tb927.11.11730
AGOF: nucleotide binding
AGOC: null
AGOP: null
PGOF: null
PGOC: null
PGOP: null

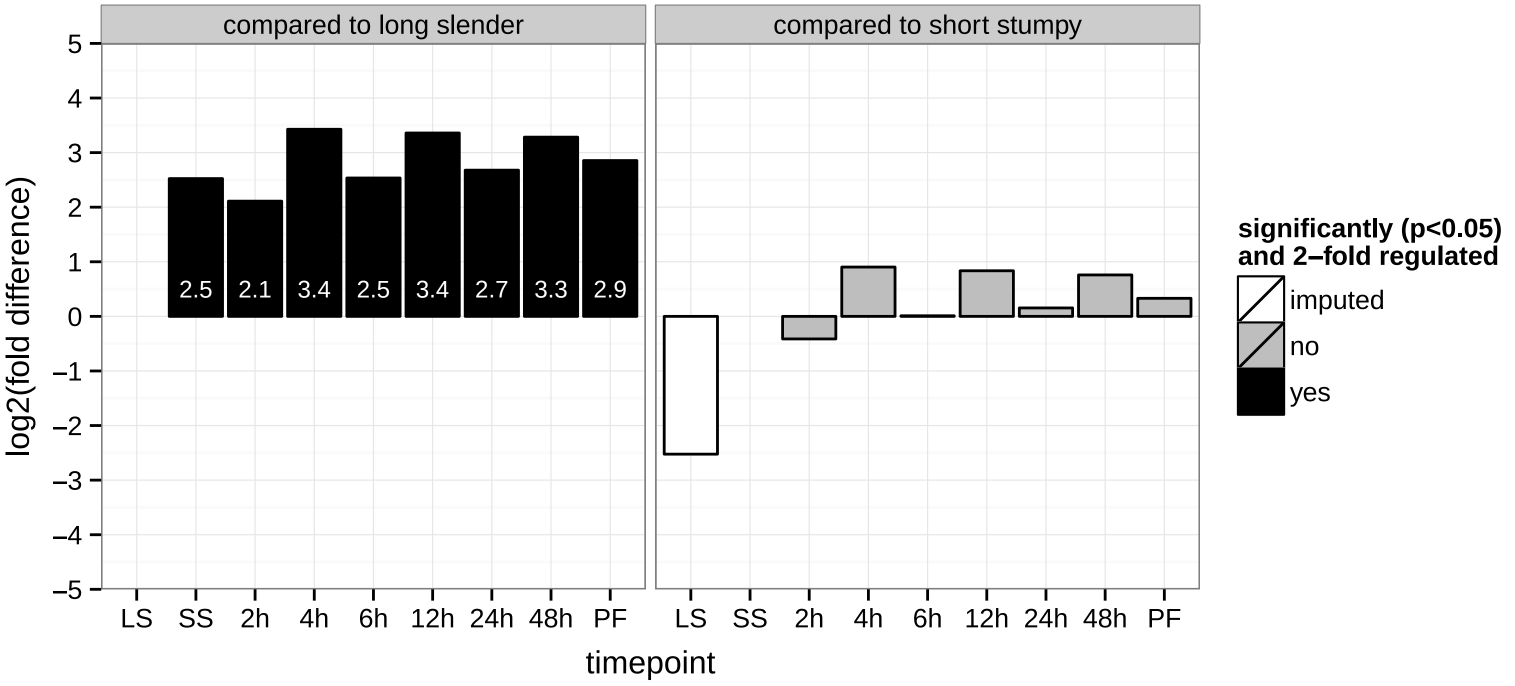

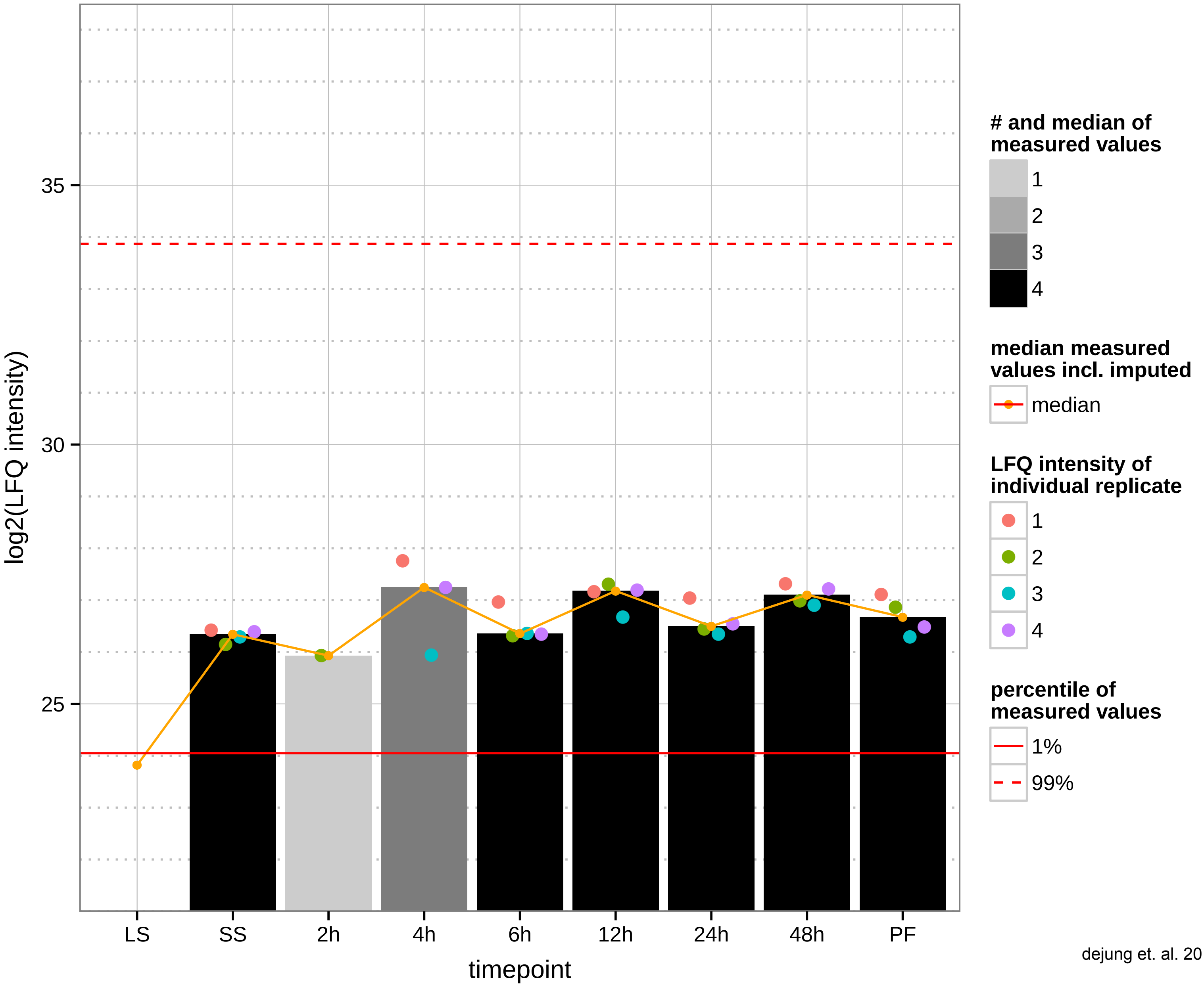

dejung et. al. 2015

NA, splicing factor 3B subunit 1, putative
Tb927.11.11850
AGOF: NA, null
AGOC: NA, null
AGOP: NA, null
PGOF: NA, binding, protein binding
PGOC: NA, null
PGOP: NA, null

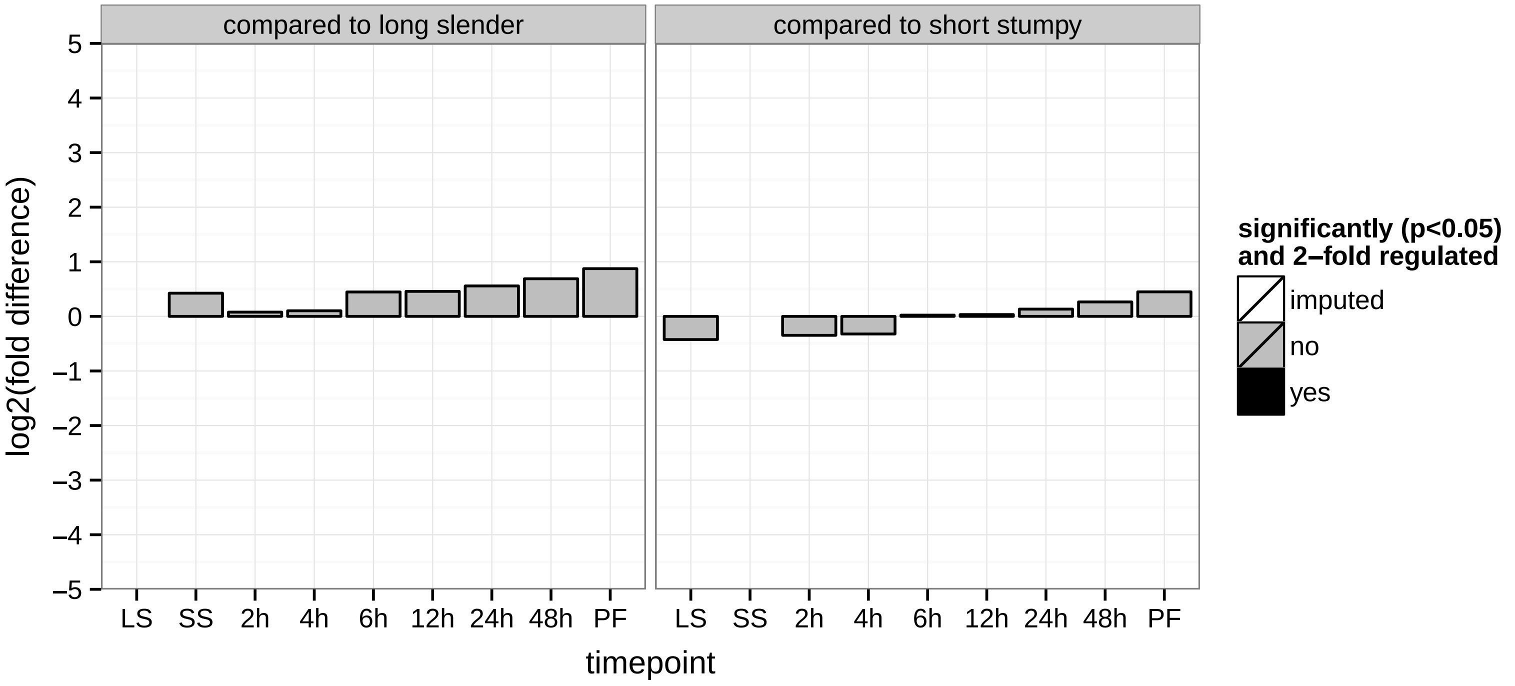

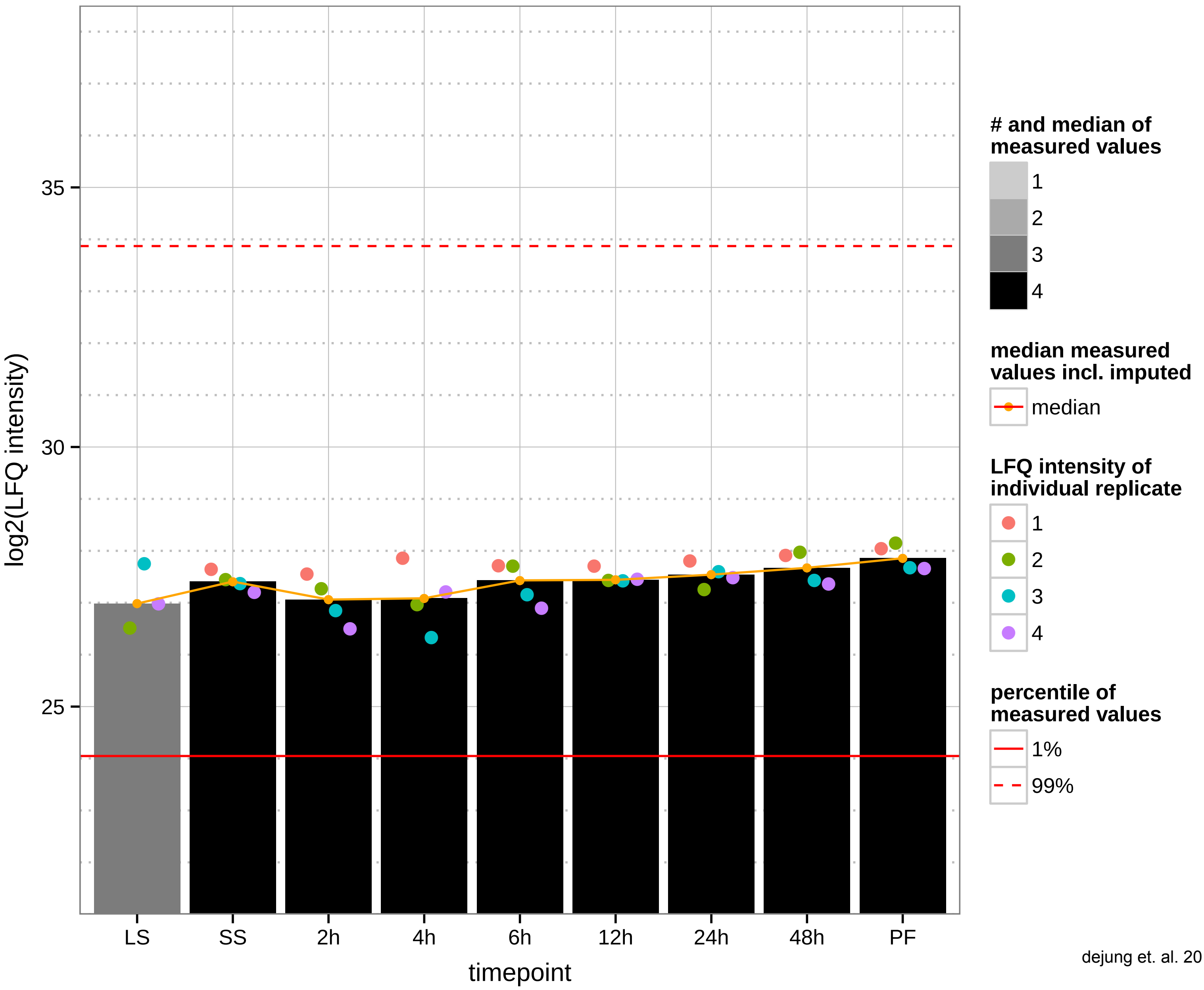

dejung et. al. 2015

hypothetical protein, conserved
Tb927.11.11870
AGOF: null
AGOC: mitochondrion
AGOP: null
PGOF: null
PGOC: null
PGOP: null

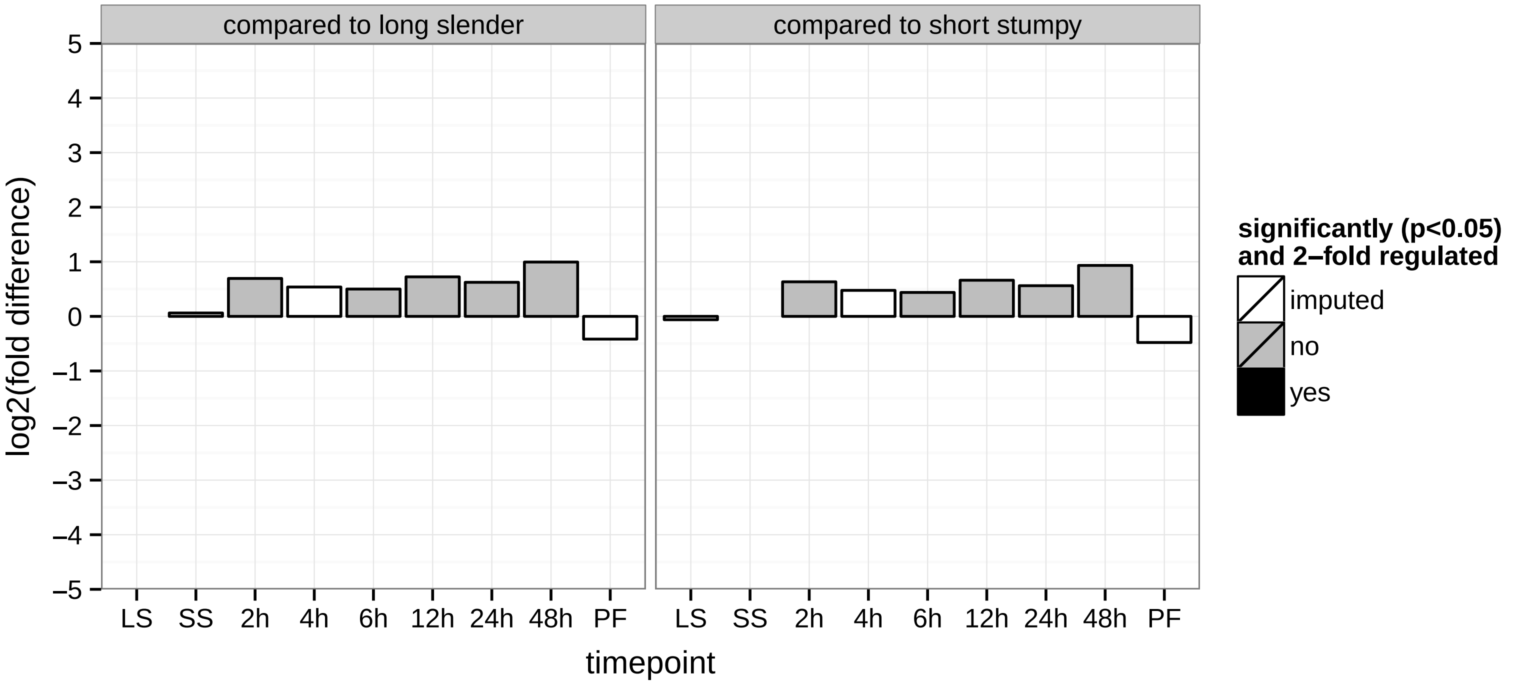

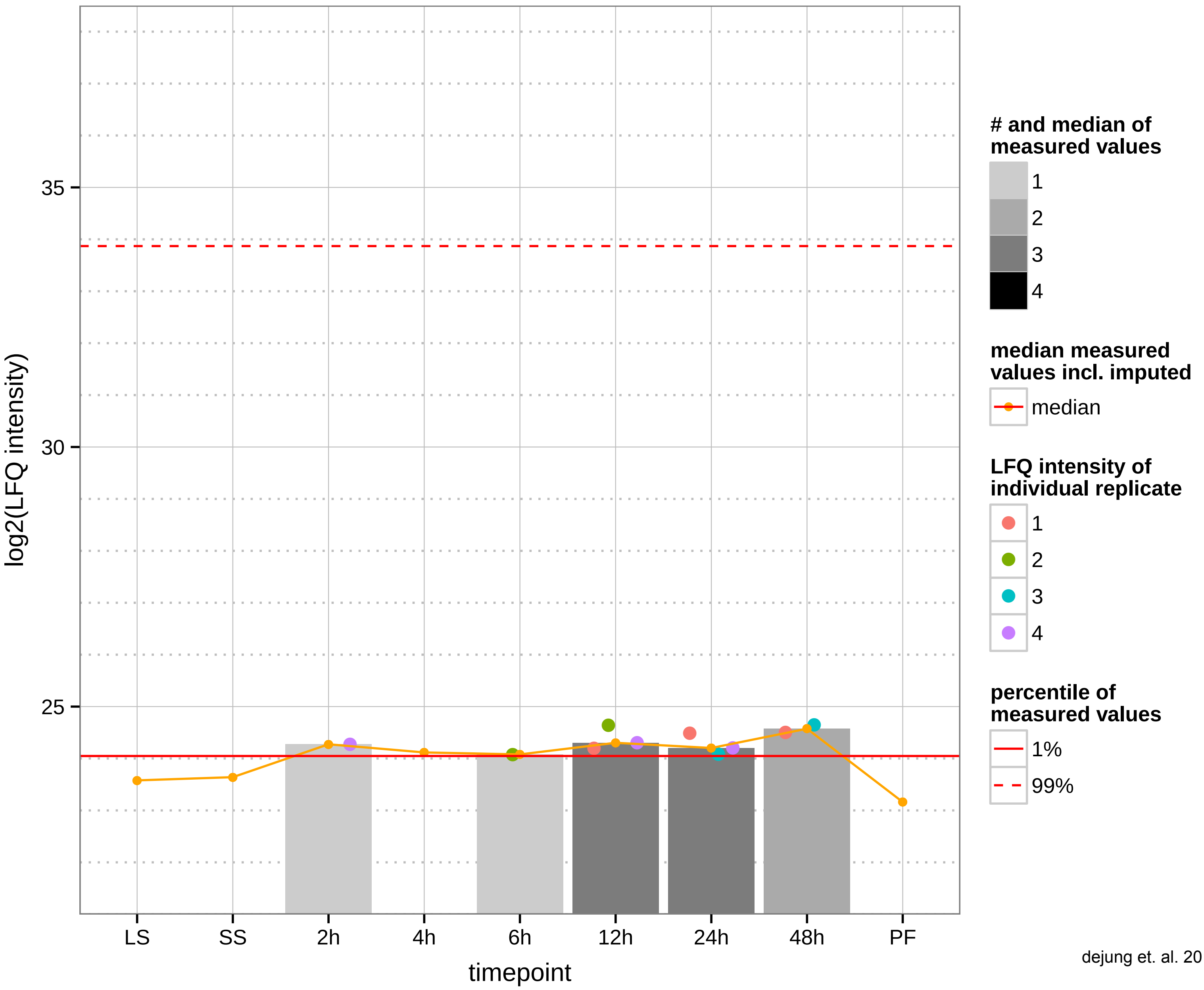

dejung et. al. 2015

conserved protein
Tb927.11.11880
AGOF: null
AGOC: null
AGOP: null
PGOF: protein binding
PGOC: null
PGOP: null

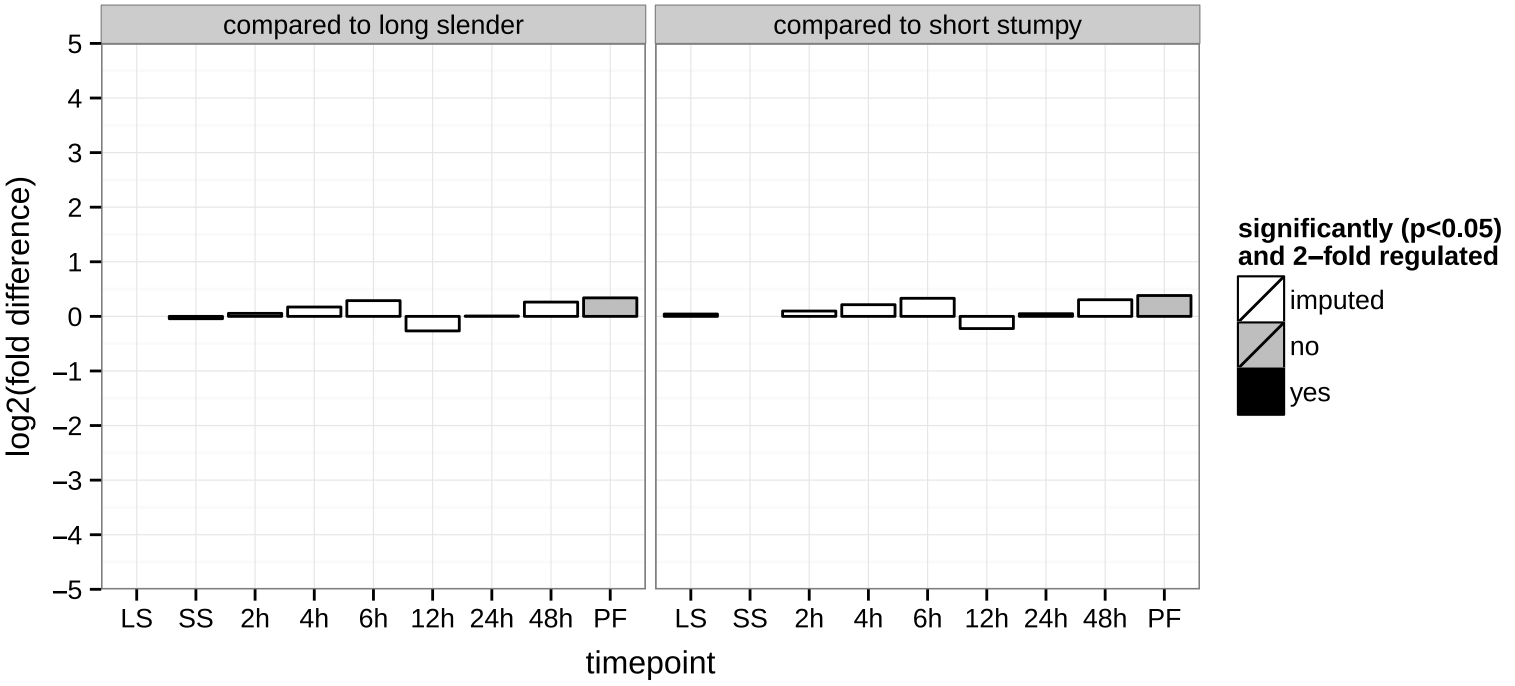

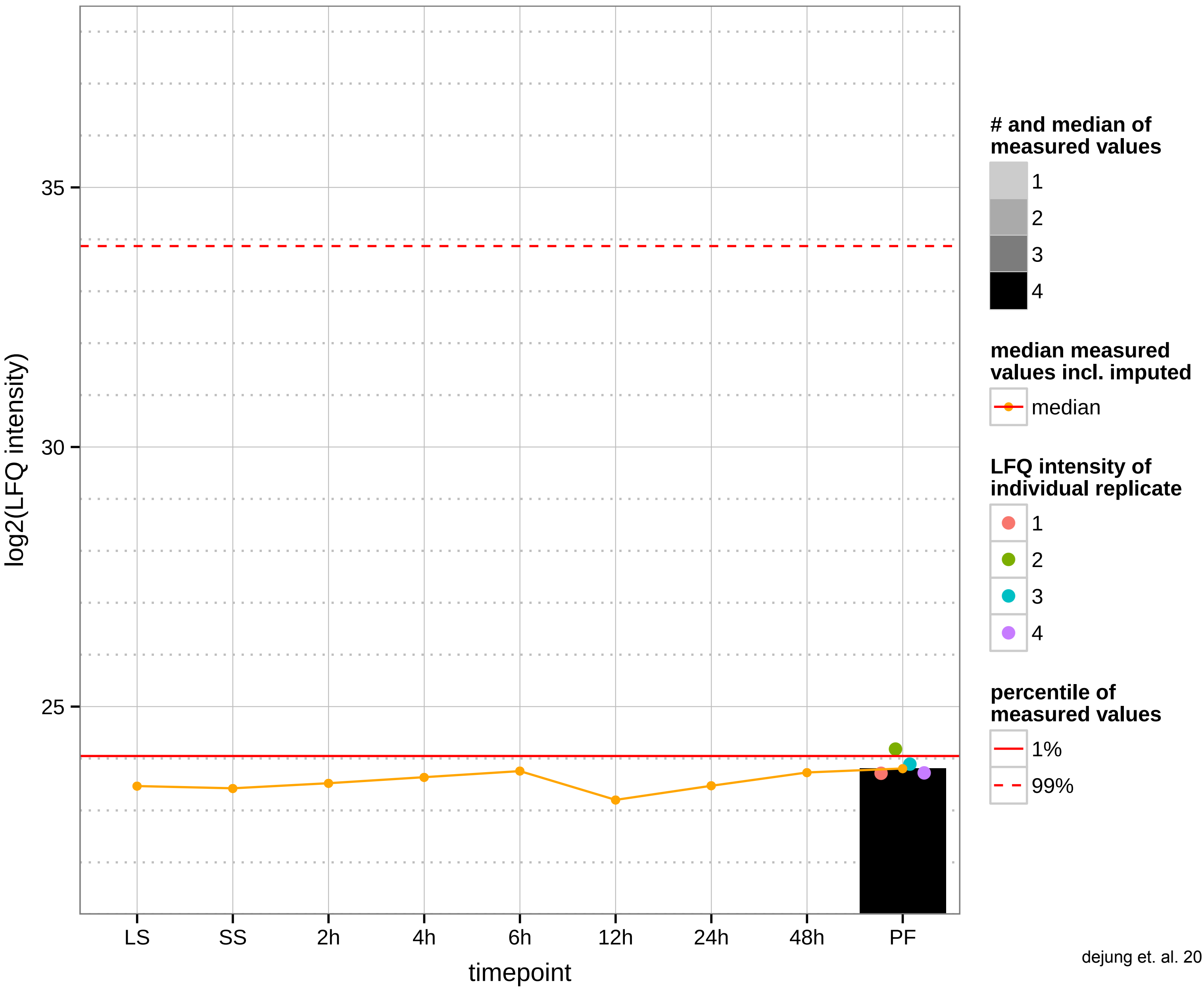

dejung et. al. 2015

coatomer gamma subunit, putative, cytosolic coat protein, putative (TbCoatomerGamma)
Tb927.11.11900
AGOF: structural molecule activity
AGOC: COPI vesicle coat, Golgi–associated vesicle
AGOP: intracellular protein transport, retrograde vesicle–mediated transport, Golgi to ER
PGOF: binding, structural molecule activity
PGOC: Golgi–associated vesicle, membrane coat
PGOP: intracellular protein transport, vesicle–mediated transport

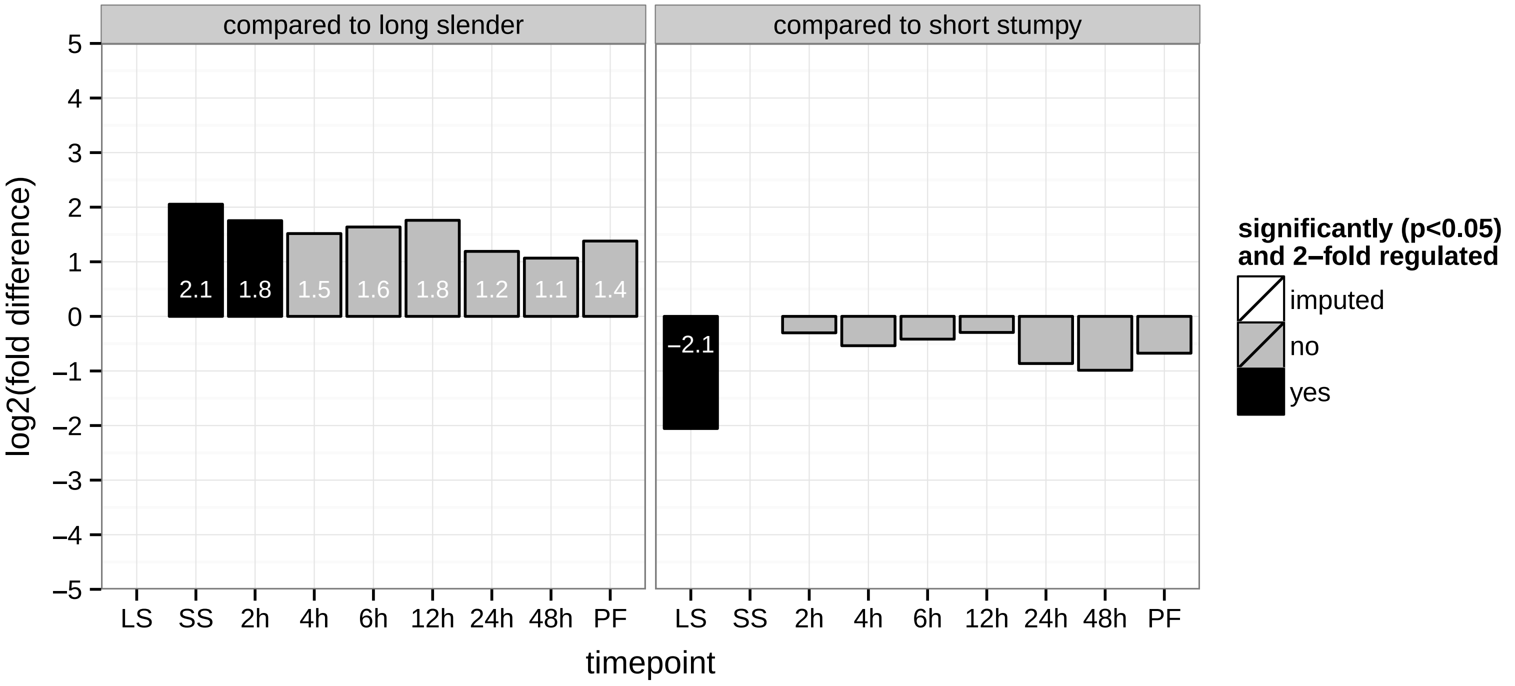

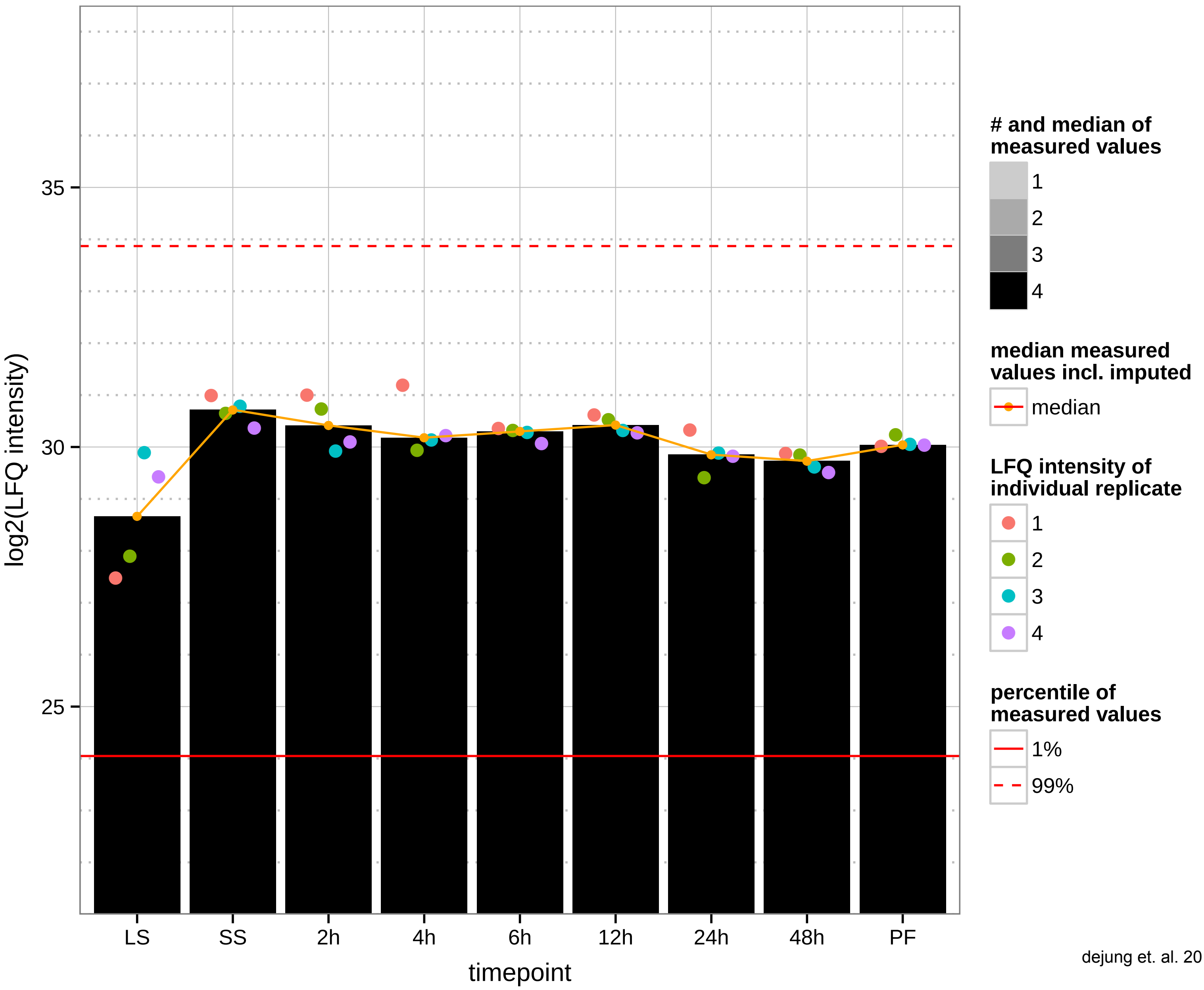

dejung et. al. 2015

hypothetical protein, conserved
Tb927.11.1210
AGOF: null
AGOC: null
AGOP: null
PGOF: null
PGOC: null
PGOP: null

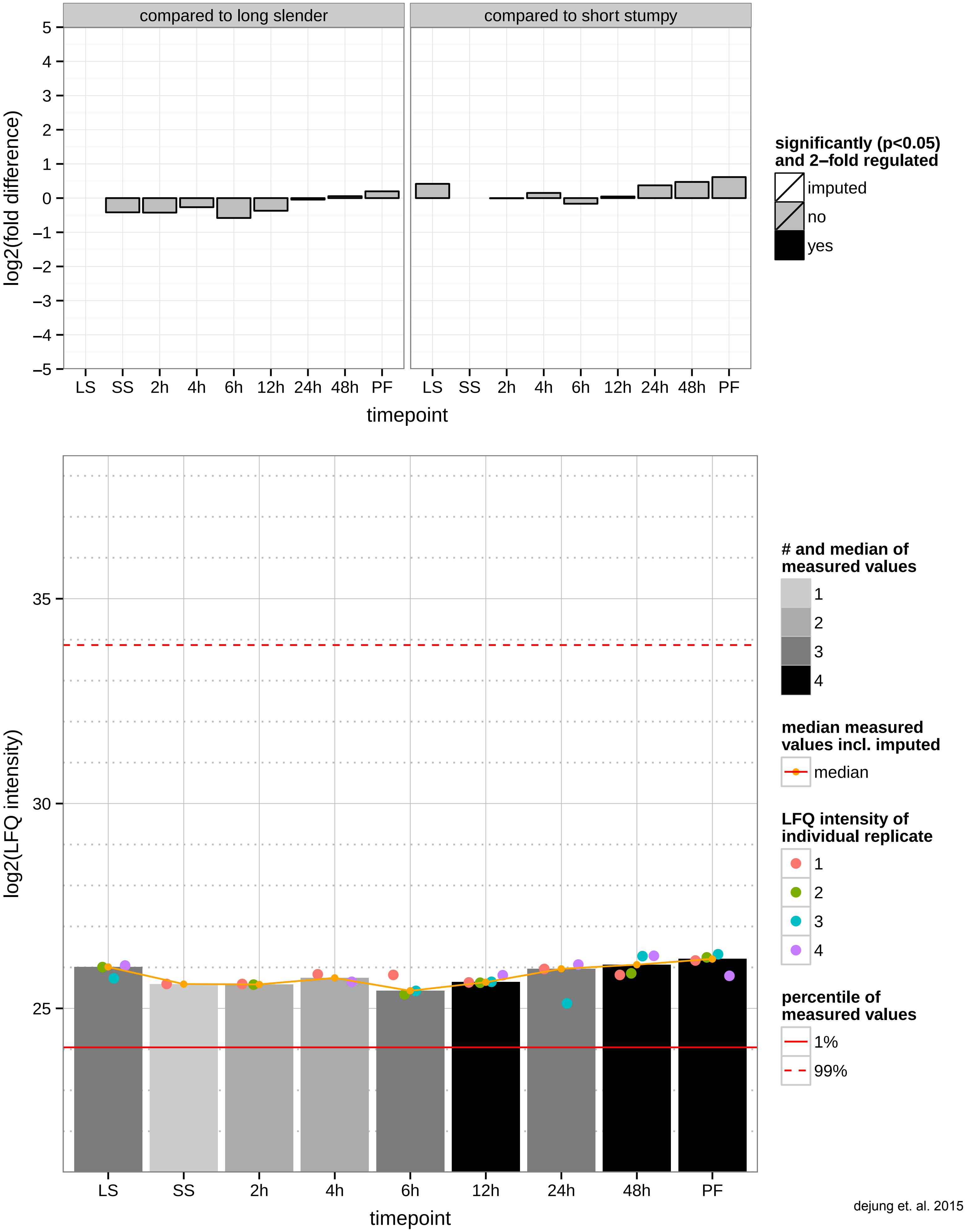

dejung et. al. 2015

hypothetical protein, conserved
Tb927.11.12210
AGOF: null
AGOC: null
AGOP: null
PGOF: null
PGOC: null
PGOP: null

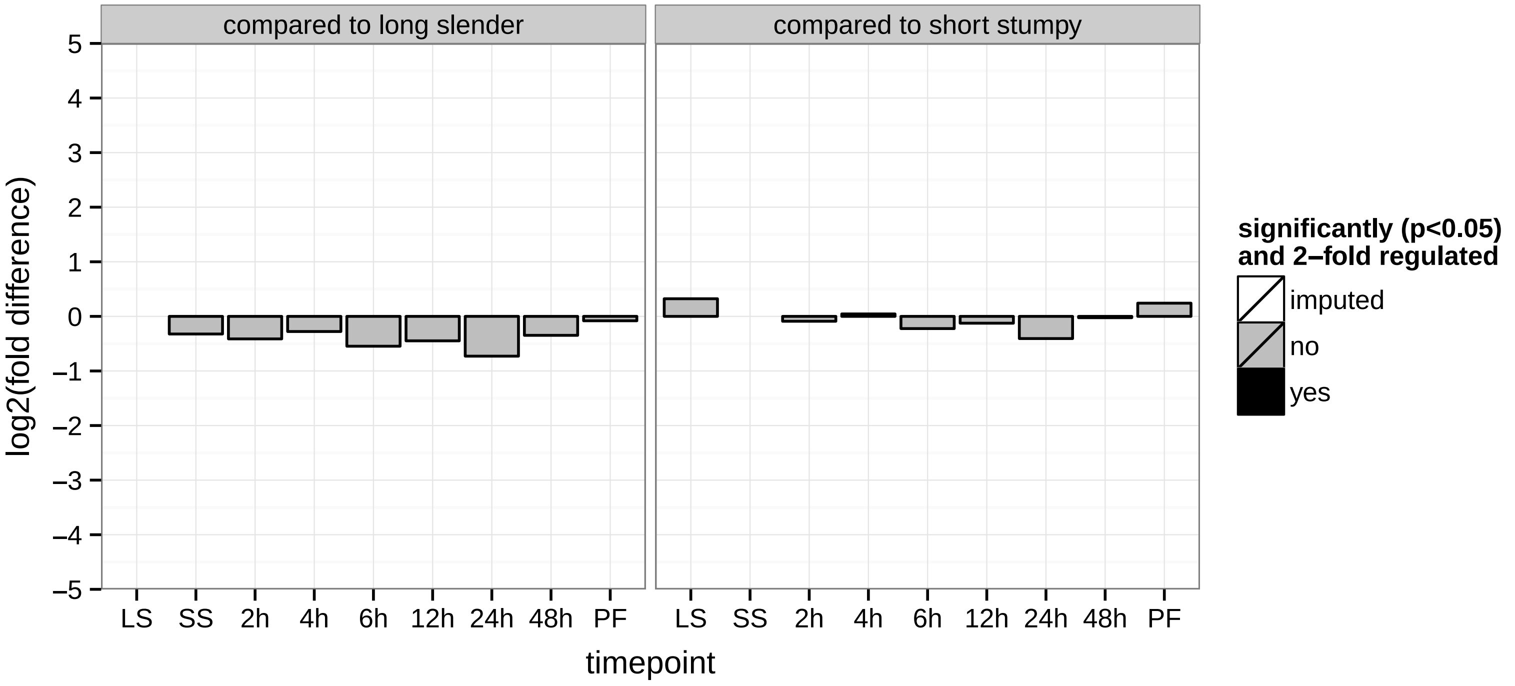

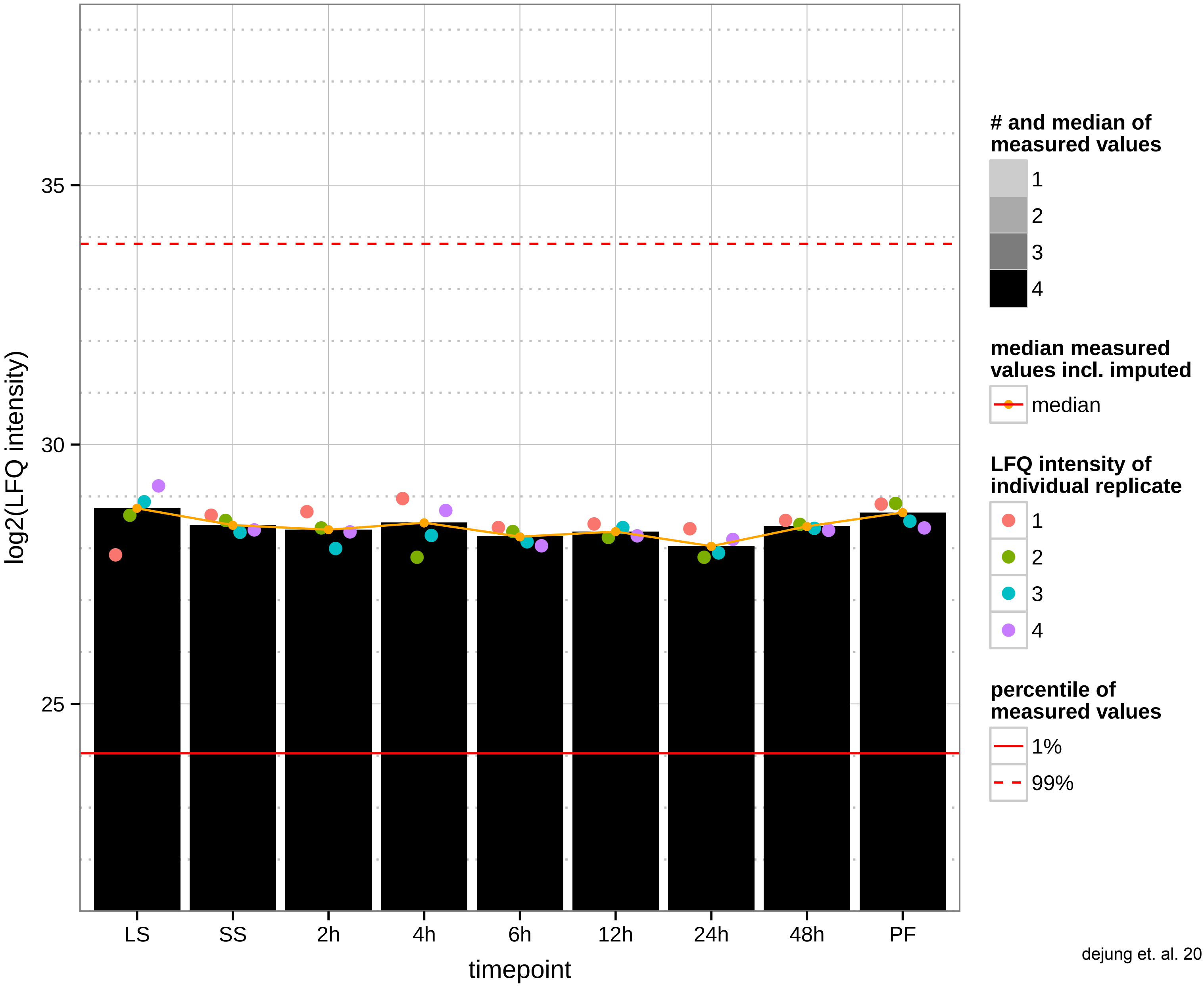

dejung et. al. 2015

catalytic subunit of the vacuolar transporter chaperone 4
Tb927.11.12220
AGOF: metal ion binding
AGOC: vacuolar transporter chaperone complex
AGOP: polyphosphate biosynthetic process
PGOF: null
PGOC: null
PGOP: null

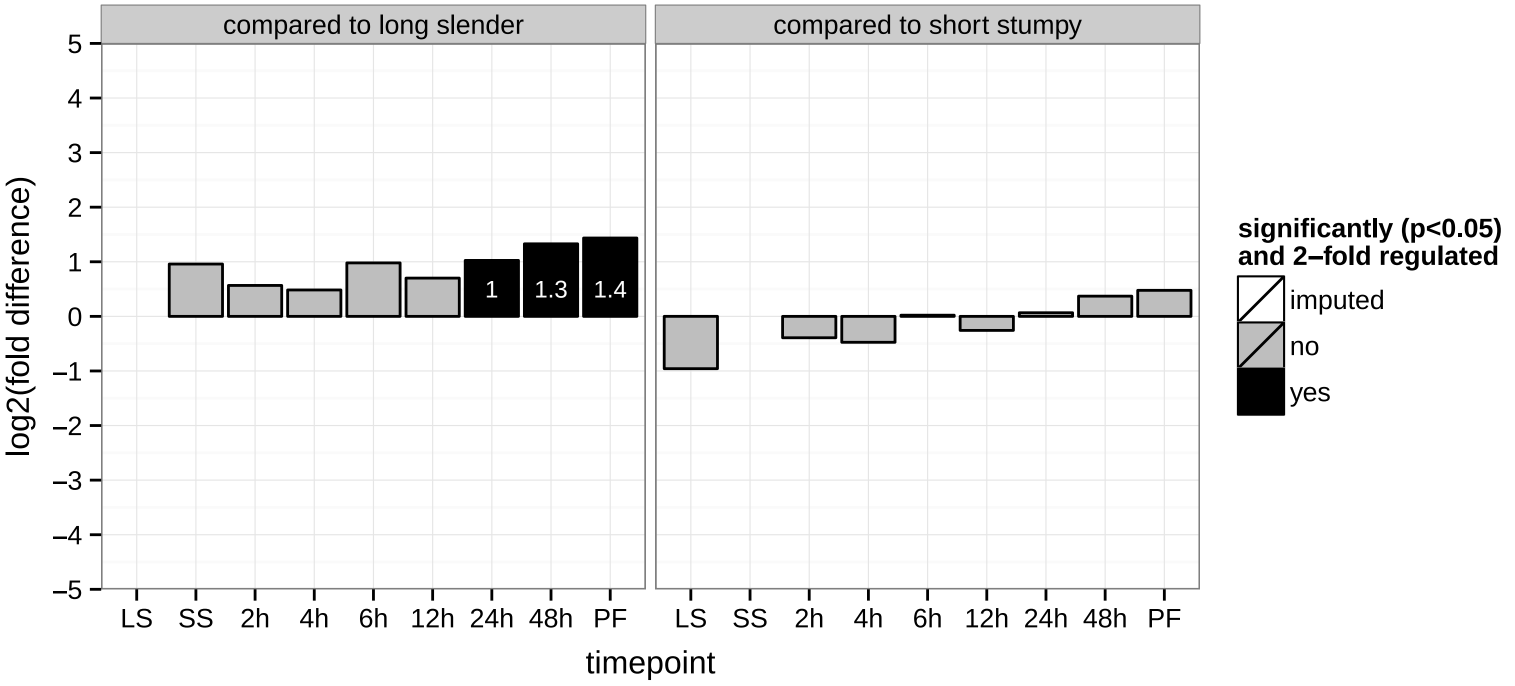

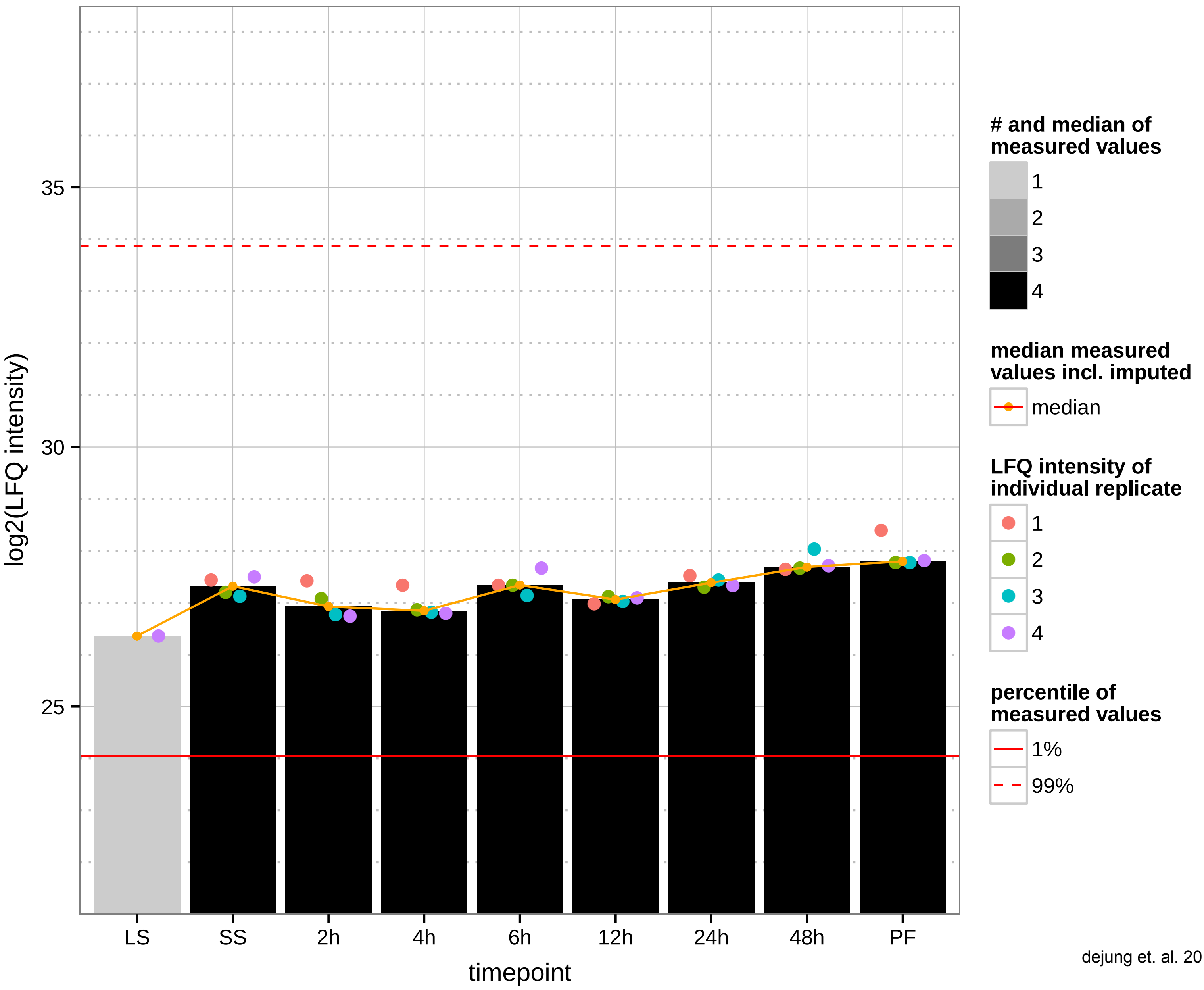

dejung et. al. 2015

hypothetical protein, conserved
Tb927.11.12260
AGOF: null
AGOC: null
AGOP: null
PGOF: null
PGOC: null
PGOP: null

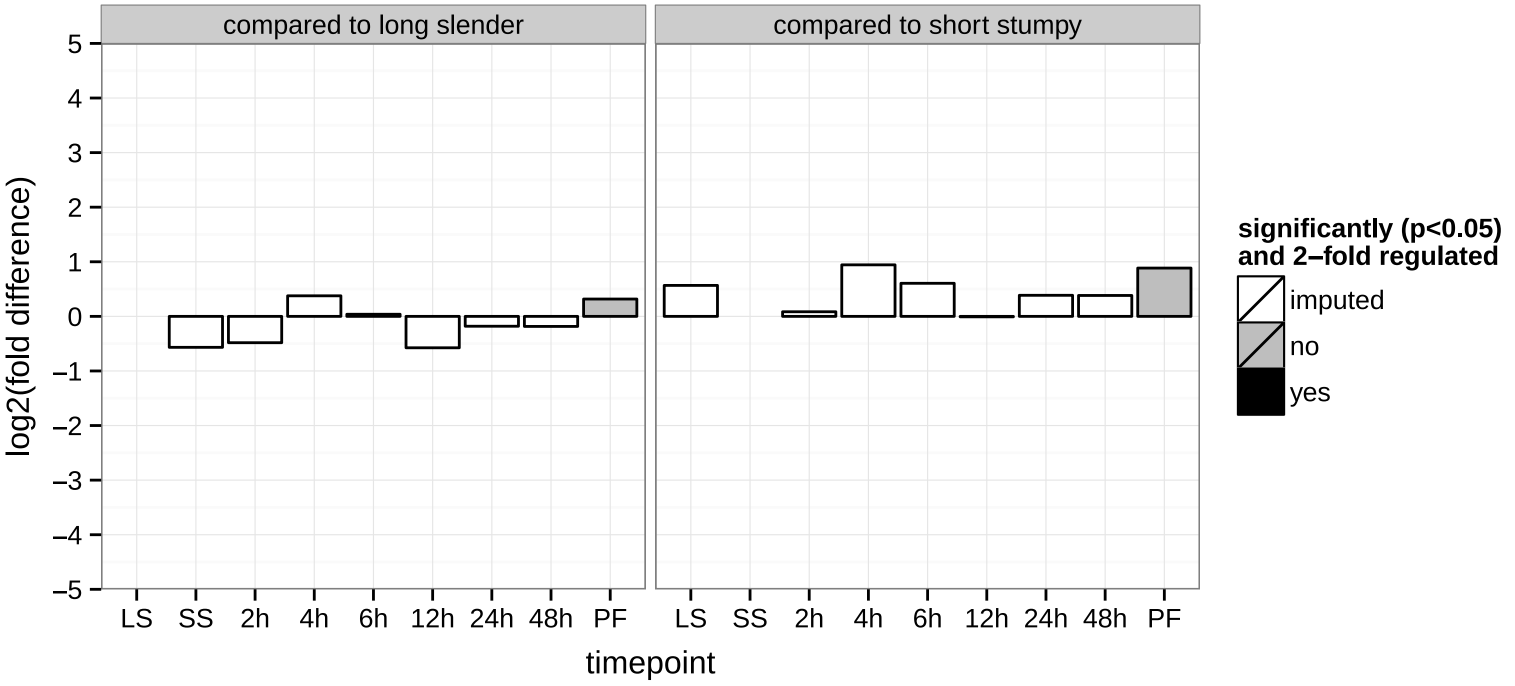

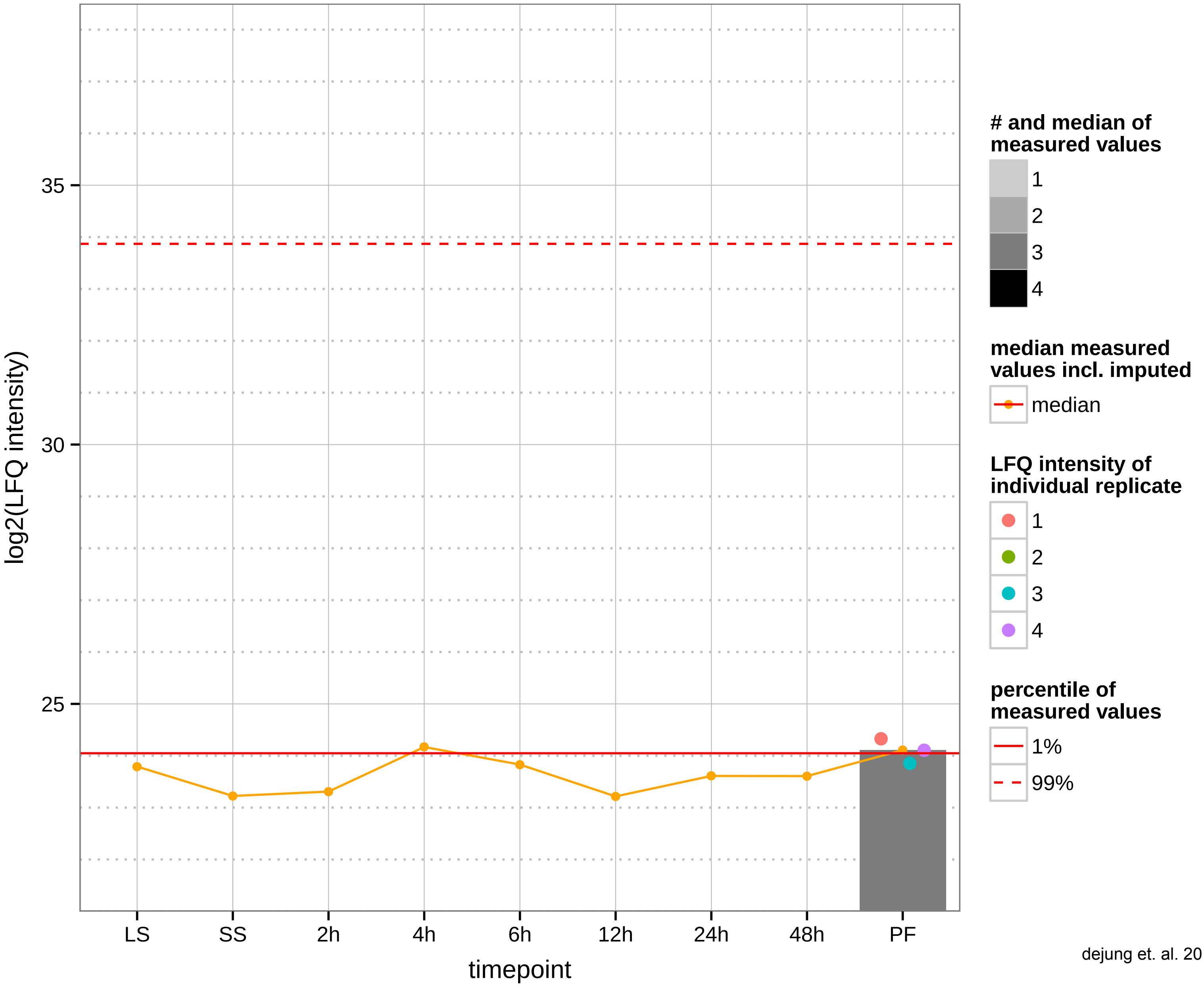

dejung et. al. 2015

hypothetical protein, conserved
Tb927.11.12380
AGOF: null
AGOC: null
AGOP: null
PGOF: null
PGOC: null
PGOP: null

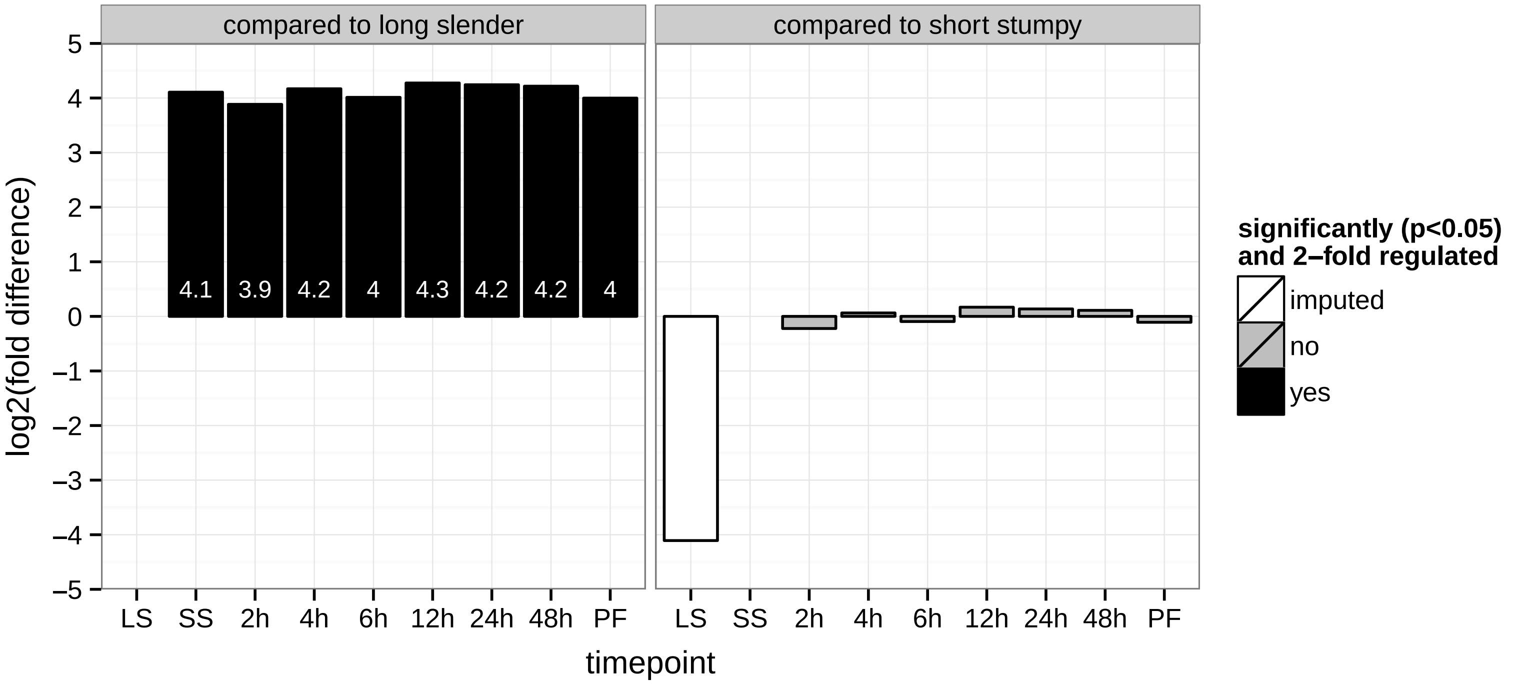

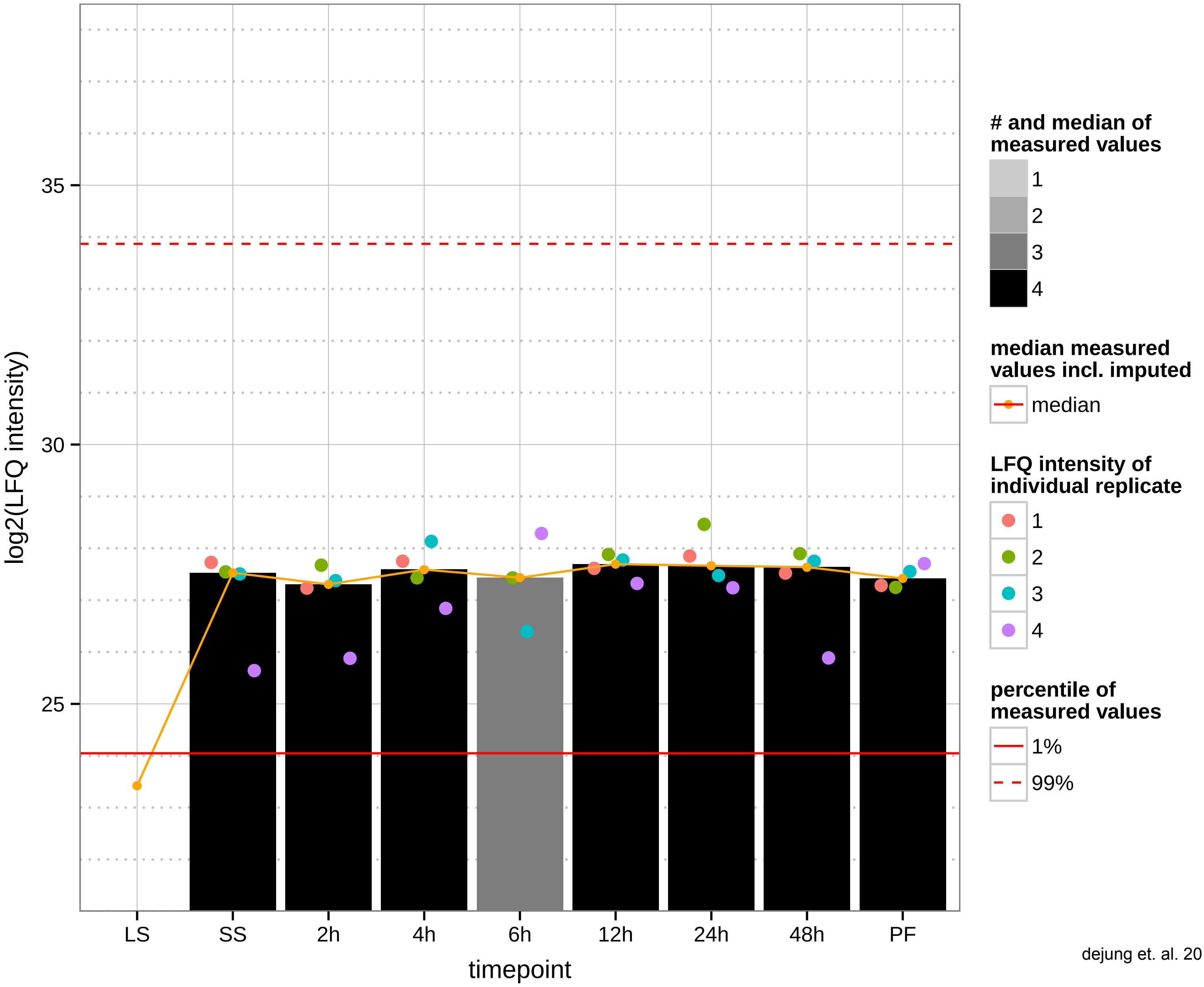

dejung et. al. 2015

hypothetical protein, conserved
Tb927.11.12470
AGOF: null
AGOC: null
AGOP: null
PGOF: null
PGOC: null
PGOP: null

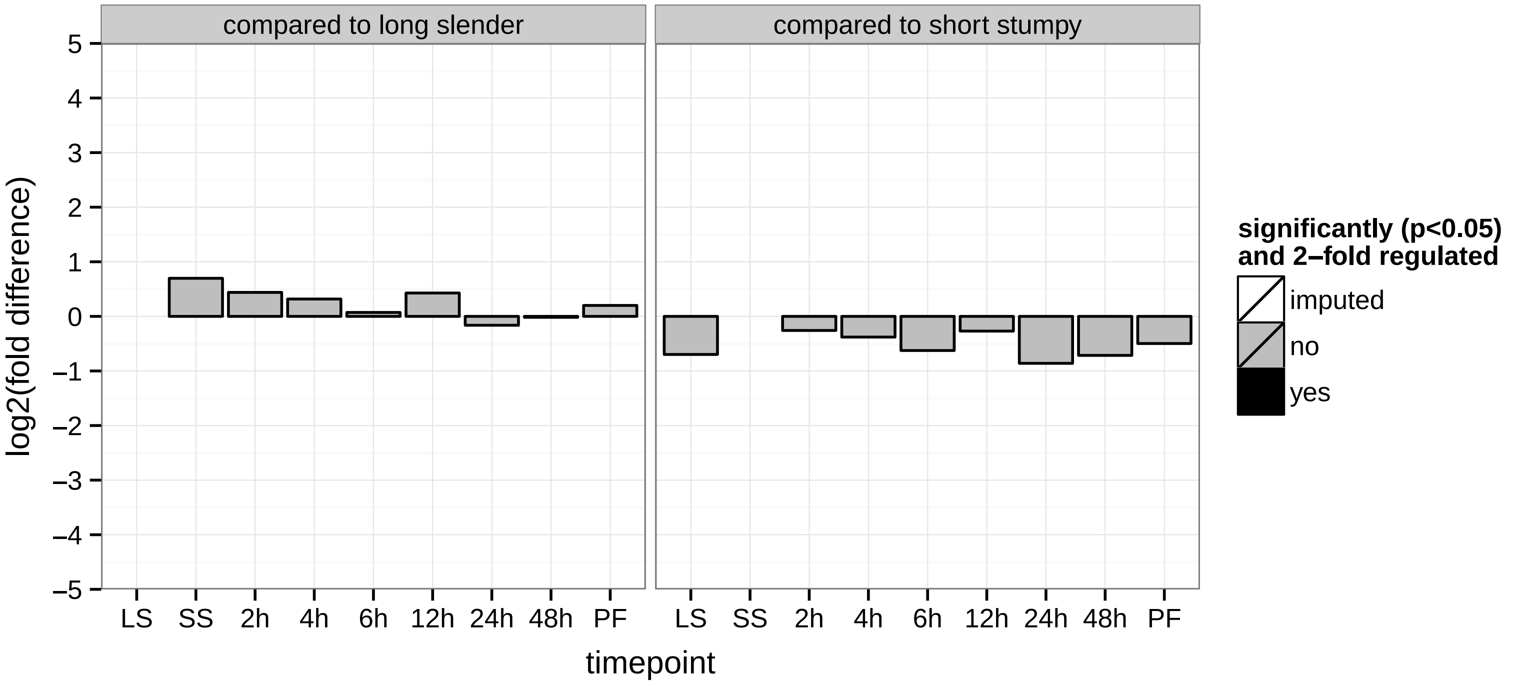

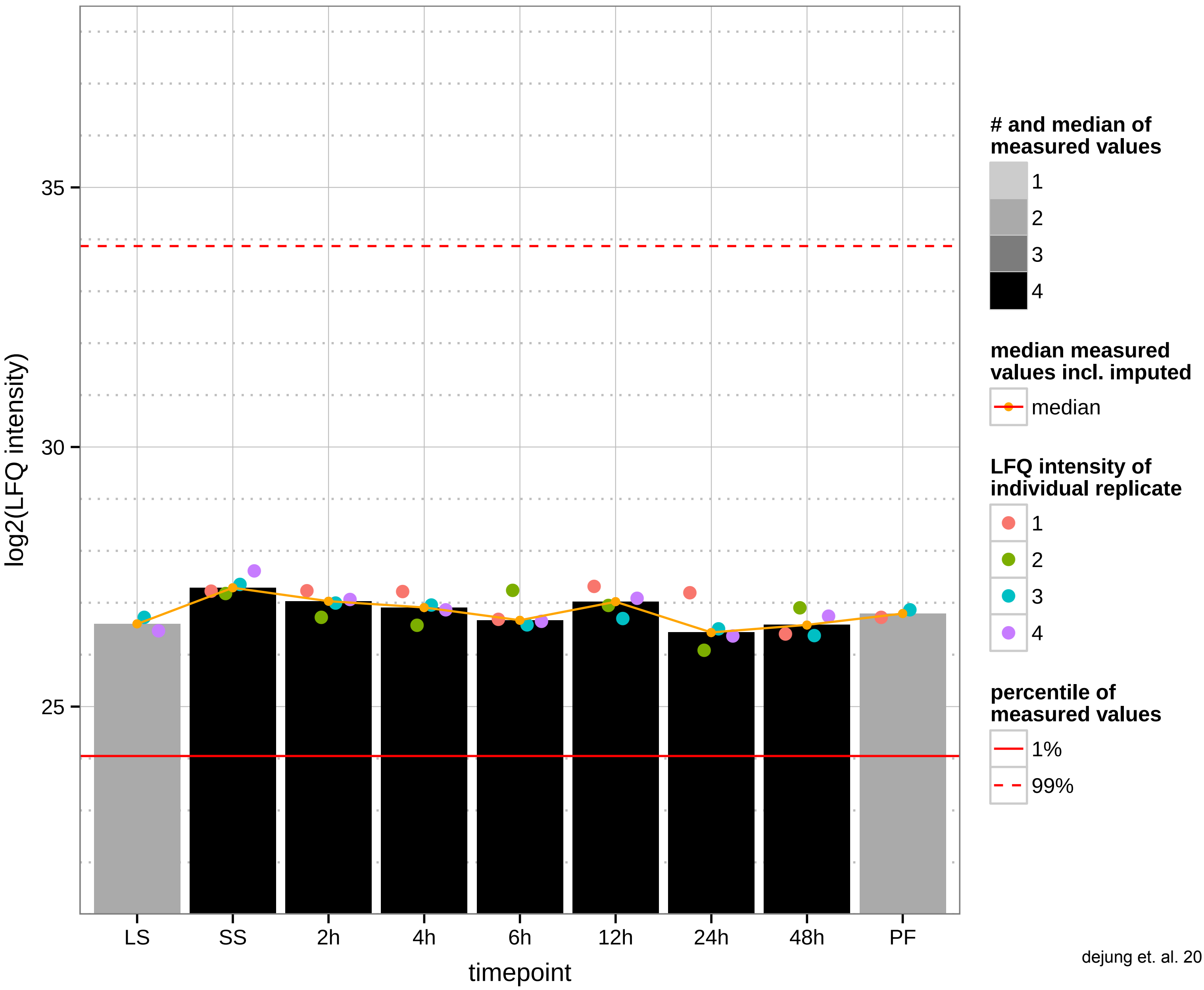

dejung et. al. 2015

kinetoplastid−specific phospho−protein phosphatase, putative
Tb927.11.12480
AGOF: hydrolase activity, protein serine/threonine phosphatase activity
AGOC: null
AGOP: protein dephosphorylation
PGOF: hydrolase activity
PGOC: null
PGOP: null

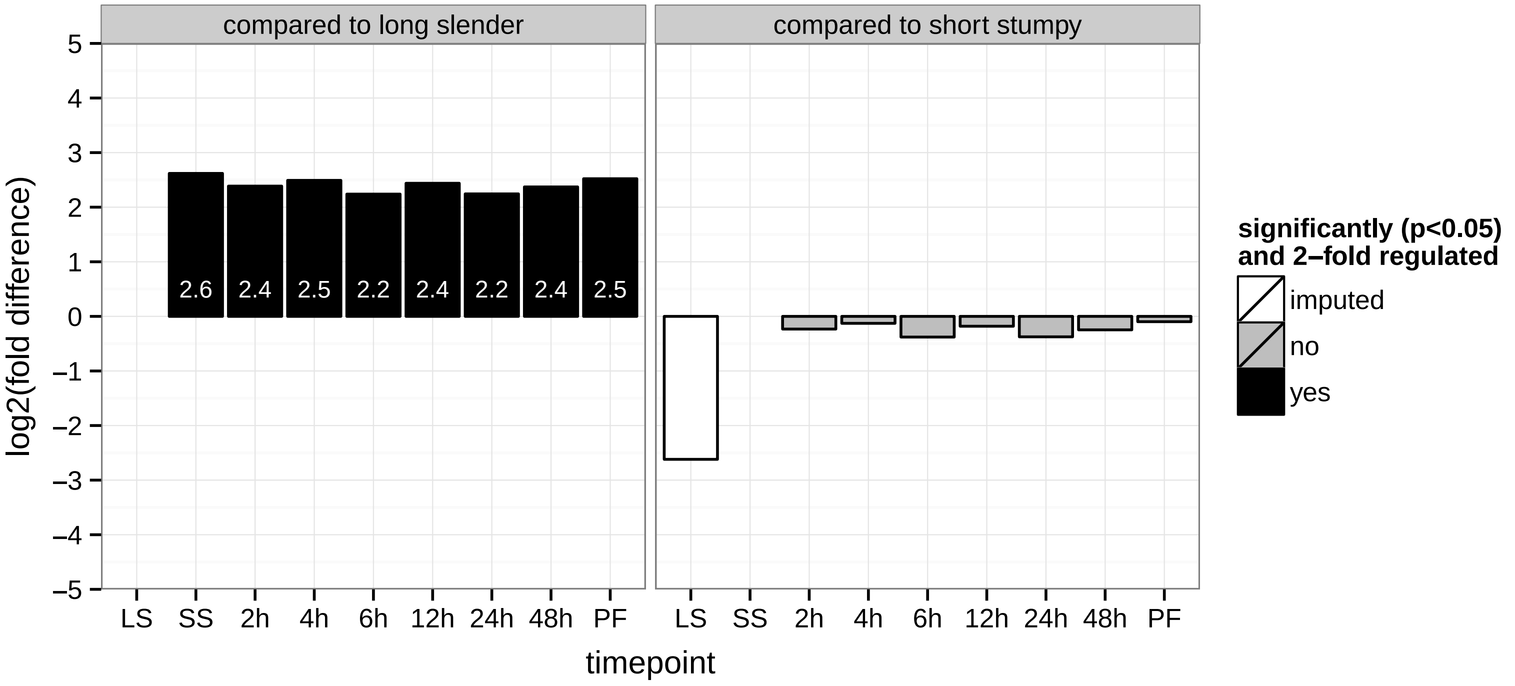

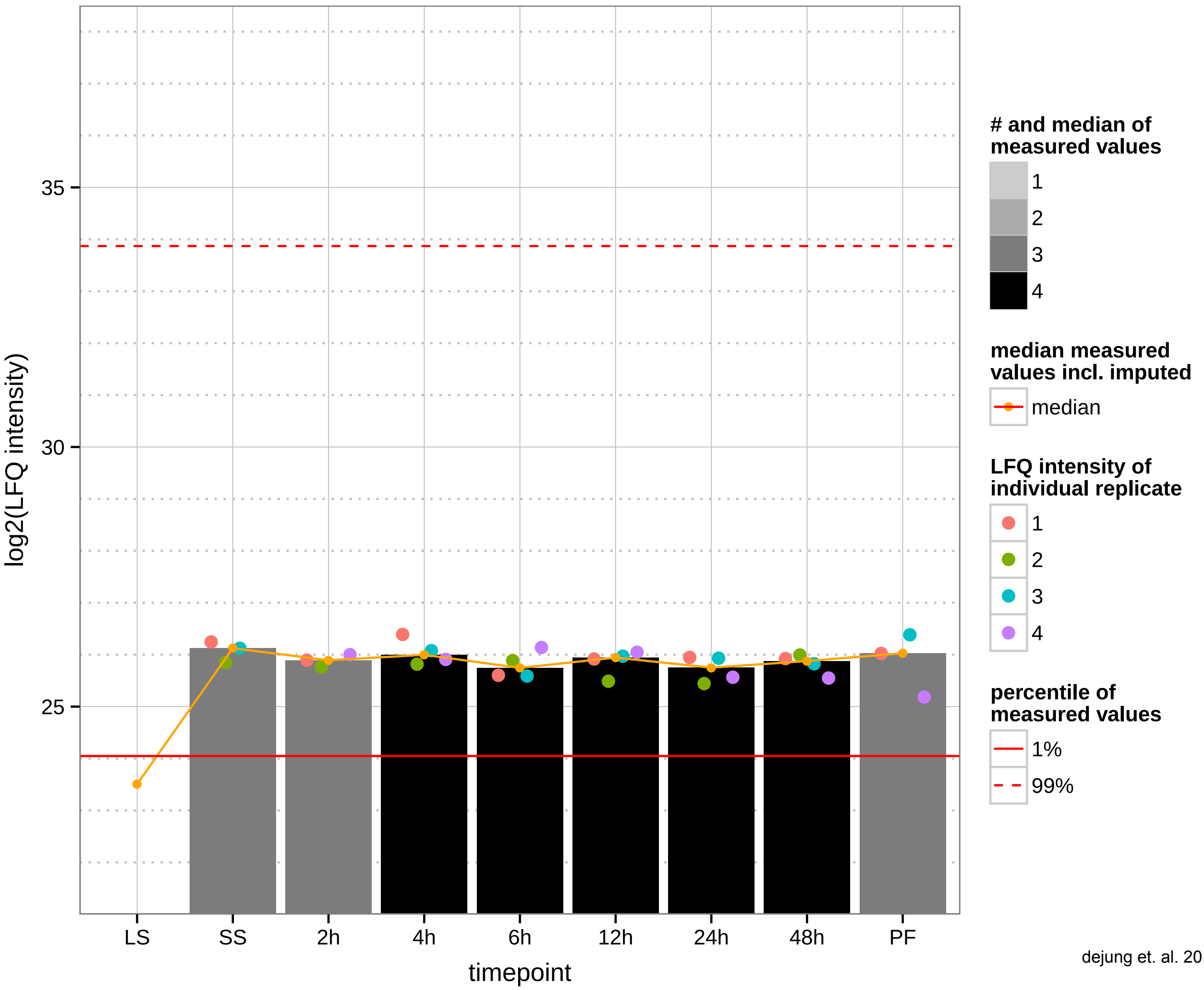

dejung et. al. 2015

hypothetical protein, conserved
Tb927.11.12490
AGOF: null
AGOC: null
AGOP: null
PGOF: null
PGOC: null
PGOP: null

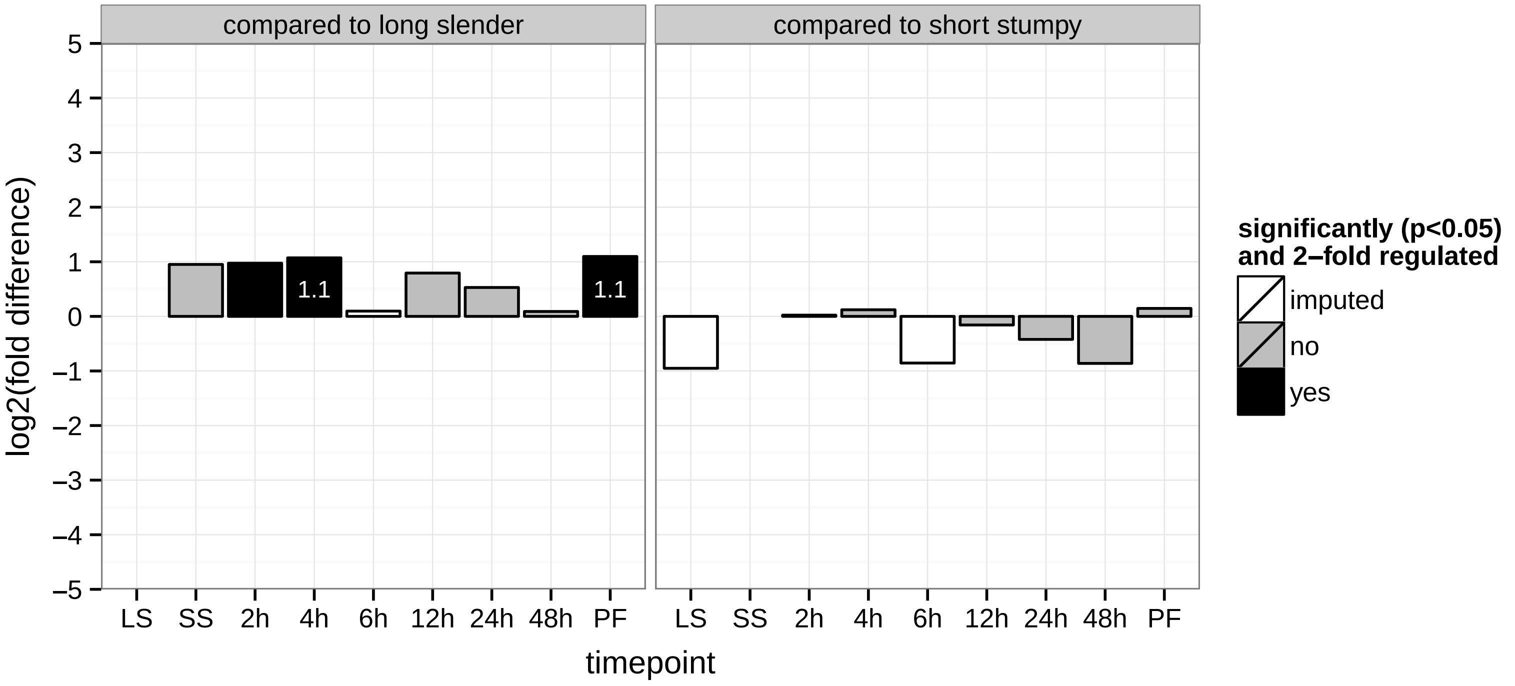

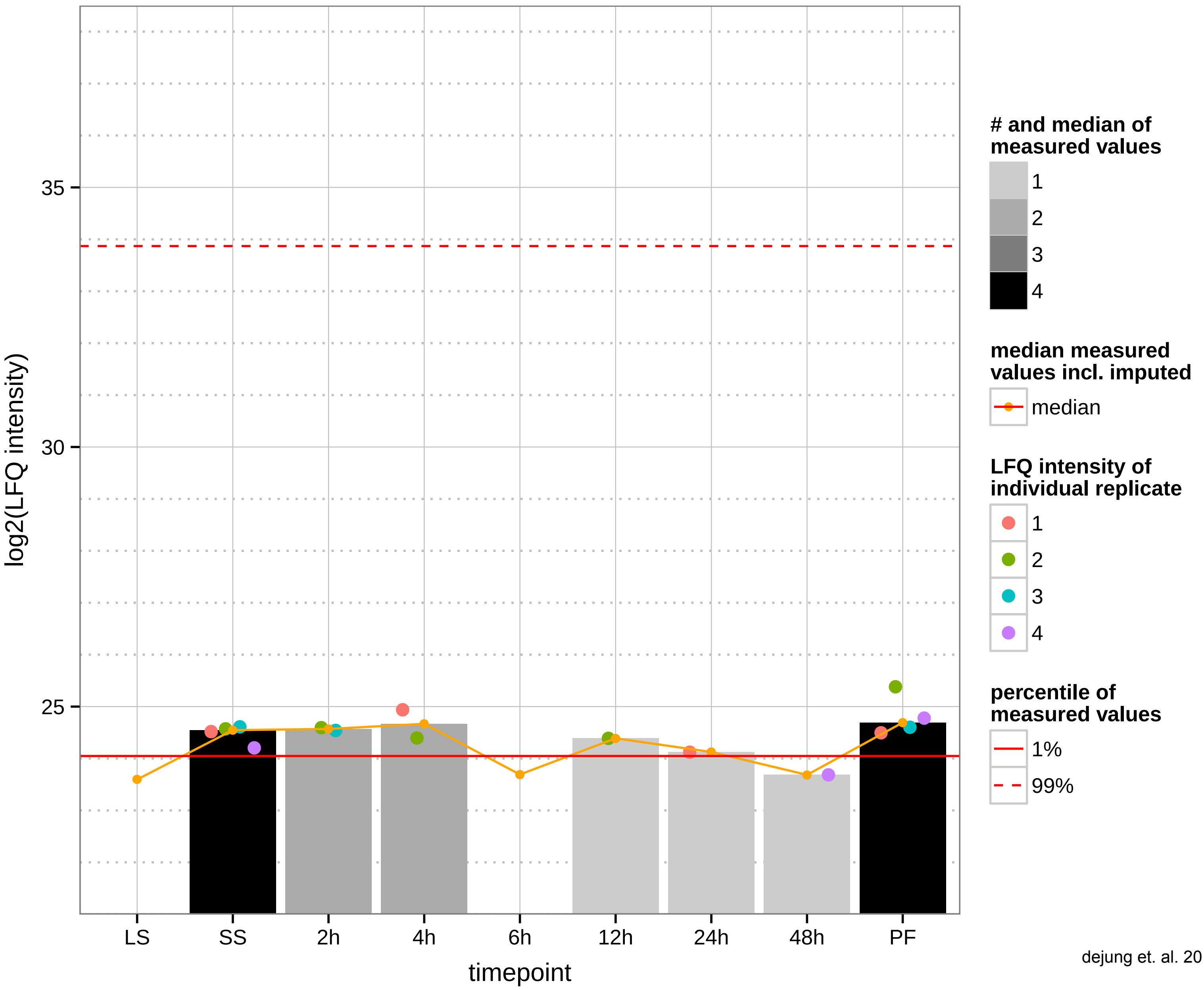

dejung et. al. 2015

hypothetical protein, conserved
Tb927.11.12530
AGOF: null
AGOC: null
AGOP: null
PGOF: null
PGOC: null
PGOP: null

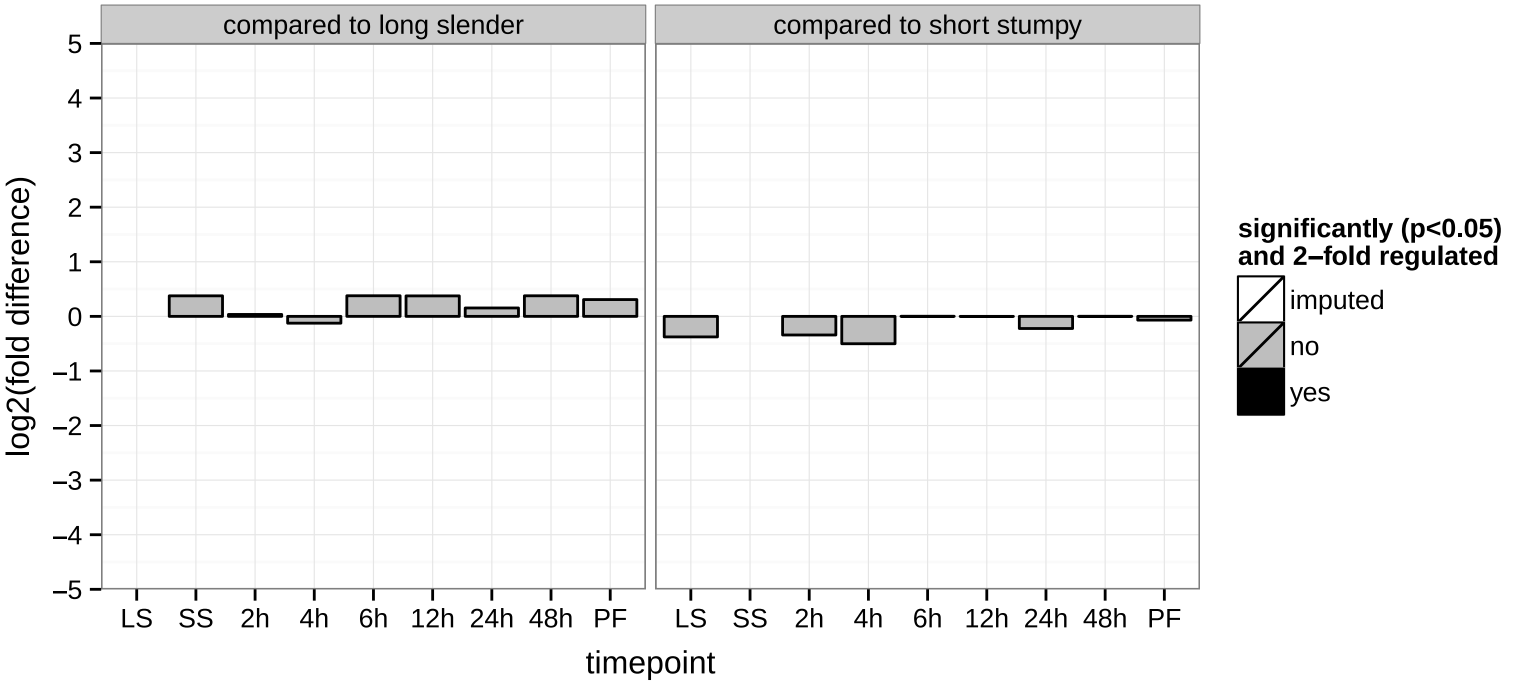

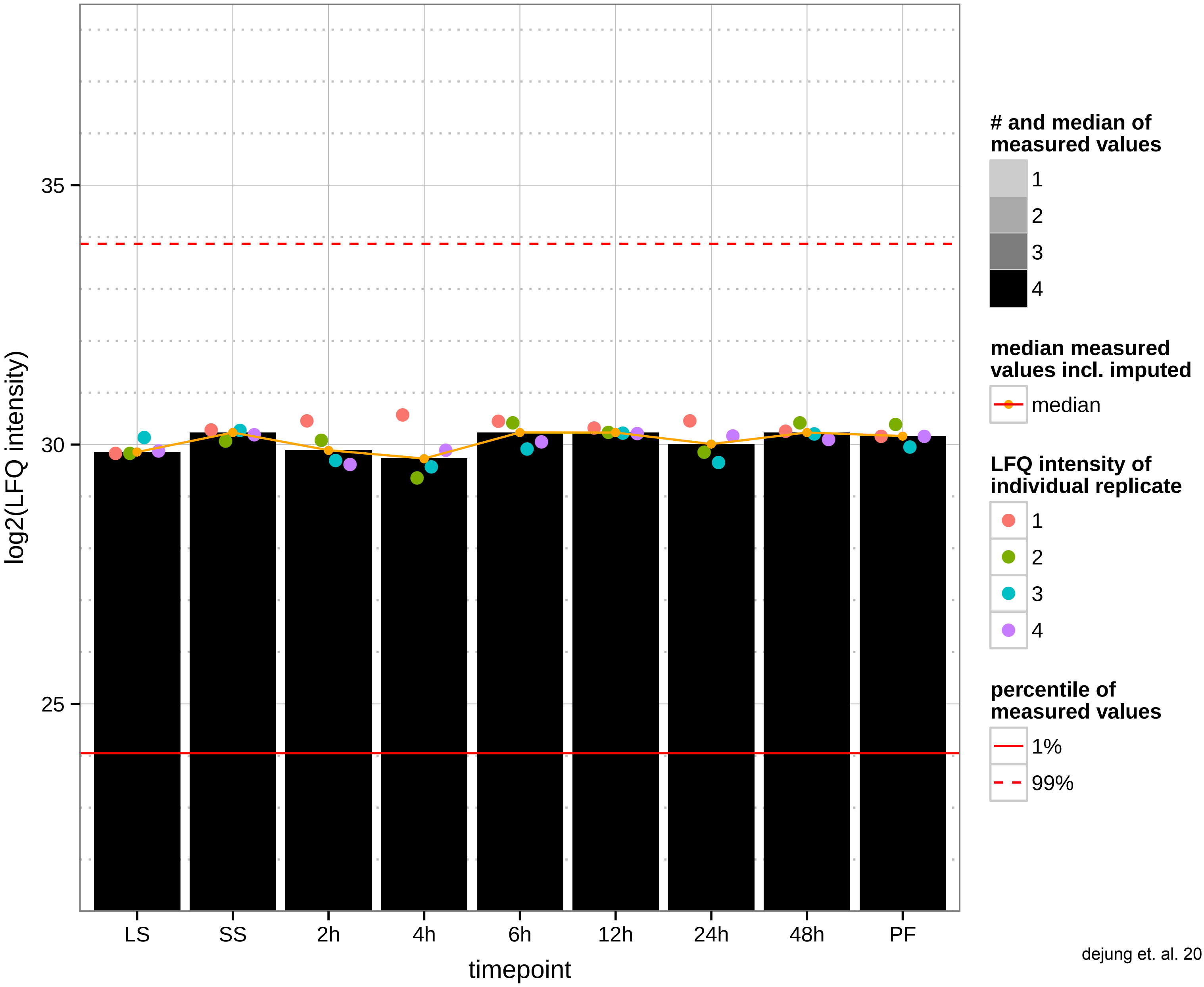

dejung et. al. 2015

leucine−rich repeat protein (LRRP), putative
Tb927.11.12540
AGOF: null
AGOC: null
AGOP: null
PGOF: null
PGOC: null
PGOP: null

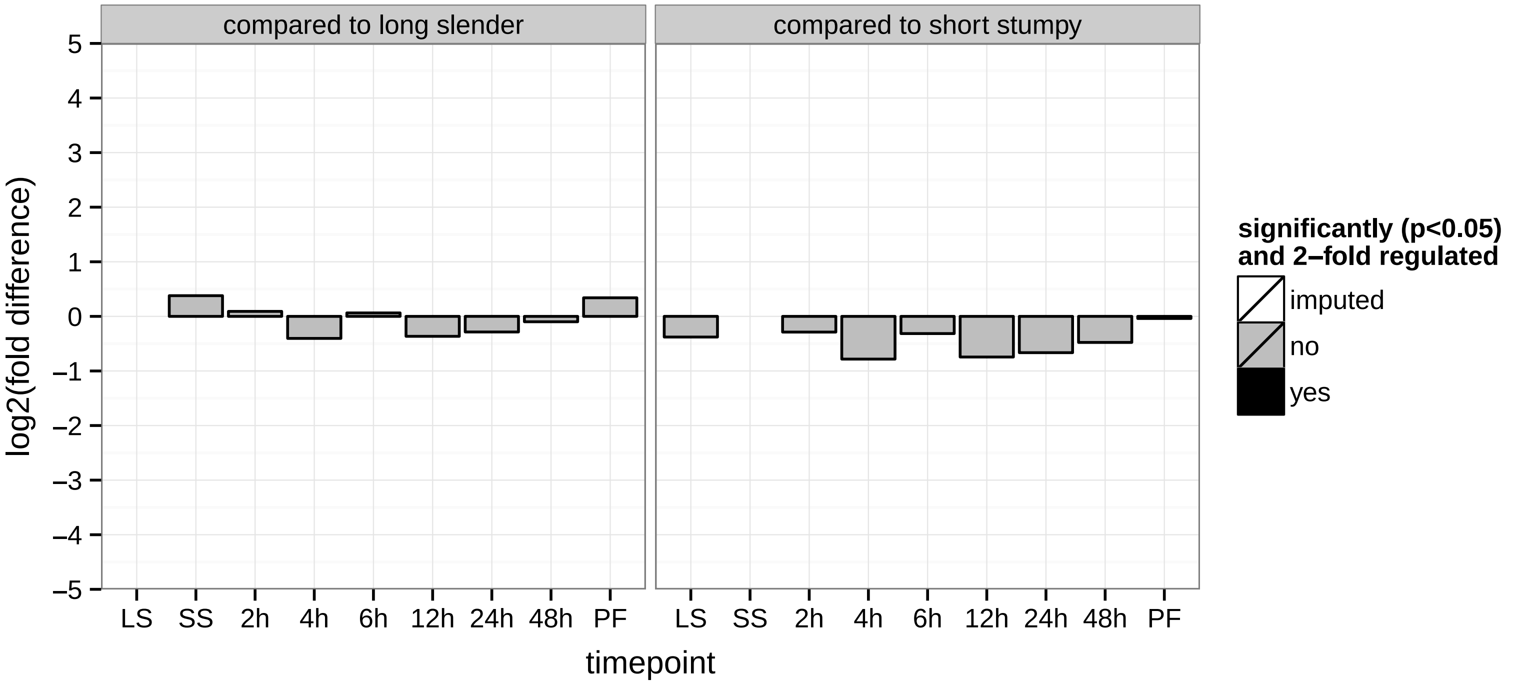

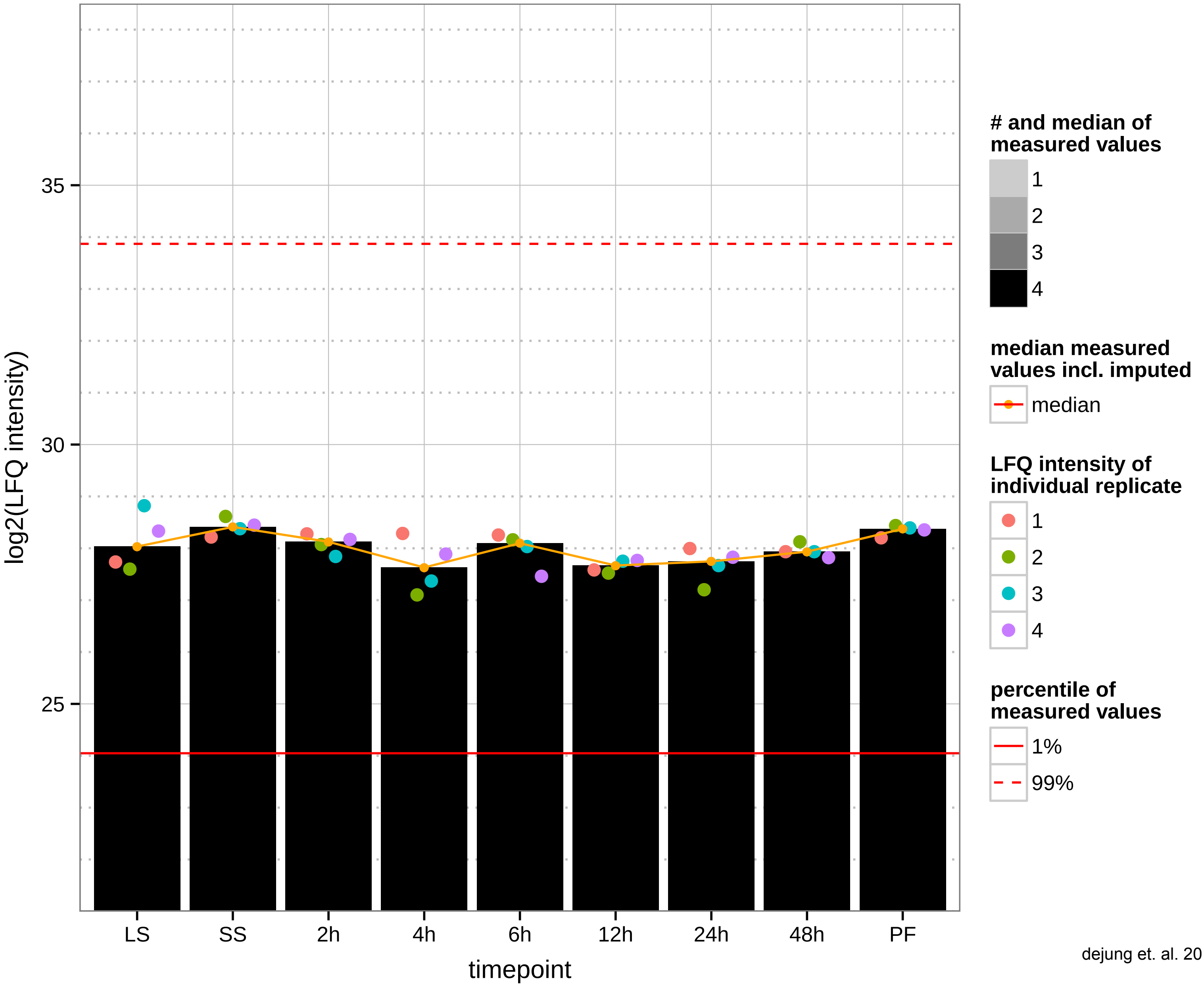

dejung et. al. 2015

hypothetical protein, conserved
Tb927.11.12590
AGOF: null
AGOC: null
AGOP: null
PGOF: null
PGOC: null
PGOP: null

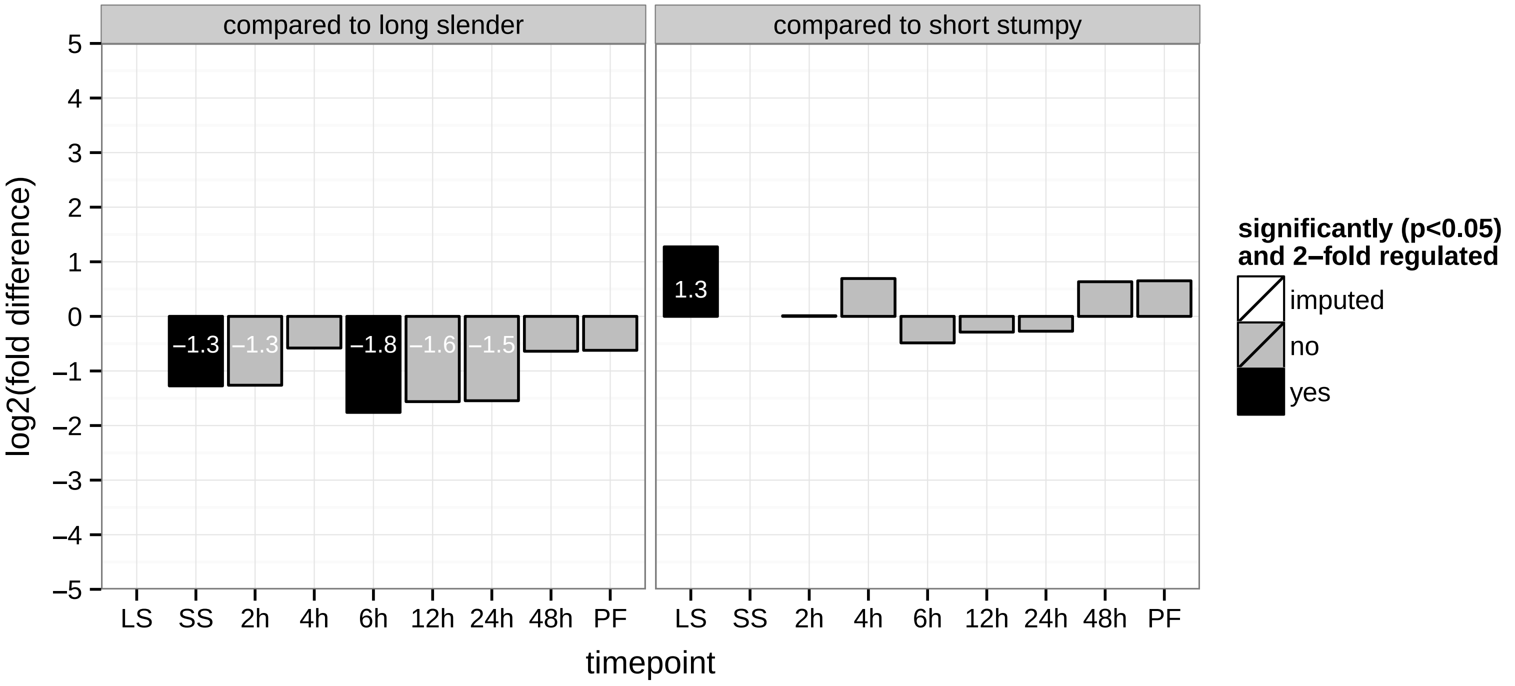

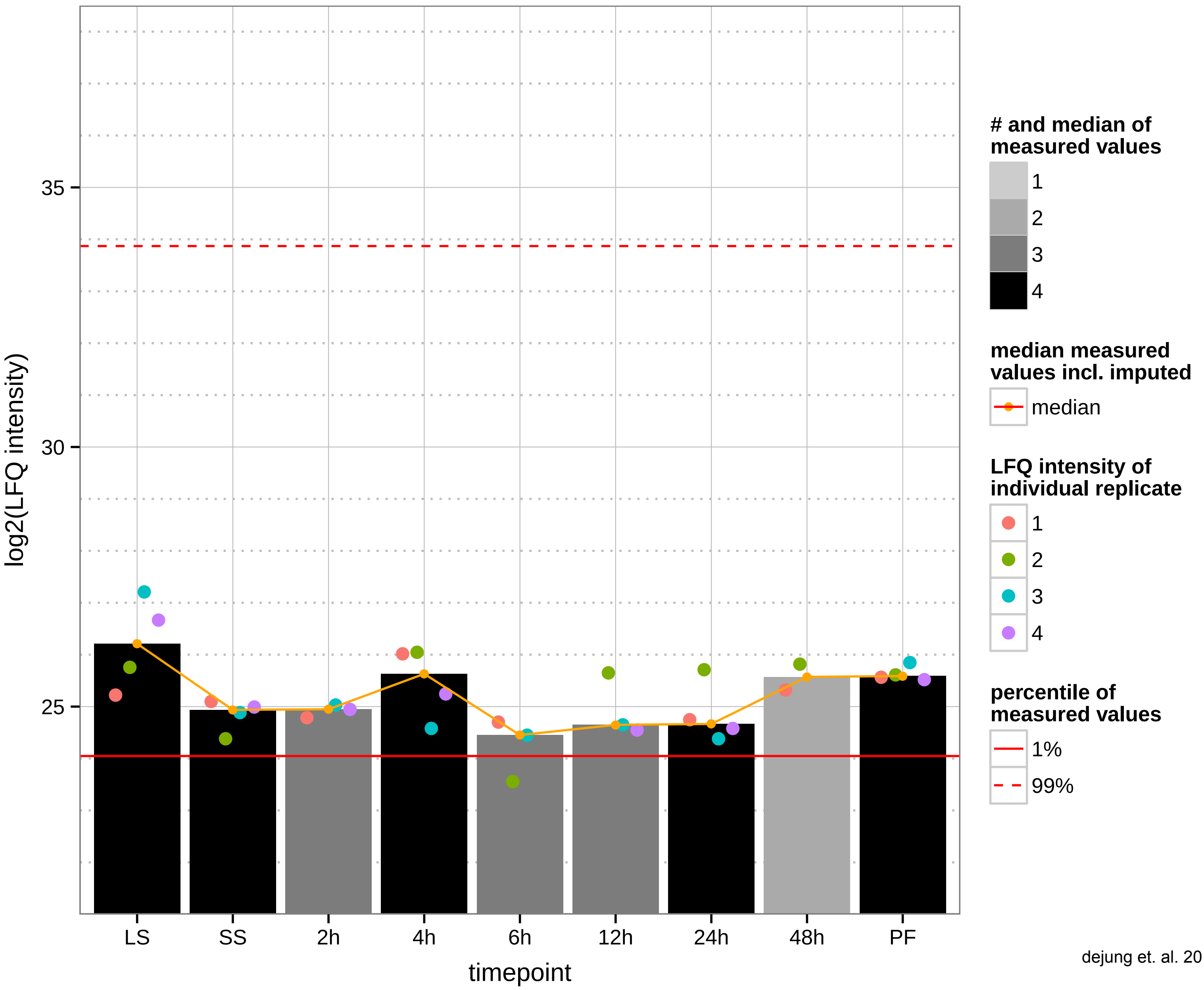

dejung et. al. 2015

hypothetical protein, conserved
Tb927.11.12630
AGOF: null
AGOC: null
AGOP: null
PGOF: null
PGOC: null
PGOP: null

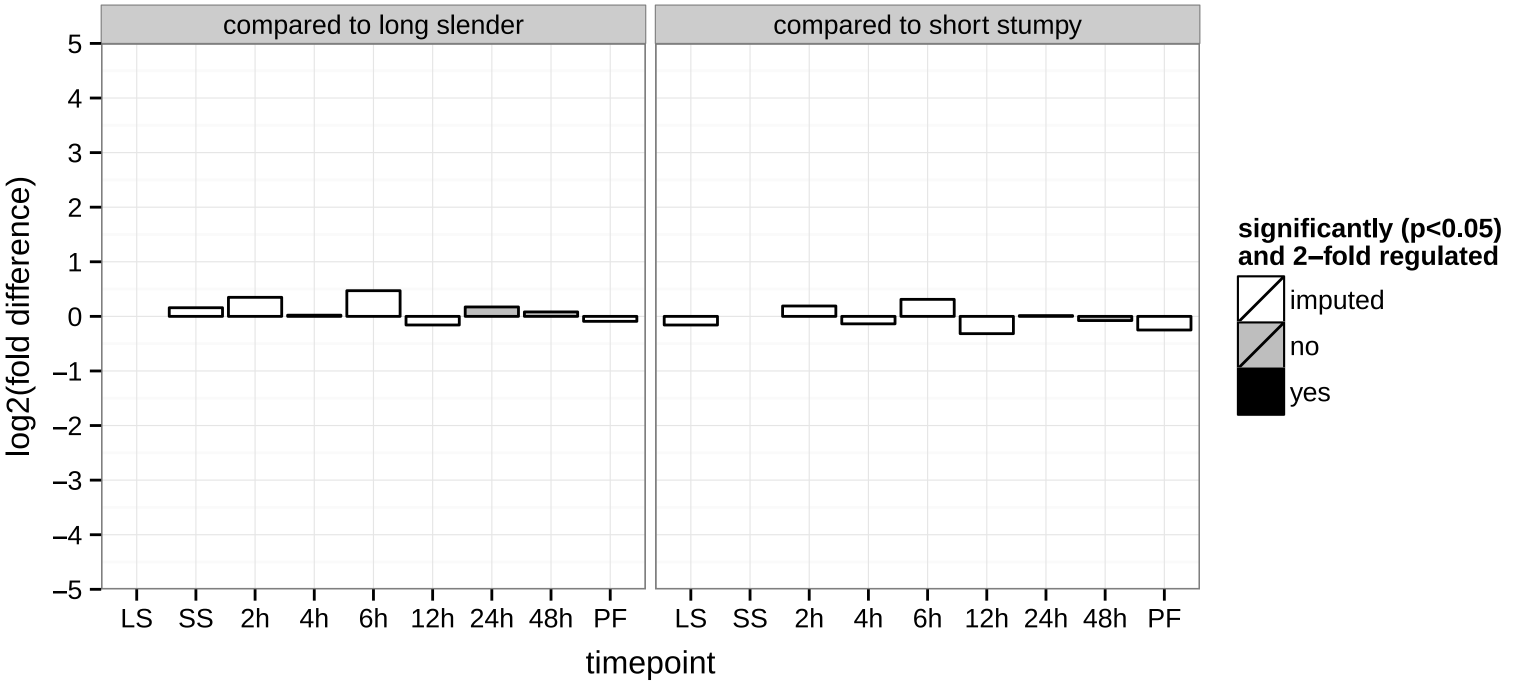

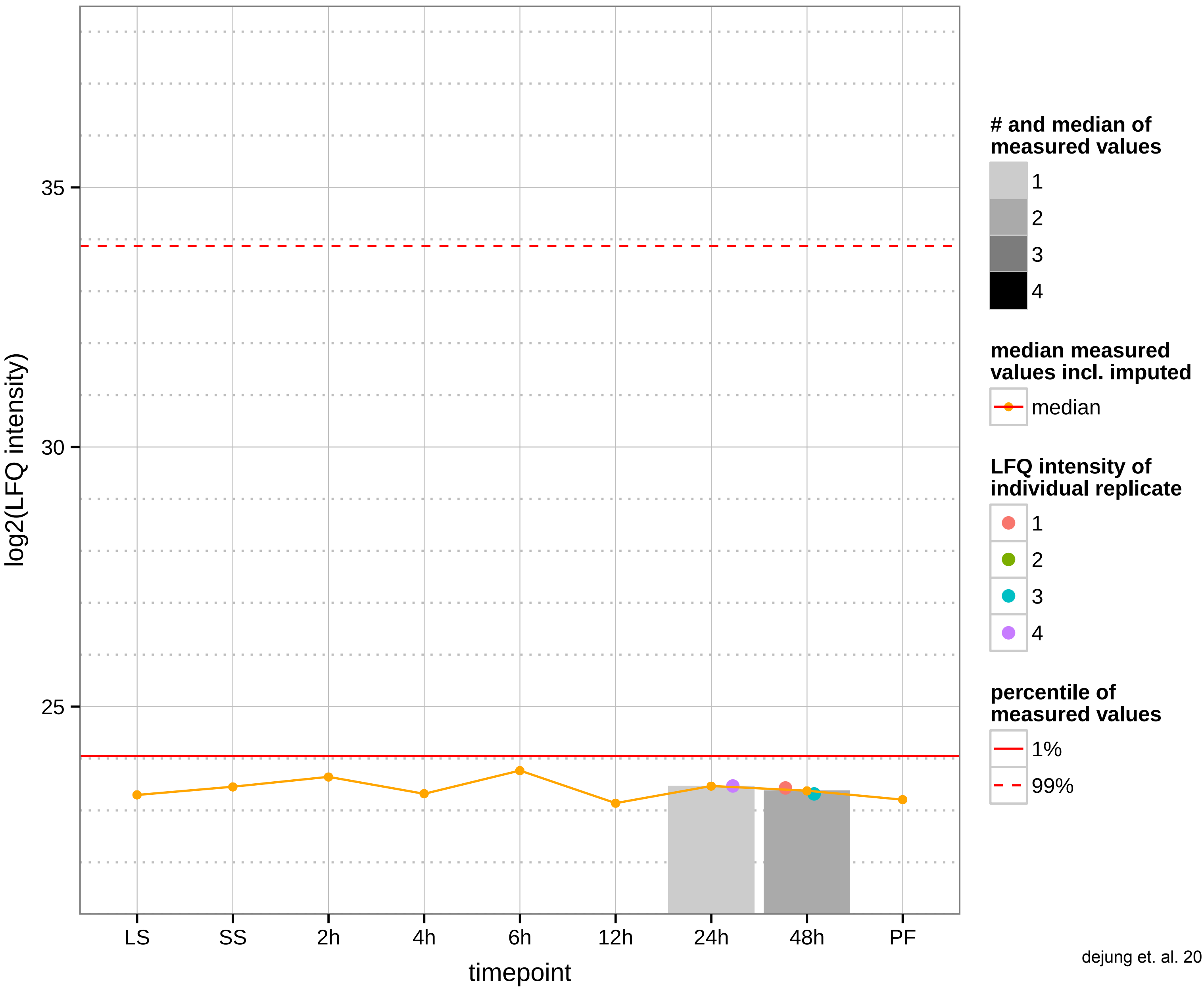

dejung et. al. 2015

hypothetical protein, conserved
Tb927.11.12640
AGOF: null
AGOC: null
AGOP: null
PGOF: Rho guanyl–nucleotide exchange factor activity, phospholipid binding, protein binding
PGOC: intracellular
PGOP: regulation of Rho protein signal transduction

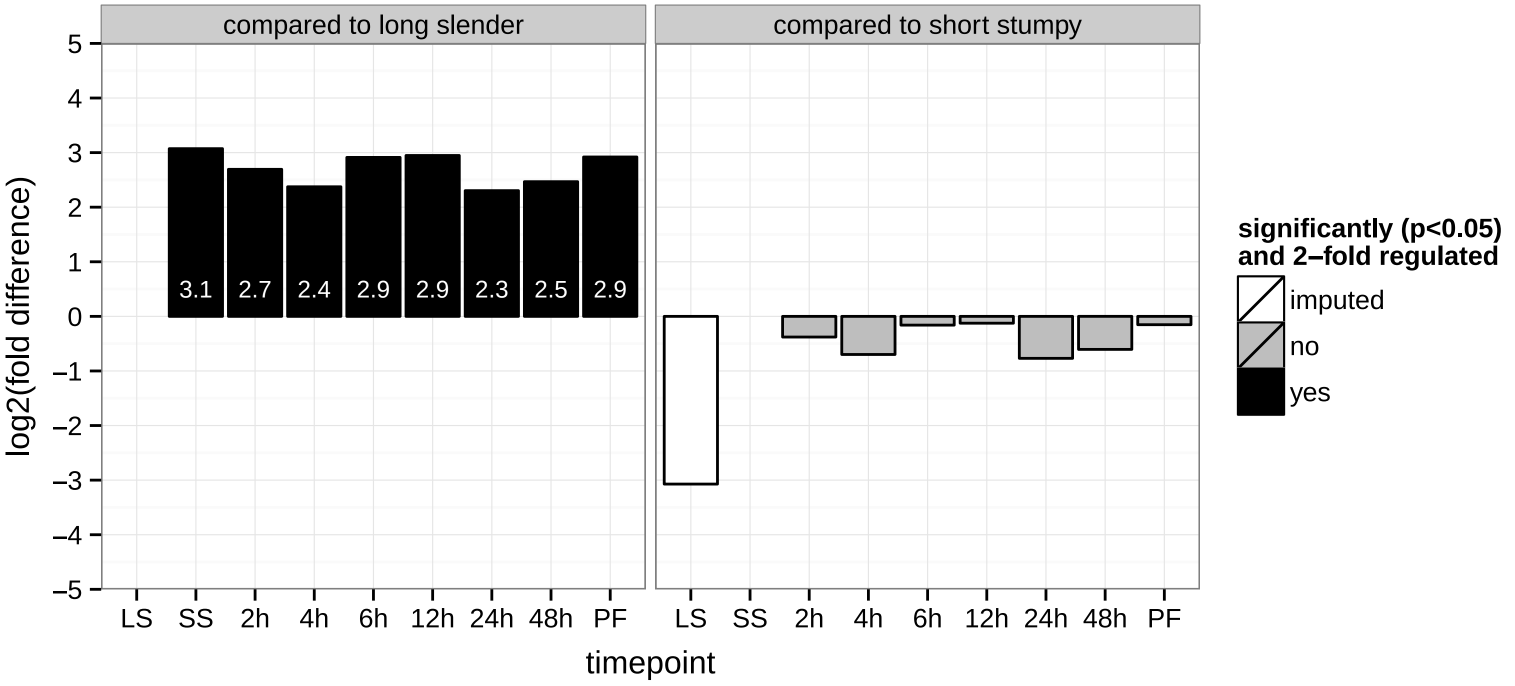

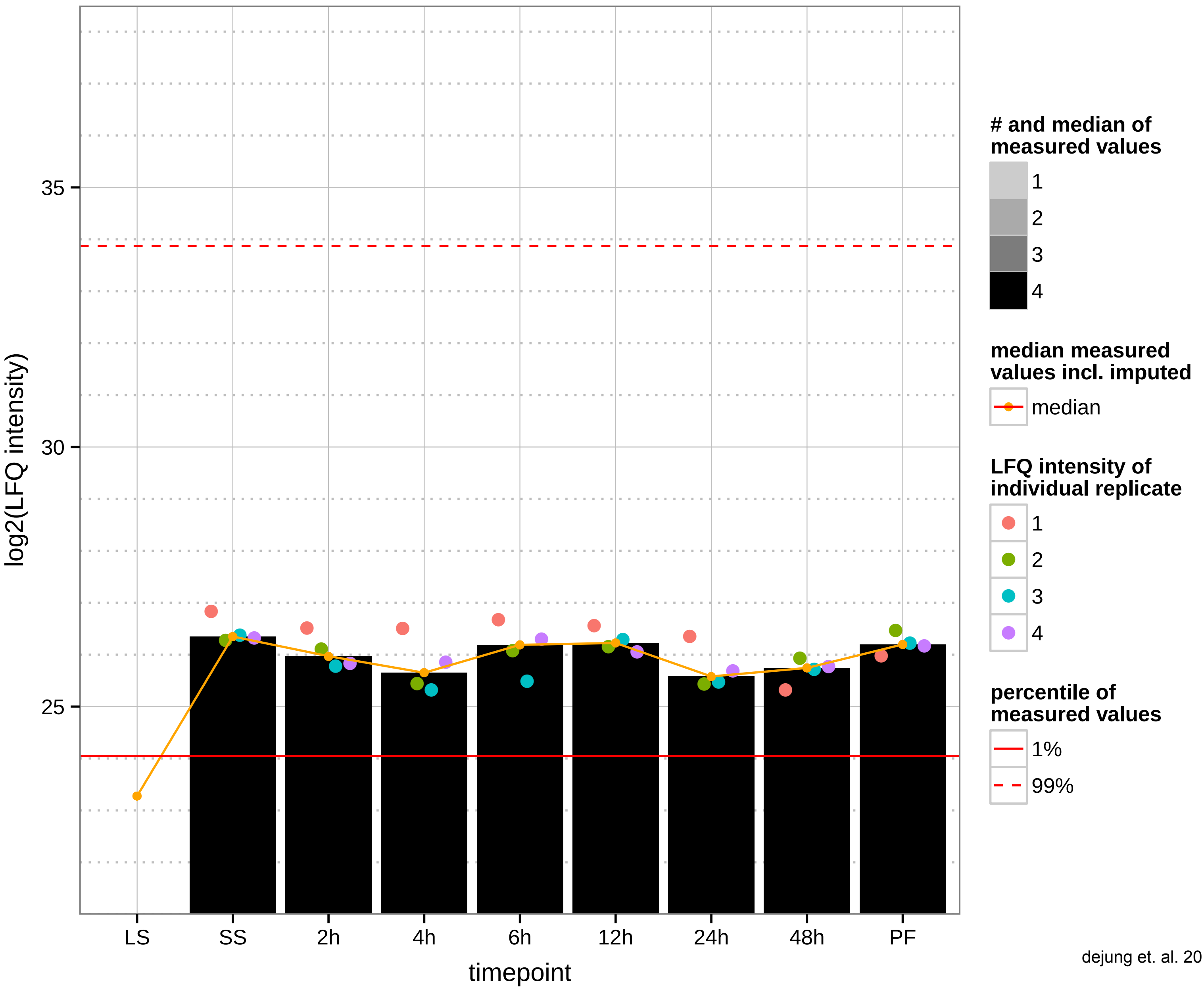

dejung et. al. 2015

hypothetical protein, conserved
Tb927.11.12910
AGOF: null
AGOC: null
AGOP: null
PGOF: unfolded protein binding
PGOC: prefoldin complex
PGOP: protein folding

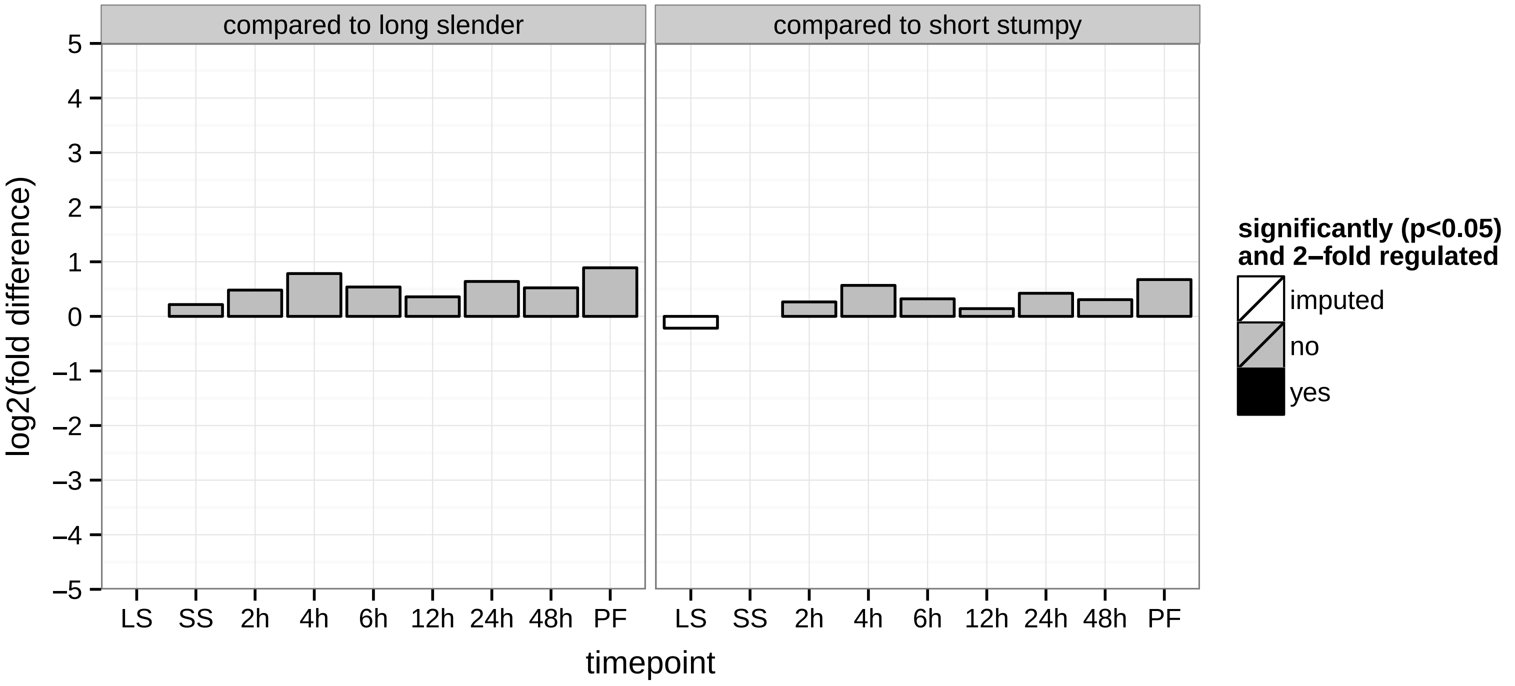

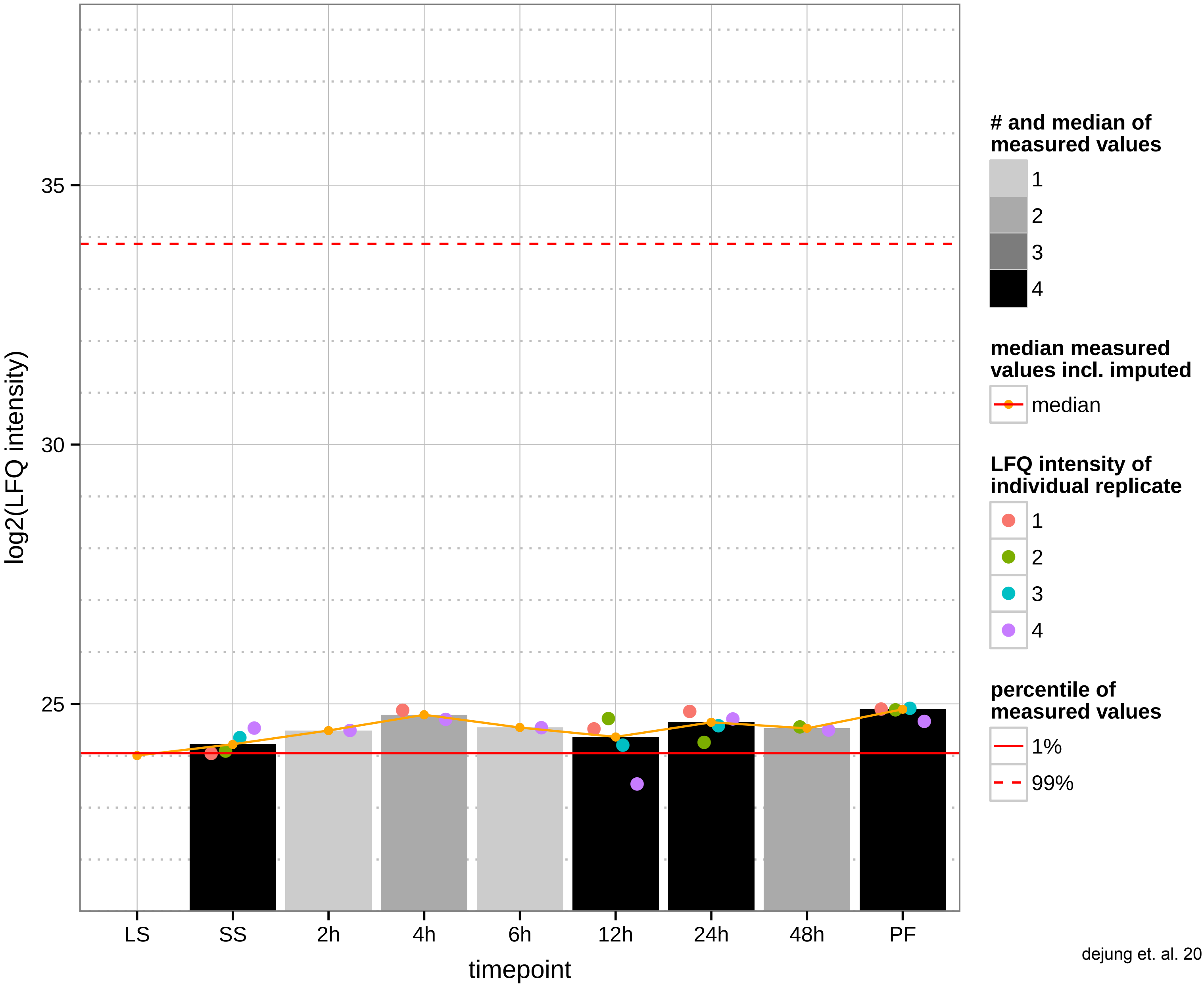

dejung et. al. 2015

hypothetical protein, conserved
Tb927.11.12920
AGOF: ATP binding, nucleoside−triphosphatase activity
AGOC: null
AGOP: null
PGOF: nucleoside−triphosphatase activity, nucleotide binding
PGOC: null
PGOP: null

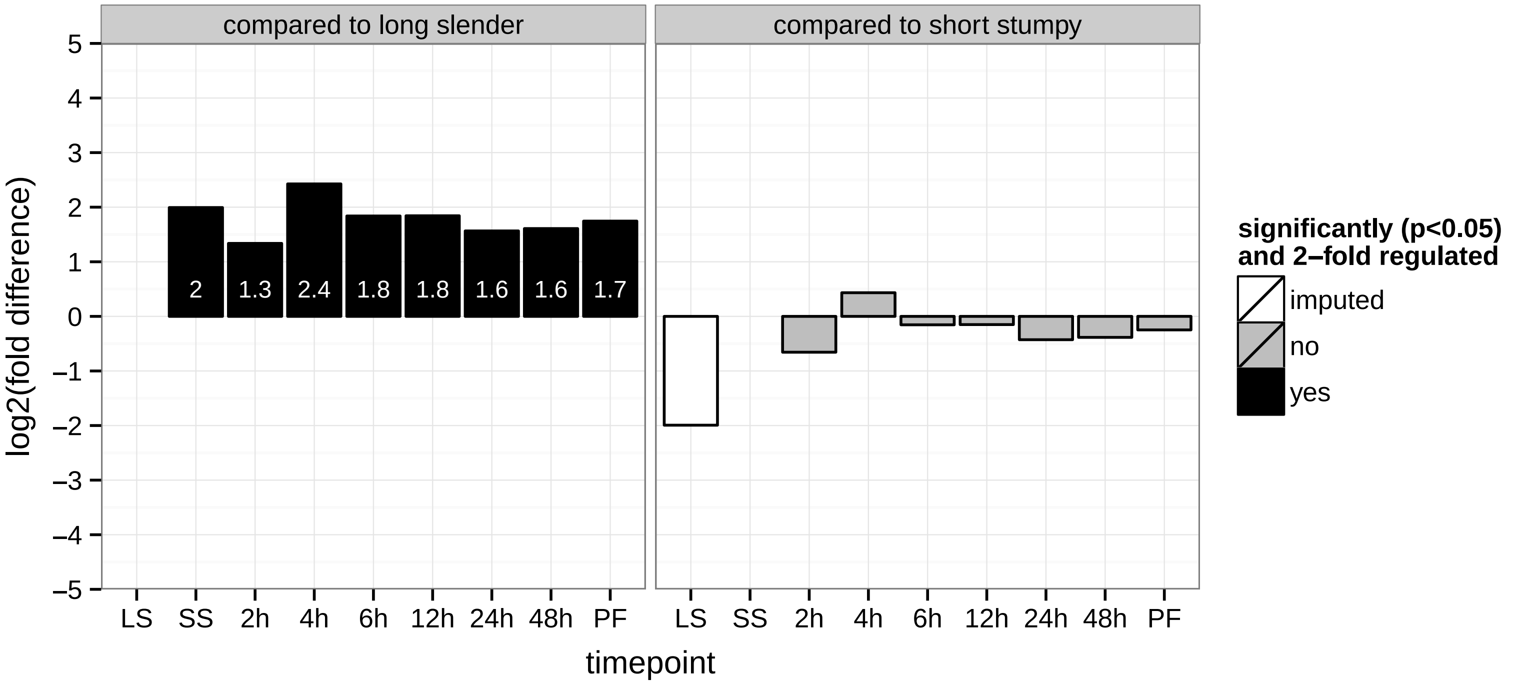

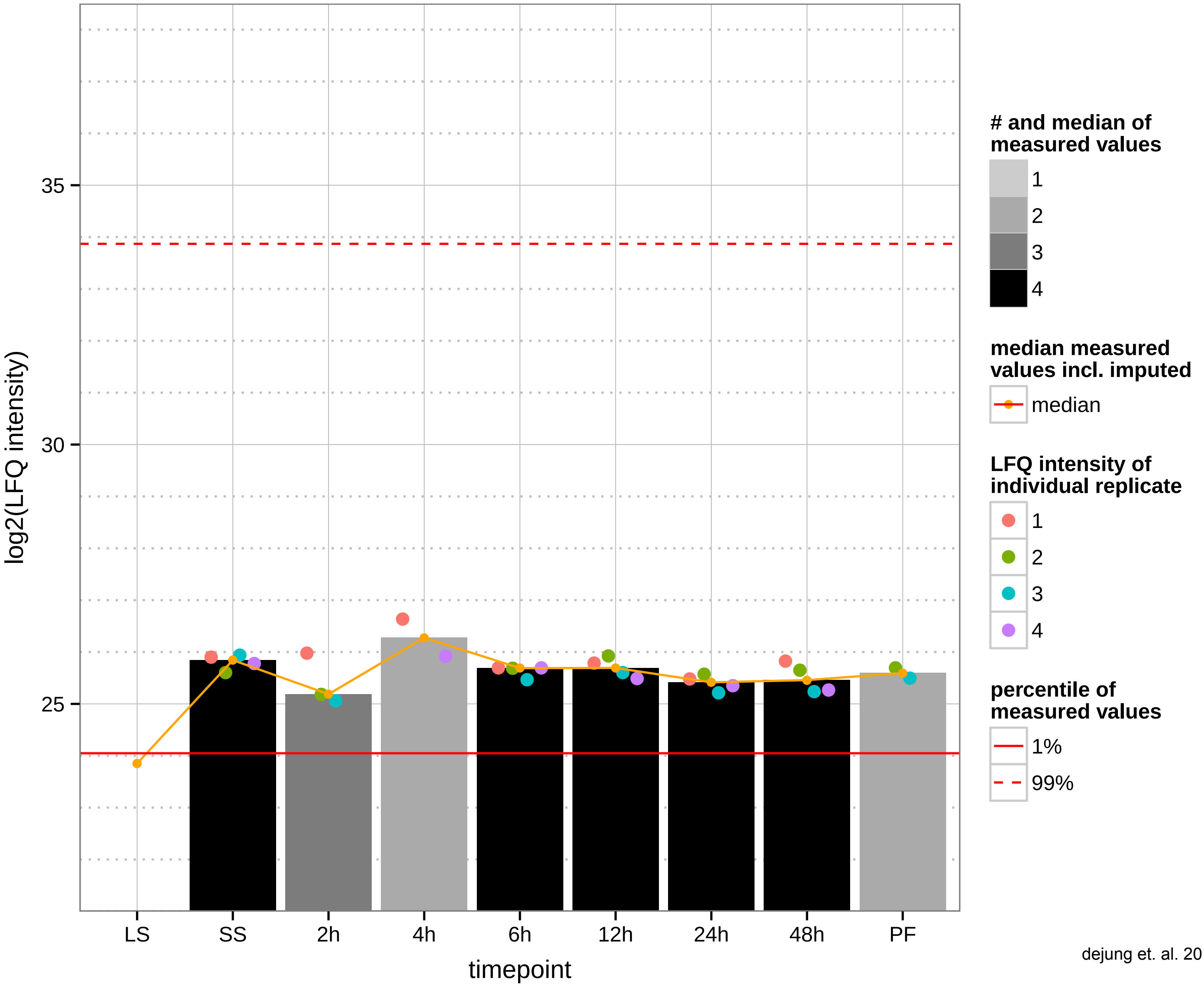

dejung et. al. 2015

hypothetical protein, conserved
Tb927.11.12940
AGOF: null
AGOC: null
AGOP: null
PGOF: null
PGOC: null
PGOP: null

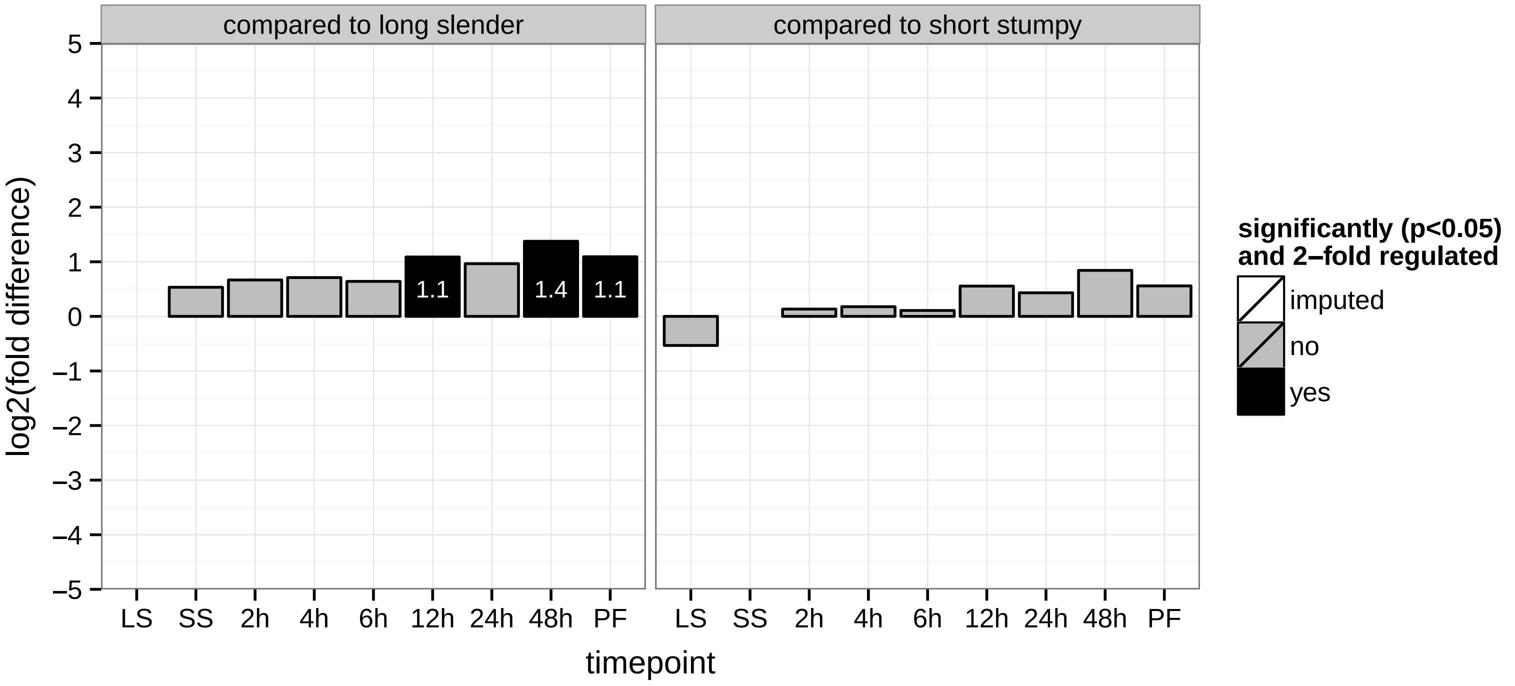

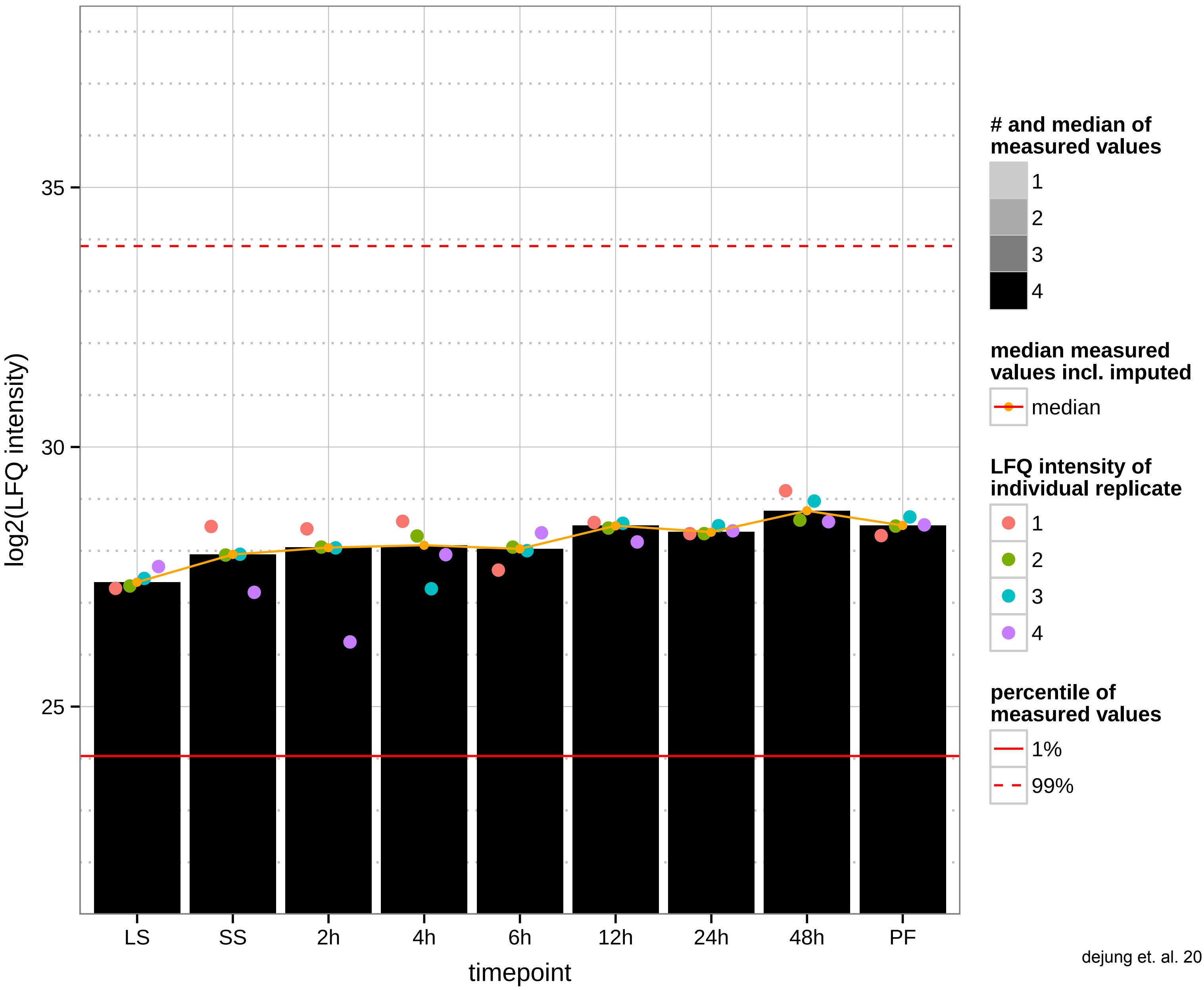

dejung et. al. 2015

hypothetical protein
Tb927.11.13010
AGOF: null
AGOC: null
AGOP: null
PGOF: null
PGOC: null
PGOP: null

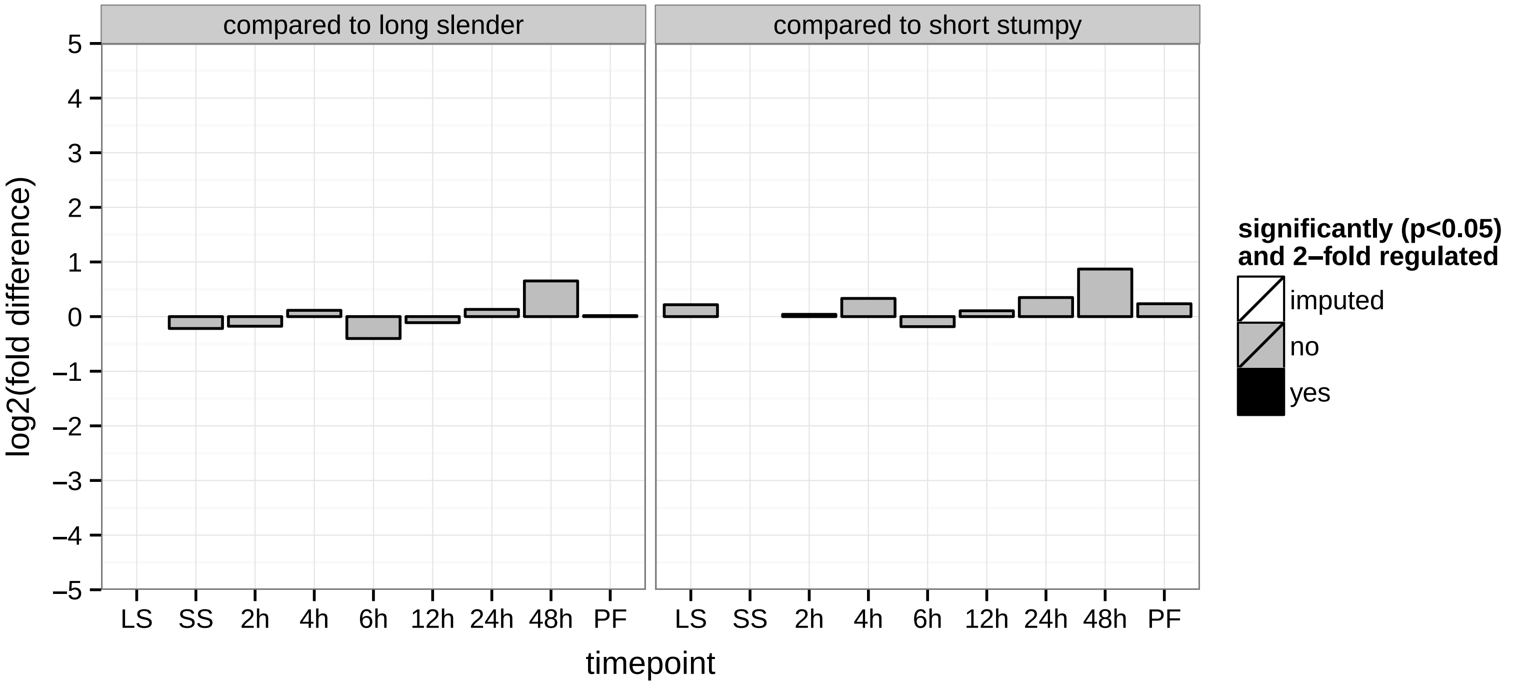

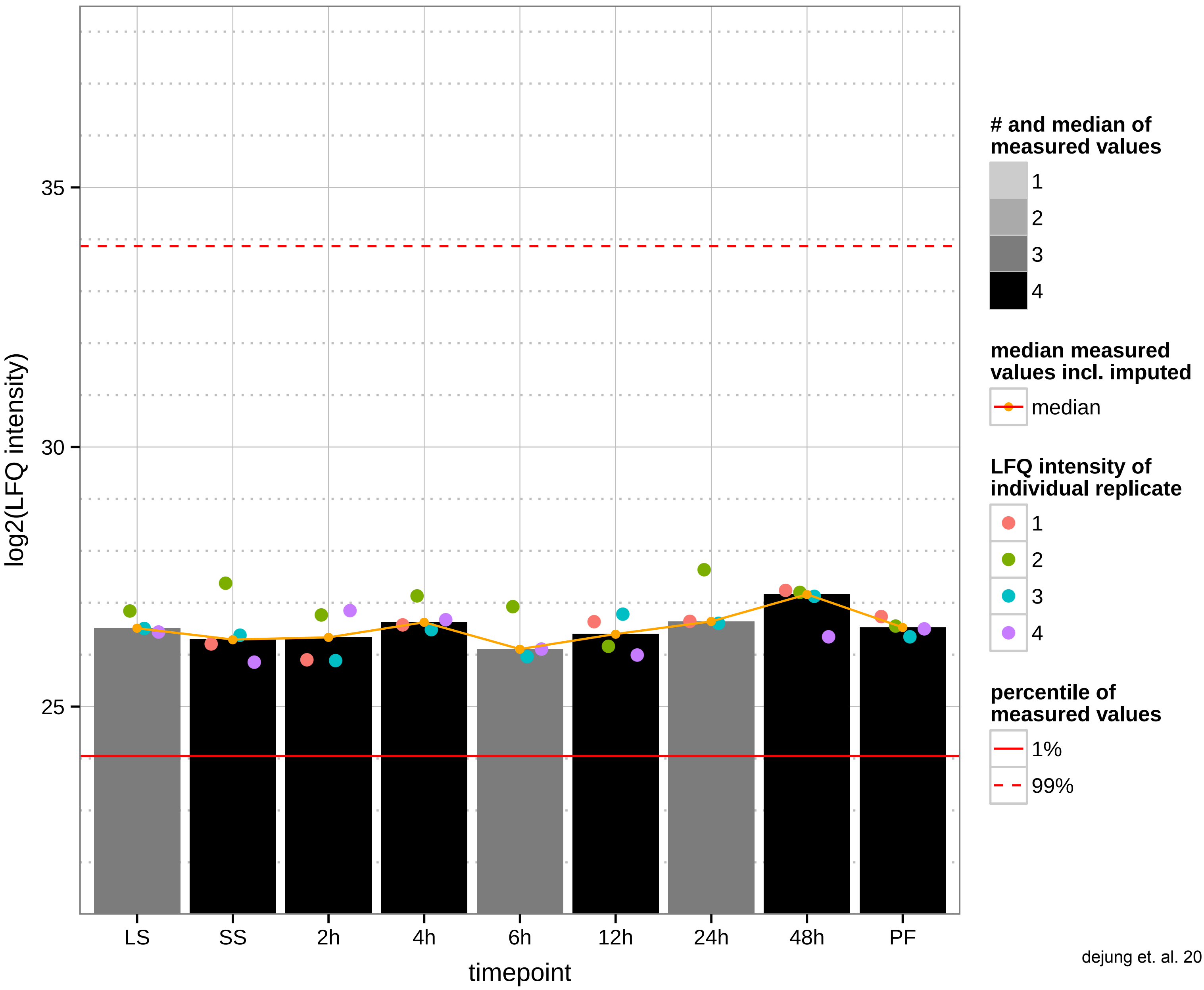

dejung et. al. 2015

calmodulin
Tb927.11.13050;Tb927.11.13040;Tb927.11.13030;Tb927.11.13020
AGOF: calcium ion binding
AGOC: cilium, contractile vacuole, cytosol
AGOP: null
PGOF: null, calcium ion binding
PGOC: null
PGOP: null

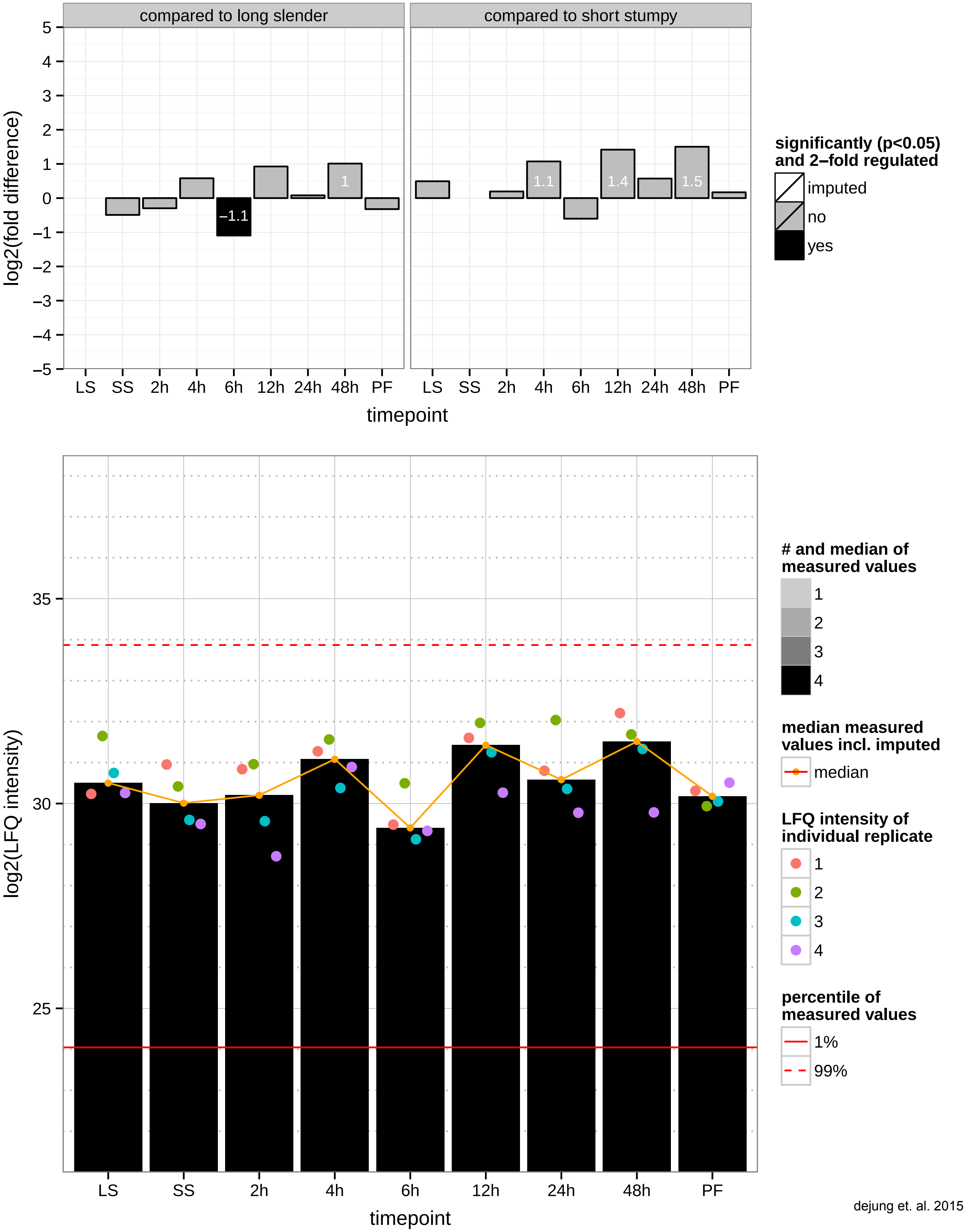

dejung et. al. 2015

hypothetical protein, conserved
Tb927.11.13070
AGOF: null
AGOC: null
AGOP: null
PGOF: transferase activity, transferase activity, transferring selenium–containing groups
PGOC: null
PGOP: null

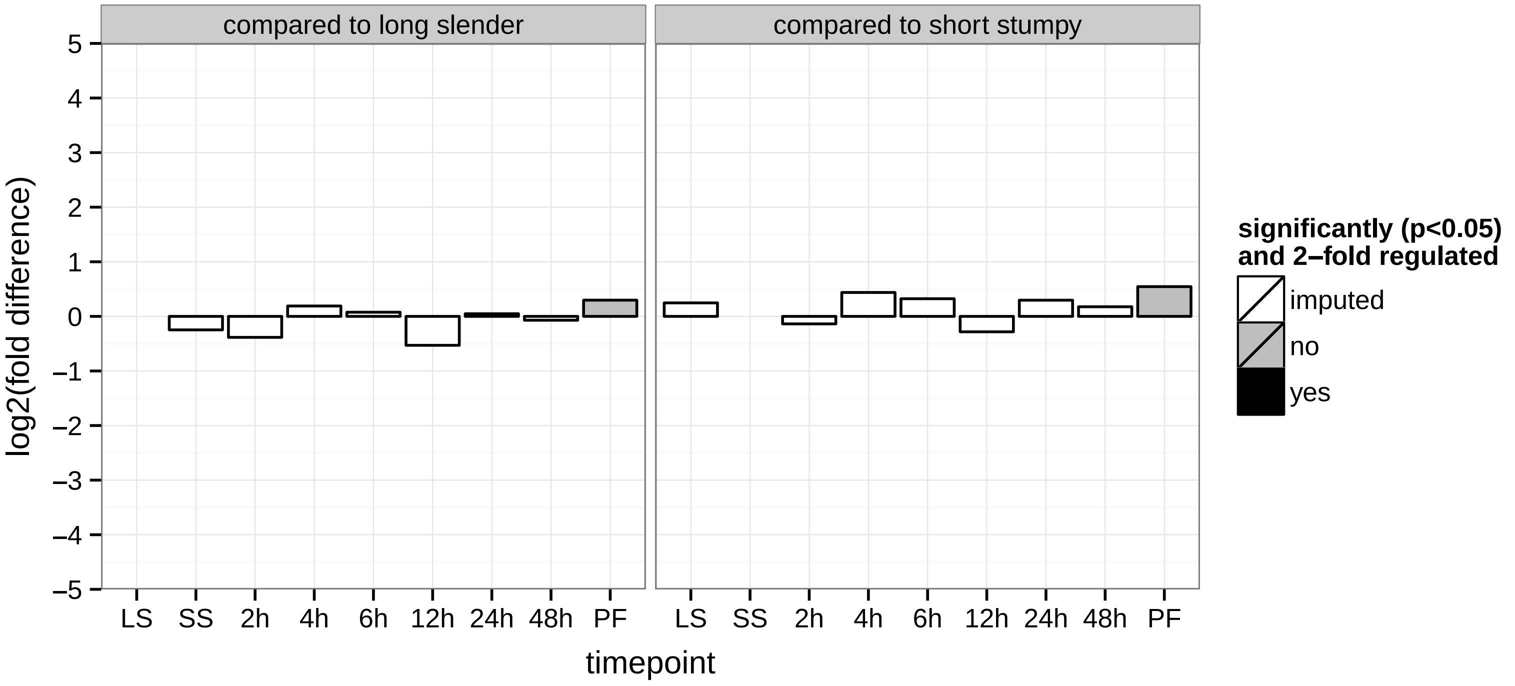

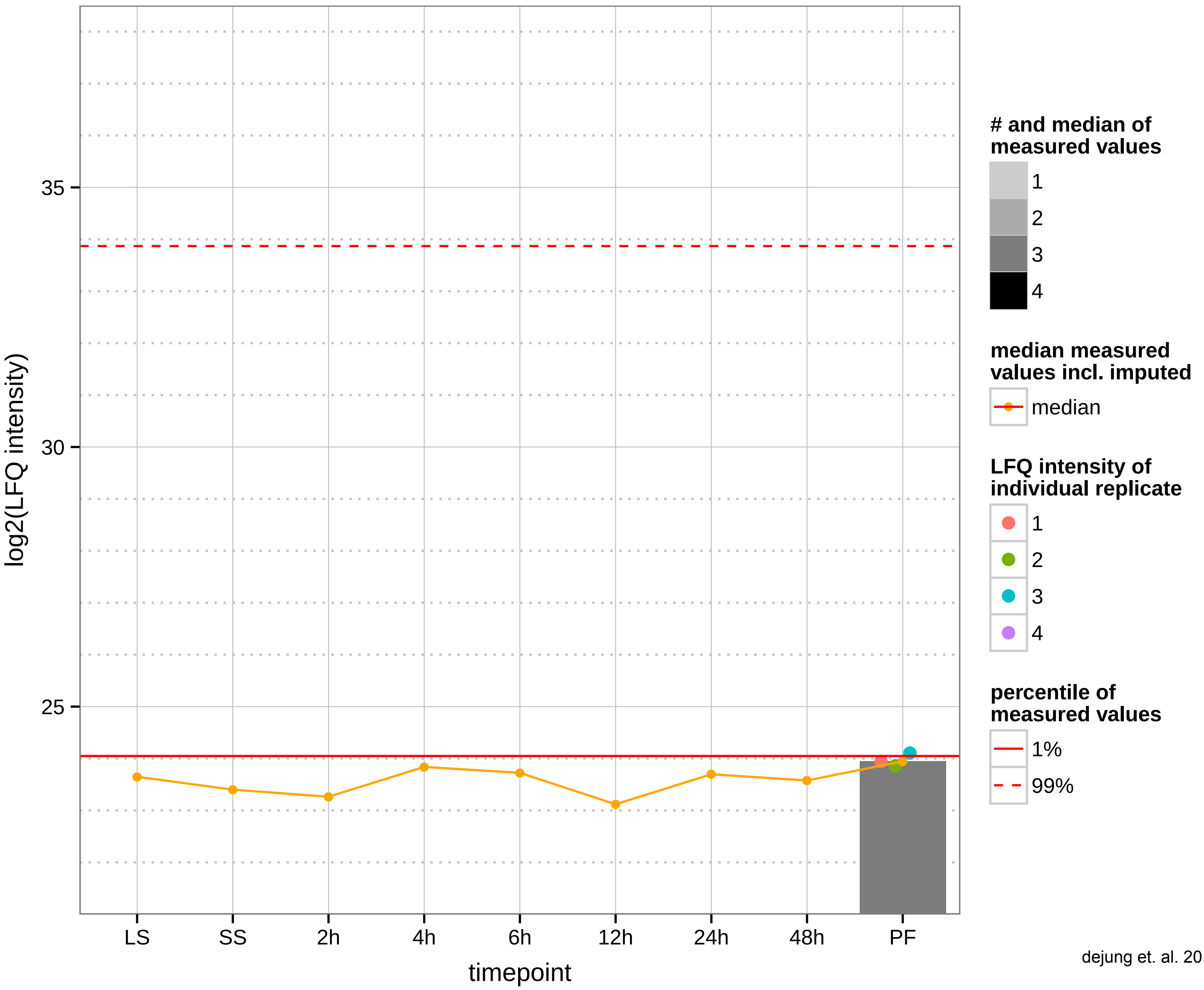

dejung et. al. 2015

hypothetical protein, conserved
Tb927.11.13080
AGOF: null
AGOC: null
AGOP: null
PGOF: null
PGOC: null
PGOP: null

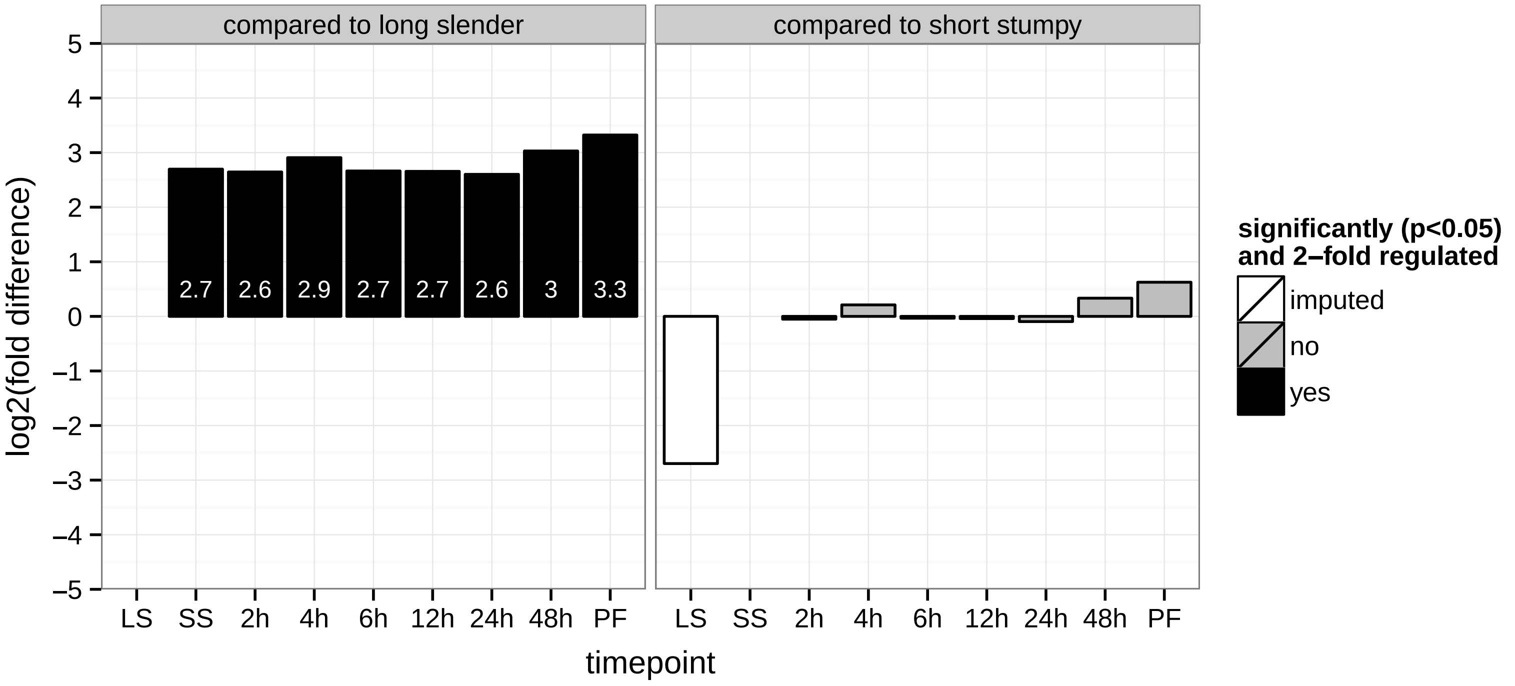

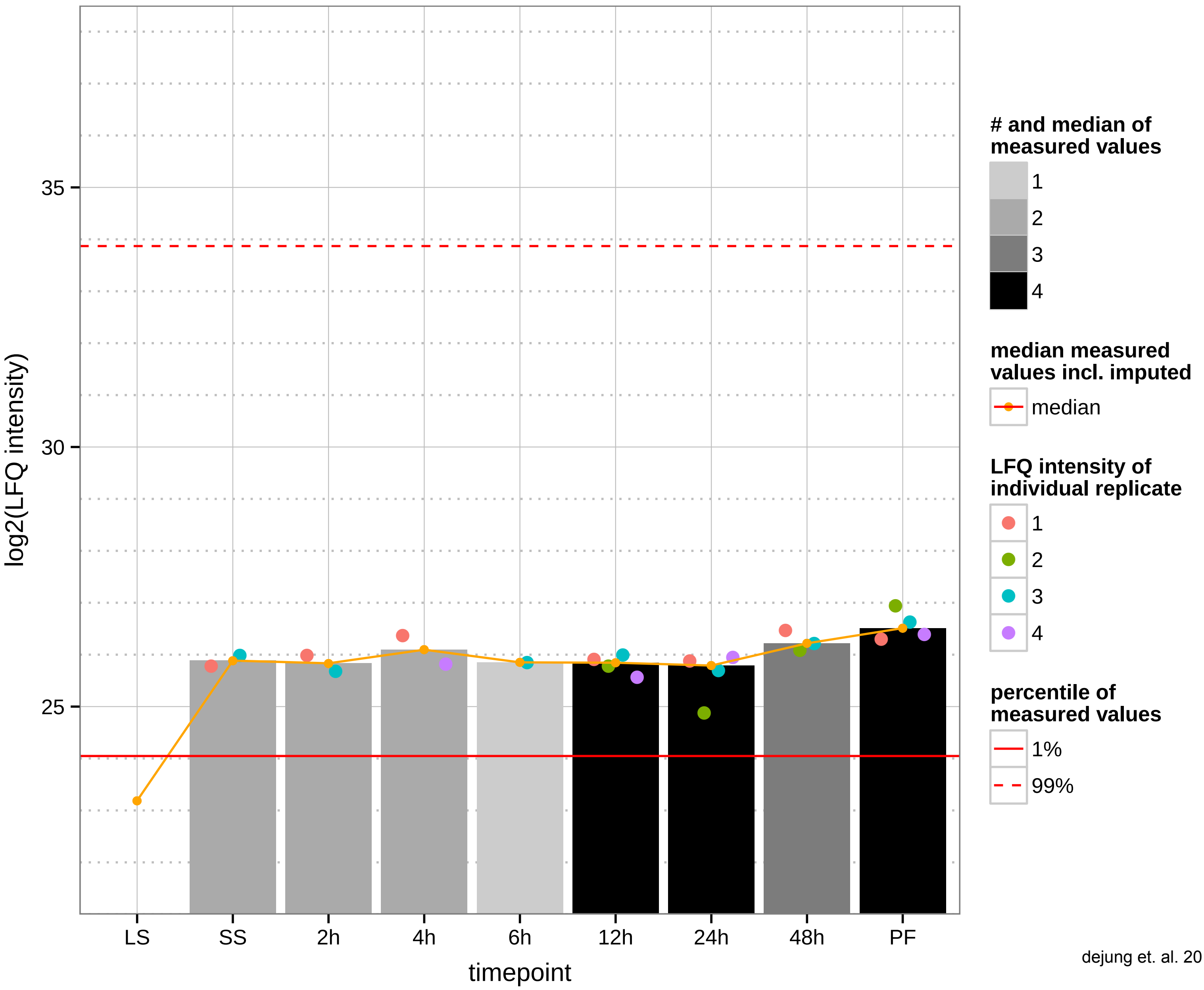

dejung et. al. 2015

elongation factor 1 gamma, putative
Tb927.11.13190;Tb927.11.13090
AGOF: translation elongation factor activity
AGOC: eukaryotic translation elongation factor 1 complex
AGOP: translational elongation
PGOF: protein binding, translation elongation factor activity
PGOC: eukaryotic translation elongation factor 1 complex
PGOP: translational elongation

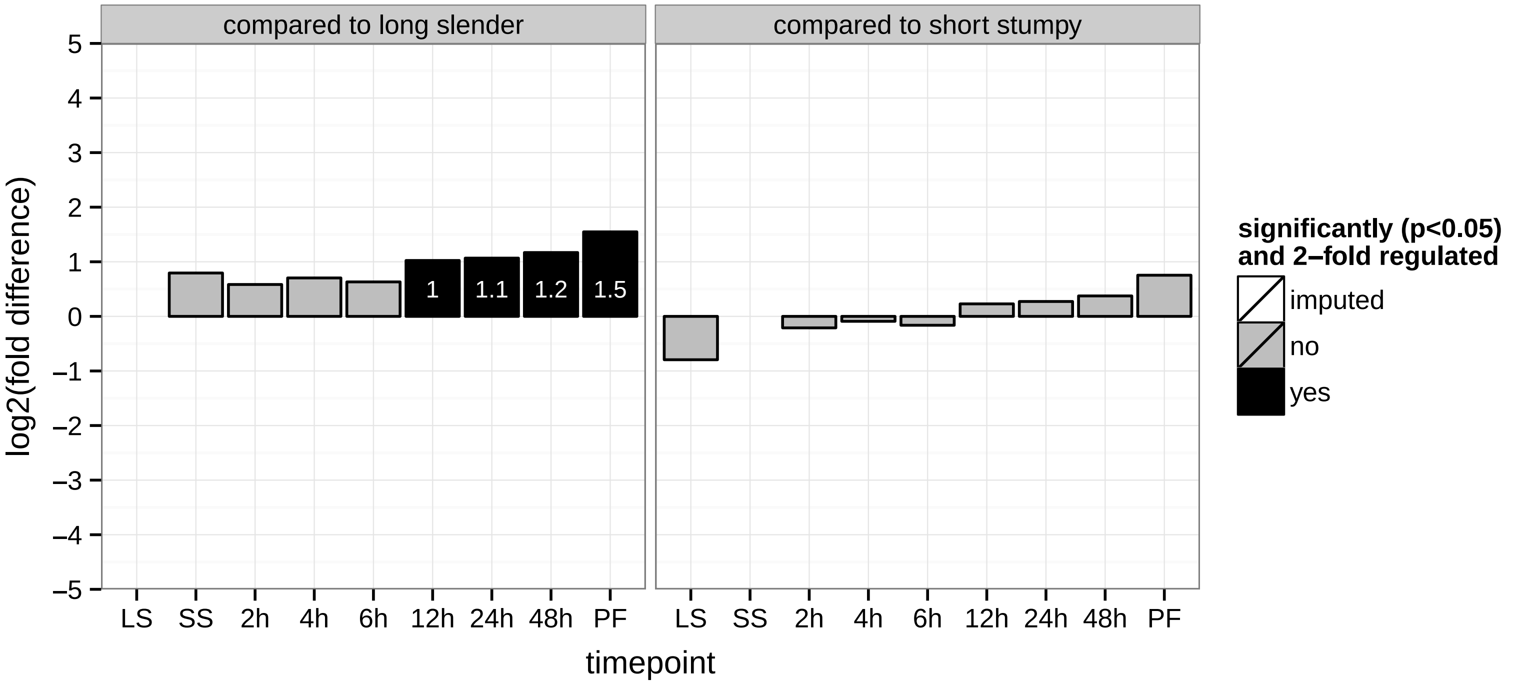

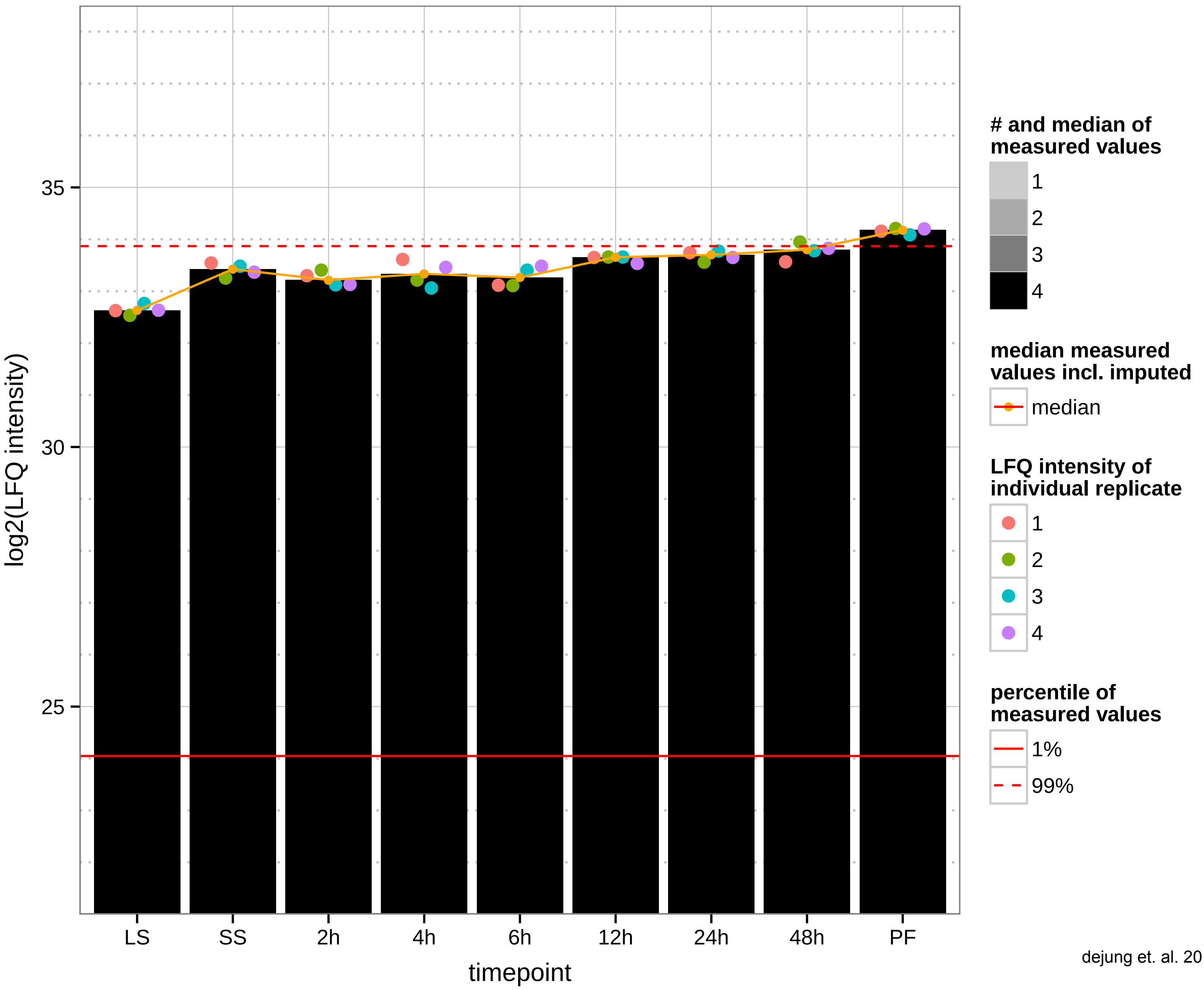

dejung et. al. 2015

hypothetical protein, conserved
Tb927.11.13270
AGOF: null
AGOC: null
AGOP: null
PGOF: null
PGOC: null
PGOP: null

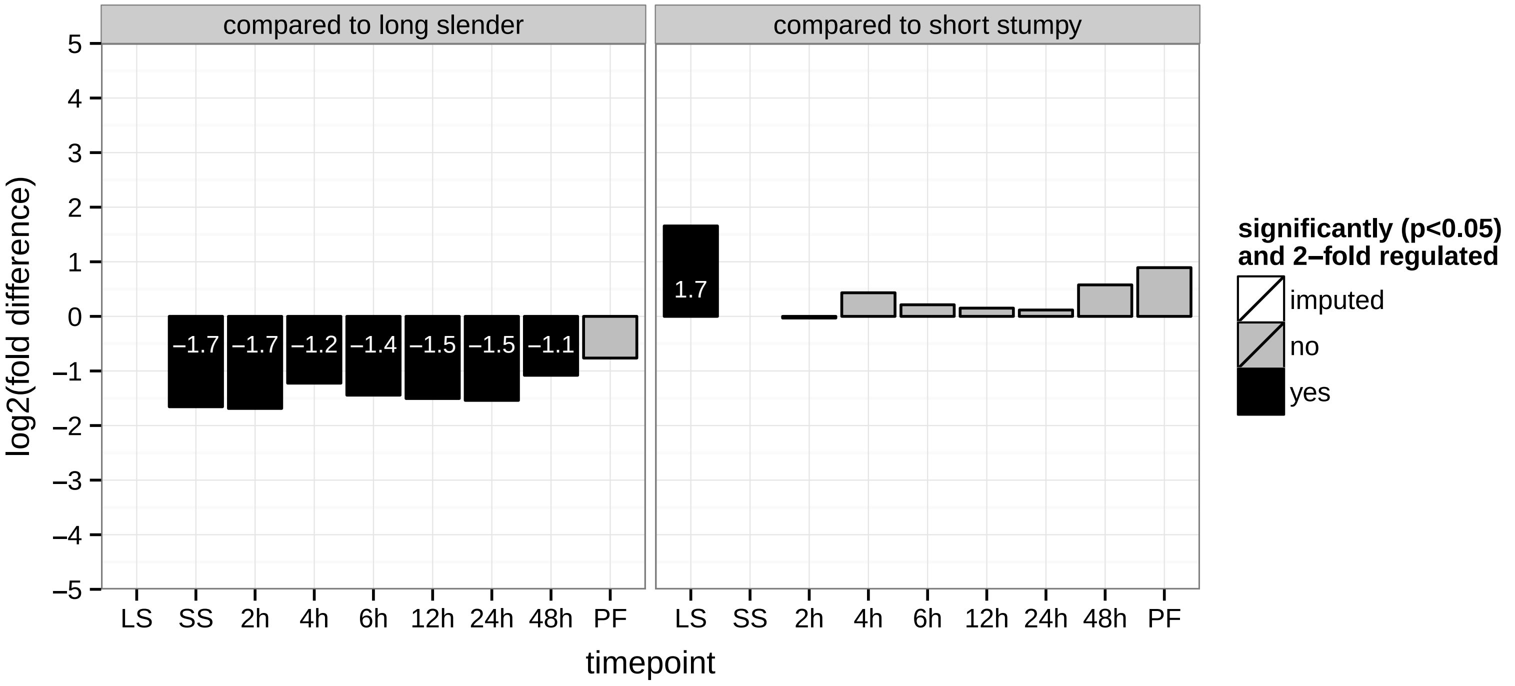

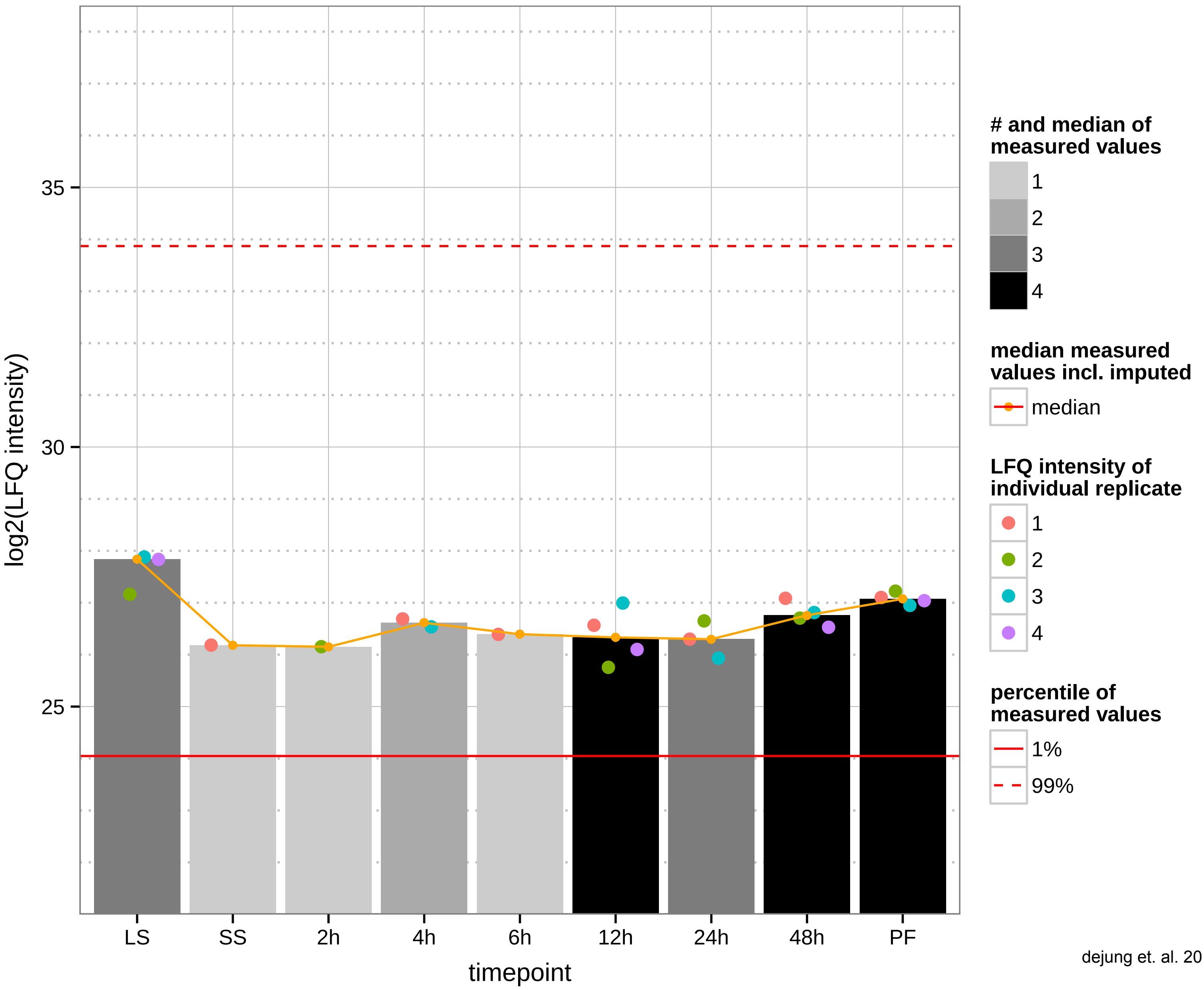

dejung et. al. 2015

mitochondrial RNA binding protein 2 (GBP25)
Tb927.11.13280
AGOF: RNA binding
AGOC: mitochondrion
AGOP: RNA modification
PGOF: DNA binding
PGOC: null
PGOP: regulation of transcription, DNA–dependent

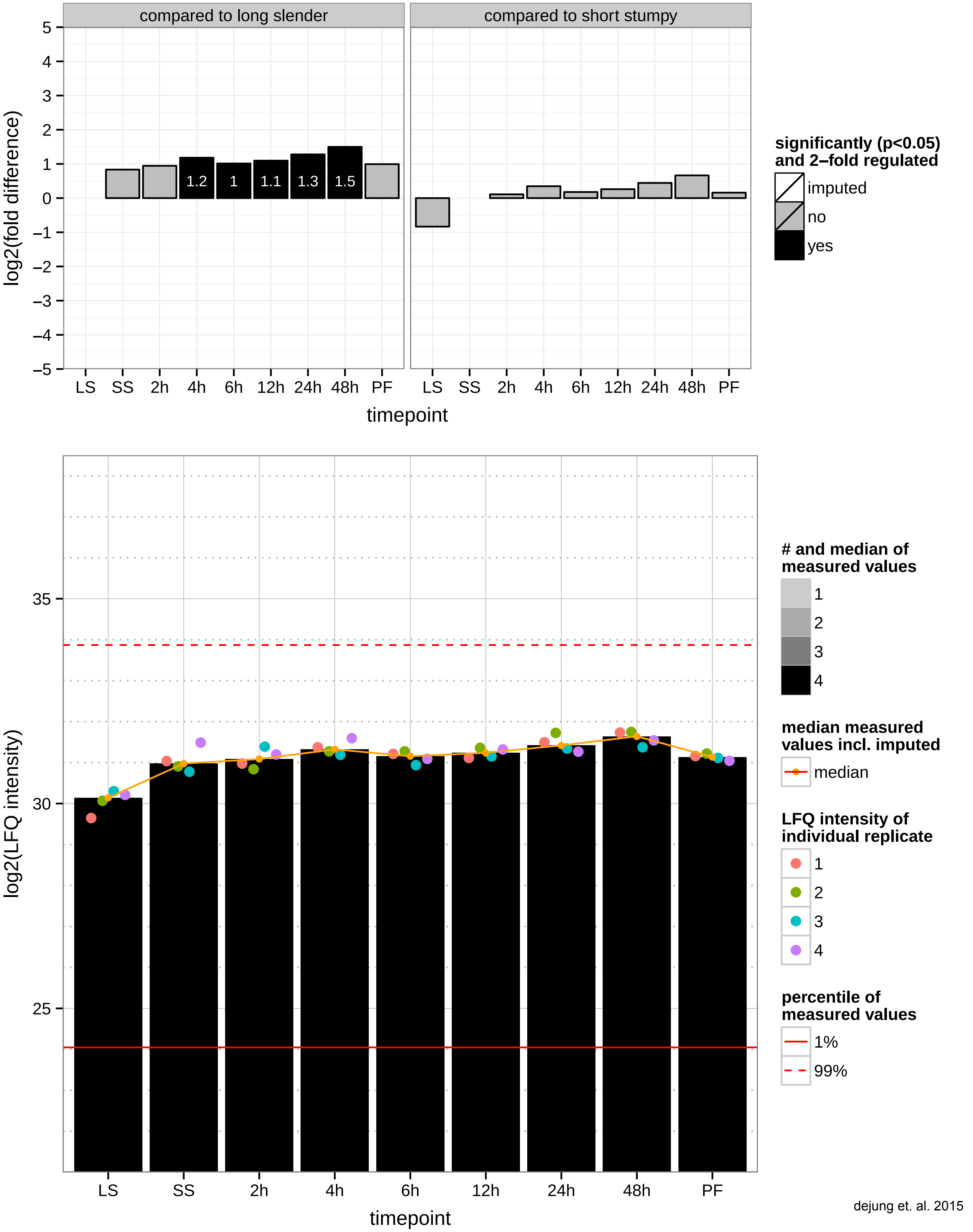

dejung et. al. 2015

hypothetical protein, conserved
Tb927.11.1330
AGOF: null
AGOC: null
AGOP: null
PGOF: null
PGOC: null
PGOP: null

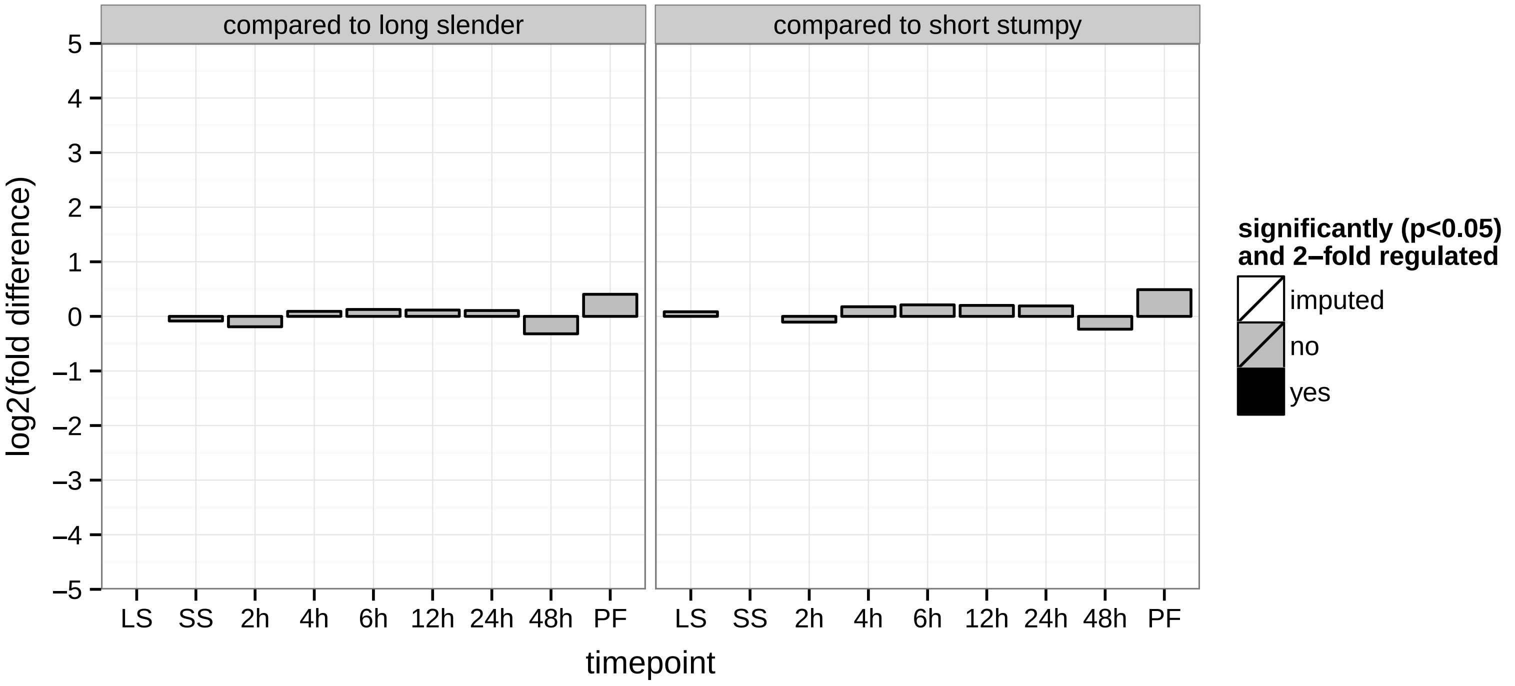

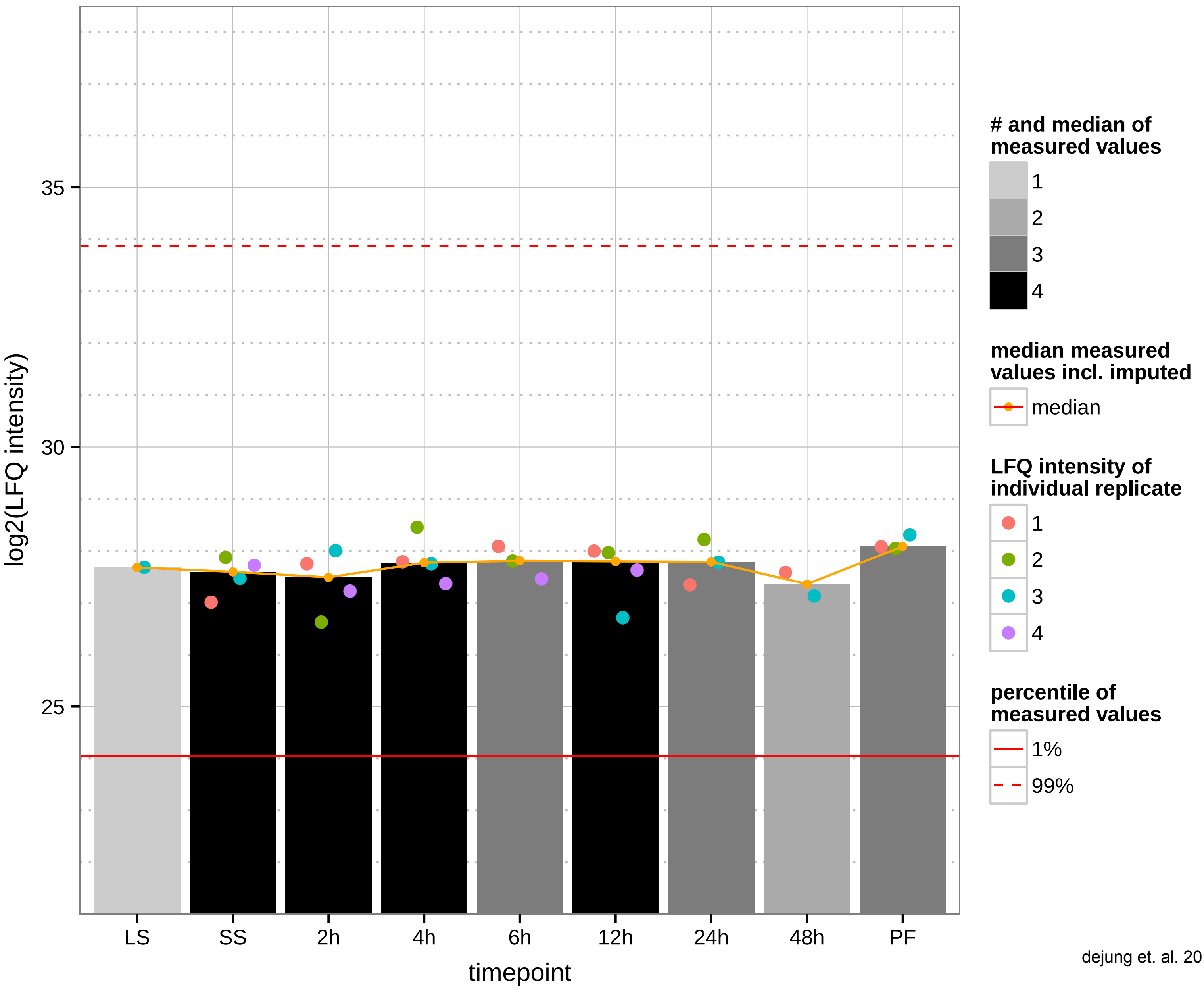

dejung et. al. 2015

hypothetical protein, conserved
Tb927.11.13300
AGOF: null
AGOC: null
AGOP: null
PGOF: null
PGOC: null
PGOP: null

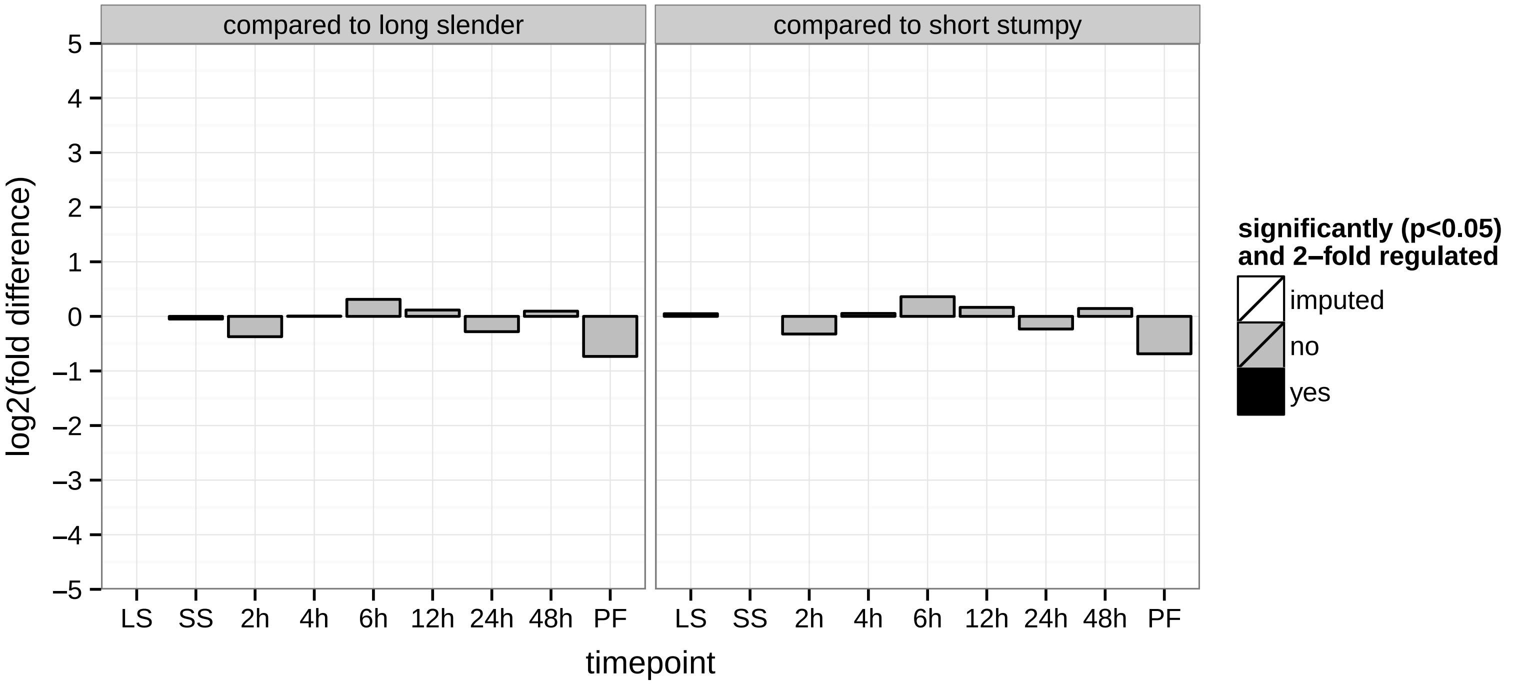

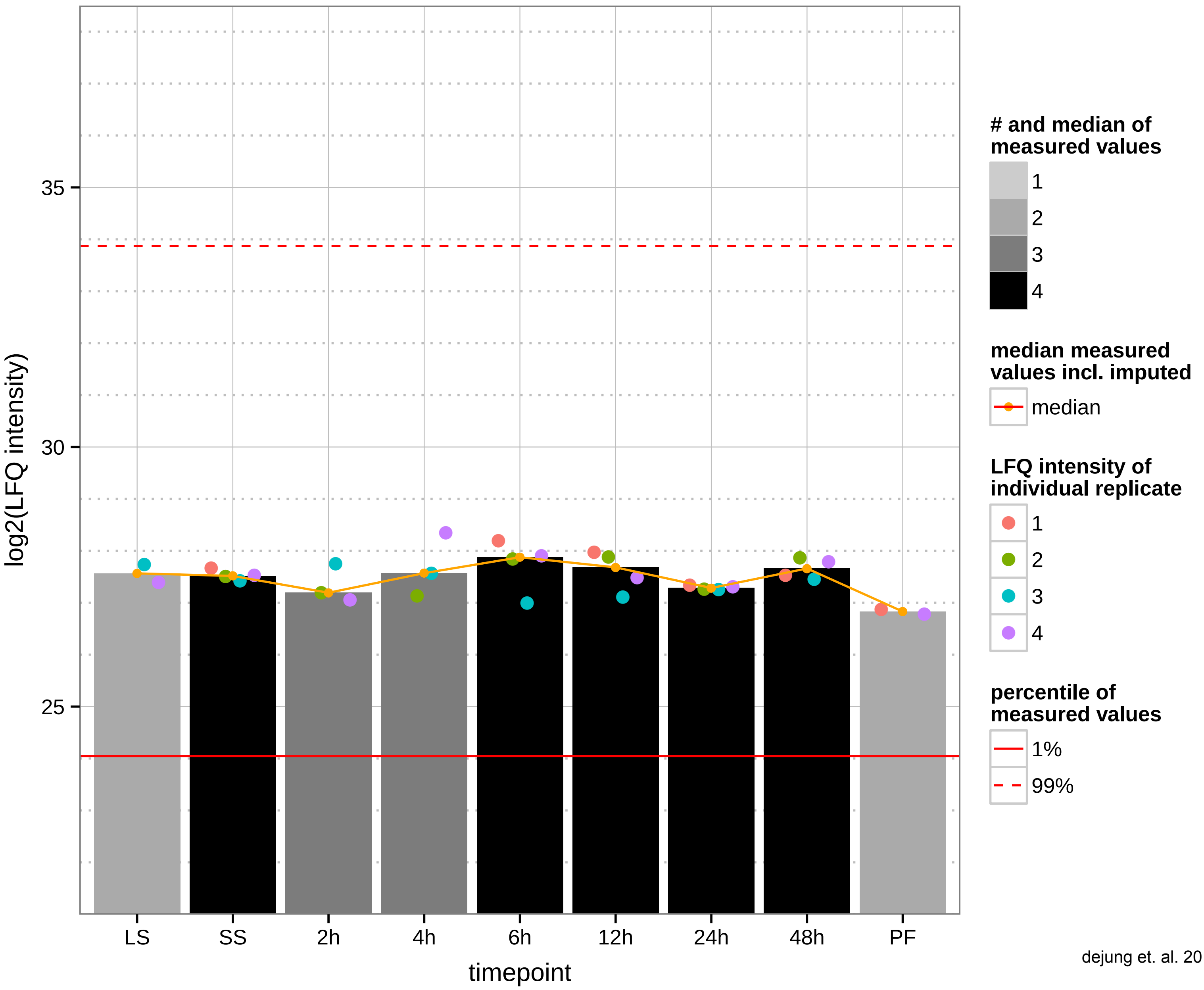

dejung et. al. 2015

AAA ATPase, putative
Tb927.11.13360
AGOF: ATP binding, ATPase activity
AGOC: null
AGOP: protein metabolic process
PGOF: ATP binding, nucleoside–triphosphatase activity, nucleotide binding
PGOC: null
PGOP: null

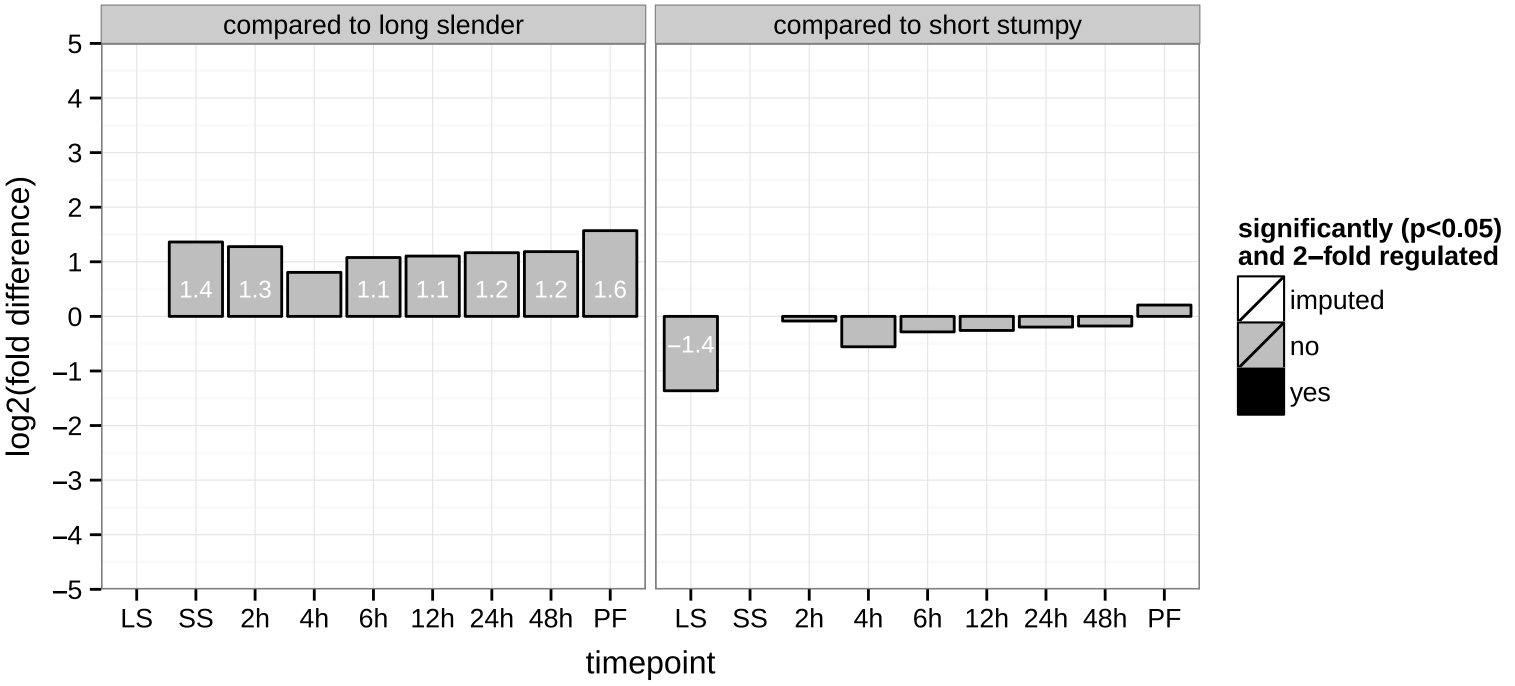

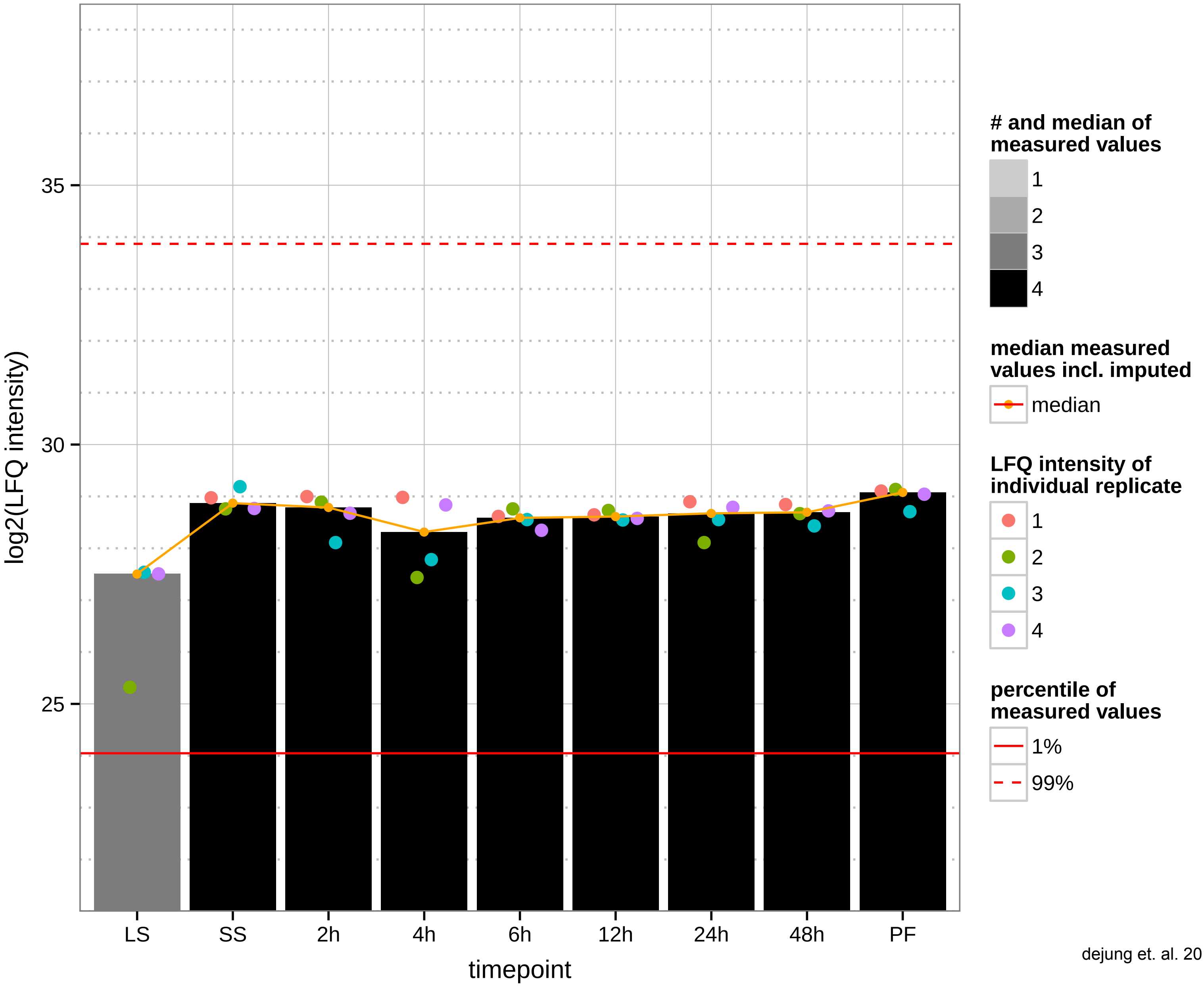

dejung et. al. 2015

hypothetical protein, conserved
Tb927.11.13400
AGOF: null
AGOC: null
AGOP: null
PGOF: protein binding
PGOC: null
PGOP: null

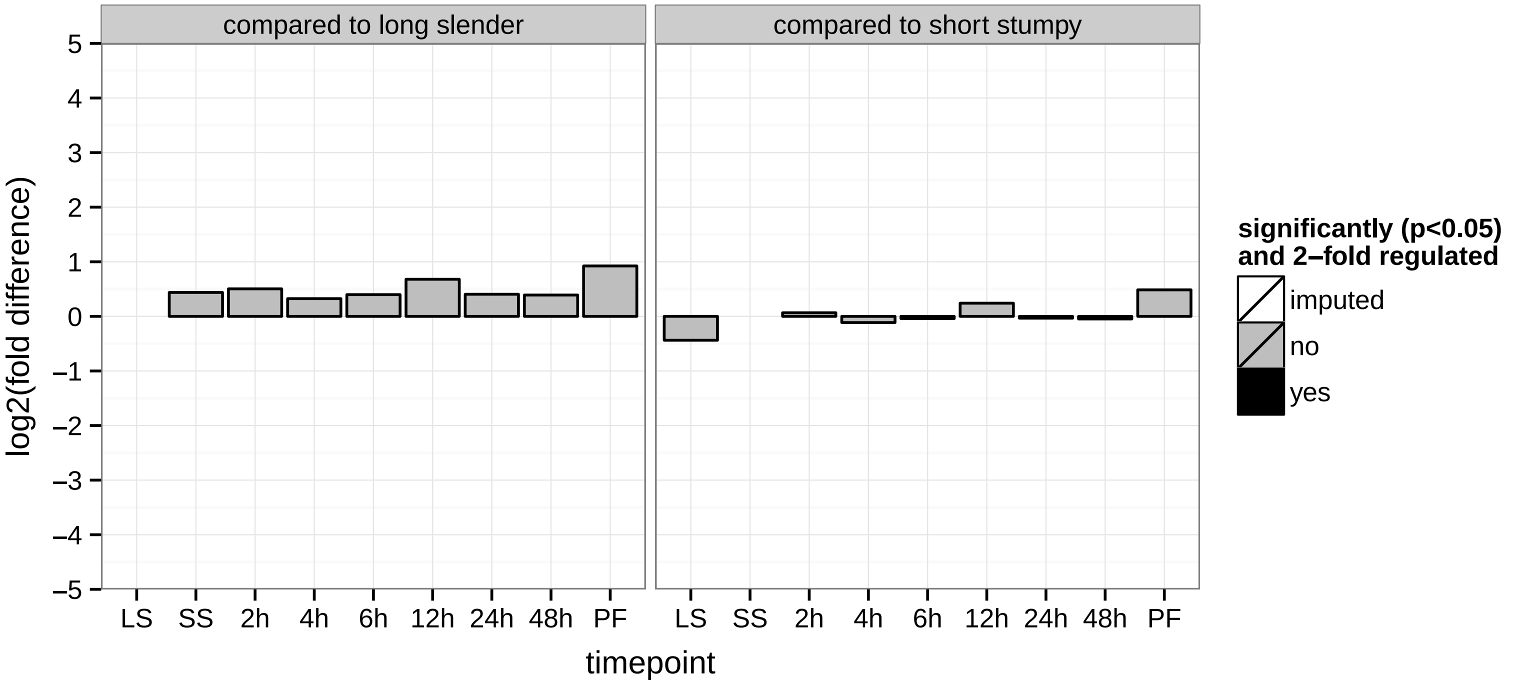

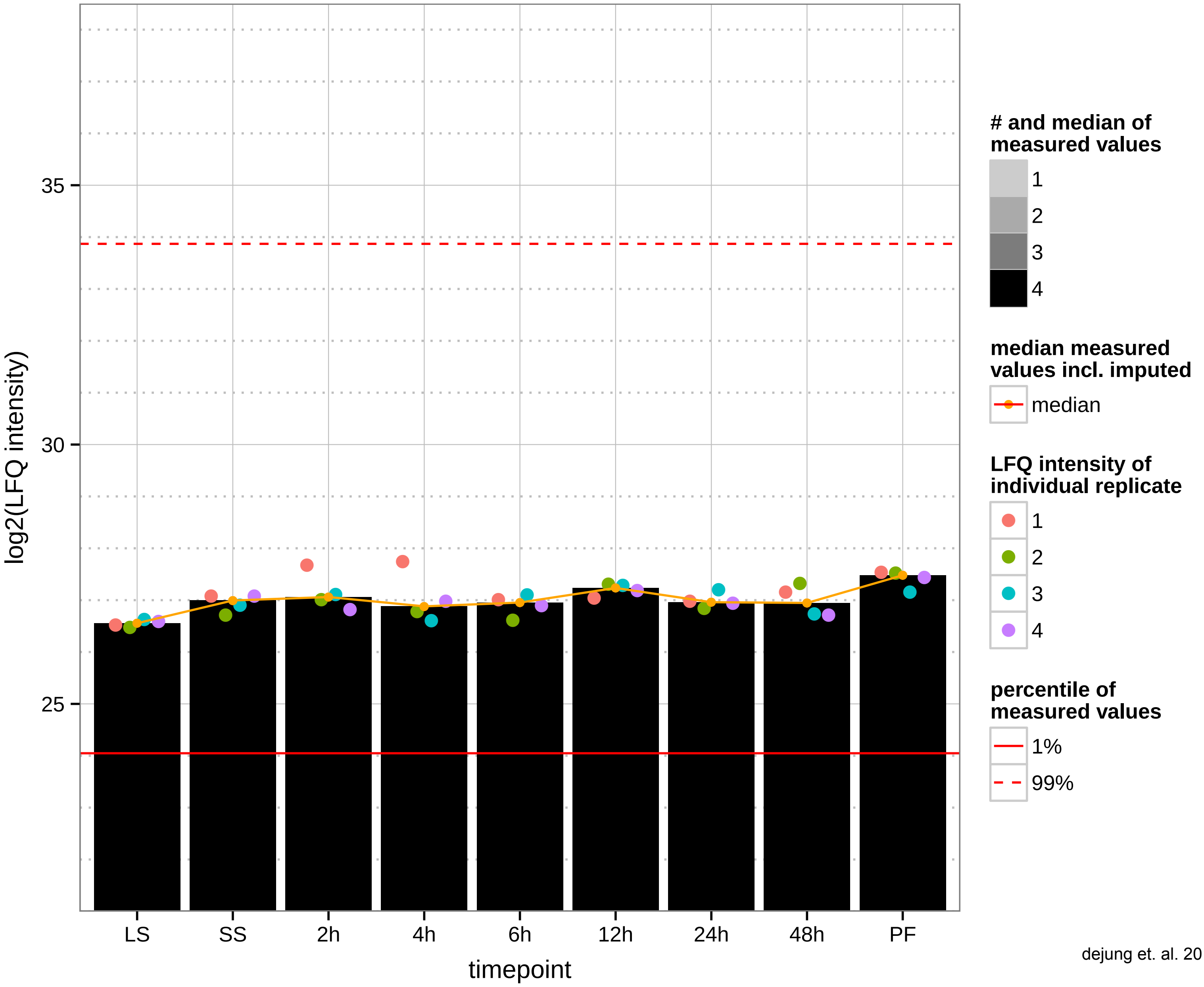

dejung et. al. 2015

hypothetical protein, conserved
Tb927.11.13470
AGOF: null
AGOC: null
AGOP: null
PGOF: null
PGOC: null
PGOP: null

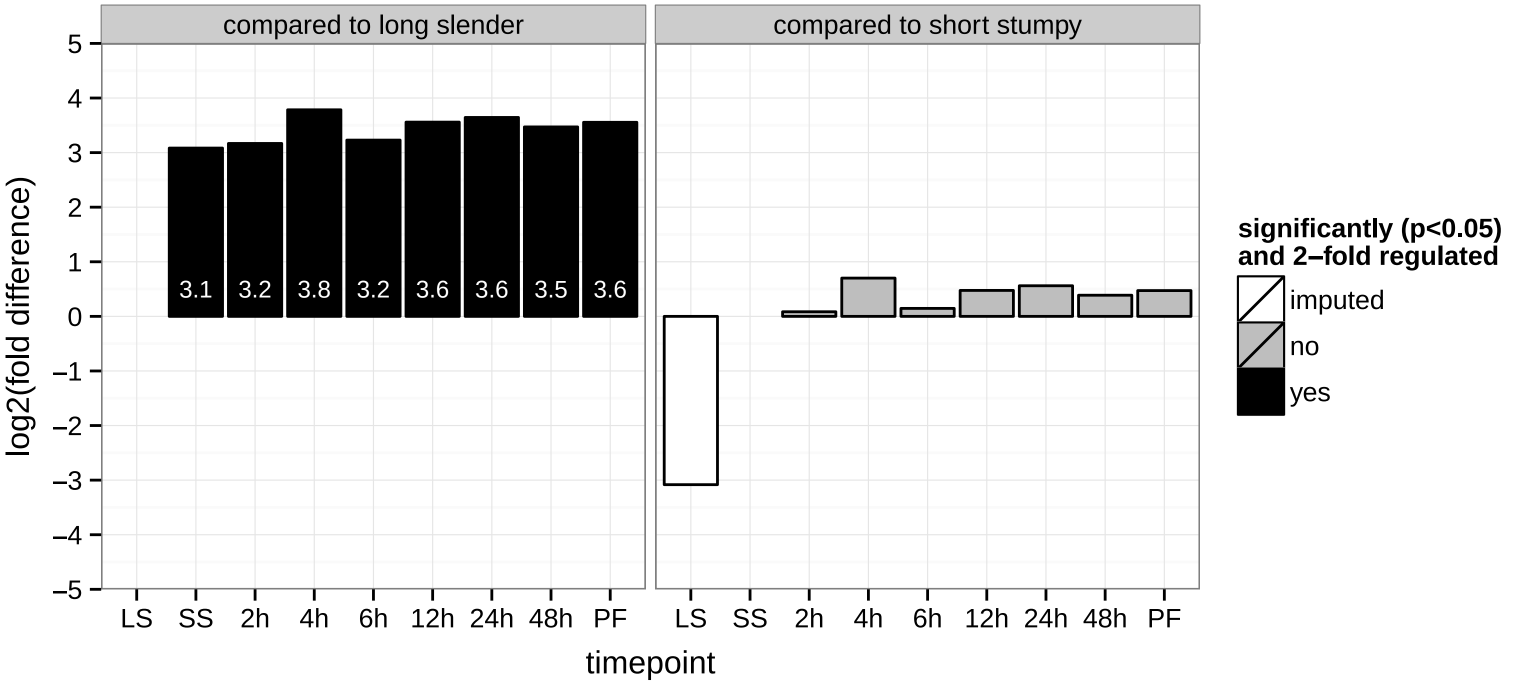

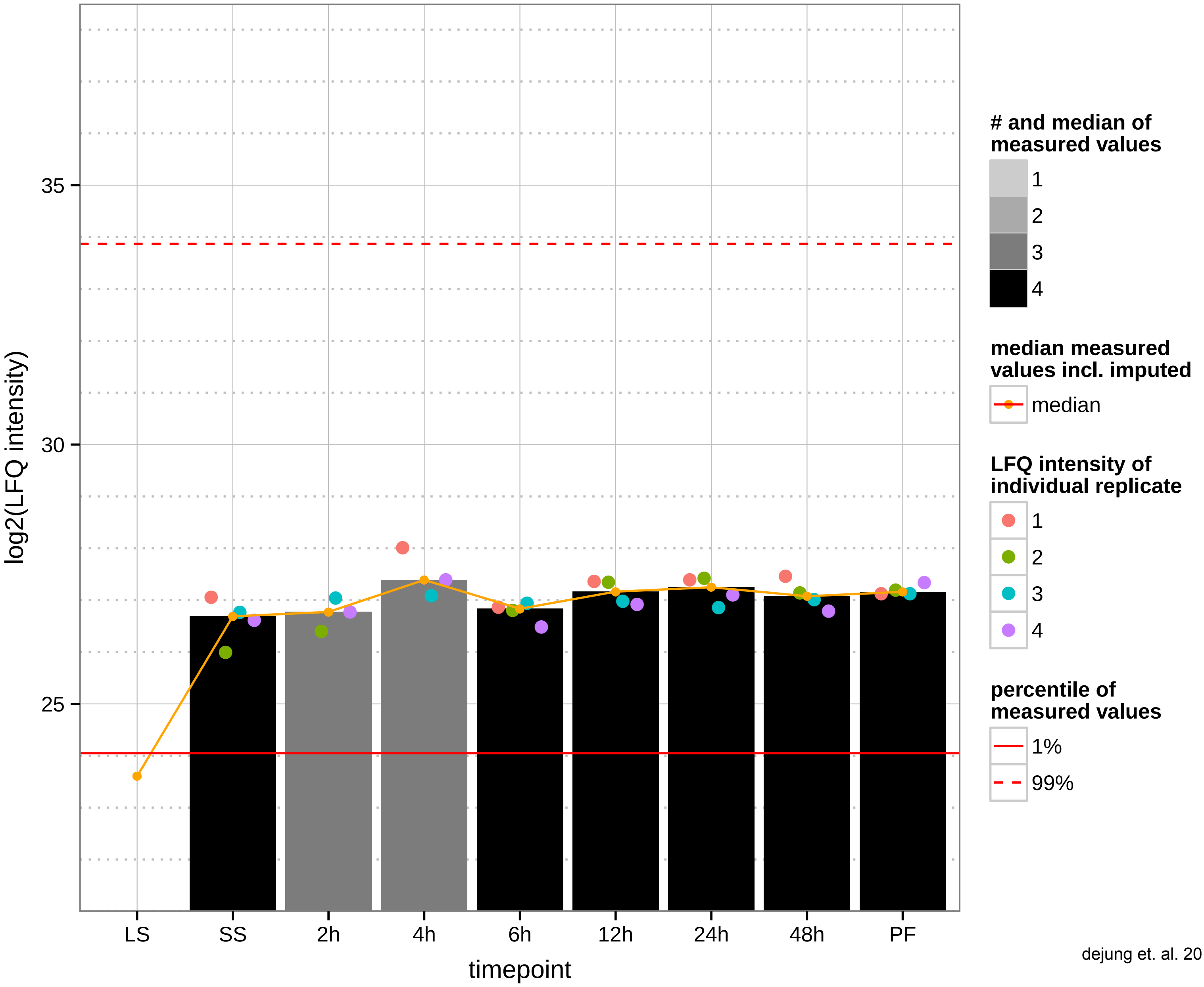

dejung et. al. 2015

par1
Tb927.11.13500
AGOF: calmodulin binding
AGOC: motile cilium
AGOP: null
PGOF: calmodulin binding
PGOC: microtubule–based flagellum
PGOP: null

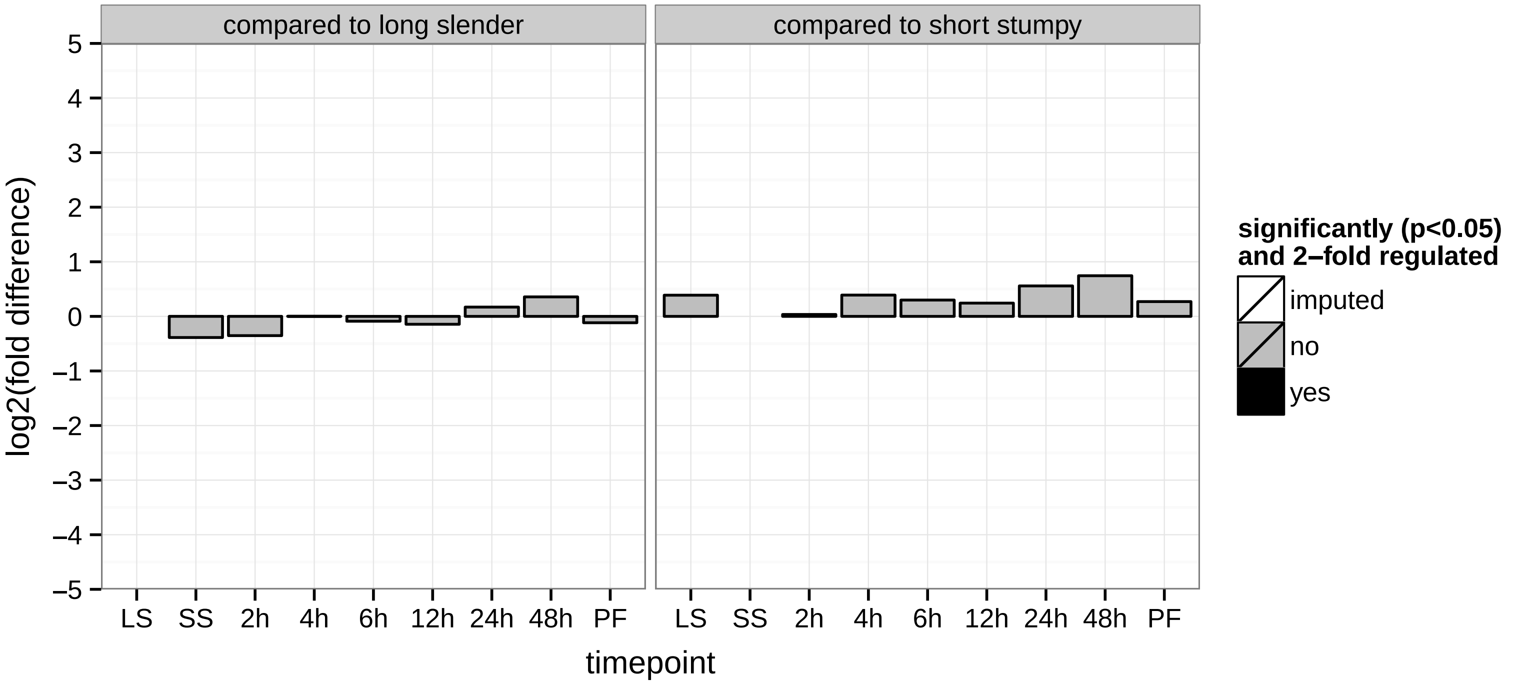

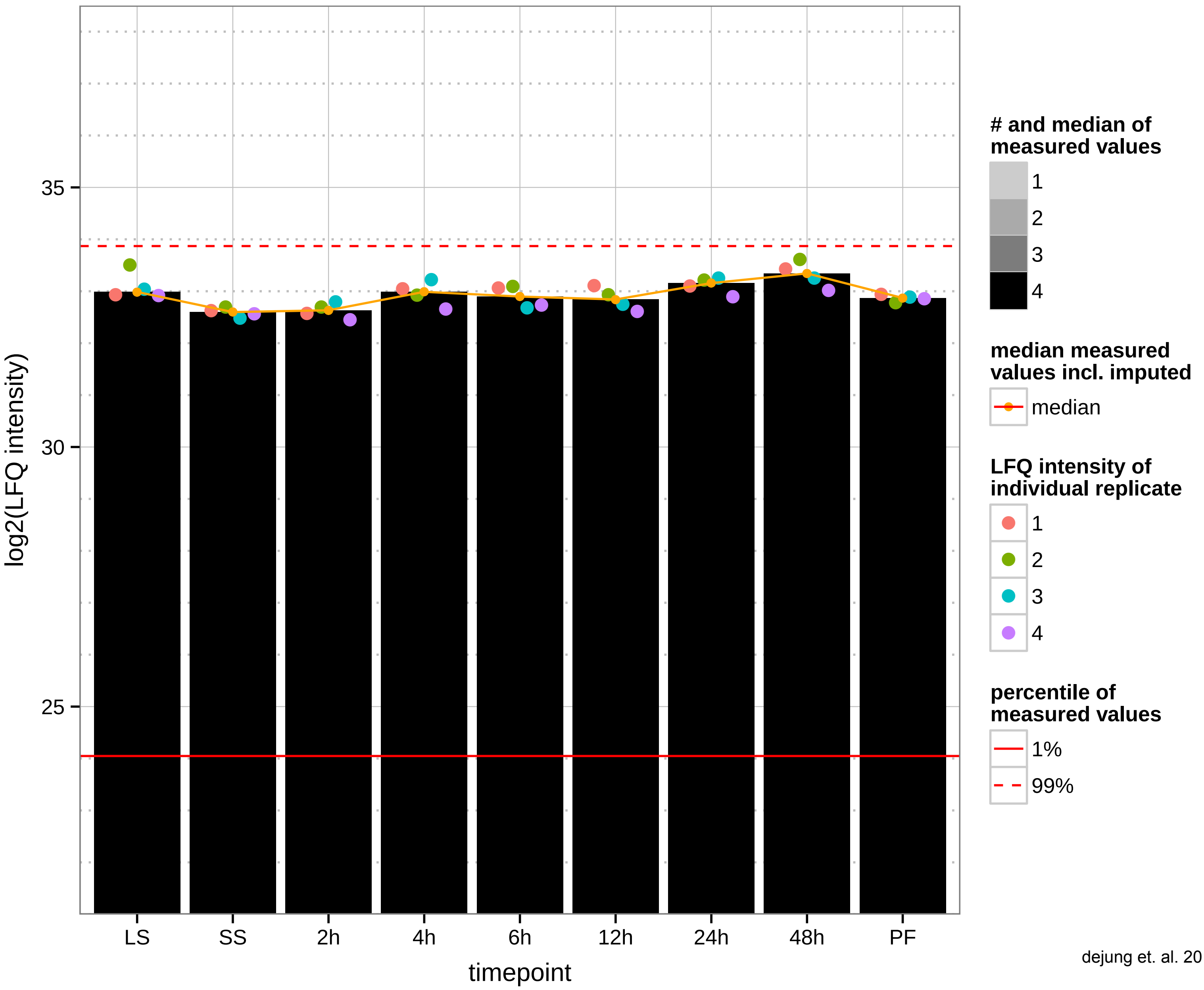

dejung et. al. 2015

hypothetical protein, conserved
Tb927.11.13540
AGOF: null
AGOC: null
AGOP: null
PGOF: null
PGOC: null
PGOP: null

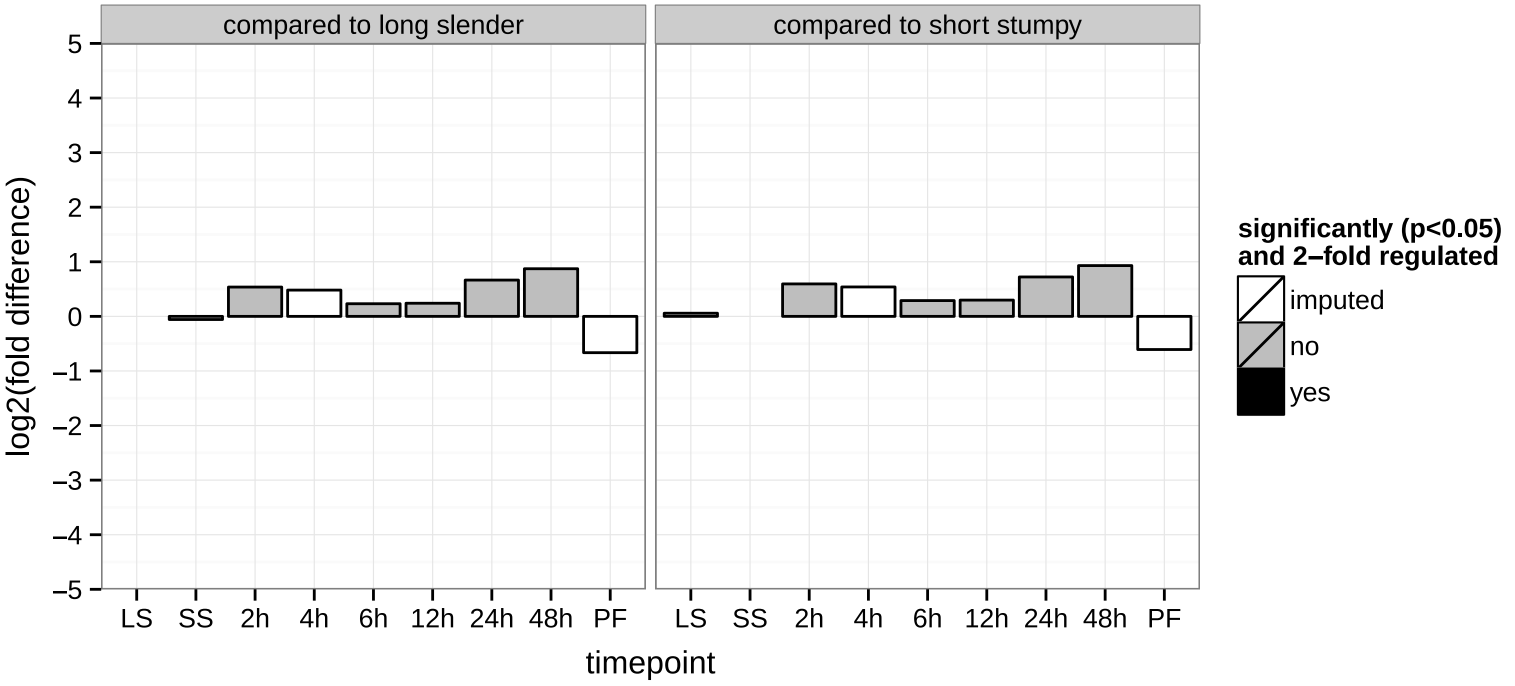

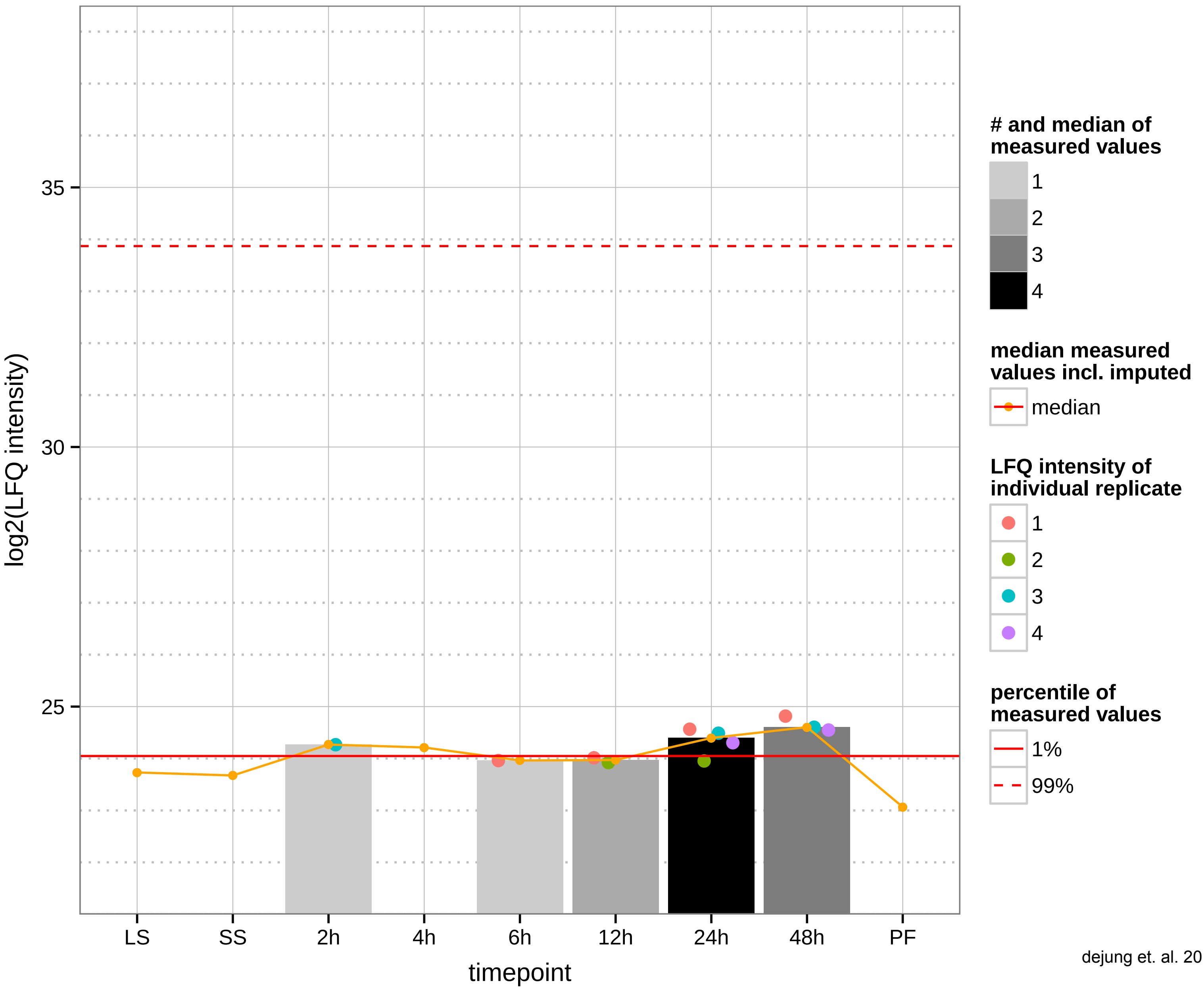

dejung et. al. 2015

ubiquinone biosynthesis protein–like protein
Tb927.11.13580
AGOF: null
AGOC: integral to membrane, mitochondrion
AGOP: ubiquinone biosynthetic process
PGOF: null
PGOC: null
PGOP: ubiquinone biosynthetic process

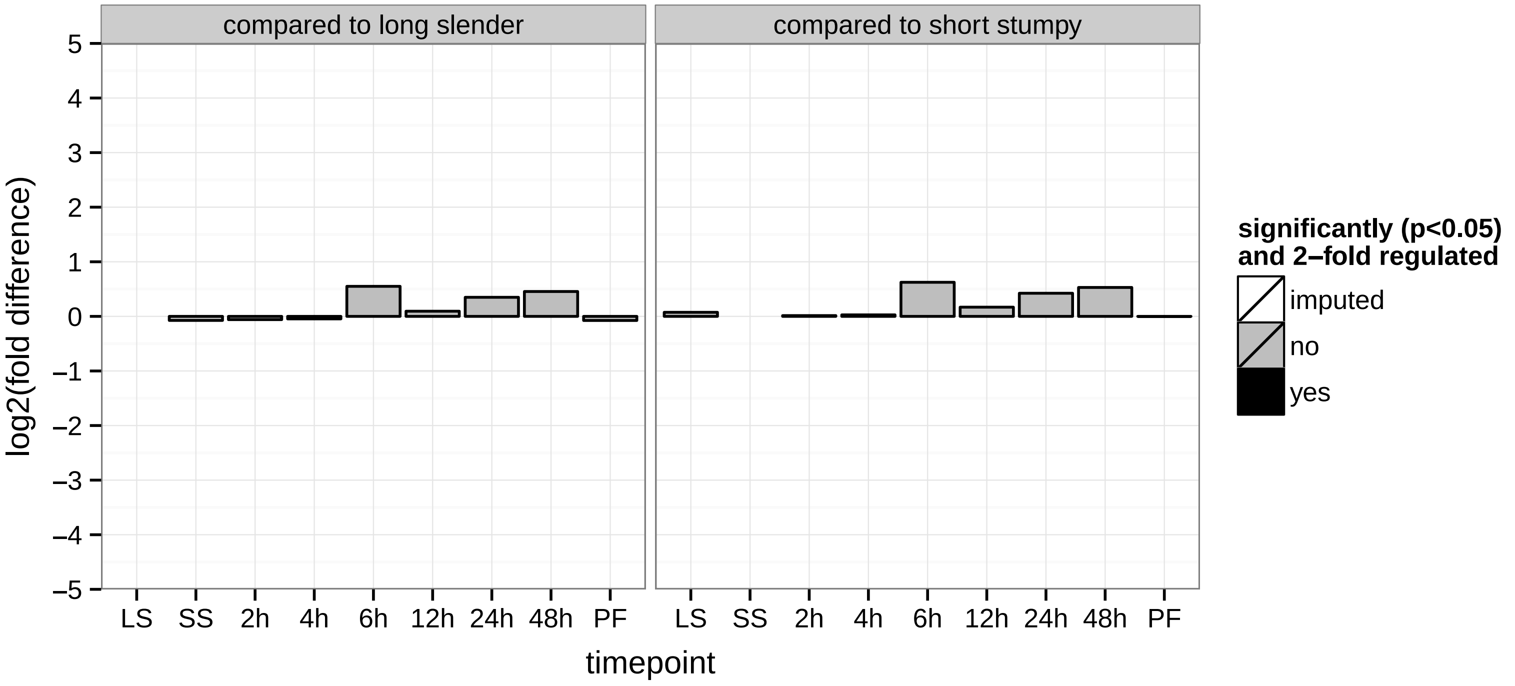

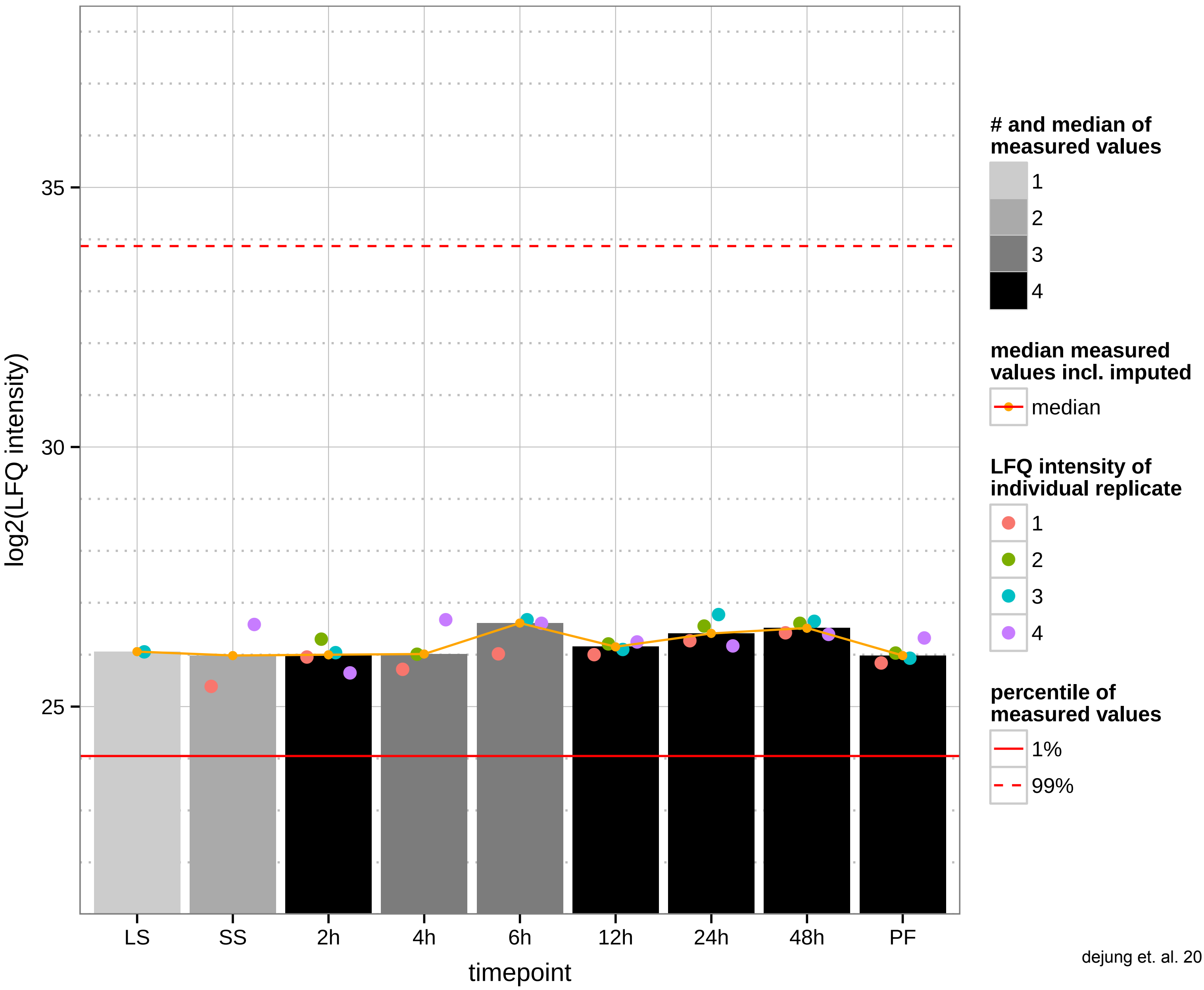

dejung et. al. 2015

hypothetical protein, conserved
Tb927.11.13670
AGOF: null
AGOC: null
AGOP: null
PGOF: null
PGOC: null
PGOP: null

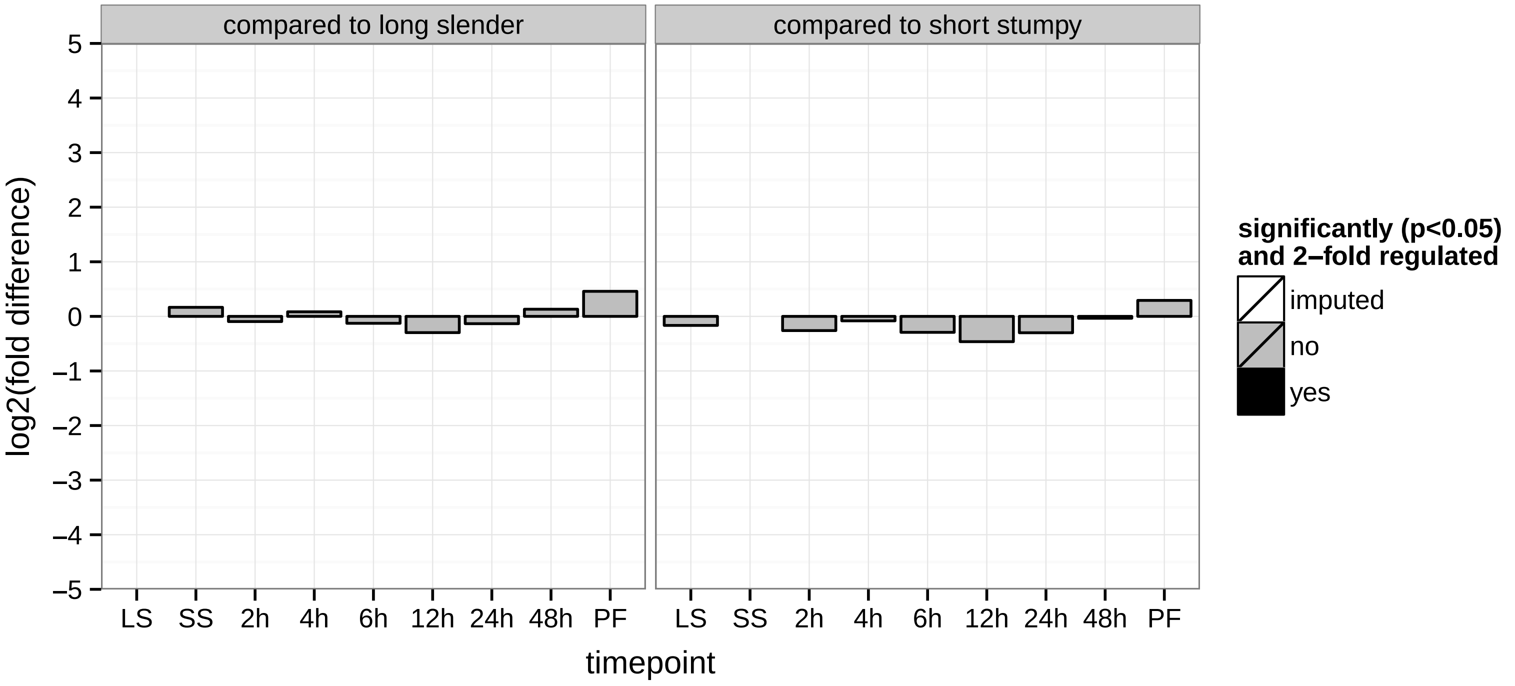

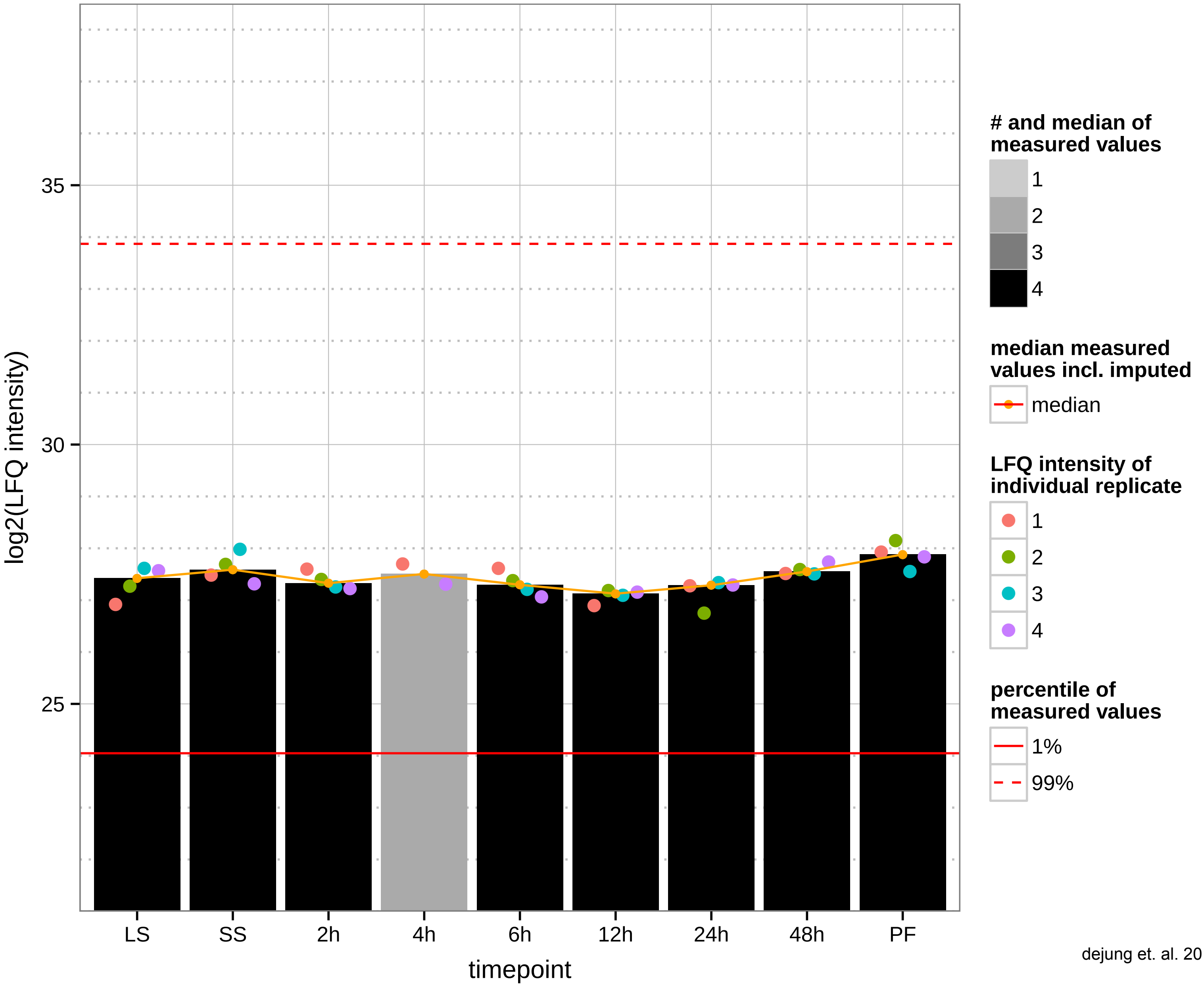

dejung et. al. 2015

Ras−related protein Rab4 (RAB4)
Tb927.11.13750
AGOF: GTP binding, GTPase activity
AGOC: intracellular
AGOP: intracellular protein transport, nucleocytoplasmic transport, small GTPase mediated signal transduction
PGOF: GTP binding, GTPase activity
PGOC: intracellular, membrane
PGOP: GTP catabolic process, intracellular protein transport, nucleocytoplasmic transport, protein transport, signal transducti

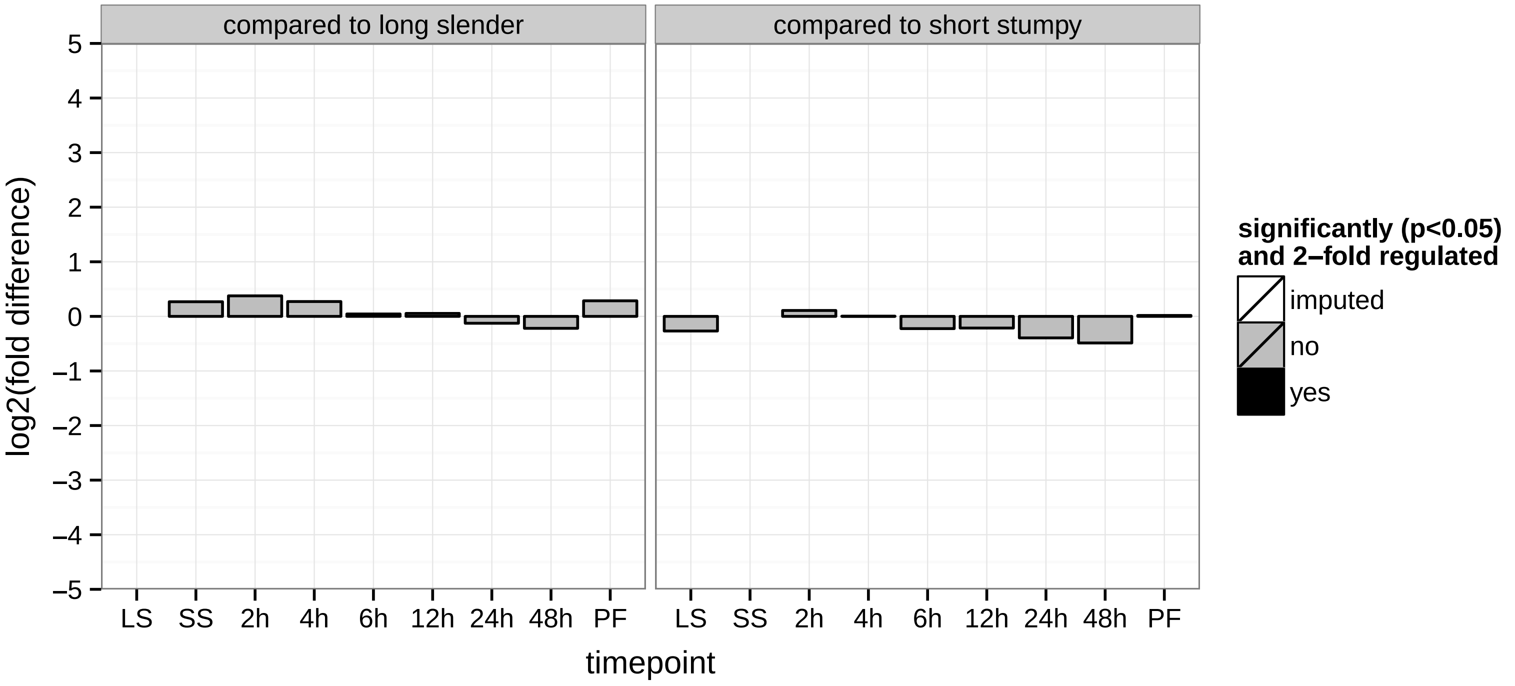

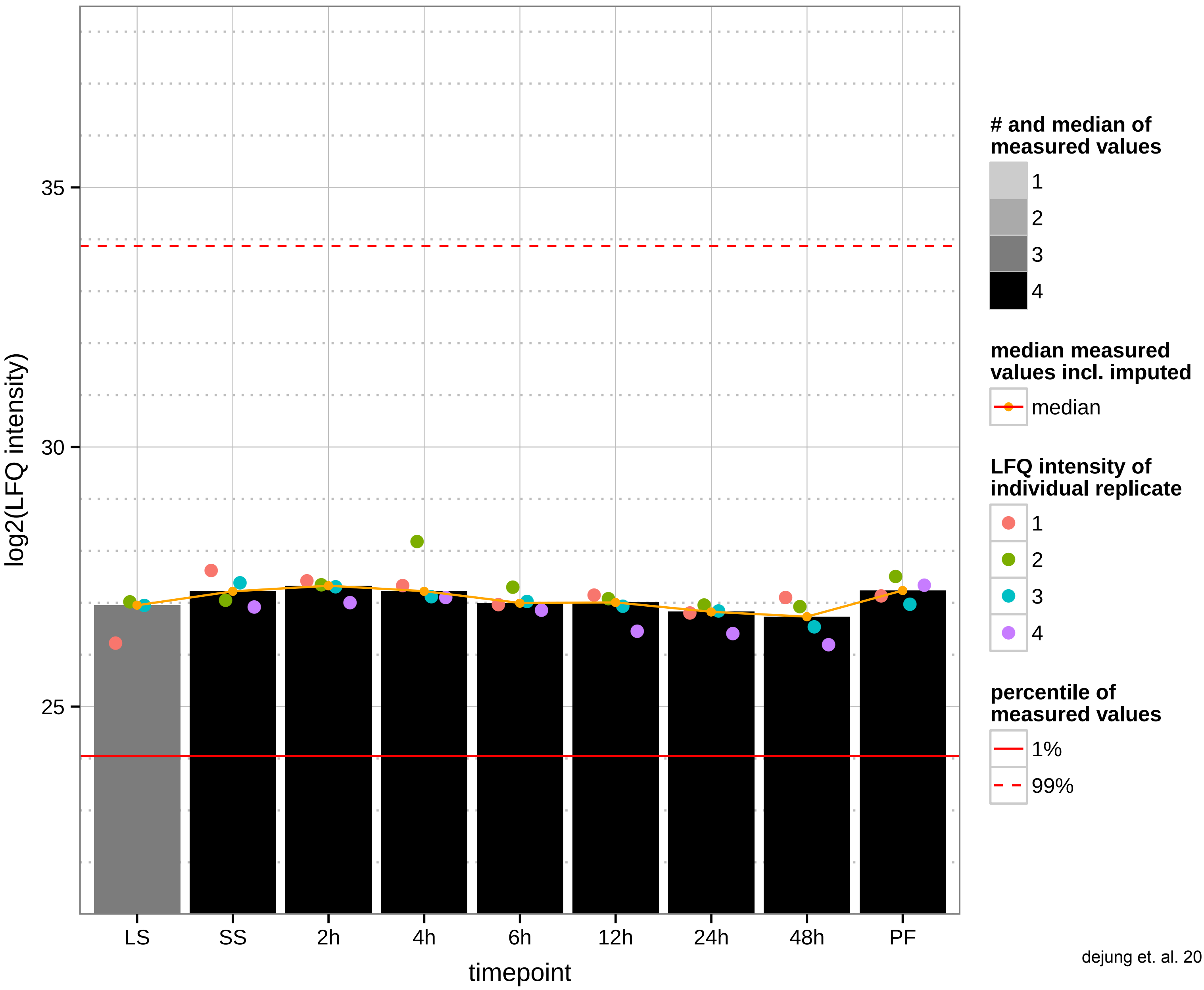

dejung et. al. 2015

hypothetical protein, conserved
Tb927.11.13770
AGOF: null
AGOC: membrane
AGOP: null
PGOF: null
PGOC: membrane
PGOP: null

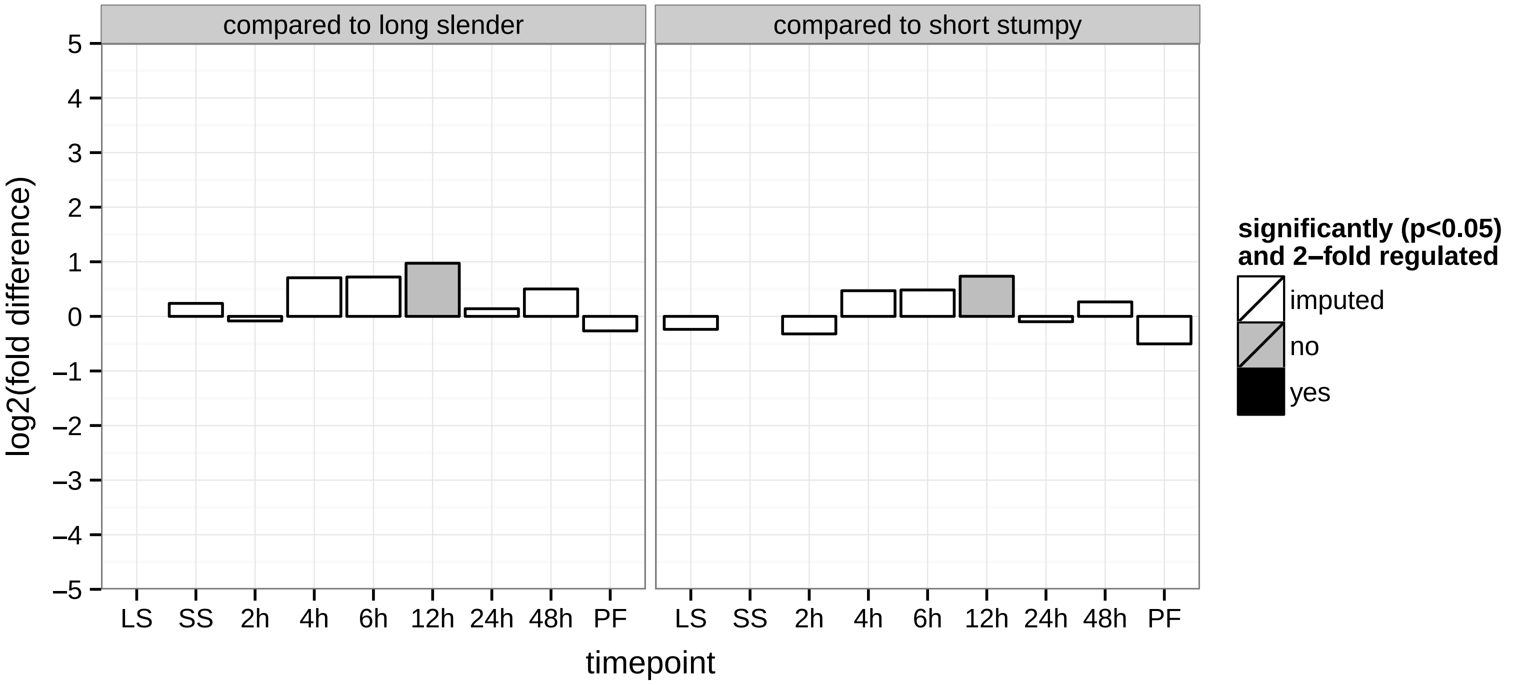

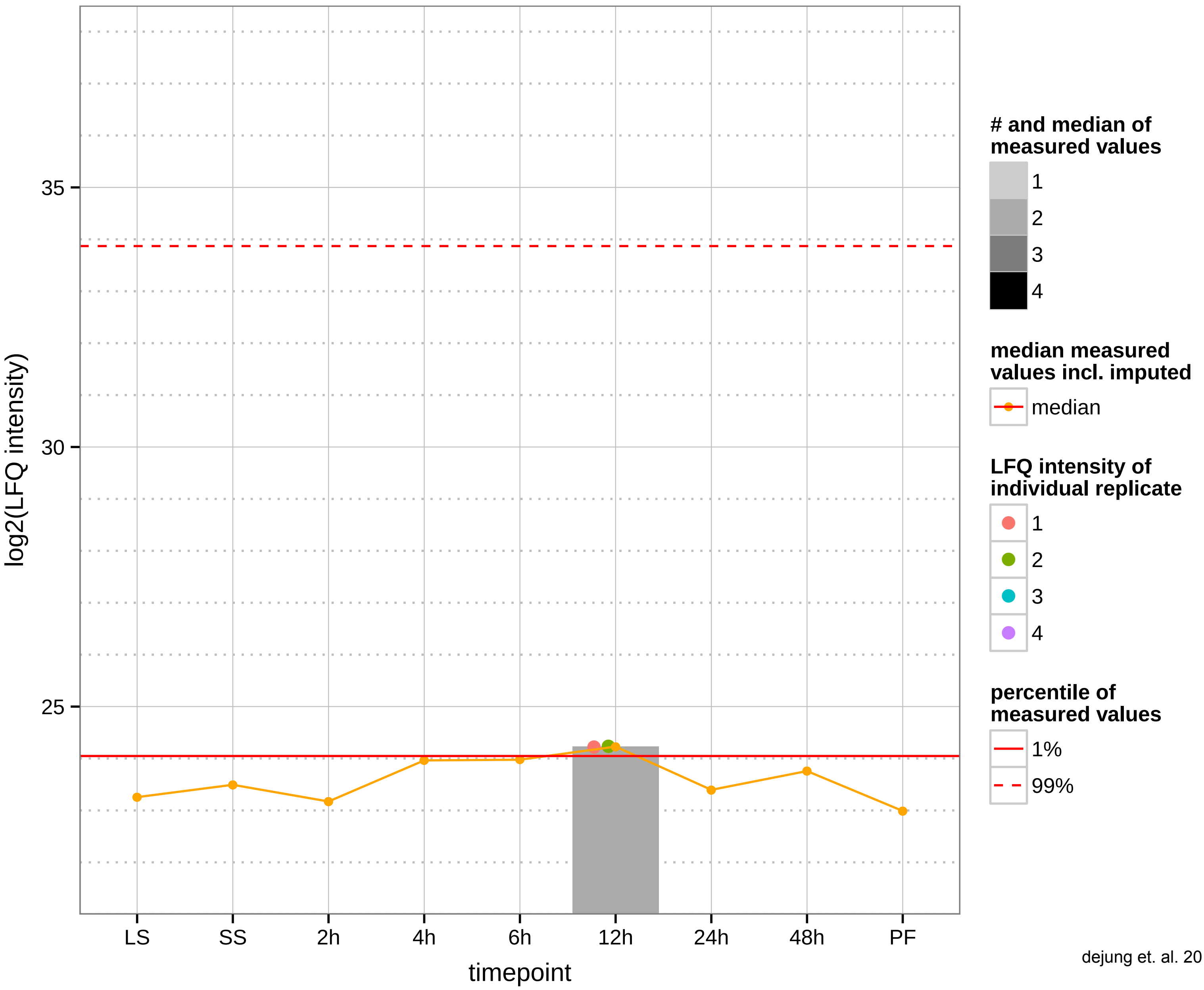

dejung et. al. 2015

profilin
Tb927.11.13780
AGOF: actin binding
AGOC: null
AGOP: actin cytoskeleton organization, cytoskeleton organization
PGOF: actin binding
PGOC: actin cytoskeleton
PGOP: cytoskeleton organization

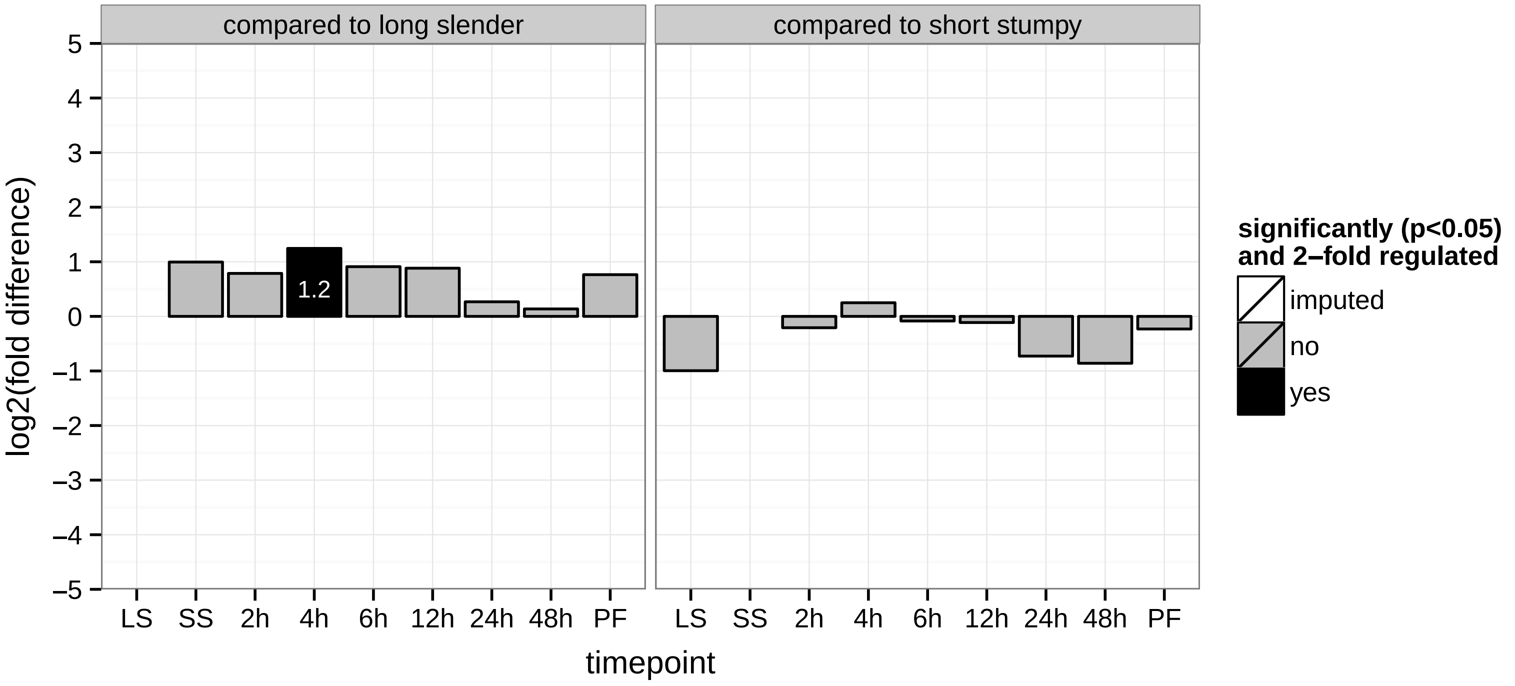

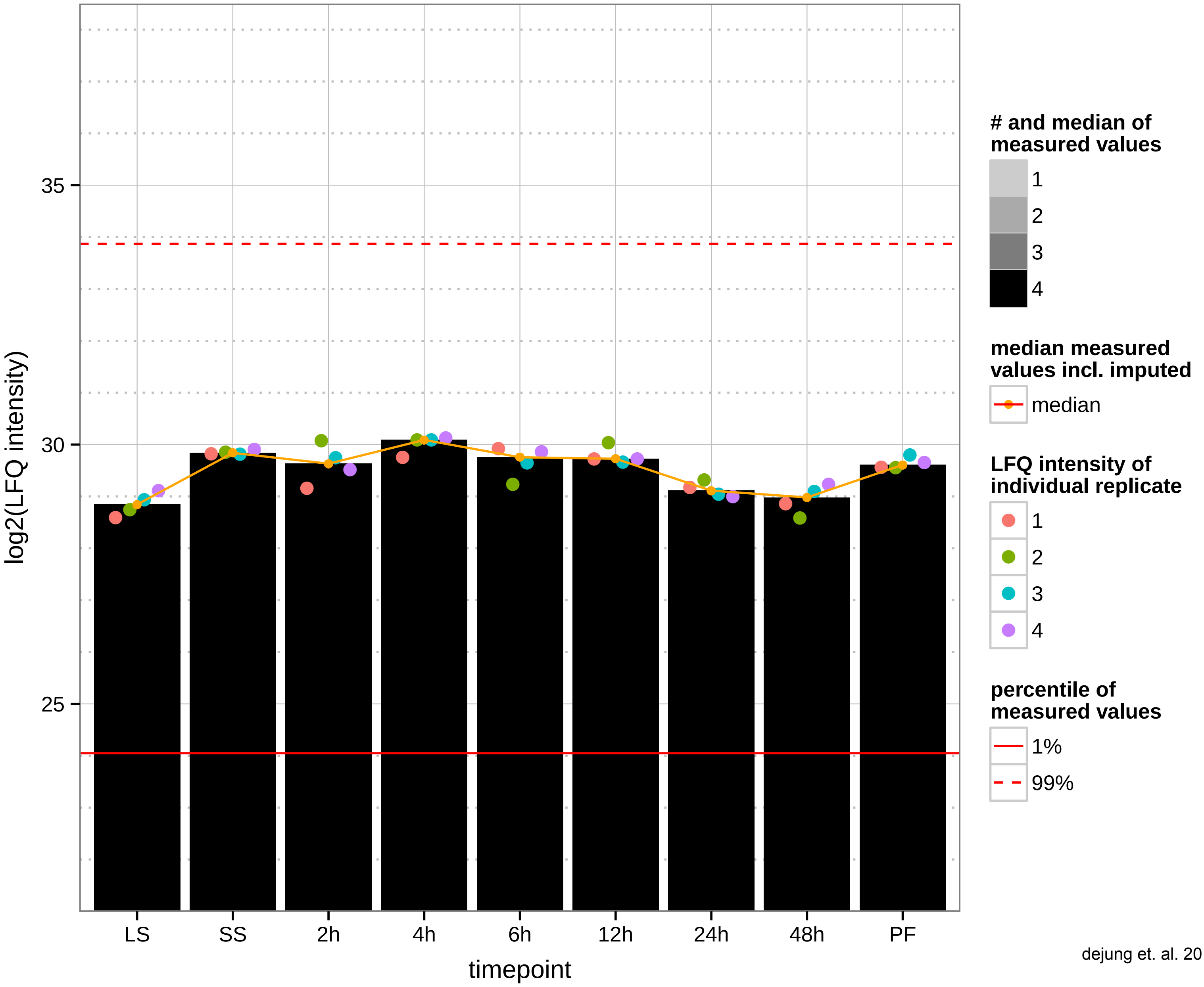

dejung et. al. 2015

ATP−dependent DEAD/H RNA helicase, putative, ATP− dependent RNA helicase
Tb927.11.13810
AGOF: ATP binding, ATP−dependent helicase activity, helicase activity, nucleic acid binding
AGOC: null
AGOP: nucleobase−containing compound metabolic process
PGOF: ATP binding, ATP−dependent helicase activity, helicase activity, nucleic acid binding
PGOC: null
PGOP: null

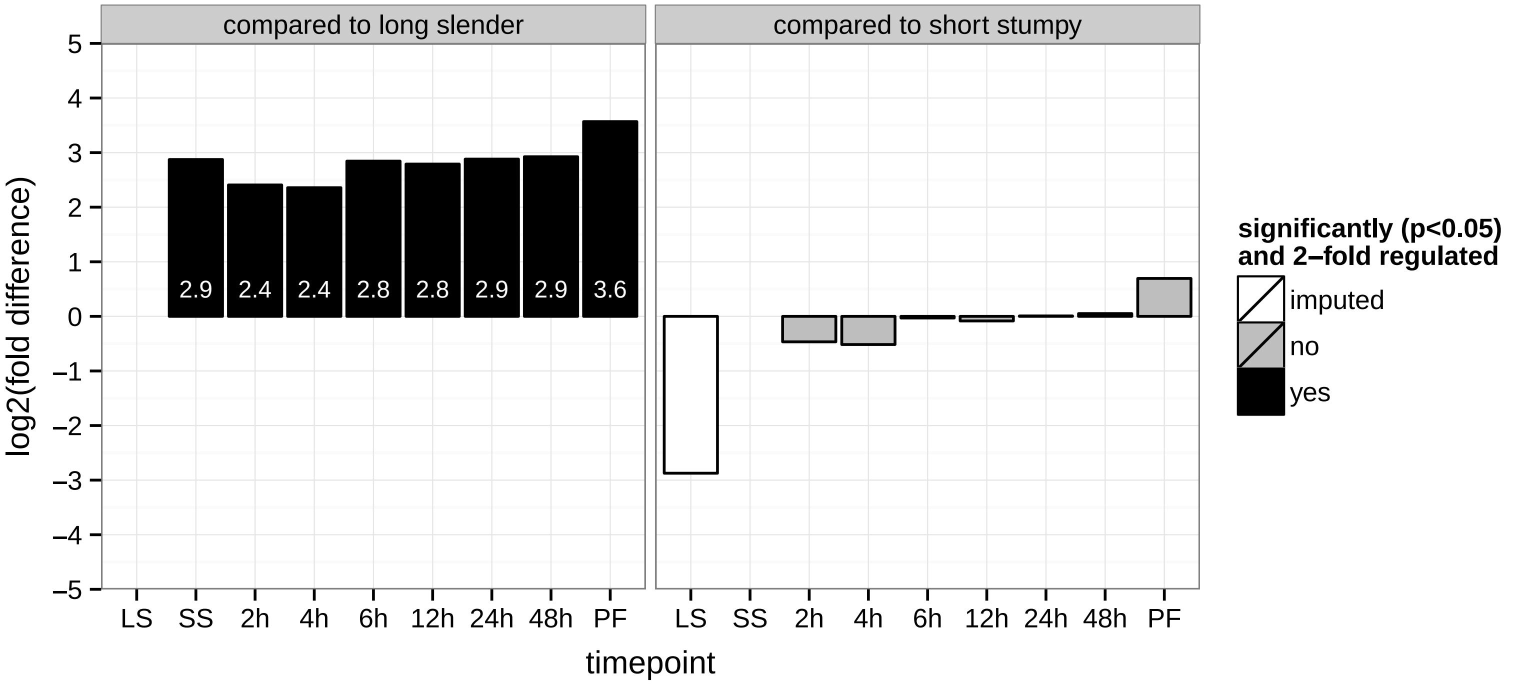

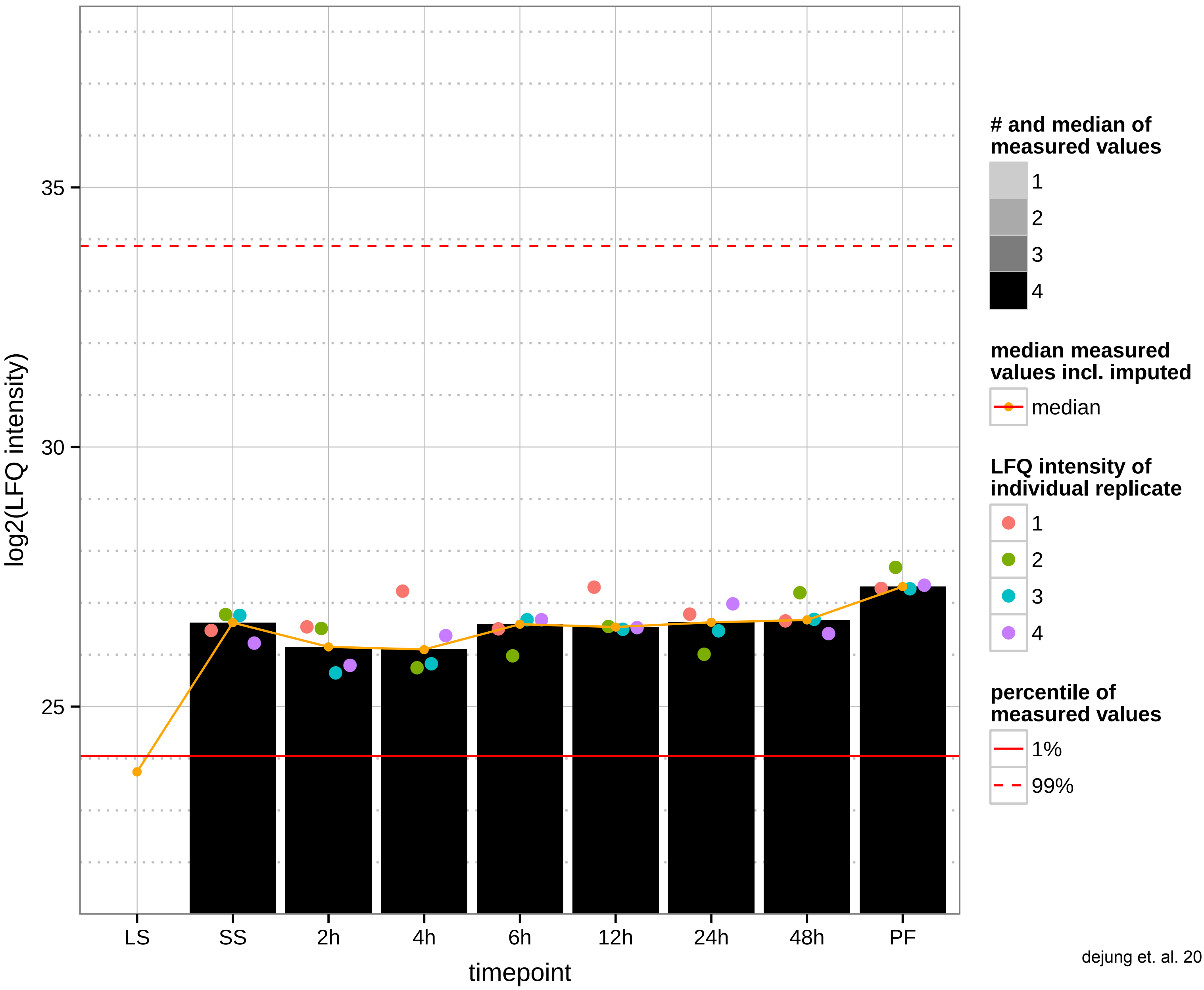

dejung et. al. 2015

hypothetical protein, conserved
Tb927.11.13820
AGOF: null
AGOC: null
AGOP: null
PGOF: null
PGOC: null
PGOP: null

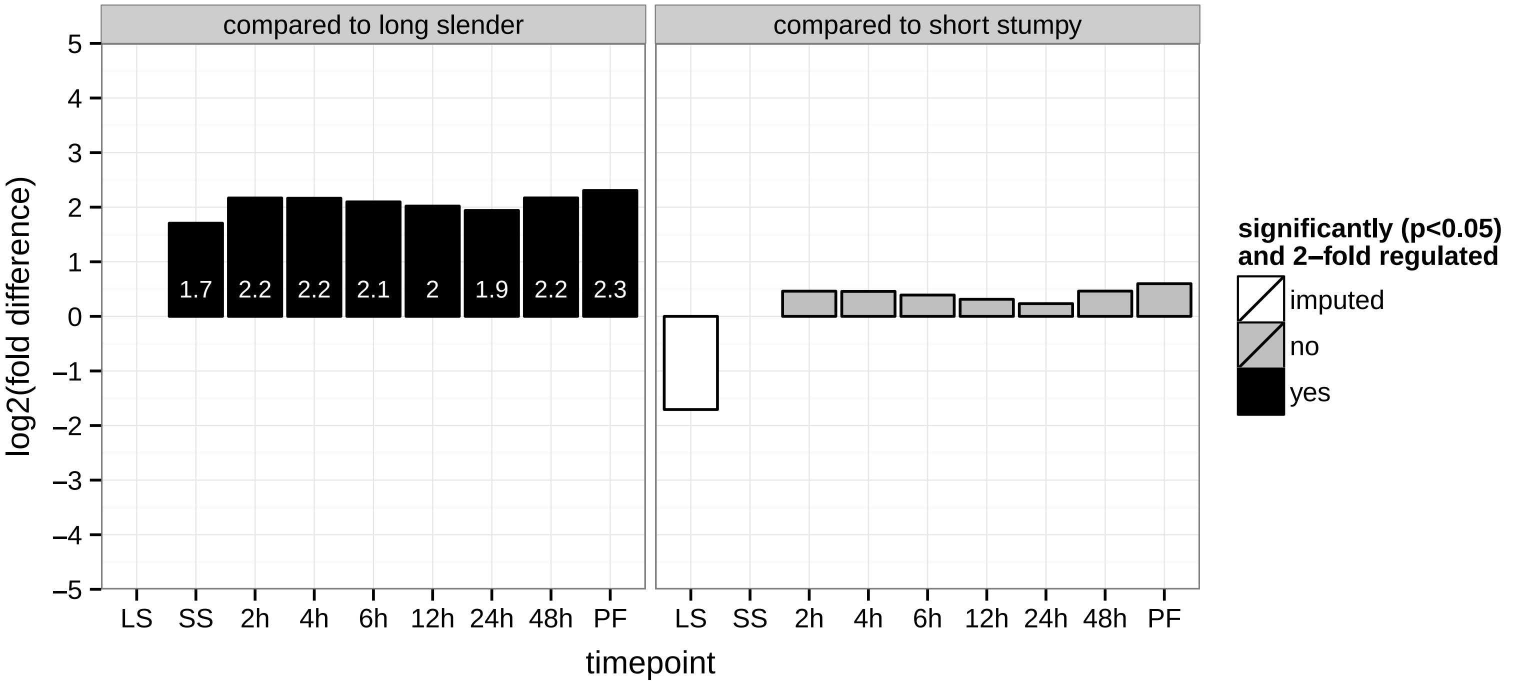

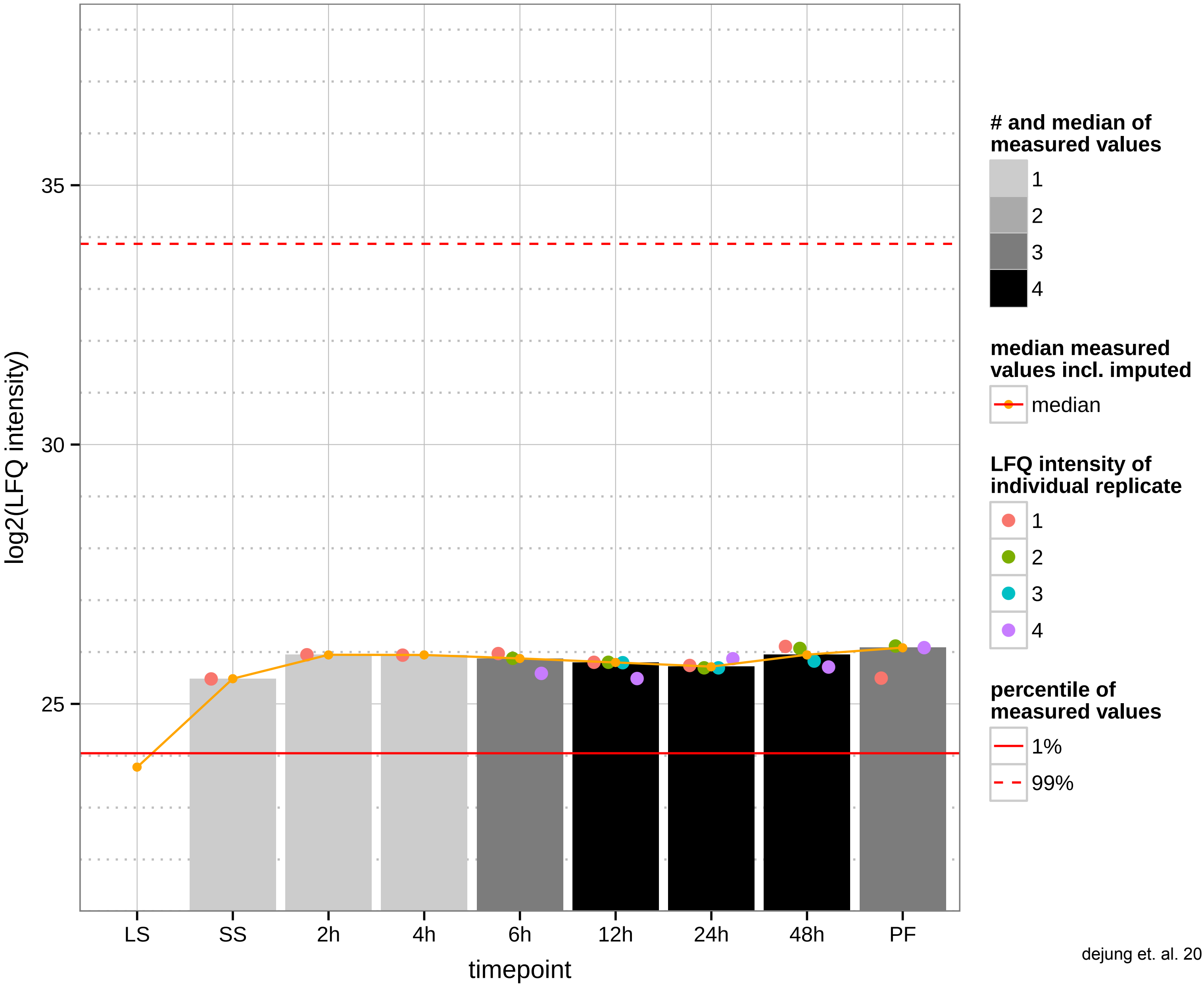

dejung et. al. 2015

hypothetical protein, conserved
Tb927.11.13860
AGOF: null
AGOC: null
AGOP: null
PGOF: null
PGOC: null
PGOP: null

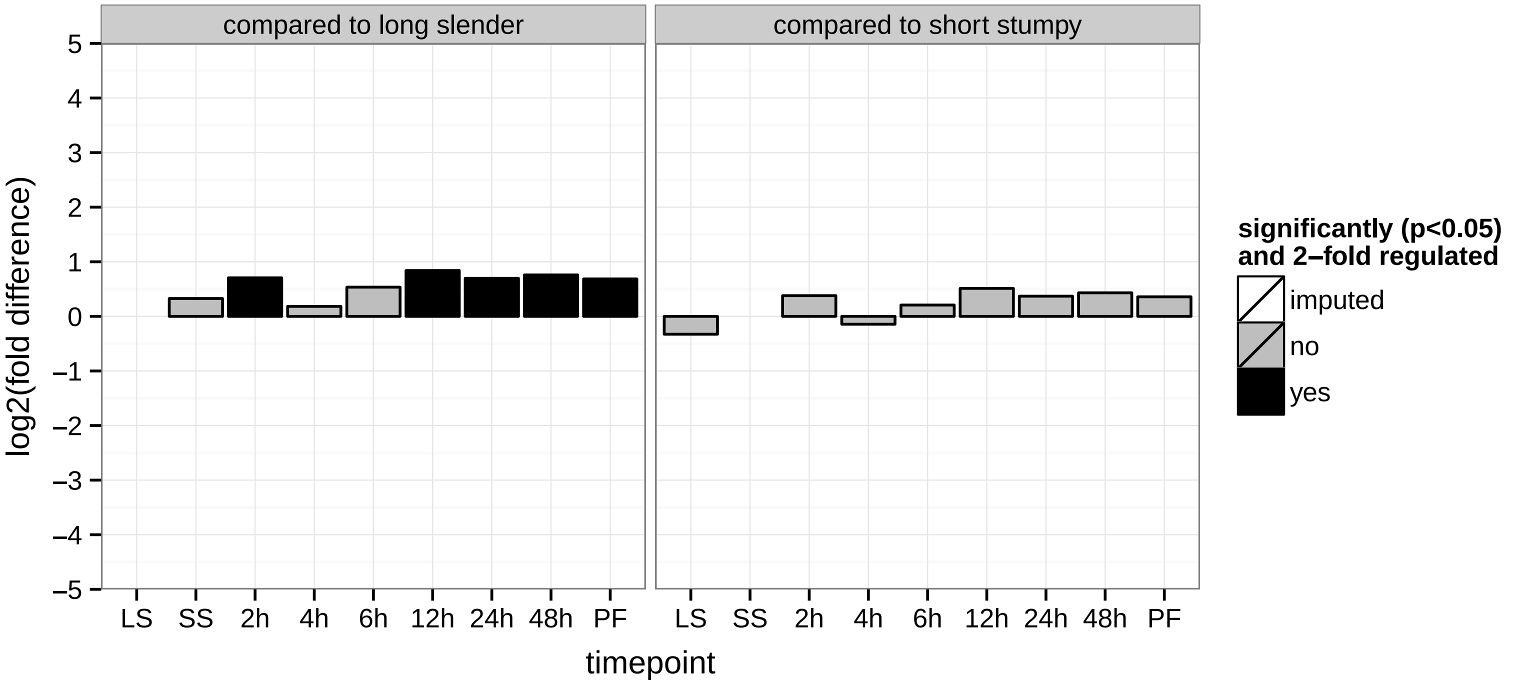

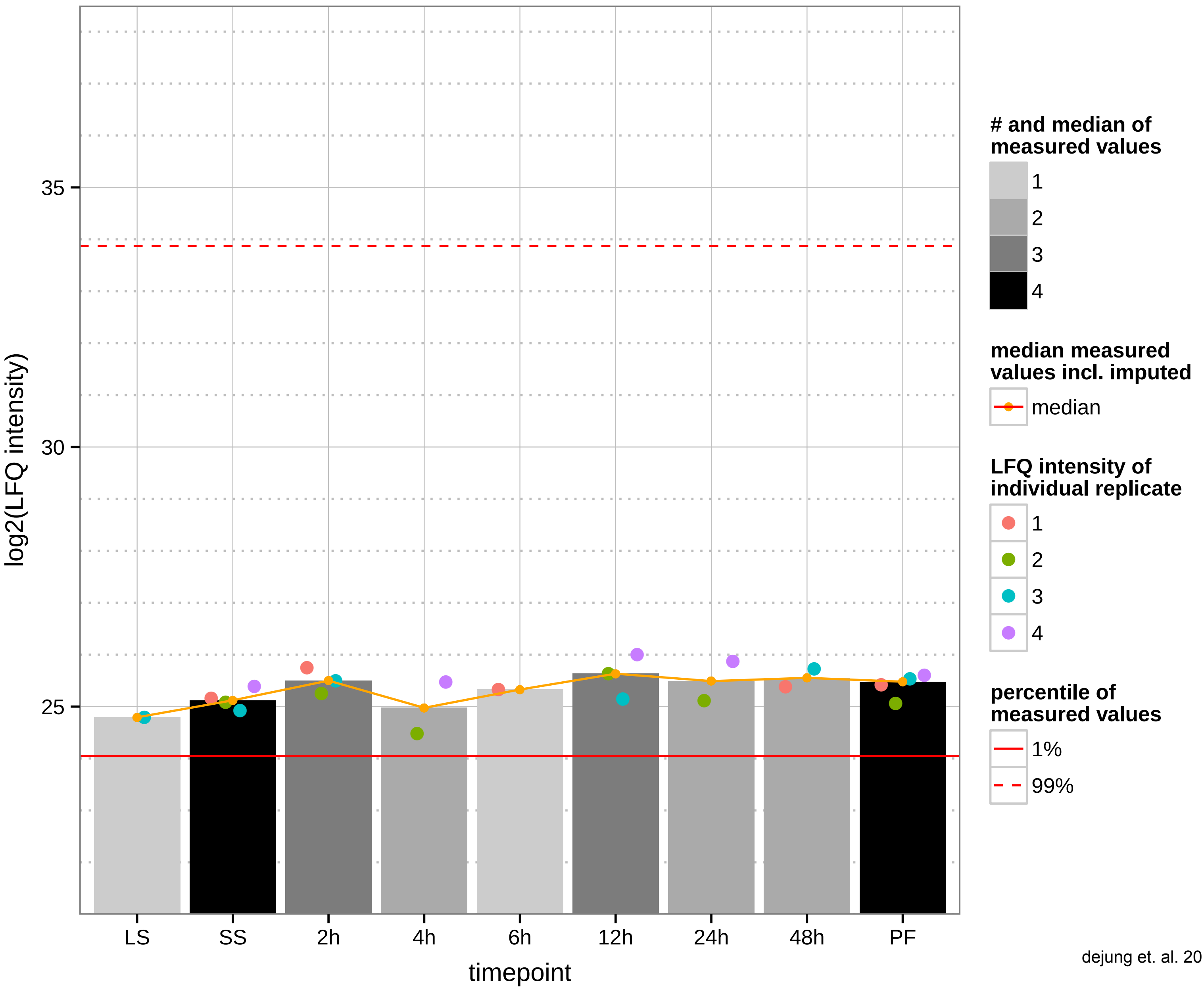

dejung et. al. 2015

class I transcription factor A, subunit 1 (CITFA−1)
Tb927.11.1390
AGOF: null
AGOC: null
AGOP: null
PGOF: null
PGOC: null
PGOP: null

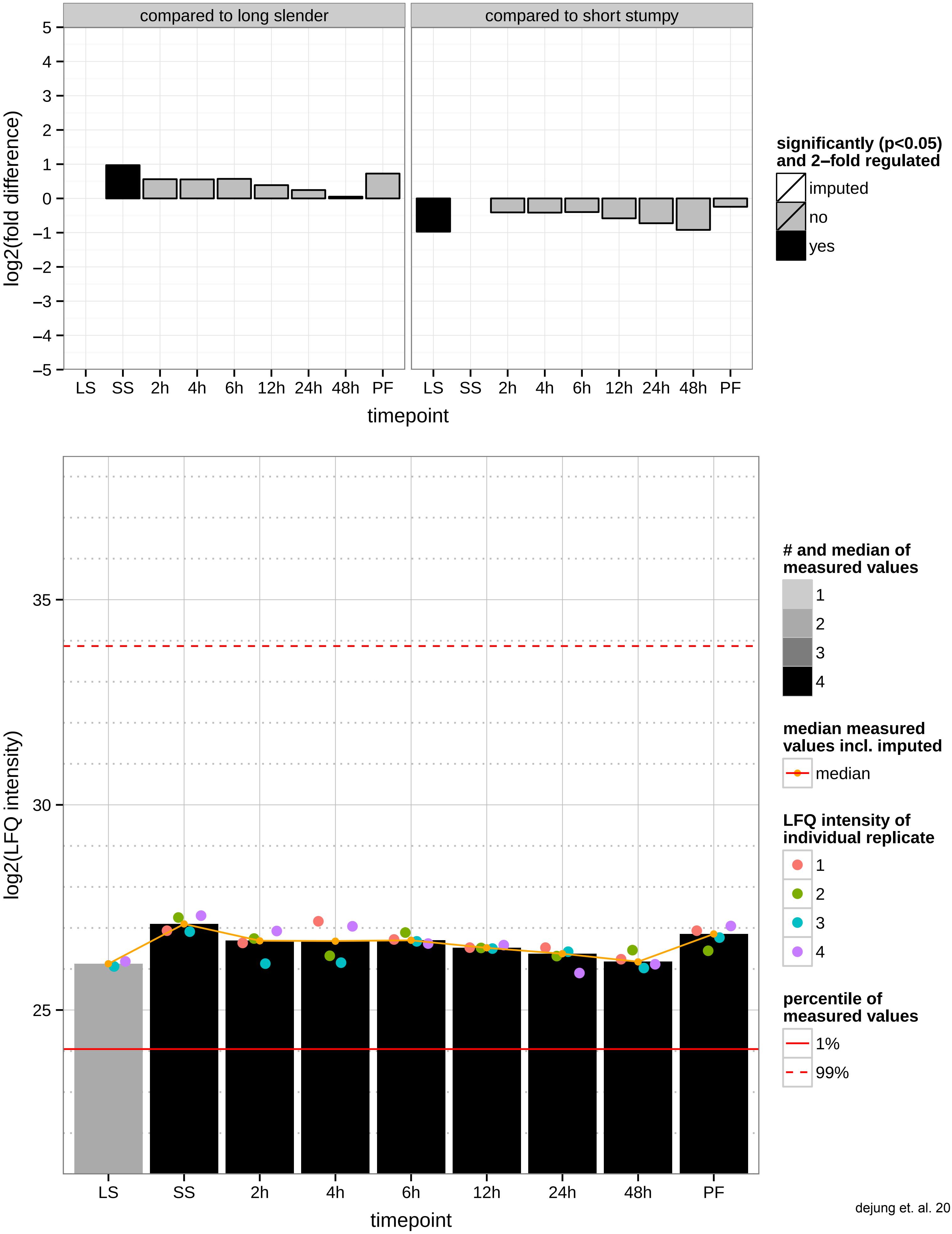

dejung et. al. 2015

centrin, putative
Tb927.11.13900
AGOF: calcium ion binding
AGOC: cilium basal body
AGOP: null
PGOF: calcium ion binding
PGOC: null
PGOP: null

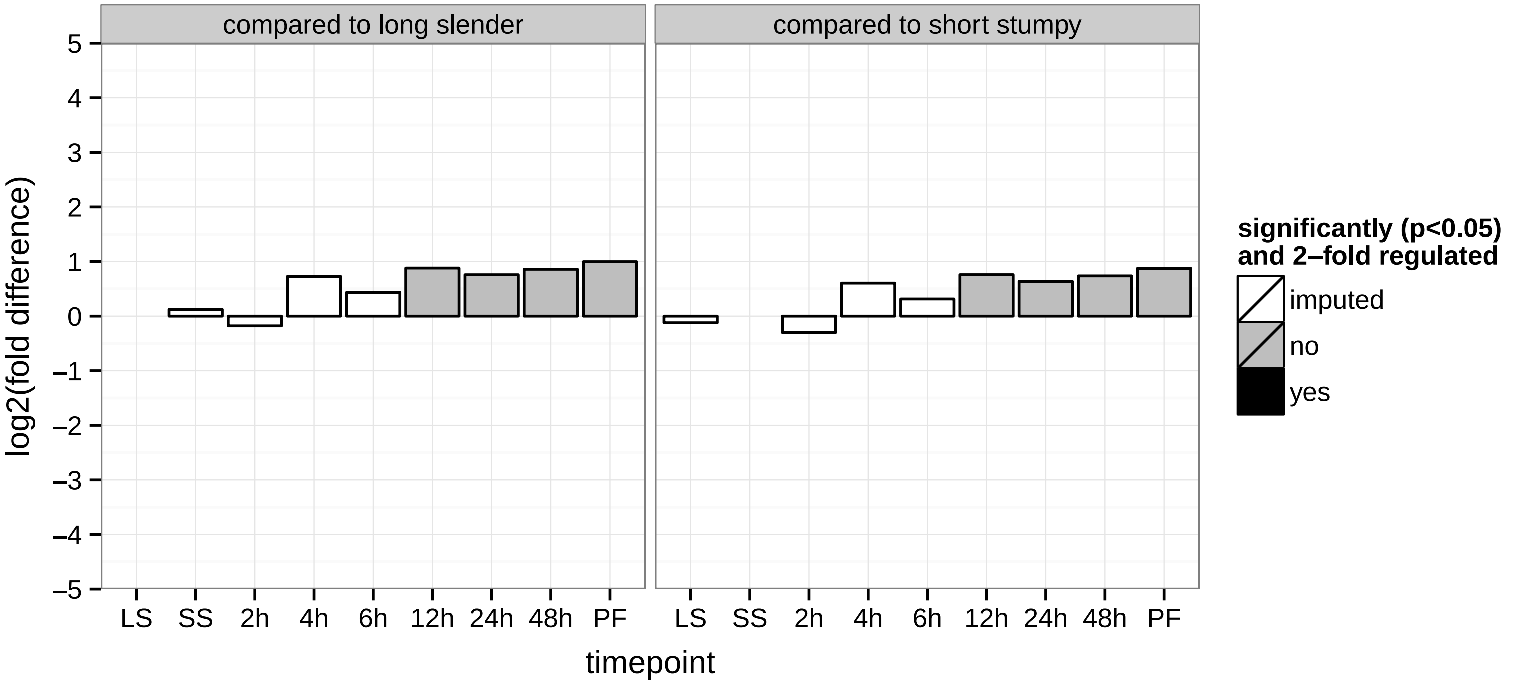

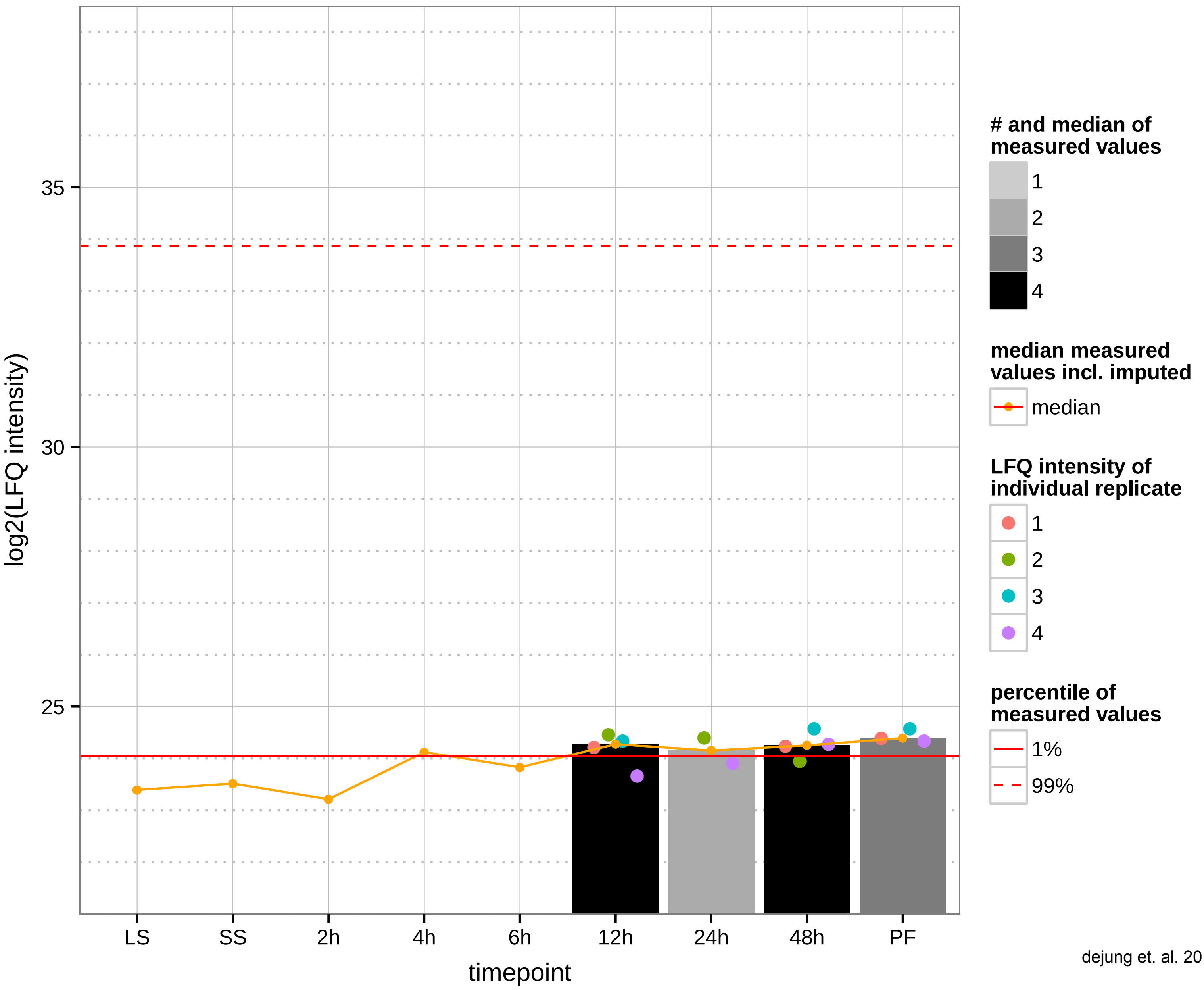

dejung et. al. 2015

hypothetical protein, conserved
Tb927.11.1400
AGOF: null
AGOC: mitochondrion
AGOP: null
PGOF: null
PGOC: null
PGOP: null

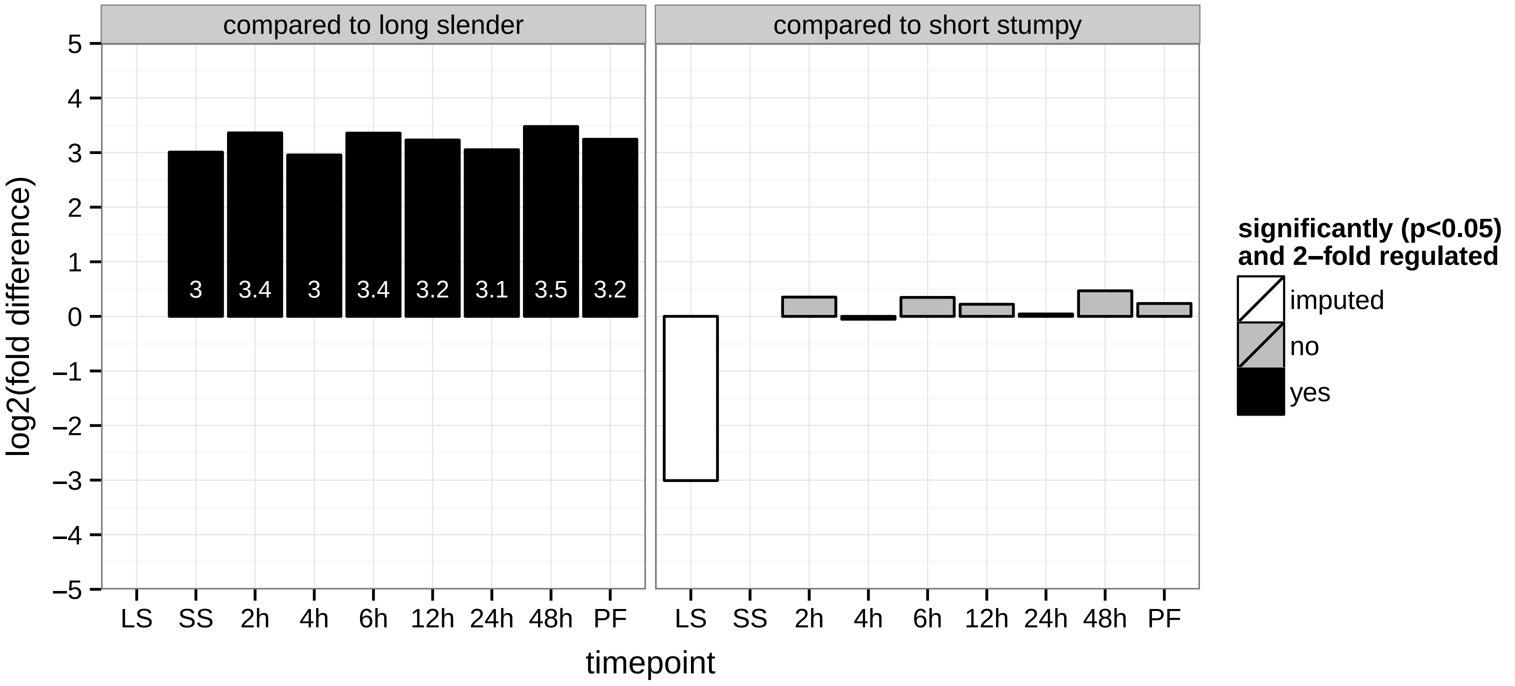

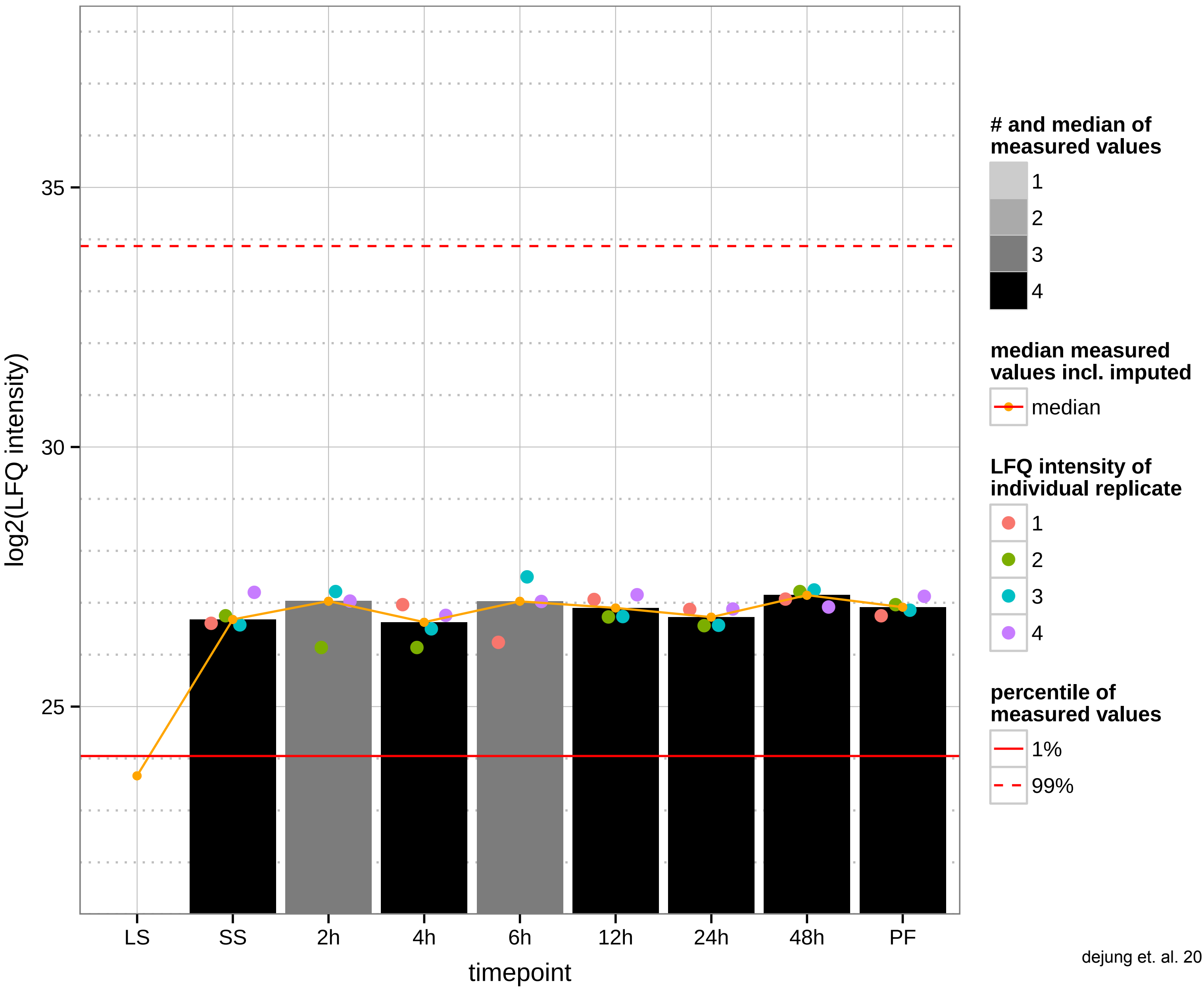

dejung et. al. 2015

NRBD1, p34 (NRBD1), RNA–binding protein (NRBD2)
Tb927.11.14020;Tb927.11.14000
AGOF: RNA binding, protein binding
AGOC: nucleus
AGOP: RNA processing
PGOF: nucleic acid binding
PGOC: null
PGOP: null

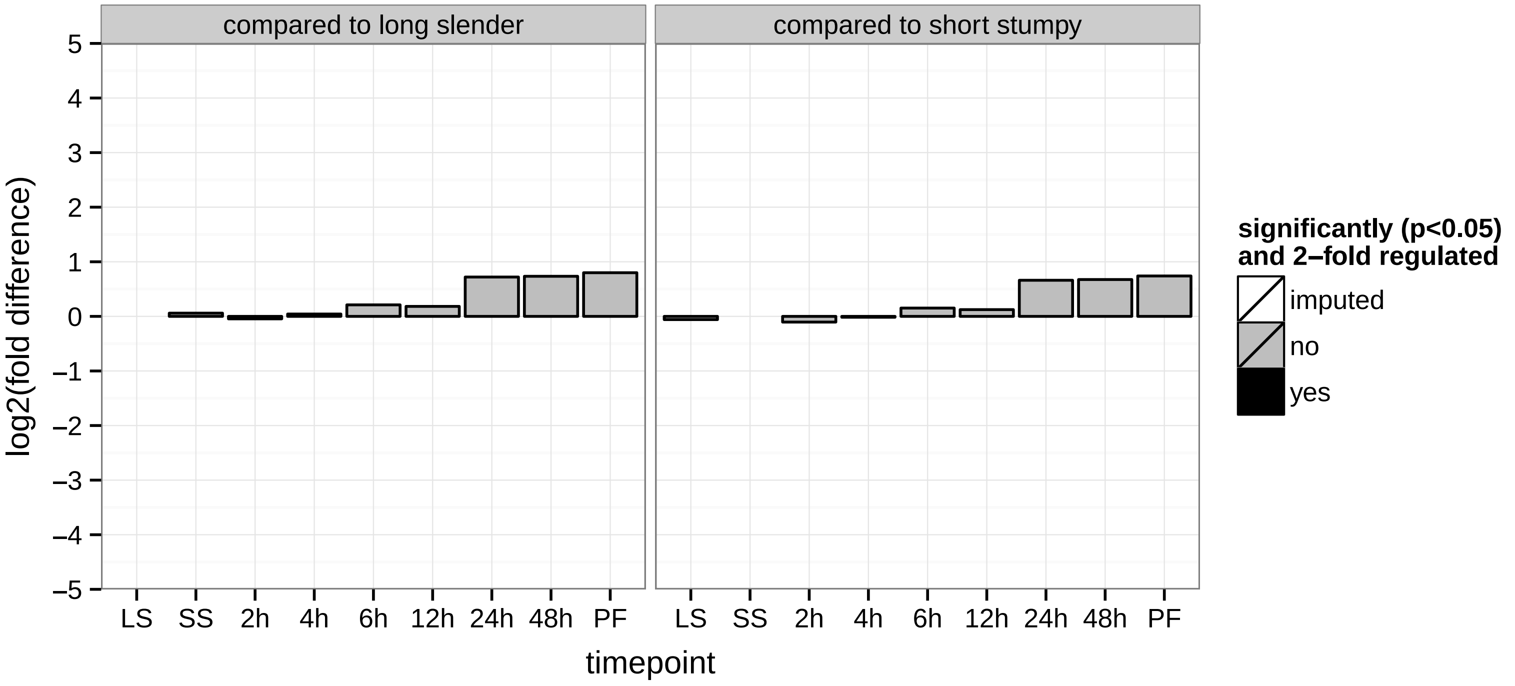

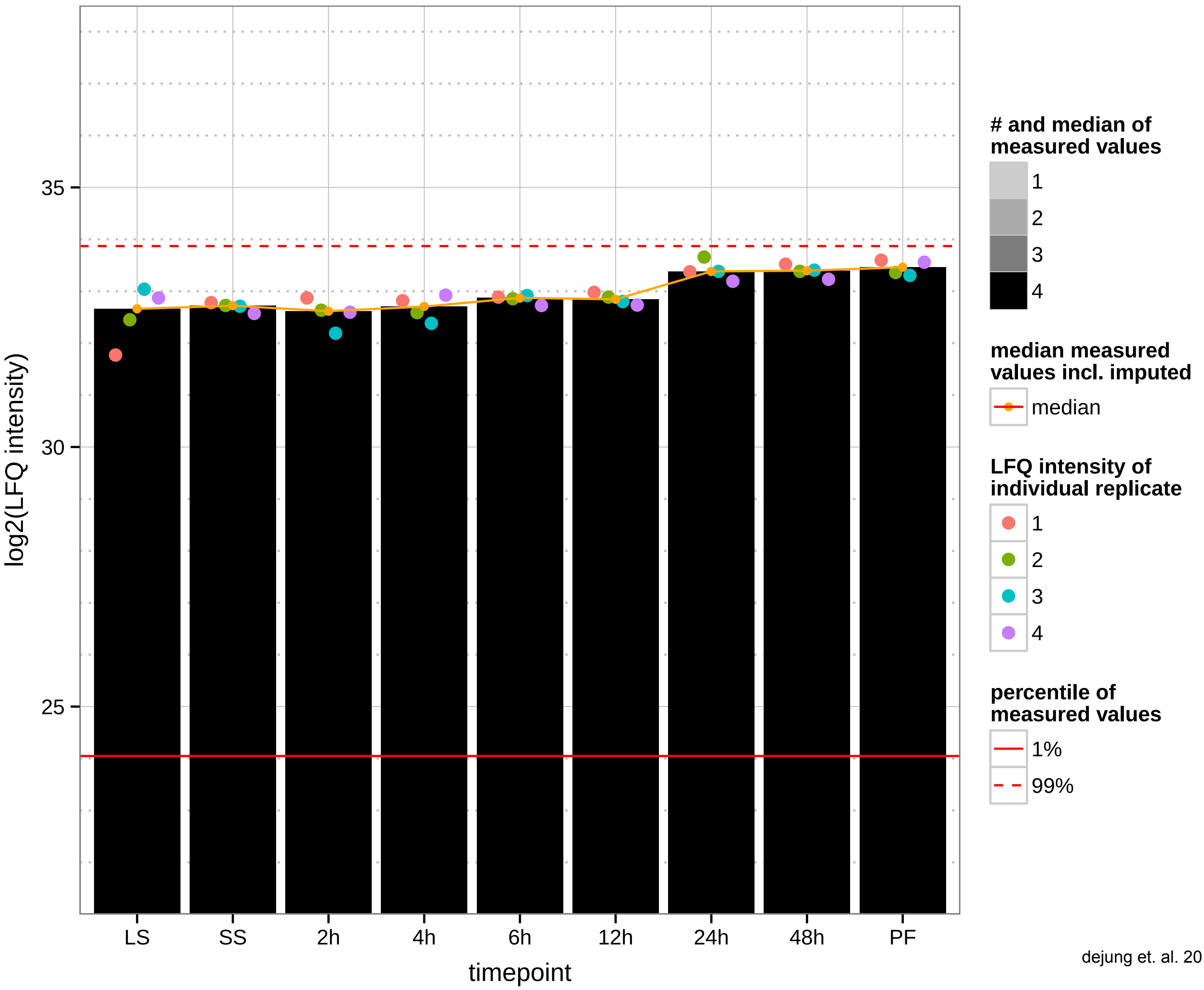

dejung et. al. 2015

cyclin 2, G1 cyclin (CYC2)
Tb927.11.14080
AGOF: cyclin binding, cyclin–dependent protein kinase regulator activity, protein binding
AGOC: cyclin–dependent protein kinase holoenzyme complex
AGOP: cell cycle
PGOF: protein kinase binding
PGOC: null
PGOP: regulation of cyclin–dependent protein kinase activity

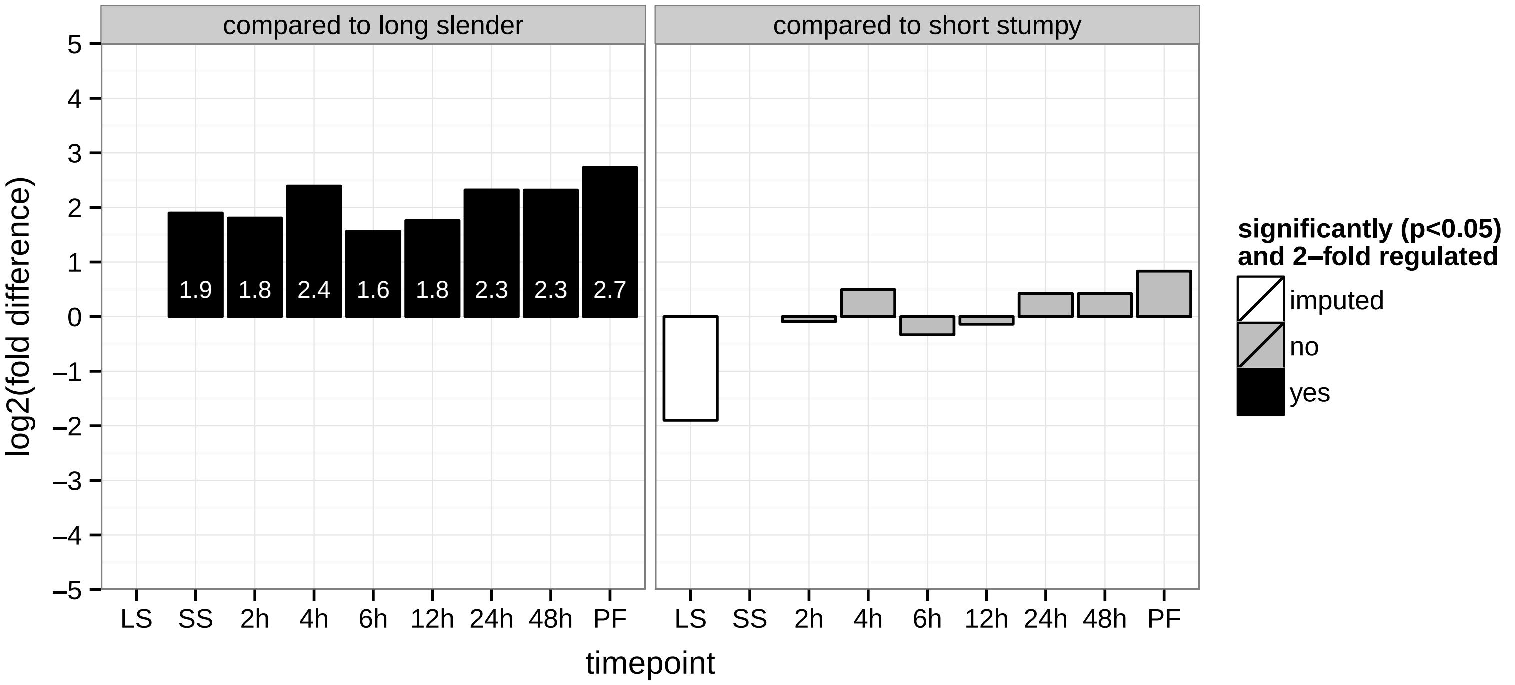

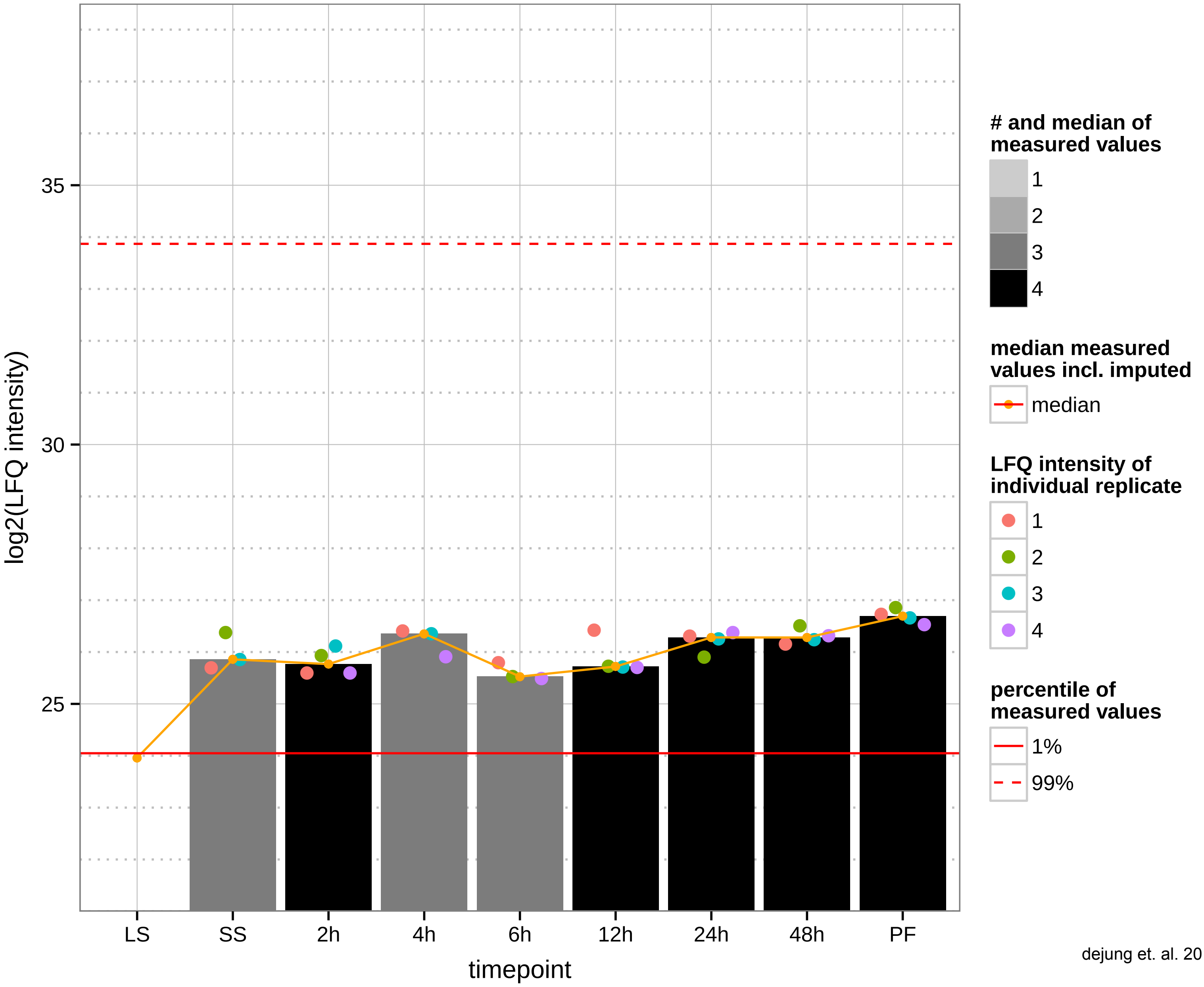

dejung et. al. 2015

hypothetical protein, conserved
Tb927.11.14090
AGOF: null
AGOC: null
AGOP: null
PGOF: null
PGOC: null
PGOP: null

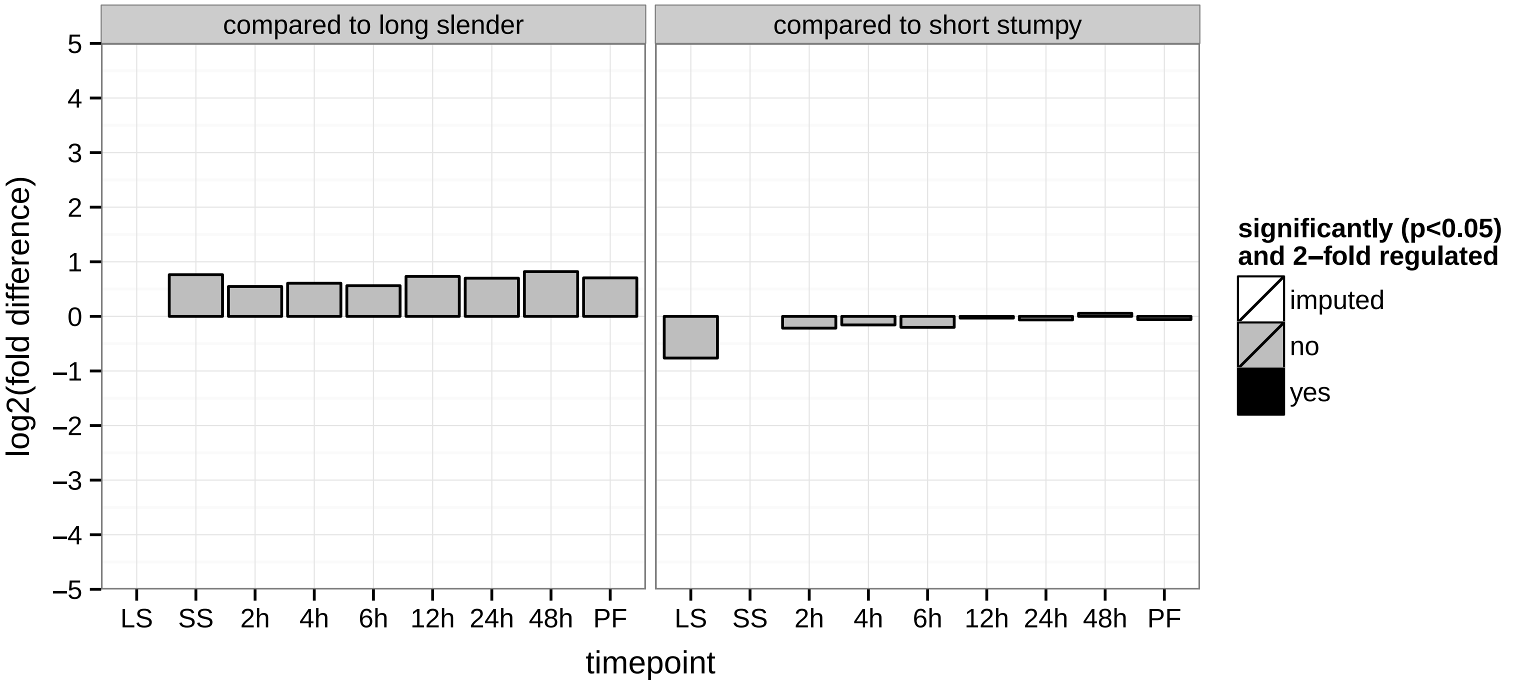

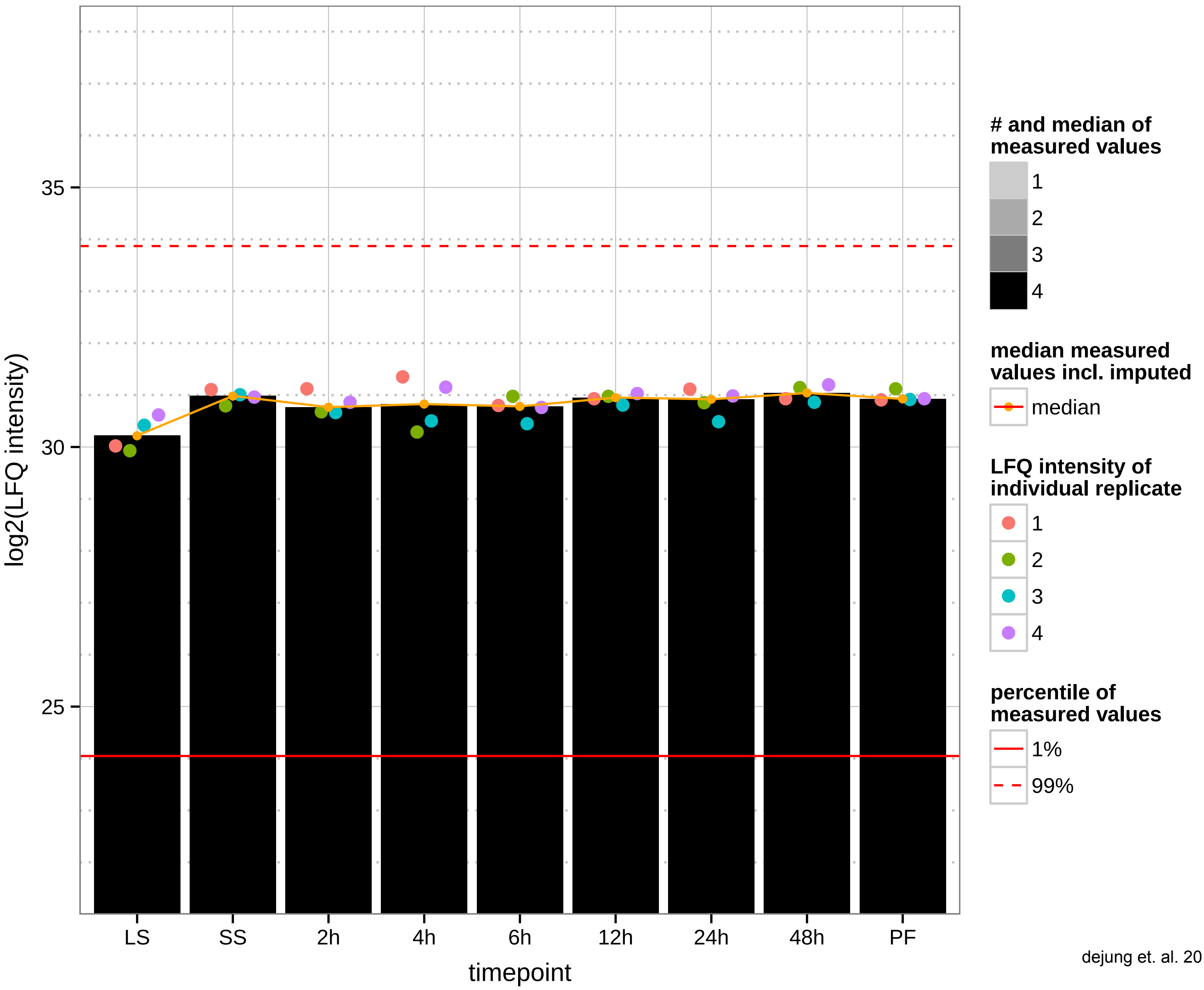

dejung et. al. 2015

class I transcription factor A, subunit 3 (CITFA−3)
Tb927.11.1410
AGOF: null
AGOC: null
AGOP: null
PGOF: null
PGOC: null
PGOP: null

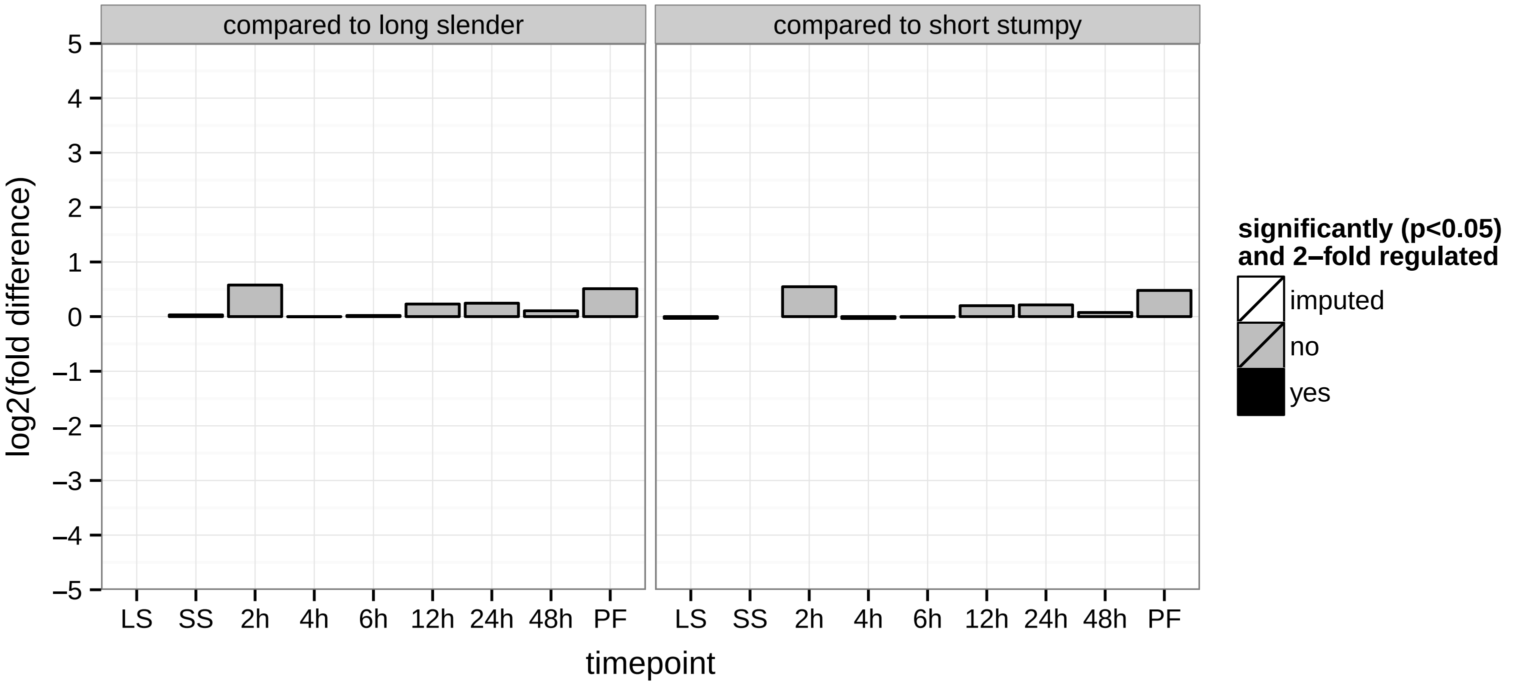

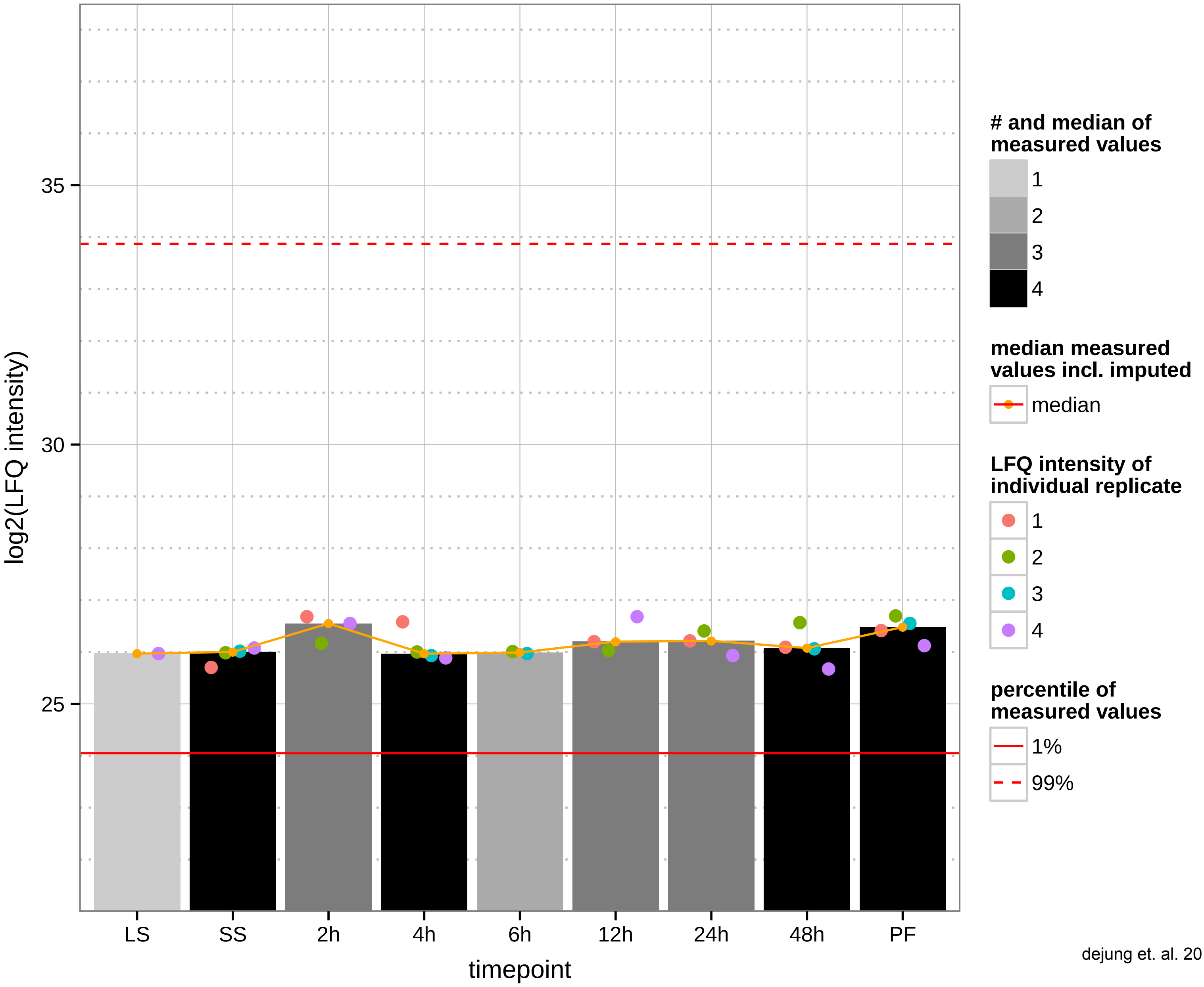

dejung et. al. 2015

DRBD4 (DRBD4)
Tb927.11.14100
AGOF: RNA binding
AGOC: null
AGOP: RNA processing
PGOF: nucleic acid binding
PGOC: null
PGOP: null

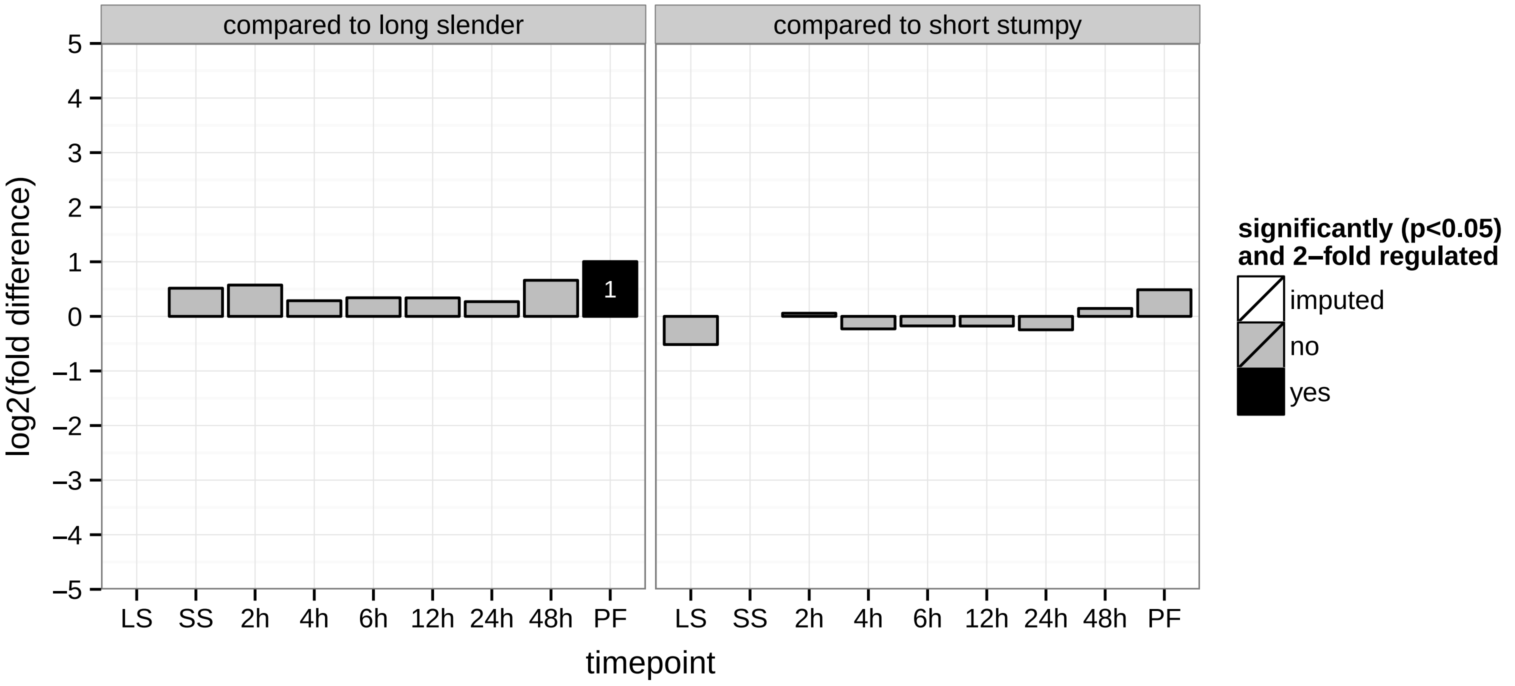

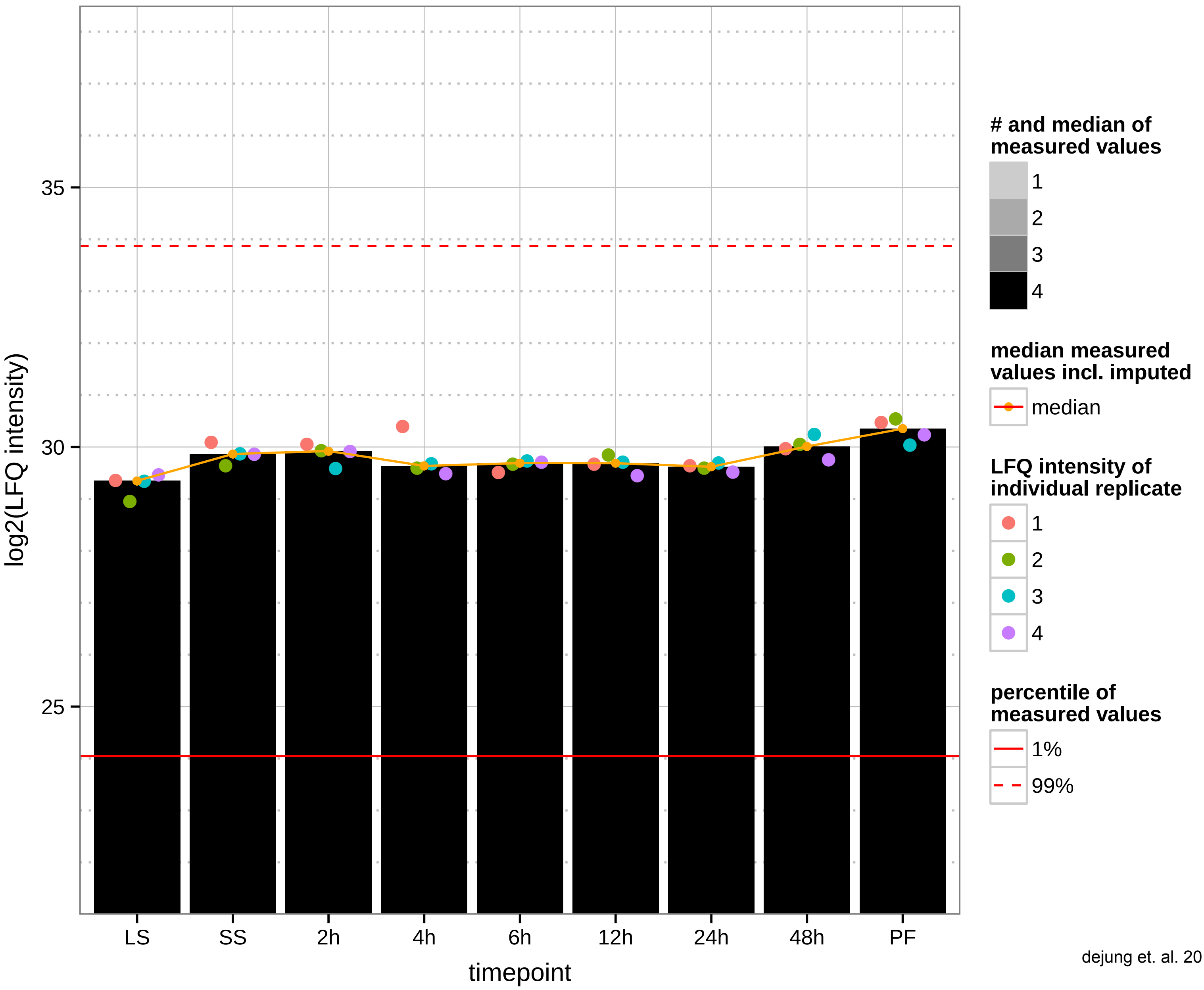

dejung et. al. 2015

phenylalanyl–tRNA synthetase alpha chain, putative
Tb927.11.14120
AGOF: ATP binding, phenylalanine–tRNA ligase activity, tRNA binding
AGOC: cytoplasm
AGOP: phenylalanyl–tRNA aminoacylation, translation
PGOF: ATP binding, aminoacyl–tRNA ligase activity, nucleotide binding, phenylalanine–tRNA ligase activity, tRNA binding
PGOC: cytoplasm
PGOP: phenylalanyl–tRNA aminoacylation, tRNA aminoacylation, tRNA aminoacylation for protein translation

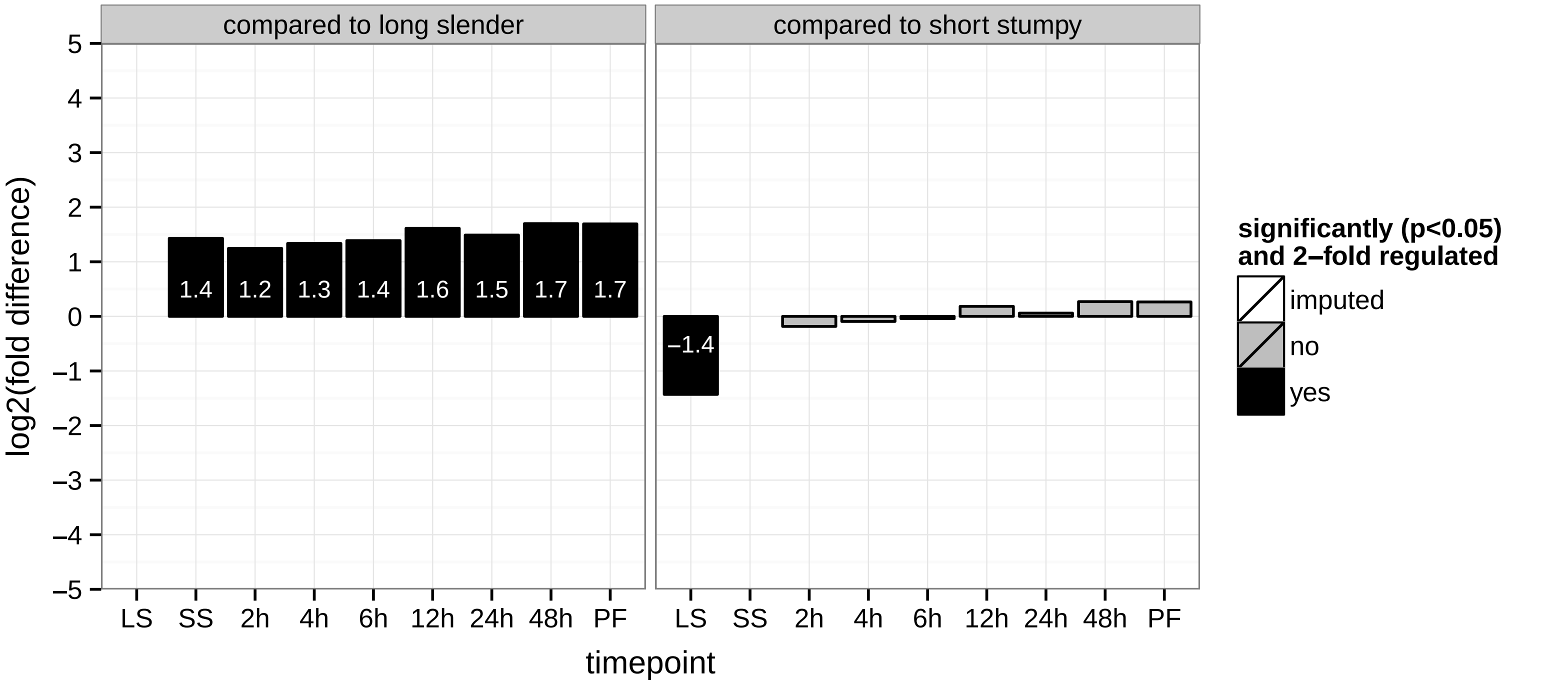

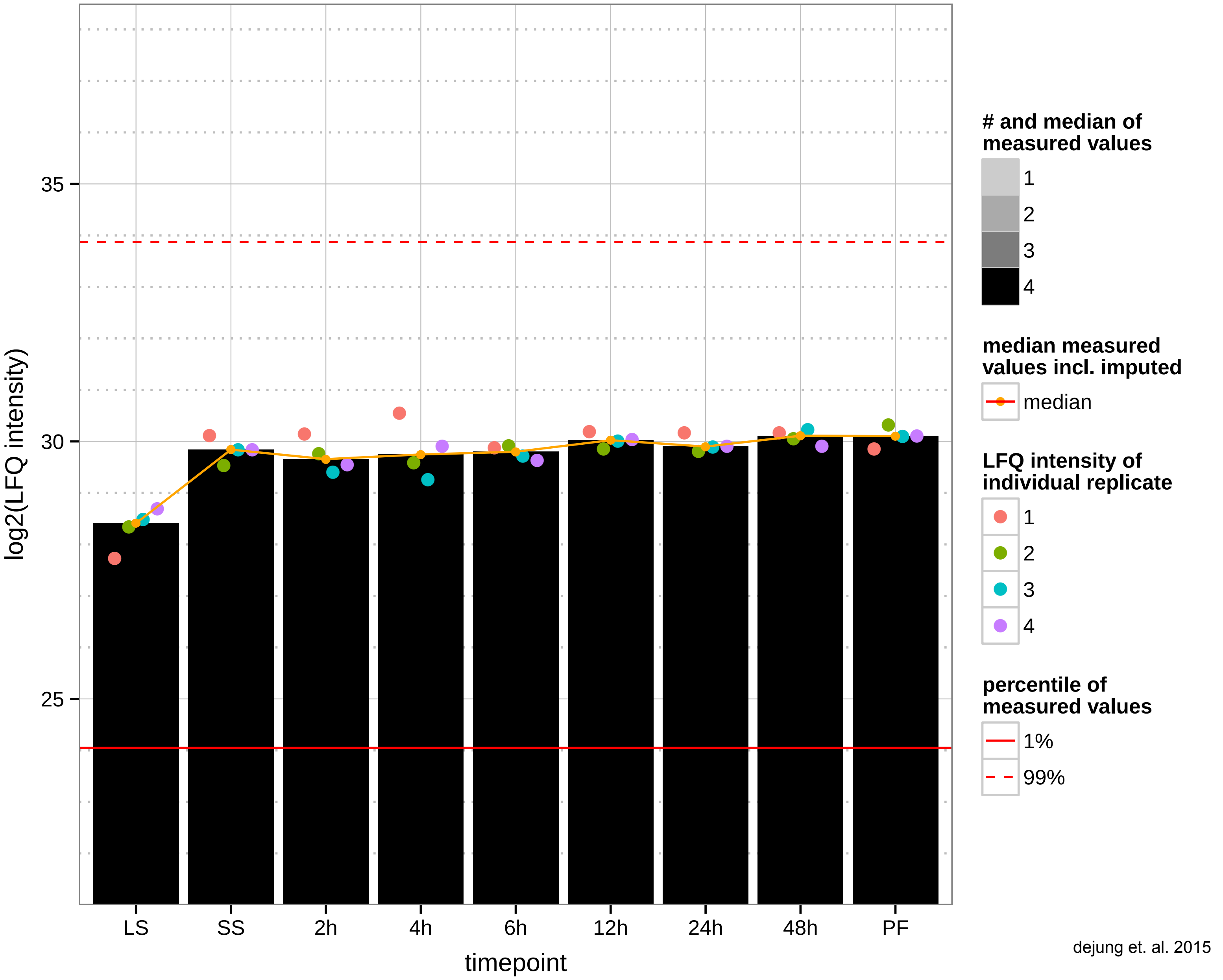

dejung et. al. 2015

ethanolamine−phosphate cytidylyltransferase (TbECT)
Tb927.11.14140
AGOF: ethanolamine−phosphate cytidylyltransferase activity, nucleotidyltransferase activity
AGOC: cytosol
AGOP: ethanolamine biosynthetic process, phosphatidylethanolamine biosynthetic process, phospholipid biosynthetic process
PGOF: catalytic activity, nucleotidyltransferase activity
PGOC: null
PGOP: biosynthetic process

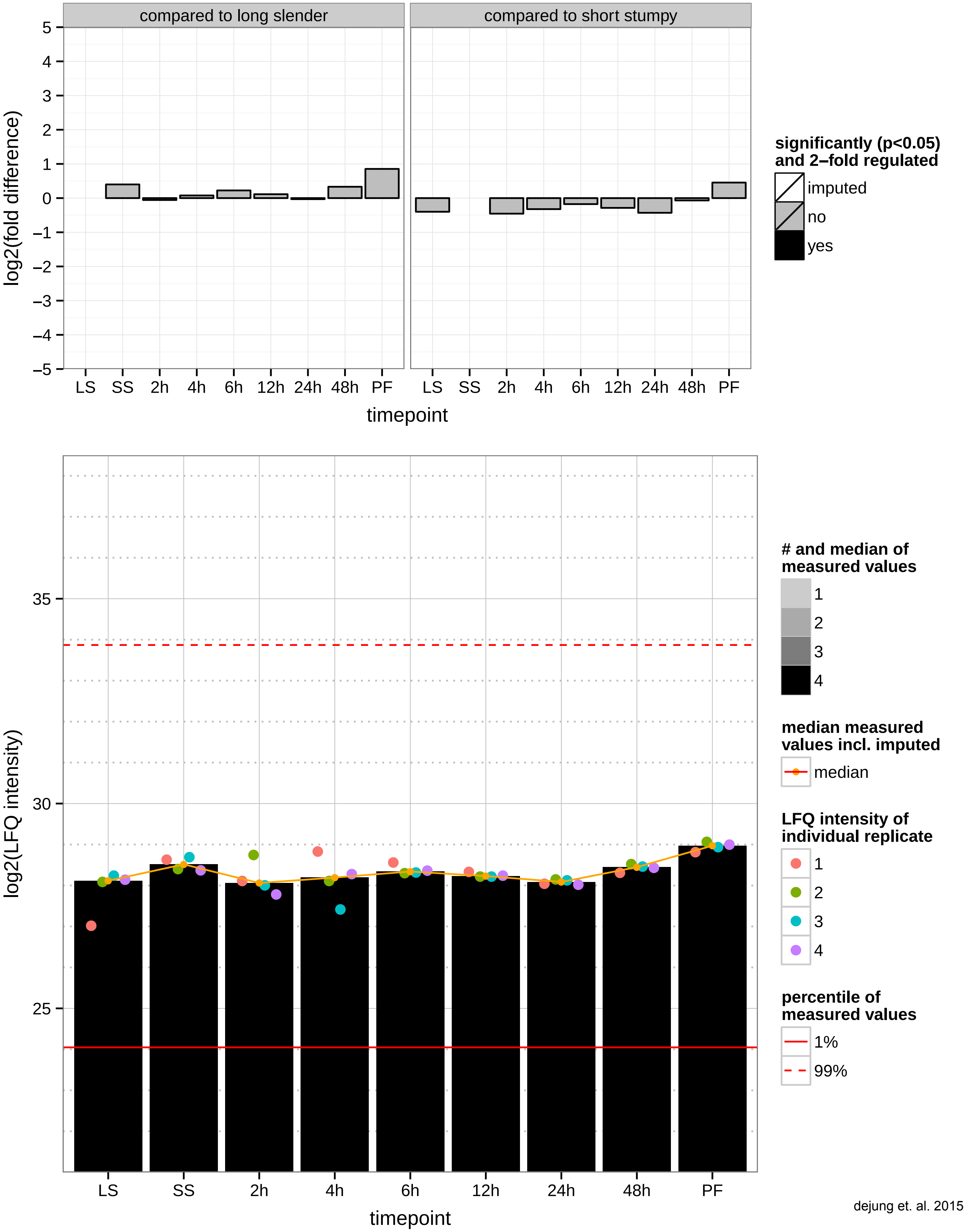

dejung et. al. 2015

hypothetical protein, conserved
Tb927.11.14190
AGOF: hydrolase activity, acting on ester bonds, nucleic acid binding
AGOC: RNA–induced silencing complex
AGOP: RNA interference
PGOF: hydrolase activity, acting on ester bonds, nucleic acid binding
PGOC: RNA–induced silencing complex
PGOP: gene silencing by RNA

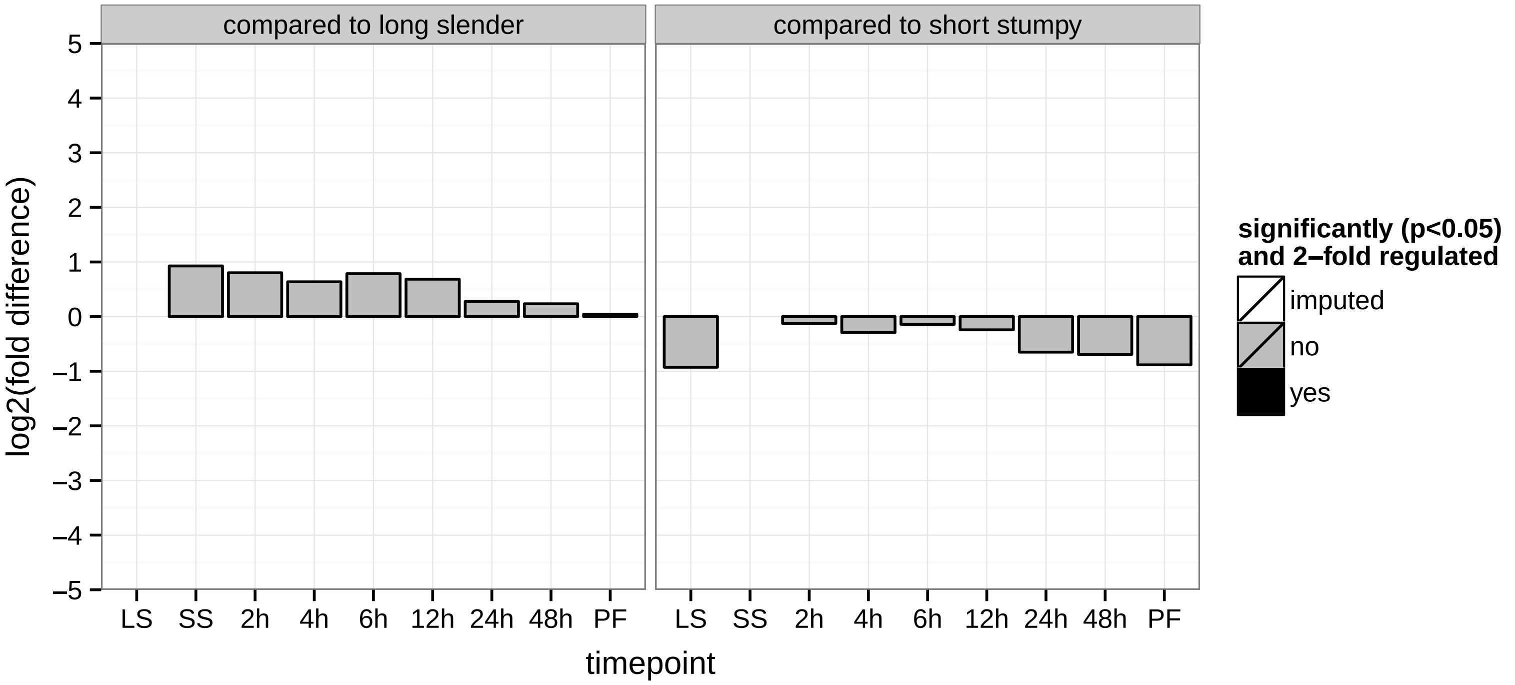

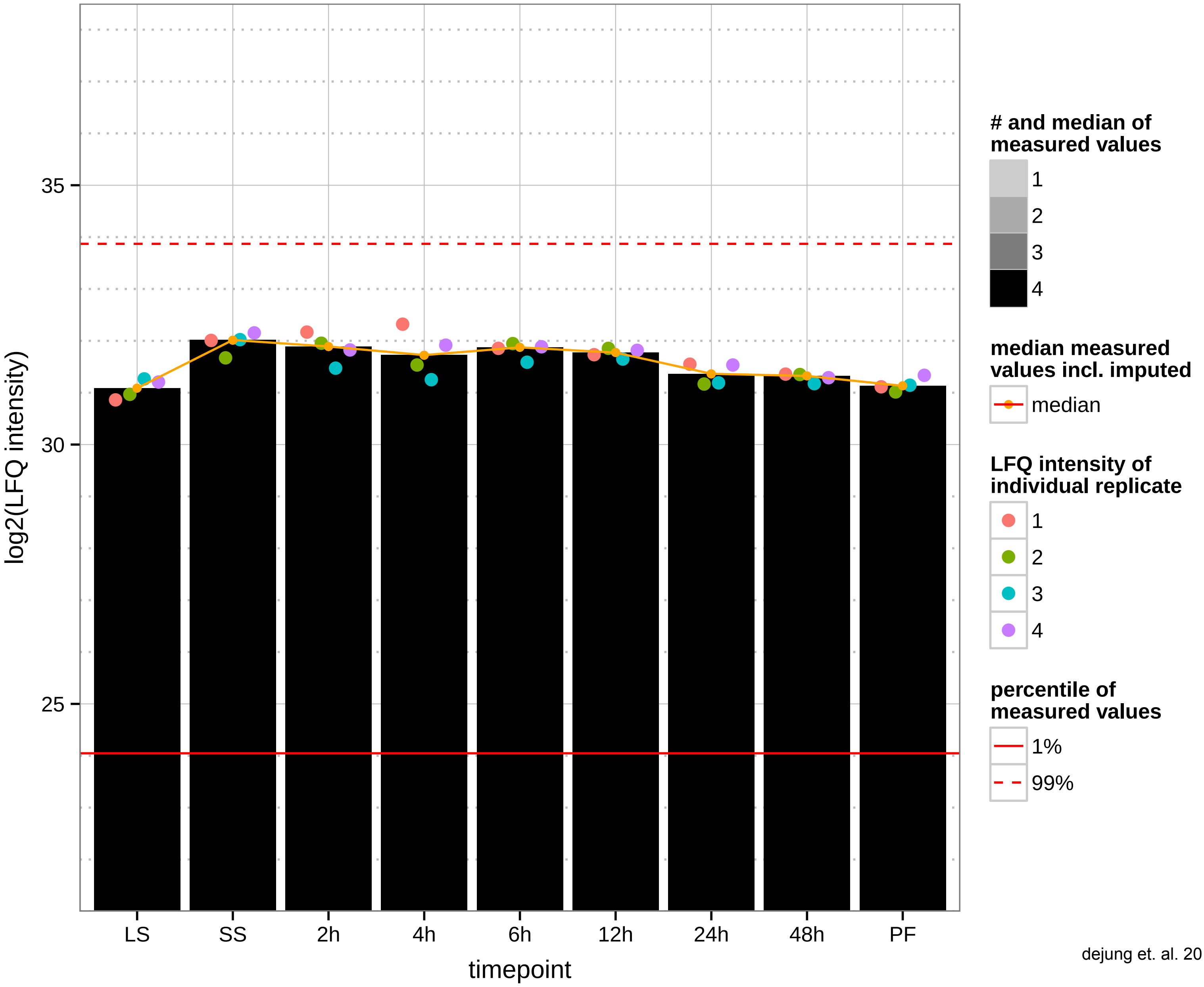

dejung et. al. 2015

hypothetical protein, conserved
Tb927.11.1420
AGOF: null
AGOC: null
AGOP: null
PGOF: protein binding
PGOC: null
PGOP: null

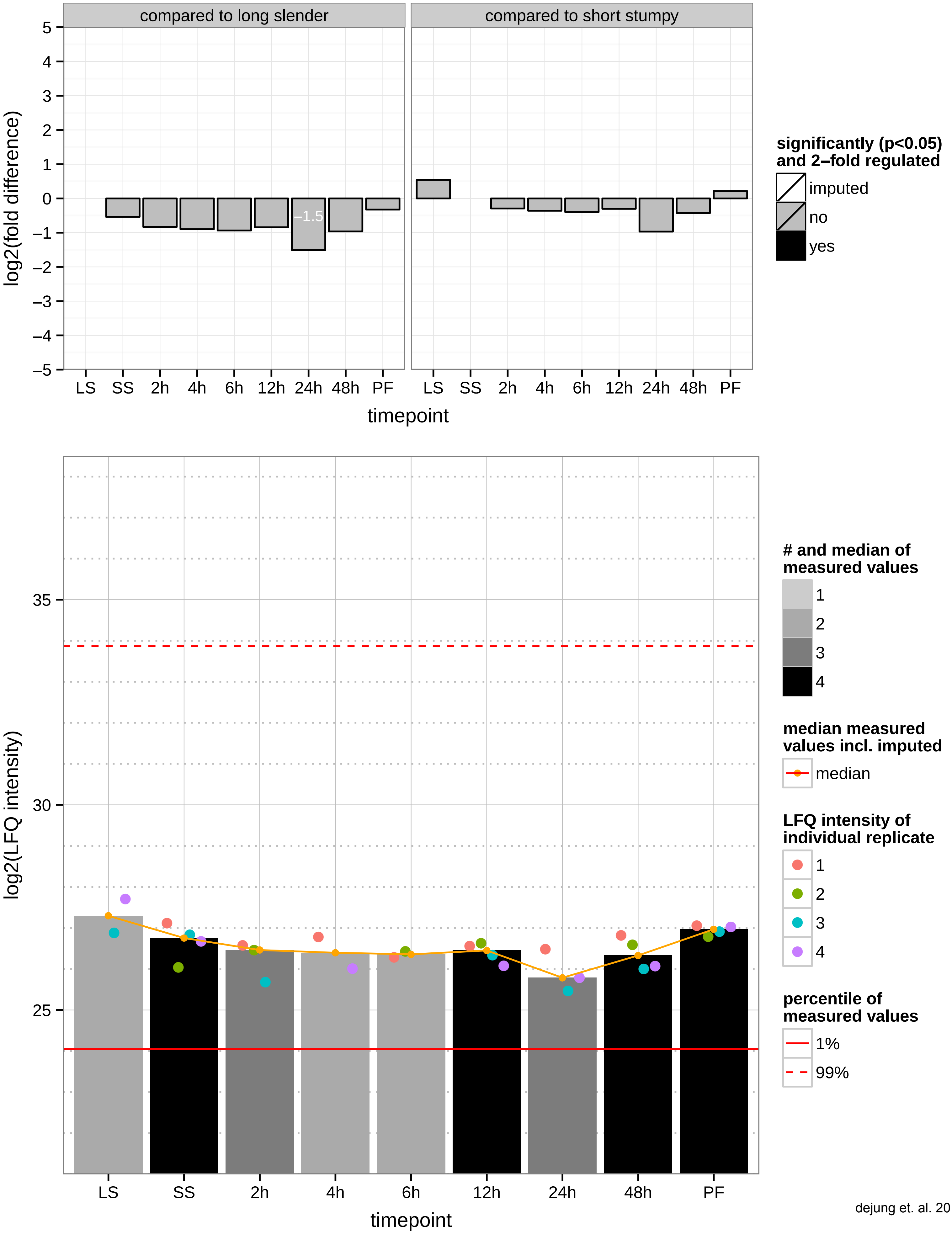

dejung et. al. 2015

T–complex protein 1, epsilon subunit, putative (TCP–1–epsilon)
Tb927.11.14250
AGOF: ATP binding, unfolded protein binding
AGOC: chaperonin–containing T–complex
AGOP: cellular protein metabolic process, regulation of cell cycle
PGOF: ATP binding, unfolded protein binding
PGOC: null
PGOP: cellular protein metabolic process, protein folding

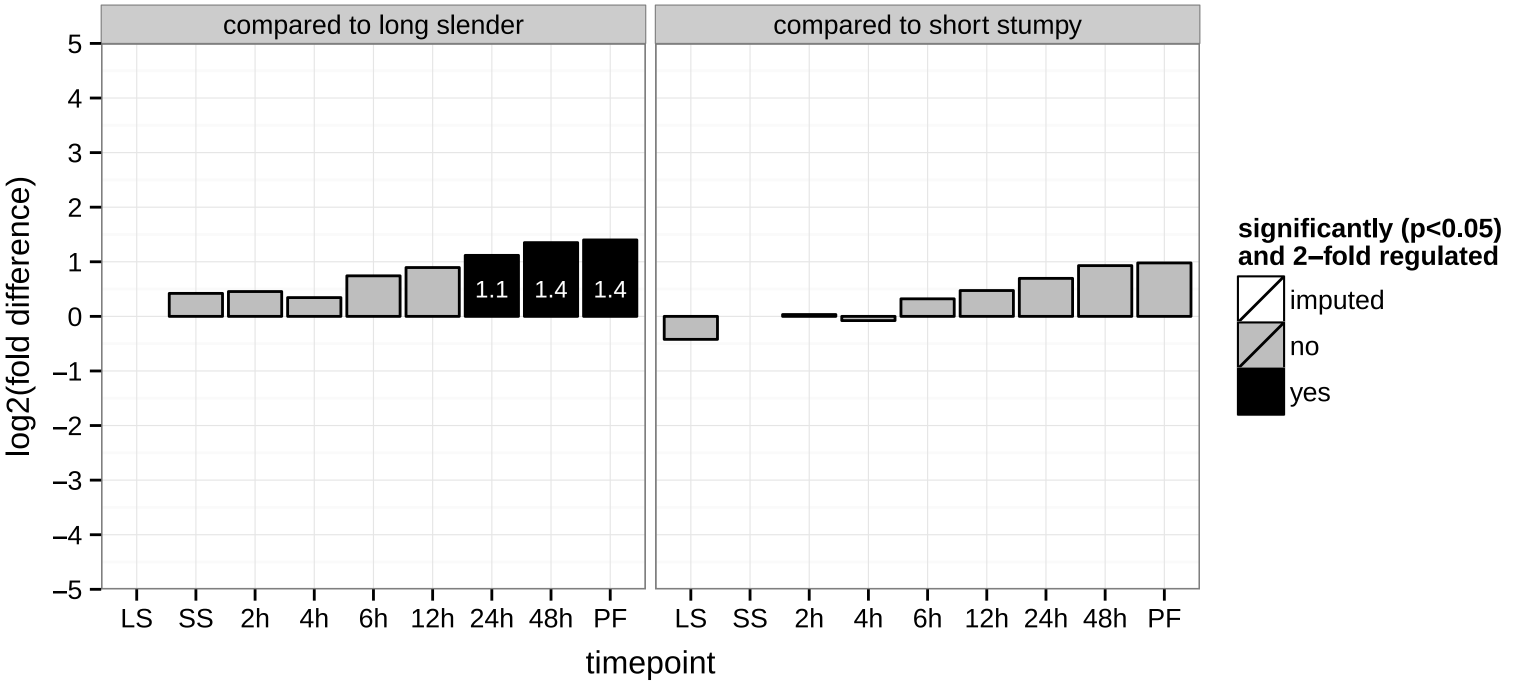

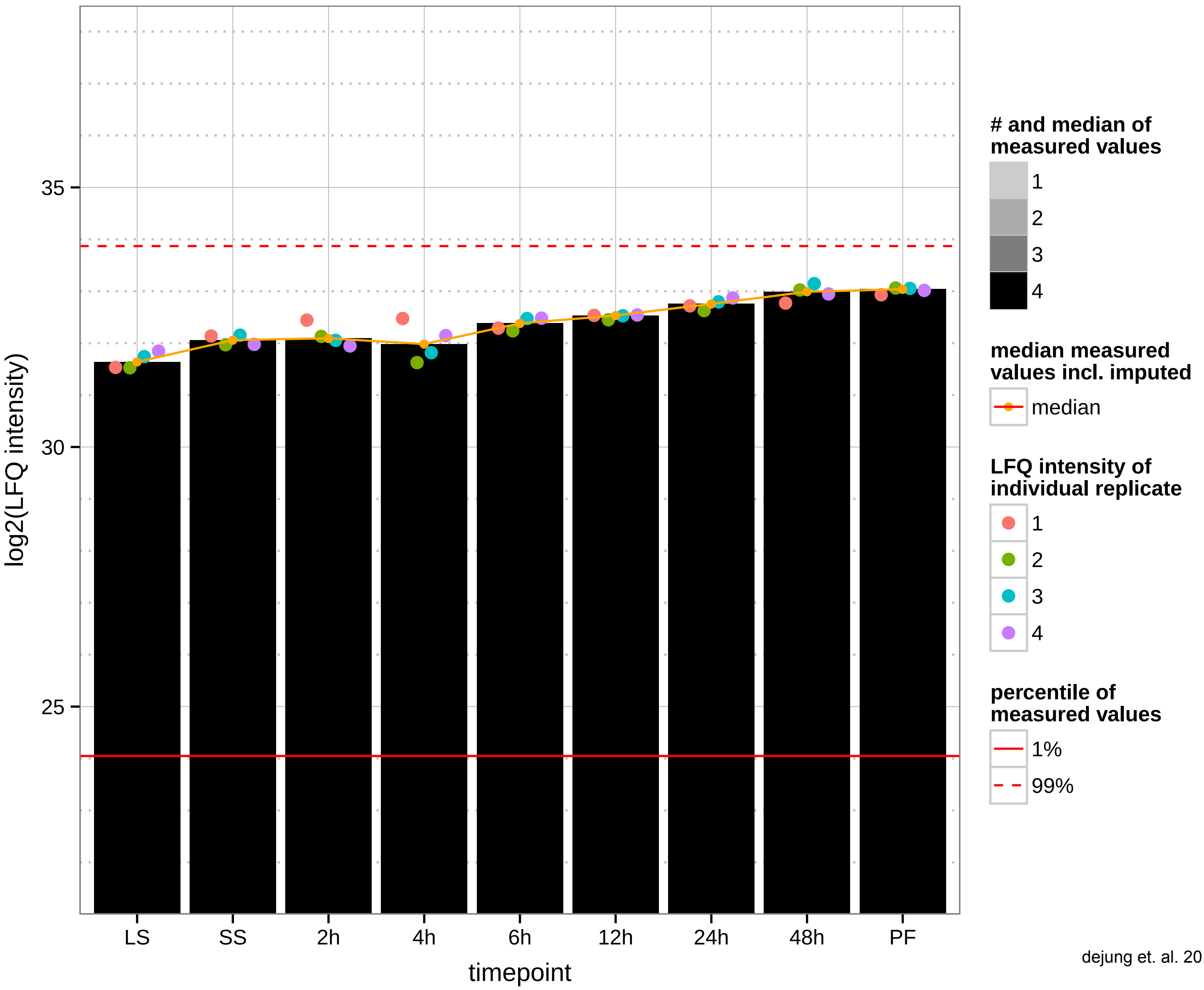

dejung et. al. 2015

septum formation protein MAF homologue, putative
Tb927.11.14280
AGOF: null
AGOC: cytoplasm
AGOP: barrier septum assembly
PGOF: null
PGOC: cytoplasm
PGOP: null

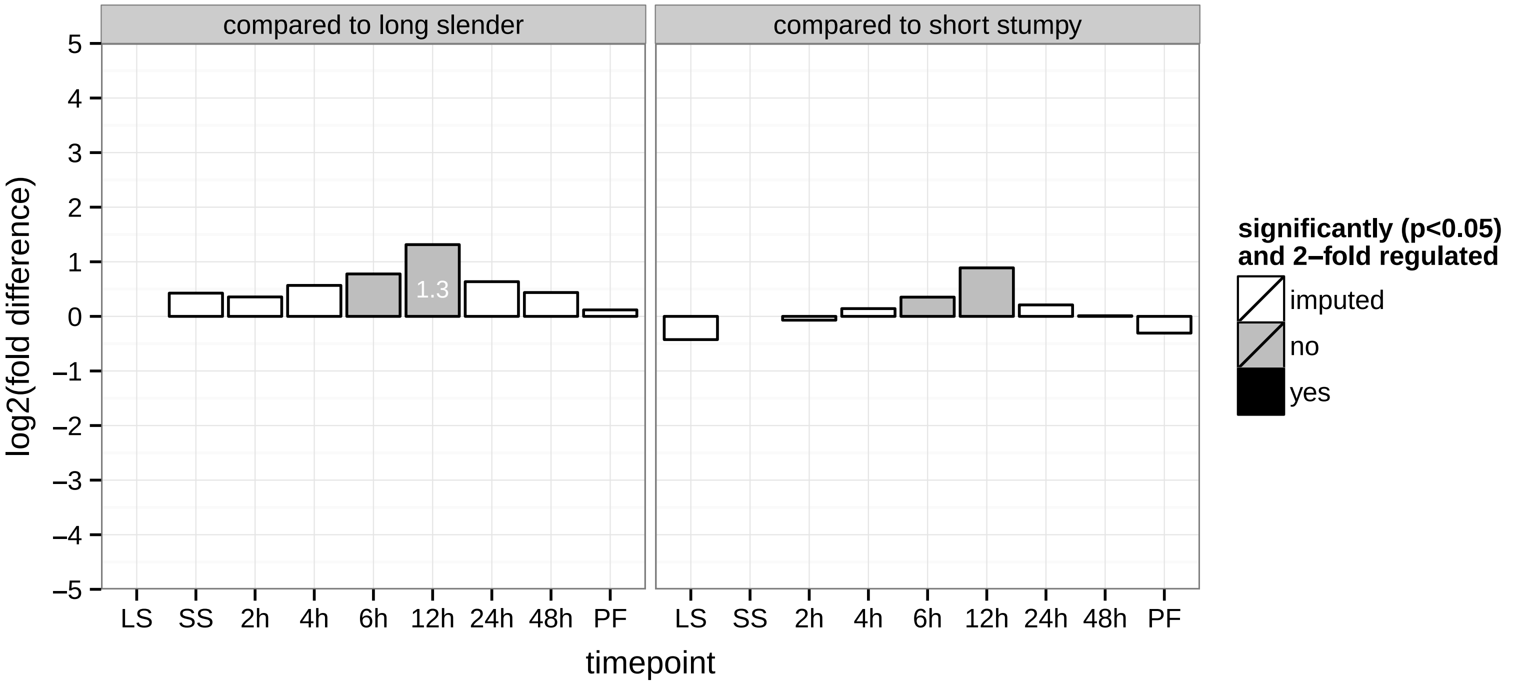

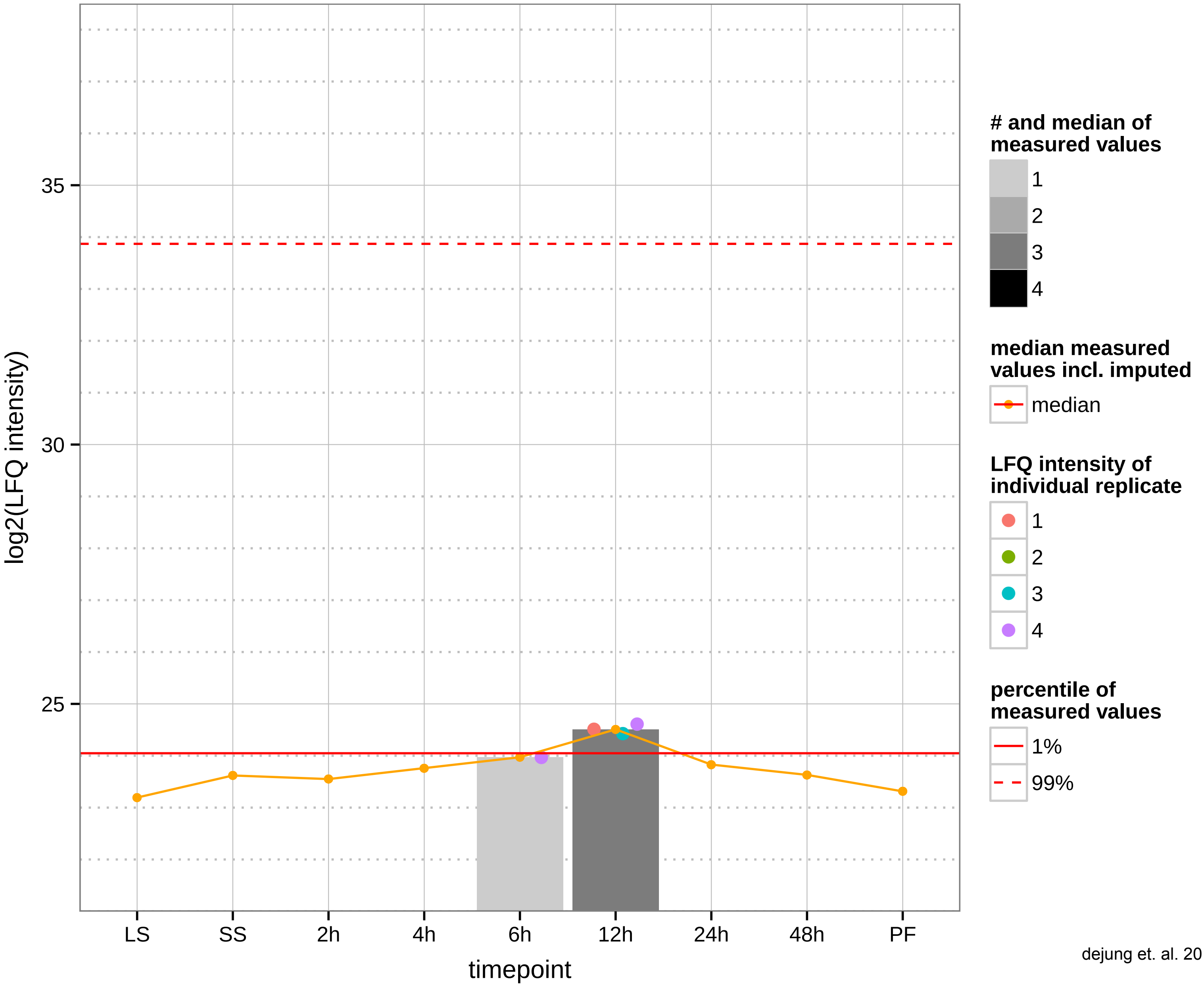

dejung et. al. 2015

hypothetical protein, conserved
Tb927.11.1430
AGOF: null
AGOC: null
AGOP: ciliary or flagellar motility
PGOF: null
PGOC: null
PGOP: null

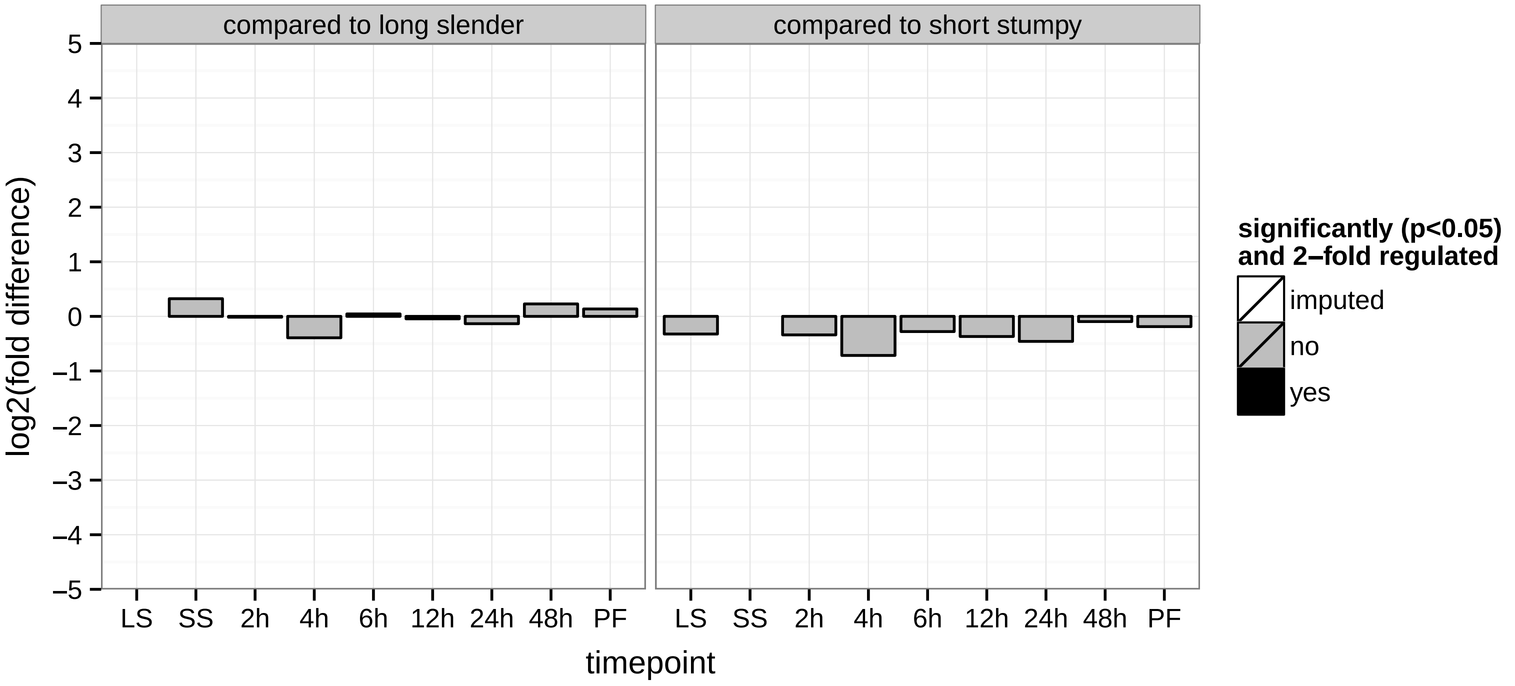

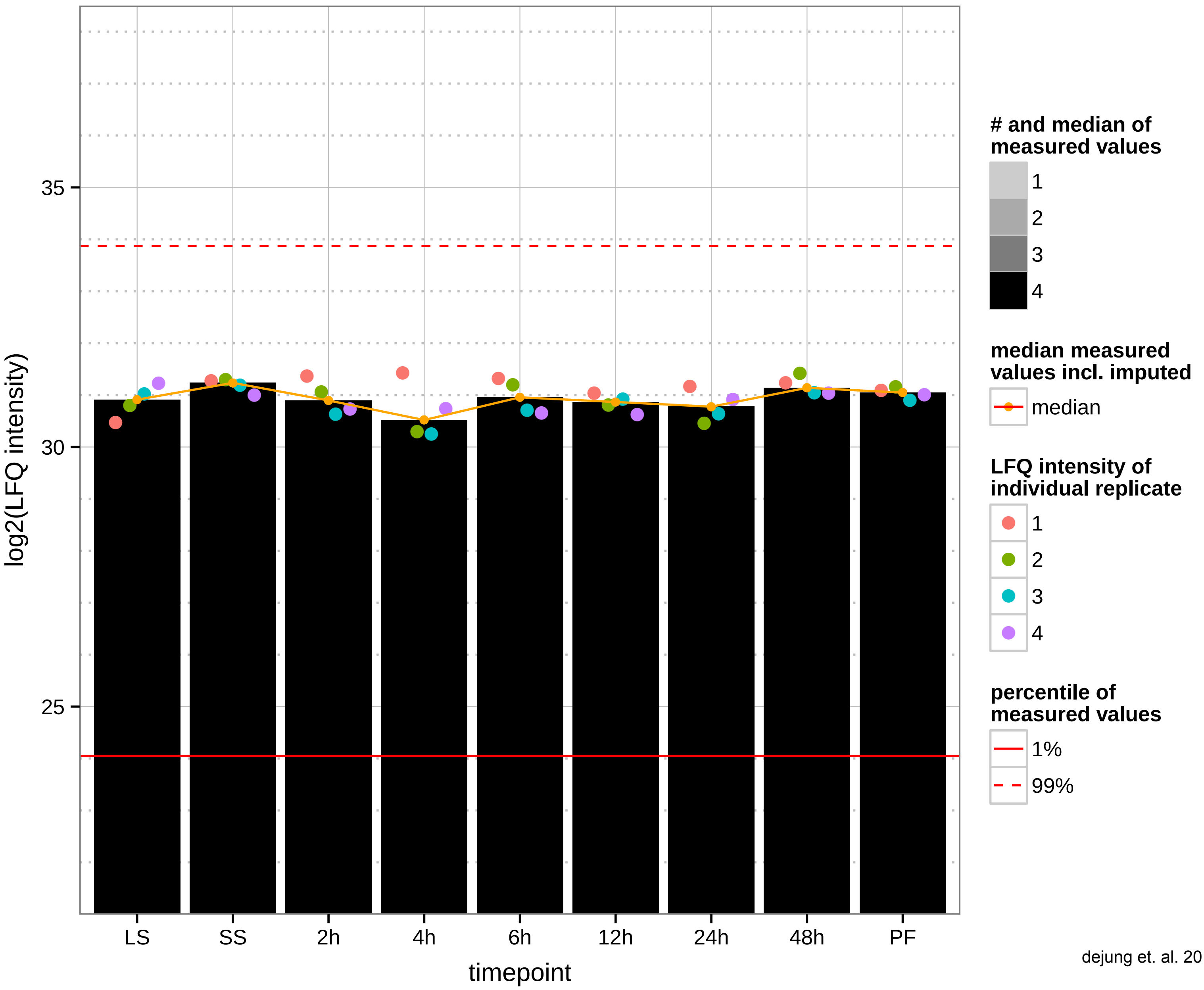

dejung et. al. 2015

dynein intermediate chain IC70, putative
Tb927.11.14300
AGOF: microtubule motor activity, motor activity
AGOC: dynein complex
AGOP: null
PGOF: protein binding
PGOC: null
PGOP: null

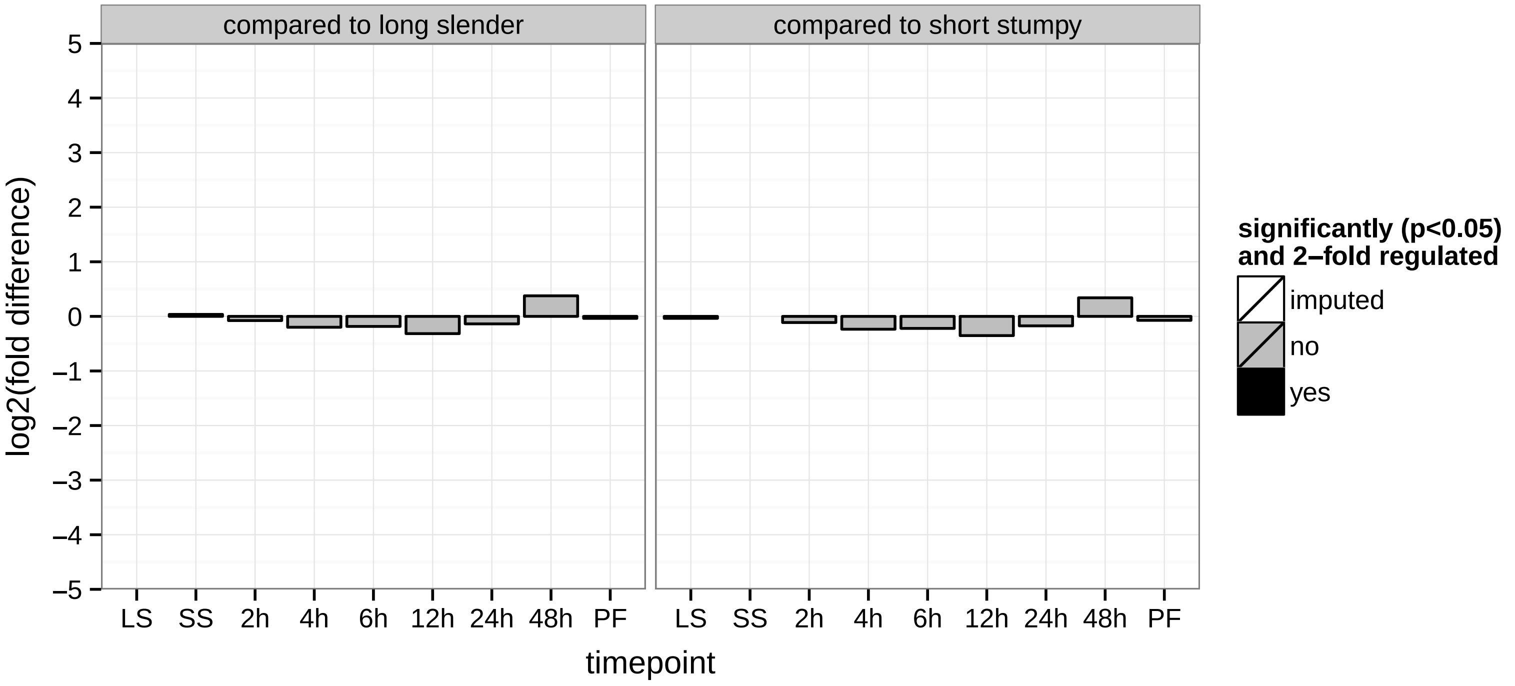

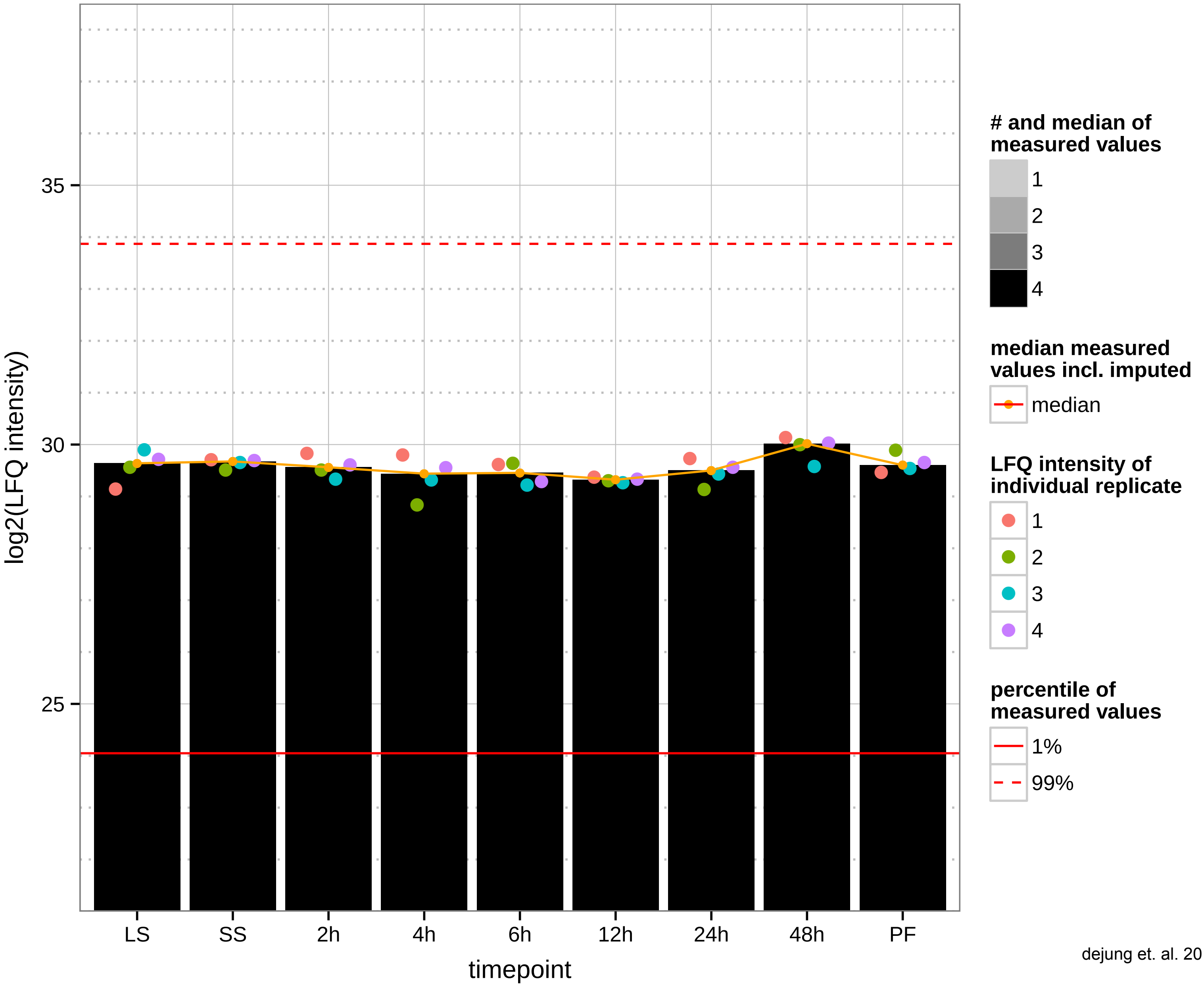

dejung et. al. 2015

small nuclear ribonucleoprotein Sm–G (Sm–G)
Tb927.11.14310
AGOF: null
AGOC: nucleus, small nucleolar ribonucleoprotein complex
AGOP: RNA splicing, RNA splicing, via transesterification reactions, nuclear mRNA trans splicing, SL addition
PGOF: null
PGOC: null
PGOP: null

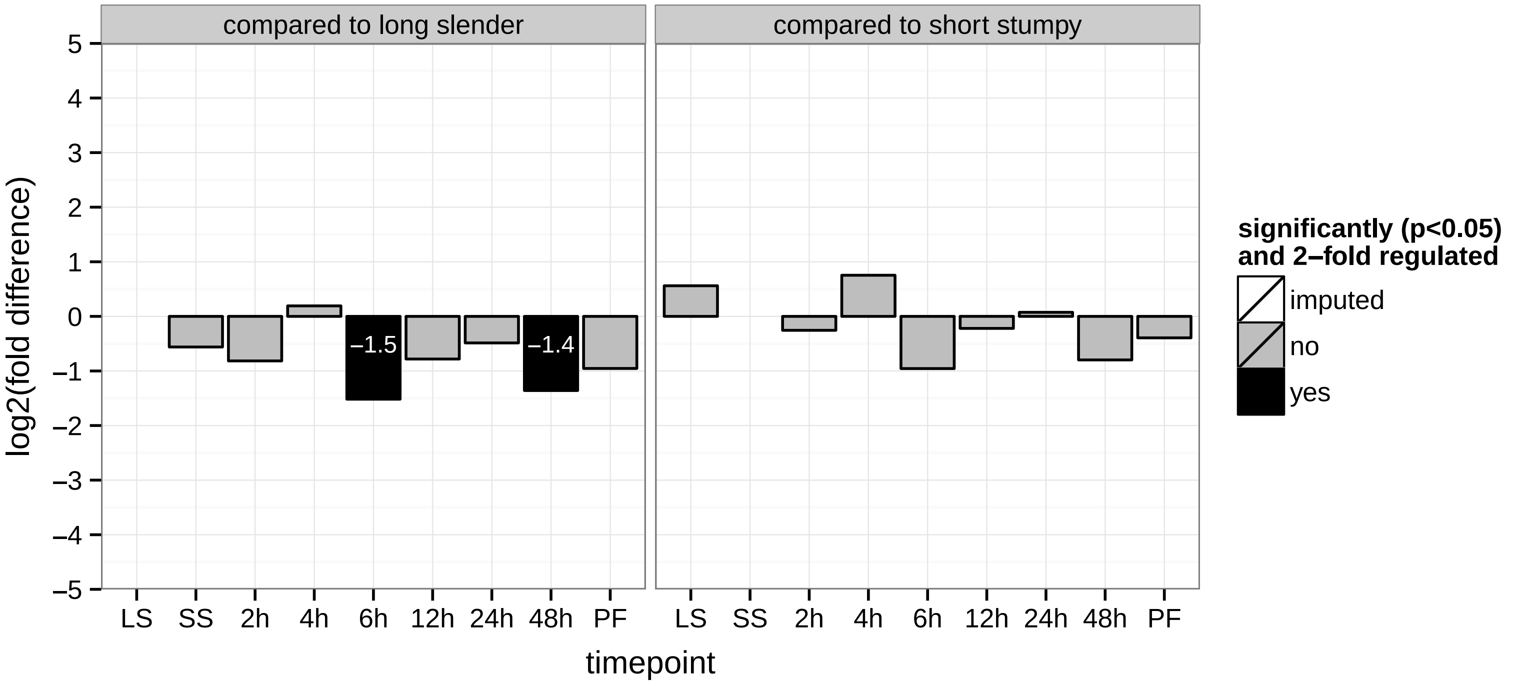

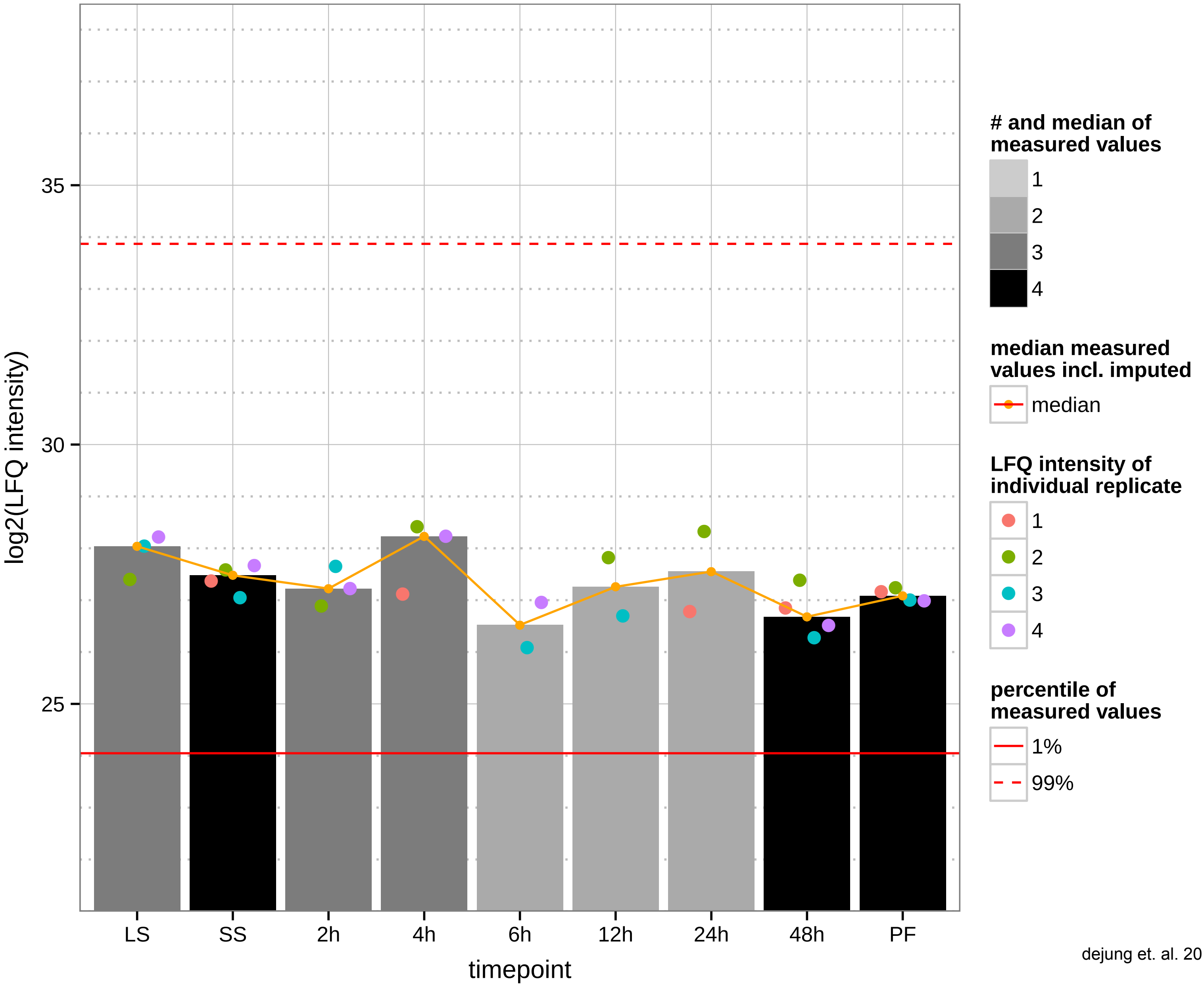

dejung et. al. 2015

hypothetical protein, conserved
Tb927.11.14330
AGOF: acid–amino acid ligase activity, zinc ion binding
AGOC: intracellular
AGOP: cellular protein modification process
PGOF: acid–amino acid ligase activity, binding, zinc ion binding
PGOC: intracellular
PGOP: cellular protein modification process

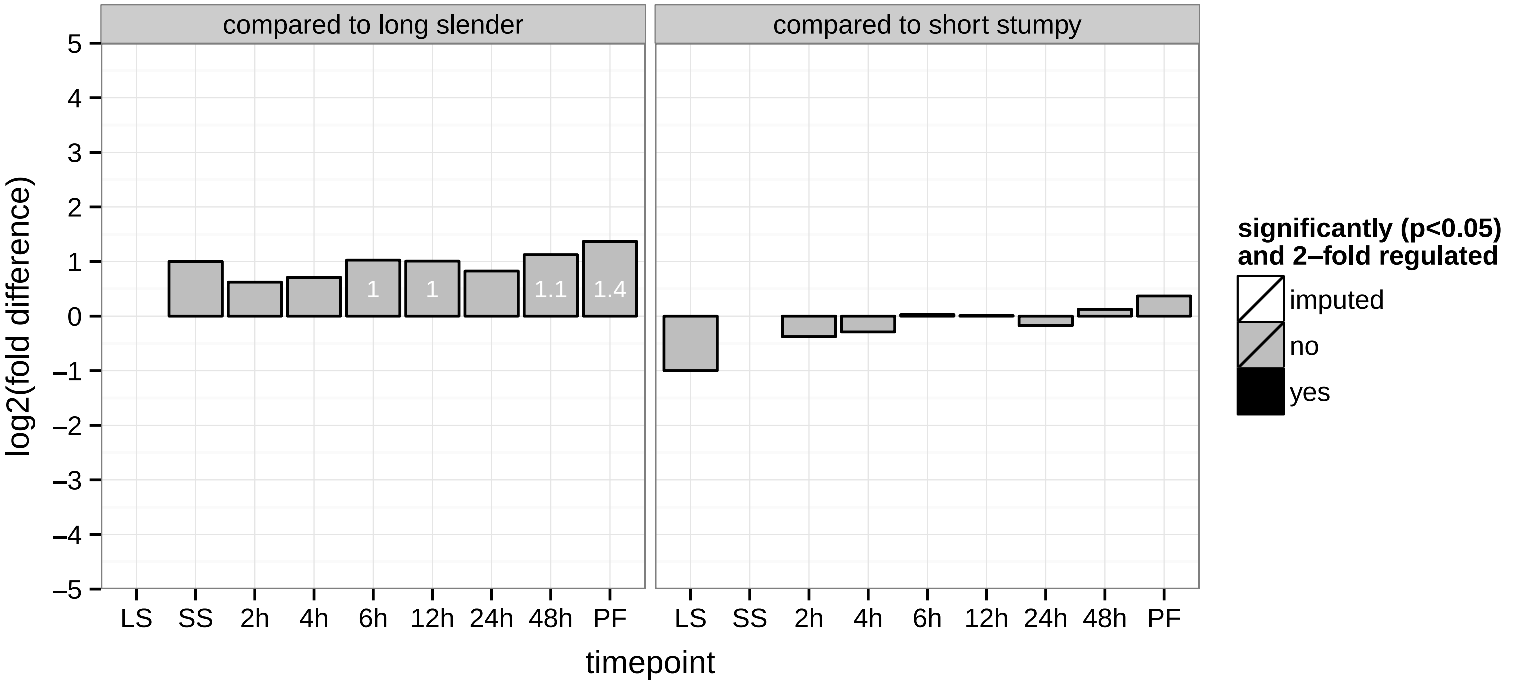

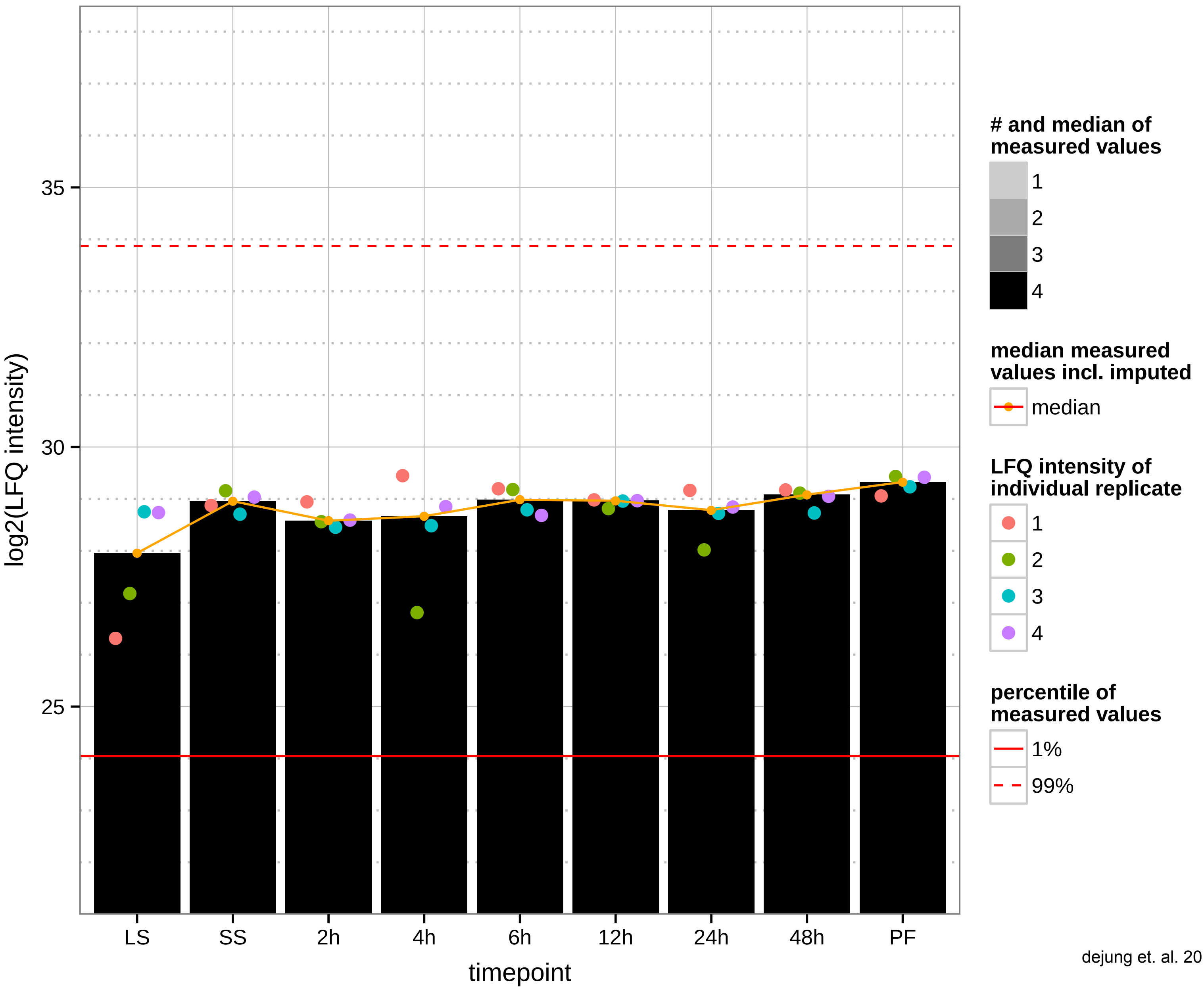

dejung et. al. 2015

exportin 1 (XPO1)
Tb927.11.14340
AGOF: protein transporter activity
AGOC: cytoplasm, nuclear pore, nucleus
AGOP: intracellular protein transport, nuclear export, protein import into nucleus, docking
PGOF: binding, protein transporter activity
PGOC: null
PGOP: intracellular protein transport

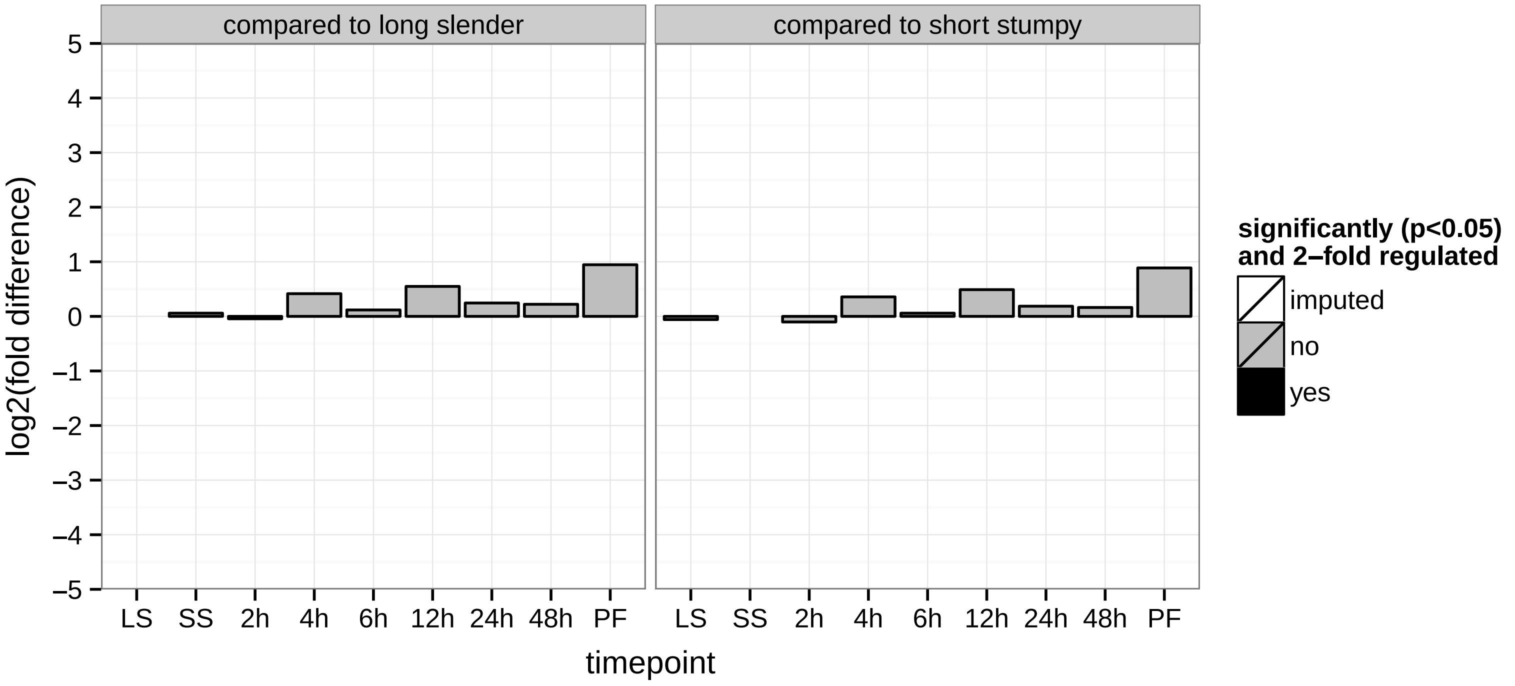

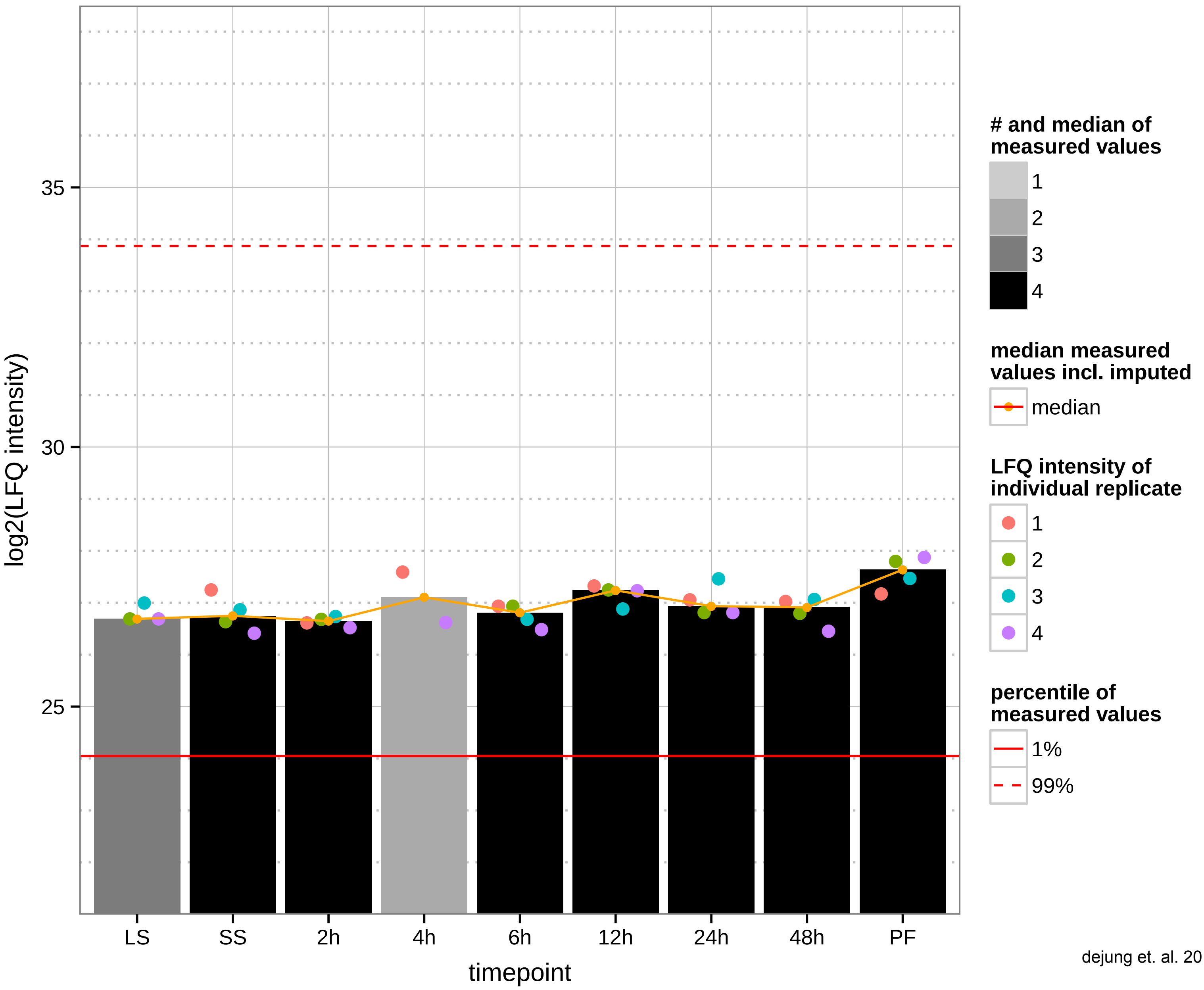

dejung et. al. 2015

proteasome regulatory non–ATP–ase subunit
Tb927.11.14430
AGOF: endopeptidase activity
AGOC: proteasome regulatory particle
AGOP: proteolysis
PGOF: null
PGOC: null
PGOP: null

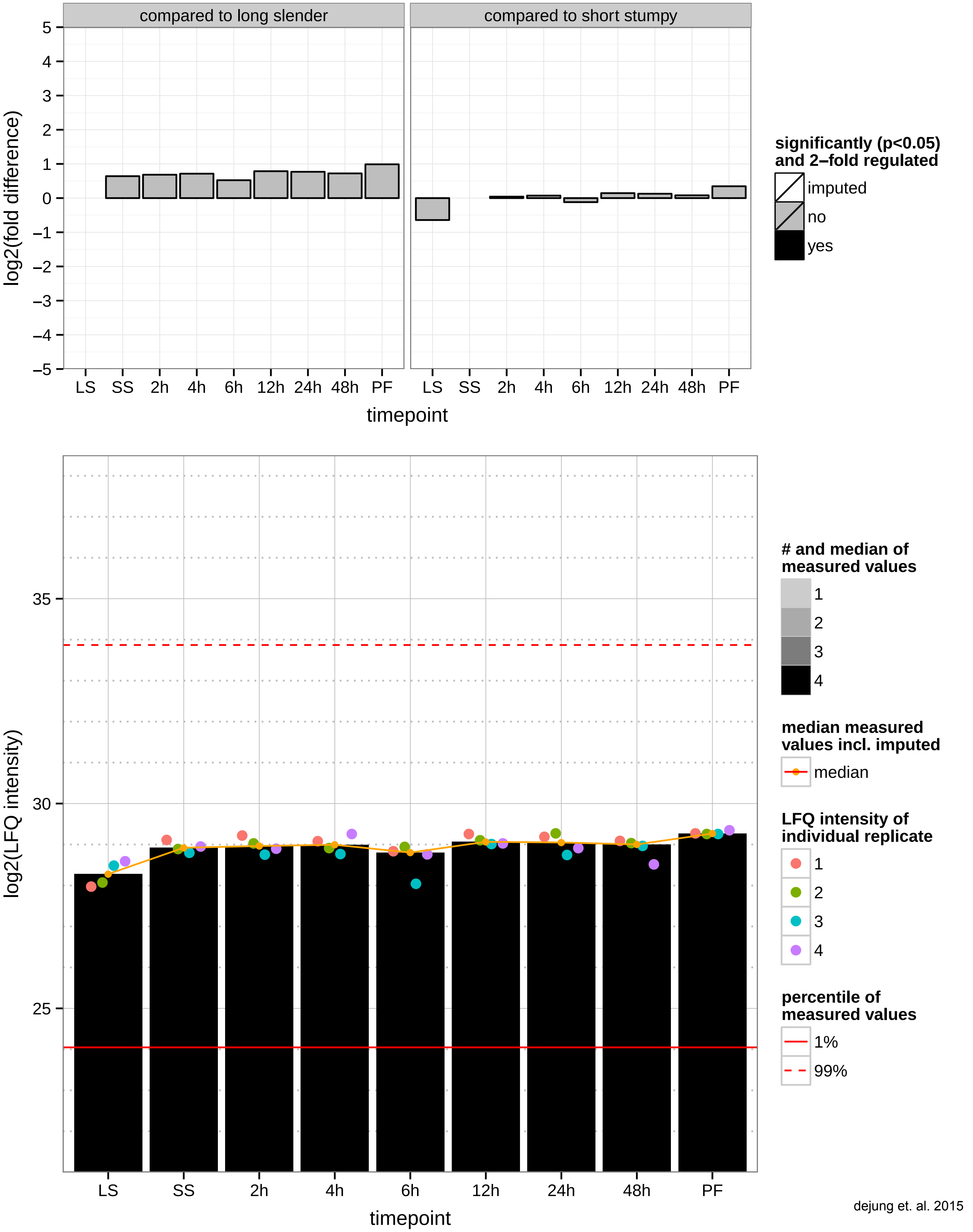

ADP–ribosylation factor GTPase activating protein 1, putative
Tb927.11.14460
AGOF: ARF GTPase activator activity, DNA binding, zinc ion binding
AGOC: nucleus
AGOP: regulation of ARF GTPase activity
PGOF: ARF GTPase activator activity, zinc ion binding
PGOC: null
PGOP: regulation of ARF GTPase activity

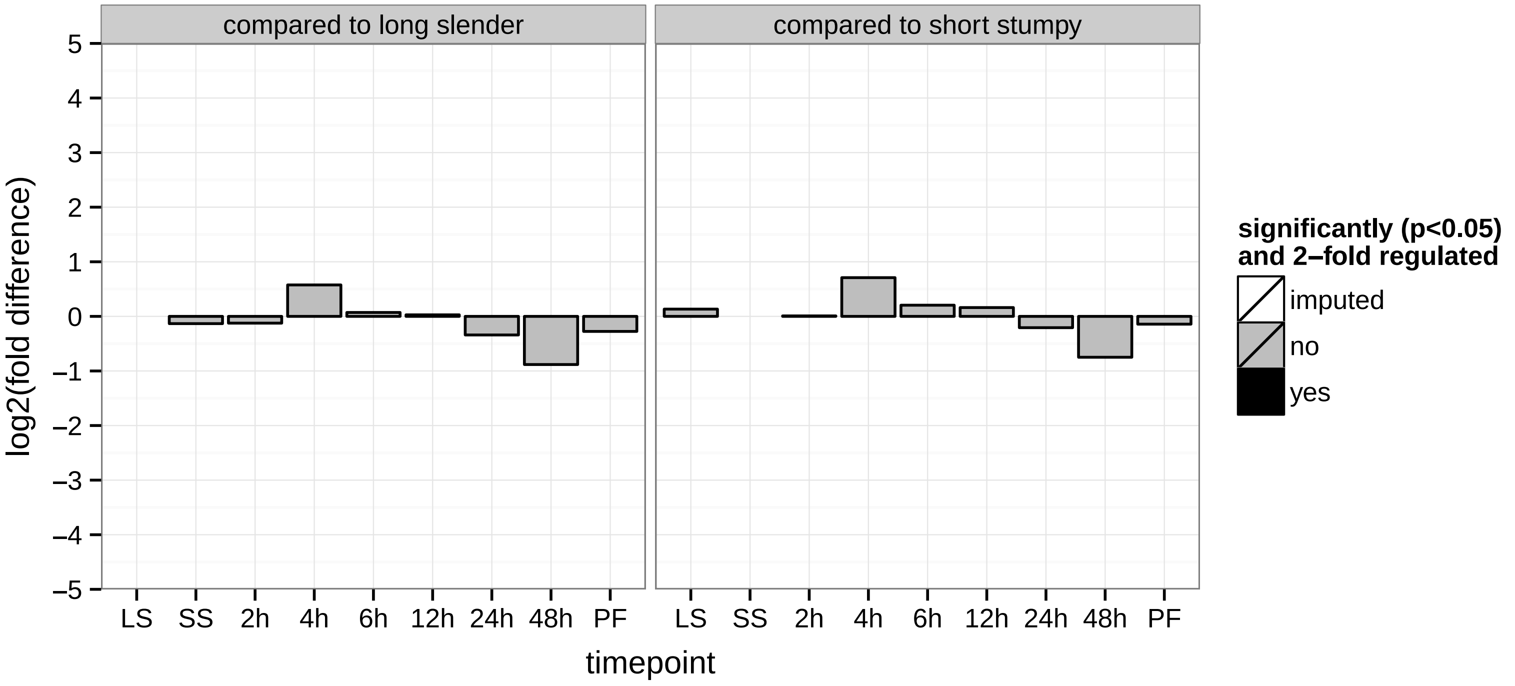

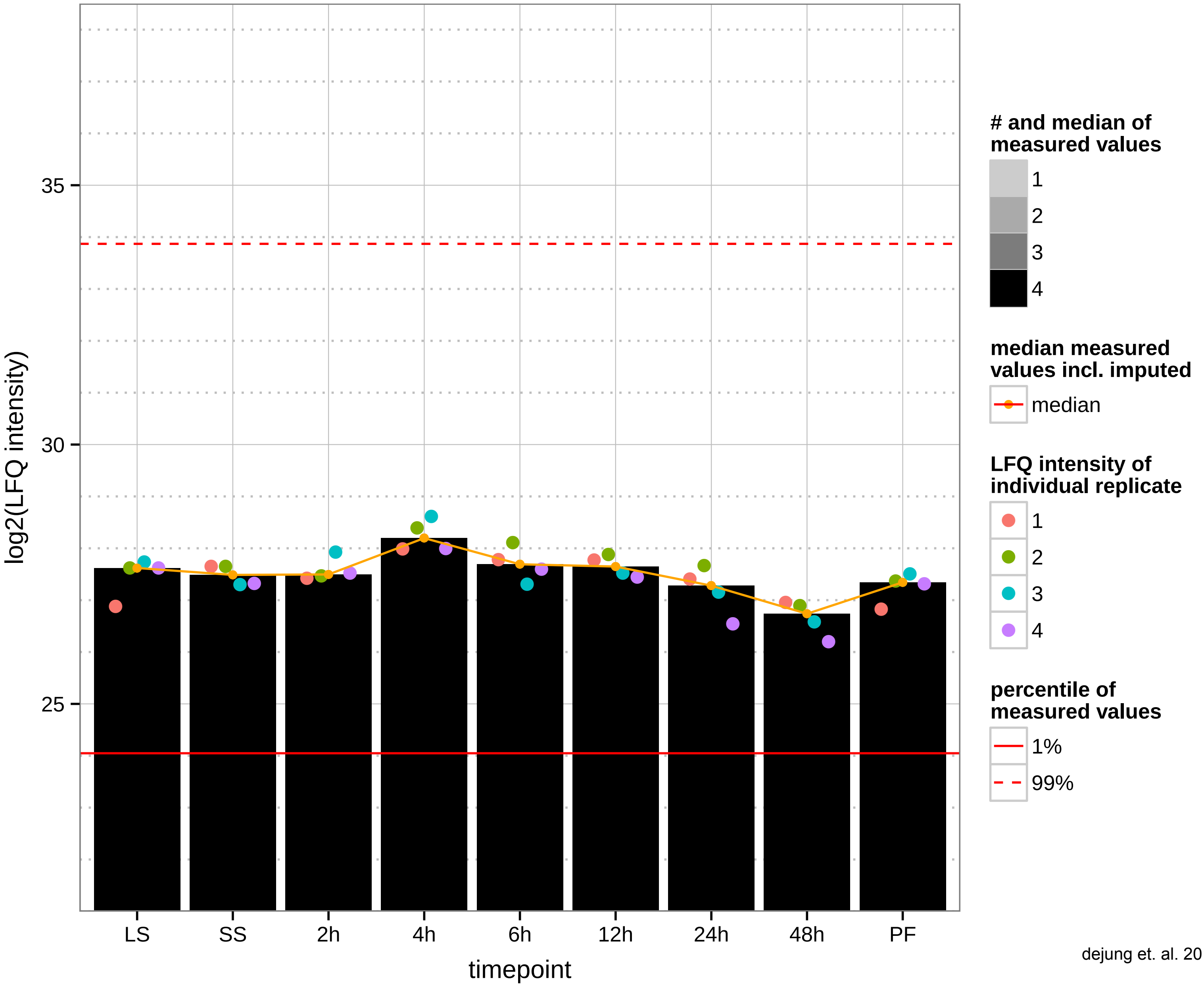

dejung et. al. 2015

RNA polymerase subunit, putative (RPB7)
Tb927.11.14490
AGOF: DNA–directed RNA polymerase activity
AGOC: null
AGOP: transcription, DNA–dependent
PGOF: DNA–directed RNA polymerase activity
PGOC: null
PGOP: transcription, DNA–dependent

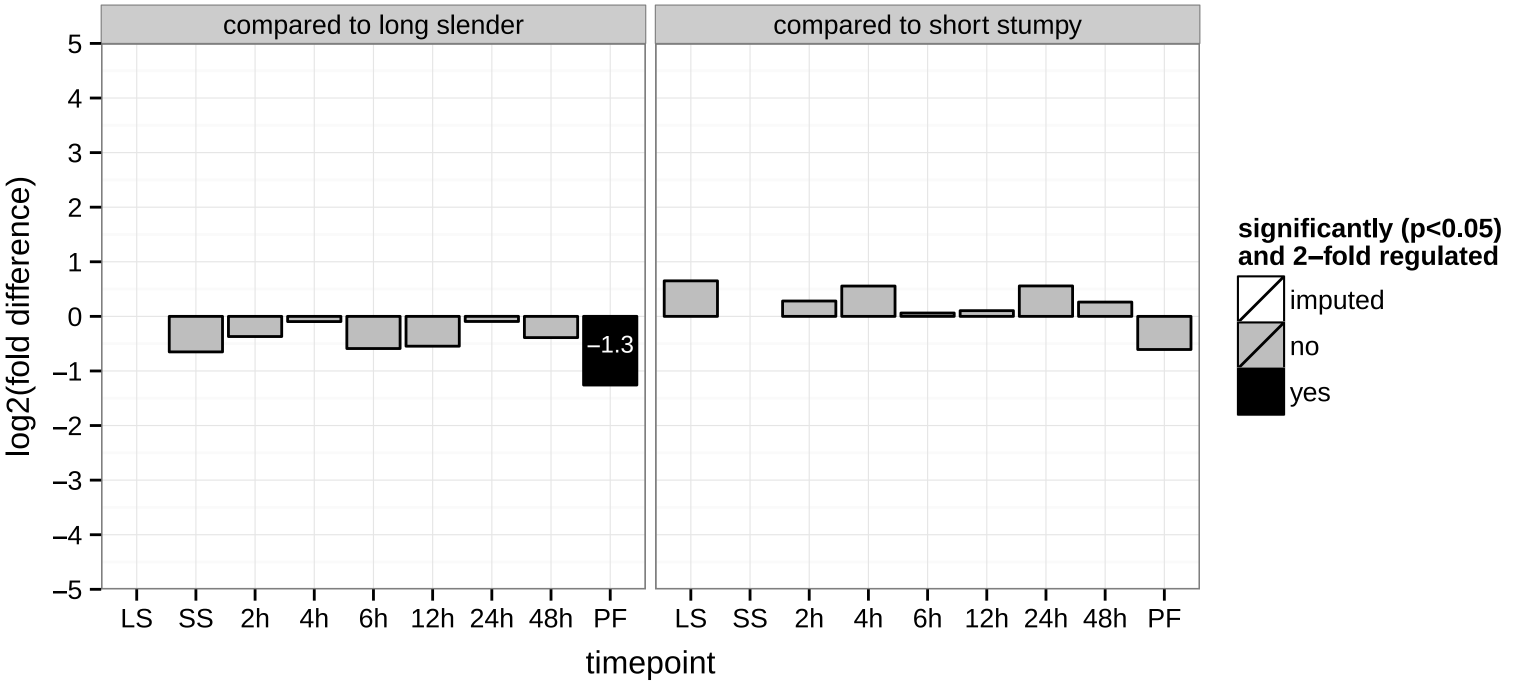

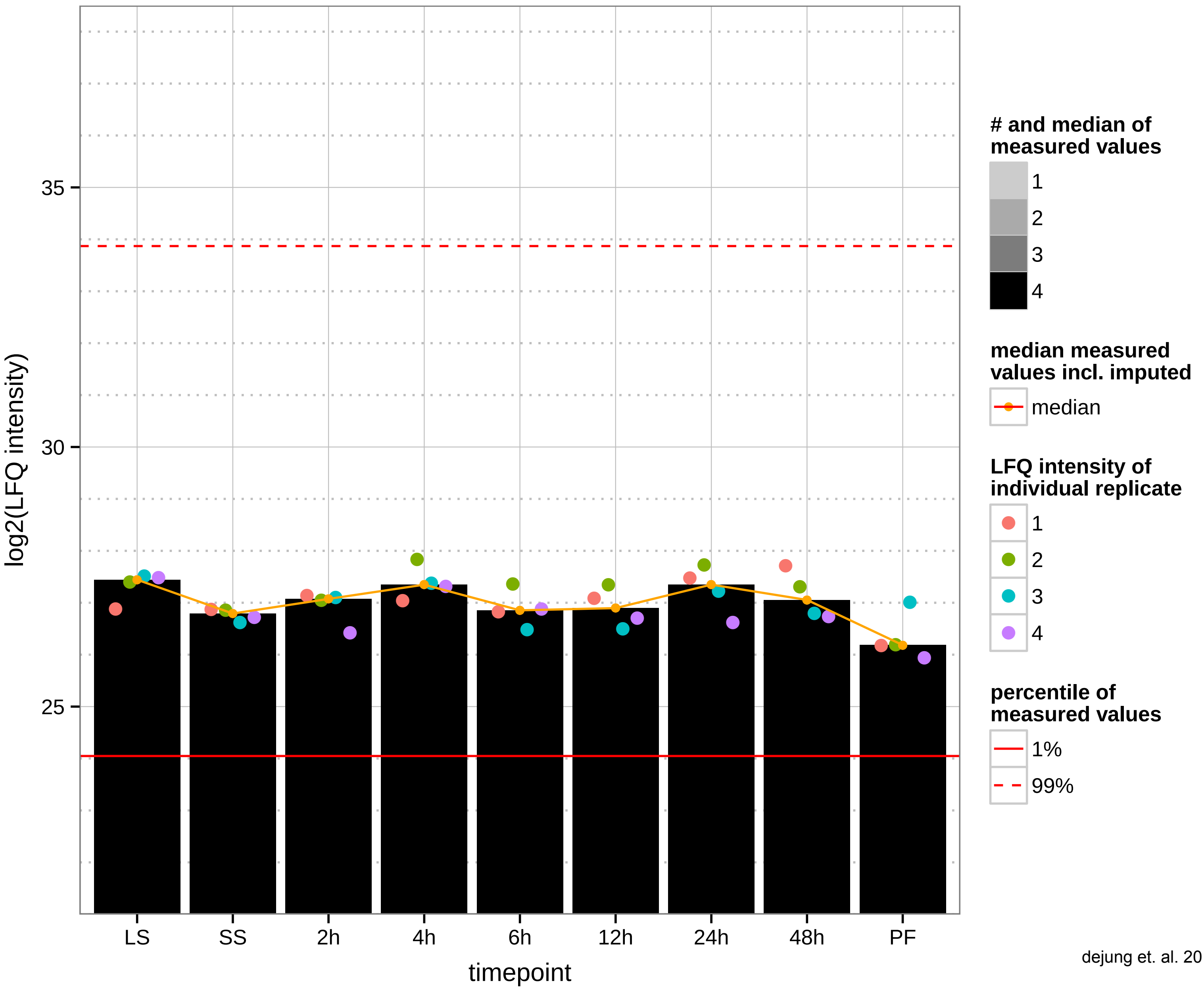

dejung et. al. 2015

2–oxoglutarate dehydrogenase E1 component, putative
Tb927.11.1450
AGOF: oxoglutarate dehydrogenase (succinyl–transferring) activity, thiamine pyrophosphate binding
AGOC: mitochondrion, oxoglutarate dehydrogenase complex
AGOP: glycolysis, lysine catabolic process, tricarboxylic acid cycle, tryptophan metabolic process
PGOF: oxidoreductase activity, acting on the aldehyde or oxo group of donors, disulfide as acceptor, oxoglutarate dehydrogen
PGOC: null
PGOP: metabolic process, oxidation–reduction process, tricarboxylic acid cycle

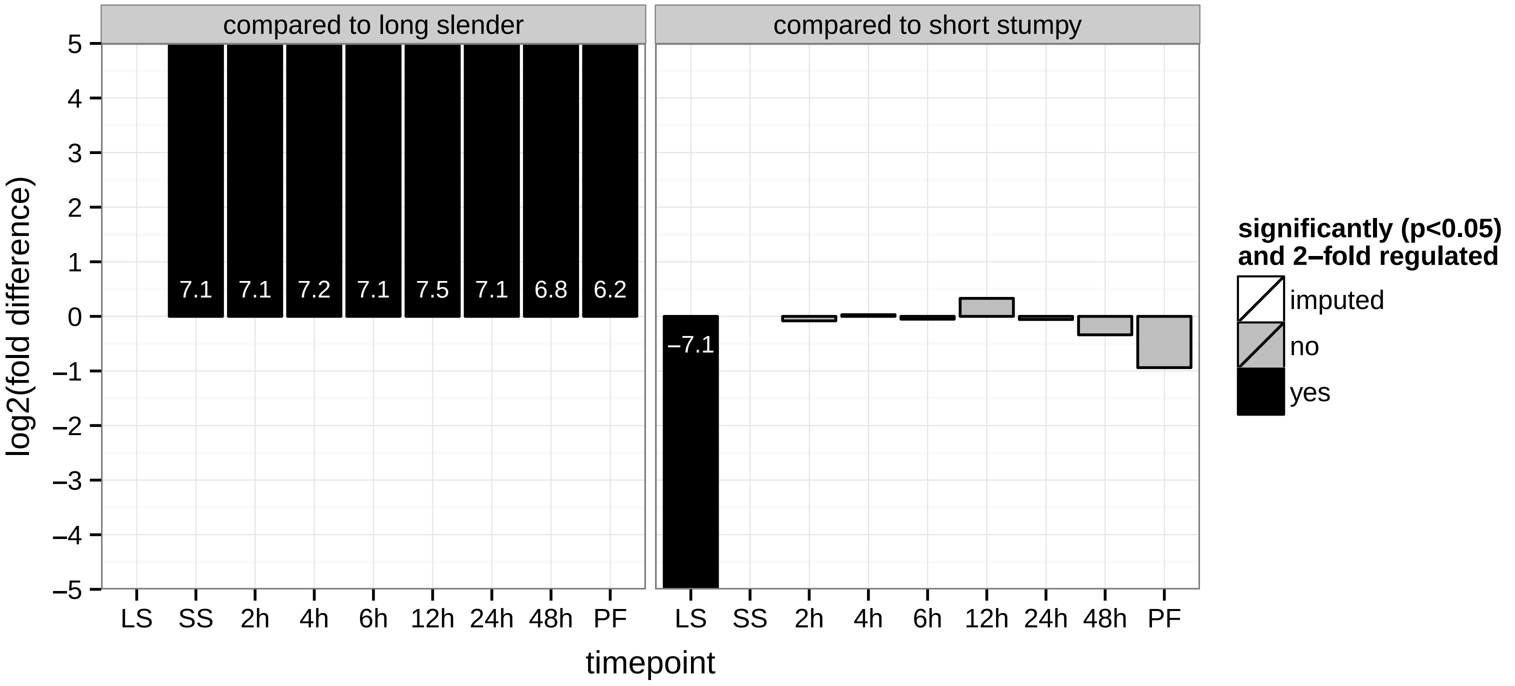

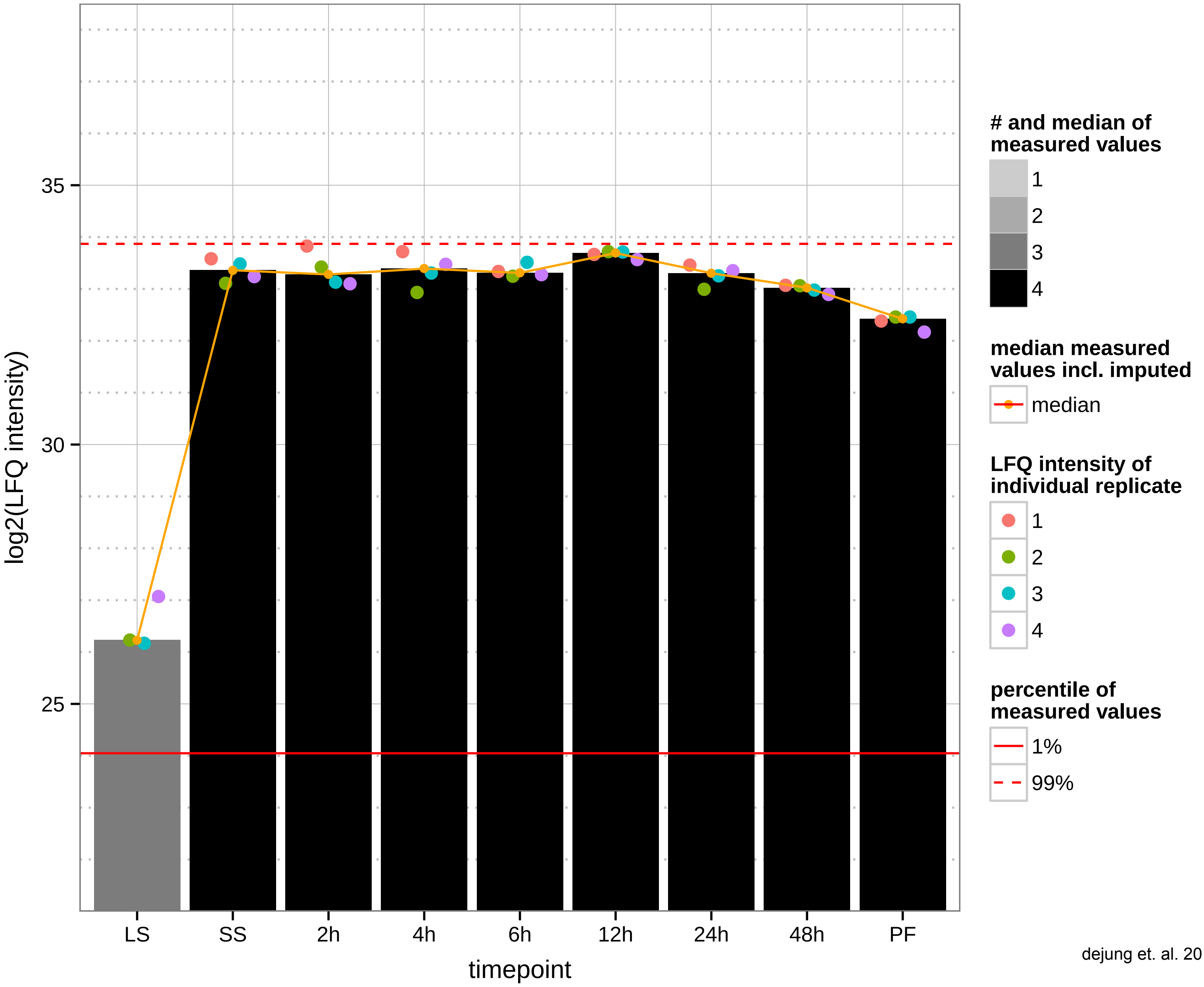

dejung et. al. 2015

cleavage and polyadenylation specificity factor–like protein, putative
Tb927.11.14560
AGOF: nucleic acid binding
AGOC: nucleus
AGOP: mRNA cleavage, mRNA polyadenylation
PGOF: nucleic acid binding
PGOC: nucleus
PGOP: null

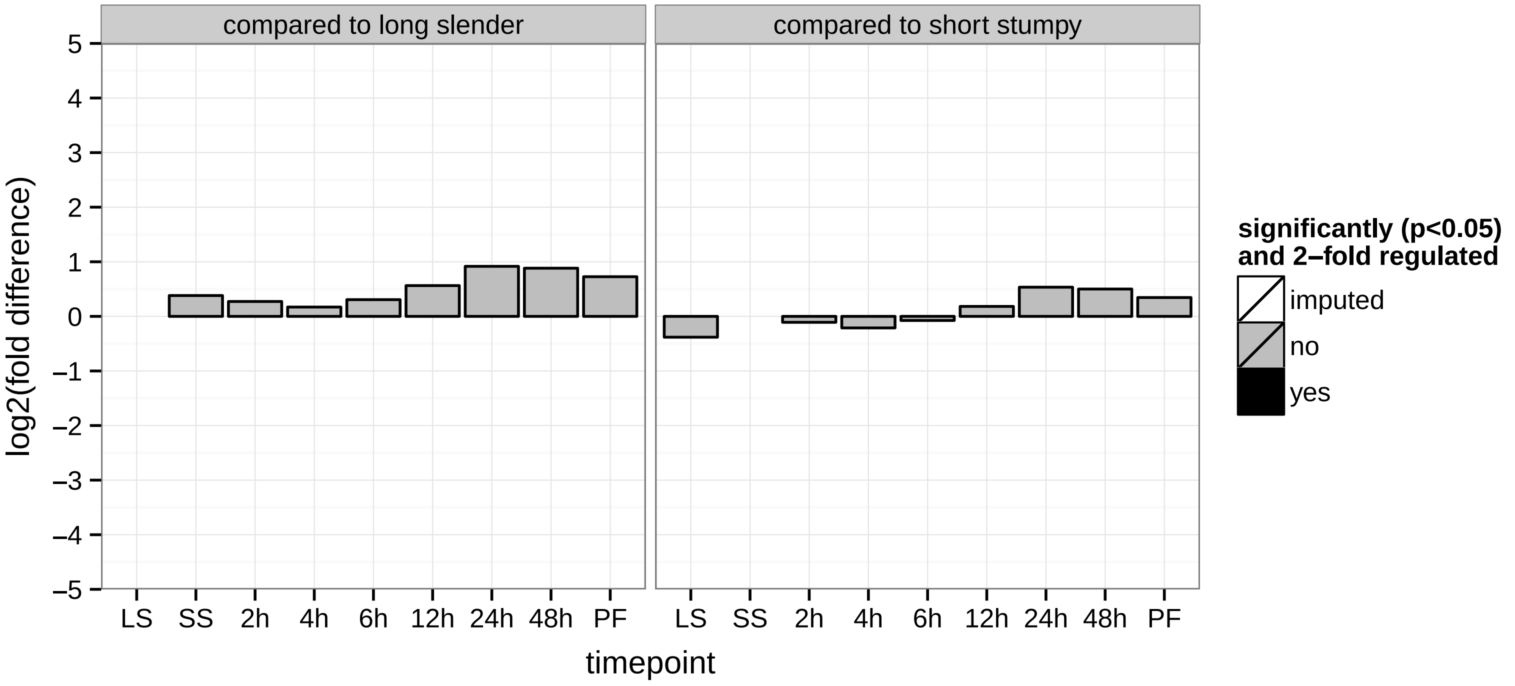

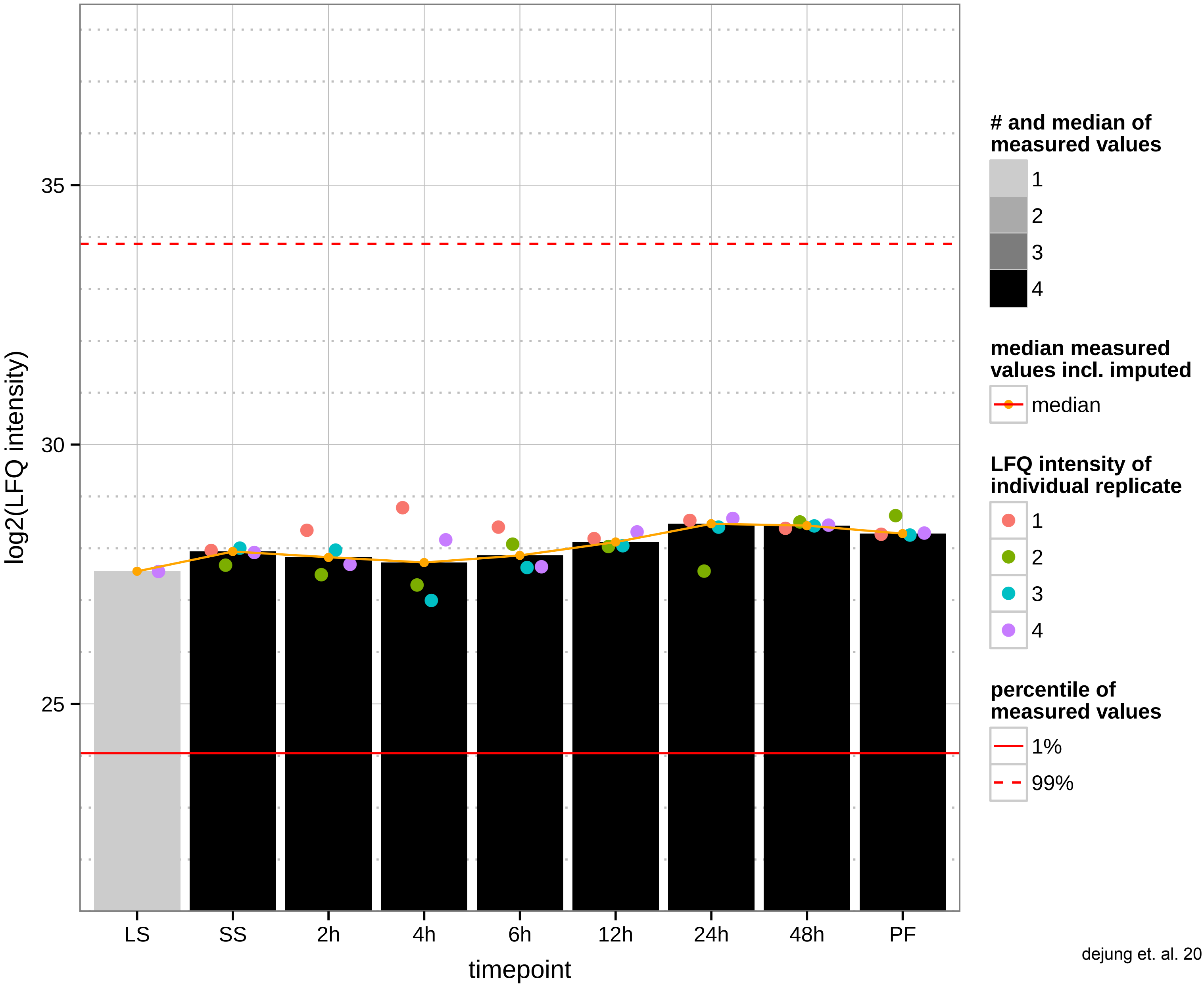

dejung et. al. 2015

NAD+ synthase, putative
Tb927.11.14870
AGOF: ATP binding, NAD+ synthase (glutamine−hydrolyzing) activity
AGOC: null
AGOP: NAD biosynthetic process
PGOF: ATP binding, NAD+ synthase (glutamine−hydrolyzing) activity
PGOC: null
PGOP: NAD biosynthetic process

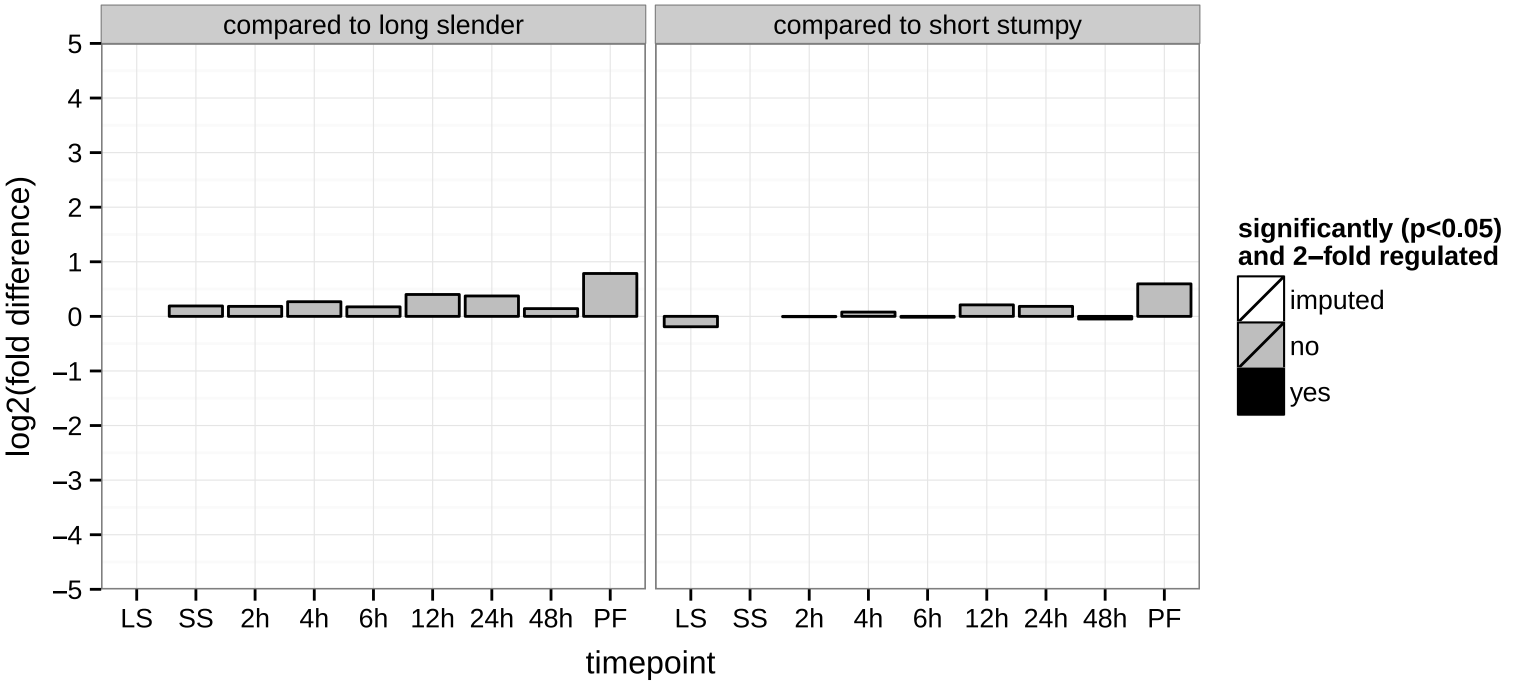

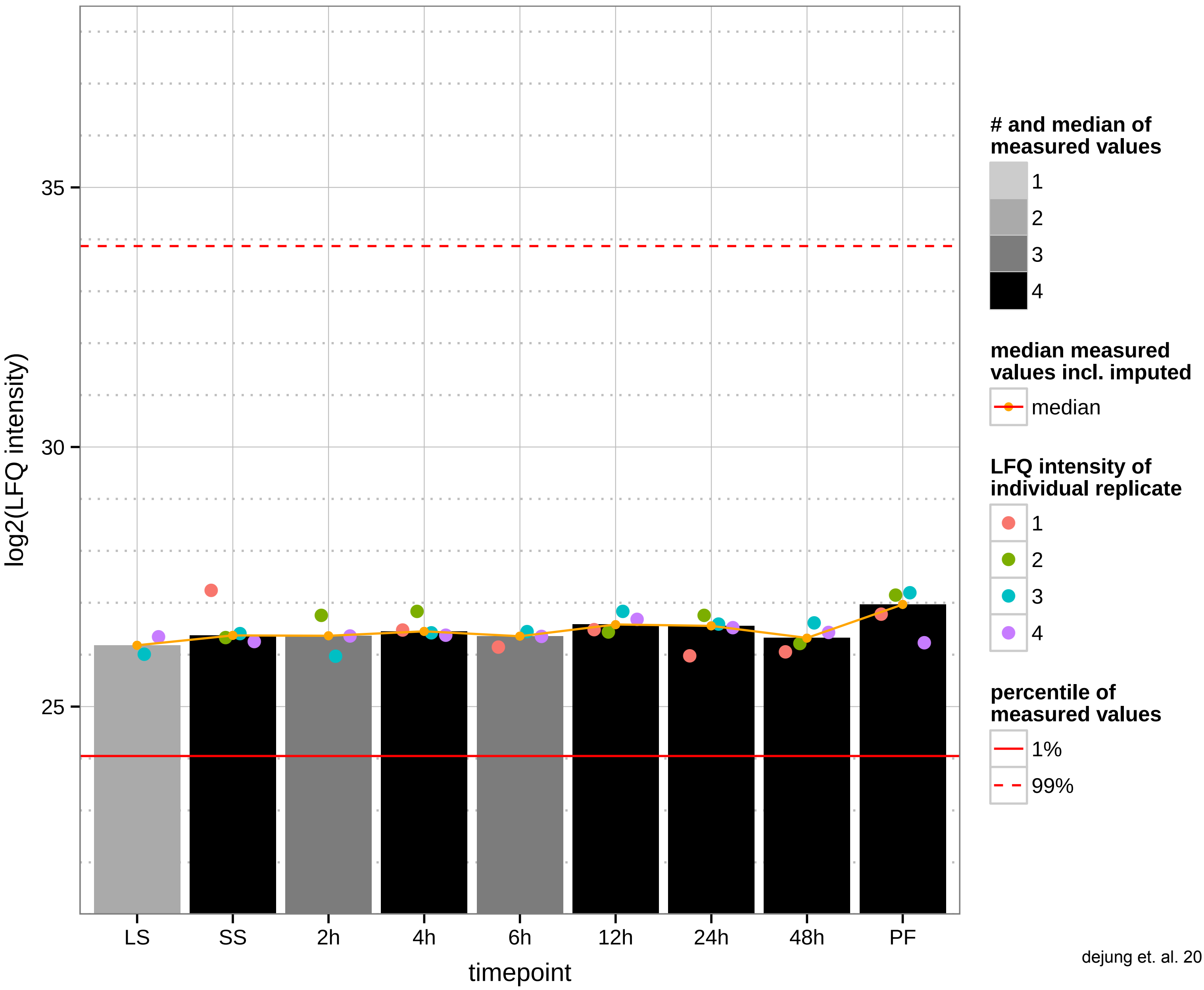

dejung et. al. 2015

hypothetical protein, conserved
Tb927.11.14890
AGOF: DNA binding, DNA−directed DNA polymerase activity
AGOC: nucleus
AGOP: DNA replication
PGOF: DNA binding, DNA−directed DNA polymerase activity
PGOC: null
PGOP: DNA replication

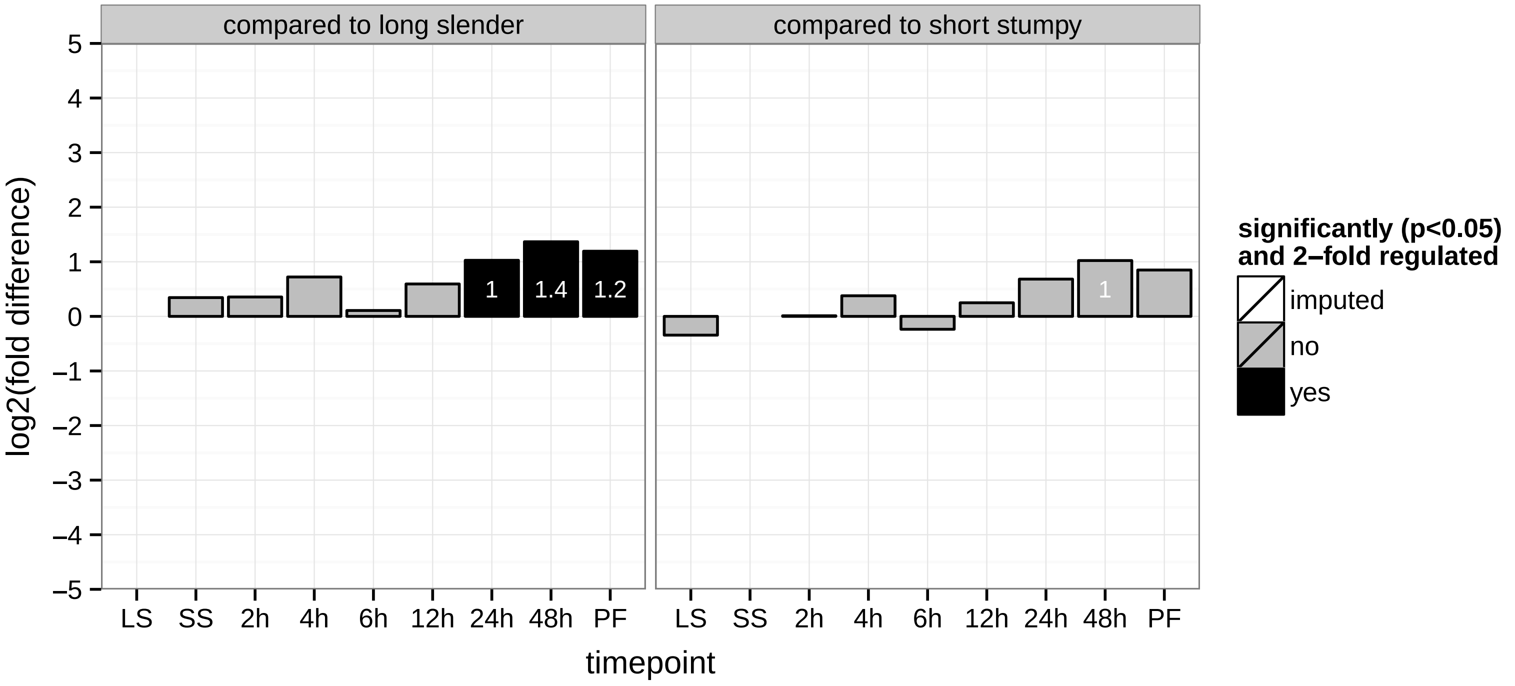

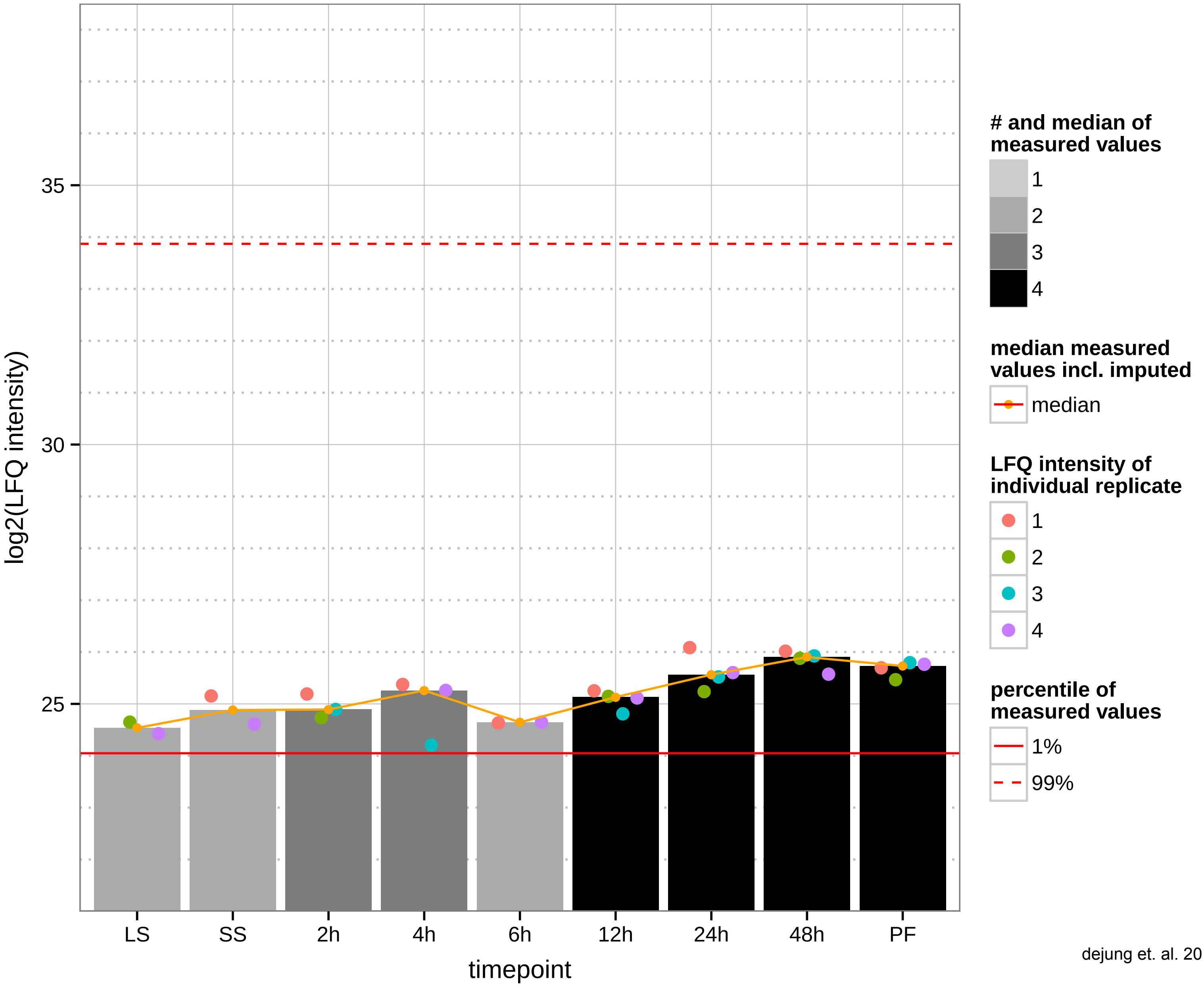

dejung et. al. 2015

coatomer epsilon subunit, putative, cytosolic coat protein
Tb927.11.14900
AGOF: protein transporter activity, structural molecule activity
AGOC: COPI vesicle coat, cytoplasm, membrane
AGOP: intracellular protein transport, retrograde vesicle–mediated transport, Golgi to ER
PGOF: structural molecule activity
PGOC: COPI vesicle coat
PGOP: retrograde vesicle–mediated transport, Golgi to ER

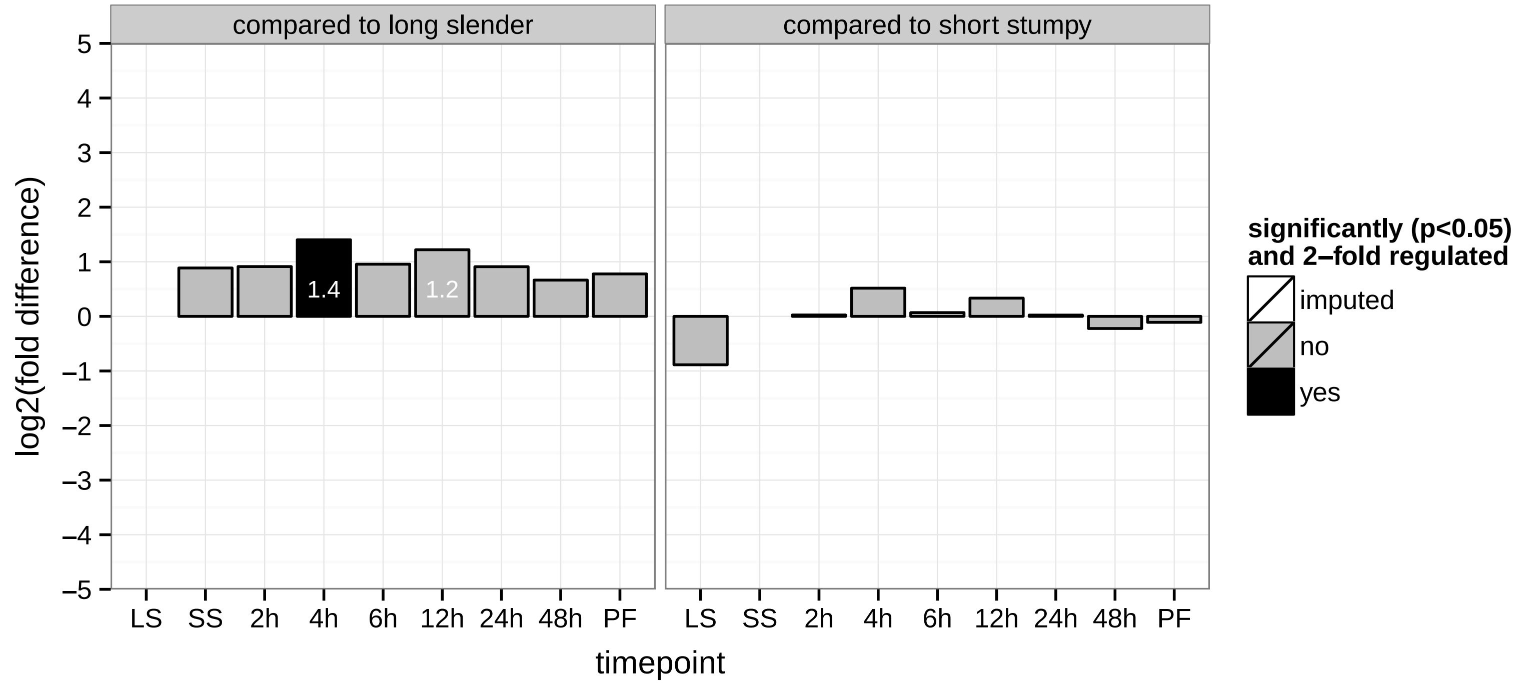

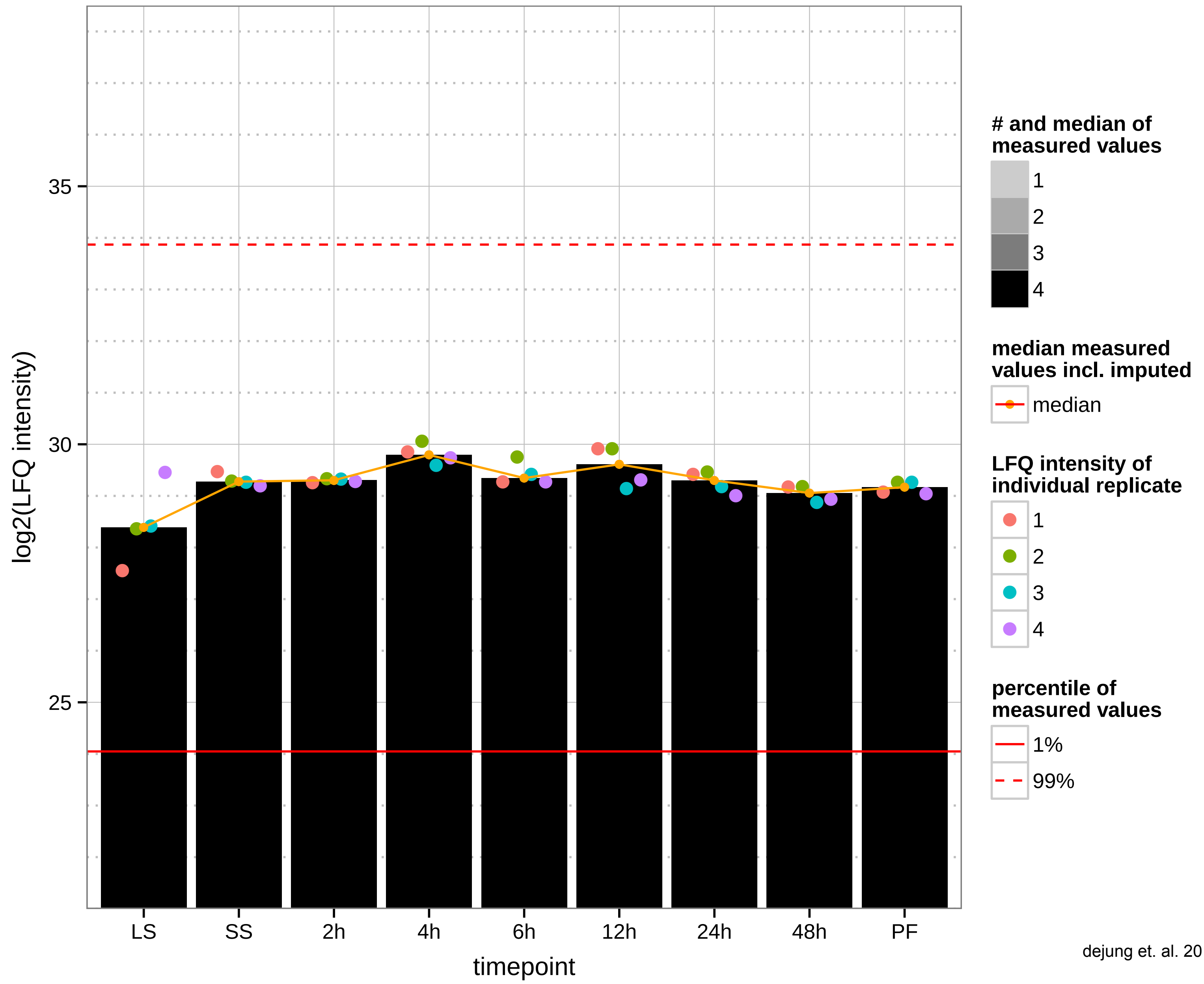

dejung et. al. 2015

protein phosphatase 2C, putative
Tb927.11.14910
AGOF: phosphatase activity
AGOC: integral to membrane
AGOP: dephosphorylation
PGOF: catalytic activity
PGOC: null
PGOP: null

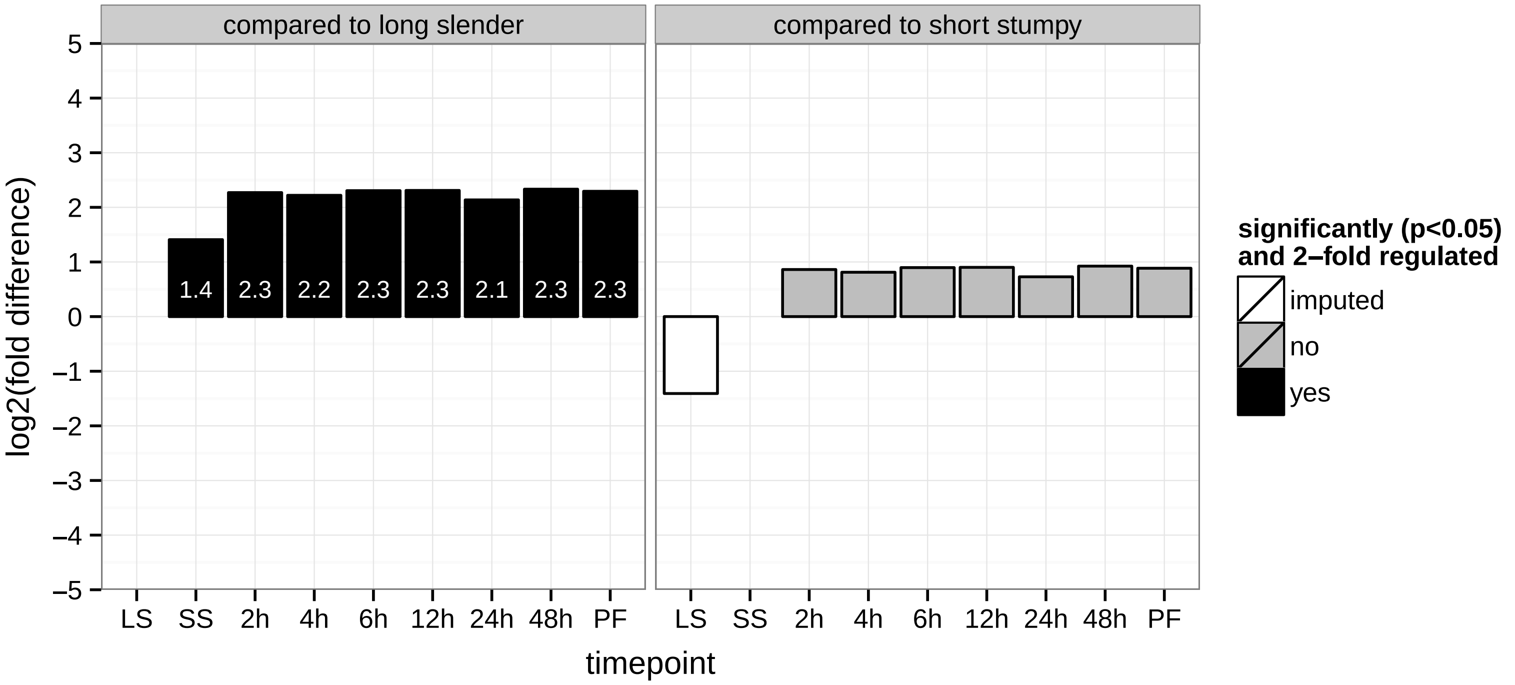

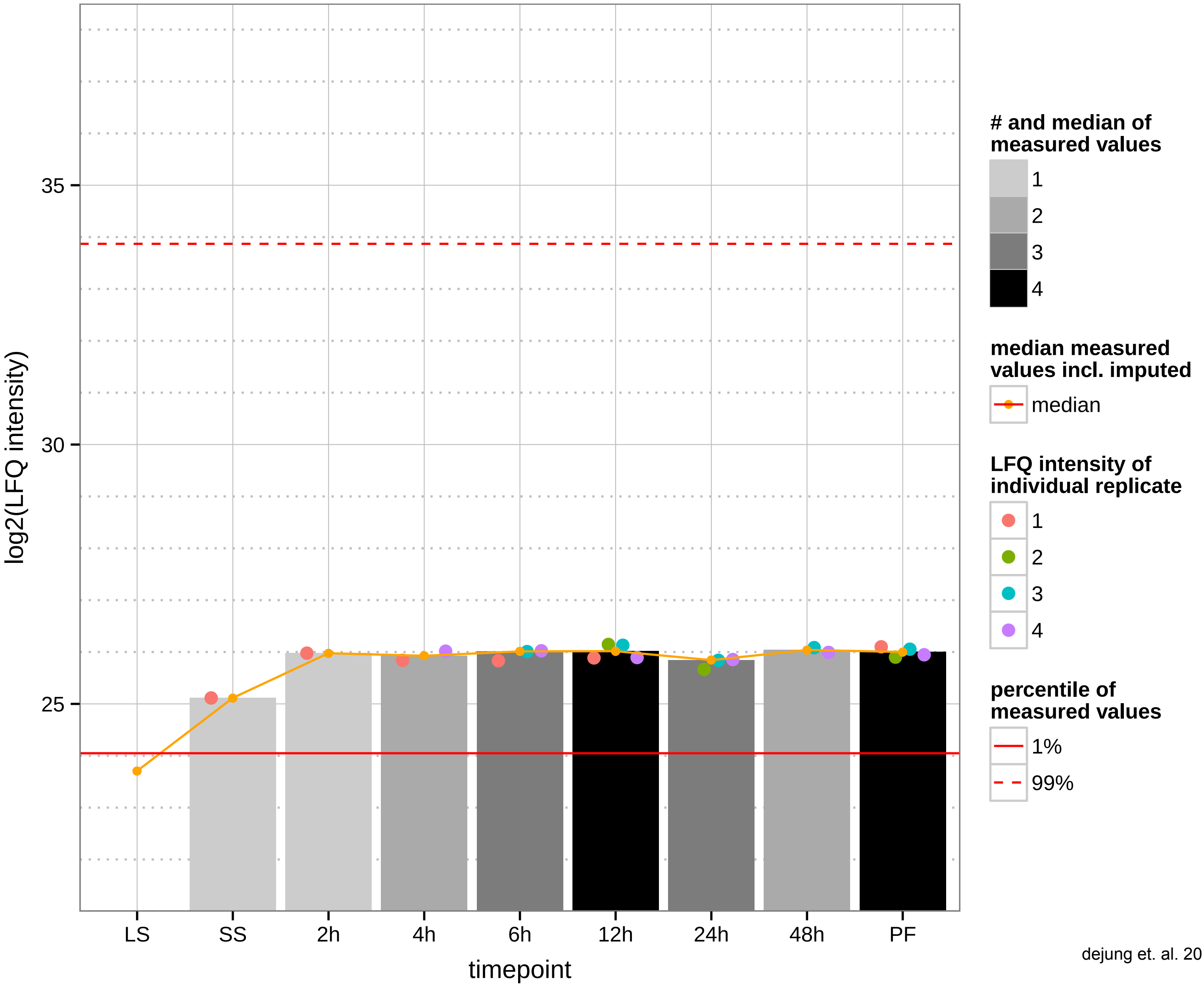

dejung et. al. 2015

hypothetical protein, conserved
Tb927.11.14970
AGOF: null
AGOC: null
AGOP: null
PGOF: null
PGOC: null
PGOP: null

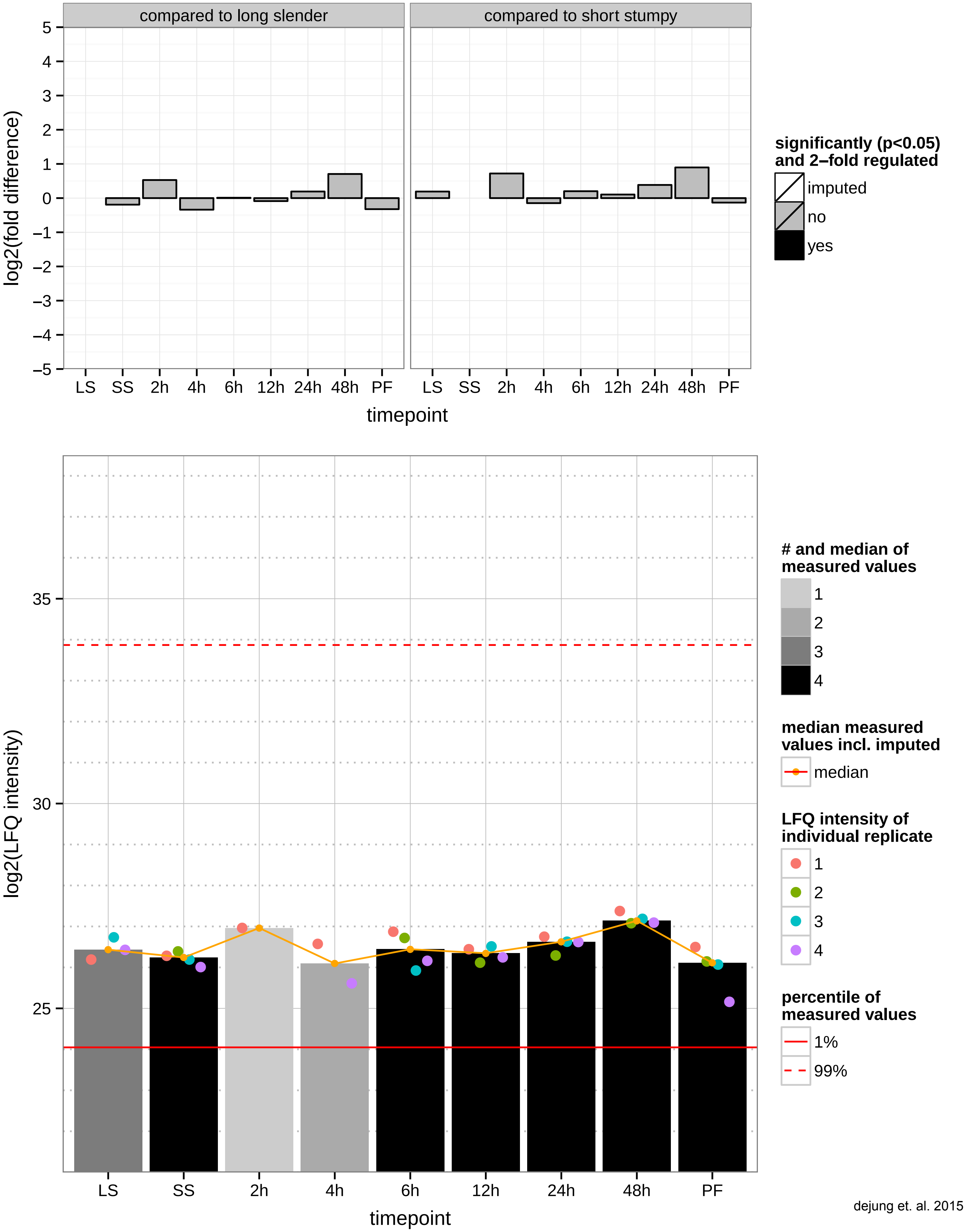

chaperonin HSP60, mitochondrial precursor, putative, heat shock protein 60 (Cpn60)
Tb927.11.15040
AGOF: ATP binding, unfolded protein binding
AGOC: mitochondrion
AGOP: protein complex assembly, protein folding
PGOF: ATP binding
PGOC: cytoplasm
PGOP: cellular protein metabolic process, protein refolding

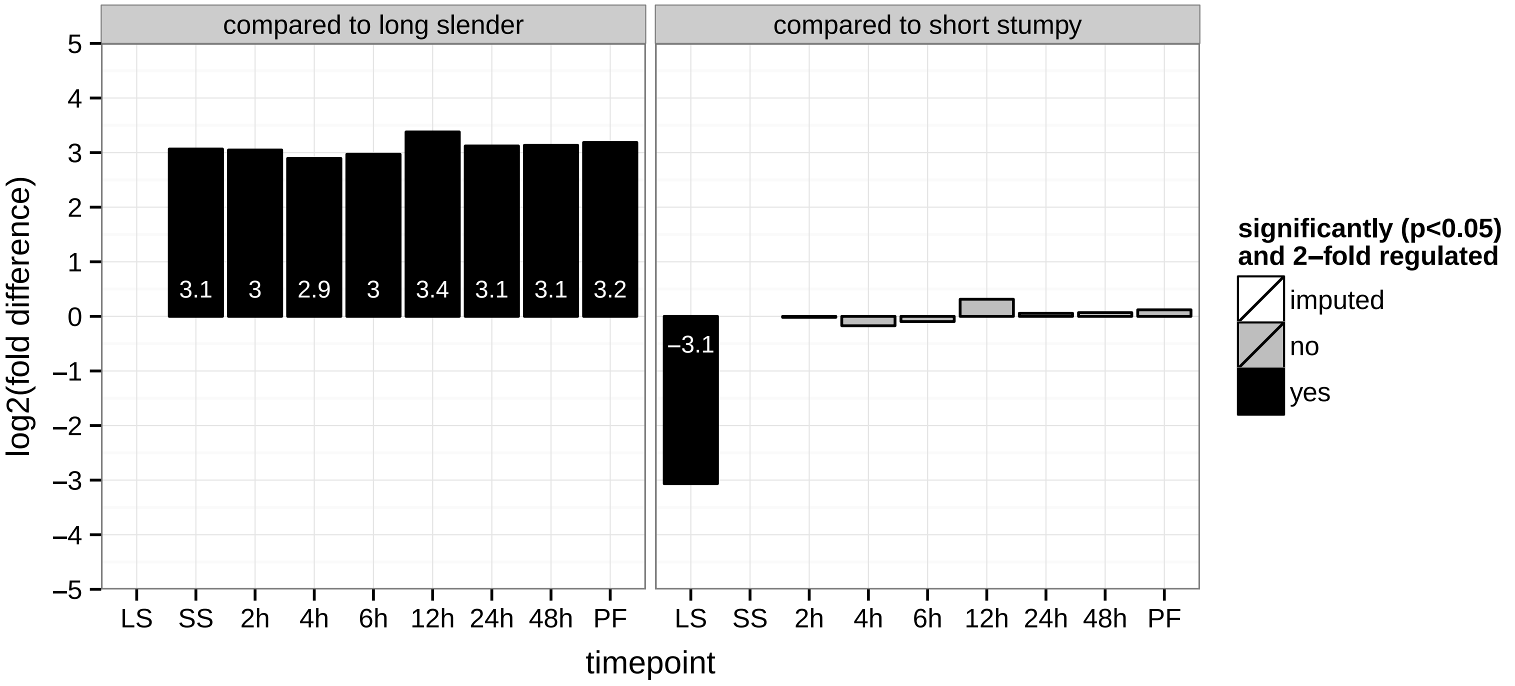

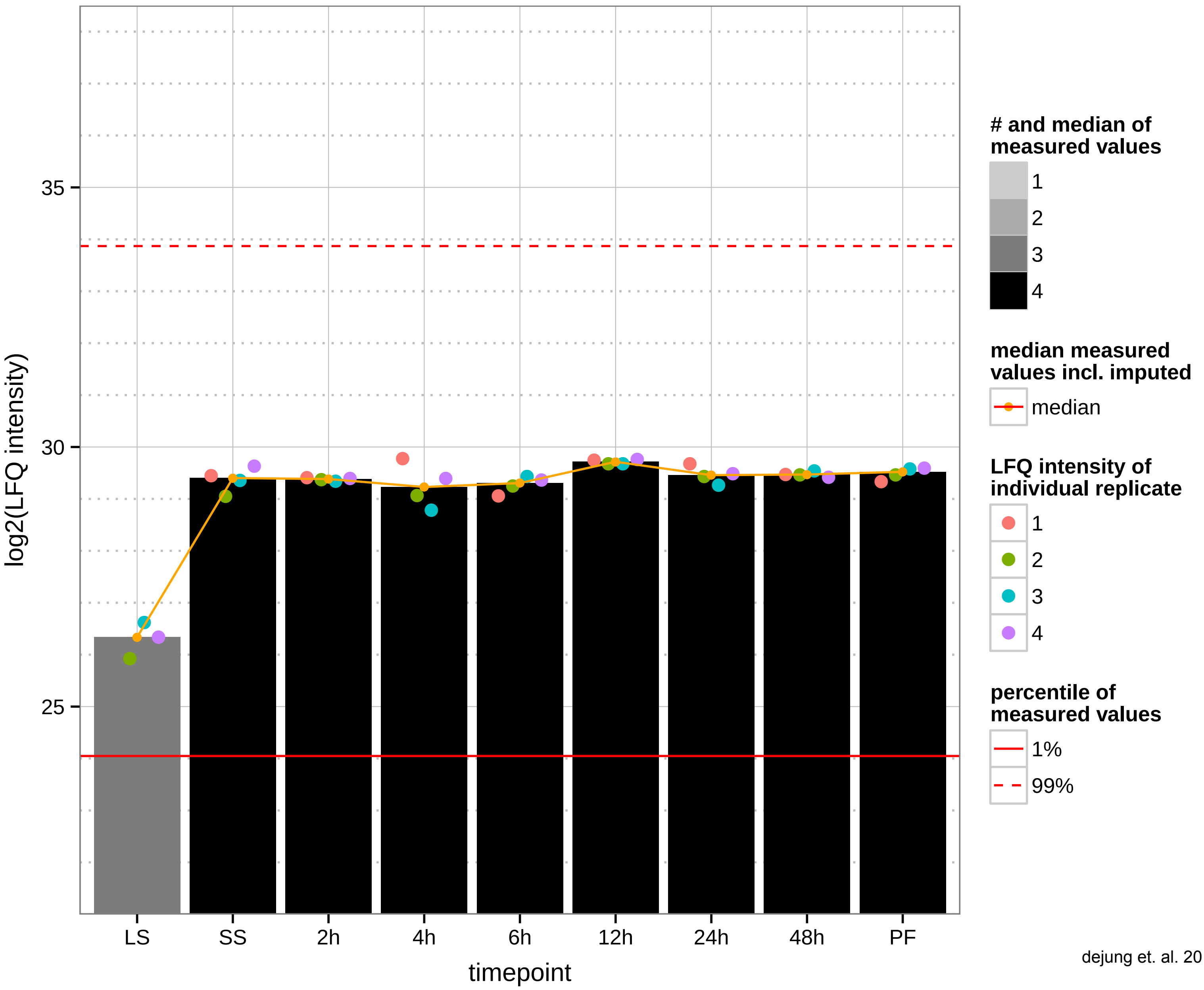

dejung et. al. 2015

hypothetical protein, conserved
Tb927.11.15070
AGOF: null
AGOC: mitochondrion
AGOP: null
PGOF: null
PGOC: null
PGOP: null

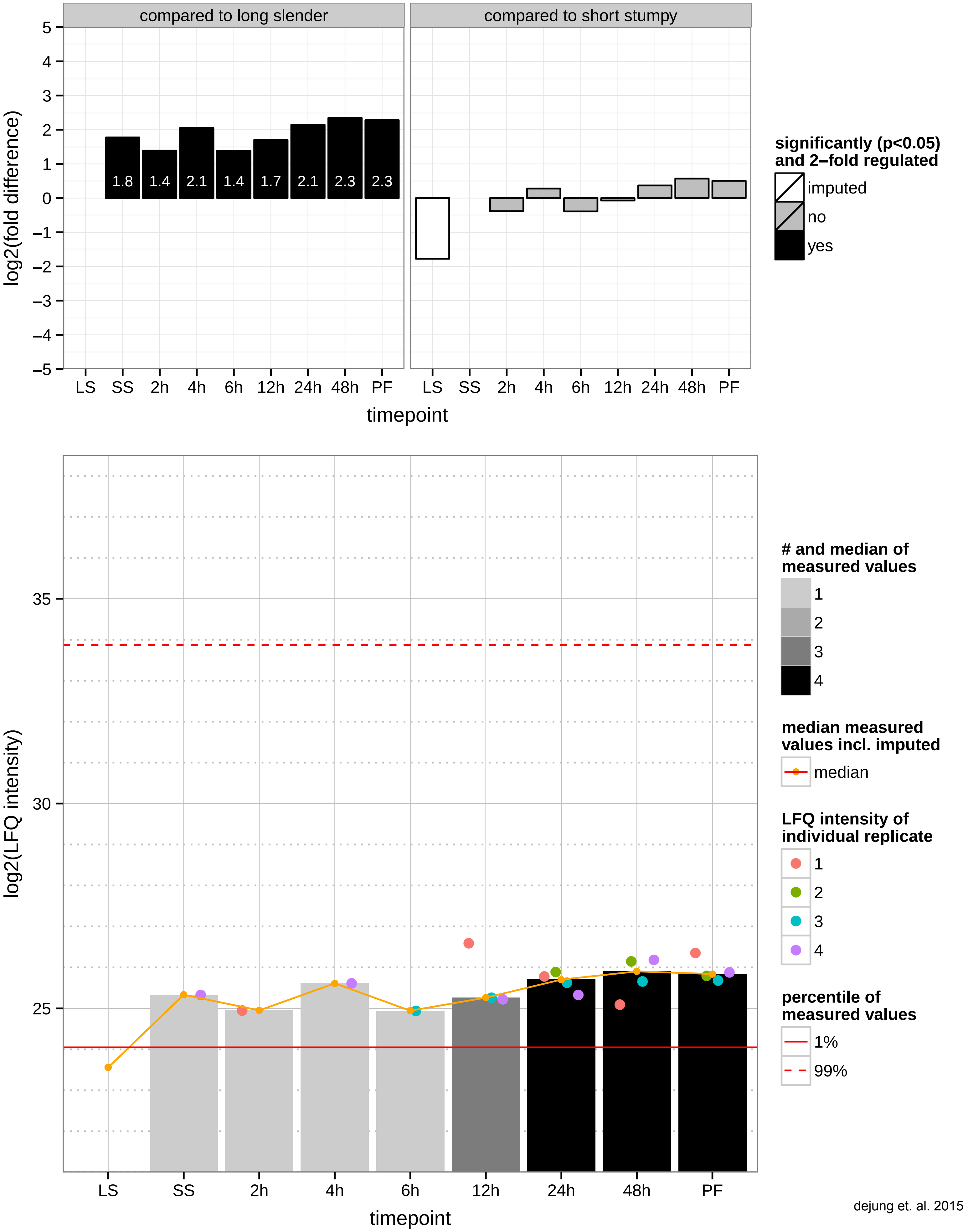

dejung et. al. 2015

Tb5.20
Tb927.11.15100
AGOF: null
AGOC: null
AGOP: null
PGOF: null
PGOC: null
PGOP: null

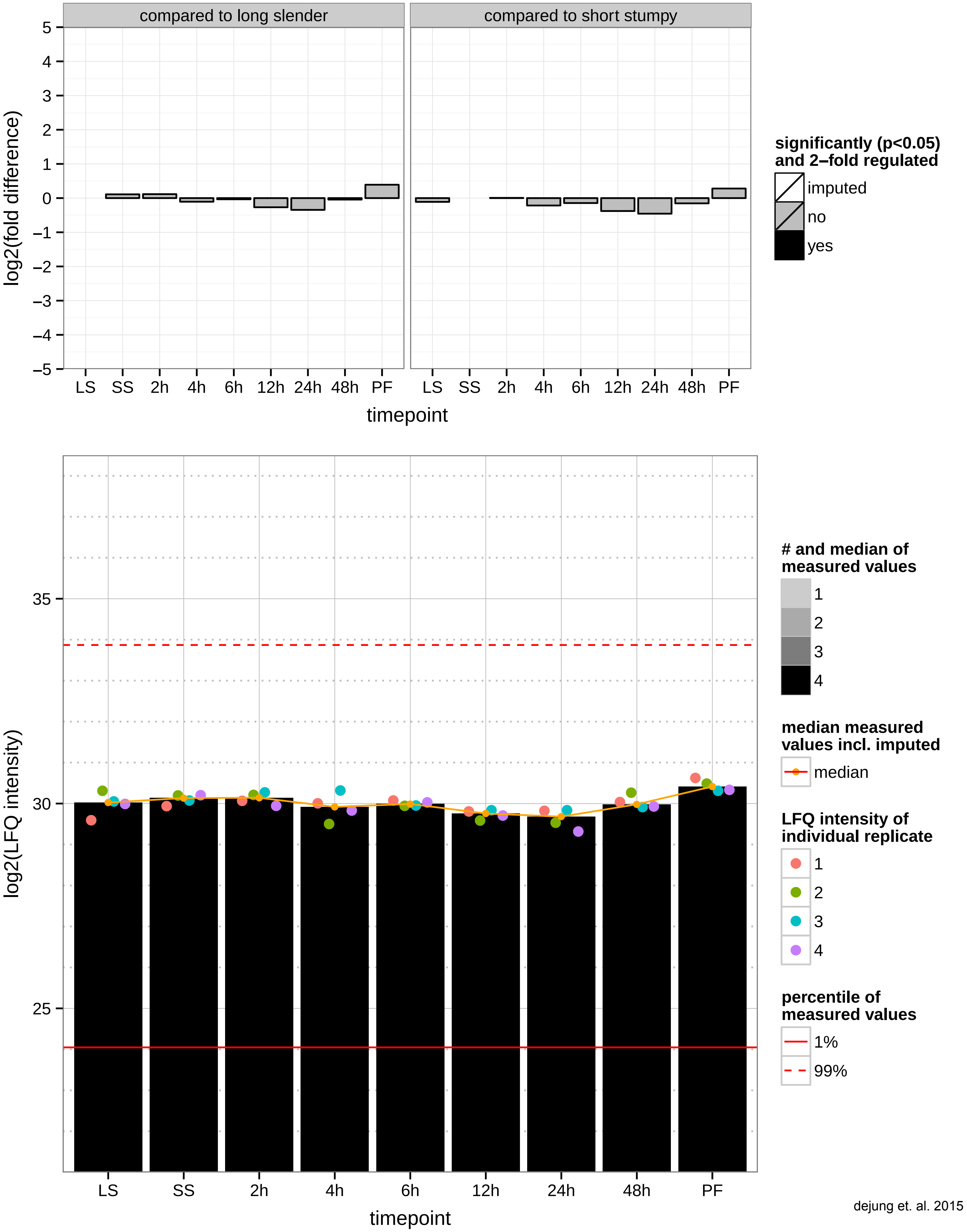

dejung et. al. 2015

chaperone protein DNAj, putative, heat shock protein–like protein
Tb927.11.15130
AGOF: heat shock protein binding, unfolded protein binding
AGOC: null
AGOP: protein folding
PGOF: heat shock protein binding, unfolded protein binding
PGOC: null
PGOP: protein folding

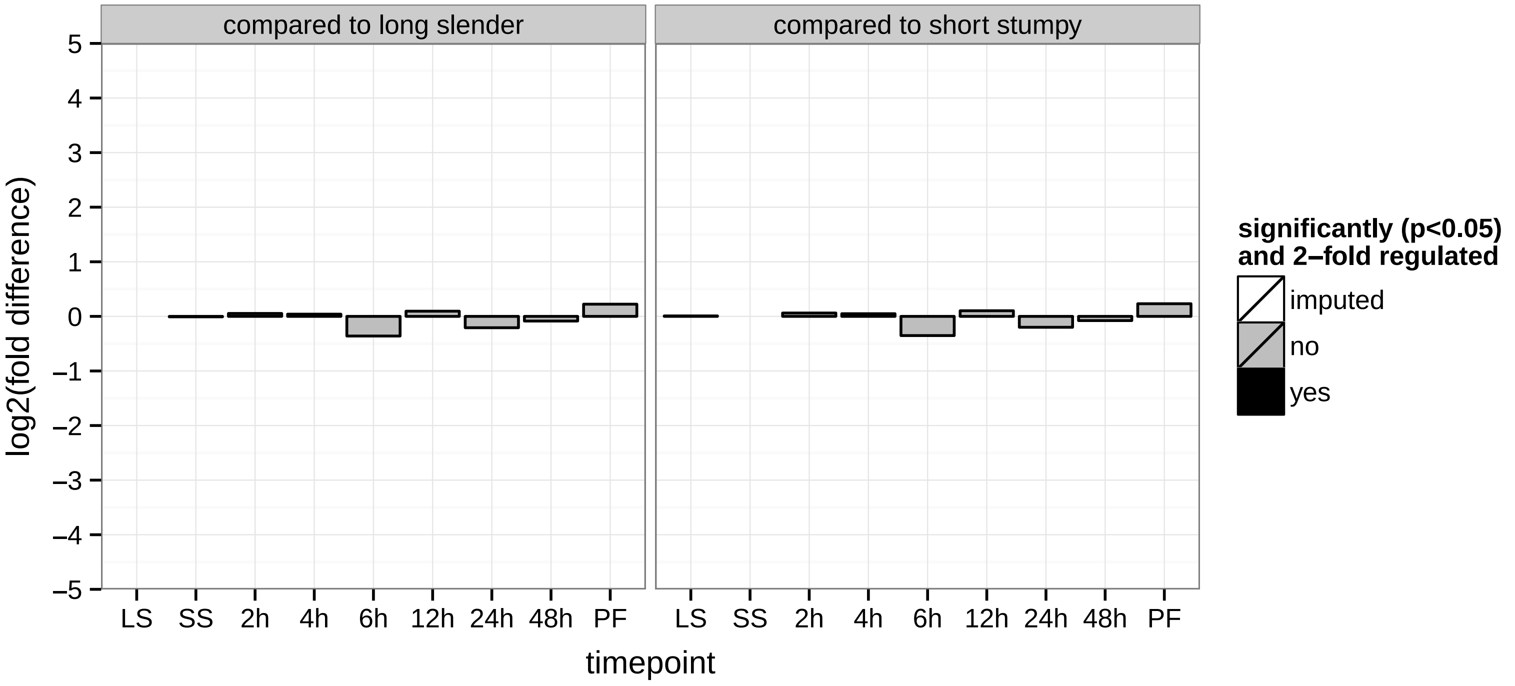

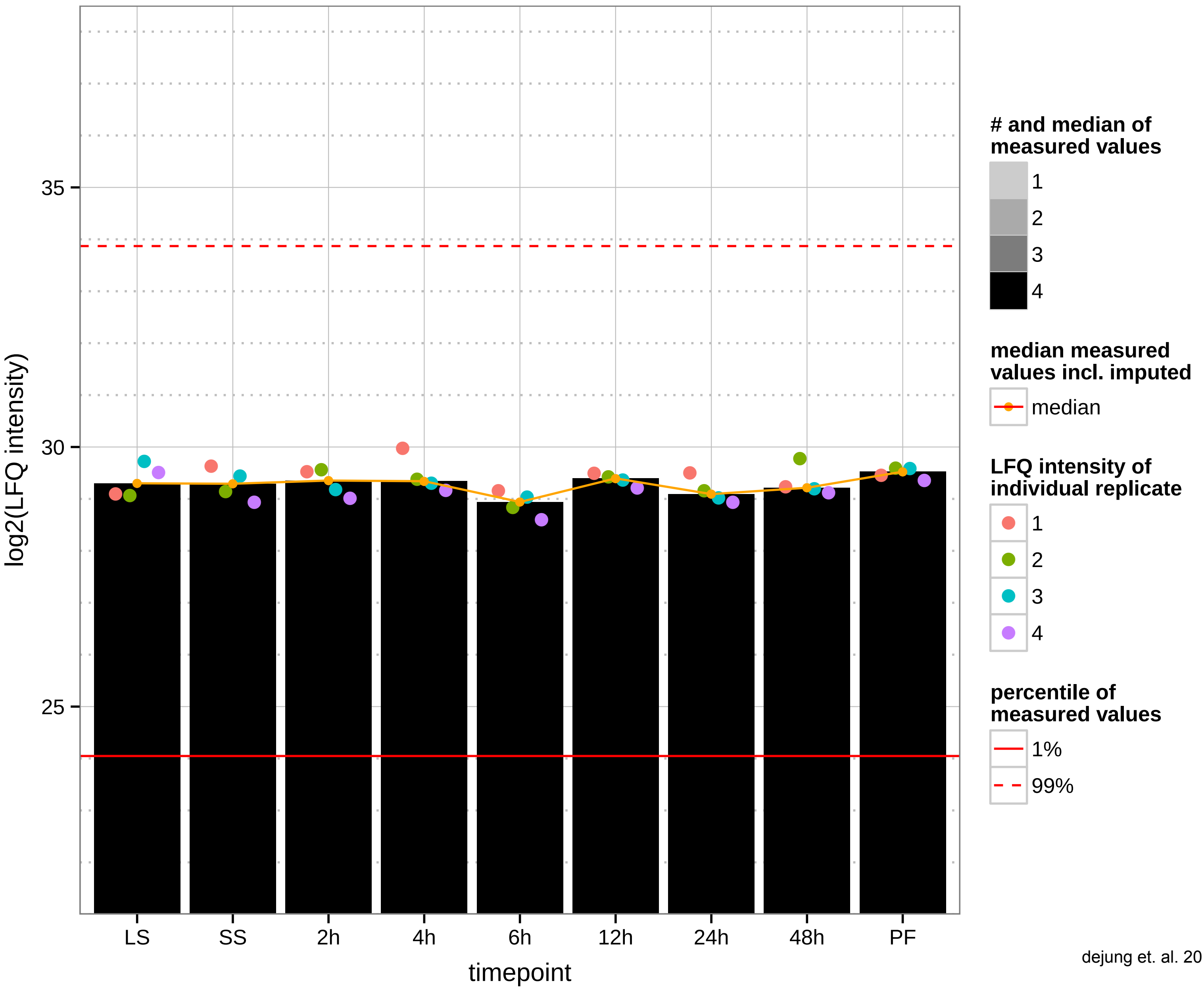

dejung et. al. 2015

1–acyl–sn–glycerol–3–phosphate acyltransferase, putative
Tb927.11.15150
AGOF: 1–acylglycerol–3–phosphate O–acyltransferase activity
AGOC: integral to membrane
AGOP: metabolic process
PGOF: transferase activity, transferring acyl groups
PGOC: null
PGOP: metabolic process

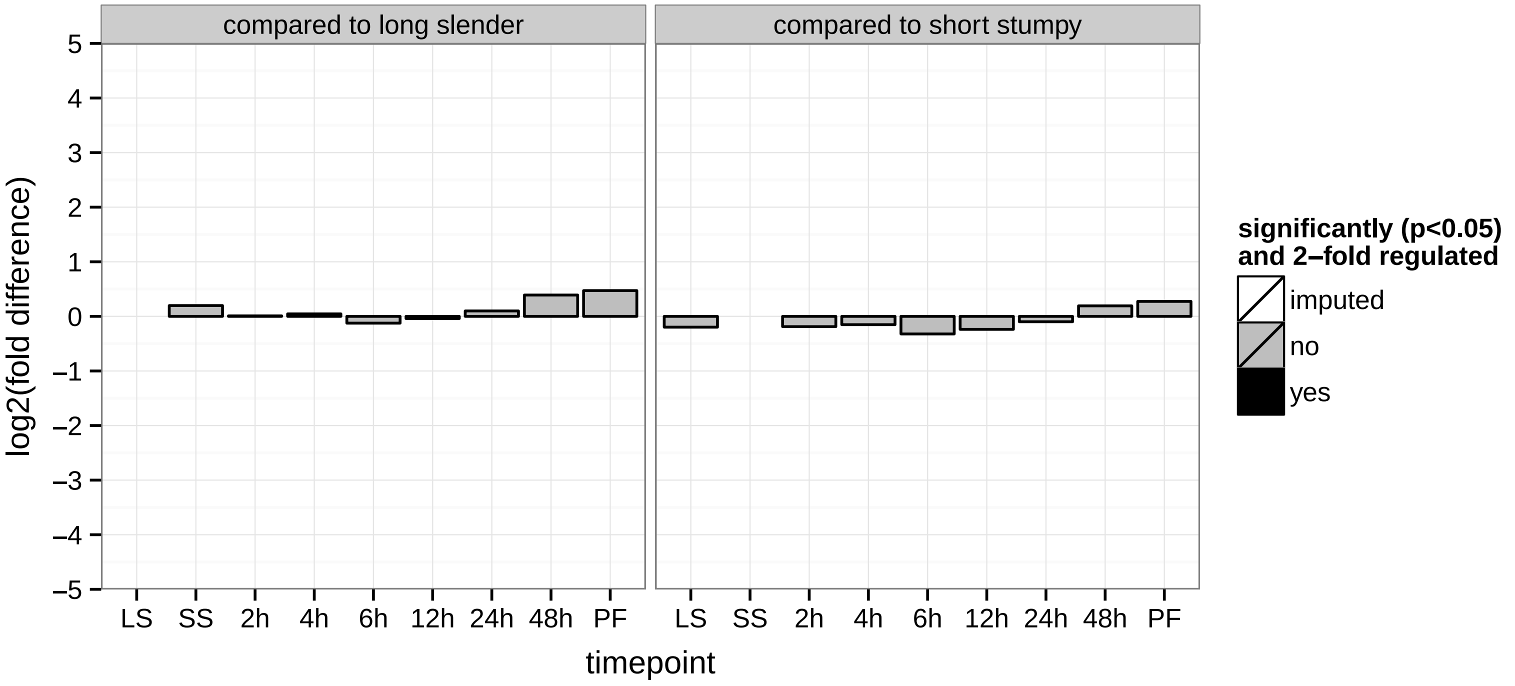

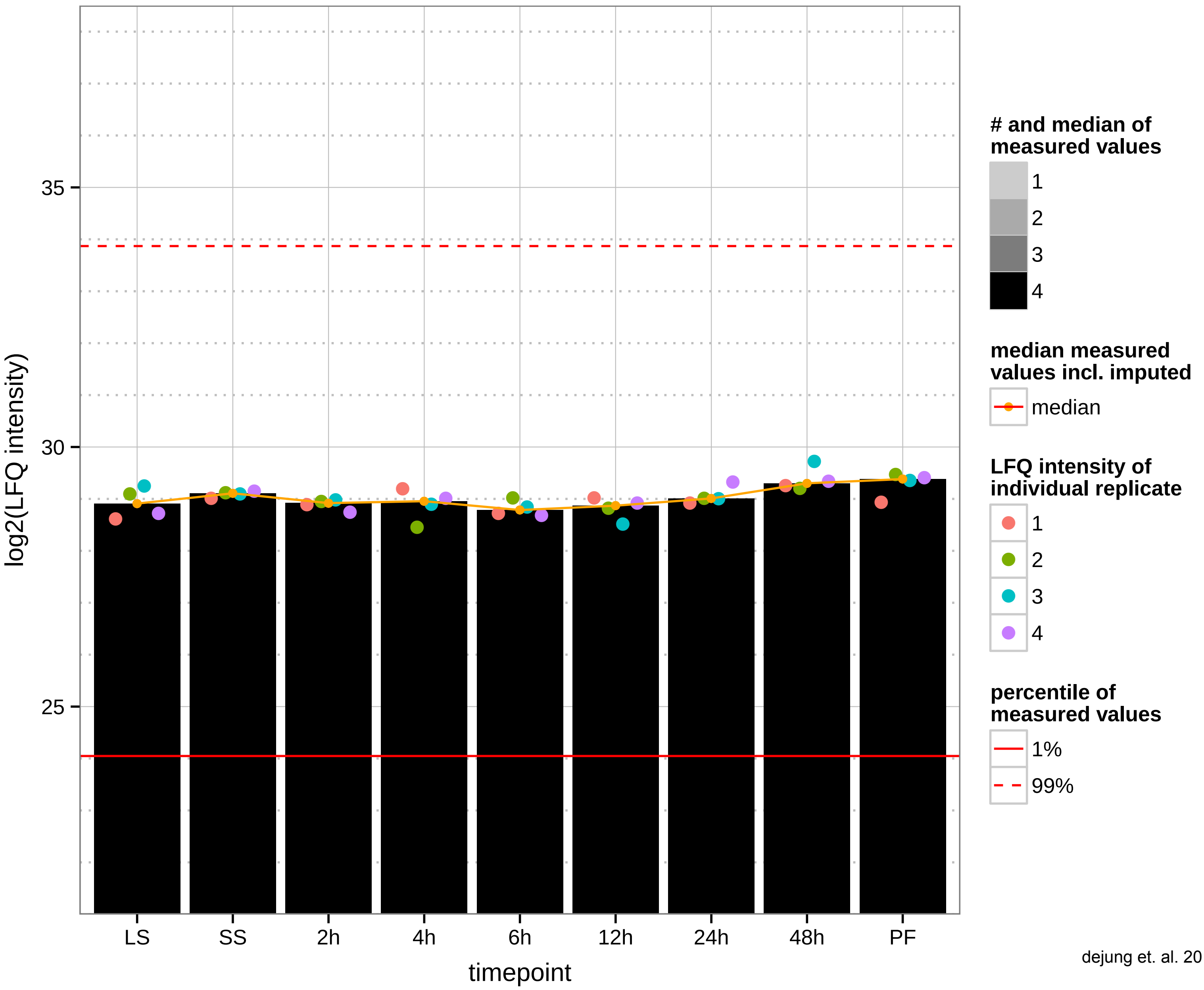

dejung et. al. 2015

hypothetical protein, conserved
Tb927.11.15180
AGOF: null
AGOC: null
AGOP: null
PGOF: null
PGOC: null
PGOP: null

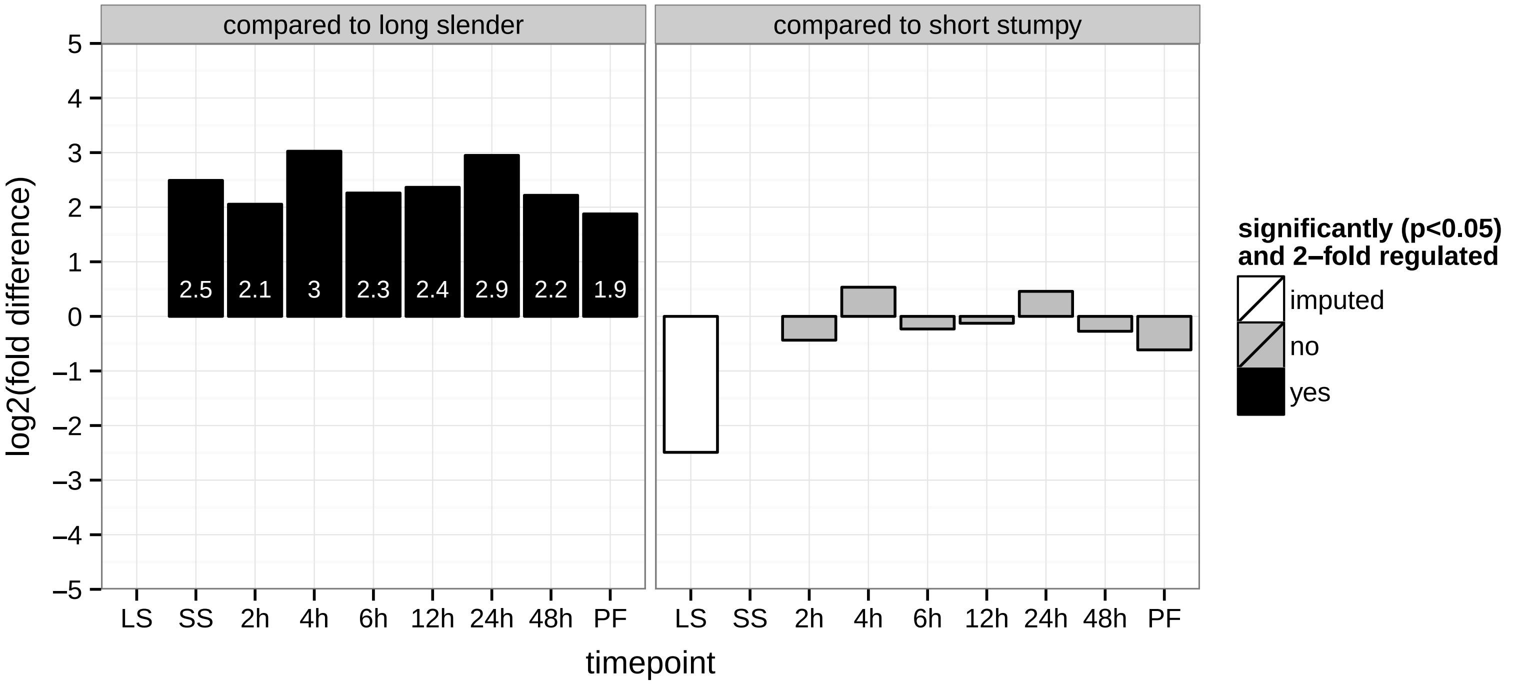

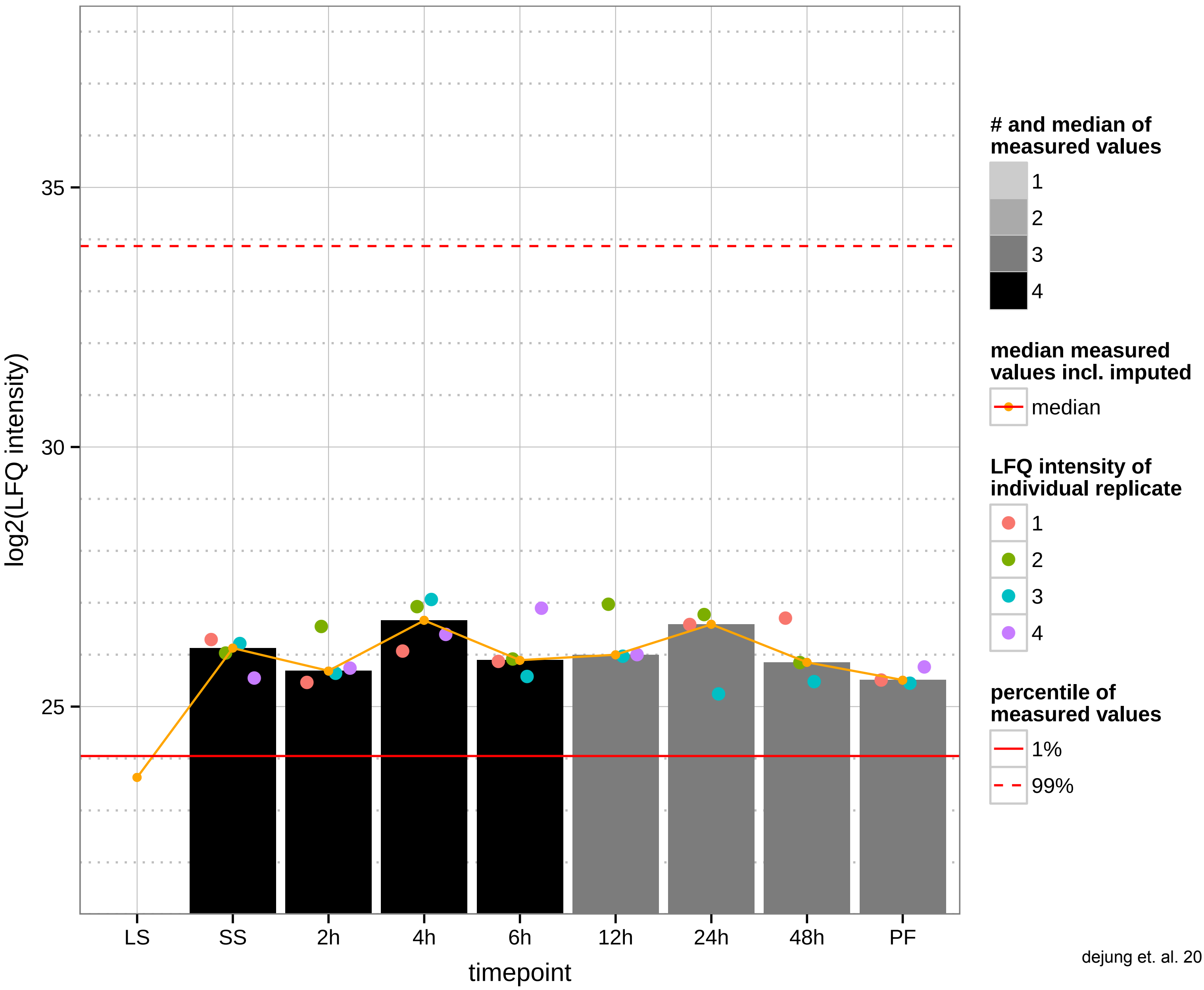

dejung et. al. 2015

hypothetical protein, conserved
Tb927.11.15190
AGOF: null
AGOC: null
AGOP: null
PGOF: null
PGOC: null
PGOP: null

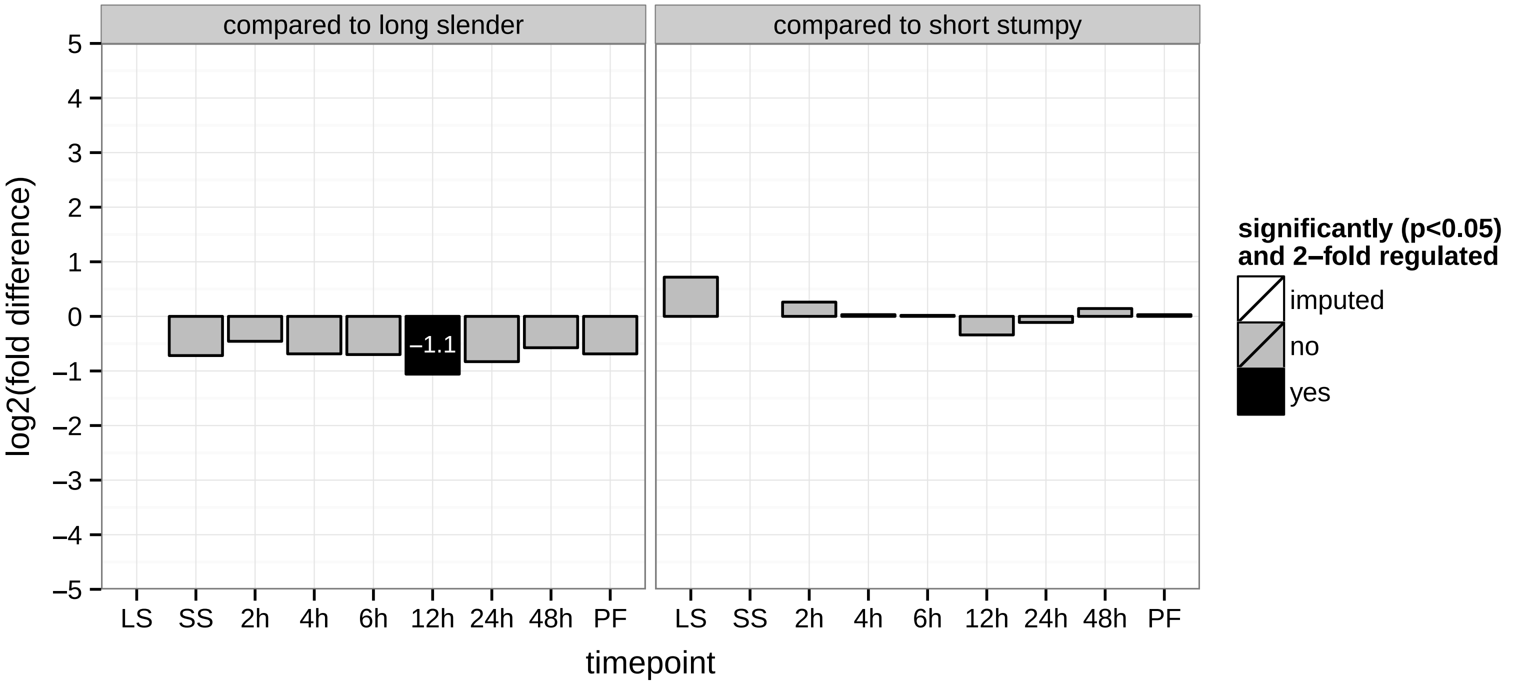

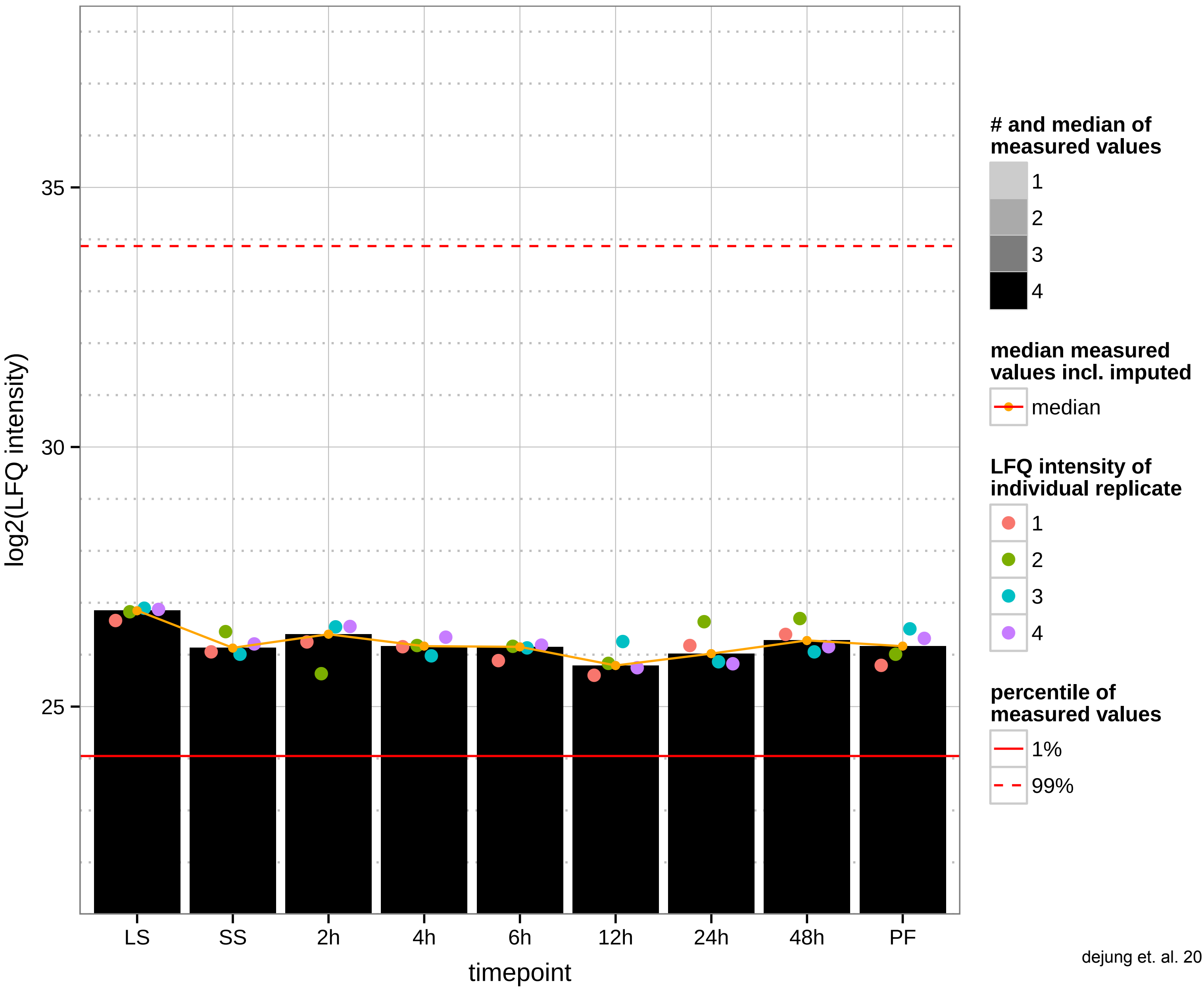

dejung et. al. 2015

cytosolic coat protein, putative
Tb927.11.15230
AGOF: protein transmembrane transporter activity
AGOC: ER to Golgi transport vesicle membrane, integral to membrane
AGOP: ER to Golgi vesicle–mediated transport
PGOF: null
PGOC: integral to membrane
PGOP: transport

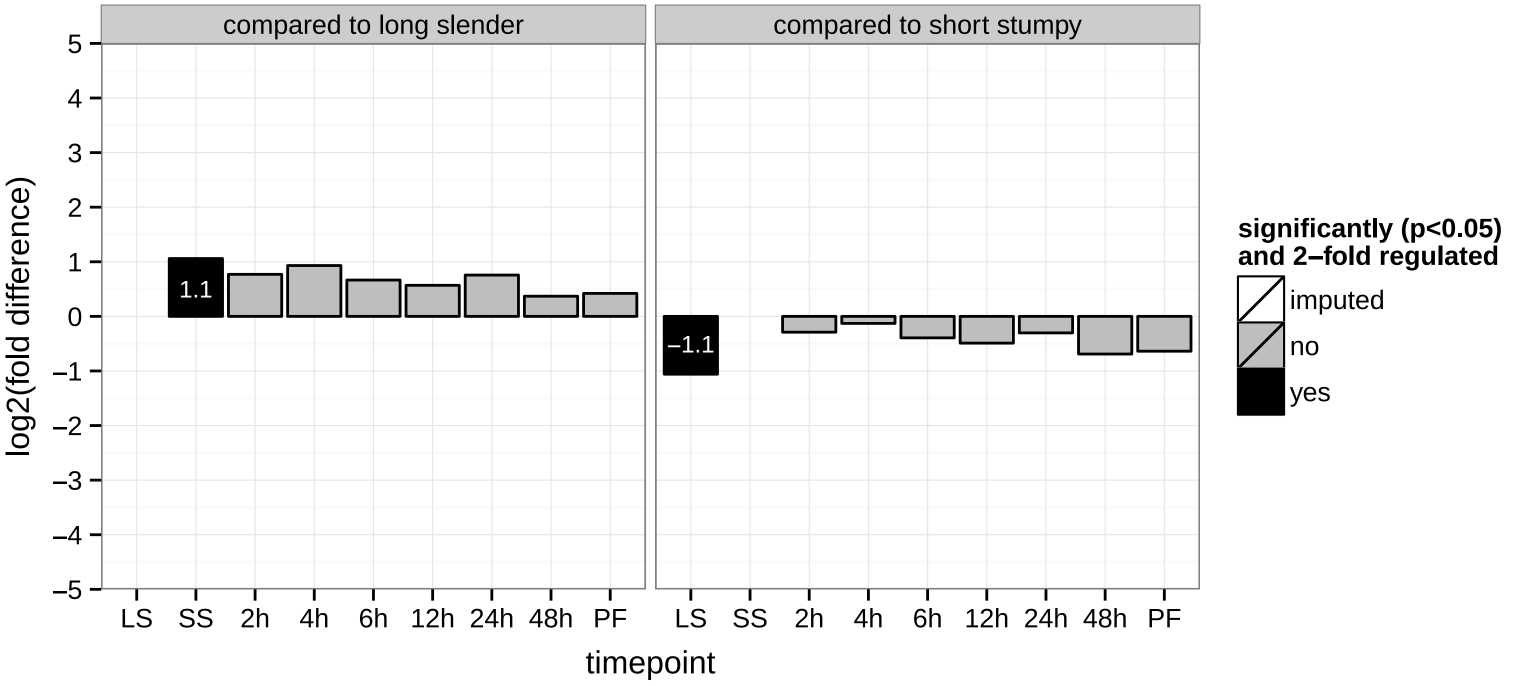

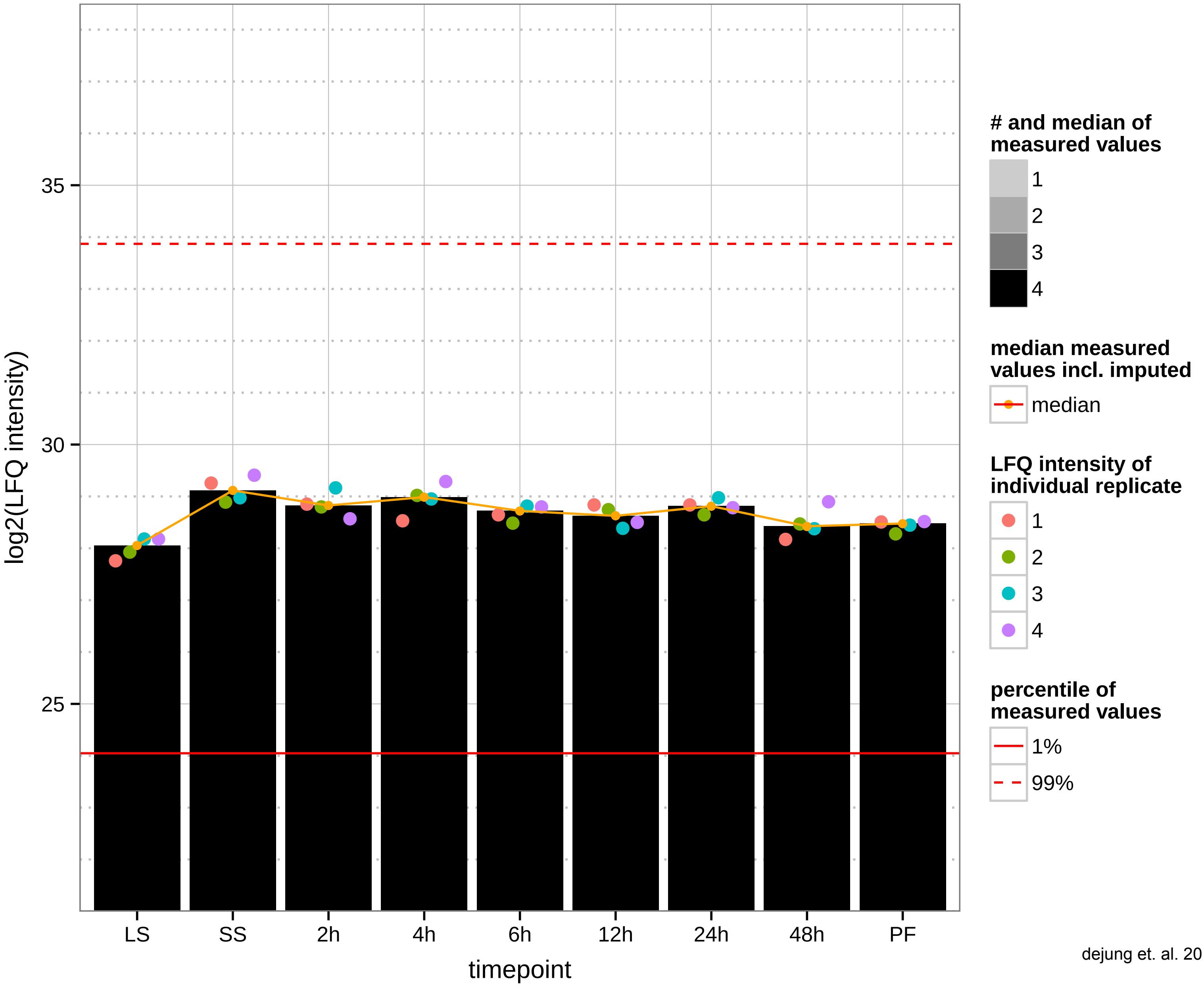

dejung et. al. 2015

hypothetical protein, conserved
Tb927.11.15270
AGOF: nucleotidyltransferase activity
AGOC: null
AGOP: biosynthetic process
PGOF: nucleotidyltransferase activity
PGOC: null
PGOP: biosynthetic process

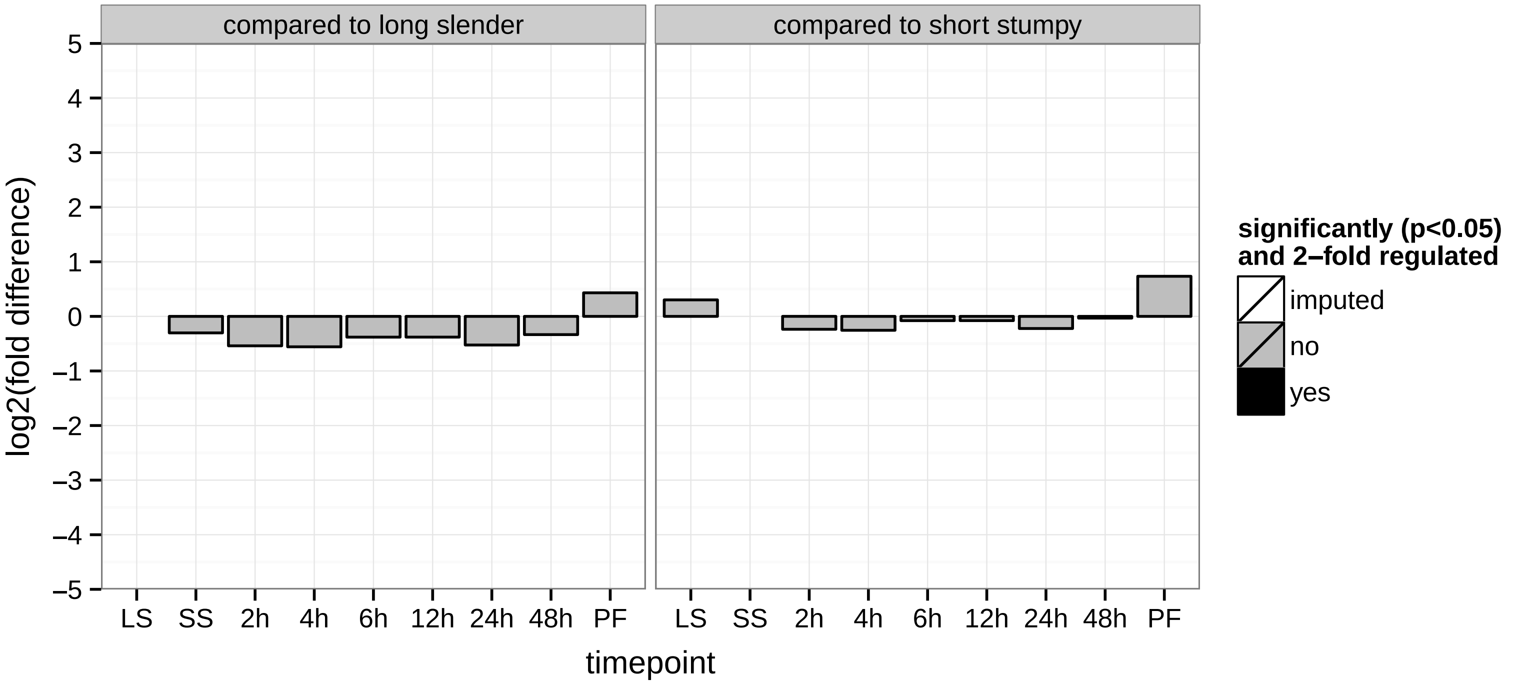

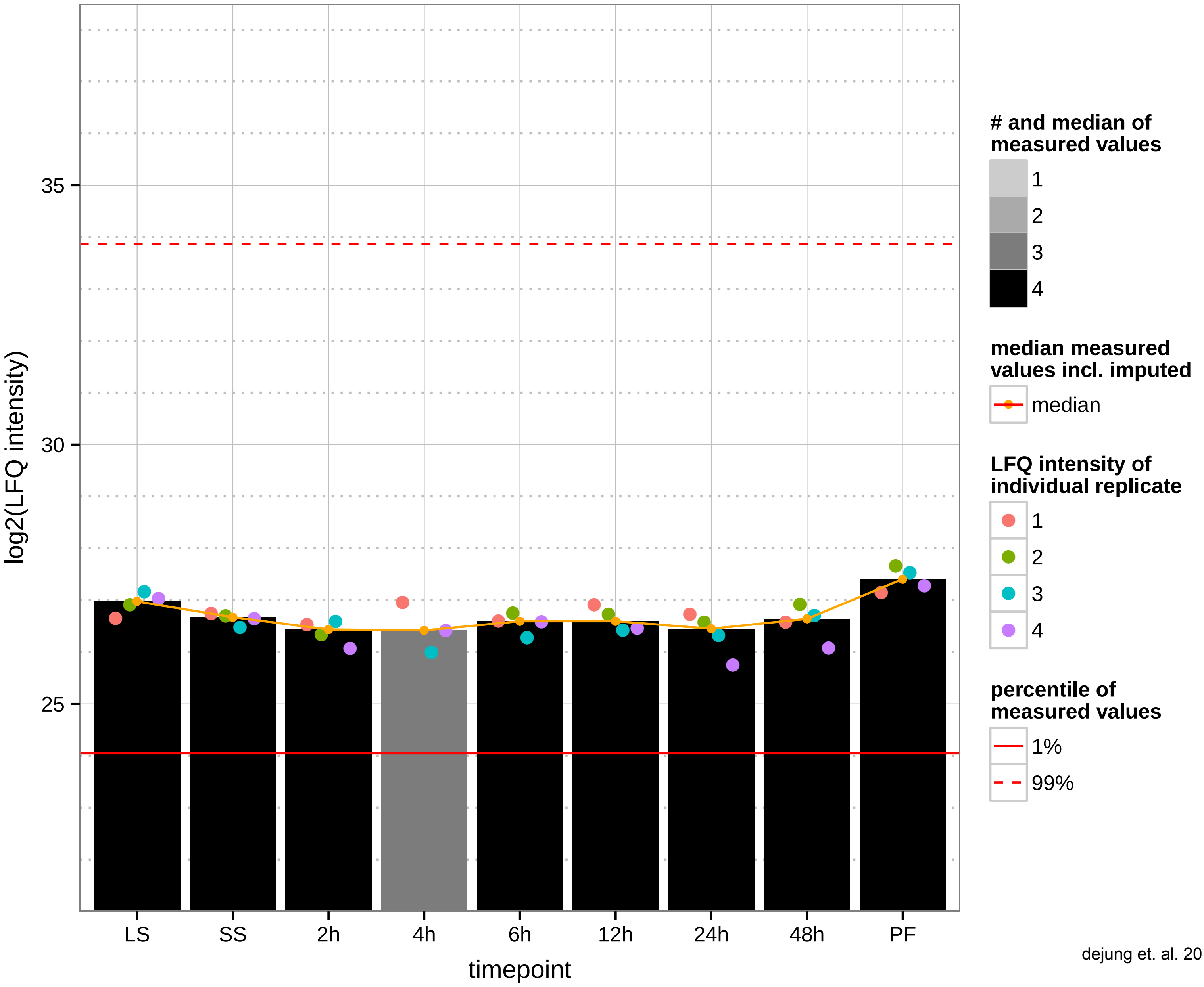

dejung et. al. 2015

isovaleryl–coA dehydrogenase, putative
Tb927.11.1540
AGOF: acyl–CoA dehydrogenase activity, oxidoreductase activity
AGOC: mitochondrion
AGOP: metabolic process
PGOF: acyl–CoA dehydrogenase activity, oxidoreductase activity, acting on the CH–CH group of donors
PGOC: null
PGOP: metabolic process, oxidation–reduction process

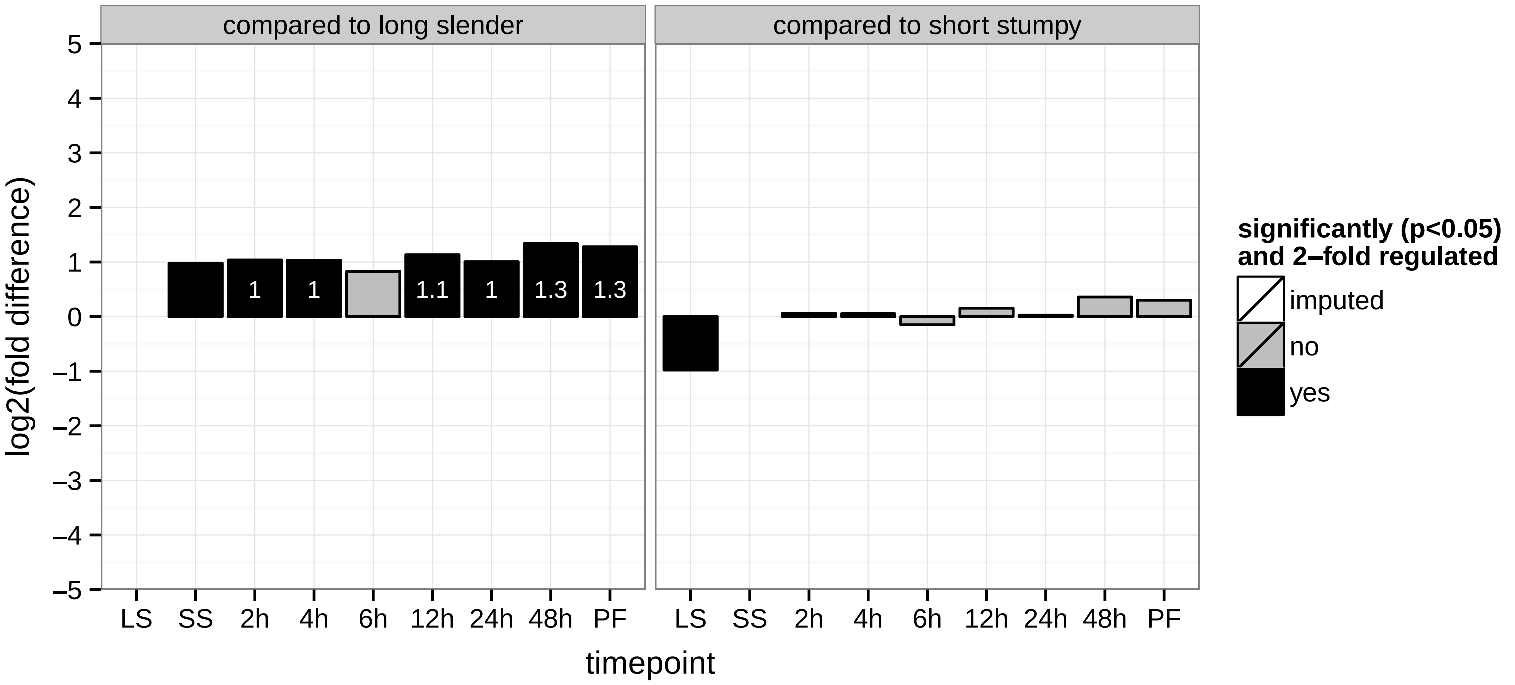

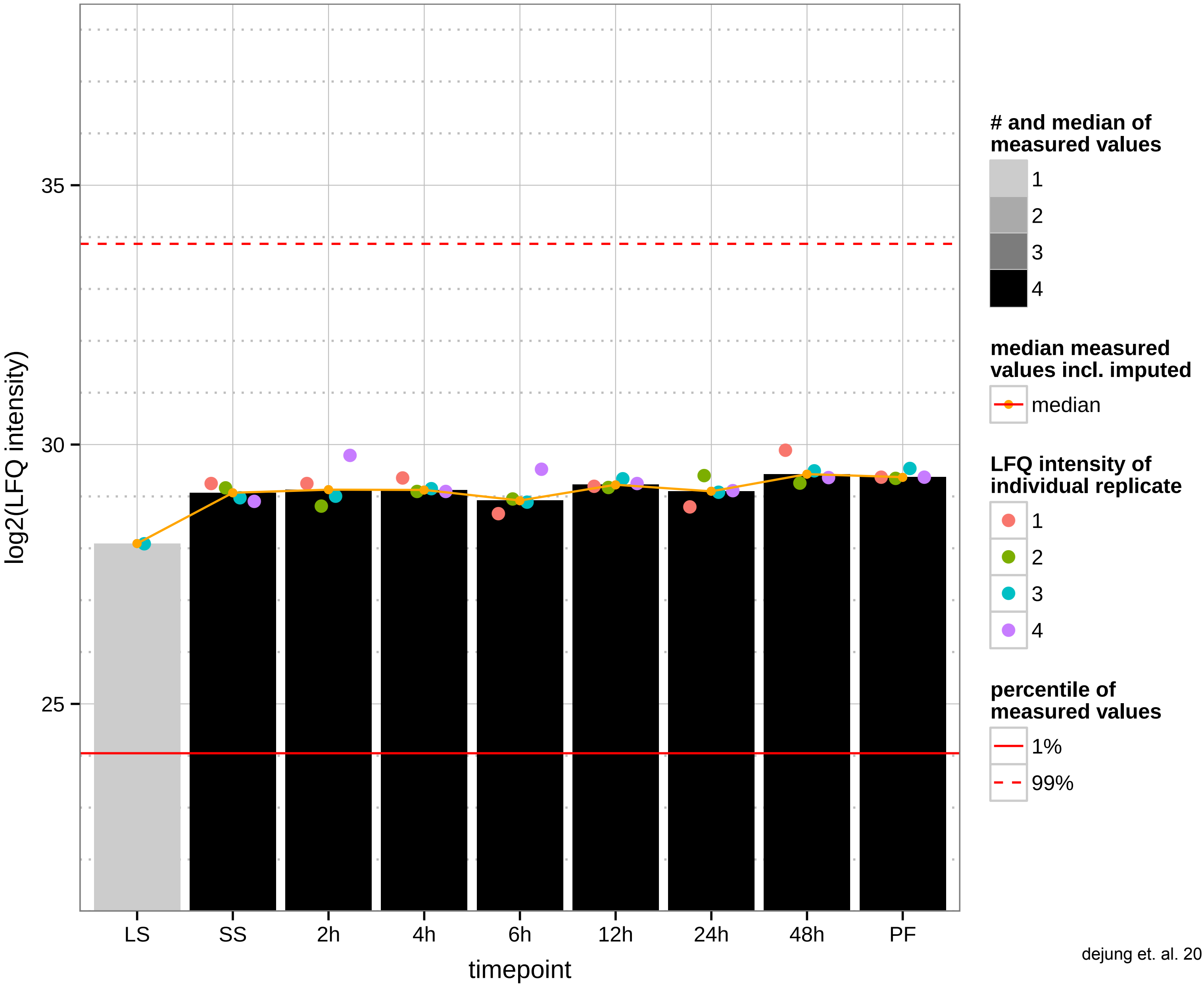

dejung et. al. 2015

hypothetical protein, conserved
Tb927.11.15420
AGOF: translation initiation factor activity
AGOC: nucleus
AGOP: translational initiation
PGOF: binding
PGOC: null
PGOP: null

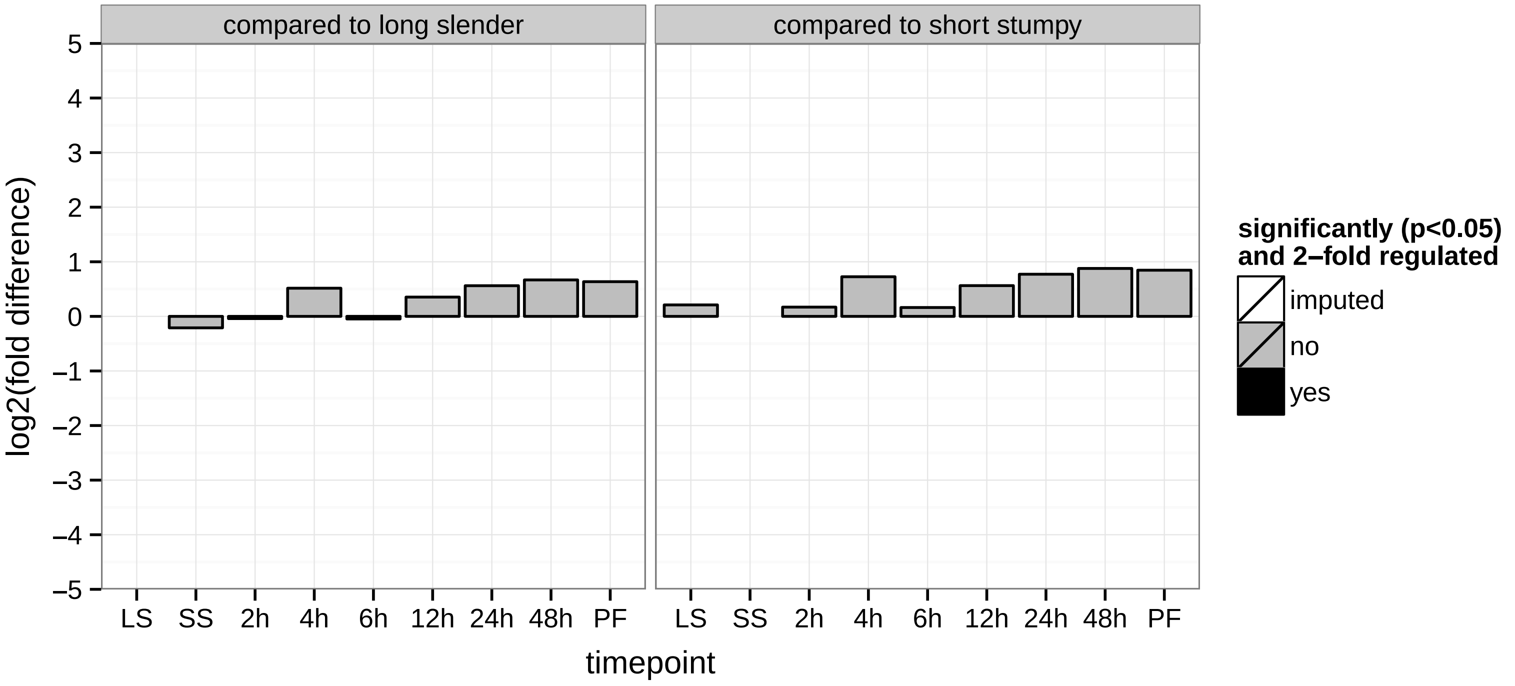

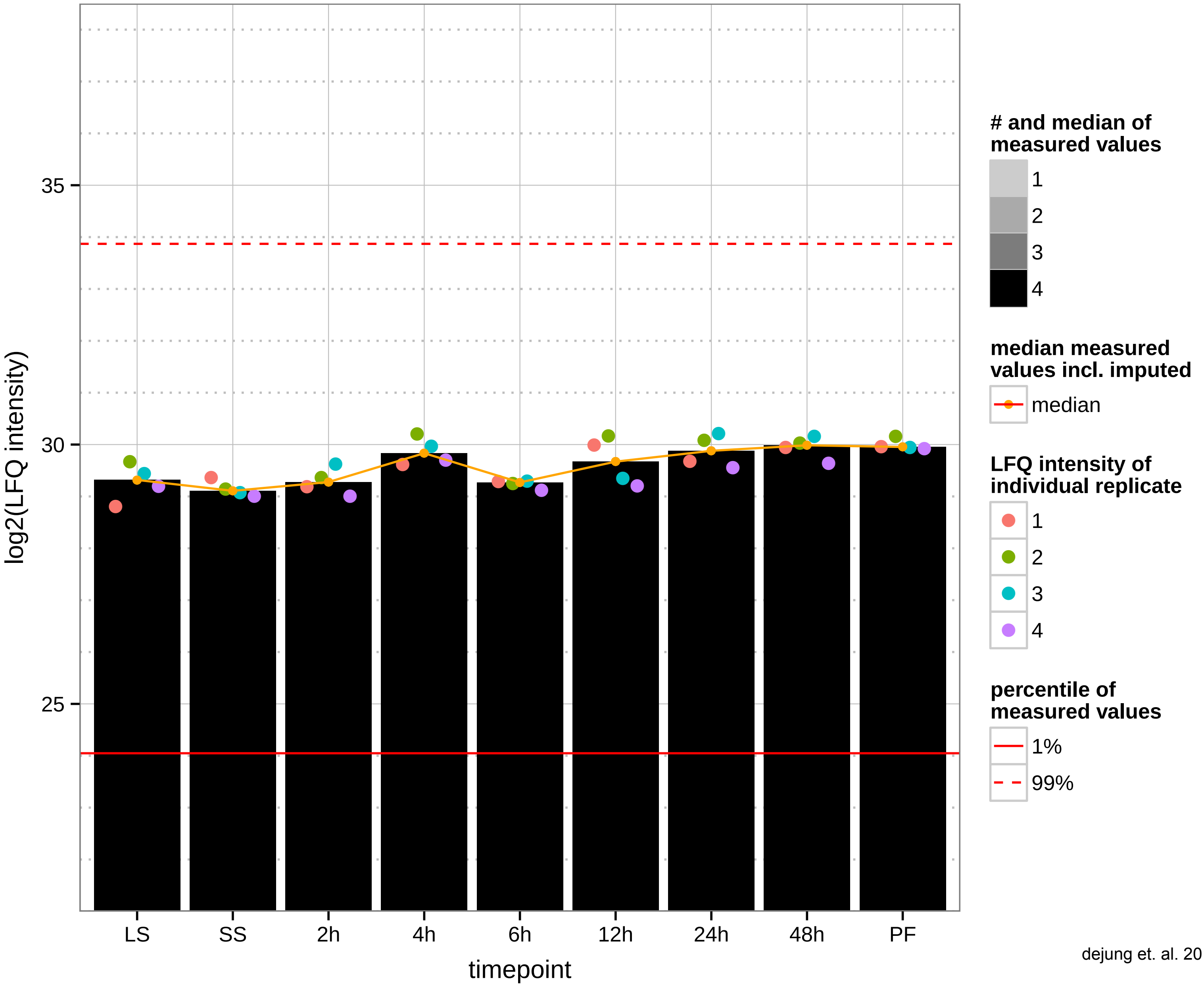

dejung et. al. 2015

U5 small nuclear ribonucleoprotein component, putative, U5 snrnp−specific protein
Tb927.11.15430
AGOF: GTP binding, GTPase activity
AGOC: nucleus
AGOP: translation
PGOF: GTP binding, GTPase activity
PGOC: null
PGOP: null

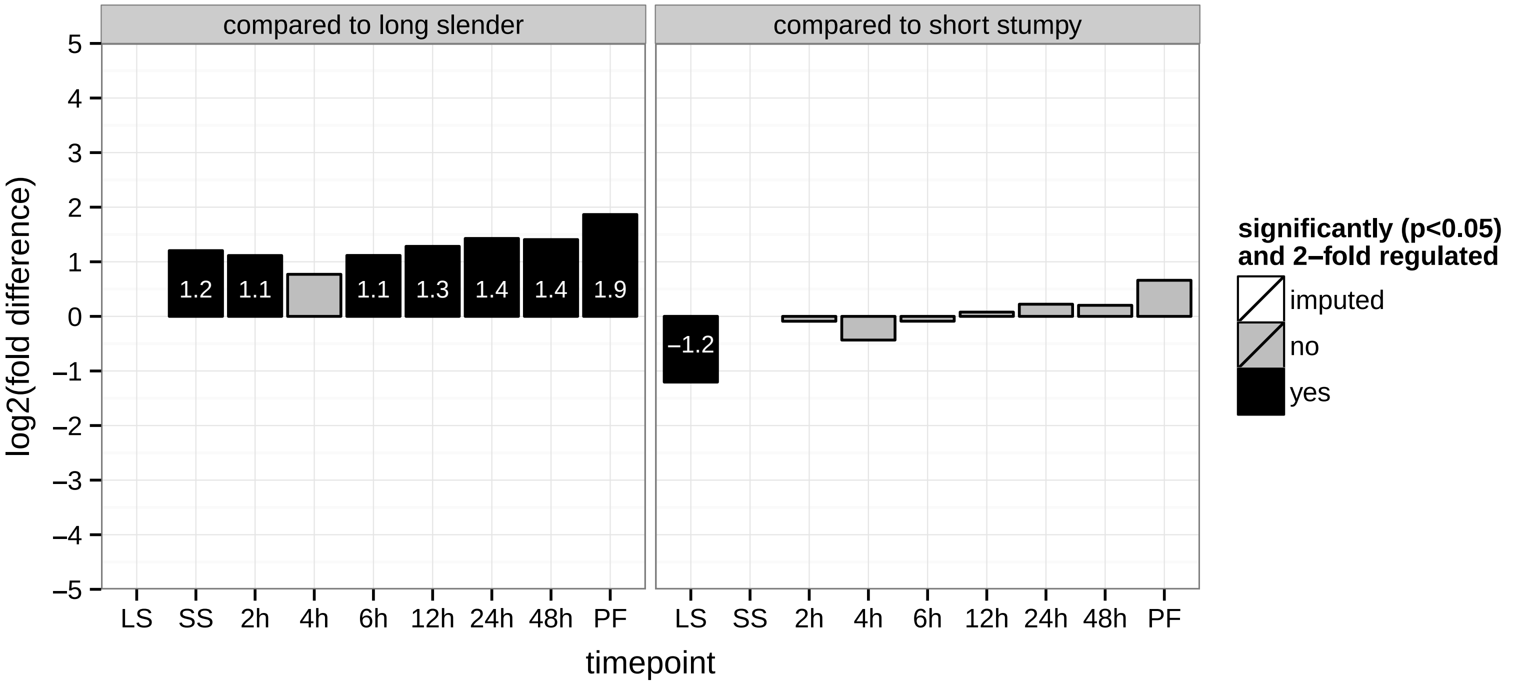

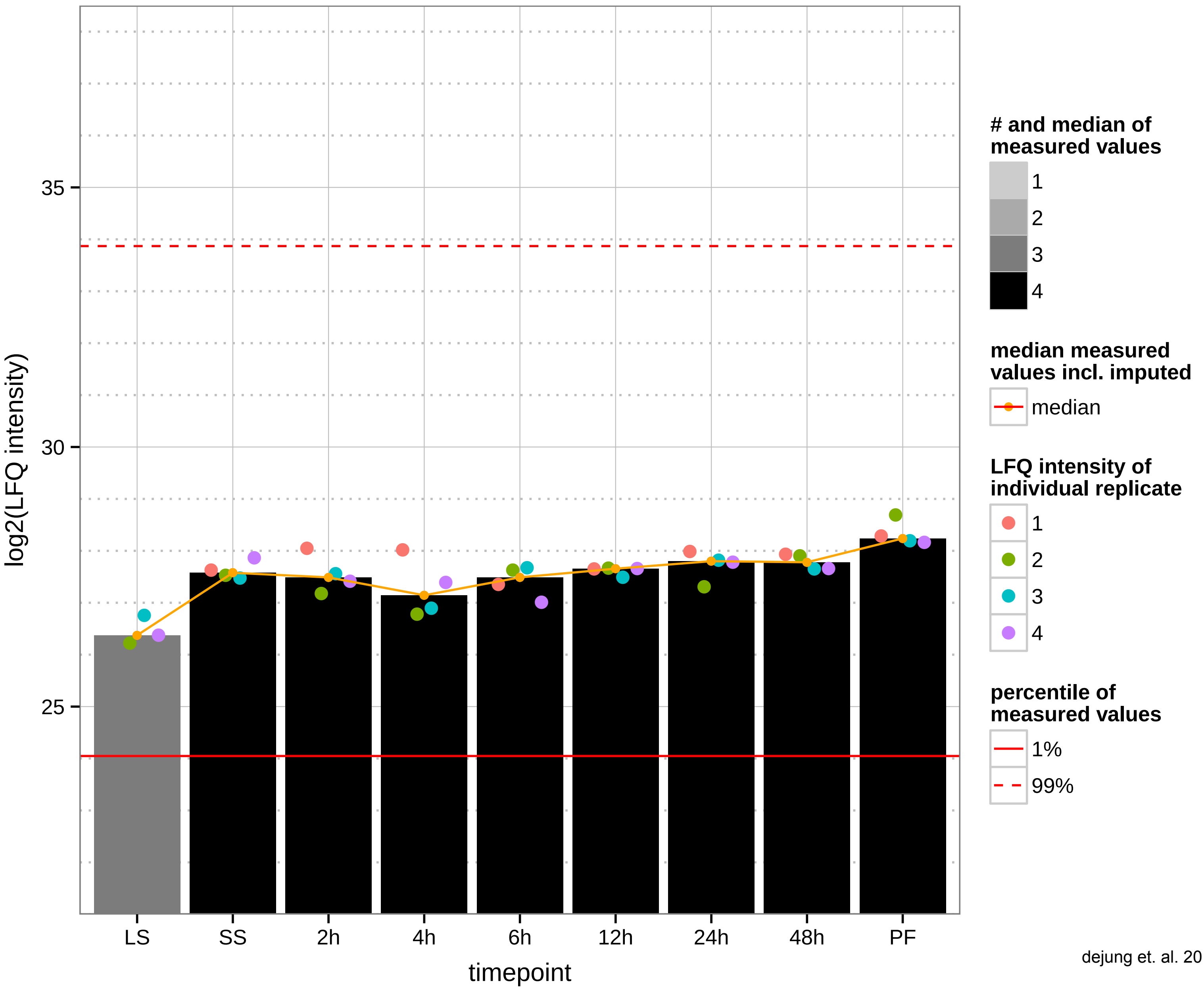

dejung et. al. 2015

heat shock protein Hsp20, putative
Tb927.11.15480
AGOF: null
AGOC: null
AGOP: null
PGOF: null
PGOC: null
PGOP: null

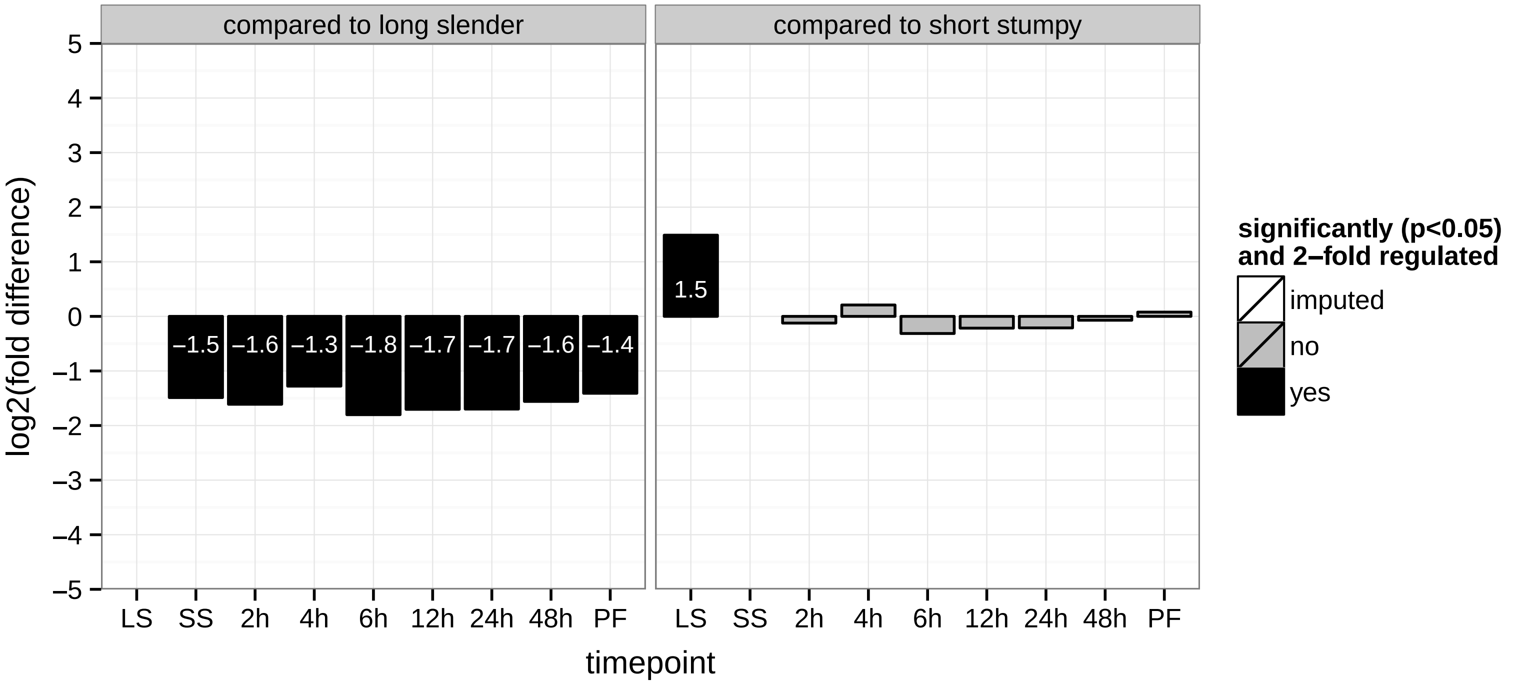

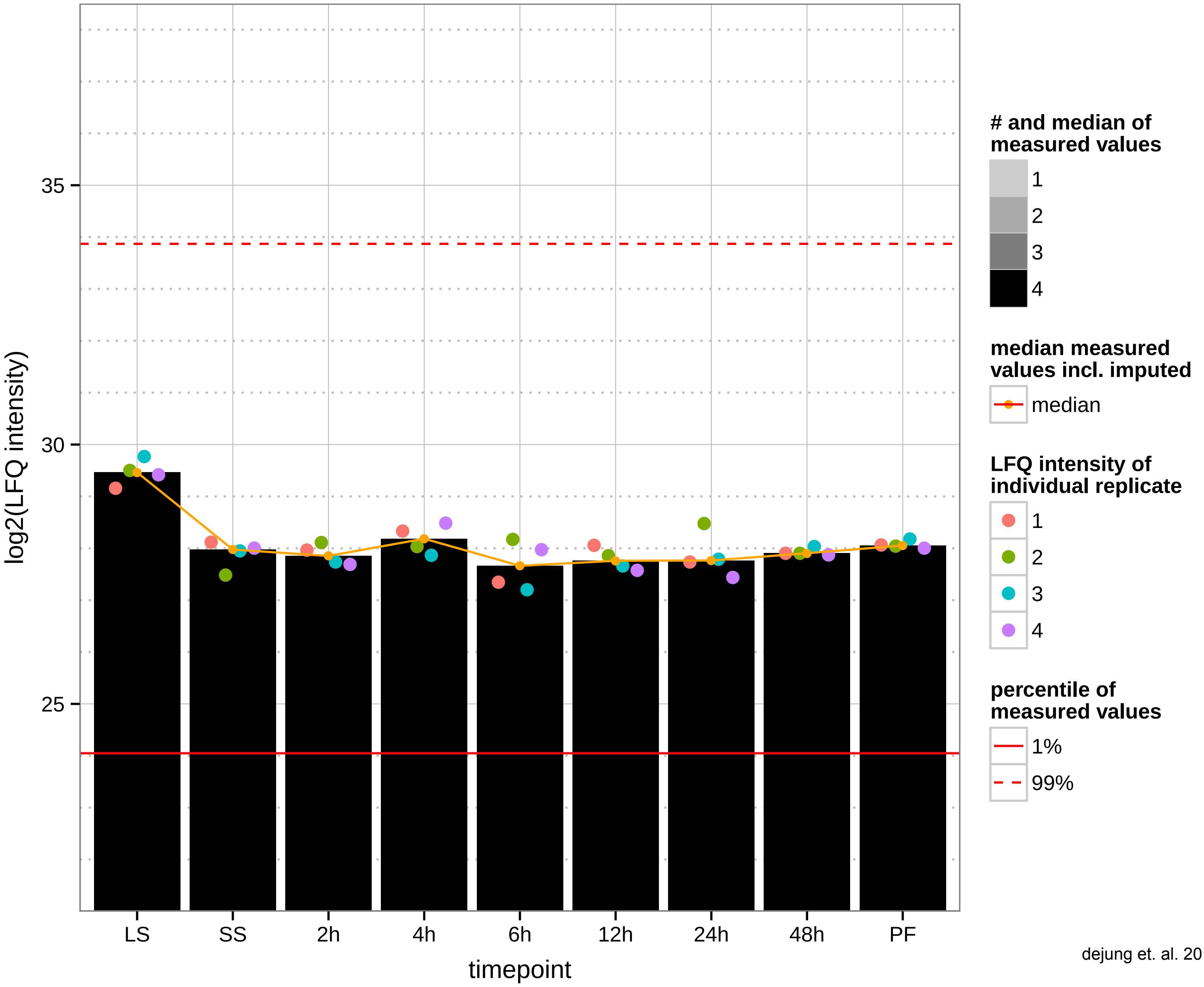

dejung et. al. 2015

Nucleoporin (TbNup53a)
Tb927.11.15560
AGOF: structural constituent of nuclear pore
AGOC: nuclear pore
AGOP: null
PGOF: structural constituent of nuclear pore
PGOC: nuclear pore
PGOP: null

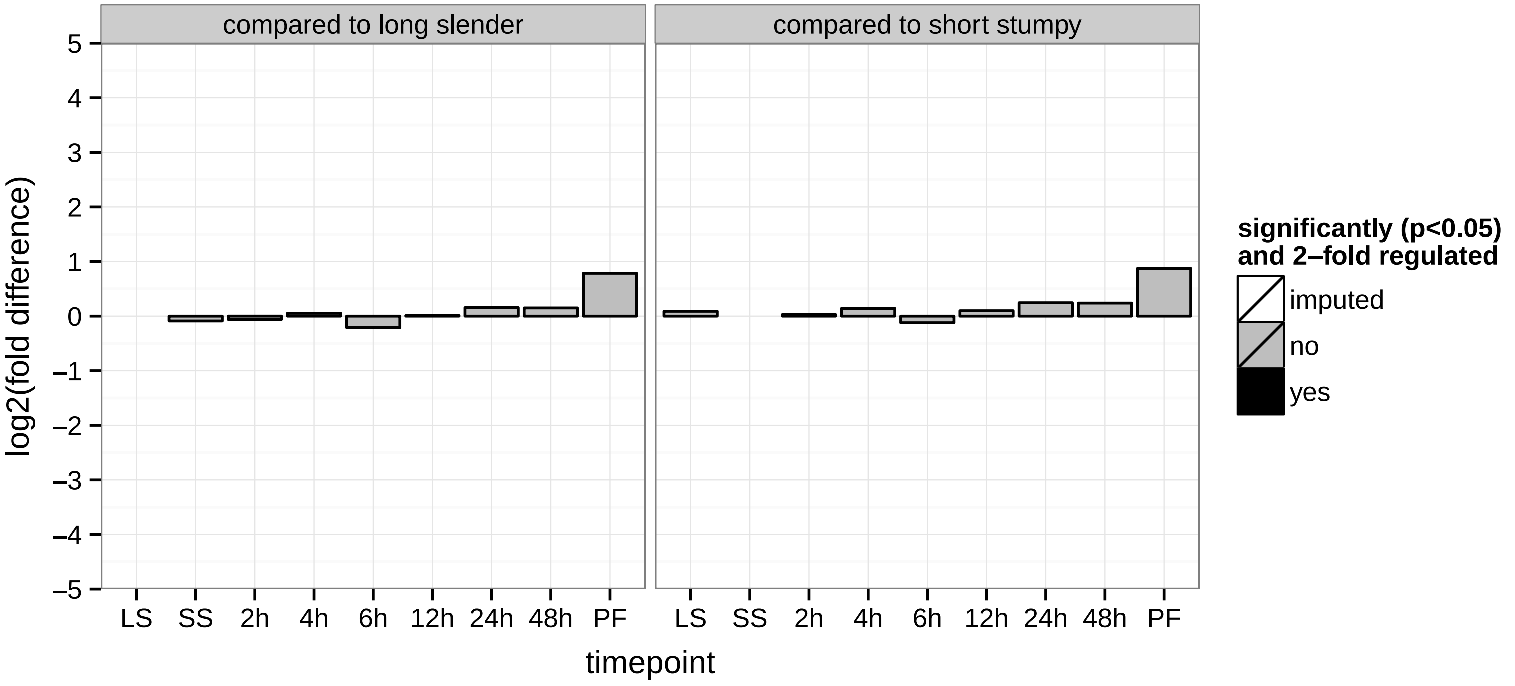

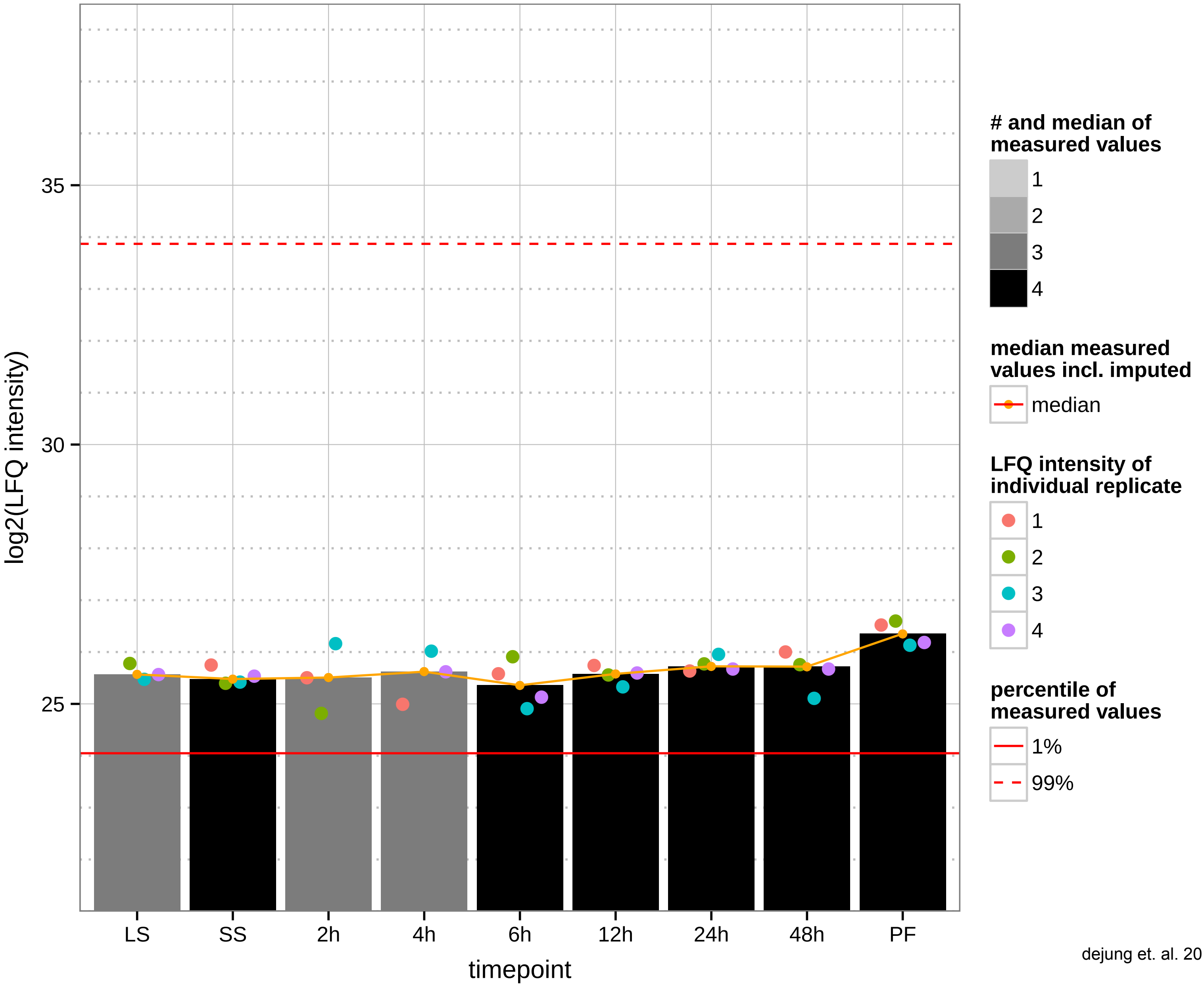

dejung et. al. 2015

1, 2–Dihydroxy–3–keto–5–methylthiopentene dioxygenase, putative
Tb927.11.1560
AGOF: dioxygenase activity, metal ion binding
AGOC: cytosol
AGOP: L–methionine salvage from methylthioadenosine
PGOF: acireductone dioxygenase [iron(II)–requiring] activity
PGOC: null
PGOP: oxidation–reduction process

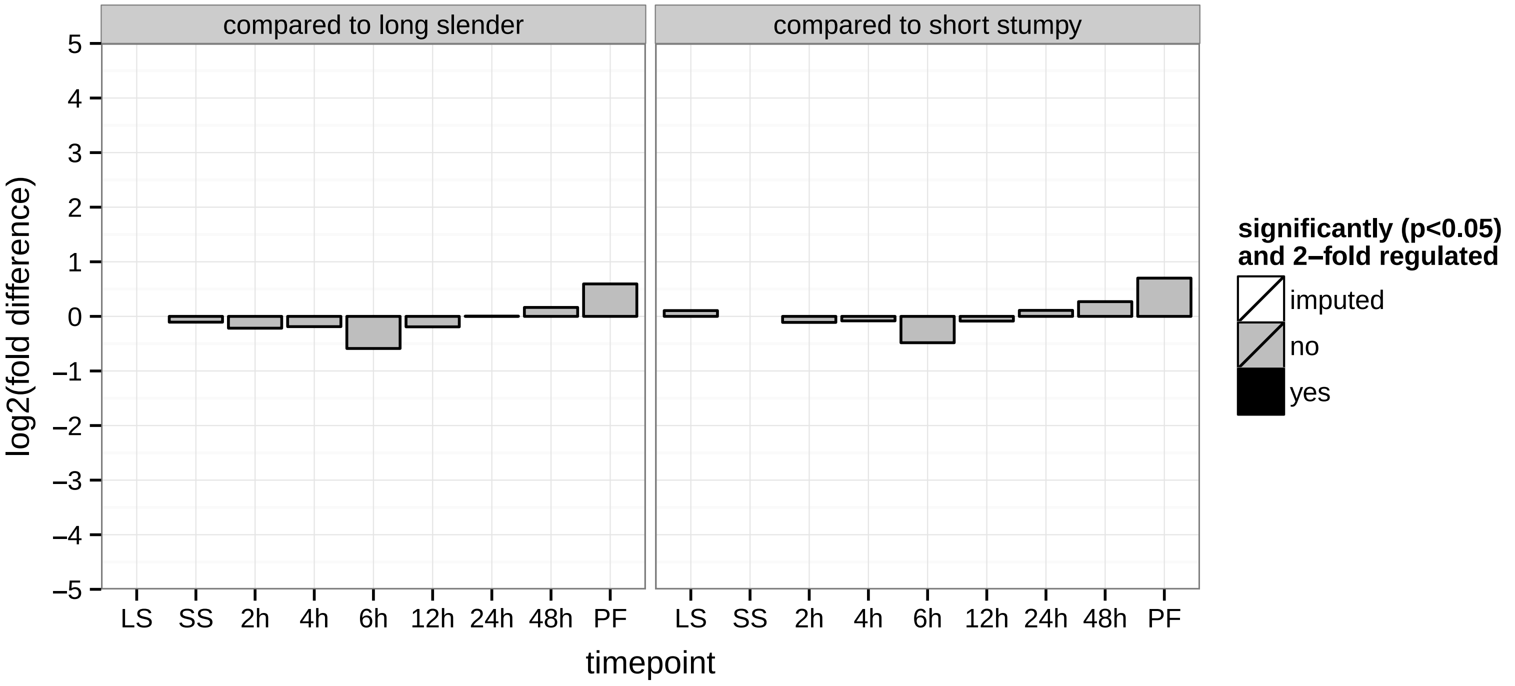

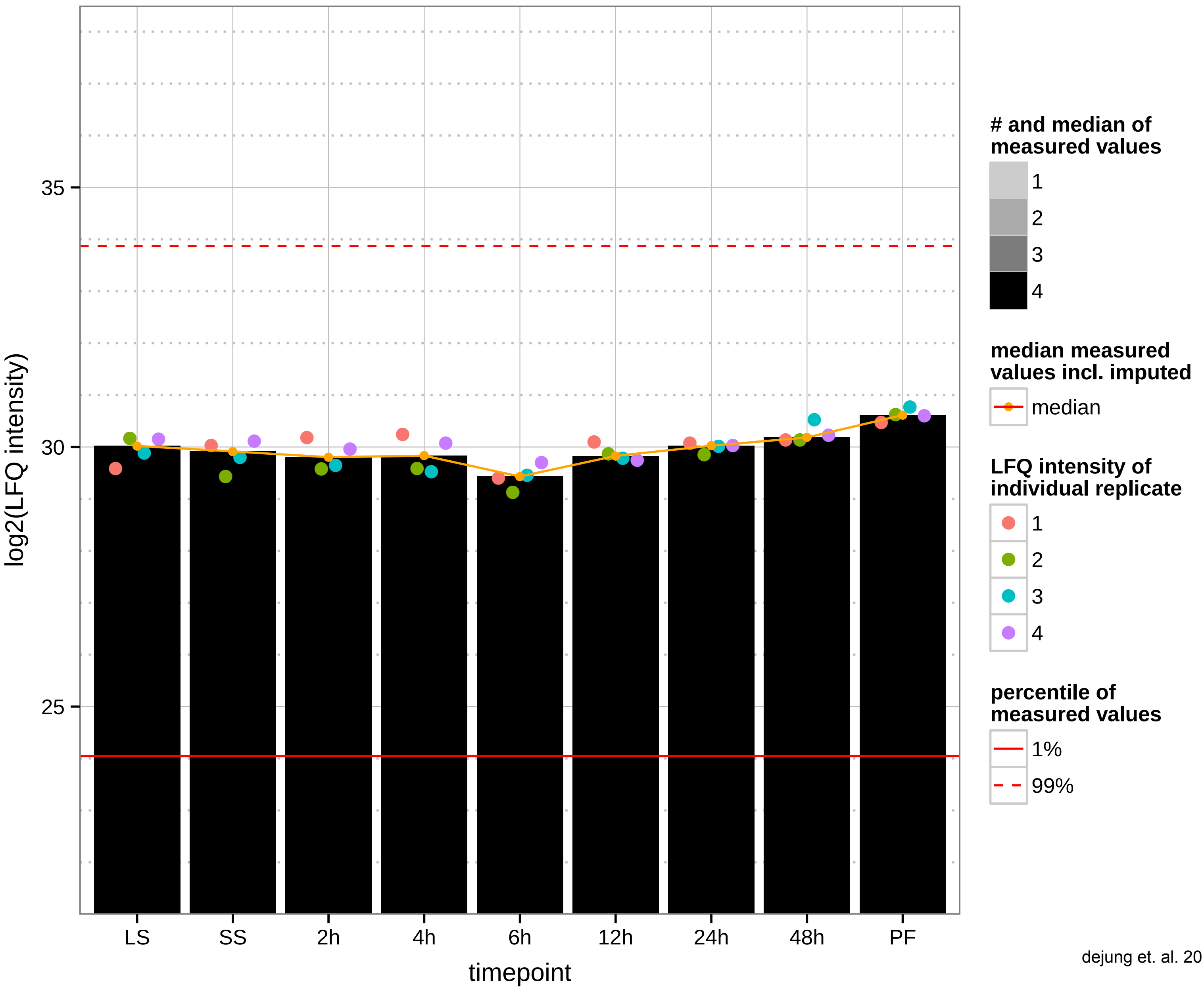

dejung et. al. 2015

mitochondrial RNA binding complex 1 subunit, NUDIX hydrolase, putative (MERS1)
Tb927.11.15640
AGOF: RNA binding, hydrolase activity
AGOC: mitochondrion
AGOP: cytidine to uridine editing, mRNA stabilization
PGOF: hydrolase activity
PGOC: null
PGOP: null

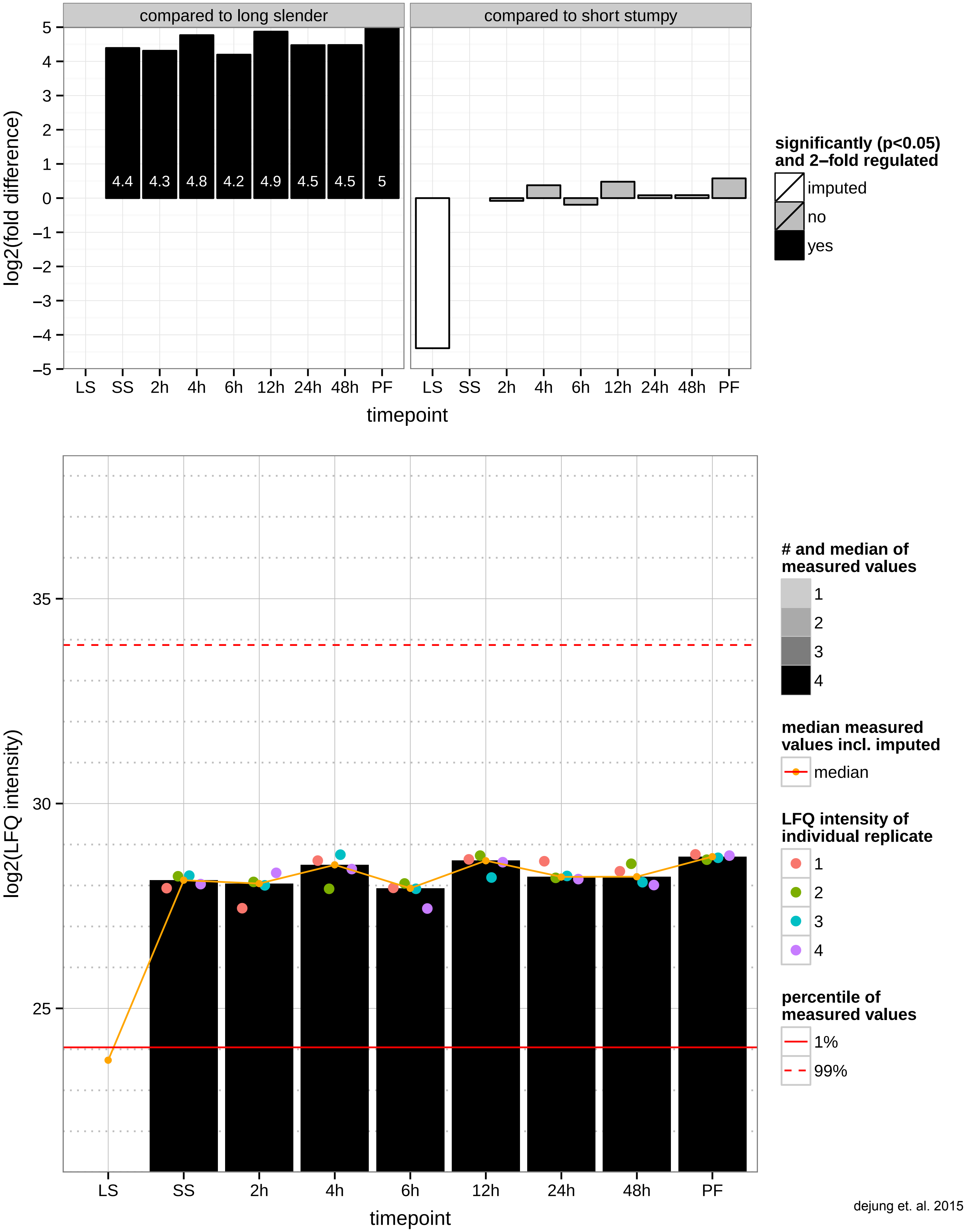

dejung et. al. 2015

hypothetical protein, conserved
Tb927.11.15680
AGOF: null
AGOC: null
AGOP: null
PGOF: null
PGOC: null
PGOP: null

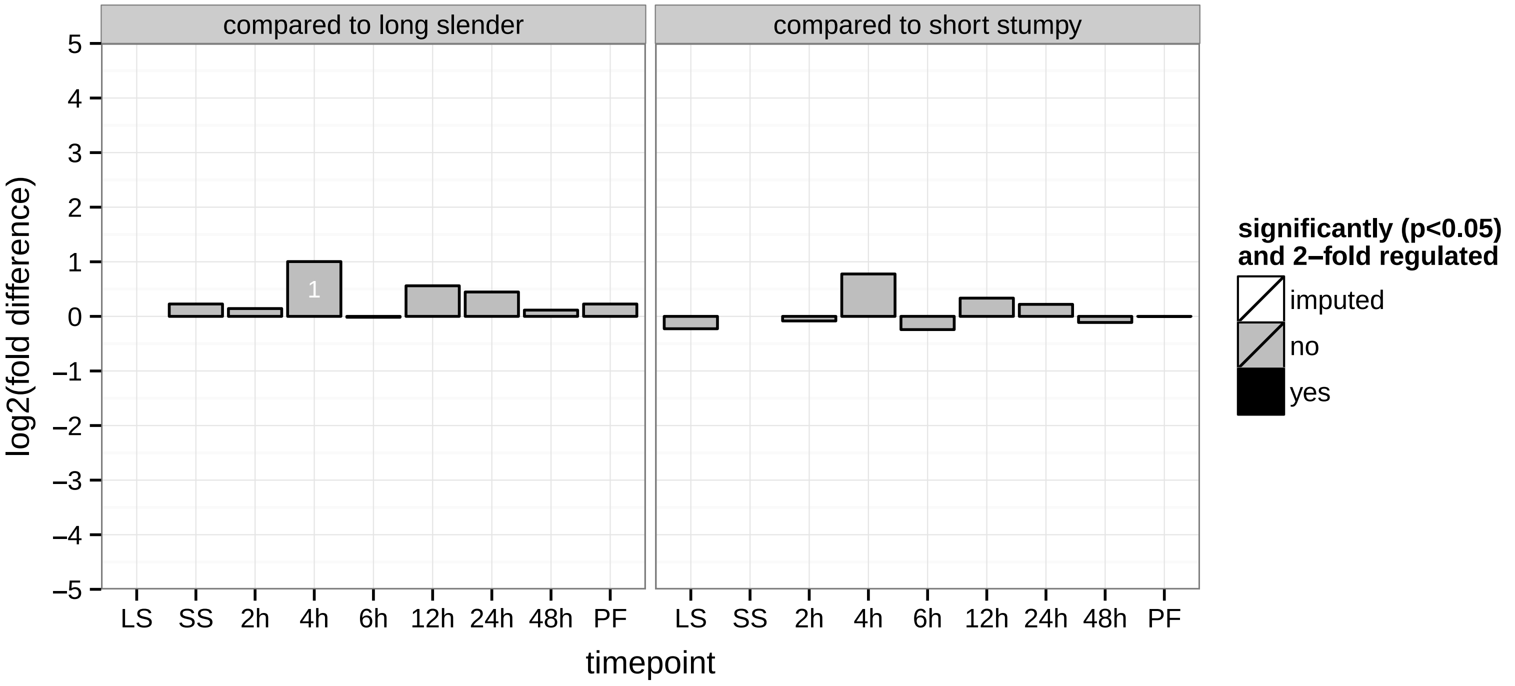

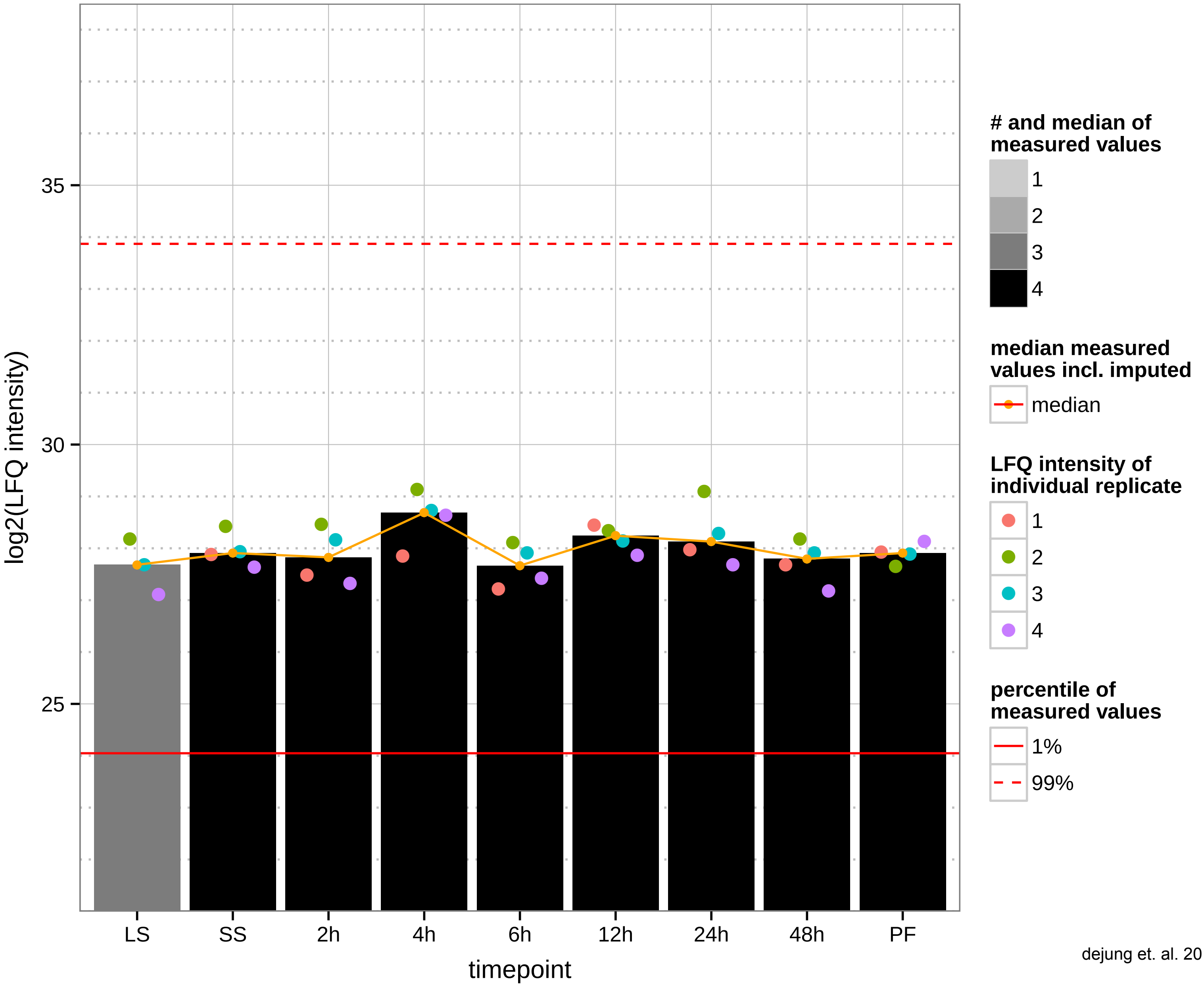

dejung et. al. 2015

U5 snRNP–associated 102 kDa protein, putative
Tb927.11.15690
AGOF: null
AGOC: null
AGOP: null
PGOF: null
PGOC: null
PGOP: null

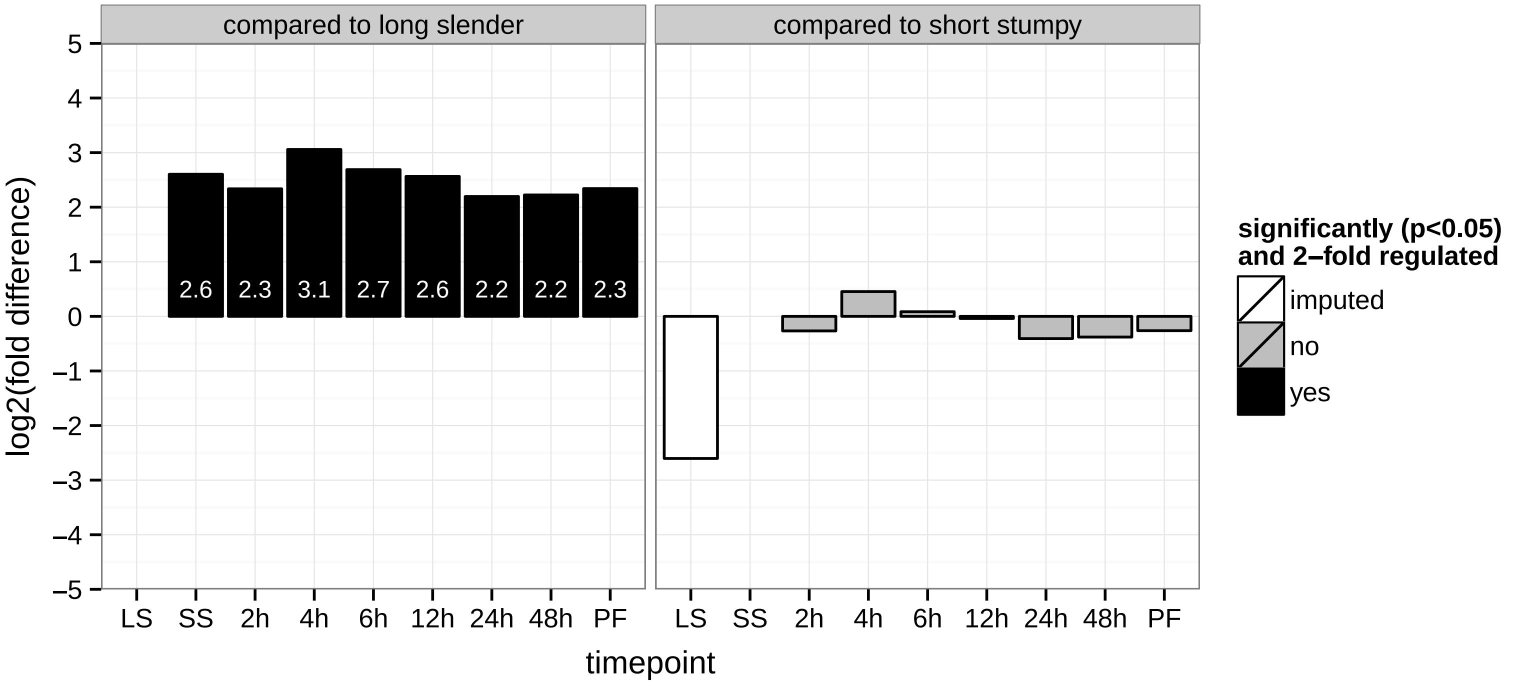

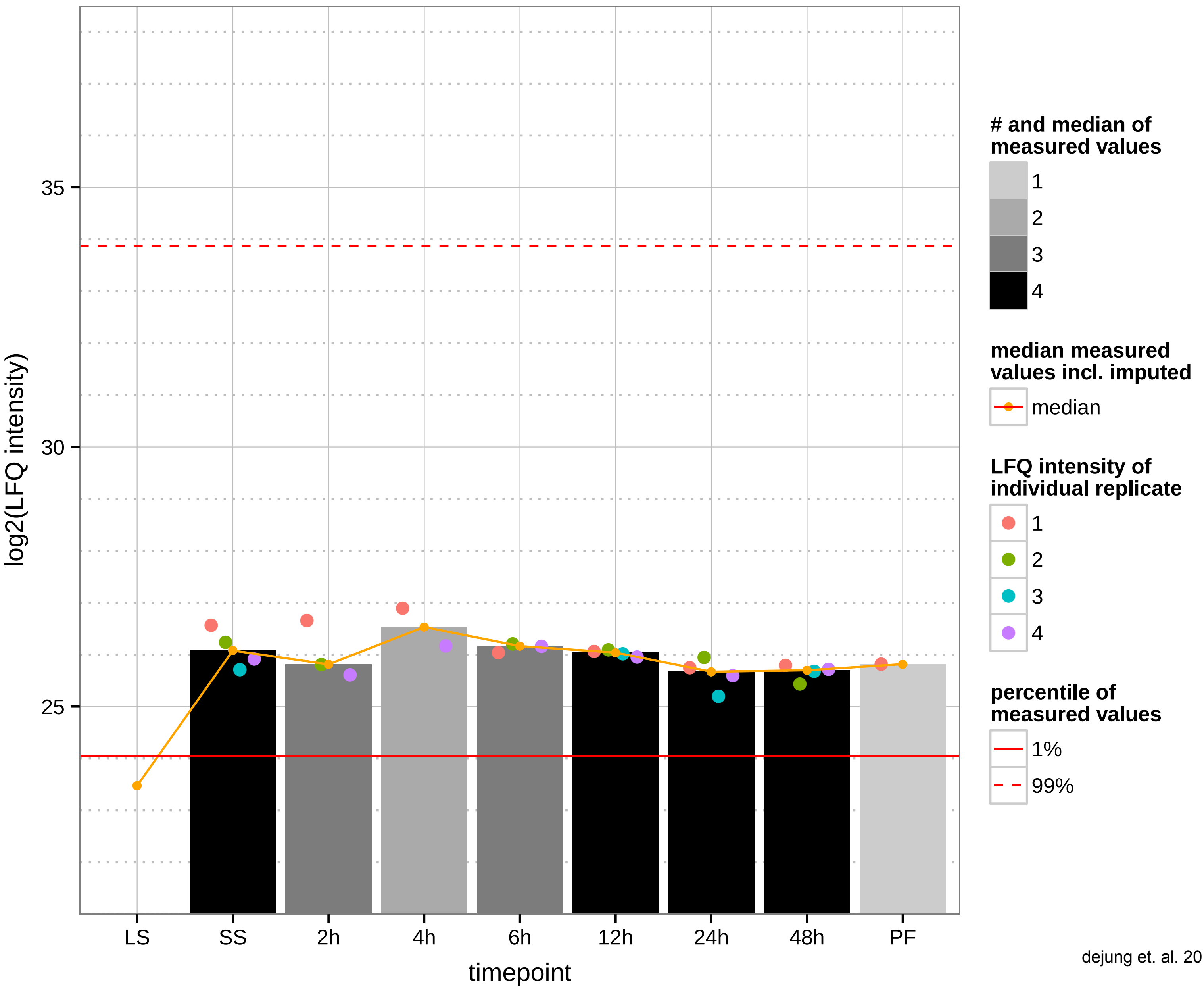

dejung et. al. 2015

hypothetical protein, conserved
Tb927.11.15730
AGOF: null
AGOC: mitochondrion
AGOP: null
PGOF: null
PGOC: null
PGOP: null

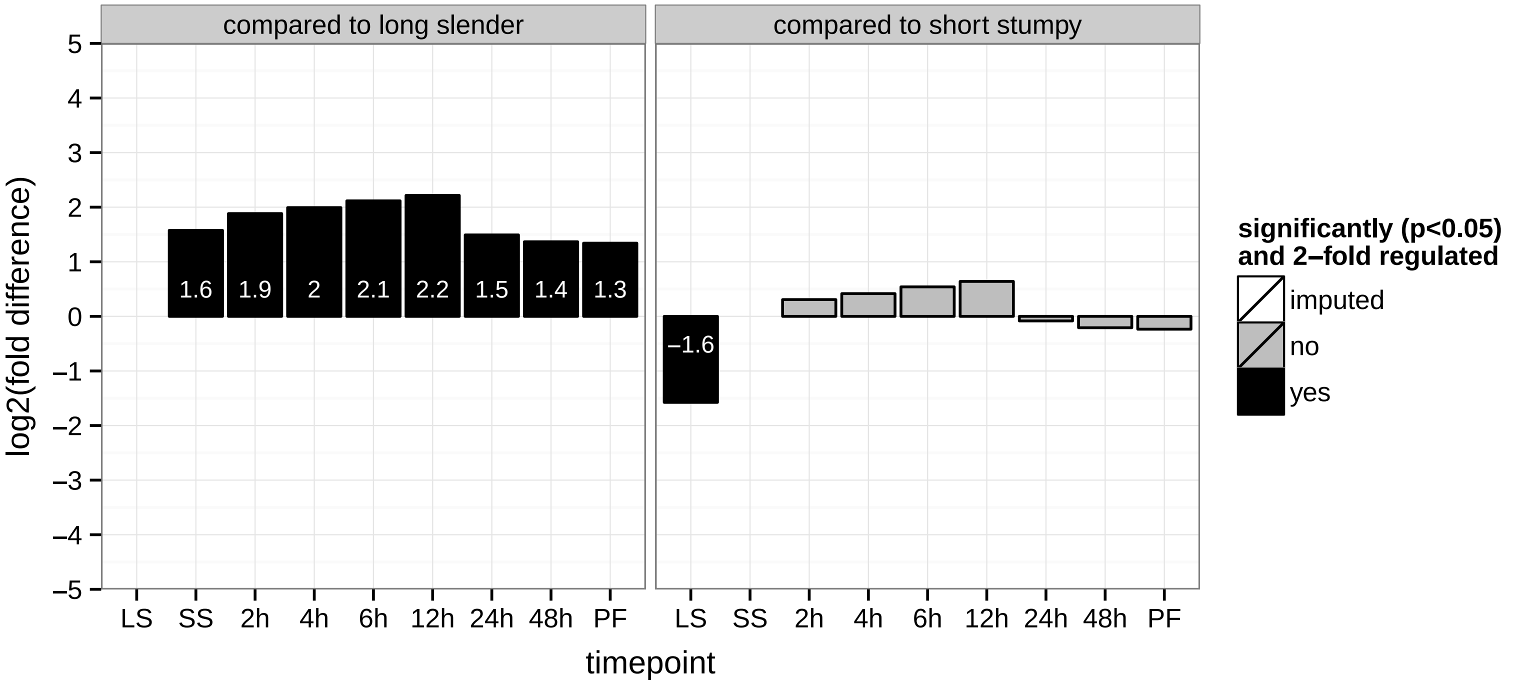

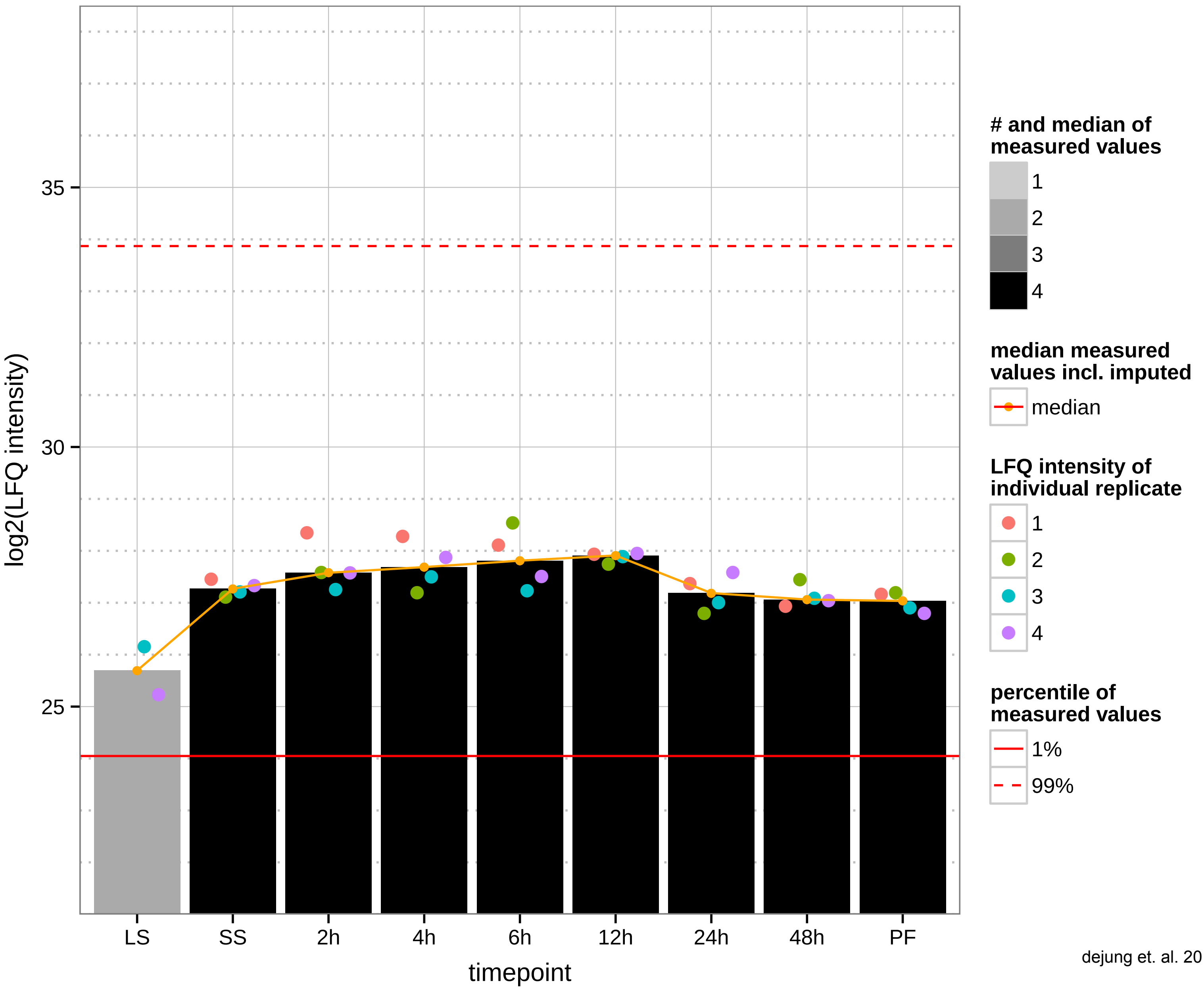

dejung et. al. 2015

AMP deaminase, putative
Tb927.11.15750
AGOF: AMP deaminase activity
AGOC: null
AGOP: generation of precursor metabolites and energy, purine ribonucleoside monophosphate biosynthetic process
PGOF: deaminase activity
PGOC: null
PGOP: purine ribonucleoside monophosphate biosynthetic process

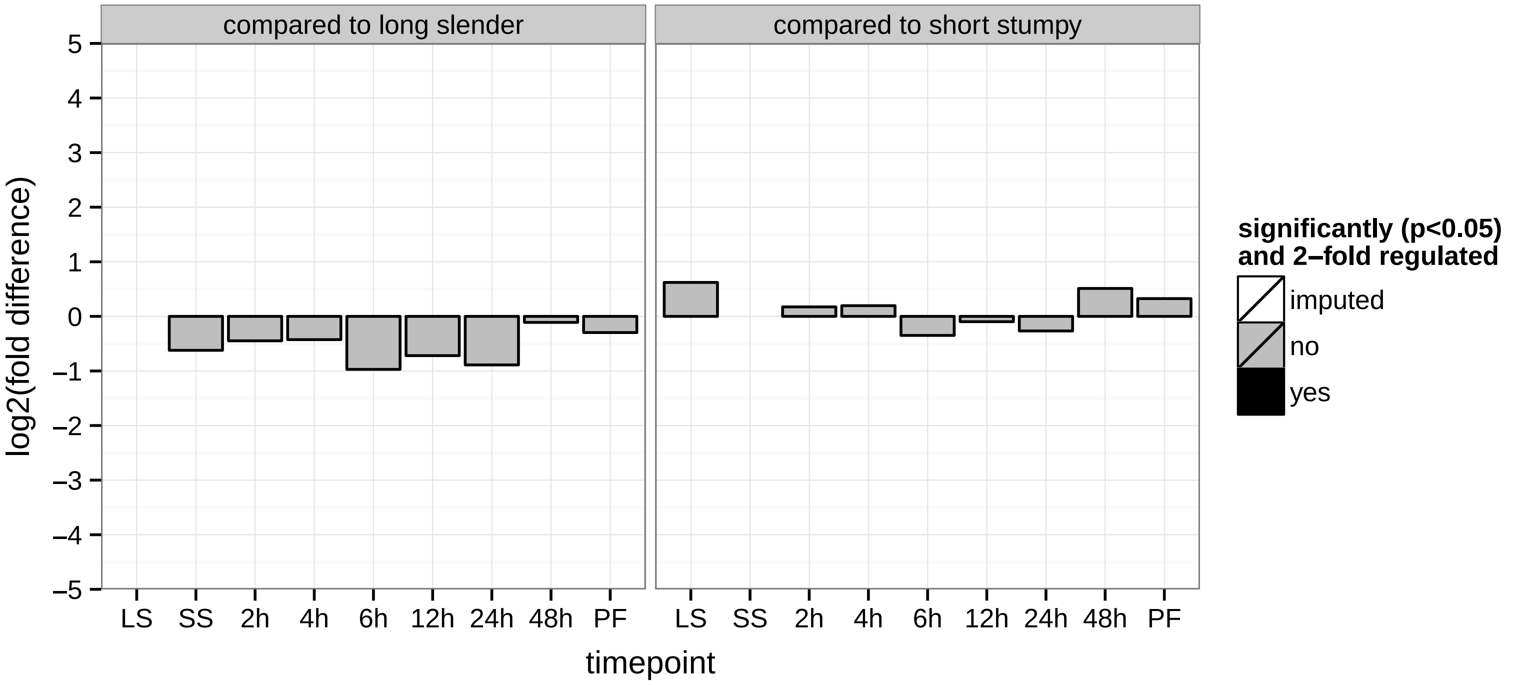

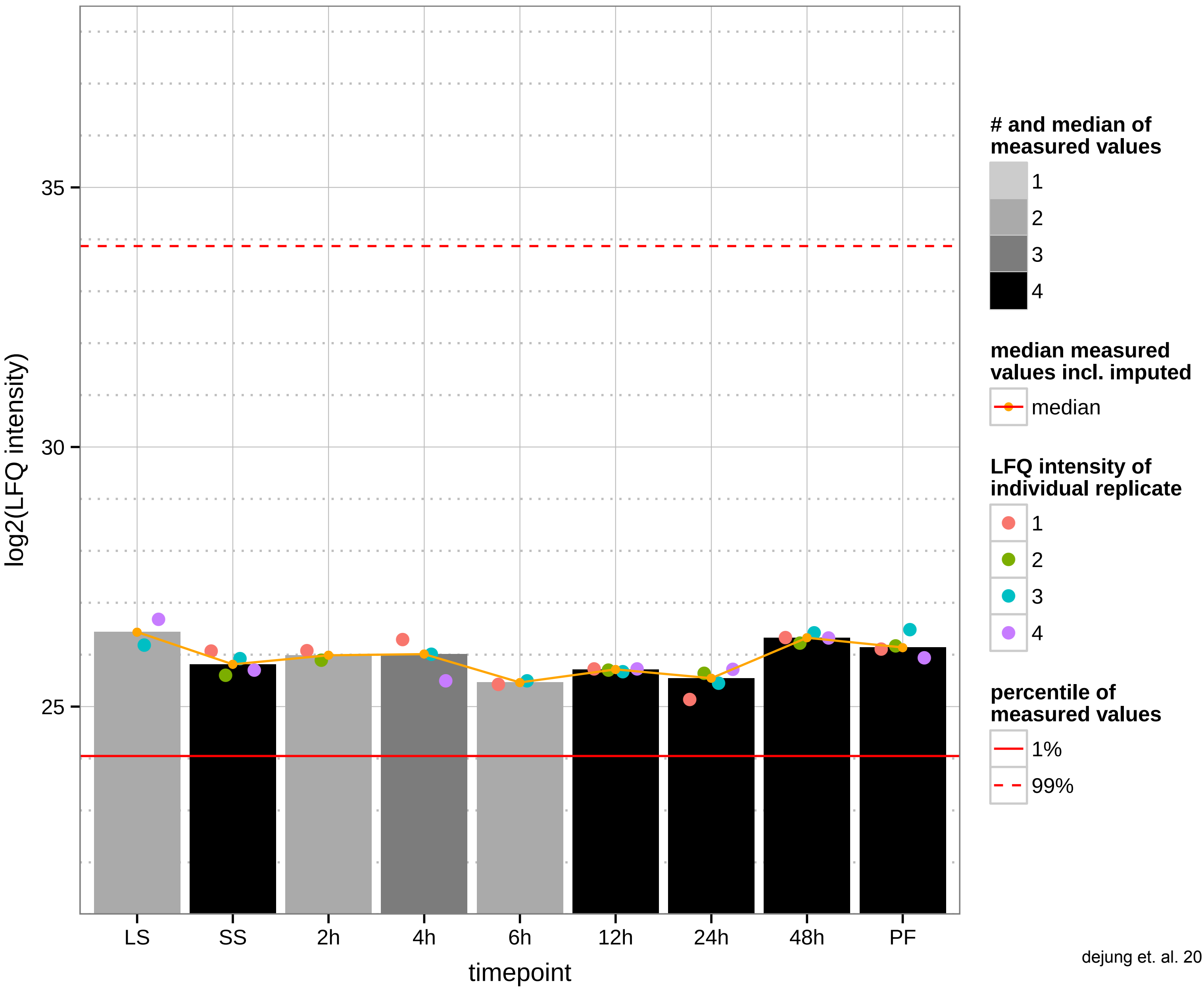

dejung et. al. 2015

variant surface glycoprotein (VSG), putative, RNA methyltransferase
Tb927.11.15830;Tb11.v5.0683
AGOF: null, RNA binding, RNA methyltransferase activity
AGOC: null
AGOP: null, RNA processing, tRNA methylation
PGOF: RNA binding, RNA methyltransferase activity
PGOC: null
PGOP: RNA processing

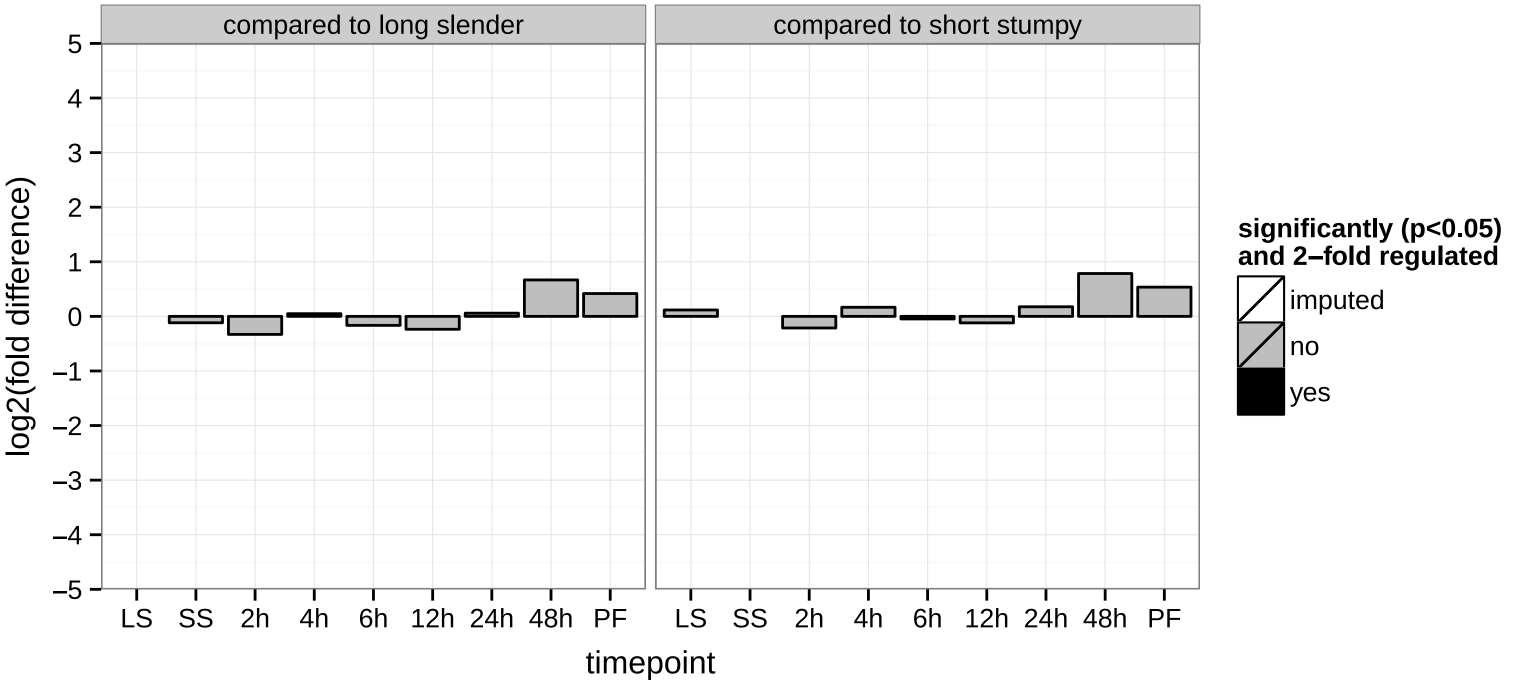

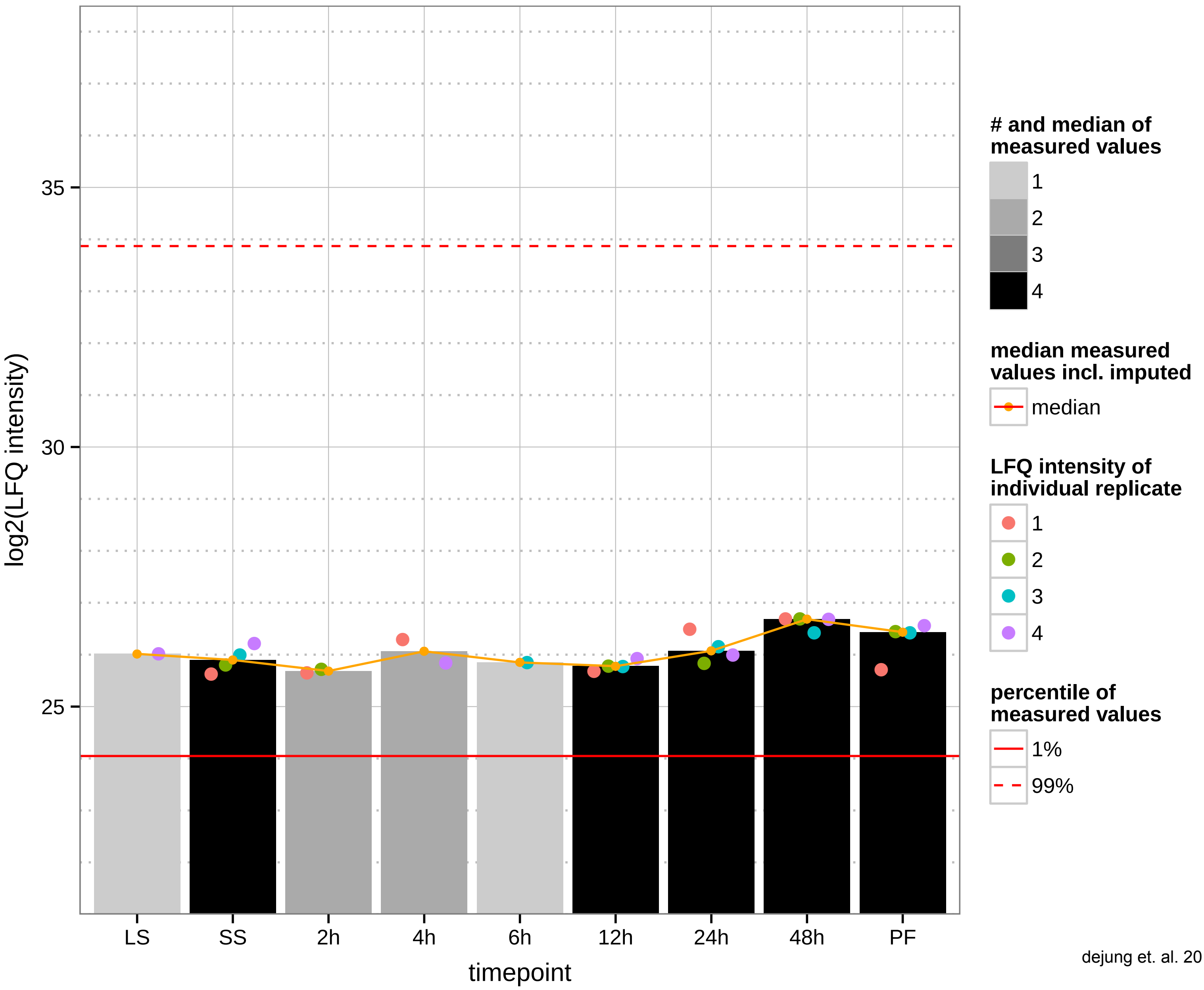

dejung et. al. 2015

60S ribosomal protein L27, putative
Tb927.11.15900;Tb927.11.15880
AGOF: structural constituent of ribosome
AGOC: intracellular, ribosome
AGOP: translation
PGOF: structural constituent of ribosome, null
PGOC: intracellular, ribosome, null
PGOP: translation, null

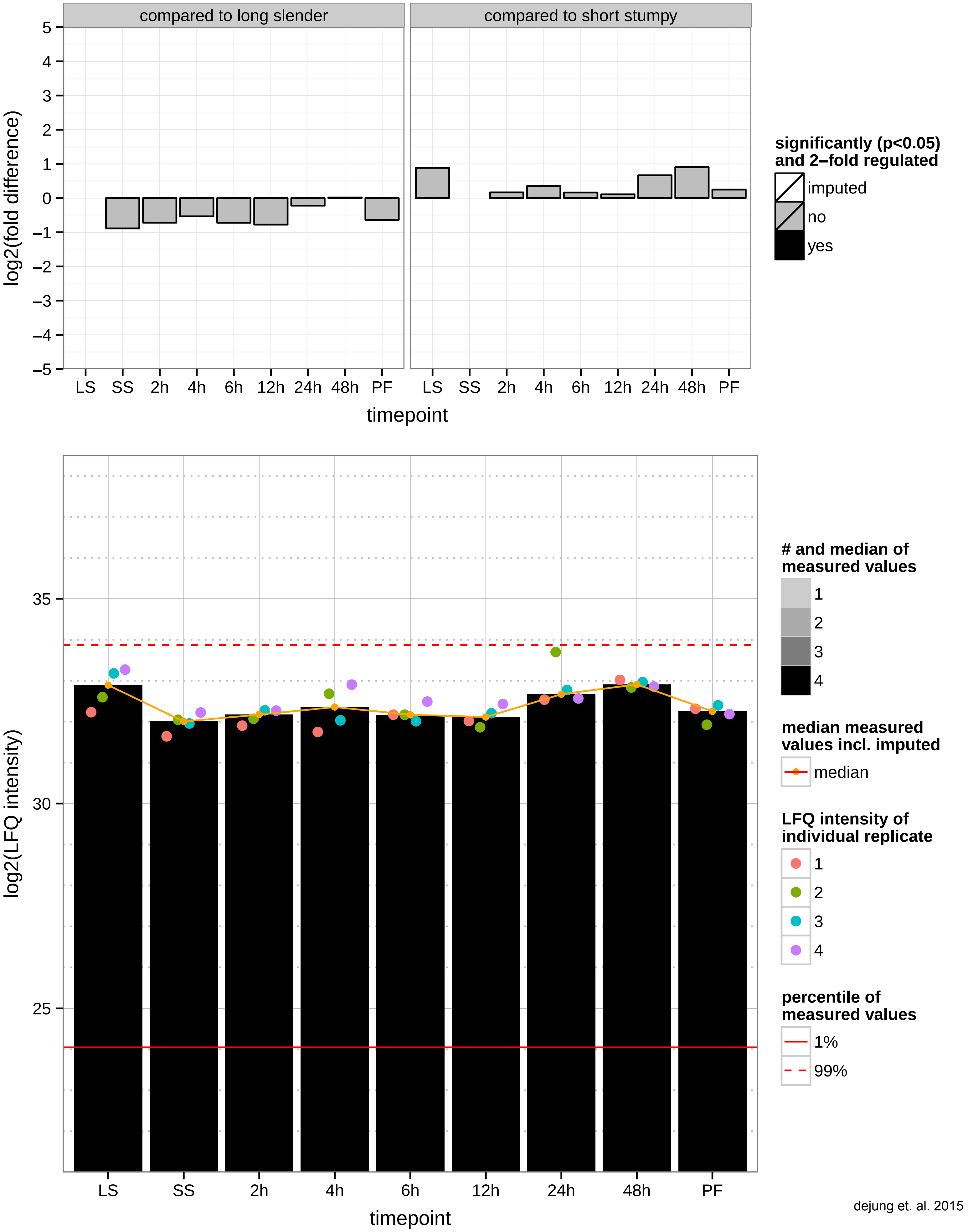

dejung et. al. 2015

hypothetical protein, conserved
Tb927.11.15930
AGOF: null
AGOC: cis–Golgi network
AGOP: ER to Golgi vesicle–mediated transport
PGOF: null
PGOC: cis–Golgi network
PGOP: ER to Golgi vesicle–mediated transport, transport

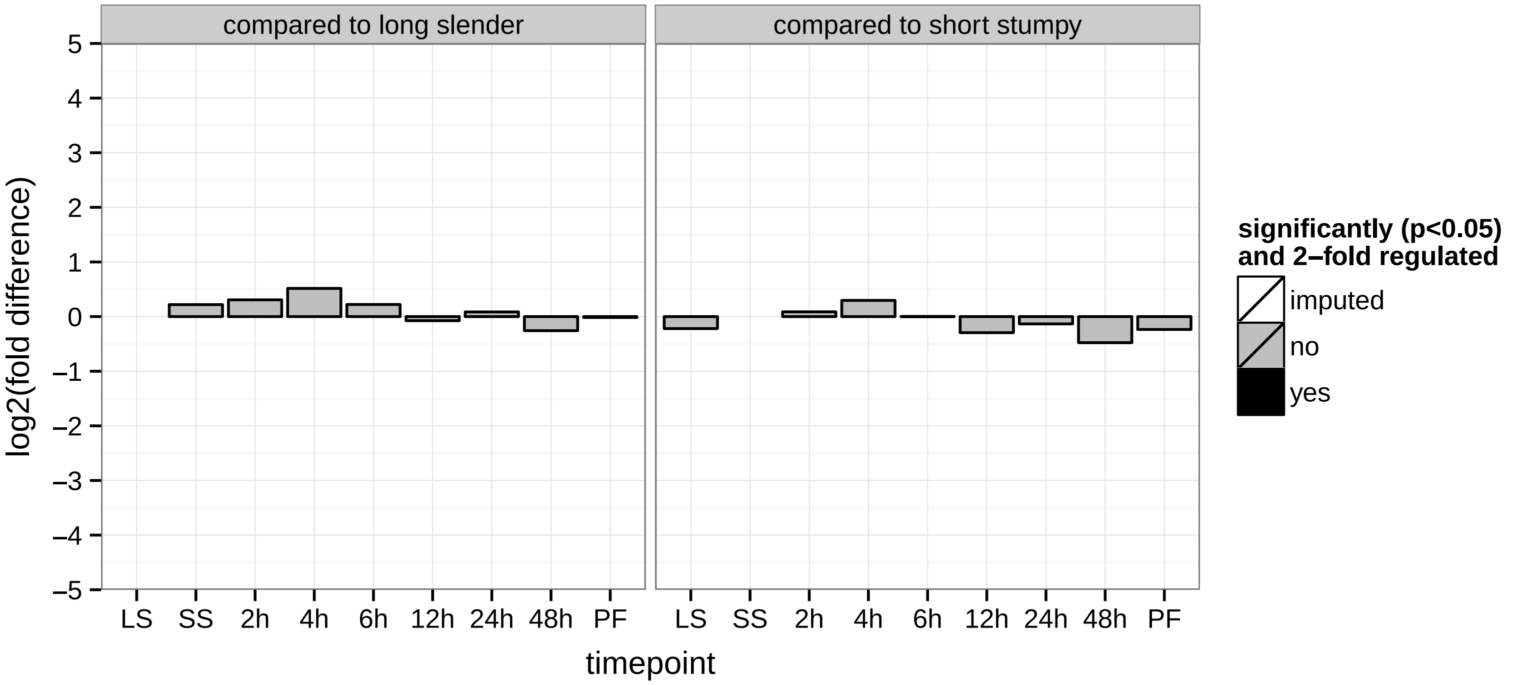

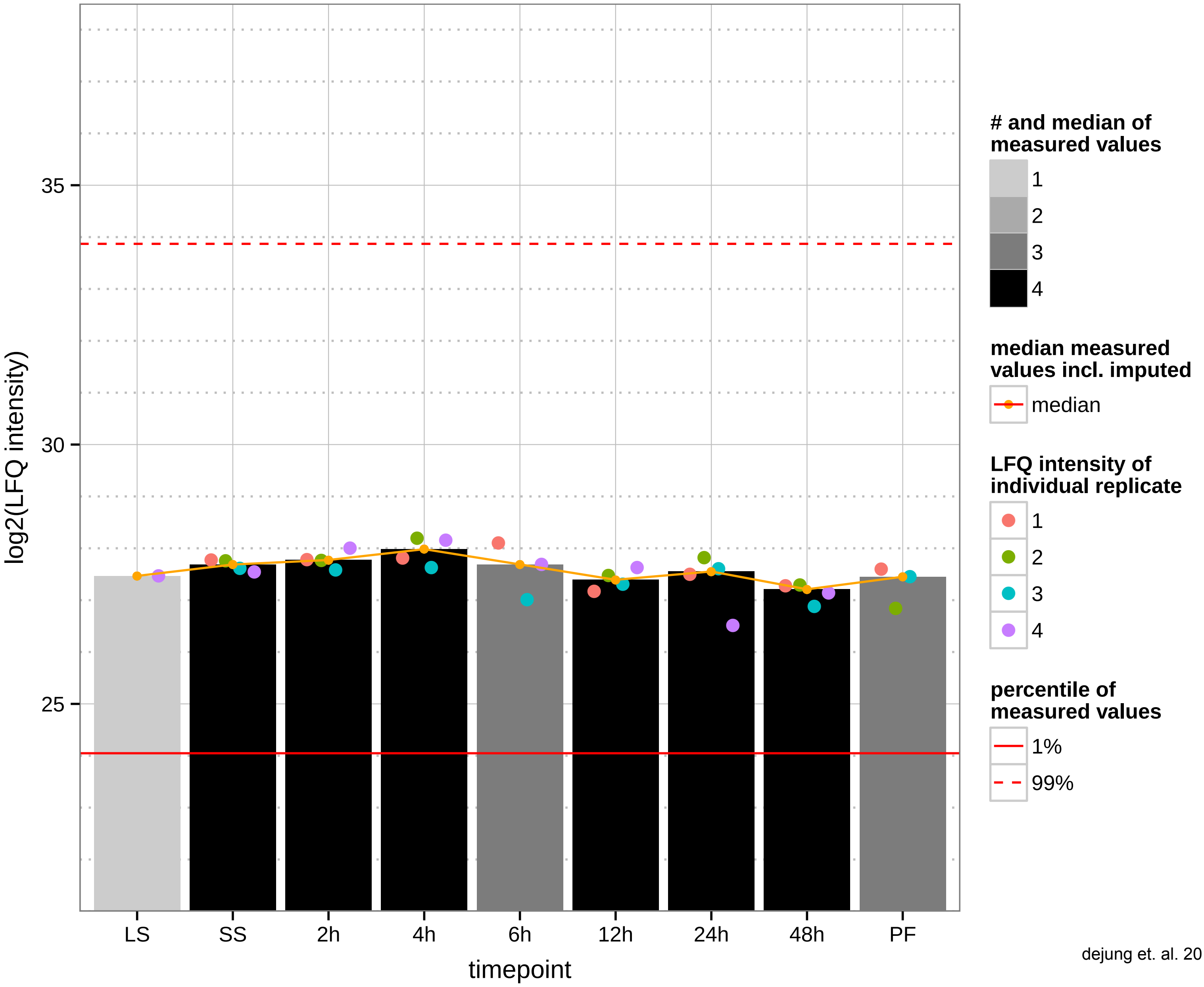

dejung et. al. 2015

nucleoporin Nup109 (TbNup109)
Tb927.11.15990
AGOF: null
AGOC: nuclear pore
AGOP: null
PGOF: null
PGOC: null
PGOP: null

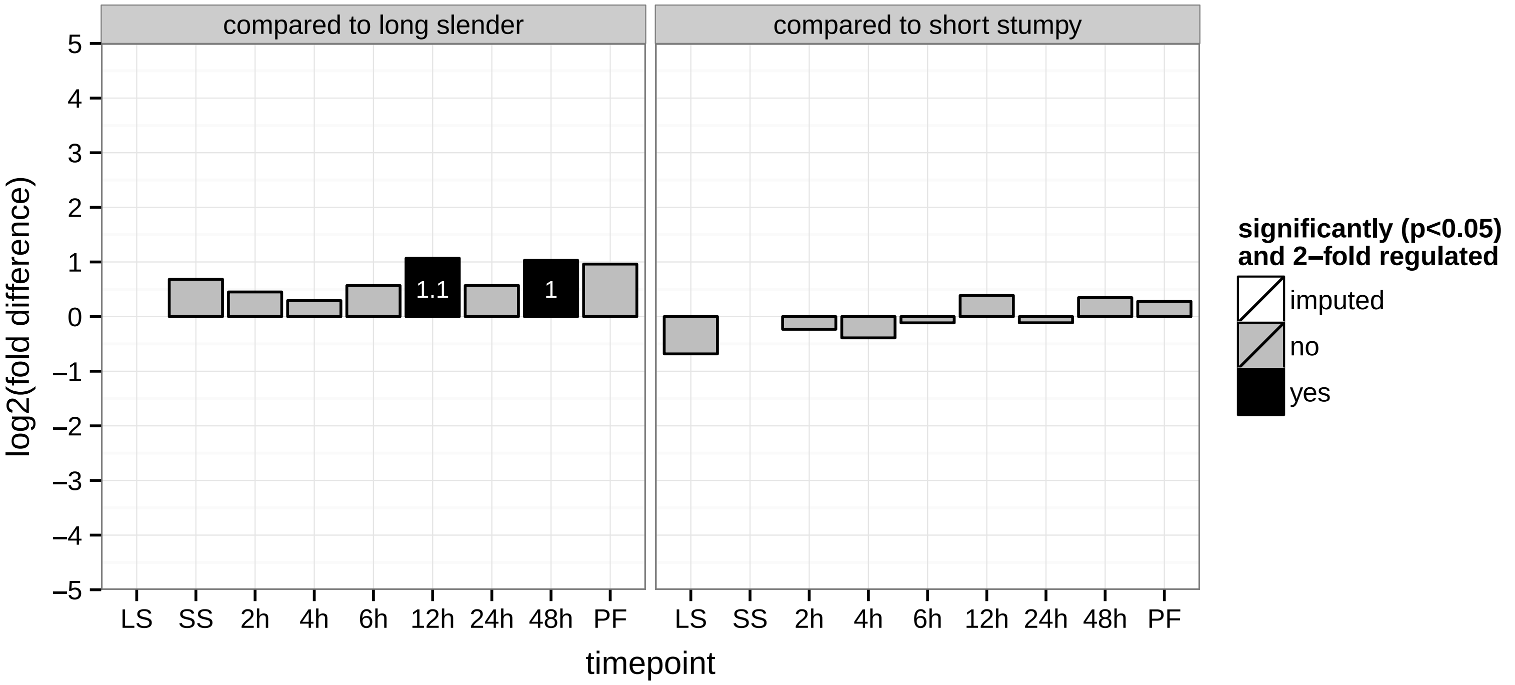

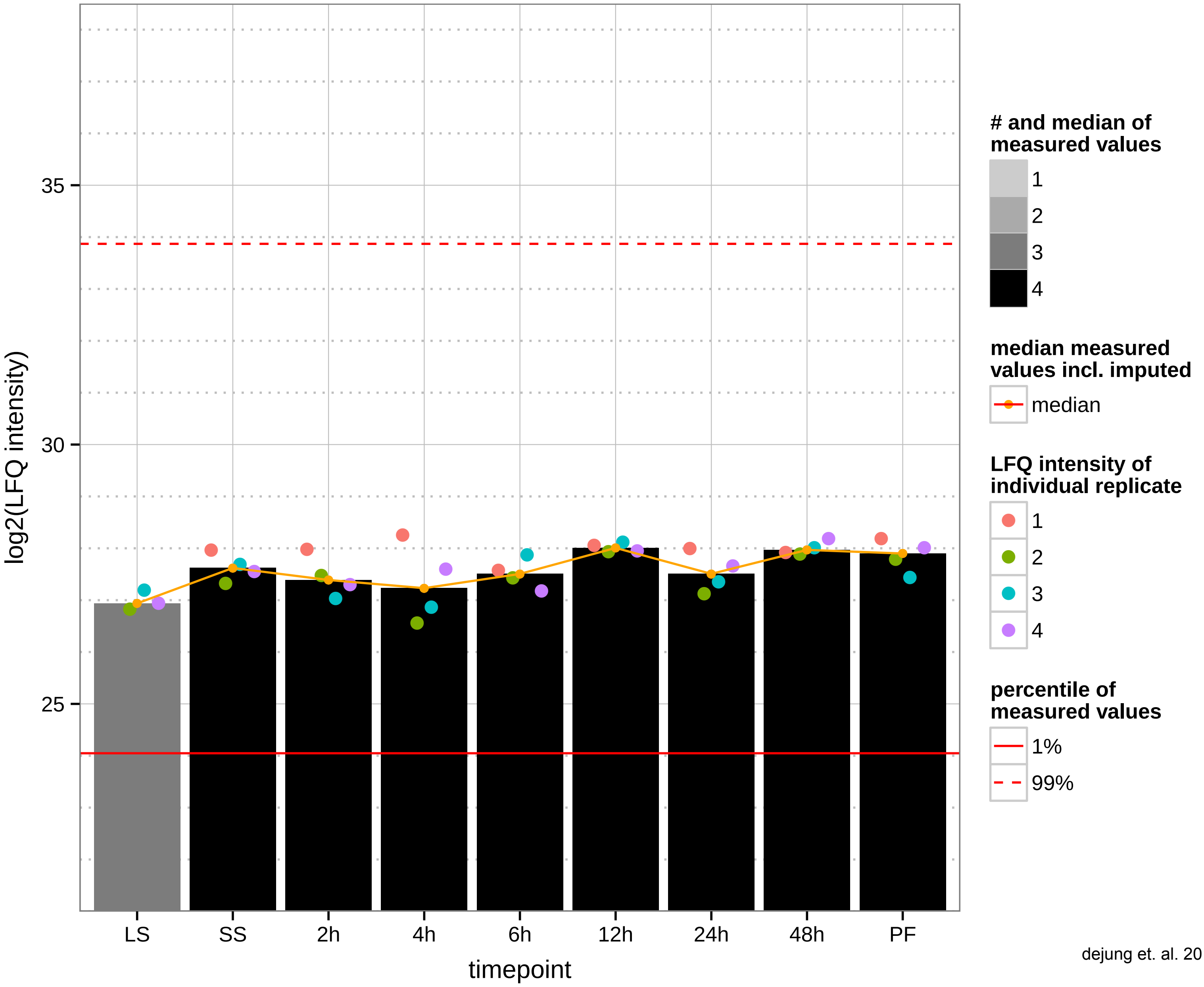

dejung et. al. 2015

proteasome regulatory non–ATP–ase subunit 7 (RPN7)
Tb927.11.16030
AGOF: endopeptidase activity
AGOC: proteasome regulatory particle
AGOP: ubiquitin–dependent protein catabolic process
PGOF: protein binding
PGOC: null
PGOP: null

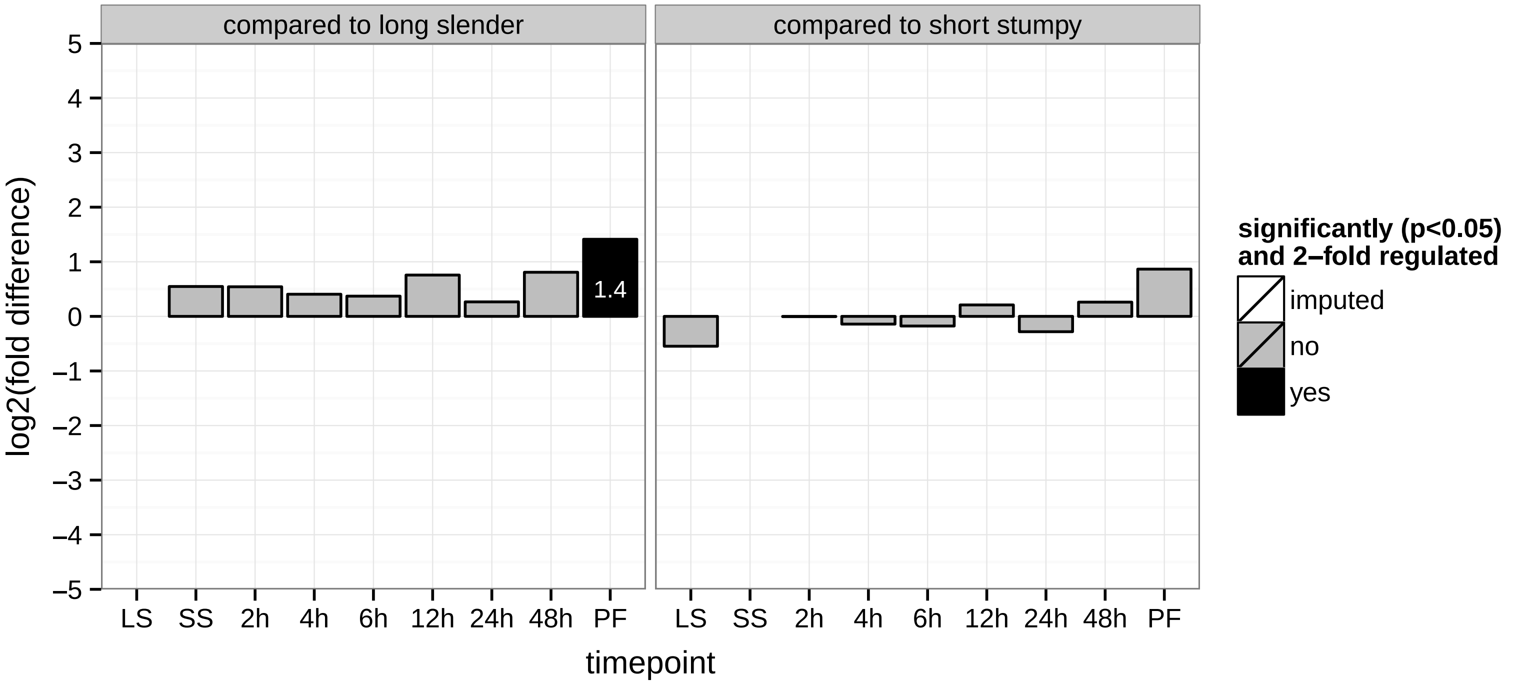

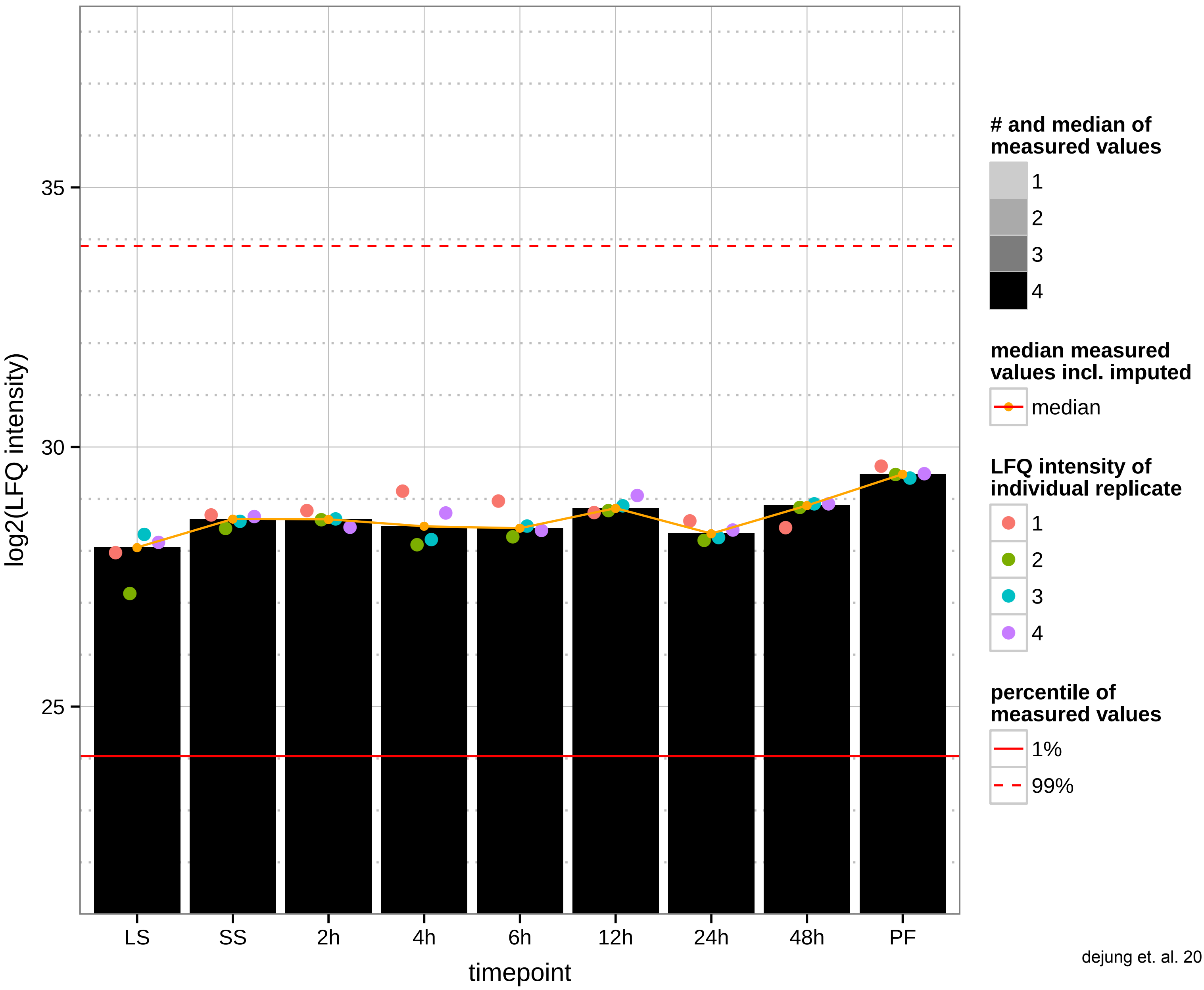

dejung et. al. 2015

outer dynein arm docking complex, putative (DC2)
Tb927.11.16090
AGOF: null
AGOC: axonemal dynein complex
AGOP: axonemal dynein complex assembly
PGOF: null
PGOC: null
PGOP: null

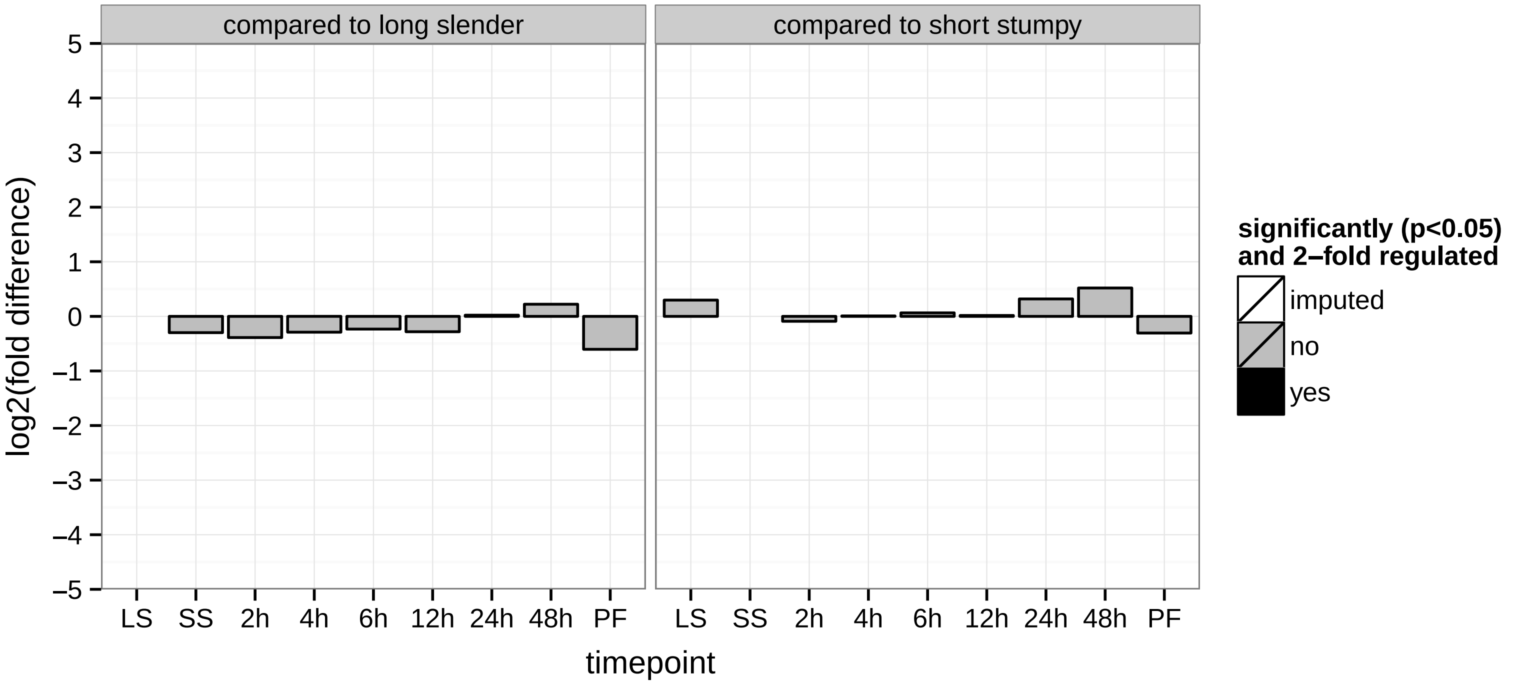

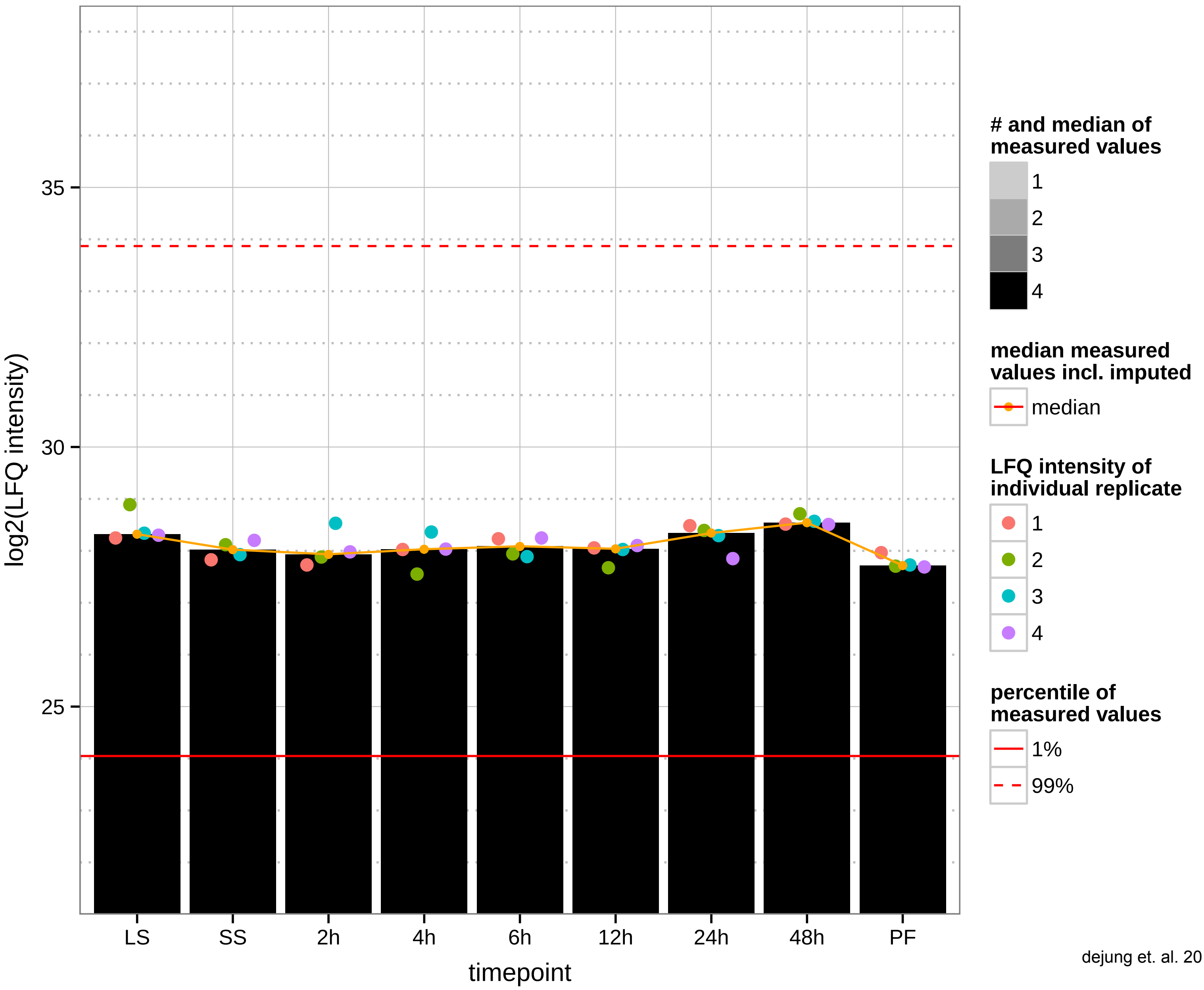

dejung et. al. 2015

ubiquitin carboxyl–terminal hydrolase, putative
Tb927.11.16100
AGOF: cysteine–type endopeptidase activity, ubiquitin thiolesterase activity
AGOC: null
AGOP: ubiquitin–dependent protein catabolic process
PGOF: ubiquitin thiolesterase activity
PGOC: null
PGOP: ubiquitin–dependent protein catabolic process

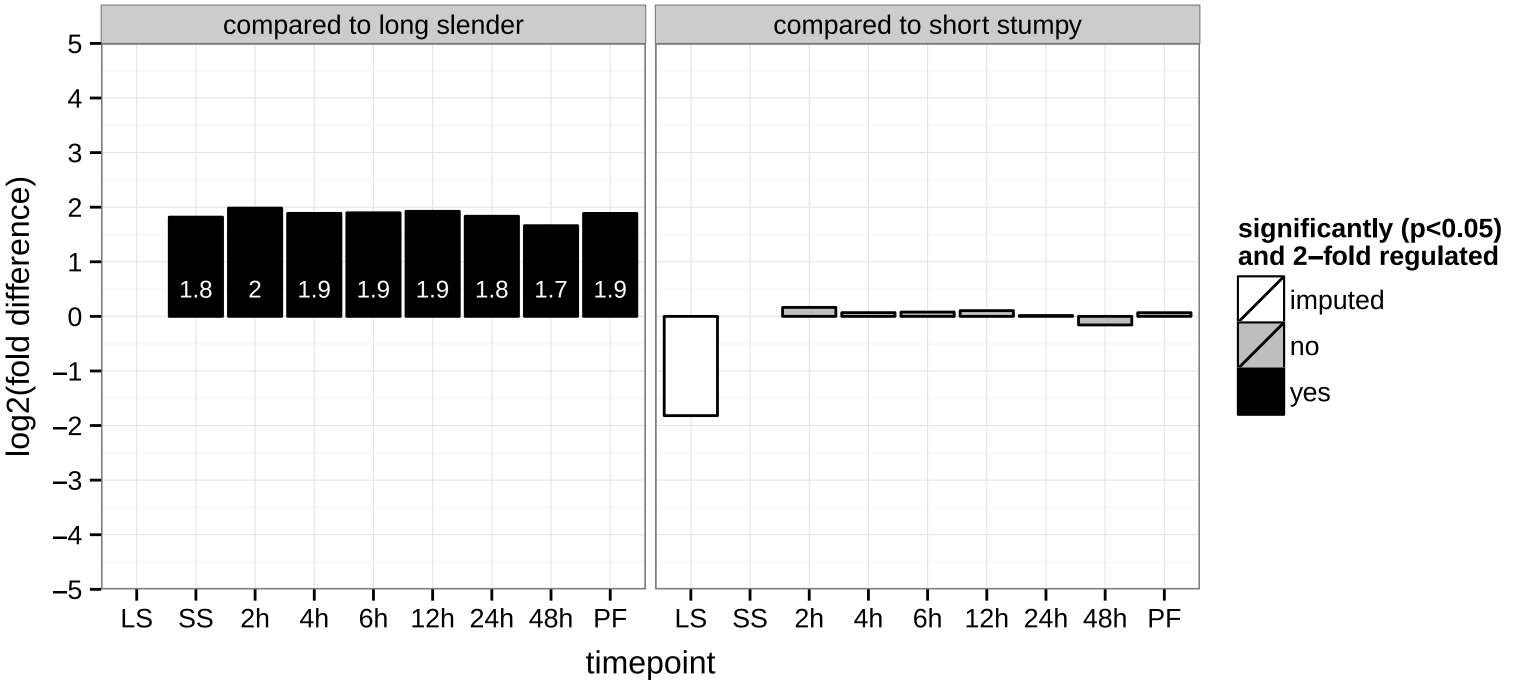

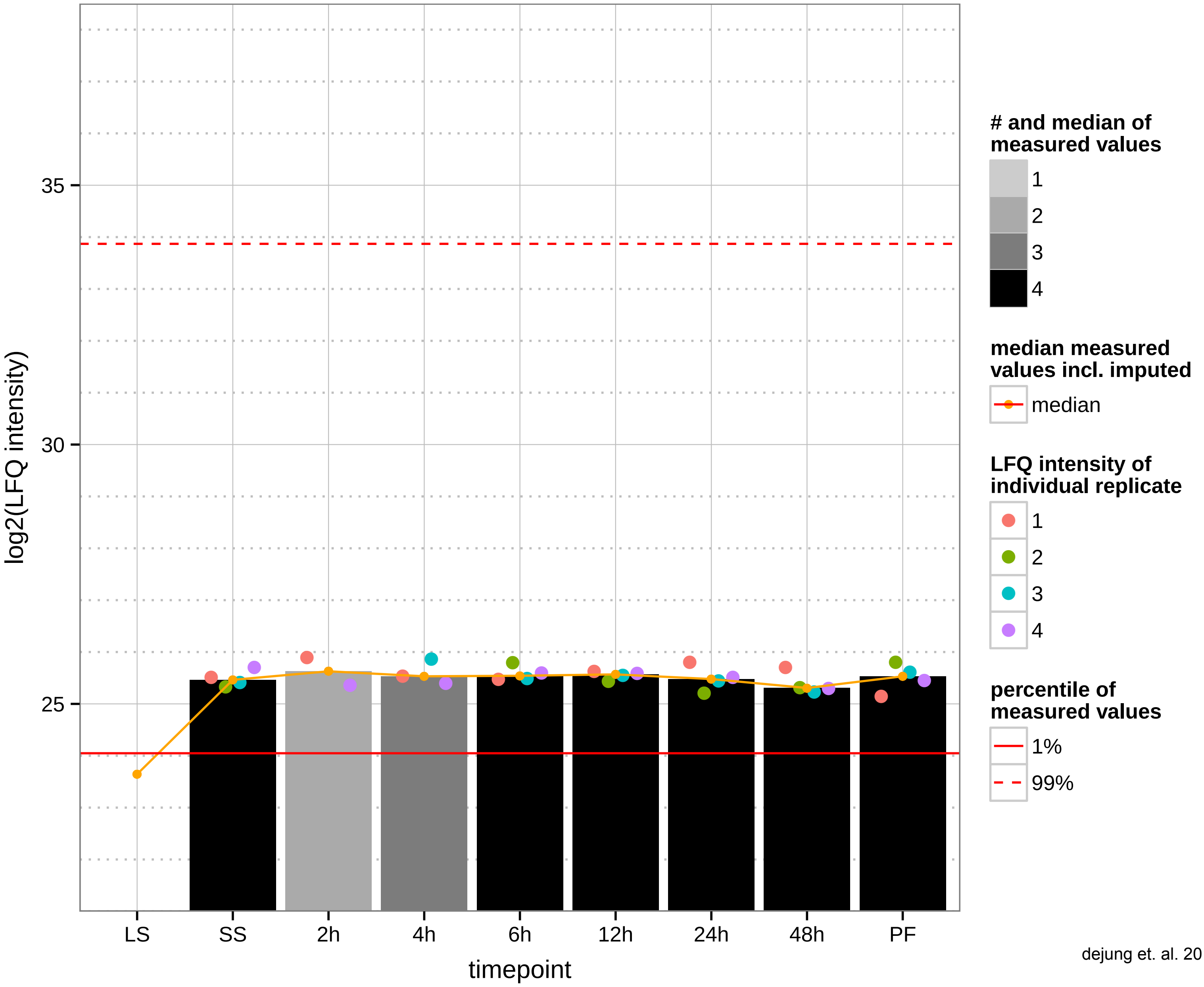

dejung et. al. 2015

integrin alpha chain protein, putative, FG−GAP repeat protein
Tb927.11.16110
AGOF: null
AGOC: integral to membrane, integrin complex
AGOP: cell−matrix adhesion
PGOF: null
PGOC: null
PGOP: null

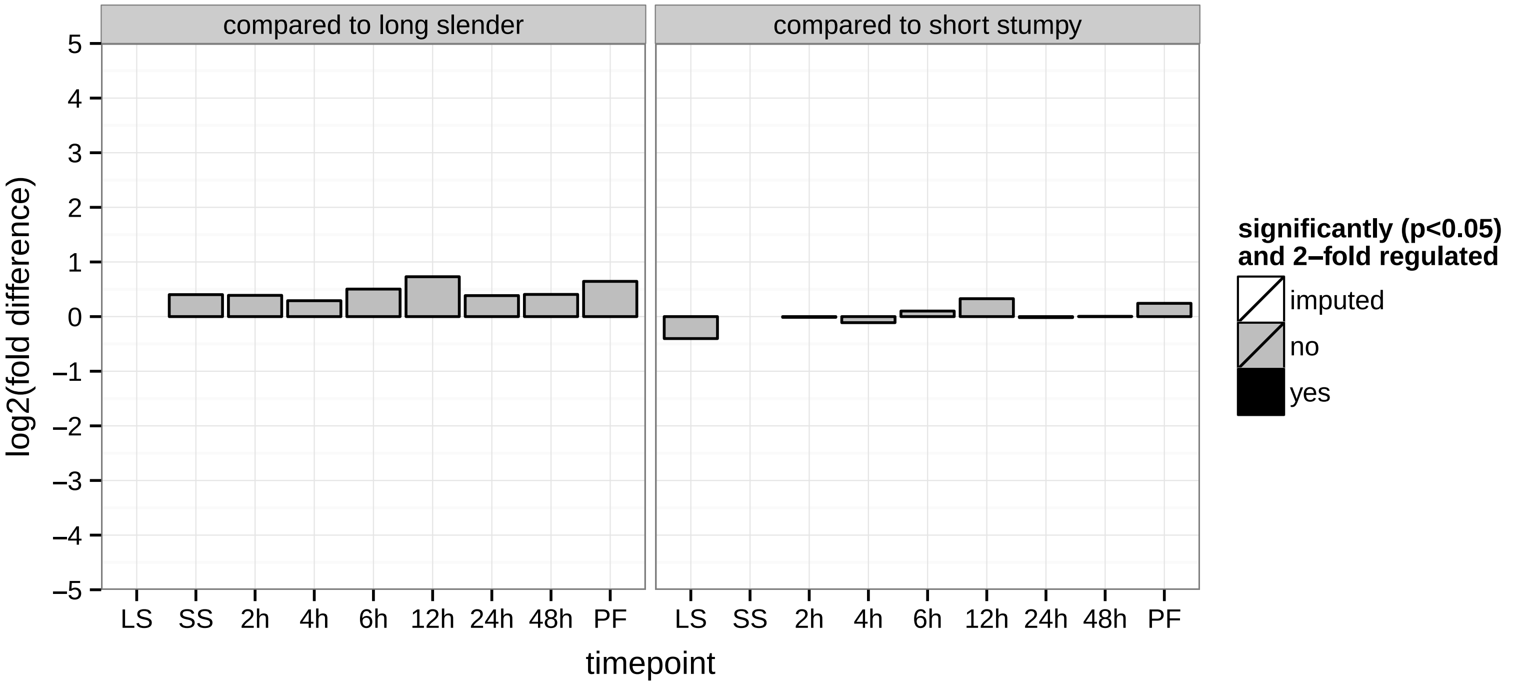

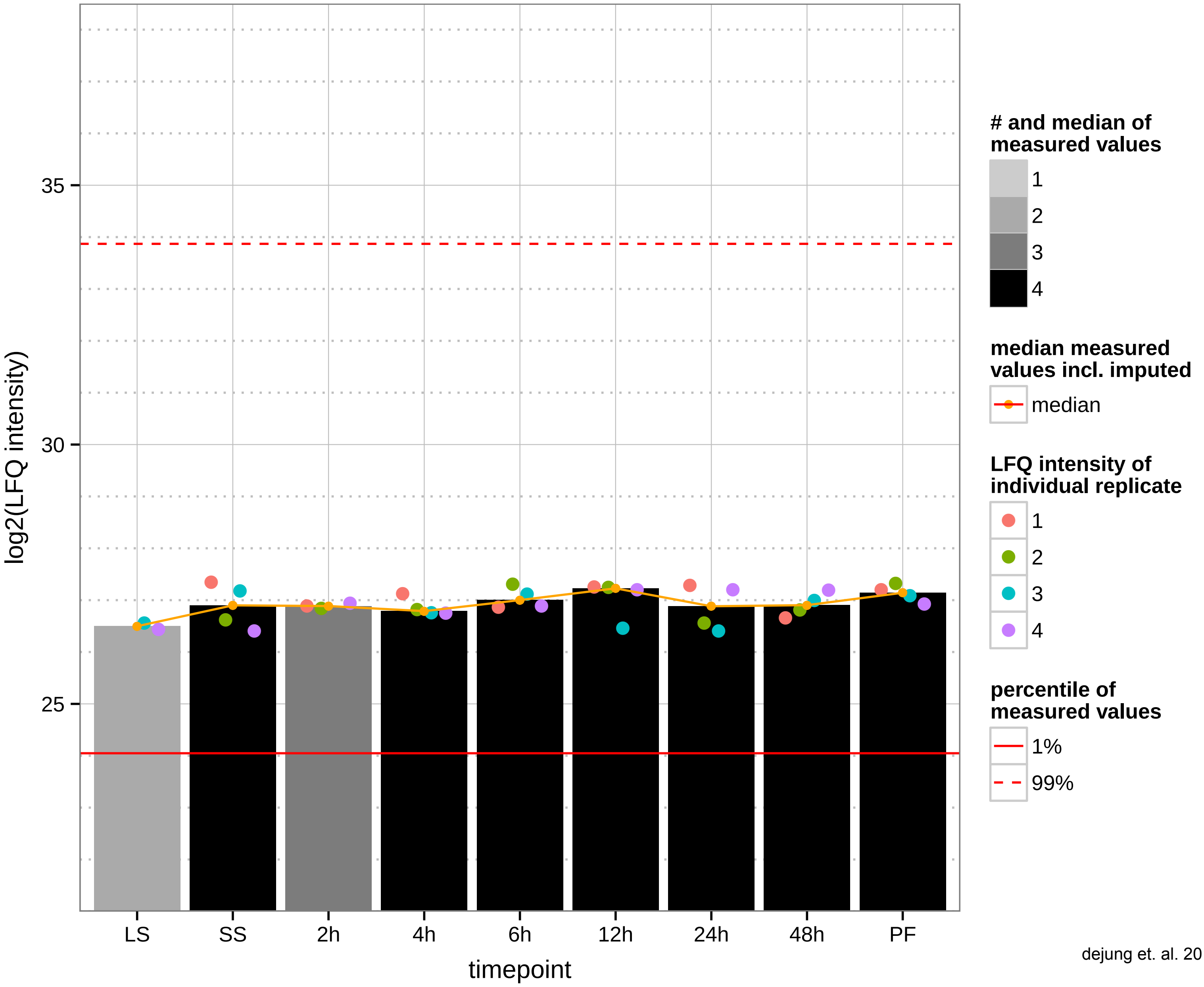

dejung et. al. 2015

nucleoside diphosphate kinase (NDPK)
Tb927.11.16130
AGOF: ATP binding, nucleoside diphosphate kinase activity
AGOC: nucleus
AGOP: CTP biosynthetic process, GTP biosynthetic process, UTP biosynthetic process
PGOF: ATP binding, nucleoside diphosphate kinase activity
PGOC: null
PGOP: CTP biosynthetic process, GTP biosynthetic process, UTP biosynthetic process, nucleoside diphosphate phosphoryla

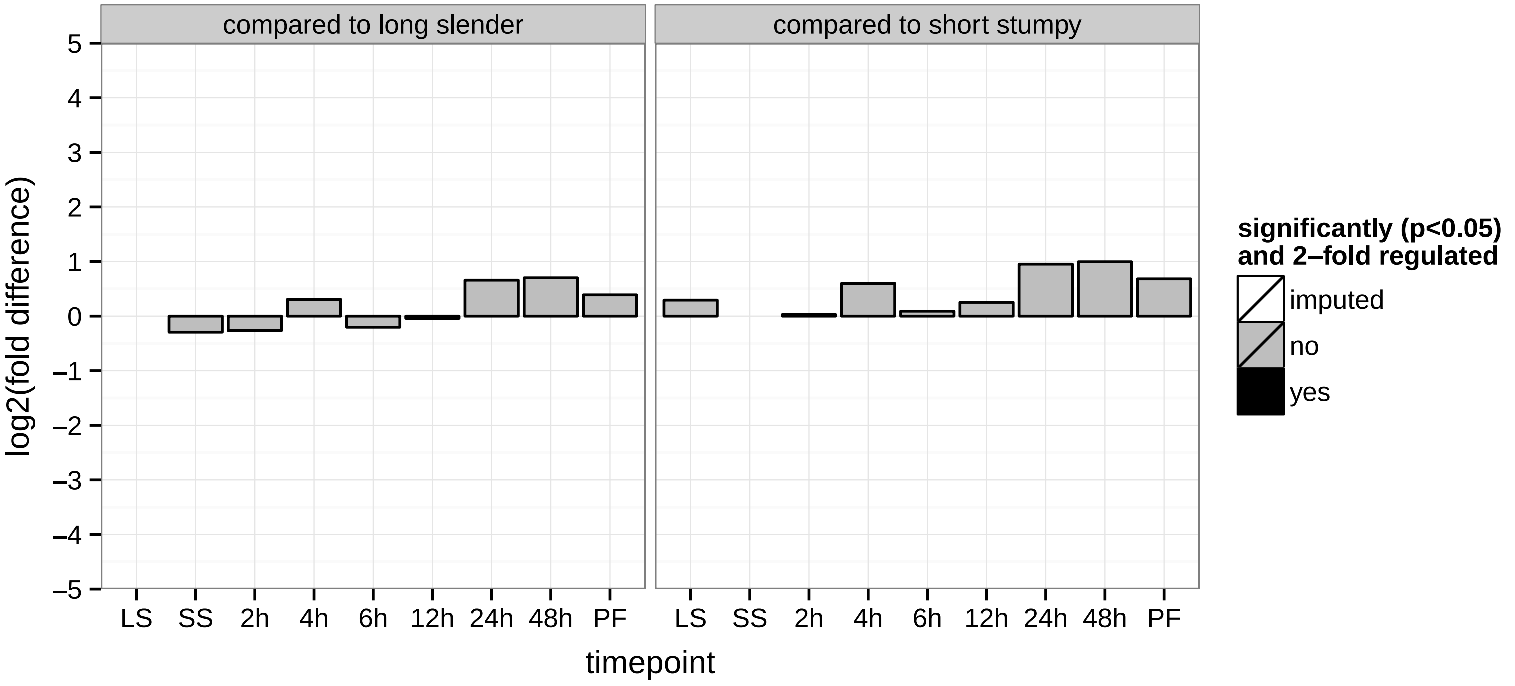

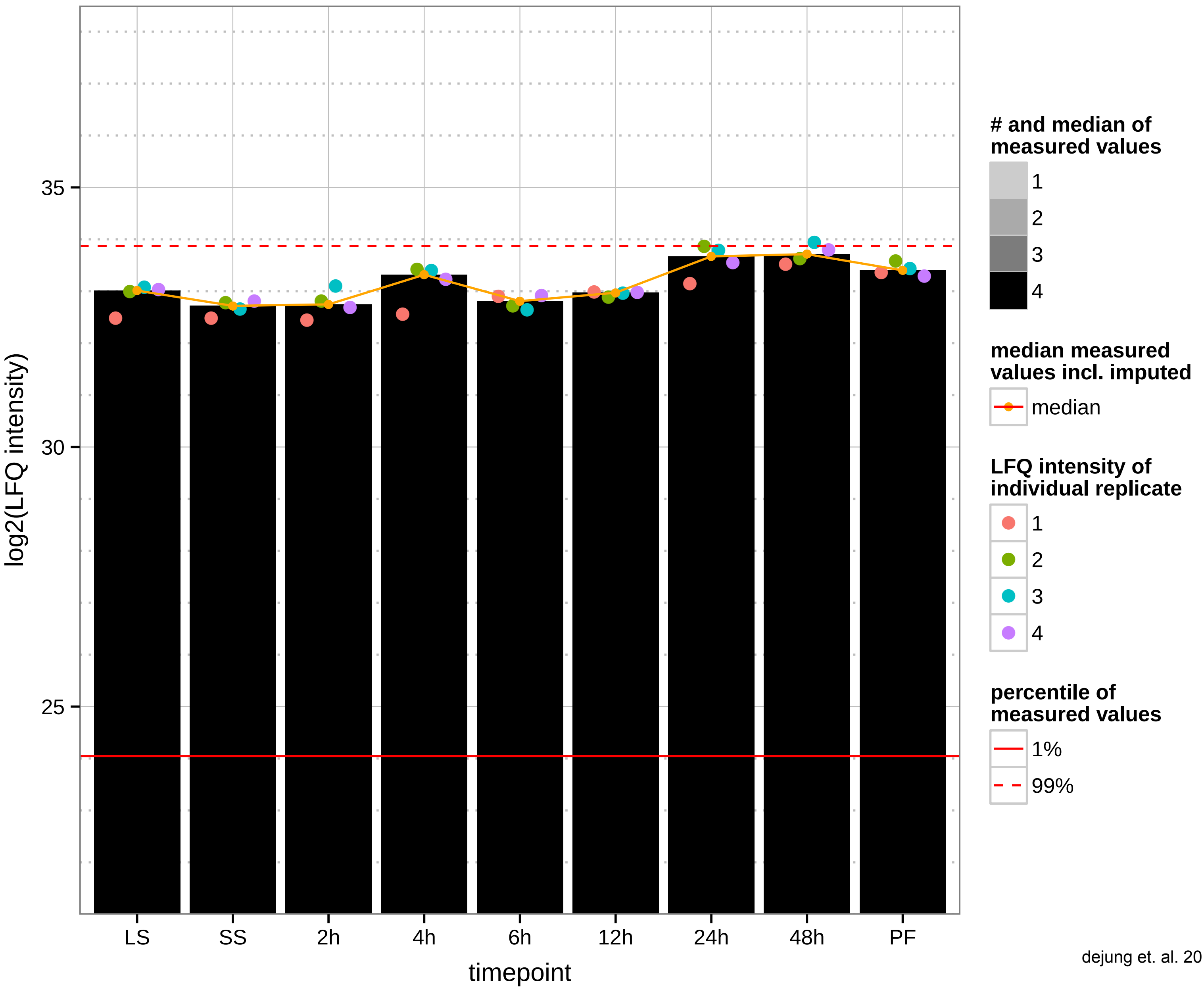

dejung et. al. 2015

hypothetical protein, conserved
Tb927.11.1620
AGOF: null
AGOC: mitochondrion
AGOP: null
PGOF: null
PGOC: null
PGOP: null

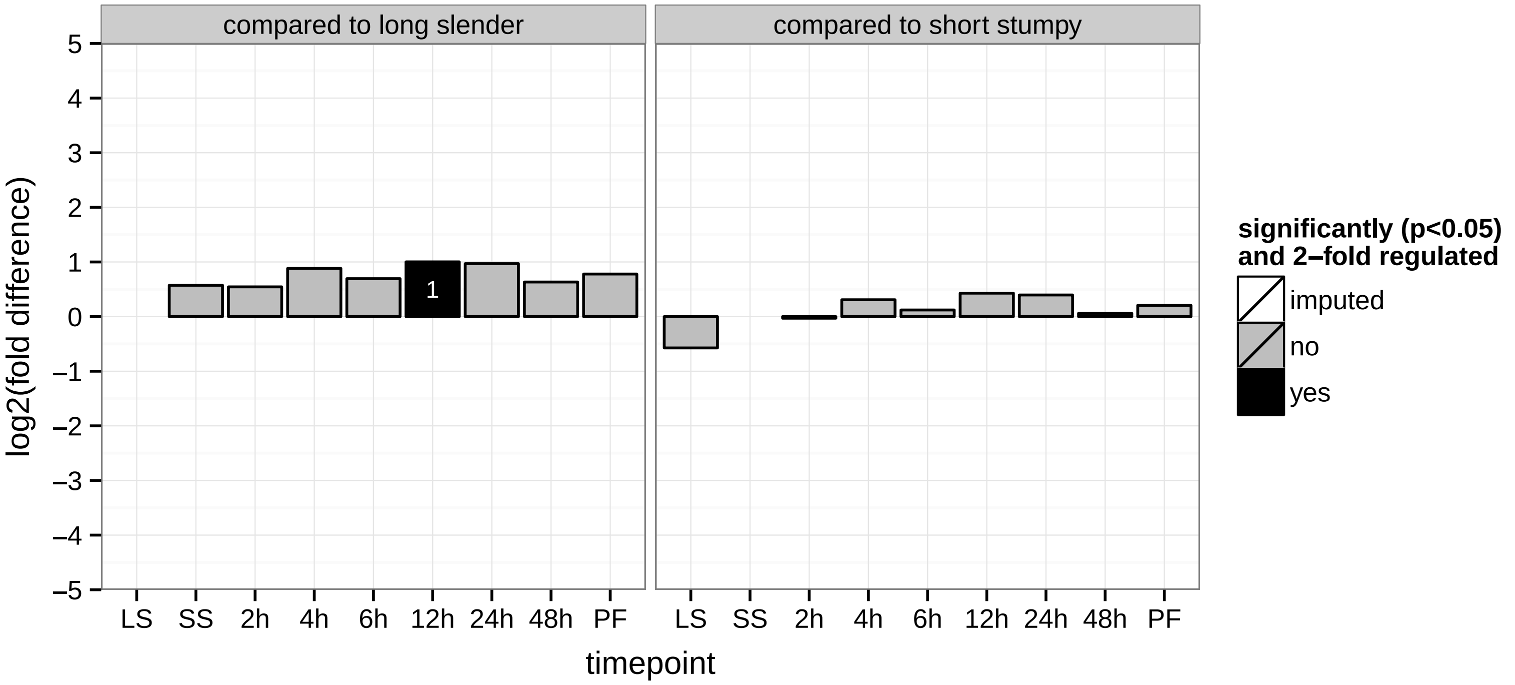

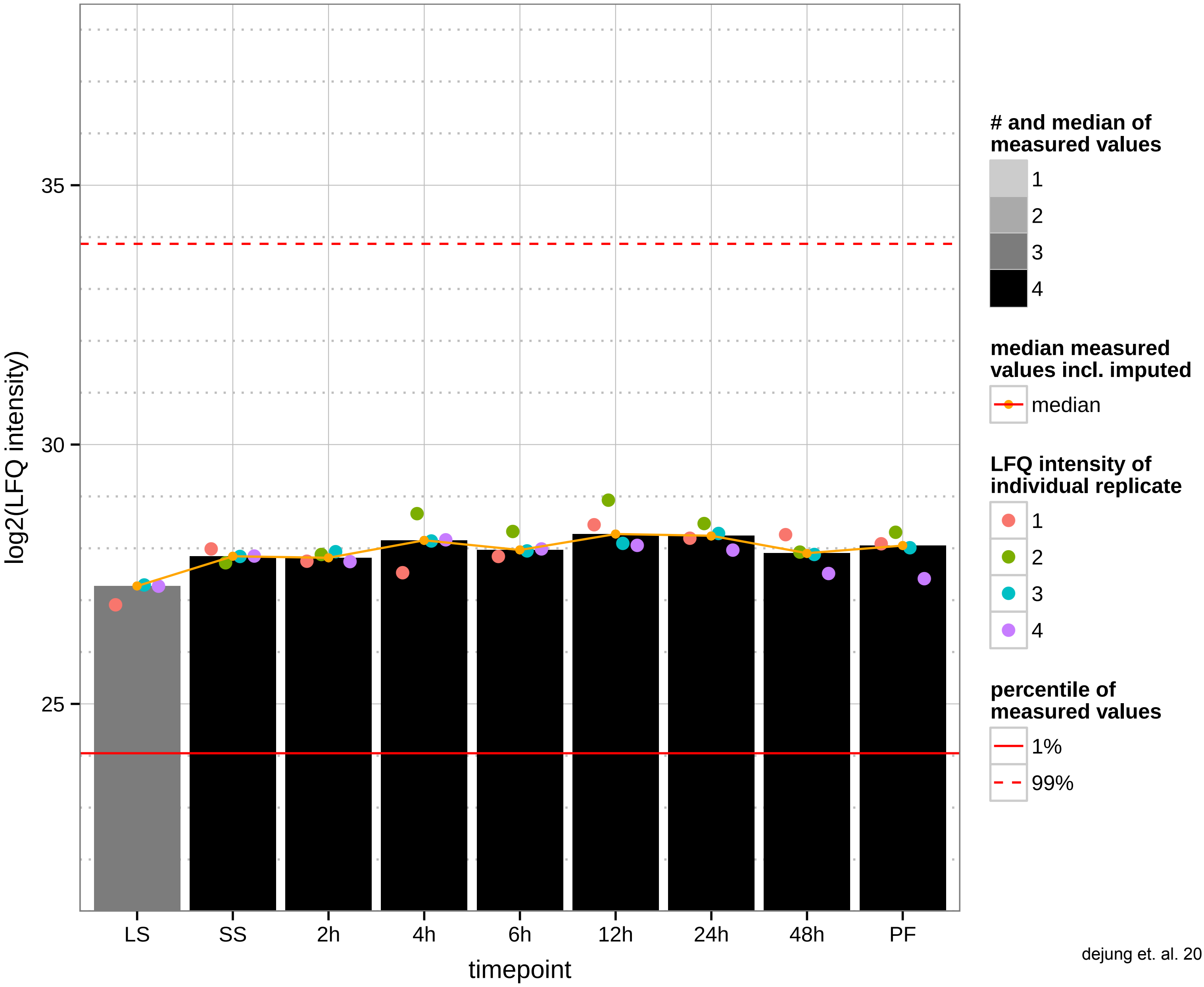

dejung et. al. 2015

hypothetical protein, conserved
Tb927.11.16350
AGOF: null
AGOC: null
AGOP: null
PGOF: null
PGOC: membrane
PGOP: intracellular signal transduction

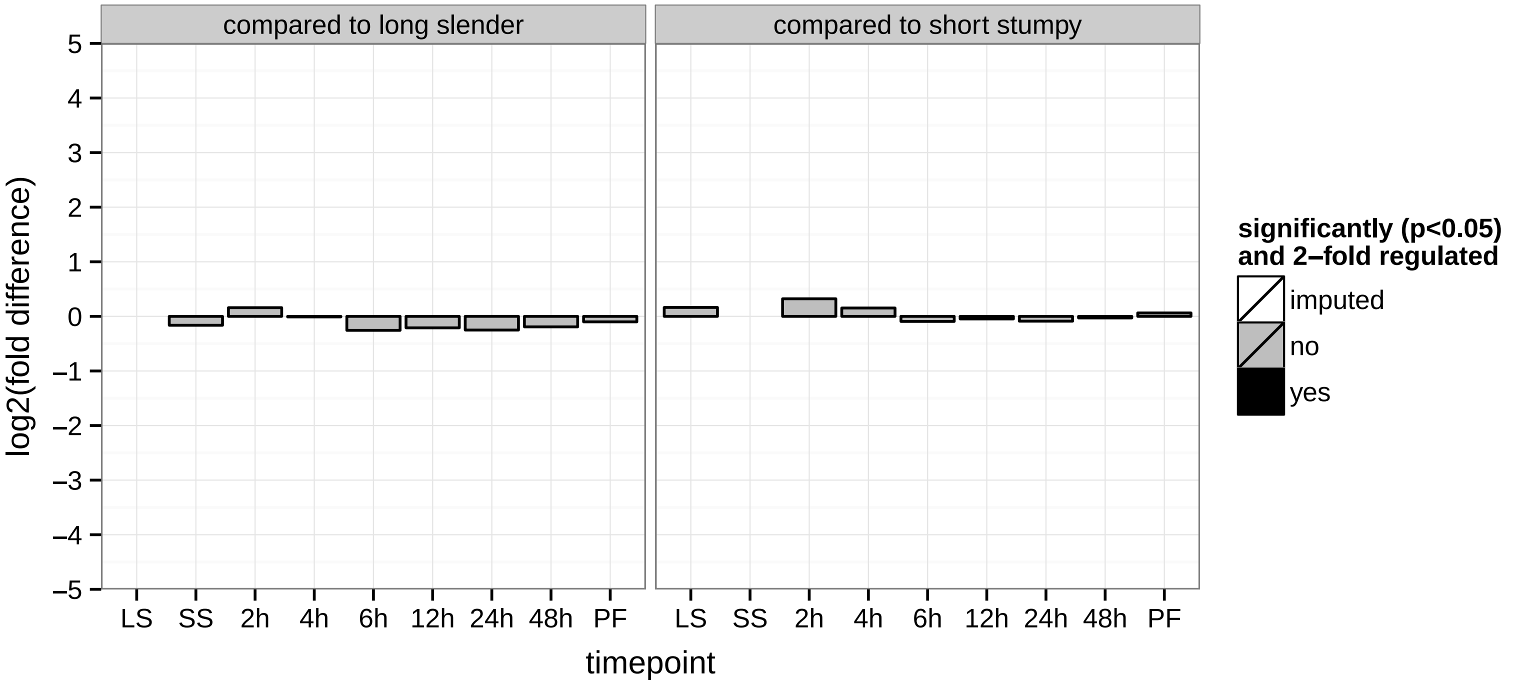

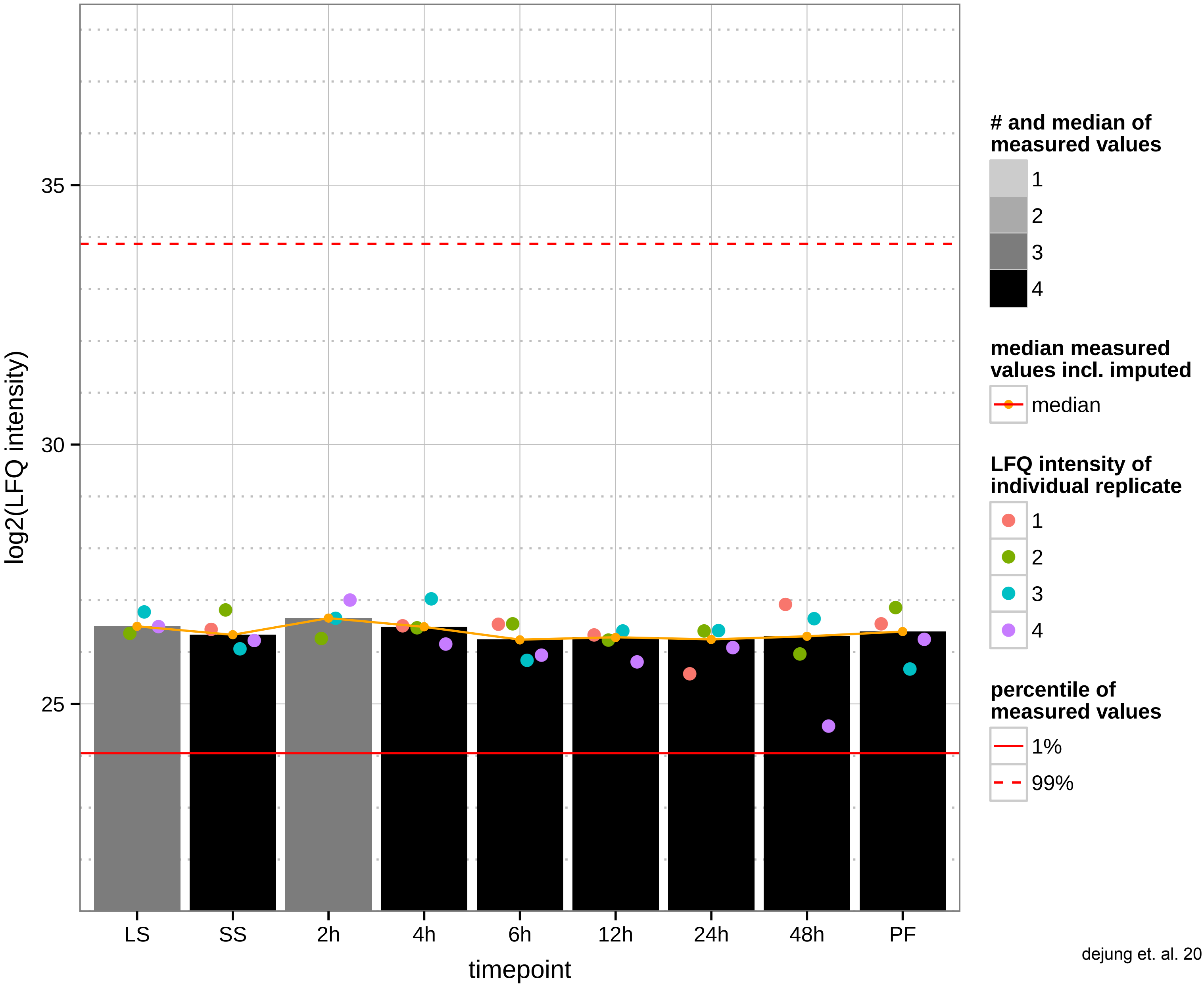

dejung et. al. 2015

hypothetical protein, conserved
Tb927.11.16370
AGOF: null
AGOC: null
AGOP: null
PGOF: null
PGOC: null
PGOP: null

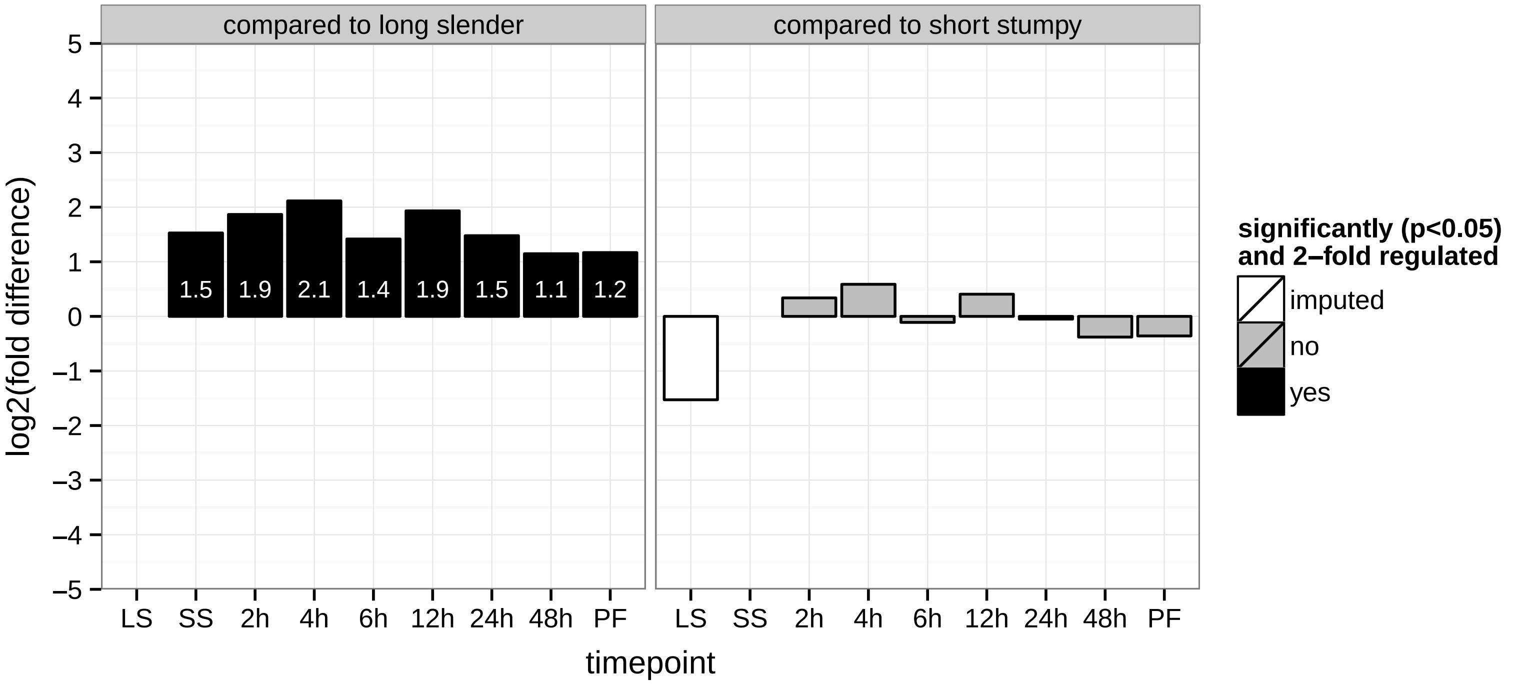

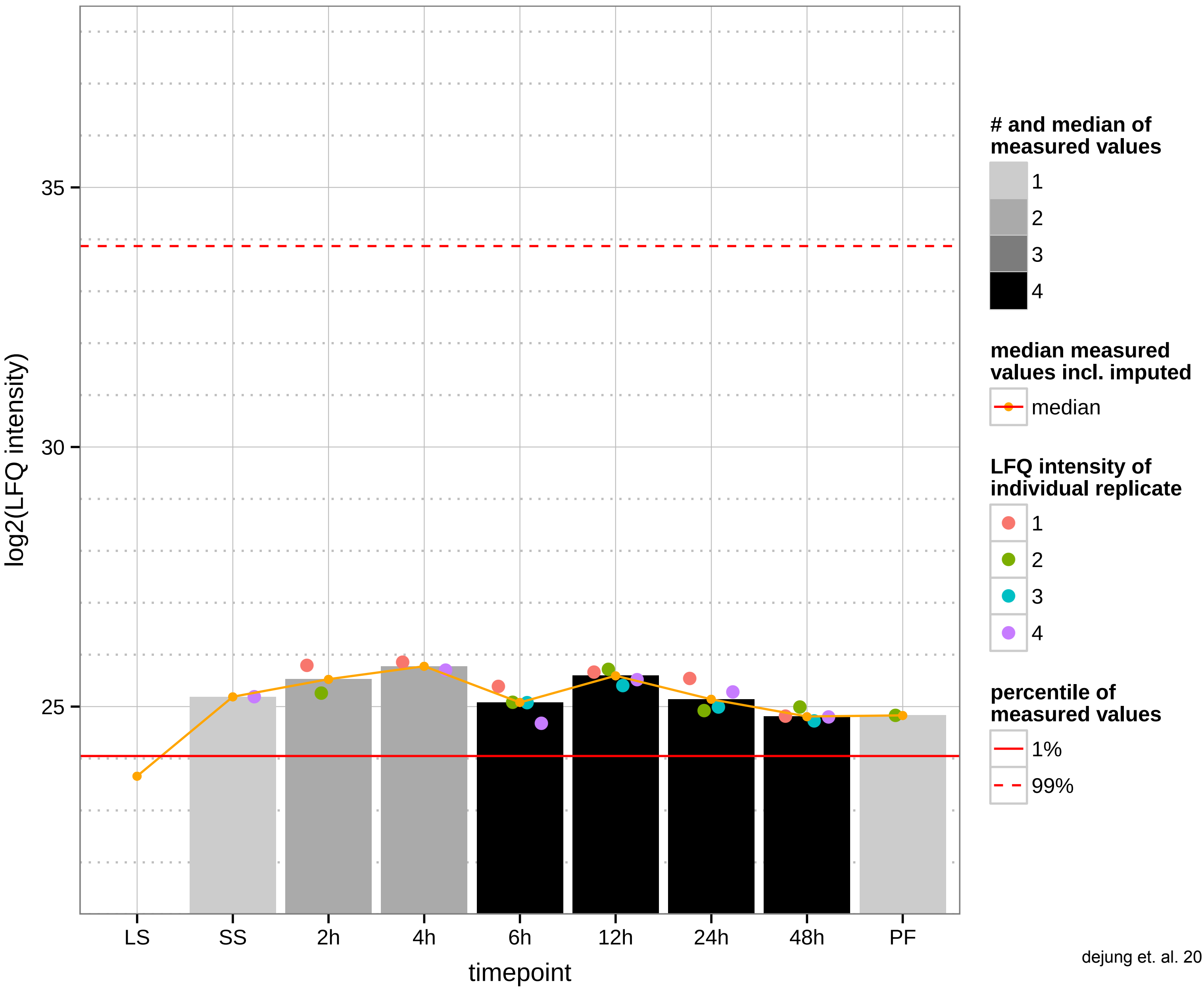

dejung et. al. 2015

hypothetical protein, conserved
Tb927.11.16380
AGOF: null
AGOC: mitochondrion
AGOP: protein folding
PGOF: protein binding
PGOC: null
PGOP: null

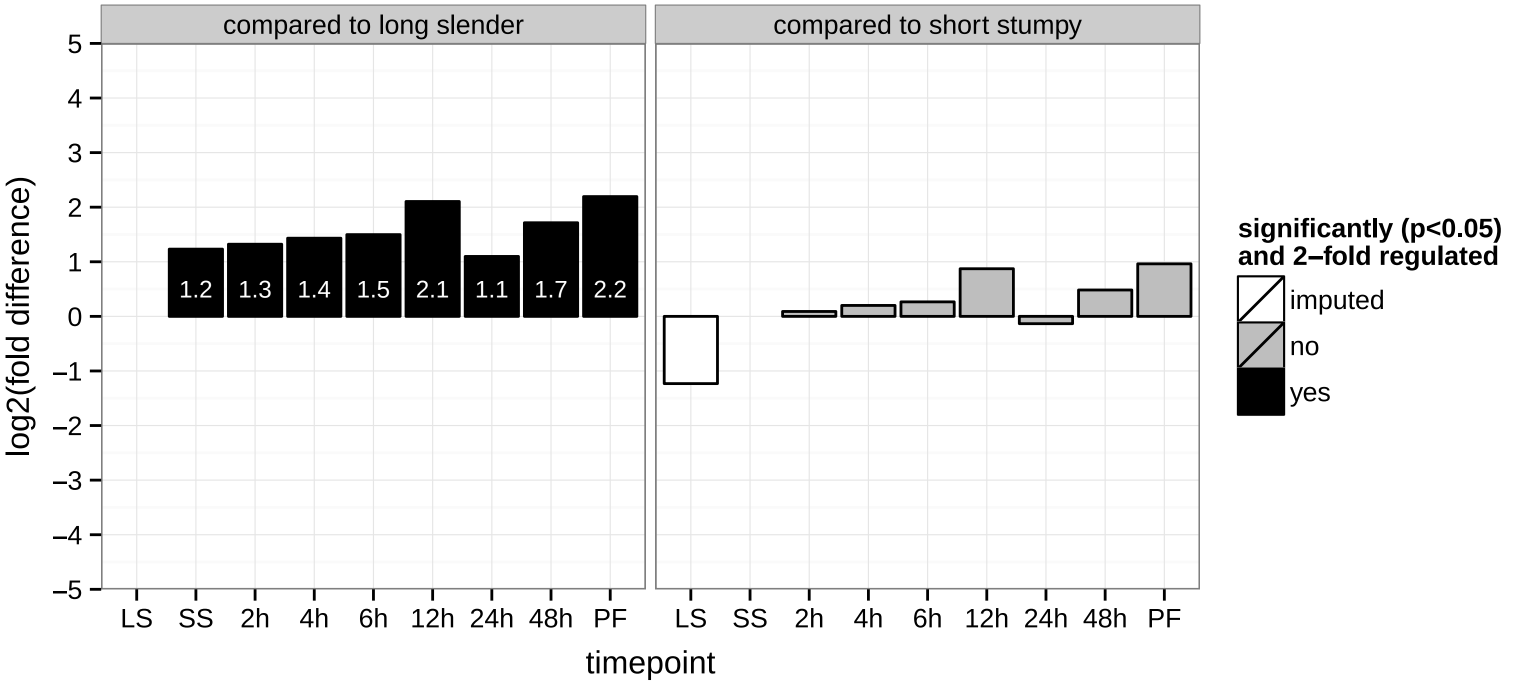

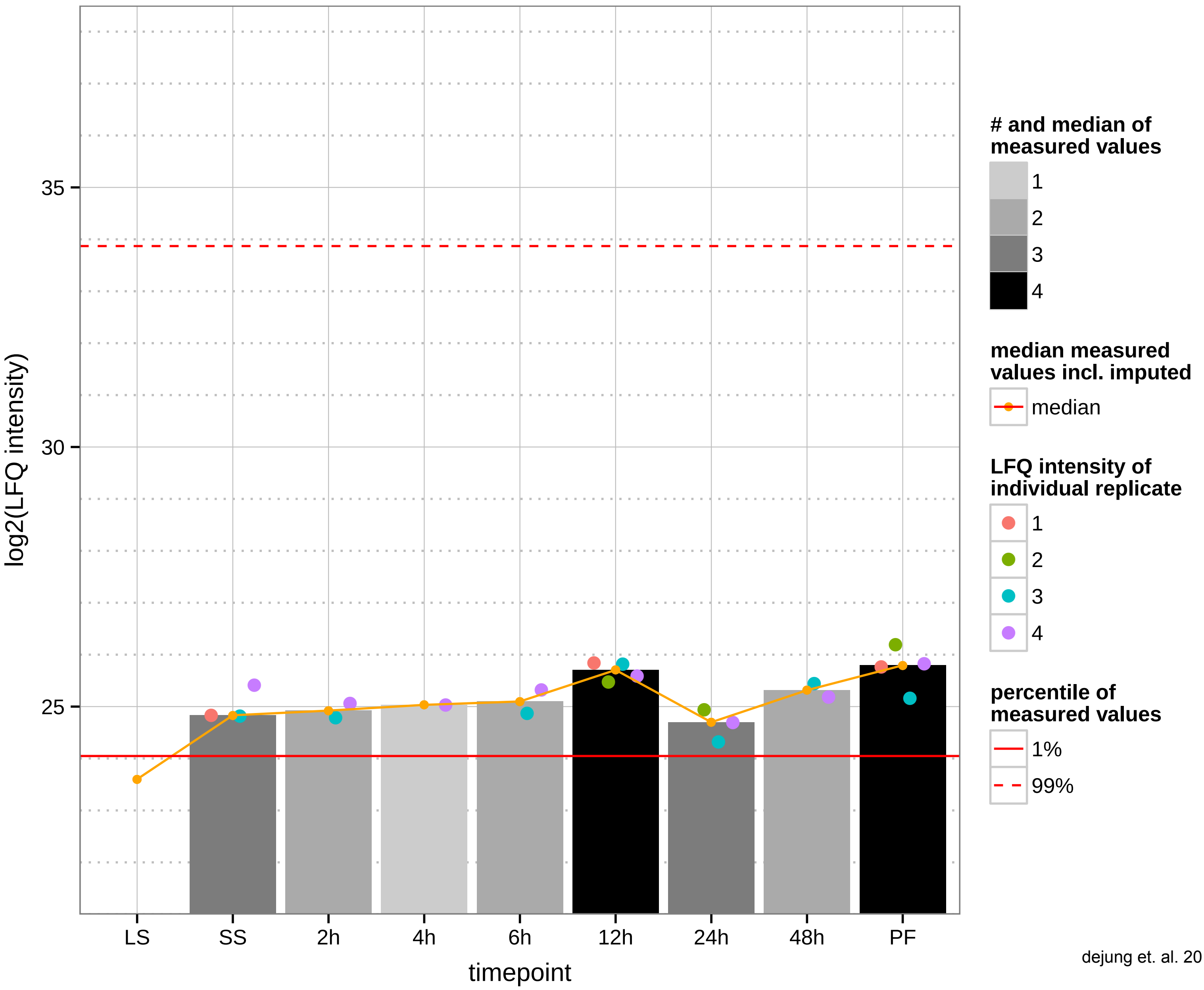

dejung et. al. 2015

kinetoplast–associated protein 3, putative (KAP3)
Tb927.11.16400
AGOF: DNA binding
AGOC: kinetoplast, mitochondrial matrix, mitochondrion
AGOP: DNA metabolic process, DNA packaging
PGOF: null
PGOC: null
PGOP: null

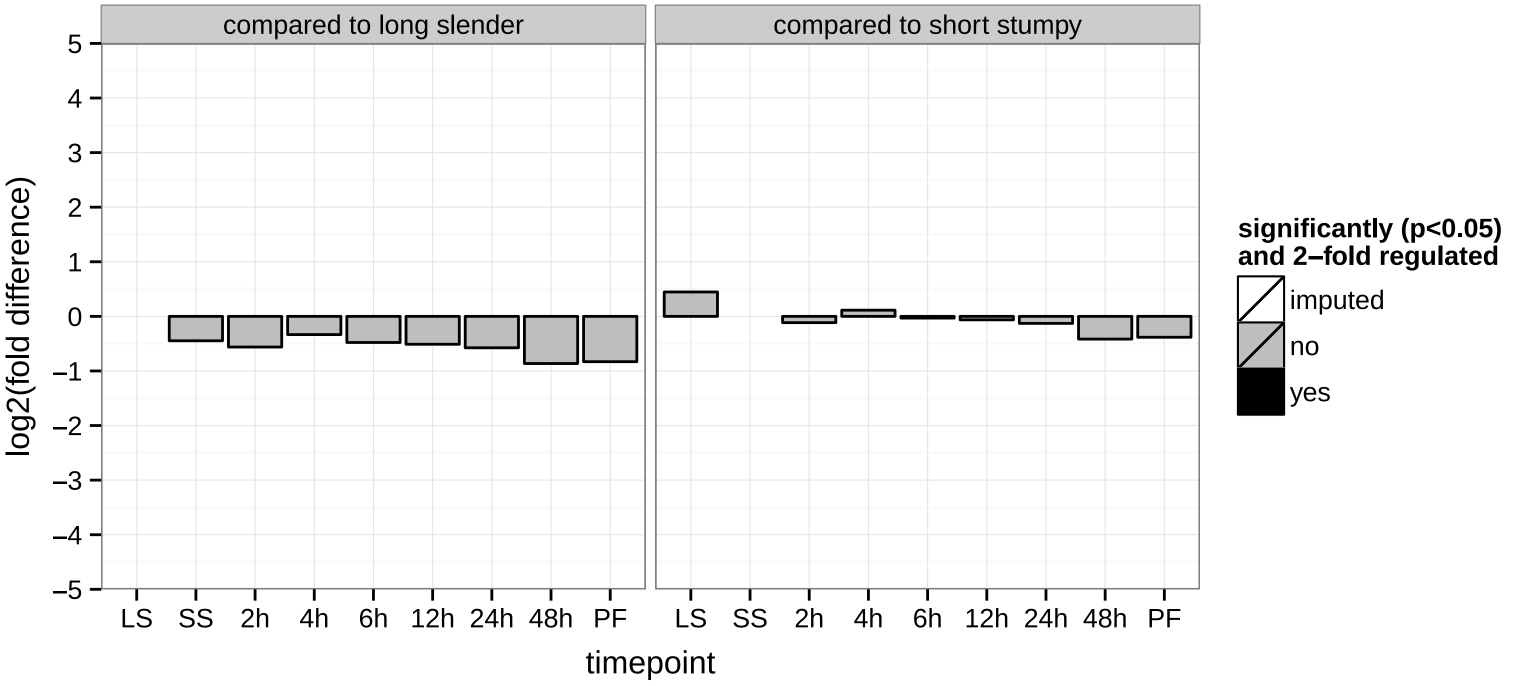

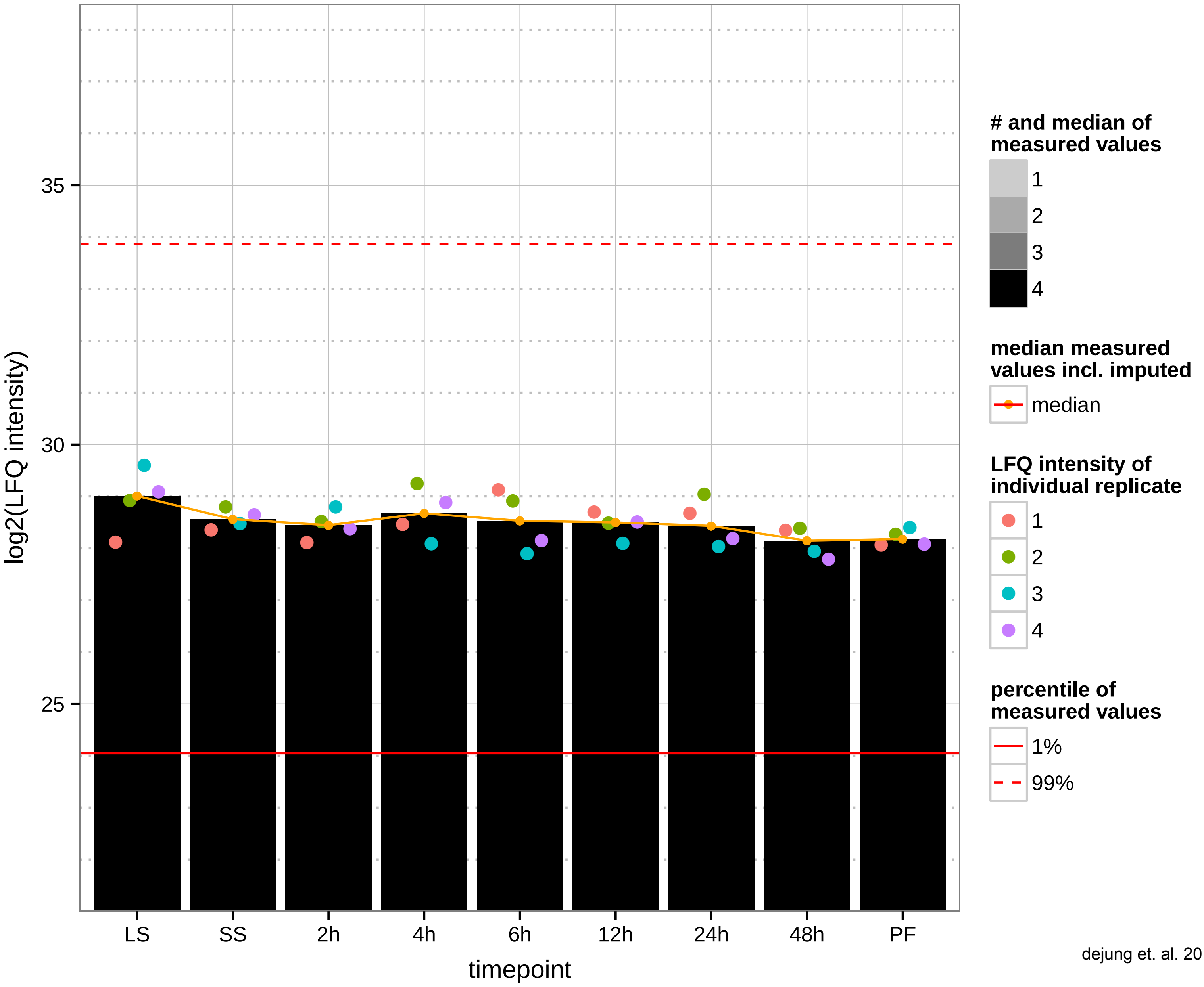

dejung et. al. 2015

hypothetical protein, conserved
Tb927.11.16460
AGOF: null
AGOC: null
AGOP: null
PGOF: null
PGOC: null
PGOP: null

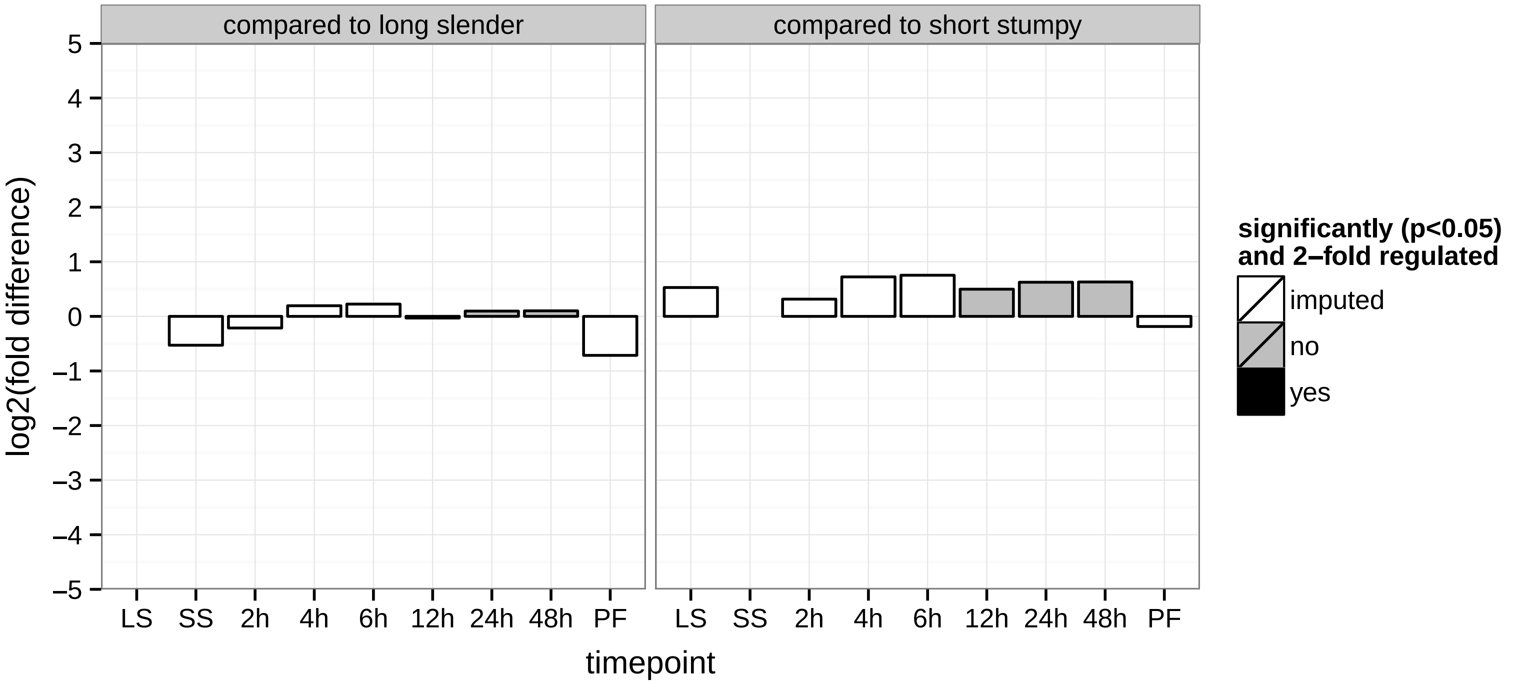

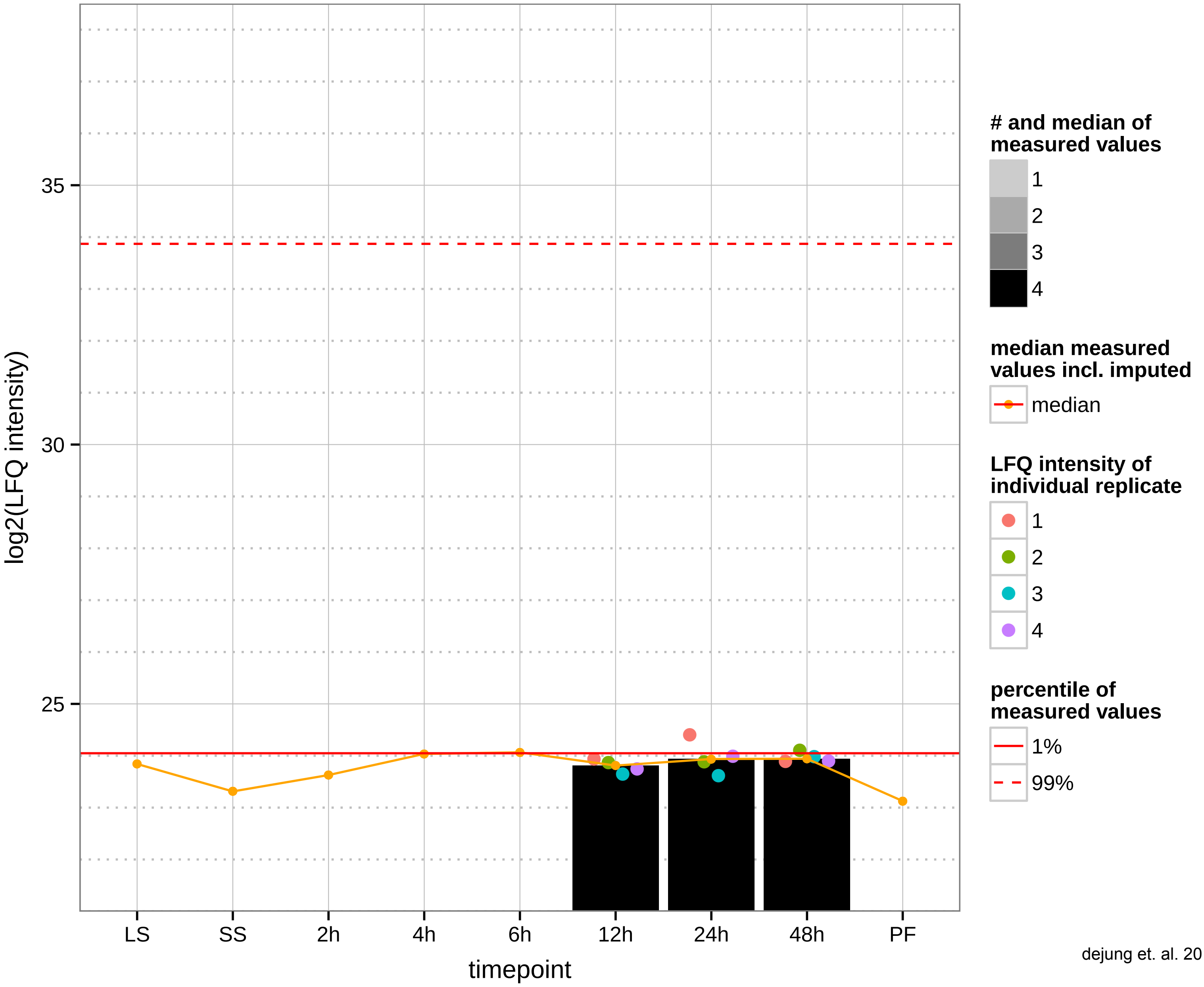

dejung et. al. 2015

hypothetical protein, conserved
Tb927.11.16470
AGOF: null
AGOC: null
AGOP: null
PGOF: null
PGOC: null
PGOP: null

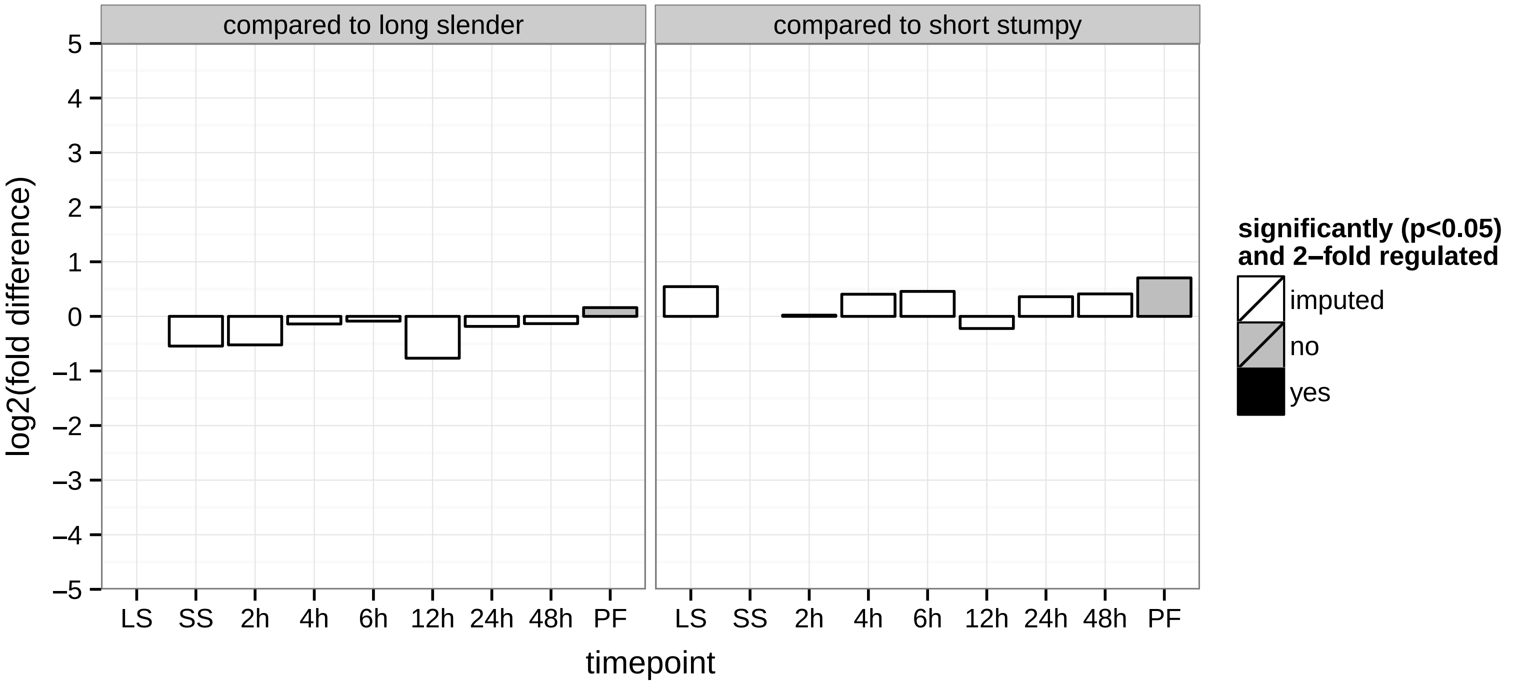

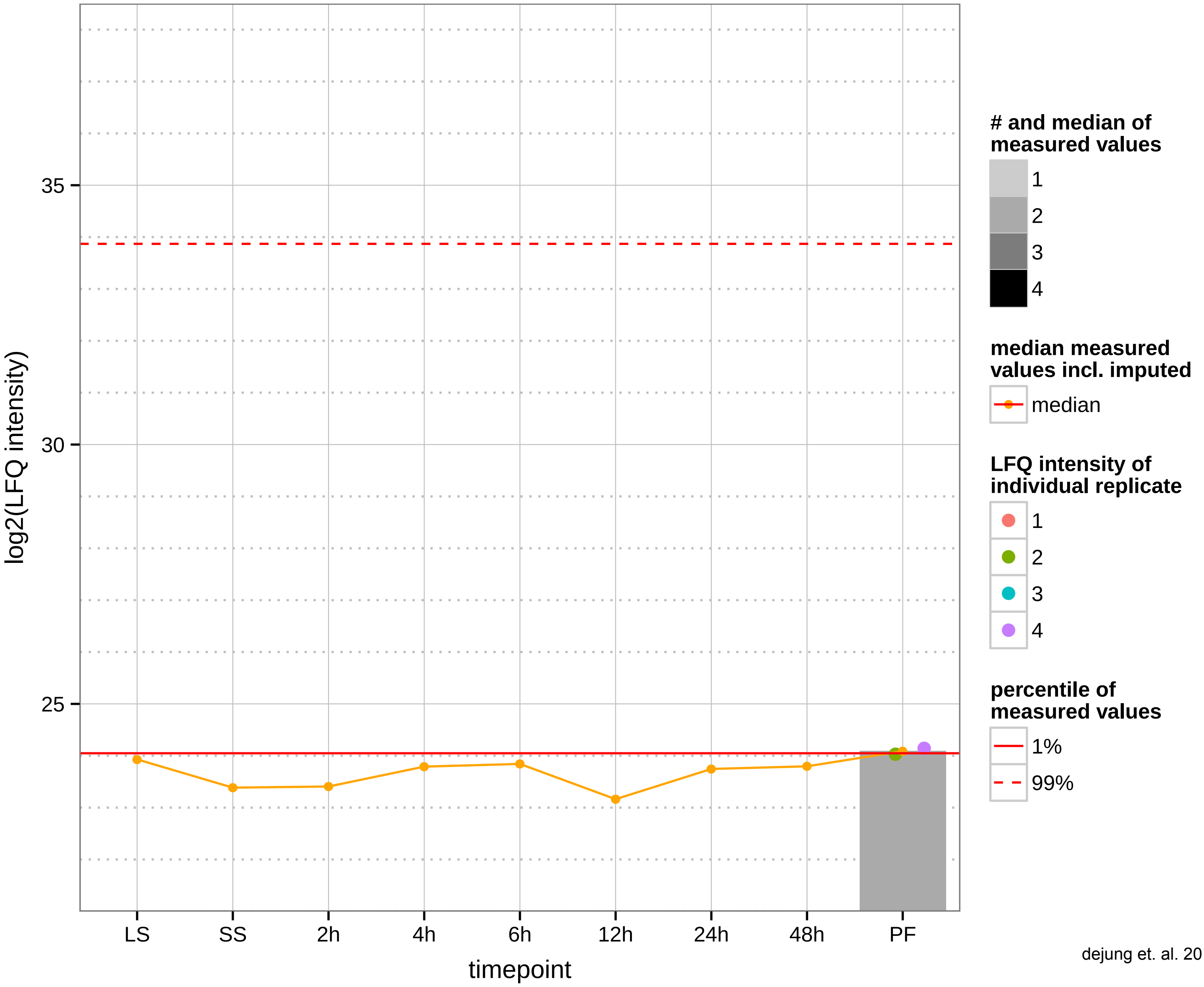

dejung et. al. 2015

vesicular transport protein (CDC48 homologue), putative
Tb927.11.1660
AGOF: ATP binding, ATPase activity
AGOC: null
AGOP: regulation of transcription, DNA–dependent, two–component signal transduction system (phosphorelay)
PGOF: ATP binding, nucleoside–triphosphatase activity, nucleotide binding
PGOC: null
PGOP: null

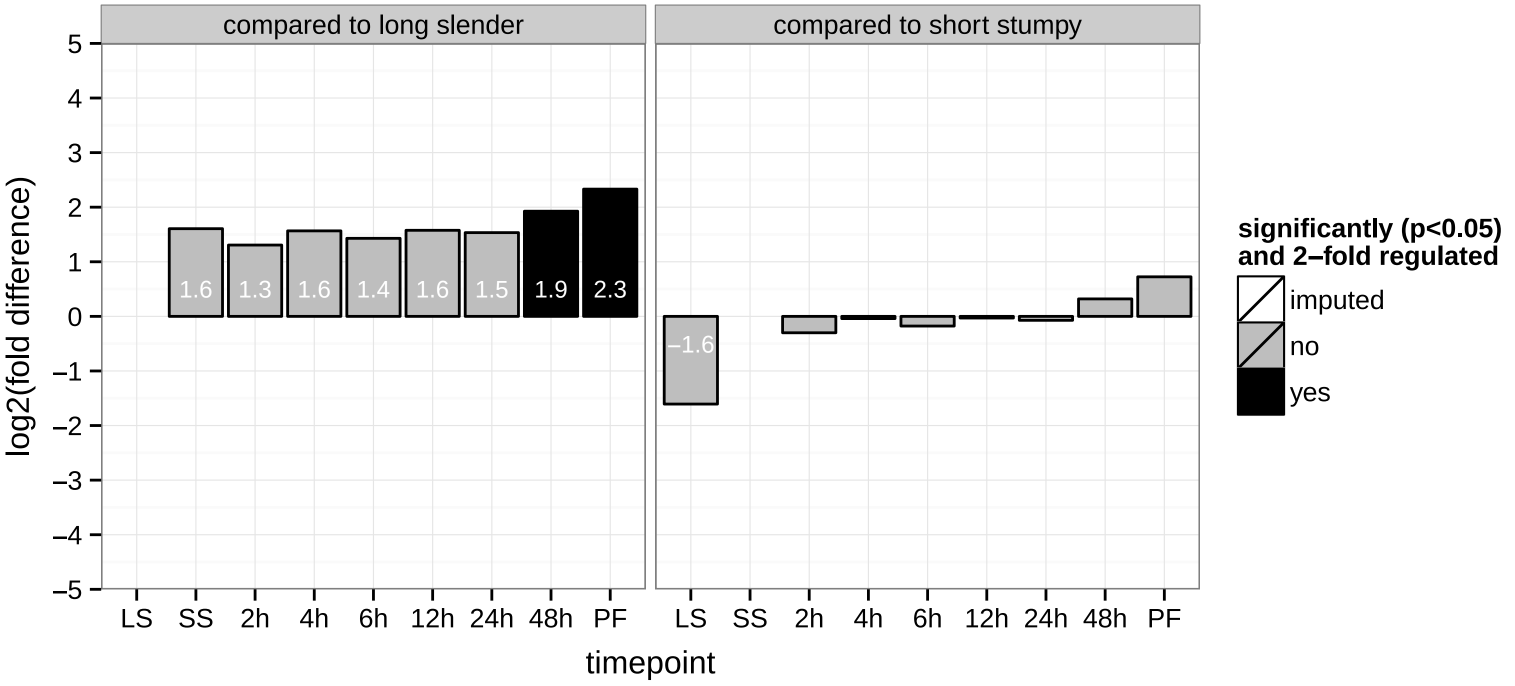

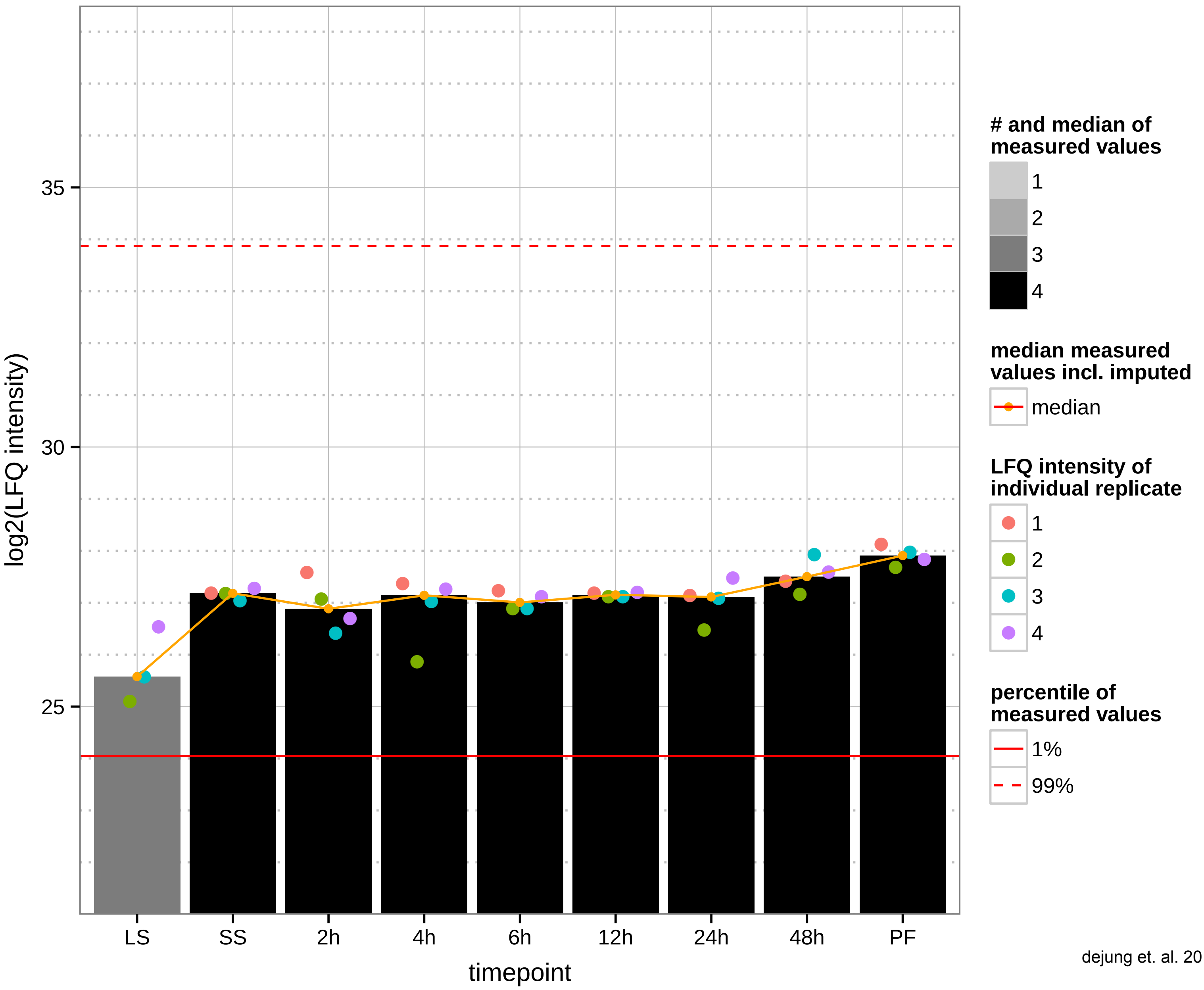

dejung et. al. 2015

exosome–associated protein 2 (EAP2)
Tb927.11.16600
AGOF: 3'–5'–exoribonuclease activity, RNA binding
AGOC: cytoplasmic exosome (RNase complex), nuclear exosome (RNase complex)
AGOP: RNA processing, rRNA processing
PGOF: null
PGOC: null
PGOP: null

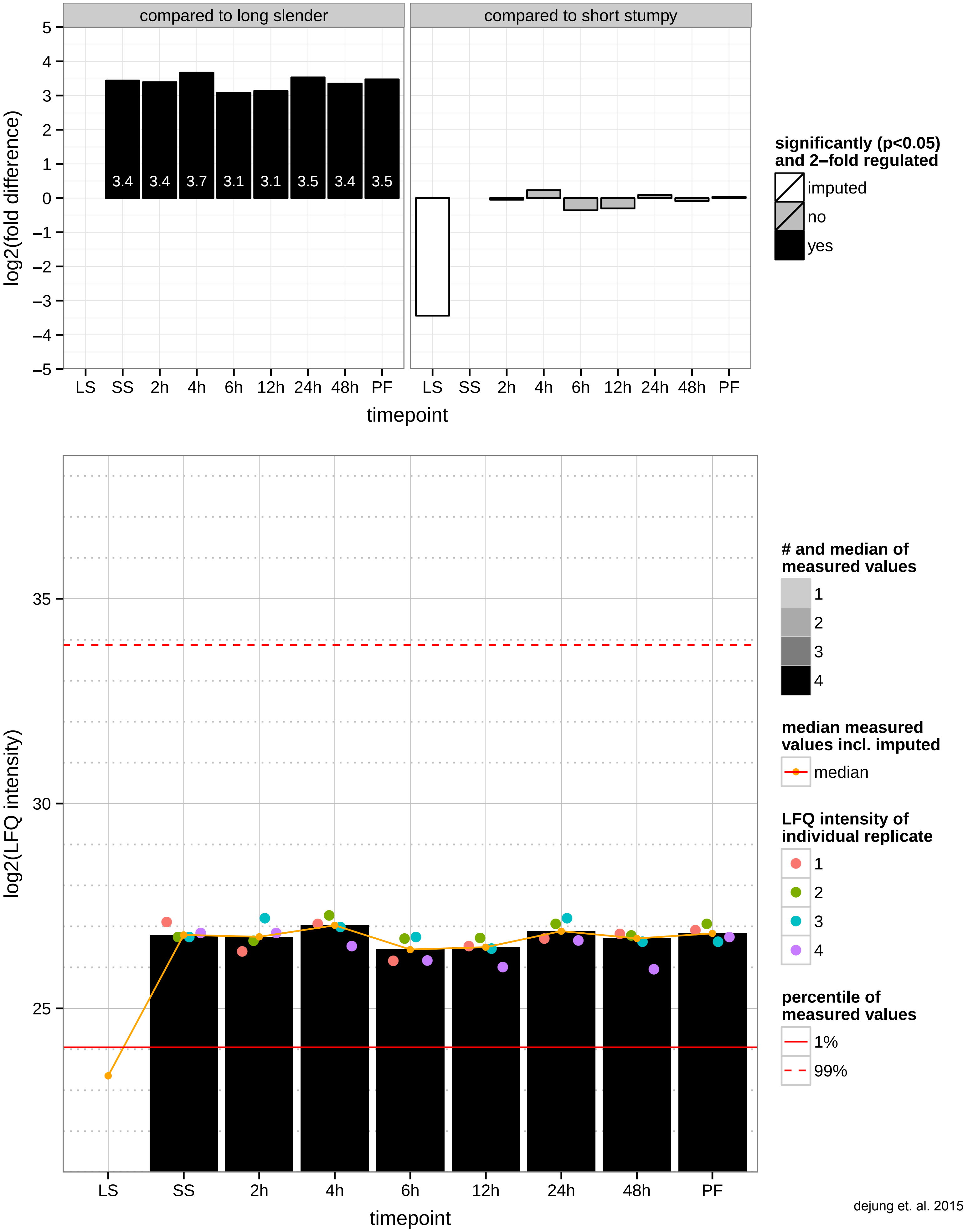

dejung et. al. 2015

hypothetical protein, conserved
Tb927.11.16630
AGOF: null
AGOC: integral to membrane
AGOP: null
PGOF: null
PGOC: integral to membrane
PGOP: transmembrane transport

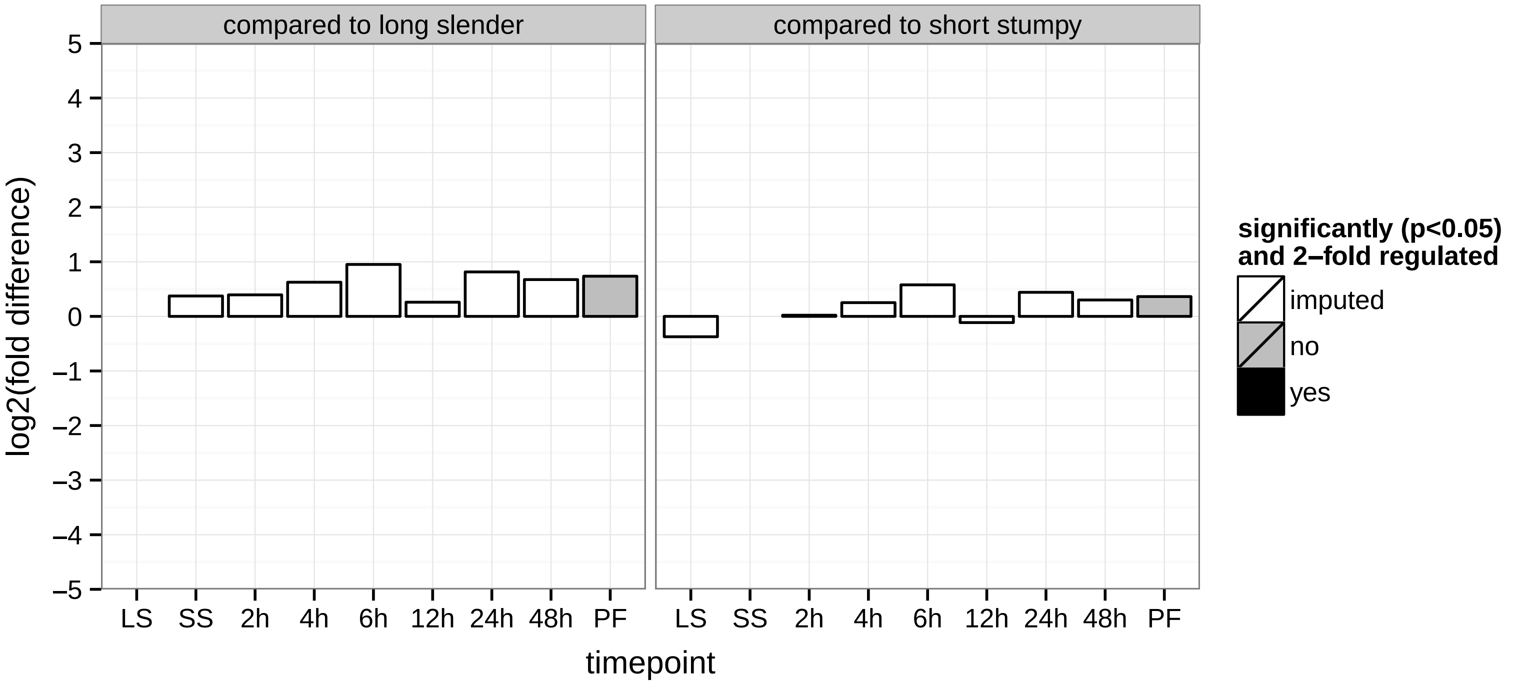

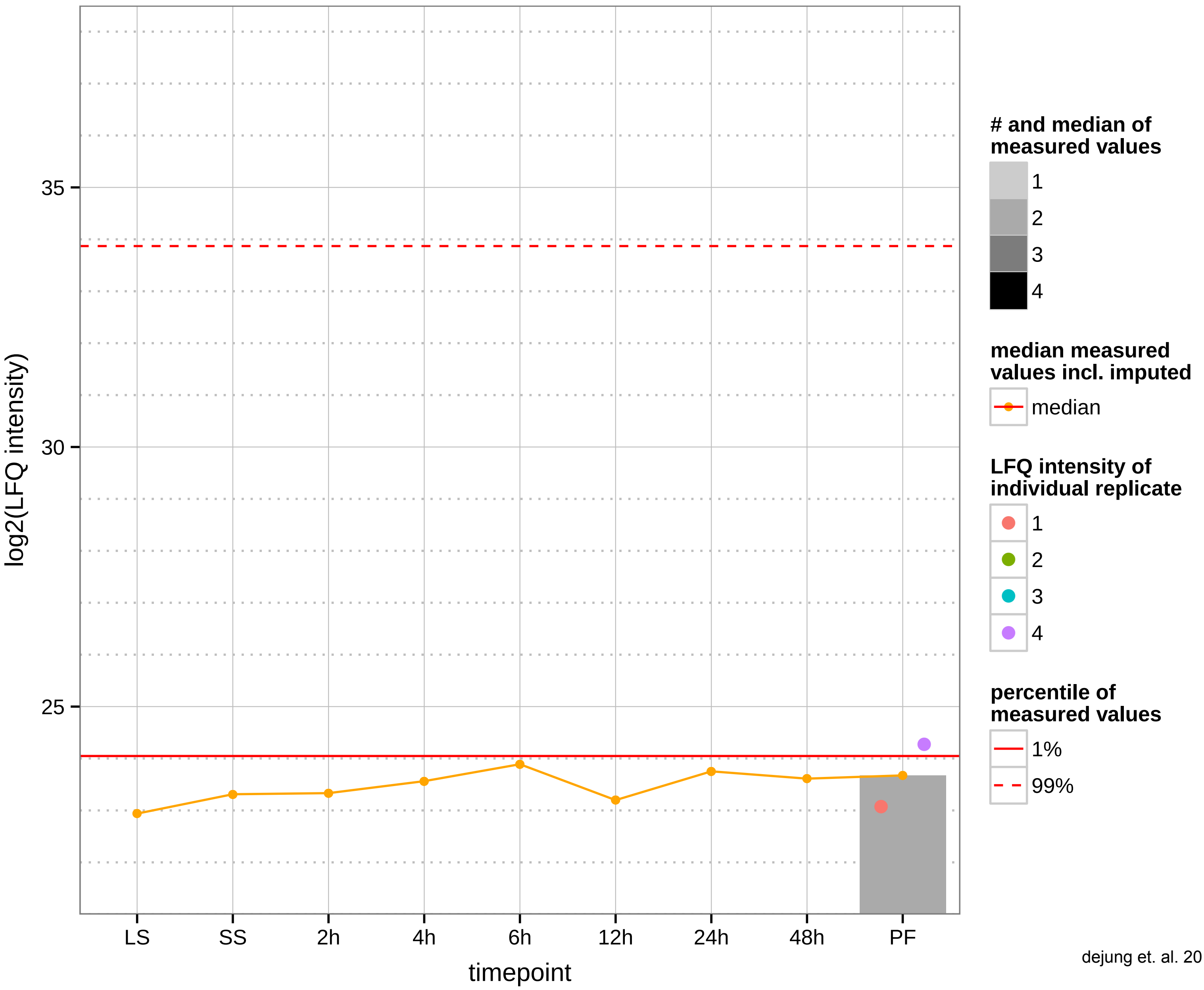

dejung et. al. 2015

hypothetical protein, conserved
Tb927.11.16660
AGOF: null
AGOC: null
AGOP: null
PGOF: binding
PGOC: null
PGOP: null

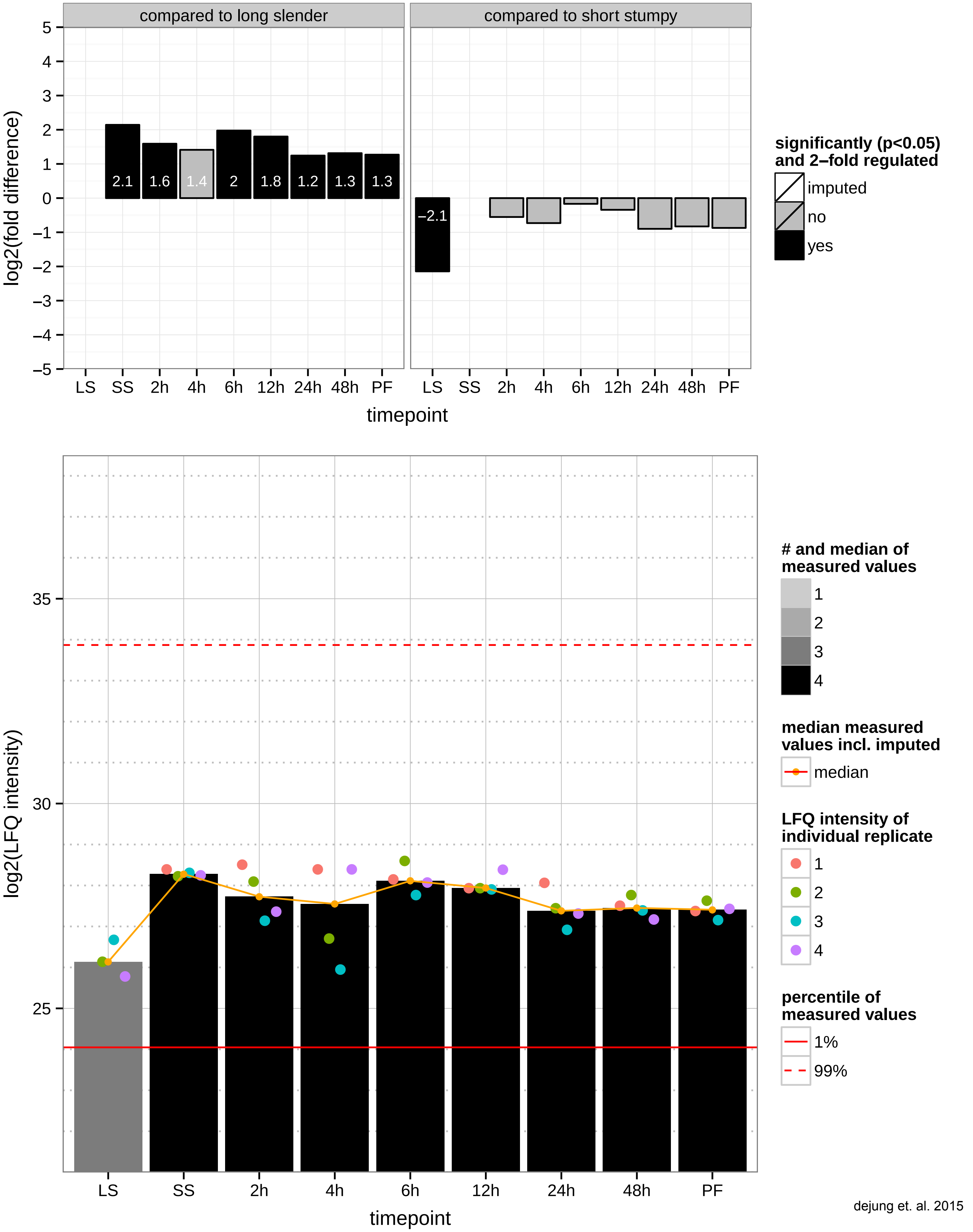

dejung et. al. 2015

cysteine desulfurase
Tb927.11.1670
AGOF: cysteine desulfurase activity, transaminase activity
AGOC: mitochondrion
AGOP: iron-sulfur cluster assembly, protein complex assembly, sulfur amino acid metabolic process, tRNA wobble position ur
PGOF: null
PGOC: null
PGOP: metabolic process

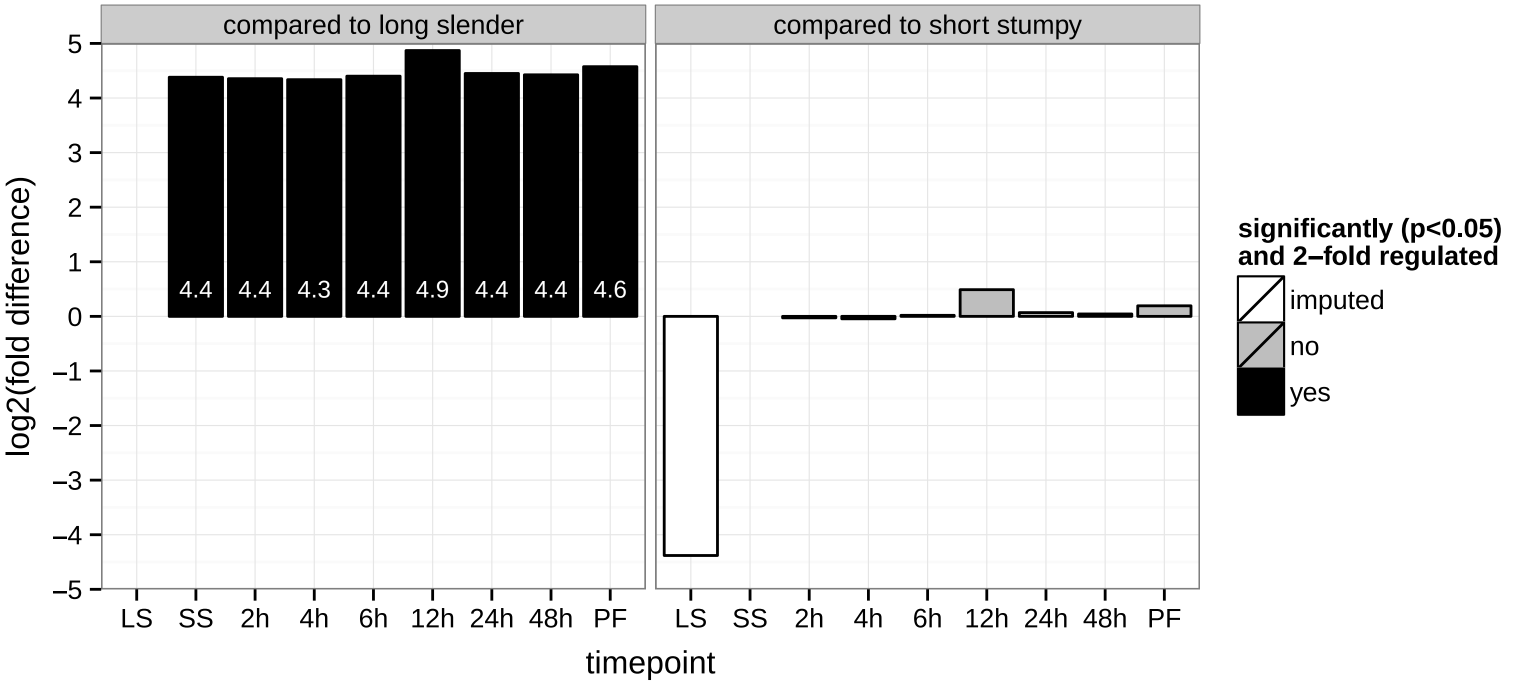

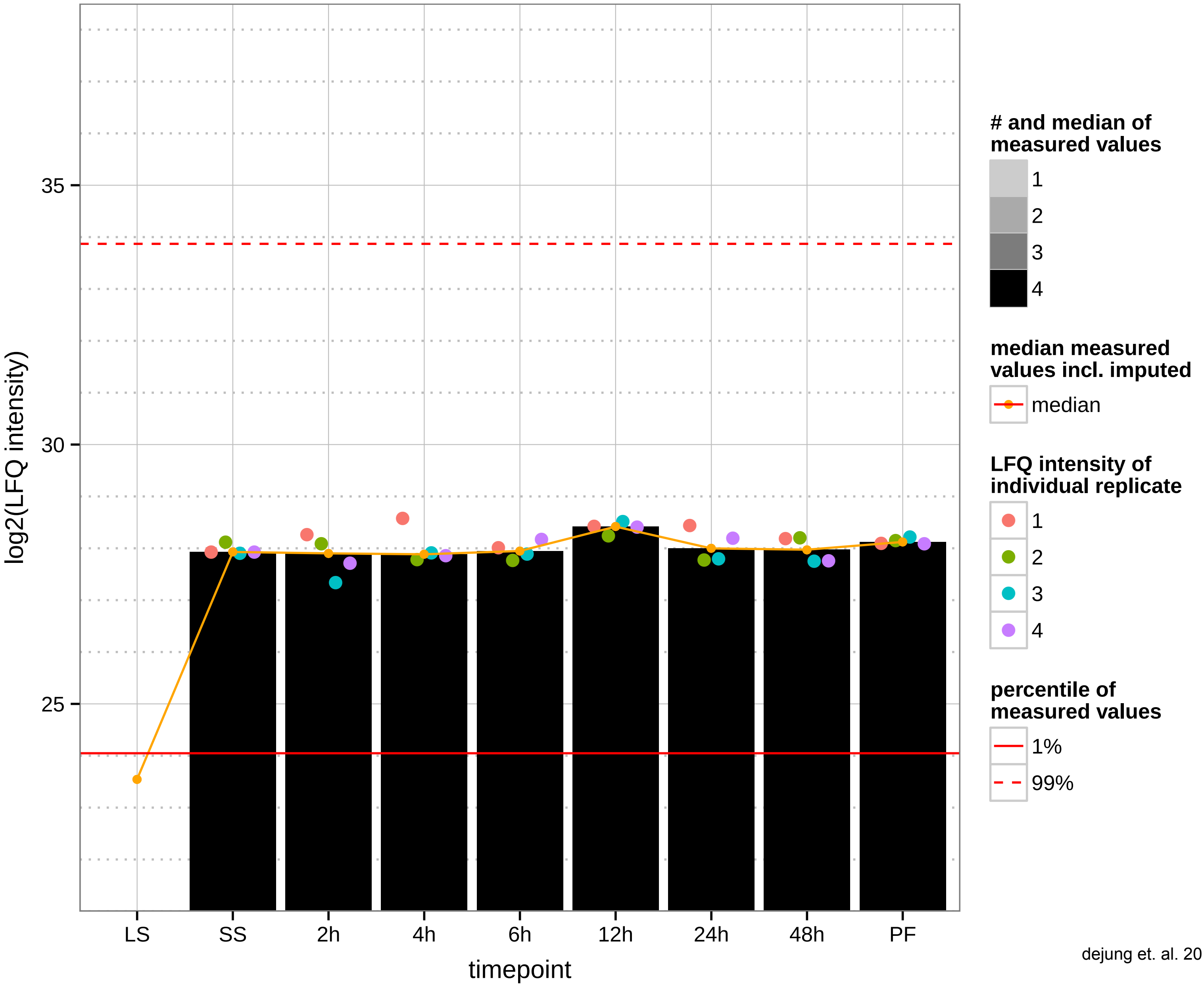

dejung et. al. 2015

hypothetical protein, conserved
Tb927.11.16700
AGOF: null
AGOC: null
AGOP: null
PGOF: null
PGOC: null
PGOP: null

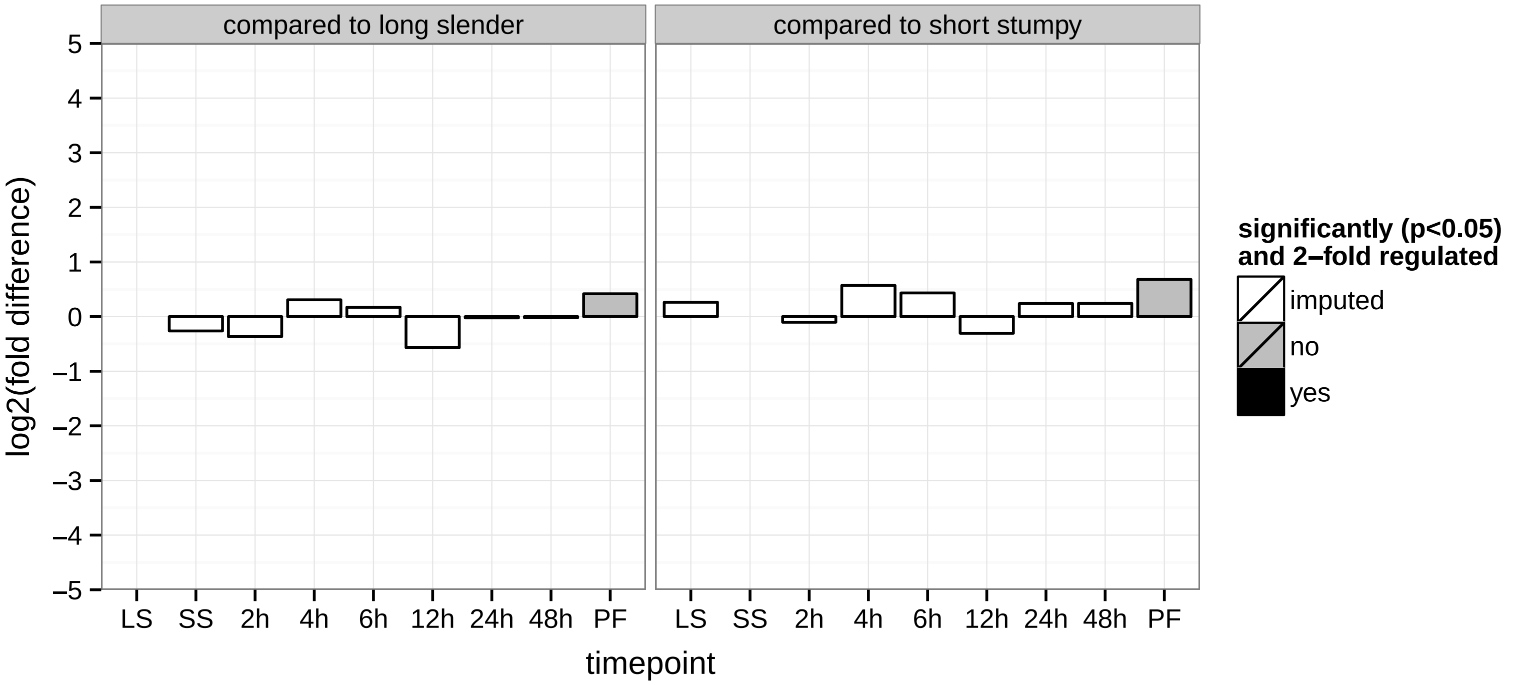

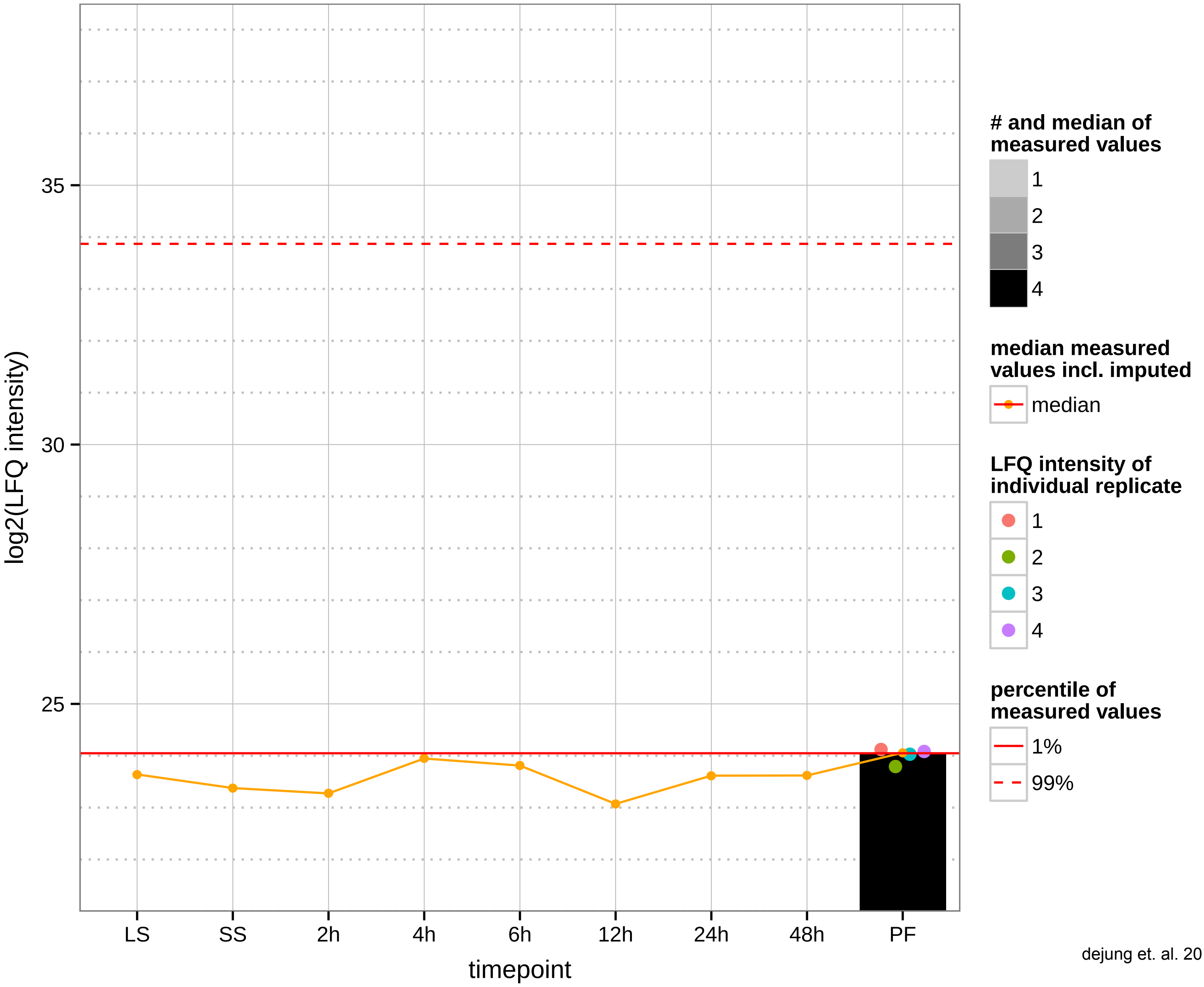

dejung et. al. 2015

dihydrolipoyl dehydrogenase (GCVL−2)
Tb927.11.16730
AGOF: dihydrolipoyl dehydrogenase activity, disulfide oxidoreductase activity, flavin adenine dinucleotide binding
AGOC: cytoplasm, mitochondrial inner membrane, mitochondrion
AGOP: cell redox homeostasis, oxidation−reduction process
PGOF: dihydrolipoyl dehydrogenase activity, flavin adenine dinucleotide binding, oxidoreductase activity
PGOC: cytoplasm
PGOP: cell redox homeostasis, oxidation−reduction process

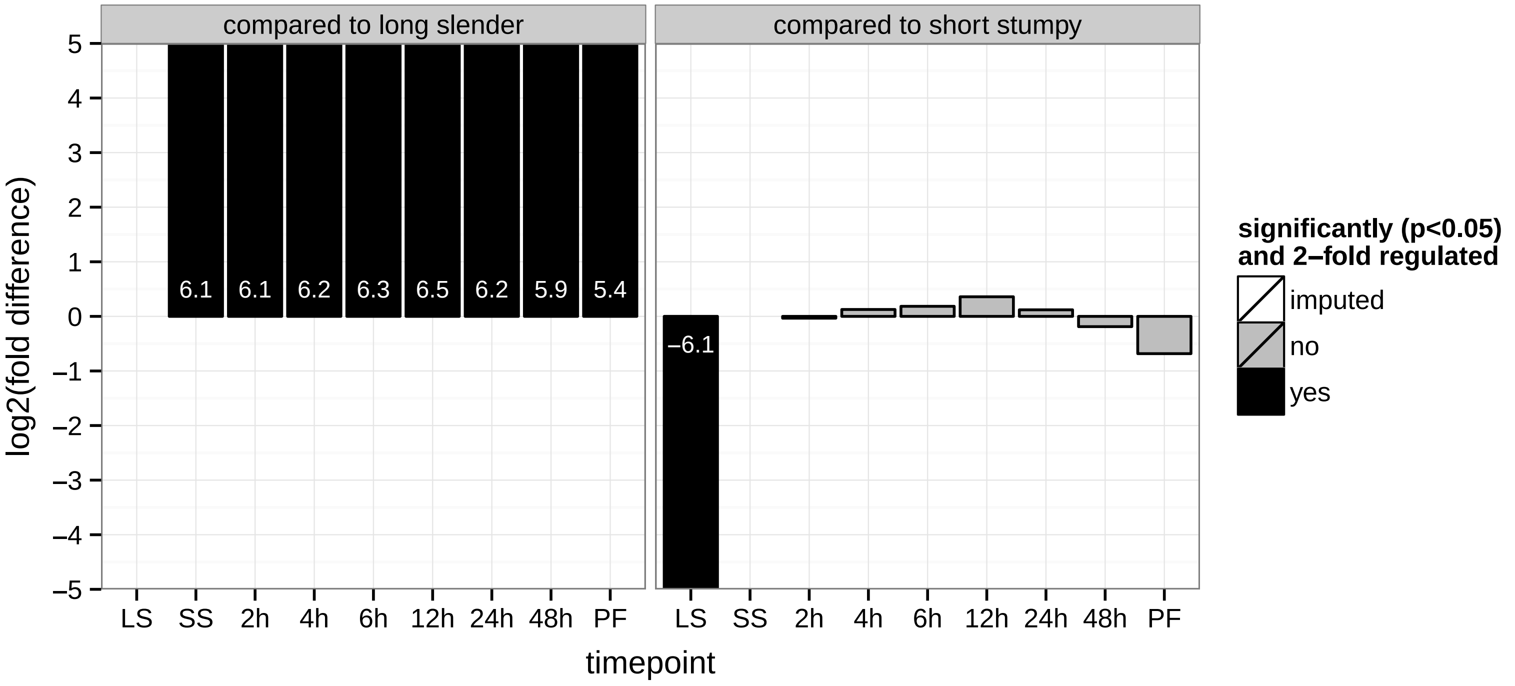

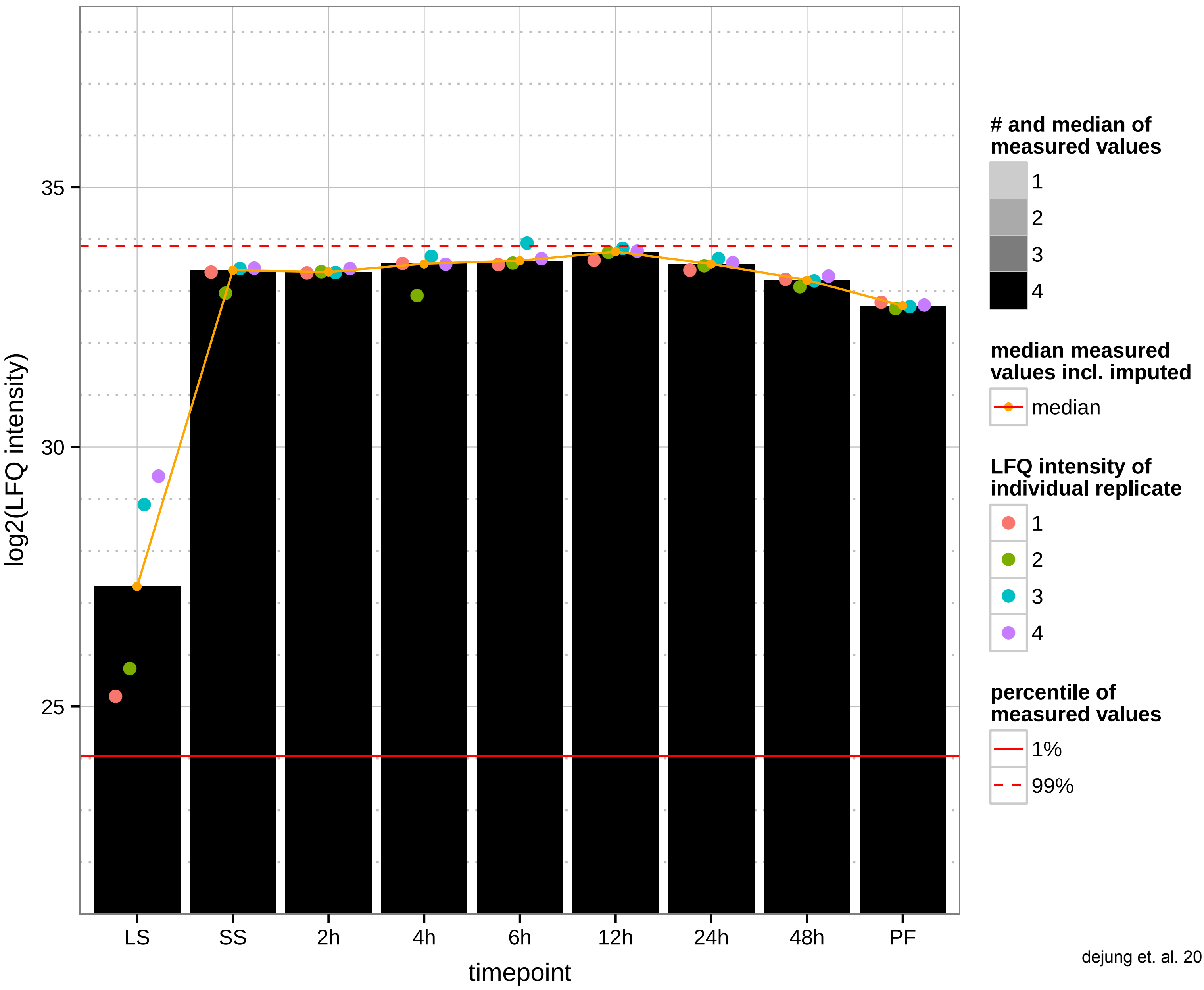

dejung et. al. 2015

chaperone protein DNAj, putative
Tb927.11.16740
AGOF: chaperone binding, heat shock protein binding, unfolded protein binding
AGOC: null
AGOP: protein folding
PGOF: heat shock protein binding, unfolded protein binding
PGOC: null
PGOP: protein folding

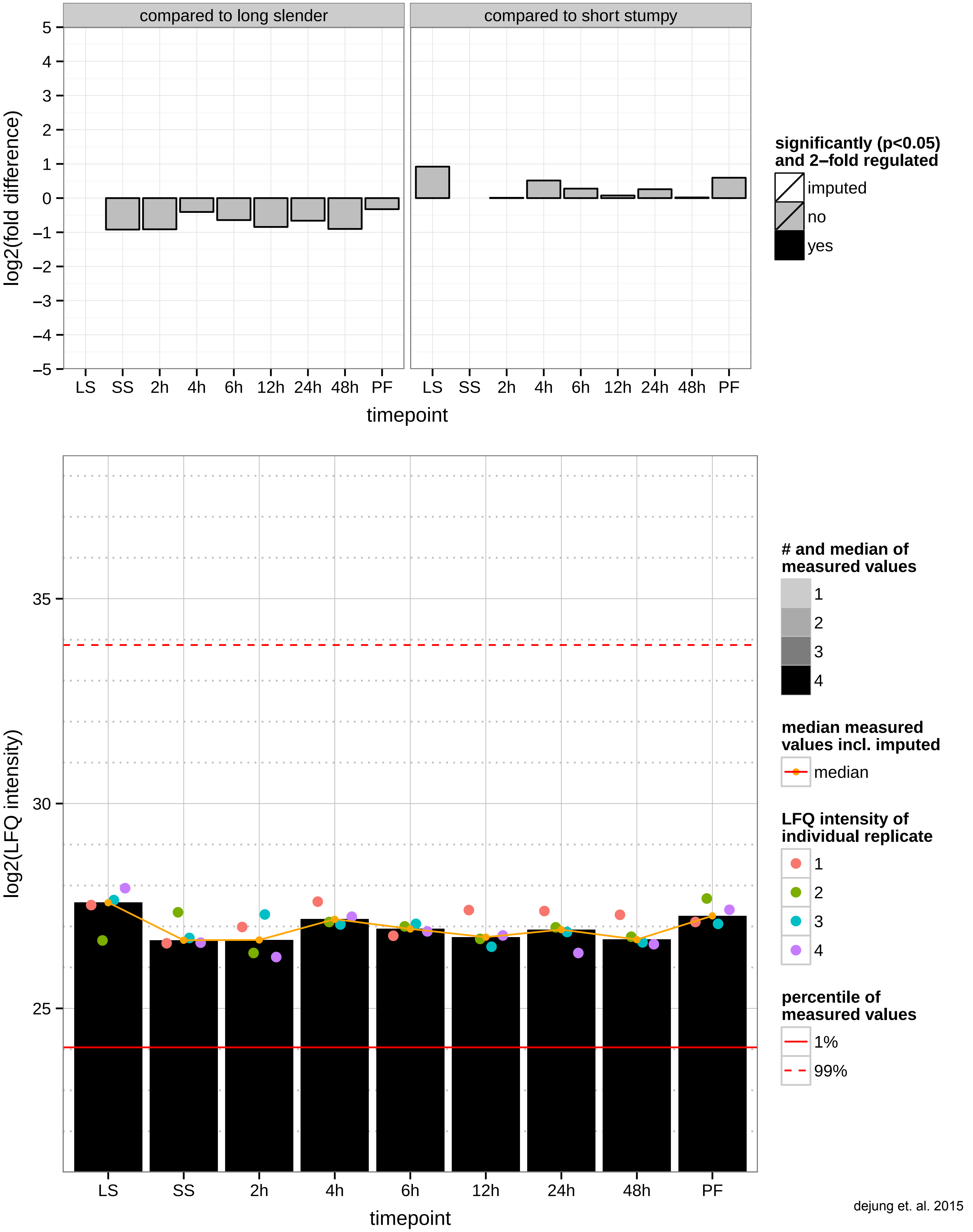

dejung et. al. 2015

T−complex protein 1, alpha subunit, putative (TCP−1−alpha)
Tb927.11.16760
AGOF: ATP binding, unfolded protein binding
AGOC: chaperonin−containing T−complex
AGOP: protein folding, regulation of cell cycle
PGOF: ATP binding
PGOC: null
PGOP: cellular protein metabolic process

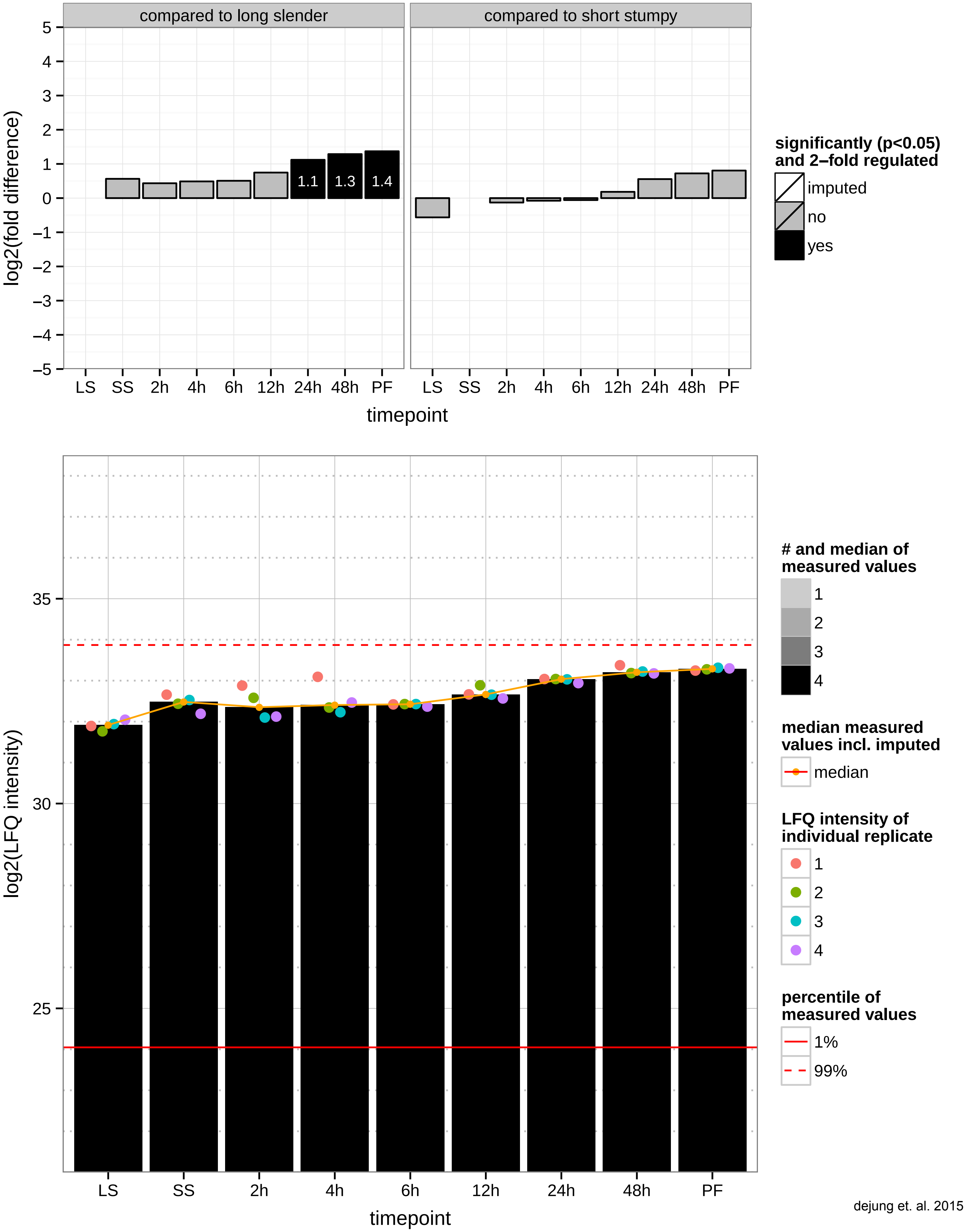

dejung et. al. 2015

glucosamine–6–phosphate isomerase, putative
Tb927.11.16770
AGOF: glucosamine–6–phosphate deaminase activity
AGOC: null
AGOP: N–acetylglucosamine catabolic process, carbohydrate metabolic process
PGOF: glucosamine–6–phosphate deaminase activity
PGOC: null
PGOP: N–acetylglucosamine metabolic process, carbohydrate metabolic process

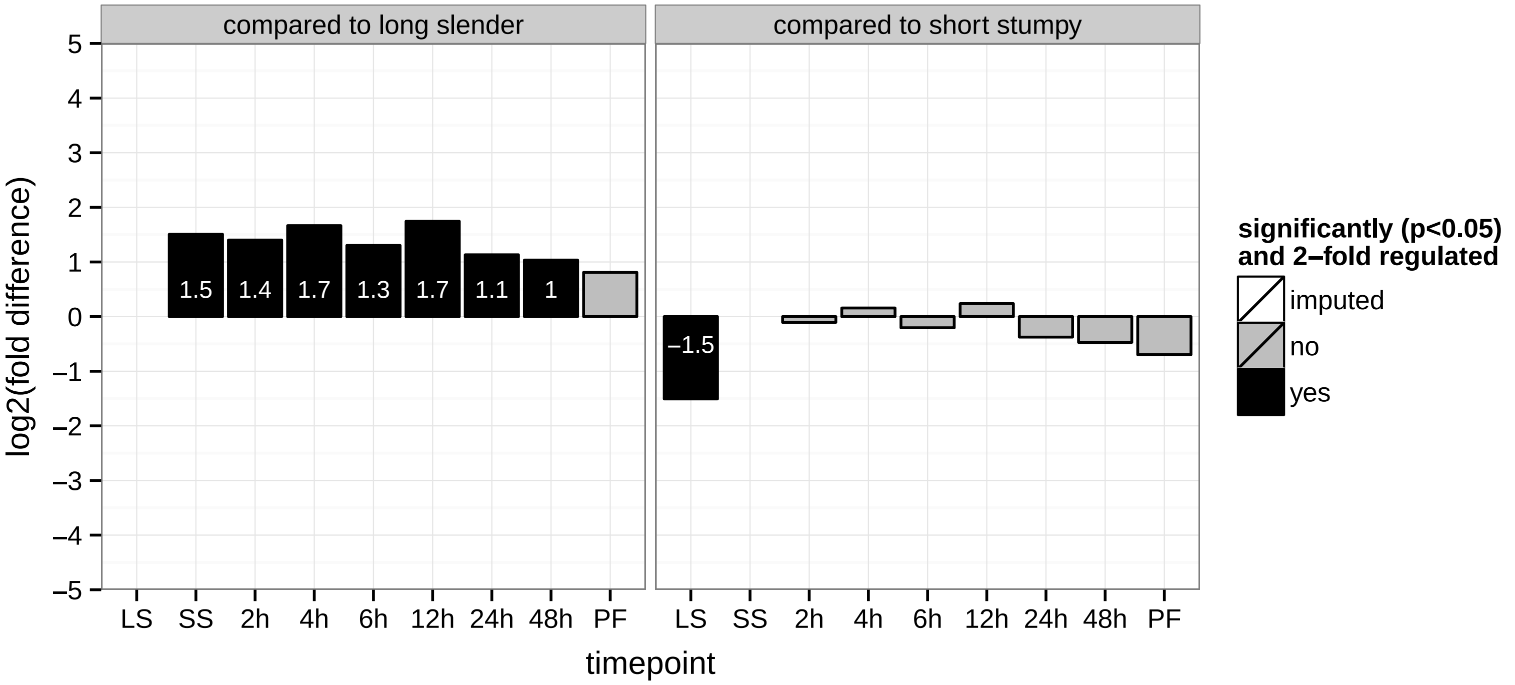

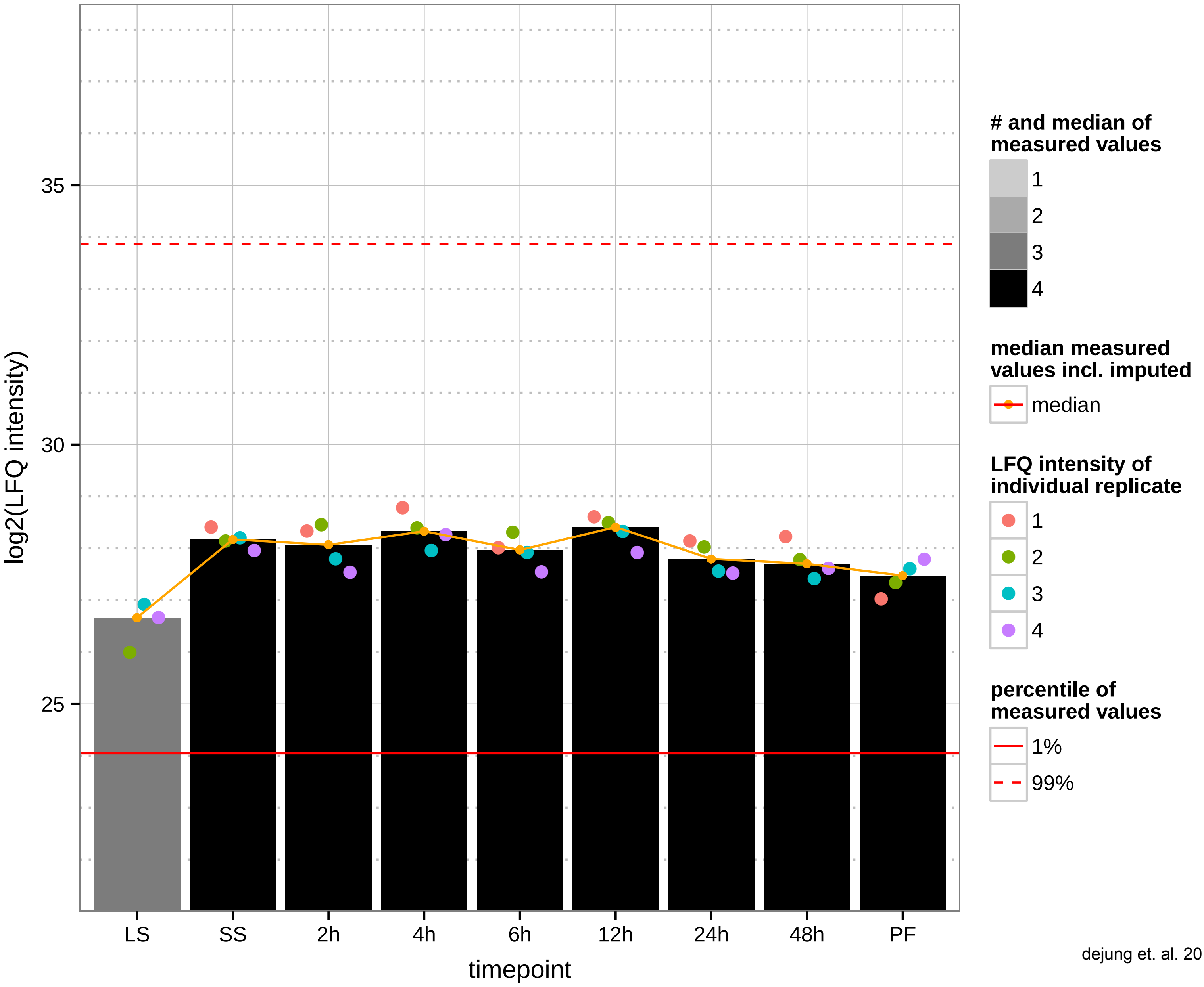

dejung et. al. 2015

mitogen–activated protein kinase (ECK1)
Tb927.11.16790
AGOF: ATP binding, protein kinase activity, protein serine/threonine kinase activity, protein tyrosine kinase activity
AGOC: null
AGOP: protein phosphorylation
PGOF: ATP binding, protein kinase activity, transferase activity, transferring phosphorus–containing groups
PGOC: null
PGOP: protein phosphorylation

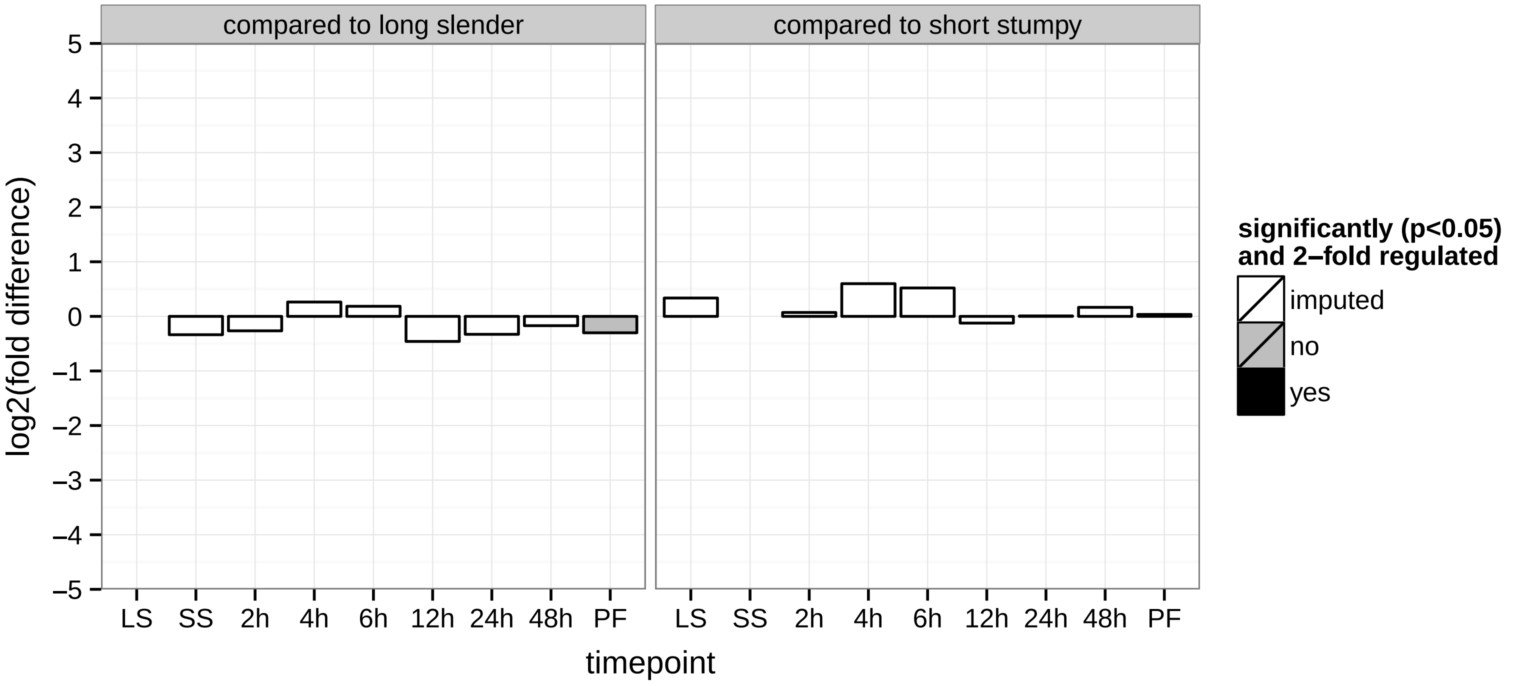

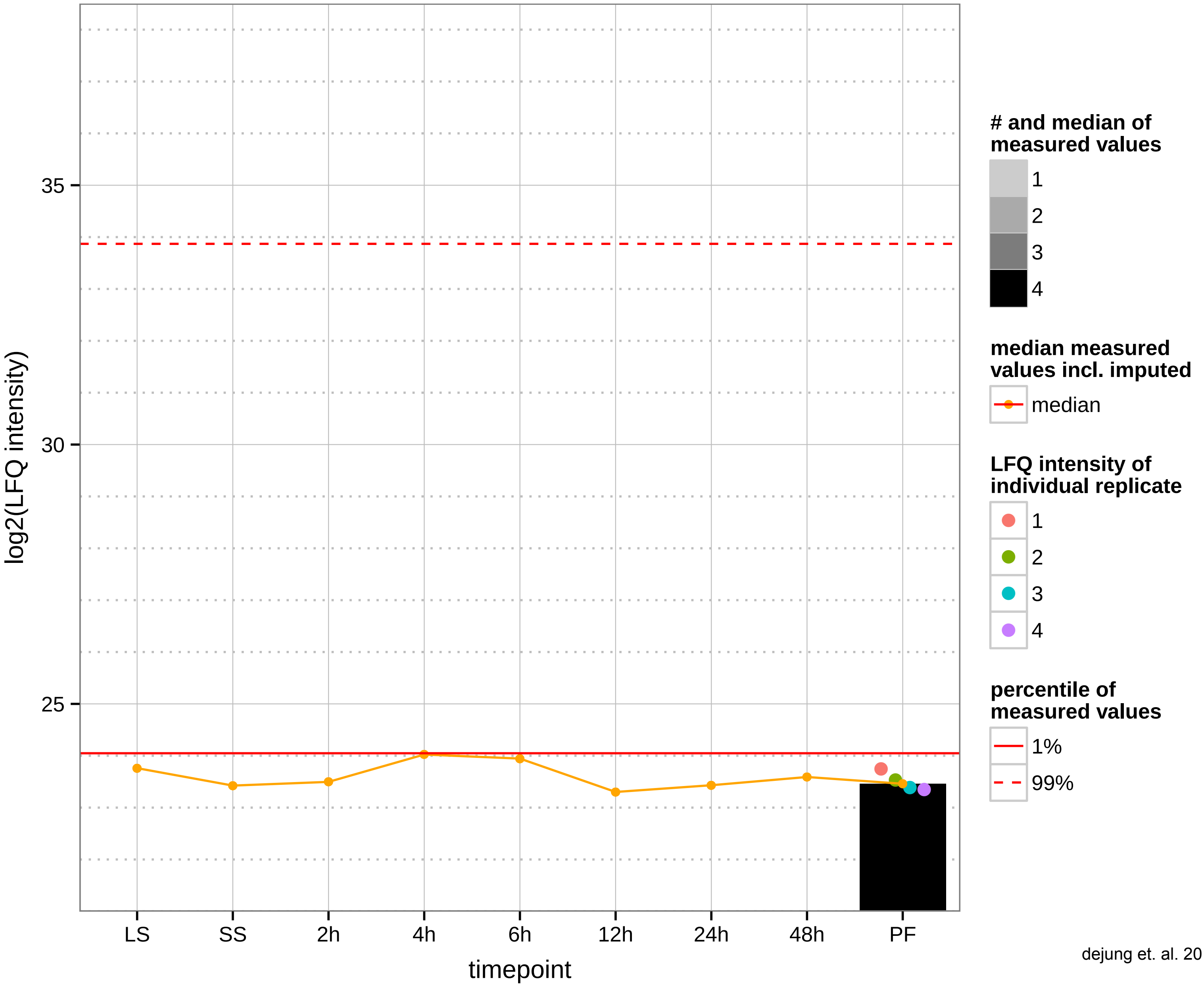

dejung et. al. 2015

vesicular–fusion protein SEC18, putative
Tb927.11.1680
AGOF: ATP binding, nucleoside–triphosphatase activity
AGOC: null
AGOP: ER to Golgi vesicle–mediated transport, Golgi vesicle transport, vesicle–mediated transport
PGOF: ATP binding, nucleoside–triphosphatase activity, nucleotide binding
PGOC: null
PGOP: null

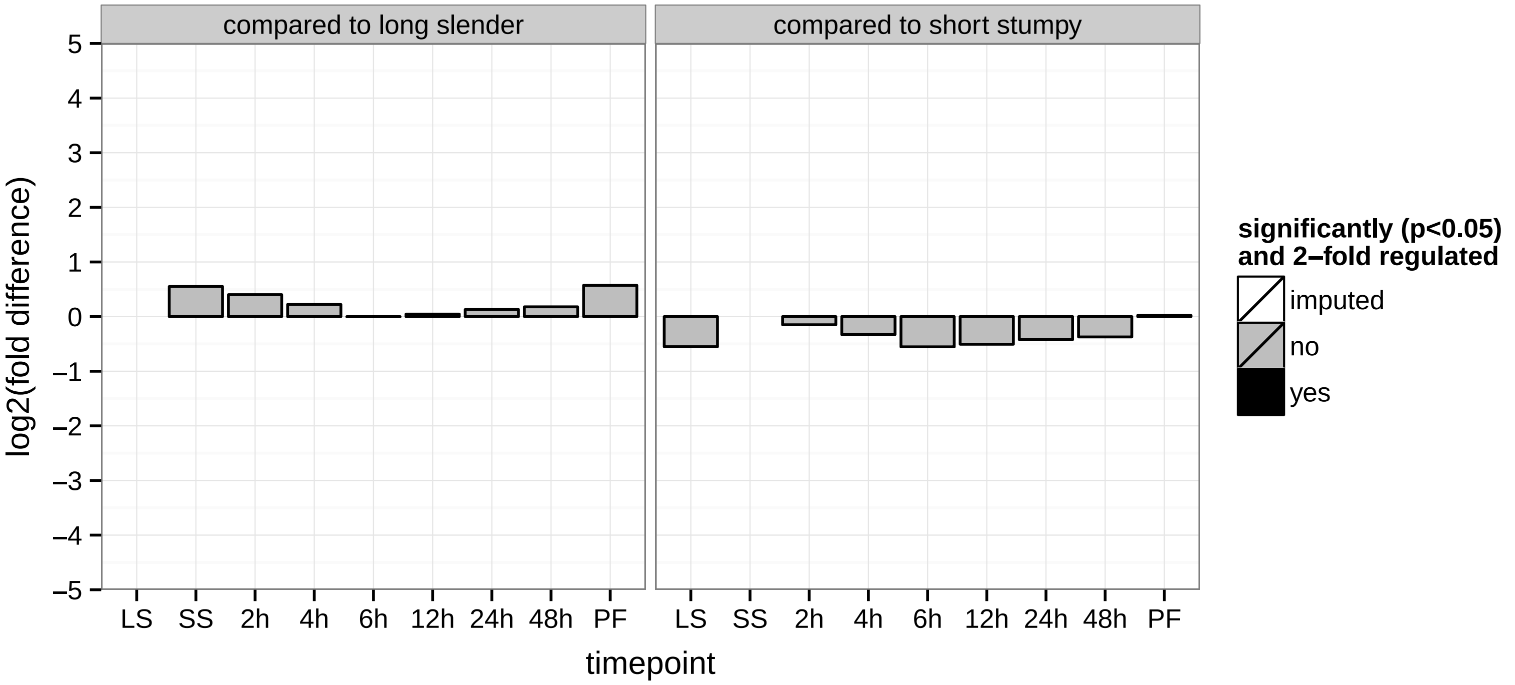

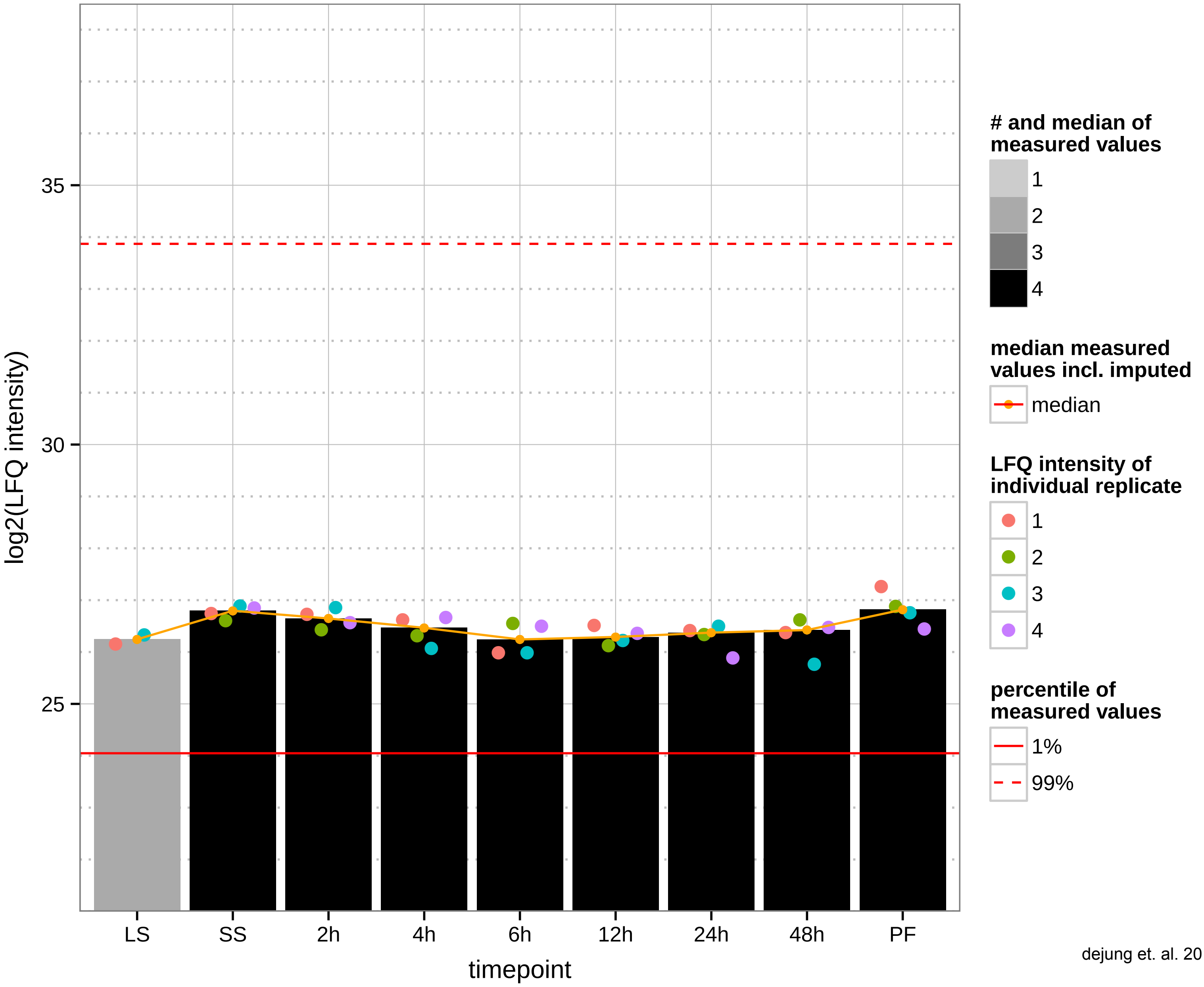

dejung et. al. 2015

dynein light intermediate chain D1bLIC, putative
Tb927.11.16810
AGOF: null
AGOC: null
AGOP: null
PGOF: GTP binding
PGOC: intracellular
PGOP: small GTPase mediated signal transduction

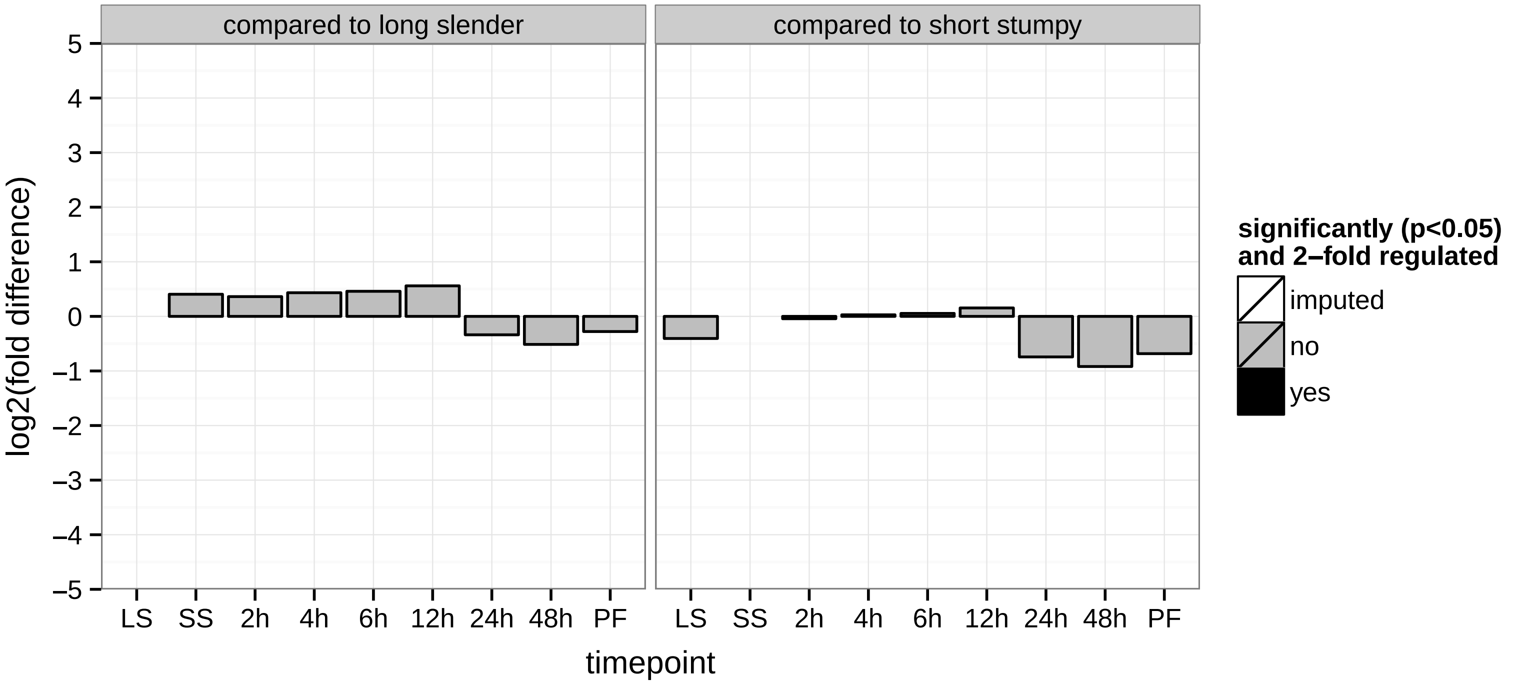

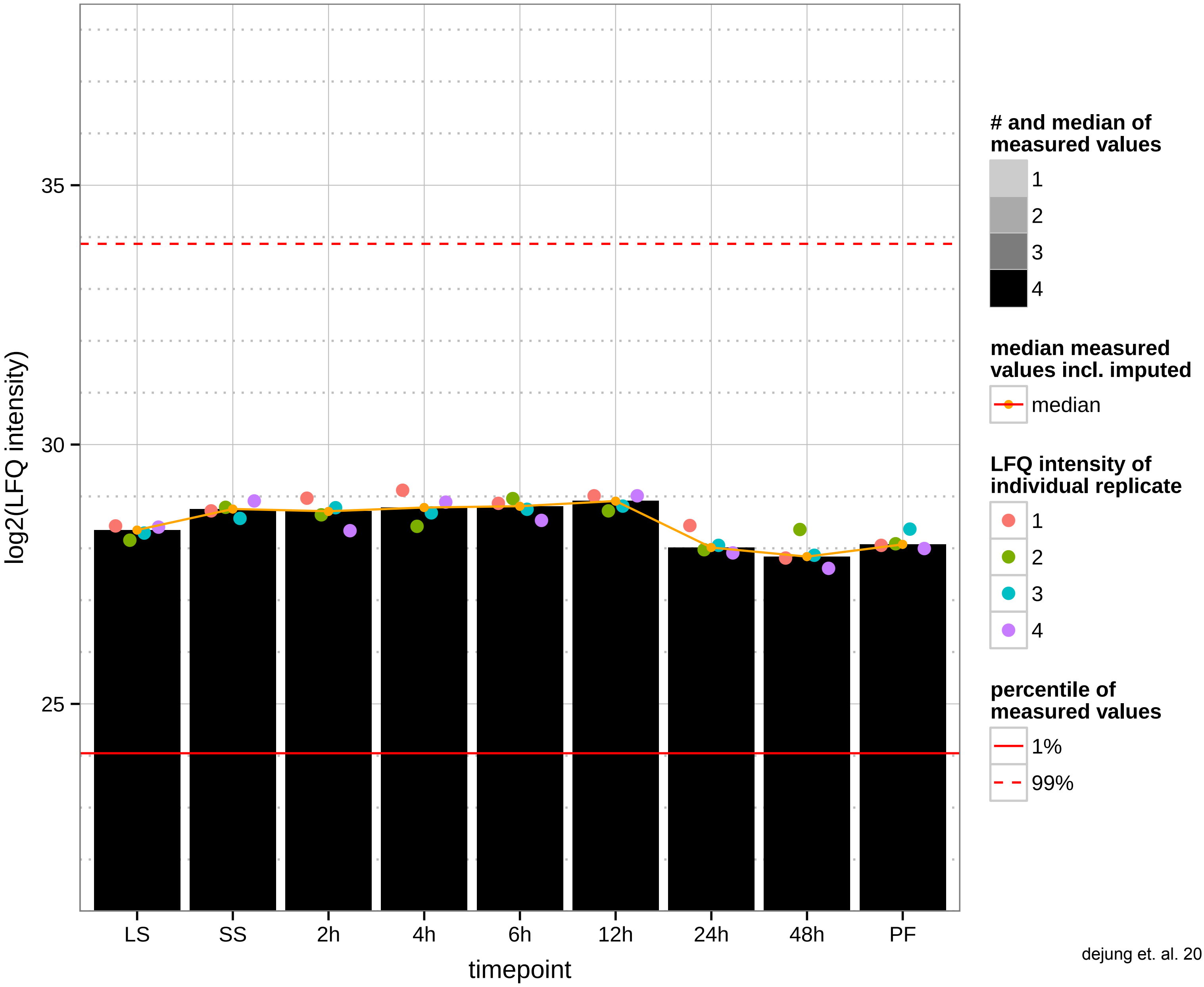

dejung et. al. 2015

Rab–like 5, small G protein (RABL5)
Tb927.11.16830
AGOF: GTP binding
AGOC: null
AGOP: cilium assembly
PGOF: GTP binding
PGOC: intracellular
PGOP: small GTPase mediated signal transduction

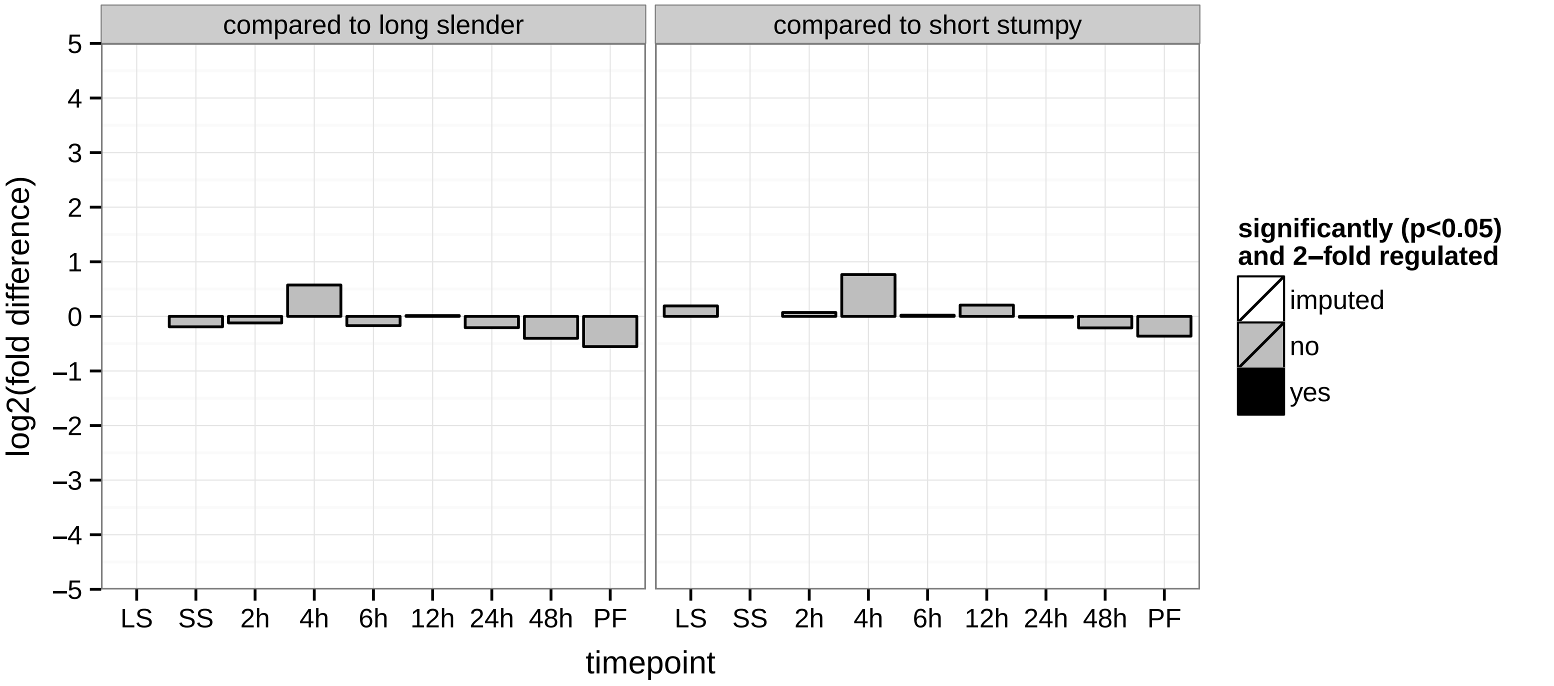

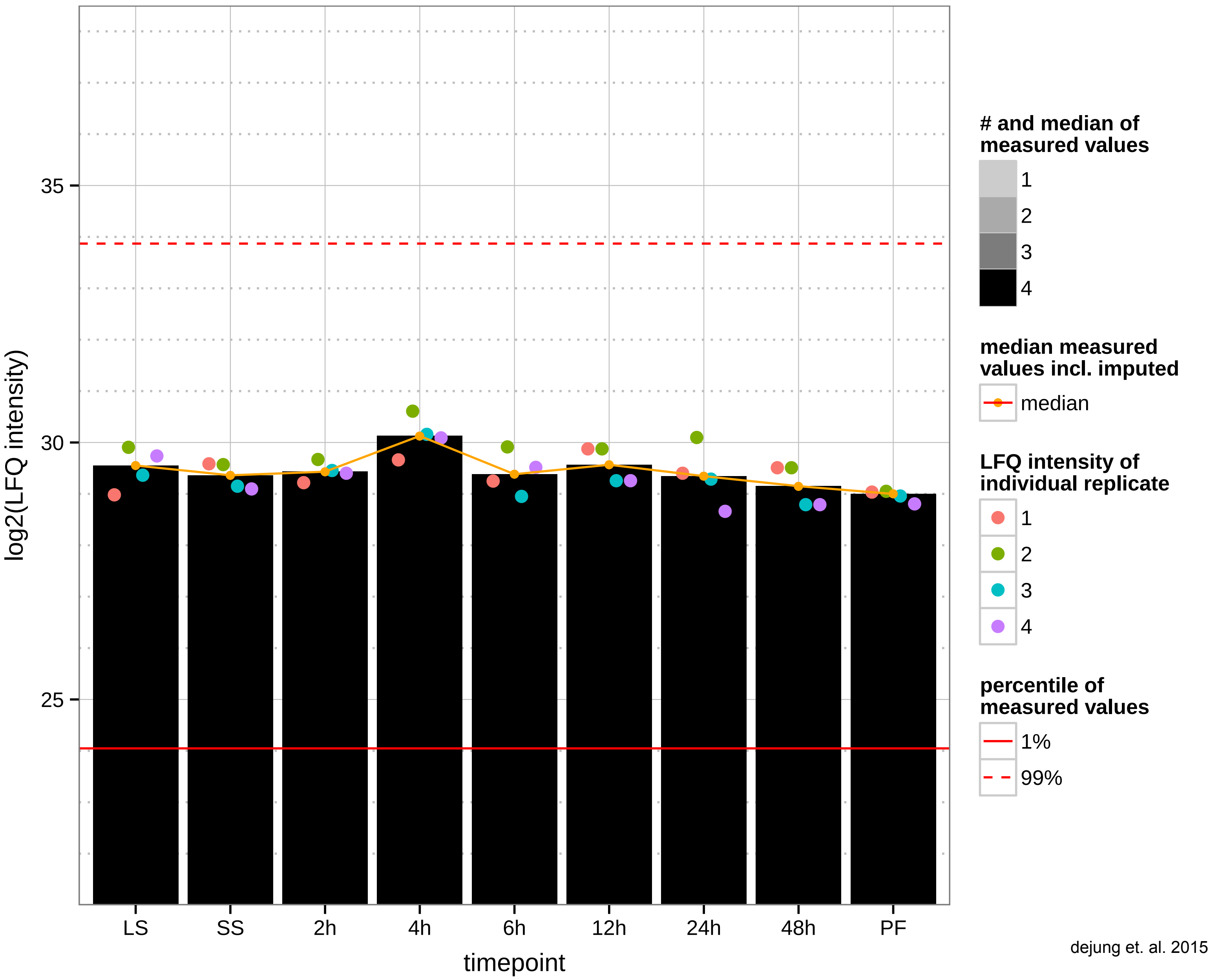

dejung et. al. 2015

hypothetical protein, conserved
Tb927.11.16850
AGOF: null
AGOC: null
AGOP: null
PGOF: null
PGOC: null
PGOP: null

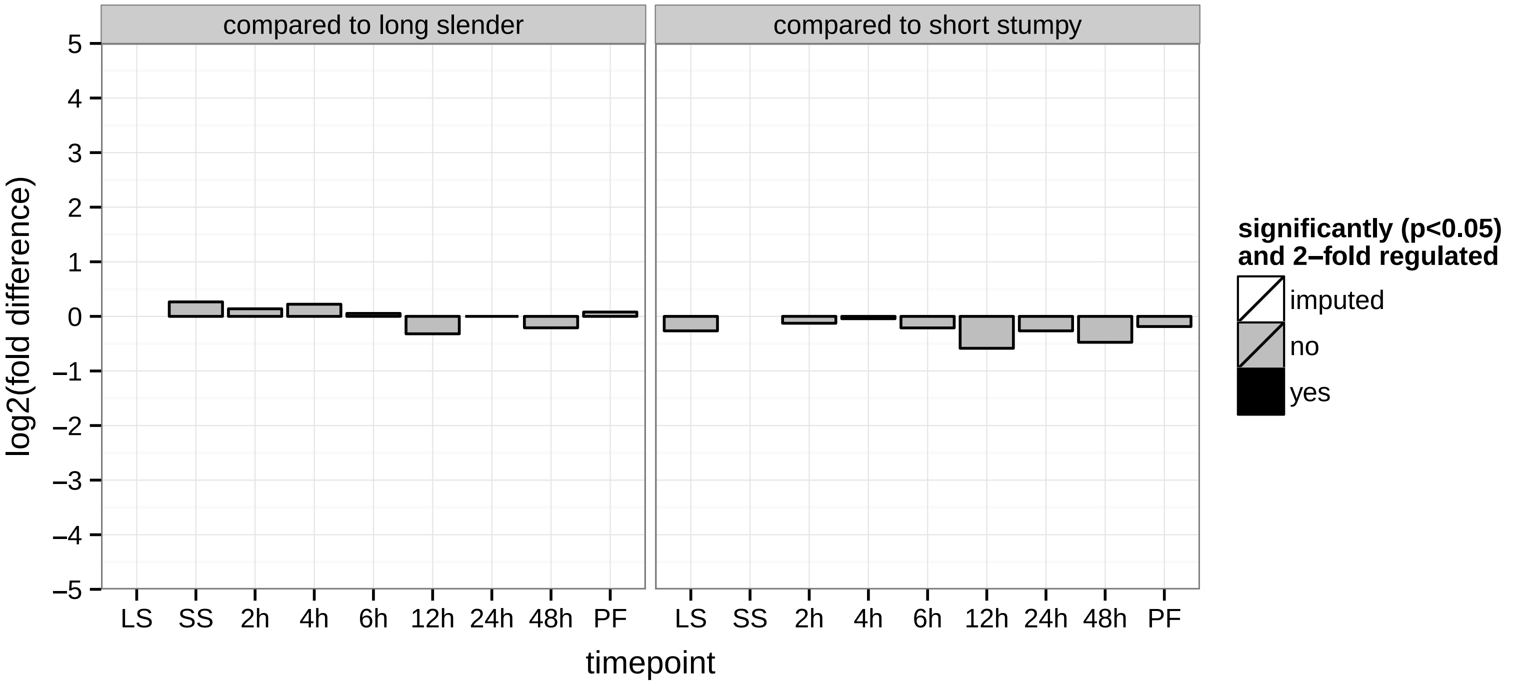

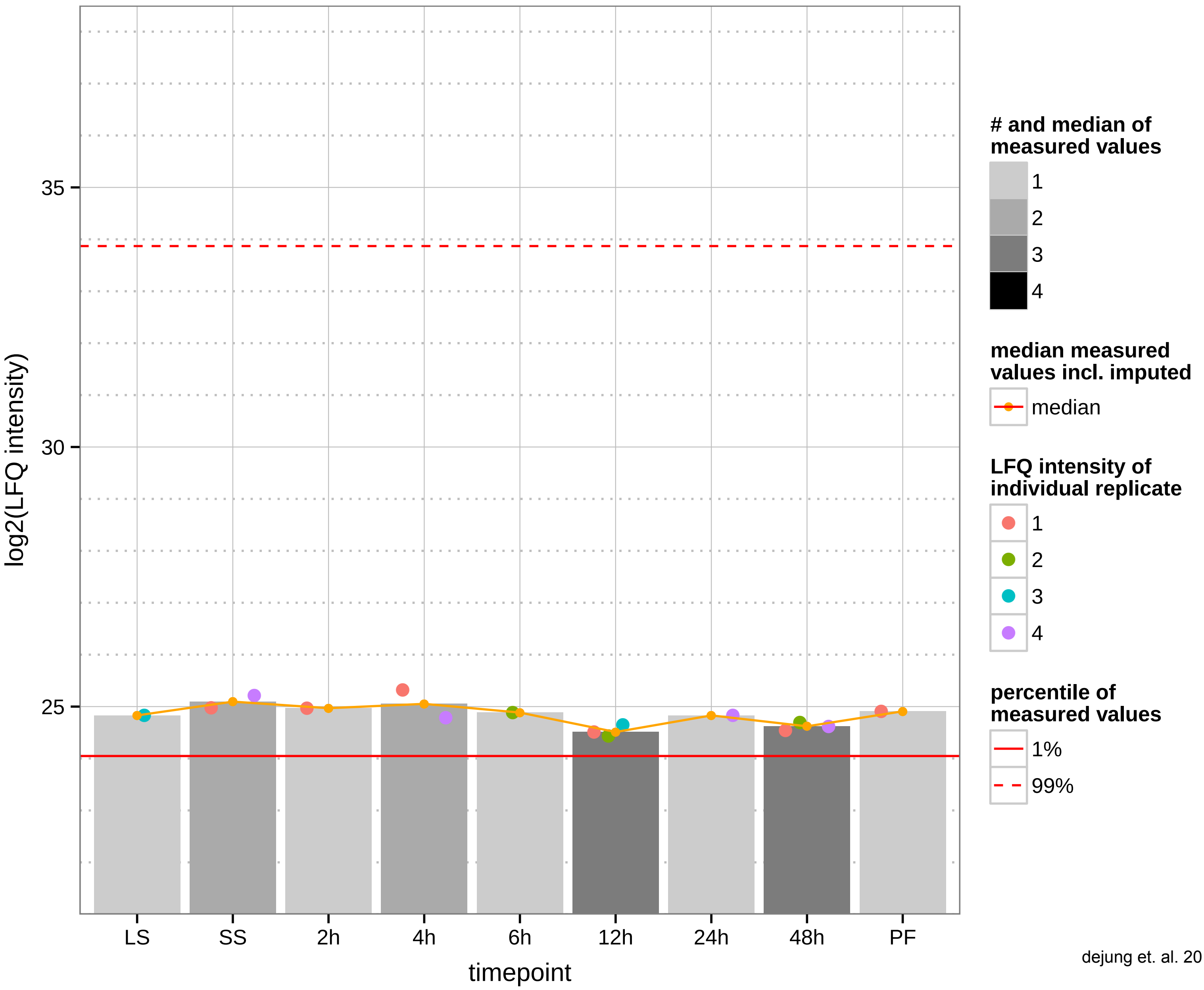

dejung et. al. 2015

mitochondrial RNA binding protein (MRB8620)
Tb927.11.16860
AGOF: RNA binding
AGOC: mitochondrion
AGOP: RNA metabolic process
PGOF: null
PGOC: null
PGOP: null

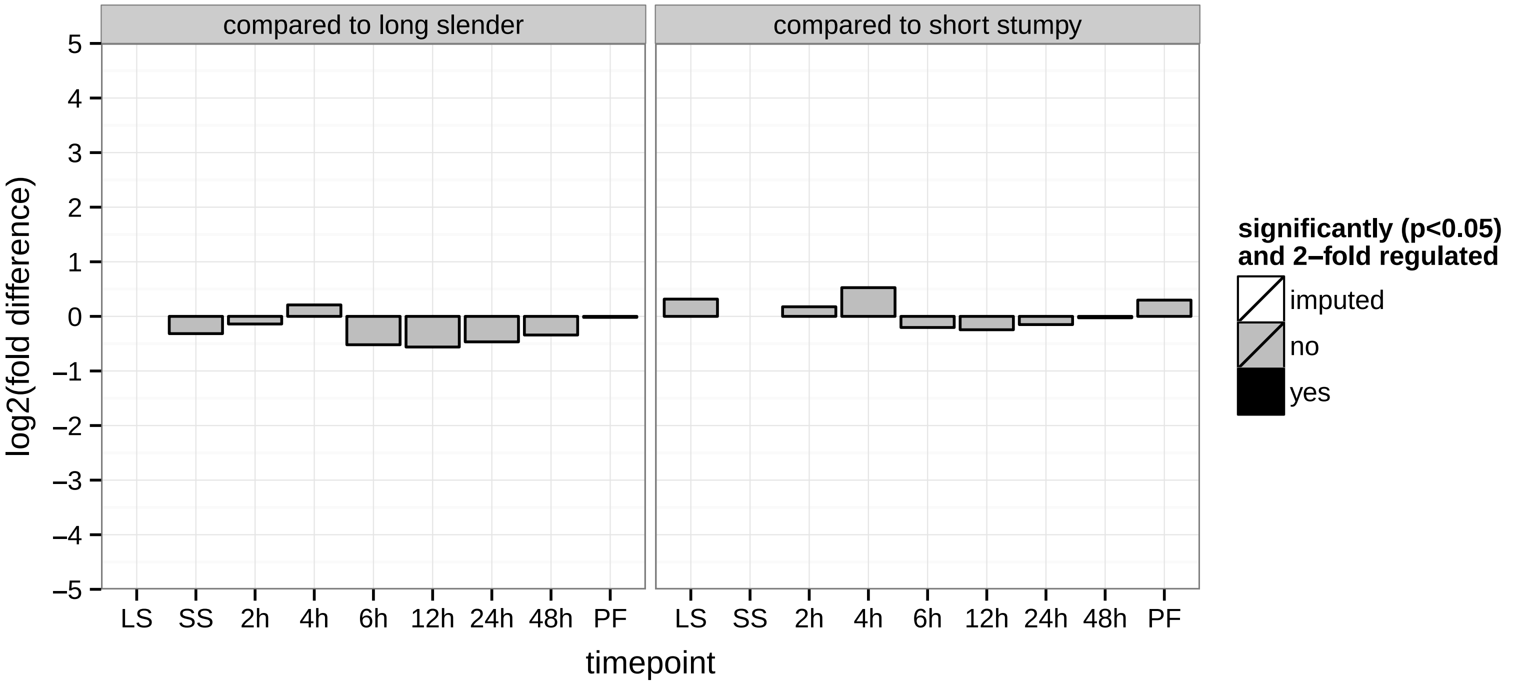

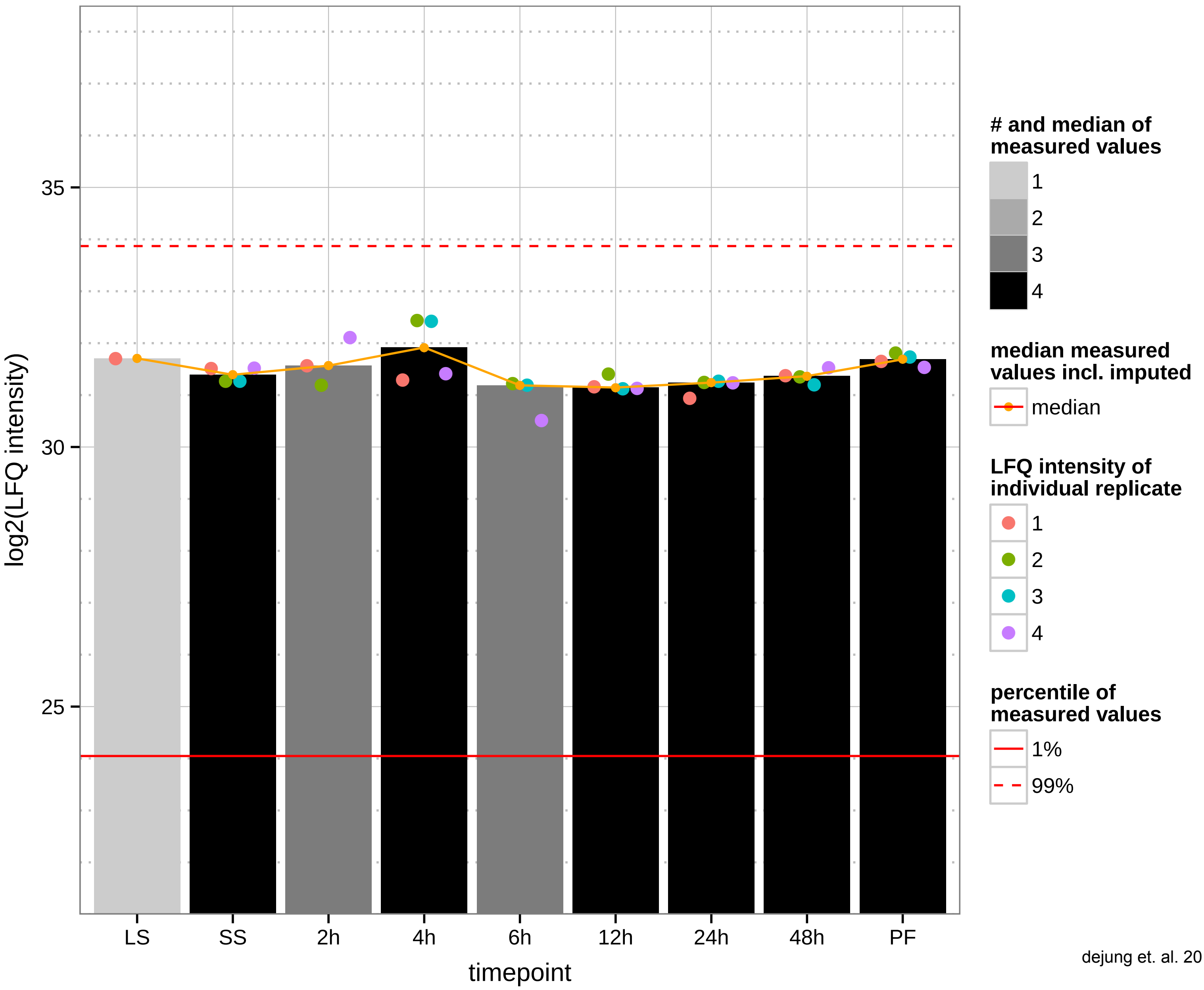

dejung et. al. 2015

leucine–rich repeat protein (LRRP), putative
Tb927.11.16880
AGOF: null
AGOC: null
AGOP: null
PGOF: null
PGOC: null
PGOP: null

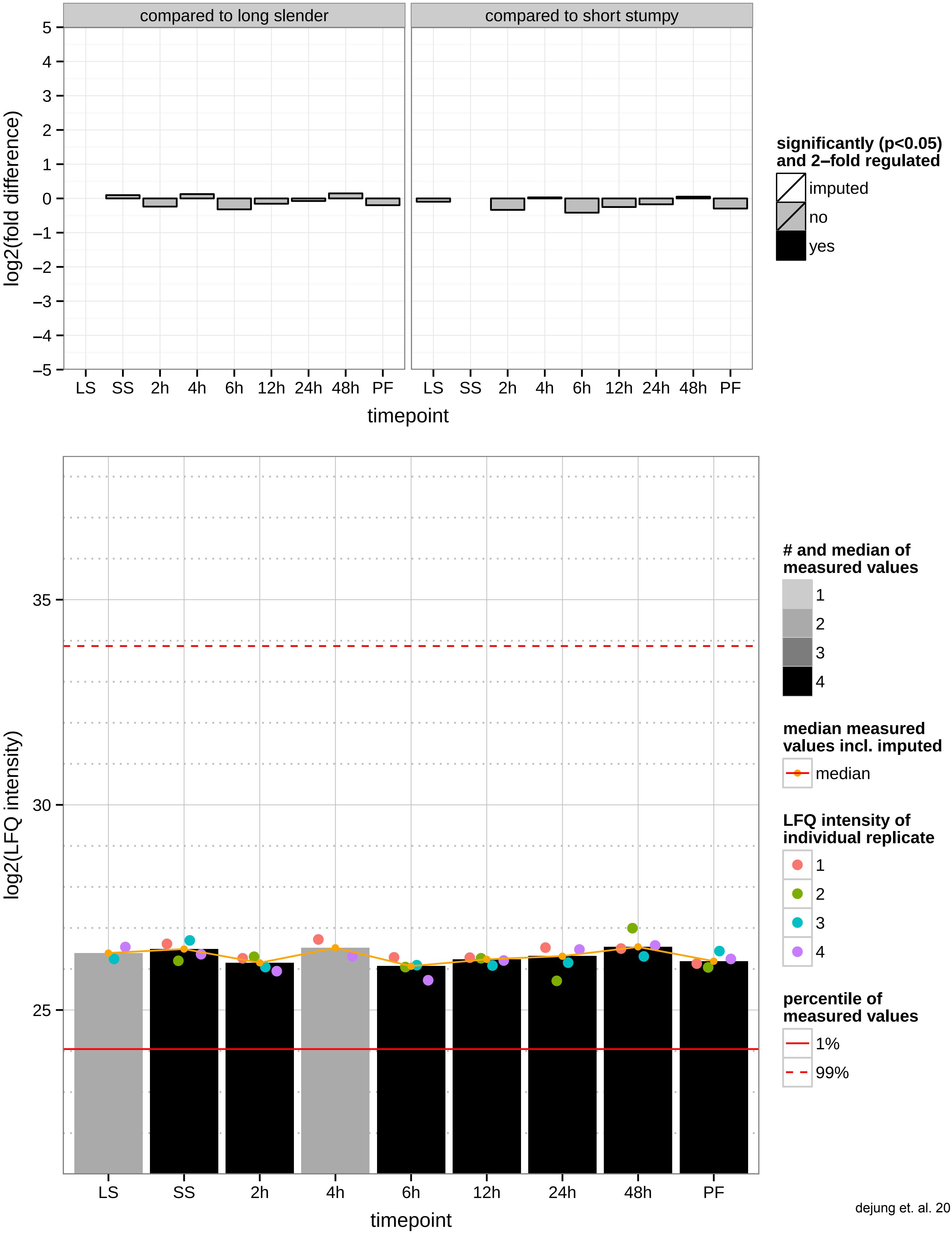

dejung et. al. 2015

hypothetical protein, conserved
Tb927.11.16890
AGOF: null
AGOC: null
AGOP: null
PGOF: protein binding
PGOC: null
PGOP: null

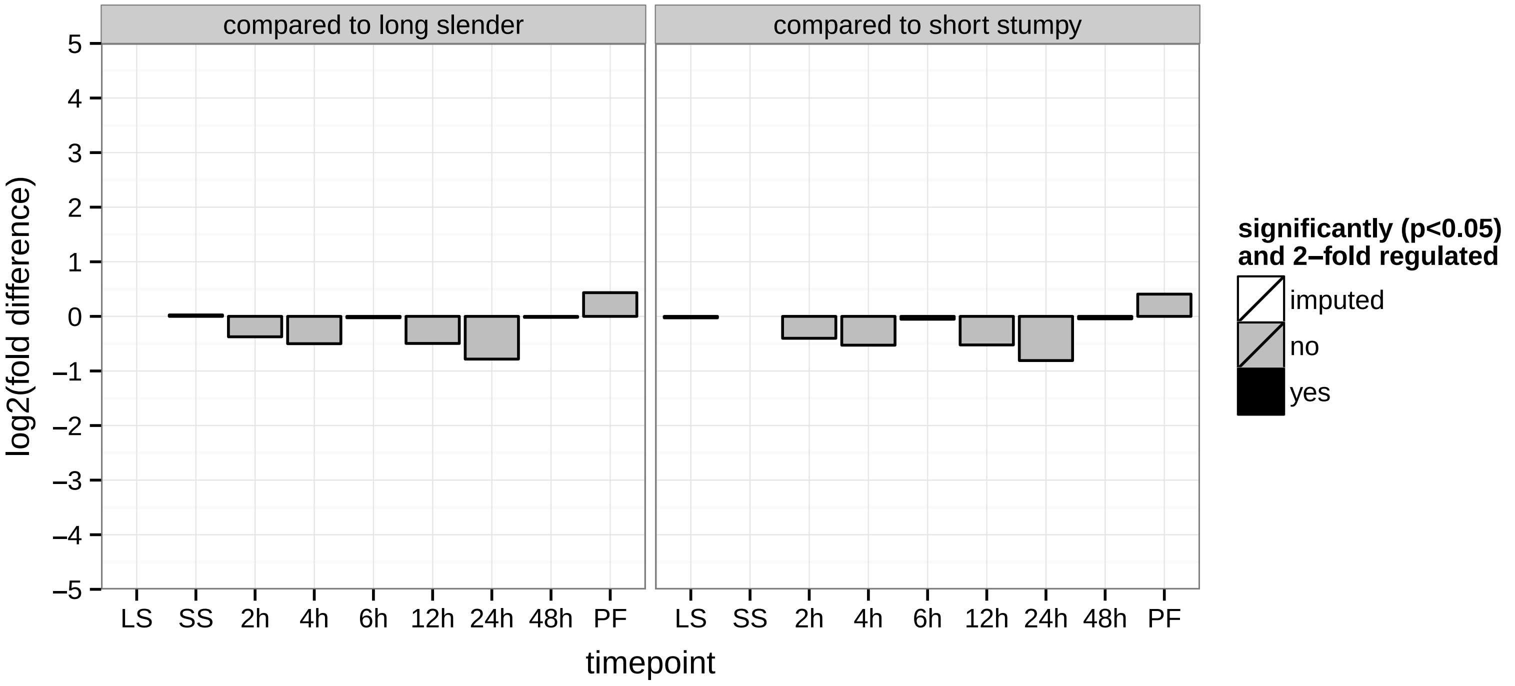

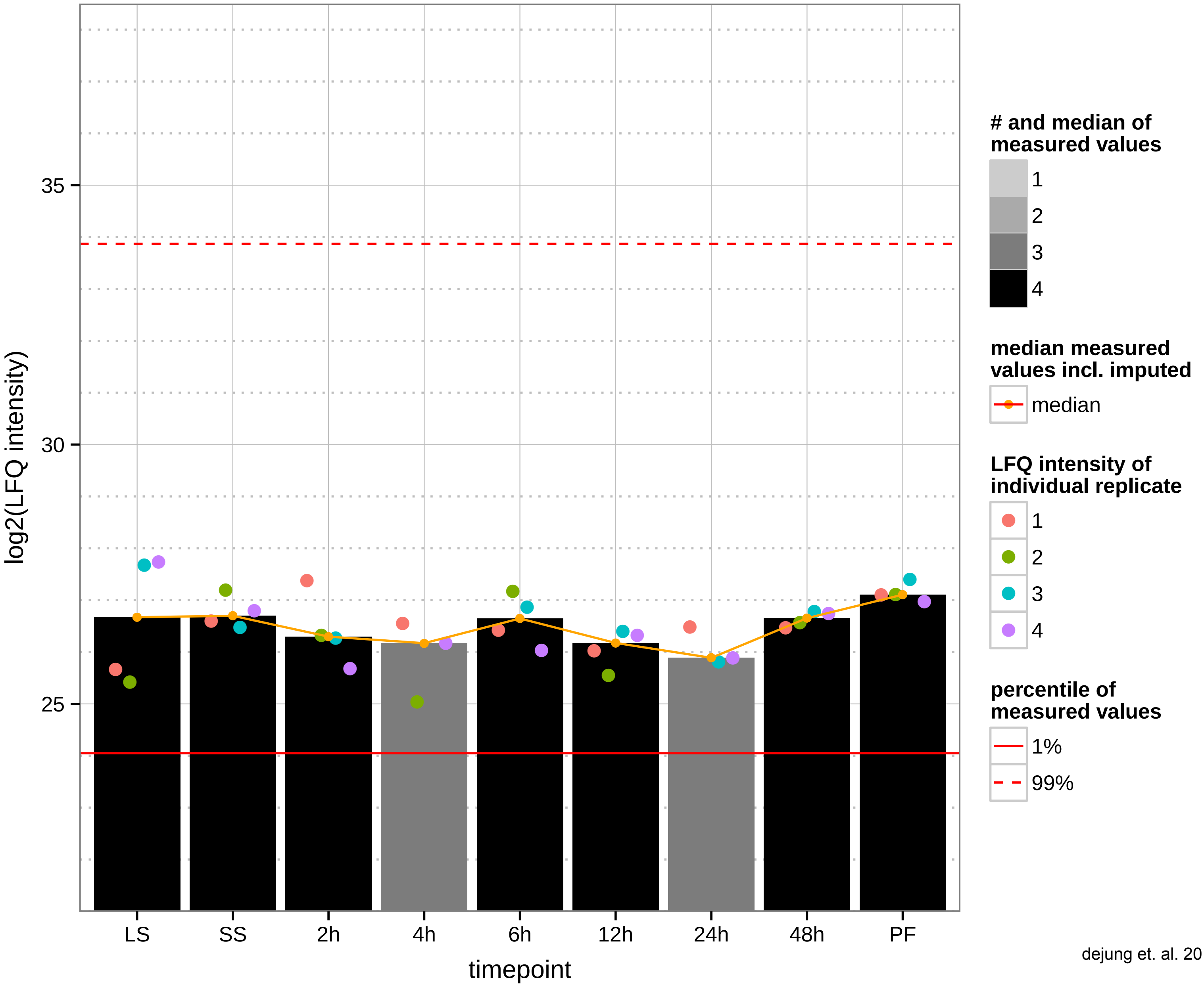

dejung et. al. 2015

protein phosphatase 4, putative
Tb927.11.16970
AGOF: phosphoric ester hydrolase activity, protein serine/threonine phosphatase activity
AGOC: protein phosphatase type 2A complex
AGOP: phosphate–containing compound metabolic process
PGOF: hydrolase activity
PGOC: null
PGOP: null

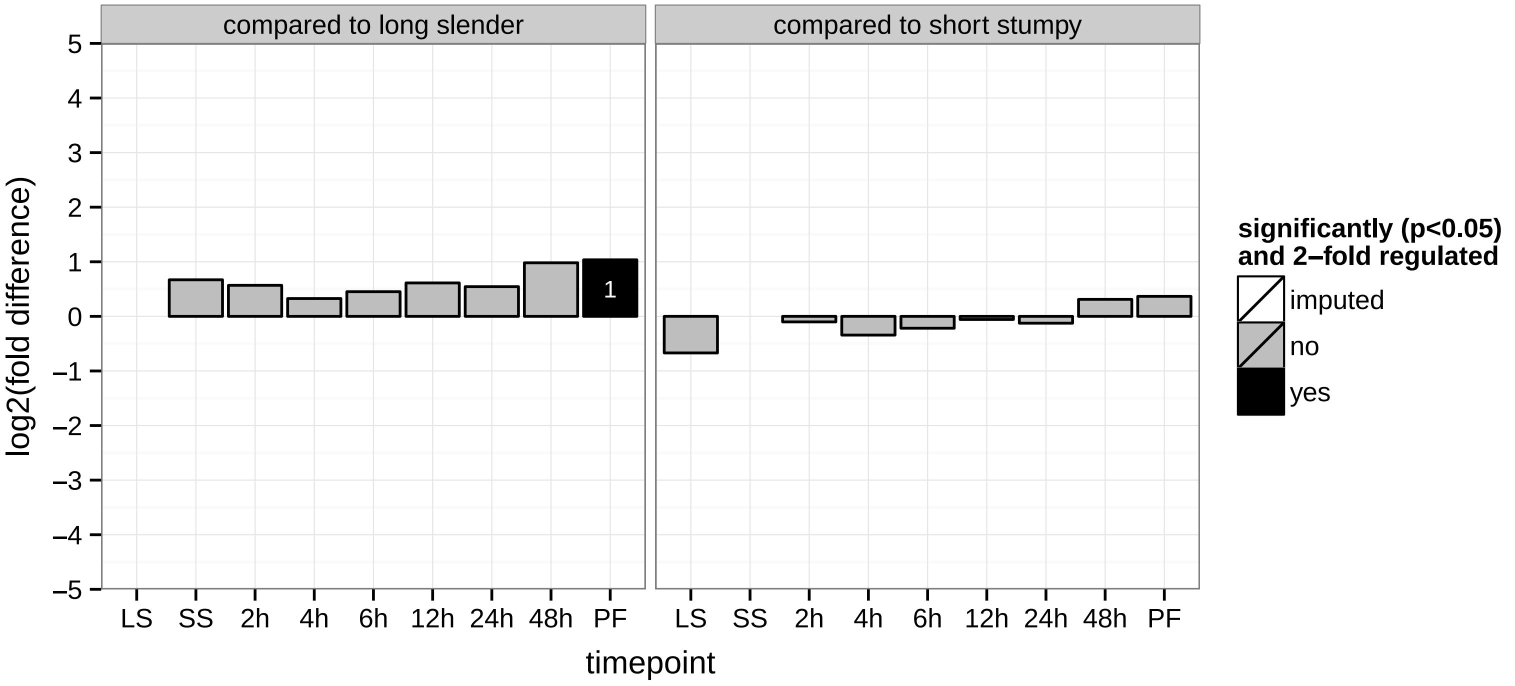

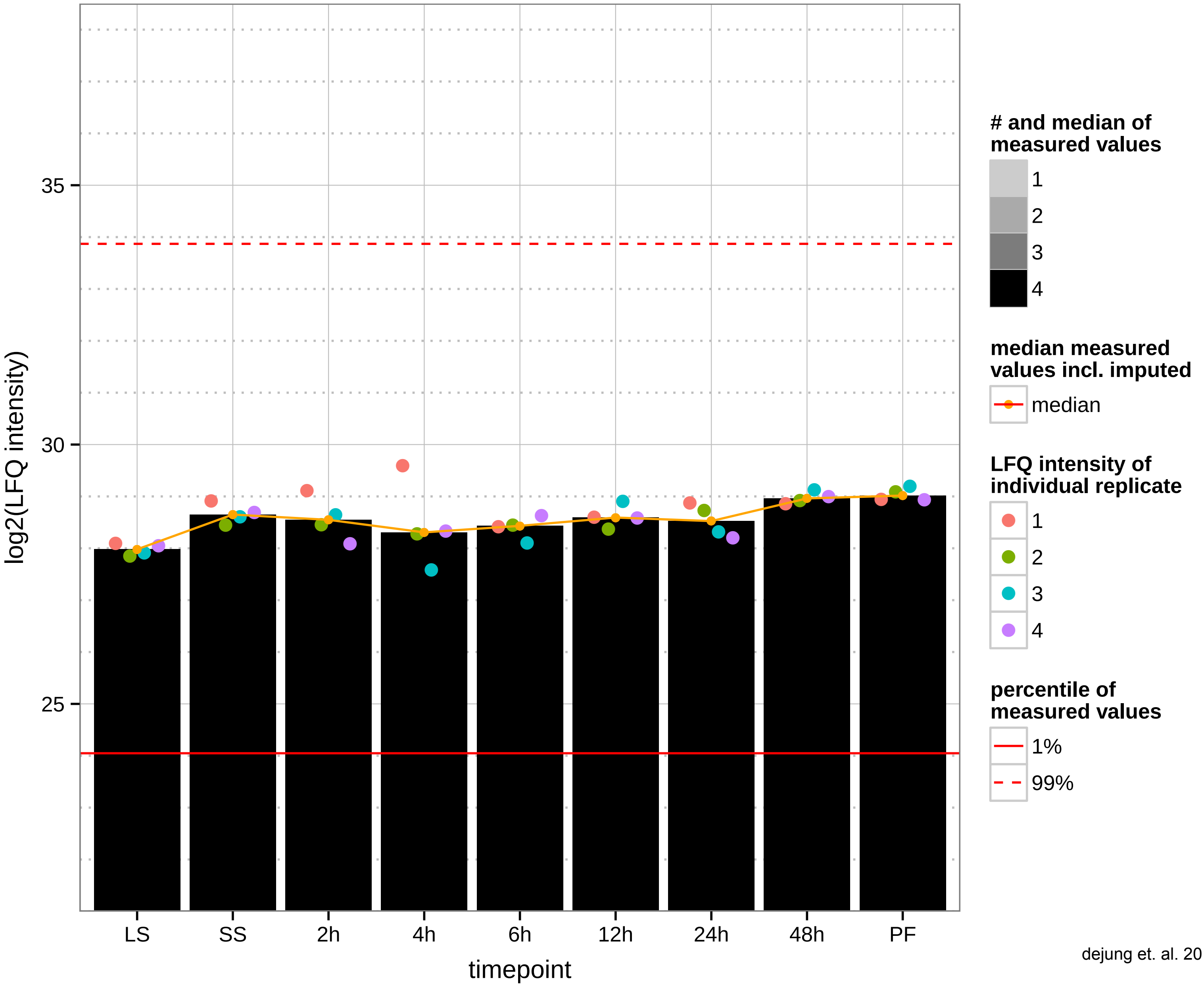

dejung et. al. 2015

hypothetical protein, conserved
Tb927.11.1700
AGOF: null
AGOC: mitochondrion
AGOP: null
PGOF: null
PGOC: null
PGOP: null

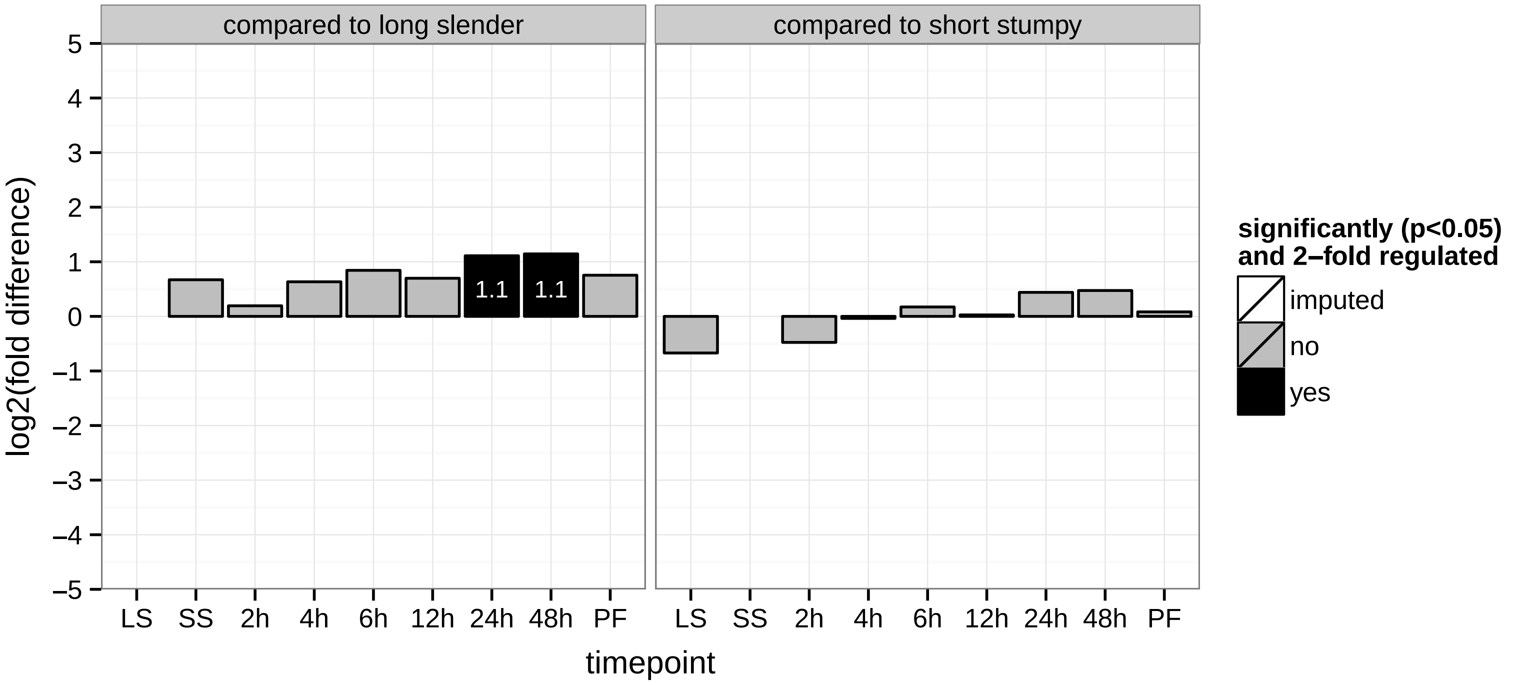

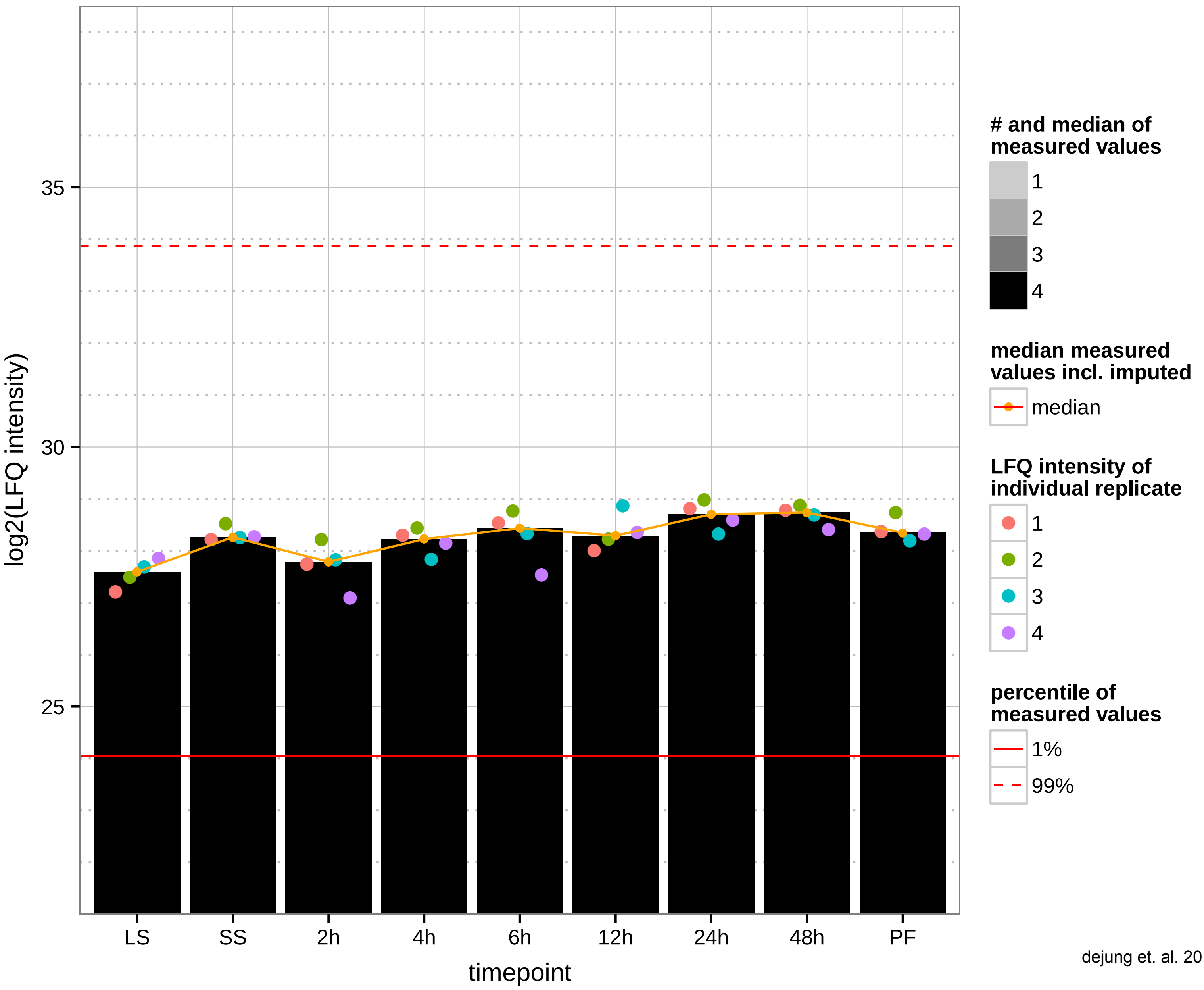

dejung et. al. 2015

hypothetical protein, conserved, leucine−rich repeat protein (LRRP), putative
Tb927.11.17000;Tb11.v5.0404
AGOF: null
AGOC: null
AGOP: null
PGOF: null, protein binding
PGOC: null
PGOP: null

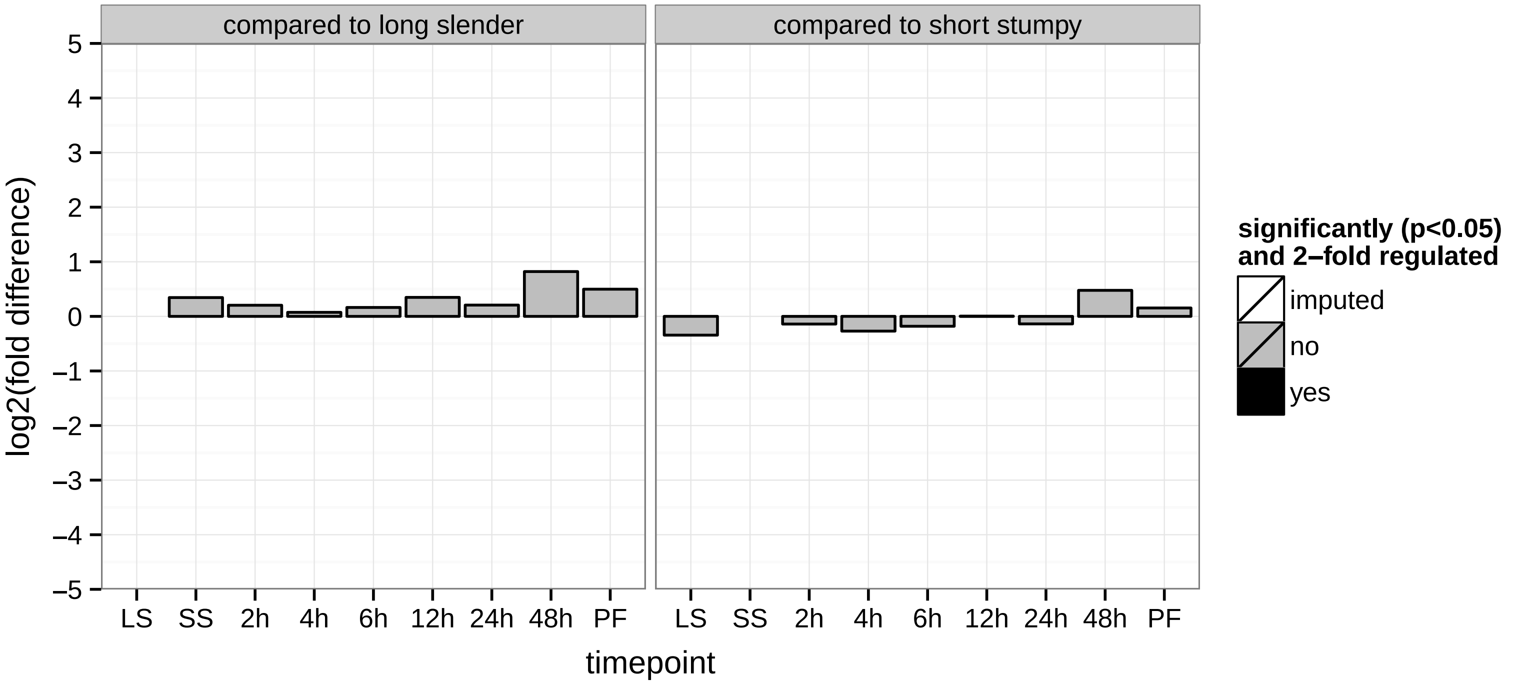

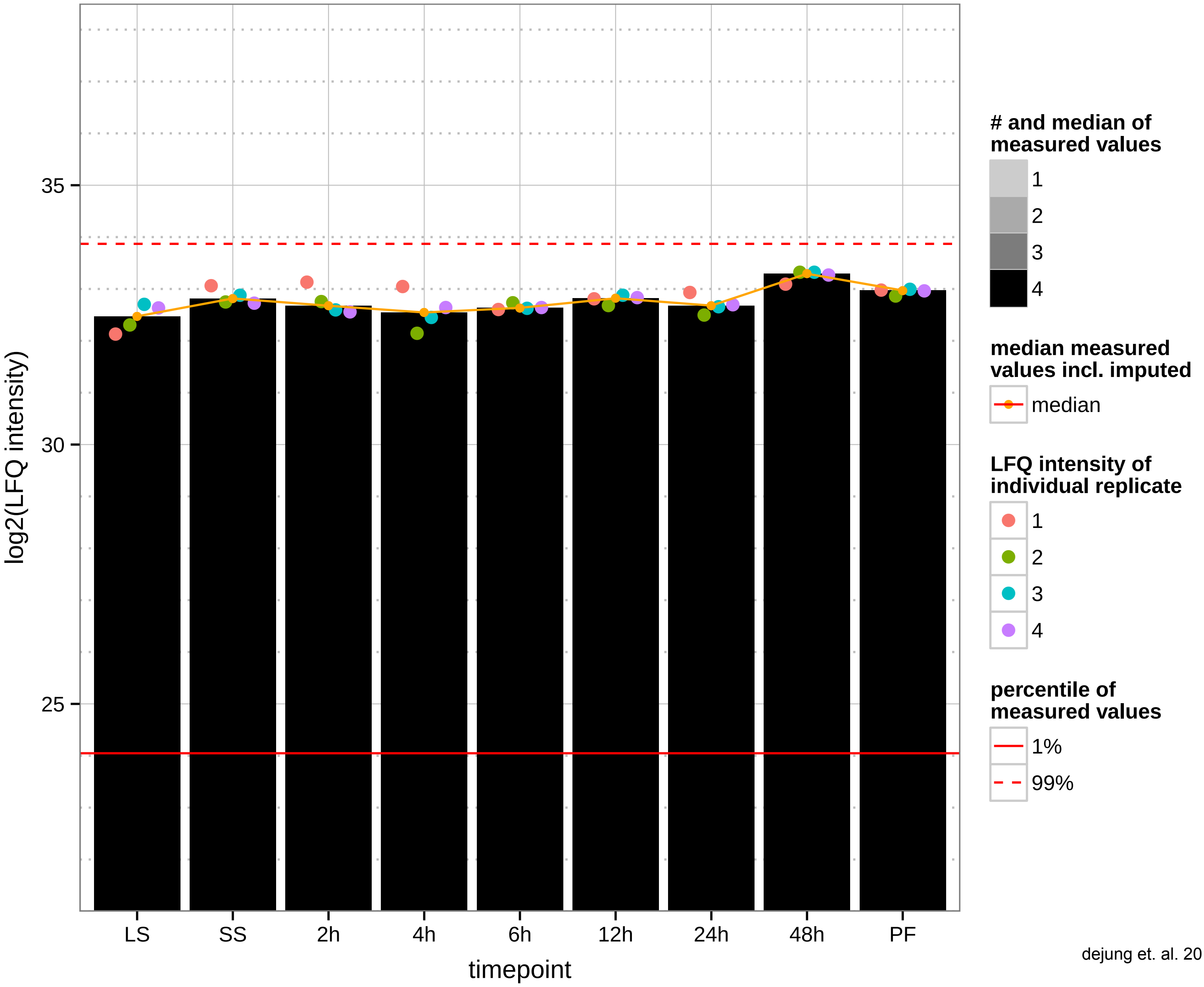

dejung et. al. 2015

mitochondrial RNA binding protein 1, gBP21, MRP1 (GBP21)
Tb927.11.1710
AGOF: RNA binding
AGOC: mitochondrion
AGOP: mRNA modification
PGOF: DNA binding
PGOC: null
PGOP: regulation of transcription, DNA−dependent

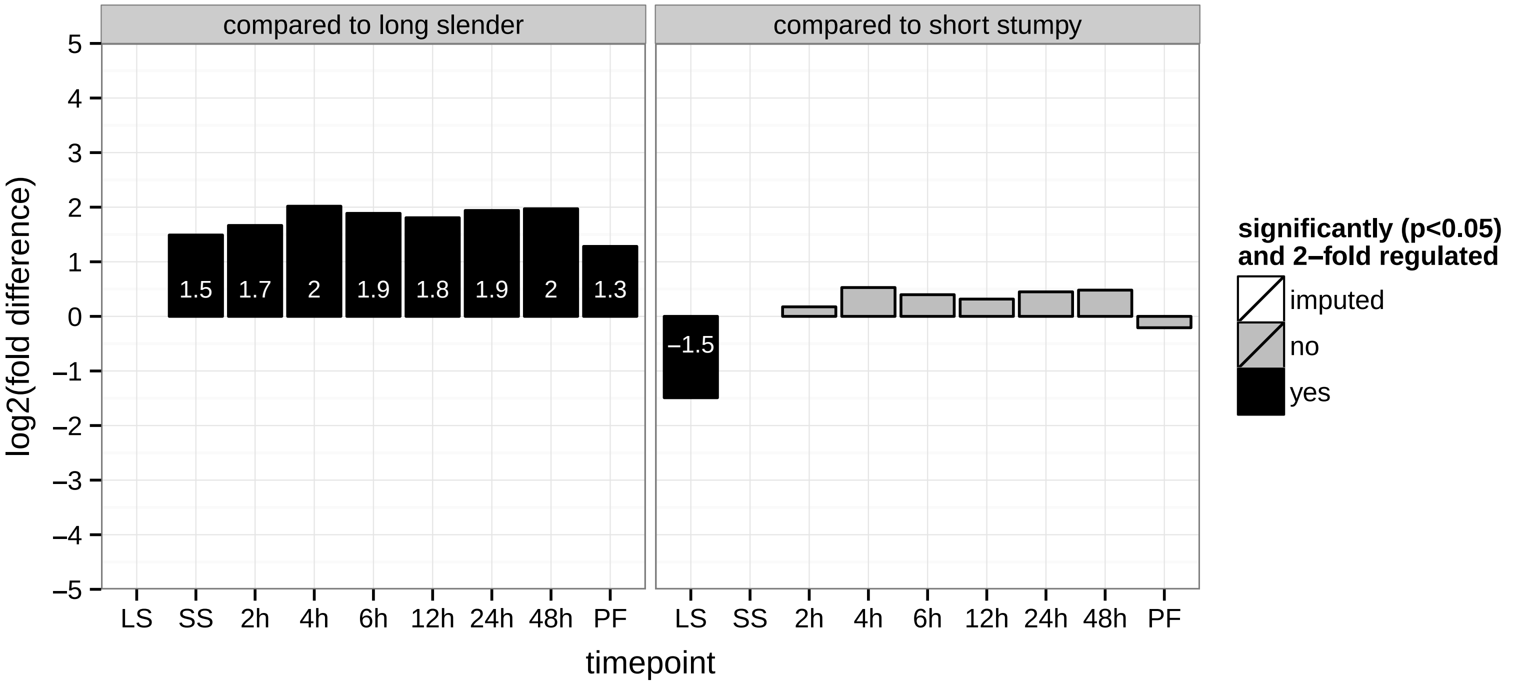

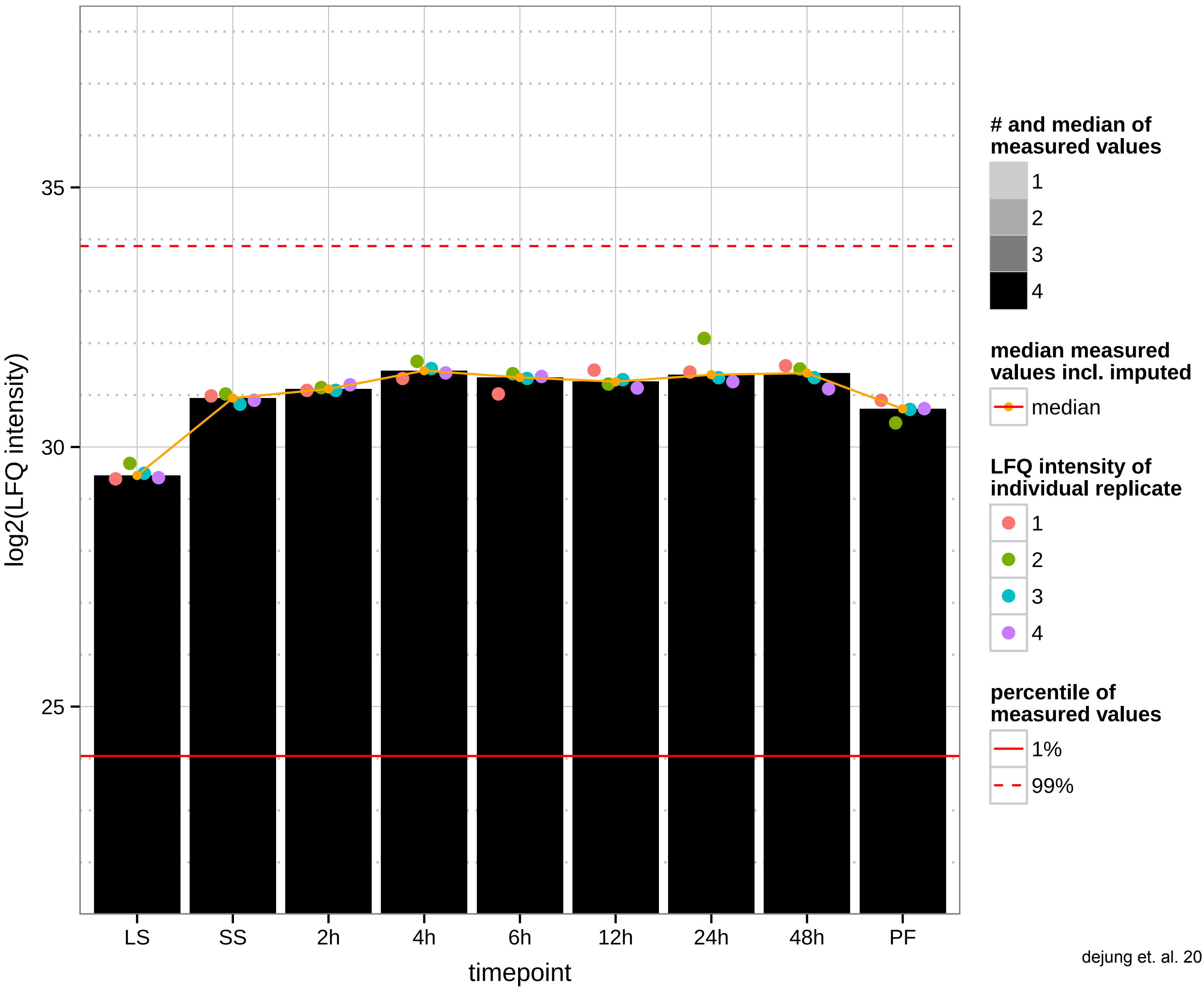

dejung et. al. 2015

intraflagellar transport protein IFT88 (IFT88)
Tb927.11.1740
AGOF: null
AGOC: motile cilium
AGOP: cilium assembly, establishment of cell polarity, regulation of cell size
PGOF: protein binding
PGOC: null
PGOP: null

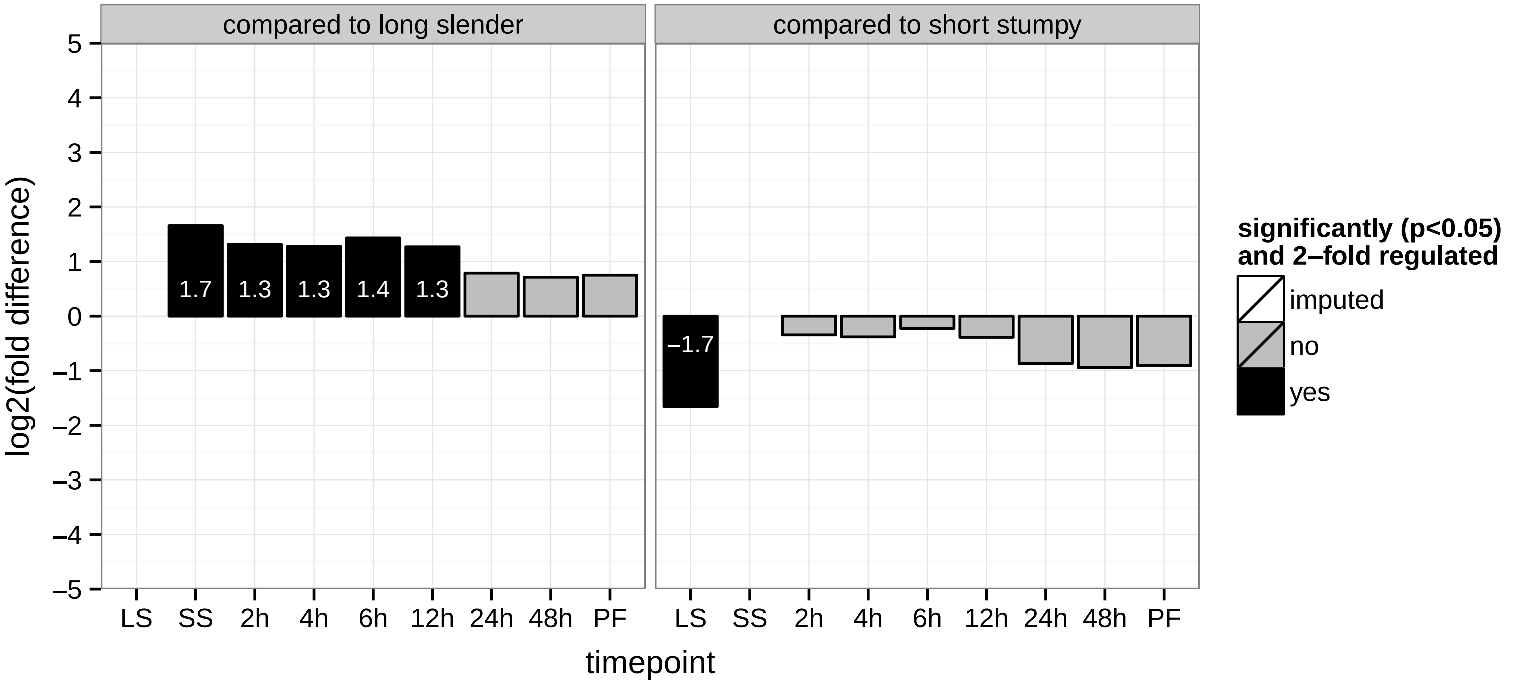

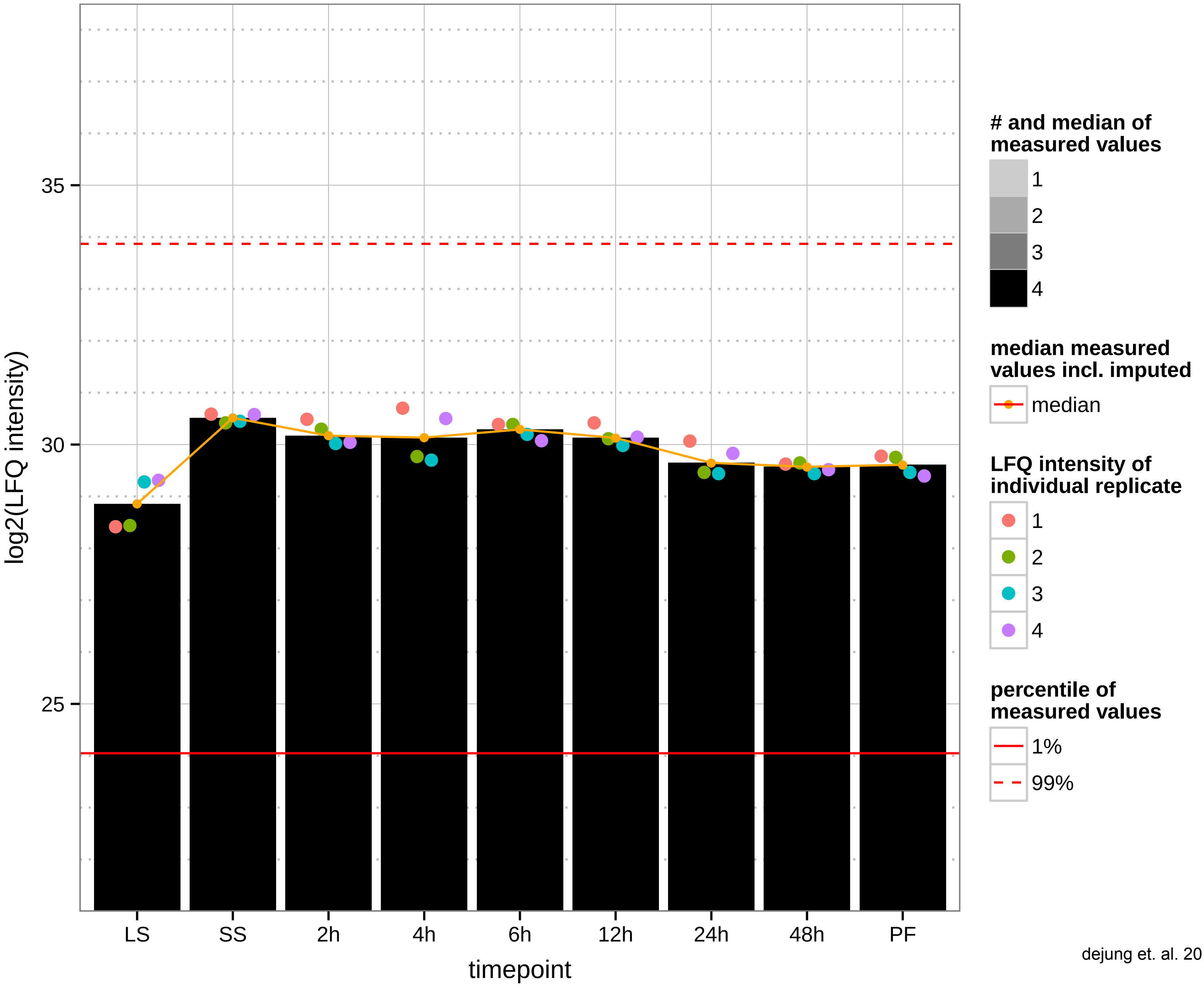

dejung et. al. 2015

hypothetical protein, conserved (pseudogene), translation initiation factor eIF2B delta subunit, putative, eIF−2B GDP−GTP exc
Tb927.11.1820;Tb11.1400
AGOF: null, GTP binding, translation initiation factor activity
AGOC: null, eukaryotic translation initiation factor 2B complex
AGOP: cellular metabolic process, regulation of translation, translational initiation
PGOF: null
PGOC: null
PGOP: cellular metabolic process

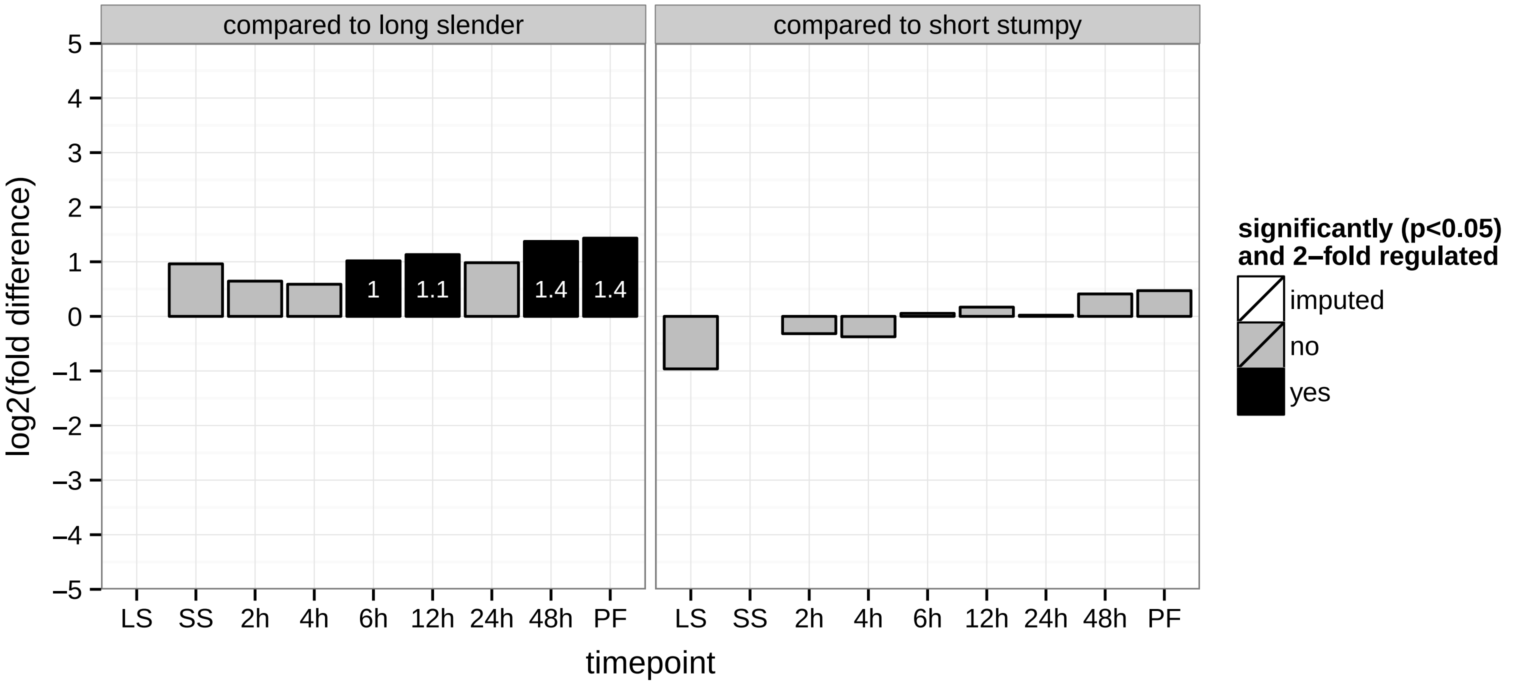

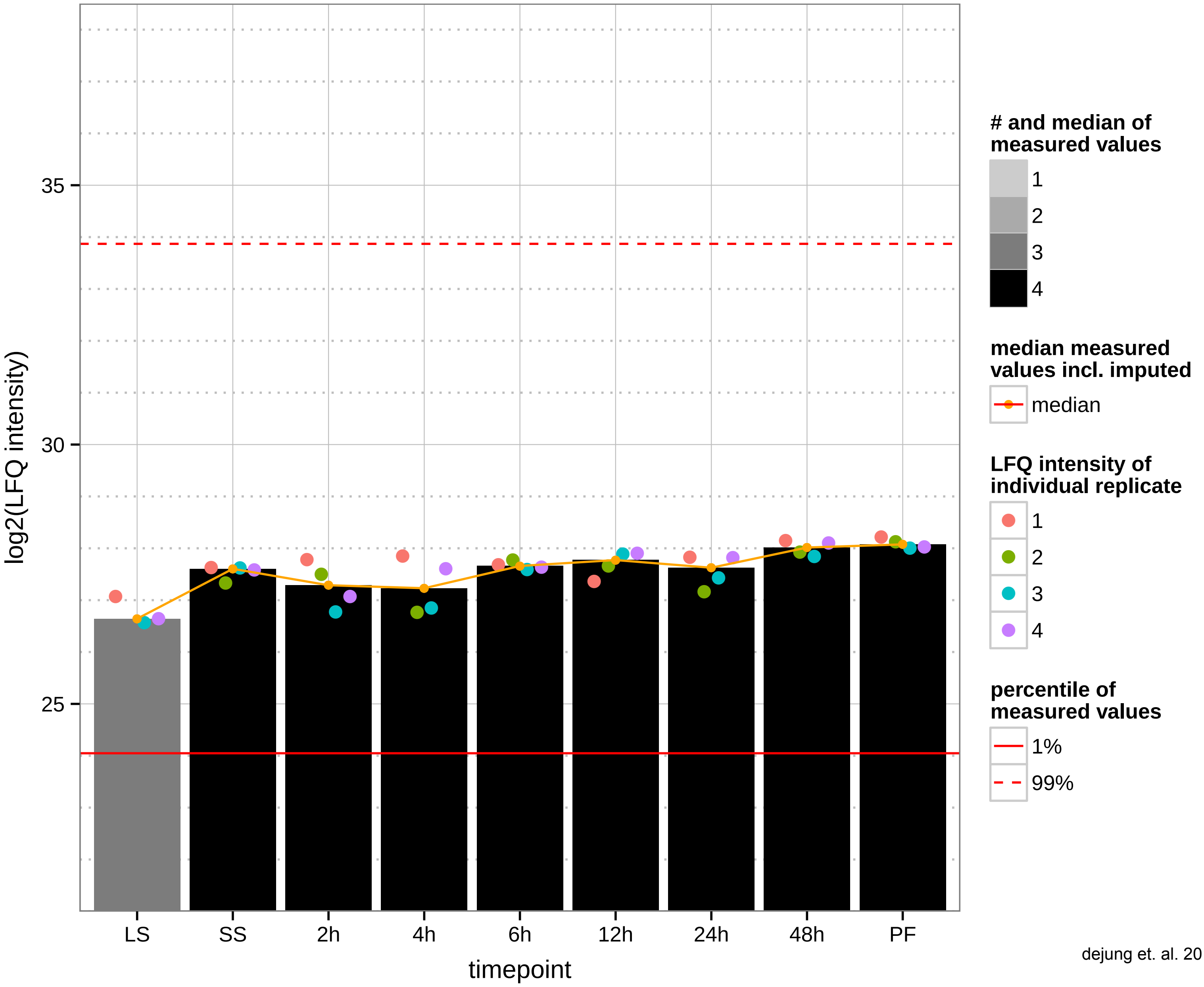

dejung et. al. 2015

hypothetical protein, conserved
Tb927.11.1890
AGOF: null
AGOC: null
AGOP: null
PGOF: protein binding
PGOC: null
PGOP: null

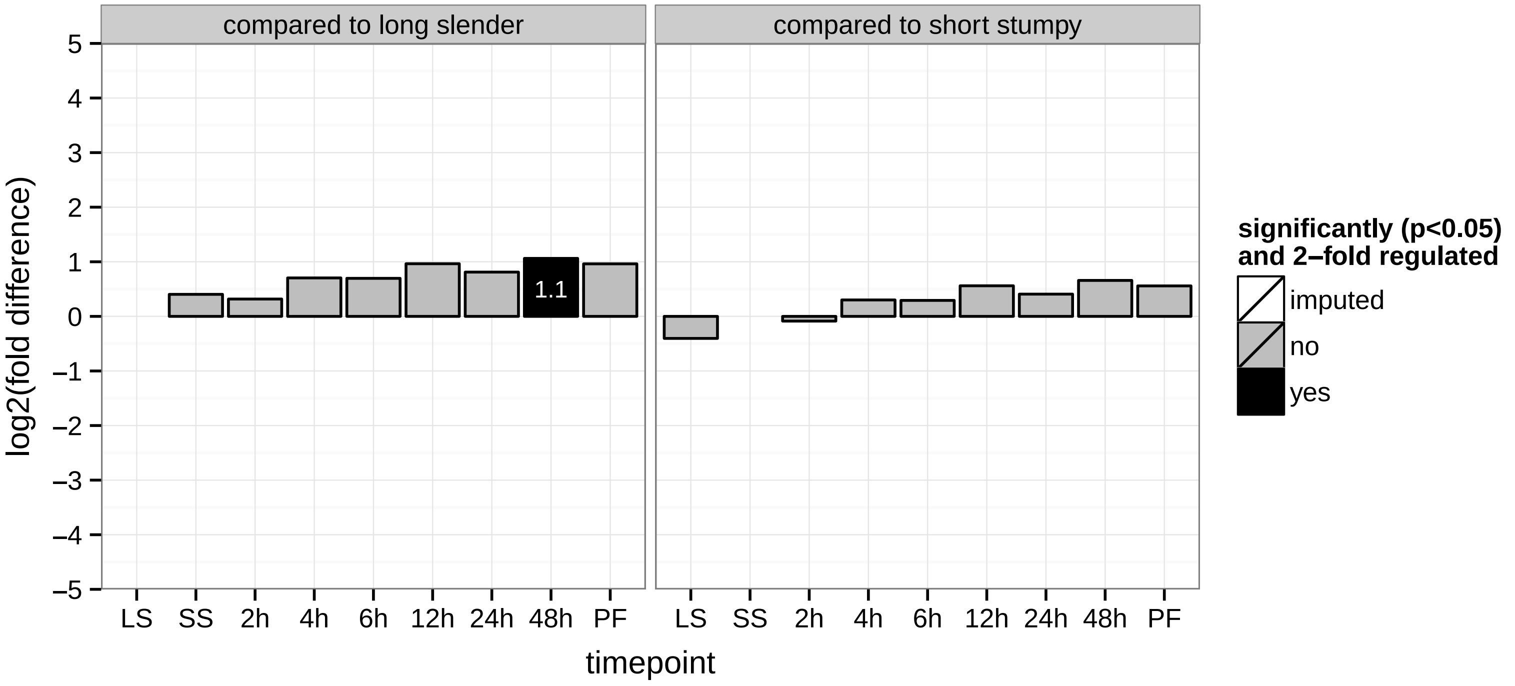

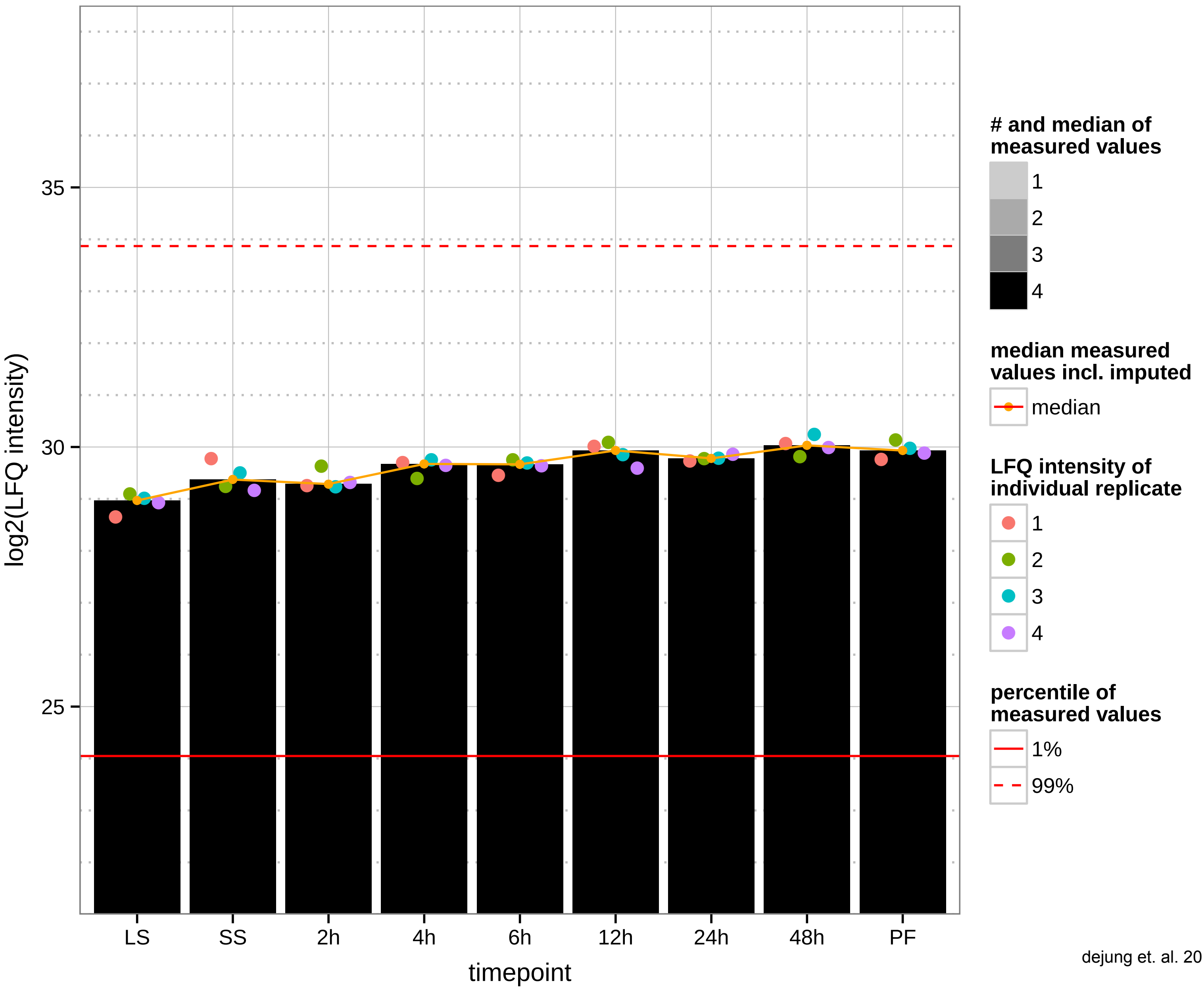

dejung et. al. 2015

T–complex protein 1, beta subunit, putative
Tb927.11.1900
AGOF: ATP binding, unfolded protein binding
AGOC: chaperonin–containing T–complex
AGOP: protein folding, regulation of cell cycle
PGOF: ATP binding, unfolded protein binding
PGOC: null
PGOP: cellular protein metabolic process, protein folding

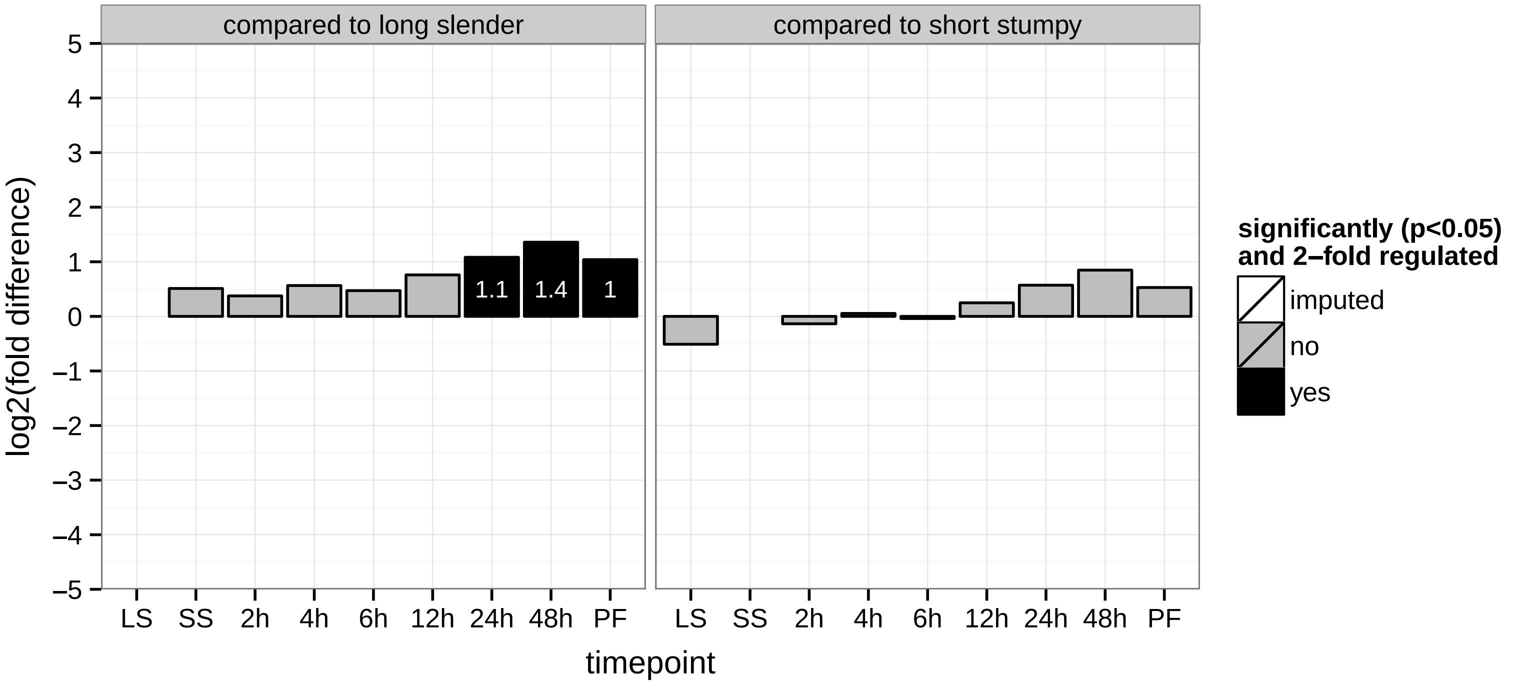

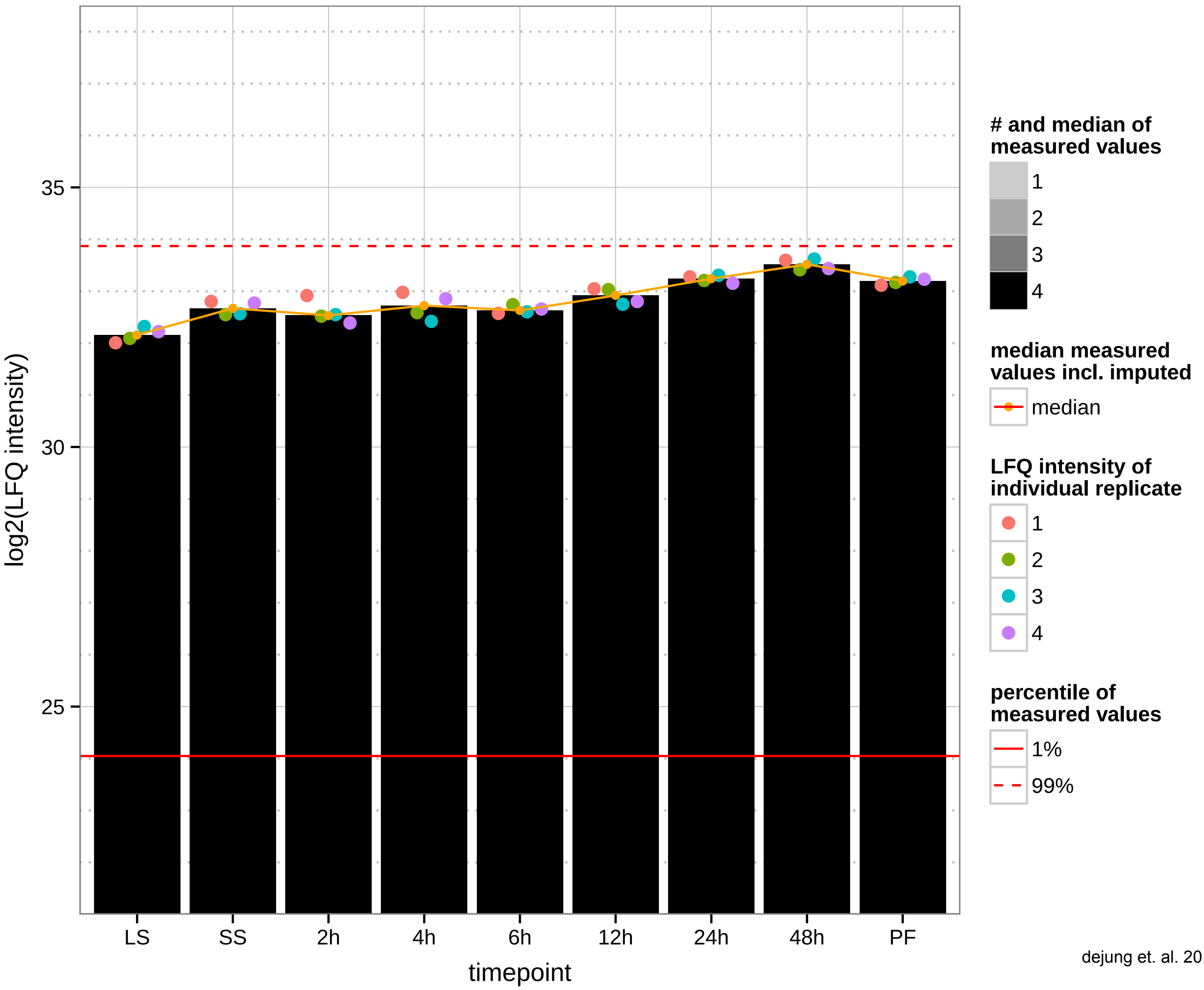

dejung et. al. 2015

ubiquitin carboxyl–terminal hydrolase, putative, cysteine peptidase, Clan CA, family C19
Tb927.11.1930
AGOF: cysteine–type endopeptidase activity, ubiquitin thiolesterase activity
AGOC: null
AGOP: ubiquitin–dependent protein catabolic process
PGOF: ubiquitin thiolesterase activity
PGOC: null
PGOP: ubiquitin–dependent protein catabolic process

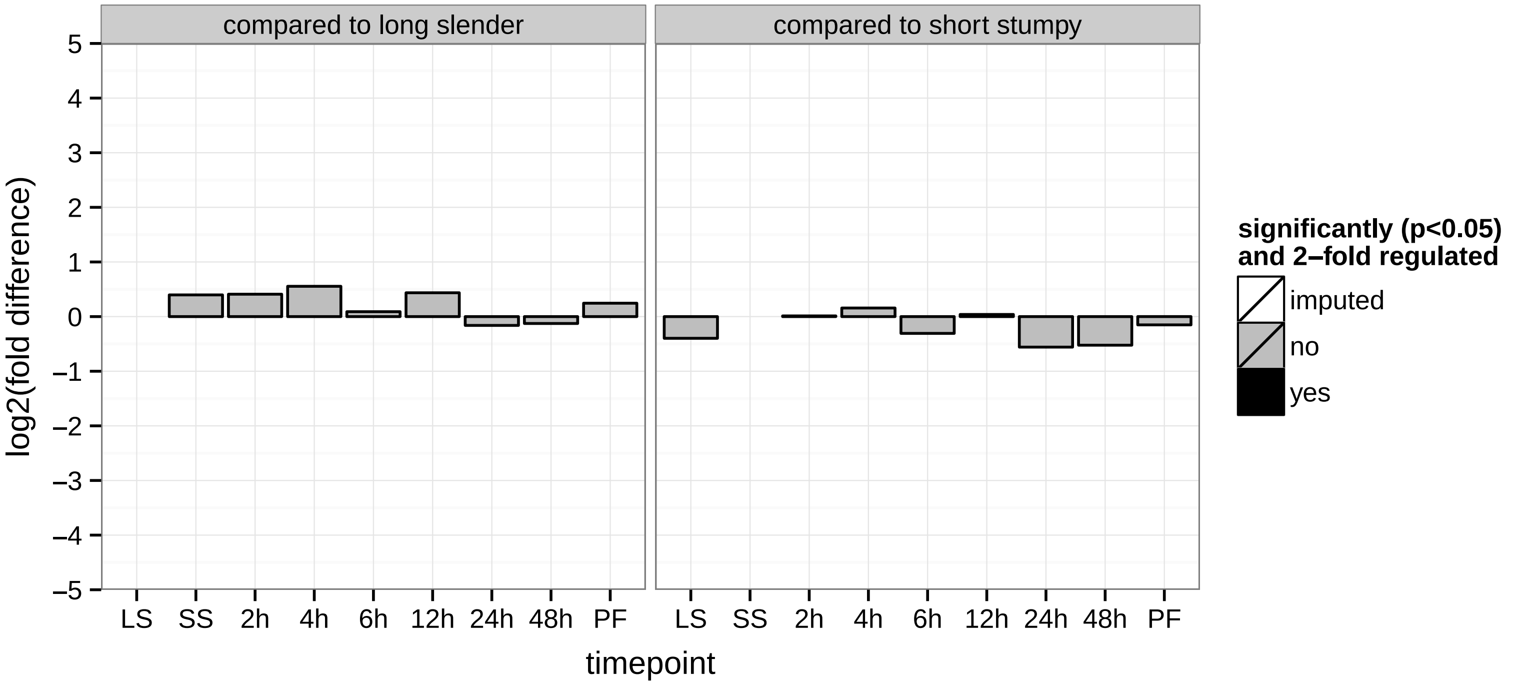

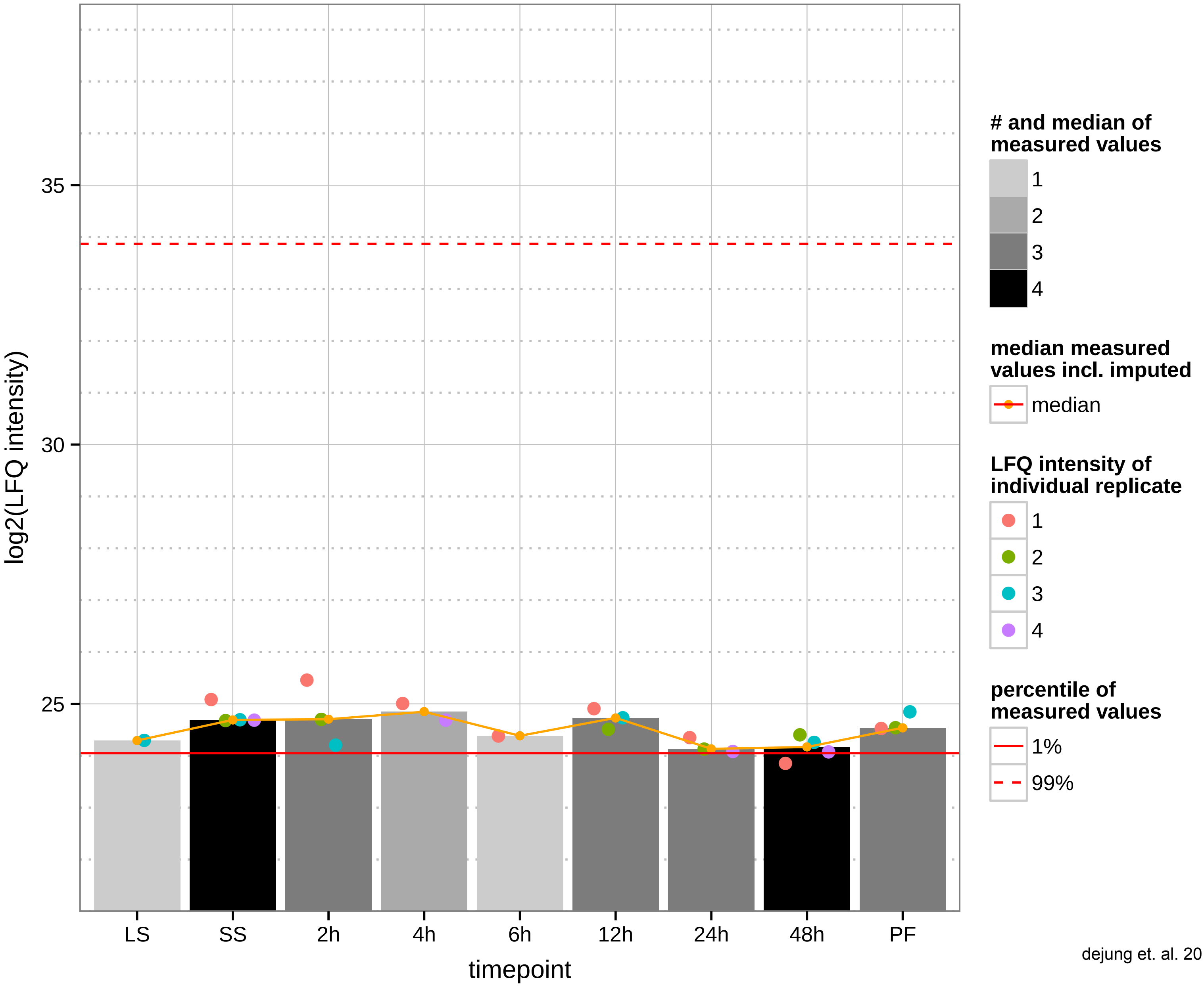

dejung et. al. 2015

leucine–rich repeat protein (LRRP), putative
Tb927.11.1960
AGOF: null
AGOC: null
AGOP: null
PGOF: protein binding
PGOC: null
PGOP: null

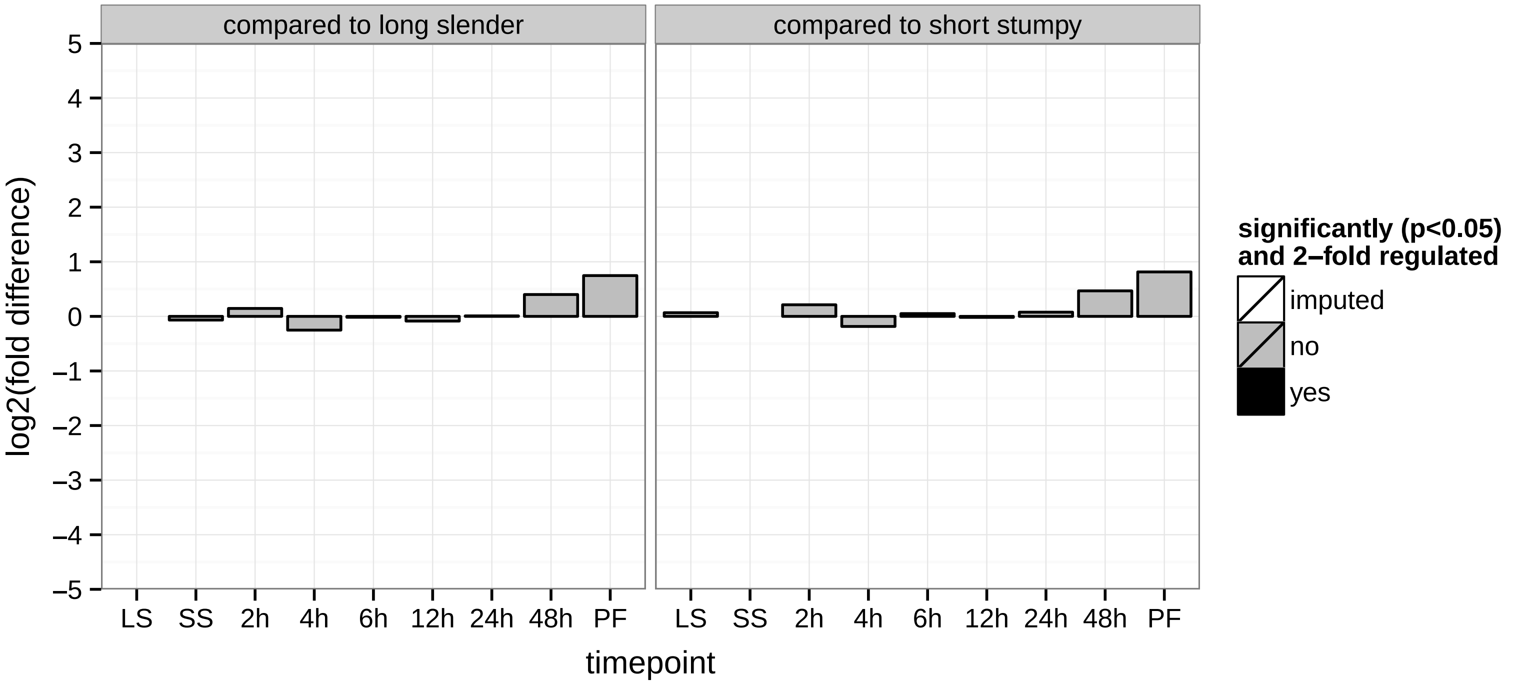

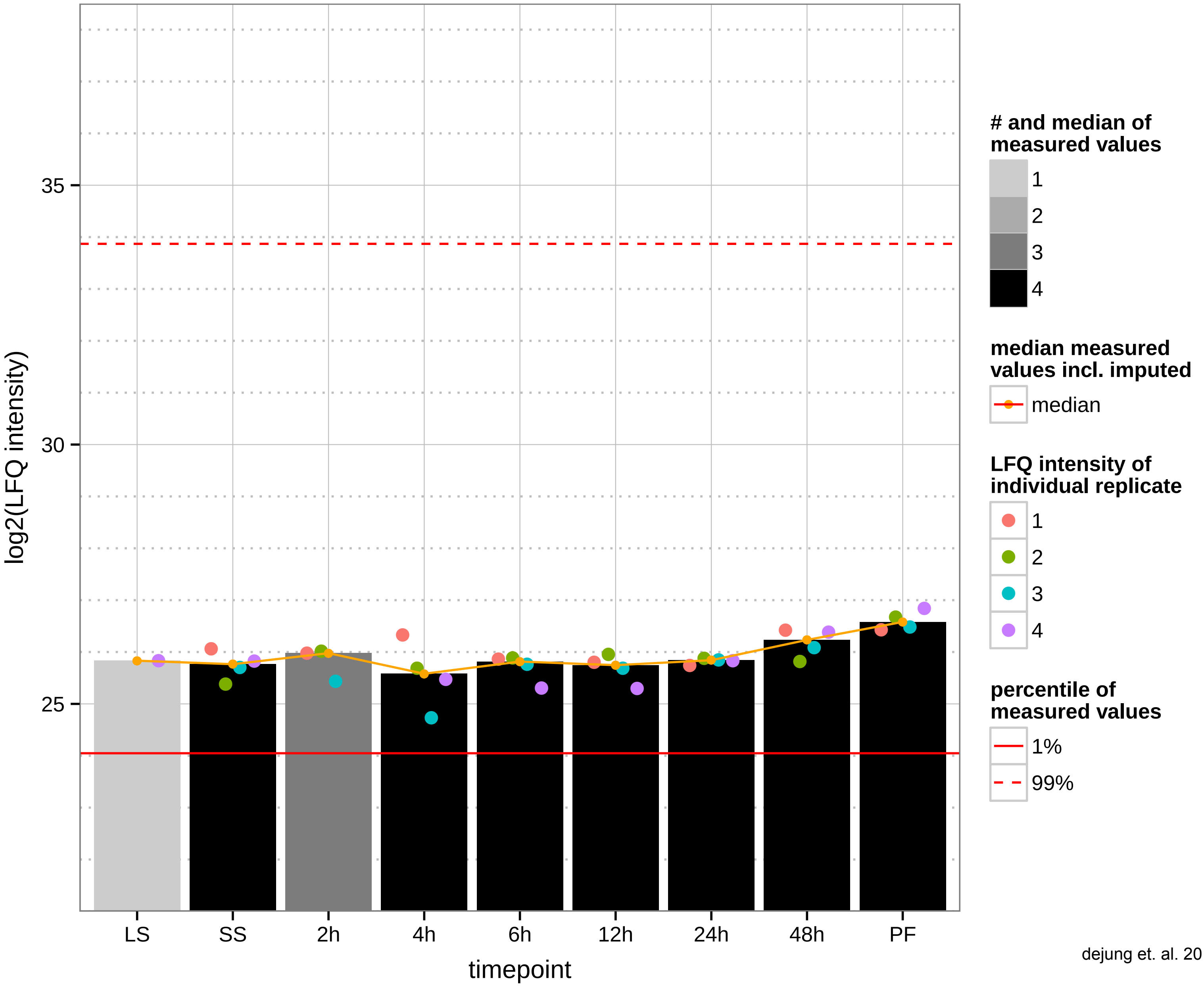

dejung et. al. 2015

hypothetical protein, conserved
Tb927.11.2030
AGOF: null
AGOC: null
AGOP: null
PGOF: null
PGOC: null
PGOP: null

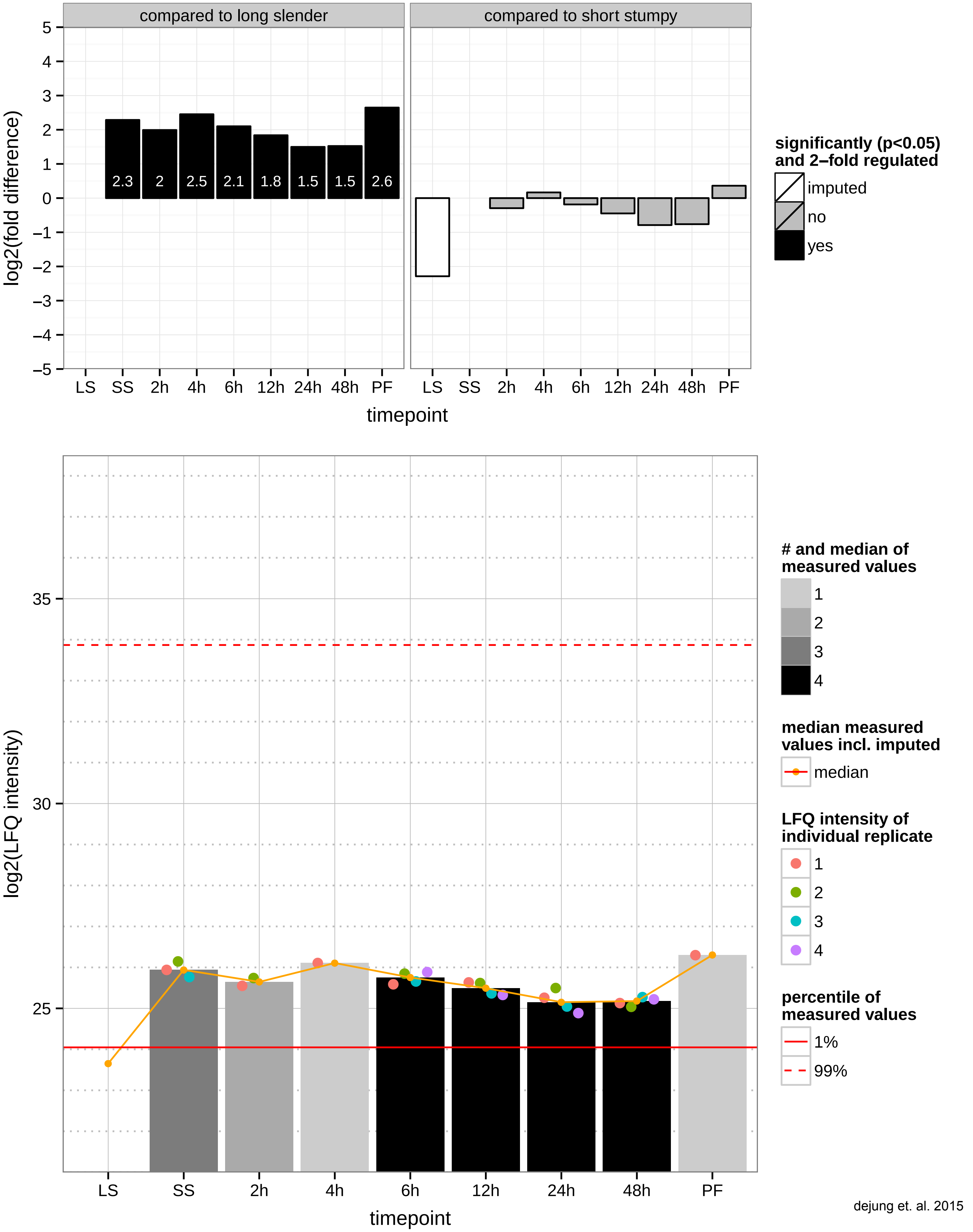

dejung et. al. 2015

60S acidic ribosomal subunit protein, putative
Tb927.11.2060;Tb927.11.2050
AGOF: structural constituent of ribosome
AGOC: intracellular, large ribosomal subunit, ribosome
AGOP: ribosome biogenesis, translation, translational elongation
PGOF: null, structural constituent of ribosome
PGOC: null, intracellular, ribosome
PGOP: null, ribosome biogenesis, translational elongation

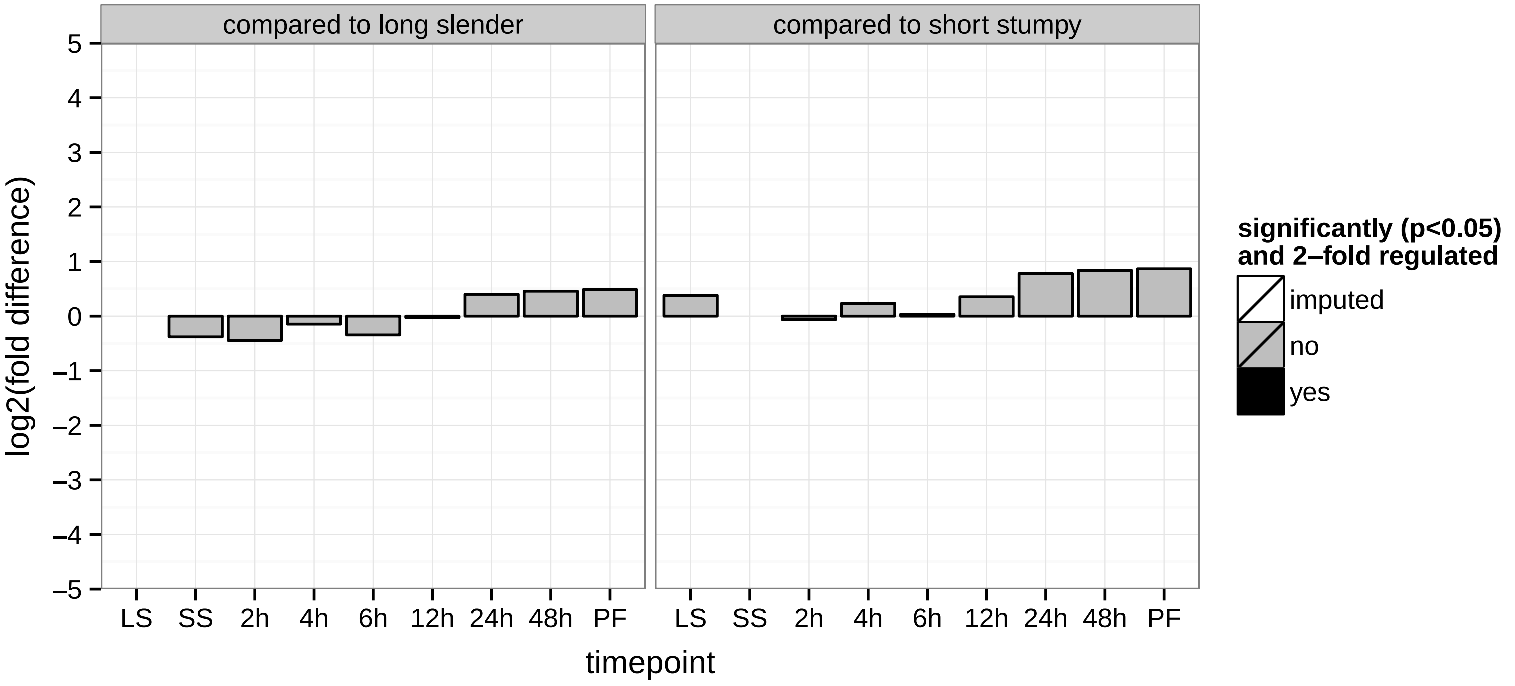

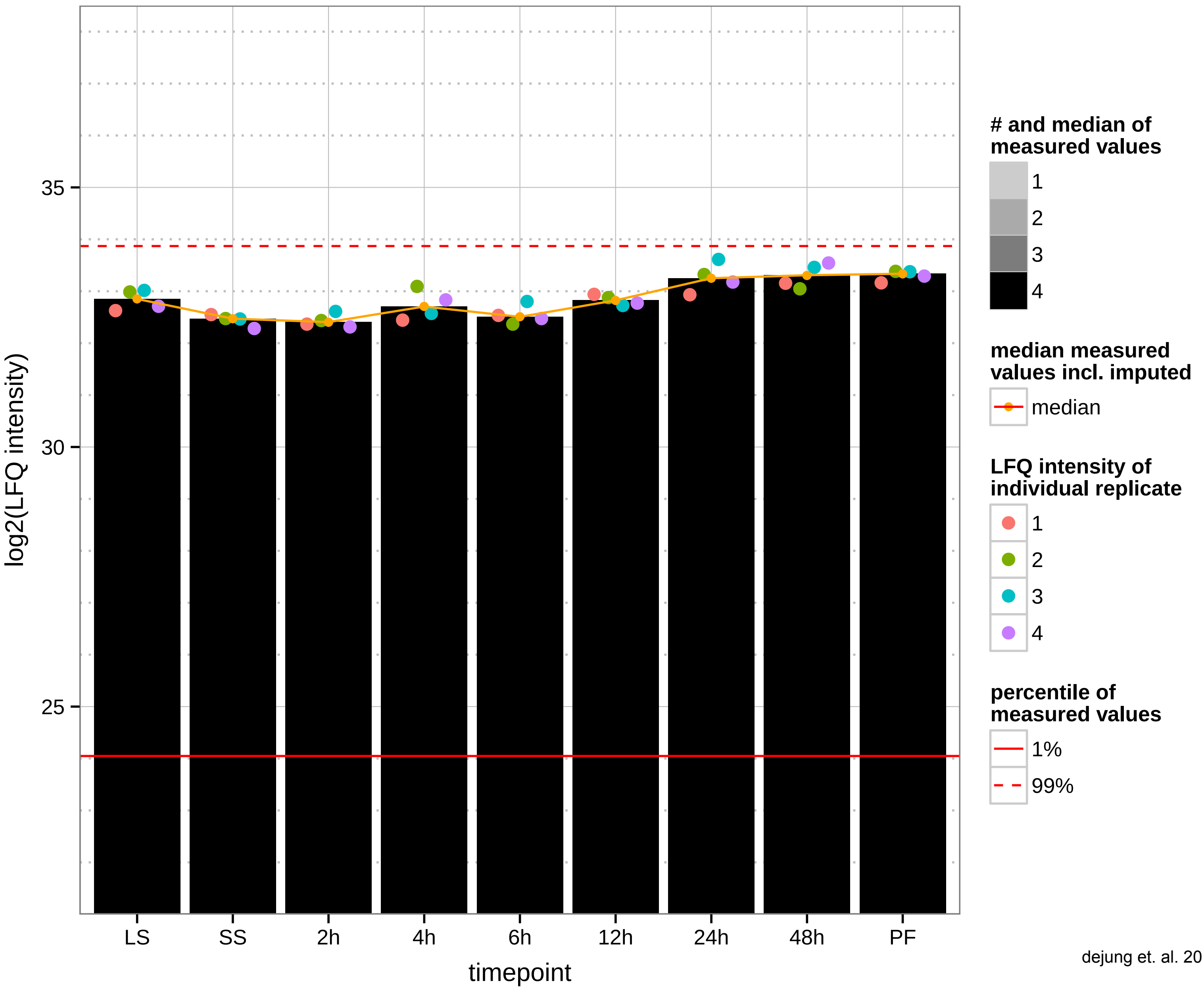

dejung et. al. 2015

hypothetical protein, conserved
Tb927.11.2080
AGOF: null
AGOC: null
AGOP: null
PGOF: null
PGOC: null
PGOP: null

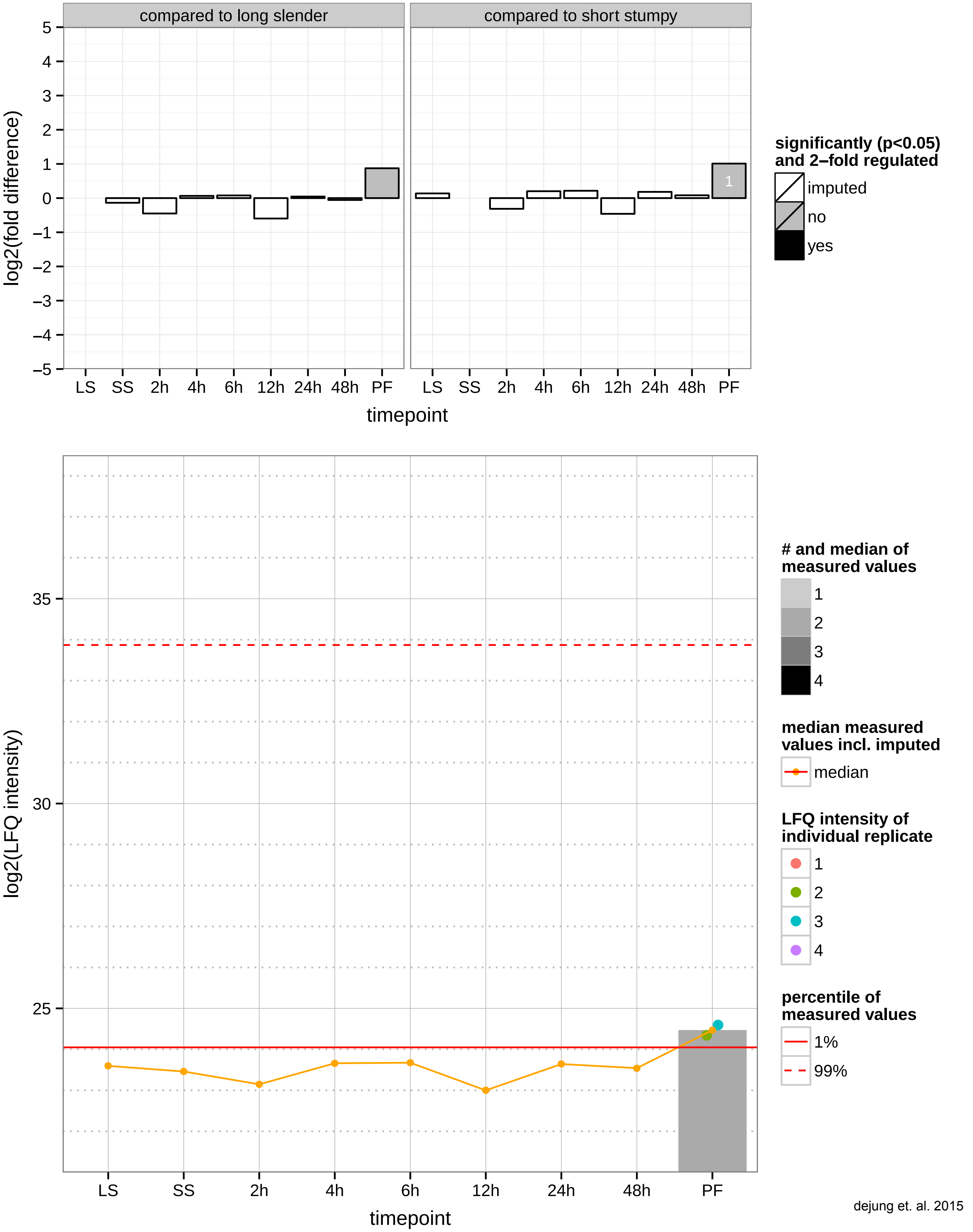

choline kinase (EK1)
Tb927.11.2090
AGOF: ethanolamine kinase activity
AGOC: cytosol
AGOP: phosphatidylethanolamine biosynthetic process
PGOF: phosphotransferase activity, alcohol group as acceptor, transferase activity, transferring phosphorus–containing groups
PGOC: null
PGOP: null

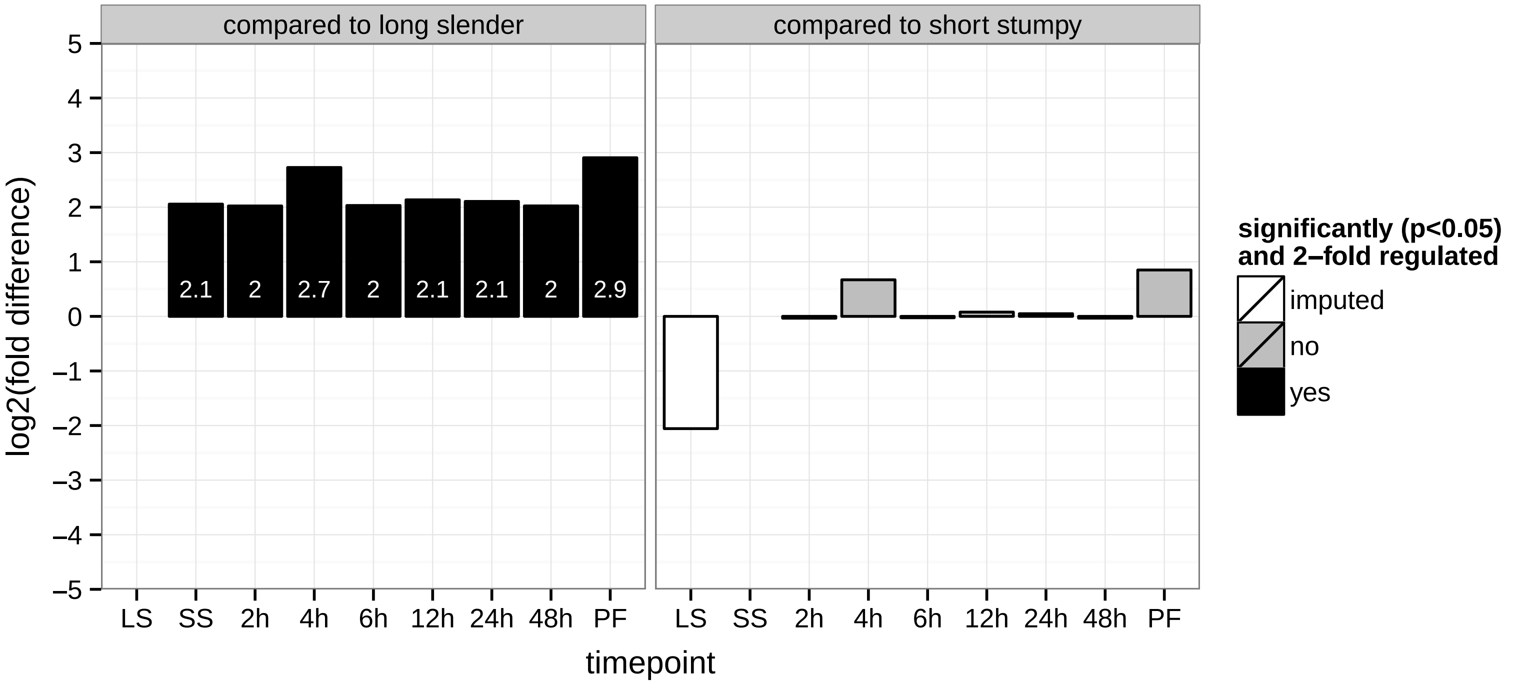

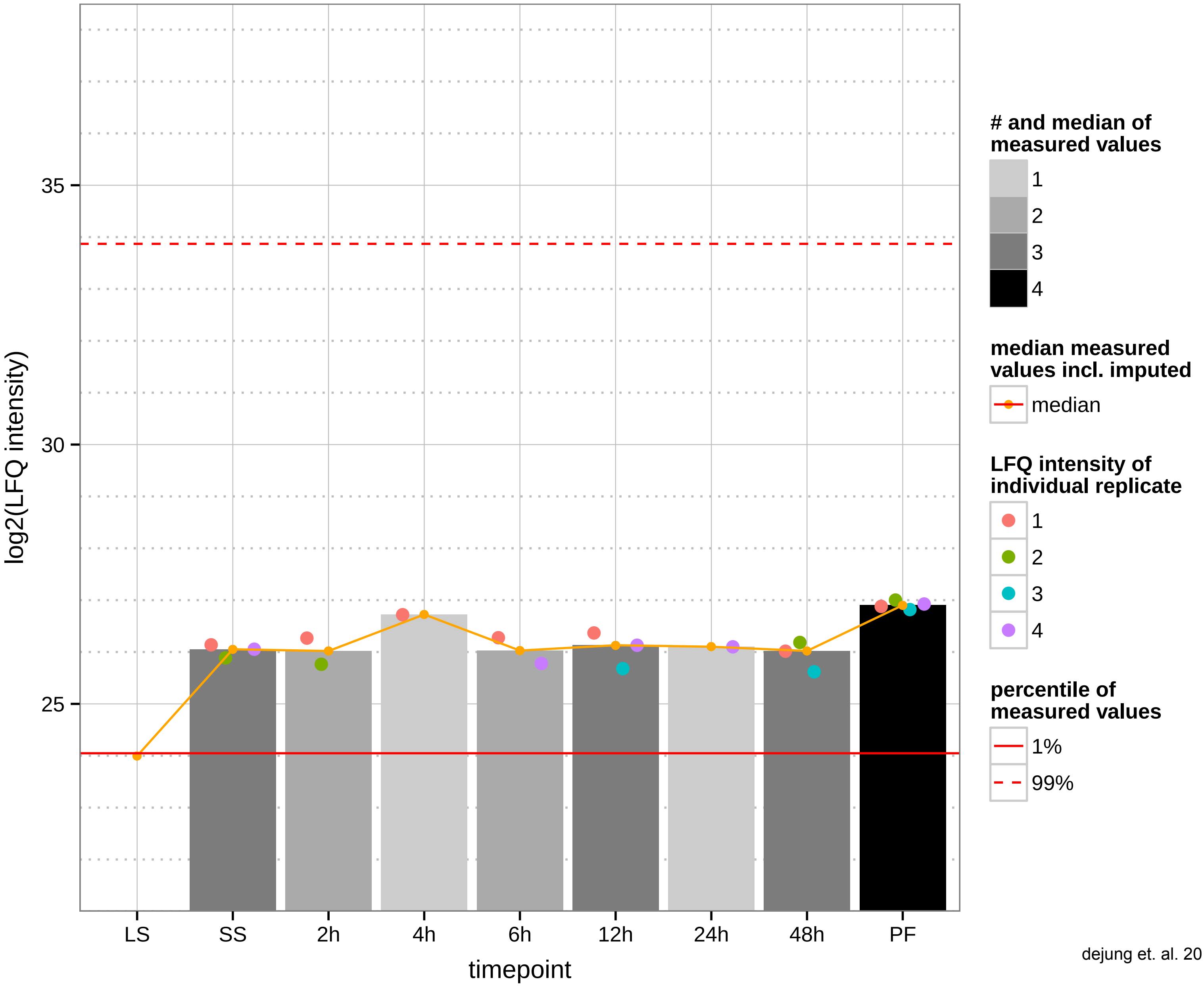

dejung et. al. 2015

hypothetical protein, conserved
Tb927.11.210
AGOF: null
AGOC: null
AGOP: null
PGOF: null
PGOC: null
PGOP: null

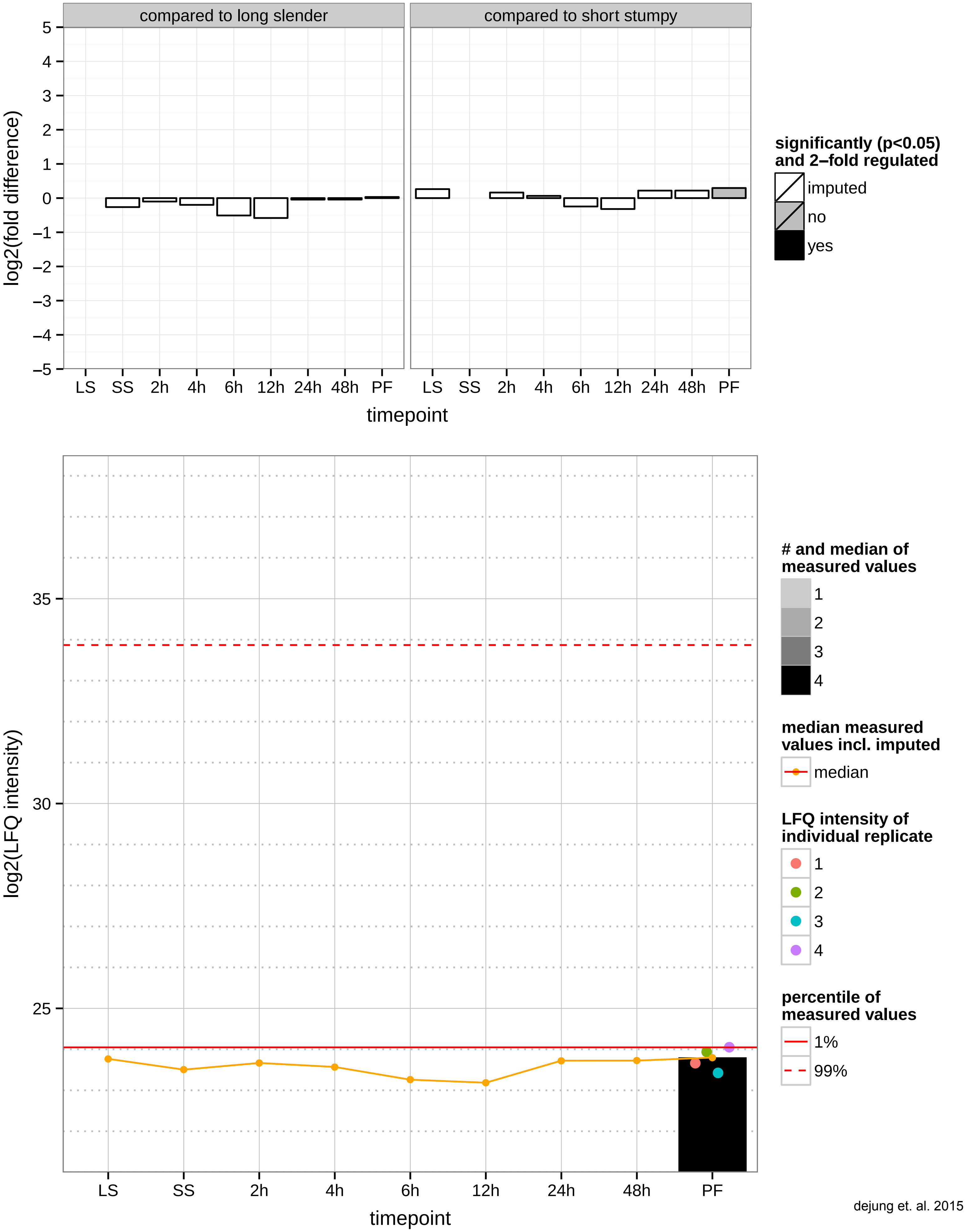

dejung et. al. 2015

hypothetical protein, conserved
Tb927.11.2110
AGOF: null
AGOC: null
AGOP: protein transport
PGOF: null
PGOC: null
PGOP: protein transport

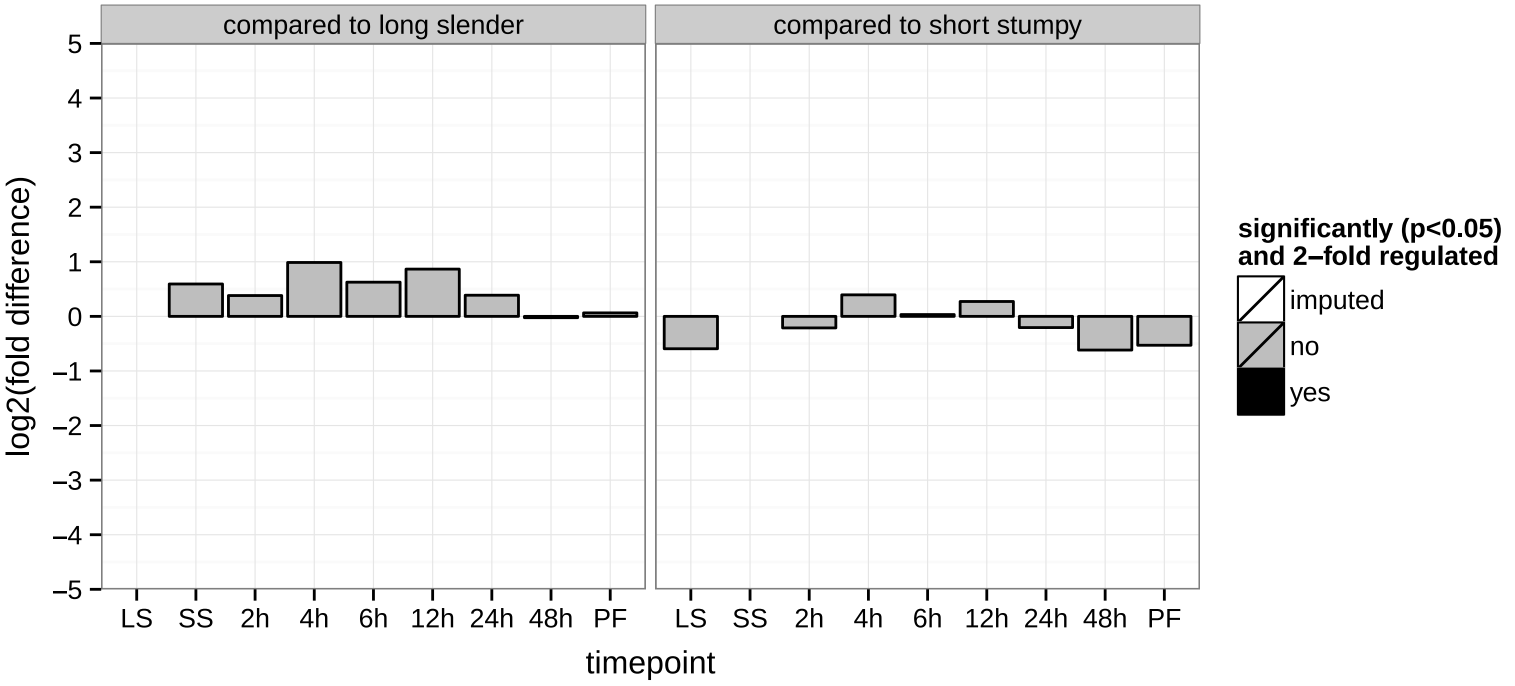

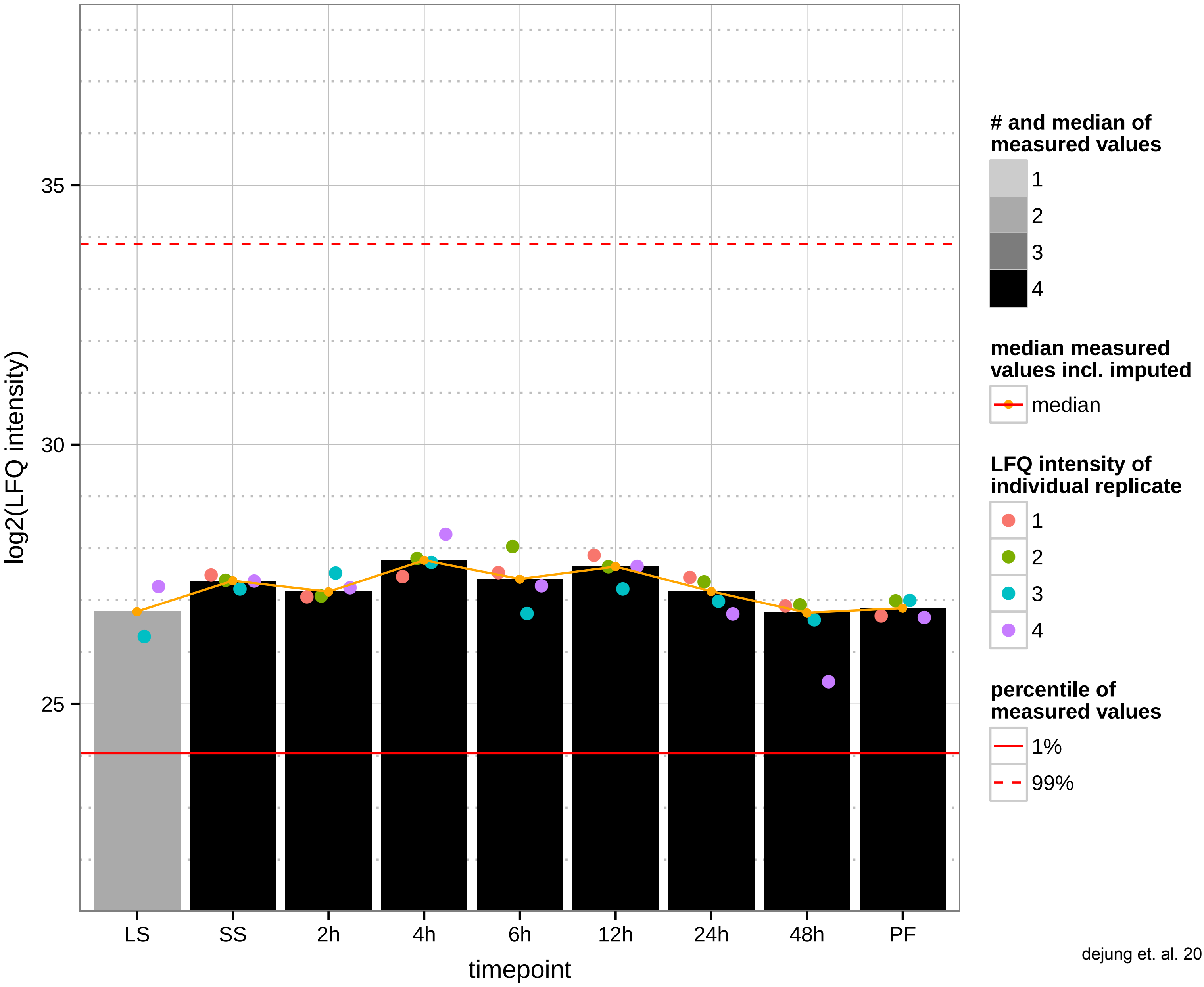

dejung et. al. 2015

proteasome regulatory non–ATP–ase subunit 3 (RPN3)
Tb927.11.2130
AGOF: endopeptidase activity, enzyme regulator activity
AGOC: proteasome complex, proteasome regulatory particle
AGOP: regulation of protein catabolic process, ubiquitin–dependent protein catabolic process
PGOF: enzyme regulator activity, protein binding
PGOC: proteasome complex
PGOP: regulation of protein catabolic process

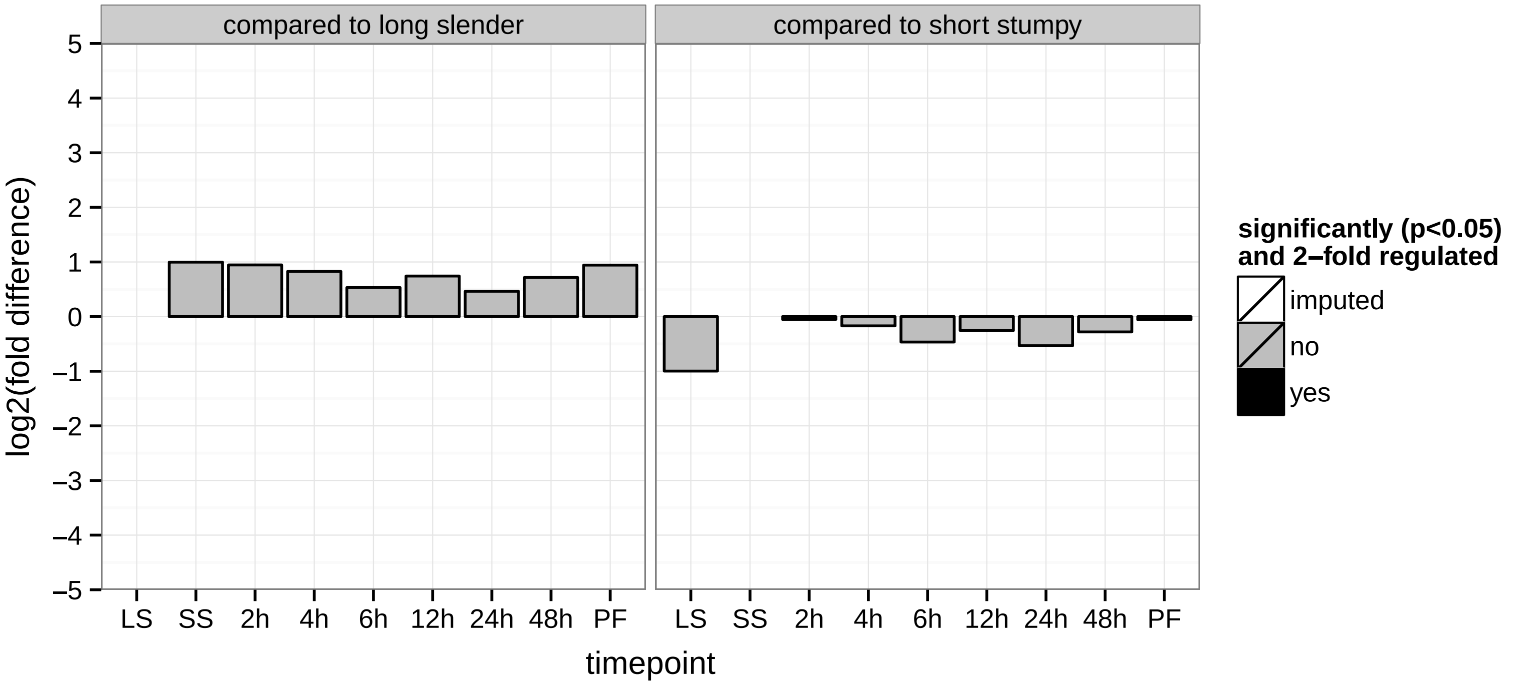

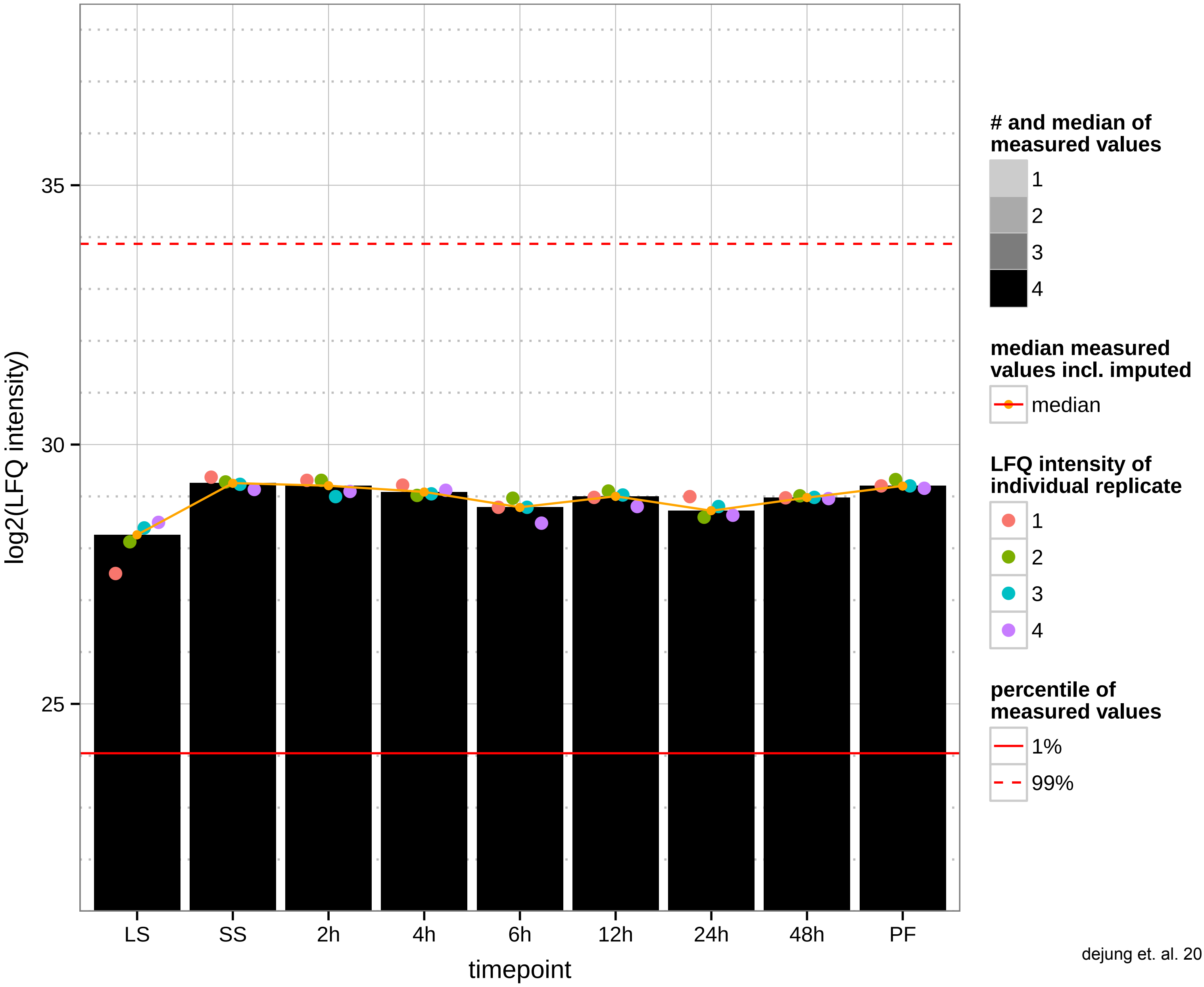

dejung et. al. 2015

hypothetical protein, conserved
Tb927.11.2140
AGOF: null
AGOC: null
AGOP: null
PGOF: null
PGOC: null
PGOP: null

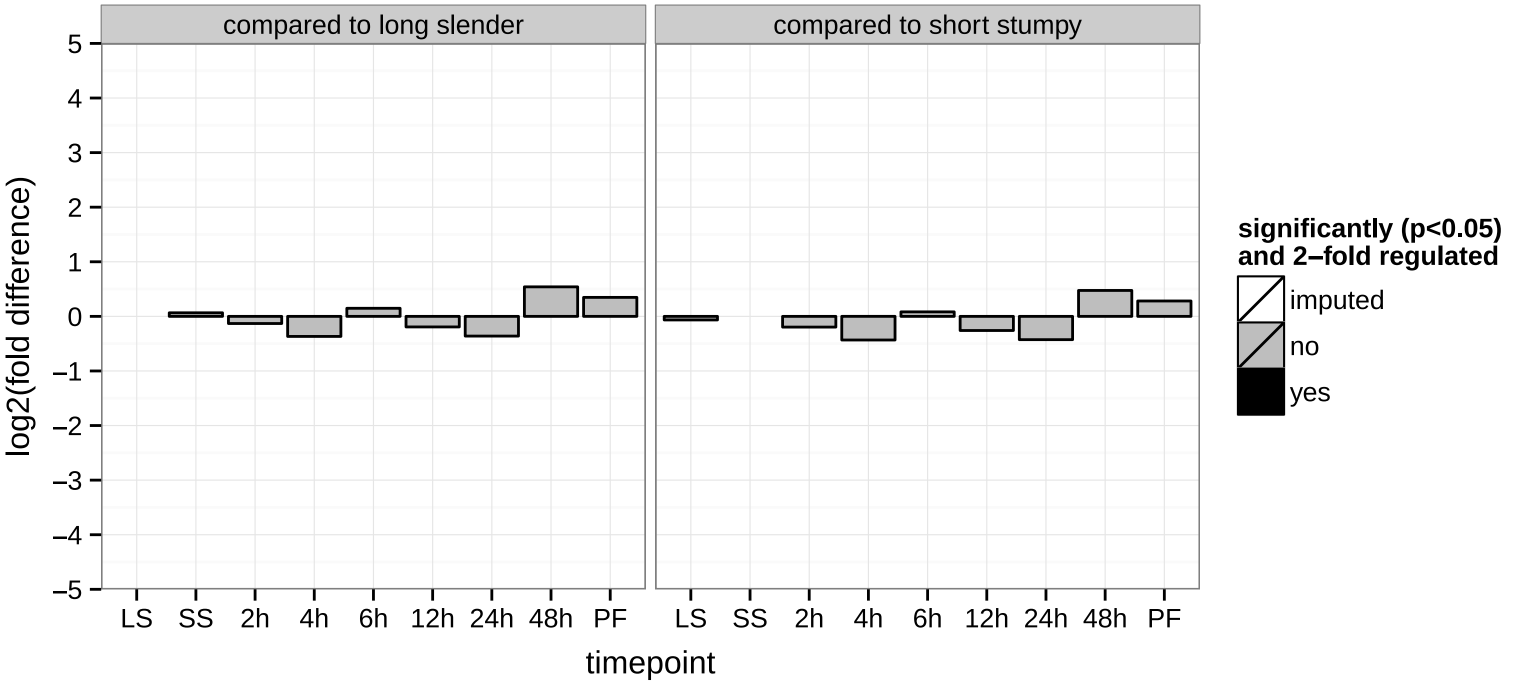

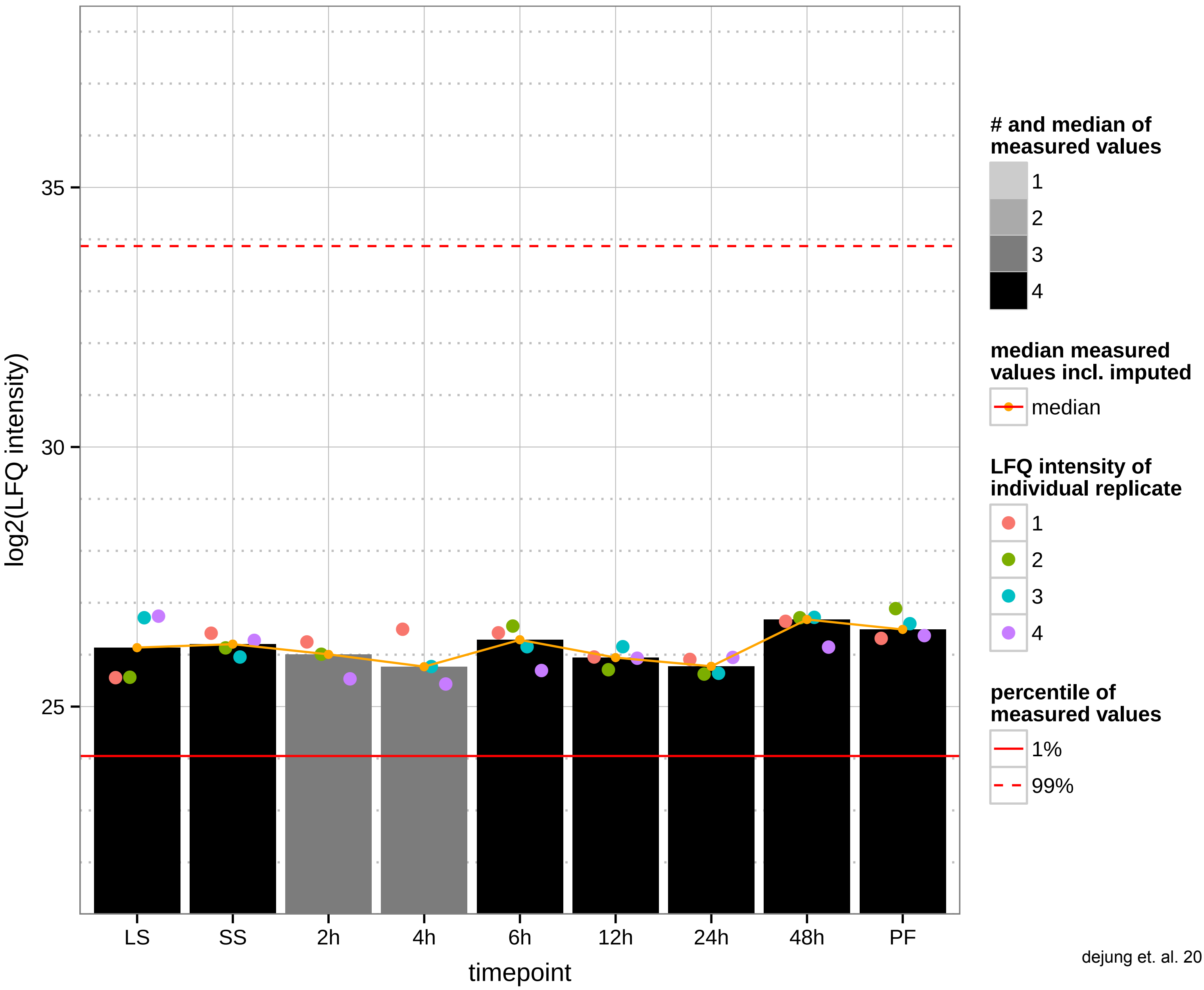

dejung et. al. 2015

hypothetical protein, conserved
Tb927.11.2190
AGOF: null
AGOC: null
AGOP: null
PGOF: null
PGOC: null
PGOP: null

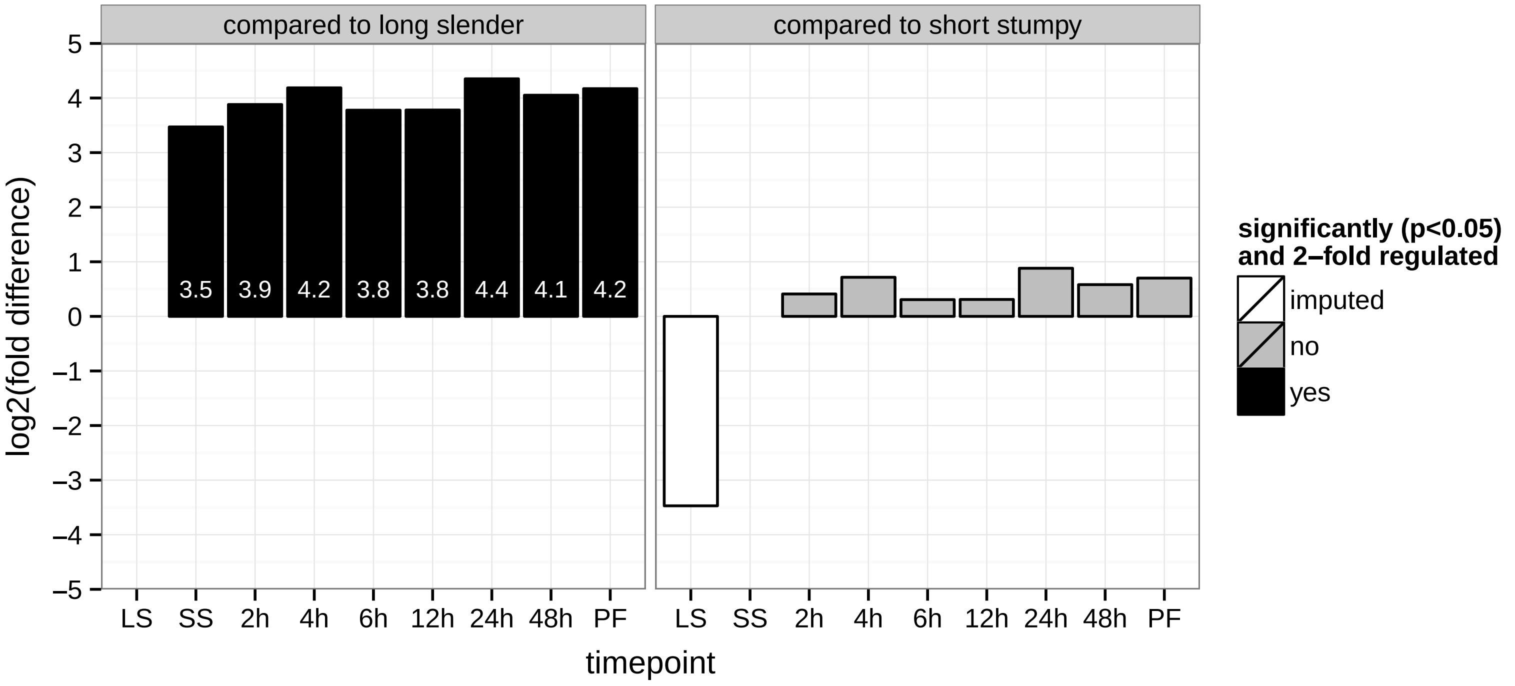

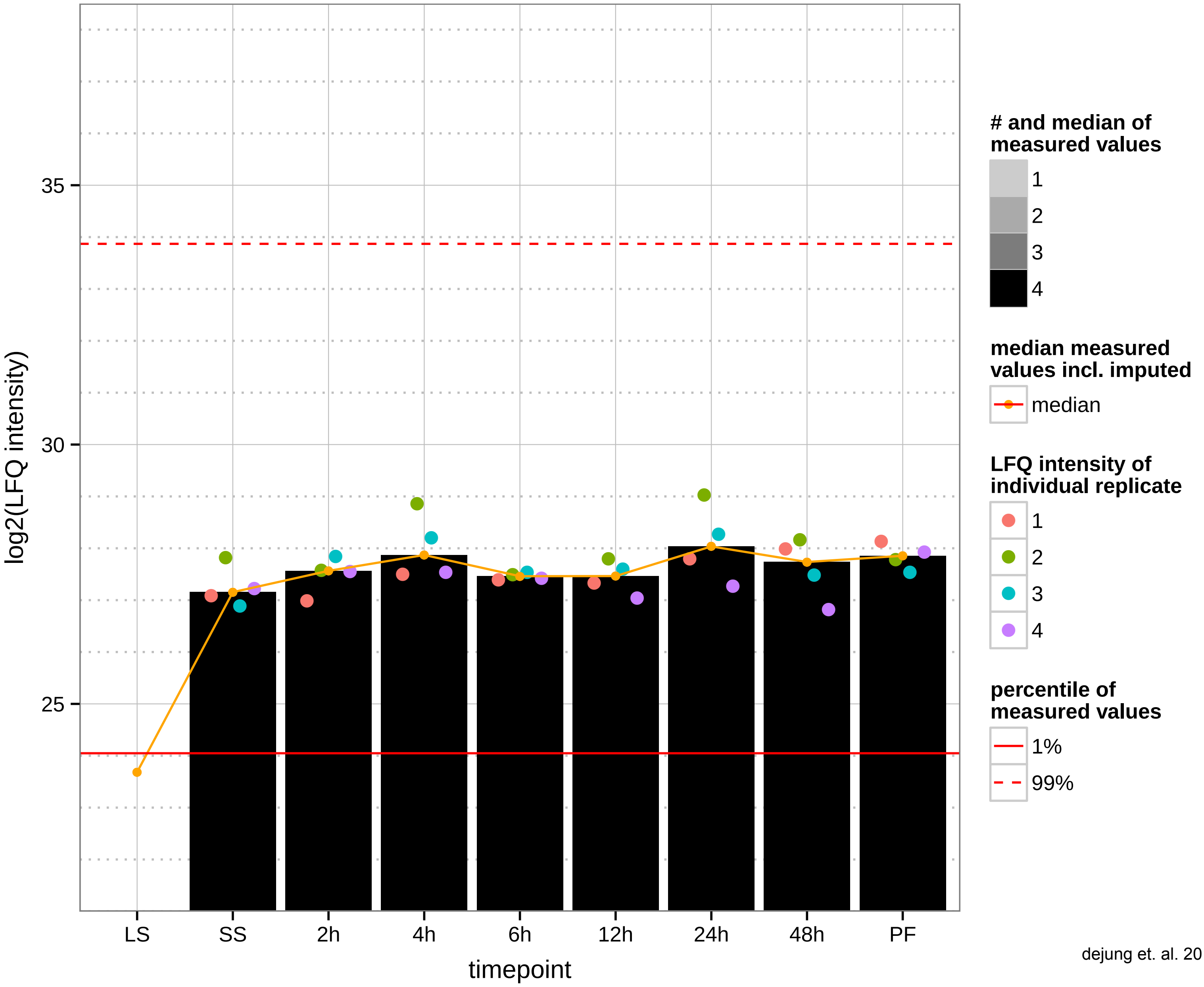

dejung et. al. 2015

eukaryotic translation initiation factor 4e, putative (eIF4E)
Tb927.11.2260
AGOF: RNA binding, translation initiation factor activity
AGOC: cytoplasm
AGOP: translational initiation
PGOF: RNA binding, translation initiation factor activity
PGOC: cytoplasm
PGOP: translational initiation

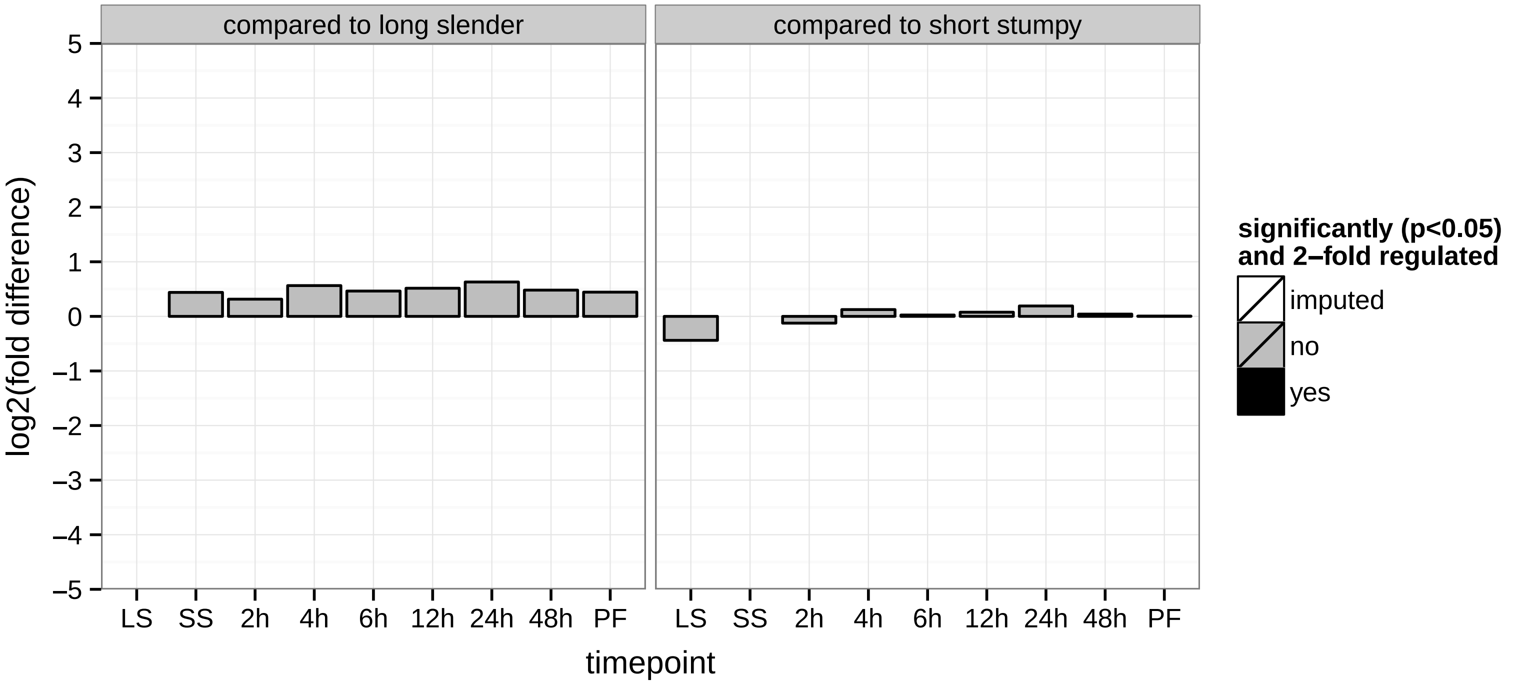

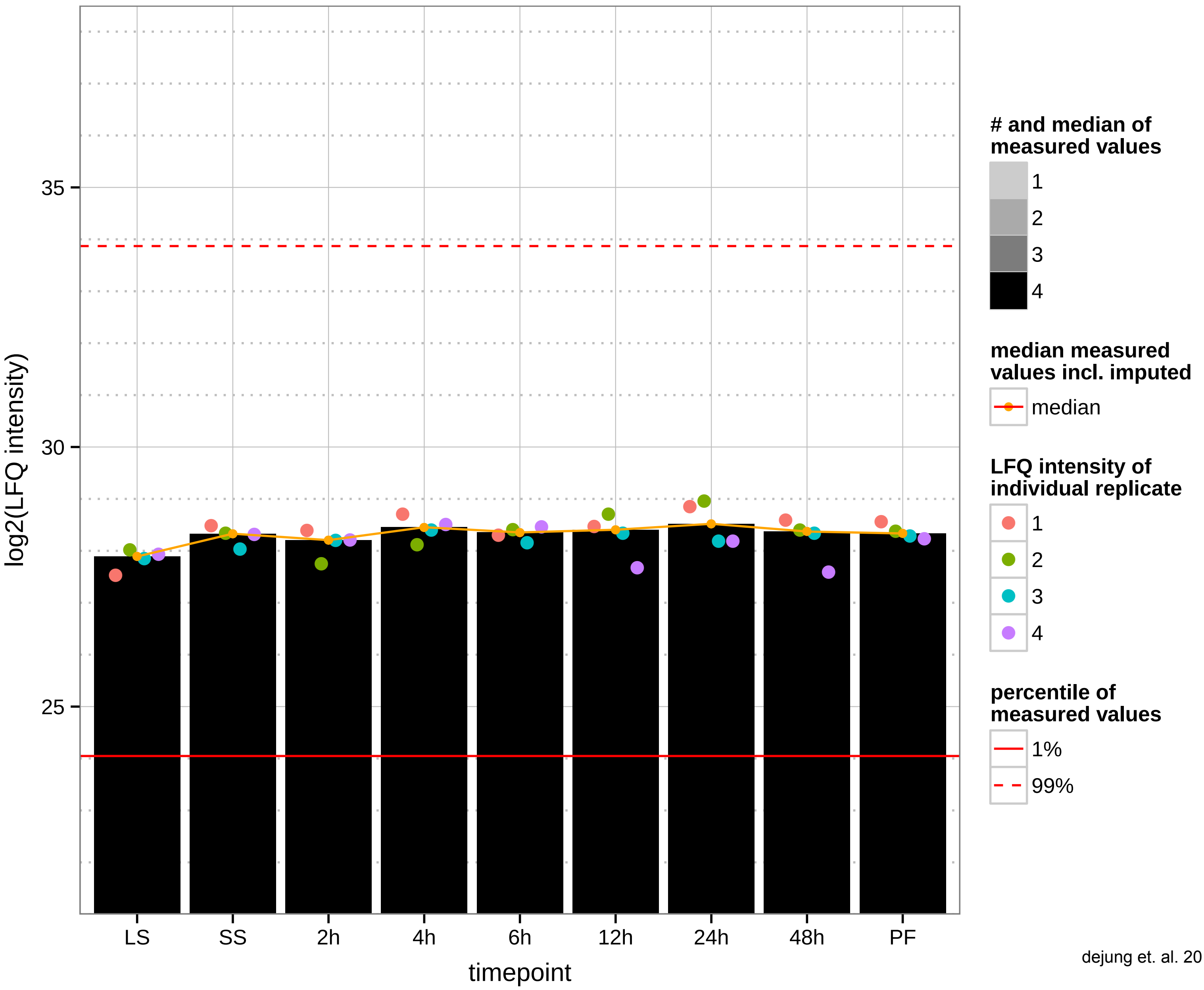

dejung et. al. 2015

dynein intermediate chain IC140 (IDA7), putative
Tb927.11.2270
AGOF: null
AGOC: null
AGOP: ciliary or flagellar motility
PGOF: protein binding
PGOC: null
PGOP: null

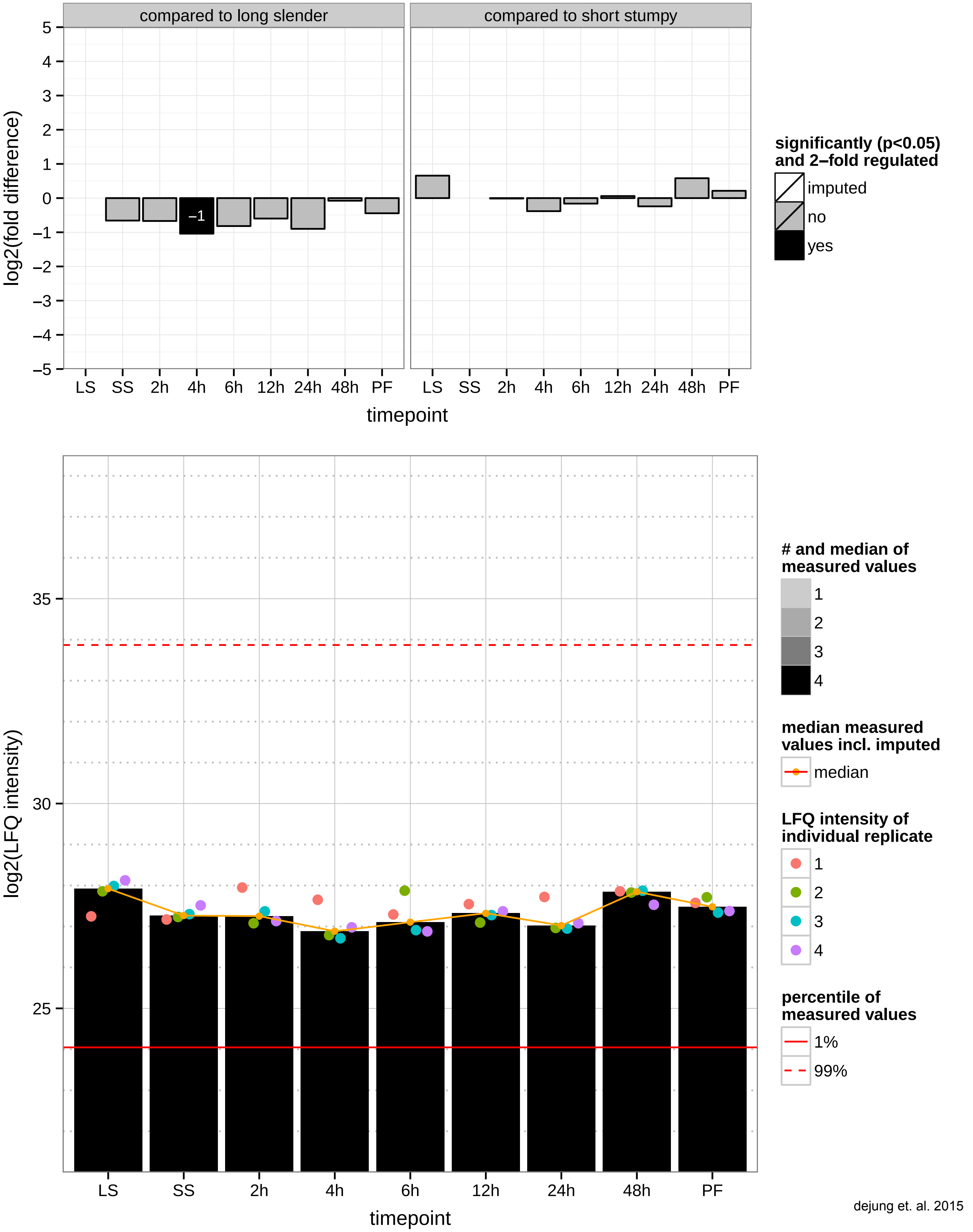

dejung et. al. 2015

hypothetical protein, conserved
Tb927.11.2280
AGOF: null
AGOC: null
AGOP: null
PGOF: protein binding
PGOC: null
PGOP: null

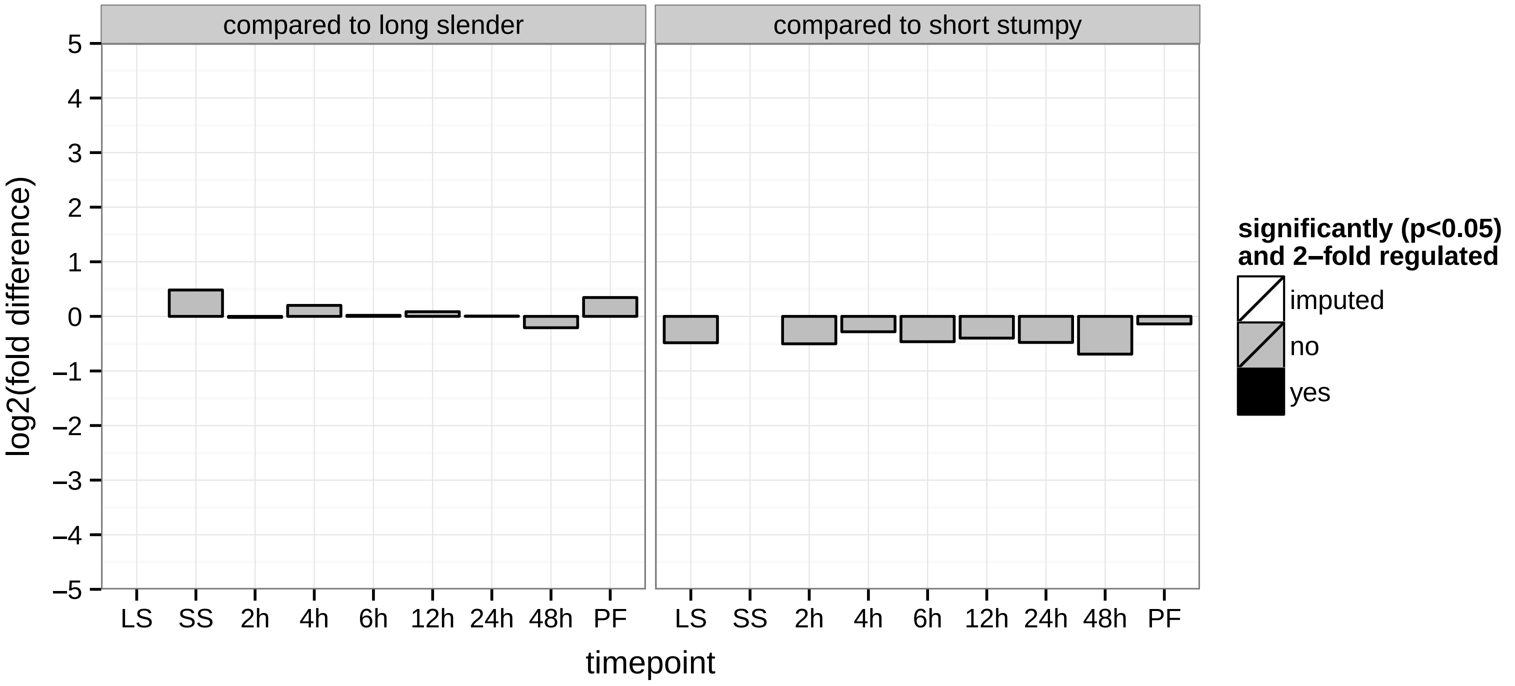

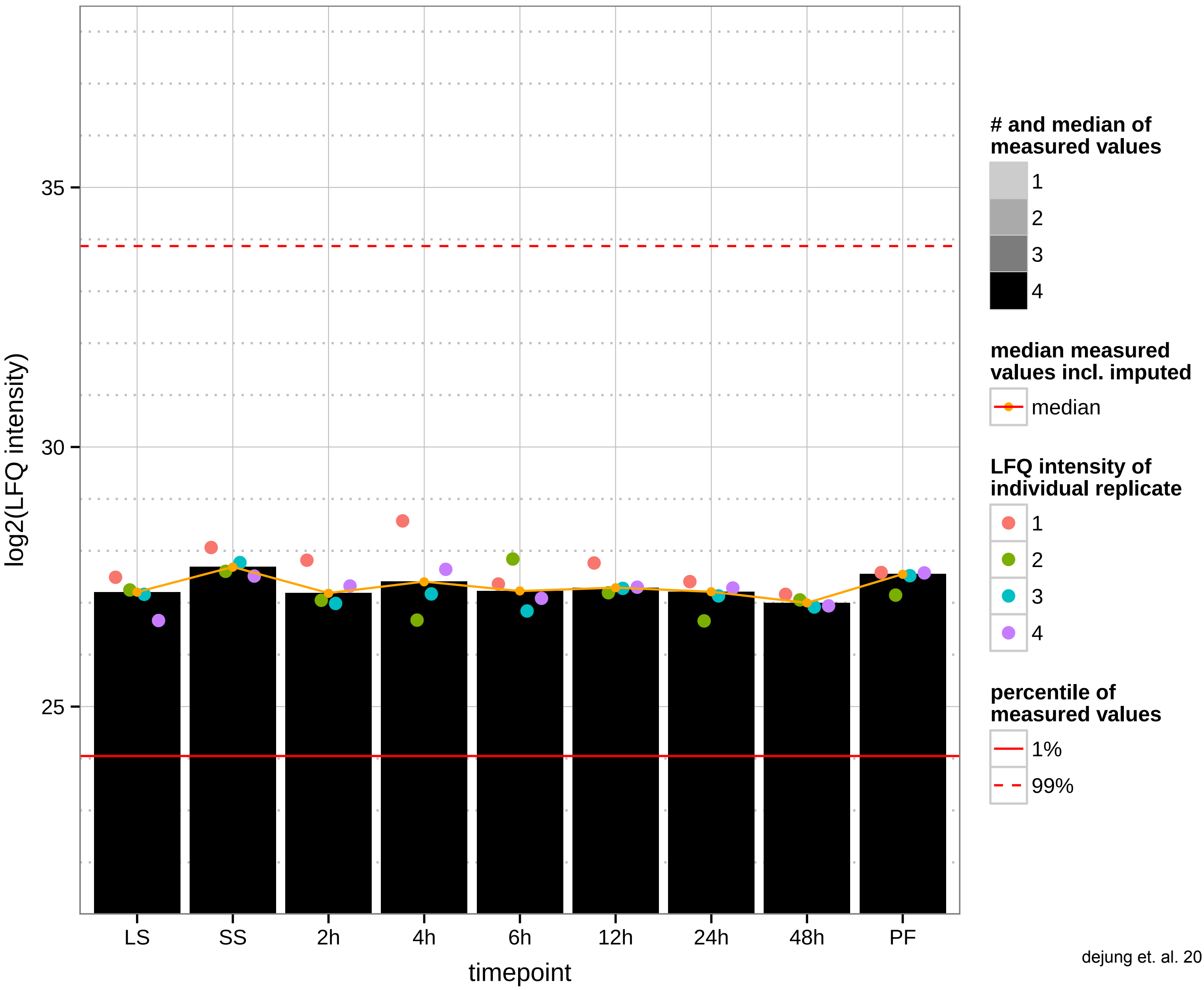

dejung et. al. 2015

cleavage and polyadenylation specificity factor, putative
Tb927.11.230
AGOF: mRNA 3'–UTR binding
AGOC: mRNA cleavage and polyadenylation specificity factor complex
AGOP: mRNA cleavage, mRNA polyadenylation
PGOF: null
PGOC: null
PGOP: null

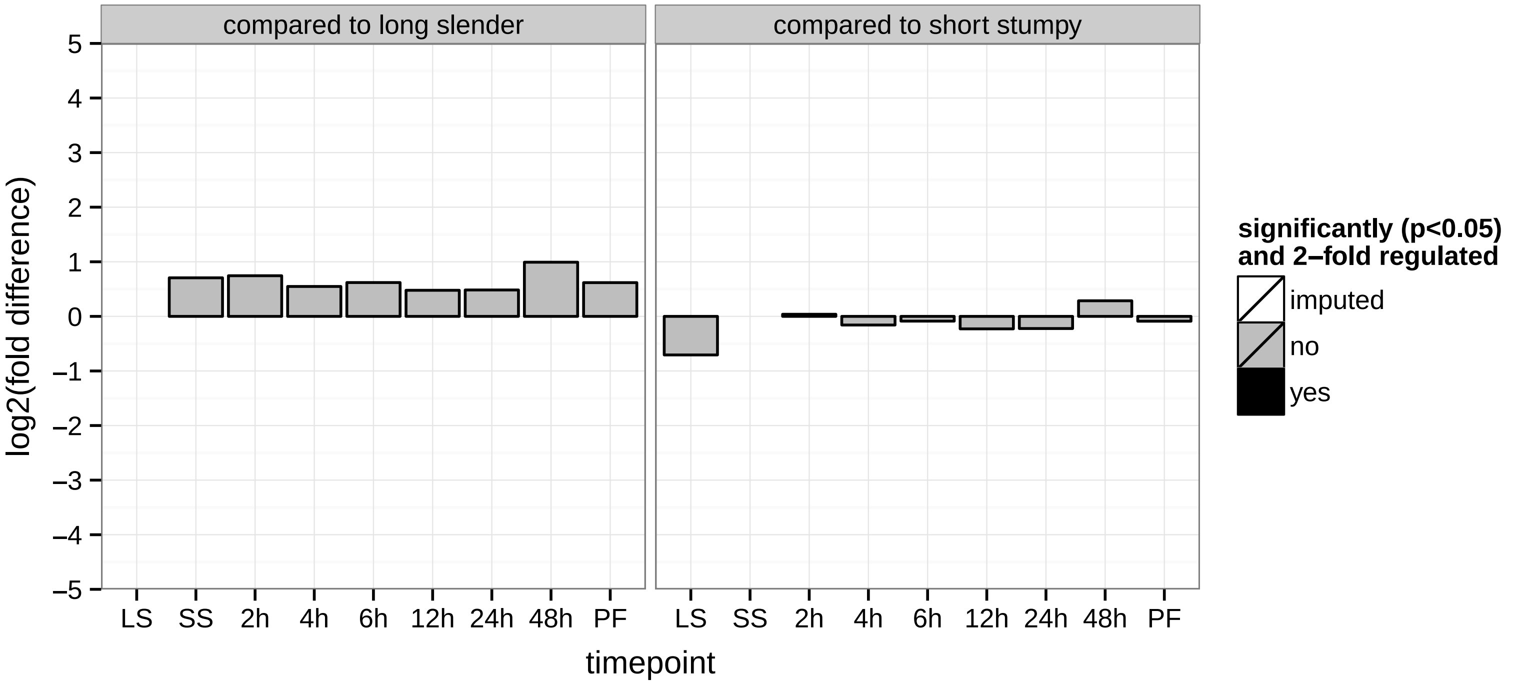

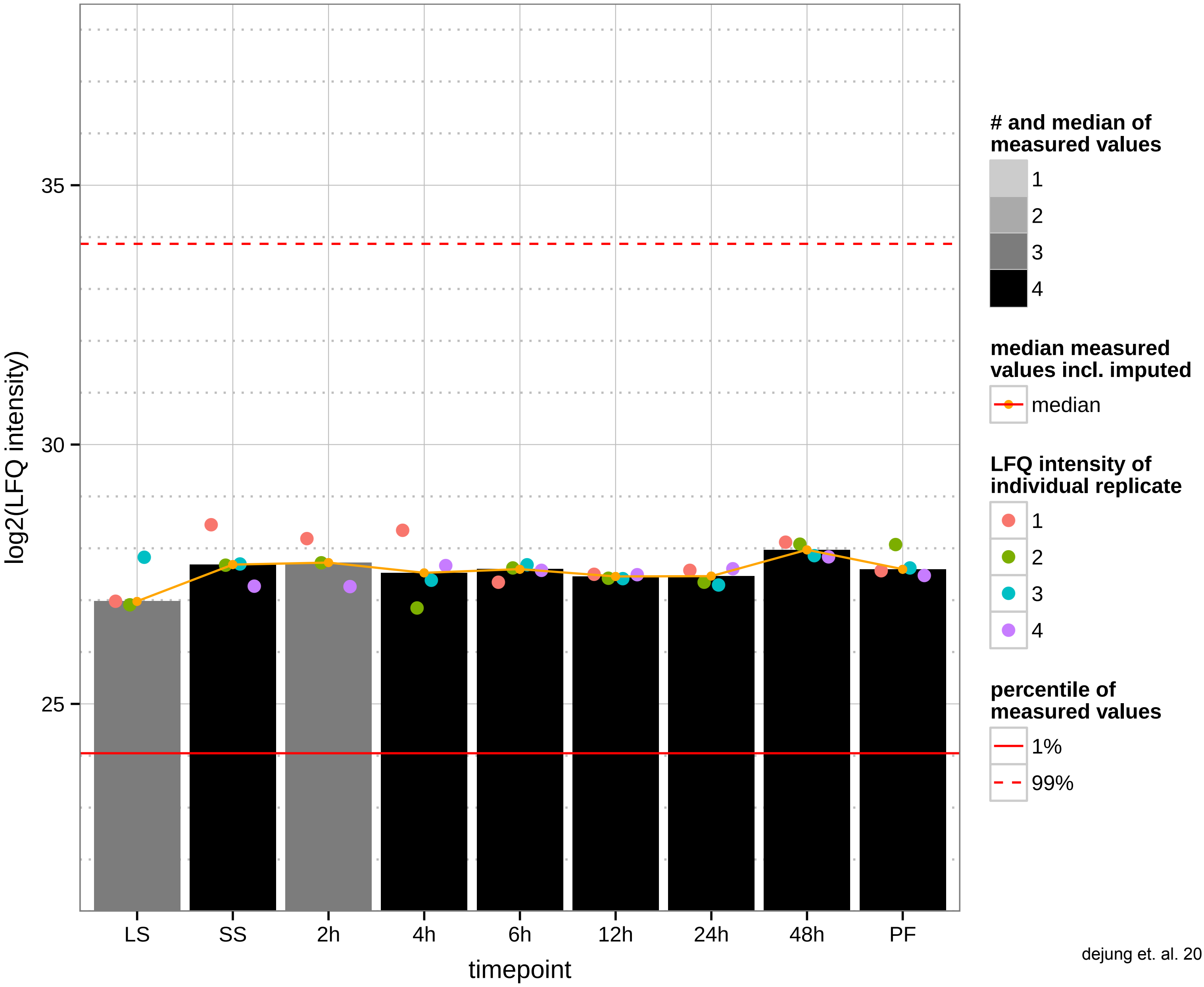

dejung et. al. 2015

eukaryotic peptide chain release factor subunit 1, putative (ERF1)
Tb927.11.2300
AGOF: translation release factor activity, translation release factor activity, codon specific
AGOC: null
AGOP: translation, translational termination
PGOF: translation release factor activity, codon specific
PGOC: cytoplasm
PGOP: translational termination

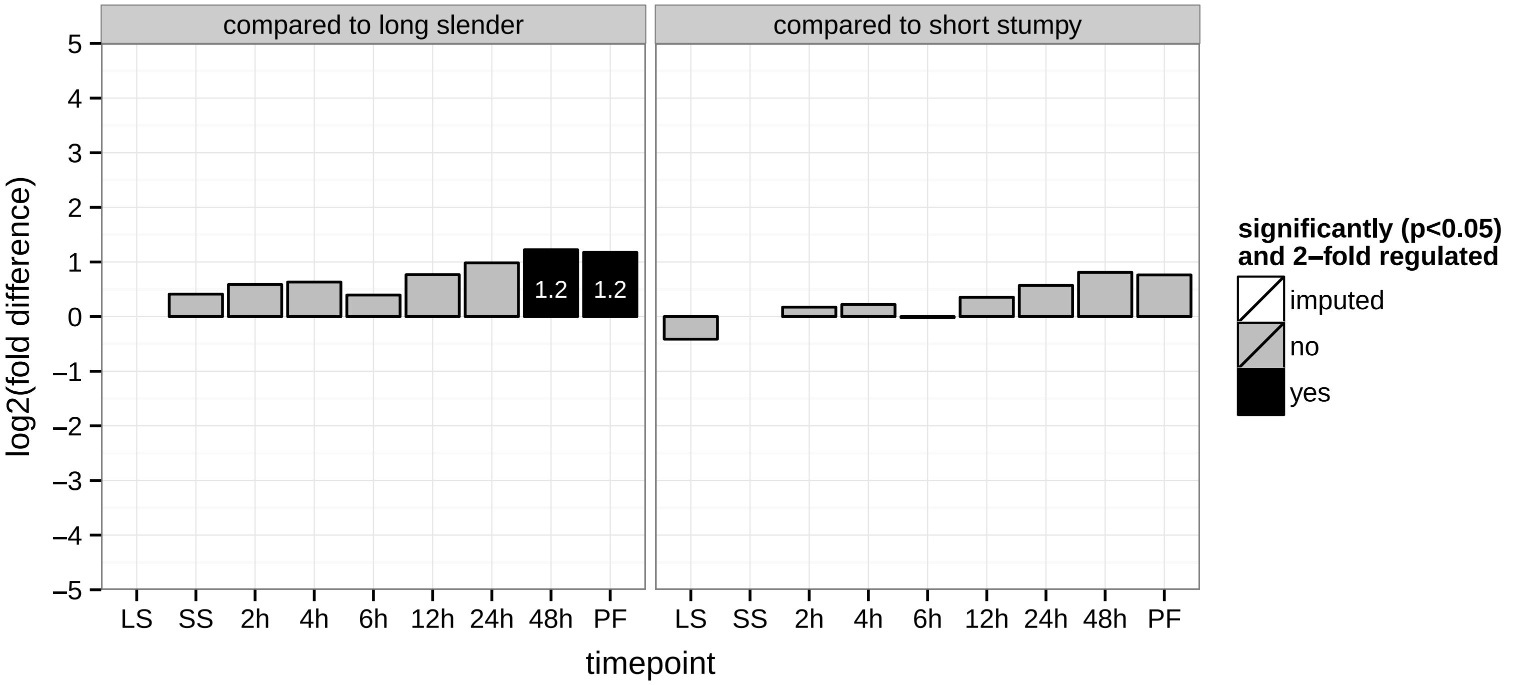

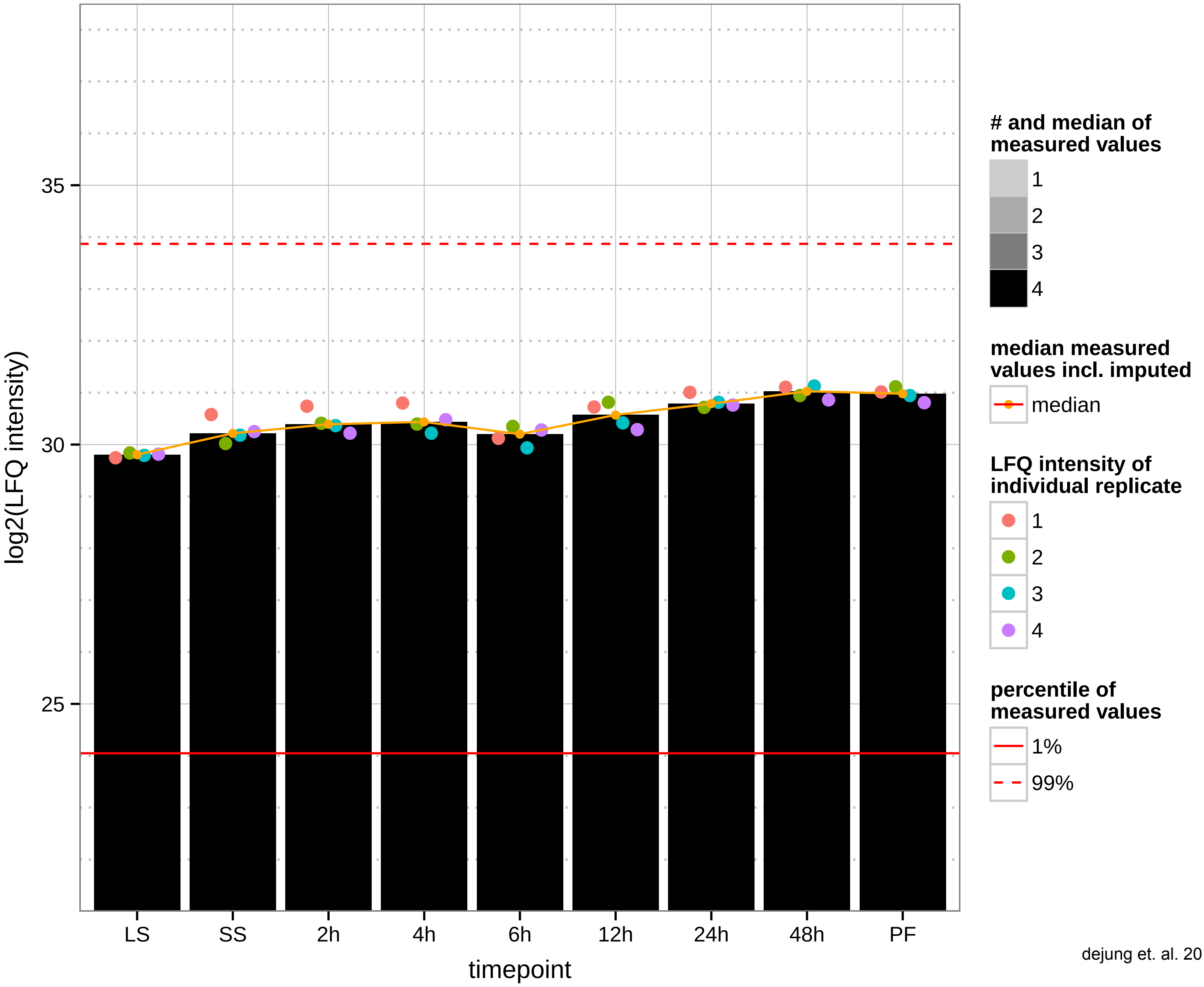

dejung et. al. 2015

hypothetical protein, conserved
Tb927.11.2340
AGOF: null
AGOC: null
AGOP: null
PGOF: null
PGOC: null
PGOP: null

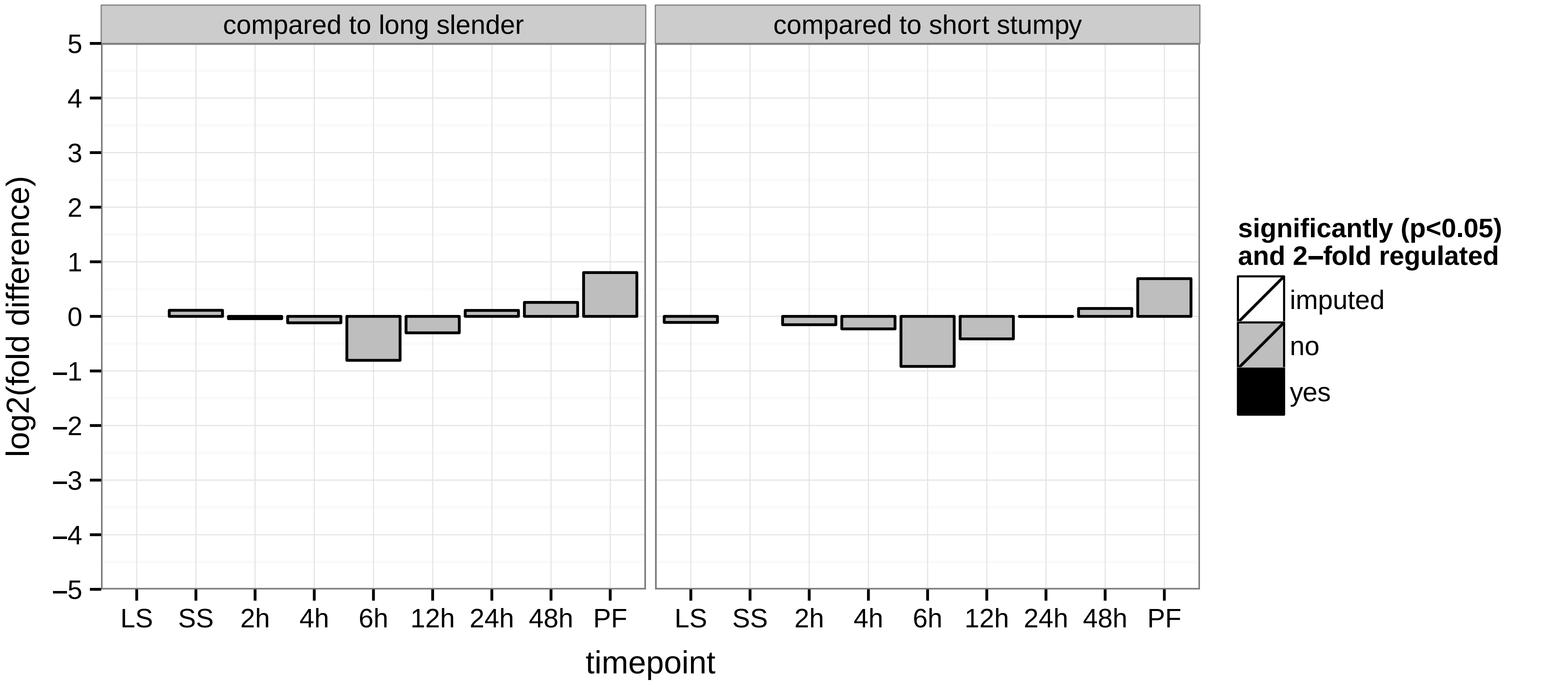

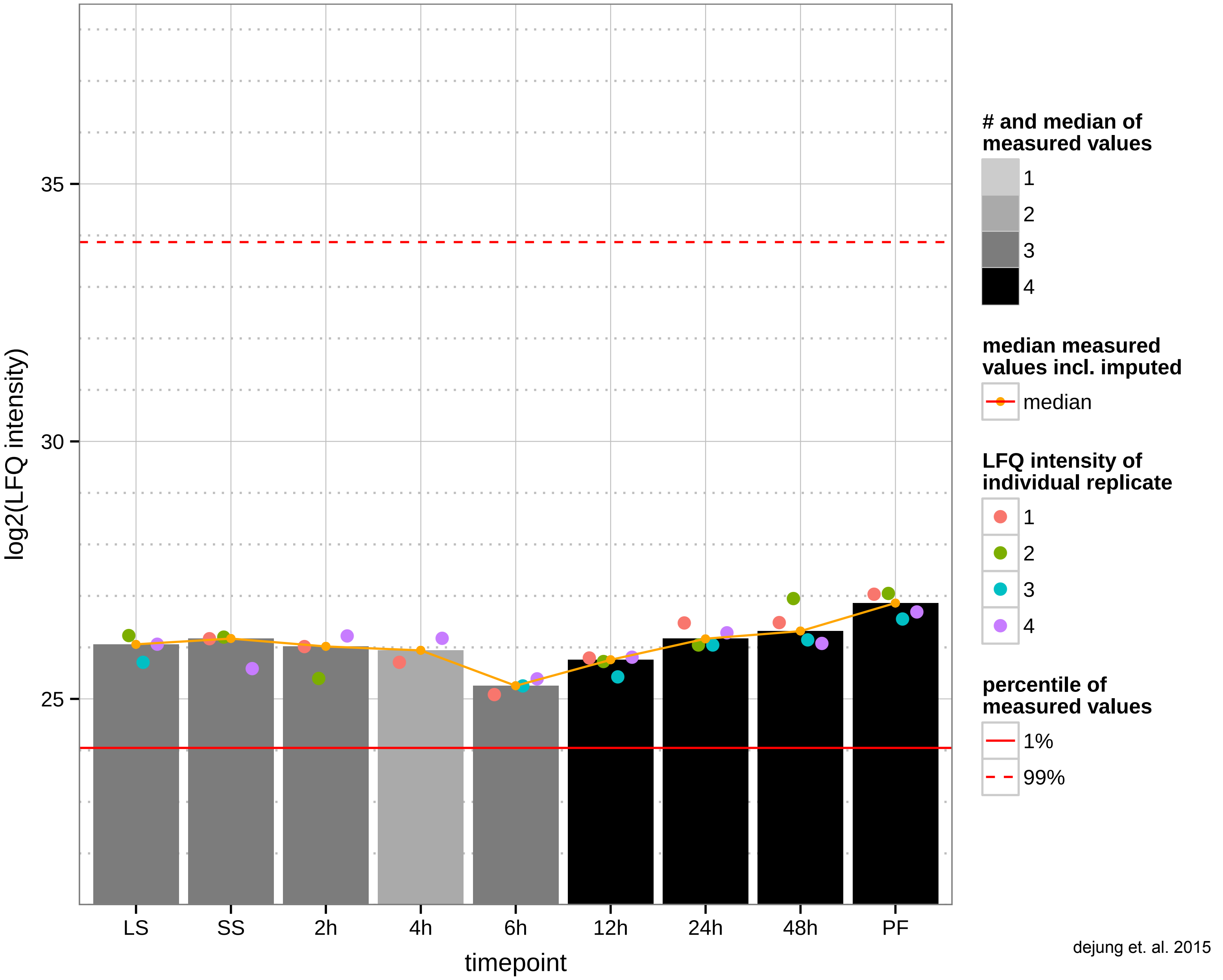

dejung et. al. 2015

phenylalanyl–tRNA synthetase (beta subunit), putative
Tb927.11.2360
AGOF: ATP binding, RNA binding, magnesium ion binding, phenylalanine–tRNA ligase activity
AGOC: cytoplasm, phenylalanine–tRNA ligase complex
AGOP: phenylalanyl–tRNA aminoacylation, translation
PGOF: ATP binding, RNA binding, magnesium ion binding, nucleotide binding, phenylalanine–tRNA ligase activity
PGOC: cytoplasm
PGOP: phenylalanyl–tRNA aminoacylation

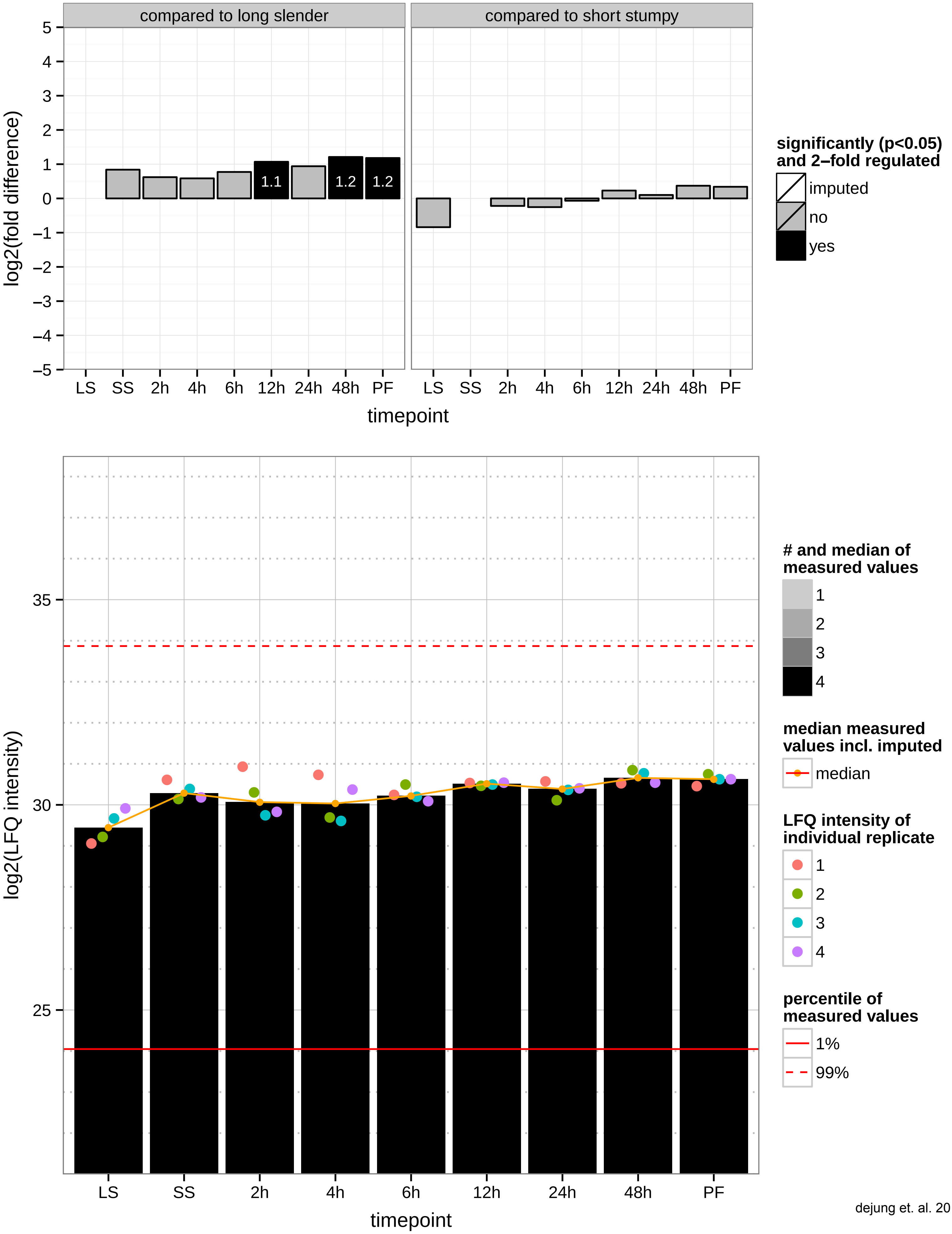

dejung et. al. 2015

hypothetical protein, conserved
Tb927.11.2370
AGOF: RNA binding, zinc ion binding
AGOC: null
AGOP: null
PGOF: nucleic acid binding, protein binding, zinc ion binding
PGOC: null
PGOP: null

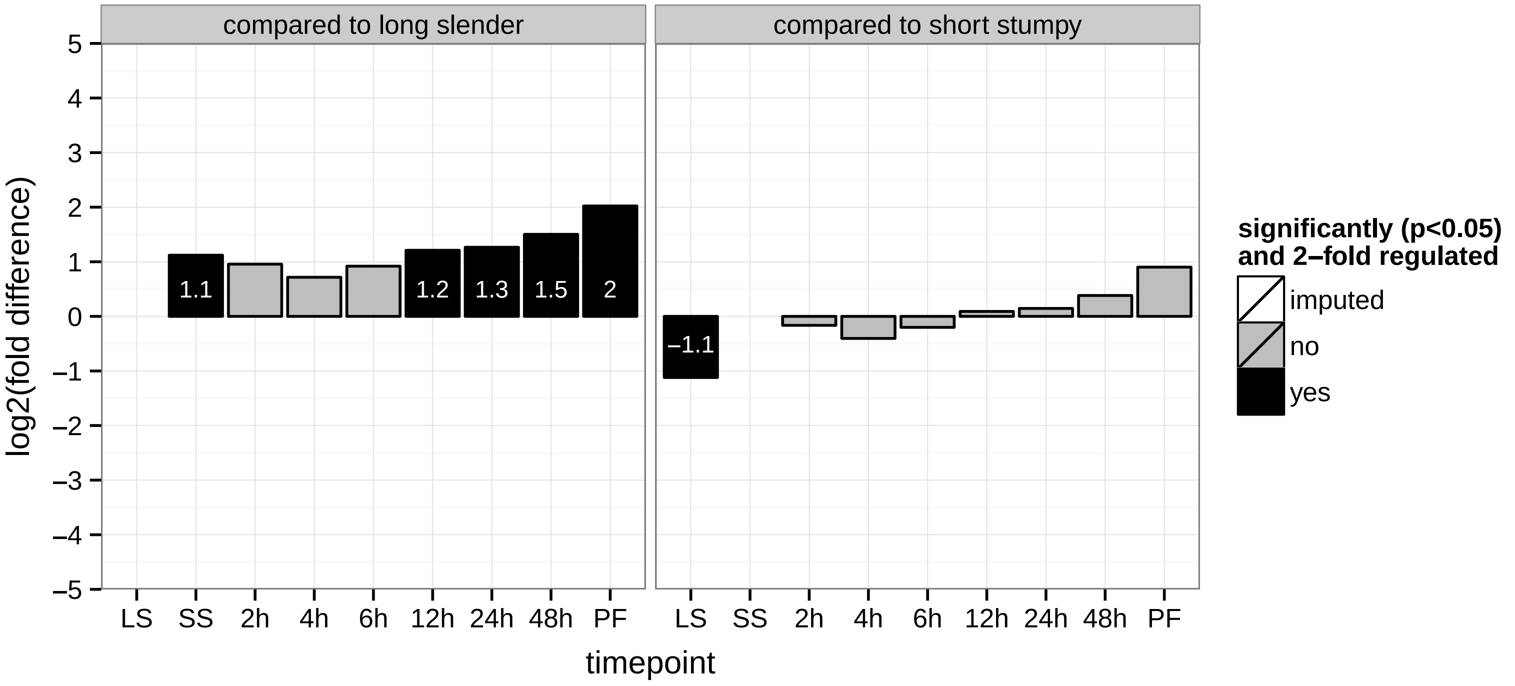

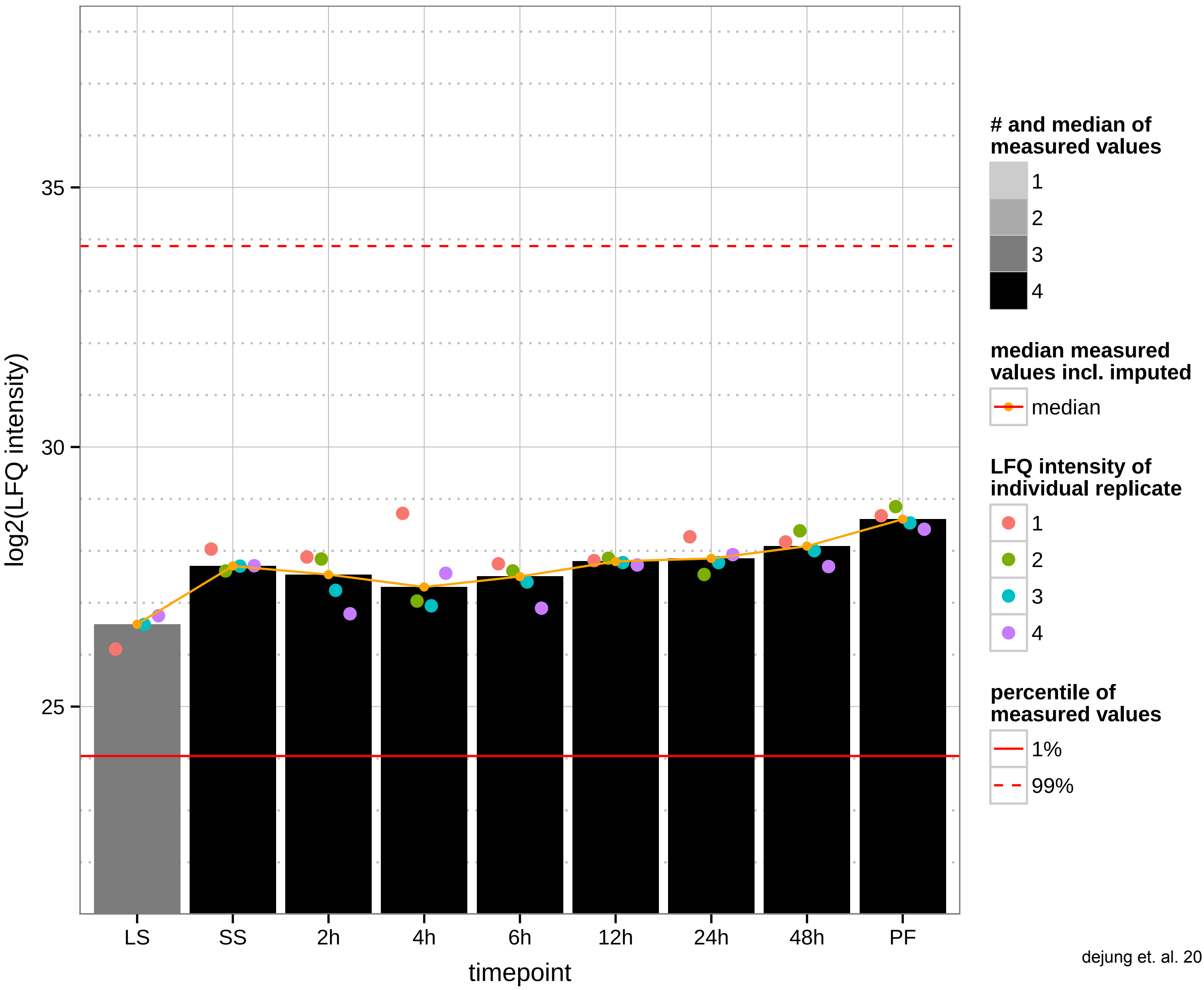

dejung et. al. 2015

phosphoglycerate kinase, putative
Tb927.11.2380
AGOF: phosphoglycerate kinase activity
AGOC: integral to membrane
AGOP: glycolysis
PGOF: phosphoglycerate kinase activity
PGOC: null
PGOP: glycolysis

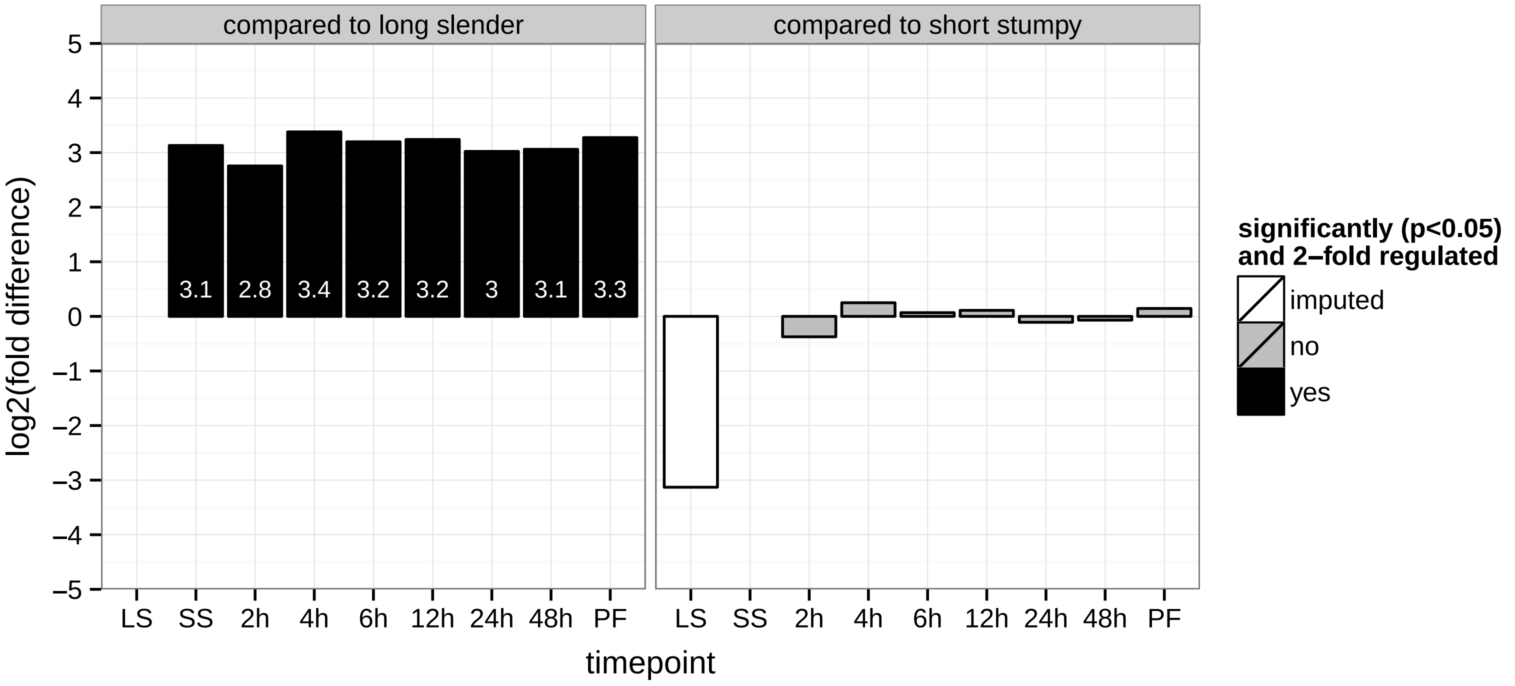

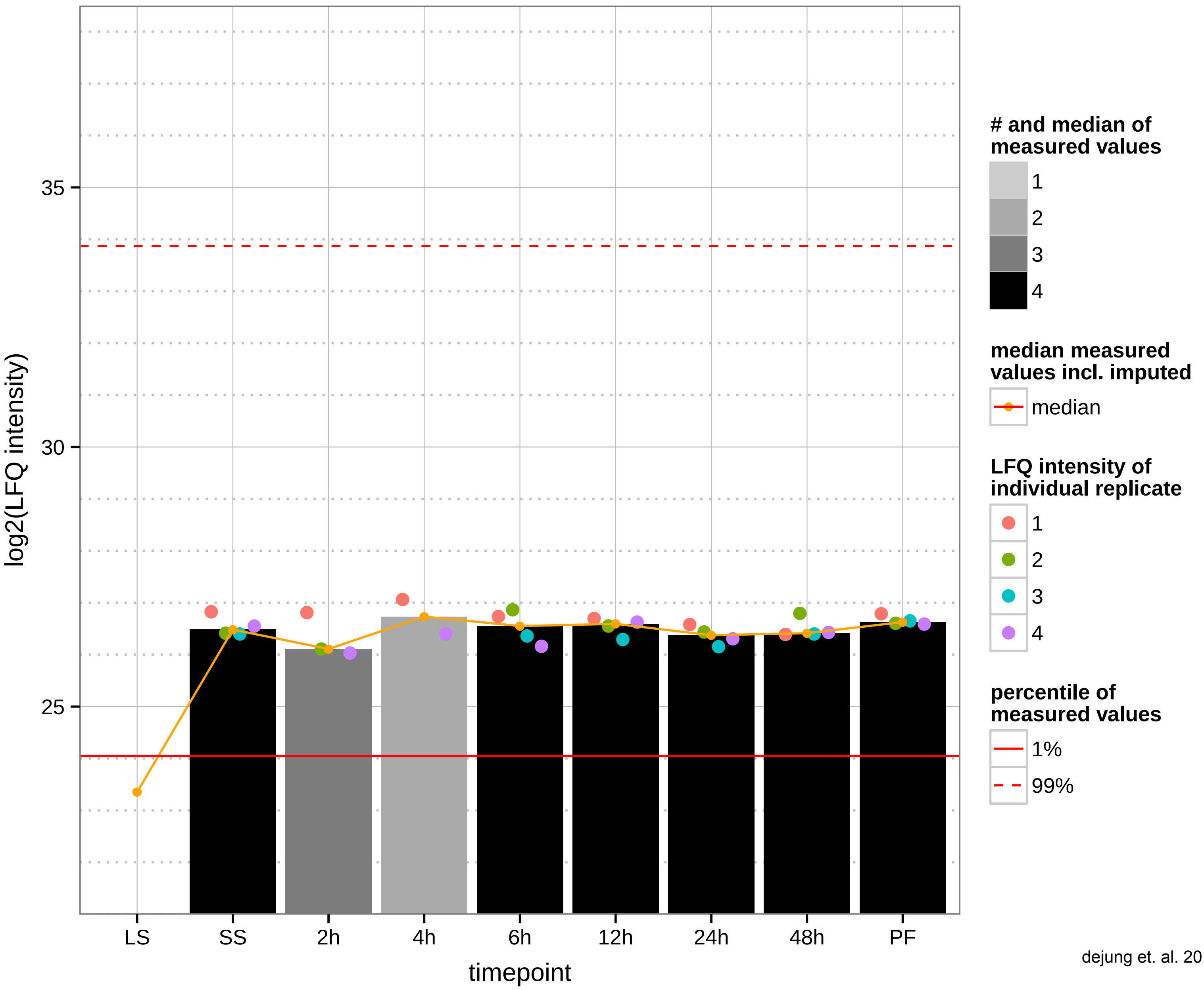

dejung et. al. 2015

UDP−N−acetylglucosamine pyrophosphorylase, putative
Tb927.11.2520
AGOF: nucleotidyltransferase activity
AGOC: null
AGOP: UDP−glucose metabolic process
PGOF: nucleotidyltransferase activity
PGOC: null
PGOP: metabolic process

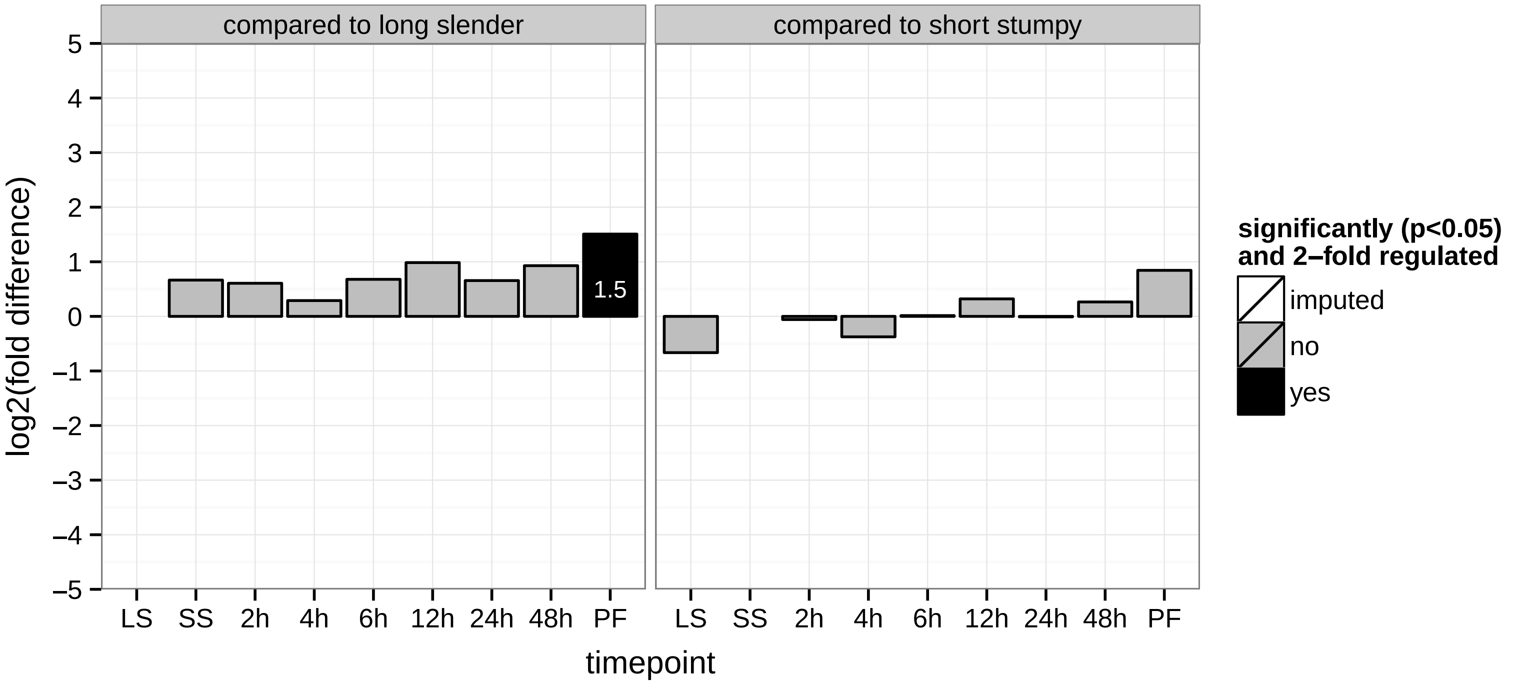

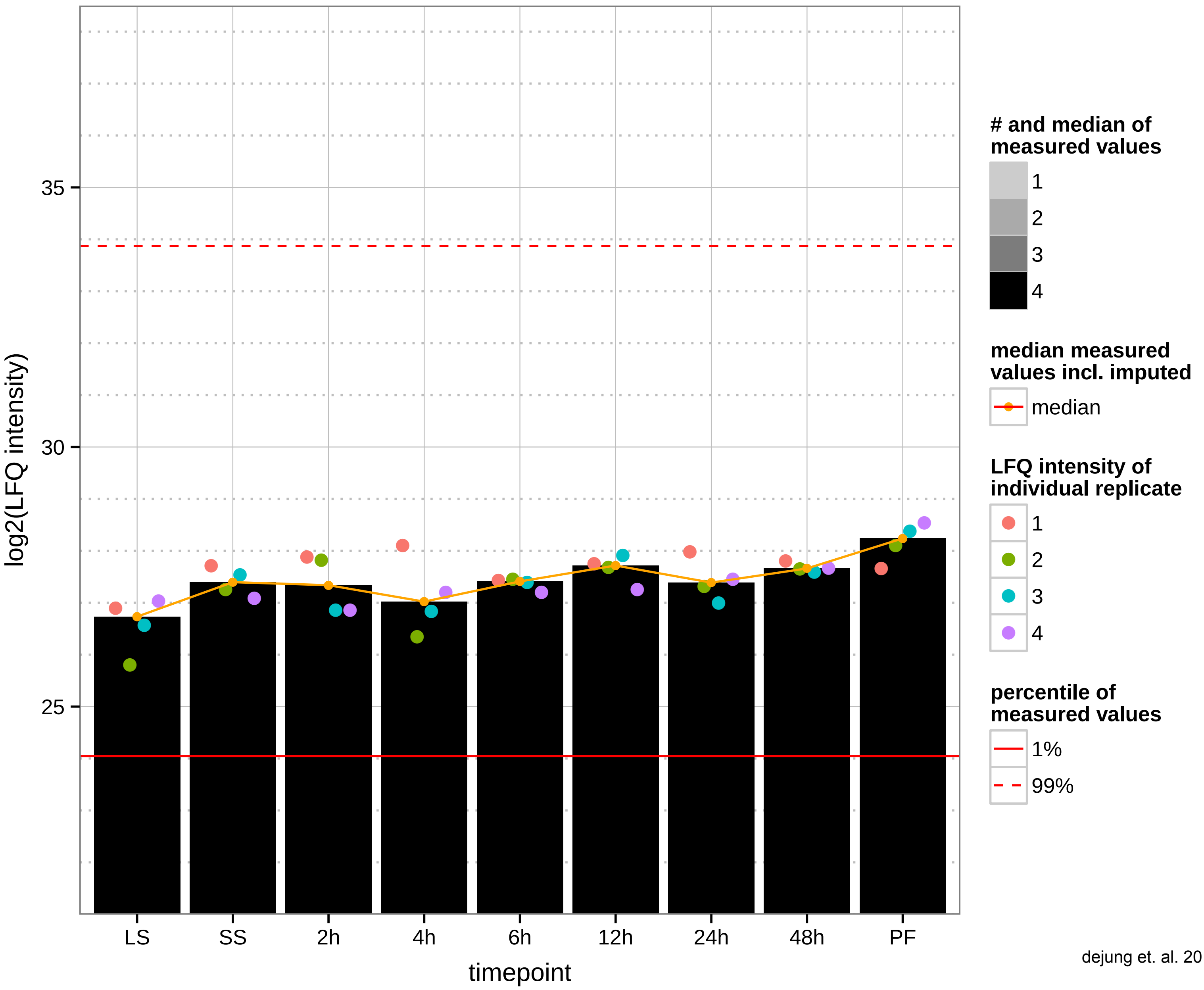

dejung et. al. 2015

hypothetical protein, conserved
Tb927.11.2540
AGOF: null
AGOC: null
AGOP: null
PGOF: null
PGOC: null
PGOP: null

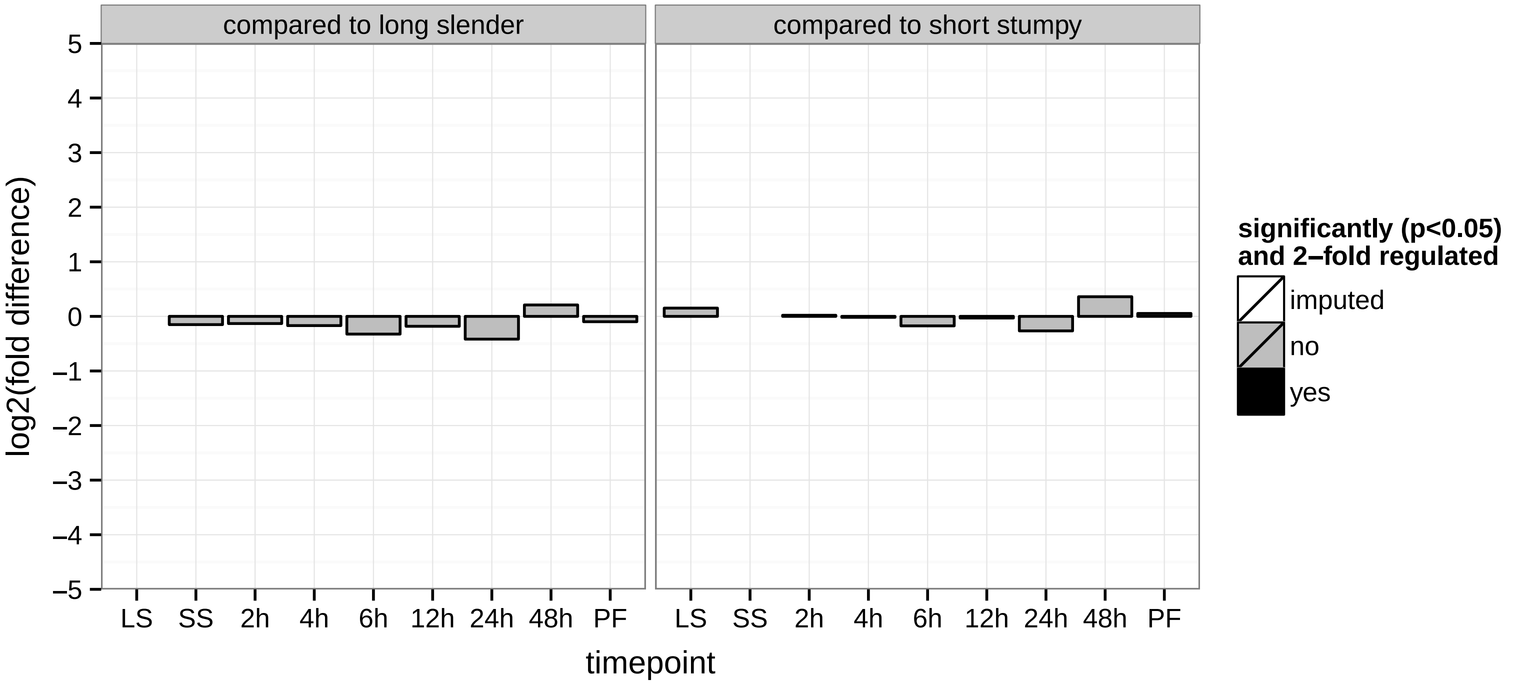

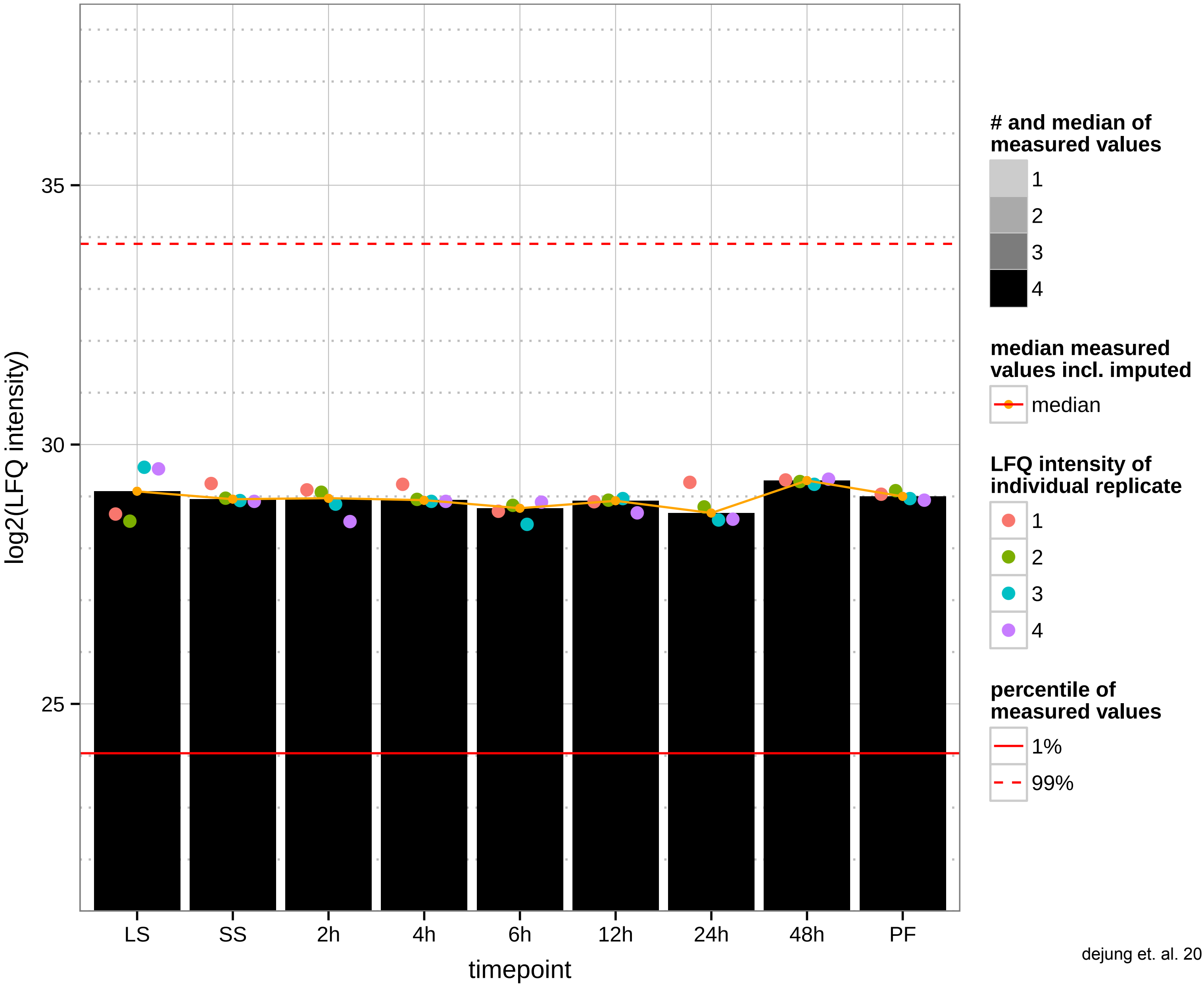

dejung et. al. 2015

hypothetical protein, conserved
Tb927.11.2570
AGOF: null
AGOC: null
AGOP: null
PGOF: null
PGOC: null
PGOP: null

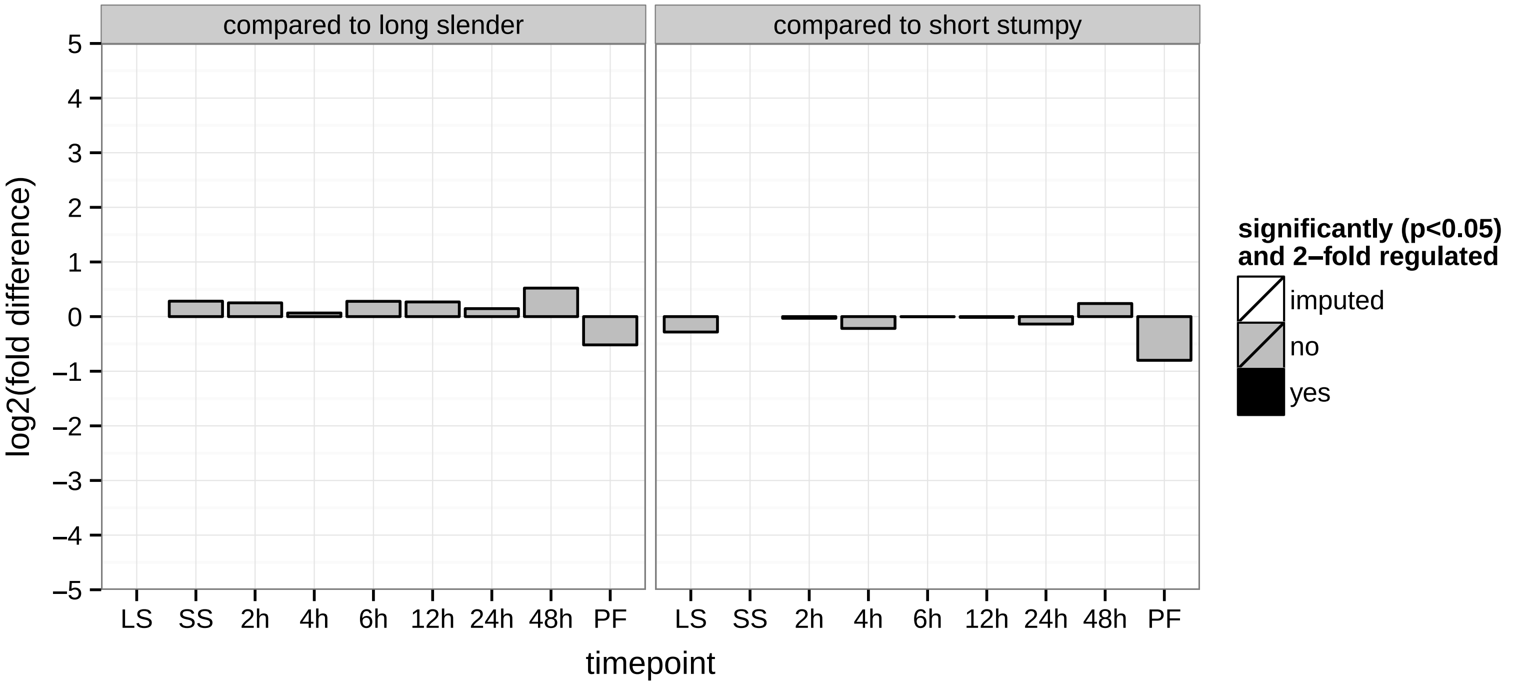

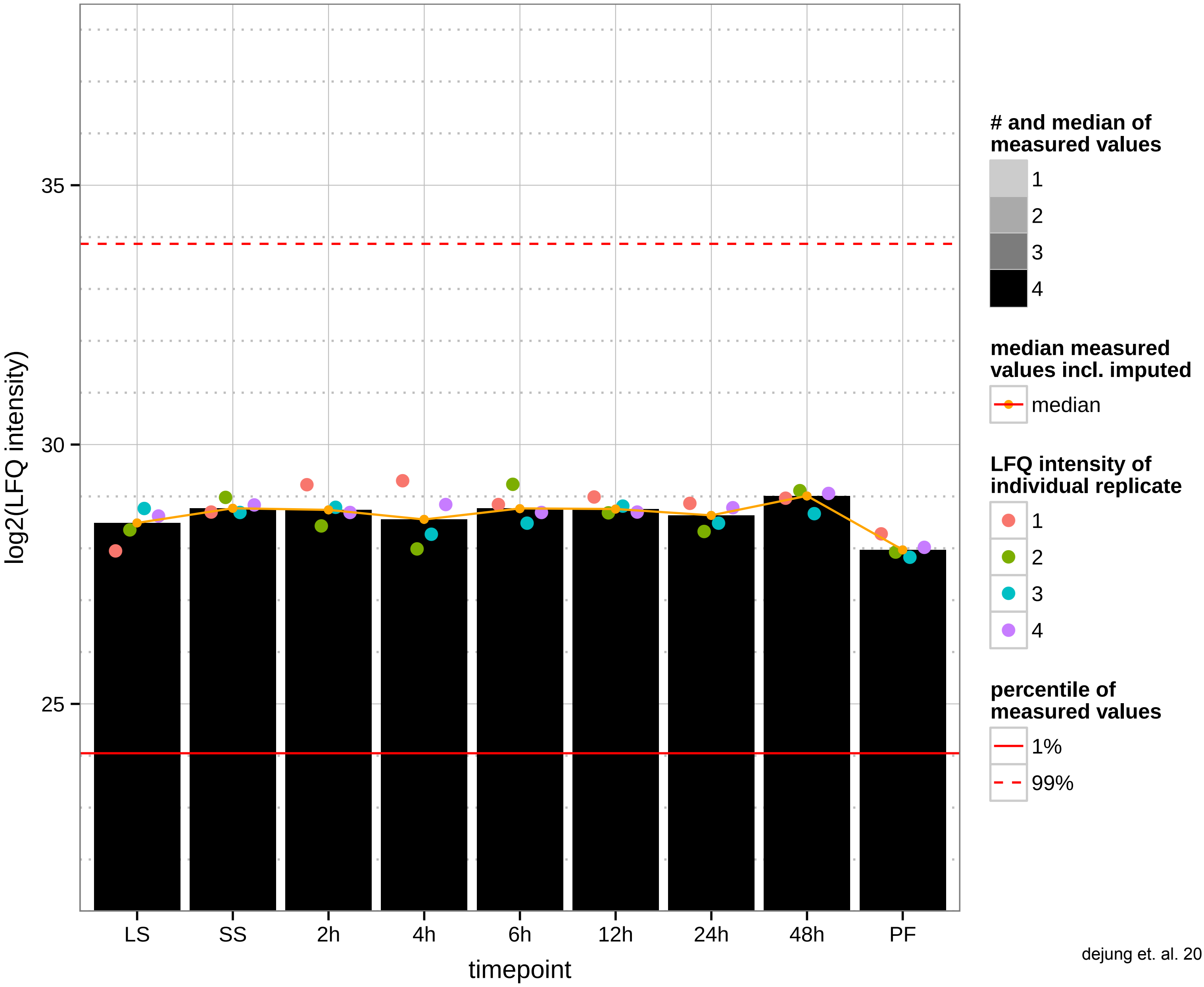

dejung et. al. 2015

predicted WD40 repeat protein (PIFTF6)
Tb927.11.260
AGOF: null
AGOC: null
AGOP: null
PGOF: protein binding
PGOC: null
PGOP: null

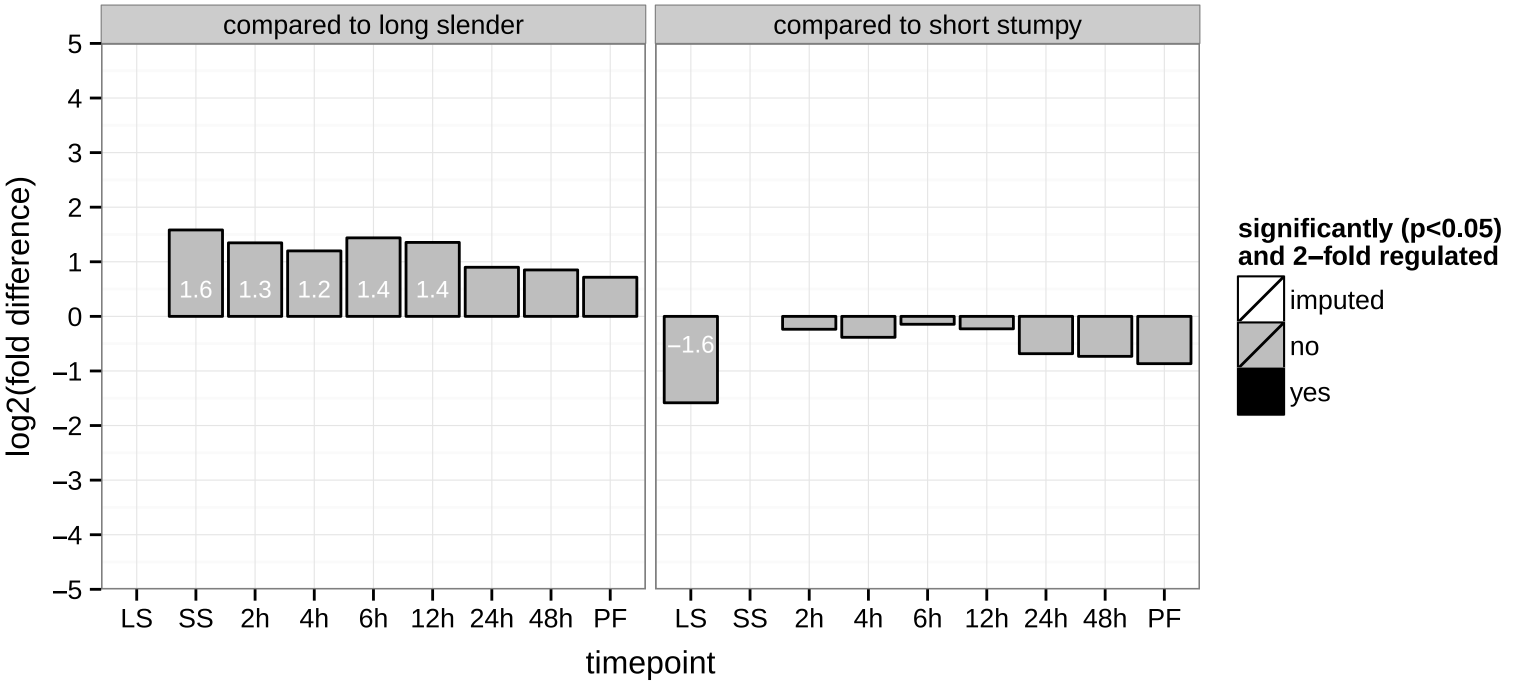

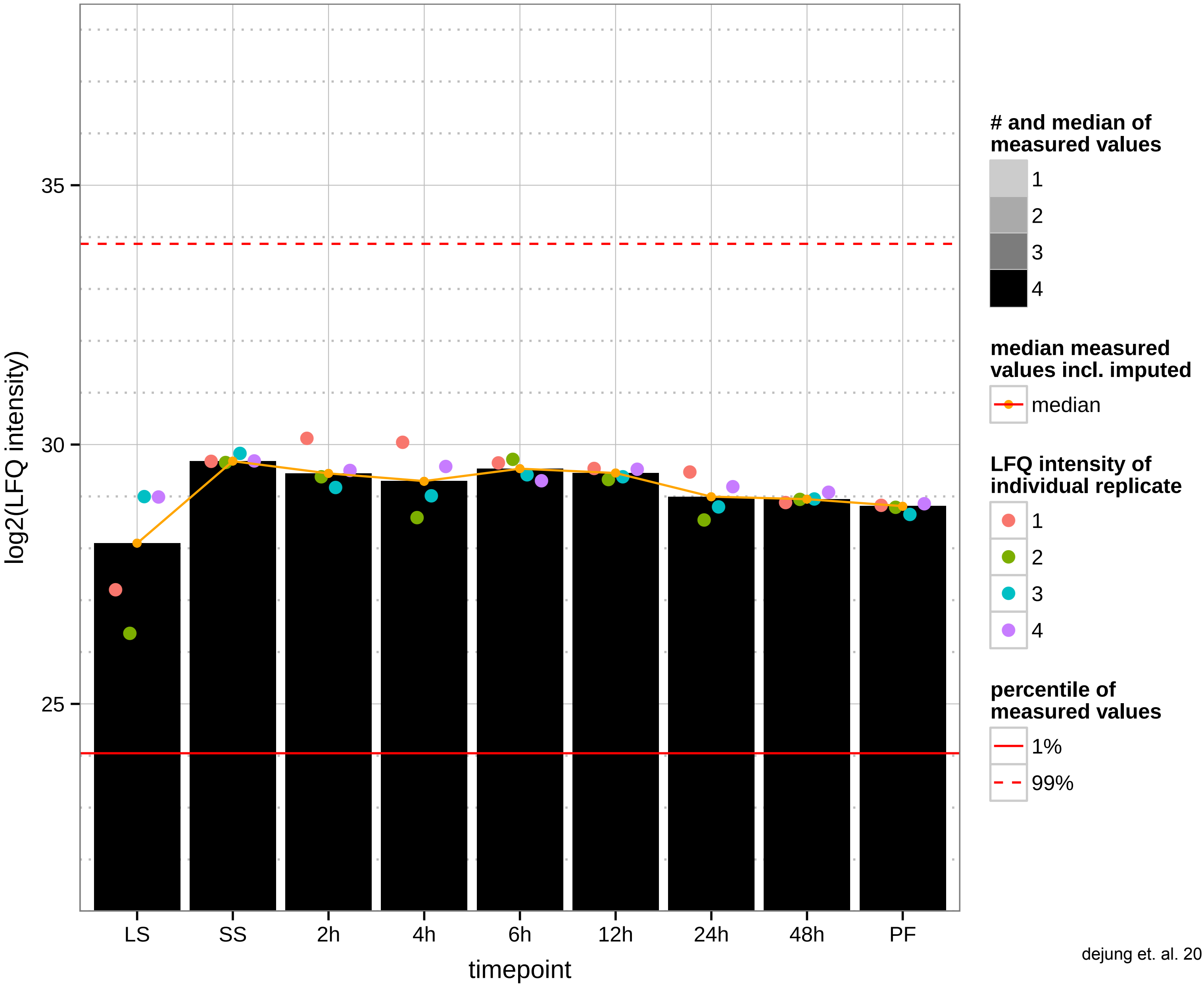

dejung et. al. 2015

hypothetical protein, conserved
Tb927.11.2600
AGOF: null
AGOC: null
AGOP: null
PGOF: null
PGOC: null
PGOP: null

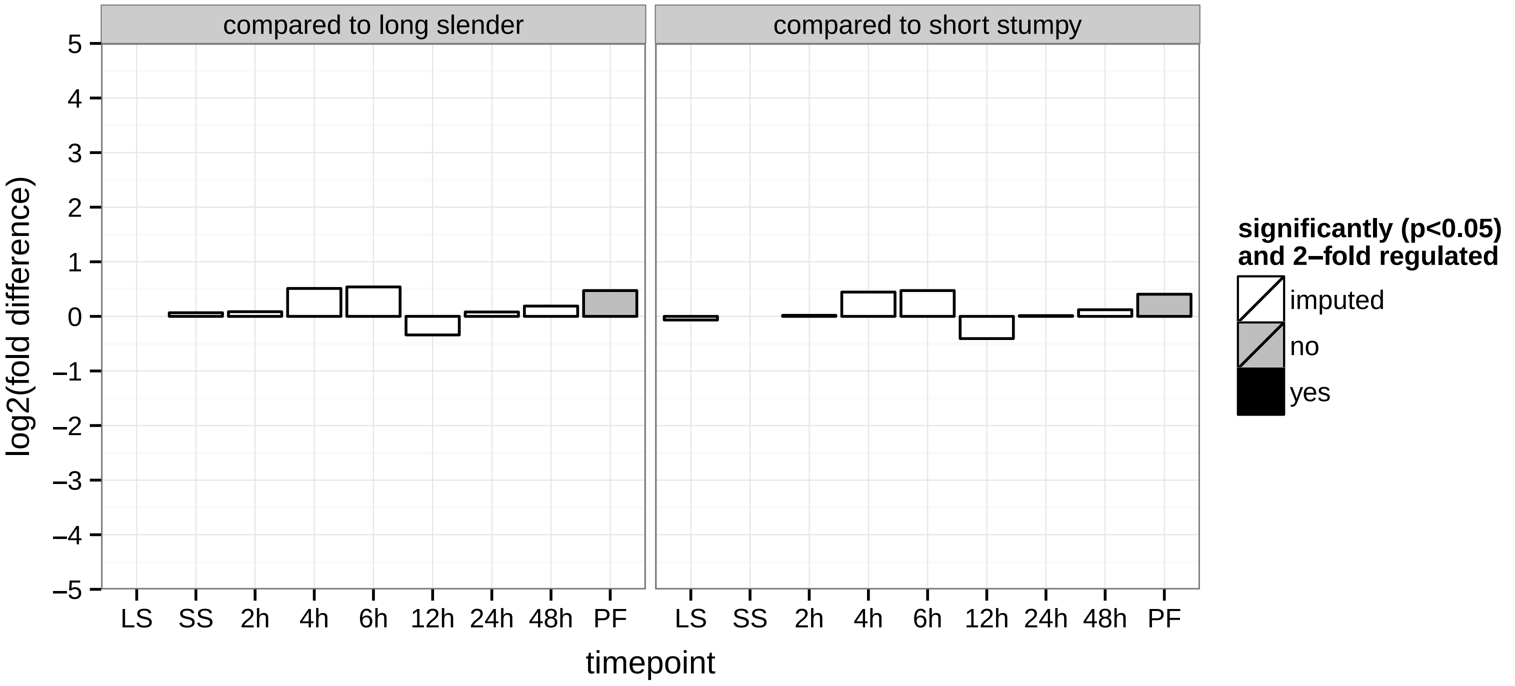

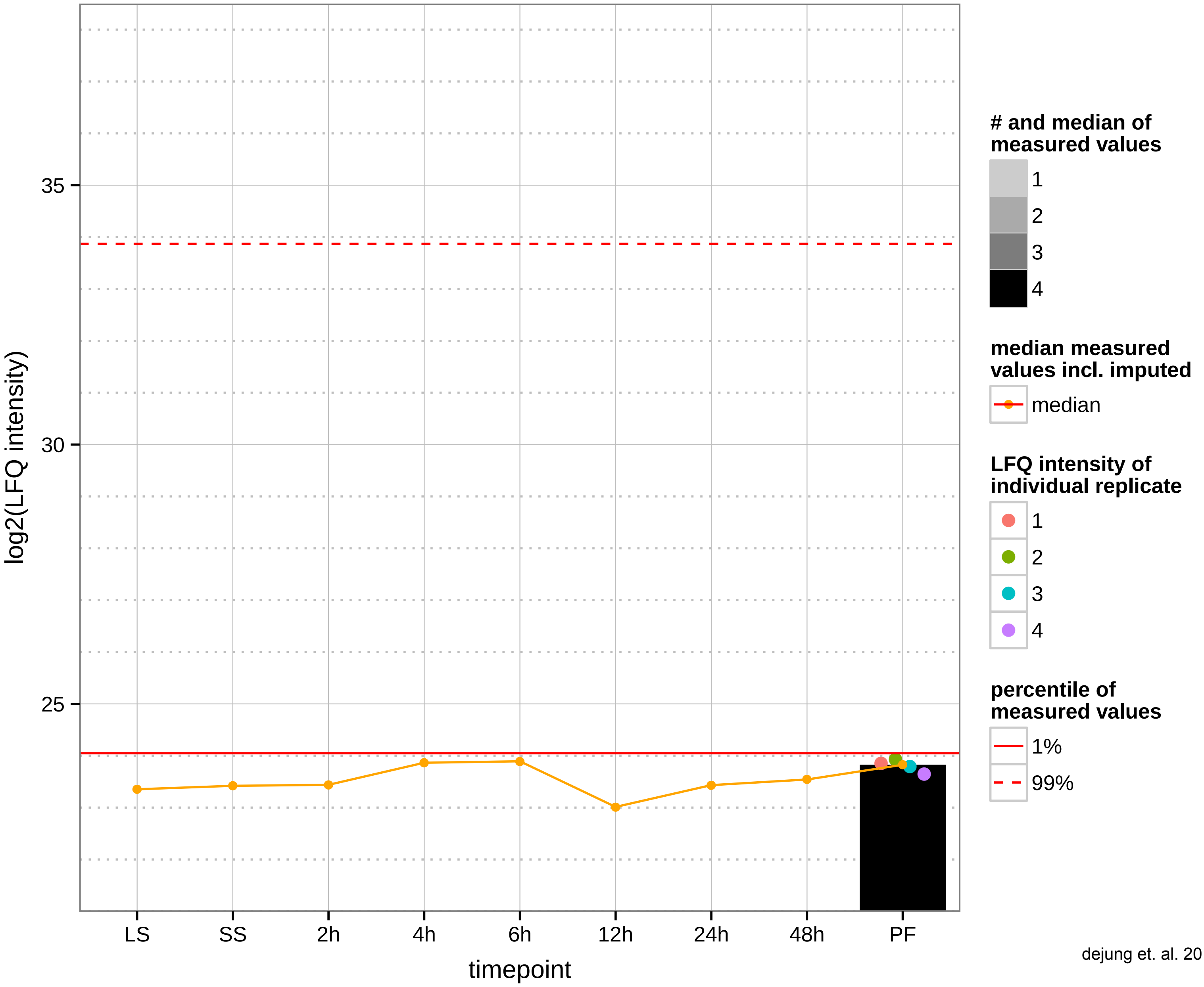

dejung et. al. 2015

hypothetical protein, conserved
Tb927.11.2610
AGOF: null
AGOC: null
AGOP: null
PGOF: null
PGOC: null
PGOP: null

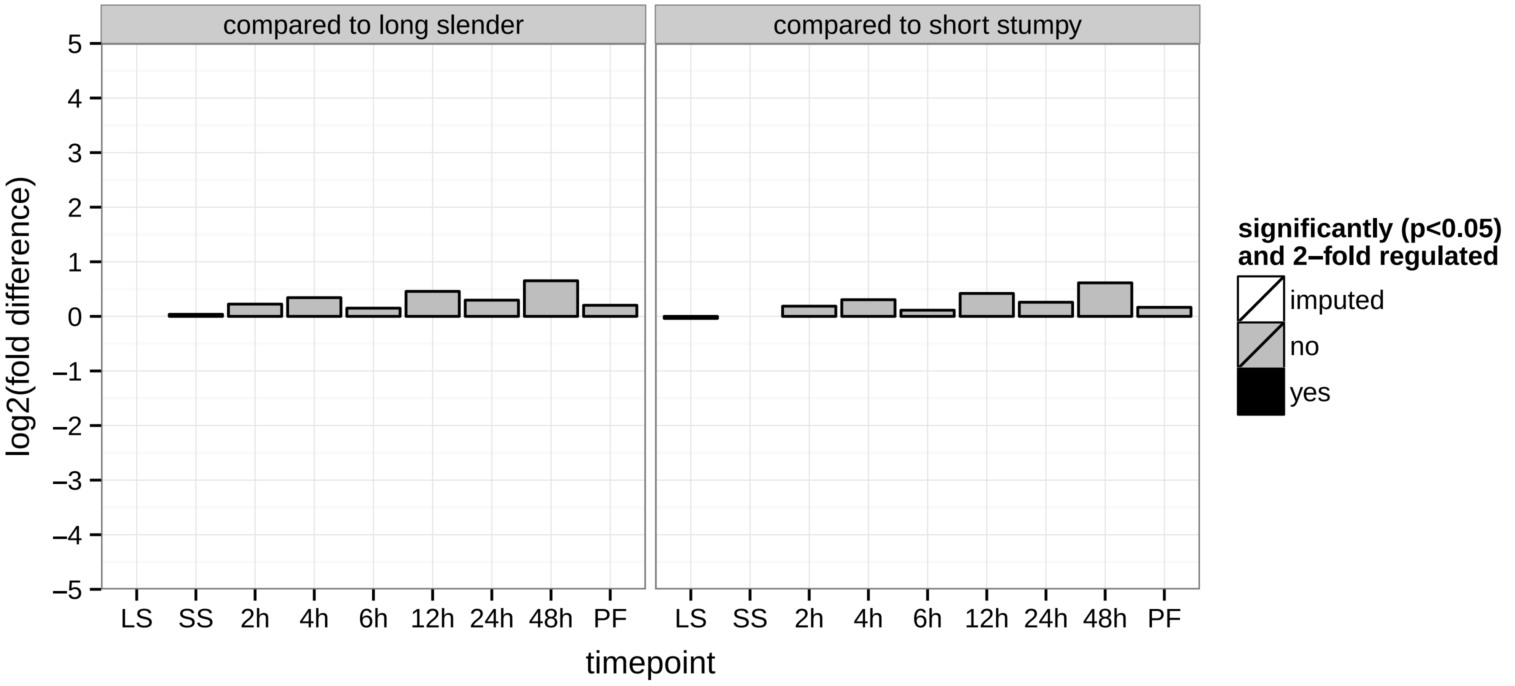

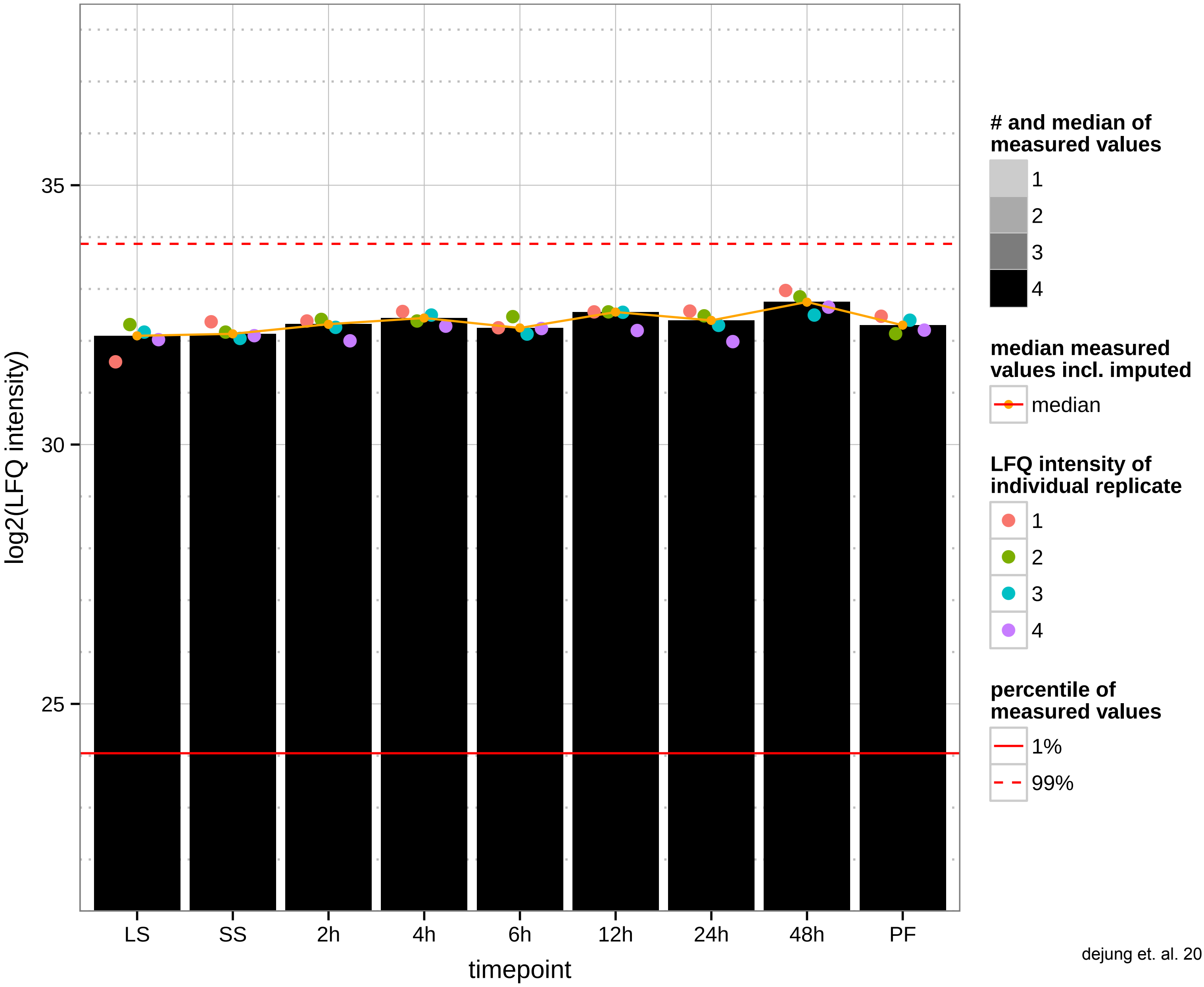

dejung et. al. 2015

hypothetical protein, conserved
Tb927.11.2620
AGOF: null
AGOC: null
AGOP: null
PGOF: null
PGOC: null
PGOP: null

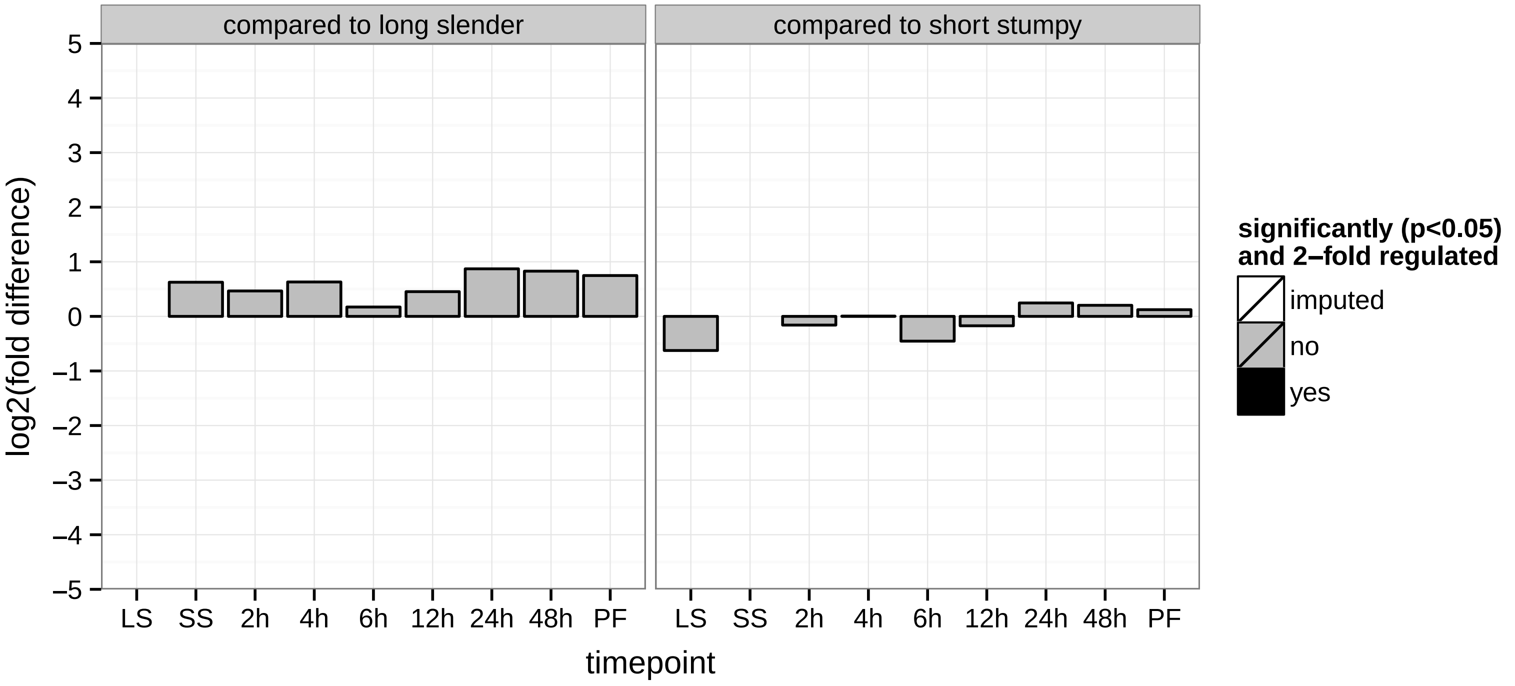

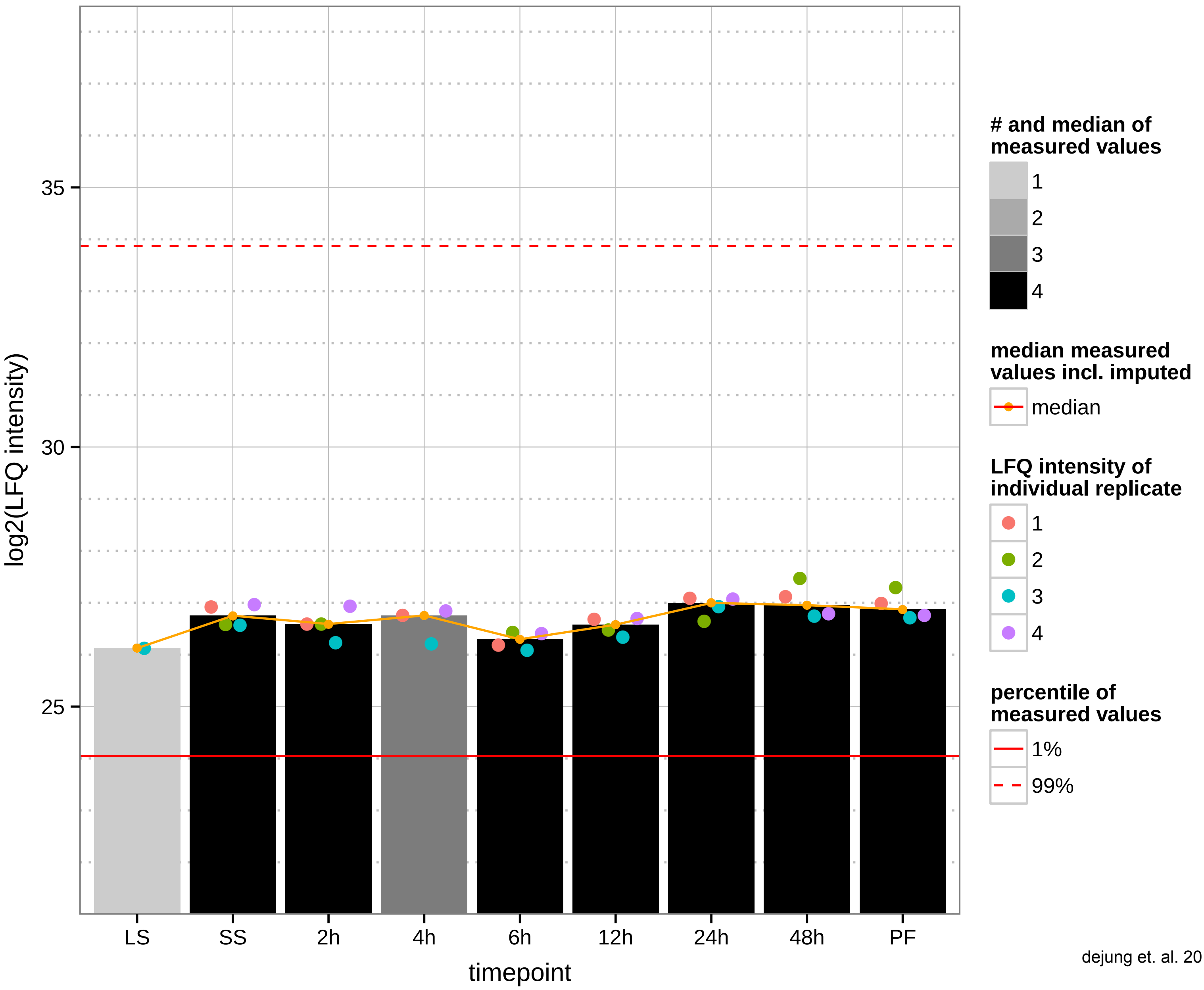

dejung et. al. 2015

ras–like small GTPase, putative (TbNST)
Tb927.11.2640
AGOF: GTP binding, GTPase activity
AGOC: intracellular
AGOP: null
PGOF: GTP binding
PGOC: null
PGOP: null

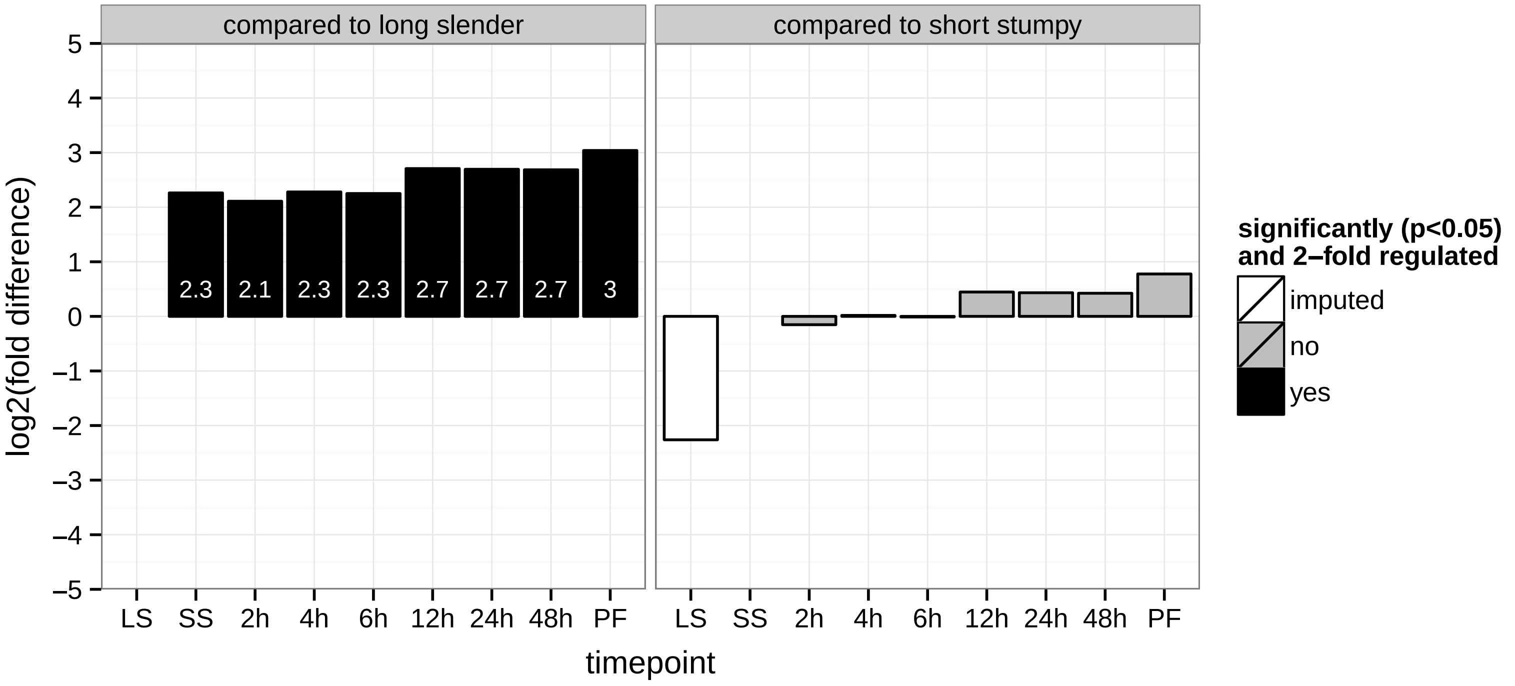

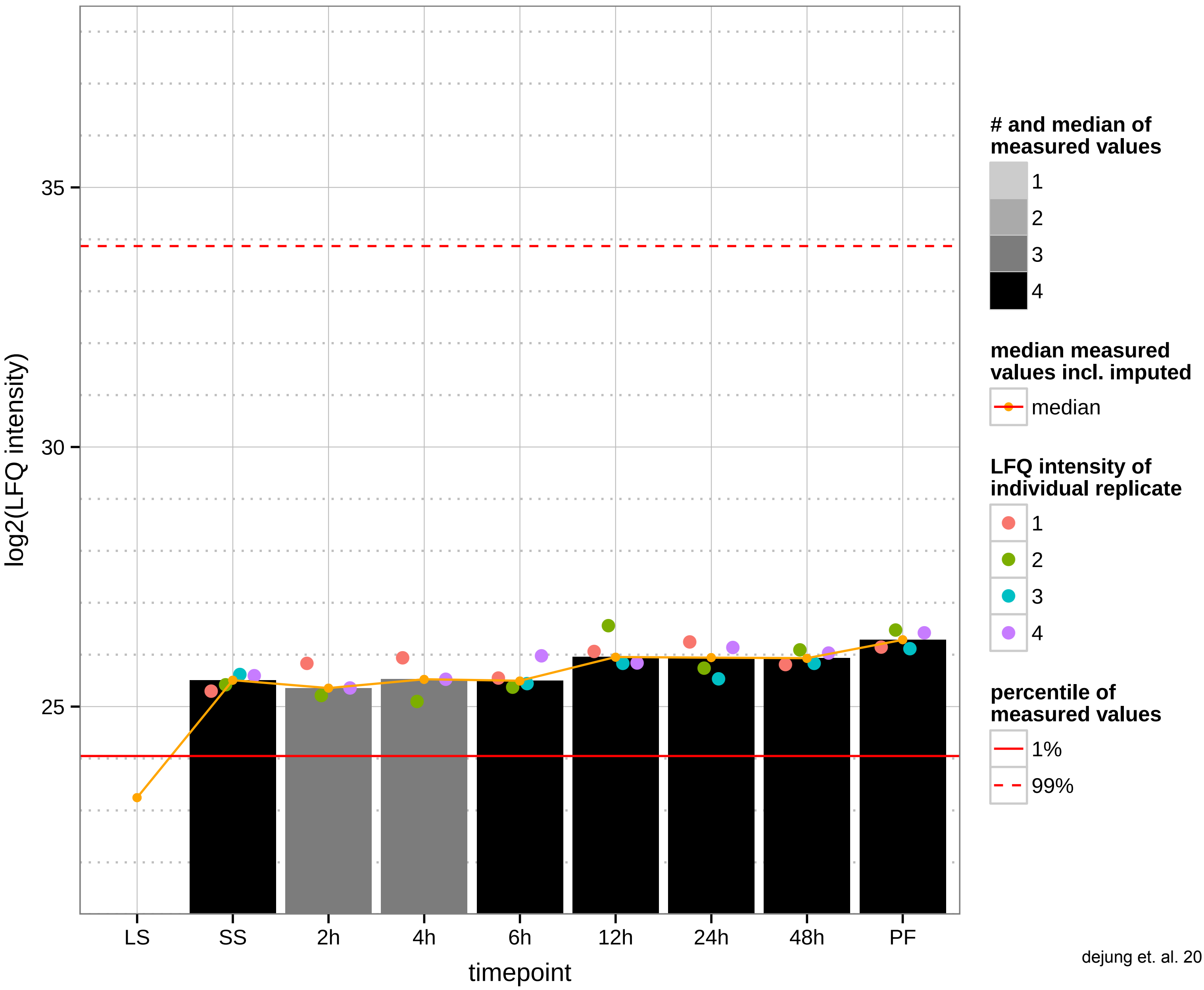

dejung et. al. 2015

heat shock protein 84, putative
Tb927.11.2650
AGOF: ATP binding, ATPase activity, coupled, unfolded protein binding
AGOC: mitochondrion
AGOP: protein folding, response to stress
PGOF: ATP binding, unfolded protein binding
PGOC: null
PGOP: protein folding, response to stress

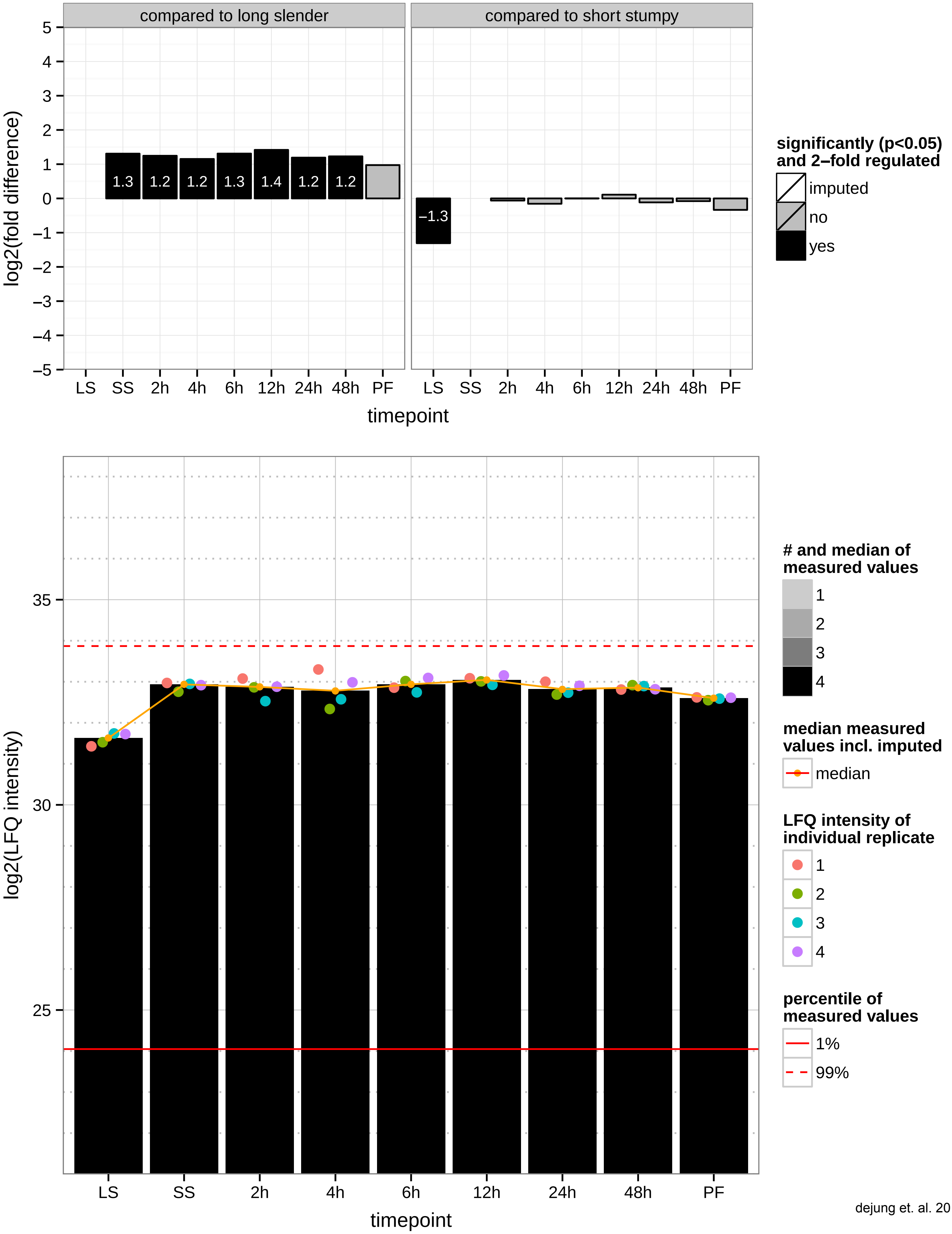

dejung et. al. 2015

Nucleoporin (TbNup59)
Tb927.11.2670
AGOF: null
AGOC: null
AGOP: null
PGOF: null
PGOC: null
PGOP: null

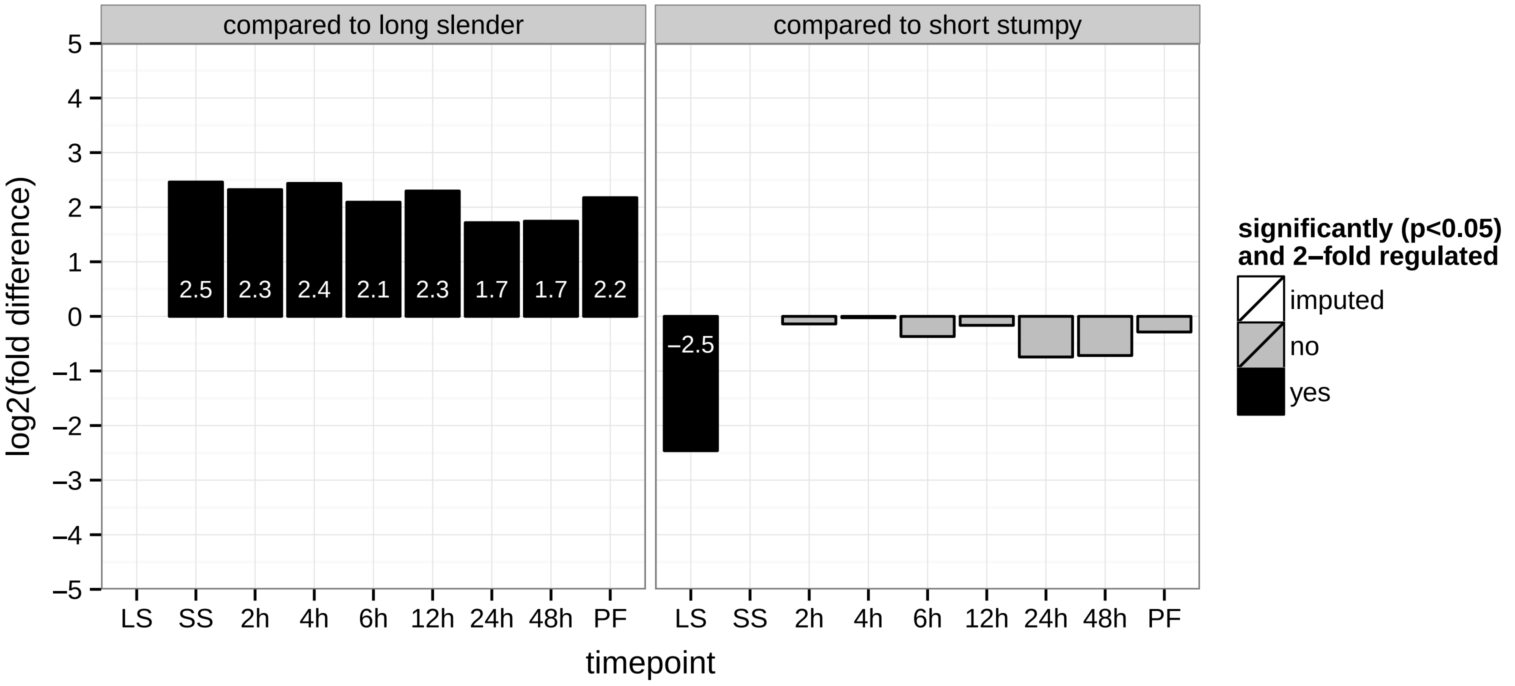

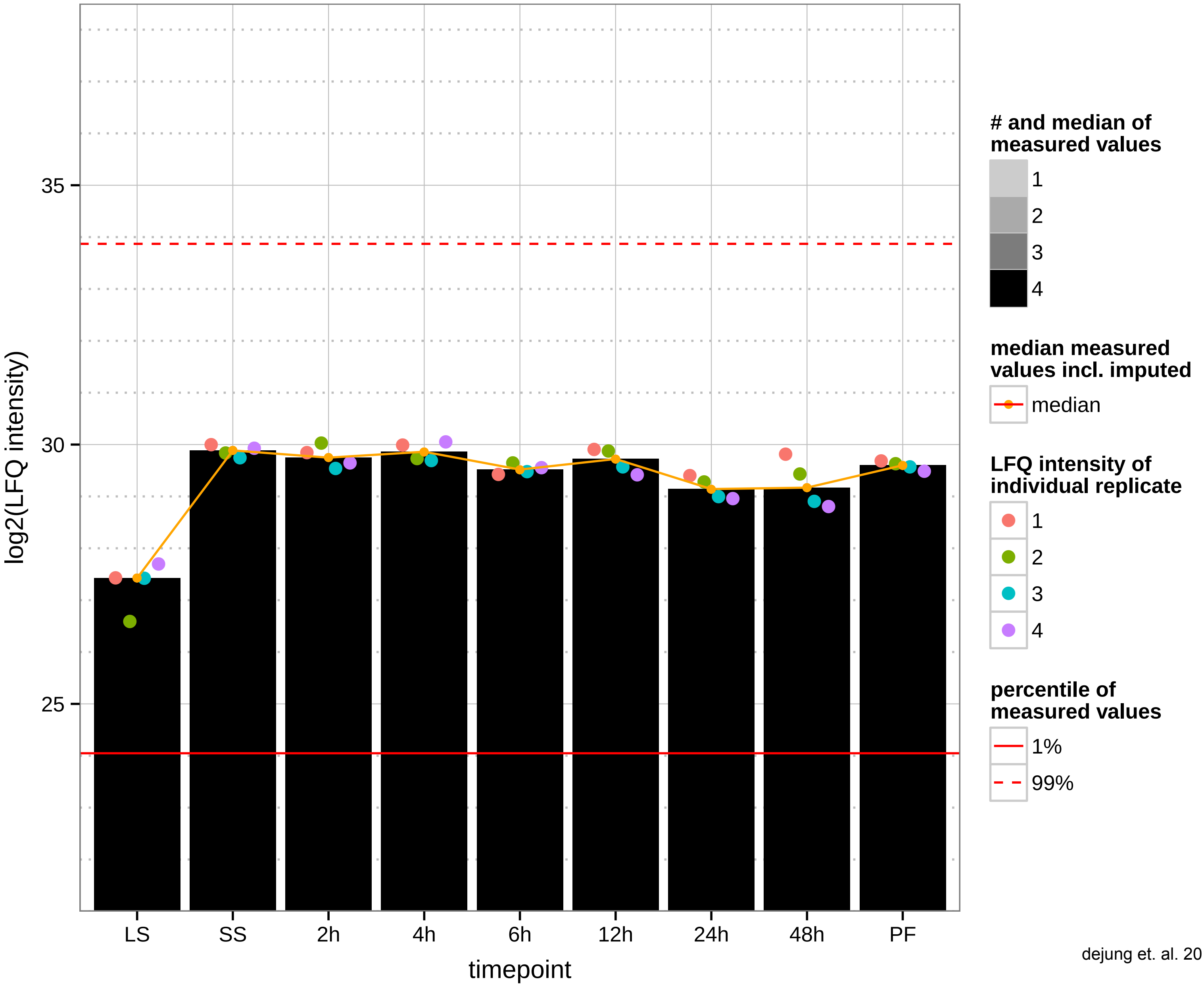

dejung et. al. 2015

hypothetical protein, conserved
Tb927.11.2740
AGOF: zinc ion binding
AGOC: null
AGOP: null
PGOF: zinc ion binding
PGOC: null
PGOP: null

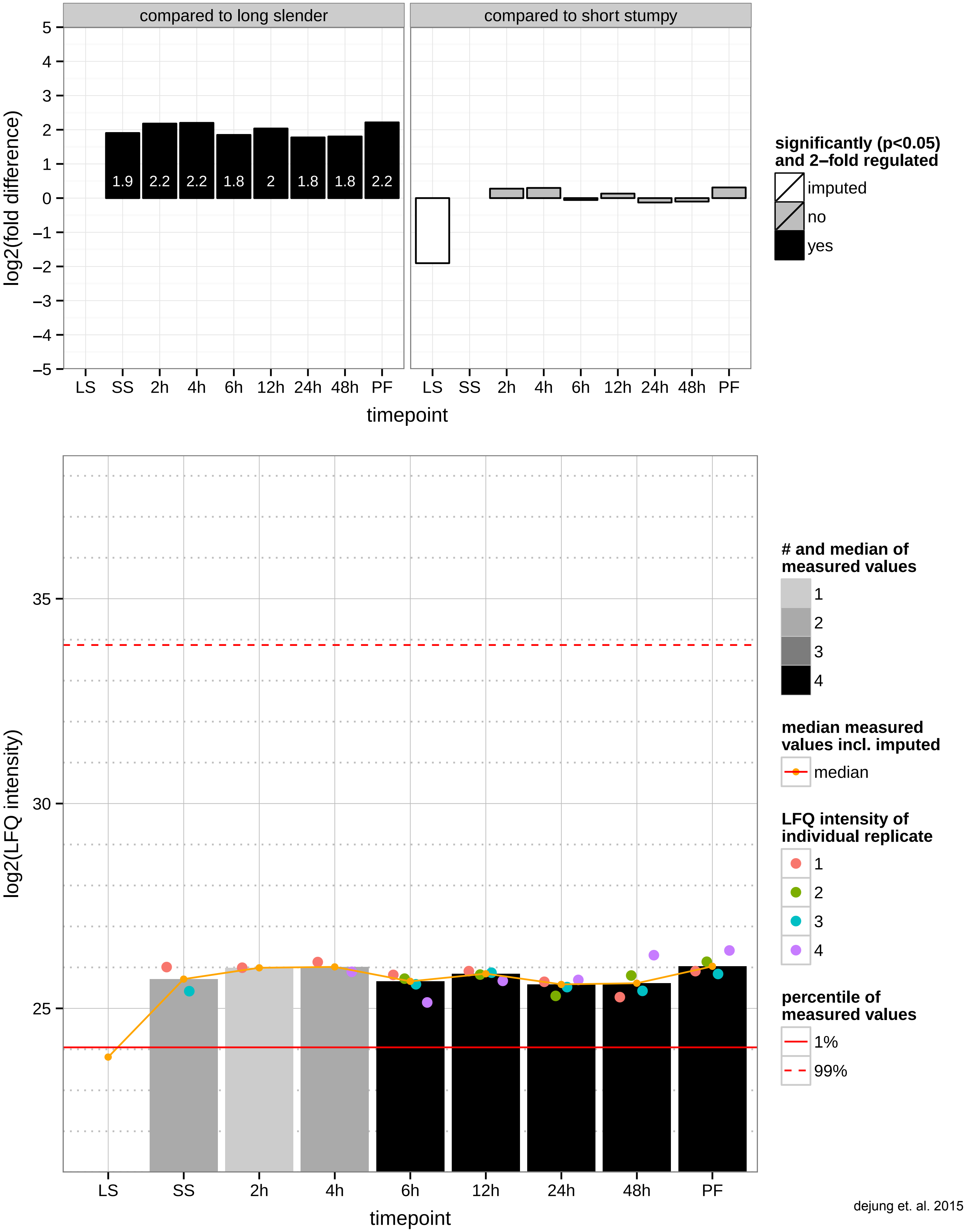

dejung et. al. 2015

hypothetical protein, conserved
Tb927.11.2760
AGOF: null
AGOC: null
AGOP: null
PGOF: null
PGOC: null
PGOP: null

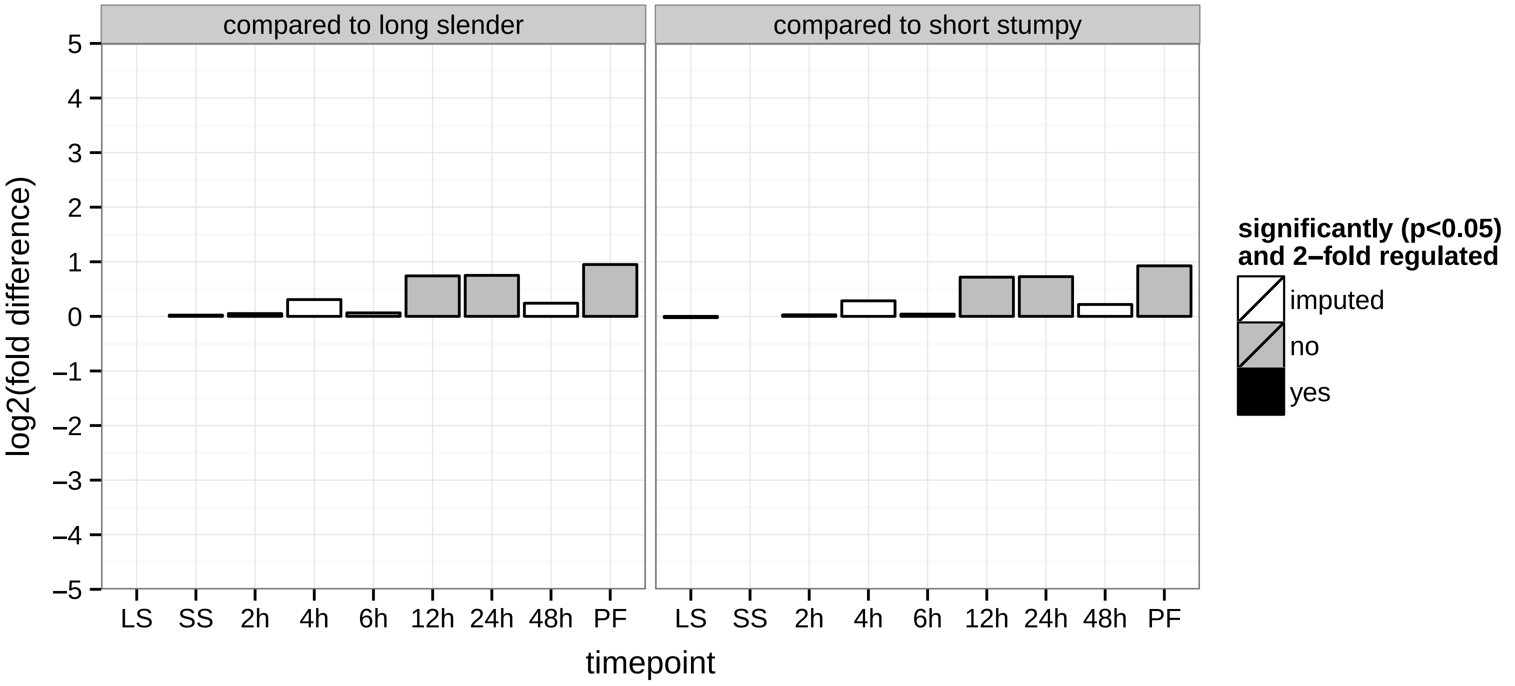

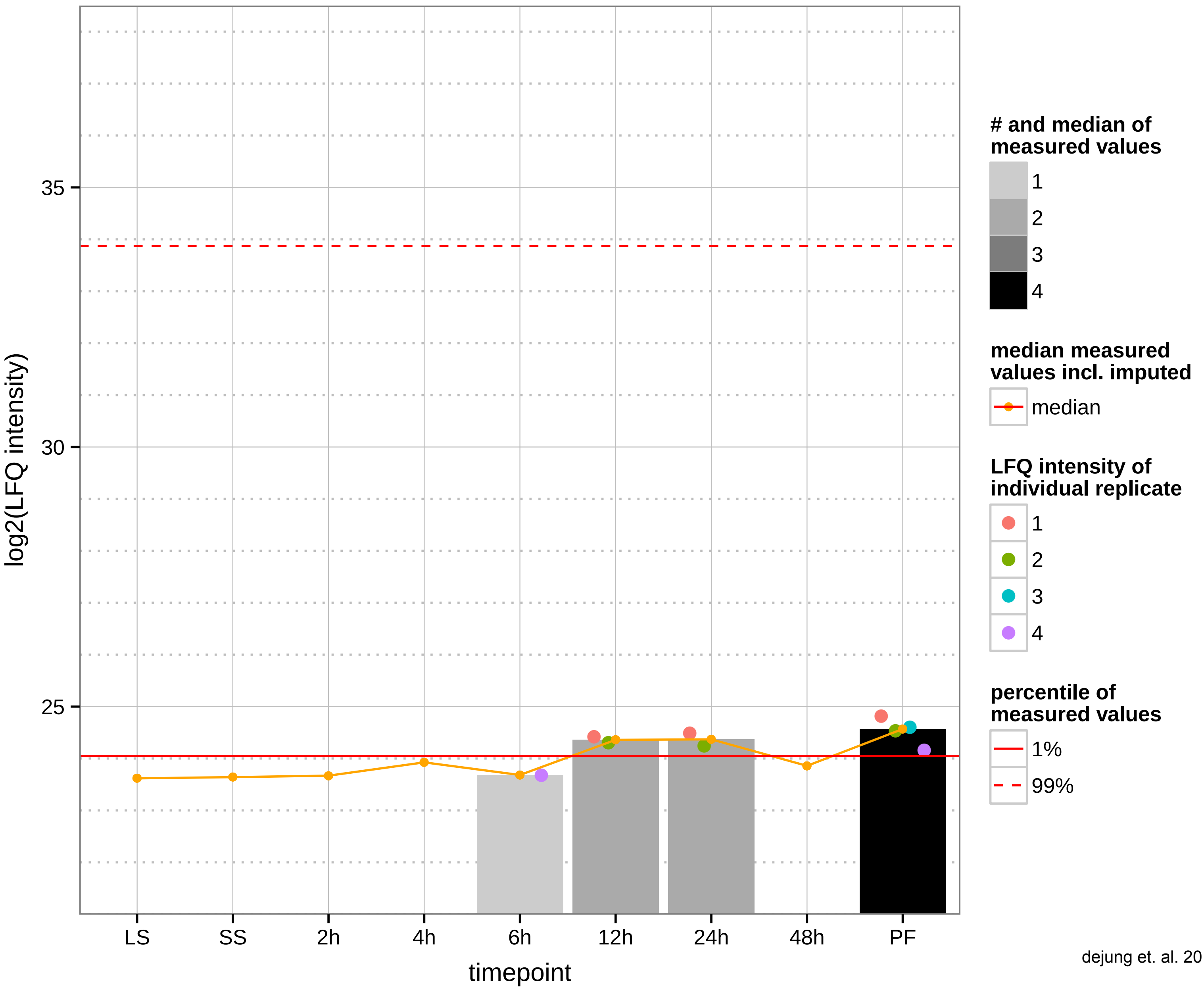

dejung et. al. 2015

hypothetical protein, conserved
Tb927.11.2770
AGOF: null
AGOC: null
AGOP: null
PGOF: null
PGOC: null
PGOP: null

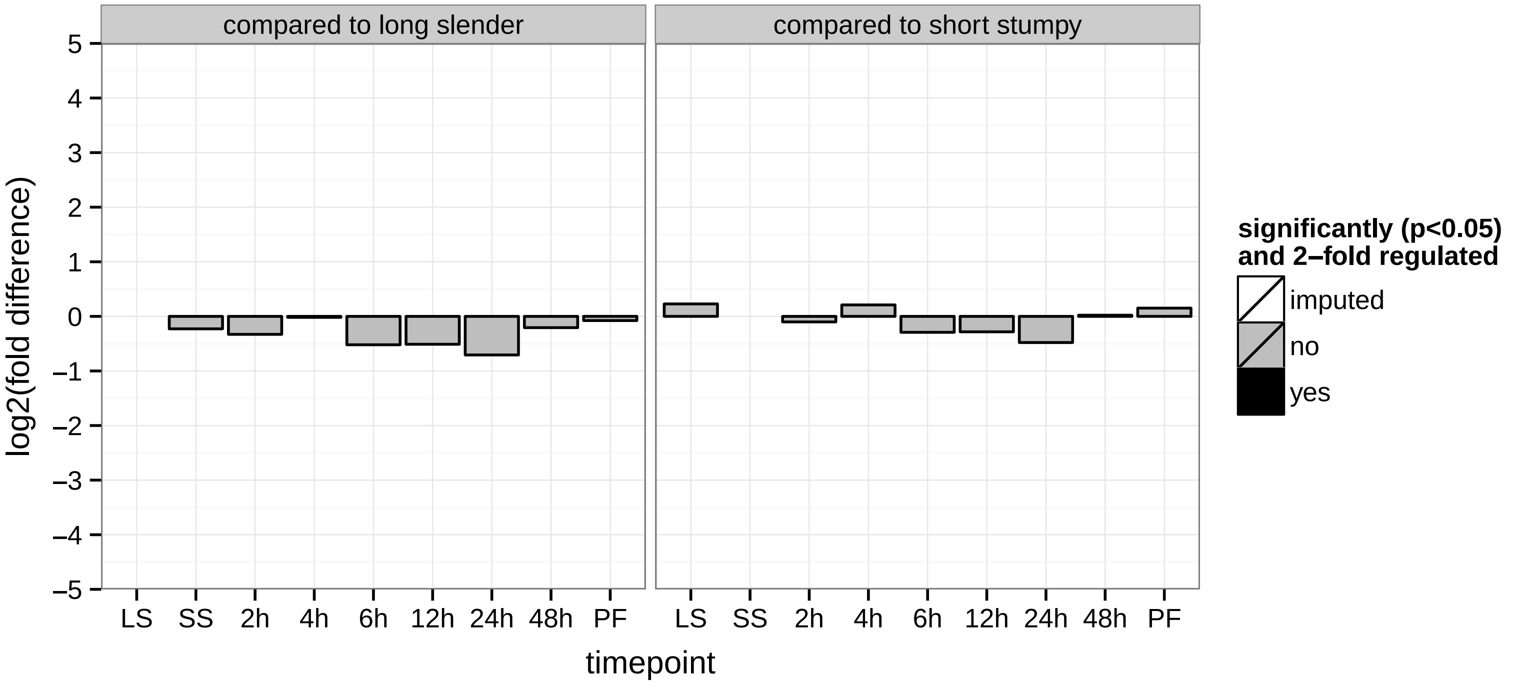

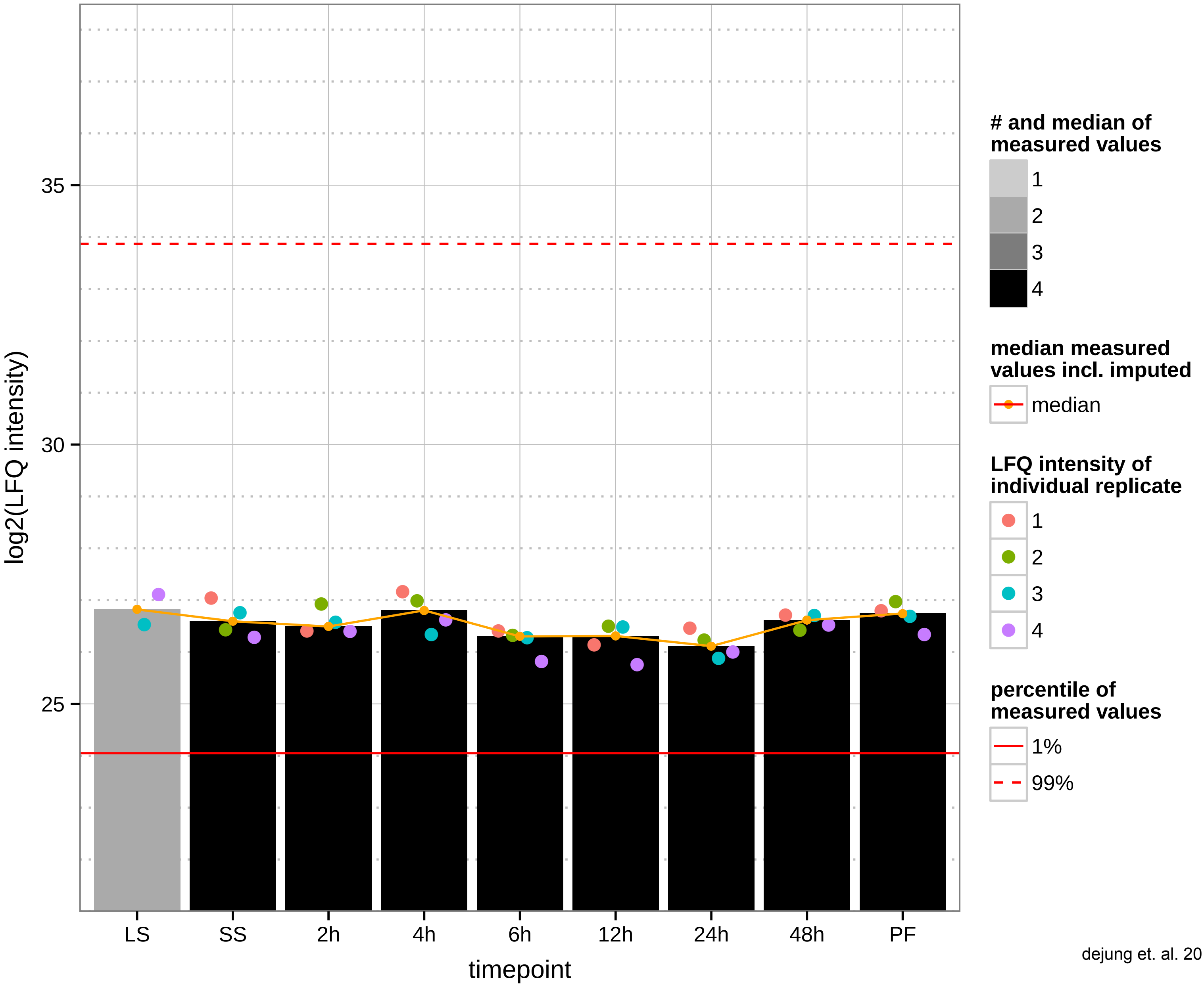

dejung et. al. 2015

hypothetical protein, conserved
Tb927.11.2790
AGOF: null
AGOC: null
AGOP: null
PGOF: null
PGOC: null
PGOP: null

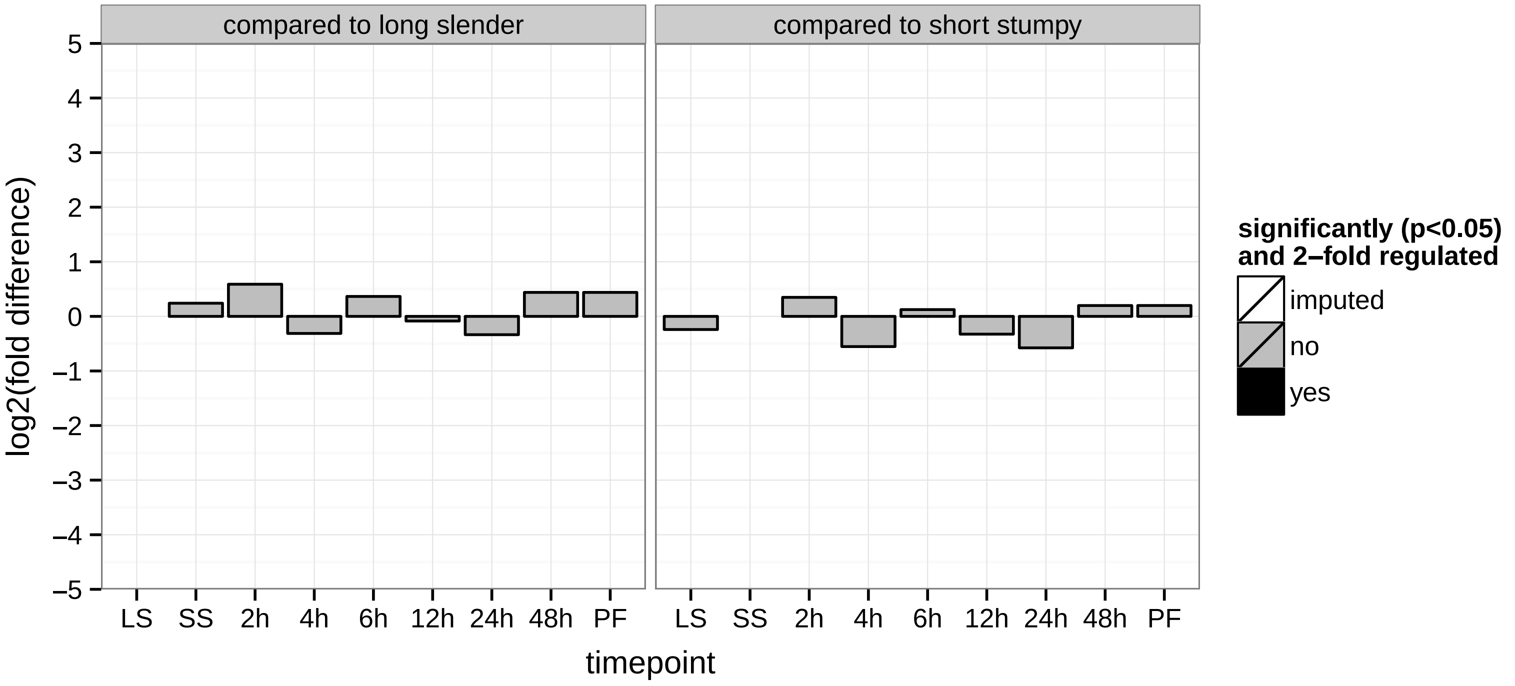

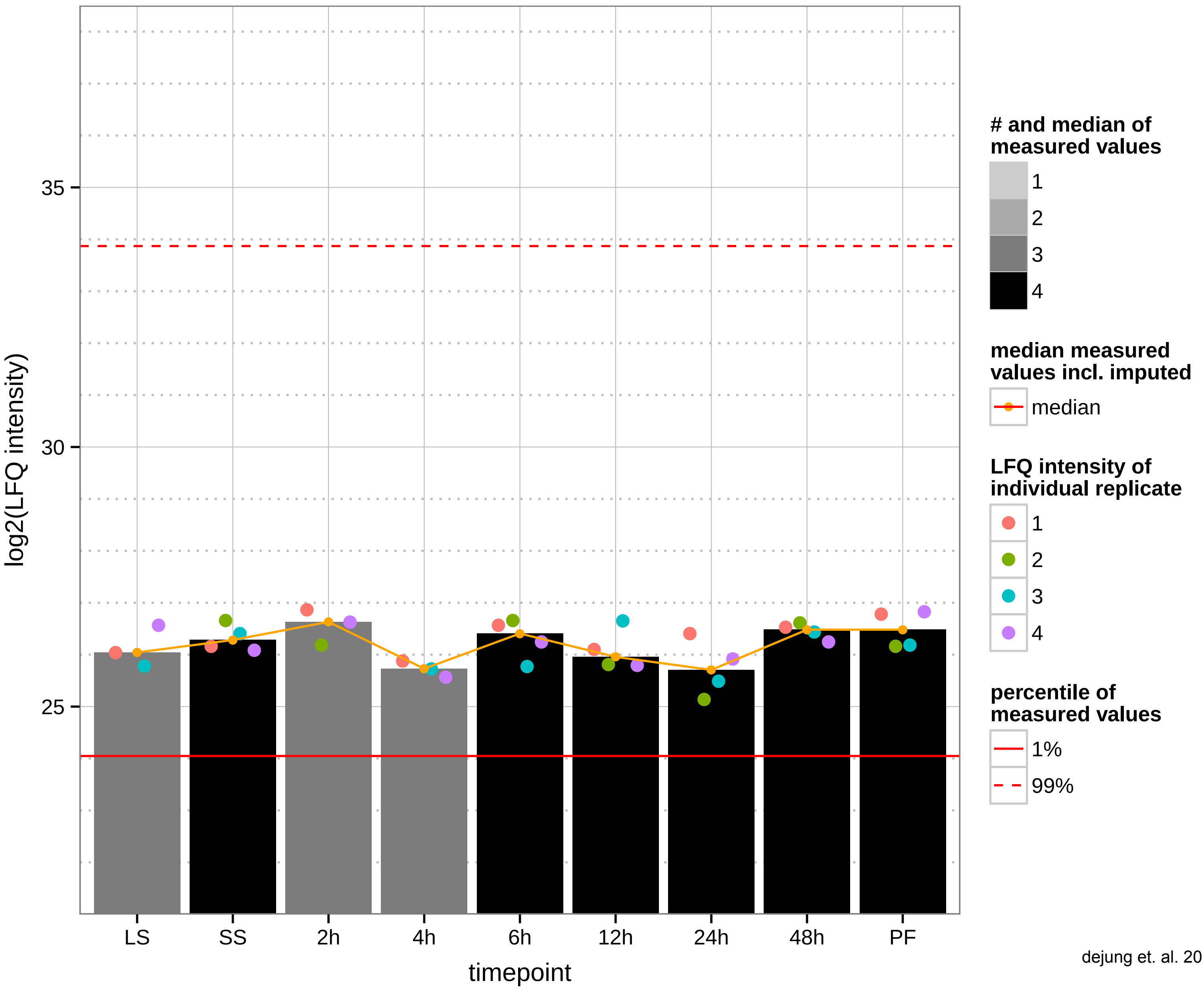

dejung et. al. 2015

hypothetical protein, conserved
Tb927.11.280
AGOF: null
AGOC: null
AGOP: null
PGOF: null
PGOC: null
PGOP: null

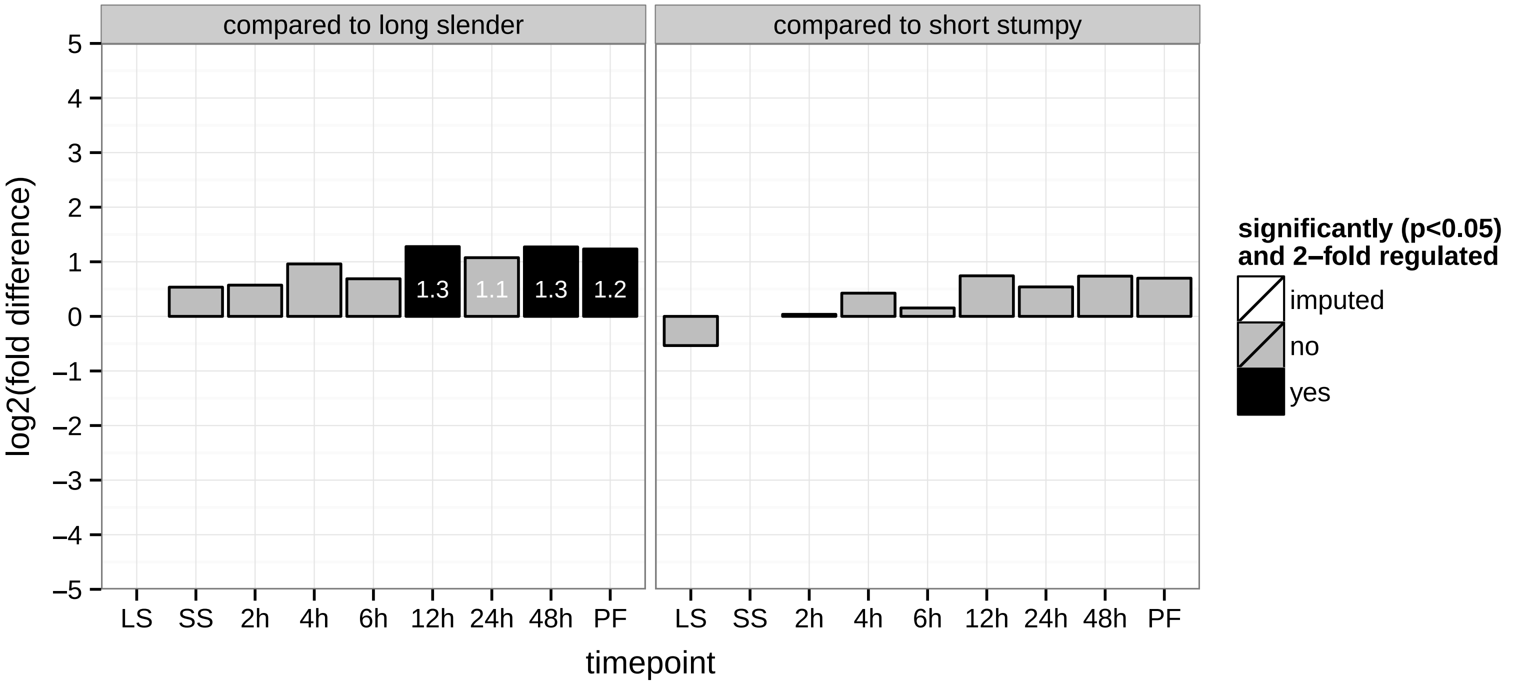

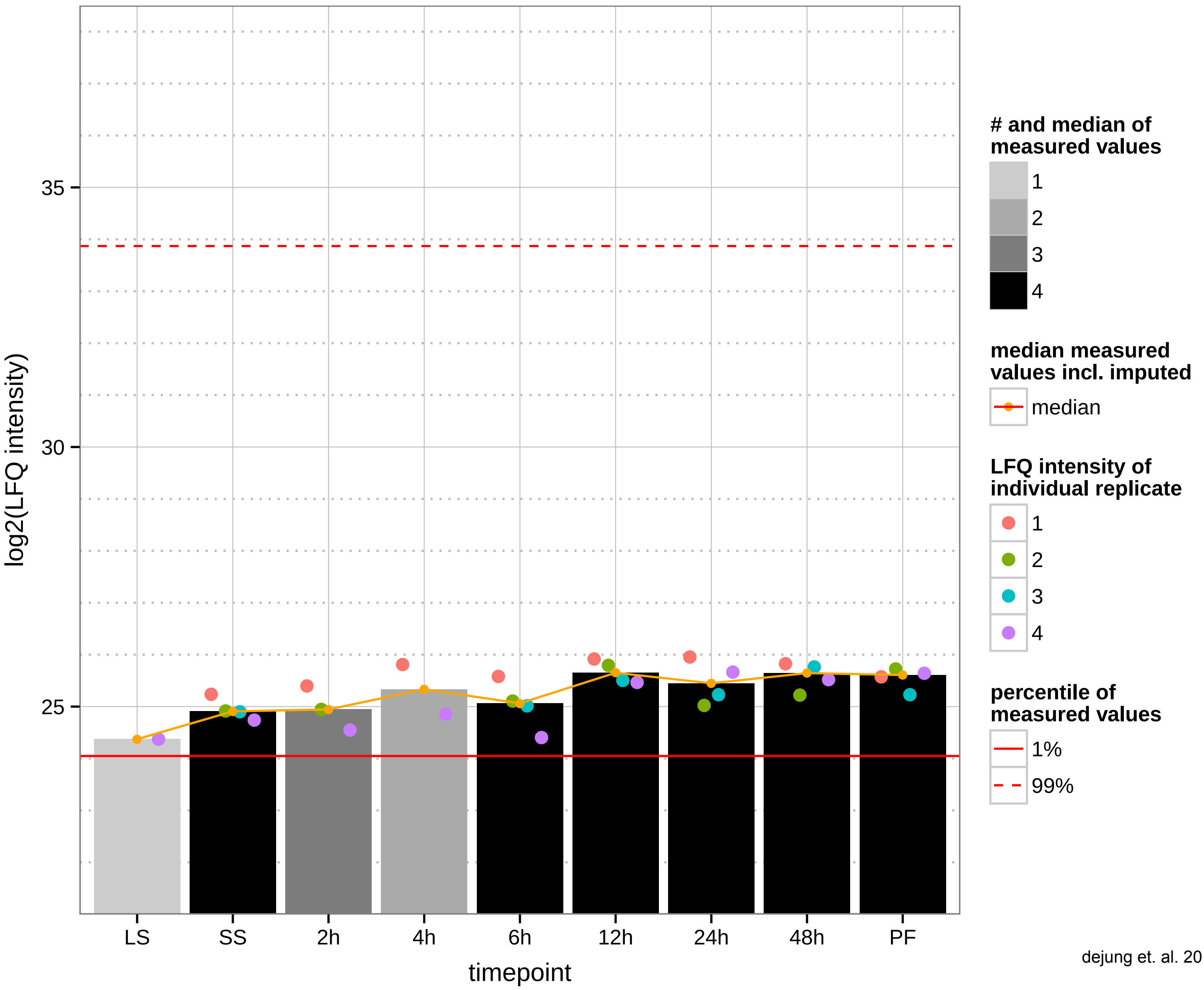

dejung et. al. 2015

kinesin, putative (KIN–A)
Tb927.11.2880
AGOF: ATP binding, microtubule motor activity, motor activity, protein binding
AGOC: chromosome passenger complex, kinesin complex, microtubule associated complex, nucleus, spindle midzone
AGOP: chromosome segregation, cytokinesis, microtubule–based movement, spindle assembly
PGOF: ATP binding, microtubule motor activity
PGOC: null
PGOP: microtubule–based movement

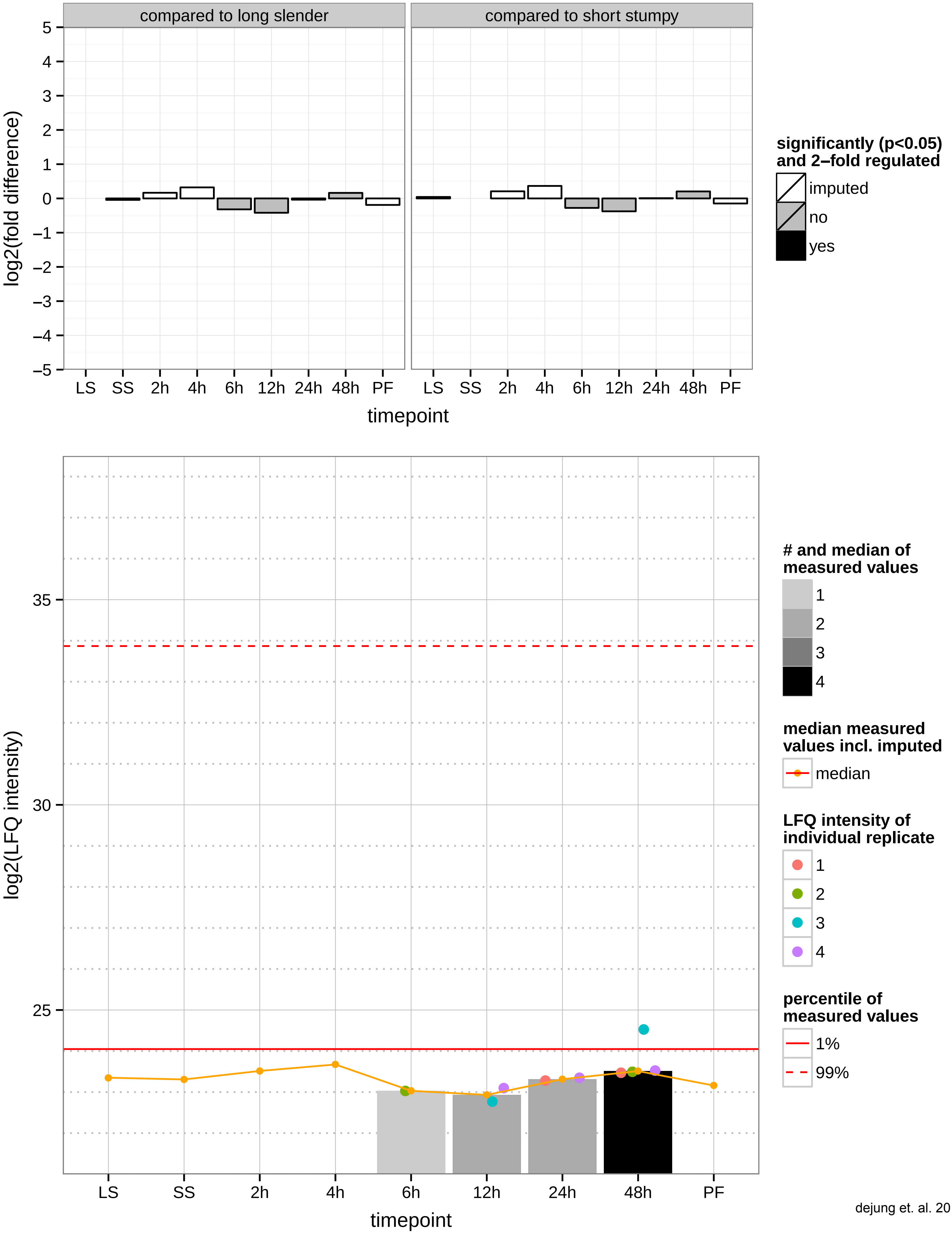

dejung et. al. 2015

phosphoglycerate mutase, putative (iPGAM)
Tb927.11.2910
AGOF: cobalt ion binding, phosphoglycerate mutase activity
AGOC: mitochondrion
AGOP: glycolysis
PGOF: null
PGOC: null
PGOP: null

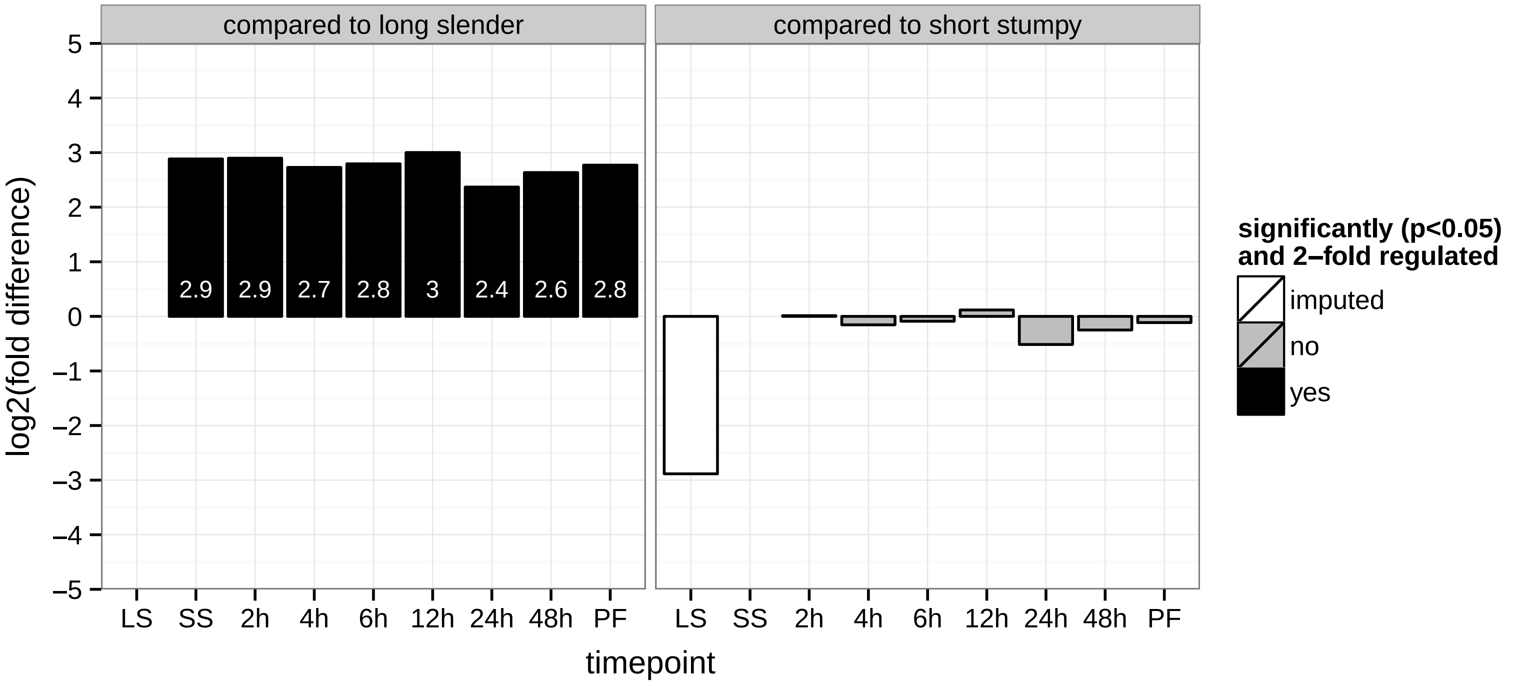

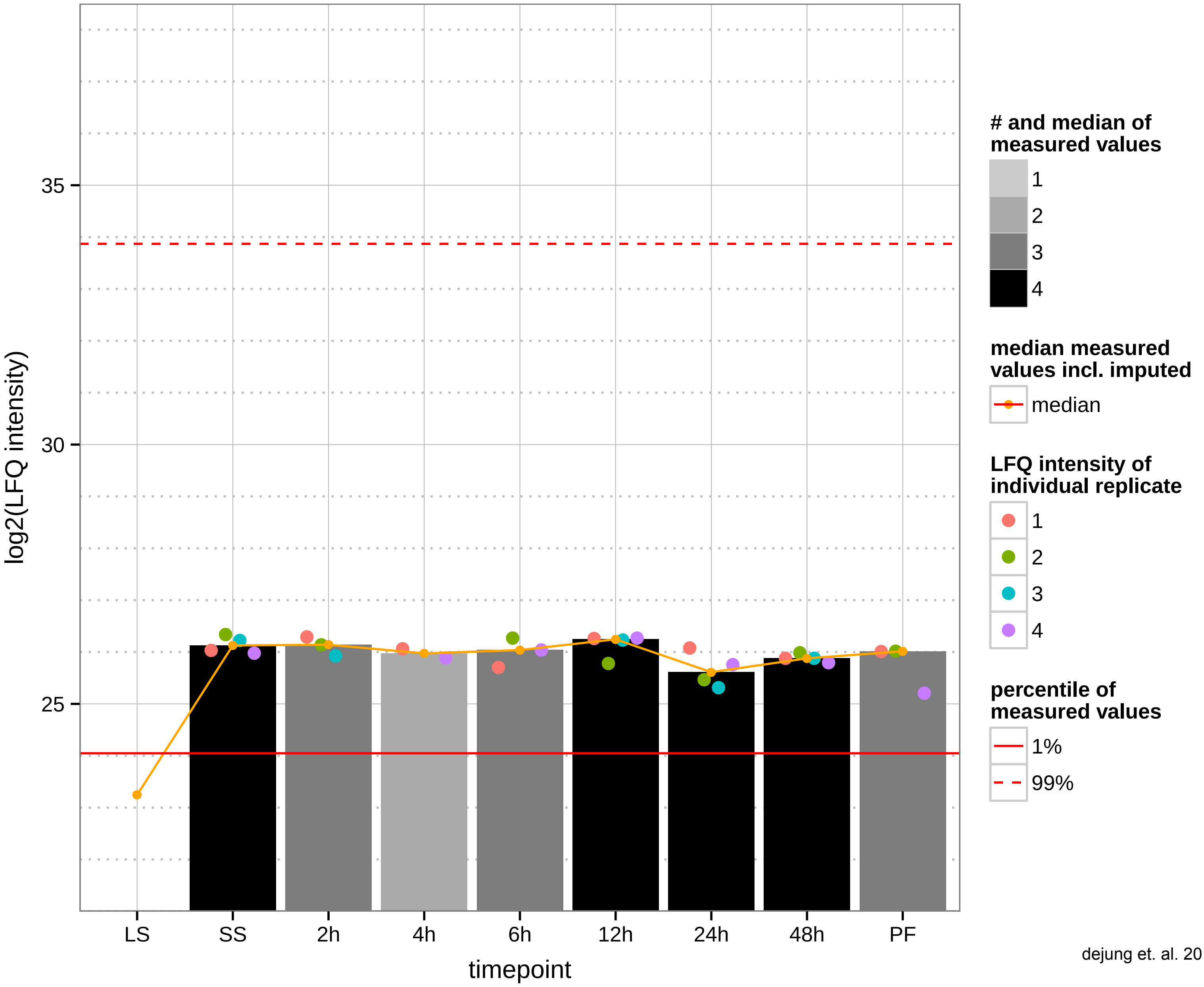

dejung et. al. 2015

Nucleoporin (TbNup89)
Tb927.11.2950
AGOF: null
AGOC: nuclear pore
AGOP: null
PGOF: null
PGOC: null
PGOP: null

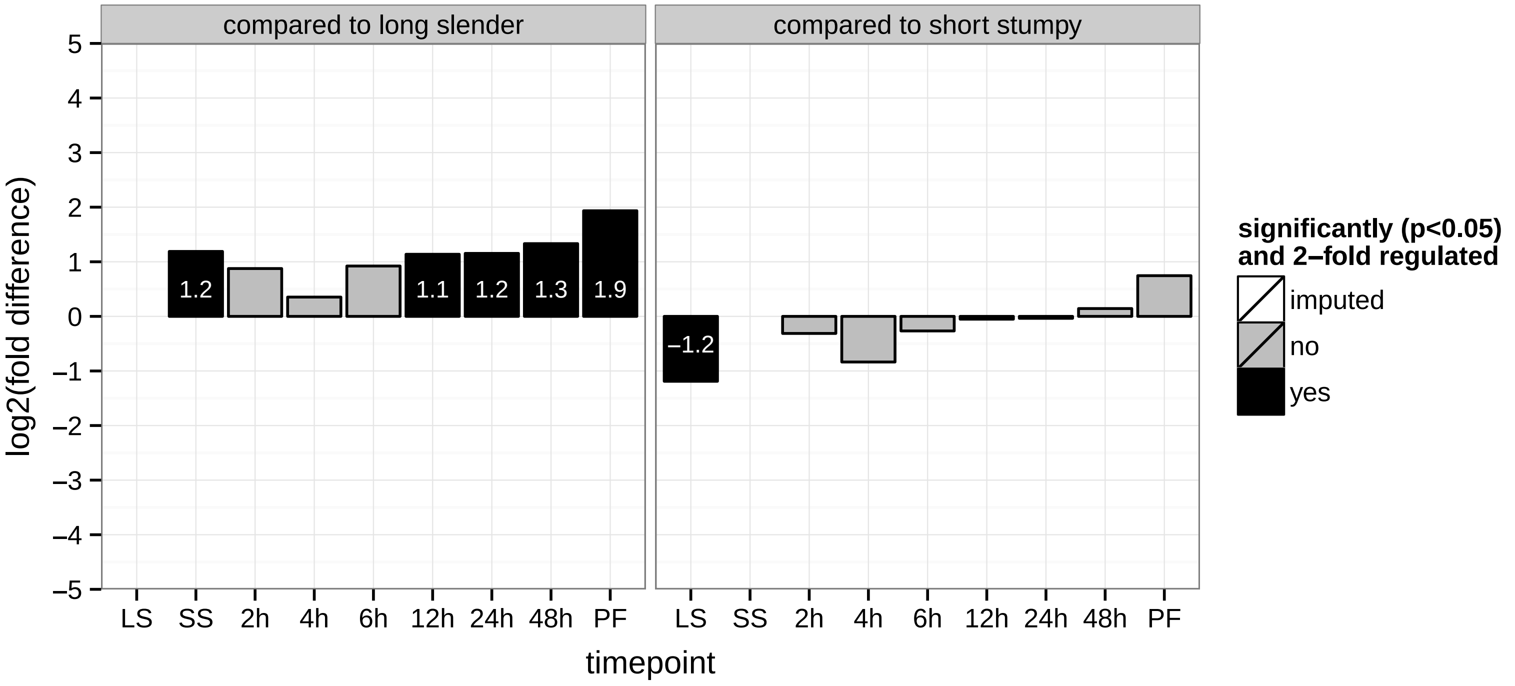

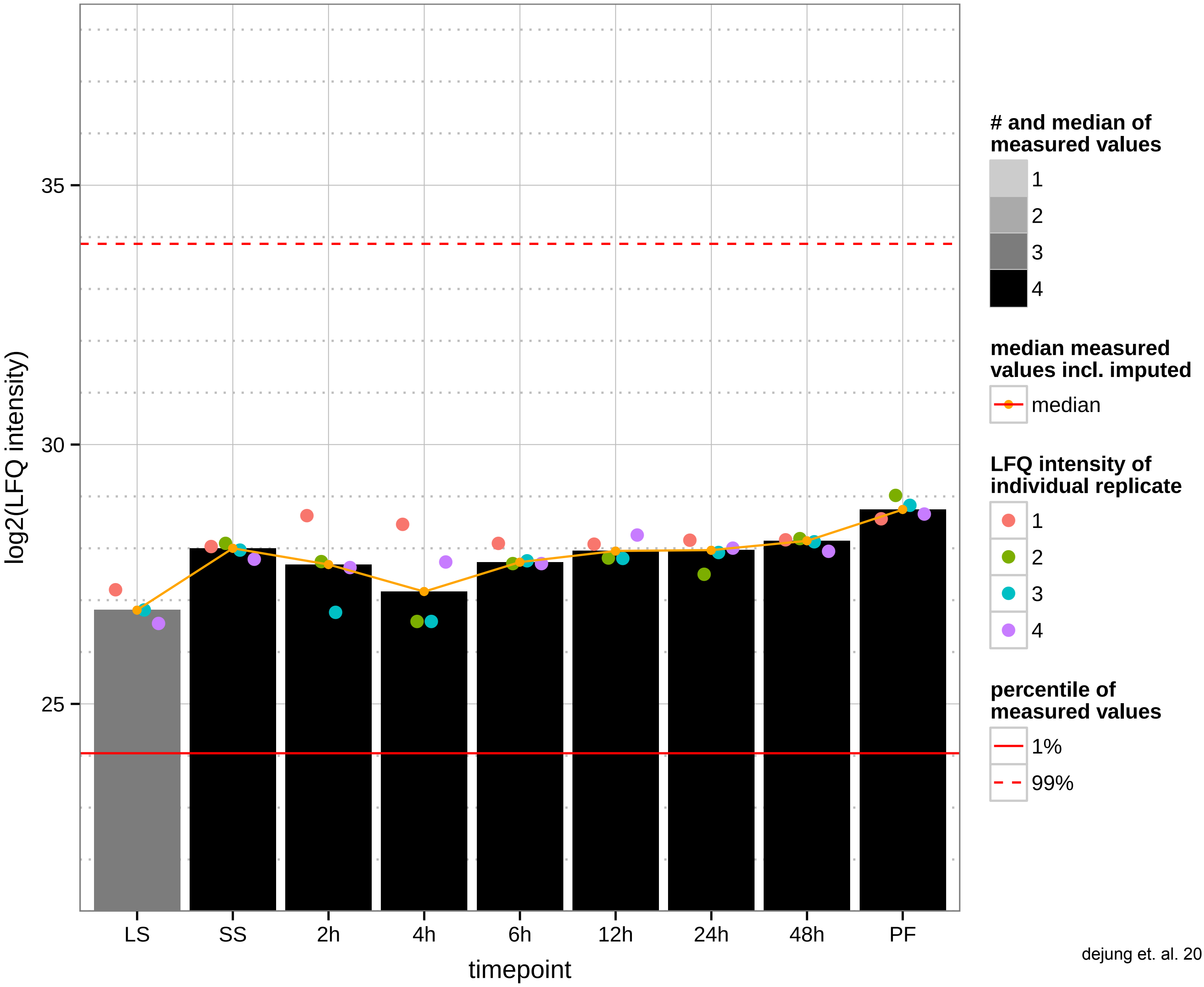

dejung et. al. 2015

hypothetical protein, conserved
Tb927.11.2970
AGOF: null
AGOC: null
AGOP: null
PGOF: binding
PGOC: null
PGOP: null

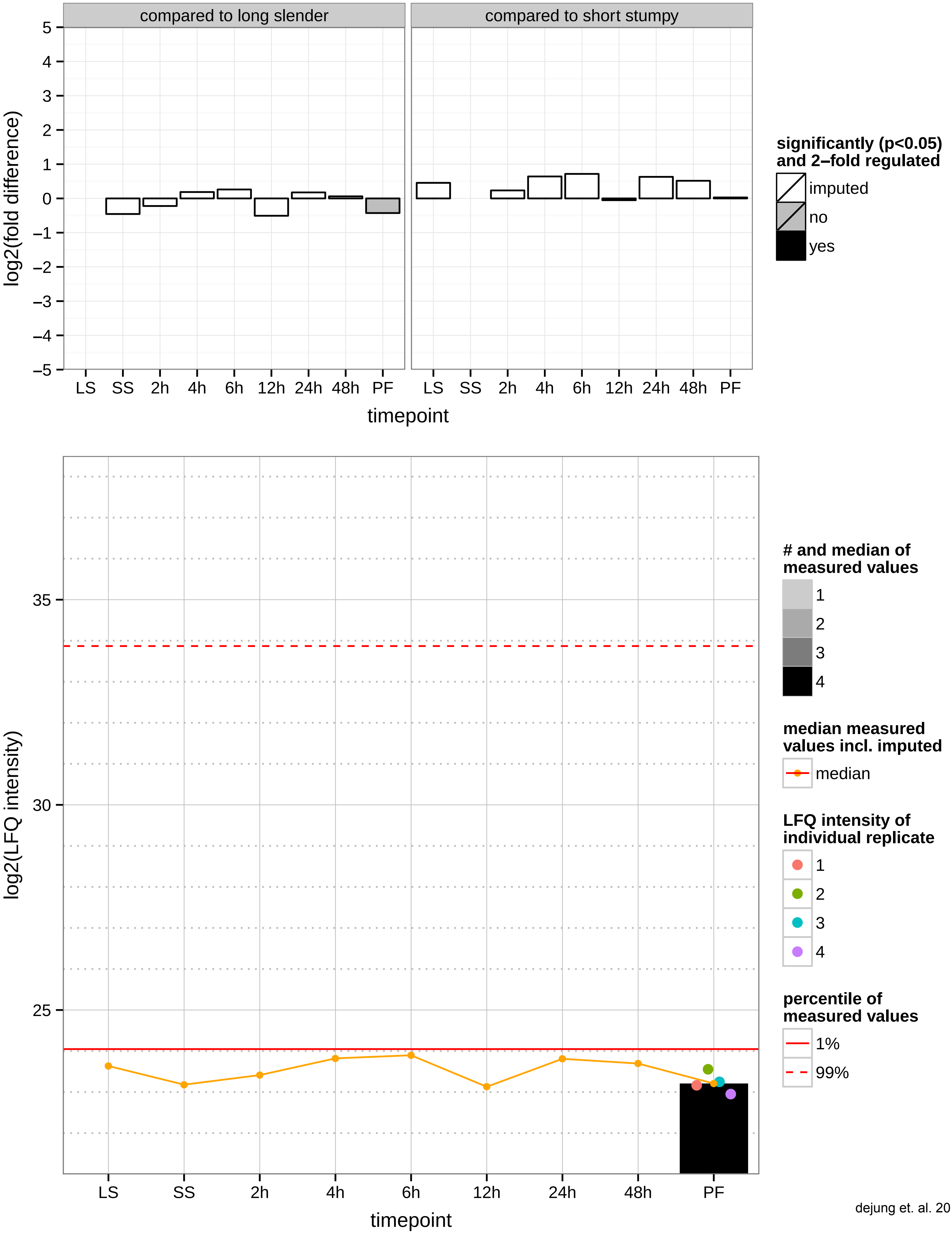

dejung et. al. 2015

phosphoribosylpyrophosphate synthetase, putative (PRS)
Tb927.11.3030
AGOF: ATP binding, magnesium ion binding, ribose phosphate diphosphokinase activity
AGOC: integral to membrane
AGOP: nucleoside metabolic process, purine ribonucleotide biosynthetic process
PGOF: magnesium ion binding, ribose phosphate diphosphokinase activity
PGOC: null
PGOP: nucleoside metabolic process, nucleotide biosynthetic process

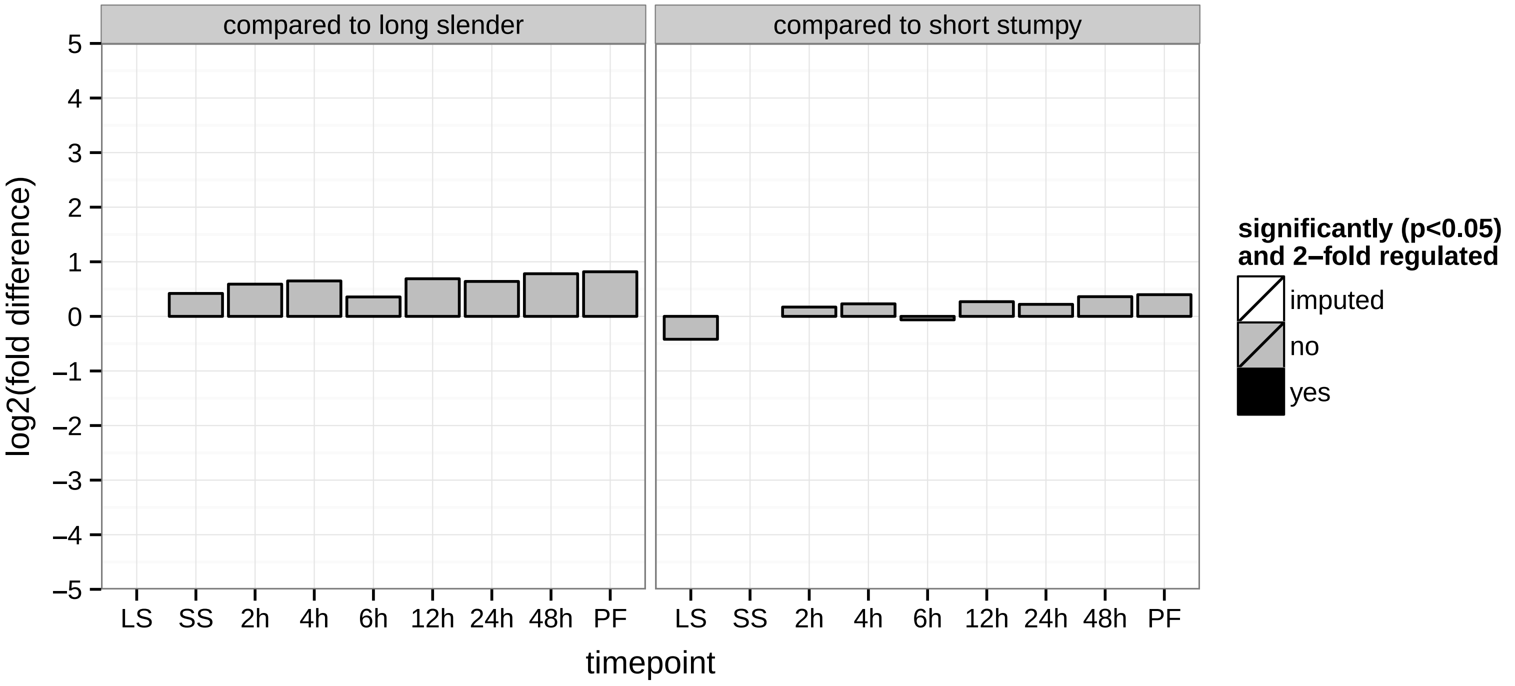

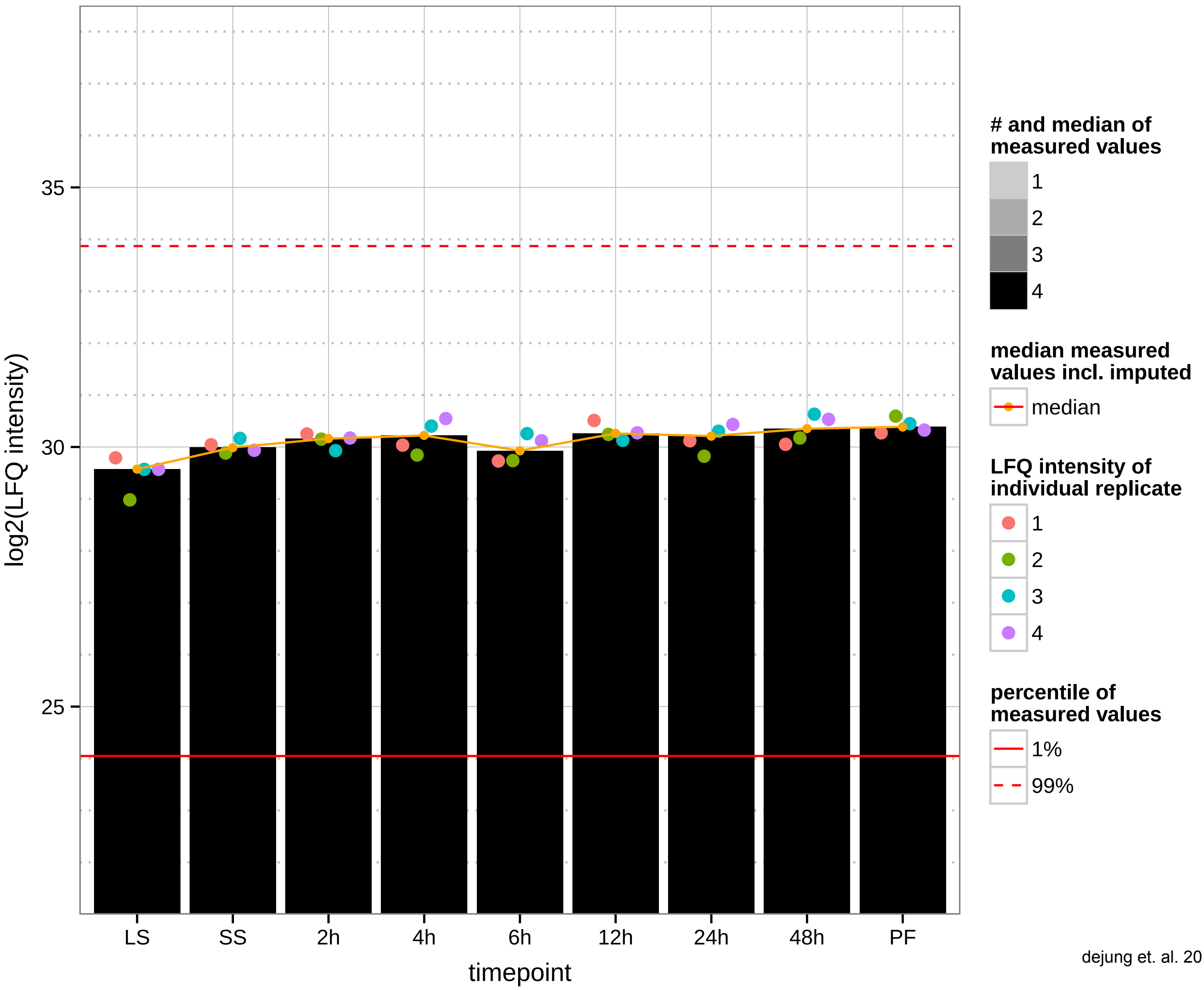

dejung et. al. 2015

hypothetical protein, conserved
Tb927.11.3160
AGOF: null
AGOC: null
AGOP: null
PGOF: null
PGOC: null
PGOP: null

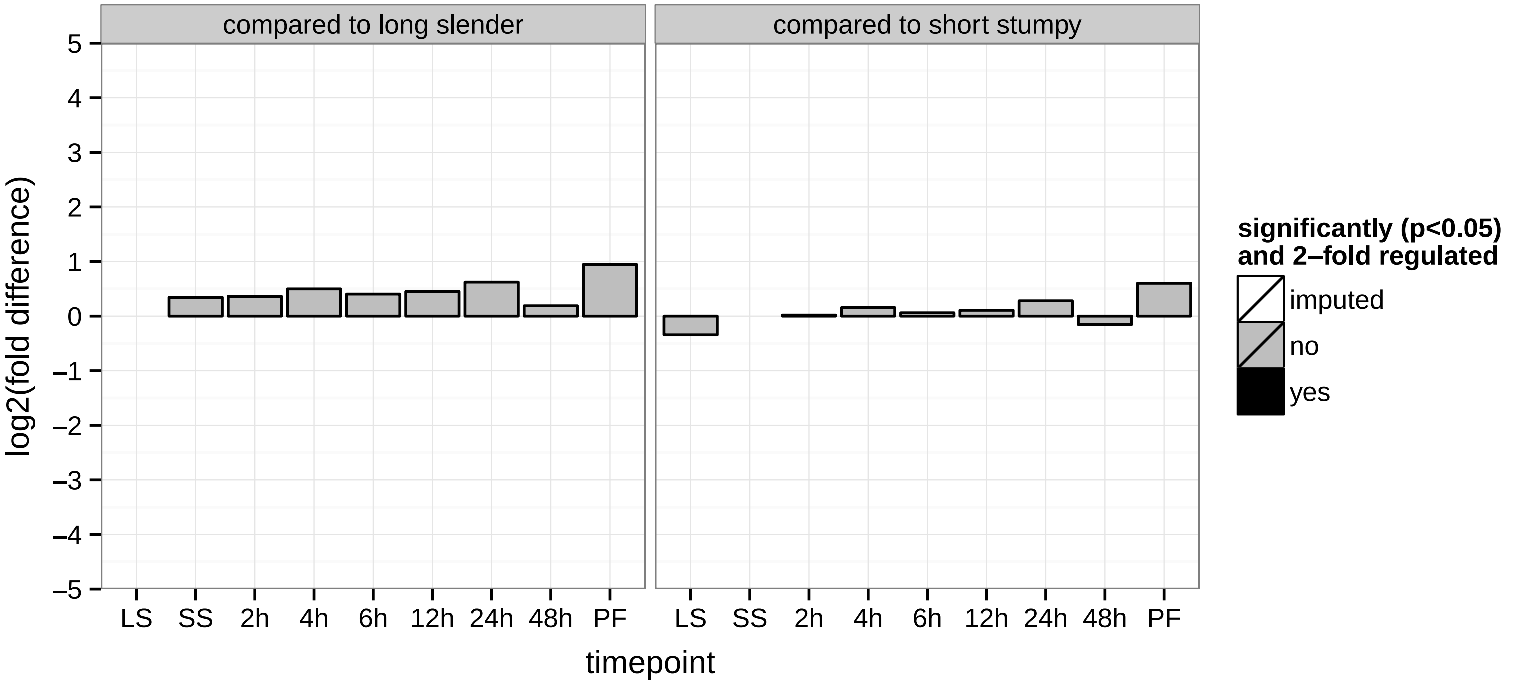

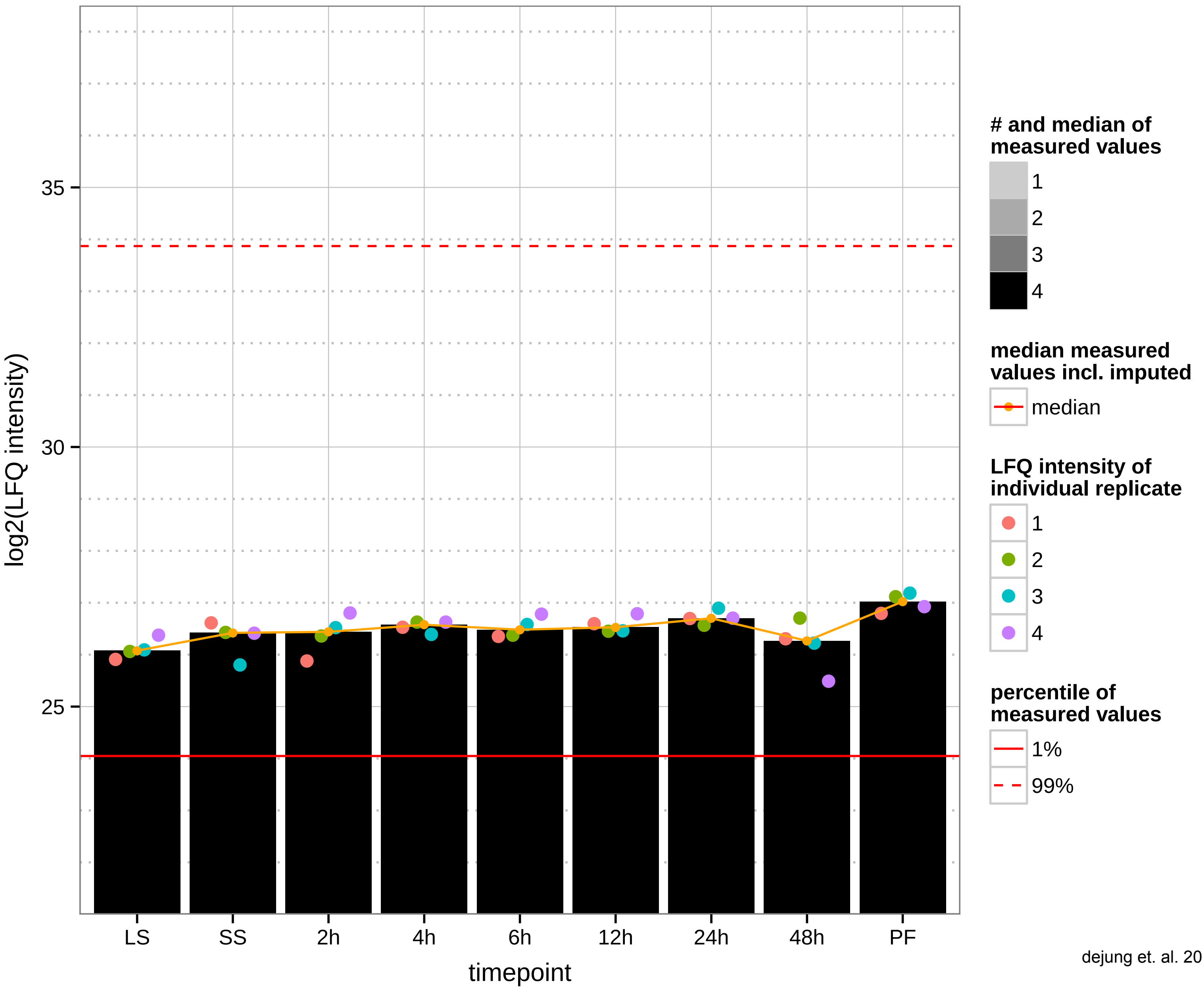

dejung et. al. 2015

hypothetical protein, conserved
Tb927.11.3190
AGOF: null
AGOC: null
AGOP: null
PGOF: binding, protein binding
PGOC: null
PGOP: null

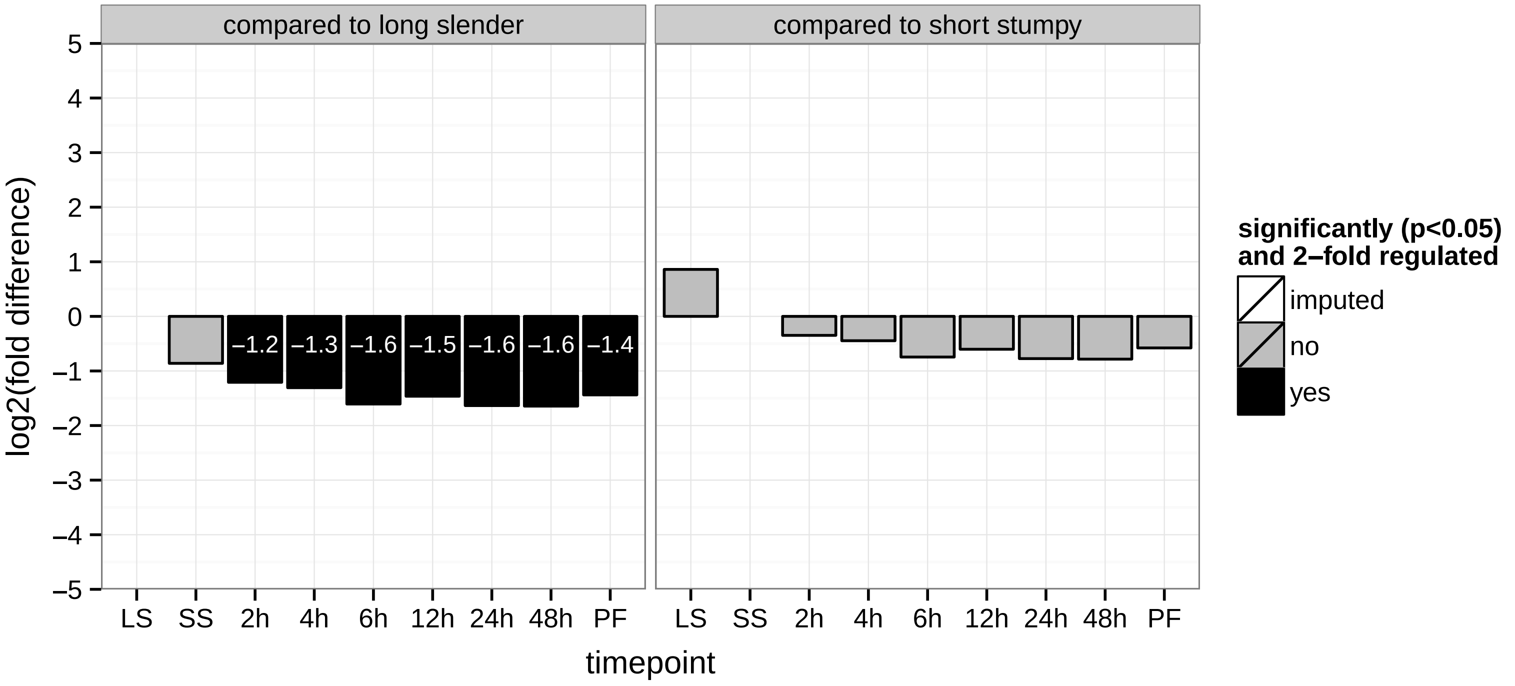

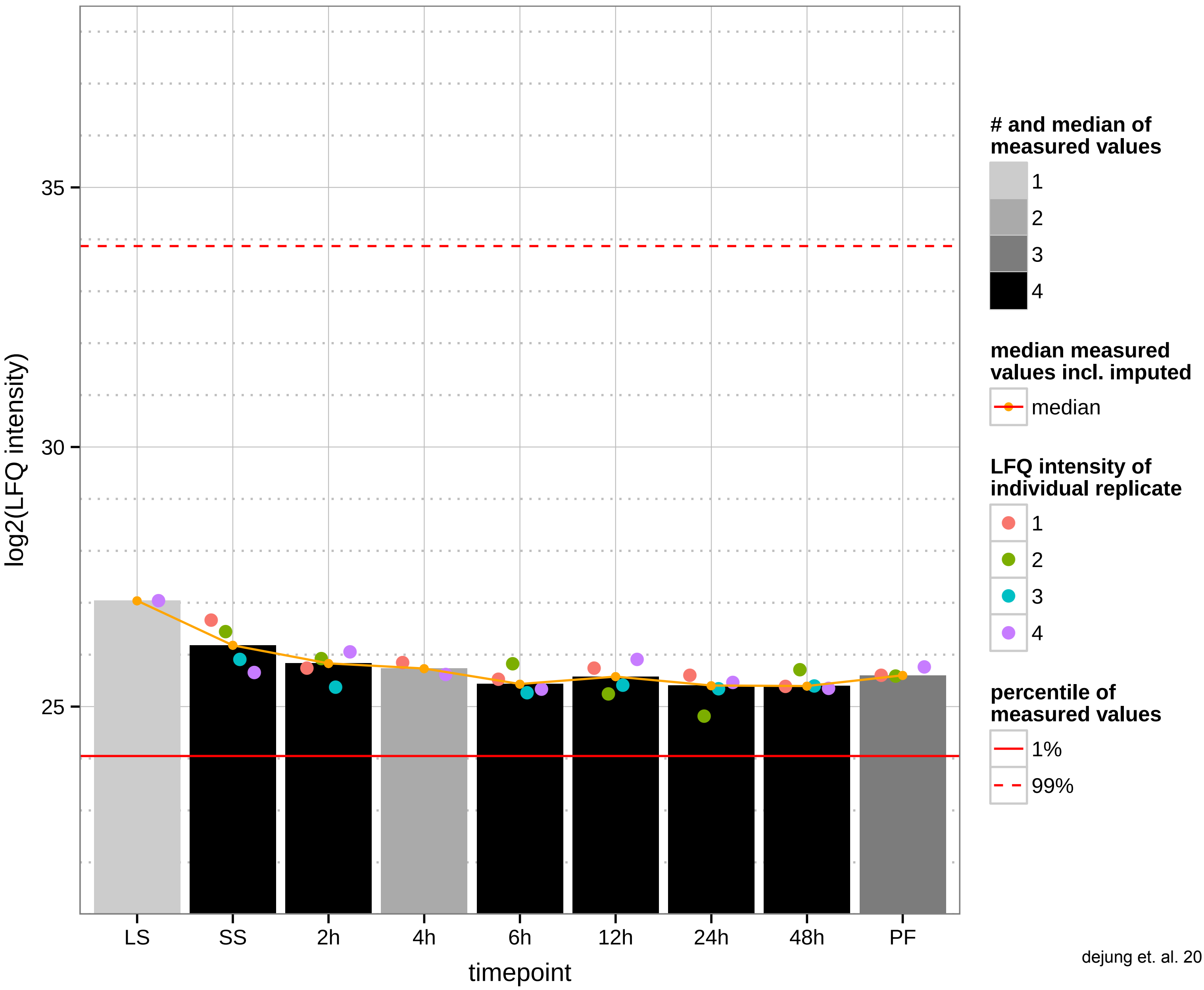

dejung et. al. 2015

dynein light chain 1, putative
Tb927.11.320
AGOF: microtubule motor activity
AGOC: microtubule associated complex
AGOP: microtubule–based process
PGOF: null
PGOC: microtubule associated complex
PGOP: microtubule–based process

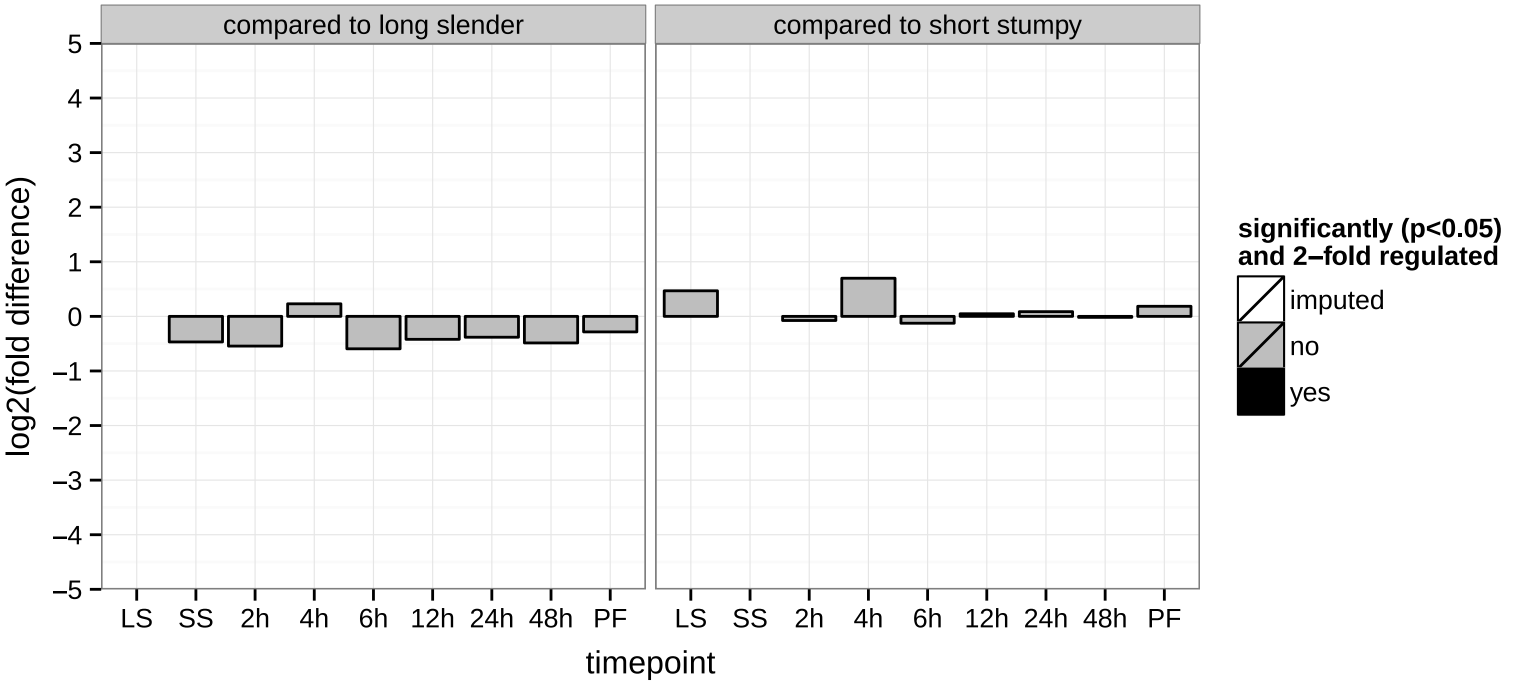

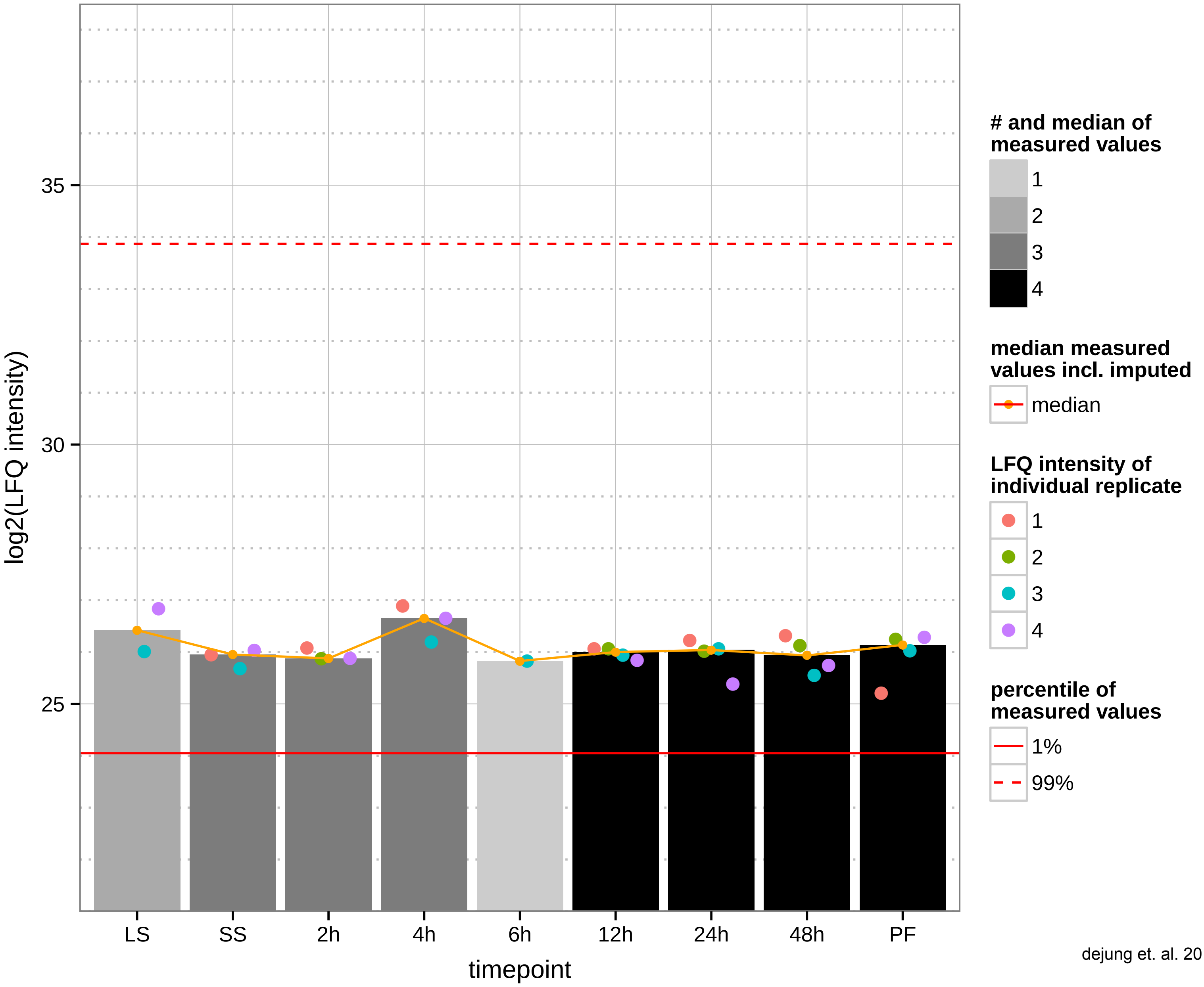

dejung et. al. 2015

T–complex protein 1, zeta subunit, putative (TCP–1–zeta)
Tb927.11.3240
AGOF: ATP binding, unfolded protein binding
AGOC: chaperonin–containing T–complex
AGOP: protein folding, regulation of cell cycle
PGOF: ATP binding, unfolded protein binding
PGOC: null
PGOP: cellular protein metabolic process, protein folding

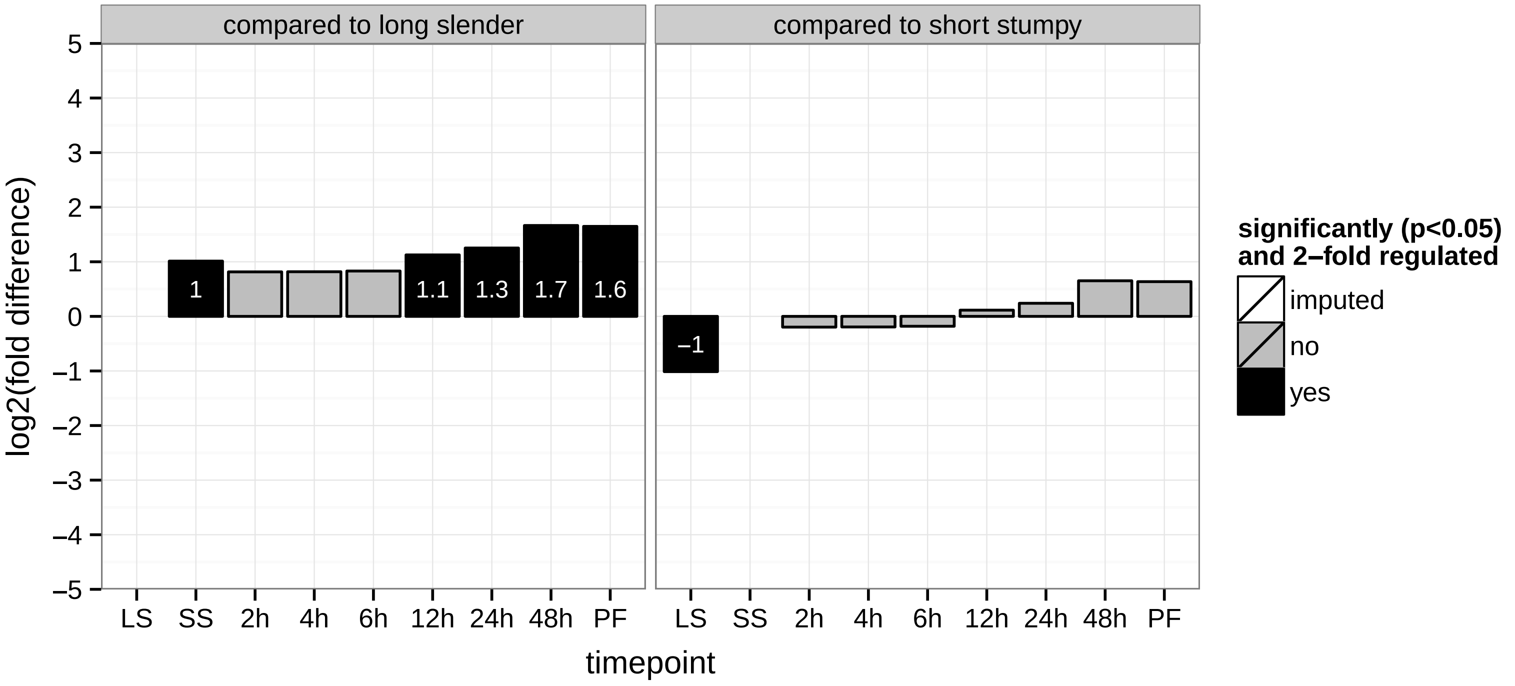

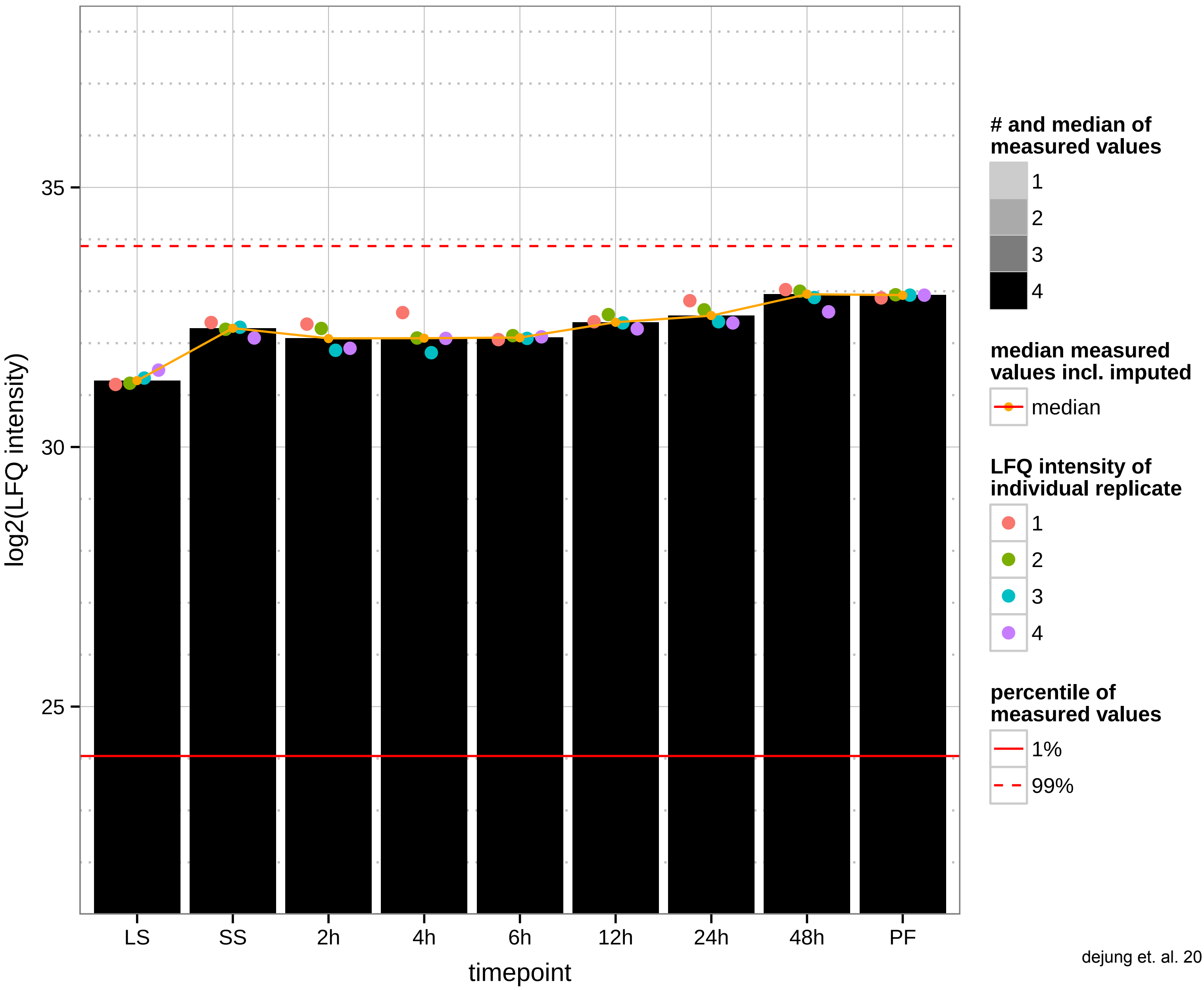

dejung et. al. 2015

dynein heavy chain, putative
Tb927.11.3250
AGOF: ATP binding, ATPase activity, microtubule motor activity
AGOC: dynein complex
AGOP: microtubule−based movement
PGOF: ATP binding, ATPase activity, microtubule motor activity, nucleoside−triphosphatase activity, nucleotide binding
PGOC: dynein complex
PGOP: microtubule−based movement

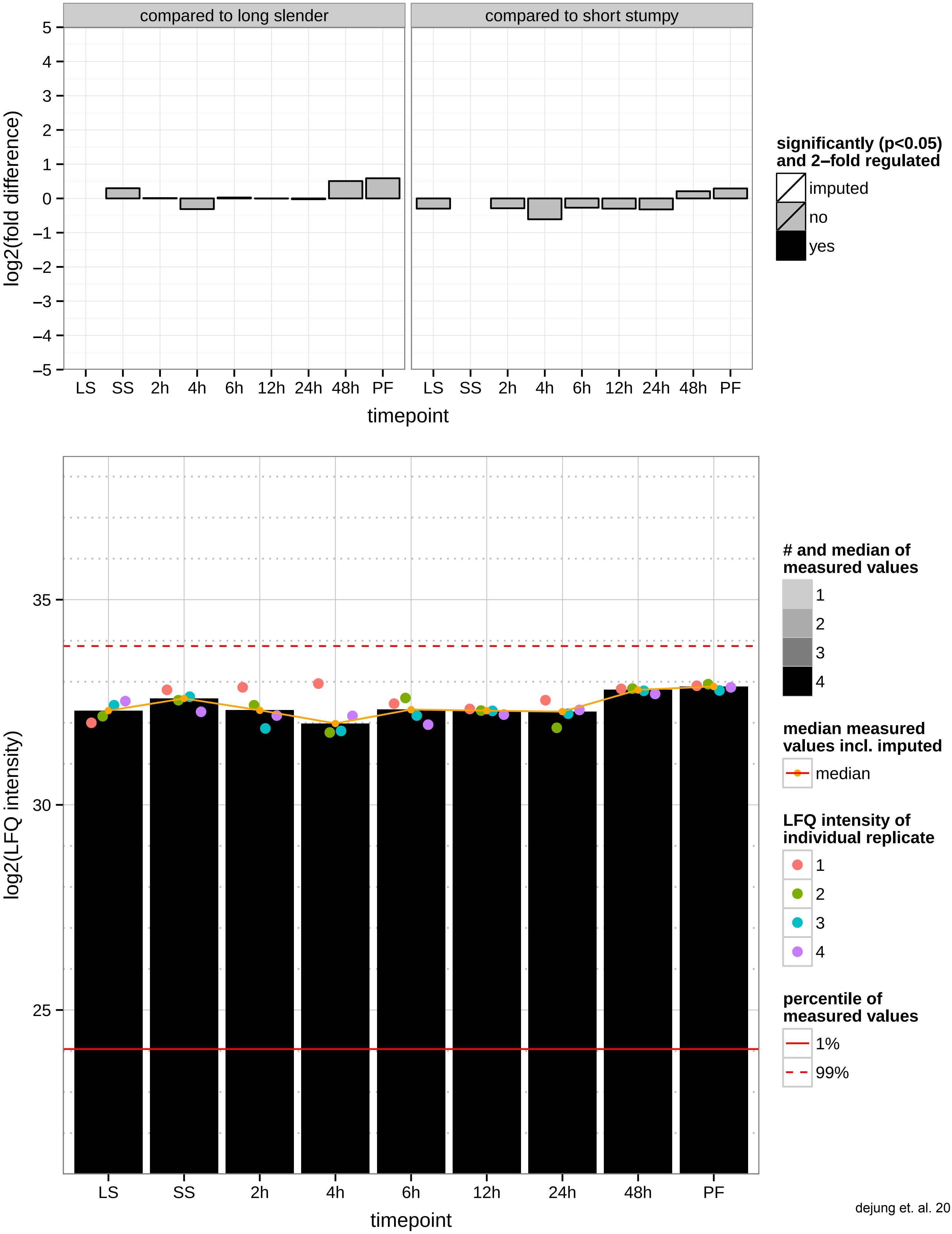

dejung et. al. 2015

hypothetical protein, conserved
Tb927.11.3300
AGOF: null
AGOC: mitochondrial membrane
AGOP: null
PGOF: null
PGOC: null
PGOP: null

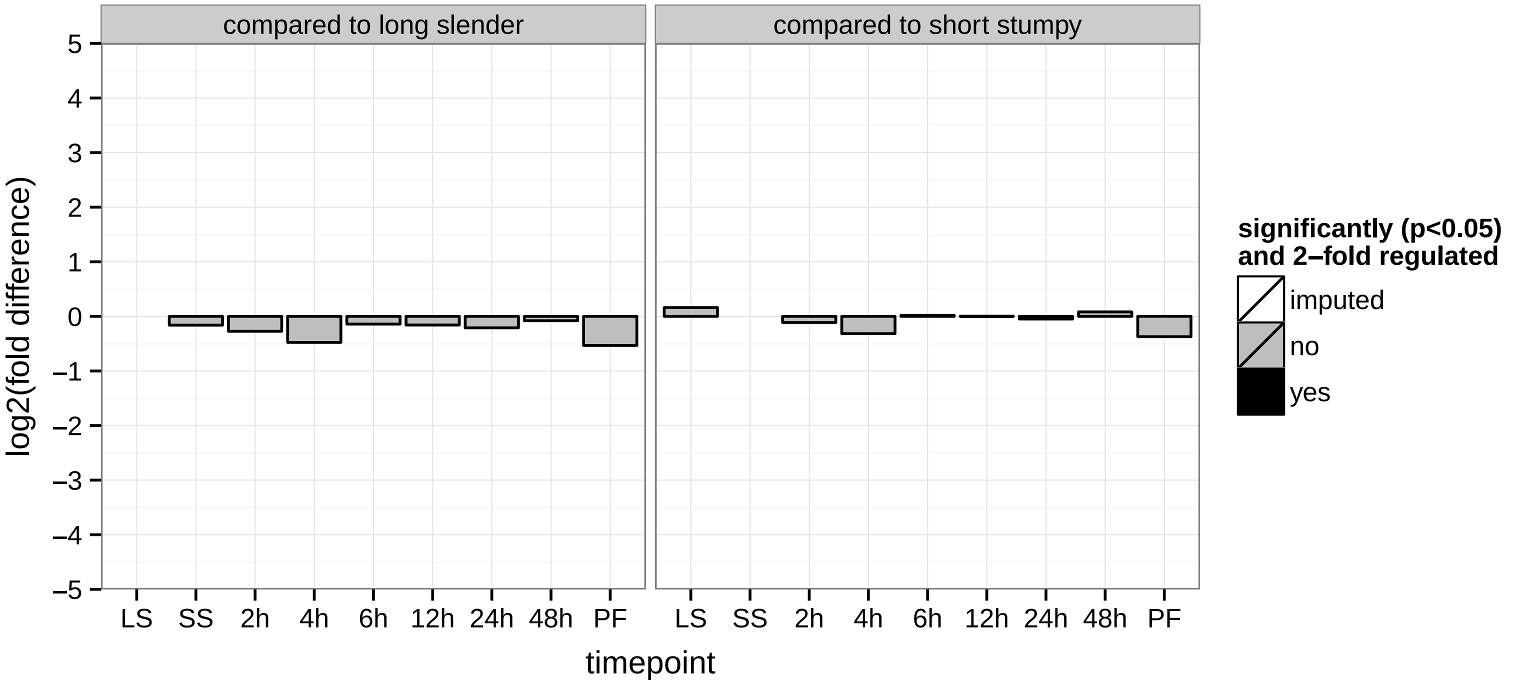

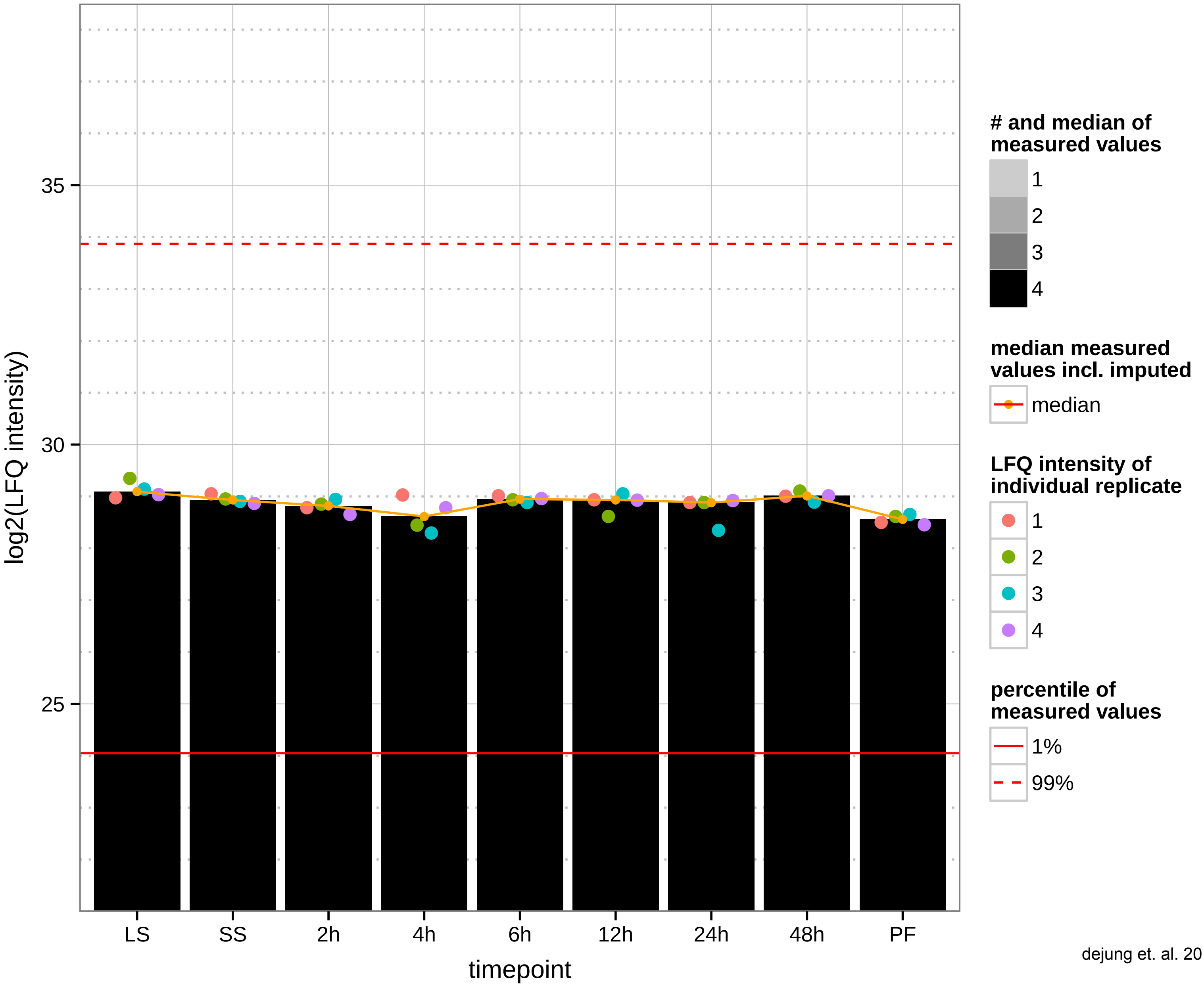

dejung et. al. 2015

hypothetical protein, conserved
Tb927.11.3360
AGOF: ATP binding
AGOC: null
AGOP: ciliary or flagellar motility
PGOF: ATP binding, protein binding
PGOC: null
PGOP: null

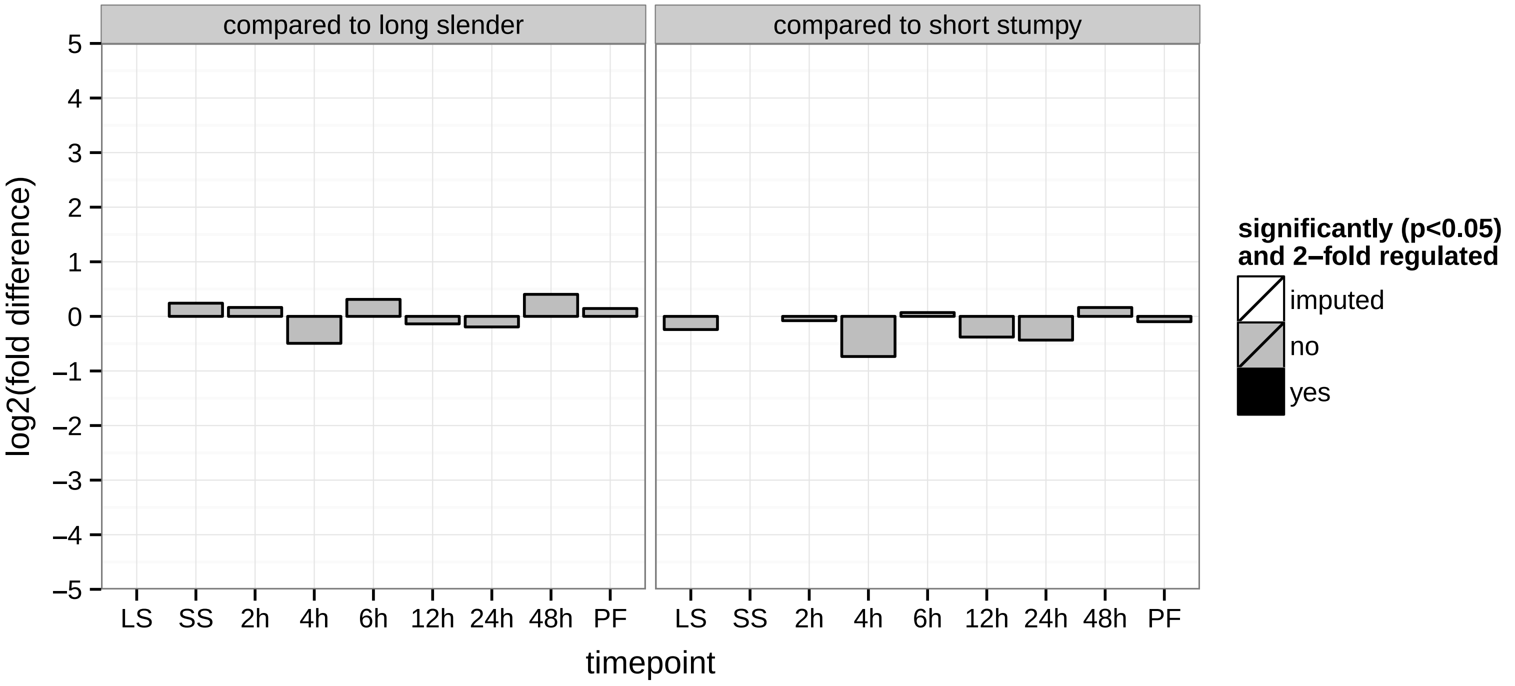

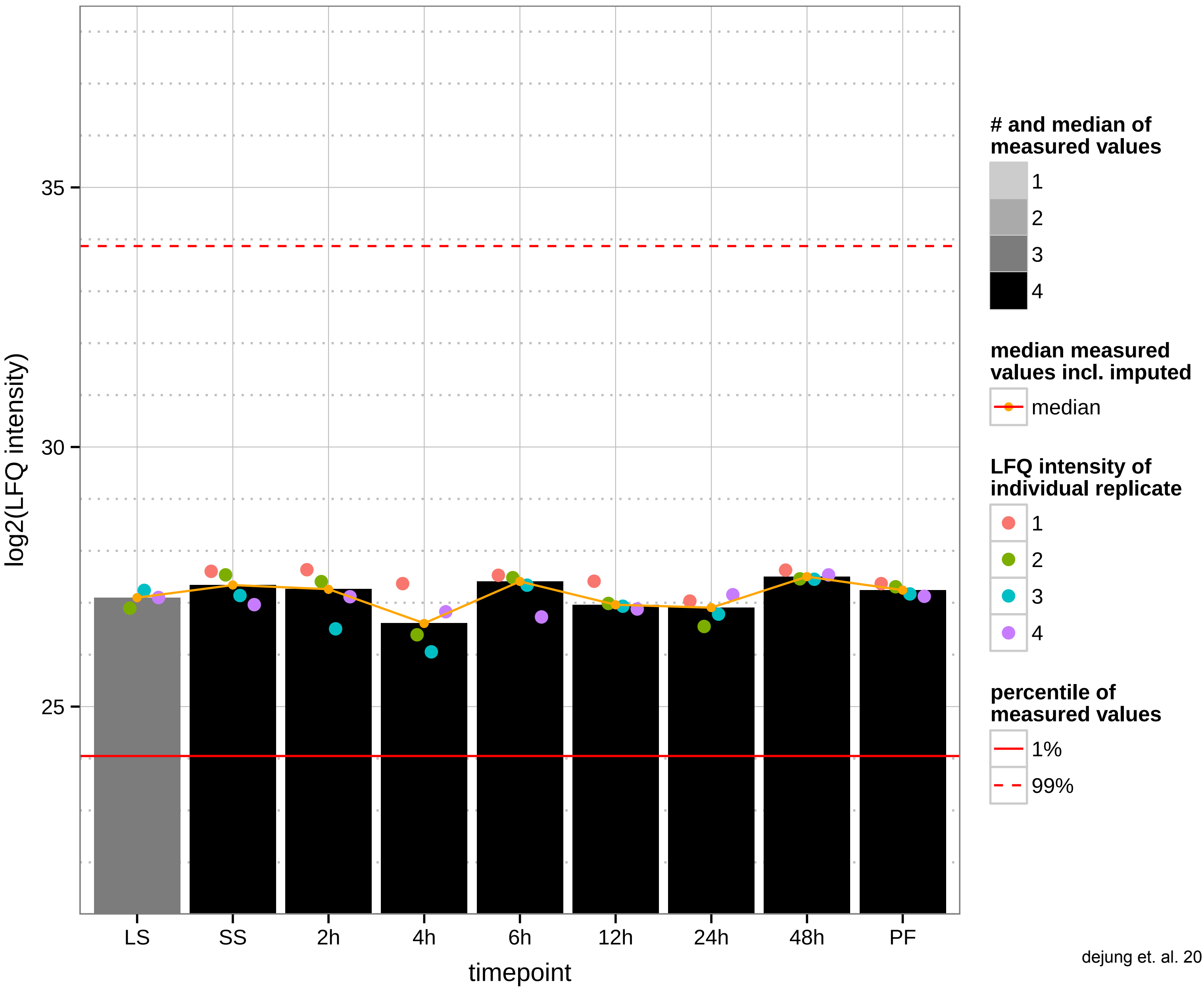

dejung et. al. 2015

Ran–binding protein 1, putative
Tb927.11.3380
AGOF: GDP–dissociation inhibitor activity
AGOC: cytoplasm, nucleus
AGOP: nuclear transport
PGOF: null
PGOC: null
PGOP: intracellular transport

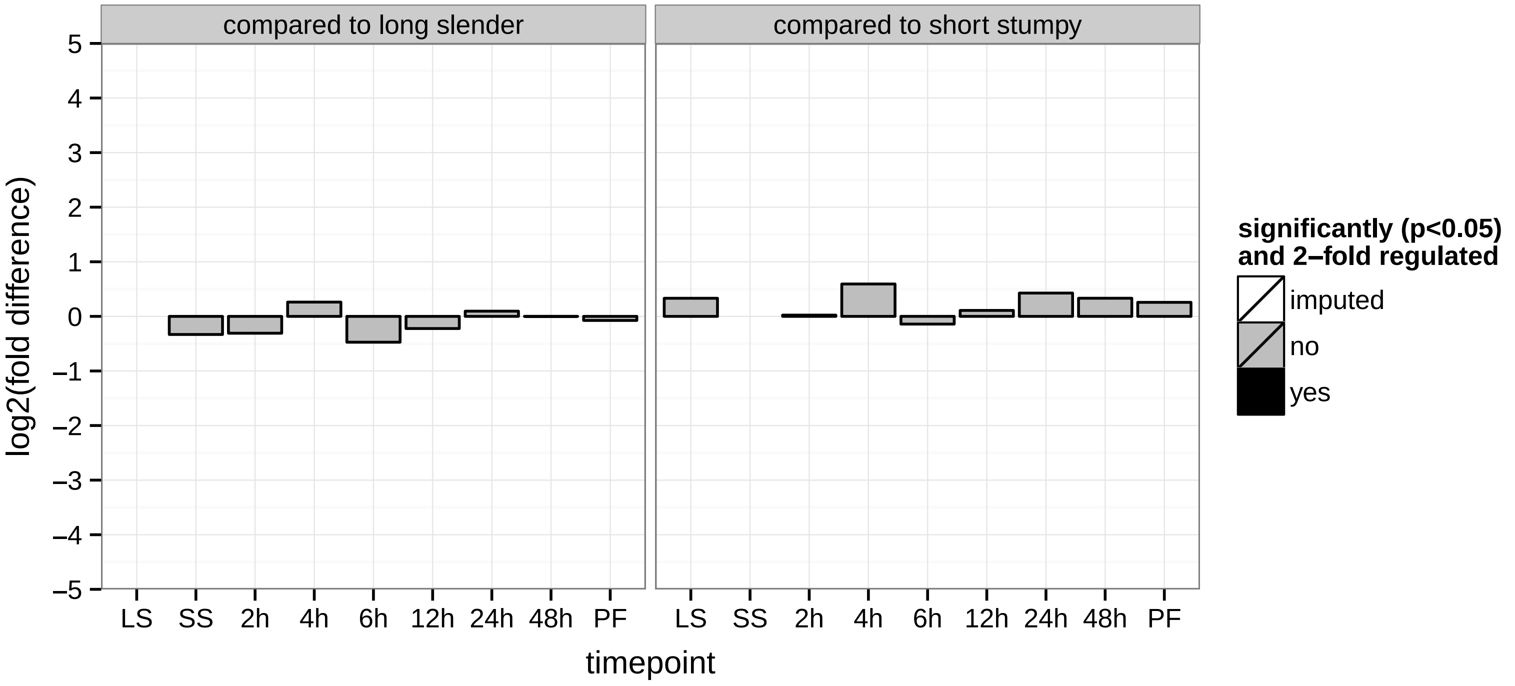

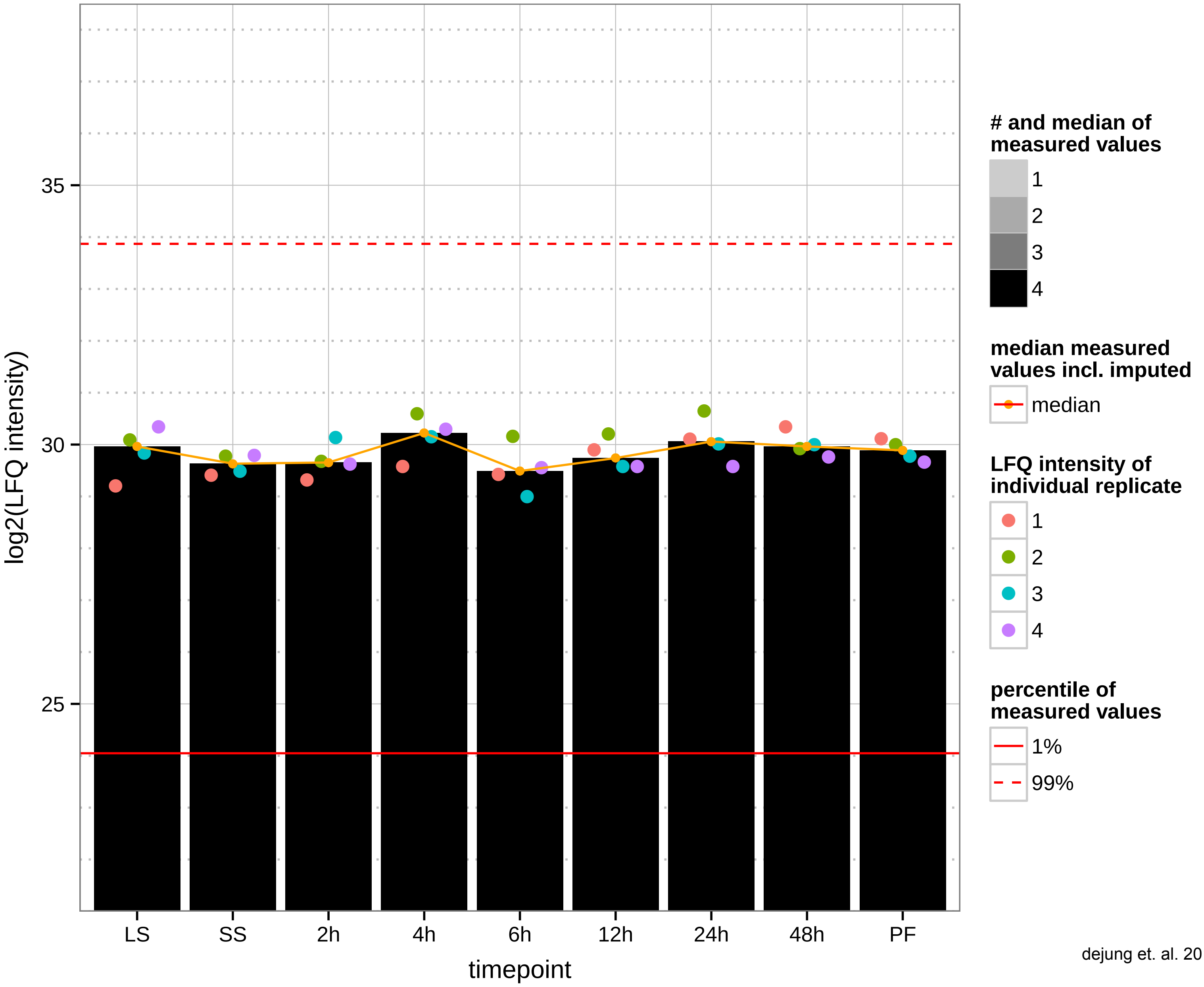

dejung et. al. 2015

hypothetical protein, conserved
Tb927.11.3390
AGOF: acid–amino acid ligase activity
AGOC: intracellular
AGOP: cellular protein modification process
PGOF: acid–amino acid ligase activity, protein binding
PGOC: intracellular
PGOP: cellular protein modification process

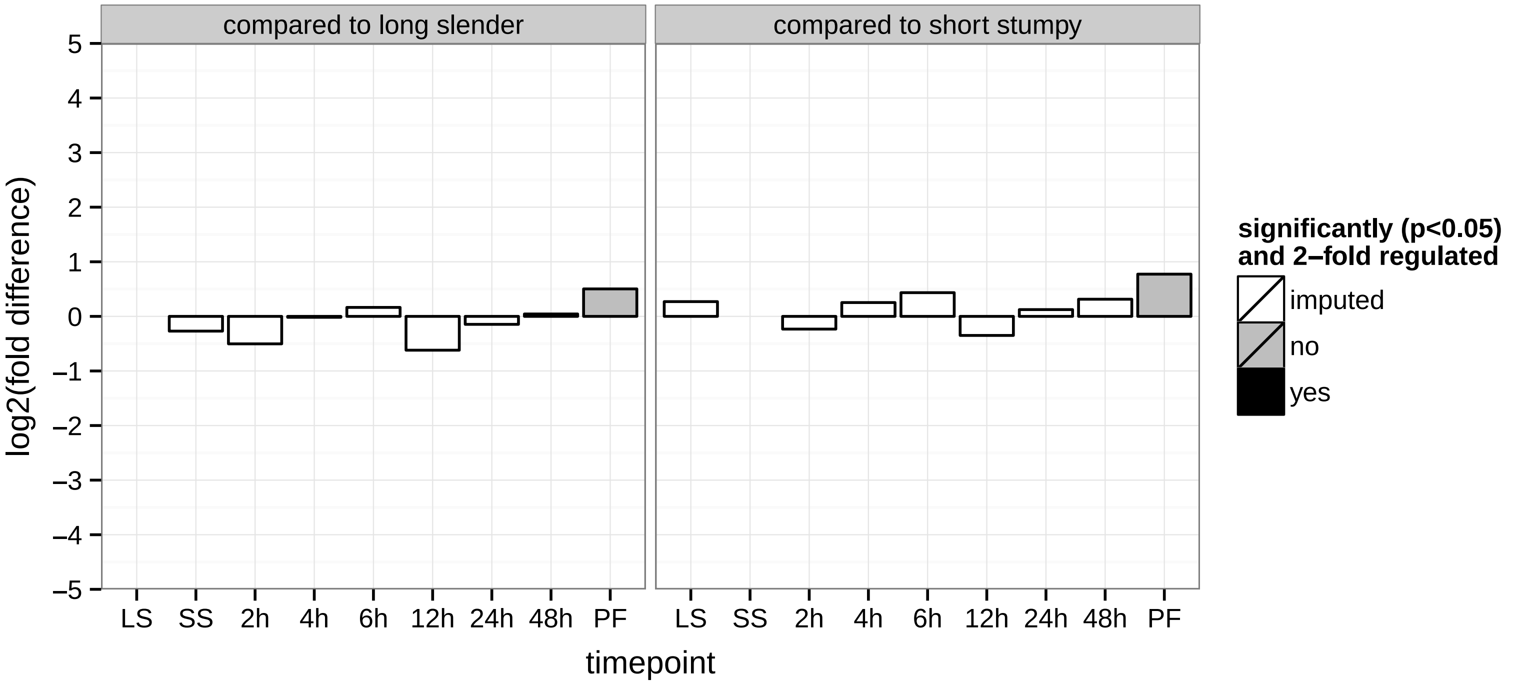

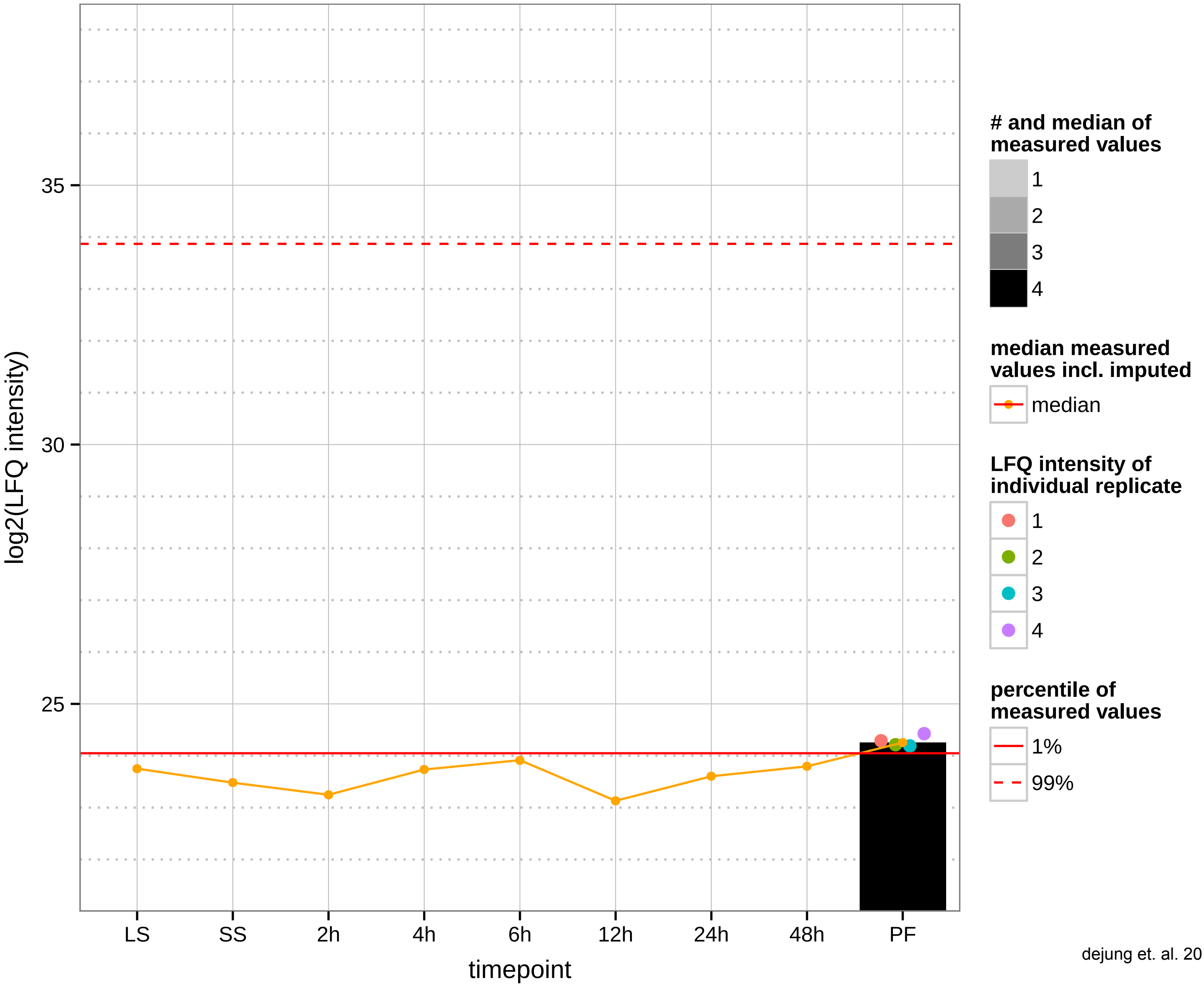

dejung et. al. 2015

hypothetical protein, conserved
Tb927.11.3480
AGOF: null
AGOC: null
AGOP: null
PGOF: DNA–directed RNA polymerase activity
PGOC: nucleus
PGOP: transcription, DNA–dependent

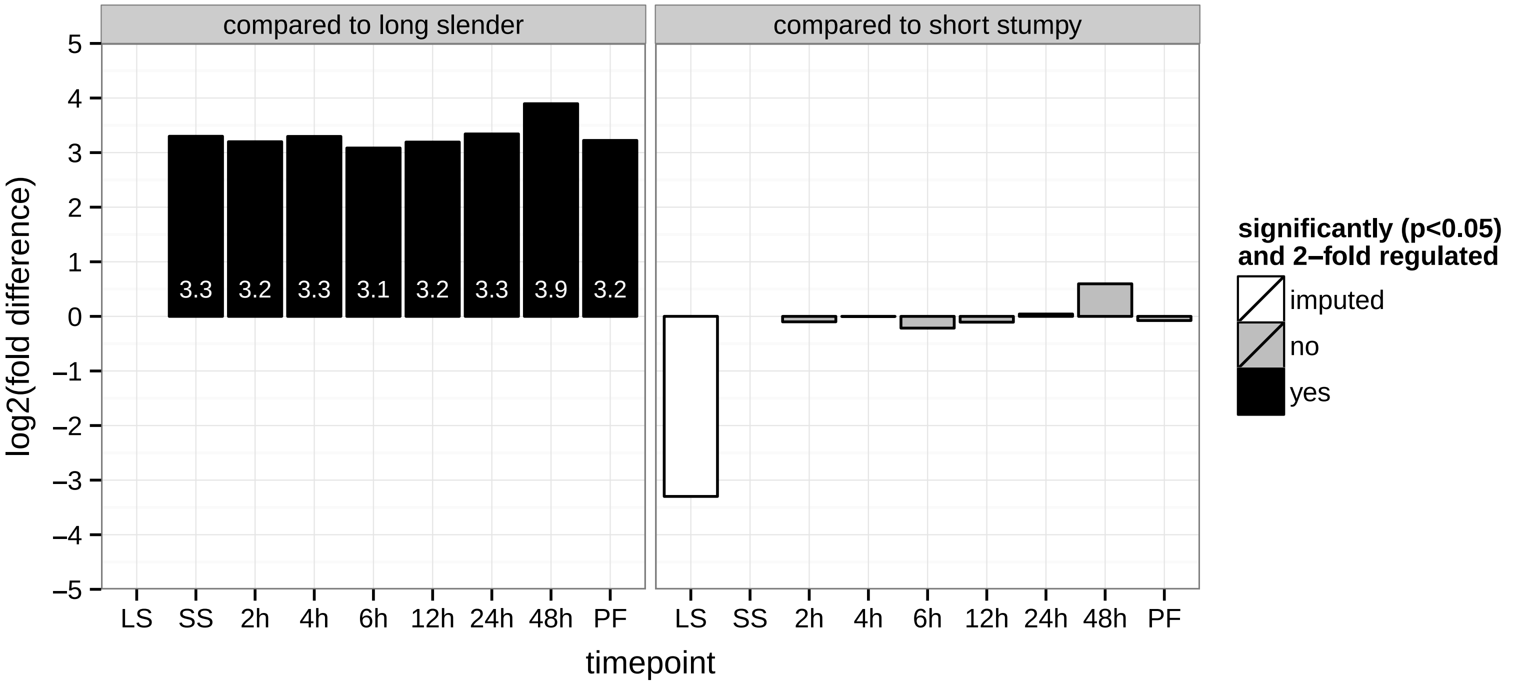

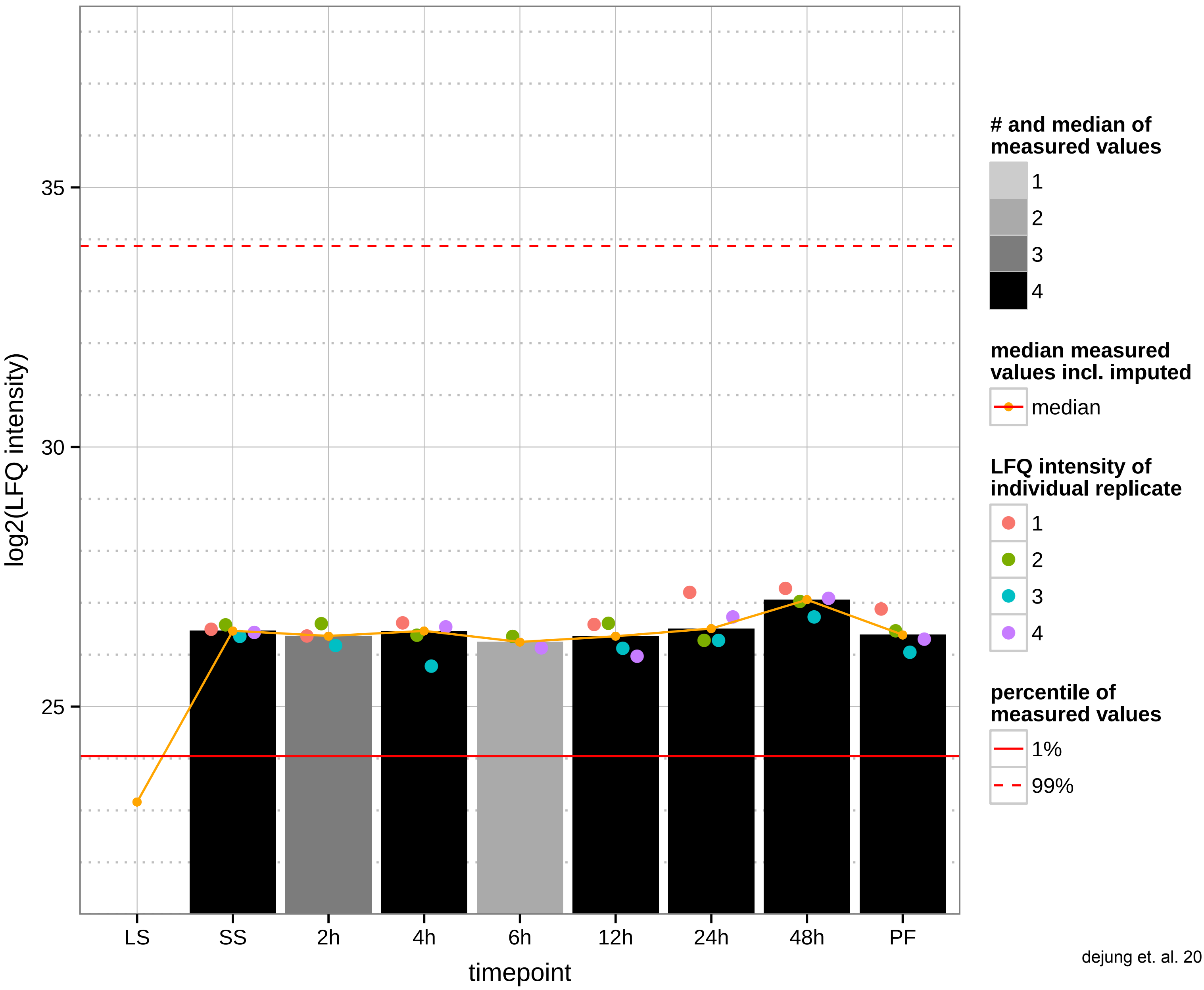

dejung et. al. 2015

hypothetical protein, conserved
Tb927.11.3490
AGOF: null
AGOC: null
AGOP: null
PGOF: null
PGOC: null
PGOP: null

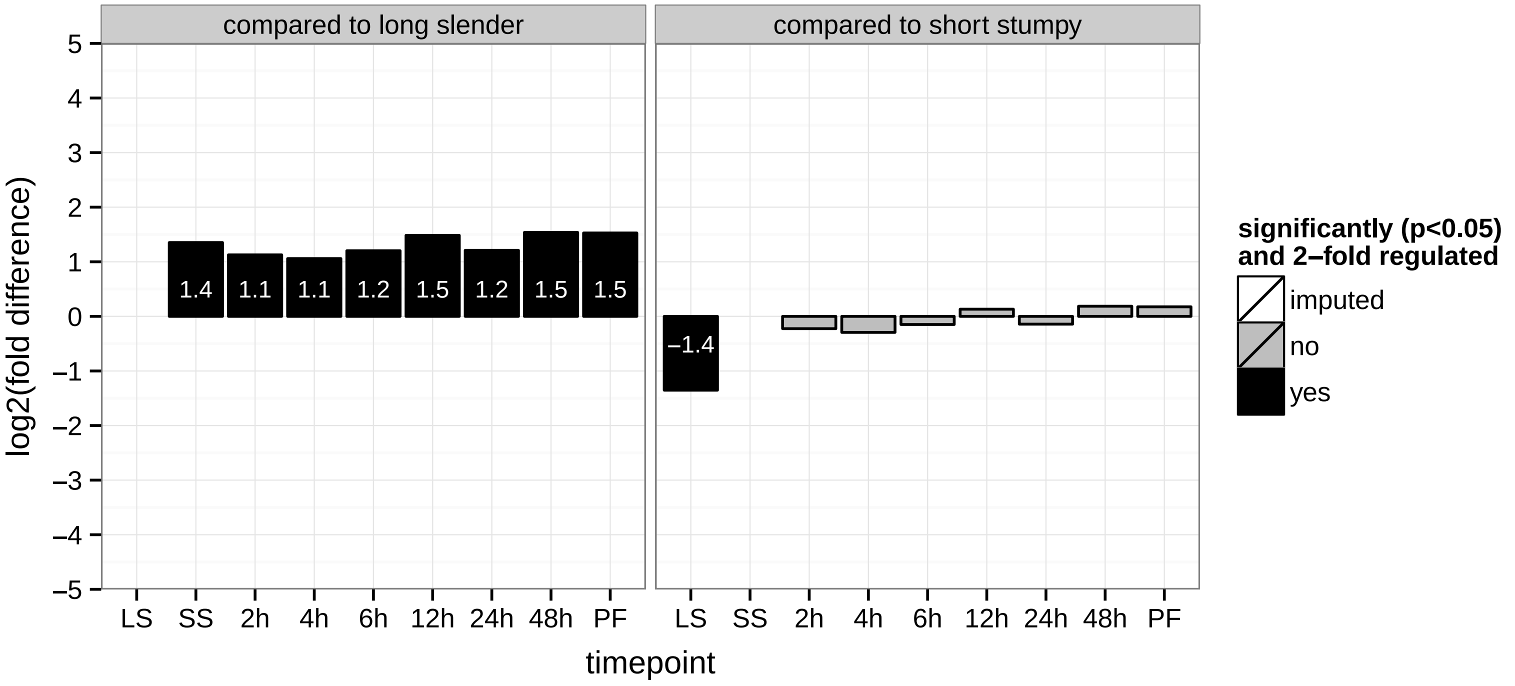

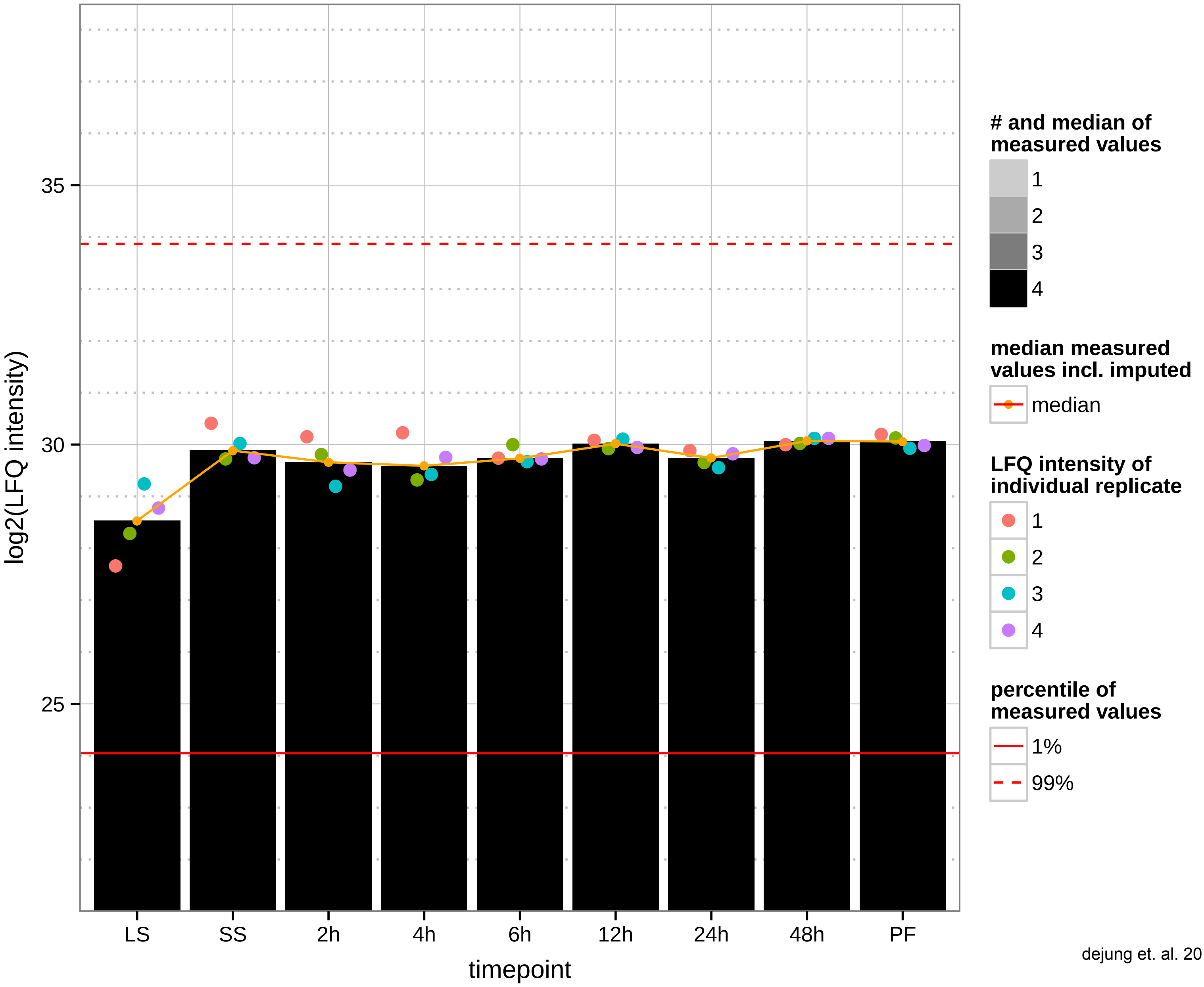

dejung et. al. 2015

hypothetical protein, conserved
Tb927.11.3500
AGOF: catalytic activity
AGOC: null
AGOP: metabolic process
PGOF: null
PGOC: null
PGOP: null

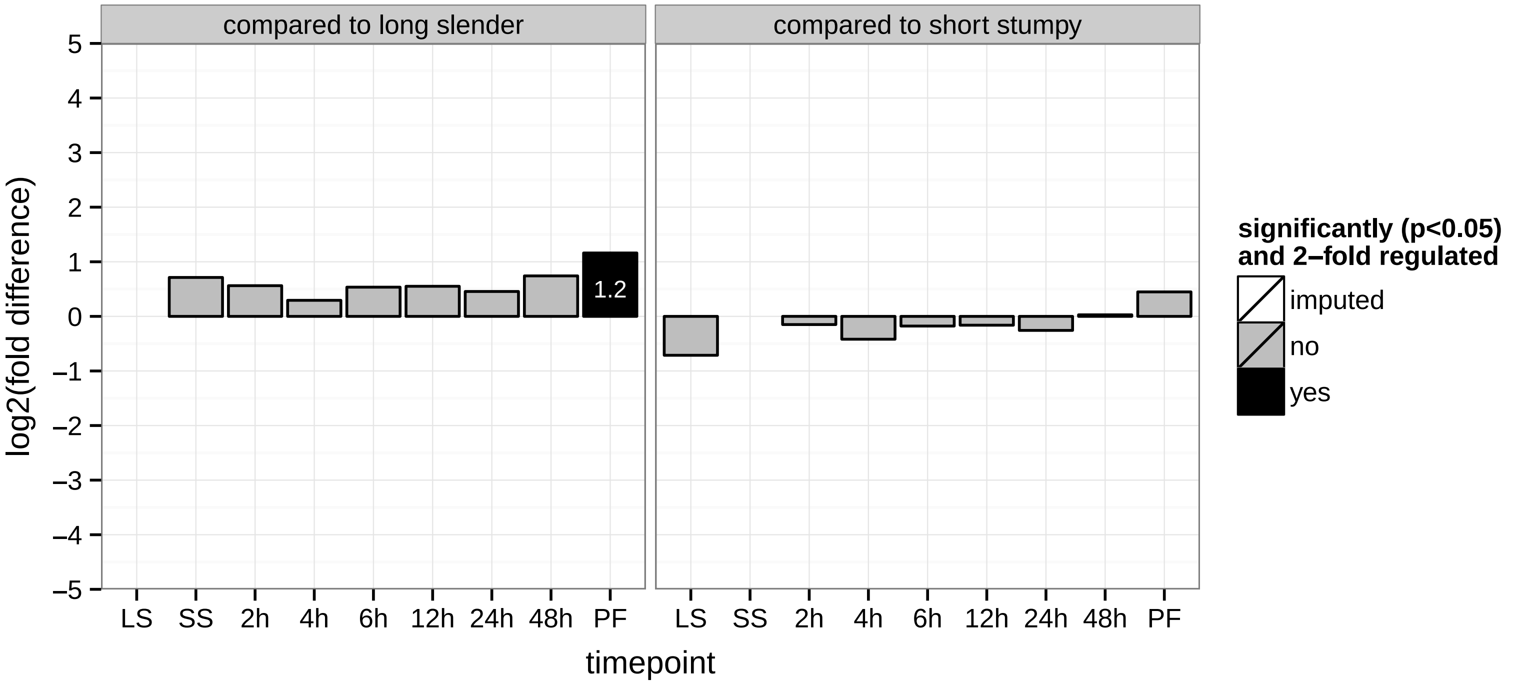

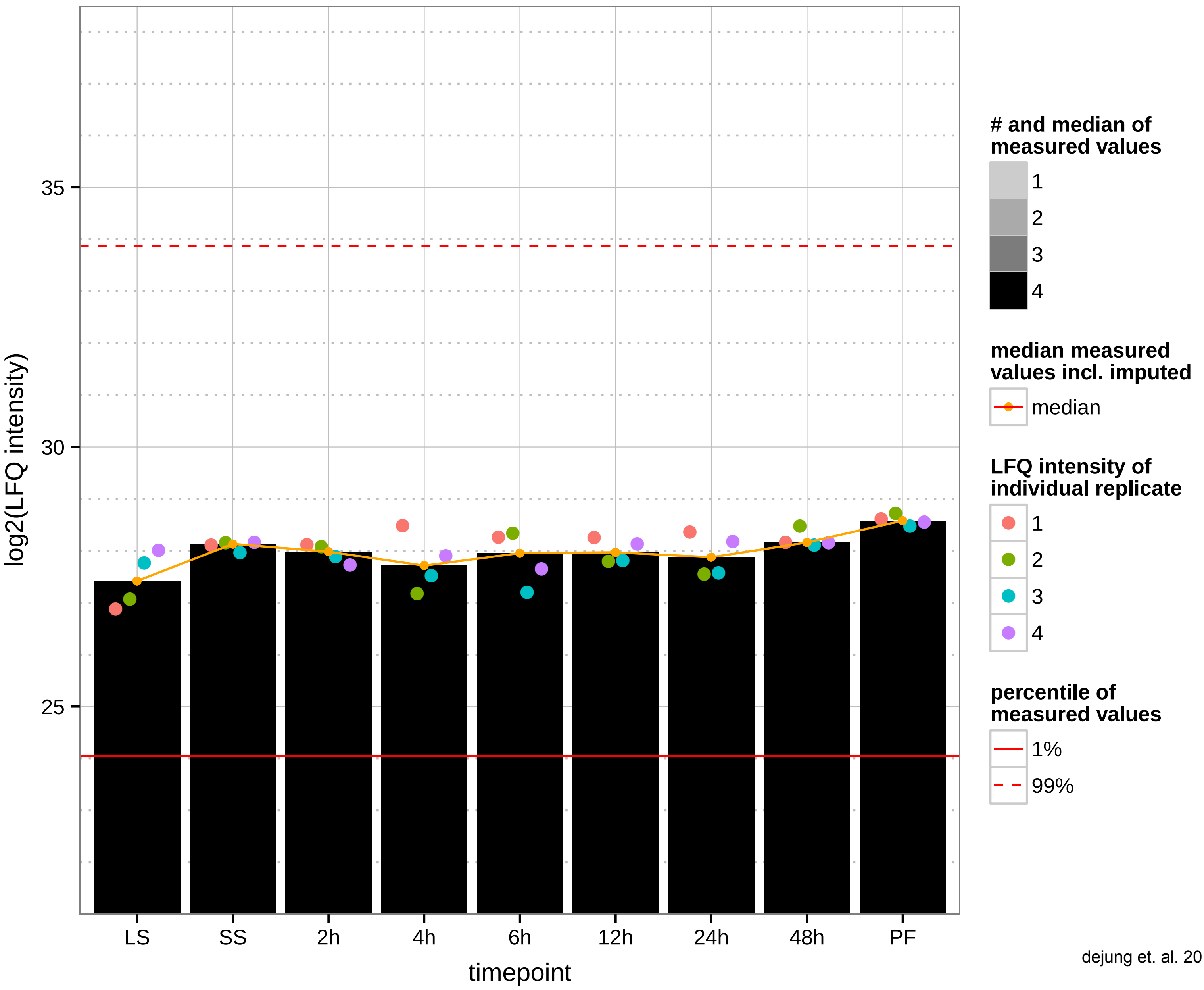

dejung et. al. 2015

hypothetical protein, conserved
Tb927.11.3510
AGOF: null
AGOC: null
AGOP: protein folding
PGOF: null
PGOC: null
PGOP: protein folding

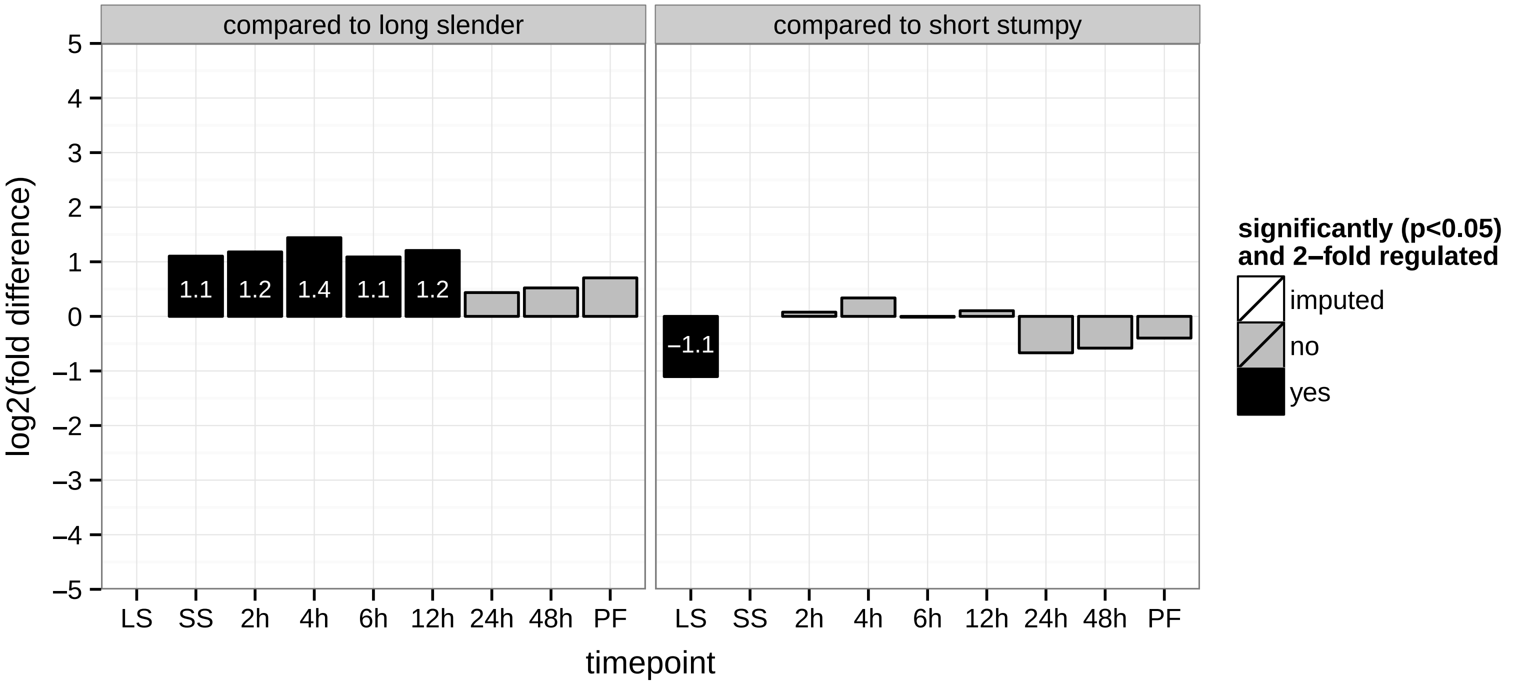

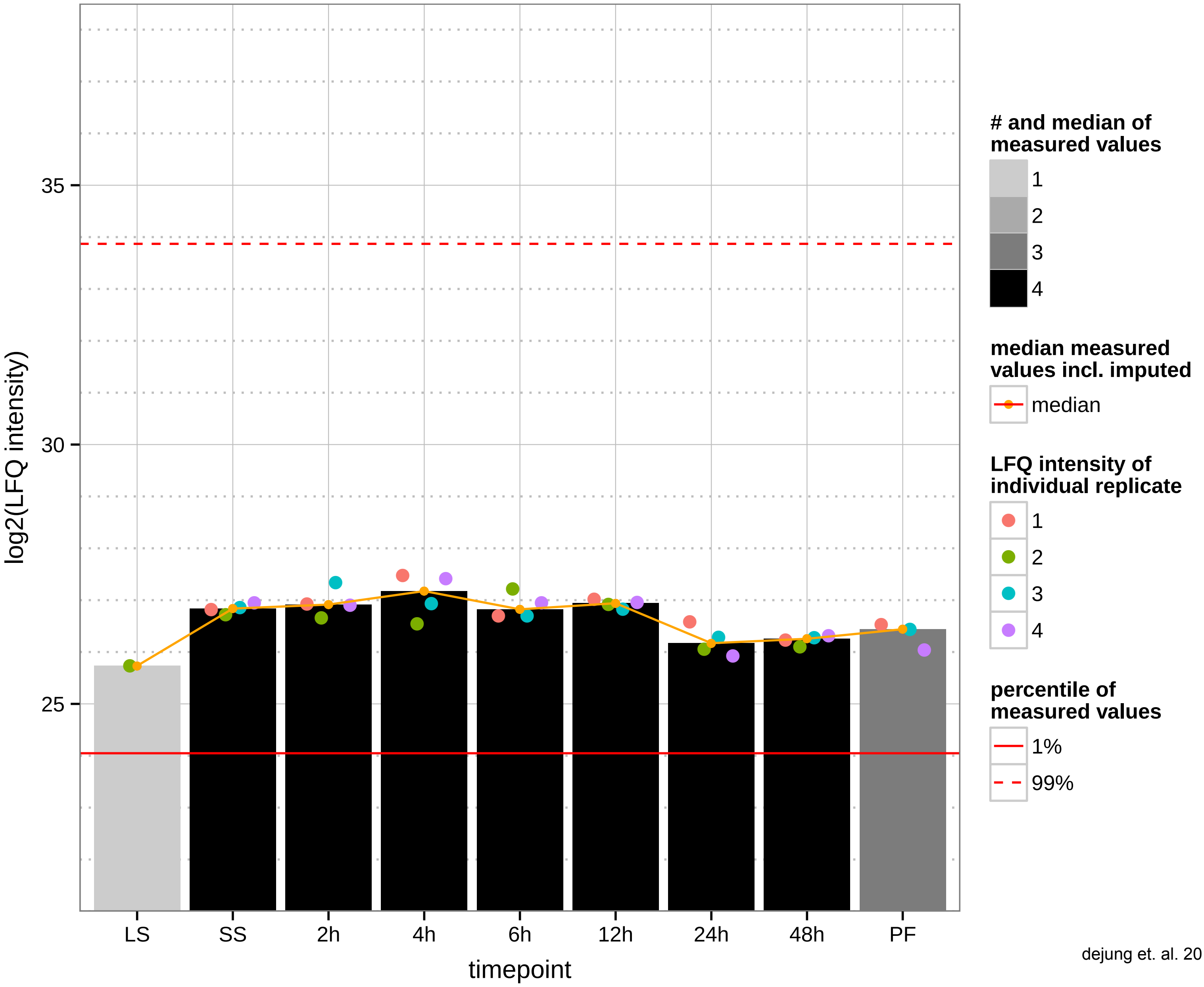

dejung et. al. 2015

hypothetical protein, conserved
Tb927.11.3540
AGOF: null
AGOC: null
AGOP: null
PGOF: null
PGOC: null
PGOP: null

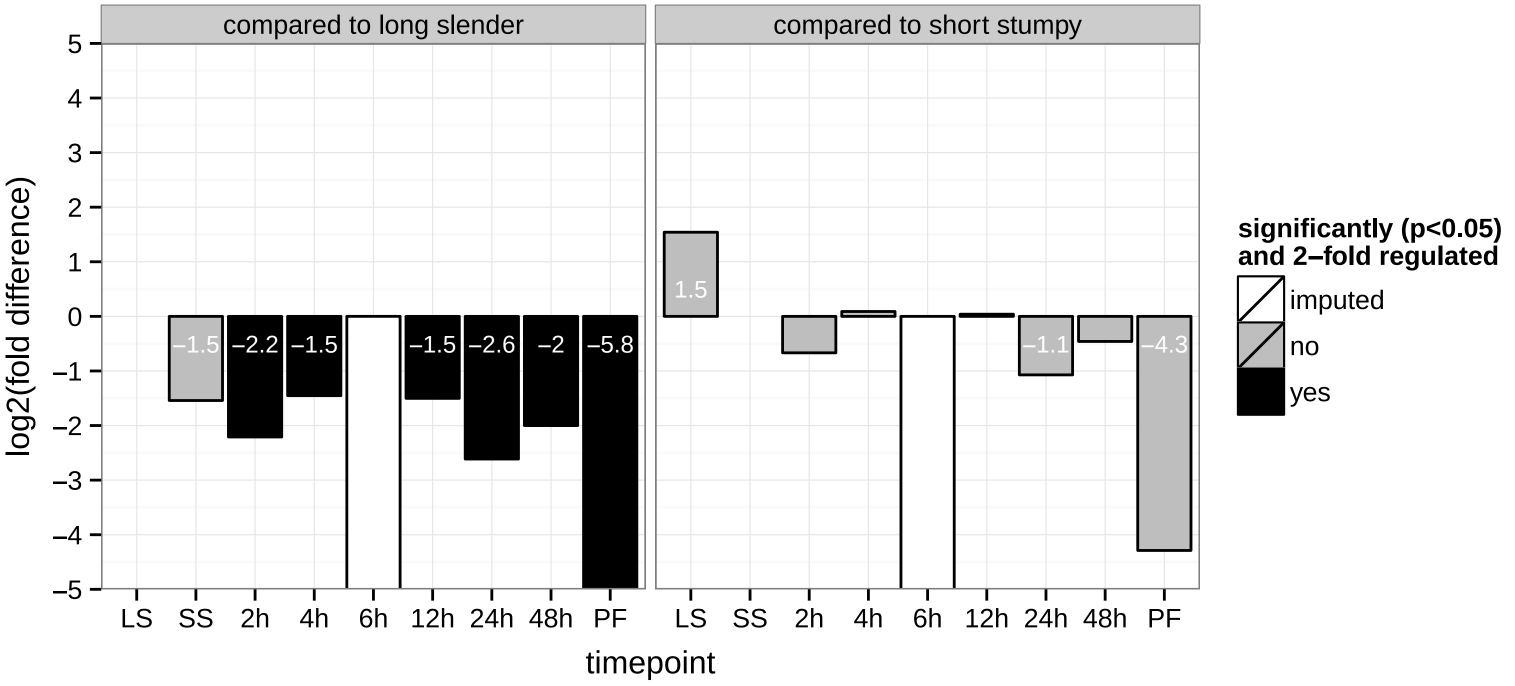

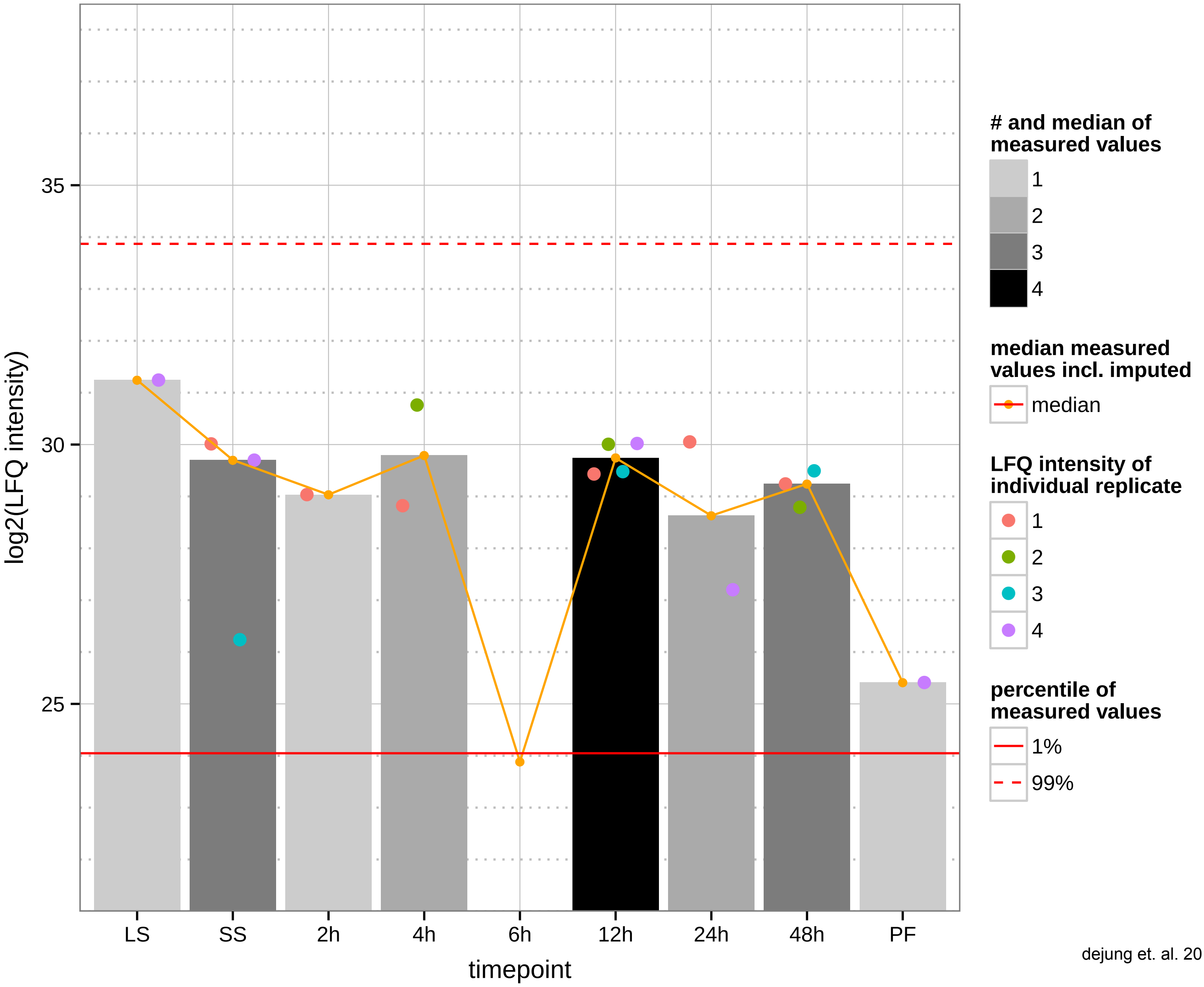

dejung et. al. 2015

XPA–interacting protein, putative
Tb927.11.3550
AGOF: GTP binding, nucleoside–triphosphatase activity
AGOC: null
AGOP: small GTPase mediated signal transduction
PGOF: nucleoside–triphosphatase activity, nucleotide binding
PGOC: null
PGOP: null

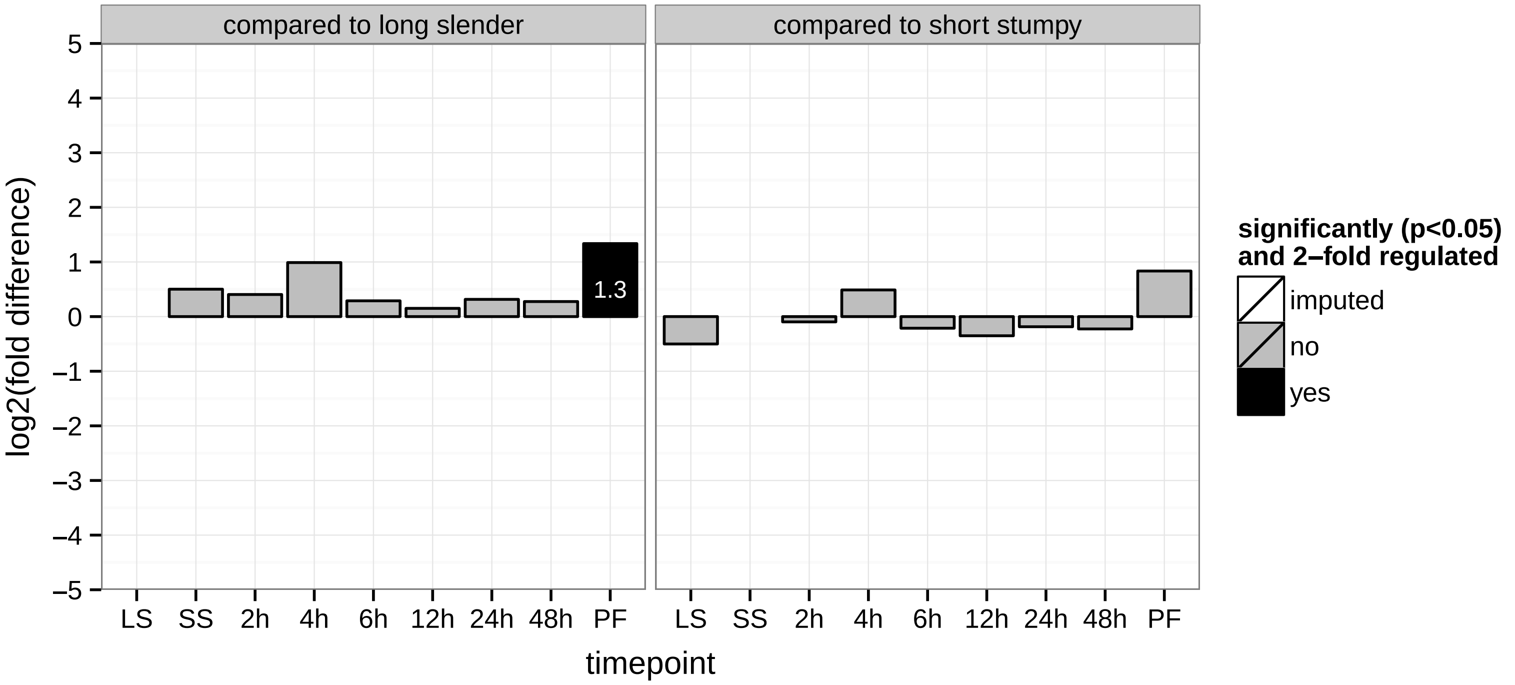

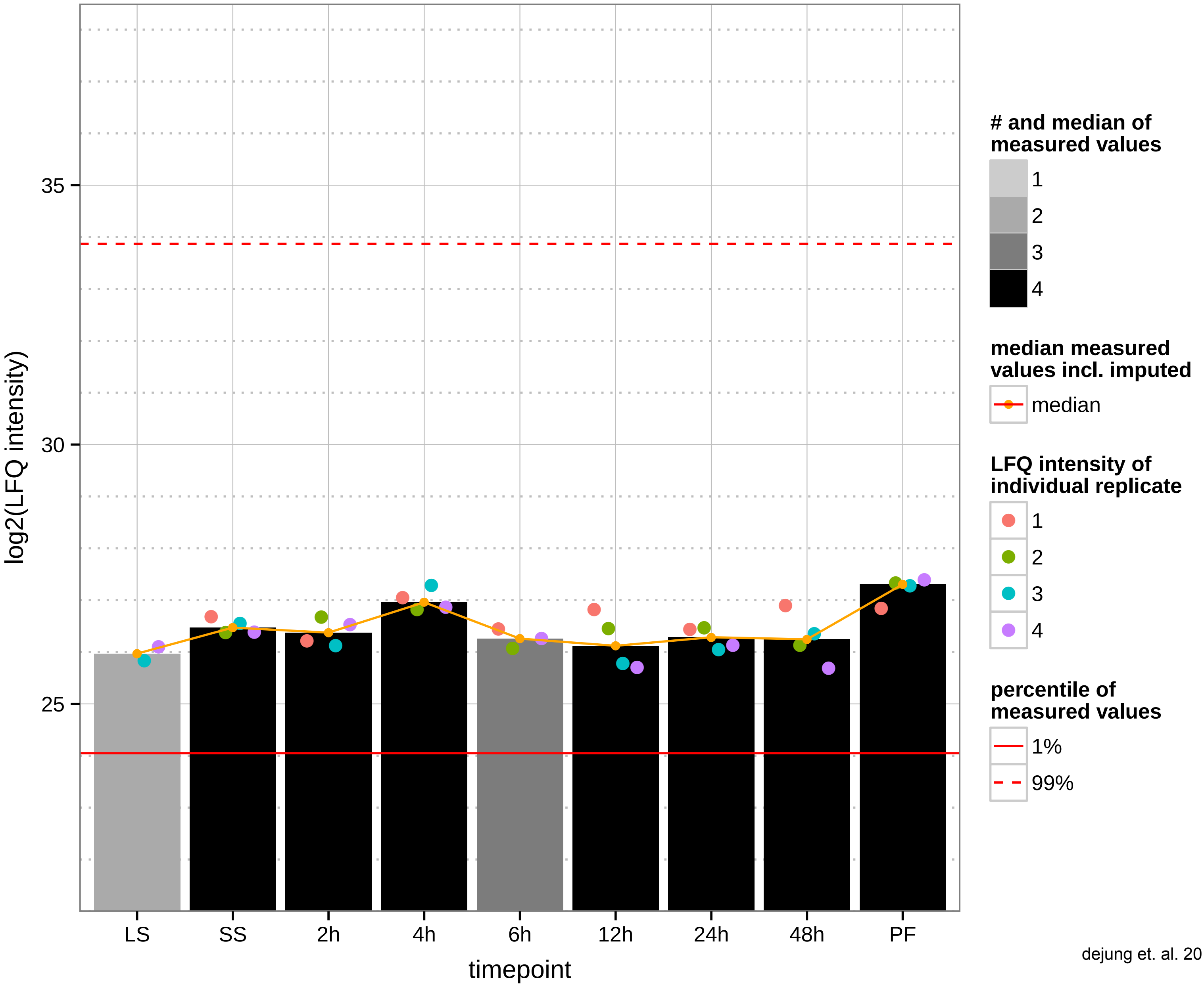

dejung et. al. 2015

aminopeptidase, putative, metallo–peptidase, Clan MA(E) Family M1
Tb927.11.3570
AGOF: metalloexopeptidase activity, metallopeptidase activity, zinc ion binding
AGOC: null
AGOP: proteolysis
PGOF: metallopeptidase activity, zinc ion binding
PGOC: null
PGOP: null

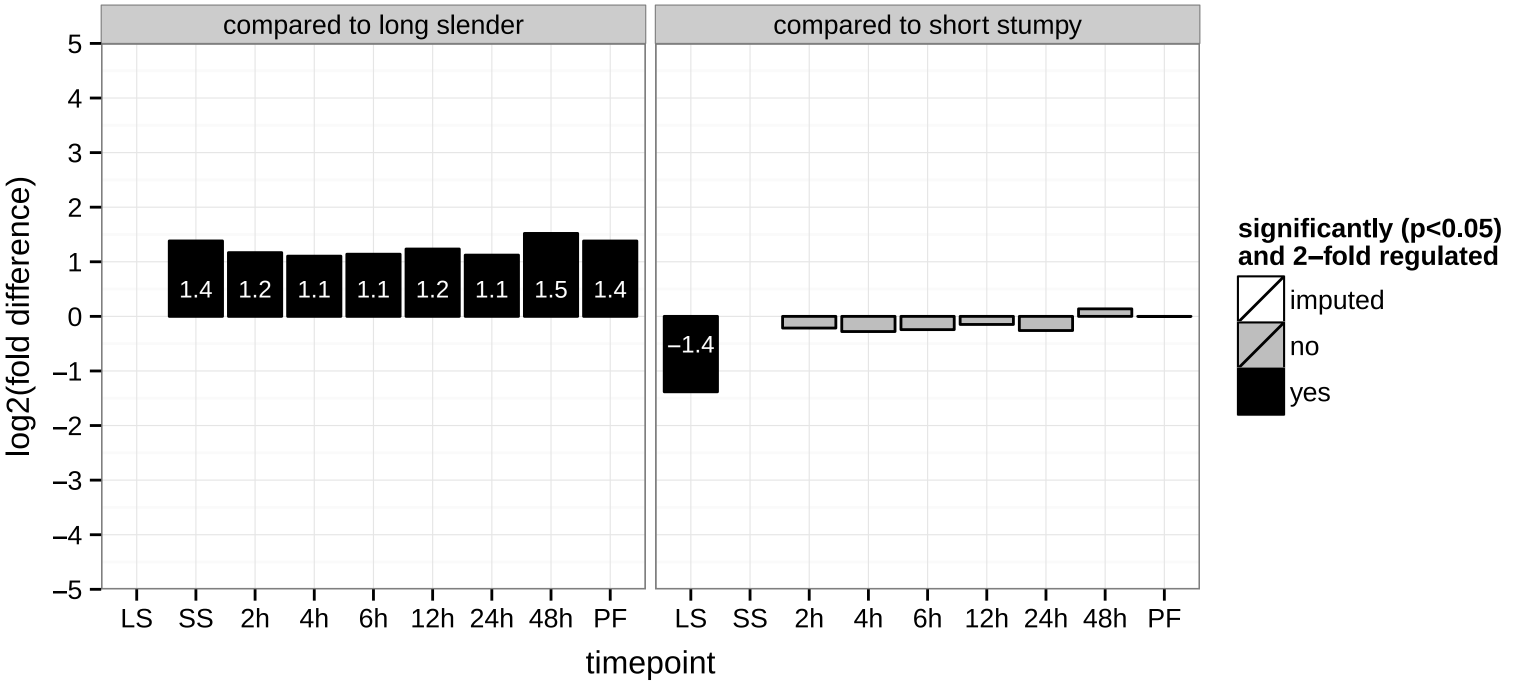

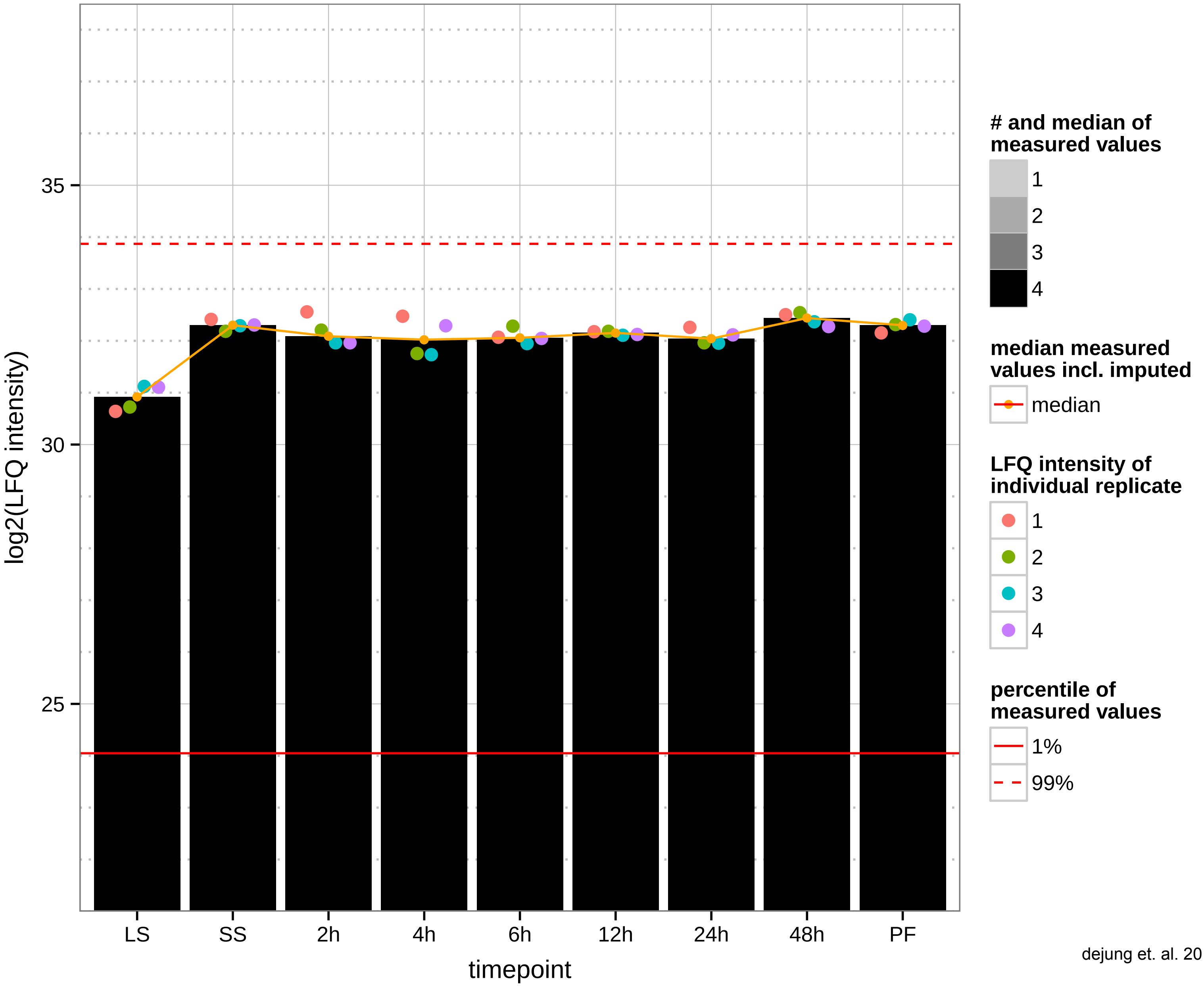

dejung et. al. 2015

hypothetical protein, conserved
Tb927.11.3580
AGOF: ATP binding, thiamine diphosphokinase activity
AGOC: null
AGOP: thiamine diphosphate biosynthetic process
PGOF: ATP binding, thiamine diphosphokinase activity
PGOC: null
PGOP: thiamine diphosphate biosynthetic process

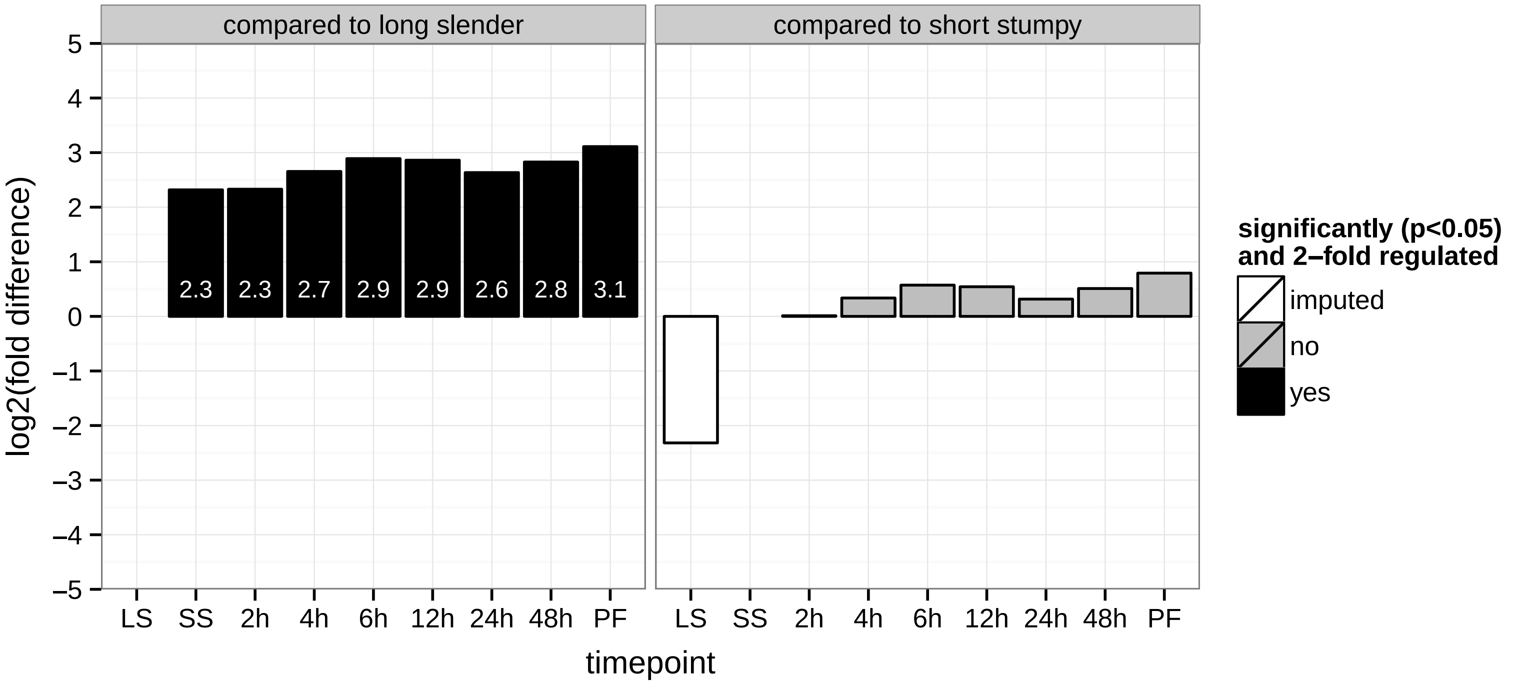

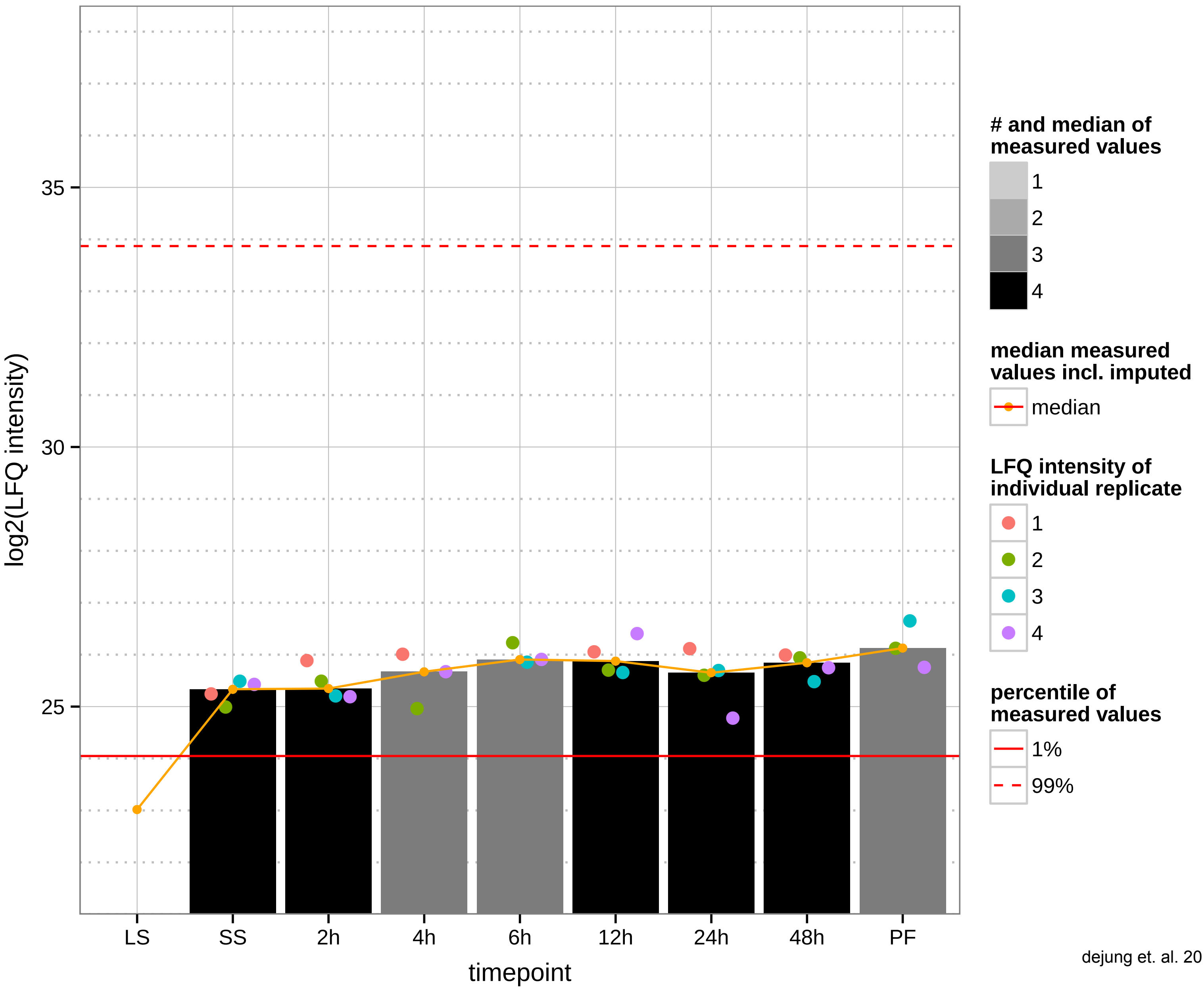

dejung et. al. 2015

Dynein light chain Tctex–type, putative
Tb927.11.3660
AGOF: microtubule motor activity
AGOC: microtubule associated complex
AGOP: microtubule–based process
PGOF: null
PGOC: null
PGOP: null

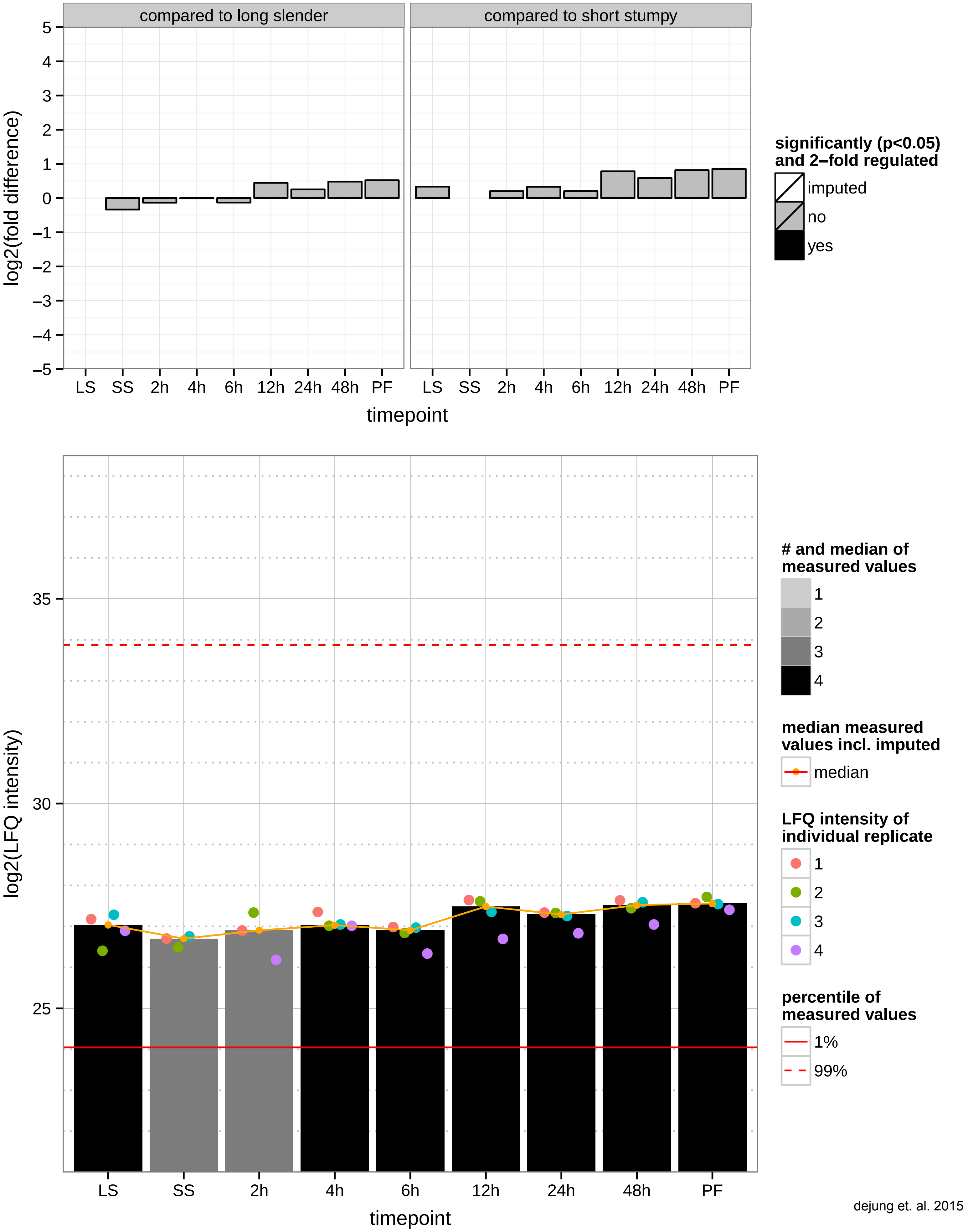

dejung et. al. 2015

repressor activator protein 1 (RAP1)
Tb927.11.370
AGOF: null
AGOC: nuclear chromosome, telomeric region, nucleus
AGOP: null
PGOF: null
PGOC: null
PGOP: null

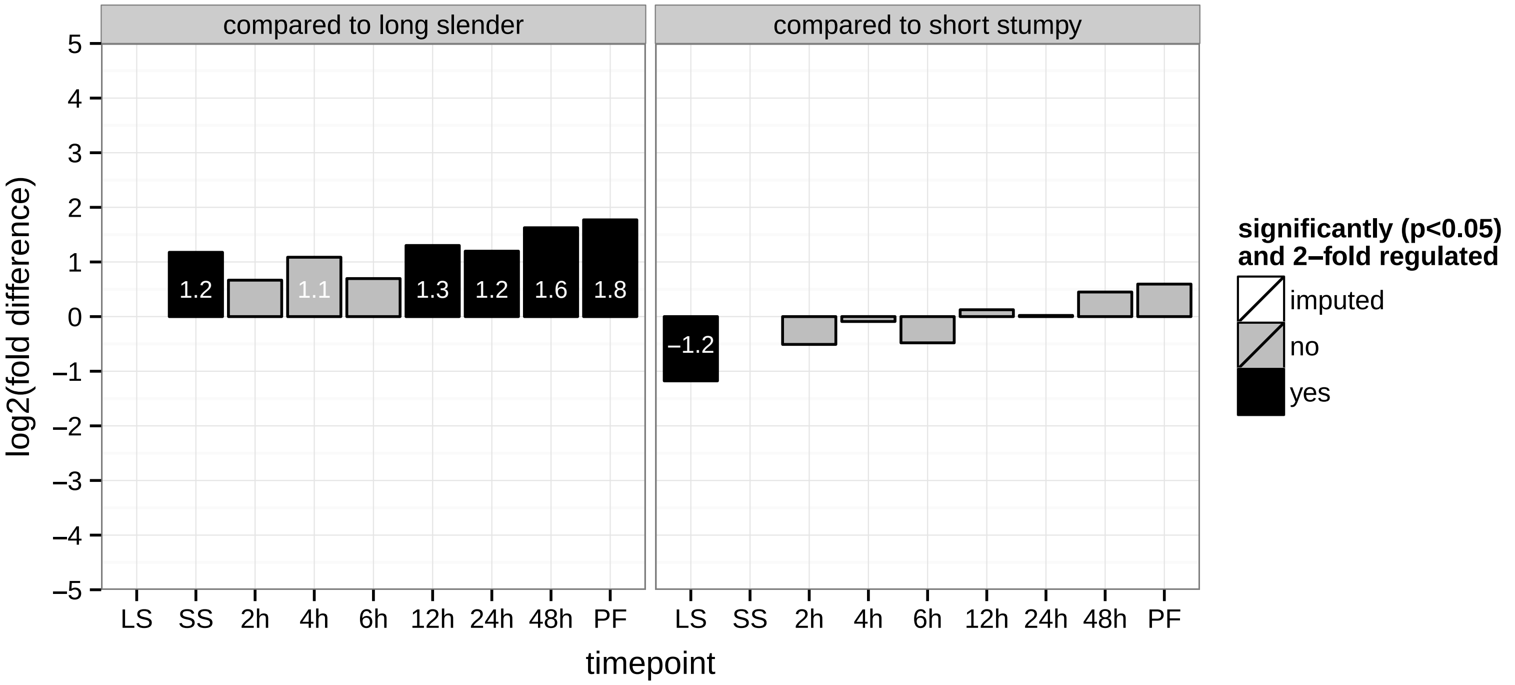

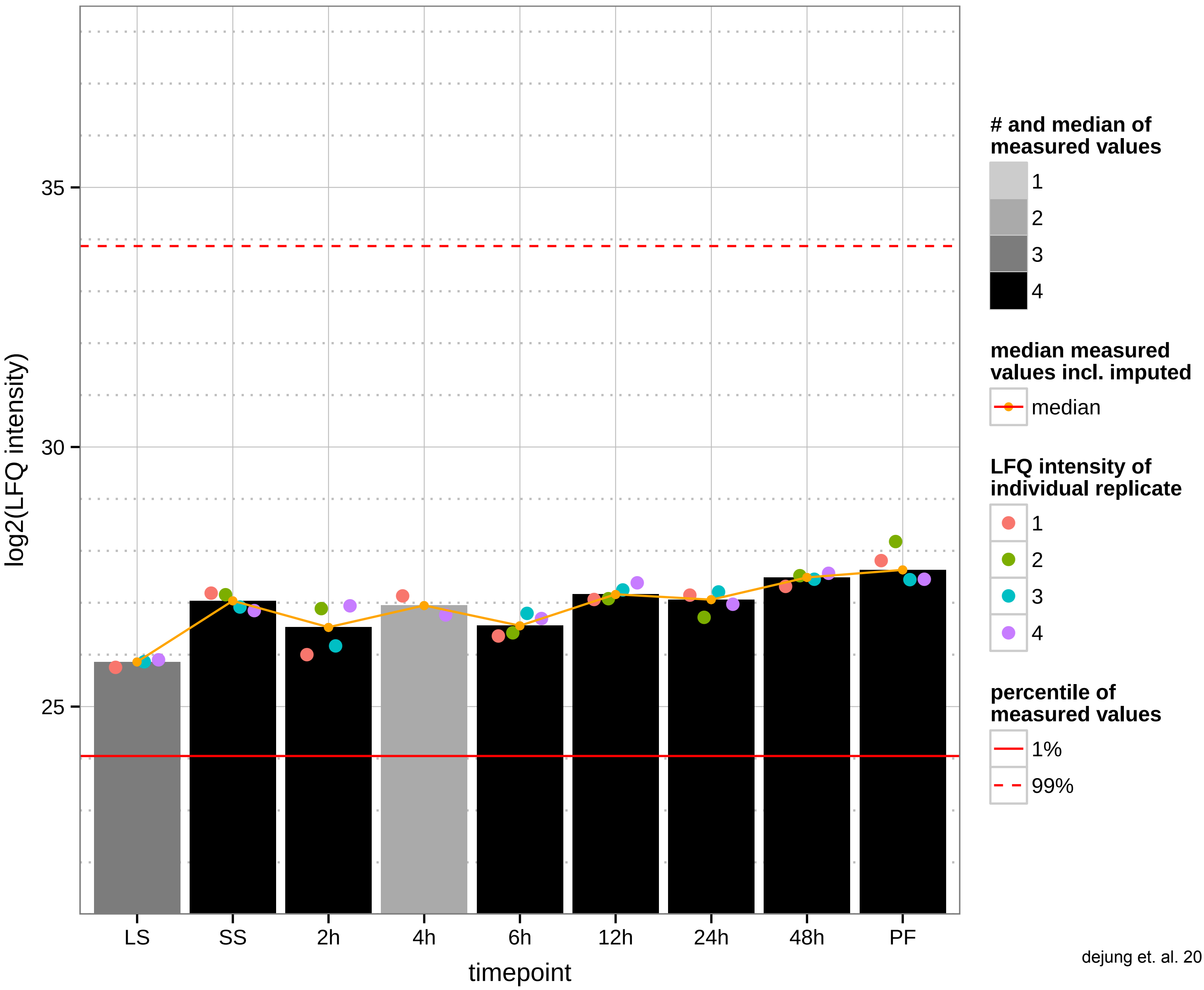

dejung et. al. 2015

hypothetical protein, conserved
Tb927.11.3700
AGOF: null
AGOC: mitochondrion
AGOP: null
PGOF: null
PGOC: null
PGOP: null

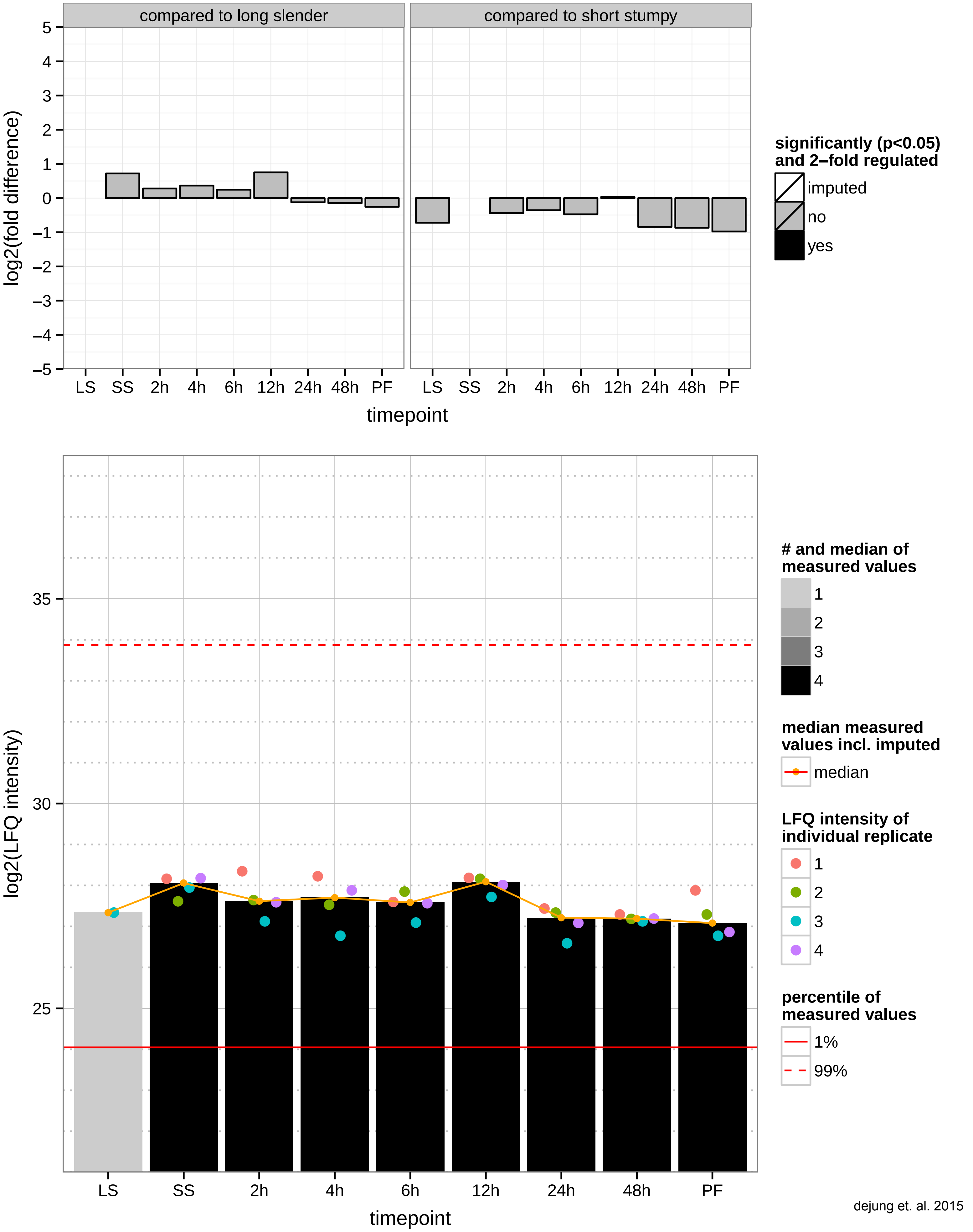

dejung et. al. 2015

hypothetical protein, conserved
Tb927.11.3710
AGOF: null
AGOC: null
AGOP: null
PGOF: null
PGOC: null
PGOP: null

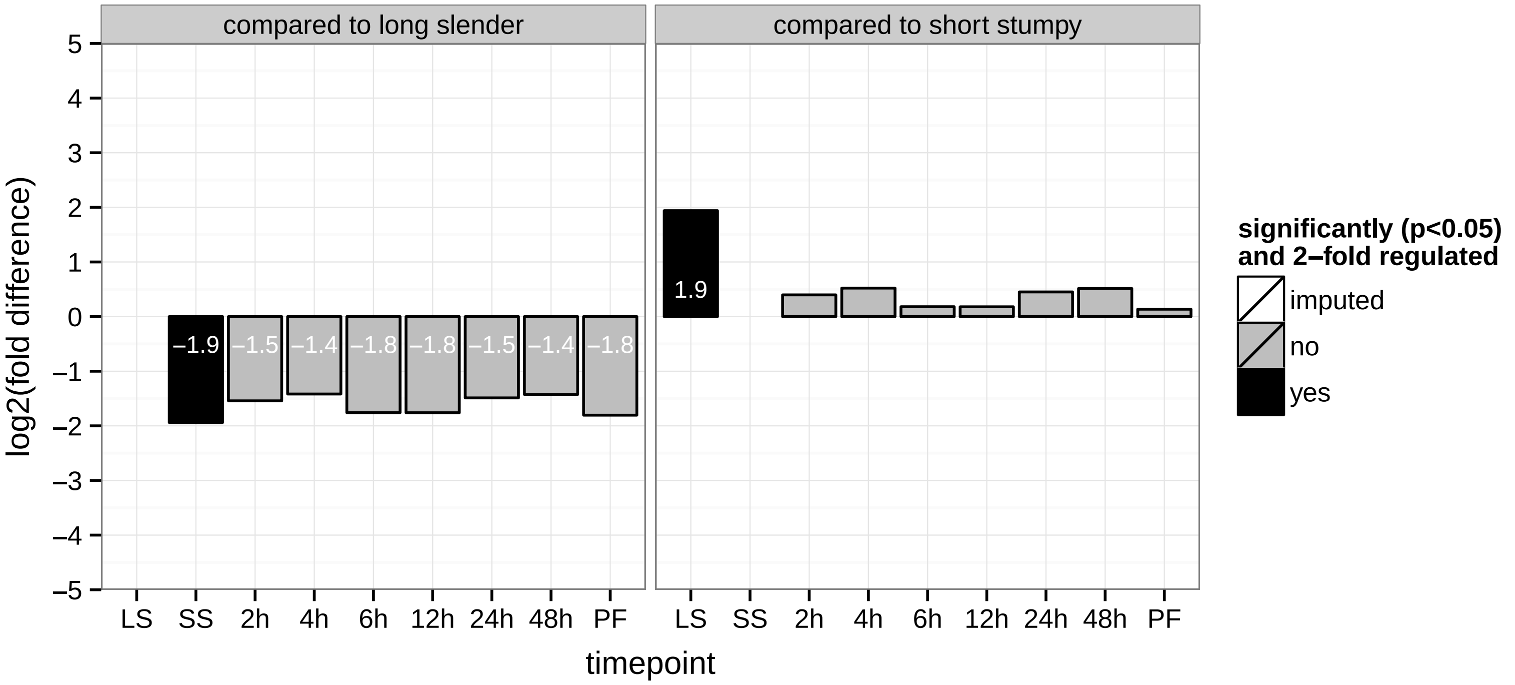

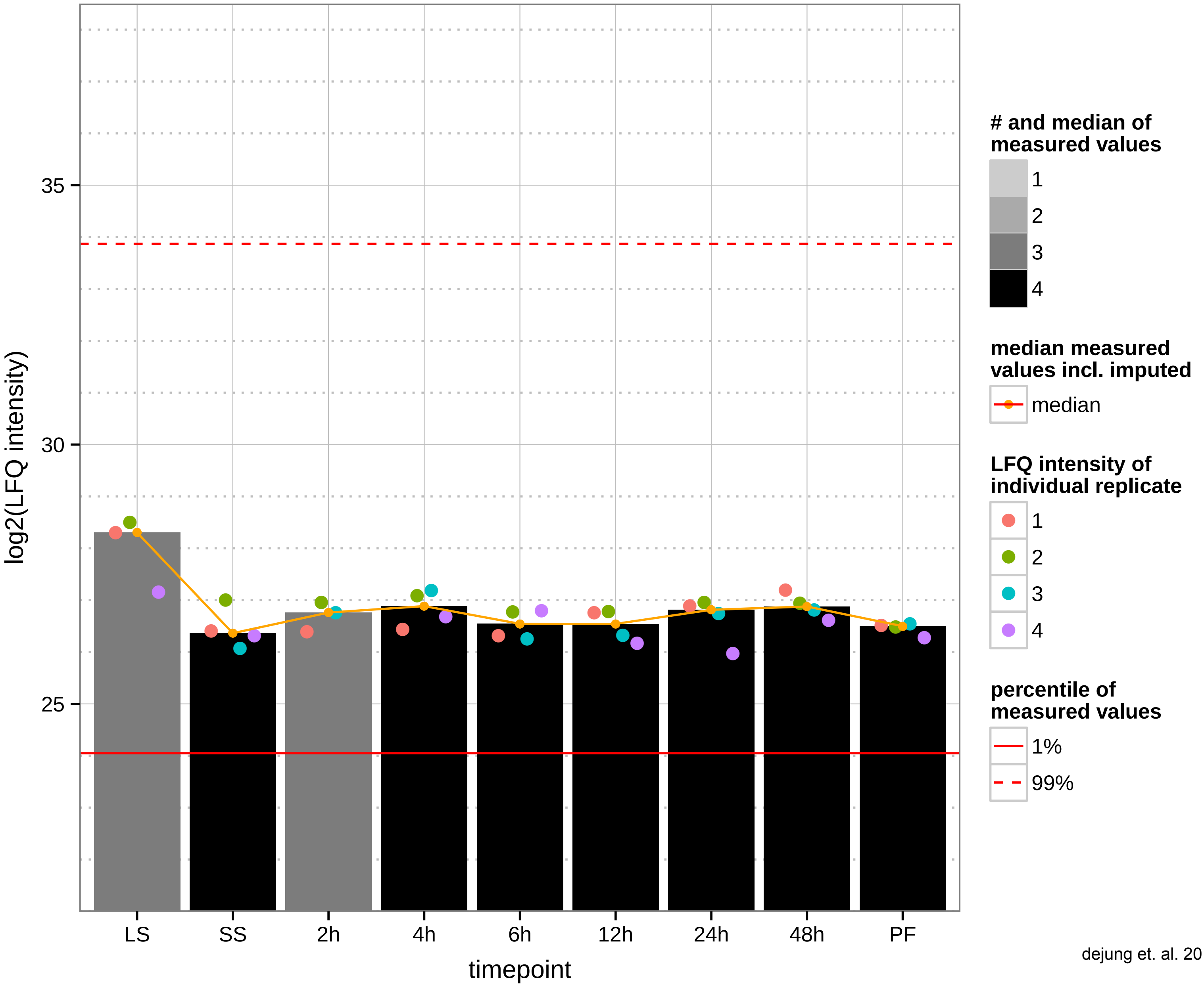

dejung et. al. 2015

leucyl–tRNA synthetase, putative (LeuRS)
Tb927.11.3730
AGOF: ATP binding, leucine–tRNA ligase activity
AGOC: cytoplasm
AGOP: leucyl–tRNA aminoacylation, translation
PGOF: ATP binding, aminoacyl–tRNA editing activity, aminoacyl–tRNA ligase activity, leucine–tRNA ligase activity, nucleotide
PGOC: cytoplasm
PGOP: leucyl–tRNA aminoacylation, tRNA aminoacylation for protein translation

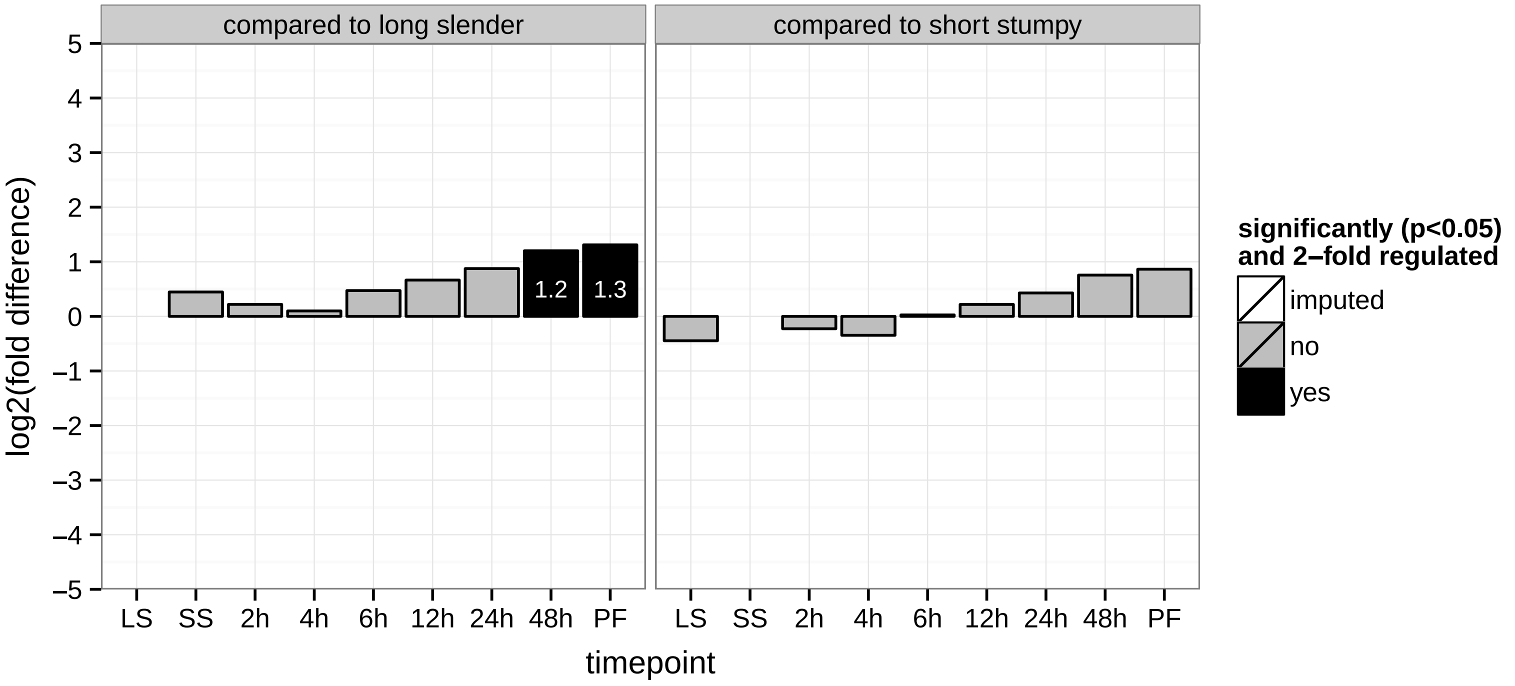

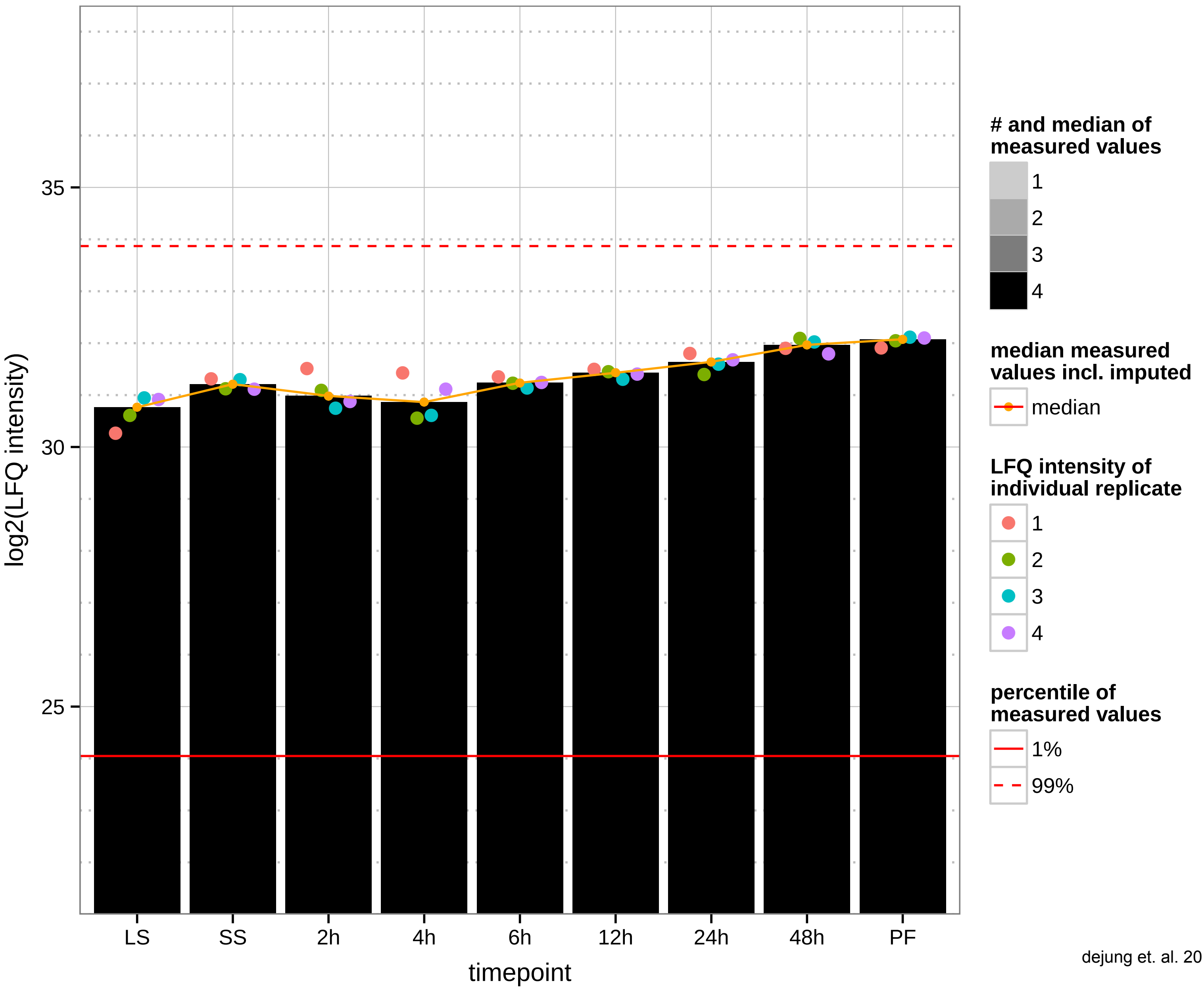

dejung et. al. 2015

proteasome regulatory ATPase subunit 2 (RPT2)
Tb927.11.3740
AGOF: ATP binding, ATPase activity
AGOC: cytoplasm, nucleus, proteasome regulatory particle
AGOP: ubiquitin–dependent protein catabolic process
PGOF: ATP binding, hydrolase activity, nucleoside–triphosphatase activity, nucleotide binding
PGOC: cytoplasm
PGOP: protein catabolic process

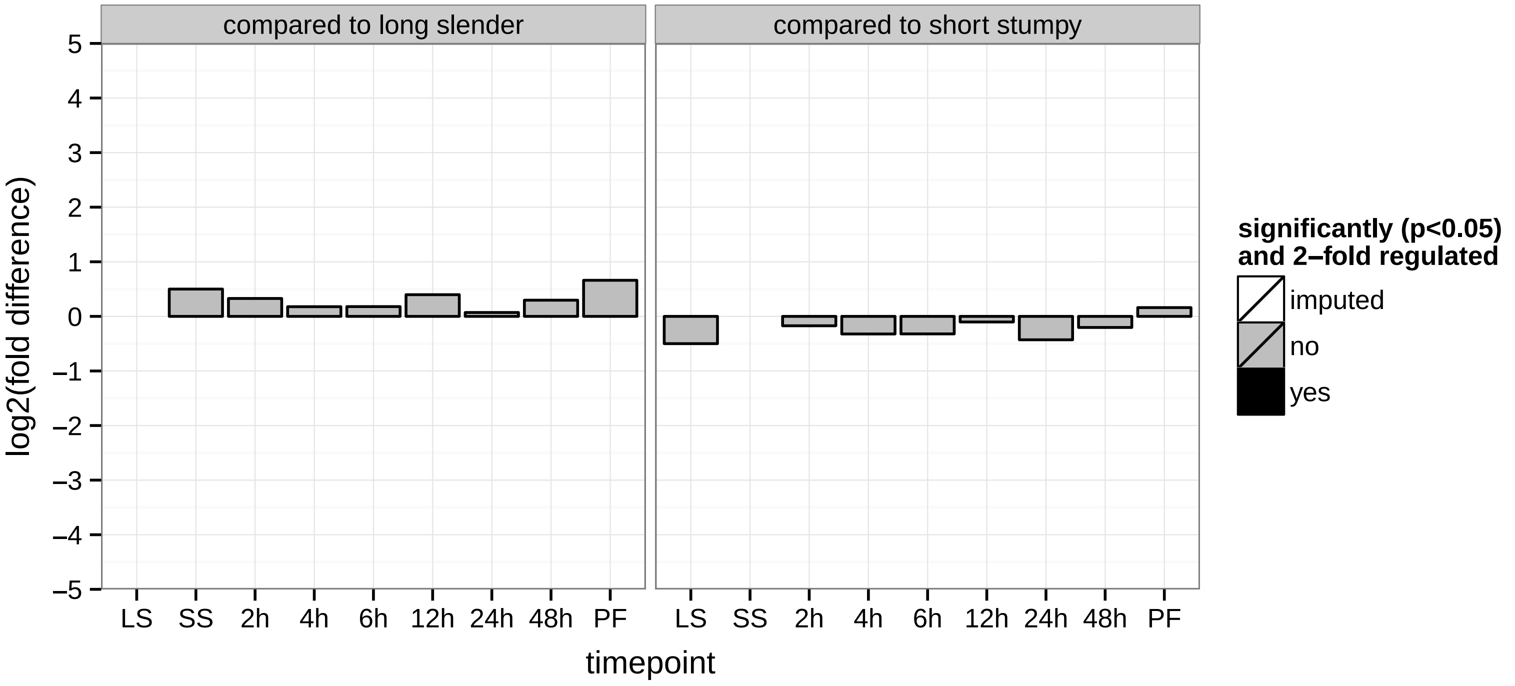

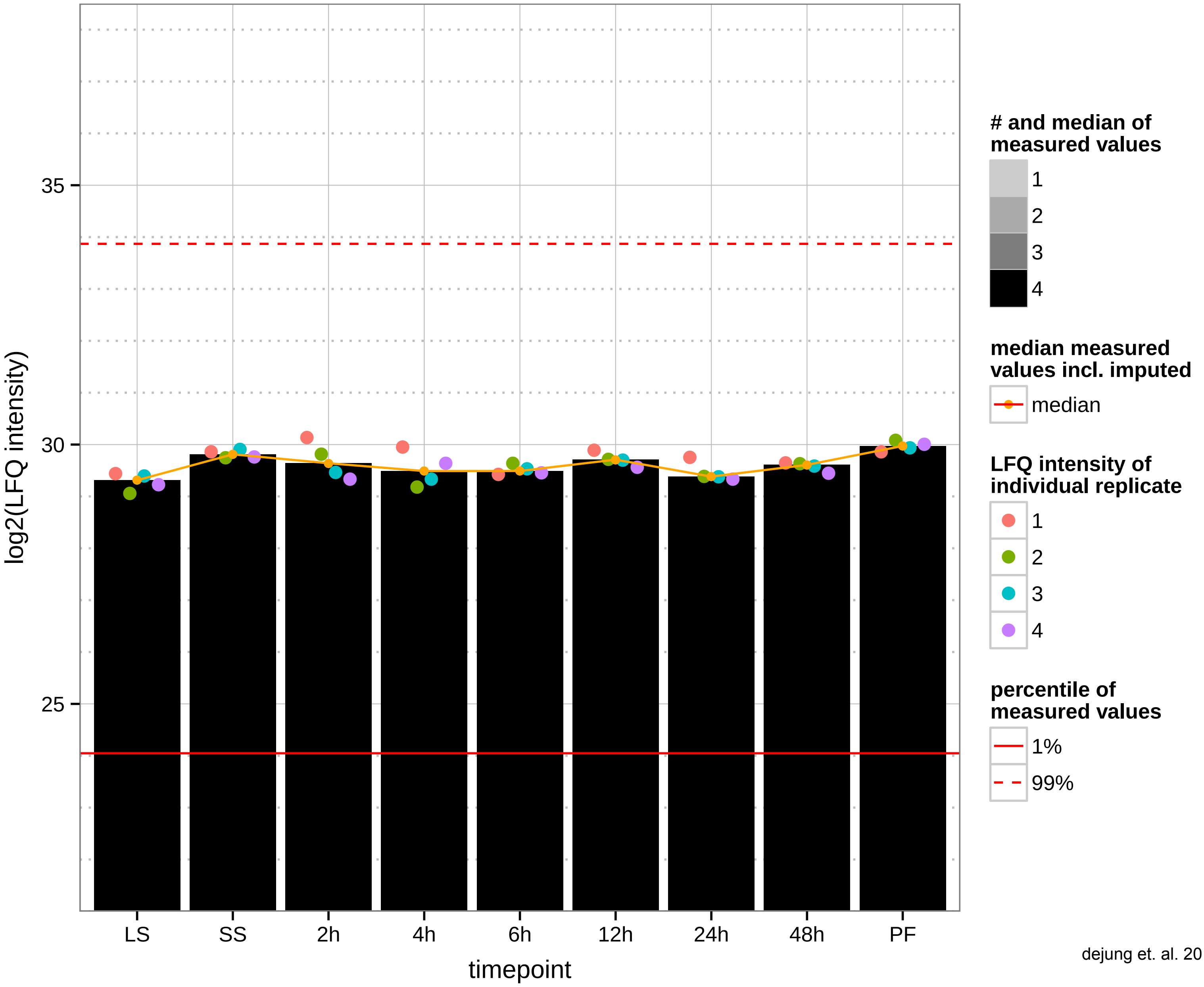

dejung et. al. 2015

hypothetical protein, conserved
Tb927.11.3770
AGOF: null
AGOC: null
AGOP: null
PGOF: null
PGOC: null
PGOP: null

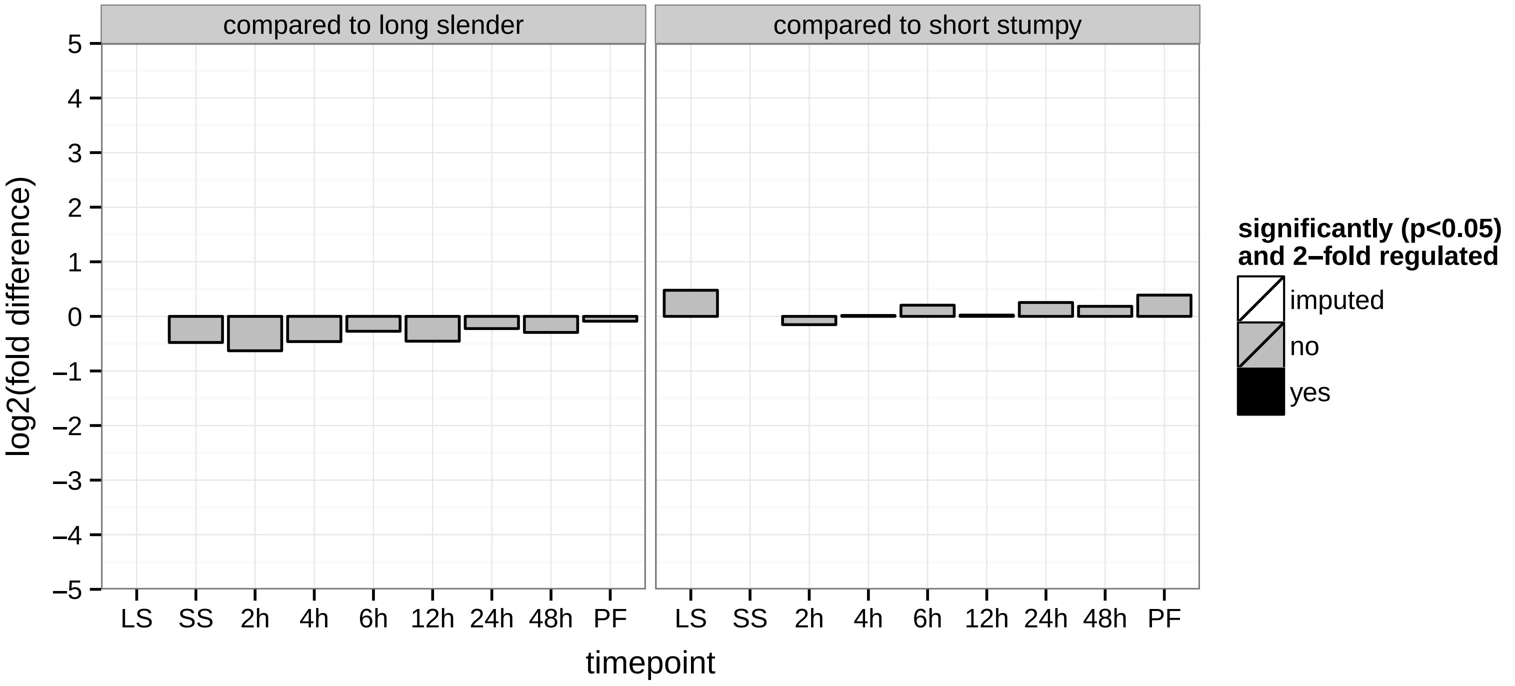

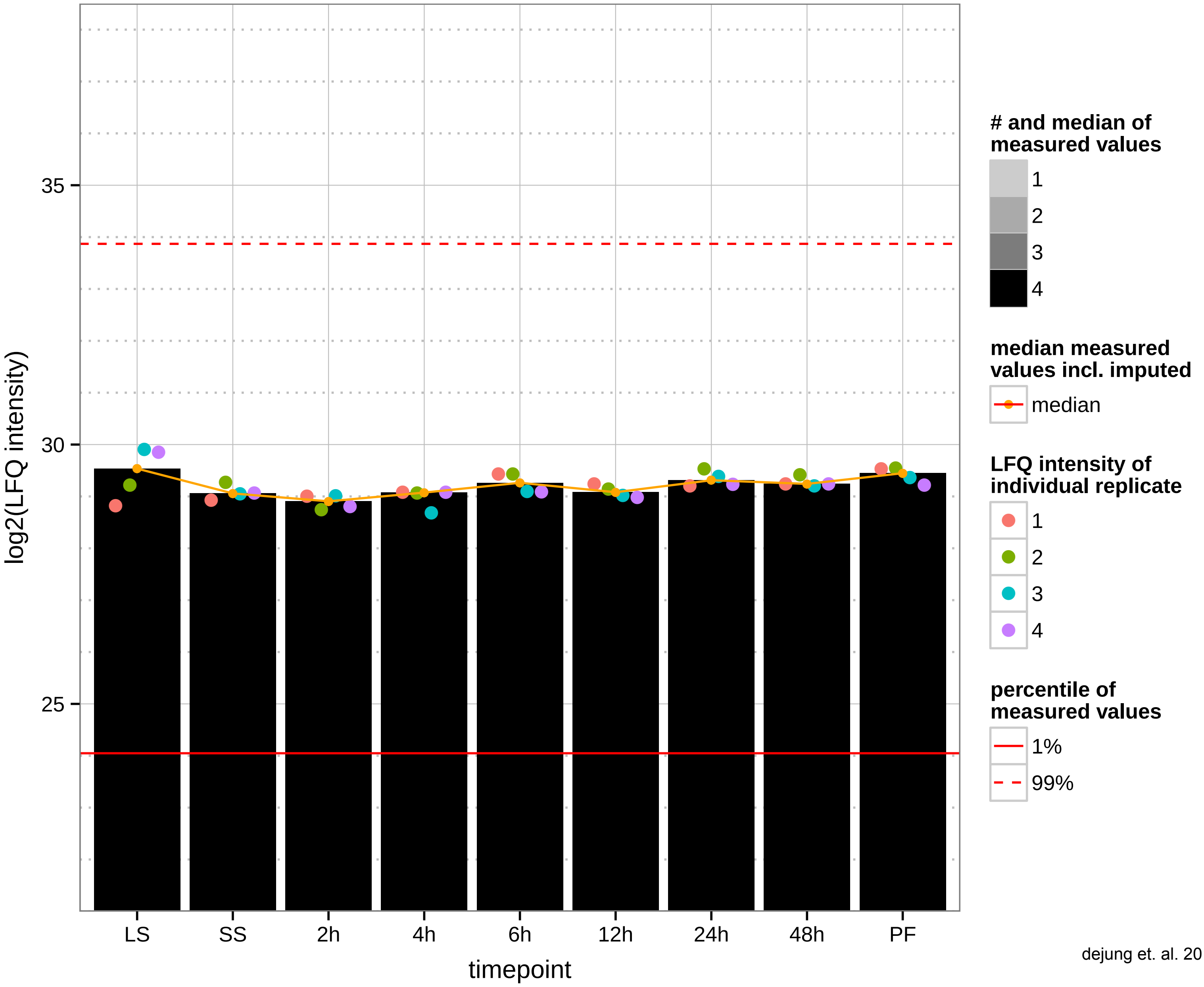

dejung et. al. 2015

hypothetical protein, conserved
Tb927.11.3790
AGOF: null
AGOC: null
AGOP: null
PGOF: null
PGOC: null
PGOP: null

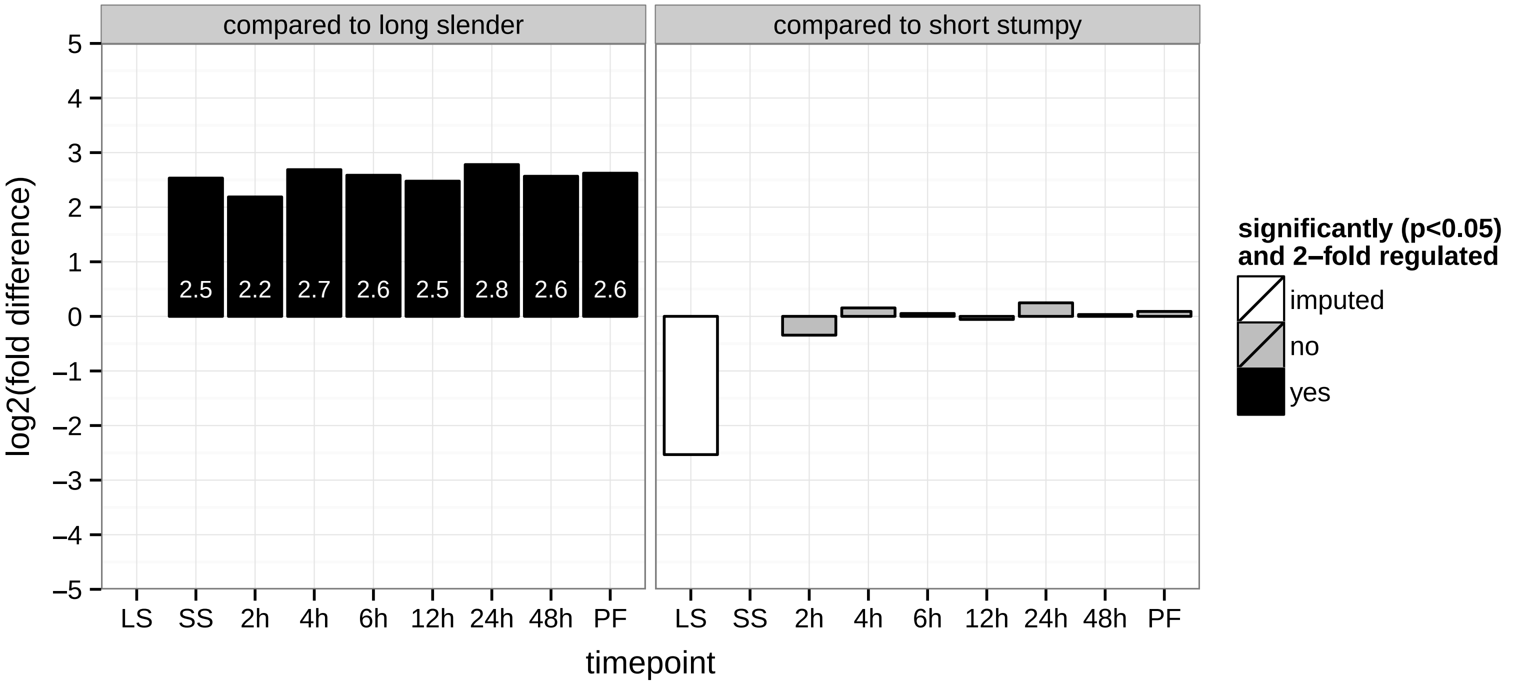

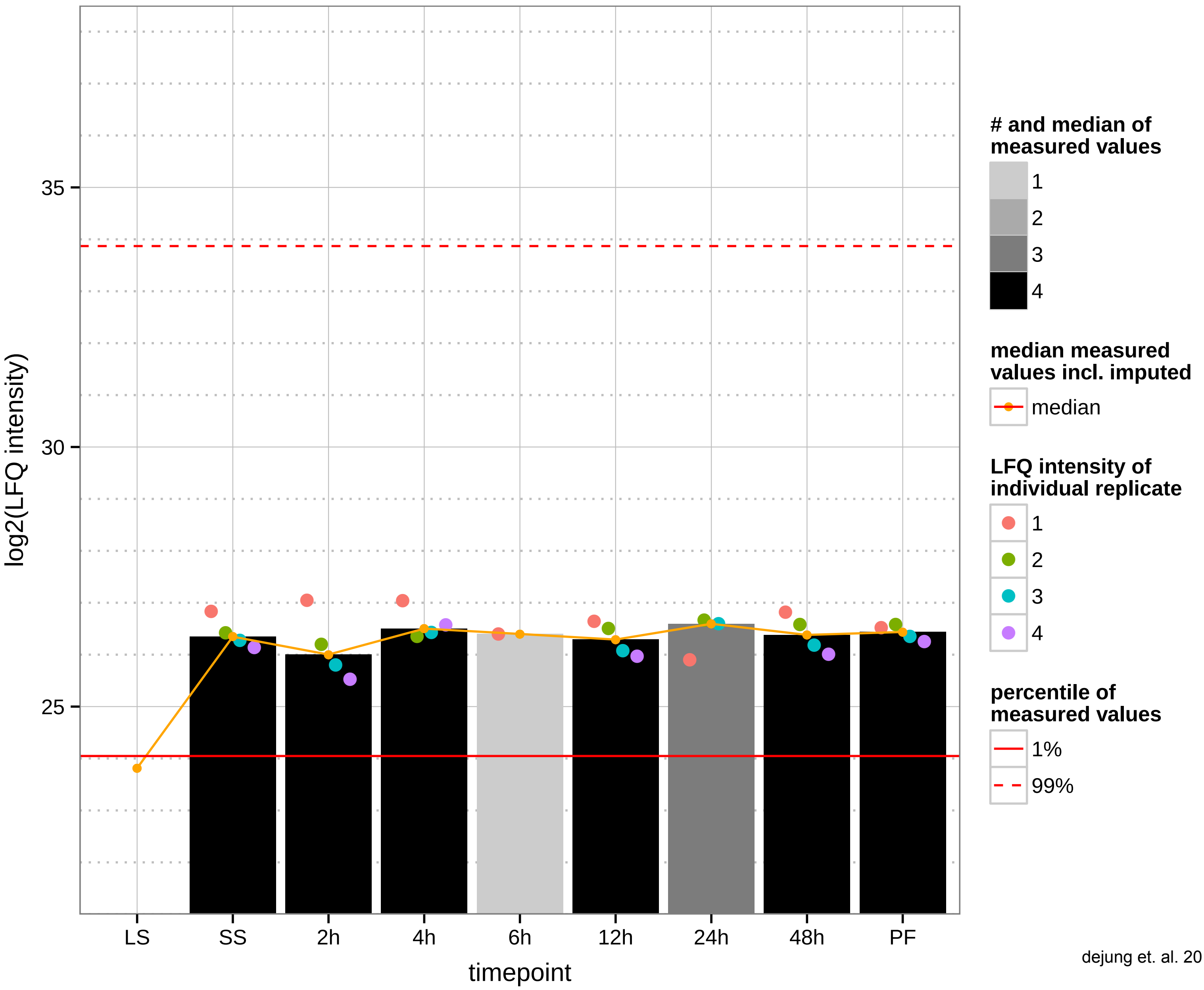

dejung et. al. 2015

hypothetical protein, conserved
Tb927.11.3830
AGOF: null
AGOC: null
AGOP: null
PGOF: null
PGOC: null
PGOP: null

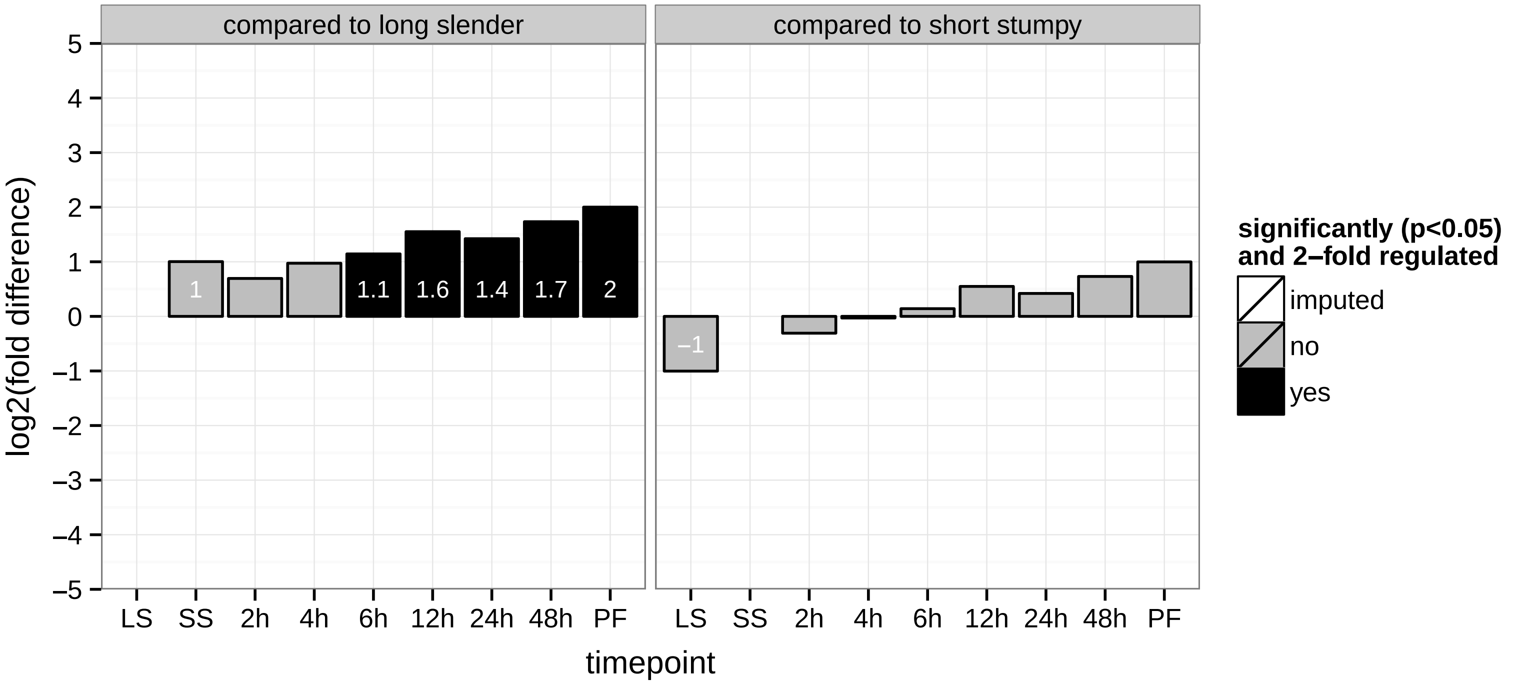

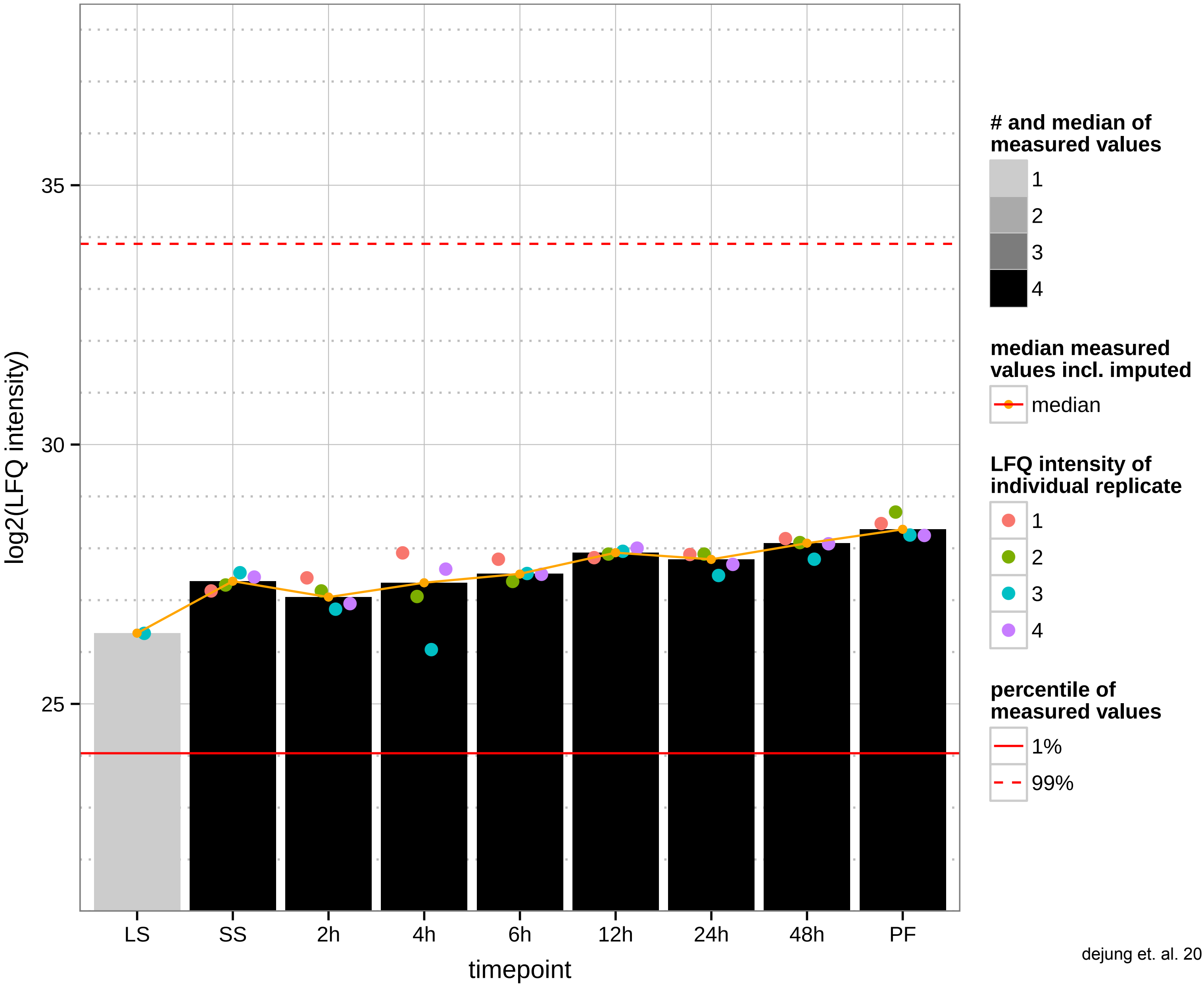

dejung et. al. 2015

actin, putative (TRYPARP)
Tb927.11.3880
AGOF: structural constituent of cytoskeleton
AGOC: actin cytoskeleton
AGOP: cytoskeleton organization
PGOF: null
PGOC: null
PGOP: null

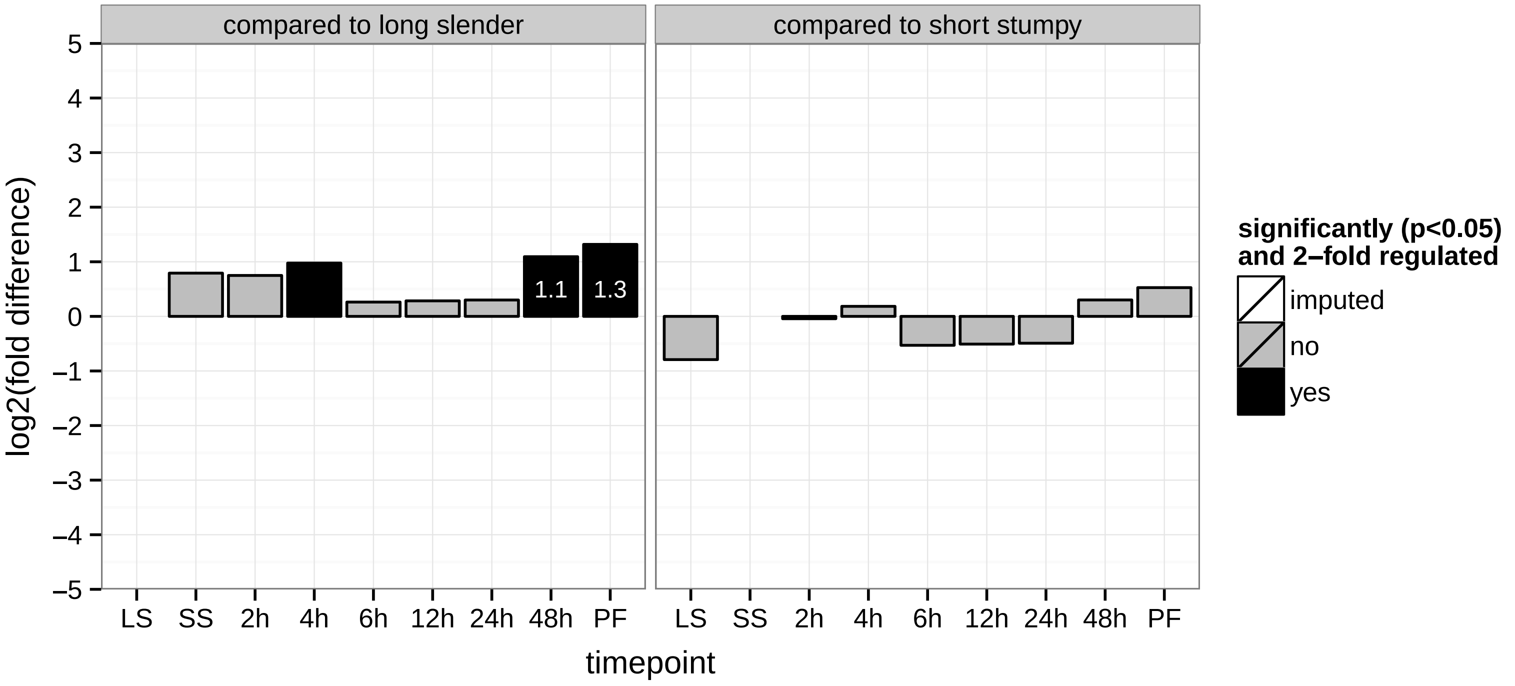

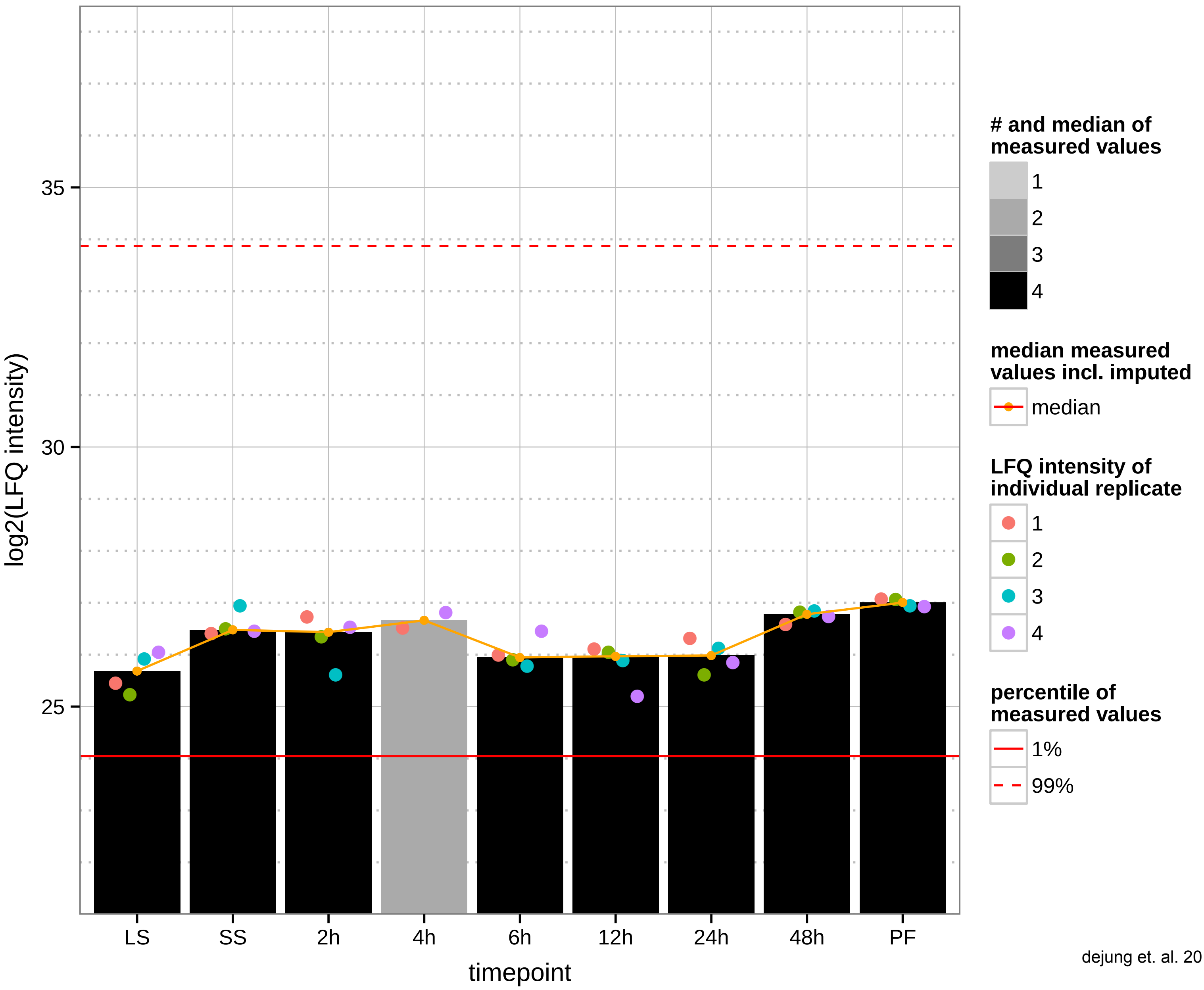

dejung et. al. 2015

hypothetical protein, conserved
Tb927.11.3910
AGOF: null
AGOC: null
AGOP: null
PGOF: null
PGOC: null
PGOP: null

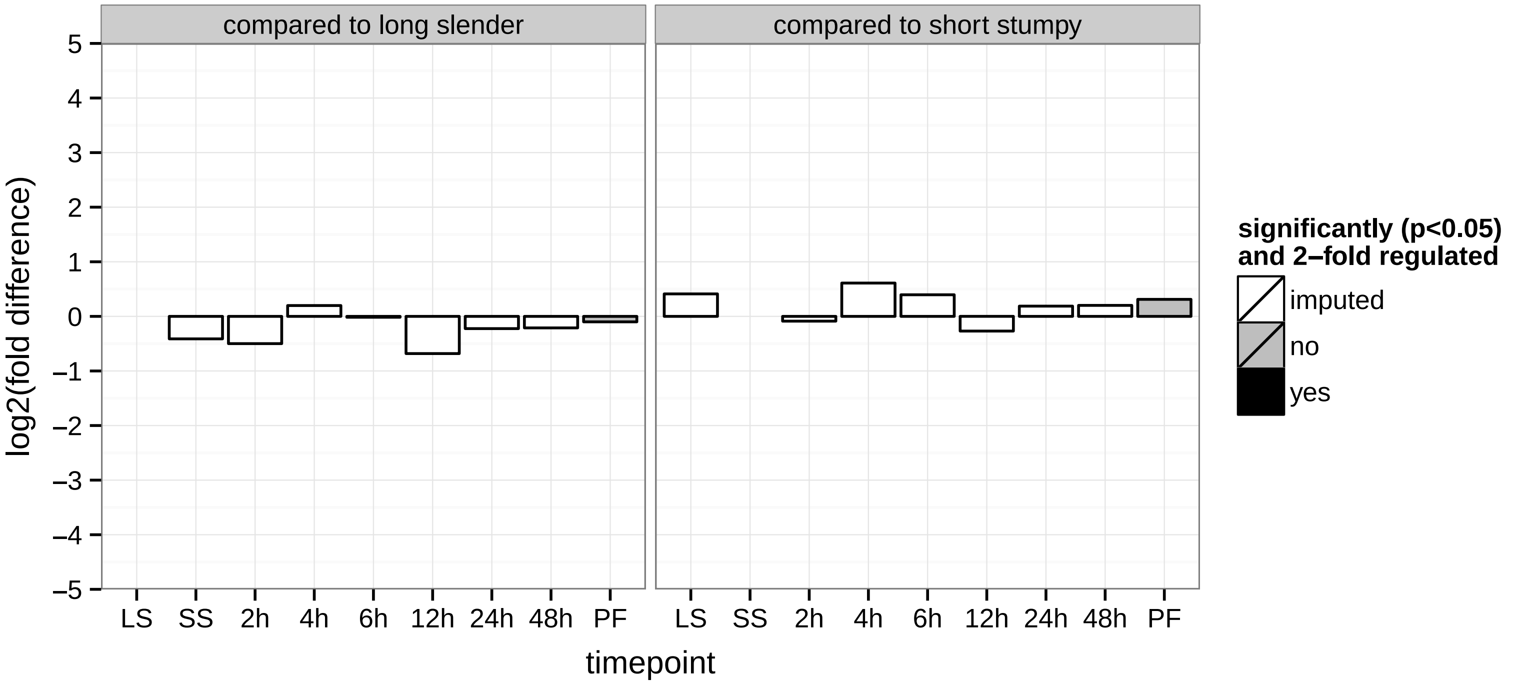

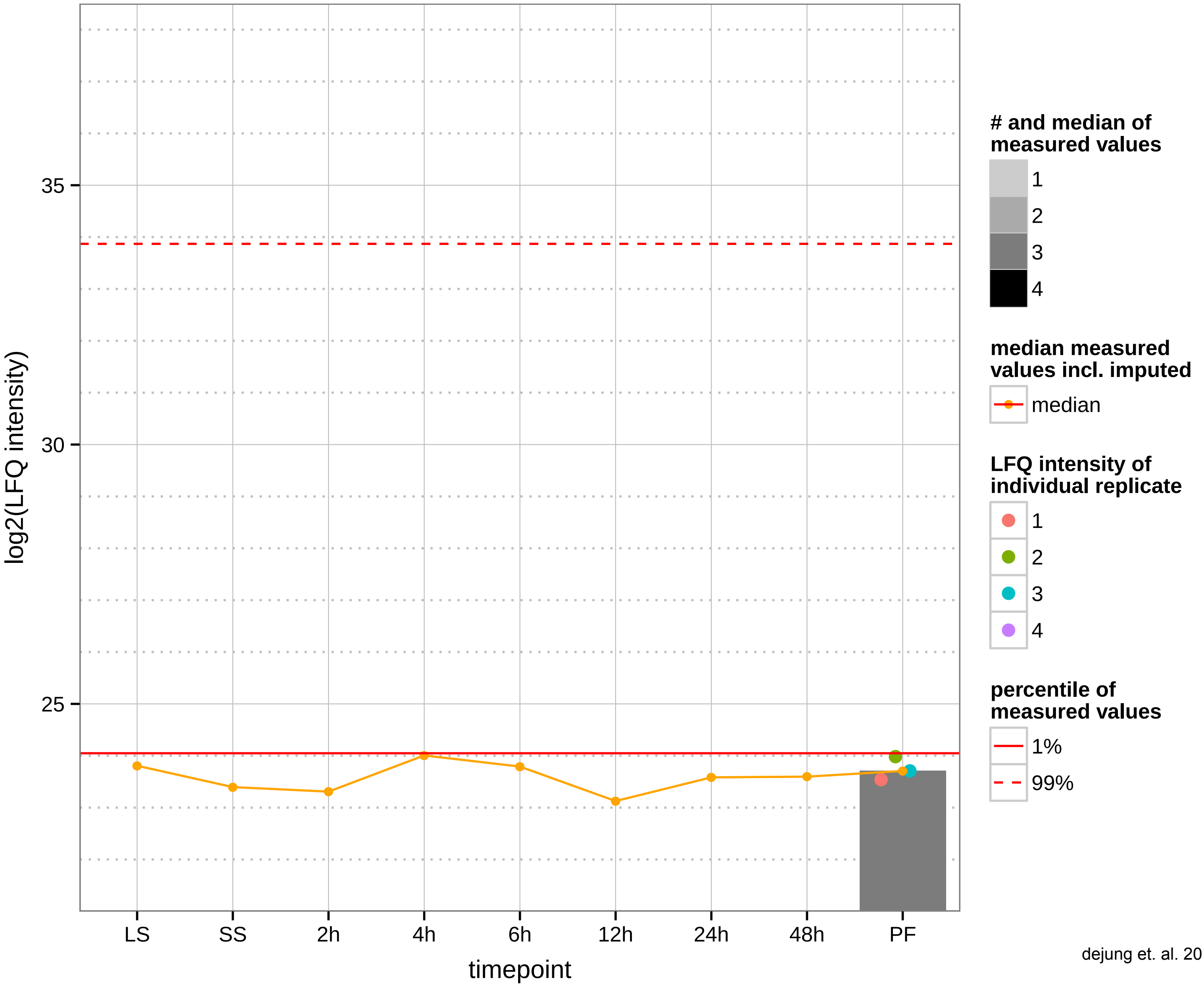

dejung et. al. 2015

hypothetical protein, conserved
Tb927.11.3940
AGOF: null
AGOC: mitochondrion
AGOP: null
PGOF: null
PGOC: null
PGOP: metabolic process

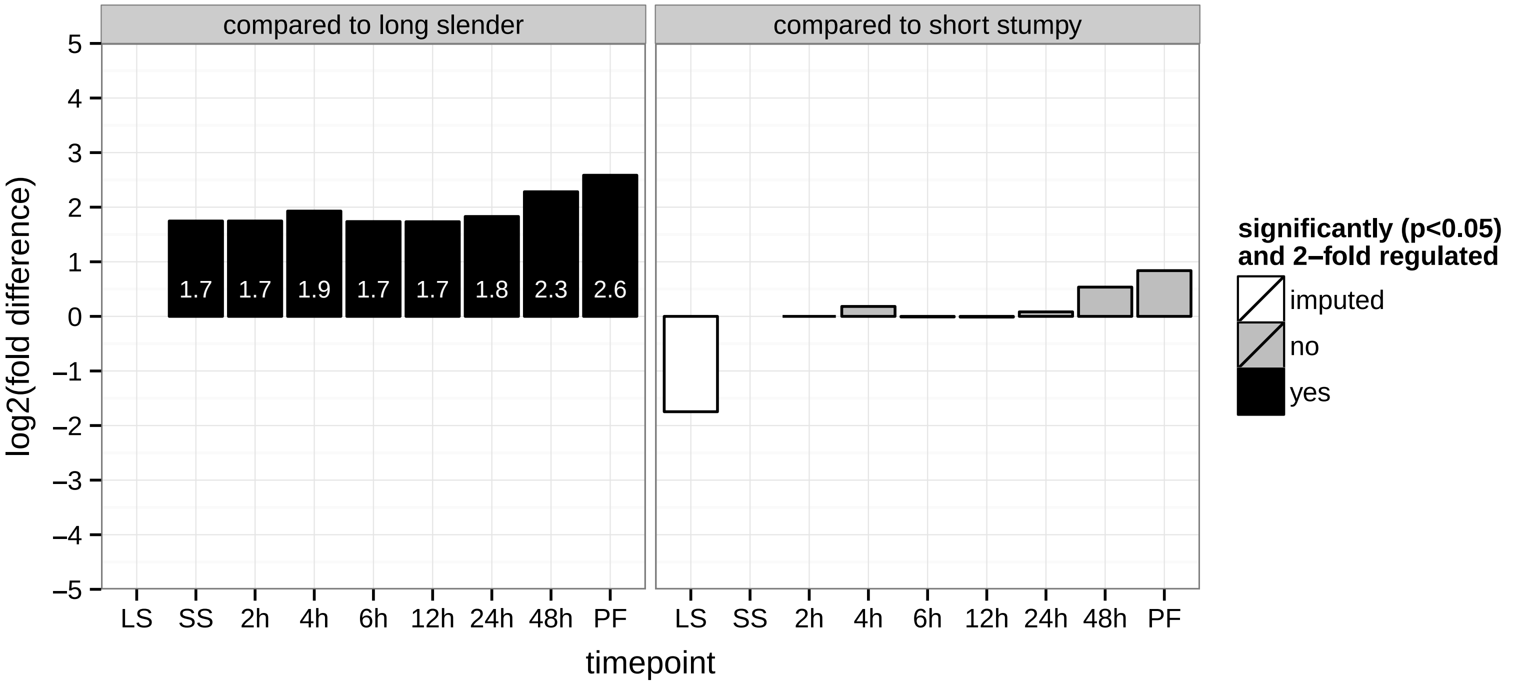

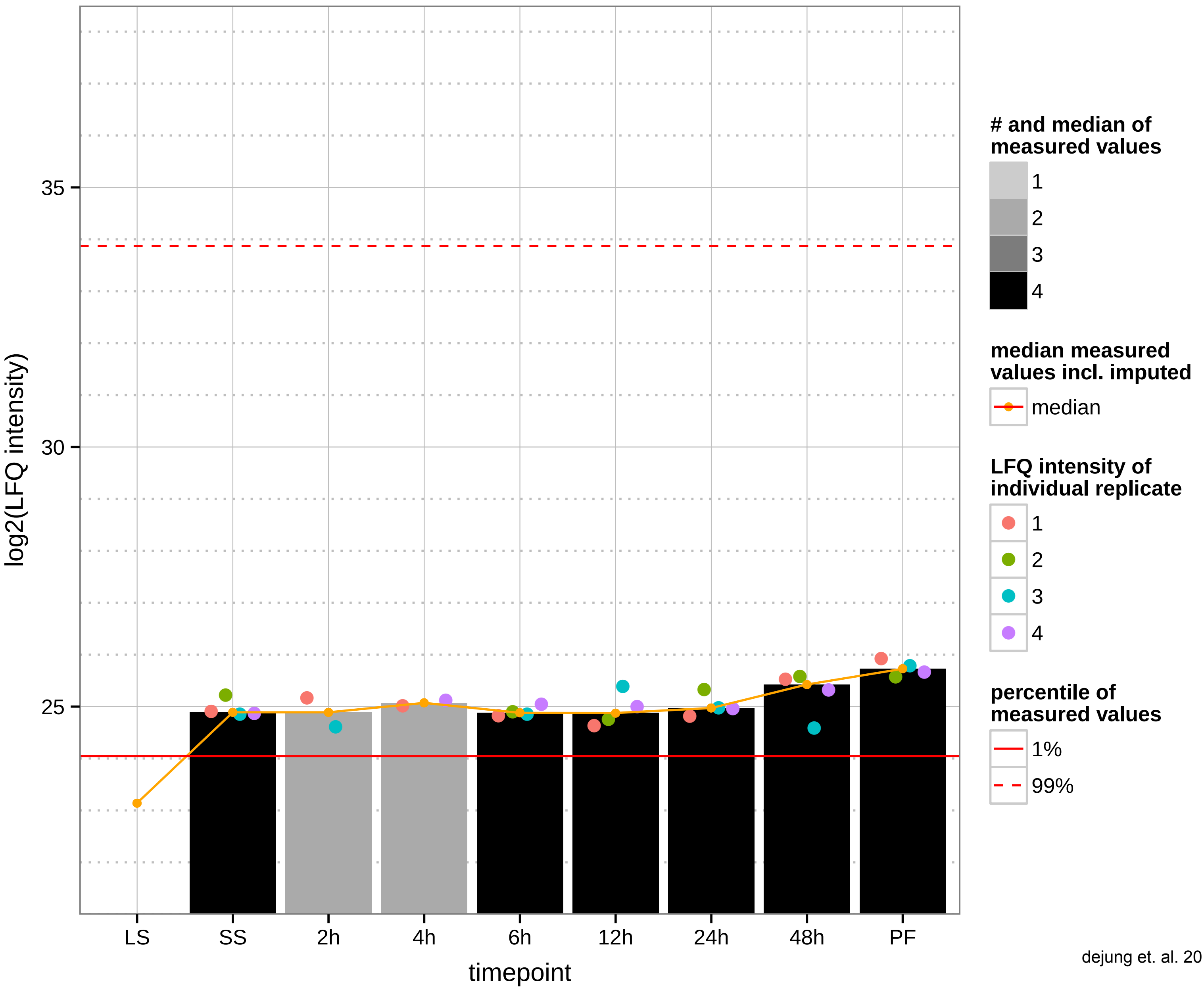

dejung et. al. 2015

hypothetical protein, conserved
Tb927.11.3970
AGOF: RNA binding
AGOC: null
AGOP: null
PGOF: nucleic acid binding, zinc ion binding
PGOC: null
PGOP: null

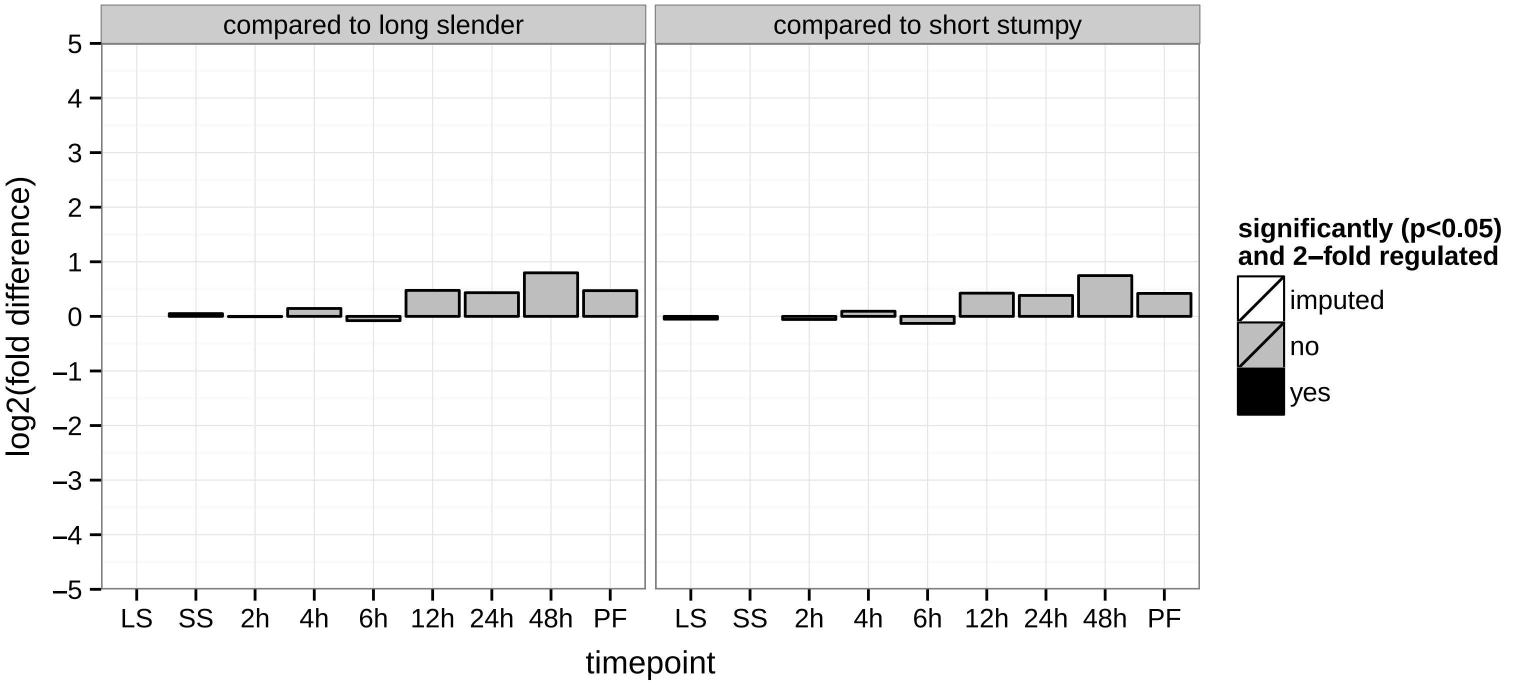

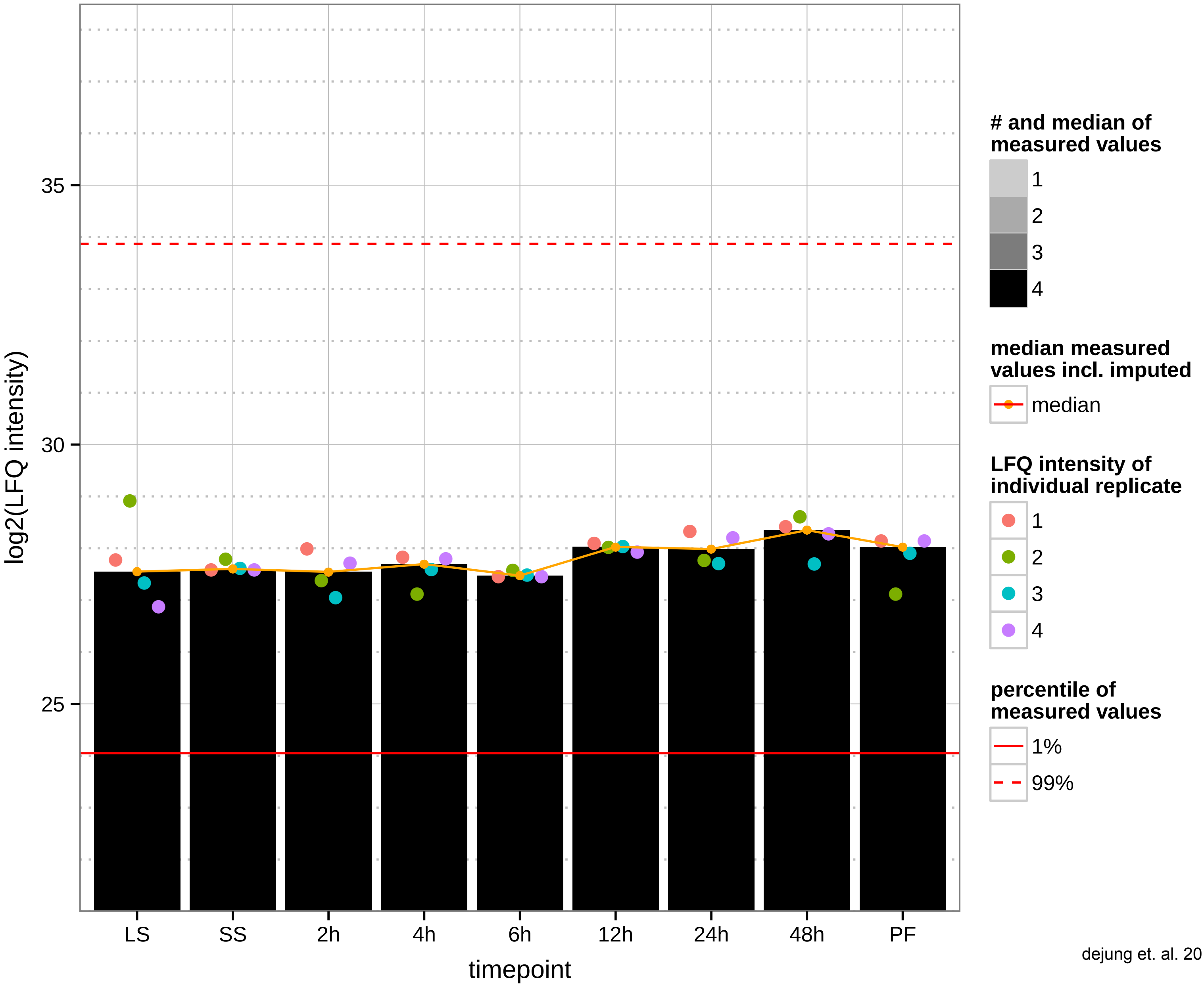

dejung et. al. 2015

hypothetical protein, conserved
Tb927.11.400
AGOF: null
AGOC: null
AGOP: null
PGOF: null
PGOC: null
PGOP: null

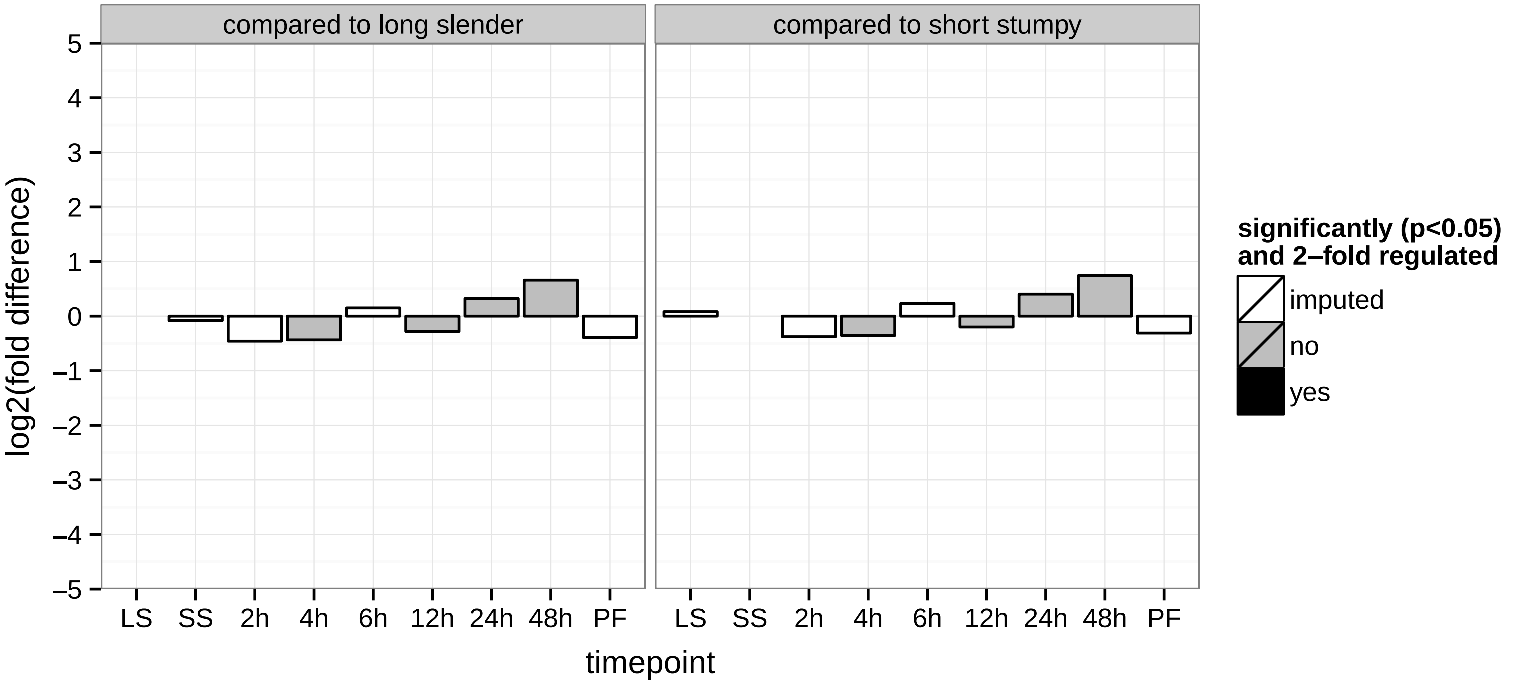

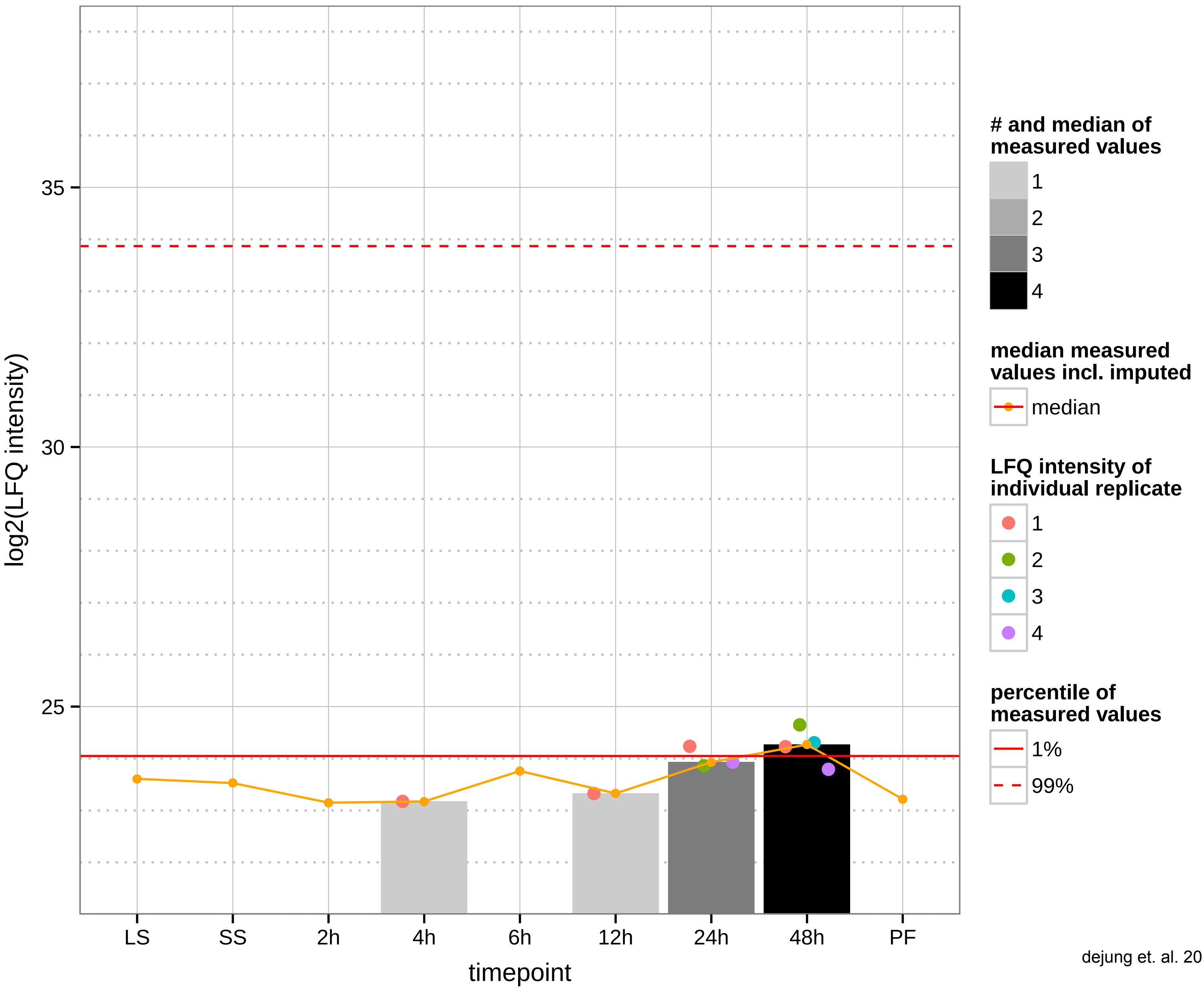

dejung et. al. 2015

hypothetical protein, conserved
Tb927.11.4000
AGOF: null
AGOC: null
AGOP: null
PGOF: null
PGOC: null
PGOP: null

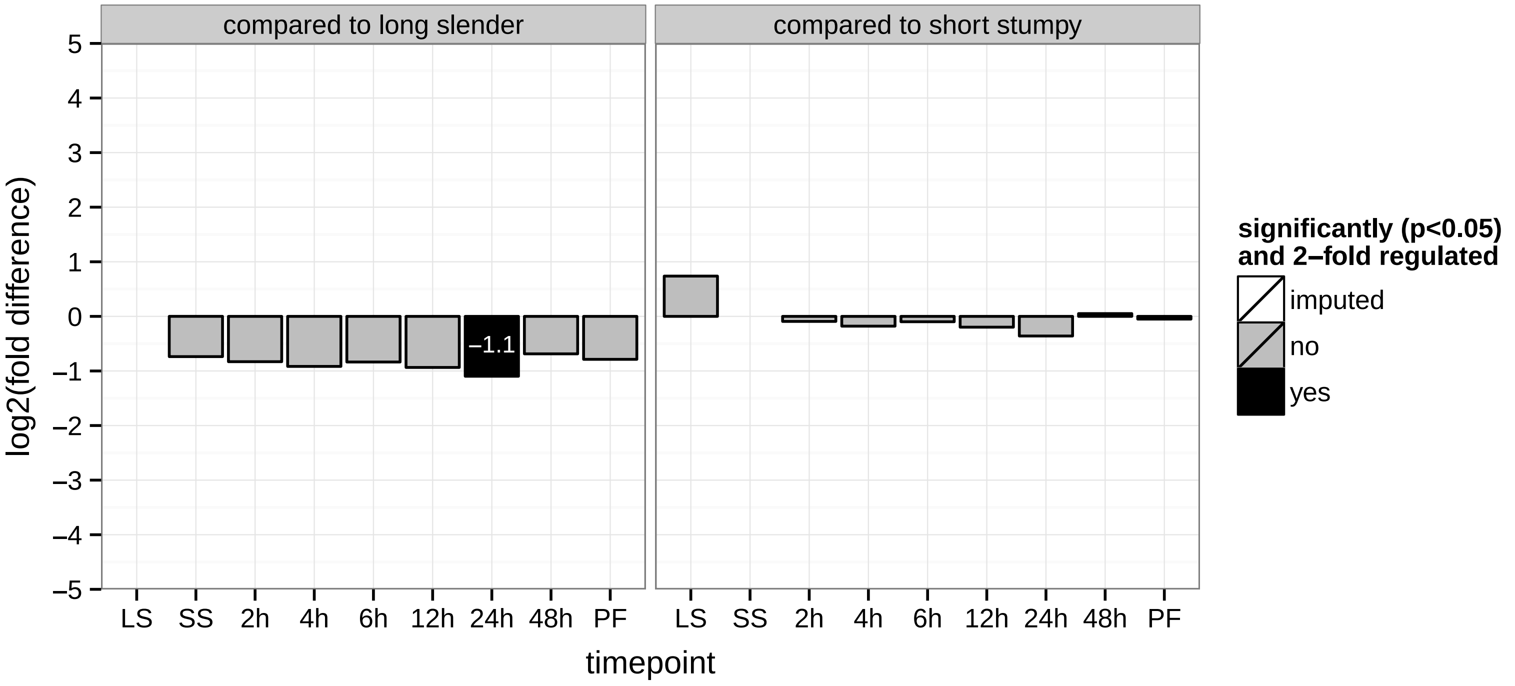

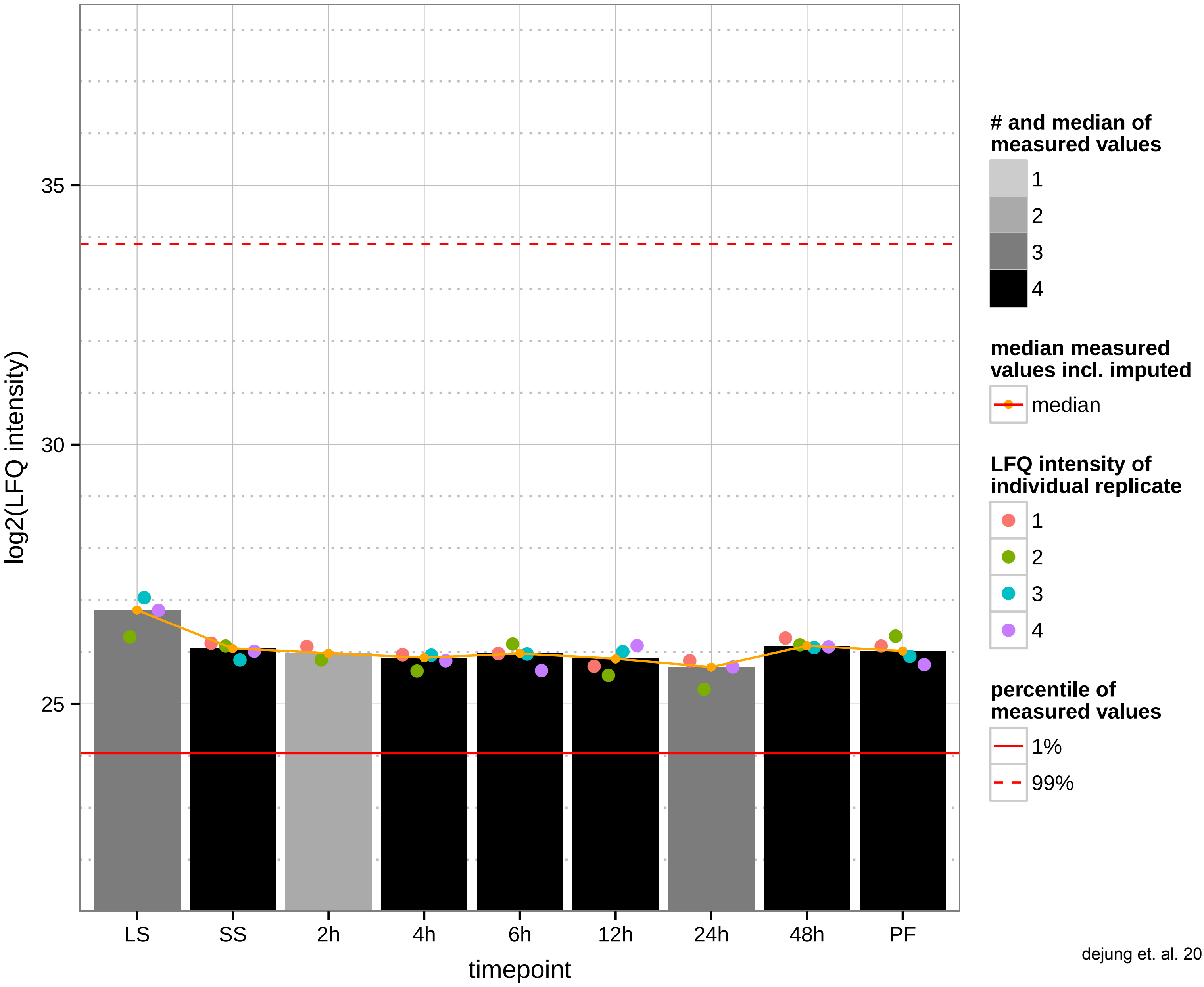

dejung et. al. 2015

ubiquitin–like protein, putative
Tb927.11.4130
AGOF: null
AGOC: null
AGOP: null
PGOF: protein binding
PGOC: null
PGOP: null

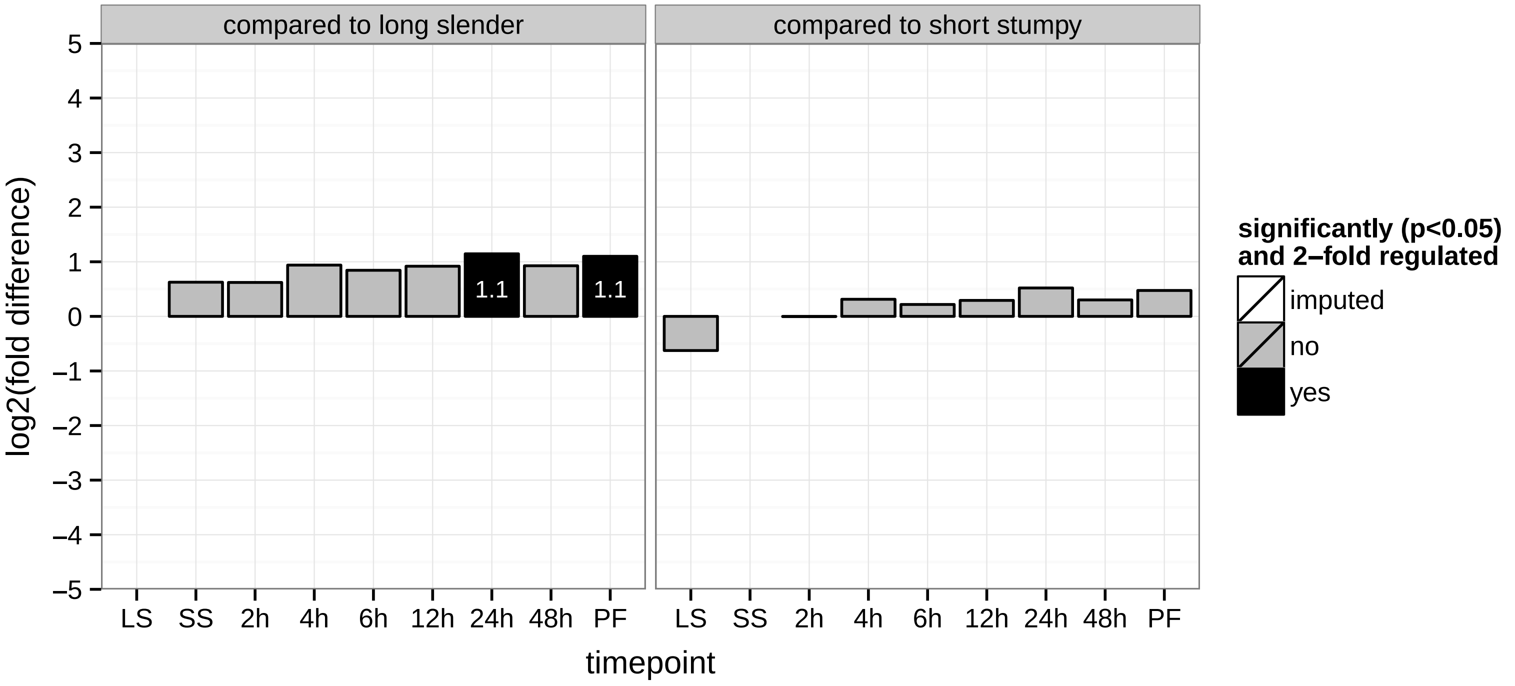

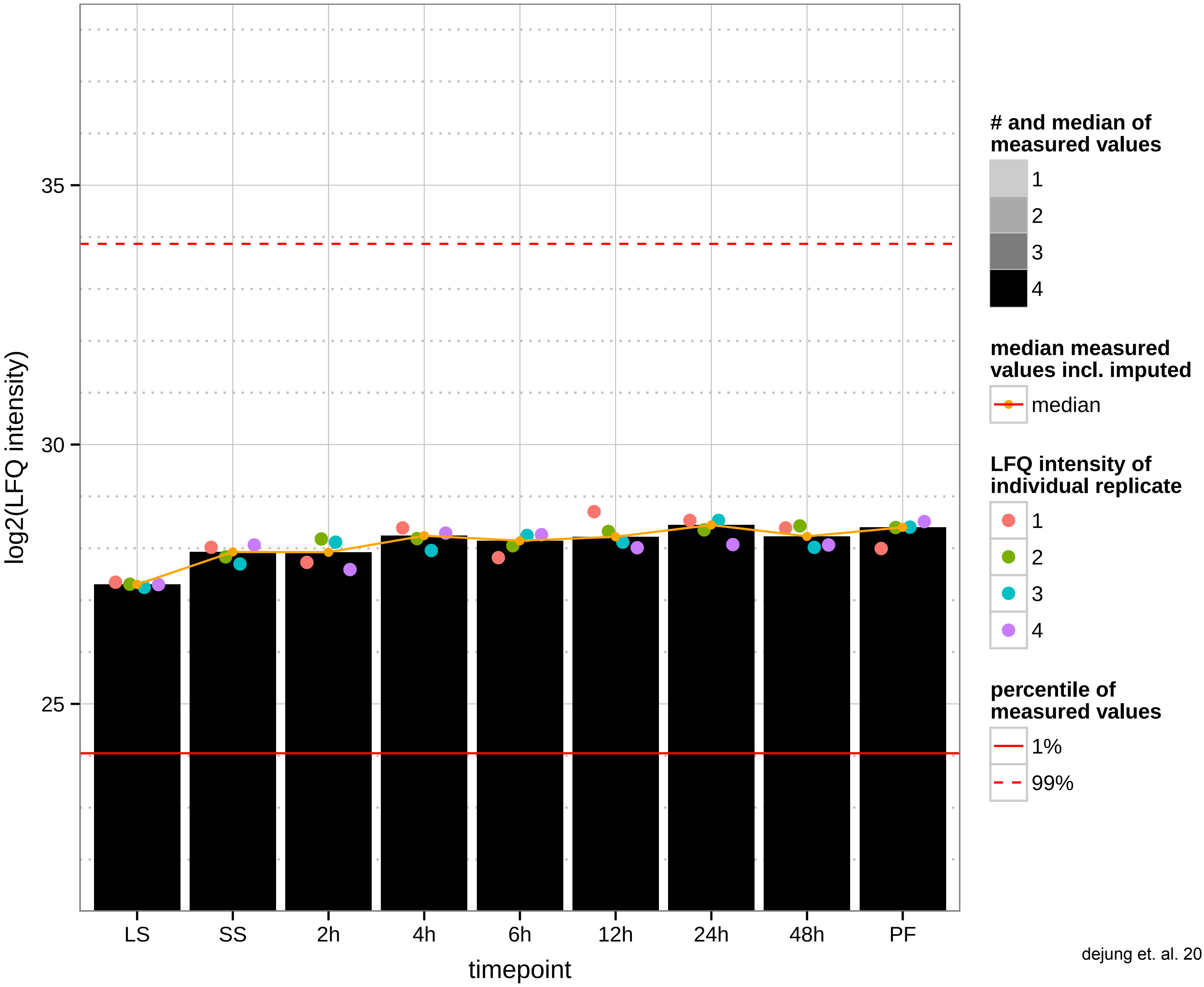

dejung et. al. 2015

lectin, putative, mannose−specific lectin
Tb927.11.4200
AGOF: mannose binding
AGOC: integral to membrane, membrane
AGOP: protein secretion
PGOF: null
PGOC: membrane
PGOP: null

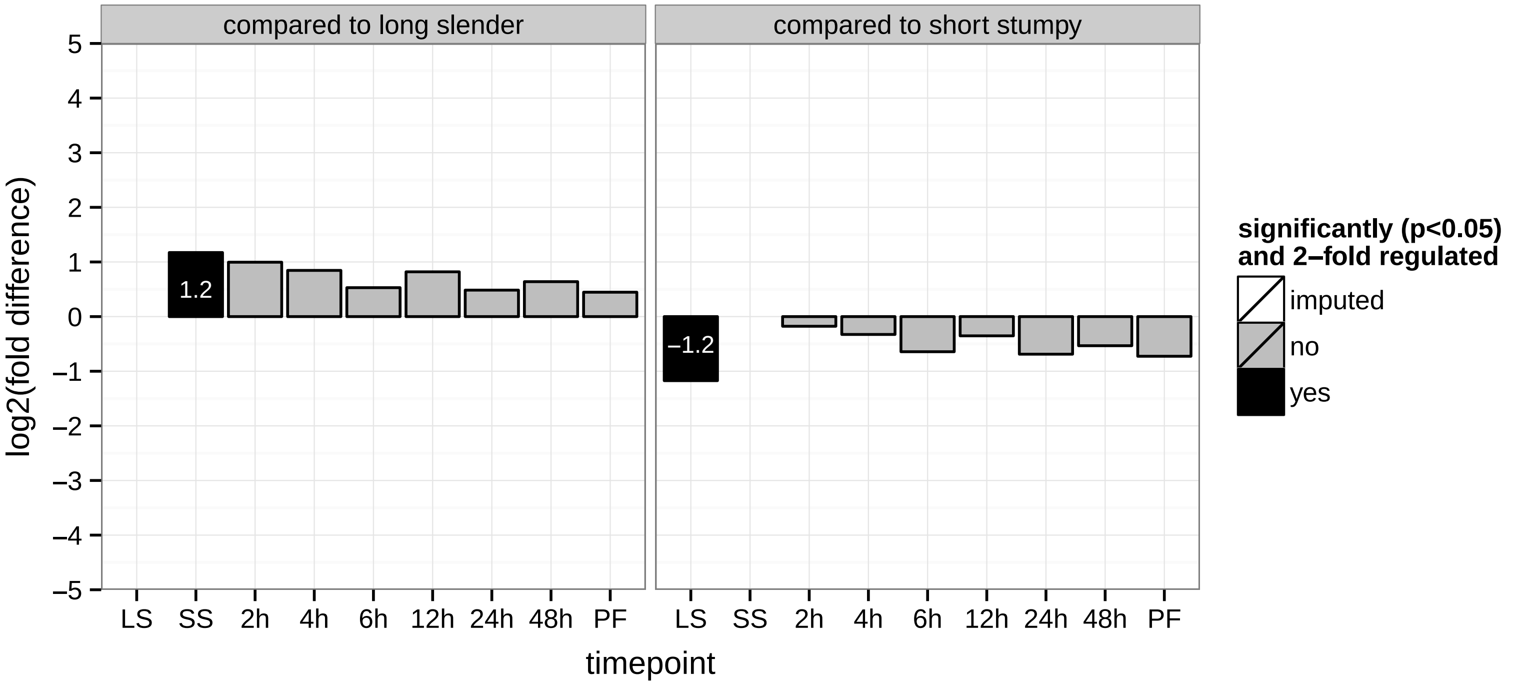

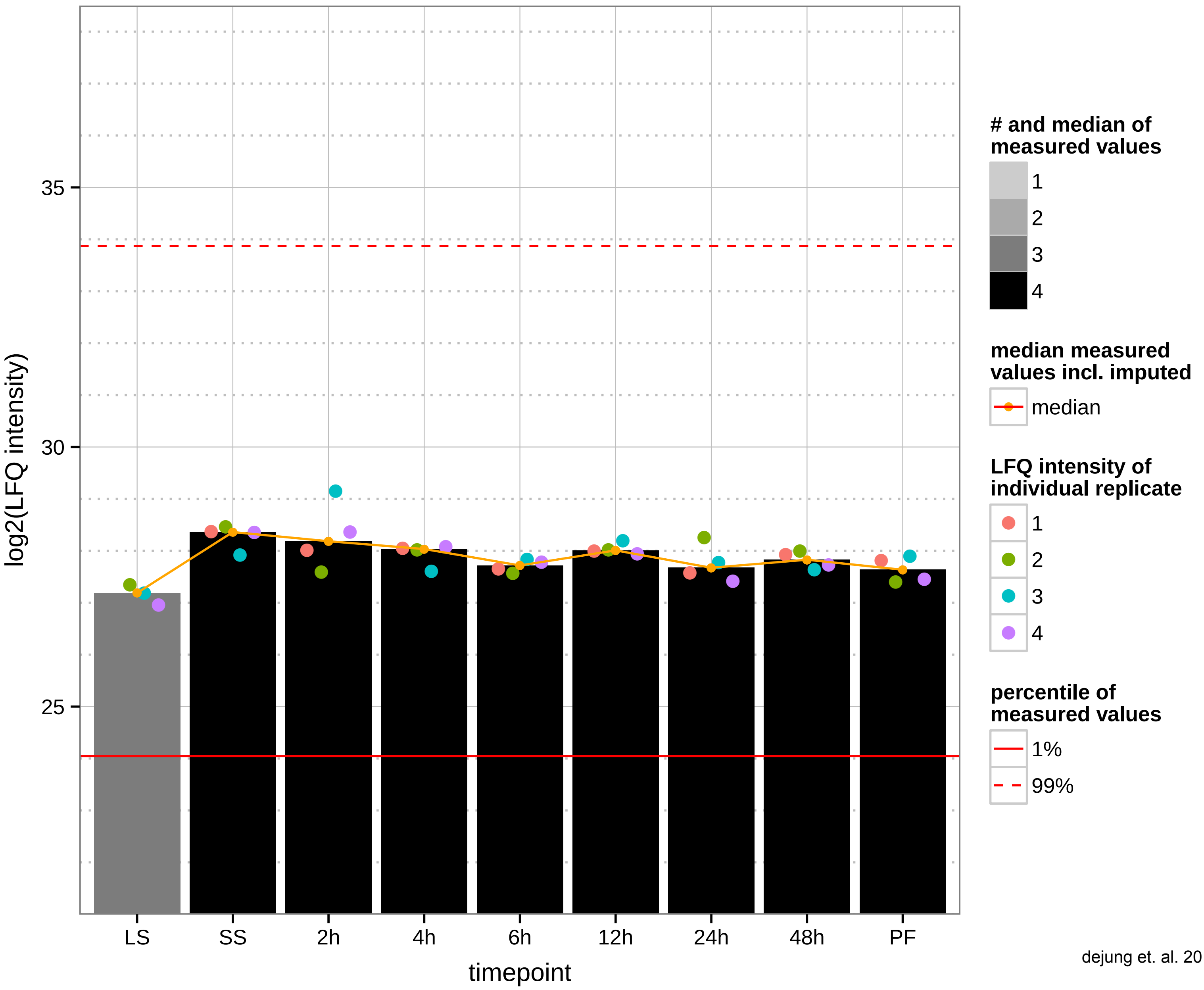

dejung et. al. 2015

hypothetical protein, conserved
Tb927.11.4210
AGOF: ubiquitin–protein ligase activity
AGOC: ubiquitin ligase complex
AGOP: protein ubiquitination
PGOF: binding, ubiquitin–protein ligase activity
PGOC: ubiquitin ligase complex
PGOP: protein ubiquitination

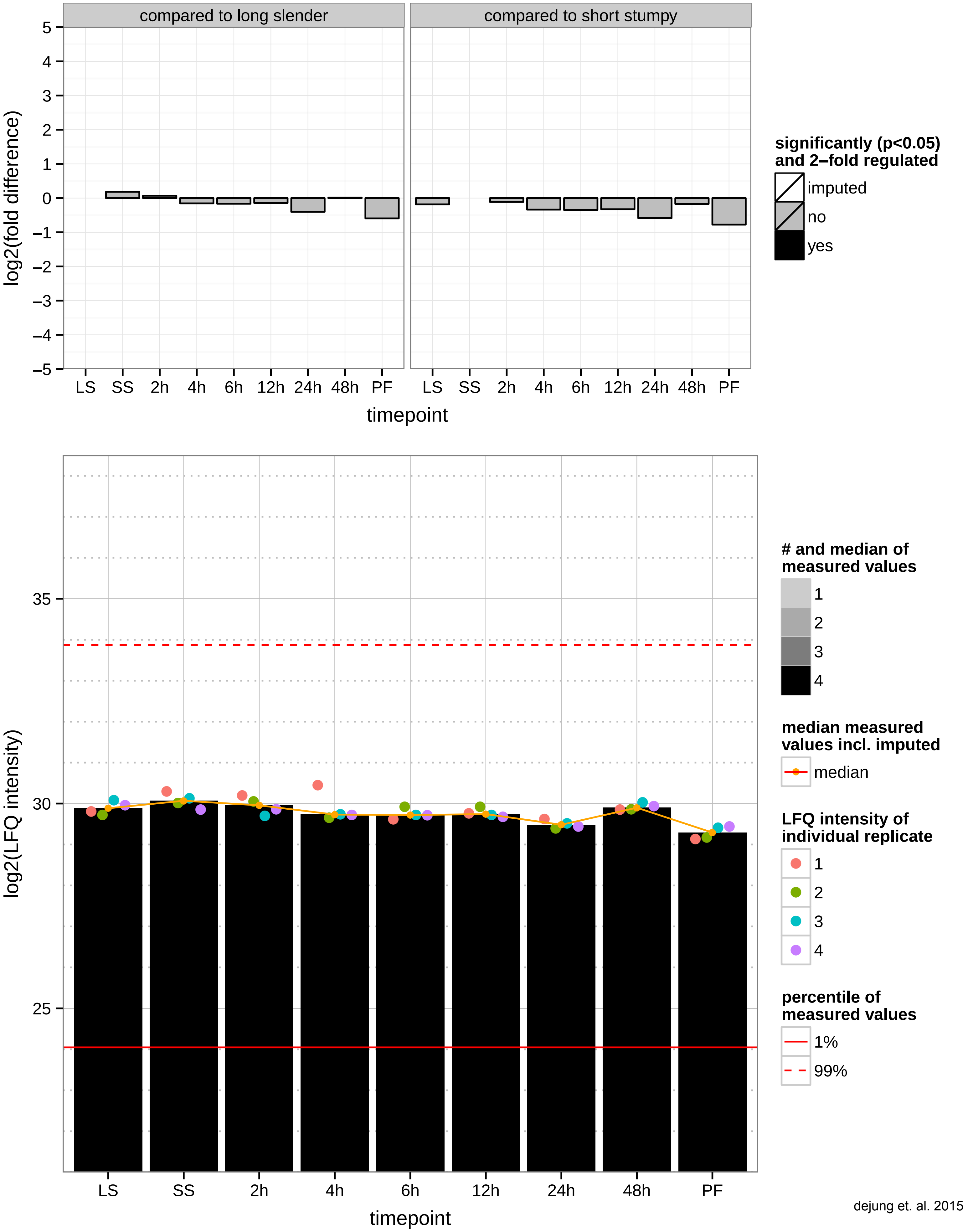

dejung et. al. 2015

hypothetical protein, conserved
Tb927.11.4230
AGOF: null
AGOC: null
AGOP: null
PGOF: binding
PGOC: null
PGOP: null

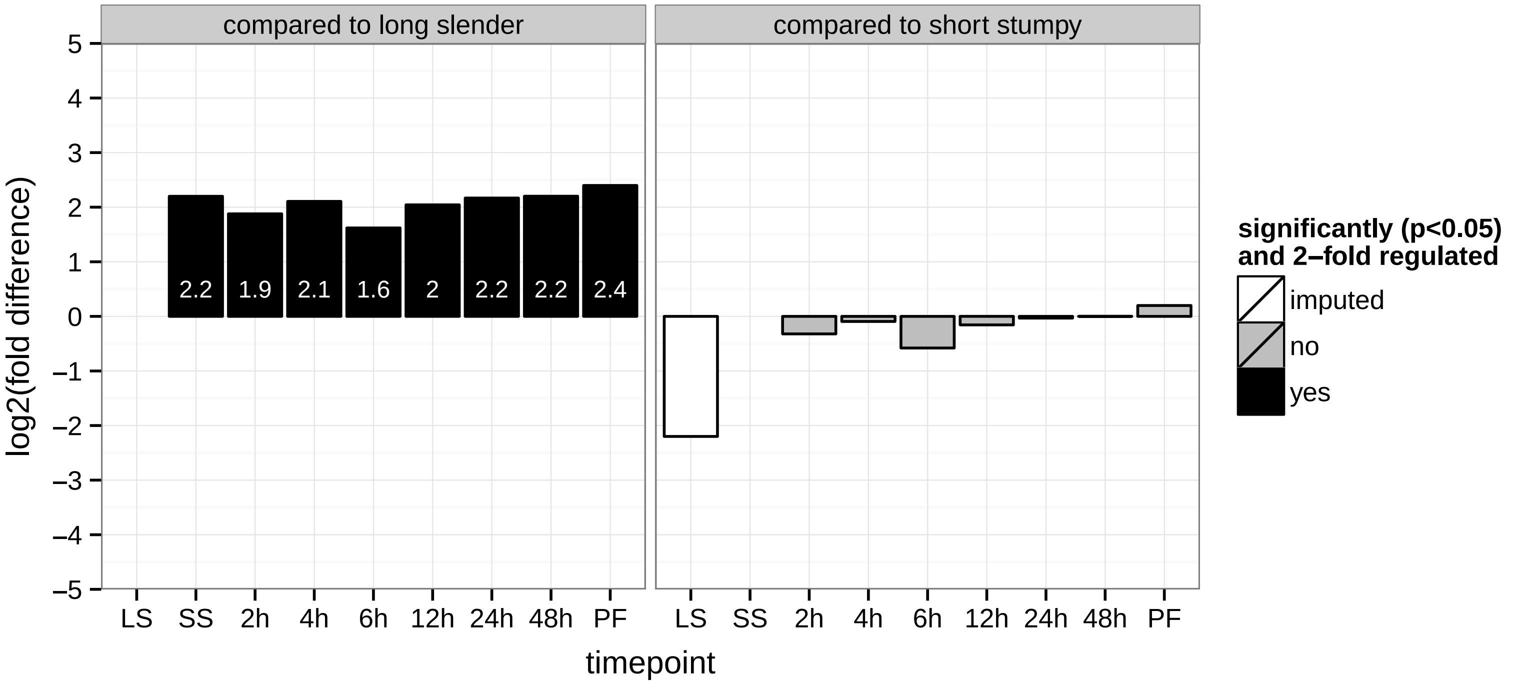

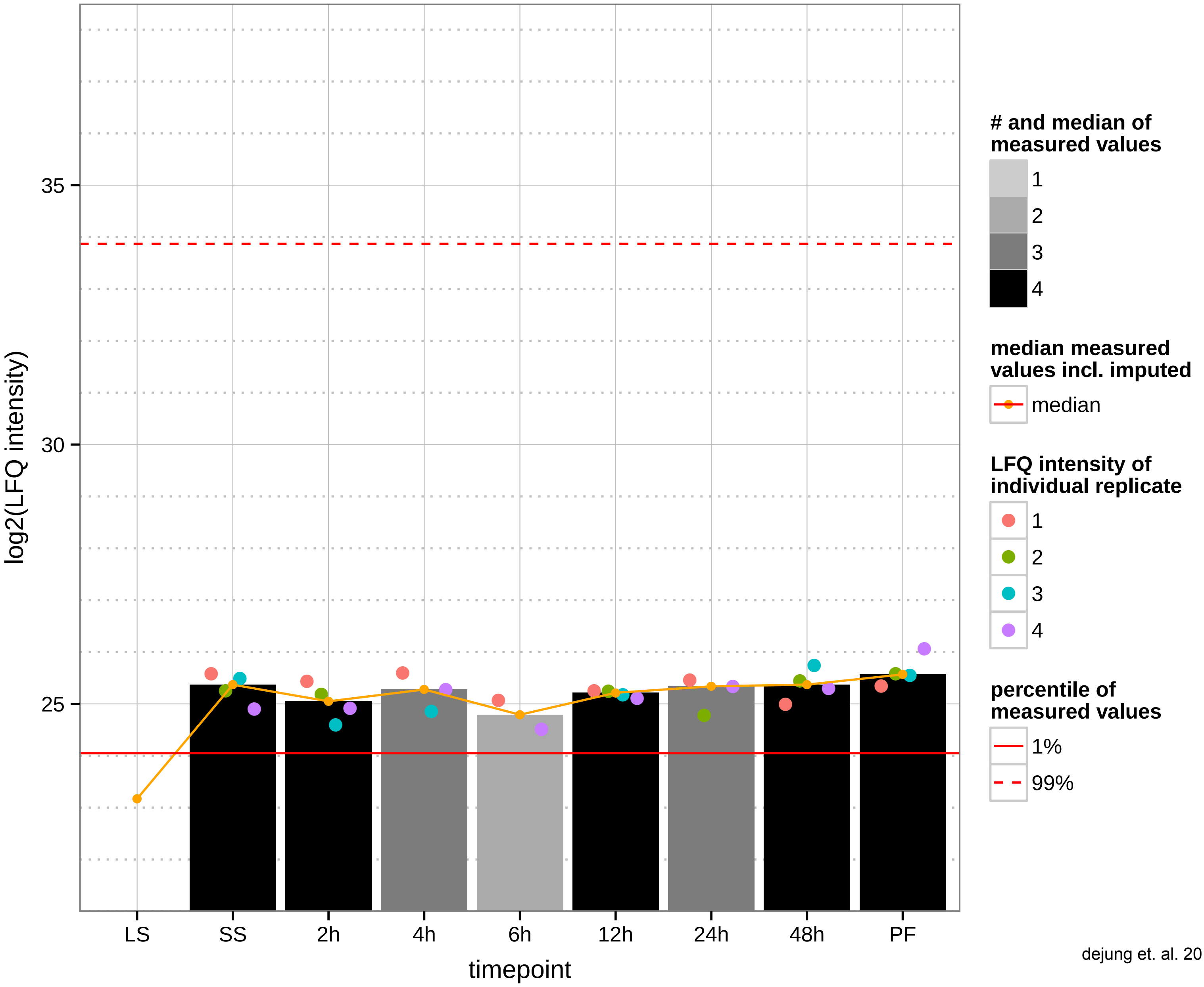

dejung et. al. 2015

hypothetical protein, conserved
Tb927.11.4280
AGOF: null
AGOC: null
AGOP: null
PGOF: protein binding
PGOC: null
PGOP: null

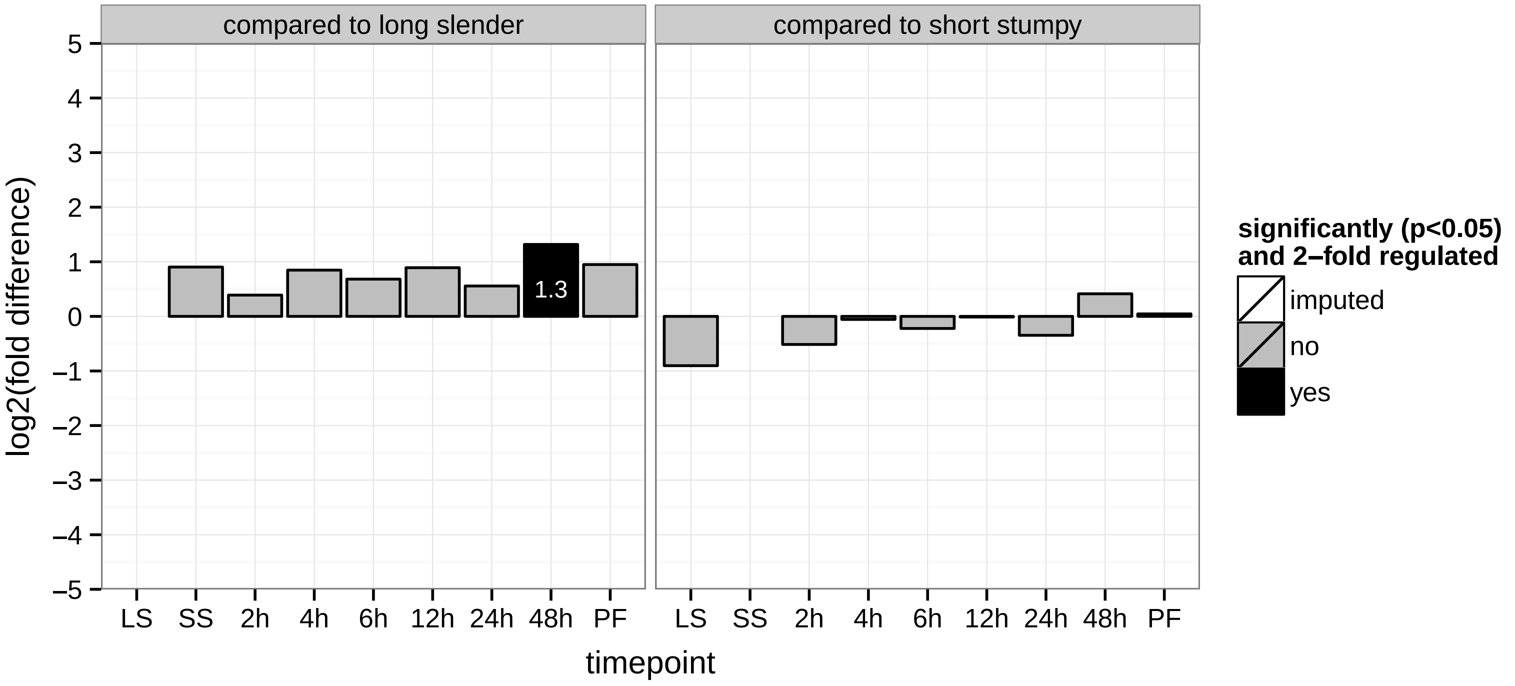

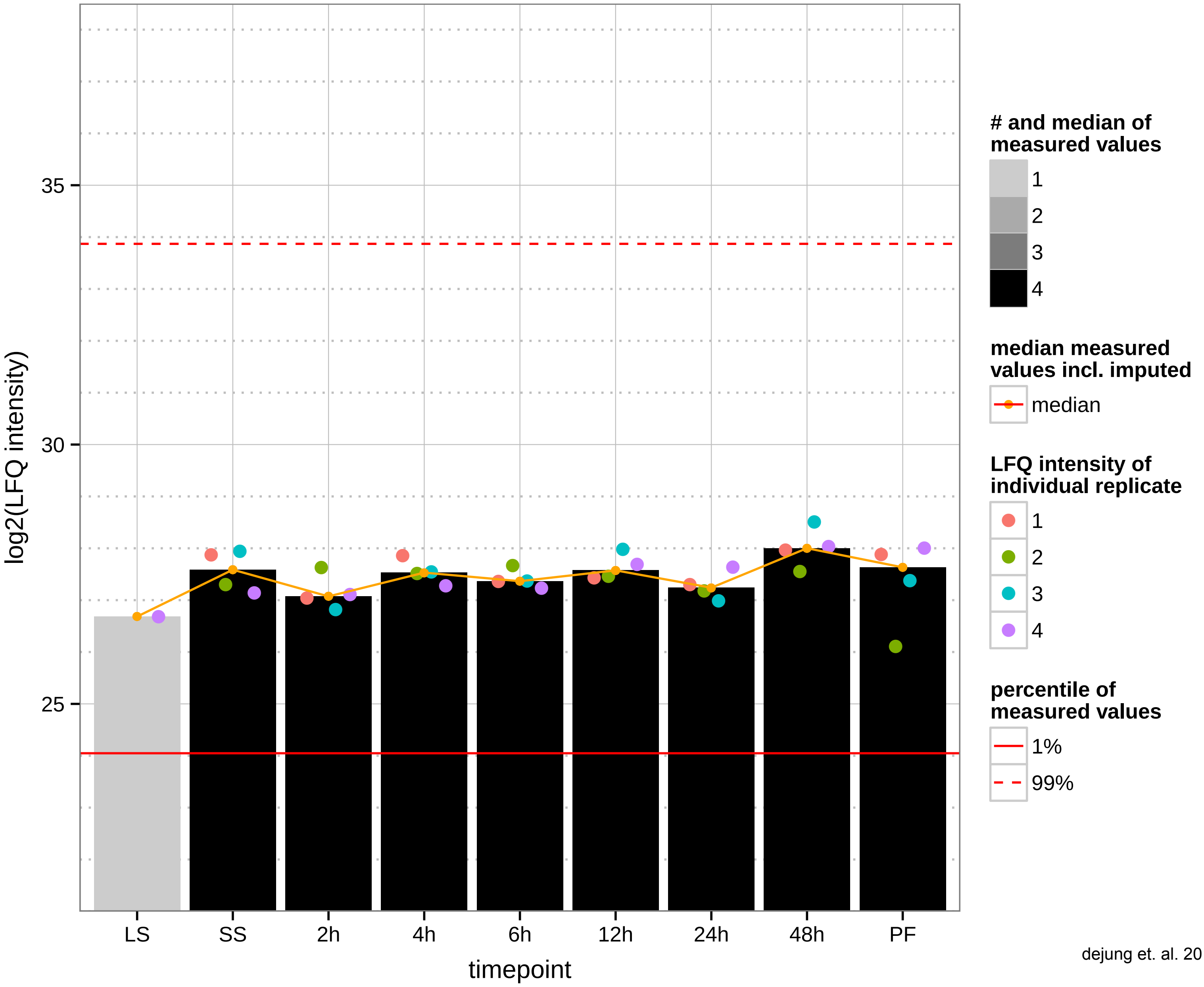

dejung et. al. 2015

40S ribosomal protein S12 (RPS12)
Tb927.11.4290
AGOF: structural constituent of ribosome
AGOC: intracellular, ribosome
AGOP: translation
PGOF: null
PGOC: null
PGOP: null

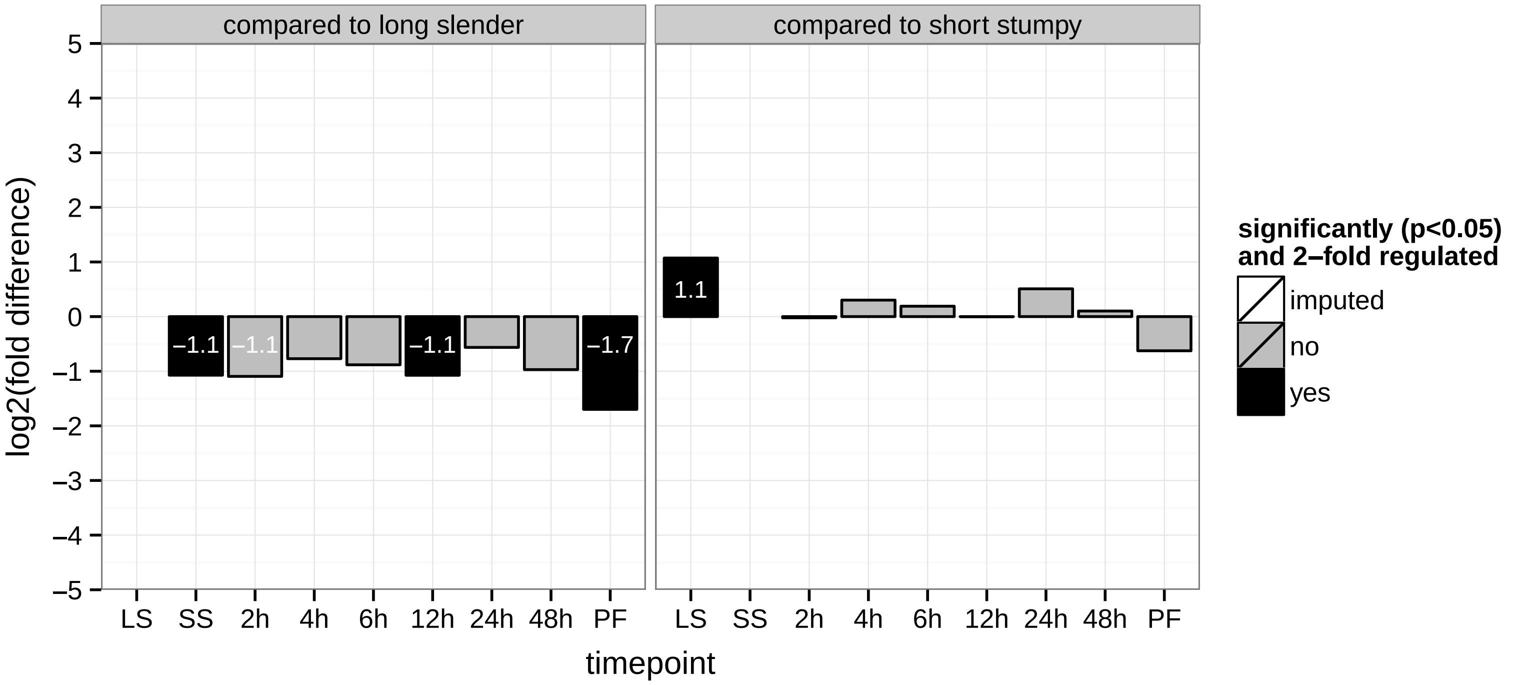

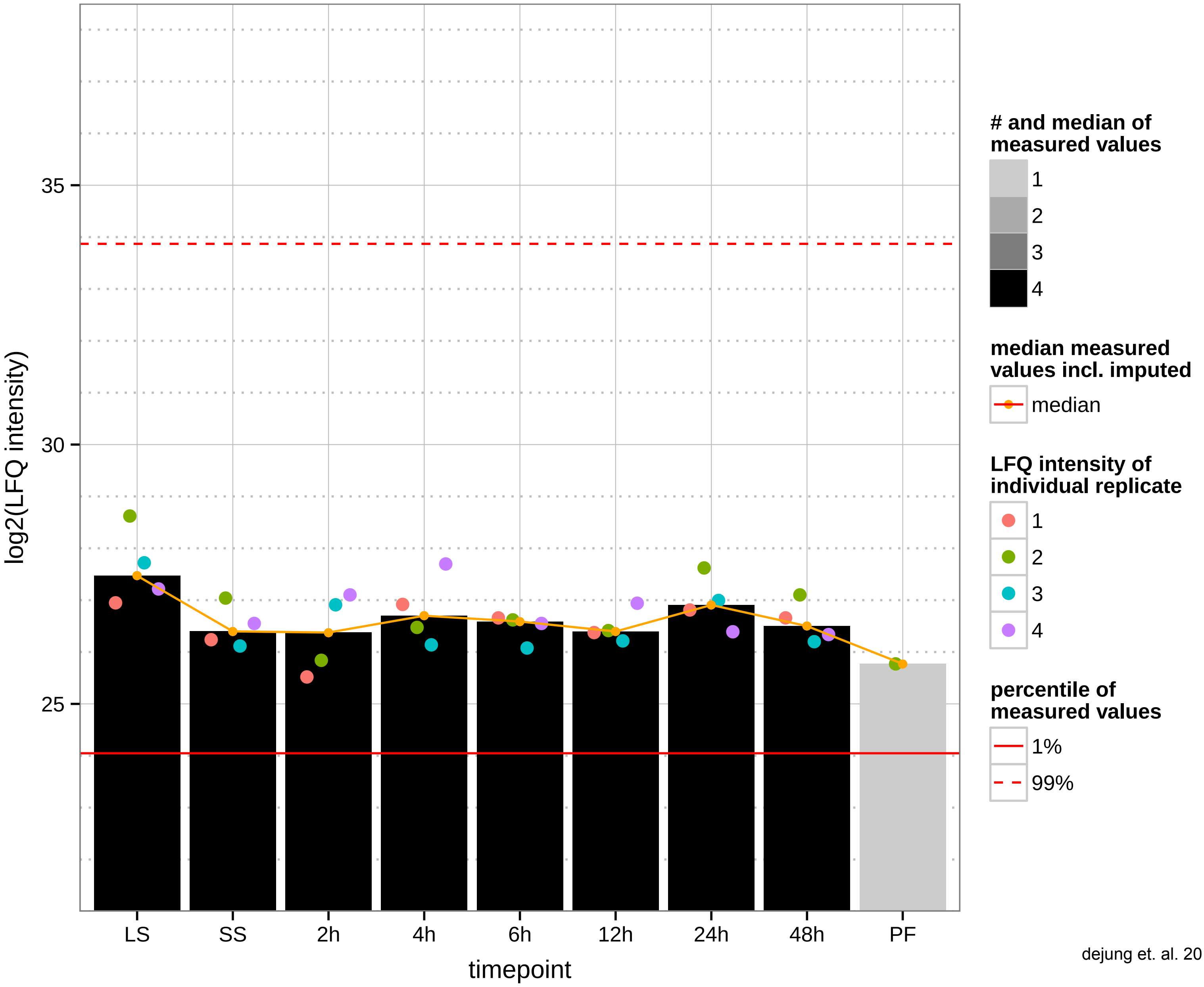

dejung et. al. 2015

hypothetical protein, conserved
Tb927.11.4320
AGOF: null
AGOC: mitochondrion
AGOP: null
PGOF: null
PGOC: null
PGOP: null

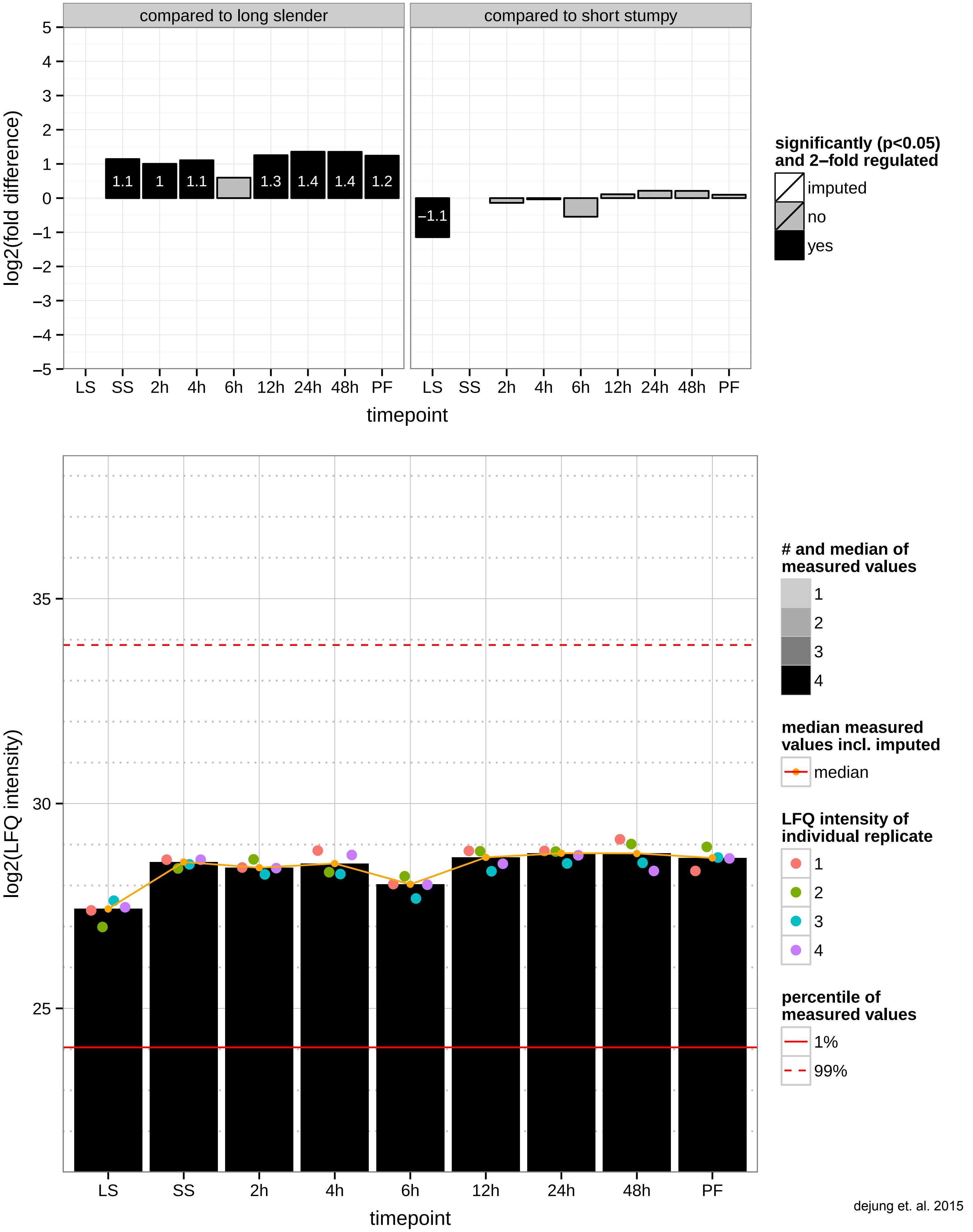

dejung et. al. 2015

hypothetical protein, conserved
Tb927.11.4360
AGOF: null
AGOC: null
AGOP: null
PGOF: protein binding
PGOC: null
PGOP: null

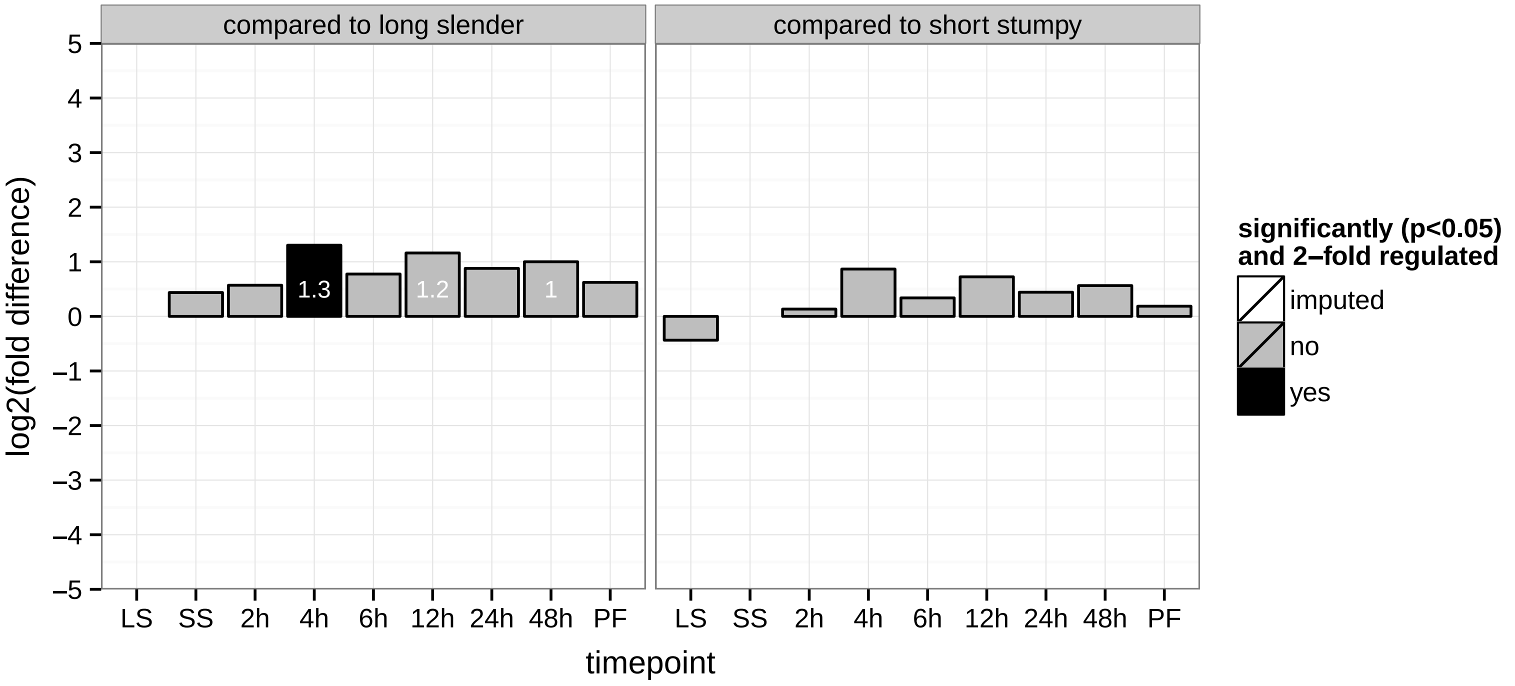

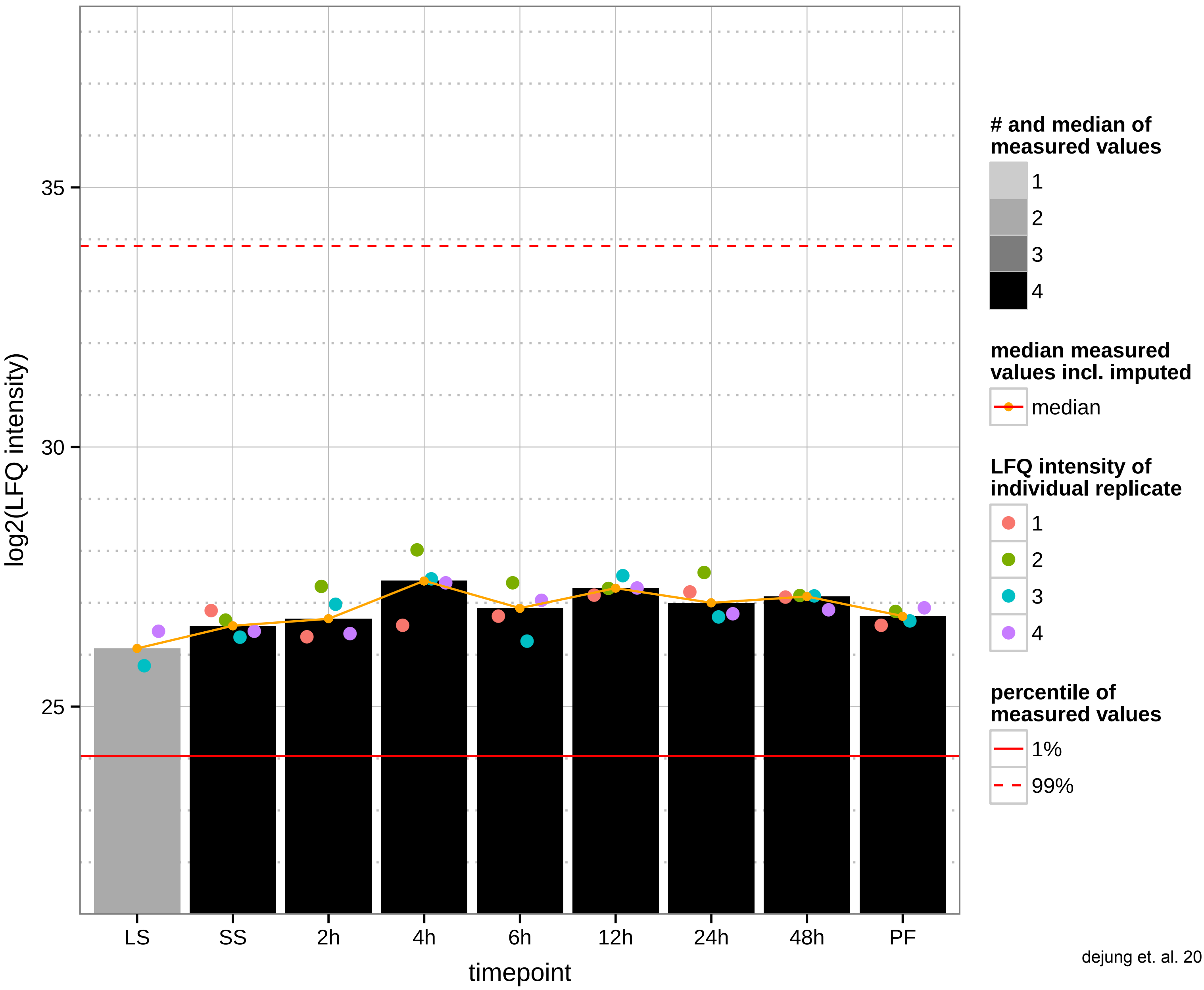

dejung et. al. 2015

ALBA–Domain Protein (ALBA2)
Tb927.11.4450
AGOF: nucleic acid binding
AGOC: cytoplasm, cytoplasmic stress granule
AGOP: null
PGOF: nucleic acid binding
PGOC: null
PGOP: null

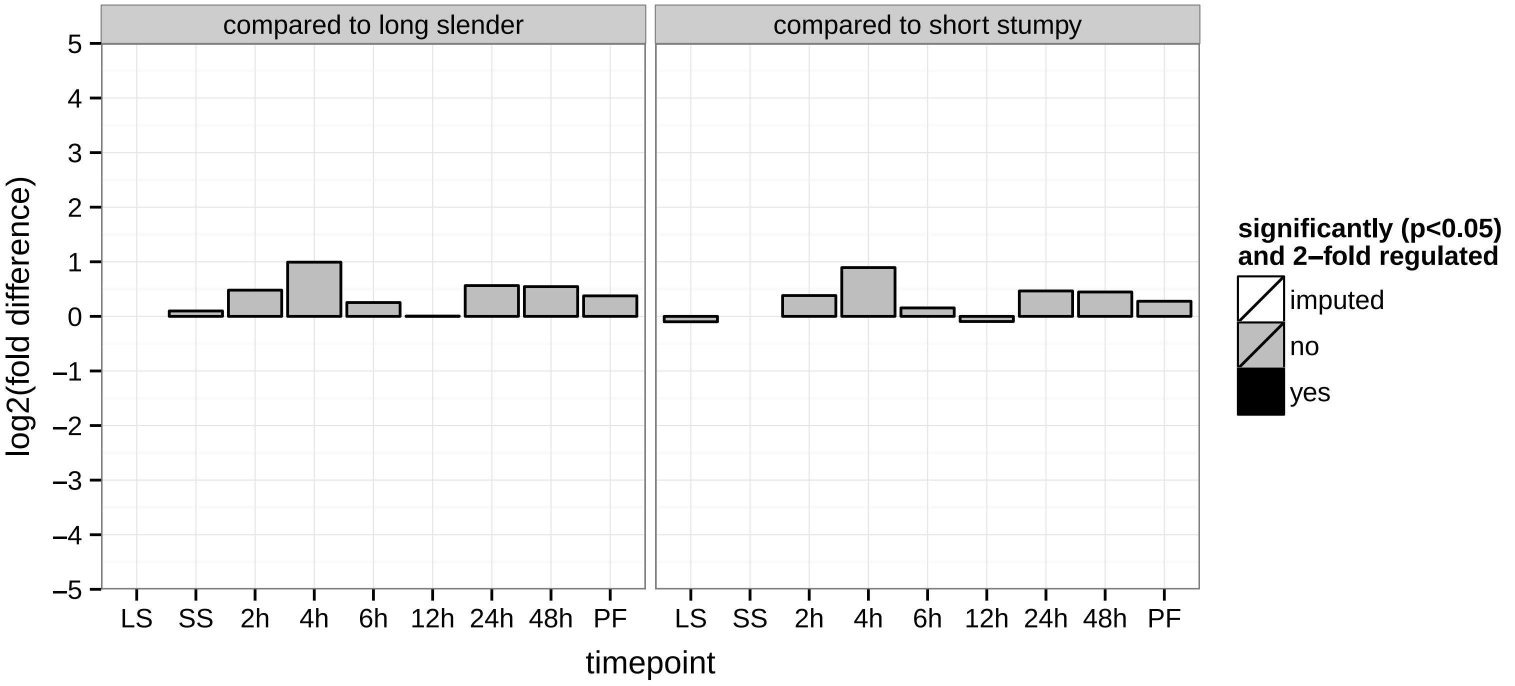

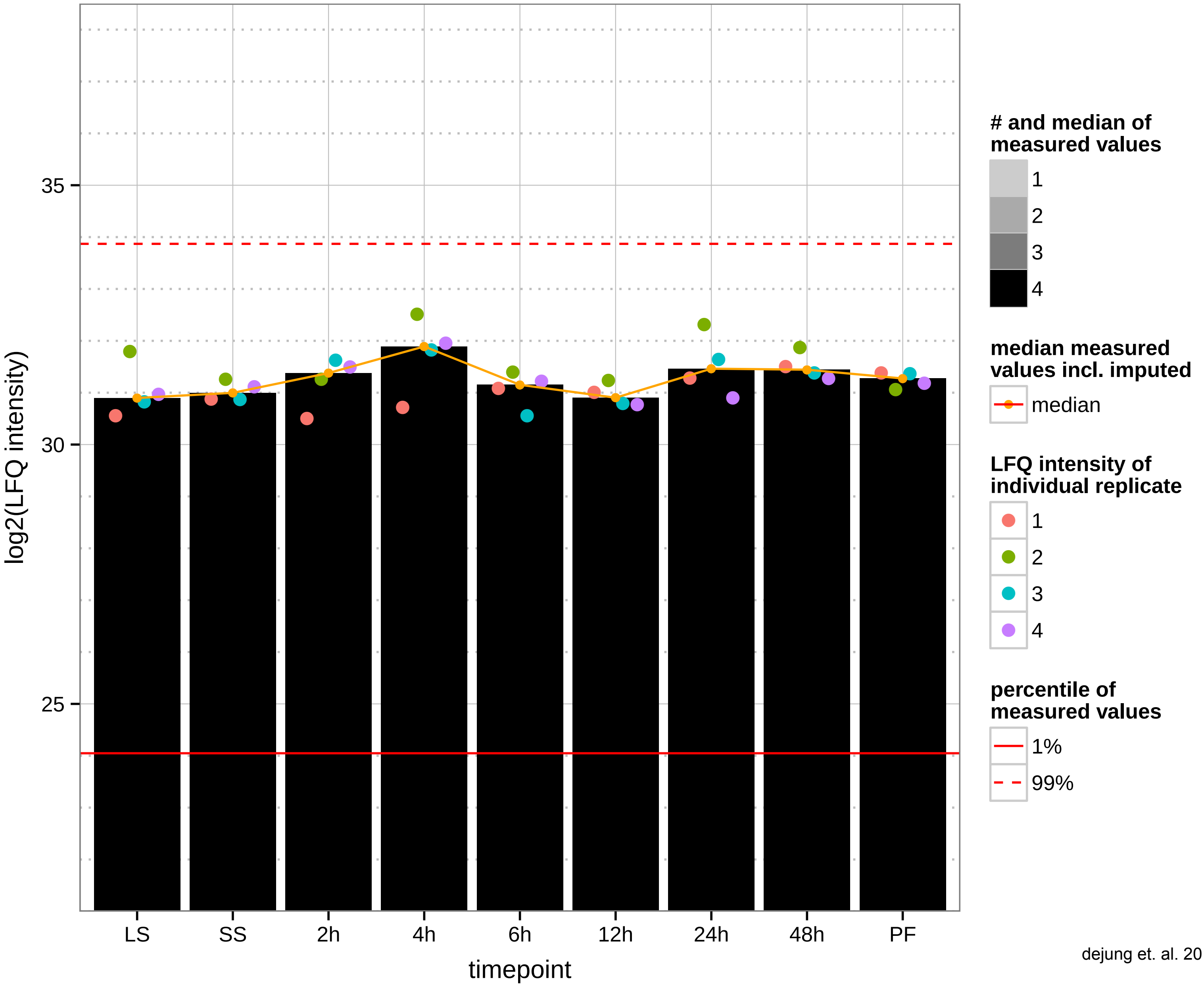

dejung et. al. 2015

ALBA–Domain Protein (ALBA1)
Tb927.11.4460
AGOF: nucleic acid binding
AGOC: cytoplasm, cytoplasmic stress granule
AGOP: null
PGOF: nucleic acid binding
PGOC: null
PGOP: null

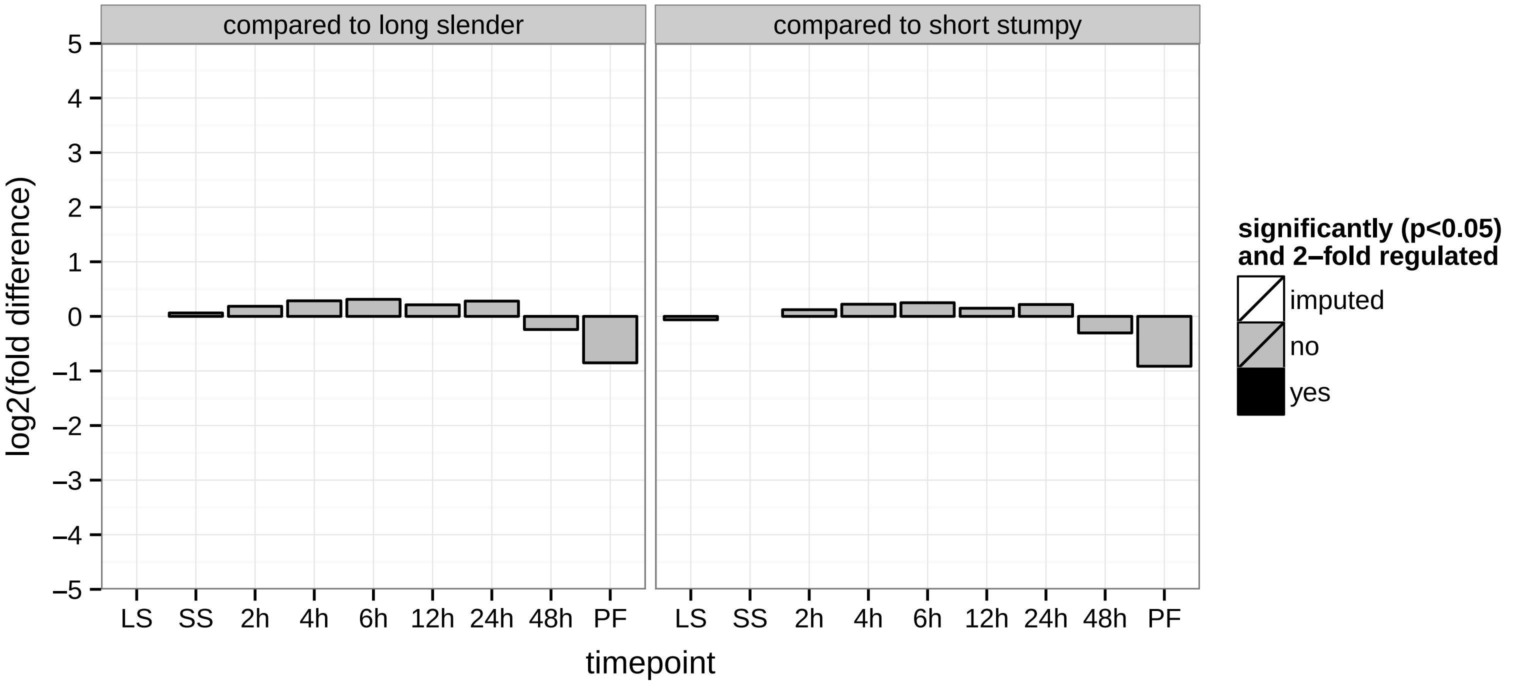

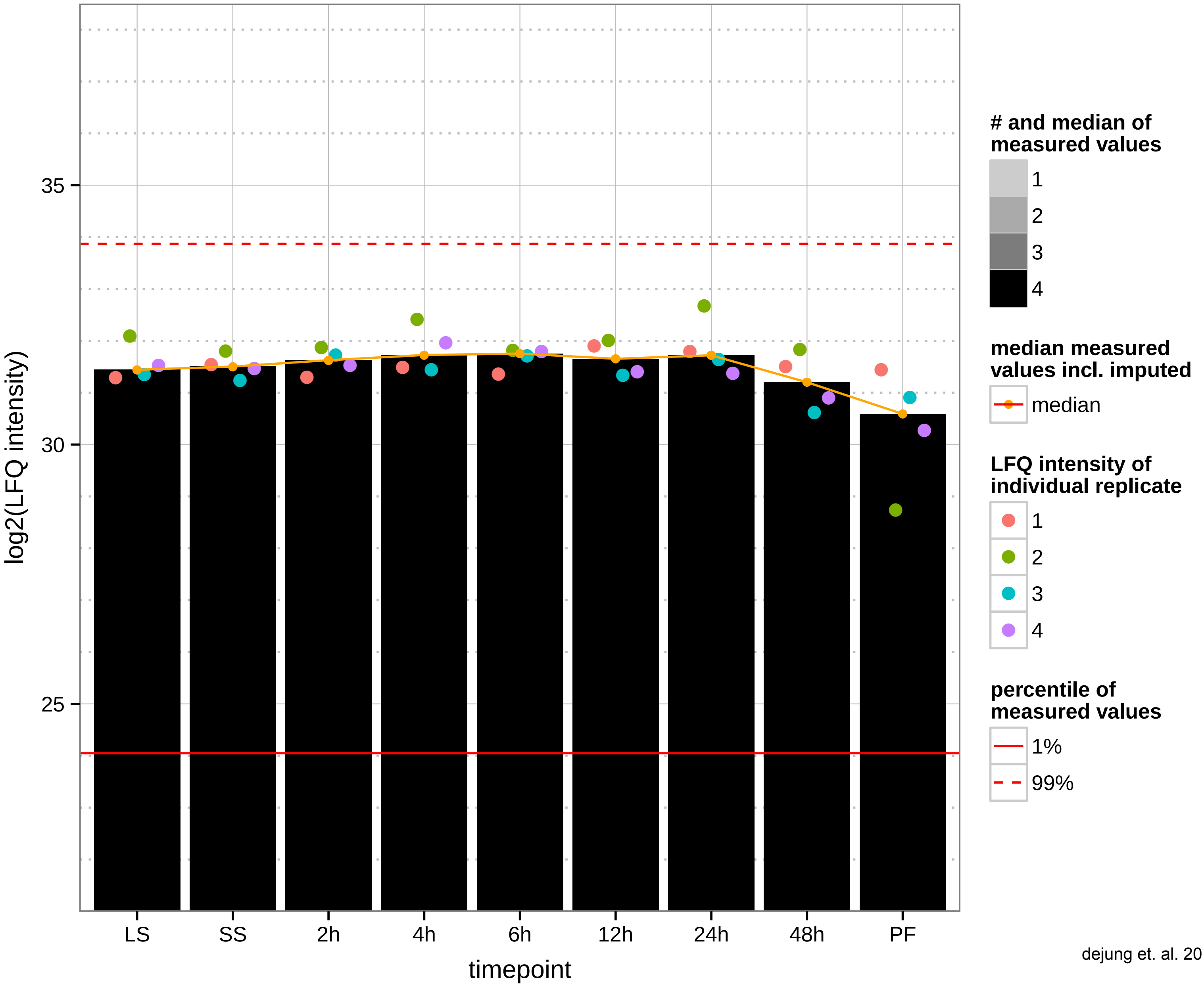

dejung et. al. 2015

radial spoke protein RSP4/6, putative
Tb927.11.4480
AGOF: null
AGOC: radial spoke
AGOP: ciliary or flagellar motility
PGOF: null
PGOC: null
PGOP: null

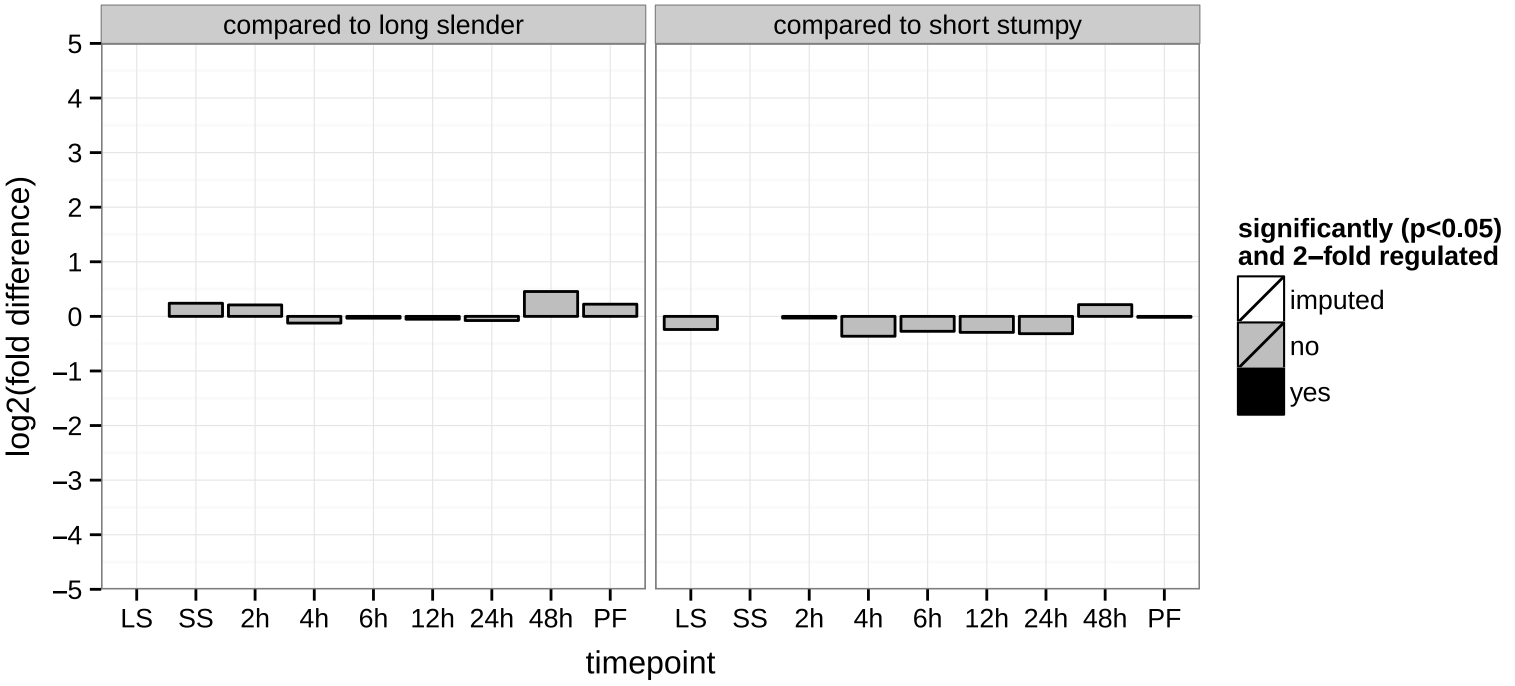

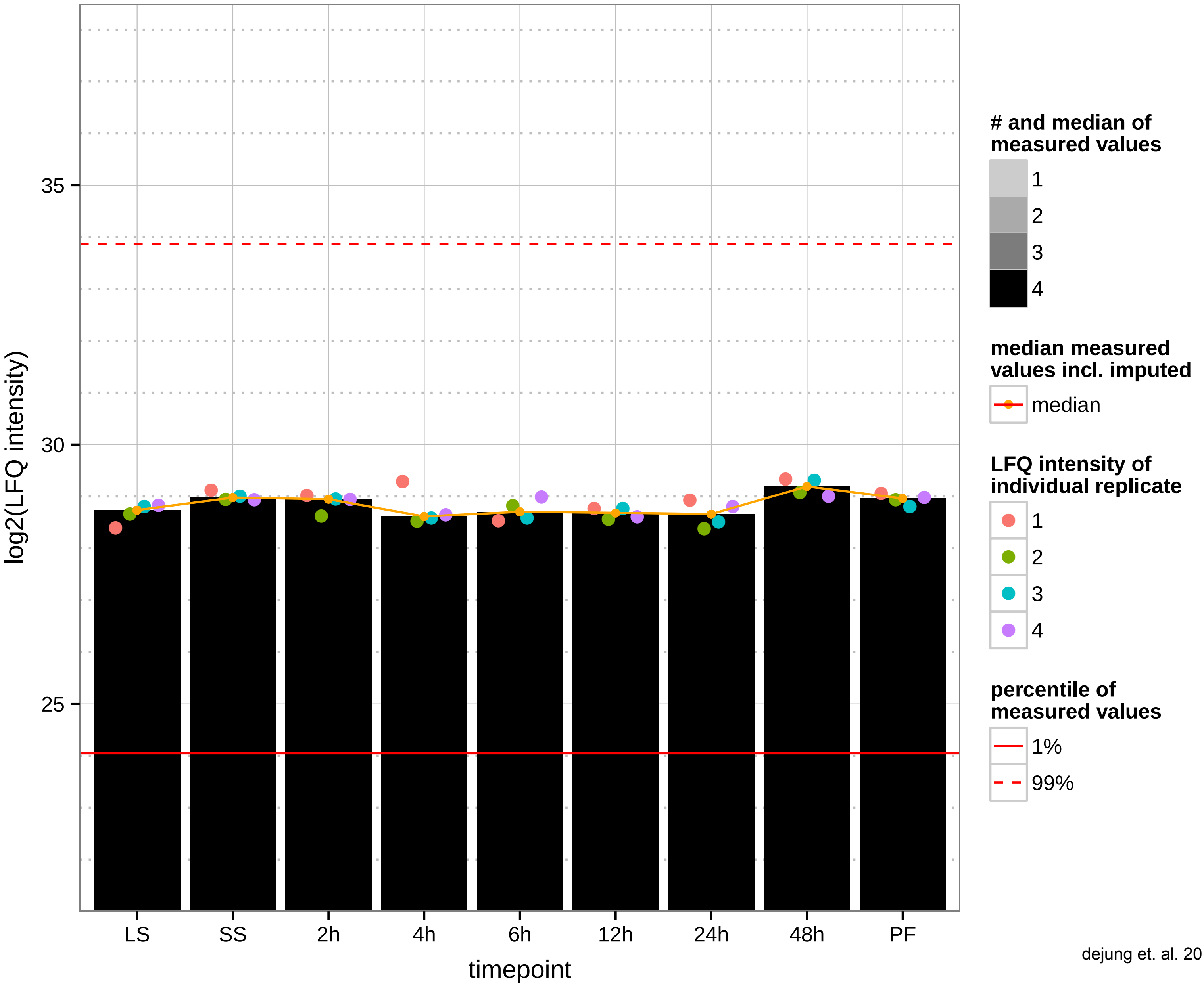

dejung et. al. 2015

hypothetical protein, conserved
Tb927.11.450
AGOF: null
AGOC: null
AGOP: null
PGOF: null
PGOC: null
PGOP: null

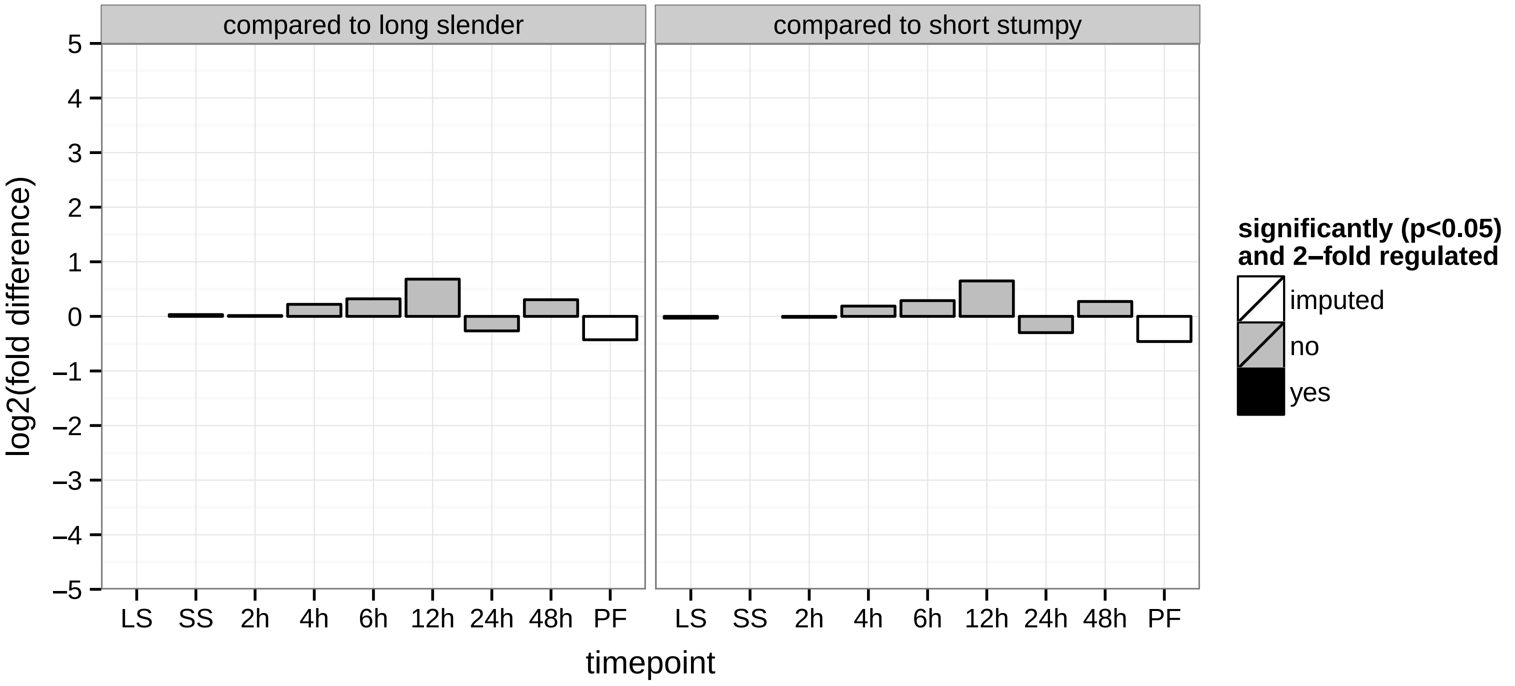

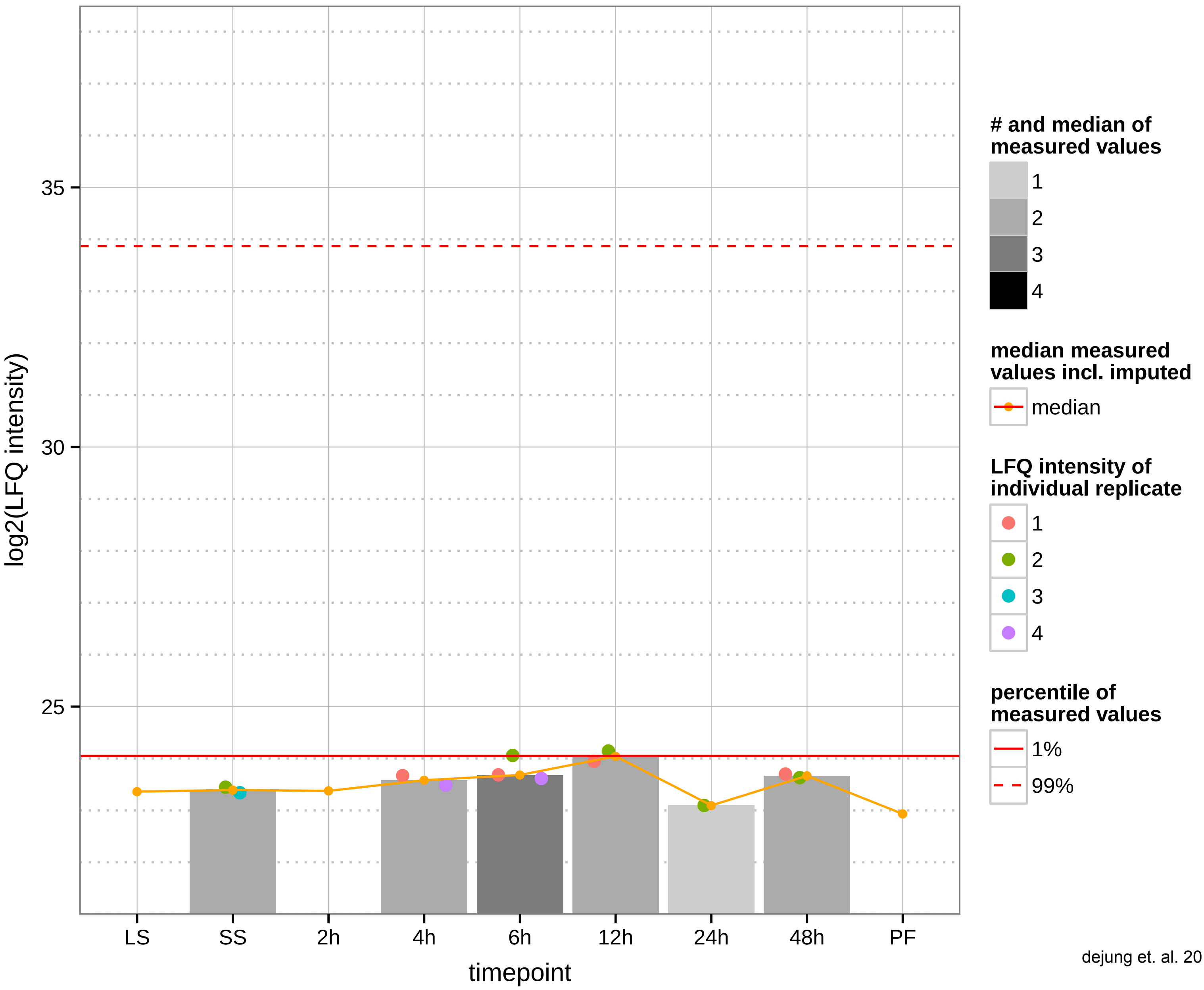

dejung et. al. 2015

hypothetical protein, conserved
Tb927.11.4510
AGOF: null
AGOC: null
AGOP: null
PGOF: binding
PGOC: null
PGOP: null

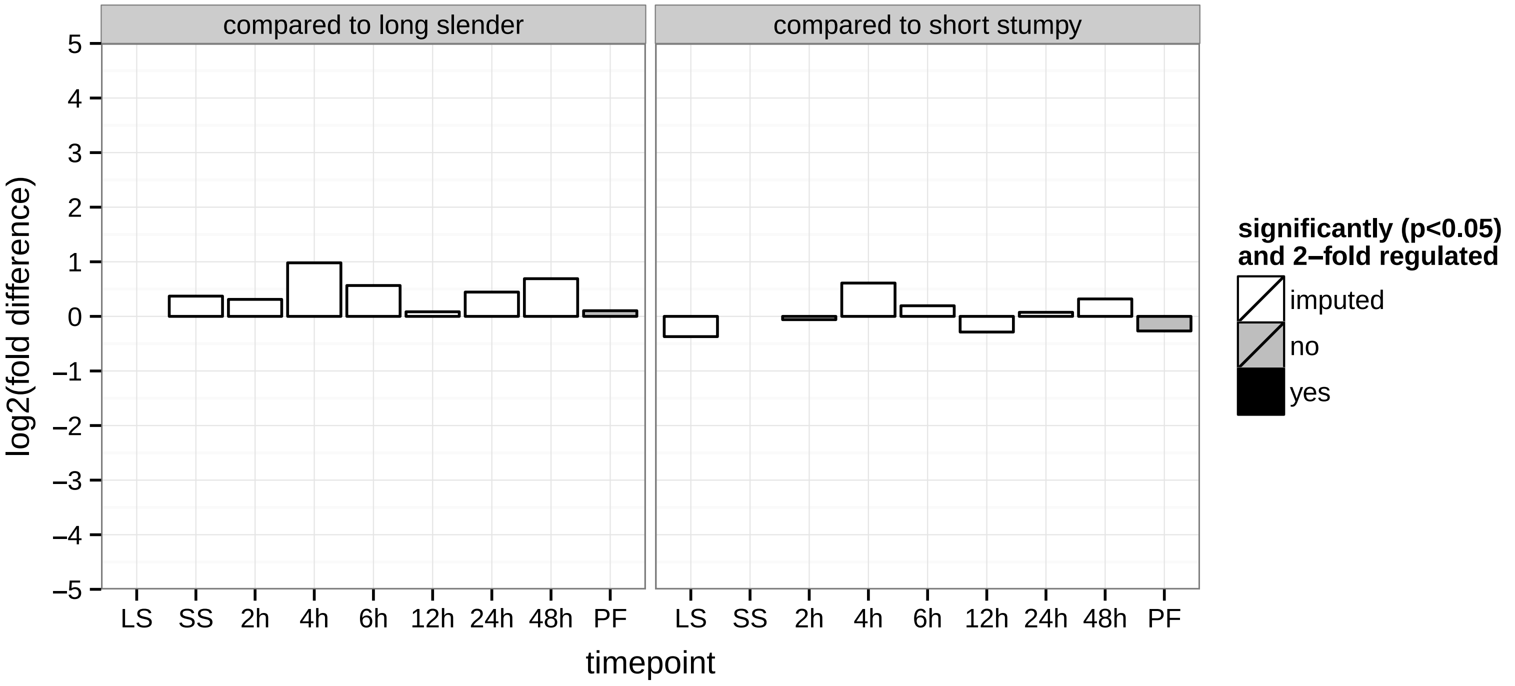

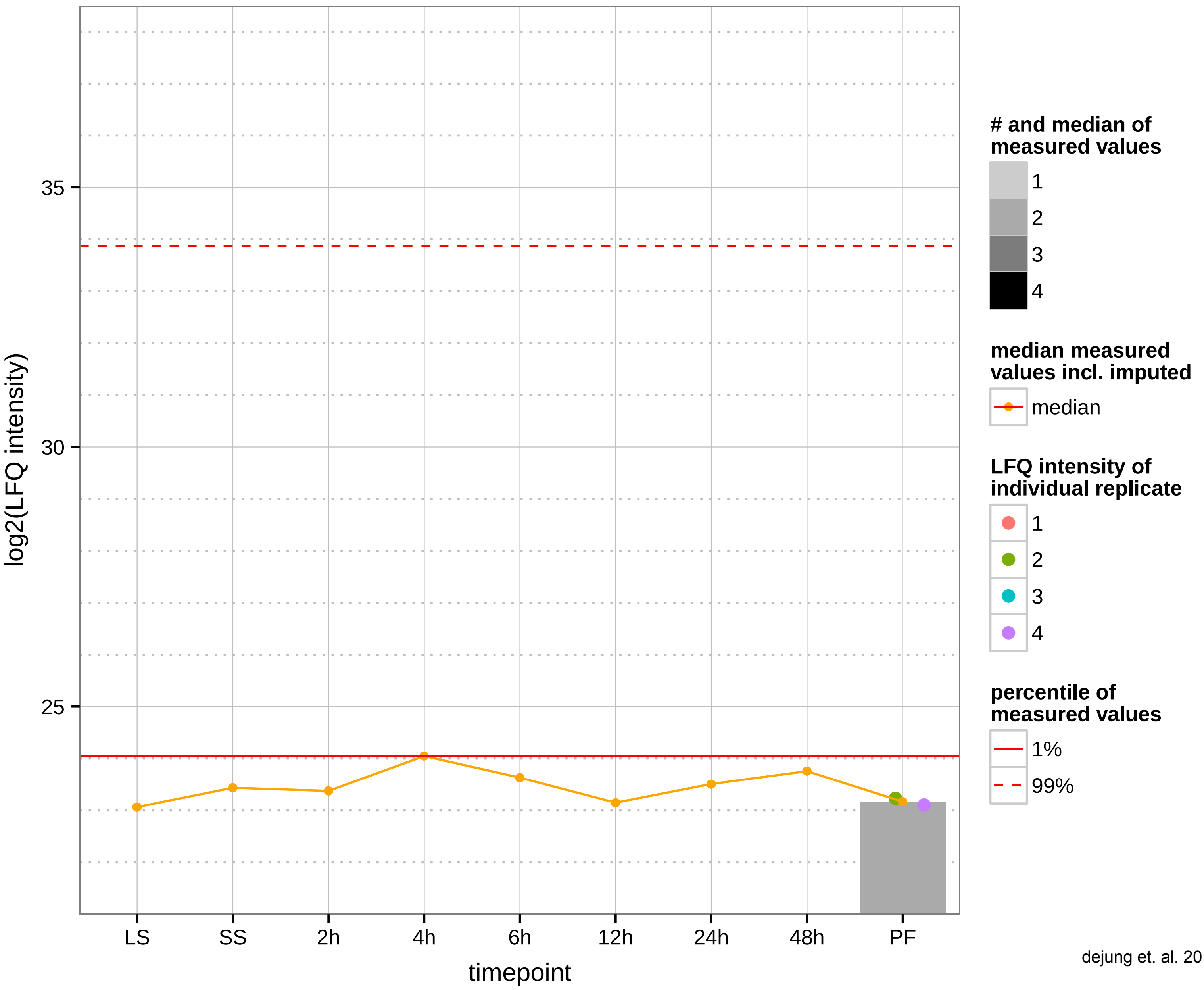

dejung et. al. 2015

hypothetical protein, conserved
Tb927.11.4810
AGOF: null
AGOC: mitochondrion
AGOP: null
PGOF: null
PGOC: null
PGOP: null

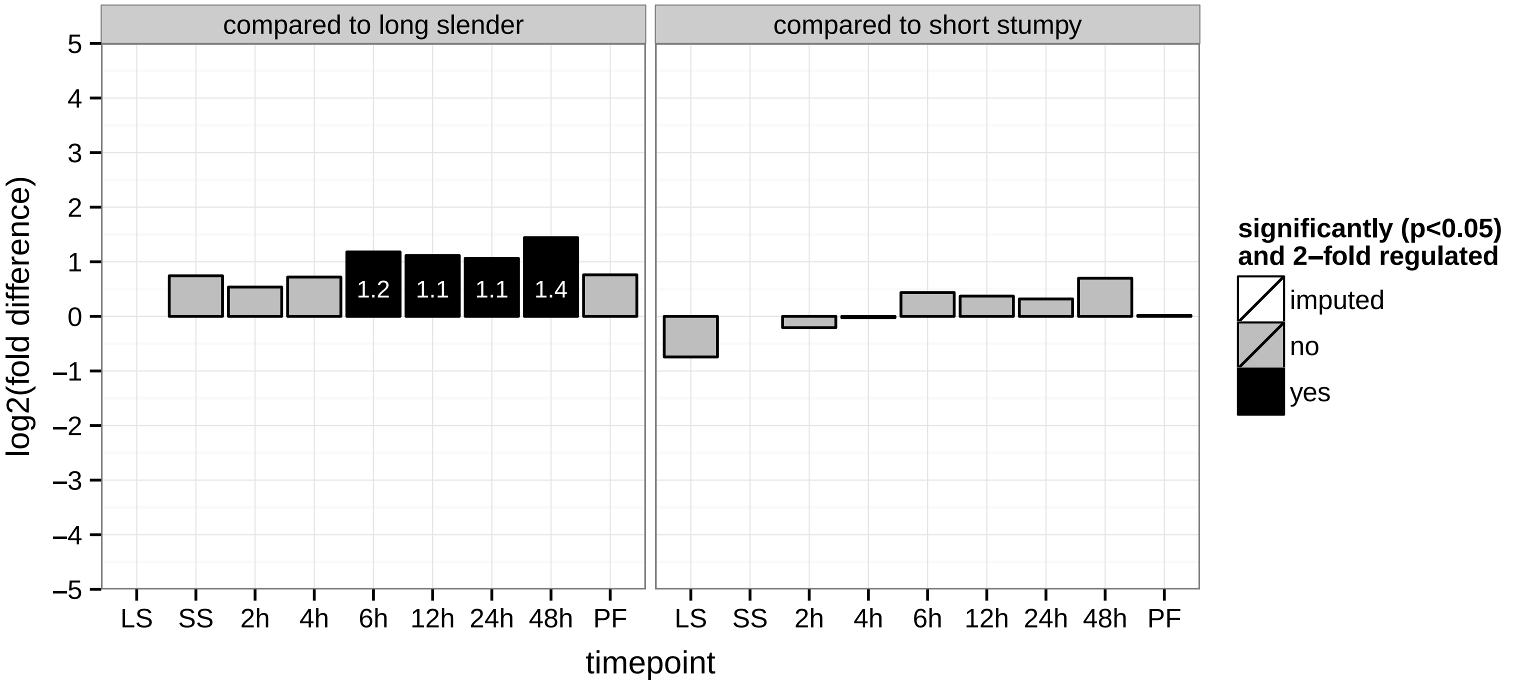

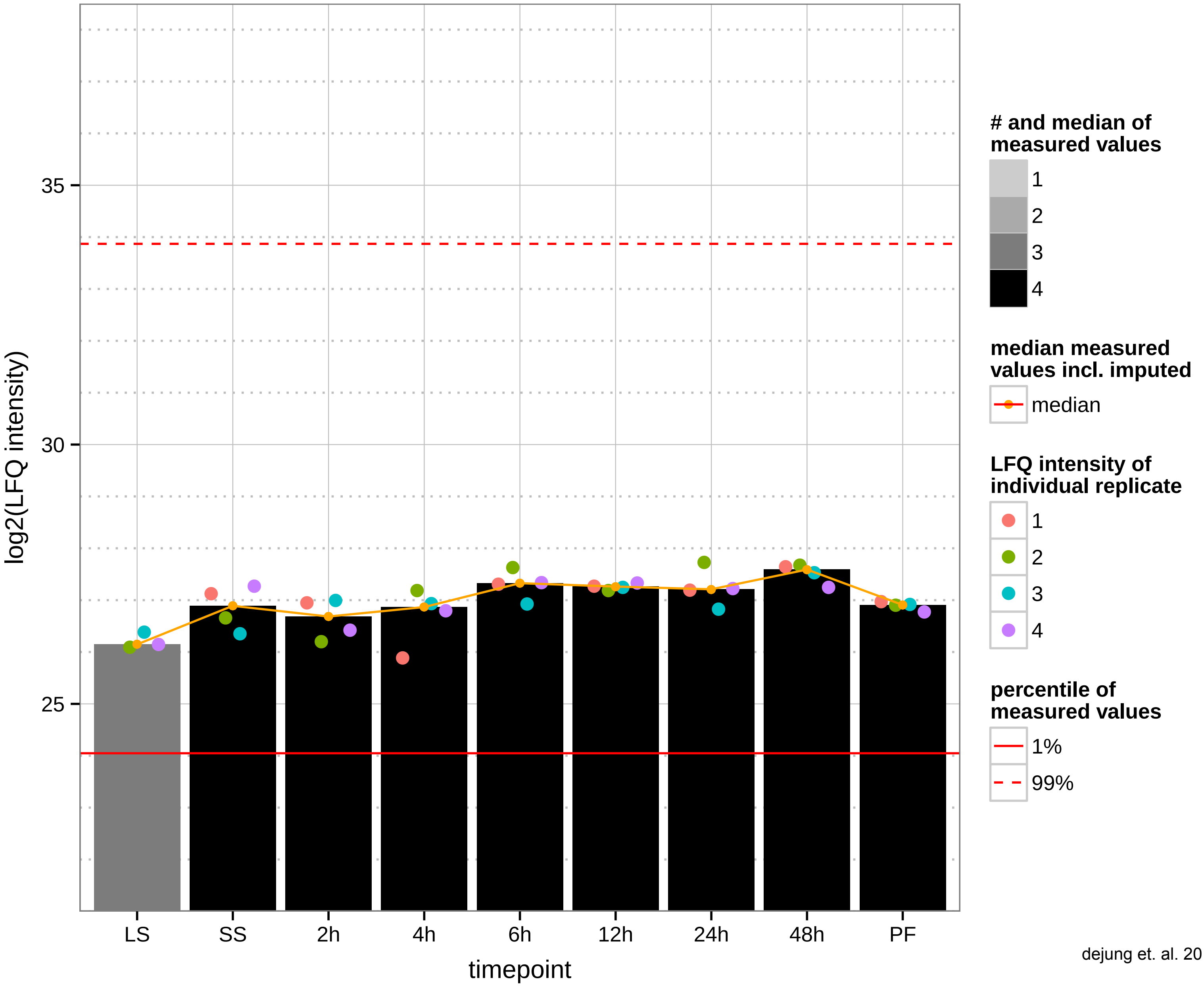

dejung et. al. 2015

hypothetical protein, conserved
Tb927.11.4880
AGOF: null
AGOC: null
AGOP: null
PGOF: null
PGOC: null
PGOP: null

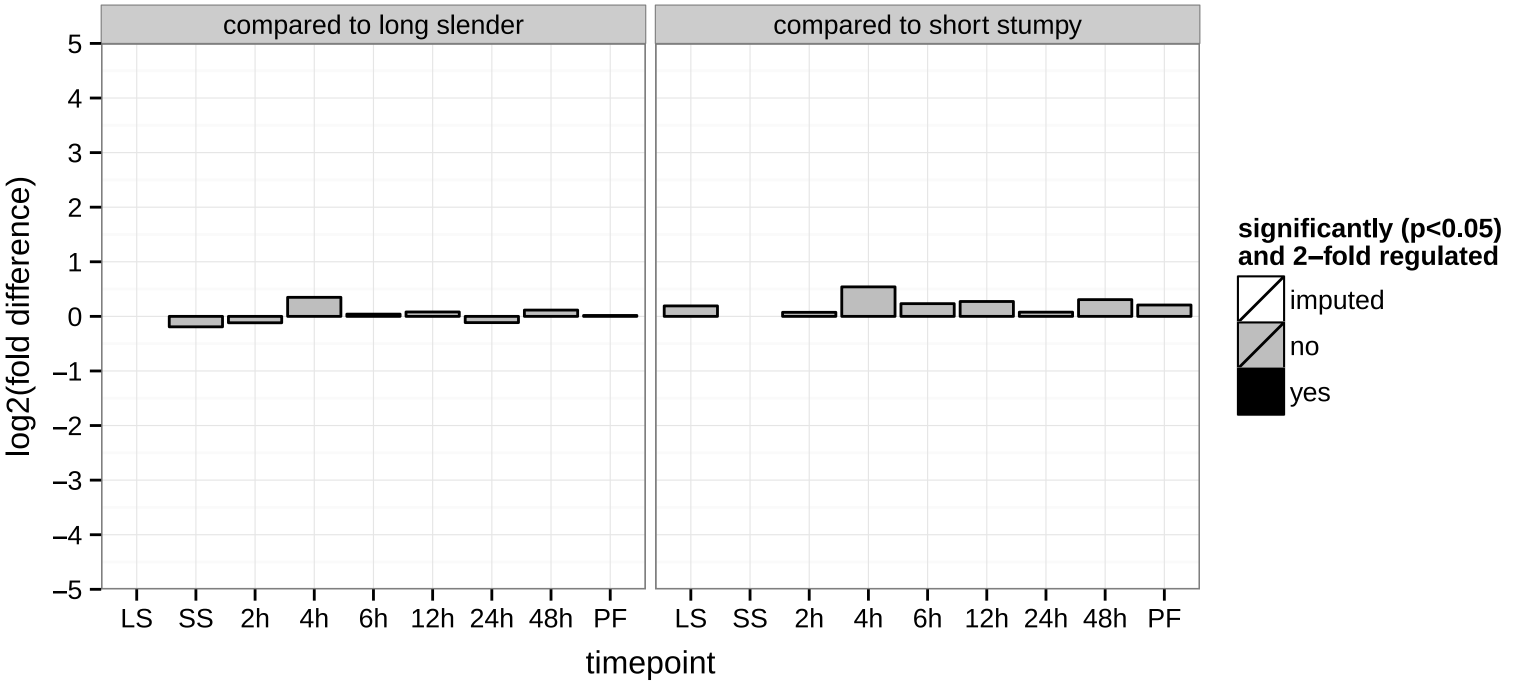

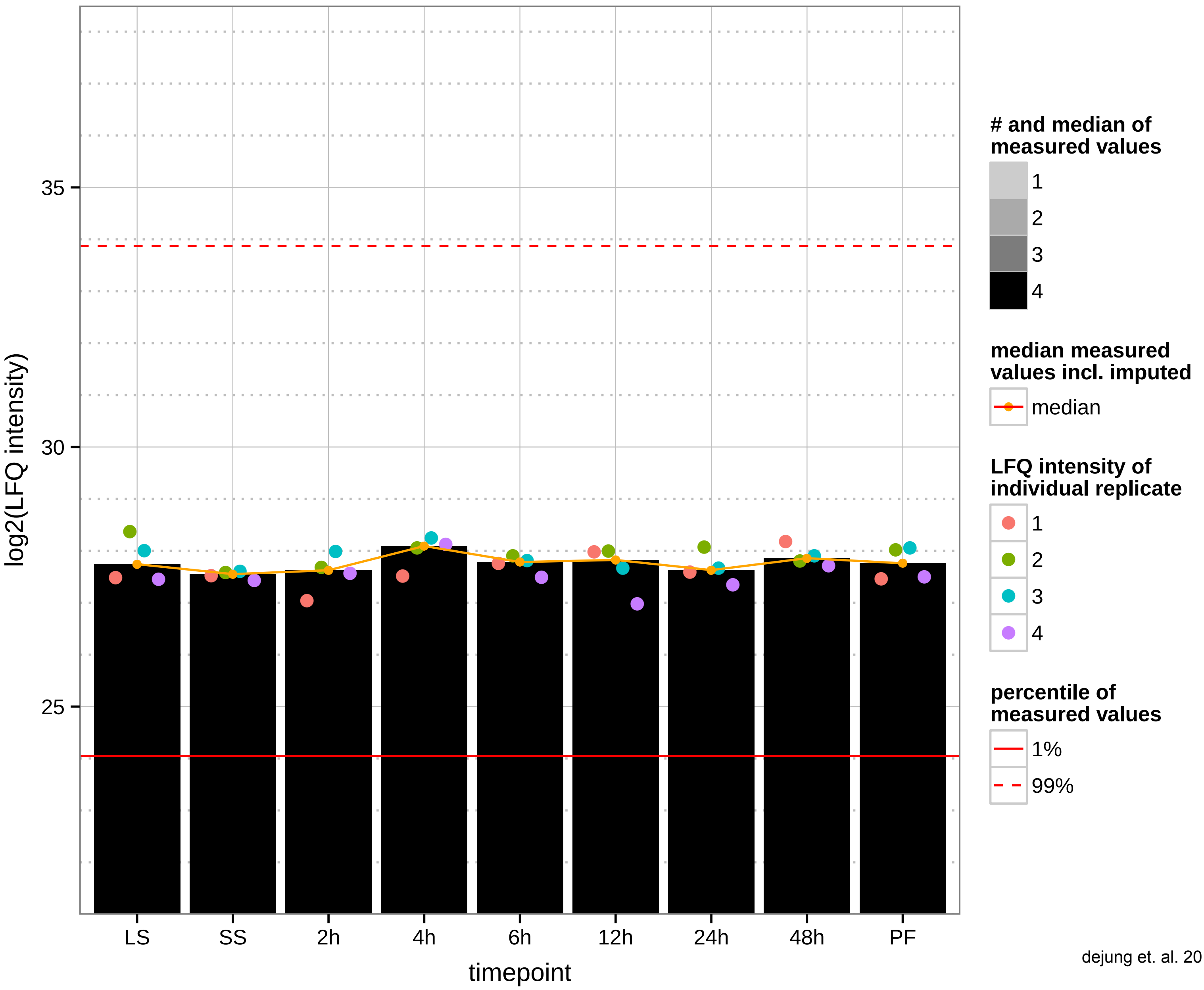

dejung et. al. 2015

hypothetical protein, conserved
Tb927.11.4890
AGOF: mRNA (nucleoside−2'−O−)−methyltransferase activity
AGOC: null
AGOP: 7−methylguanosine mRNA capping
PGOF: null
PGOC: null
PGOP: null

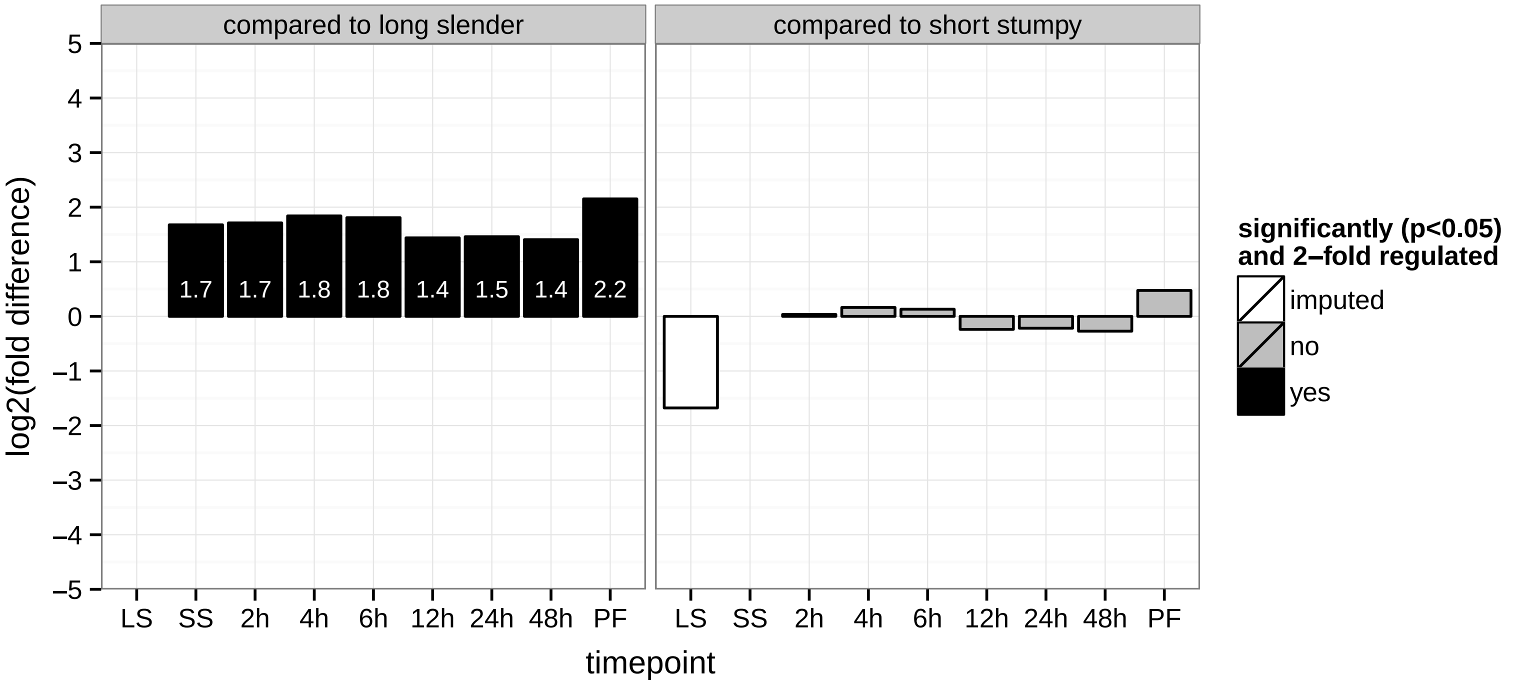

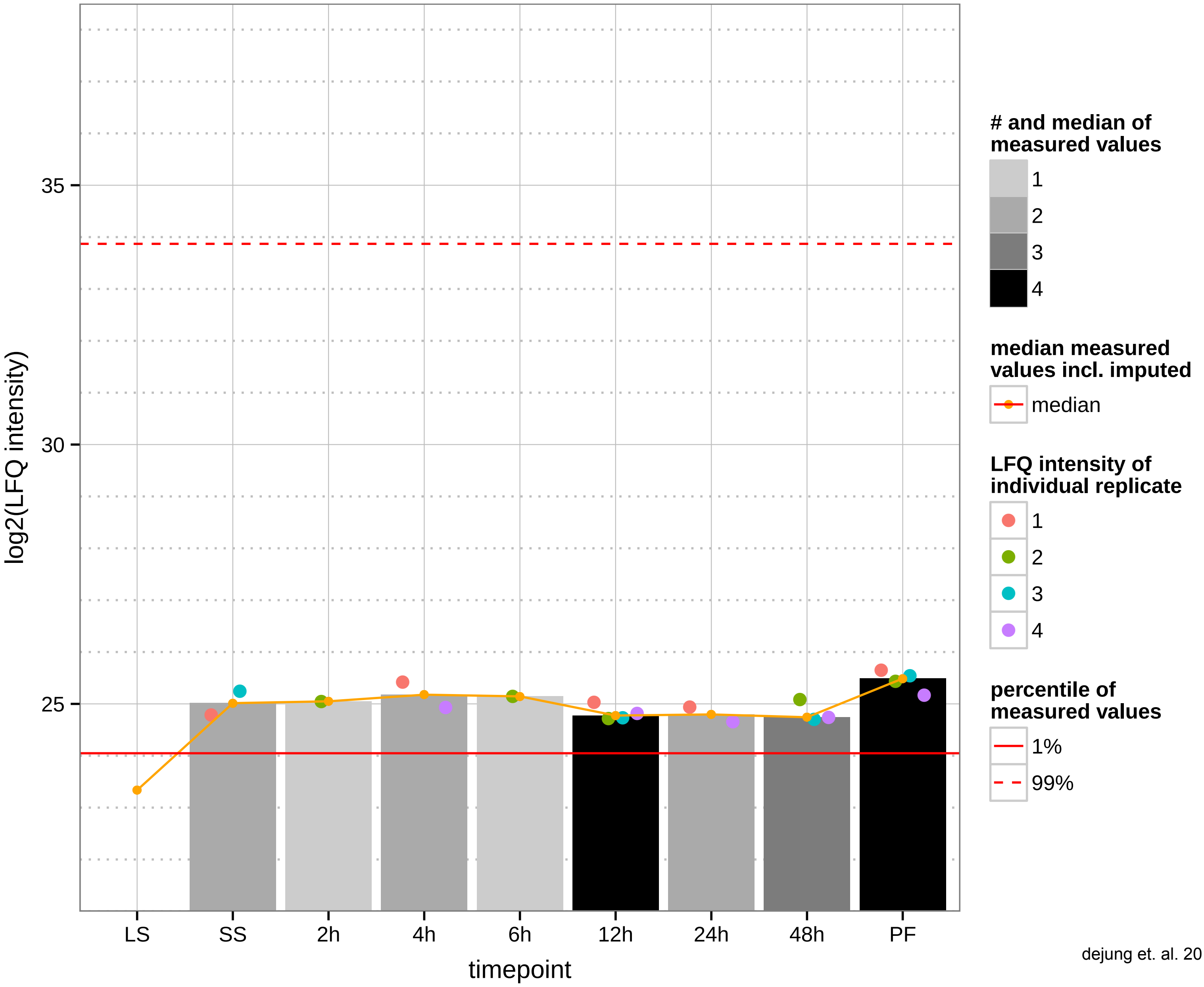

dejung et. al. 2015

guanine nucleotide–binding beta subunit–like protein, G–protein (beta)–like protein, beta transducin–like protein
Tb927.11.4900
AGOF: null
AGOC: null
AGOP: null
PGOF: protein binding
PGOC: null
PGOP: null

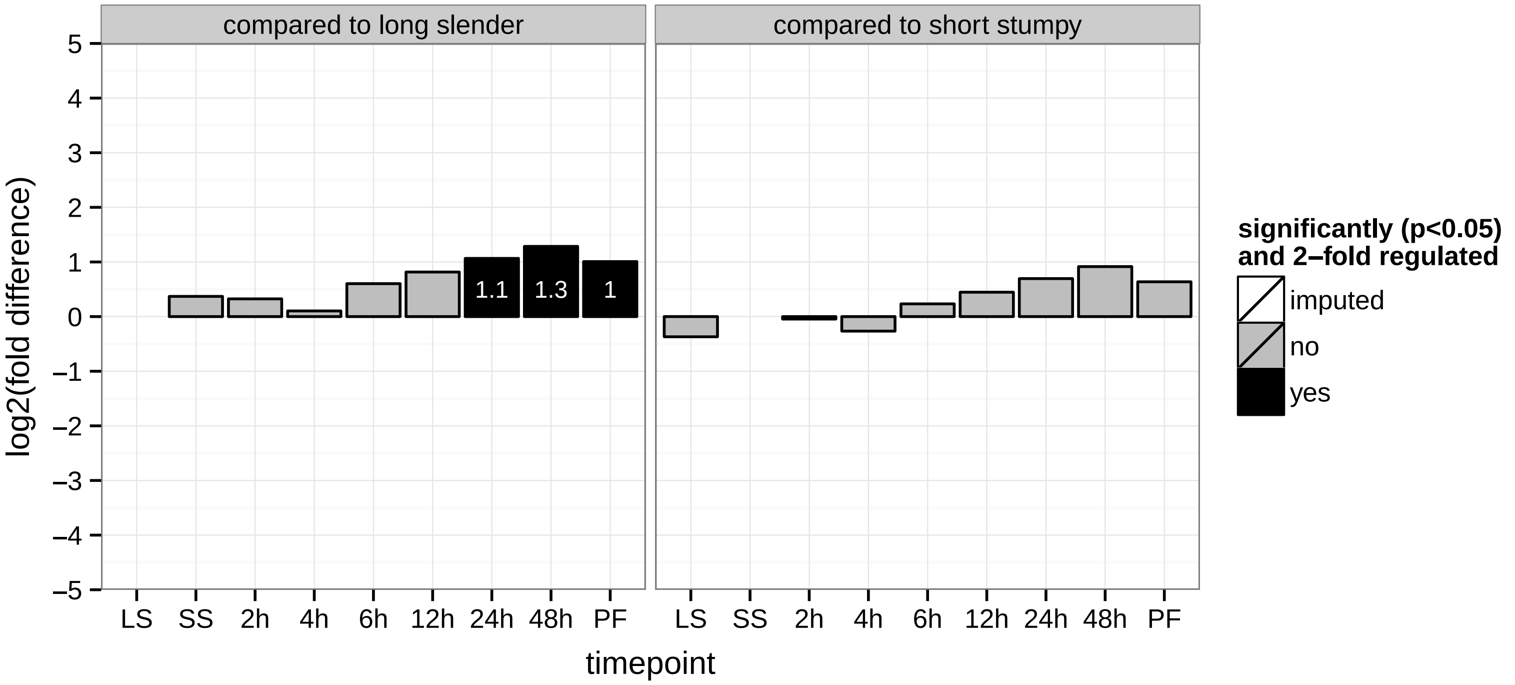

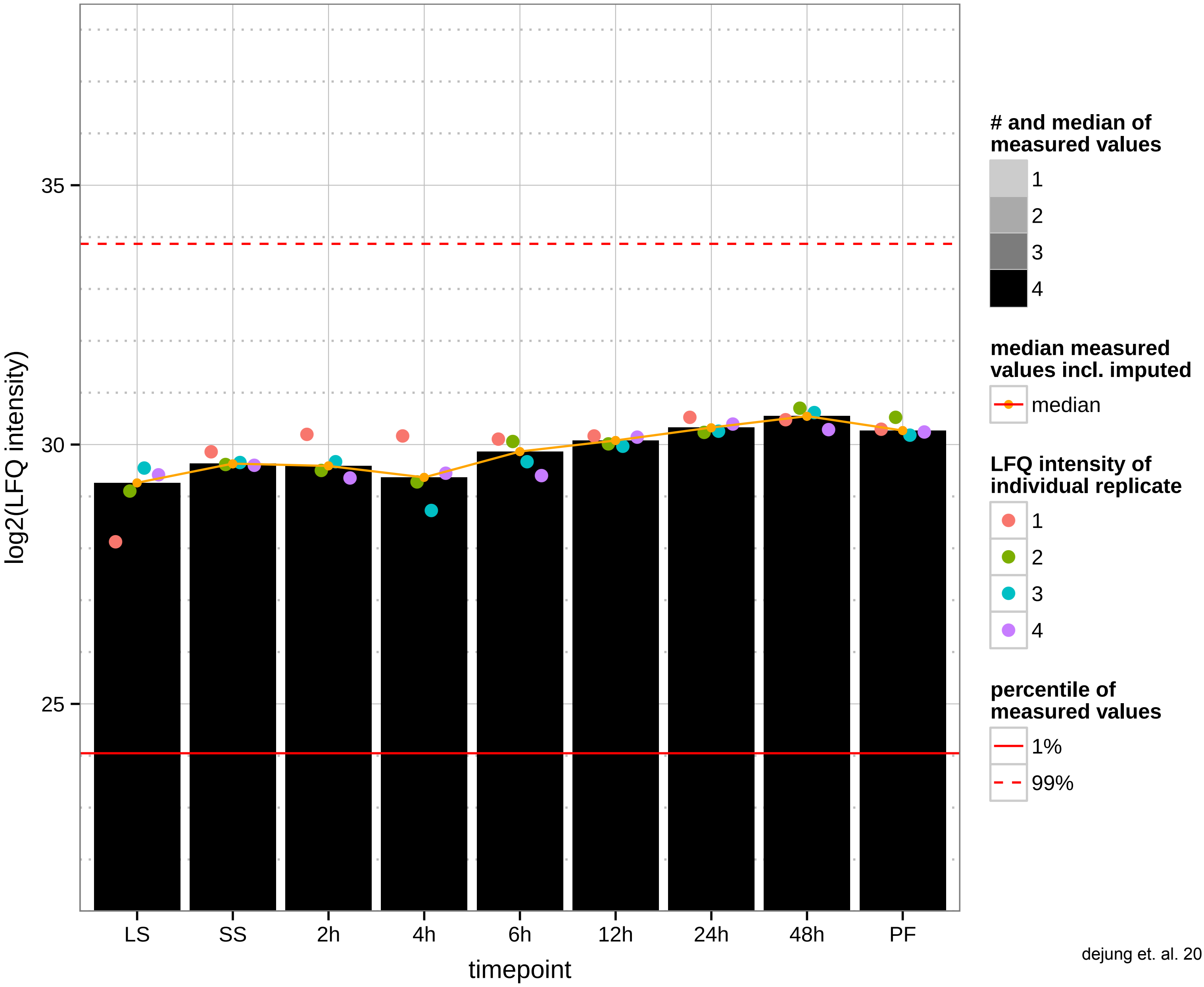

dejung et. al. 2015

hypothetical protein, conserved
Tb927.11.4920
AGOF: null
AGOC: null
AGOP: null
PGOF: null
PGOC: null
PGOP: null

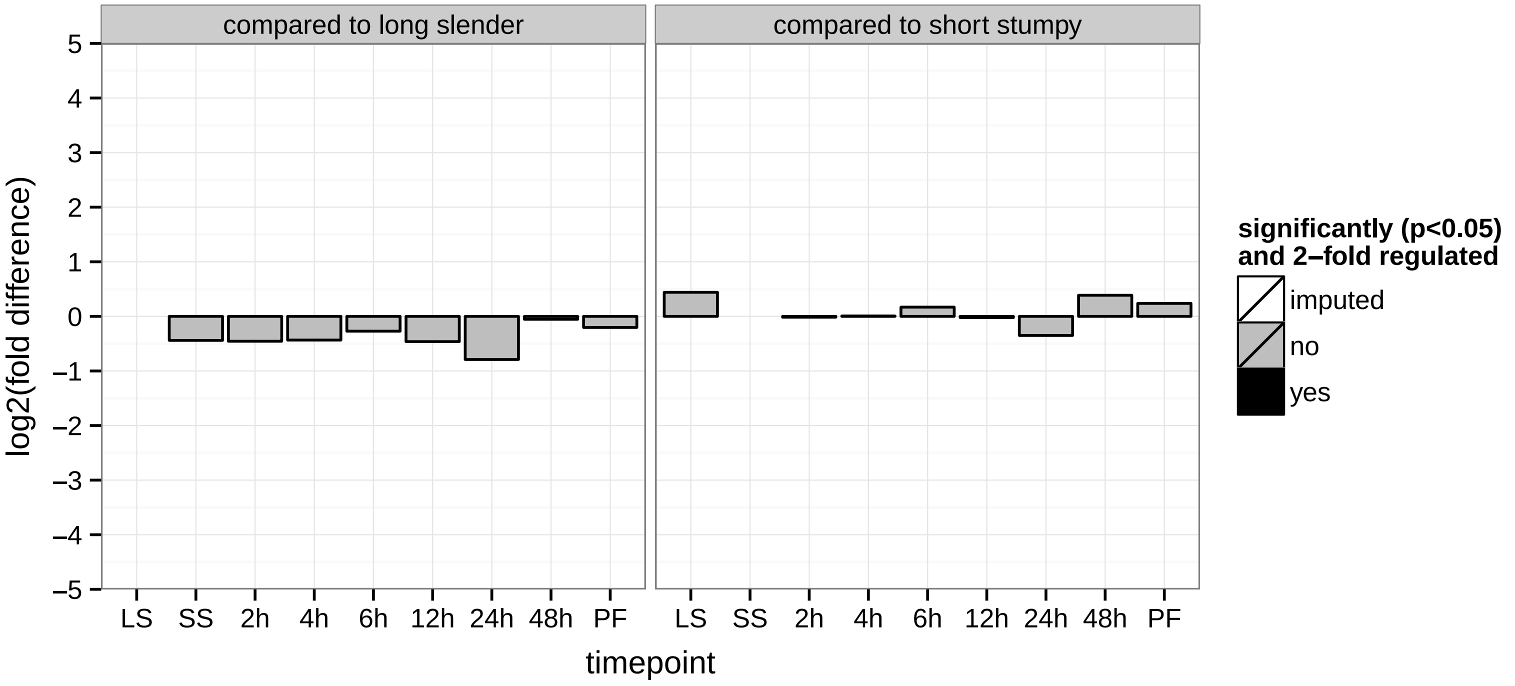

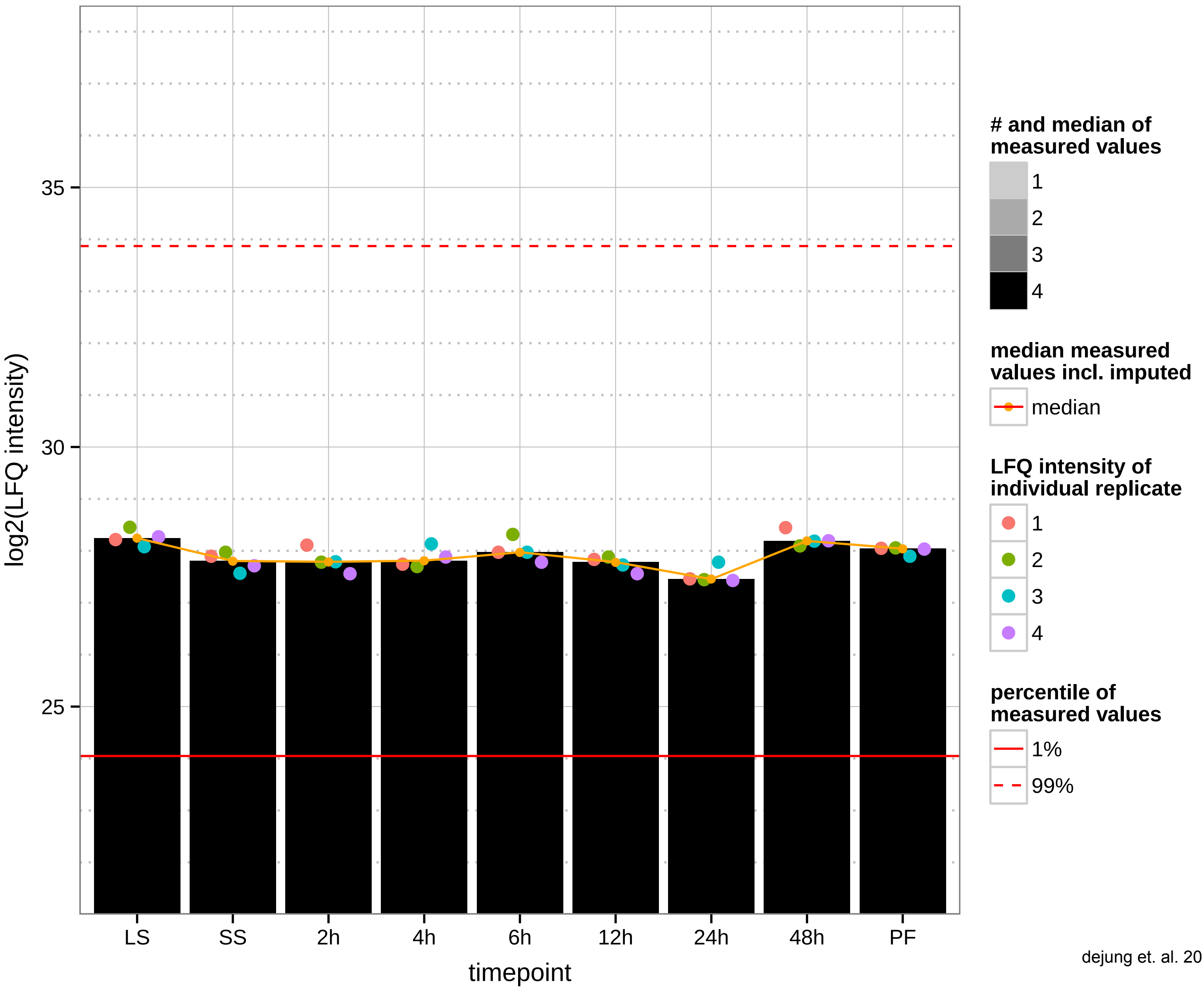

dejung et. al. 2015

hypothetical protein, conserved
Tb927.11.4950
AGOF: null
AGOC: null
AGOP: null
PGOF: binding
PGOC: null
PGOP: null

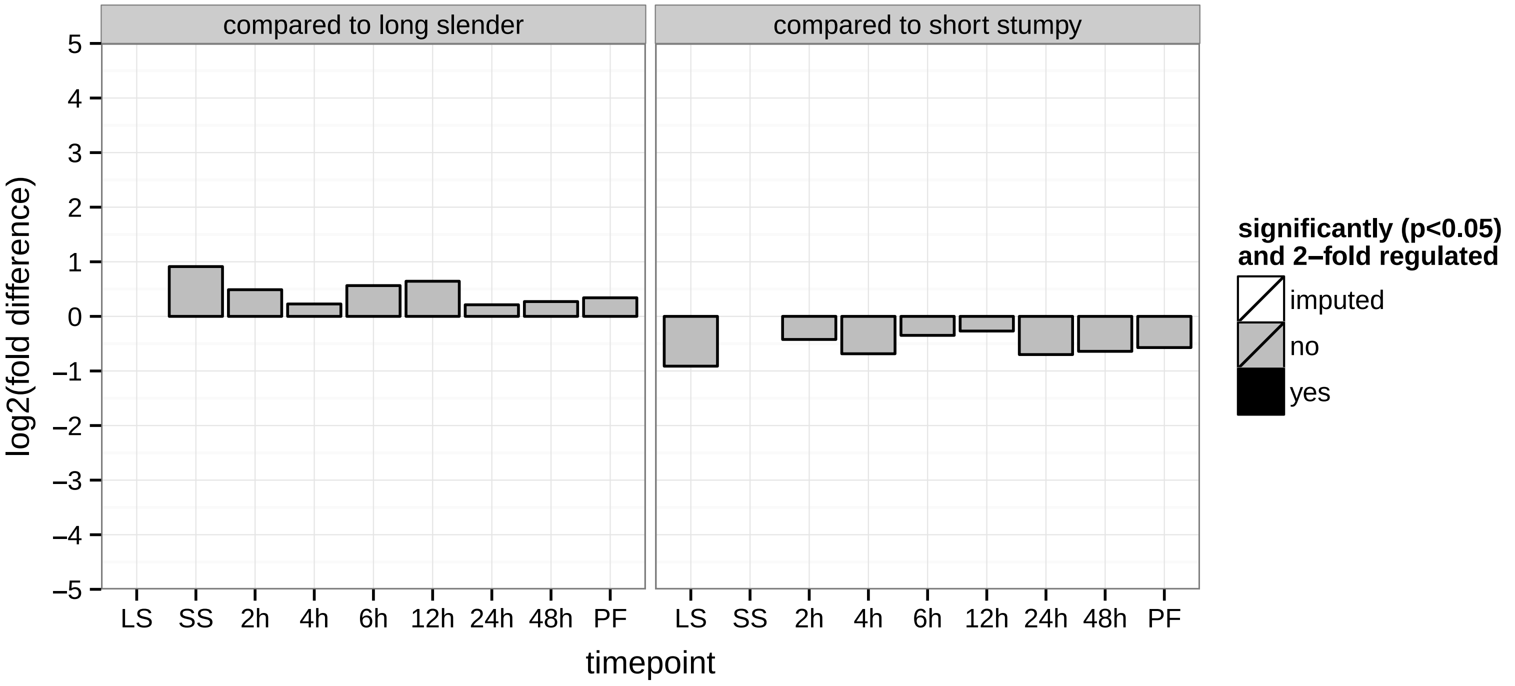

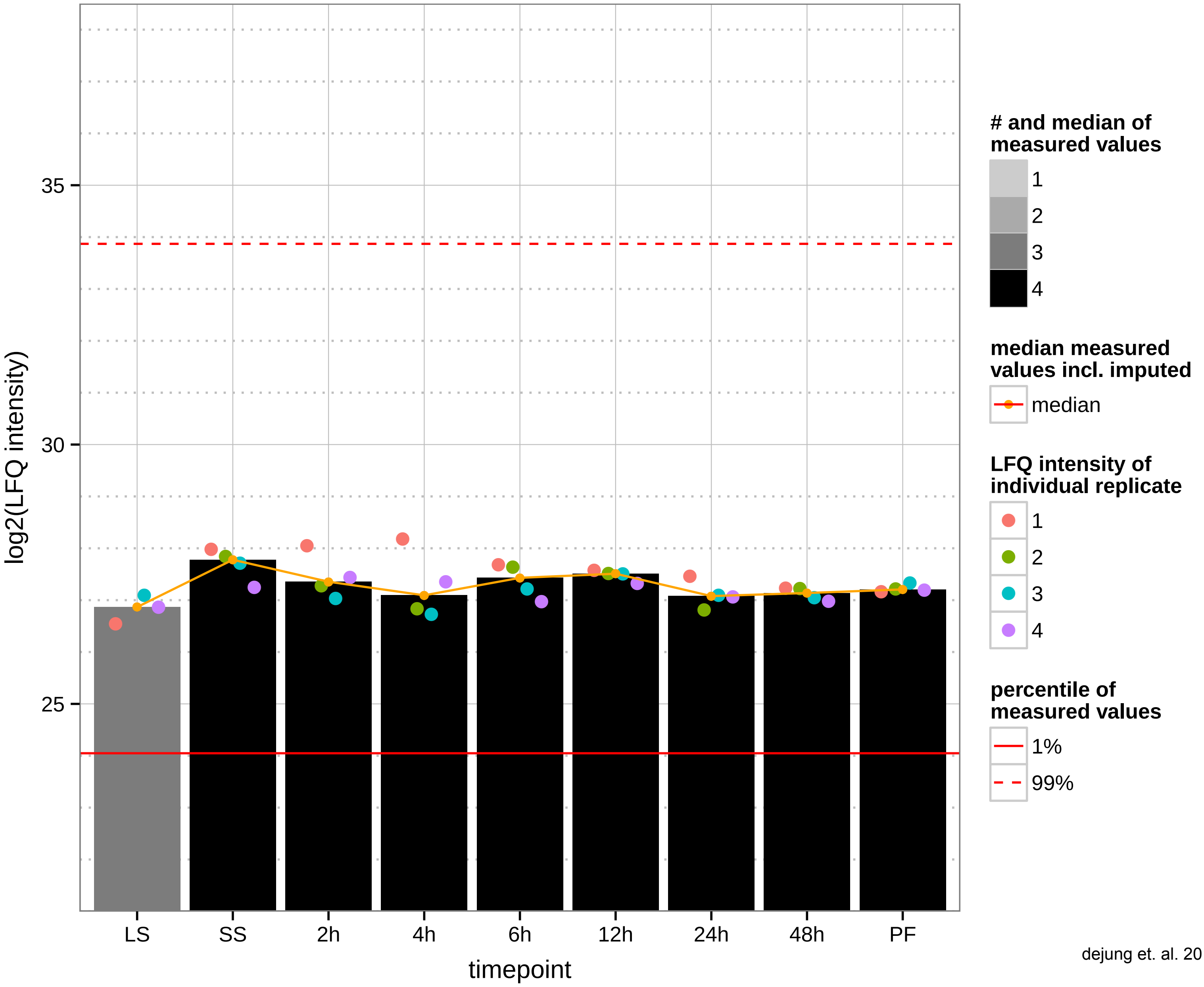

dejung et. al. 2015

predicted WD40 repeat protein
Tb927.11.4970
AGOF: null
AGOC: null
AGOP: null
PGOF: protein binding
PGOC: null
PGOP: null

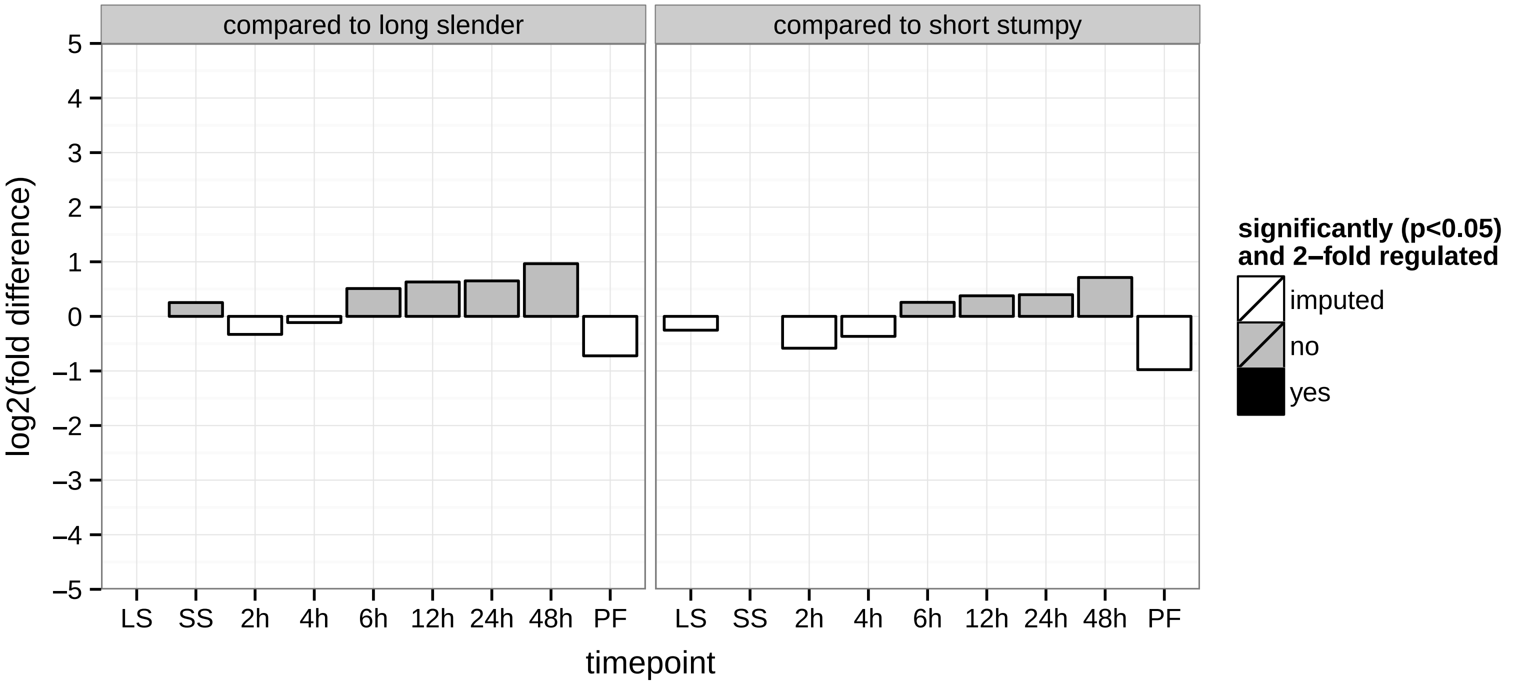

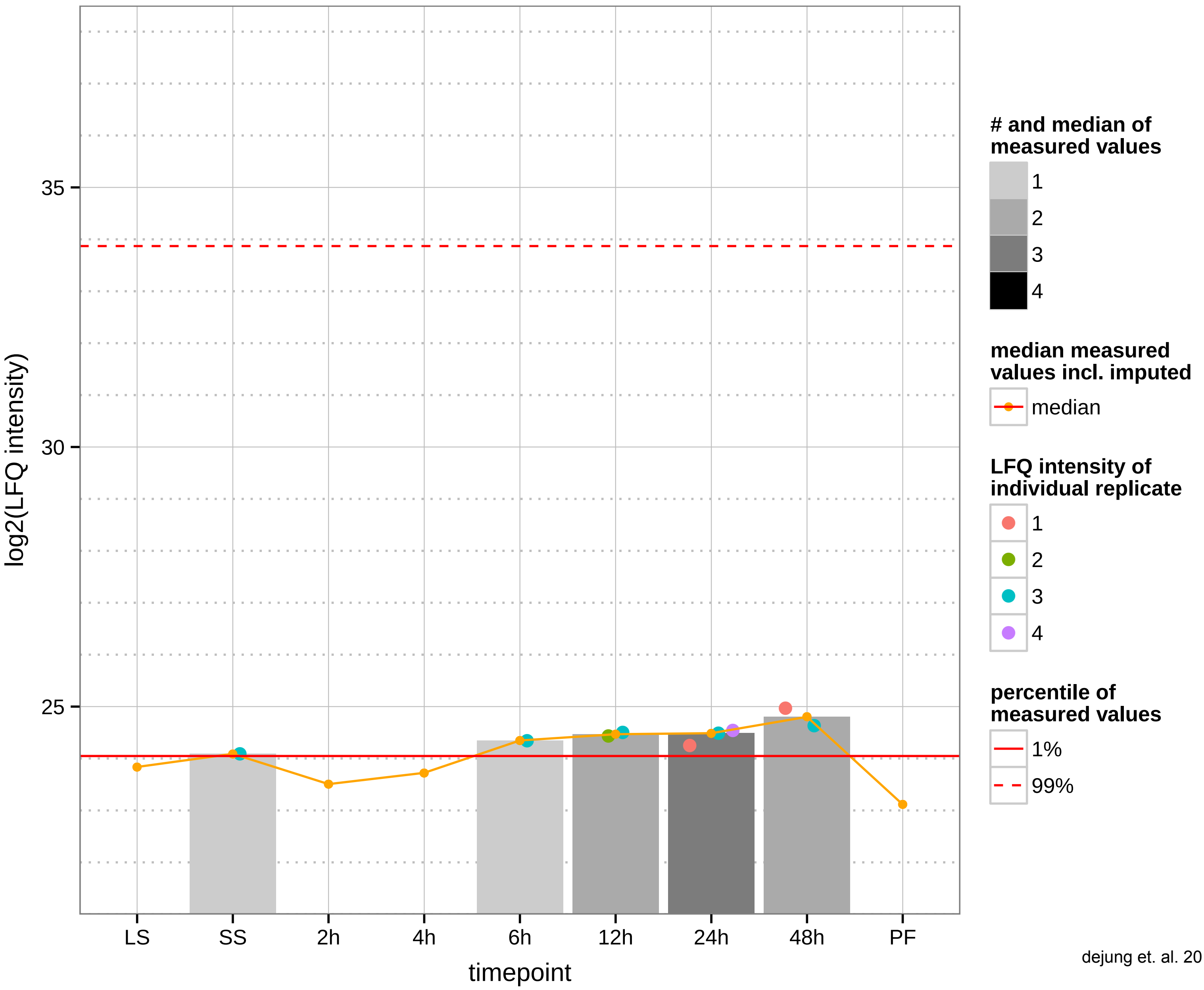

dejung et. al. 2015

ATP–dependent DEAD/H RNA helicase, putative, ATP– dependent RNA helicase
Tb927.11.4980
AGOF: ATP binding, ATP–dependent helicase activity, helicase activity, nucleic acid binding
AGOC: null
AGOP: nucleobase–containing compound metabolic process
PGOF: ATP binding, ATP–dependent helicase activity, helicase activity, nucleic acid binding
PGOC: null
PGOP: null

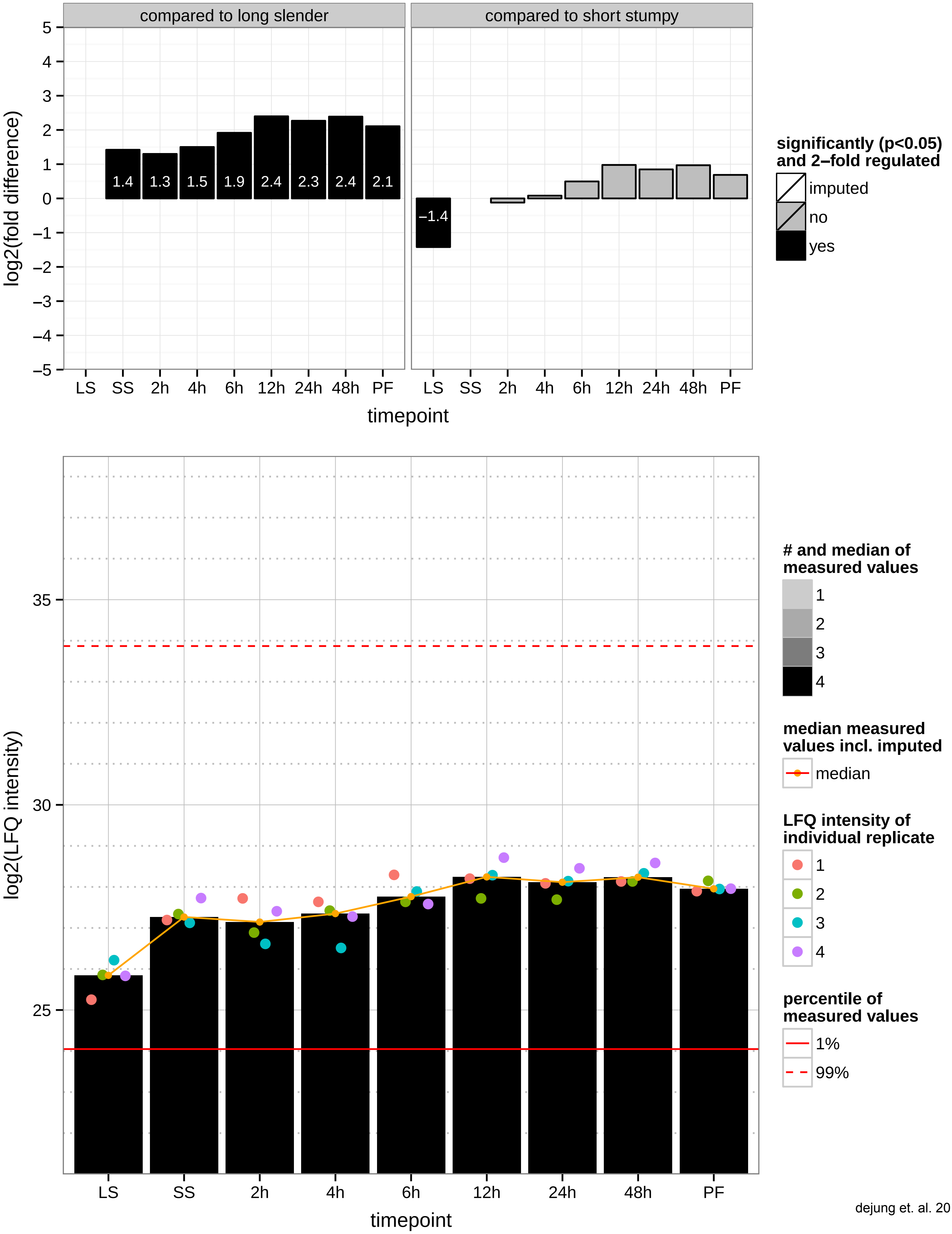

dejung et. al. 2015

dynein intermediate chain IC78, putative
Tb927.11.5000
AGOF: motor activity
AGOC: cytoskeleton, dynein complex
AGOP: microtubule–based movement
PGOF: protein binding
PGOC: null
PGOP: null

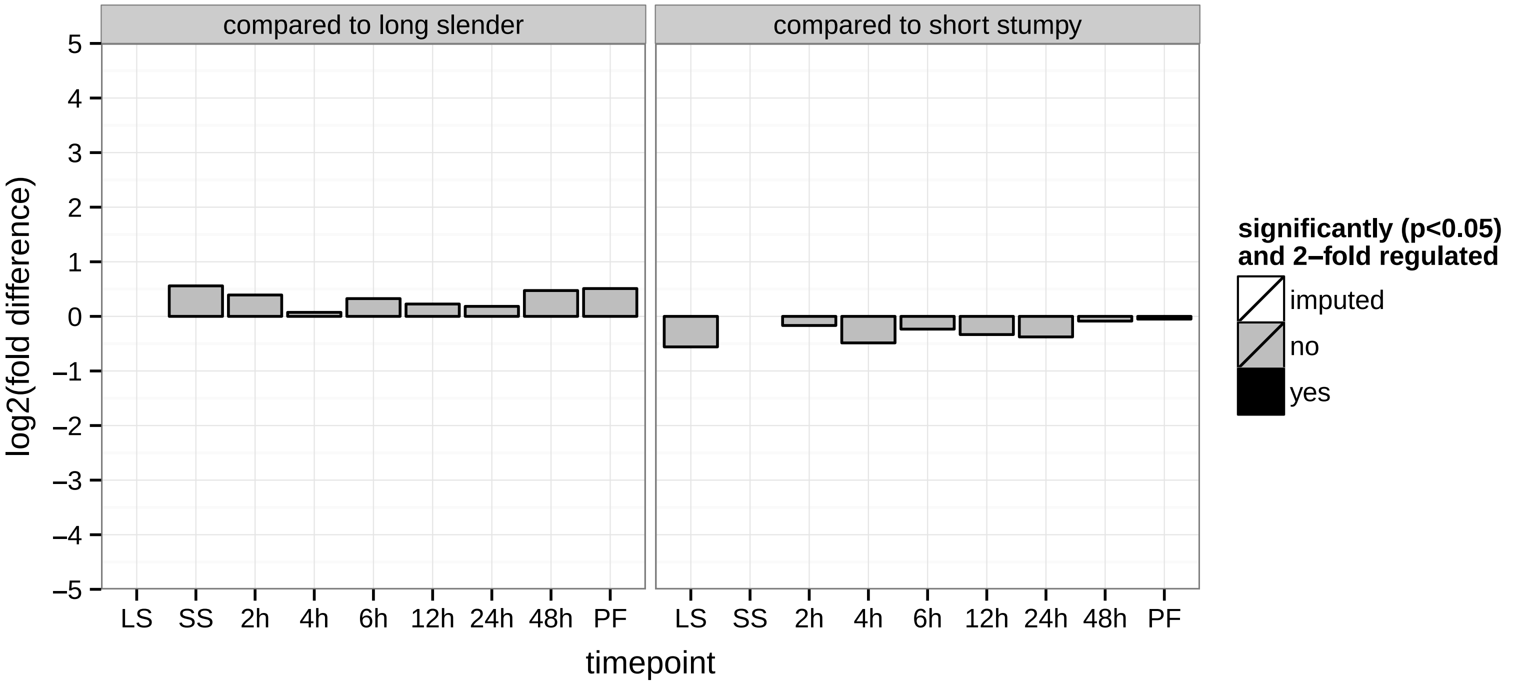

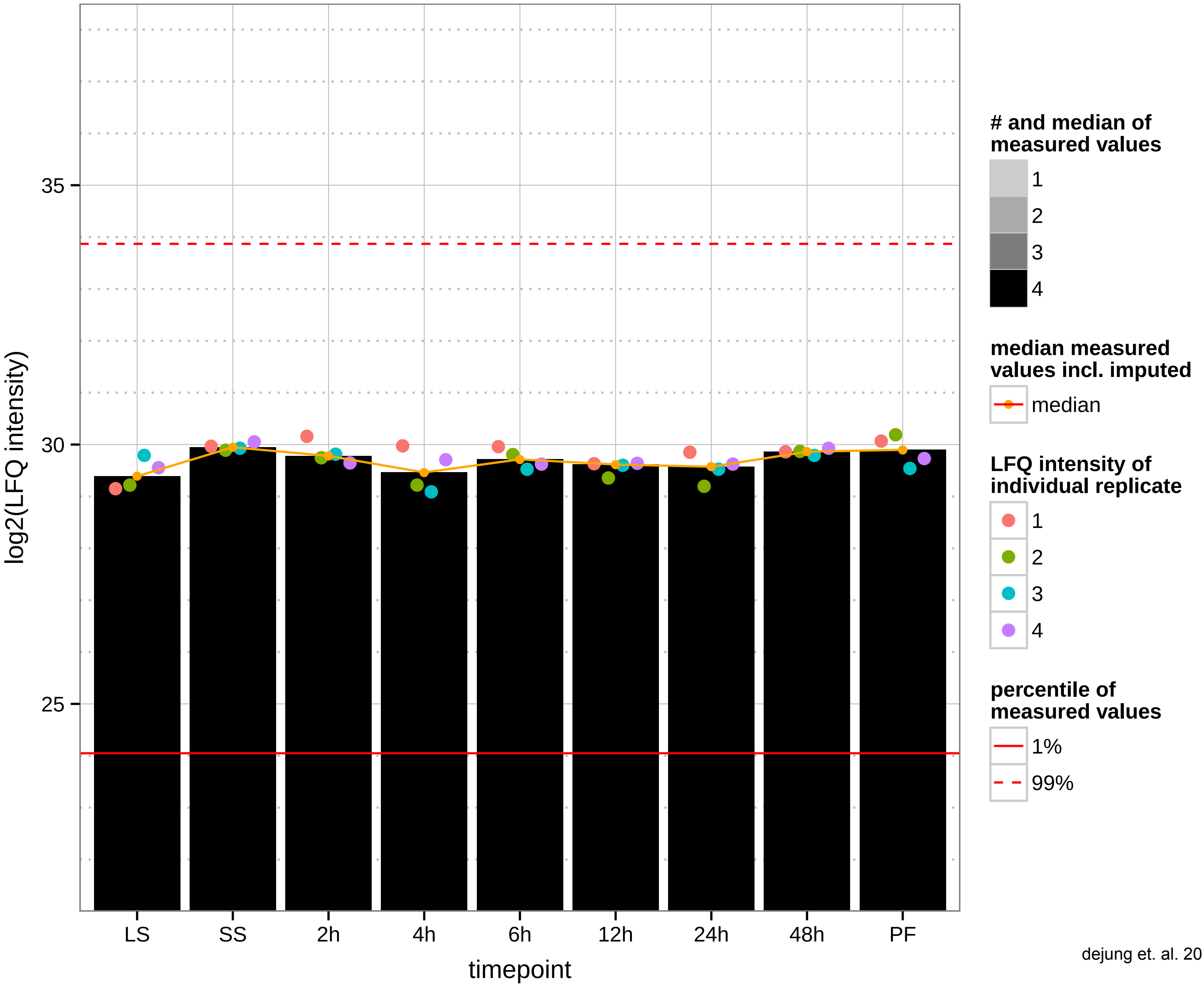

dejung et. al. 2015

hypothetical protein, conserved
Tb927.11.5030
AGOF: null
AGOC: mitochondrion
AGOP: null
PGOF: null
PGOC: null
PGOP: null

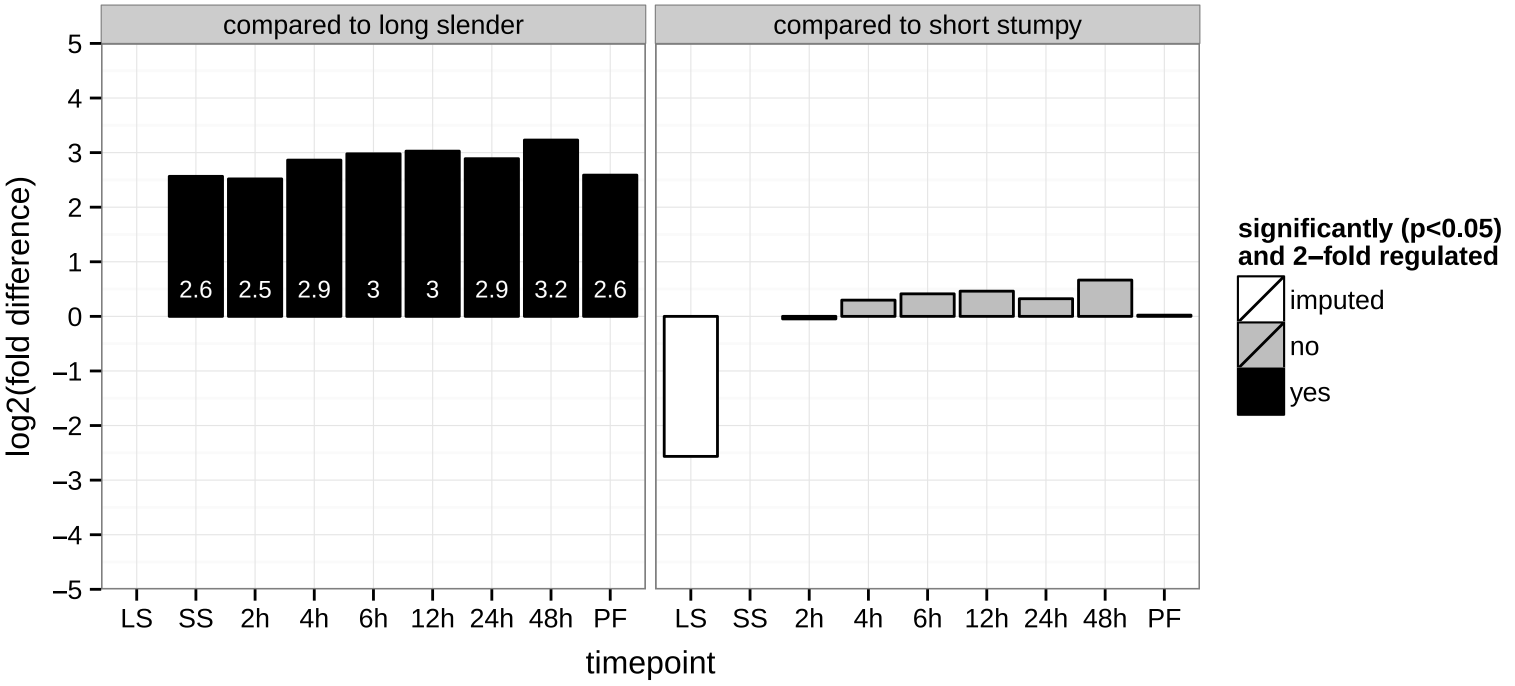

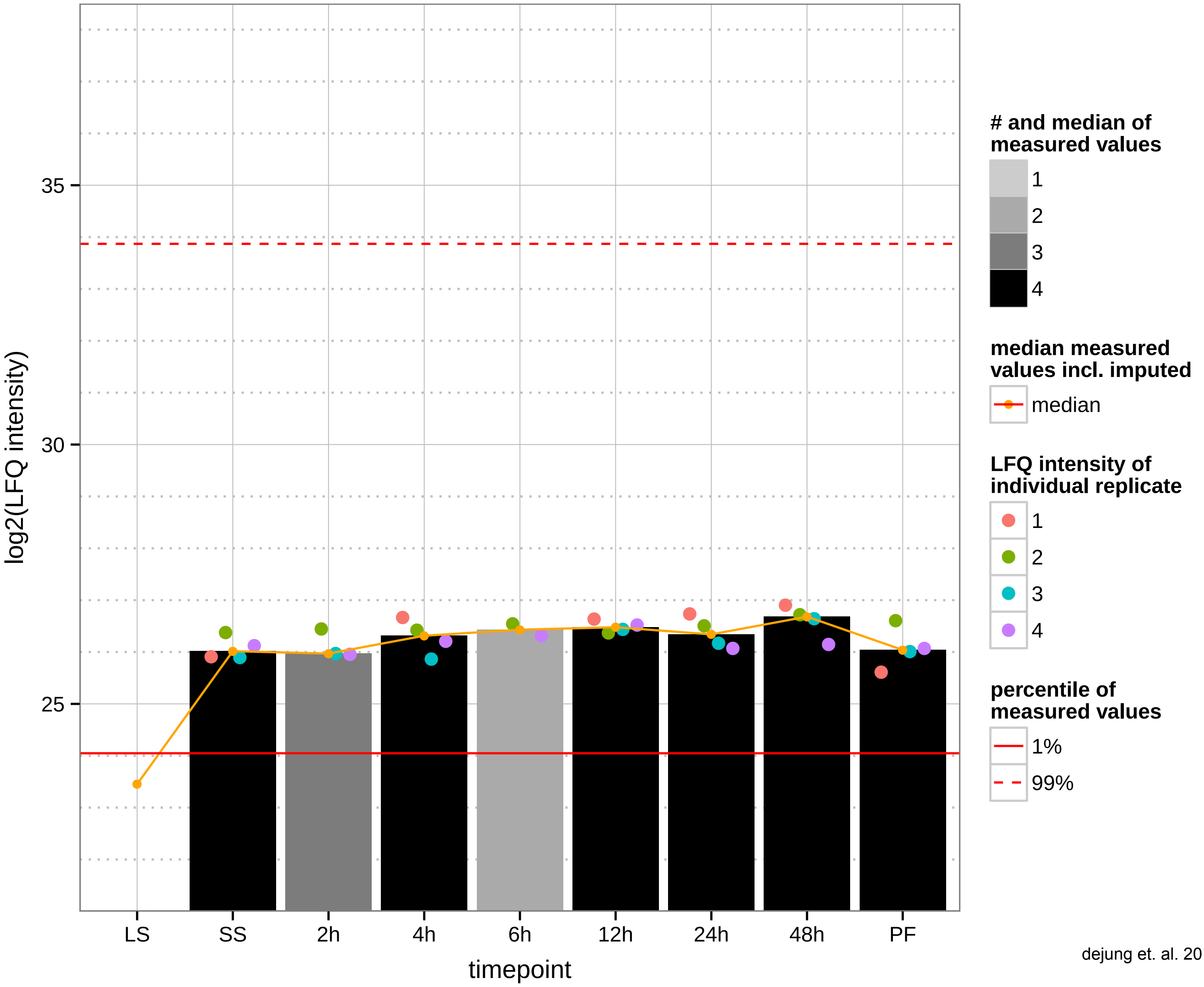

dejung et. al. 2015

leucine−rich repeat protein (LRRP), putative
Tb927.11.5040
AGOF: null
AGOC: null
AGOP: null
PGOF: protein binding
PGOC: null
PGOP: null

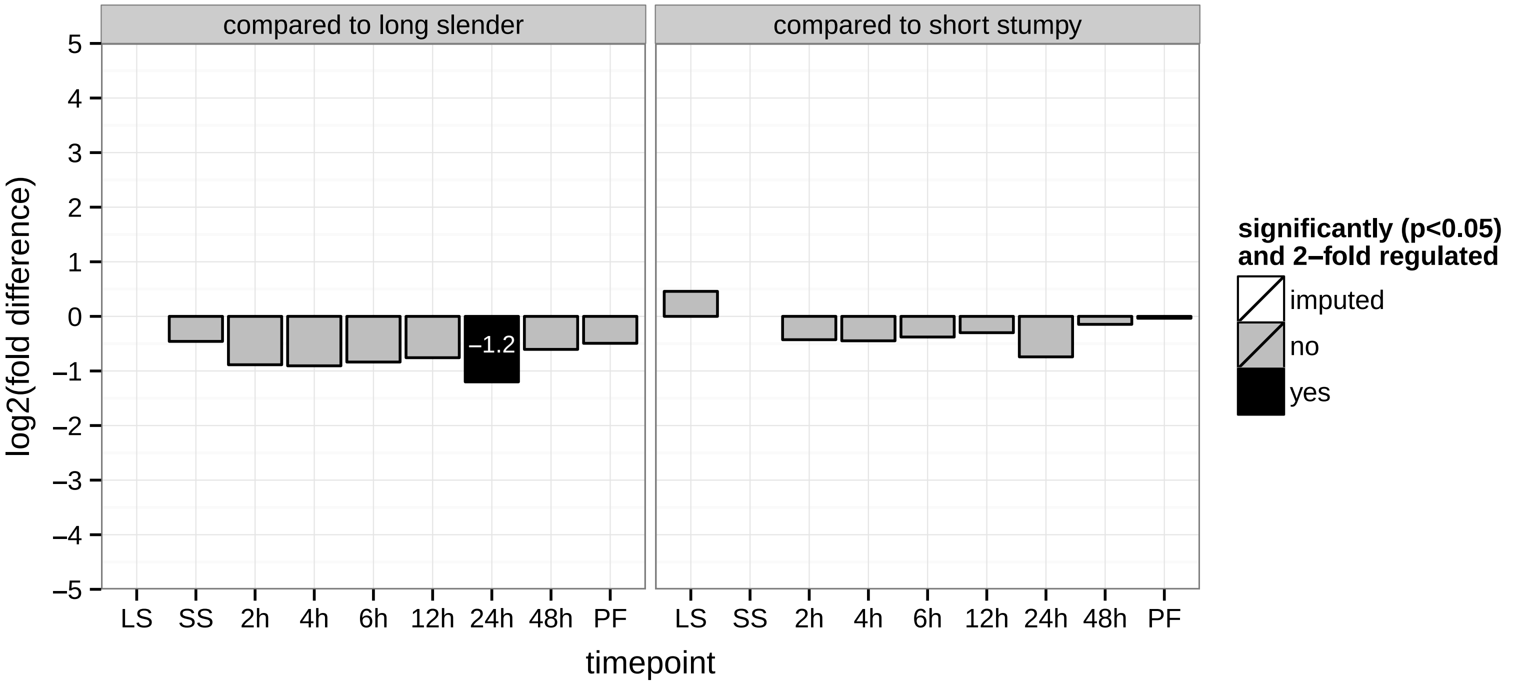

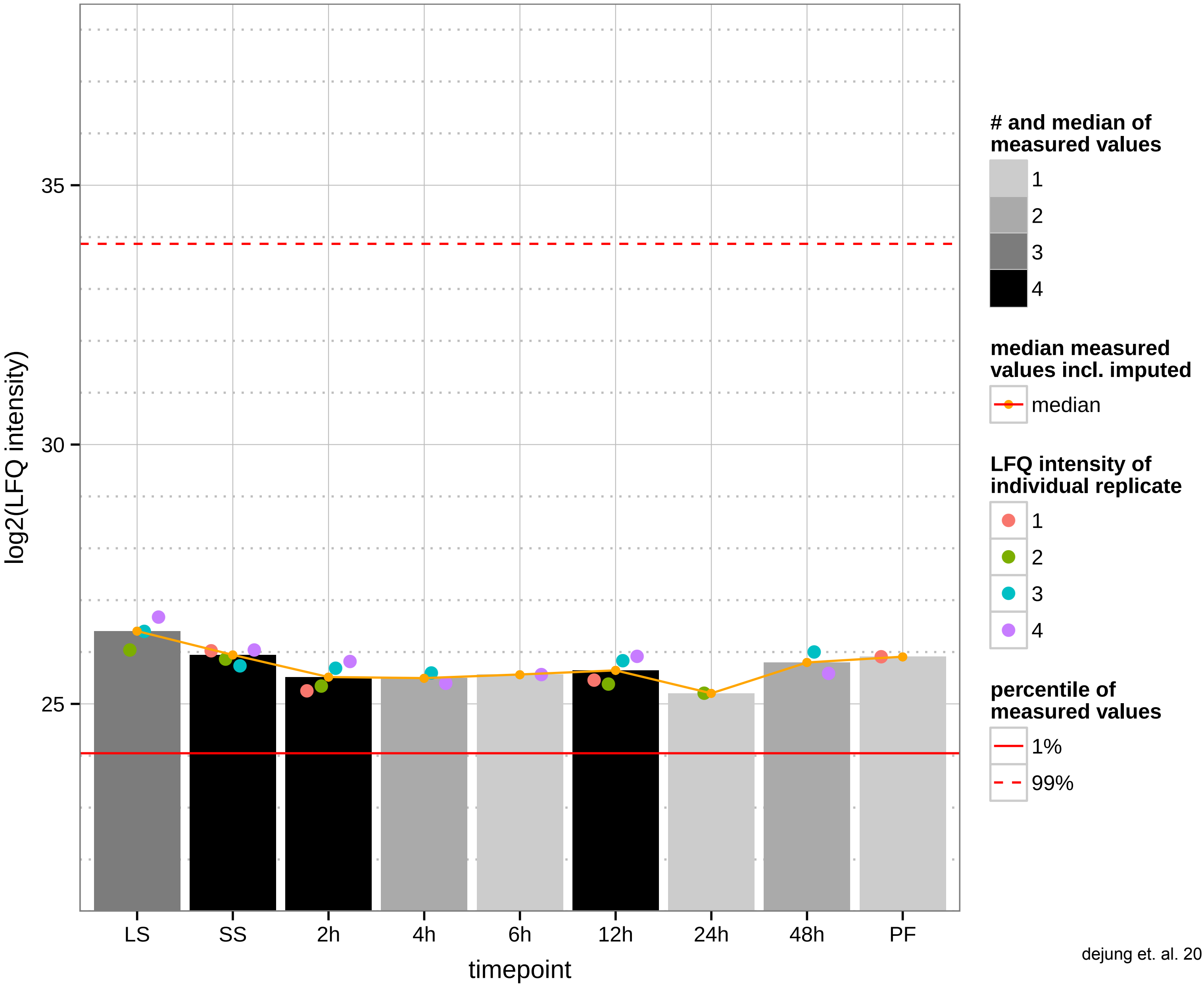

dejung et. al. 2015

fumarate hydratase, class I (FHm)
Tb927.11.5050
AGOF: fumarate hydratase activity
AGOC: mitochondrion
AGOP: fumarate metabolic process, generation of precursor metabolites and energy
PGOF: fumarate hydratase activity, hydro–lyase activity, lyase activity
PGOC: null
PGOP: generation of precursor metabolites and energy

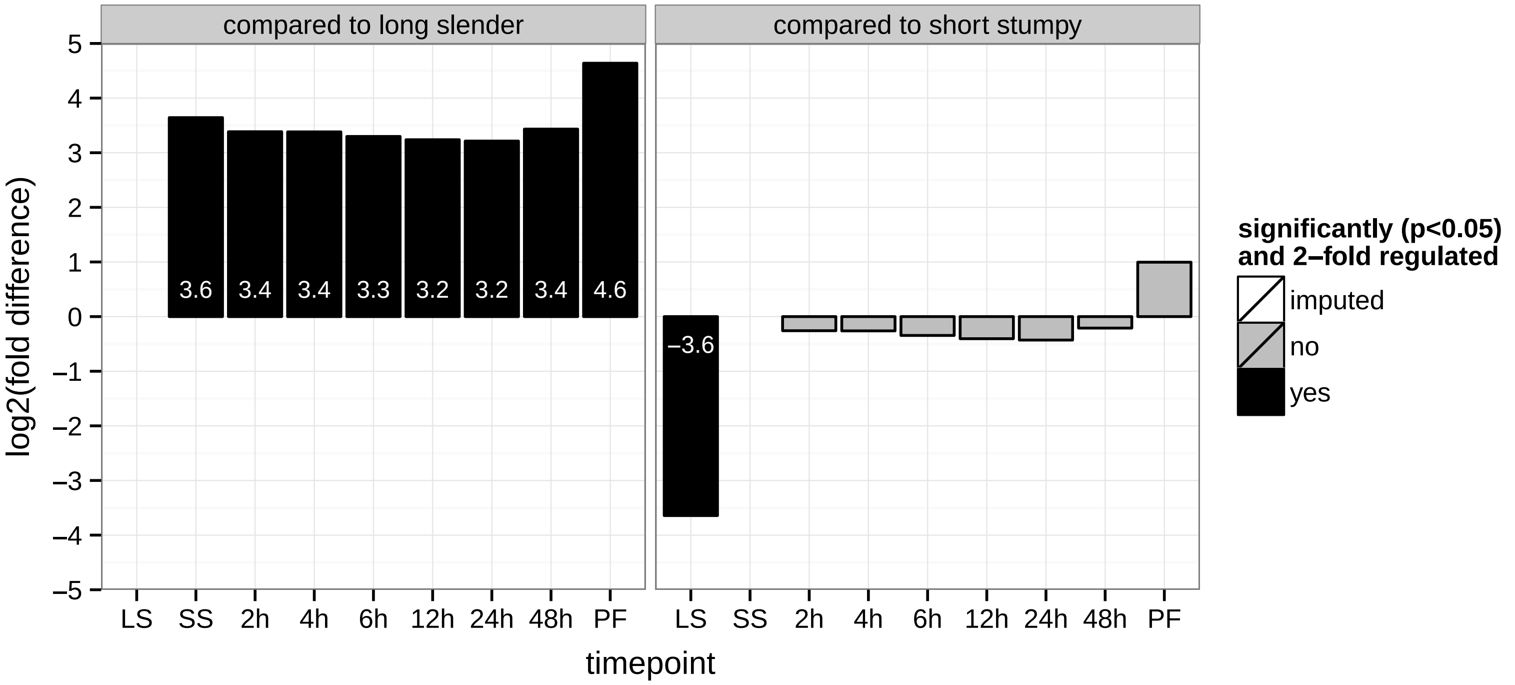

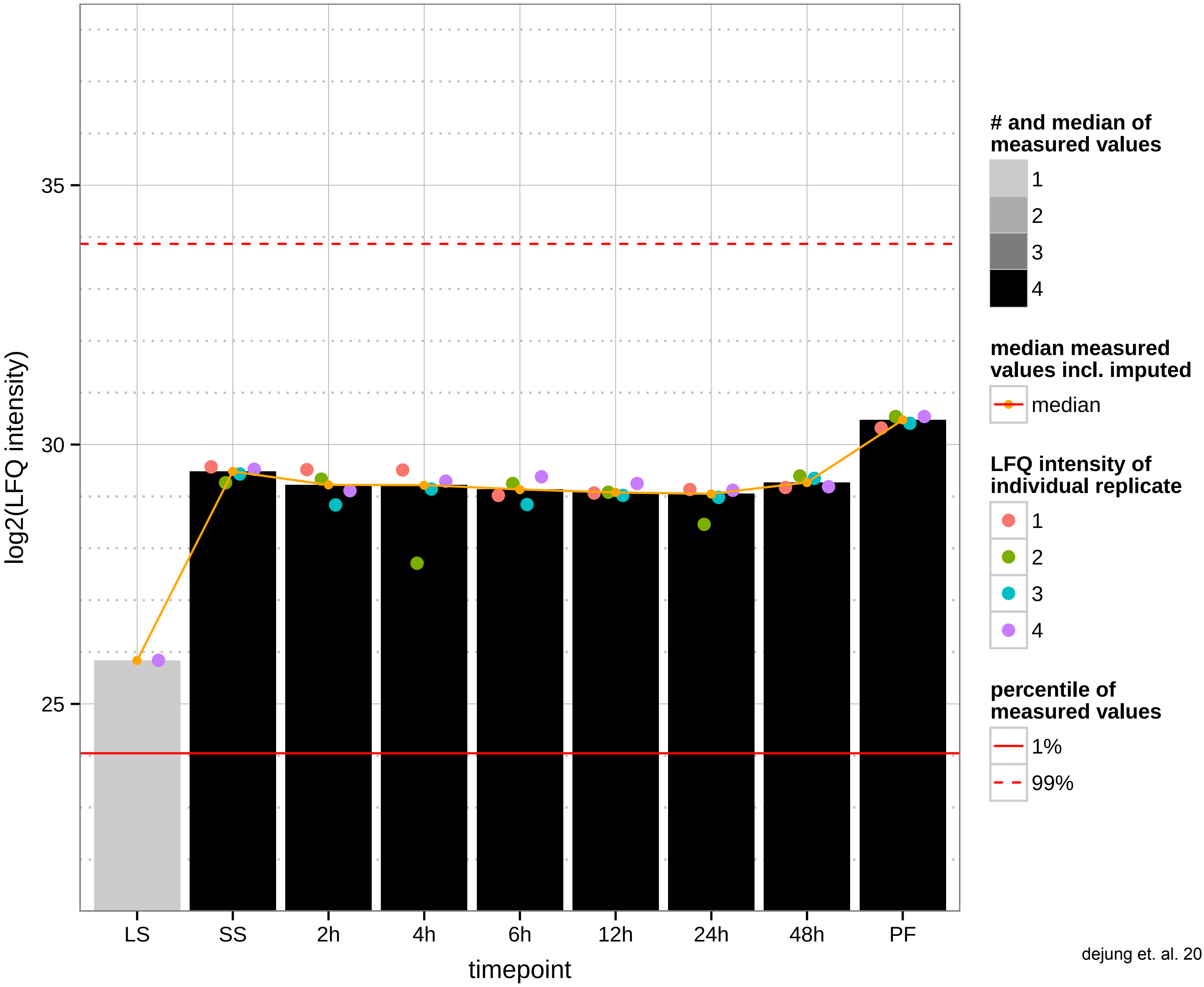

dejung et. al. 2015

hypothetical protein, conserved
Tb927.11.5230
AGOF: null
AGOC: null
AGOP: null
PGOF: null
PGOC: null
PGOP: null

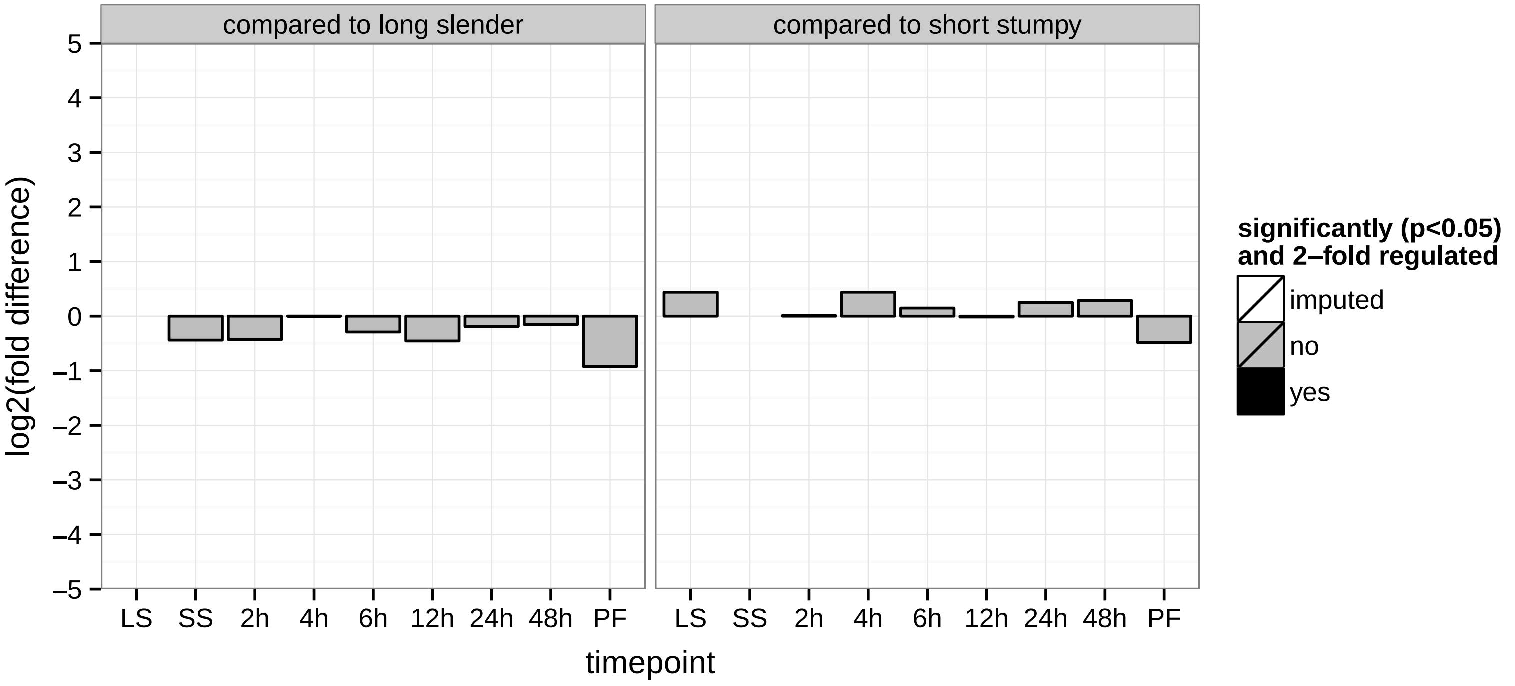

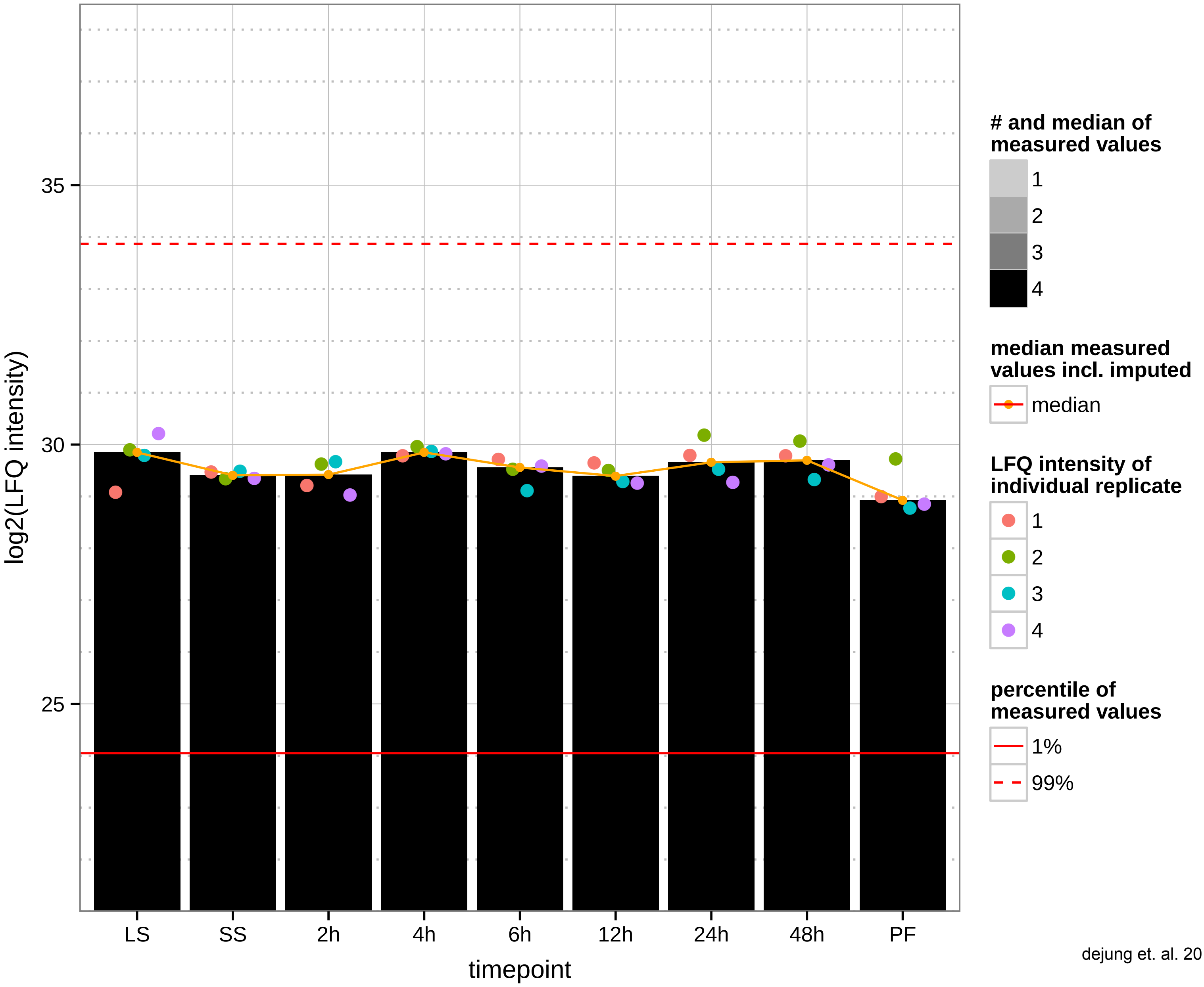

dejung et. al. 2015

mitochondrial carrier protein (MCP9)
Tb927.11.5290
AGOF: null
AGOC: integral to membrane, membrane, mitochondrial inner membrane, mitochondrion
AGOP: transport
PGOF: null
PGOC: null
PGOP: null

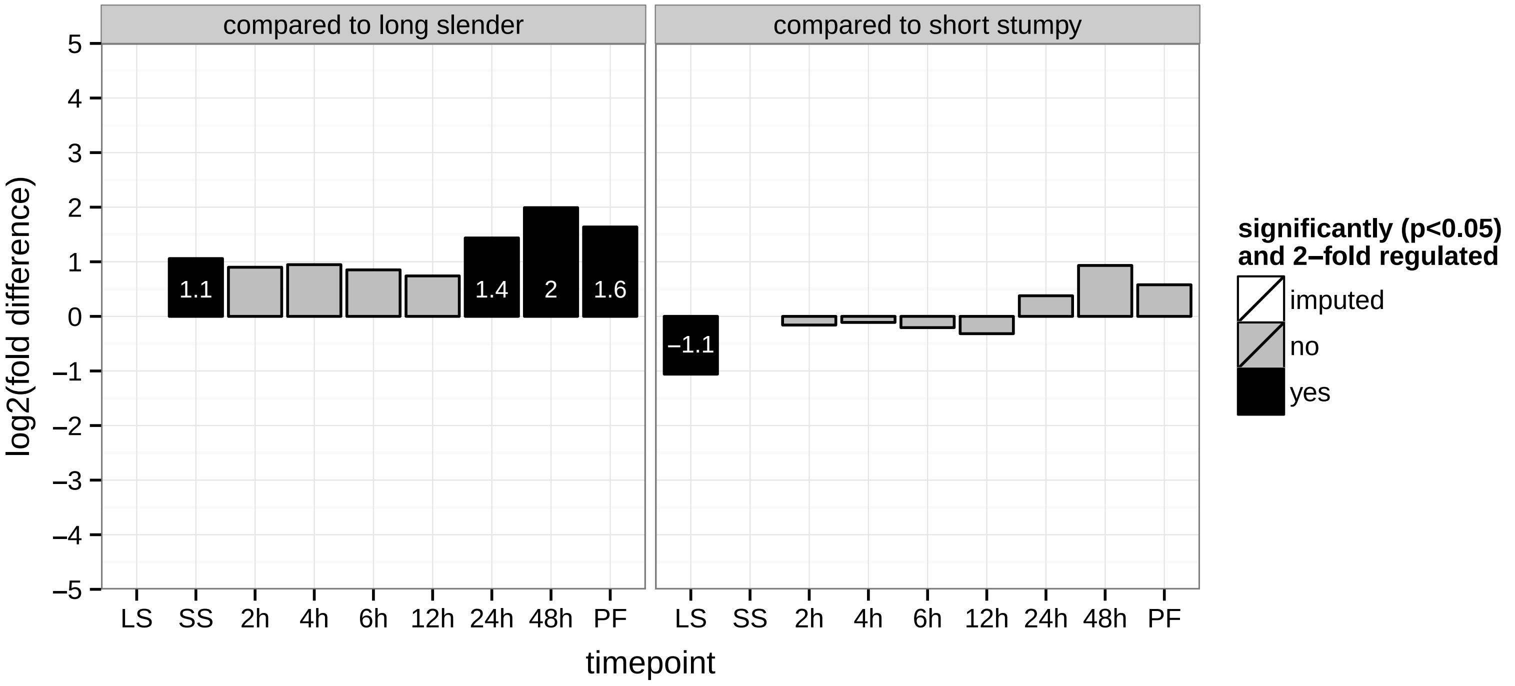

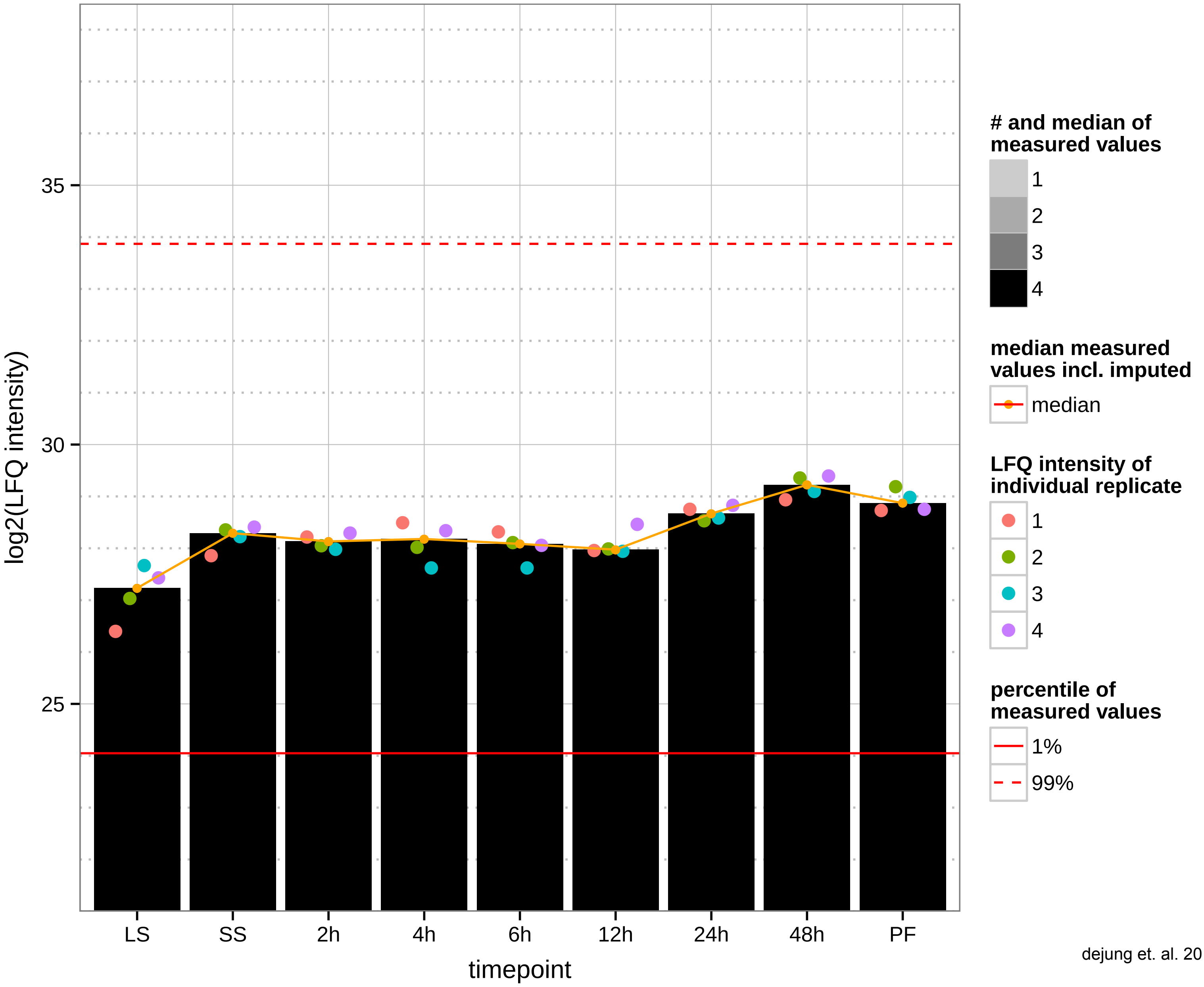

dejung et. al. 2015

RNA–binding protein, putative (RBP3)
Tb927.11.530
AGOF: RNA binding
AGOC: cytoplasm
AGOP: null
PGOF: nucleic acid binding
PGOC: null
PGOP: null

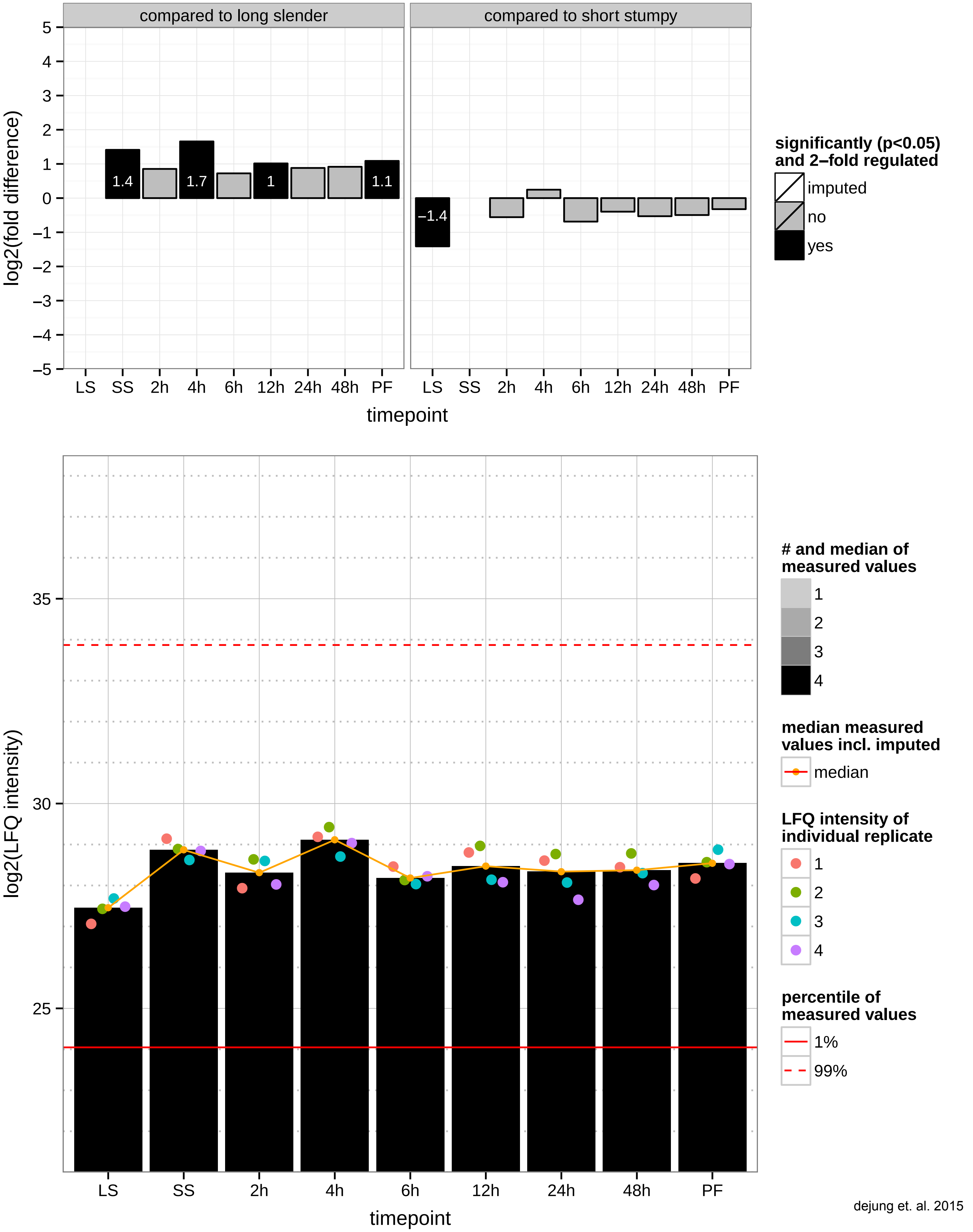

dejung et. al. 2015

kinesin, putative, mitotic centromere–associated kinesin, putative (Kif13–3)
Tb927.11.5300
AGOF: ATP binding, microtubule motor activity, motor activity
AGOC: cell part, kinesin complex, microtubule associated complex
AGOP: microtubule–based movement
PGOF: ATP binding, microtubule motor activity
PGOC: null
PGOP: microtubule–based movement

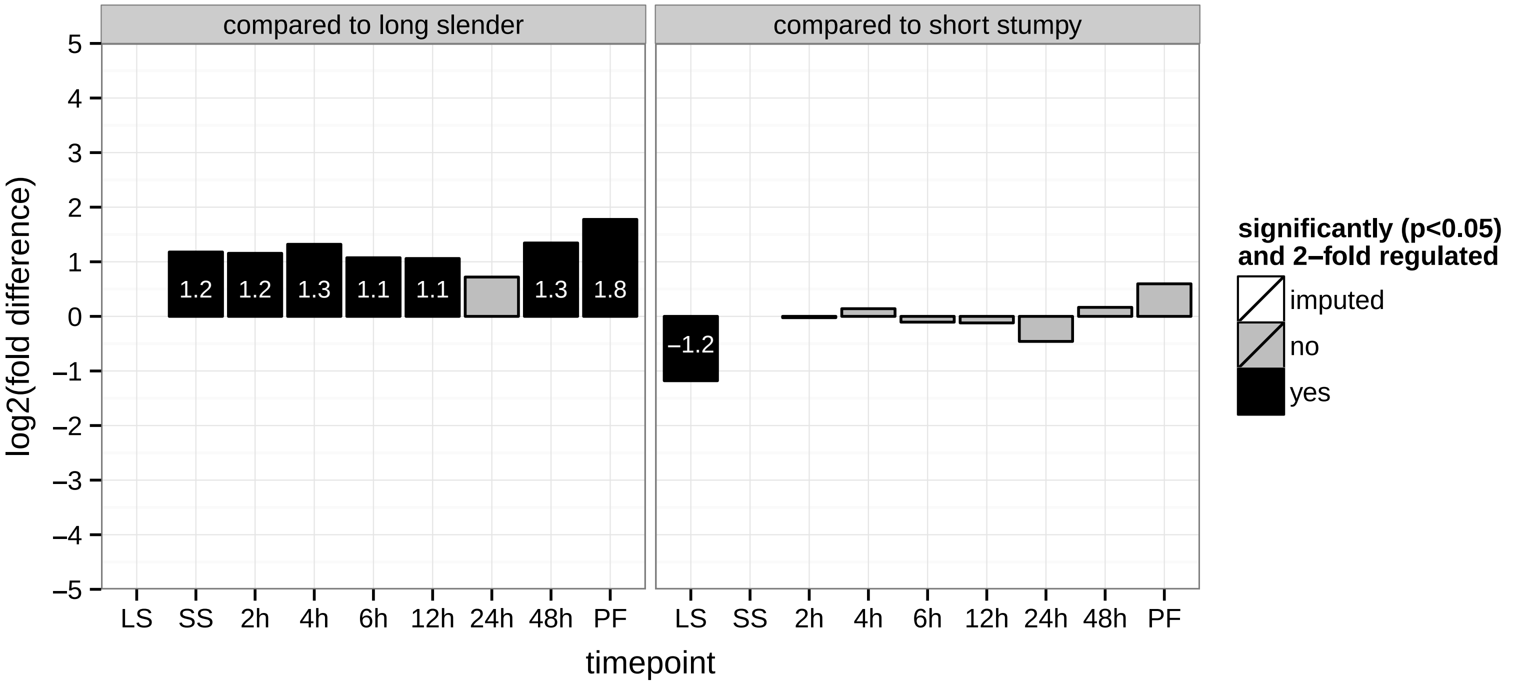

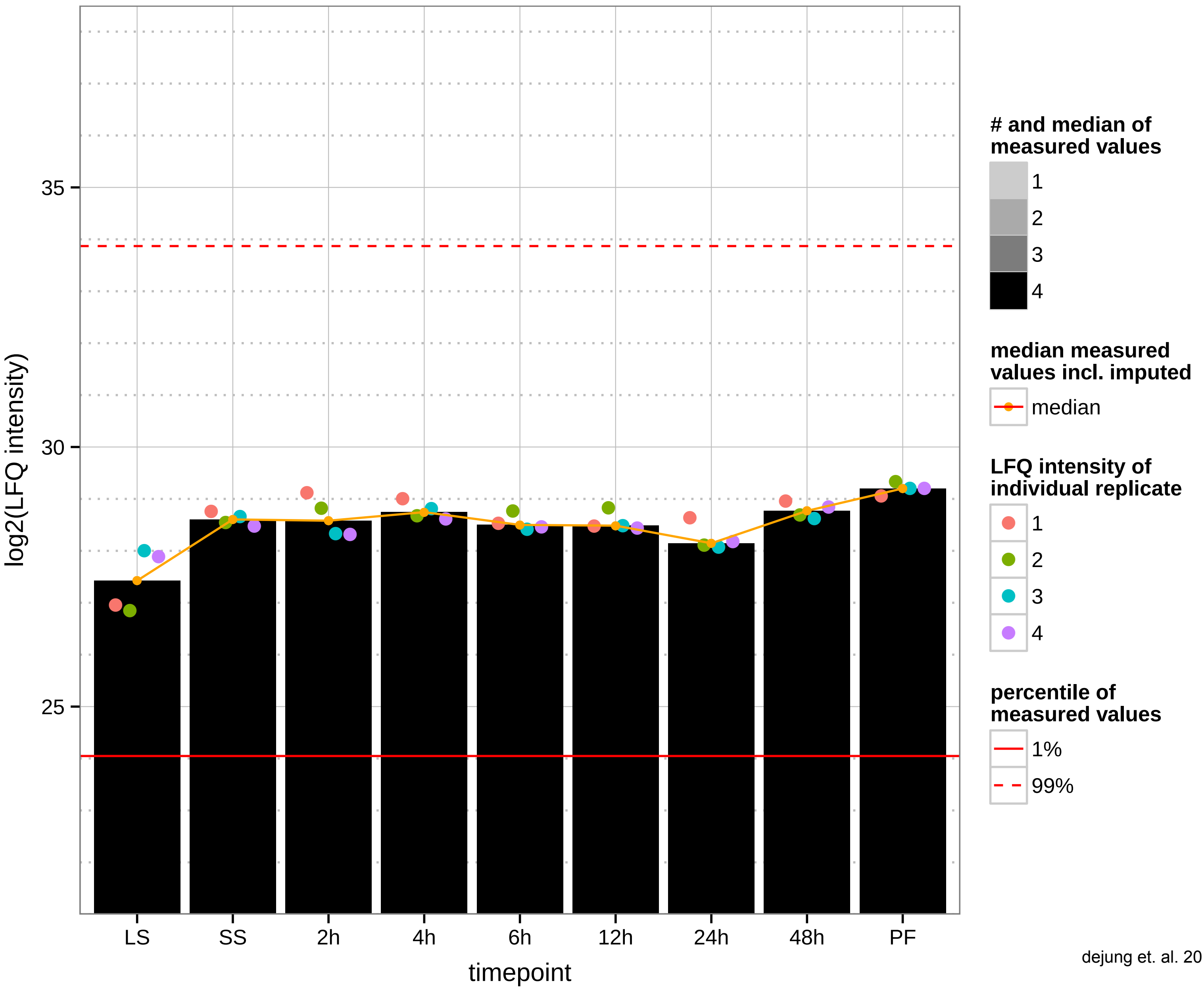

dejung et. al. 2015

ABC transporter, putative (ABCB10)
Tb927.11.540
AGOF: ATP binding, ATPase activity, coupled to transmembrane movement of substances
AGOC: ATP–binding cassette (ABC) transporter complex, integral to membrane, mitochondrial inner membrane
AGOP: transport
PGOF: ATP binding, ATPase activity, coupled to transmembrane movement of substances, nucleoside–triphosphatase activity
PGOC: integral to membrane
PGOP: transmembrane transport, transport

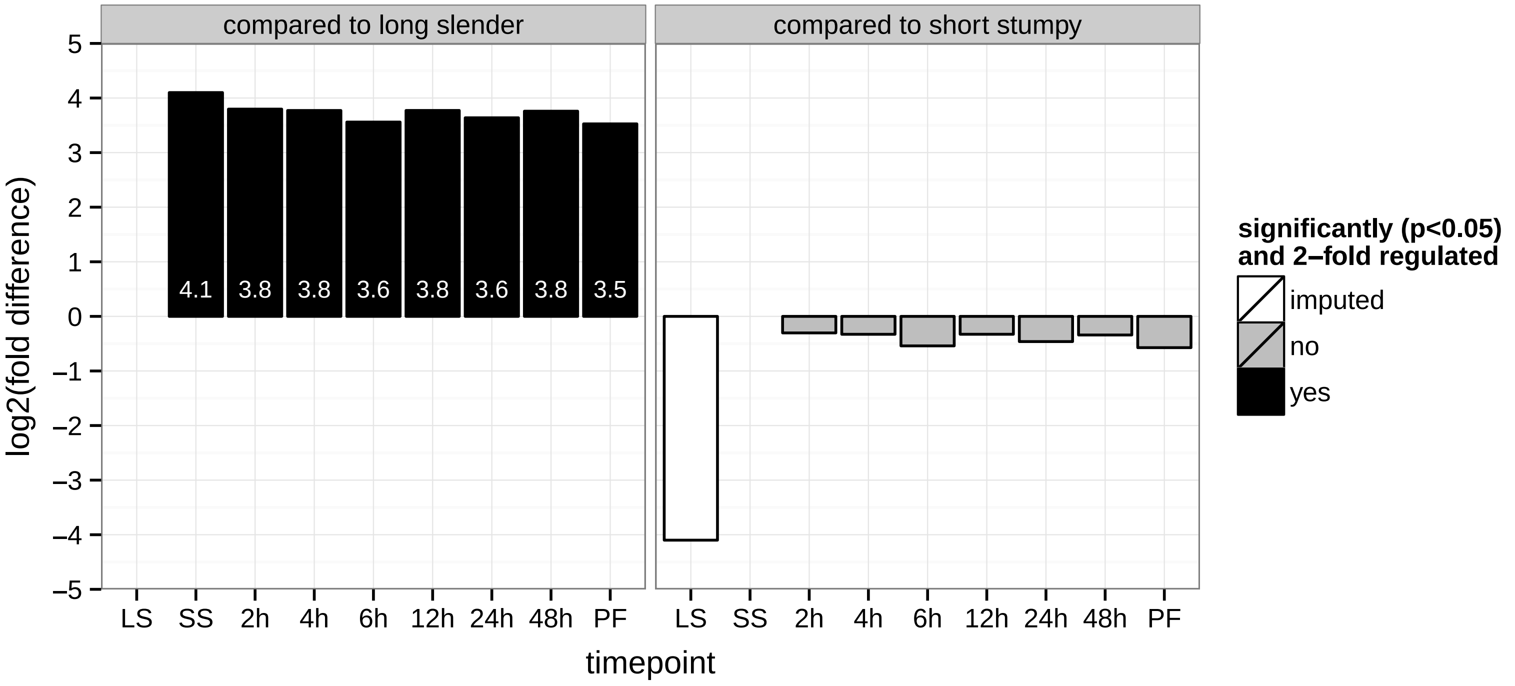

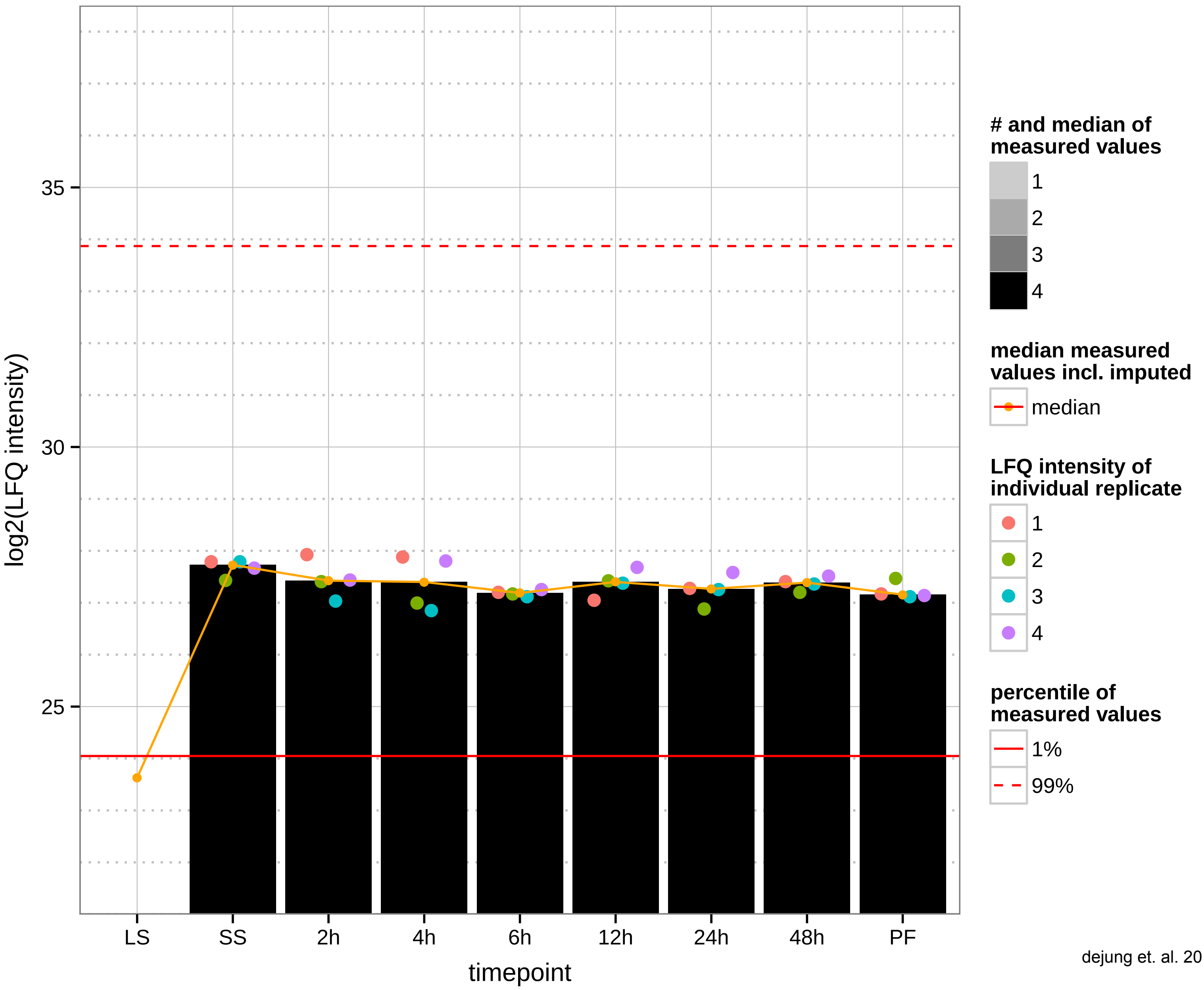

dejung et. al. 2015

signal recognition particle 54 kDa (SRP54)
Tb927.11.5400
AGOF: 7S RNA binding, GTP binding, GTPase activity, RNA binding
AGOC: signal recognition particle, endoplasmic reticulum targeting
AGOP: SRP–dependent cotranslational protein targeting to membrane, protein targeting
PGOF: 7S RNA binding, GTP binding, nucleoside–triphosphatase activity, nucleotide binding
PGOC: signal recognition particle
PGOP: SRP–dependent cotranslational protein targeting to membrane

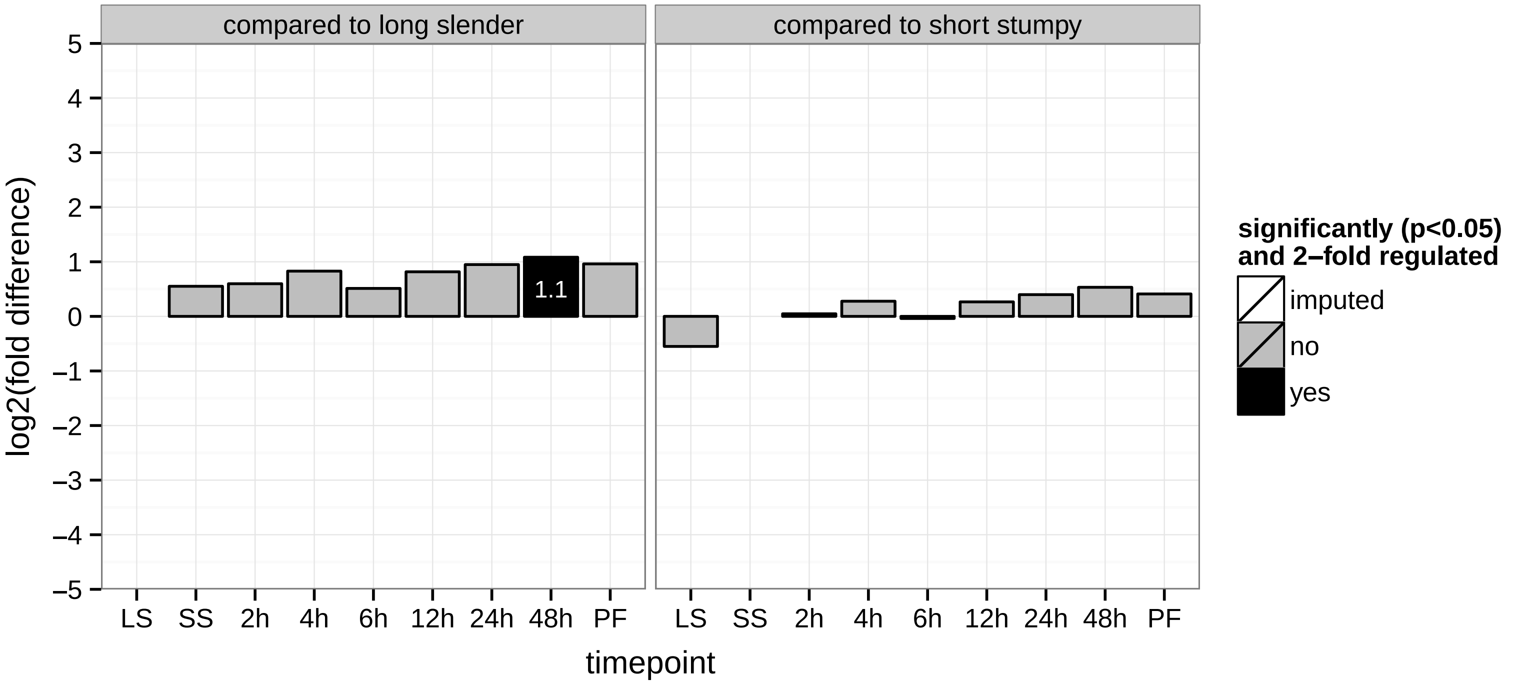

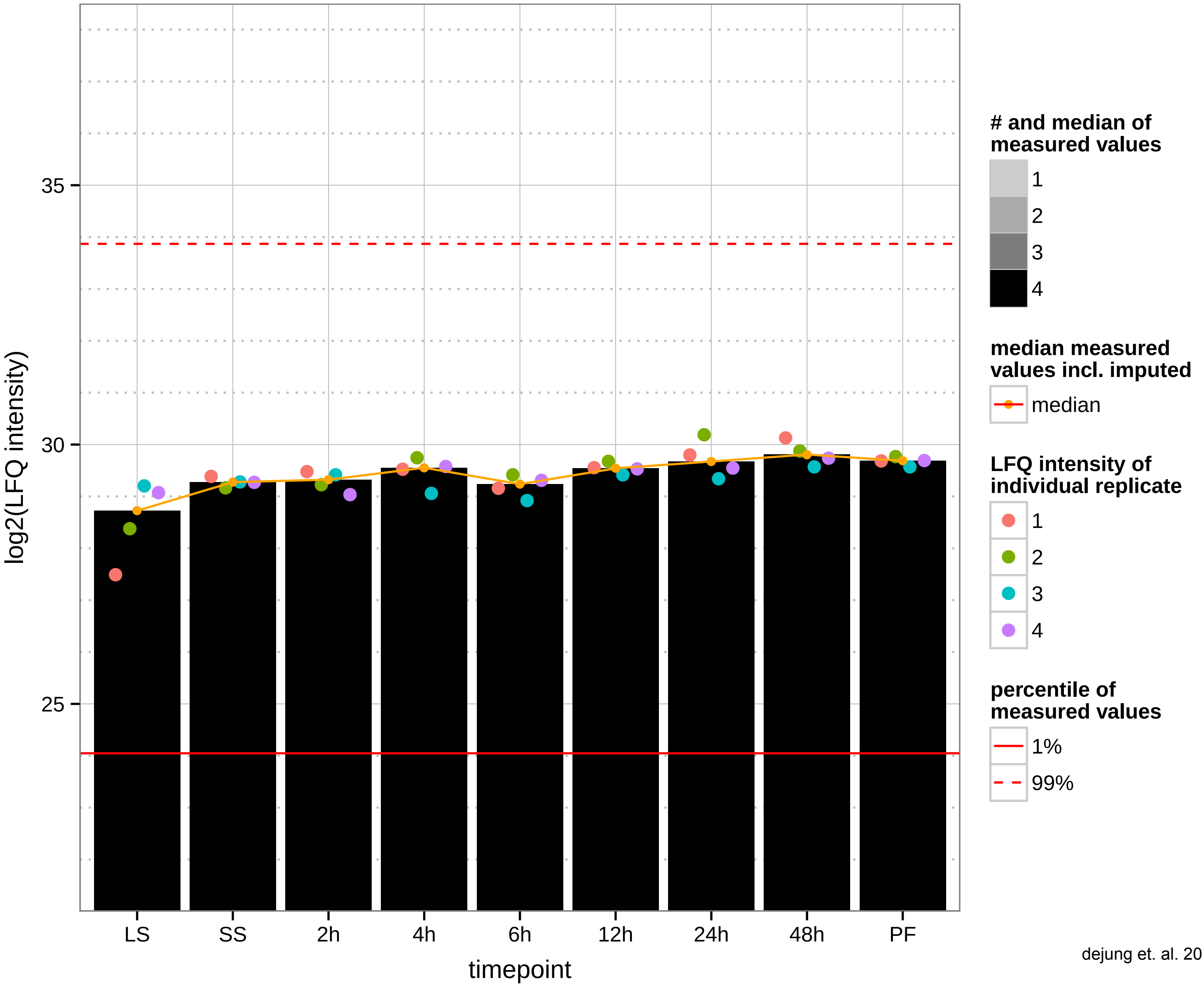

dejung et. al. 2015

hypothetical protein, conserved
Tb927.11.5420
AGOF: null
AGOC: null
AGOP: null
PGOF: null
PGOC: null
PGOP: null

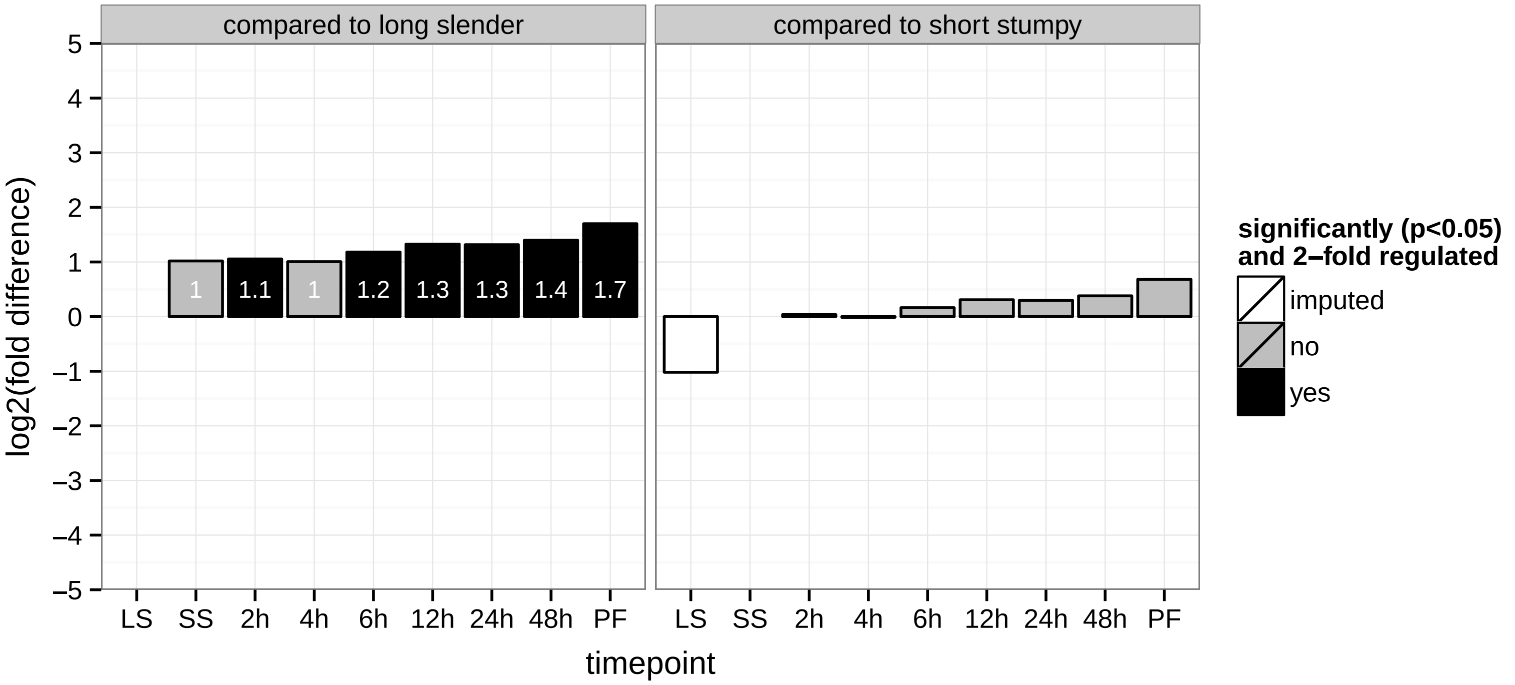

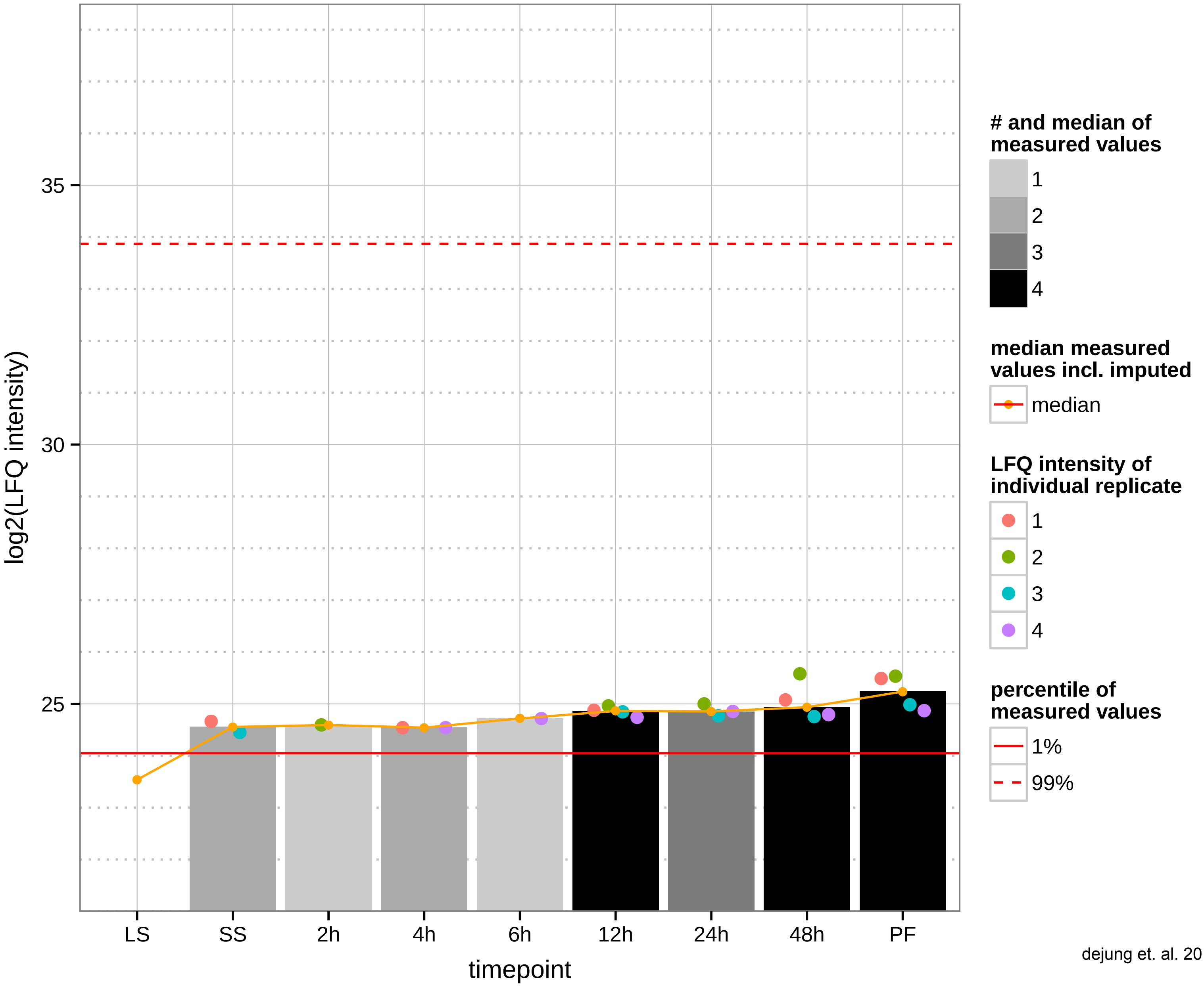

dejung et. al. 2015

malic enzyme
Tb927.11.5450
AGOF: NAD binding, NADP+ binding, malic enzyme activity, oxidoreductase activity, acting on the CH–OH group of donors, N
AGOC: mitochondrion
AGOP: malate metabolic process
PGOF: NAD binding, malate dehydrogenase (oxaloacetate–decarboxylating) activity, malic enzyme activity, oxidoreductase ac
PGOC: null
PGOP: malate metabolic process, oxidation–reduction process

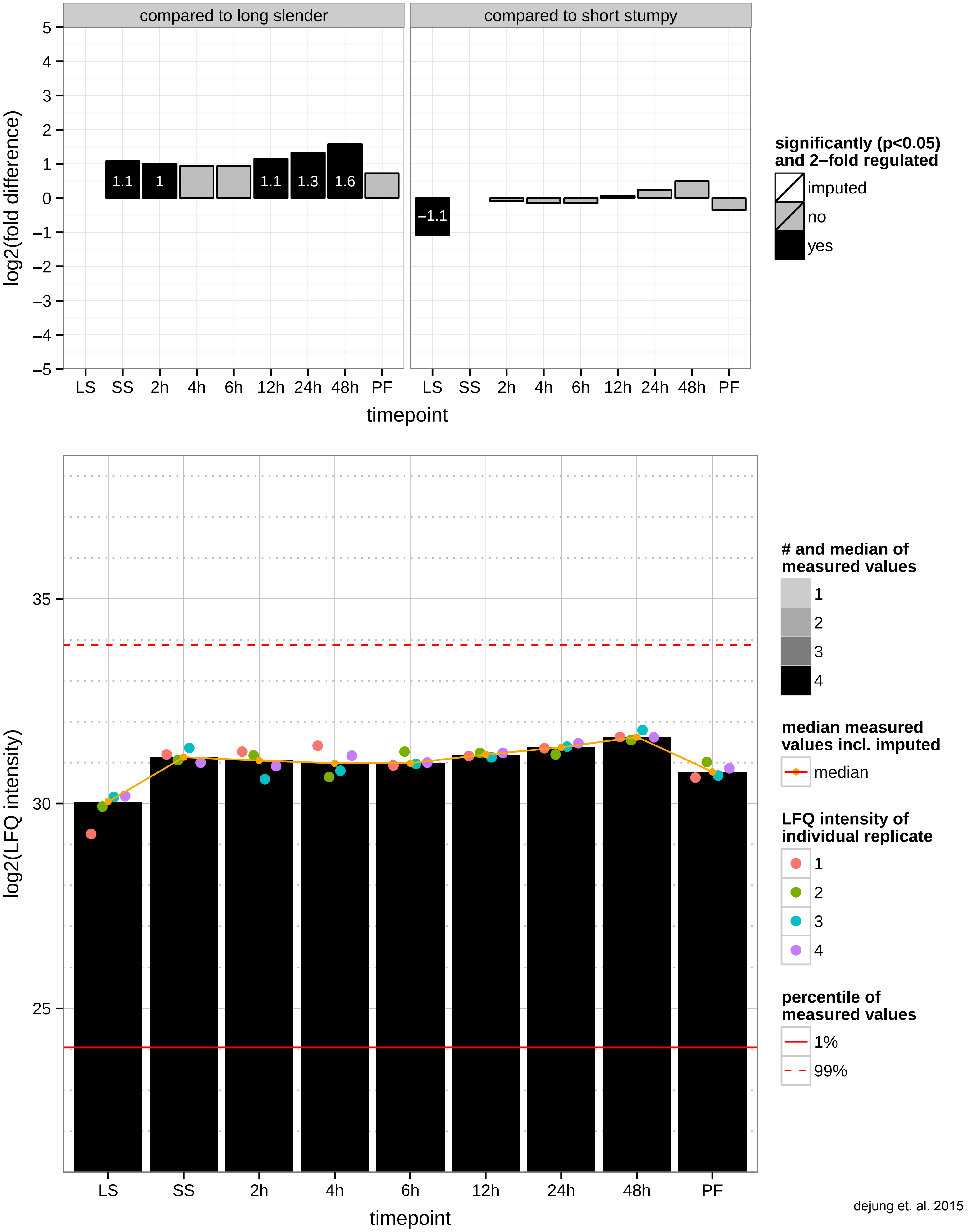

dejung et. al. 2015

hypothetical protein SCD6.10
Tb927.11.550
AGOF: null
AGOC: cytoplasm
AGOP: null
PGOF: null
PGOC: null
PGOP: null

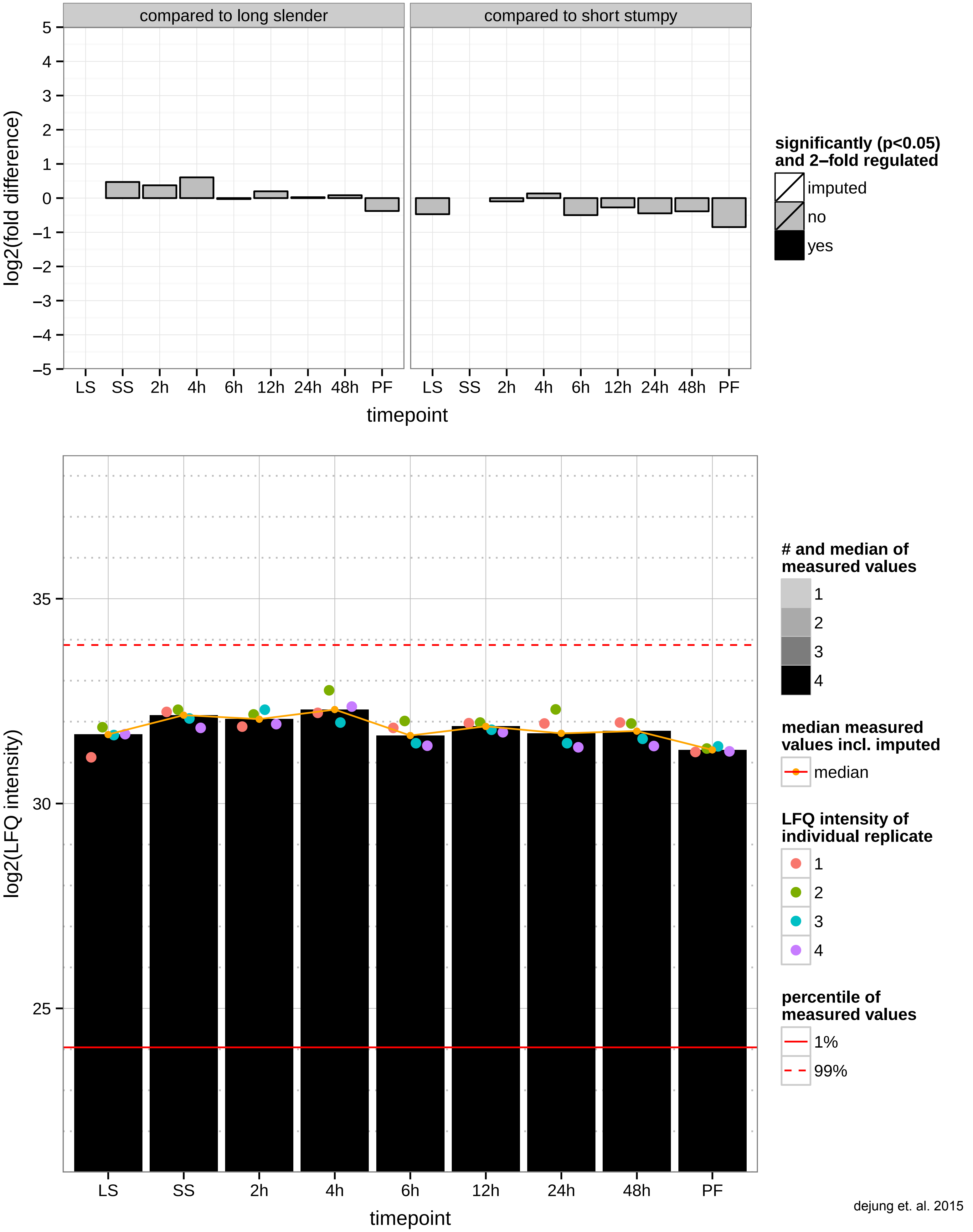

dejung et. al. 2015

dynein light chain p28, axonemal, putative
Tb927.11.5510
AGOF: microtubule motor activity
AGOC: axonemal dynein complex
AGOP: cellular component movement
PGOF: null
PGOC: null
PGOP: null

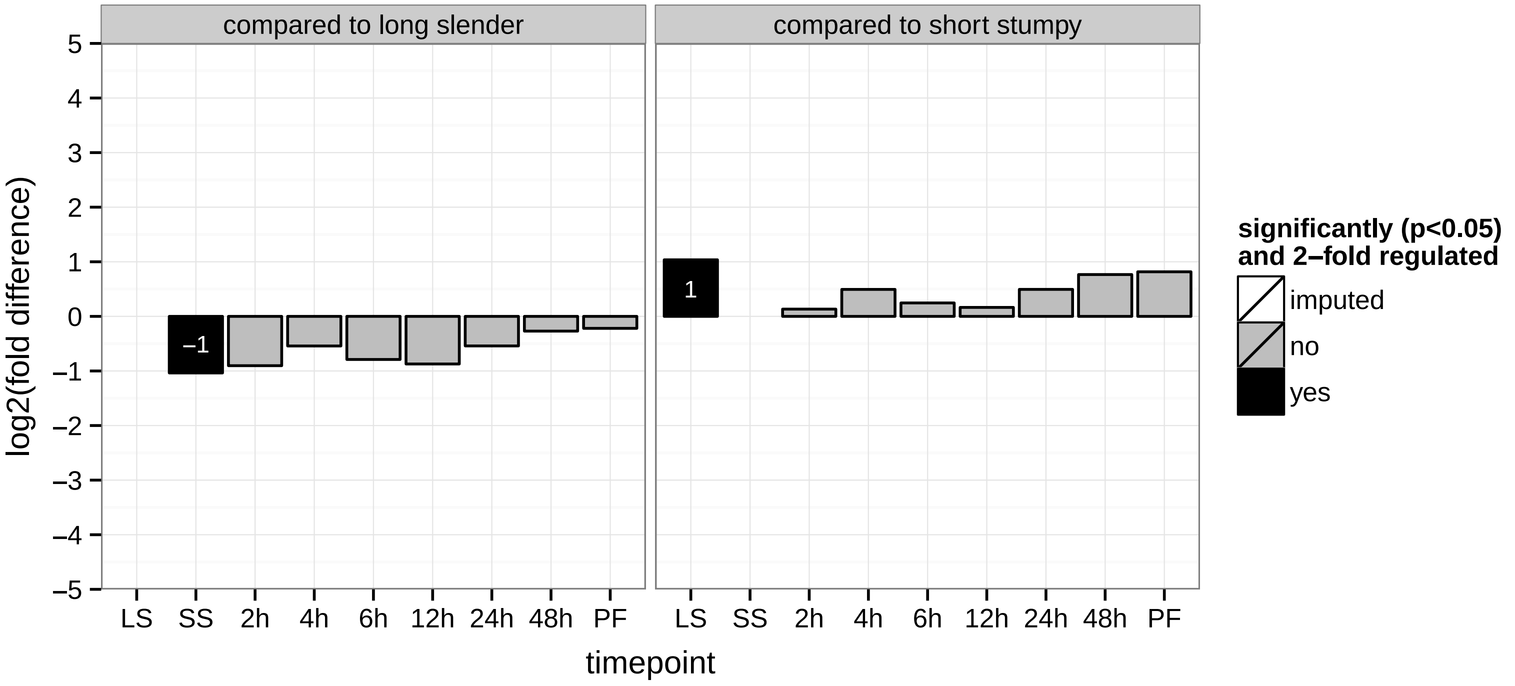

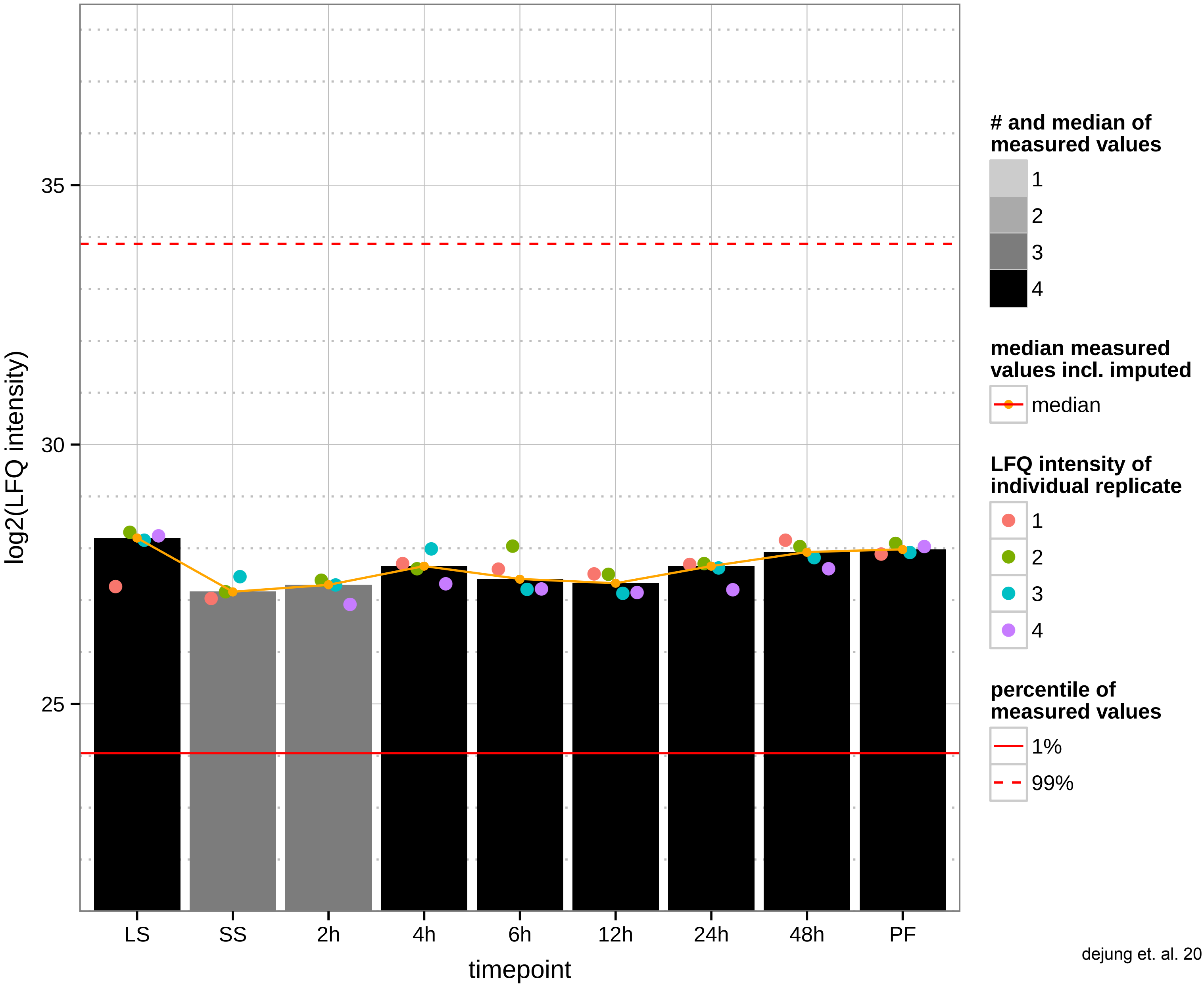

dejung et. al. 2015

triosephosphate isomerase (TIM)
Tb927.11.5520
AGOF: triose−phosphate isomerase activity
AGOC: glycosome
AGOP: glycolysis
PGOF: triose−phosphate isomerase activity
PGOC: null
PGOP: metabolic process

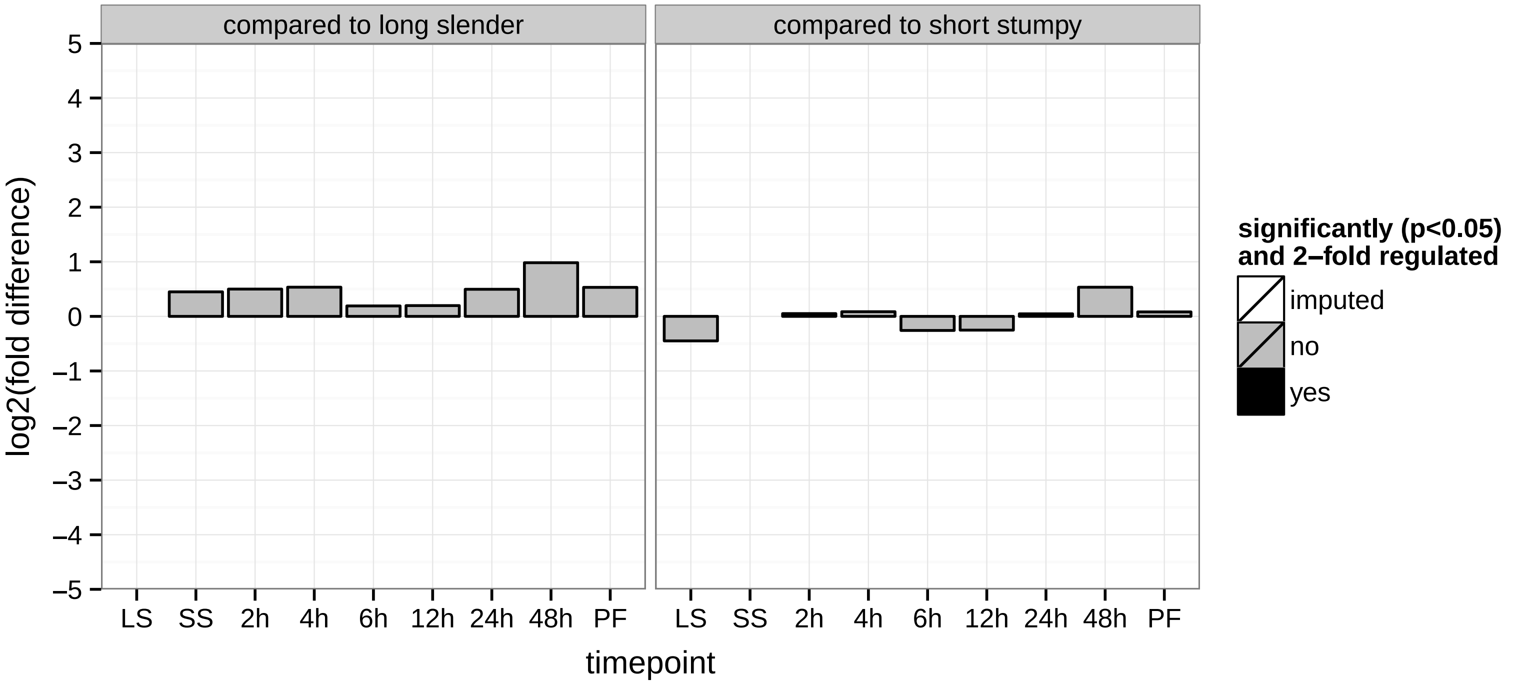

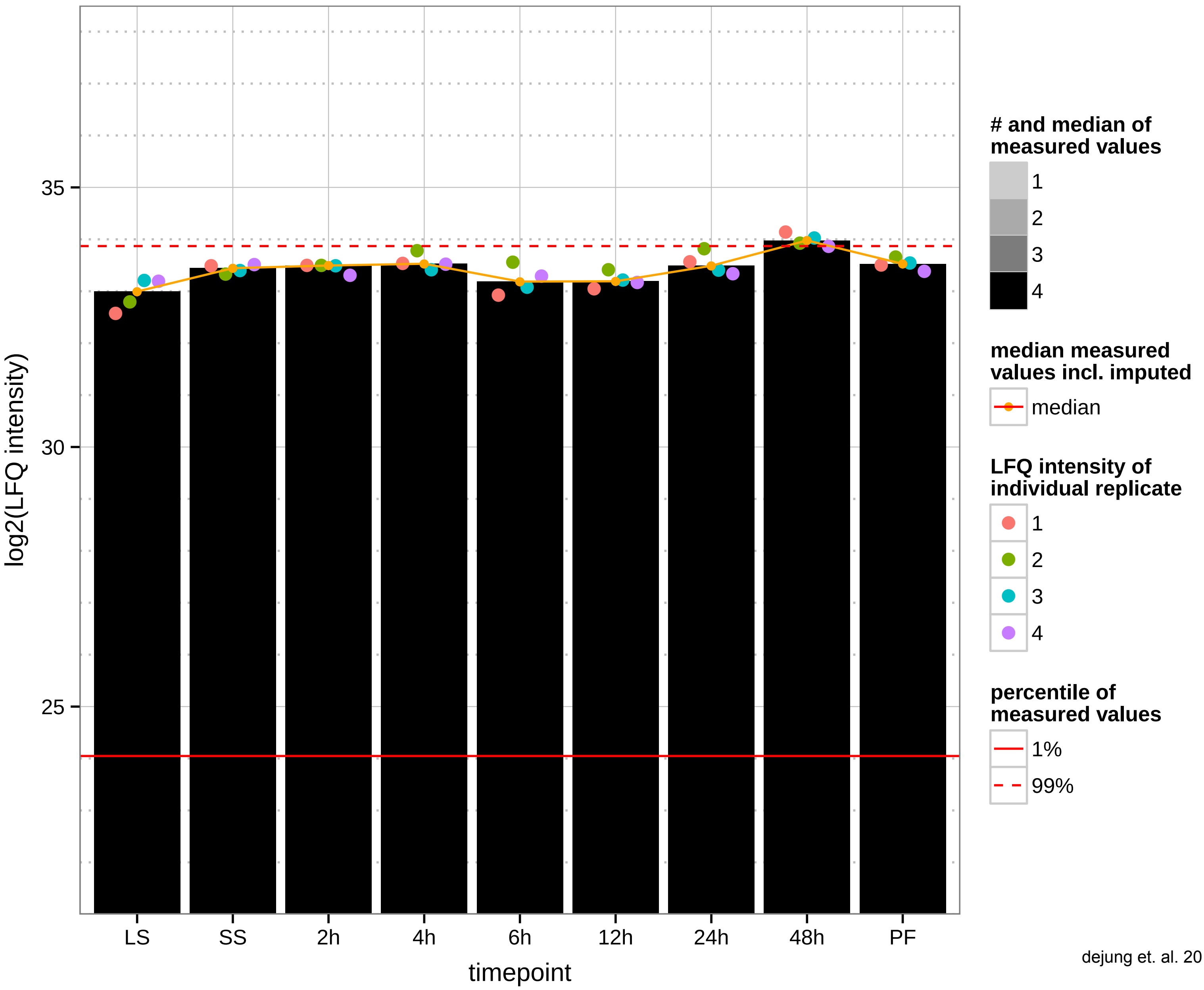

dejung et. al. 2015

hypothetical protein, conserved
Tb927.11.5590
AGOF: null
AGOC: null
AGOP: null
PGOF: null
PGOC: null
PGOP: null

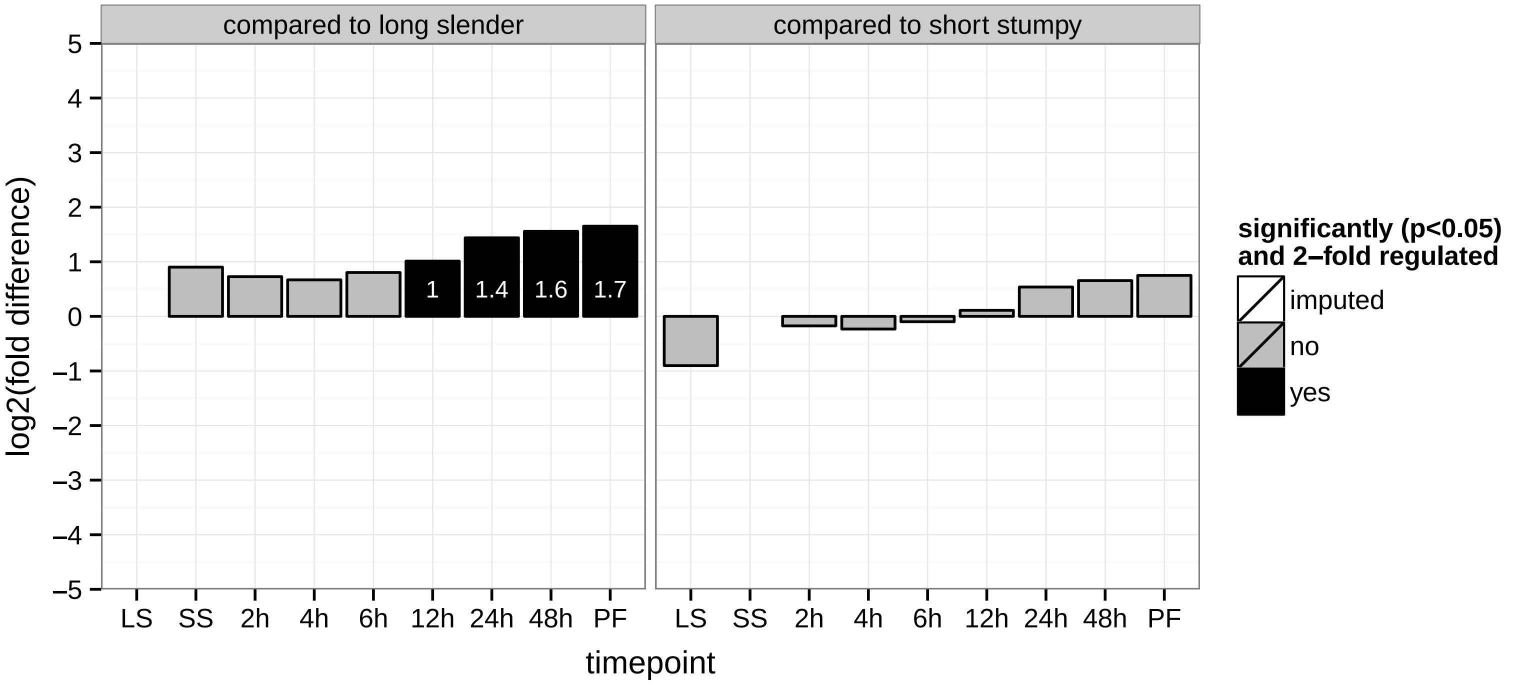

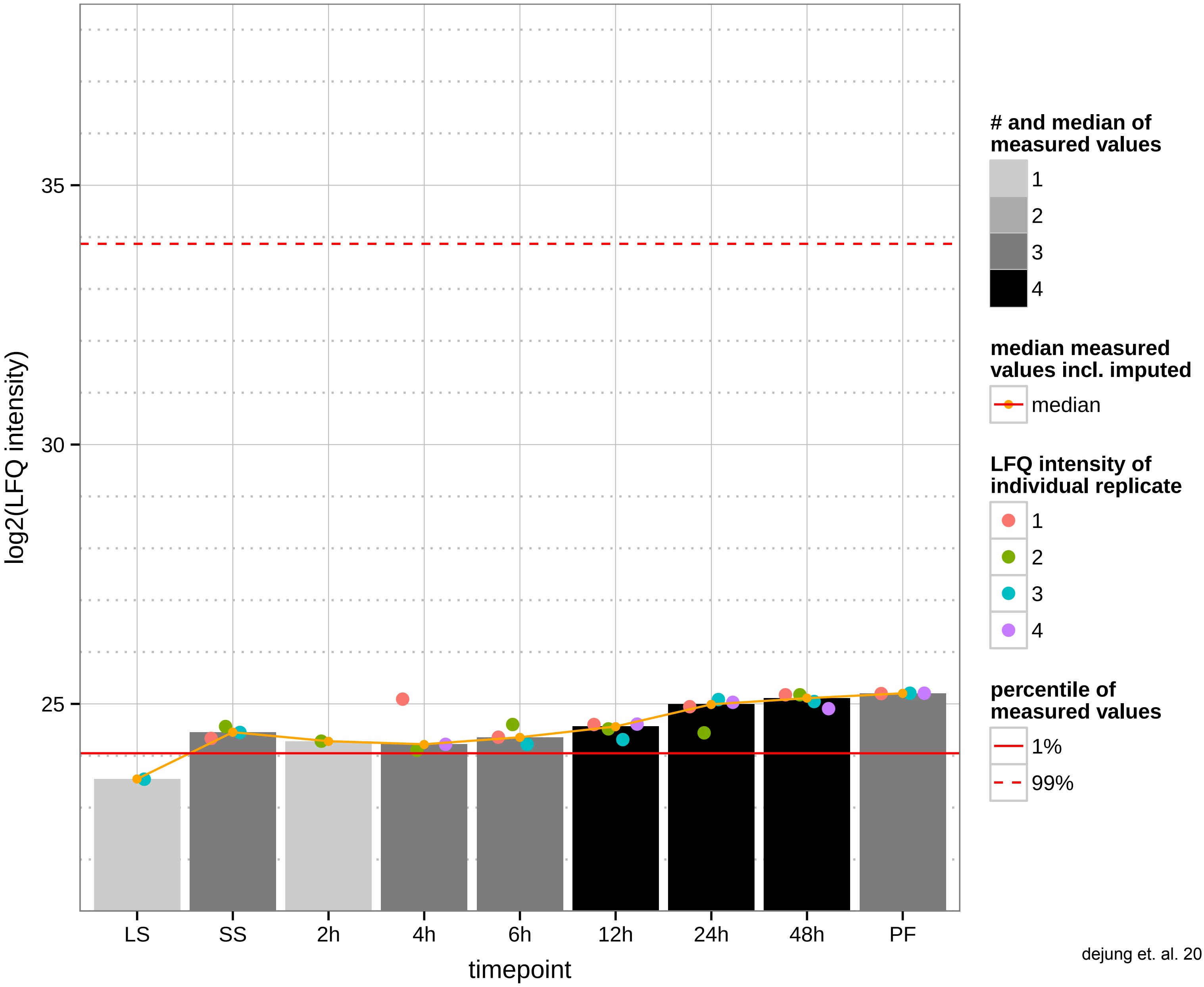

dejung et. al. 2015

replication factor C, subunit 1, putative, replication factor C large subunit
Tb927.11.5650
AGOF: ATP binding, ATP−dependent peptidase activity, DNA binding, DNA clamp loader activity, serine−type endopeptidase a
AGOC: DNA replication factor C complex
AGOP: DNA replication, proteolysis
PGOF: ATP binding, DNA binding, DNA clamp loader activity, nucleoside−triphosphatase activity, nucleotide binding
PGOC: DNA replication factor C complex
PGOP: DNA replication

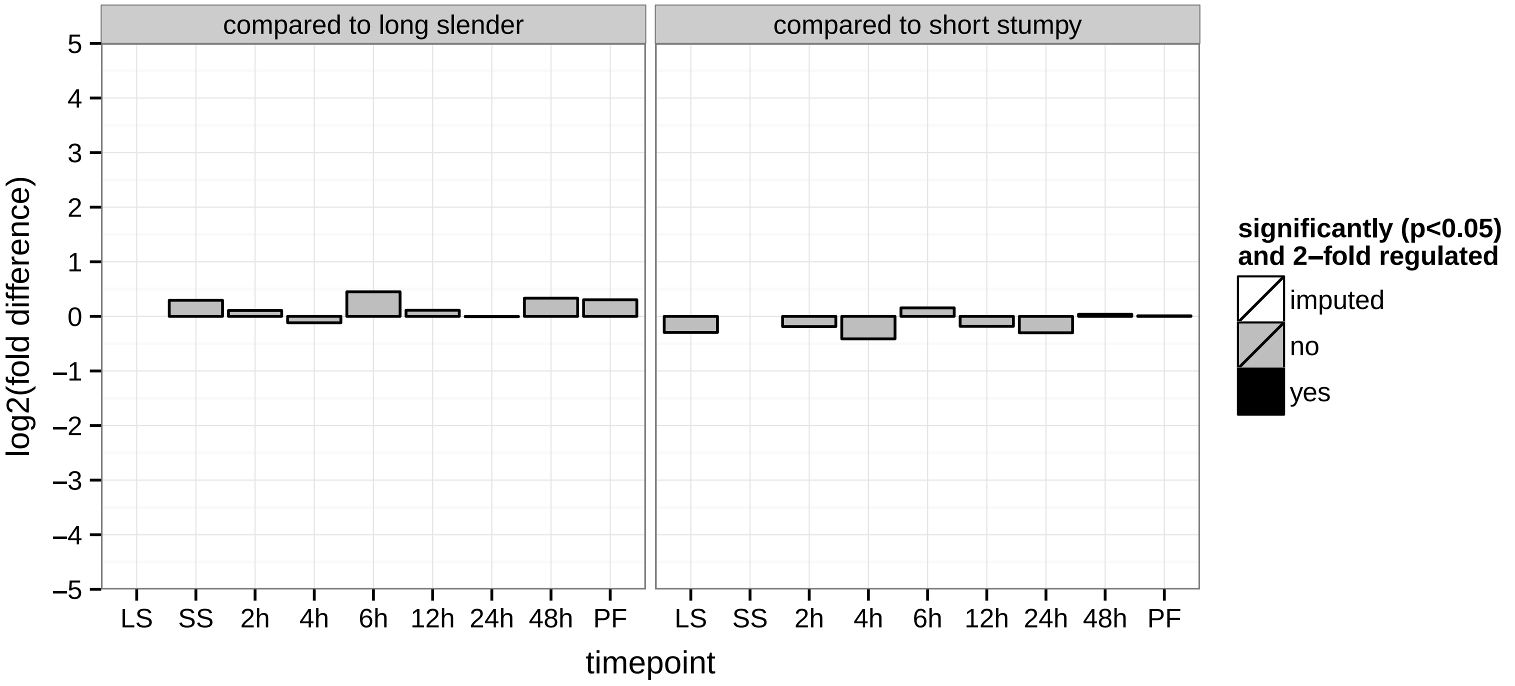

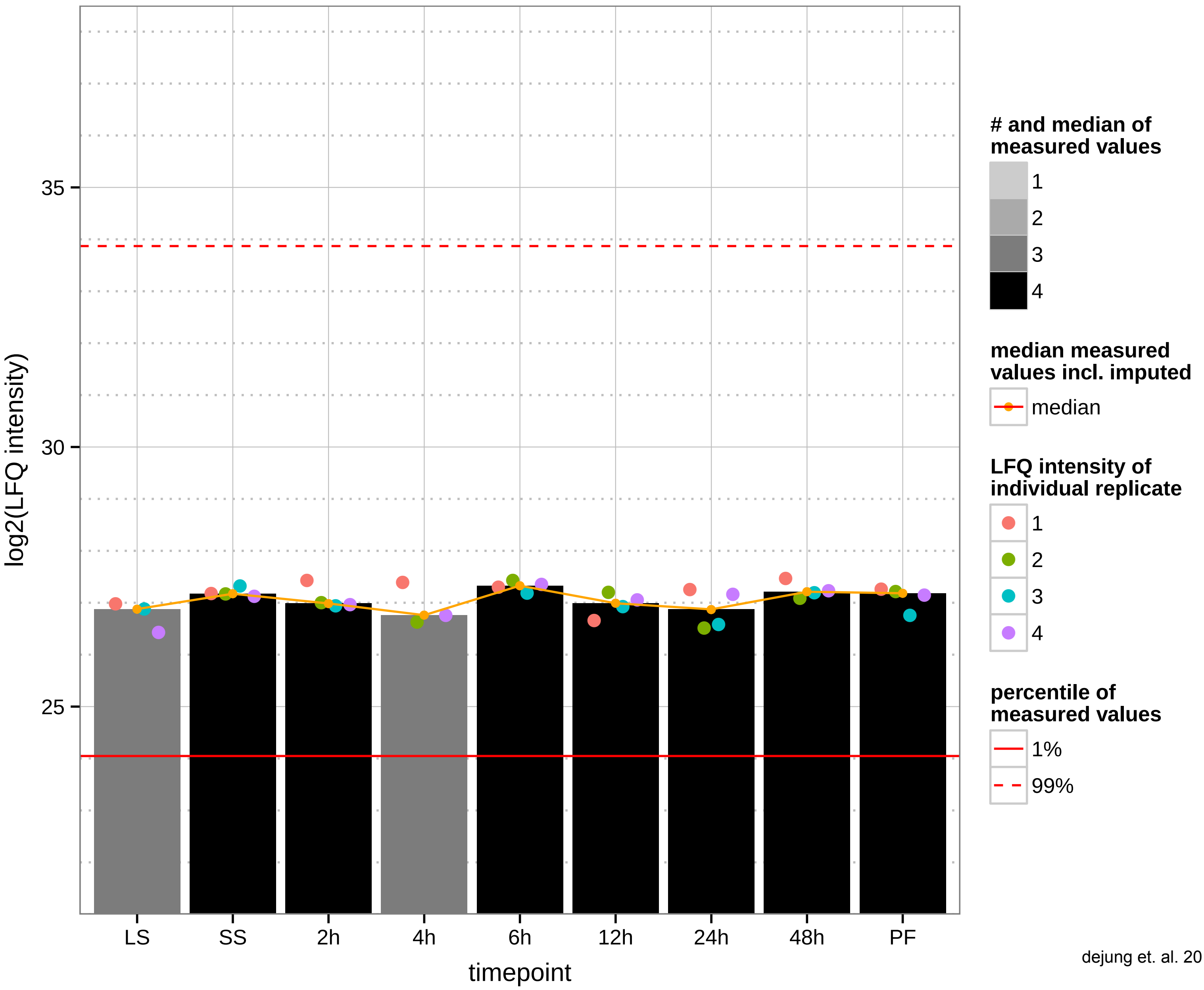

dejung et. al. 2015

hypothetical protein, conserved
Tb927.11.5700
AGOF: null
AGOC: null
AGOP: null
PGOF: null
PGOC: null
PGOP: null

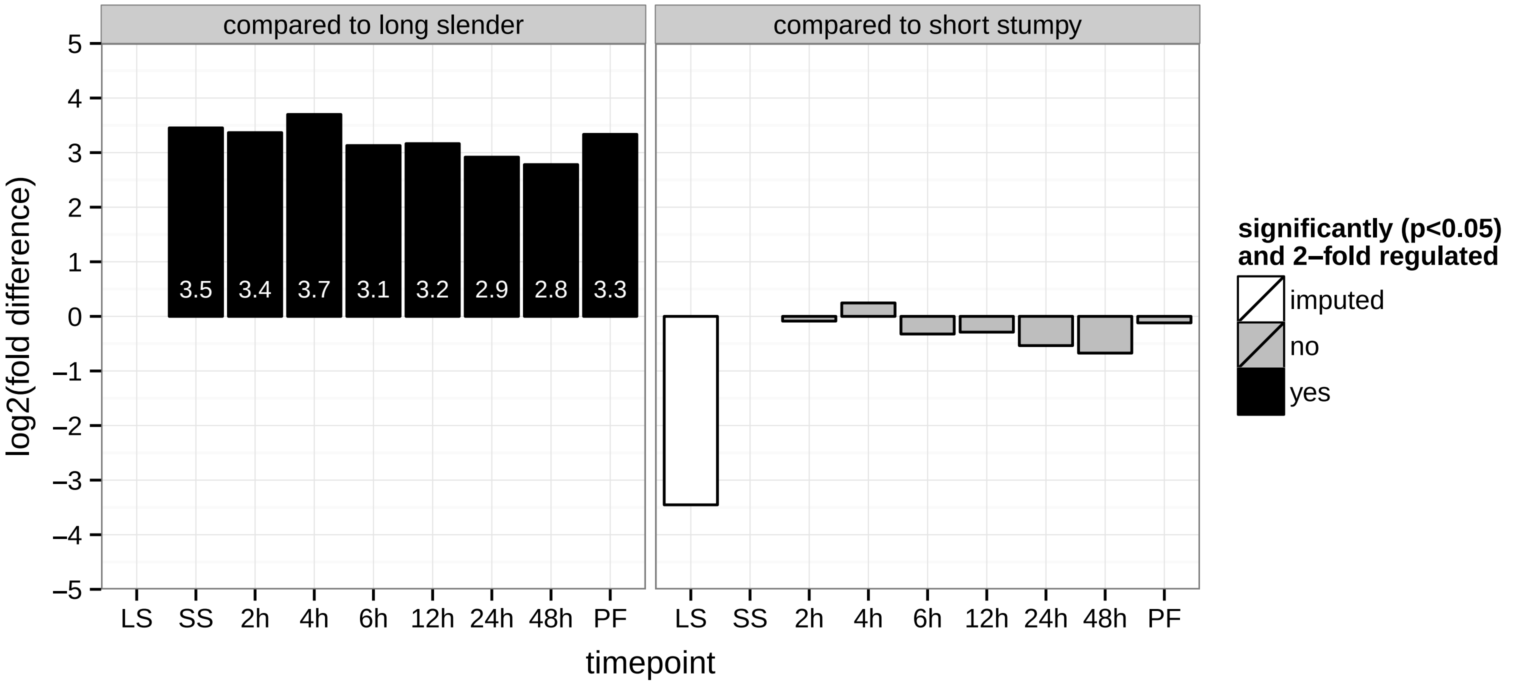

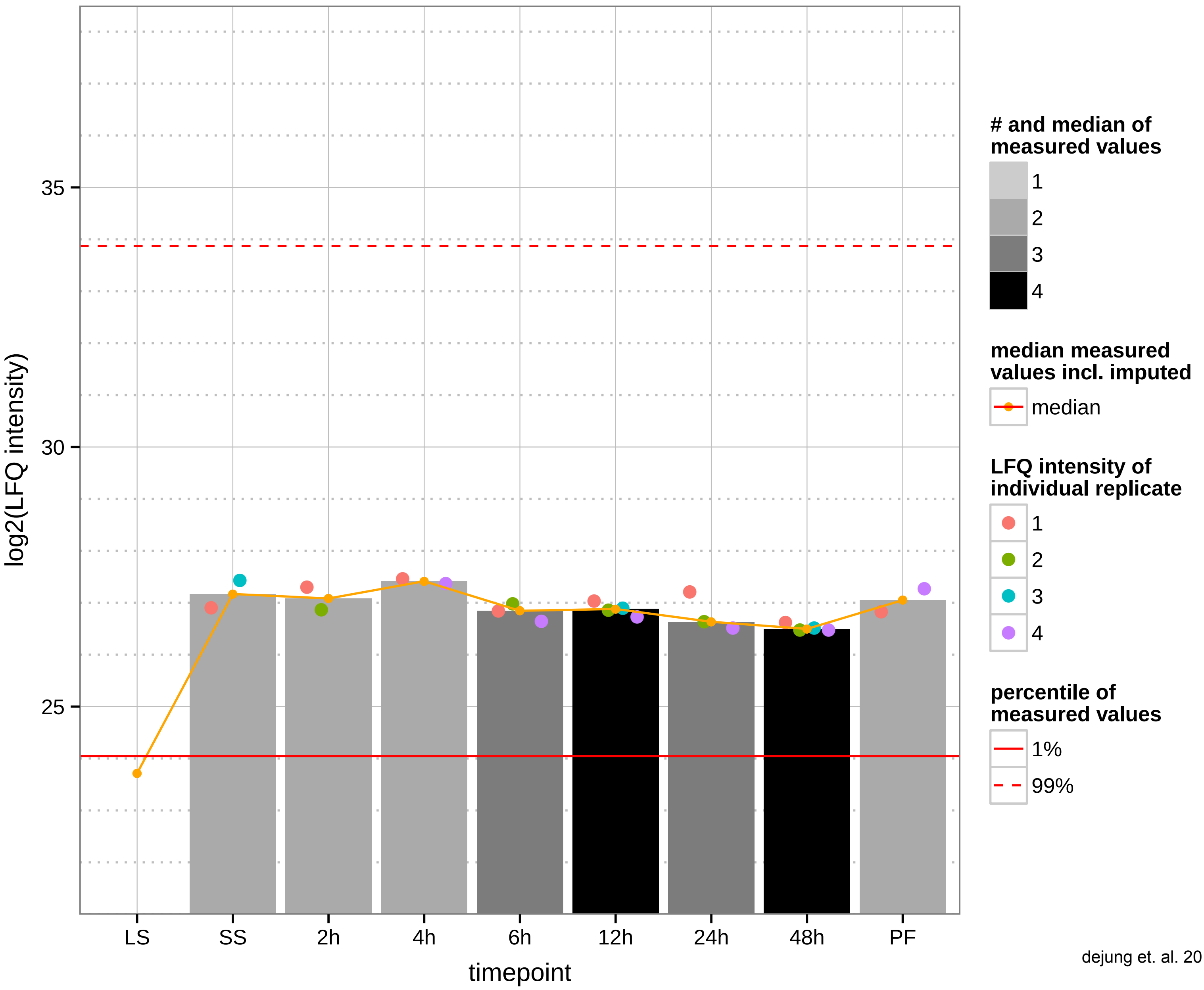

dejung et. al. 2015

pre–mRNA splicing factor, putative, ATP– dependent RNA helicase
Tb927.11.5730
AGOF: ATP binding, ATP–dependent RNA helicase activity, nucleic acid binding
AGOC: null
AGOP: RNA splicing, RNA splicing, via transesterification reactions, nuclear mRNA splicing, via spliceosome
PGOF: ATP binding, ATP–dependent helicase activity, helicase activity, nucleic acid binding
PGOC: null
PGOP: null

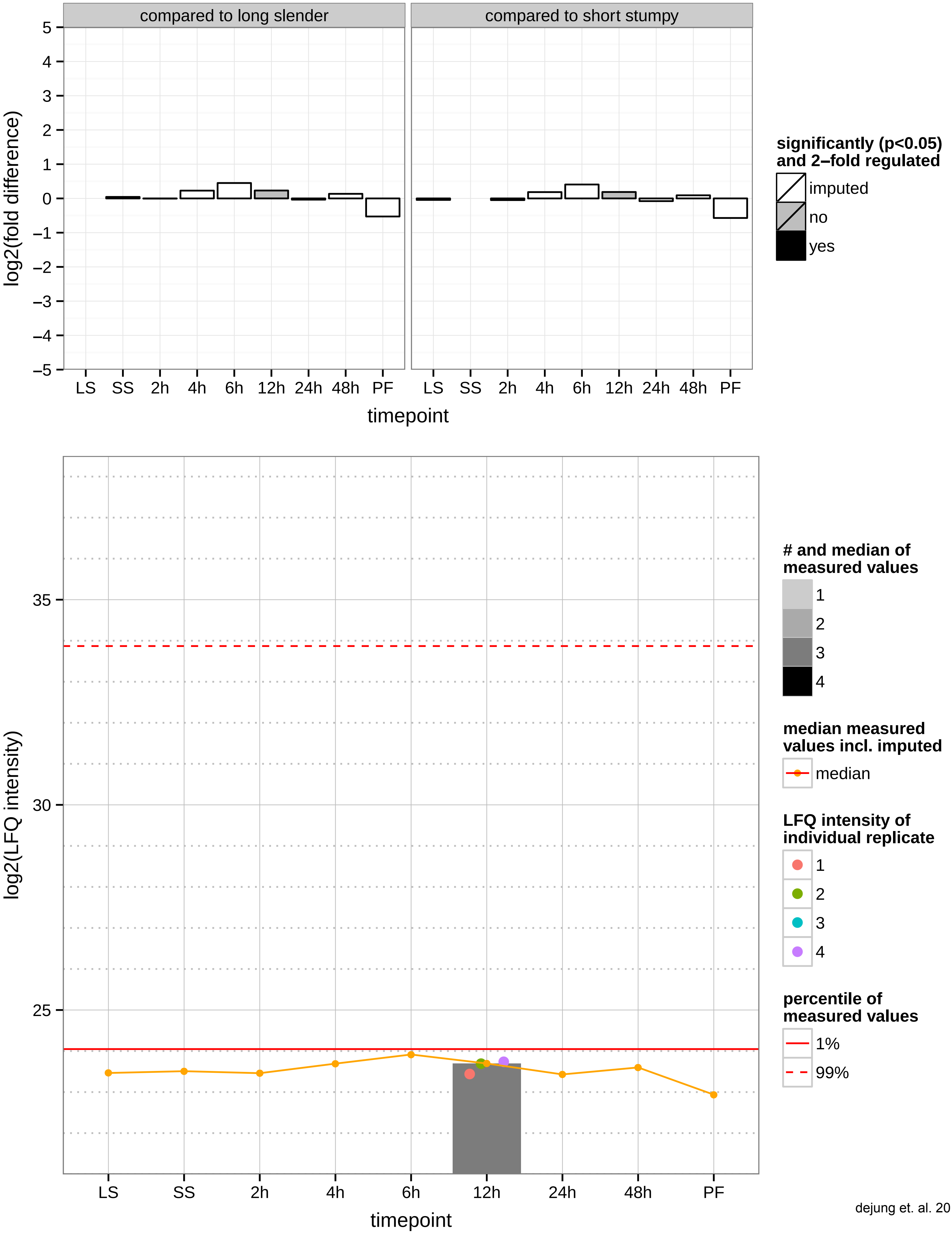

dejung et. al. 2015

hypothetical protein, conserved
Tb927.11.5890
AGOF: null
AGOC: null
AGOP: null
PGOF: null
PGOC: null
PGOP: null

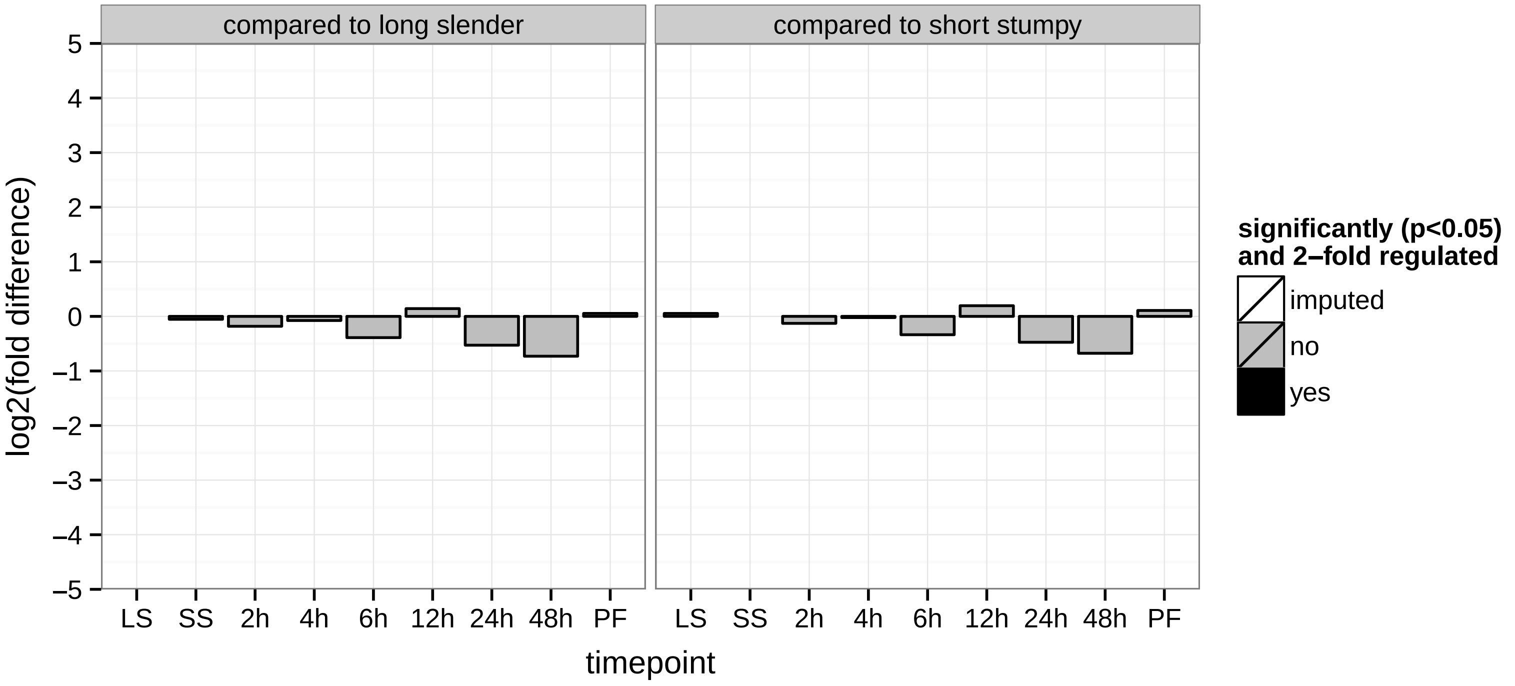

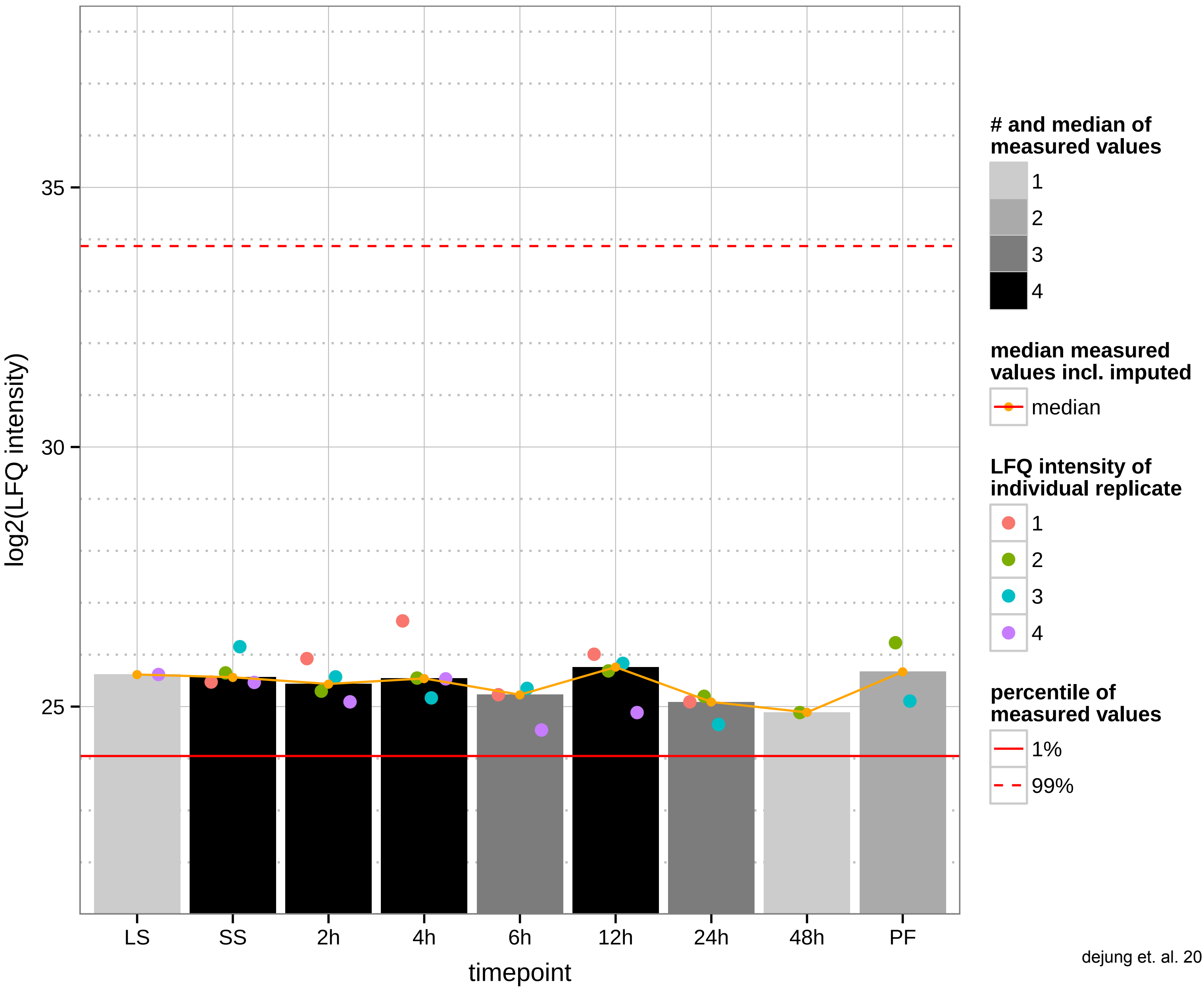

dejung et. al. 2015

conserved protein, unknown function
Tb927.11.6010
AGOF: null
AGOC: mitochondrion
AGOP: null
PGOF: null
PGOC: null
PGOP: null

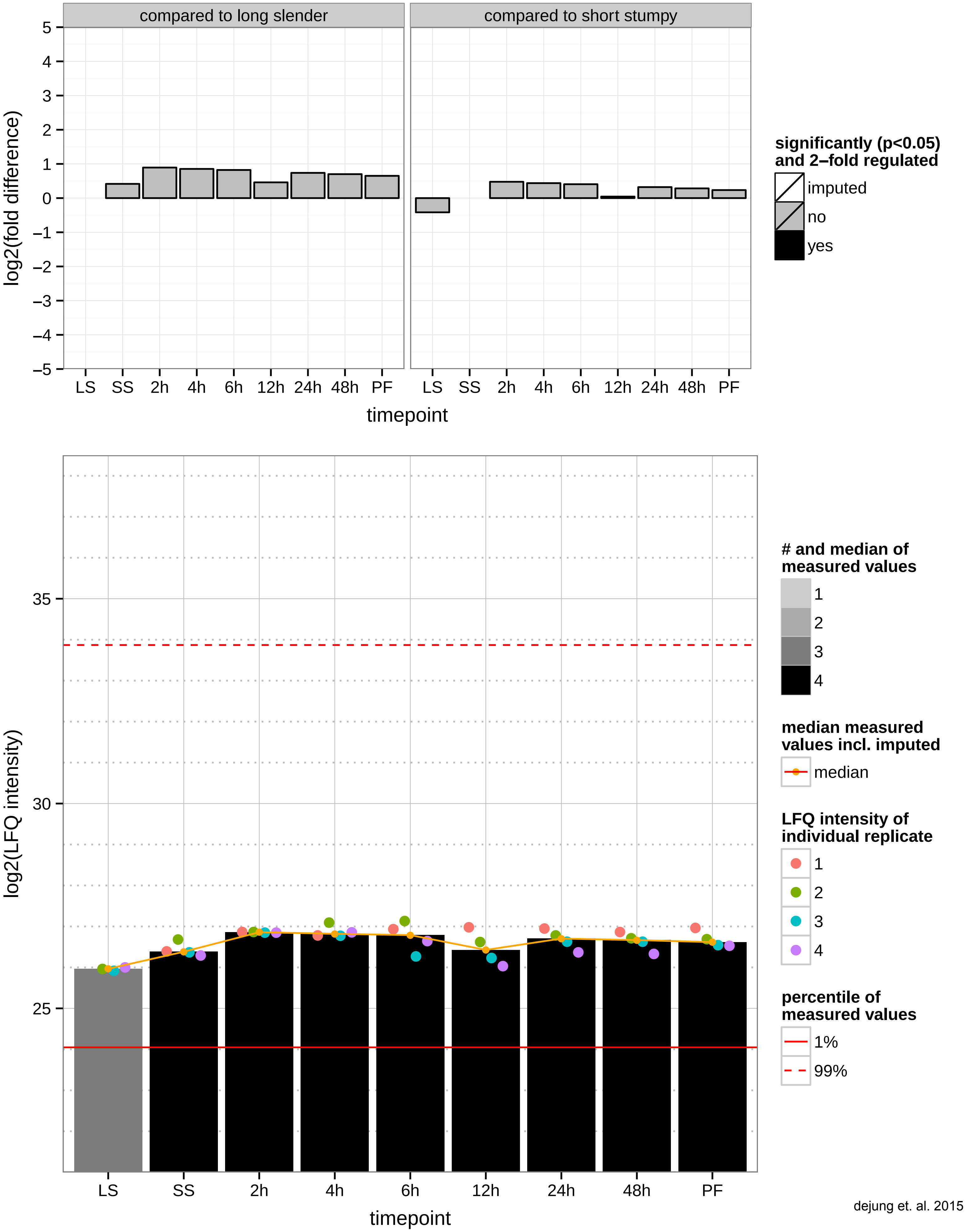

dejung et. al. 2015

hypothetical protein, conserved
Tb927.11.6050
AGOF: null
AGOC: null
AGOP: null
PGOF: null
PGOC: null
PGOP: null

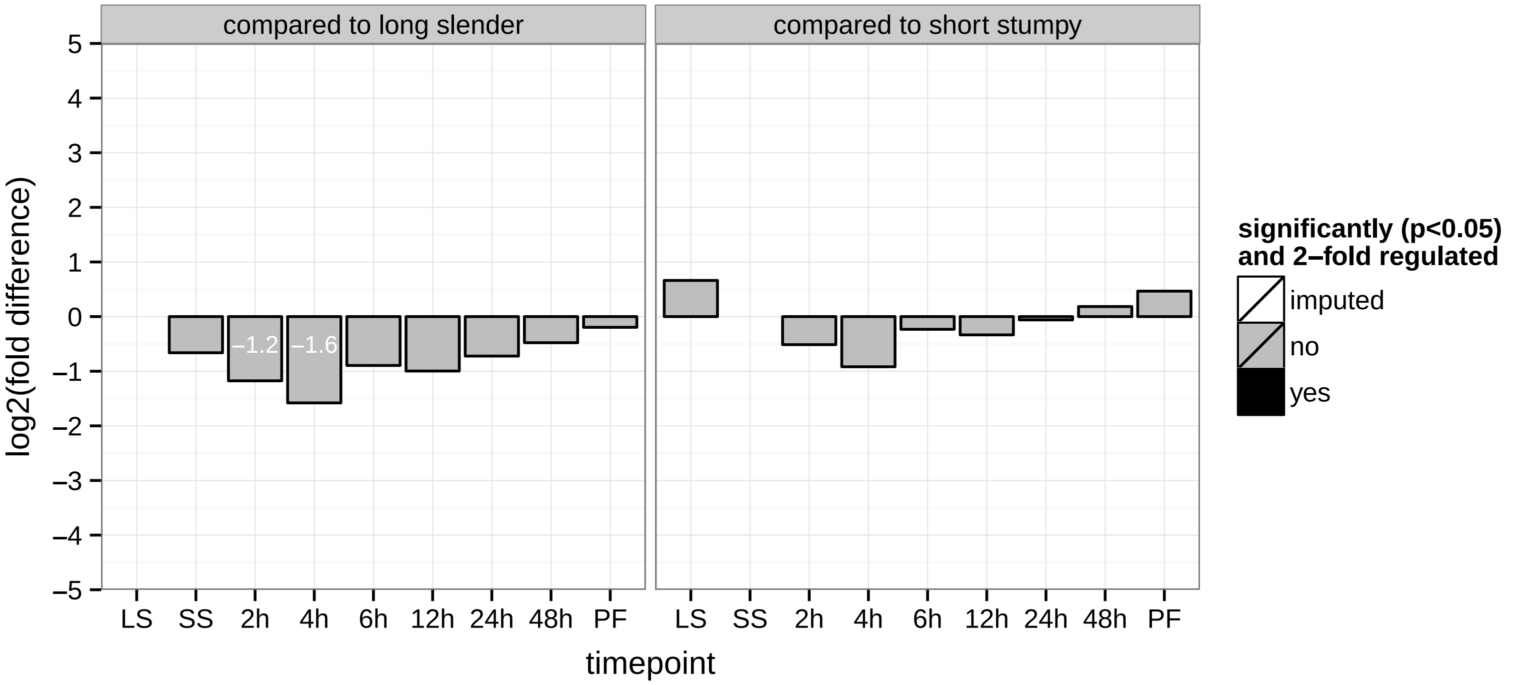

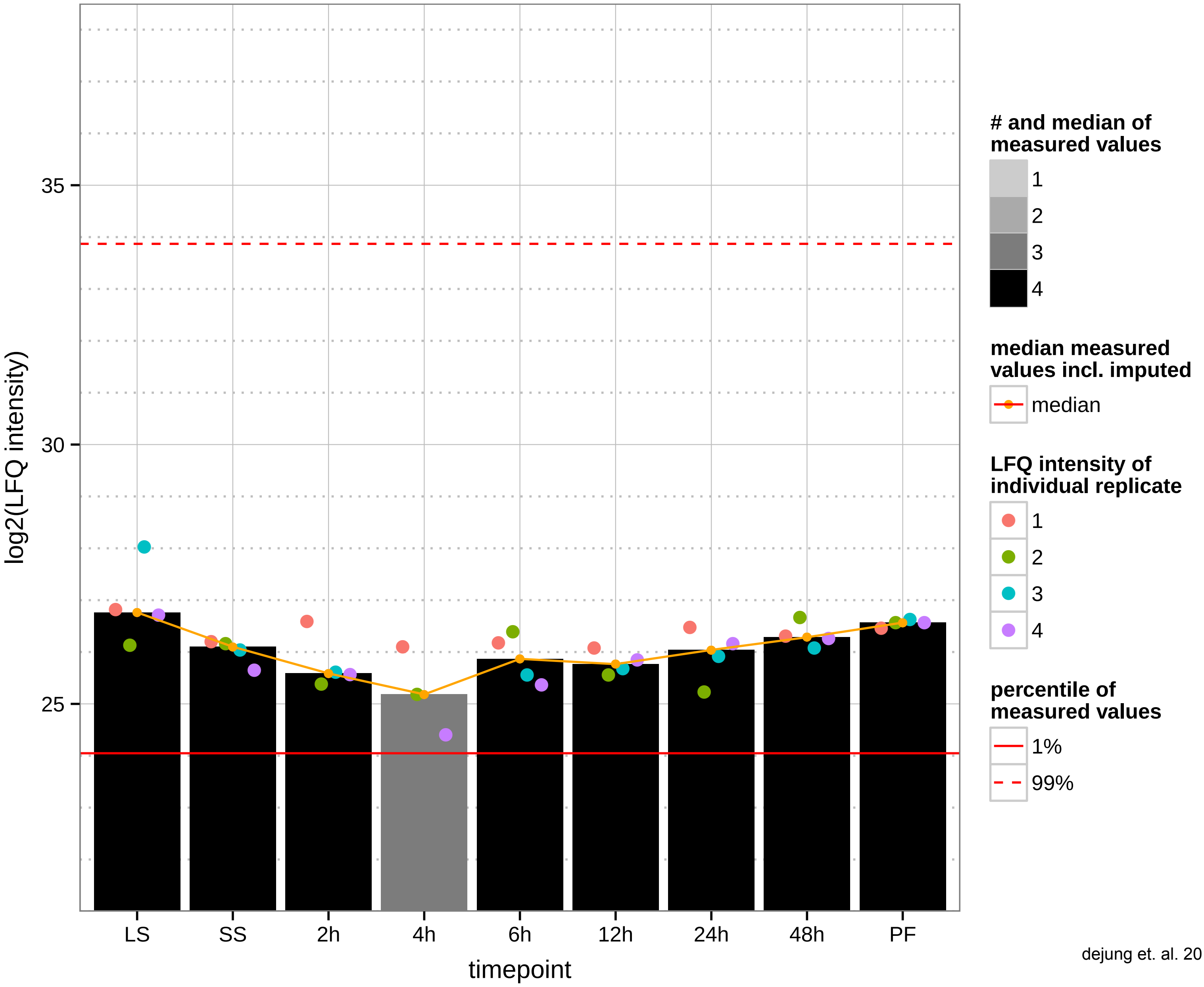

dejung et. al. 2015

hypothetical protein, conserved
Tb927.11.6070
AGOF: null
AGOC: null
AGOP: null
PGOF: binding
PGOC: null
PGOP: null

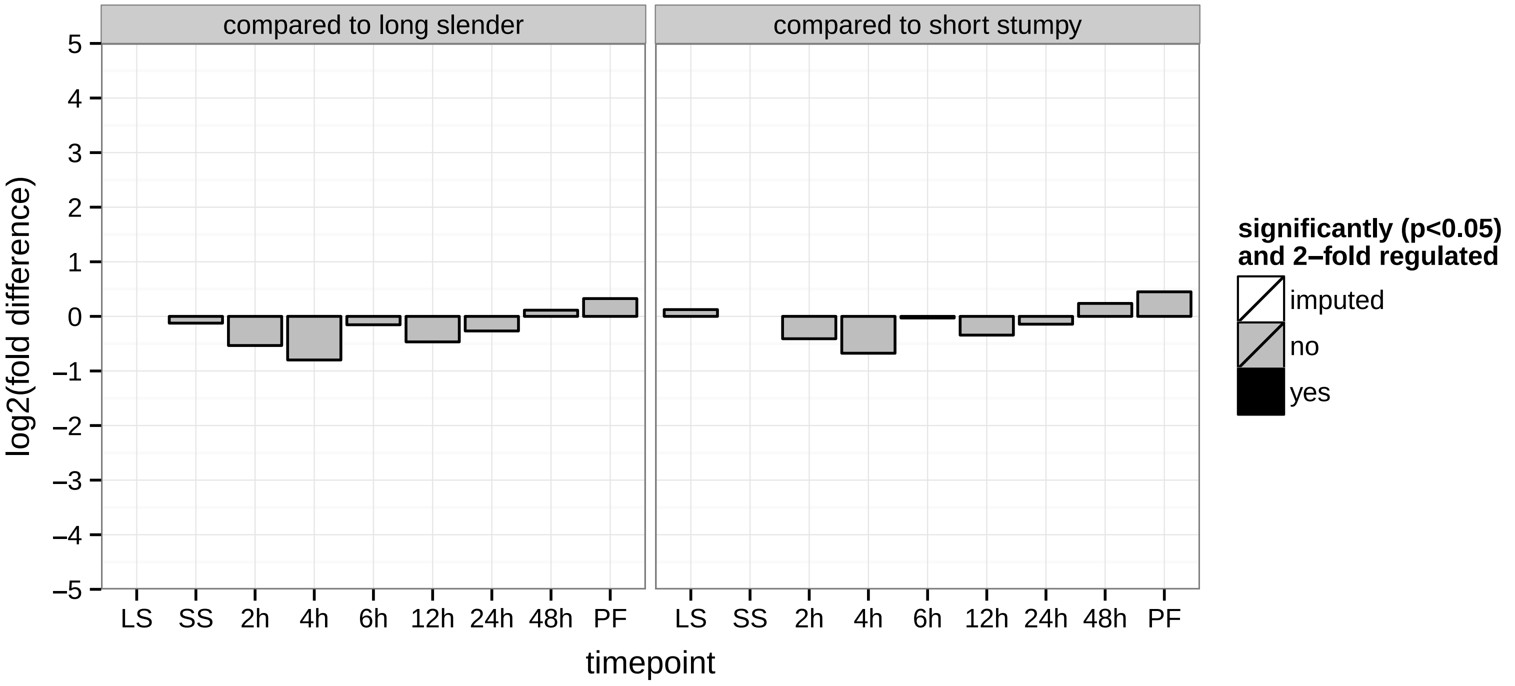

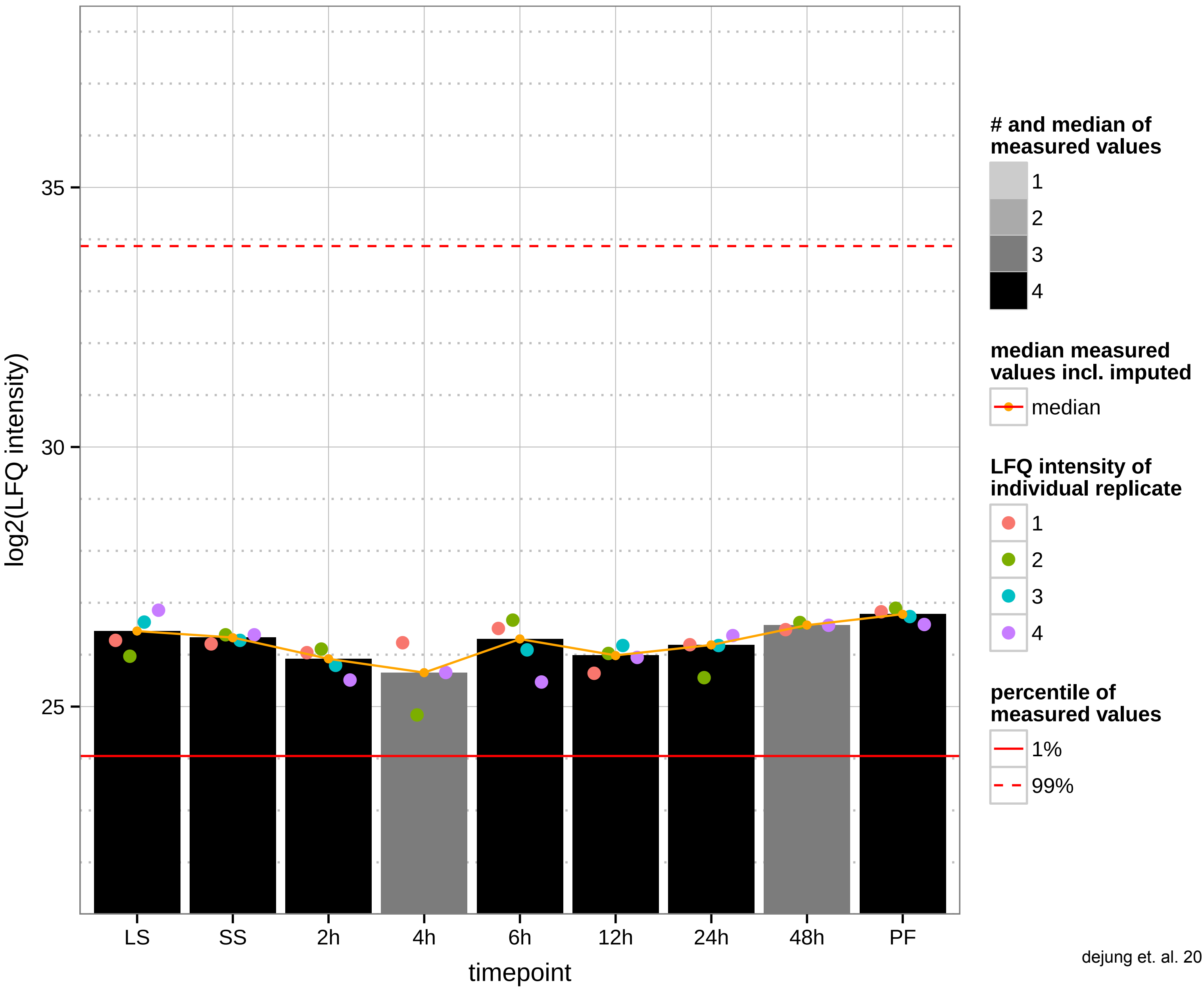

dejung et. al. 2015

hypothetical protein, conserved
Tb927.11.610
AGOF: null
AGOC: null
AGOP: null
PGOF: null
PGOC: null
PGOP: null

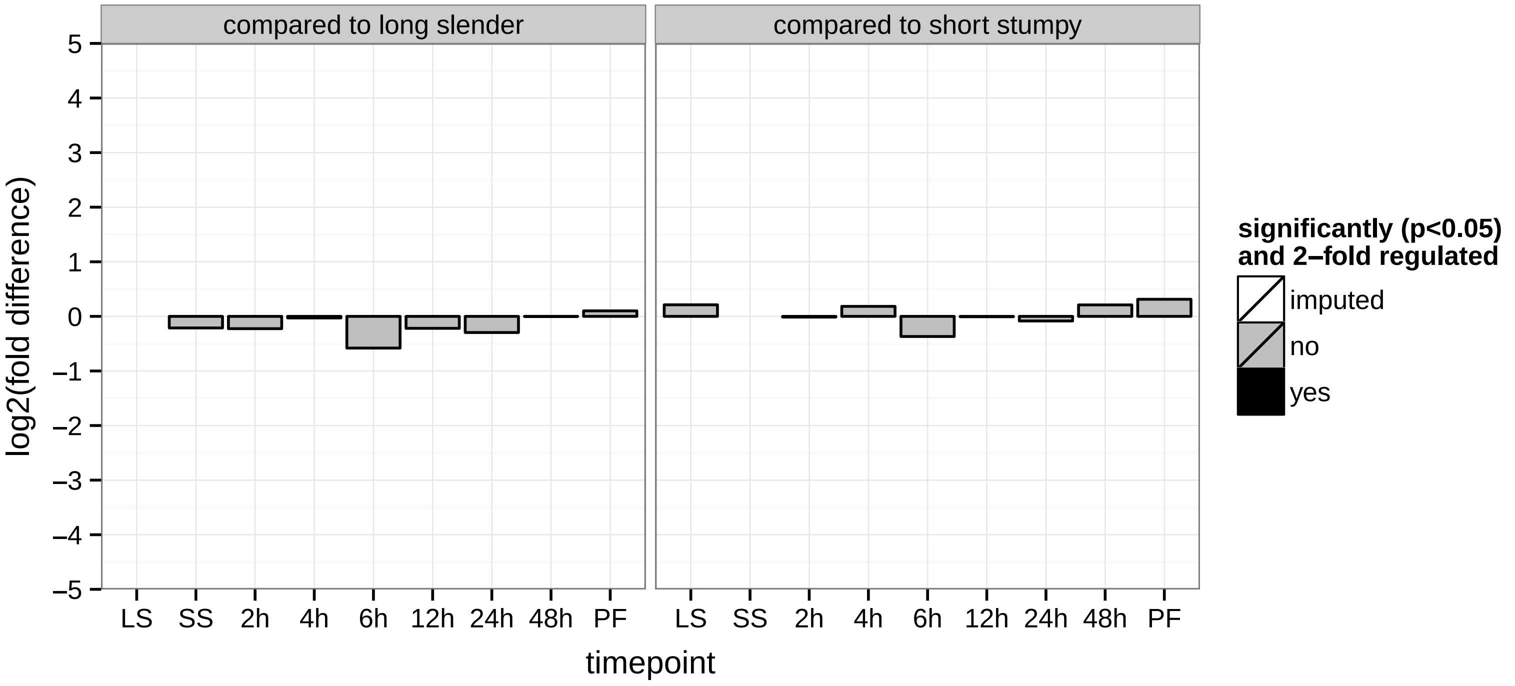

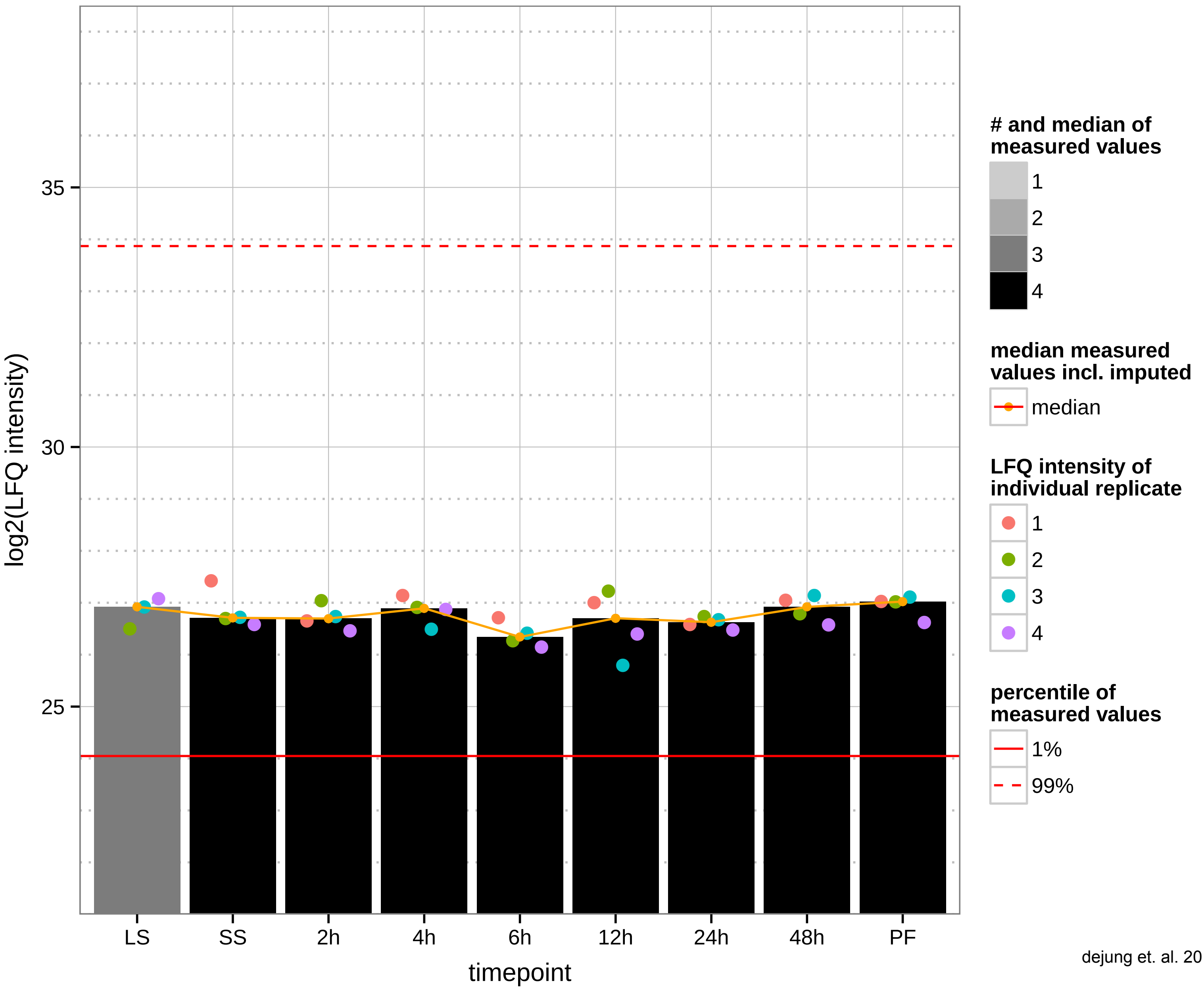

dejung et. al. 2015

SKP1–like protein
Tb927.11.6130
AGOF: null
AGOC: null
AGOP: ubiquitin–dependent protein catabolic process
PGOF: null
PGOC: null
PGOP: ubiquitin–dependent protein catabolic process

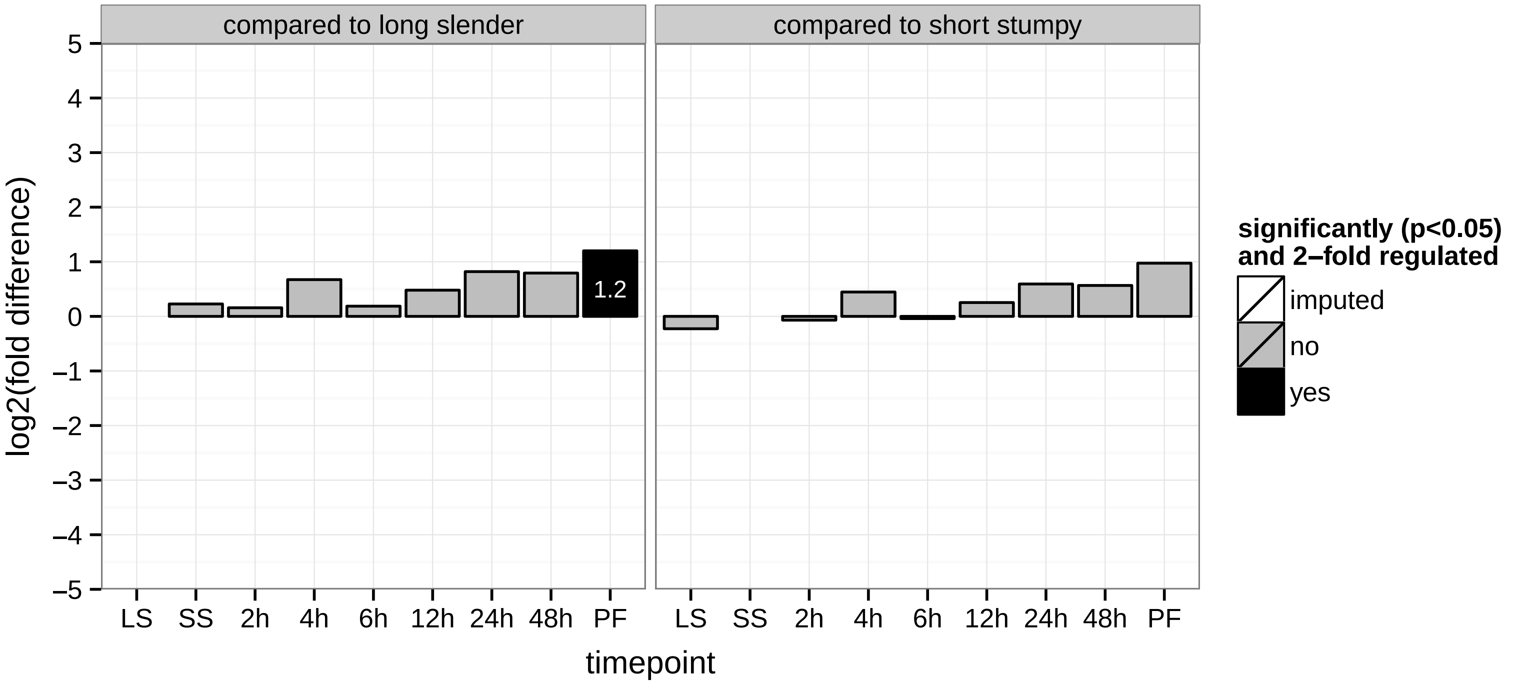

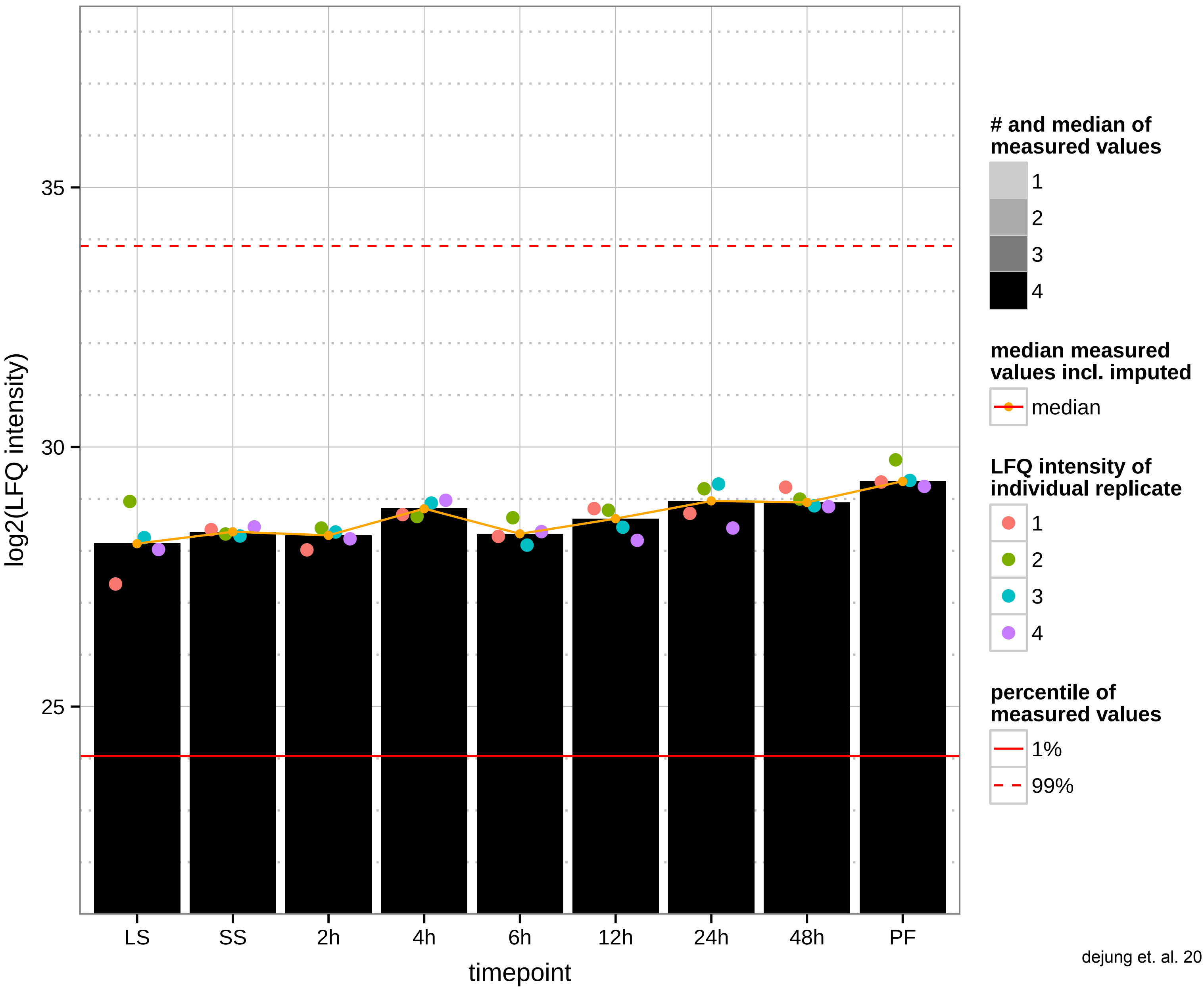

dejung et. al. 2015

40S ribosomal protein S15A, putative
Tb927.11.6140;Tb927.3.4360
AGOF: structural constituent of ribosome
AGOC: intracellular, organellar small ribosomal subunit, ribosome
AGOP: translation
PGOF: structural constituent of ribosome
PGOC: intracellular, ribosome
PGOP: translation

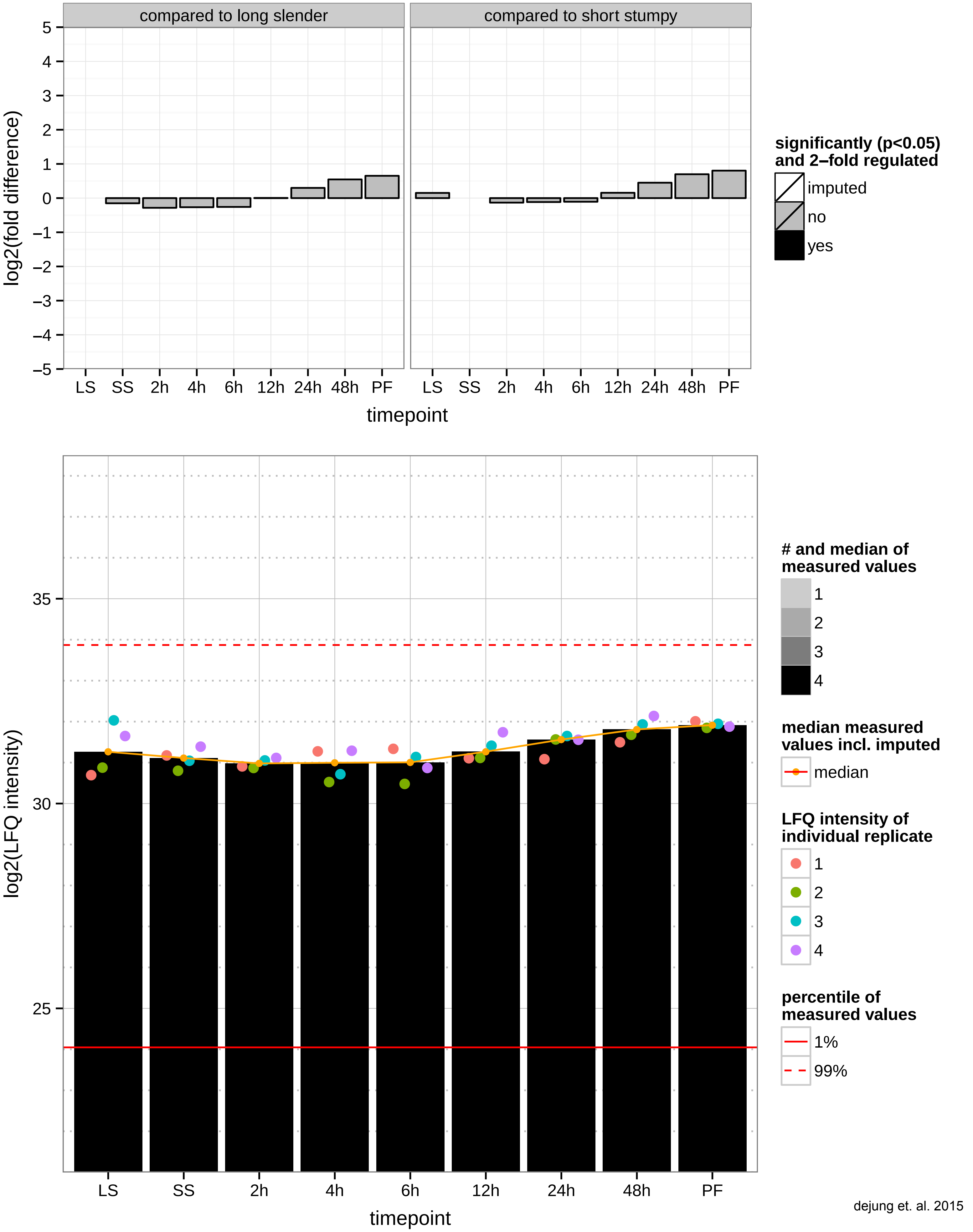

dejung et. al. 2015

protein transport protein SEC31, putative, cytosolic coat protein
Tb927.11.6170
AGOF: null
AGOC: null
AGOP: transport
PGOF: protein binding
PGOC: null
PGOP: null

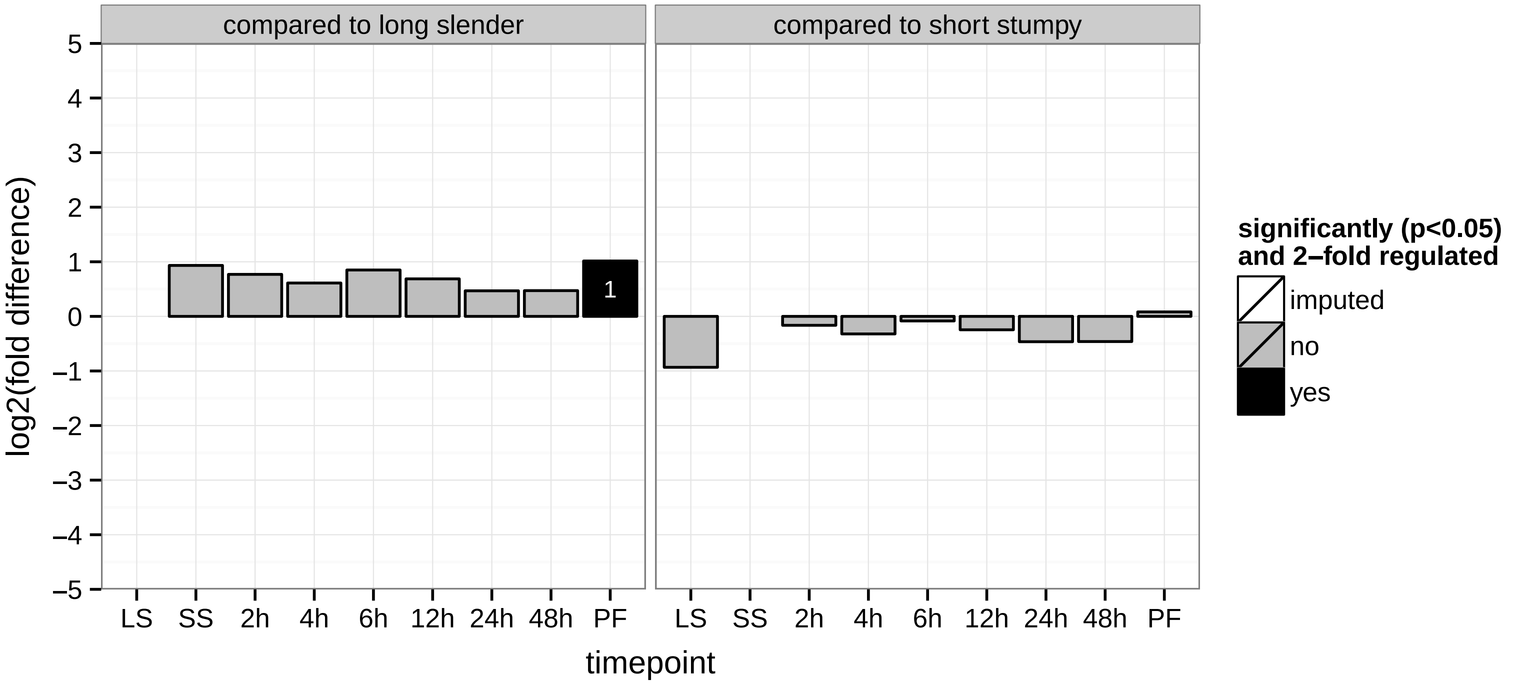

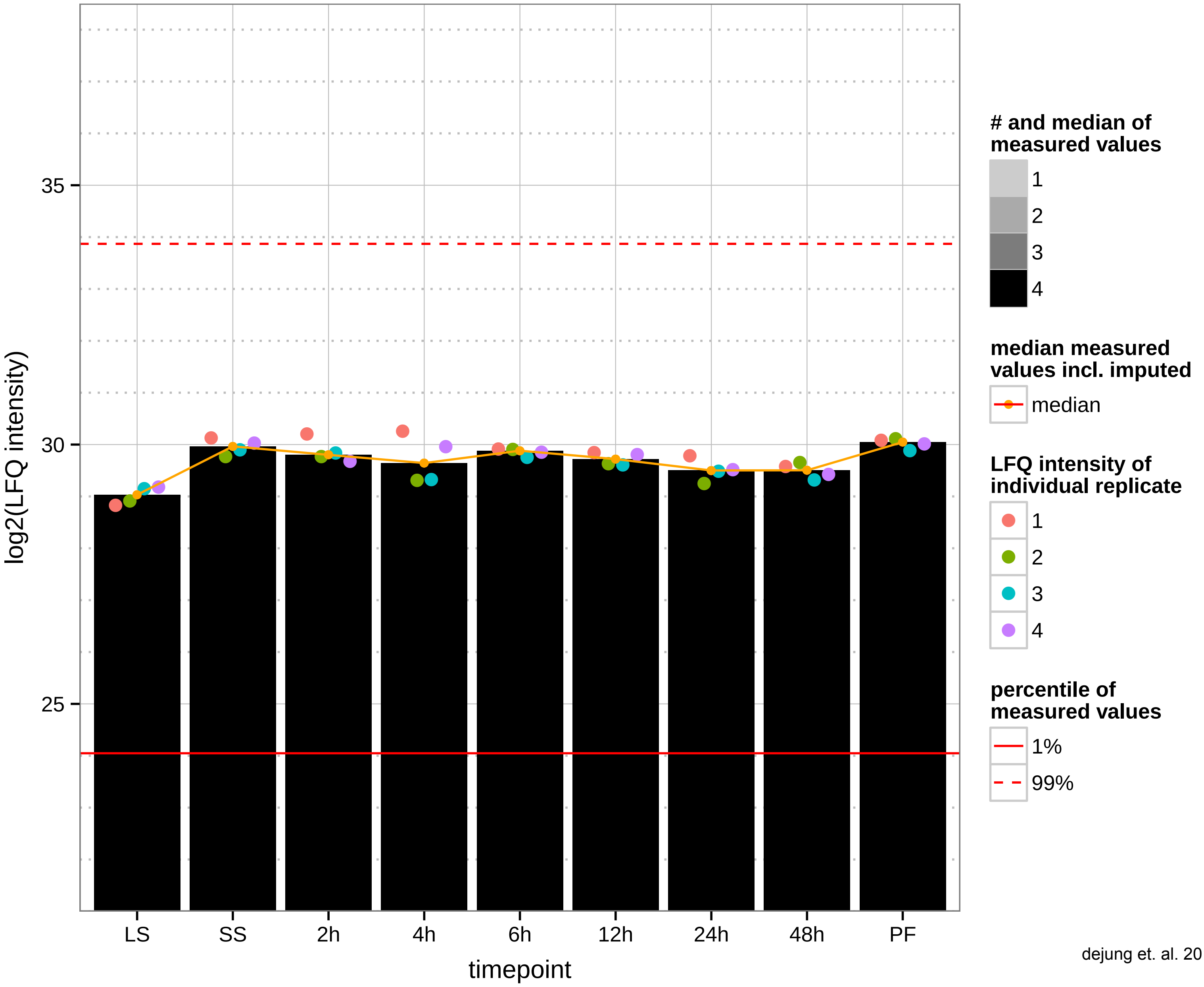

dejung et. al. 2015

RNA polymerase I second largest subunit (RPA135)
Tb927.11.630
AGOF: DNA binding, DNA−directed RNA polymerase activity, ribonucleoside binding
AGOC: nucleus
AGOP: transcription, DNA−dependent
PGOF: DNA binding, DNA−directed RNA polymerase activity
PGOC: nucleus
PGOP: transcription, DNA−dependent

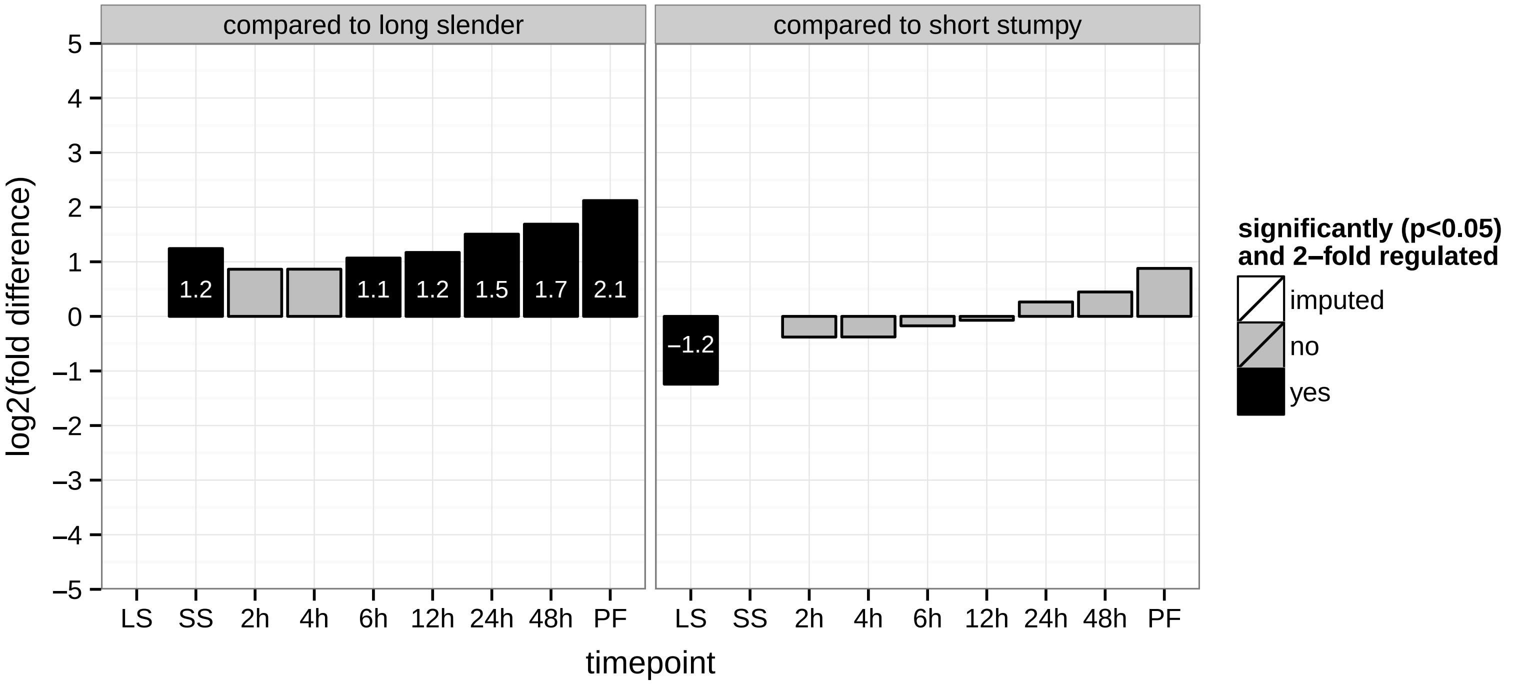

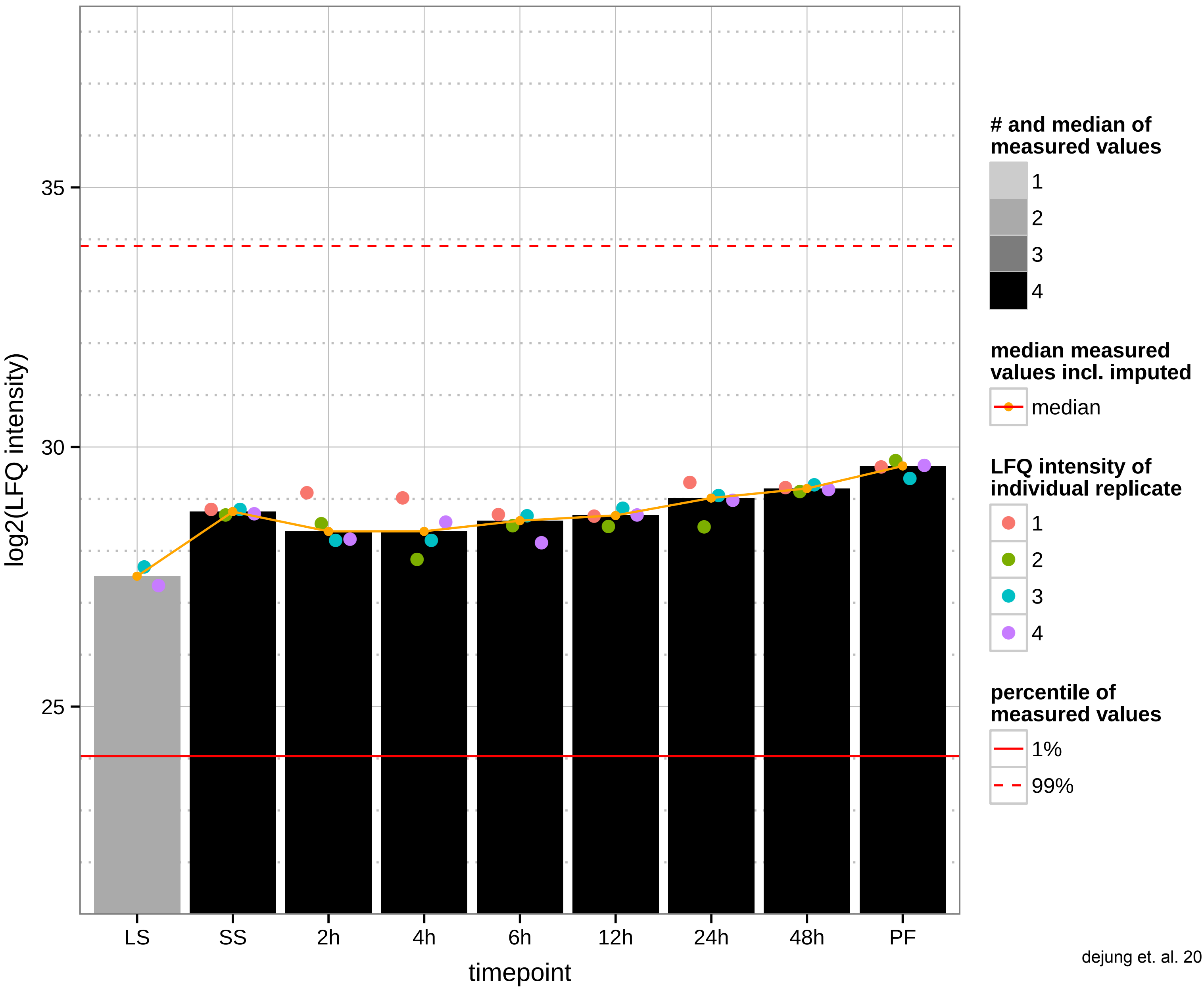

dejung et. al. 2015

hypothetical protein, conserved
Tb927.11.6310
AGOF: null
AGOC: null
AGOP: null
PGOF: null
PGOC: null
PGOP: null

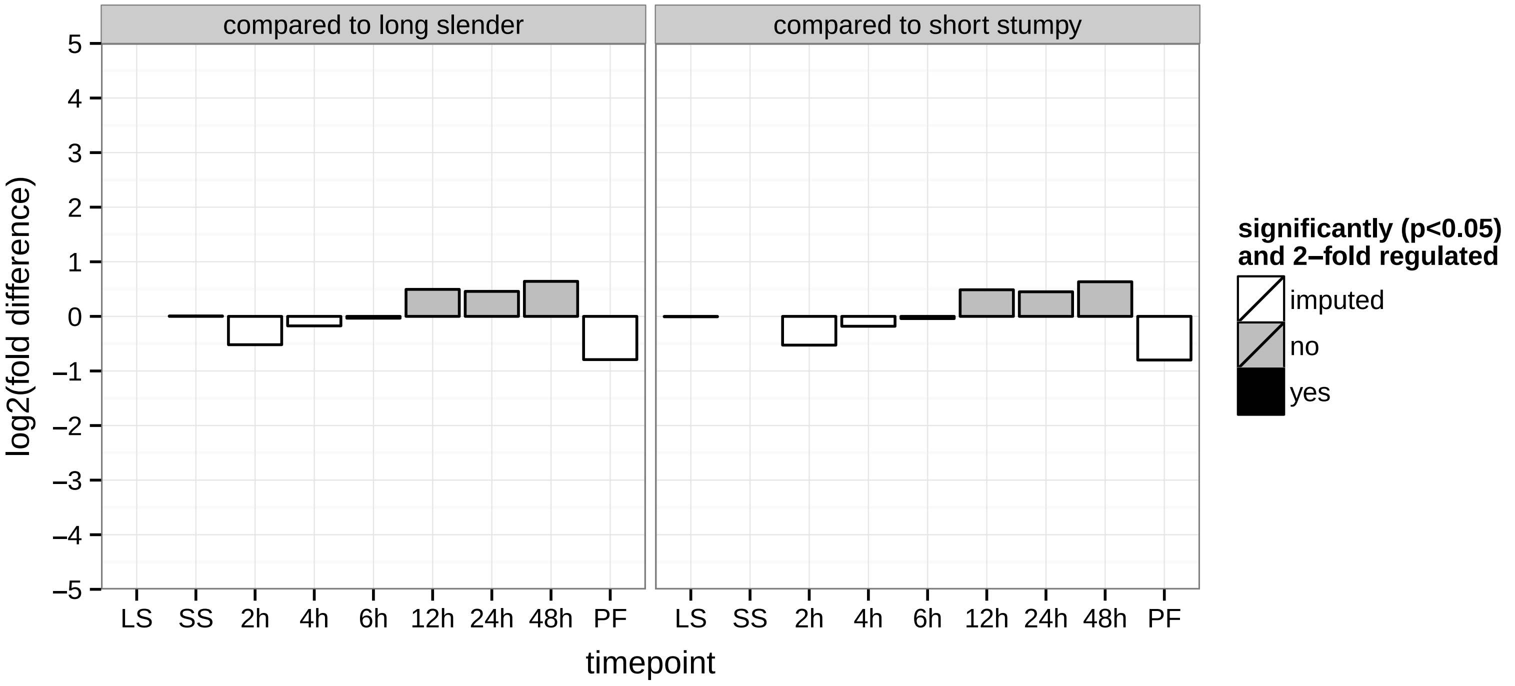

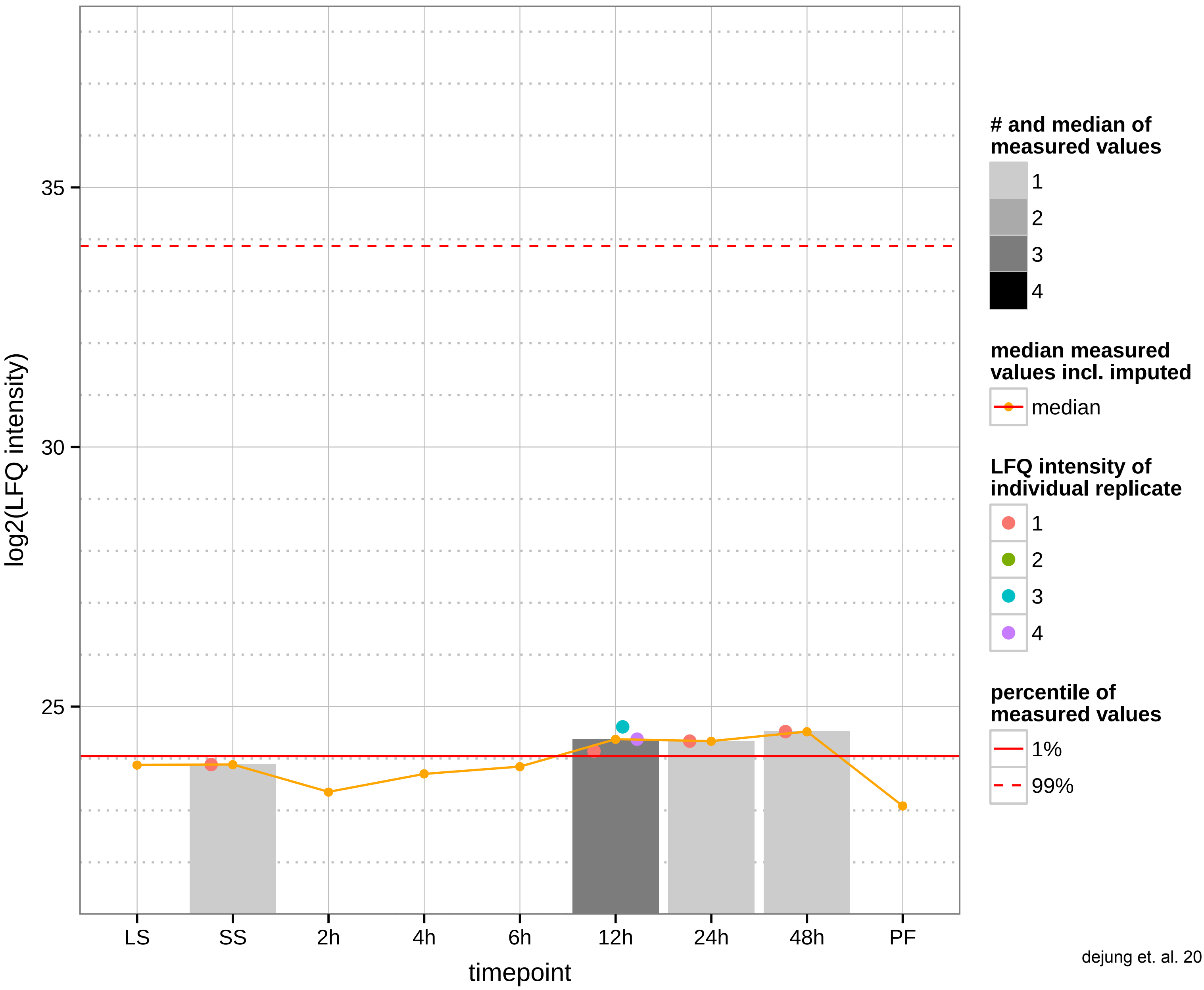

dejung et. al. 2015

MRB1−associated protein, zinc finger ccch and cchc domain−containing protein
Tb927.11.6320
AGOF: null
AGOC: null
AGOP: null
PGOF: nucleic acid binding, zinc ion binding
PGOC: null
PGOP: null

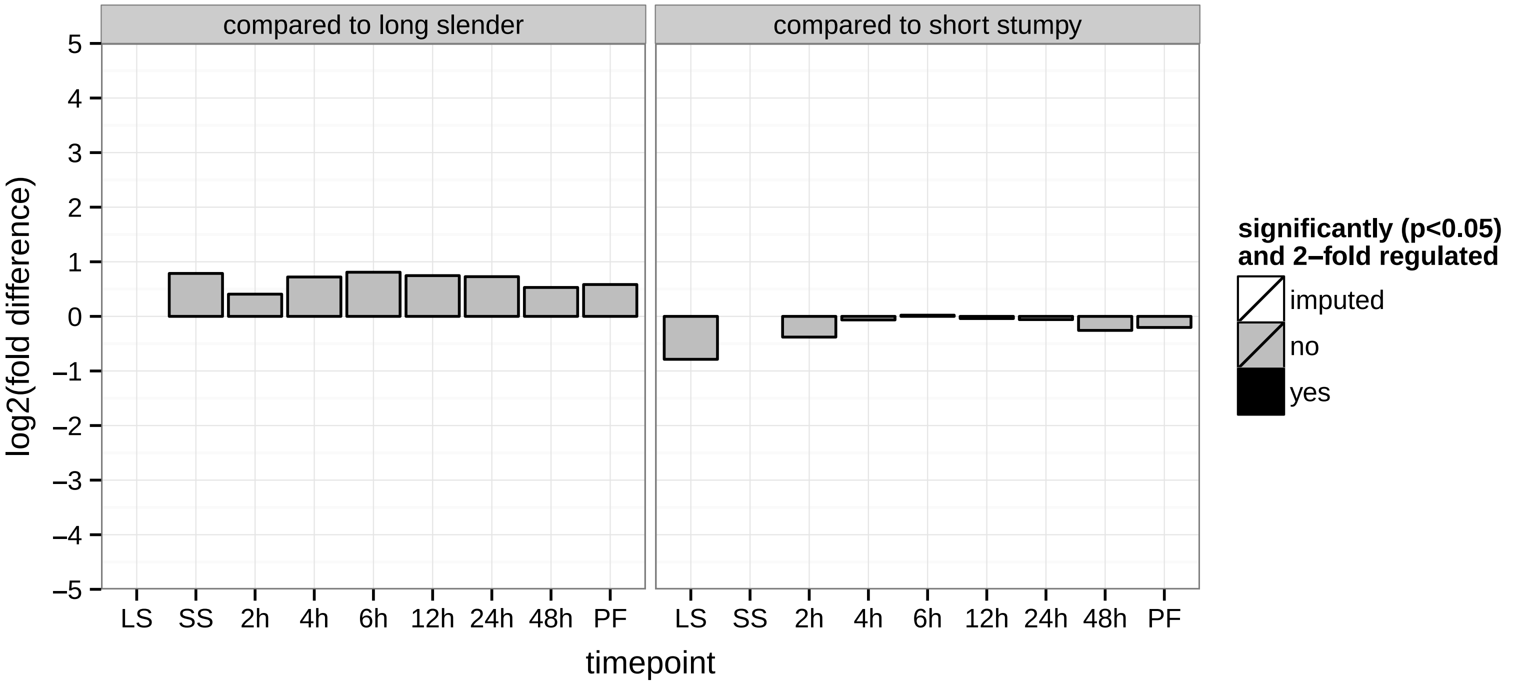

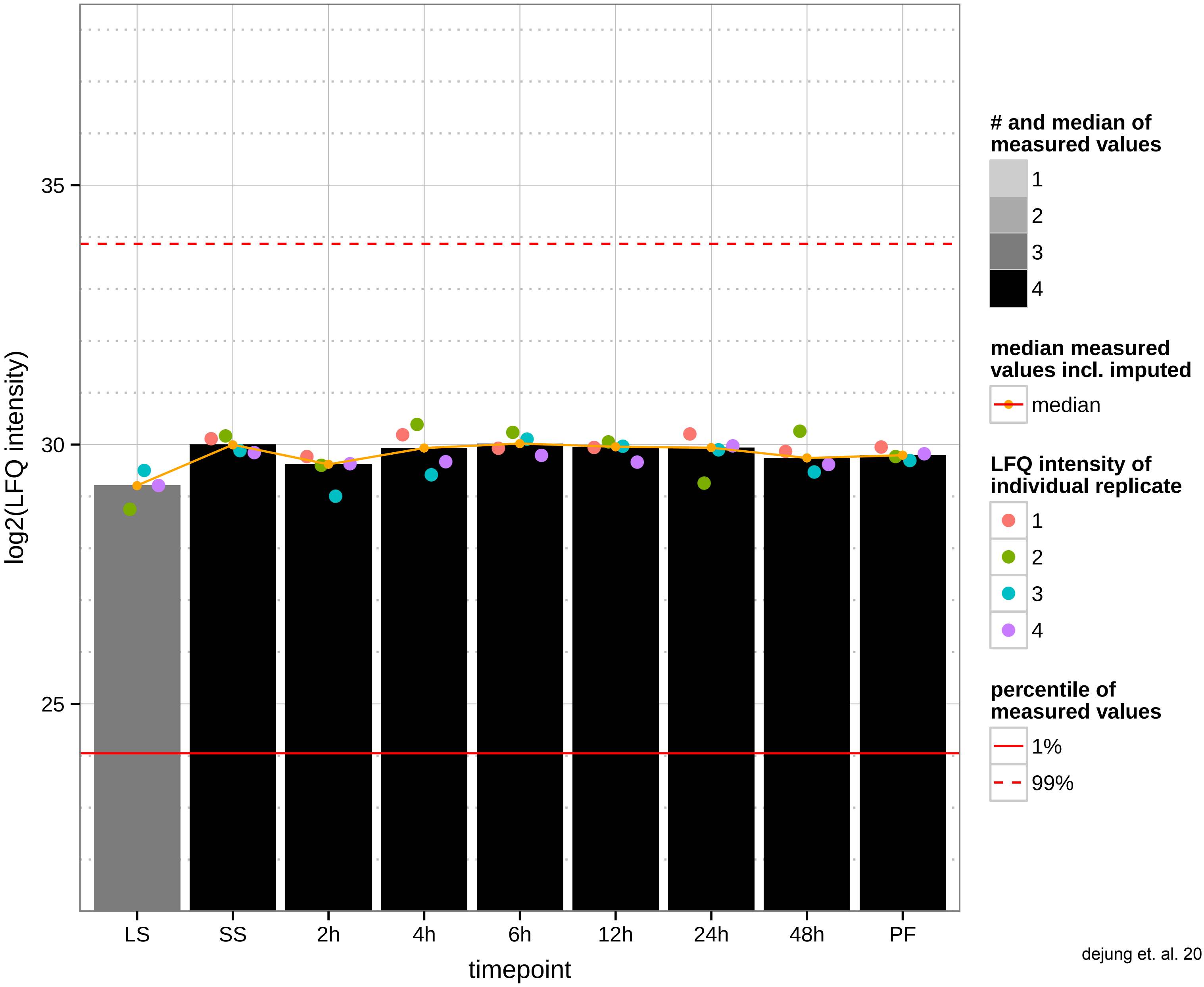

dejung et. al. 2015

60S ribosomal protein L24, putative
Tb927.11.6360
AGOF: structural constituent of ribosome
AGOC: intracellular, ribosome
AGOP: translation
PGOF: null
PGOC: null
PGOP: null

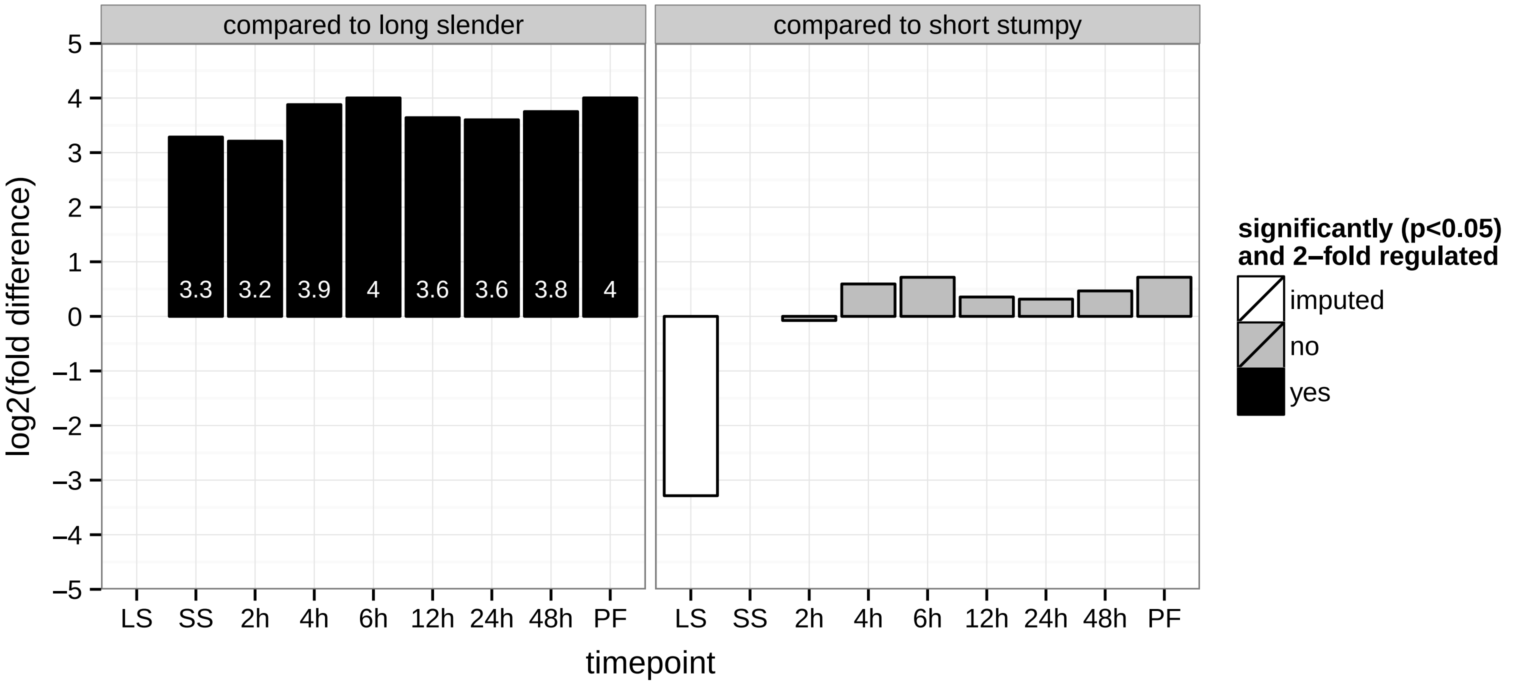

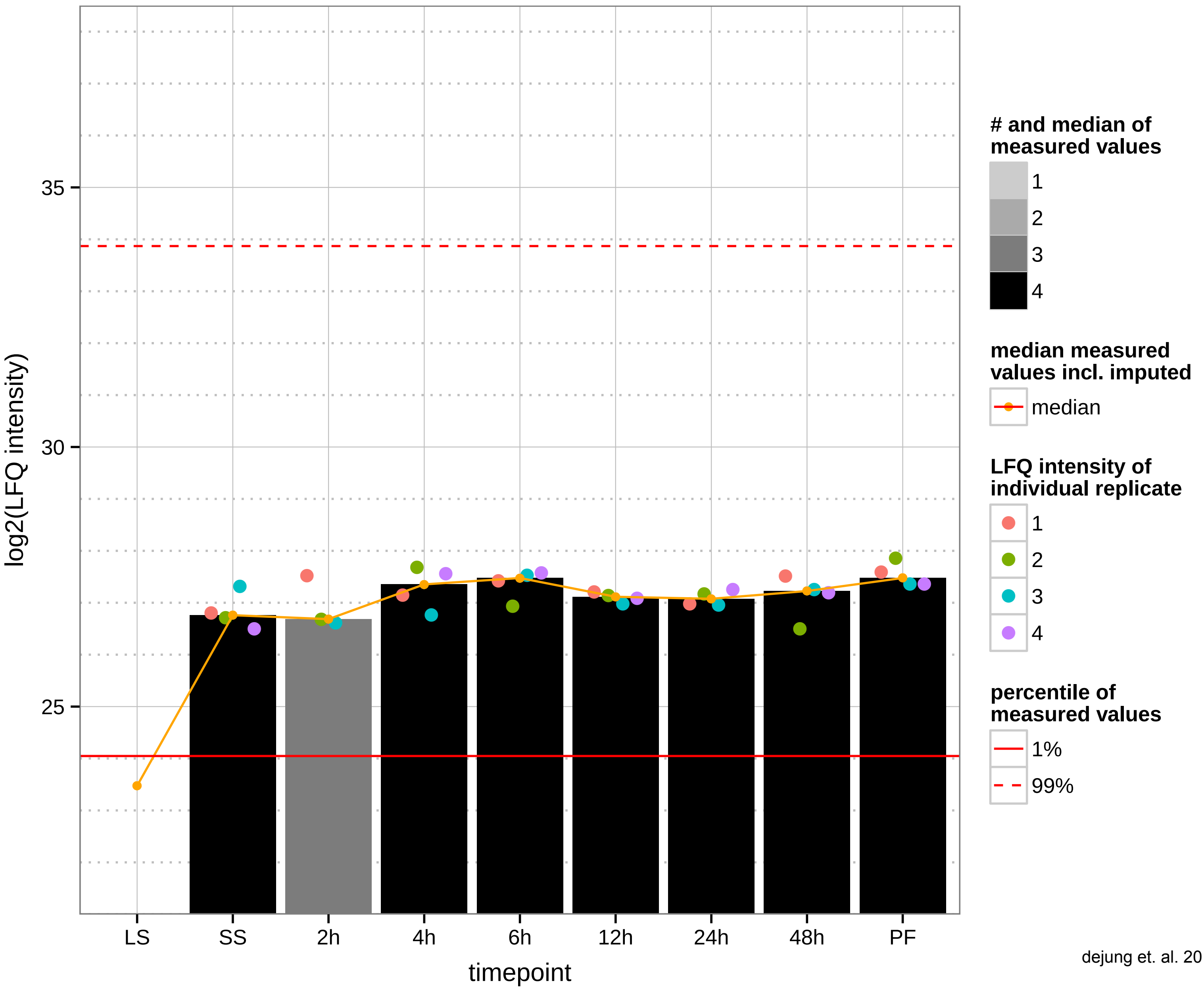

dejung et. al. 2015

leucine–rich repeat protein (LRRP), putative
Tb927.11.6370
AGOF: null
AGOC: null
AGOP: null
PGOF: protein binding
PGOC: null
PGOP: null

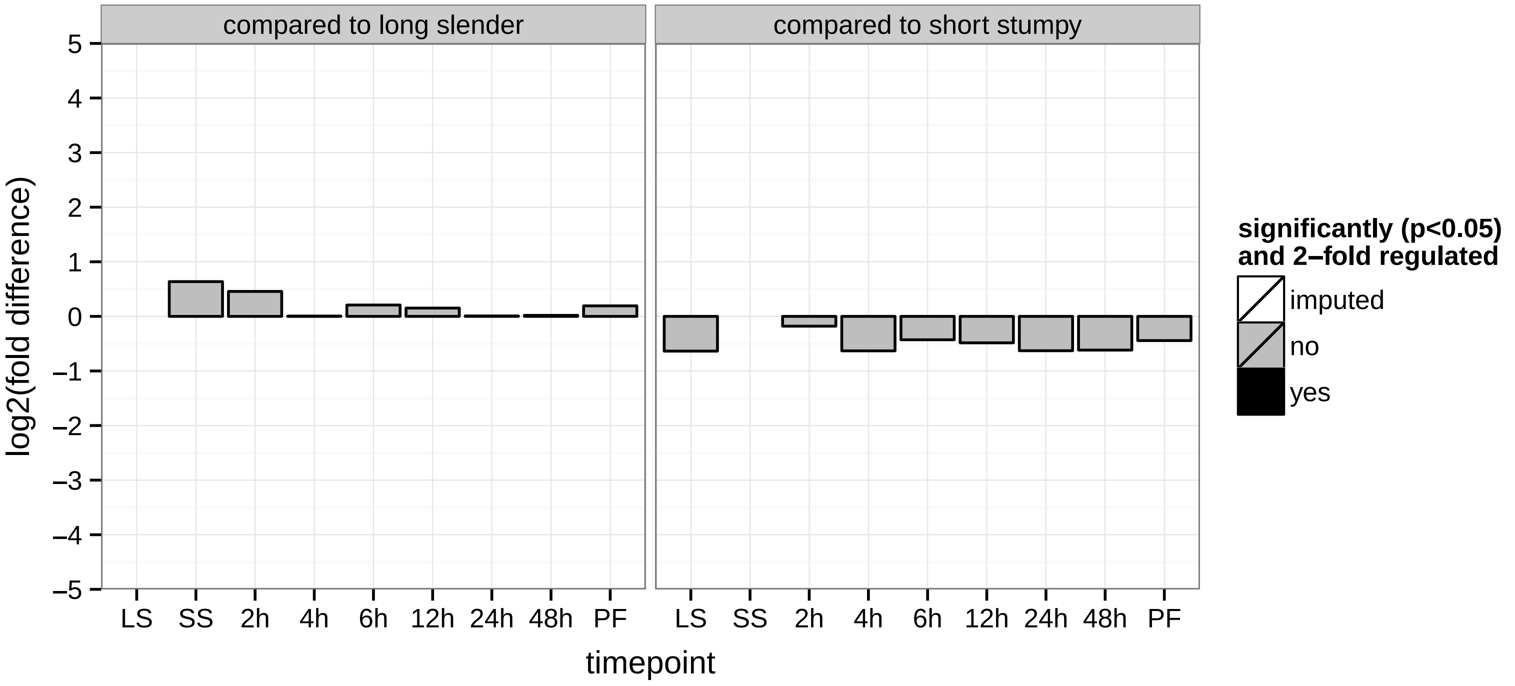

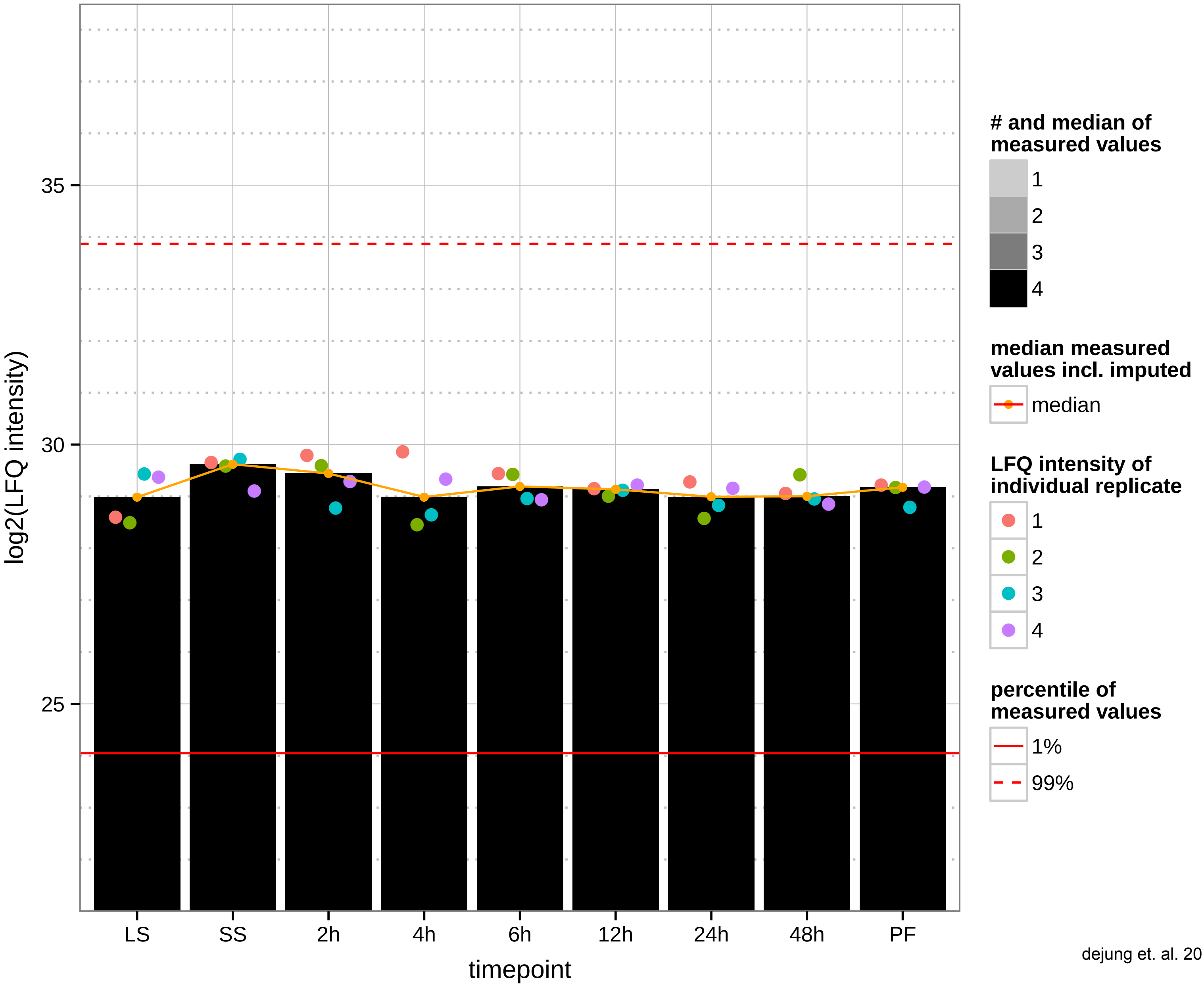

dejung et. al. 2015

kinesin, putative
Tb927.11.6400
AGOF: ATP binding, microtubule motor activity, motor activity
AGOC: kinesin complex, microtubule associated complex
AGOP: microtubule–based movement
PGOF: ATP binding, microtubule motor activity
PGOC: null
PGOP: microtubule–based movement

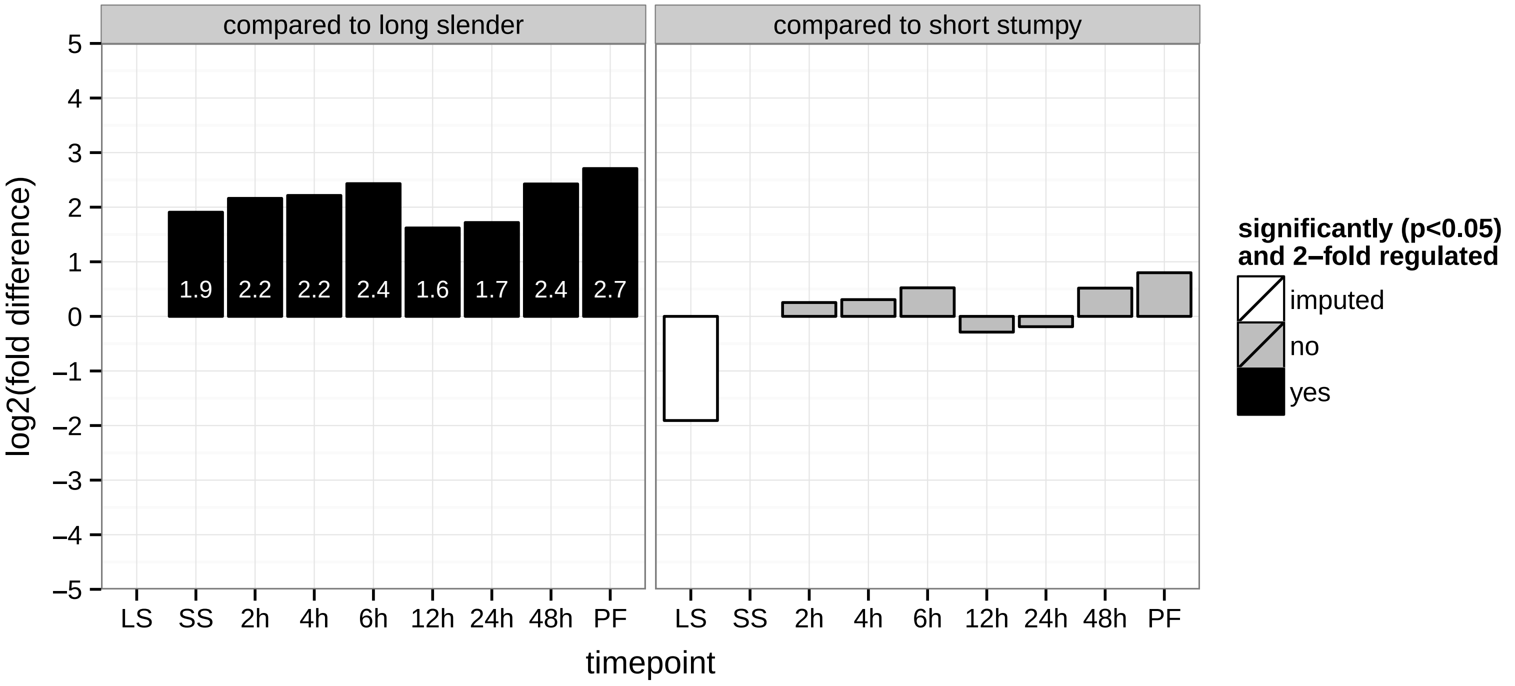

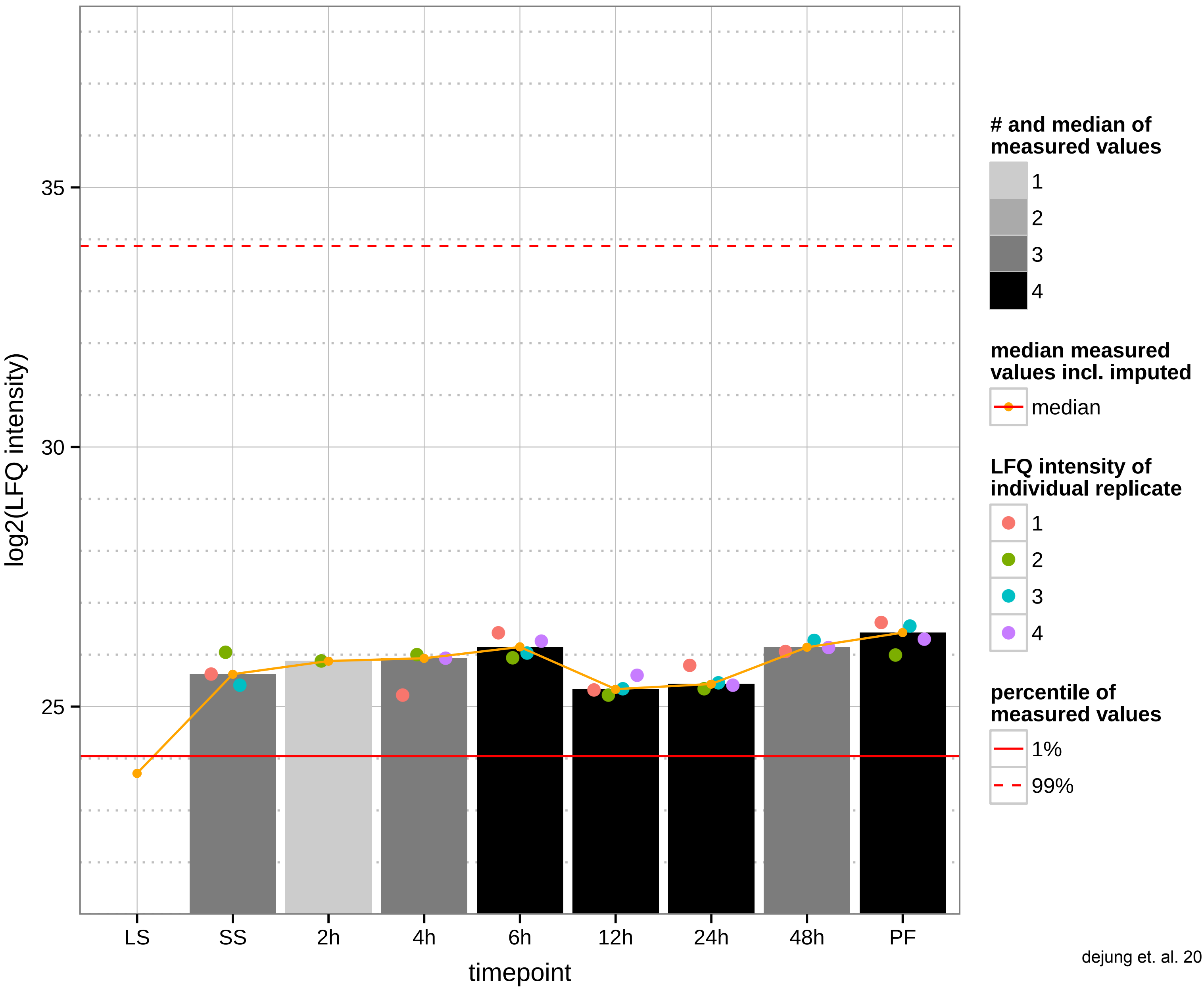

dejung et. al. 2015

hypothetical protein, conserved
Tb927.11.6460
AGOF: null
AGOC: null
AGOP: null
PGOF: null
PGOC: null
PGOP: null

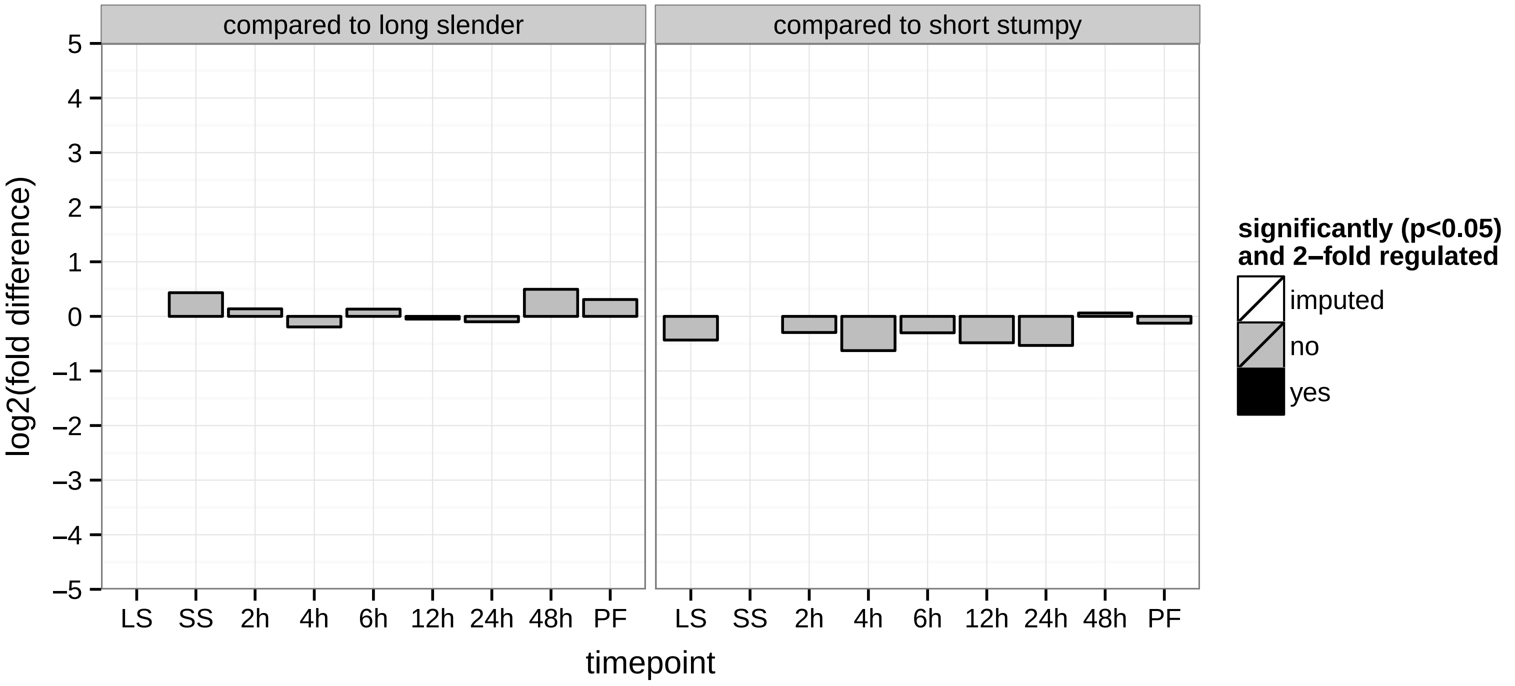

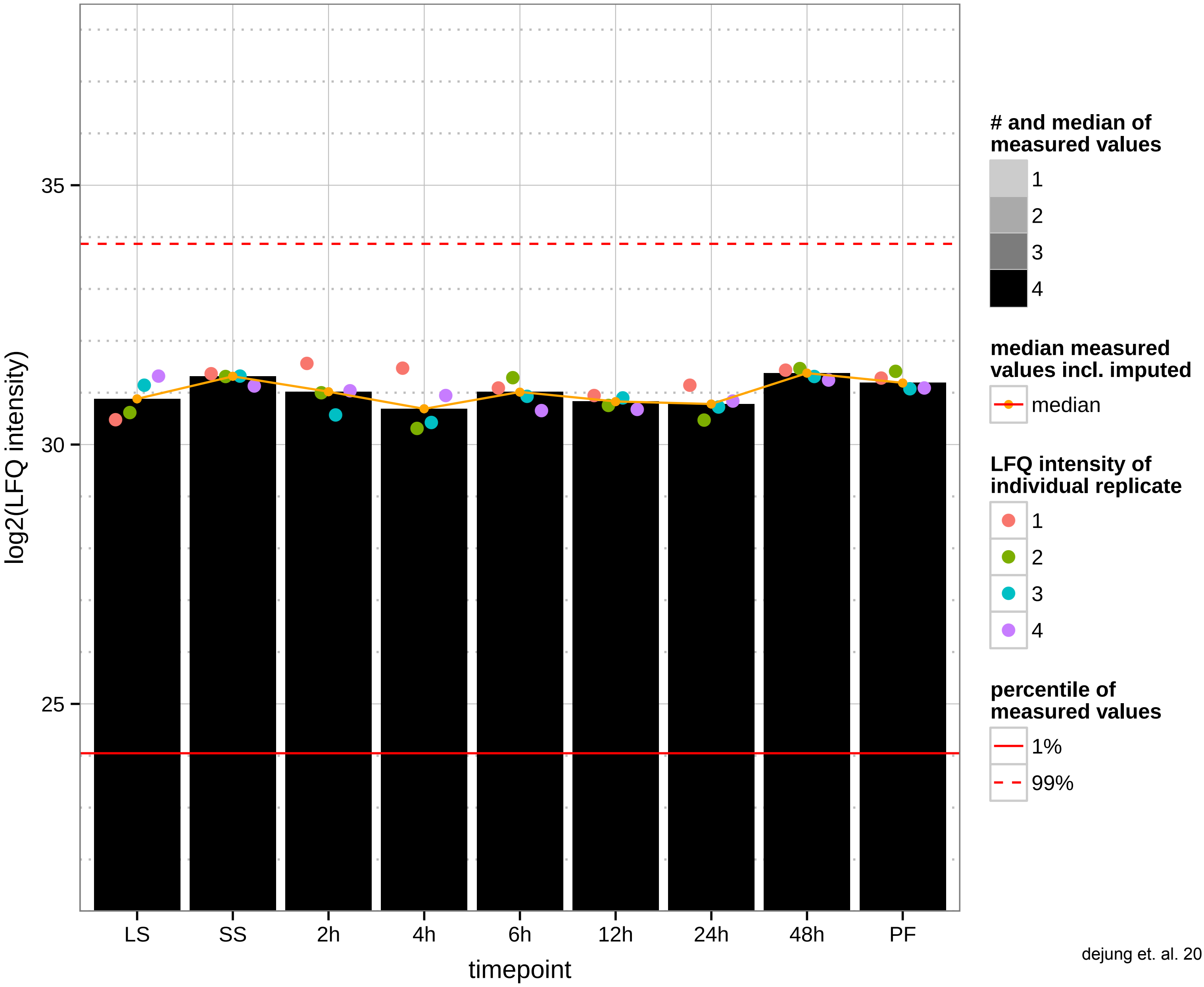

dejung et. al. 2015

hypothetical protein, conserved
Tb927.11.6470
AGOF: null
AGOC: mitochondrion
AGOP: null
PGOF: null
PGOC: null
PGOP: null

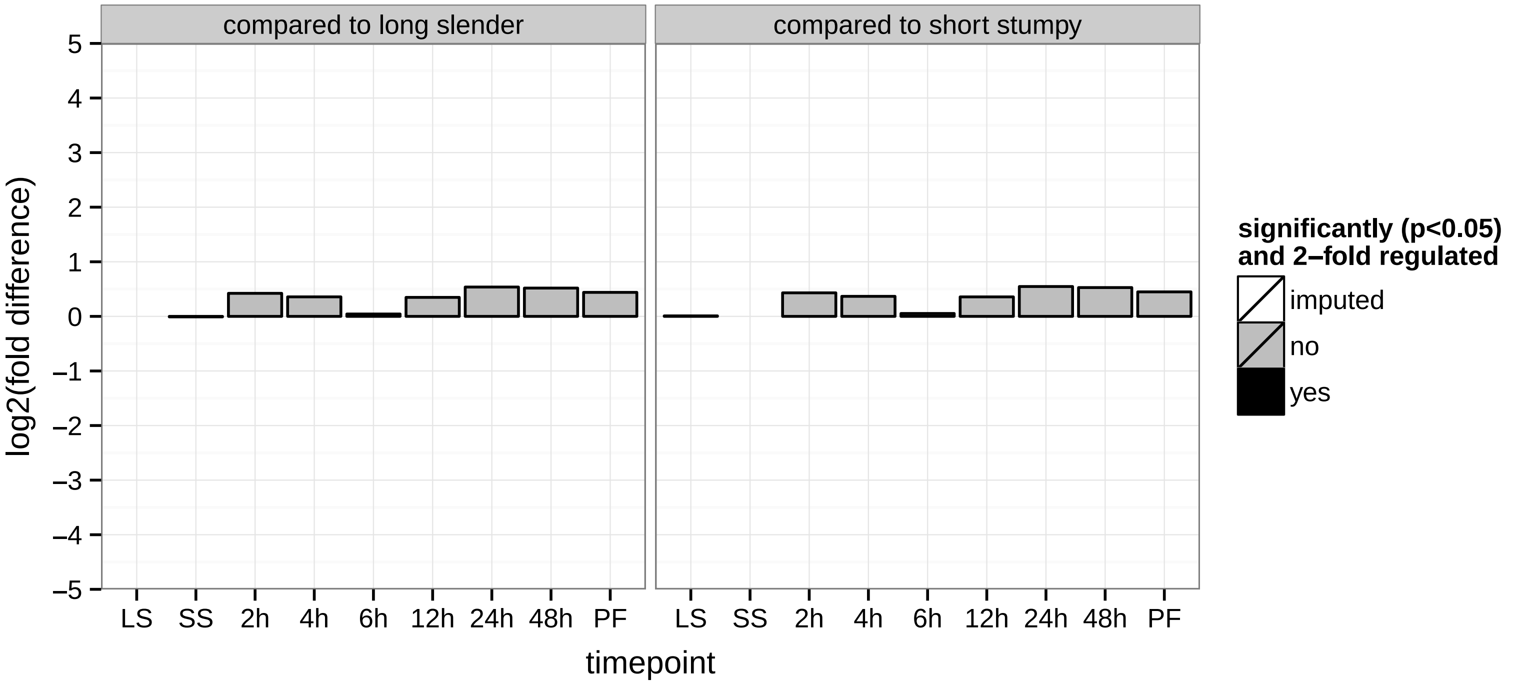

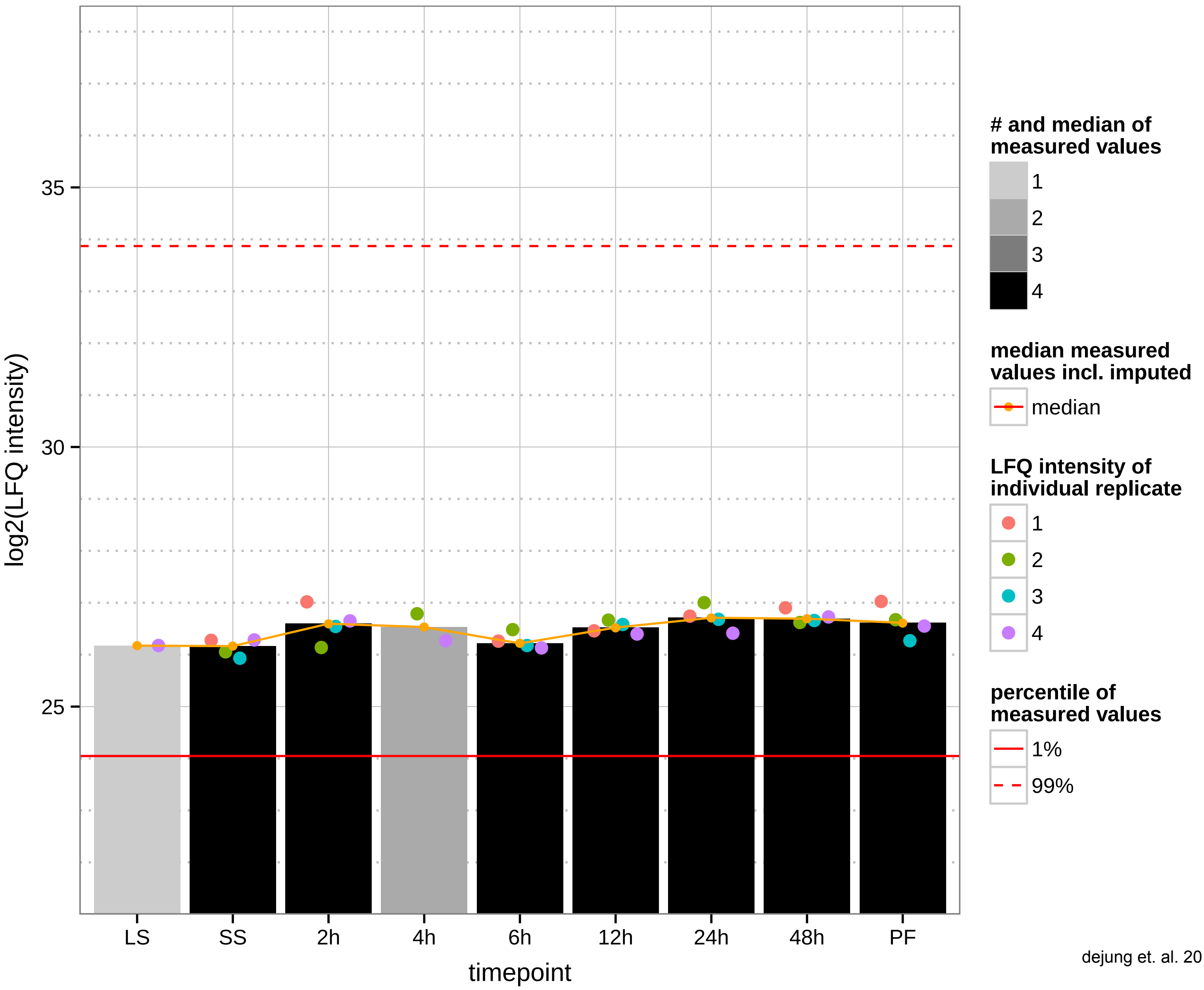

dejung et. al. 2015

hypothetical protein, conserved
Tb927.11.6530
AGOF: null
AGOC: null
AGOP: null
PGOF: null
PGOC: null
PGOP: null

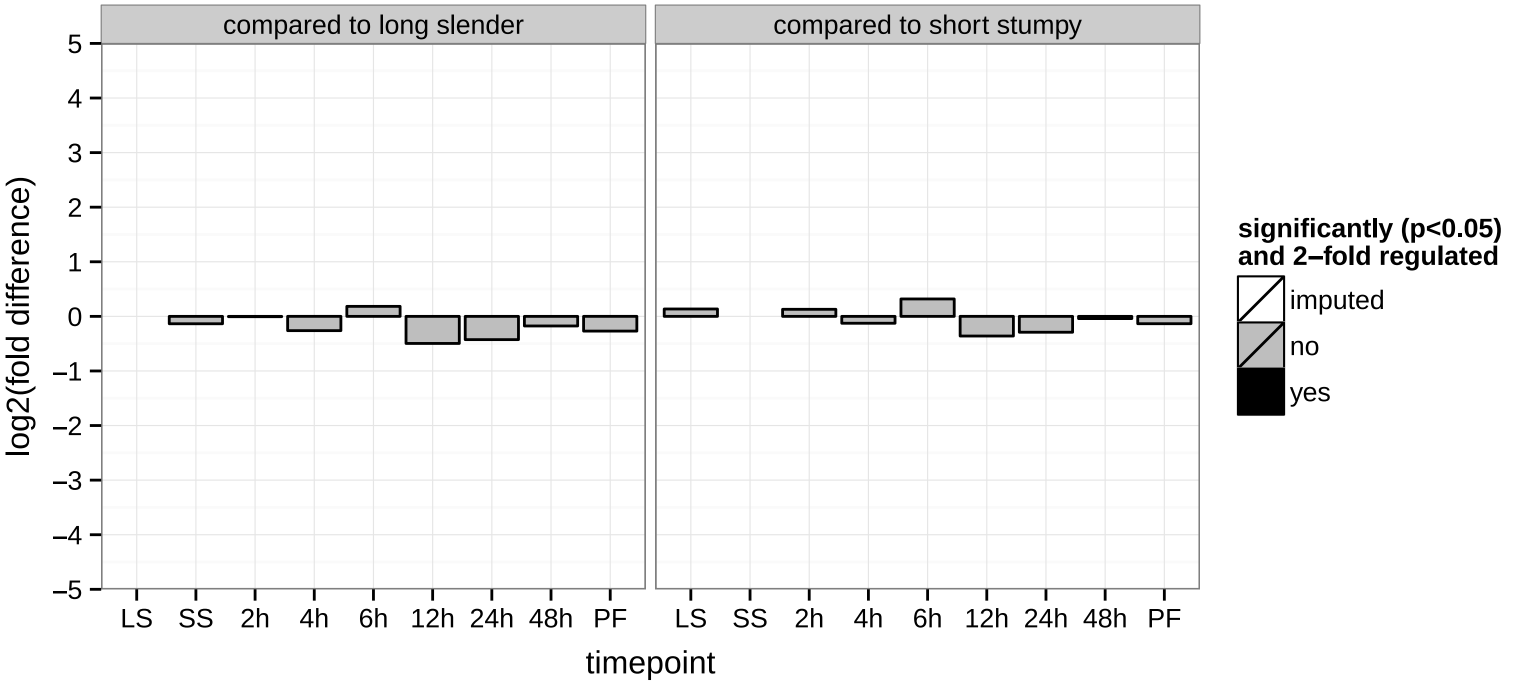

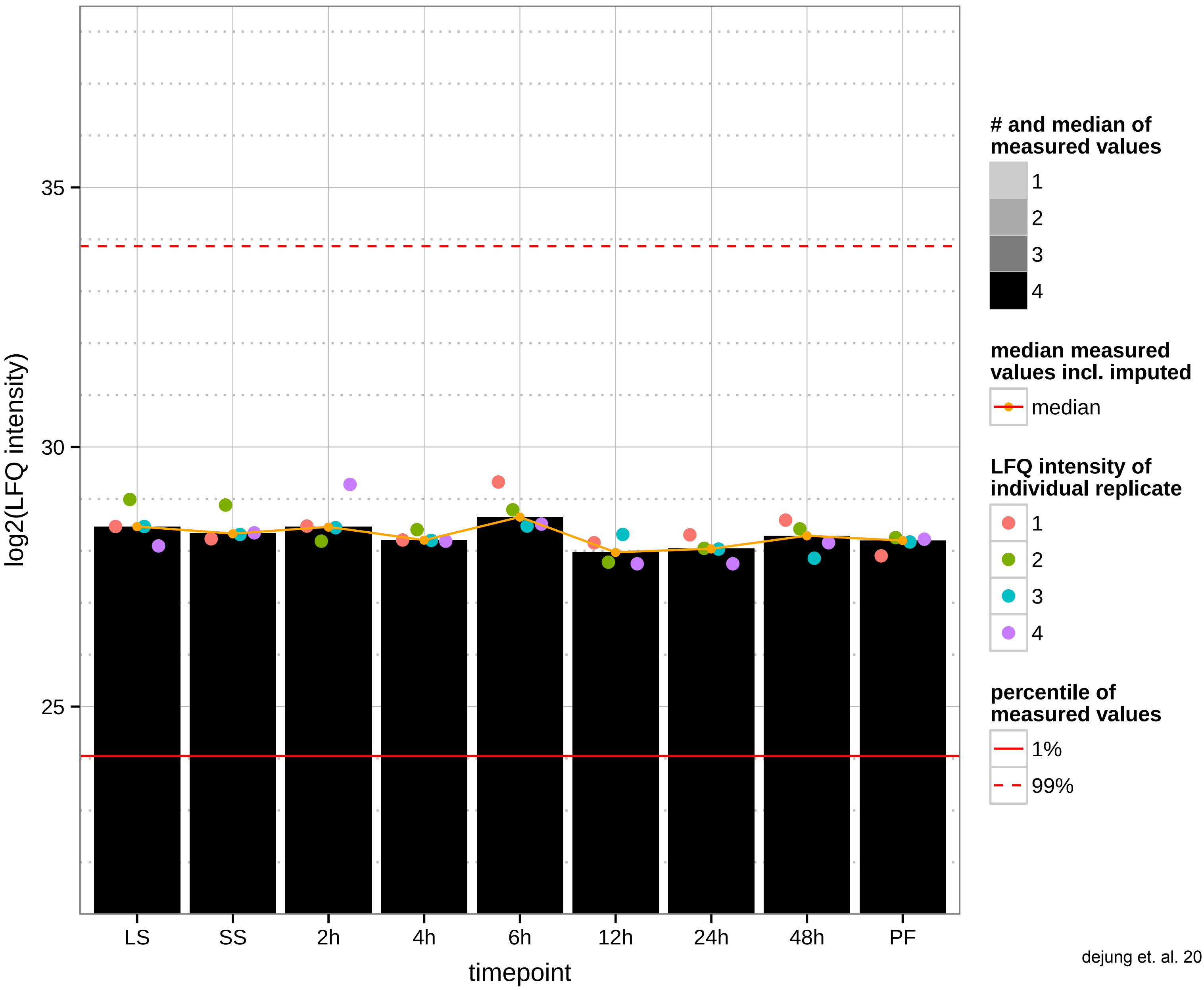

dejung et. al. 2015

hypothetical protein, conserved
Tb927.11.6600
AGOF: null
AGOC: null
AGOP: modulation of development of symbiont involved in interaction with host, quorum sensing involved in interaction with ho
PGOF: null
PGOC: null
PGOP: null

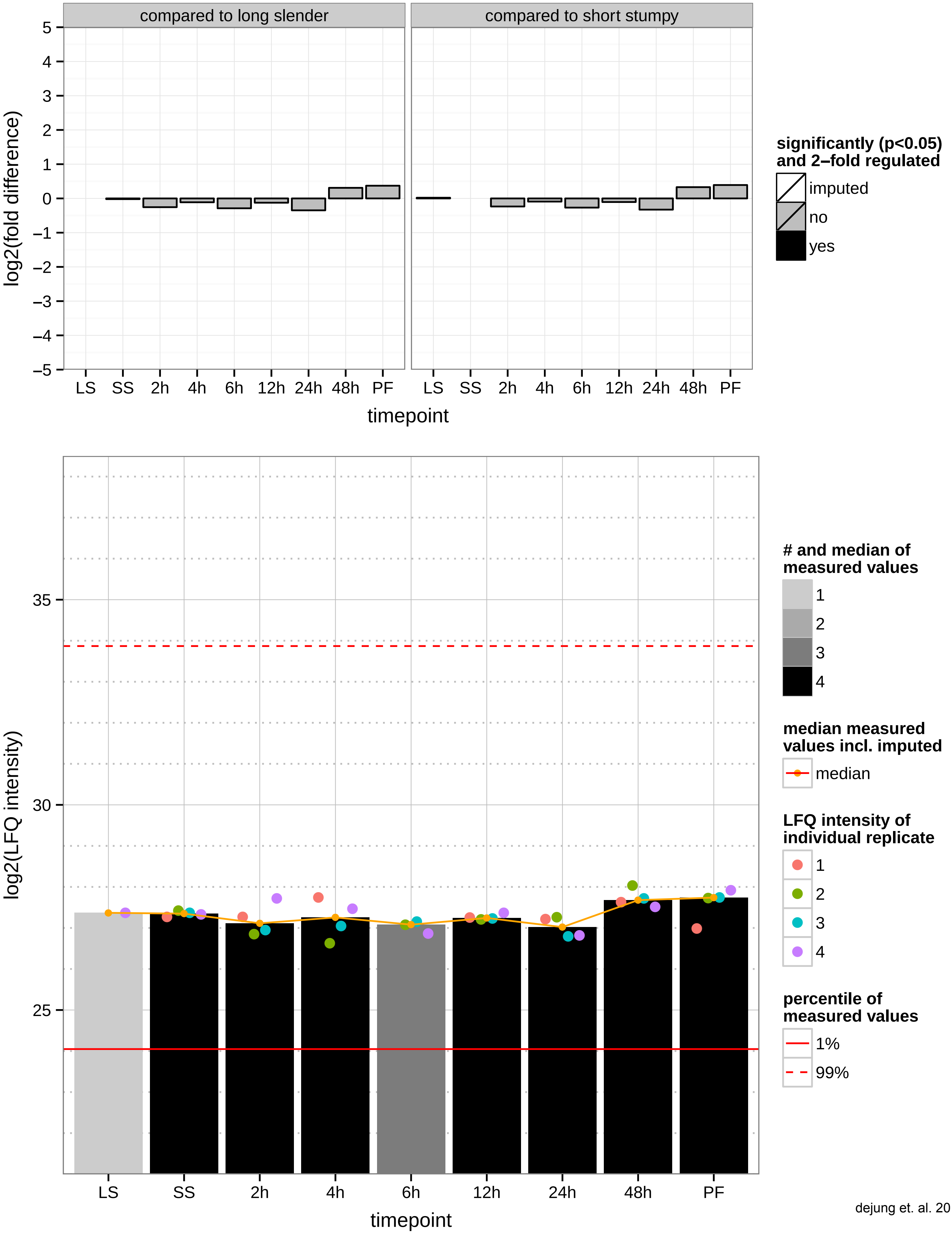

dejung et. al. 2015

hypothetical protein, conserved
Tb927.11.6610
AGOF: null
AGOC: null
AGOP: null
PGOF: null
PGOC: null
PGOP: null

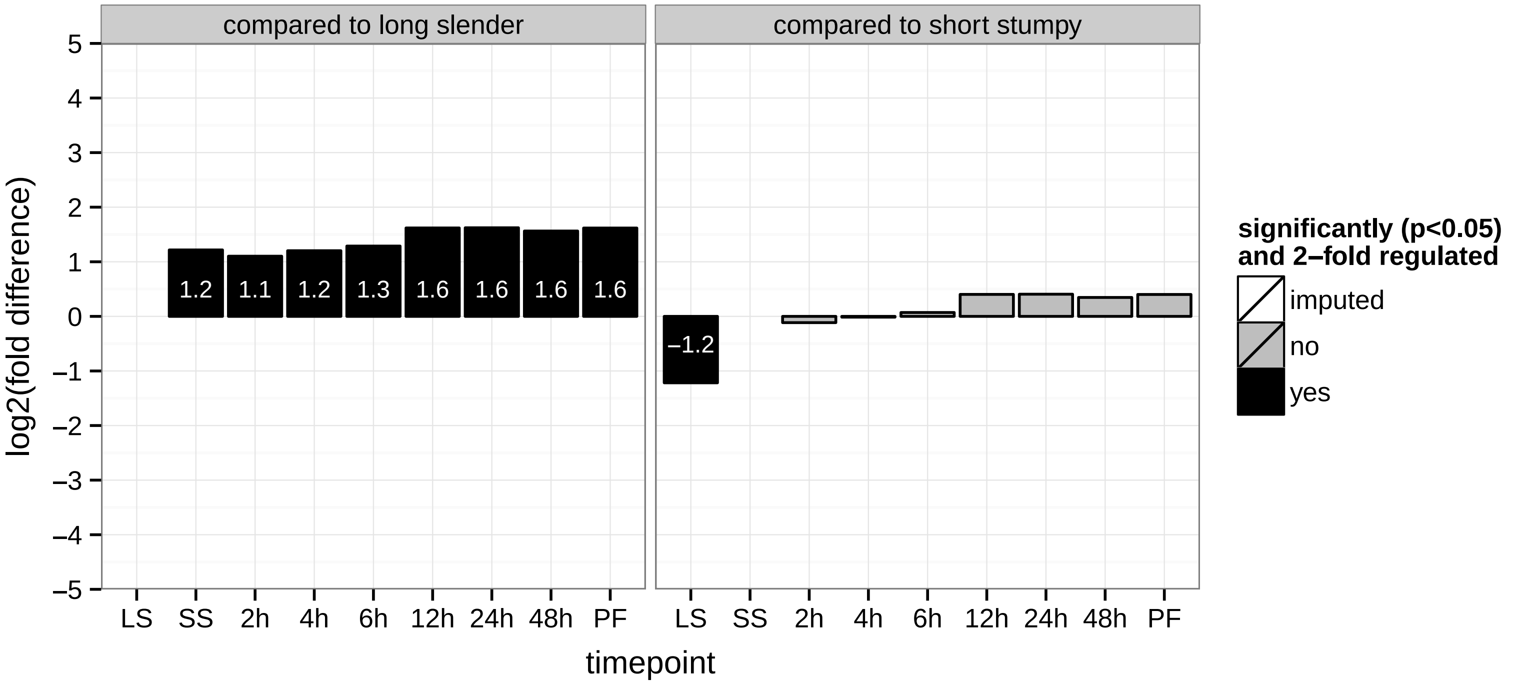

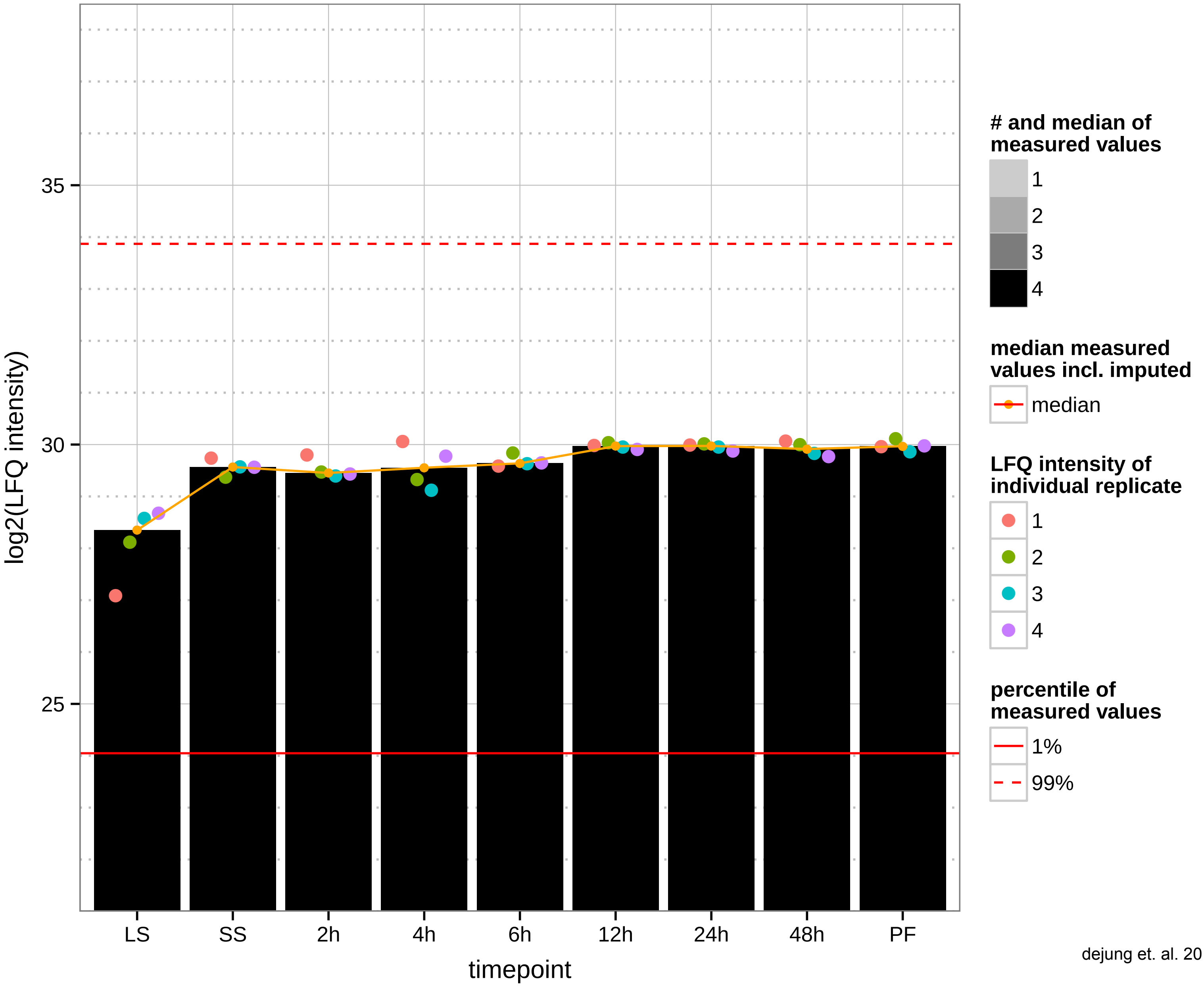

dejung et. al. 2015

3–methylcrotonoyl–CoA carboxylase beta subunit, putative
Tb927.11.6630
AGOF: methylcrotonoyl–CoA carboxylase activity
AGOC: mitochondrion
AGOP: null
PGOF: ligase activity
PGOC: null
PGOP: null

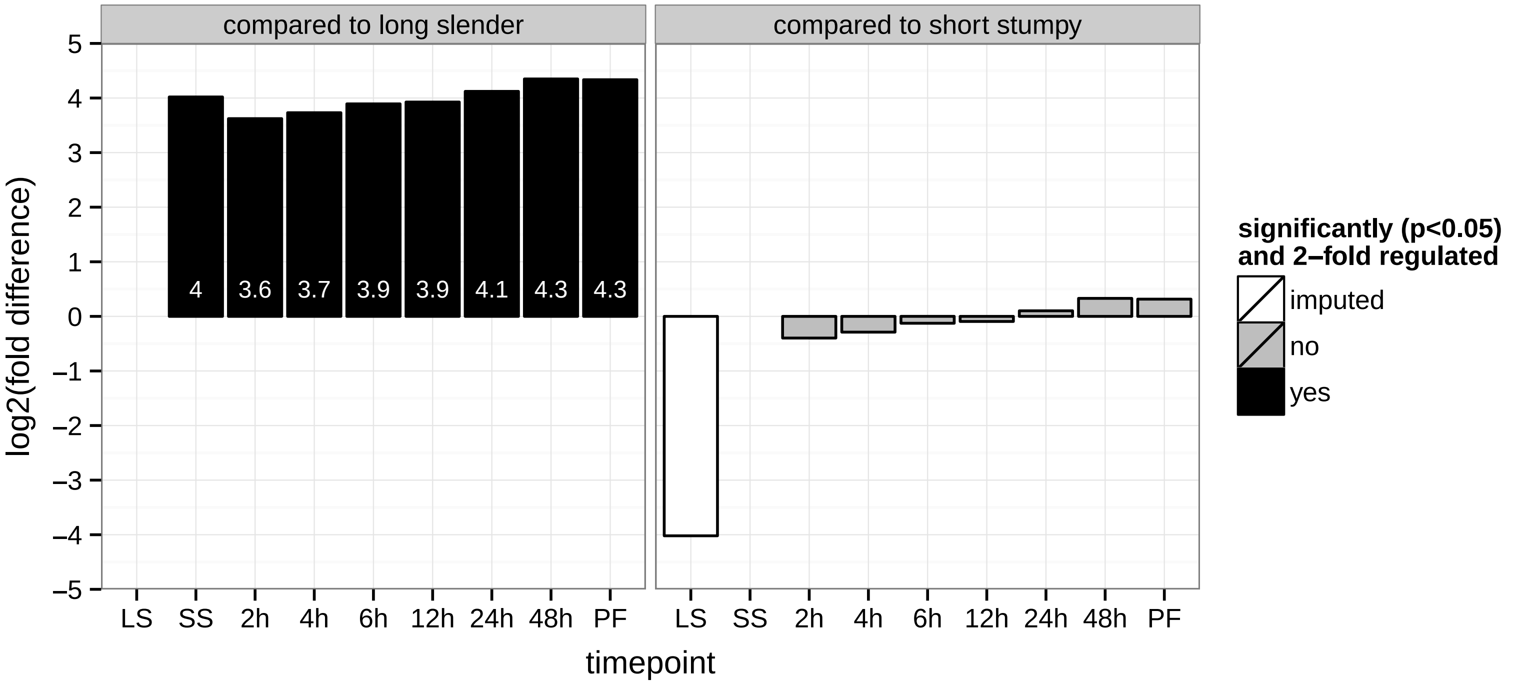

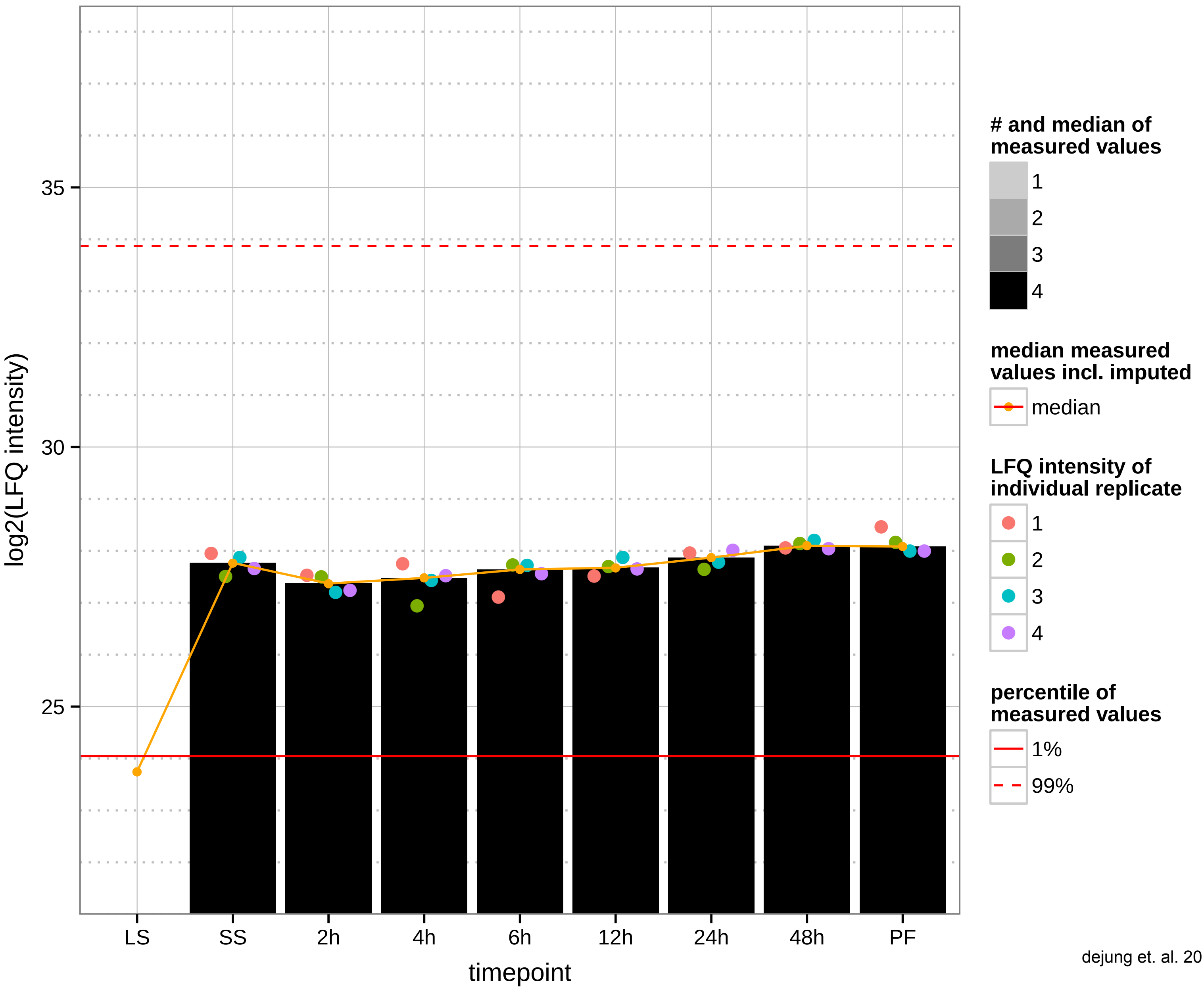

dejung et. al. 2015

epsin, putative, chrXI additional, unordered contigs, epsinR
Tb927.11.670;Tb11.0890
AGOF: null, clathrin binding
AGOC: null, clathrin coat of coated pit, cytosol, endosome membrane, flagellar pocket
AGOP: null, receptor–mediated endocytosis
PGOF: null
PGOC: null
PGOP: null

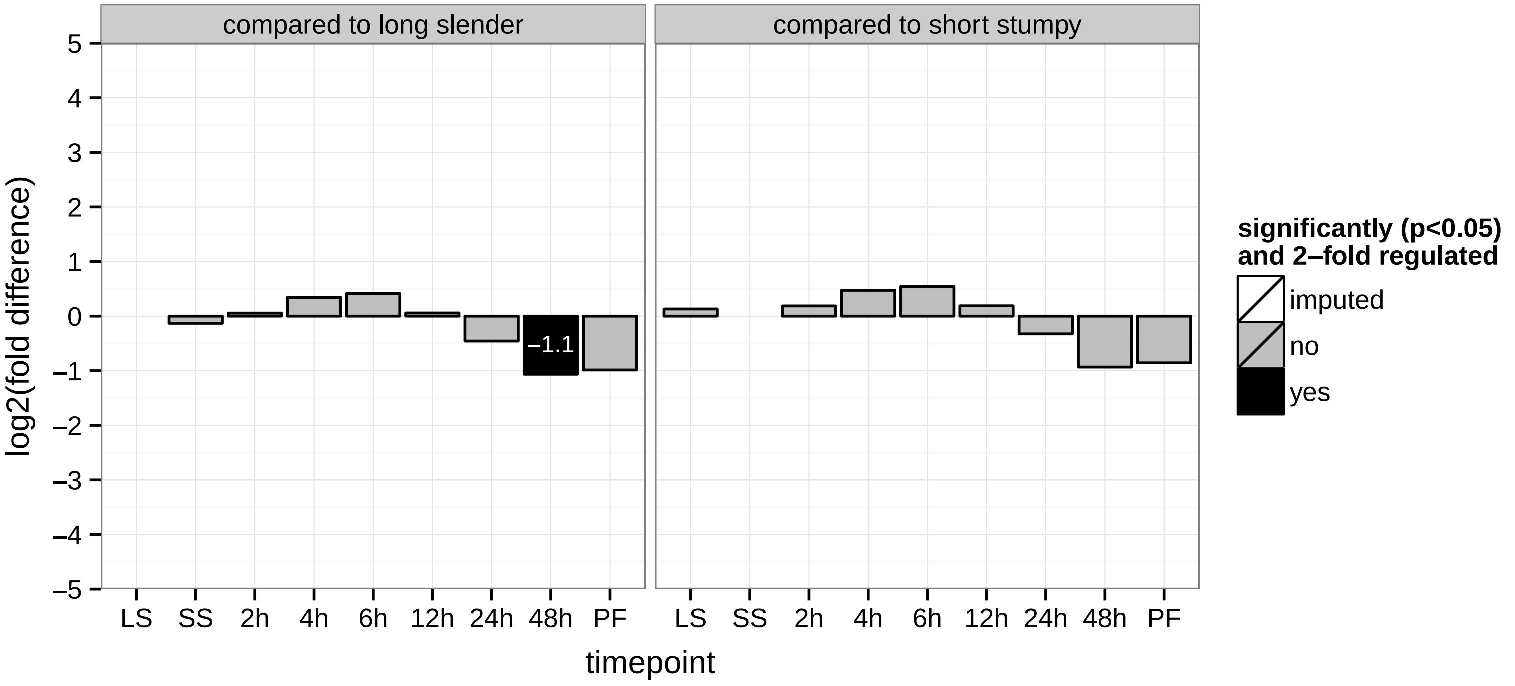

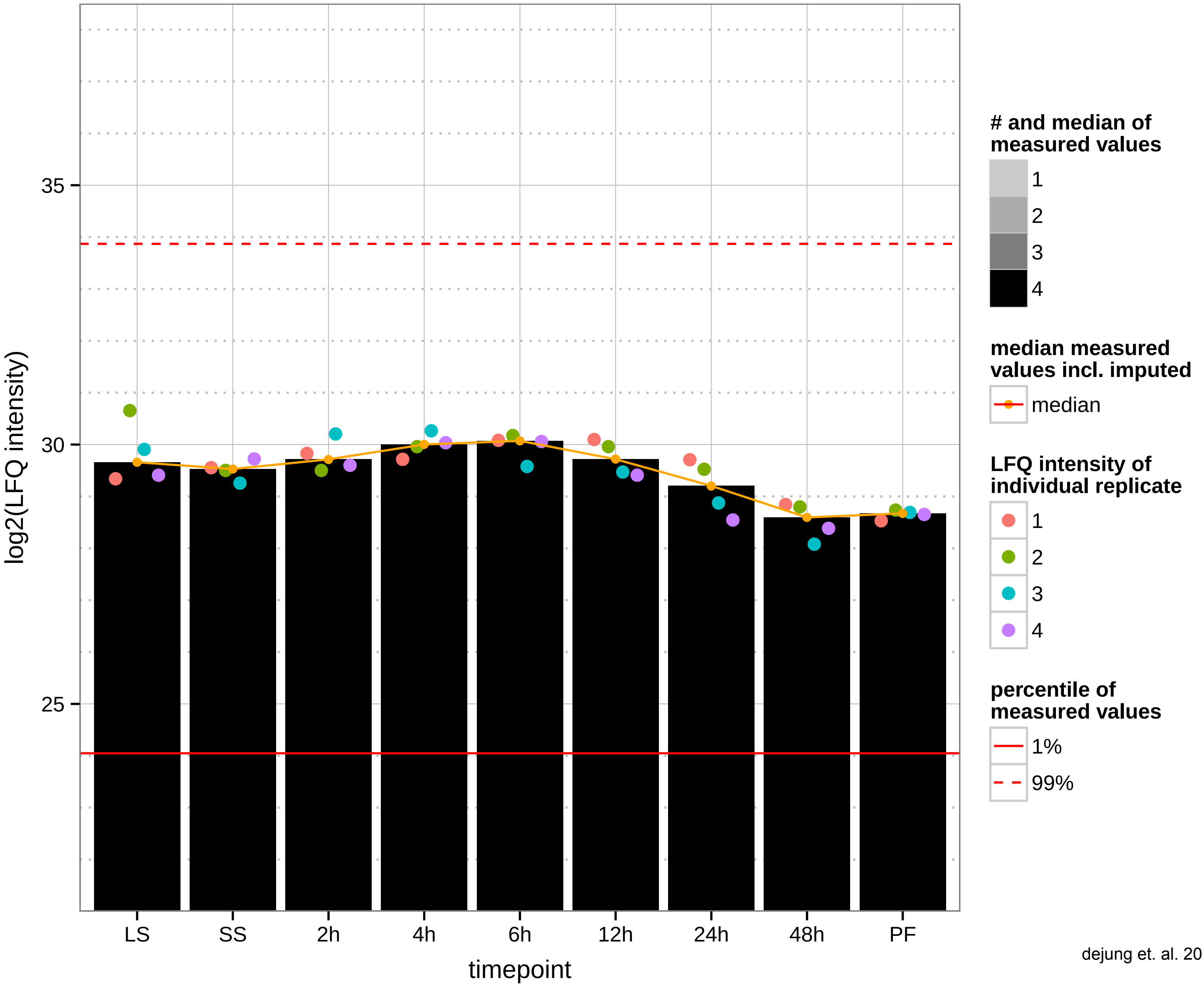

dejung et. al. 2015

predicted tetratricopeptide repeat (TPR) protein
Tb927.11.6710
AGOF: null
AGOC: null
AGOP: null
PGOF: protein binding
PGOC: null
PGOP: null

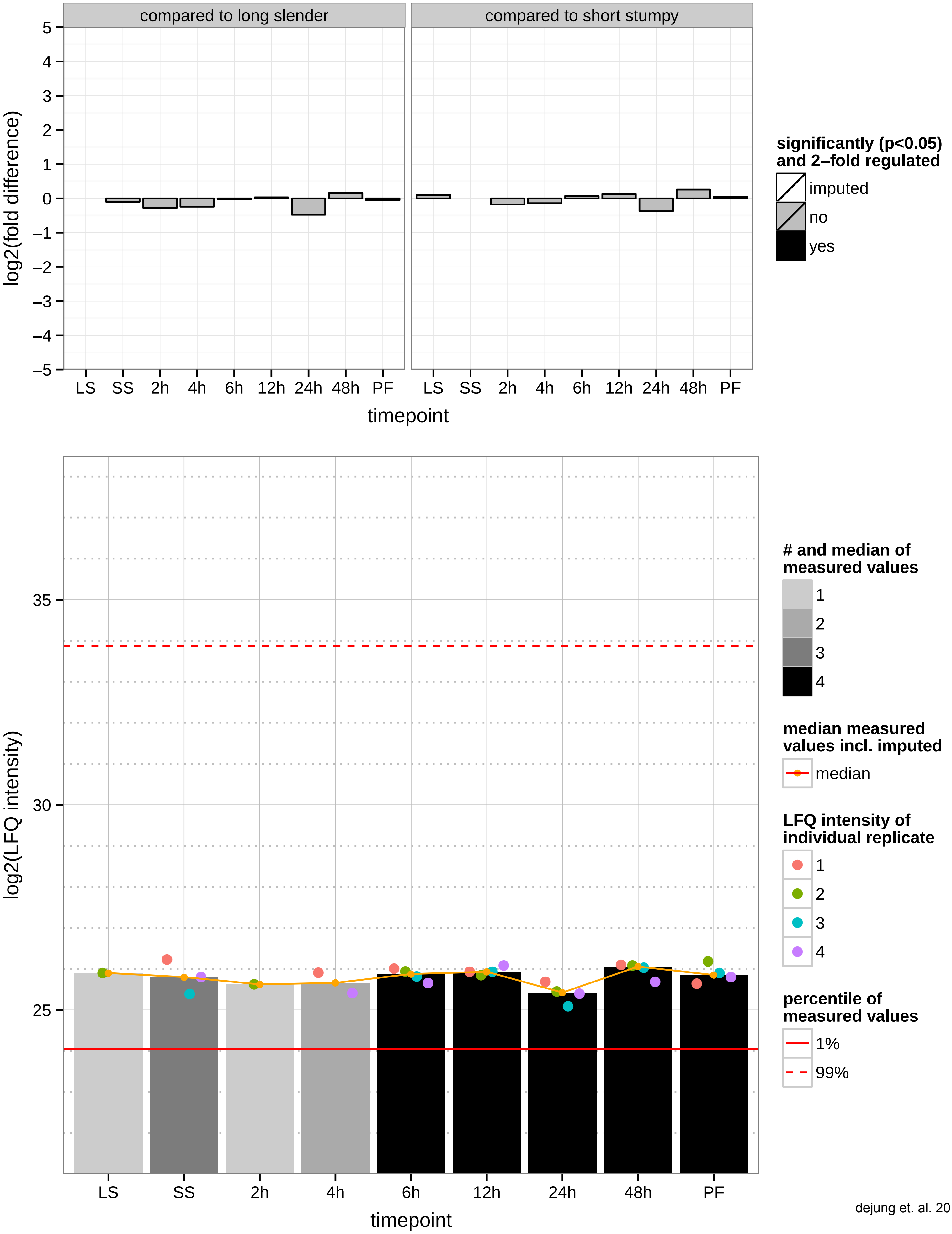

dejung et. al. 2015

chromatin binding protein, putative, regulator of chromatin condensation, putative (RCC1)
Tb927.11.6780
AGOF: Ran GTPase binding, chromatin binding
AGOC: null
AGOP: chromosome organization
PGOF: null
PGOC: null
PGOP: null

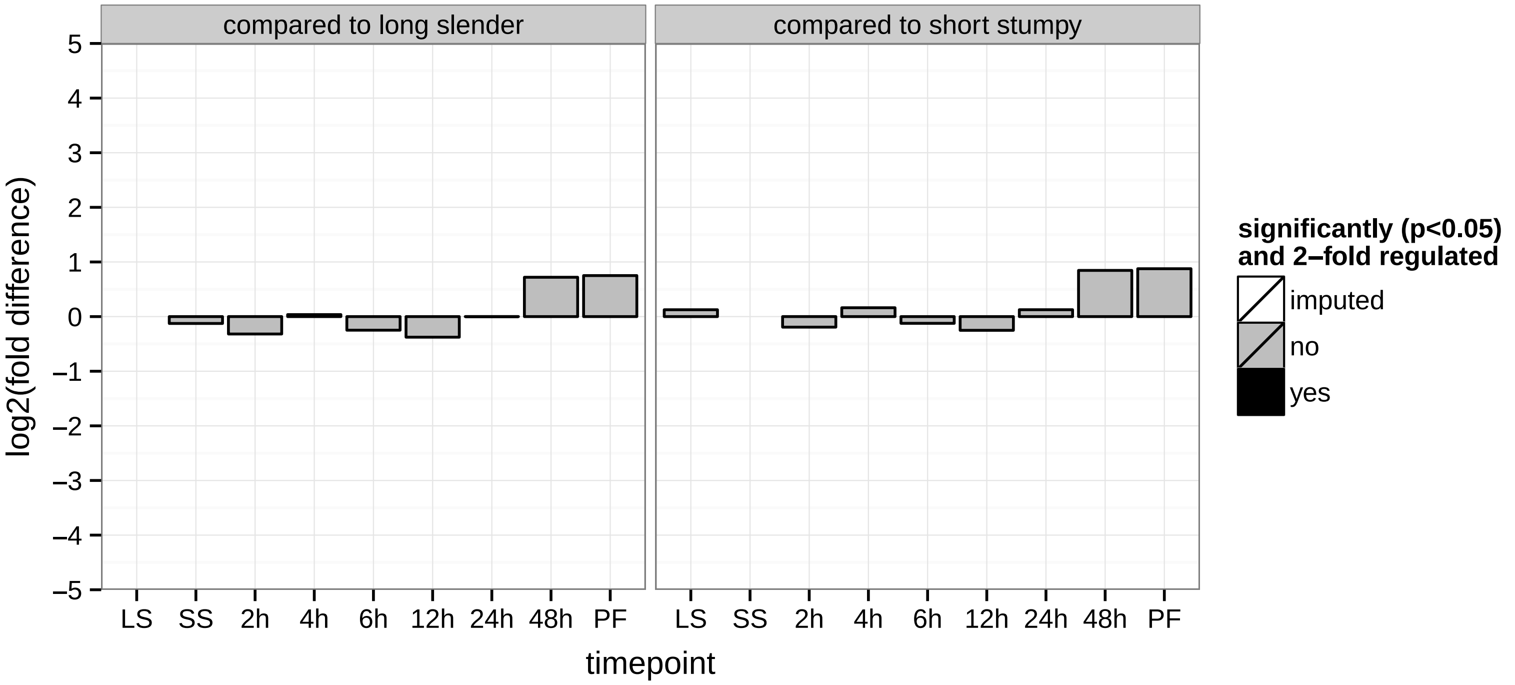

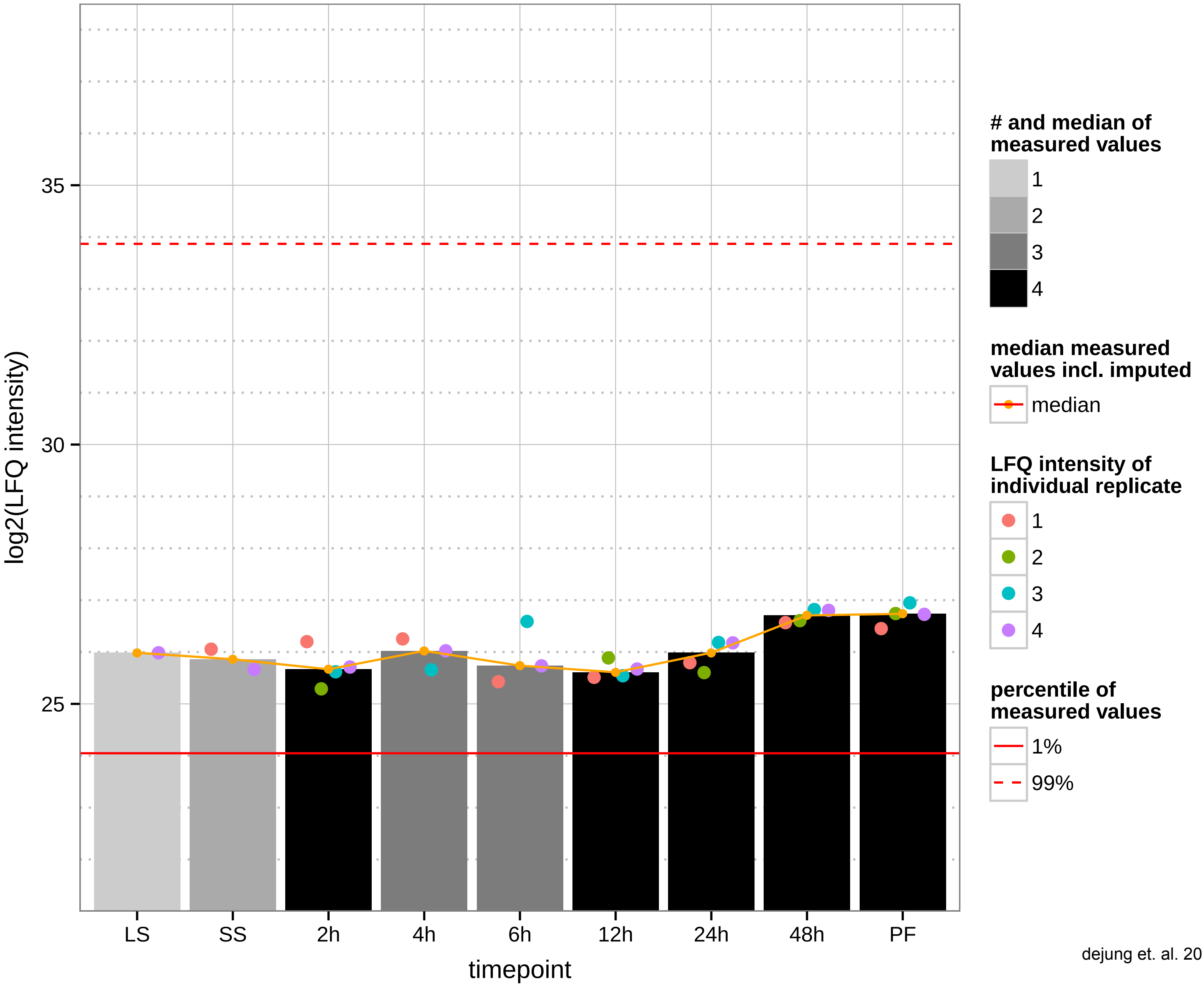

dejung et. al. 2015

predicted WD40 repeat protein
Tb927.11.6790
AGOF: null
AGOC: nucleus
AGOP: rRNA processing
PGOF: protein binding
PGOC: null
PGOP: rRNA processing, regulation of cell cycle

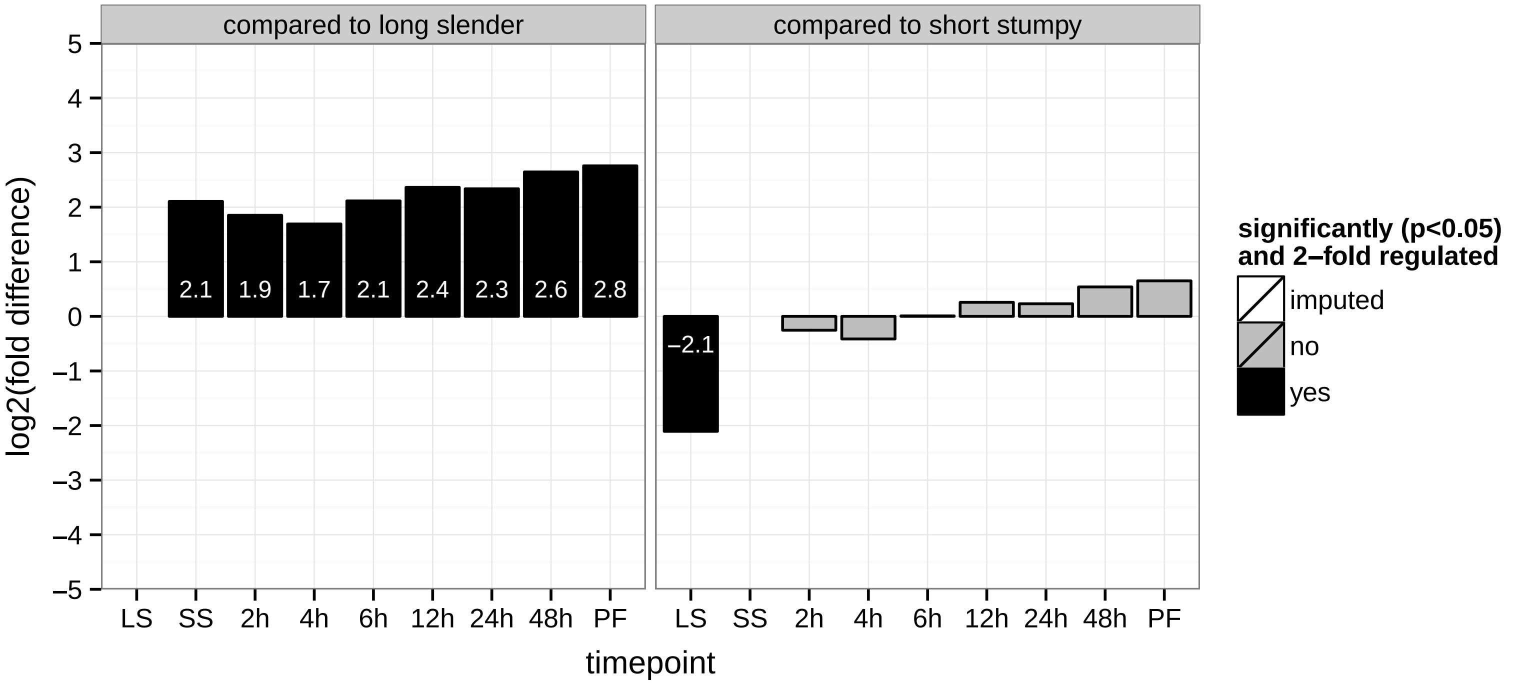

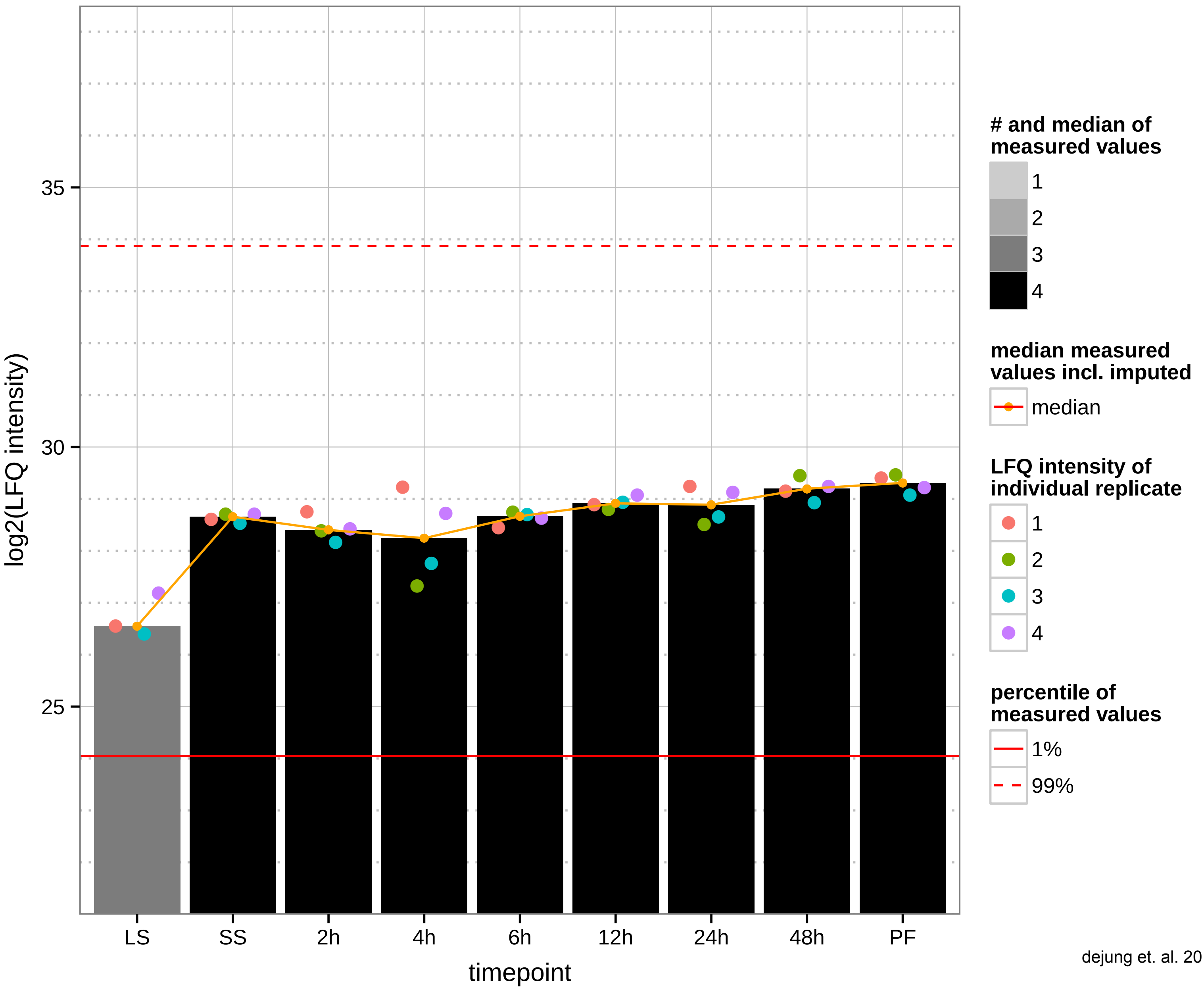

dejung et. al. 2015

hypothetical protein, conserved
Tb927.11.6820;Tb11.v5.0900
AGOF: null
AGOC: null
AGOP: null
PGOF: null
PGOC: null
PGOP: null

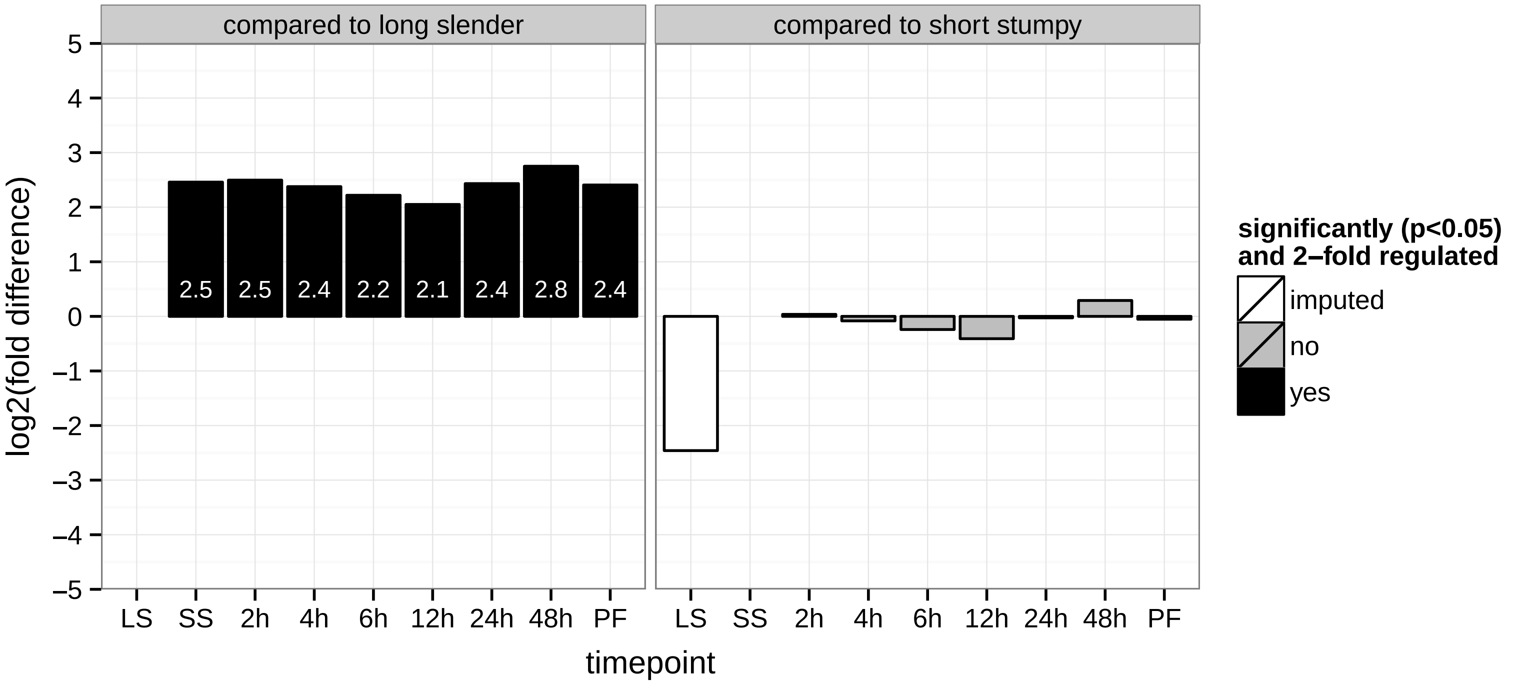

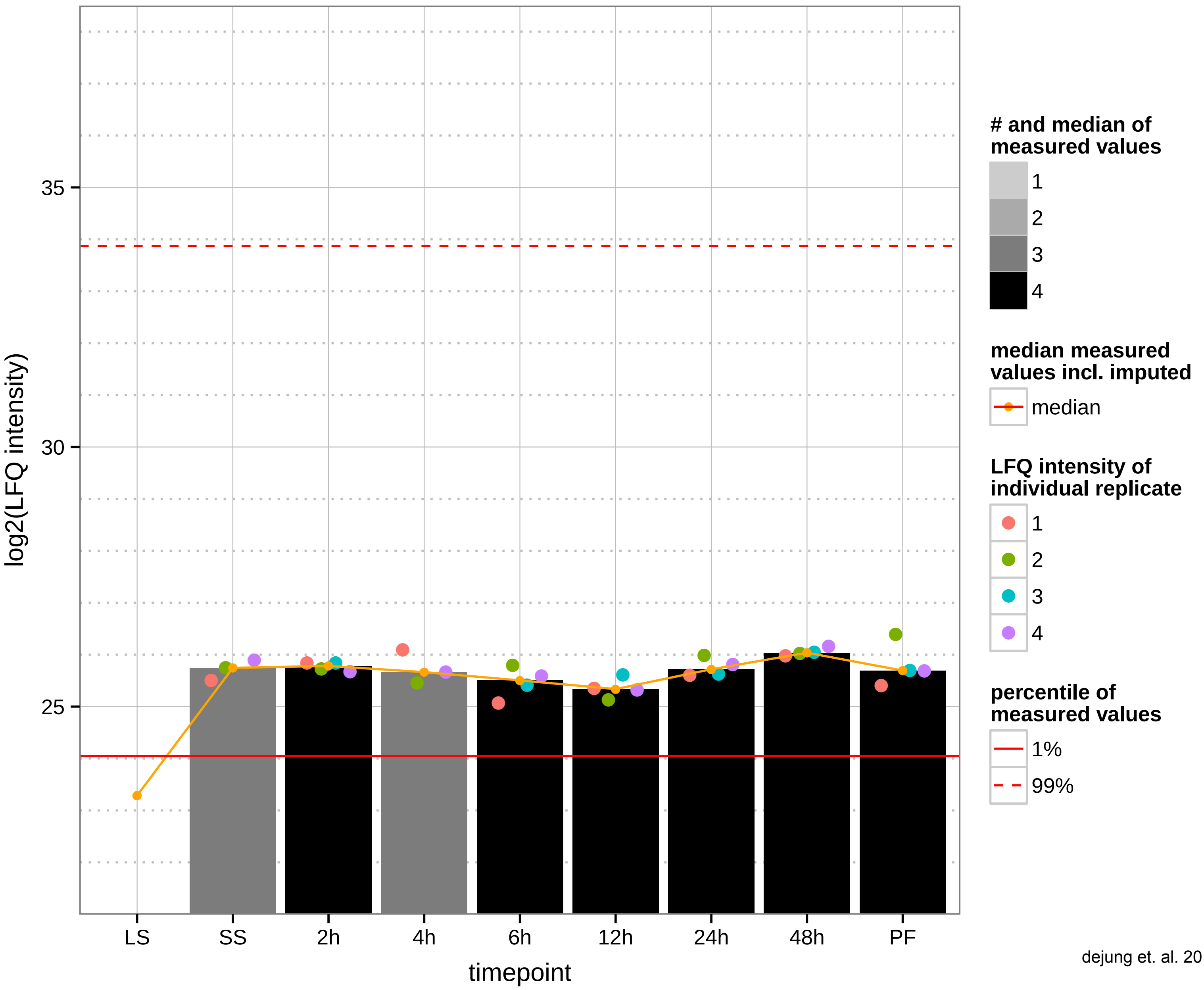

dejung et. al. 2015

14–3–3 protein
Tb927.11.6870
AGOF: protein binding, protein domain specific binding, protein phosphorylated amino acid binding
AGOC: eukaryotic translation initiation factor 4F complex, nucleus
AGOP: DNA damage checkpoint, endocytosis, regulation of mitosis
PGOF: protein domain specific binding
PGOC: null
PGOP: null

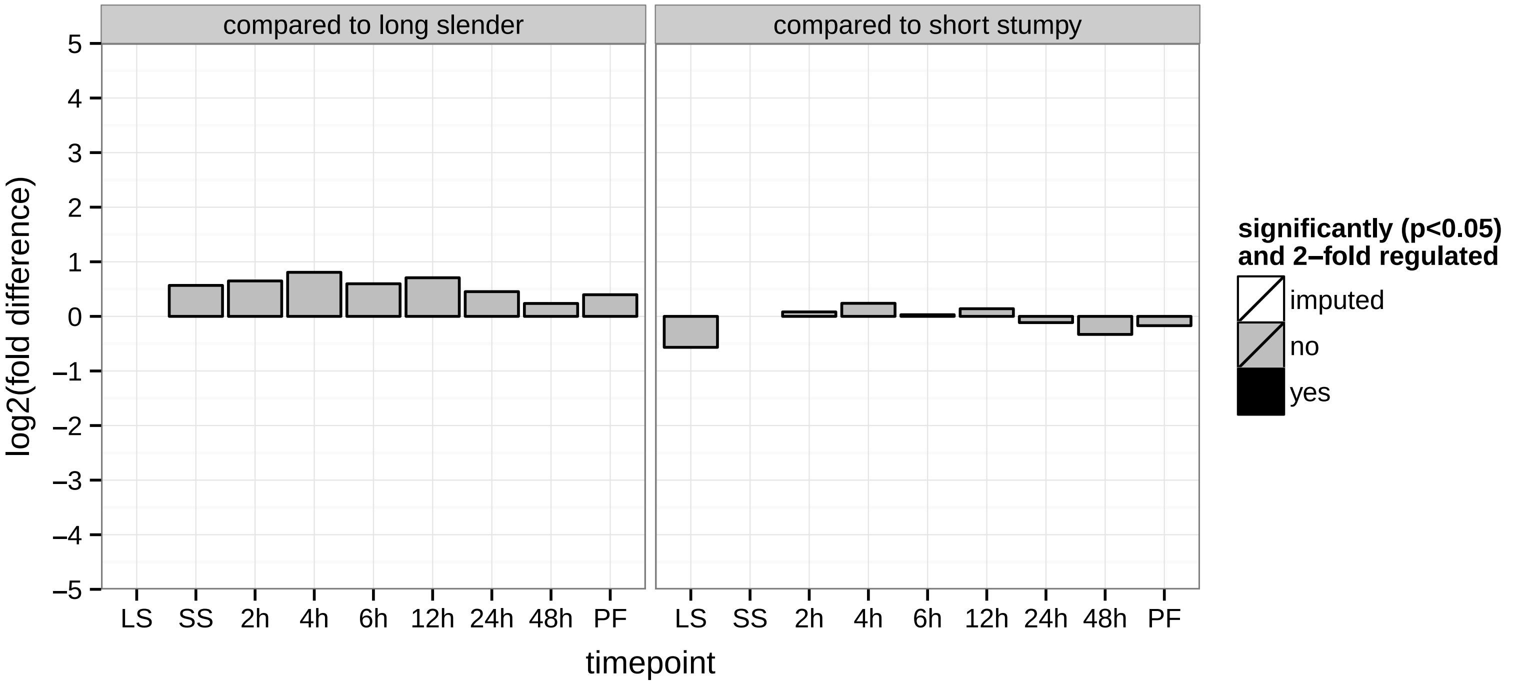

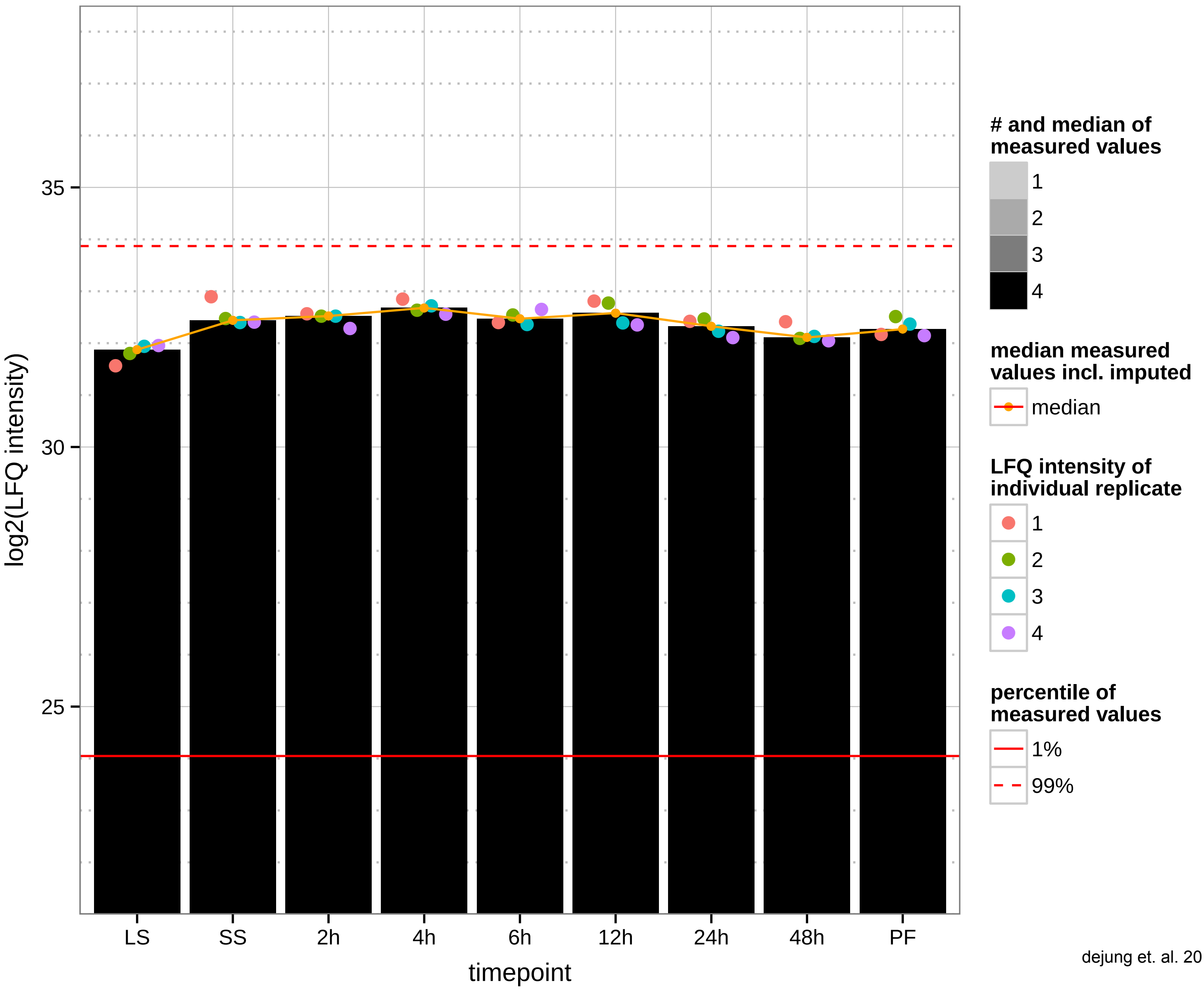

dejung et. al. 2015

proteasome alpha 7 subunit, putative (PSA4)
Tb927.11.7020
AGOF: threonine–type endopeptidase activity
AGOC: cytosol, nucleus, proteasome core complex
AGOP: ubiquitin–dependent protein catabolic process
PGOF: endopeptidase activity, threonine–type endopeptidase activity
PGOC: proteasome core complex, proteasome core complex, alpha–subunit complex
PGOP: proteolysis involved in cellular protein catabolic process, ubiquitin–dependent protein catabolic process

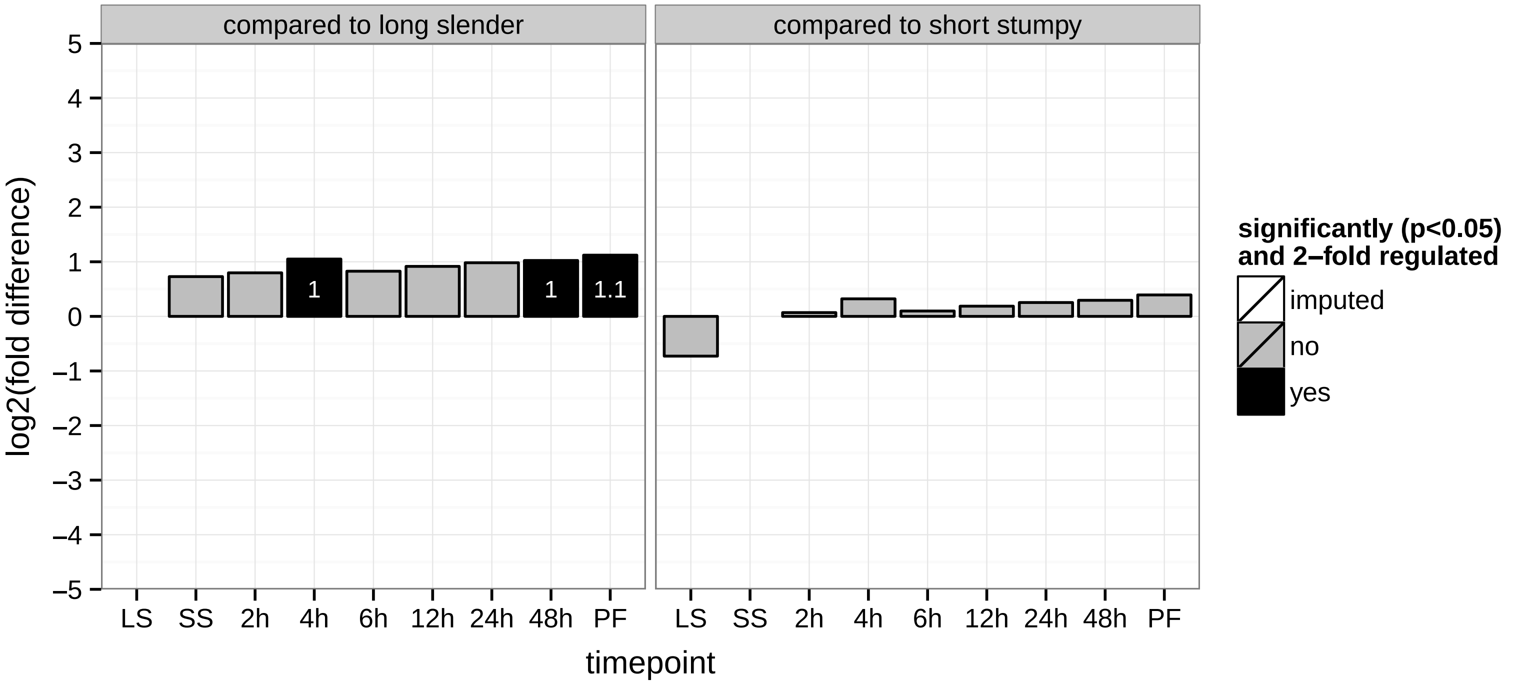

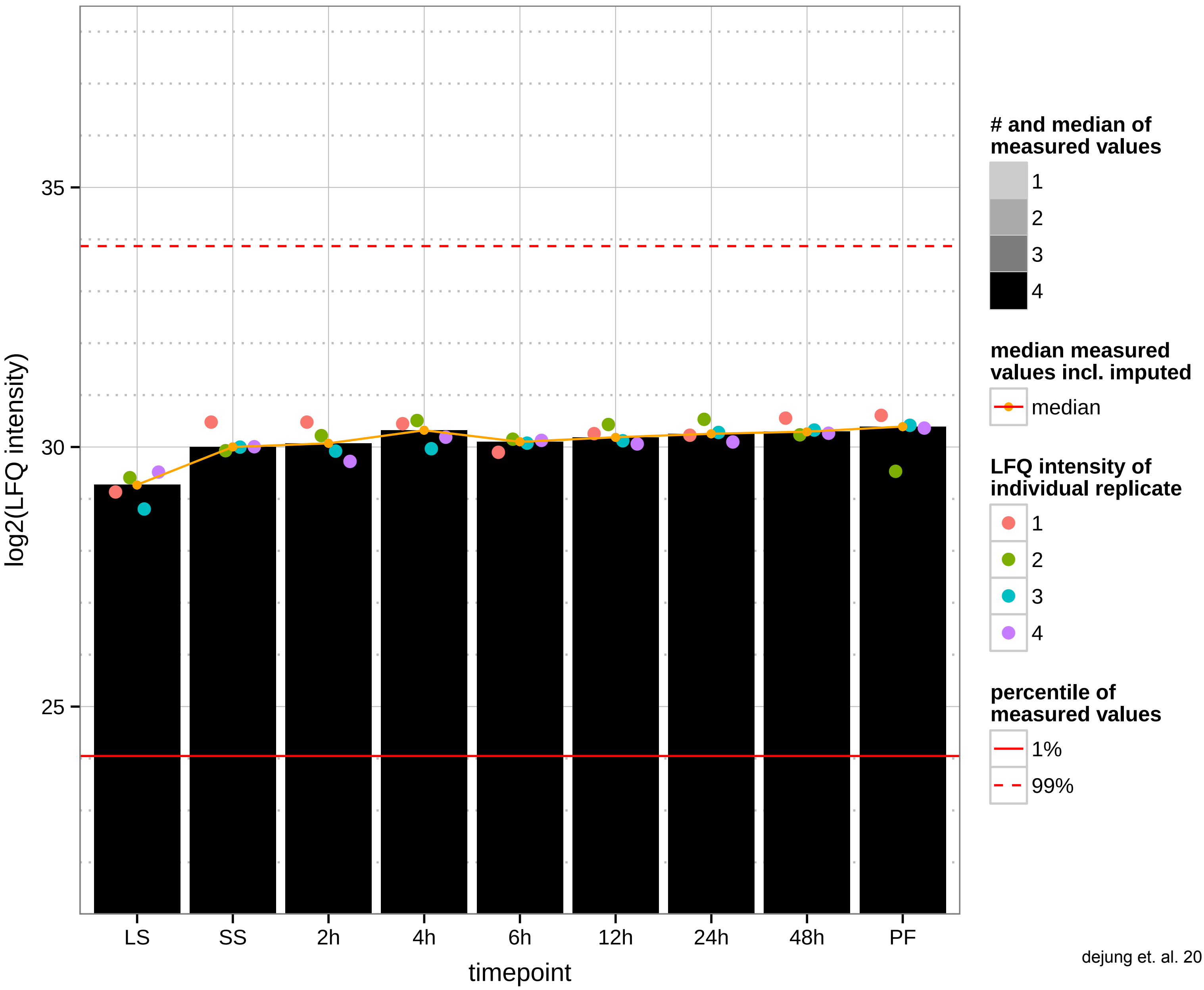

dejung et. al. 2015

acidocalcisomal pyrophosphatase
Tb927.11.7060
AGOF: inorganic diphosphatase activity, magnesium ion binding, pyrophosphatase activity
AGOC: cytoplasm, membrane
AGOP: metabolic process, phosphate−containing compound metabolic process
PGOF: inorganic diphosphatase activity, magnesium ion binding
PGOC: cytoplasm
PGOP: phosphate−containing compound metabolic process

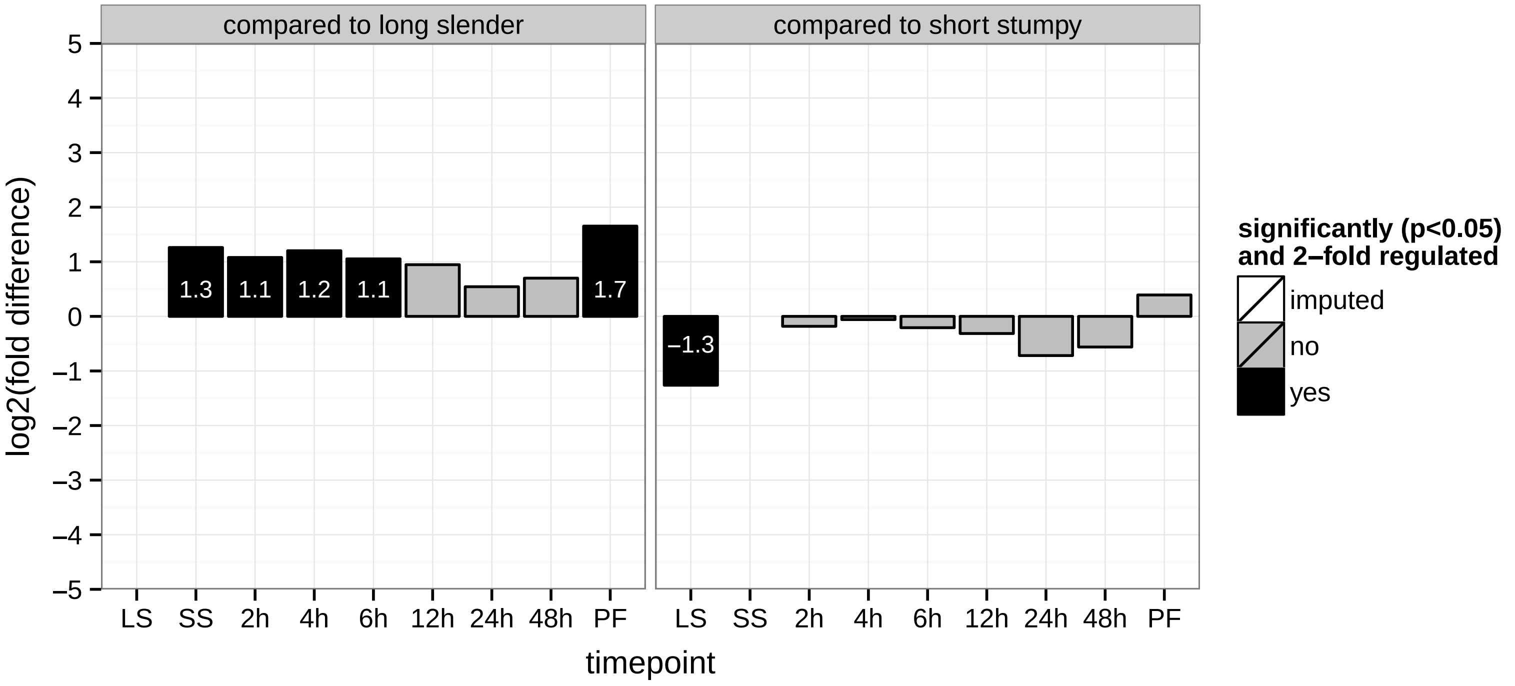

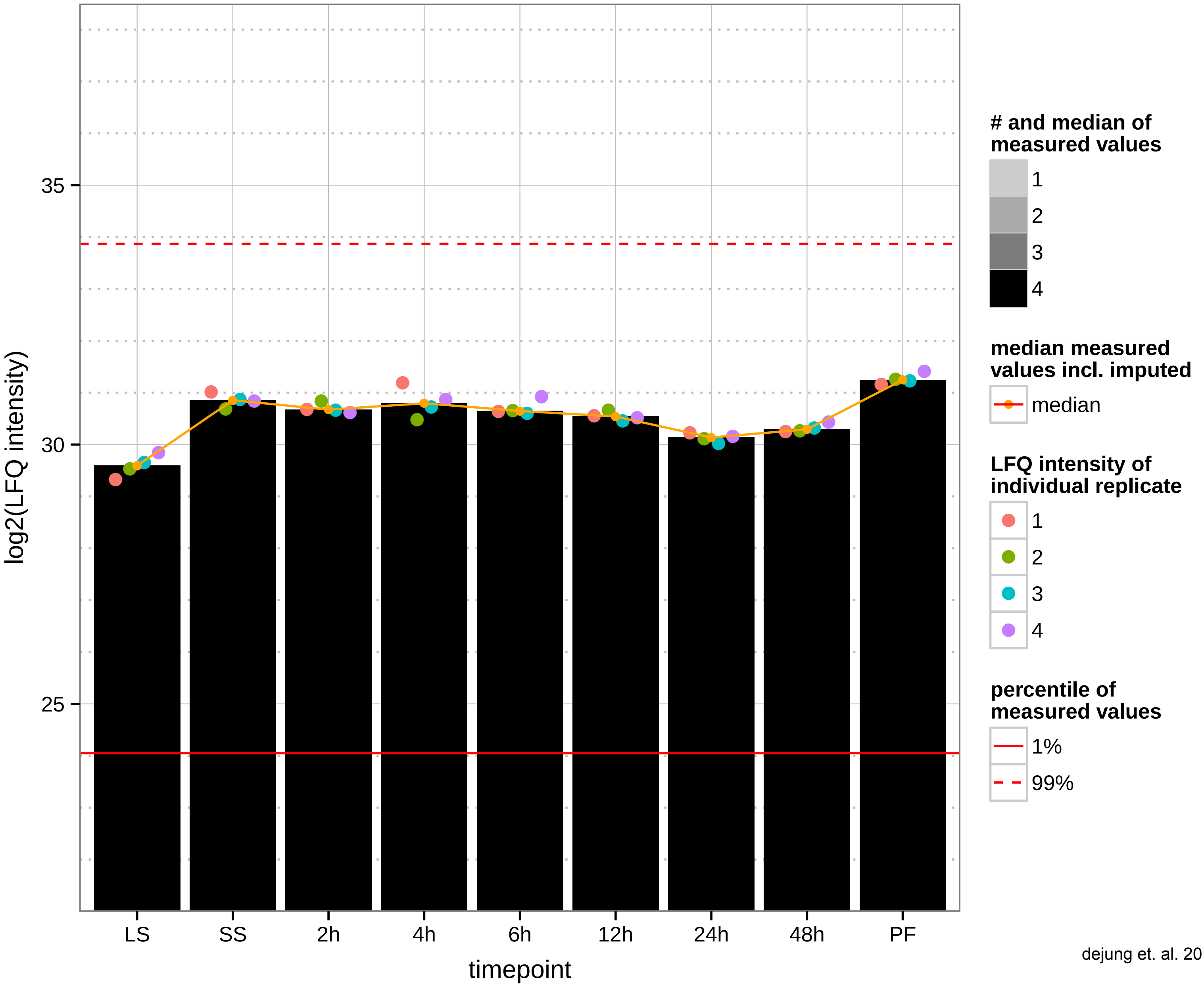

dejung et. al. 2015

cytoplasmic translation machinery associated protein, putative
Tb927.11.7100
AGOF: RNA binding
AGOC: cytosol, ribosome
AGOP: ribosome biogenesis
PGOF: RNA binding
PGOC: null
PGOP: null

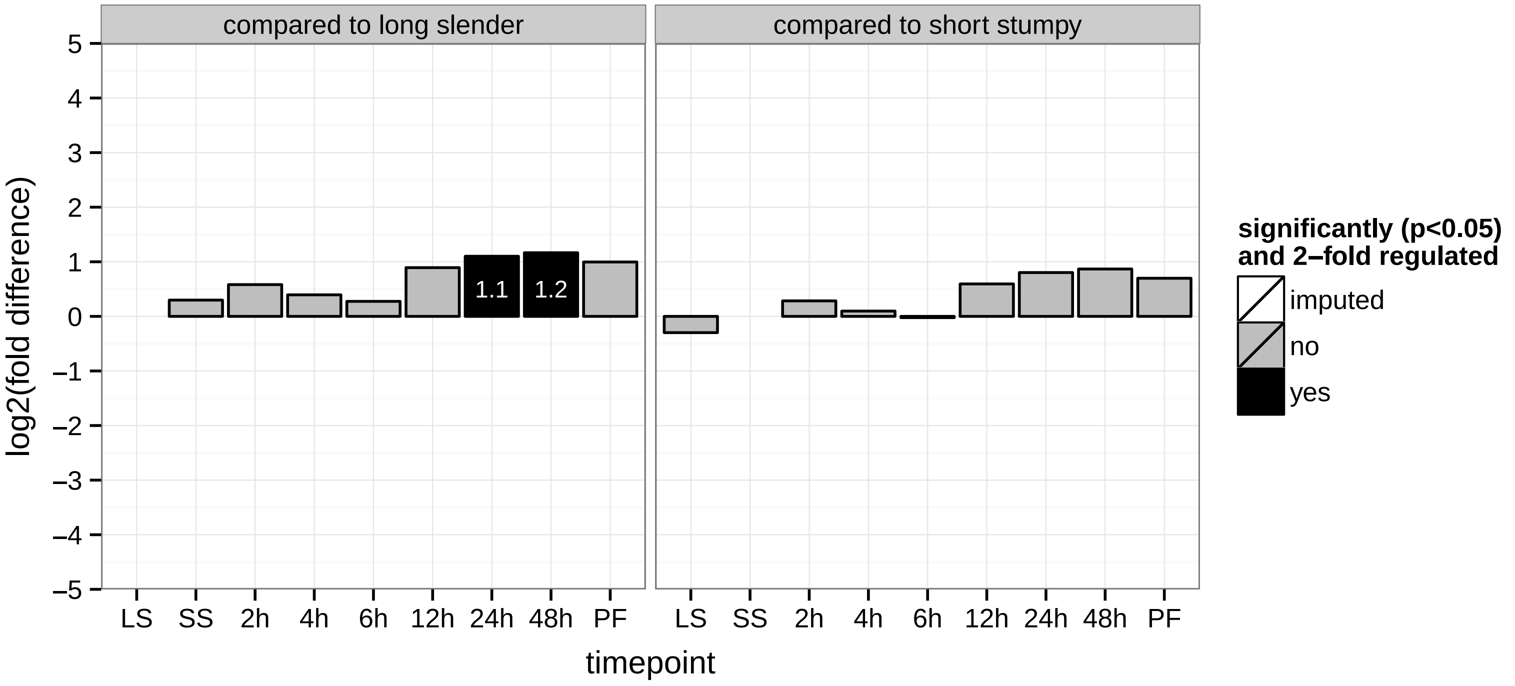

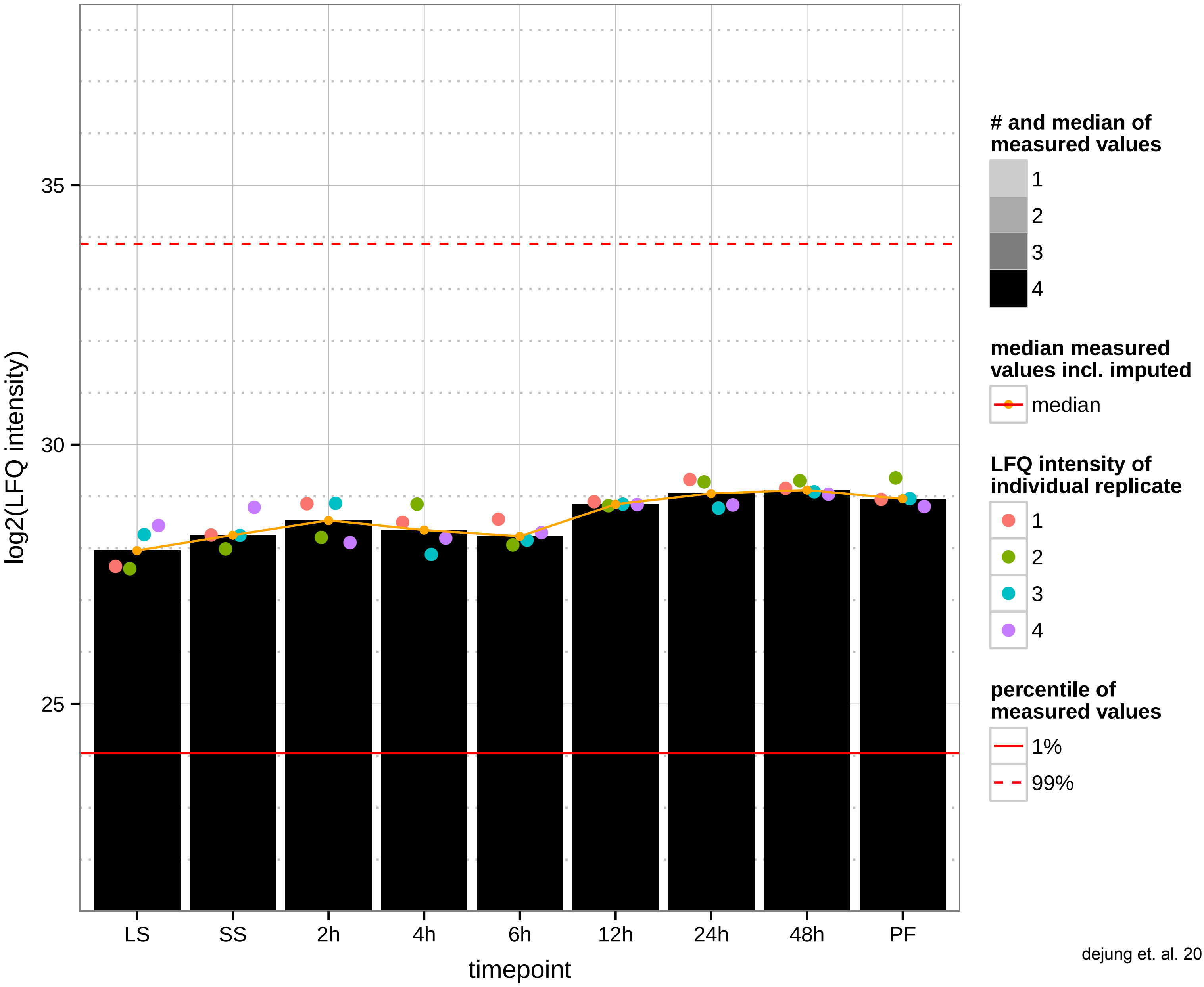

dejung et. al. 2015

cell cycle sequence binding phosphoprotein (RBP45), putative
Tb927.11.7140
AGOF: null
AGOC: null
AGOP: null
PGOF: null
PGOC: null
PGOP: null

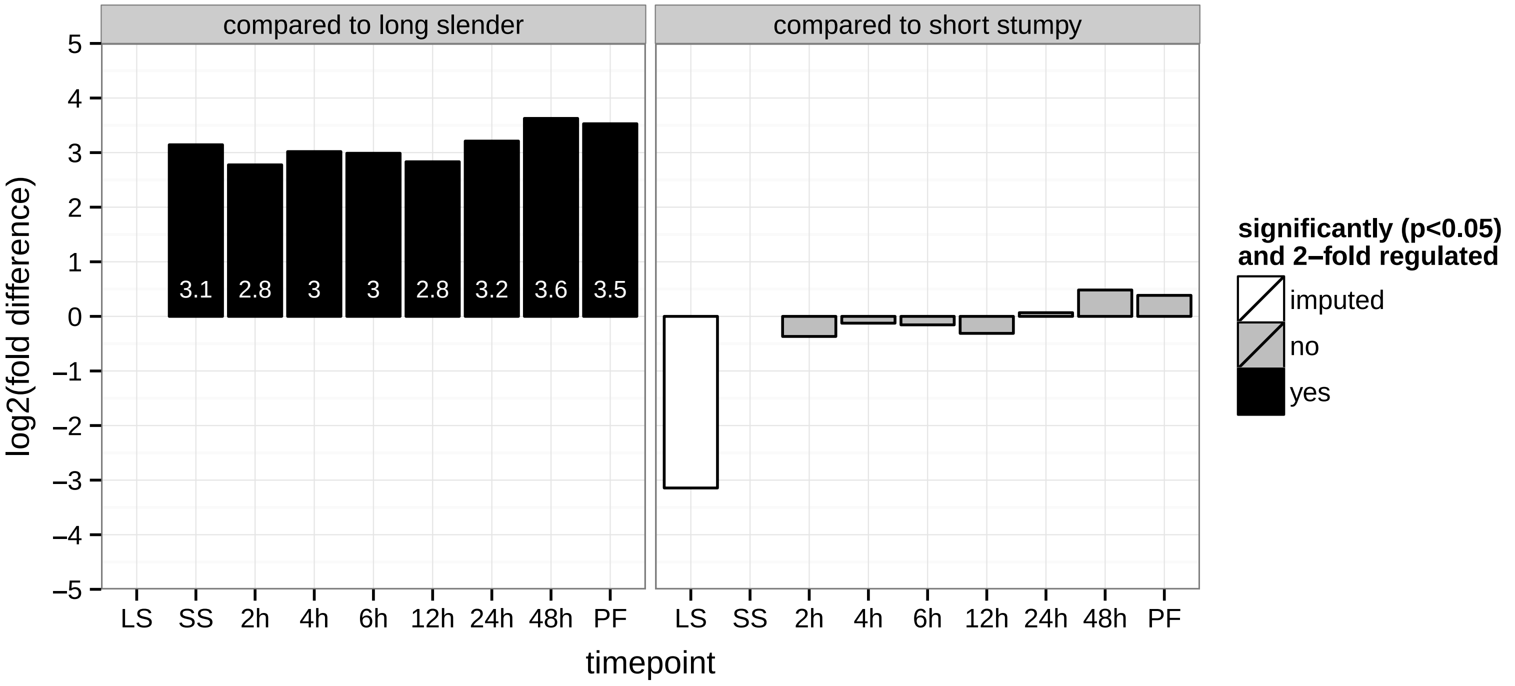

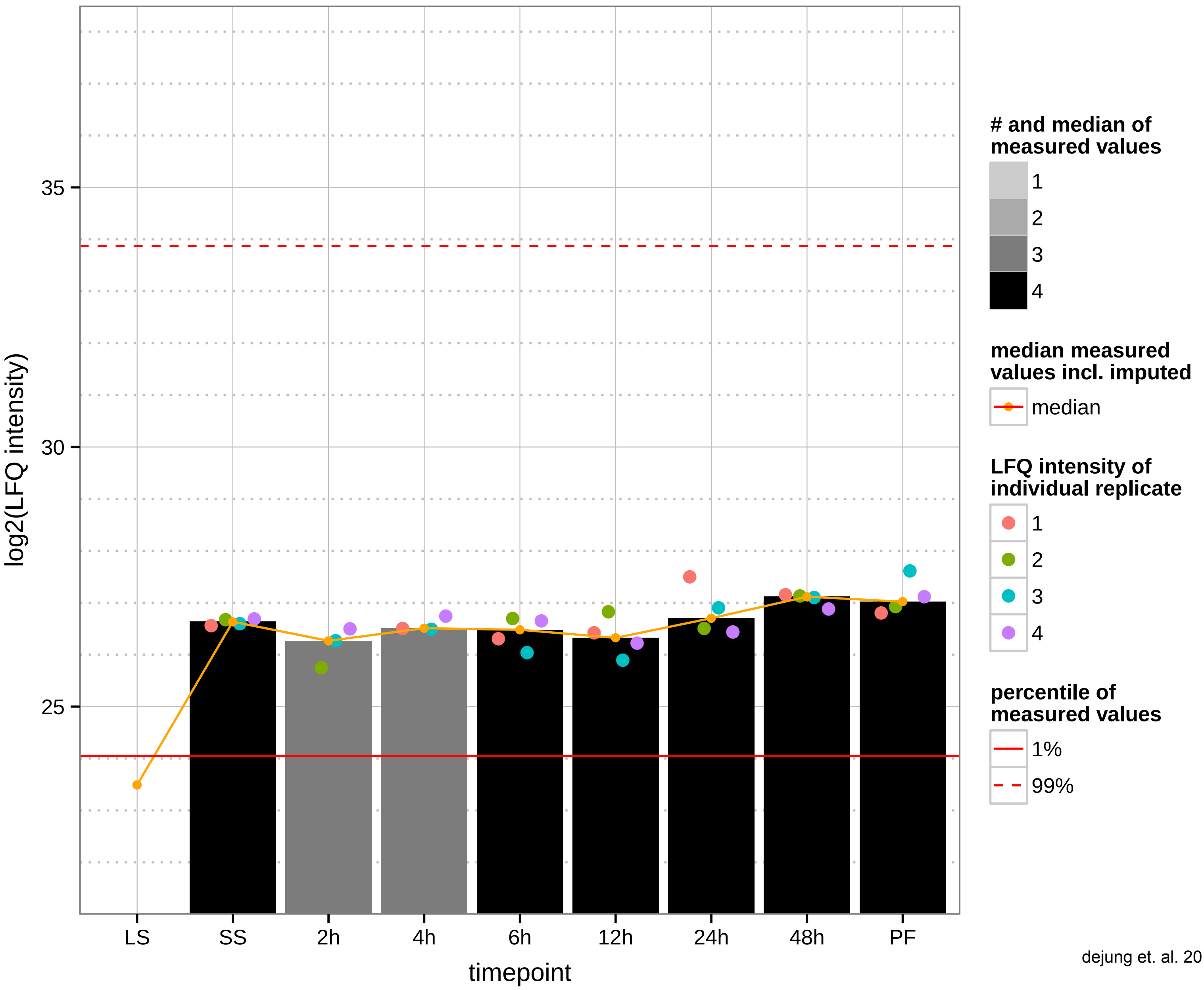

dejung et. al. 2015

NGG1 interacting factor 3–like
Tb927.11.7150
AGOF: null
AGOC: null
AGOP: null
PGOF: null
PGOC: null
PGOP: null

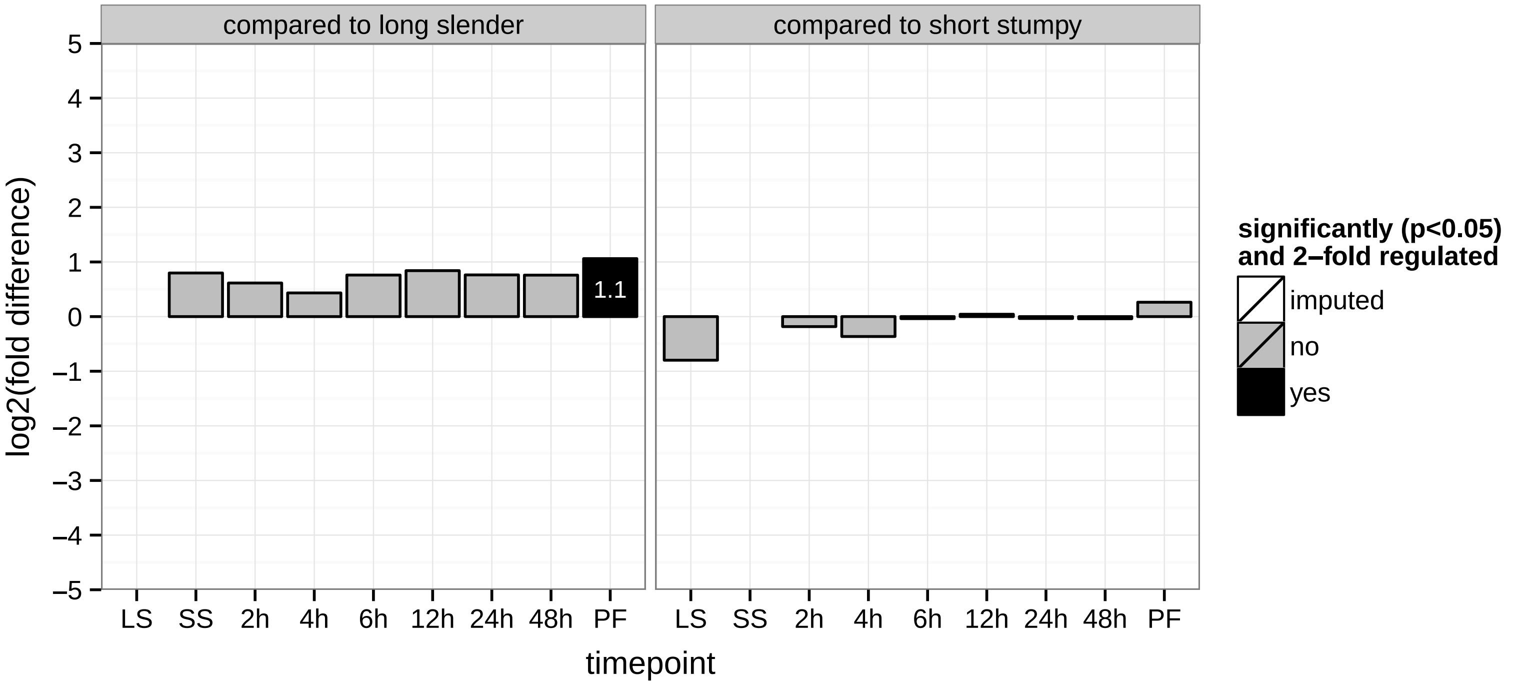

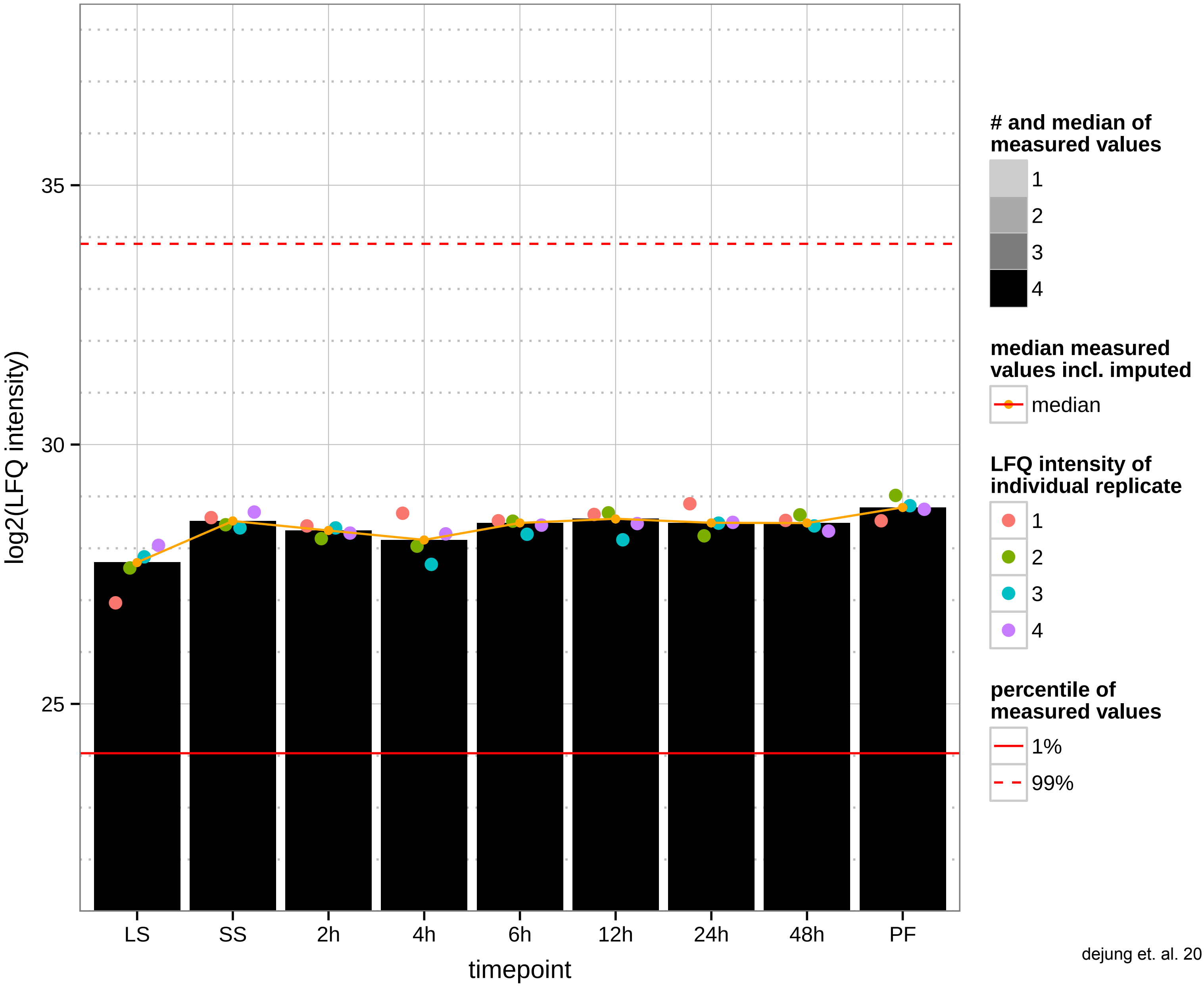

dejung et. al. 2015

seryl–tRNA synthetase
Tb927.11.7170
AGOF: ATP binding, serine–tRNA ligase activity
AGOC: cytoplasm, cytosol, mitochondrion
AGOP: seryl–tRNA aminoacylation, translation
PGOF: ATP binding, aminoacyl–tRNA ligase activity, nucleotide binding, serine–tRNA ligase activity
PGOC: cytoplasm
PGOP: seryl–tRNA aminoacylation, tRNA aminoacylation for protein translation

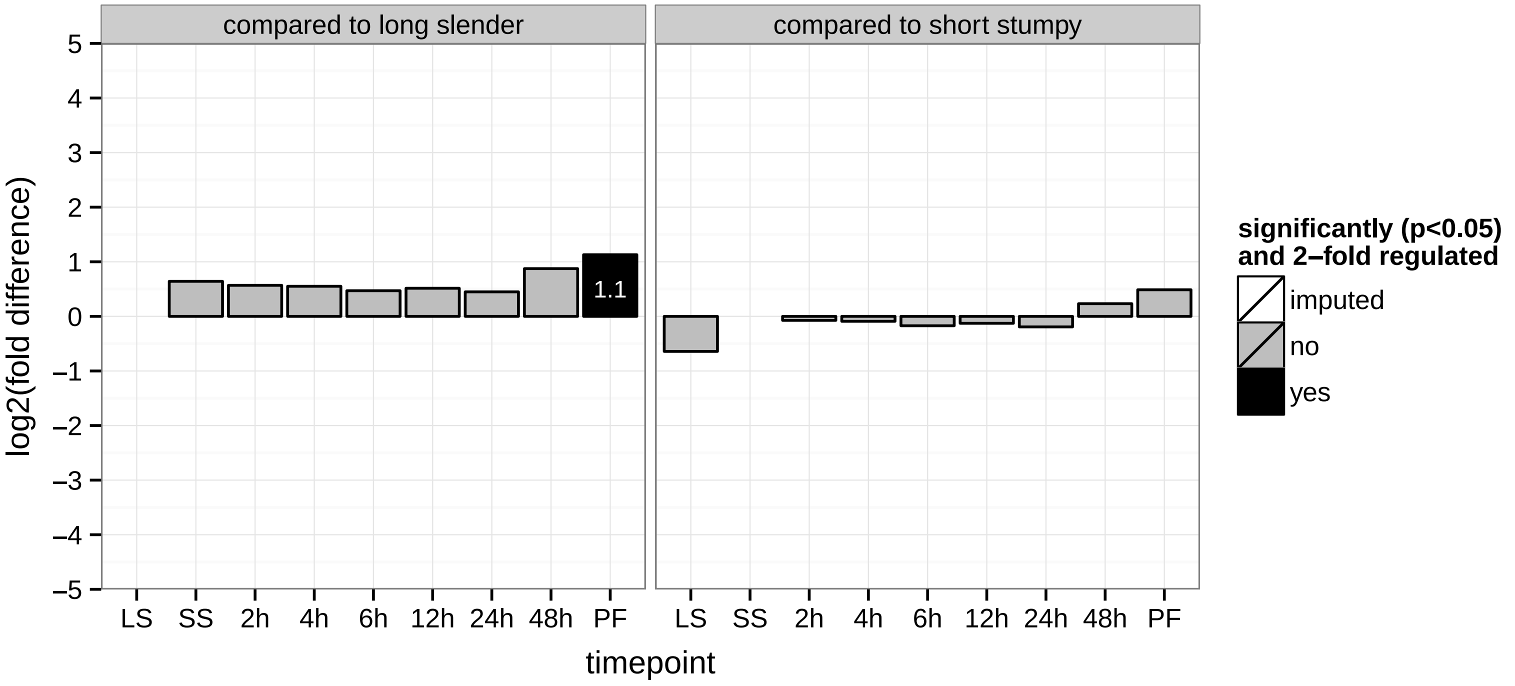

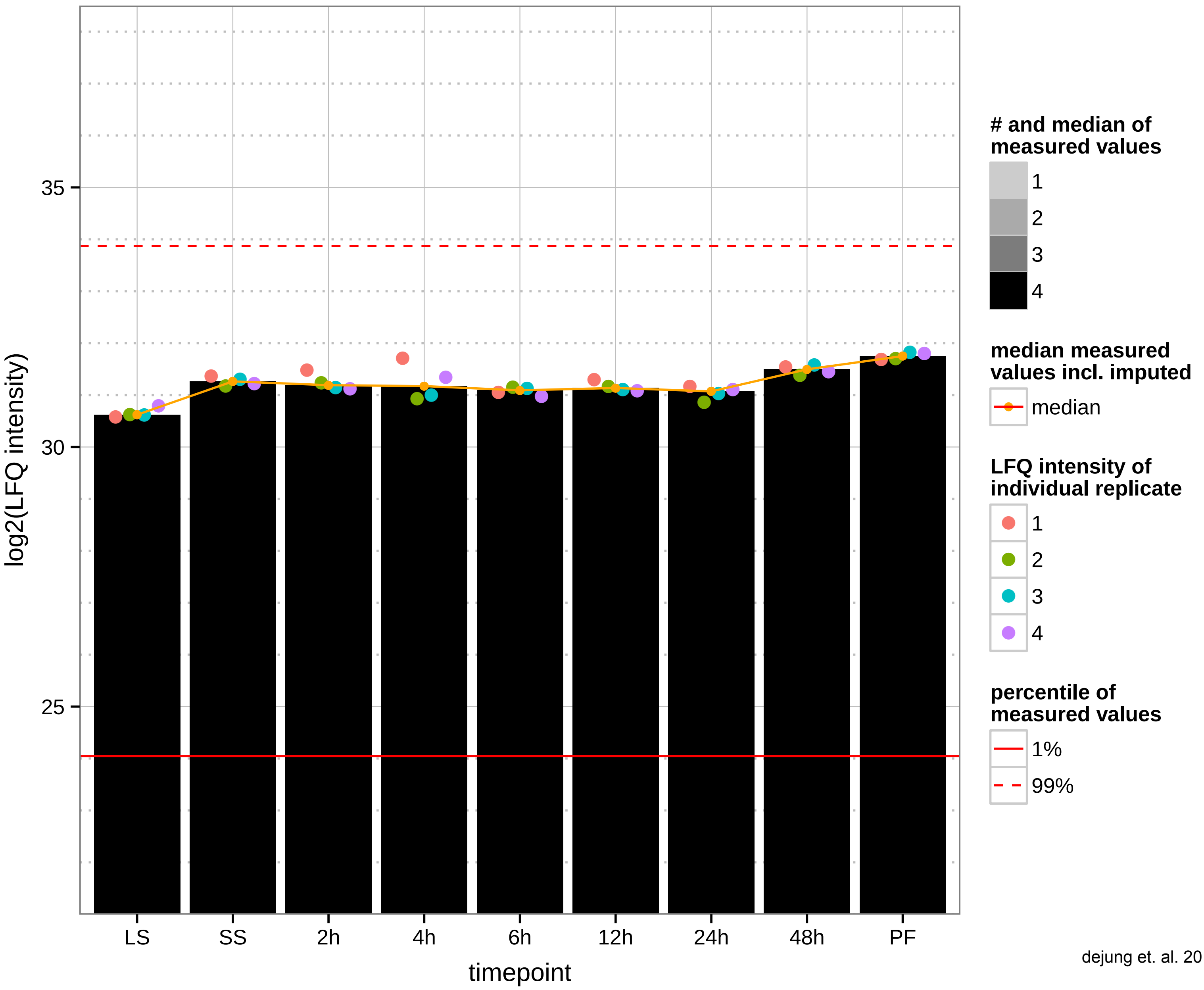

dejung et. al. 2015

hypothetical protein
Tb927.11.7190
AGOF: null
AGOC: null
AGOP: null
PGOF: null
PGOC: null
PGOP: null

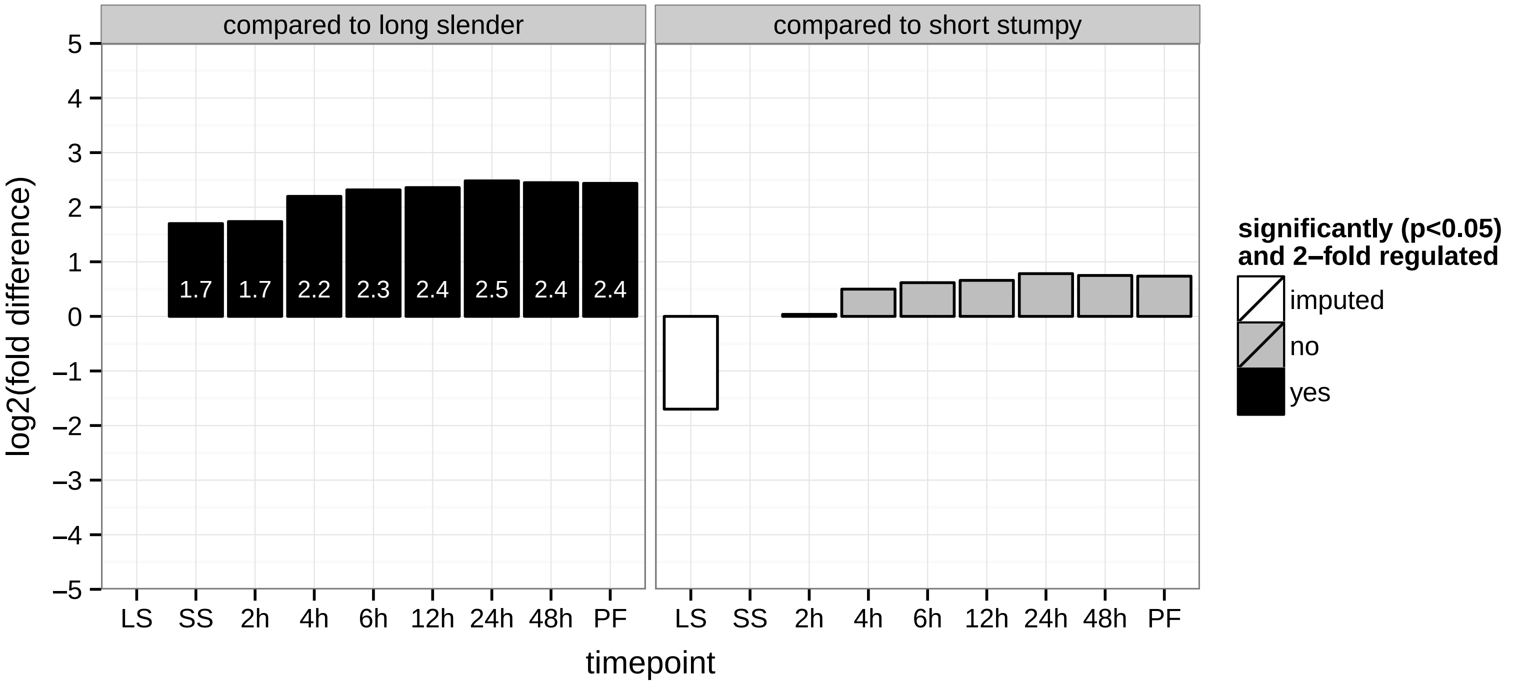

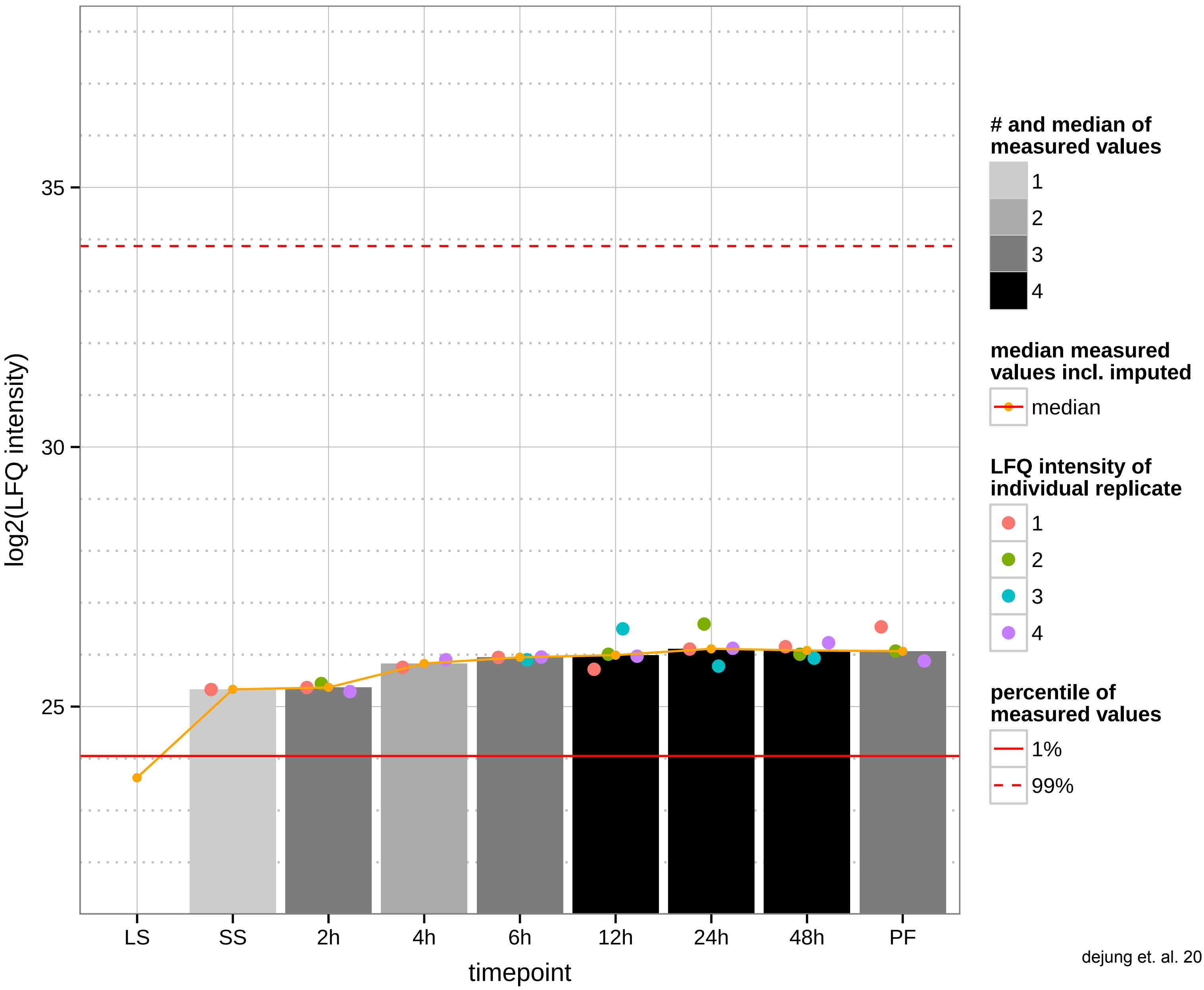

dejung et. al. 2015

hypothetical protein, chrXI additional, unordered contigs, conserved
Tb927.11.720;Tb11.0840
AGOF: null
AGOC: null
AGOP: null
PGOF: null
PGOC: null
PGOP: null

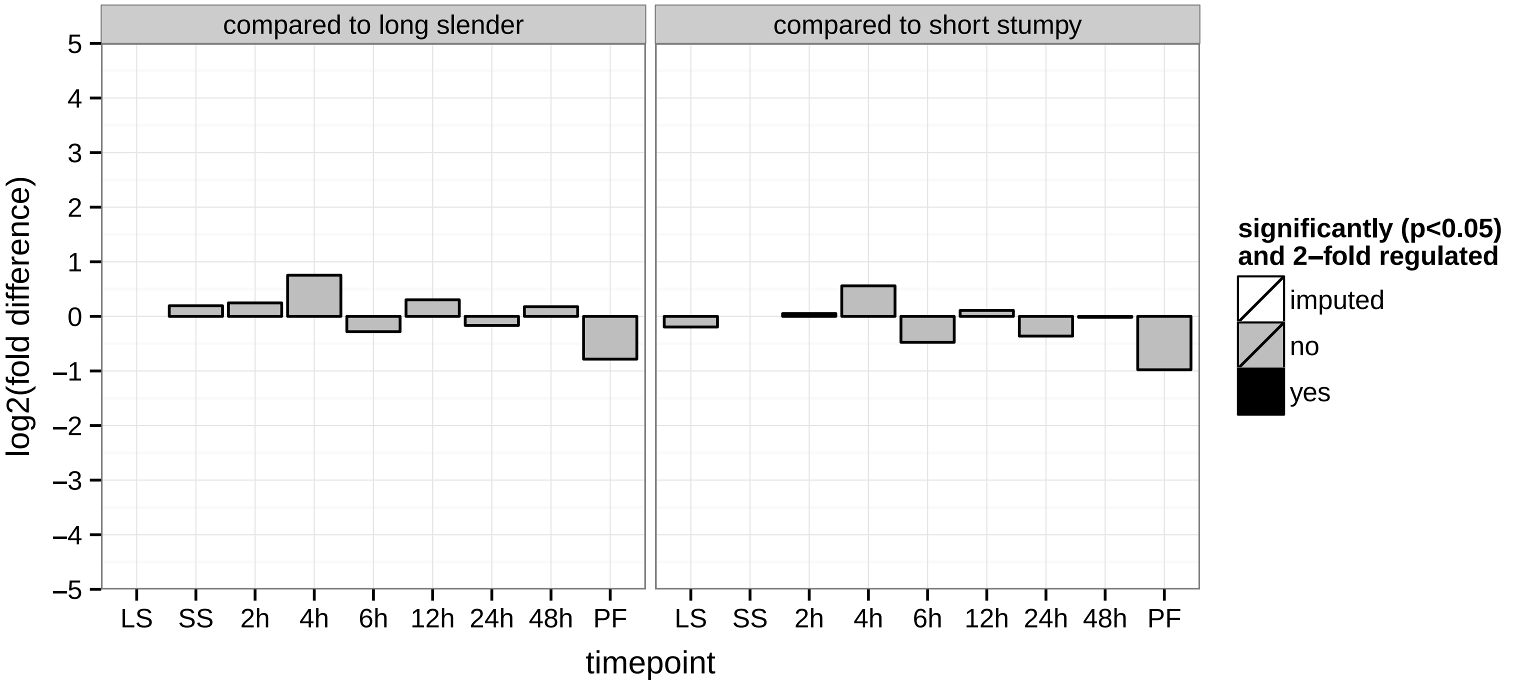

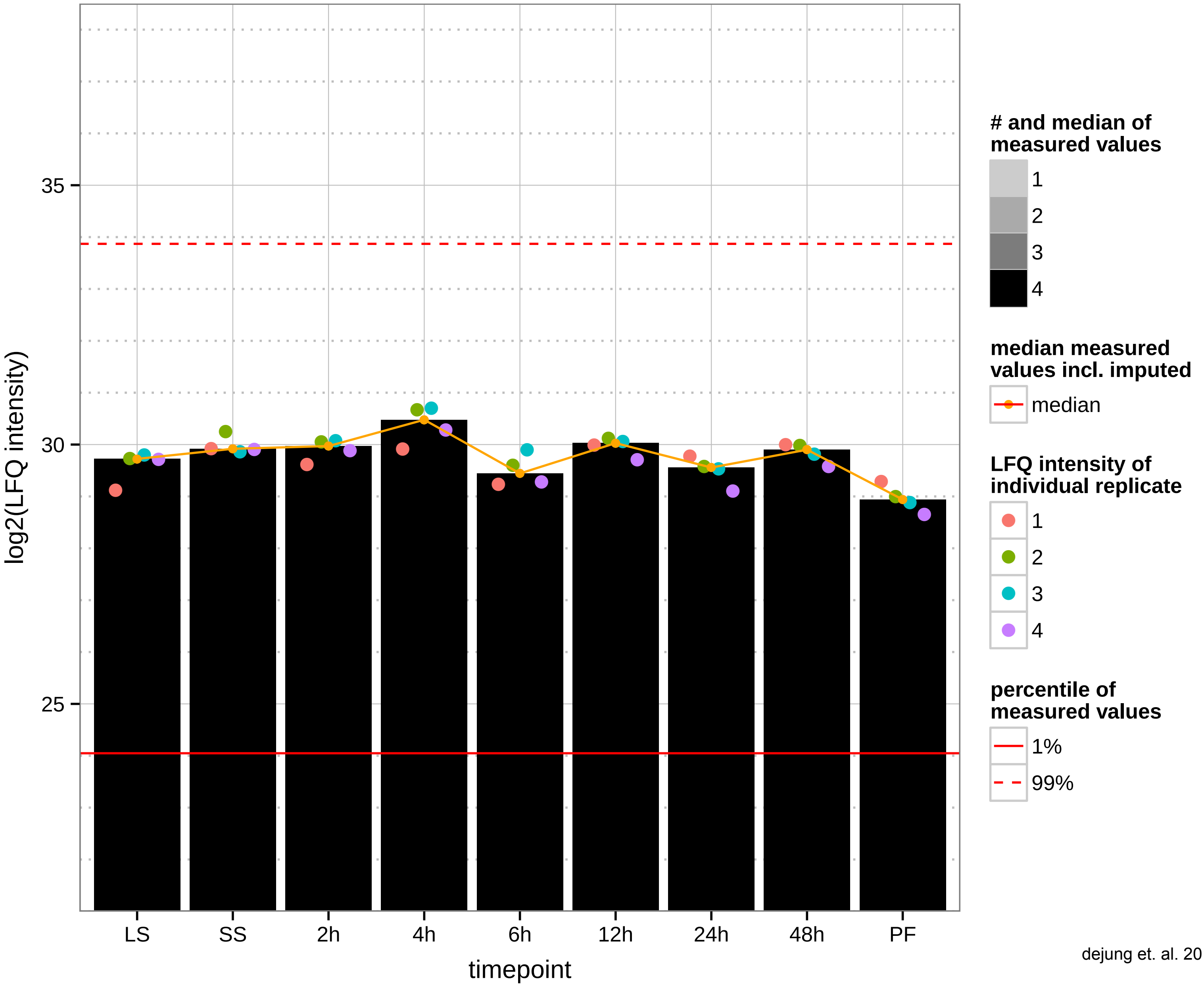

dejung et. al. 2015

histone H2B variant, putative (H2BVAR)
Tb927.11.7350
AGOF: DNA binding
AGOC: nucleosome, nucleus
AGOP: DNA replication, nucleosome assembly
PGOF: DNA binding
PGOC: nucleosome, nucleus
PGOP: nucleosome assembly

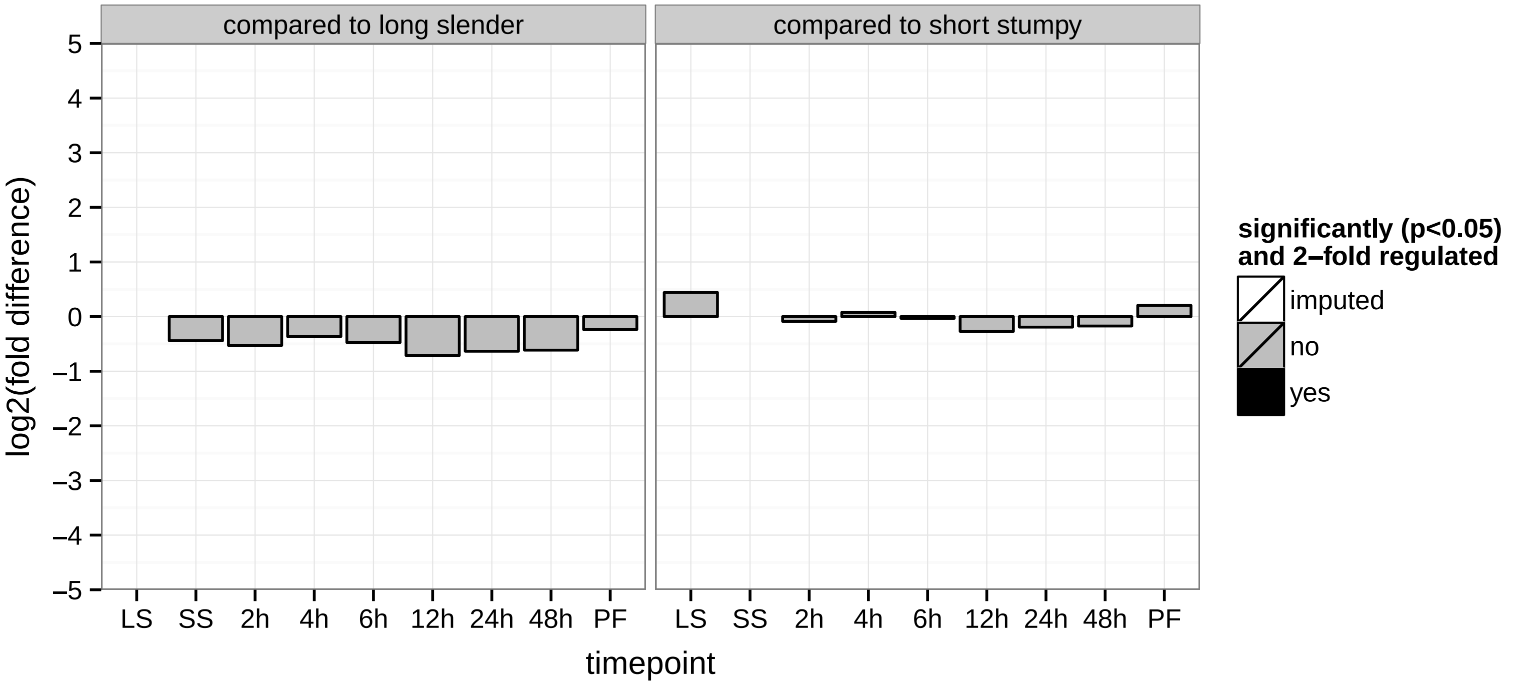

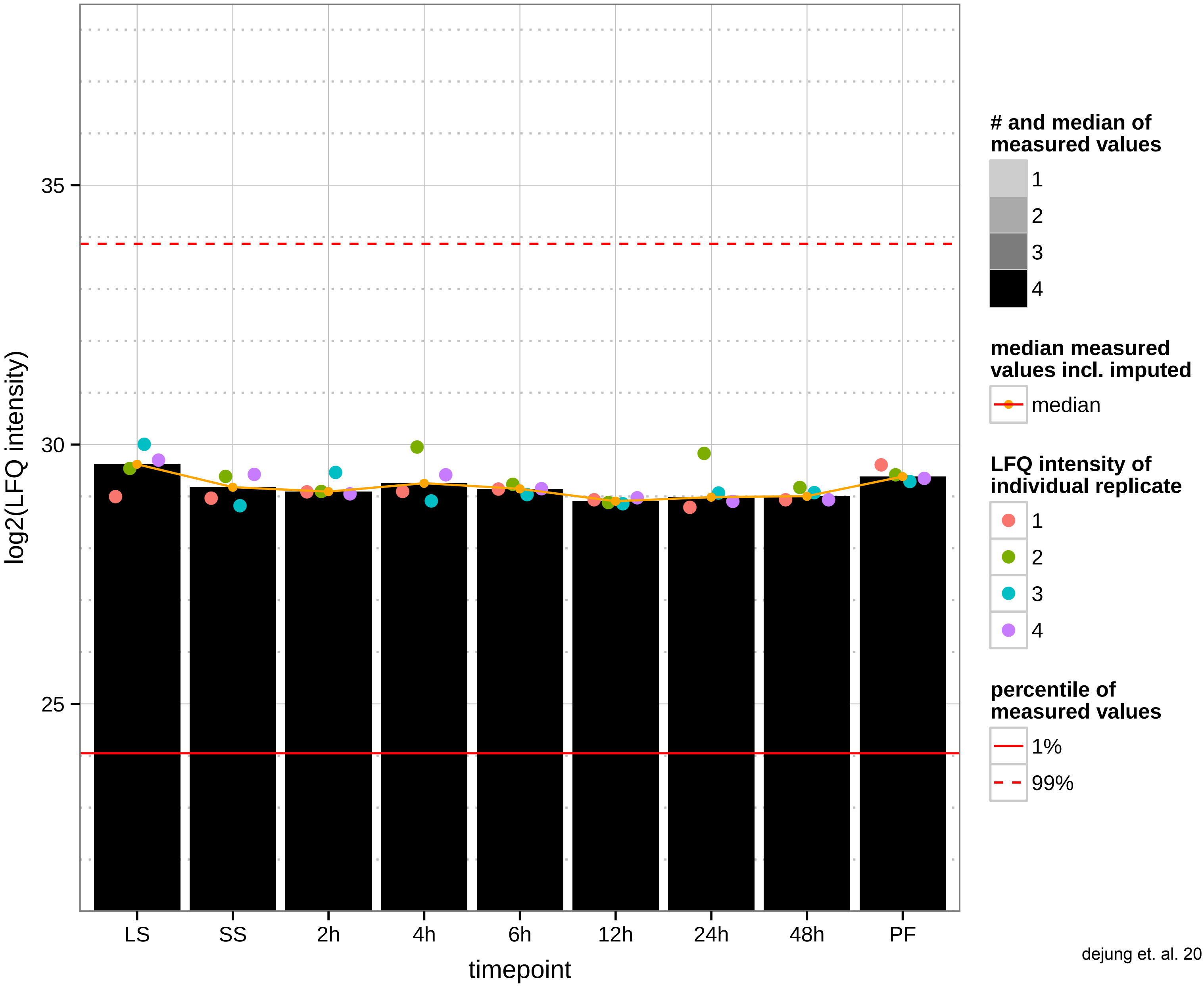

dejung et. al. 2015

hypothetical protein, conserved
Tb927.11.7390
AGOF: null
AGOC: mitochondrion
AGOP: null
PGOF: null
PGOC: null
PGOP: null

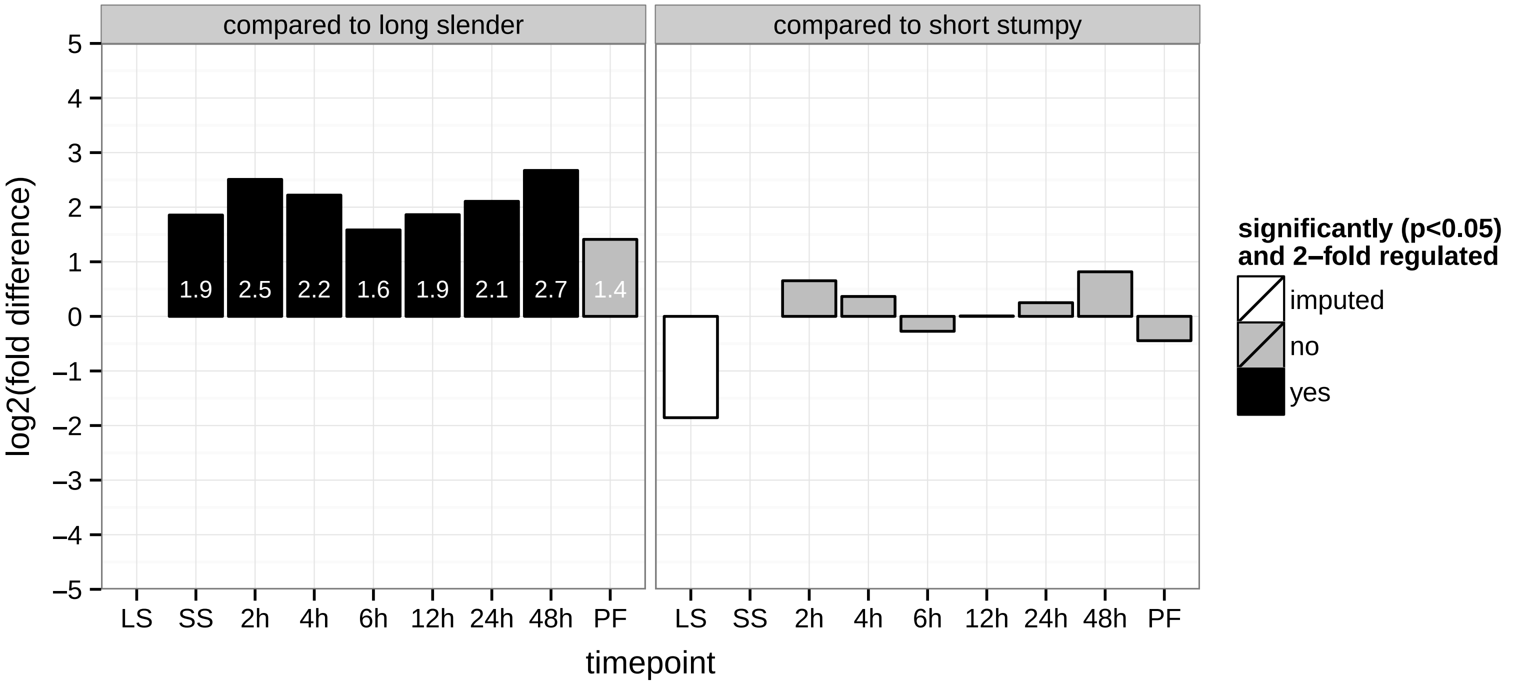

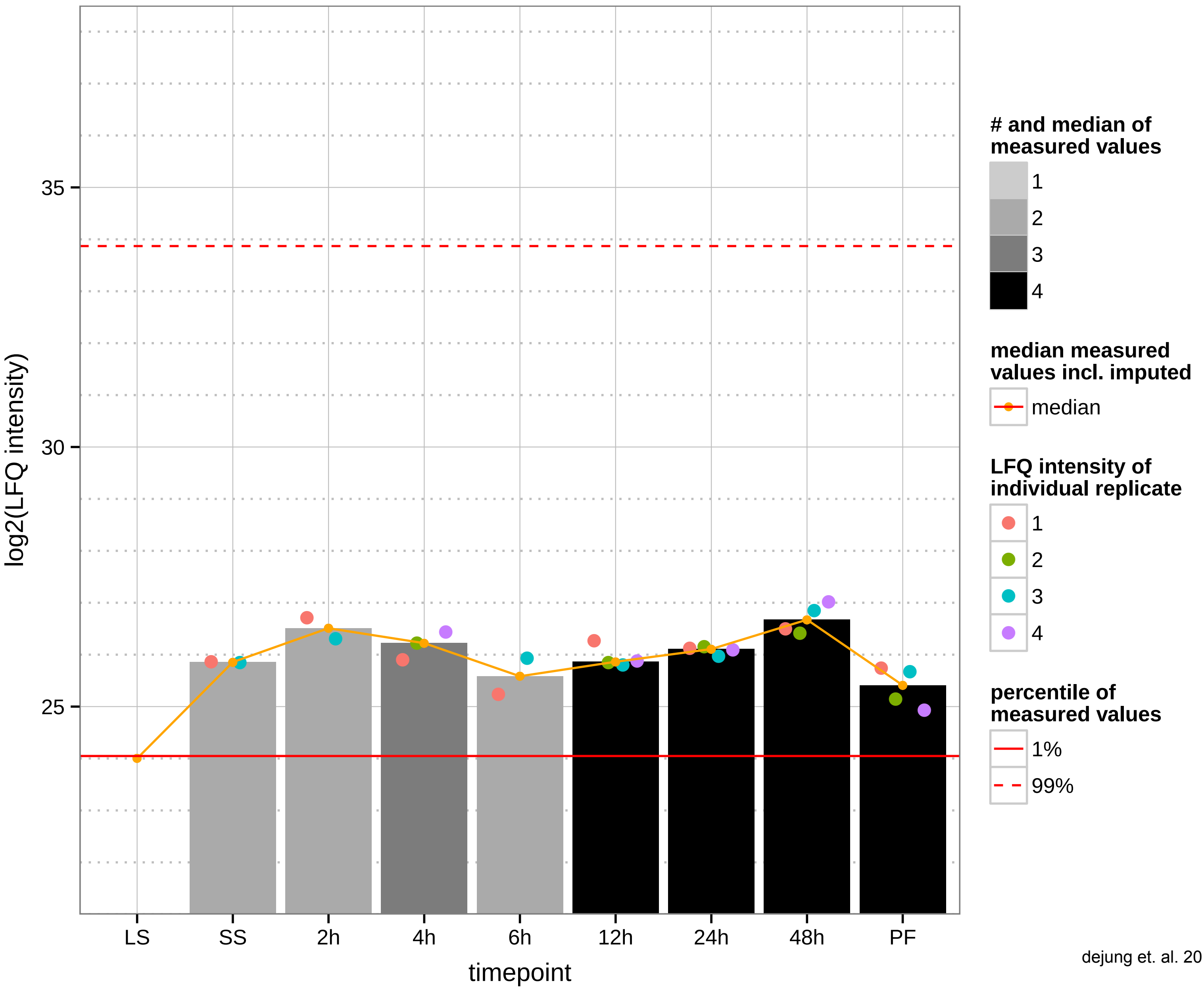

dejung et. al. 2015

hypothetical protein, conserved
Tb927.11.7430
AGOF: null
AGOC: null
AGOP: ubiquitin–dependent protein catabolic process
PGOF: null
PGOC: null
PGOP: ubiquitin–dependent protein catabolic process

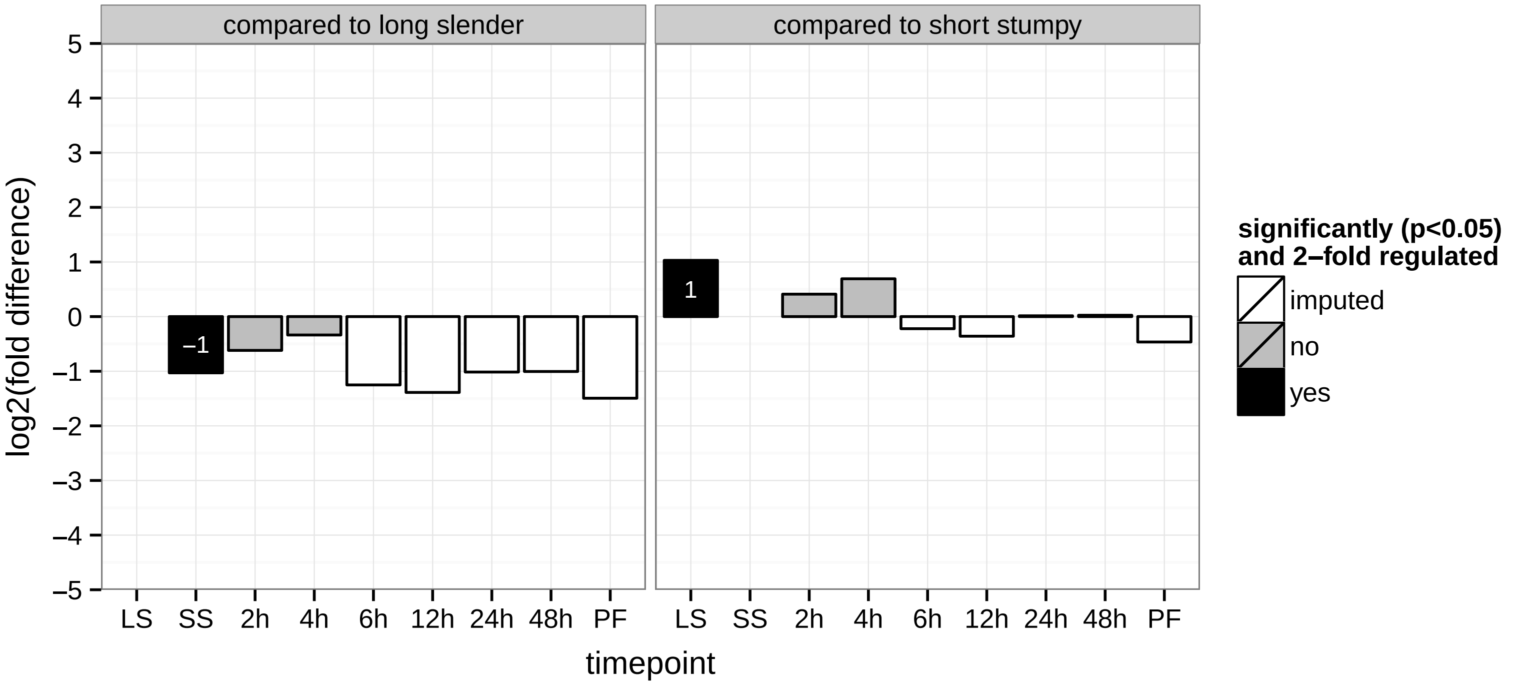

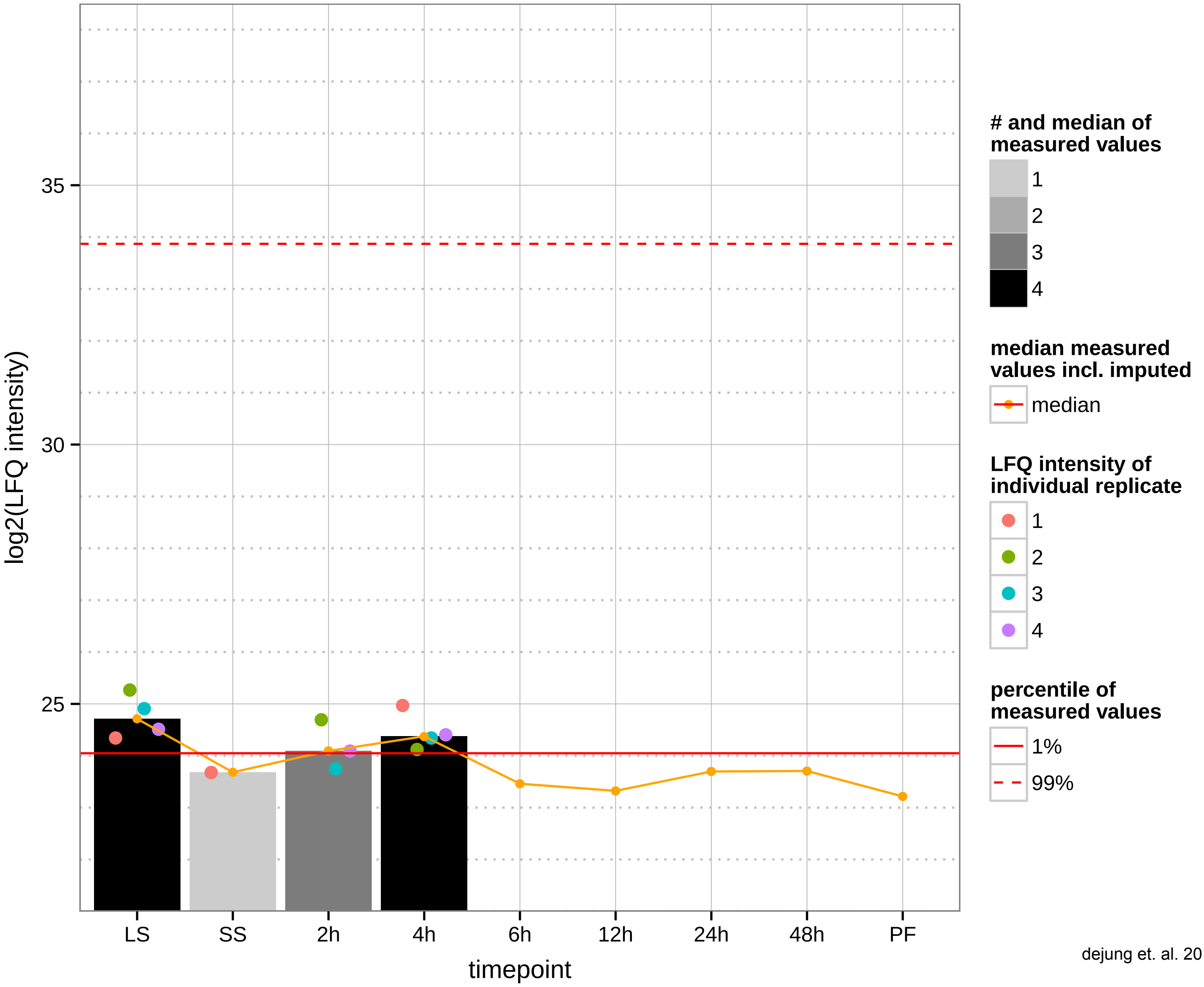

dejung et. al. 2015

hypothetical protein, conserved
Tb927.11.7440
AGOF: null
AGOC: null
AGOP: null
PGOF: null
PGOC: null
PGOP: ubiquitin–dependent protein catabolic process

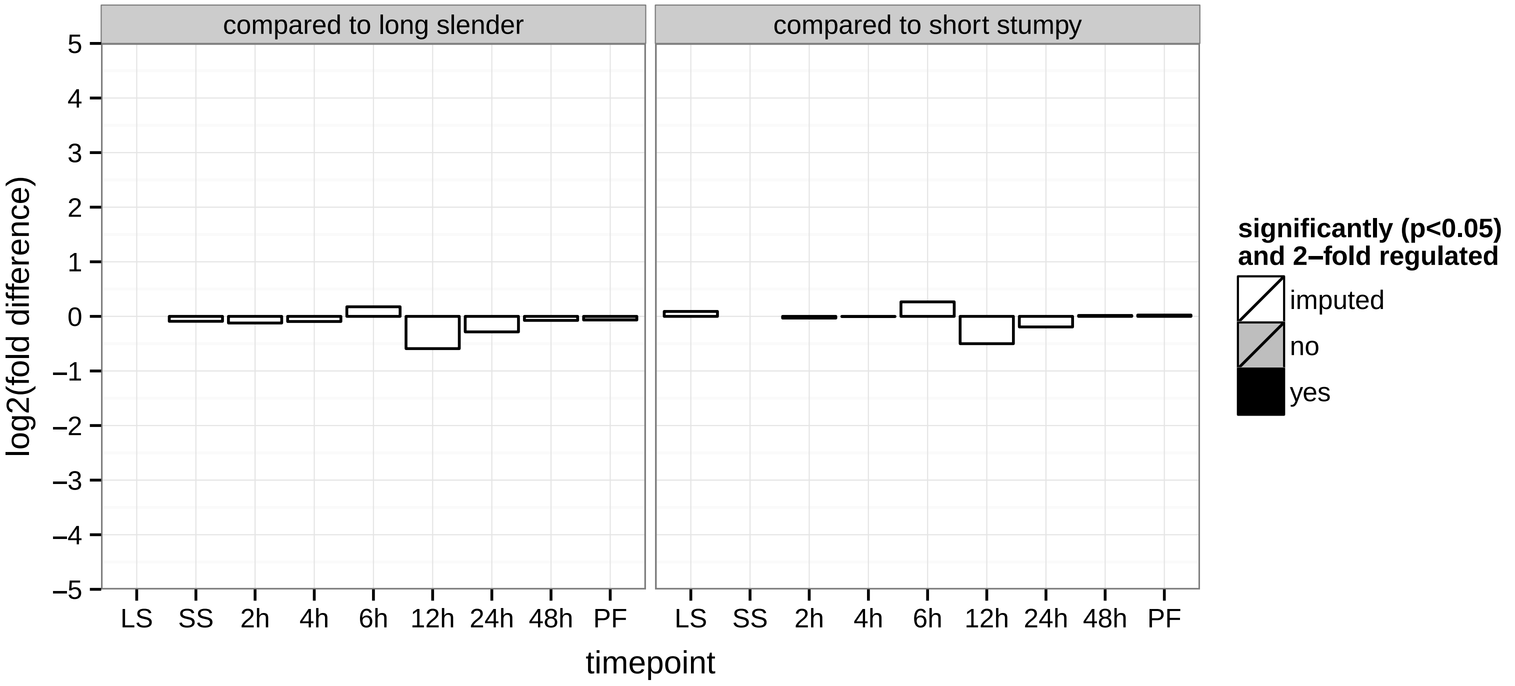

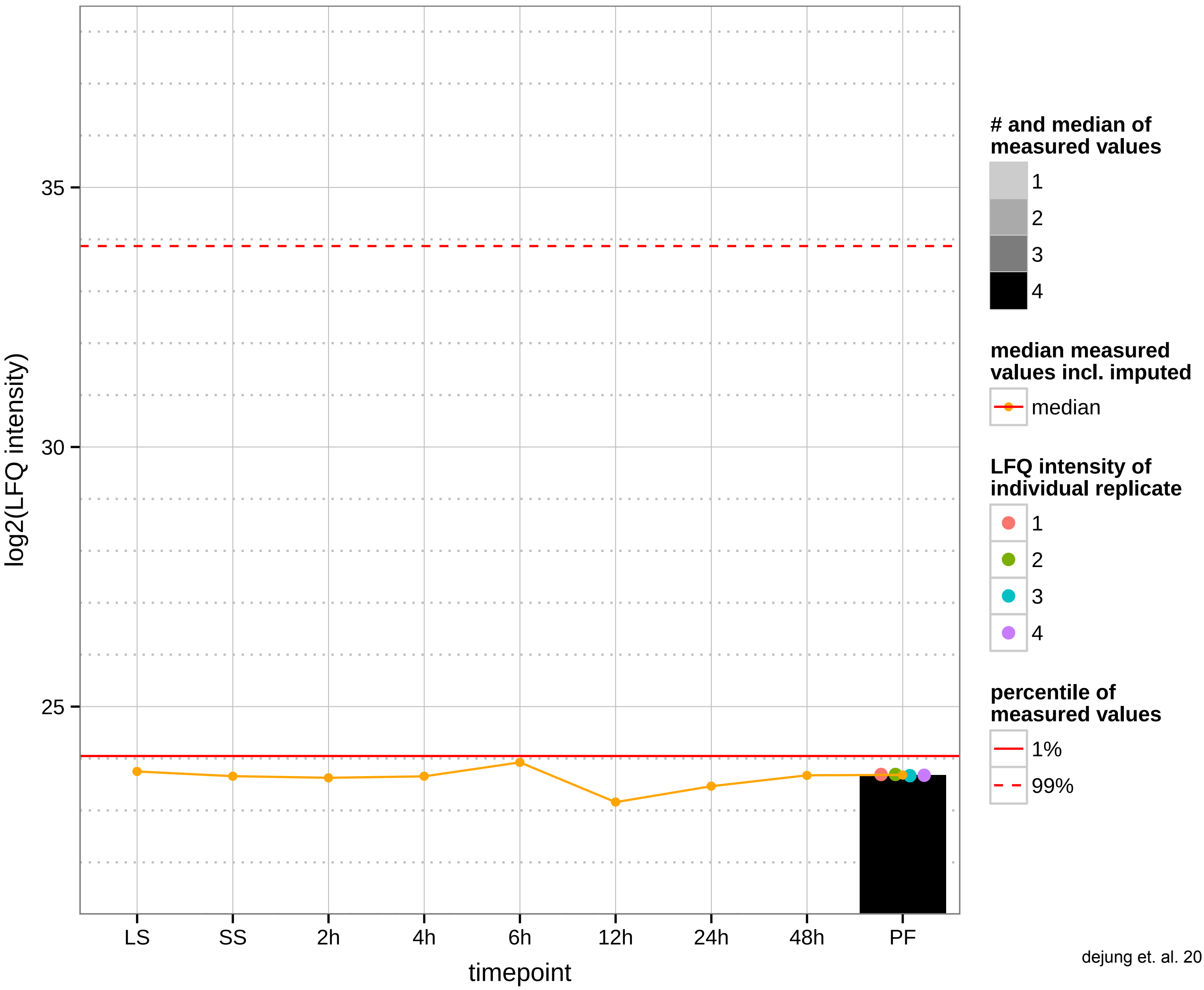

dejung et. al. 2015

glucose−regulated protein 78, putative, luminal binding protein 1 (BiP)
Tb927.11.7510;Tb927.11.7460
AGOF: ATP binding
AGOC: endoplasmic reticulum
AGOP: SRP−dependent cotranslational protein targeting to membrane, protein folding
PGOF: null
PGOC: null
PGOP: null

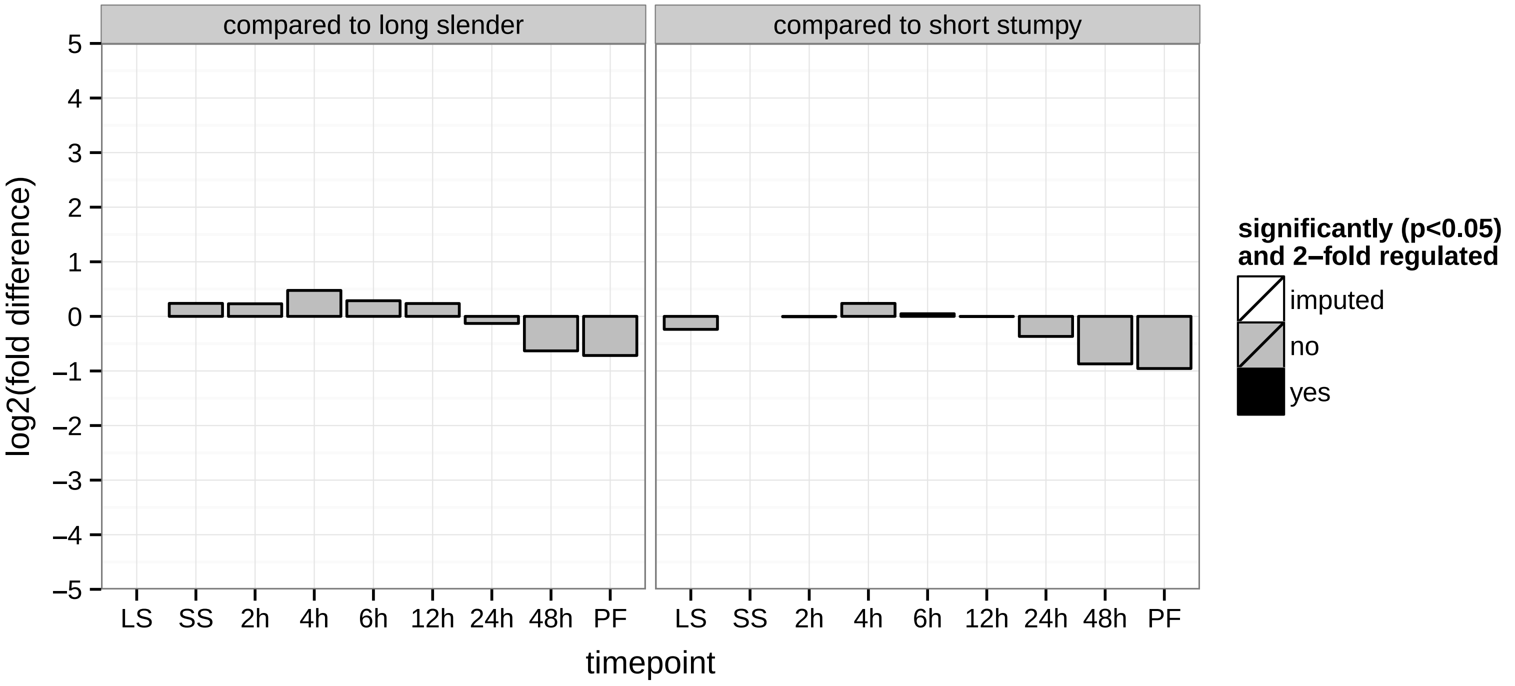

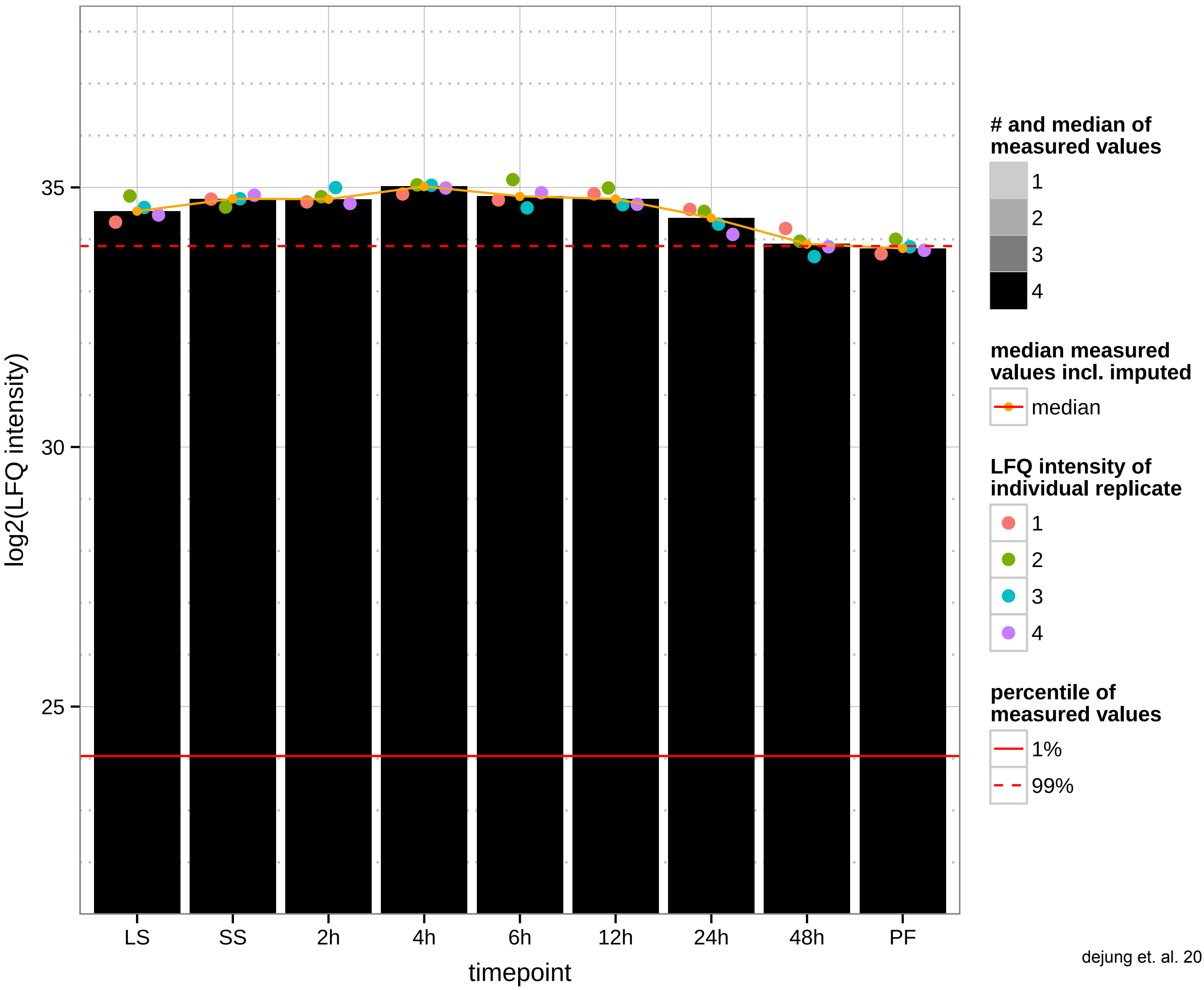

dejung et. al. 2015

hypothetical protein, conserved
Tb927.11.7470
AGOF: null
AGOC: null
AGOP: null
PGOF: null
PGOC: null
PGOP: null

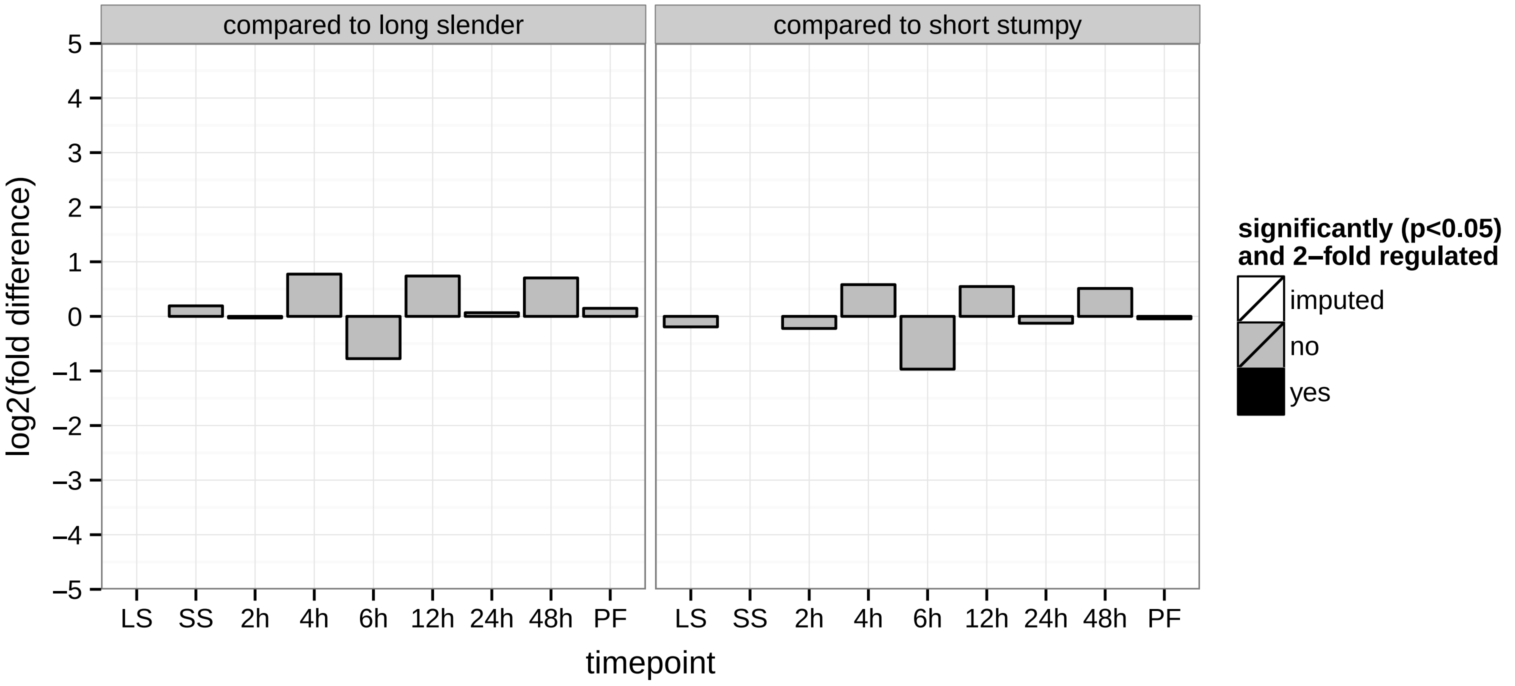

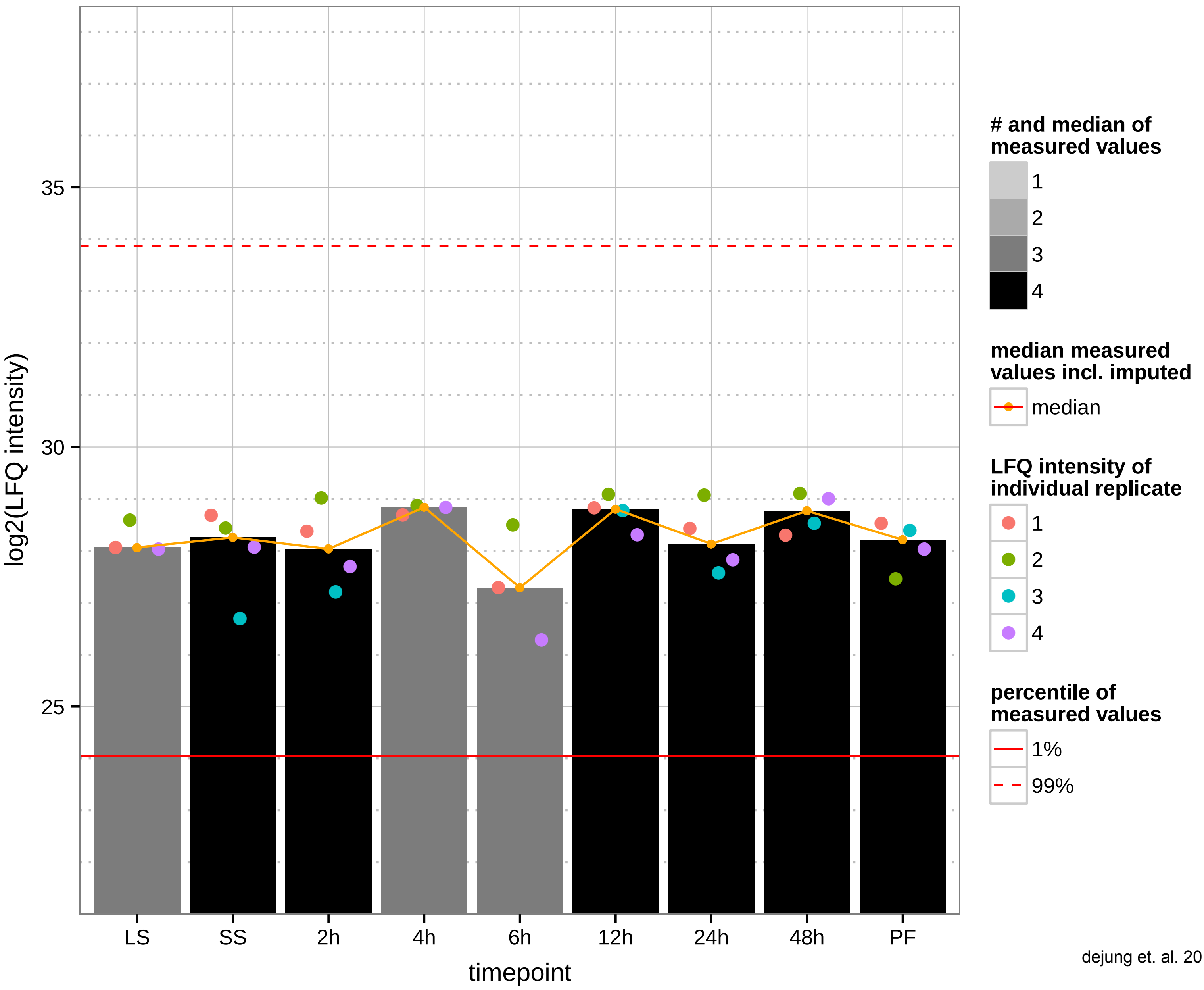

dejung et. al. 2015

long−chain−fatty−acid−CoA ligase, putative
Tb927.11.7530
AGOF: catalytic activity
AGOC: null
AGOP: metabolic process
PGOF: catalytic activity
PGOC: null
PGOP: metabolic process

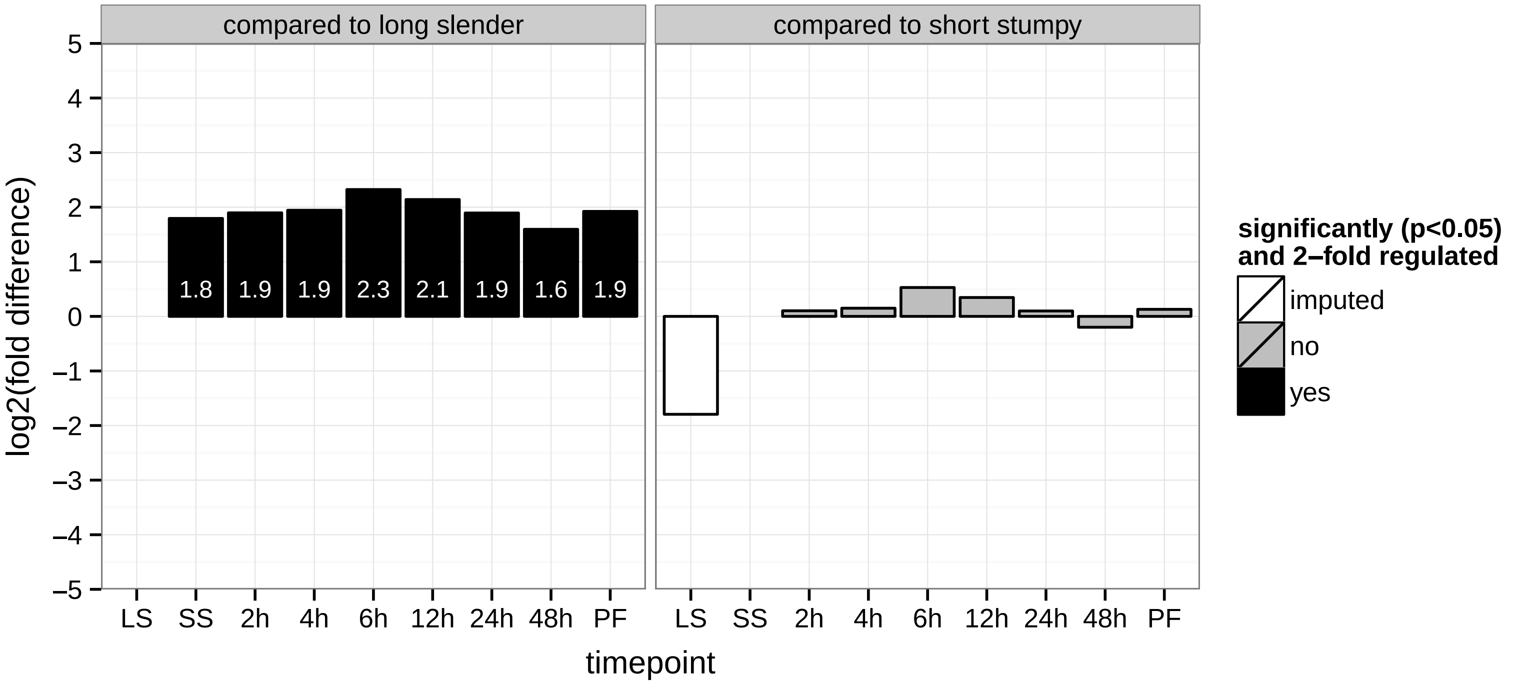

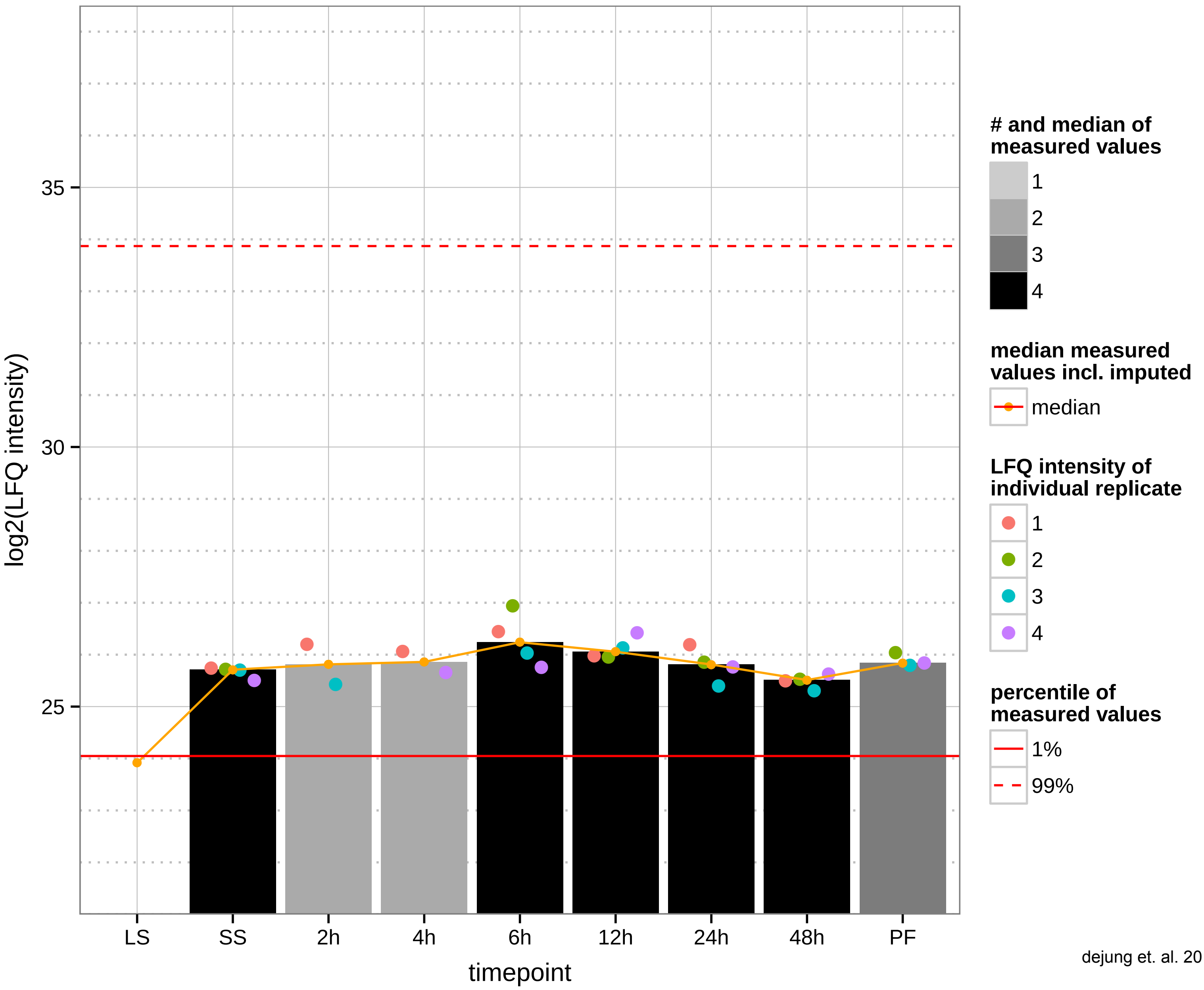

dejung et. al. 2015

predicted WD40 repeat protein
Tb927.11.7560
AGOF: null
AGOC: null
AGOP: ciliary or flagellar motility
PGOF: protein binding
PGOC: null
PGOP: null

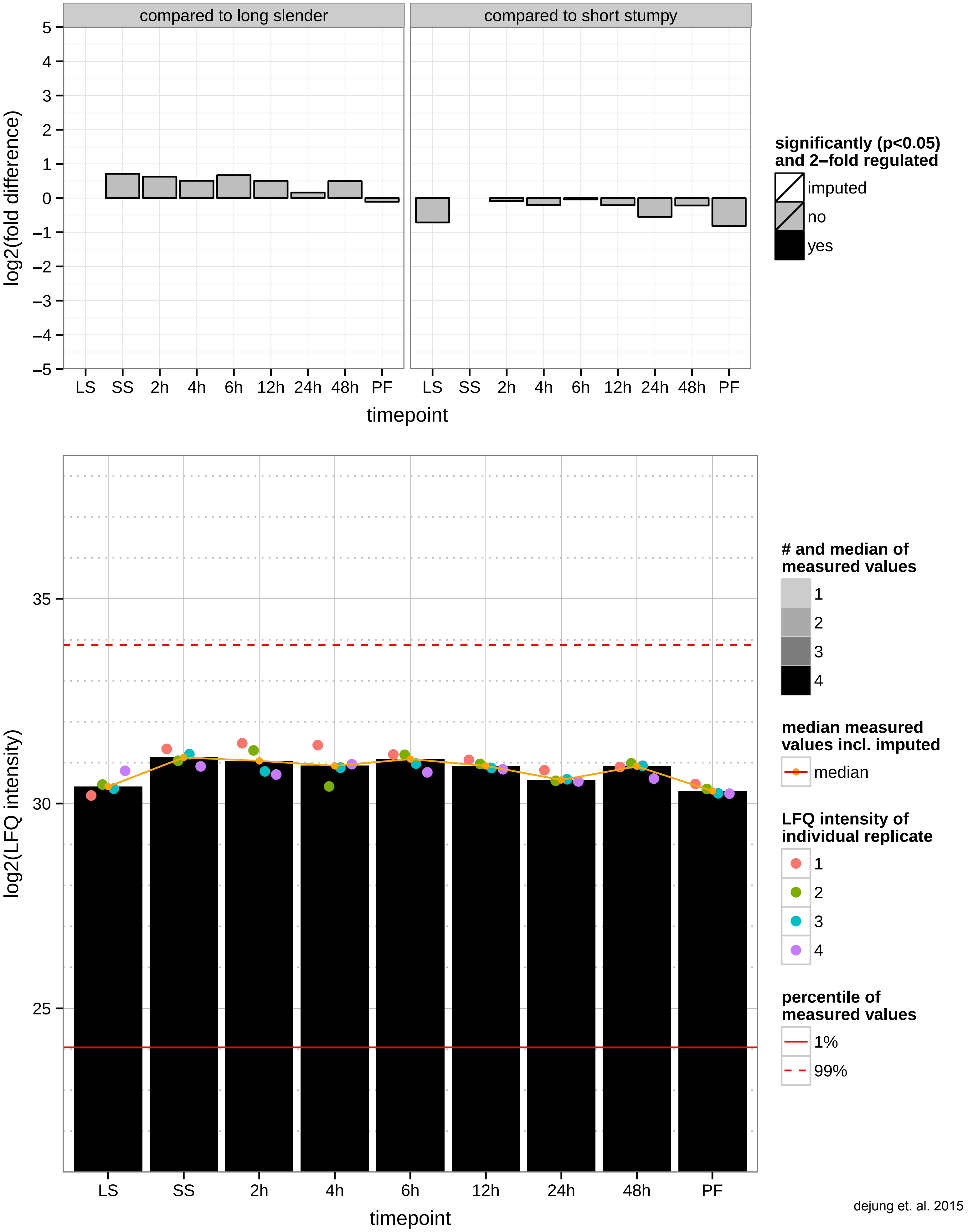

dejung et. al. 2015

protein phosphatase 2C, putative
Tb927.11.760
AGOF: protein serine/threonine phosphatase activity
AGOC: protein serine/threonine phosphatase complex
AGOP: modulation of development of symbiont involved in interaction with host, protein dephosphorylation, quorum sensing i
PGOF: catalytic activity
PGOC: null
PGOP: null

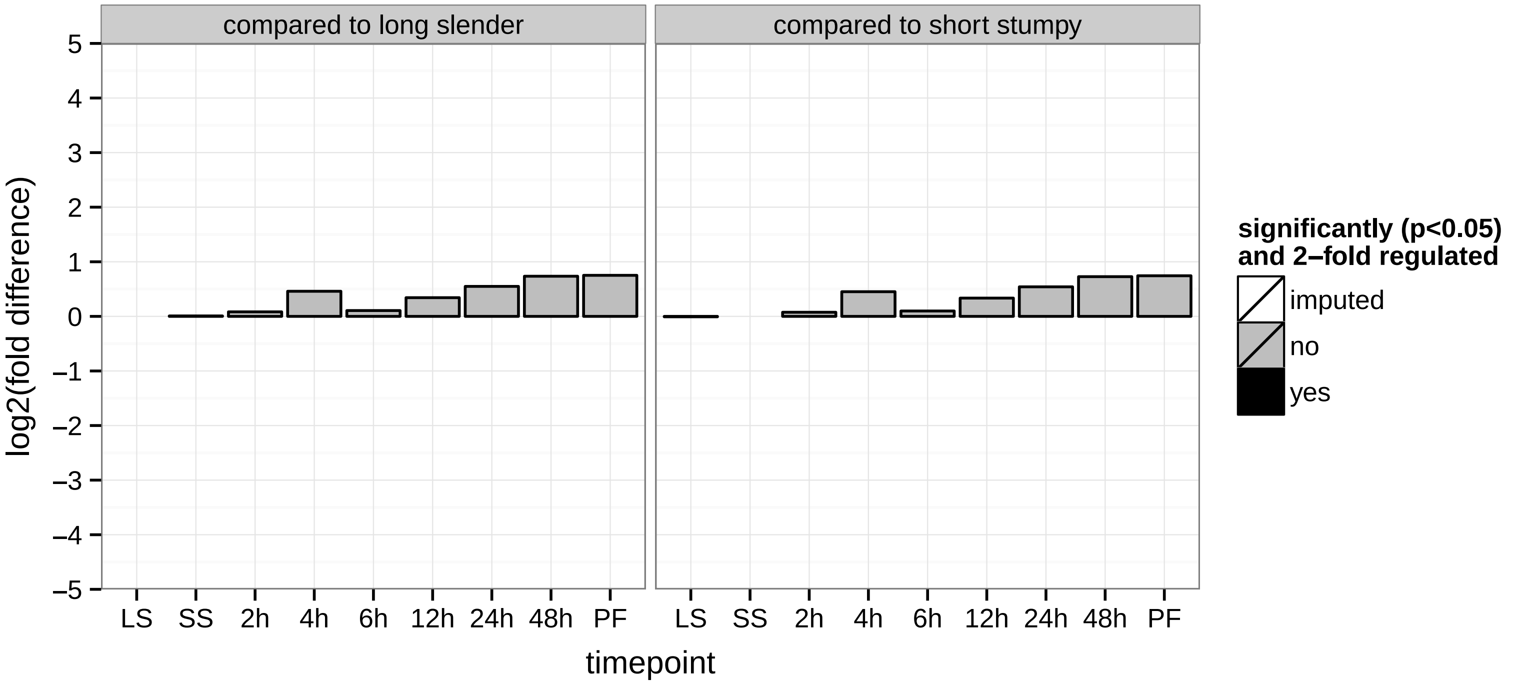

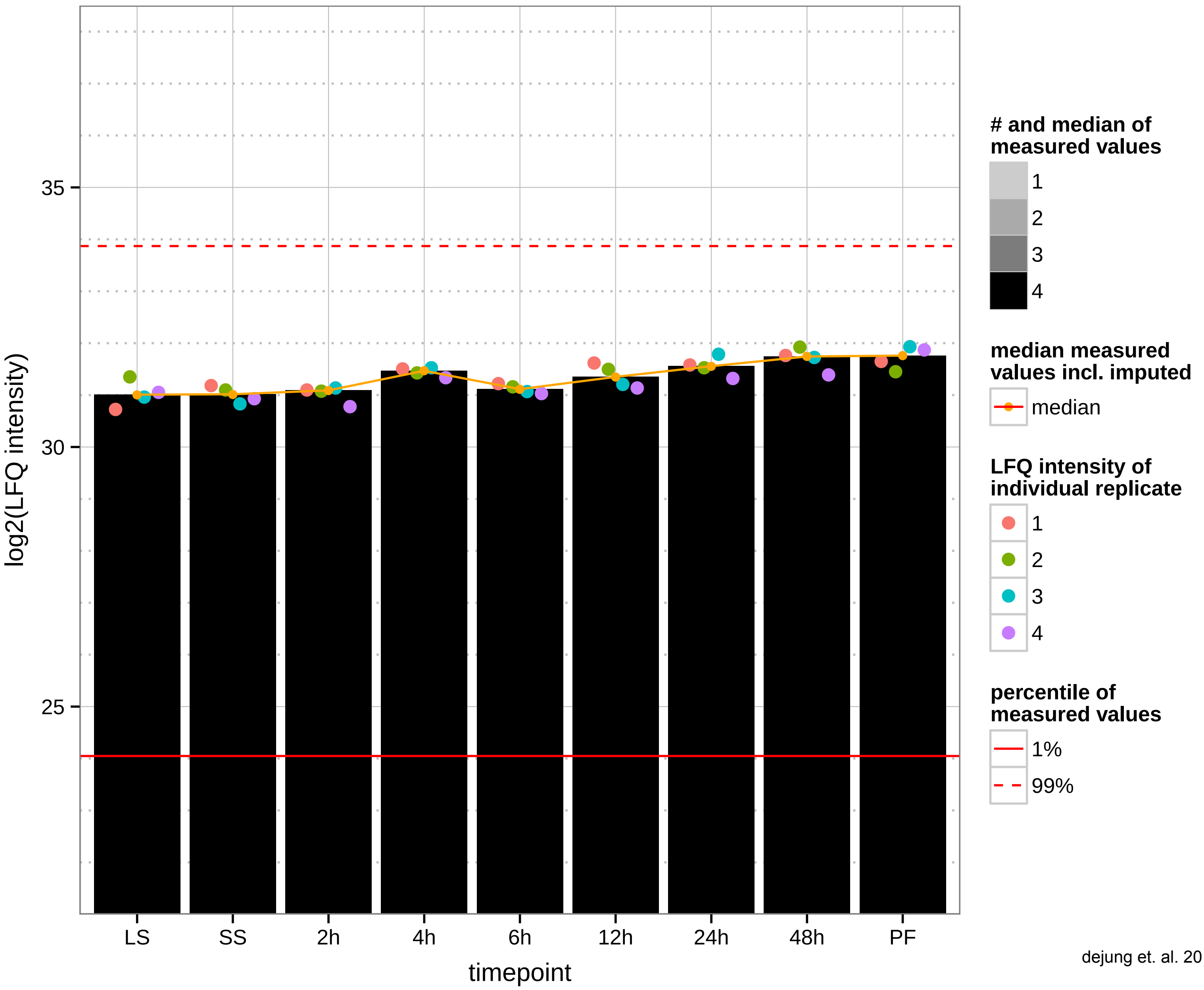

dejung et. al. 2015

dynein light chain, putative, dynein Tctex2 family
Tb927.11.7740
AGOF: microtubule motor activity
AGOC: microtubule associated complex
AGOP: microtubule−based process
PGOF: null
PGOC: null
PGOP: null

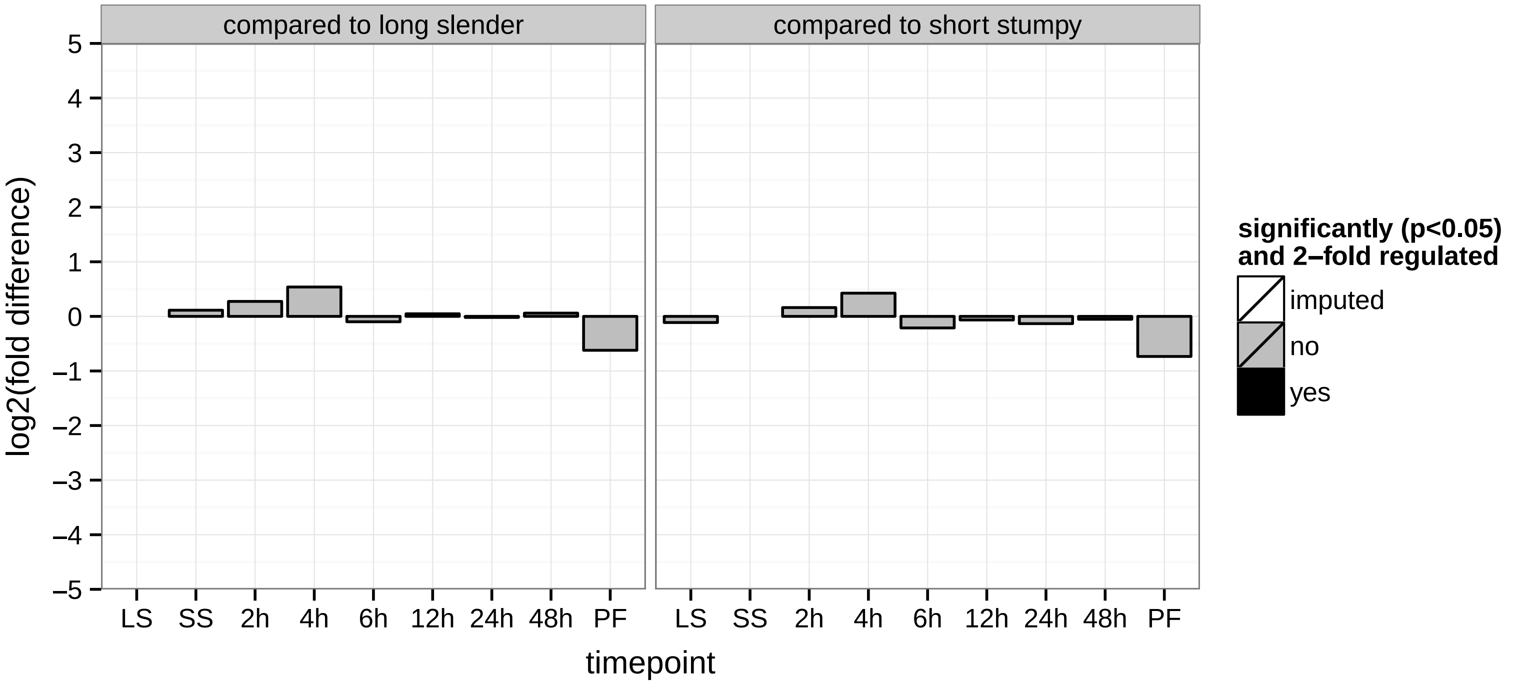

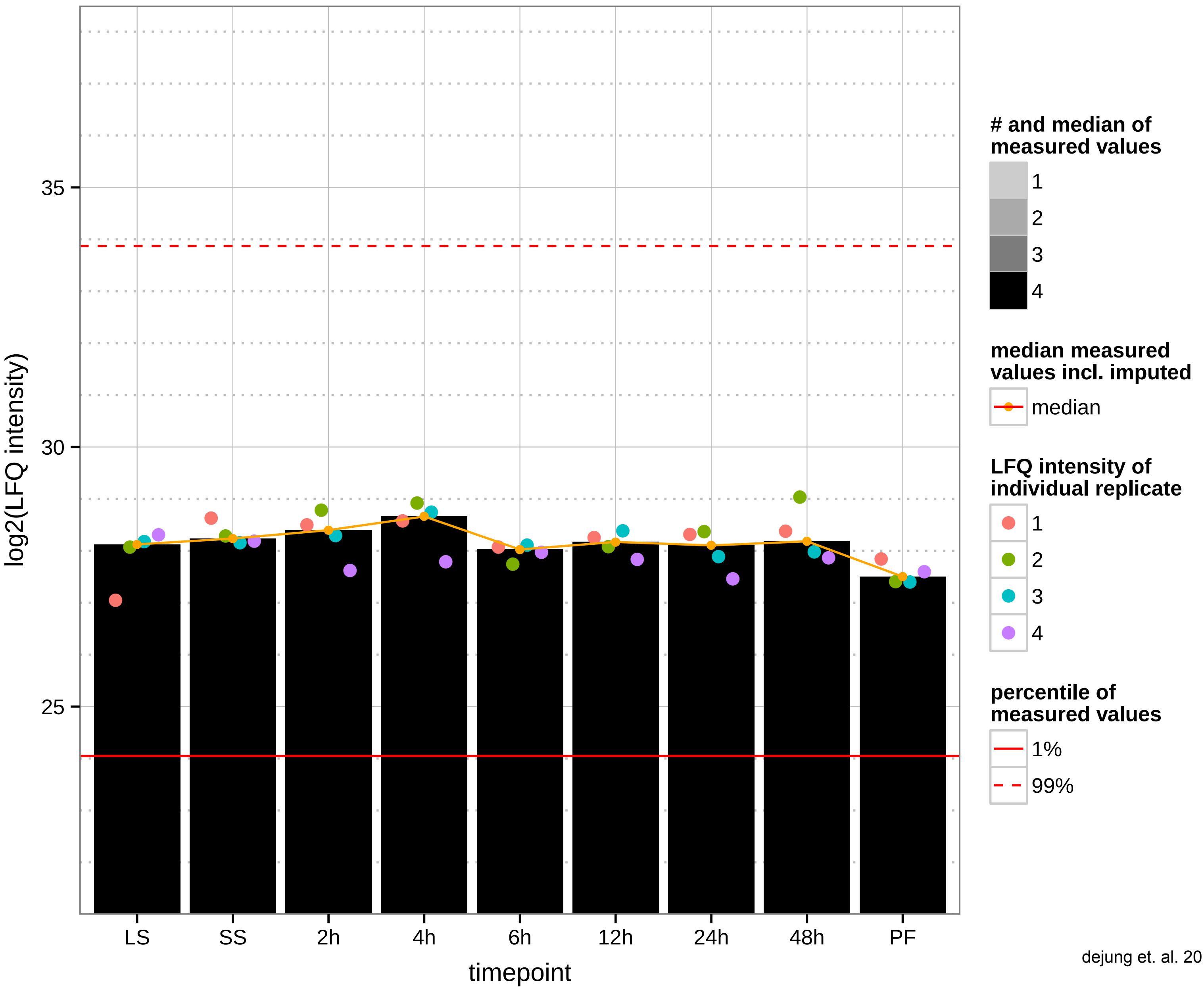

dejung et. al. 2015

hypothetical protein, conserved
Tb927.11.7800
AGOF: null
AGOC: integral to membrane
AGOP: null
PGOF: null
PGOC: null
PGOP: null

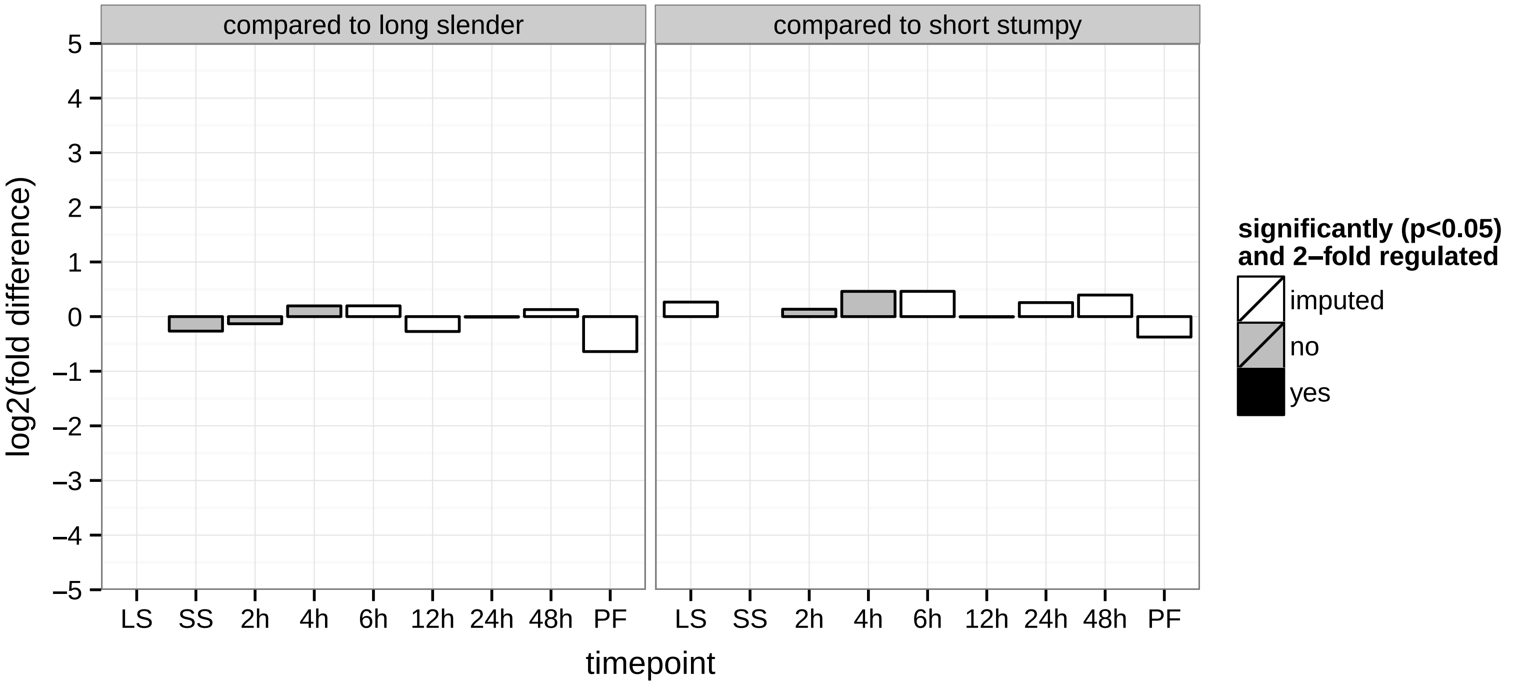

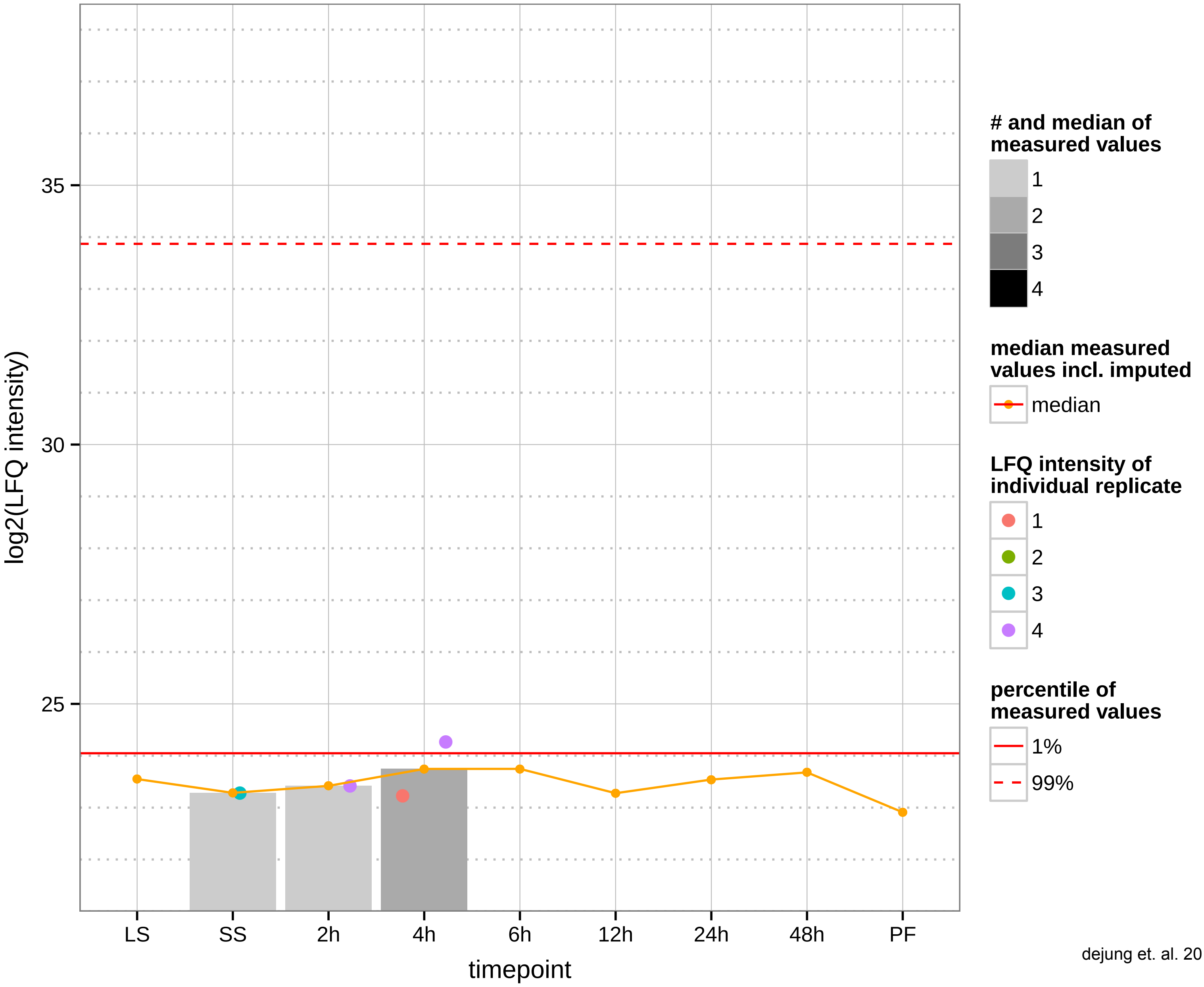

dejung et. al. 2015

ribonucleoside–diphosphate reductase large chain (RNR1)
Tb927.11.7840
AGOF: ATP binding, ribonucleoside–diphosphate reductase activity, thioredoxin disulfide as acceptor
AGOC: ribonucleoside–diphosphate reductase complex
AGOP: DNA replication, oxidation–reduction process
PGOF: ATP binding, ribonucleoside–diphosphate reductase activity, thioredoxin disulfide as acceptor
PGOC: ribonucleoside–diphosphate reductase complex
PGOP: DNA replication, oxidation–reduction process

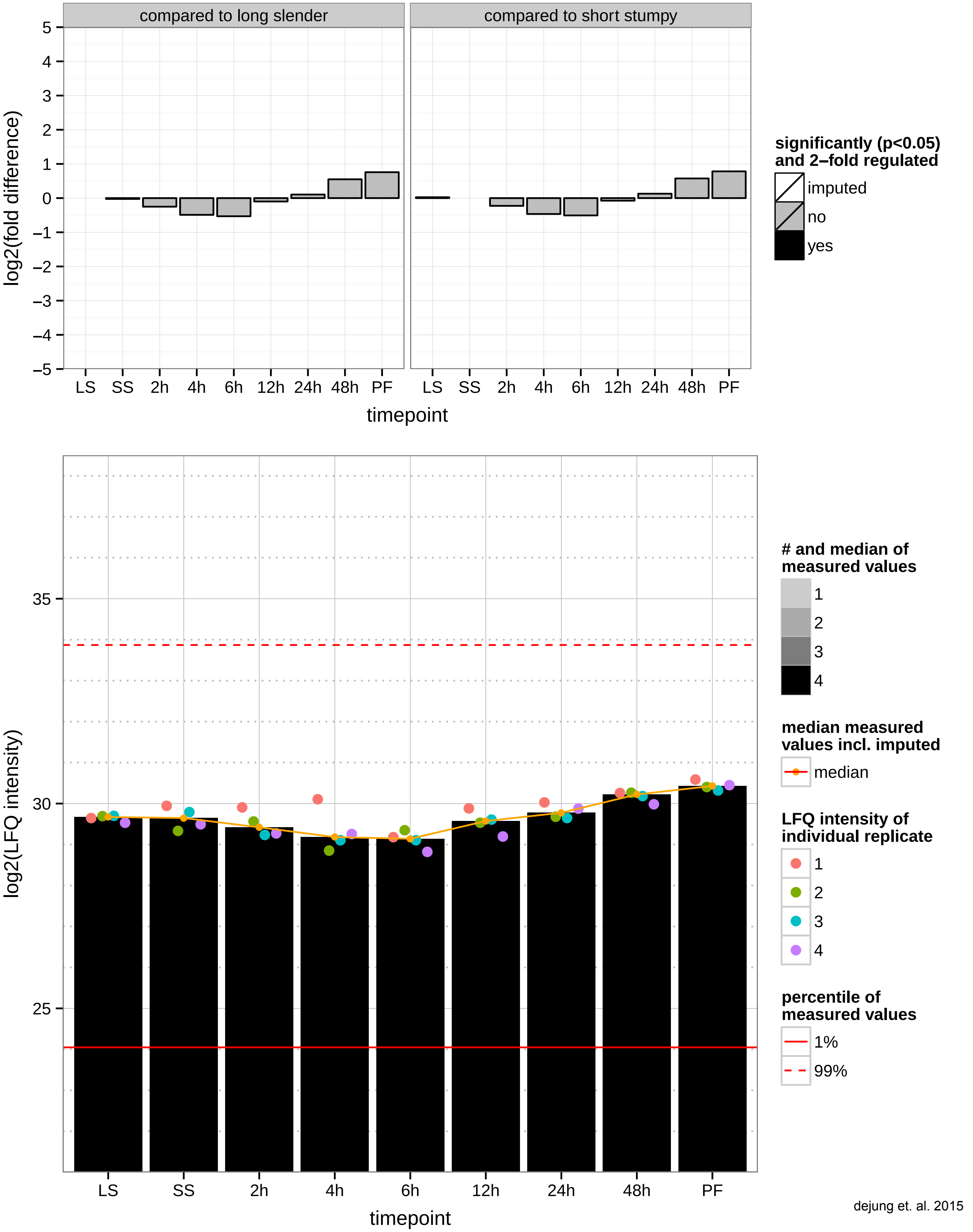

dejung et. al. 2015

hypothetical protein, conserved
Tb927.11.7850
AGOF: null
AGOC: null
AGOP: null
PGOF: null
PGOC: null
PGOP: null

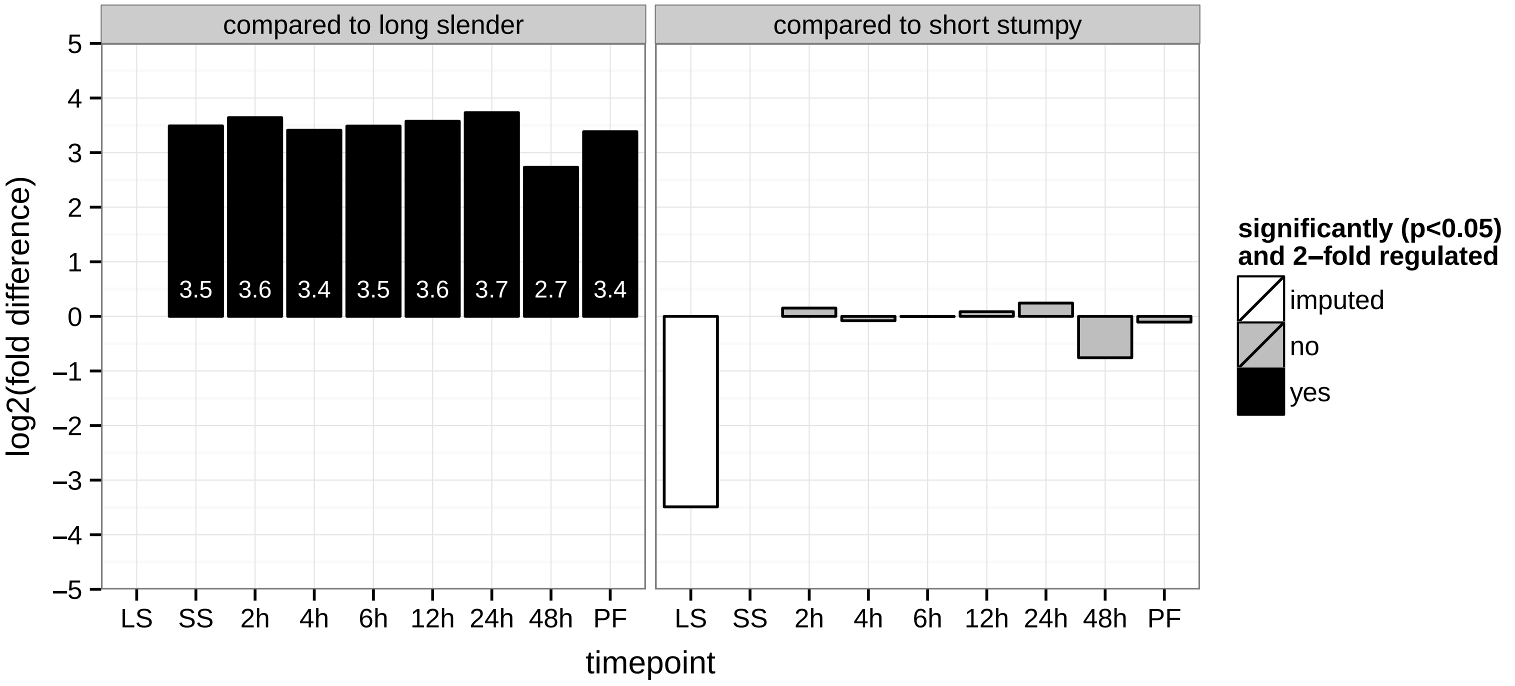

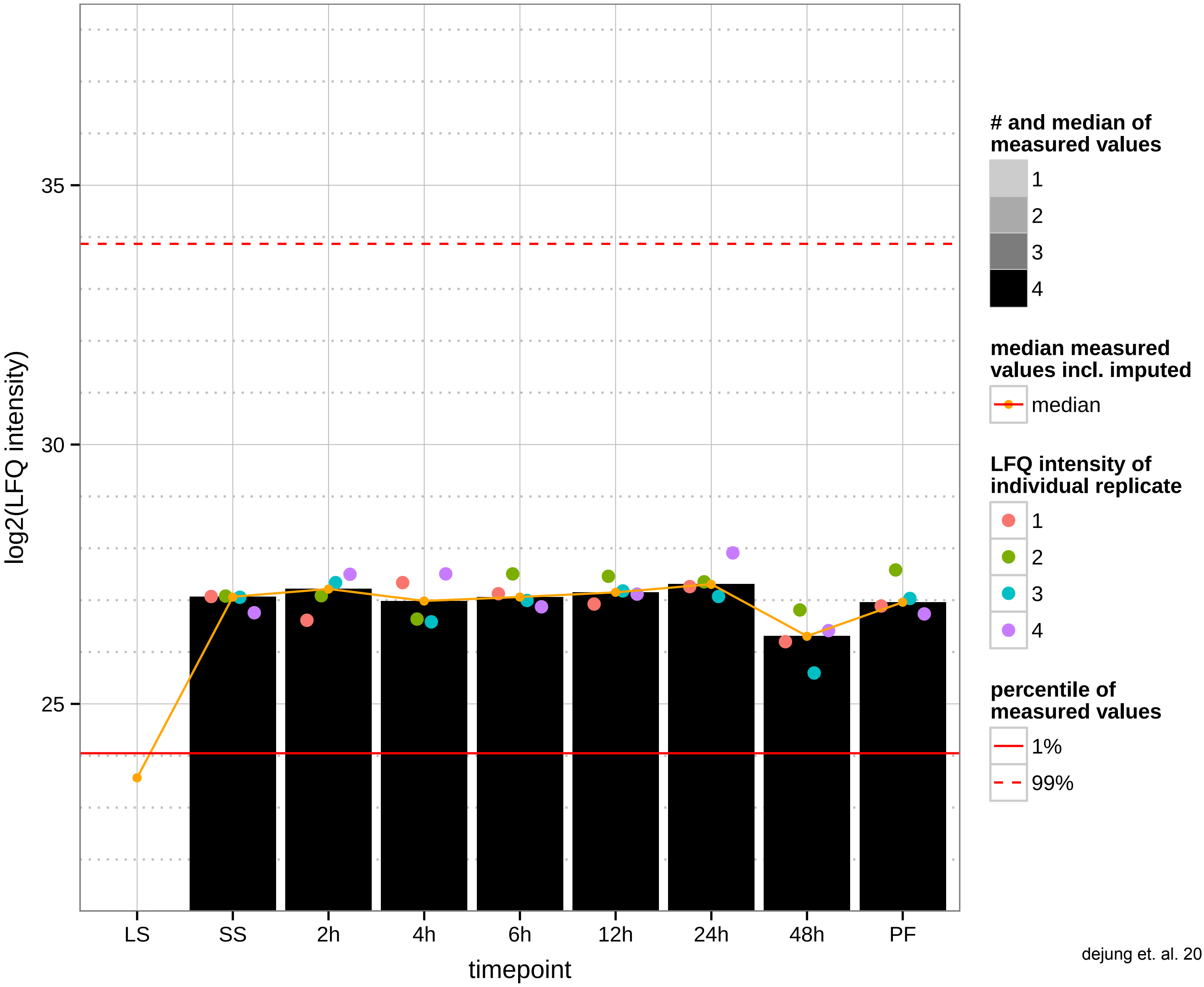

dejung et. al. 2015

zinc finger CCCH domain containing protein 44 (ZC3H44)
Tb927.11.7890
AGOF: RNA binding
AGOC: null
AGOP: null
PGOF: nucleic acid binding, zinc ion binding
PGOC: null
PGOP: null

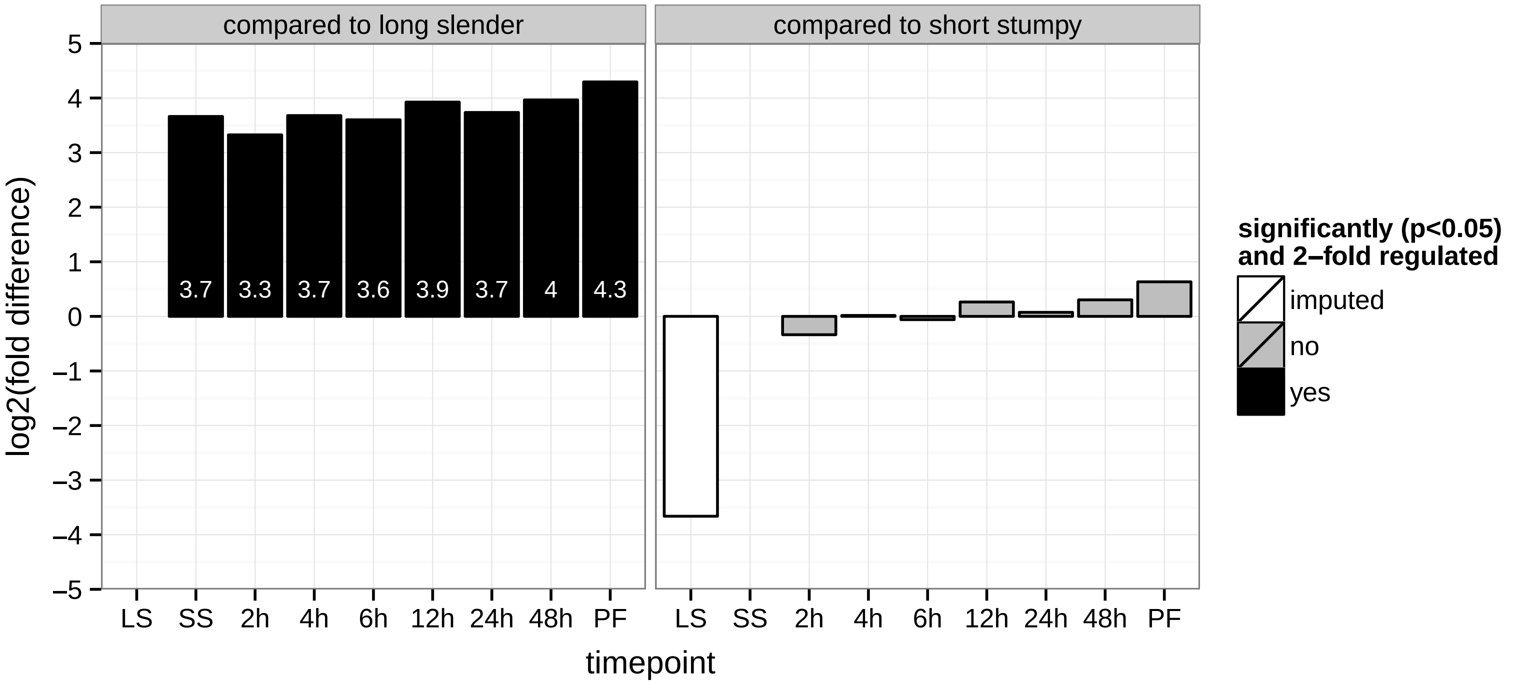

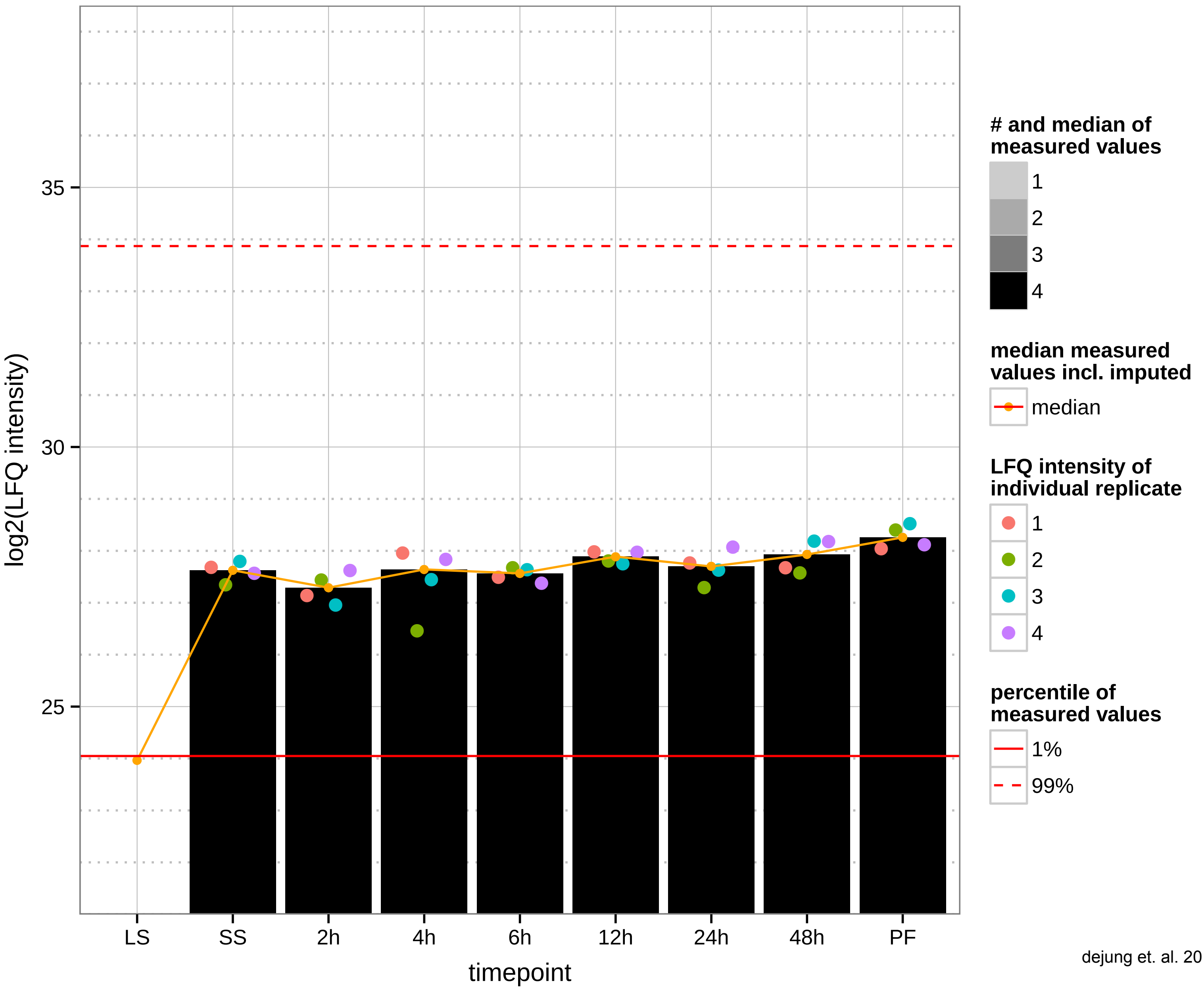

dejung et. al. 2015

RNA polymerase B subunit RPB8, putative (RPB8)
Tb927.11.7930
AGOF: DNA−directed RNA polymerase activity
AGOC: null
AGOP: transcription, DNA−dependent
PGOF: null
PGOC: null
PGOP: transcription, DNA−dependent

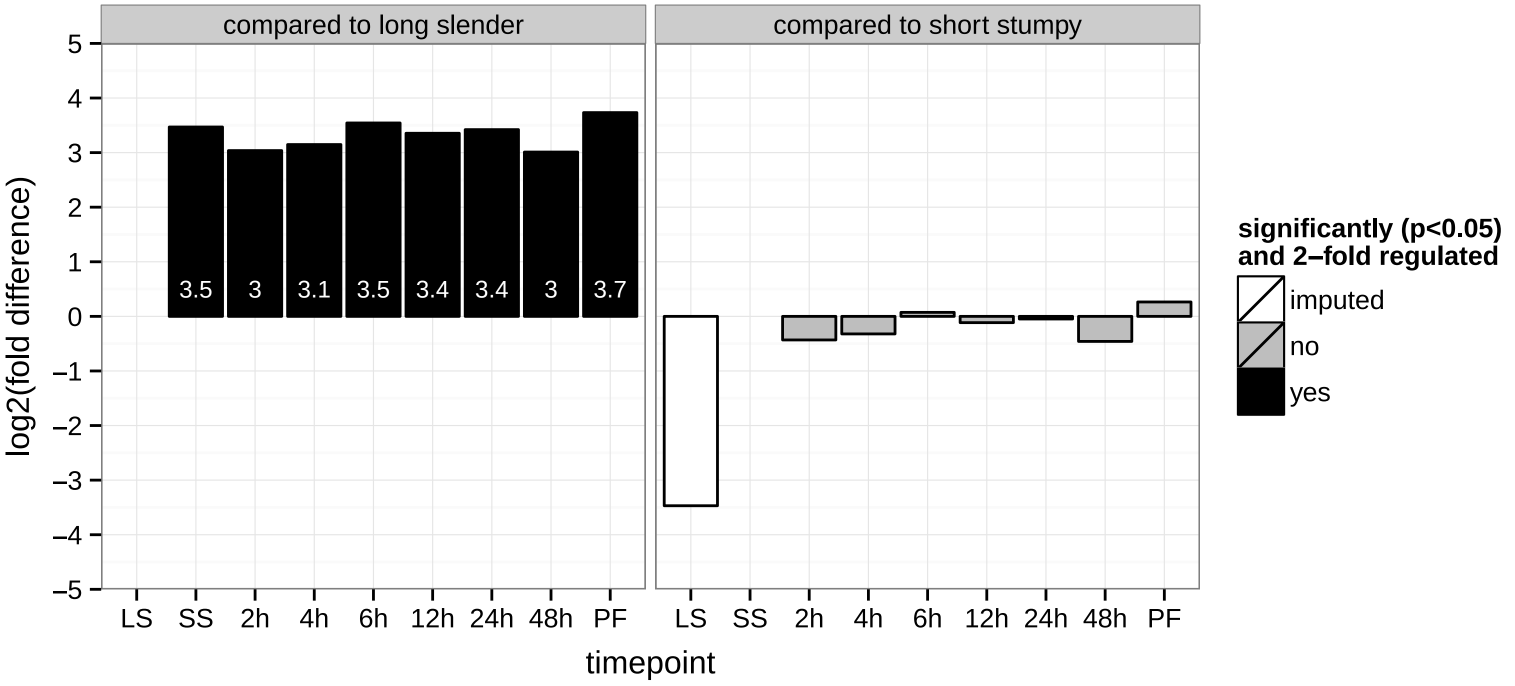

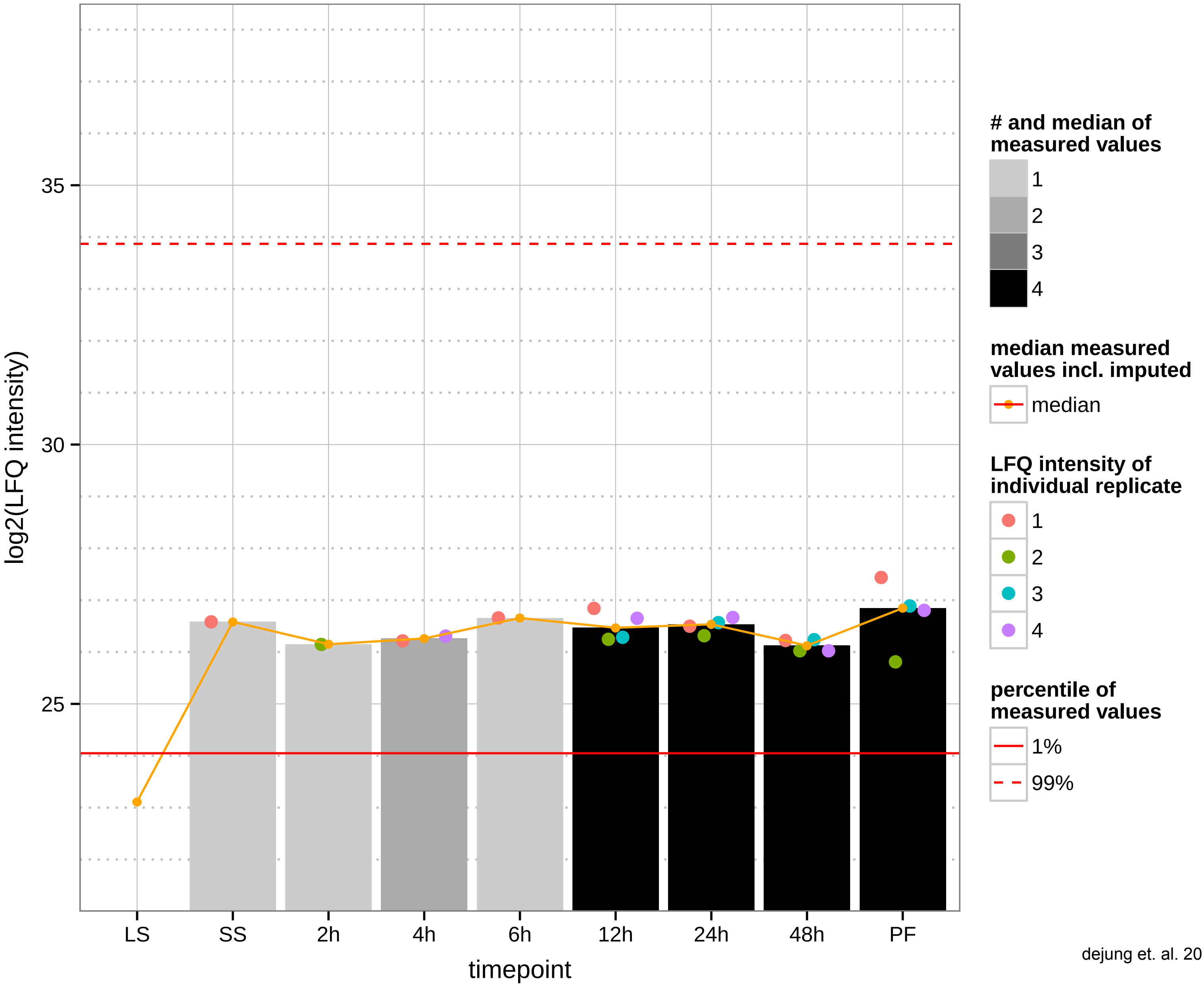

dejung et. al. 2015

kinetoplast poly(A) polymerase 1, KPAP1
Tb927.11.7960
AGOF: polynucleotide adenylyltransferase activity
AGOC: mitochondrion
AGOP: mRNA polyadenylation
PGOF: null
PGOC: null
PGOP: null

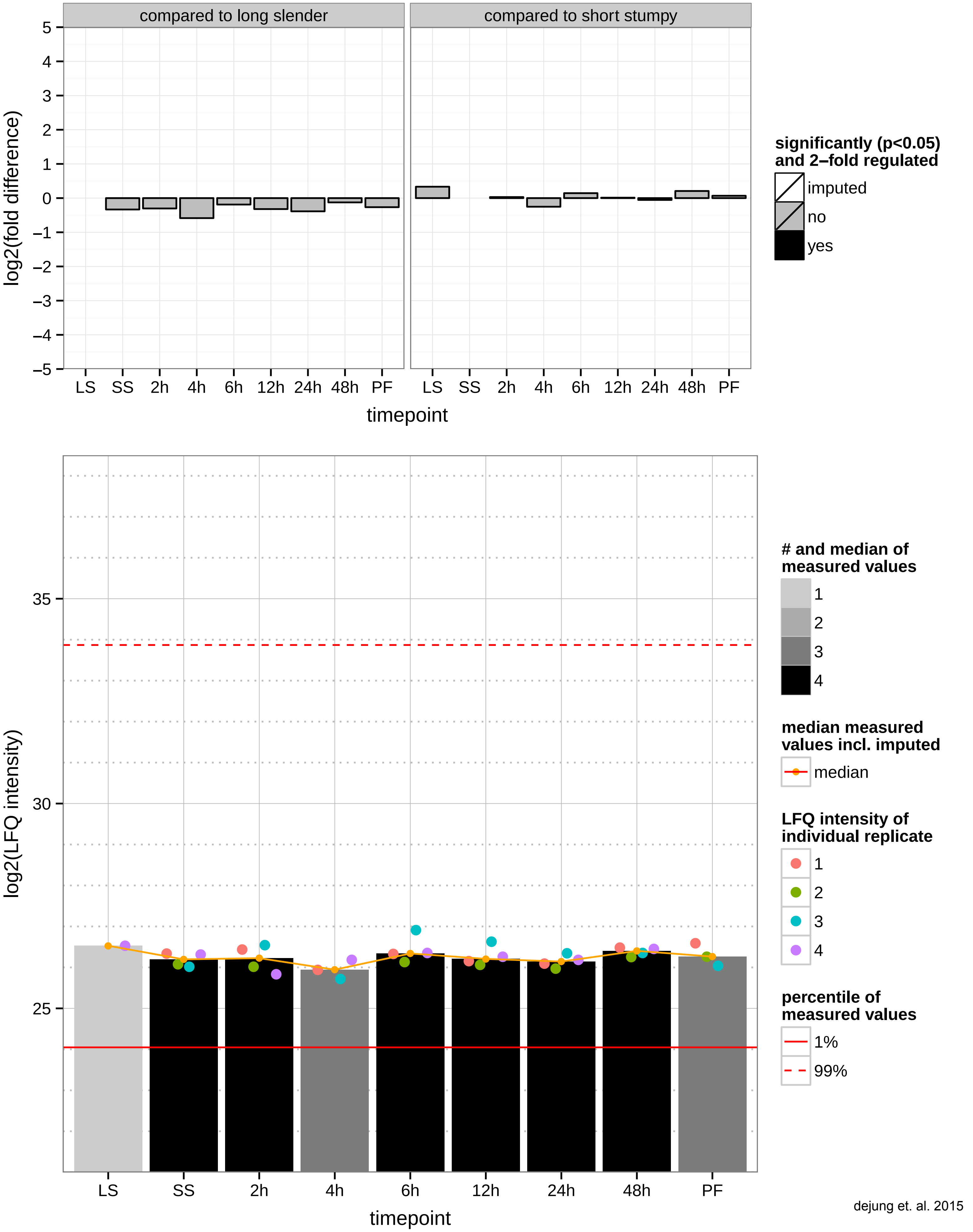

dejung et. al. 2015

hypothetical protein, conserved
Tb927.11.8030
AGOF: null
AGOC: null
AGOP: null
PGOF: null
PGOC: null
PGOP: null

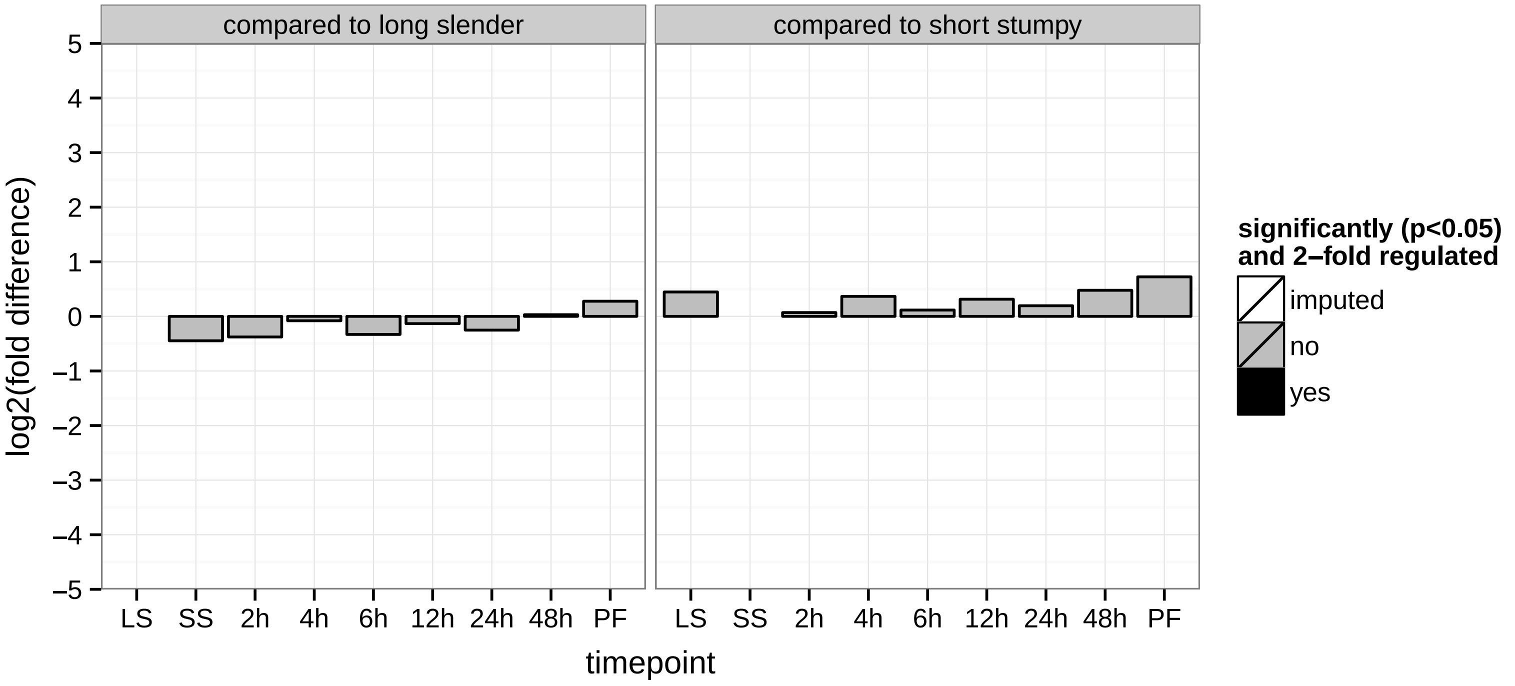

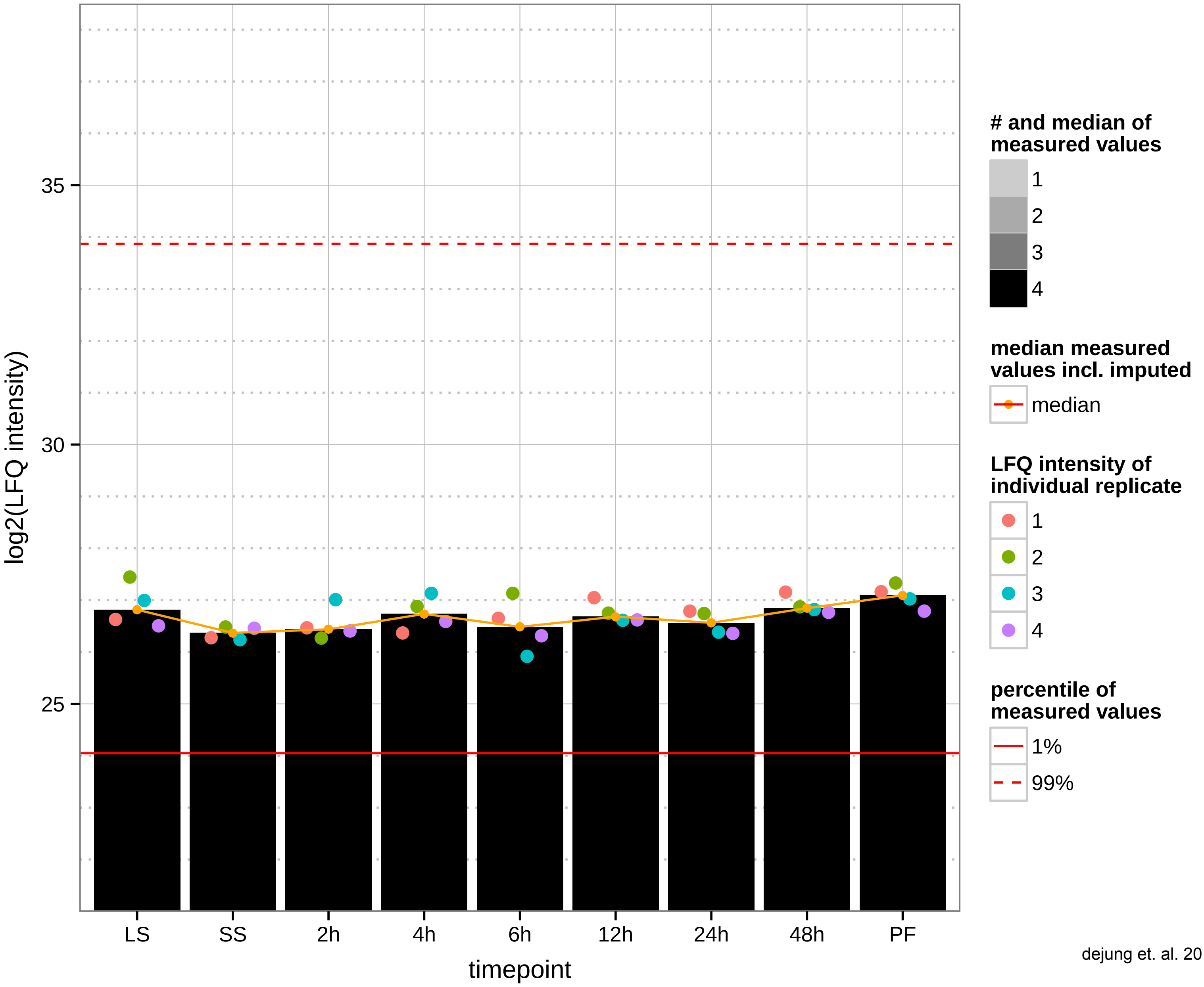

dejung et. al. 2015

hypothetical protein, conserved
Tb927.11.8050
AGOF: null
AGOC: nucleus
AGOP: gene silencing
PGOF: null
PGOC: nucleus
PGOP: gene silencing

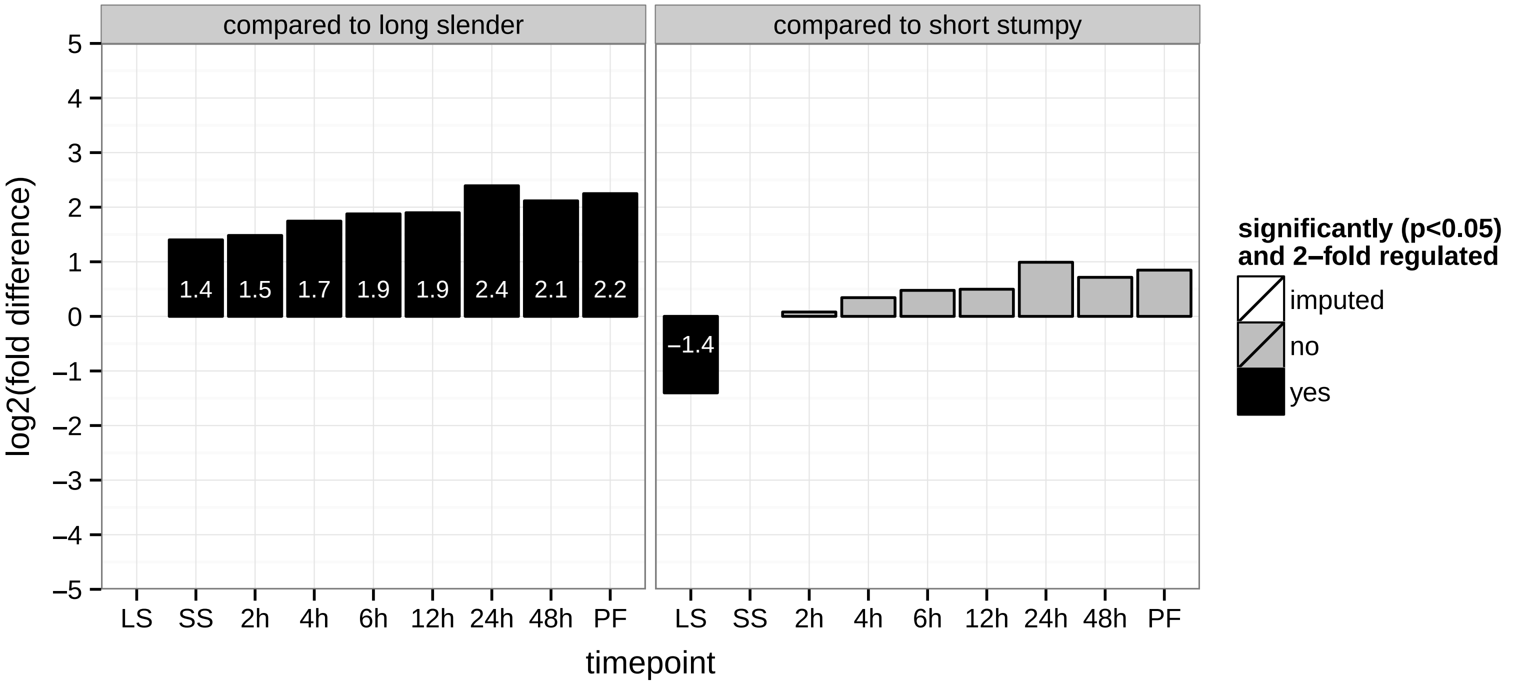

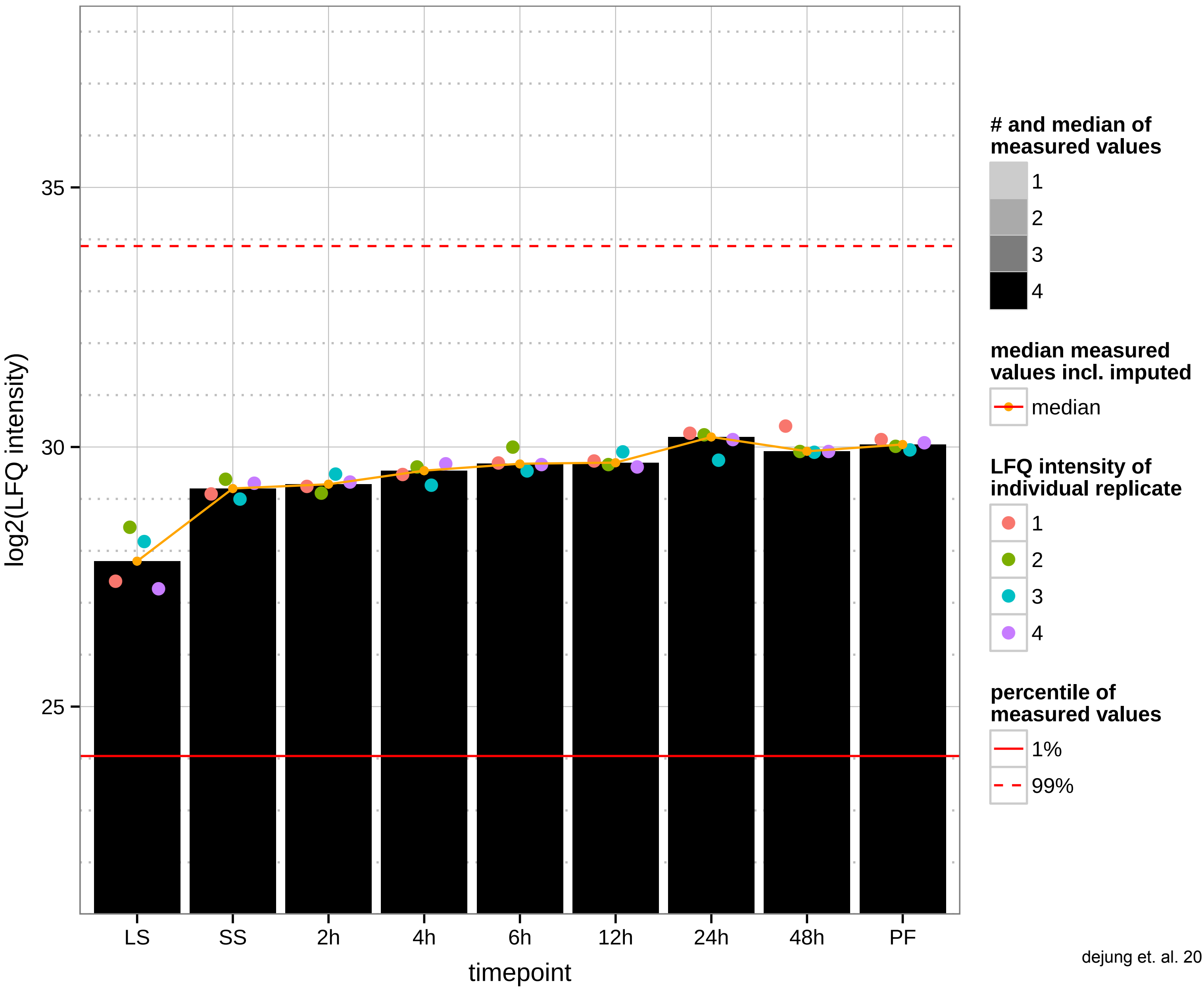

dejung et. al. 2015

hypothetical protein, conserved
Tb927.11.8080
AGOF: voltage−gated potassium channel activity
AGOC: membrane, voltage−gated potassium channel complex
AGOP: potassium ion transport
PGOF: protein binding
PGOC: null
PGOP: protein homooligomerization

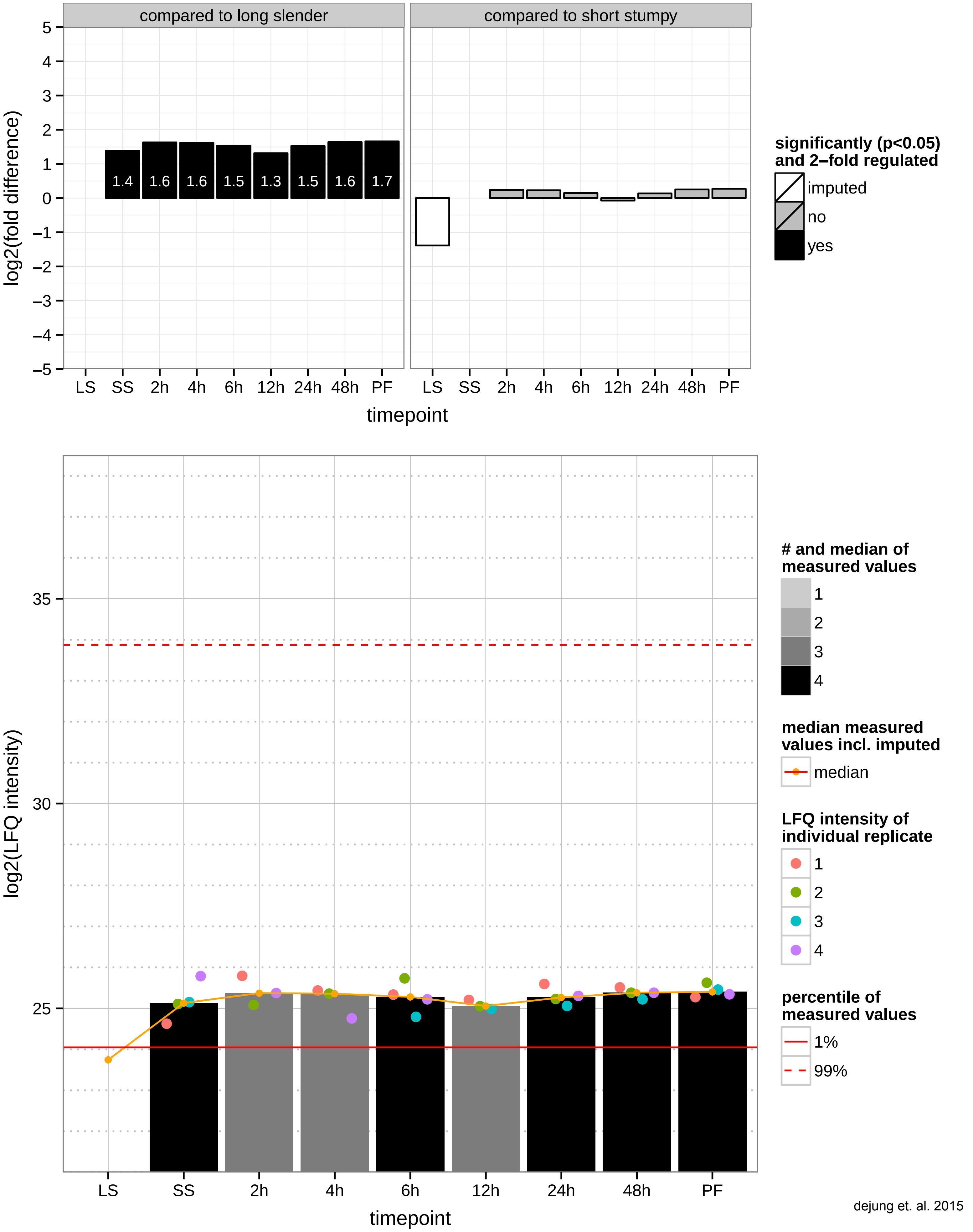

dejung et. al. 2015

protein transport protein SEC13, putative
Tb927.11.8120
AGOF: null
AGOC: null
AGOP: intracellular protein transport
PGOF: protein binding
PGOC: null
PGOP: null

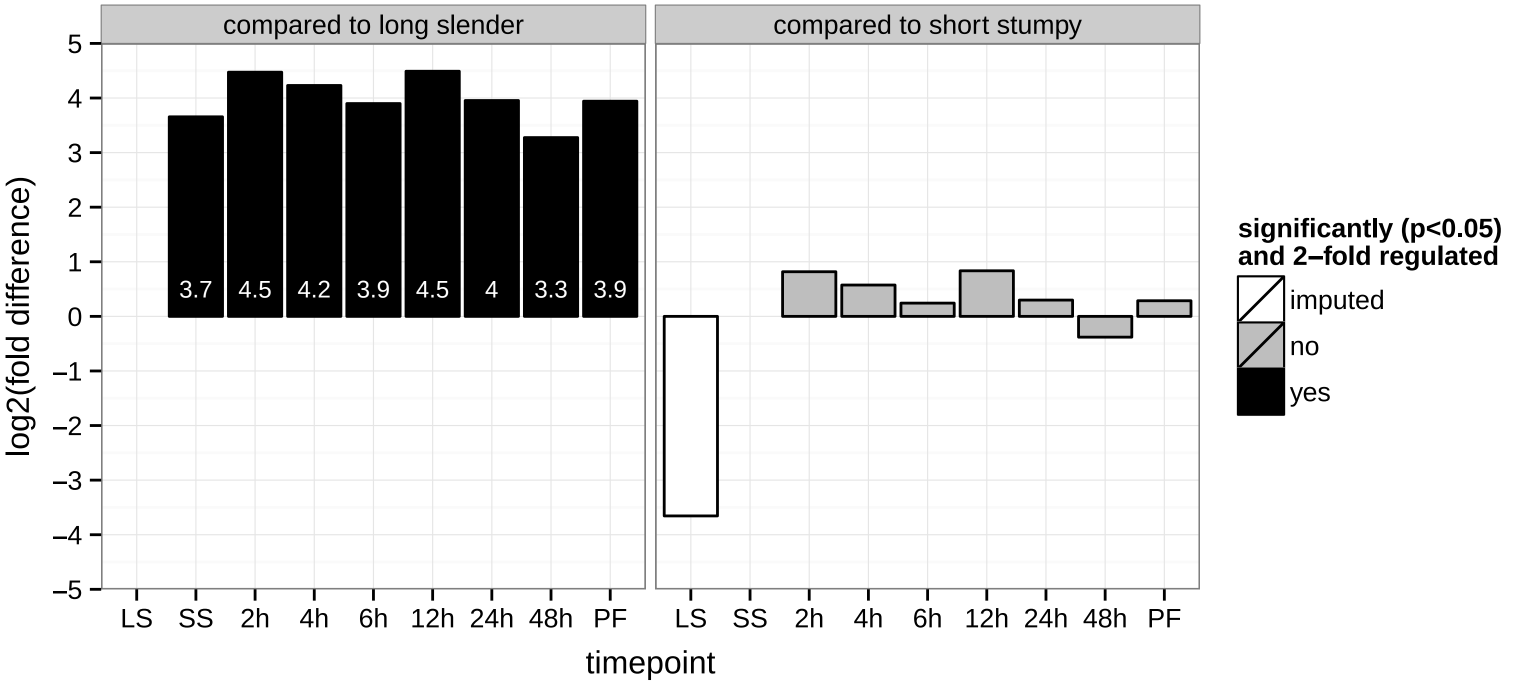

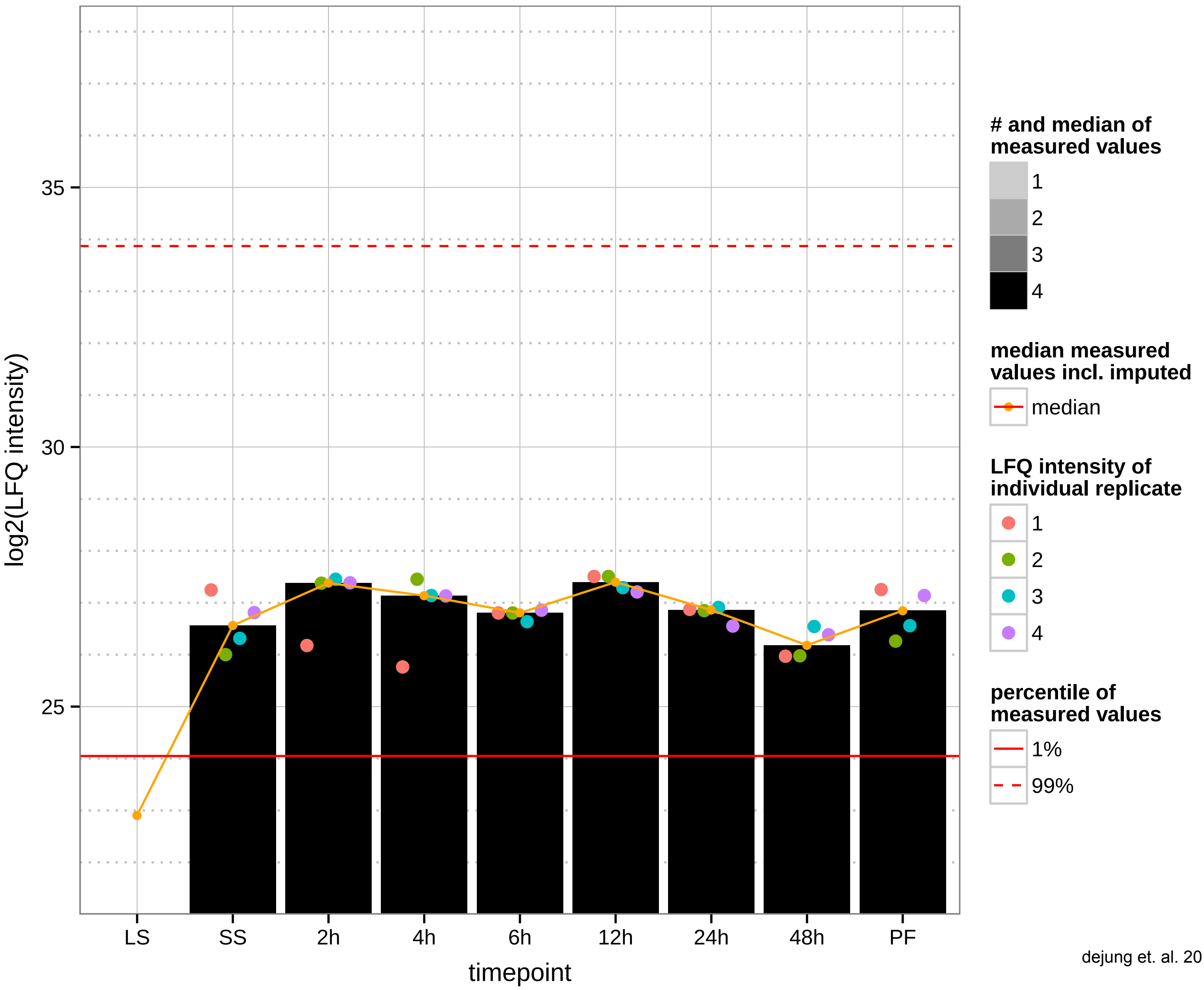

dejung et. al. 2015

ER lumen retaining receptor protein, putative
Tb927.11.8140
AGOF: ER retention sequence binding
AGOC: integral to membrane
AGOP: protein retention in ER lumen
PGOF: ER retention sequence binding
PGOC: integral to membrane
PGOP: protein retention in ER lumen

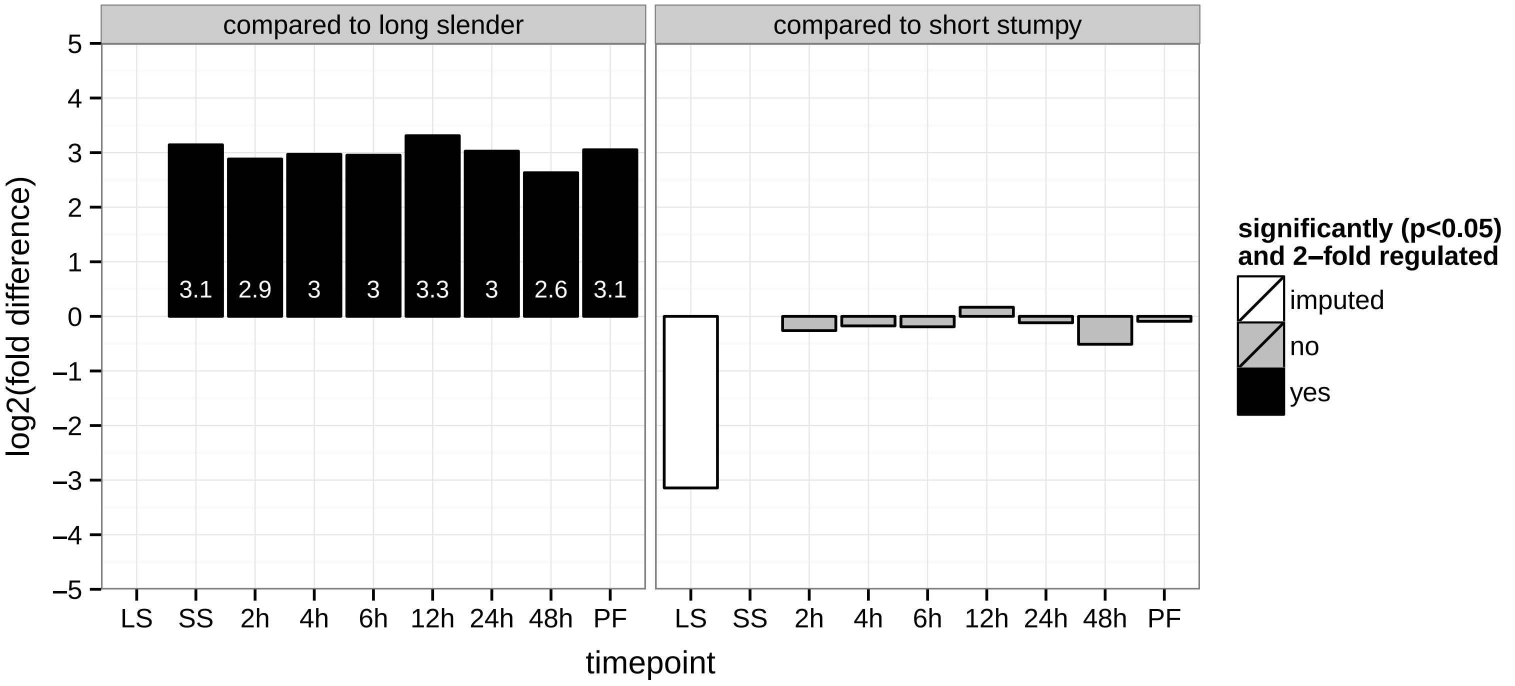

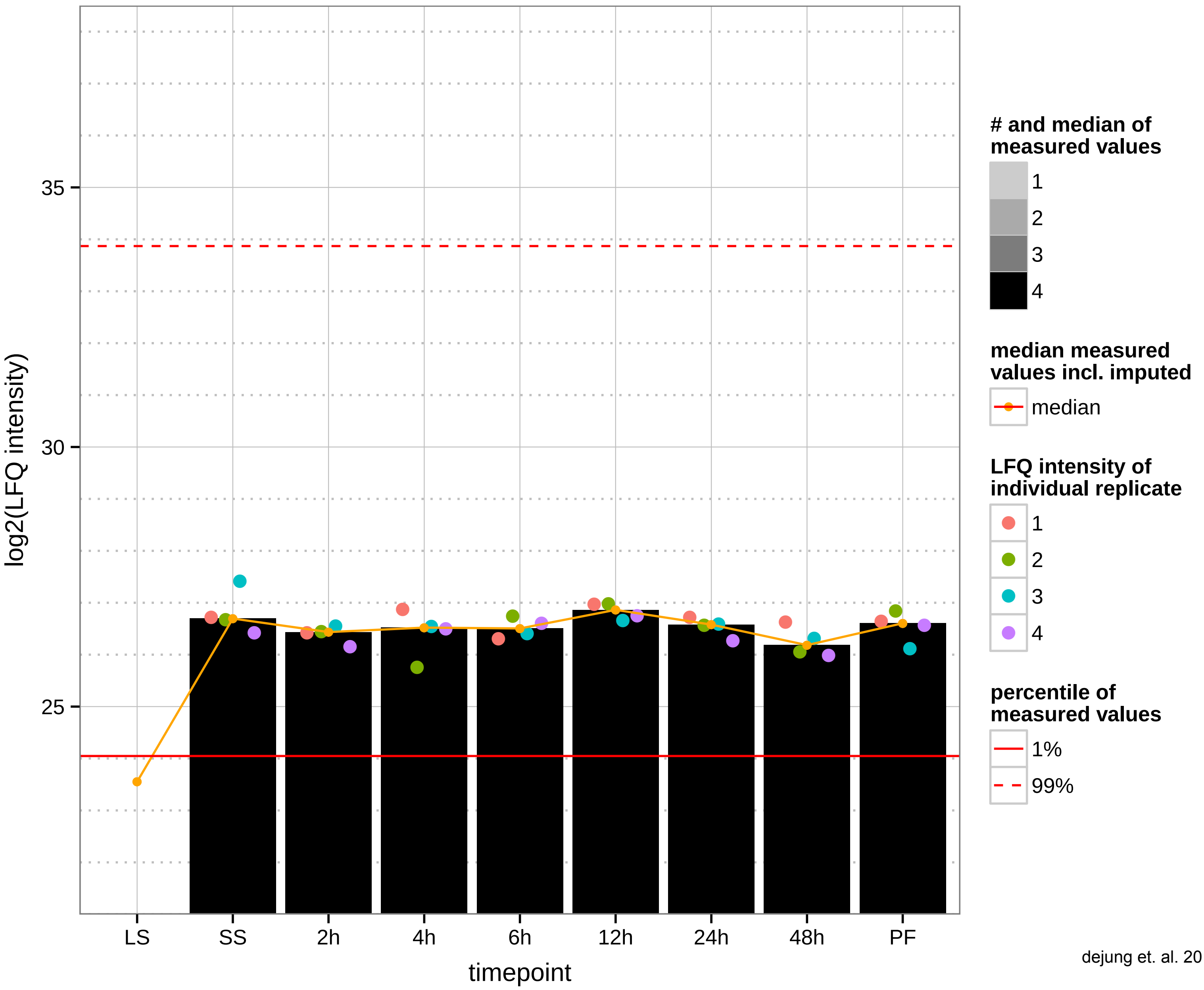

dejung et. al. 2015

protein kinase, putative
Tb927.11.8150
AGOF: null, ATP binding, protein tyrosine kinase activity
AGOC: null
AGOP: null, protein phosphorylation
PGOF: binding, ATP binding, binding, protein kinase activity, transferase activity, transferring phosphorus–containing groups
PGOC: null
PGOP: null, protein phosphorylation

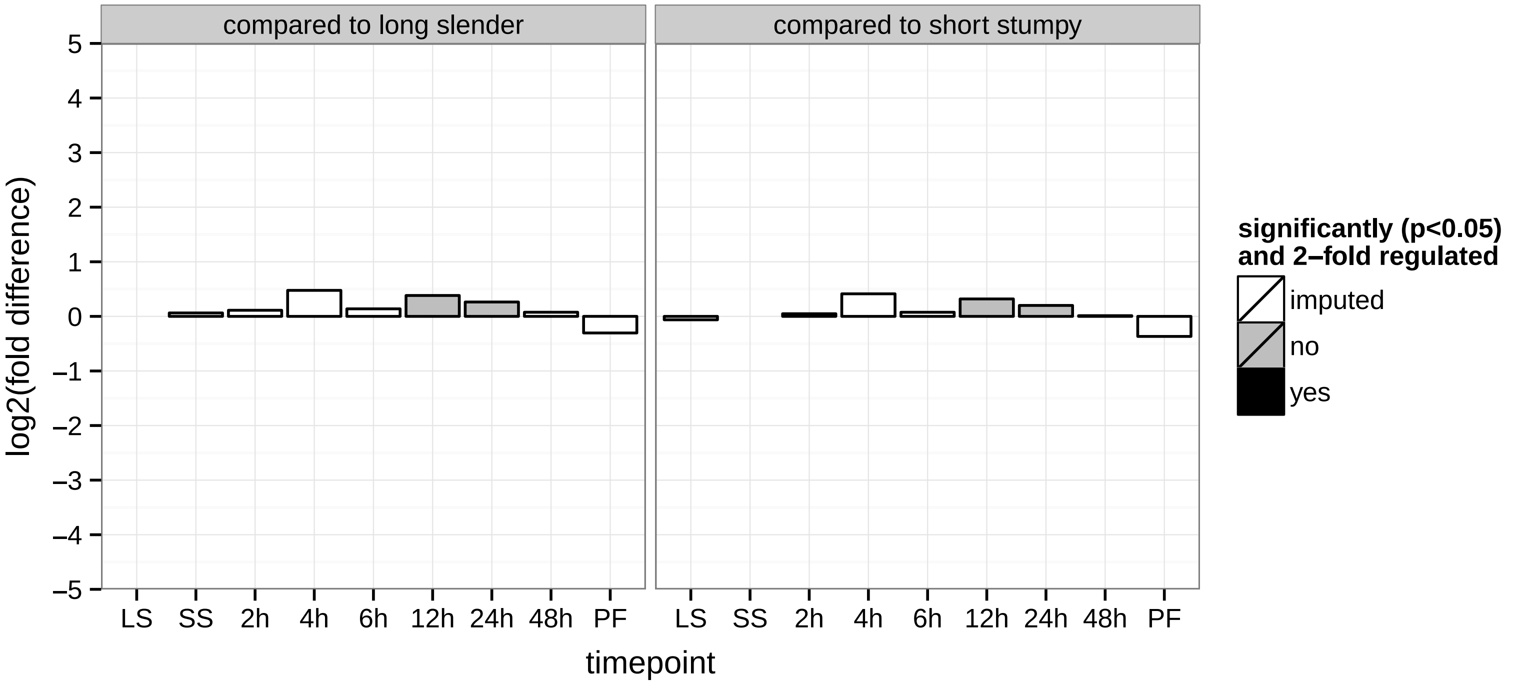

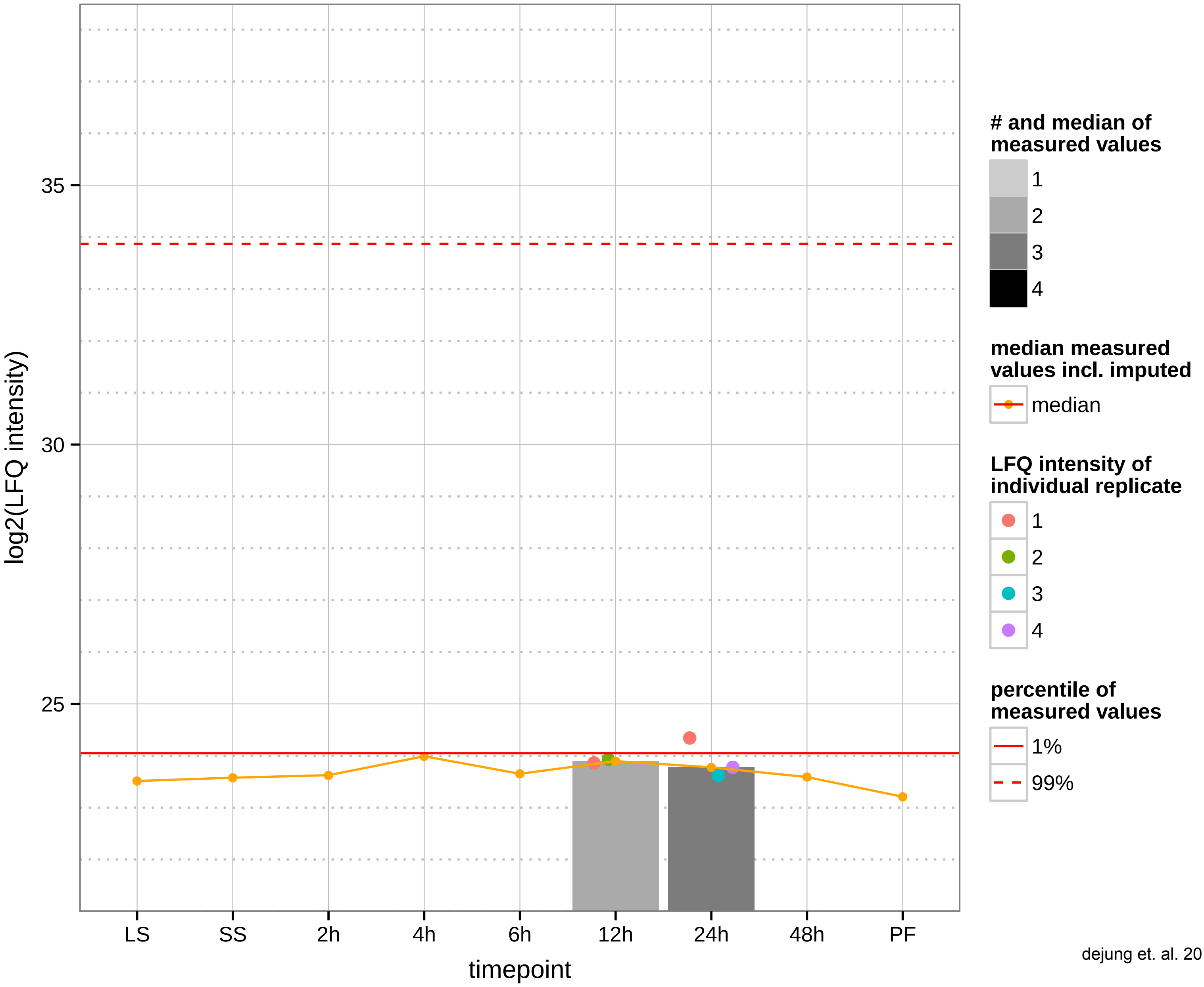

dejung et. al. 2015

dynein heavy chain, putative
Tb927.11.8160
AGOF: ATP binding, ATPase activity, microtubule motor activity
AGOC: dynein complex
AGOP: microtubule−based movement
PGOF: microtubule motor activity
PGOC: dynein complex
PGOP: microtubule−based movement

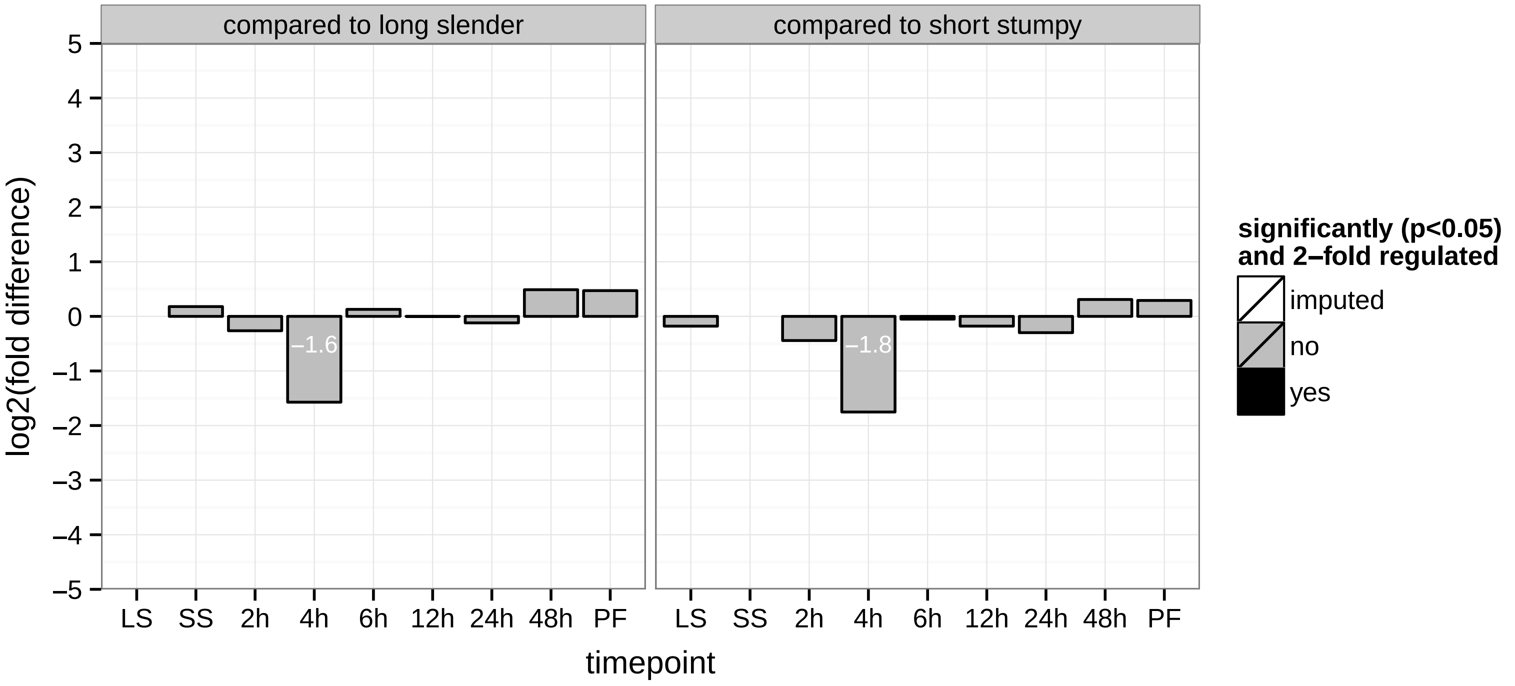

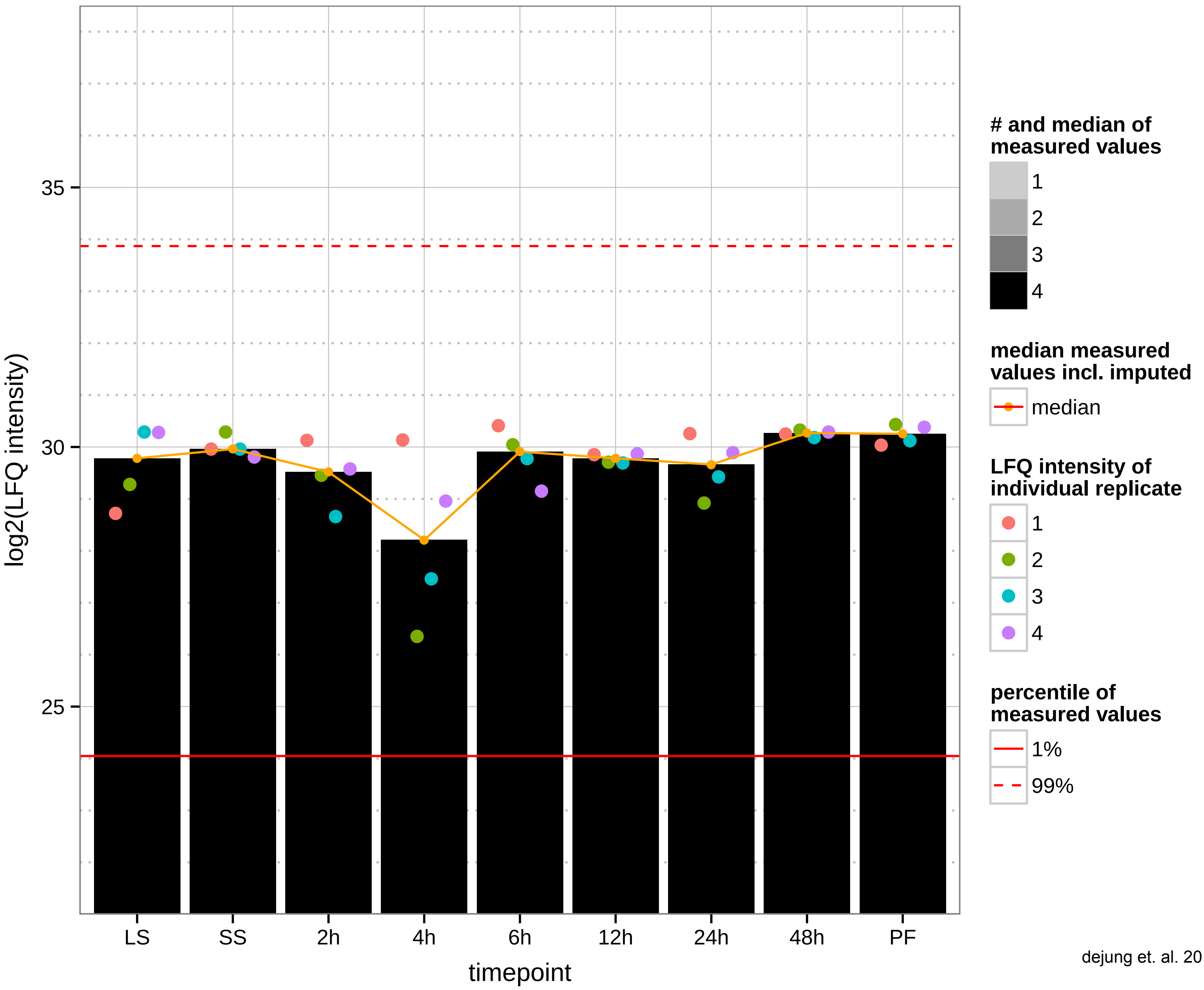

dejung et. al. 2015

carbonic anhydrase–like protein
Tb927.11.8260
AGOF: carbonate dehydratase activity, zinc ion binding
AGOC: null
AGOP: one–carbon metabolic process
PGOF: null
PGOC: null
PGOP: null

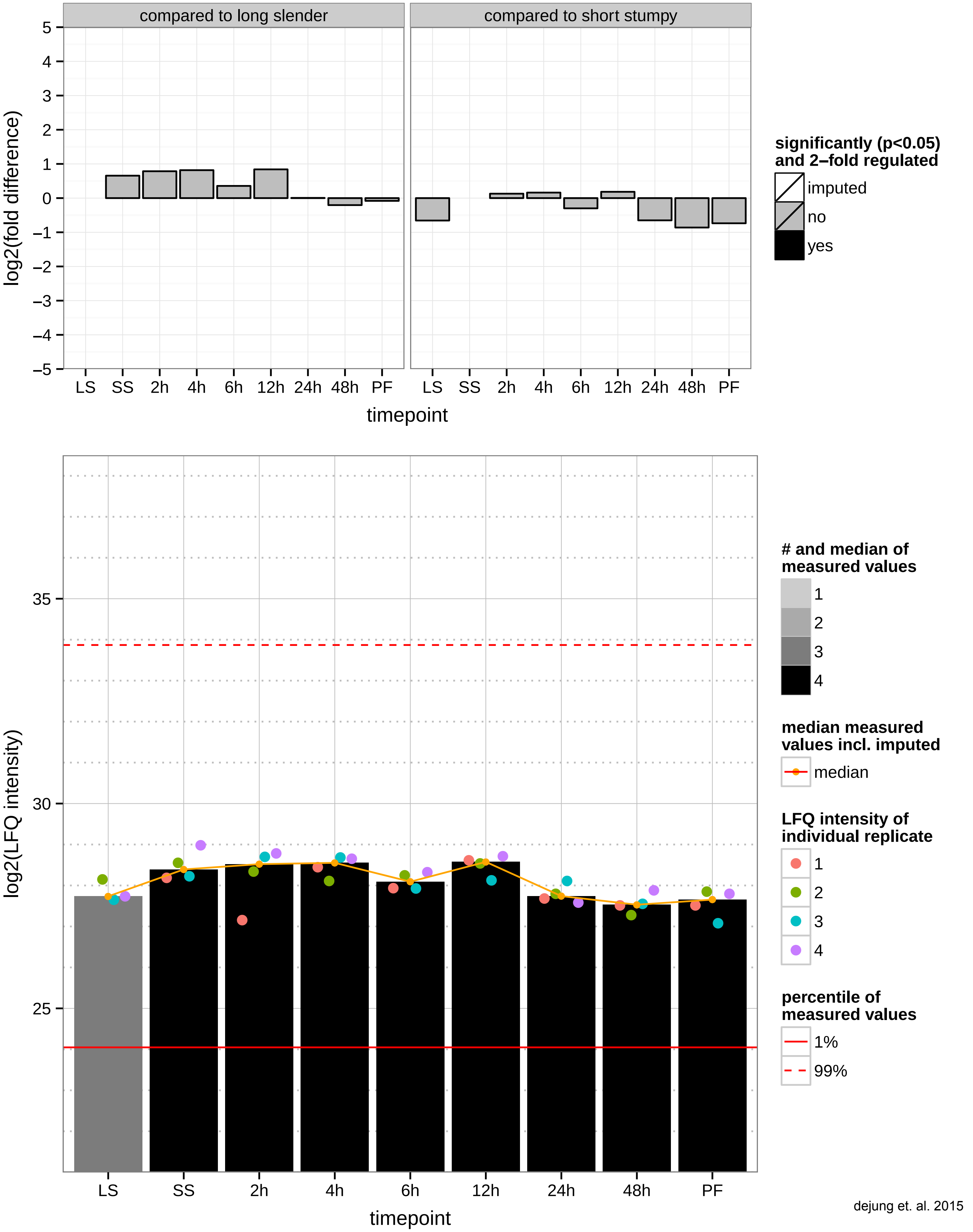

dejung et. al. 2015

hypothetical protein, conserved
Tb927.11.8300
AGOF: null
AGOC: null
AGOP: null
PGOF: null
PGOC: null
PGOP: null

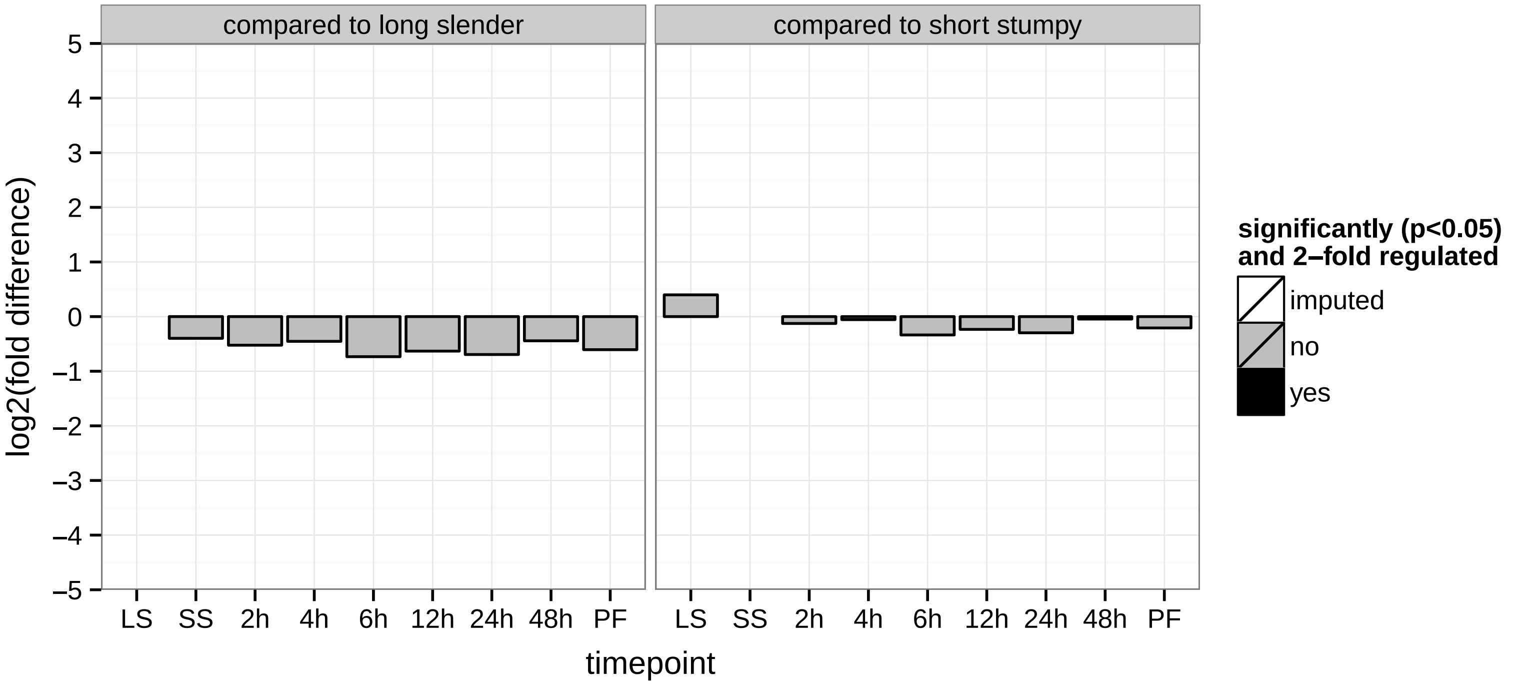

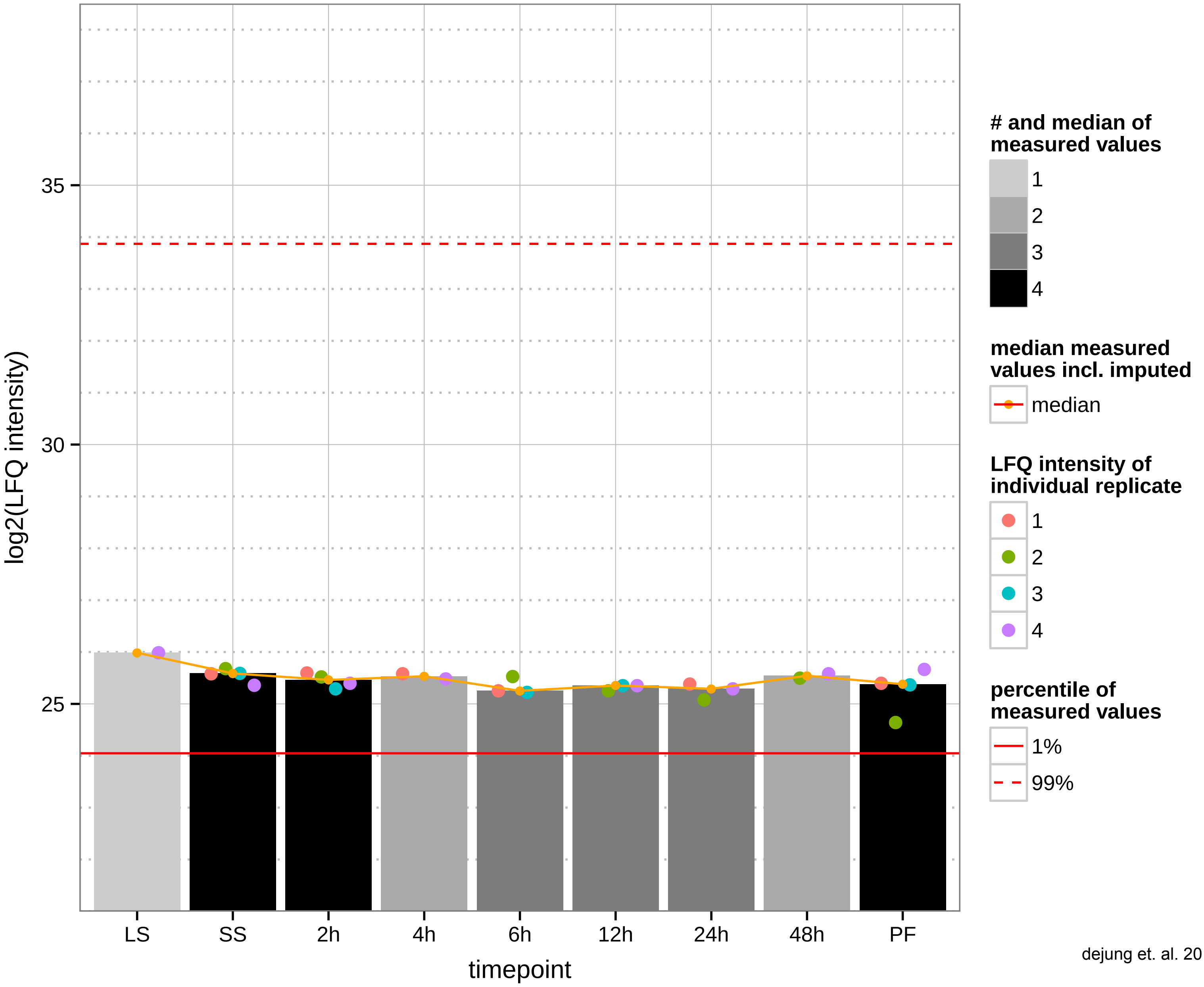

dejung et. al. 2015

class I transcription factor A, subunit 4 (CITFA–4)
Tb927.11.8310
AGOF: null
AGOC: null
AGOP: null
PGOF: null
PGOC: null
PGOP: null

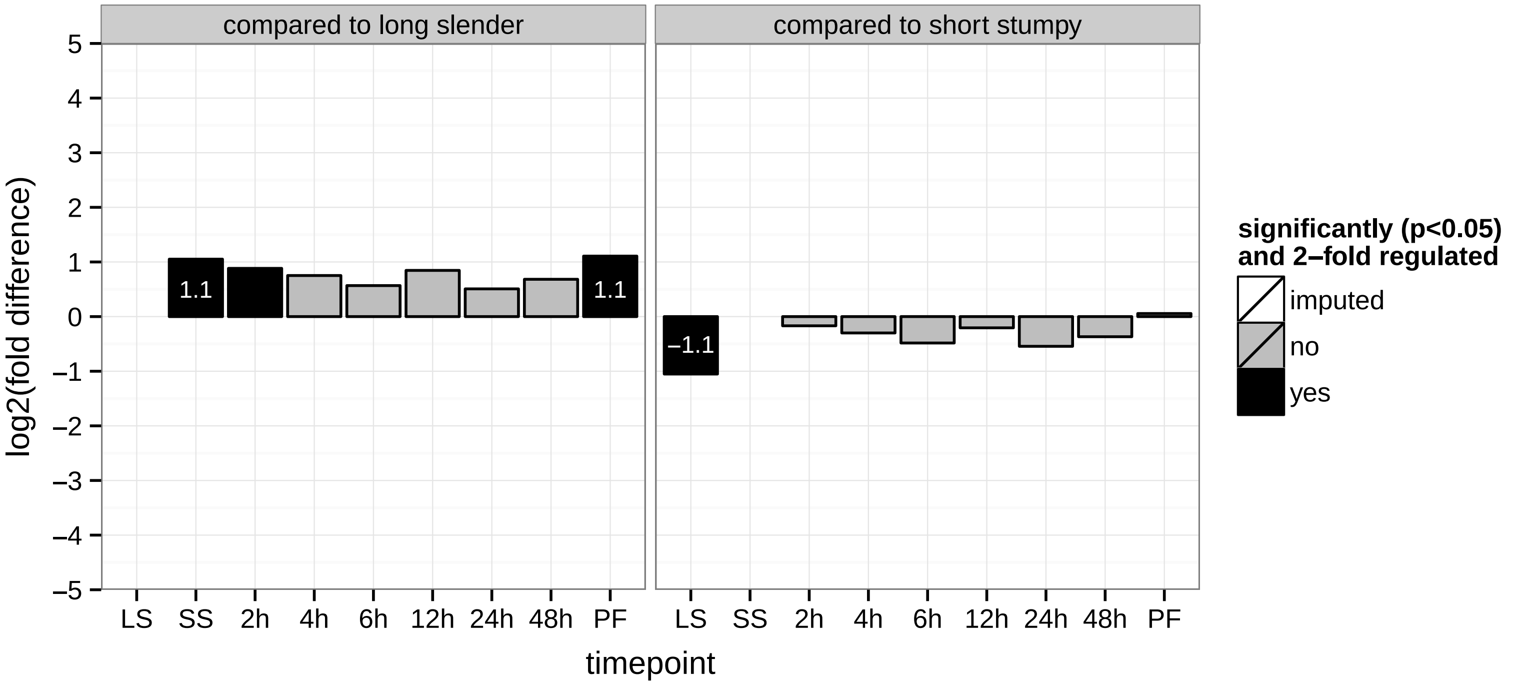

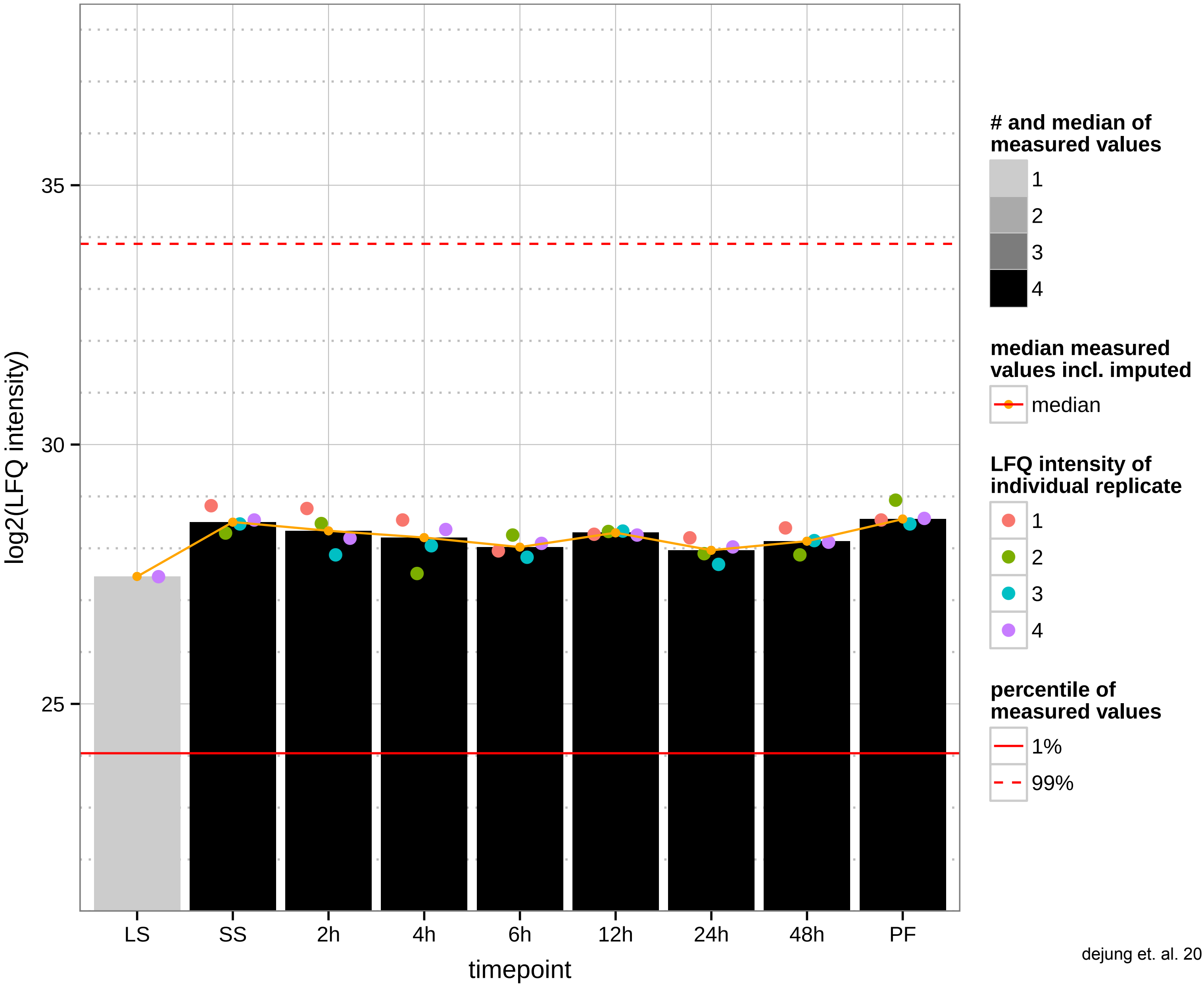

dejung et. al. 2015

hypothetical protein, conserved
Tb927.11.8390
AGOF: null
AGOC: null
AGOP: null
PGOF: null
PGOC: null
PGOP: null

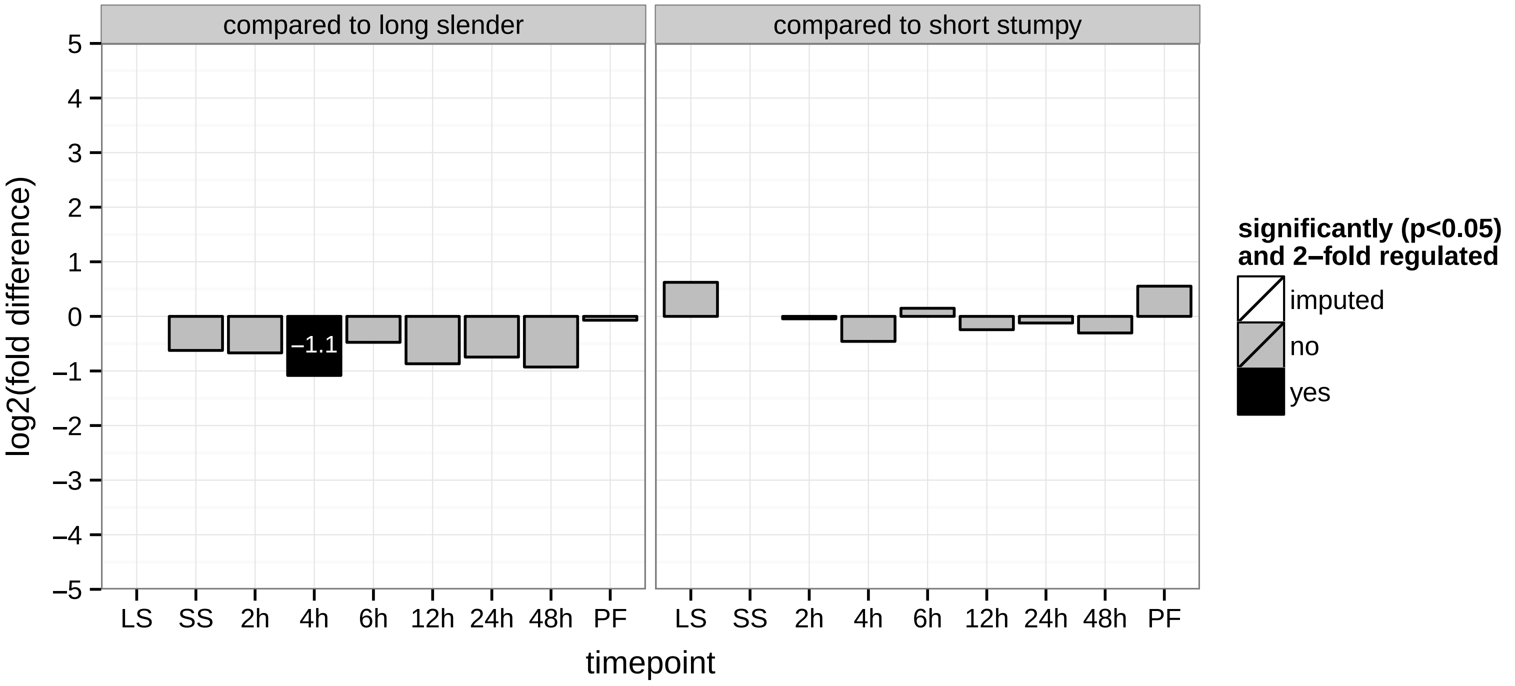

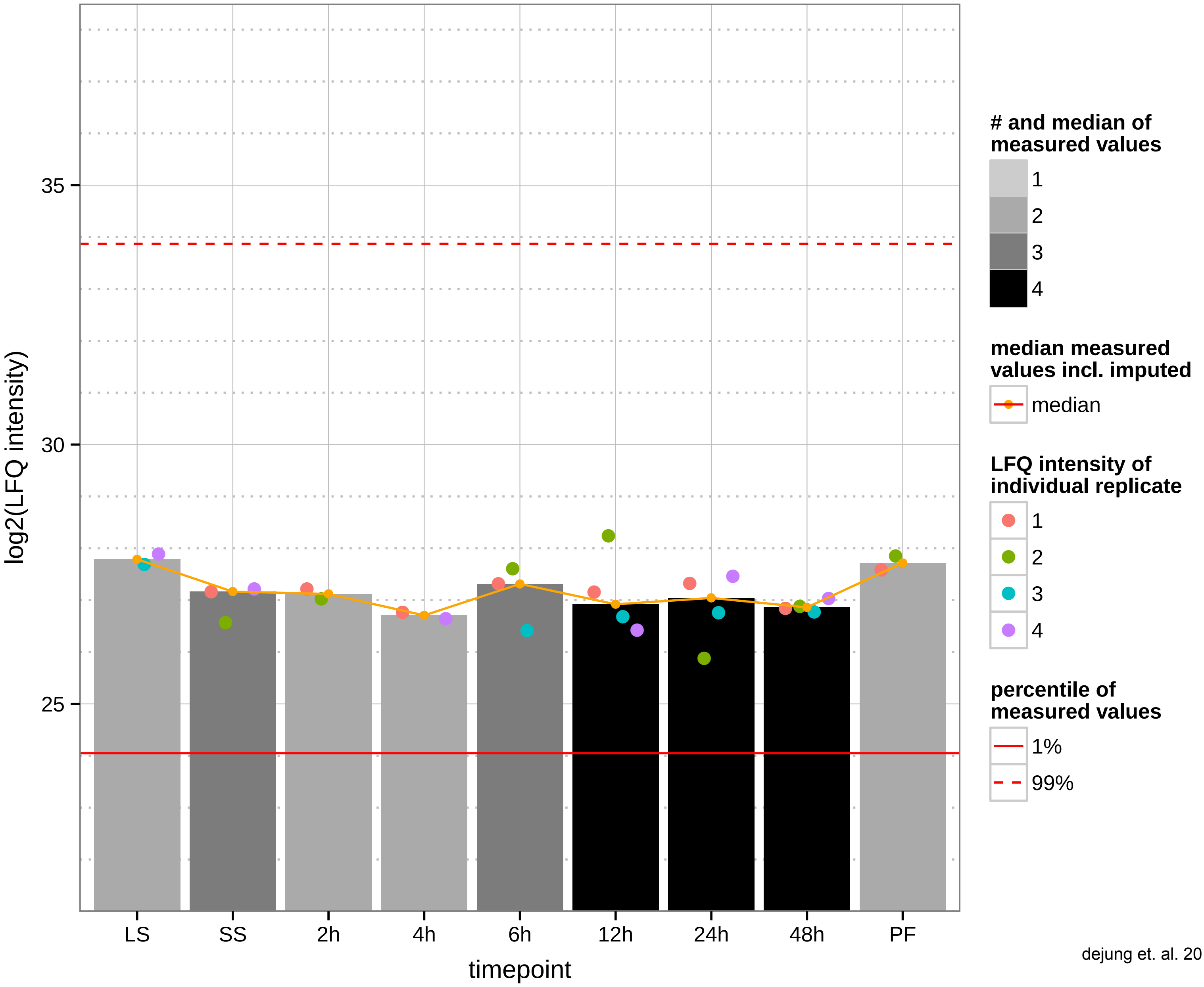

dejung et. al. 2015

hypothetical protein, conserved
Tb927.11.8450
AGOF: null
AGOC: null
AGOP: null
PGOF: null
PGOC: null
PGOP: null

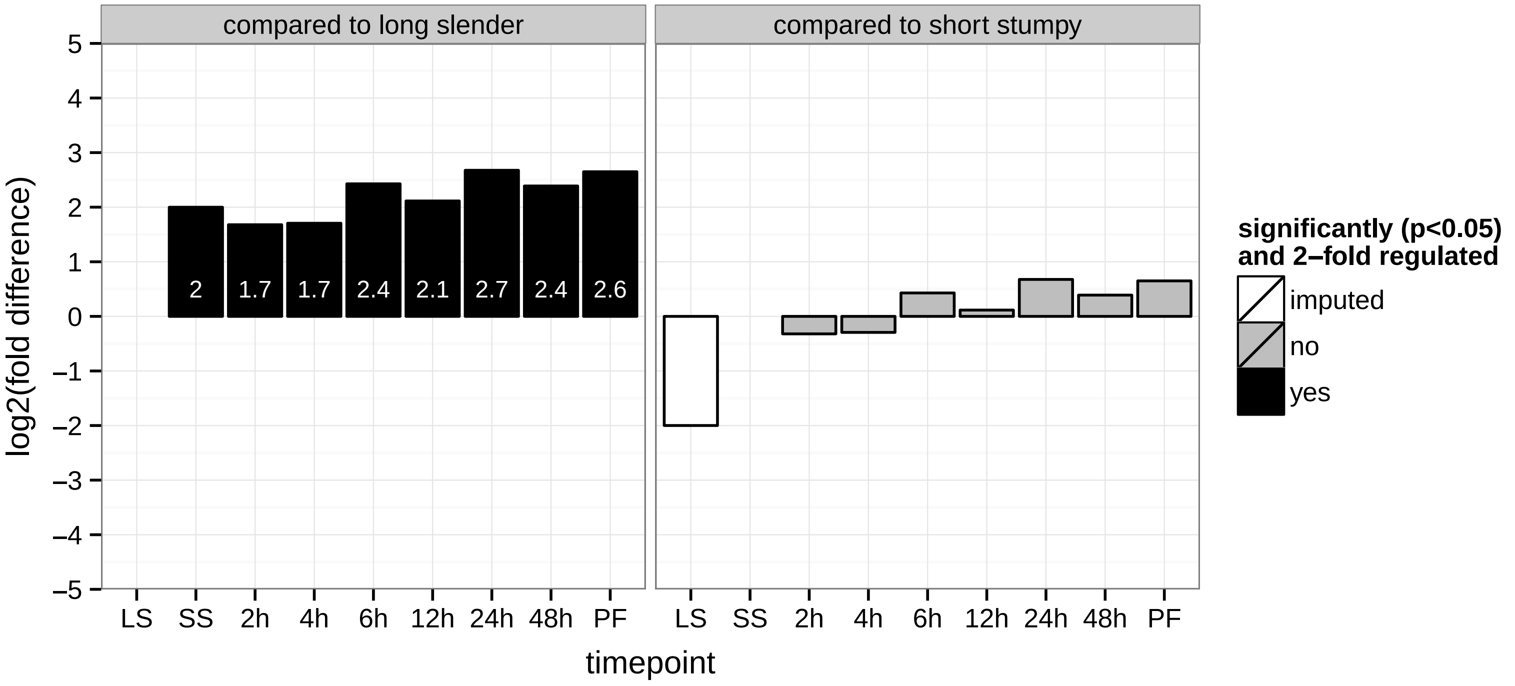

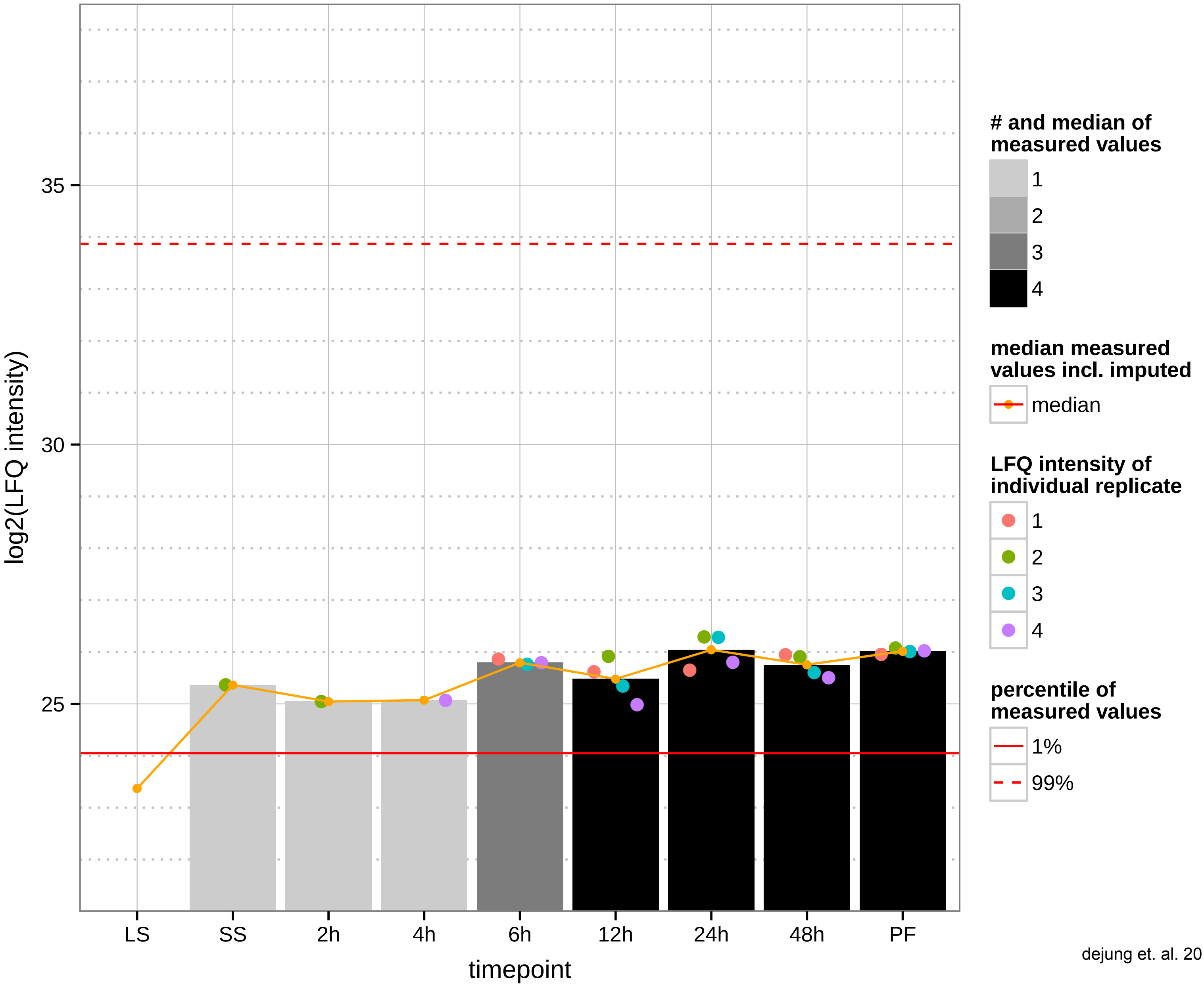

dejung et. al. 2015

ATP−dependent RNA helicase FAL1, putative
Tb927.11.8770
AGOF: ATP binding, ATP−dependent helicase activity, helicase activity, nucleic acid binding
AGOC: nucleolus
AGOP: maturation of SSU−rRNA from tricistronic rRNA transcript (SSU−rRNA, 5.8S rRNA, LSU−rRNA)
PGOF: ATP binding, ATP−dependent helicase activity, helicase activity, nucleic acid binding
PGOC: null
PGOP: null

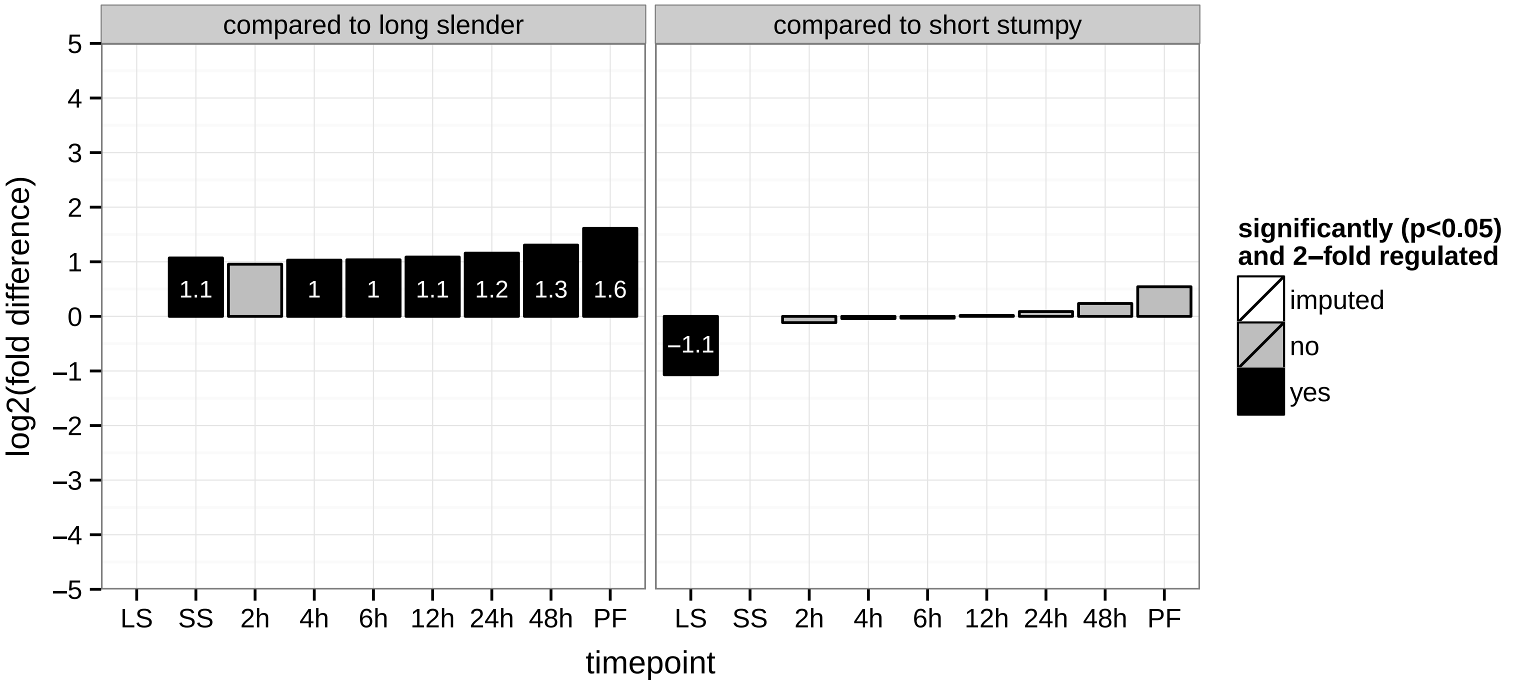

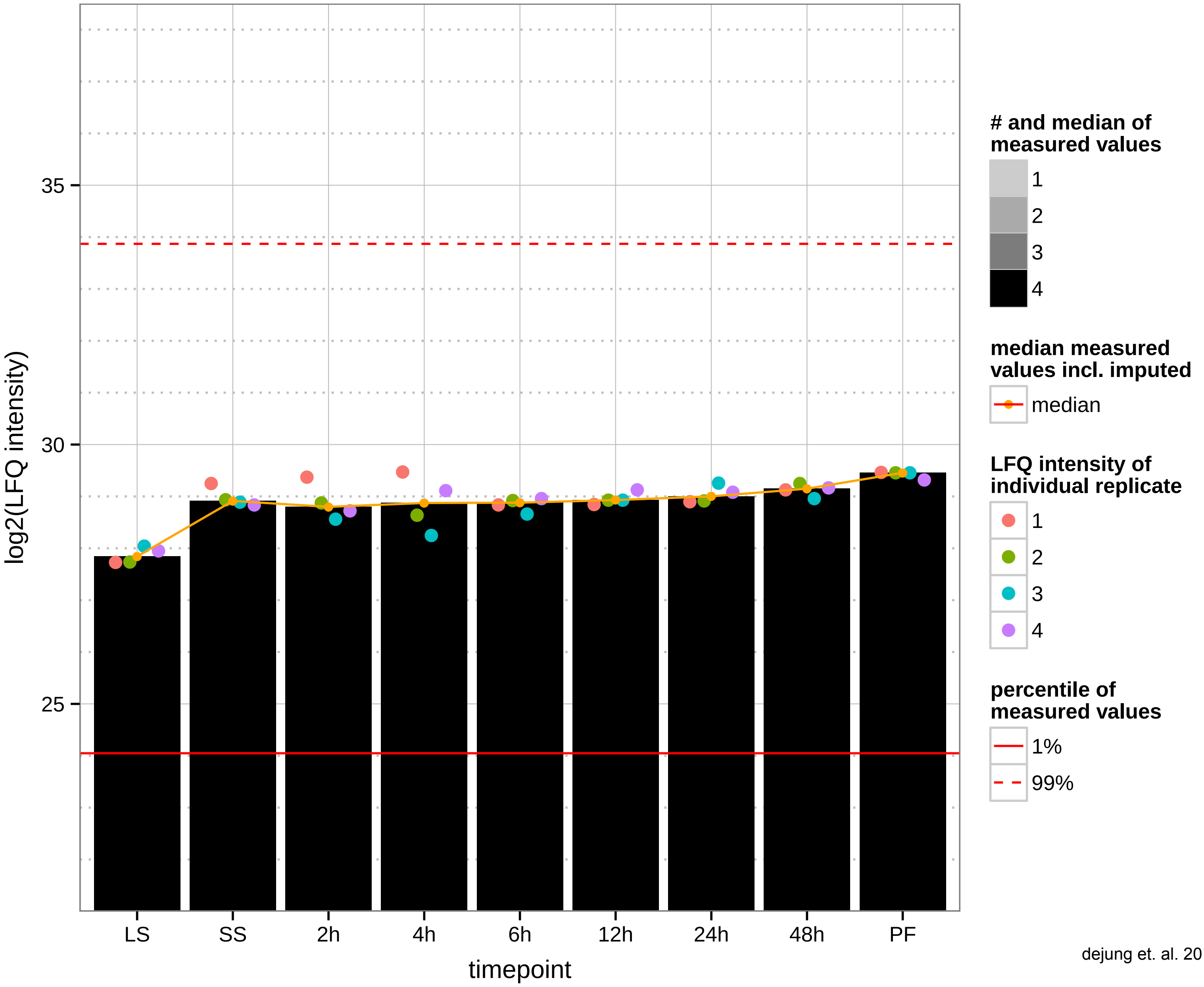

dejung et. al. 2015

cyclophilin a, cyclophilin type peptidyl–prolyl cis–trans isomerase (CYPA)
Tb927.11.880
AGOF: peptidyl–prolyl cis–trans isomerase activity
AGOC: cilium, cytosol
AGOP: protein folding
PGOF: peptidyl–prolyl cis–trans isomerase activity
PGOC: null
PGOP: protein folding

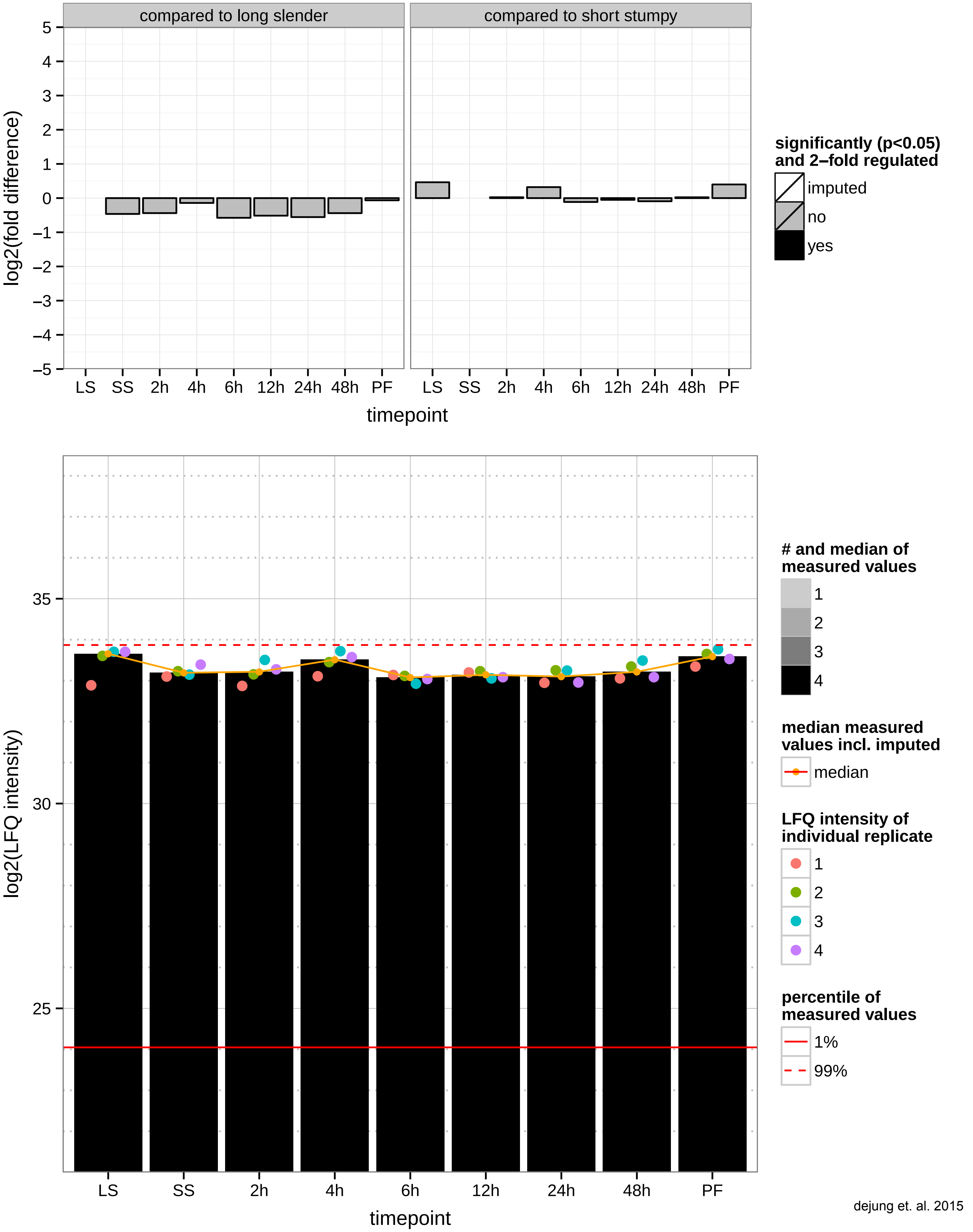

dejung et. al. 2015

hypothetical protein, conserved
Tb927.11.8800
AGOF: null
AGOC: mitochondrial inner membrane, mitochondrion
AGOP: null
PGOF: null
PGOC: null
PGOP: null

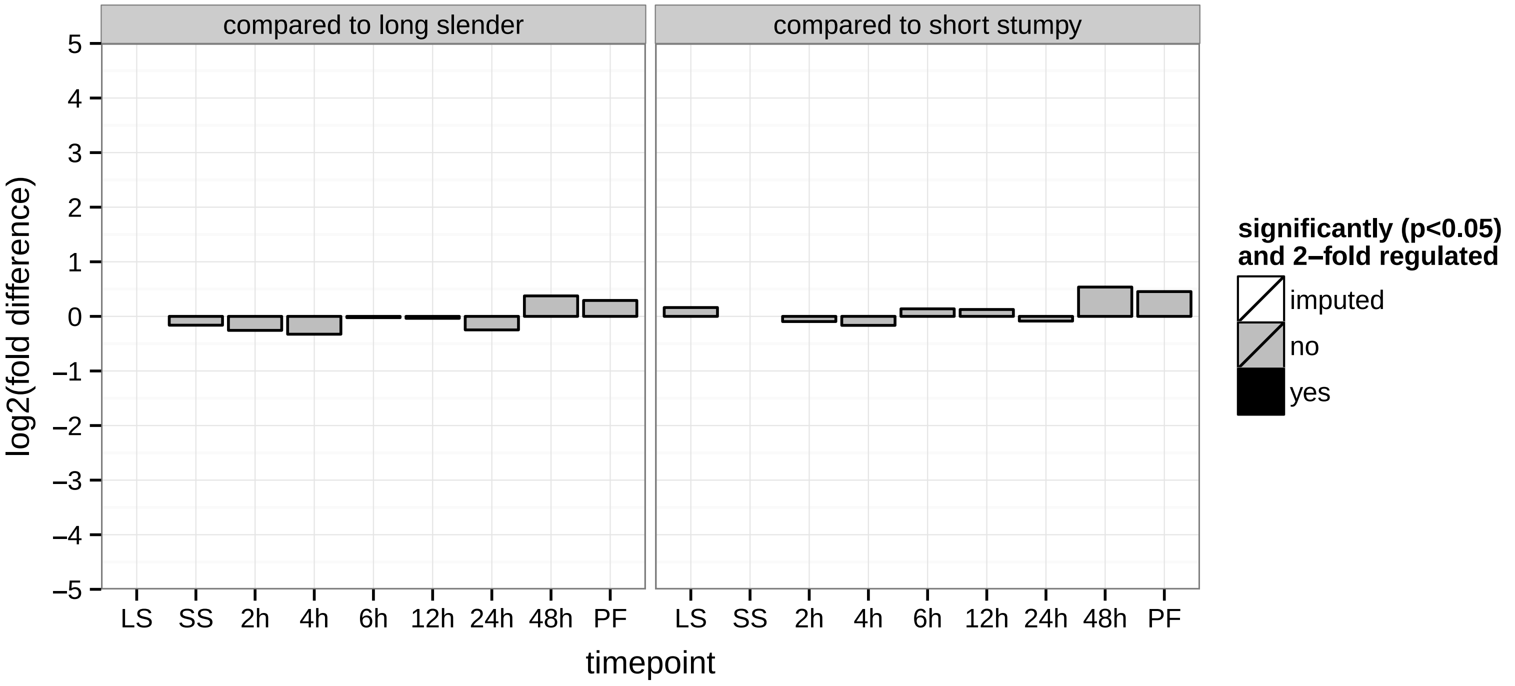

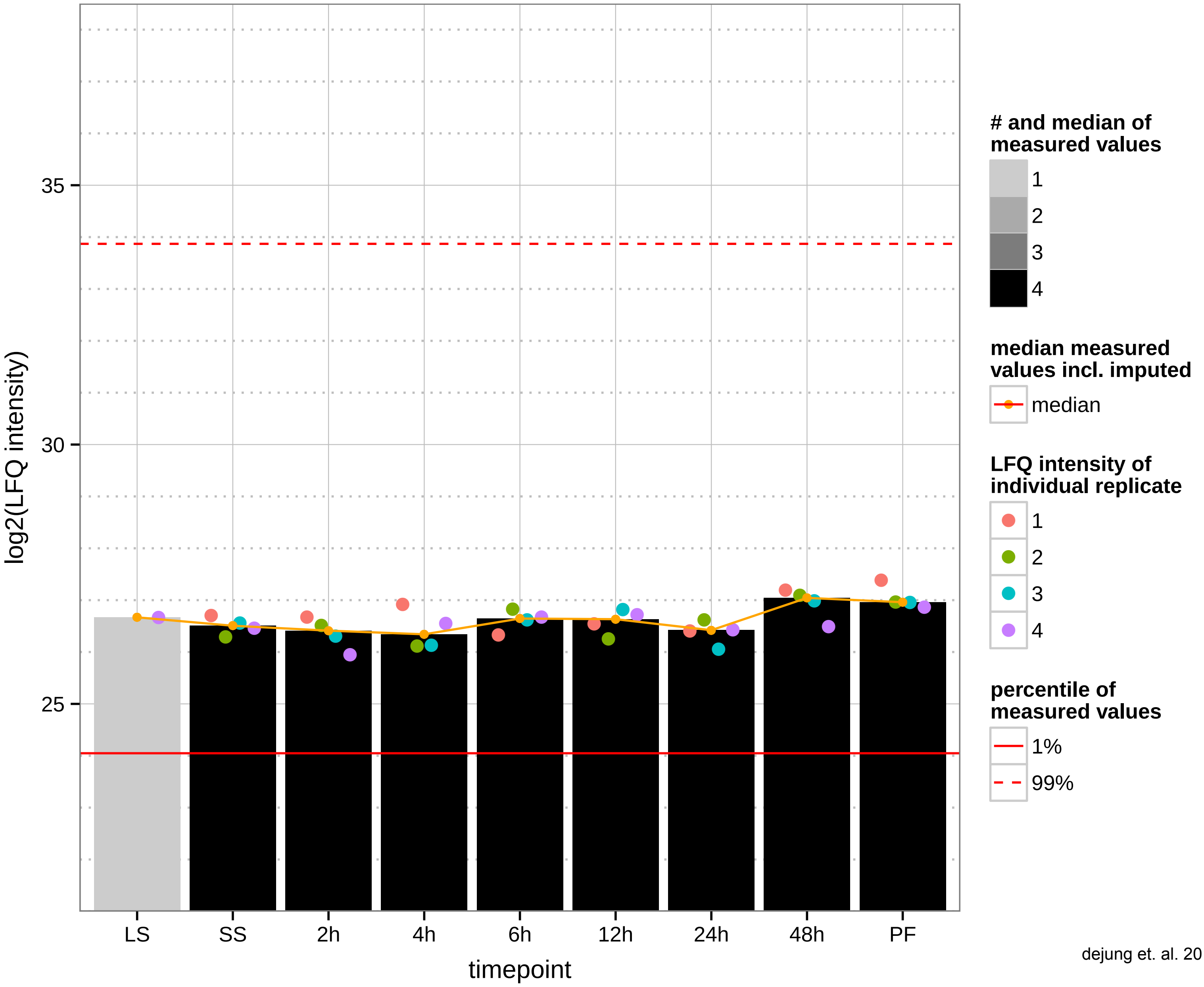

dejung et. al. 2015

DNA–dependent RNA polymerases, putative (RPC19)
Tb927.11.8890
AGOF: DNA binding, DNA–directed RNA polymerase activity, protein dimerization activity
AGOC: nucleus
AGOP: transcription, DNA–dependent
PGOF: DNA binding, DNA–directed RNA polymerase activity
PGOC: null
PGOP: transcription, DNA–dependent

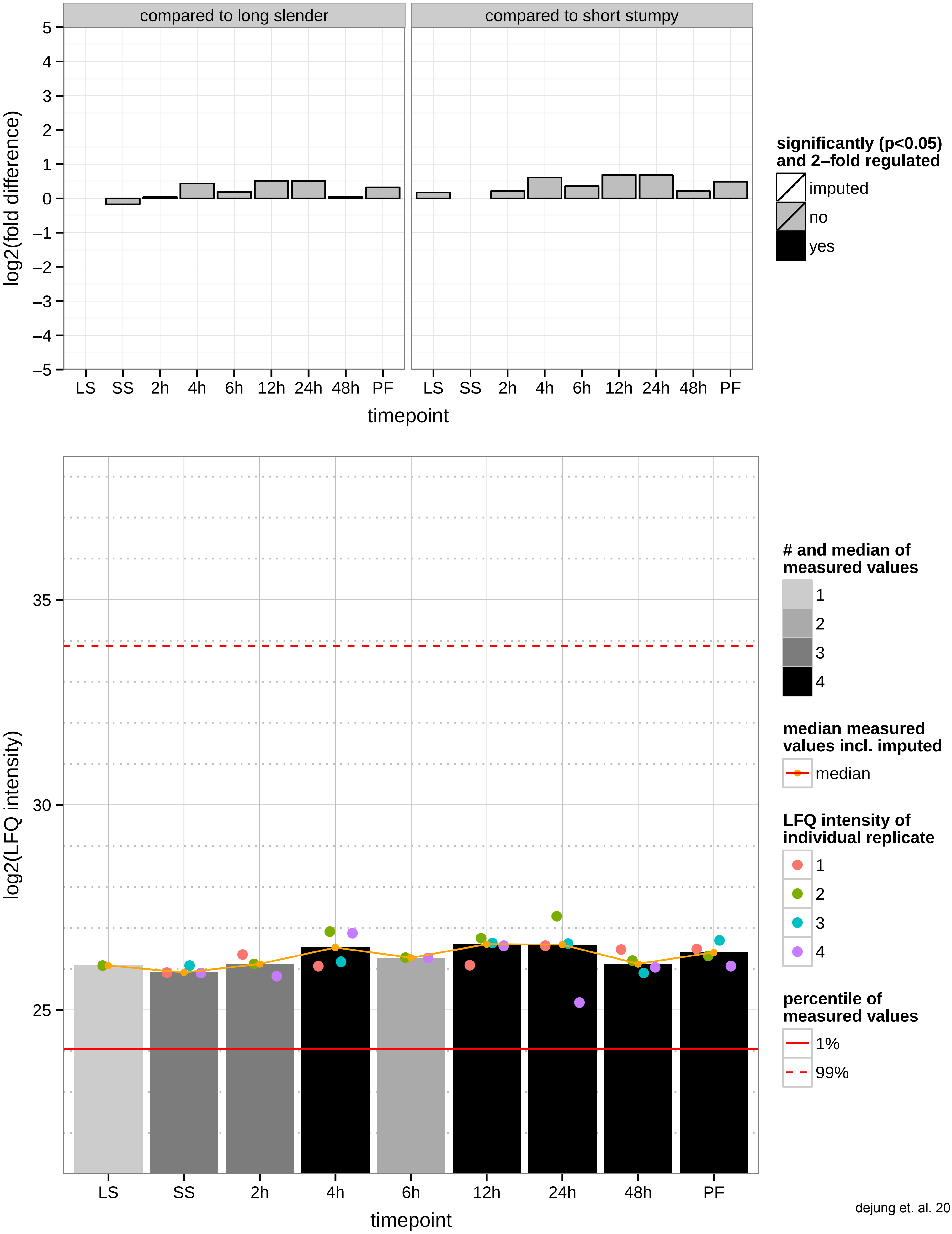

dejung et. al. 2015

hypothetical protein, conserved
Tb927.11.8900
AGOF: null
AGOC: null
AGOP: null
PGOF: null
PGOC: null
PGOP: null

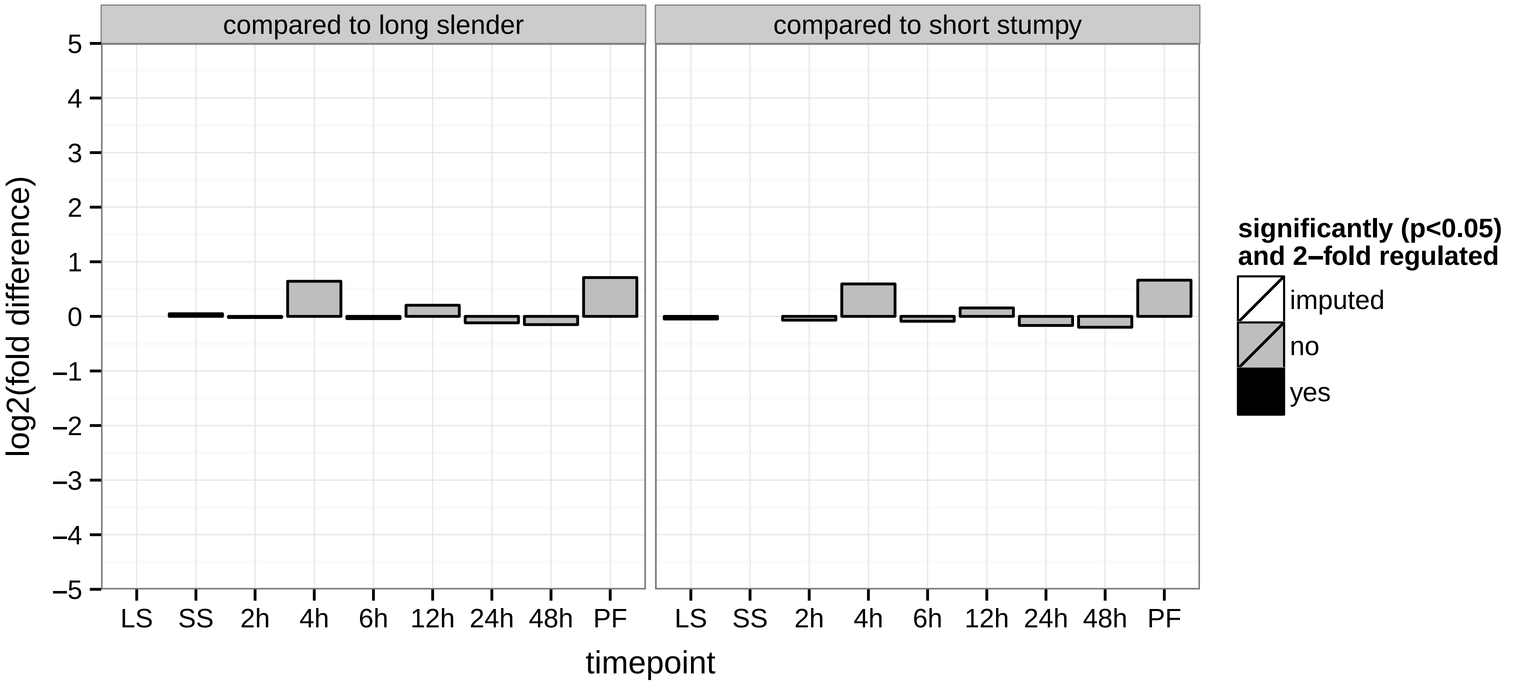

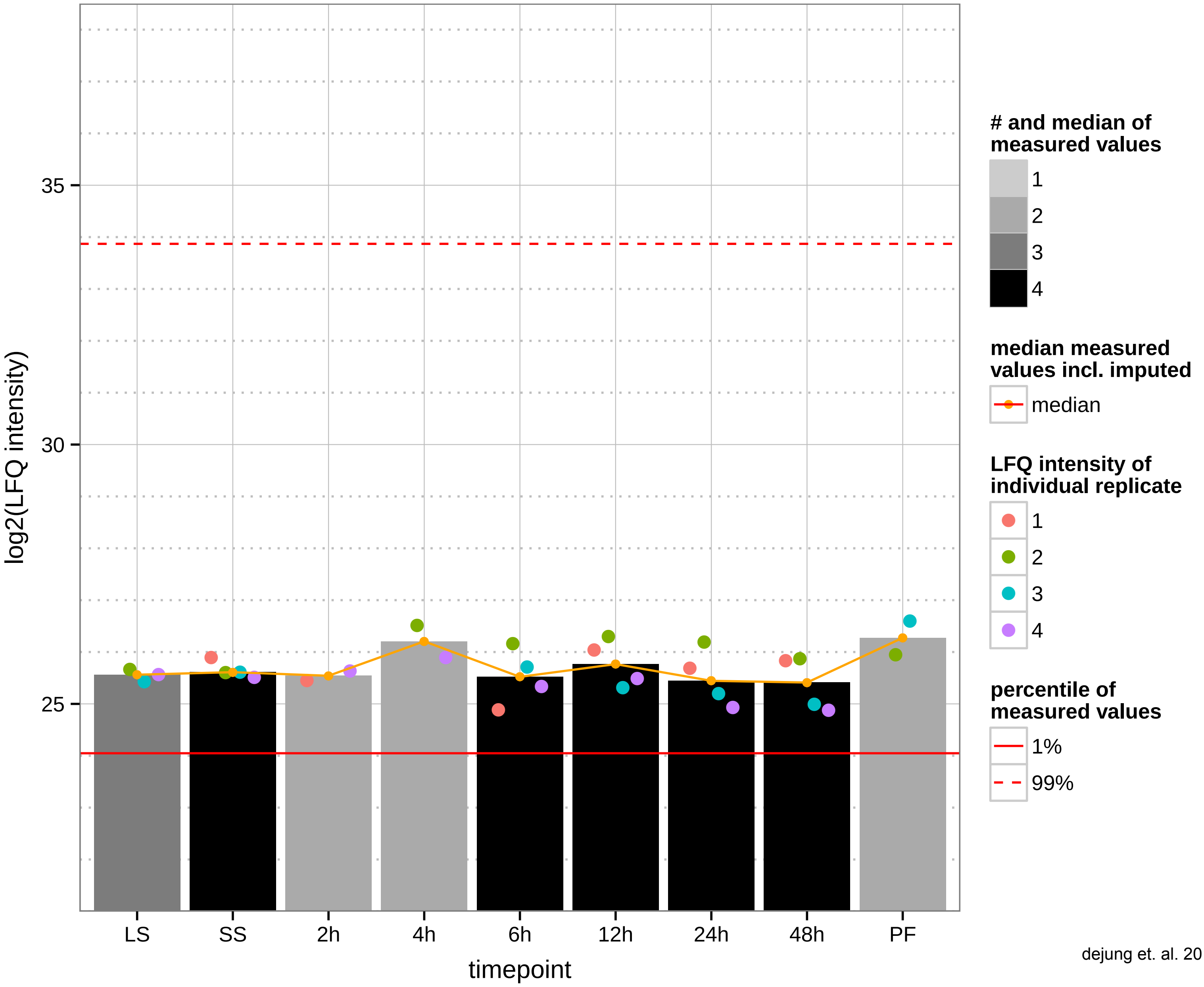

dejung et. al. 2015

leucine rich repeat (TbLRRP1)
Tb927.11.8950
AGOF: null
AGOC: null
AGOP: null
PGOF: protein binding
PGOC: null
PGOP: null

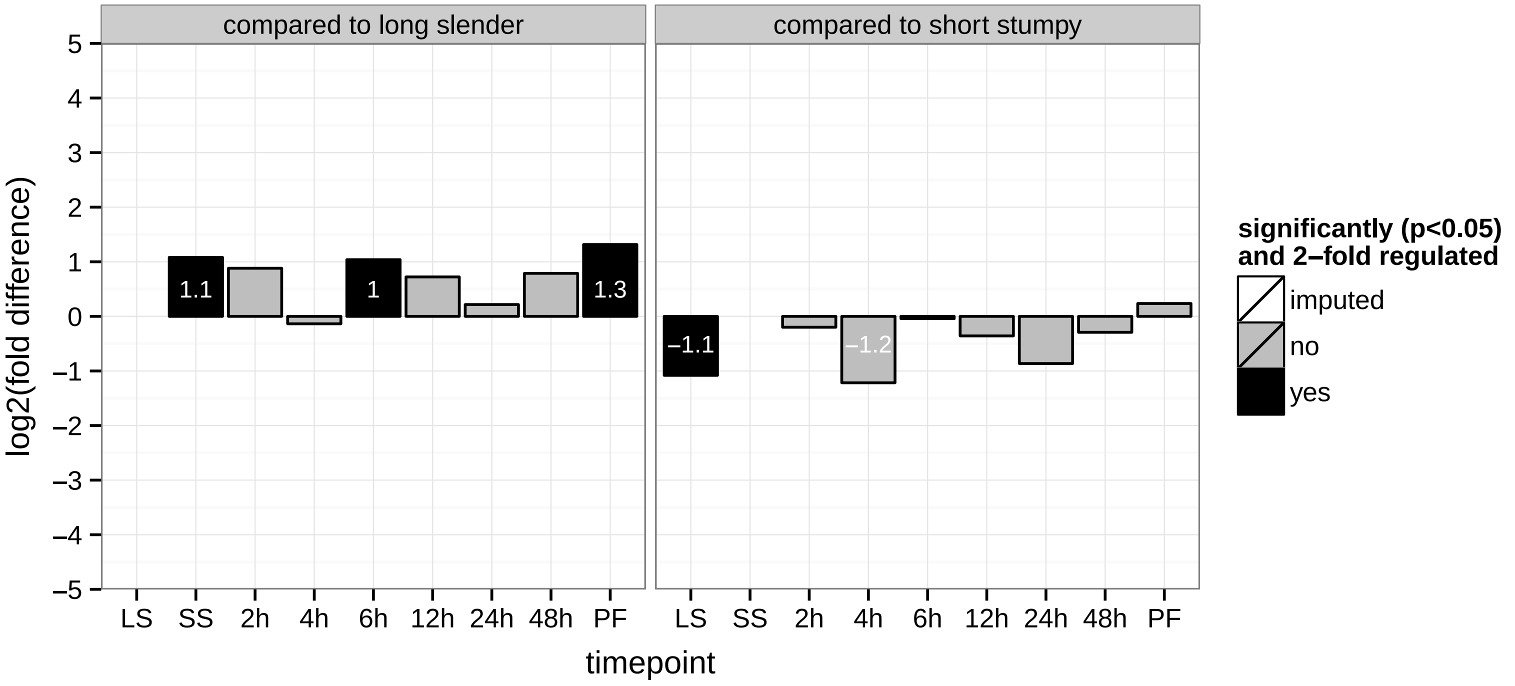

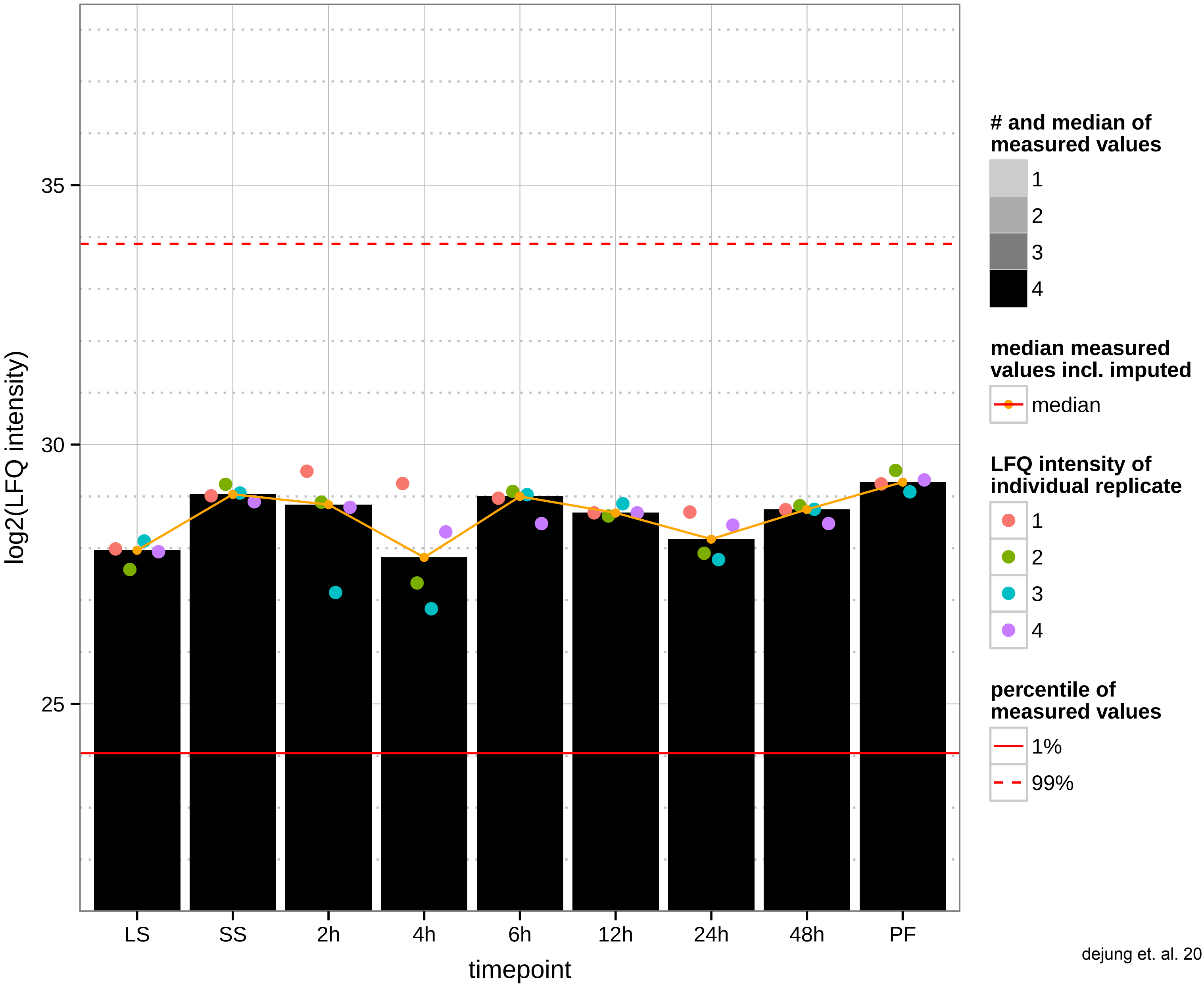

dejung et. al. 2015

ribose 5−phosphate isomerase, putative
Tb927.11.8970
AGOF: ribose−5−phosphate isomerase activity
AGOC: null
AGOP: pentose−phosphate shunt, pentose−phosphate shunt, non−oxidative branch
PGOF: ribose−5−phosphate isomerase activity
PGOC: null
PGOP: carbohydrate metabolic process, pentose−phosphate shunt

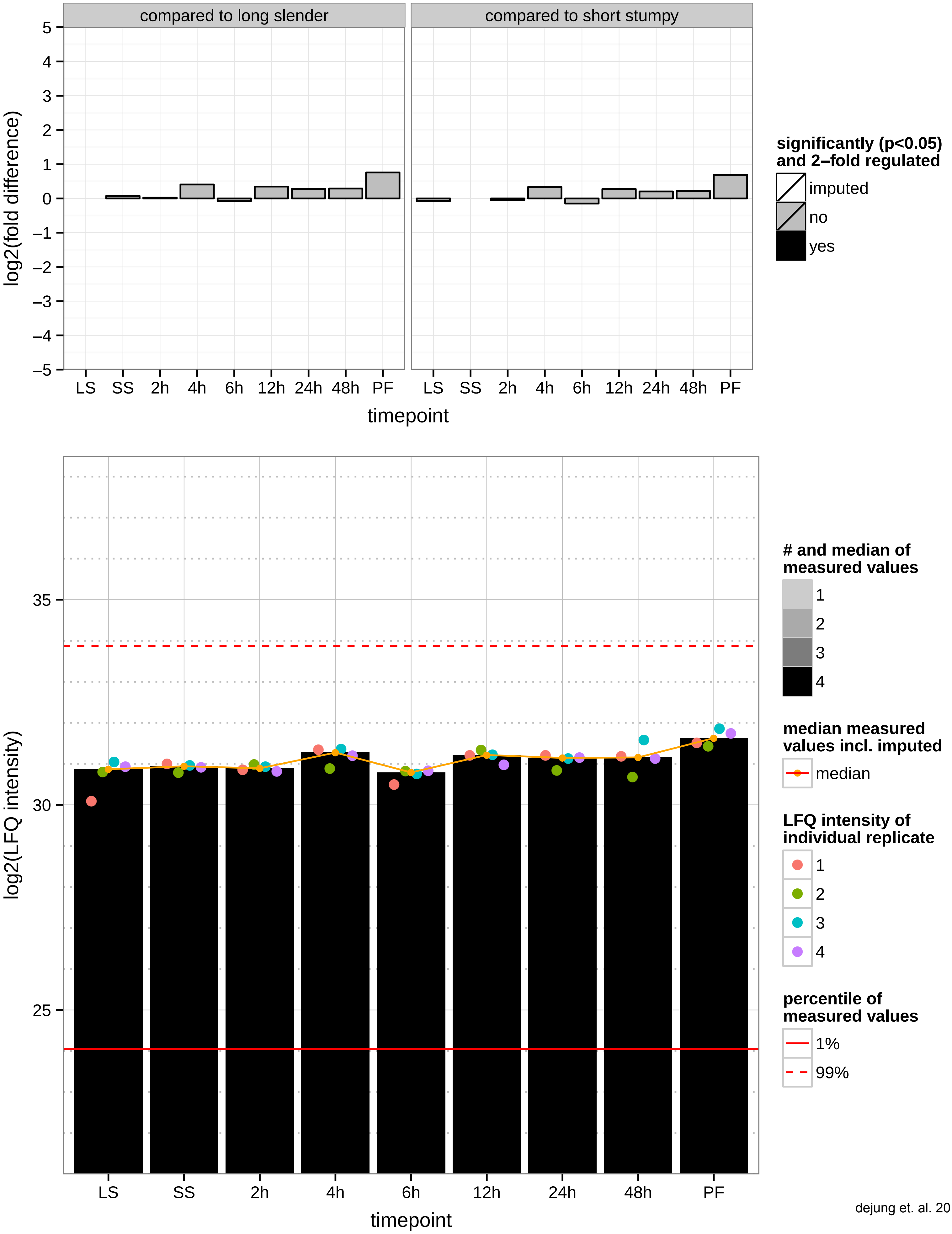

dejung et. al. 2015

Elongation factor G 2, mitochondrial, putative (EF−G2)
Tb927.11.8980
AGOF: GTP binding, GTPase activity
AGOC: mitochondrion
AGOP: null
PGOF: GTP binding, GTPase activity
PGOC: null
PGOP: null

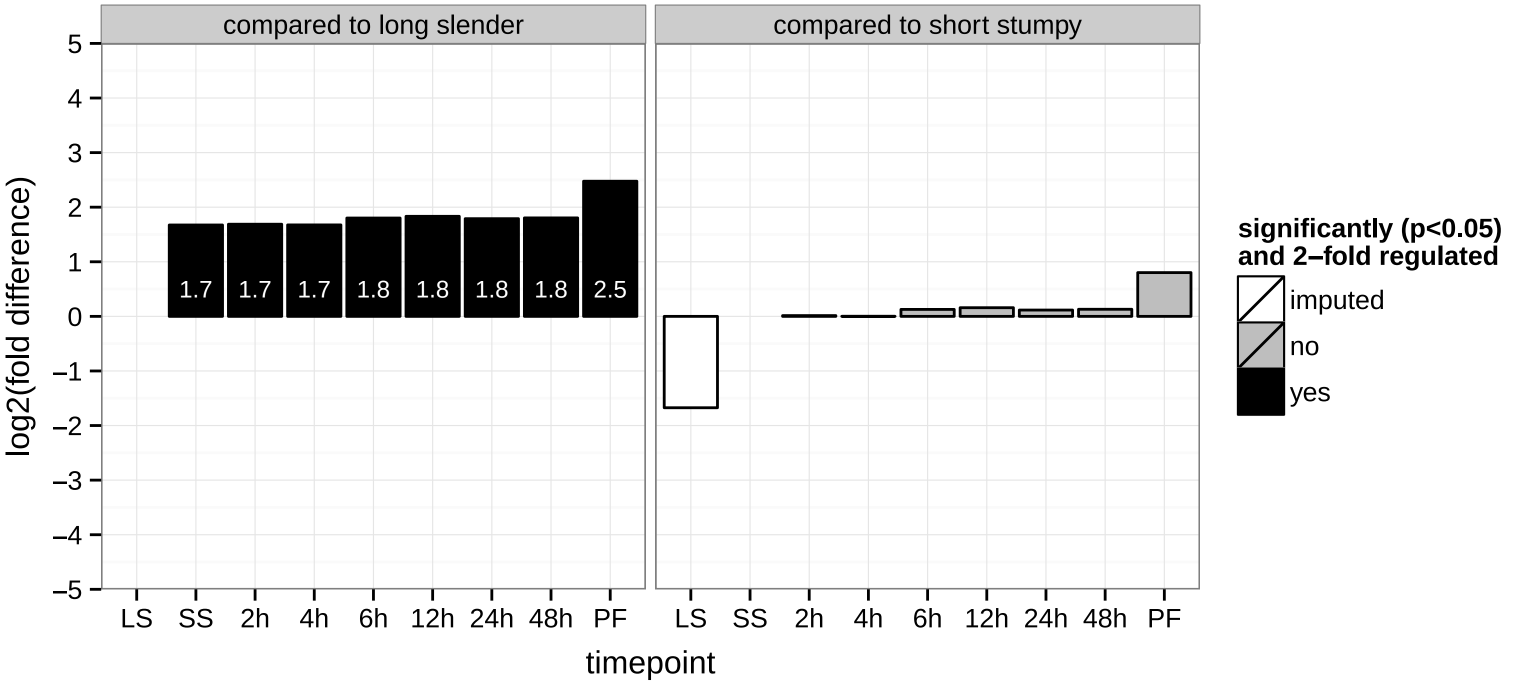

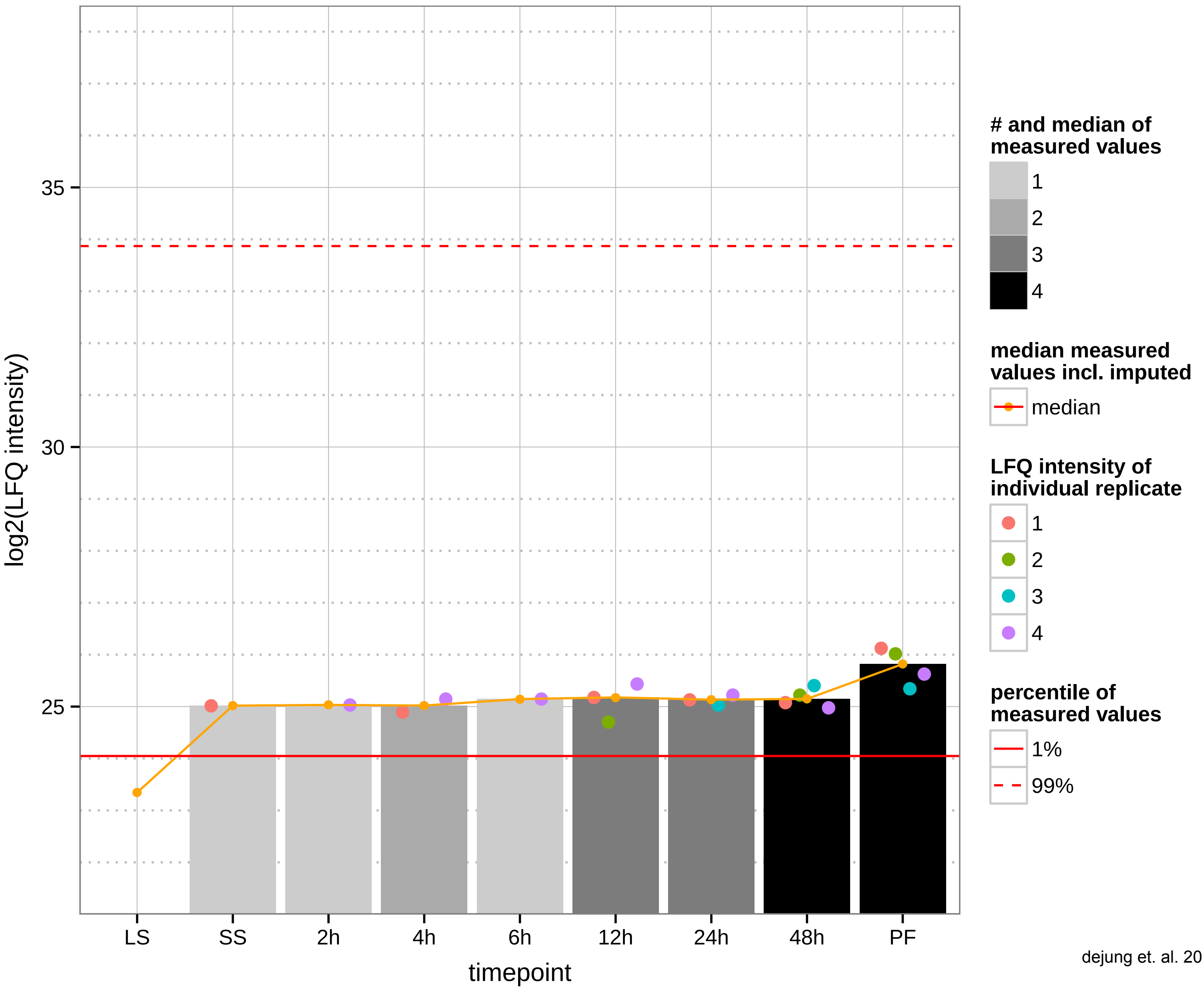

dejung et. al. 2015

isocitrate dehydrogenase, putative (IDH)
Tb927.11.900
AGOF: NAD binding, isocitrate dehydrogenase (NADP+) activity, magnesium ion binding
AGOC: null
AGOP: isocitrate metabolic process, oxidation−reduction process
PGOF: isocitrate dehydrogenase (NADP+) activity, oxidoreductase activity, acting on the CH−OH group of donors, NAD or NA
PGOC: null
PGOP: isocitrate metabolic process, oxidation−reduction process

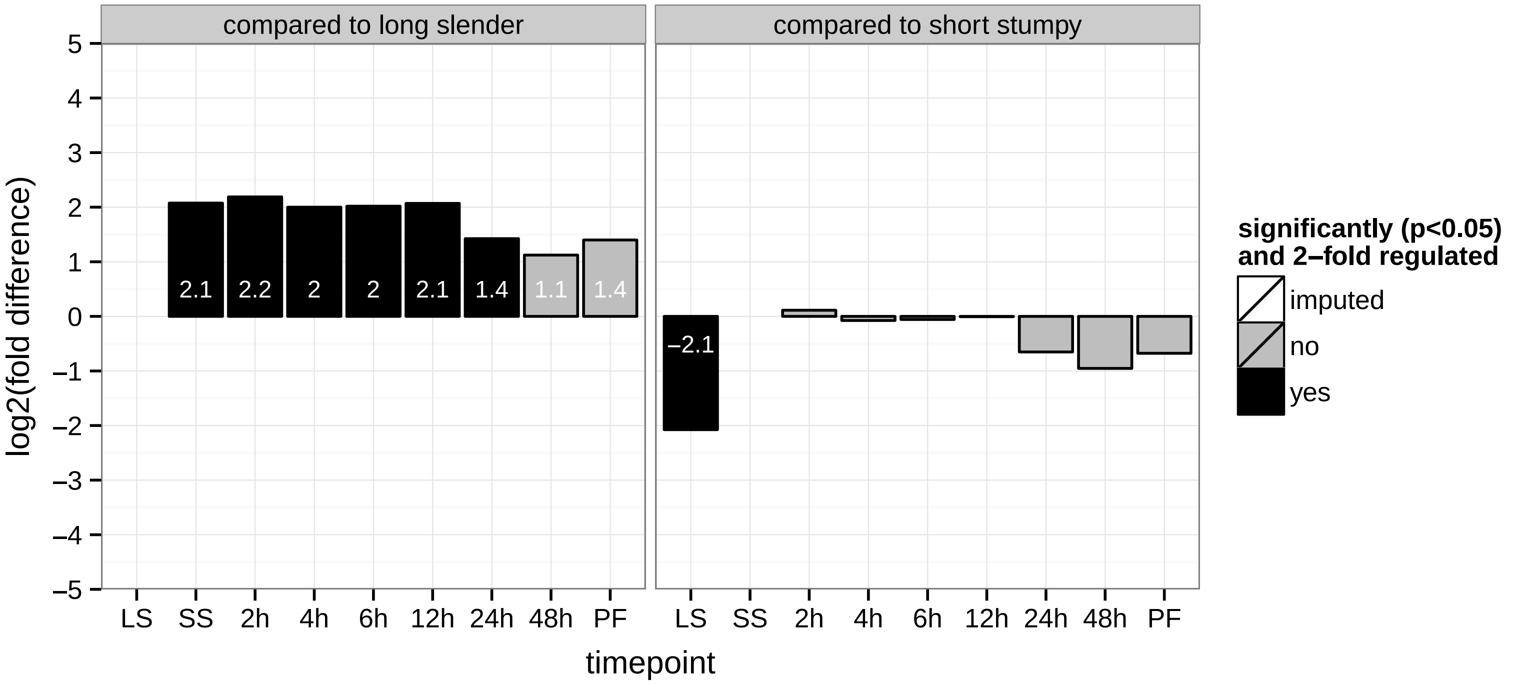

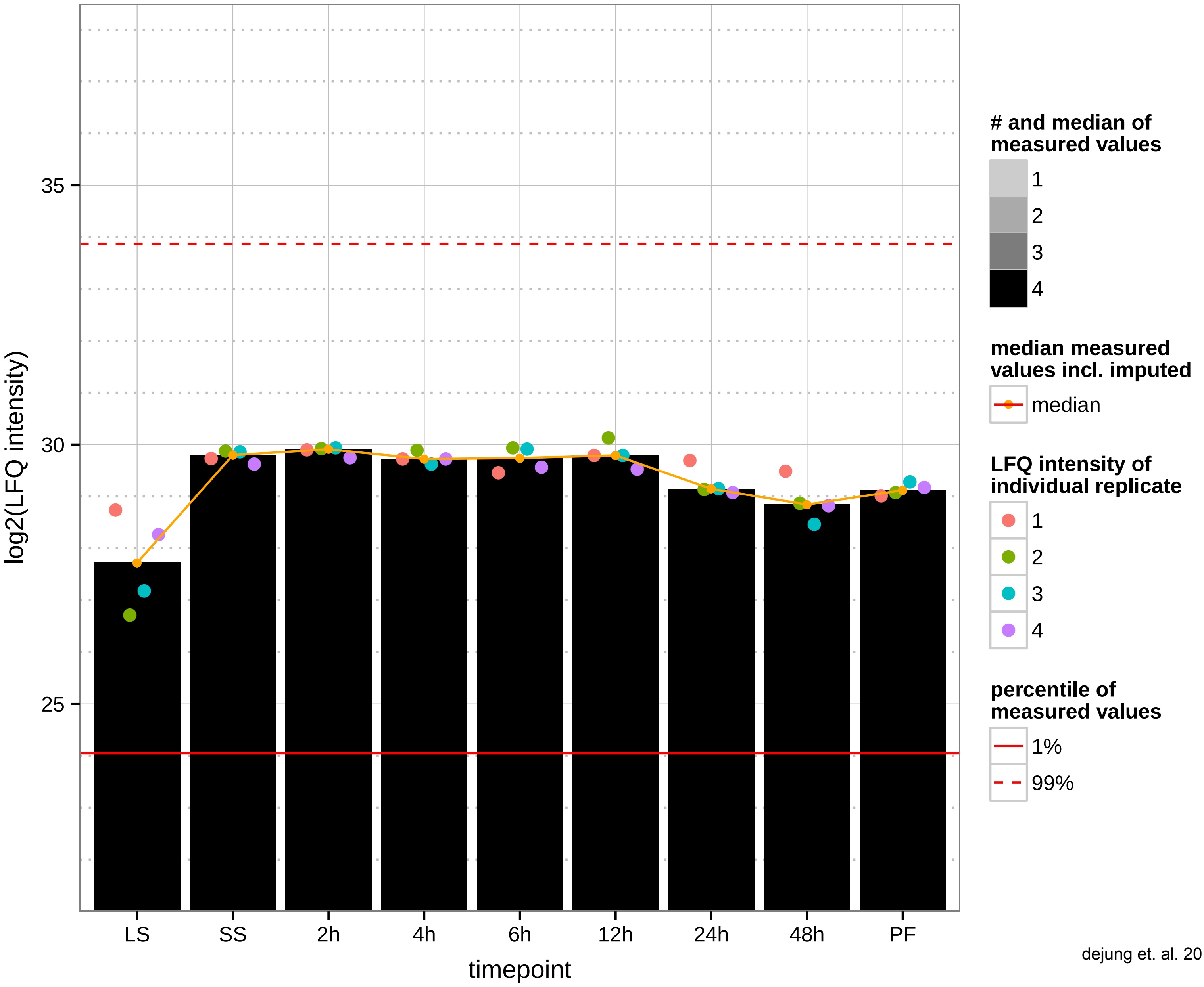

dejung et. al. 2015

hypothetical protein, conserved
Tb927.11.9050
AGOF: null
AGOC: null
AGOP: null
PGOF: null
PGOC: null
PGOP: null

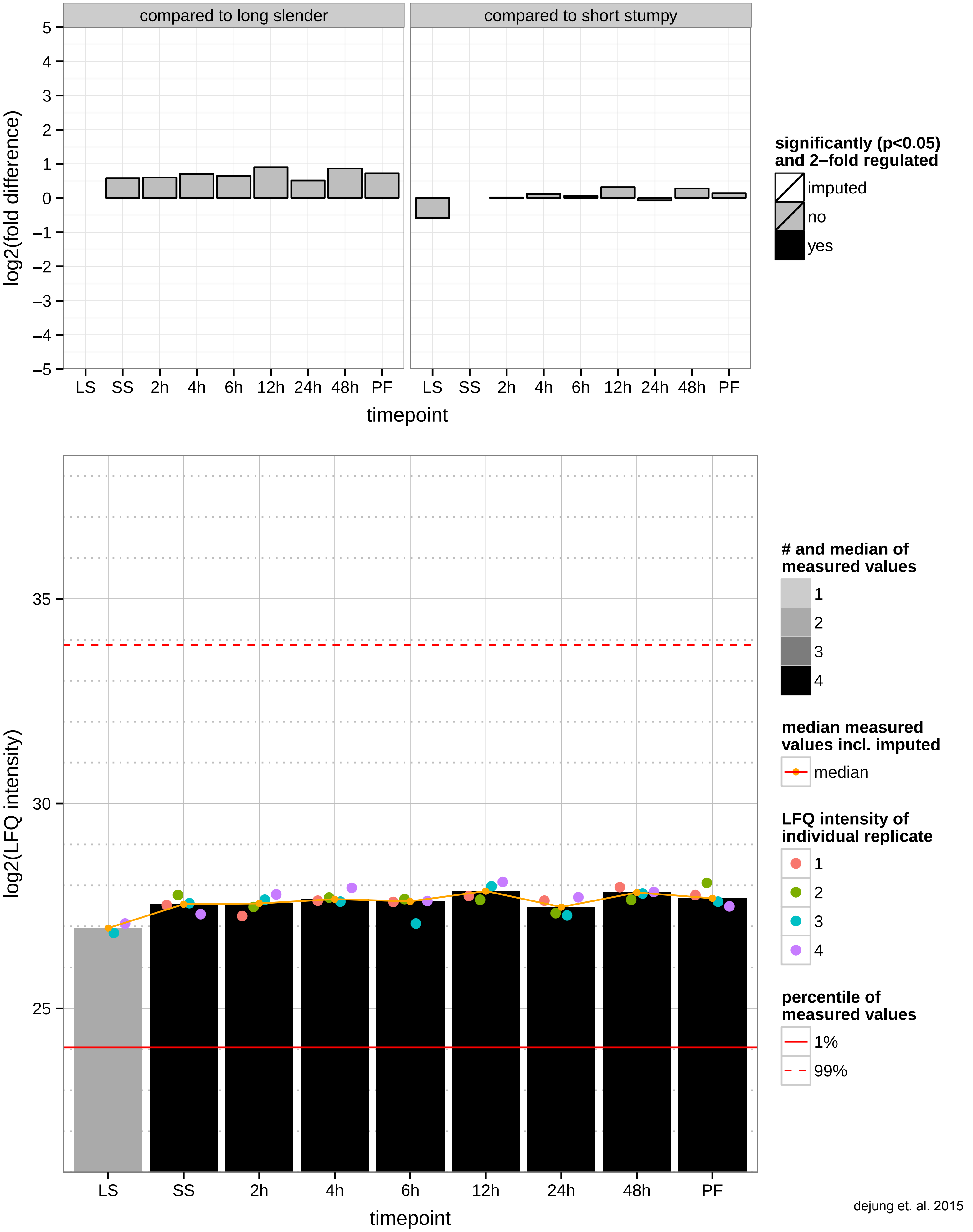

dejung et. al. 2015

hypothetical protein, conserved
Tb927.11.9080
AGOF: nucleotide binding
AGOC: nucleus
AGOP: ribosome biogenesis
PGOF: null
PGOC: nucleus
PGOP: ribosome biogenesis

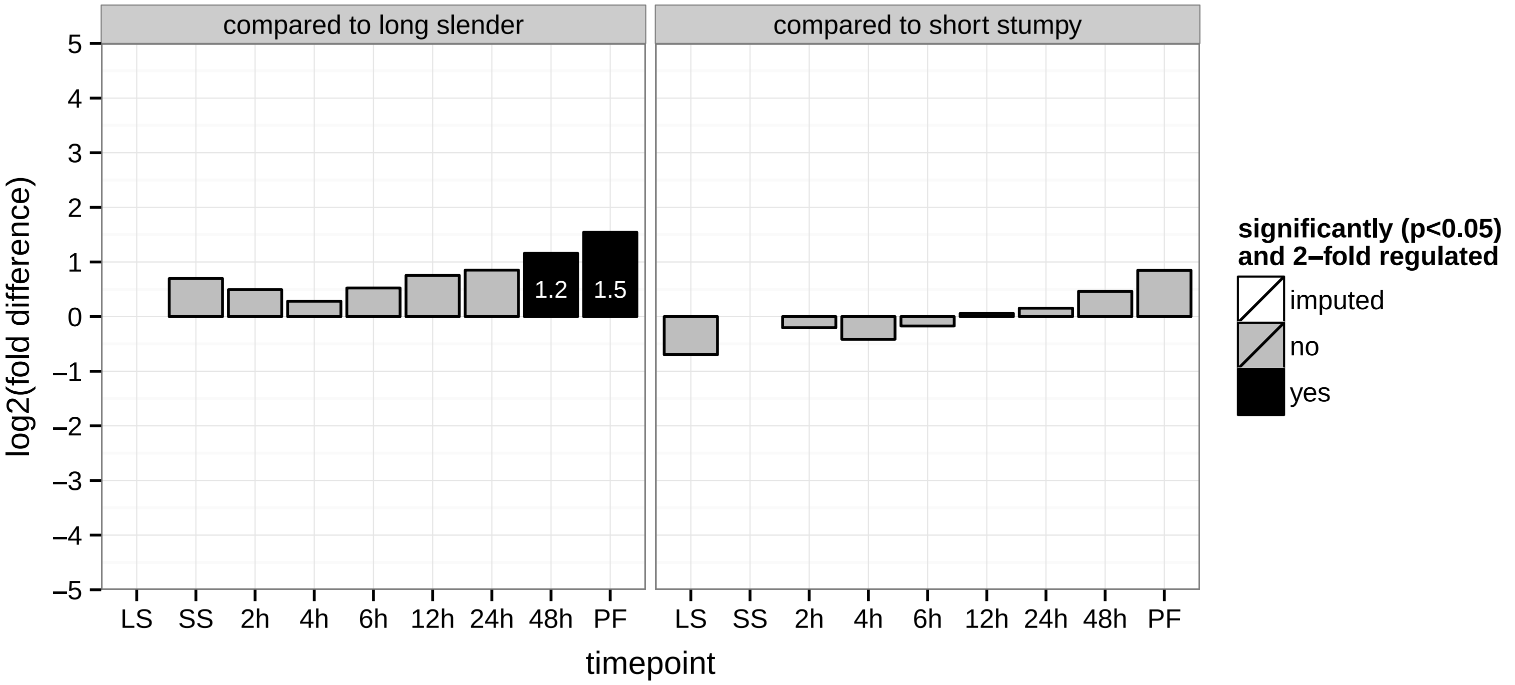

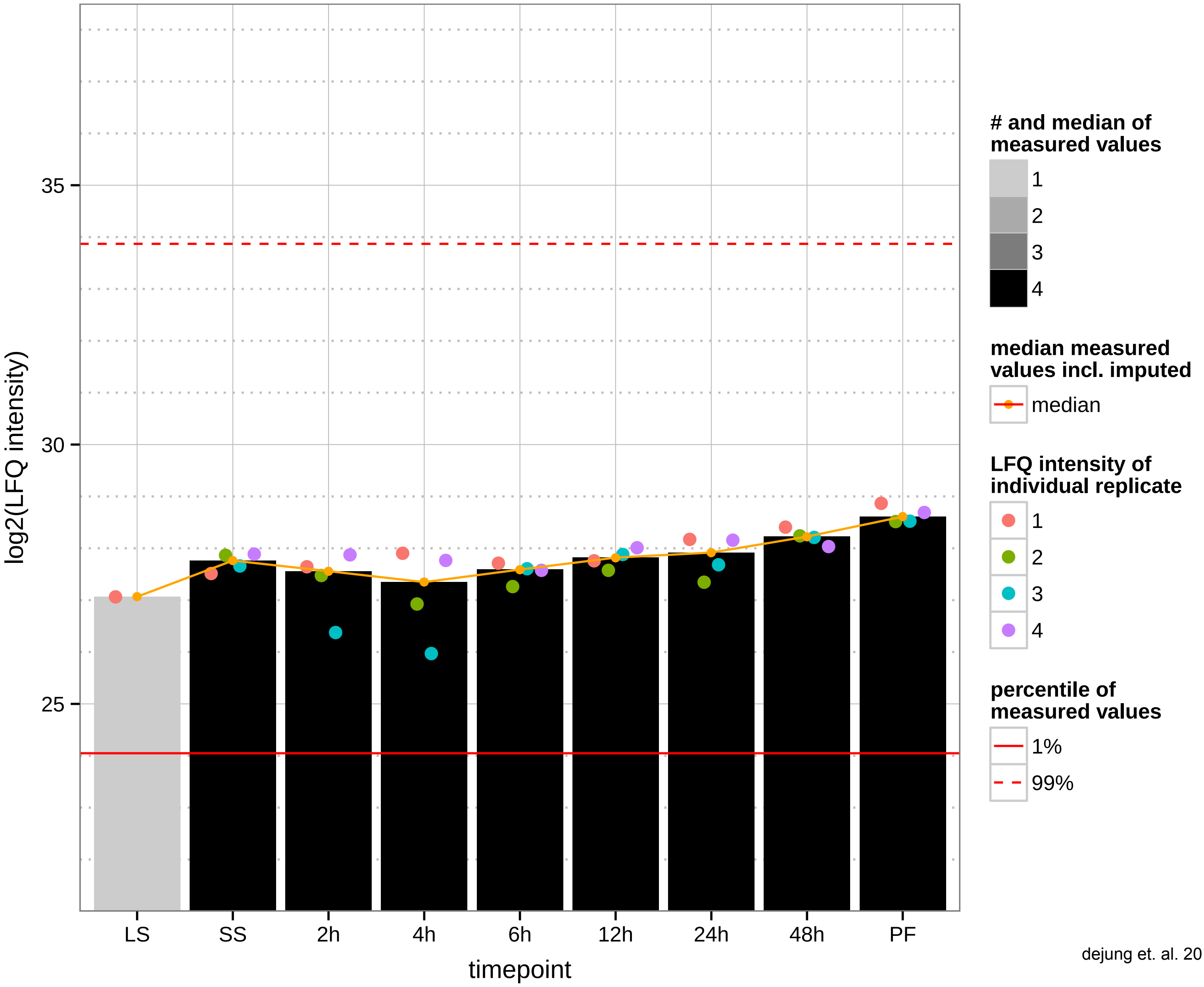

dejung et. al. 2015

DNA topoisomerase III, putative
Tb927.11.9170
AGOF: DNA binding, DNA topoisomerase type I activity
AGOC: chromosome
AGOP: DNA modification, DNA topological change, DNA unwinding involved in replication
PGOF: DNA binding, DNA topoisomerase activity
PGOC: chromosome
PGOP: DNA topological change

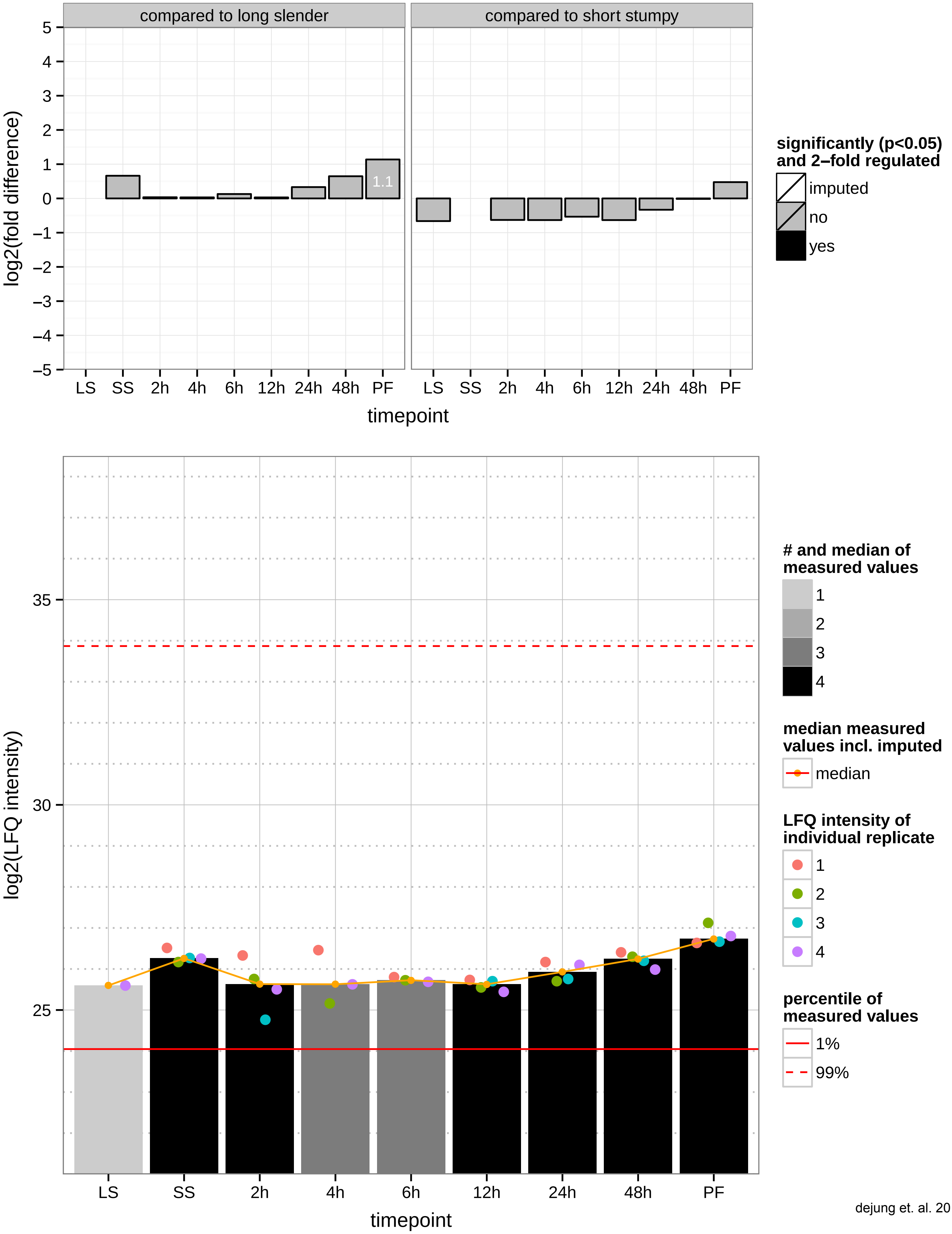

dejung et. al. 2015

ADP–ribosylation factor GTPase activating protein, putative, putative (Cnt–b5)
Tb927.11.9180
AGOF: ARF GTPase activator activity, DNA binding, zinc ion binding
AGOC: nucleus
AGOP: ER to Golgi vesicle–mediated transport, regulation of ARF GTPase activity
PGOF: ARF GTPase activator activity, zinc ion binding
PGOC: null
PGOP: regulation of ARF GTPase activity

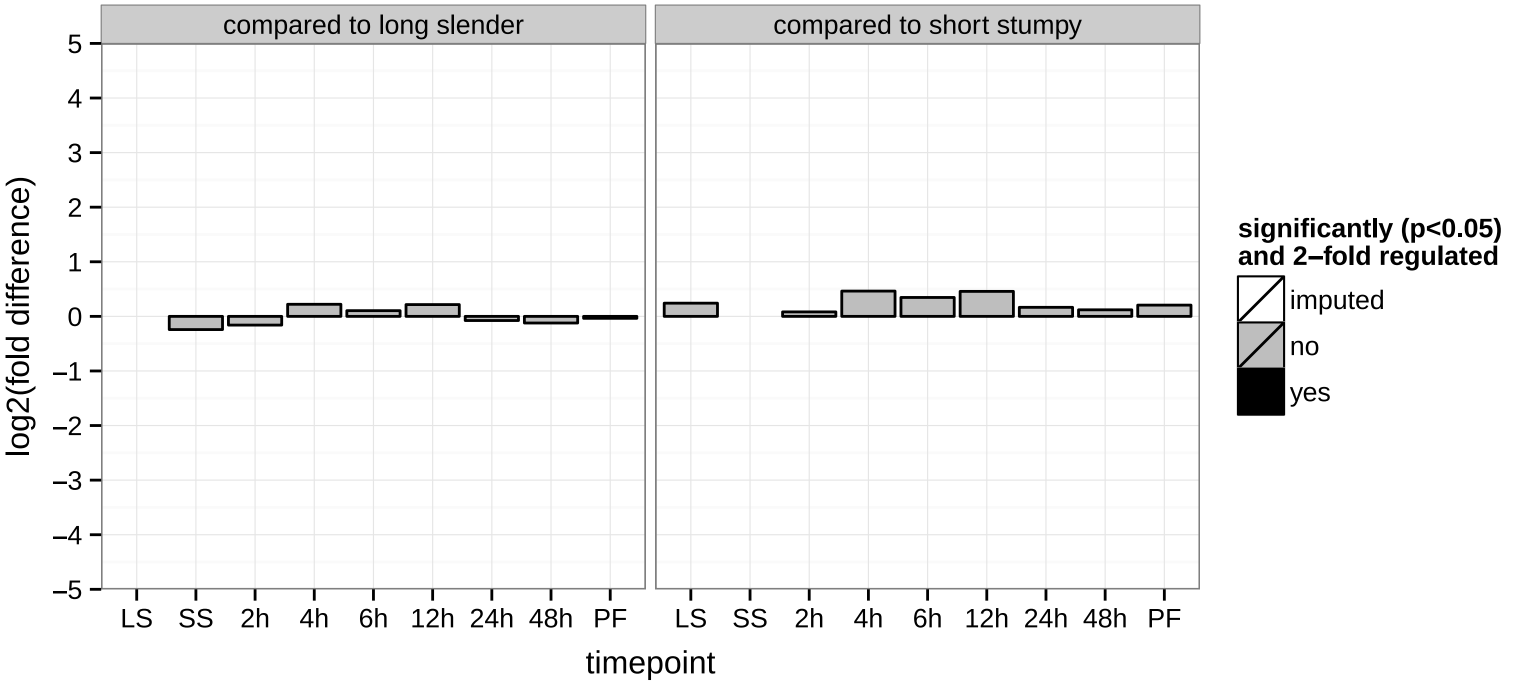

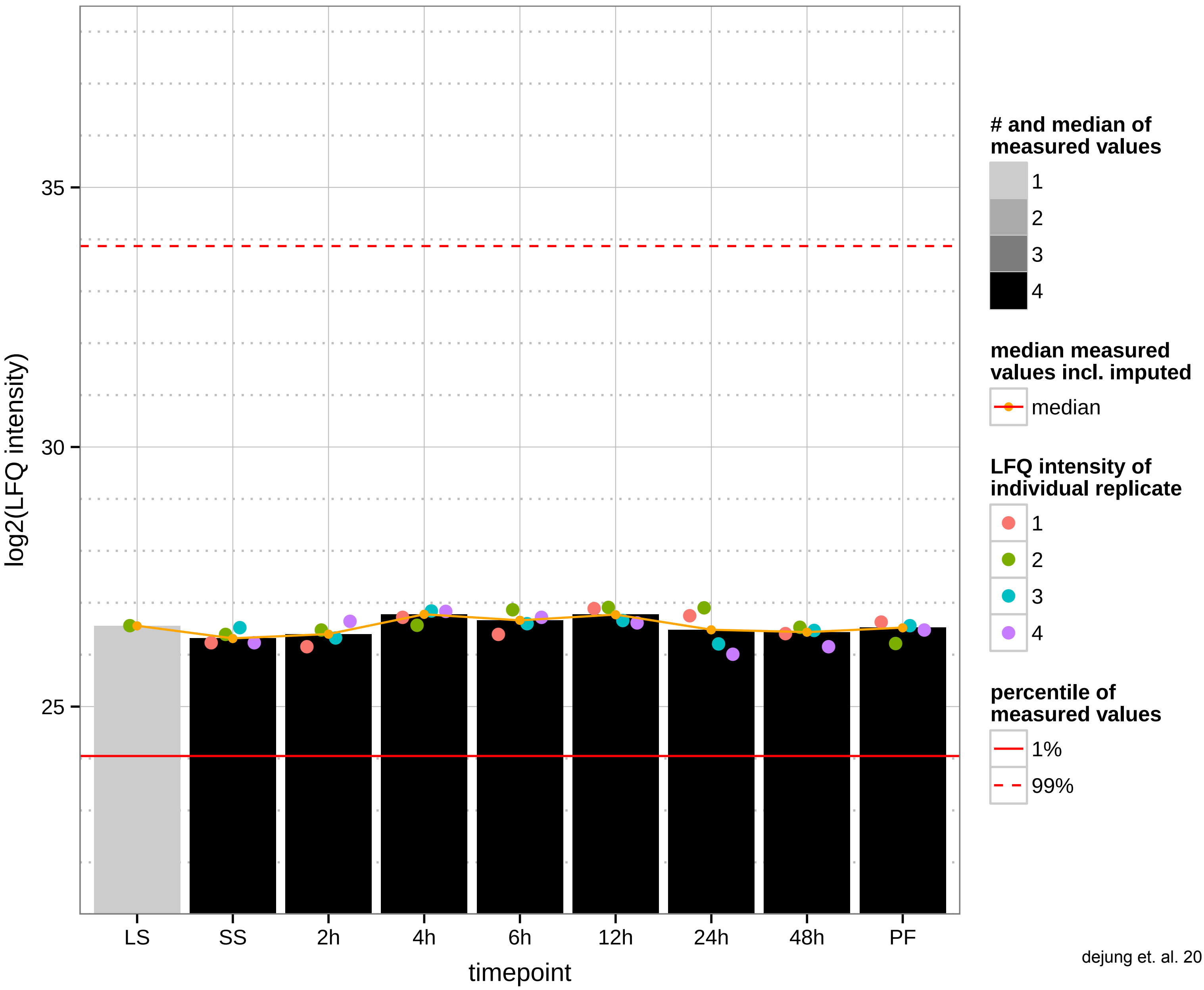

dejung et. al. 2015

protein kinase, putative
Tb927.11.9190
AGOF: ATP binding, protein kinase activity, protein serine/threonine kinase activity
AGOC: null
AGOP: protein phosphorylation
PGOF: ATP binding, binding, protein binding, protein kinase activity, transferase activity, transferring phosphorus–containing g
PGOC: null
PGOP: protein phosphorylation

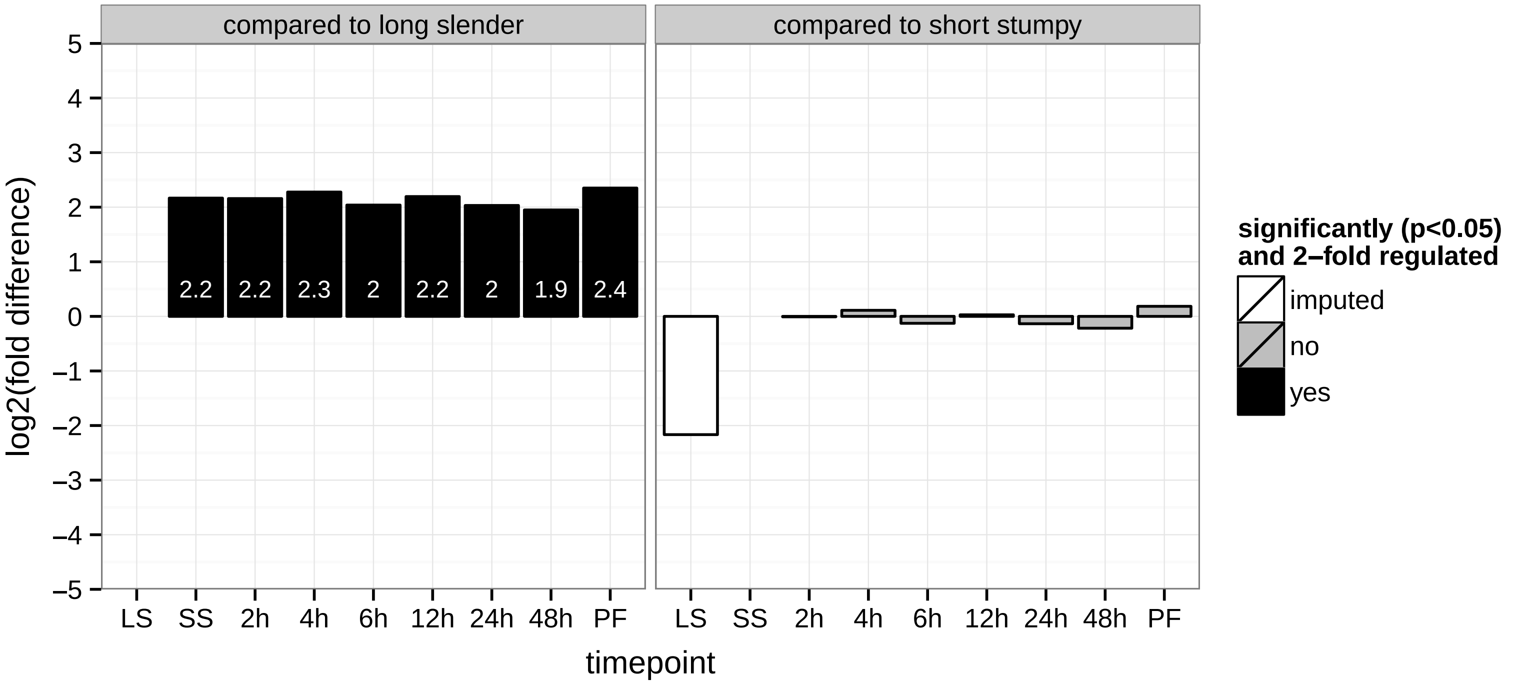

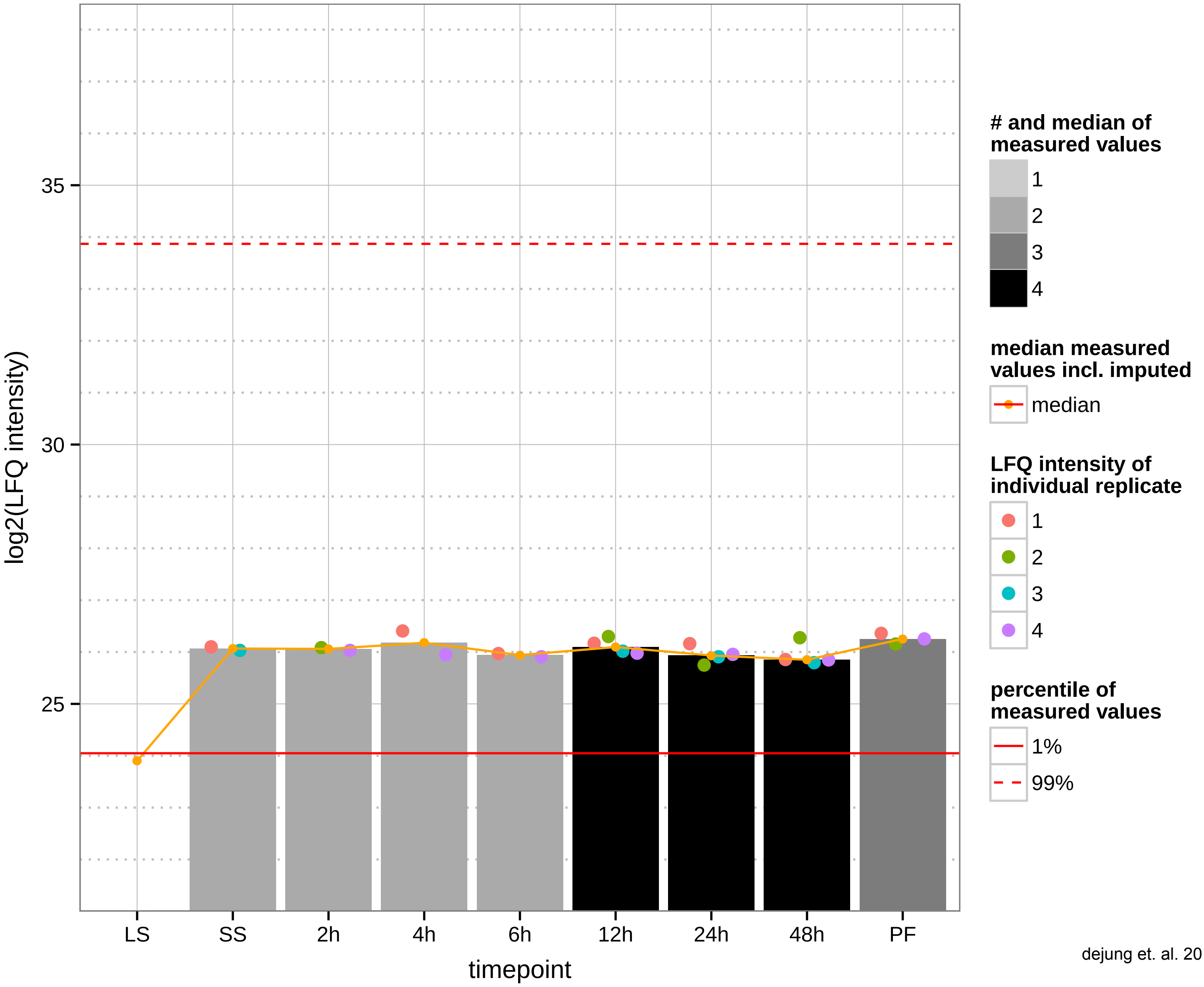

dejung et. al. 2015

proteasome regulatory non–ATP–ase subunit 2 (RPN2)
Tb927.11.9220
AGOF: endopeptidase activity, enzyme regulator activity
AGOC: proteasome complex, proteasome regulatory particle
AGOP: proteasomal ubiquitin–dependent protein catabolic process, regulation of protein catabolic process
PGOF: binding, enzyme regulator activity
PGOC: proteasome complex
PGOP: regulation of protein catabolic process

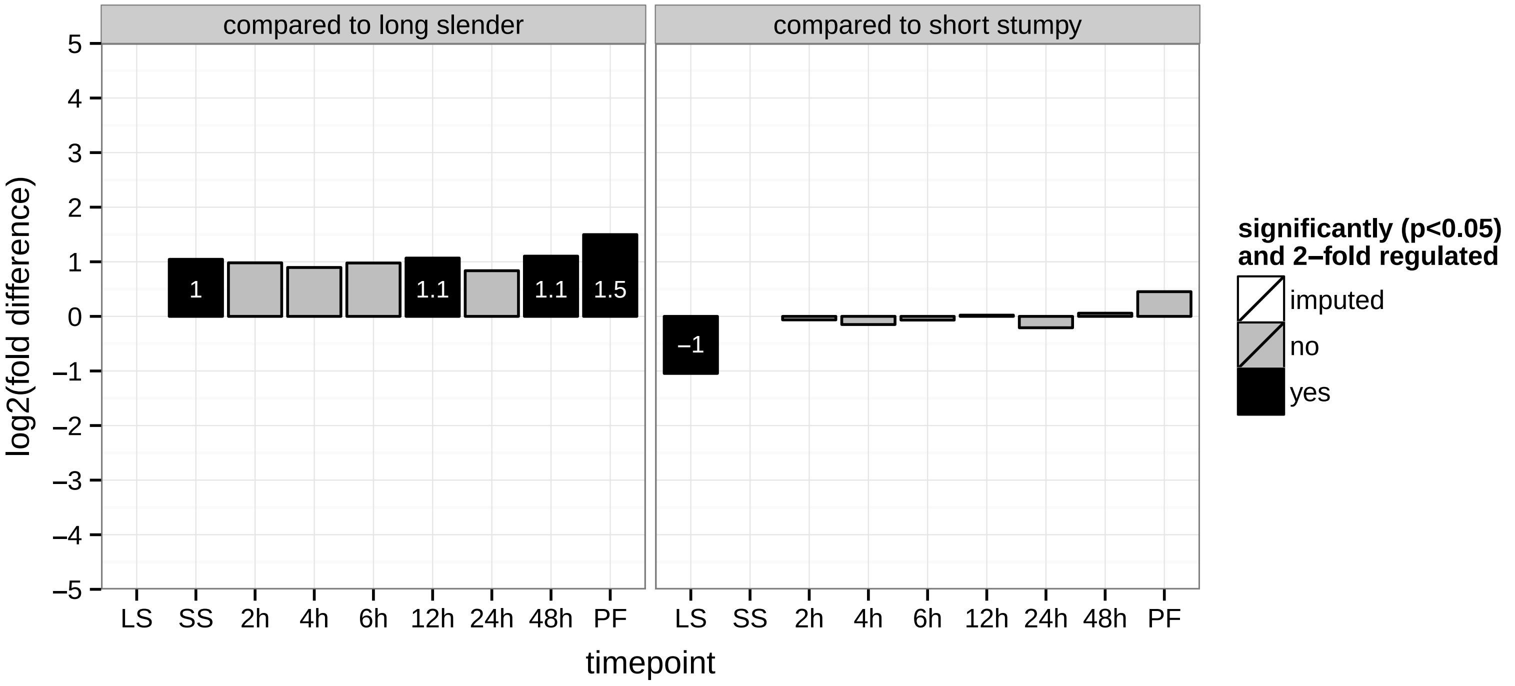

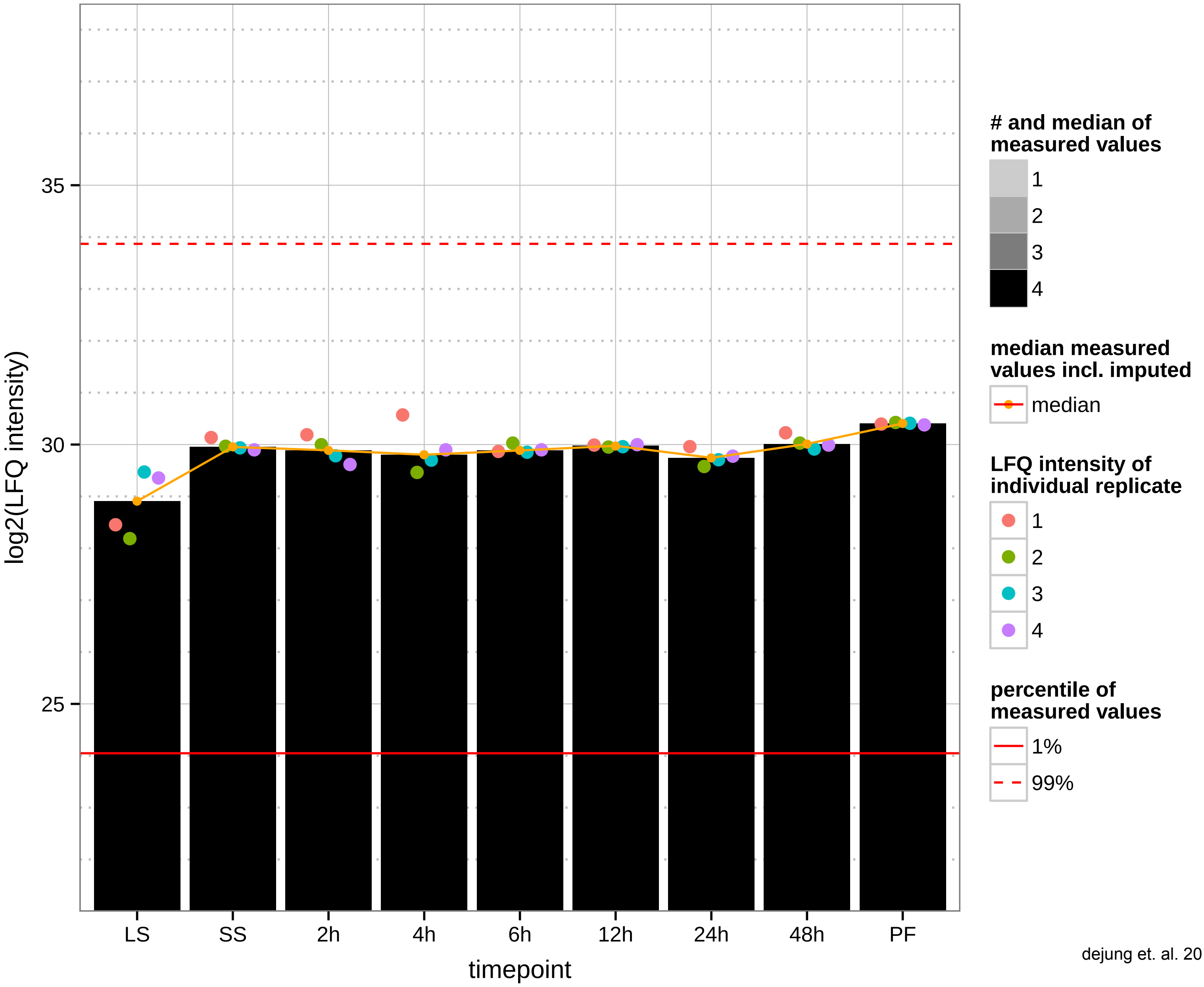

dejung et. al. 2015

hypothetical protein, conserved
Tb927.11.9300
AGOF: null
AGOC: null
AGOP: null
PGOF: null
PGOC: null
PGOP: null

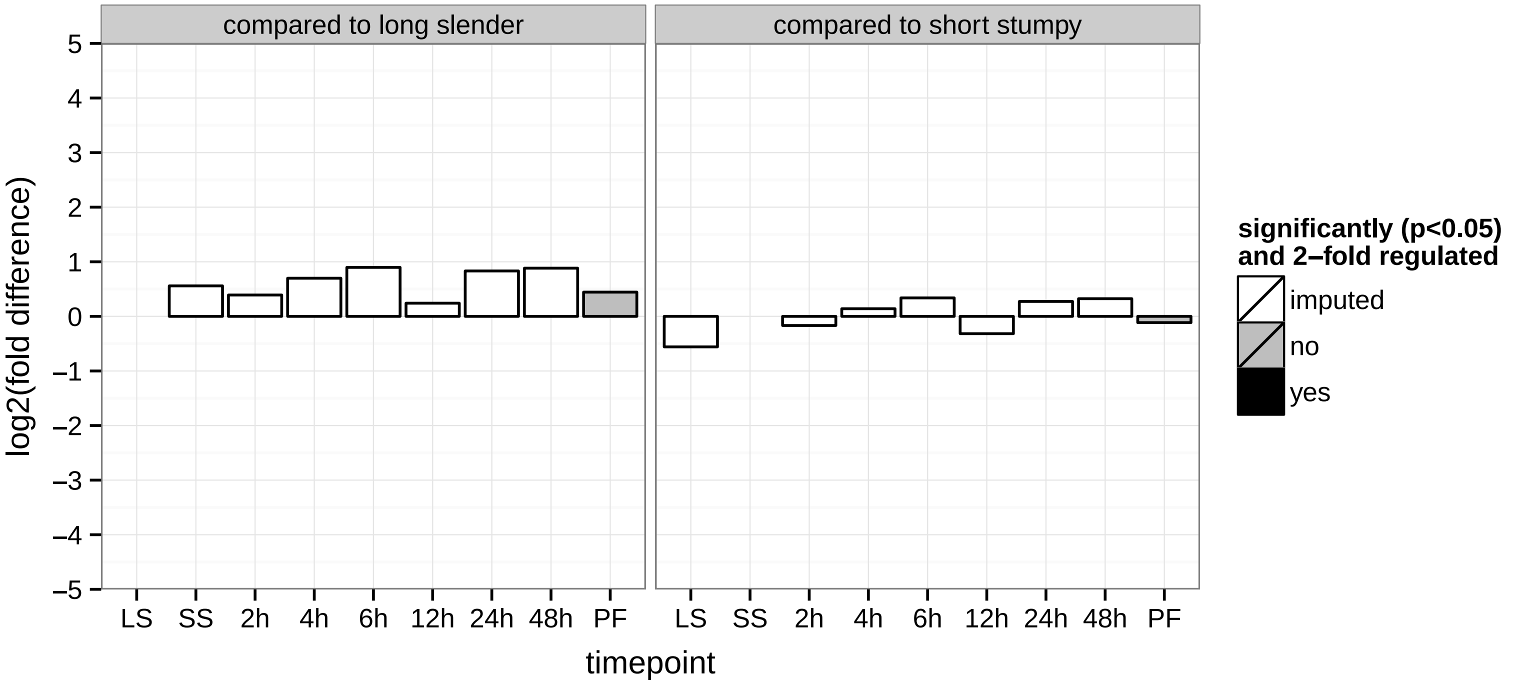

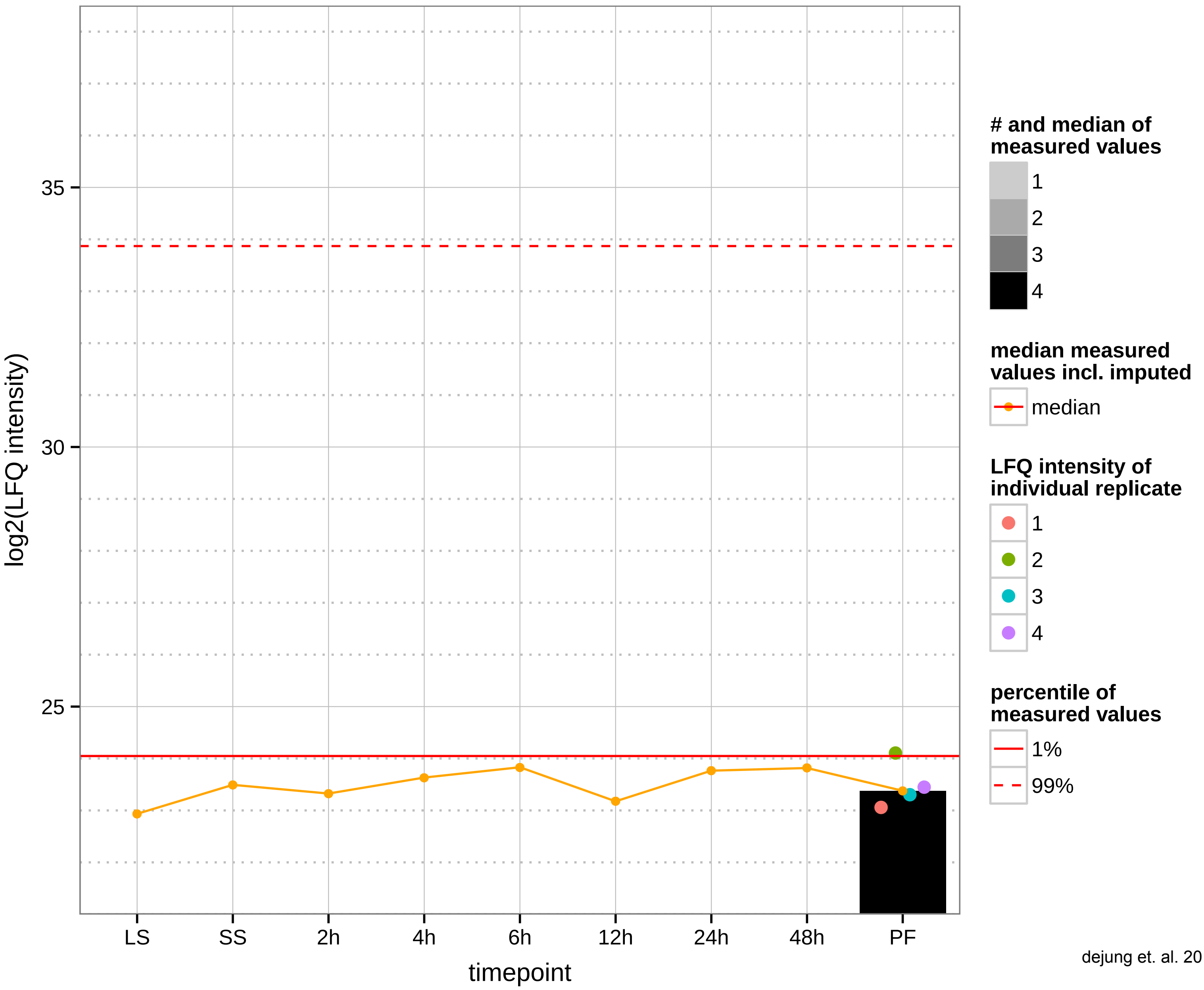

dejung et. al. 2015

hypothetical protein, conserved
Tb927.11.9400
AGOF: null
AGOC: null
AGOP: null
PGOF: null
PGOC: null
PGOP: null

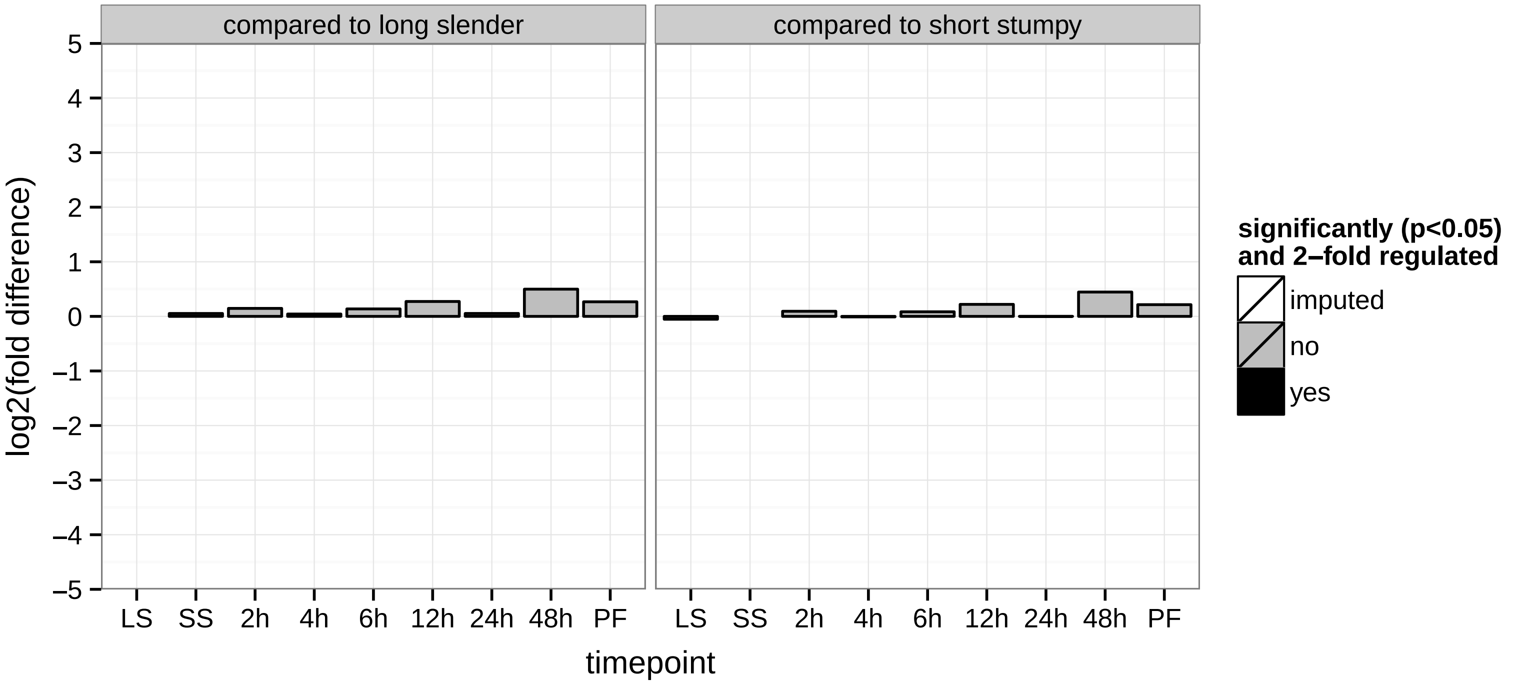

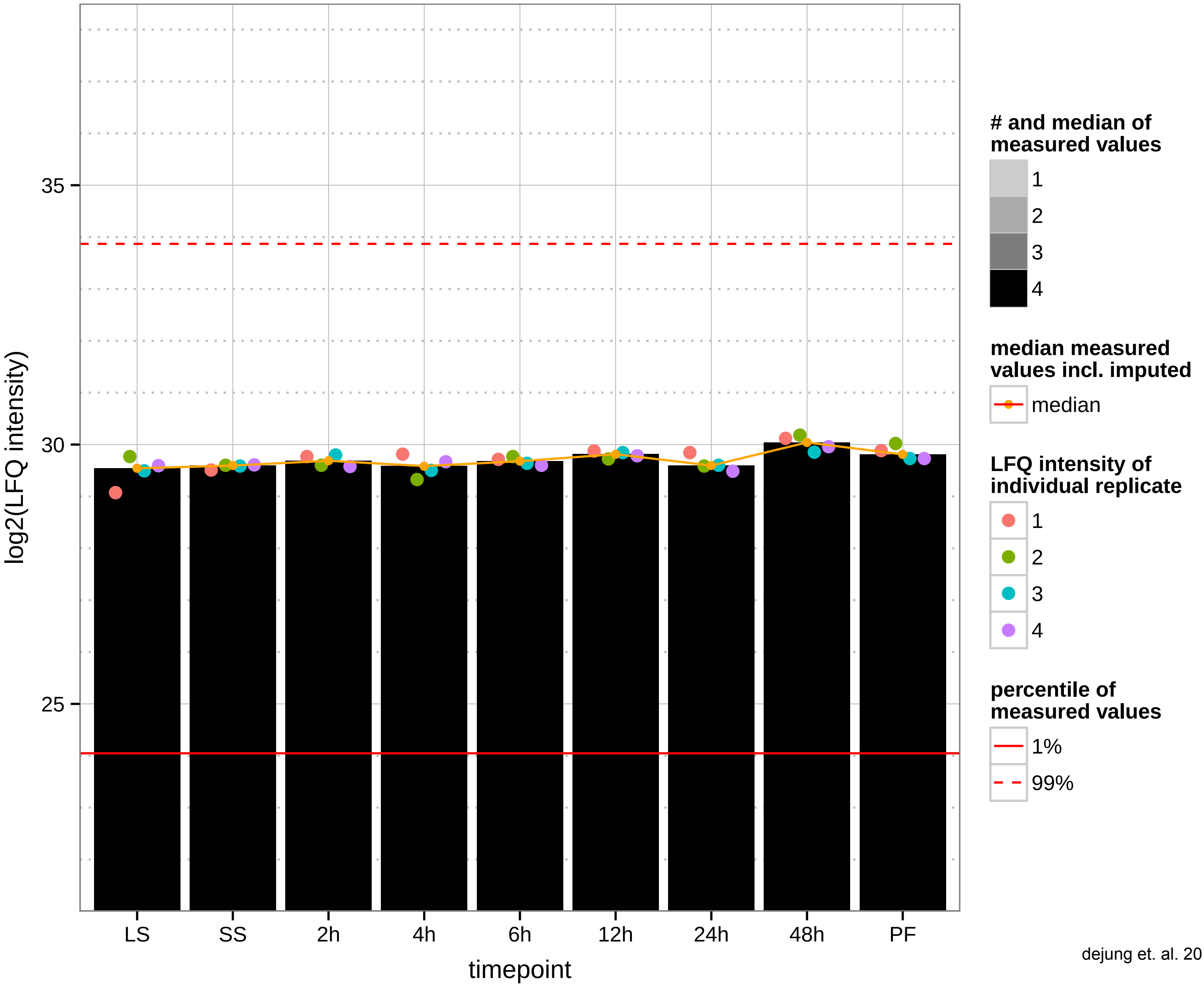

dejung et. al. 2015

TFIIH basal transcription factor subunit (p62)
Tb927.11.9430
AGOF: sequence−specific DNA binding transcription factor activity
AGOC: null
AGOP: null
PGOF: null
PGOC: null
PGOP: null

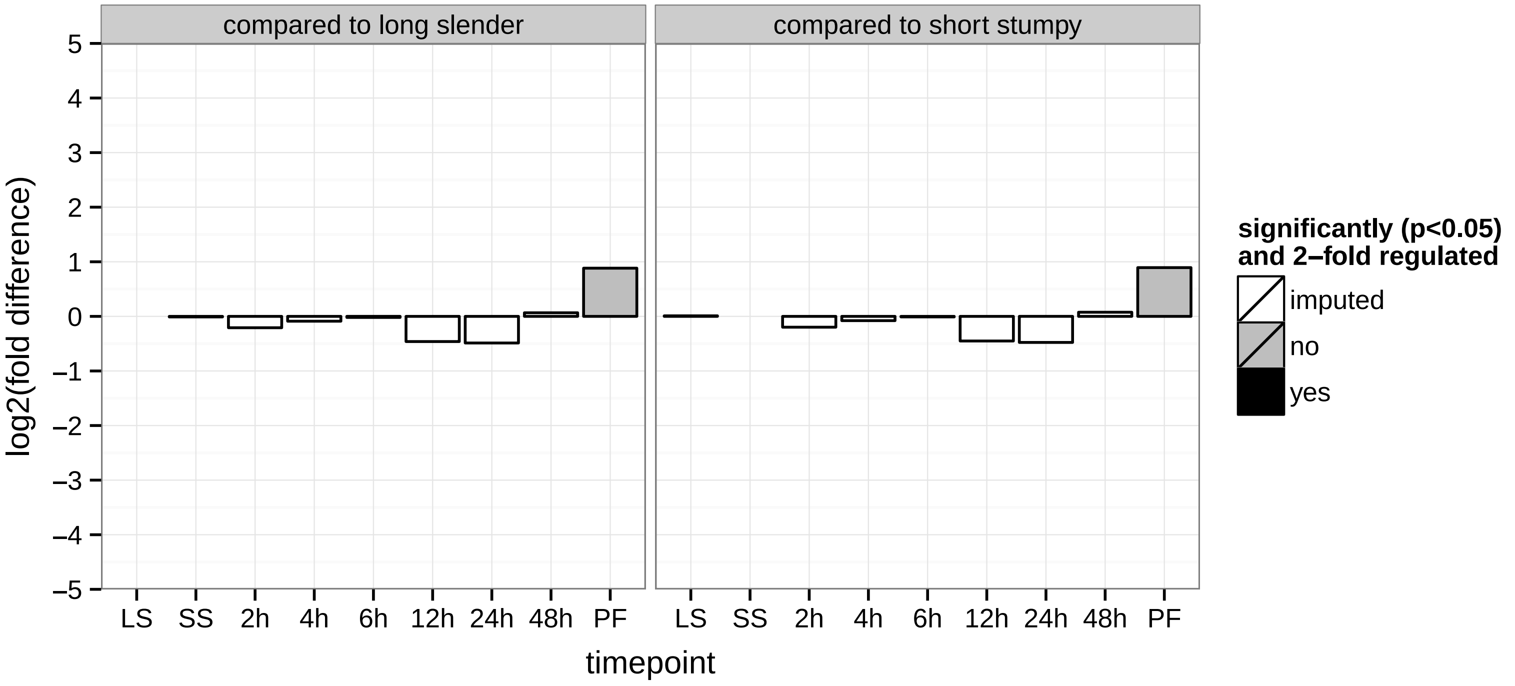

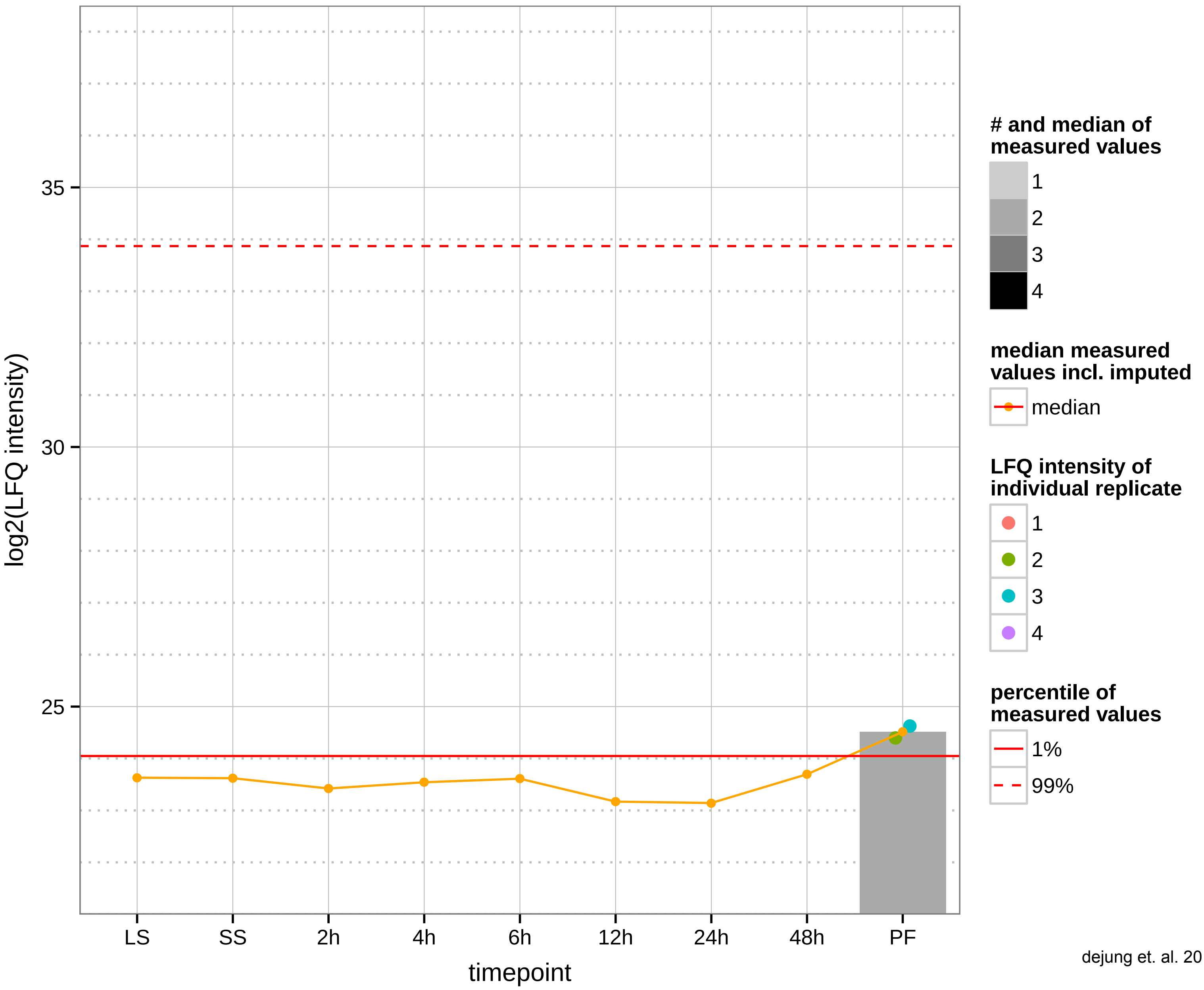

dejung et. al. 2015

hypothetical protein, conserved
Tb927.11.9440
AGOF: null
AGOC: null
AGOP: ciliary or flagellar motility
PGOF: null
PGOC: null
PGOP: null

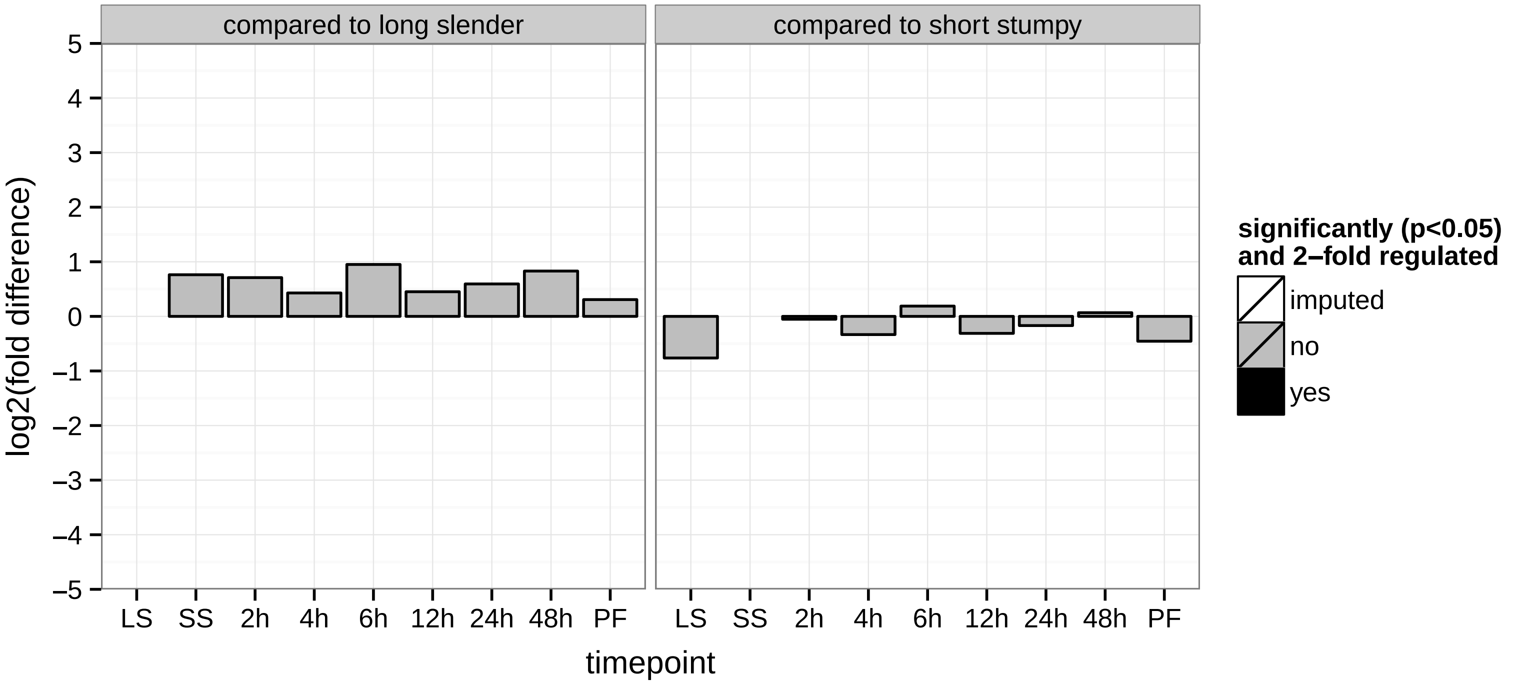

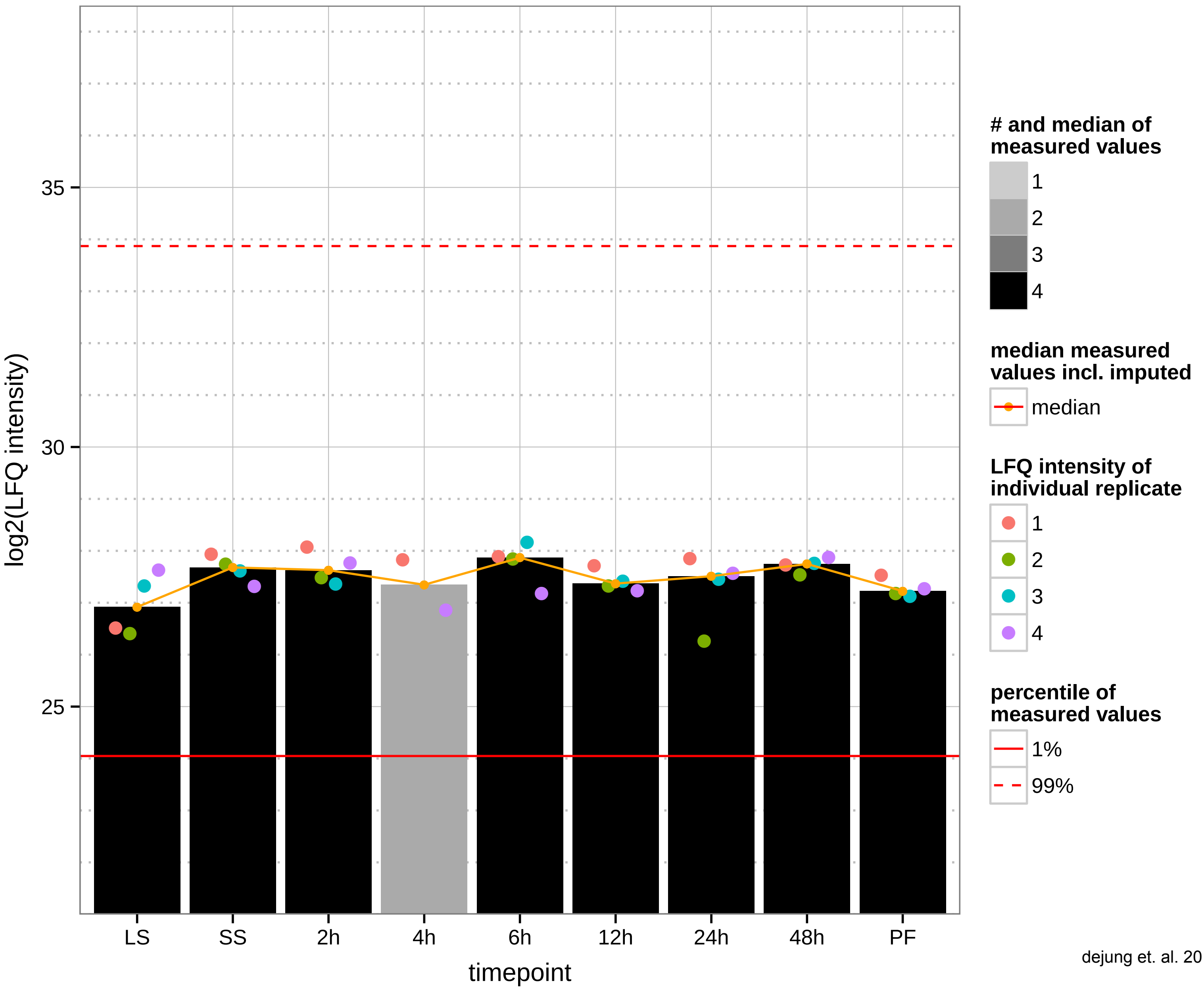

dejung et. al. 2015

hypothetical protein, conserved
Tb927.11.9490
AGOF: null
AGOC: null
AGOP: null
PGOF: null
PGOC: null
PGOP: null

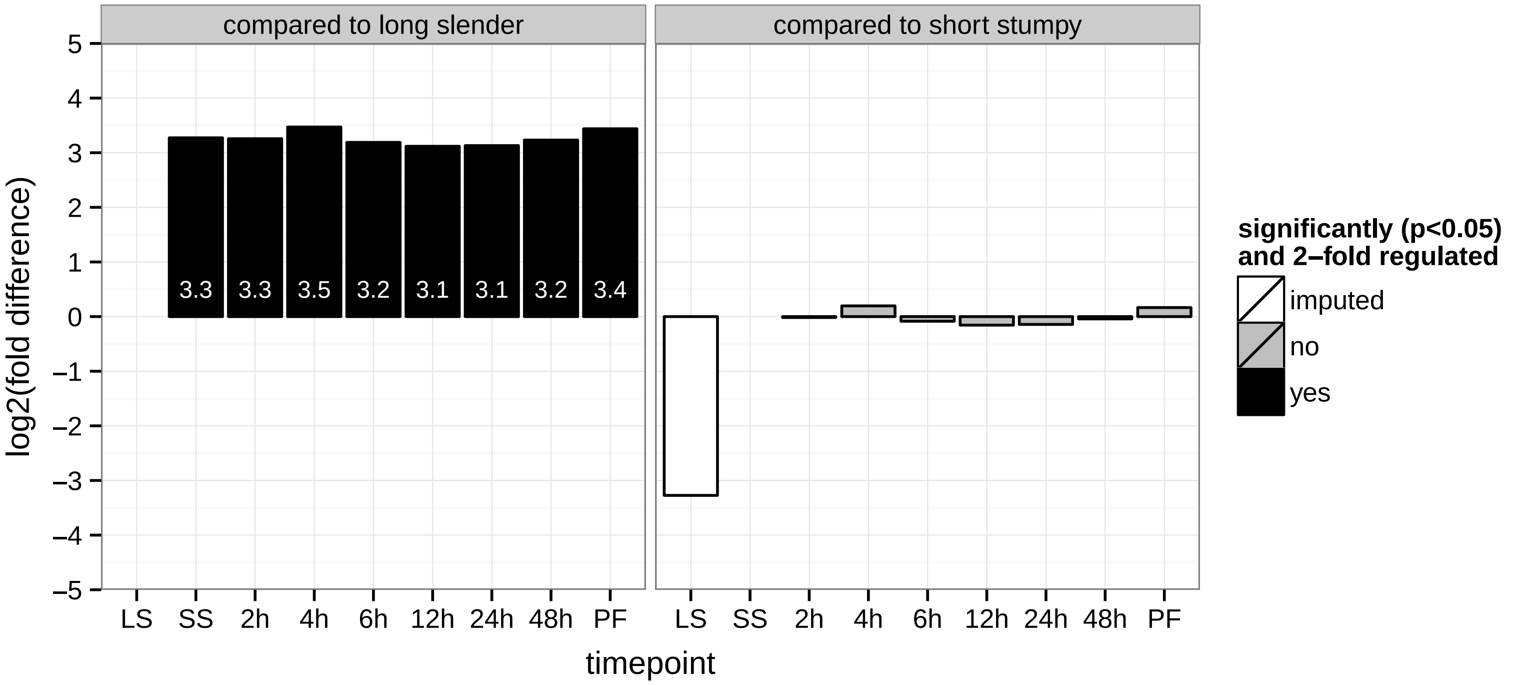

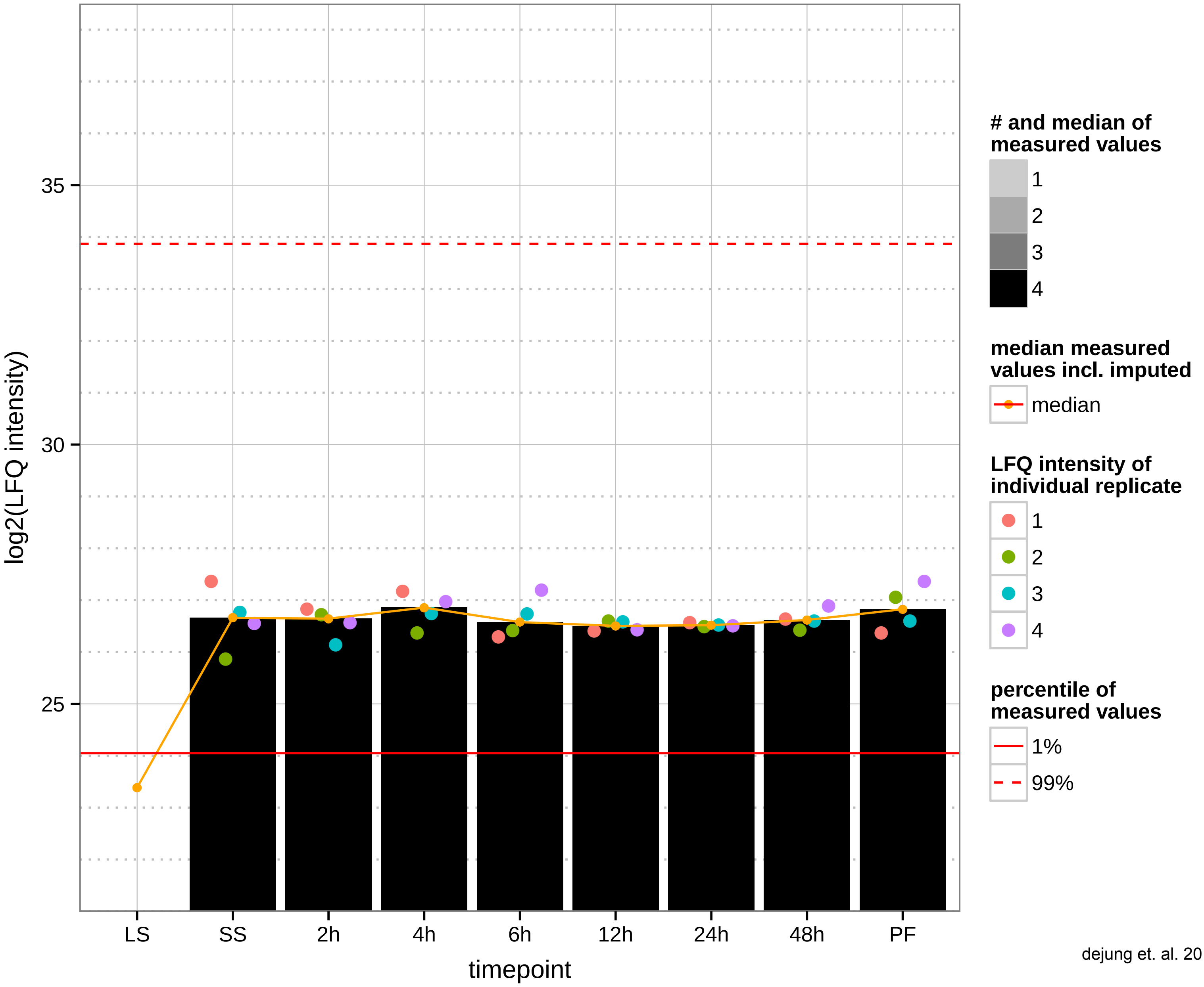

dejung et. al. 2015

14–3–3–I protein
Tb927.11.9530
AGOF: DNA binding, protein binding, protein domain specific binding
AGOC: eukaryotic translation initiation factor 4F complex
AGOP: Ras protein signal transduction, endocytosis
PGOF: protein domain specific binding
PGOC: null
PGOP: null

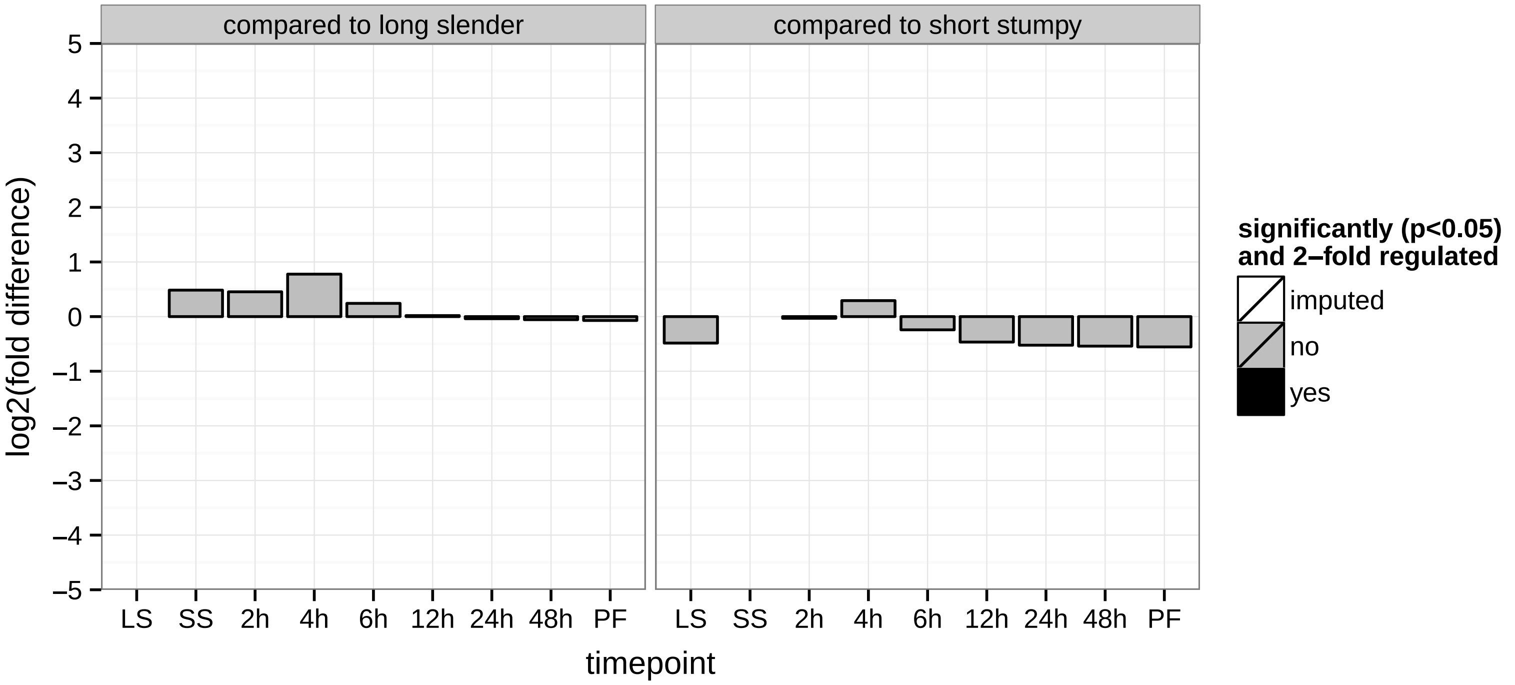

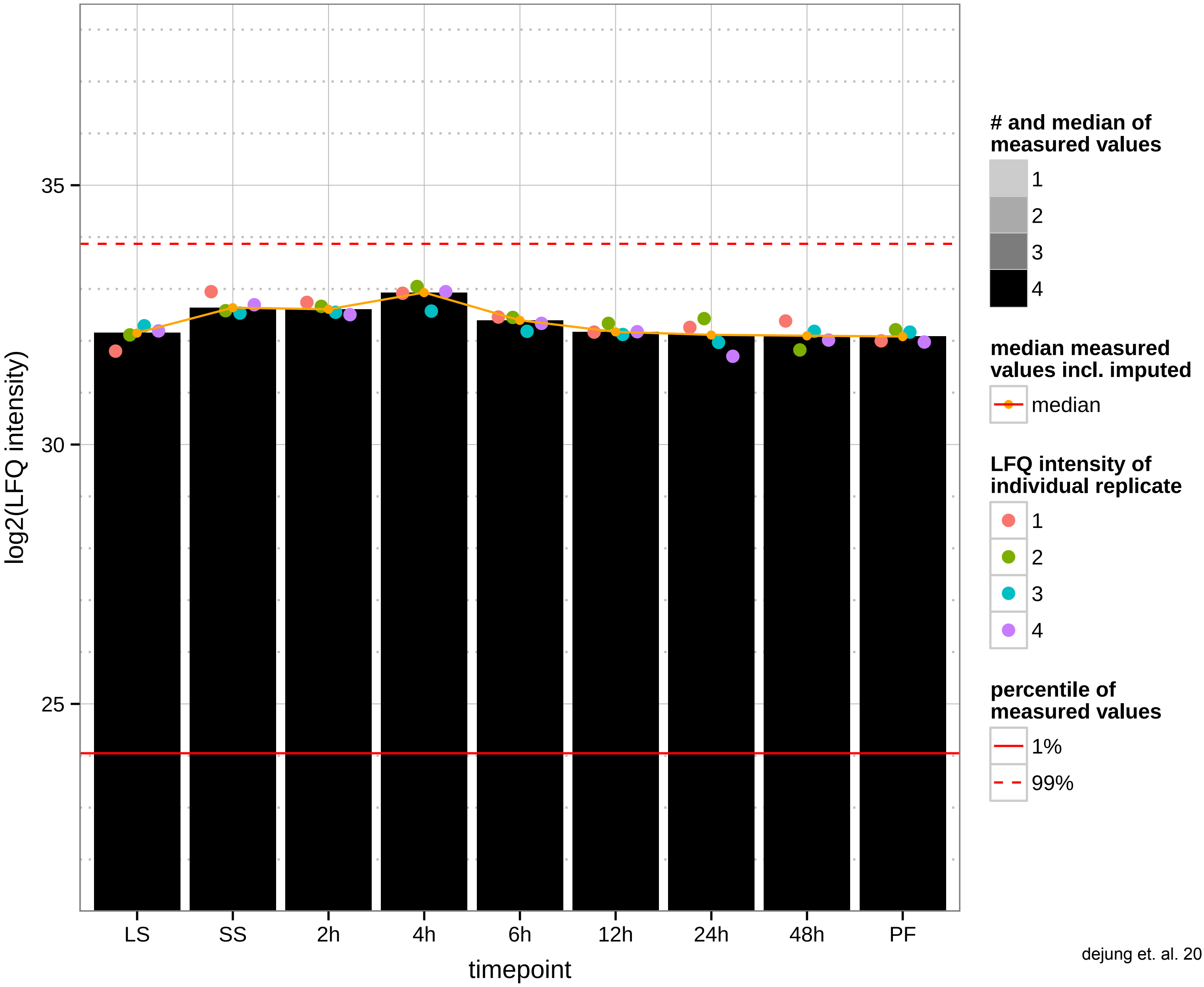

dejung et. al. 2015

replication factor C, subunit 4, putative
Tb927.11.9550
AGOF: ATP binding, DNA binding, DNA clamp loader activity
AGOC: DNA replication factor C complex
AGOP: DNA replication
PGOF: DNA binding, nucleoside–triphosphatase activity, nucleotide binding
PGOC: null
PGOP: DNA replication

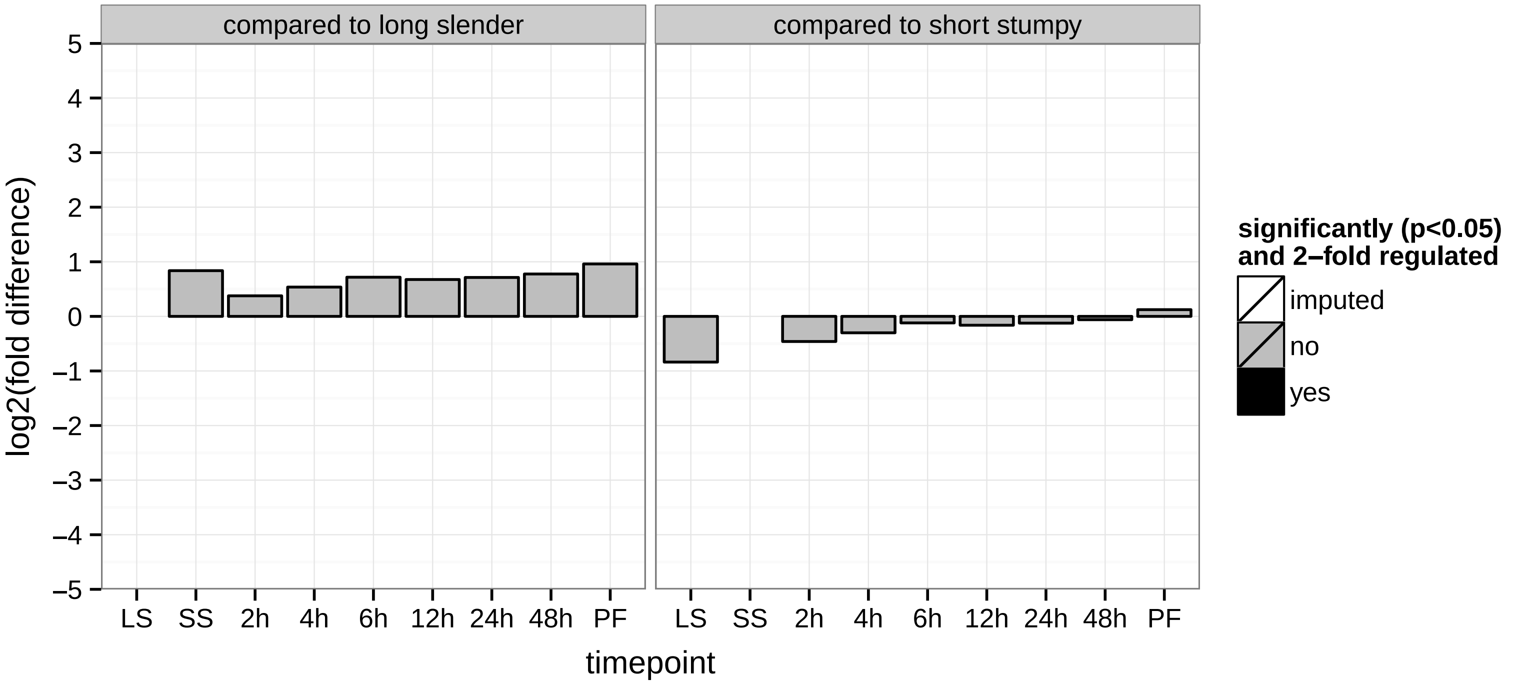

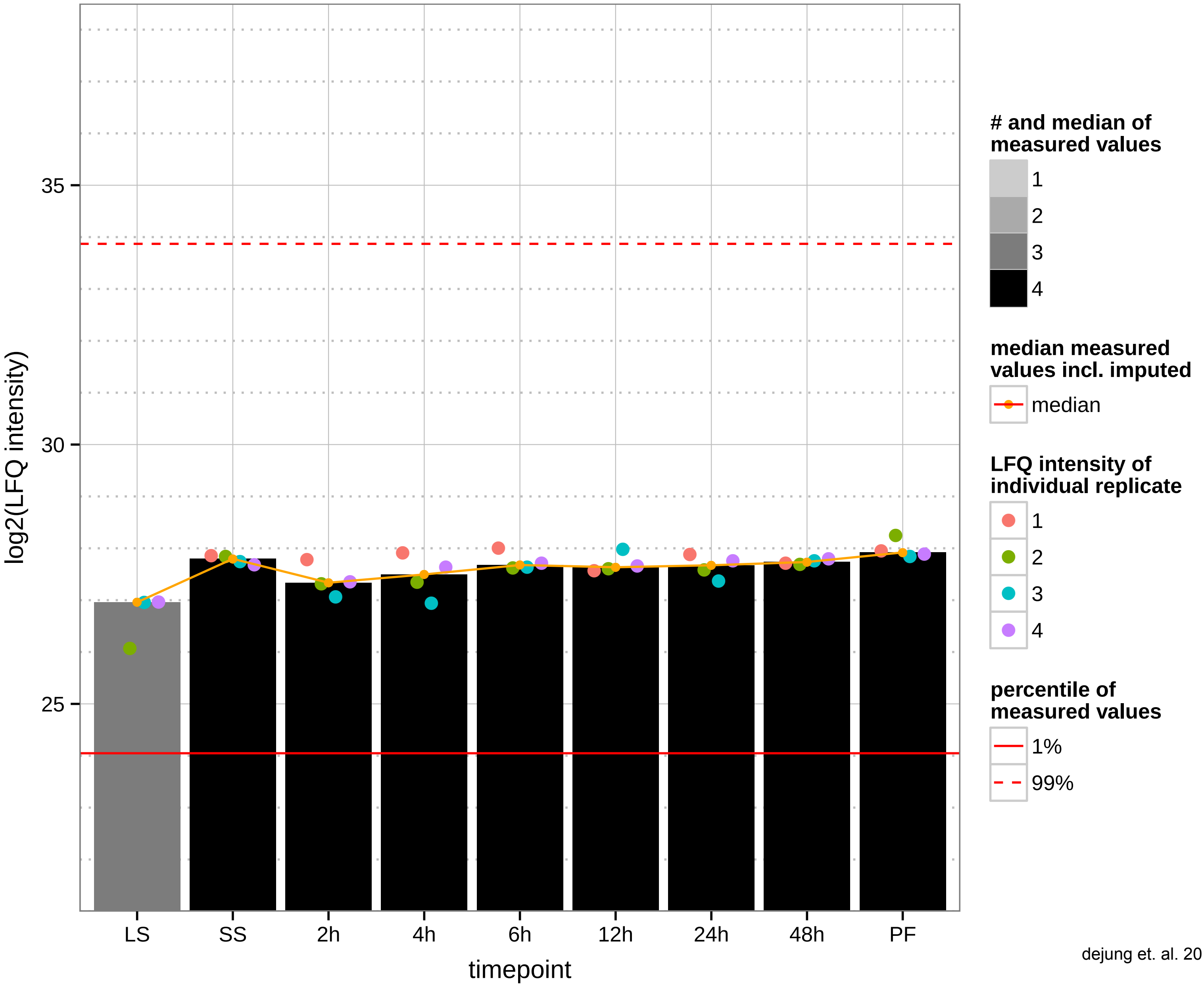

dejung et. al. 2015

eukaryotic translation initiation factor 3 subunit 2, putative (eIF−3 beta)
Tb927.11.9610
AGOF: translation initiation factor activity
AGOC: eukaryotic translation initiation factor 3 complex
AGOP: regulation of translational initiation
PGOF: protein binding
PGOC: null
PGOP: null

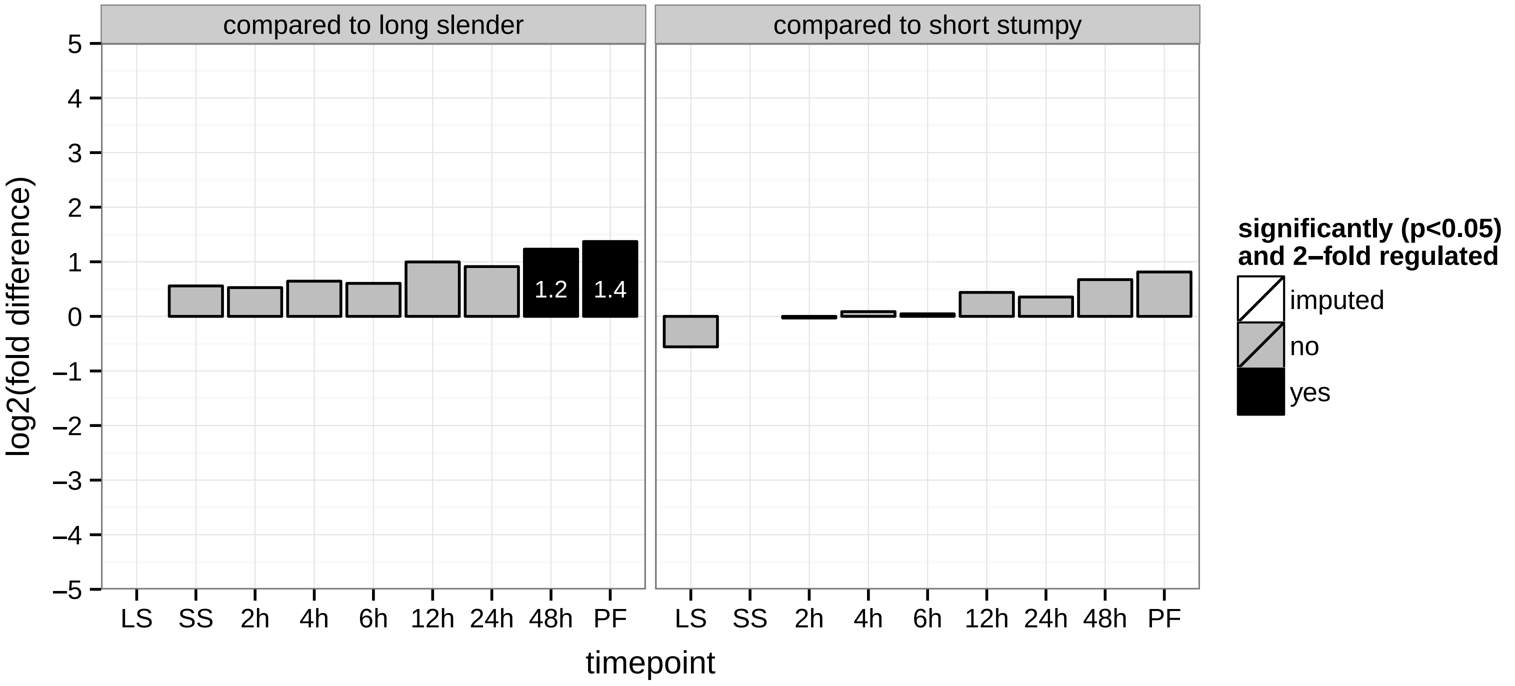

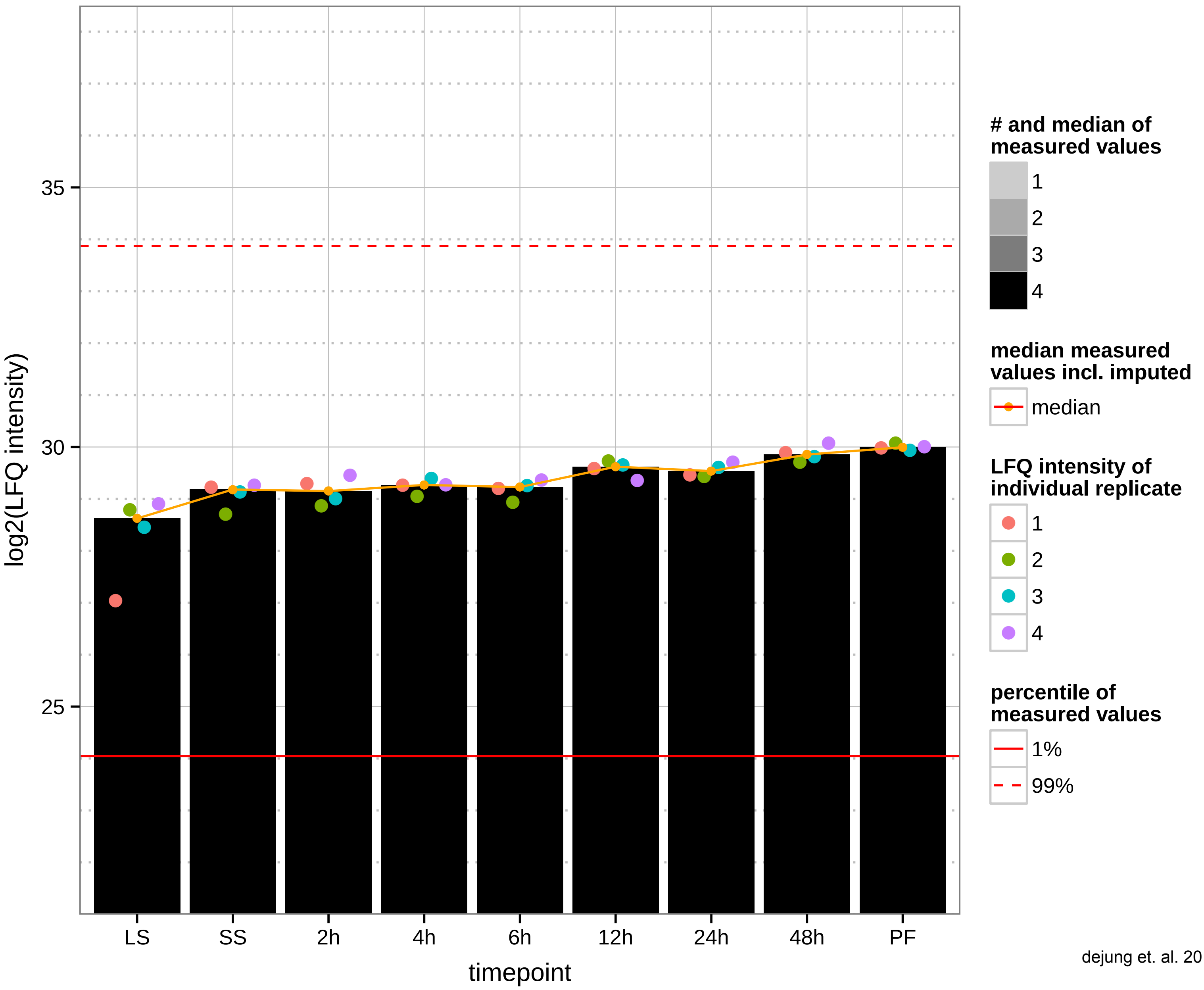

dejung et. al. 2015

glycyl–tRNA synthetase, putative
Tb927.11.9640
AGOF: ATP binding, glycine–tRNA ligase activity
AGOC: cytoplasm, glycine–tRNA ligase complex
AGOP: glycyl–tRNA aminoacylation, translation
PGOF: ATP binding, aminoacyl–tRNA ligase activity, glycine–tRNA ligase activity, nucleotide binding
PGOC: cytoplasm
PGOP: glycyl–tRNA aminoacylation, tRNA aminoacylation for protein translation

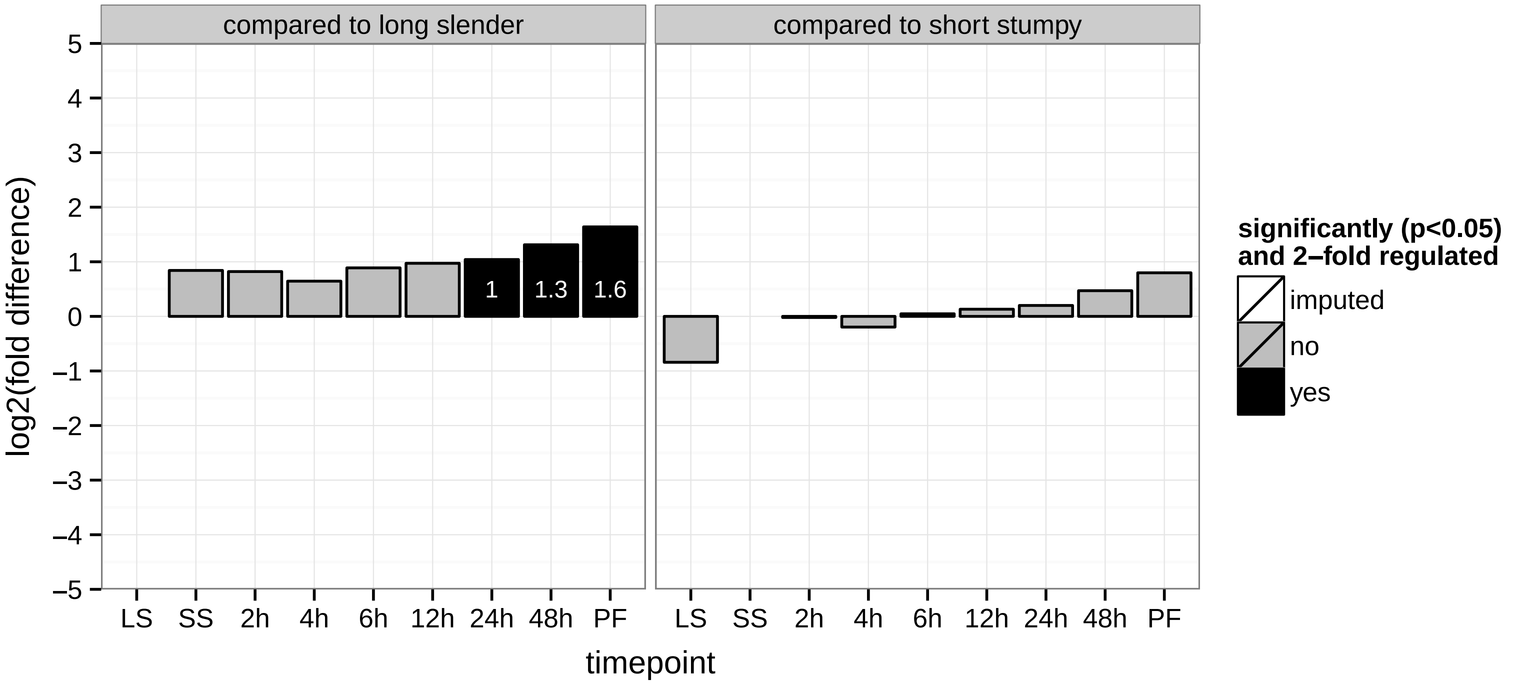

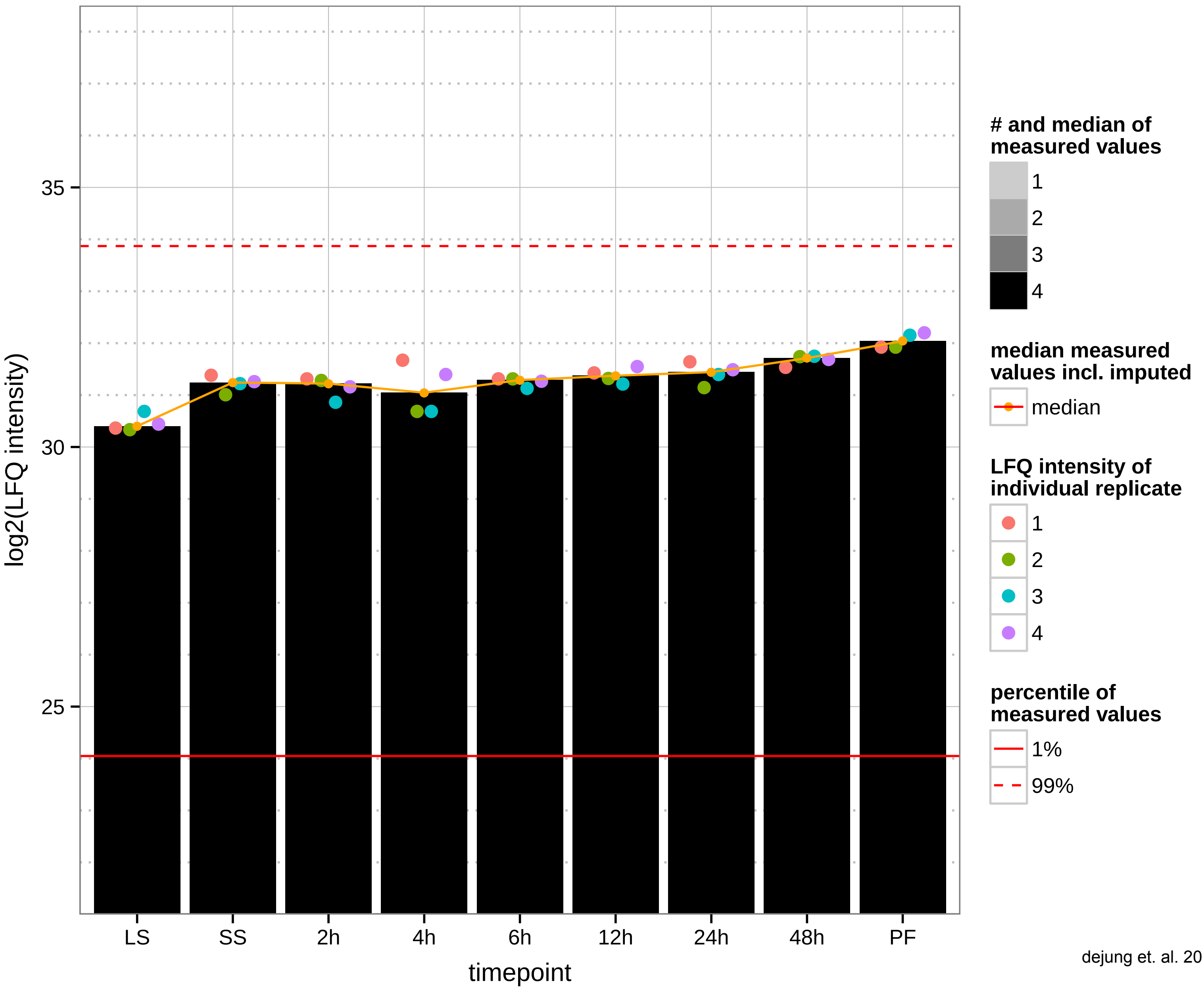

dejung et. al. 2015

40S ribosomal protein S27, putative
Tb927.11.9720
AGOF: structural constituent of ribosome
AGOC: intracellular, ribosome
AGOP: translation
PGOF: structural constituent of ribosome
PGOC: intracellular, ribosome
PGOP: translation

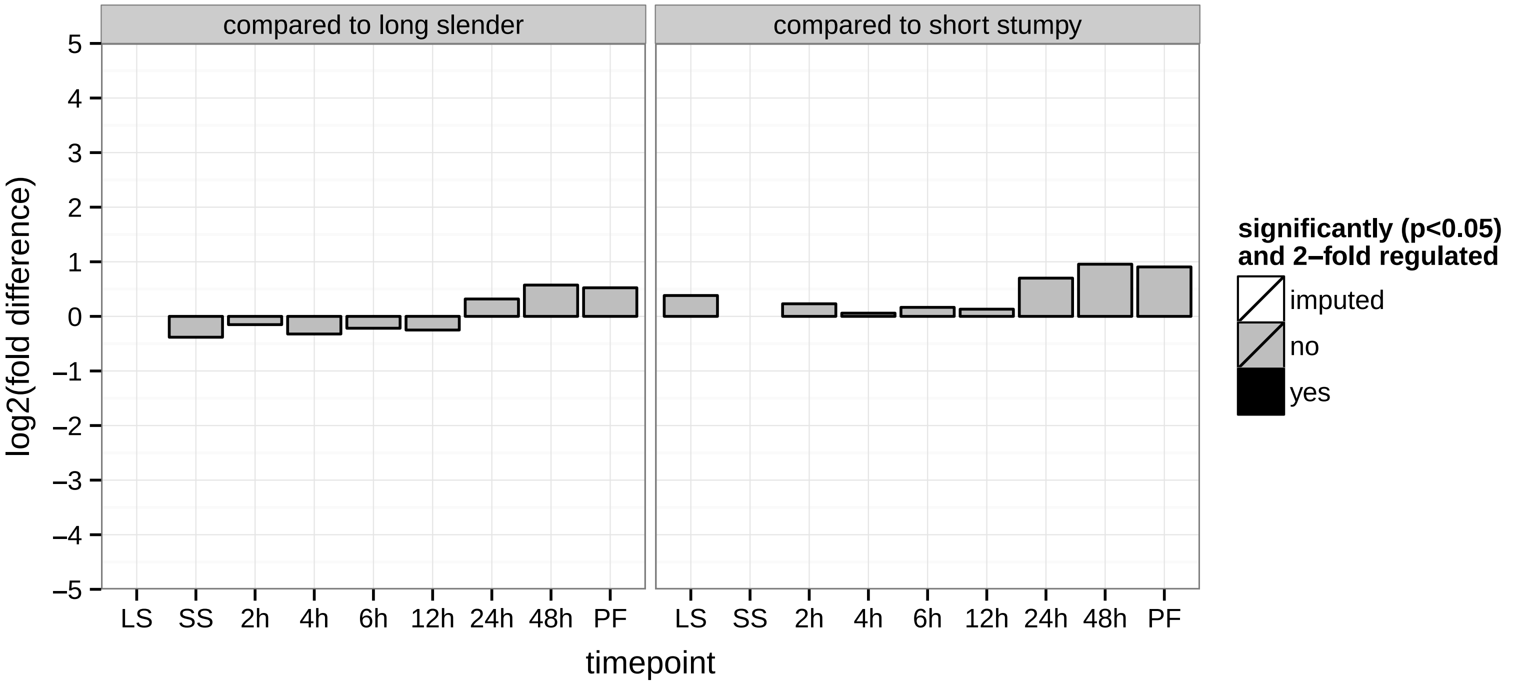

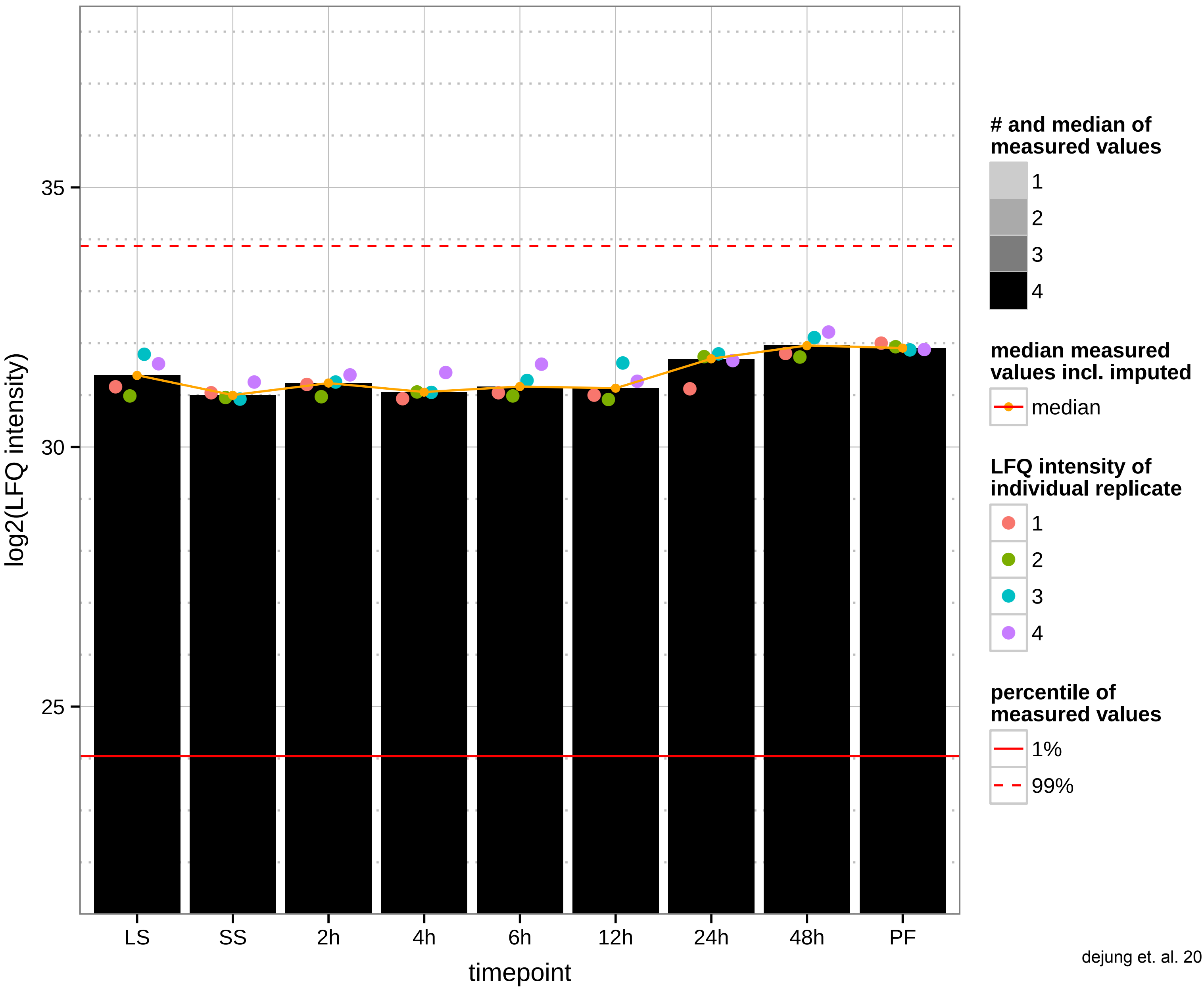

dejung et. al. 2015

hypothetical protein, conserved
Tb927.11.9760
AGOF: null
AGOC: null
AGOP: null
PGOF: null
PGOC: null
PGOP: null

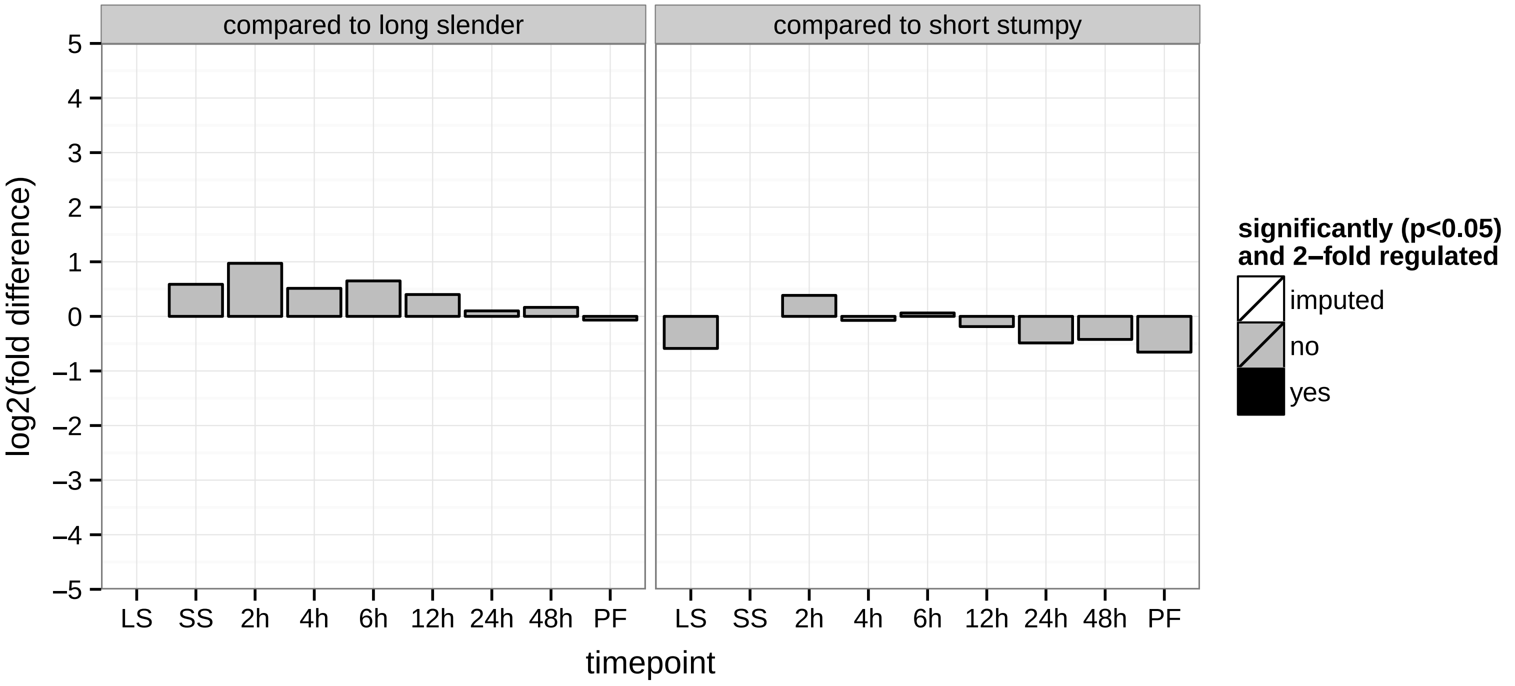

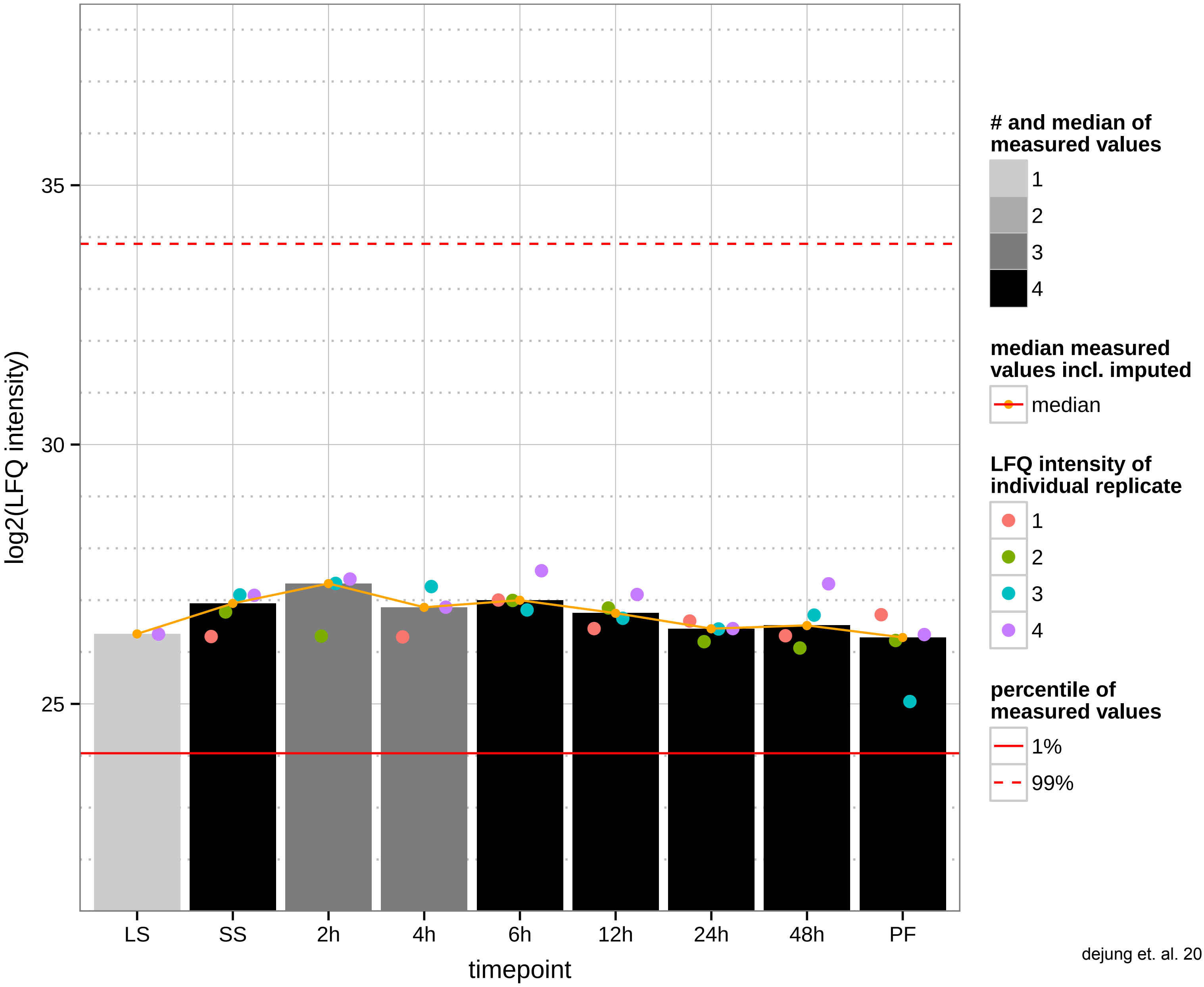

dejung et. al. 2015

Nucleoporin, serine peptidase, Clan SP, family S59, putative (TbNup158)
Tb927.11.980
AGOF: null
AGOC: membrane, nuclear pore
AGOP: protein homooligomerization, transport
PGOF: null
PGOC: nuclear pore
PGOP: transport

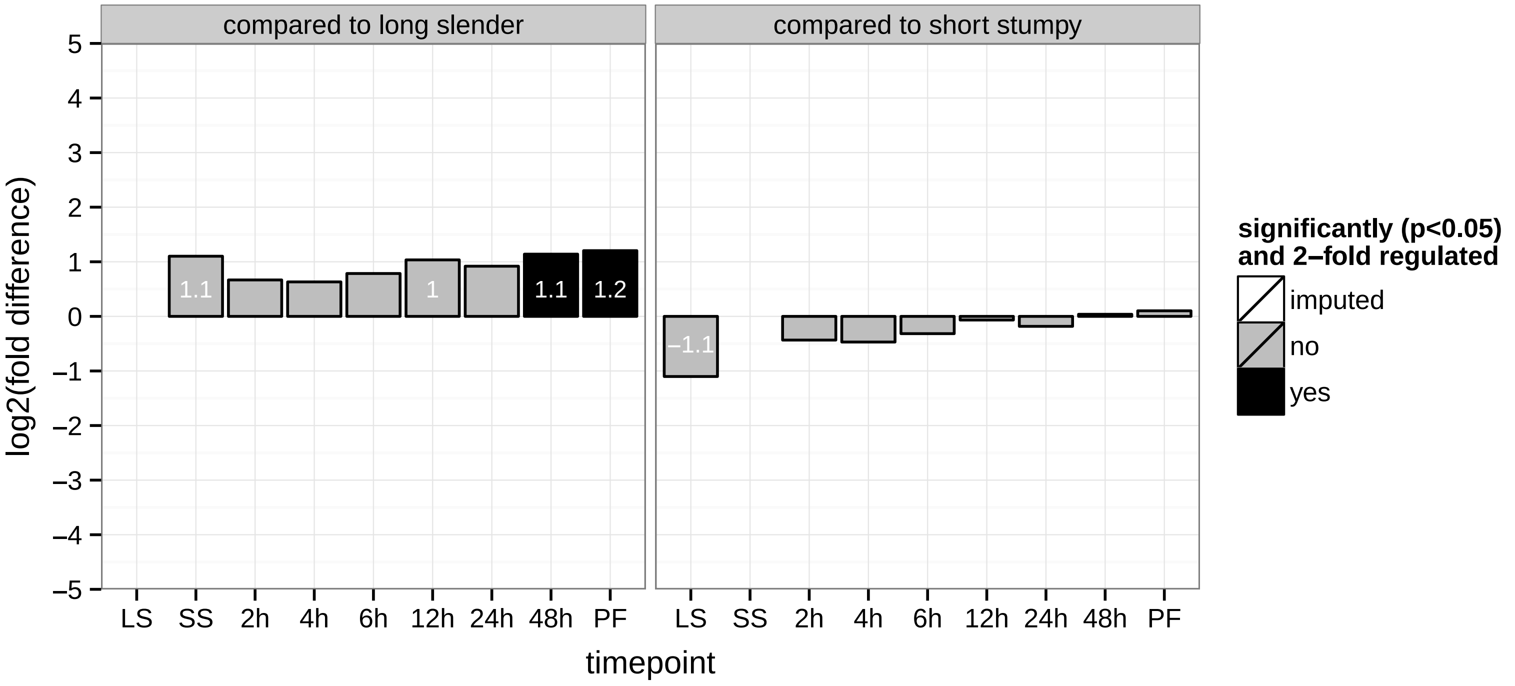

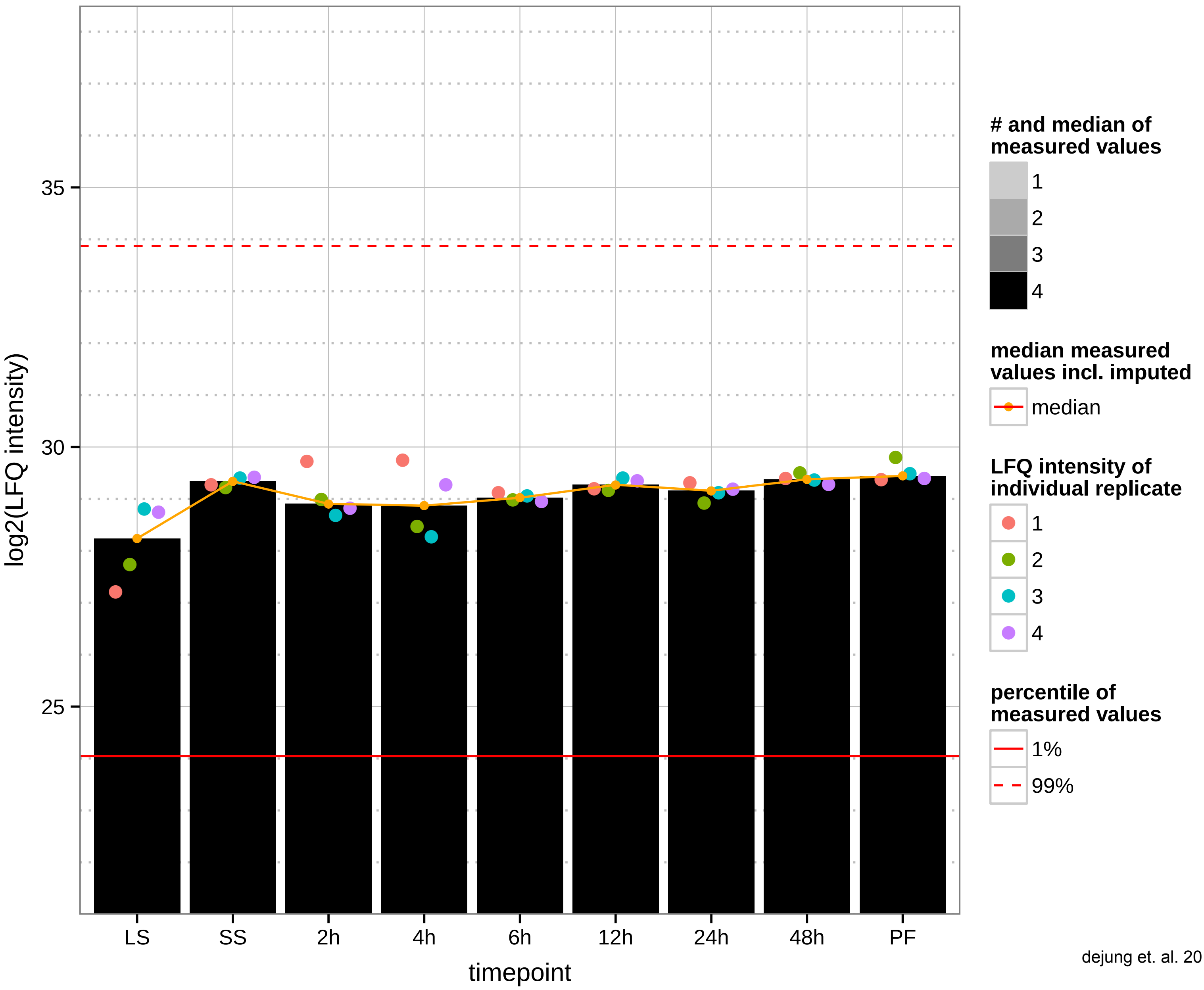

dejung et. al. 2015

hypothetical protein, conserved
Tb927.11.9860
AGOF: calcium ion binding
AGOC: null
AGOP: null
PGOF: null
PGOC: null
PGOP: null

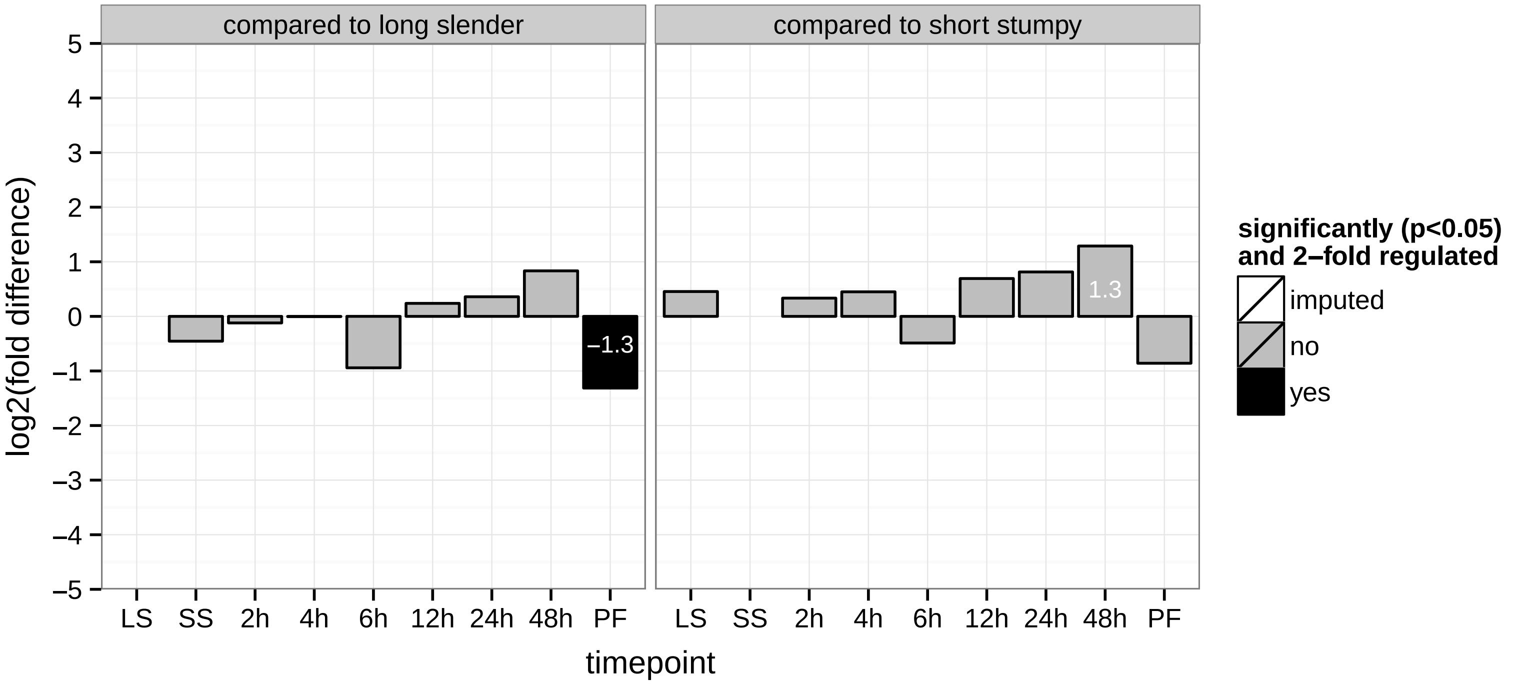

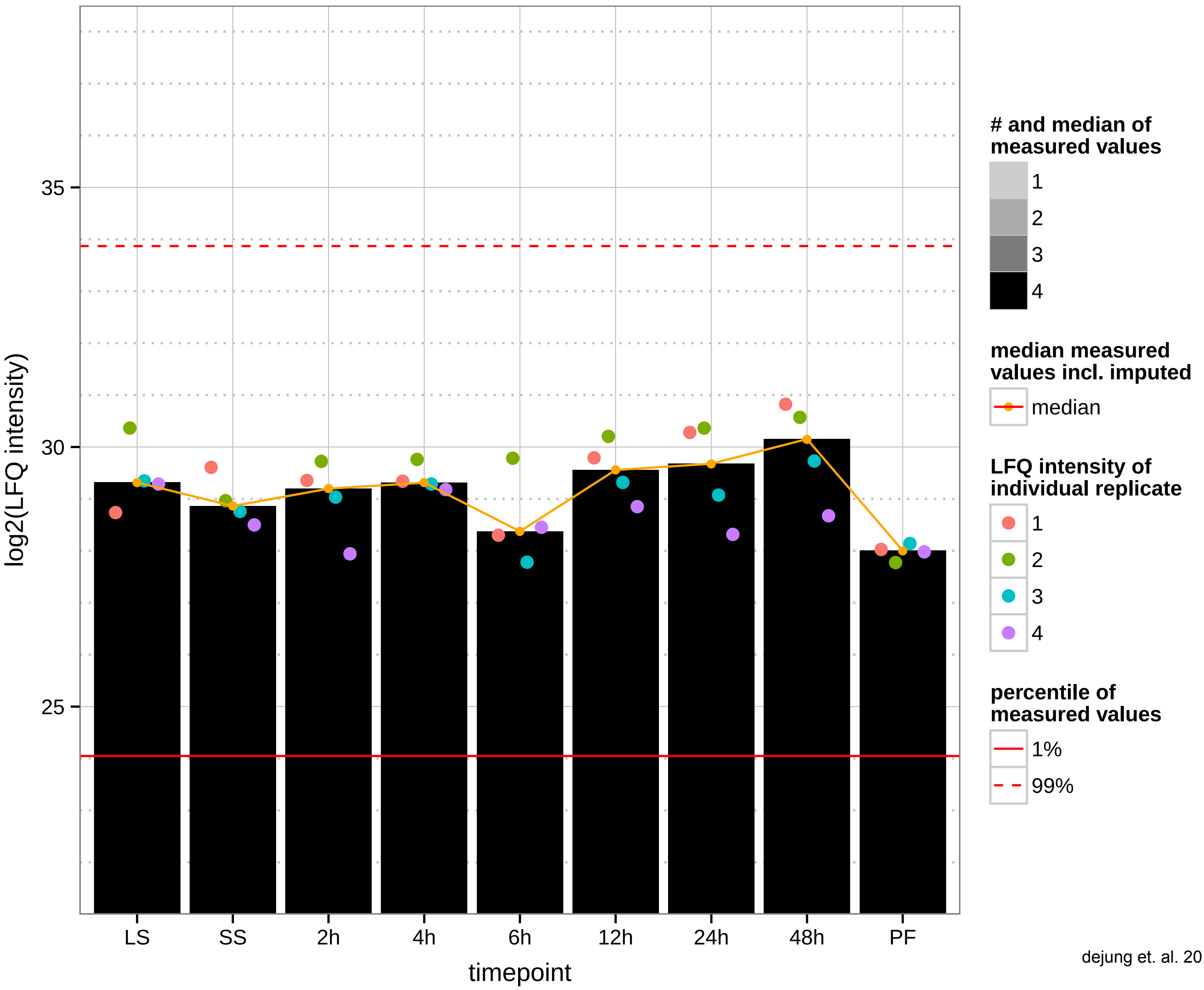

dejung et. al. 2015

hypothetical protein, conserved
Tb927.11.9880
AGOF: null
AGOC: null
AGOP: null
PGOF: null
PGOC: null
PGOP: null

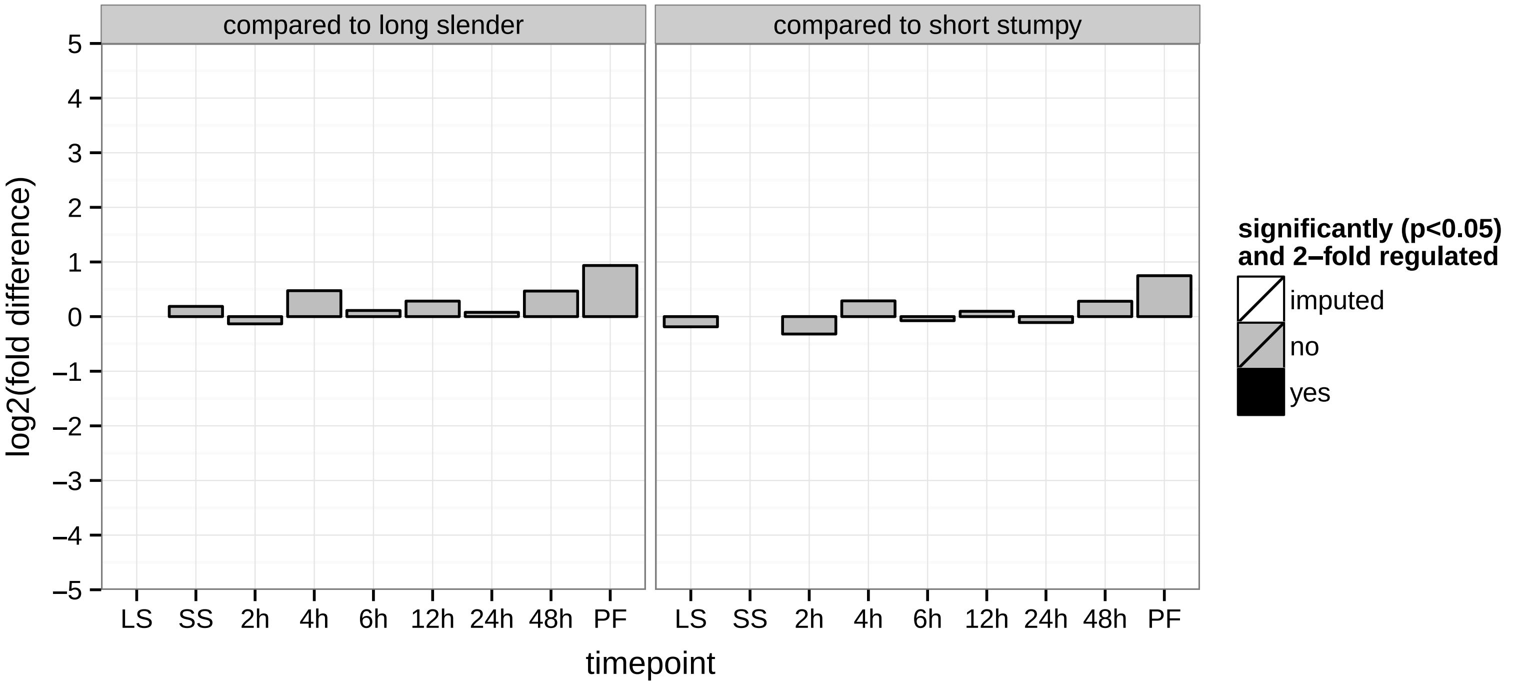

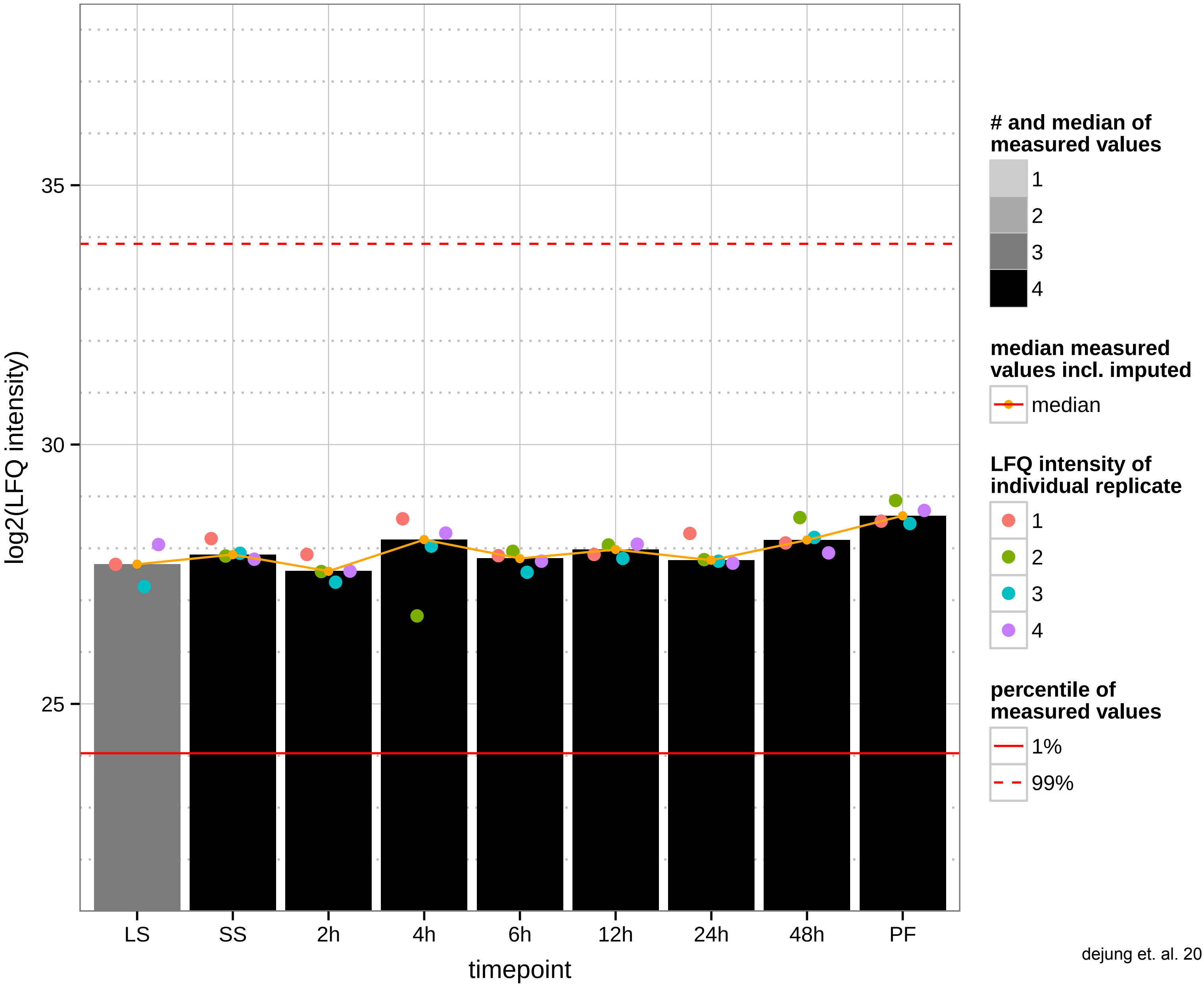

dejung et. al. 2015

NADH–ubiquinone oxidoreductase complex I subunit, putative
Tb927.11.9930
AGOF: NADH dehydrogenase (ubiquinone) activity, oxidoreductase activity
AGOC: mitochondrial respiratory chain complex I, mitochondrion
AGOP: mitochondrial electron transport, NADH to ubiquinone
PGOF: null
PGOC: null
PGOP: null

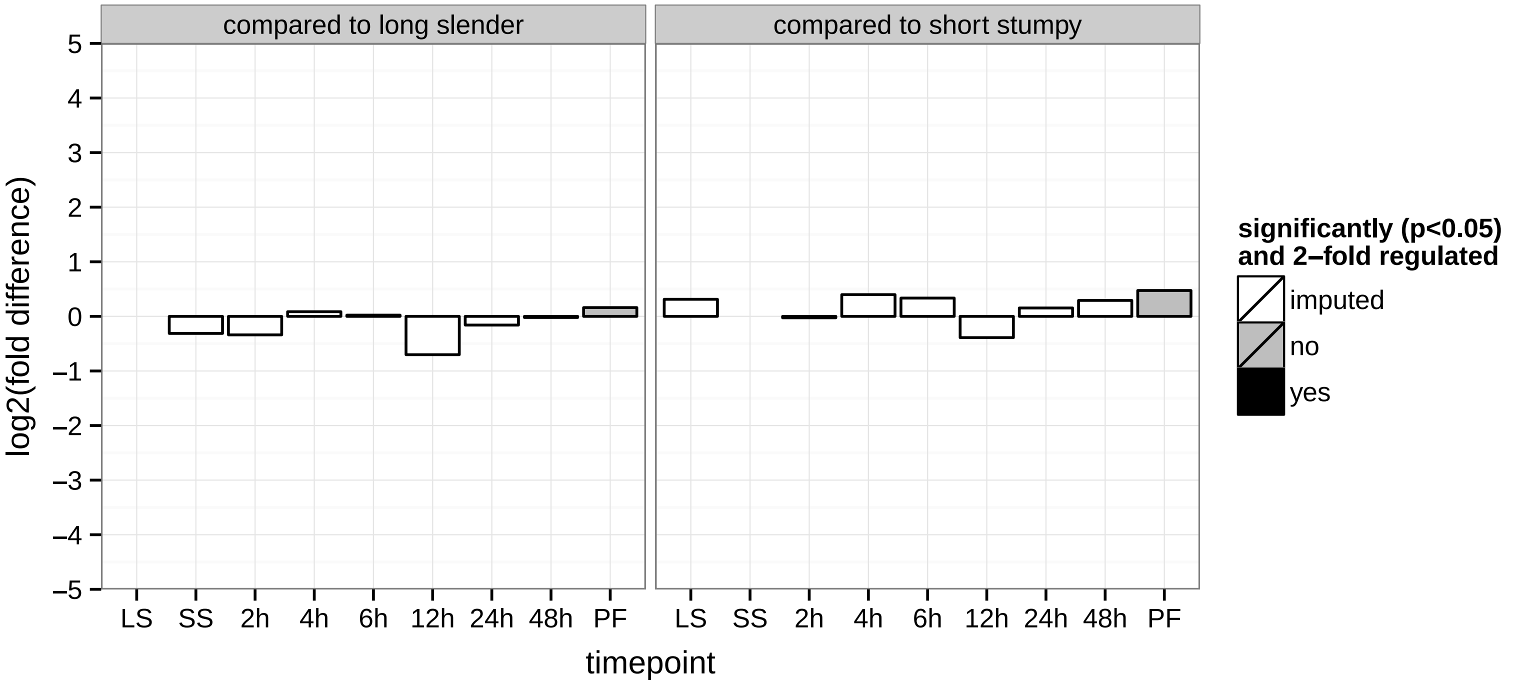

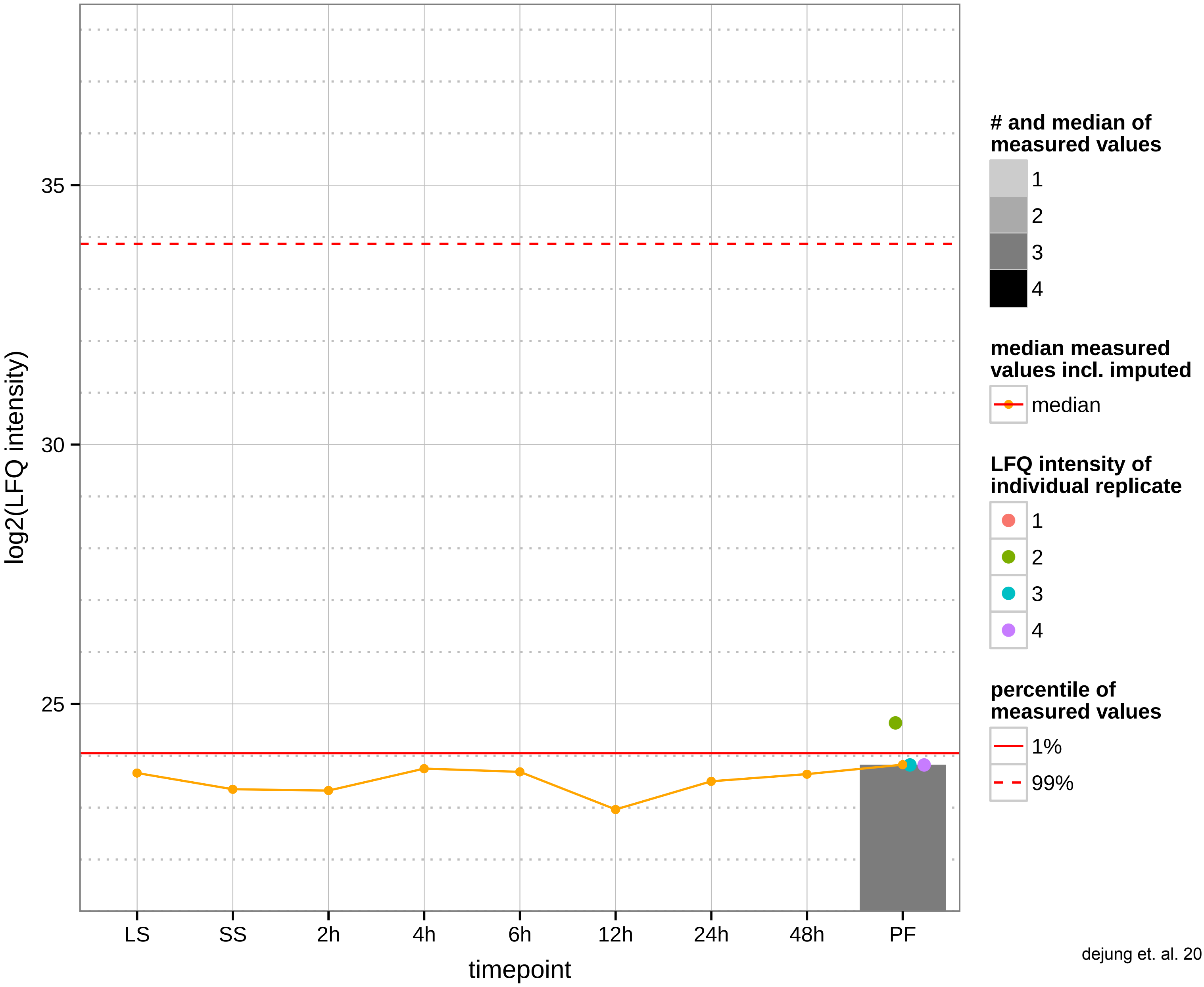

dejung et. al. 2015

retrotransposon hot spot protein 1 (RHS1), putative, retrotransposon hot spot protein (RHS, pseudogene), interrupted
Tb927.2.100
AGOF: null
AGOC: nucleus, null
AGOP: null
PGOF: null
PGOC: null
PGOP: null

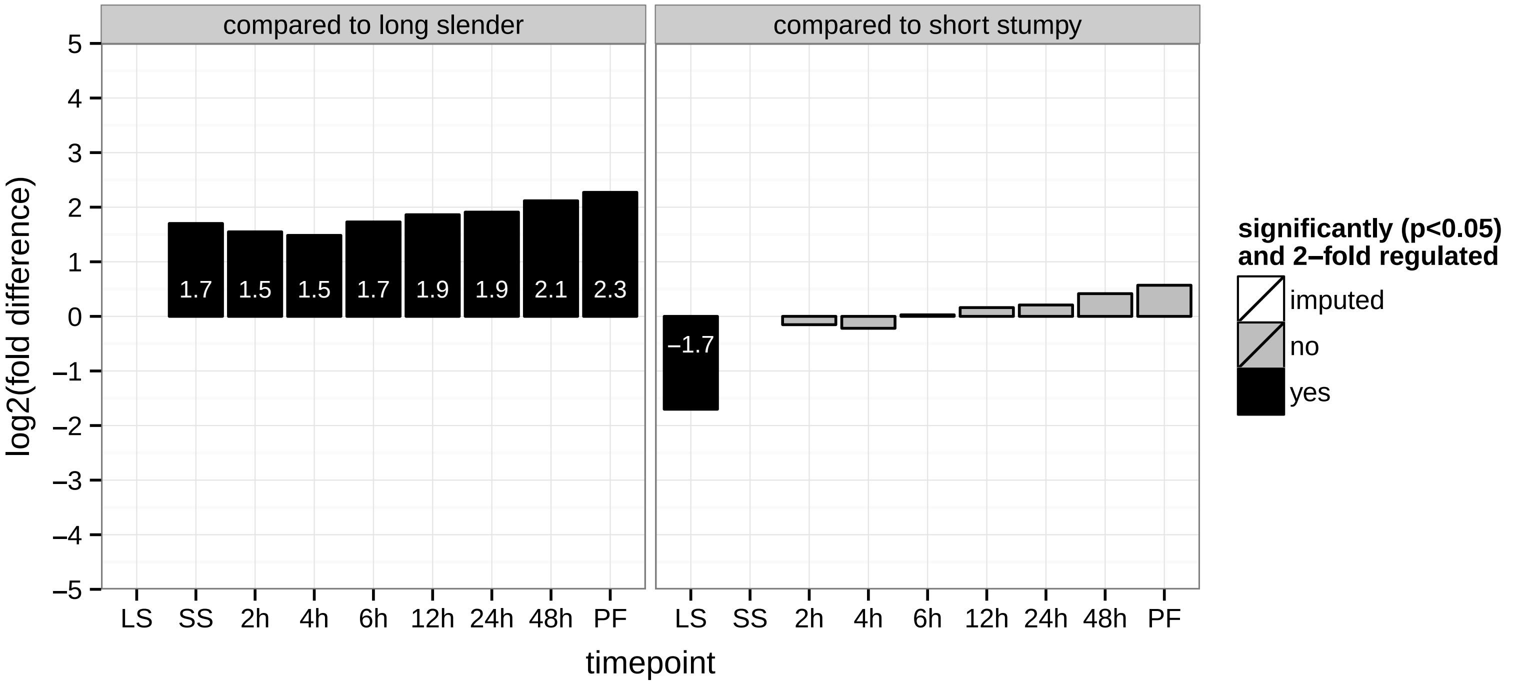

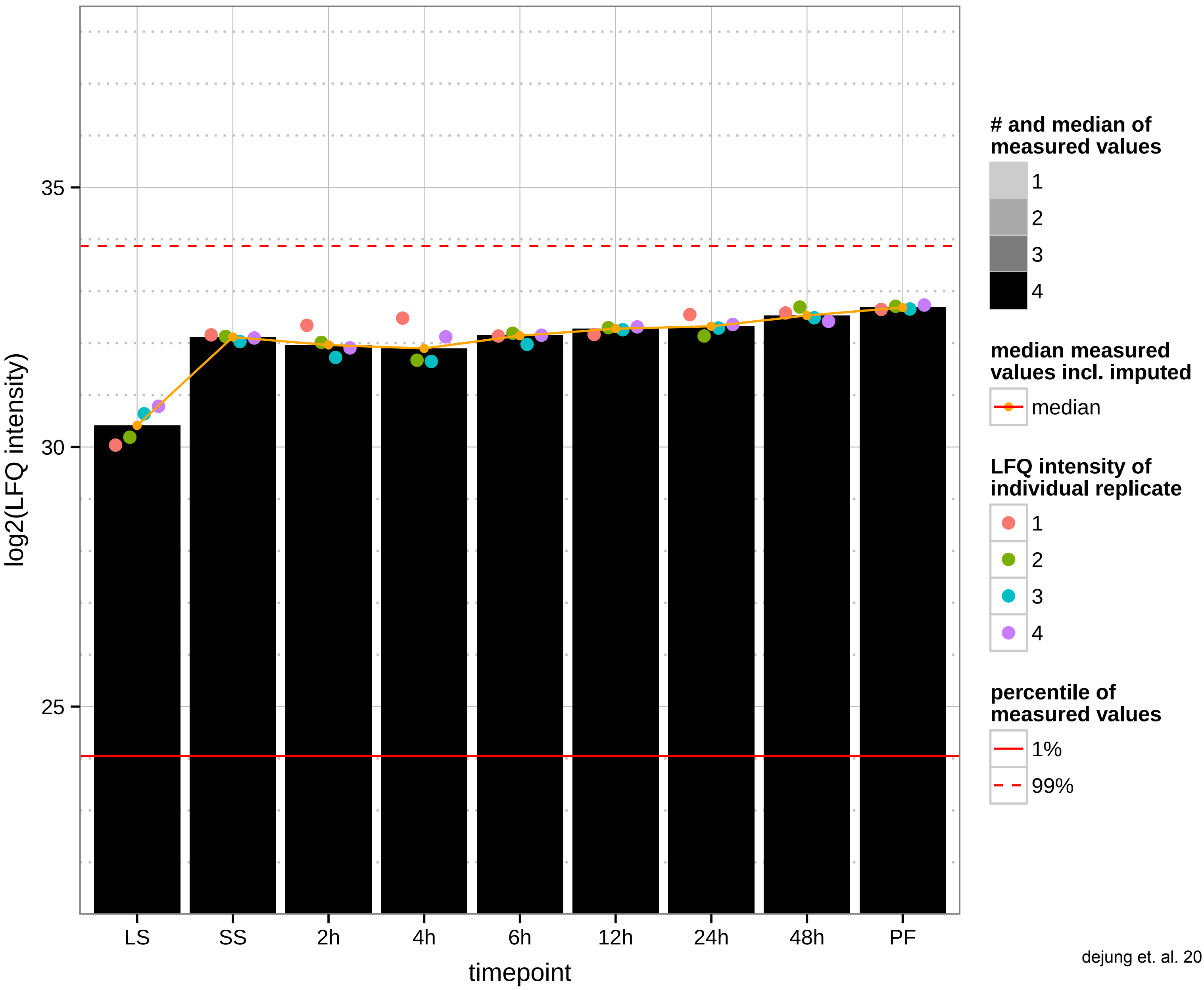

dejung et. al. 2015

retrotransposon hot spot protein (RHS, pseudogene), putative, retrotransposon hot spot protein 4 (RHS4), point mutation
Tb927.2.1120
AGOF: null
AGOC: null
AGOP: null
PGOF: null
PGOC: null
PGOP: null

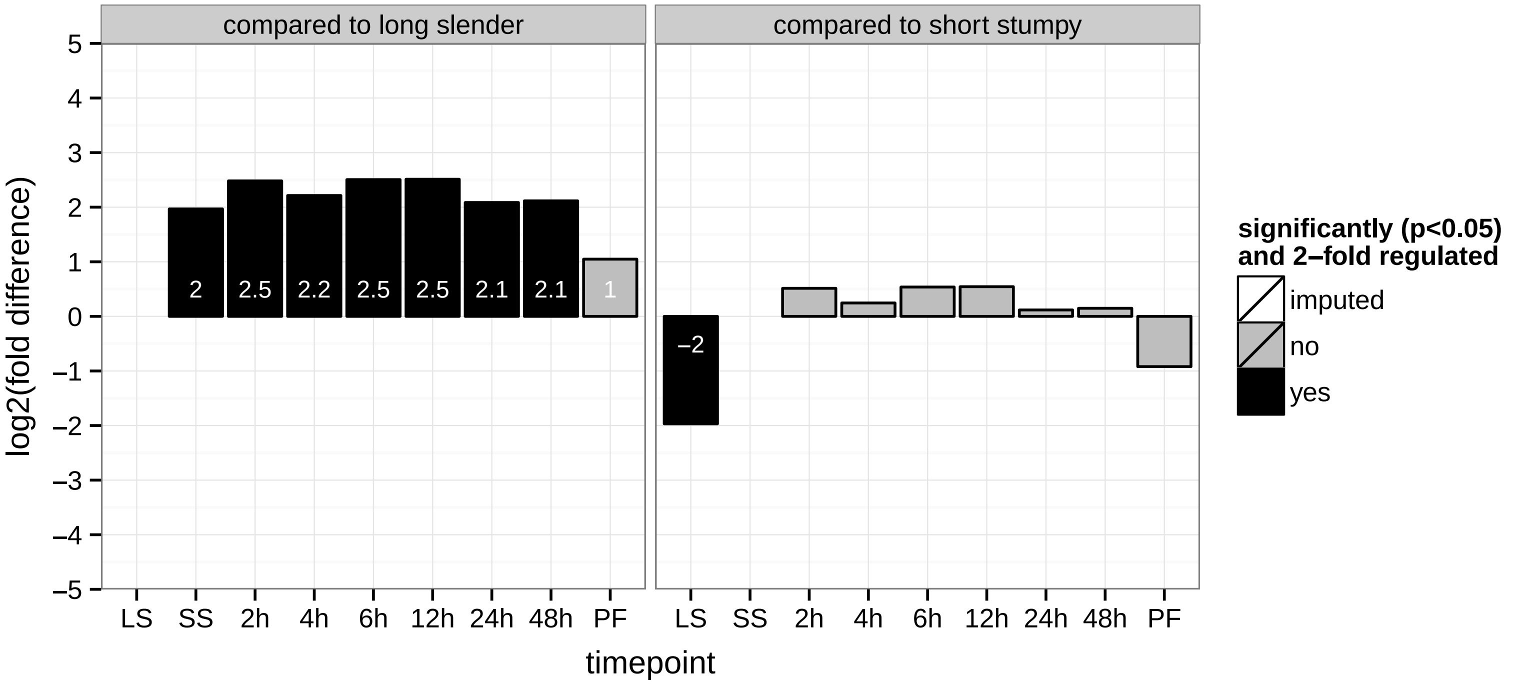

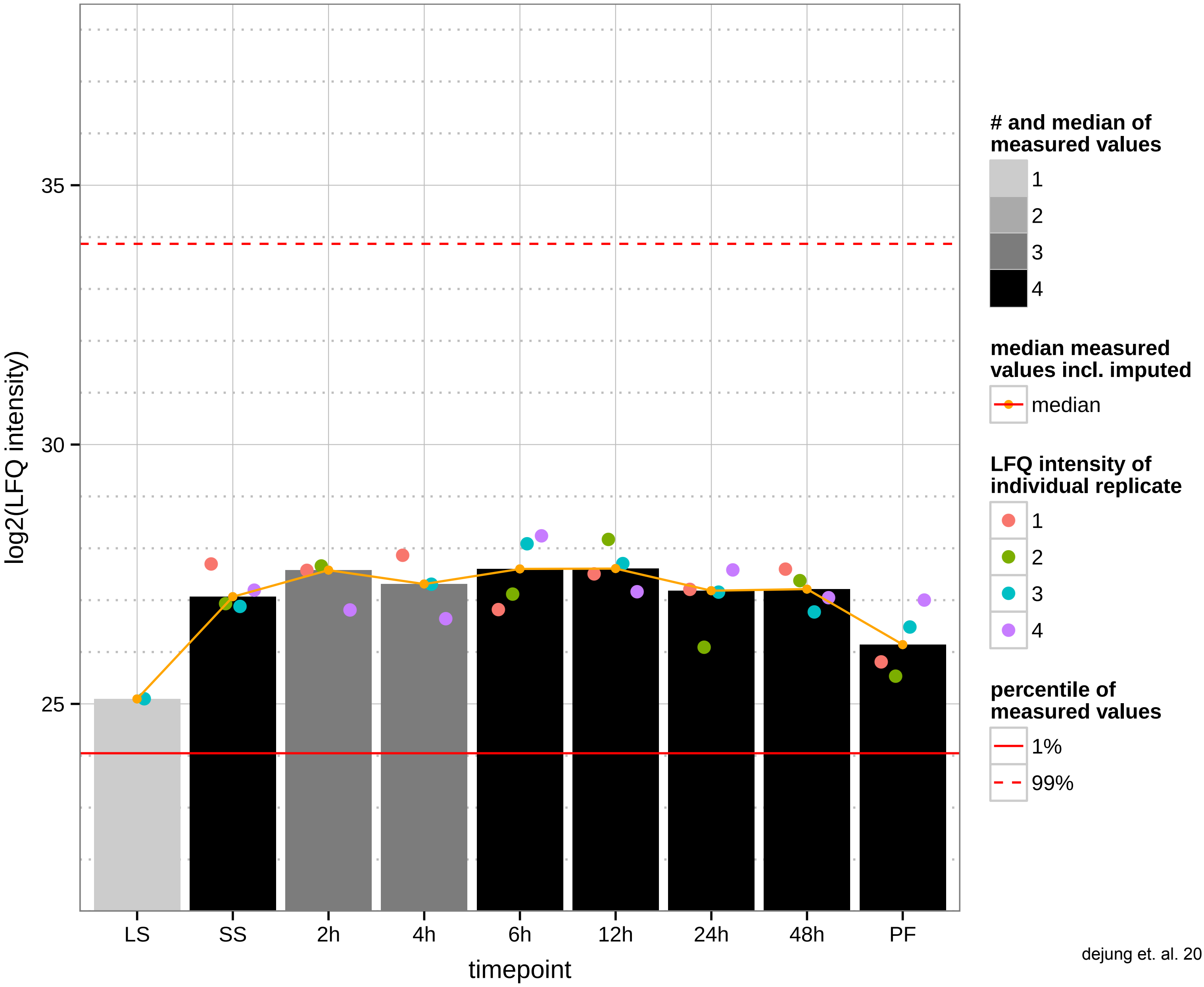

dejung et. al. 2015

retrotransposon hot spot protein 3 (RHS3), putative
Tb927.2.1180
AGOF: null
AGOC: nucleus
AGOP: null
PGOF: null
PGOC: null
PGOP: null

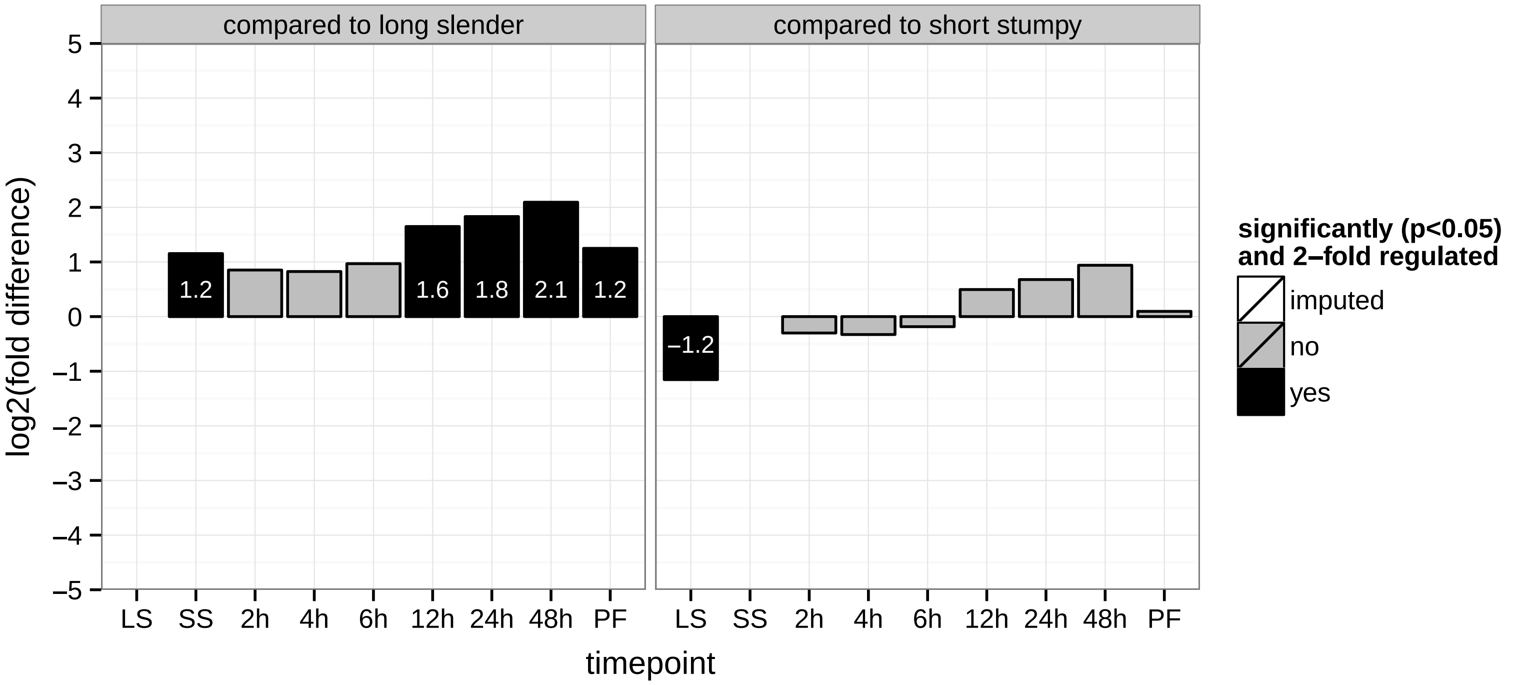

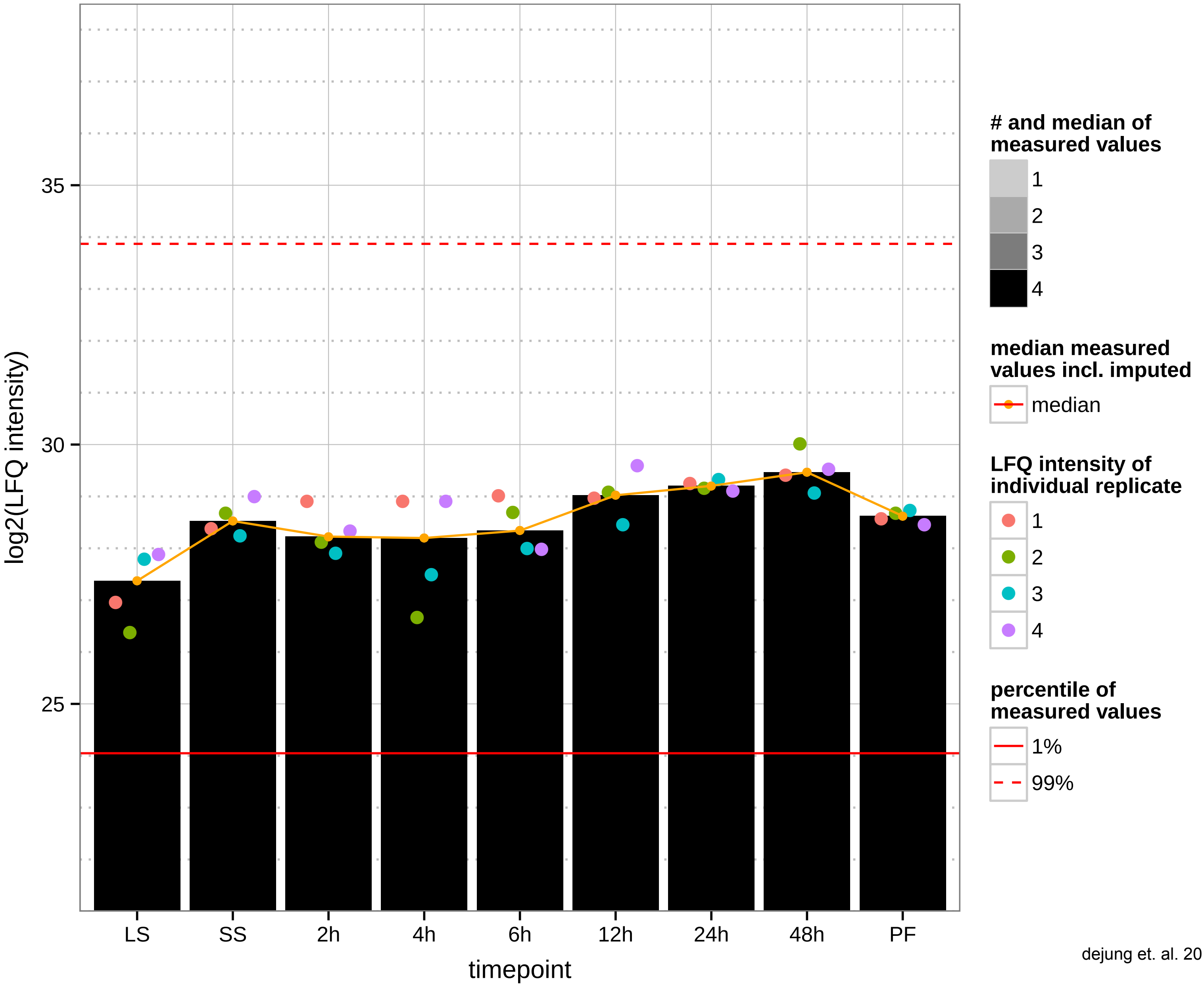

dejung et. al. 2015

retrotransposon hot spot protein 4 (RHS4), putative
Tb927.2.1210
AGOF: null
AGOC: nucleus
AGOP: null
PGOF: null
PGOC: null
PGOP: null

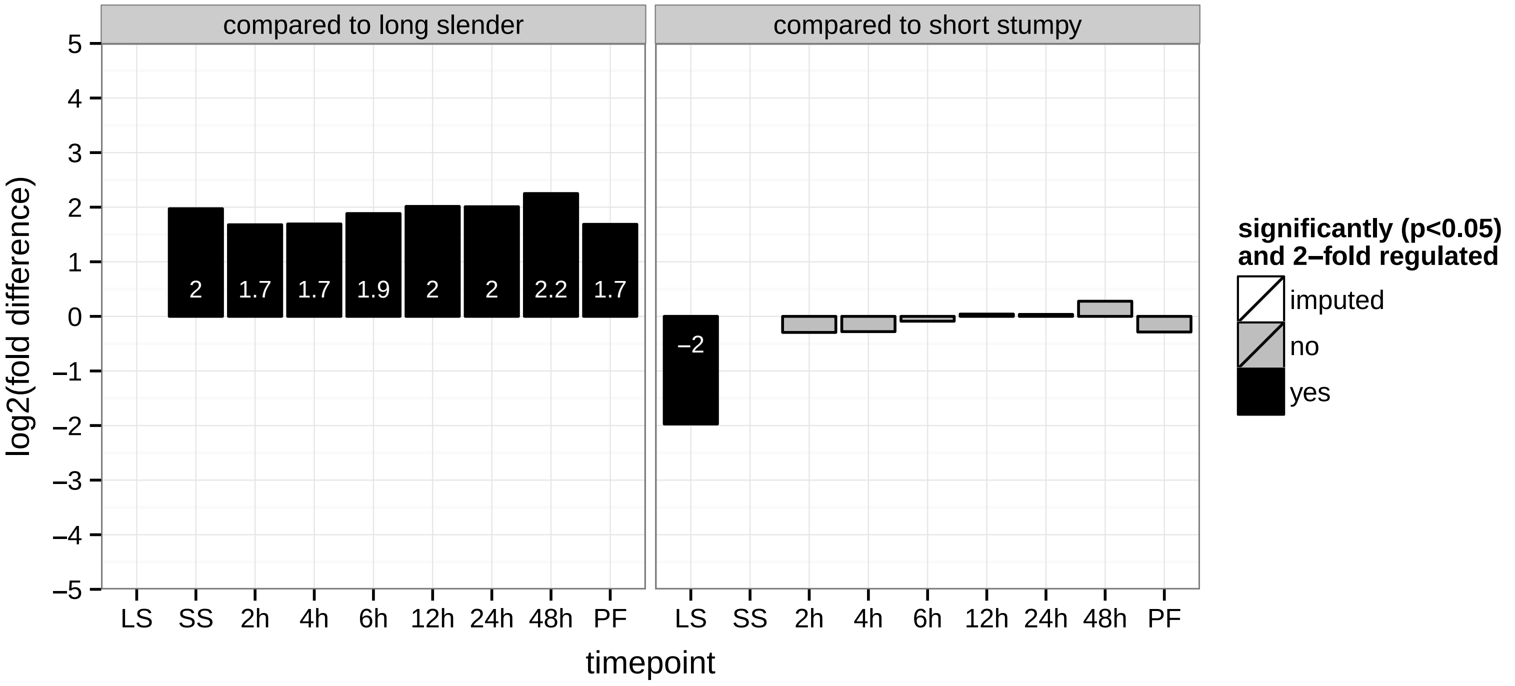

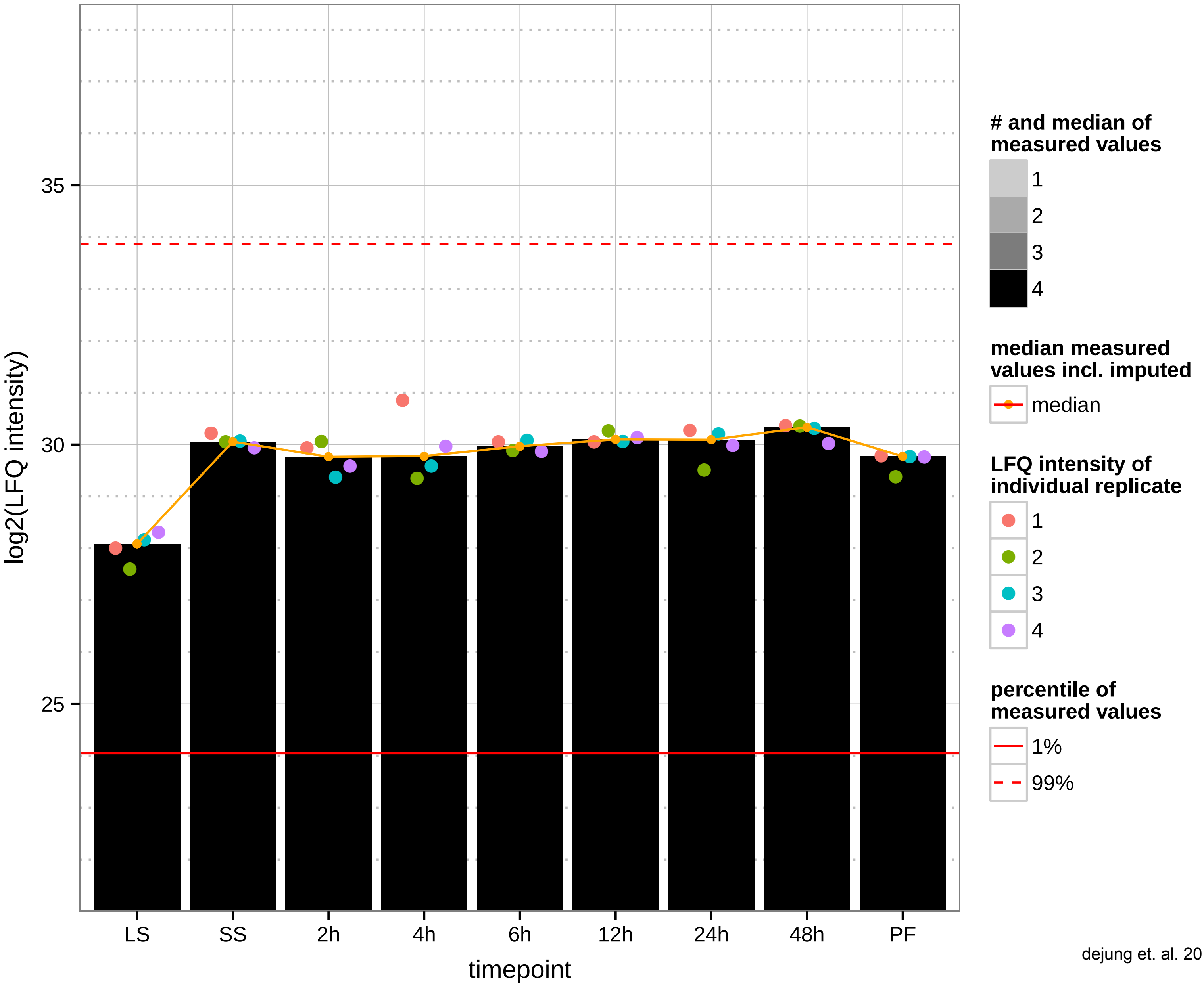

dejung et. al. 2015

cyclophilin–type peptidyl–prolyl cis–trans isomerase, putative
Tb927.2.1680;Tb927.2.1560
AGOF: peptidyl–prolyl cis–trans isomerase activity
AGOC: null
AGOP: protein folding
PGOF: peptidyl–prolyl cis–trans isomerase activity
PGOC: null
PGOP: protein folding

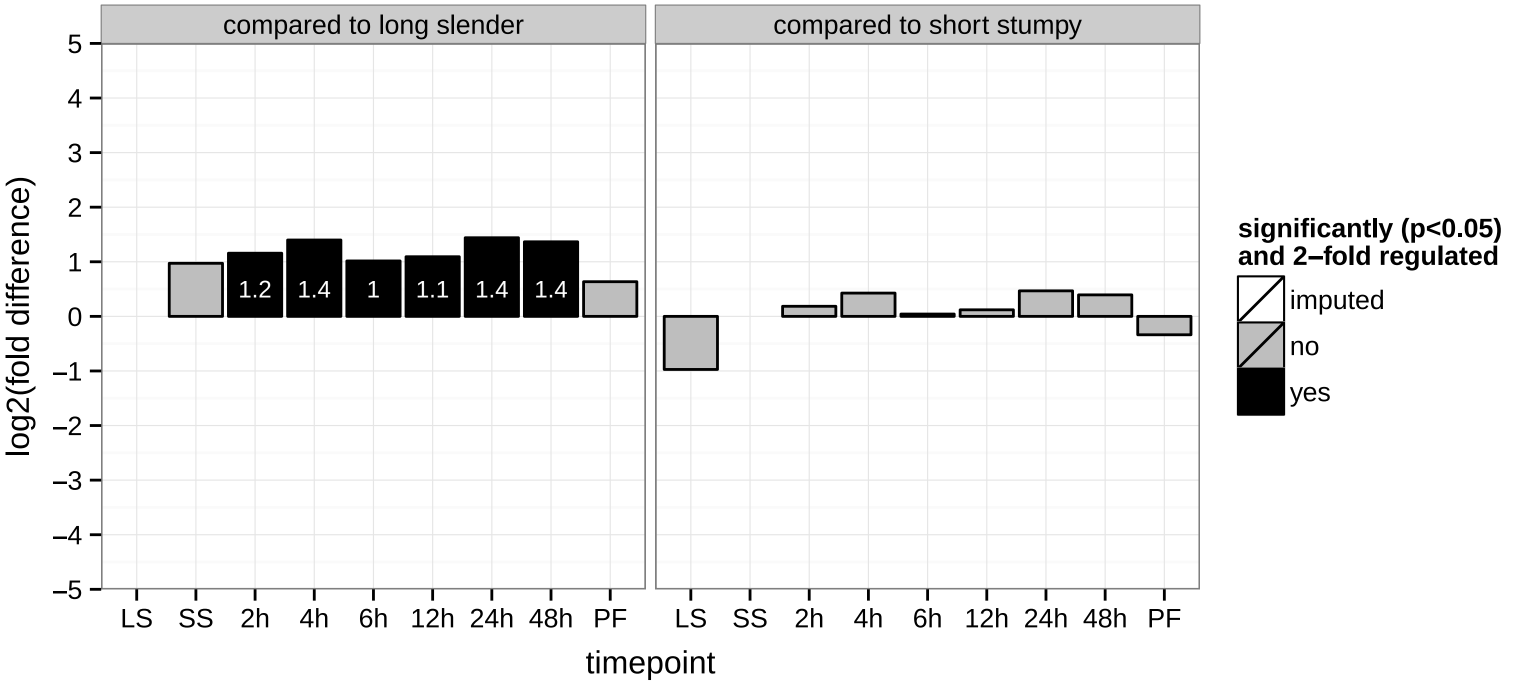

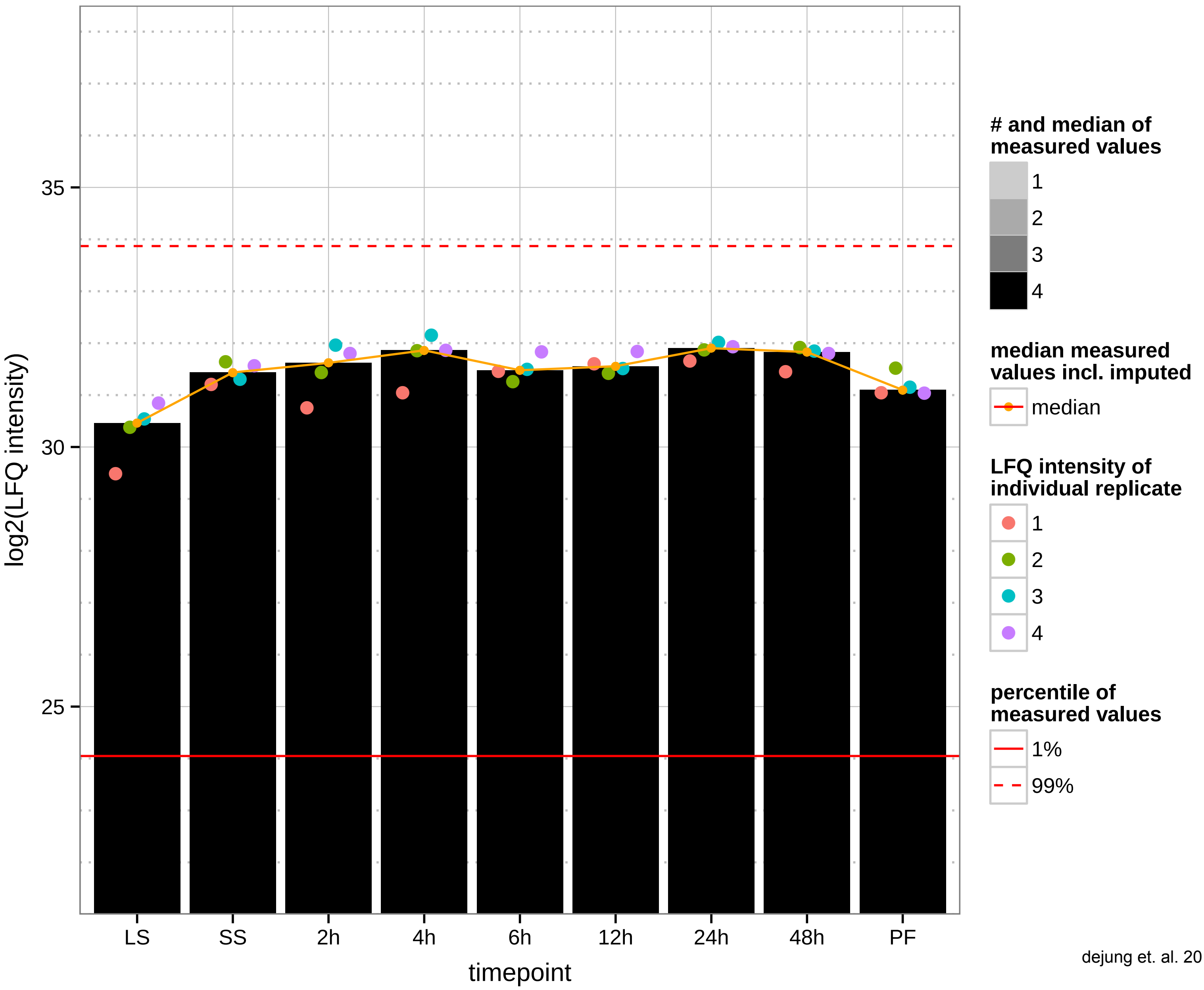

dejung et. al. 2015

transcription silencer (ISWI)
Tb927.2.1810
AGOF: ATP binding, DNA binding, helicase activity, hydrolase activity, acting on acid anhydrides, in phosphorus–containing ar
AGOC: chromatin remodeling complex, nucleus
AGOP: ATP–dependent chromatin remodeling, modulation of development of symbiont involved in interaction with host, quoru
PGOF: ATP binding, DNA binding, helicase activity, hydrolase activity, acting on acid anhydrides, in phosphorus–containing ar
PGOC: chromatin remodeling complex, nucleus
PGOP: ATP–dependent chromatin remodeling, chromatin remodeling

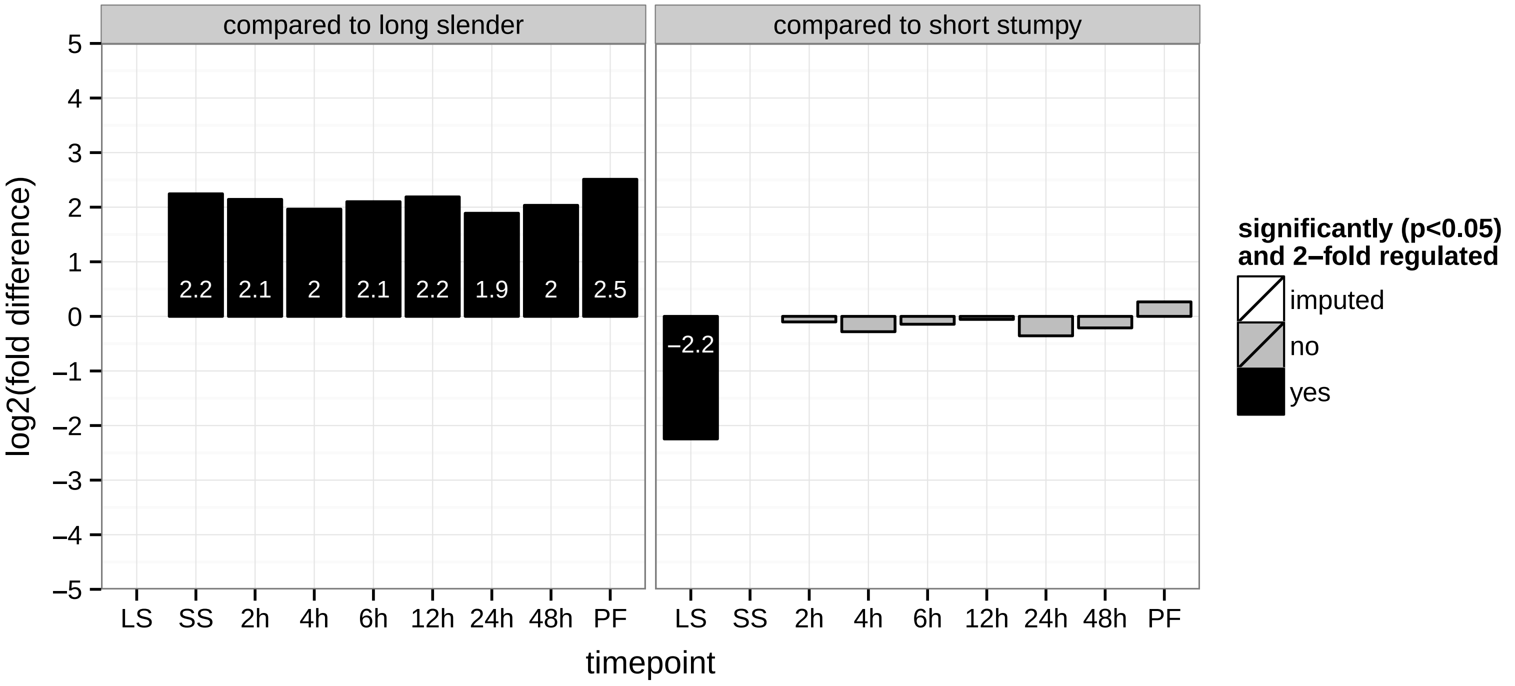

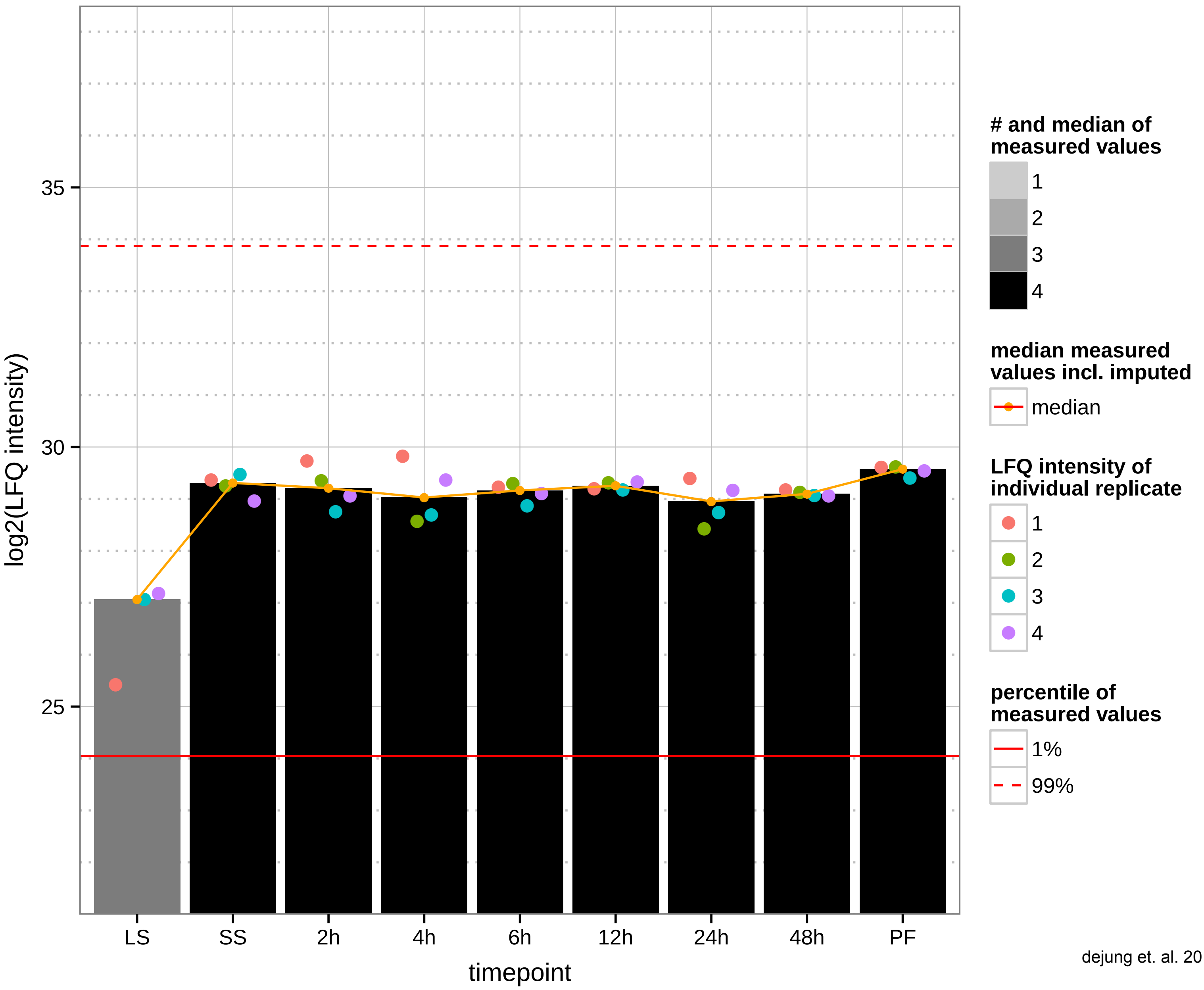

dejung et. al. 2015

E2−like ubiquitin−conjugation enzyme (ATG3)
Tb927.2.1890
AGOF: null
AGOC: null
AGOP: autophagy, protein transport
PGOF: null
PGOC: null
PGOP: null

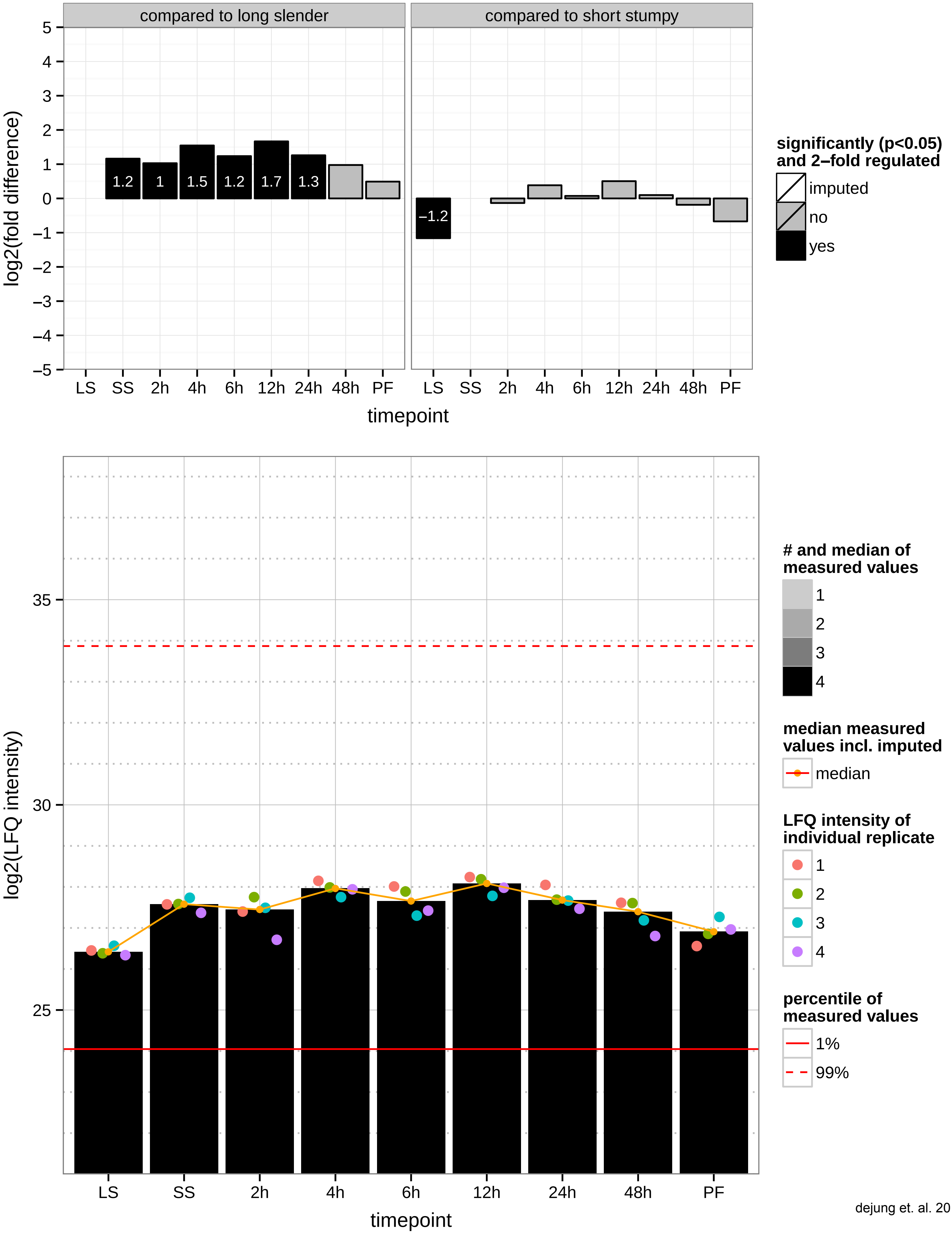

dejung et. al. 2015

paraflagellar rod component, putative (PFC11)
Tb927.2.2160
AGOF: null
AGOC: Golgi apparatus, endoplasmic reticulum
AGOP: intracellular protein transport
PGOF: null
PGOC: null
PGOP: null

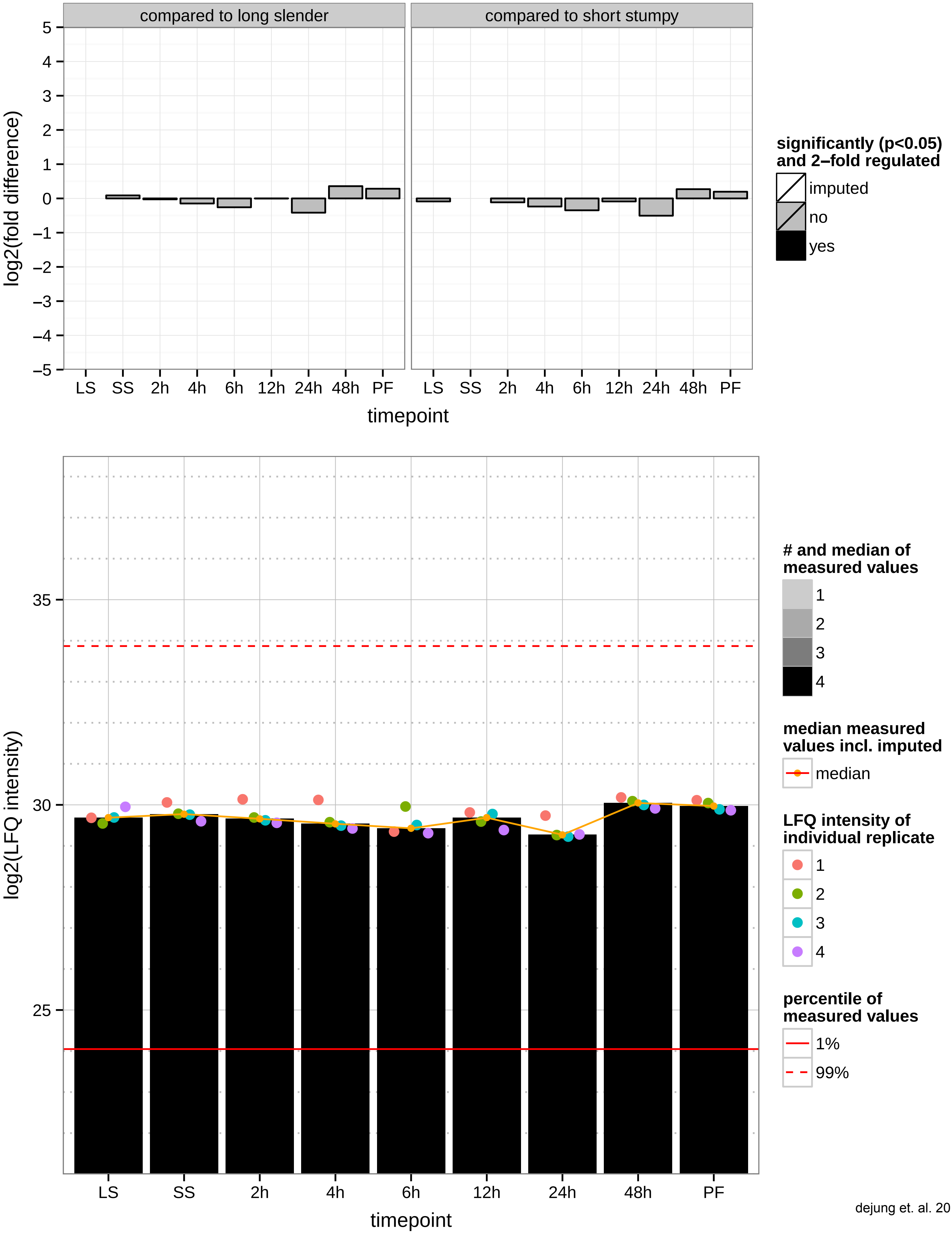

dejung et. al. 2015

hypothetical protein, conserved
Tb927.2.2230
AGOF: null
AGOC: null
AGOP: null
PGOF: null
PGOC: null
PGOP: null

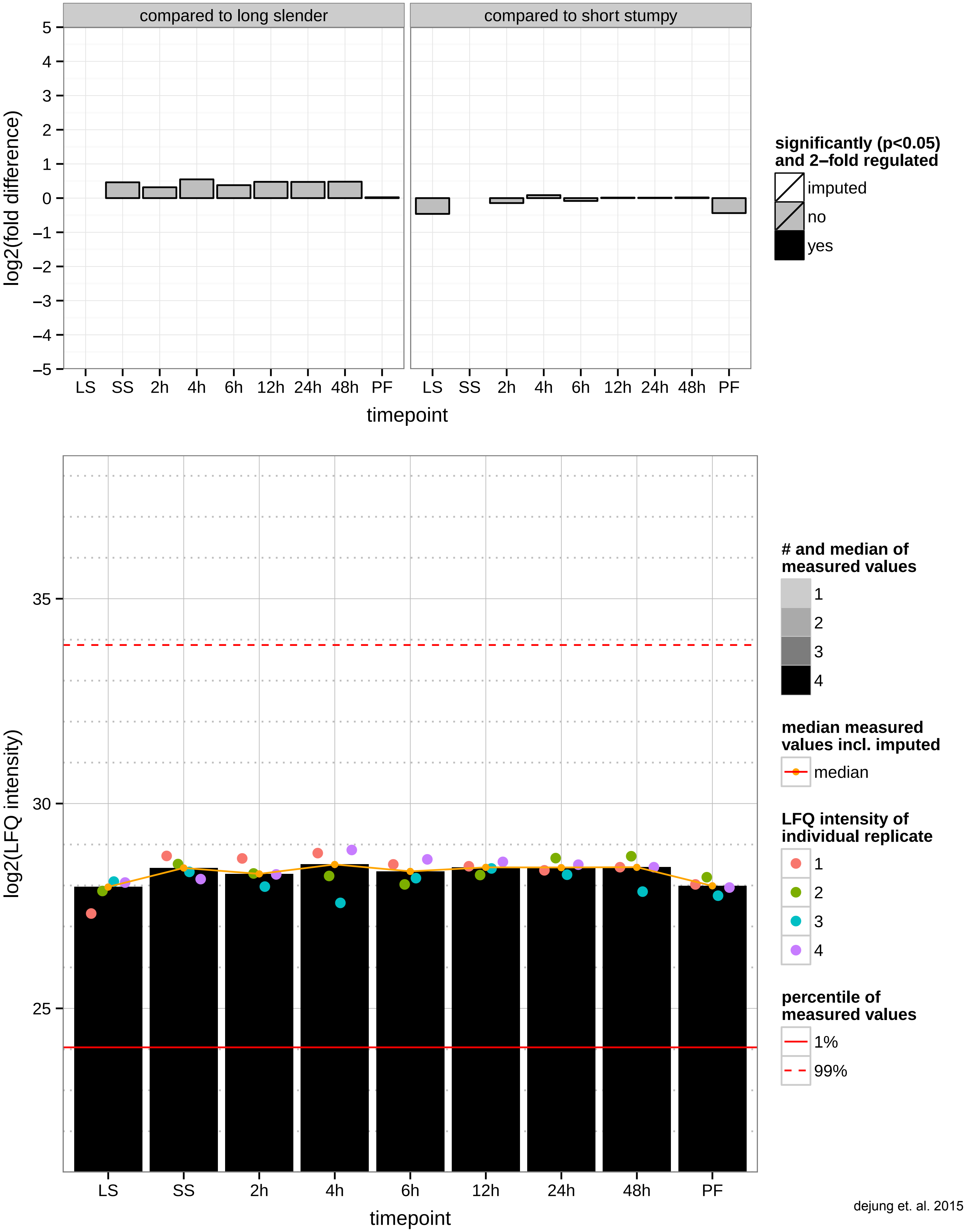

dejung et. al. 2015

tRNA exportin, putative, exportin−T
Tb927.2.2240
AGOF: tRNA binding
AGOC: nucleus
AGOP: transport
PGOF: binding
PGOC: null
PGOP: null

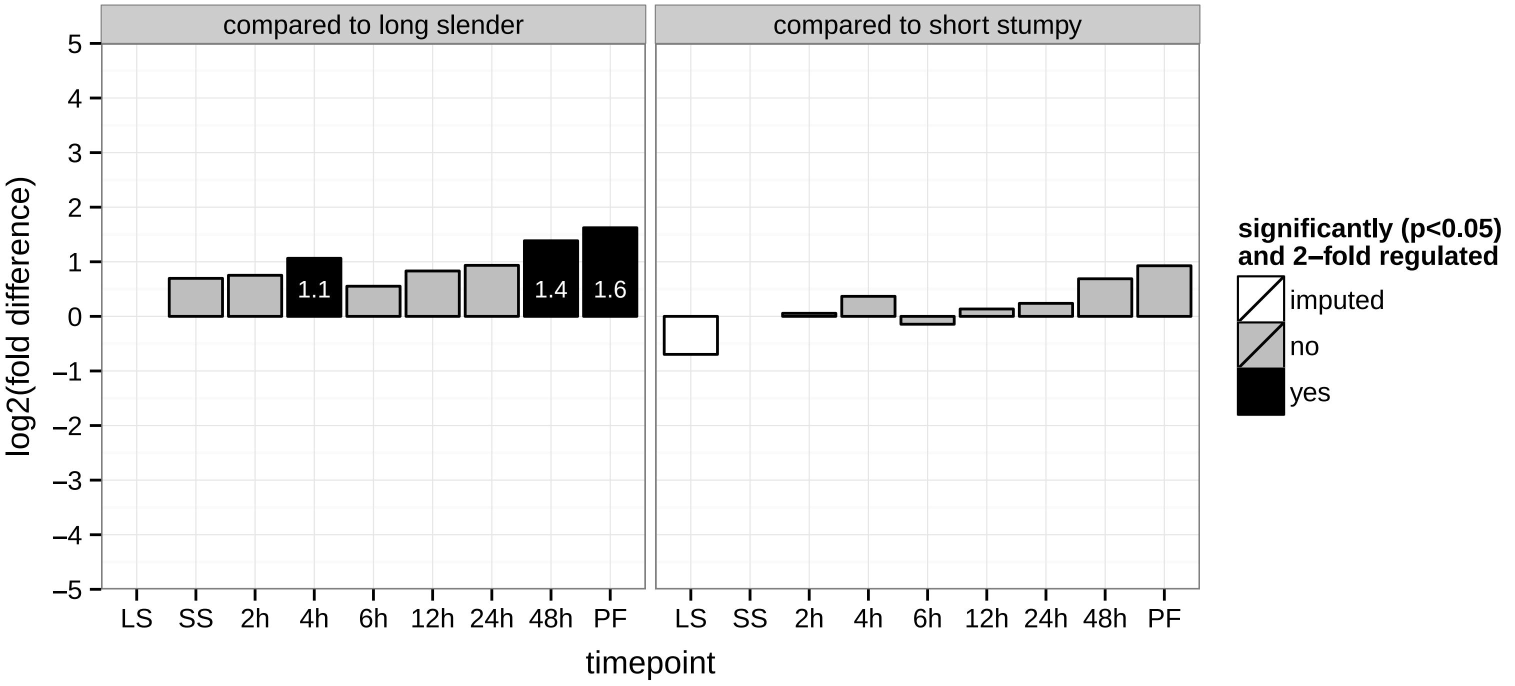

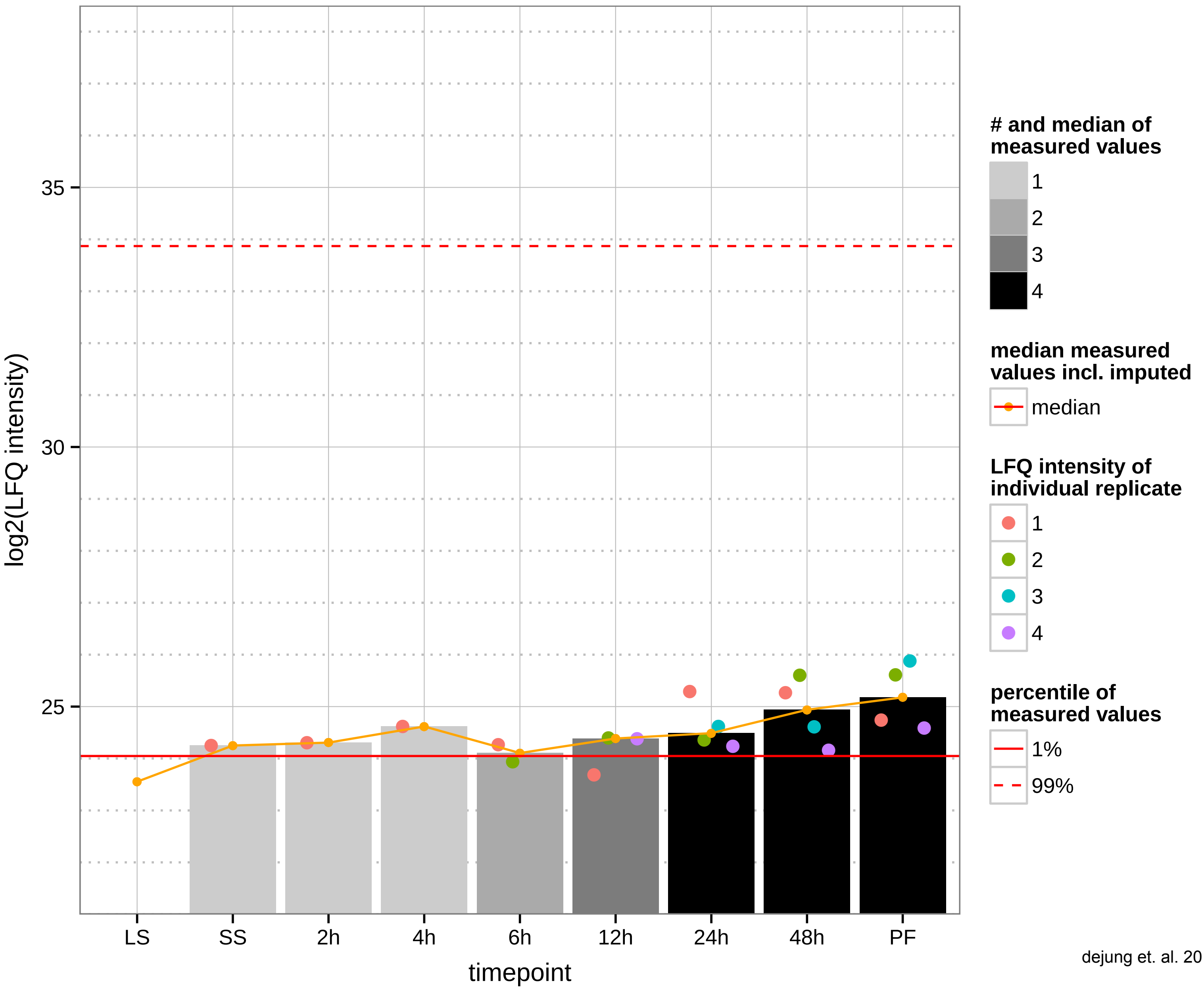

dejung et. al. 2015

hypothetical protein, conserved
Tb927.2.2360
AGOF: zinc ion binding
AGOC: null
AGOP: null
PGOF: protein binding, zinc ion binding
PGOC: null
PGOP: null

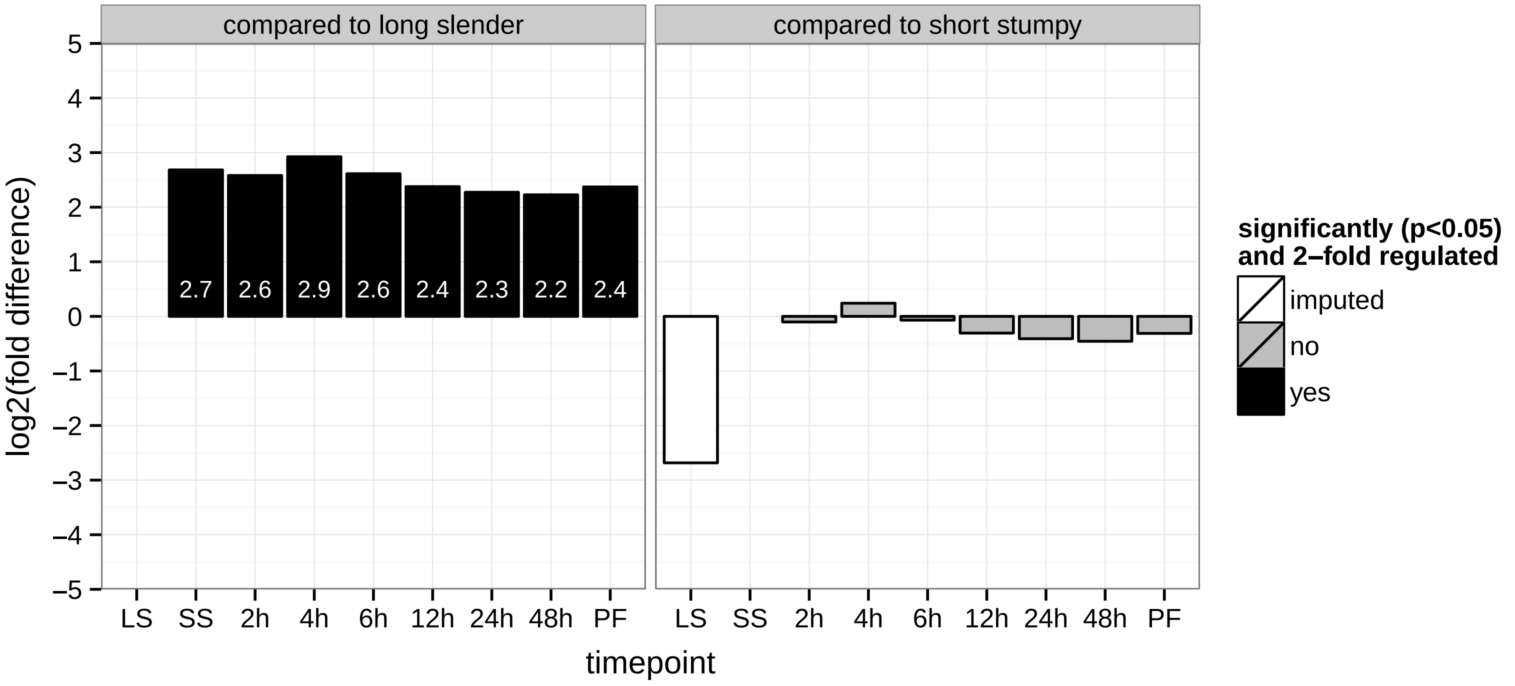

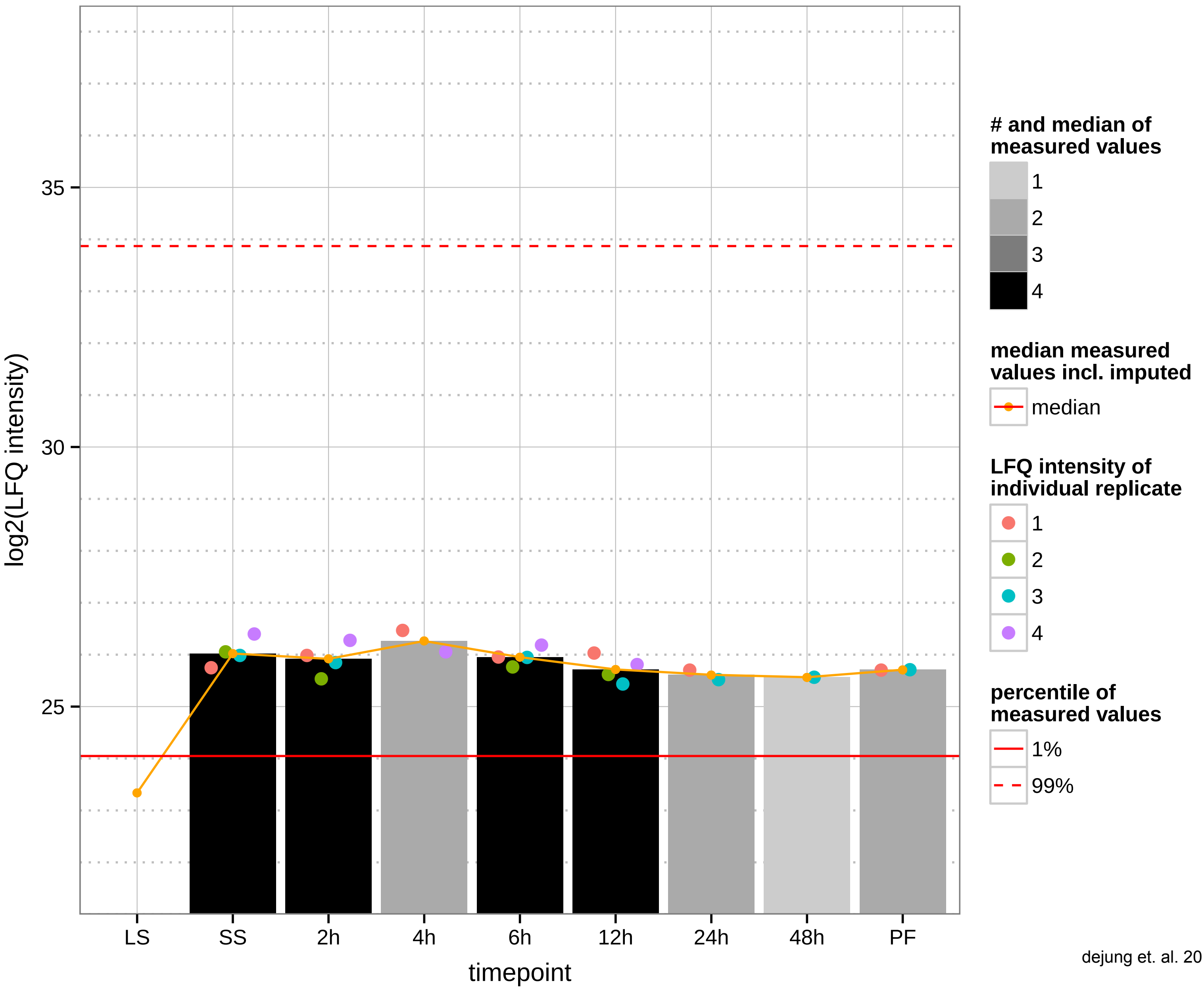

dejung et. al. 2015

proteasome regulatory non–ATPase subunit 6 (RPN6)
Tb927.2.2440
AGOF: endopeptidase activity
AGOC: proteasome regulatory particle
AGOP: ubiquitin–dependent protein catabolic process
PGOF: protein binding
PGOC: null
PGOP: null

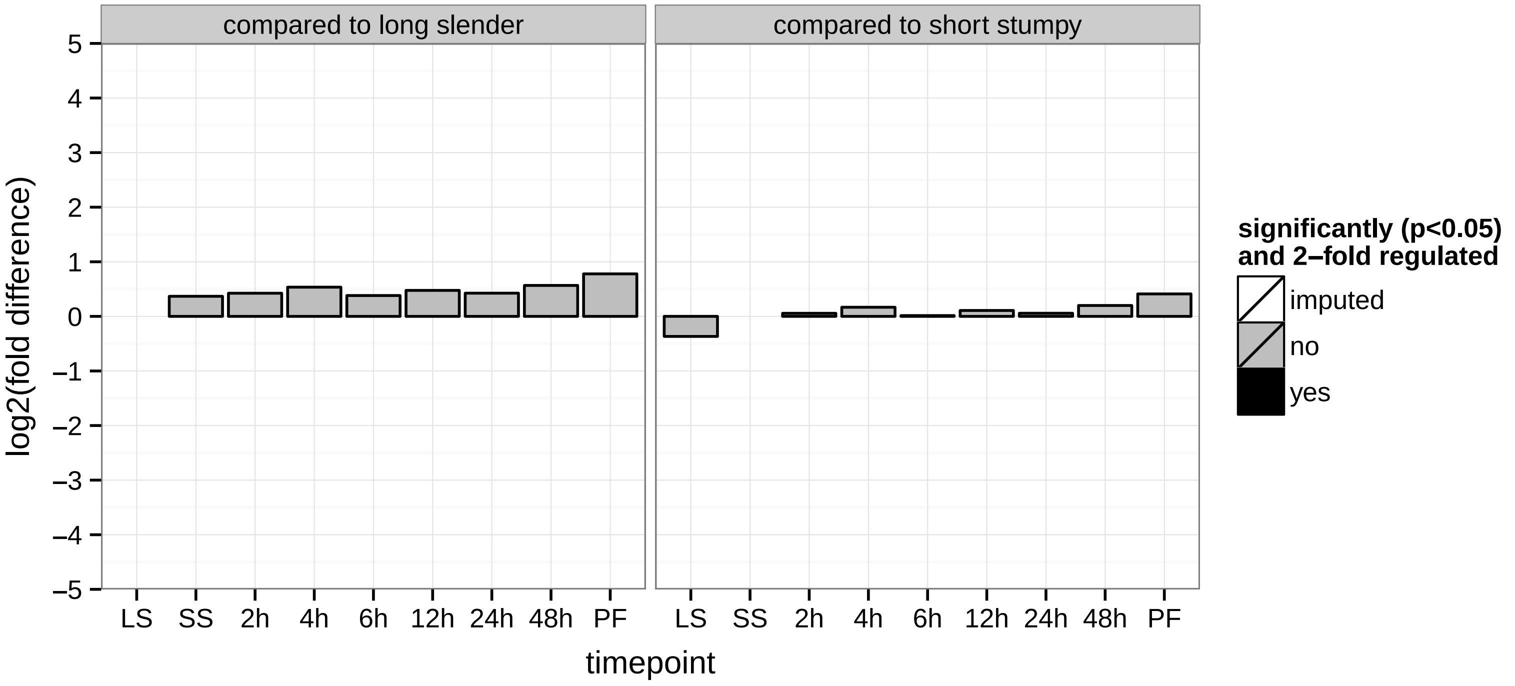

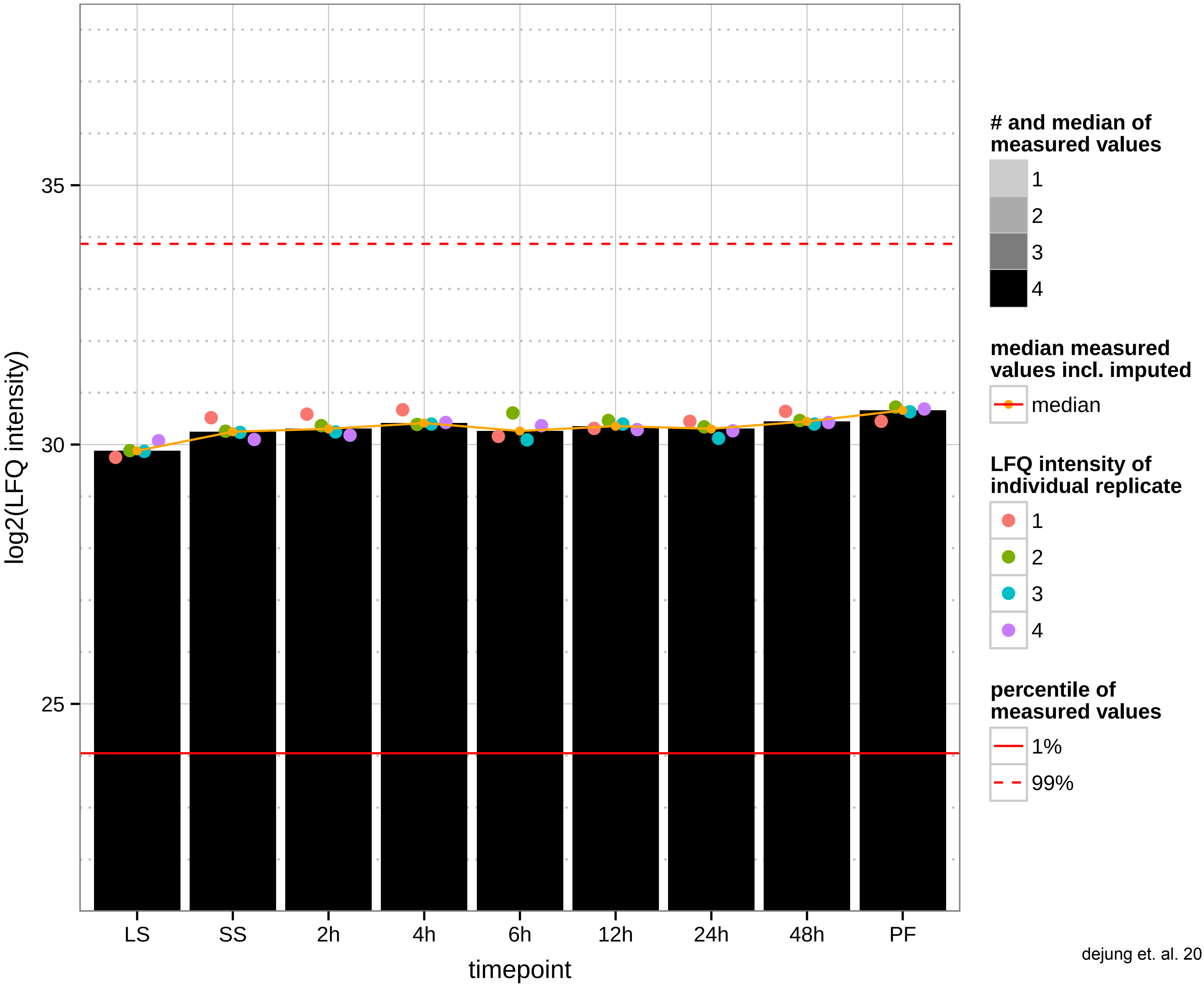

dejung et. al. 2015

hypothetical protein, conserved
Tb927.2.2880
AGOF: null
AGOC: null
AGOP: null
PGOF: protein binding
PGOC: null
PGOP: null

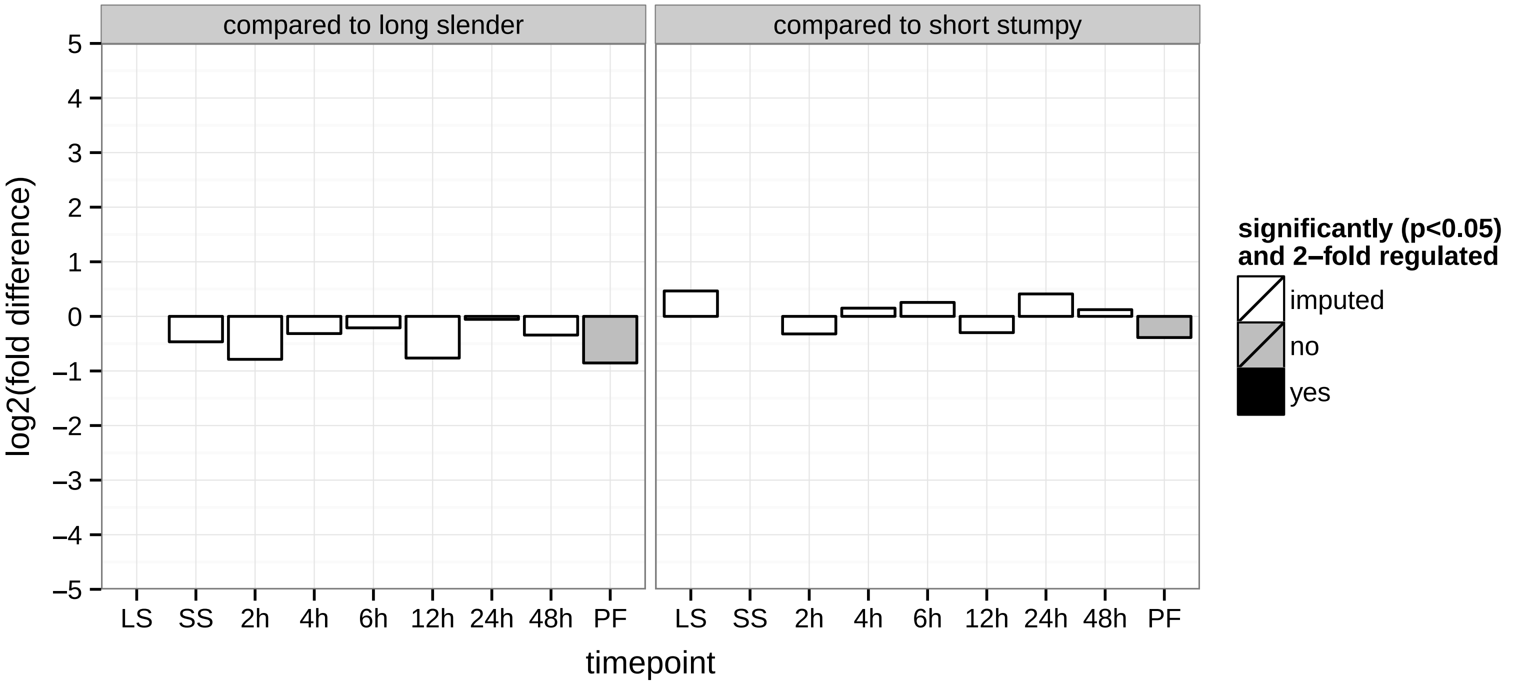

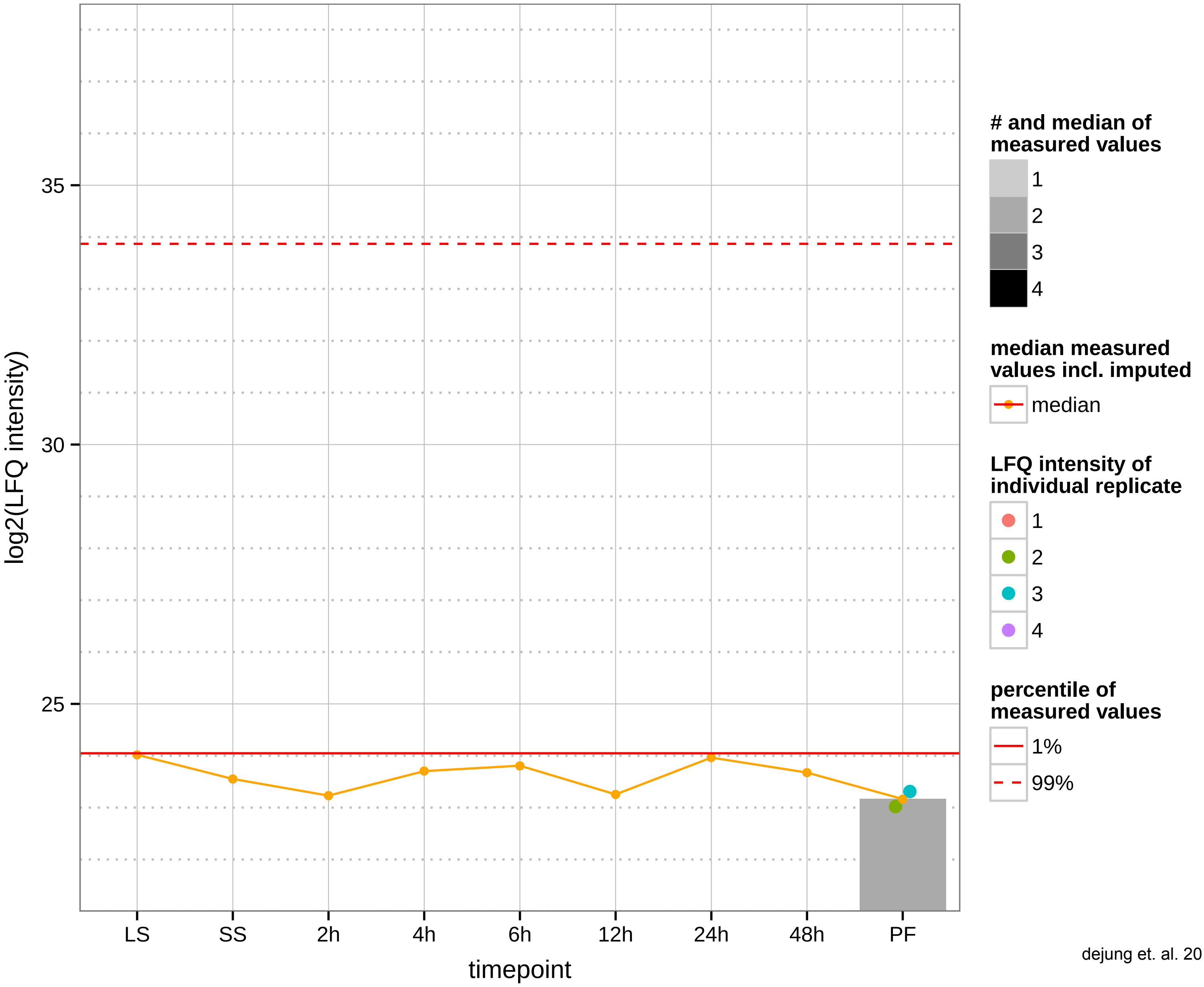

dejung et. al. 2015

hypothetical protein, conserved
Tb927.2.2920
AGOF: null
AGOC: null
AGOP: null
PGOF: null
PGOC: null
PGOP: transmembrane transport

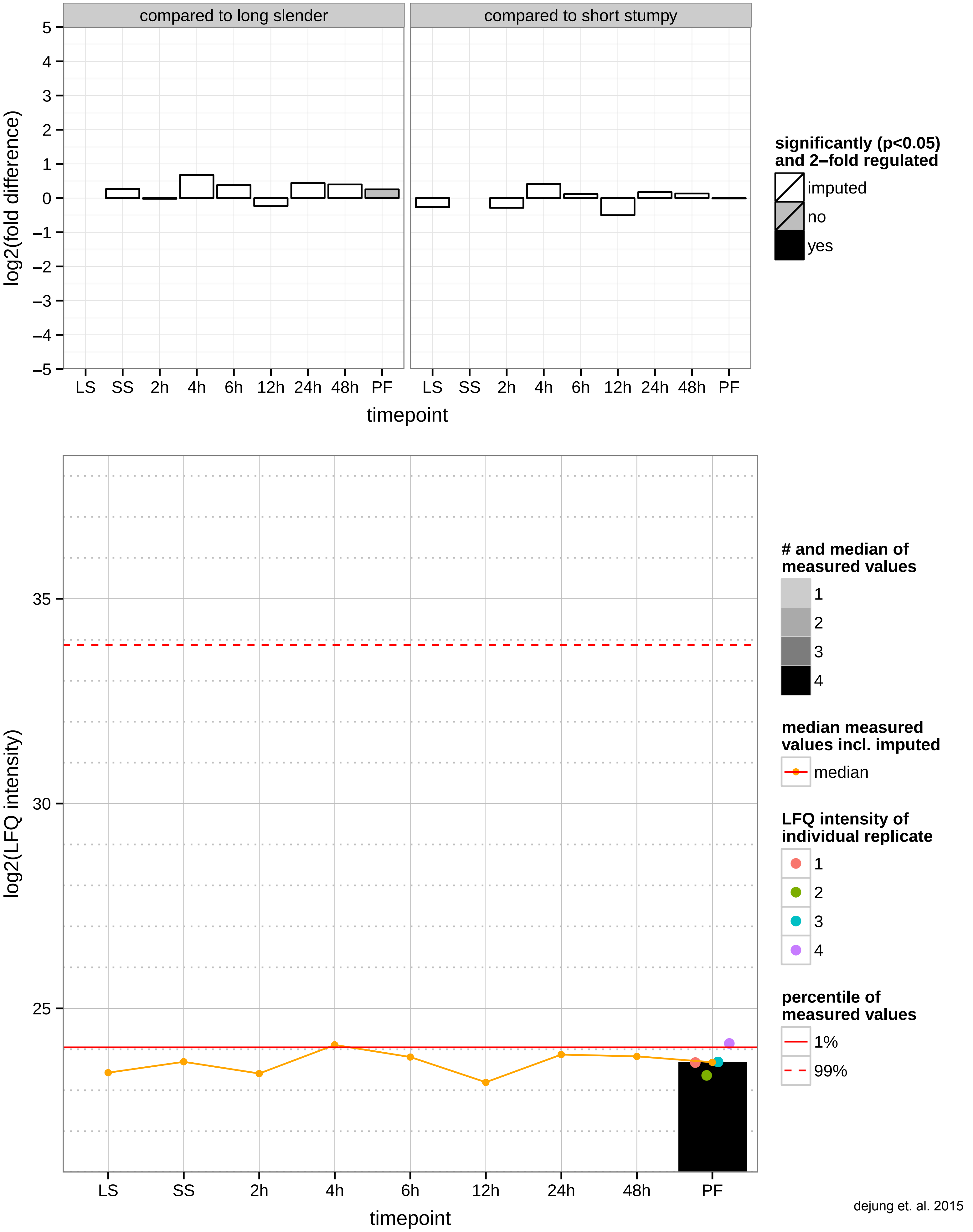

dejung et. al. 2015

hypothetical protein, conserved
Tb927.2.2940
AGOF: null
AGOC: mitochondrion
AGOP: null
PGOF: null
PGOC: null
PGOP: null

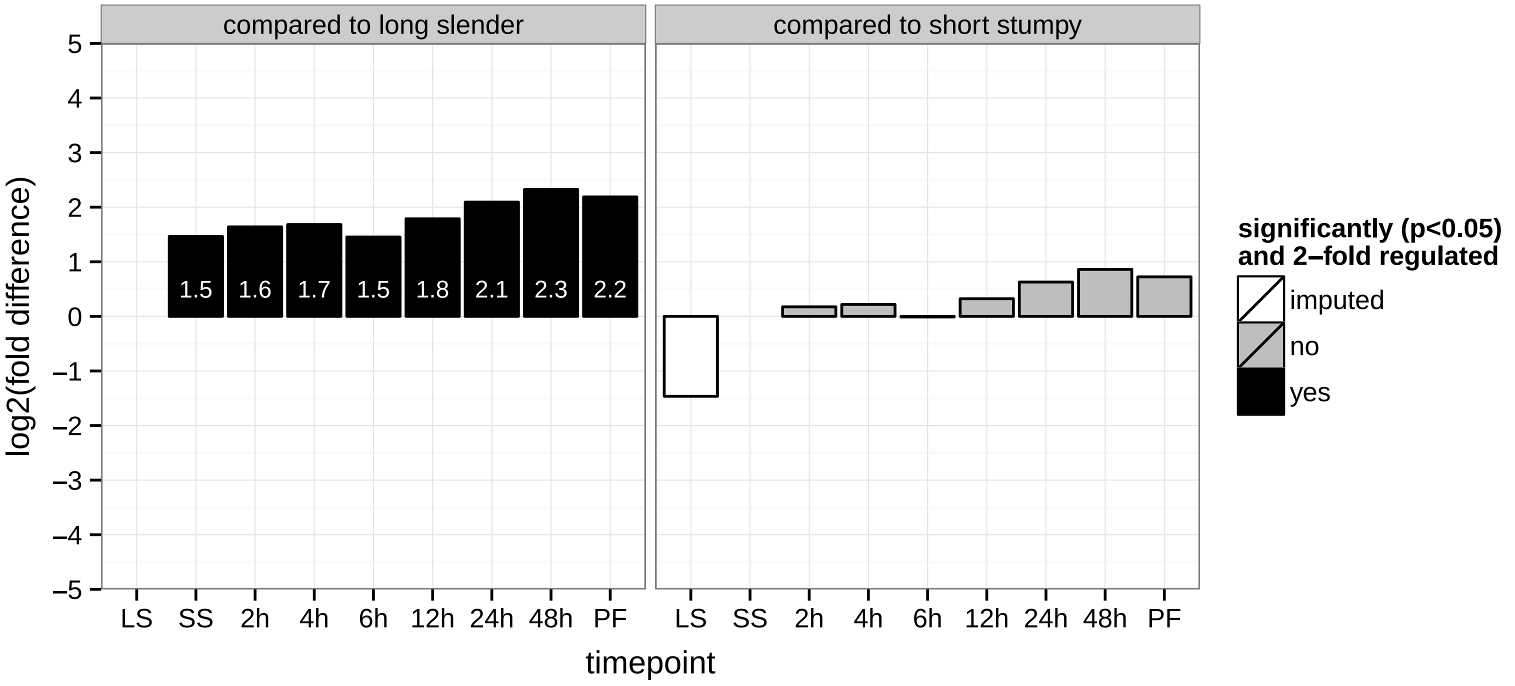

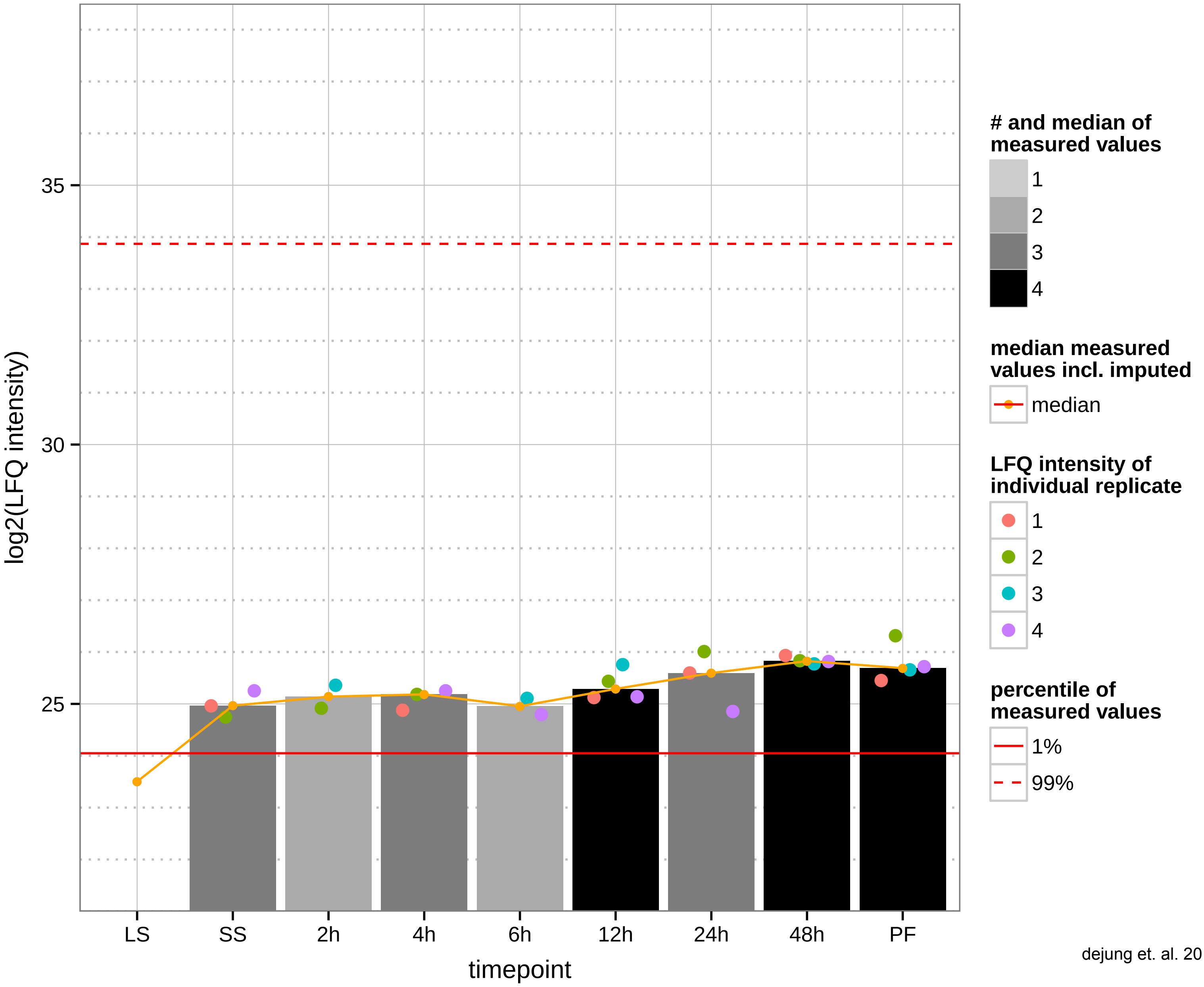

dejung et. al. 2015

mitochondrial carrier protein (MCP13)
Tb927.2.2970
AGOF: null
AGOC: mitochondrial inner membrane, mitochondrial part
AGOP: transport
PGOF: null
PGOC: null
PGOP: null

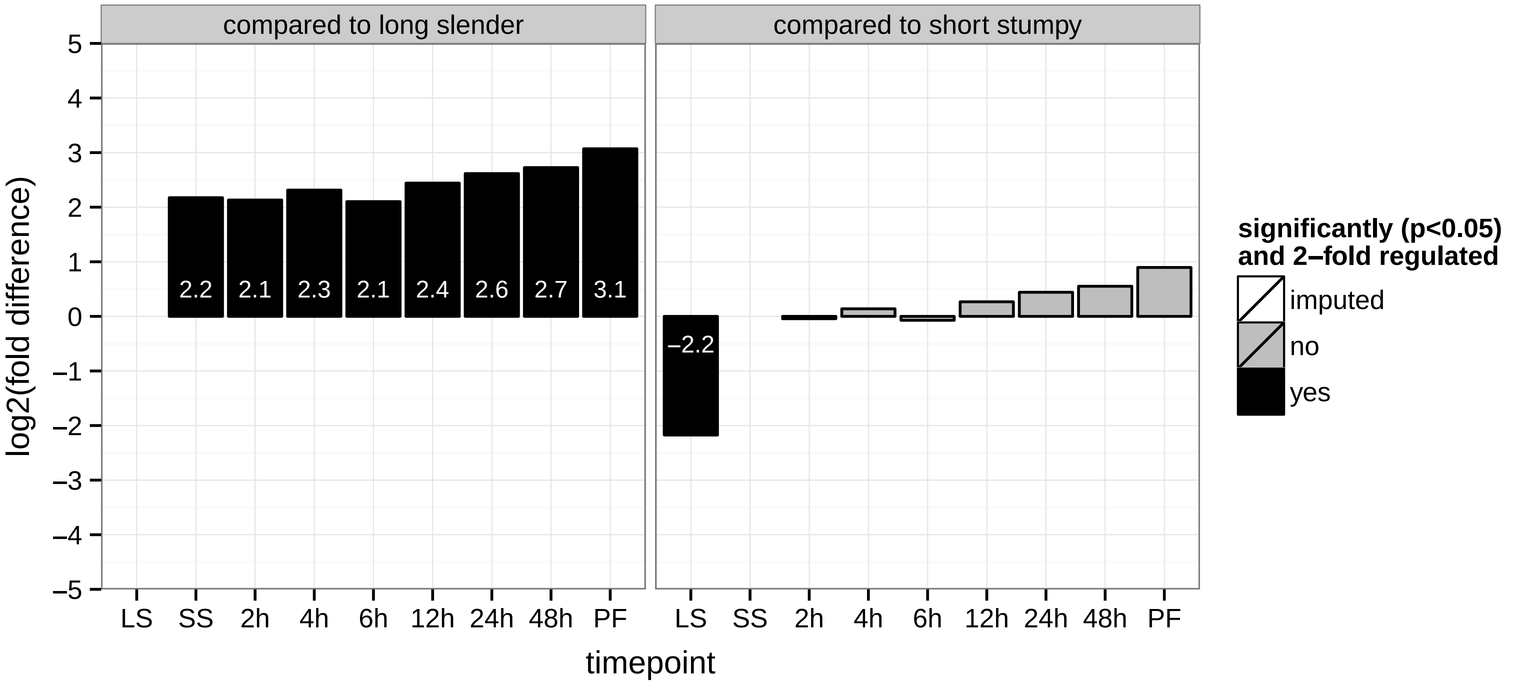

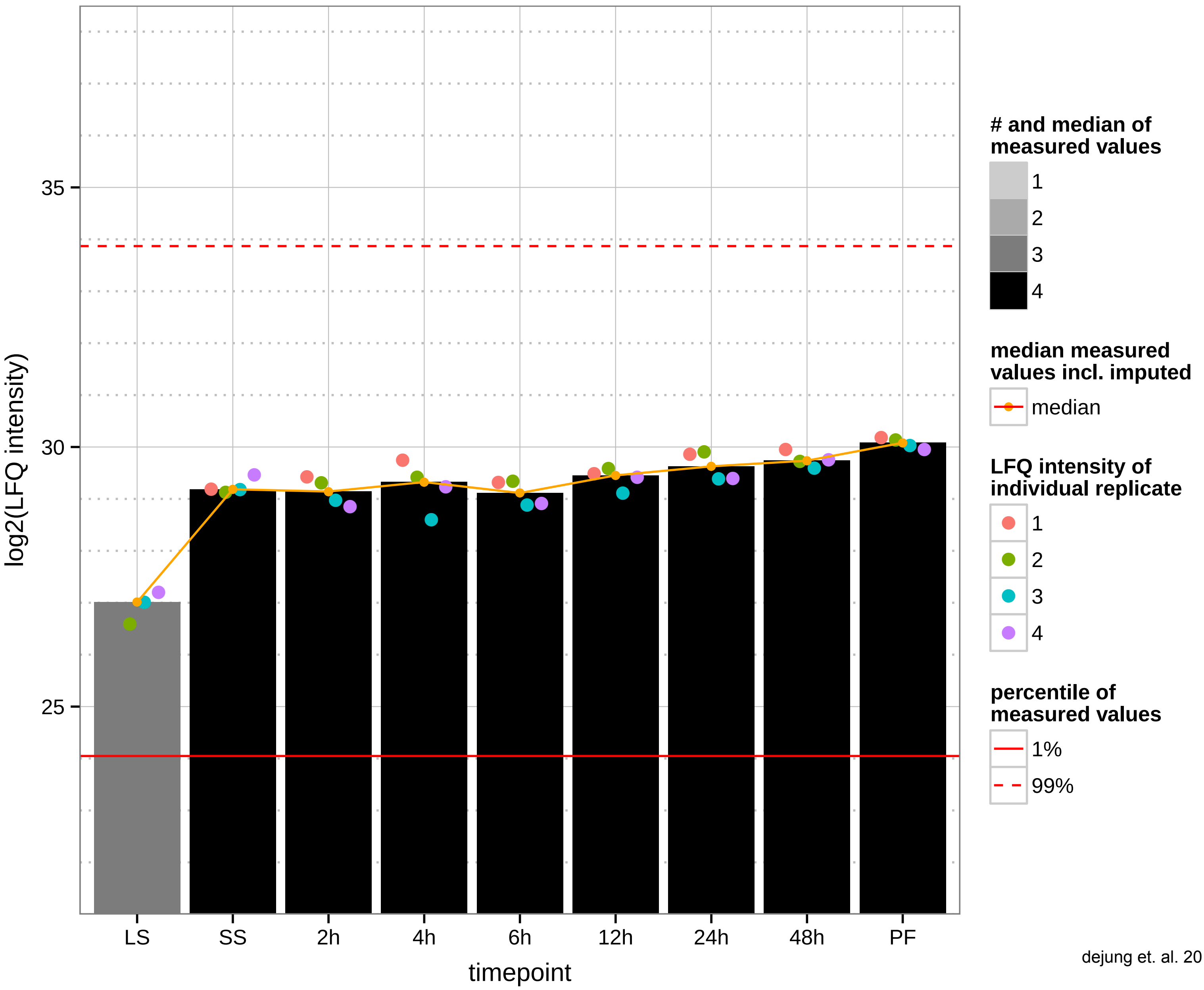

dejung et. al. 2015

hypothetical protein, conserved
Tb927.2.2990
AGOF: DNA binding, DNA−directed RNA polymerase activity
AGOC: null
AGOP: transcription, DNA−dependent
PGOF: null
PGOC: null
PGOP: null

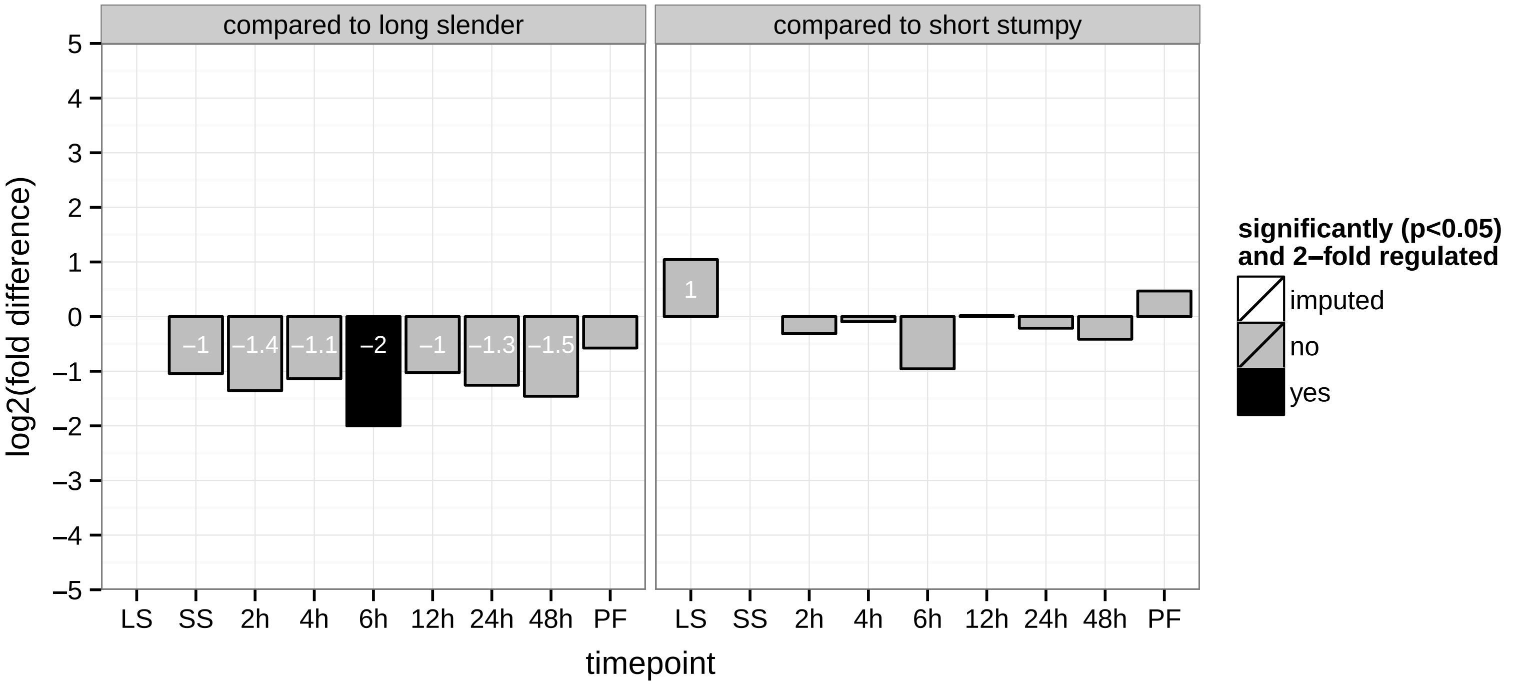

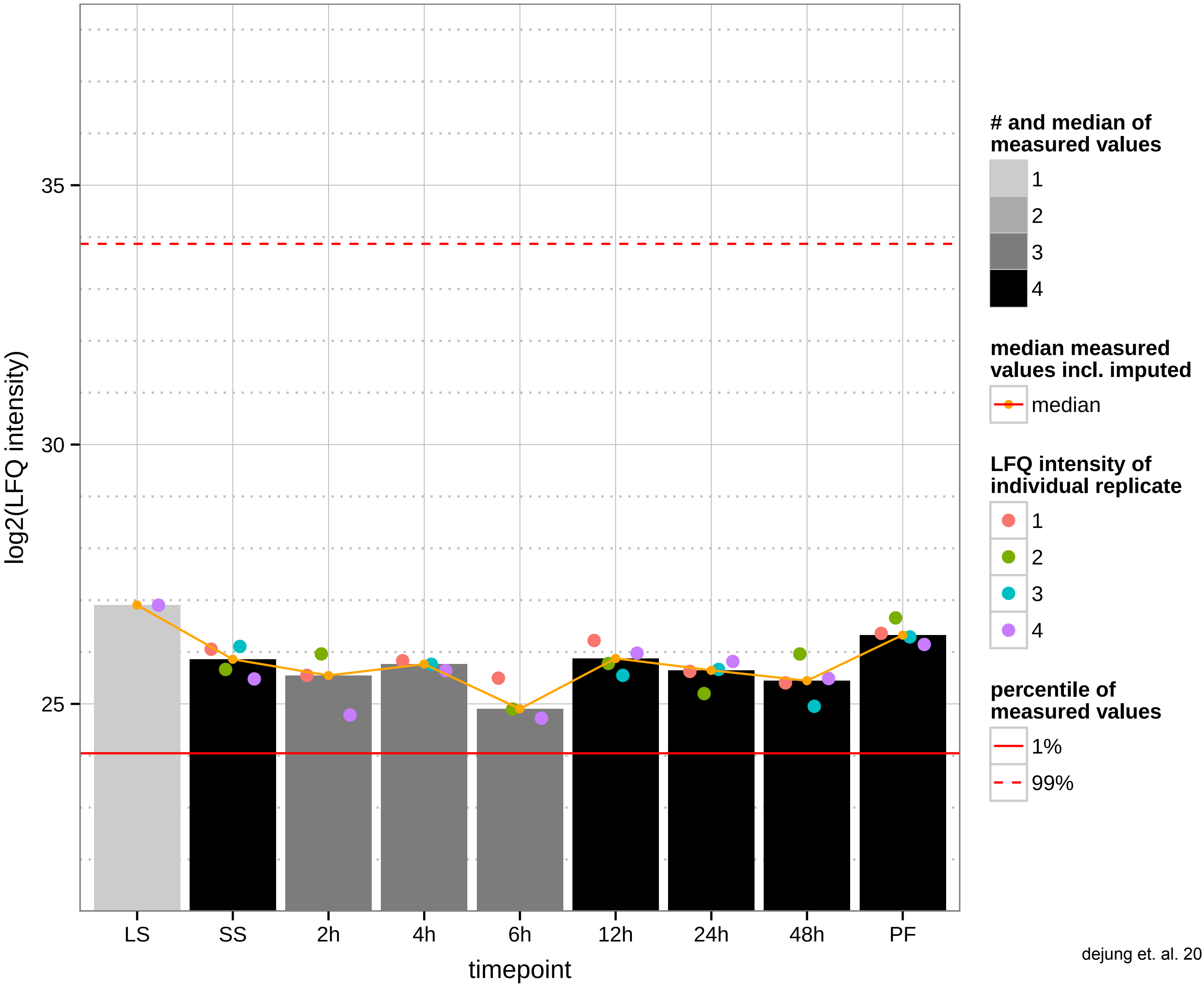

dejung et. al. 2015

ATP−dependent Clp protease subunit, heat shock protein 78 (HSP78), putative
Tb927.2.3030
AGOF: ATP binding, nucleoside−triphosphatase activity, serine−type endopeptidase activity, unfolded protein binding
AGOC: mitochondrial endopeptidase Clp complex, mitochondrion
AGOP: protein folding, proteolysis
PGOF: ATP binding, nucleoside−triphosphatase activity, nucleotide binding
PGOC: null
PGOP: null

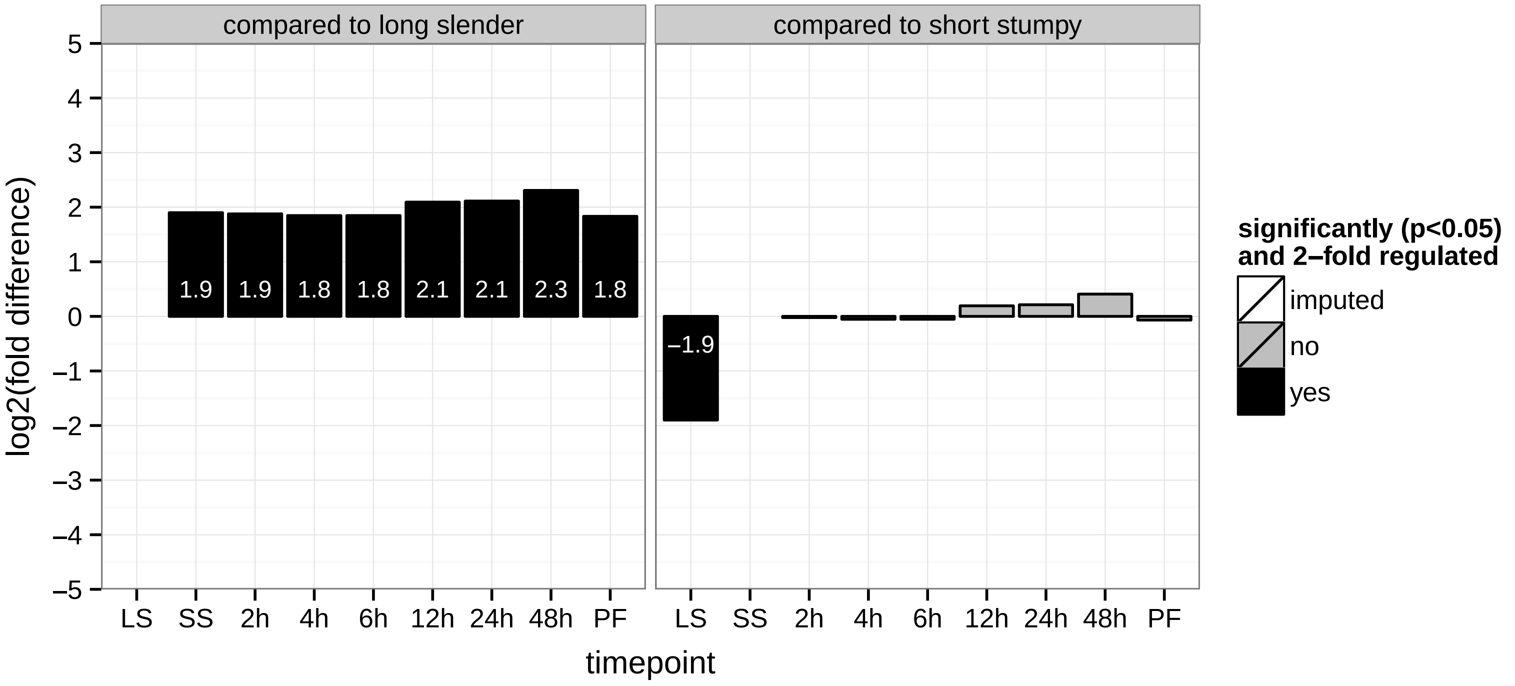

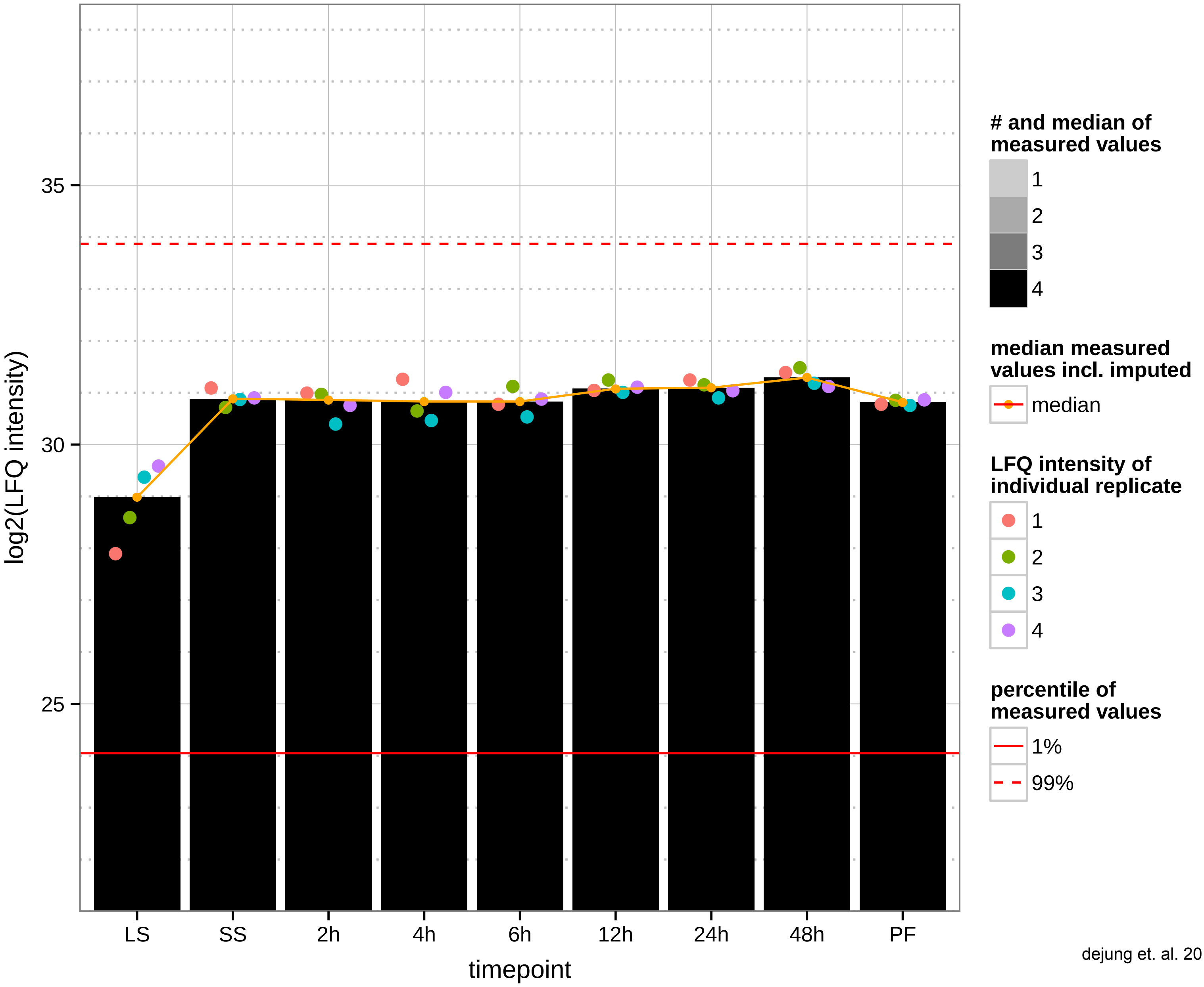

dejung et. al. 2015

hypothetical protein, conserved, kinetoplast polyadenylation/uridylation factor 1, mitochondrial RNA binding protein 1 (KPAF1)
Tb927.2.3180;Tb11.v5.0782
AGOF: null, protein binding, ribosome binding
AGOC: null, mitochondrion
AGOP: null, mRNA 3'−end processing, mRNA polyadenylation, regulation of mRNA stability
PGOF: null
PGOC: null
PGOP: null

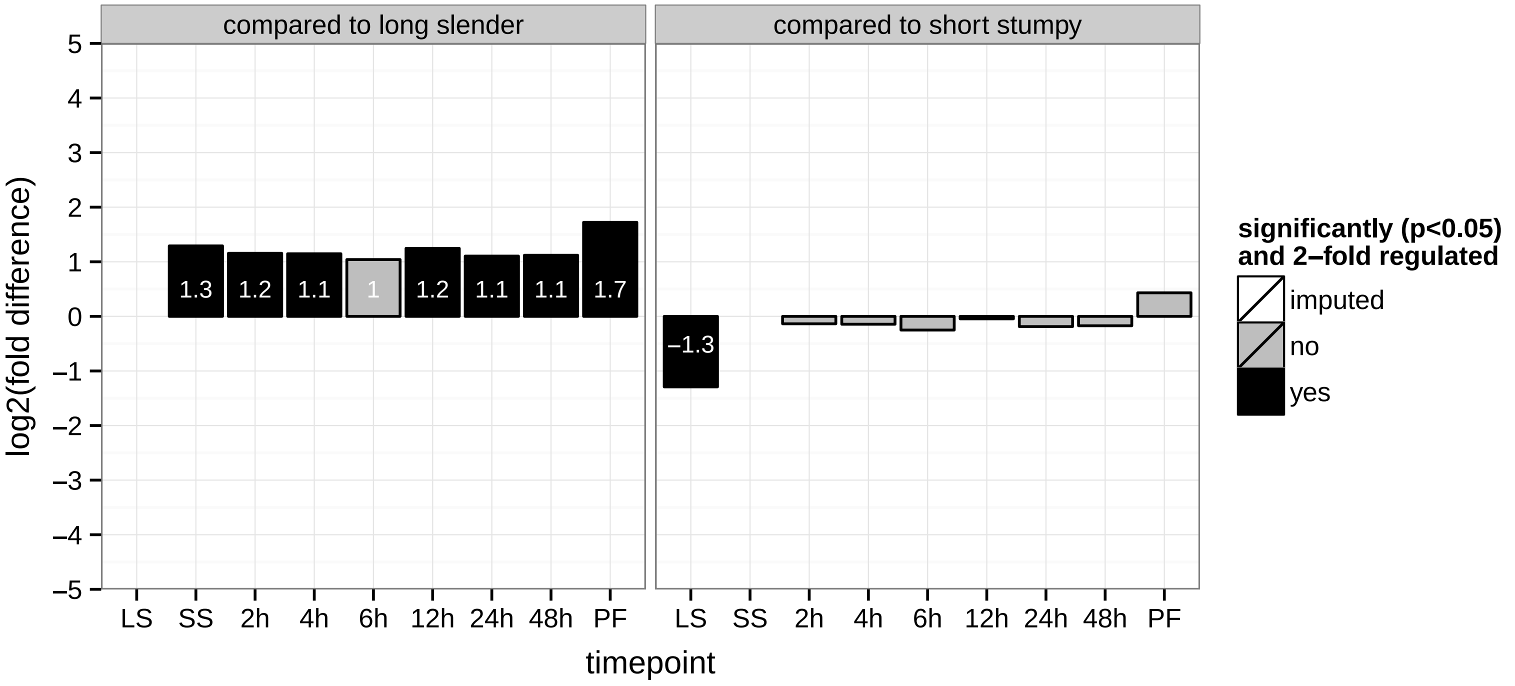

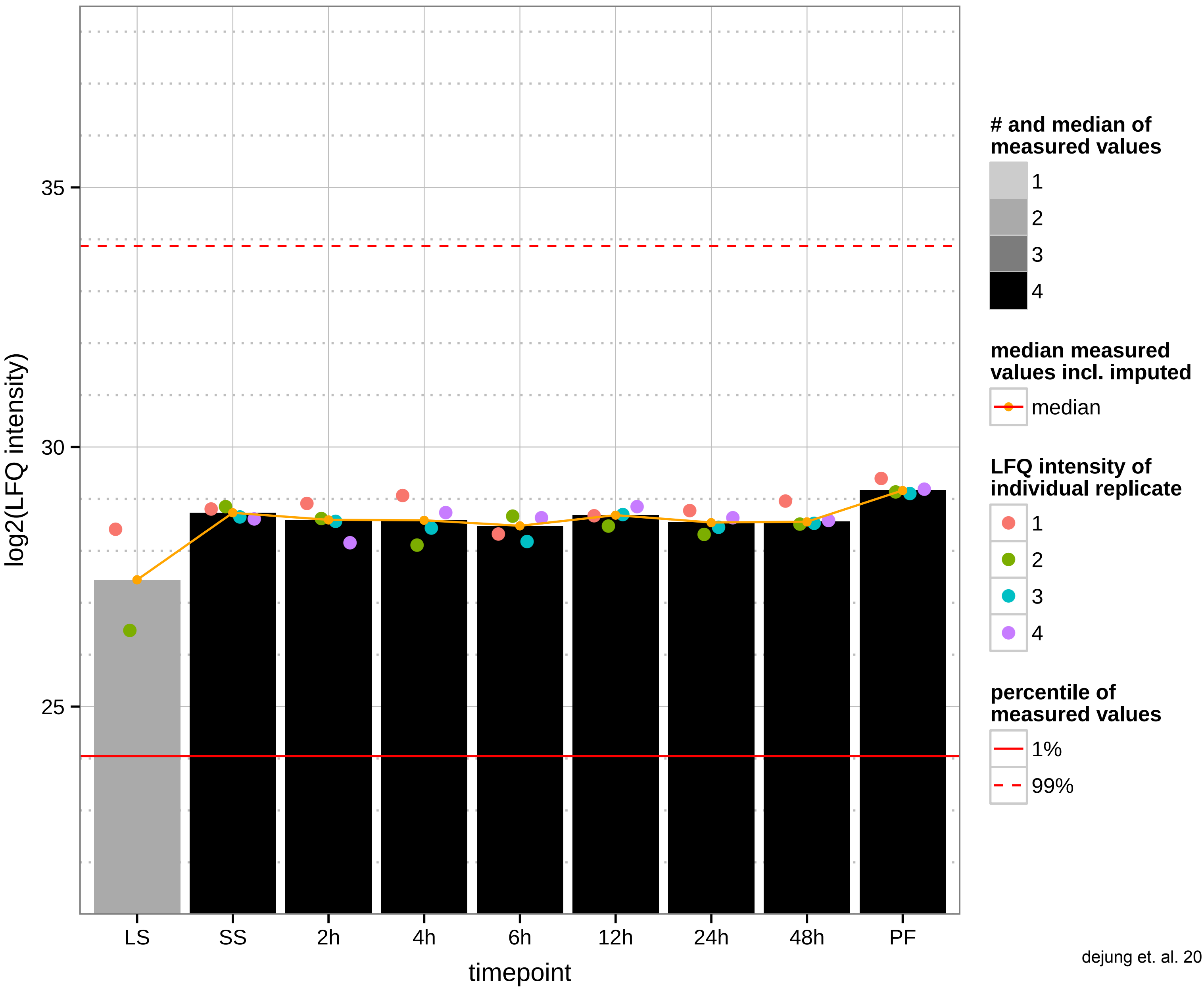

dejung et. al. 2015

transcription elongation factor s–II, putative (TFIIS2–2)
Tb927.2.3480
AGOF: DNA binding
AGOC: nucleus
AGOP: transcription, DNA–dependent
PGOF: DNA binding
PGOC: nucleus
PGOP: transcription, DNA–dependent

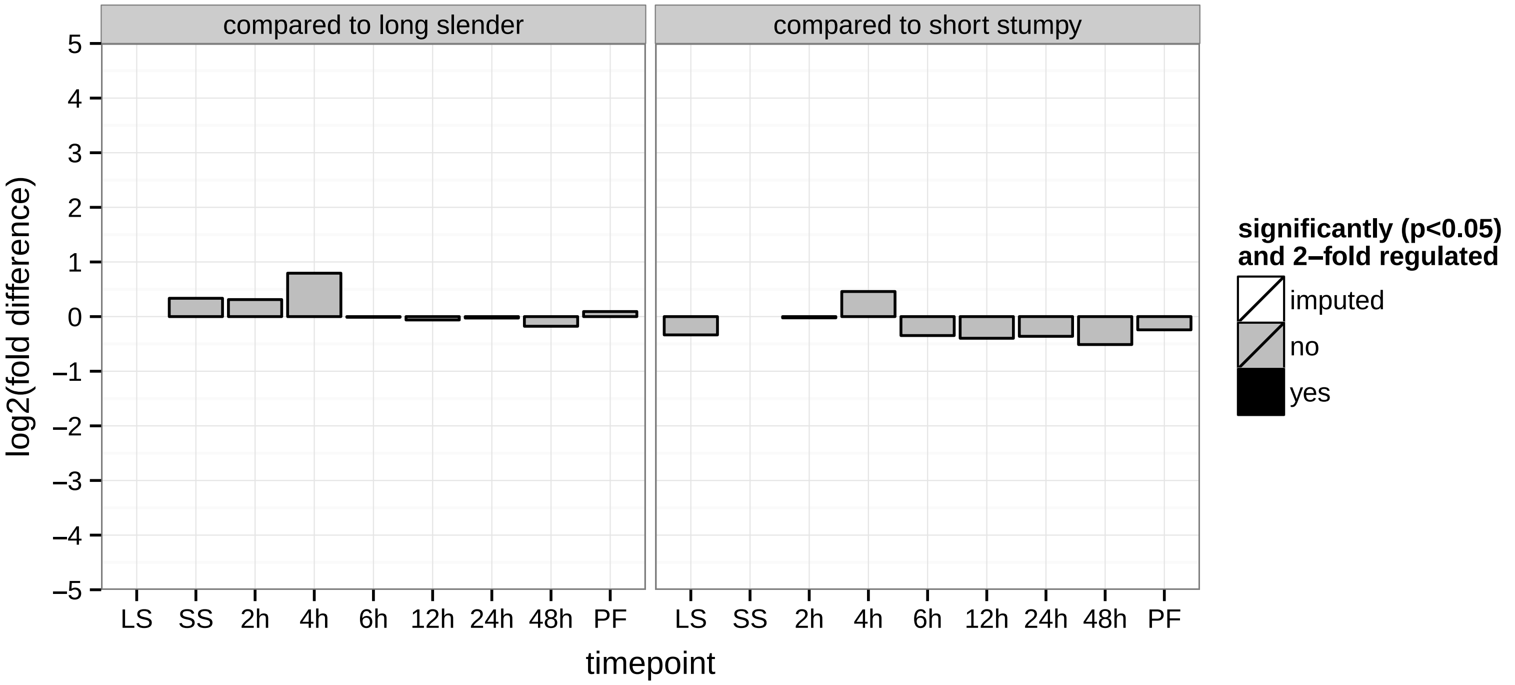

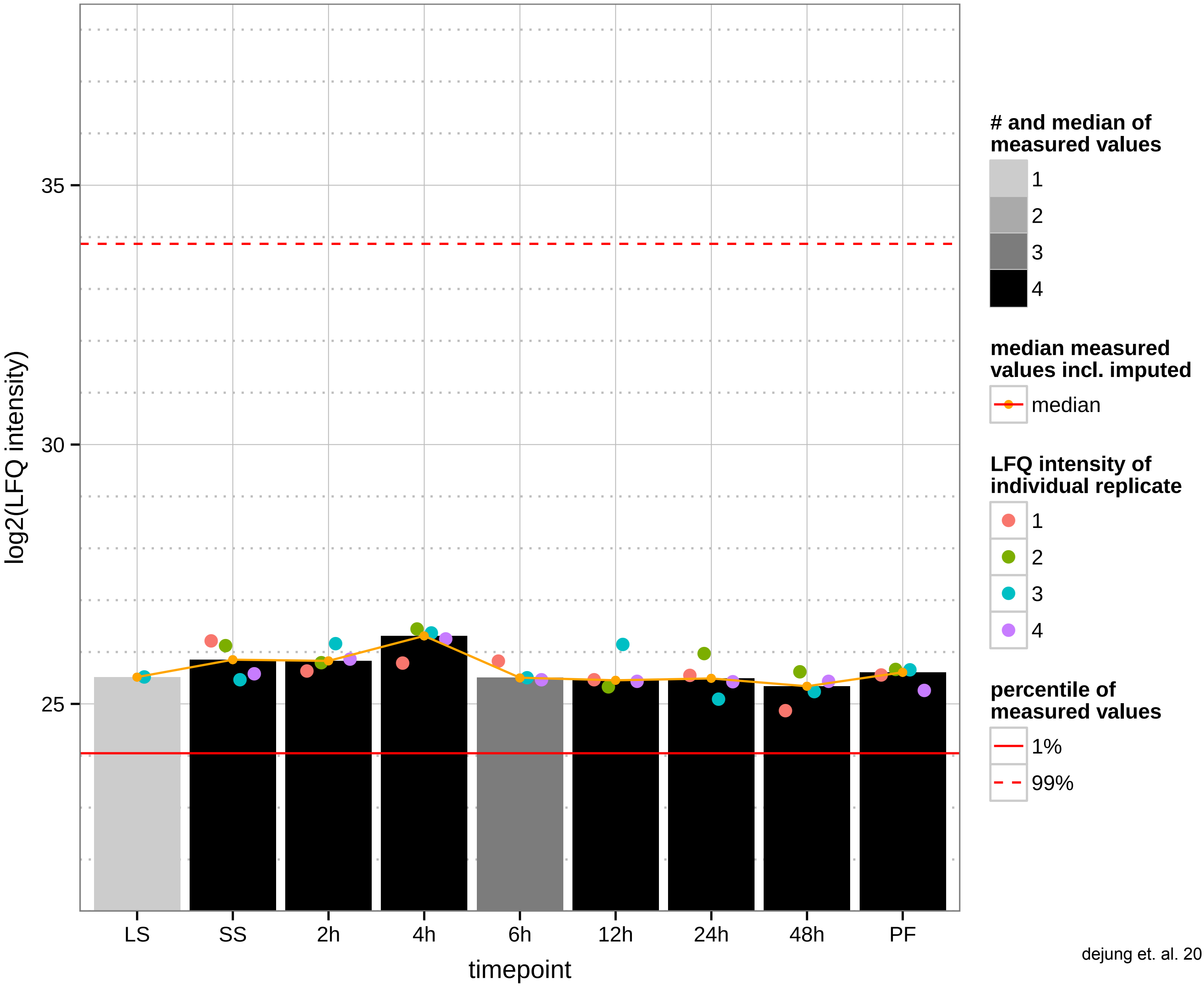

dejung et. al. 2015

transcription elongation factor s–II, putative (TFIIS2–1)
Tb927.2.3580
AGOF: DNA binding, sequence–specific DNA binding transcription factor activity, zinc ion binding
AGOC: nucleus
AGOP: DNA–dependent transcription, elongation, regulation of transcription, DNA–dependent, transcription
PGOF: DNA binding, nucleic acid binding, zinc ion binding
PGOC: nucleus
PGOP: transcription, DNA–dependent

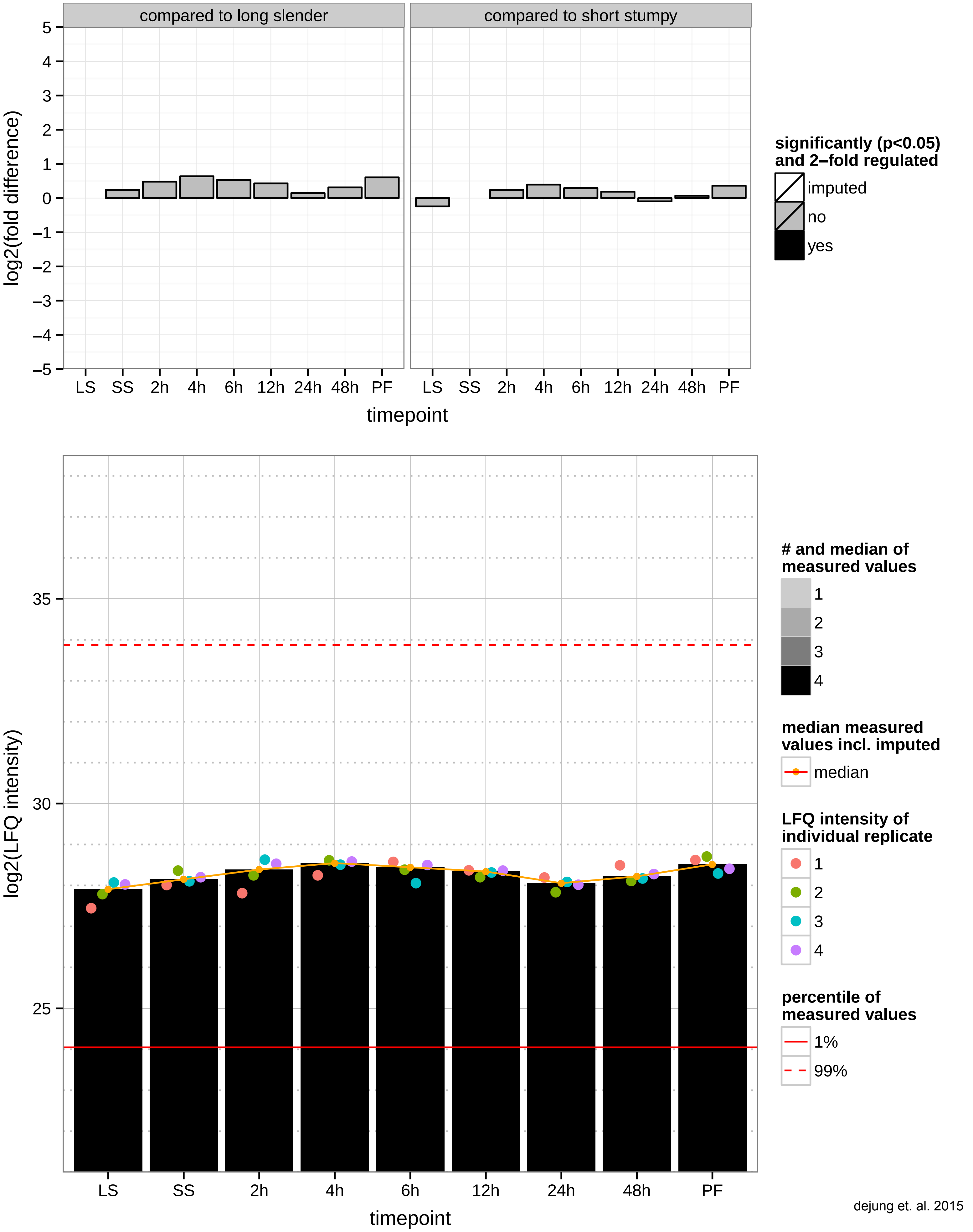

dejung et. al. 2015

retrotransposon hot spot protein 1 (RHS1), putative
Tb927.2.370
AGOF: null
AGOC: nucleus
AGOP: null
PGOF: null
PGOC: null
PGOP: null

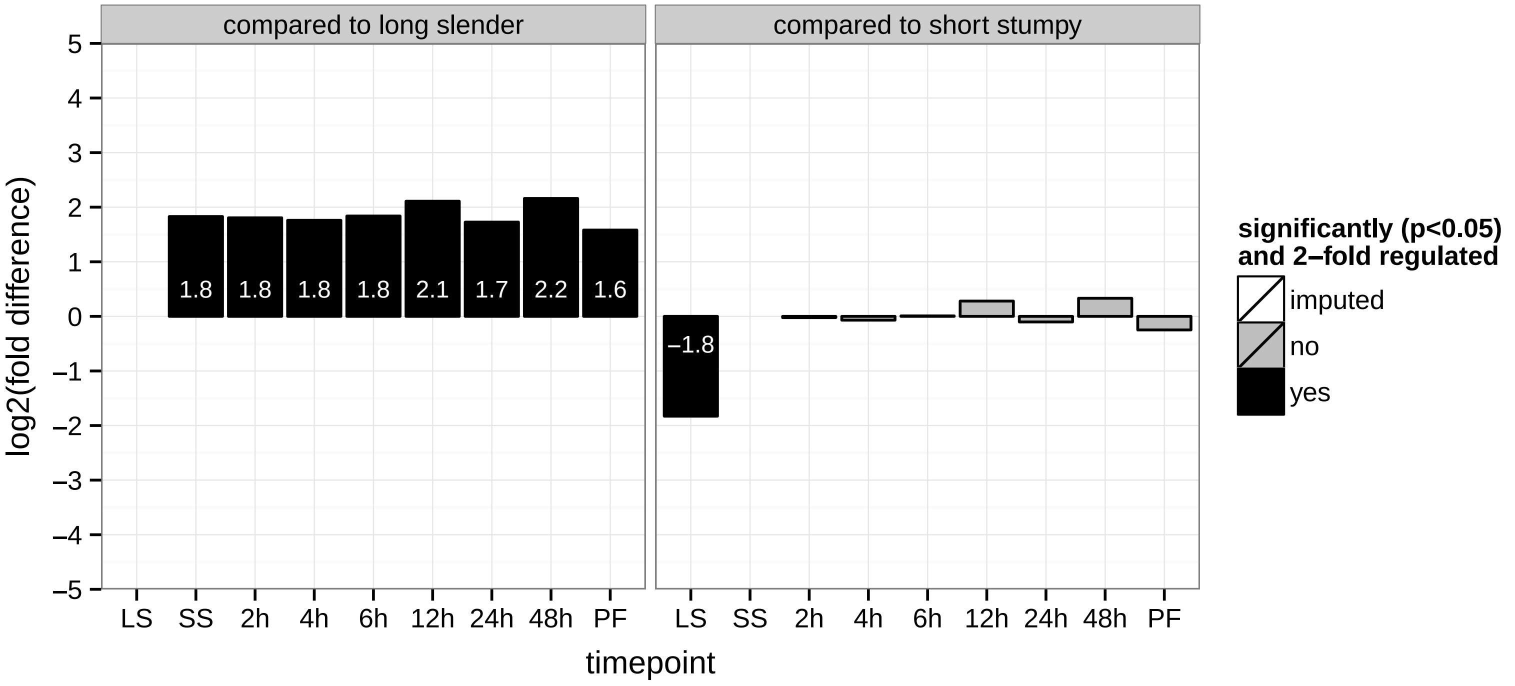

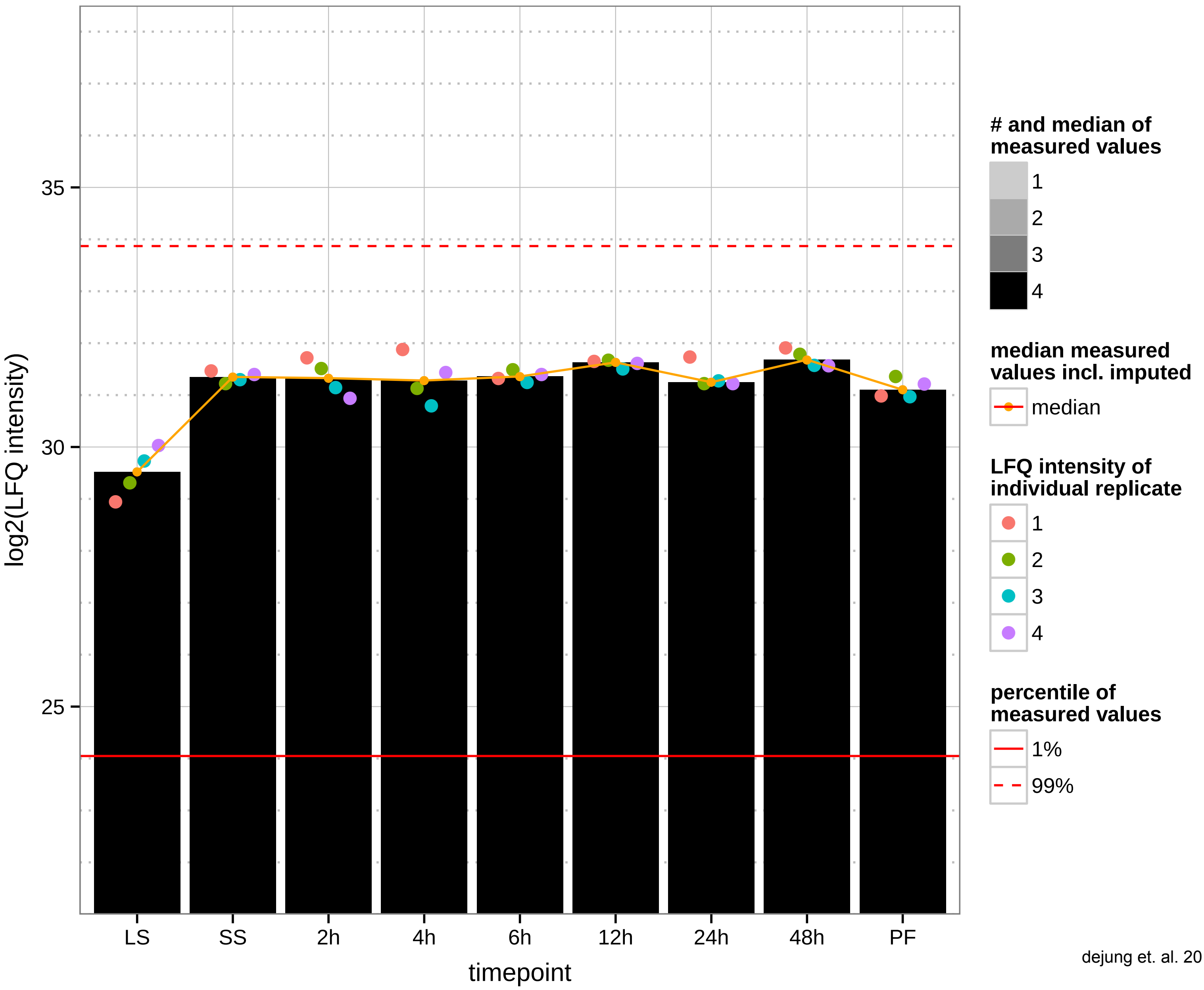

dejung et. al. 2015

retrotransposon hot spot protein 2 (RHS2), putative
Tb927.2.380
AGOF: null
AGOC: nucleus
AGOP: null
PGOF: null
PGOC: null
PGOP: null

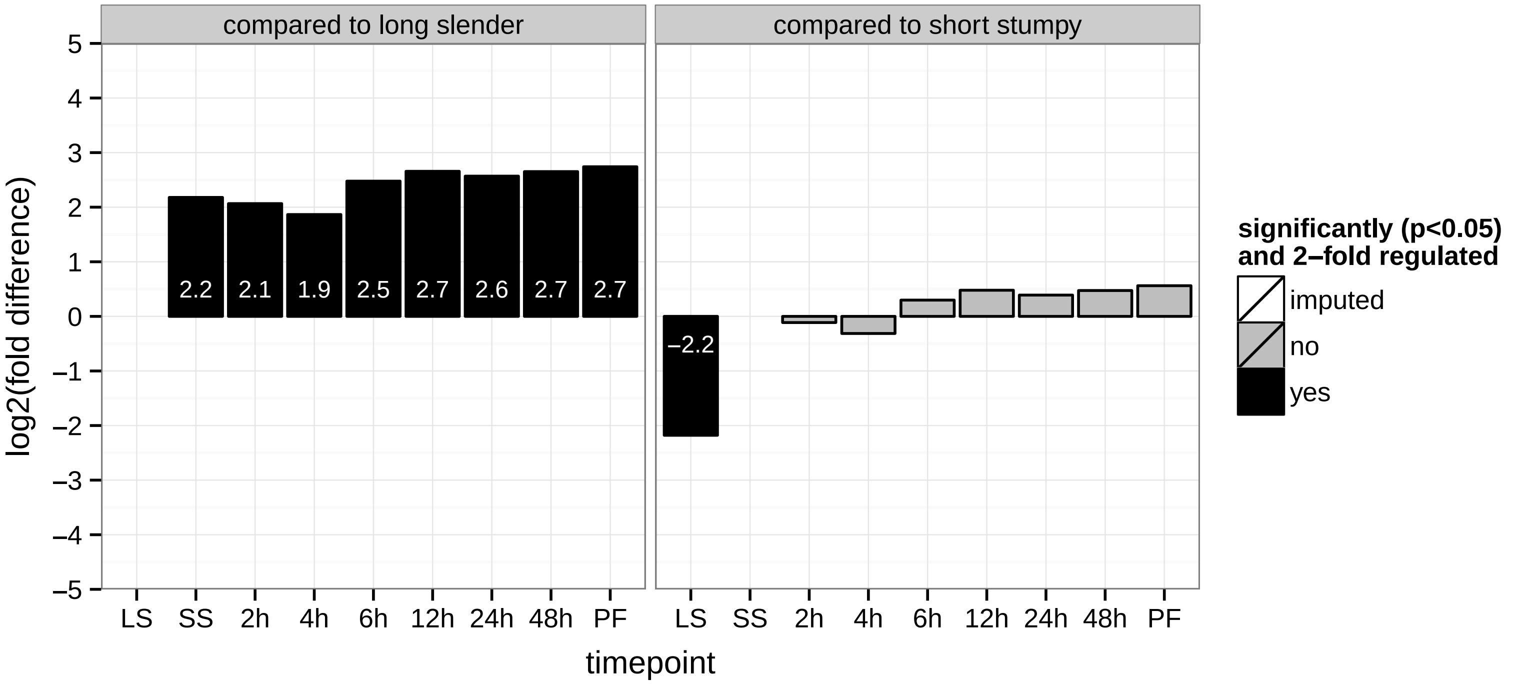

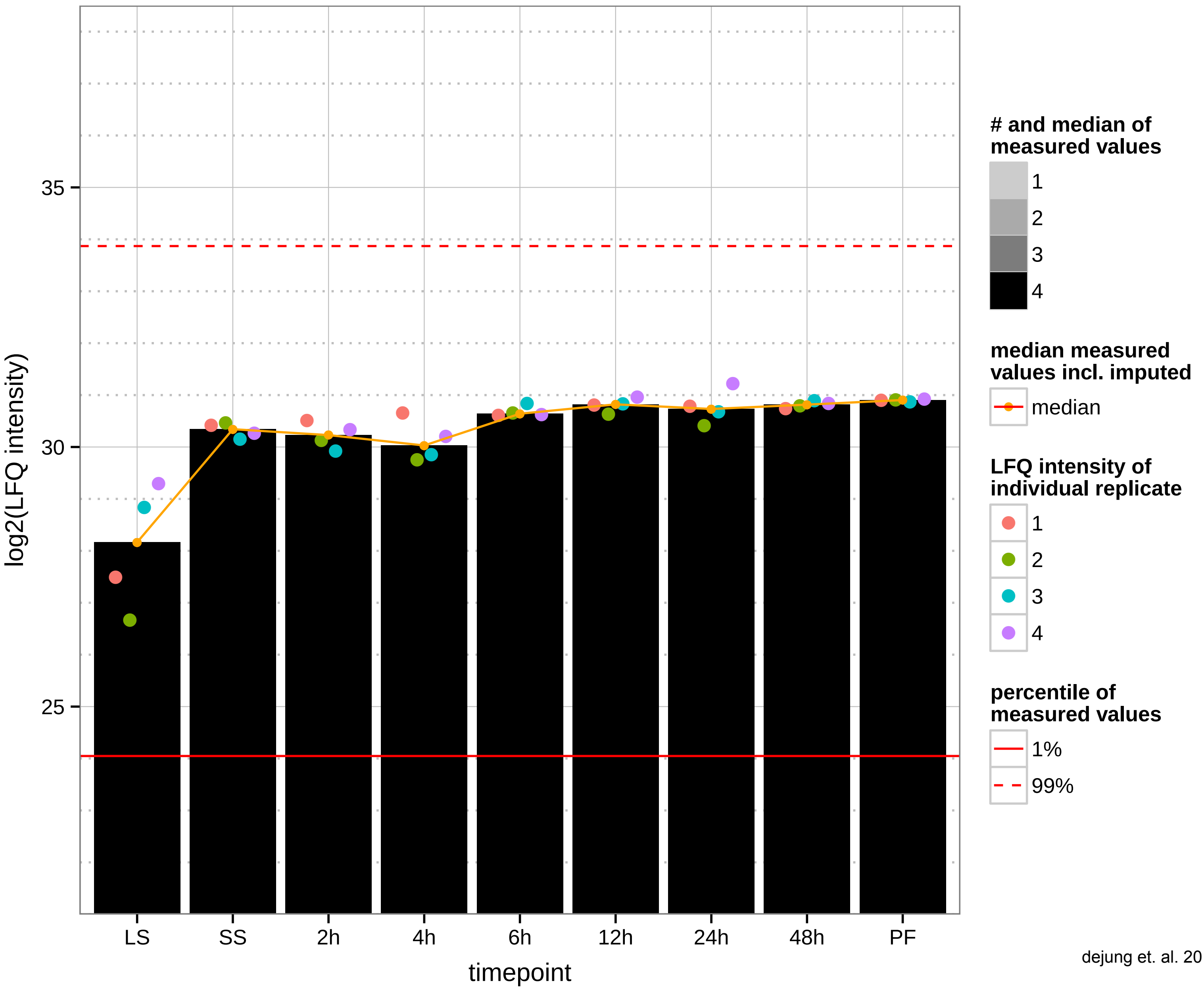

dejung et. al. 2015

guide RNA associated protein, GAP1, MRB1–associated protein
Tb927.2.3800
AGOF: mRNA binding
AGOC: mitochondrion
AGOP: mitochondrial RNA processing
PGOF: null
PGOC: null
PGOP: null

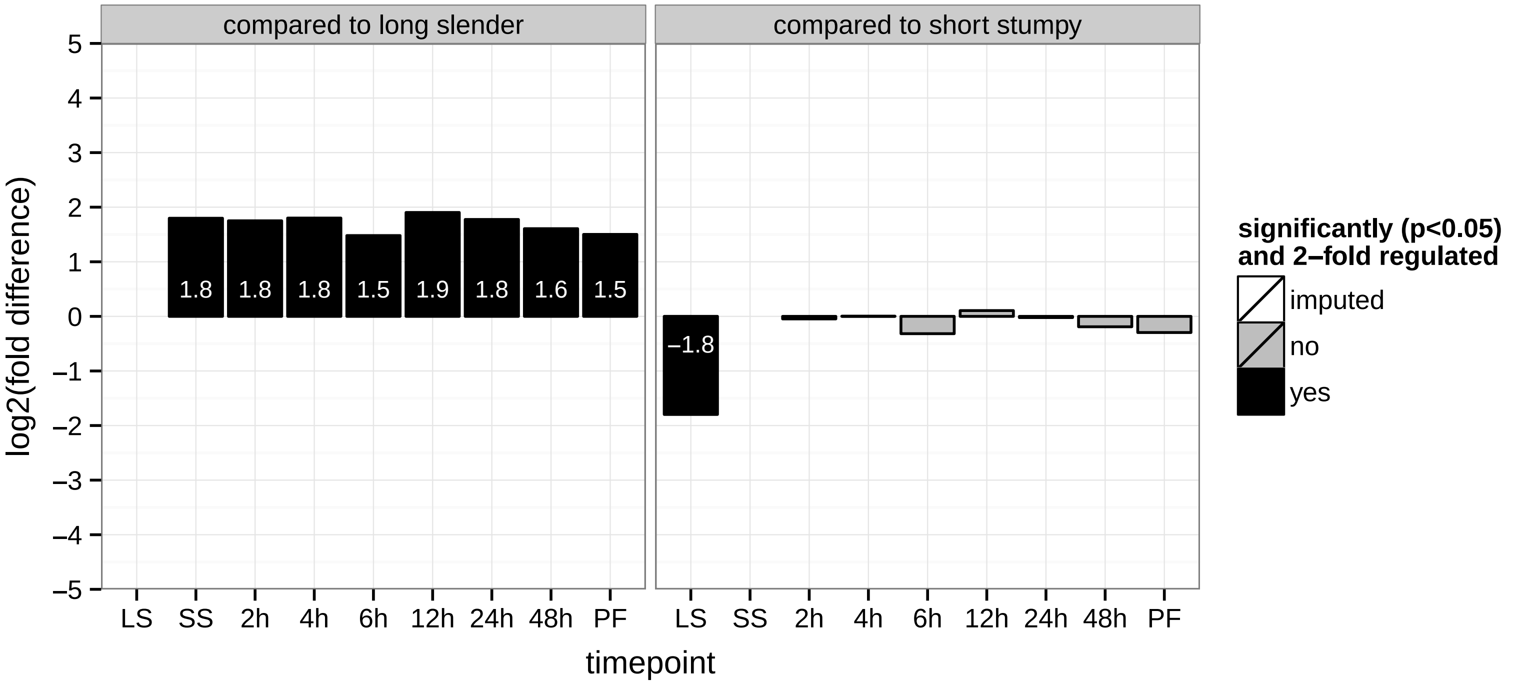

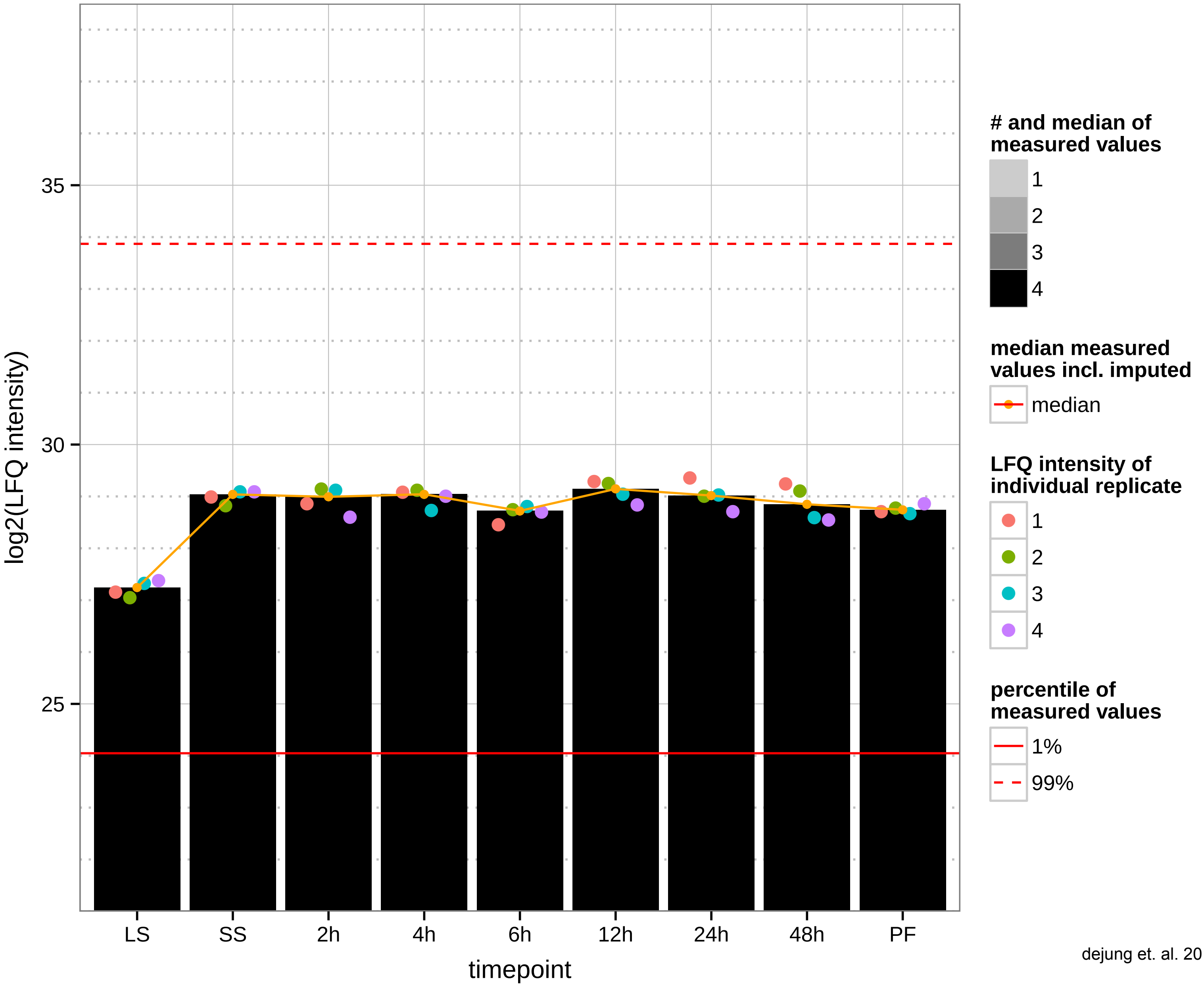

dejung et. al. 2015

retrotransposon hot spot protein 2 (RHS2), putative
Tb927.2.400
AGOF: null
AGOC: nucleus
AGOP: null
PGOF: null
PGOC: null
PGOP: null

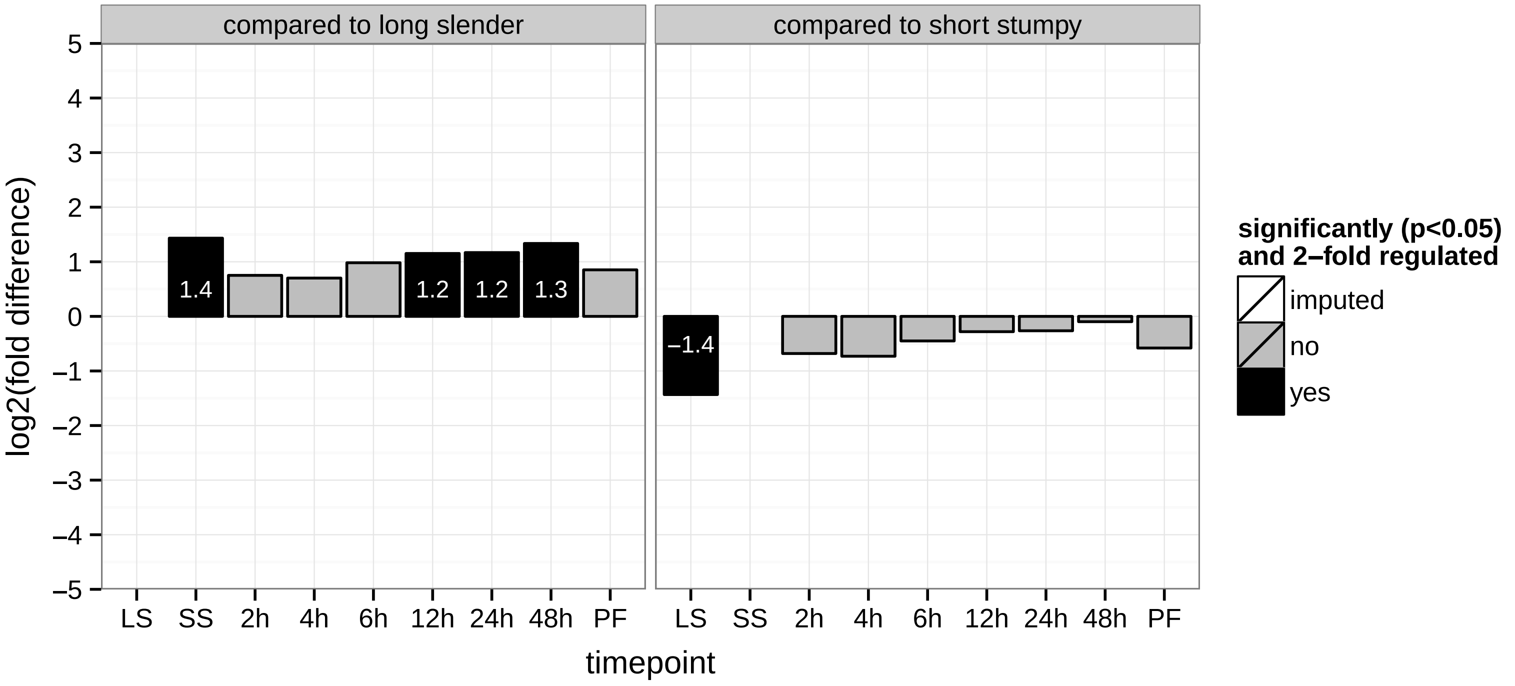

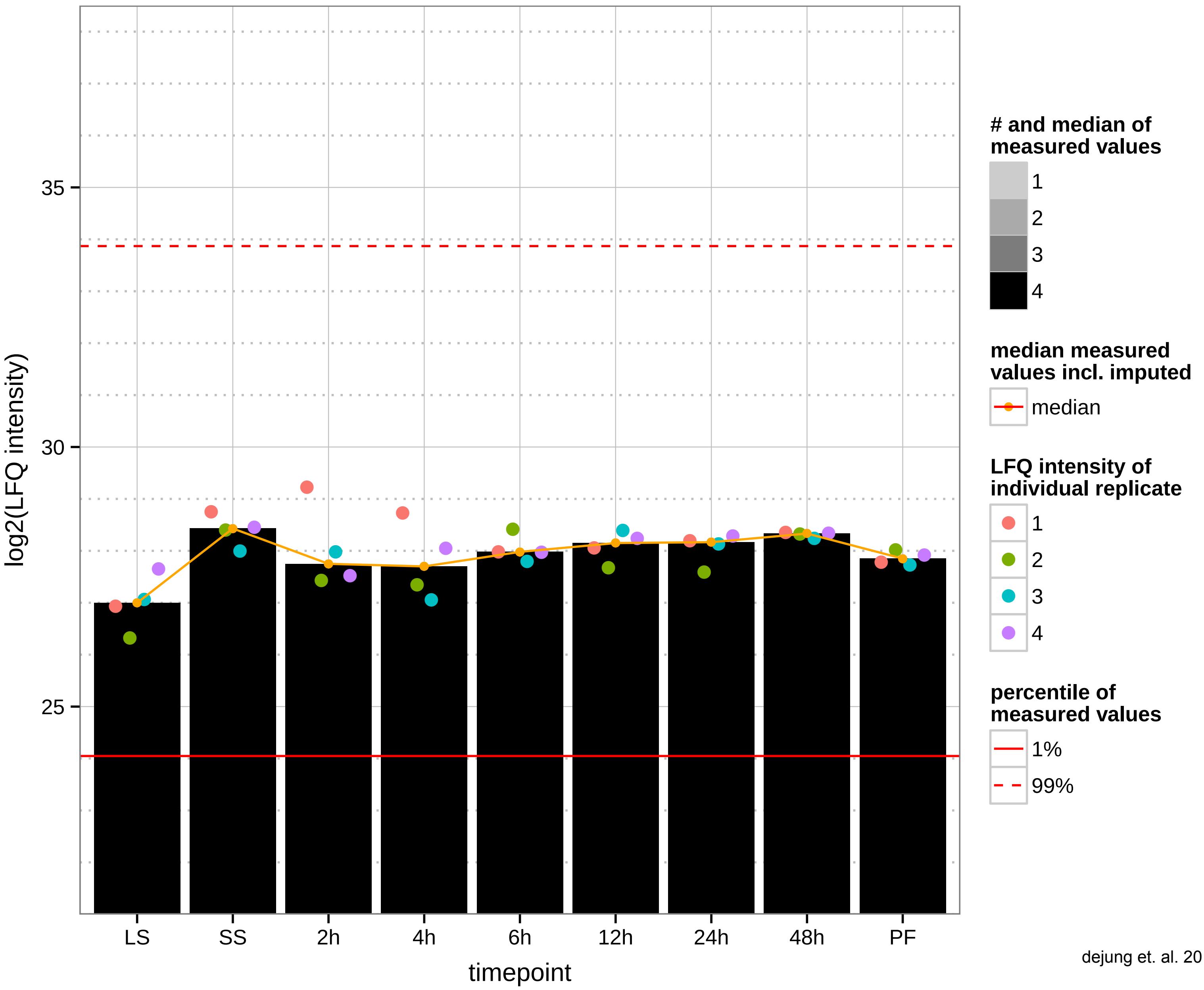

dejung et. al. 2015

dynein intermediate chain IC 138, putative
Tb927.2.4060
AGOF: motor activity
AGOC: cytoskeleton, dynein complex
AGOP: microtubule−based movement
PGOF: protein binding
PGOC: null
PGOP: null

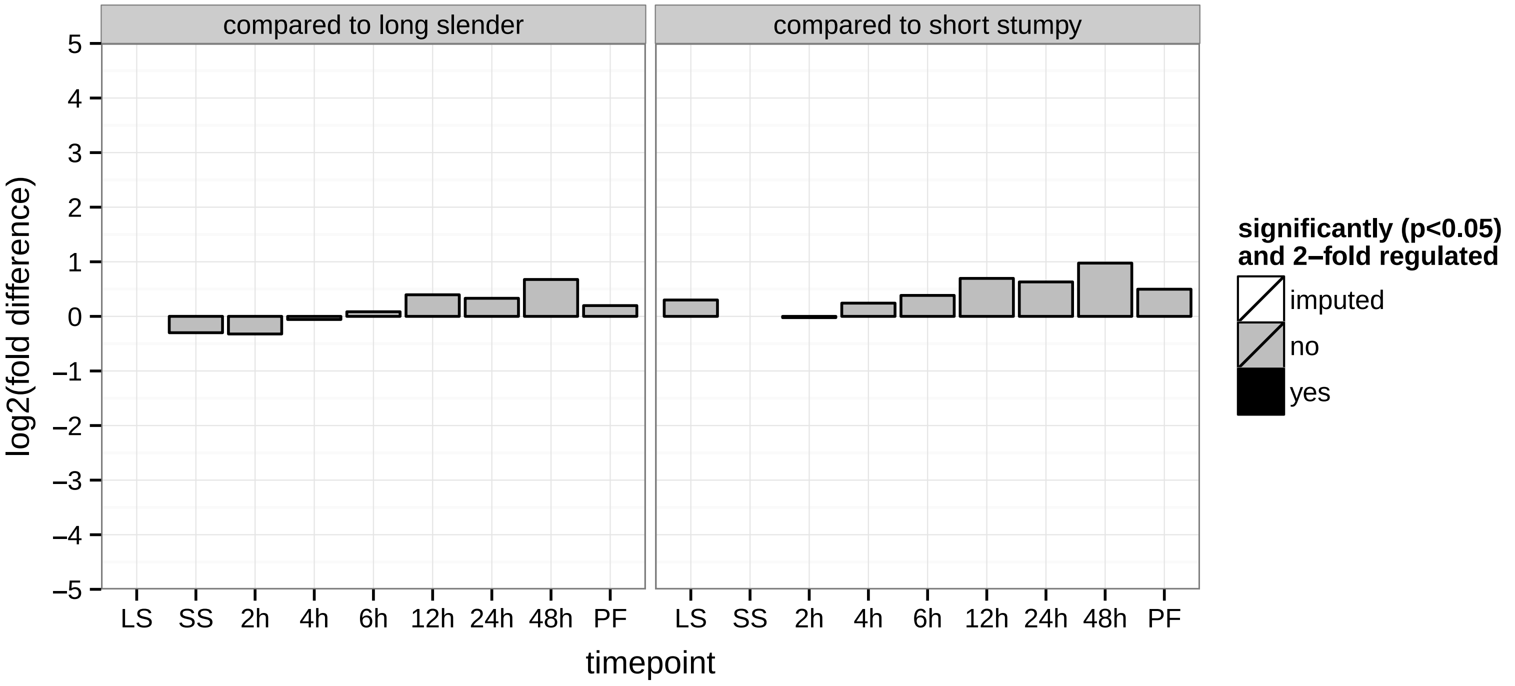

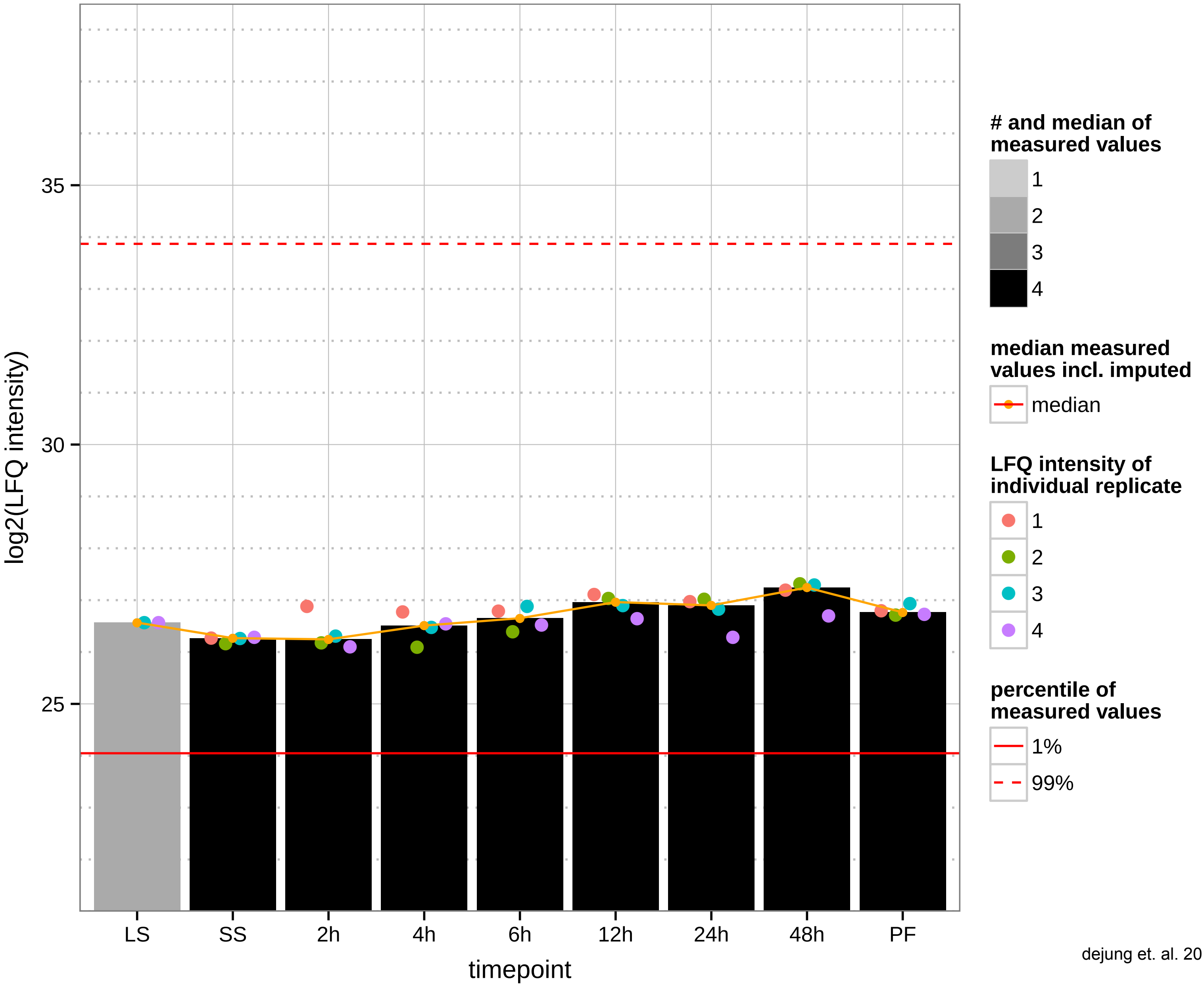

dejung et. al. 2015

hypothetical protein, conserved
Tb927.2.4090
AGOF: null
AGOC: mitochondrial inner membrane
AGOP: null
PGOF: GTP binding
PGOC: null
PGOP: SRP–dependent cotranslational protein targeting to membrane

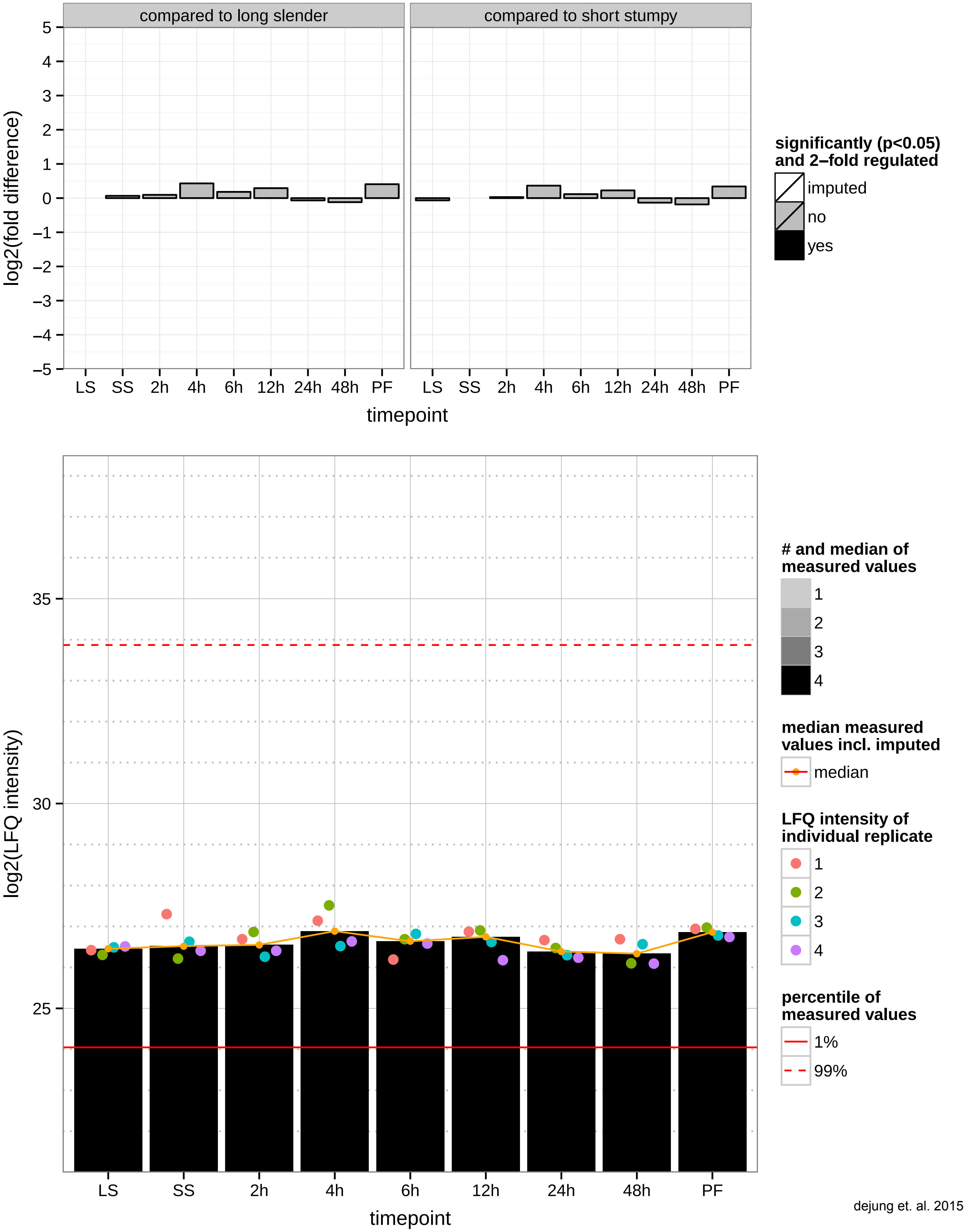

dejung et. al. 2015

mitochondrial processing peptidase alpha subunit, putative, metallo–peptidase, Clan ME, Family M16
Tb927.2.4110
AGOF: catalytic activity, metalloendopeptidase activity, zinc ion binding
AGOC: mitochondrion
AGOP: proteolysis
PGOF: catalytic activity, metal ion binding, metalloendopeptidase activity, zinc ion binding
PGOC: null
PGOP: proteolysis

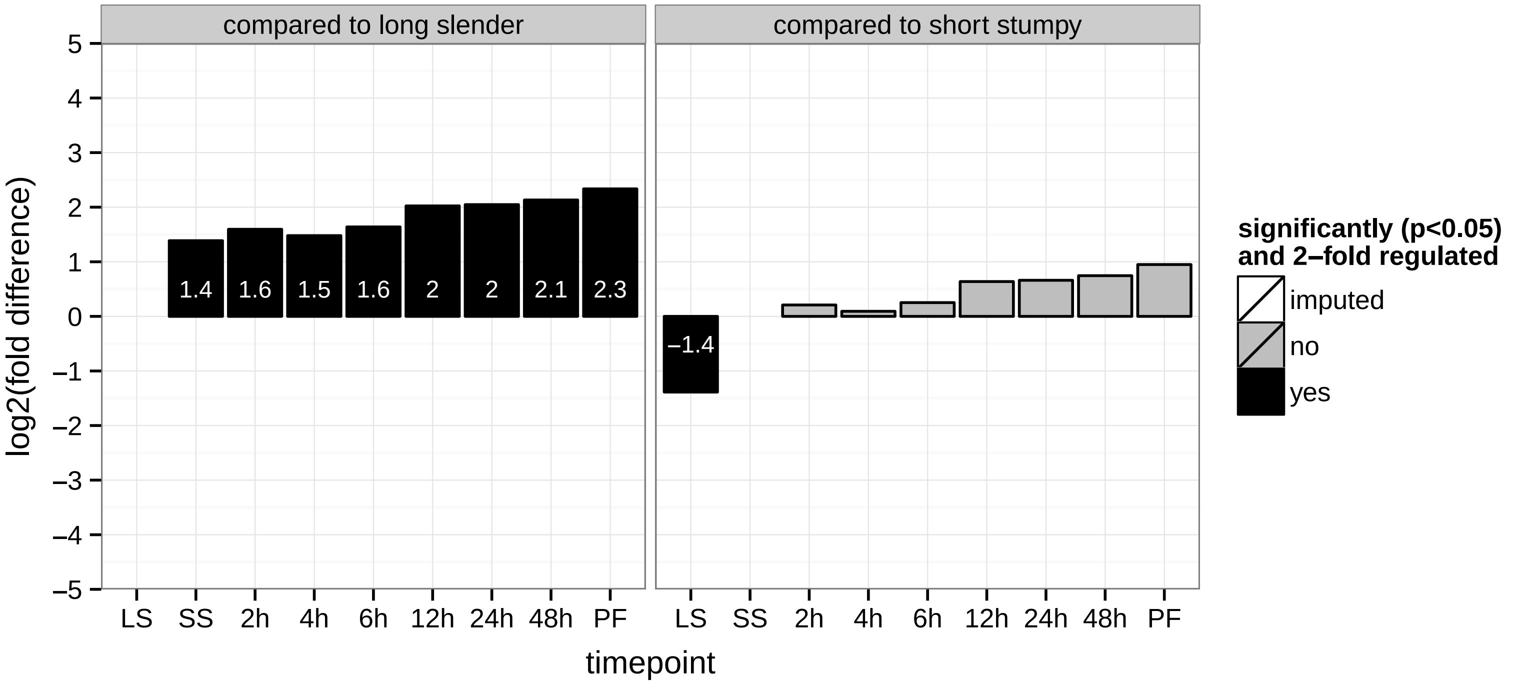

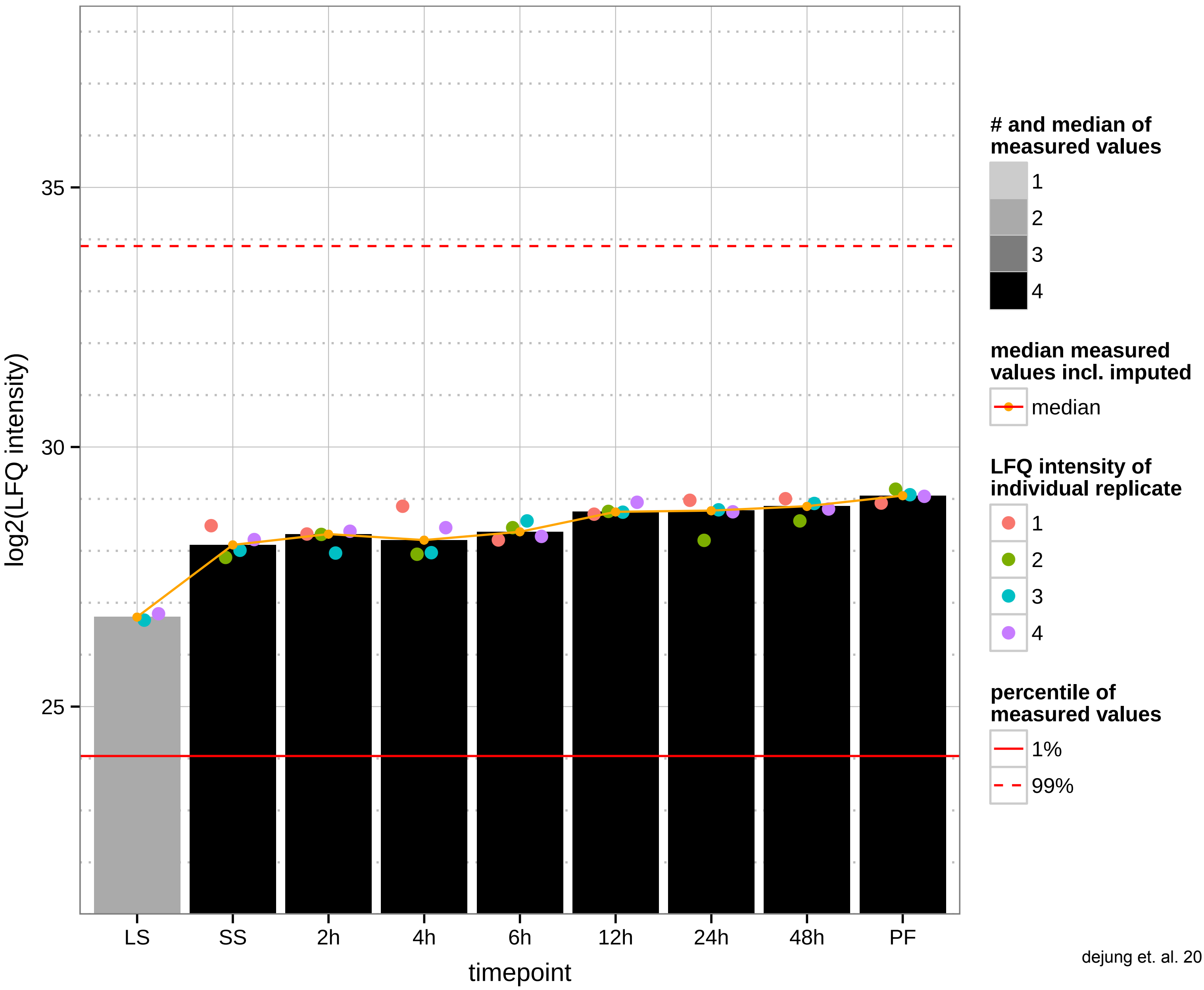

dejung et. al. 2015

paraflagellar rod protein, putative, PFR5
Tb927.2.4330;Tb11.v5.0777
AGOF: null, calmodulin binding, structural molecule activity
AGOC: null, microtubule–based flagellum
AGOP: null, ciliary or flagellar motility
PGOF: calmodulin binding, protein binding
PGOC: microtubule–based flagellum
PGOP: null

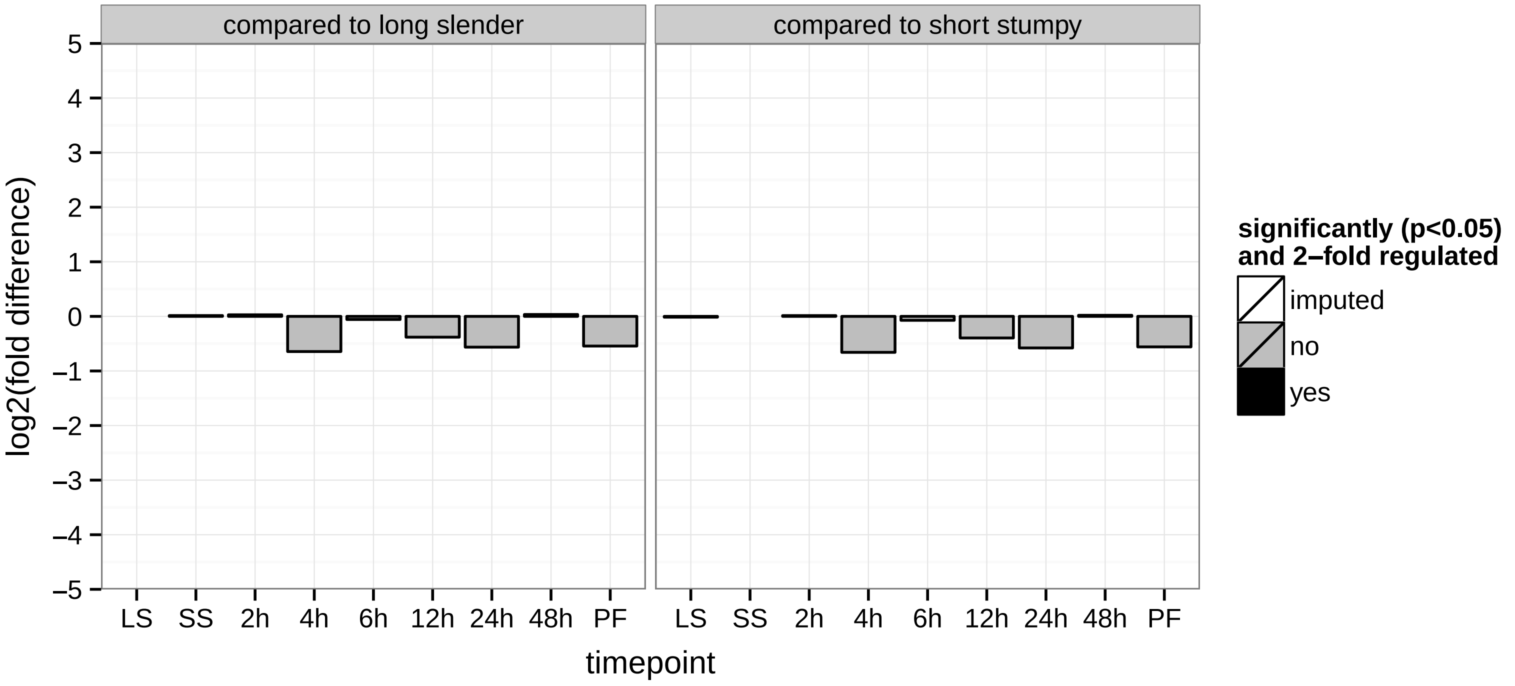

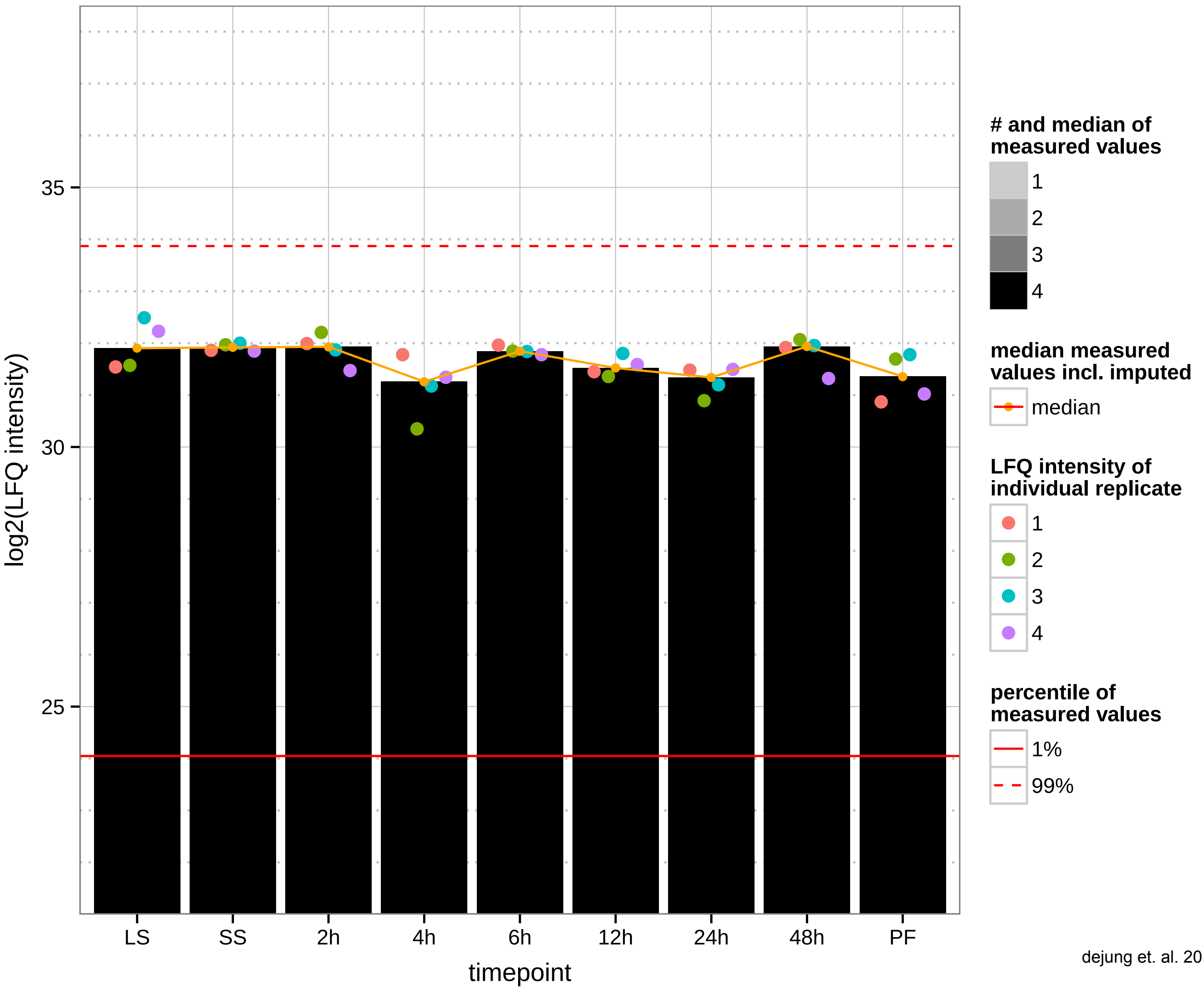

dejung et. al. 2015

retrotransposon hot spot protein 4 (RHS4), putative
Tb927.2.450
AGOF: null
AGOC: nucleus
AGOP: null
PGOF: null
PGOC: null
PGOP: null

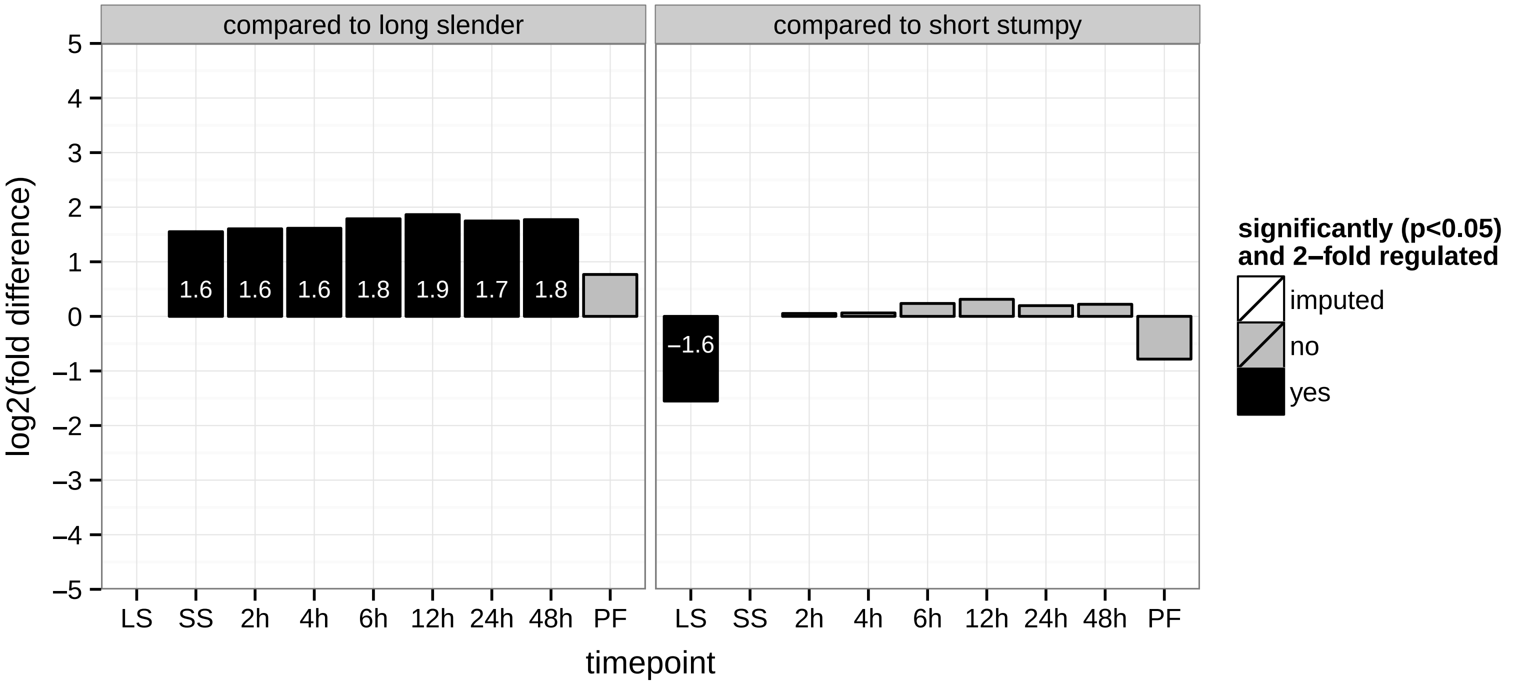

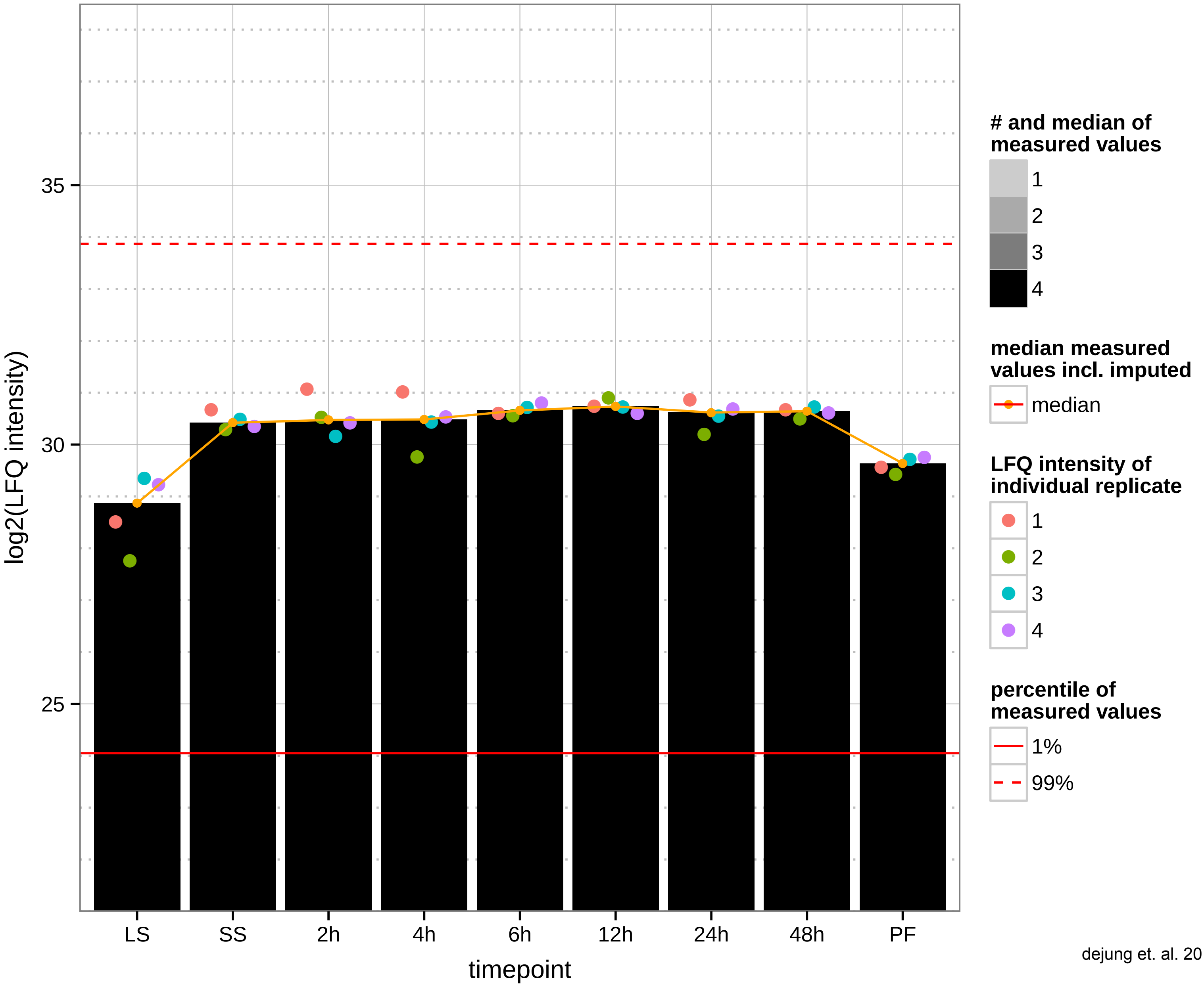

dejung et. al. 2015

hypothetical protein, conserved
Tb927.2.4520
AGOF: catalytic activity
AGOC: null
AGOP: metabolic process
PGOF: null
PGOC: null
PGOP: null

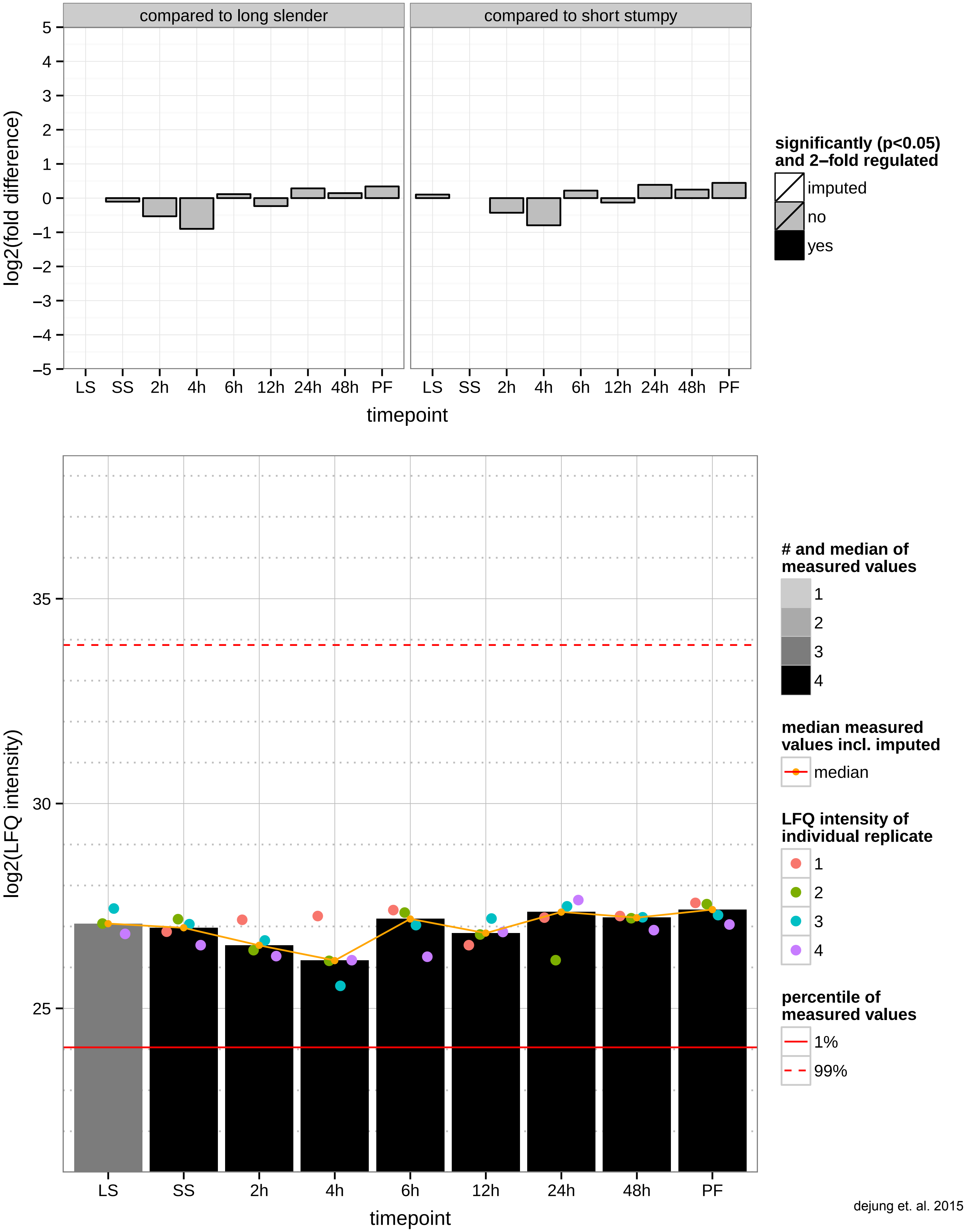

dejung et. al. 2015

hypothetical protein, conserved
Tb927.2.4530
AGOF: null
AGOC: null
AGOP: null
PGOF: null
PGOC: null
PGOP: null

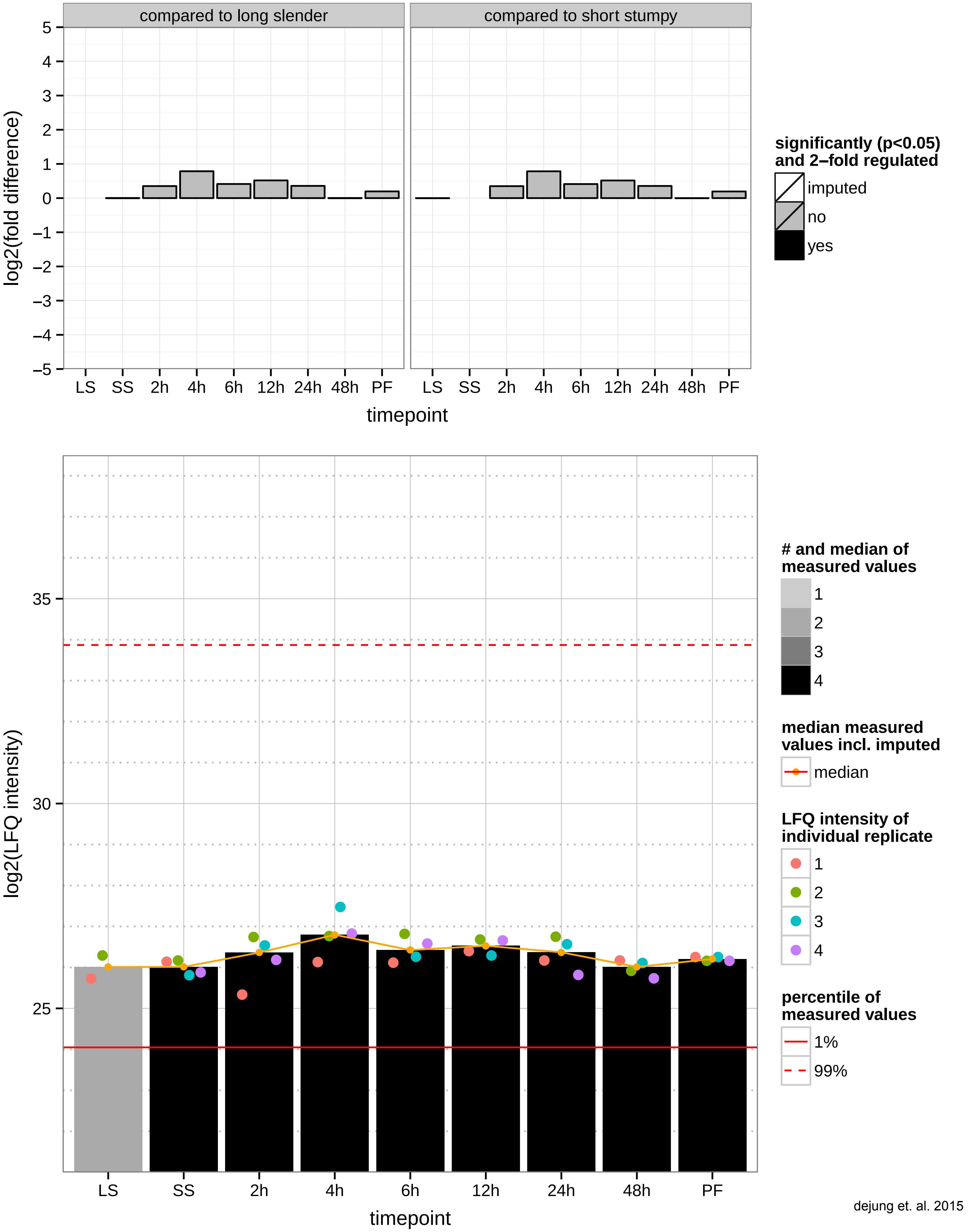

dejung et. al. 2015

Small nuclear ribonucleoprotein–associated protein B (snRNP–B) (Sm protein B) (Sm–B) (SmB), putative (TbSmB)
Tb927.2.4540
AGOF: null
AGOC: nucleus, small nucleolar ribonucleoprotein complex
AGOP: RNA splicing, RNA splicing, via transesterification reactions
PGOF: null
PGOC: null
PGOP: null

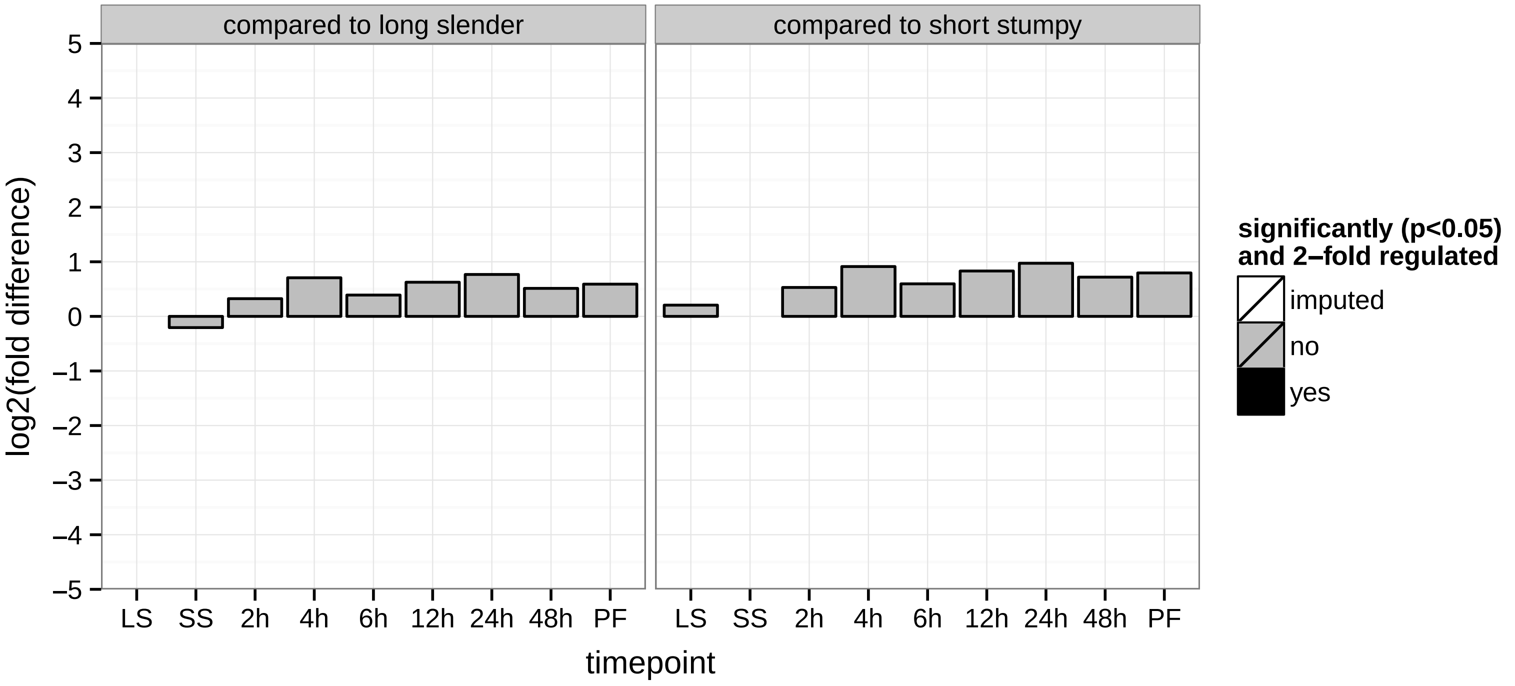

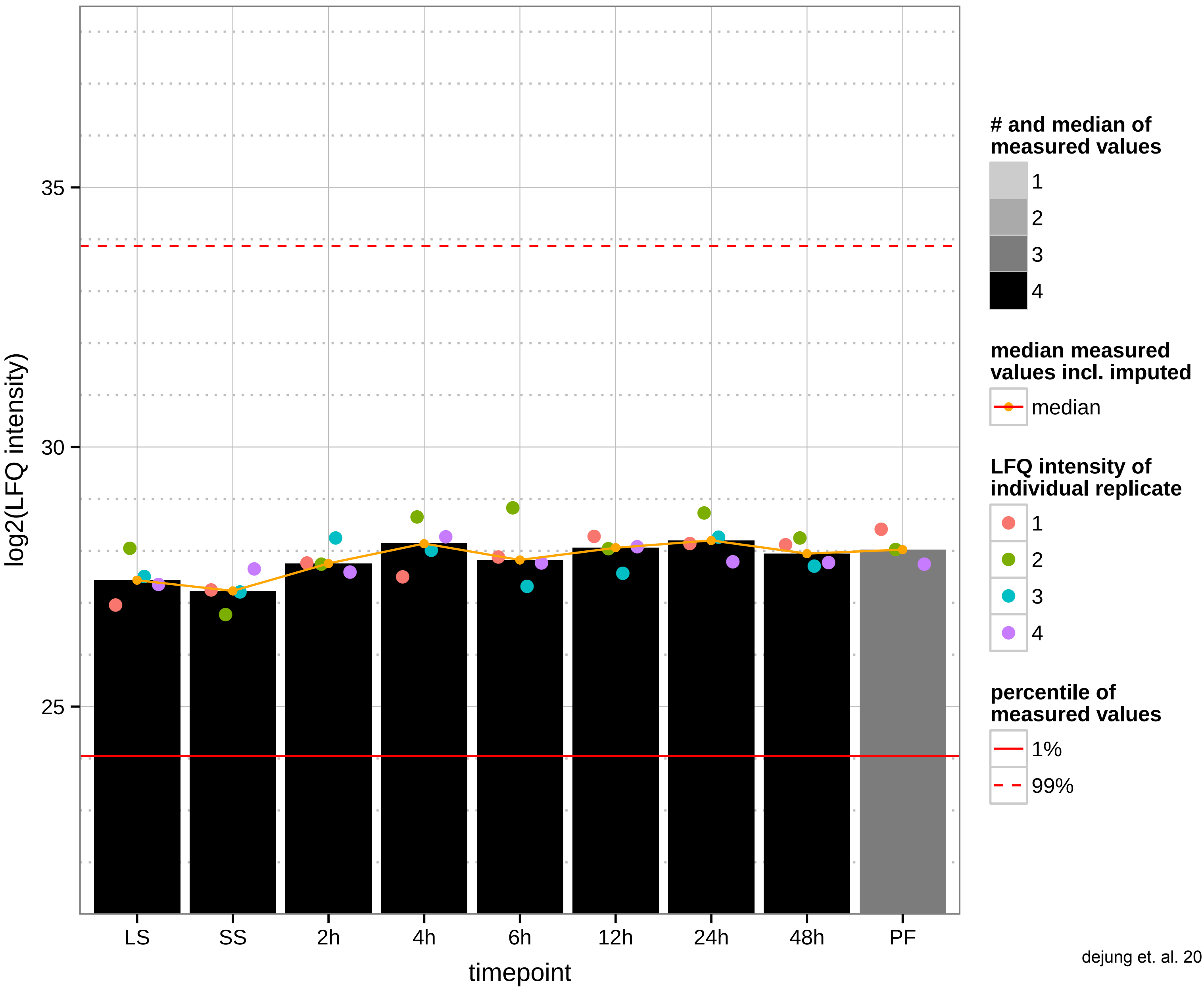

dejung et. al. 2015

UNC119 (unc119)
Tb927.2.4580
AGOF: null
AGOC: cilium
AGOP: null
PGOF: null
PGOC: null
PGOP: null

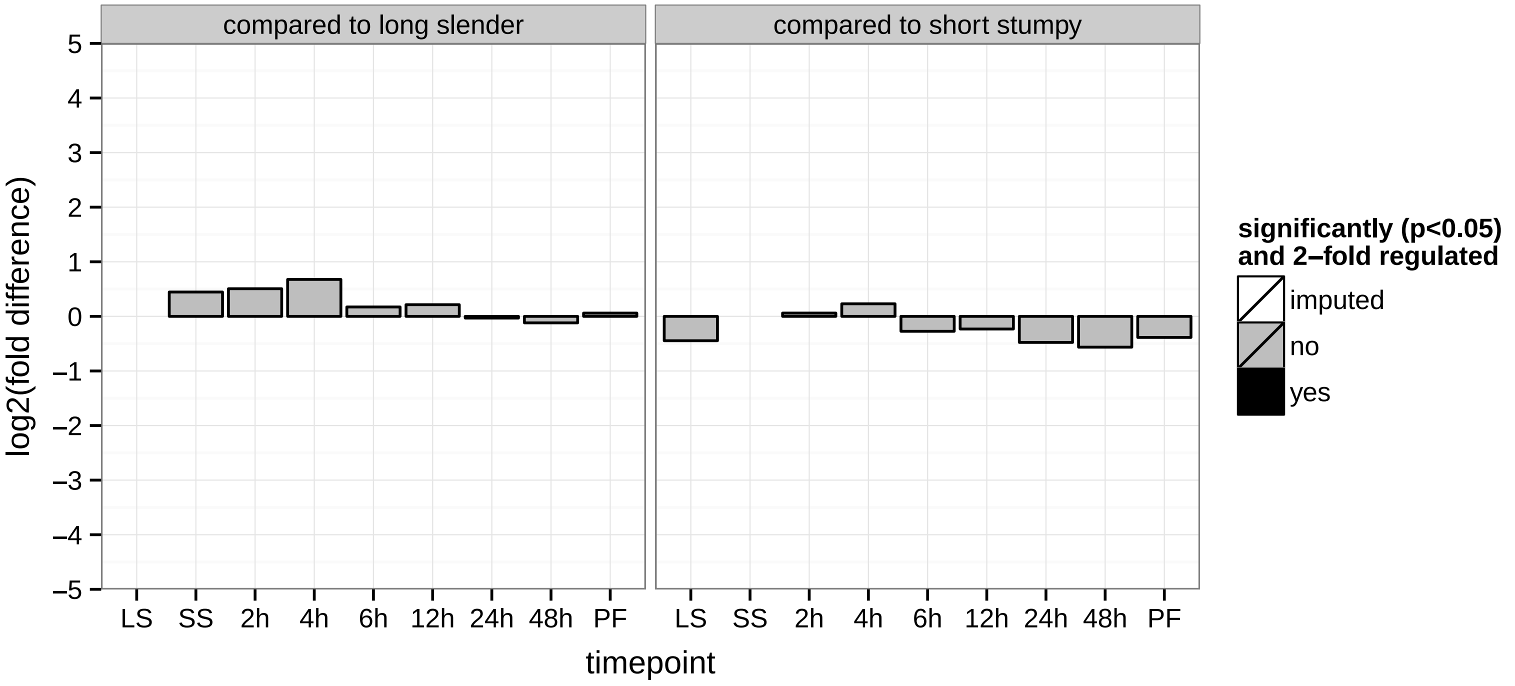

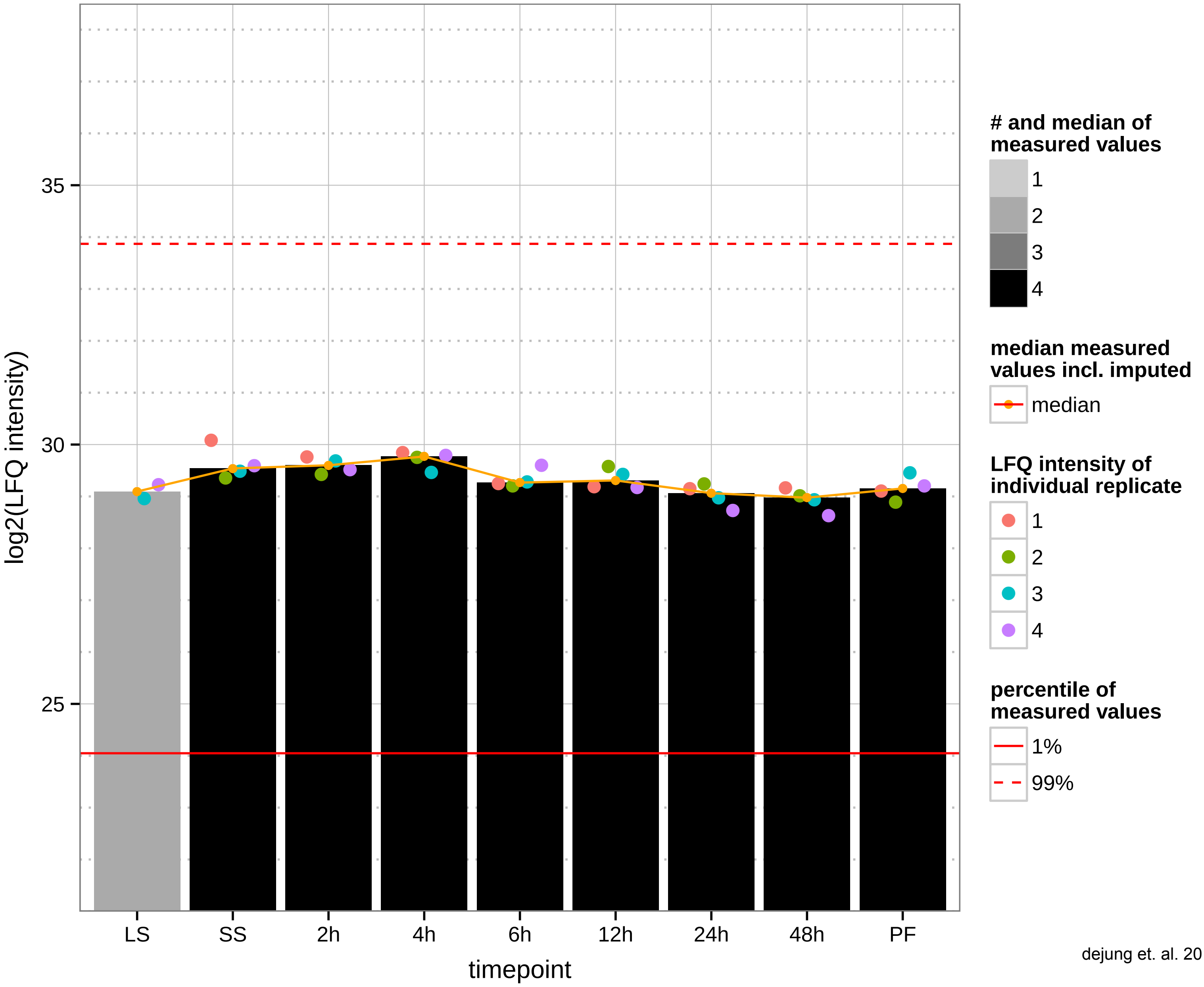

dejung et. al. 2015

RNA–binding protein, putative (TRRM1)
Tb927.2.4710
AGOF: molecular_function, nucleic acid binding, zinc ion binding
AGOC: nuclear speck
AGOP: RNA processing
PGOF: nucleic acid binding, zinc ion binding
PGOC: null
PGOP: null

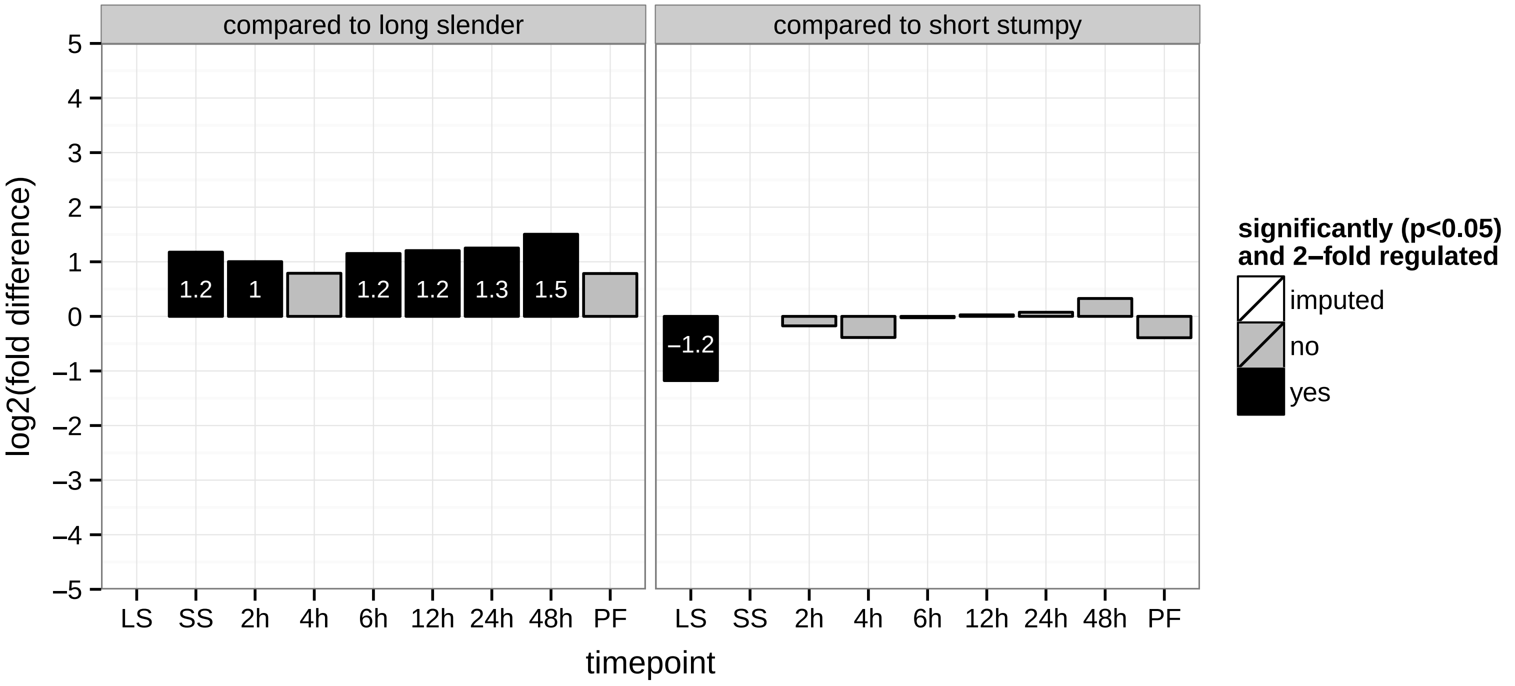

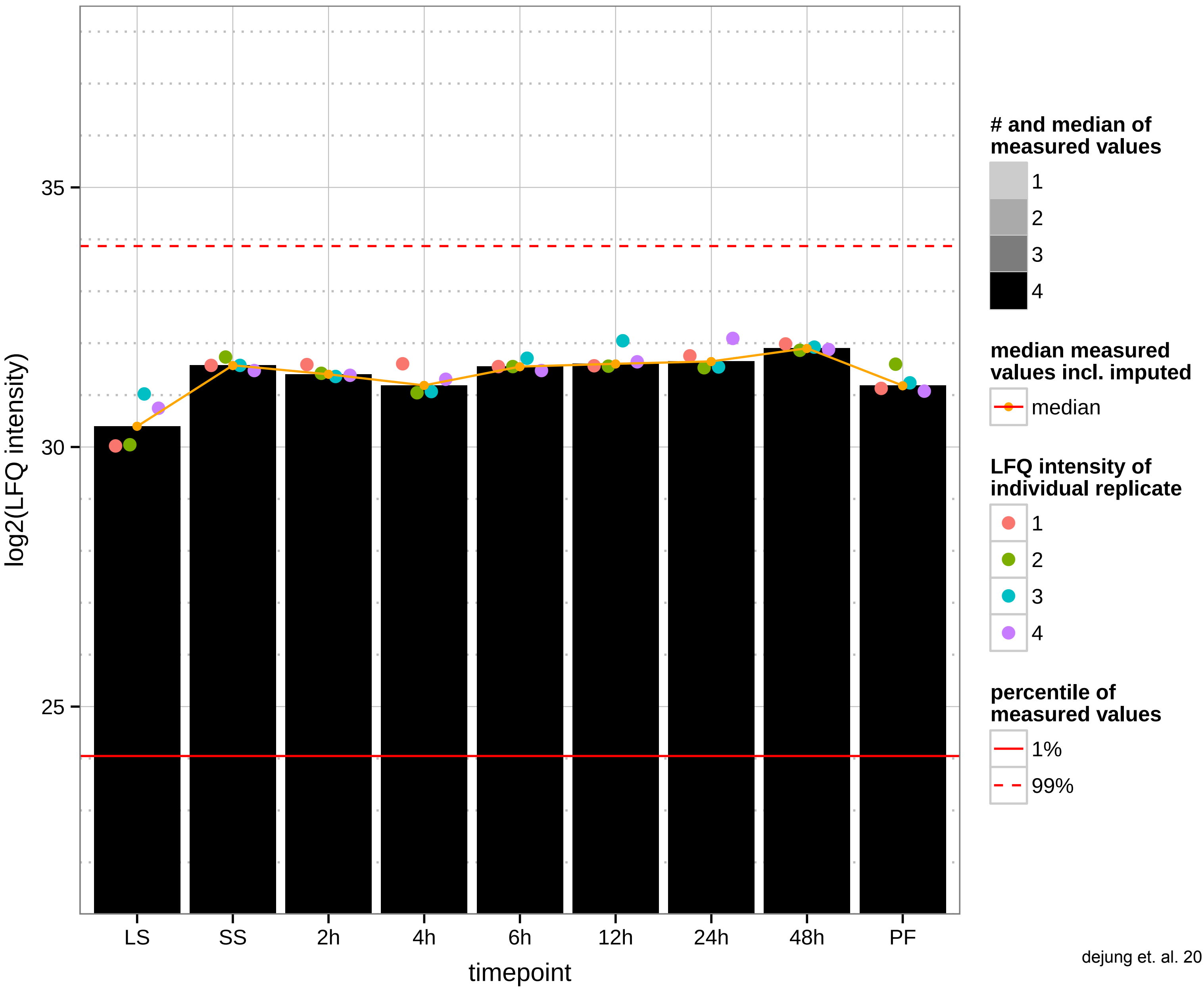

dejung et. al. 2015

hypothetical protein, conserved
Tb927.2.4800
AGOF: null
AGOC: null
AGOP: null
PGOF: null
PGOC: null
PGOP: null

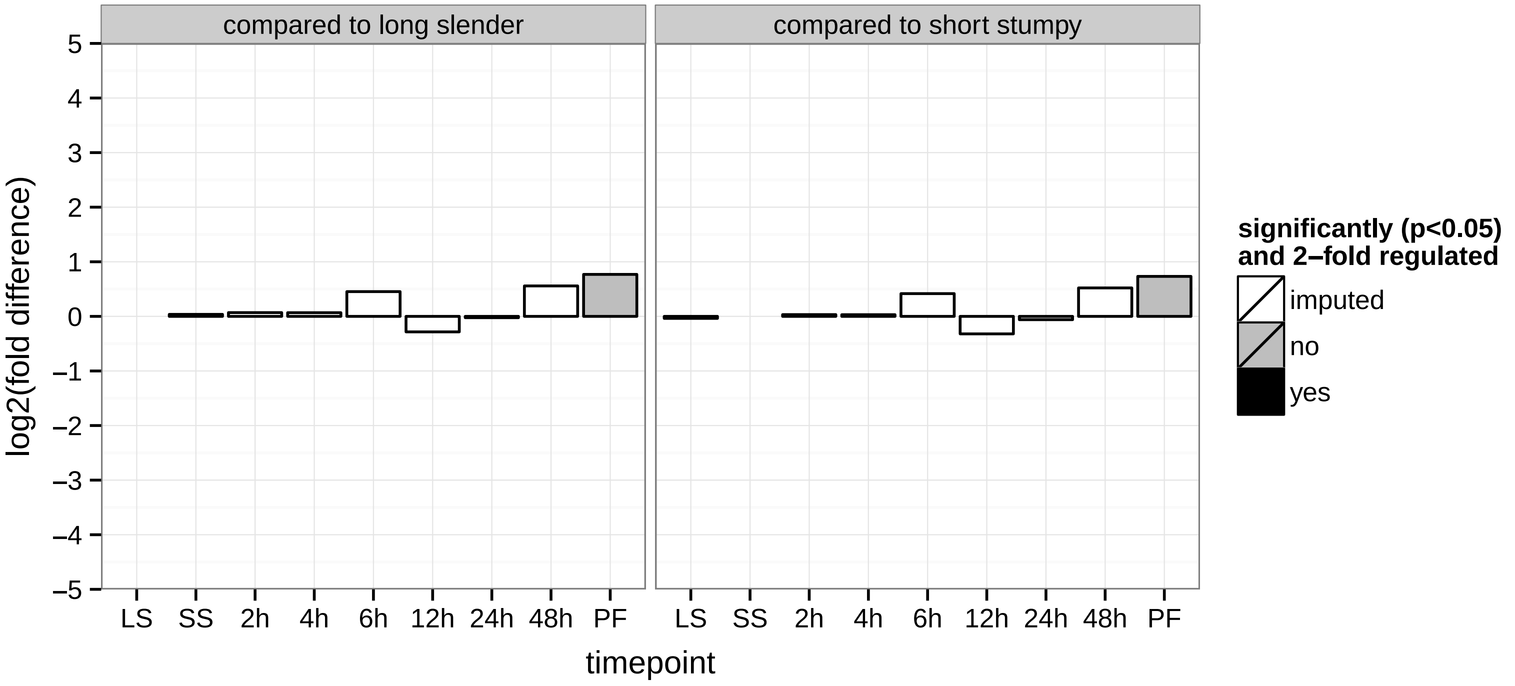

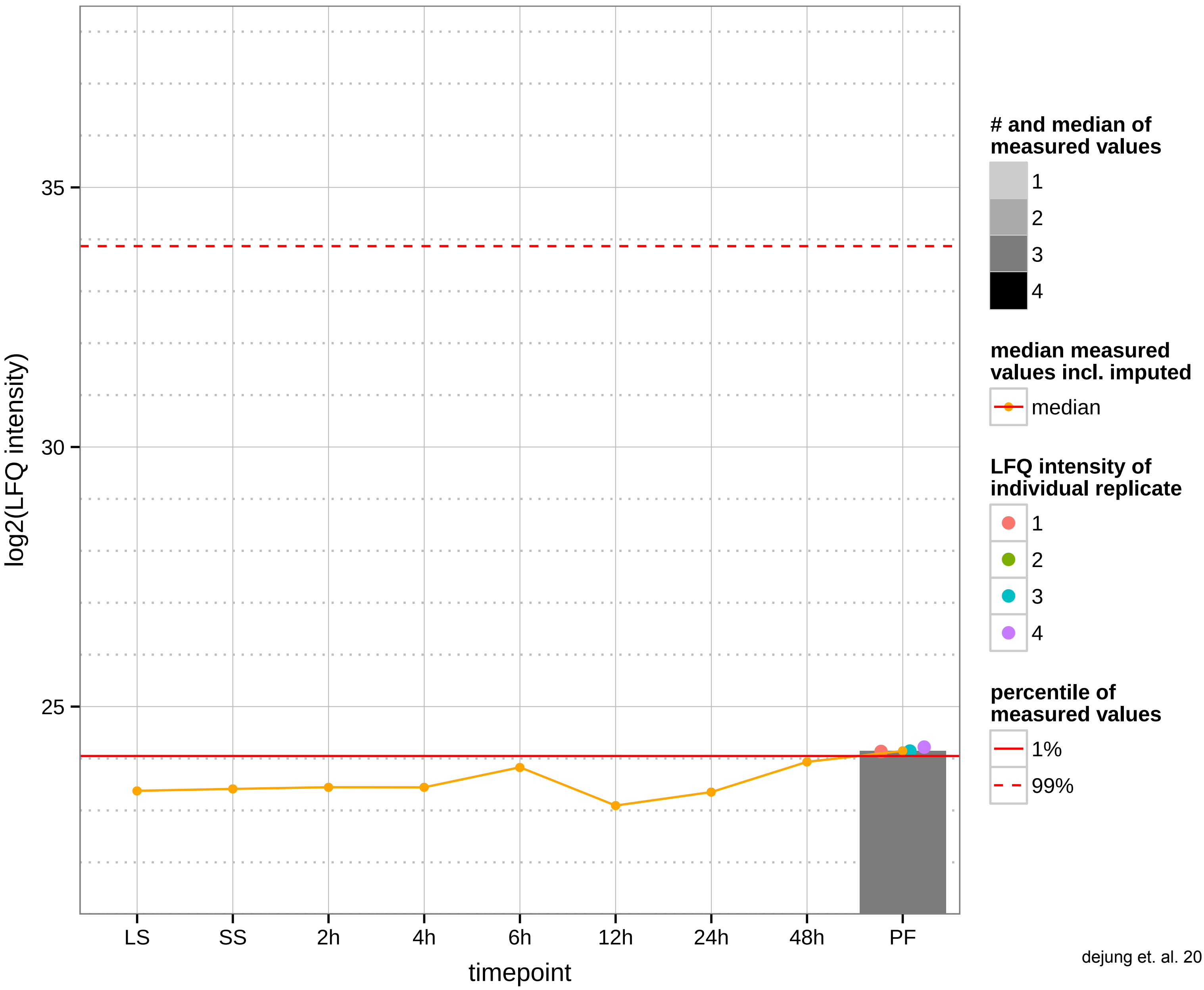

dejung et. al. 2015

hypothetical protein, conserved
Tb927.2.4810
AGOF: null
AGOC: null
AGOP: ciliary or flagellar motility
PGOF: protein binding
PGOC: null
PGOP: null

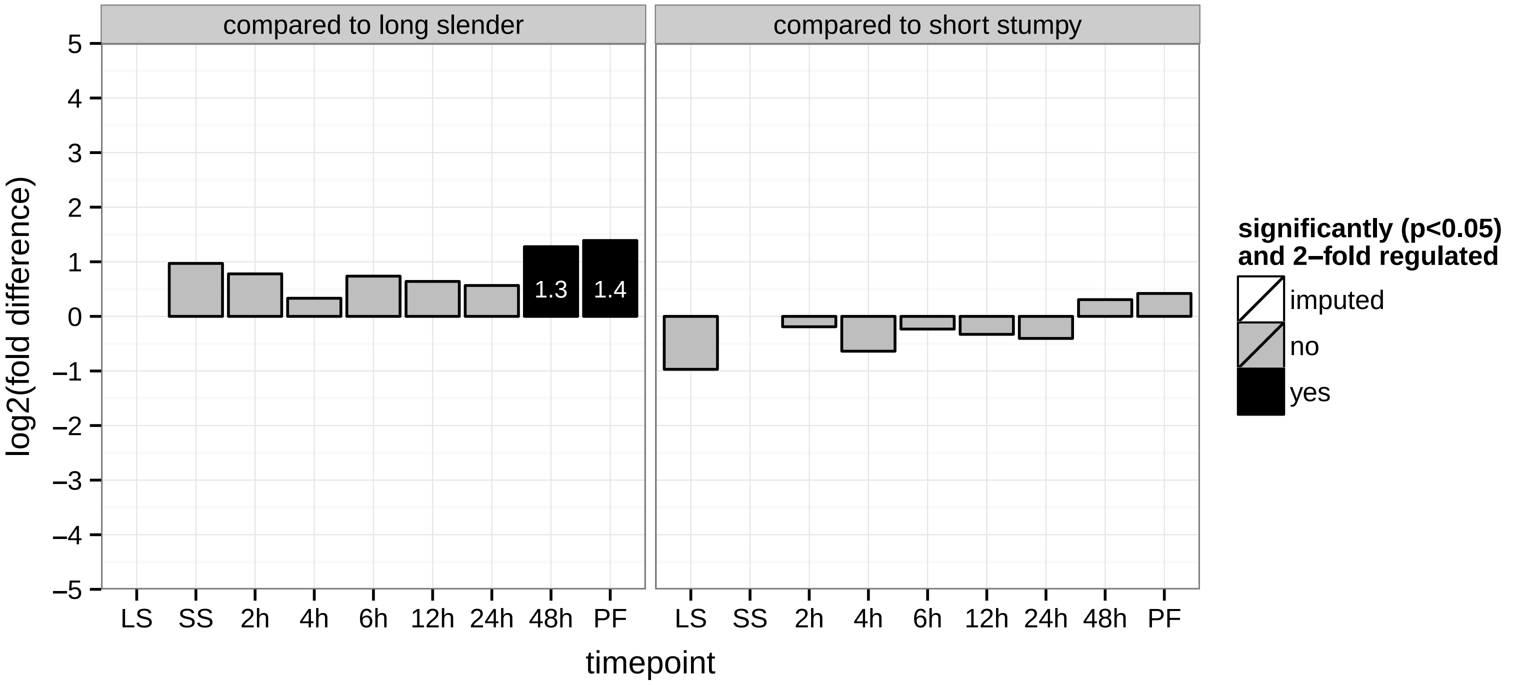

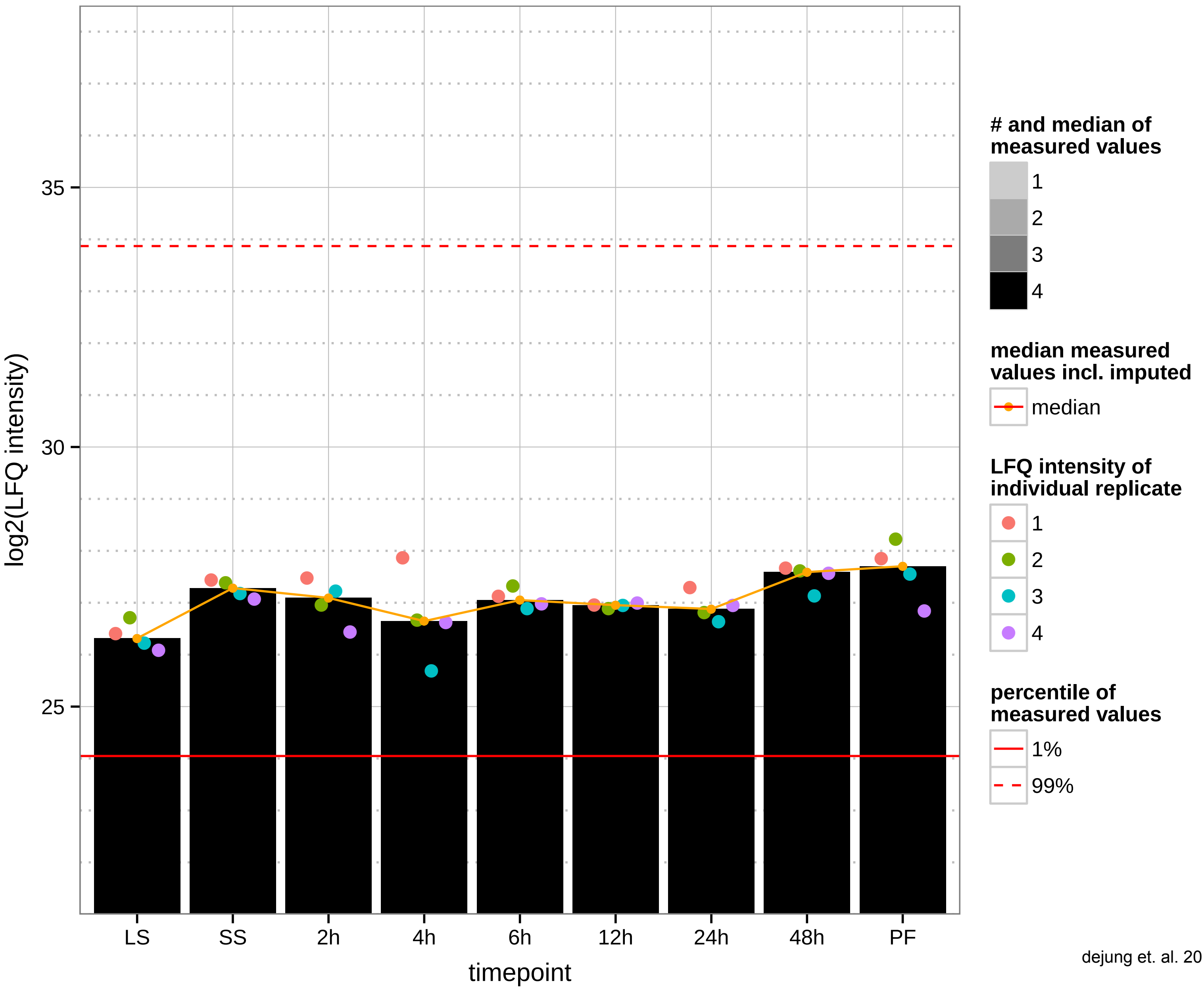

dejung et. al. 2015

kinetoplastid–specific dual specificity phosphatase, putative
Tb927.2.4870
AGOF: protein tyrosine/serine/threonine phosphatase activity
AGOC: null
AGOP: protein dephosphorylation
PGOF: phosphatase activity, protein tyrosine/serine/threonine phosphatase activity
PGOC: null
PGOP: dephosphorylation, protein dephosphorylation

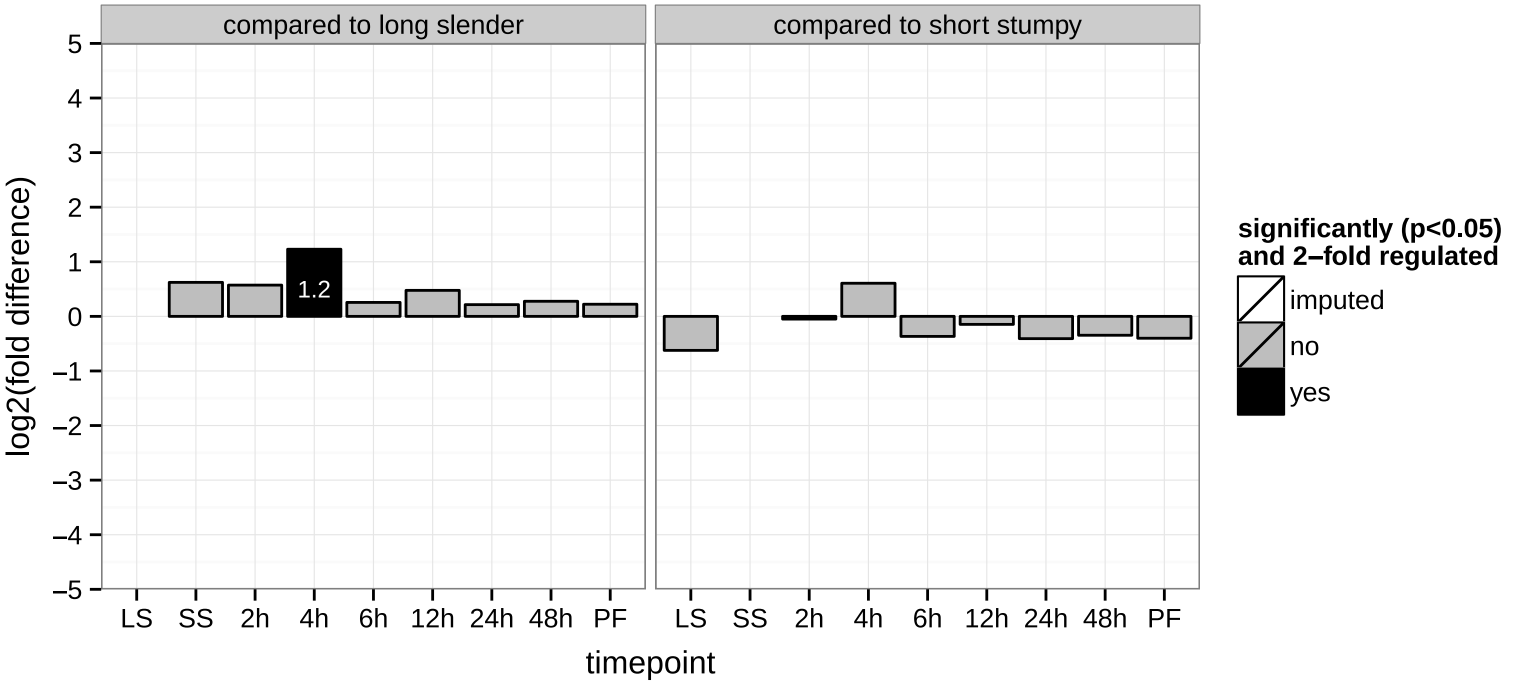

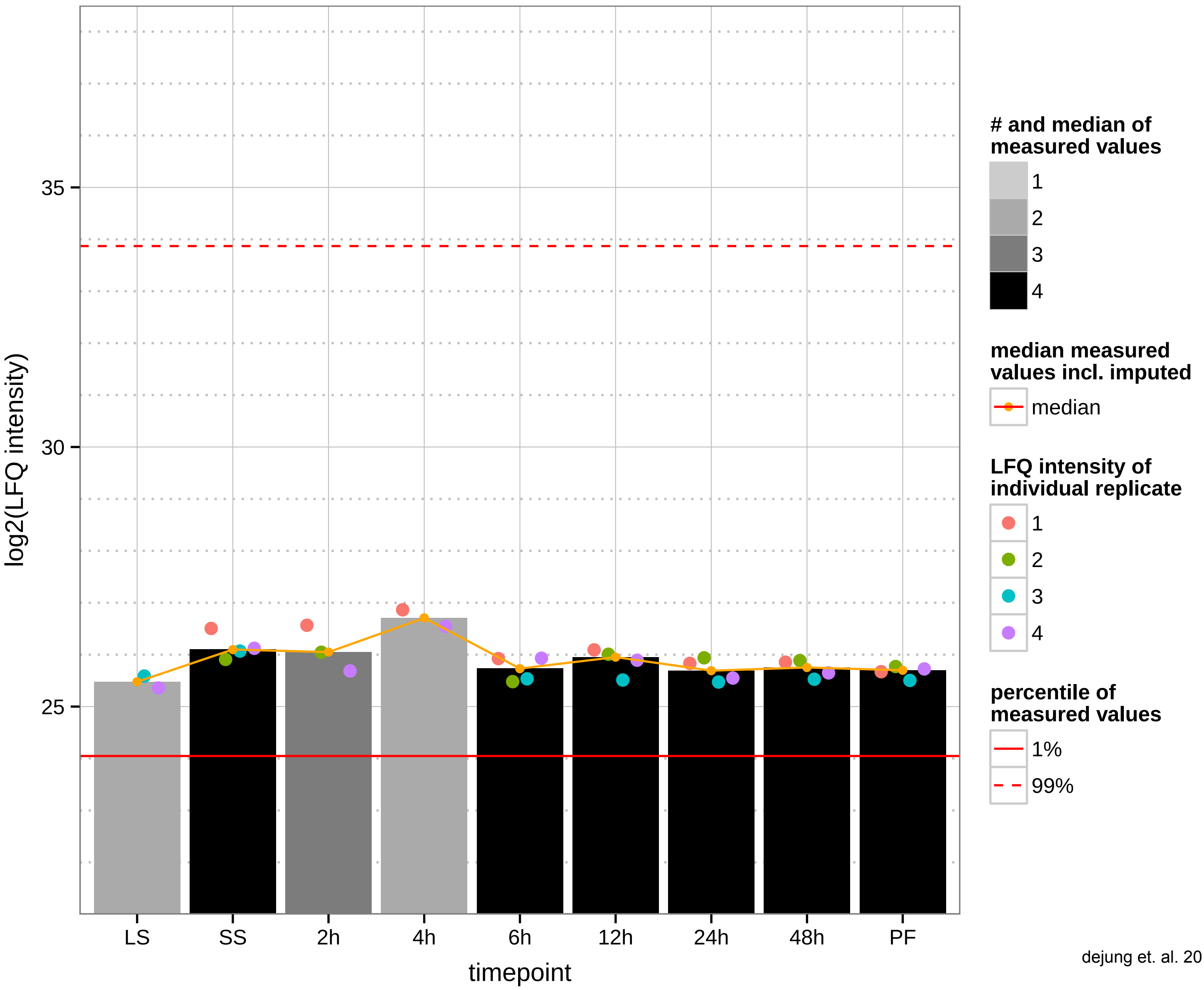

dejung et. al. 2015

esterase, putative
Tb927.2.4930
AGOF: catalytic activity, hydrolase activity, acting on ester bonds
AGOC: null
AGOP: lipid metabolic process
PGOF: null
PGOC: null
PGOP: null

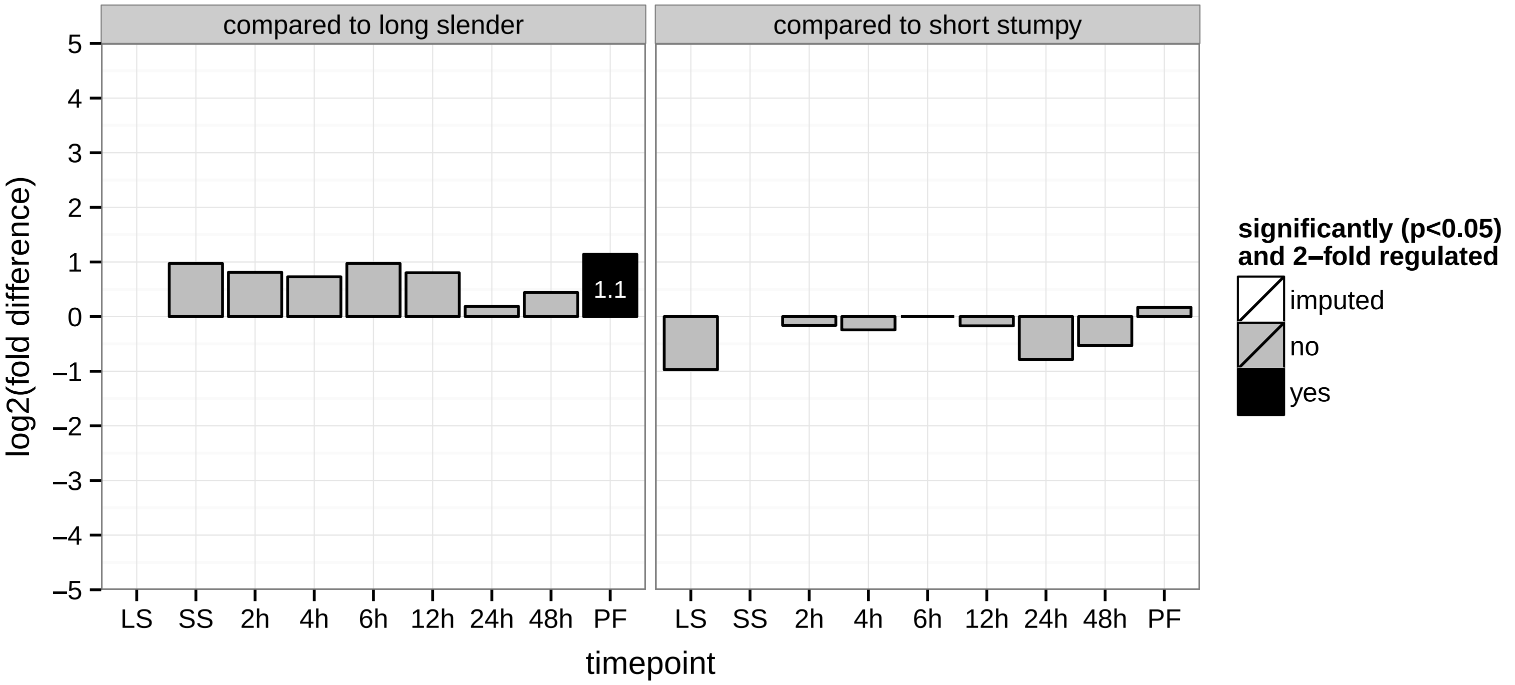

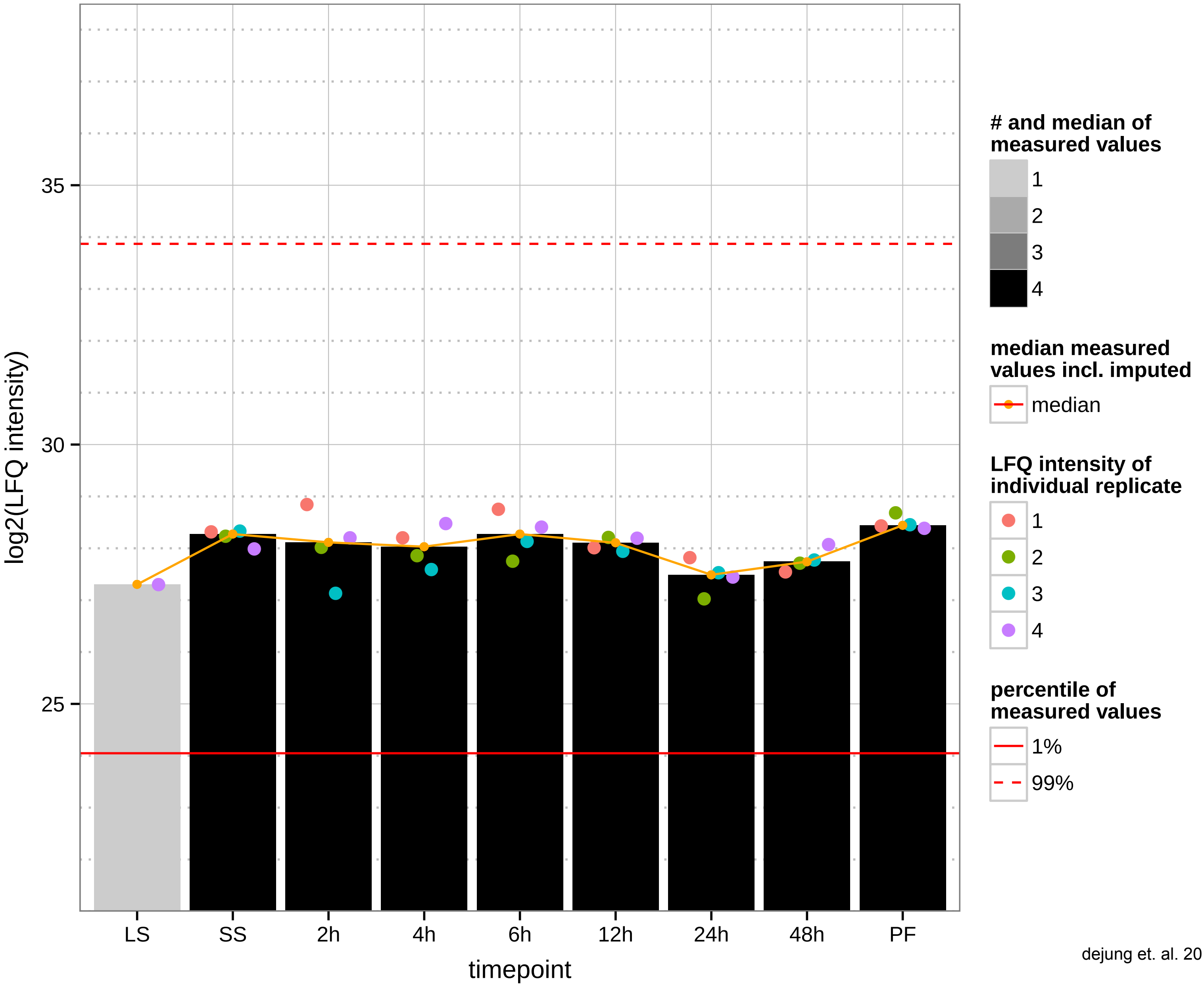

dejung et. al. 2015

transcription initiation protein, putative
Tb927.2.5030
AGOF: structural constituent of ribosome
AGOC: ribosome
AGOP: regulation of transcription, DNA–dependent
PGOF: null
PGOC: null
PGOP: null

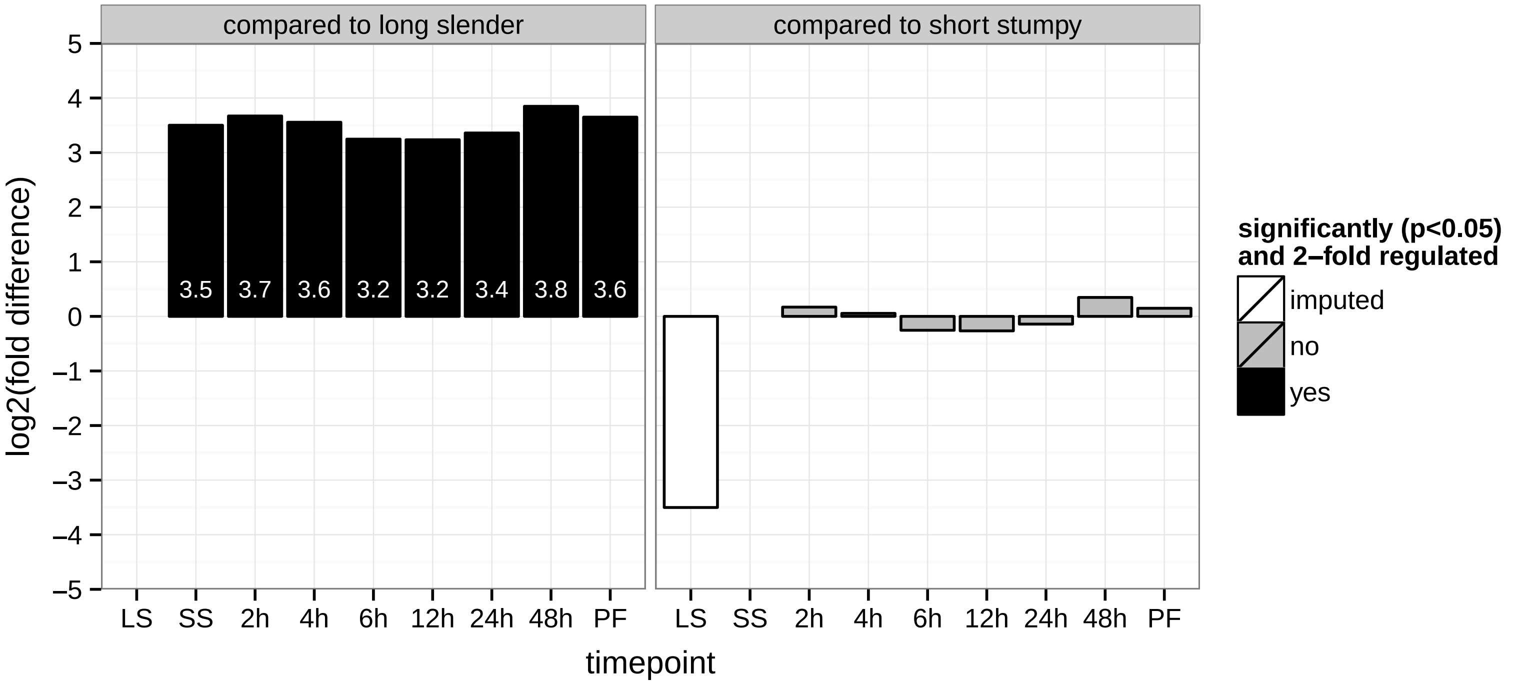

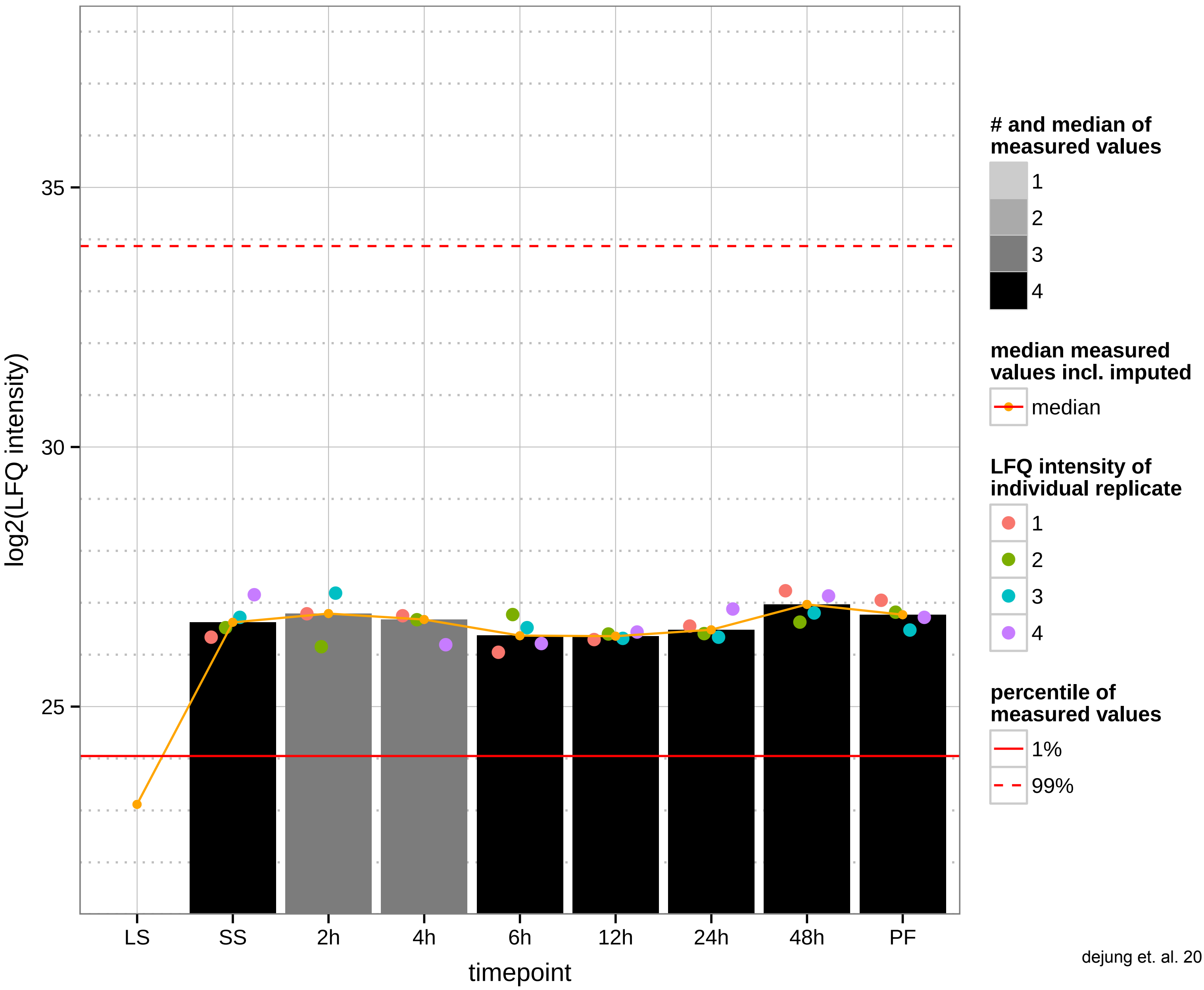

dejung et. al. 2015

GTP binding protein, putative
Tb927.2.5060
AGOF: GTP binding
AGOC: intracellular
AGOP: null
PGOF: GTP binding
PGOC: null
PGOP: null

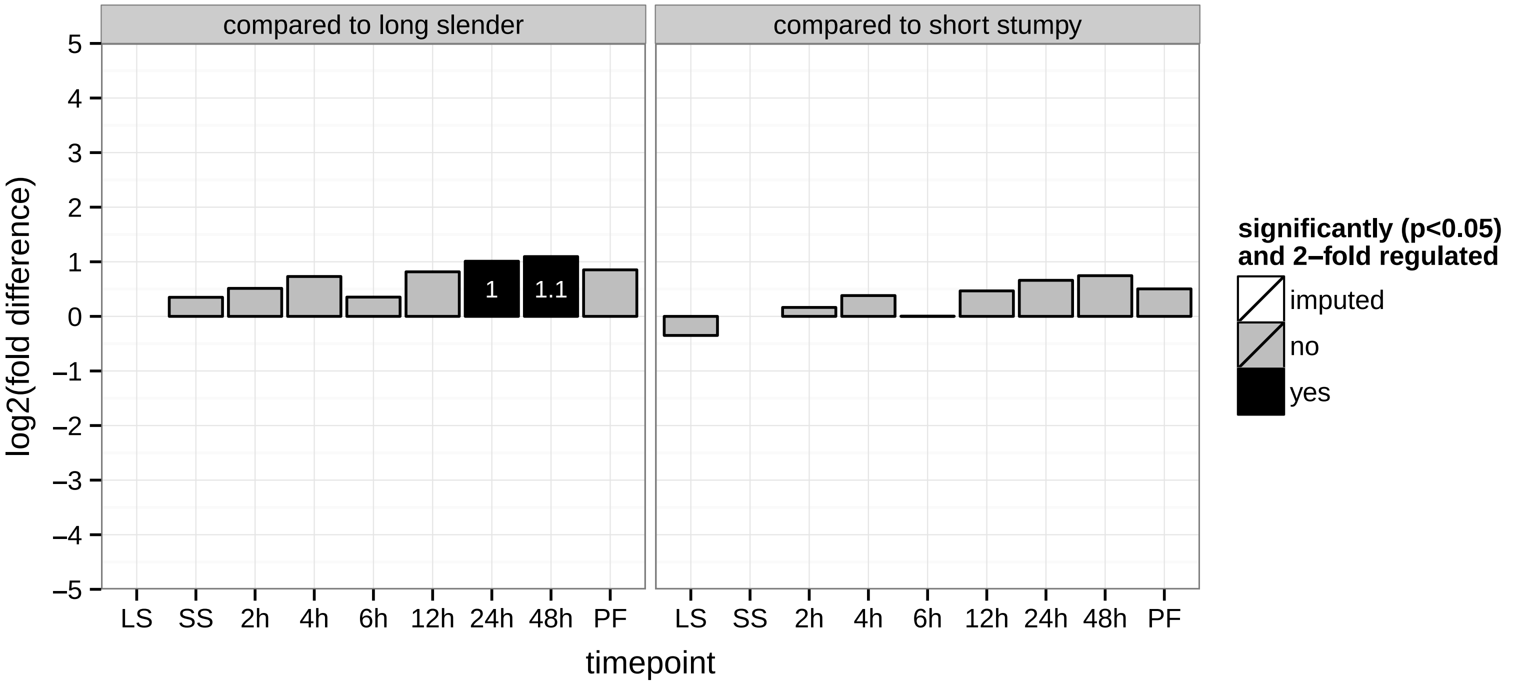

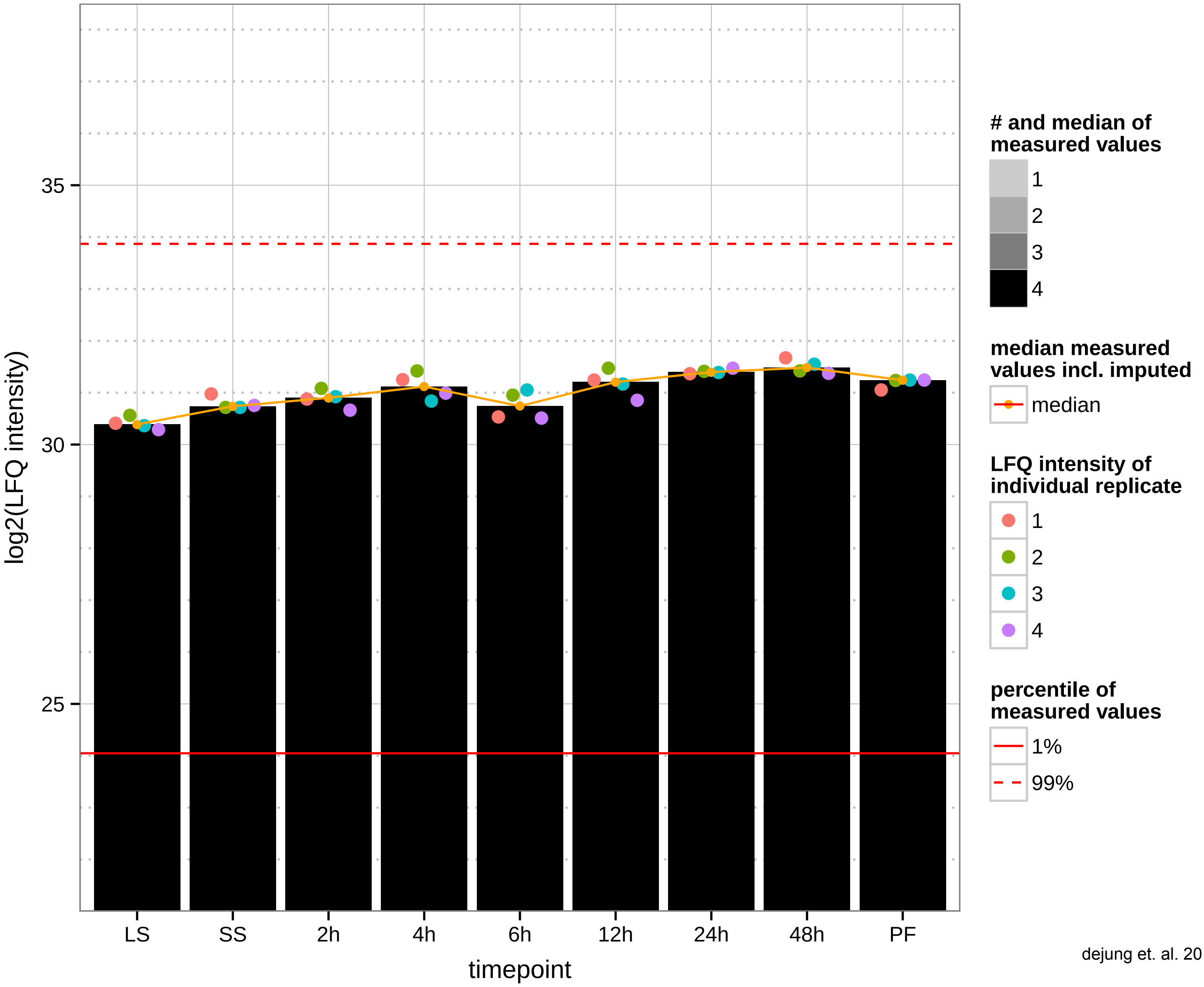

dejung et. al. 2015

retrotransposon hot spot (RHS) protein, putative, retrotransposon hot spot protein 4 (RHS4)
Tb927.2.510
AGOF: null
AGOC: null, nucleus
AGOP: null
PGOF: null
PGOC: null
PGOP: null

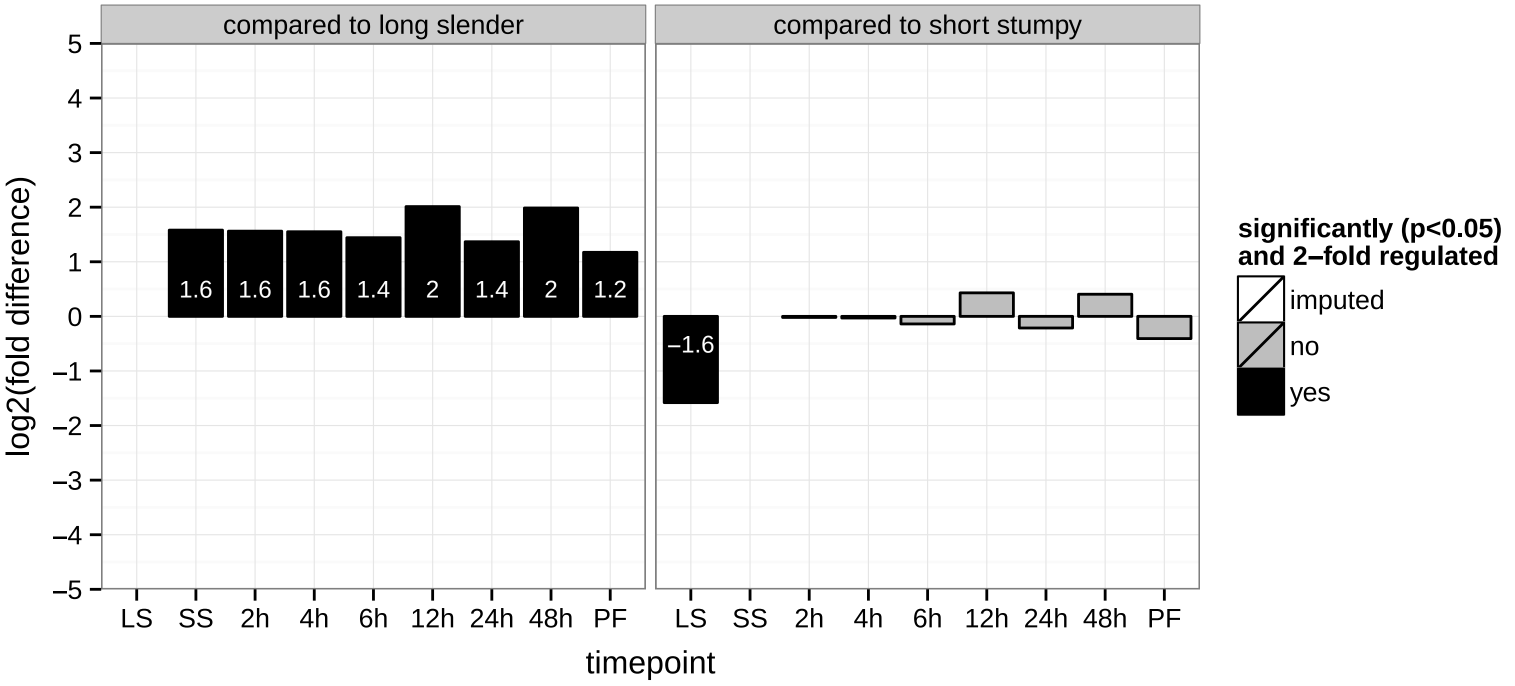

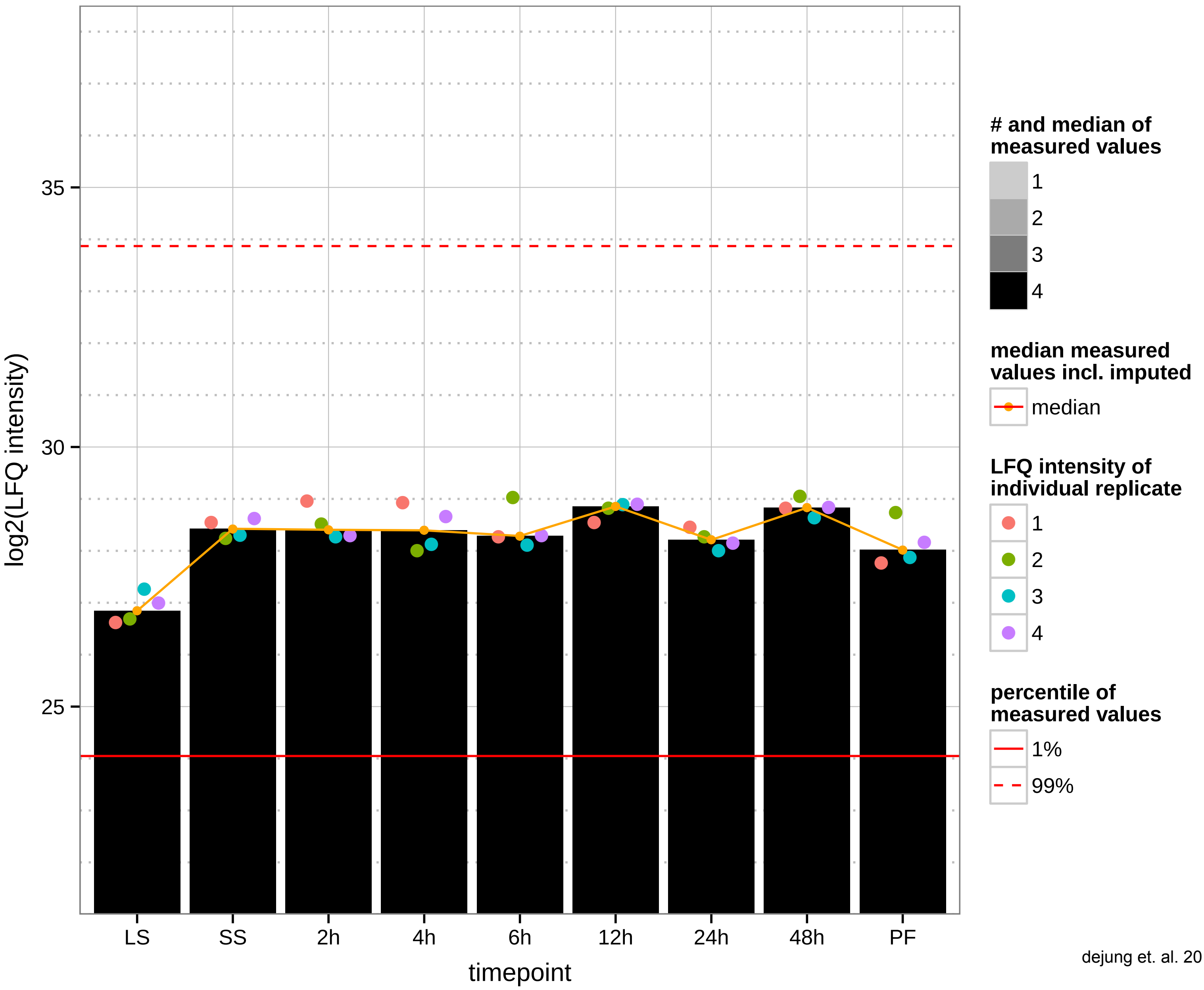

dejung et. al. 2015

TPR–repeat protein, putative
Tb927.2.5150
AGOF: null
AGOC: null
AGOP: null
PGOF: protein binding
PGOC: null
PGOP: null

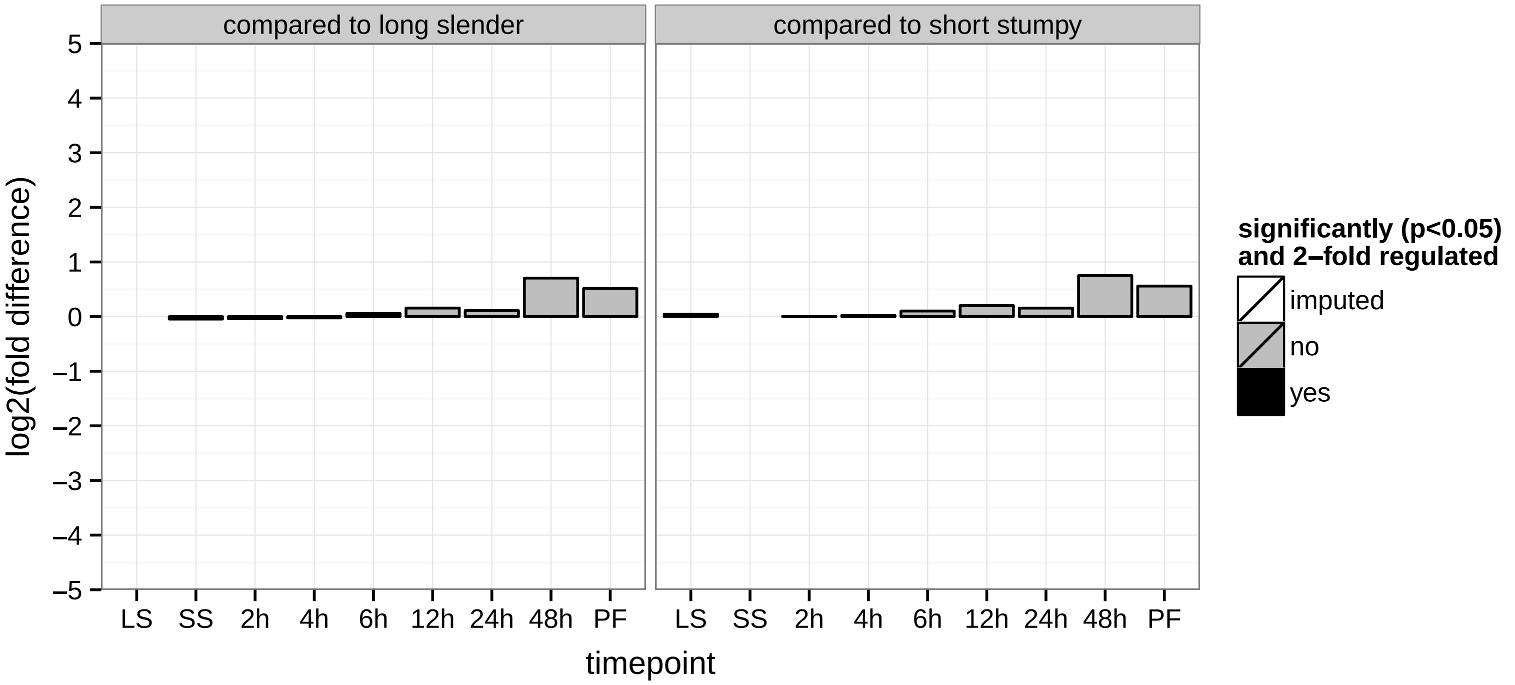

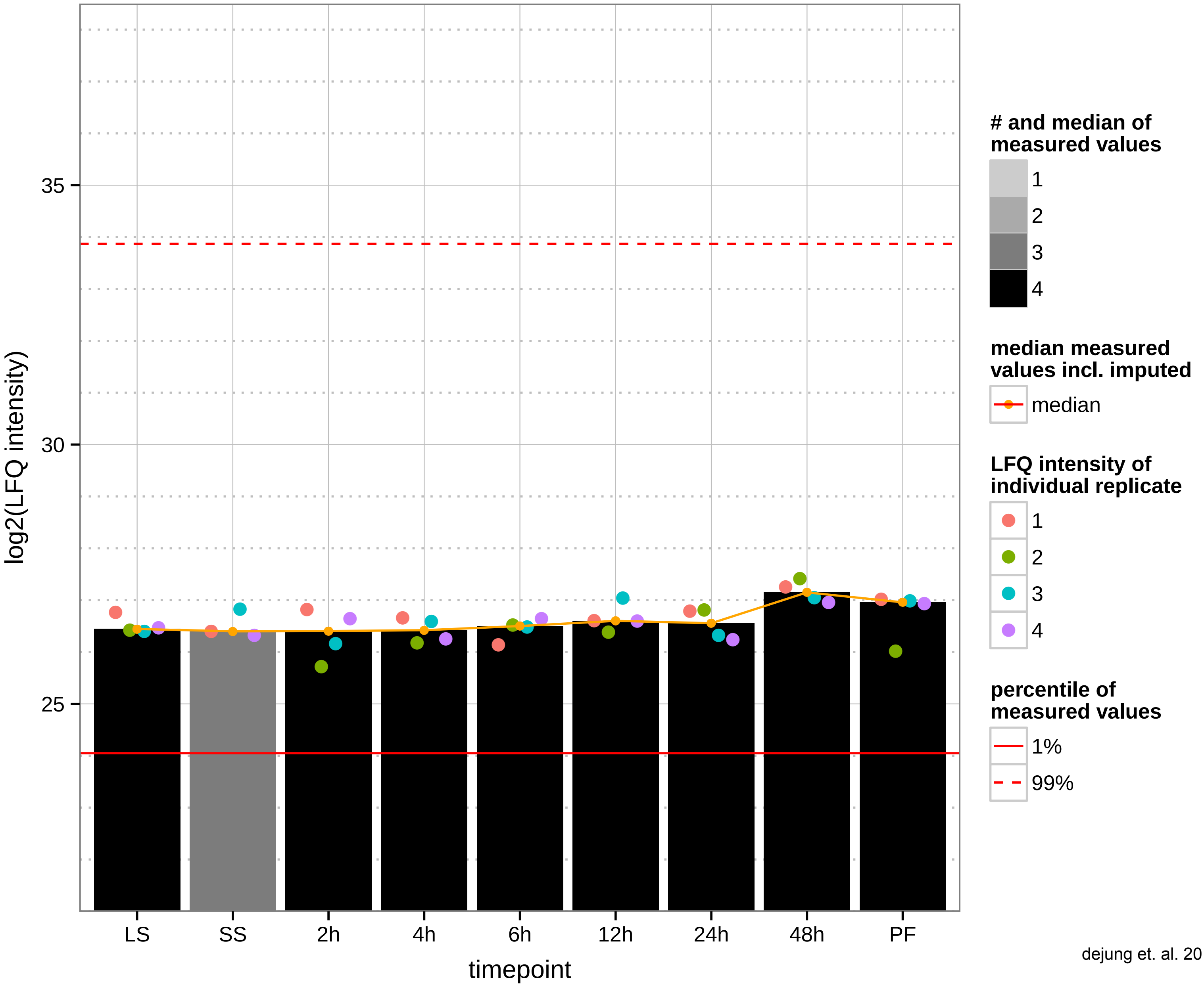

dejung et. al. 2015

chaperone protein DNAj, putative
Tb927.2.5160
AGOF: chaperone binding, heat shock protein binding, unfolded protein binding
AGOC: null
AGOP: protein folding
PGOF: heat shock protein binding, unfolded protein binding
PGOC: null
PGOP: protein folding

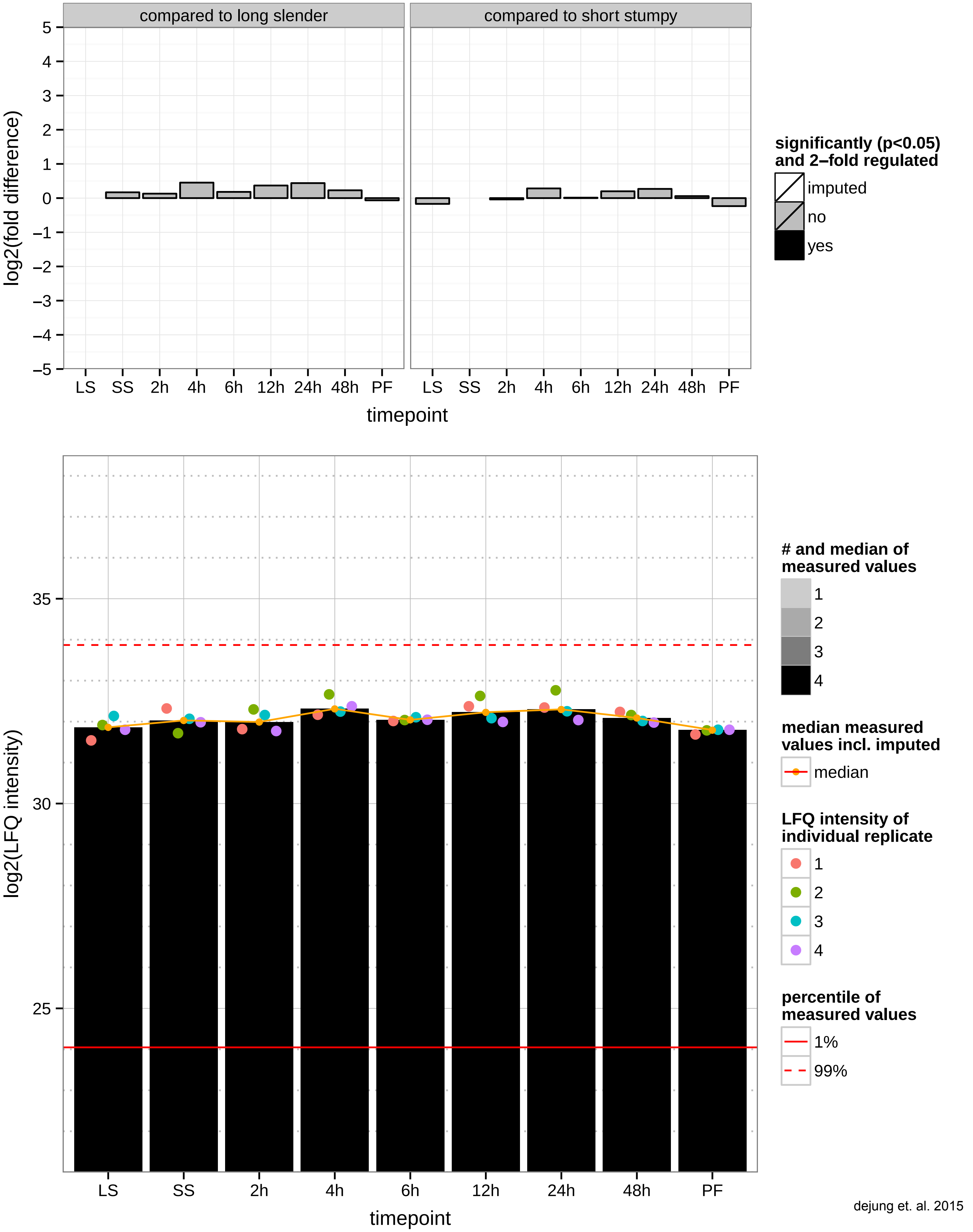

dejung et. al. 2015

PRP19–like protein, putative (TbPRP19)
Tb927.2.5240
AGOF: ubiquitin–protein ligase activity
AGOC: ubiquitin ligase complex
AGOP: protein ubiquitination
PGOF: protein binding, ubiquitin–protein ligase activity
PGOC: ubiquitin ligase complex
PGOP: protein ubiquitination

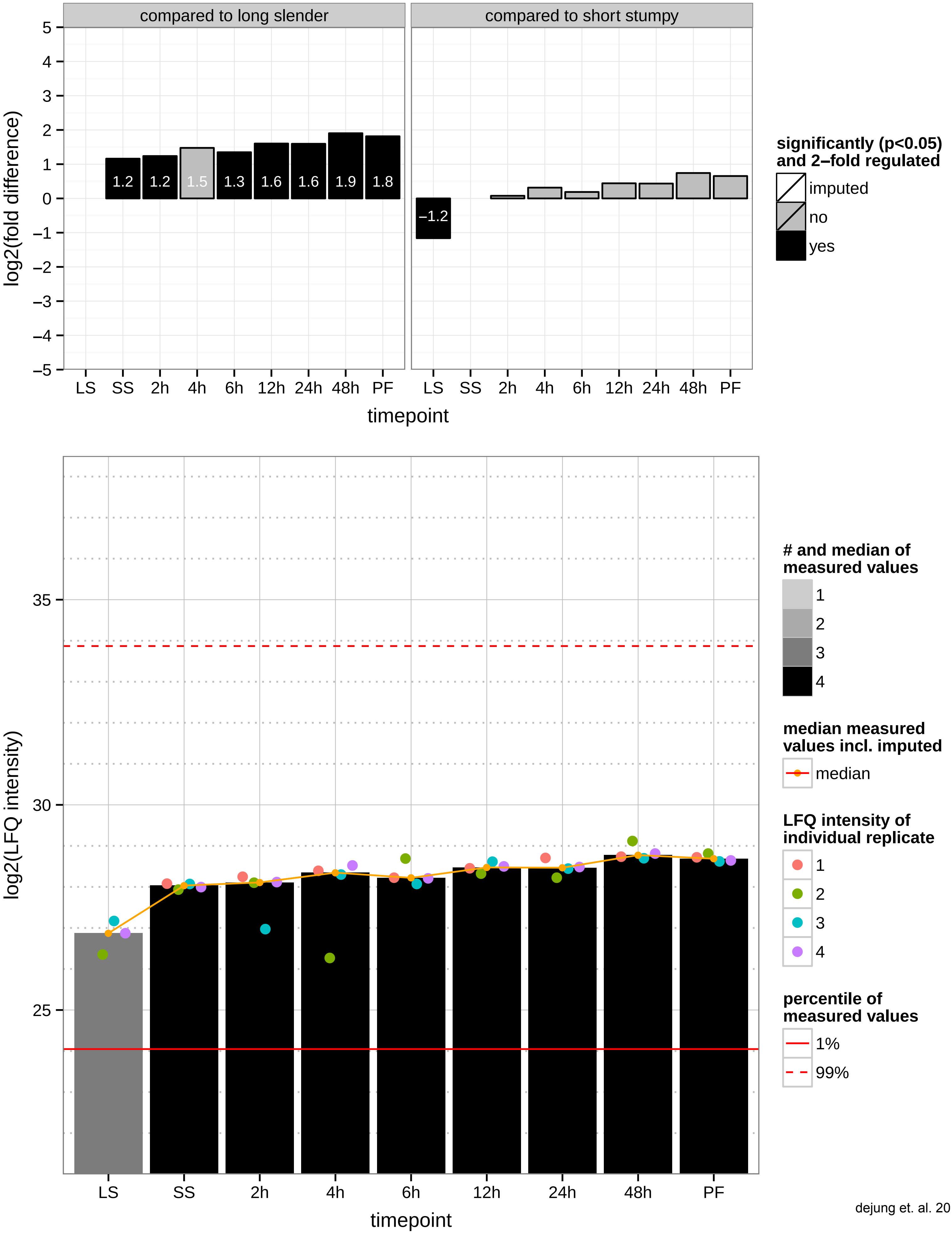

dejung et. al. 2015

dynein heavy chain, putative
Tb927.2.5270
AGOF: ATP binding, ATPase activity
AGOC: cellular_component, dynein complex
AGOP: microtubule−based movement
PGOF: microtubule motor activity, nucleoside−triphosphatase activity, nucleotide binding
PGOC: dynein complex
PGOP: microtubule−based movement

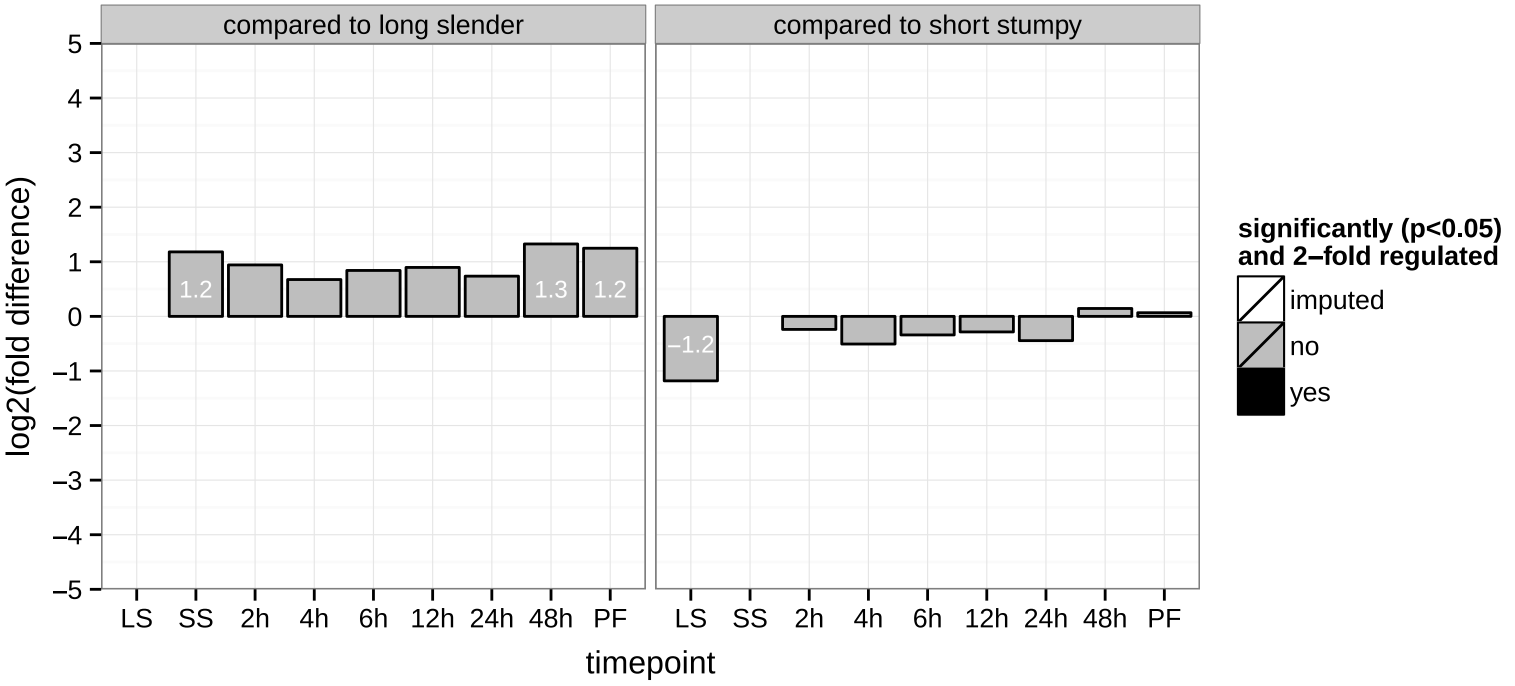

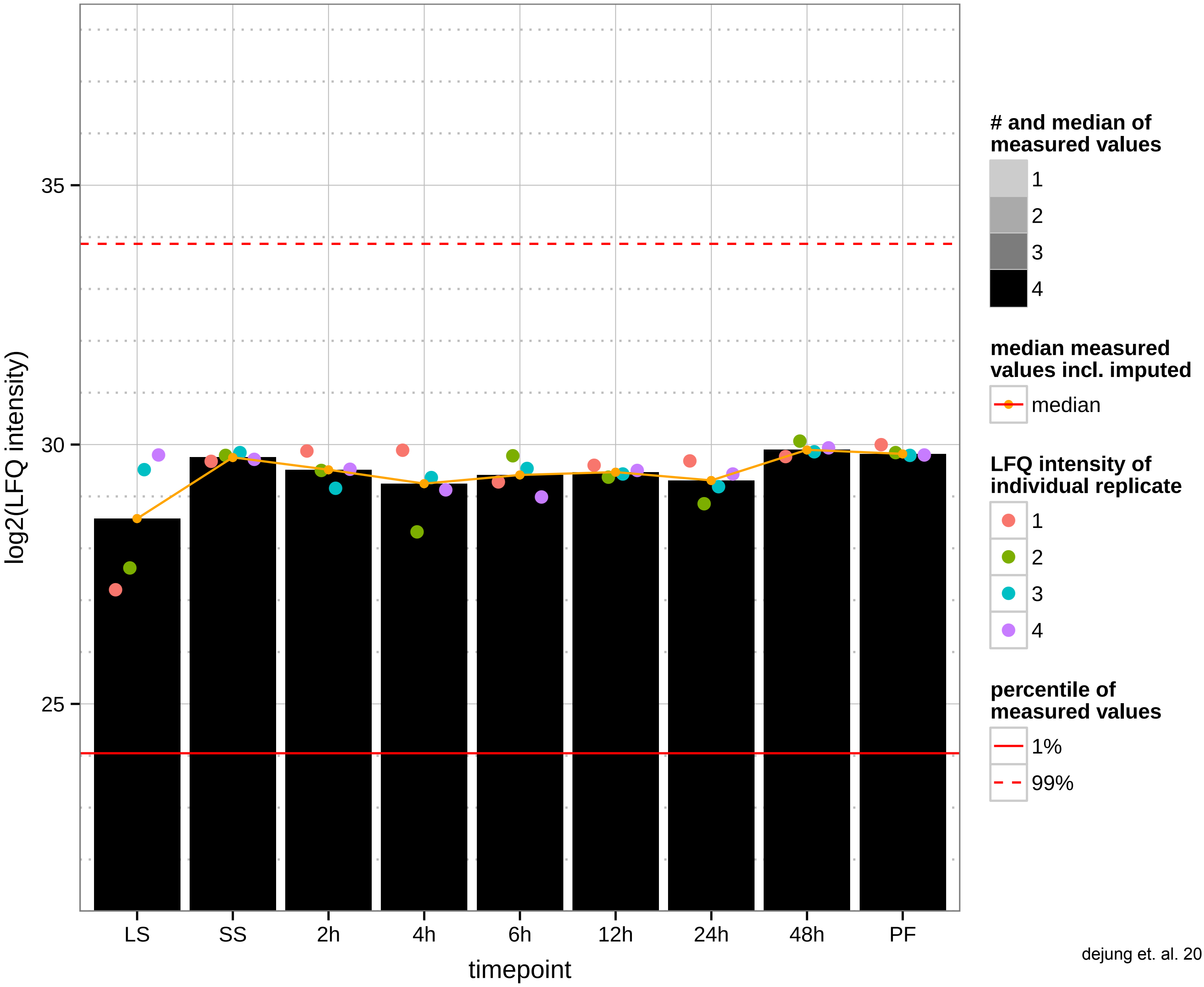

dejung et. al. 2015

adenylate kinase, putative (ADKA)
Tb927.2.5660
AGOF: ATP binding, adenylate kinase activity
AGOC: cilium part
AGOP: nucleobase–containing compound metabolic process
PGOF: ATP binding, adenylate kinase activity, cAMP–dependent protein kinase regulator activity, nucleobase–containing com
PGOC: null
PGOP: nucleobase–containing compound metabolic process, signal transduction

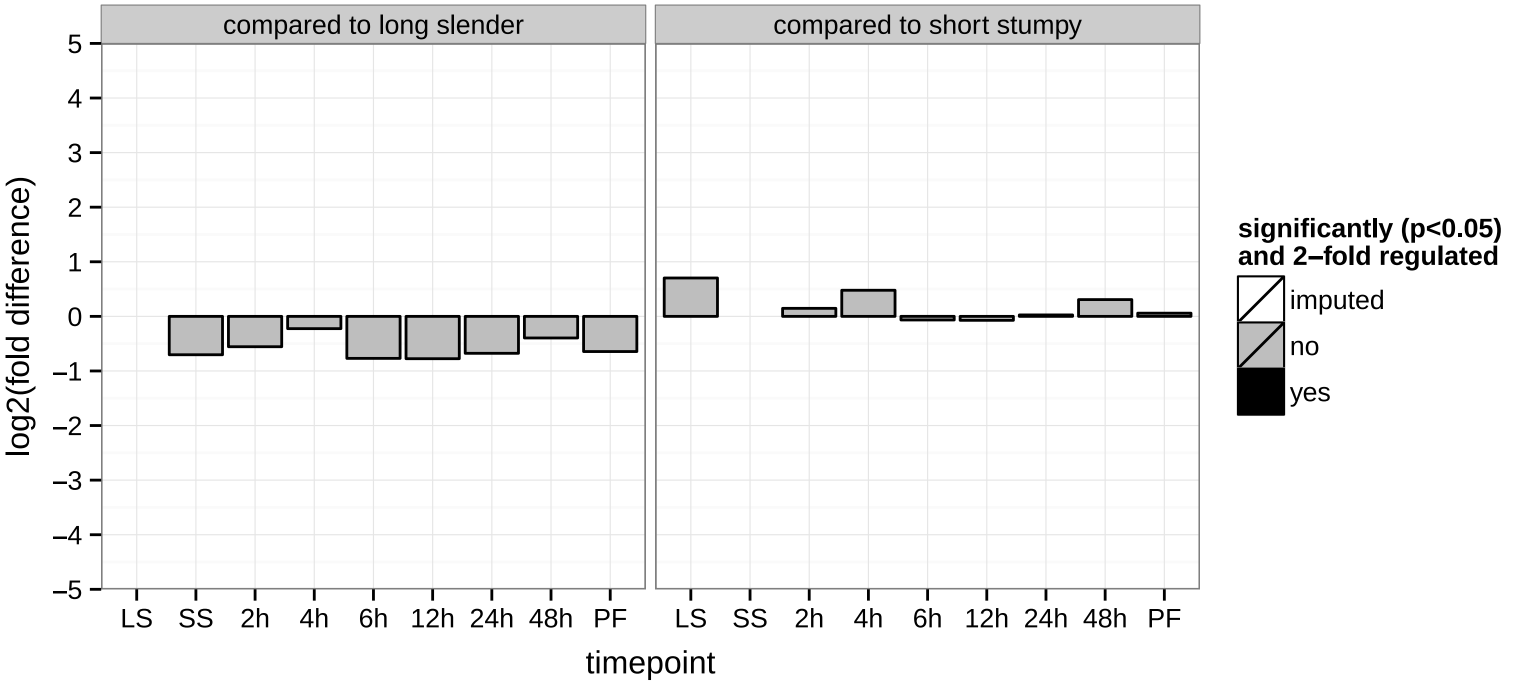

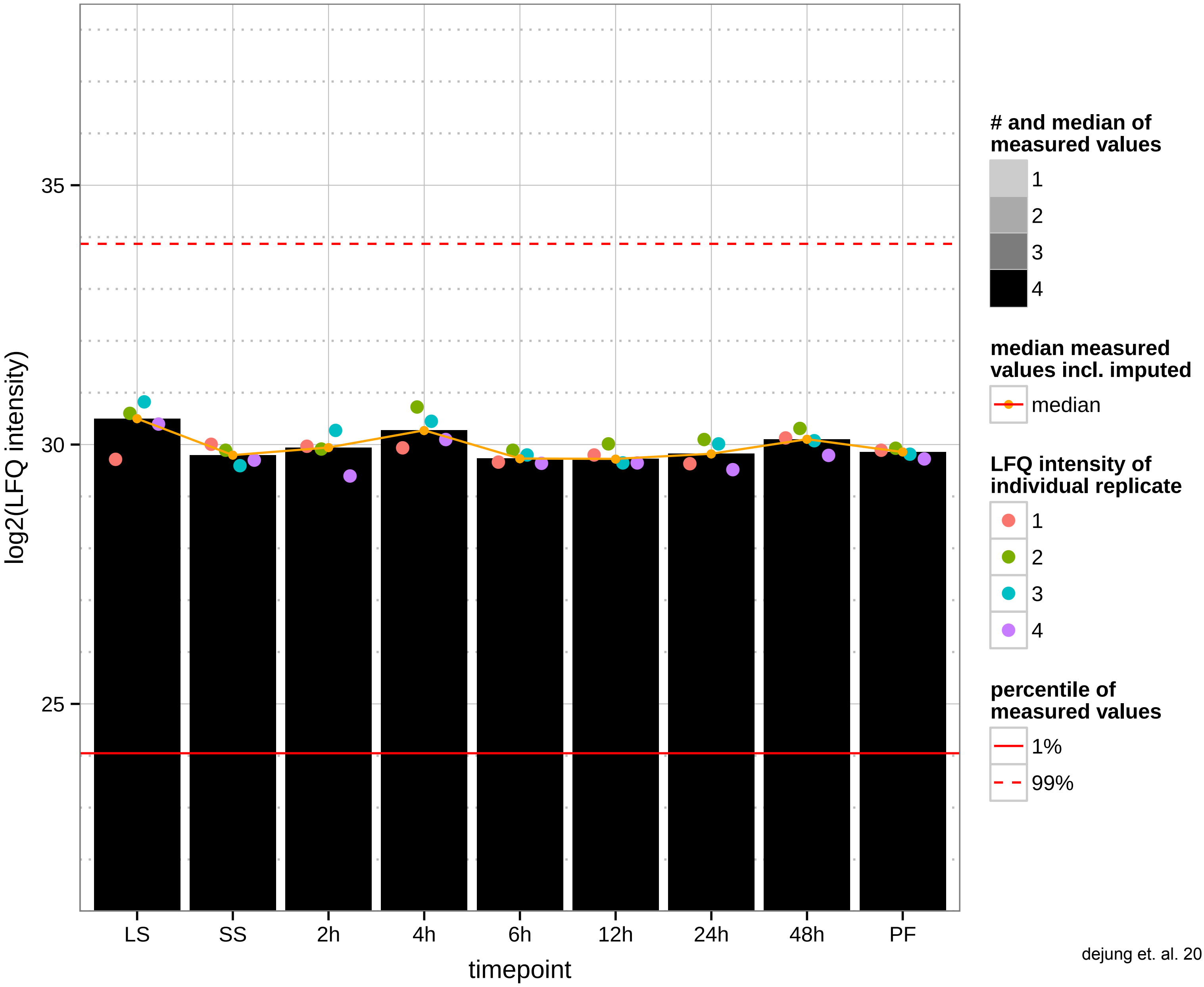

dejung et. al. 2015

hypothetical protein, conserved
Tb927.2.5760
AGOF: null
AGOC: null
AGOP: null
PGOF: null
PGOC: null
PGOP: null

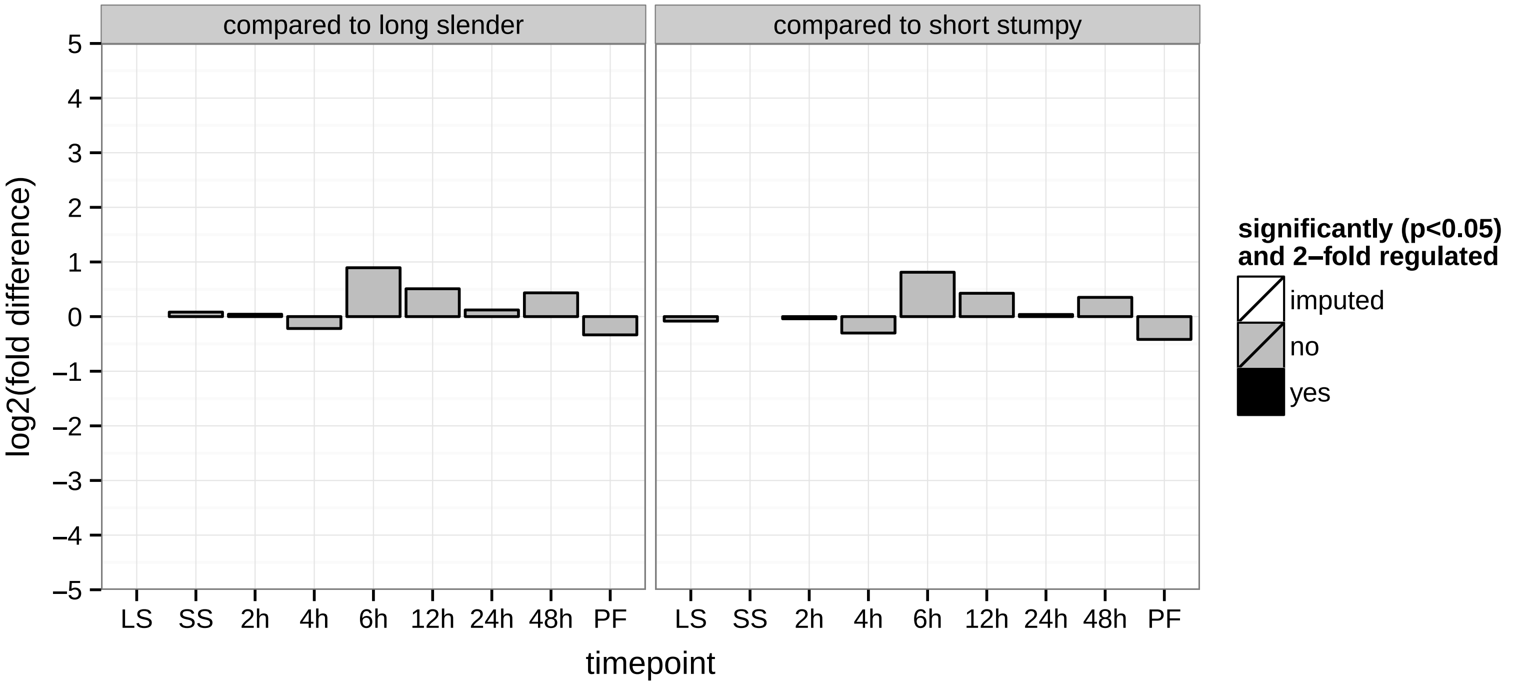

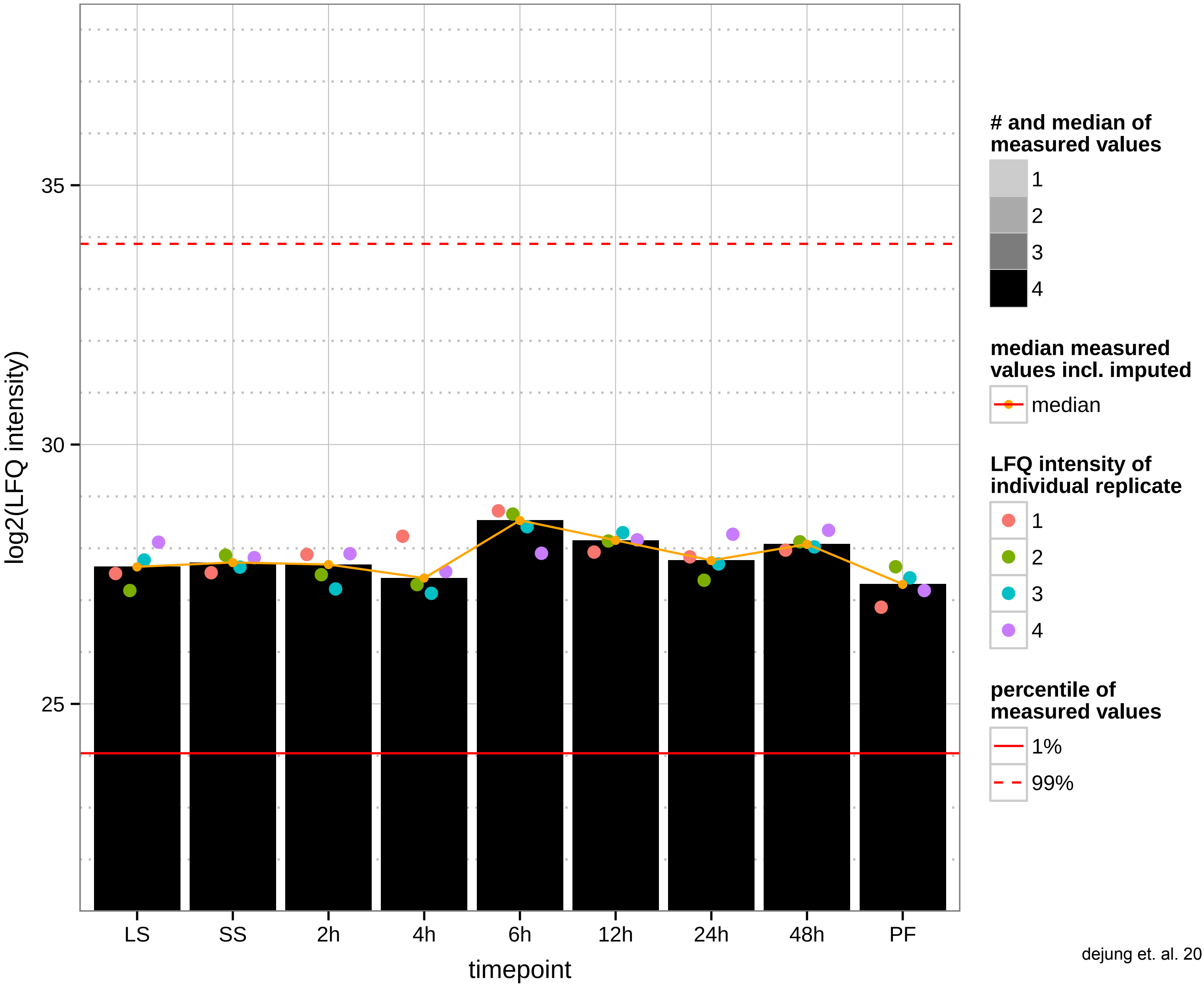

dejung et. al. 2015

hypothetical protein, conserved
Tb927.2.5810
AGOF: null
AGOC: null
AGOP: null
PGOF: nucleic acid binding
PGOC: null
PGOP: null

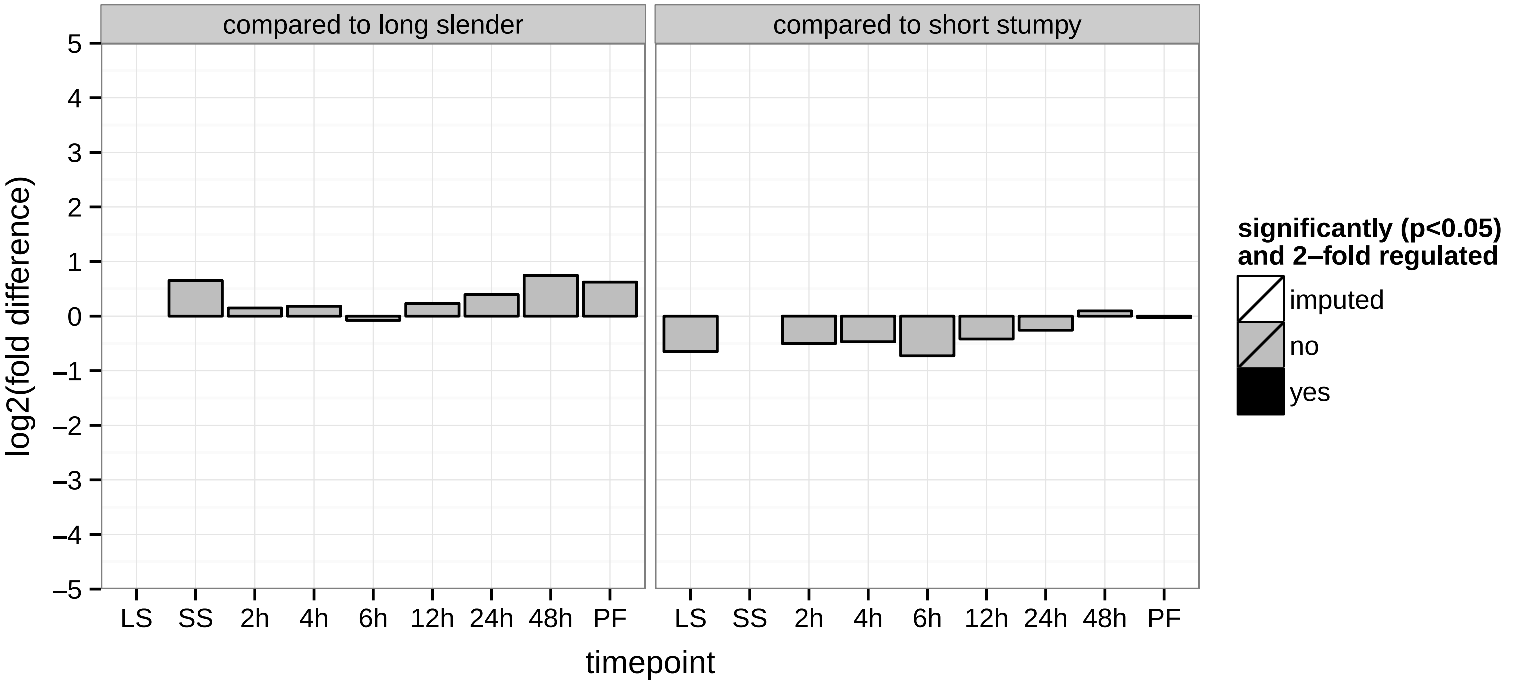

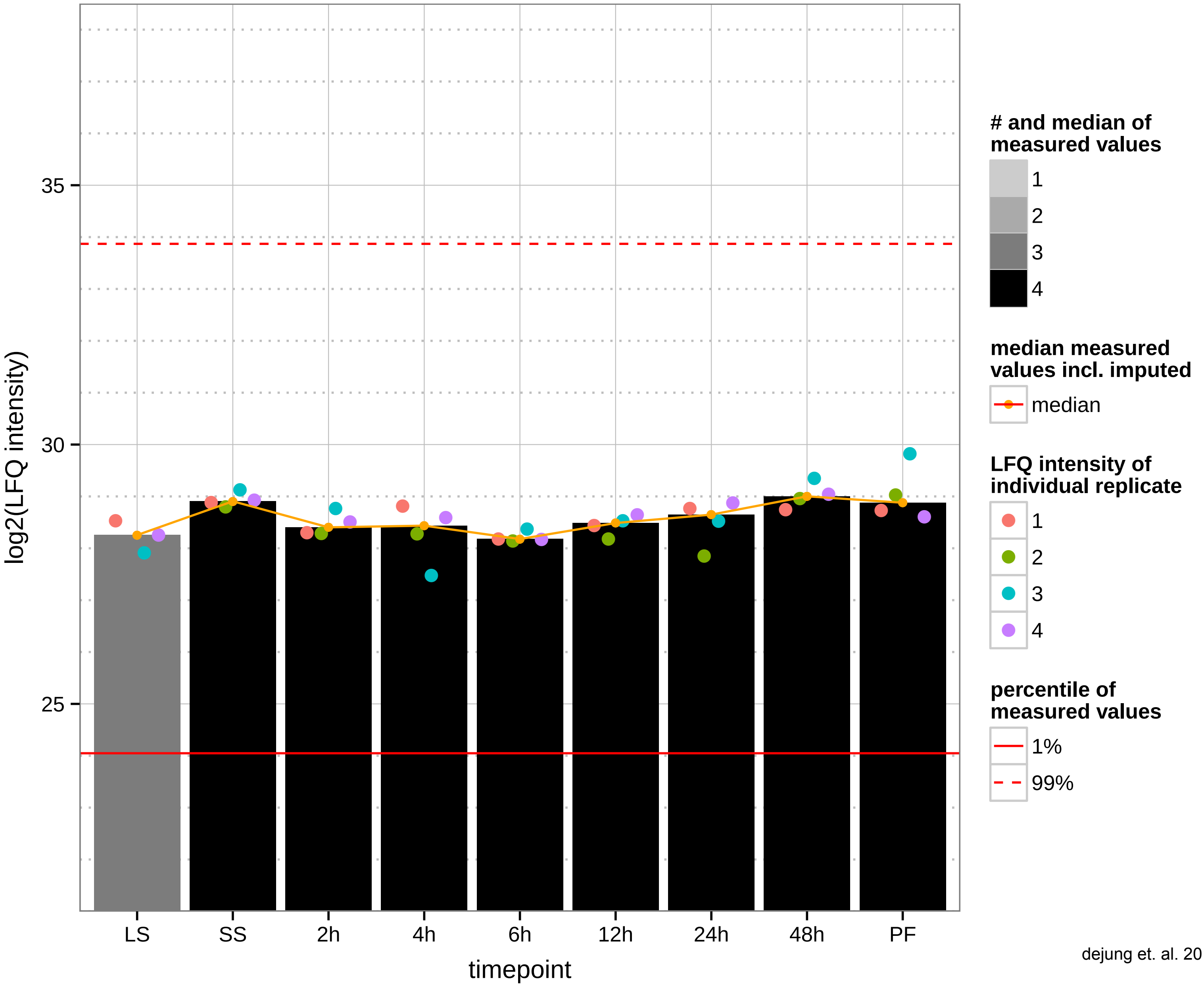

dejung et. al. 2015

hypothetical protein, conserved
Tb927.2.5820
AGOF: null
AGOC: null
AGOP: null
PGOF: binding
PGOC: null
PGOP: null

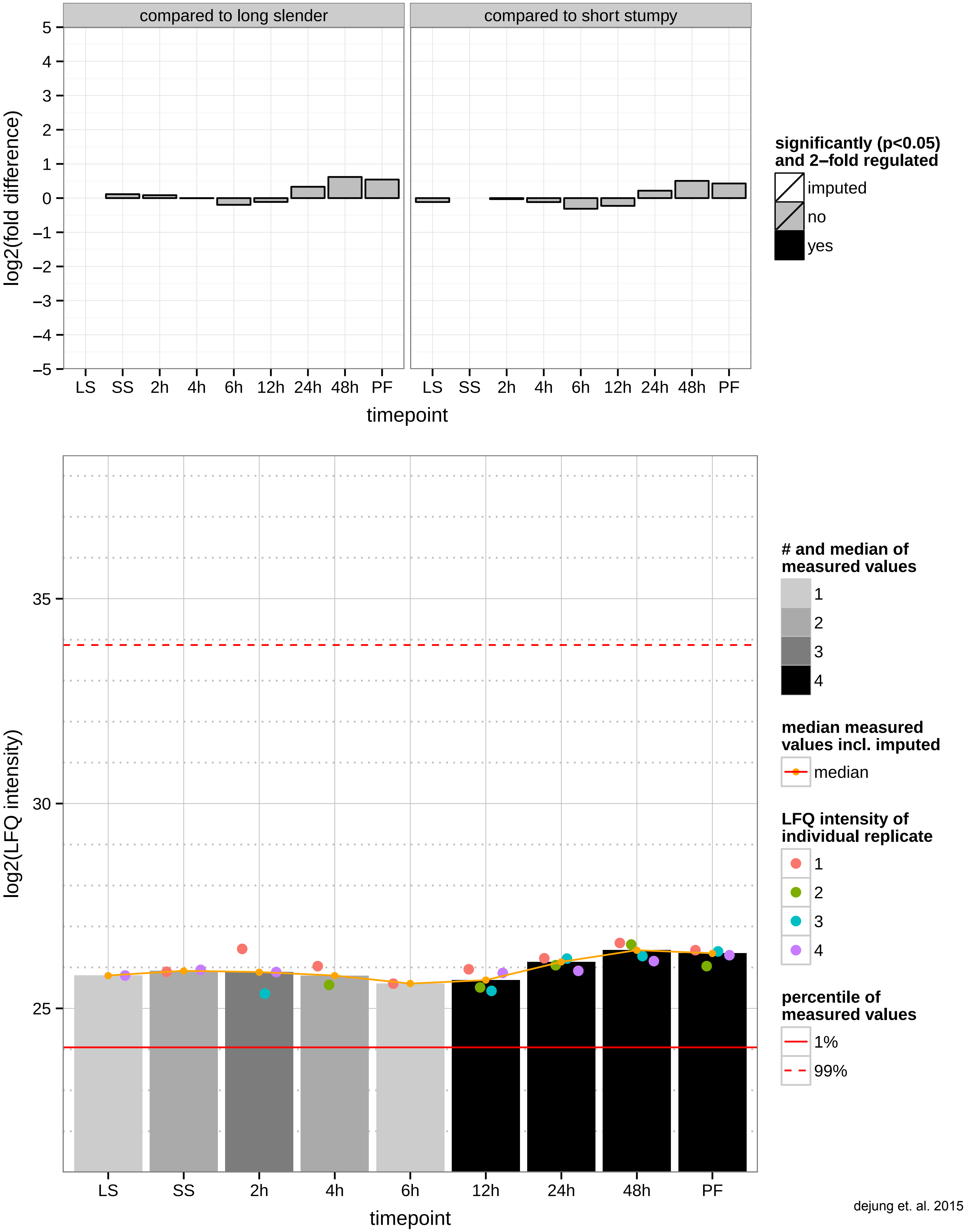

dejung et. al. 2015

small nuclear ribonucleoprotein SmD2 (Sm–D2)
Tb927.2.5850
AGOF: snRNA binding
AGOC: nucleus, small nuclear ribonucleoprotein complex, small nucleolar ribonucleoprotein complex
AGOP: nuclear mRNA trans splicing, SL addition
PGOF: null
PGOC: null
PGOP: null

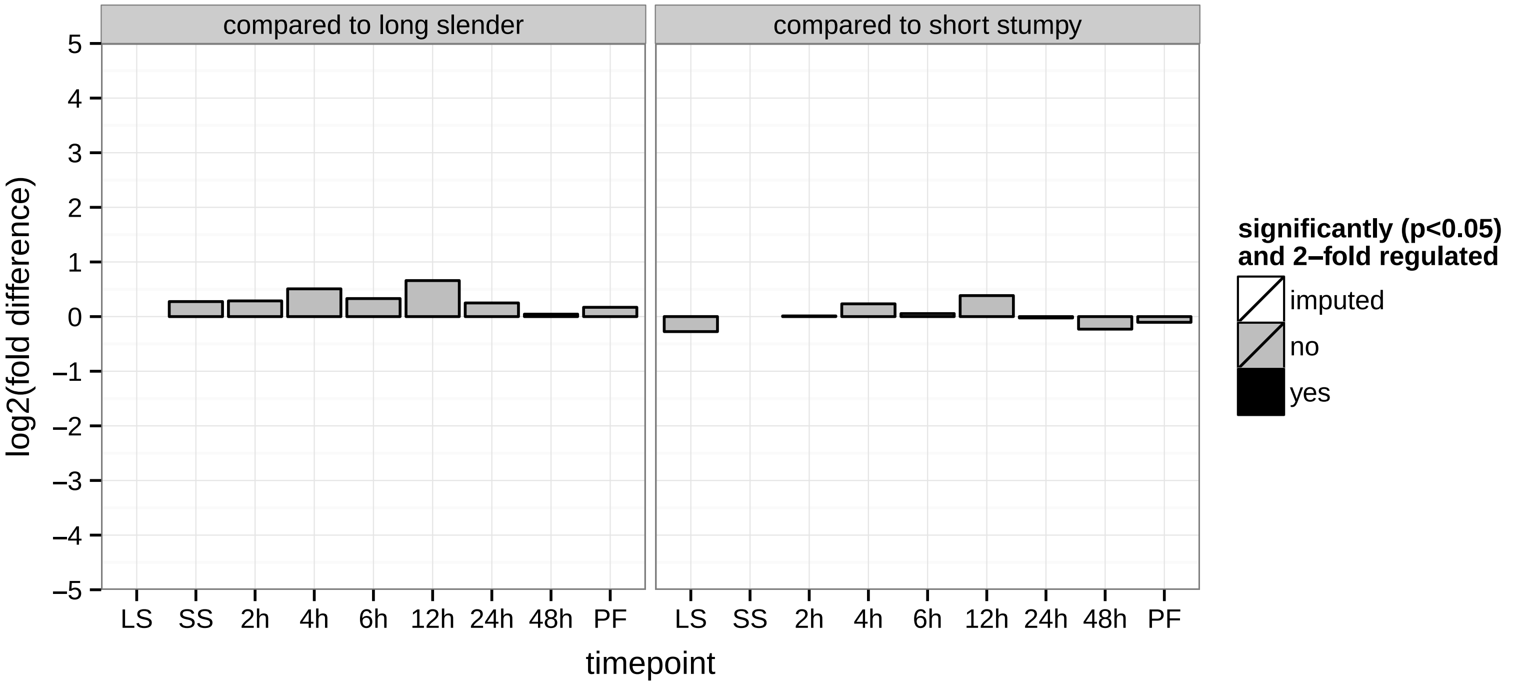

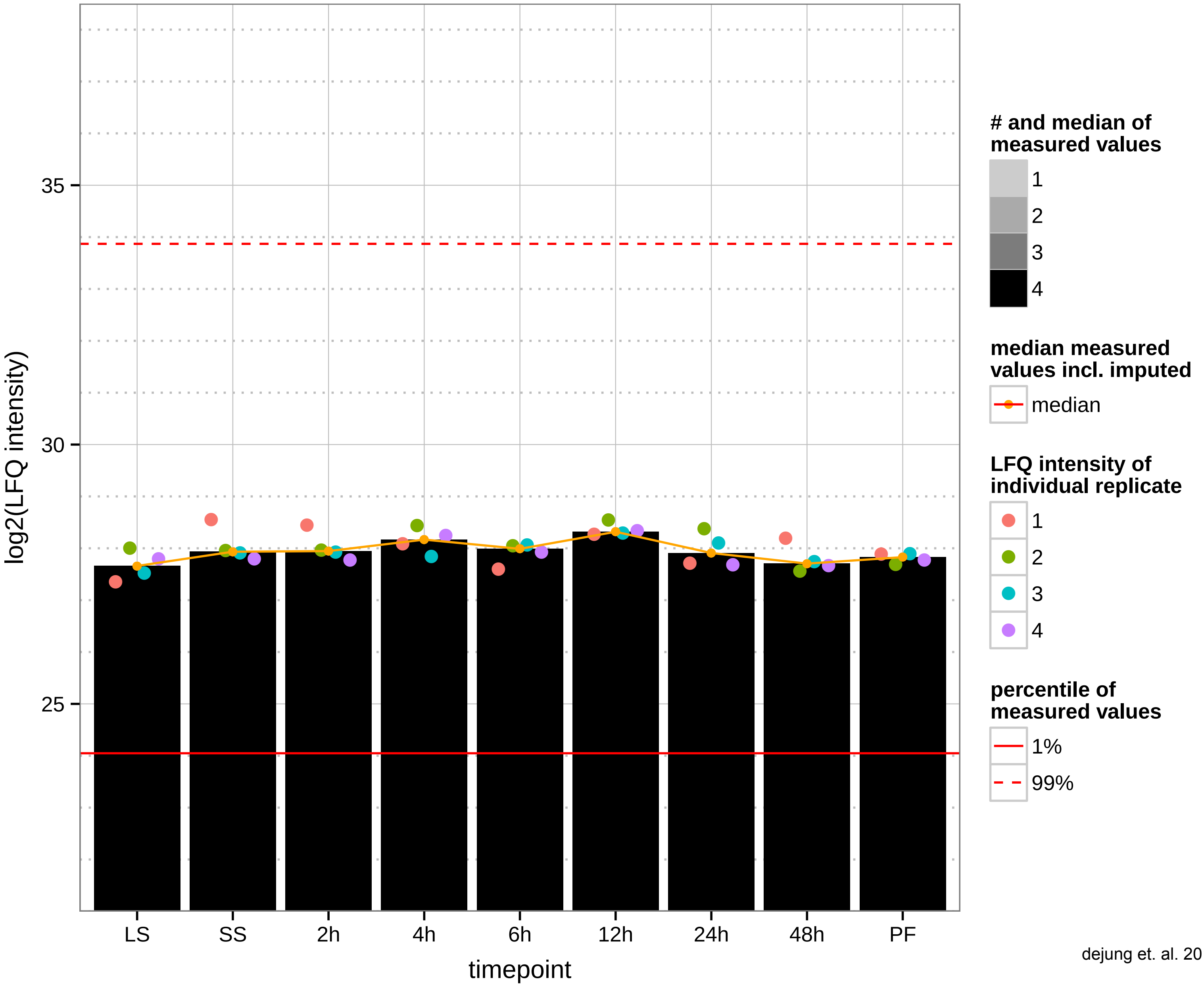

dejung et. al. 2015

beta prime COP protein
Tb927.2.6050
AGOF: structural molecule activity
AGOC: COPI vesicle coat, Golgi apparatus
AGOP: intracellular protein transport, retrograde vesicle–mediated transport, Golgi to ER
PGOF: protein binding, structural molecule activity
PGOC: membrane coat
PGOP: intracellular protein transport, vesicle–mediated transport

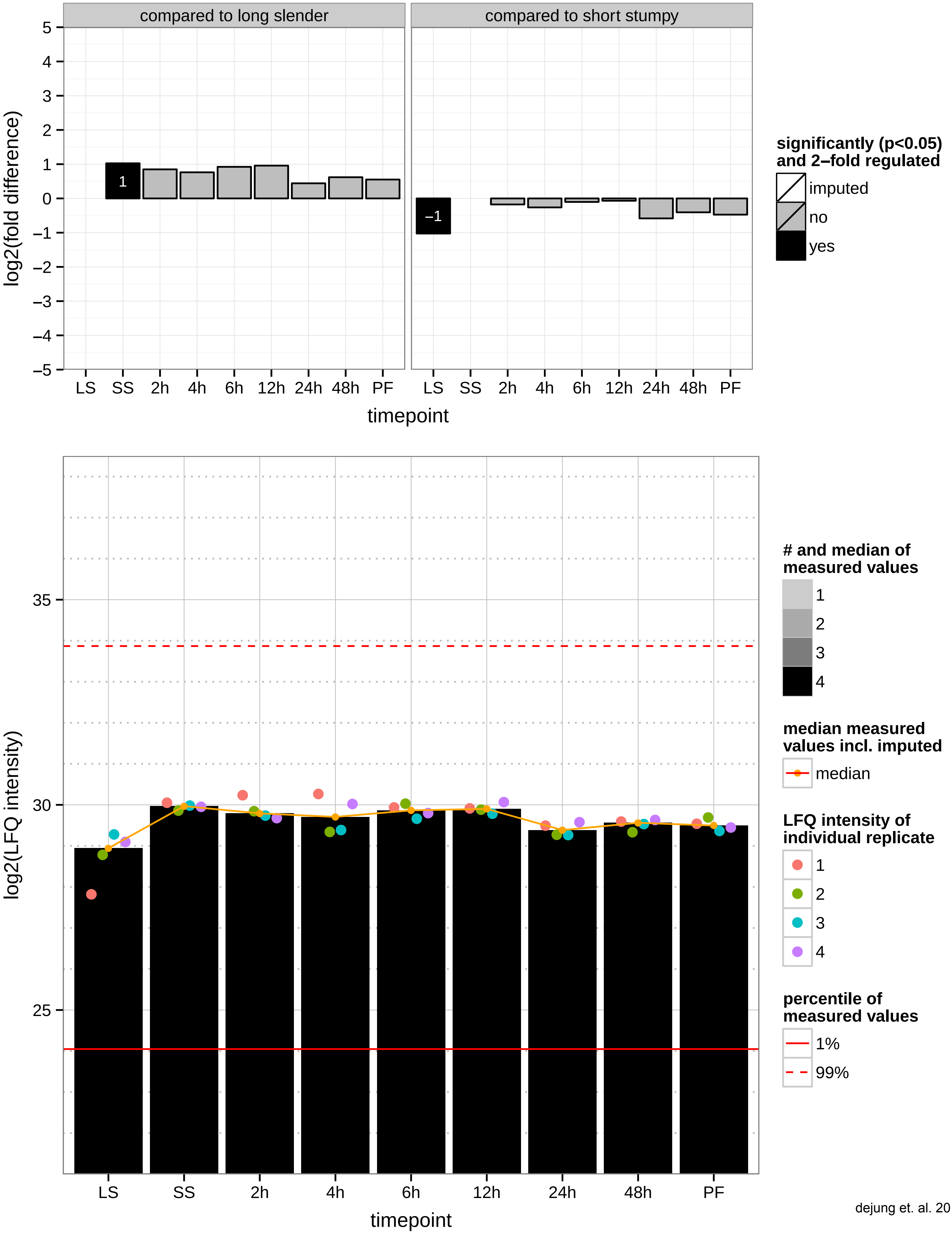

dejung et. al. 2015

retrotransposon hot spot protein (RHS, pseudogene), putative, retrotransposon hot spot protein 1 (RHS1), interrupted
Tb927.2.820
AGOF: null
AGOC: null
AGOP: null
PGOF: null
PGOC: null
PGOP: null

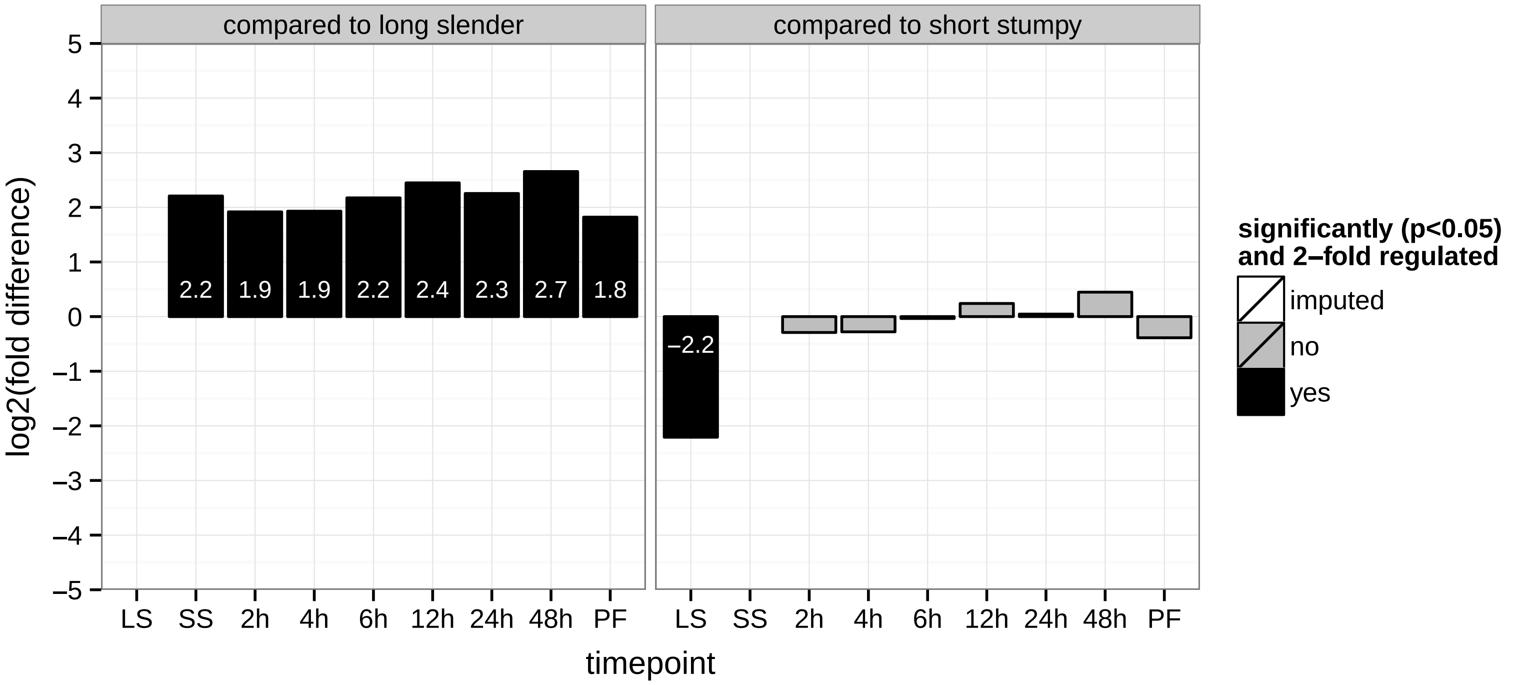

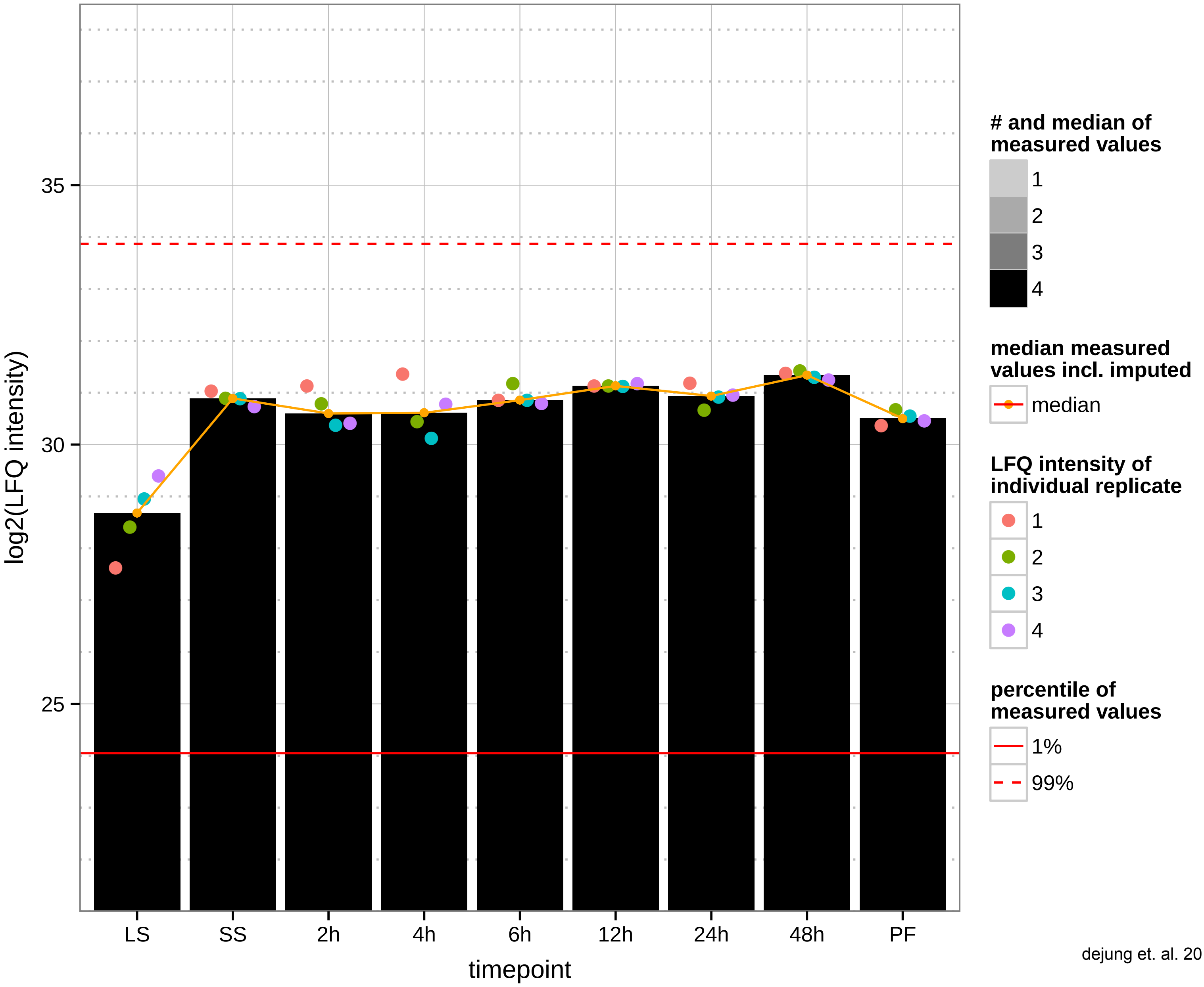

dejung et. al. 2015

hypothetical protein, conserved
Tb927.3.1010
AGOF: null
AGOC: null
AGOP: null
PGOF: null
PGOC: null
PGOP: null

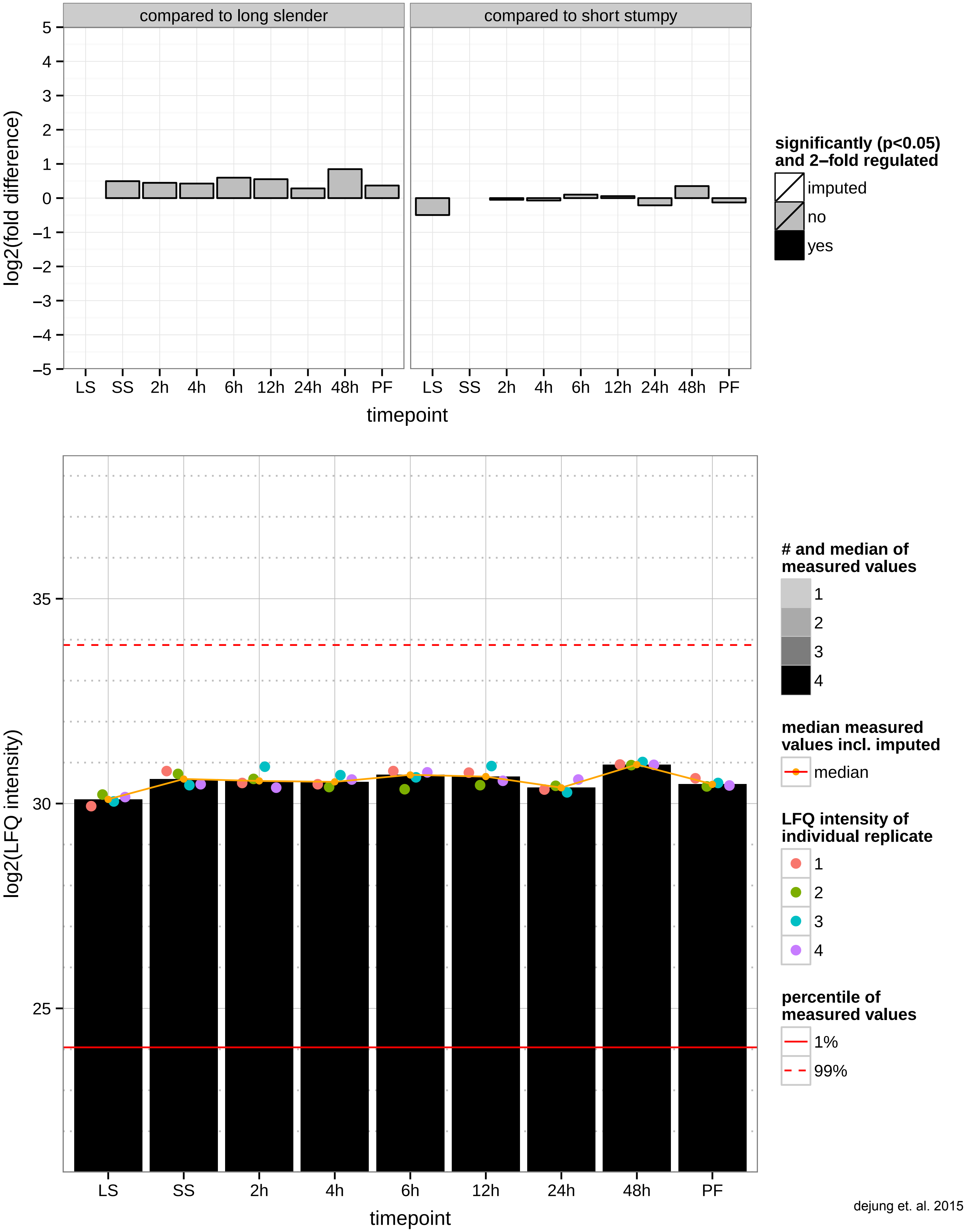

dejung et. al. 2015

unspecified product
Tb927.3.1040
AGOF: null
AGOC: null
AGOP: null
PGOF: null
PGOC: null
PGOP: null

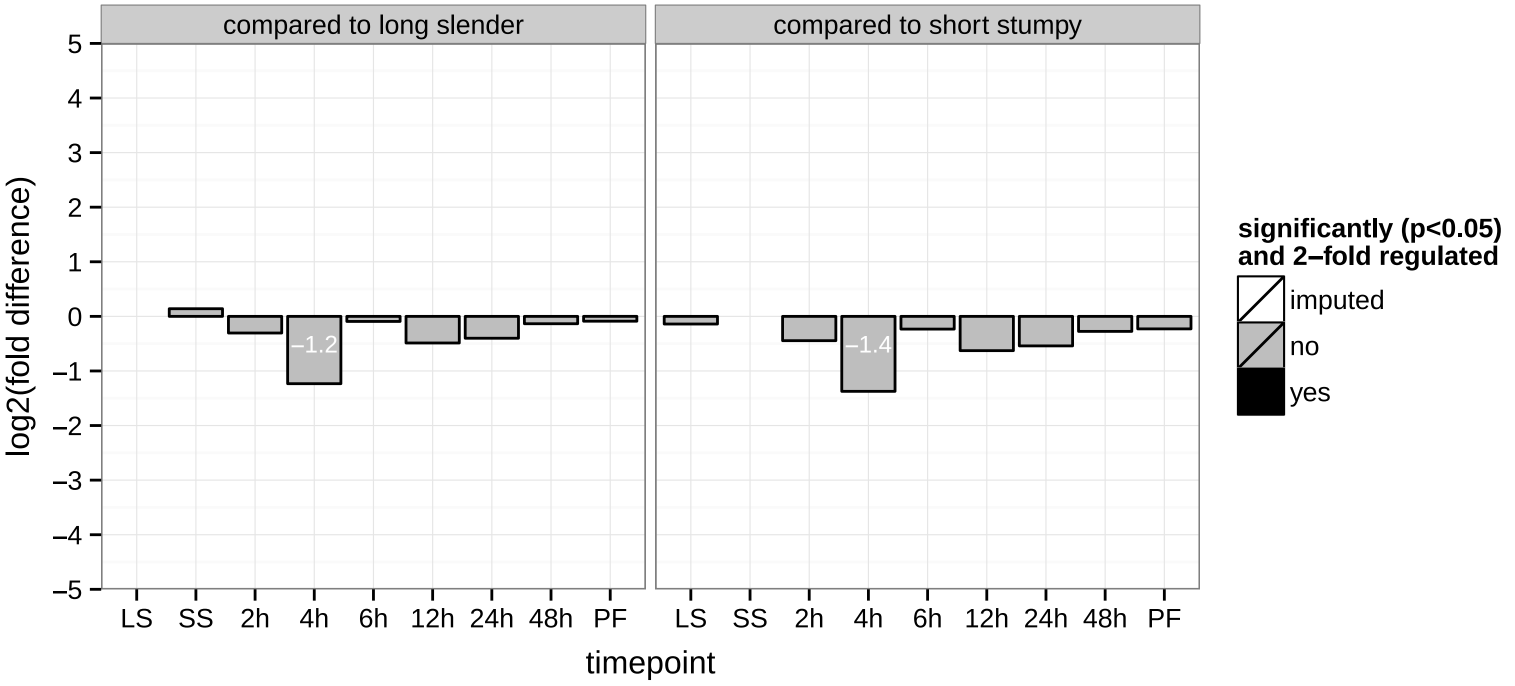

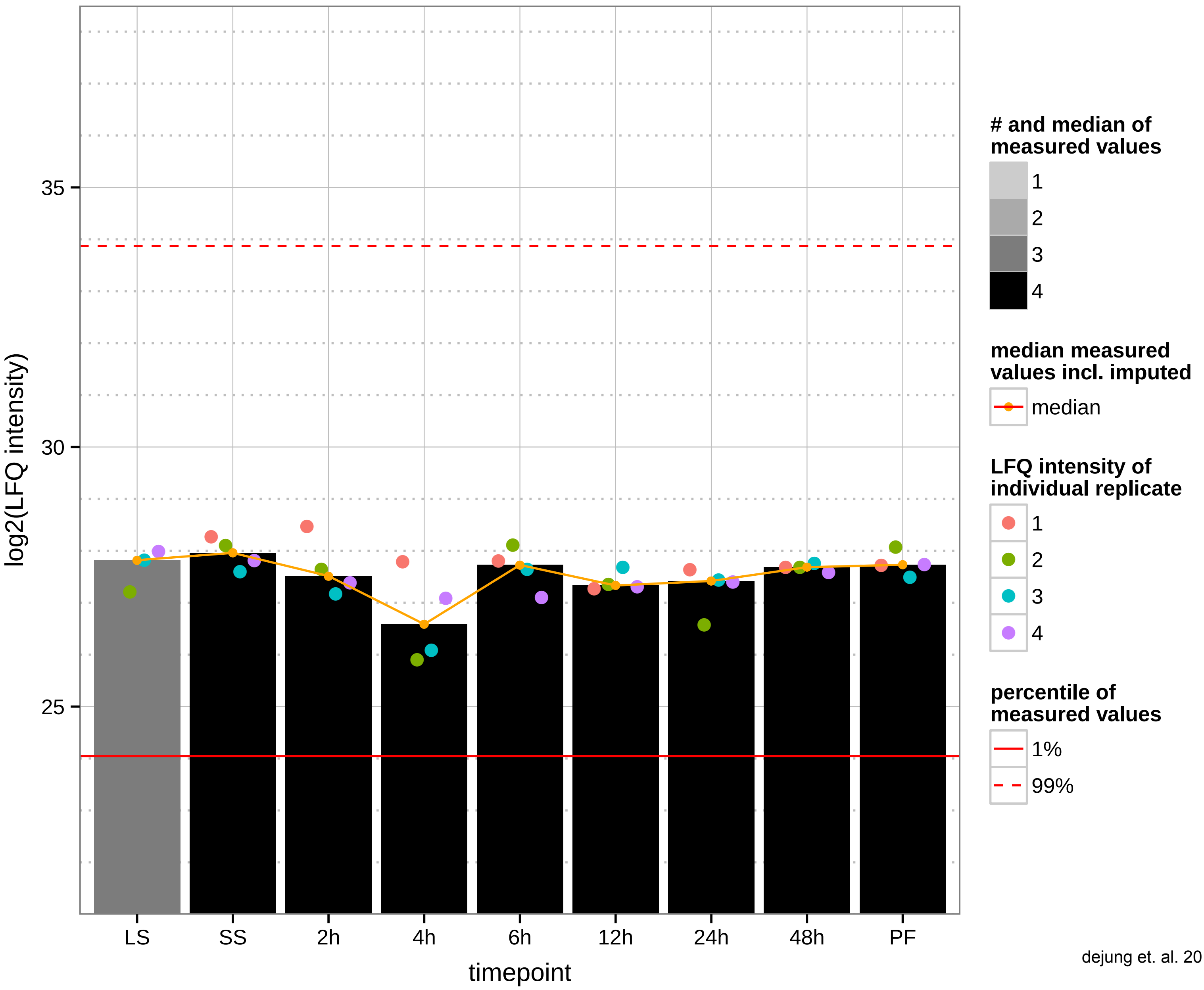

dejung et. al. 2015

cAMP response protein, putative (CARP4)
Tb927.3.1060
AGOF: null
AGOC: null
AGOP: response to drug
PGOF: null
PGOC: null
PGOP: null

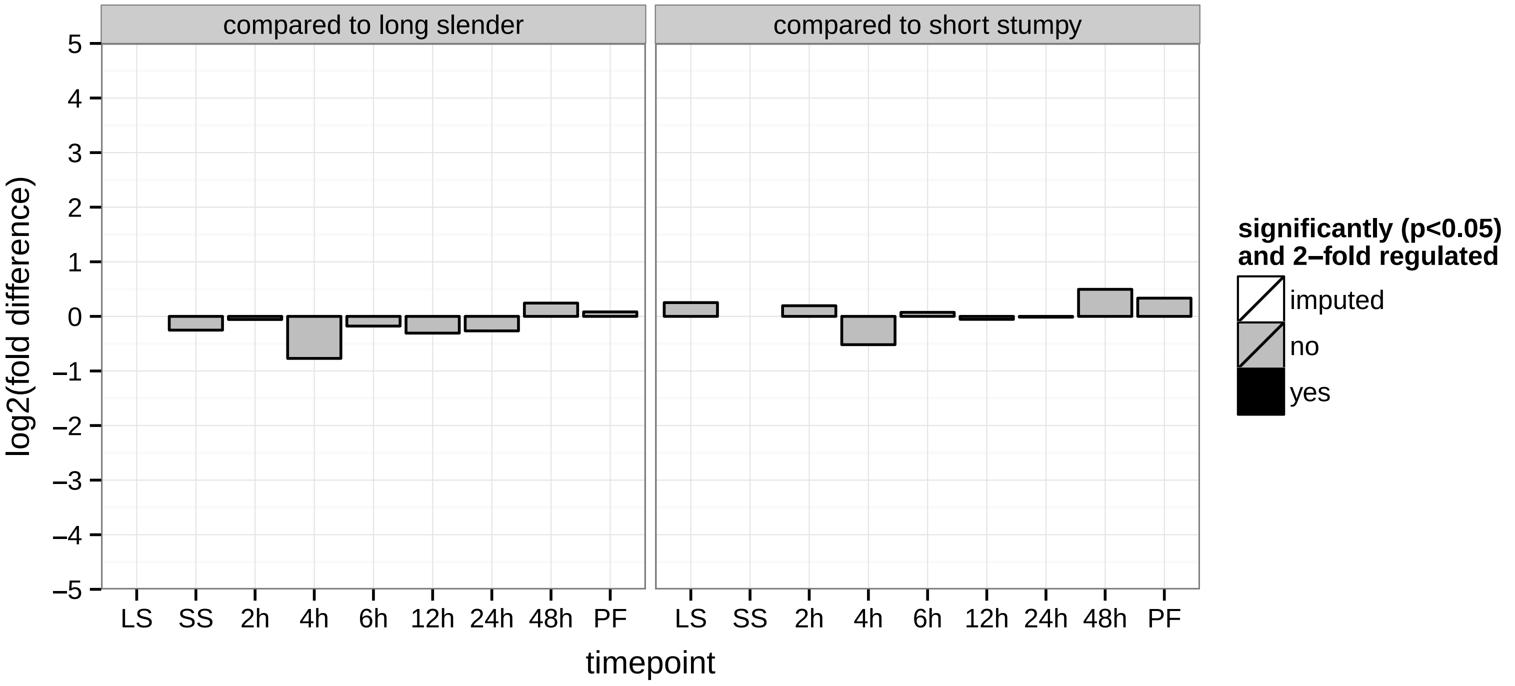

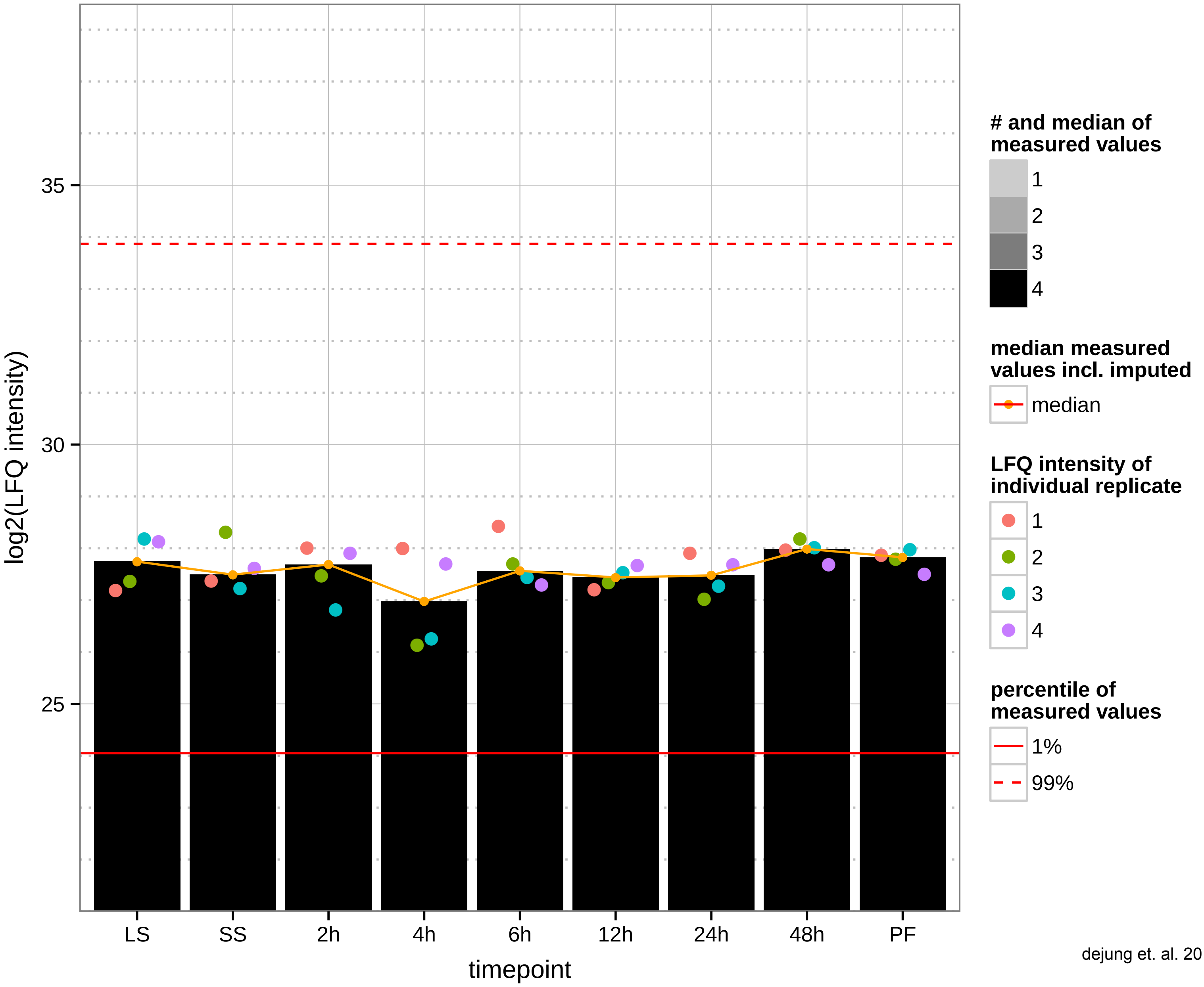

dejung et. al. 2015

hypothetical protein, conserved
Tb927.3.1080
AGOF: null
AGOC: null
AGOP: null
PGOF: null
PGOC: null
PGOP: null

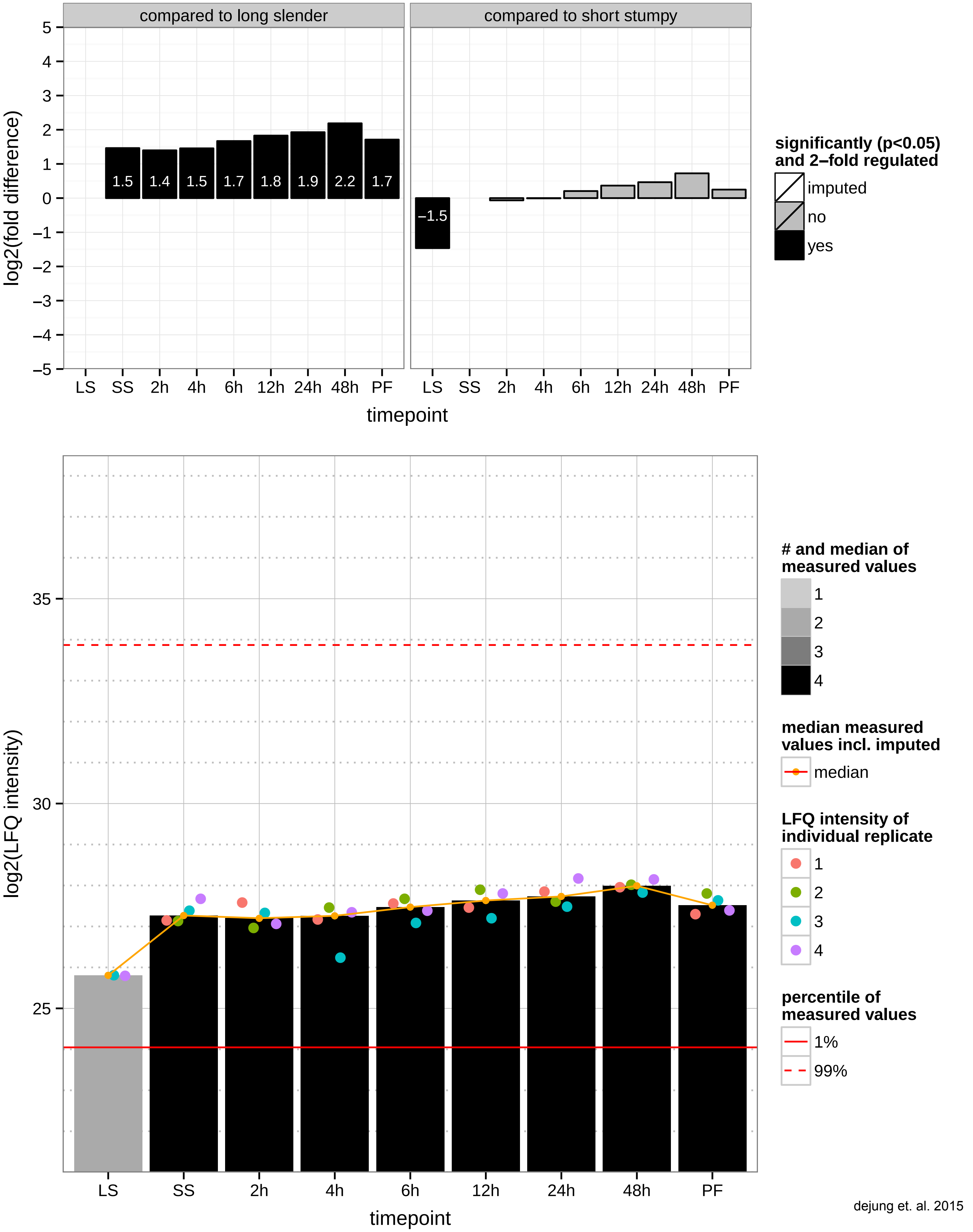

dejung et. al. 2015

GTP−binding nuclear protein rtb2, putative (rtb2)
Tb927.3.1120
AGOF: GTP binding, GTPase activity
AGOC: intracellular
AGOP: intracellular protein transport, nucleocytoplasmic transport, small GTPase mediated signal transduction
PGOF: GTP binding, GTPase activity
PGOC: intracellular, membrane
PGOP: GTP catabolic process, intracellular protein transport, nucleocytoplasmic transport, protein transport, signal transducti

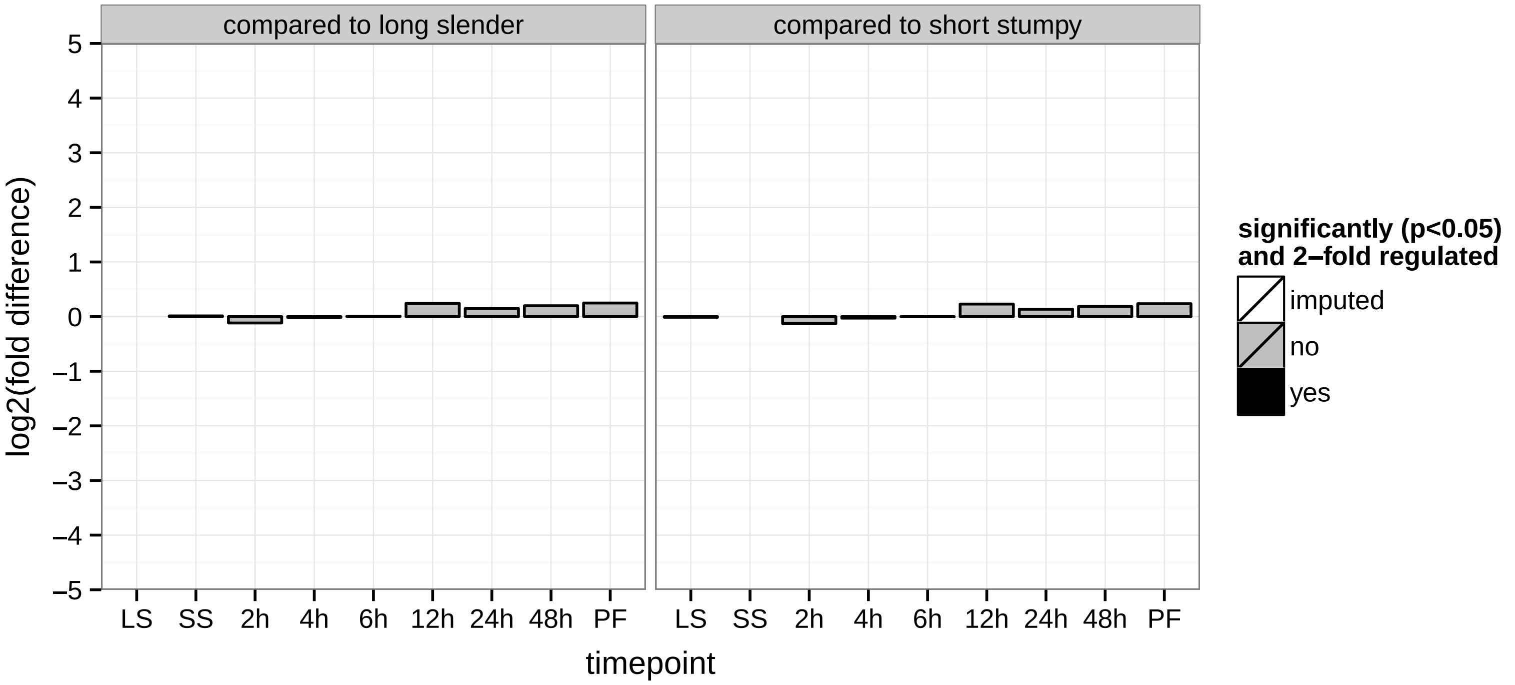

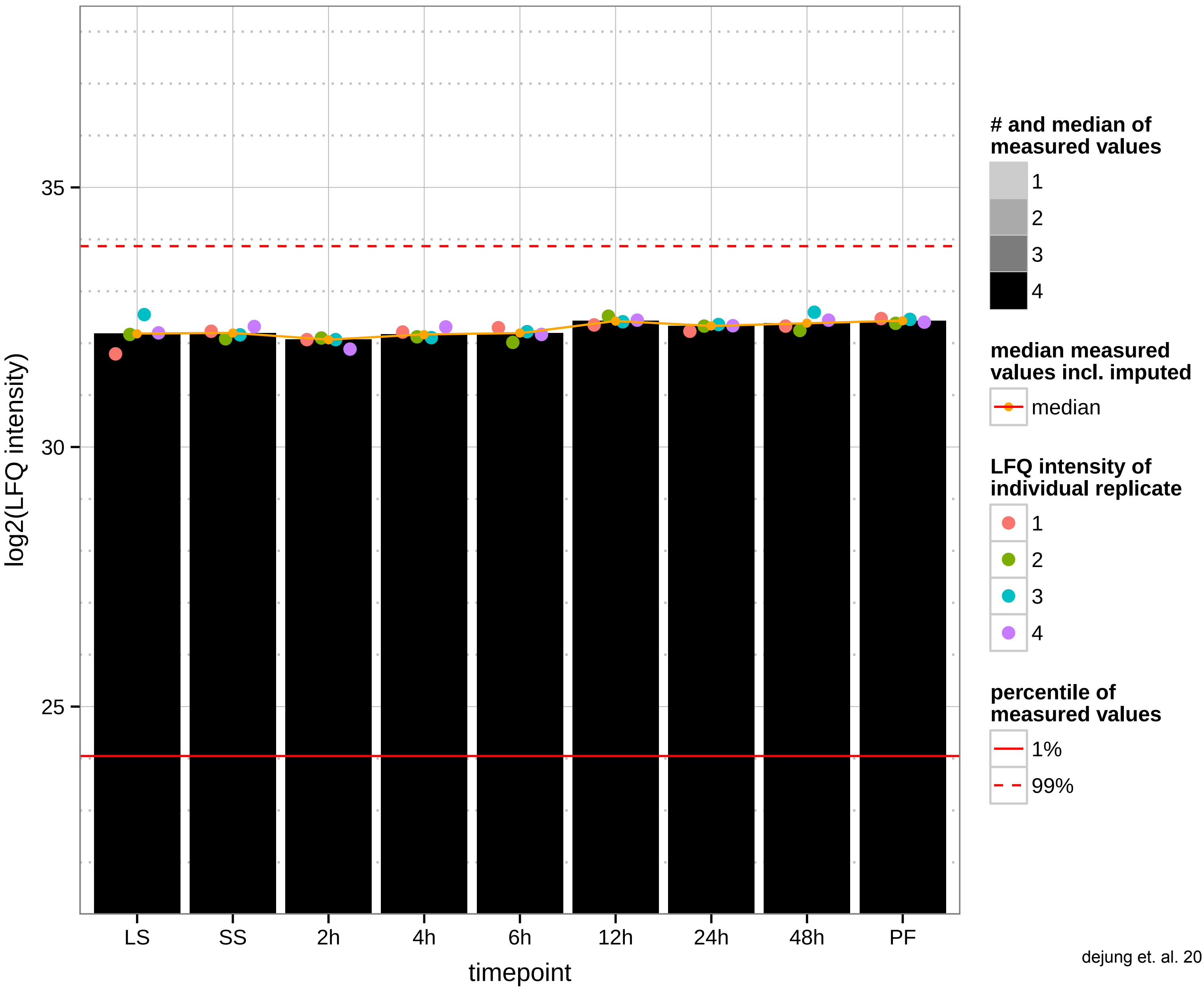

dejung et. al. 2015

hypothetical protein, conserved
Tb927.3.1150
AGOF: nucleotide binding
AGOC: null
AGOP: null
PGOF: nucleotide binding
PGOC: null
PGOP: null

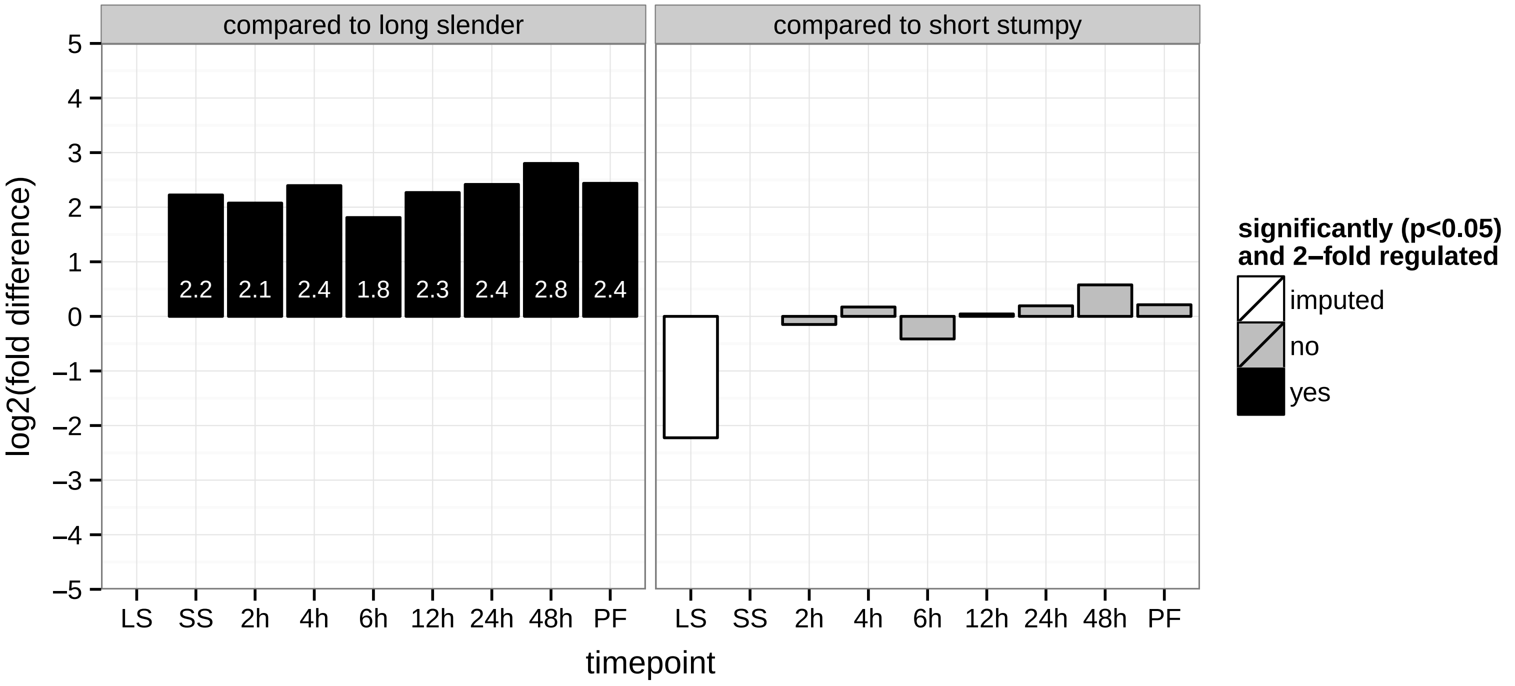

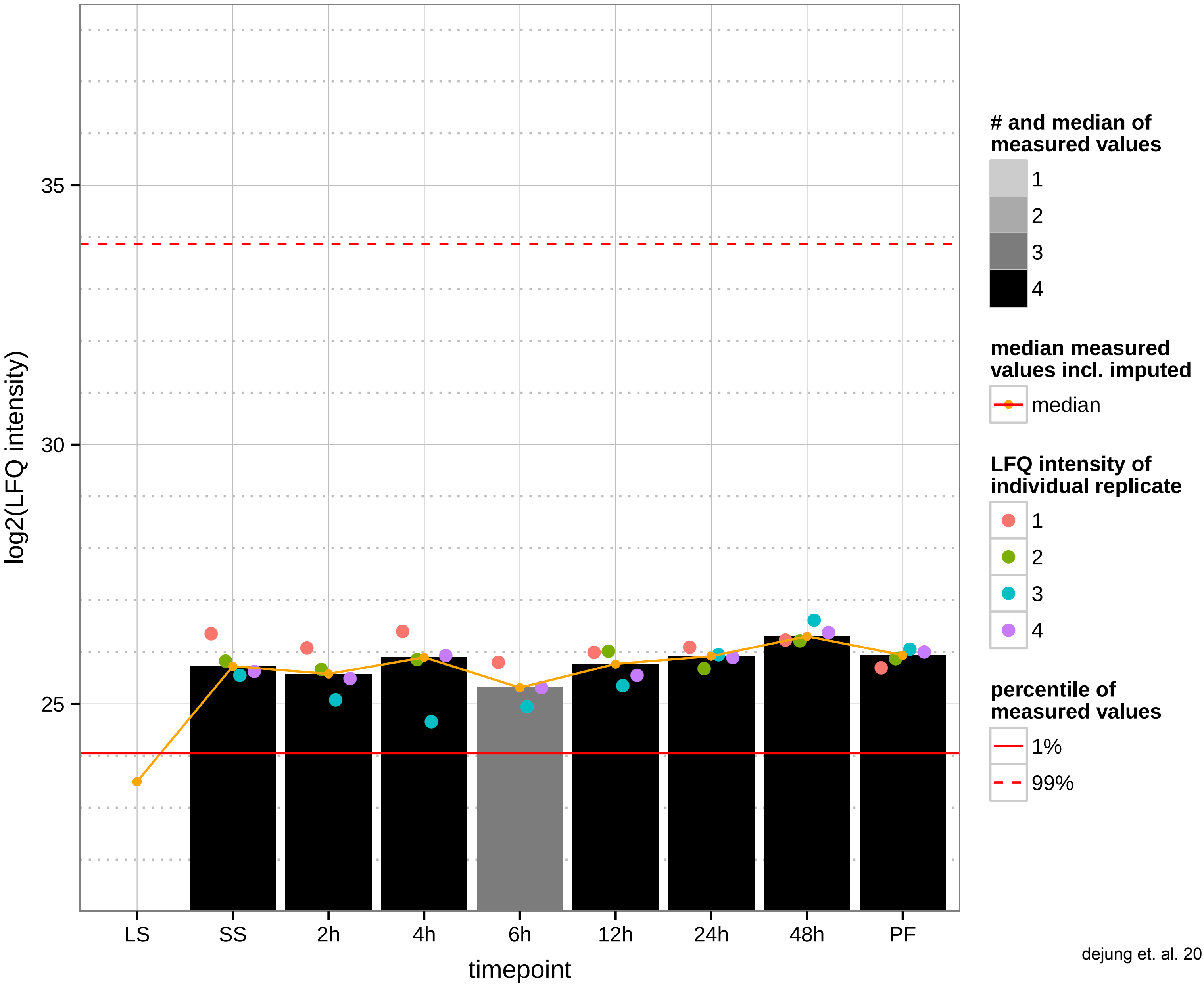

dejung et. al. 2015

hypothetical protein, conserved
Tb927.3.1160
AGOF: null
AGOC: mitochondrion
AGOP: null
PGOF: null
PGOC: null
PGOP: null

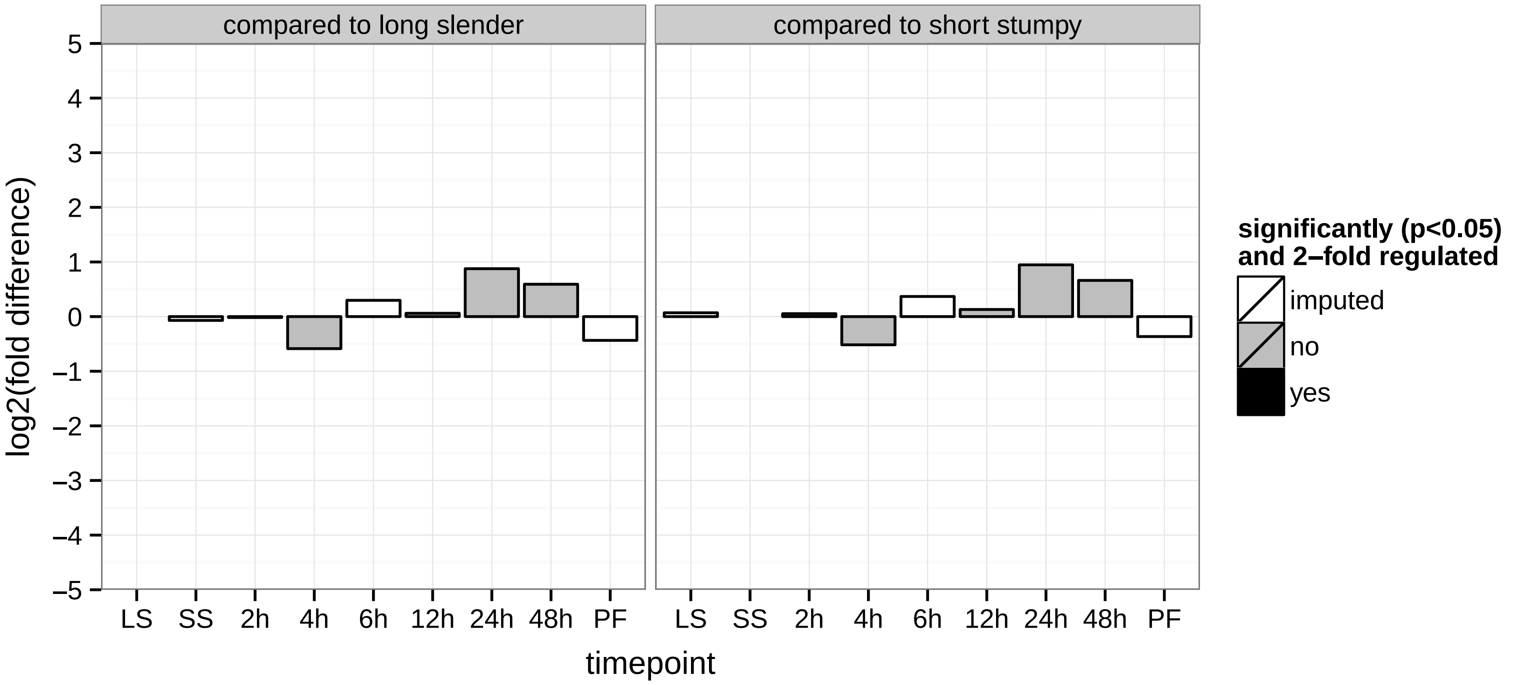

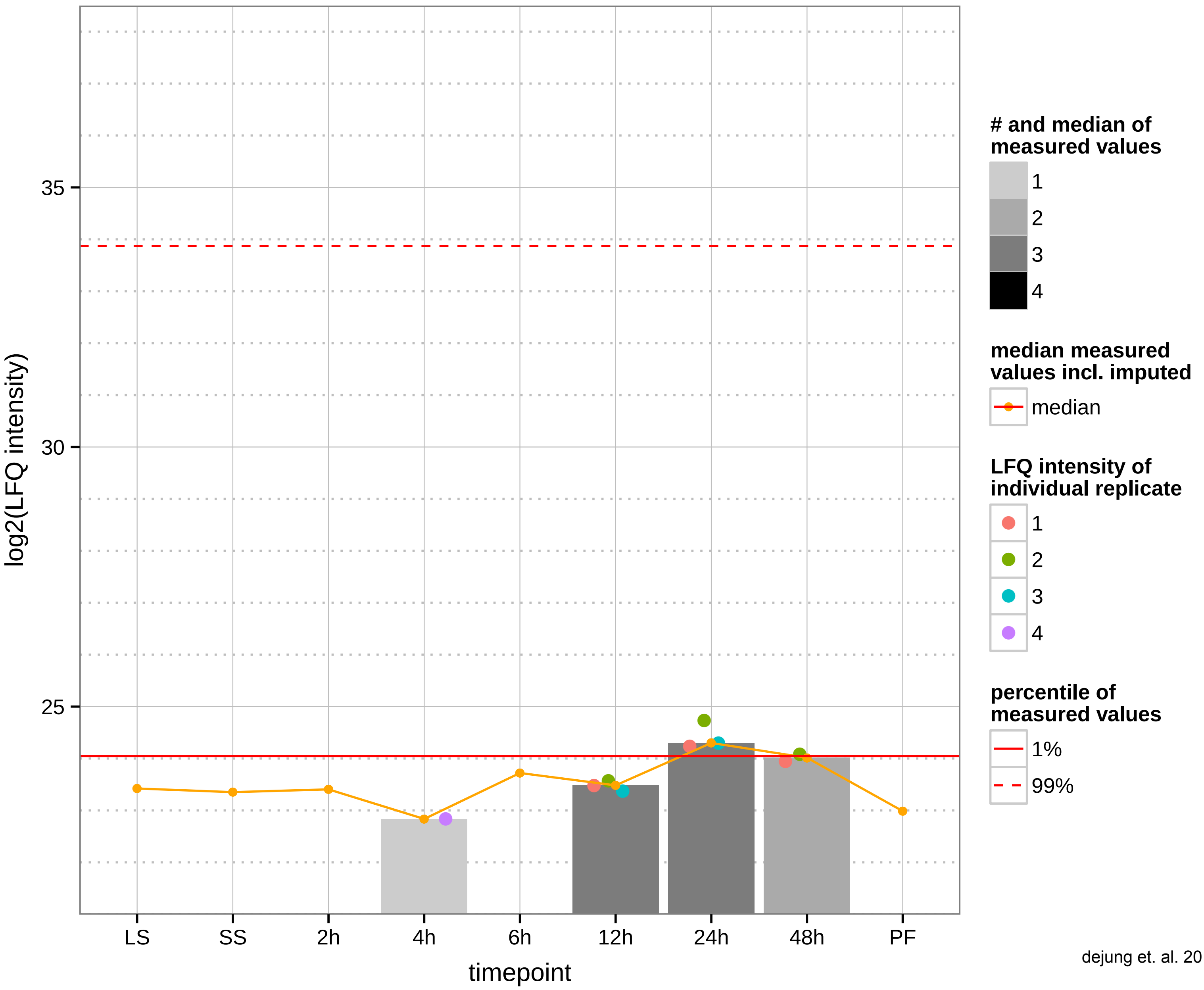

dejung et. al. 2015

hypothetical protein, conserved
Tb927.3.1170
AGOF: null
AGOC: null
AGOP: regulation of signal transduction, response to biotic stimulus
PGOF: null
PGOC: null
PGOP: regulation of signal transduction

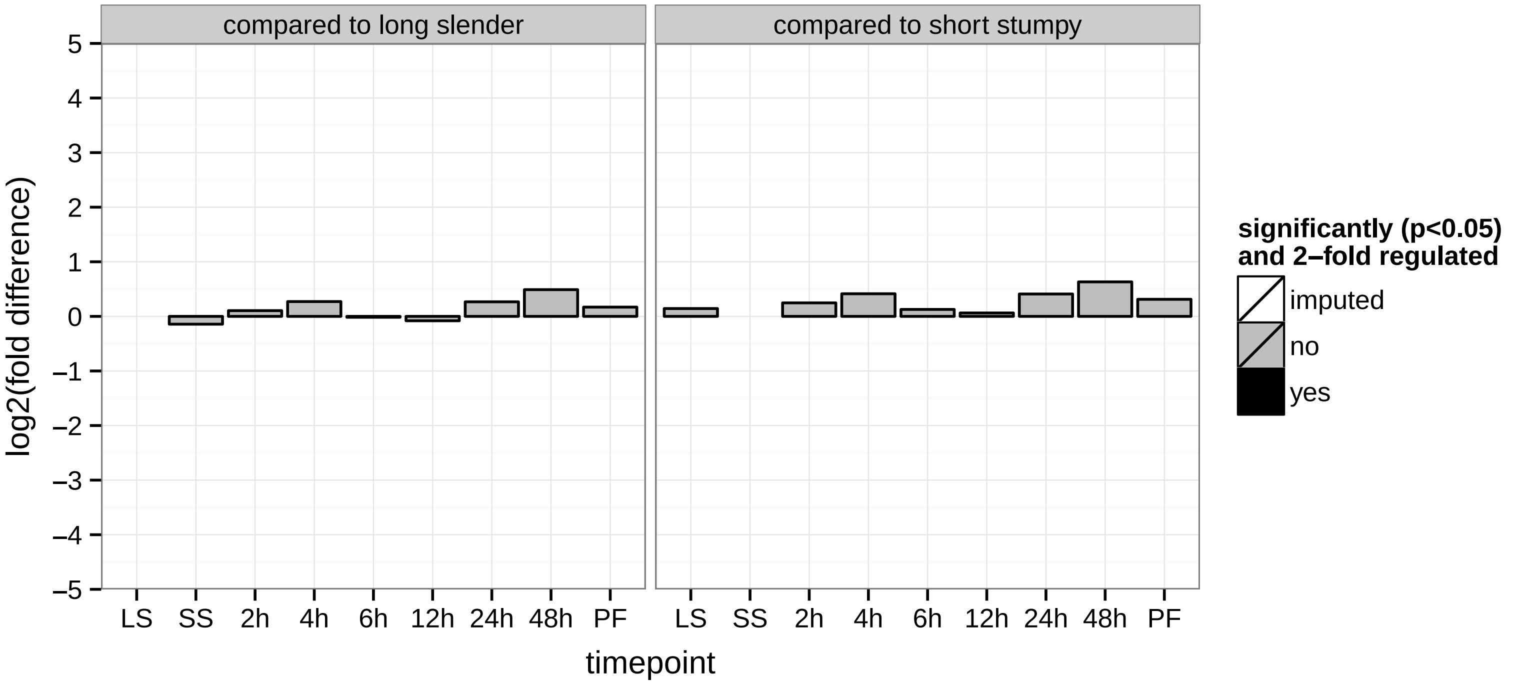

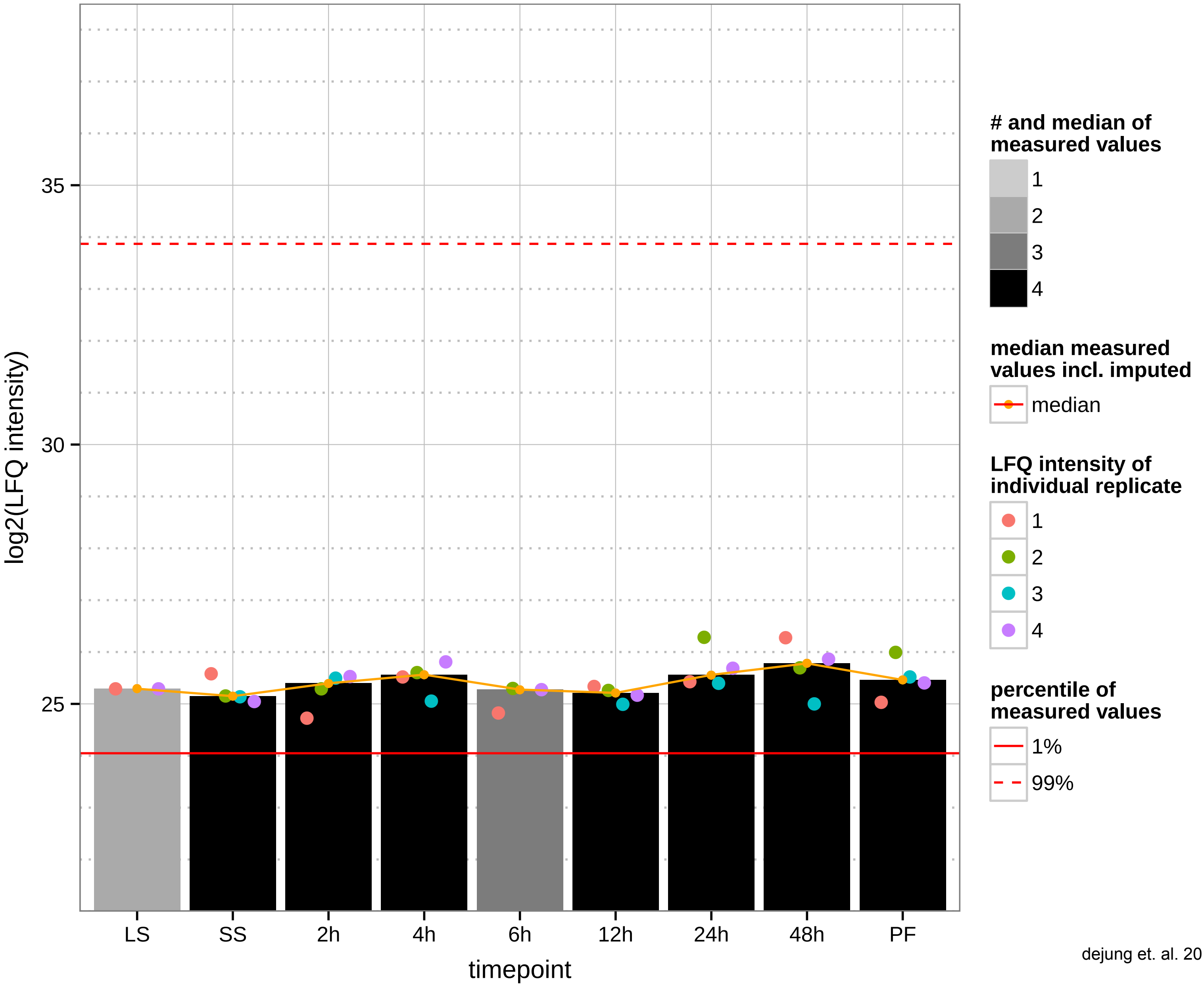

dejung et. al. 2015

hypothetical protein, conserved
Tb927.3.1180
AGOF: null
AGOC: mitochondrion
AGOP: null
PGOF: null
PGOC: null
PGOP: null

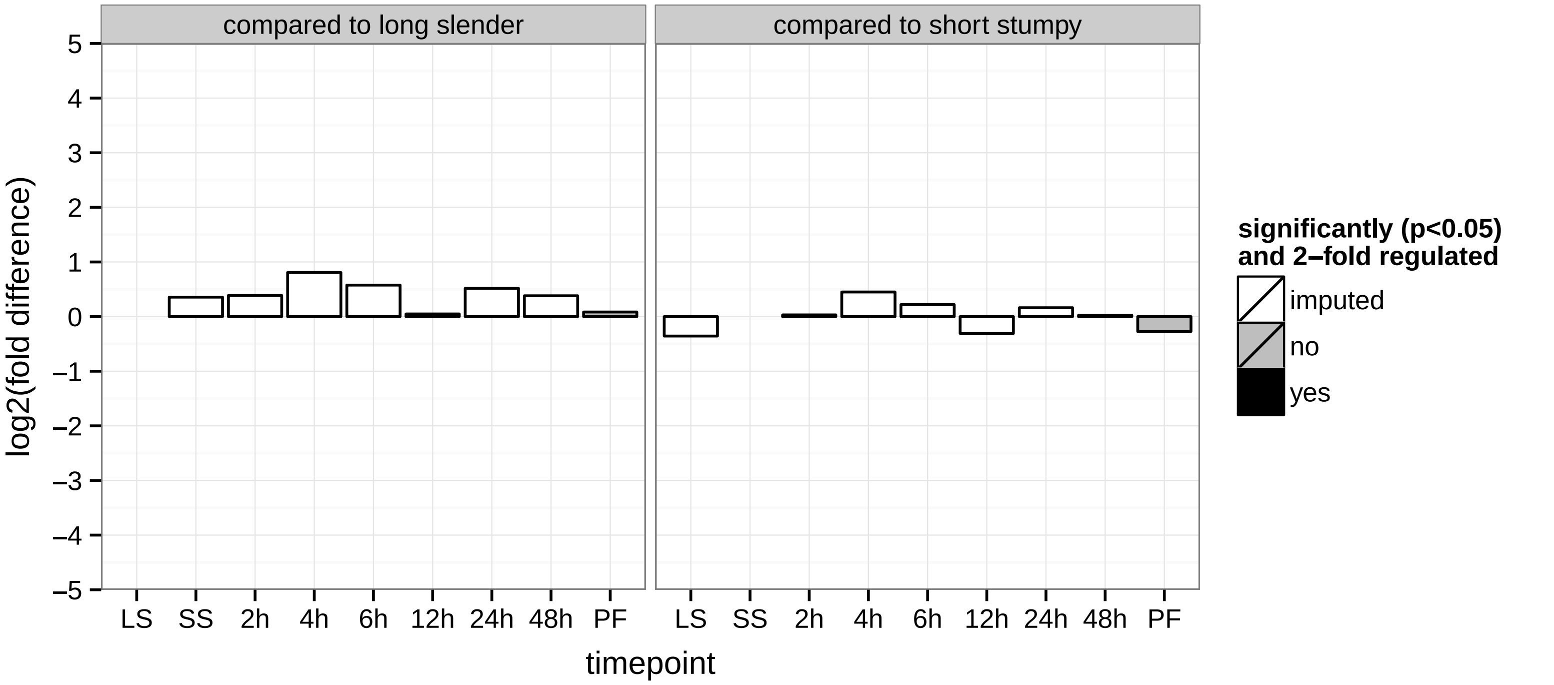

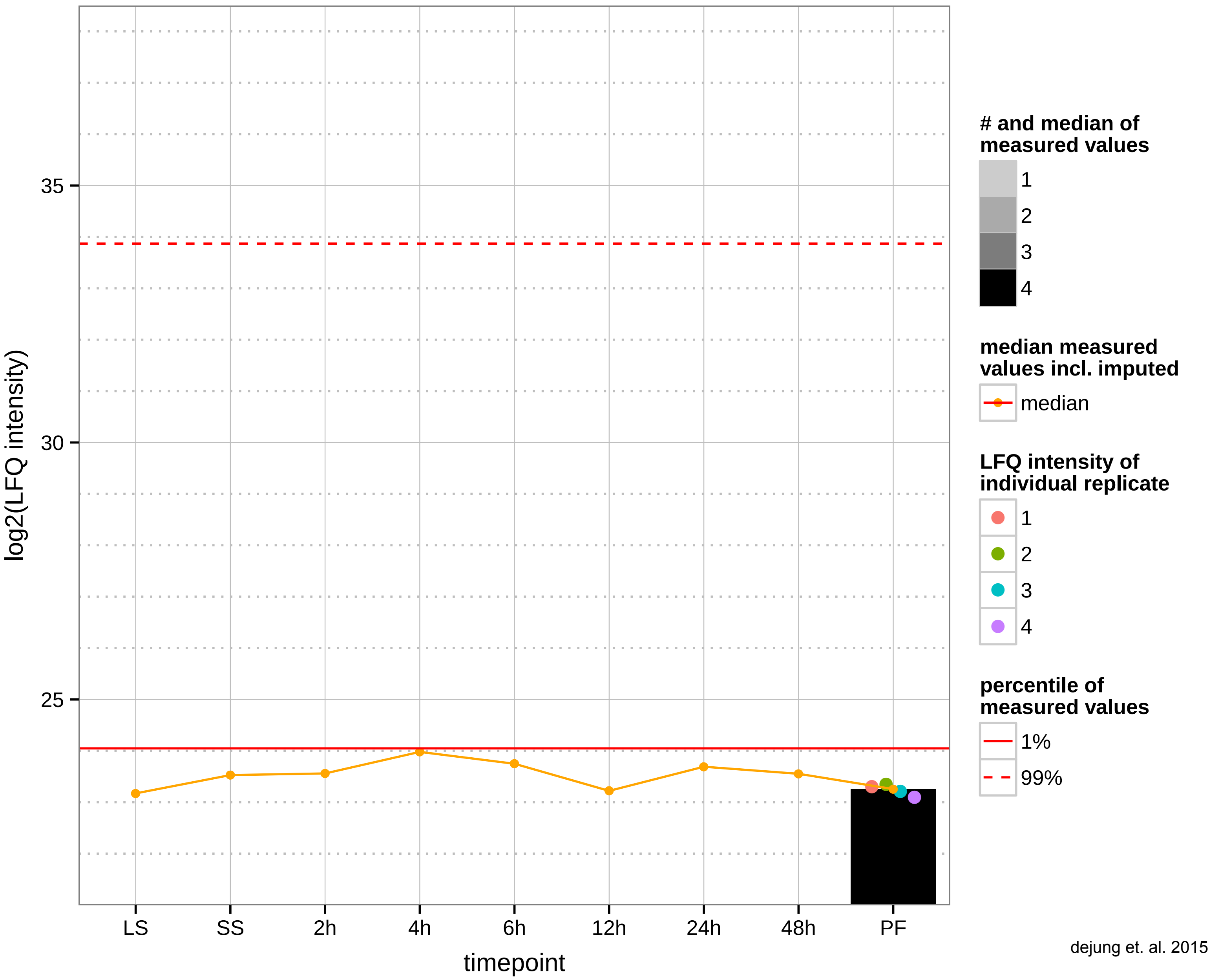

dejung et. al. 2015

hypothetical protein, conserved
Tb927.3.1190
AGOF: null
AGOC: null
AGOP: null
PGOF: null
PGOC: null
PGOP: null

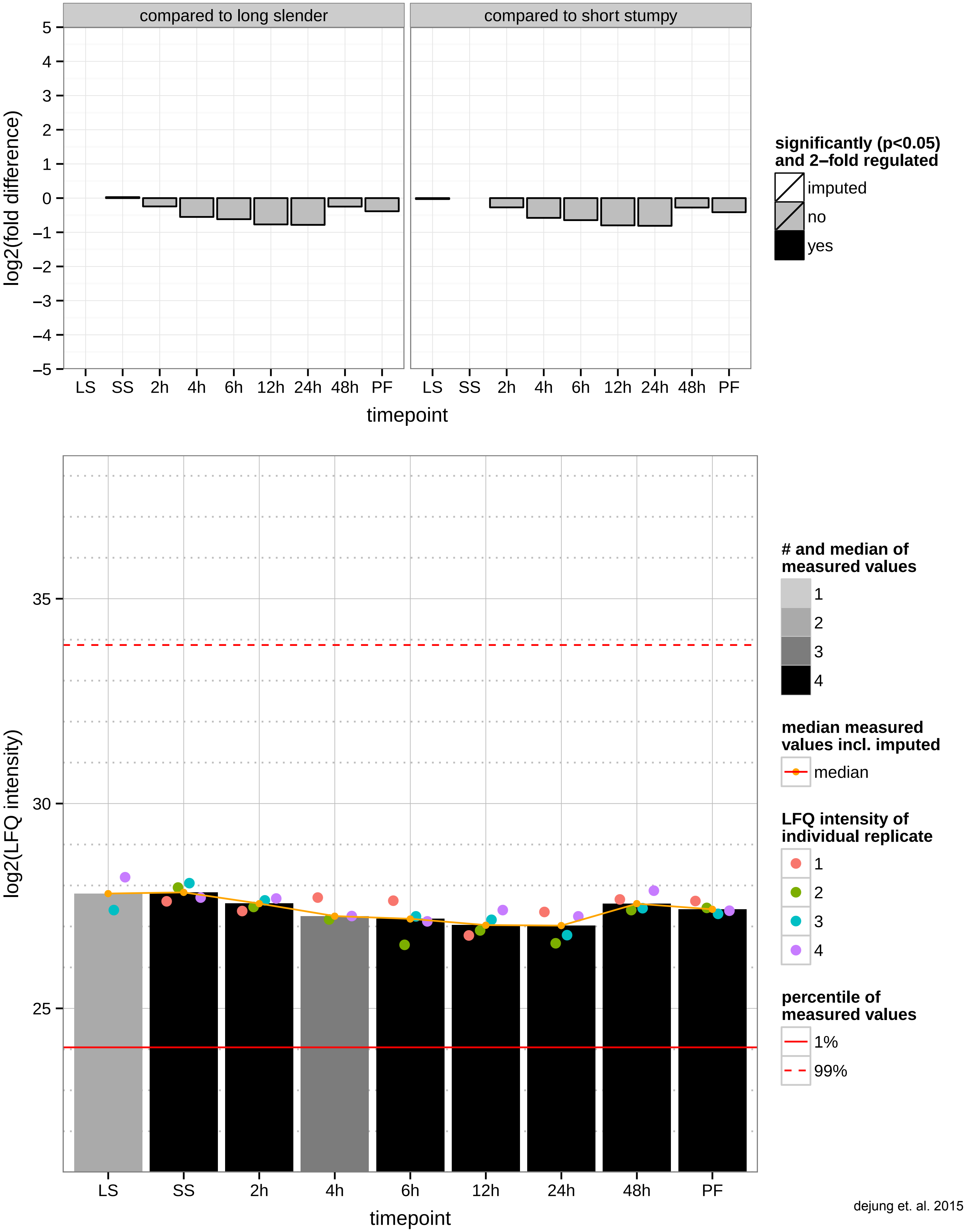

dejung et. al. 2015

protein transport protein Sec24 (SEC24.1)
Tb927.3.1210
AGOF: protein binding, zinc ion binding
AGOC: COPII vesicle coat
AGOP: ER to Golgi vesicle–mediated transport, intracellular protein transport
PGOF: zinc ion binding
PGOC: COPII vesicle coat
PGOP: ER to Golgi vesicle–mediated transport, intracellular protein transport

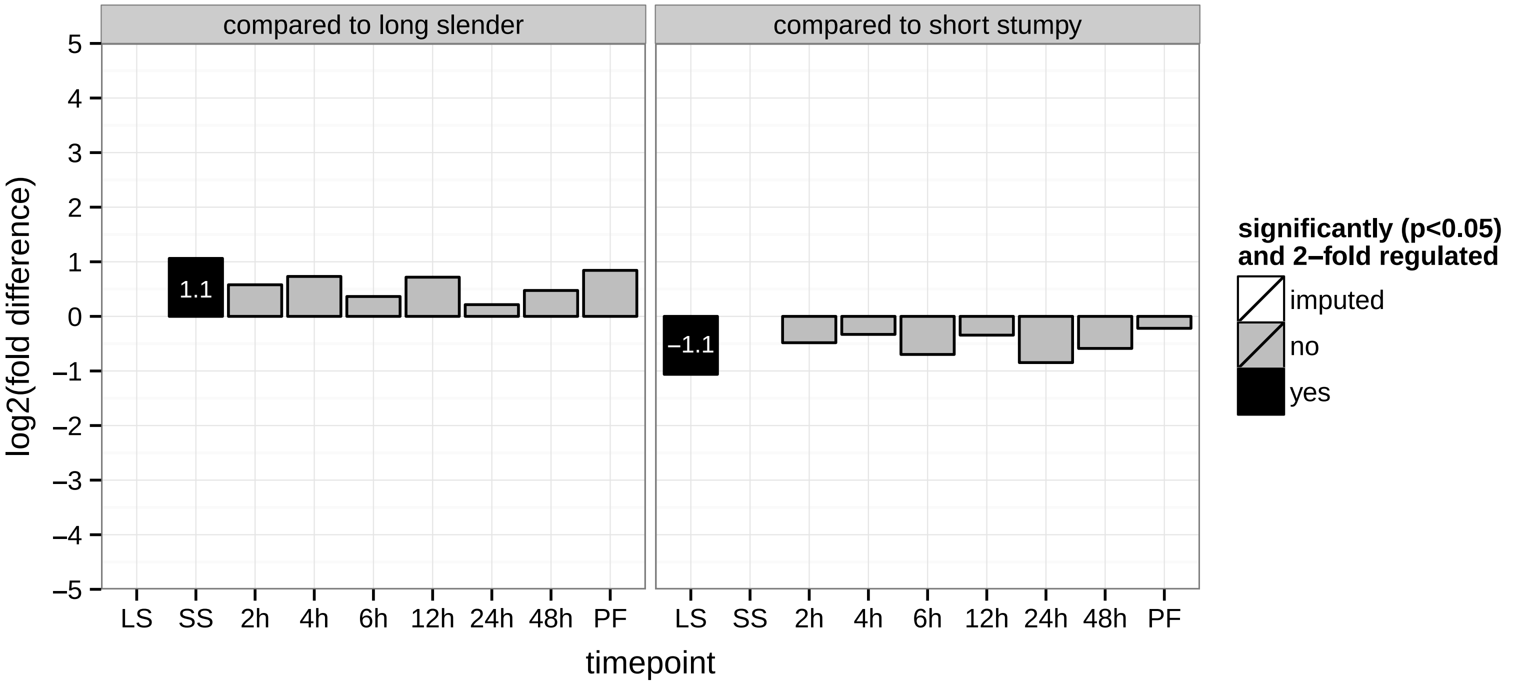

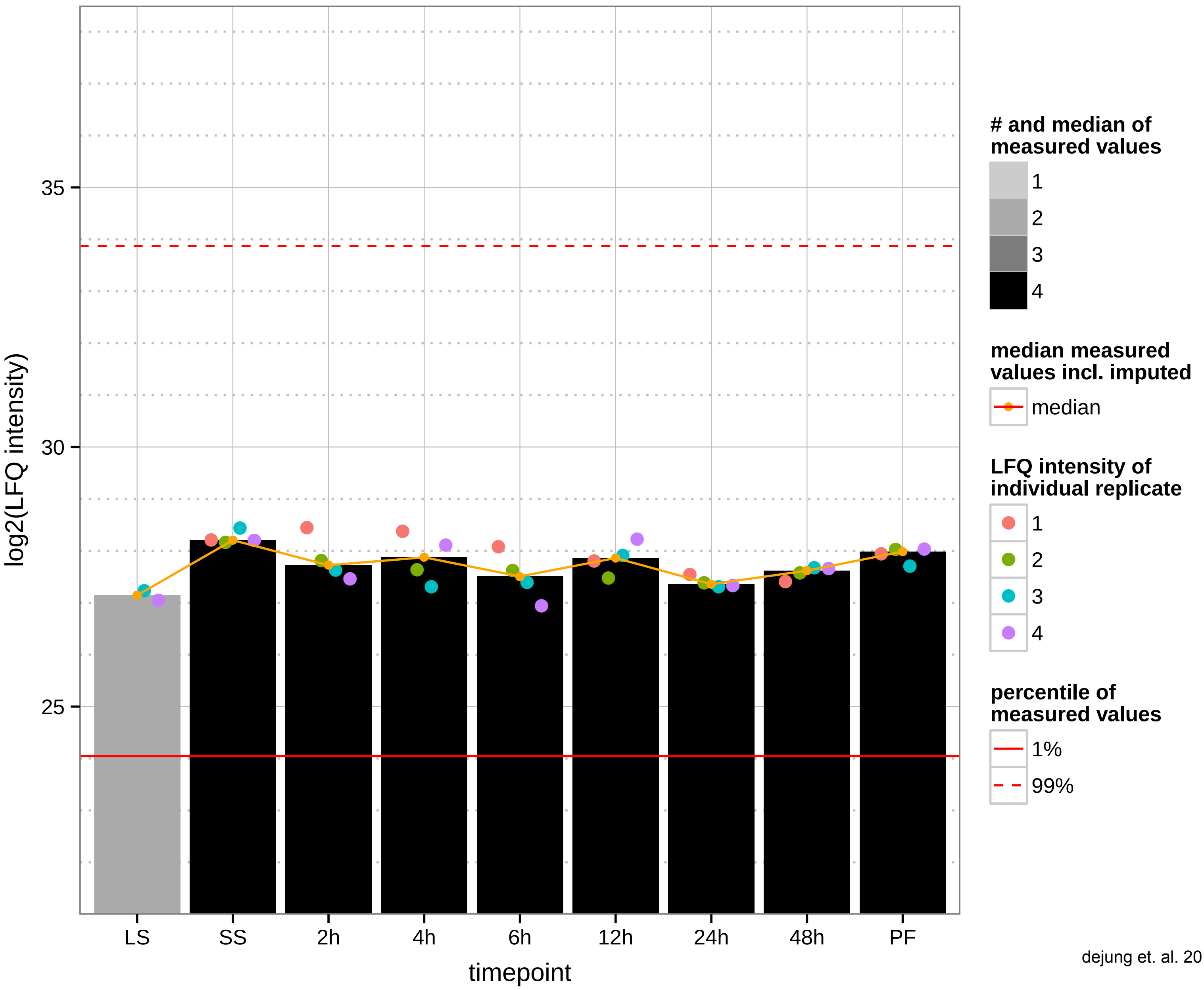

dejung et. al. 2015

hypothetical protein, conserved
Tb927.3.1270
AGOF: null
AGOC: null
AGOP: null
PGOF: null
PGOC: null
PGOP: null

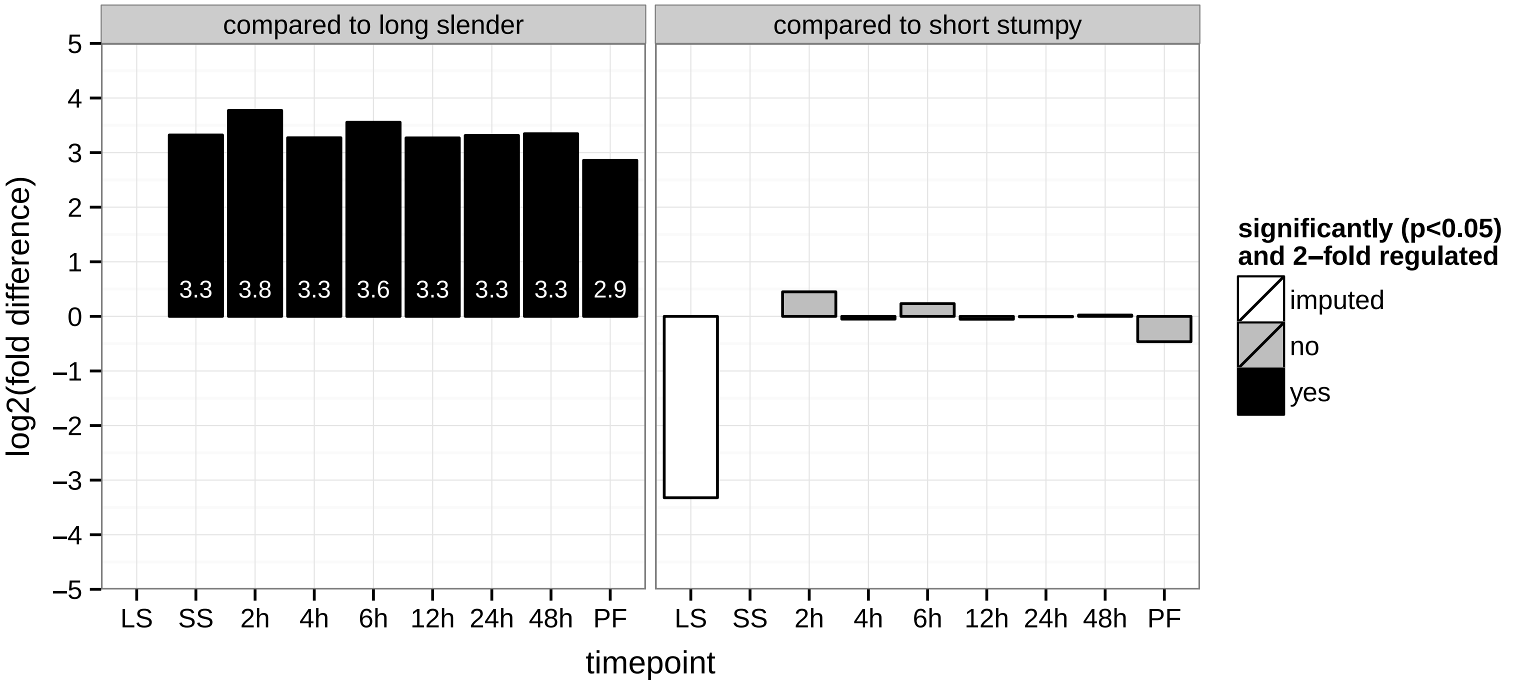

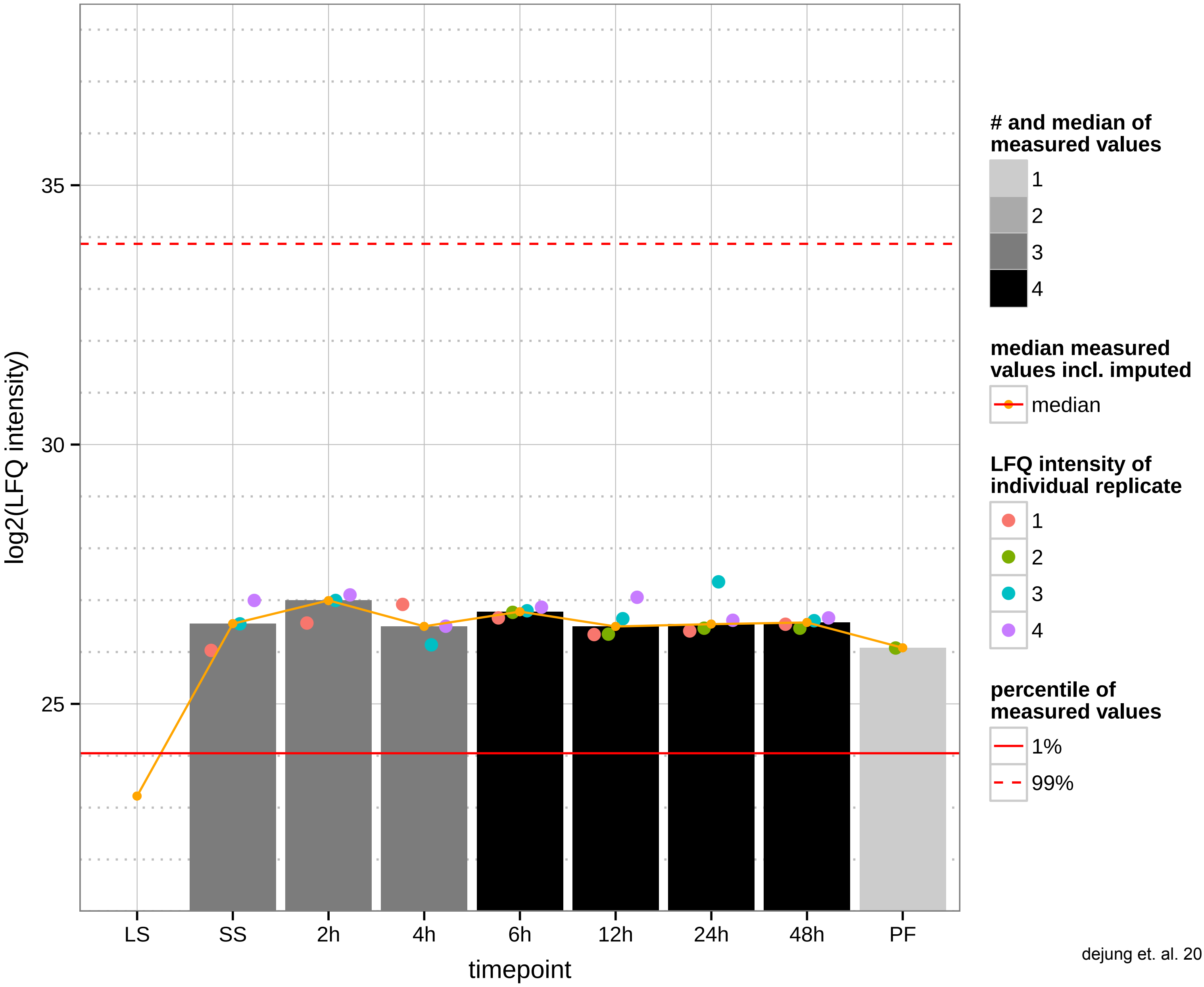

dejung et. al. 2015

hypothetical protein, conserved
Tb927.3.1280
AGOF: null
AGOC: null
AGOP: null
PGOF: null
PGOC: null
PGOP: null

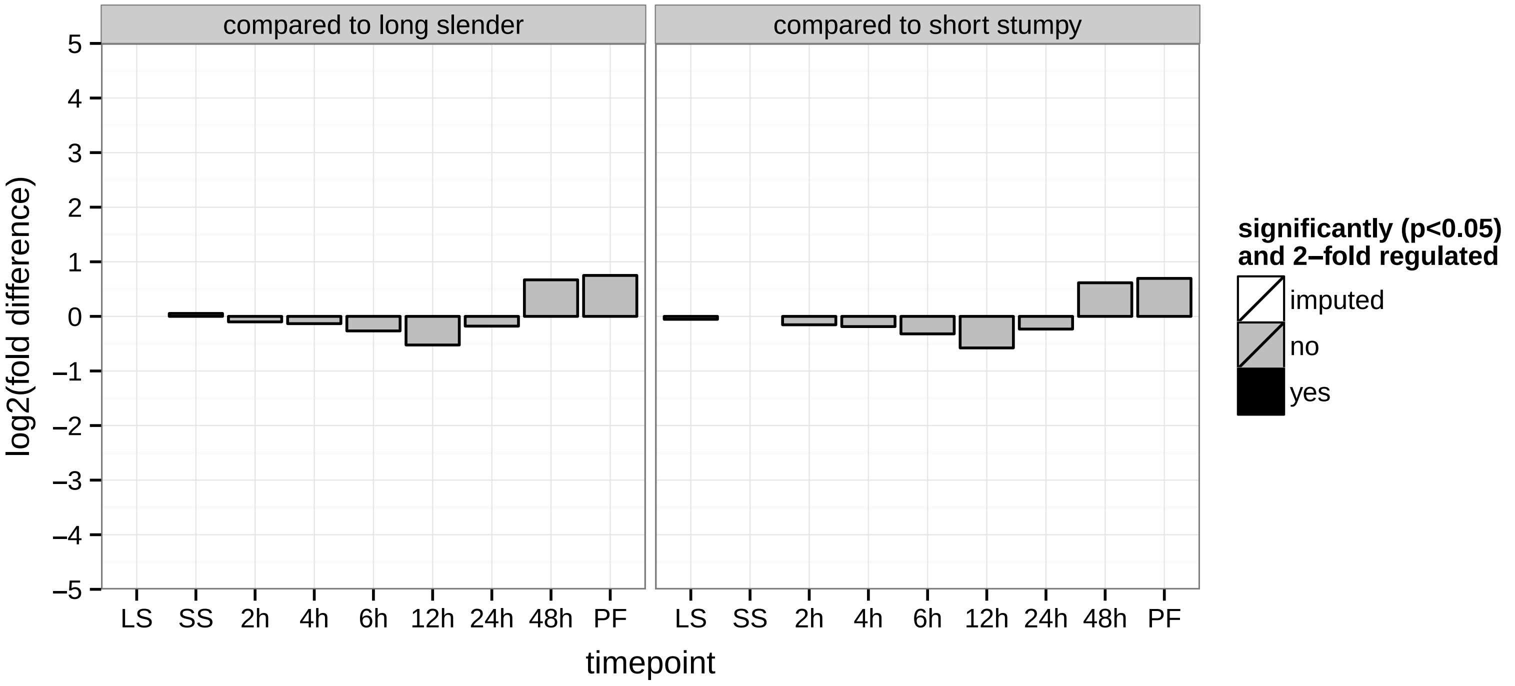

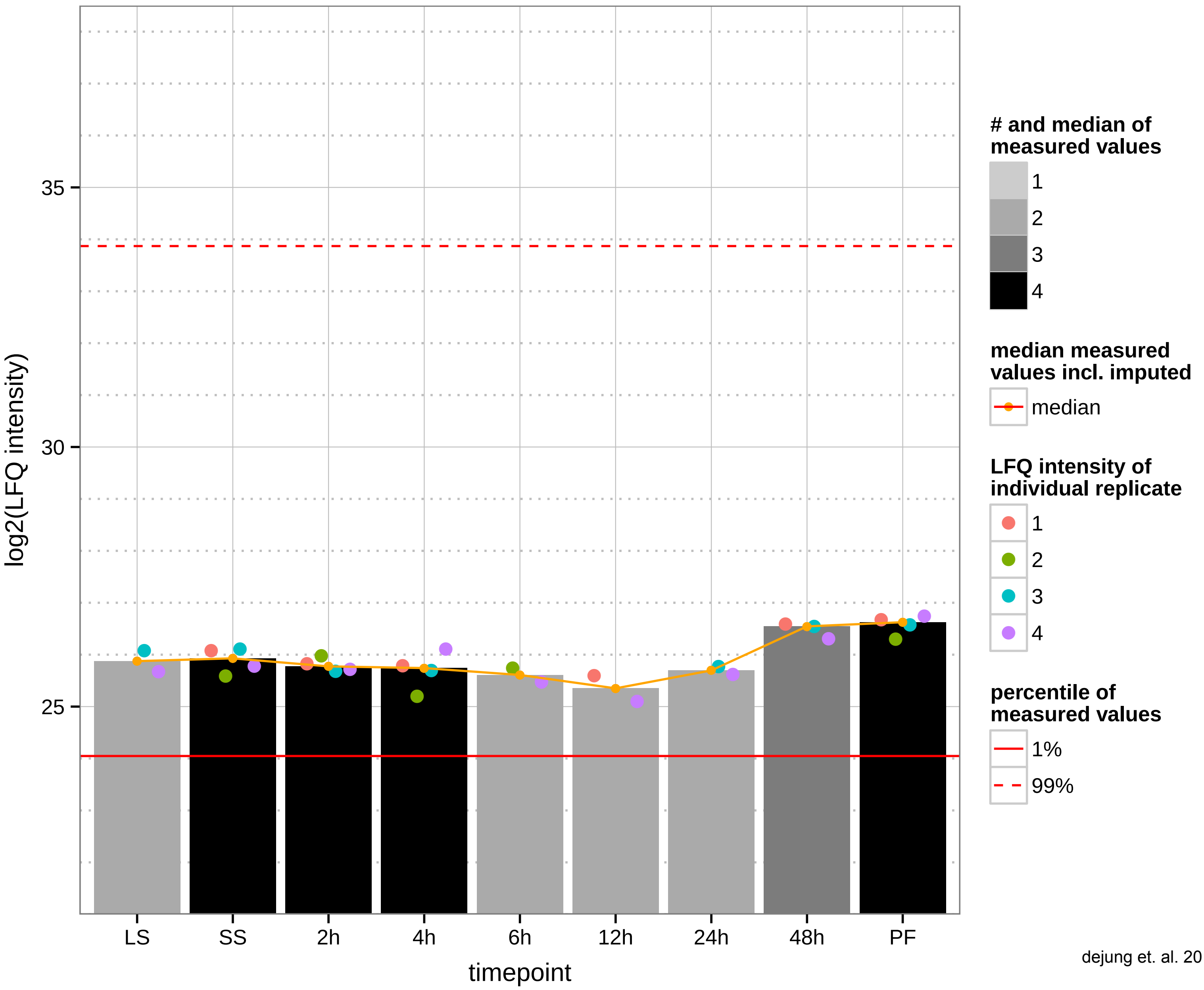

dejung et. al. 2015

cullin 4B, putative
Tb927.3.1290
AGOF: ubiquitin protein ligase binding
AGOC: cullin–RING ubiquitin ligase complex
AGOP: ubiquitin–dependent protein catabolic process
PGOF: ubiquitin protein ligase binding
PGOC: cullin–RING ubiquitin ligase complex
PGOP: ubiquitin–dependent protein catabolic process

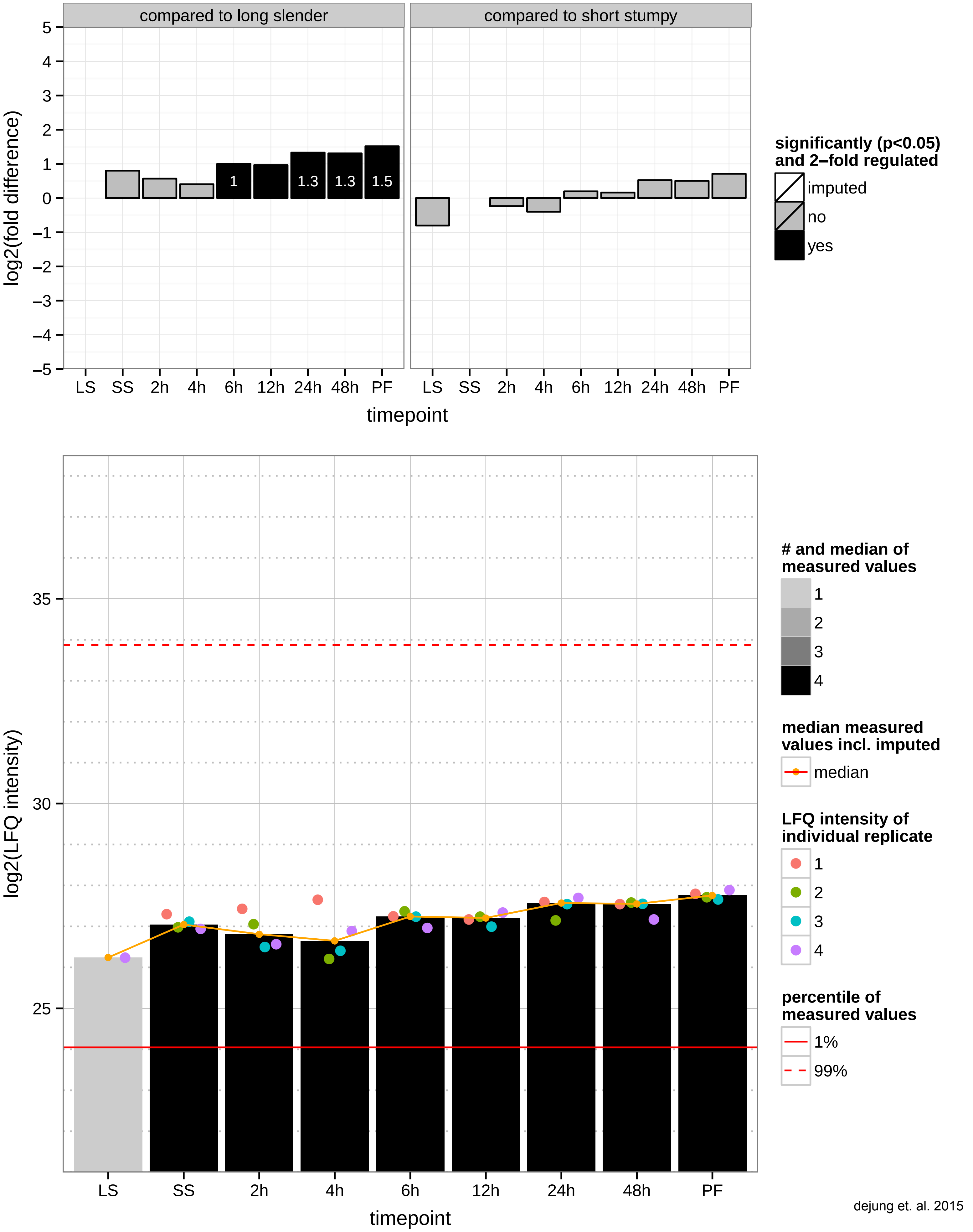

dejung et. al. 2015

hypothetical protein, conserved
Tb927.3.1300
AGOF: null
AGOC: null
AGOP: null
PGOF: null
PGOC: null
PGOP: null

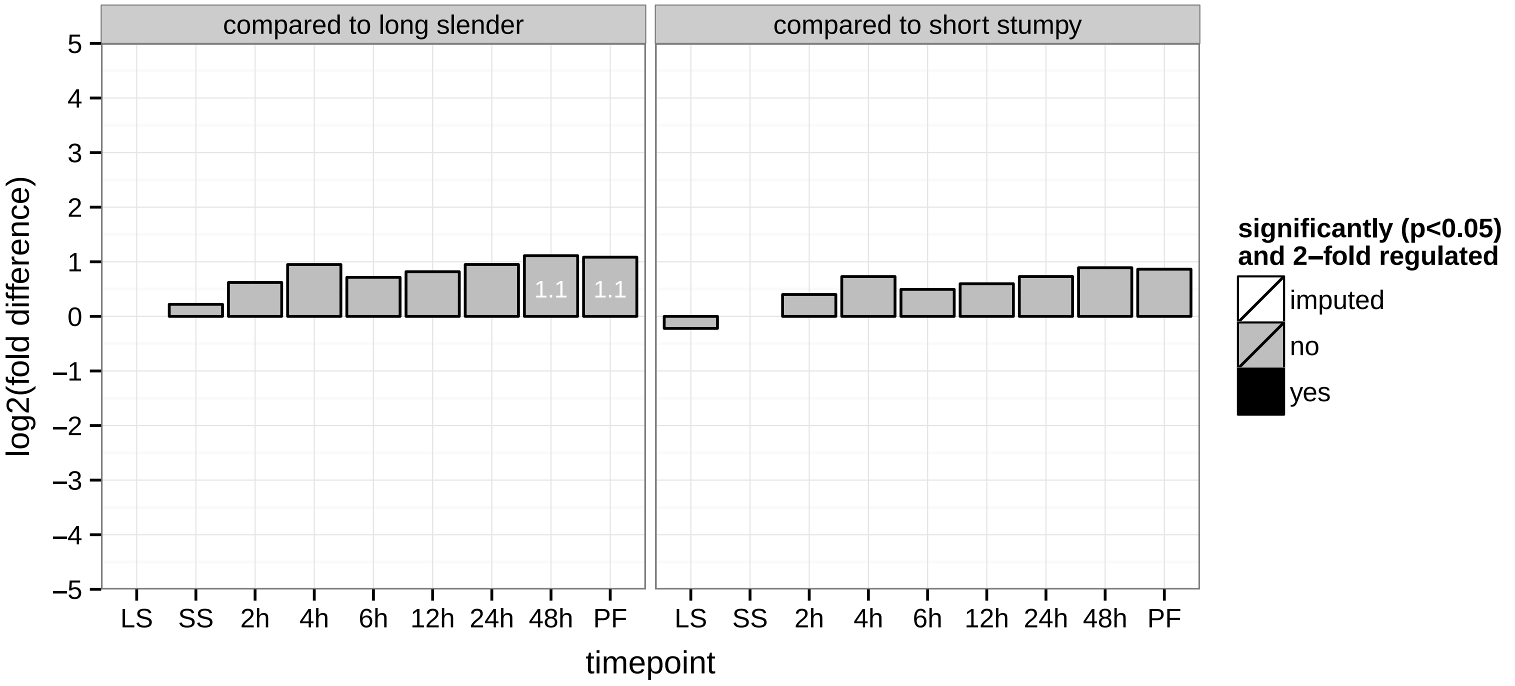

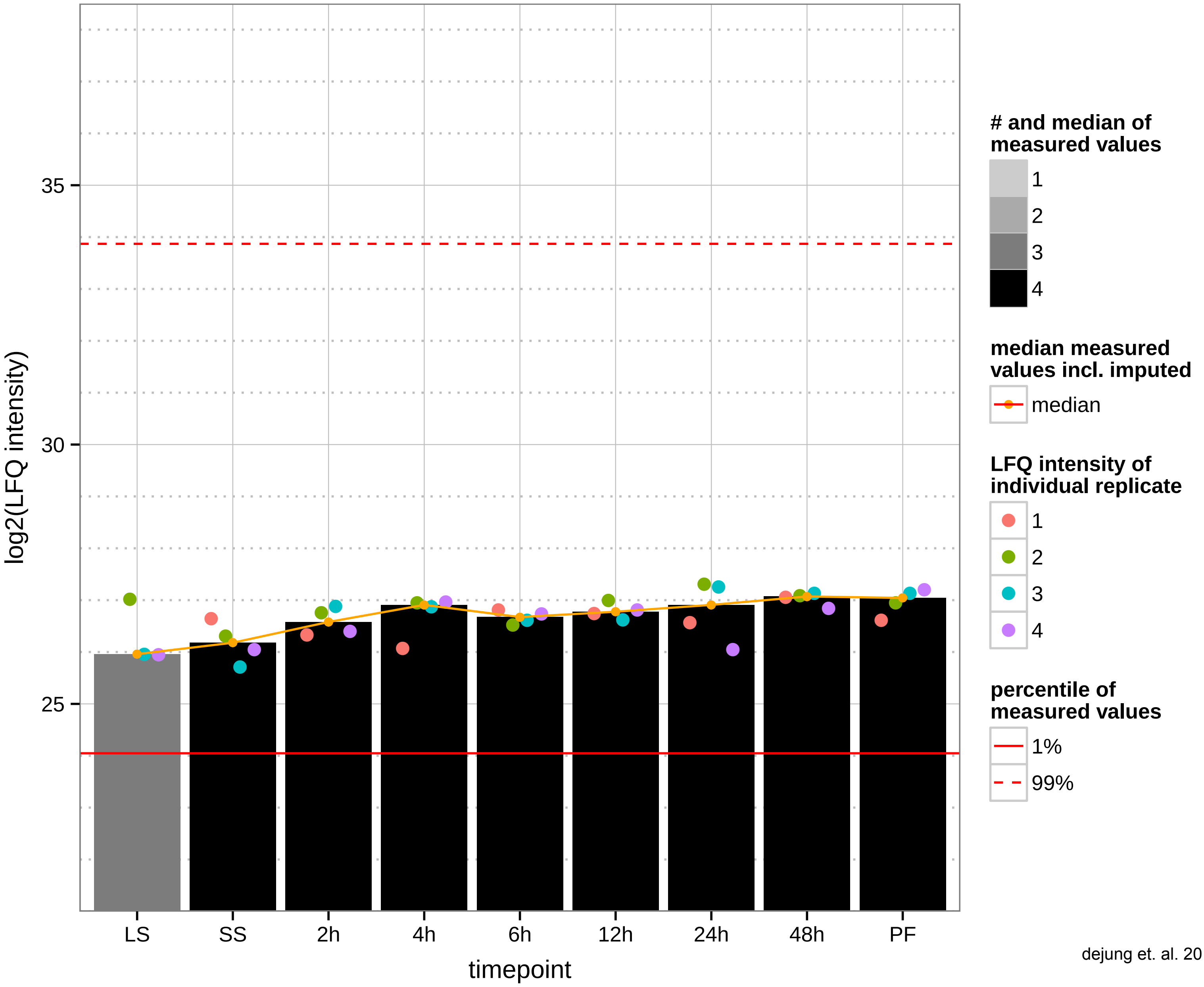

dejung et. al. 2015

Nuclear cap–binding protein subunit 66, zinc finger CCCH domain containing protein 7 (CBP66)
Tb927.3.1340
AGOF: RNA binding
AGOC: nuclear cap binding complex
AGOP: null
PGOF: null
PGOC: null
PGOP: null

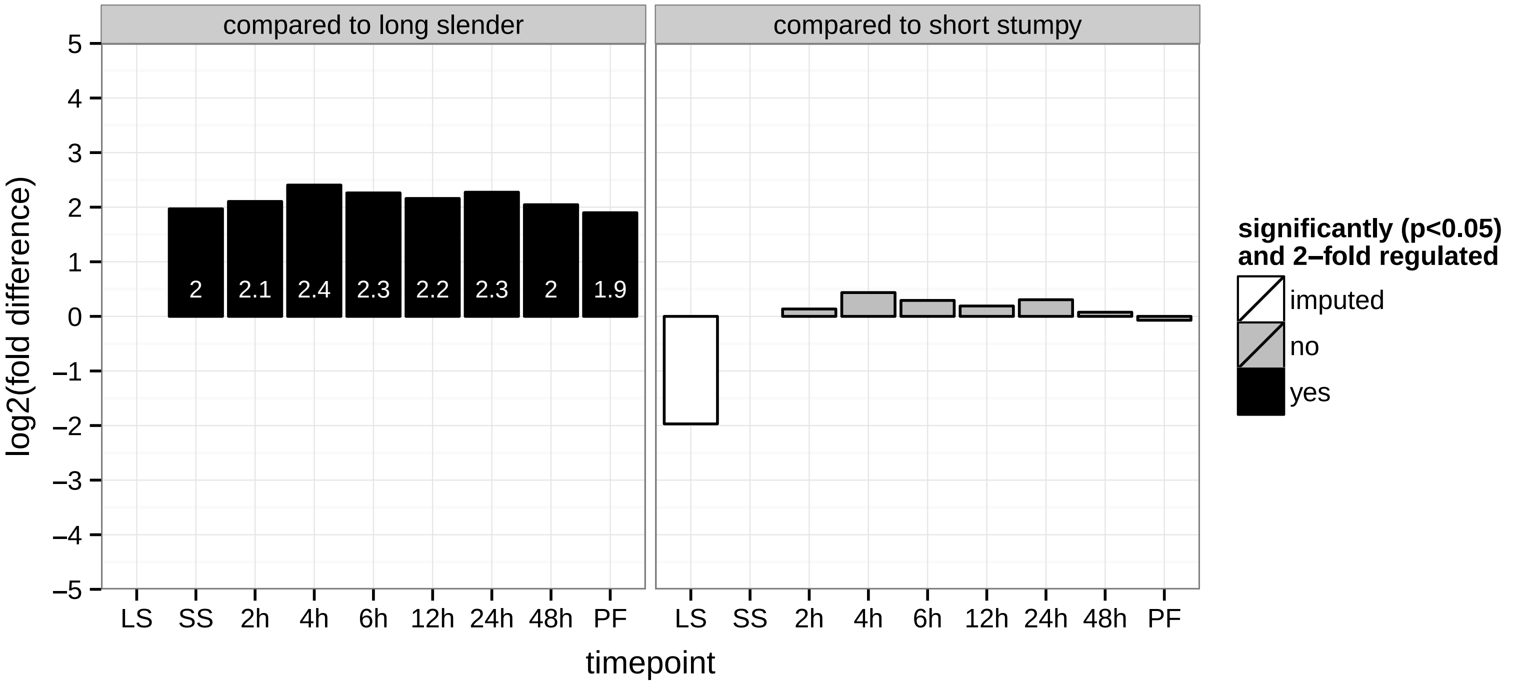

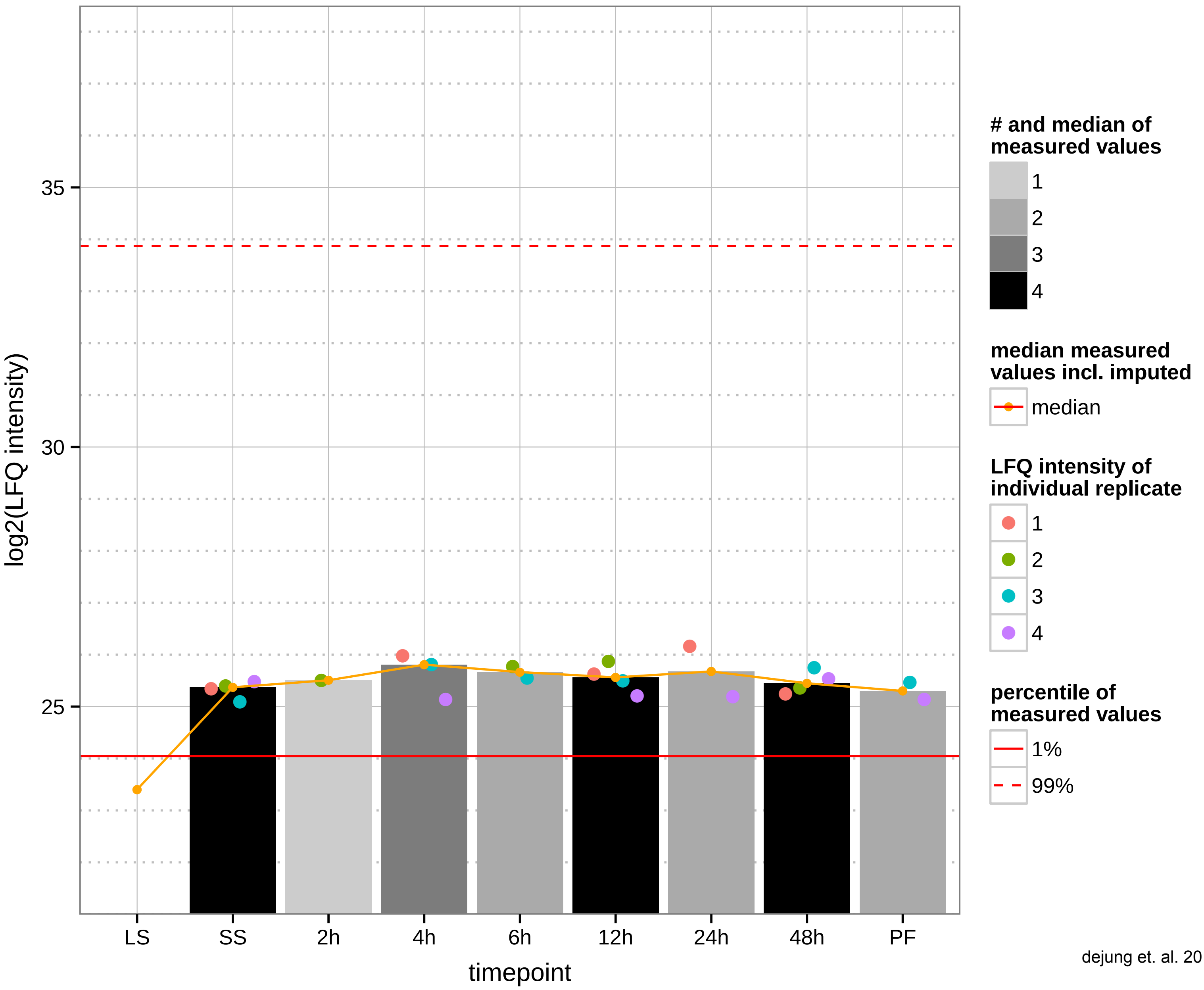

dejung et. al. 2015

chaperone protein DNAj, putative
Tb927.3.1430
AGOF: chaperone binding, heat shock protein binding, unfolded protein binding
AGOC: null
AGOP: protein folding
PGOF: heat shock protein binding, unfolded protein binding
PGOC: null
PGOP: protein folding

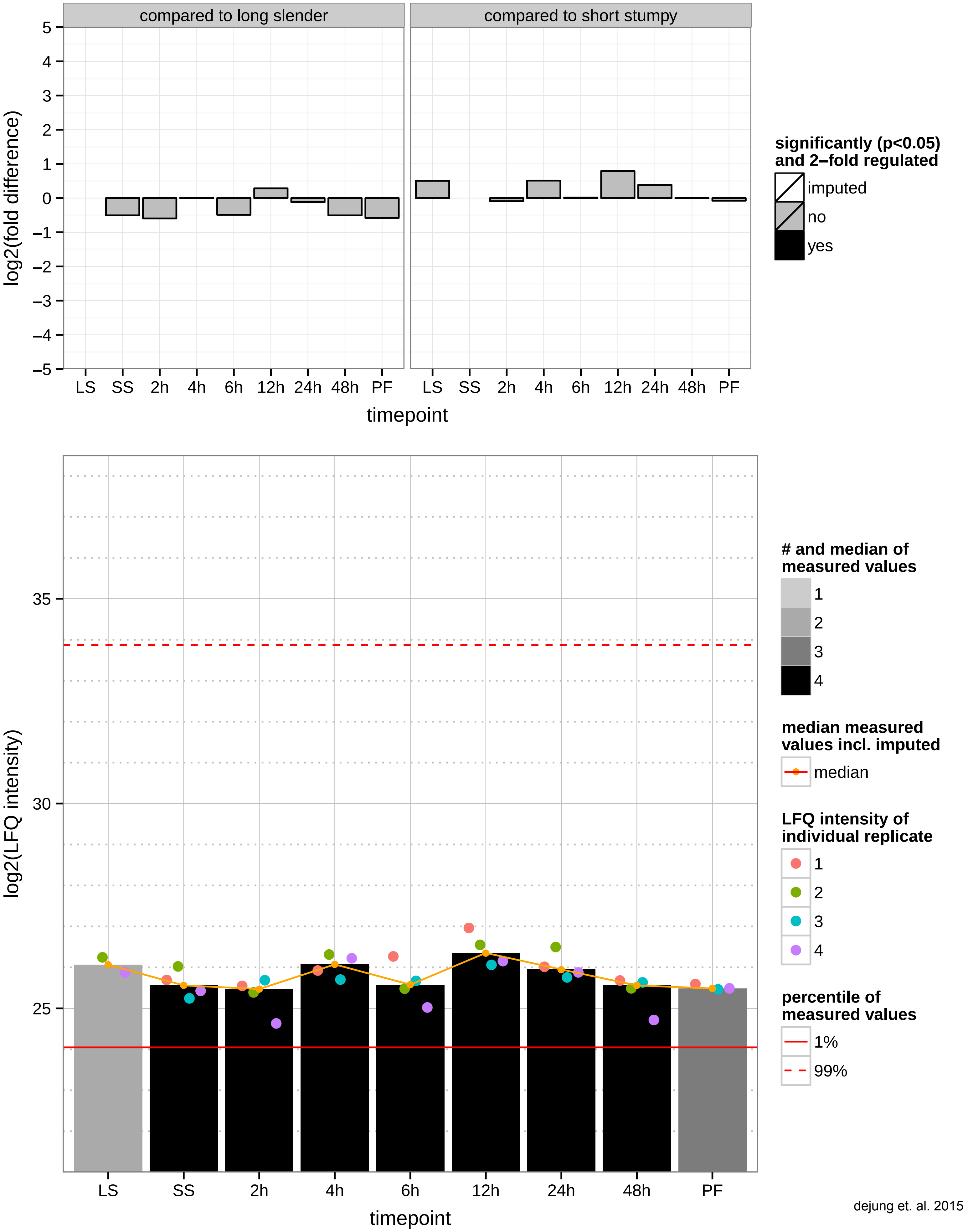

dejung et. al. 2015

hypothetical protein, conserved
Tb927.3.1530;Tb927.3.1450
AGOF: metal ion transmembrane transporter activity, null
AGOC: membrane, null
AGOP: metal ion transport, null
PGOF: metal ion transmembrane transporter activity
PGOC: membrane
PGOP: metal ion transport, transmembrane transport

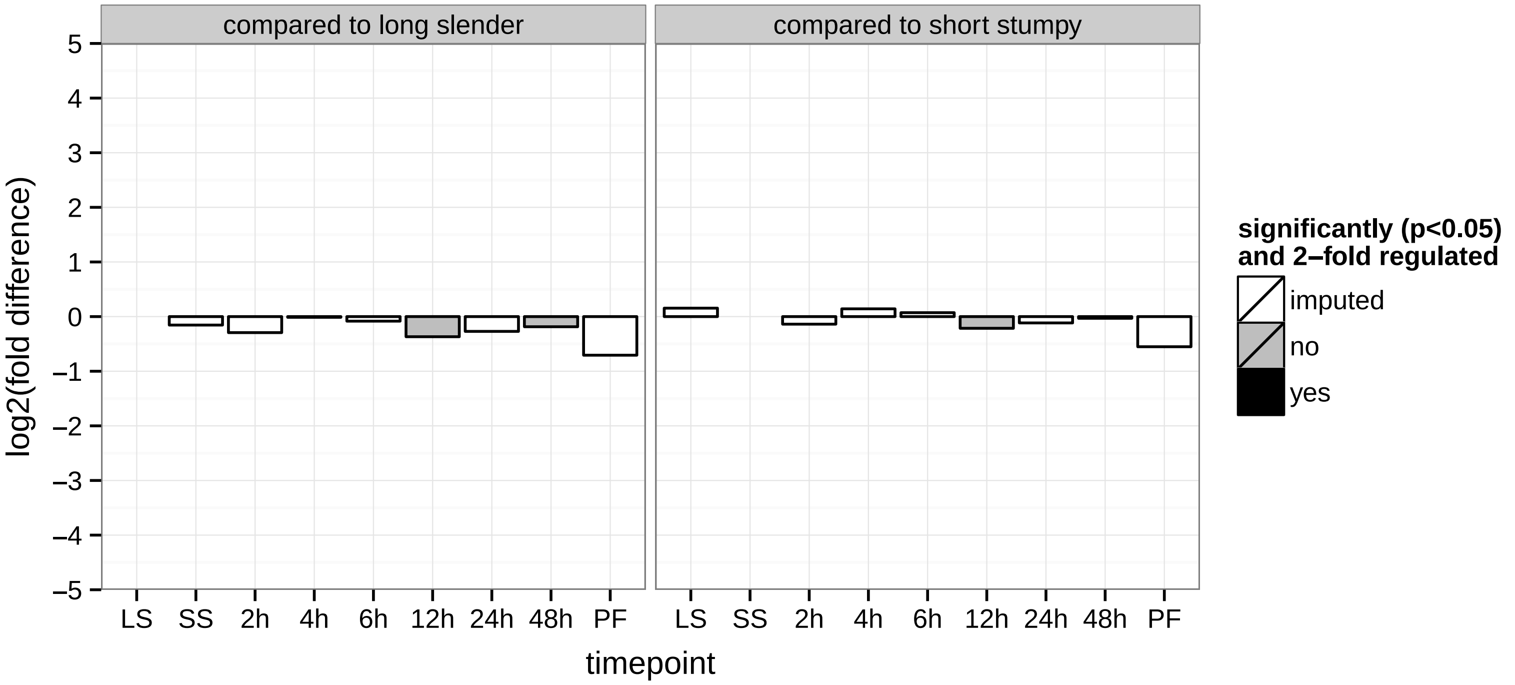

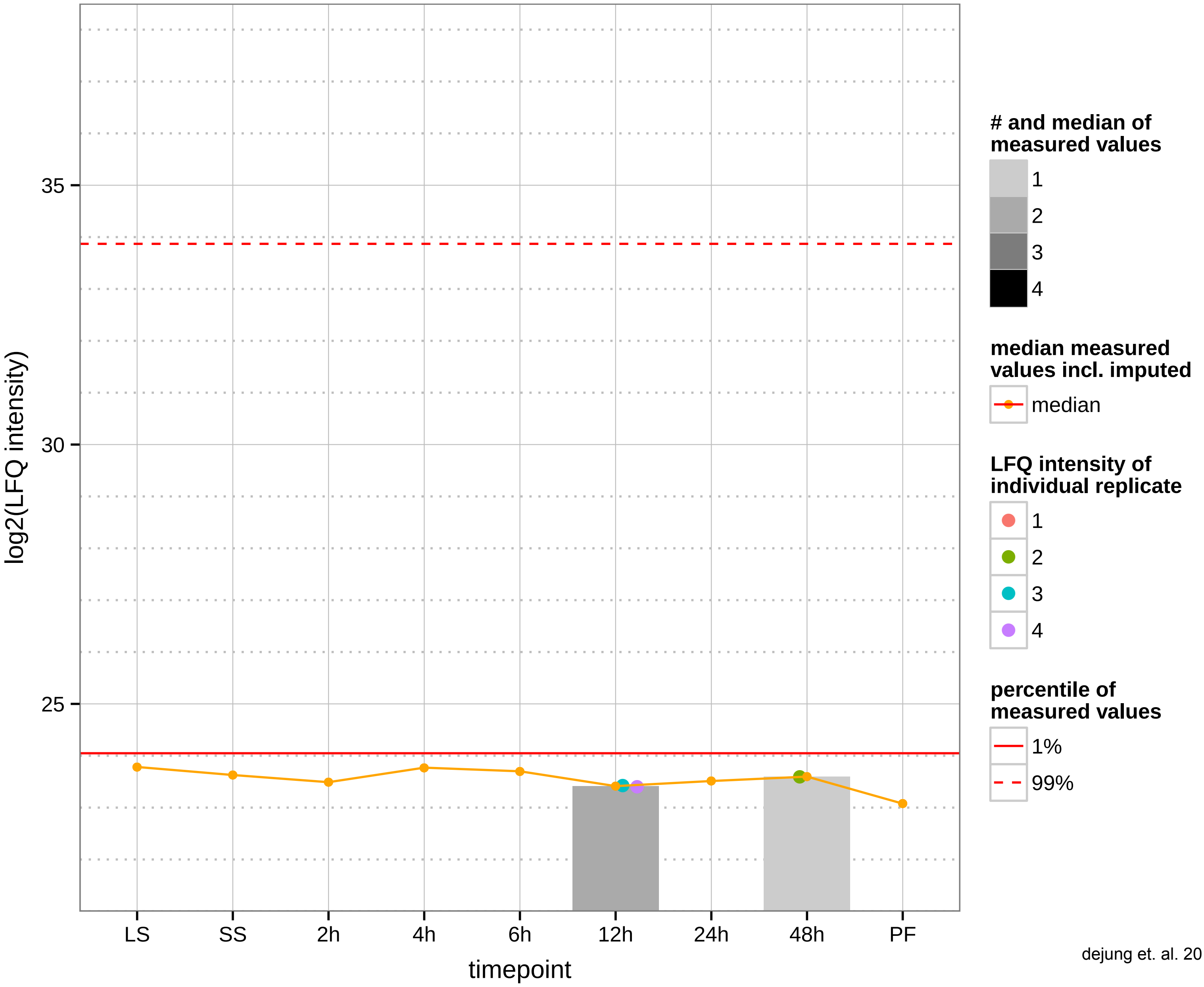

dejung et. al. 2015

protein kinase, putative
Tb927.3.1570
AGOF: ATP binding, protein serine/threonine kinase activity, protein tyrosine kinase activity
AGOC: null
AGOP: protein phosphorylation
PGOF: ATP binding, protein kinase activity, transferase activity, transferring phosphorus–containing groups
PGOC: null
PGOP: protein phosphorylation

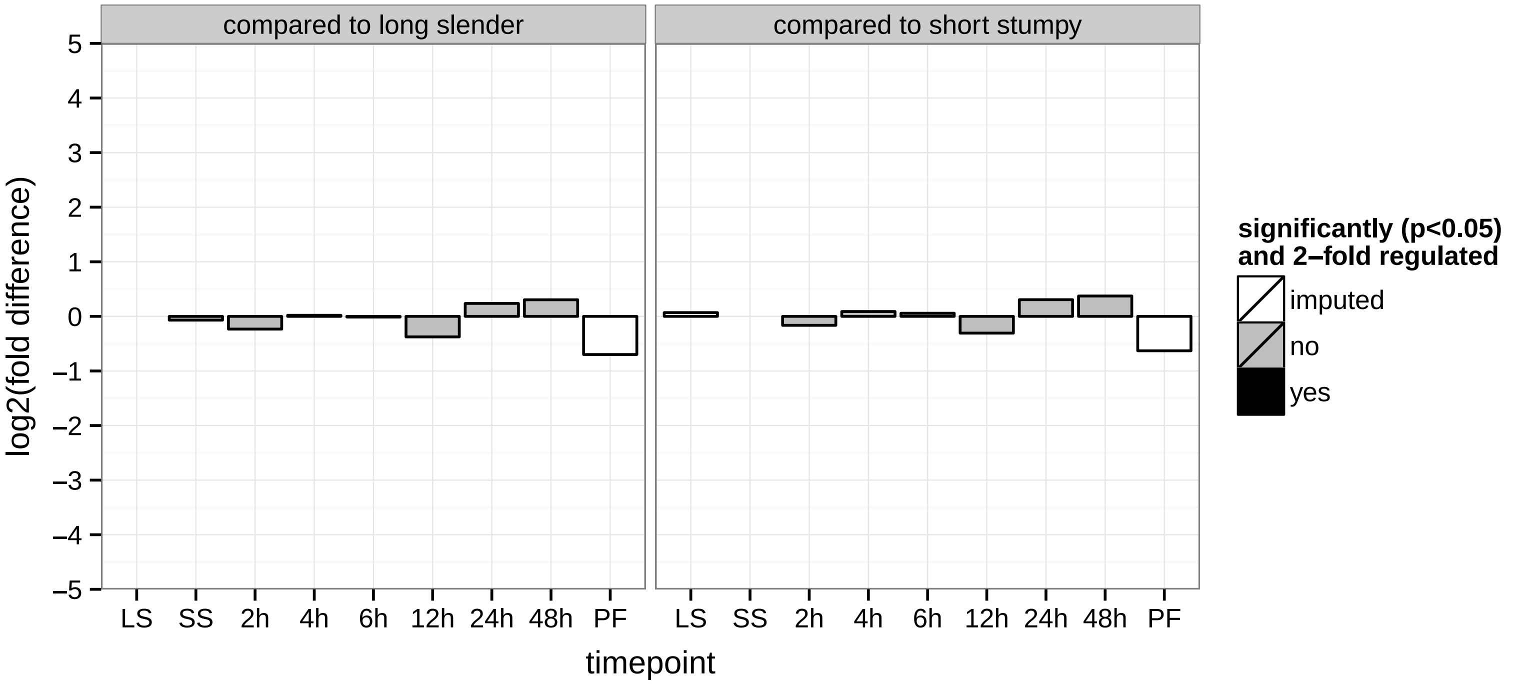

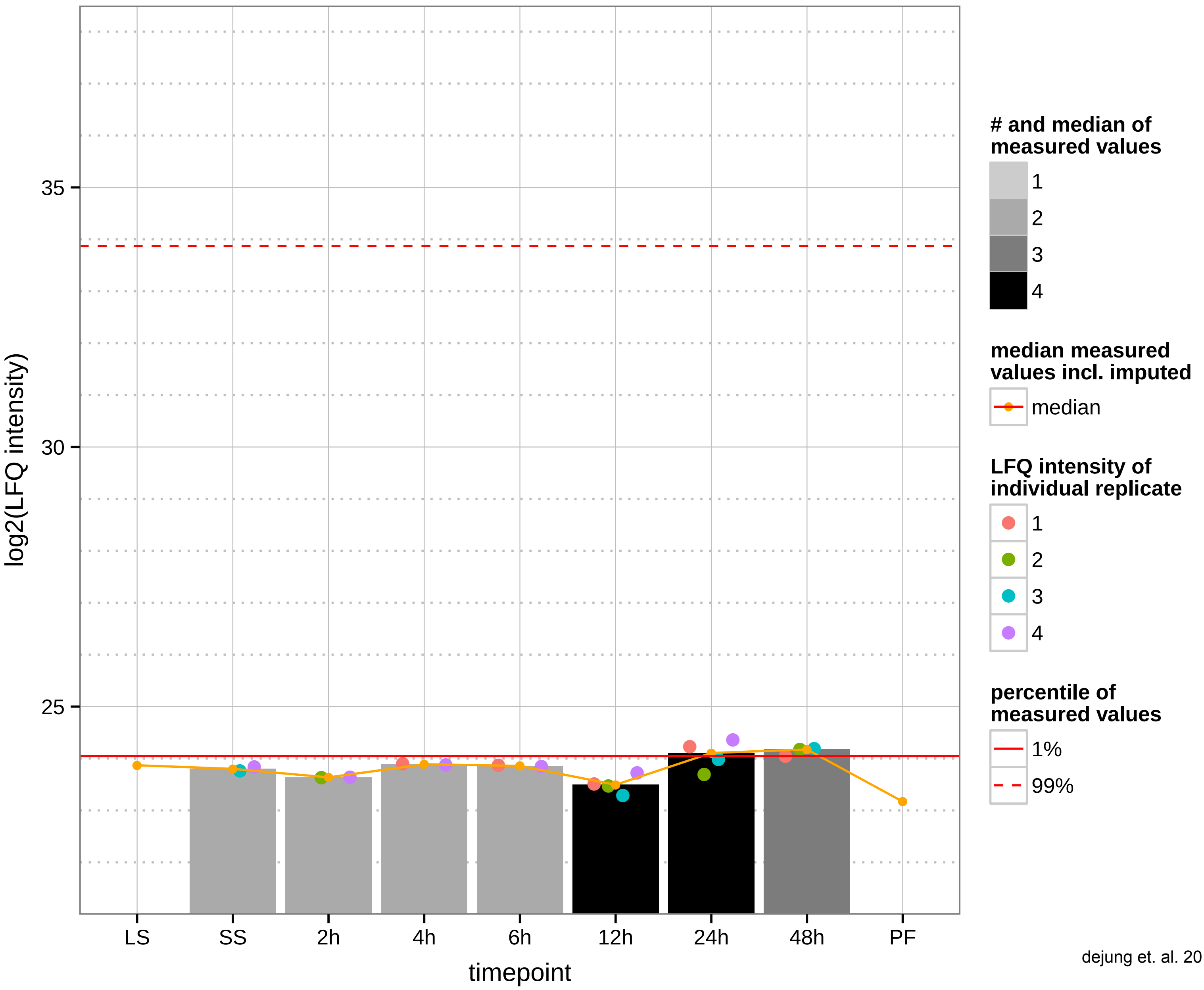

dejung et. al. 2015

mitochondrial RNA binding complex 1 subunit (MRB1590)
Tb927.3.1590
AGOF: RNA binding
AGOC: mitochondrion
AGOP: cytidine to uridine editing, mRNA stabilization
PGOF: null
PGOC: null
PGOP: null

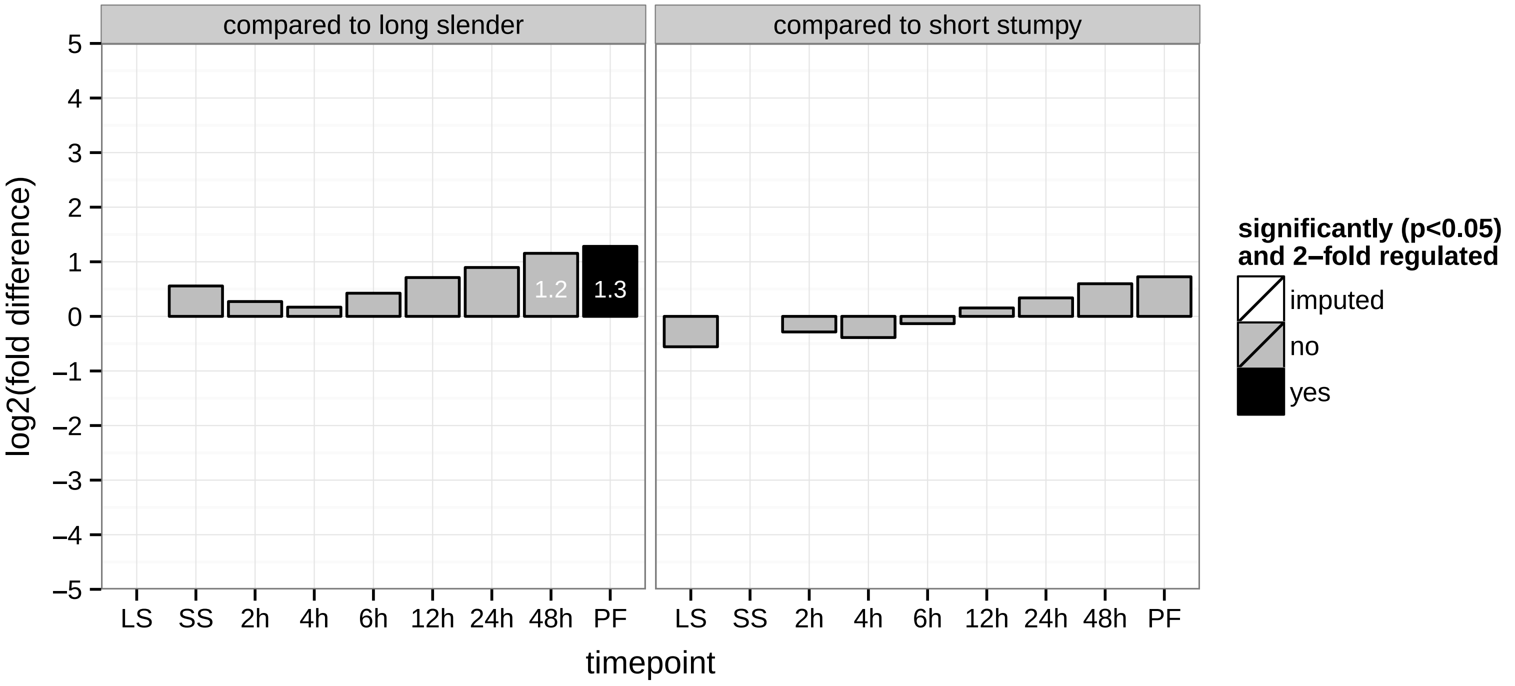

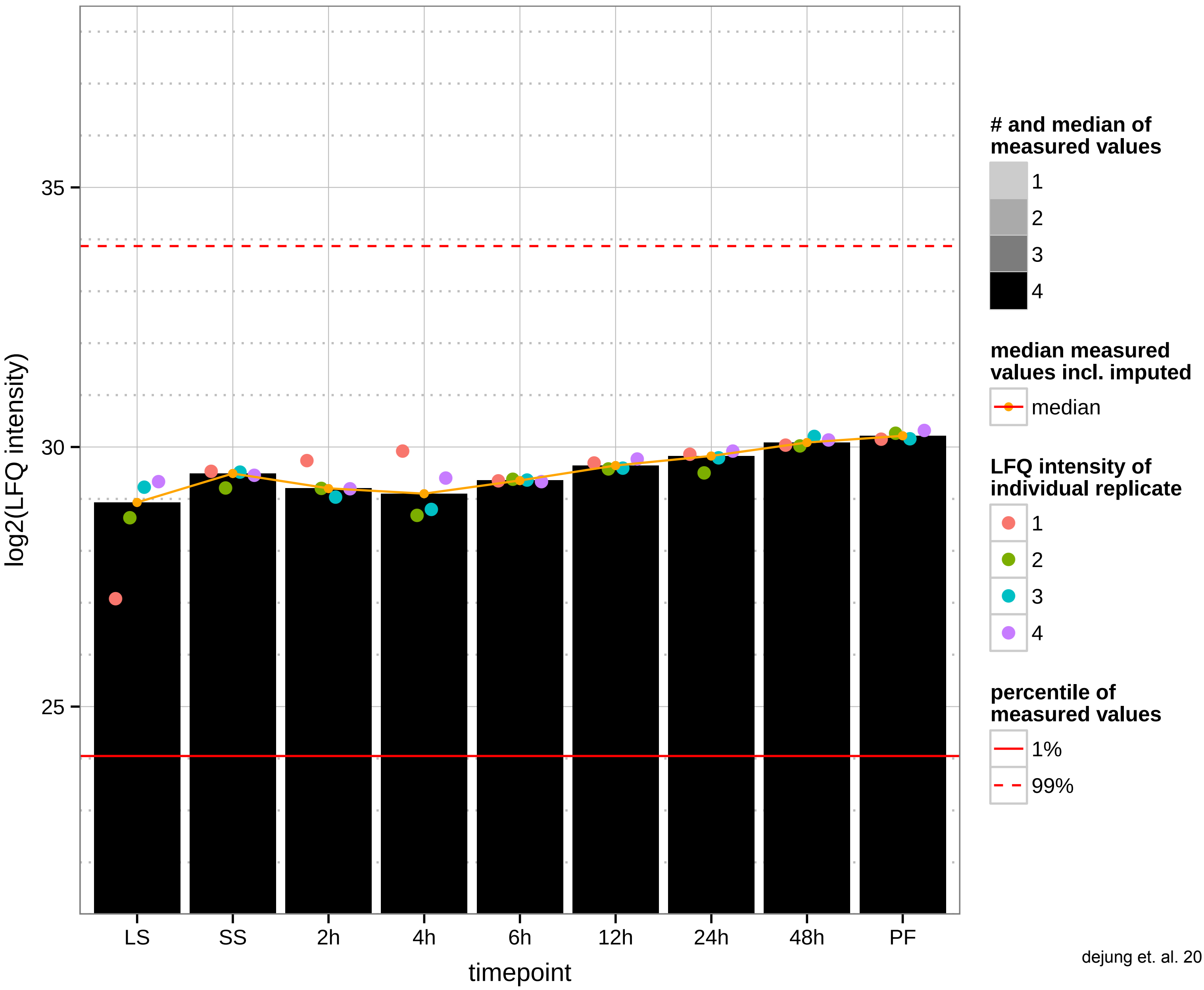

dejung et. al. 2015

hypothetical protein, conserved
Tb927.3.1620
AGOF: null
AGOC: null
AGOP: null
PGOF: null
PGOC: null
PGOP: null

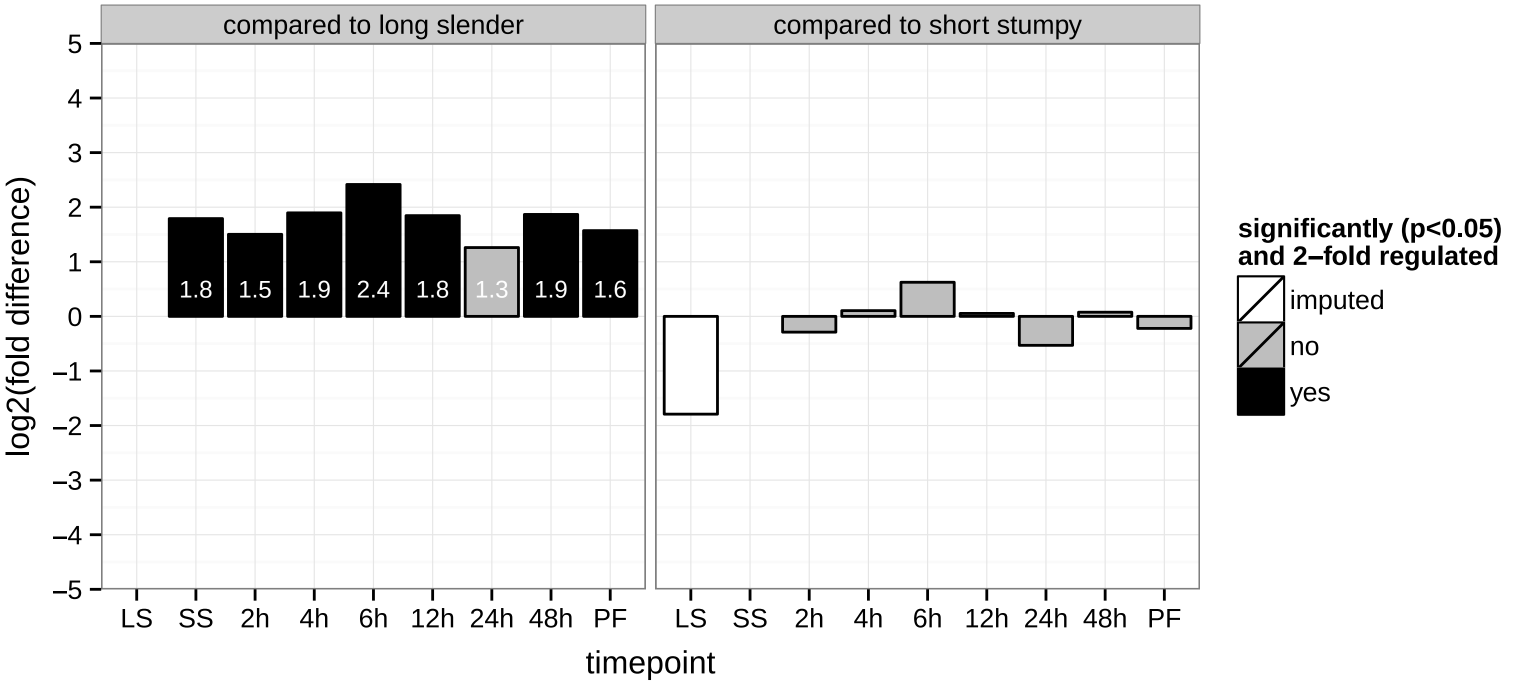

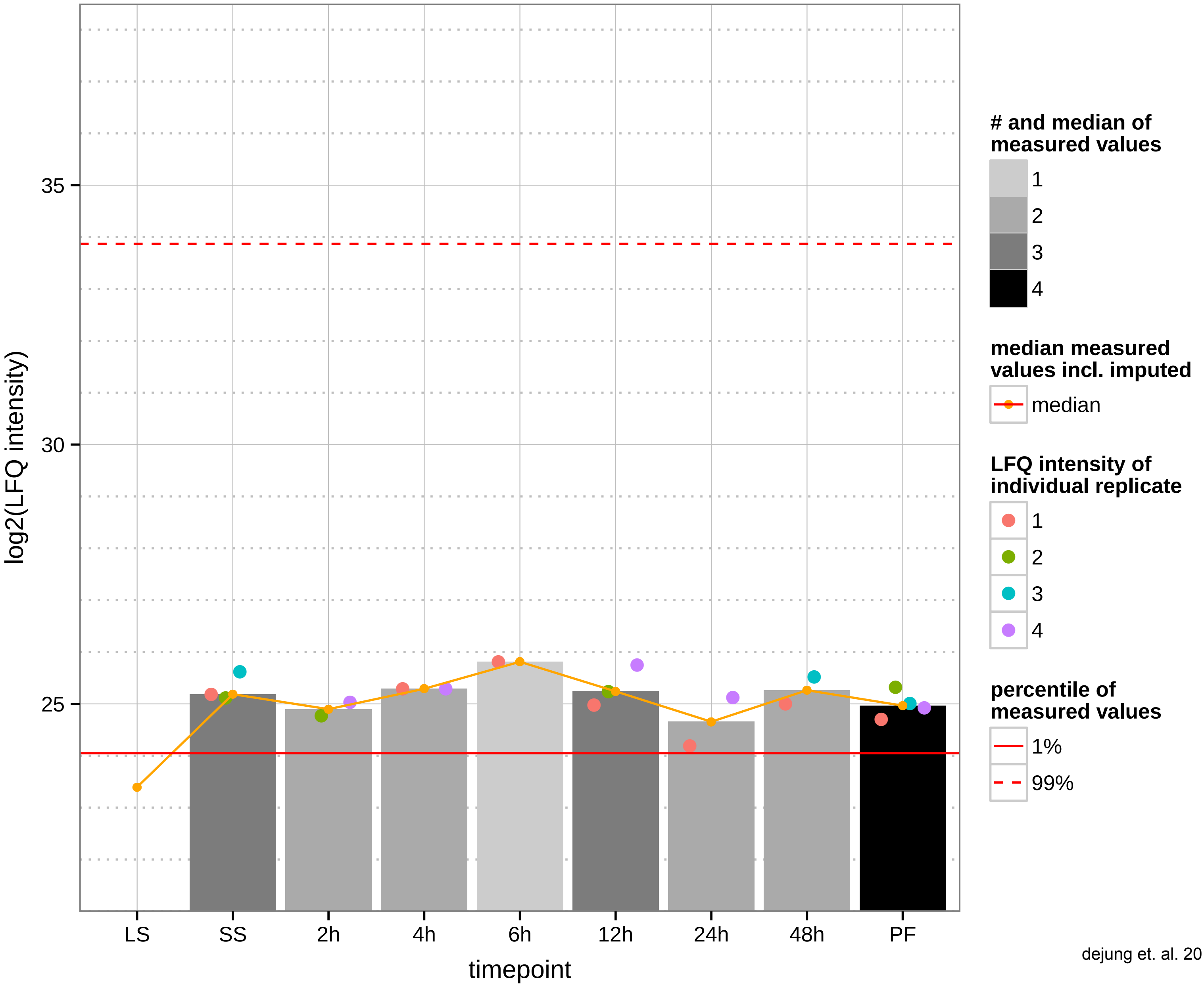

dejung et. al. 2015

hypothetical protein, conserved
Tb927.3.1670
AGOF: null
AGOC: null
AGOP: null
PGOF: protein binding
PGOC: null
PGOP: null

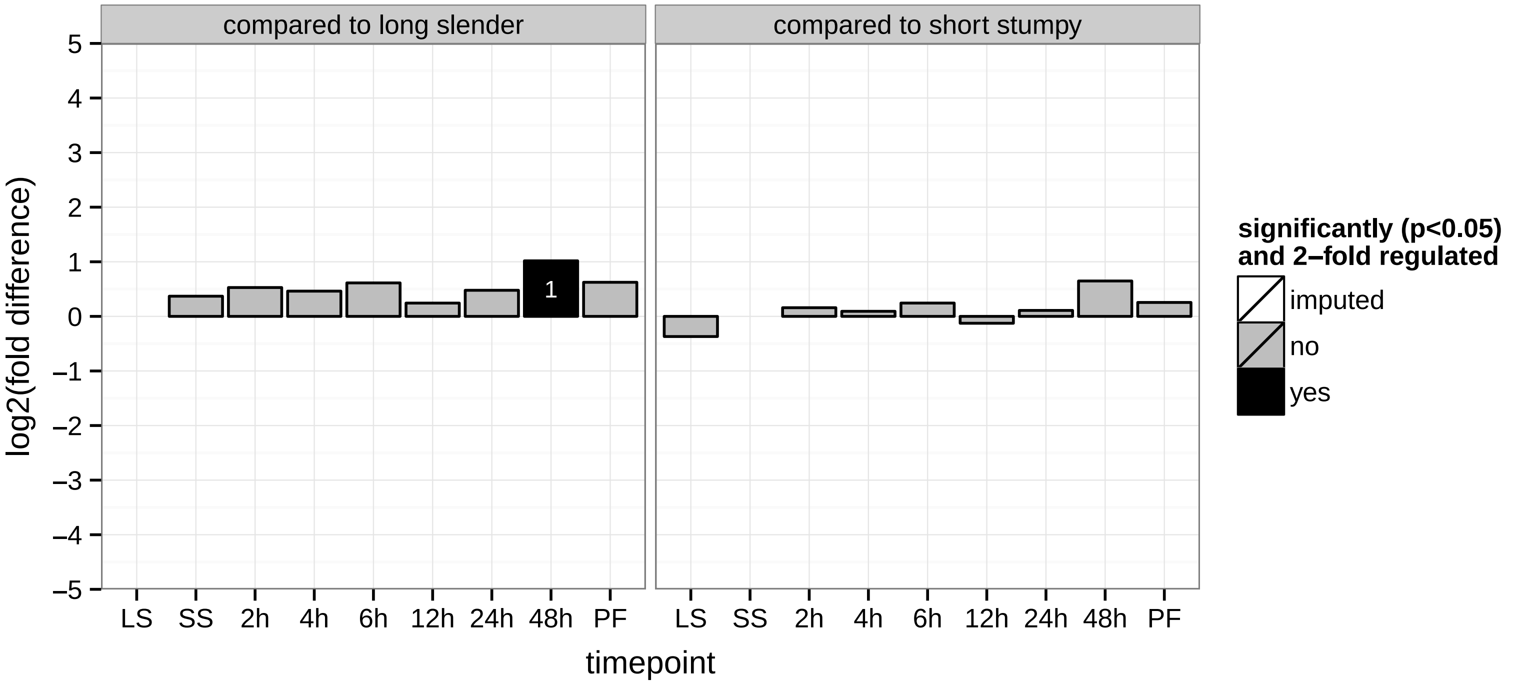

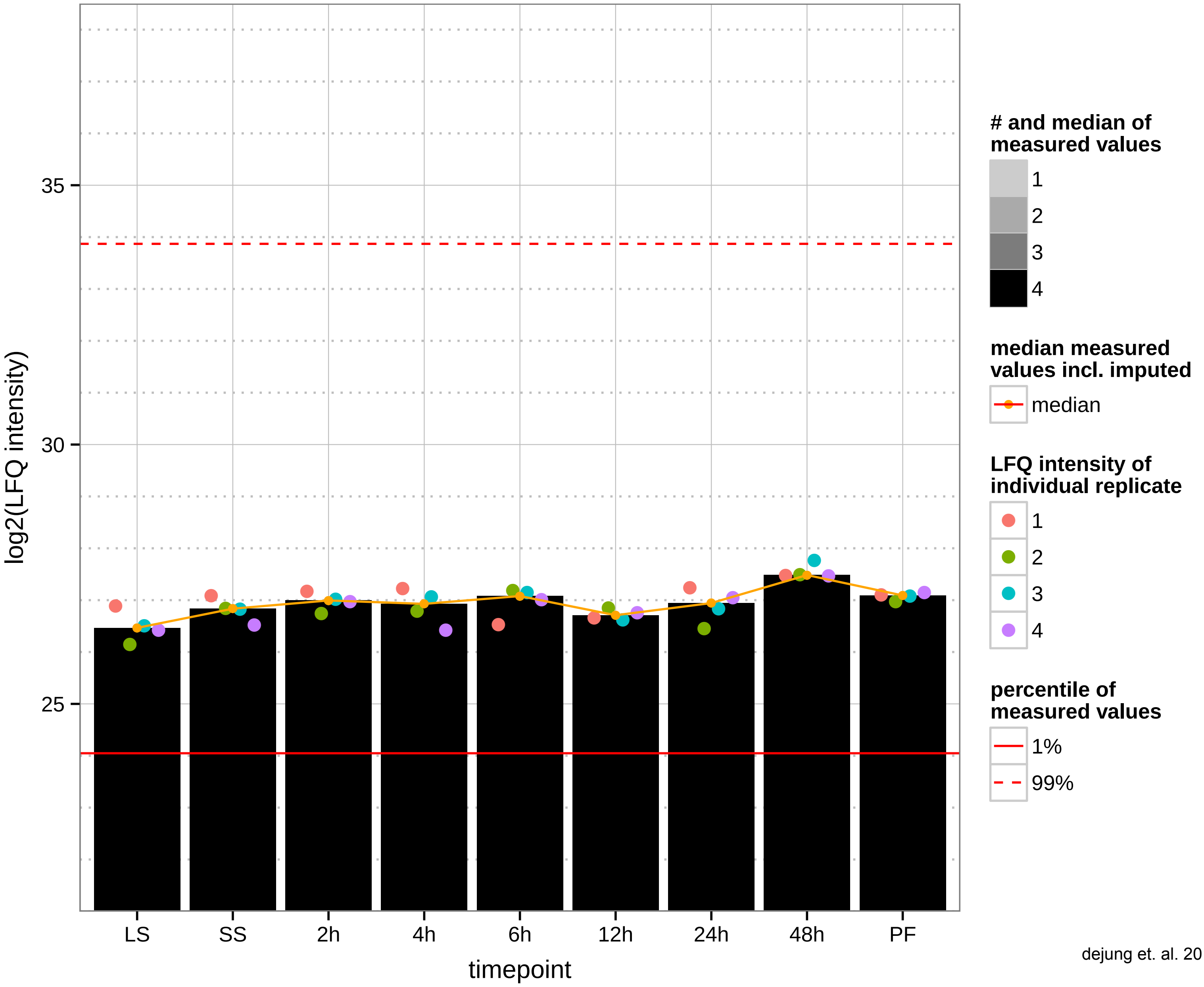

dejung et. al. 2015

diacylglycerol acyltransferase, putative
Tb927.3.1700
AGOF: diacylglycerol O−acyltransferase activity
AGOC: integral to membrane
AGOP: membrane lipid biosynthetic process
PGOF: transferase activity, transferring acyl groups other than amino−acyl groups
PGOC: null
PGOP: null

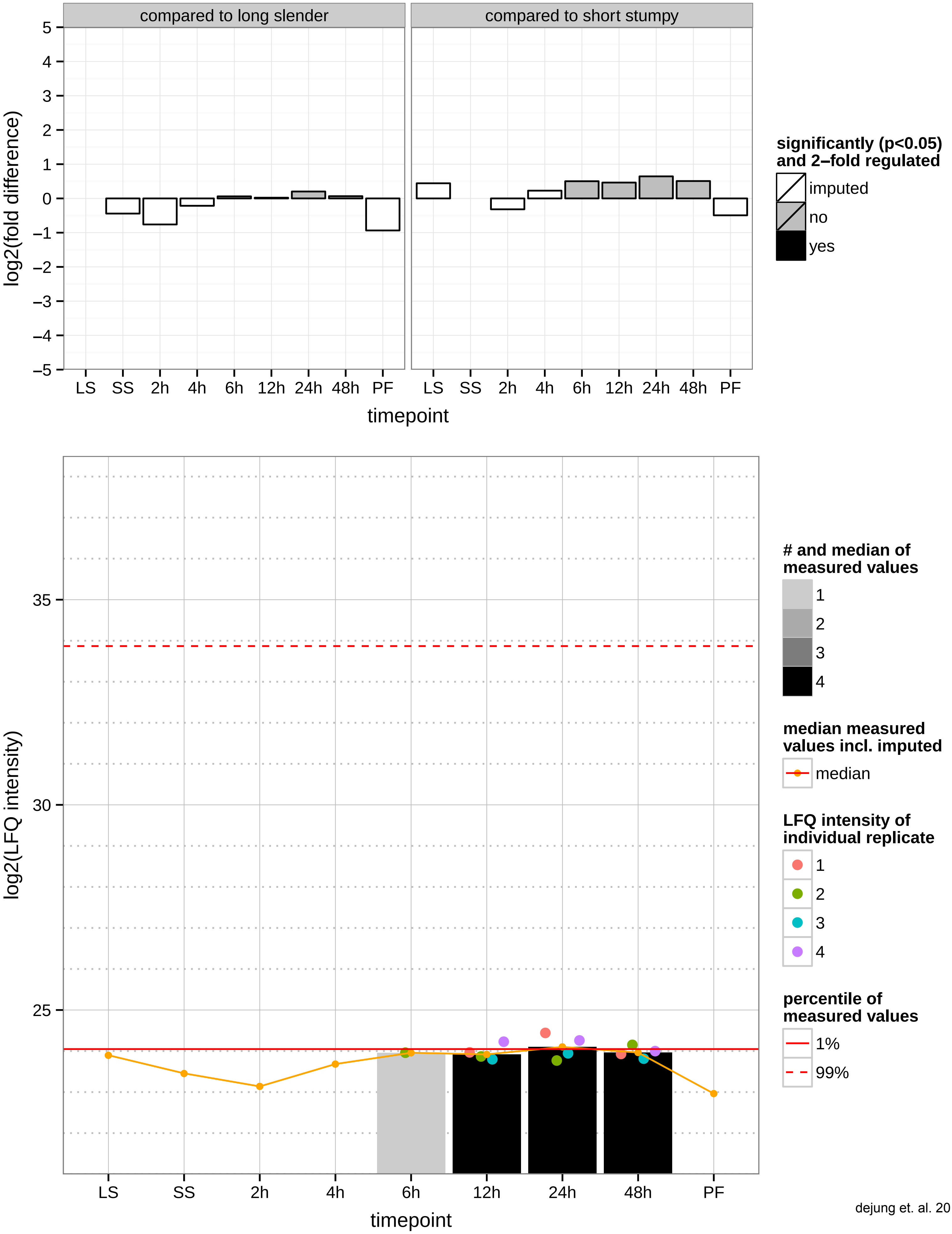

dejung et. al. 2015

hypothetical protein, conserved
Tb927.3.1740
AGOF: null
AGOC: null
AGOP: null
PGOF: null
PGOC: null
PGOP: null

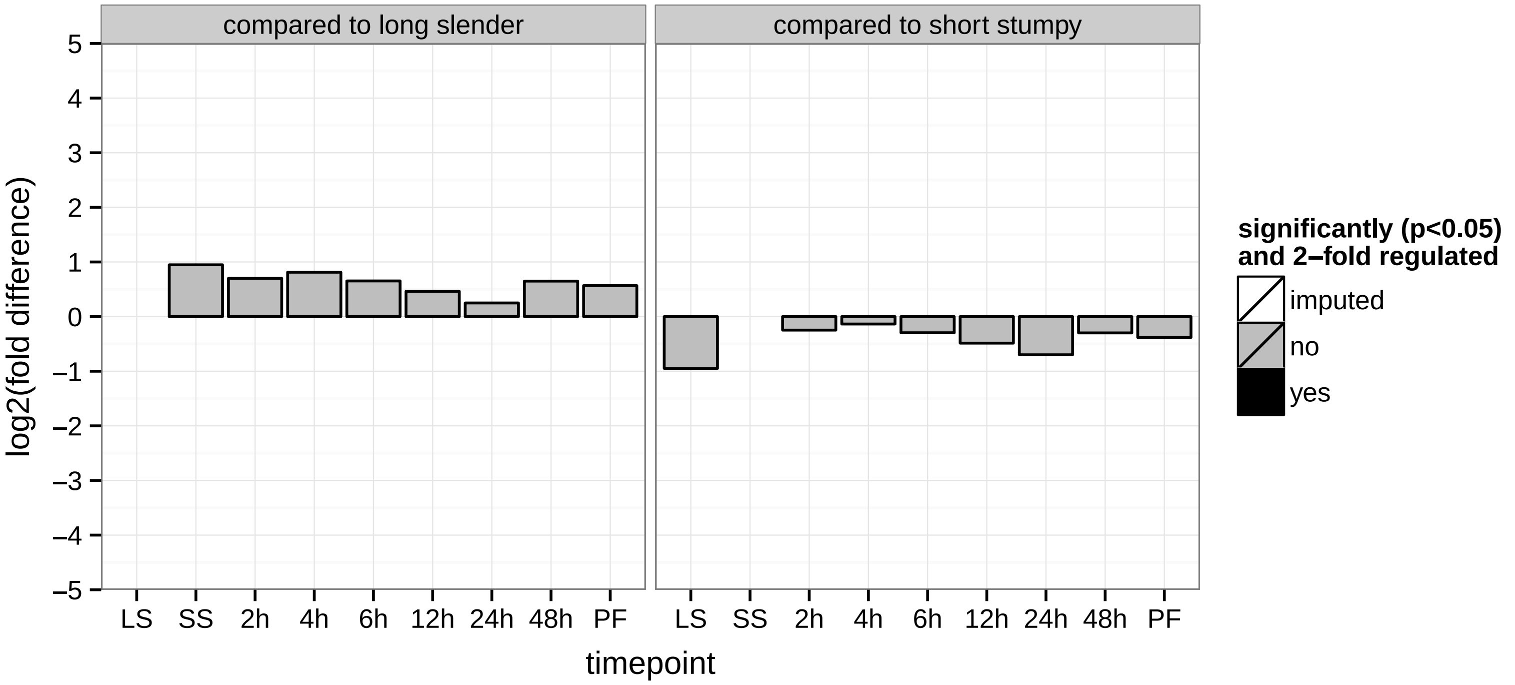

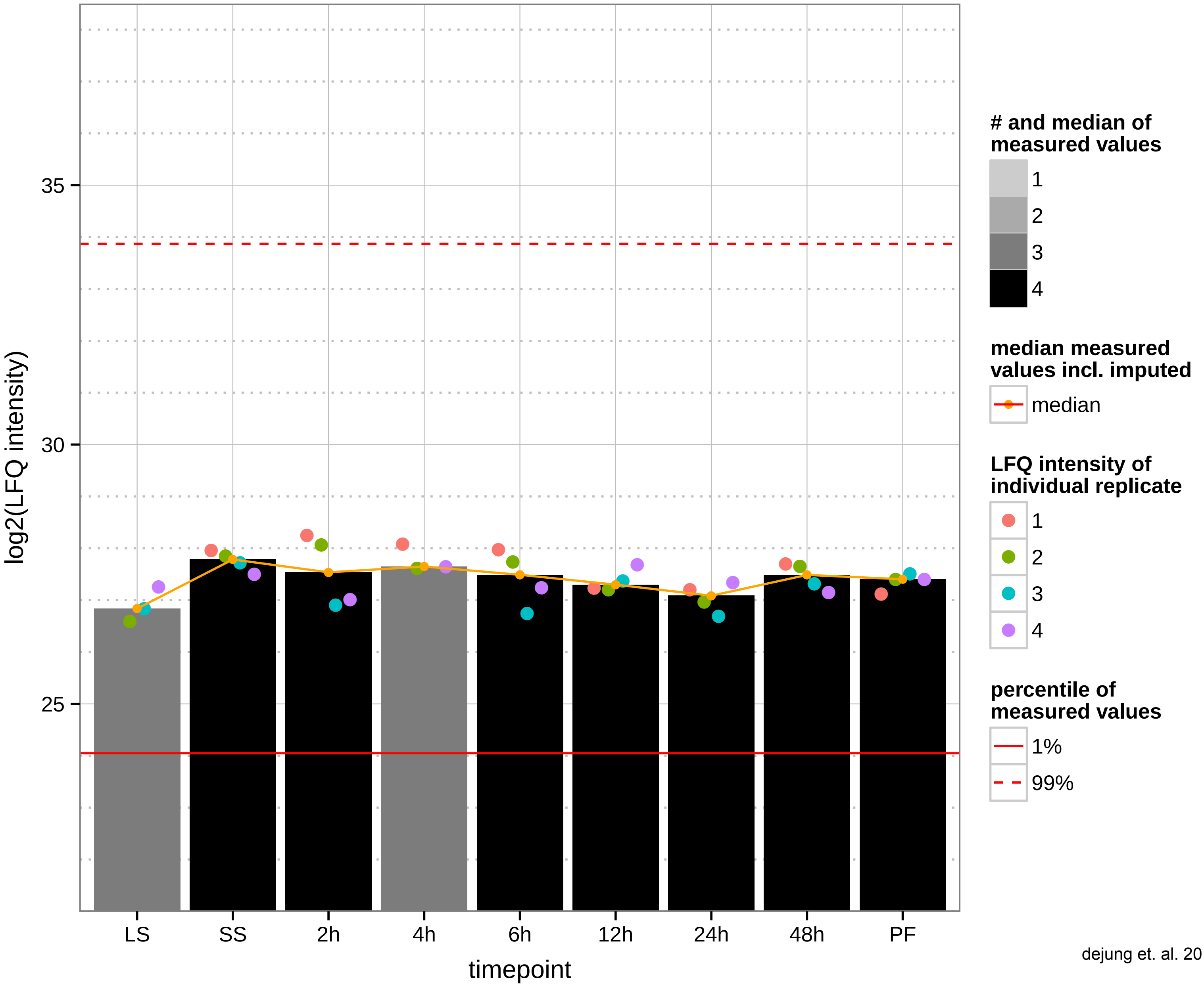

dejung et. al. 2015

chaperone protein DNAj, putative
Tb927.3.1760
AGOF: heat shock protein binding, unfolded protein binding
AGOC: mitochondrion
AGOP: protein folding
PGOF: heat shock protein binding
PGOC: null
PGOP: protein folding

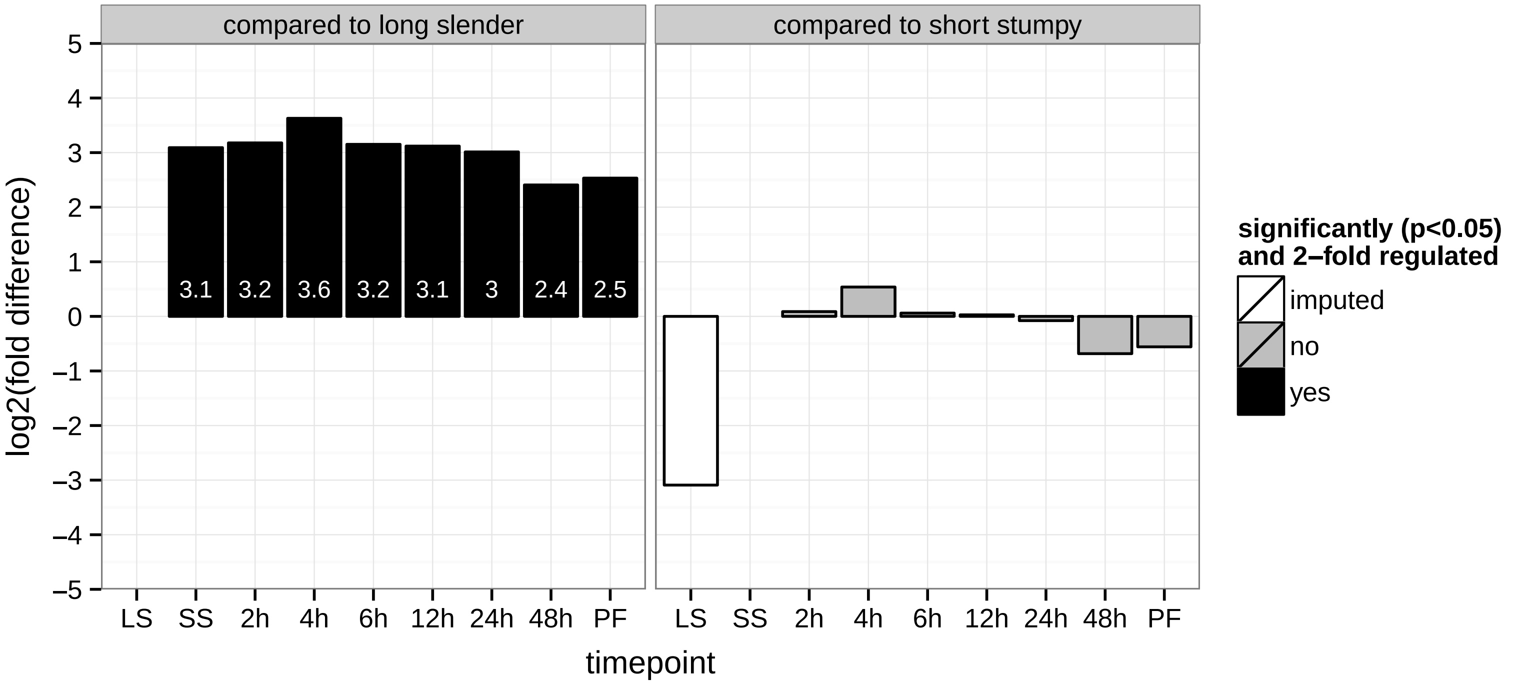

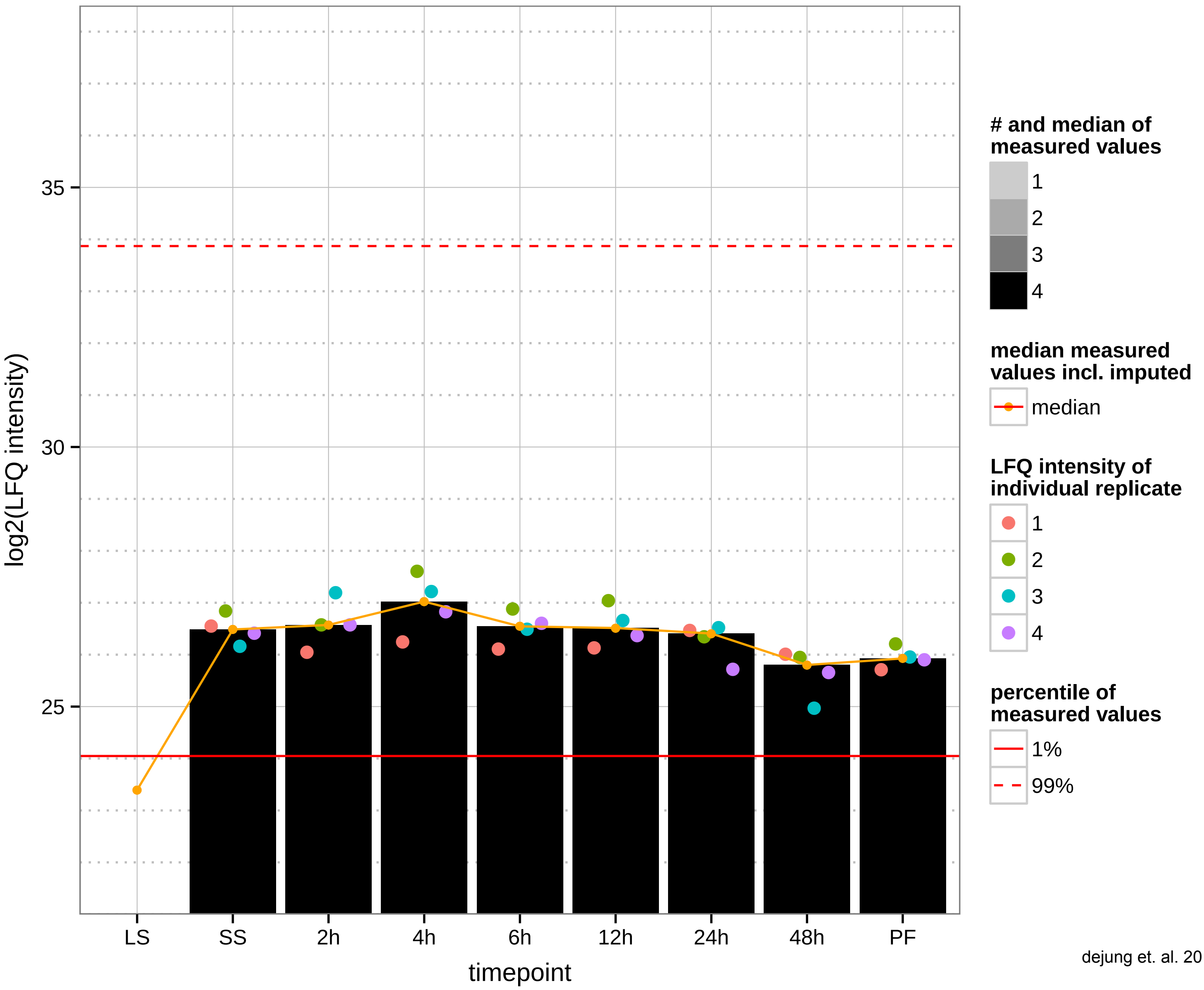

dejung et. al. 2015

pyruvate dehydrogenase E1 beta subunit, putative
Tb927.3.1790
AGOF: catalytic activity
AGOC: mitochondrial inner membrane, mitochondrion
AGOP: metabolic process
PGOF: catalytic activity
PGOC: null
PGOP: metabolic process

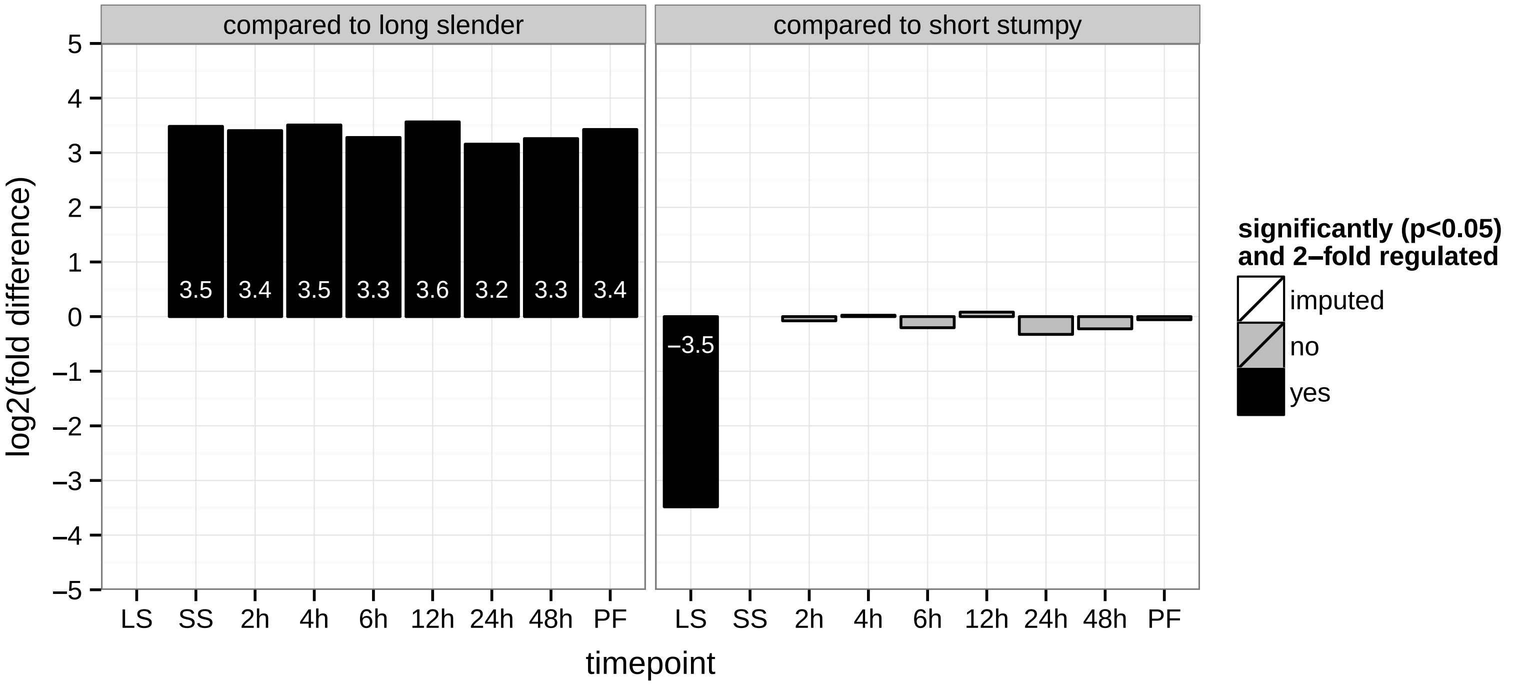

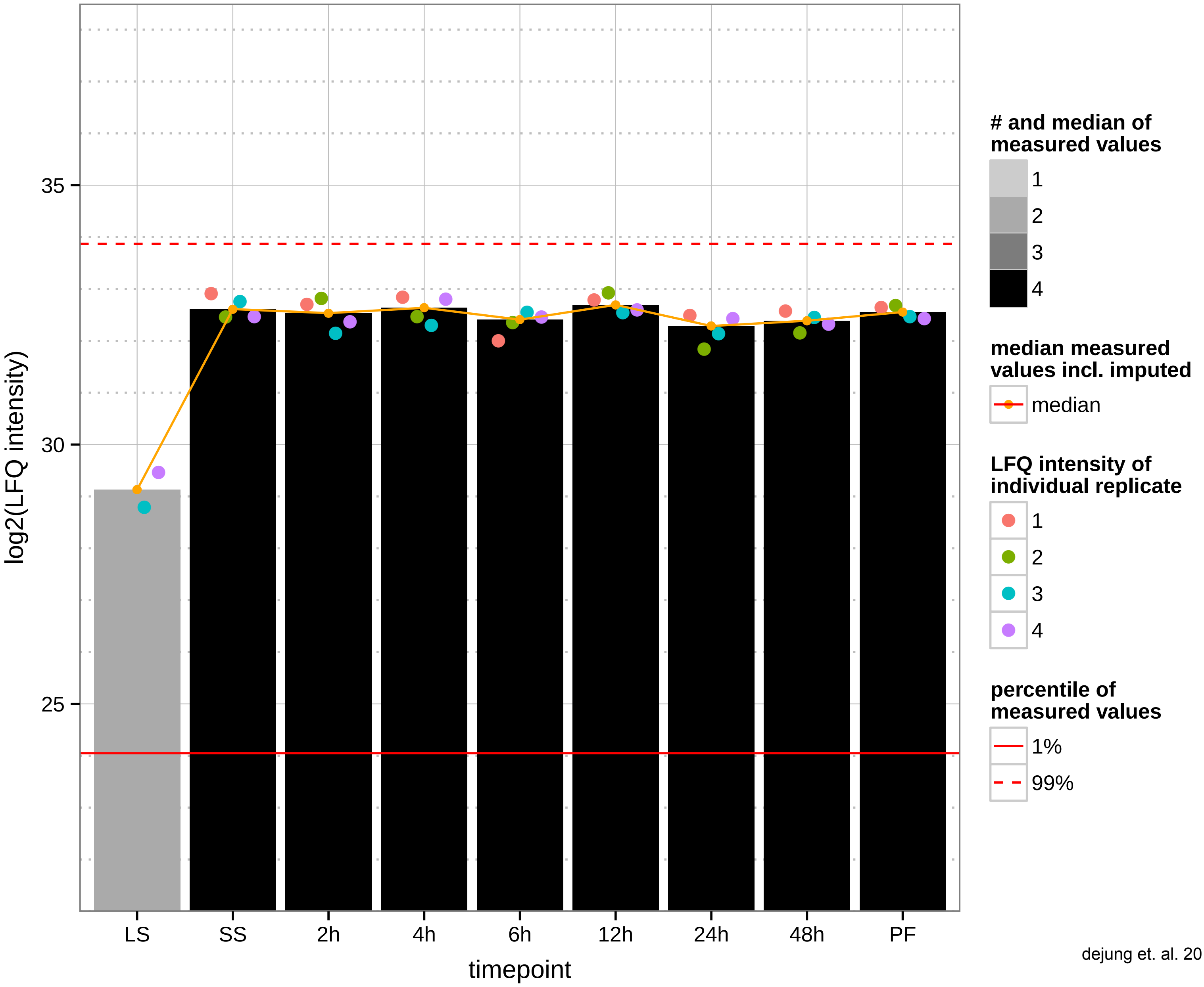

dejung et. al. 2015

mitochondrial RNA binding complex 1 subunit (MRB1820)
Tb927.3.1820
AGOF: RNA binding
AGOC: mitochondrion
AGOP: cytidine to uridine editing, mRNA stabilization
PGOF: null
PGOC: null
PGOP: null

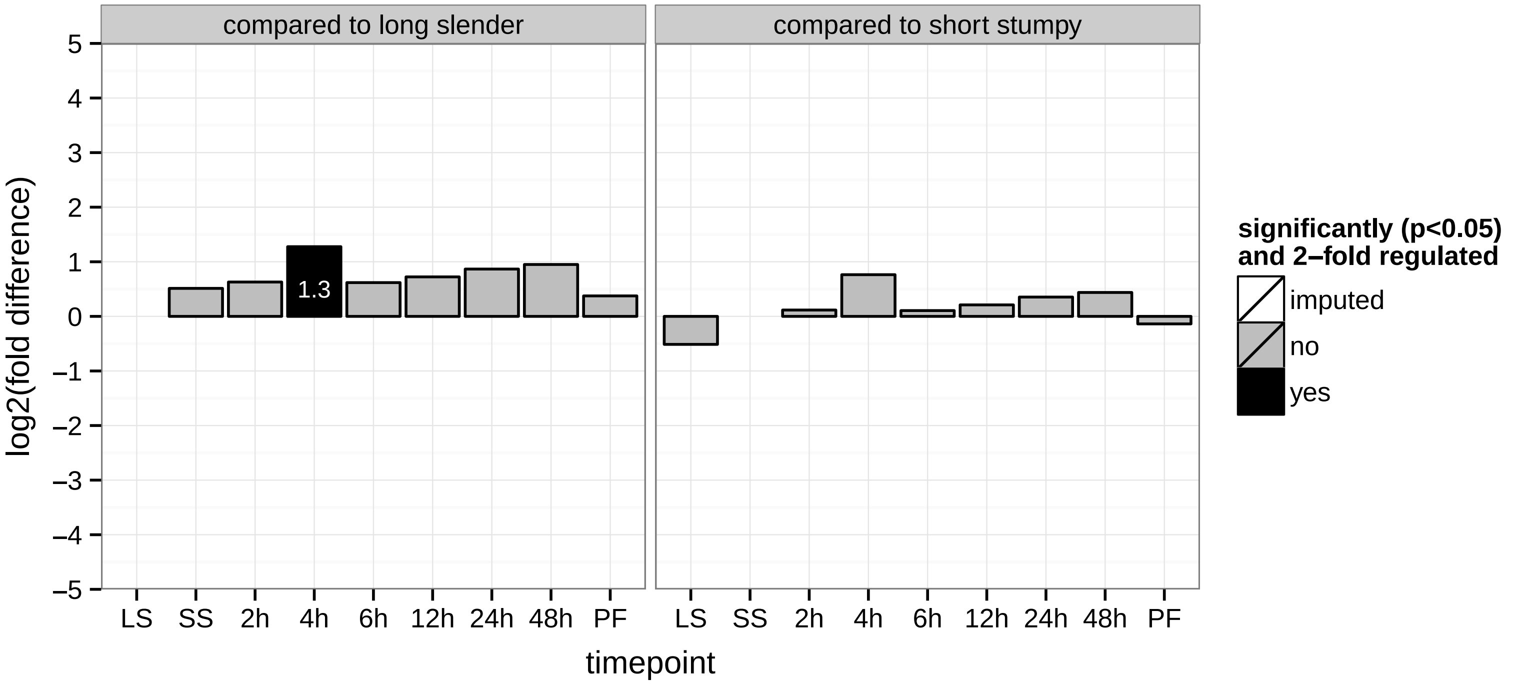

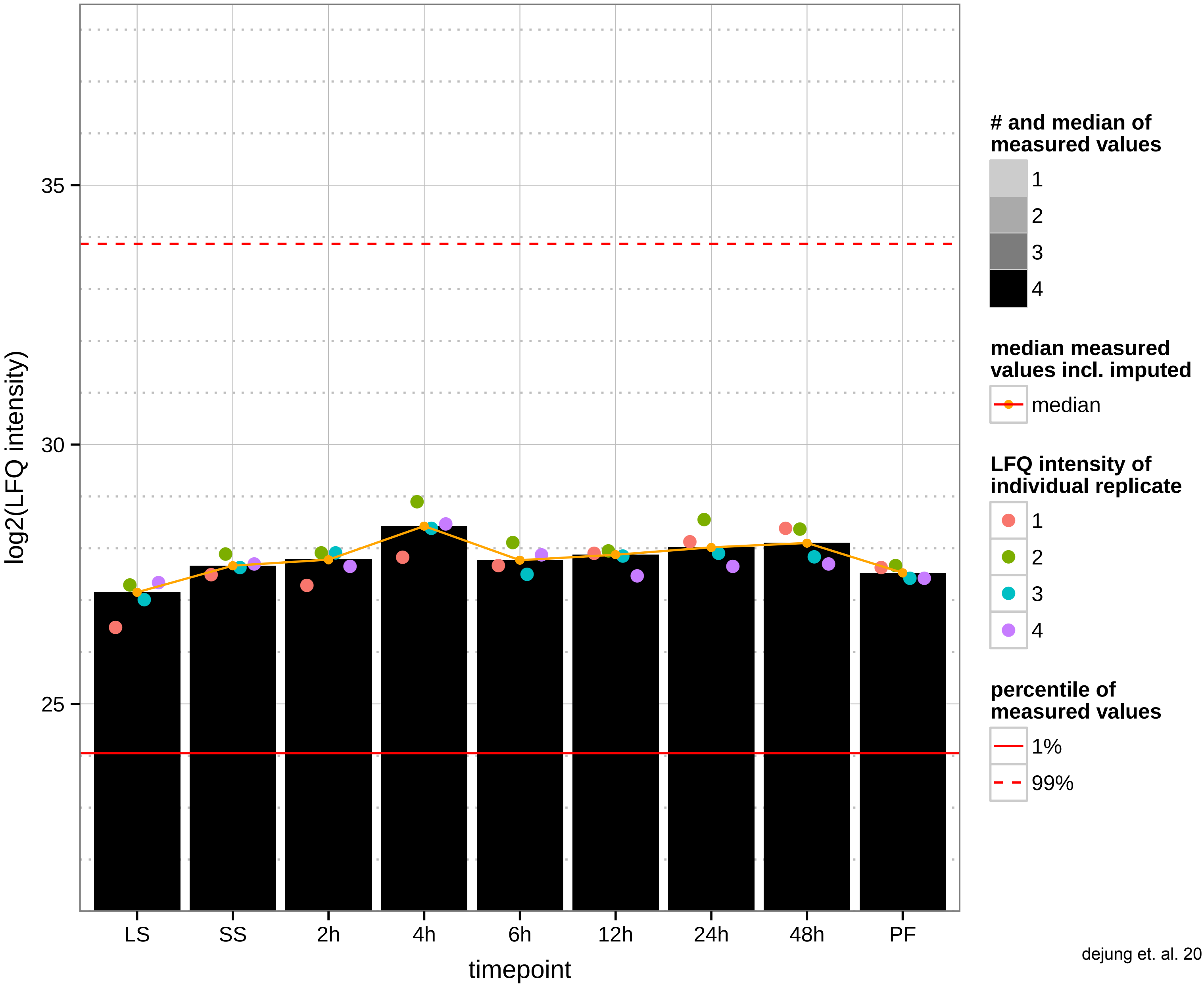

dejung et. al. 2015

3–oxo–5–alpha–steroid 4–dehydrogenase, putative, polyprenol reductase
Tb927.3.1840
AGOF: oxidoreductase activity, acting on the CH–CH group of donors
AGOC: cytoplasm, integral to membrane, mitochondrial inner membrane
AGOP: dolichol biosynthetic process, glycosylation
PGOF: oxidoreductase activity, acting on the CH–CH group of donors
PGOC: cytoplasm, integral to membrane
PGOP: lipid metabolic process

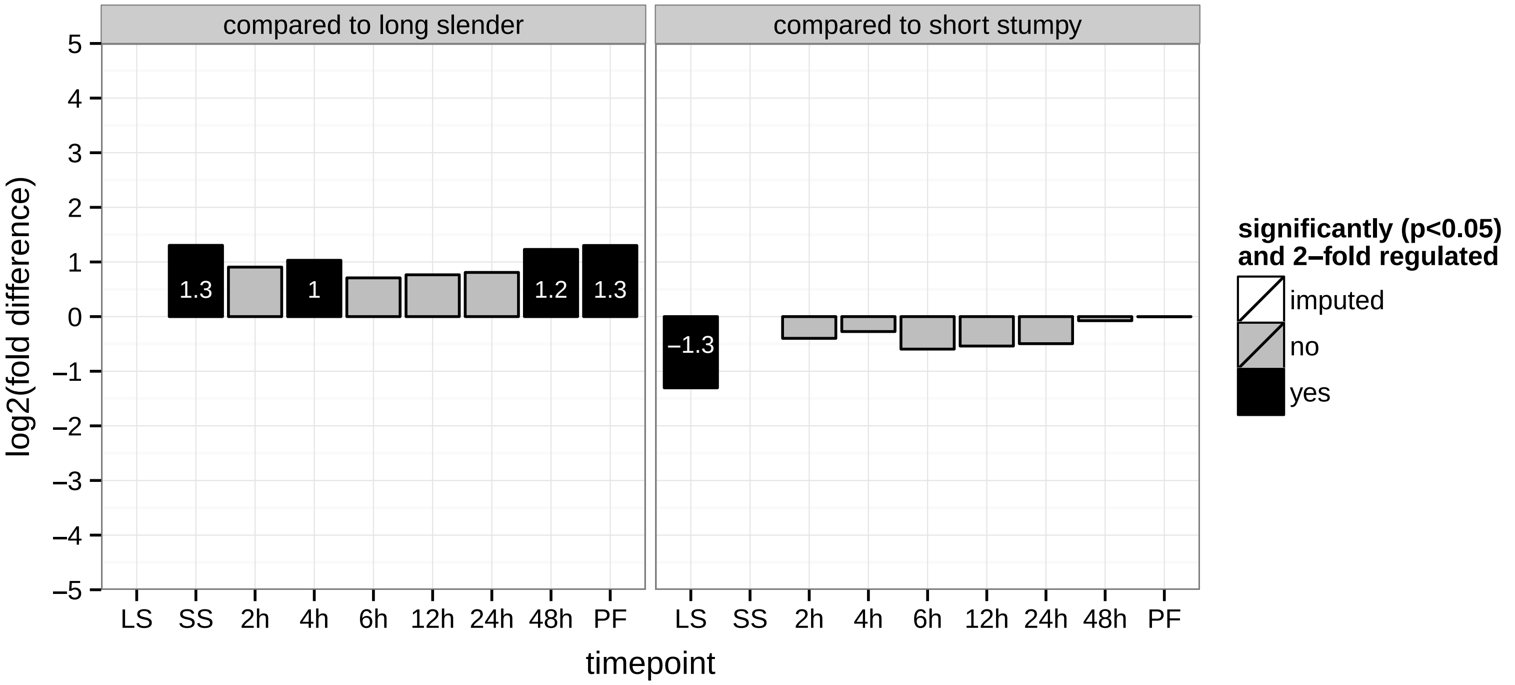

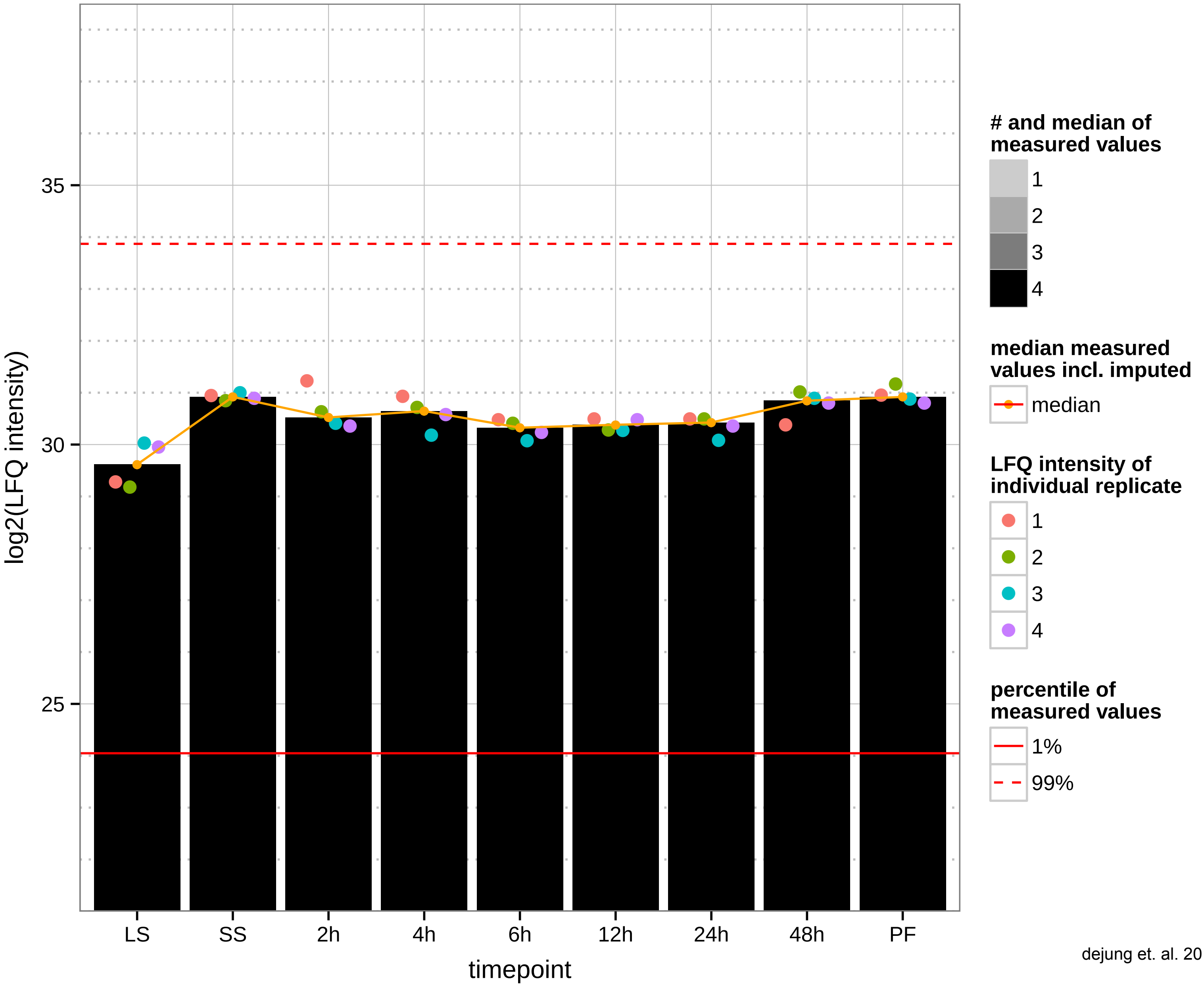

dejung et. al. 2015

hypothetical protein, conserved
Tb927.3.1860
AGOF: ATP binding, catalytic activity
AGOC: null
AGOP: null
PGOF: ATP binding, metal ion binding, protein binding
PGOC: null
PGOP: null

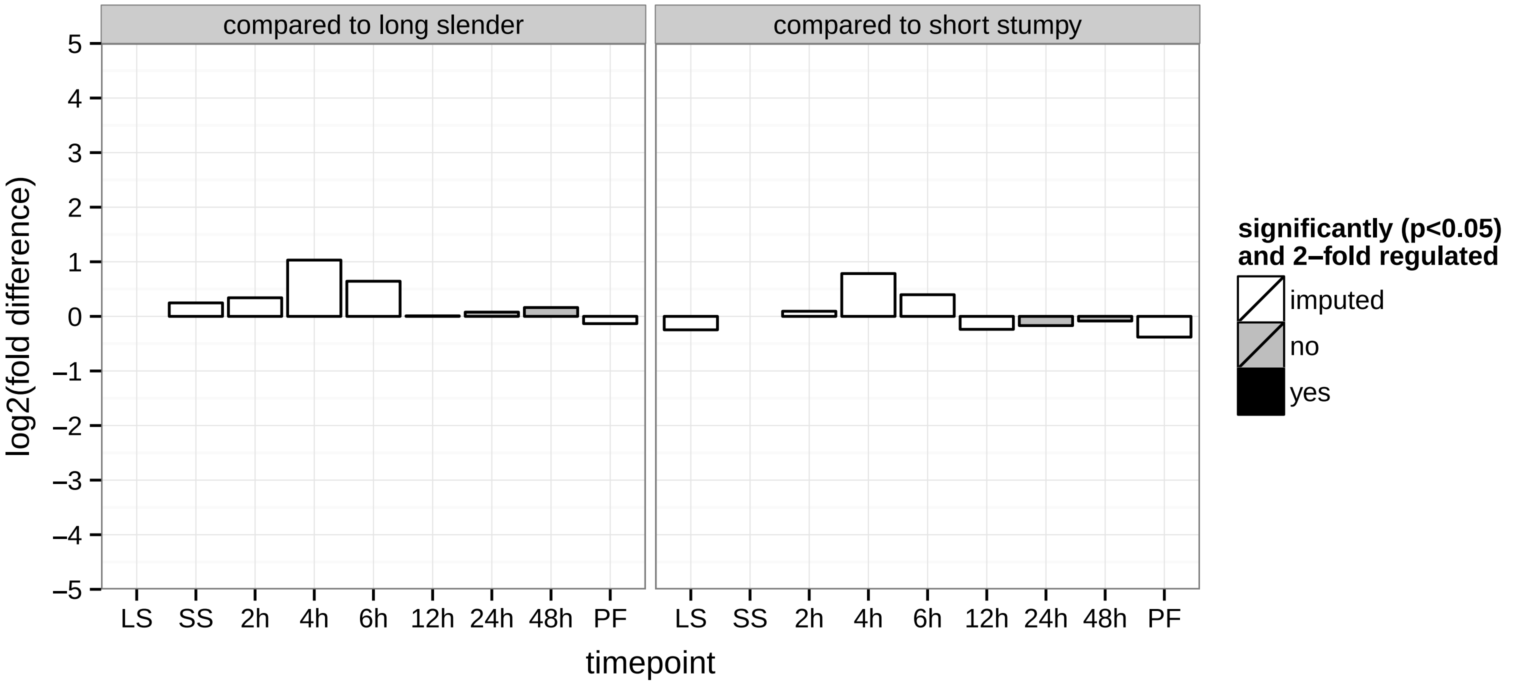

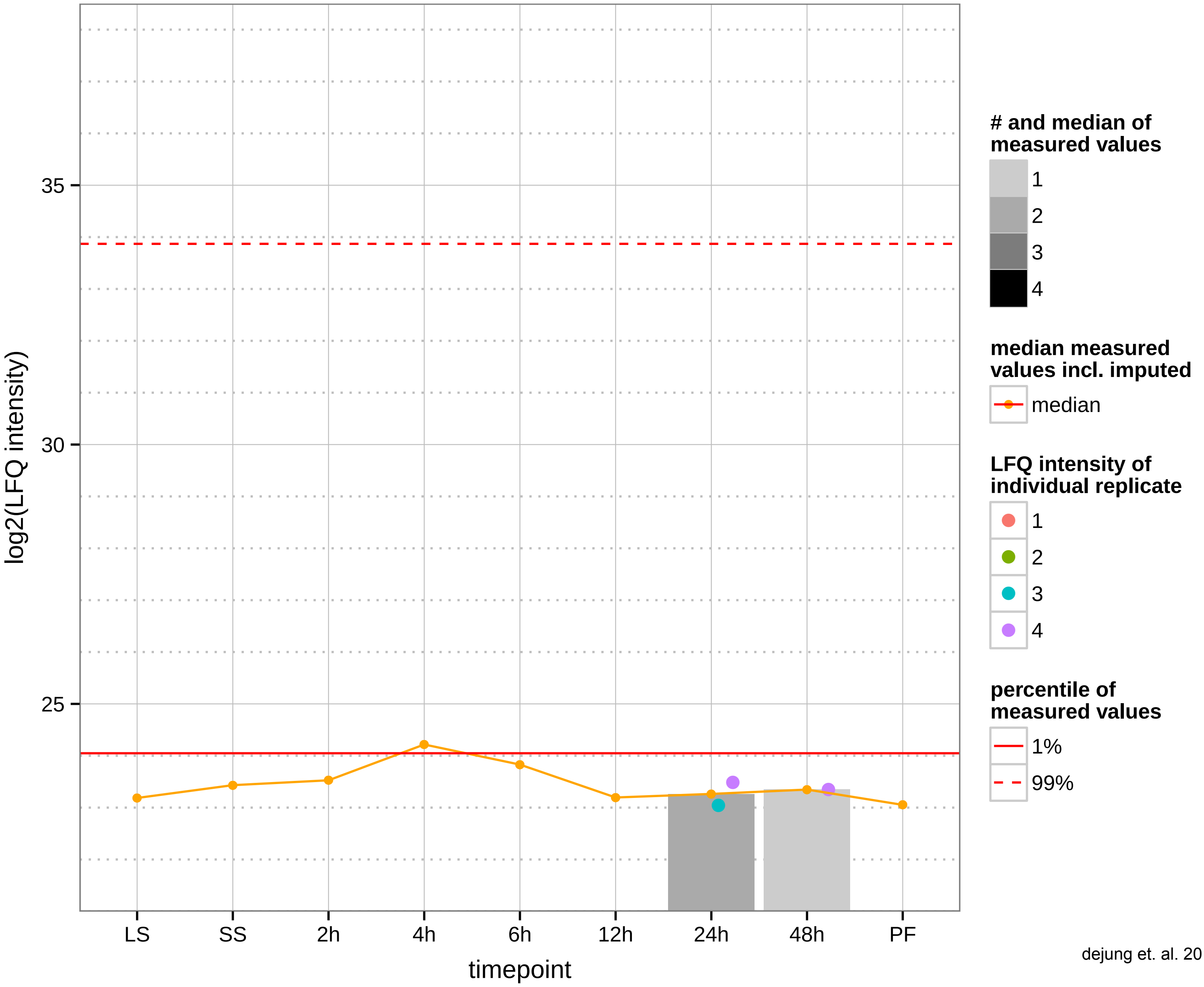

dejung et. al. 2015

hypothetical protein, conserved
Tb927.3.1900
AGOF: null
AGOC: null
AGOP: null
PGOF: null
PGOC: null
PGOP: null

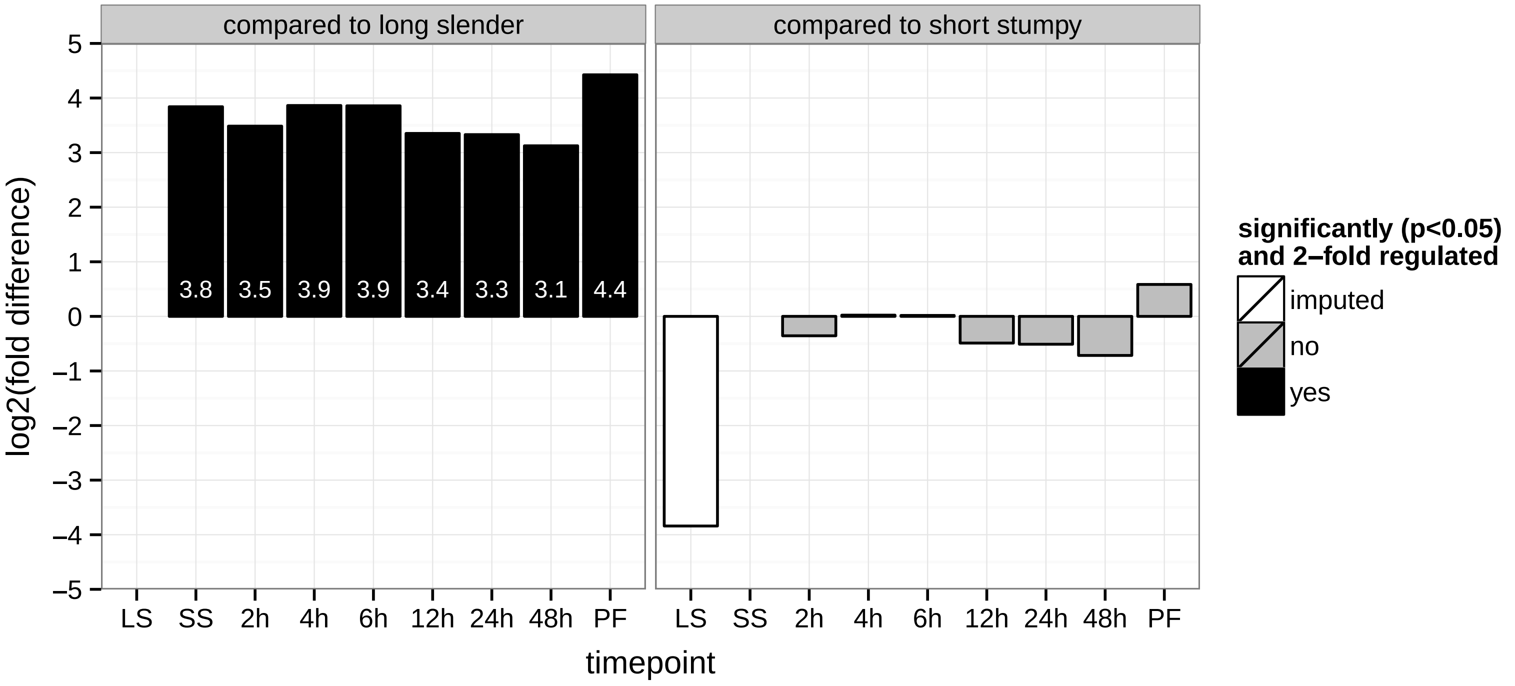

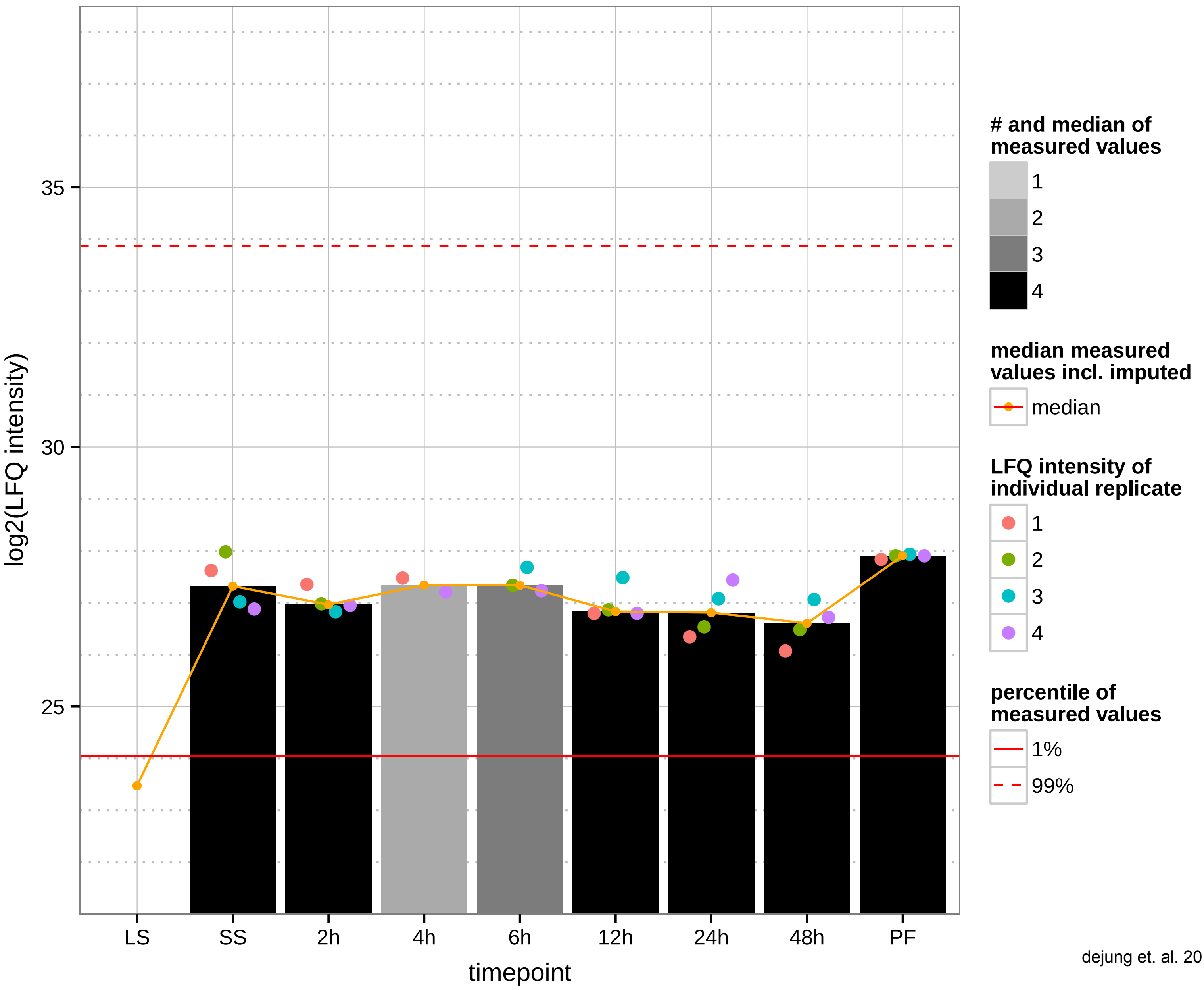

dejung et. al. 2015

NOT5 protein (NOT5)
Tb927.3.1920
AGOF: null
AGOC: nucleus
AGOP: regulation of transcription, DNA–dependent
PGOF: null
PGOC: nucleus
PGOP: regulation of transcription, DNA–dependent

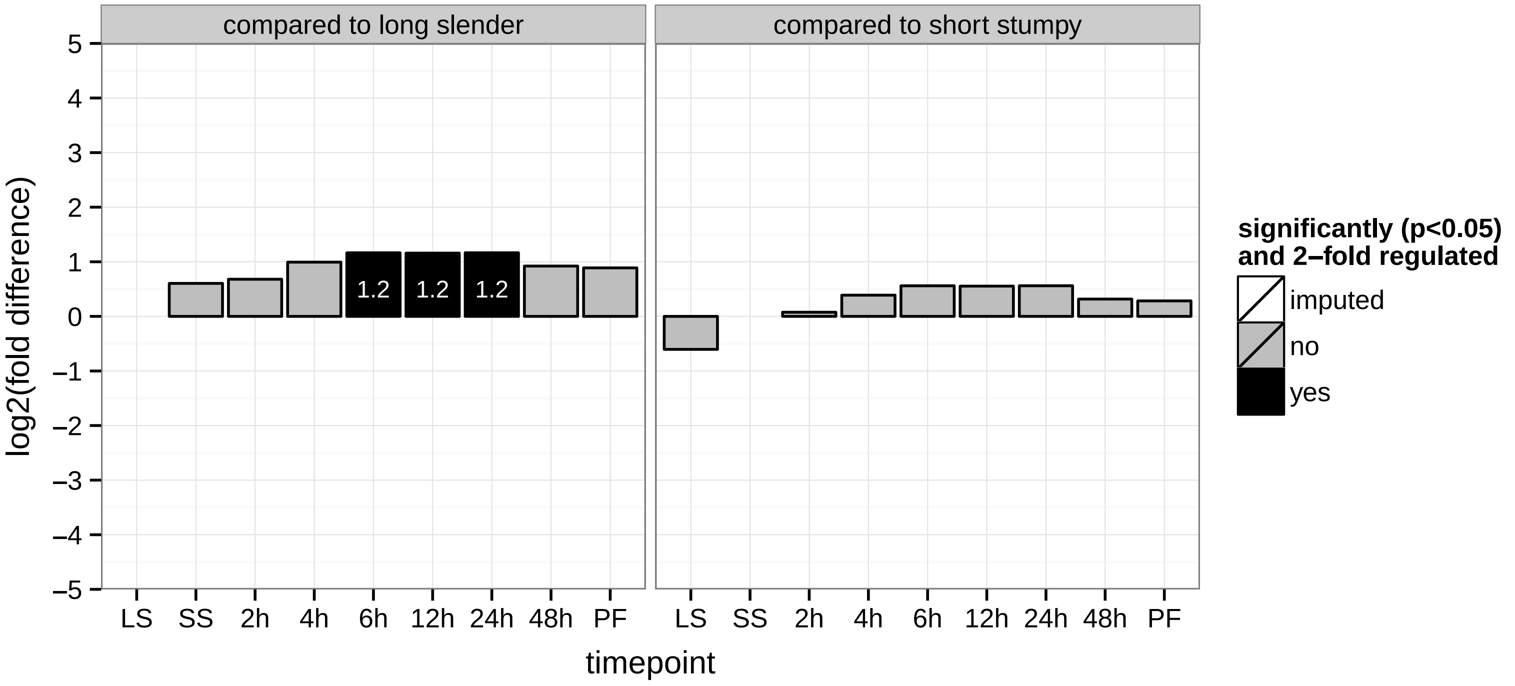

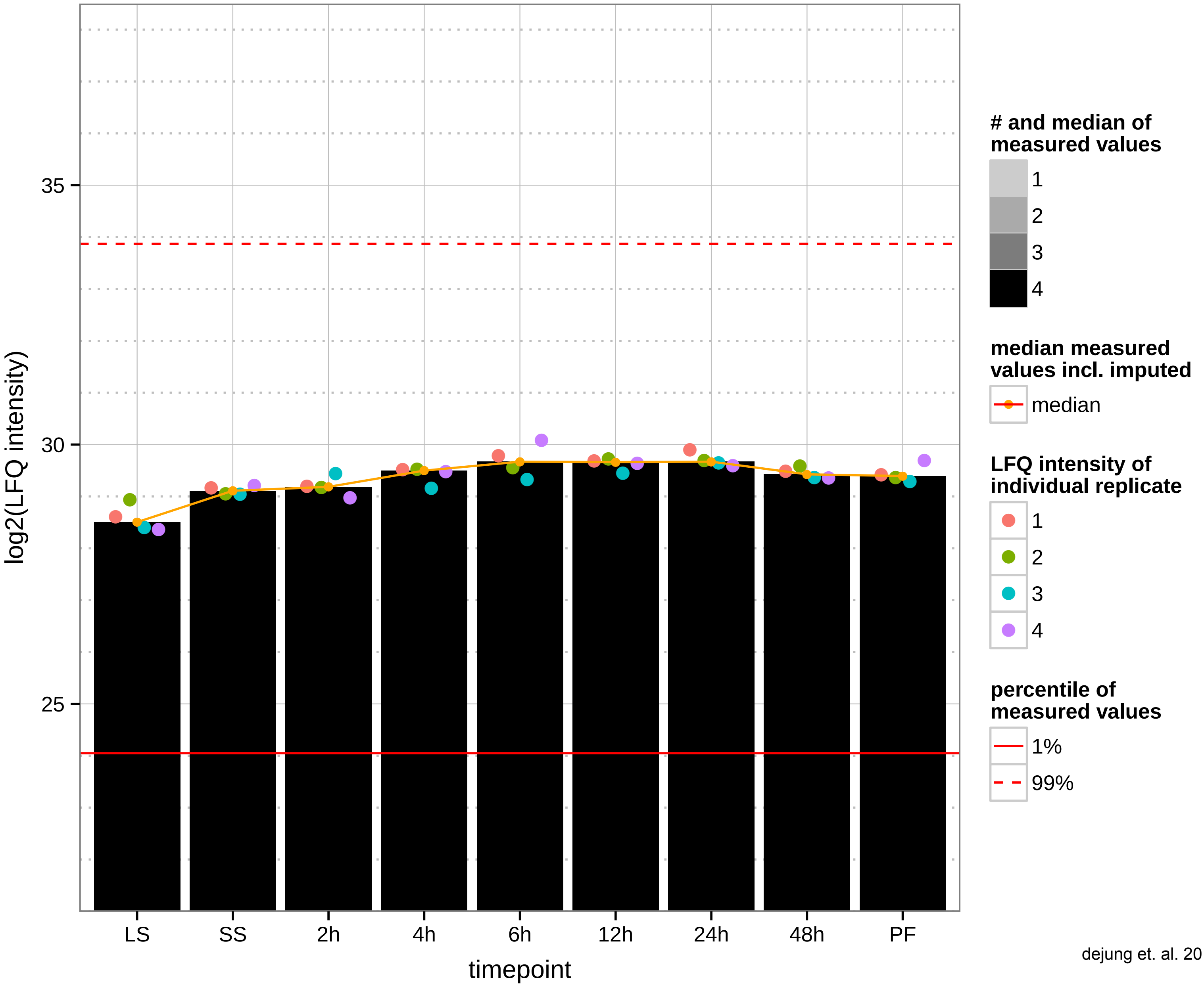

dejung et. al. 2015

hypothetical protein, conserved
Tb927.3.1990
AGOF: null
AGOC: null
AGOP: null
PGOF: protein binding
PGOC: null
PGOP: null

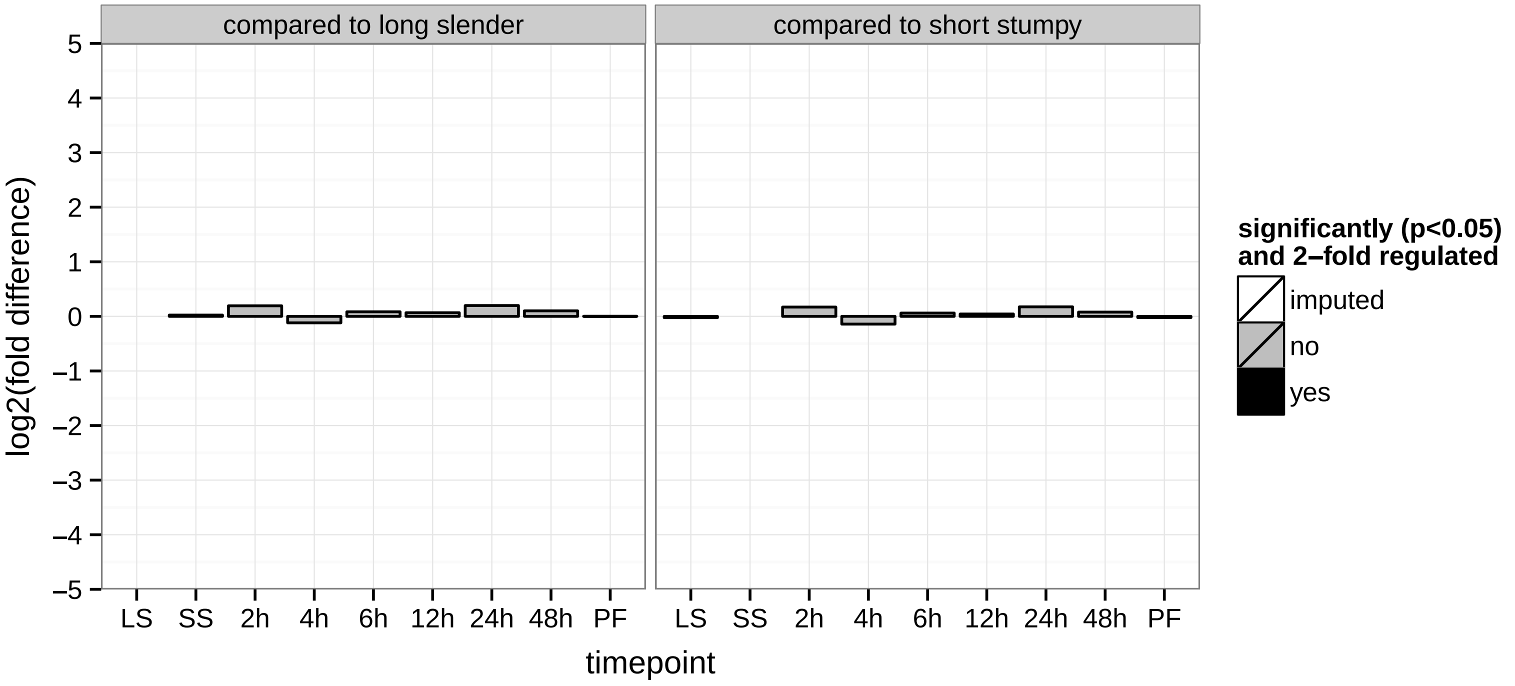

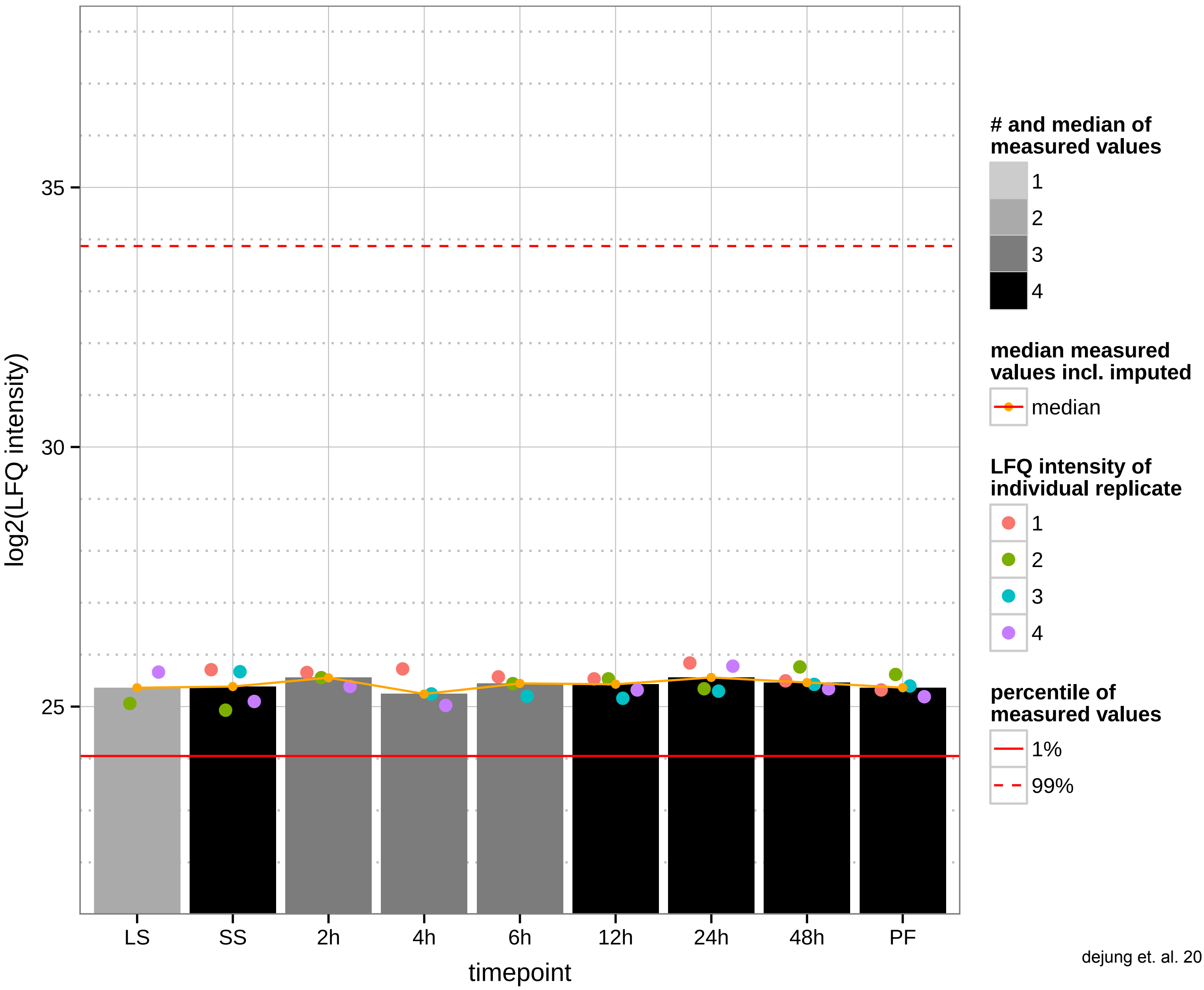

dejung et. al. 2015

hypothetical protein, conserved
Tb927.3.2050
AGOF: null
AGOC: null
AGOP: null
PGOF: null
PGOC: null
PGOP: null

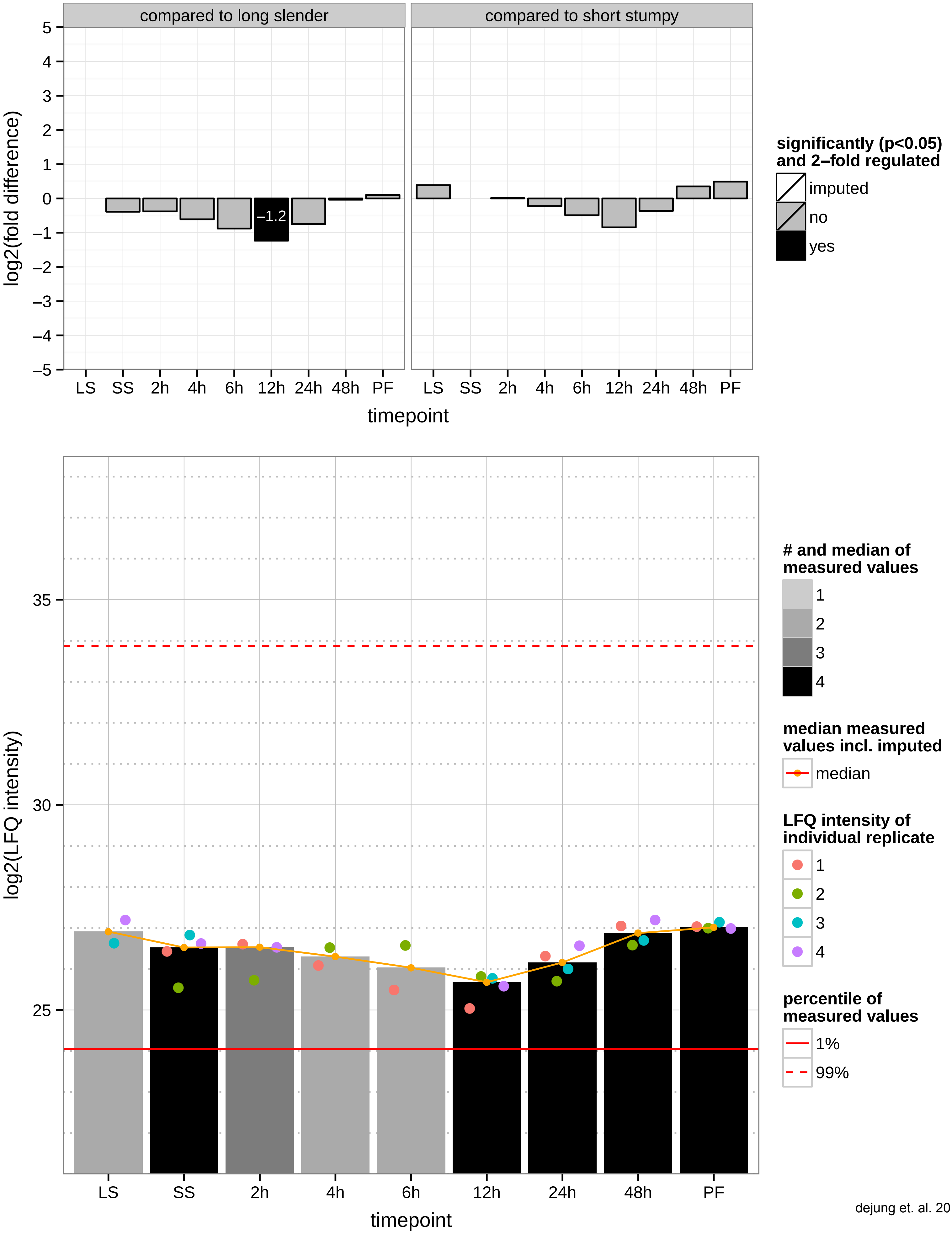

dejung et. al. 2015

aminopeptidase P1, putative, metallo–peptidase, Clan MG, Family M24
Tb927.3.2090;Tb11.v5.0697
AGOF: null, hydrolase activity
AGOC: null
AGOP: null, cellular process
PGOF: hydrolase activity
PGOC: null
PGOP: cellular process

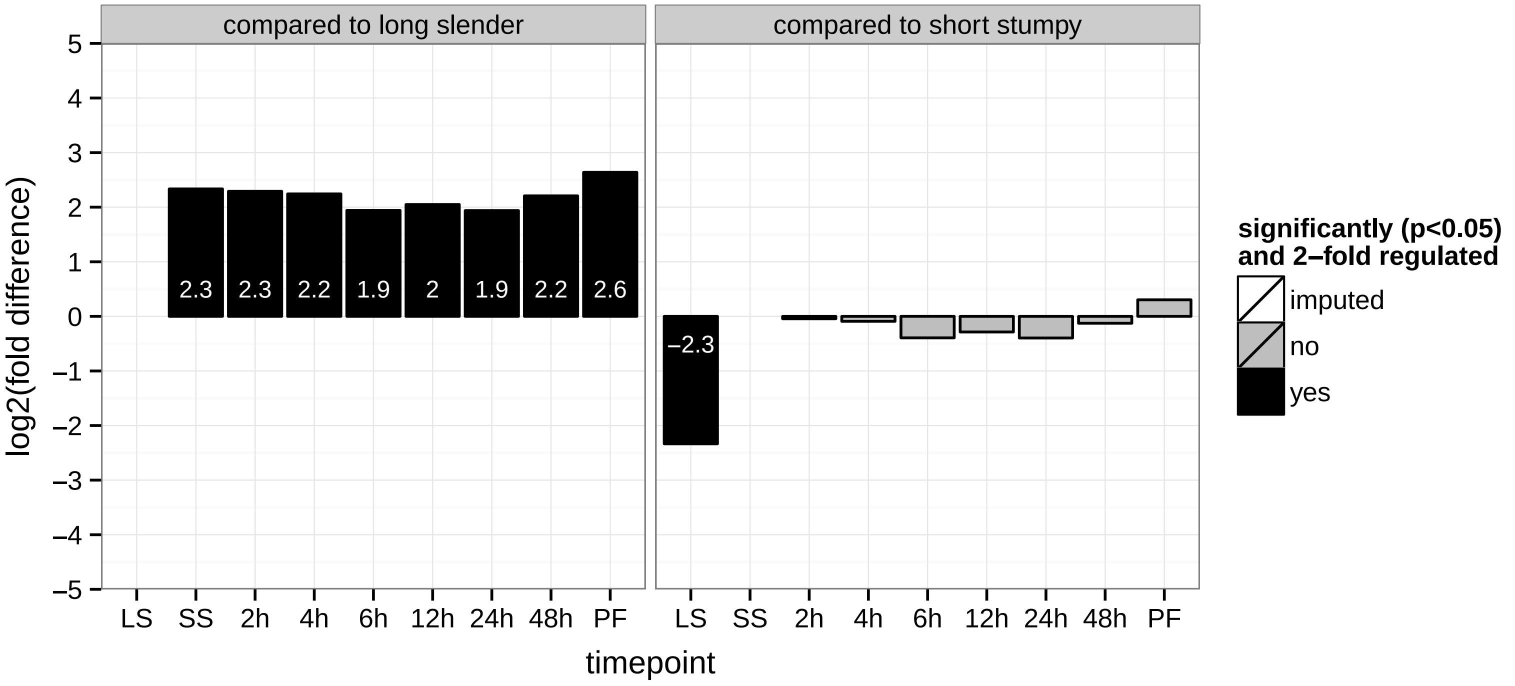

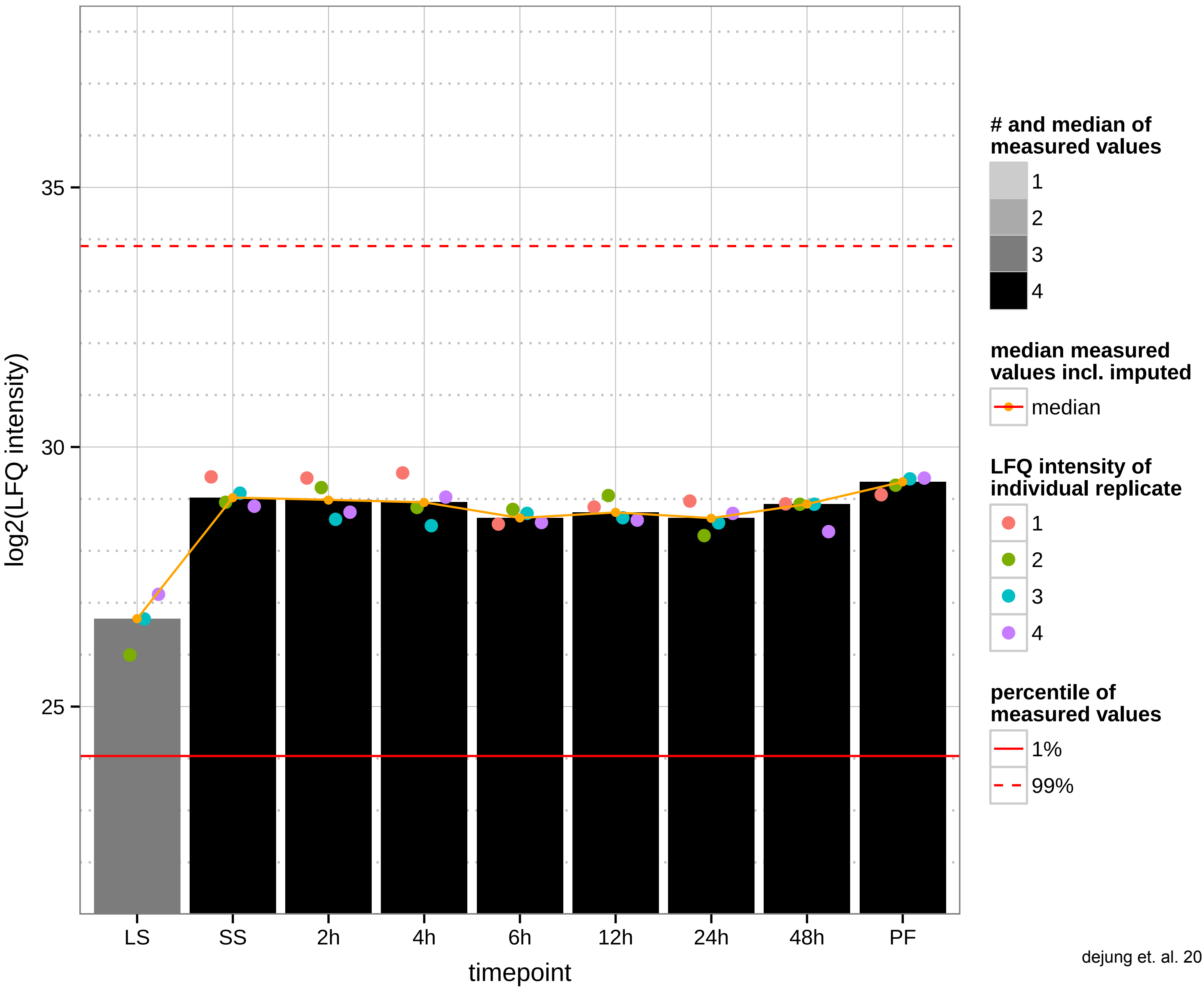

dejung et. al. 2015

hypothetical protein, conserved, TFIIF−stimulated CTD phosphatase, putative
Tb927.3.2110
AGOF: null, phosphatase activity
AGOC: null, mitochondrion
AGOP: null
PGOF: protein binding, phosphatase activity, protein binding
PGOC: null
PGOP: null

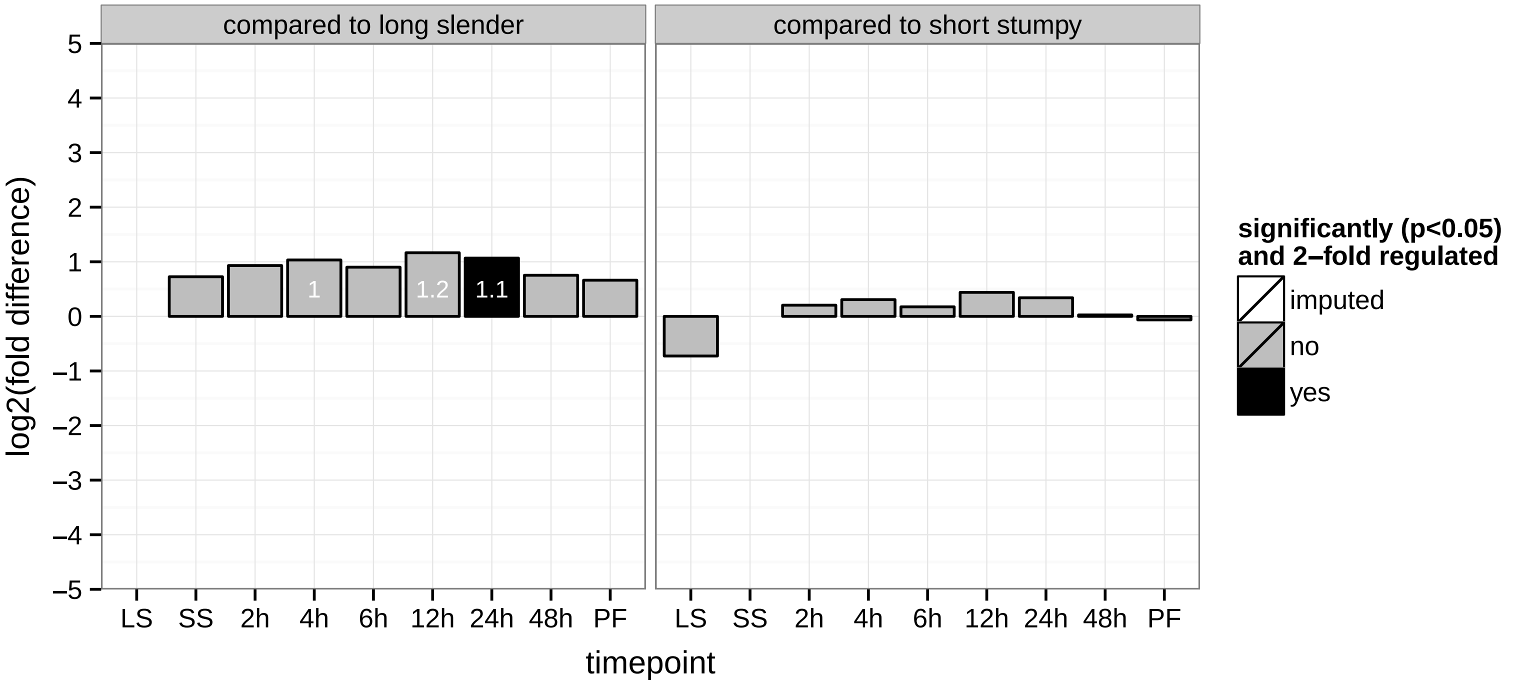

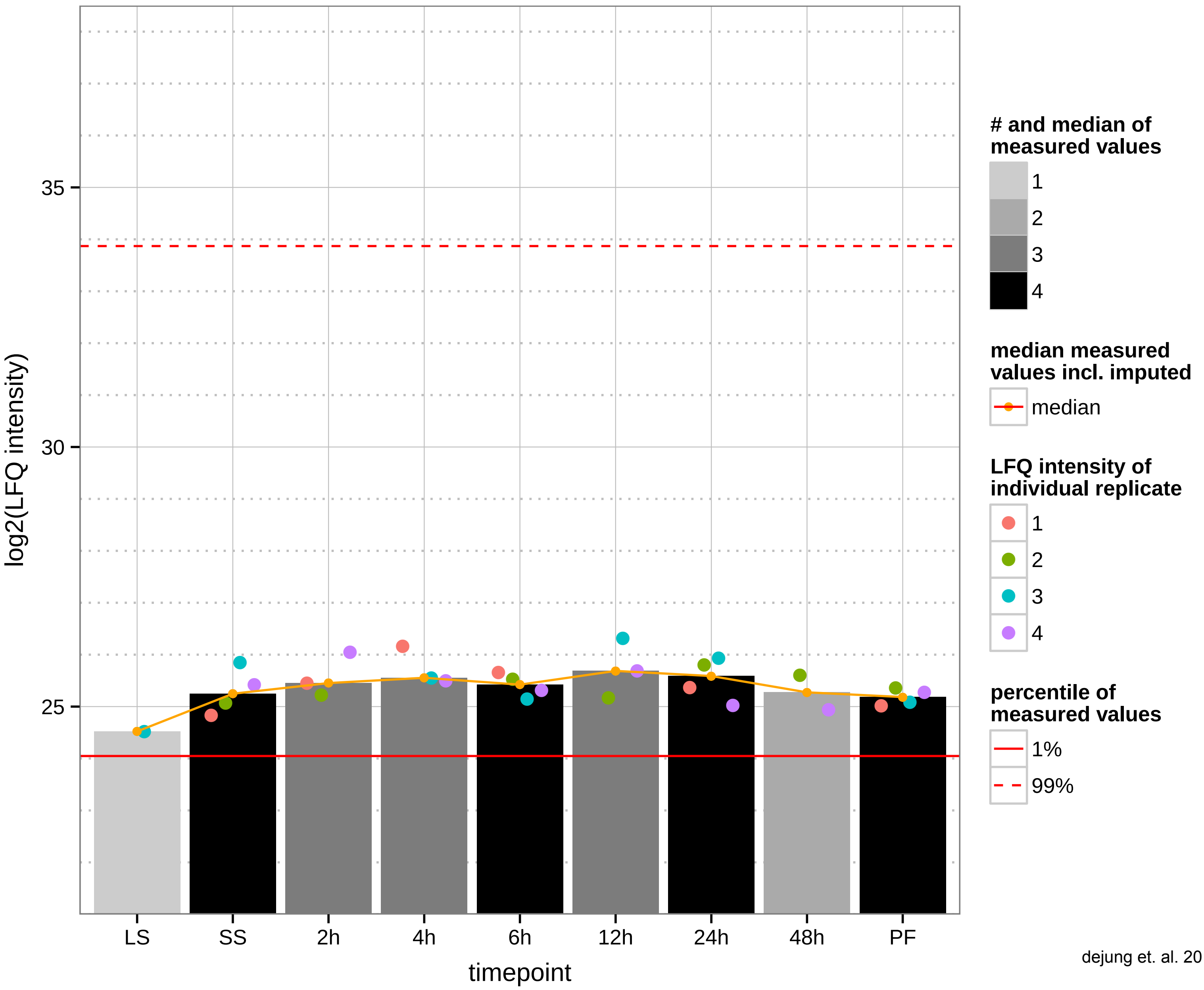

dejung et. al. 2015

RNA triphosphatase (TbCET1)
Tb927.3.2190
AGOF: nucleoside–triphosphatase activity, polynucleotide 5'–phosphatase activity
AGOC: mRNA cap methyltransferase complex
AGOP: 7–methylguanosine mRNA capping
PGOF: polynucleotide 5'–phosphatase activity, transferase activity
PGOC: null
PGOP: null

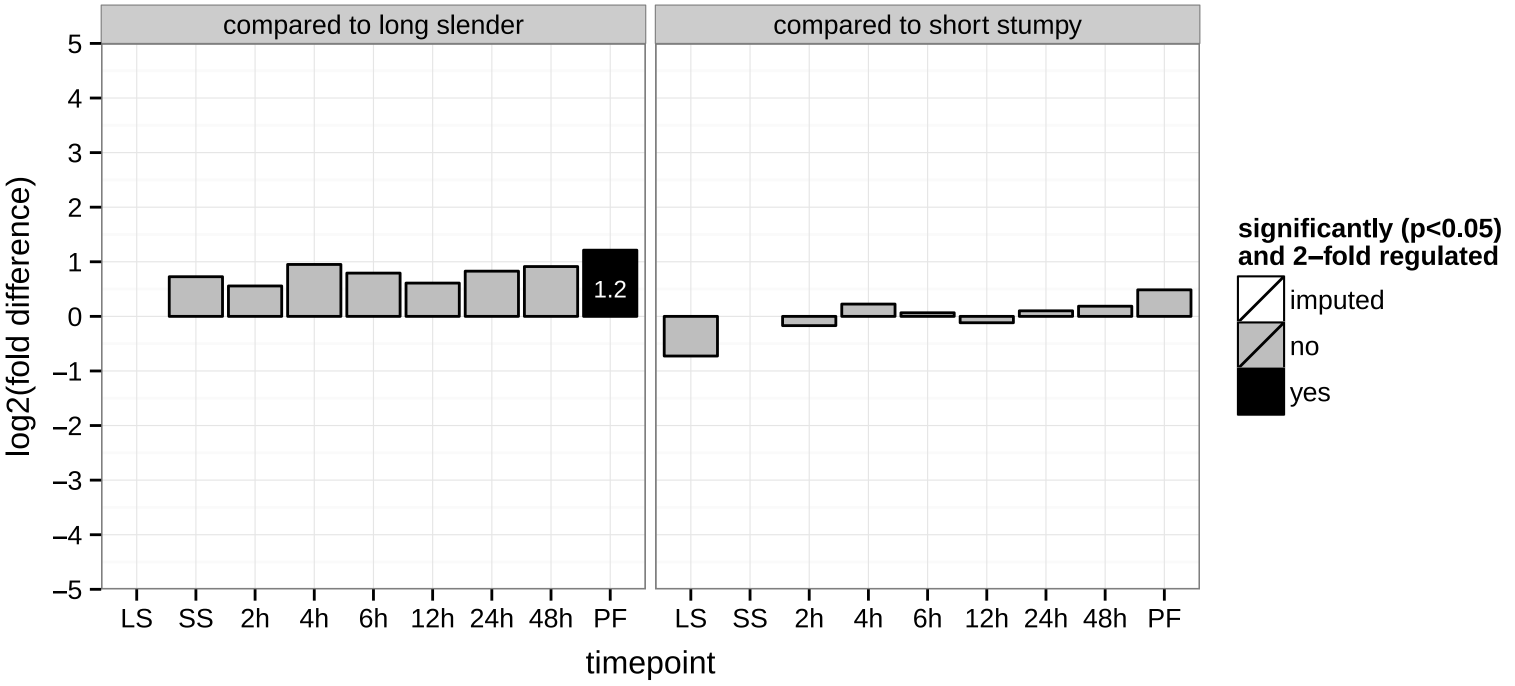

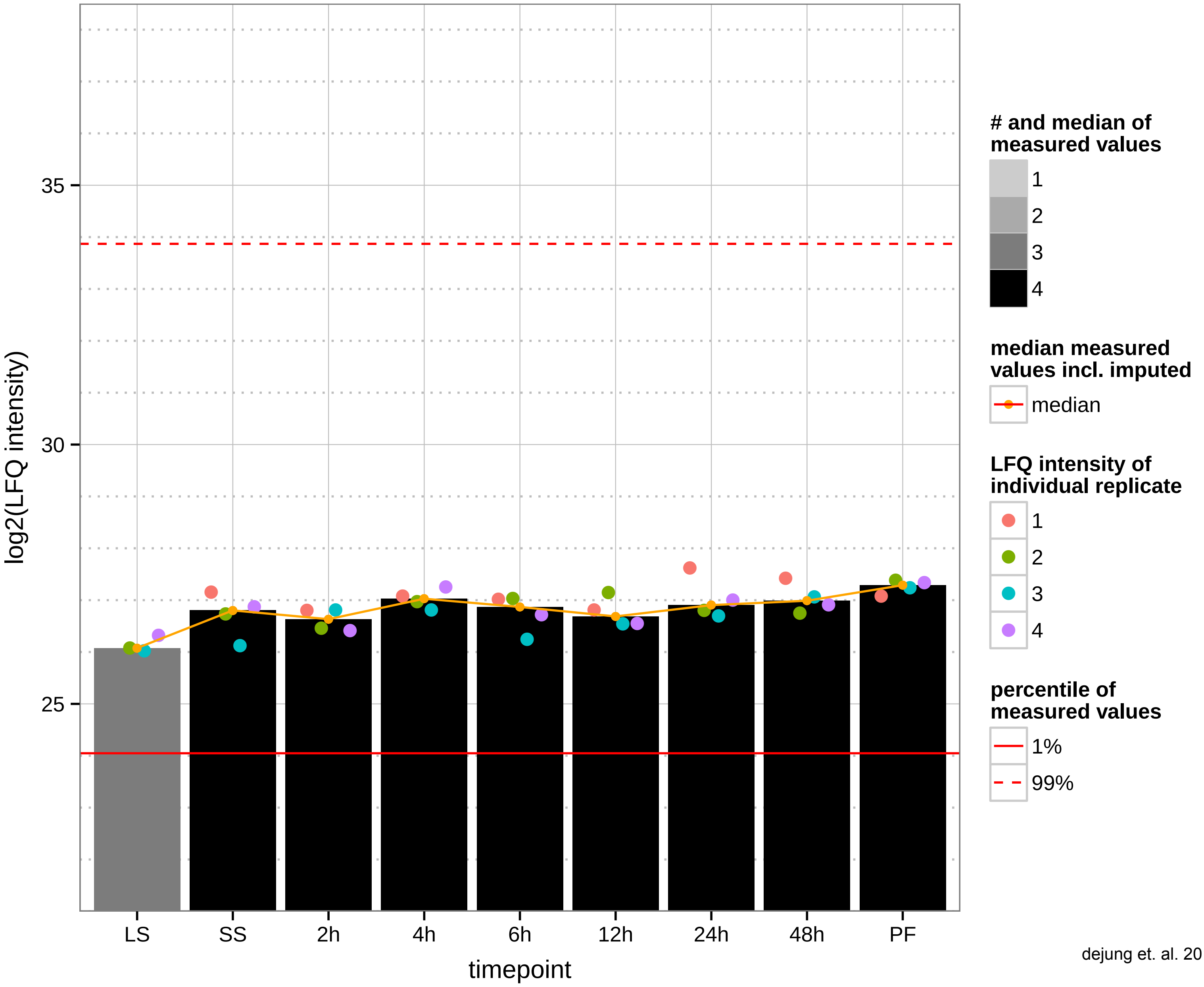

dejung et. al. 2015

hypothetical protein, conserved
Tb927.3.2220
AGOF: null
AGOC: null
AGOP: null
PGOF: null
PGOC: null
PGOP: null

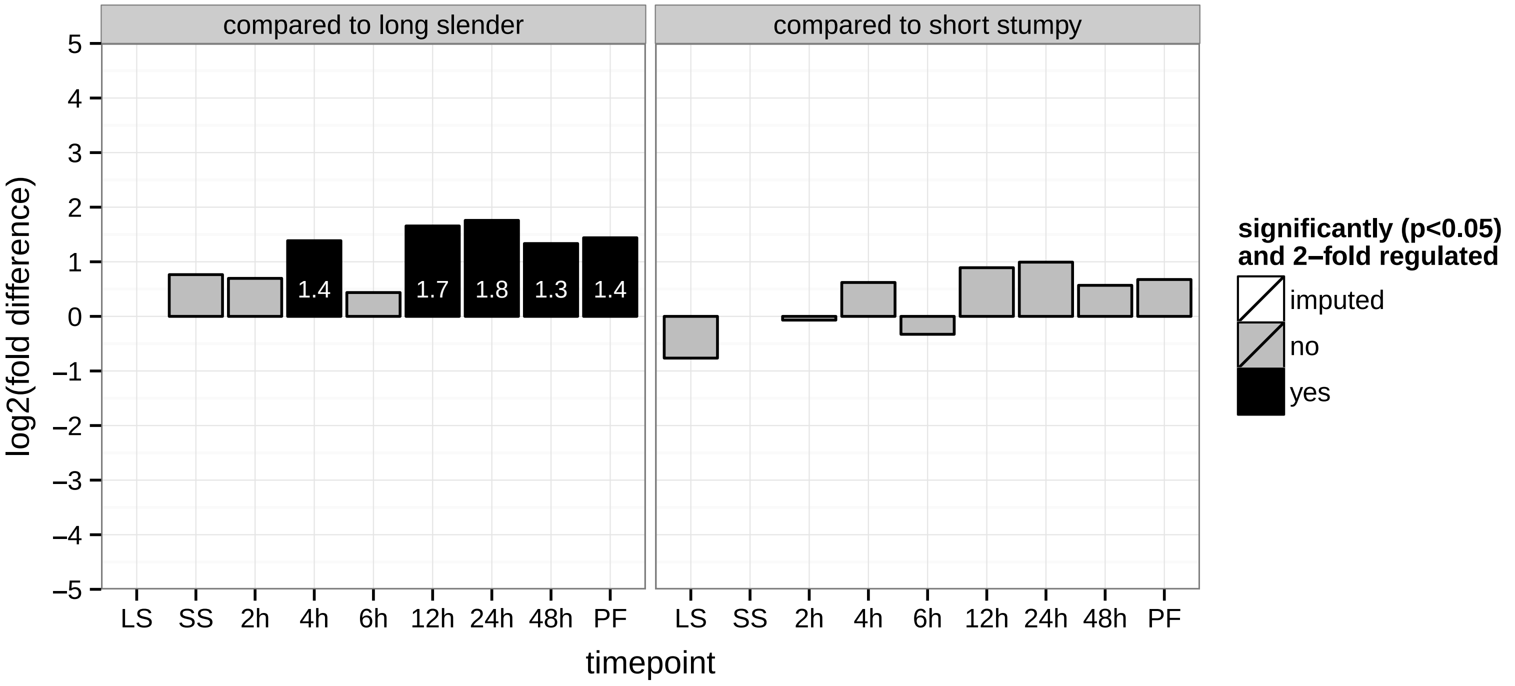

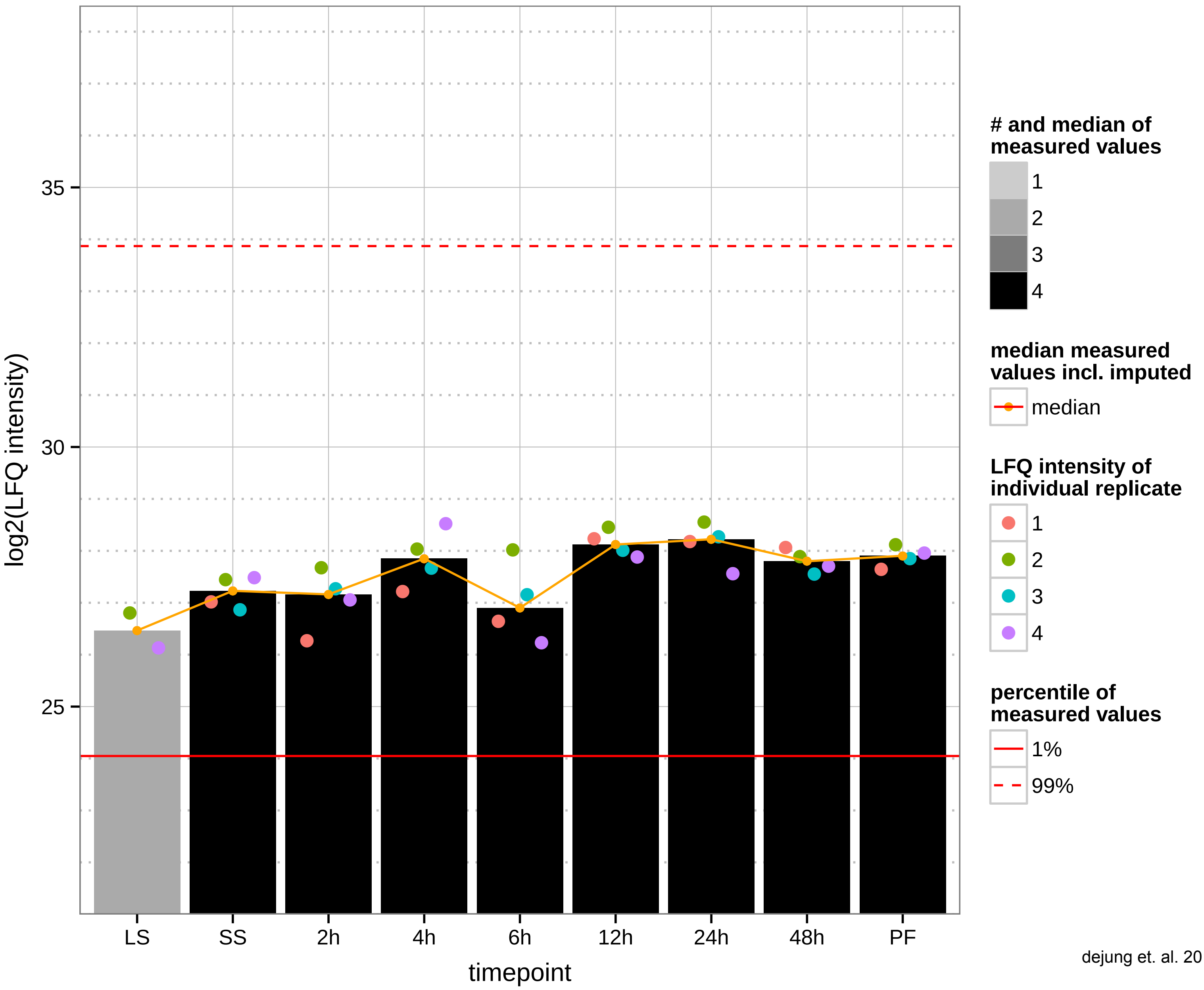

dejung et. al. 2015

succinyl–CoA synthetase alpha subunit, putative
Tb927.3.2230
AGOF: catalytic activity
AGOC: mitochondrion
AGOP: metabolic process
PGOF: catalytic activity, cofactor binding
PGOC: null
PGOP: metabolic process

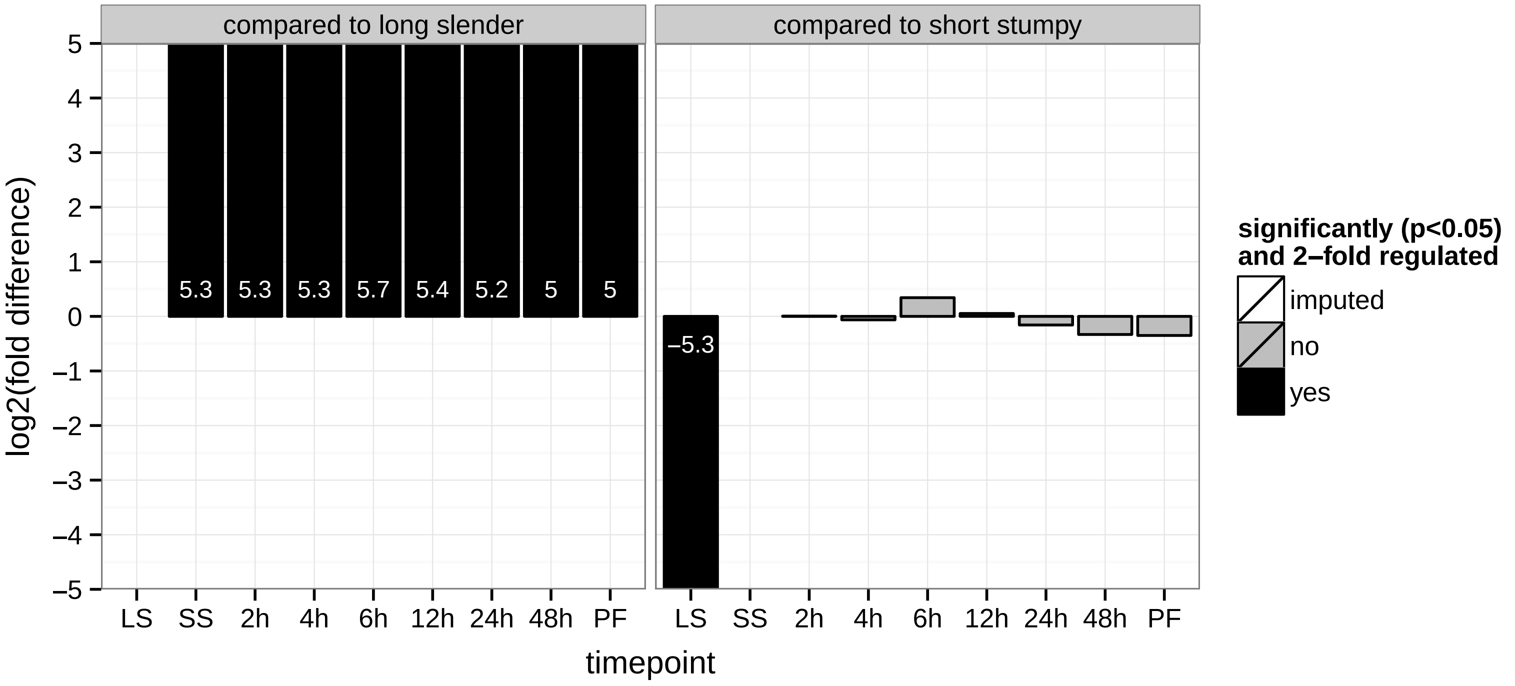

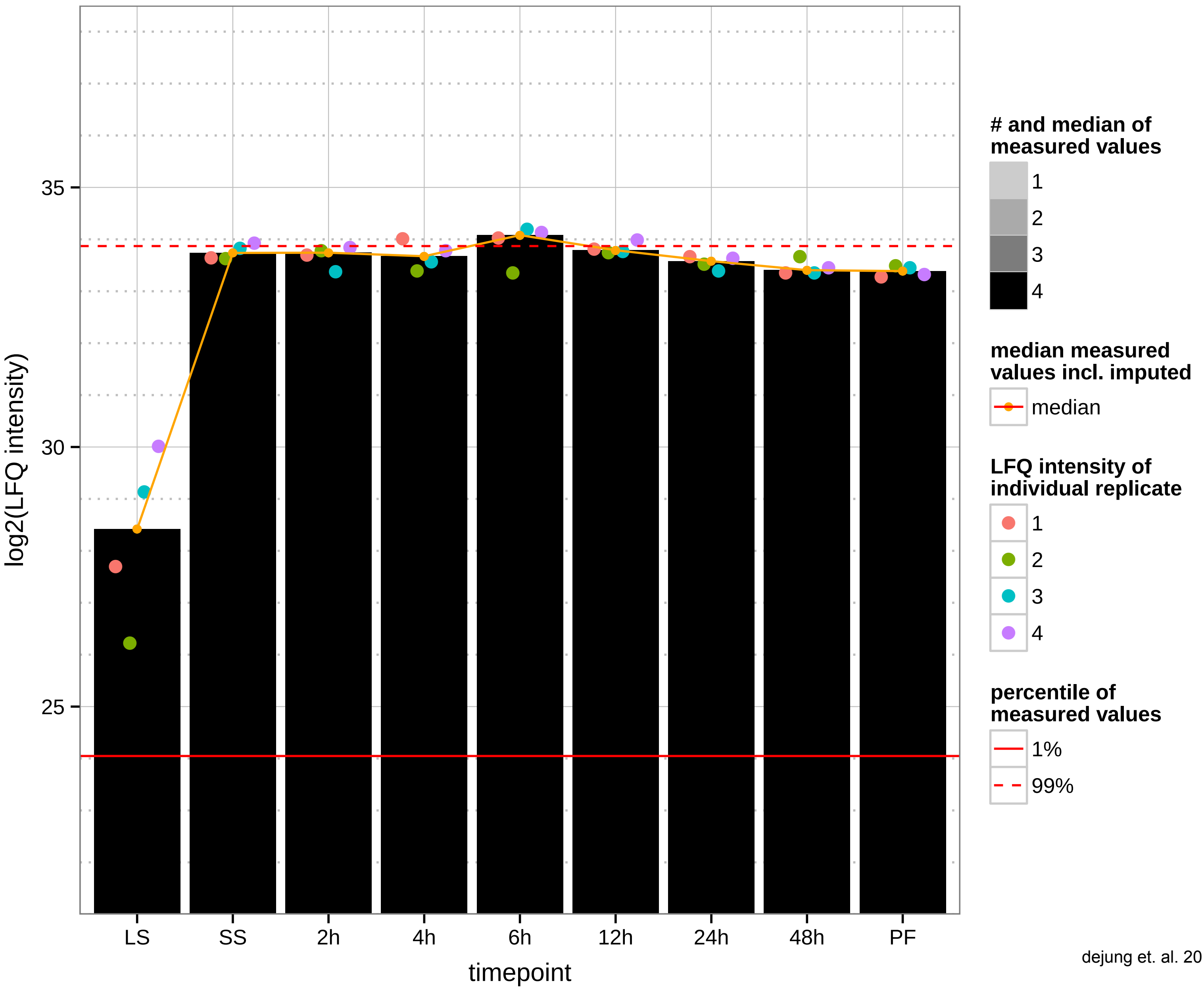

dejung et. al. 2015

hypothetical protein, conserved
Tb927.3.2270
AGOF: null
AGOC: null
AGOP: null
PGOF: null
PGOC: null
PGOP: null

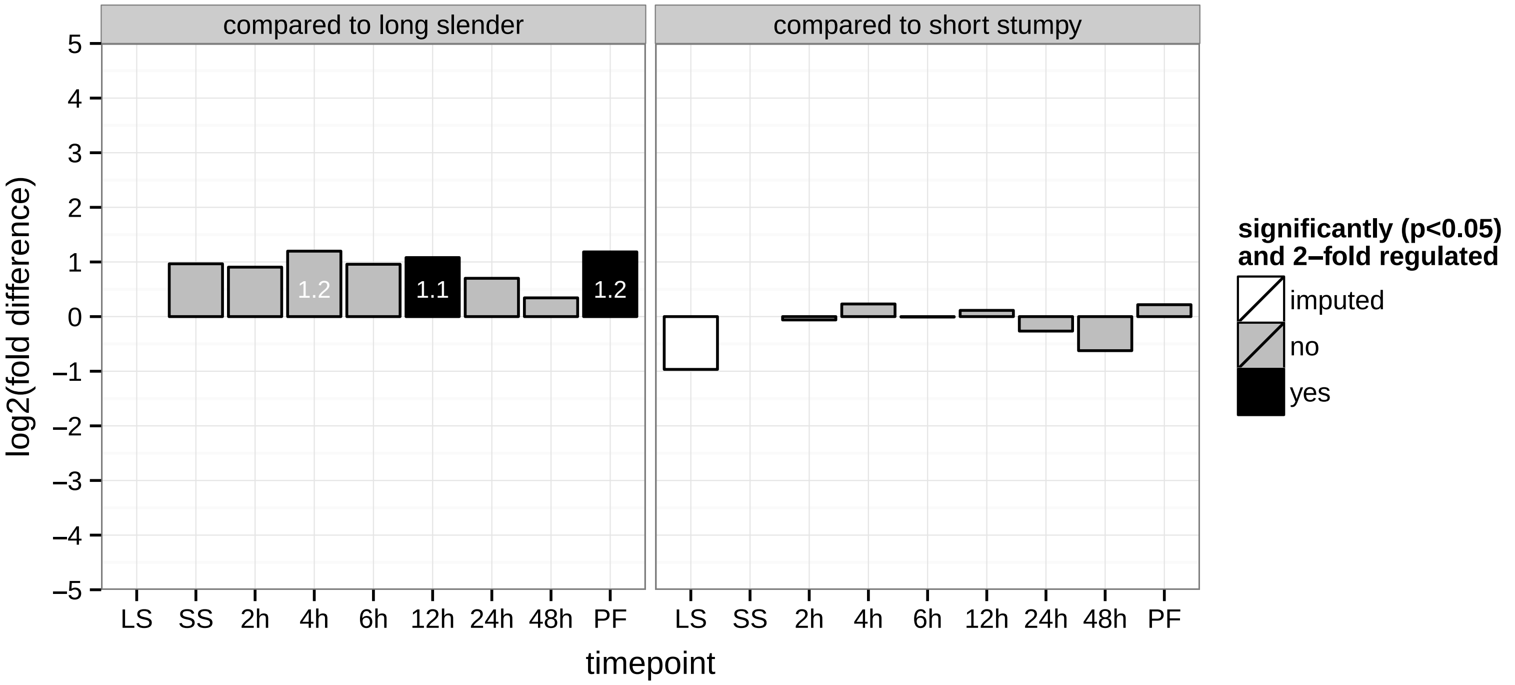

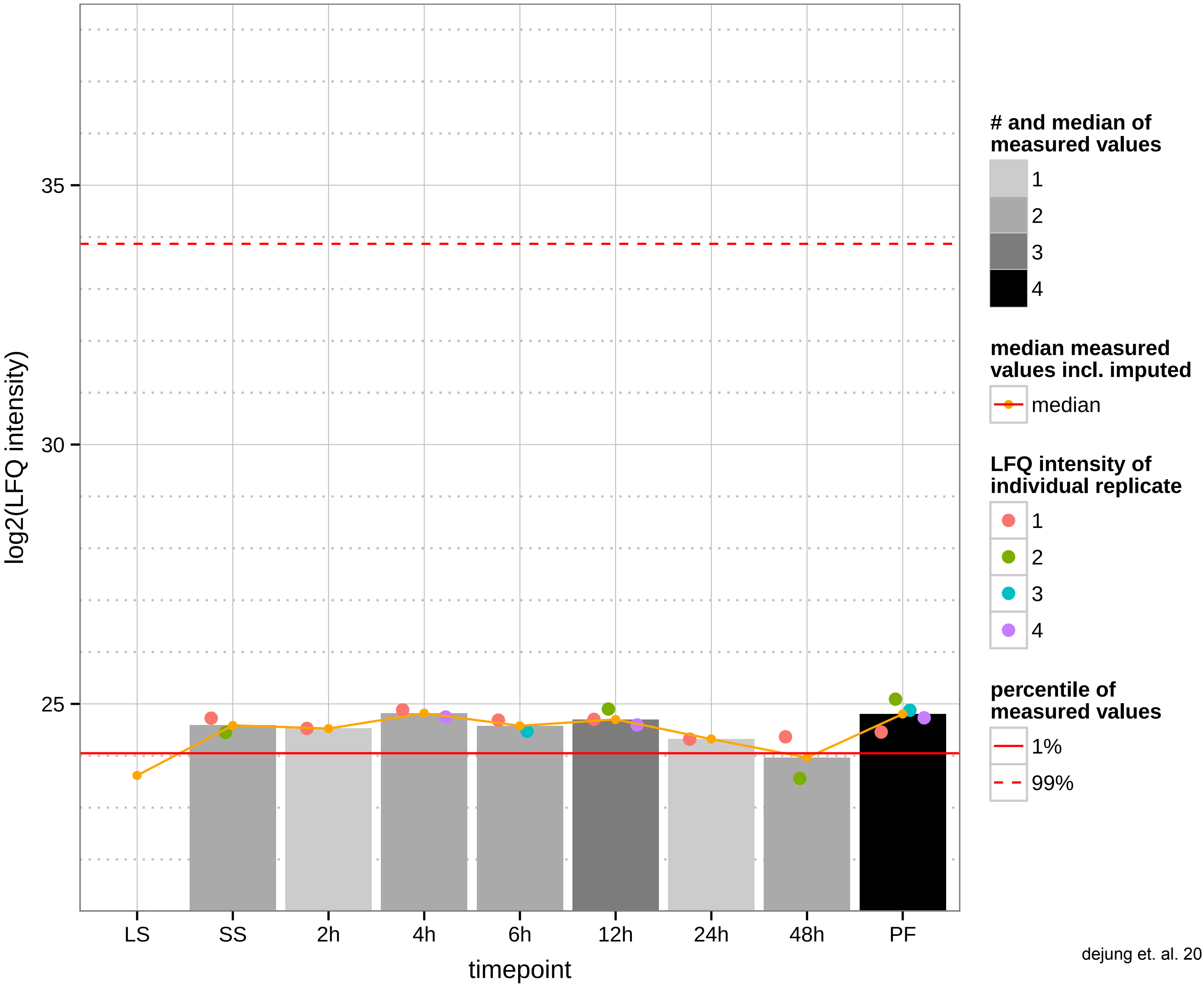

dejung et. al. 2015

PACRGA, flagellar component
Tb927.3.2310
AGOF: null
AGOC: null
AGOP: null
PGOF: binding
PGOC: null
PGOP: null

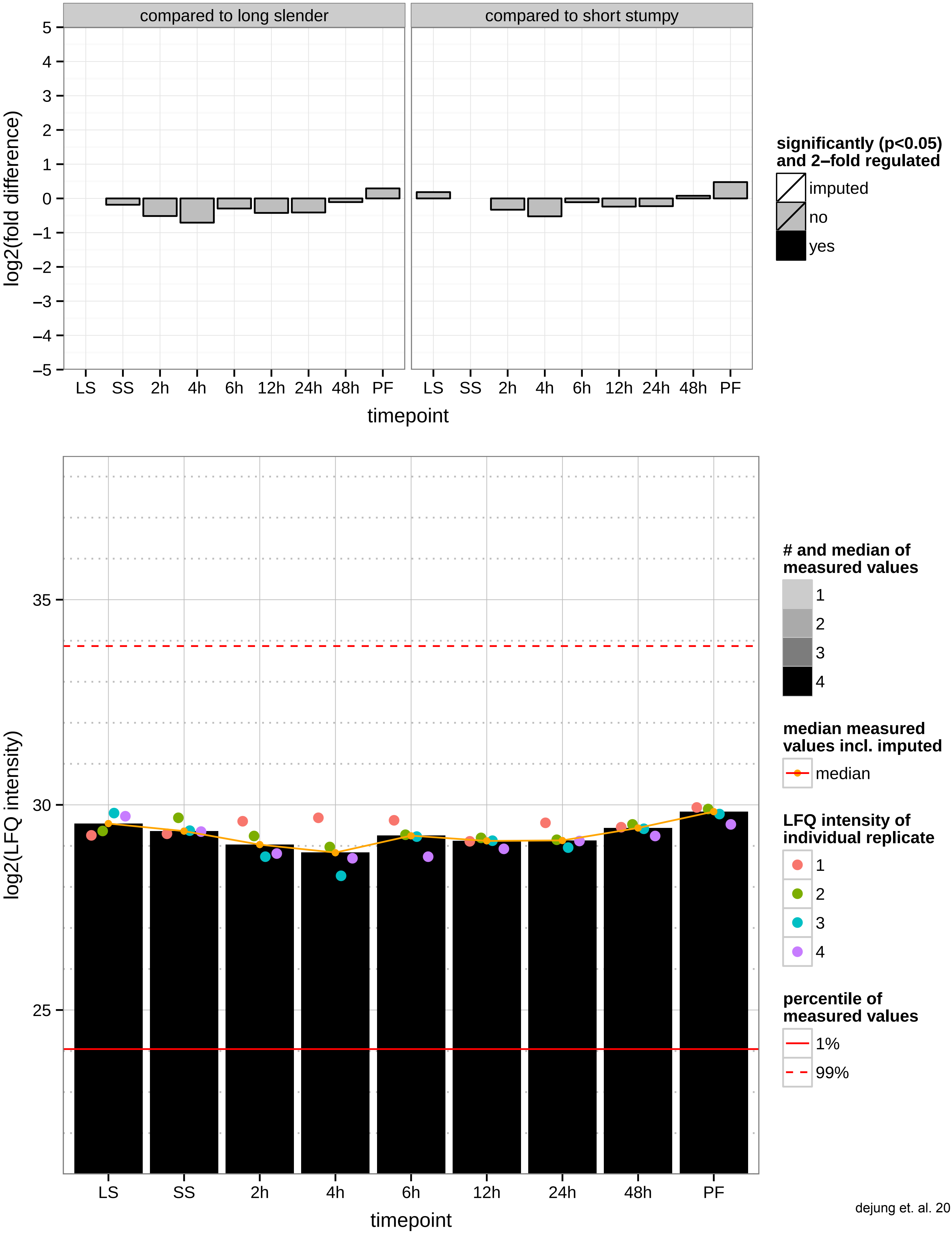

dejung et. al. 2015

hypothetical protein, conserved
Tb927.3.2420
AGOF: null
AGOC: null
AGOP: null
PGOF: peptidase activity
PGOC: integral to membrane, signal peptidase complex
PGOP: signal peptide processing

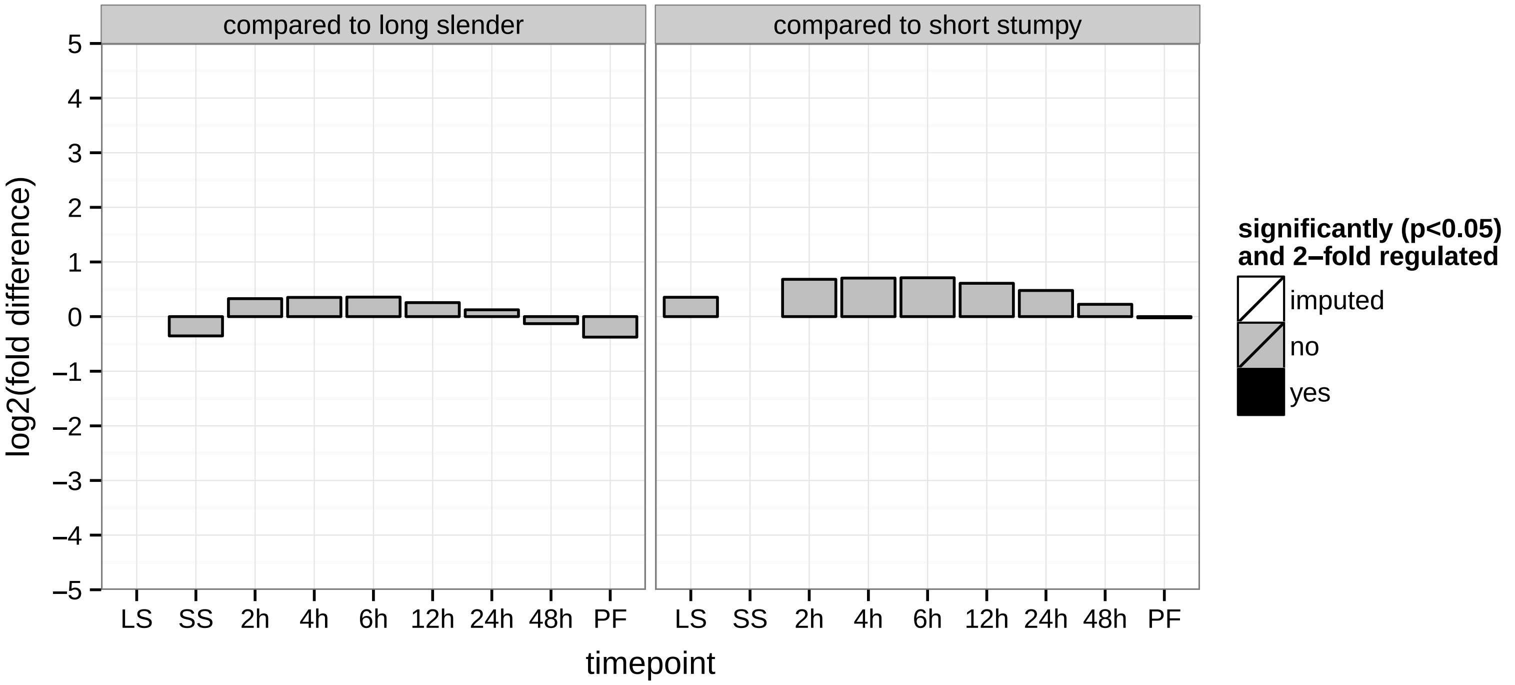

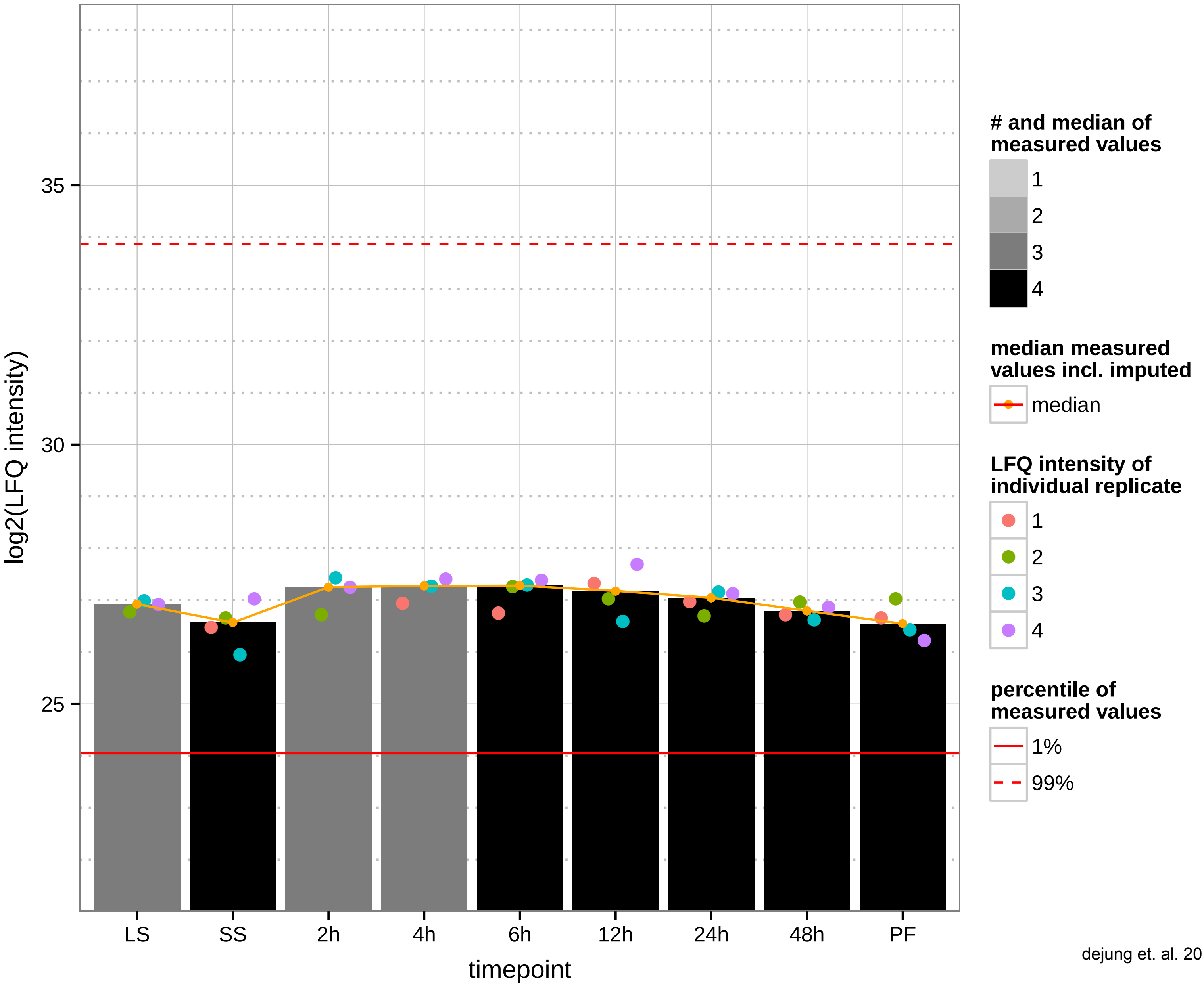

dejung et. al. 2015

hypothetical protein, conserved
Tb927.3.2430
AGOF: null
AGOC: null
AGOP: null
PGOF: null
PGOC: null
PGOP: null

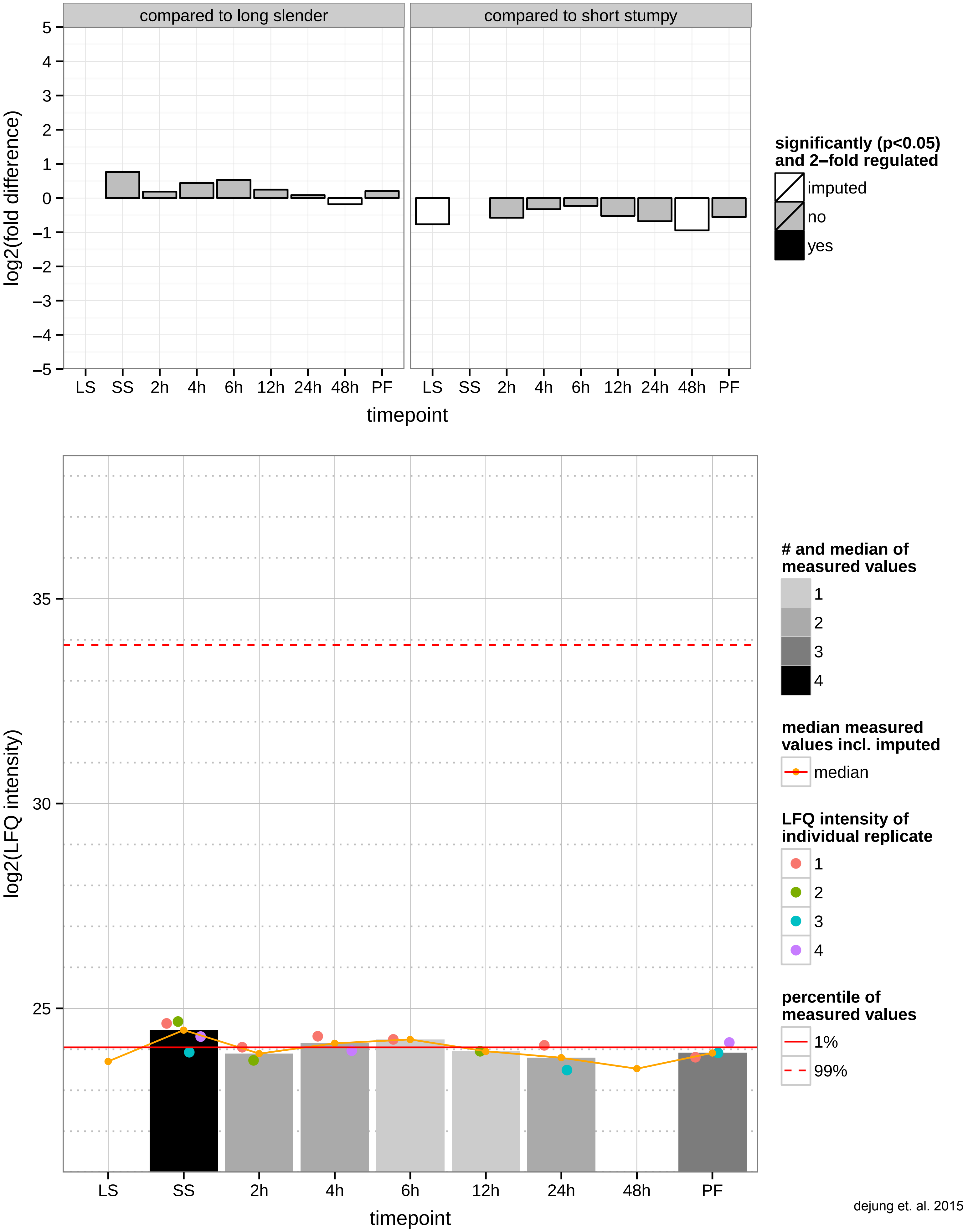

dejung et. al. 2015

serine/threonine–protein kinase, putative, protein kinase
Tb927.3.2440
AGOF: ATP binding, protein serine/threonine kinase activity, protein tyrosine kinase activity
AGOC: null
AGOP: cytokinesis after mitosis, protein phosphorylation
PGOF: ATP binding, protein kinase activity, transferase activity, transferring phosphorus–containing groups
PGOC: null
PGOP: protein phosphorylation

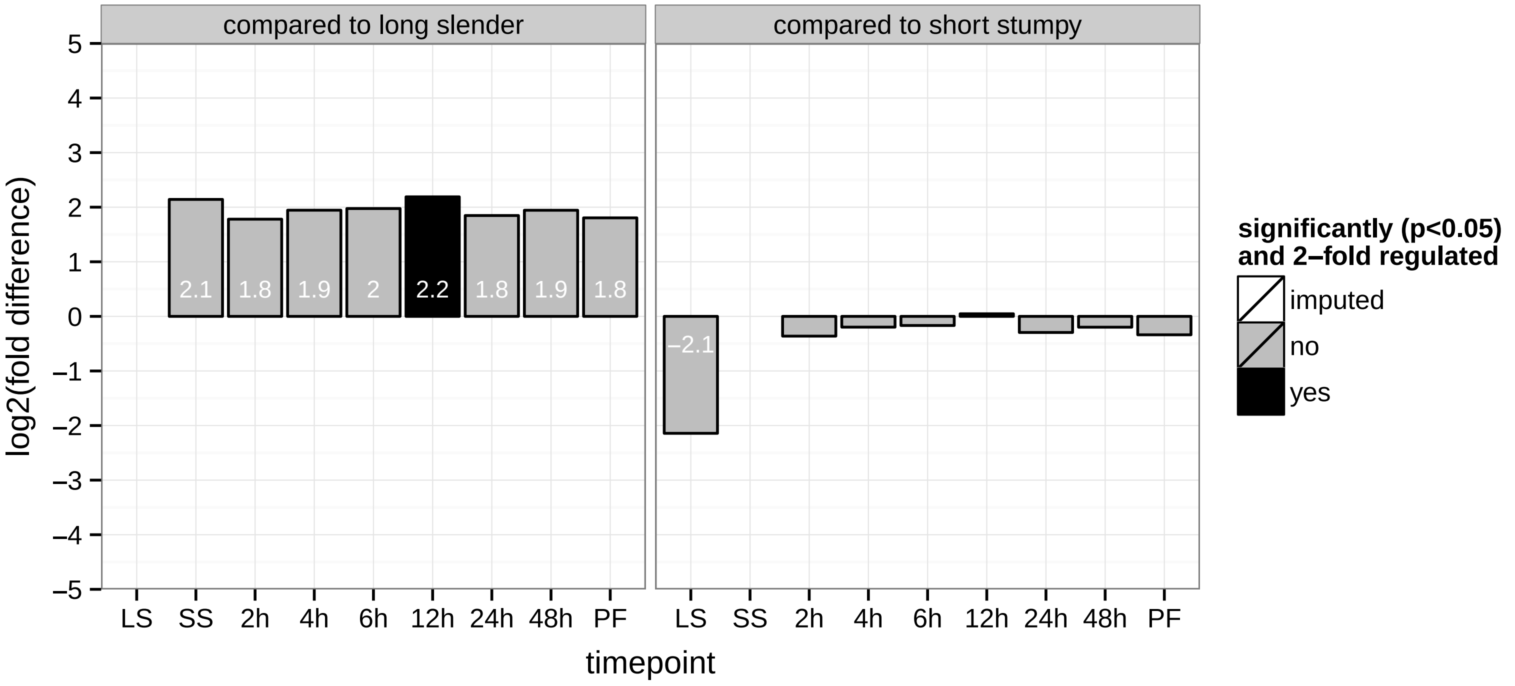

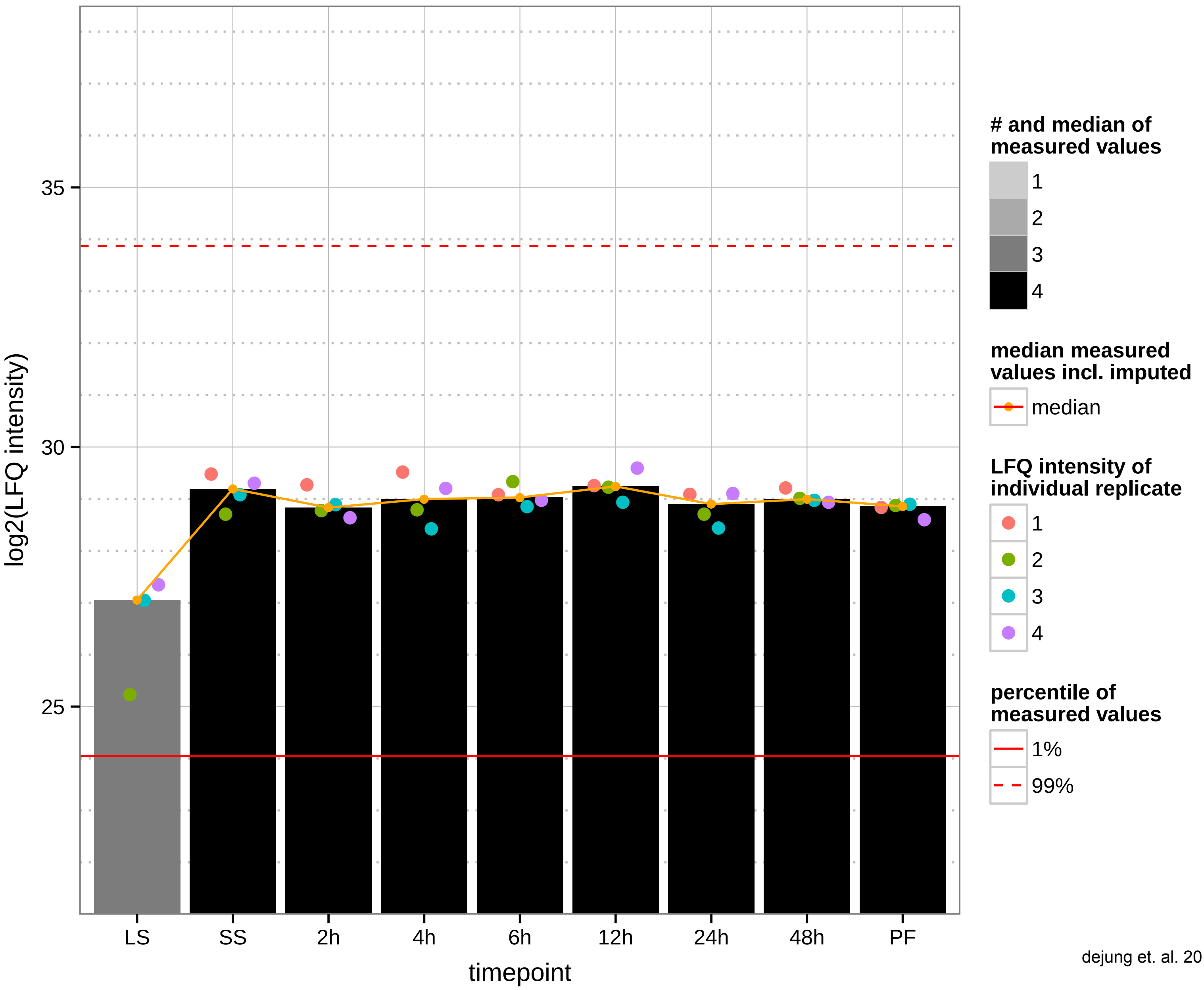

dejung et. al. 2015

hypothetical protein, conserved
Tb927.3.2490
AGOF: null
AGOC: null
AGOP: null
PGOF: DNA binding
PGOC: null
PGOP: null

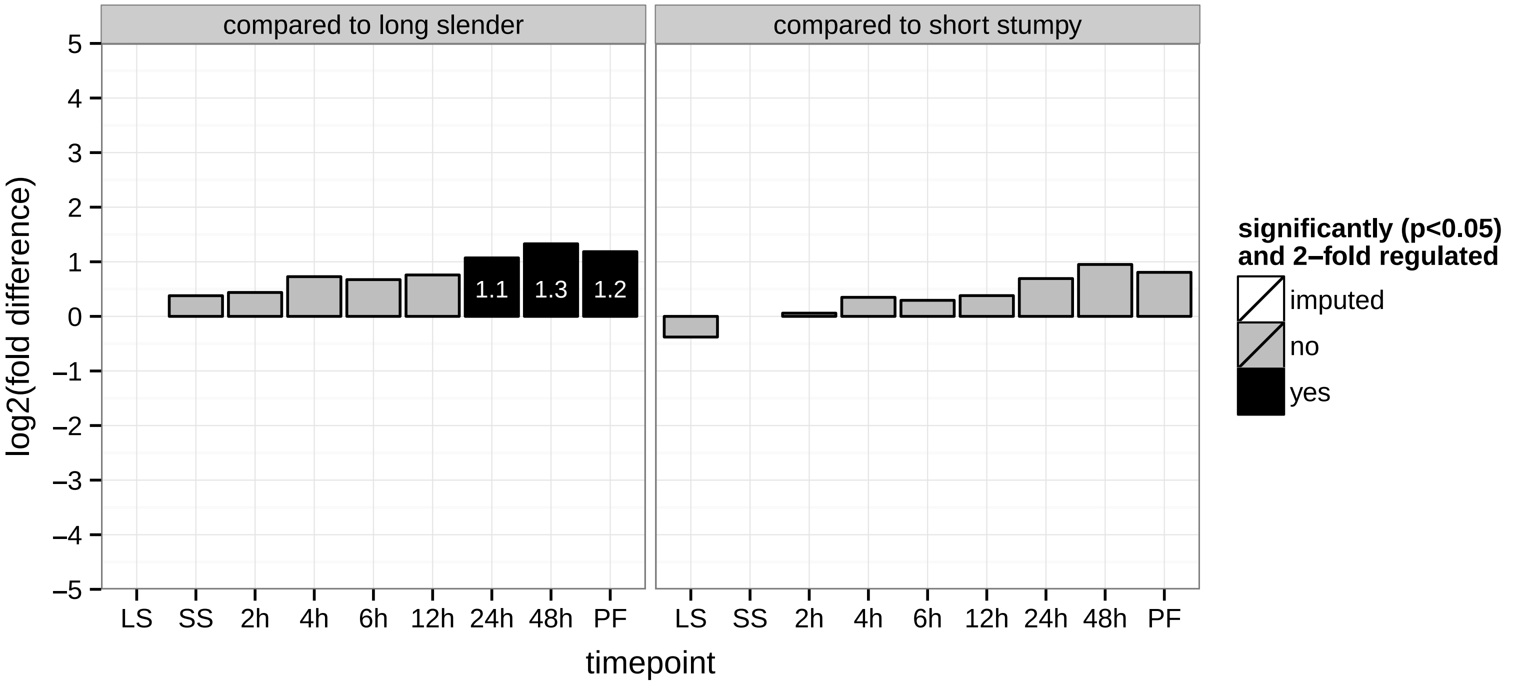

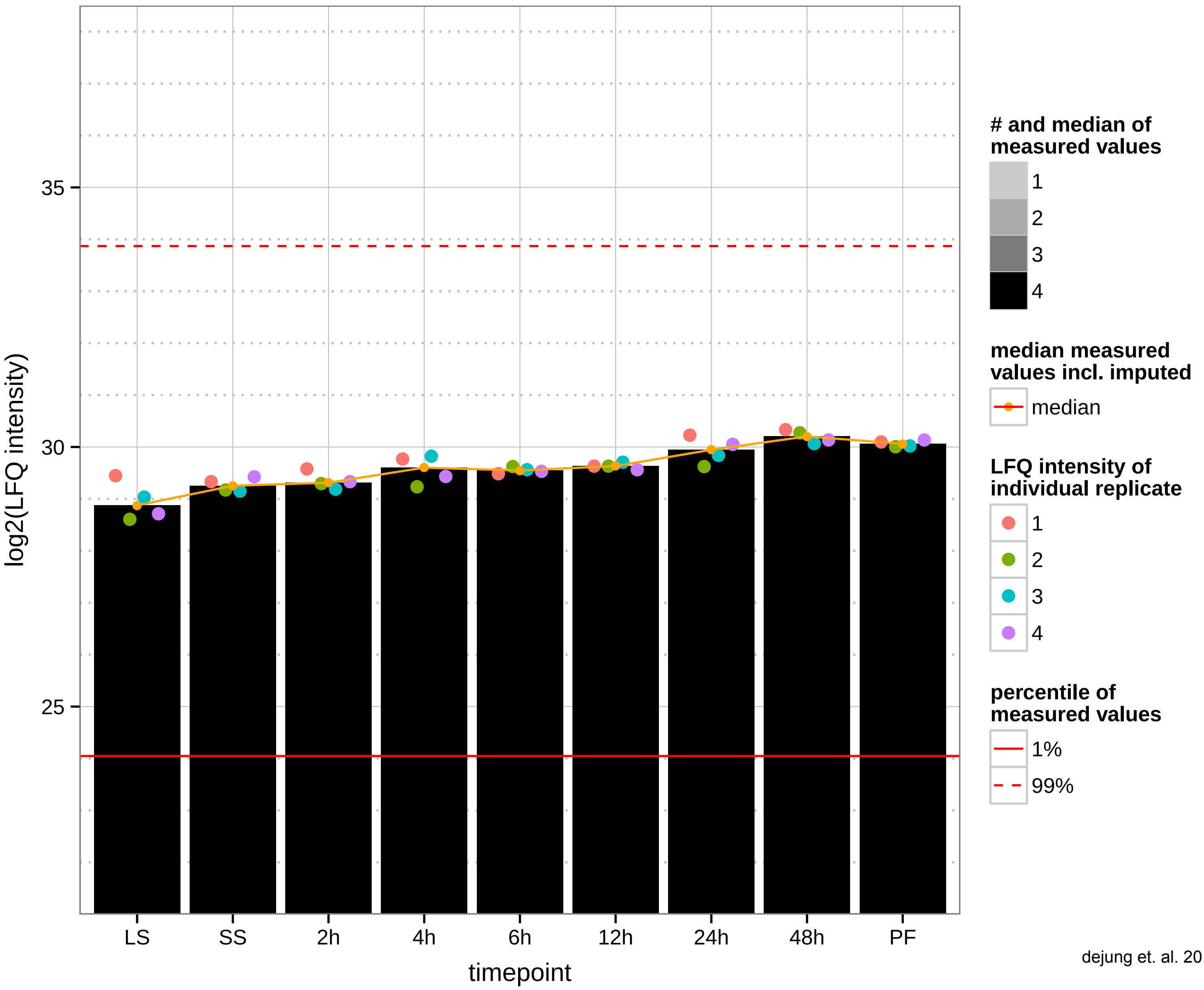

dejung et. al. 2015

hypothetical protein, conserved
Tb927.3.2700
AGOF: null
AGOC: null
AGOP: null
PGOF: null
PGOC: null
PGOP: null

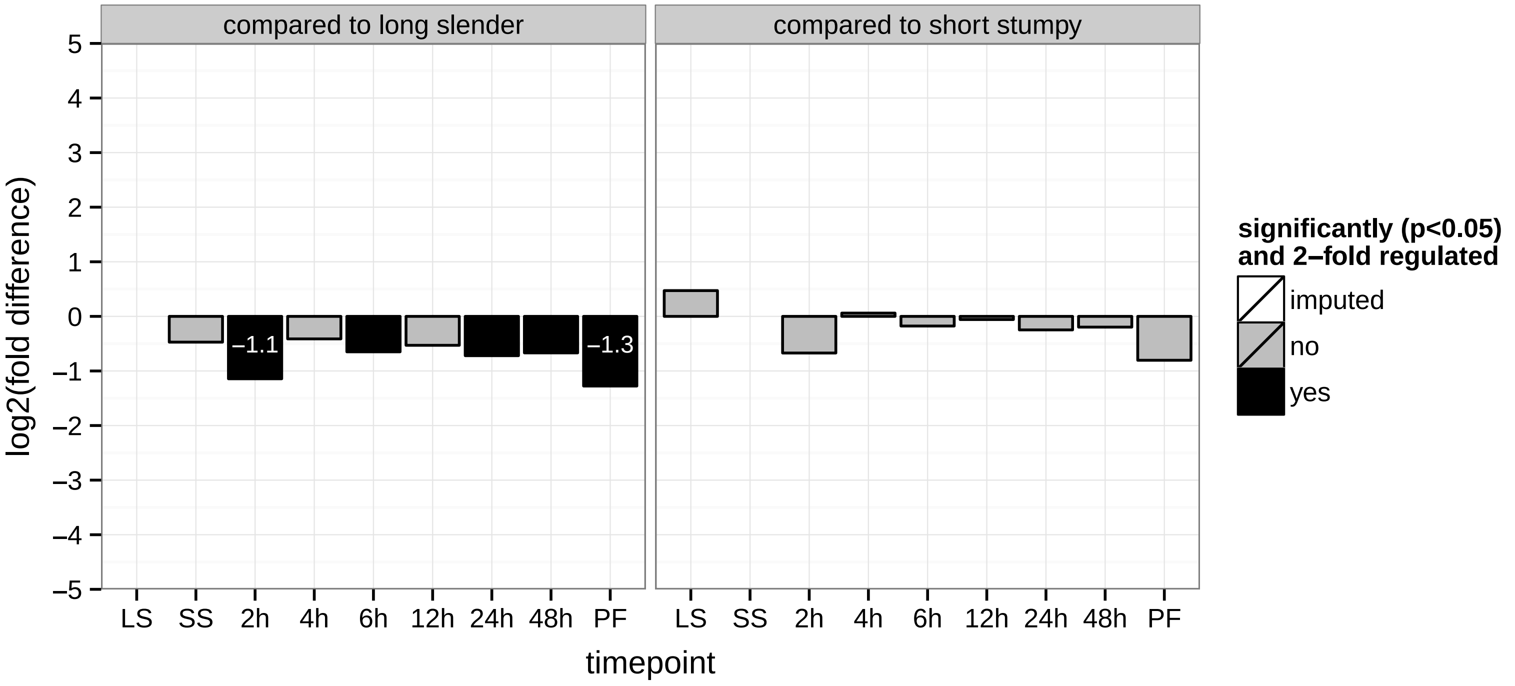

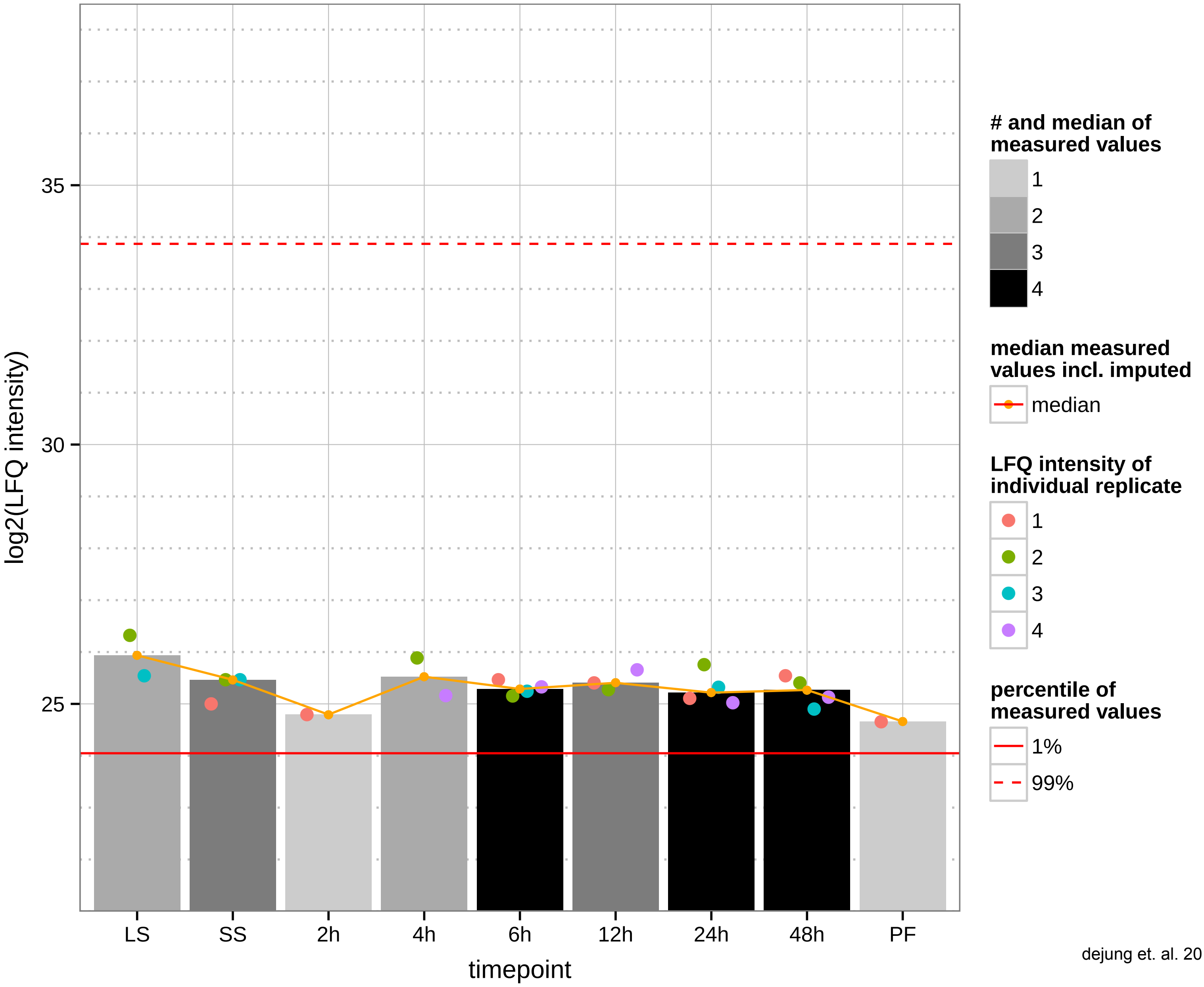

dejung et. al. 2015

hypothetical protein, conserved
Tb927.3.2810
AGOF: null
AGOC: null
AGOP: null
PGOF: null
PGOC: null
PGOP: null

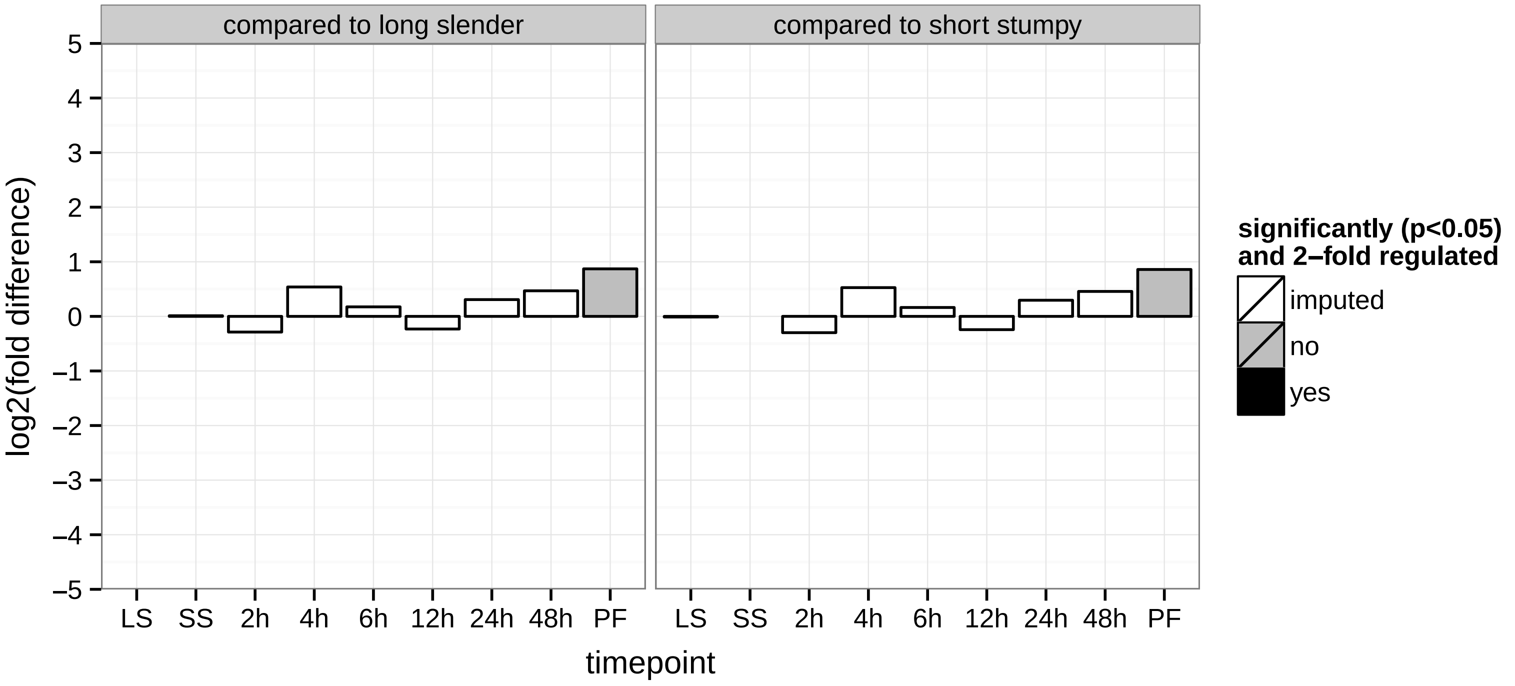

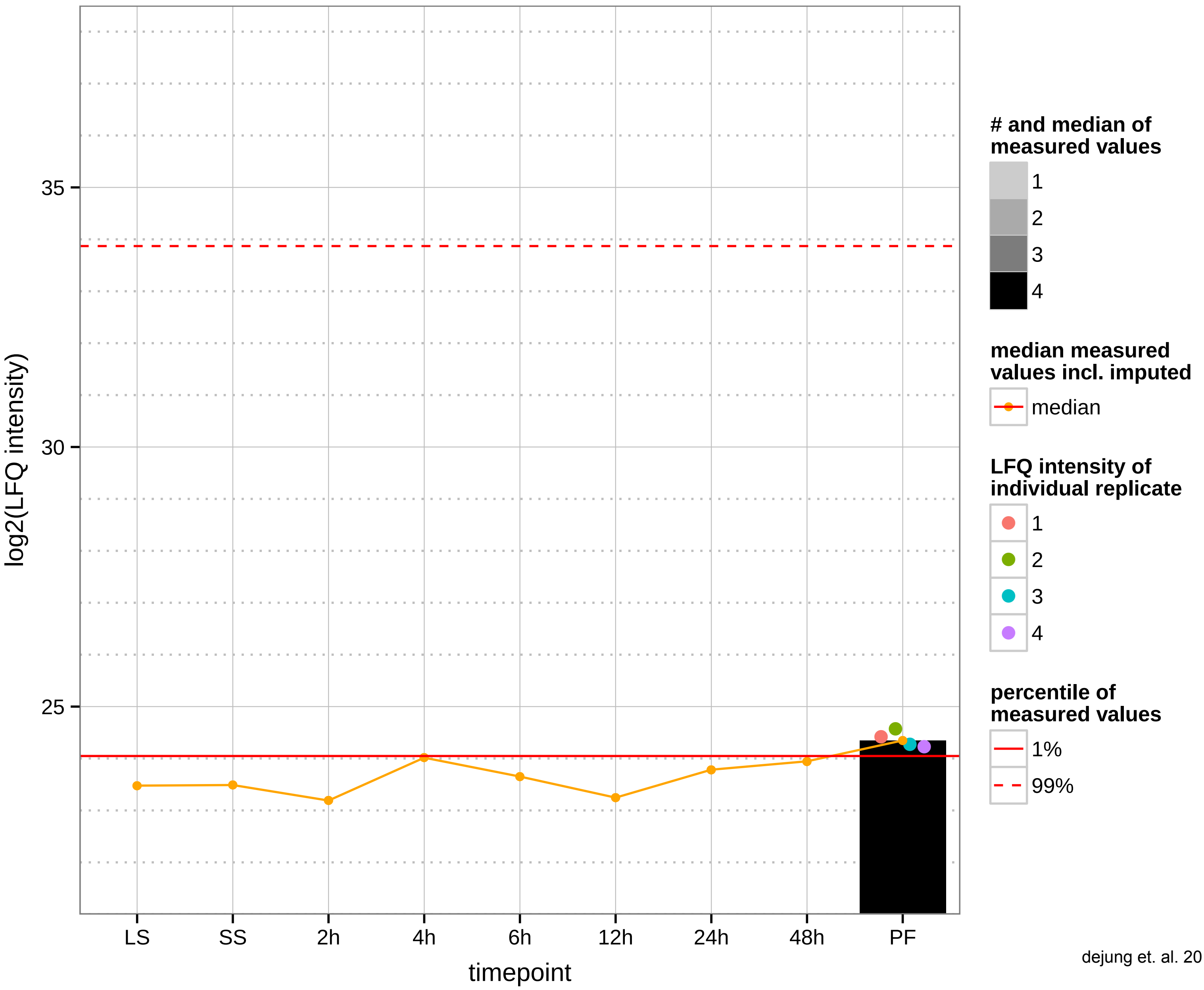

dejung et. al. 2015

hypothetical protein, conserved
Tb927.3.2830
AGOF: null
AGOC: null
AGOP: null
PGOF: null
PGOC: null
PGOP: null

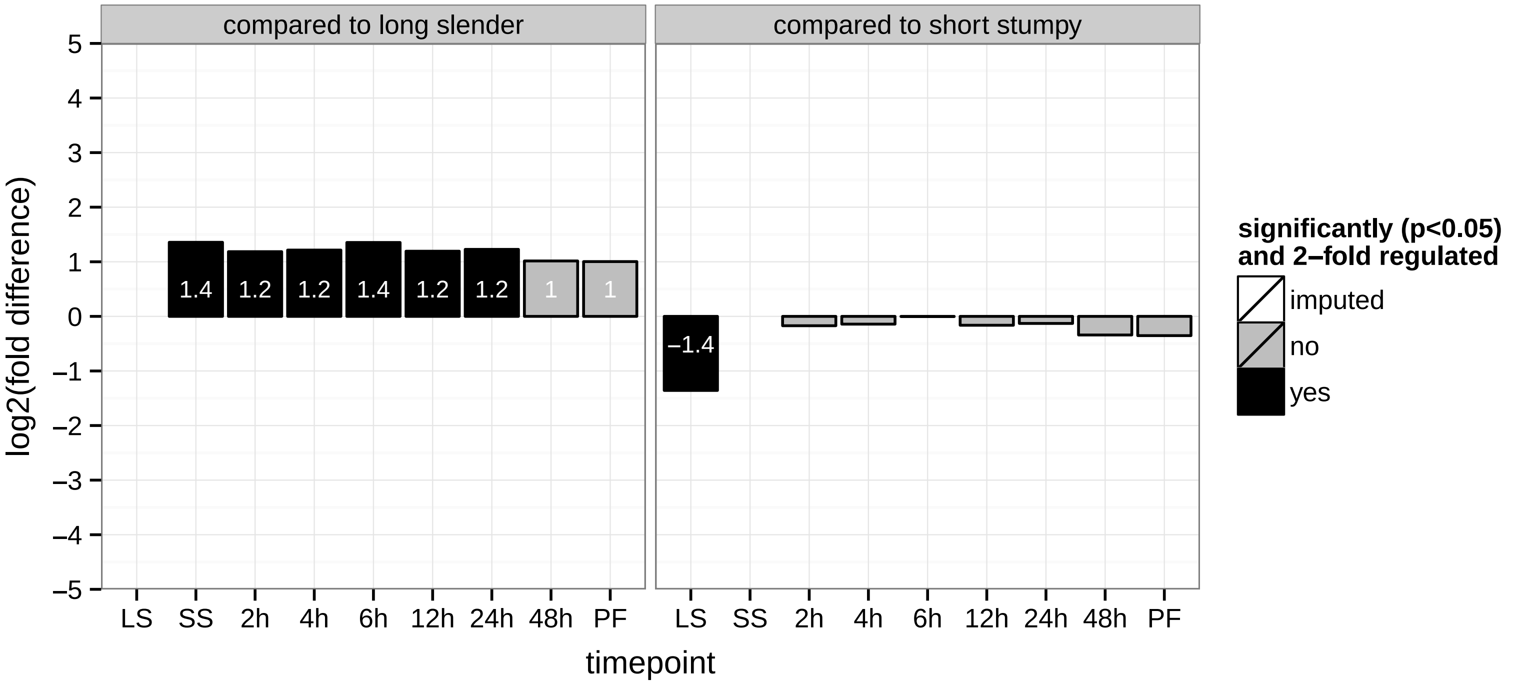

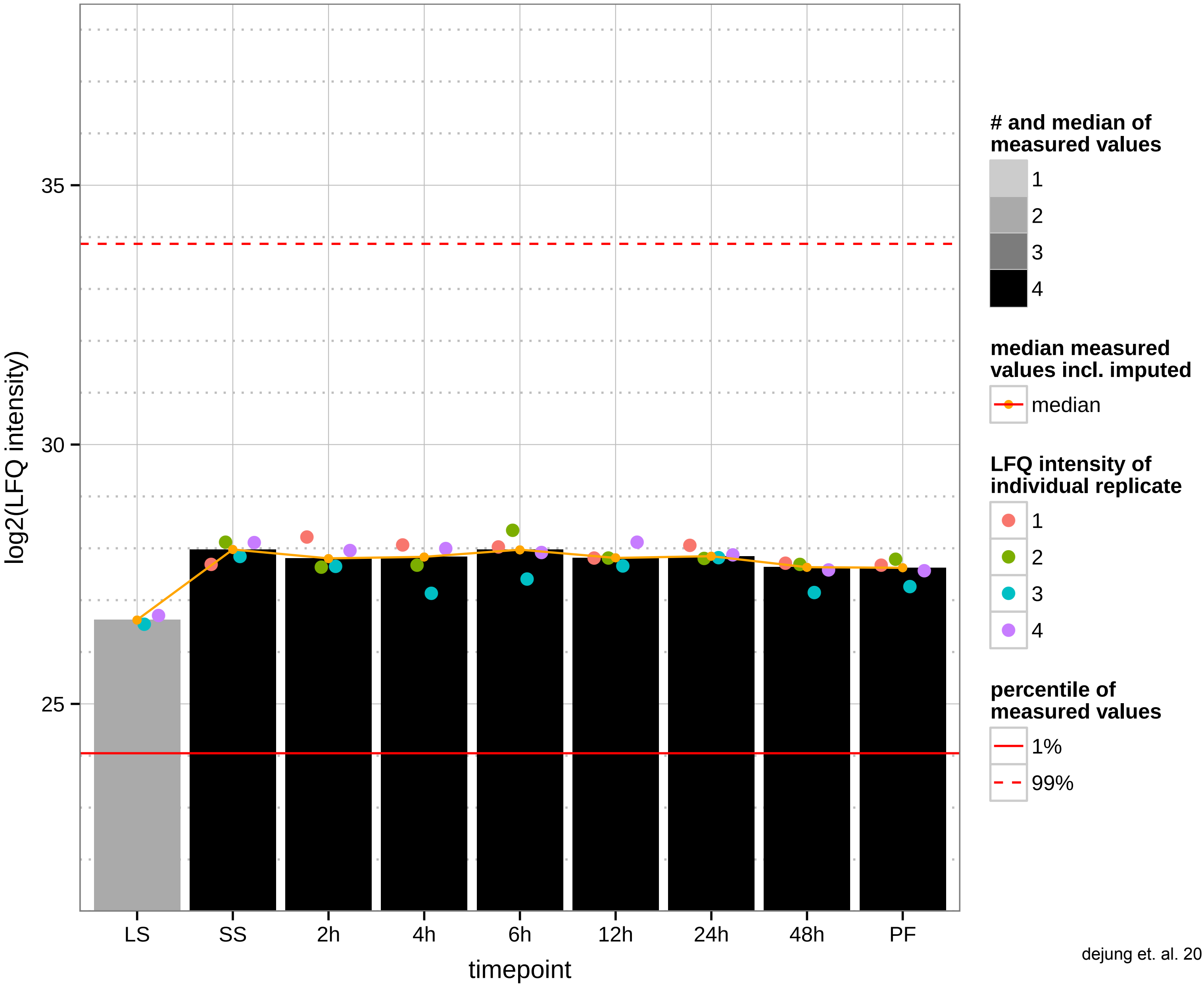

dejung et. al. 2015

inorganic pyrophosphatase, putative
Tb927.3.2840
AGOF: inorganic diphosphatase activity, magnesium ion binding
AGOC: cytoplasm
AGOP: phosphate–containing compound metabolic process
PGOF: inorganic diphosphatase activity, magnesium ion binding
PGOC: cytoplasm
PGOP: phosphate–containing compound metabolic process

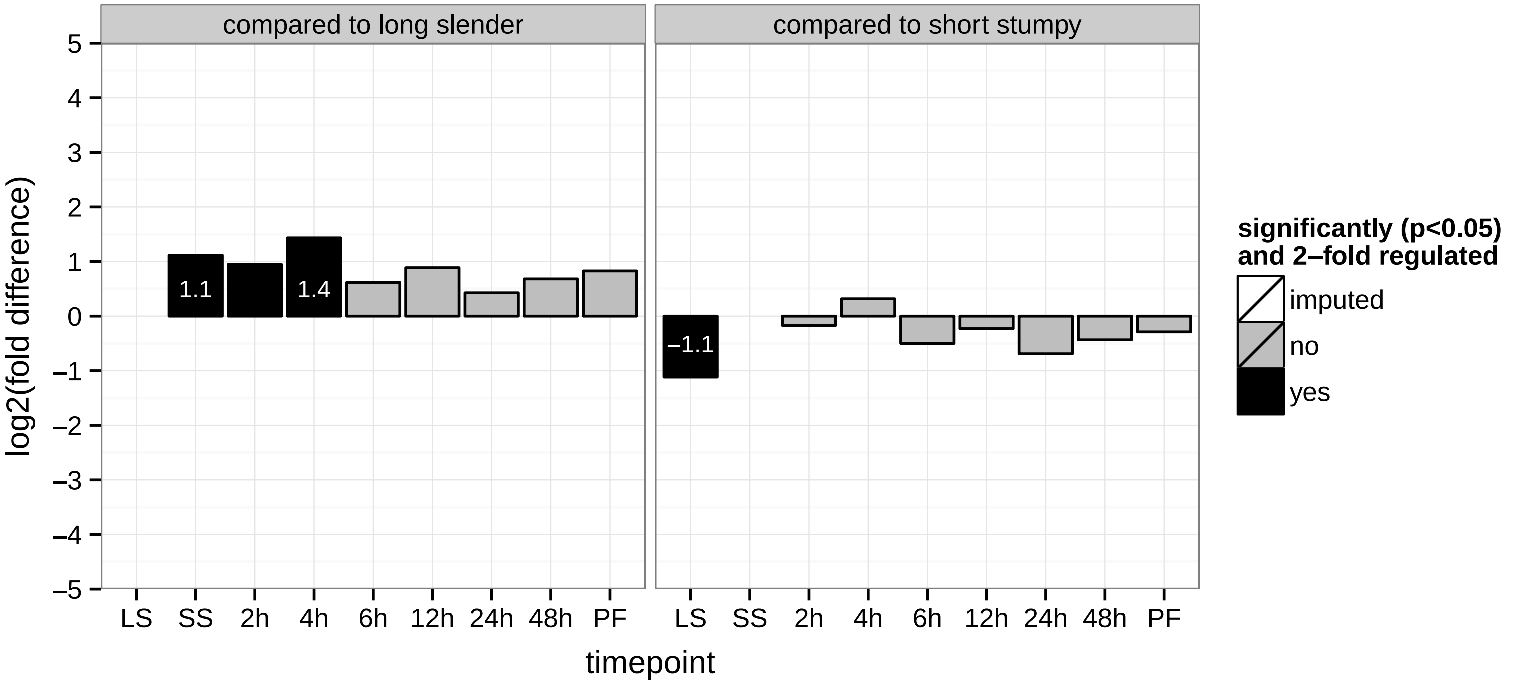

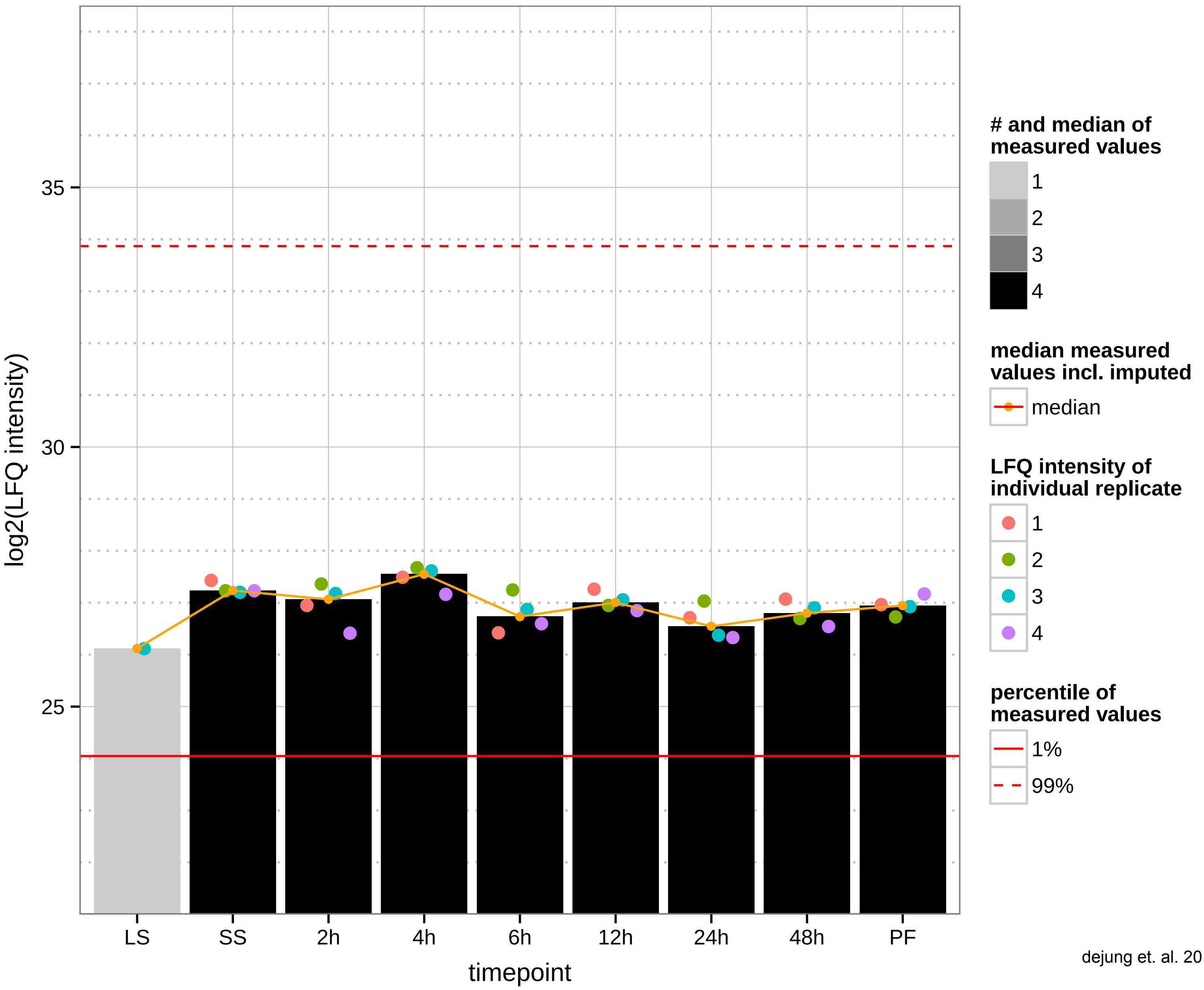

dejung et. al. 2015

radial spoke protein RSP10, putative
Tb927.3.2890
AGOF: null
AGOC: null
AGOP: null
PGOF: null
PGOC: null
PGOP: null

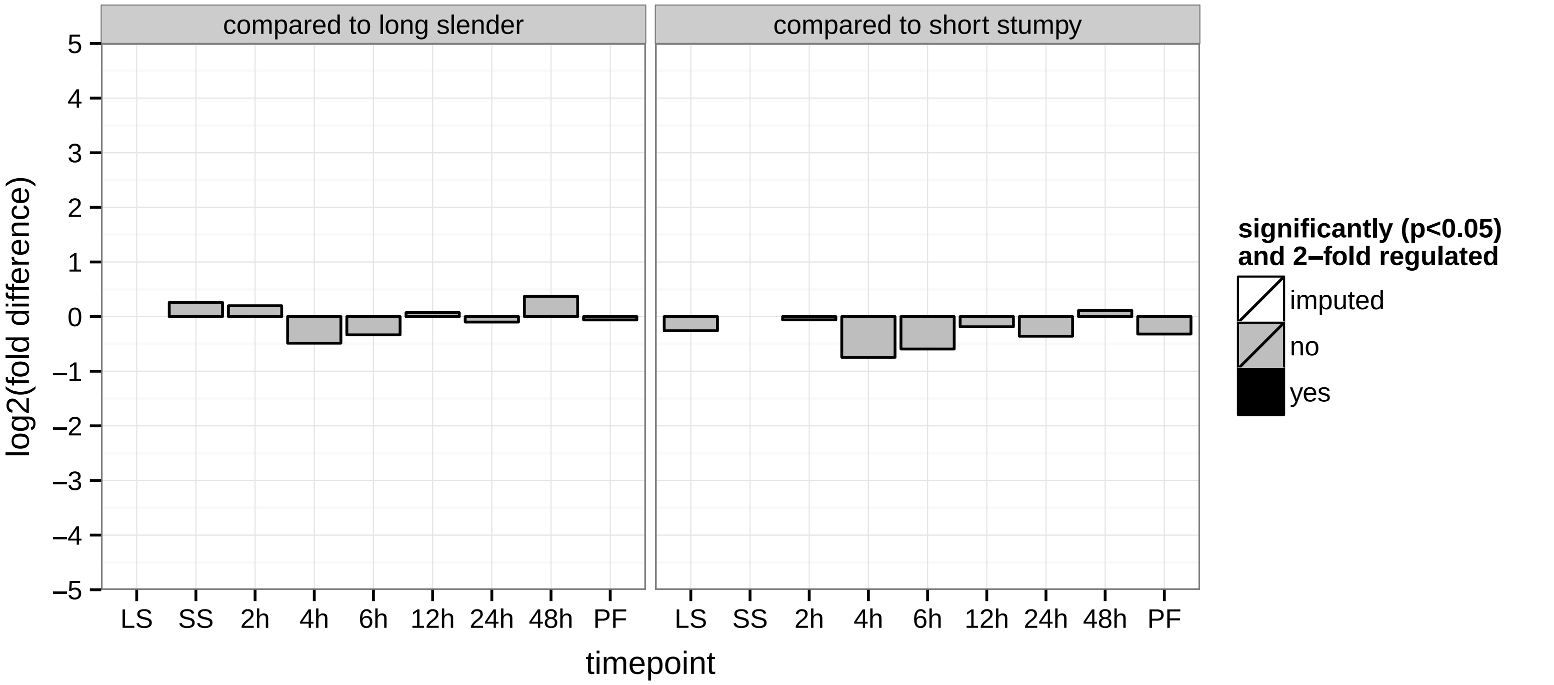

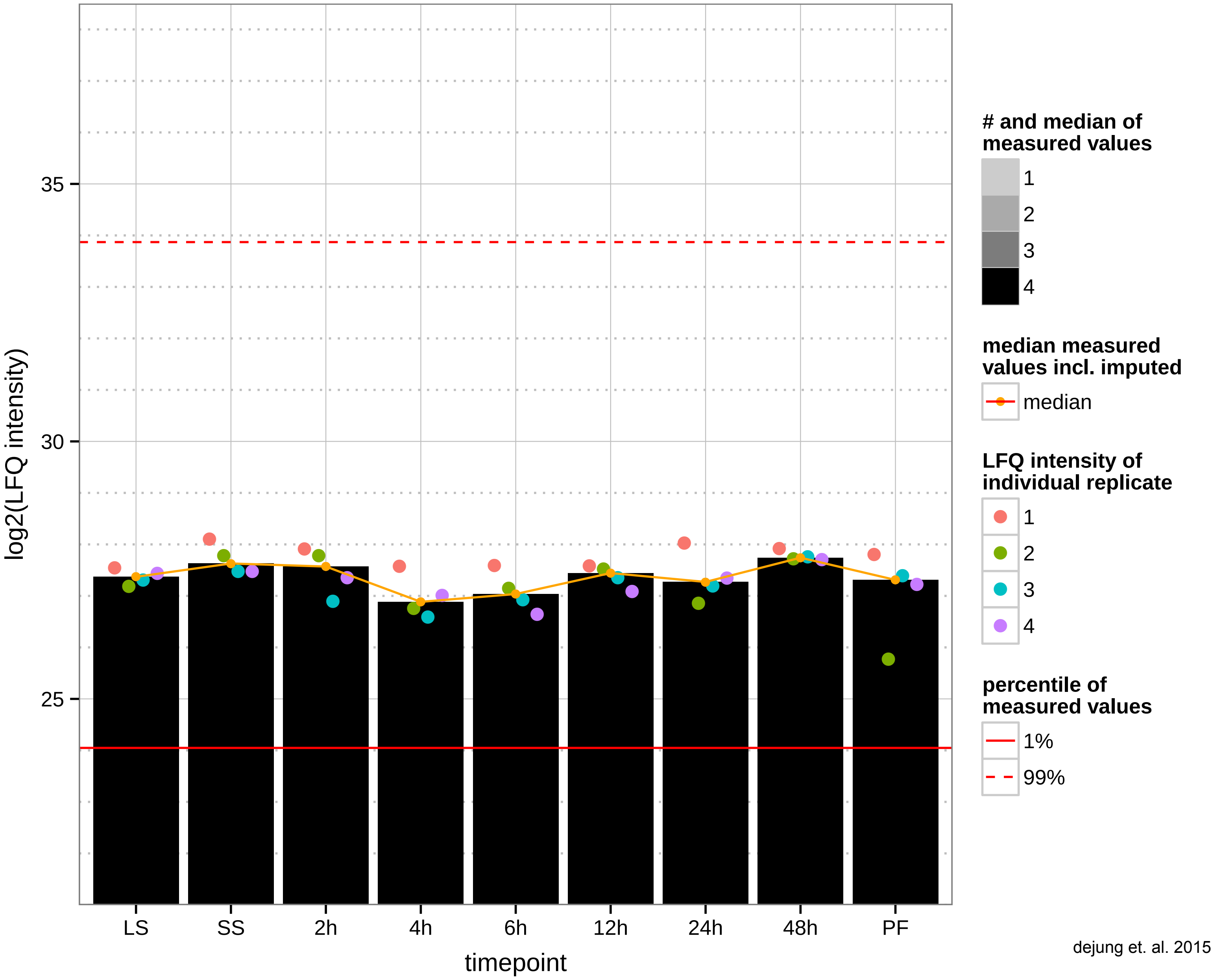

dejung et. al. 2015

ribonuclease inhibitor– like protein, leucine–rich repeat protein (LRRP), putative
Tb927.3.2950
AGOF: null
AGOC: null
AGOP: null
PGOF: null
PGOC: null
PGOP: null

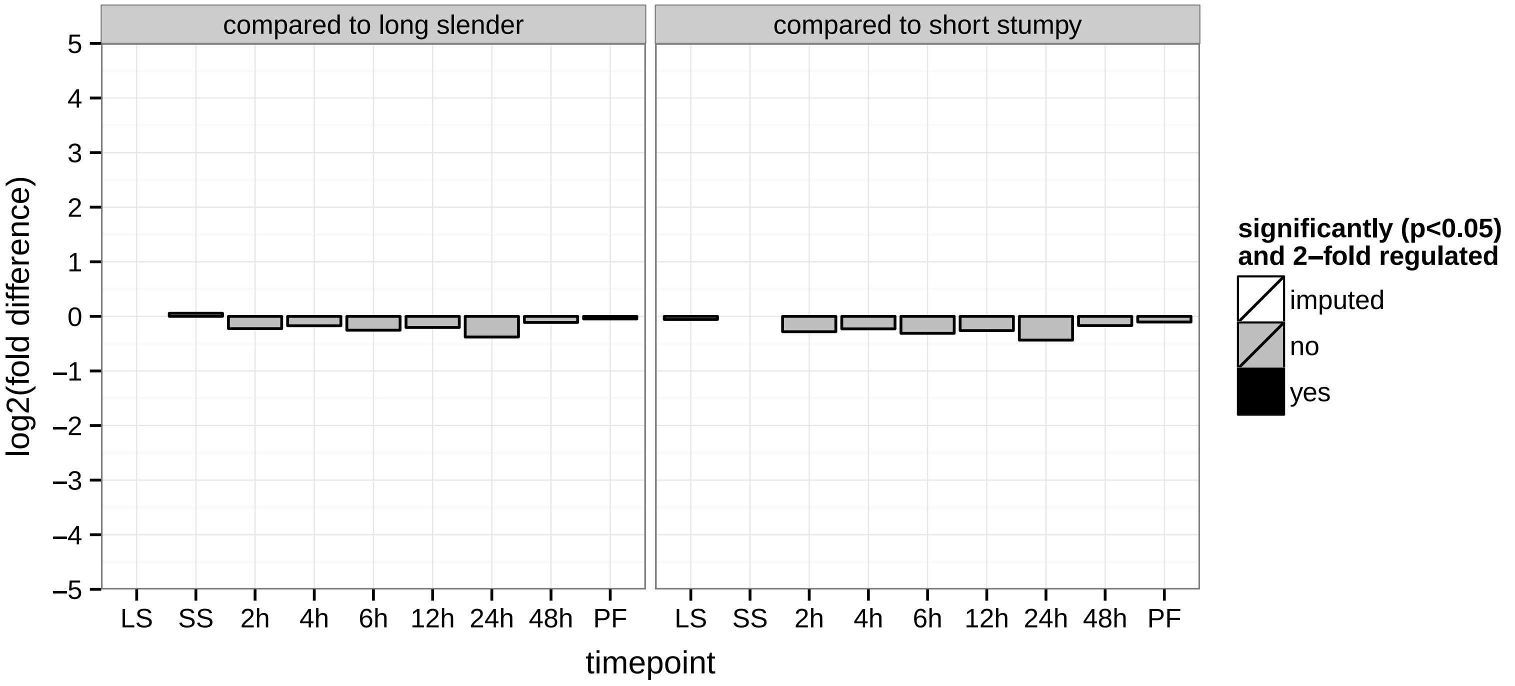

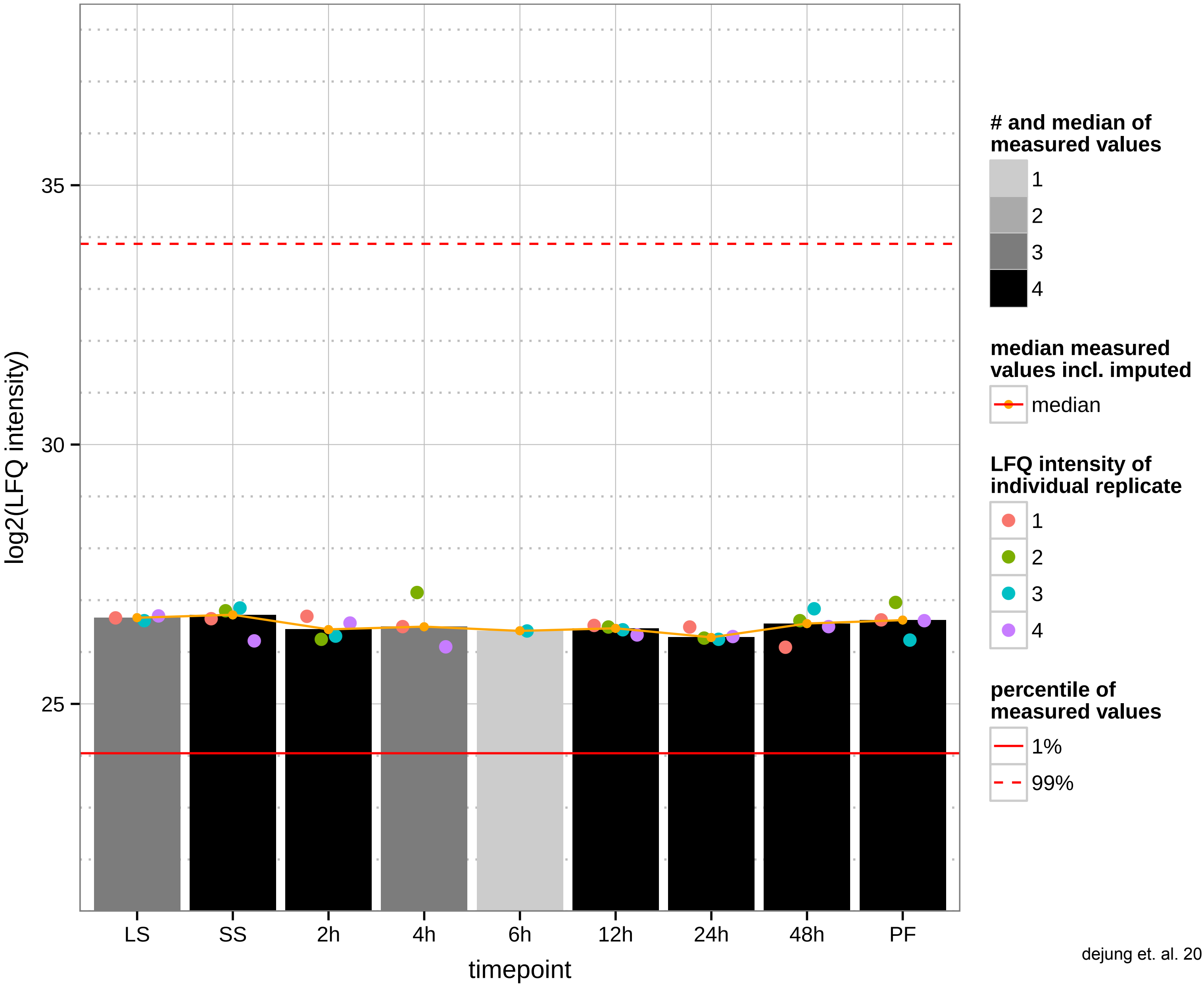

dejung et. al. 2015

inosine–adenosine–guanosine–nucleosidehydrolase, IAG–nucleoside hydrolase (IAGNH)
Tb927.3.2960
AGOF: hydrolase activity, acting on carbon–nitrogen (but not peptide) bonds
AGOC: cytosol
AGOP: purine ribonucleoside salvage
PGOF: null
PGOC: null
PGOP: null

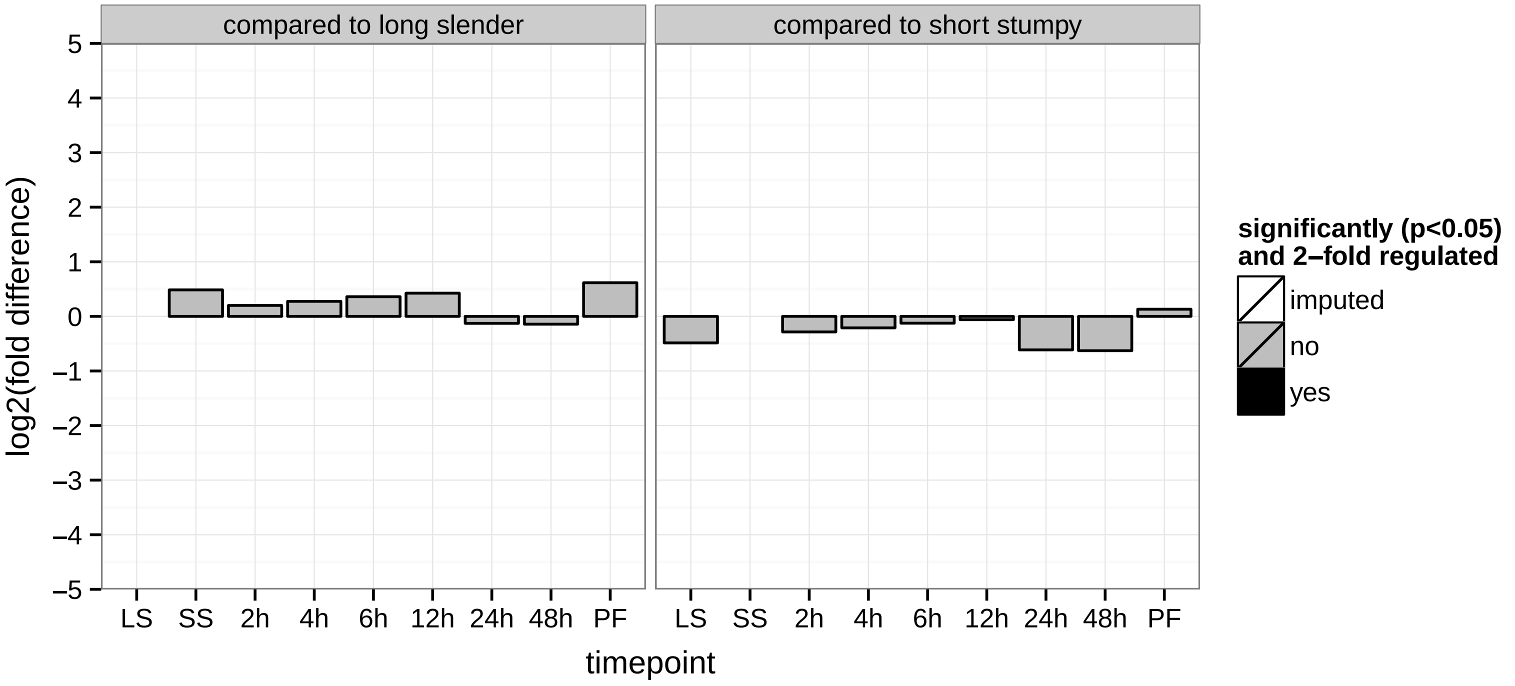

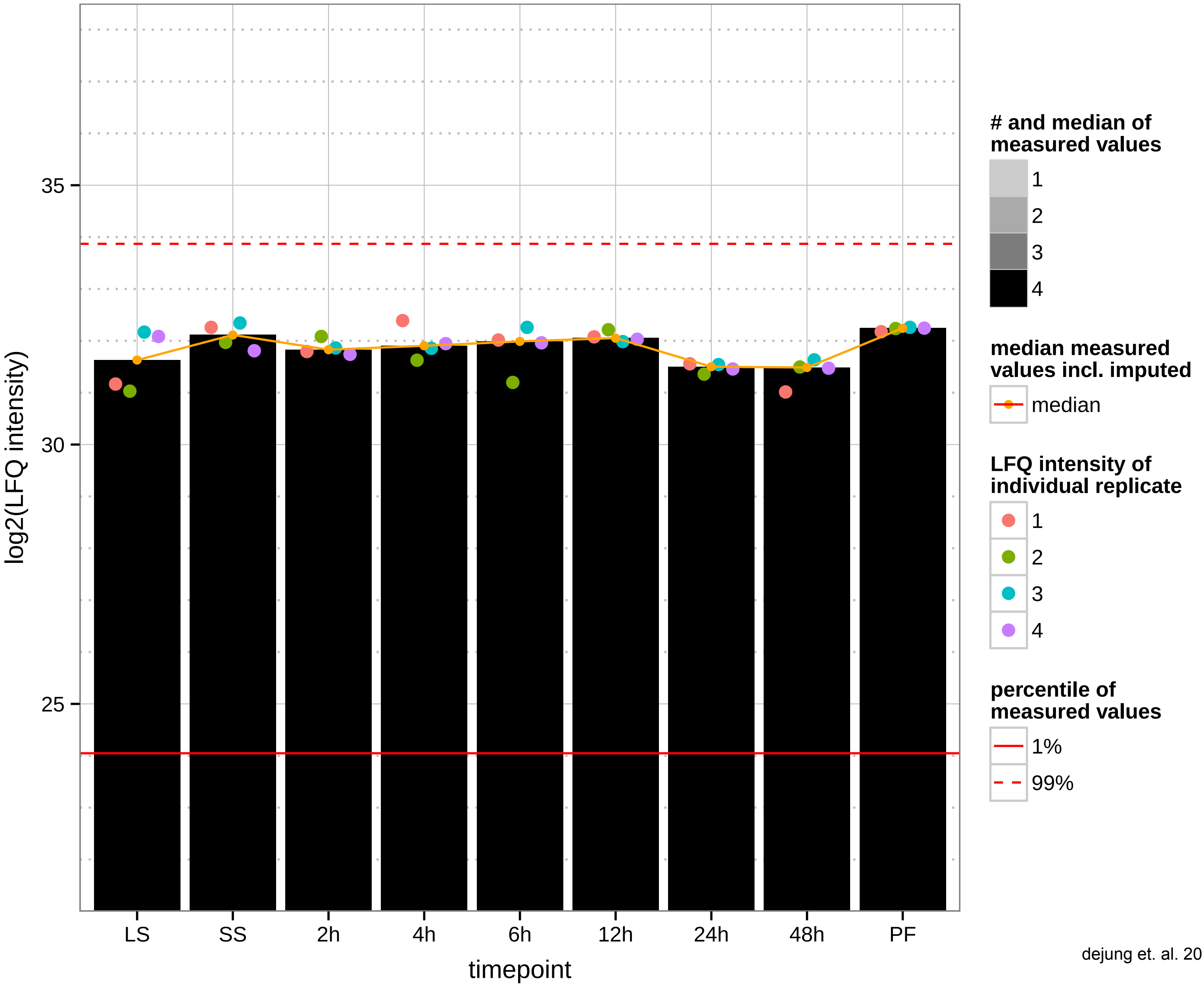

dejung et. al. 2015

mitochondrial carrier protein (MCP17)
Tb927.3.2980
AGOF: null
AGOC: mitochondrial inner membrane, mitochondrial membrane, mitochondrial part
AGOP: transport
PGOF: null
PGOC: null
PGOP: null

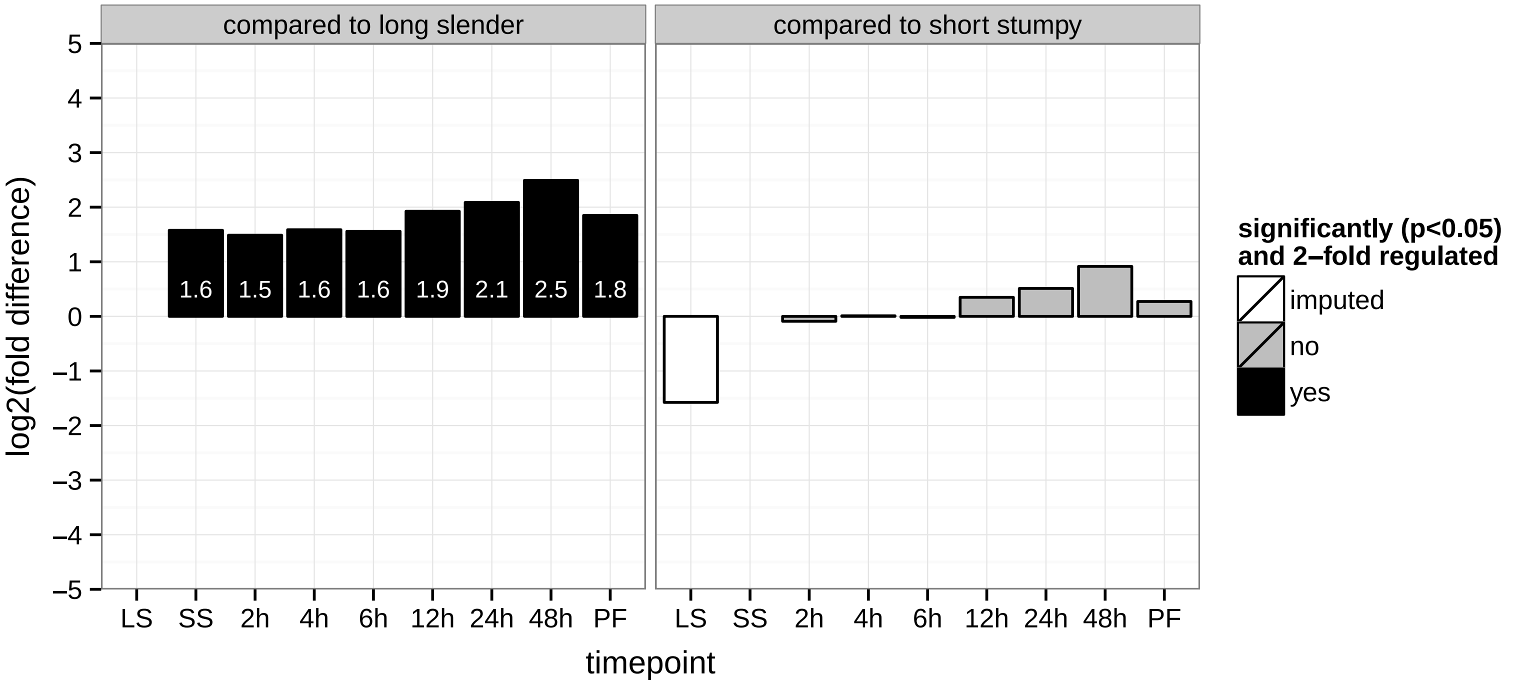

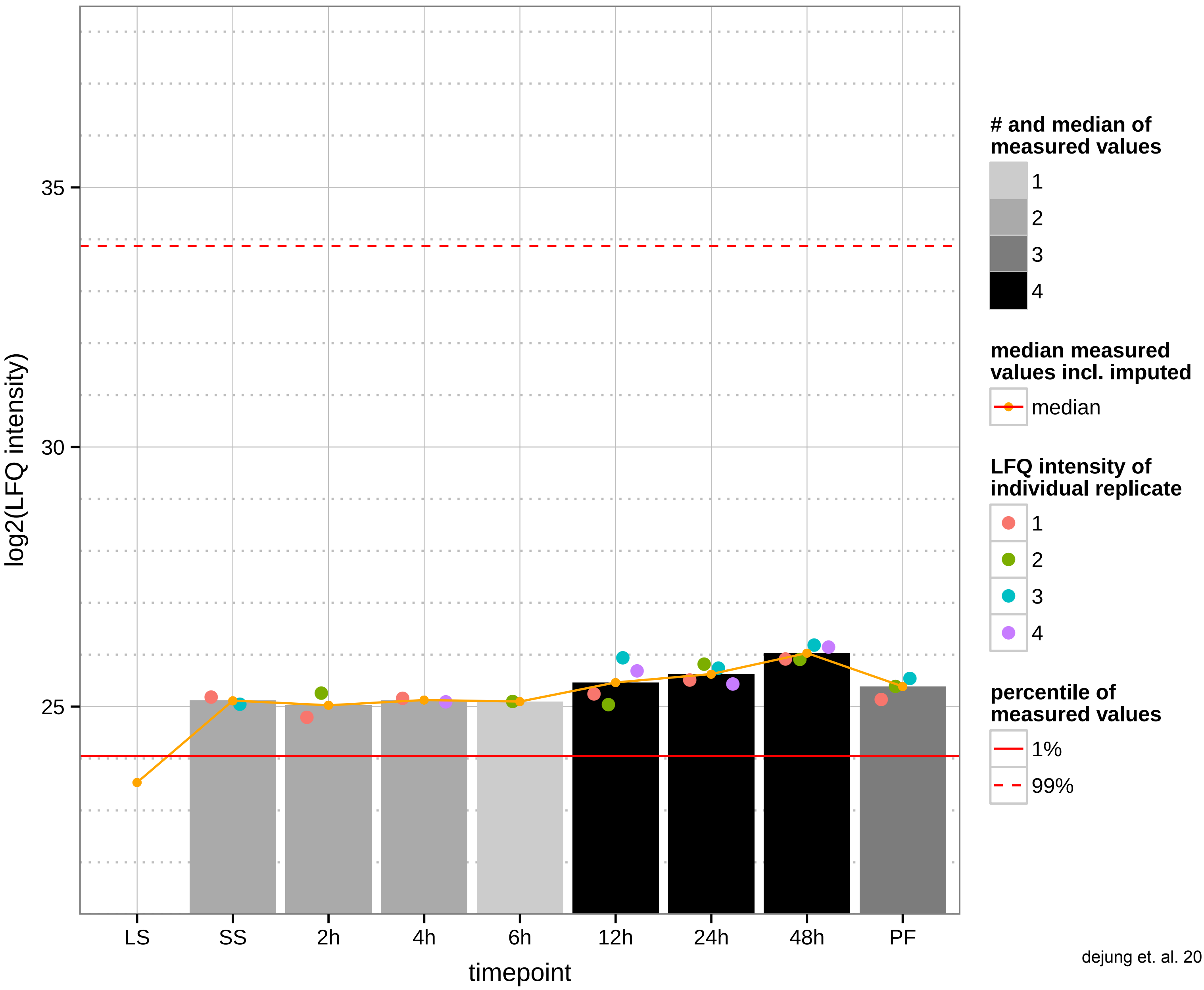

dejung et. al. 2015

hypothetical protein, conserved
Tb927.3.3040
AGOF: null
AGOC: null
AGOP: null
PGOF: null
PGOC: null
PGOP: null

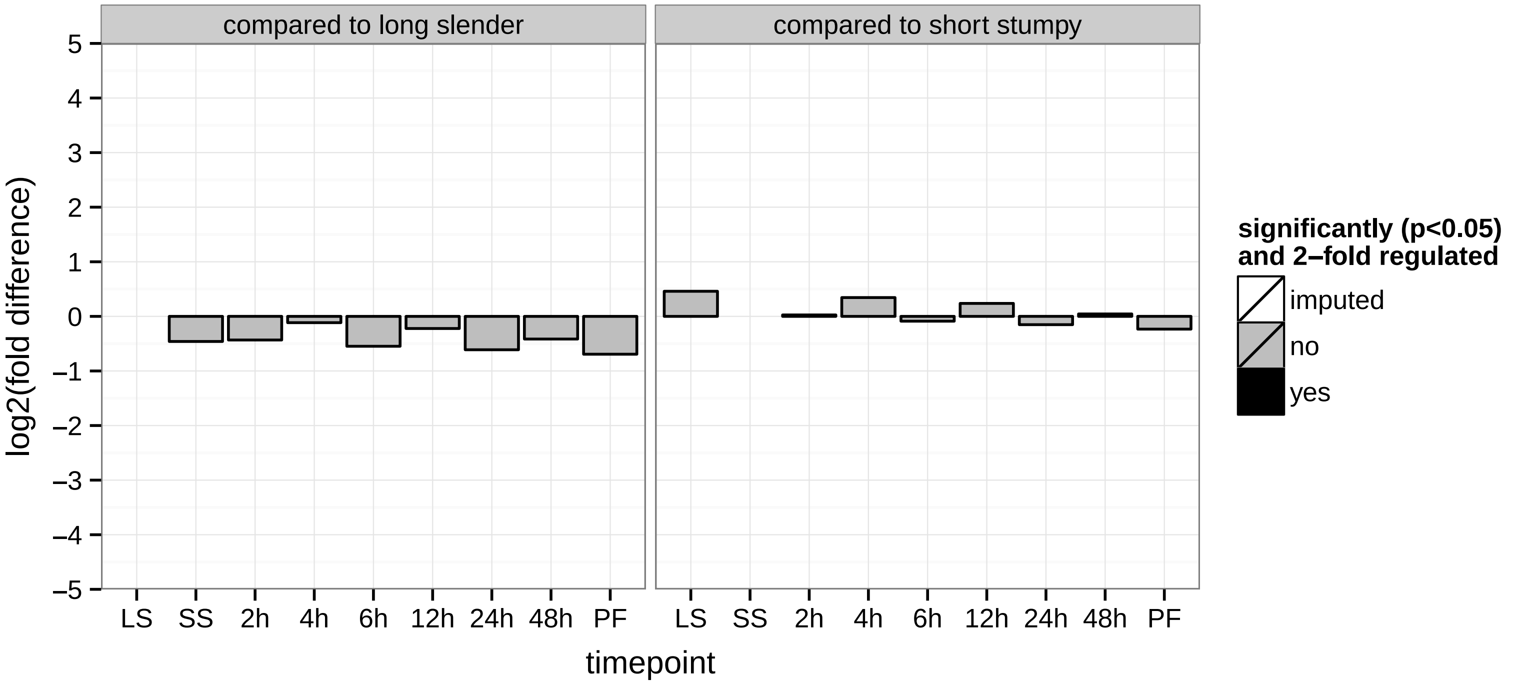

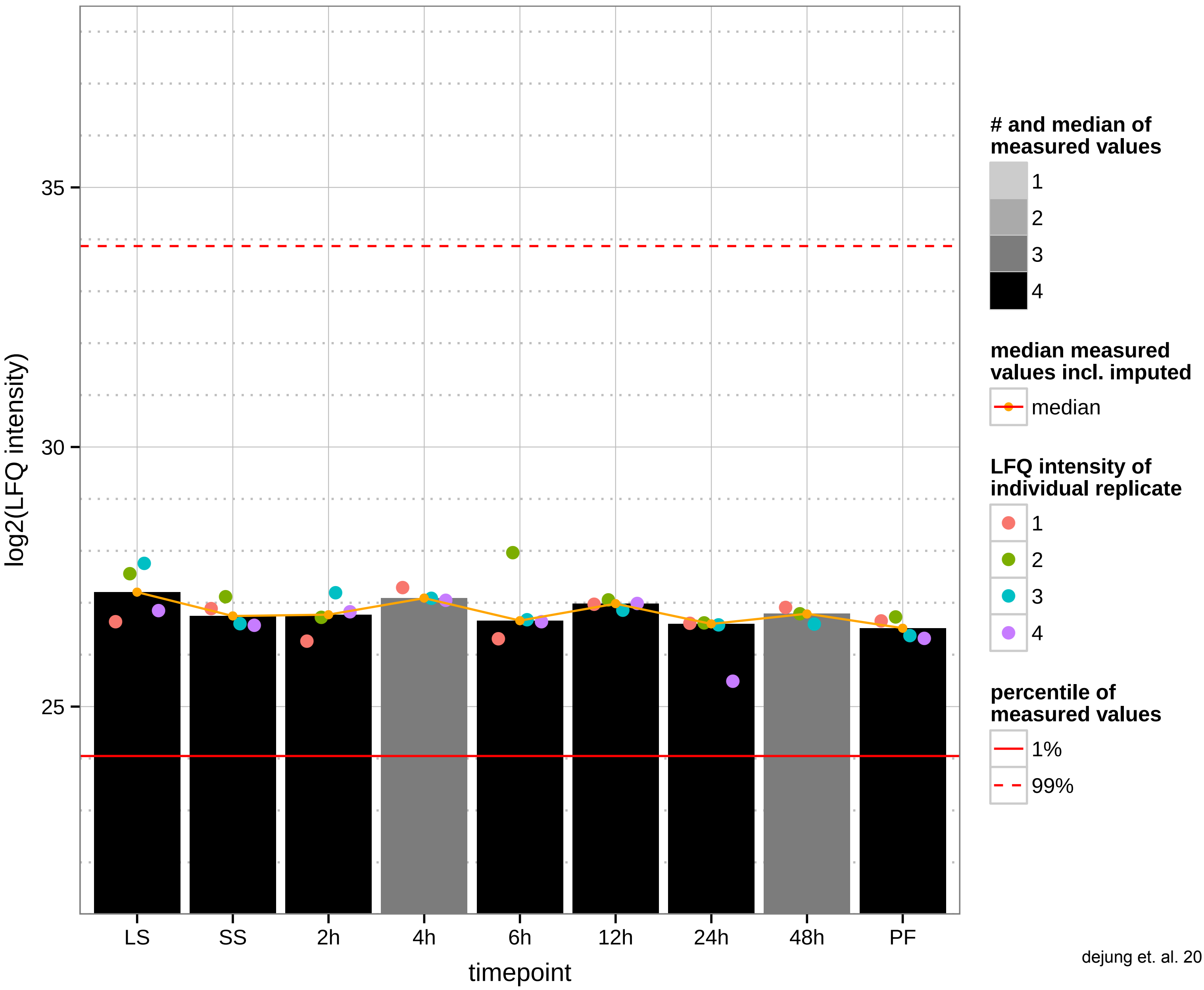

dejung et. al. 2015

hypothetical protein, conserved
Tb927.3.3120
AGOF: null
AGOC: null
AGOP: null
PGOF: binding
PGOC: null
PGOP: null

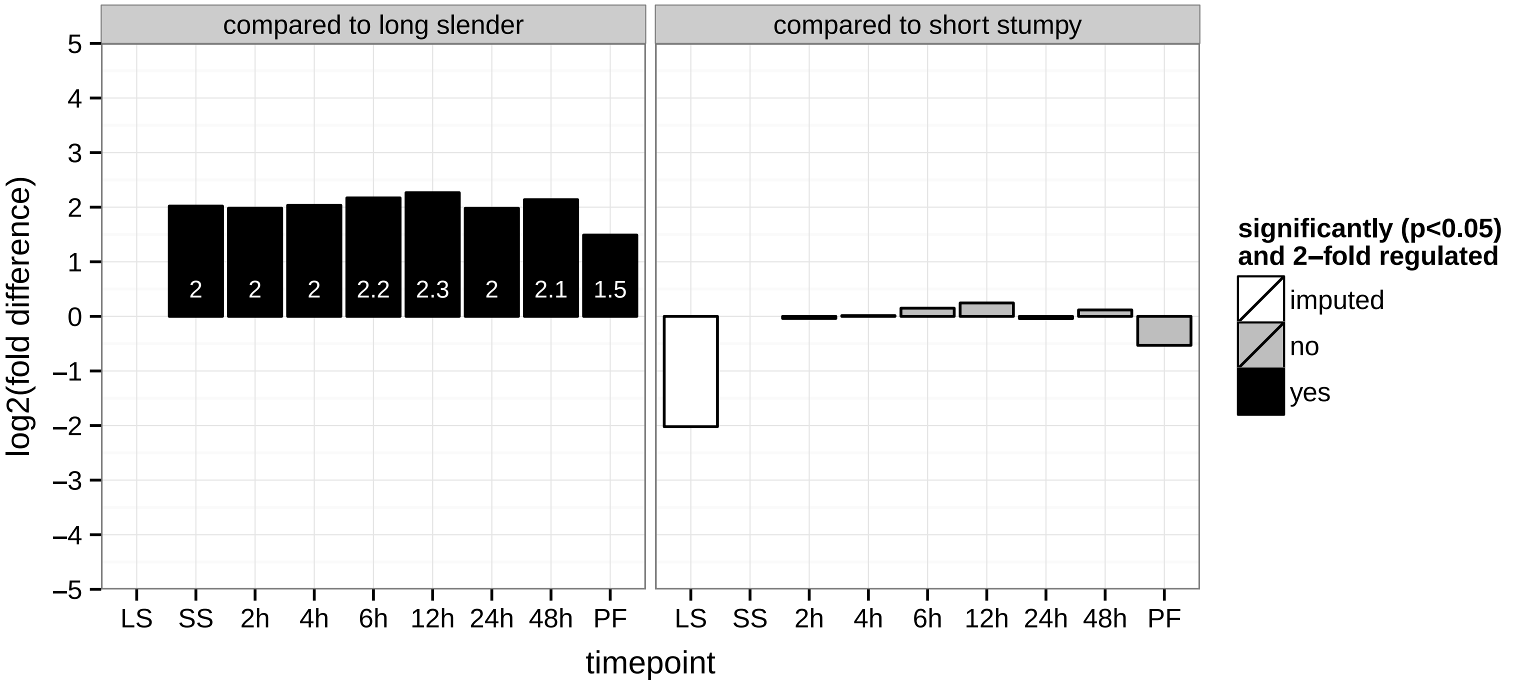

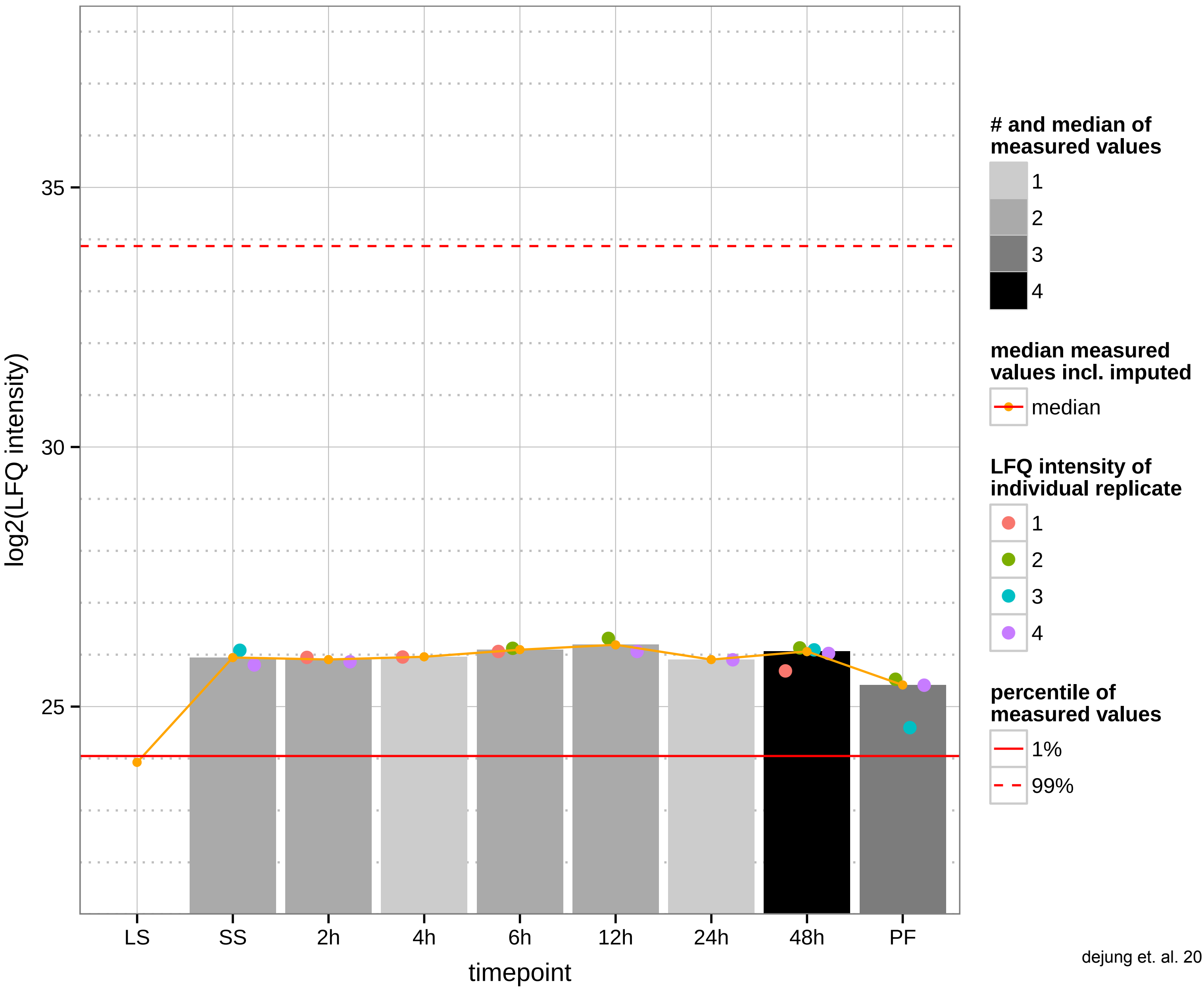

dejung et. al. 2015

poly(A) polymerase, polynucleotide adenylyltransferase (PAP)
Tb927.3.3160
AGOF: RNA binding, nucleic acid binding, nucleotidyltransferase activity, polynucleotide adenylyltransferase activity
AGOC: null
AGOP: RNA 3'–end processing, transcription, DNA–dependent
PGOF: RNA binding, nucleotidyltransferase activity, polynucleotide adenylyltransferase activity
PGOC: nucleus
PGOP: RNA 3'–end processing, RNA polyadenylation, transcription, DNA–dependent

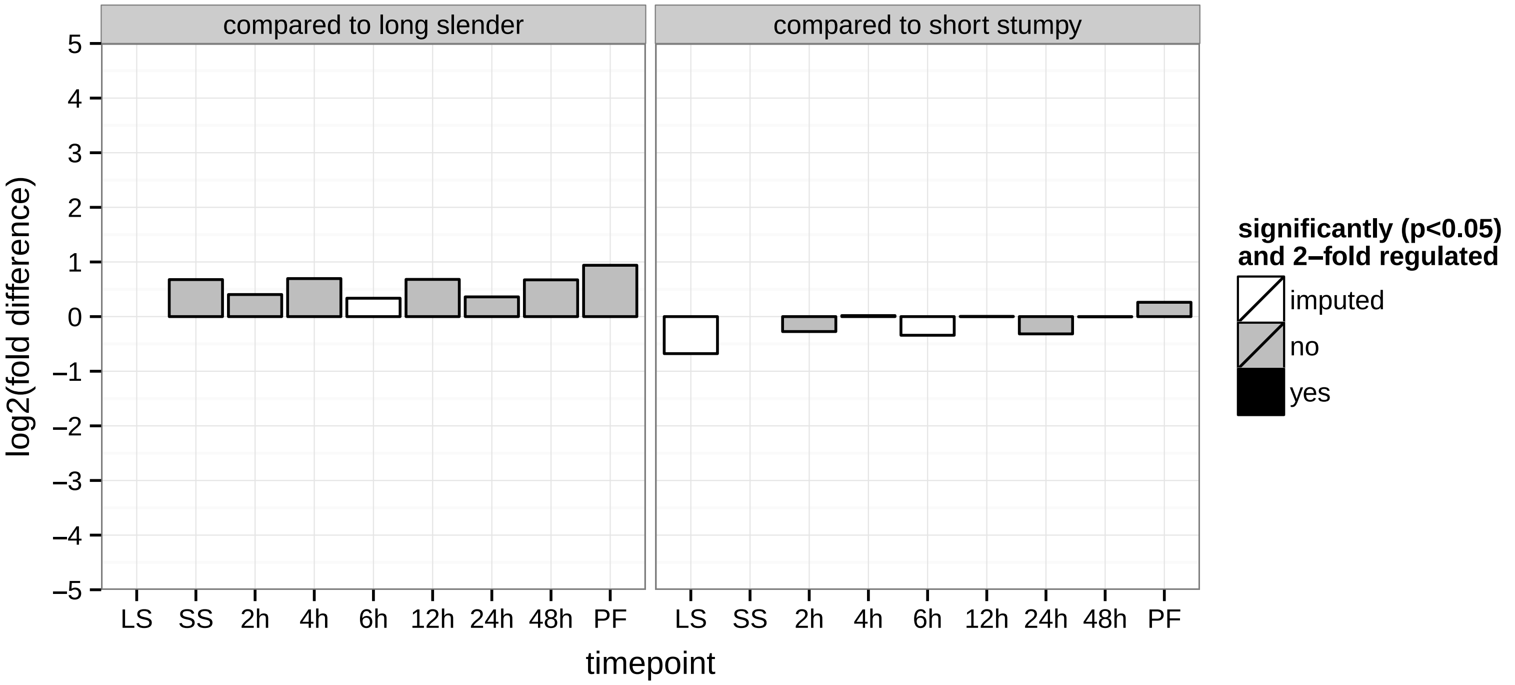

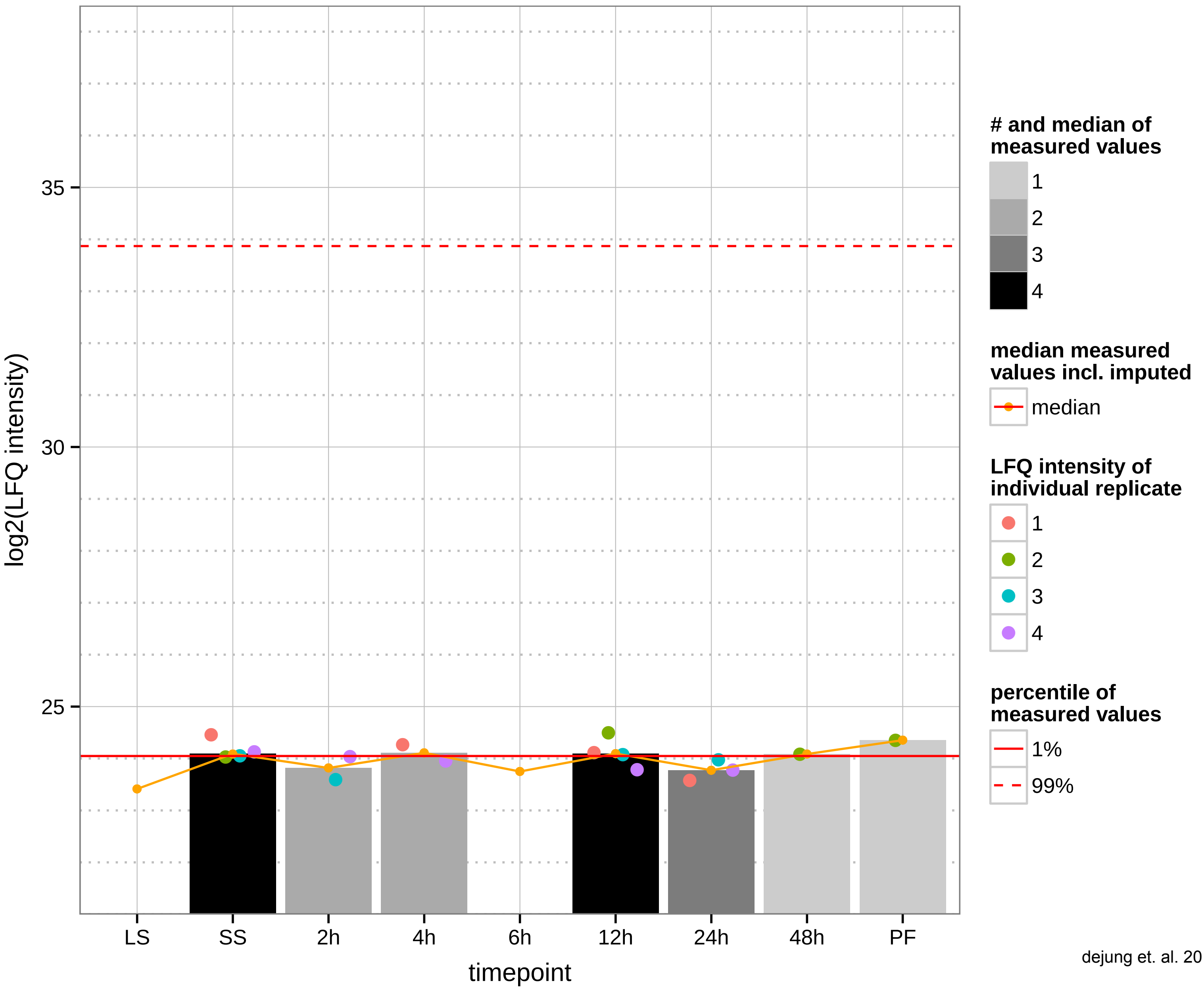

dejung et. al. 2015

hypothetical protein, conserved, Nucleoporin (TbNup98)
Tb927.3.3180;Tb11.v5.0584
AGOF: null
AGOC: null, nuclear pore
AGOP: null
PGOF: null
PGOC: null
PGOP: null

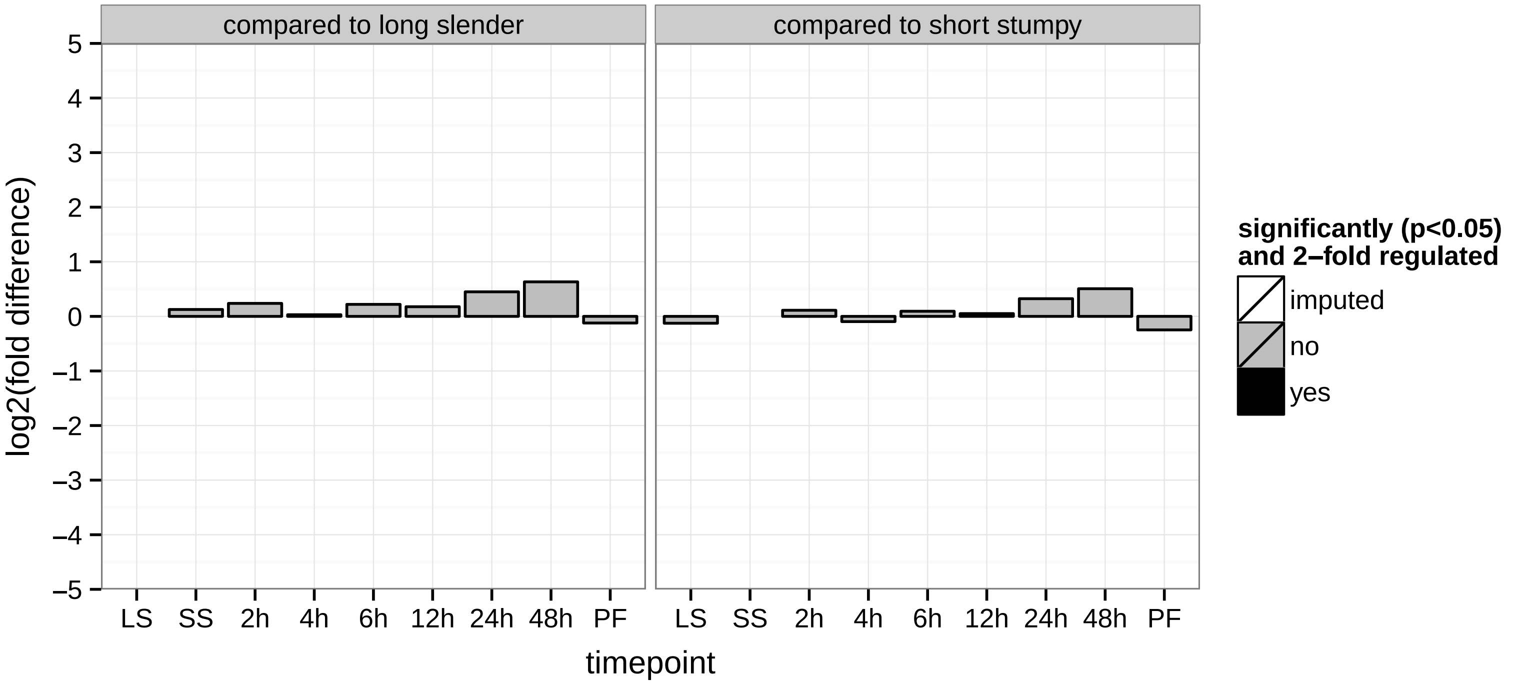

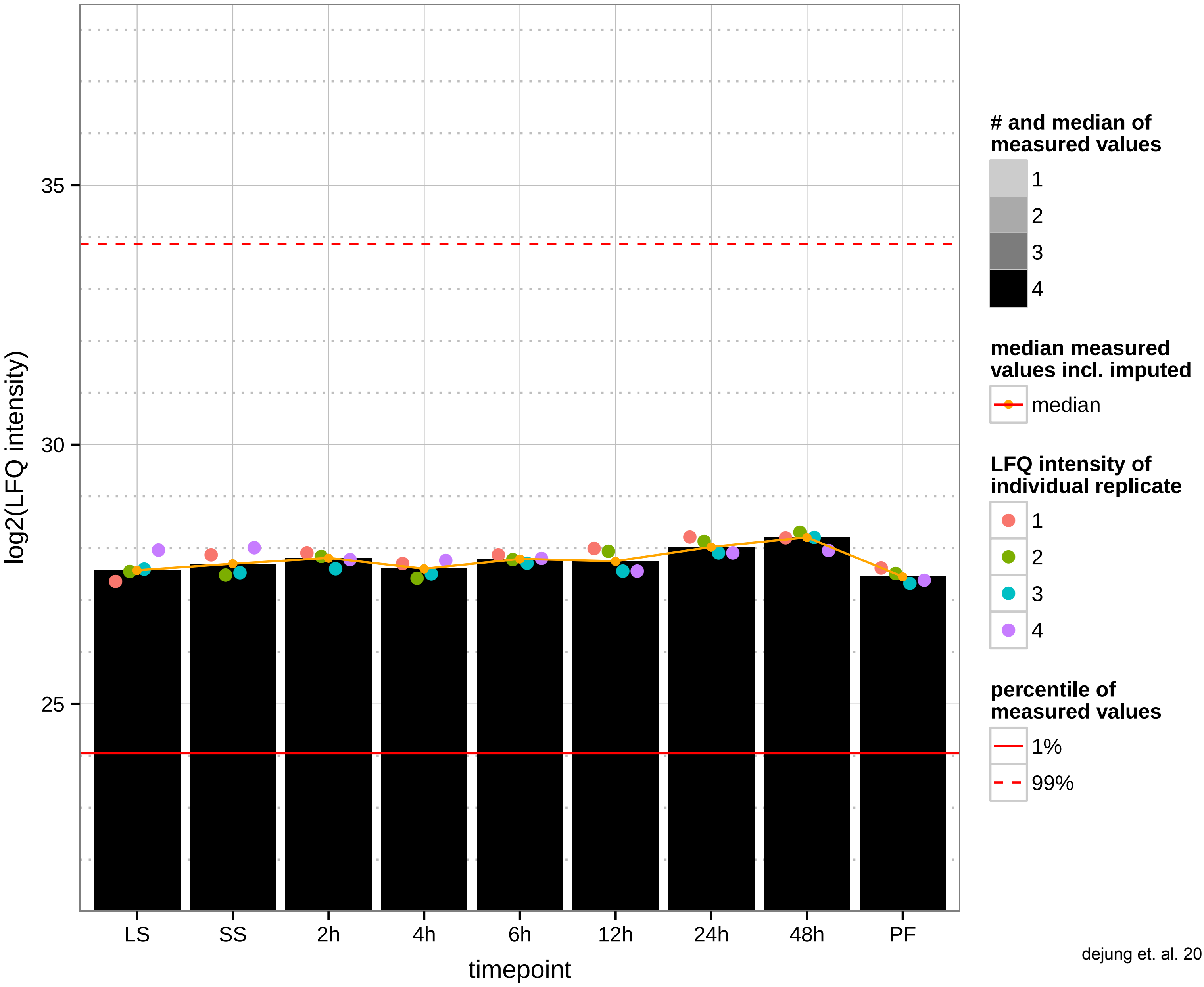

dejung et. al. 2015

hypothetical protein, conserved
Tb927.3.3200
AGOF: null
AGOC: null
AGOP: null
PGOF: null
PGOC: null
PGOP: null

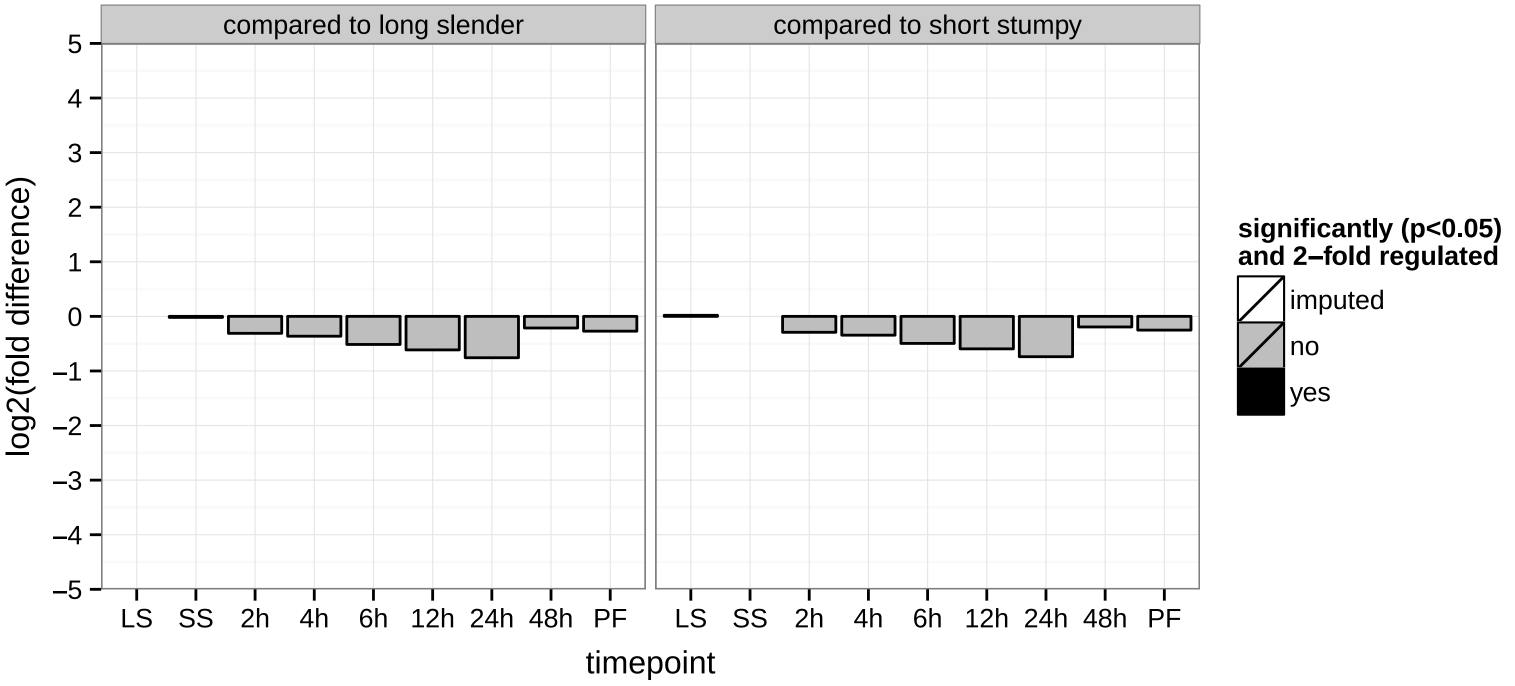

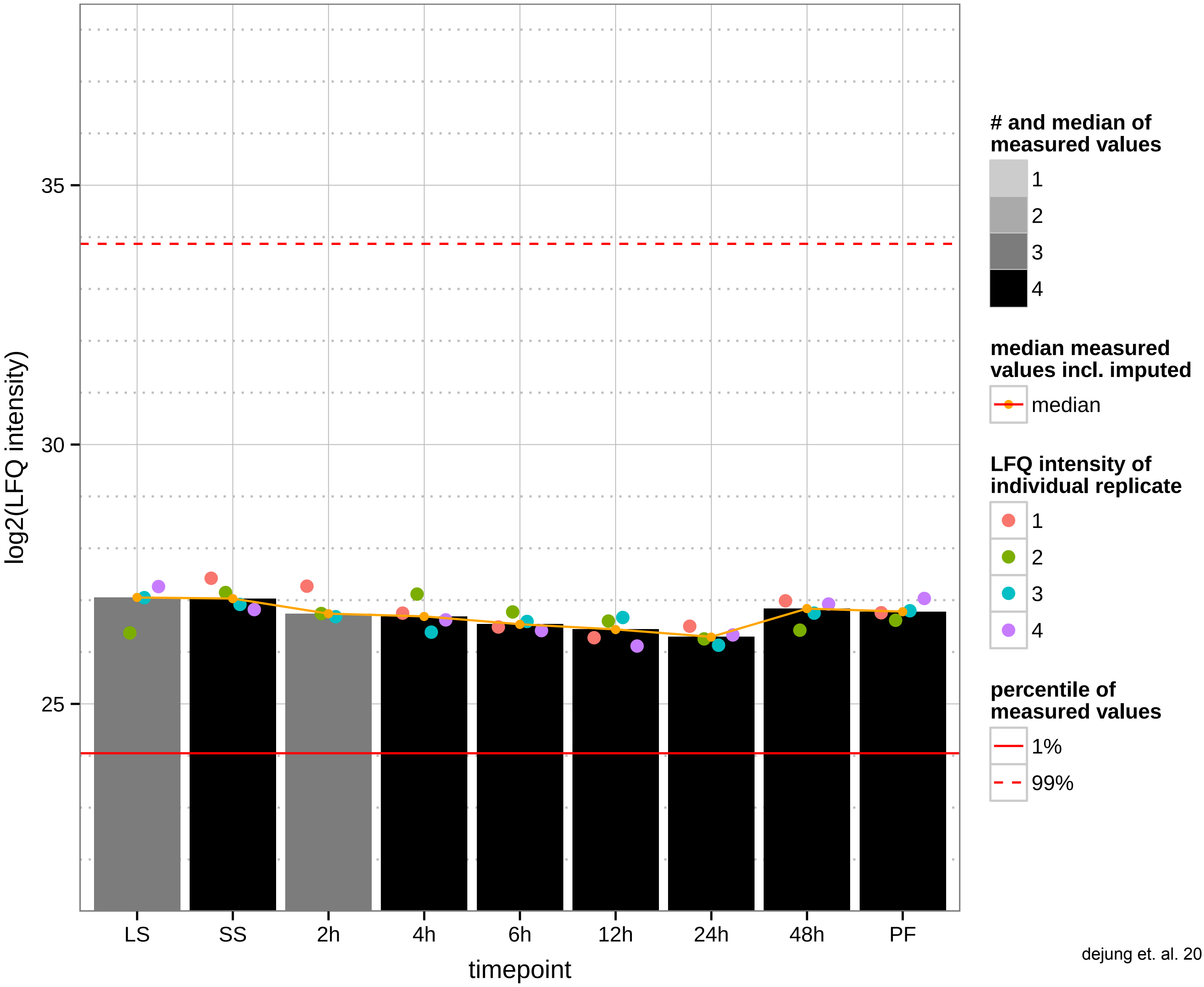

dejung et. al. 2015

RNA polymerase–associated protein CTR9, putative
Tb927.3.3220
AGOF: null
AGOC: cytoplasm, nucleus
AGOP: transcription elongation from RNA polymerase II promoter
PGOF: protein binding
PGOC: null
PGOP: null

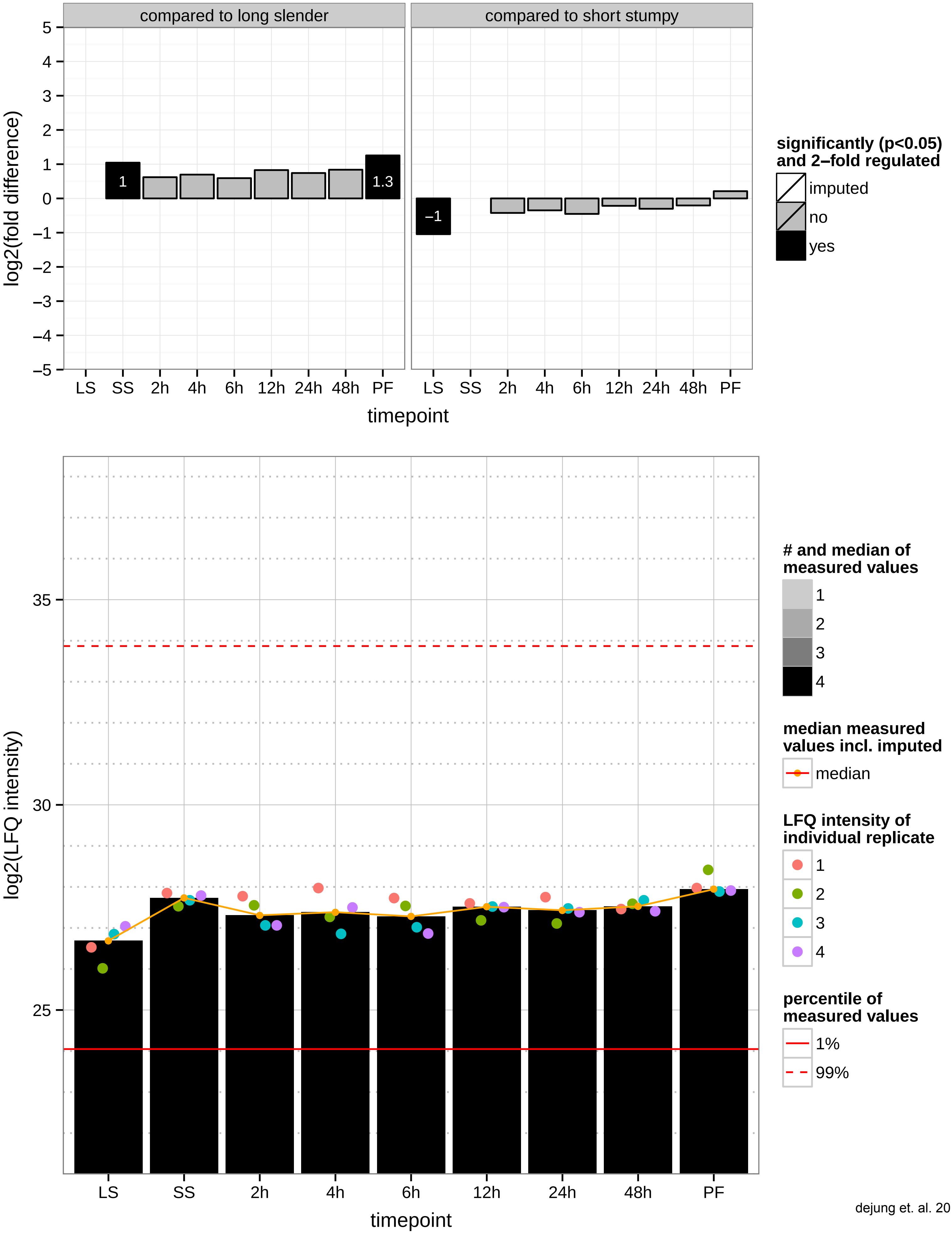

dejung et. al. 2015

hypothetical protein, conserved
Tb927.3.3230
AGOF: null
AGOC: null
AGOP: null
PGOF: null
PGOC: null
PGOP: null

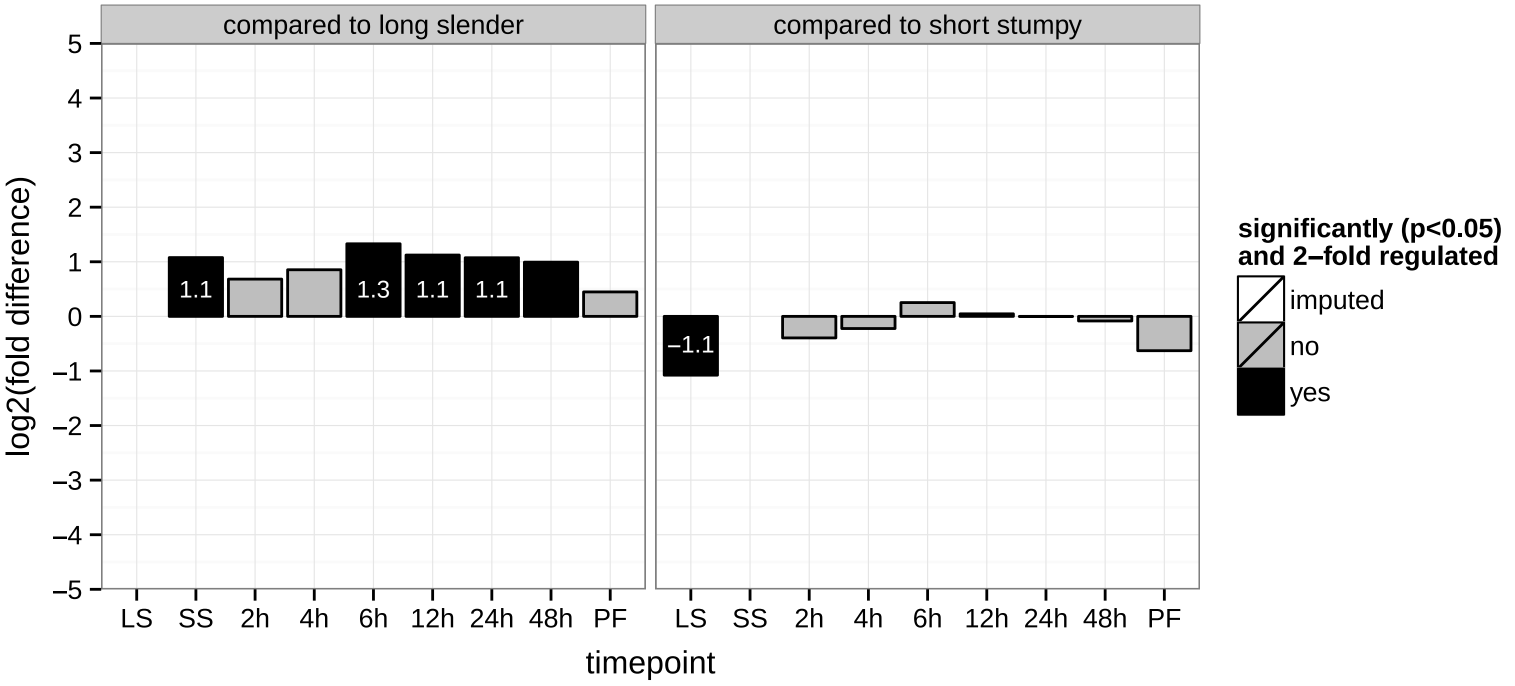

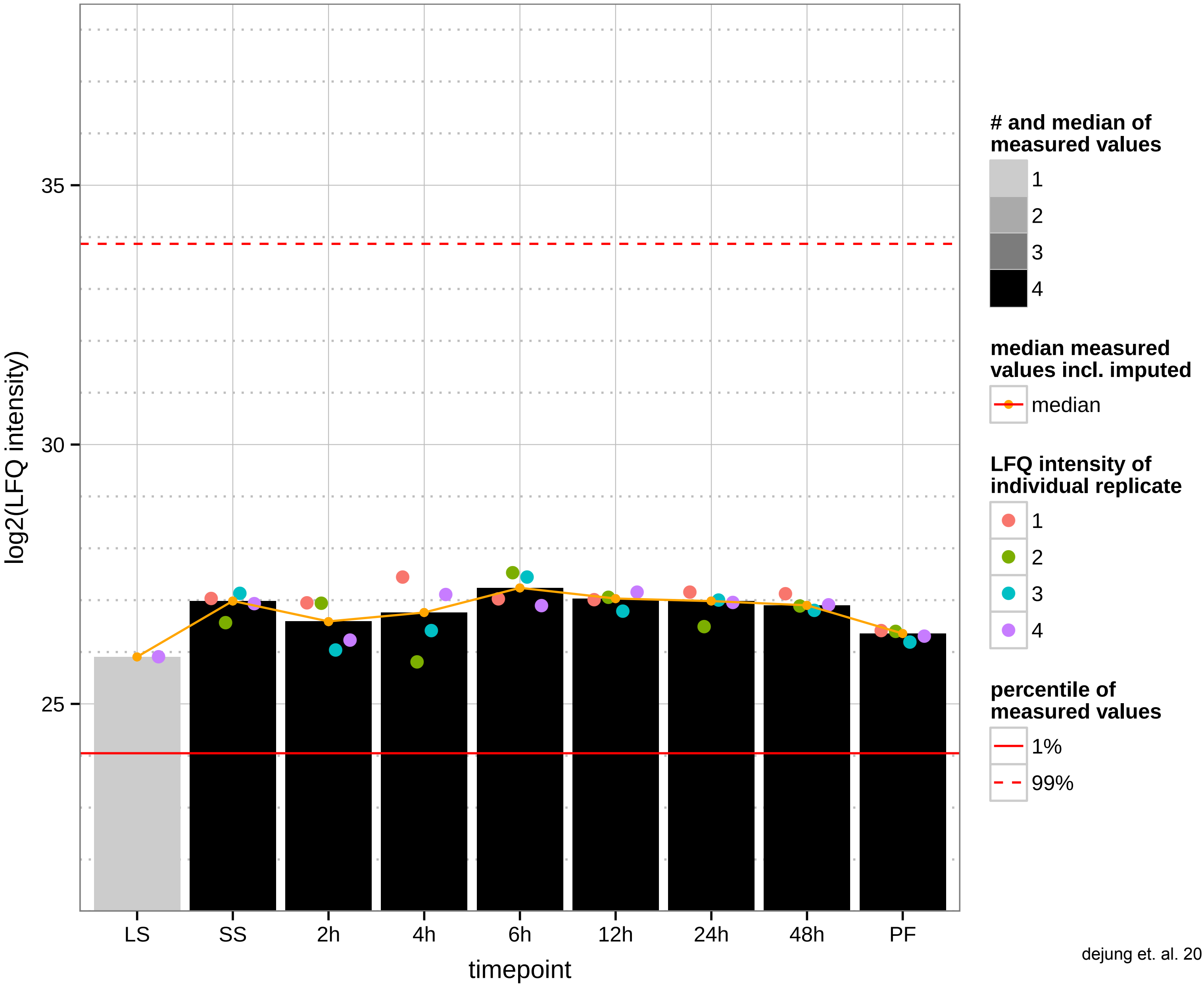

dejung et. al. 2015

vacuolar protein sorting–associated protein 4, putative (VPS4)
Tb927.3.3280
AGOF: ATP binding
AGOC: null
AGOP: null
PGOF: ATP binding, nucleoside–triphosphatase activity, nucleotide binding
PGOC: null
PGOP: null

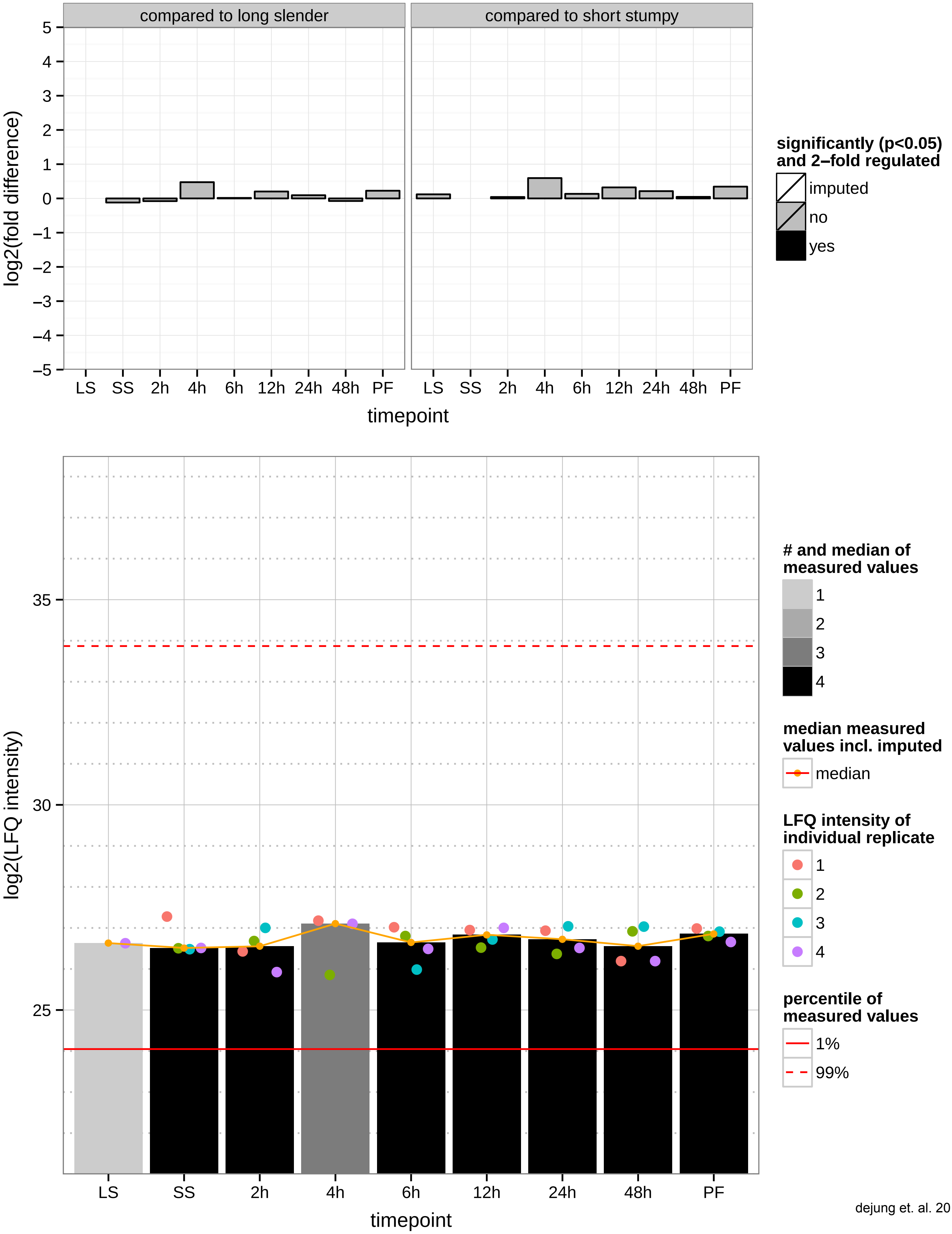

dejung et. al. 2015

hypothetical protein, conserved
Tb927.3.3300
AGOF: null
AGOC: null
AGOP: null
PGOF: null
PGOC: null
PGOP: null

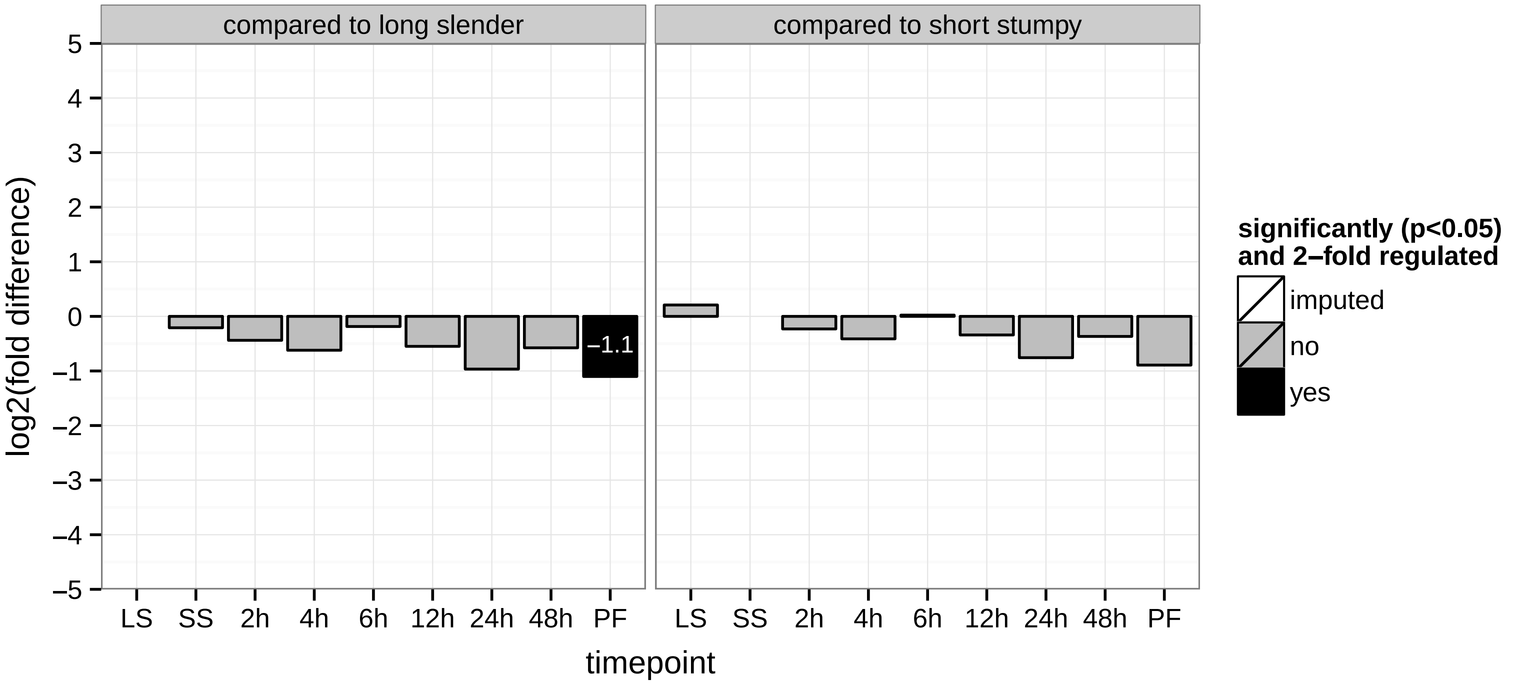

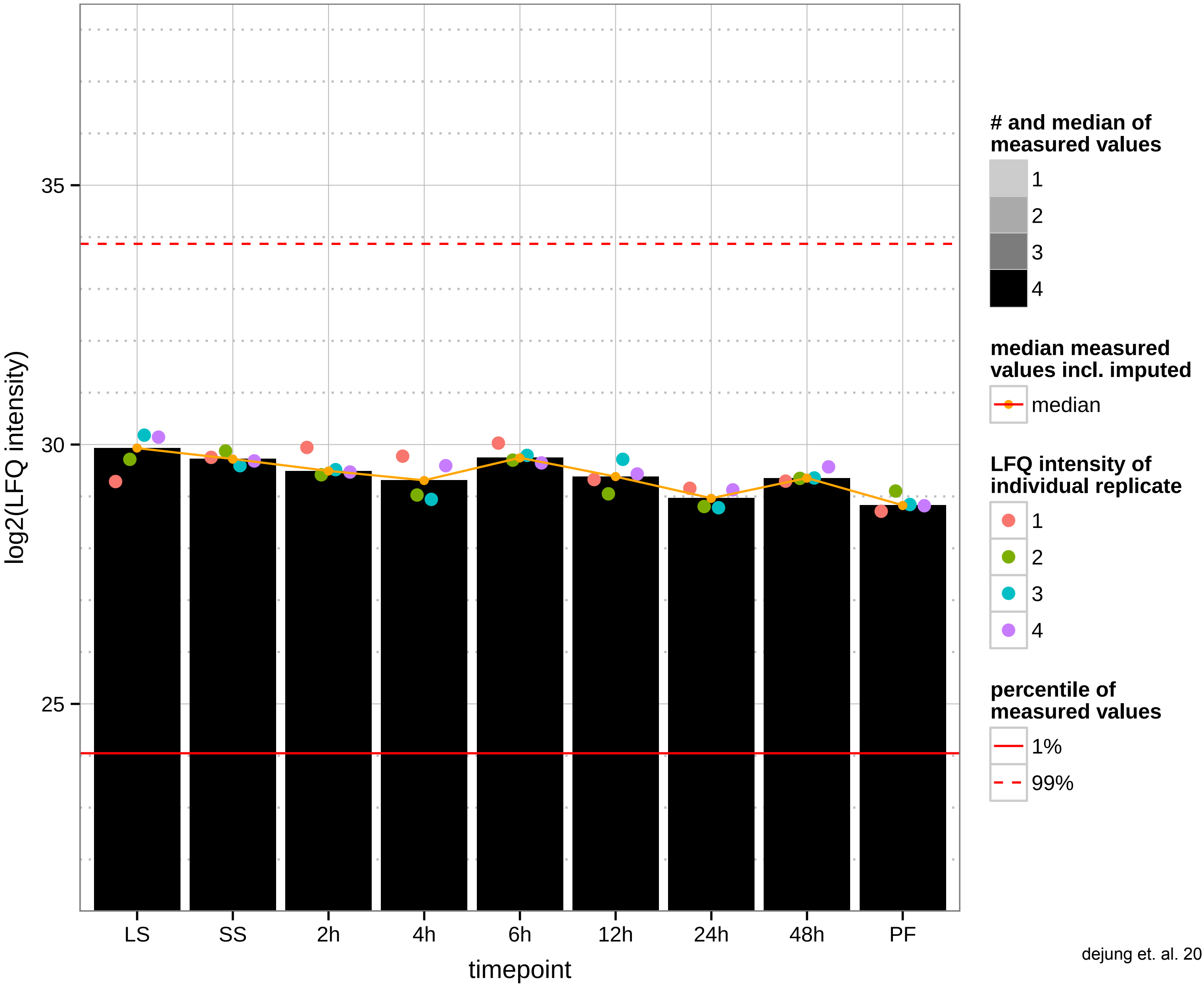

dejung et. al. 2015

heat shock protein 20, putative
Tb927.3.3330
AGOF: null
AGOC: mitochondrion
AGOP: null
PGOF: null
PGOC: null
PGOP: null

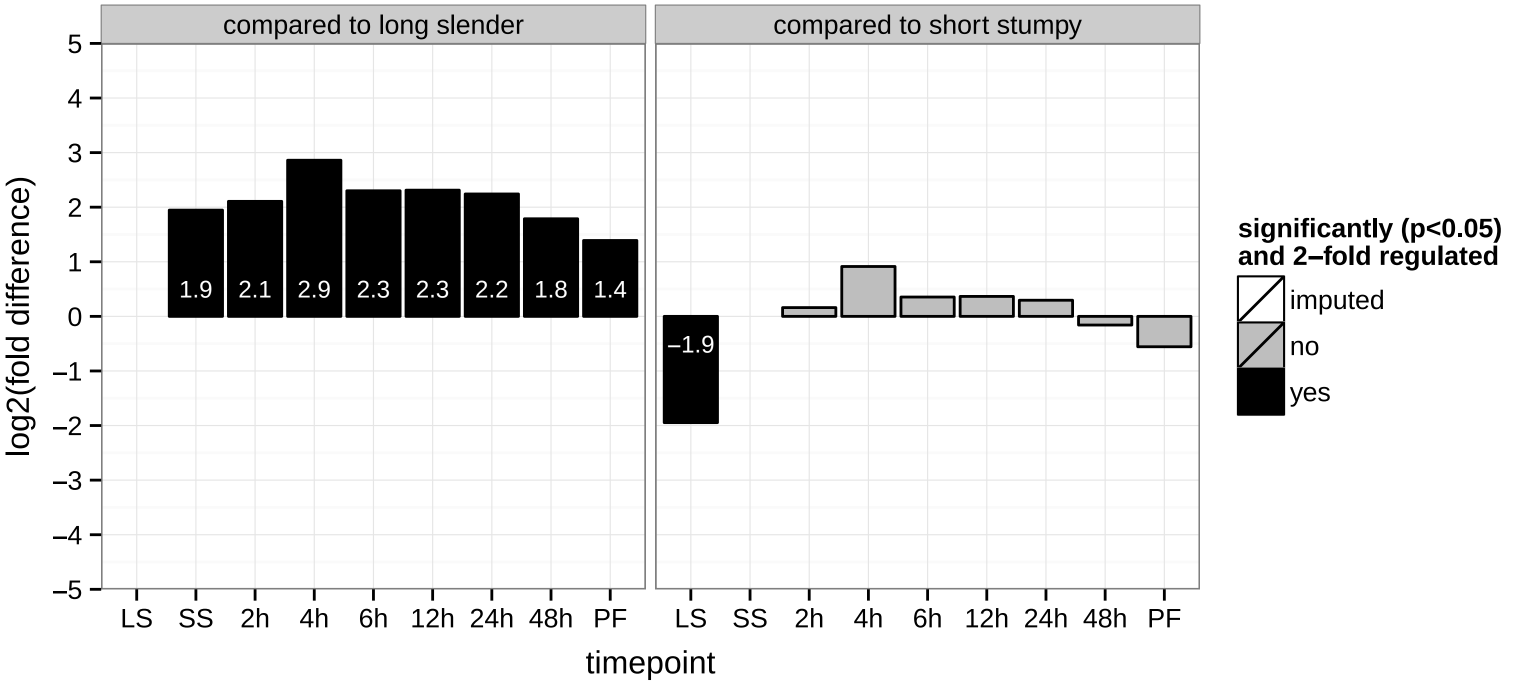

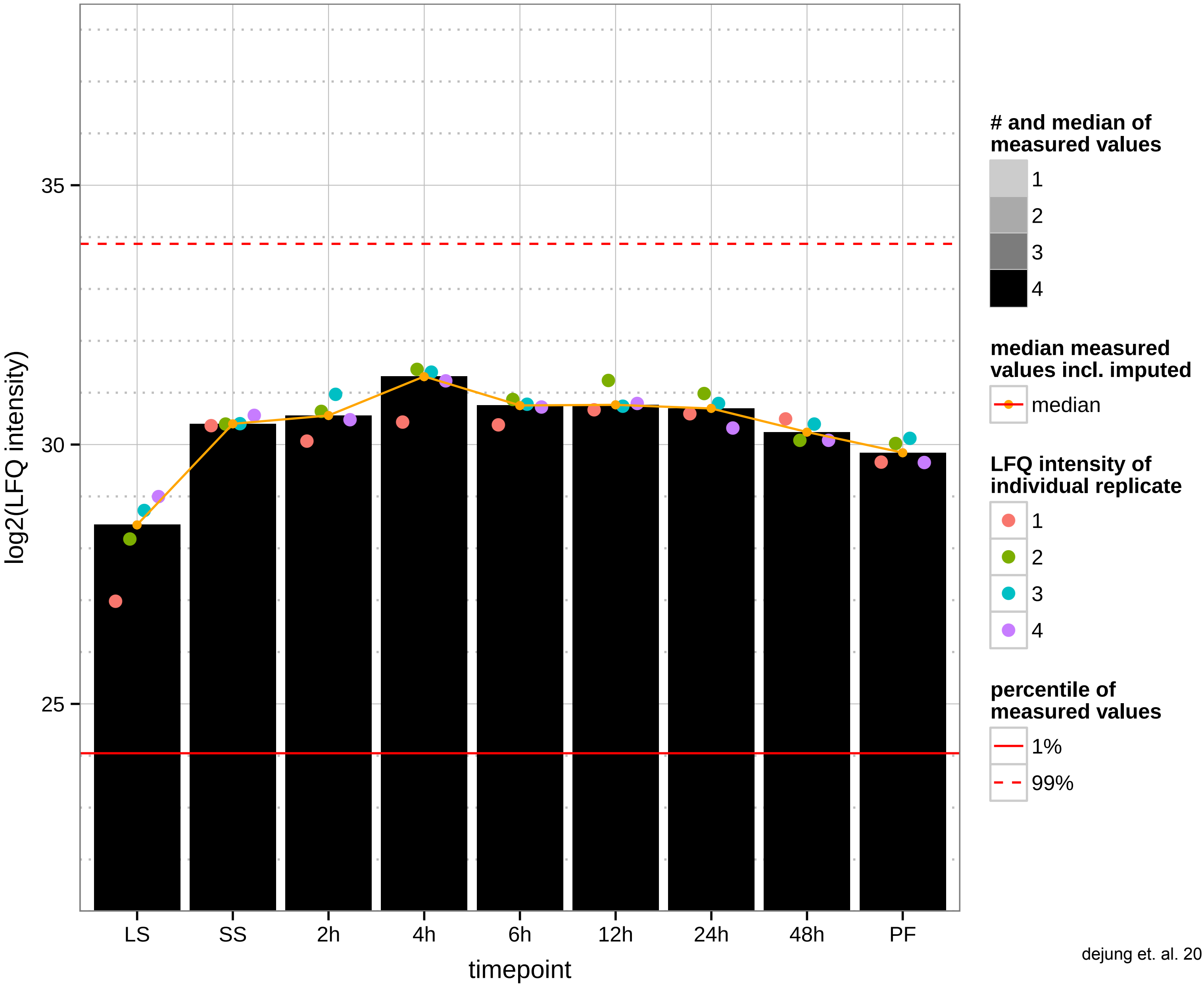

dejung et. al. 2015

aspartyl aminopeptidase, putative, metallo–peptidase, Clan MH, Family M20
Tb927.3.3410;Tb11.v5.0768
AGOF: null, aminopeptidase activity, zinc ion binding
AGOC: null, vacuole
AGOP: null, proteolysis
PGOF: aminopeptidase activity, zinc ion binding
PGOC: null
PGOP: proteolysis

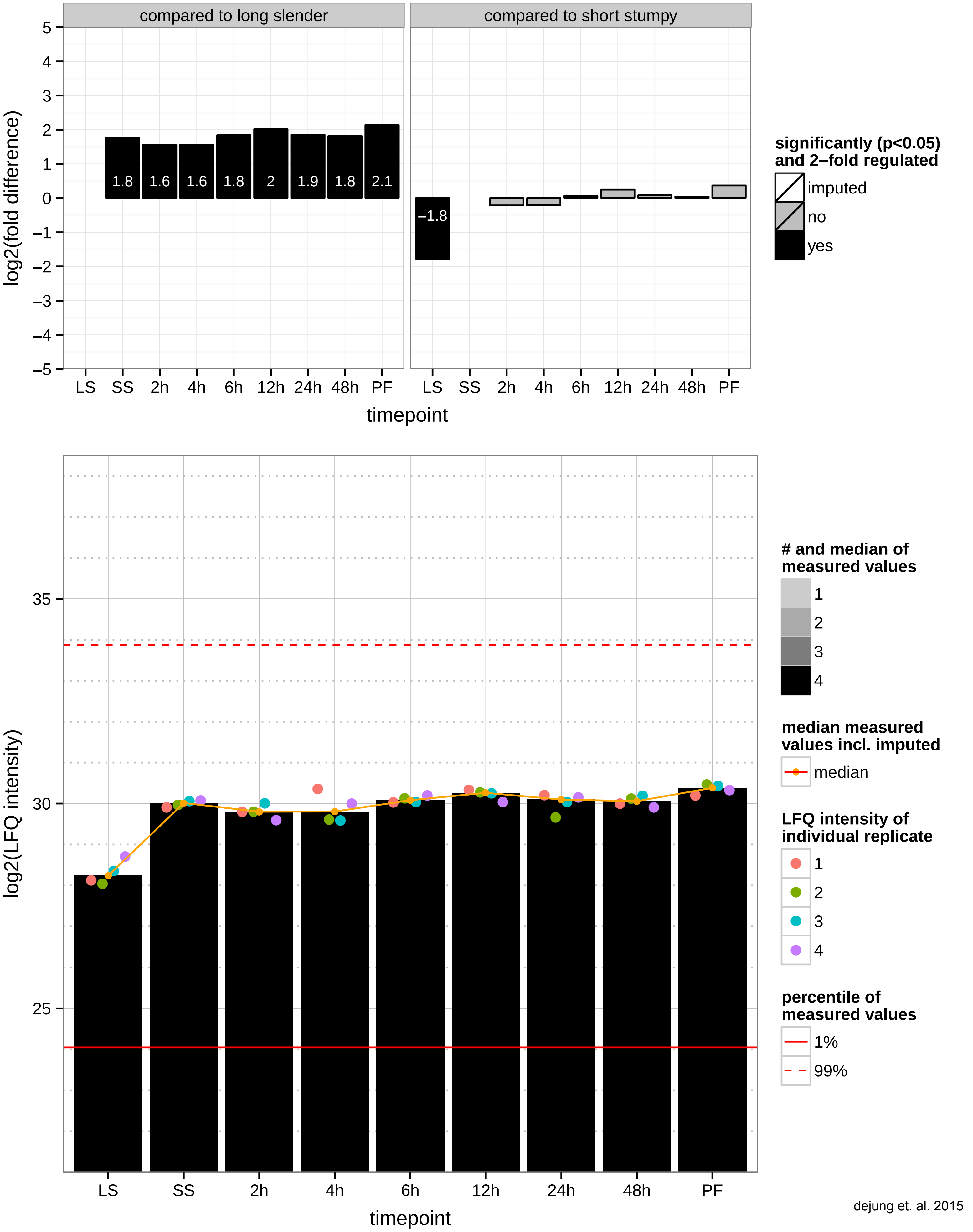

dejung et. al. 2015

ADP–ribosylation factor–like protein 3, putative (arl3)
Tb927.3.3450
AGOF: GTP binding
AGOC: cilium membrane, flagellar pocket membrane, intracellular, plasma membrane
AGOP: cell proliferation, cilium morphogenesis, dissemination or transmission of symbiont from host by vector, intracellular pr
PGOF: GTP binding
PGOC: intracellular
PGOP: intracellular protein transport, protein transport, small GTPase mediated signal transduction

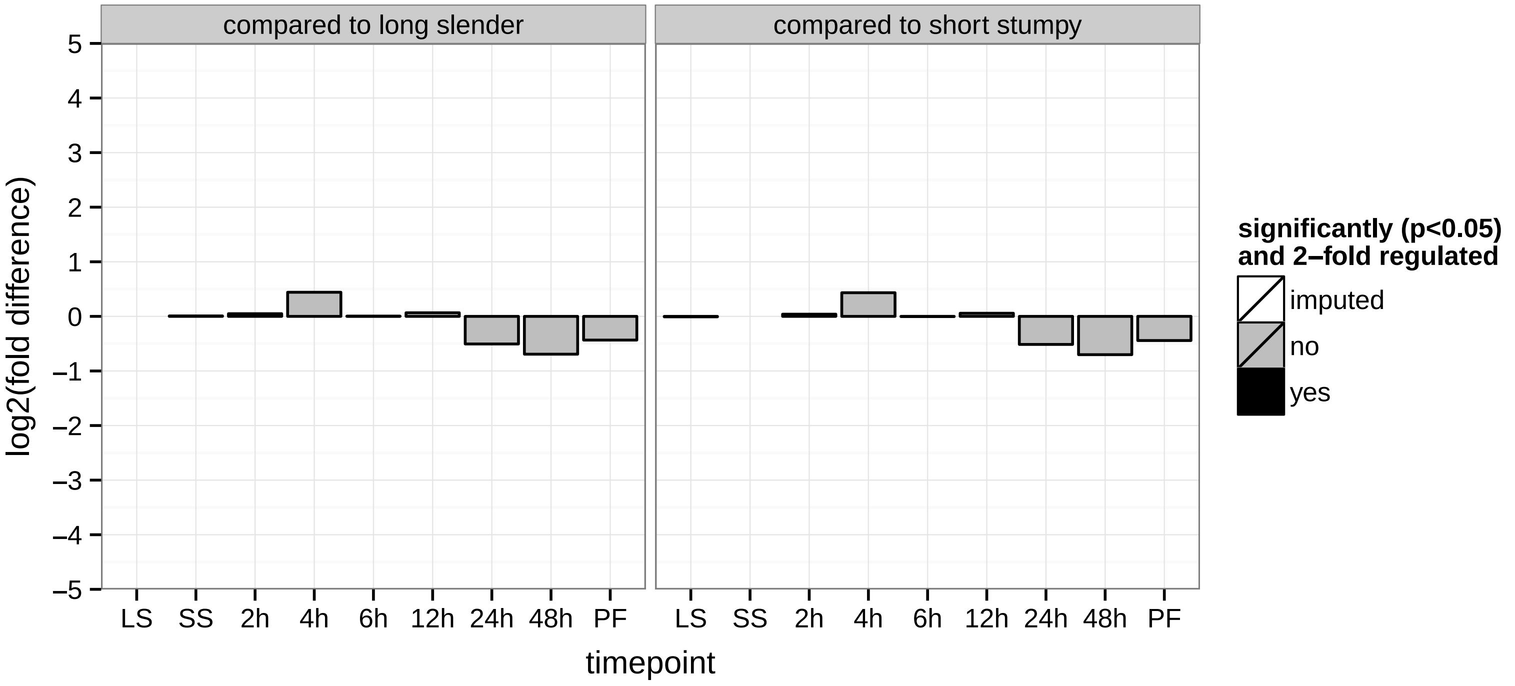

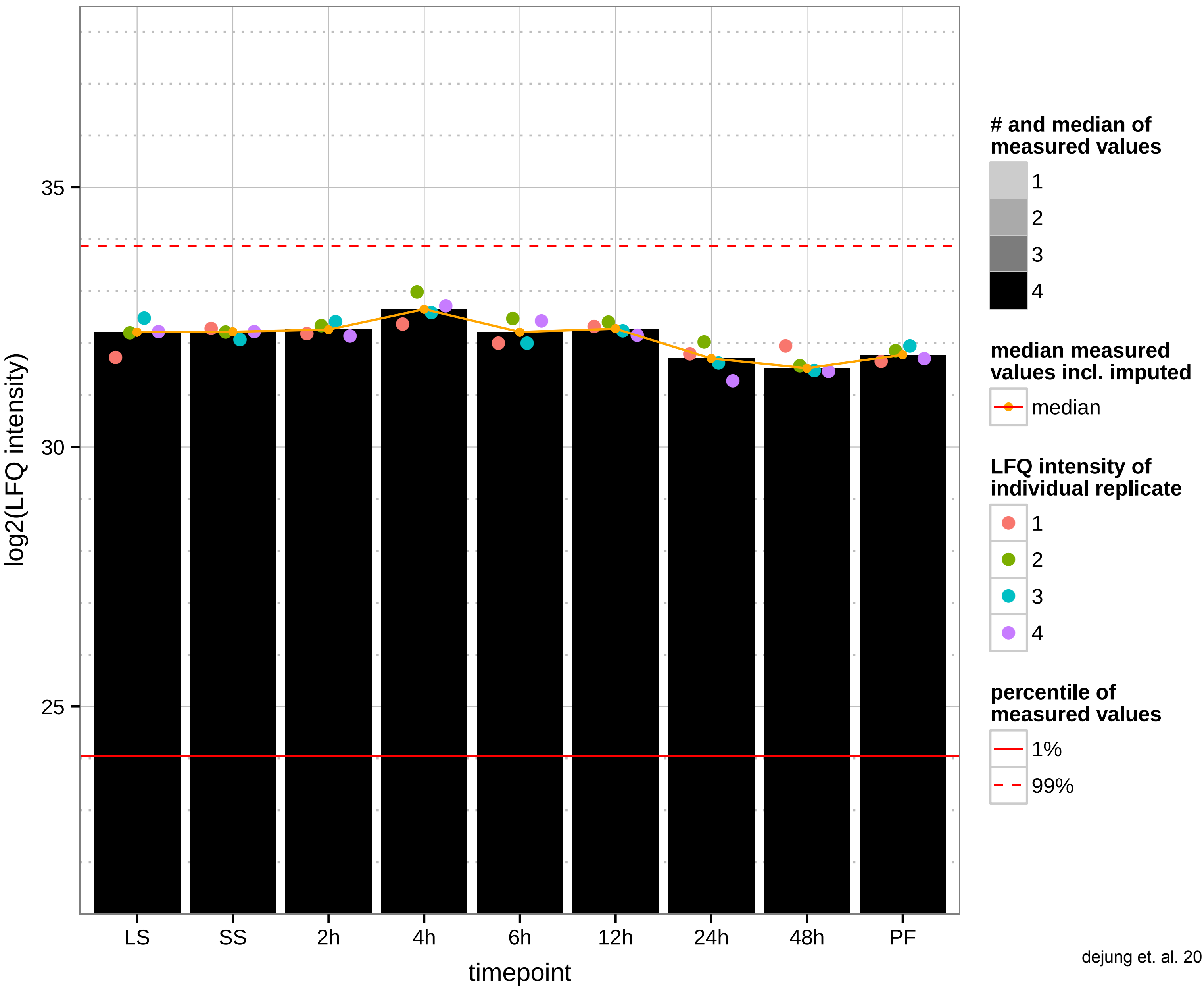

dejung et. al. 2015

high mobility group protein, putative (TDP1)
Tb927.3.3490
AGOF: DNA binding
AGOC: nucleus
AGOP: null
PGOF: protein binding
PGOC: null
PGOP: null

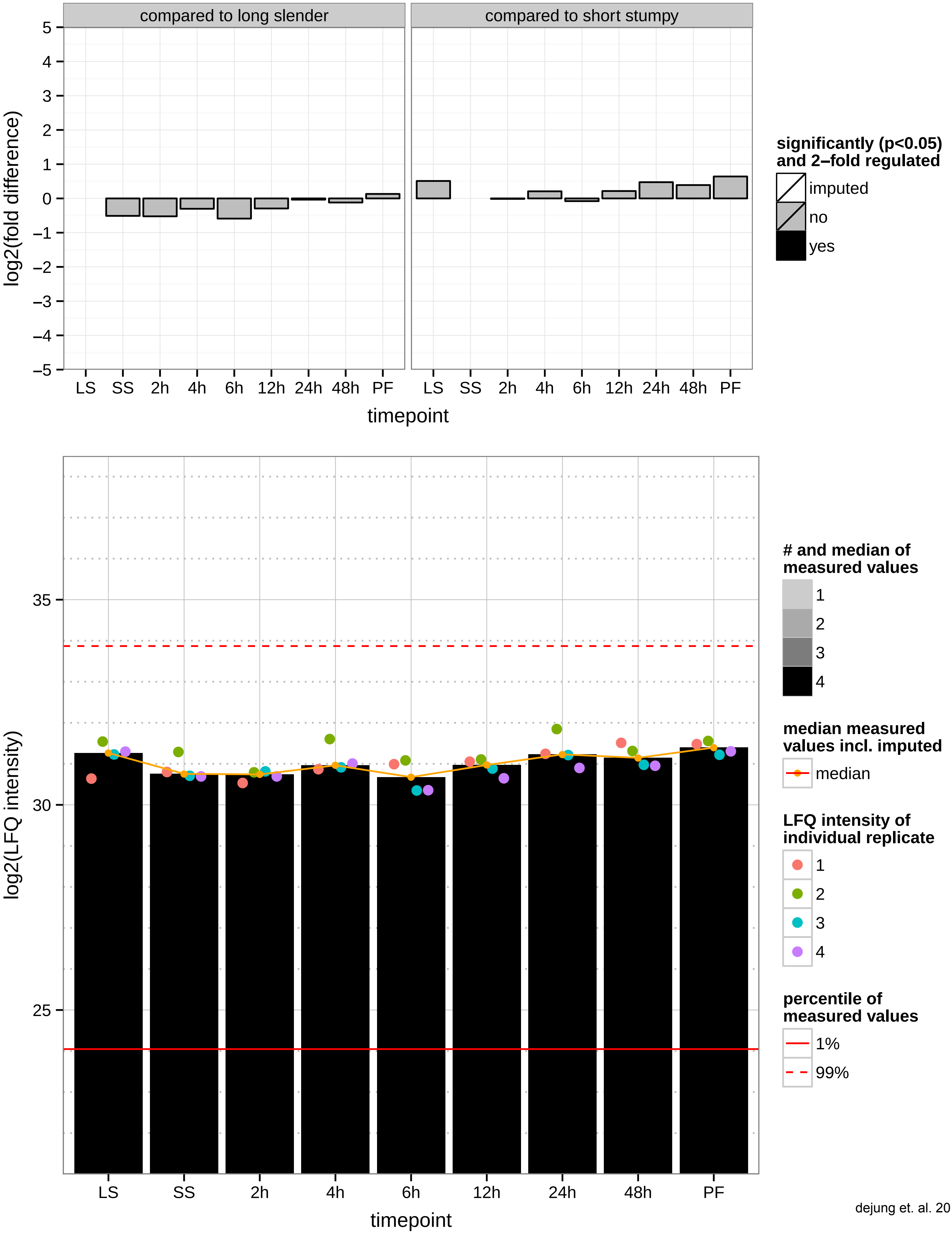

dejung et. al. 2015

hypothetical protein, conserved
Tb927.3.3520
AGOF: null
AGOC: null
AGOP: null
PGOF: null
PGOC: null
PGOP: null

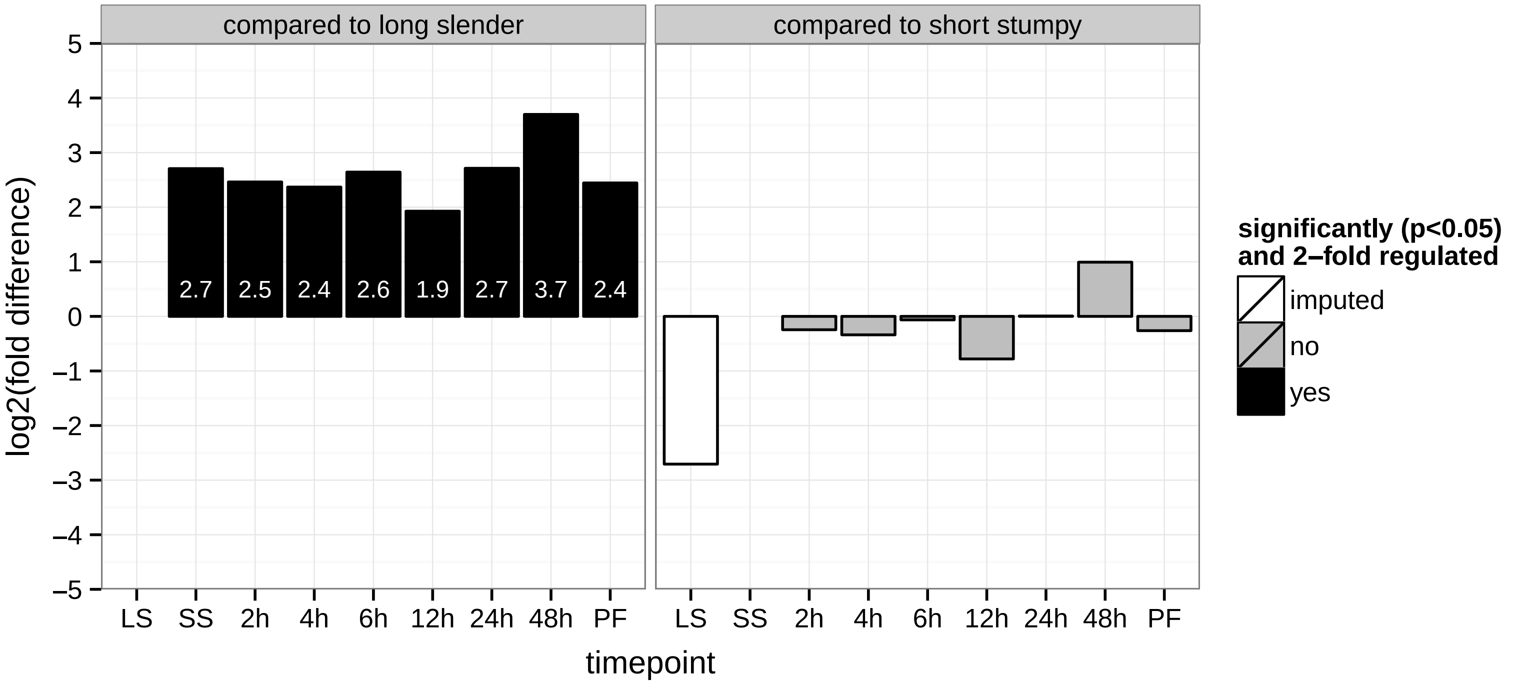

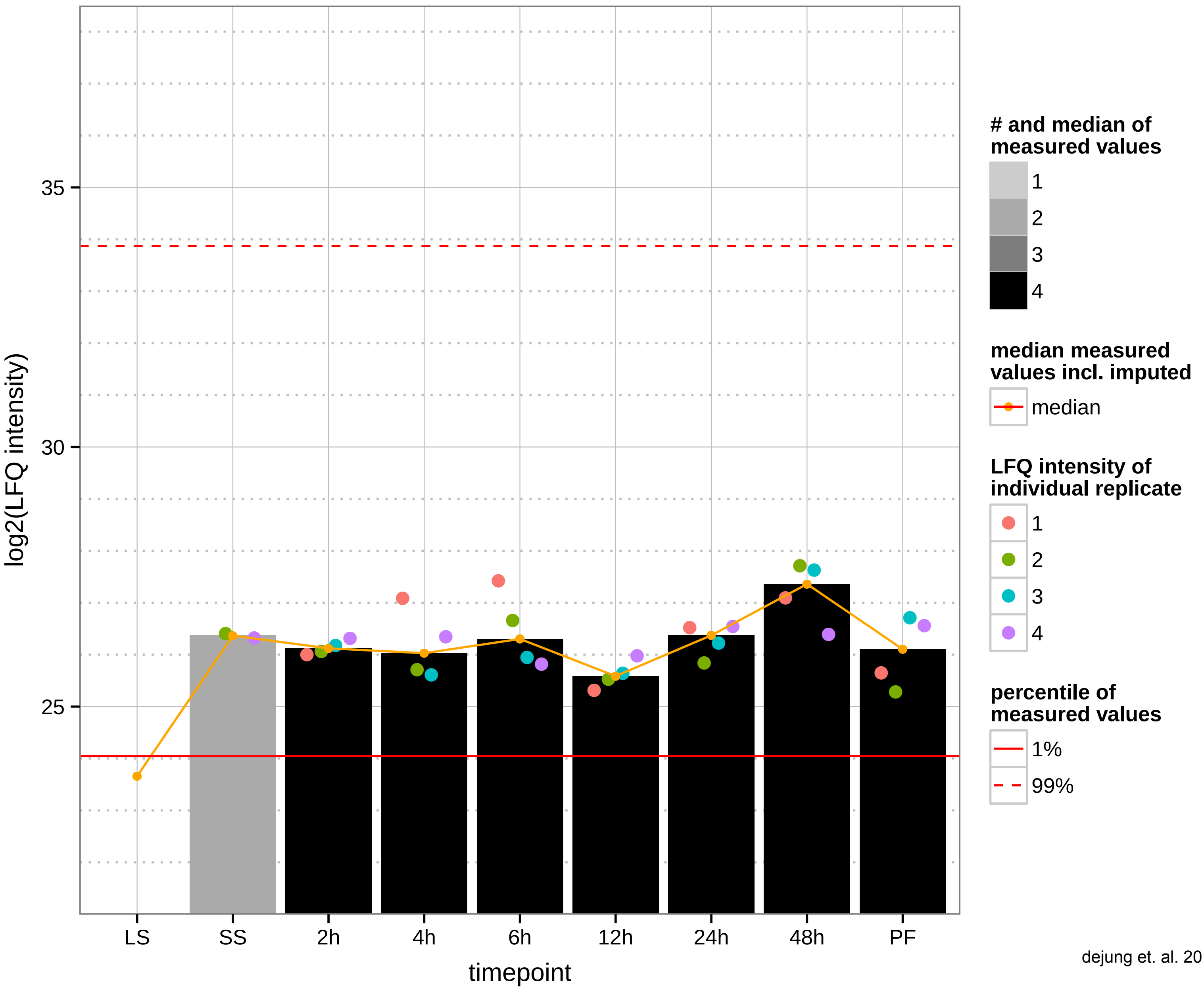

dejung et. al. 2015

Nucleoporin (TbNup53b)
Tb927.3.3540
AGOF: null
AGOC: nuclear pore
AGOP: null
PGOF: null
PGOC: null
PGOP: null

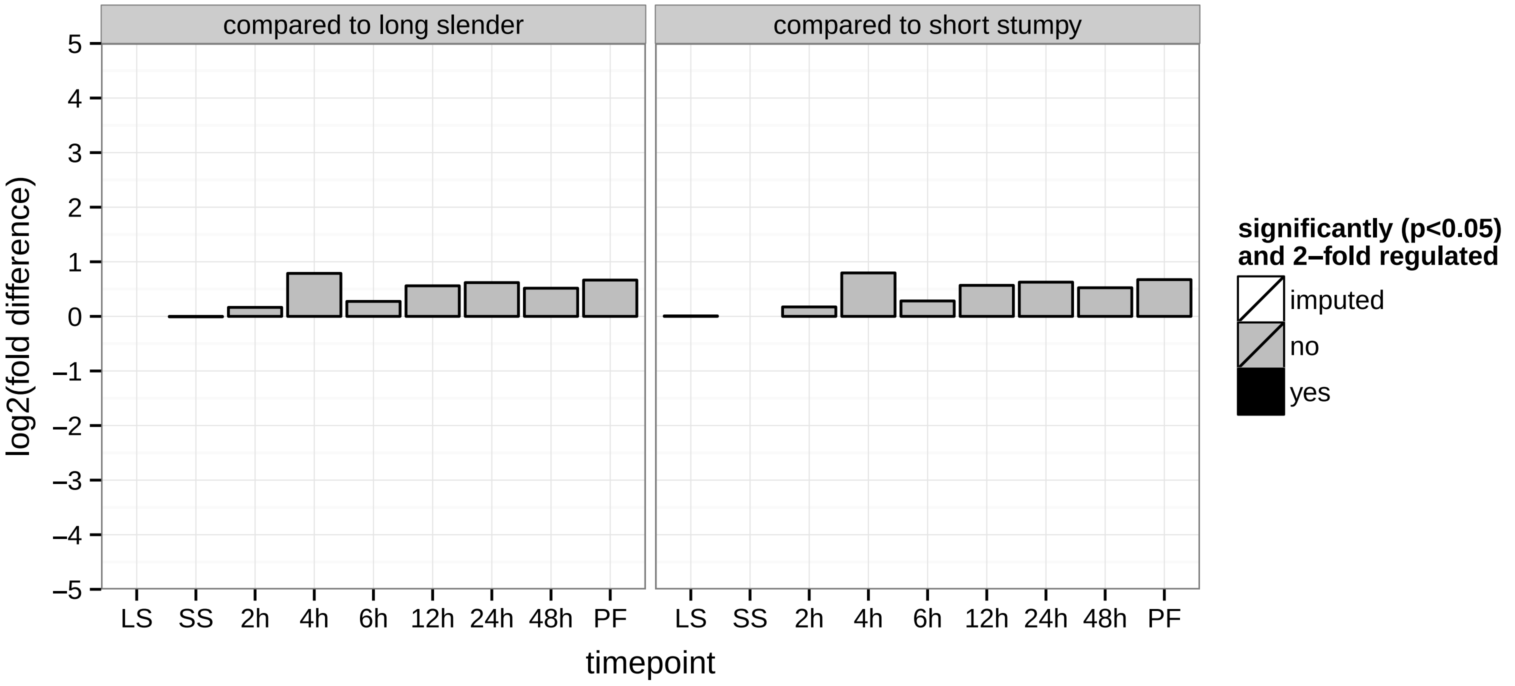

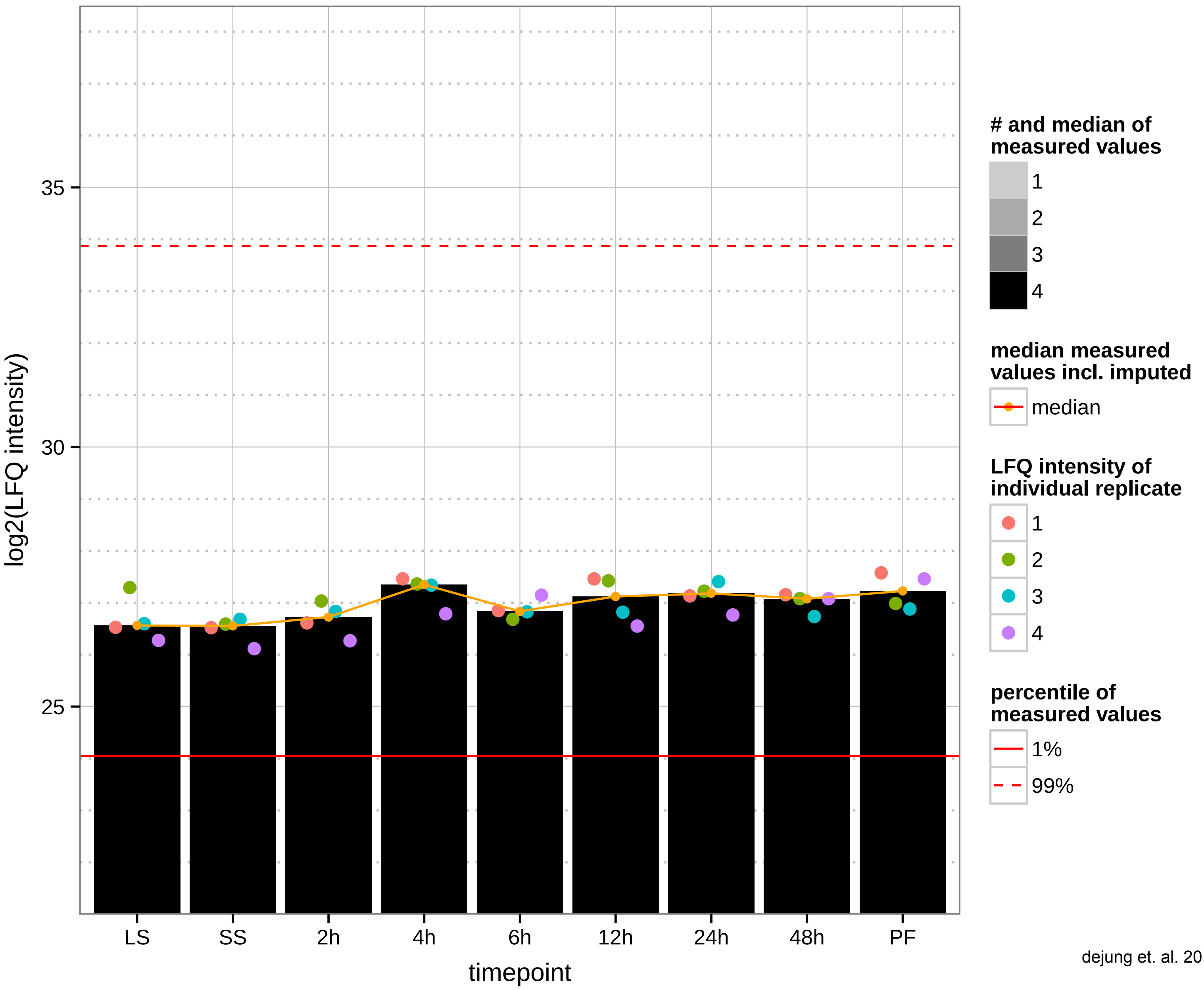

dejung et. al. 2015

heat shock protein 90, putative, lipophosphoglycan biosynthetic protein, glucose regulated protein 94, putative (LPG3)
Tb927.3.3580
AGOF: ATP binding, unfolded protein binding
AGOC: null
AGOP: protein folding, response to stress
PGOF: ATP binding, unfolded protein binding
PGOC: null
PGOP: protein folding, response to stress

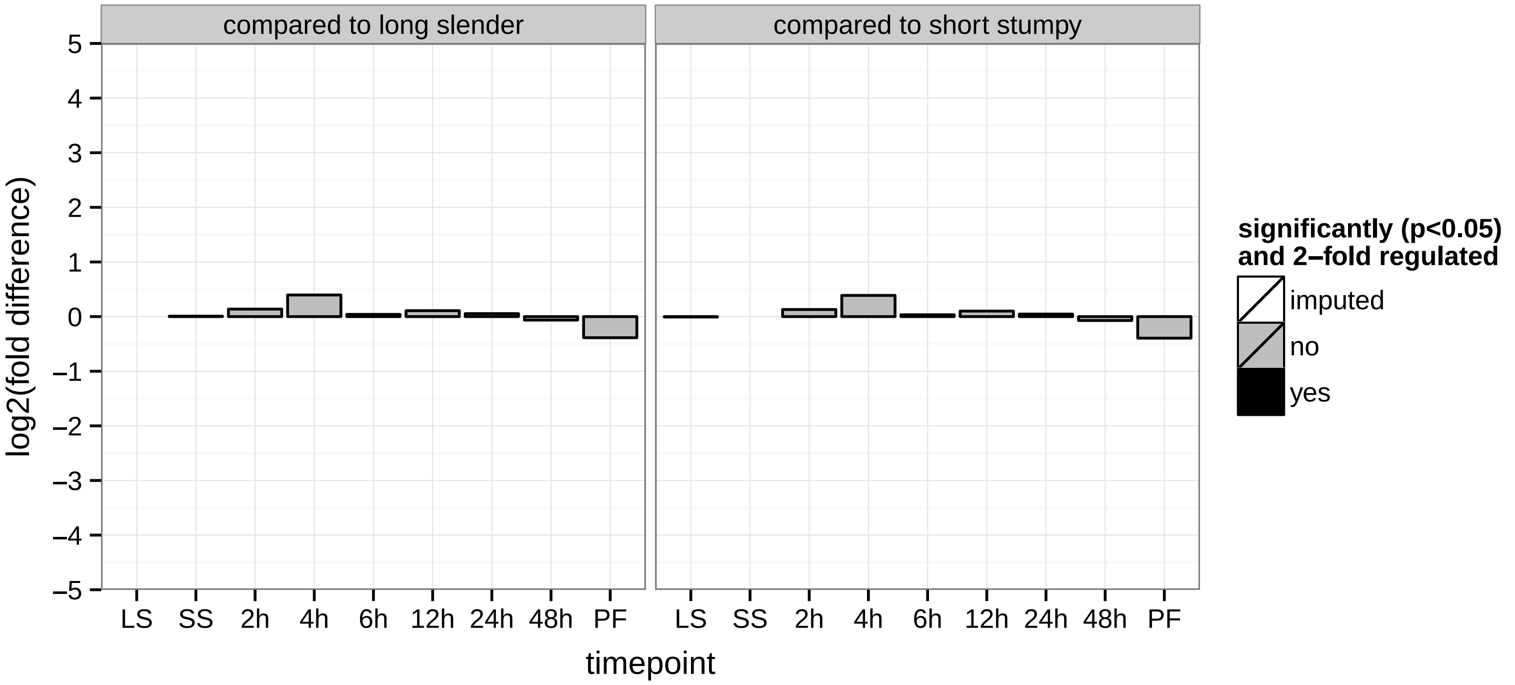

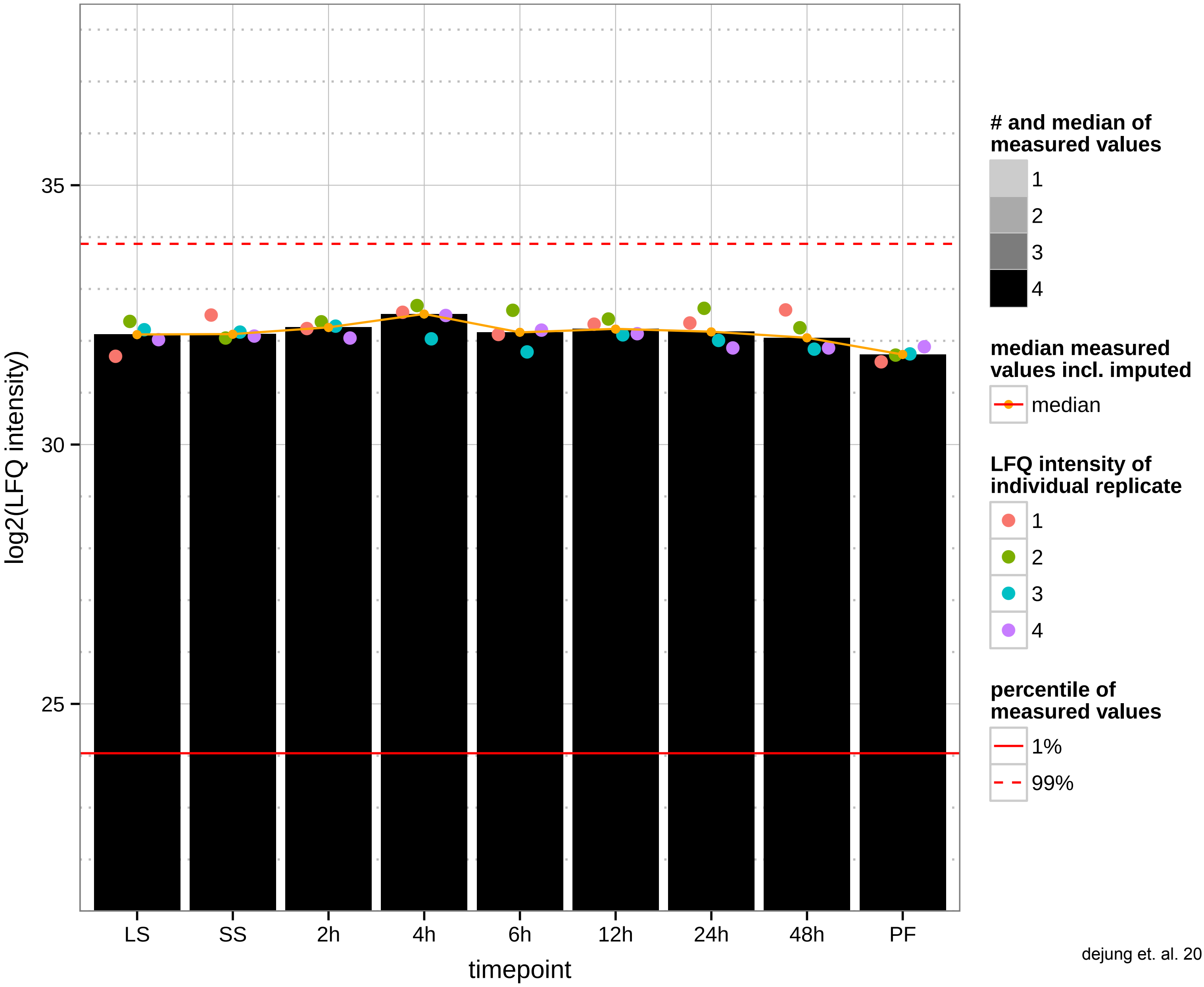

dejung et. al. 2015

U3 small nucleolar ribonucleoprotein protein MPP10, putative
Tb927.3.3590
AGOF: null
AGOC: null
AGOP: null
PGOF: null
PGOC: null
PGOP: null

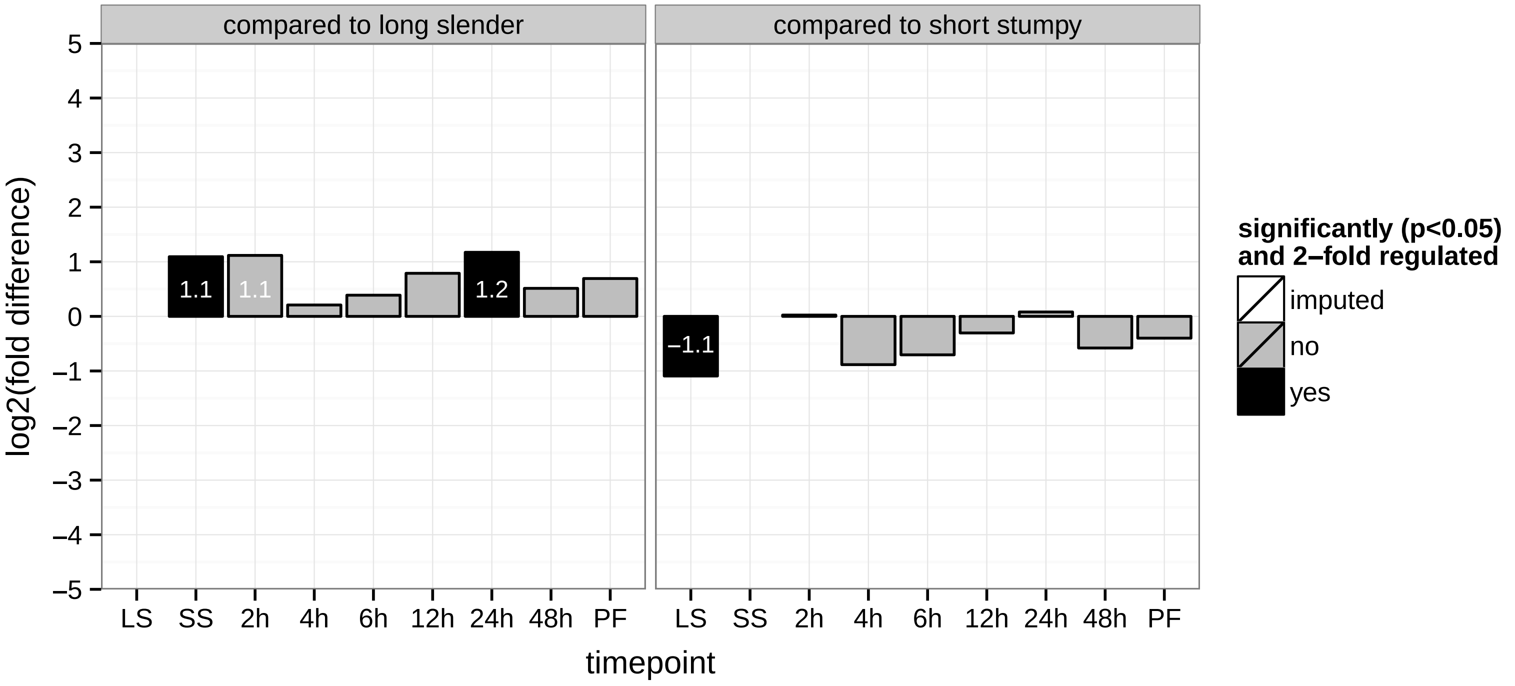

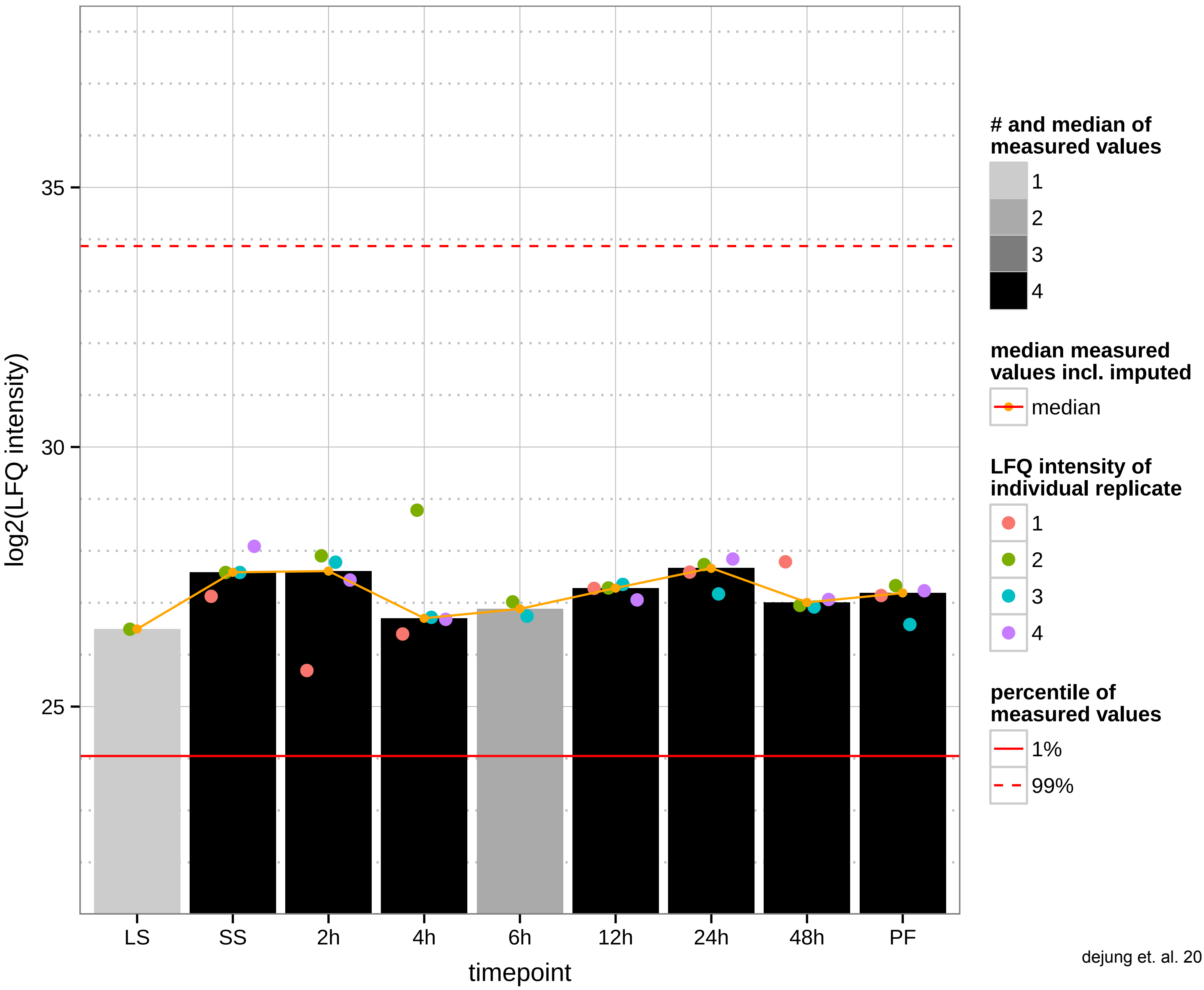

dejung et. al. 2015

flagellar radial spoke protein–like, putative
Tb927.3.3690
AGOF: null
AGOC: null
AGOP: null
PGOF: null
PGOC: null
PGOP: null

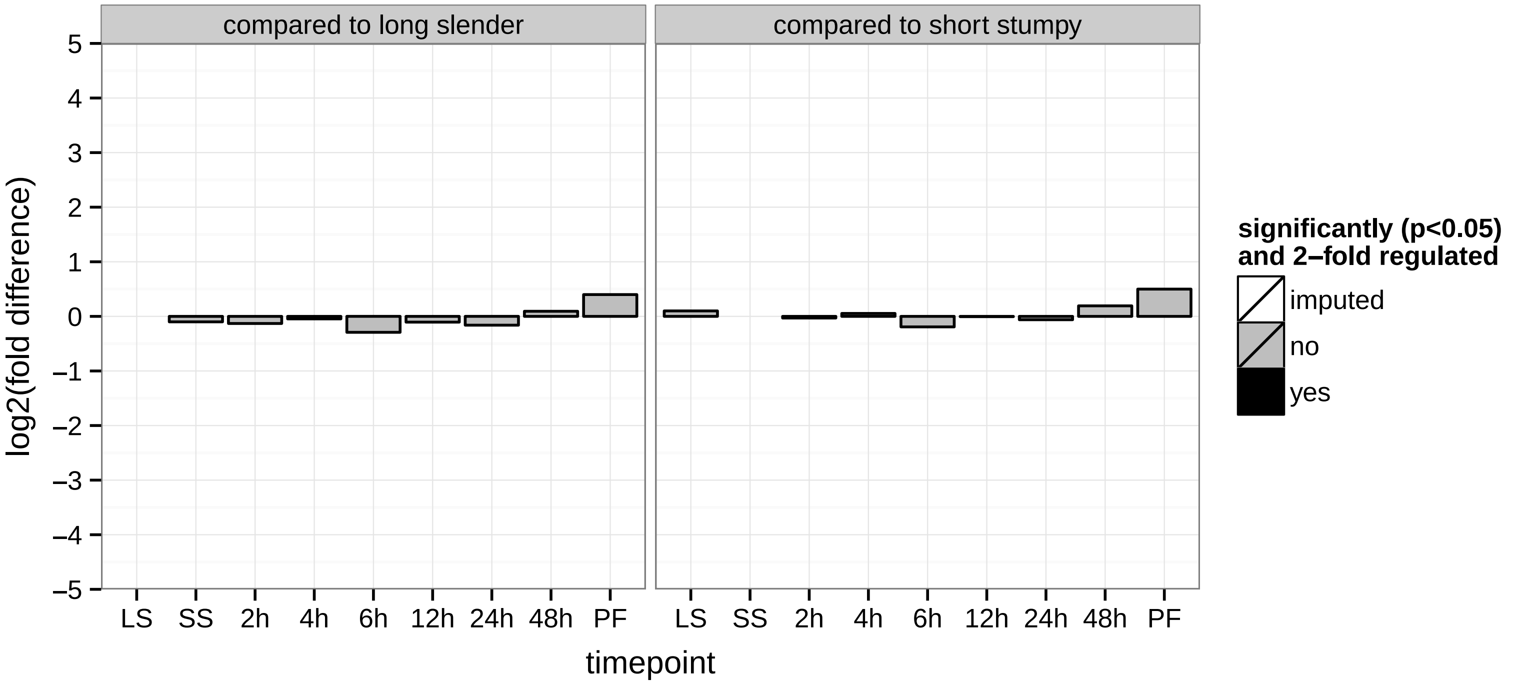

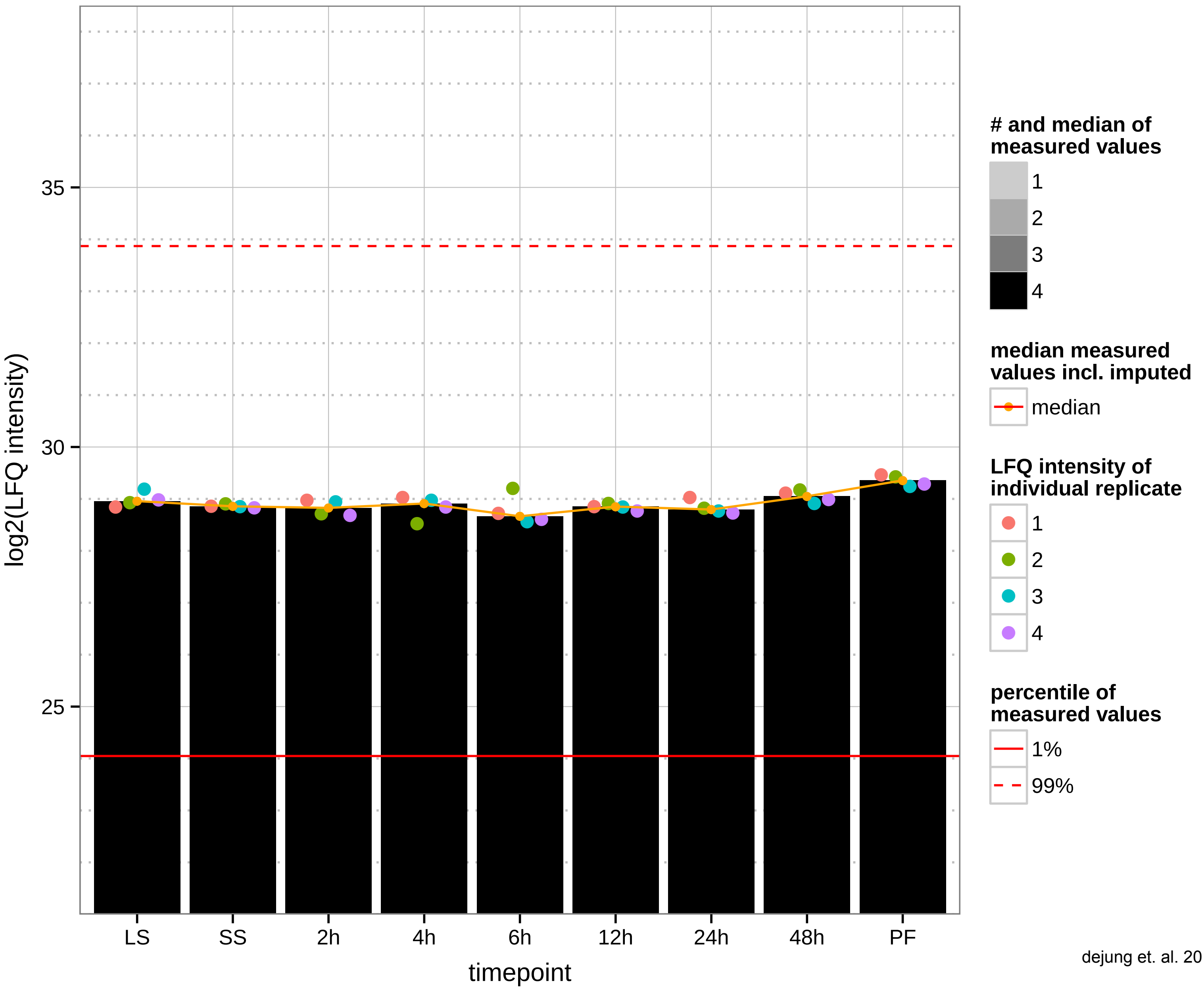

dejung et. al. 2015

paraflagellar rod component, putative (PFC7)
Tb927.3.3750
AGOF: null
AGOC: null
AGOP: null
PGOF: null
PGOC: null
PGOP: null

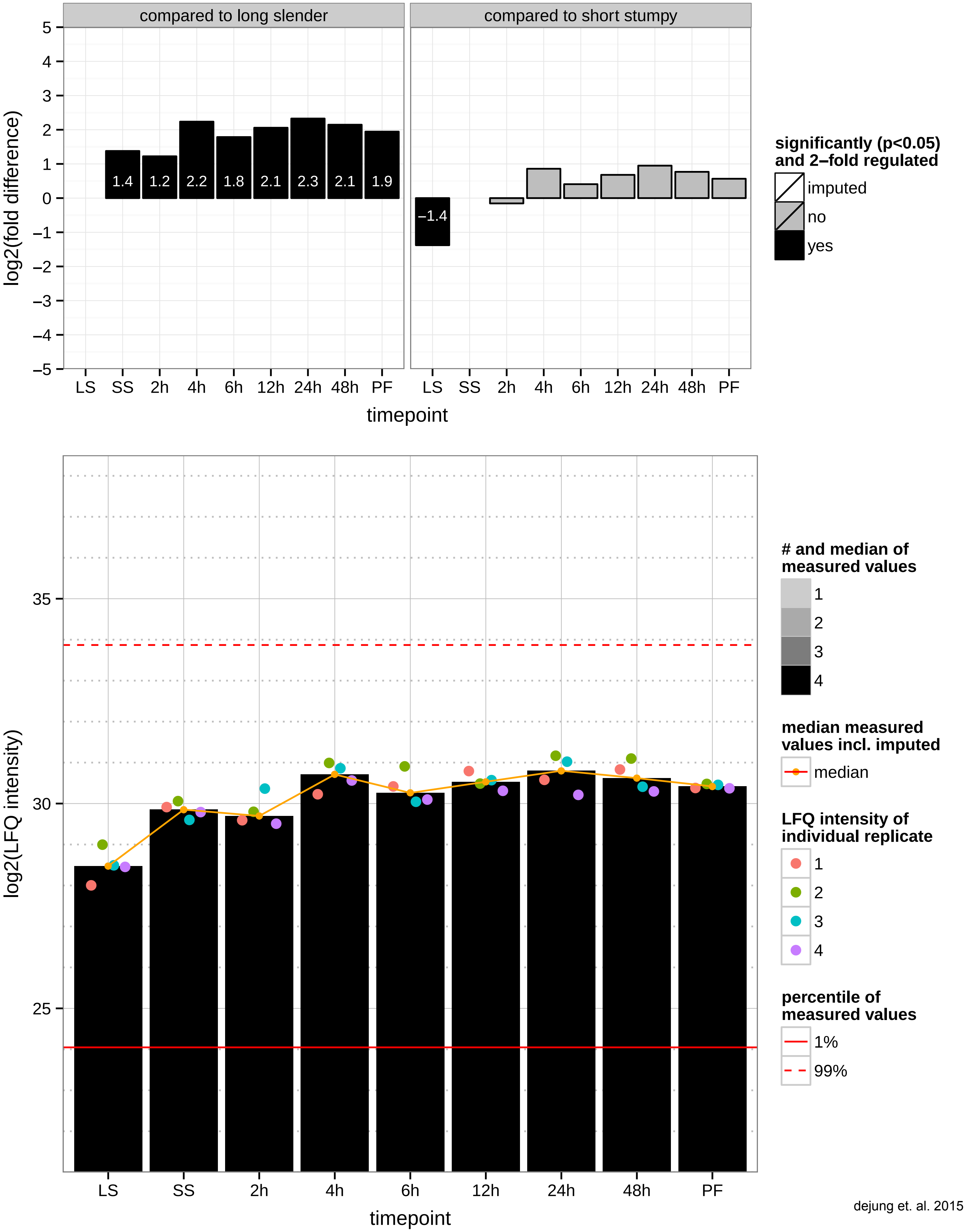

dejung et. al. 2015

tryparedoxin 1b, putative (TXN1b), tryparedoxin 1a, putative (TXN1a)
Tb927.3.3780;Tb927.3.3760
AGOF: null
AGOC: null
AGOP: hydrogen peroxide catabolic process
PGOF: null
PGOC: null
PGOP: null

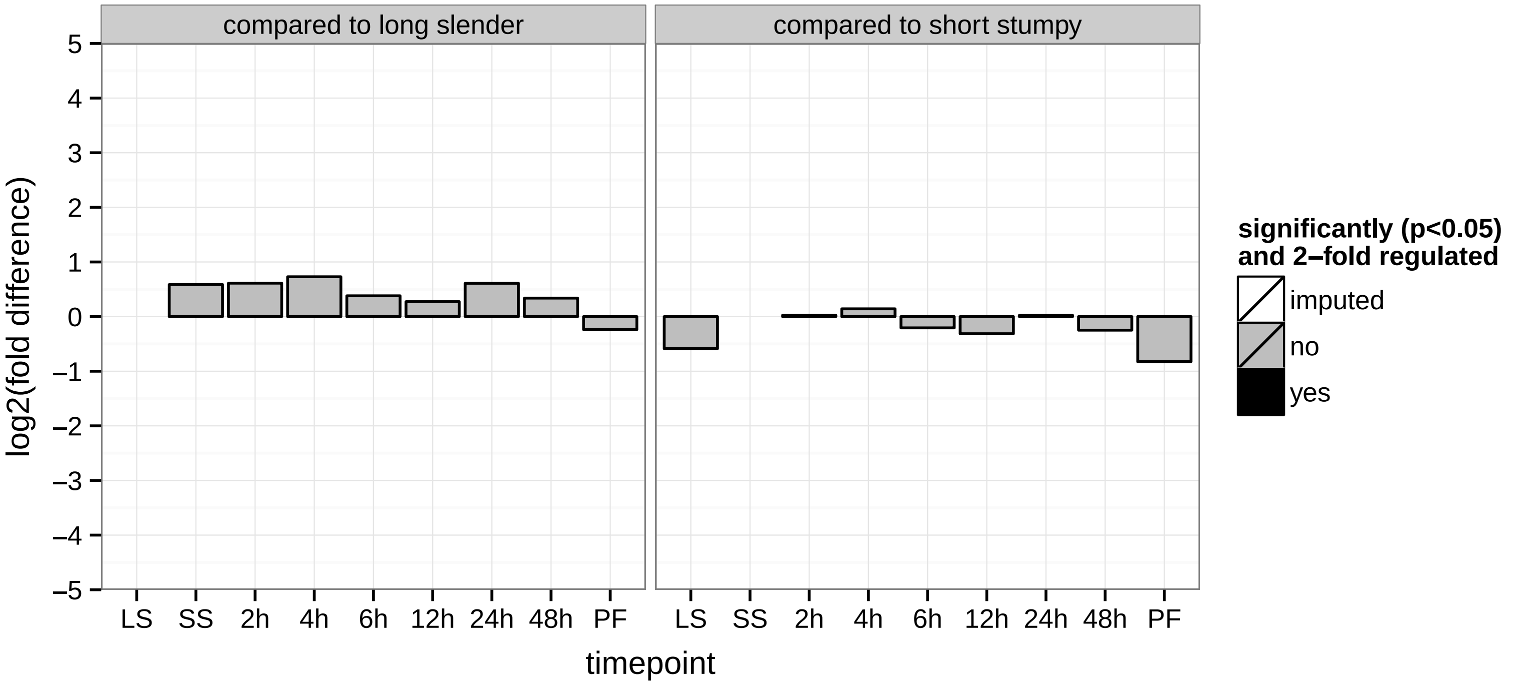

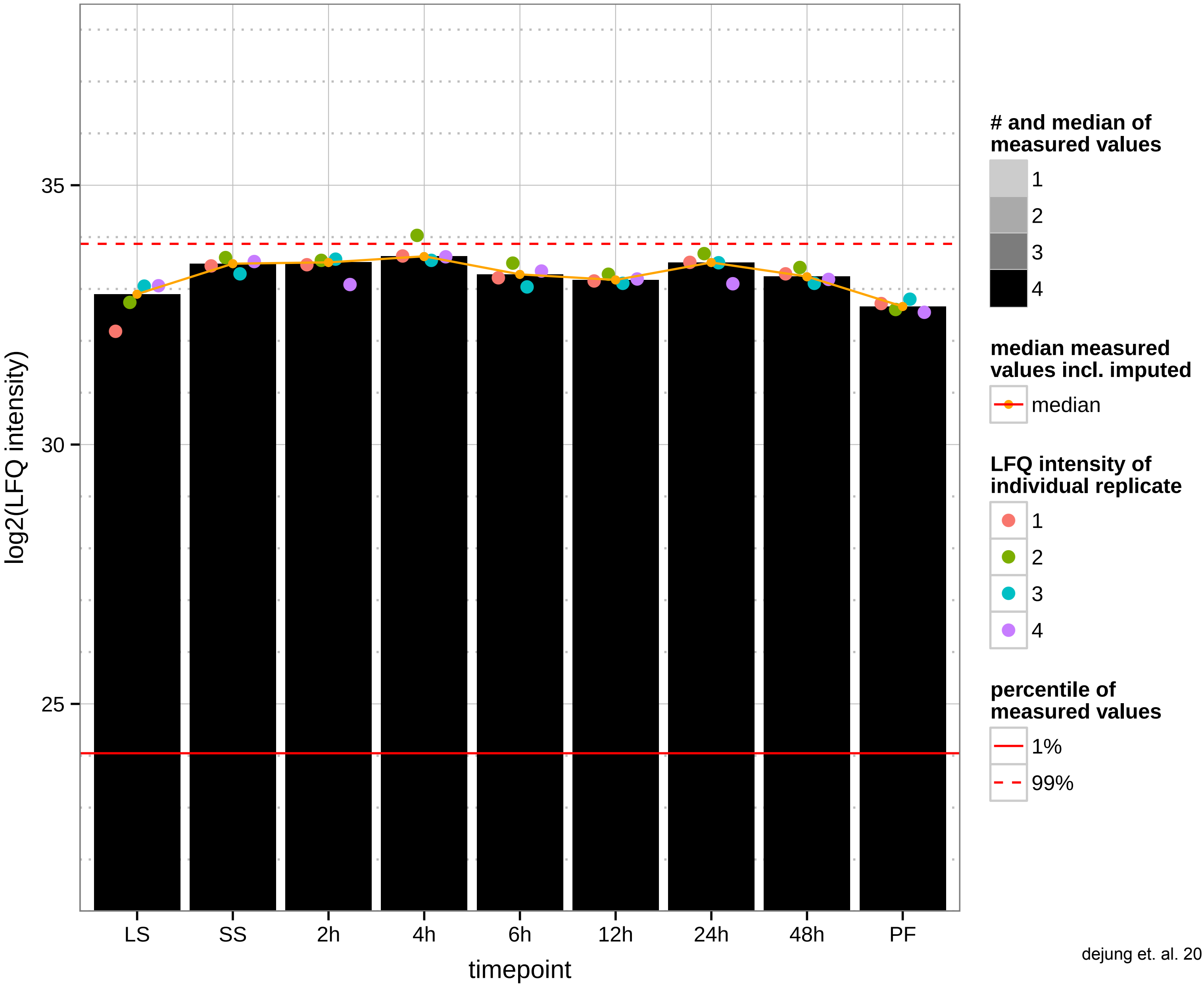

dejung et. al. 2015

hypothetical protein, conserved
Tb927.3.3820
AGOF: null
AGOC: null
AGOP: null
PGOF: null
PGOC: null
PGOP: null

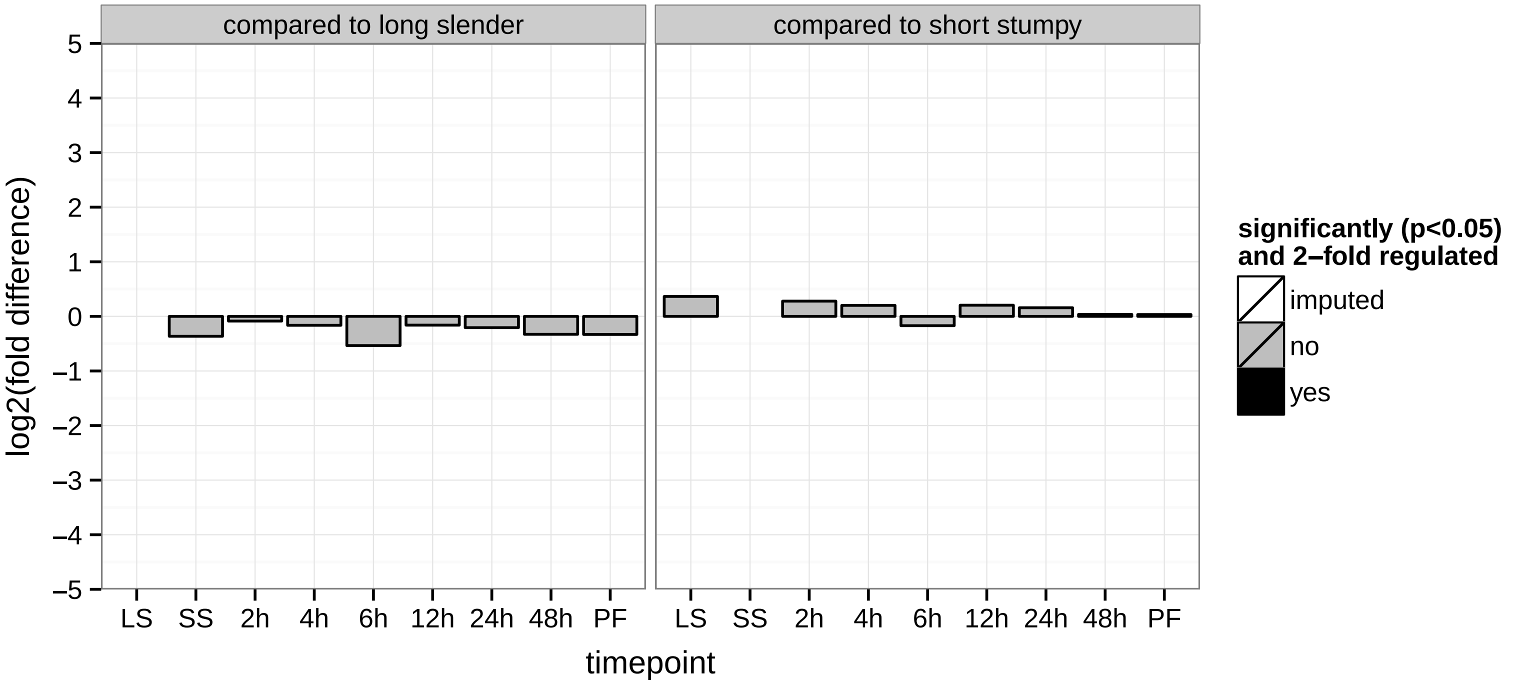

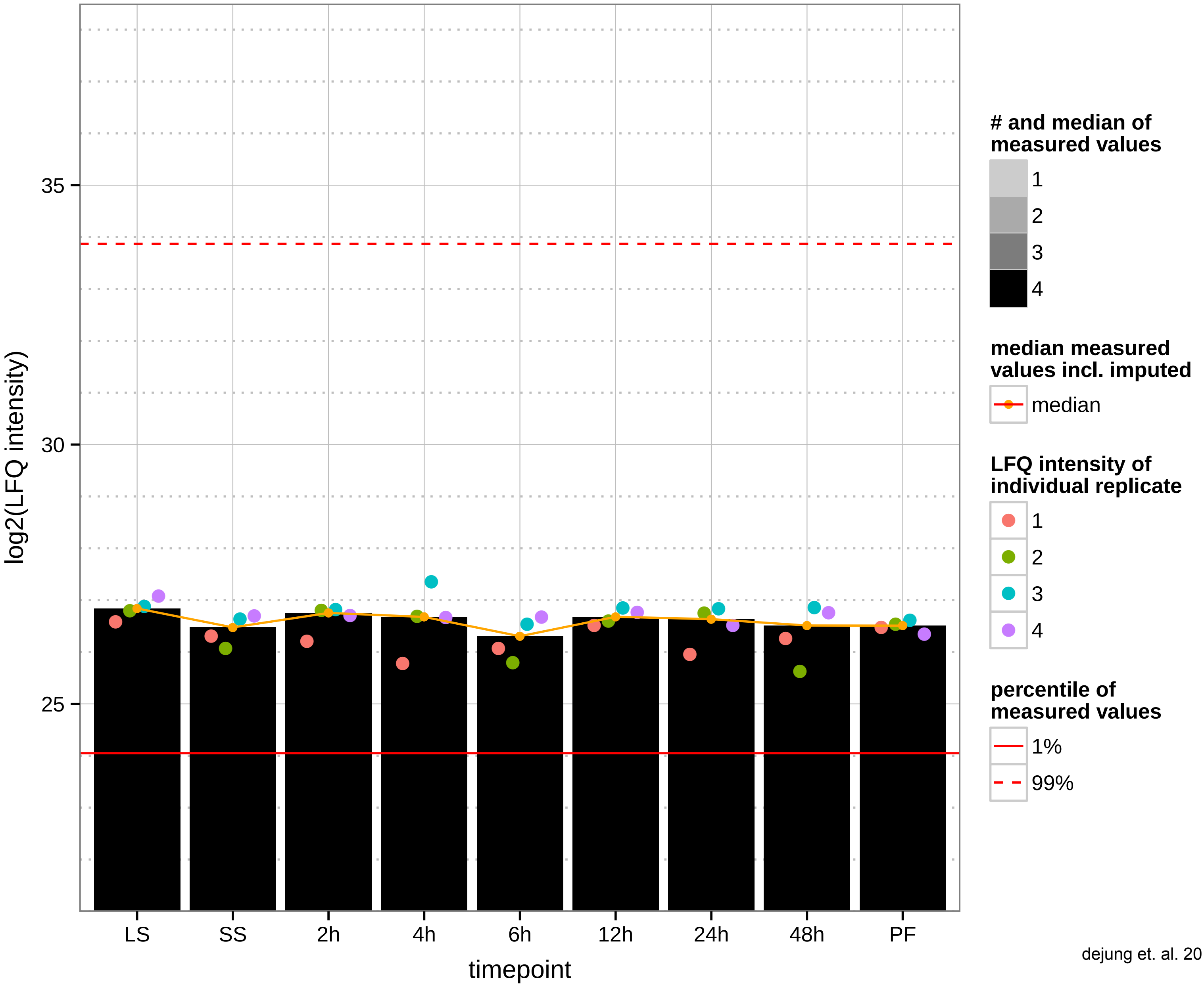

dejung et. al. 2015

hypothetical protein, conserved
Tb927.3.3830
AGOF: null
AGOC: null
AGOP: null
PGOF: null
PGOC: null
PGOP: null

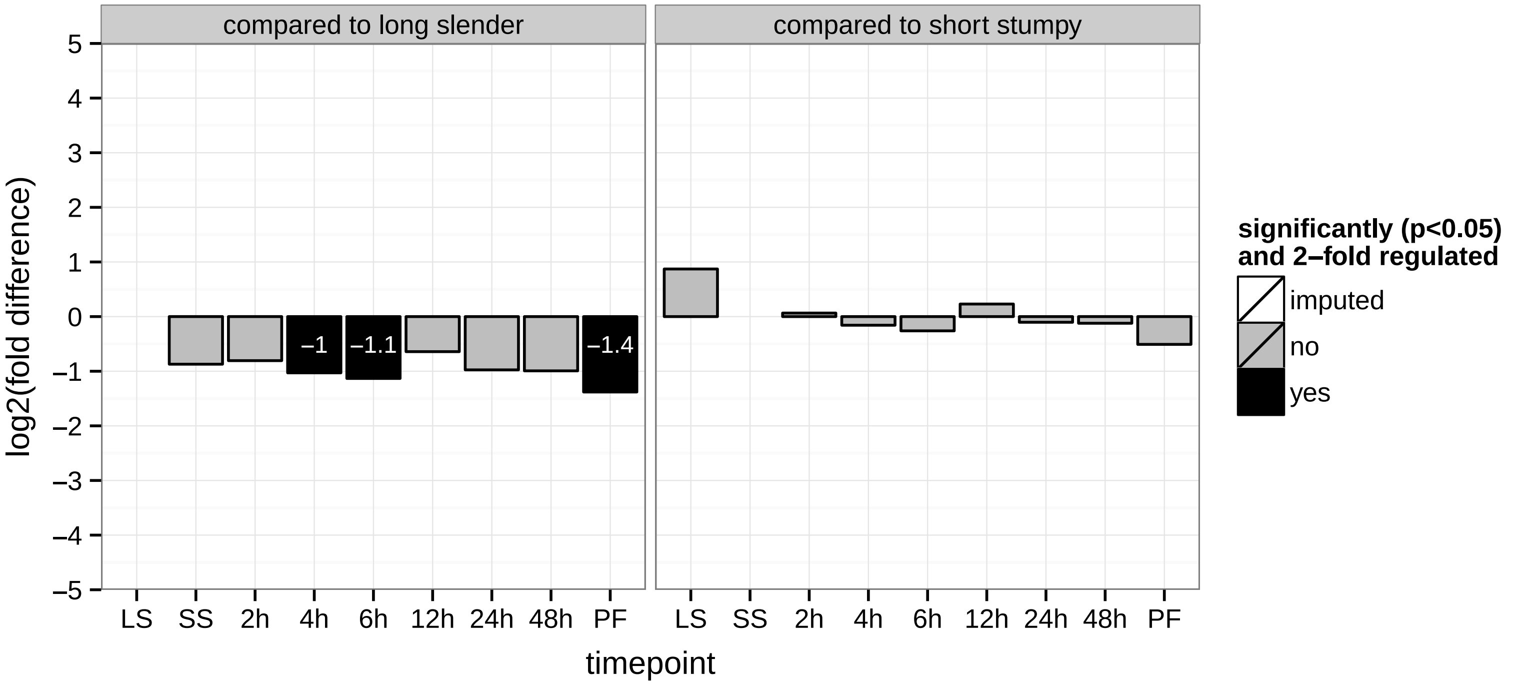

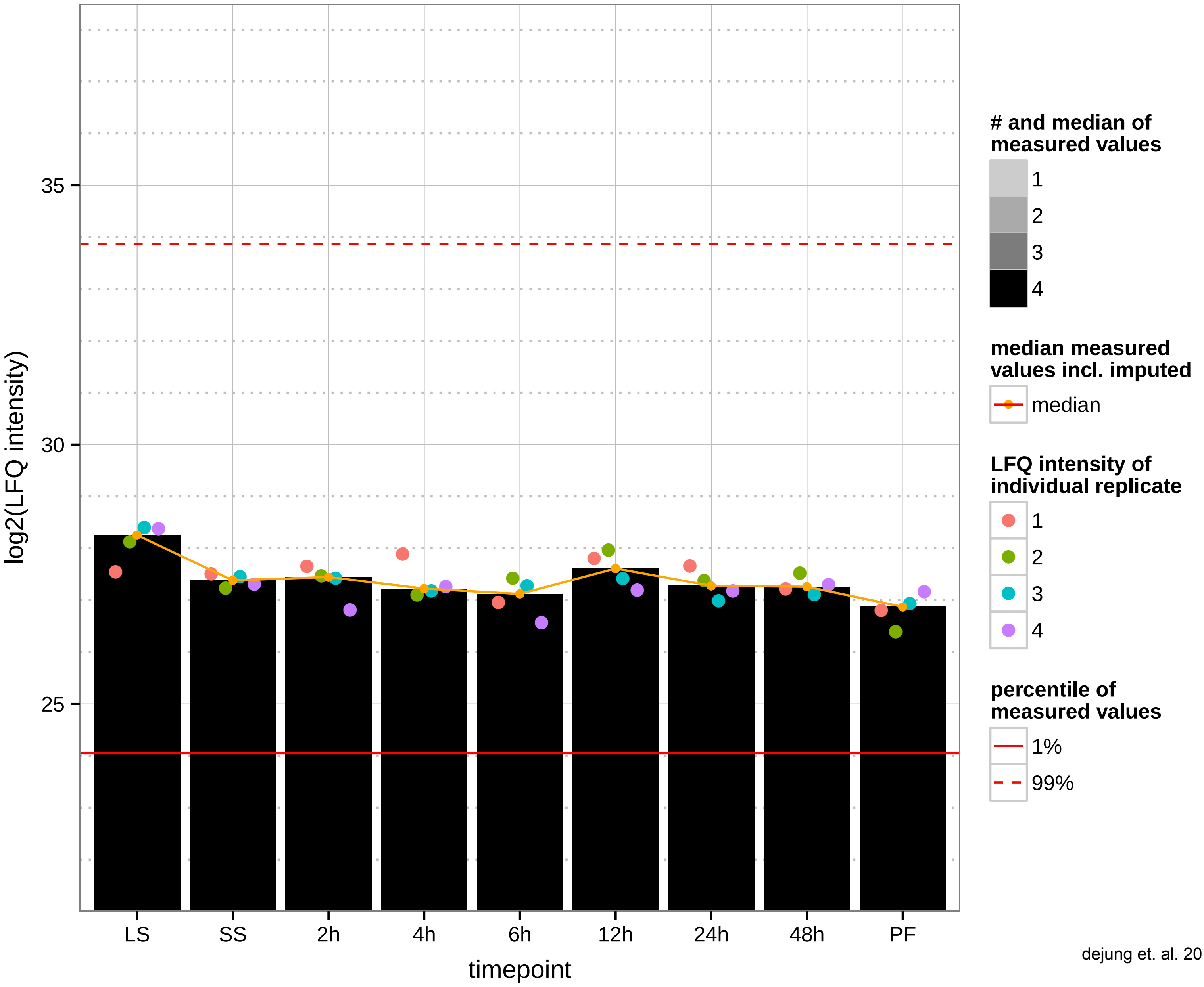

dejung et. al. 2015

COPII coat assembly protein sec16 (sec16)
Tb927.3.3850
AGOF: null
AGOC: endoplasmic reticulum exit site
AGOP: ER to Golgi vesicle–mediated transport, Golgi organization, endoplasmic reticulum organization, protein localization to
PGOF: null
PGOC: null
PGOP: null

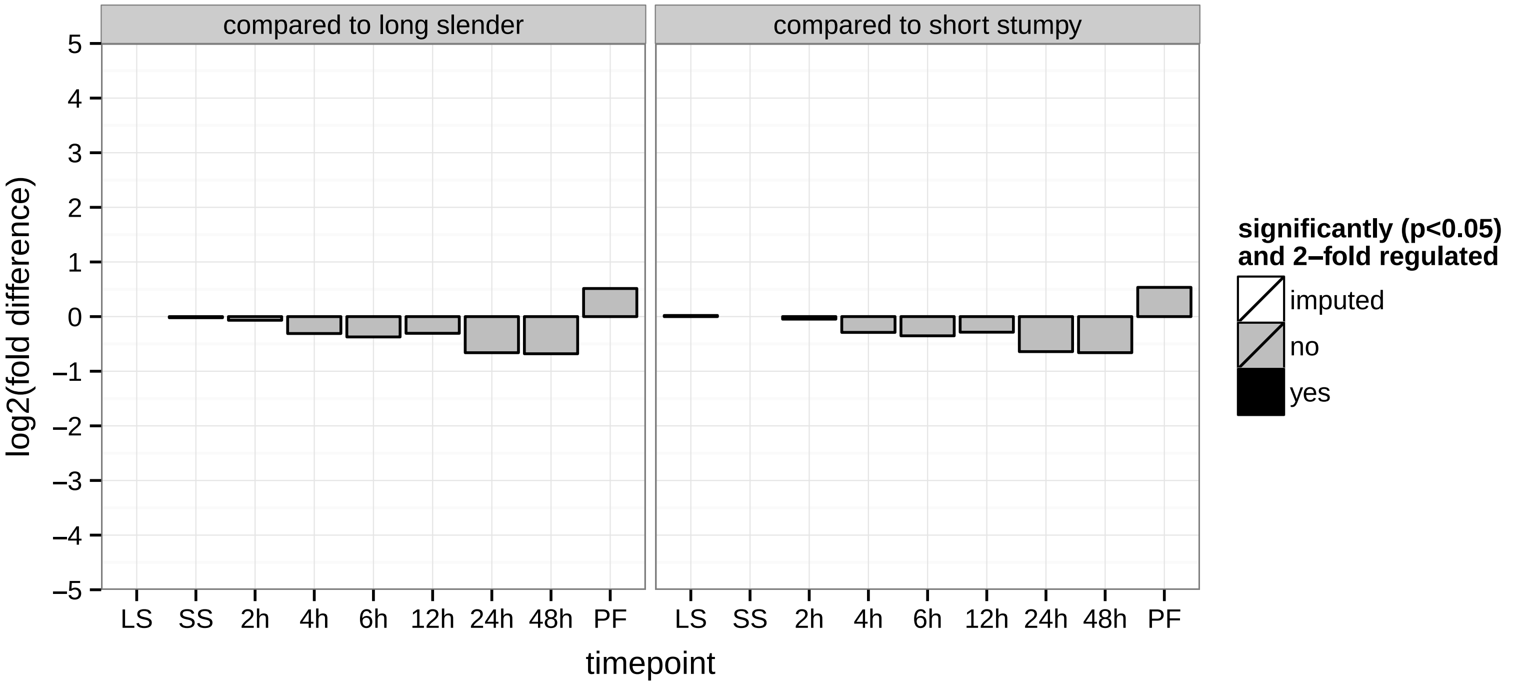

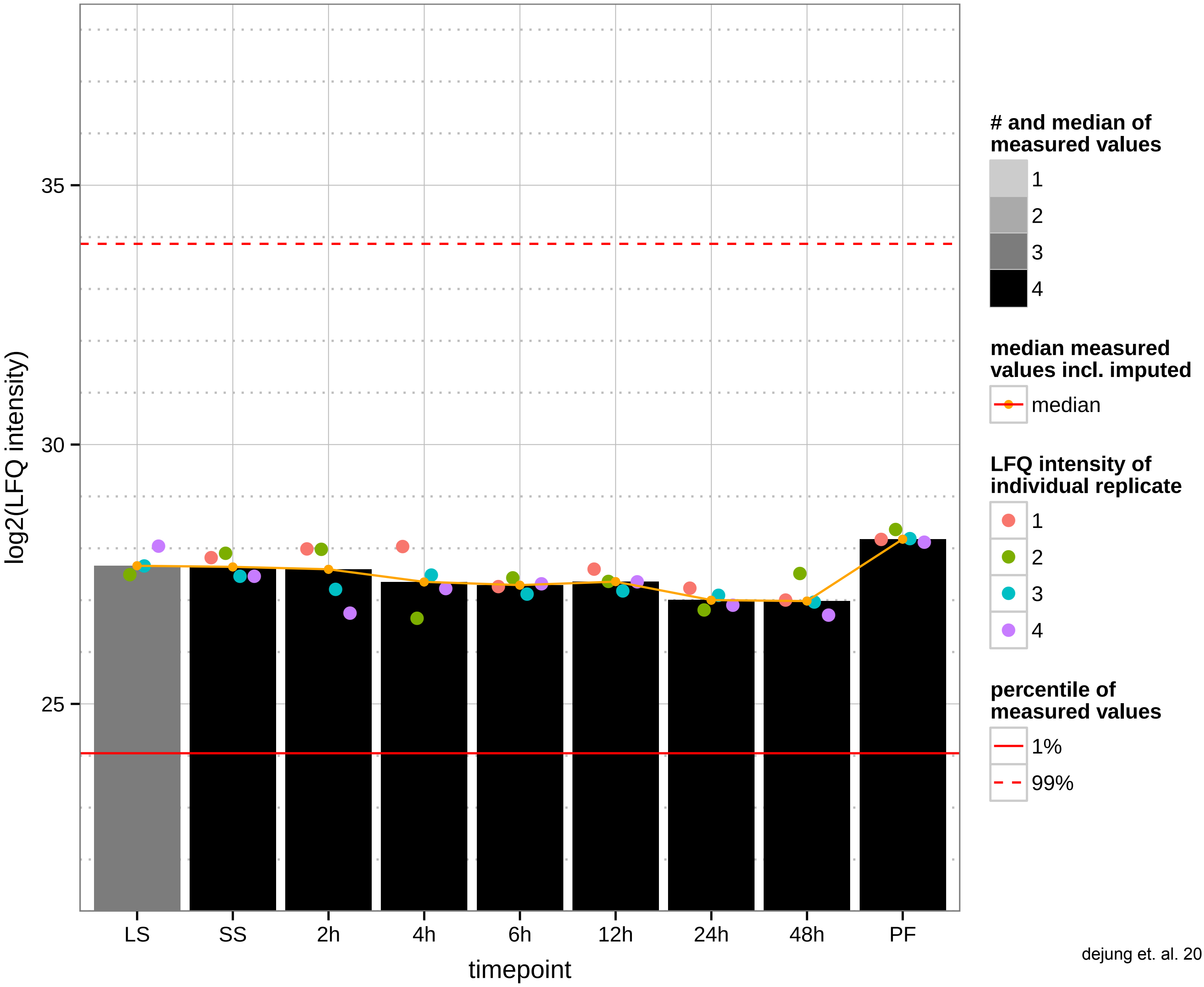

dejung et. al. 2015

carnitine O−palmitoyltransferase II, putative (CPT II)
Tb927.3.3900
AGOF: transferase activity, transferring acyl groups
AGOC: null
AGOP: null
PGOF: transferase activity, transferring acyl groups
PGOC: null
PGOP: null

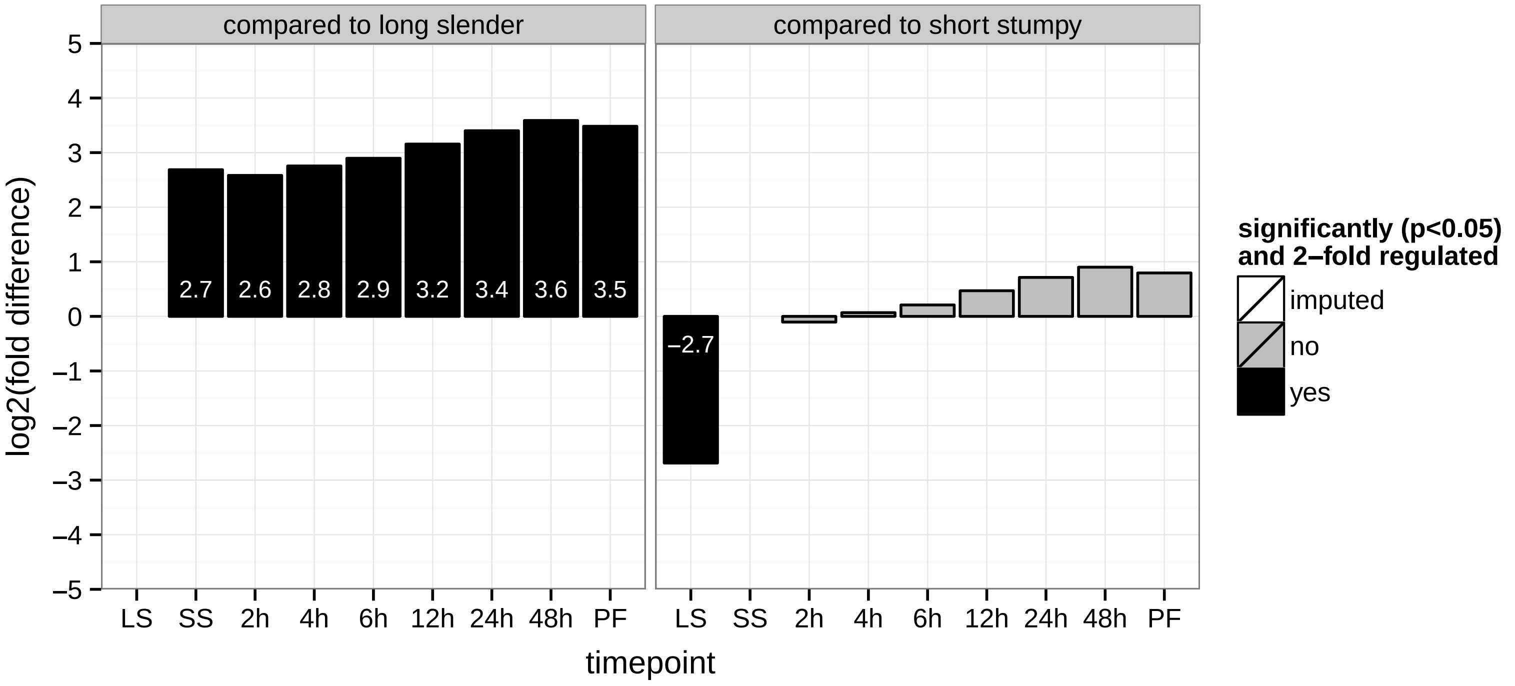

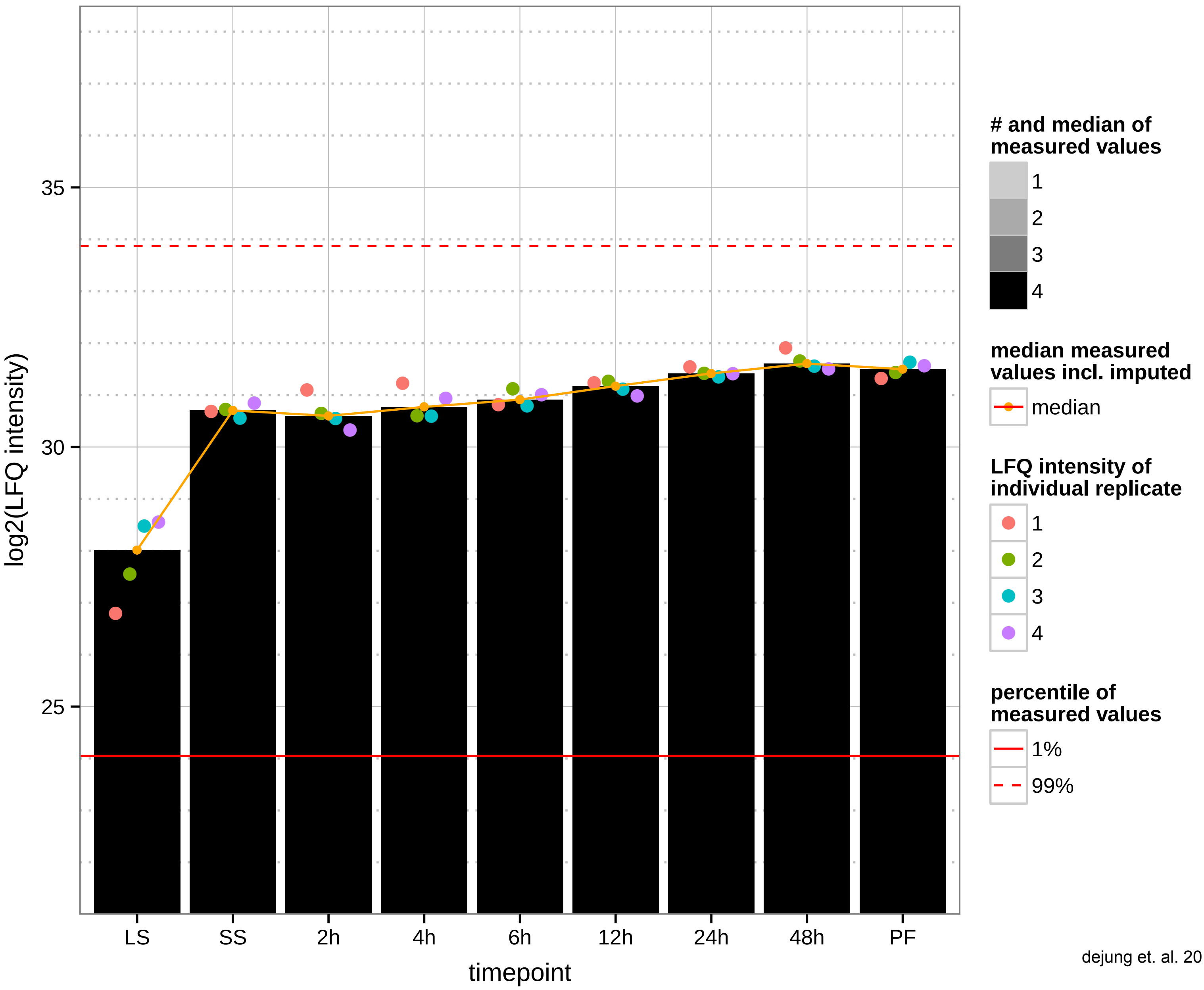

dejung et. al. 2015

clathrin coat assembly protein ap19, putative
Tb927.3.4000
AGOF: protein transporter activity
AGOC: clathrin adaptor complex, clathrin coat of trans−Golgi network vesicle
AGOP: intracellular protein transport, vesicle−mediated transport
PGOF: protein transporter activity
PGOC: null
PGOP: protein transport, transport

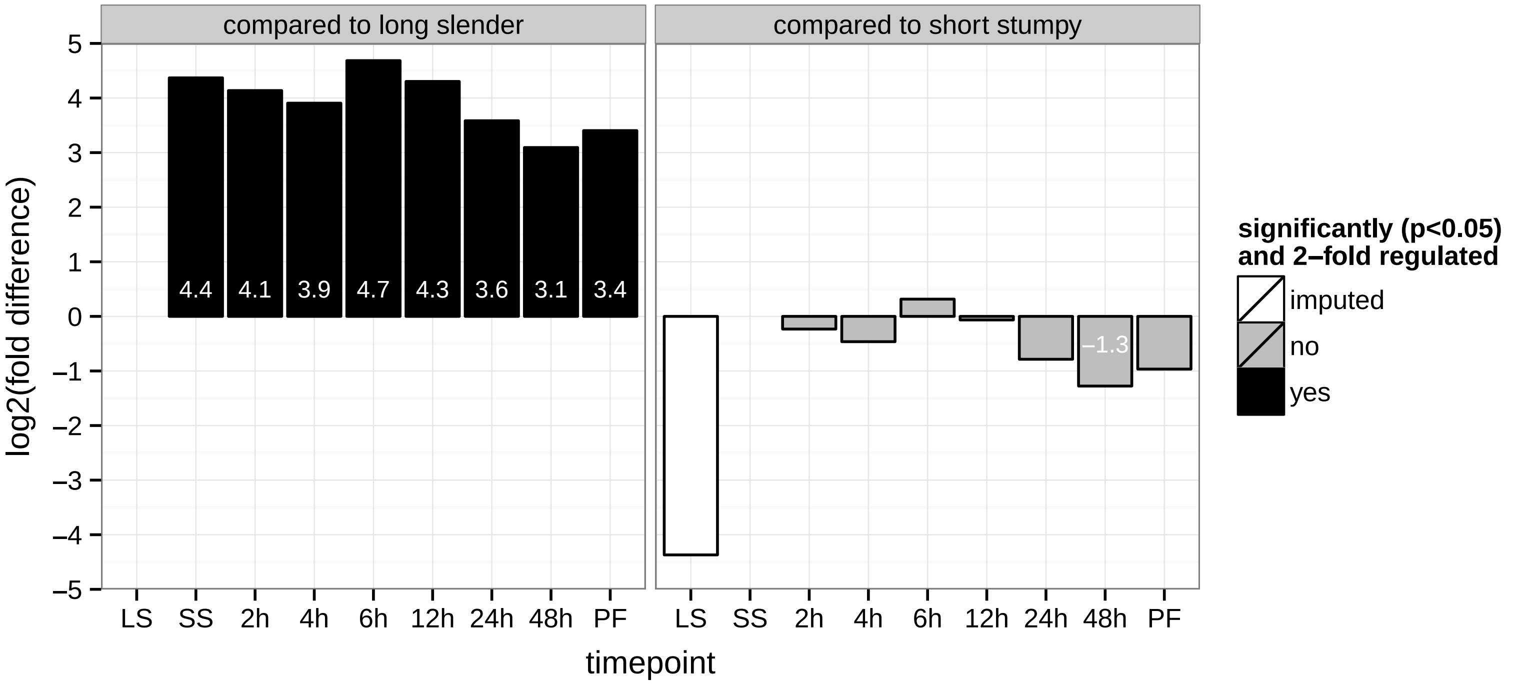

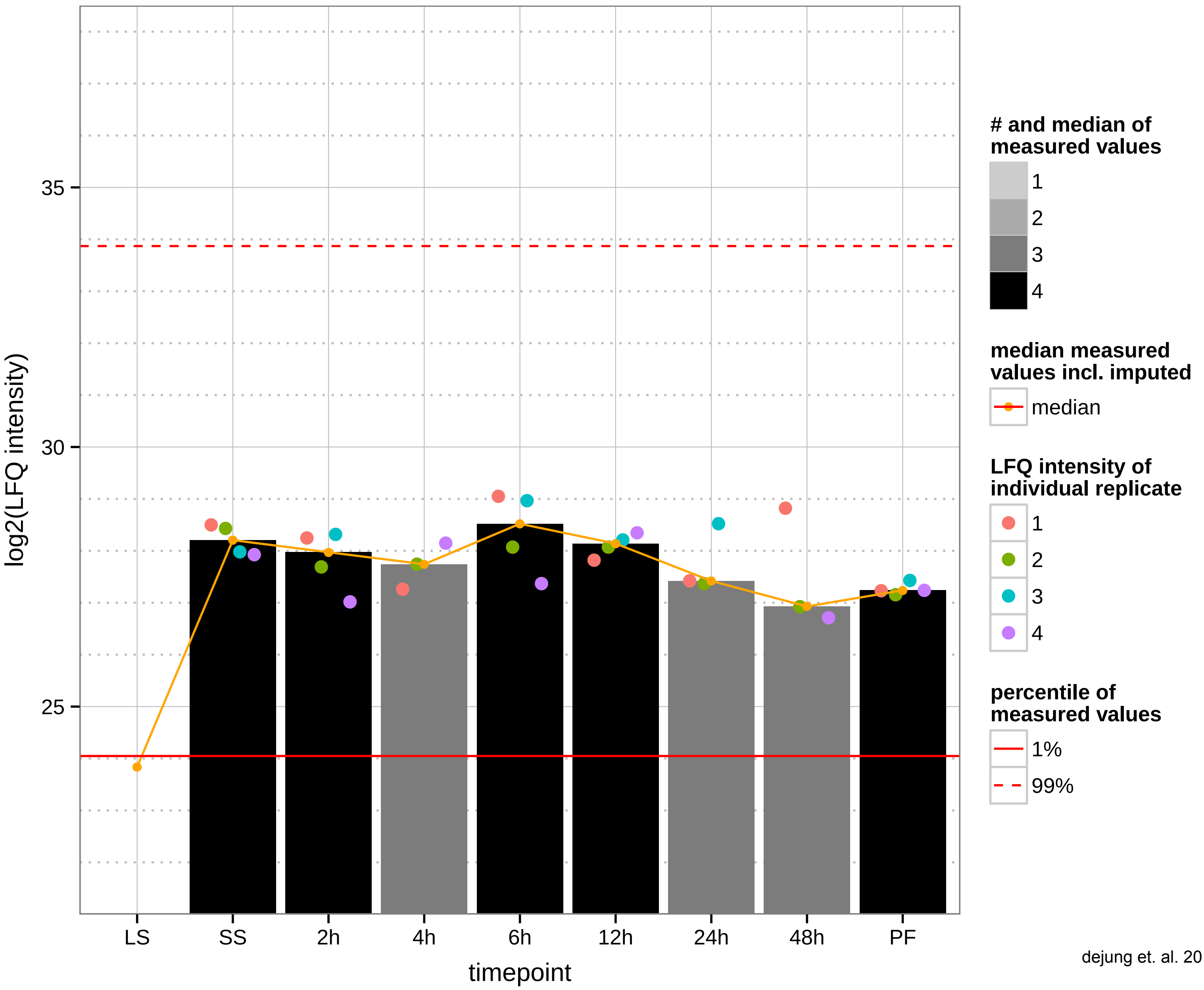

dejung et. al. 2015

phosphatidylinositol 4−kinase alpha, putative
Tb927.3.4020
AGOF: 1−phosphatidylinositol 4−kinase activity, phosphotransferase activity, alcohol group as acceptor
AGOC: cytosol
AGOP: phosphatidylinositol phosphorylation, phosphatidylinositol−mediated signaling
PGOF: binding, phosphotransferase activity, alcohol group as acceptor, transferase activity, transferring phosphorus−containin
PGOC: null
PGOP: null

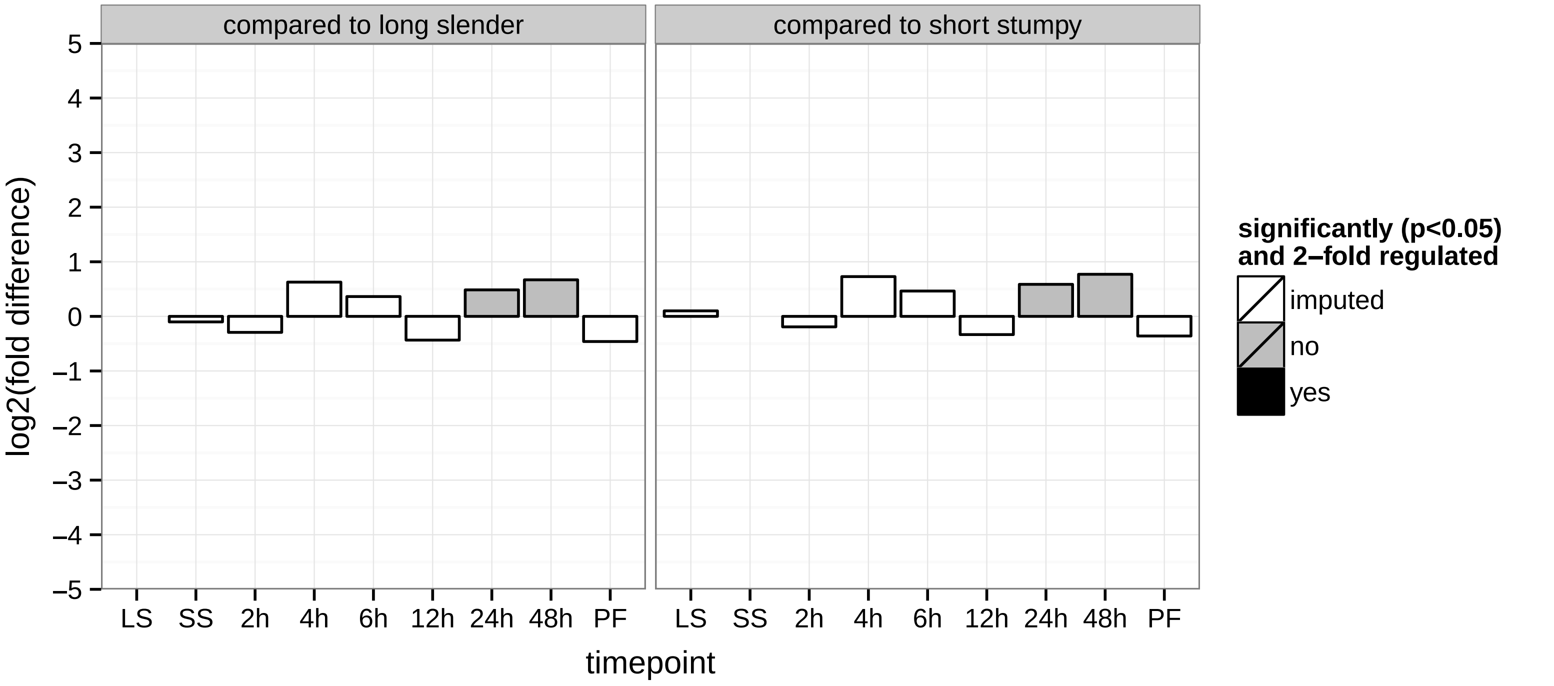

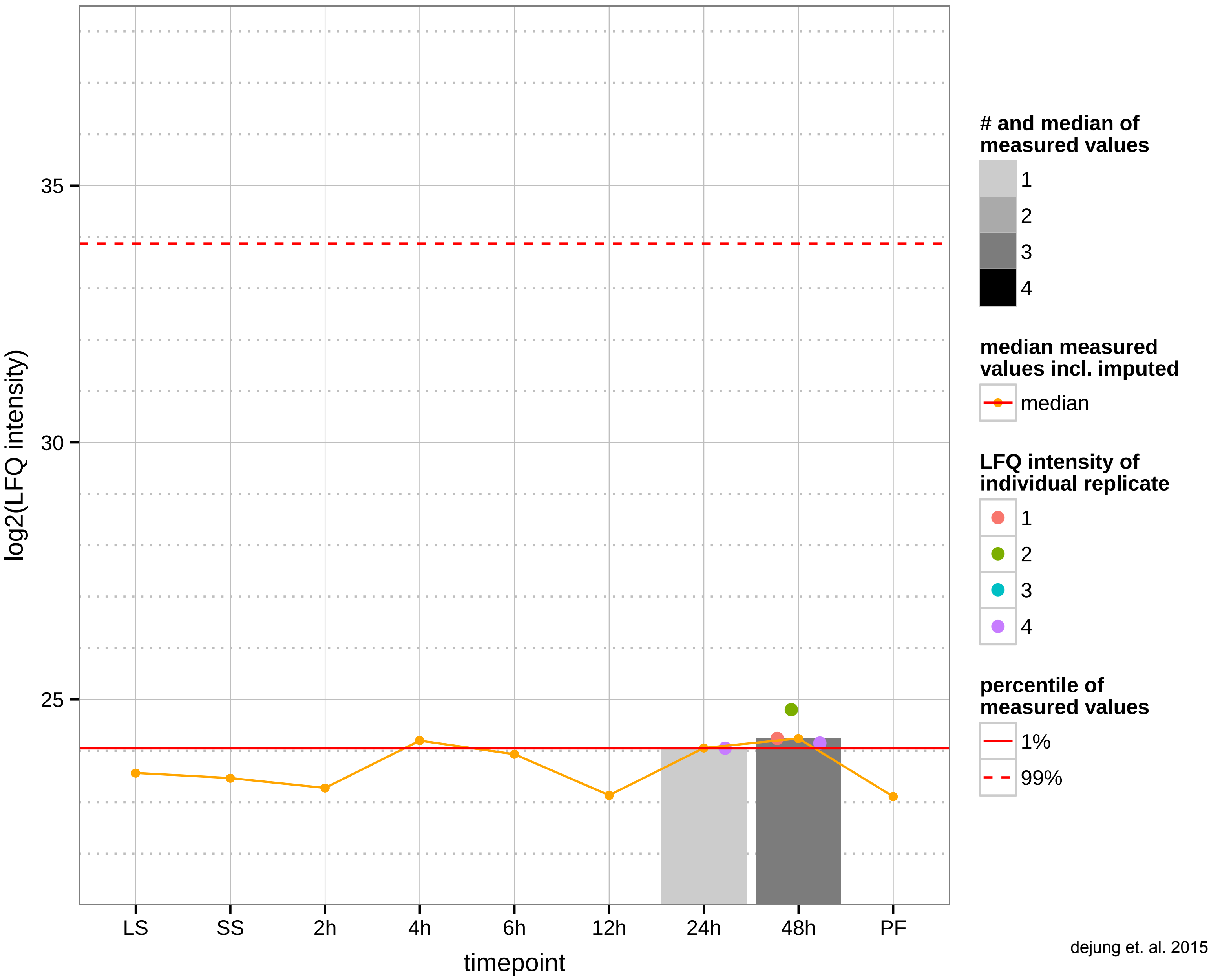

dejung et. al. 2015

hypothetical protein, conserved
Tb927.3.4030
AGOF: null
AGOC: mitochondrion
AGOP: null
PGOF: null
PGOC: null
PGOP: null

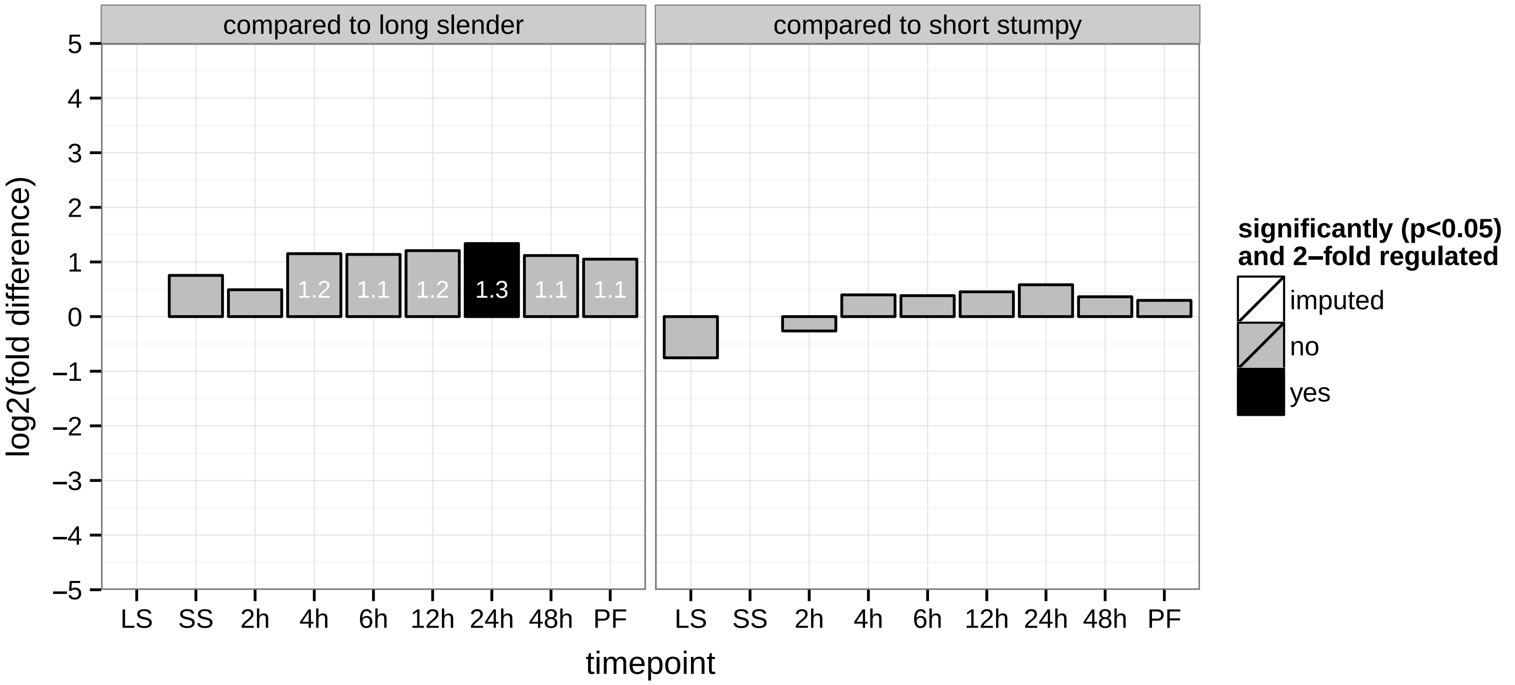

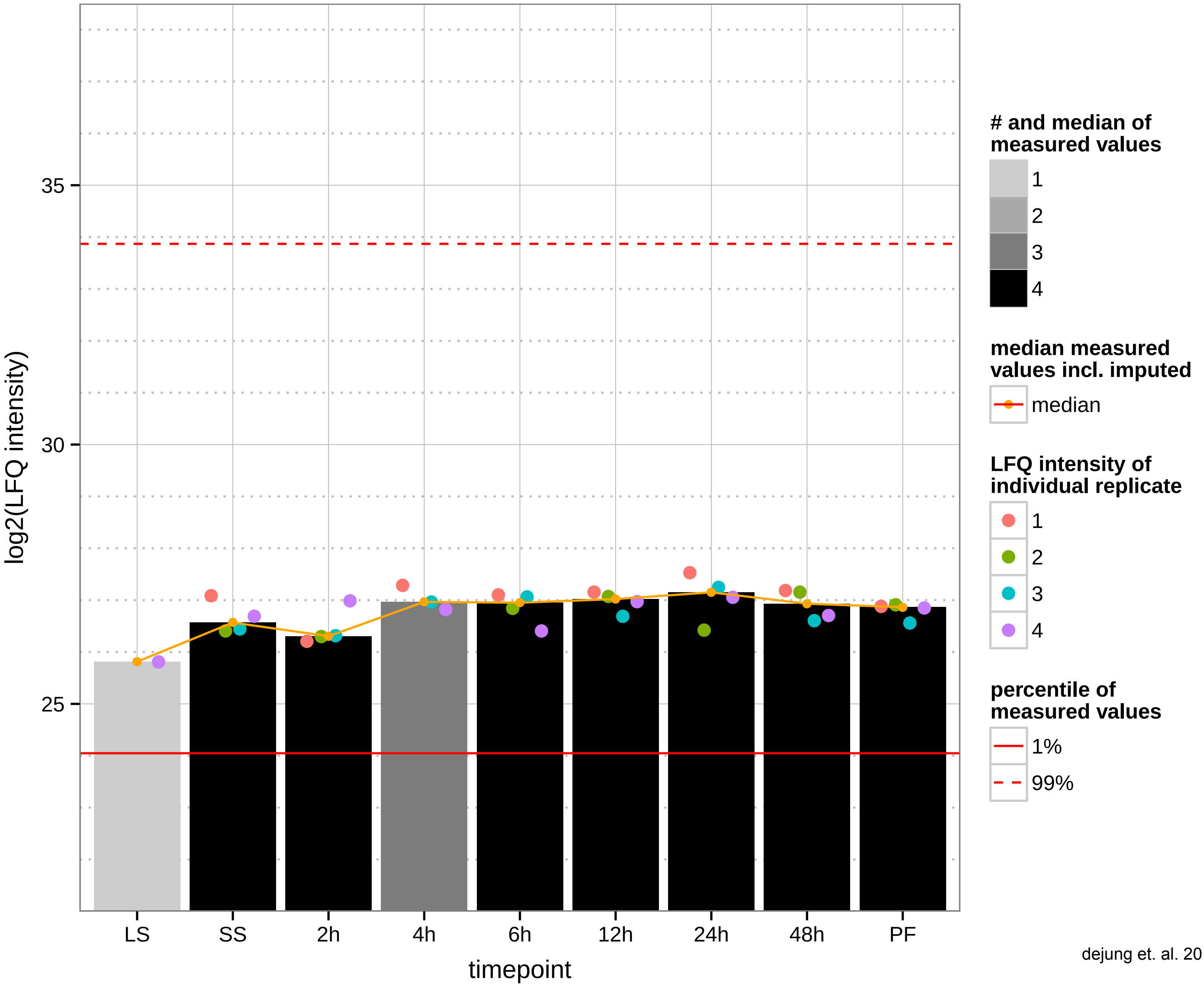

dejung et. al. 2015

hypothetical protein, conserved
Tb927.3.4040
AGOF: null
AGOC: null
AGOP: null
PGOF: protein binding
PGOC: null
PGOP: null

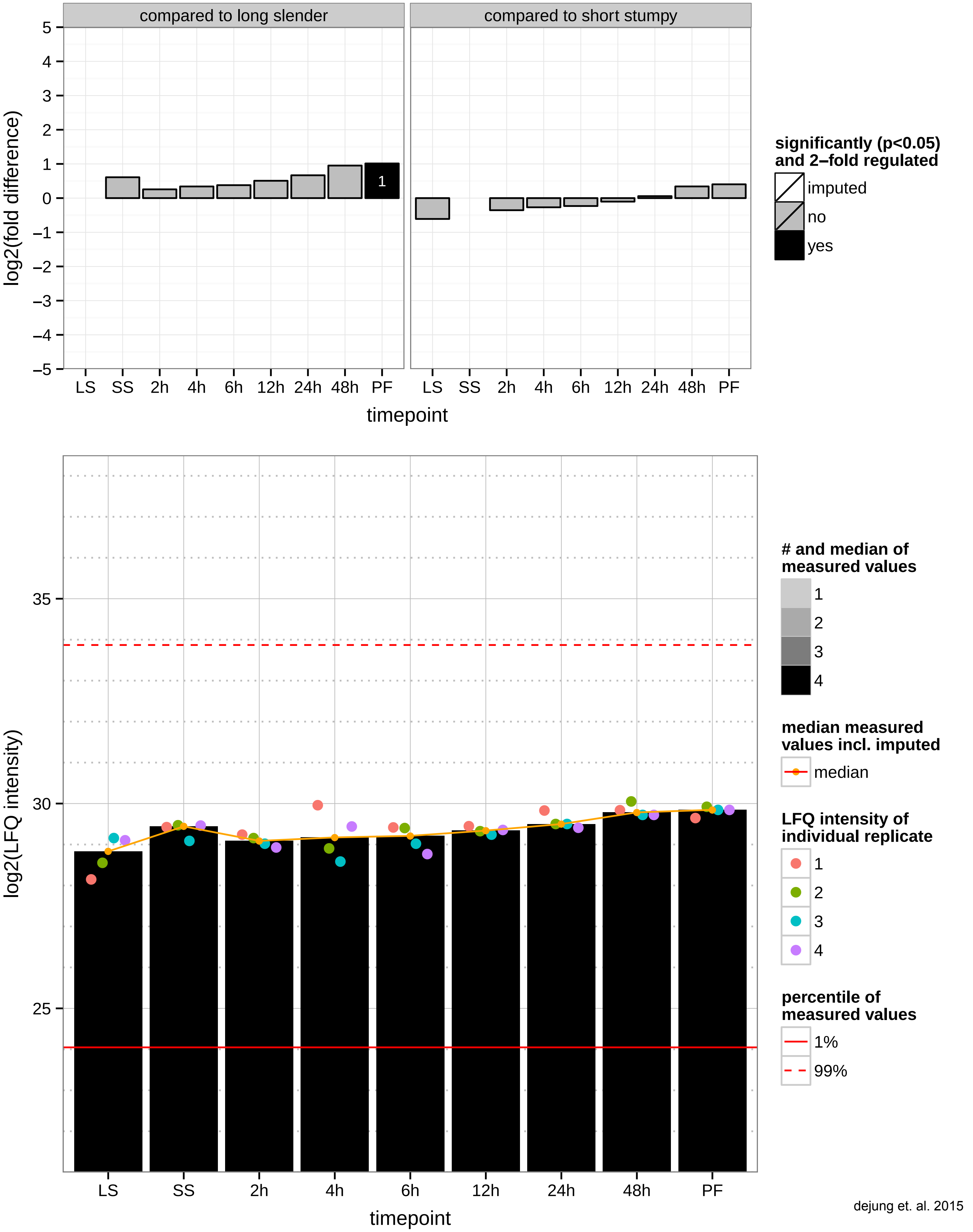

dejung et. al. 2015

hypothetical protein, conserved
Tb927.3.4120
AGOF: null
AGOC: null
AGOP: null
PGOF: null
PGOC: null
PGOP: null

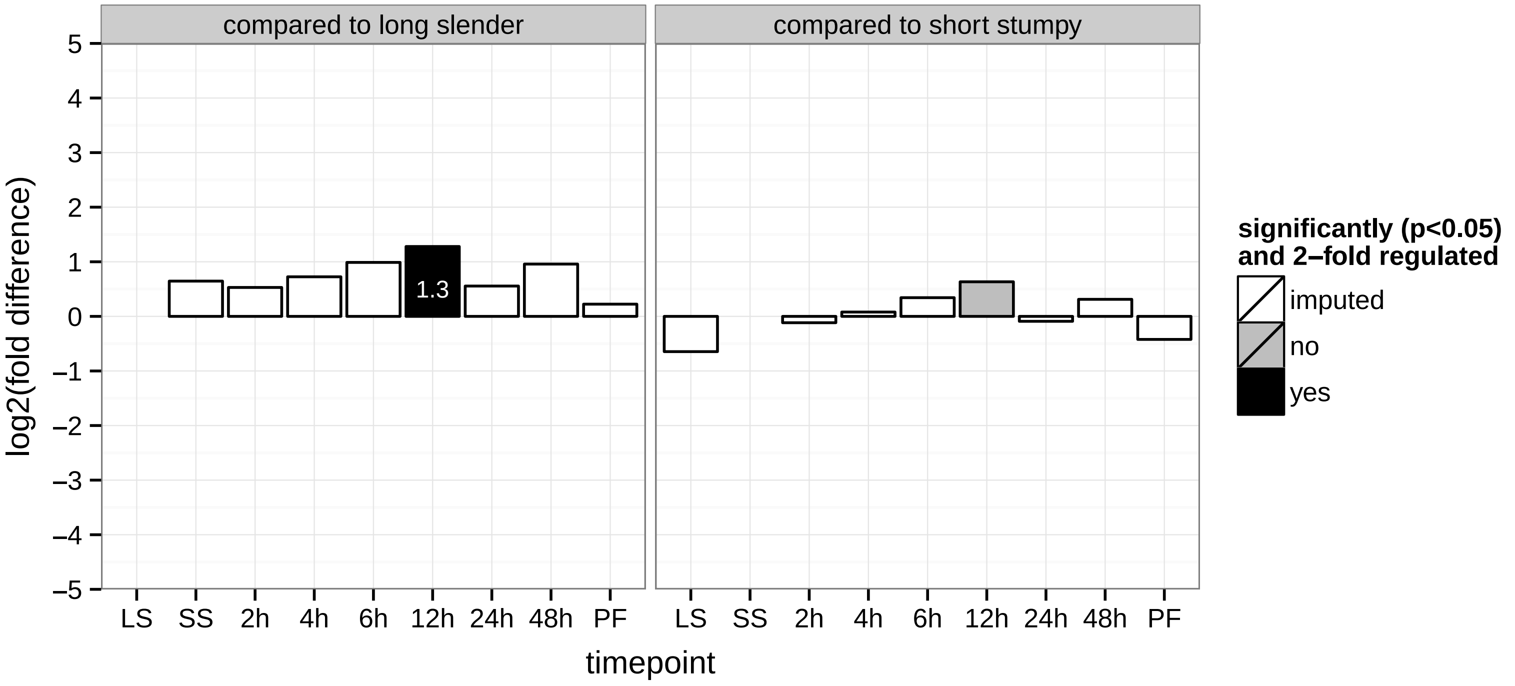

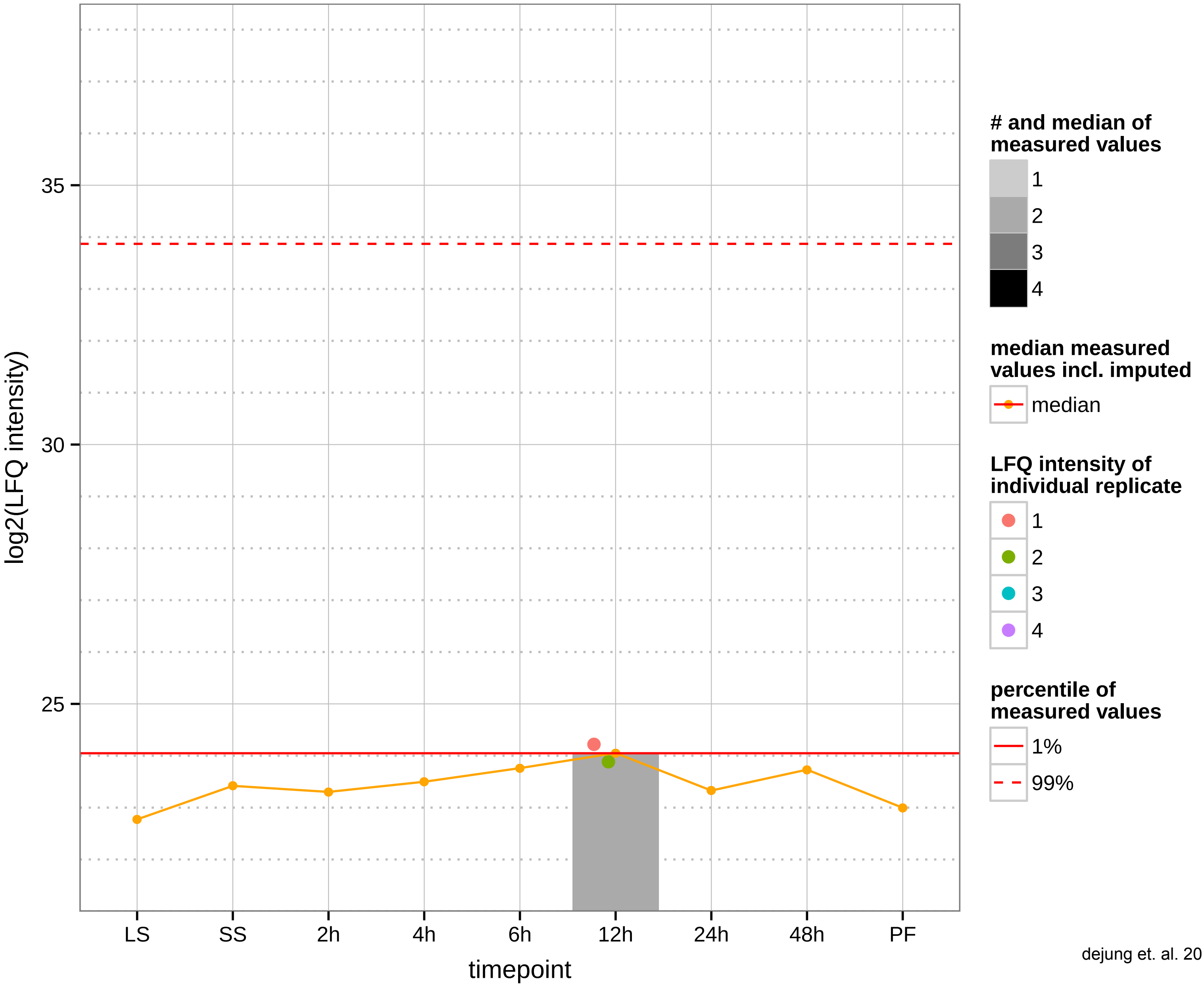

dejung et. al. 2015

hypothetical protein, conserved
Tb927.3.4140
AGOF: null
AGOC: mitochondrion
AGOP: null
PGOF: null
PGOC: null
PGOP: null

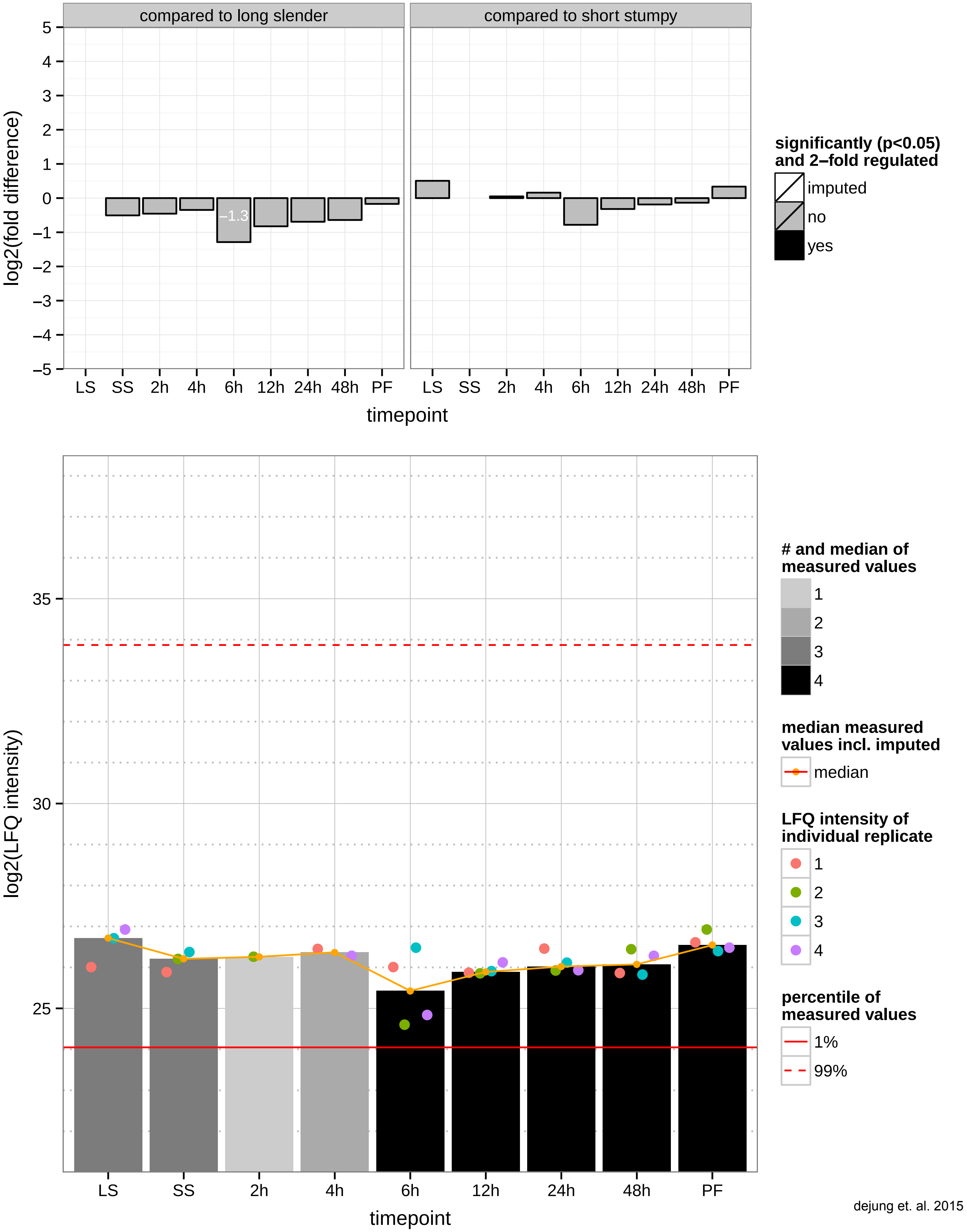

endosomal integral membrane protein, putative
Tb927.3.4190
AGOF: null
AGOC: integral to membrane
AGOP: null
PGOF: null
PGOC: integral to membrane
PGOP: null

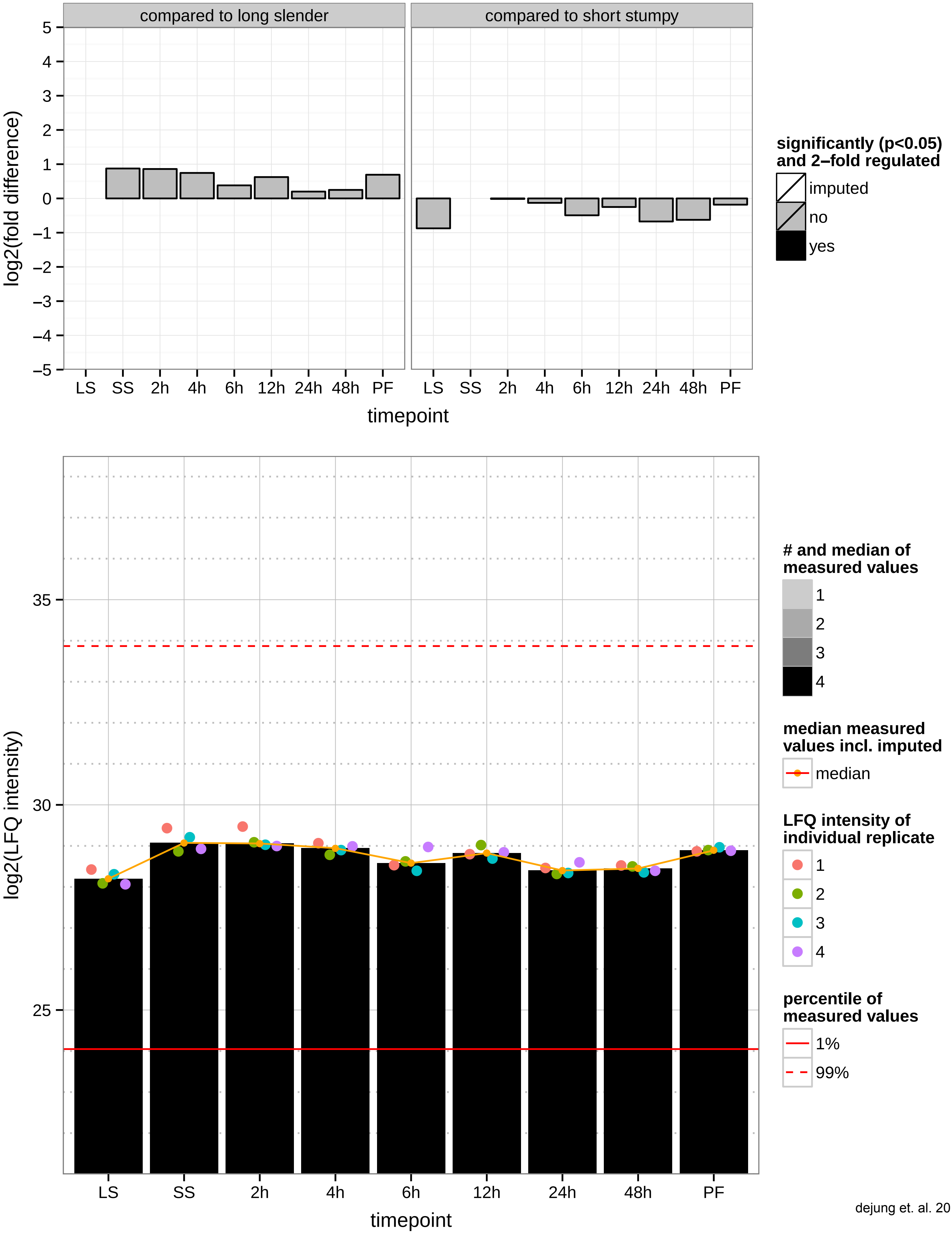

dejung et. al. 2015

Tob55, putative
Tb927.3.4380
AGOF: null
AGOC: mitochondrial outer membrane
AGOP: cellular protein complex assembly, protein complex assembly
PGOF: null
PGOC: outer membrane
PGOP: null

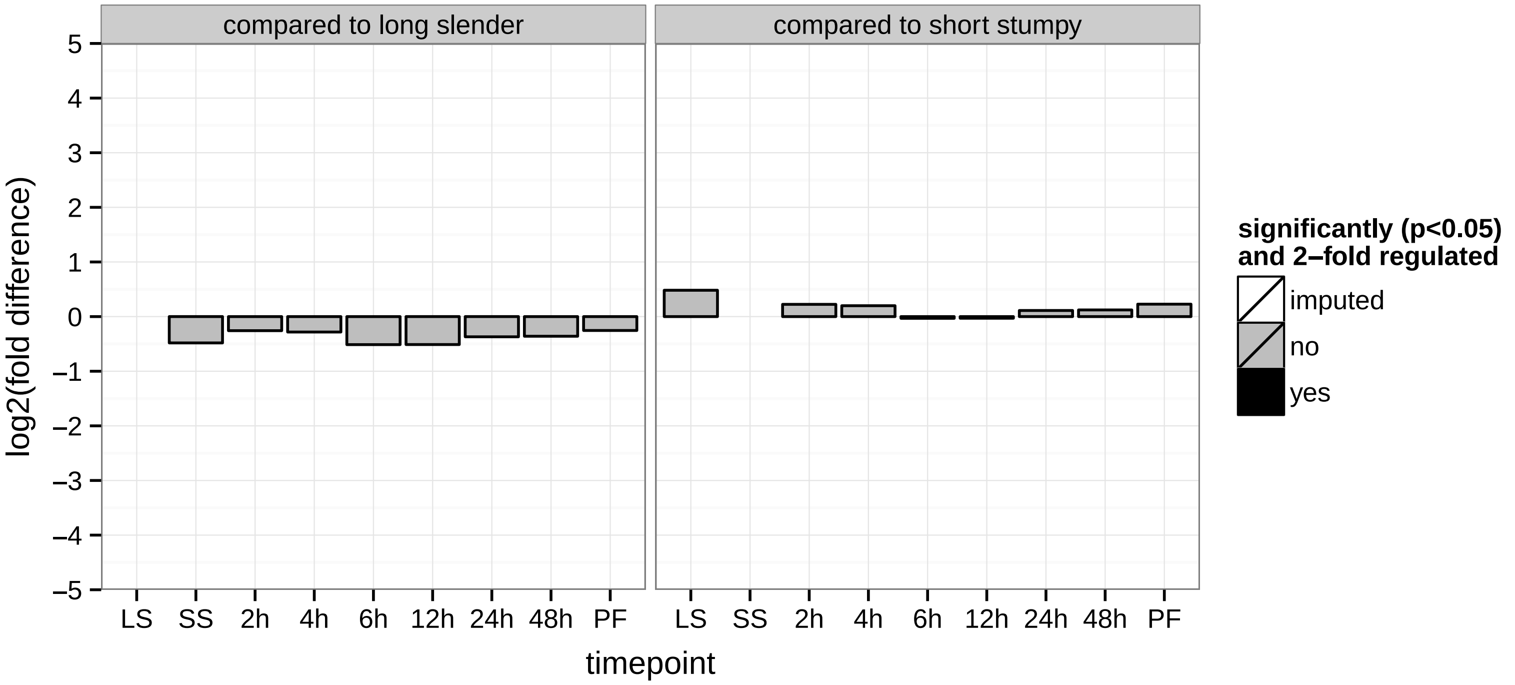

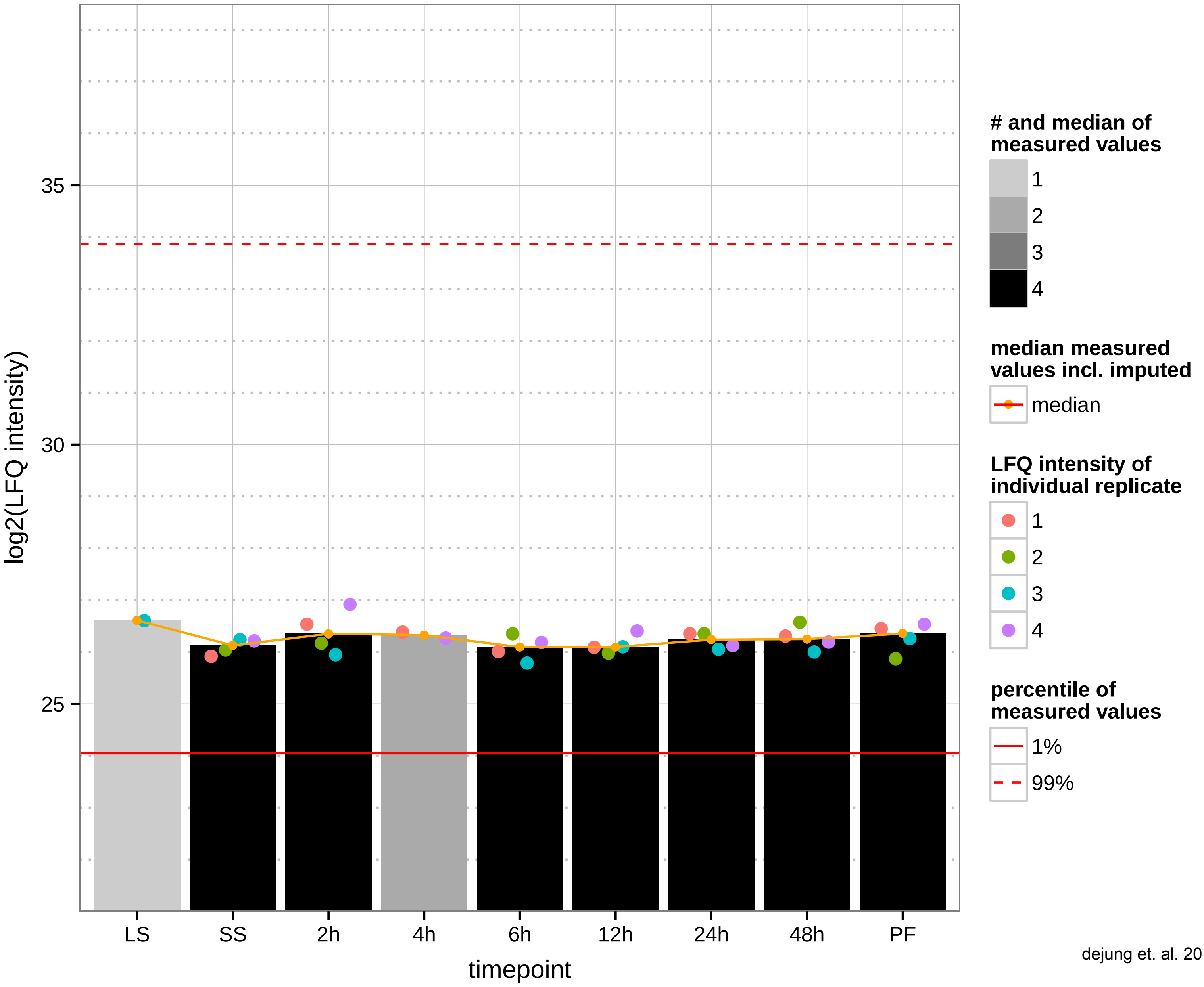

dejung et. al. 2015

hypothetical protein, conserved
Tb927.3.4550
AGOF: null
AGOC: null
AGOP: null
PGOF: null
PGOC: null
PGOP: null

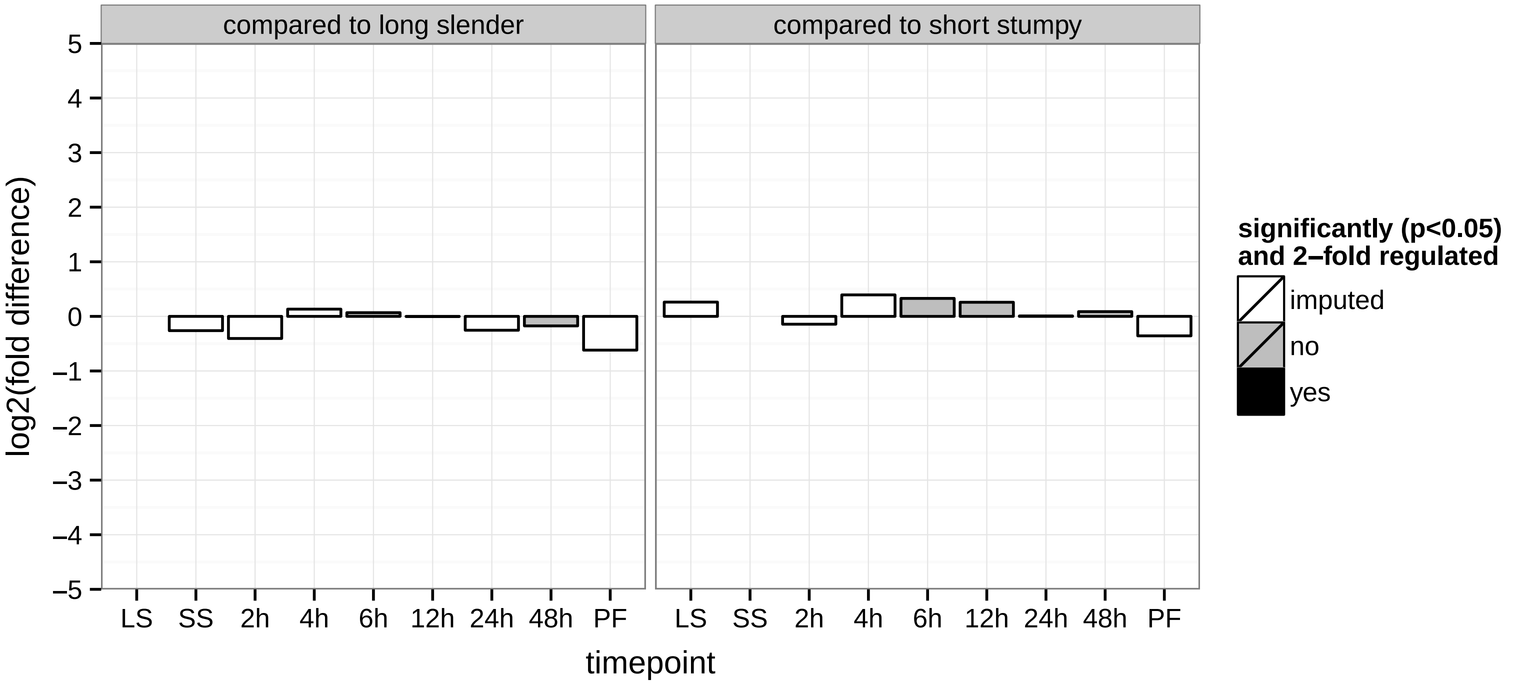

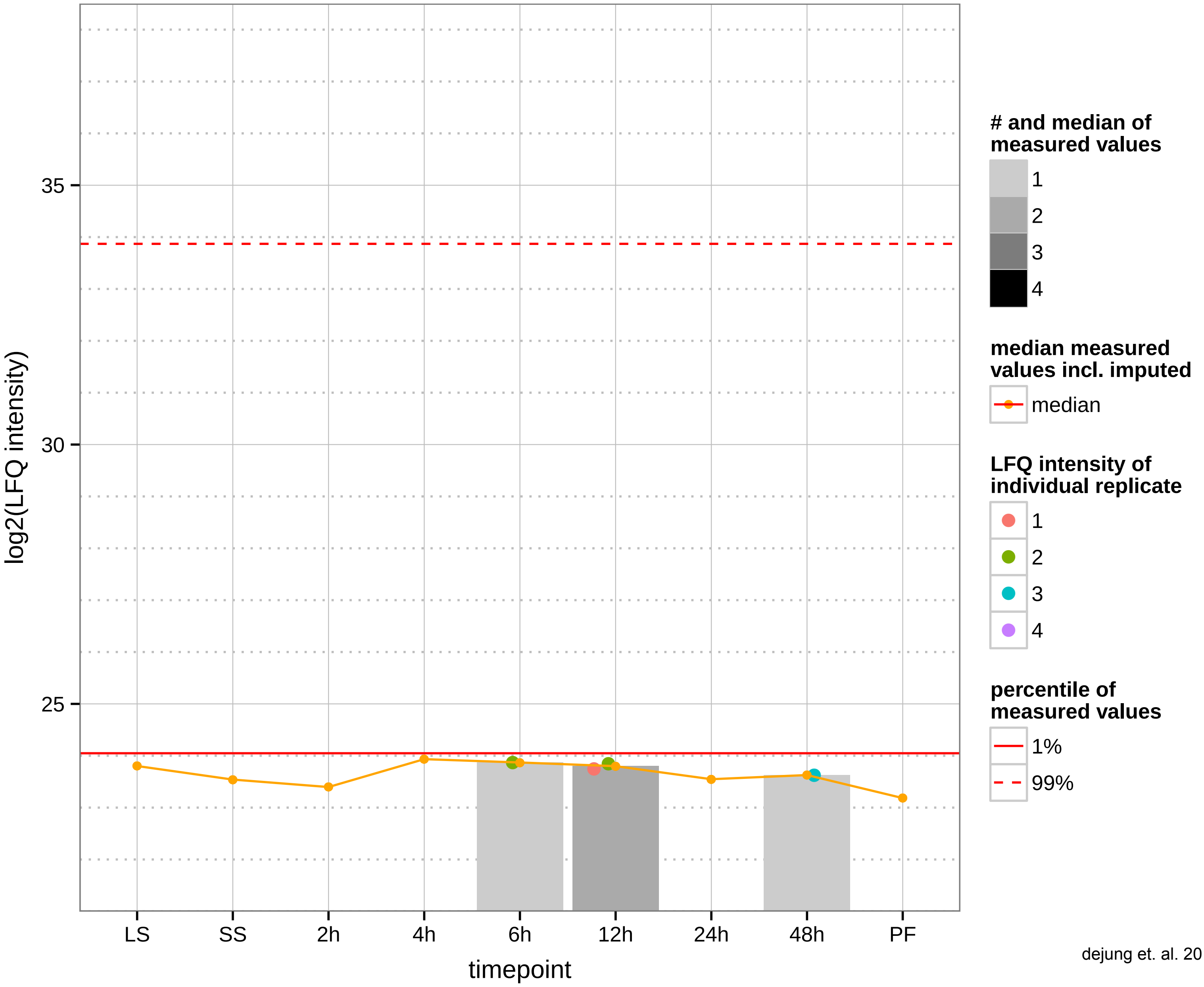

dejung et. al. 2015

hypothetical protein, conserved
Tb927.3.4600
AGOF: null
AGOC: null
AGOP: null
PGOF: binding
PGOC: null
PGOP: null

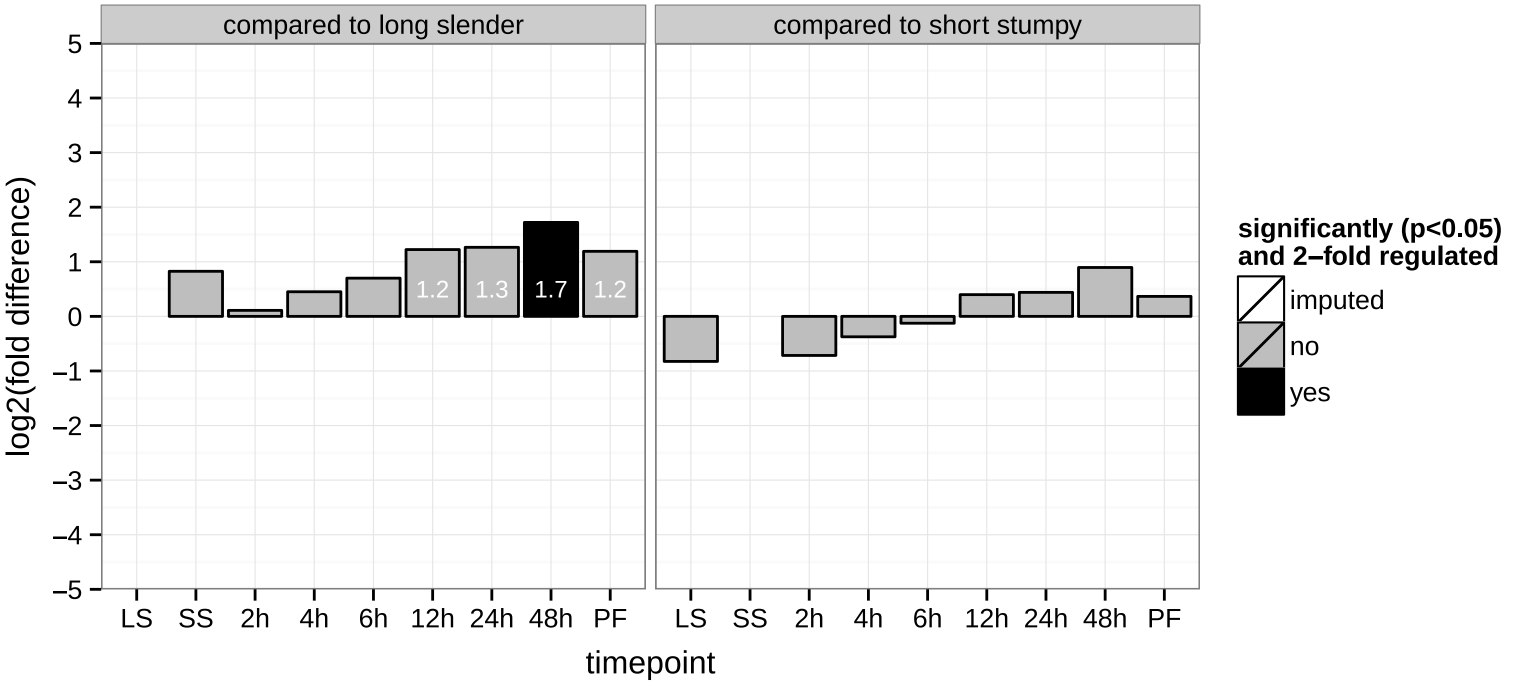

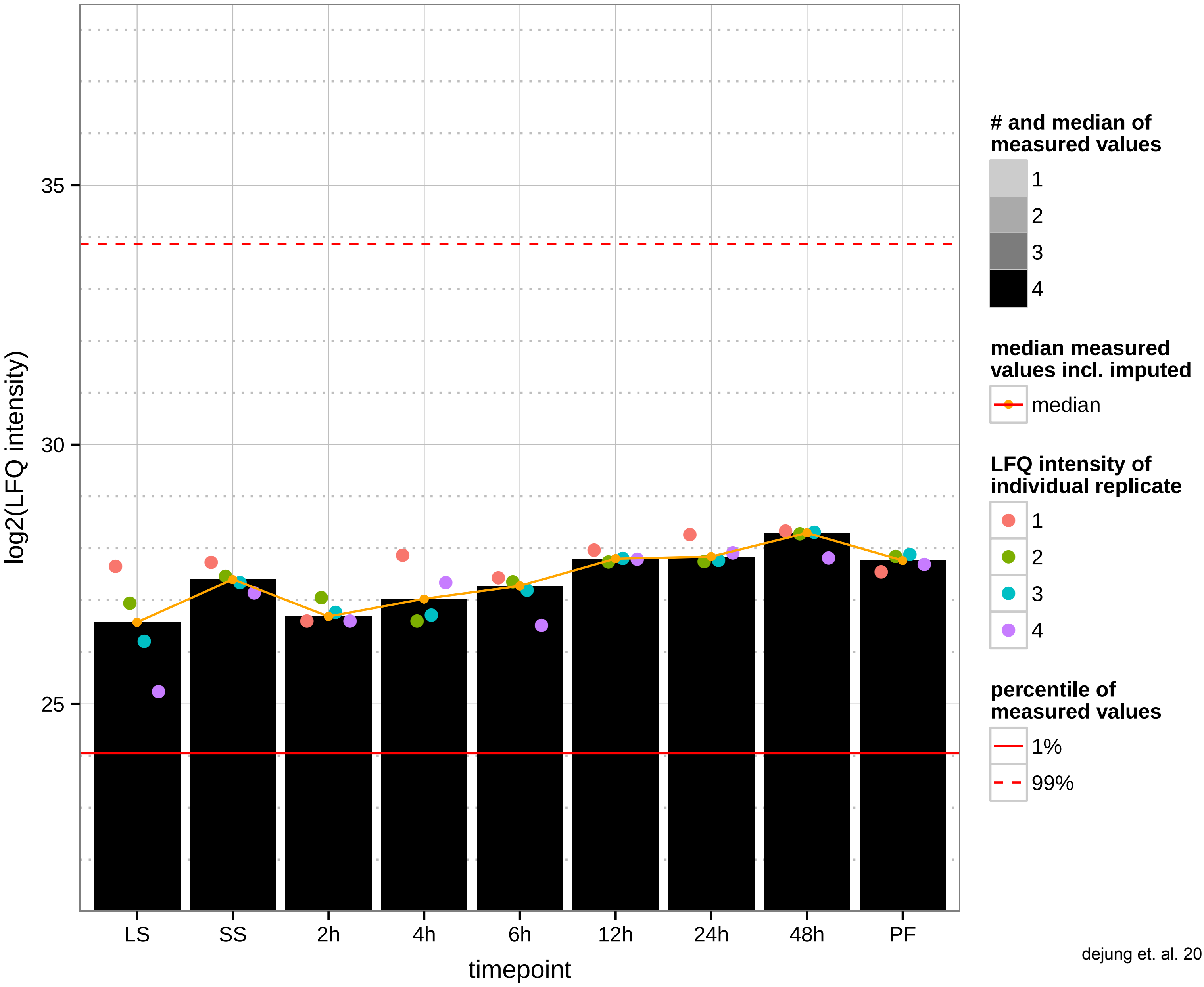

dejung et. al. 2015

hypothetical protein, conserved
Tb927.3.4640
AGOF: null
AGOC: null
AGOP: null
PGOF: null
PGOC: null
PGOP: null

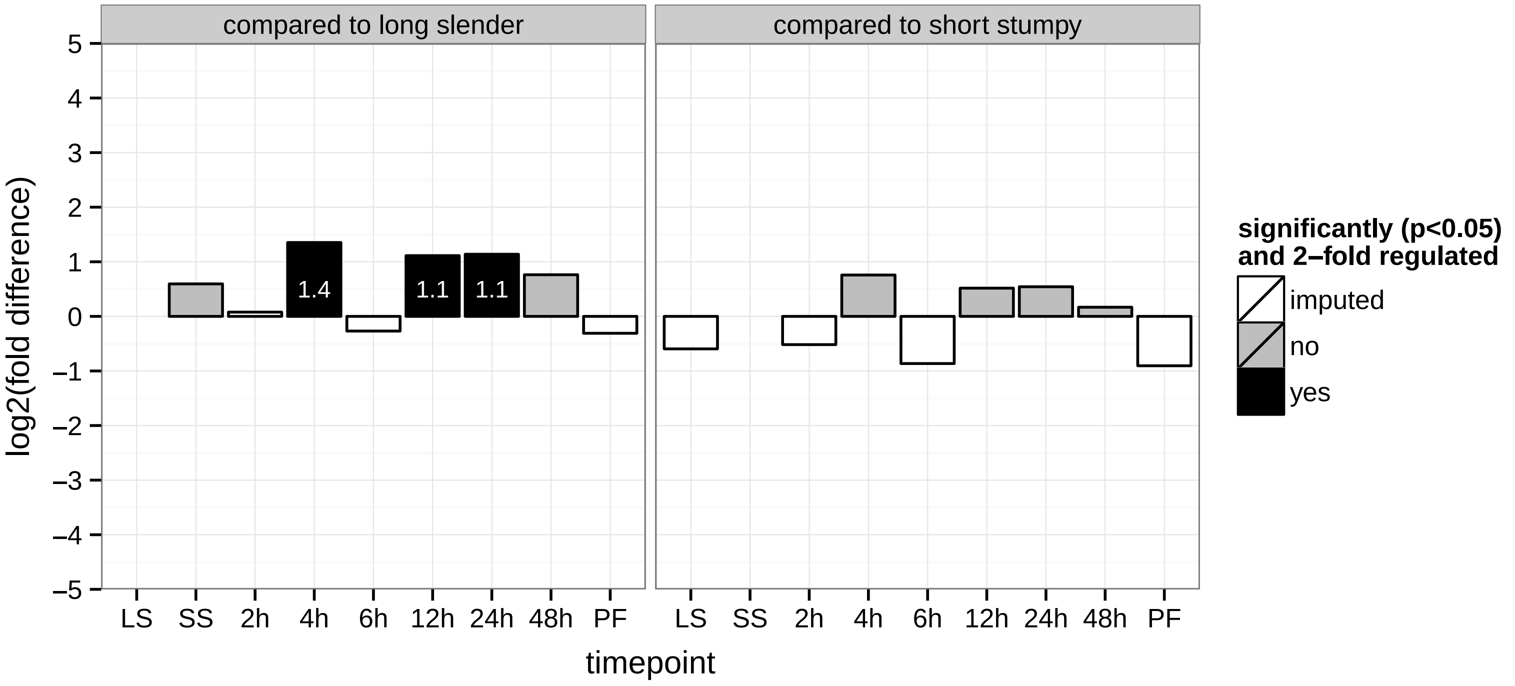

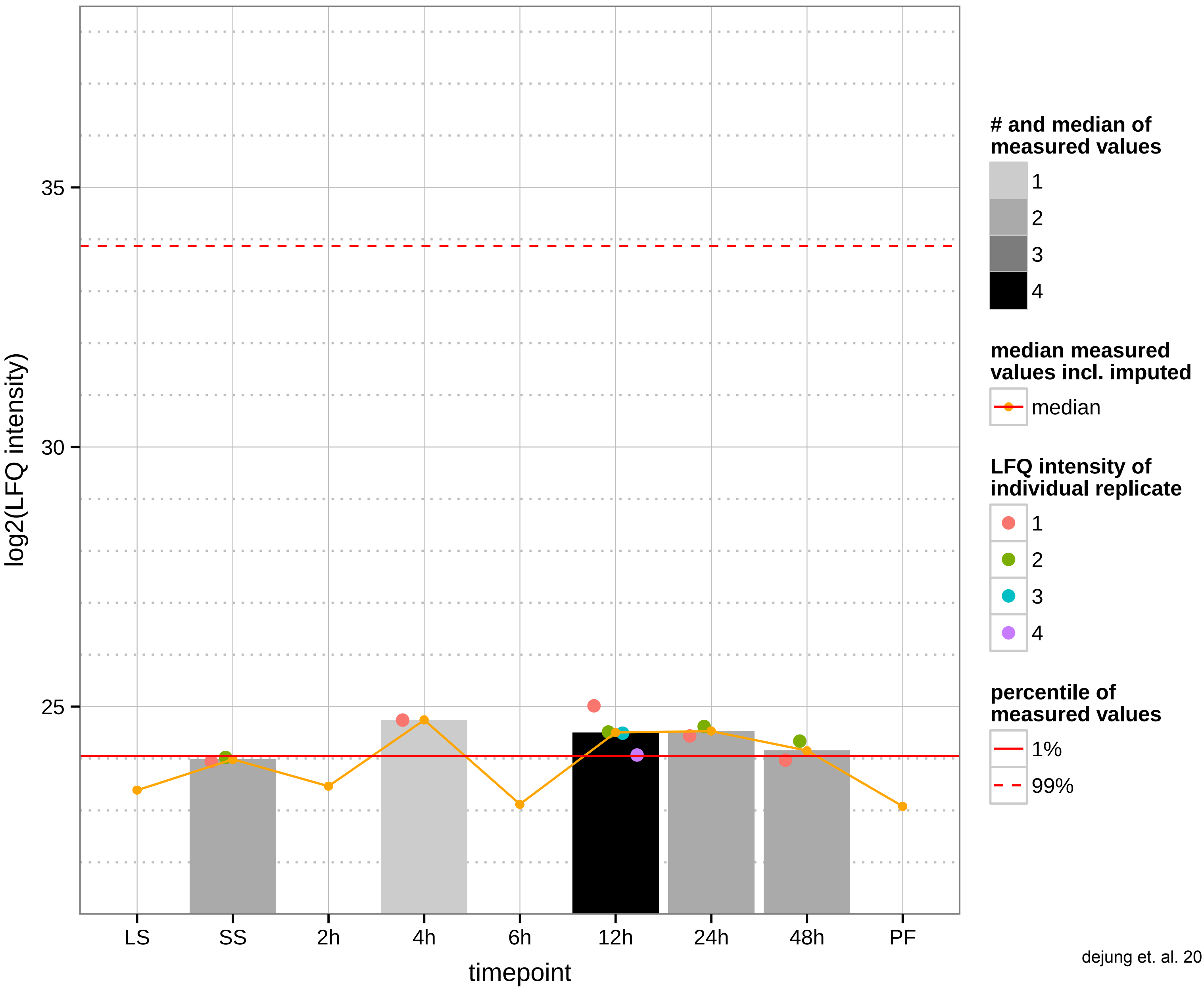

dejung et. al. 2015

aminopeptidase, putative, metallo–peptidase, Clan MA(E) Family M1
Tb927.3.4790;Tb927.3.4750
AGOF: metallopeptidase activity, zinc ion binding
AGOC: null
AGOP: proteolysis
PGOF: metallopeptidase activity, zinc ion binding
PGOC: null
PGOP: null

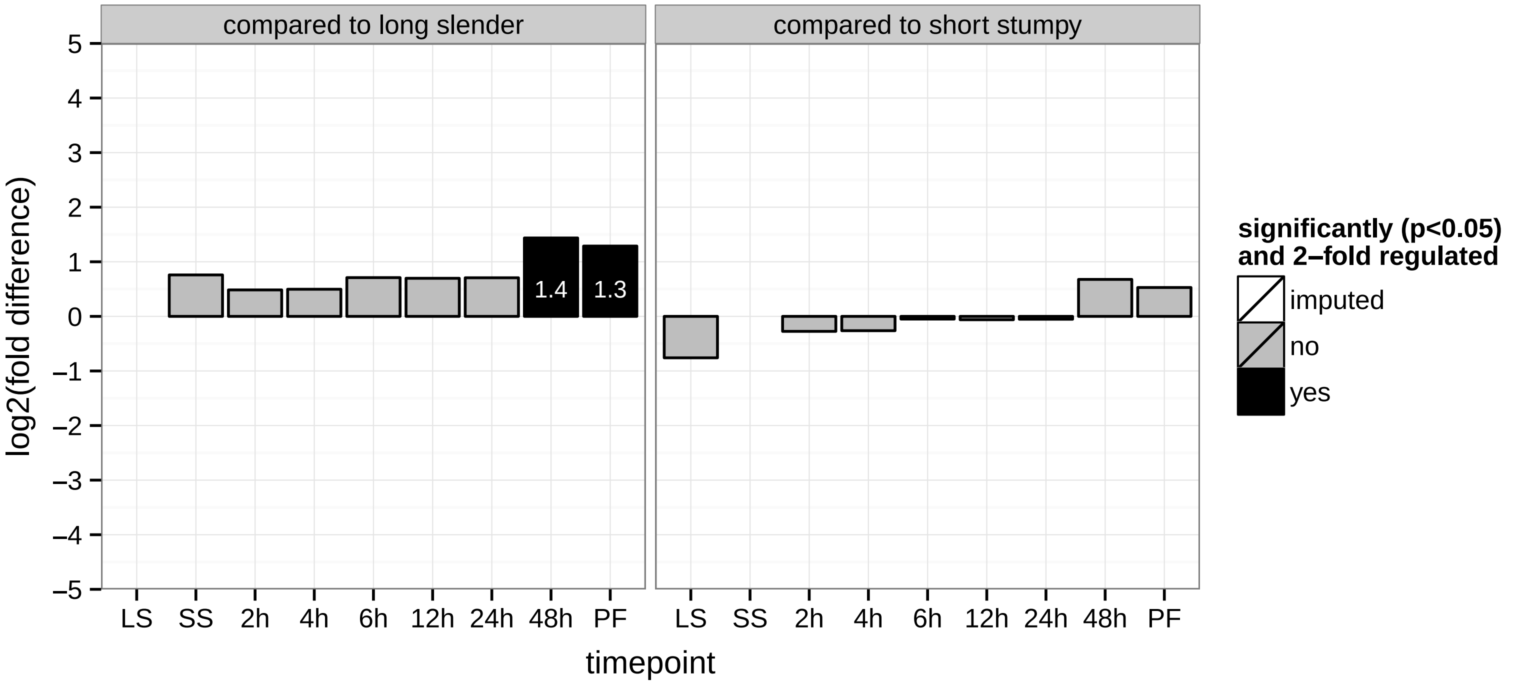

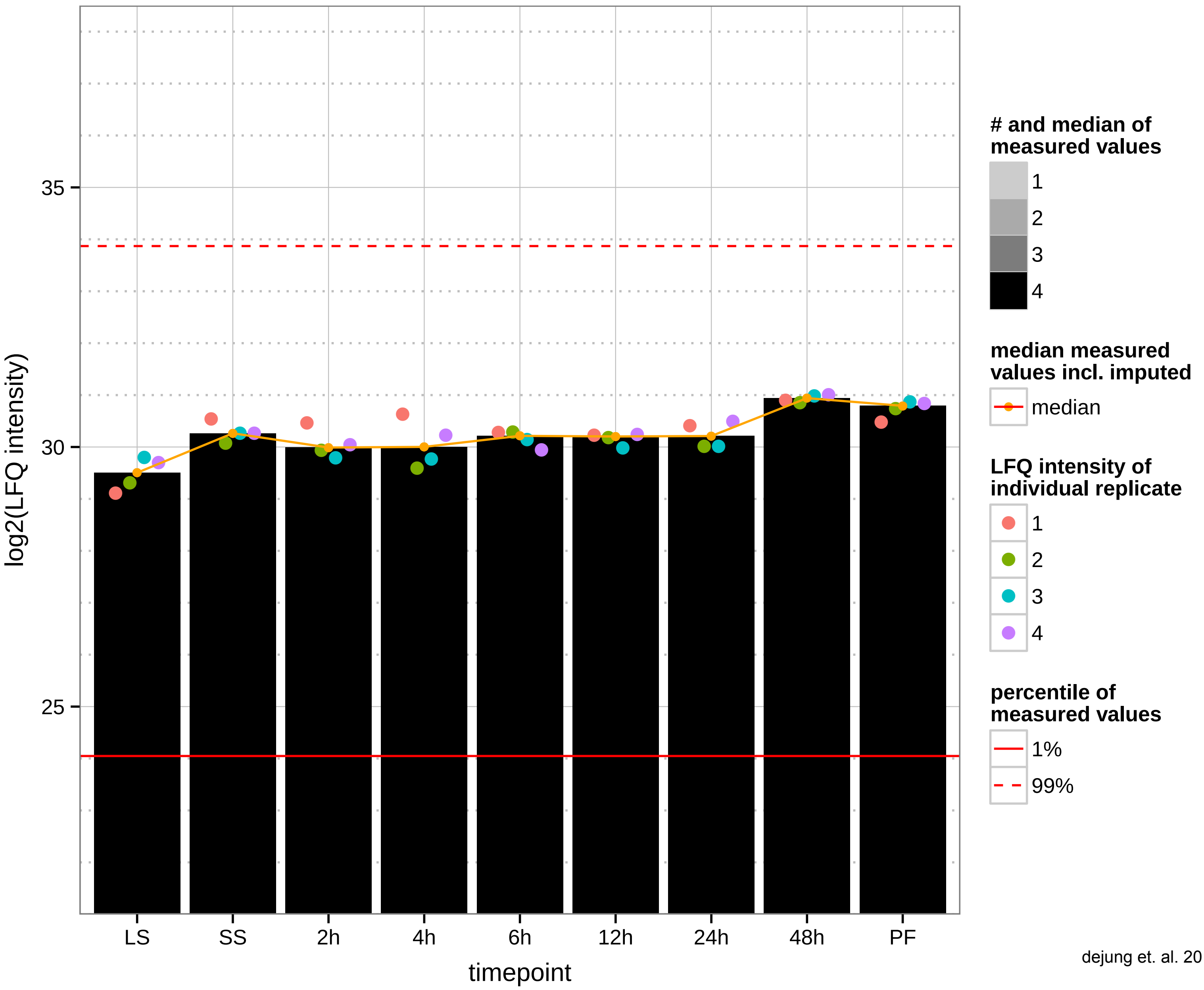

dejung et. al. 2015

dynamin, putative, vacuolar sortin protein 1
Tb927.3.4760
AGOF: GTP binding, GTPase activity
AGOC: null
AGOP: null
PGOF: GTP binding, GTPase activity
PGOC: null
PGOP: null

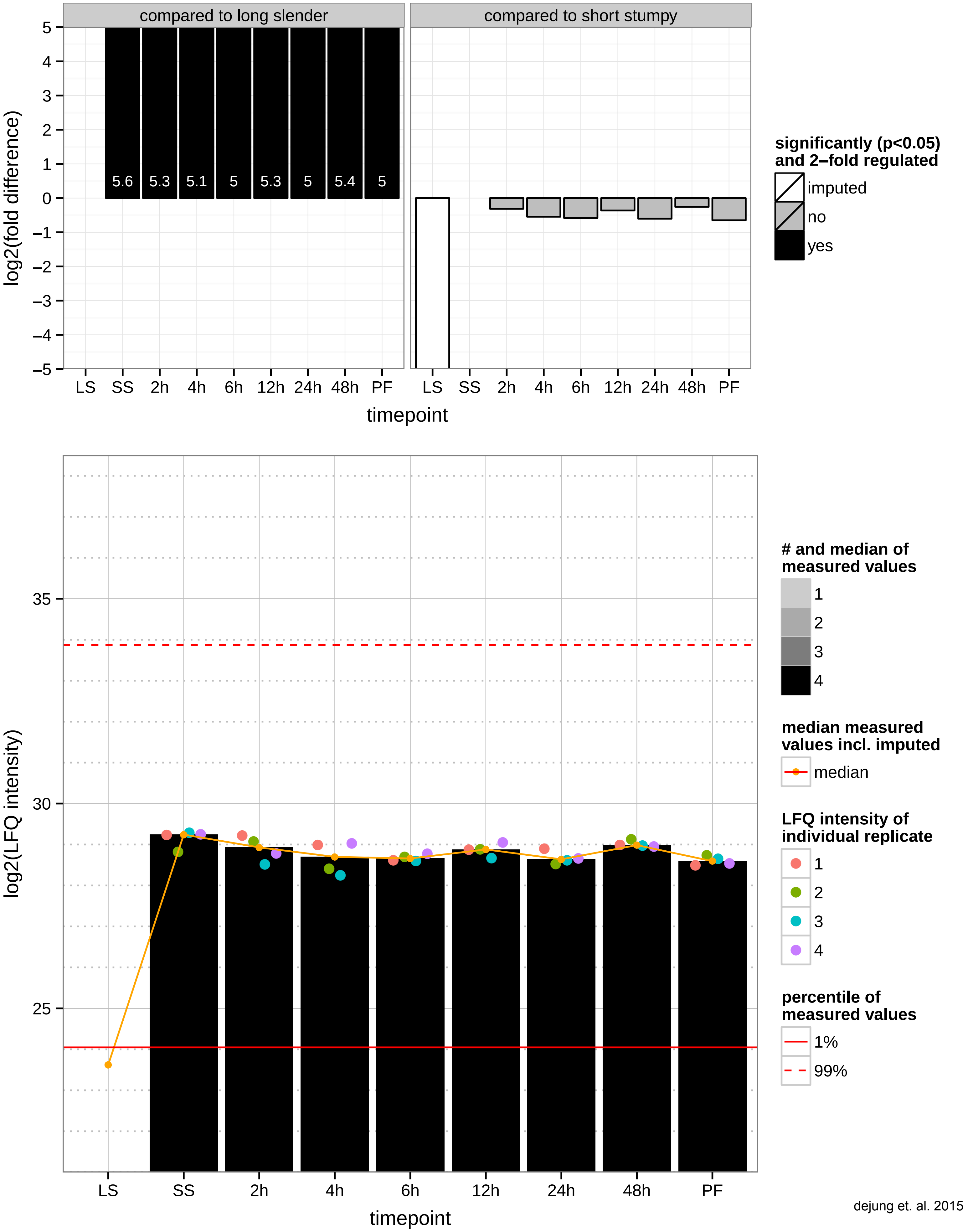

dejung et. al. 2015

acyltransferase, putative
Tb927.3.4820
AGOF: transferase activity, transferring acyl groups
AGOC: null
AGOP: metabolic process
PGOF: transferase activity, transferring acyl groups
PGOC: null
PGOP: metabolic process

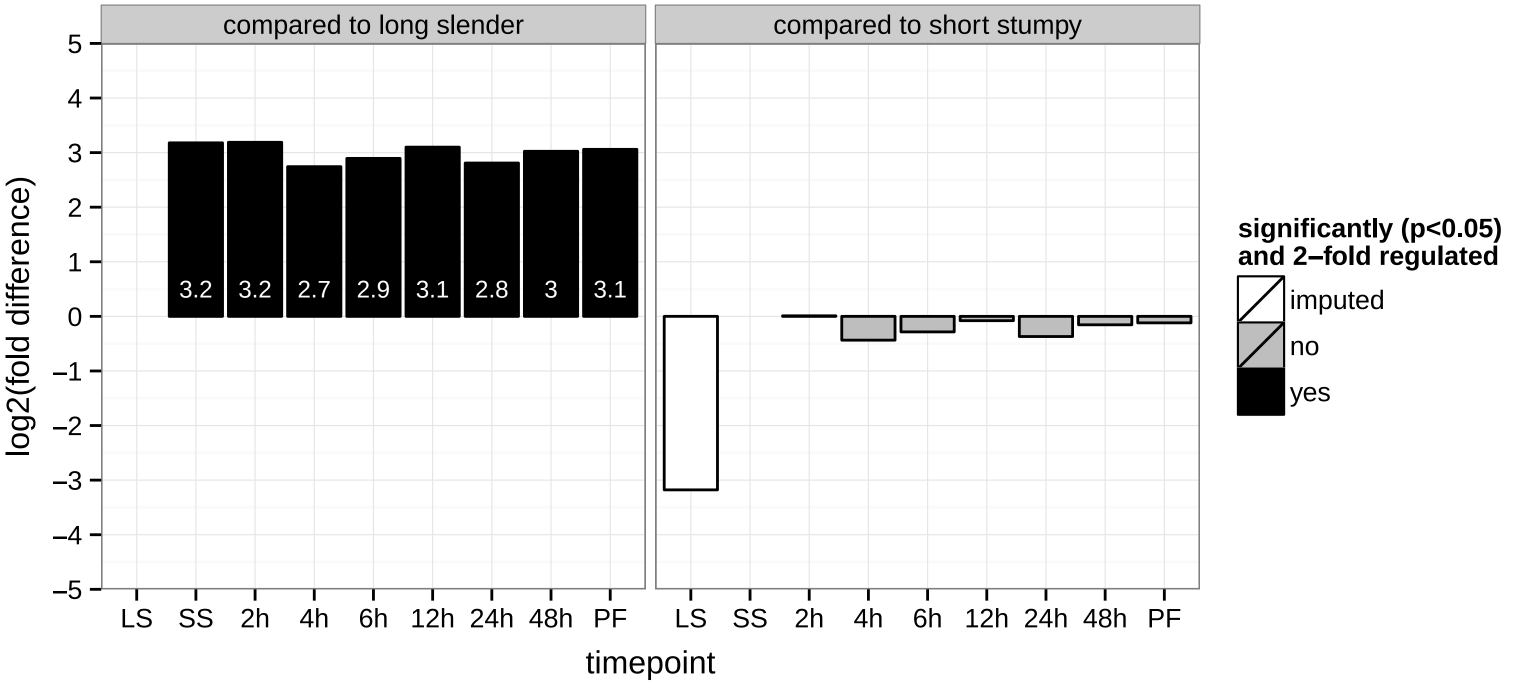

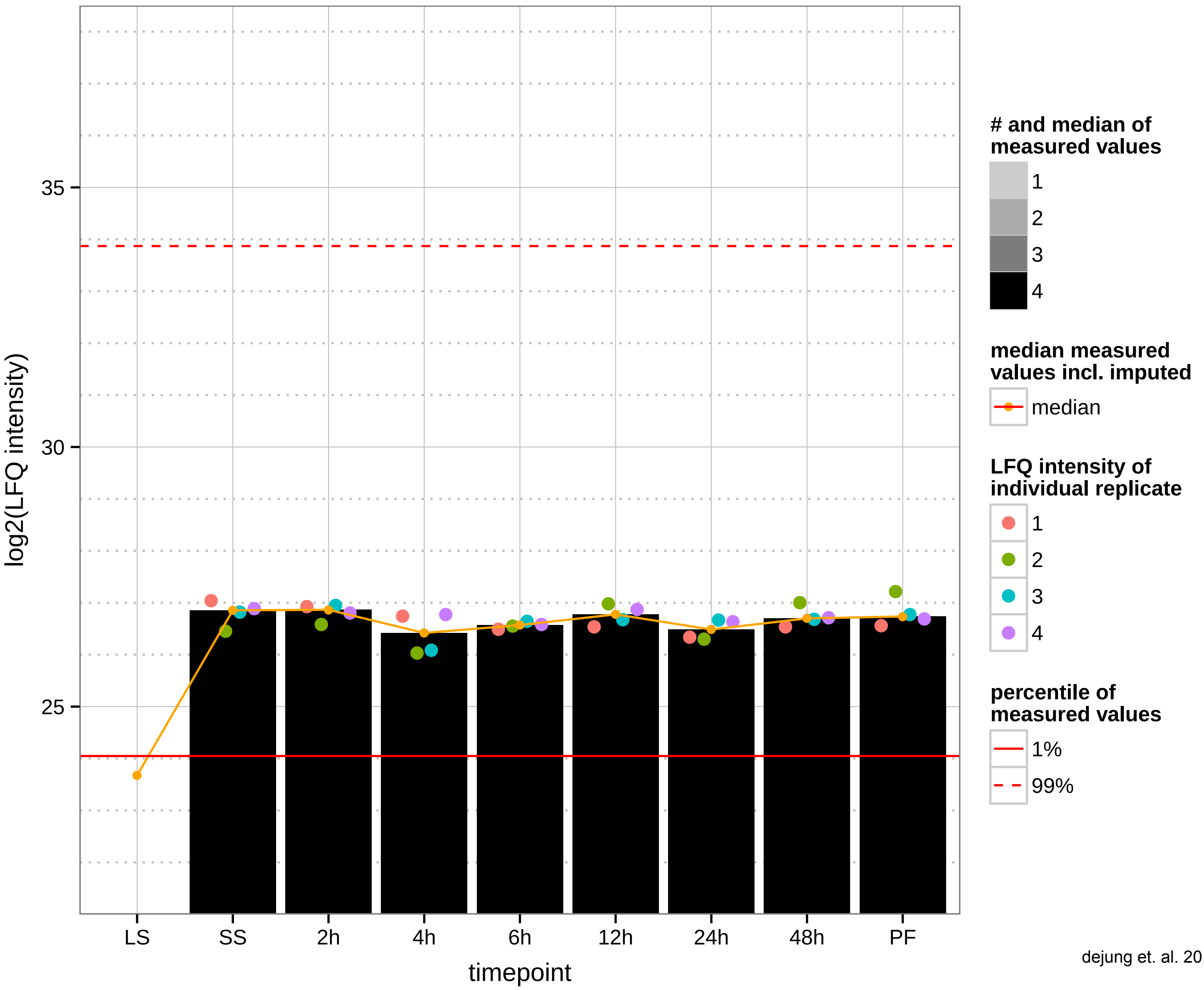

dejung et. al. 2015

mitogen–activated protein kinase kinase, putative (MKK1)
Tb927.3.4860
AGOF: ATP binding, mitogen–activated protein kinase binding, protein kinase activity, protein serine/threonine kinase activity,
AGOC: null
AGOP: protein phosphorylation
PGOF: ATP binding, protein kinase activity, transferase activity, transferring phosphorus–containing groups
PGOC: null
PGOP: protein phosphorylation

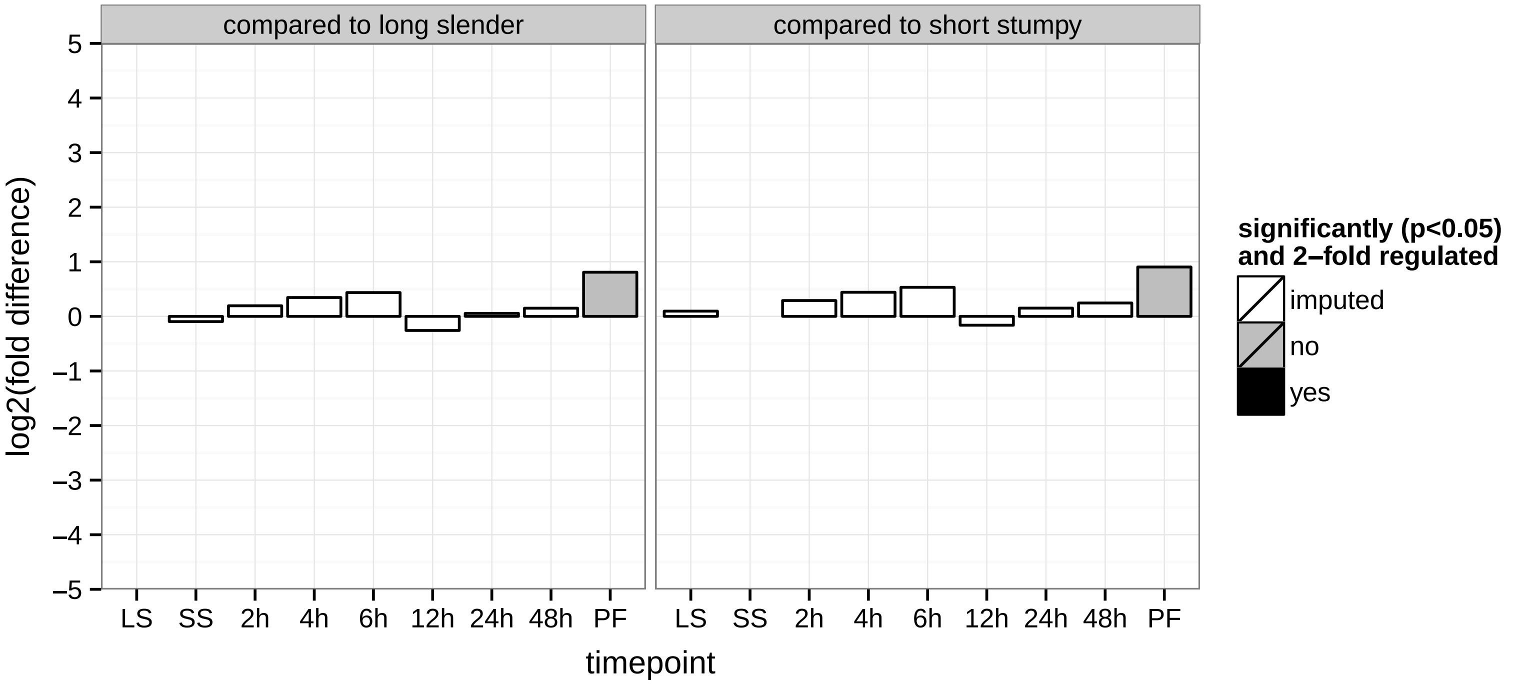

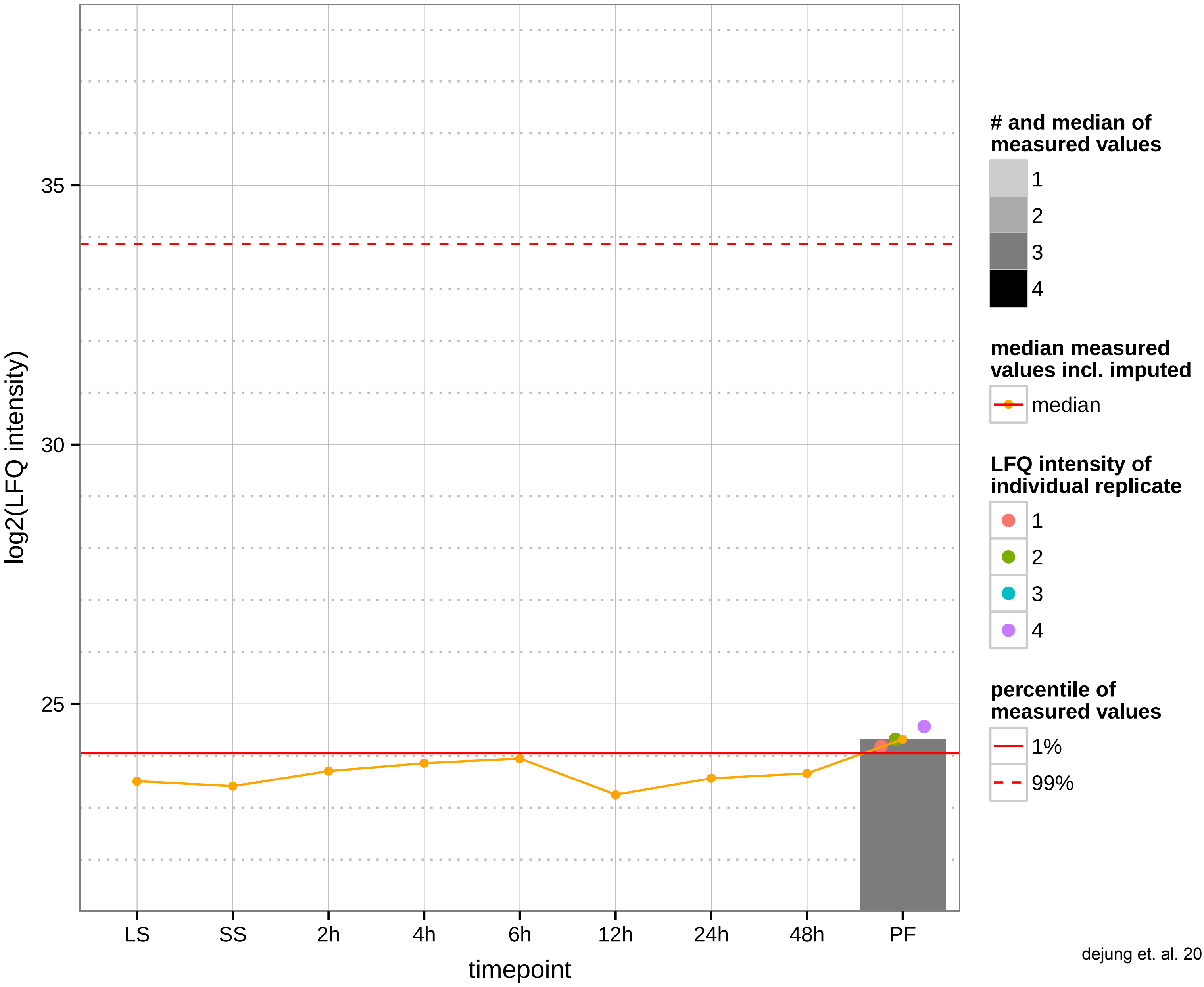

dejung et. al. 2015

LETM1 and EF−hand domain−containing protein 1, putative
Tb927.3.4920
AGOF: calcium ion binding, calcium:sodium antiporter activity
AGOC: integral to membrane, mitochondrial inner membrane
AGOP: mitochondrial calcium ion transport
PGOF: null
PGOC: null
PGOP: null

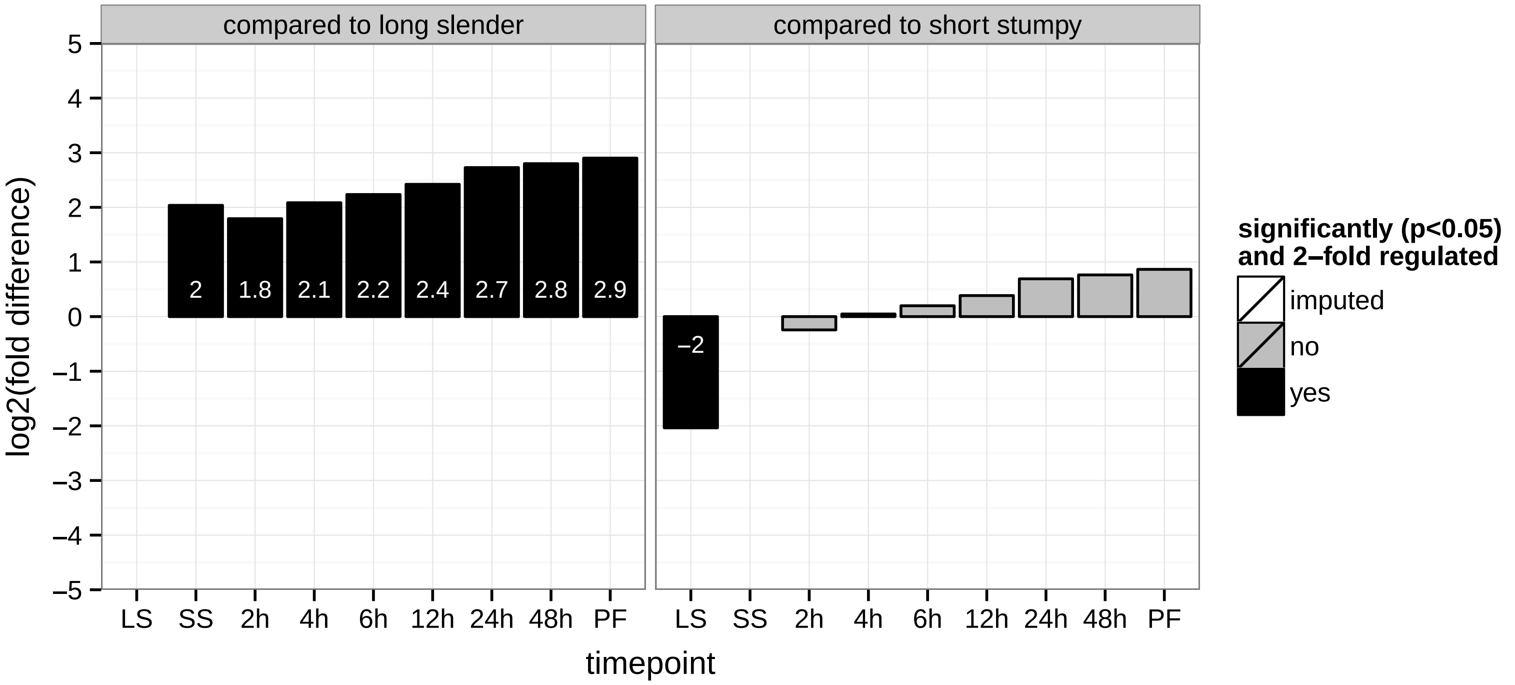

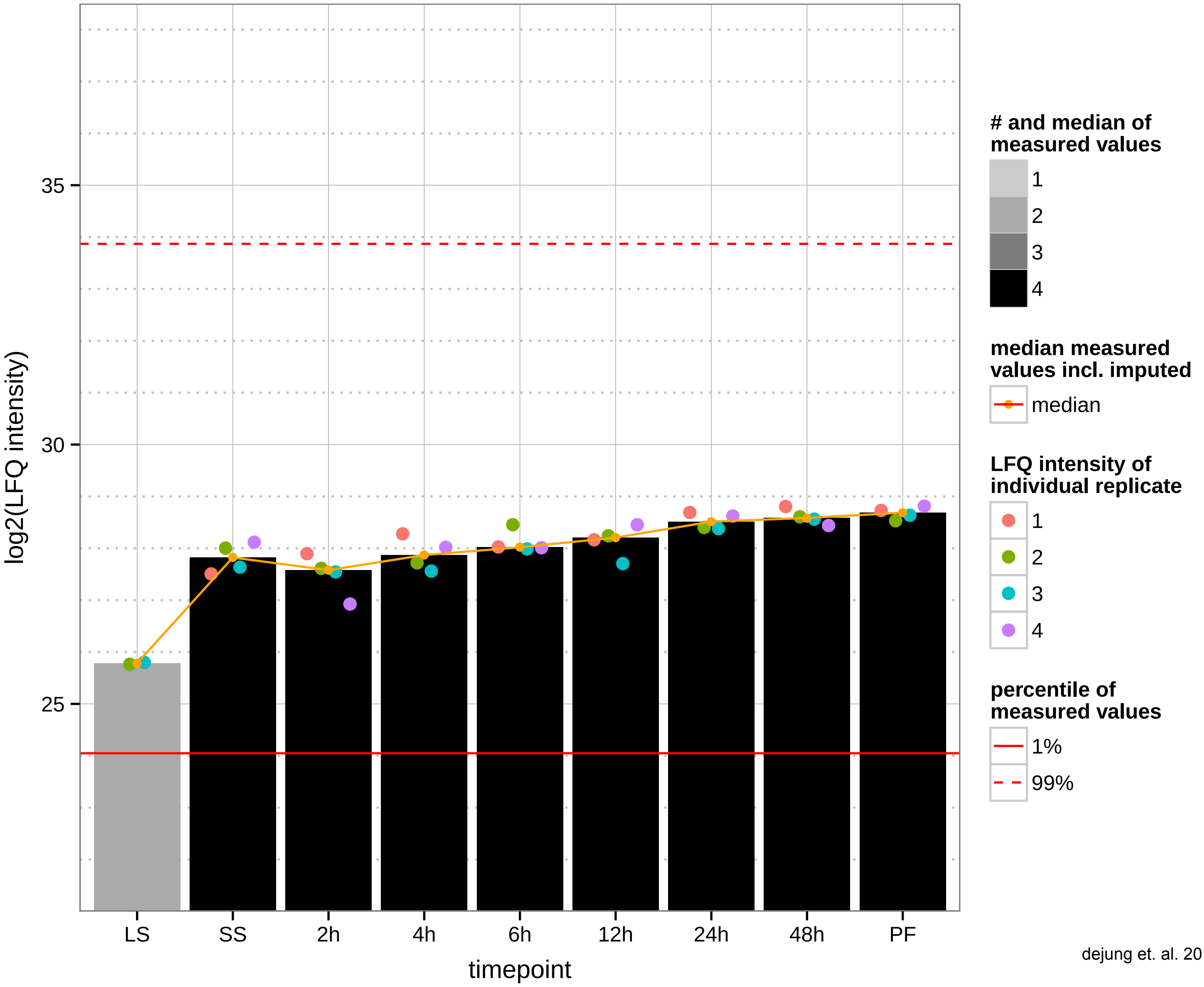

dejung et. al. 2015

5–histidylcysteine sulfoxide synthase, putative
Tb927.3.4940
AGOF: ferrous iron binding
AGOC: null
AGOP: peptidyl–cysteine oxidation, response to iron(II) ion
PGOF: null
PGOC: null
PGOP: null

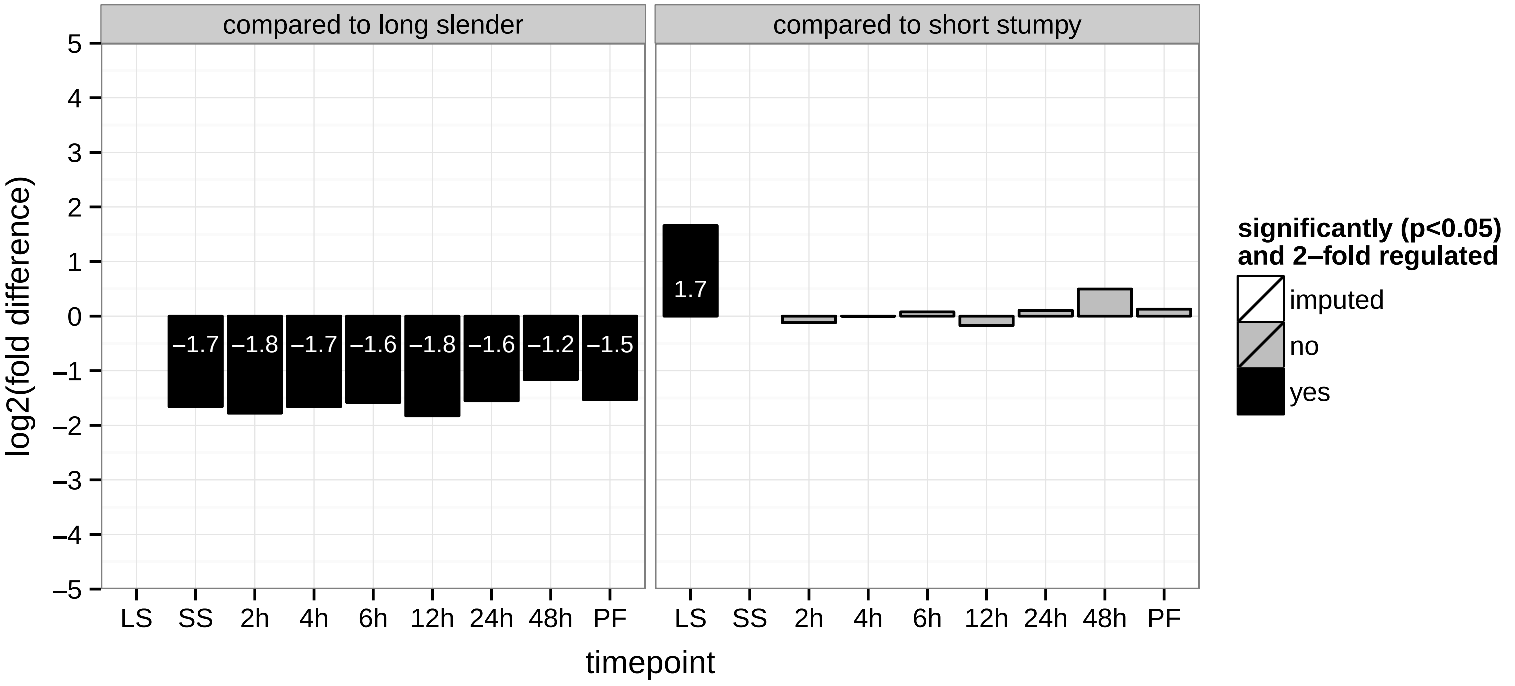

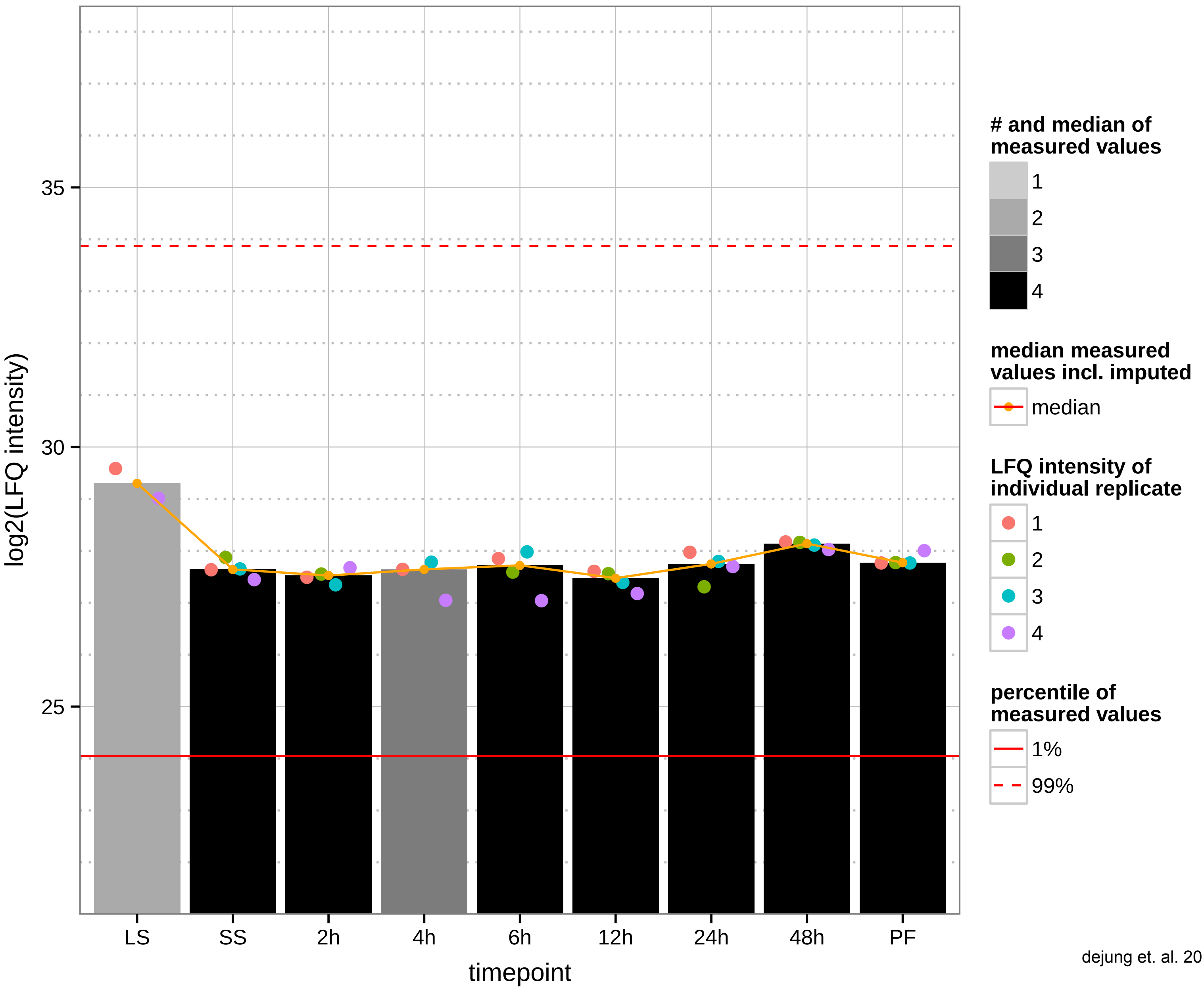

dejung et. al. 2015

hypothetical protein, conserved
Tb927.3.4970
AGOF: null
AGOC: null
AGOP: null
PGOF: null
PGOC: null
PGOP: null

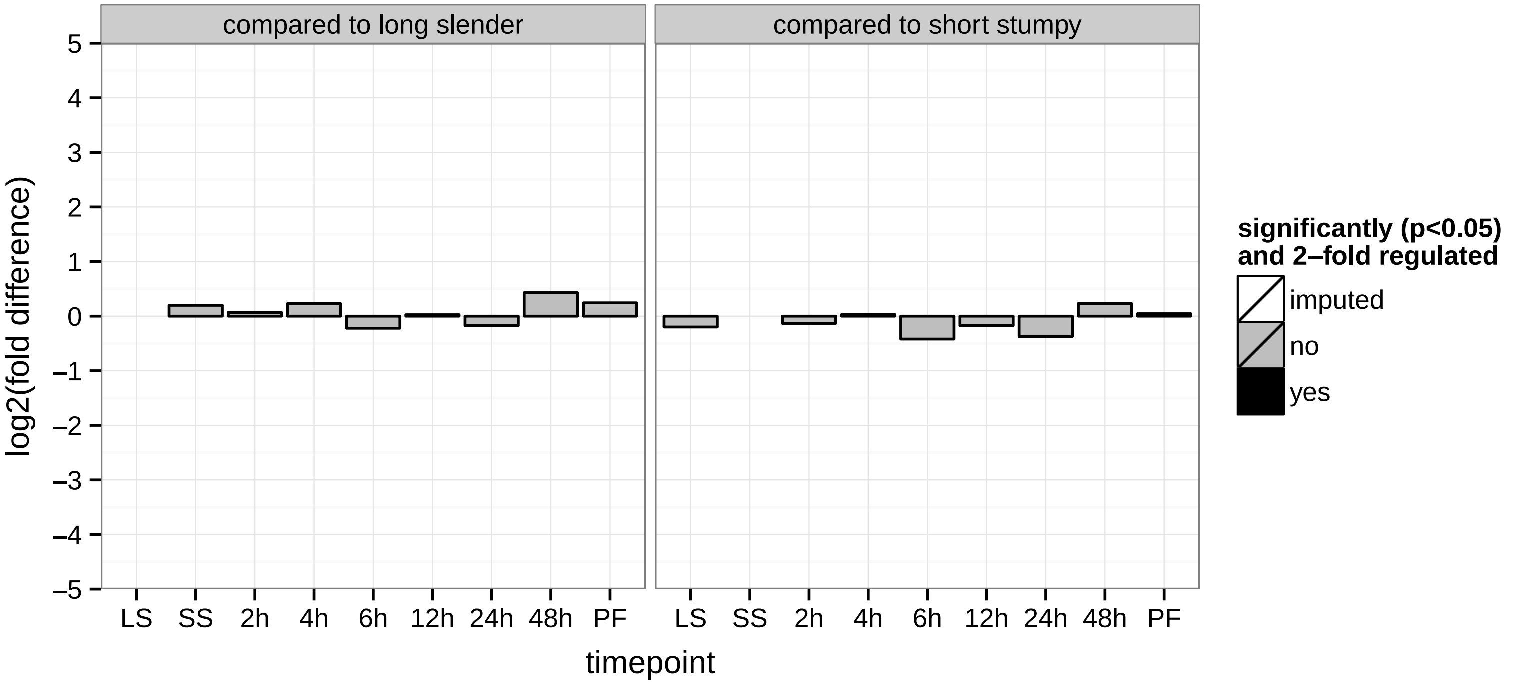

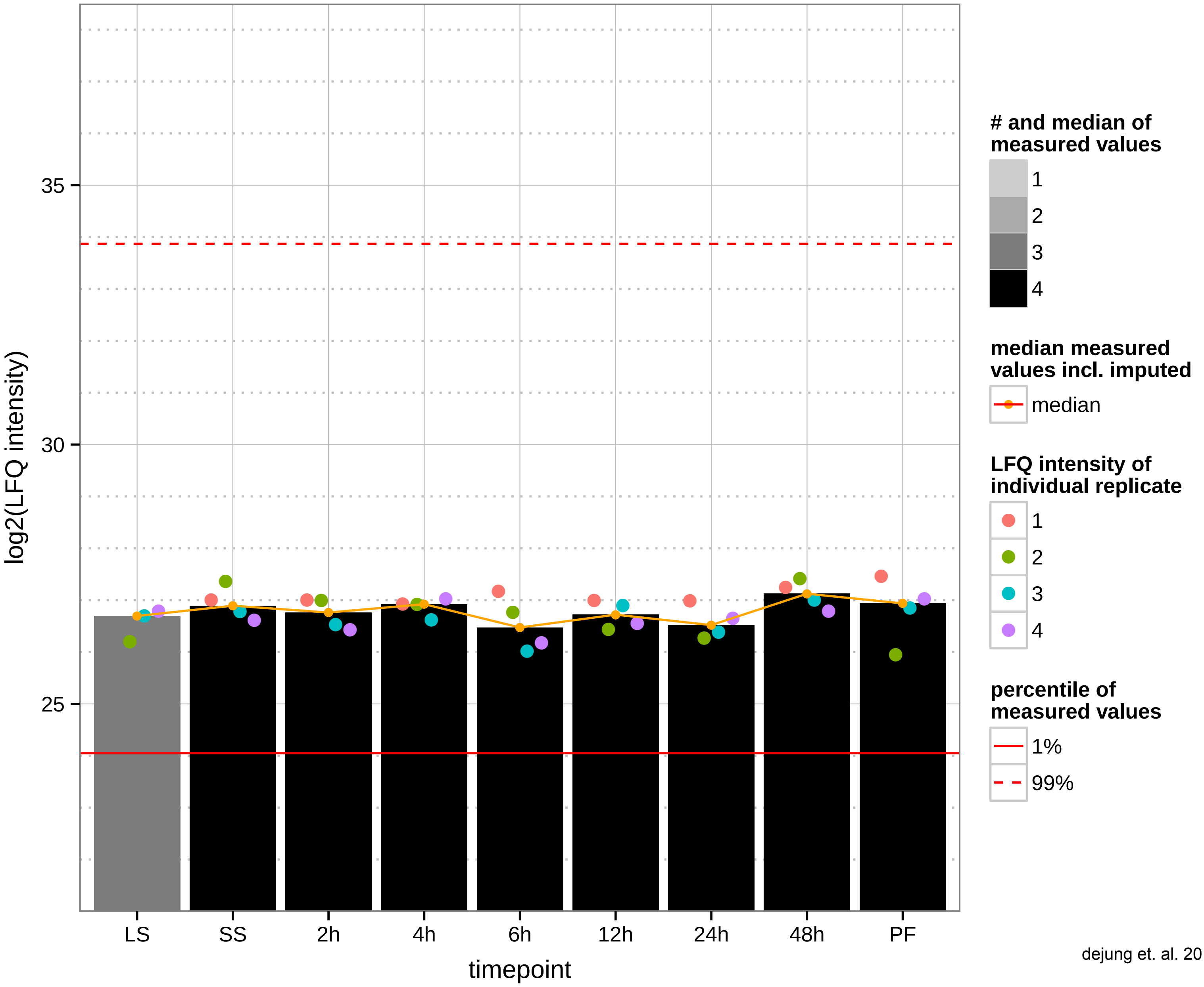

dejung et. al. 2015

oxidoreductase, putative
Tb927.3.4990
AGOF: oxidoreductase activity, zinc ion binding
AGOC: null
AGOP: metabolic process, oxidation–reduction process
PGOF: oxidoreductase activity, transferase activity, transferring acyl groups other than amino–acyl groups, zinc ion binding
PGOC: null
PGOP: oxidation–reduction process

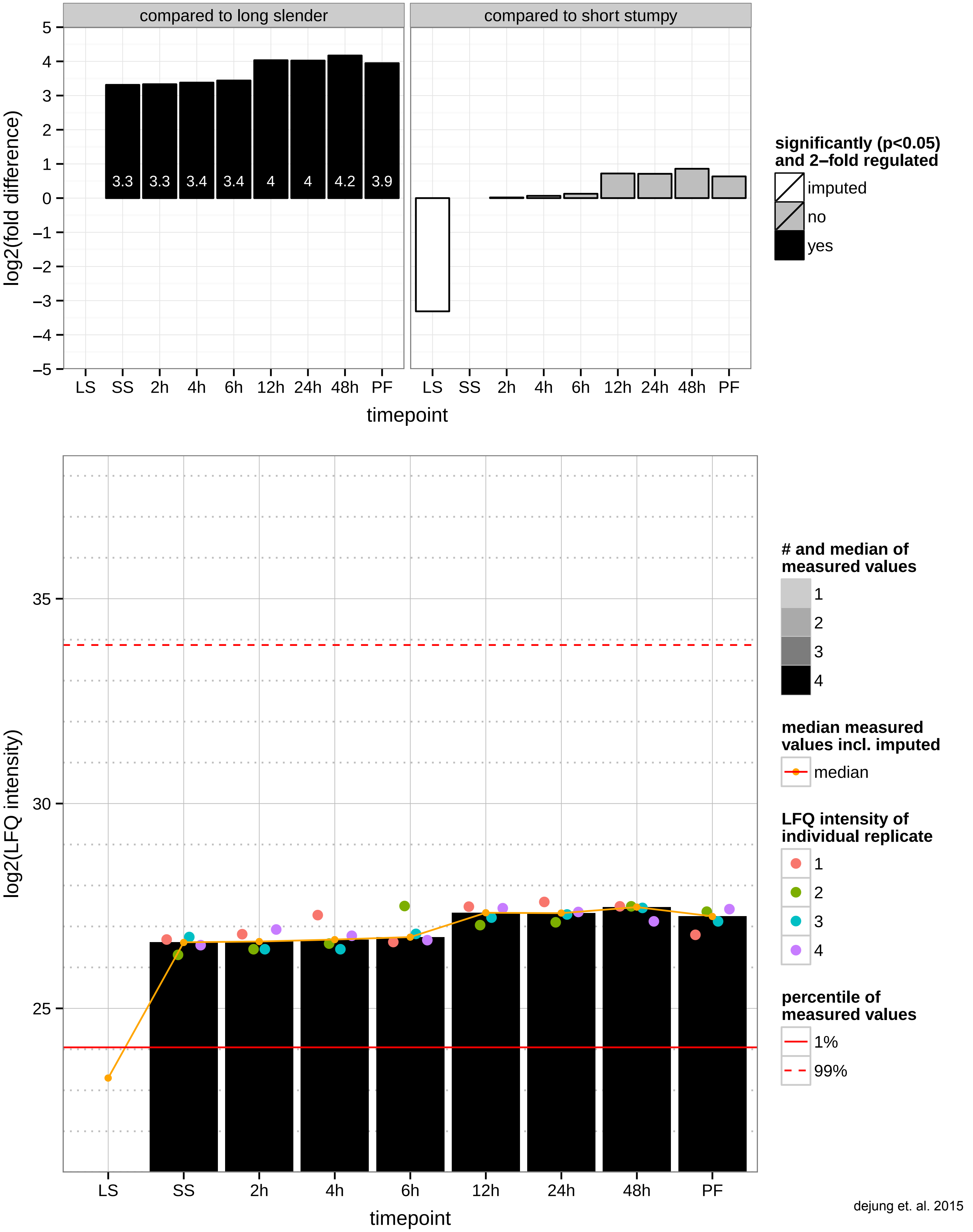

dejung et. al. 2015

hypothetical protein, conserved
Tb927.3.5010
AGOF: null
AGOC: null
AGOP: null
PGOF: null
PGOC: null
PGOP: null

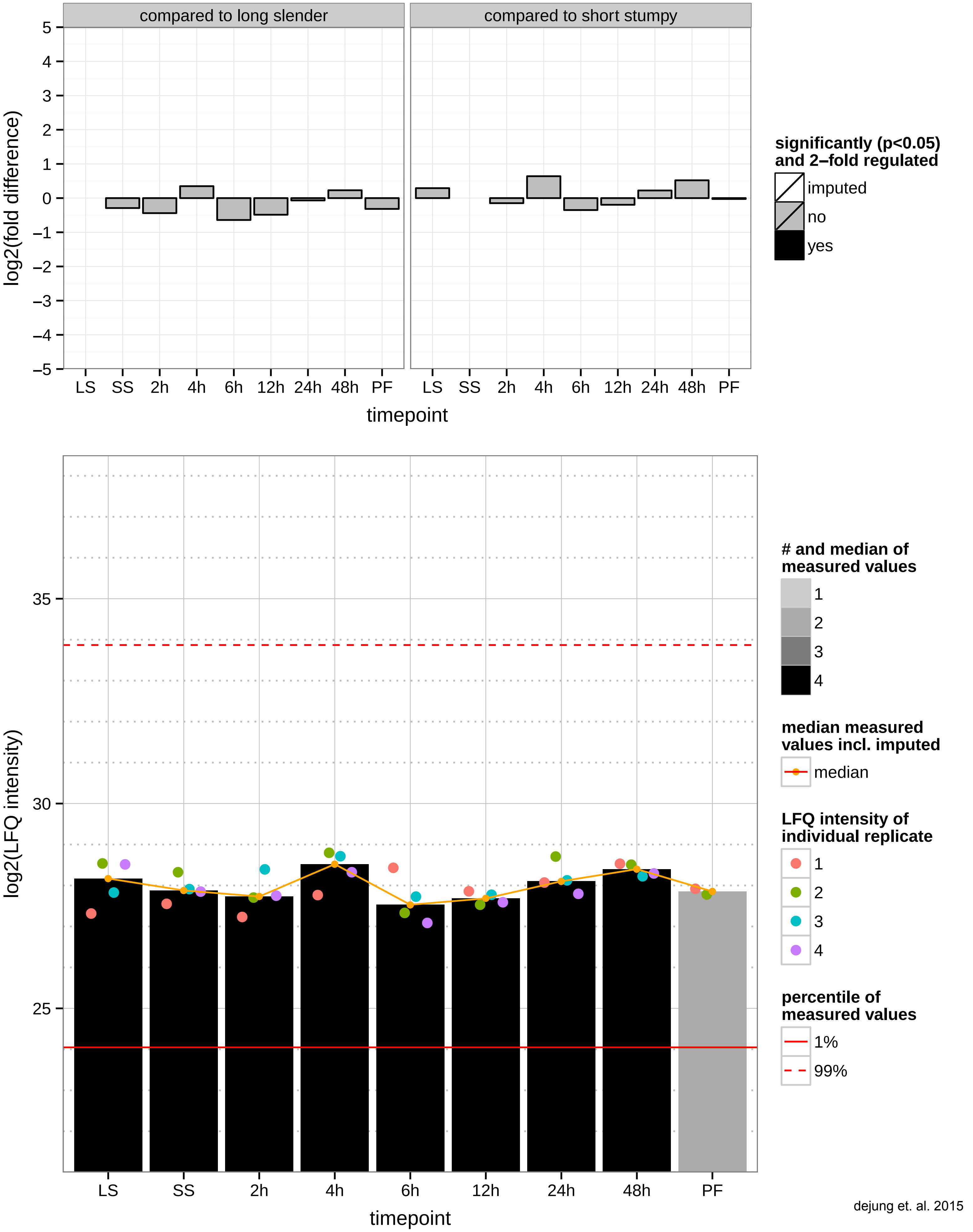

dejung et. al. 2015

hypothetical protein, conserved
Tb927.3.5020
AGOF: null
AGOC: null
AGOP: null
PGOF: null
PGOC: null
PGOP: null

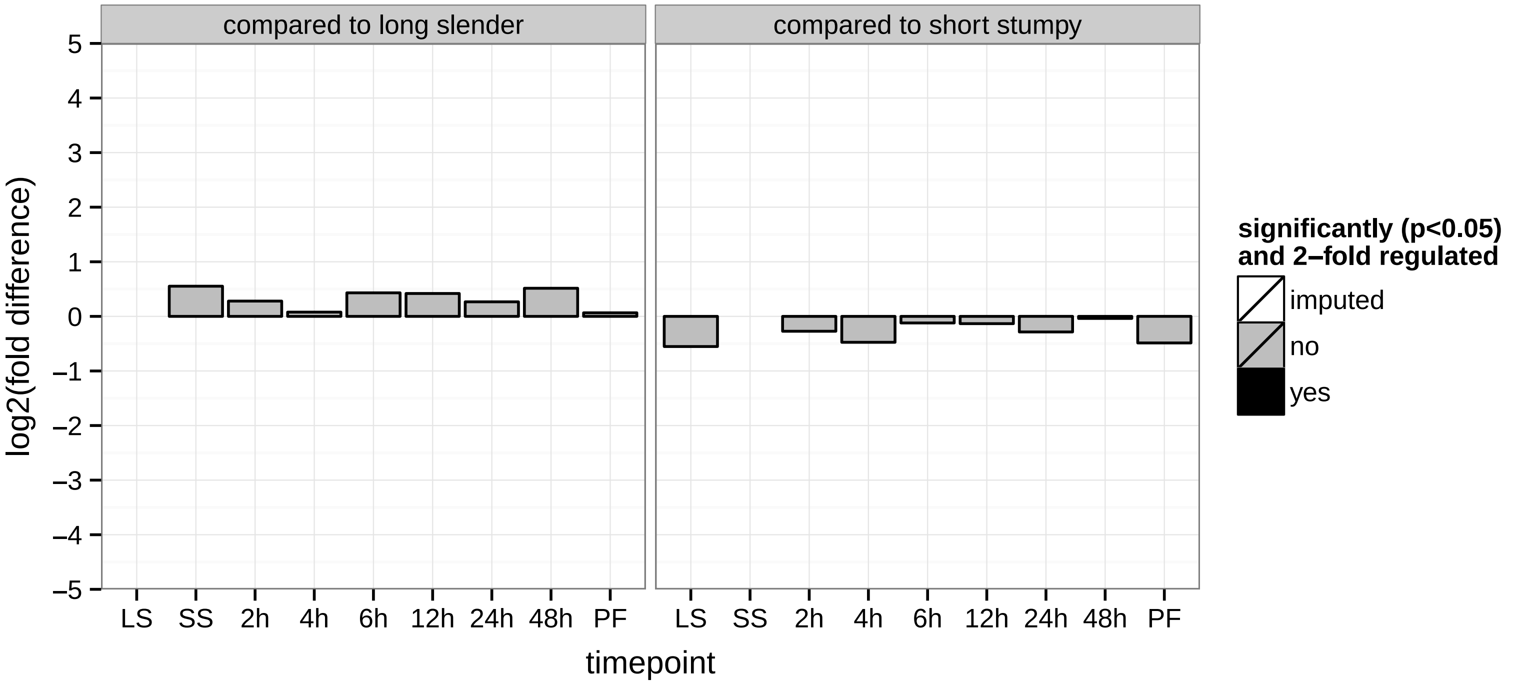

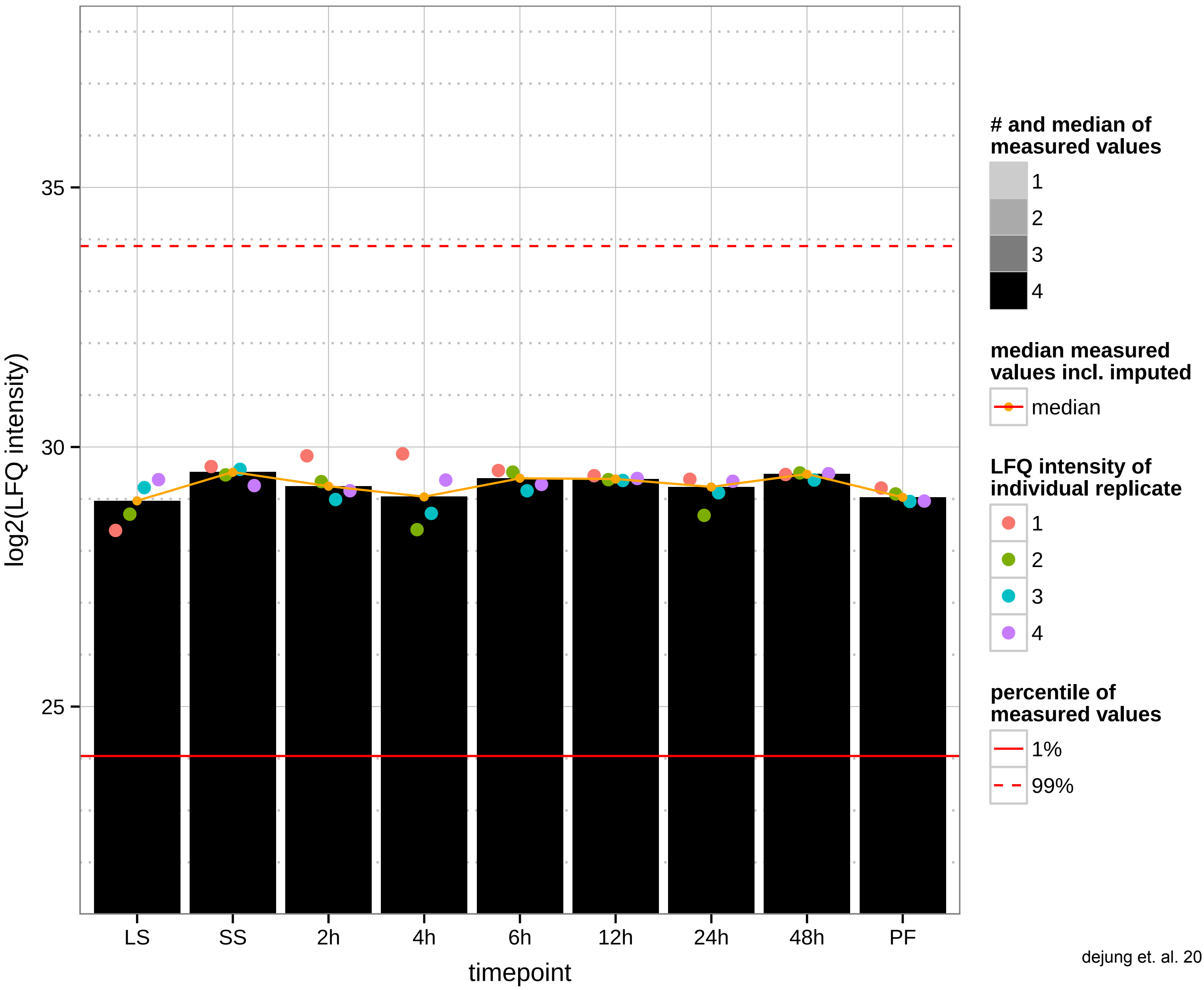

dejung et. al. 2015

hypothetical protein, conserved
Tb927.3.5060
AGOF: null
AGOC: null
AGOP: null
PGOF: protein binding
PGOC: null
PGOP: null

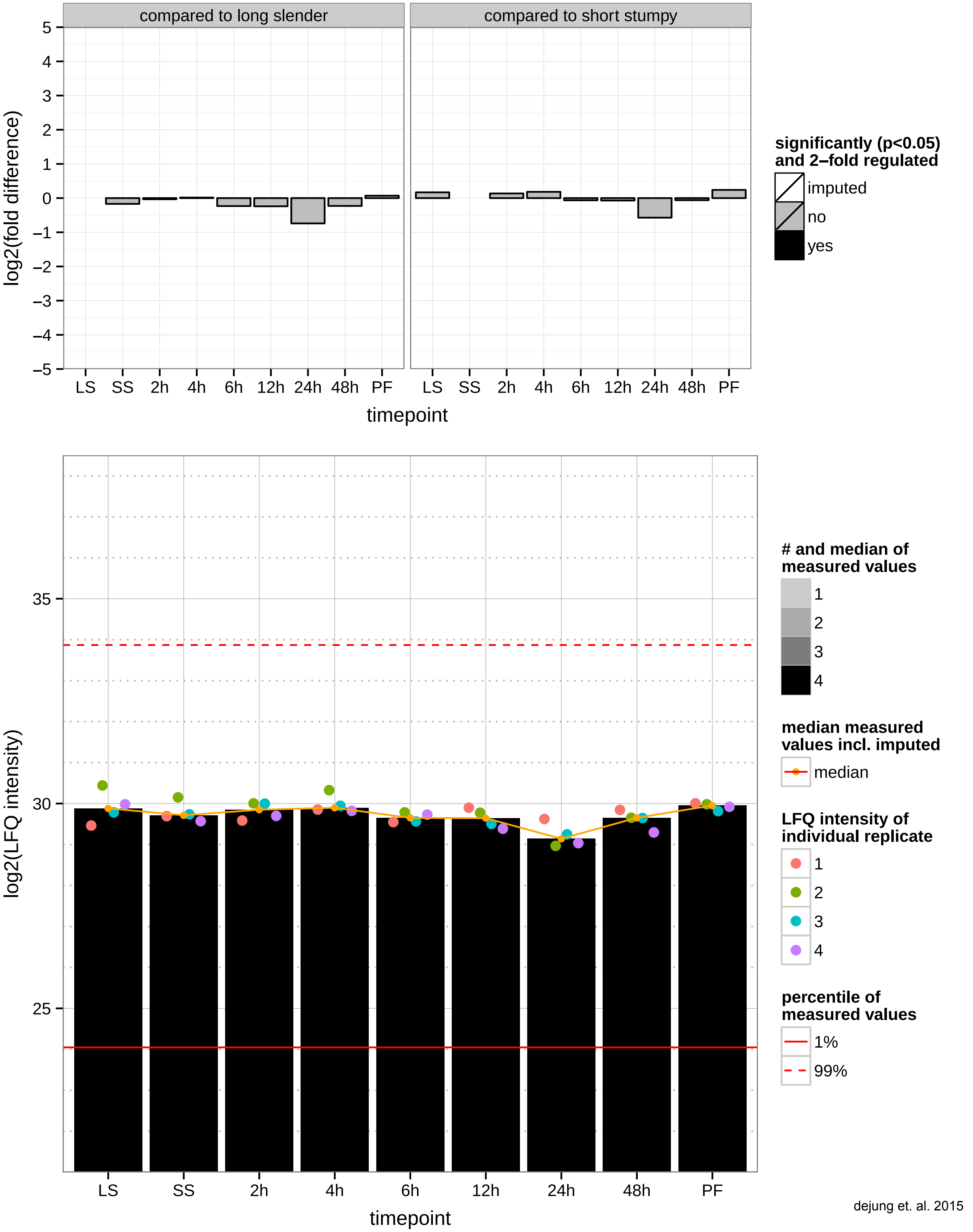

dejung et. al. 2015

hypothetical protein, conserved
Tb927.3.5070
AGOF: null
AGOC: null
AGOP: null
PGOF: null
PGOC: null
PGOP: null

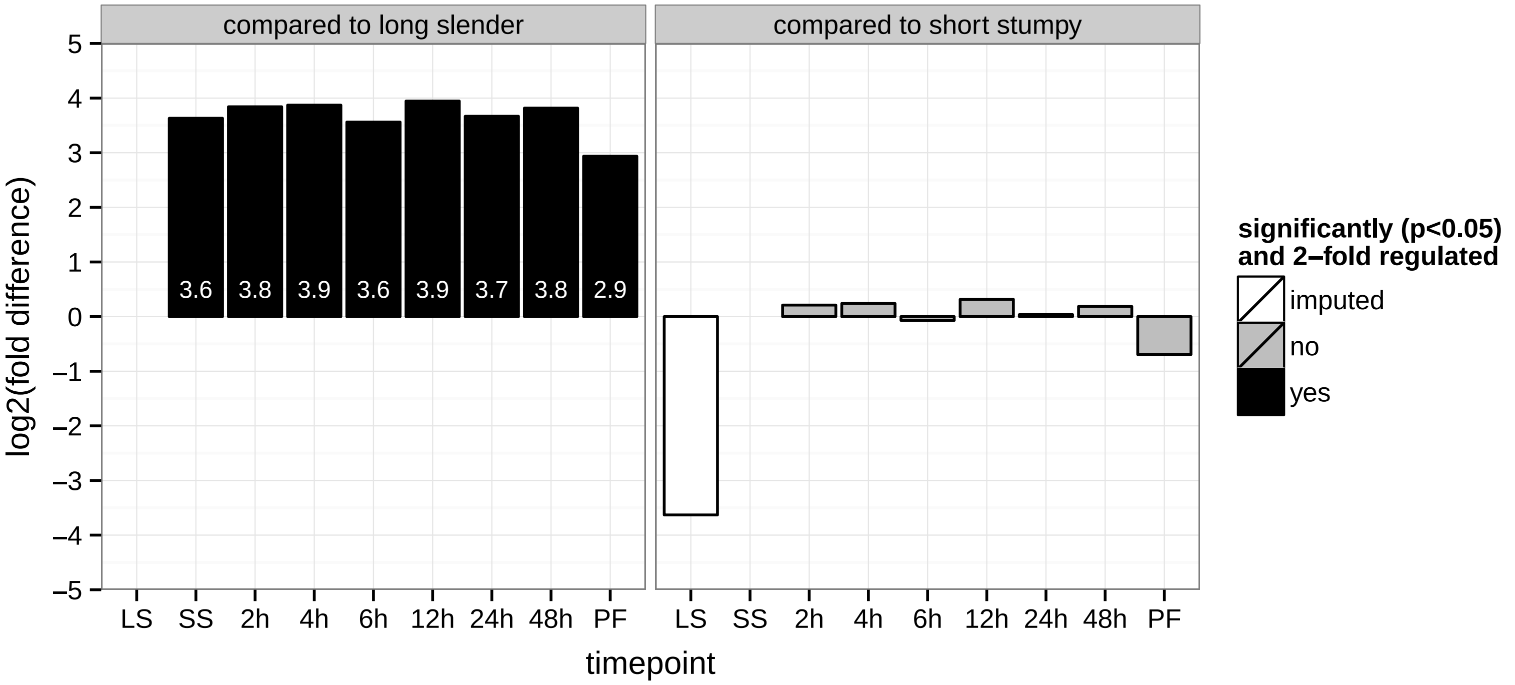

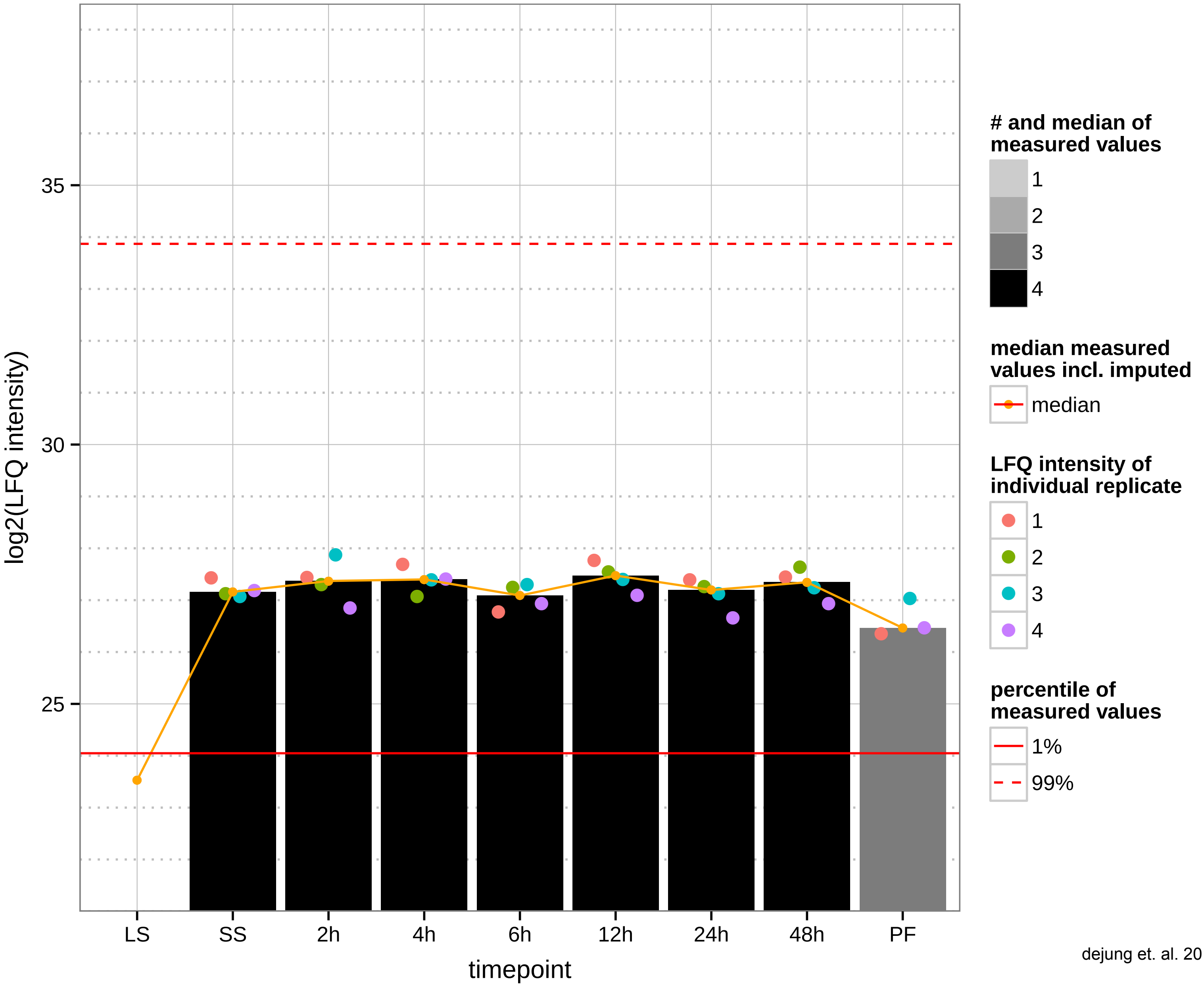

dejung et. al. 2015

exonuclease, putative
Tb927.3.5150
AGOF: exonuclease activity, nucleic acid binding
AGOC: intracellular
AGOP: null
PGOF: exonuclease activity, nucleic acid binding
PGOC: intracellular
PGOP: null

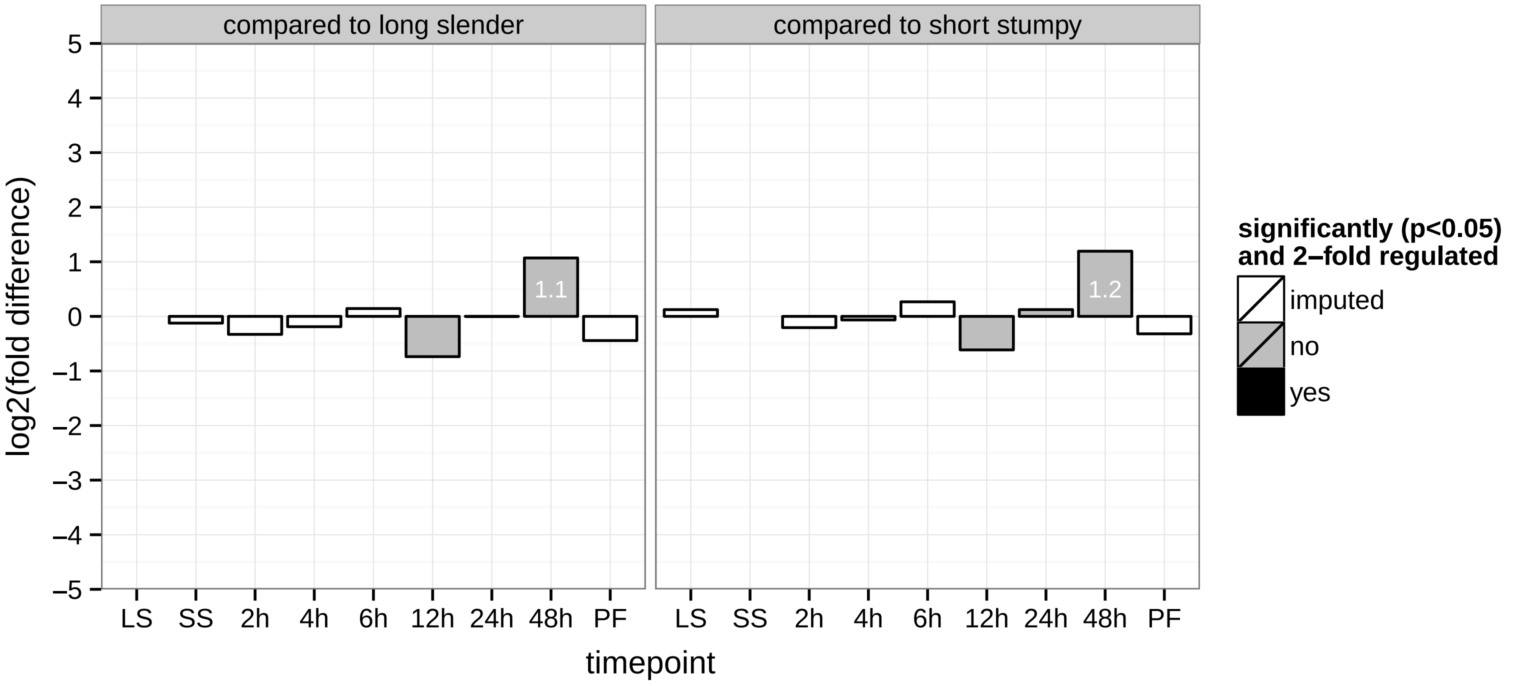

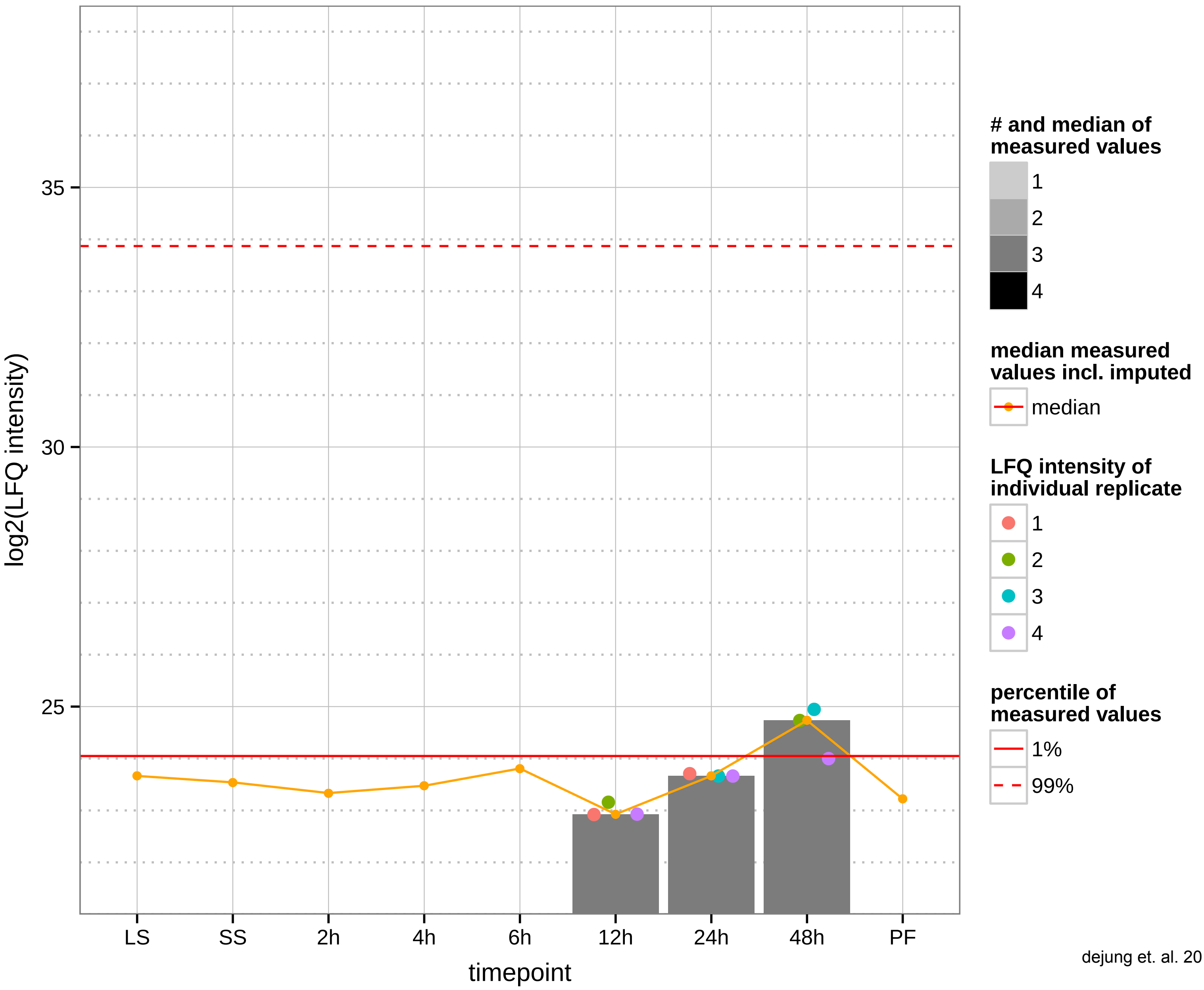

dejung et. al. 2015

cofilin/actin depolymerizing factor, putative, cofilin–like protein
Tb927.3.5180
AGOF: actin binding
AGOC: intracellular
AGOP: actin filament depolymerization, cell division, intracellular transport, microtubule basal body duplication
PGOF: actin binding
PGOC: intracellular
PGOP: null

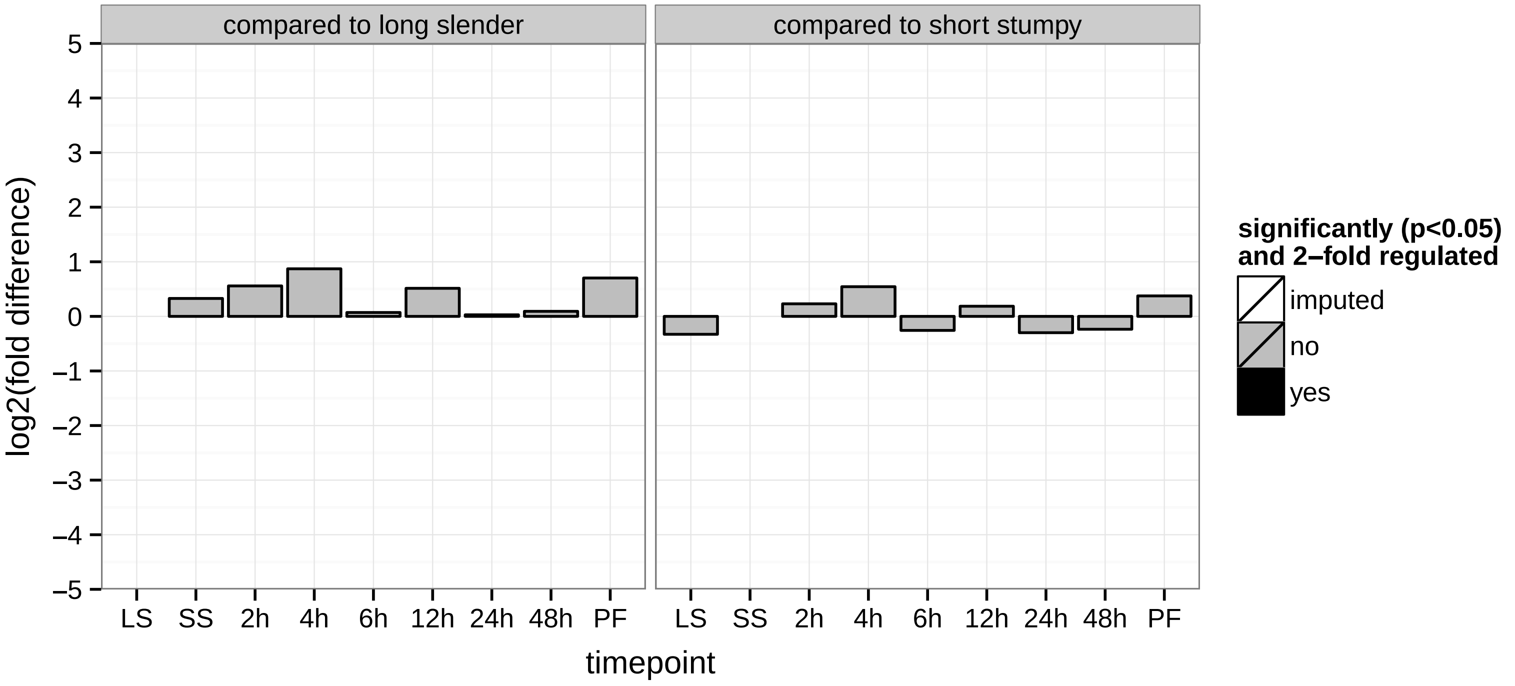

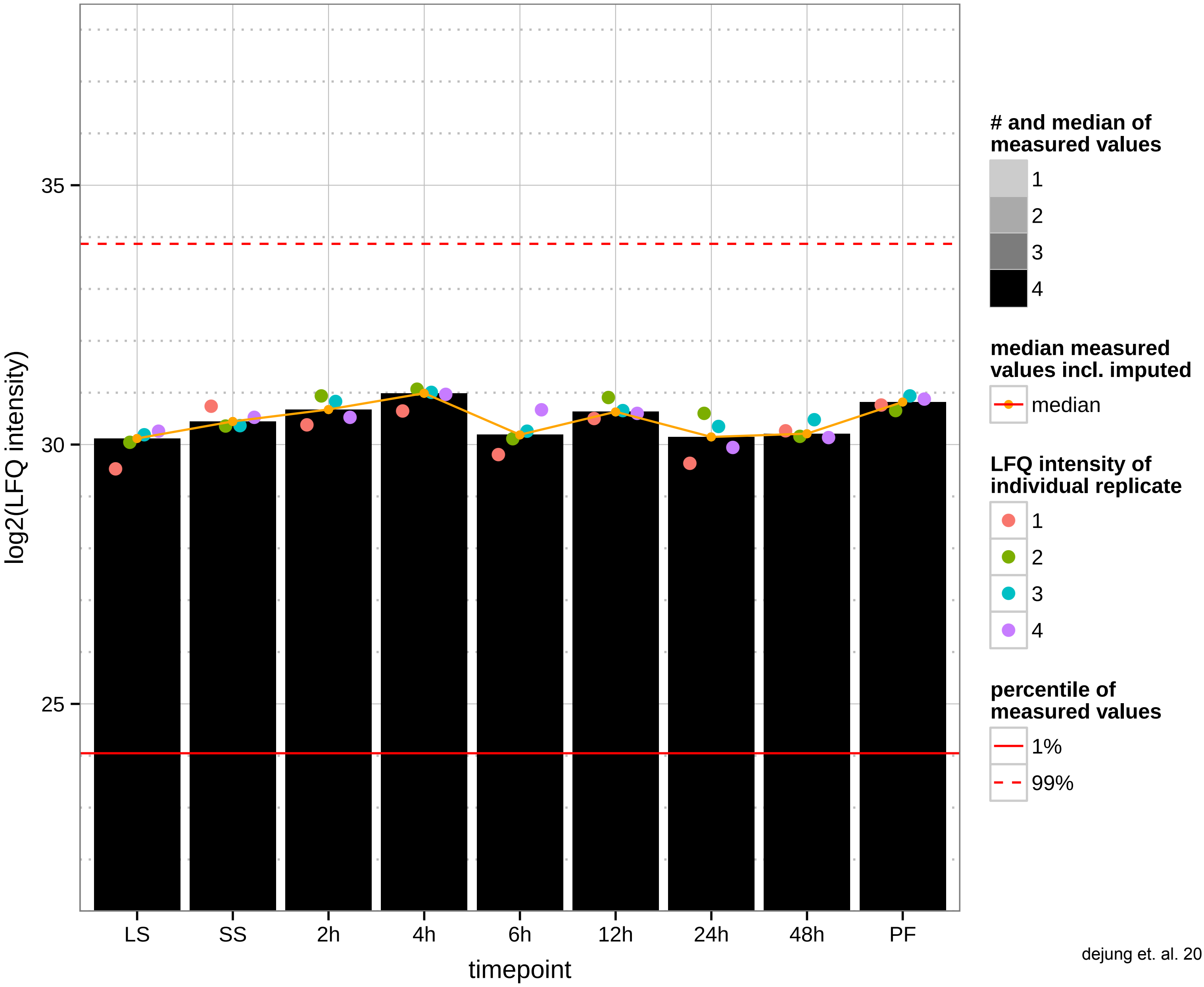

dejung et. al. 2015

DNA repair protein, putative
Tb927.3.5230
AGOF: ATP binding, DNA binding, DNA–dependent ATPase activity
AGOC: null
AGOP: DNA metabolic process
PGOF: ATP binding, DNA binding, DNA–dependent ATPase activity
PGOC: null
PGOP: DNA metabolic process

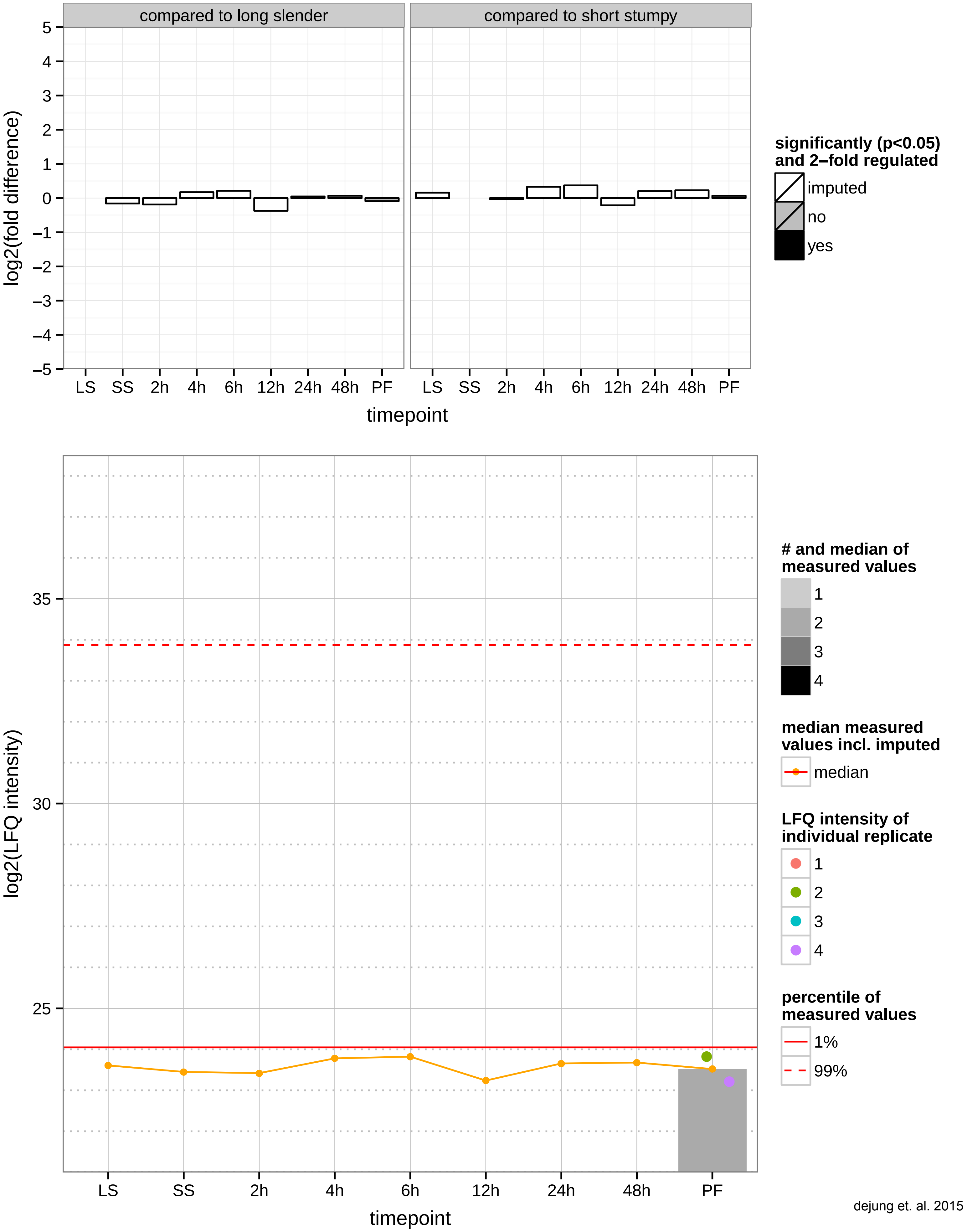

dejung et. al. 2015

Hsc70–interacting protein (Hip), putative
Tb927.3.5340
AGOF: null
AGOC: null
AGOP: null
PGOF: protein binding
PGOC: null
PGOP: null

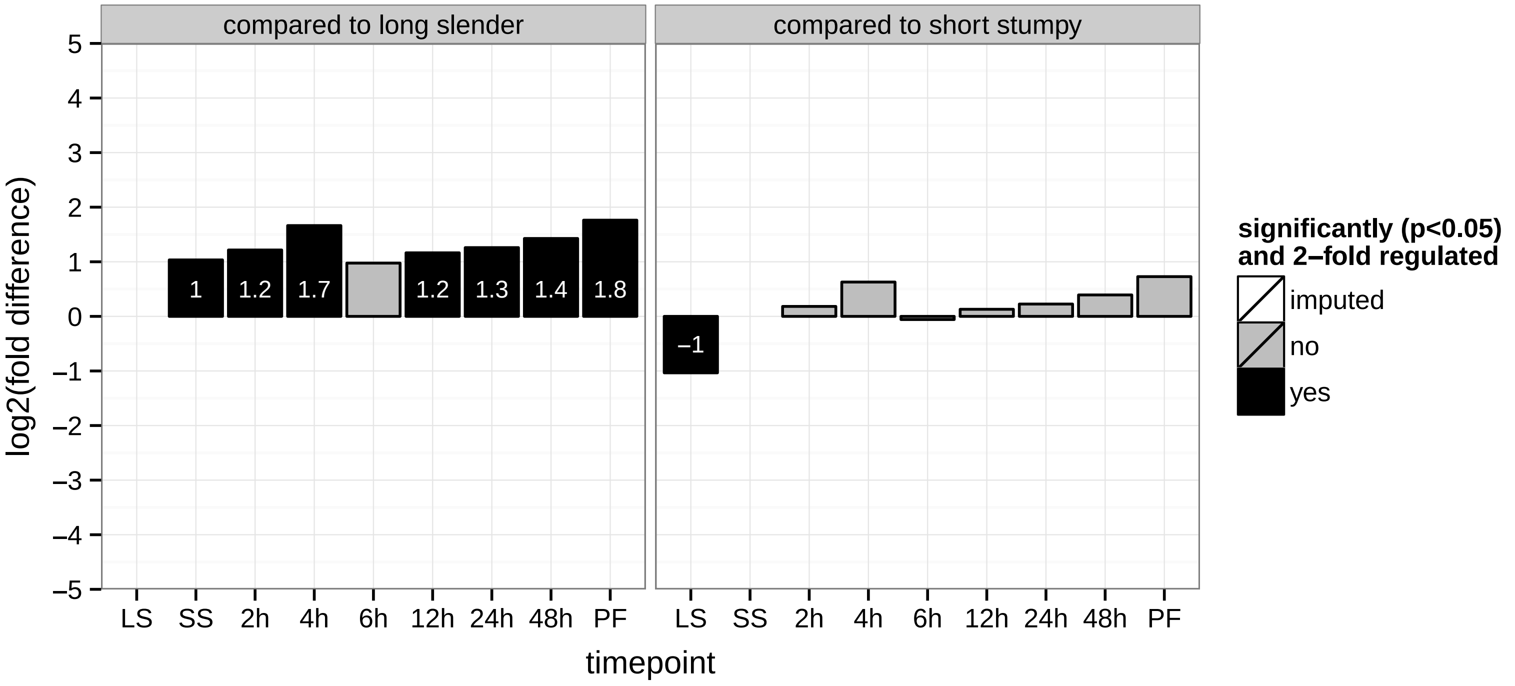

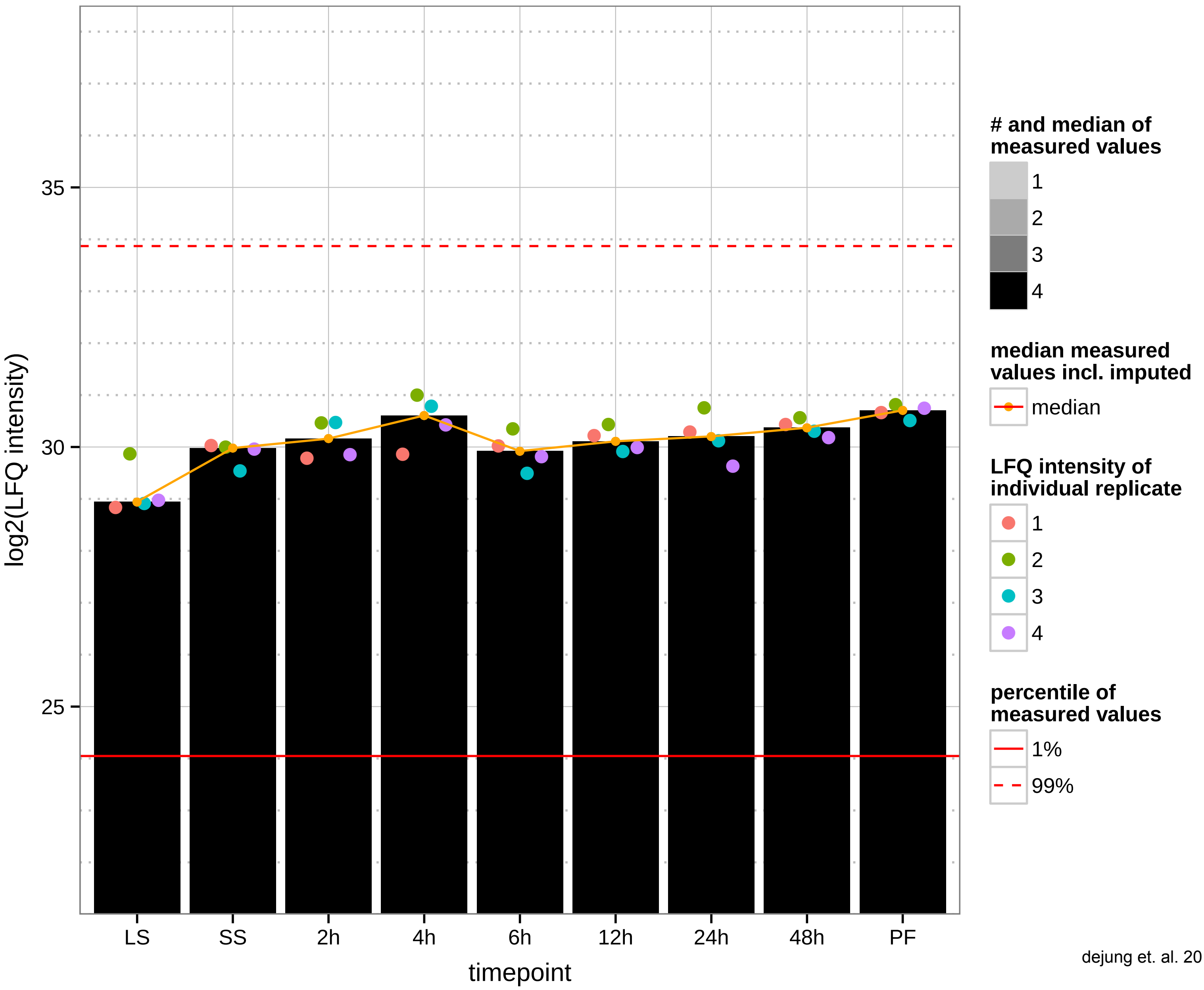

dejung et. al. 2015

hypothetical protein, conserved
Tb927.3.5370
AGOF: null
AGOC: null
AGOP: null
PGOF: null
PGOC: null
PGOP: null

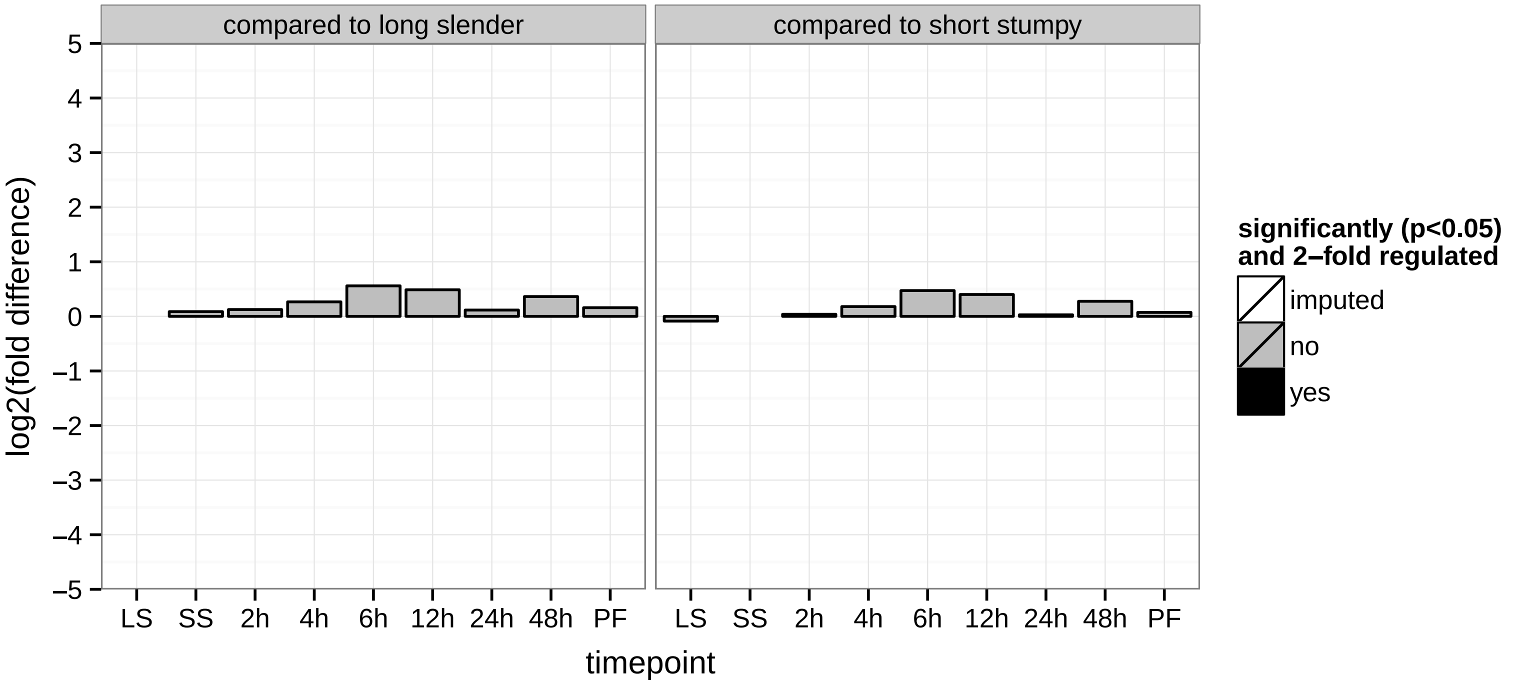

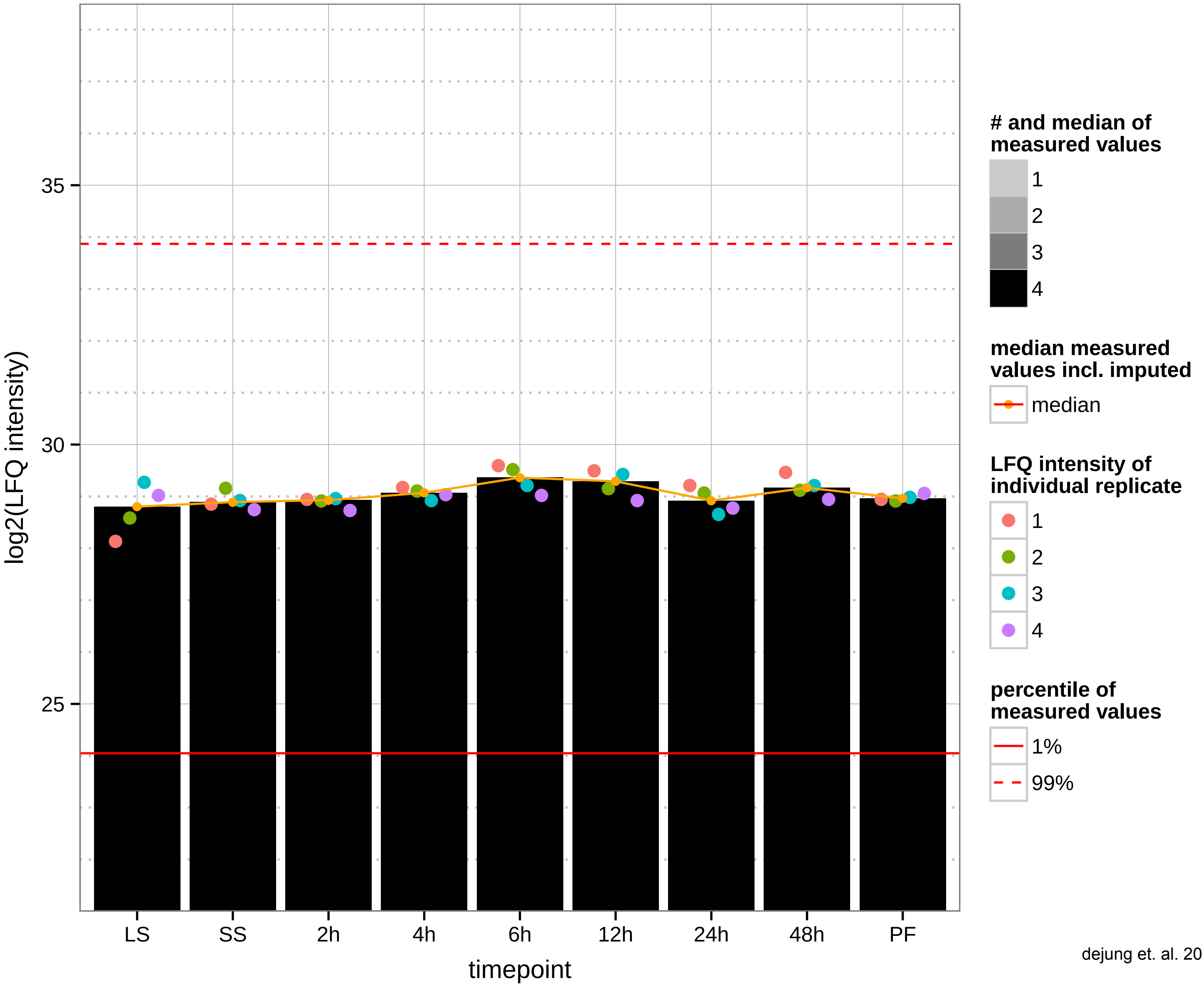

dejung et. al. 2015

protein transport protein Sec24 (SEC24.2)
Tb927.3.5420
AGOF: protein binding, zinc ion binding
AGOC: COPII vesicle coat
AGOP: ER to Golgi vesicle–mediated transport, intracellular protein transport
PGOF: zinc ion binding
PGOC: COPII vesicle coat
PGOP: ER to Golgi vesicle–mediated transport, intracellular protein transport

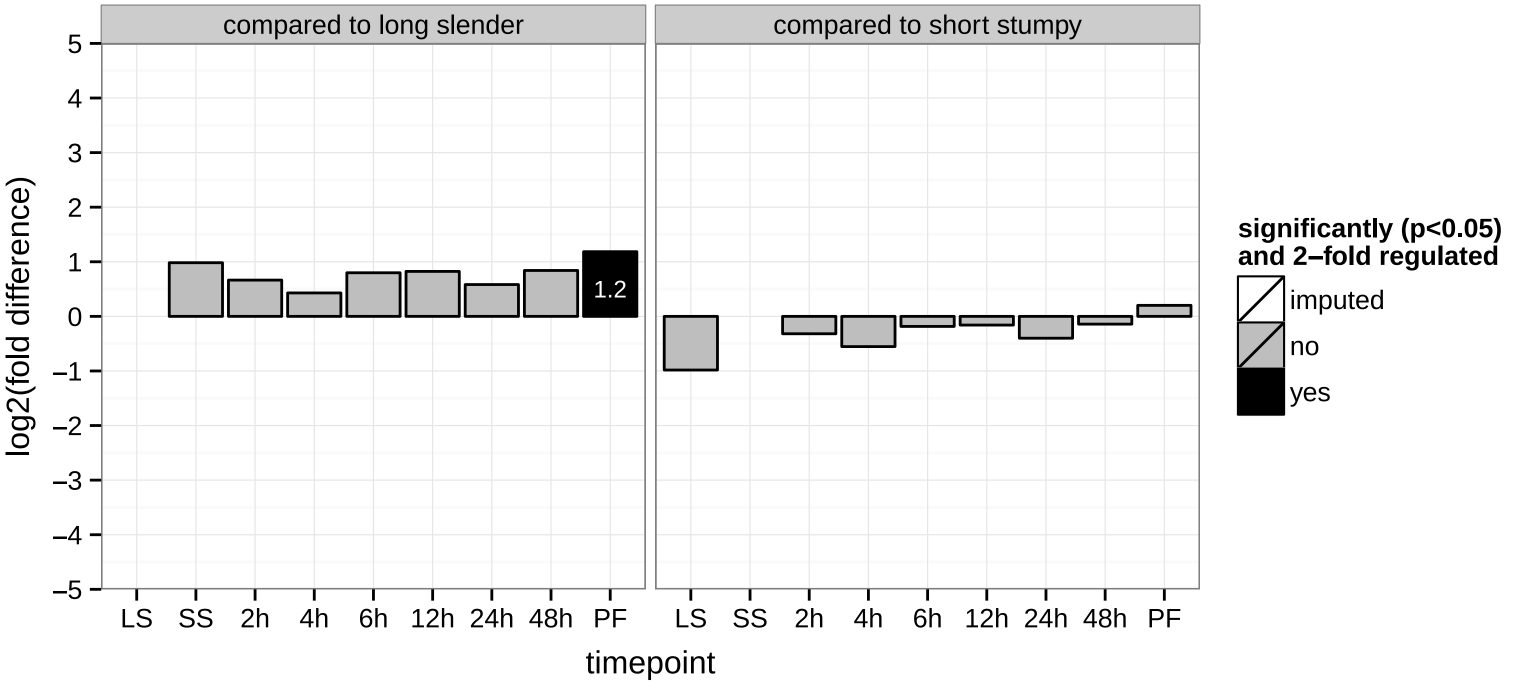

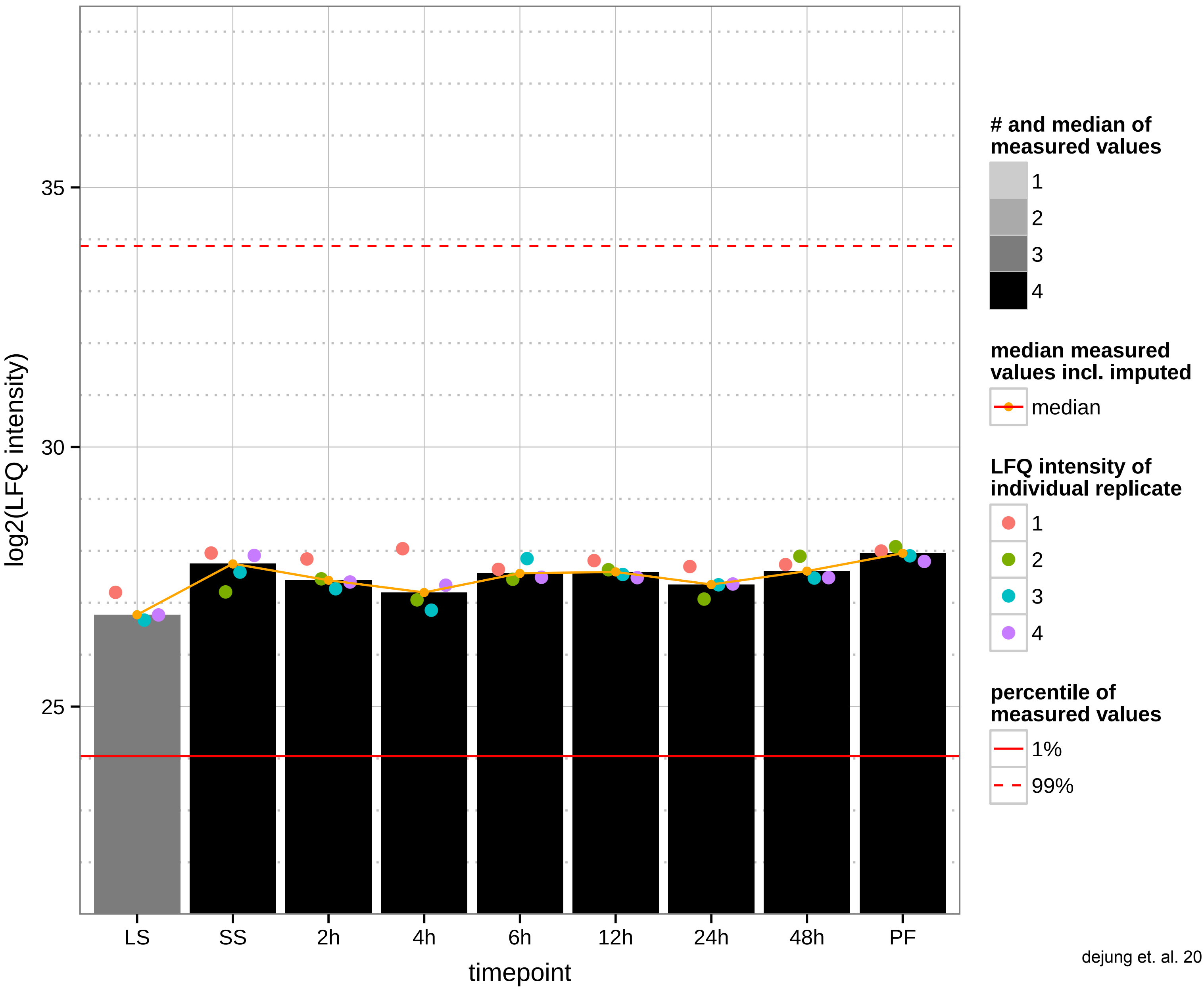

dejung et. al. 2015

SNF2 DNA repair protein, putative
Tb927.3.5440
AGOF: ATP binding, DNA binding, helicase activity
AGOC: null
AGOP: null
PGOF: ATP binding, DNA binding, helicase activity, nucleic acid binding
PGOC: null
PGOP: null

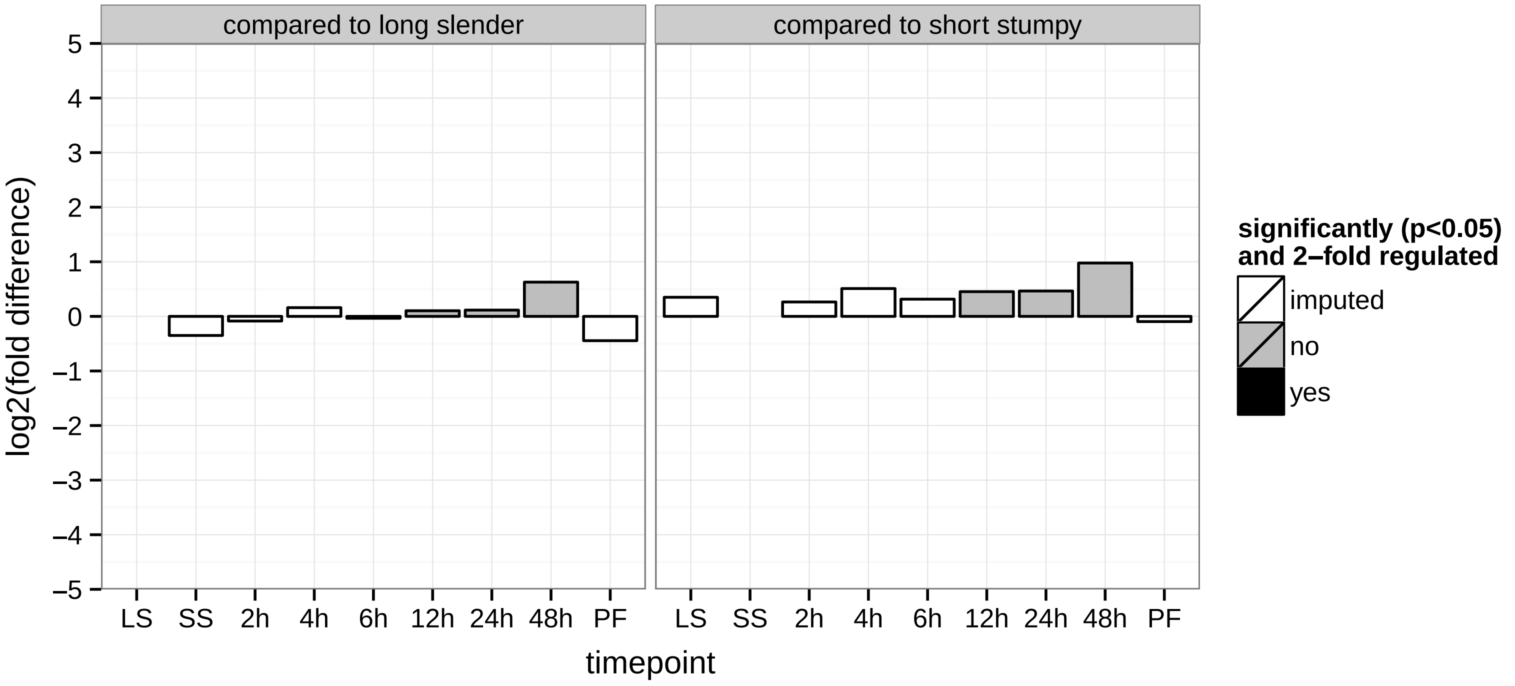

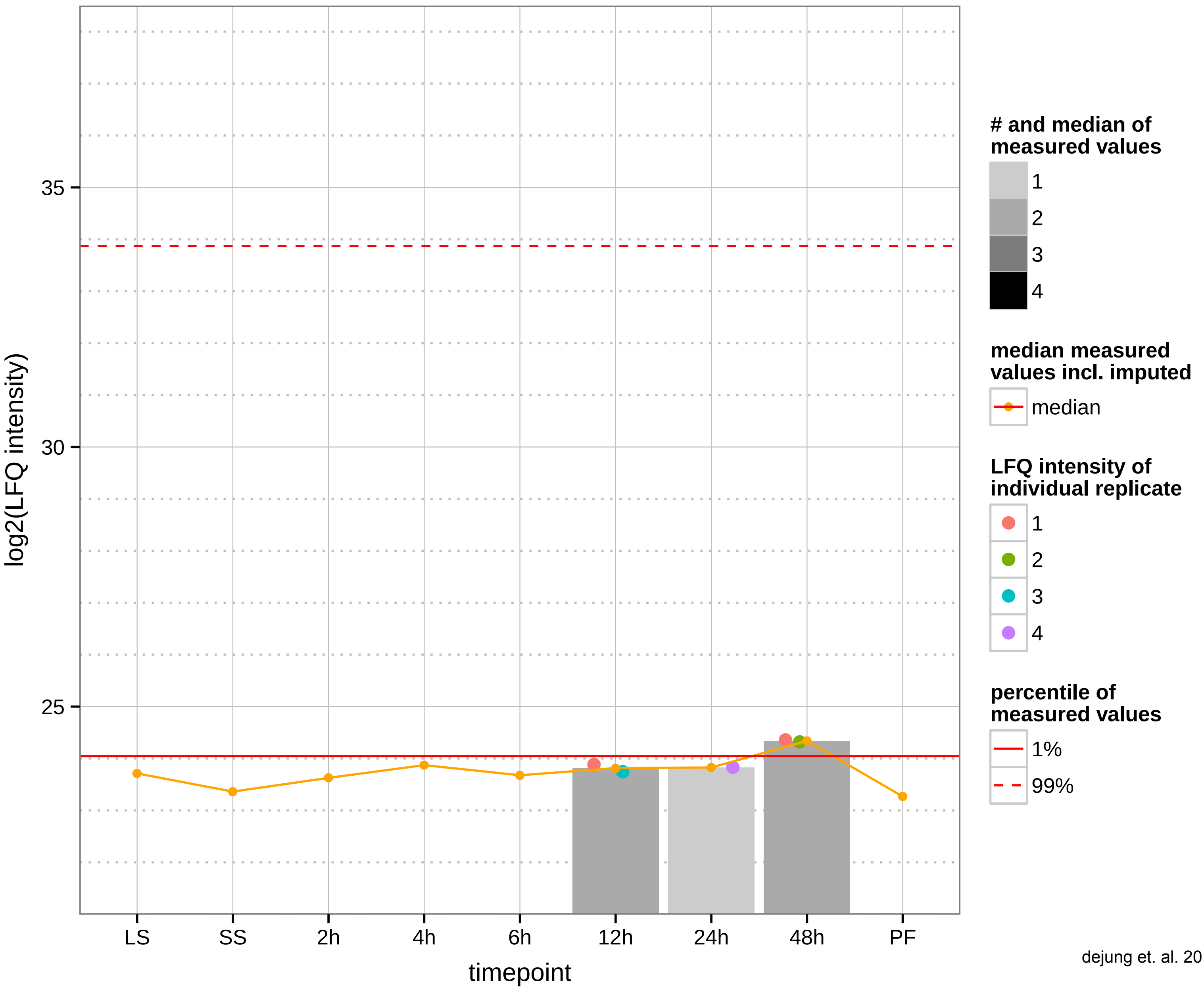

dejung et. al. 2015

DNA–directed RNA polymerase II subunit 3, putative, RNA polymerase subunit, putative (RPB3)
Tb927.3.5500
AGOF: DNA binding, DNA–directed RNA polymerase activity, protein dimerization activity
AGOC: null
AGOP: transcription, DNA–dependent
PGOF: DNA binding, DNA–directed RNA polymerase activity, protein dimerization activity
PGOC: null
PGOP: transcription, DNA–dependent

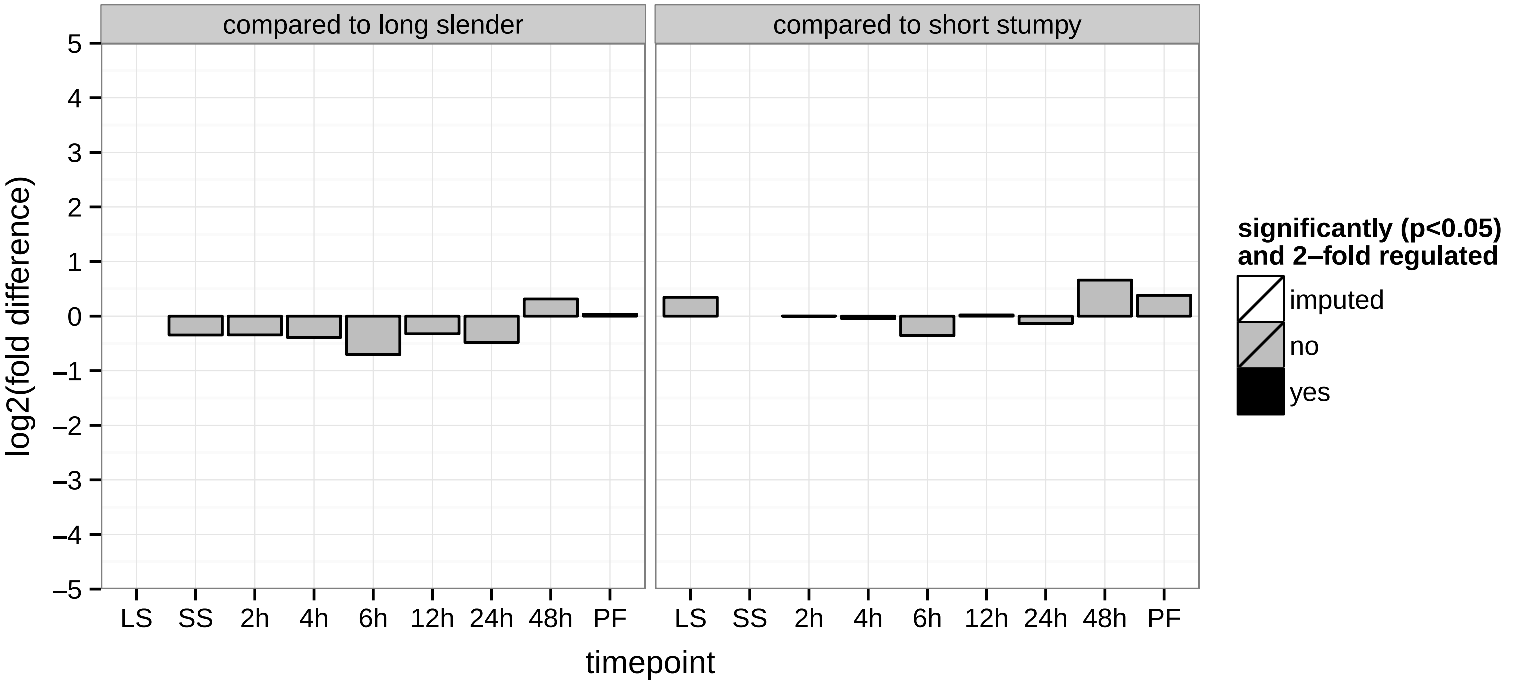

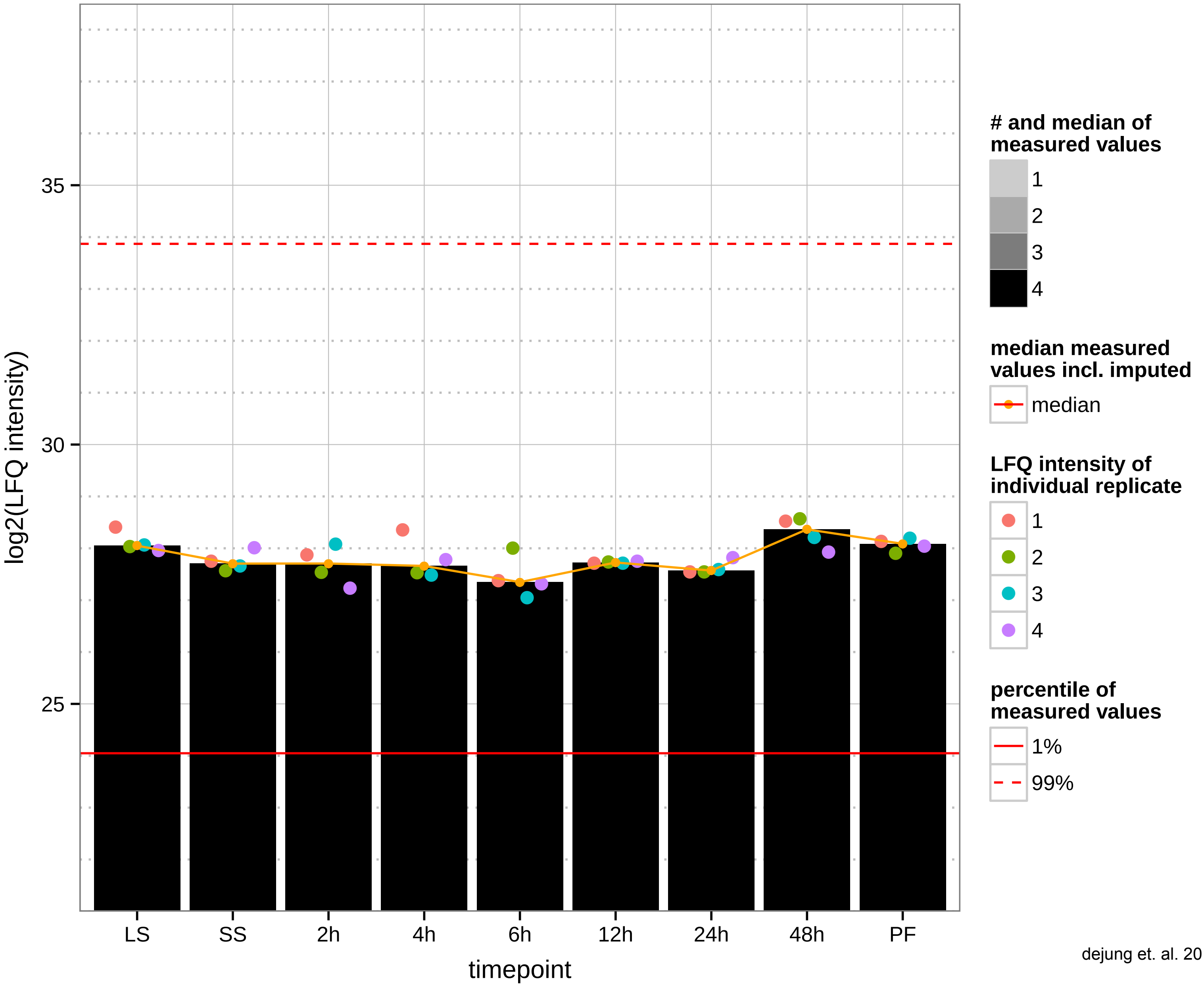

dejung et. al. 2015

NA, 26S proteasome regulatory non–ATPase subunit (RPN1)
Tb927.3.5520
AGOF: NA, enzyme regulator activity
AGOC: NA, proteasome complex
AGOP: NA, regulation of protein catabolic process
PGOF: NA, binding, enzyme regulator activity
PGOC: NA, proteasome complex
PGOP: NA, regulation of protein catabolic process

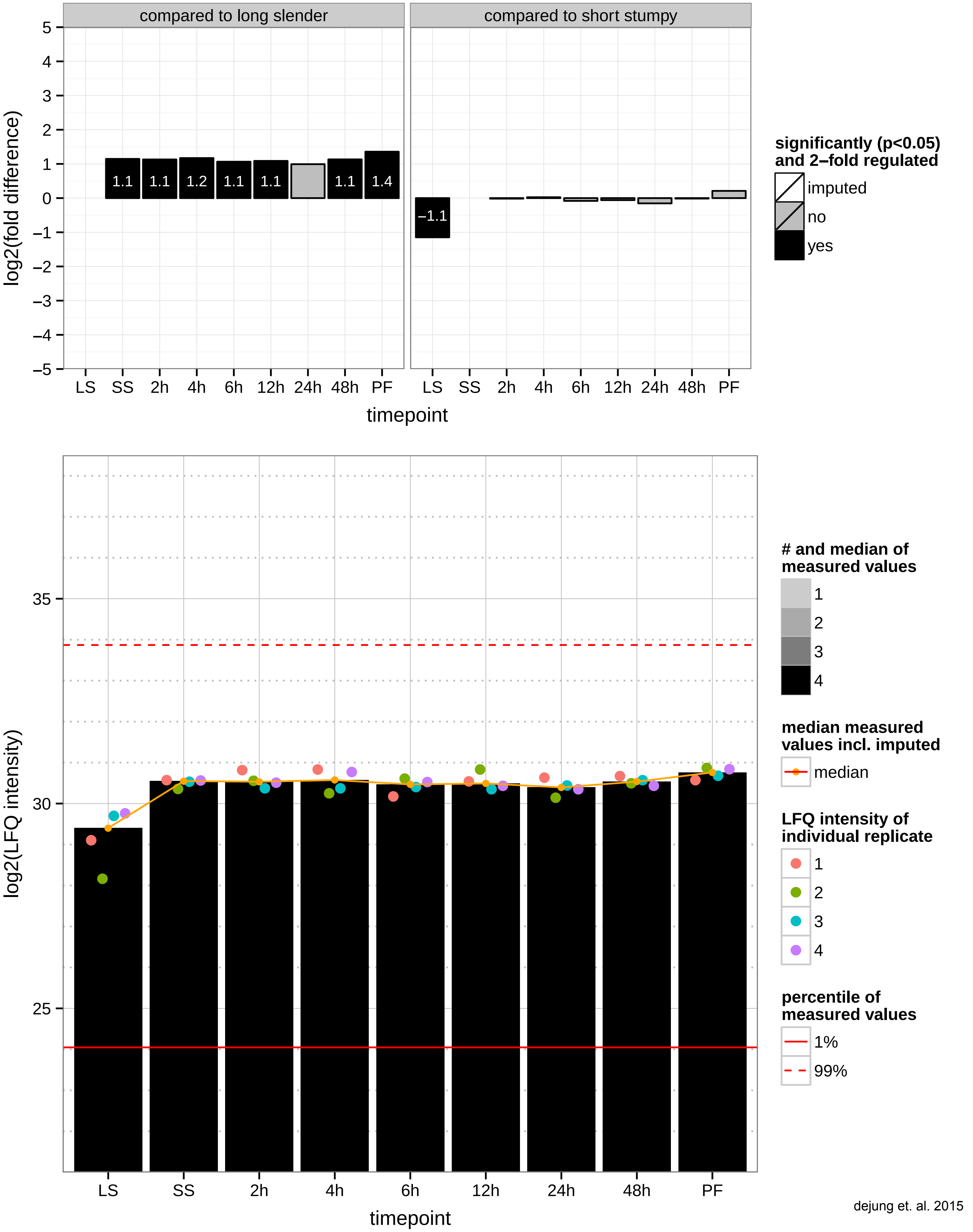

dejung et. al. 2015

Tb–292 membrane associated protein (Tb–292)
Tb927.3.5530
AGOF: null
AGOC: endocytic vesicle, flagellar pocket membrane, nuclear outer membrane
AGOP: null
PGOF: null
PGOC: null
PGOP: null

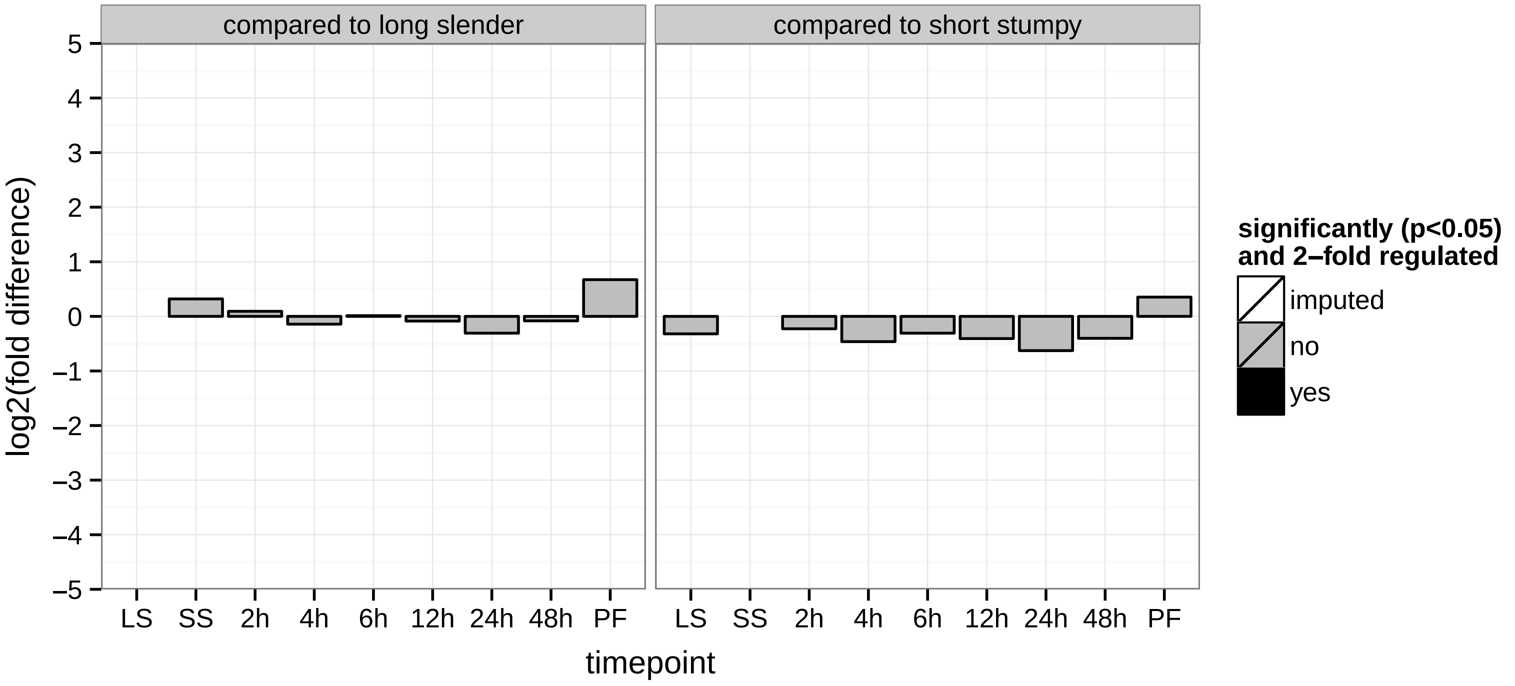

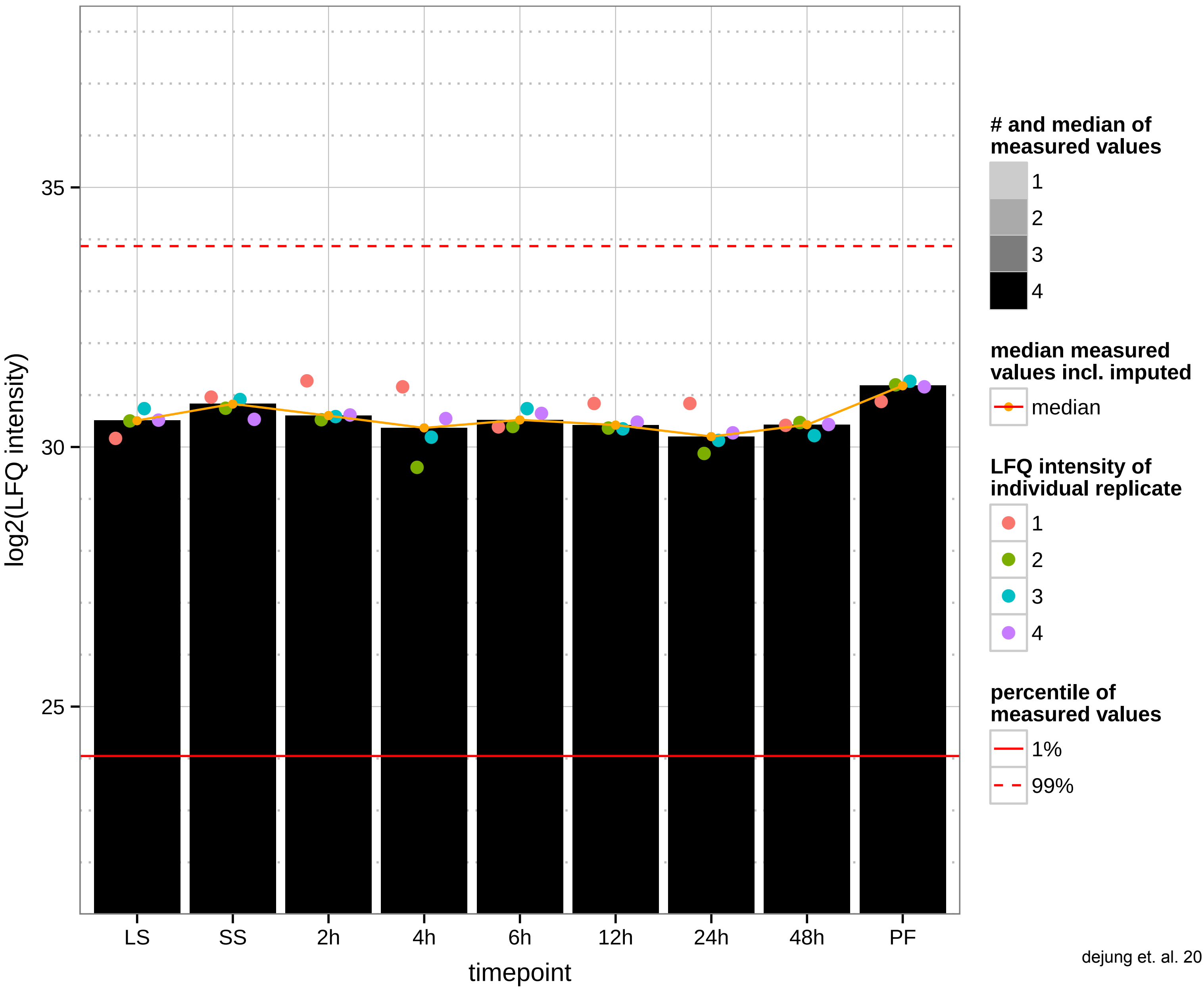

dejung et. al. 2015

tryptophanyl–tRNA synthetase
Tb927.3.5580
AGOF: ATP binding, tryptophan–tRNA ligase activity
AGOC: cytoplasm, cytosol
AGOP: translation, tryptophanyl–tRNA aminoacylation
PGOF: ATP binding, aminoacyl–tRNA ligase activity, nucleotide binding, tryptophan–tRNA ligase activity
PGOC: cytoplasm
PGOP: tRNA aminoacylation for protein translation, tryptophanyl–tRNA aminoacylation

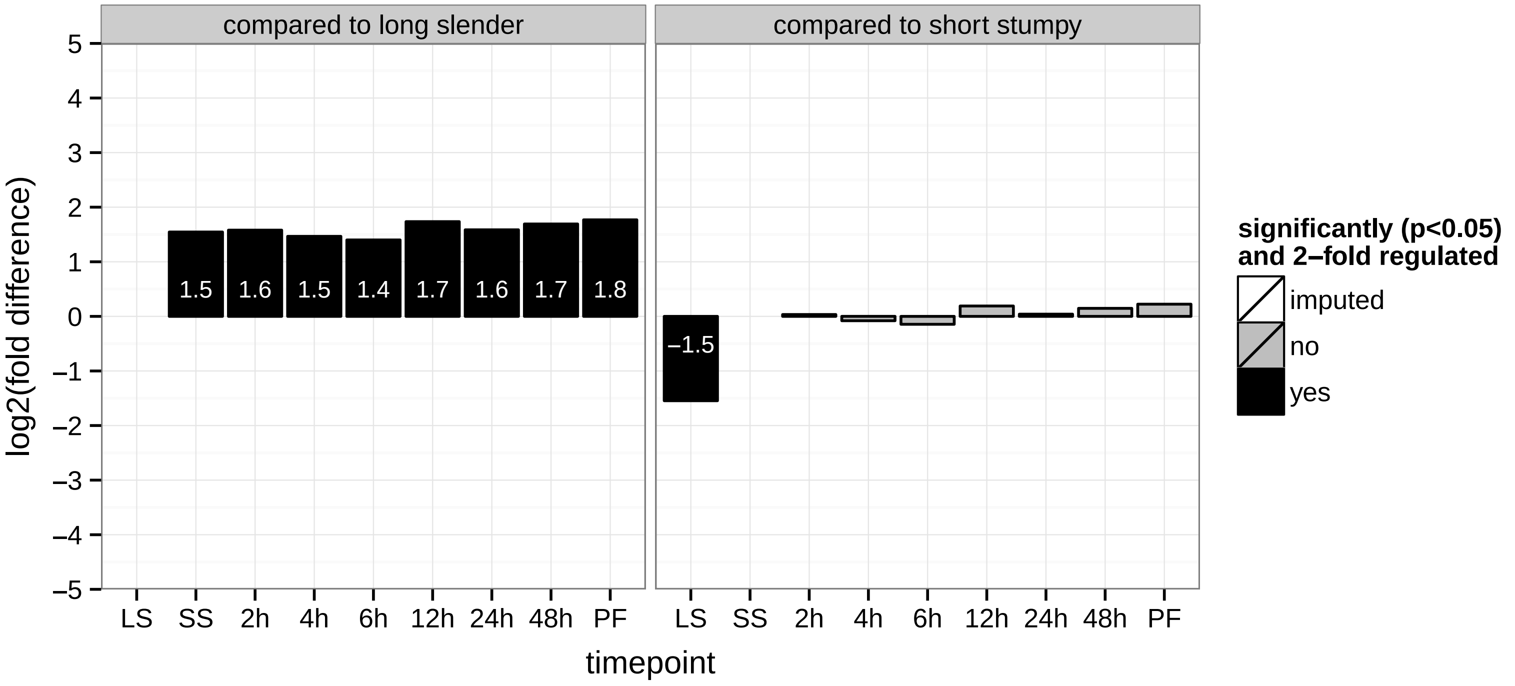

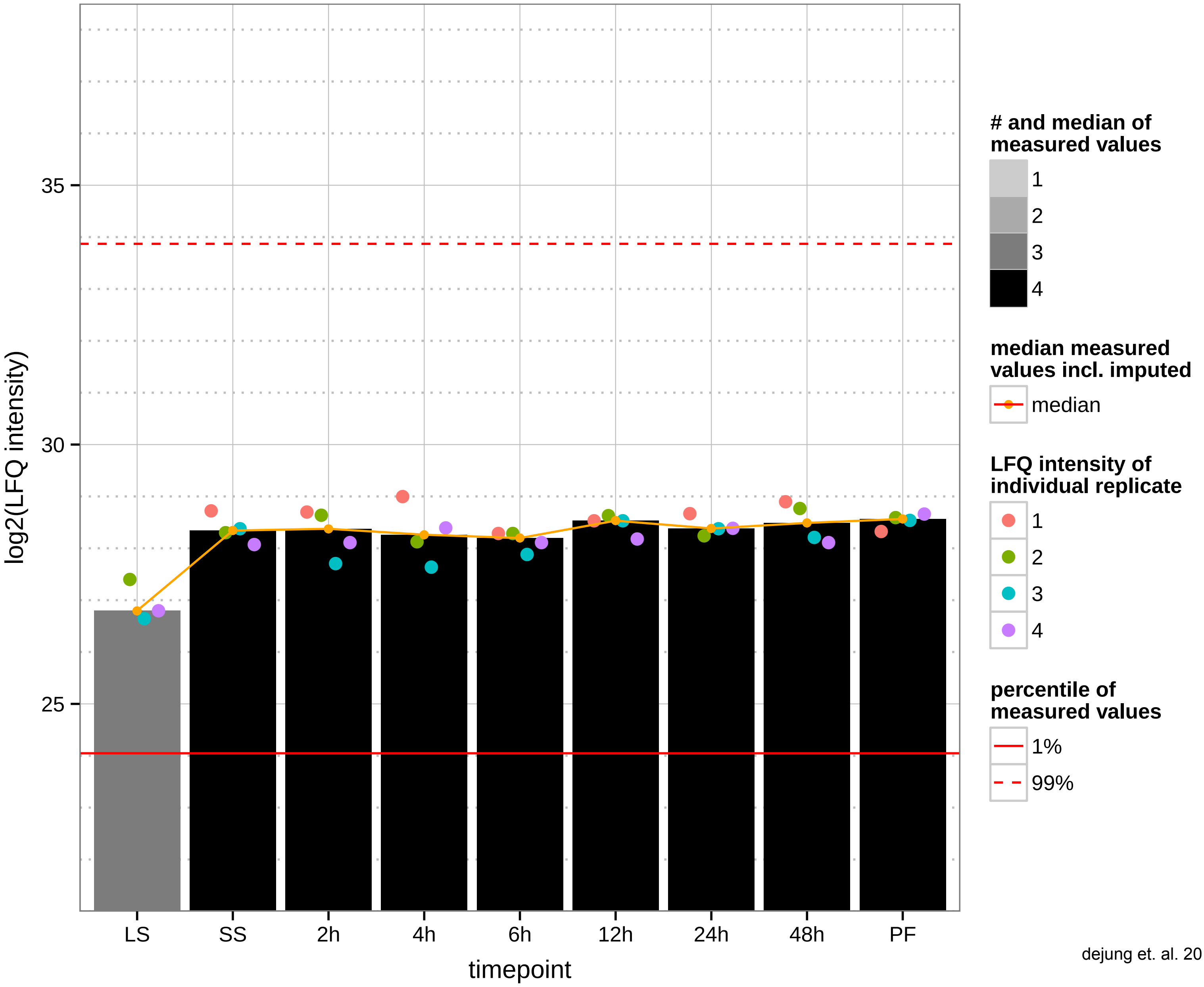

dejung et. al. 2015

hypothetical protein, conserved
Tb927.3.5620
AGOF: null
AGOC: null
AGOP: cellular process
PGOF: null
PGOC: null
PGOP: cellular process

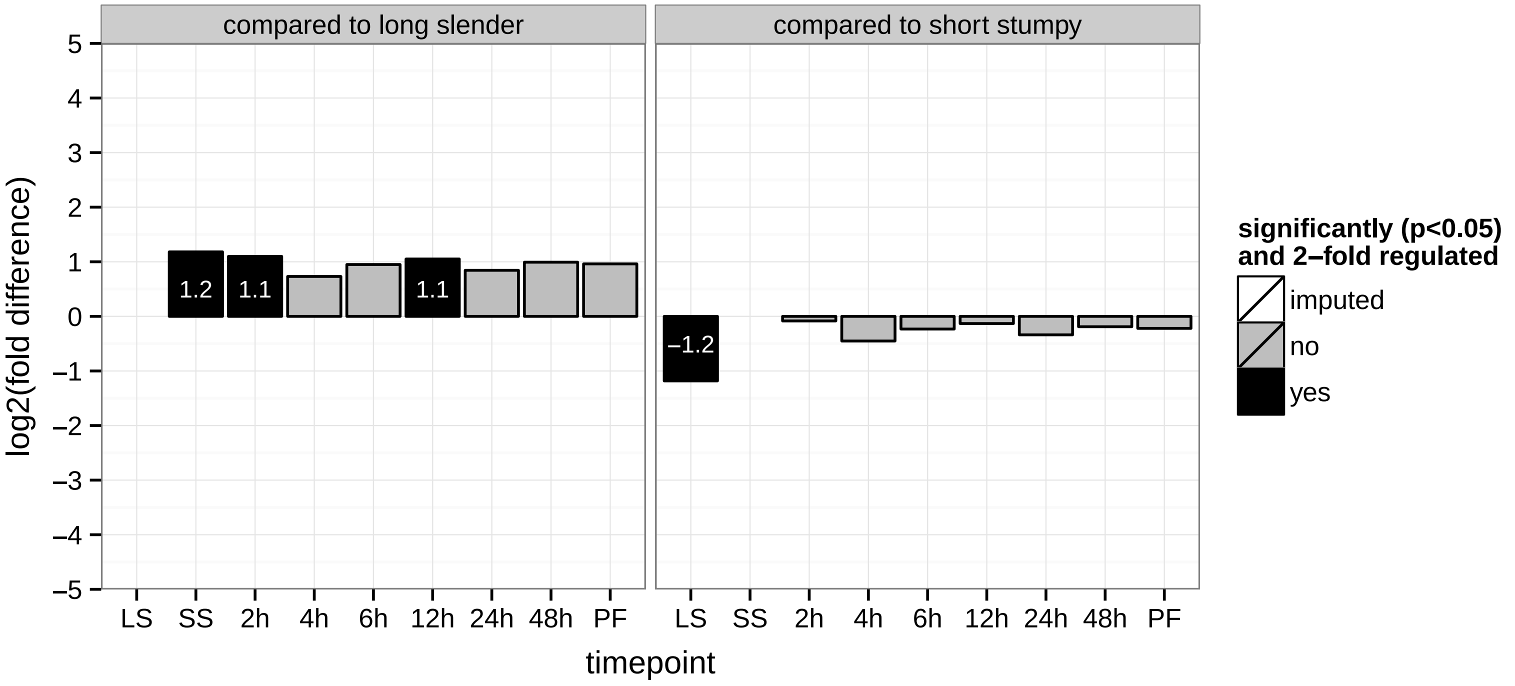

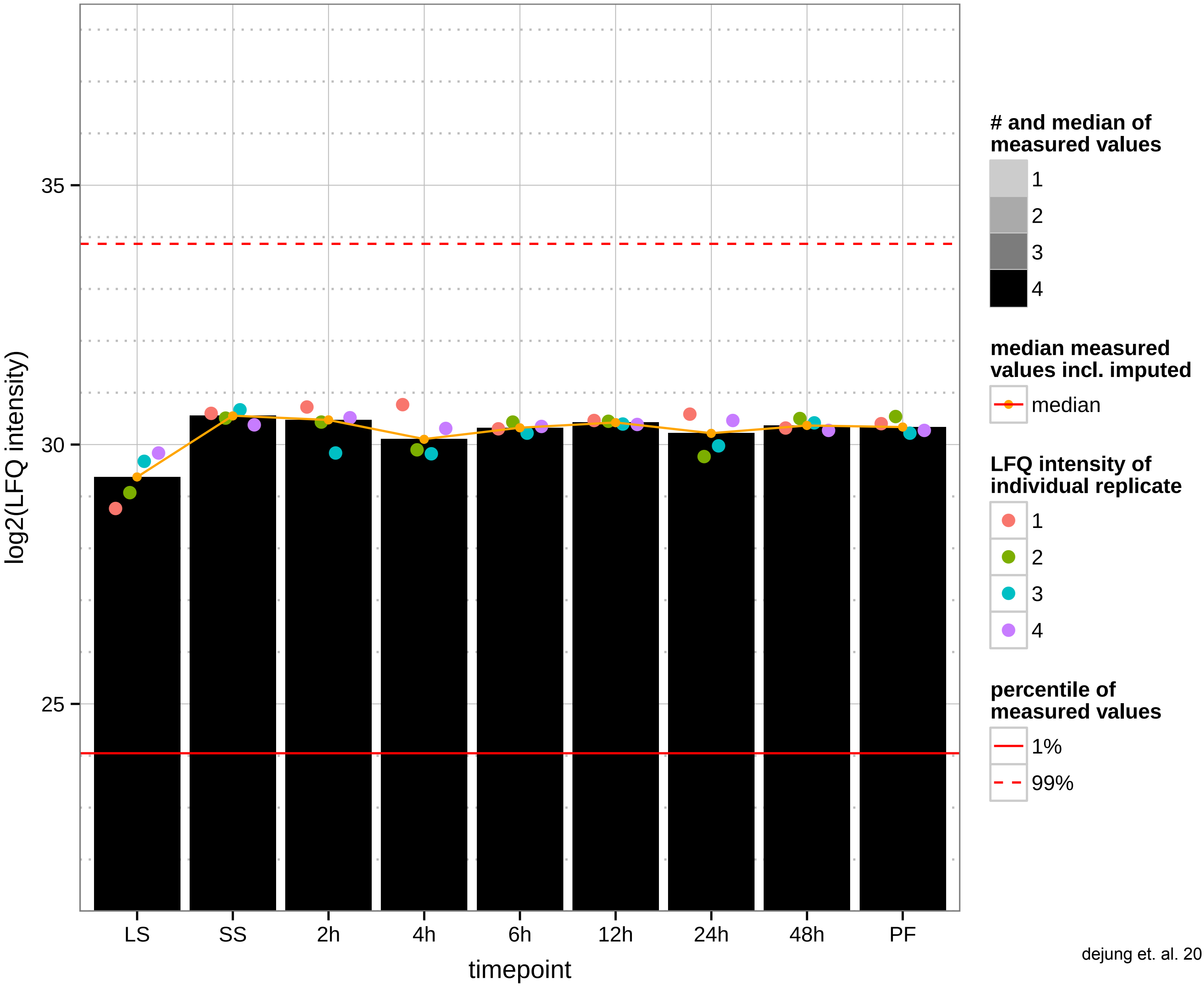

dejung et. al. 2015

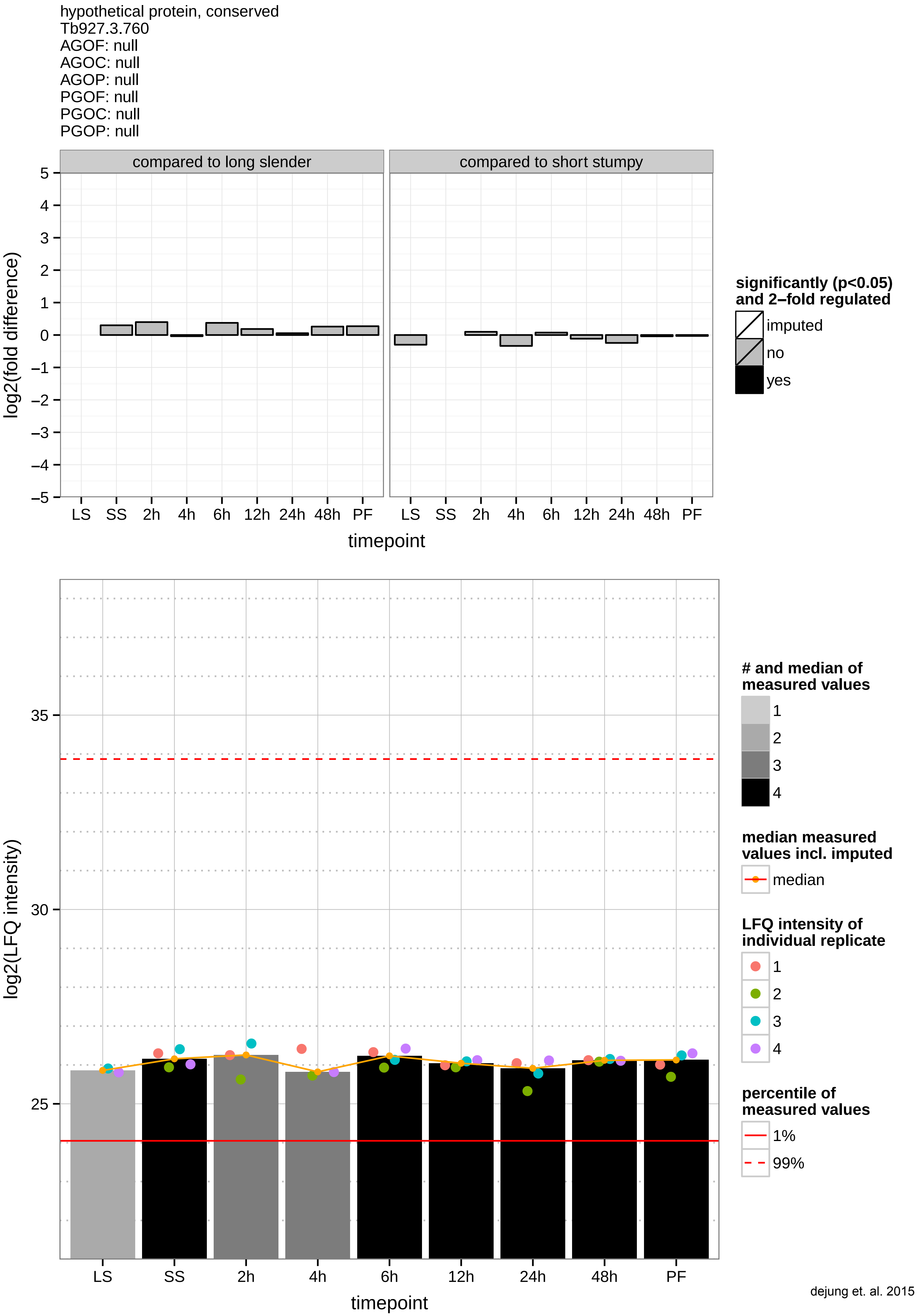
hypothetical protein, conserved
Tb927.3.760
AGOF: null
AGOC: null
AGOP: null
PGOF: null
PGOC: null
PGOP: null
compared to long slender
compared to short stumpy
log2(fold difference)
5
4
3
2
1
0
−1
−2
−3
−4
−5
LS SS 2h 4h 6h 12h 24h 48h PF
timepoint
significantly (p<0.05) and 2−fold regulated
imputed
no
yes
log2(LFQ intensity)
35
30
25
and median of measured values
1
2
3
4
median measured values incl. imputed
median
LFQ intensity of individual replicate
1
2
3
4
percentile of measured values
1%
99%
dejung et. al. 2015

proteasome alpha 7 subunit (TbPSA7)
Tb927.3.780
AGOF: endopeptidase activity, threonine–type endopeptidase activity
AGOC: proteasome core complex, proteasome core complex, alpha–subunit complex
AGOP: protein metabolic process, ubiquitin–dependent protein catabolic process
PGOF: endopeptidase activity, threonine–type endopeptidase activity
PGOC: proteasome core complex, proteasome core complex, alpha–subunit complex
PGOP: proteolysis involved in cellular protein catabolic process, ubiquitin–dependent protein catabolic process

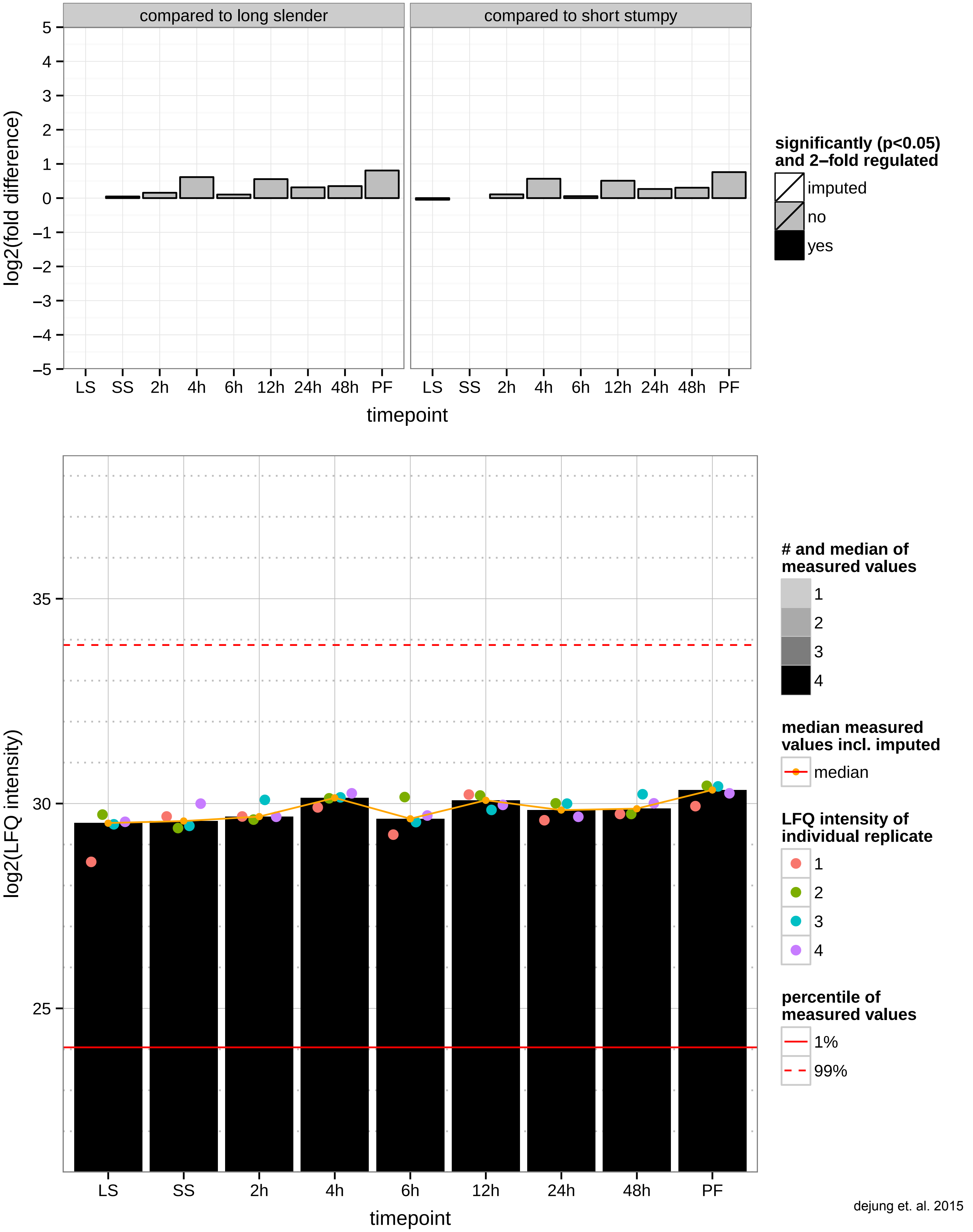

dejung et. al. 2015

hypothetical protein, conserved
Tb927.3.800
AGOF: null
AGOC: null
AGOP: null
PGOF: null
PGOC: null
PGOP: null

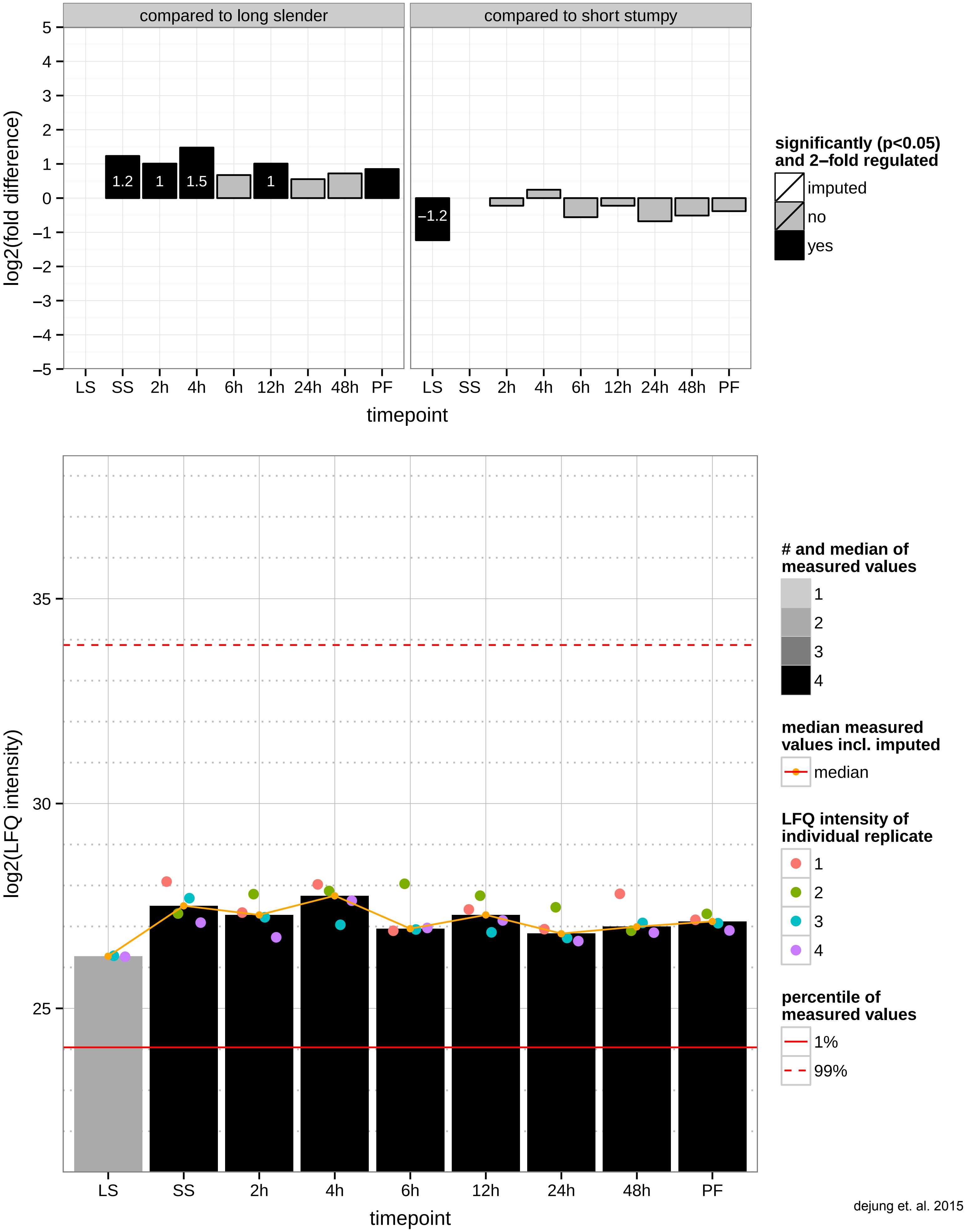

dejung et. al. 2015

hypothetical protein, conserved
Tb927.3.900
AGOF: null
AGOC: null
AGOP: null
PGOF: null
PGOC: null
PGOP: null

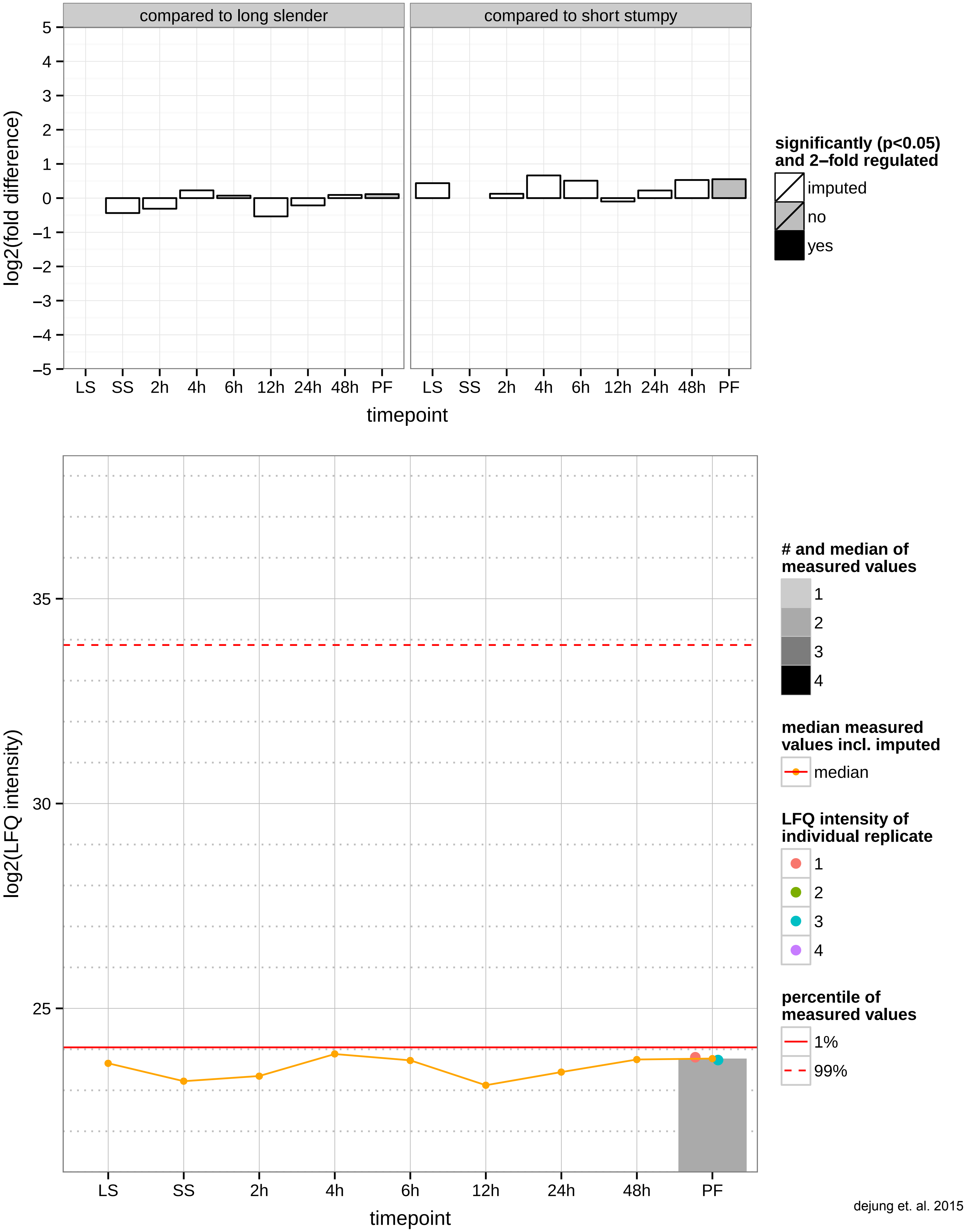

dejung et. al. 2015

gamma–tubulin, tubulin gamma chain
Tb927.3.910
AGOF: GTP binding, GTPase activity, microtubule binding
AGOC: cilium basal body, microtubule organizing center, protein complex, spindle pole
AGOP: cell growth, microtubule nucleation, protein polymerization
PGOF: GTP binding, GTPase activity
PGOC: protein complex
PGOP: GTP catabolic process, protein polymerization

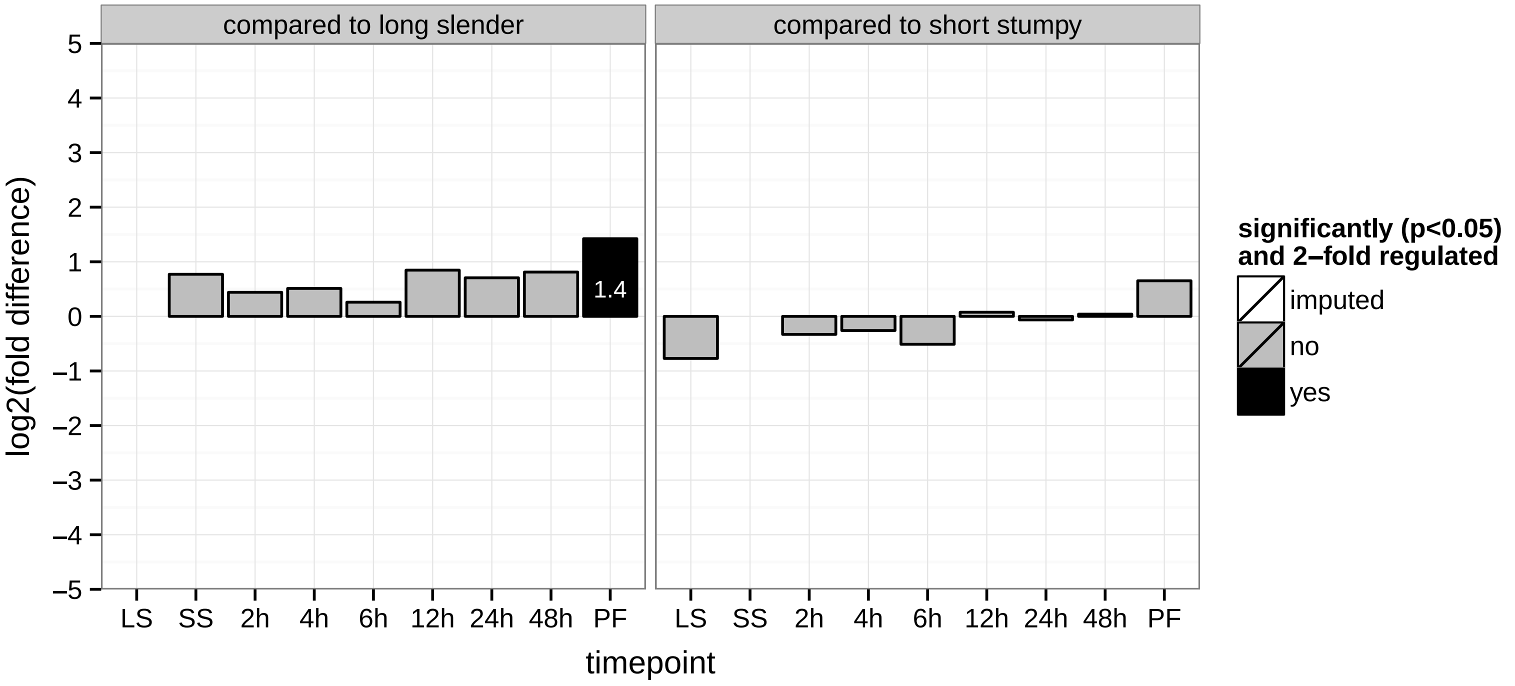

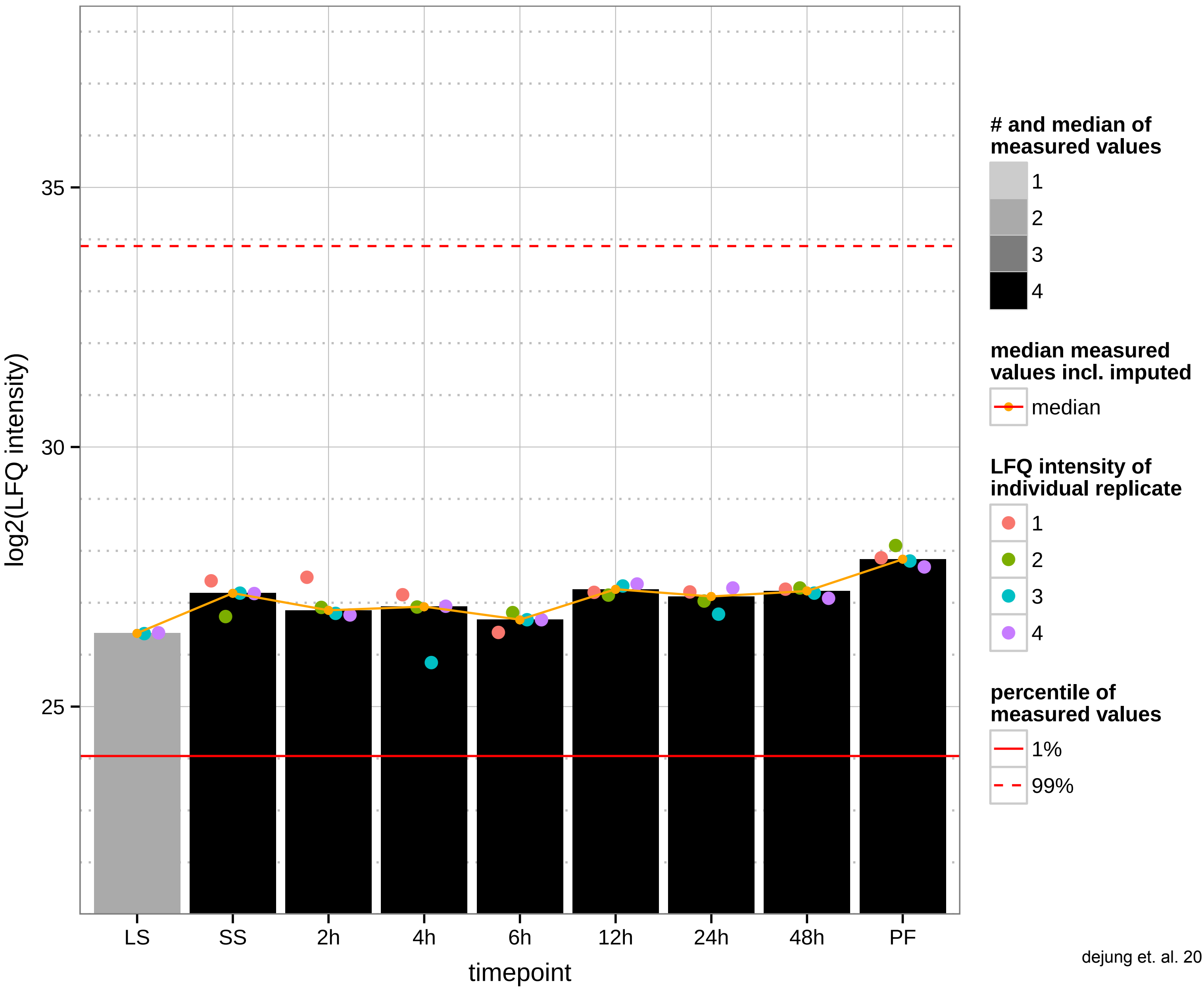

dejung et. al. 2015

dynein heavy chain, putative
Tb927.3.930
AGOF: ATP binding, ATPase activity, microtubule motor activity
AGOC: dynein complex
AGOP: microtubule−based movement
PGOF: microtubule motor activity, nucleoside−triphosphatase activity, nucleotide binding
PGOC: dynein complex
PGOP: microtubule−based movement

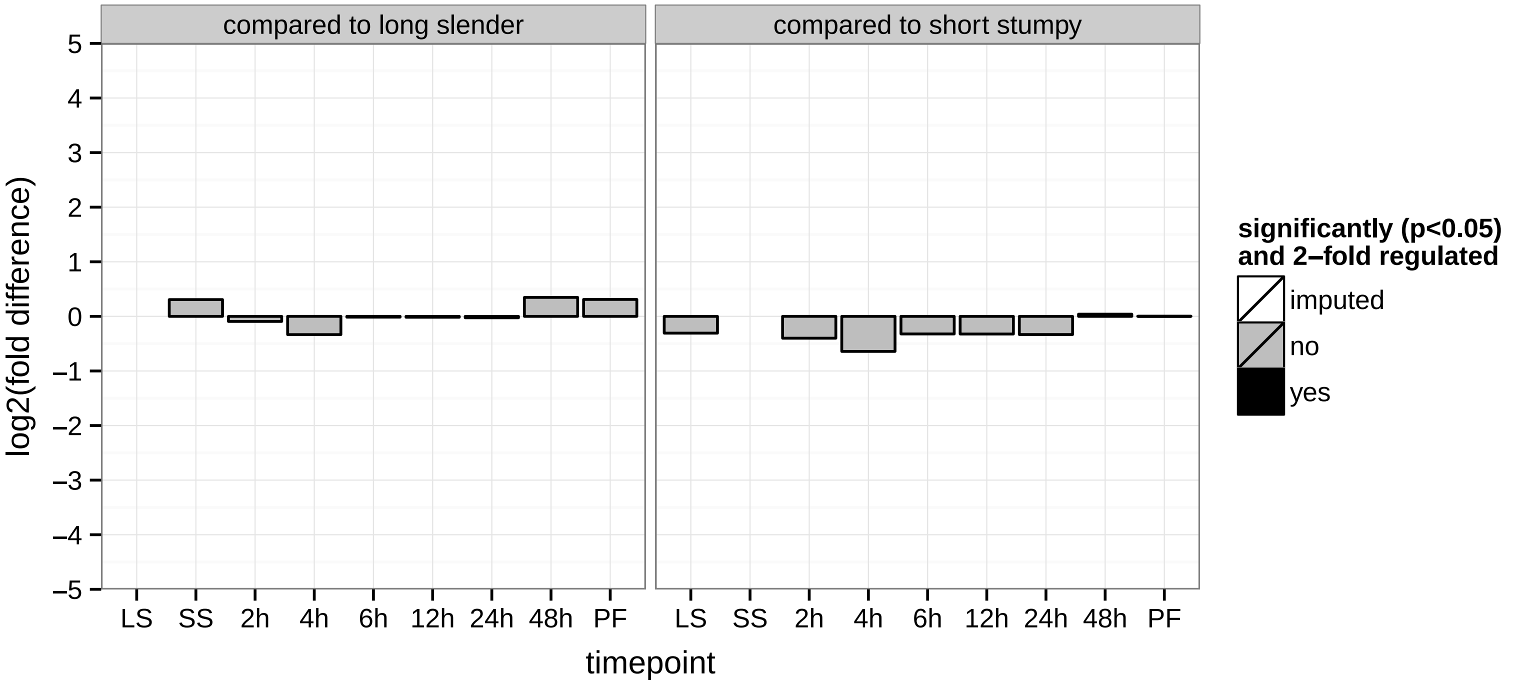

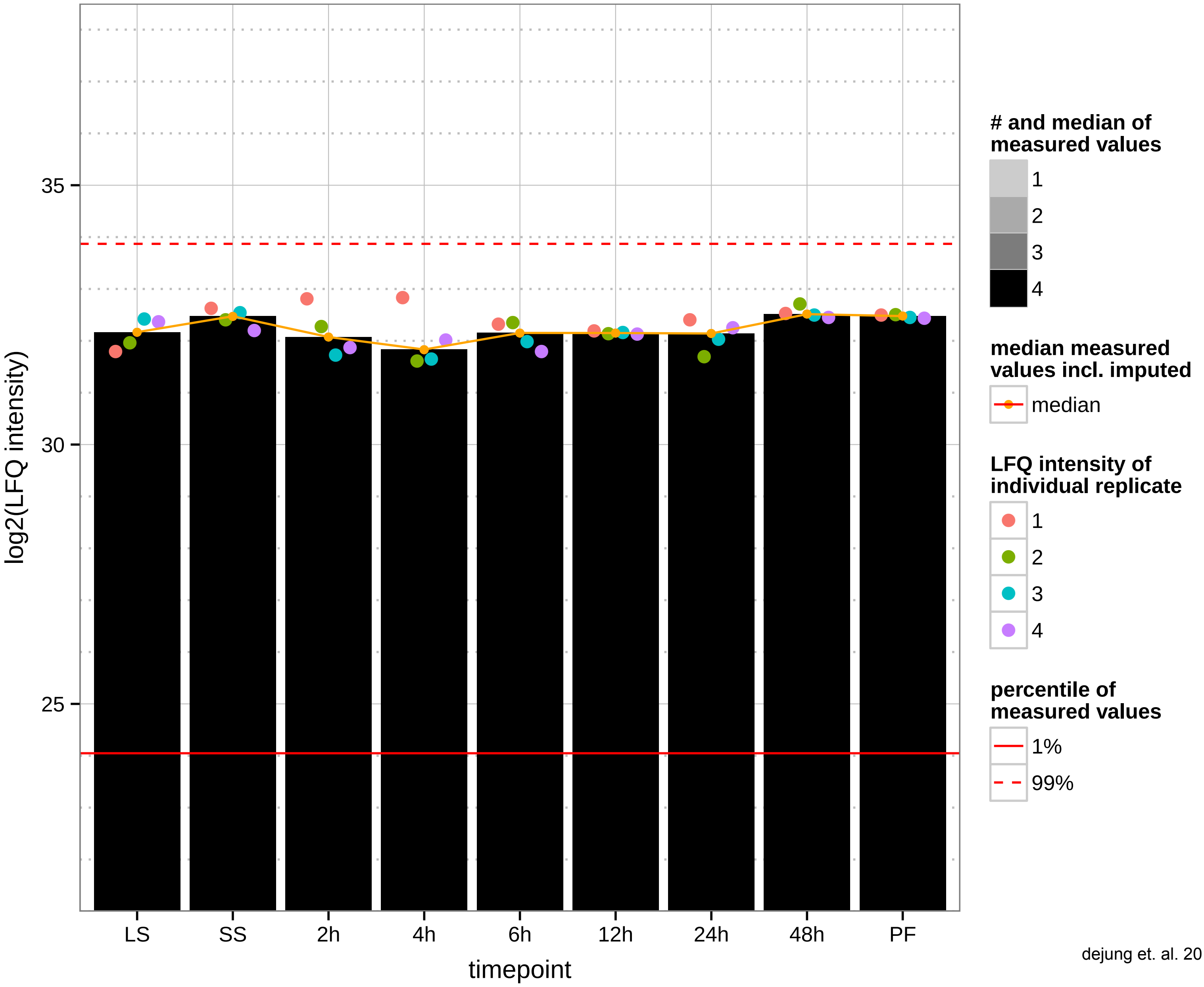

dejung et. al. 2015

hypothetical protein, conserved
Tb927.3.970
AGOF: phosphogluconate dehydrogenase (decarboxylating) activity
AGOC: mitochondrion
AGOP: pentose−phosphate shunt
PGOF: phosphogluconate dehydrogenase (decarboxylating) activity
PGOC: null
PGOP: oxidation−reduction process, pentose−phosphate shunt

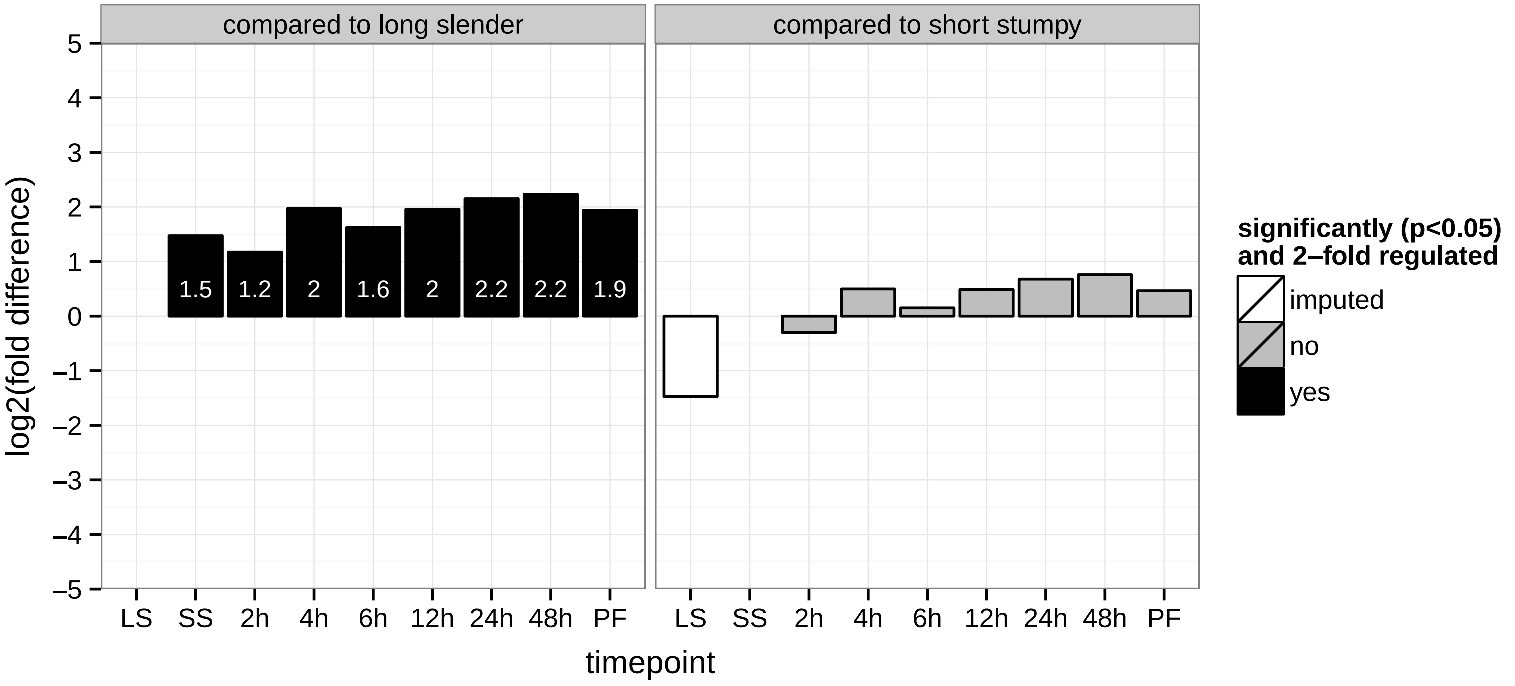

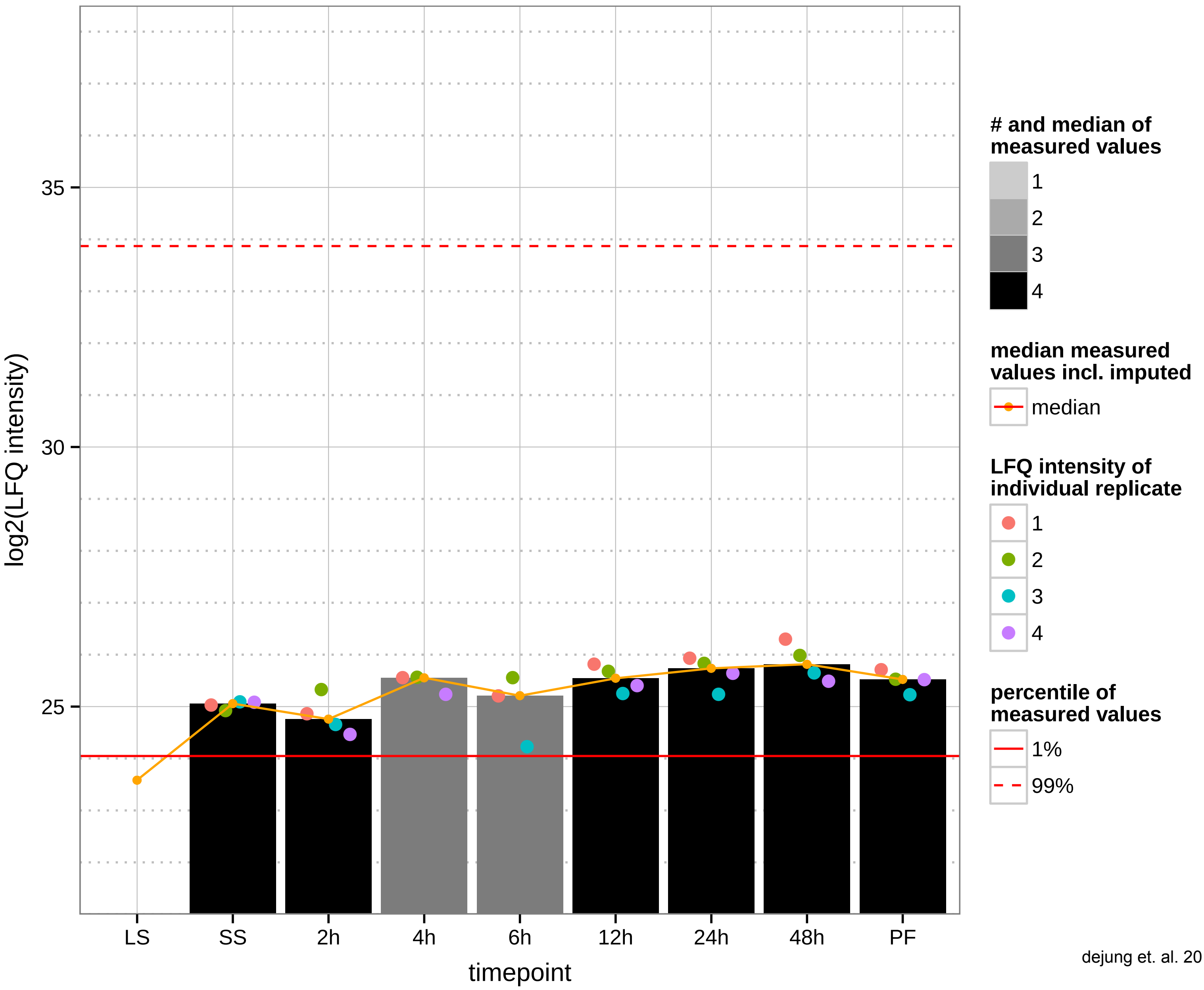

dejung et. al. 2015

hypothetical protein, conserved
Tb927.3.980
AGOF: null
AGOC: null
AGOP: null
PGOF: null
PGOC: null
PGOP: null

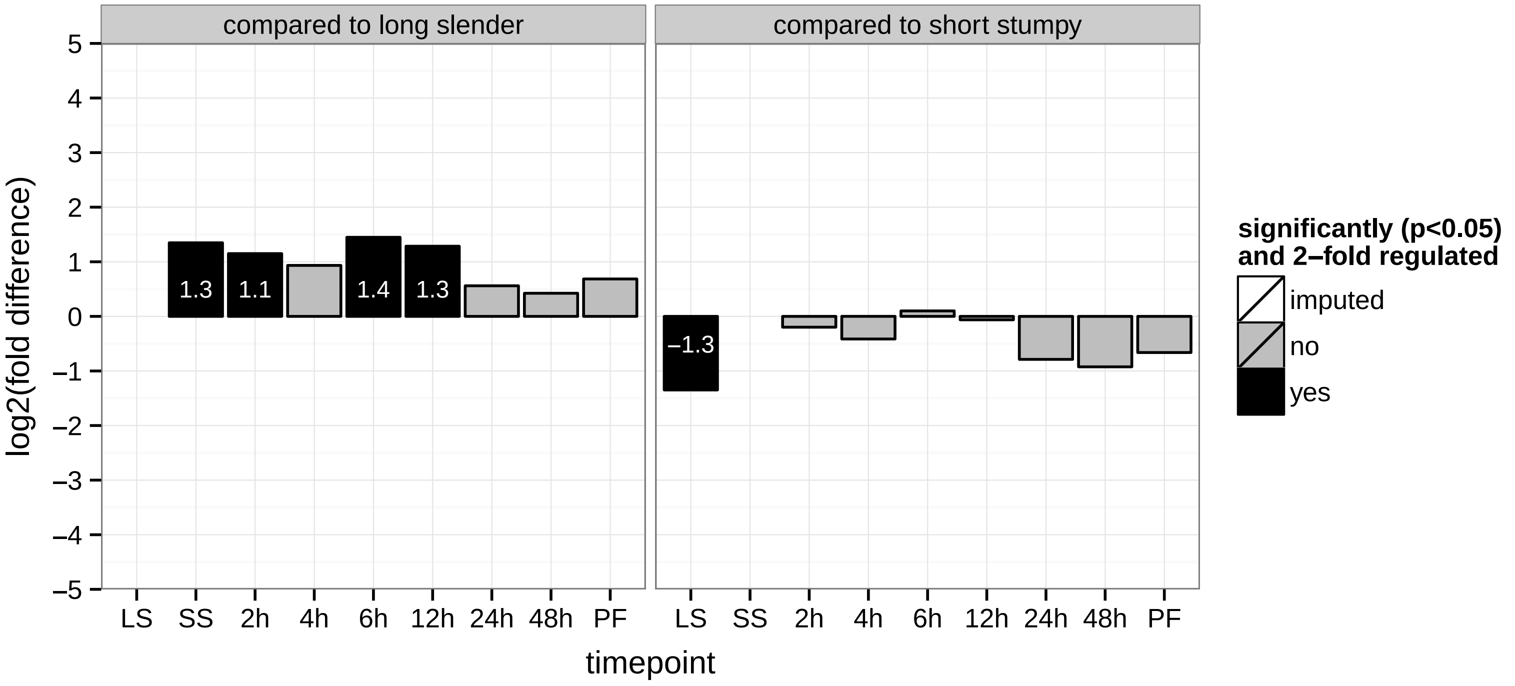

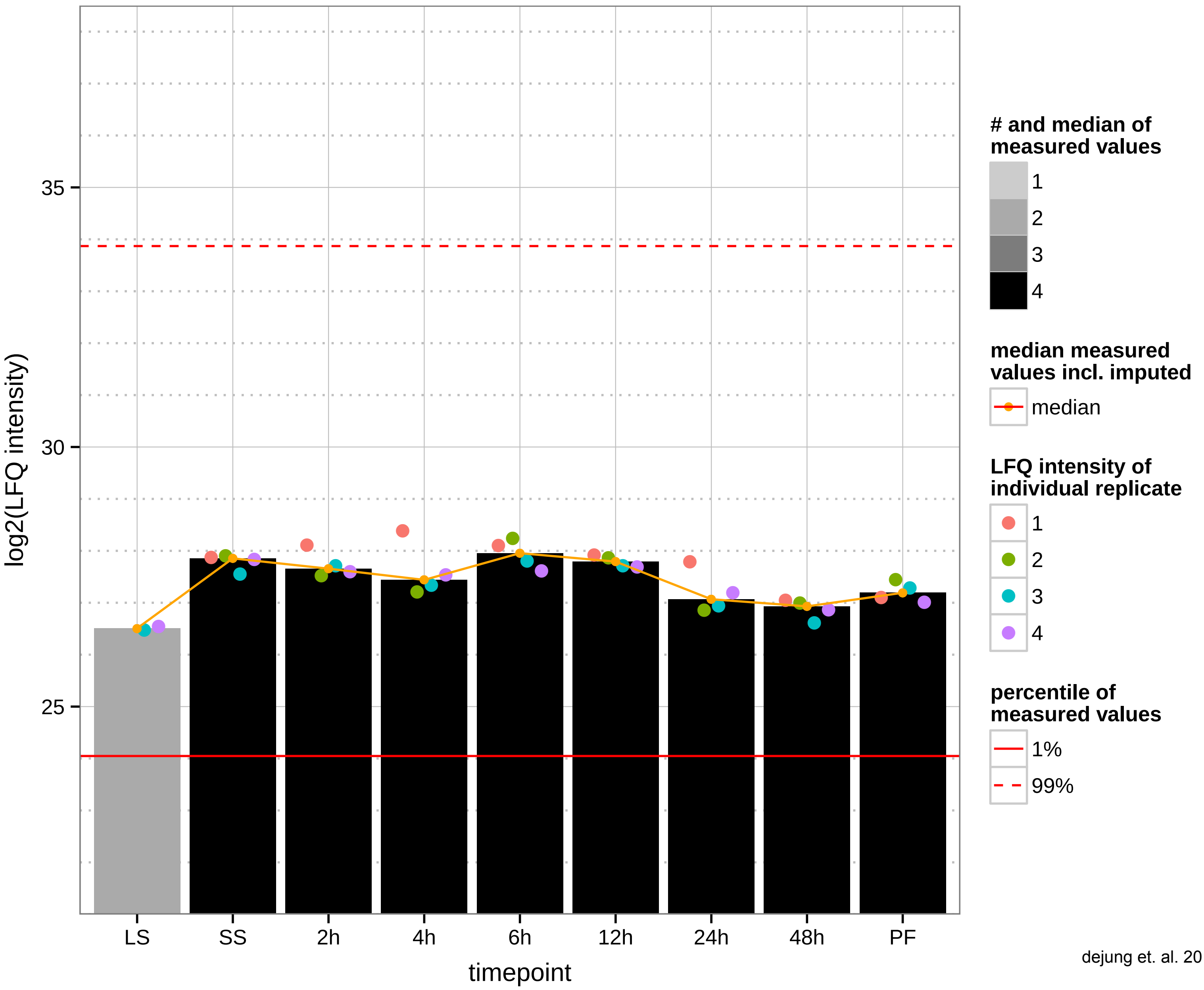

dejung et. al. 2015

hypothetical protein, conserved
Tb927.4.1040
AGOF: null
AGOC: null
AGOP: null
PGOF: null
PGOC: null
PGOP: null

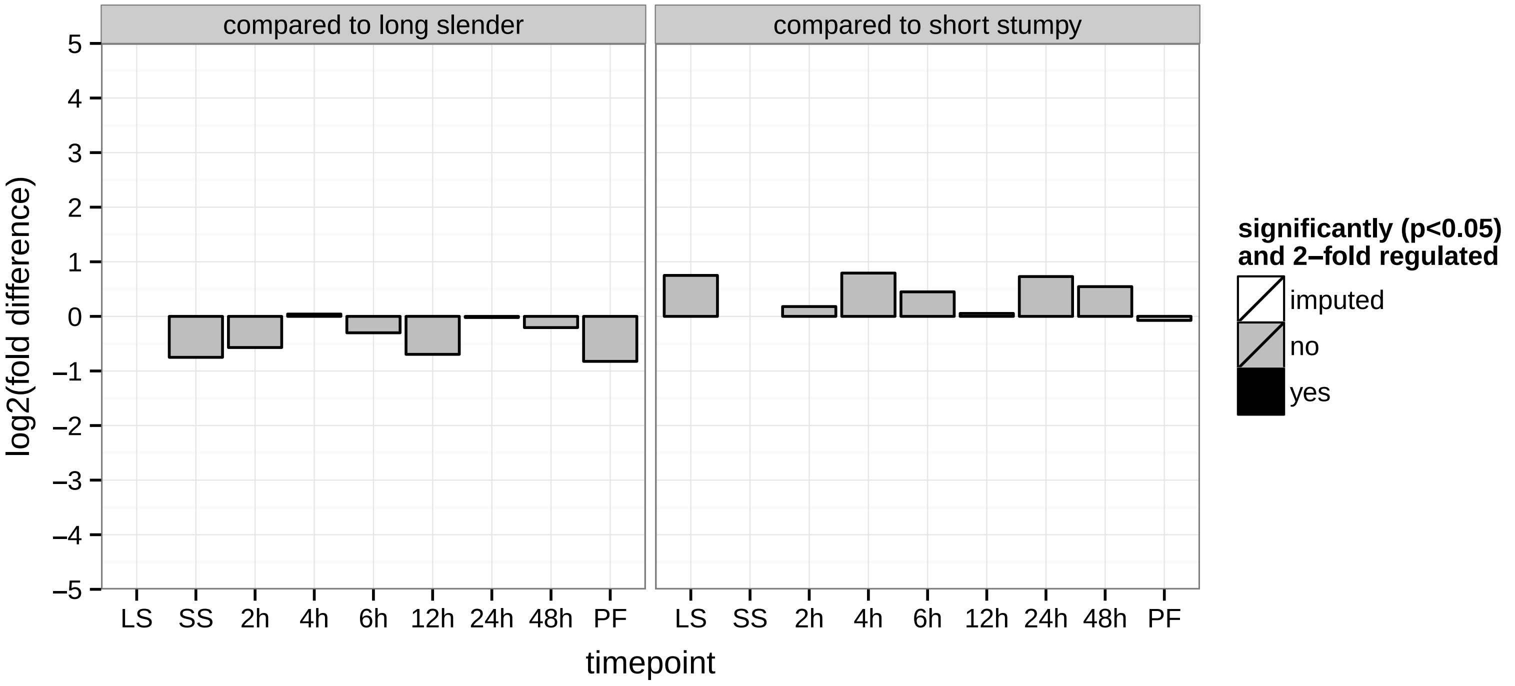

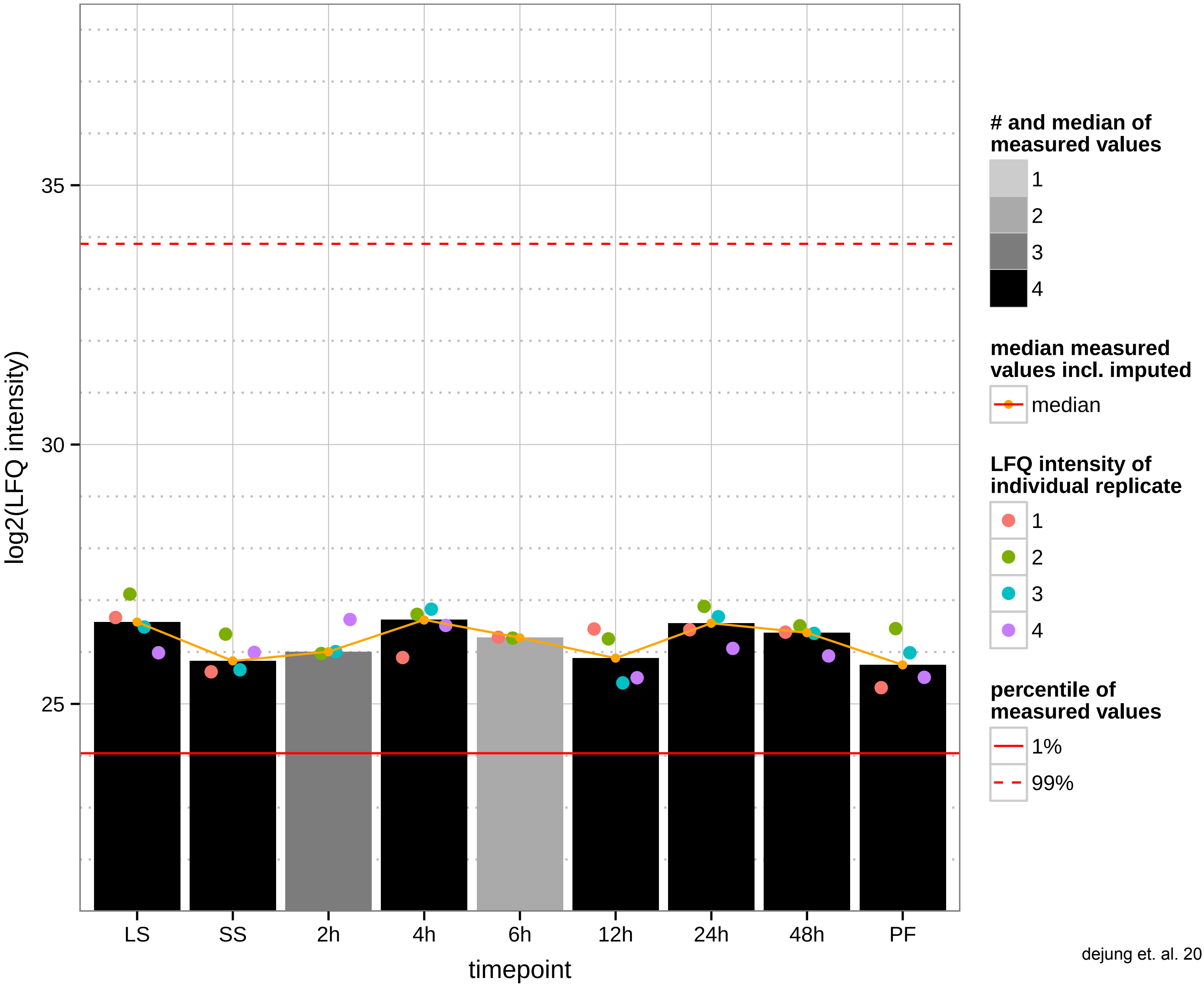

dejung et. al. 2015

V–type ATPase, A subunit, putative
Tb927.4.1080
AGOF: hydrogen ion transporting ATP synthase activity, rotational mechanism, proton–transporting ATPase activity
AGOC: proton–transporting V–type ATPase, V1 domain, proton–transporting two–sector ATPase complex
AGOP: ATP synthesis coupled proton transport
PGOF: ATP binding, hydrogen ion transporting ATP synthase activity, rotational mechanism, hydrolase activity, acting on acid
PGOC: proton–transporting V–type ATPase, V1 domain, proton–transporting two–sector ATPase complex, catalytic domain
PGOP: ATP hydrolysis coupled proton transport, ATP metabolic process, proton transport

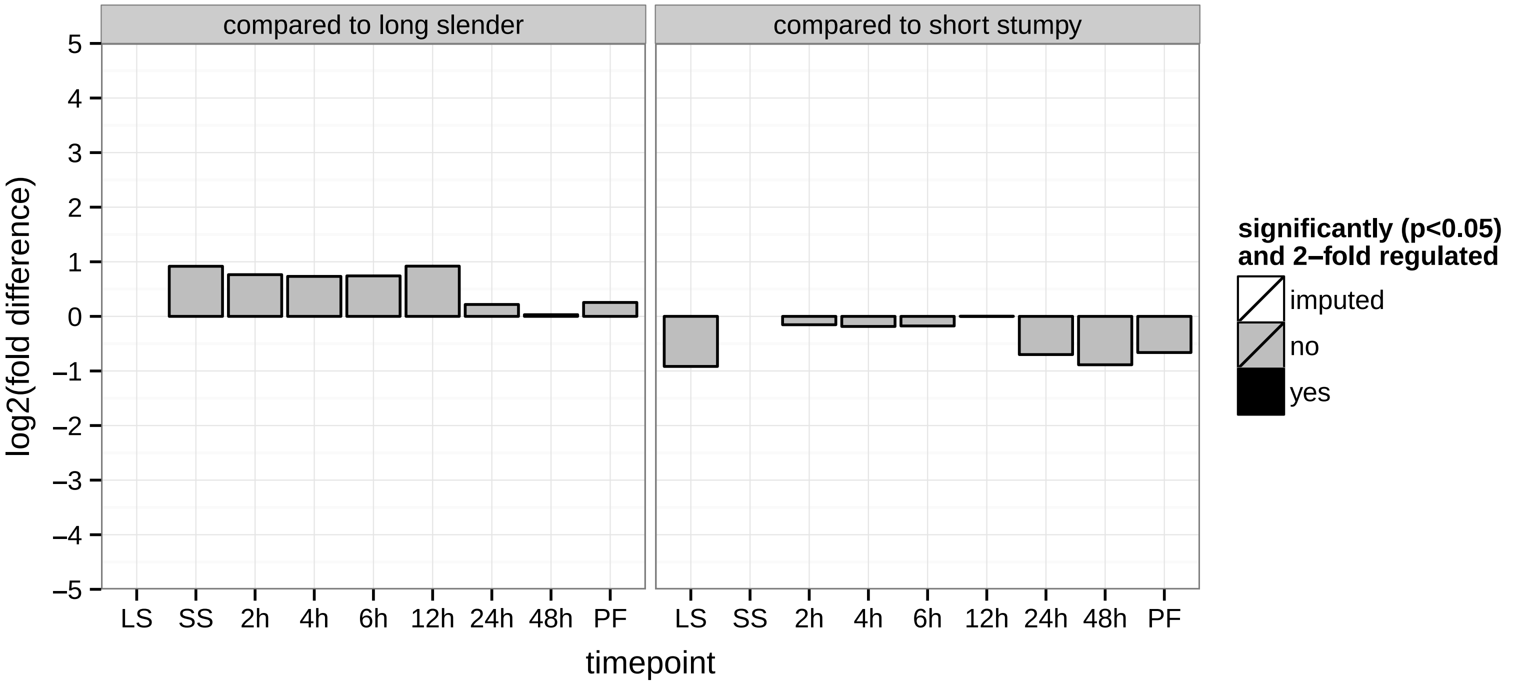

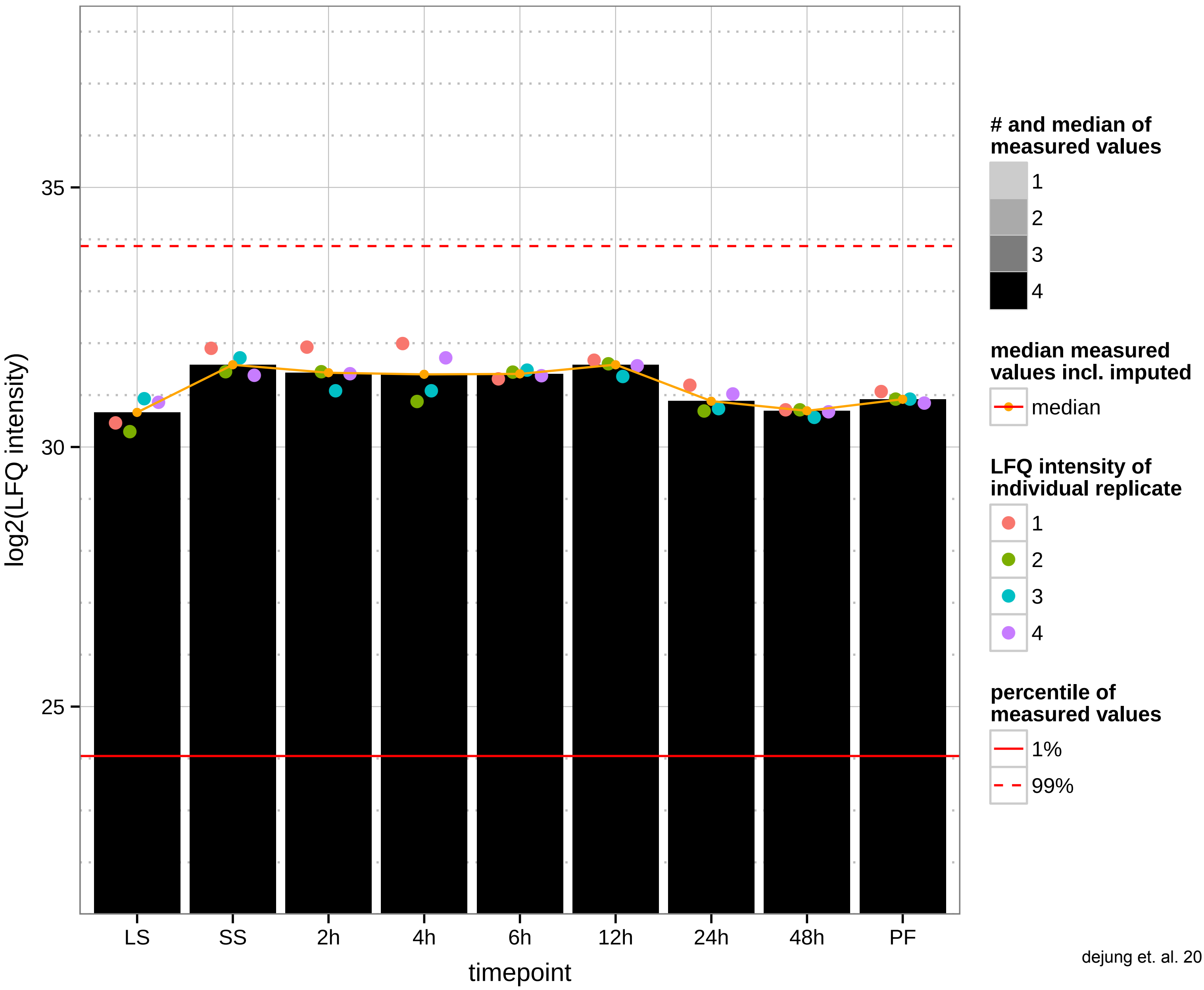

dejung et. al. 2015

retrotransposon hot spot protein (RHS, pseudogene), putative, retrotransposon hot spot protein 1 (RHS1), interrupted
Tb927.4.110
AGOF: null
AGOC: null
AGOP: null
PGOF: null
PGOC: null
PGOP: null

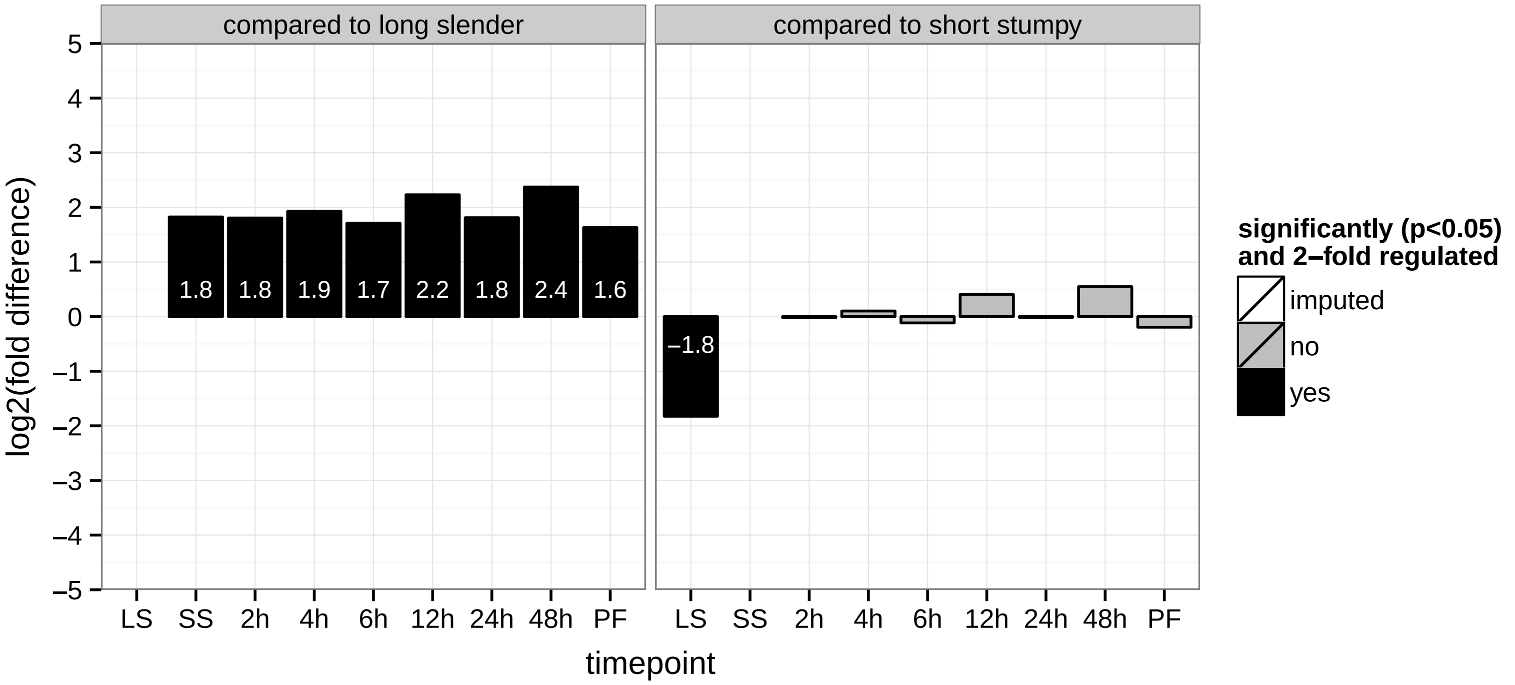

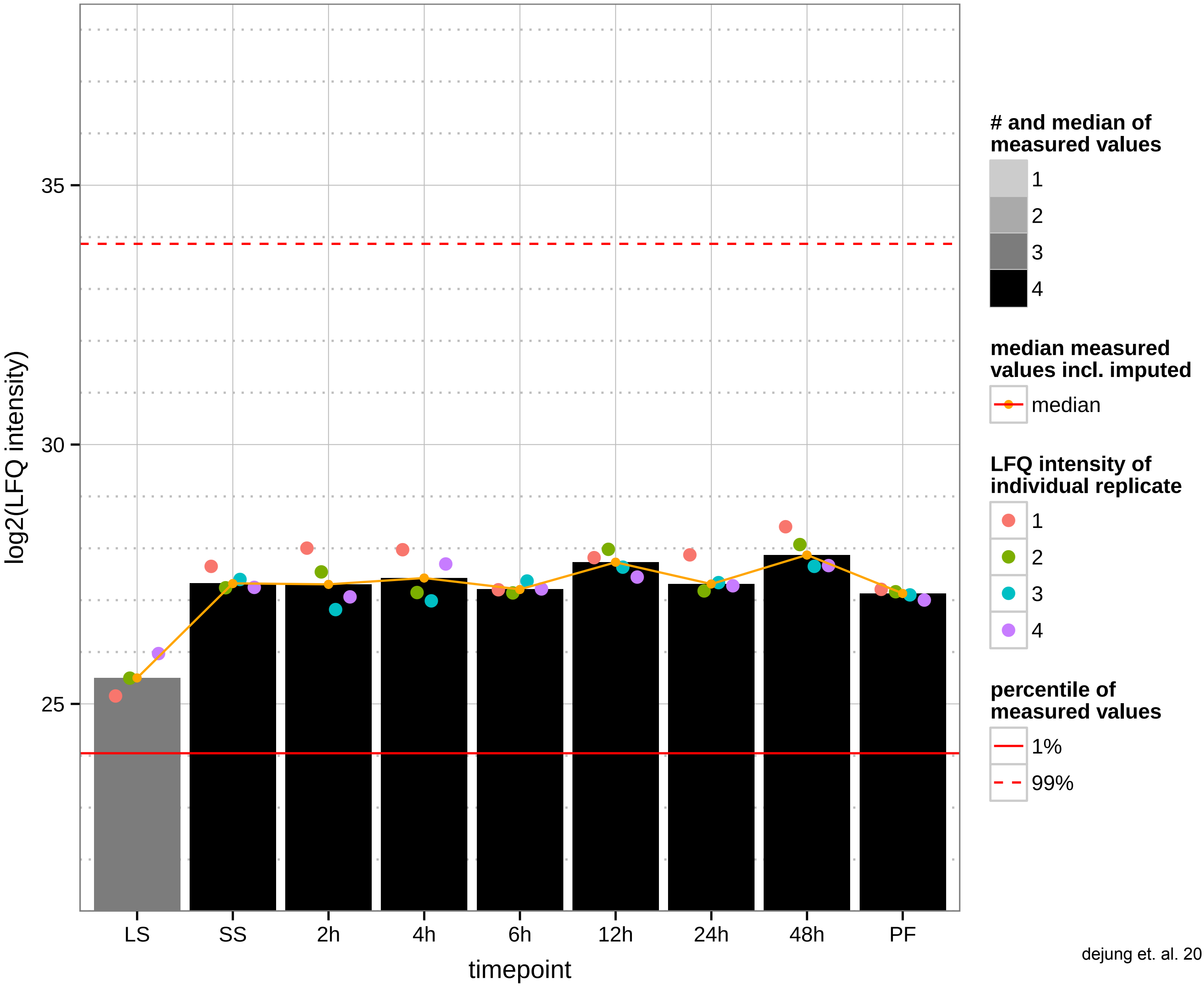

dejung et. al. 2015

phosphatidylinositol 4−kinase, putative
Tb927.4.1140
AGOF: 1−phosphatidylinositol 4−kinase activity, phosphotransferase activity, alcohol group as acceptor
AGOC: cytosol
AGOP: phosphatidylinositol phosphorylation, phosphatidylinositol−mediated signaling
PGOF: phosphotransferase activity, alcohol group as acceptor, transferase activity, transferring phosphorus−containing groups
PGOC: null
PGOP: null

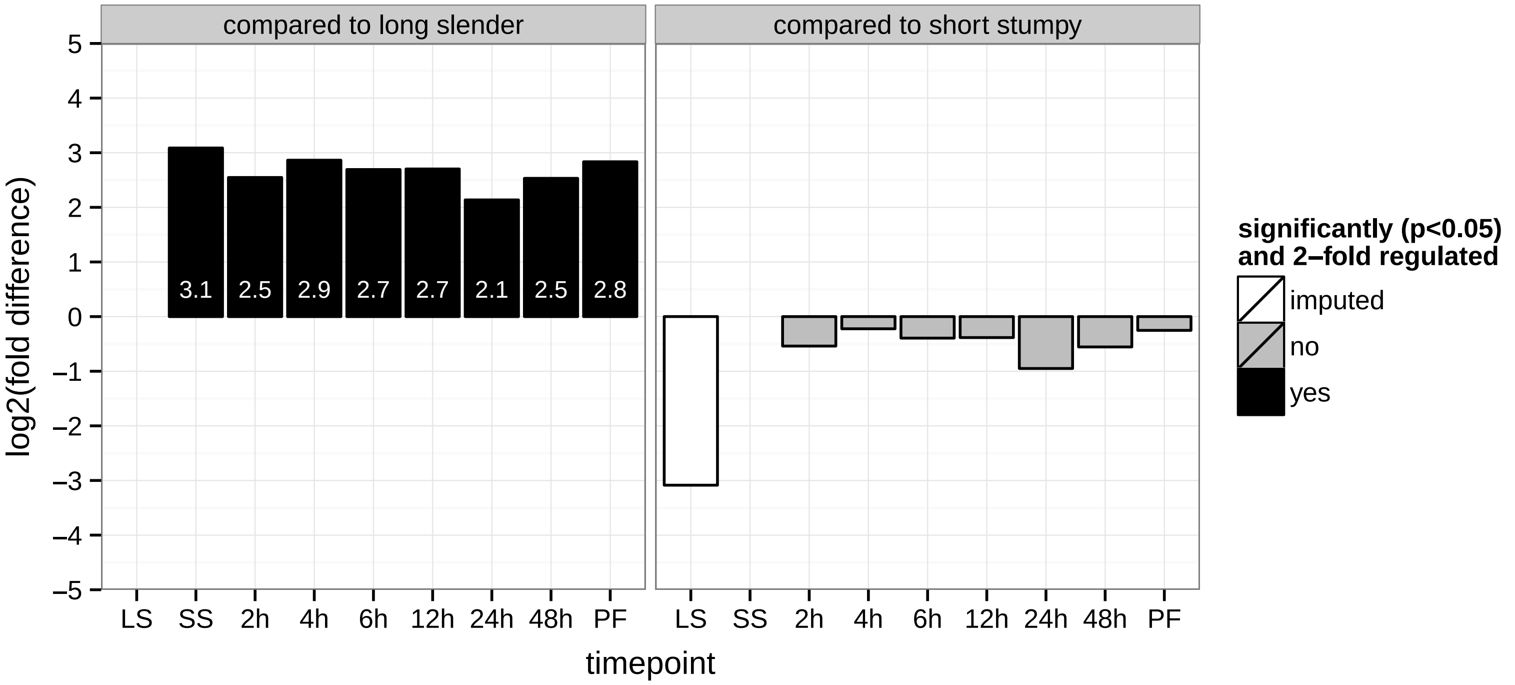

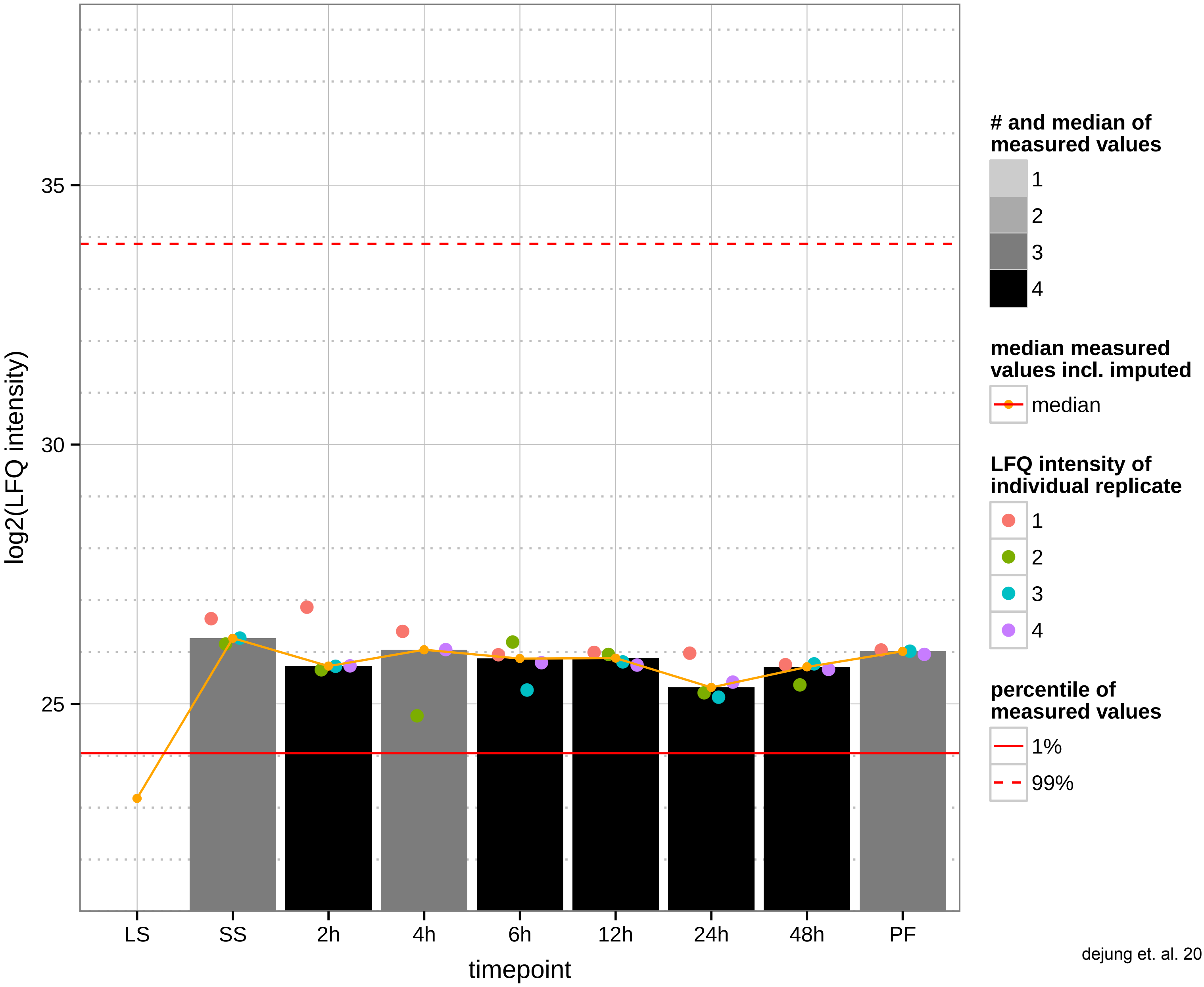

dejung et. al. 2015

hypothetical protein, conserved
Tb927.4.1150
AGOF: null
AGOC: null
AGOP: null
PGOF: null
PGOC: null
PGOP: null

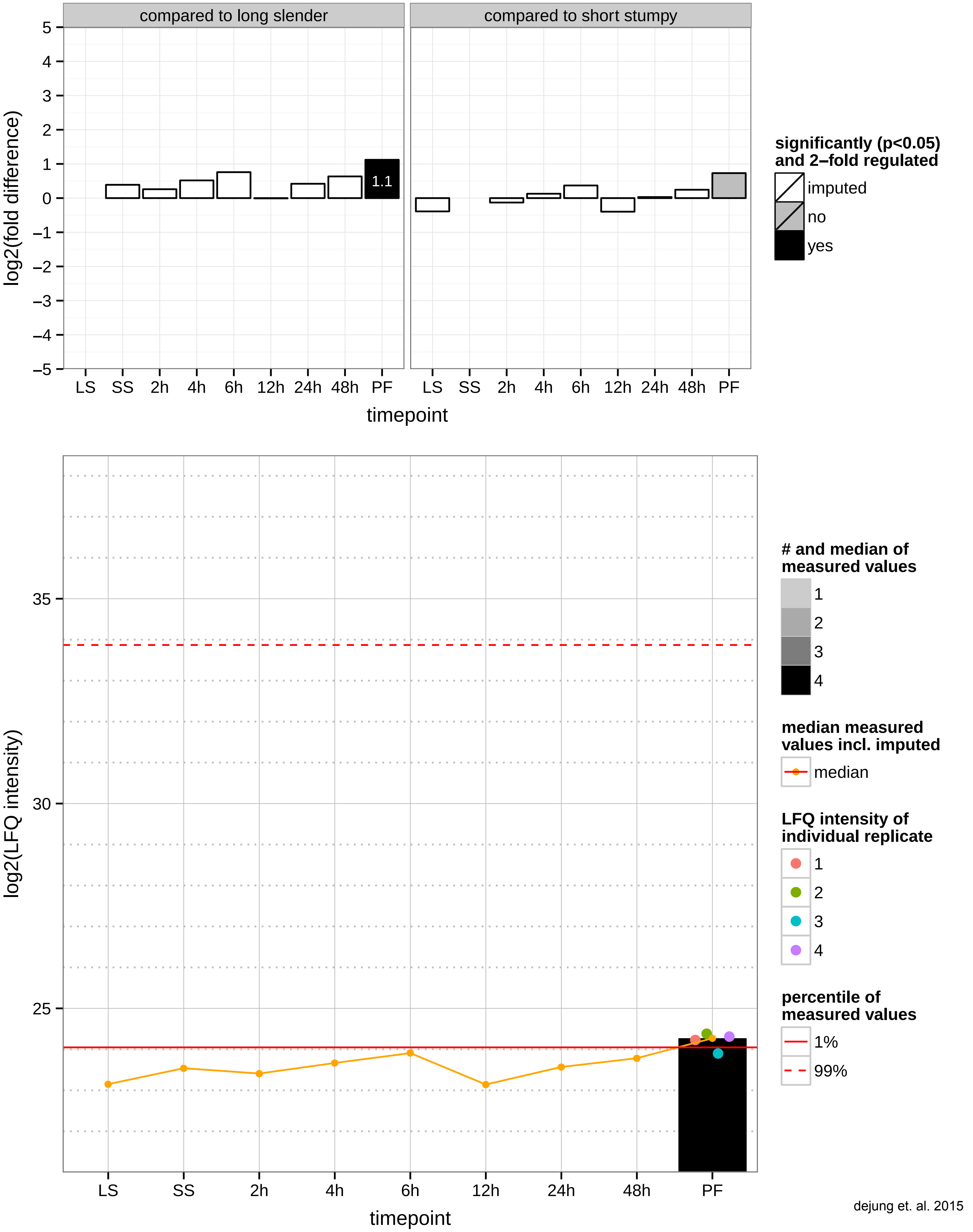

dejung et. al. 2015

ruvB–like DNA helicase, putative
Tb927.4.1270
AGOF: ATP binding, DNA helicase activity, nucleoside–triphosphatase activity
AGOC: nucleus
AGOP: null
PGOF: ATP binding, DNA helicase activity, nucleoside–triphosphatase activity, nucleotide binding
PGOC: null
PGOP: null

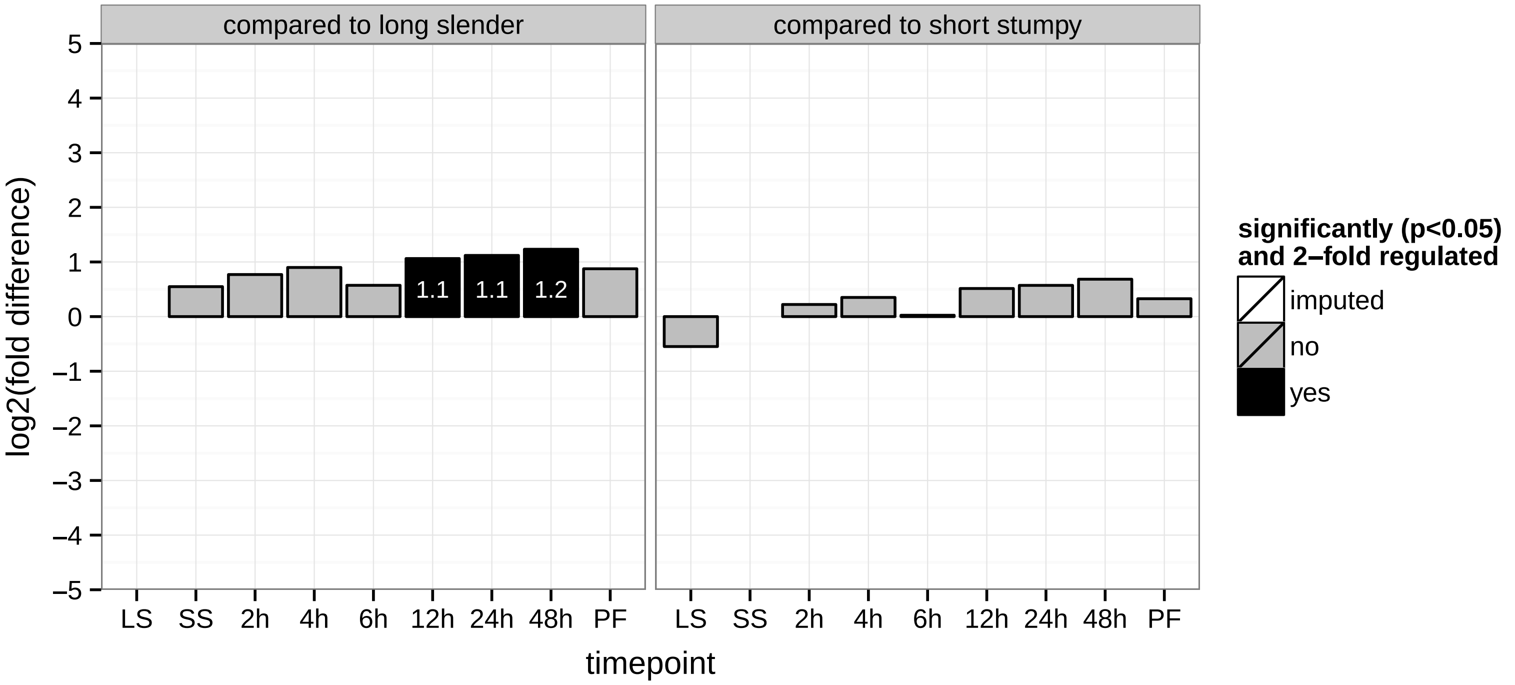

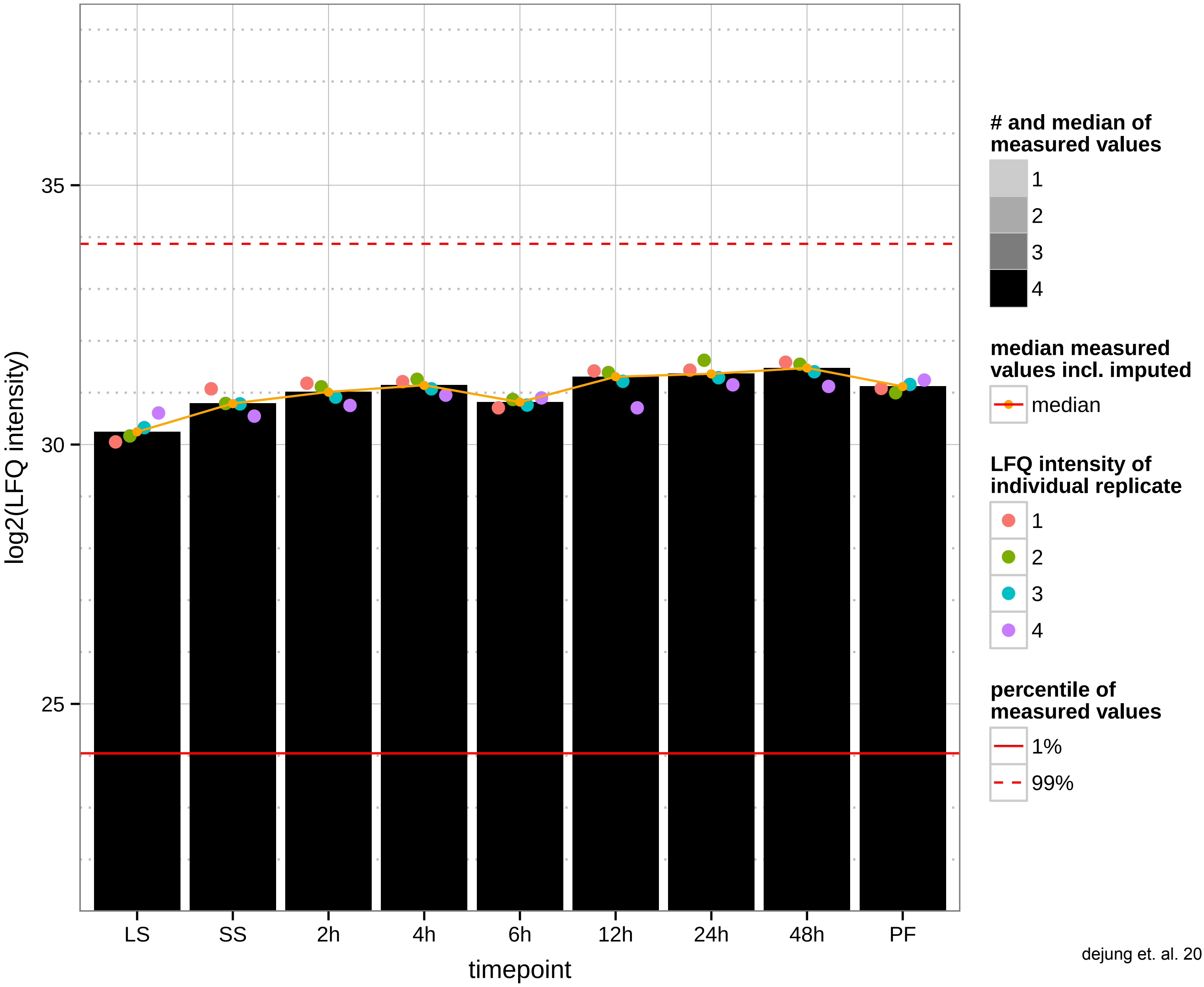

dejung et. al. 2015

amidinotransferase, putative
Tb927.4.1300
AGOF: null
AGOC: null
AGOP: null
PGOF: hydrolase activity, acting on carbon–nitrogen (but not peptide) bonds, in linear amidines
PGOC: cytoplasm
PGOP: null

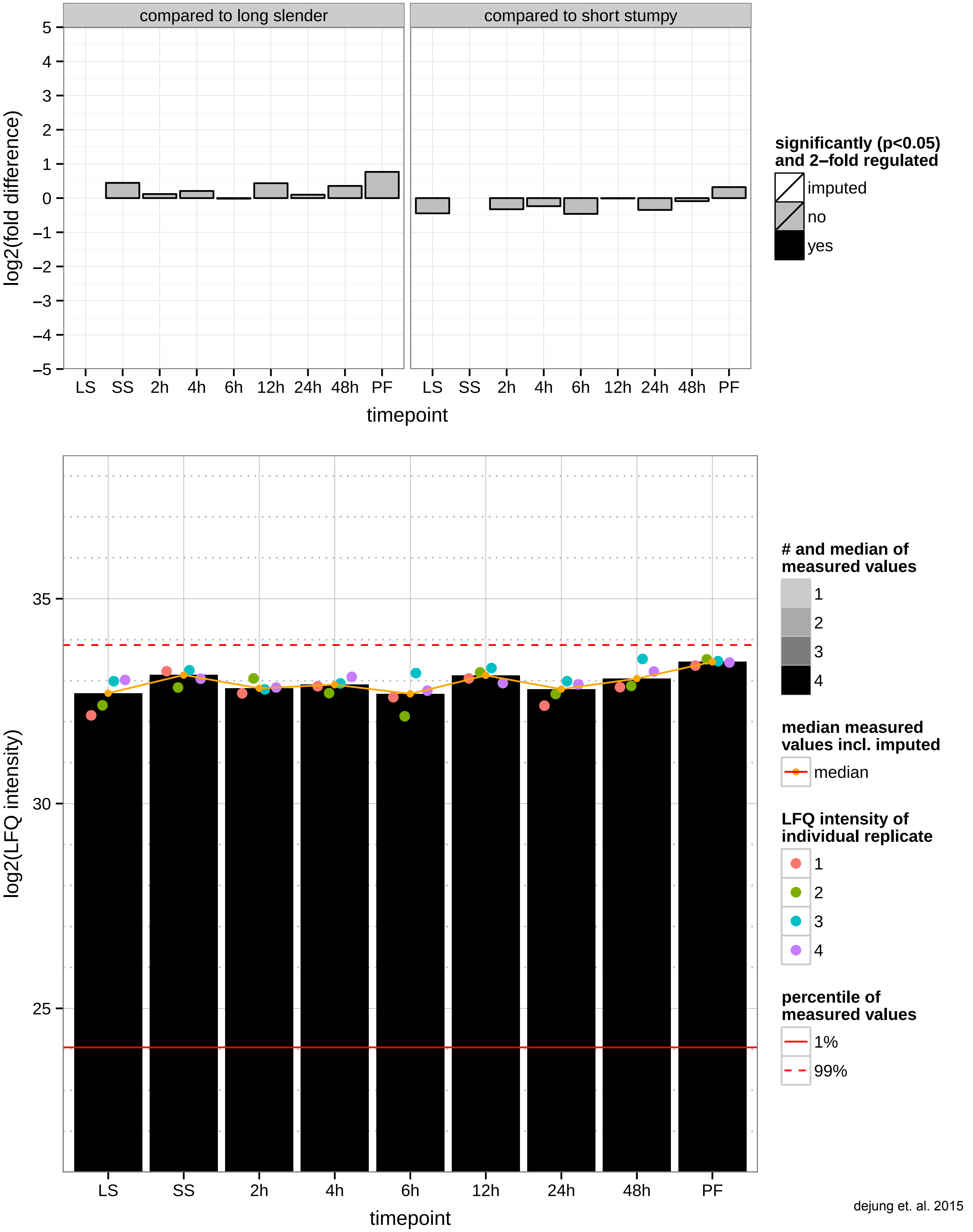

dejung et. al. 2015

cleavage and polyadenylation specificity factor subunit, putative (CPSF3)
Tb927.4.1340
AGOF: hydrolase activity
AGOC: null
AGOP: null
PGOF: hydrolase activity
PGOC: null
PGOP: null

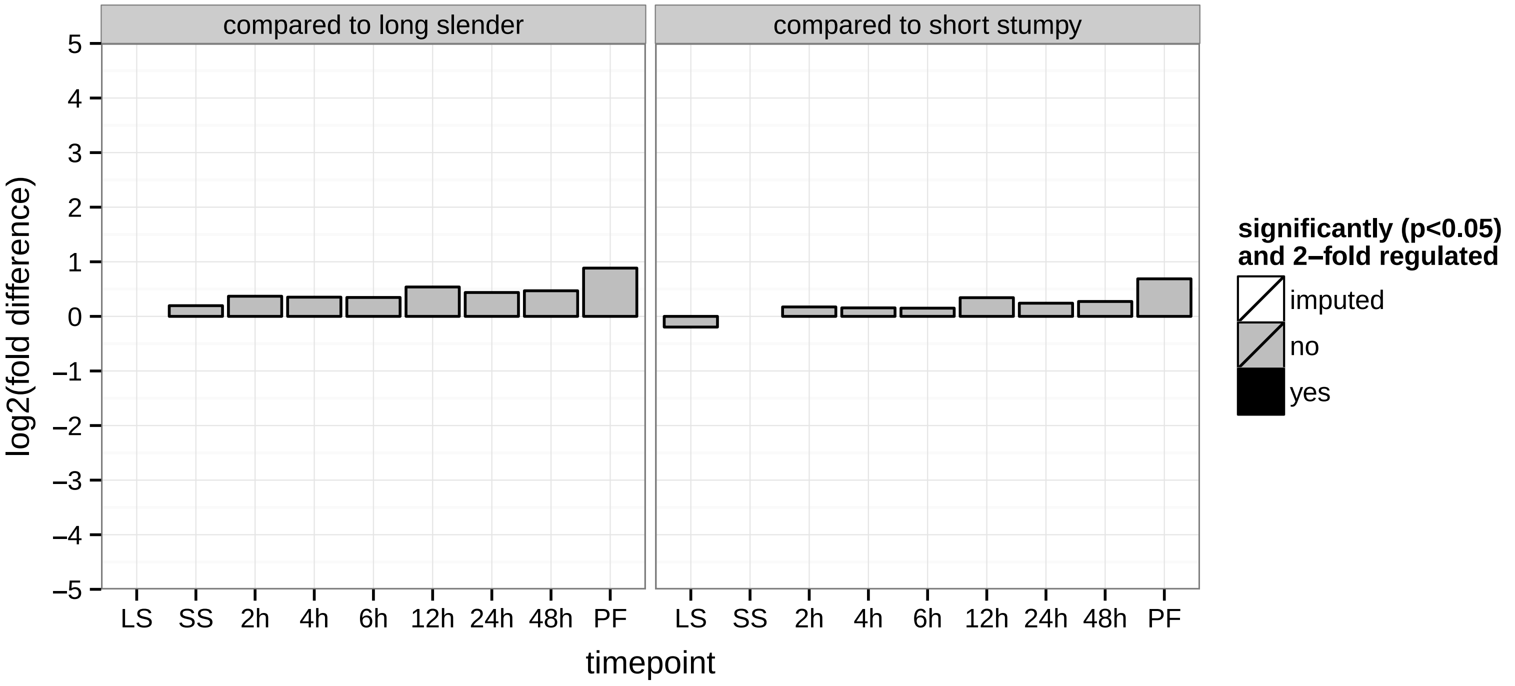

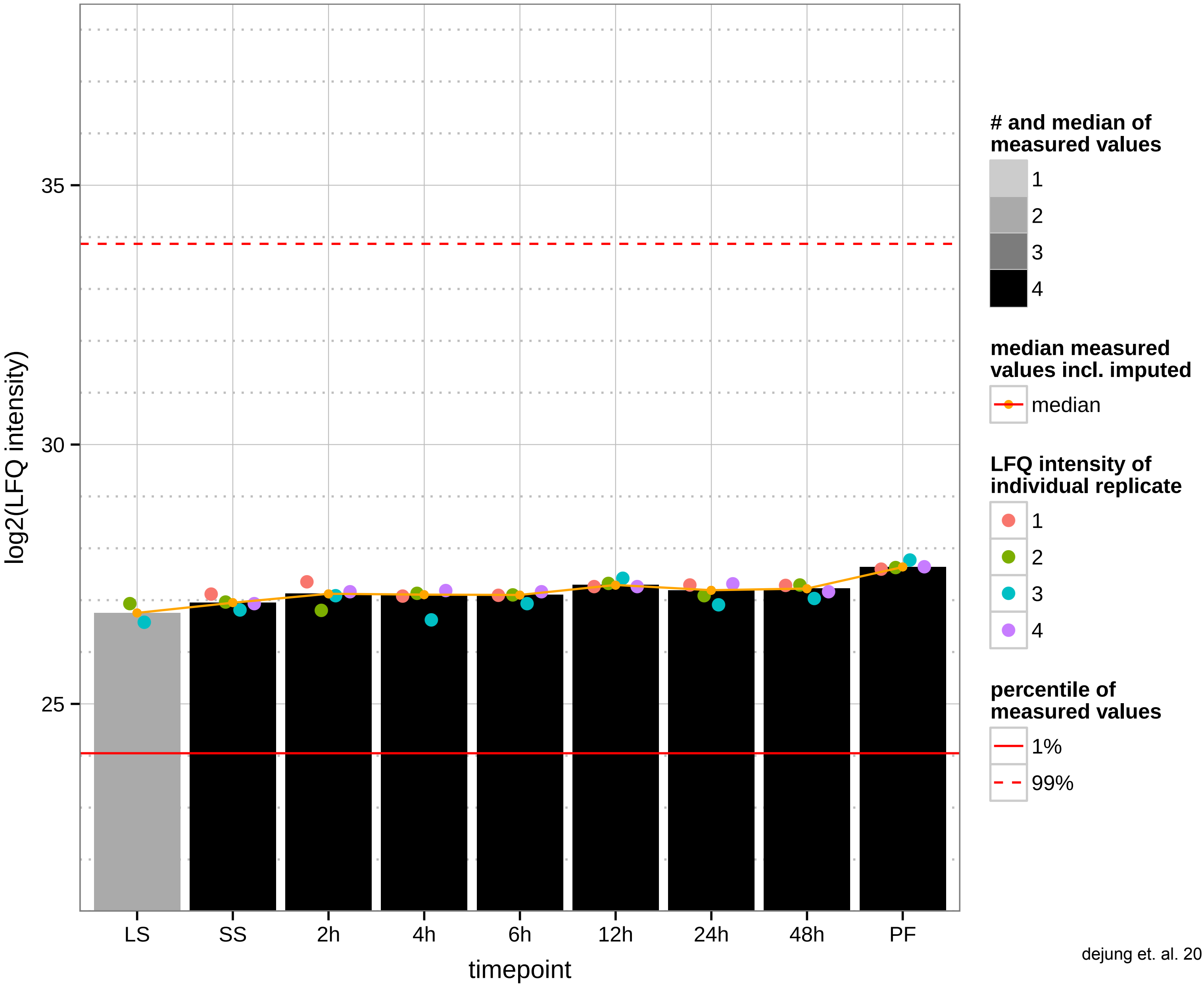

dejung et. al. 2015

hypothetical protein, conserved
Tb927.4.1420
AGOF: null
AGOC: null
AGOP: null
PGOF: null
PGOC: null
PGOP: null

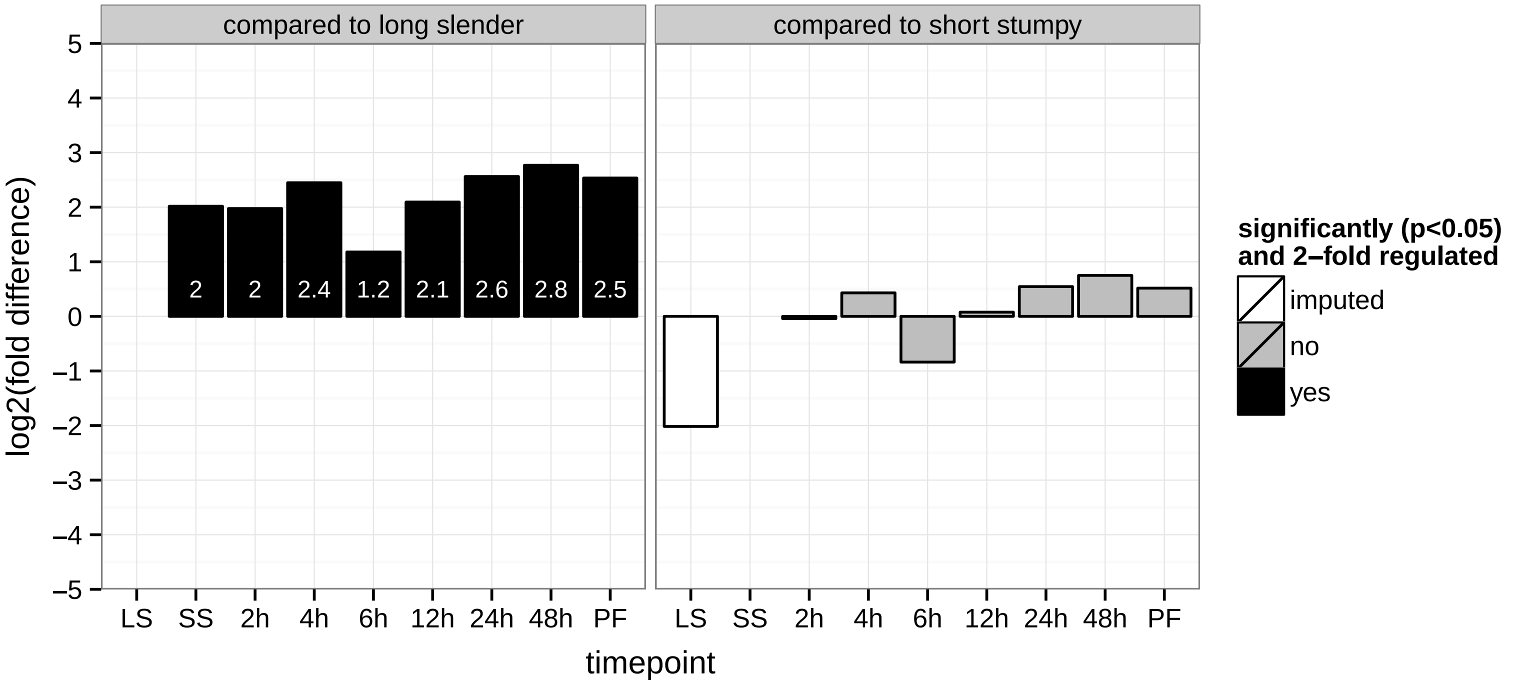

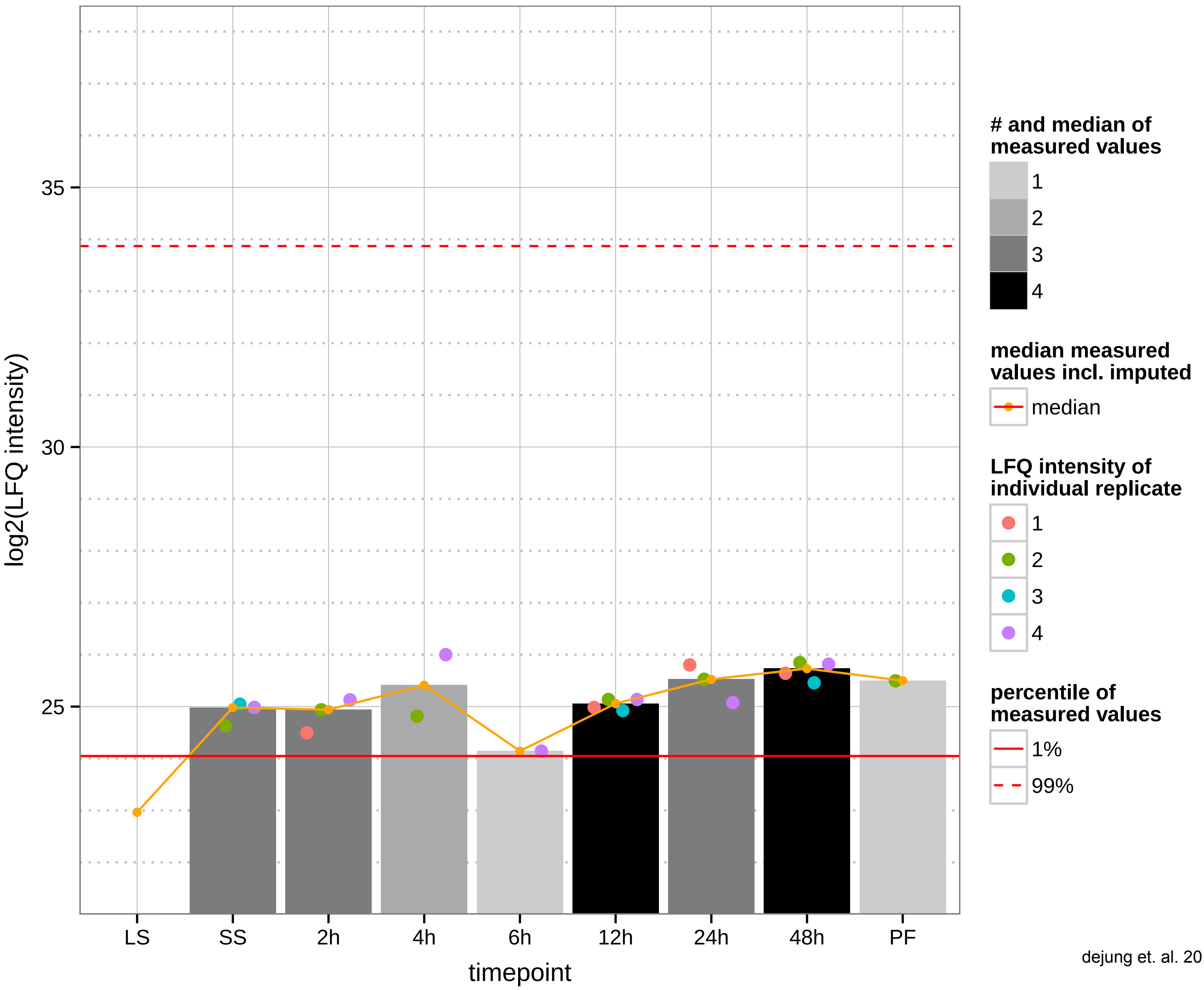

dejung et. al. 2015

phospholipid–translocating ATPase, putative
Tb927.4.1510
AGOF: ATP binding, ATPase activity, coupled to transmembrane movement of ions, phosphorylative mechanism, magnesium
AGOC: integral to membrane, membrane
AGOP: ATP biosynthetic process, phospholipid transport
PGOF: ATP binding, ATPase activity, coupled to transmembrane movement of ions, phosphorylative mechanism, hydrolase ac
PGOC: integral to membrane, membrane
PGOP: cation transport, phospholipid transport

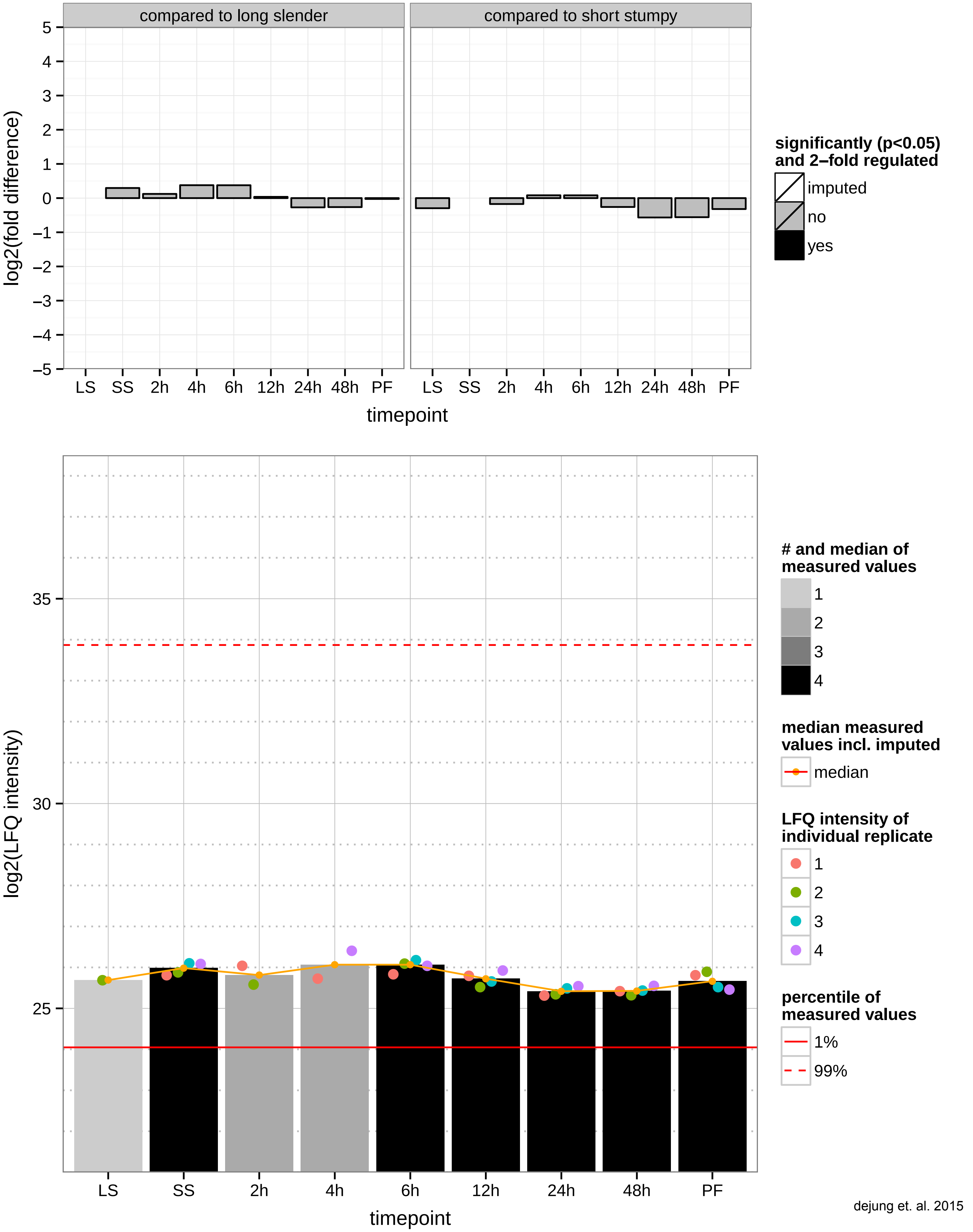

dejung et. al. 2015

hypothetical protein, conserved
Tb927.4.1540
AGOF: catalytic activity, coenzyme binding
AGOC: mitochondrion
AGOP: cellular metabolic process
PGOF: catalytic activity, coenzyme binding
PGOC: null
PGOP: cellular metabolic process

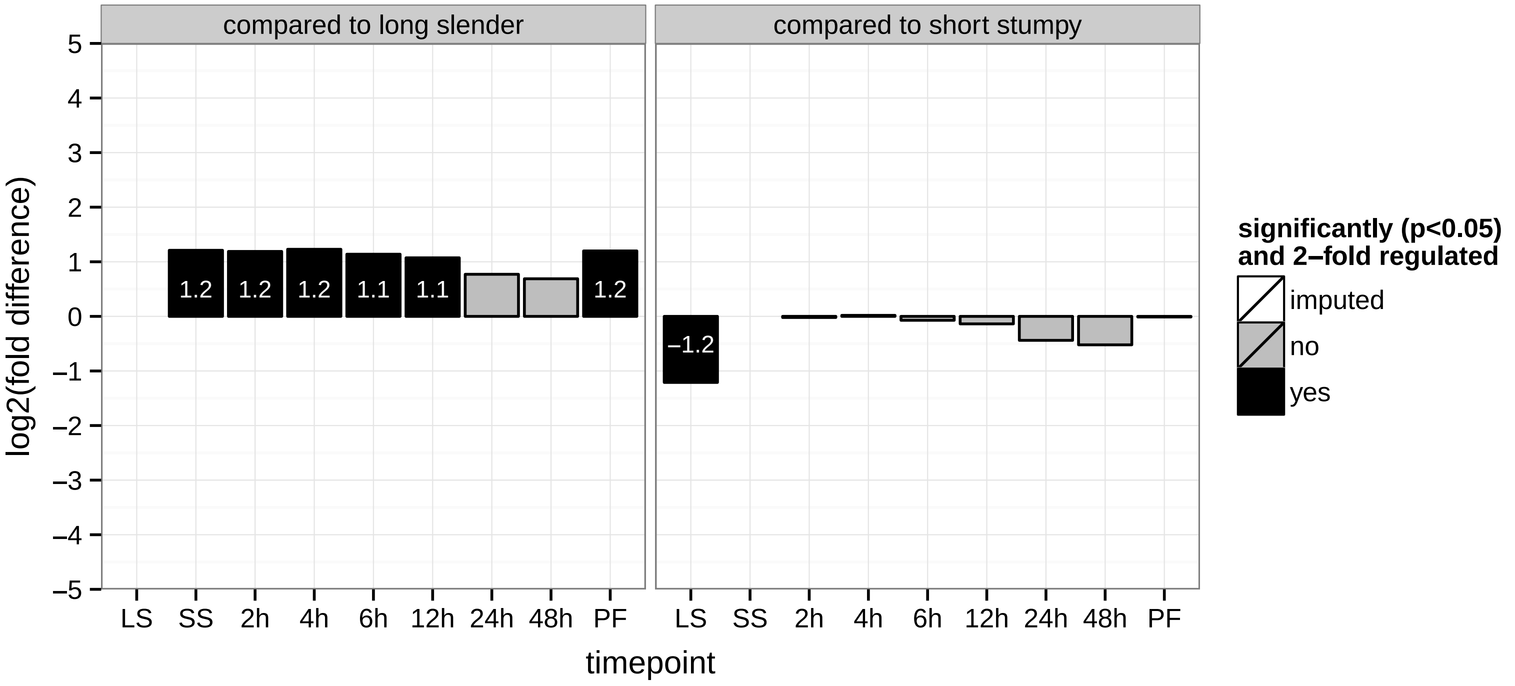

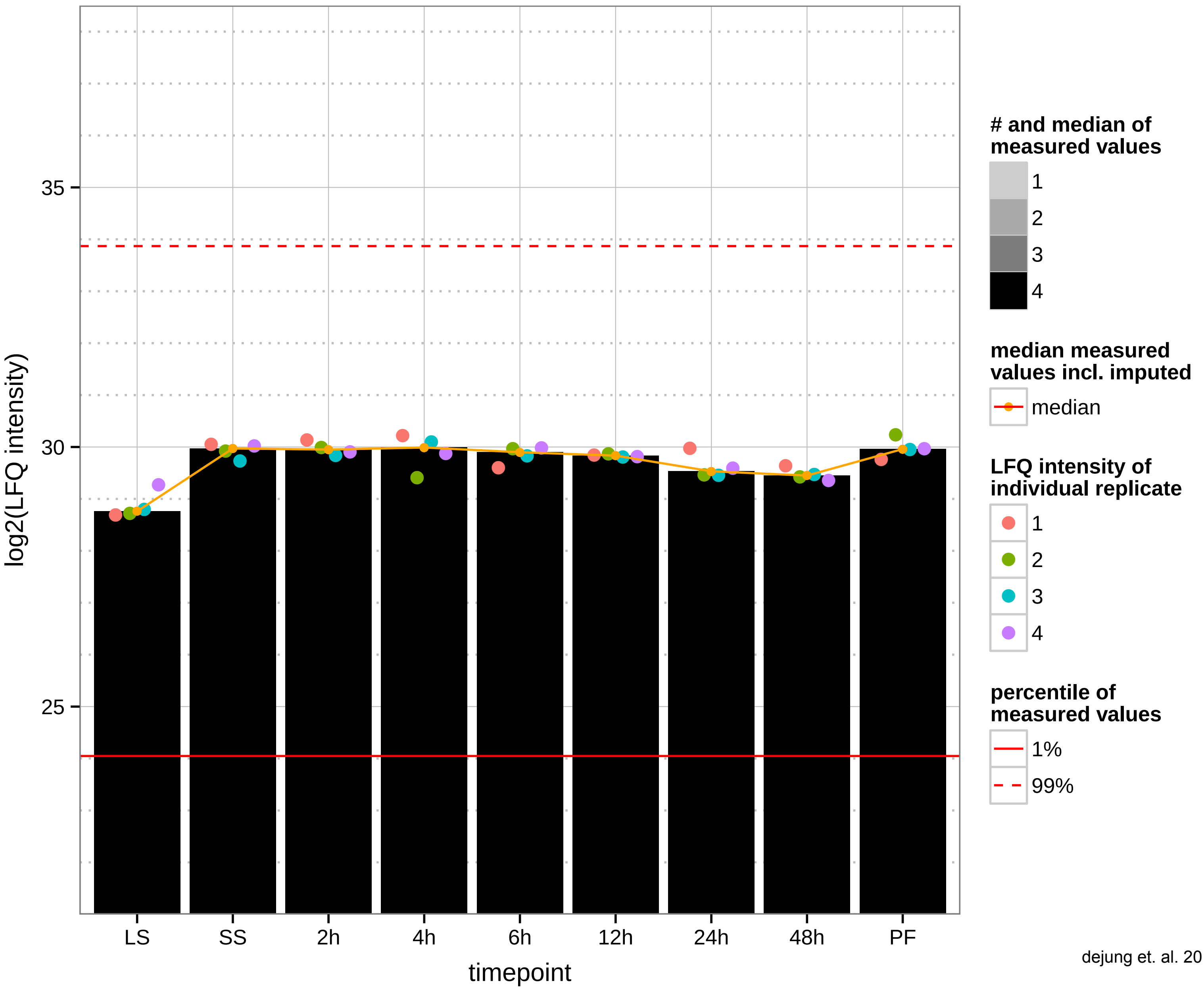

dejung et. al. 2015

hypothetical protein, conserved
Tb927.4.1600
AGOF: null
AGOC: null
AGOP: null
PGOF: null
PGOC: null
PGOP: null

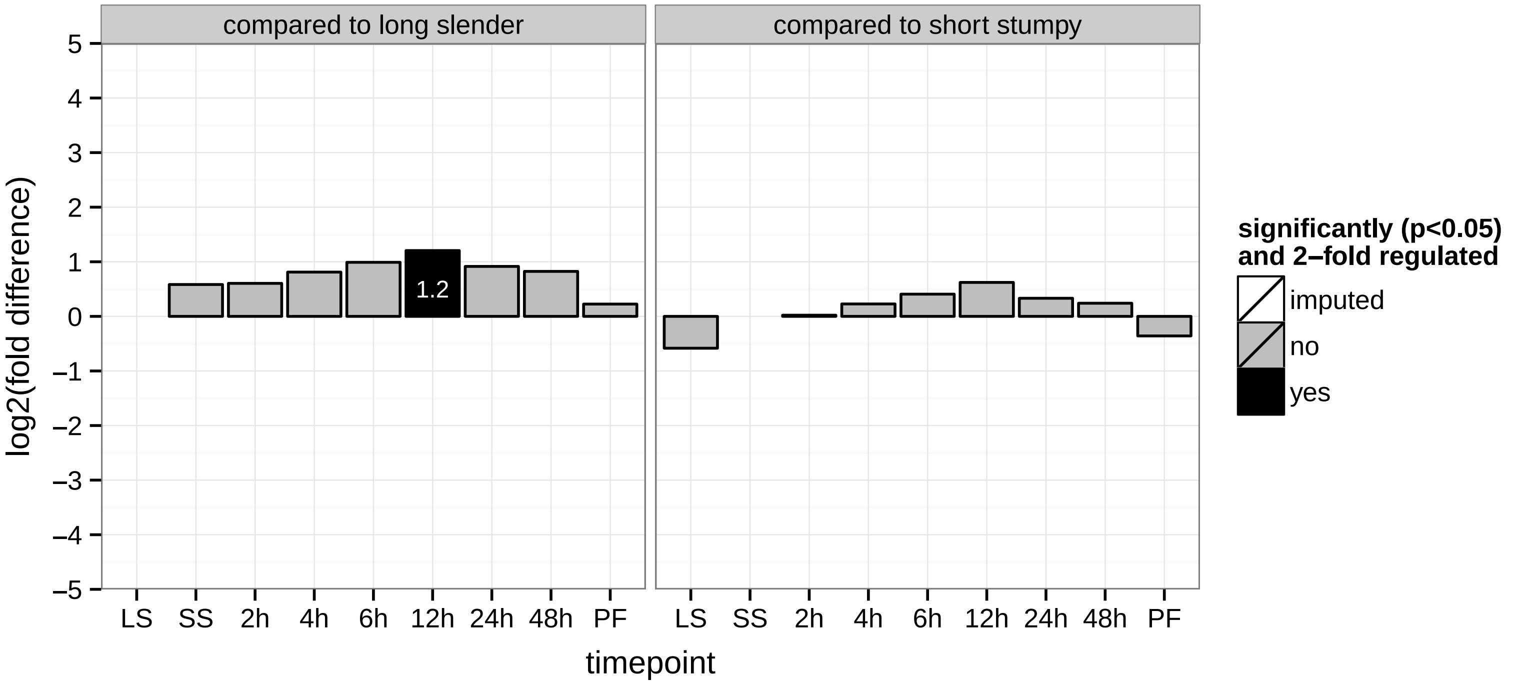

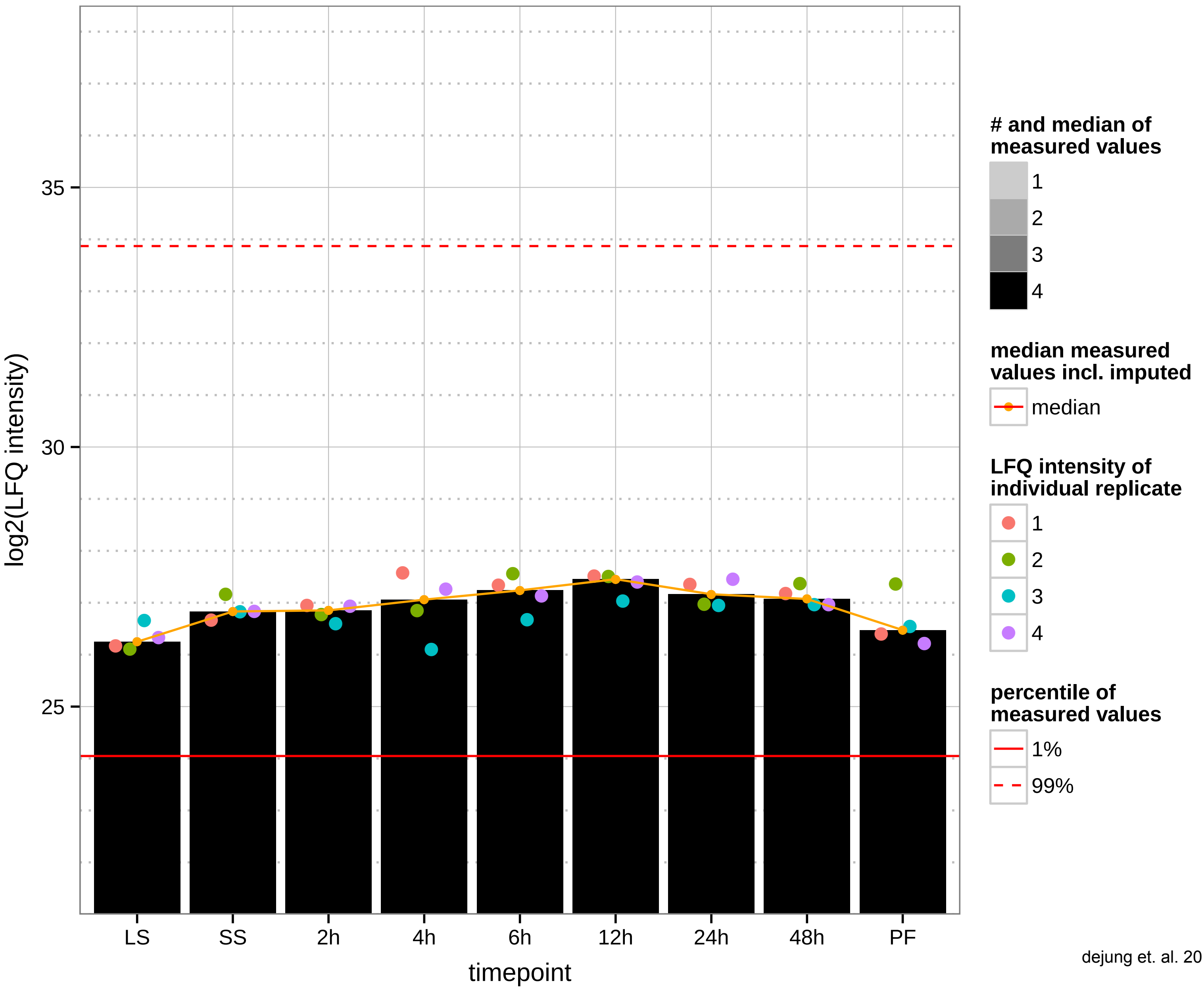

dejung et. al. 2015

ribosomal RNA processing protein 6 (RRP6)
Tb927.4.1630
AGOF: 3'–5' exonuclease activity, nucleic acid binding
AGOC: cytoplasm, cytoplasmic exosome (RNase complex), intracellular, nuclear exosome (RNase complex), nucleus
AGOP: RNA processing, cell growth, rRNA processing
PGOF: 3'–5' exonuclease activity, catalytic activity, nucleic acid binding, nucleotide binding
PGOC: intracellular, nuclear exosome (RNase complex)
PGOP: RNA processing, cellular metabolic process, nucleobase–containing compound metabolic process

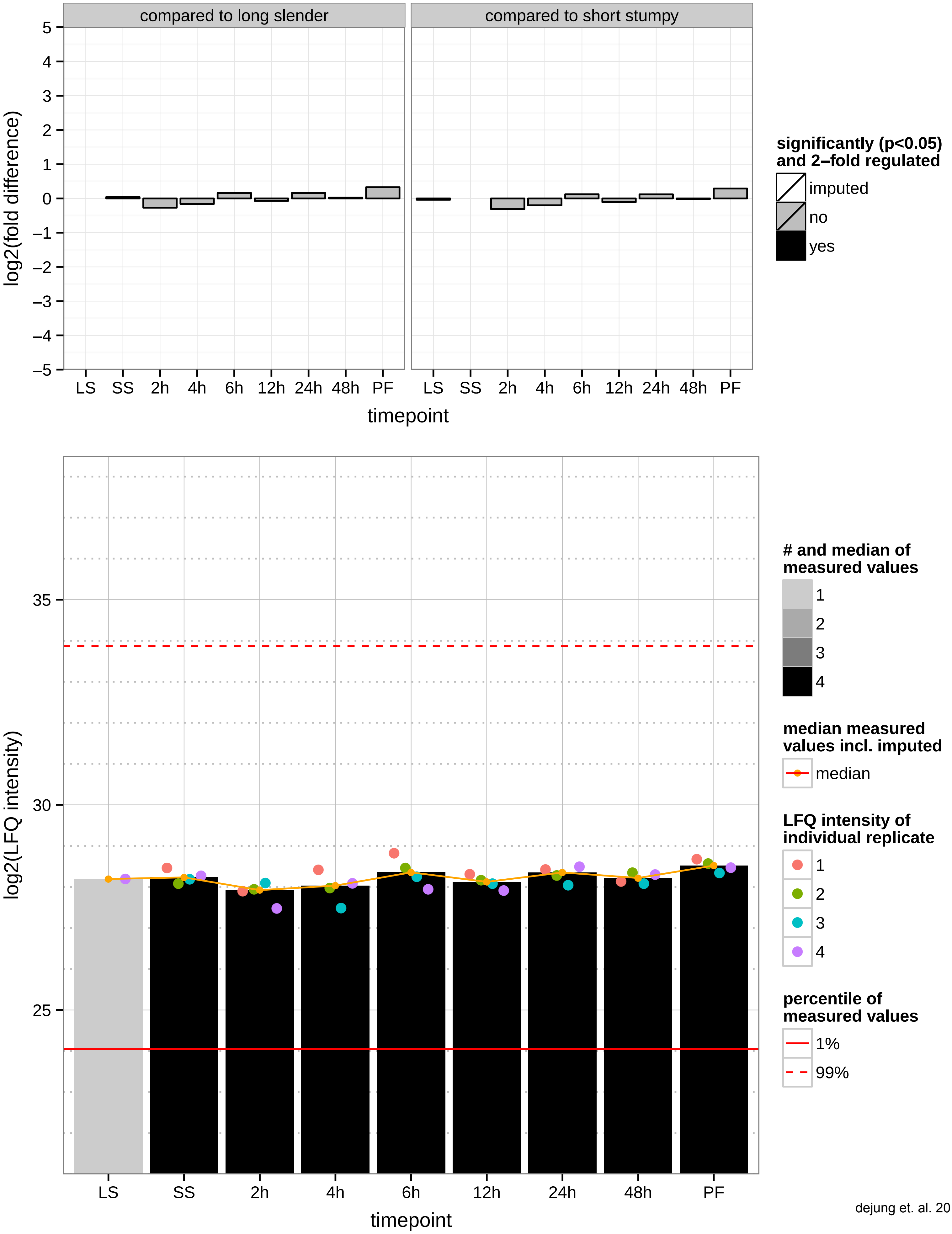

dejung et. al. 2015

mitochondrial carrier protein, putative (MCP6)
Tb927.4.1660
AGOF: null
AGOC: glycosome, mitochondrial inner membrane, mitochondrial membrane, mitochondrion
AGOP: transport
PGOF: null
PGOC: null
PGOP: null

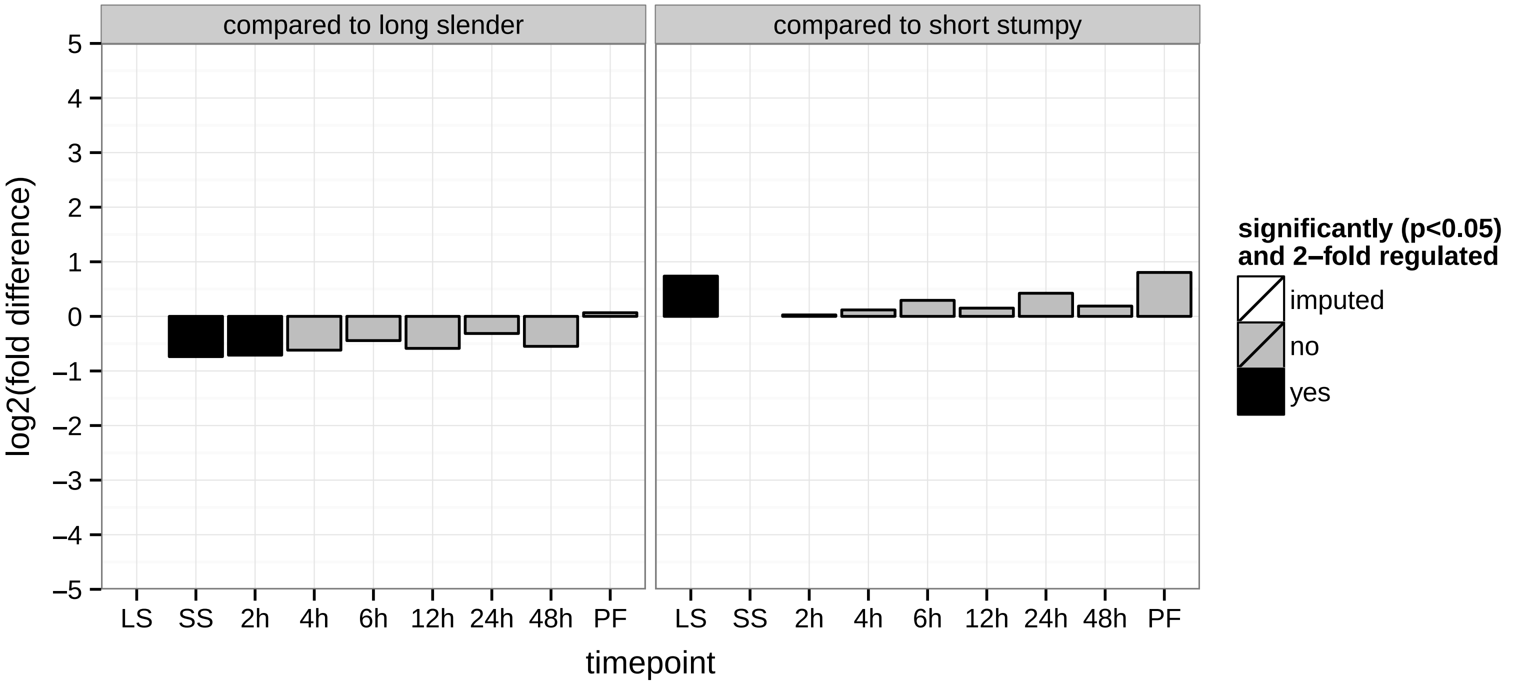

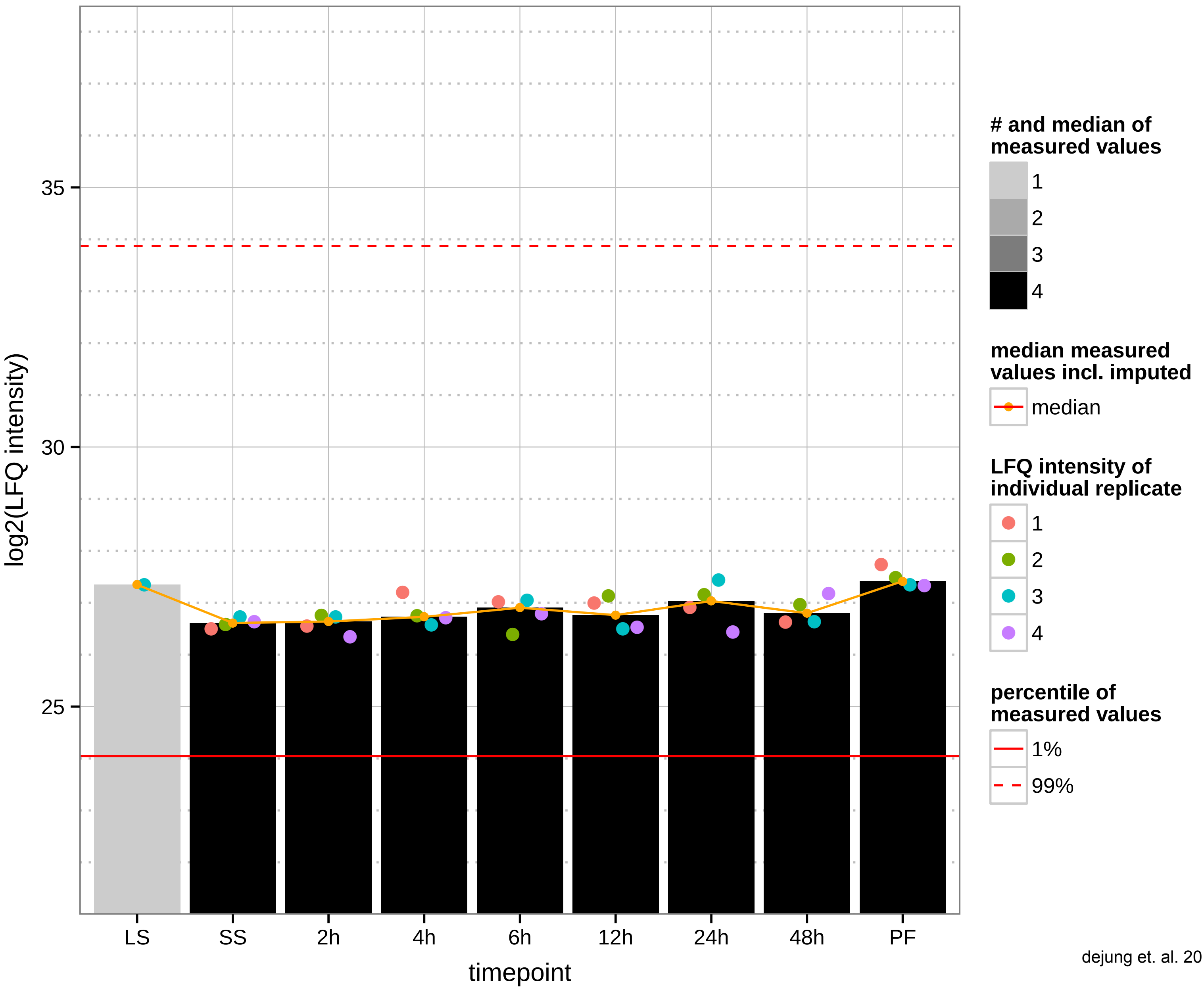

dejung et. al. 2015

ZFP family member, putative (ZC3H10)
Tb927.4.1680
AGOF: null
AGOC: null
AGOP: null
PGOF: null
PGOC: null
PGOP: null

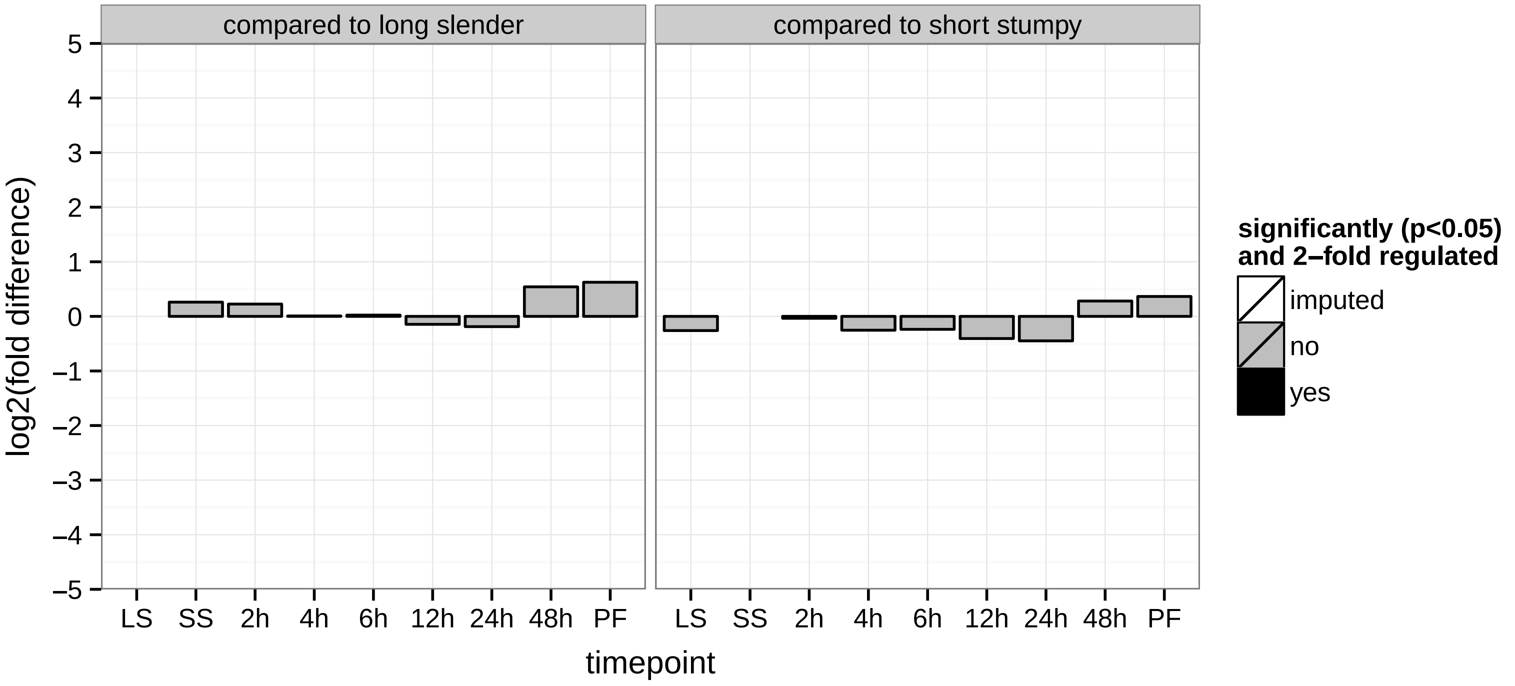

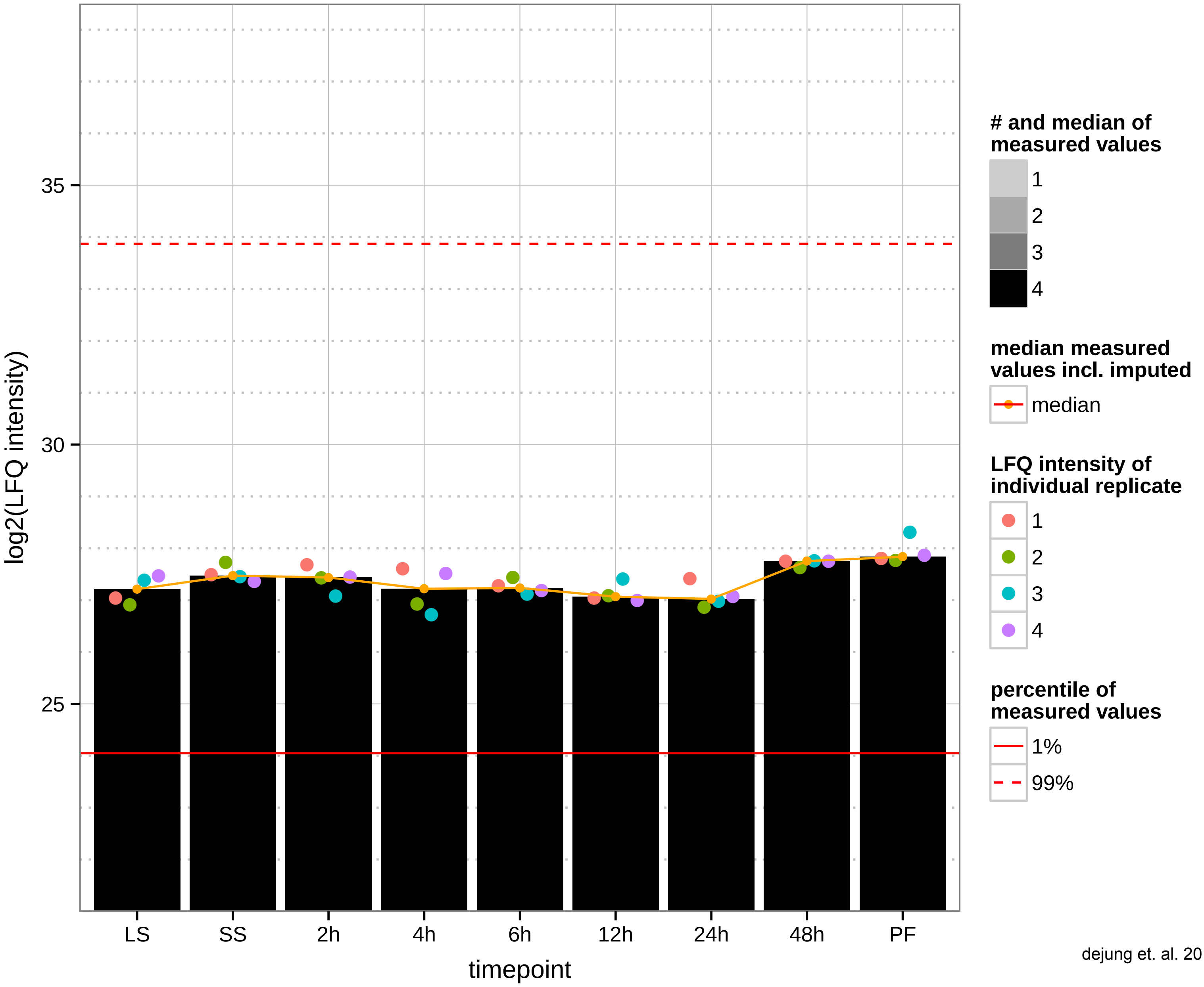

dejung et. al. 2015

nucleoside diphosphate kinase, putative
Tb927.4.1720
AGOF: ATP binding, nucleoside diphosphate kinase activity
AGOC: null
AGOP: CTP biosynthetic process, GTP biosynthetic process, UTP biosynthetic process, ciliary or flagellar motility
PGOF: ATP binding, nucleoside diphosphate kinase activity
PGOC: null
PGOP: CTP biosynthetic process, GTP biosynthetic process, UTP biosynthetic process, nucleoside diphosphate phosphoryla

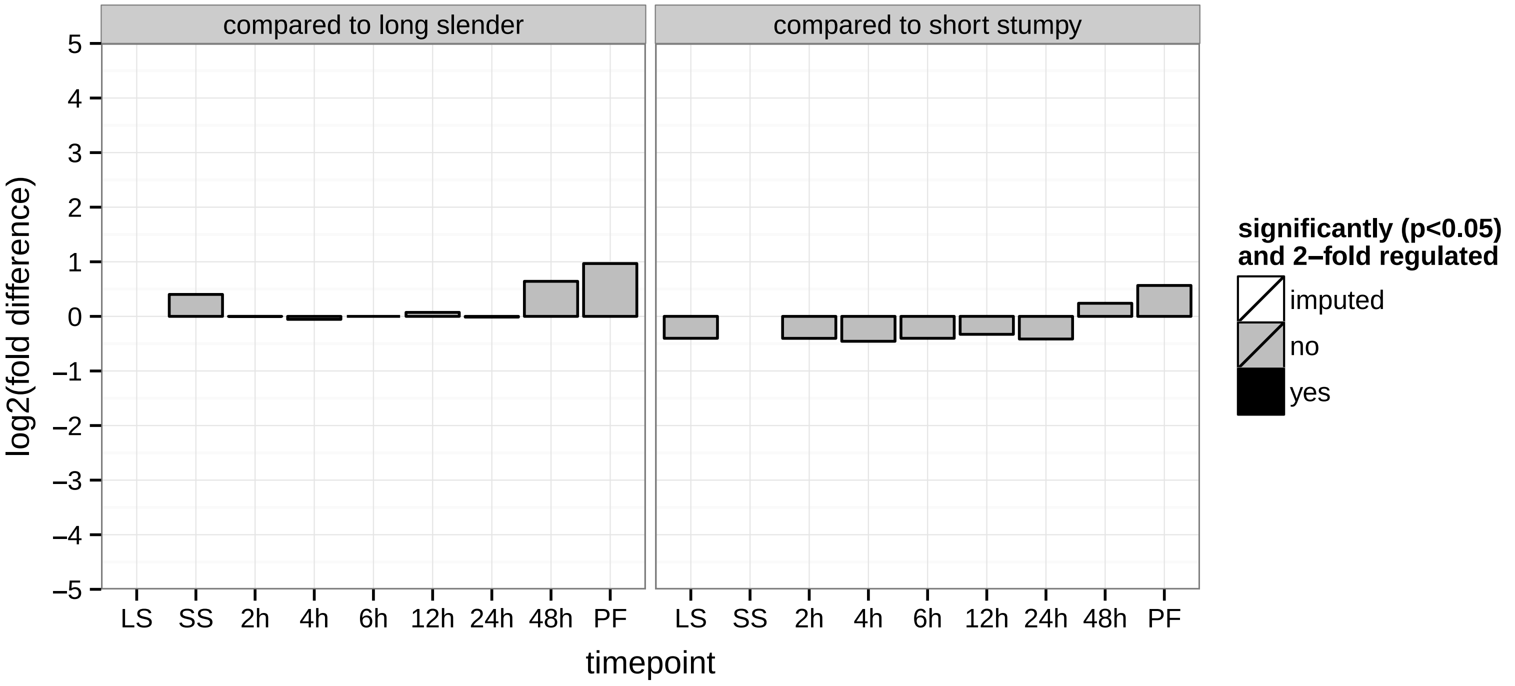

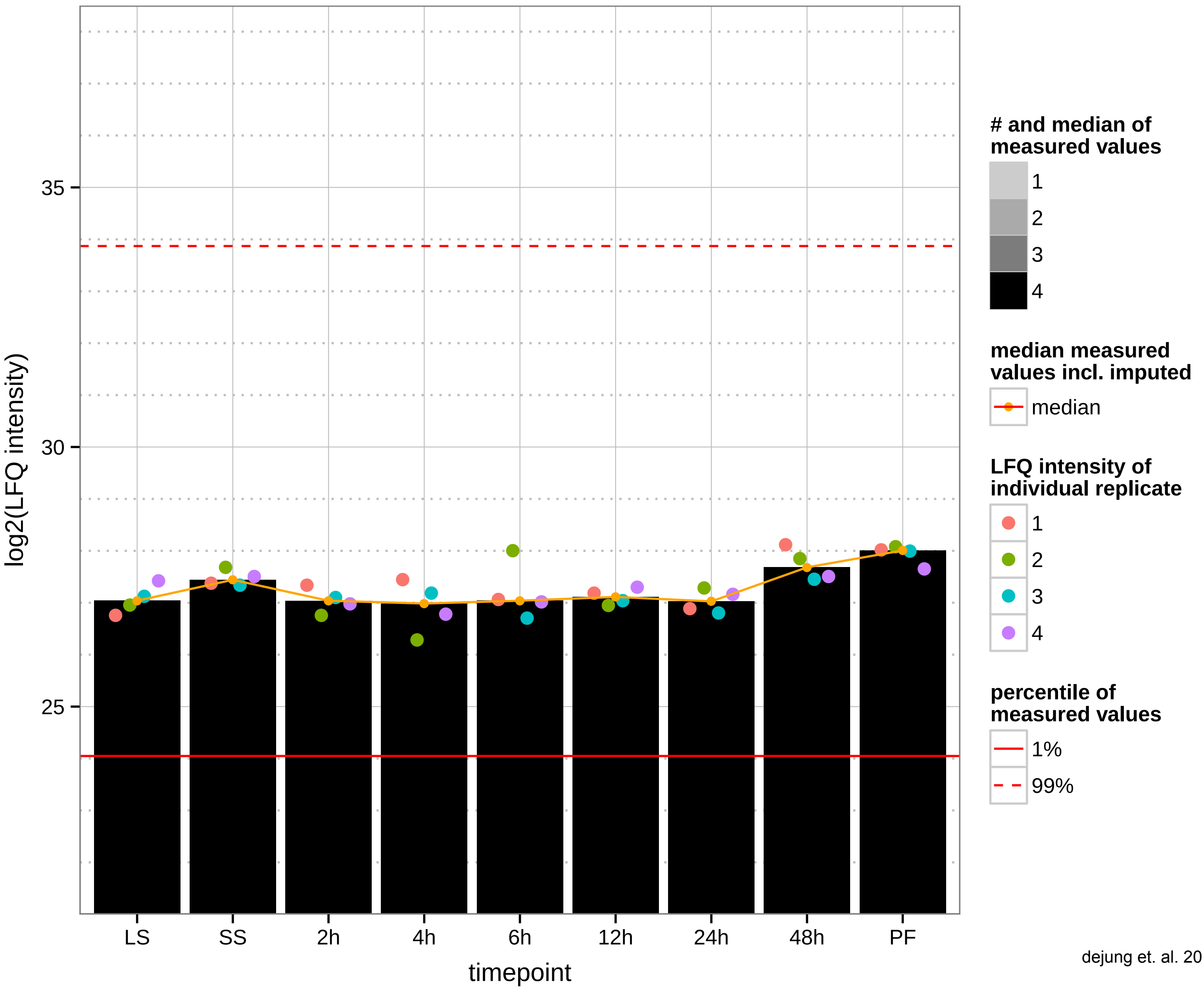

dejung et. al. 2015

hypothetical protein, conserved
Tb927.4.1740
AGOF: calcium ion binding
AGOC: null
AGOP: ciliary or flagellar motility
PGOF: null
PGOC: null
PGOP: null

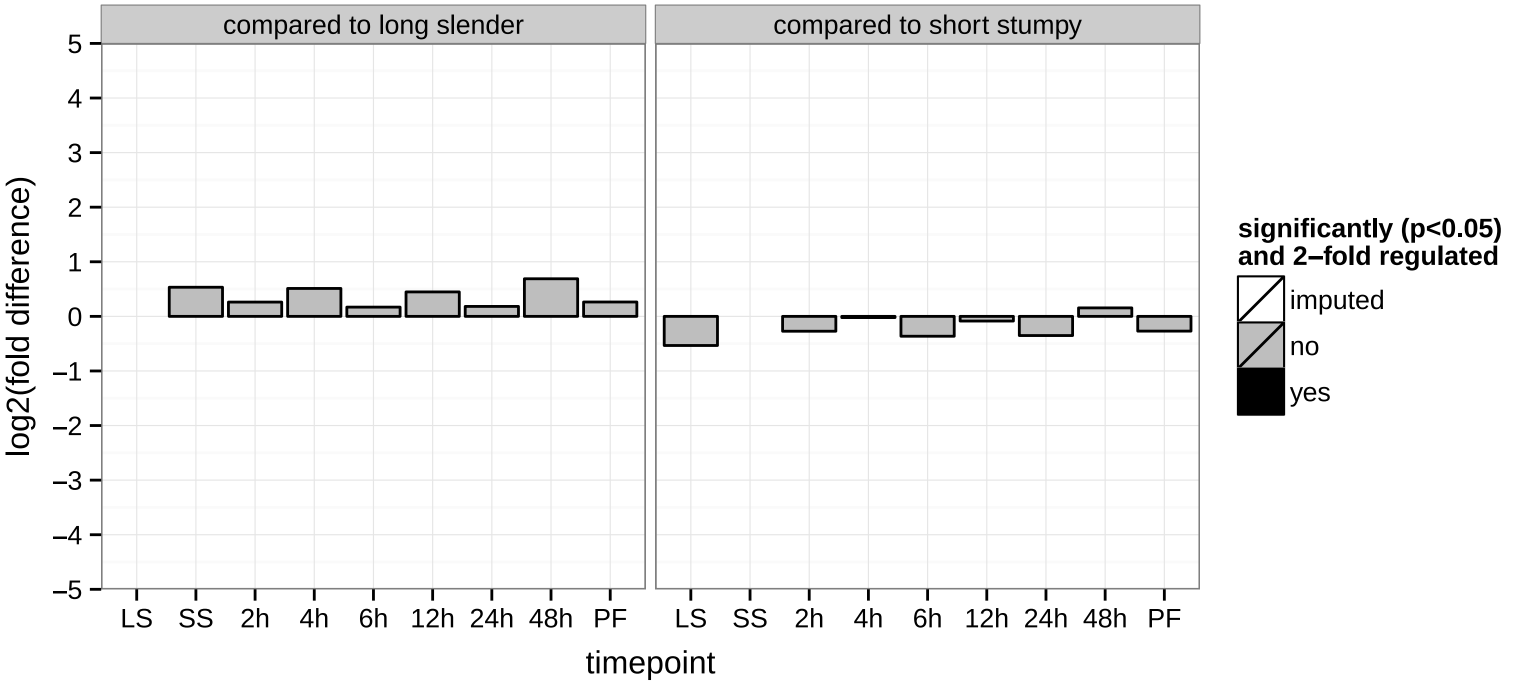

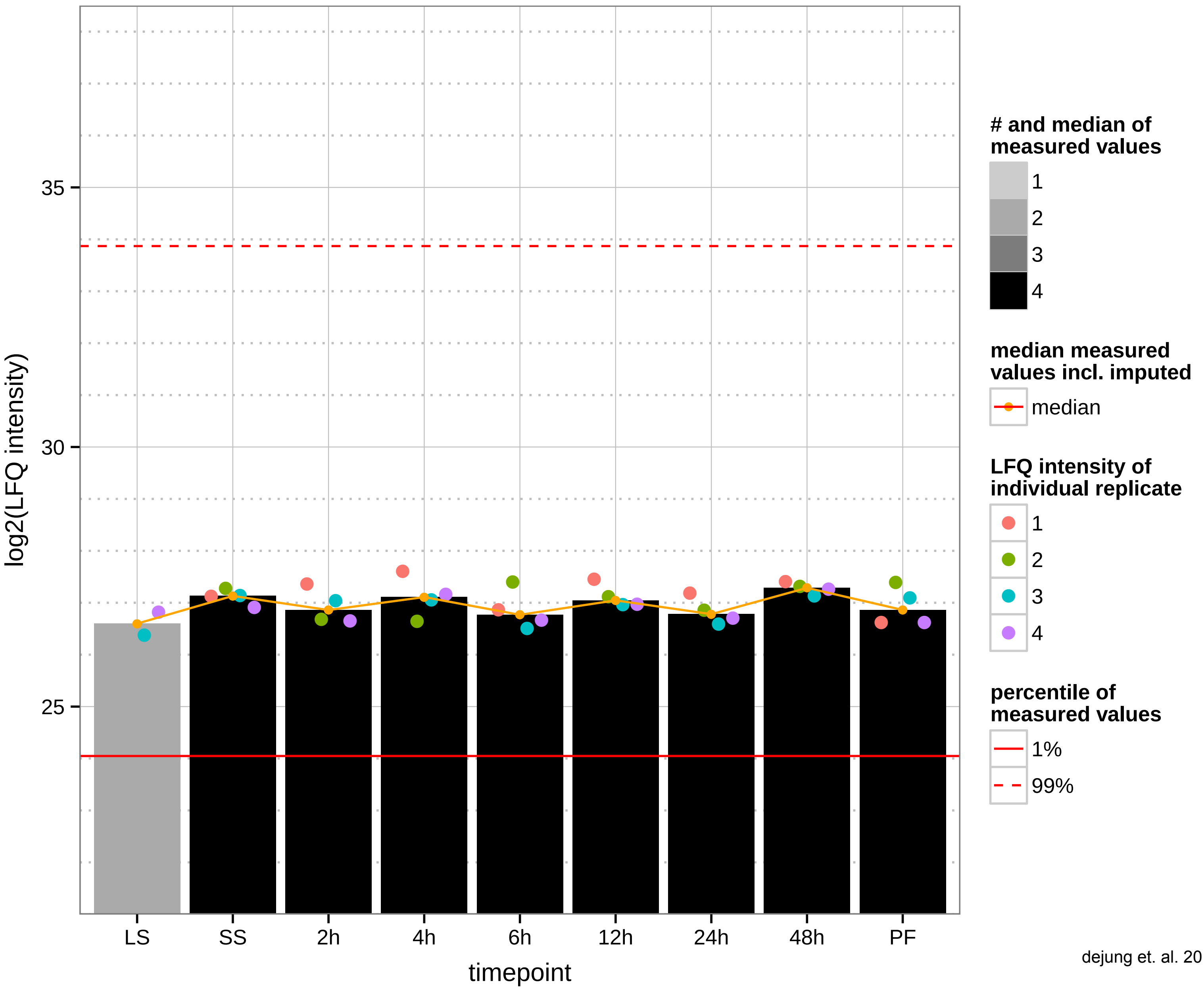

dejung et. al. 2015

ribosomal protein L3, putative, mitochondrial
Tb927.4.1800;Tb927.4.1790
AGOF: structural constituent of ribosome
AGOC: intracellular, ribosome
AGOP: translation
PGOF: structural constituent of ribosome, null
PGOC: intracellular, ribosome, null
PGOP: translation, null

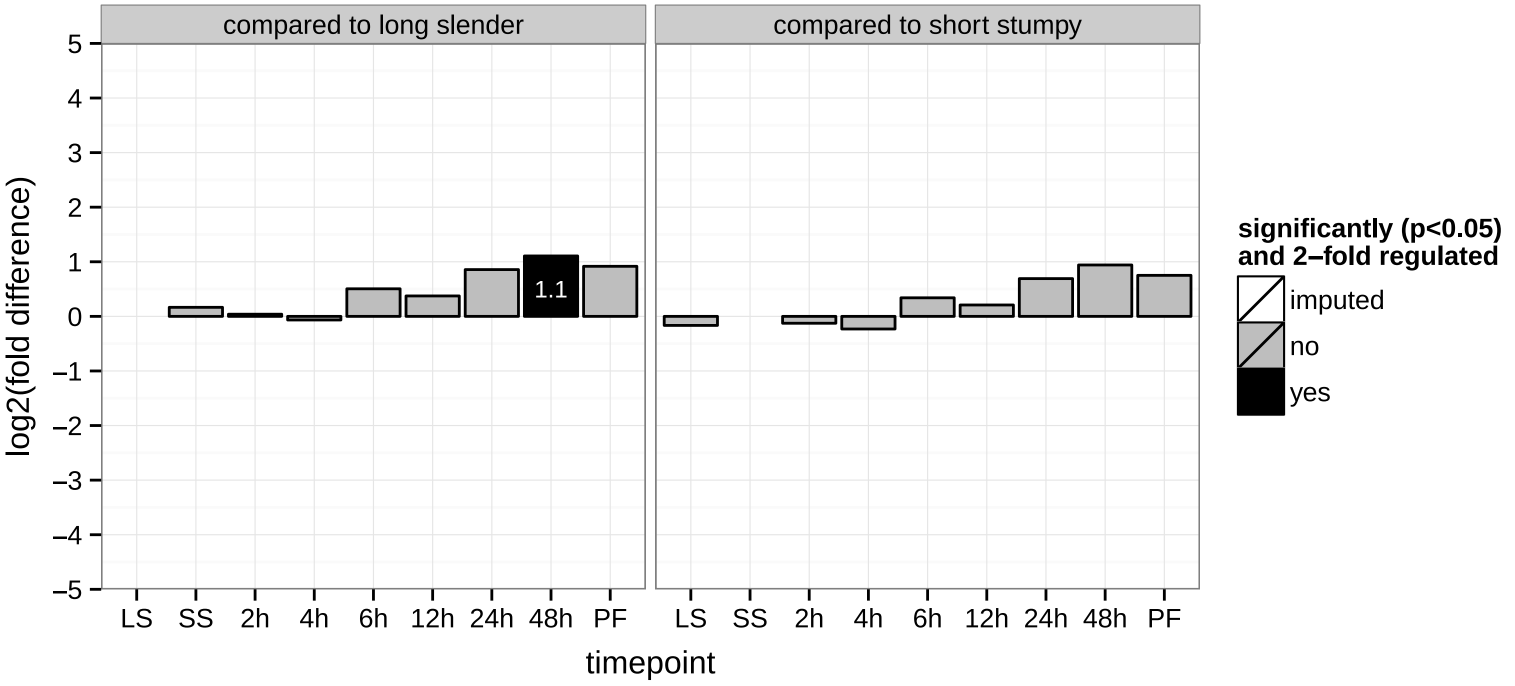

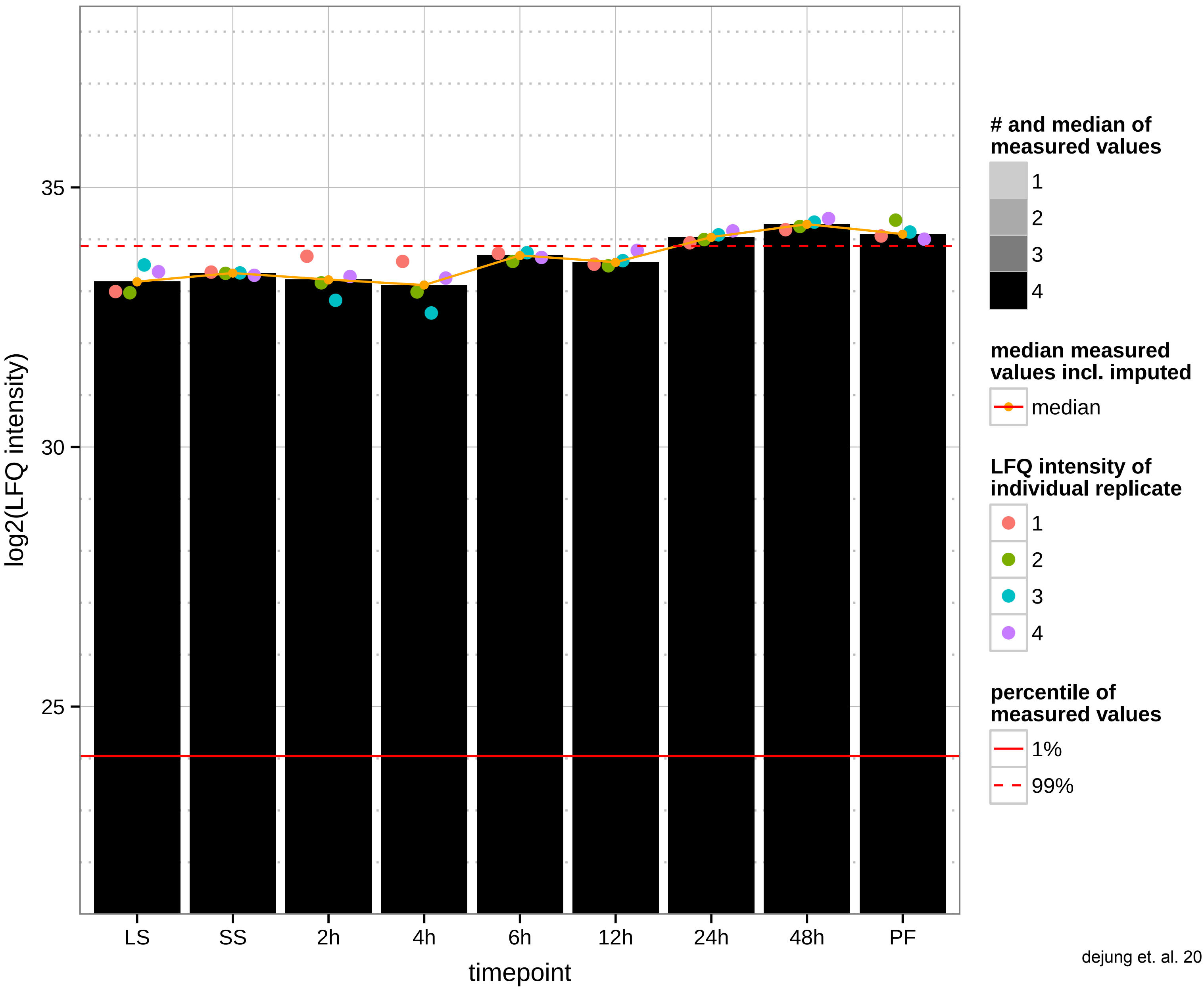

dejung et. al. 2015

zinc carboxypeptidase, putative, metallo−peptidase, Clan MC, Family M14
Tb927.4.1840
AGOF: ADP binding, ATP binding, metallocarboxypeptidase activity, zinc ion binding
AGOC: cytosol
AGOP: proteolysis
PGOF: metallocarboxypeptidase activity, zinc ion binding
PGOC: null
PGOP: proteolysis

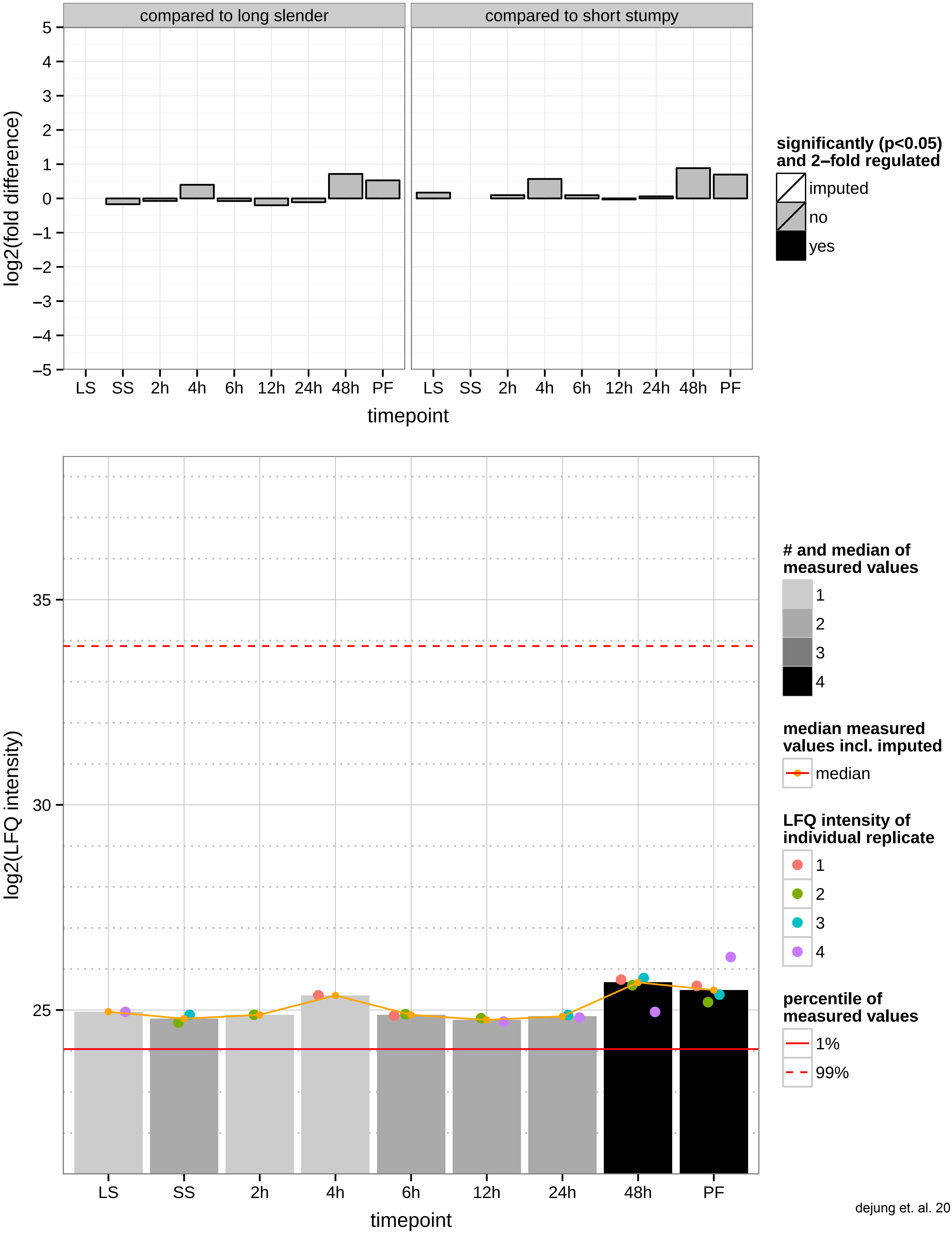

dejung et. al. 2015

hypothetical protein, conserved
Tb927.4.1850
AGOF: null
AGOC: null
AGOP: null
PGOF: null
PGOC: null
PGOP: null

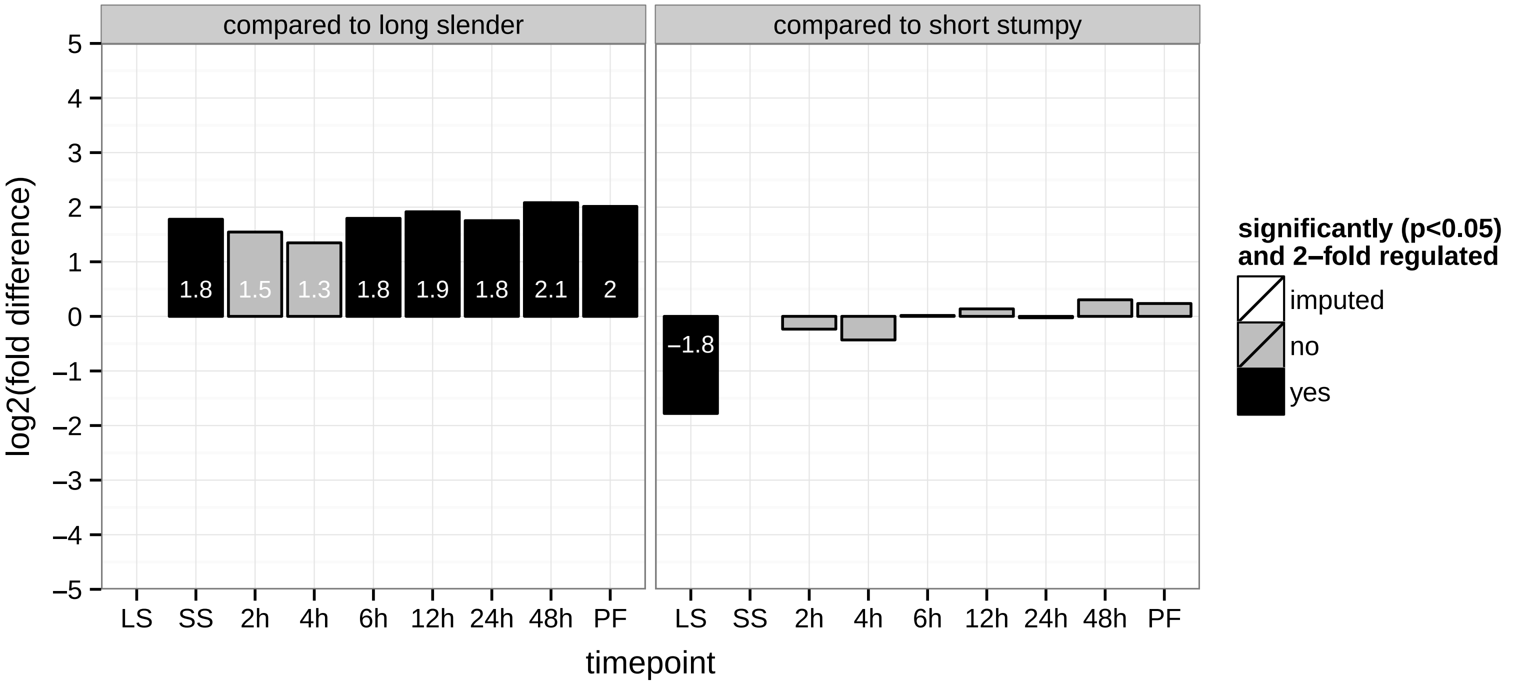

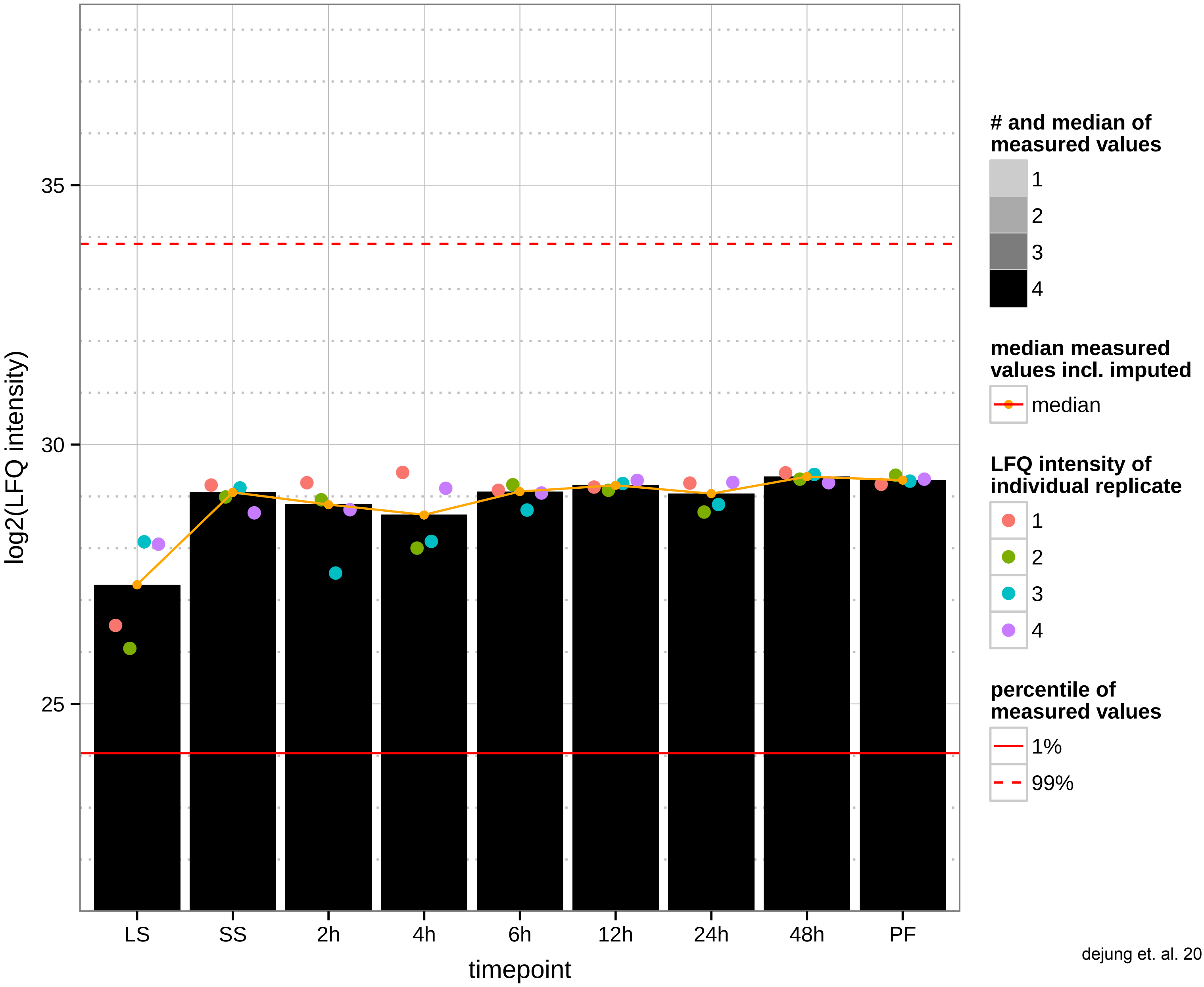

dejung et. al. 2015

ribosomal protein S19, putative
Tb927.4.1860
AGOF: structural constituent of ribosome
AGOC: intracellular, ribosome
AGOP: translation
PGOF: structural constituent of ribosome
PGOC: intracellular, ribosome
PGOP: translation

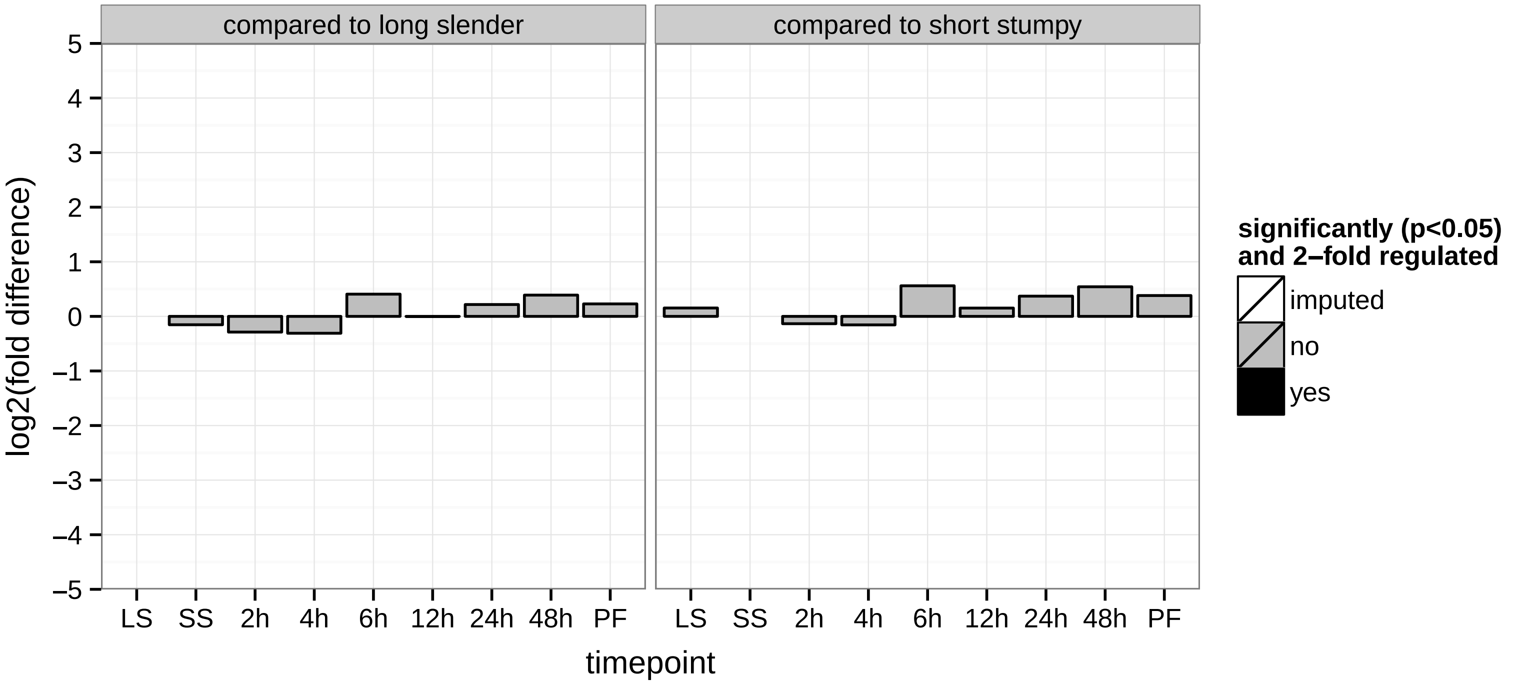

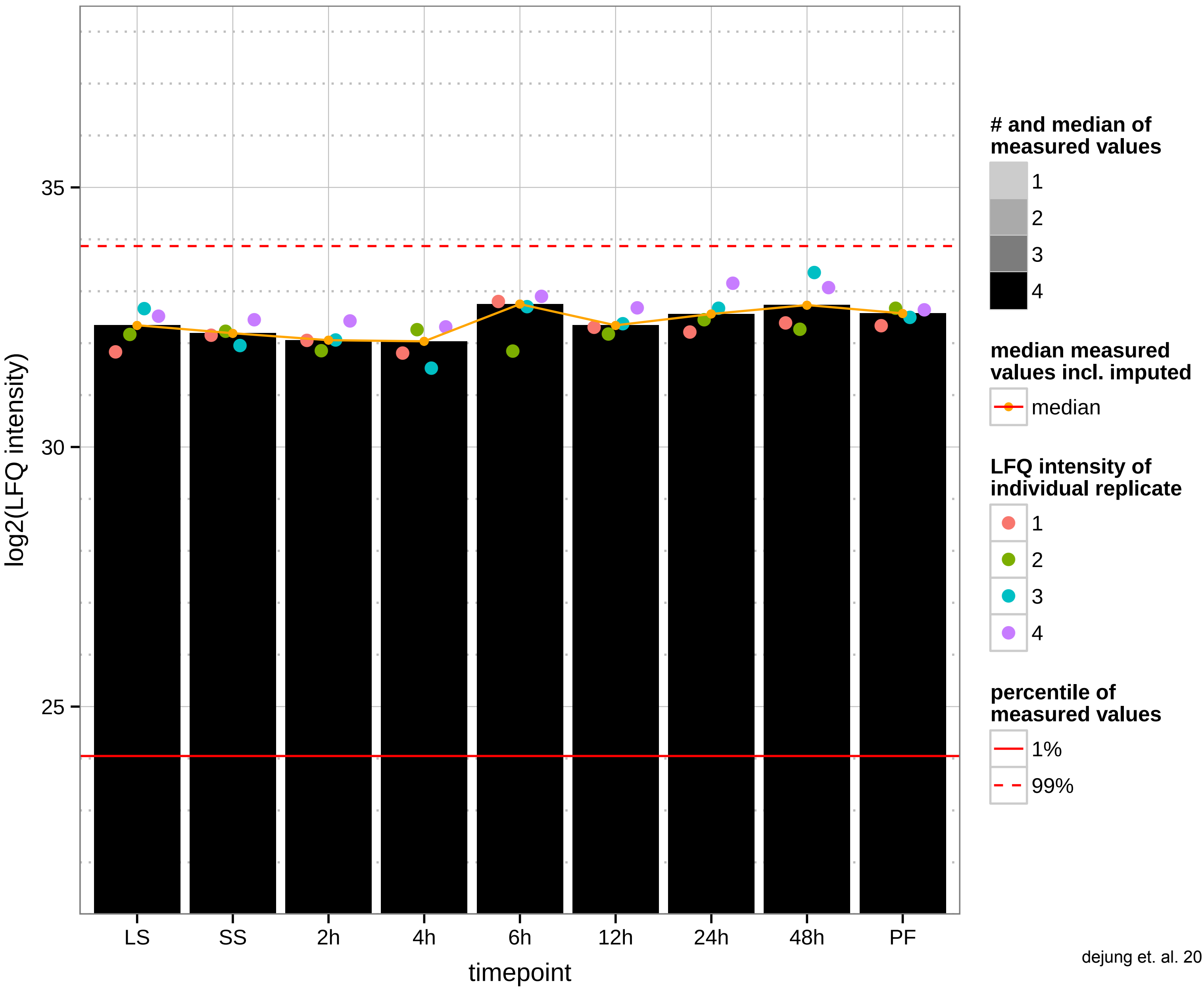

dejung et. al. 2015

hypothetical protein, conserved
Tb927.4.1890
AGOF: null
AGOC: null
AGOP: null
PGOF: null
PGOC: null
PGOP: null

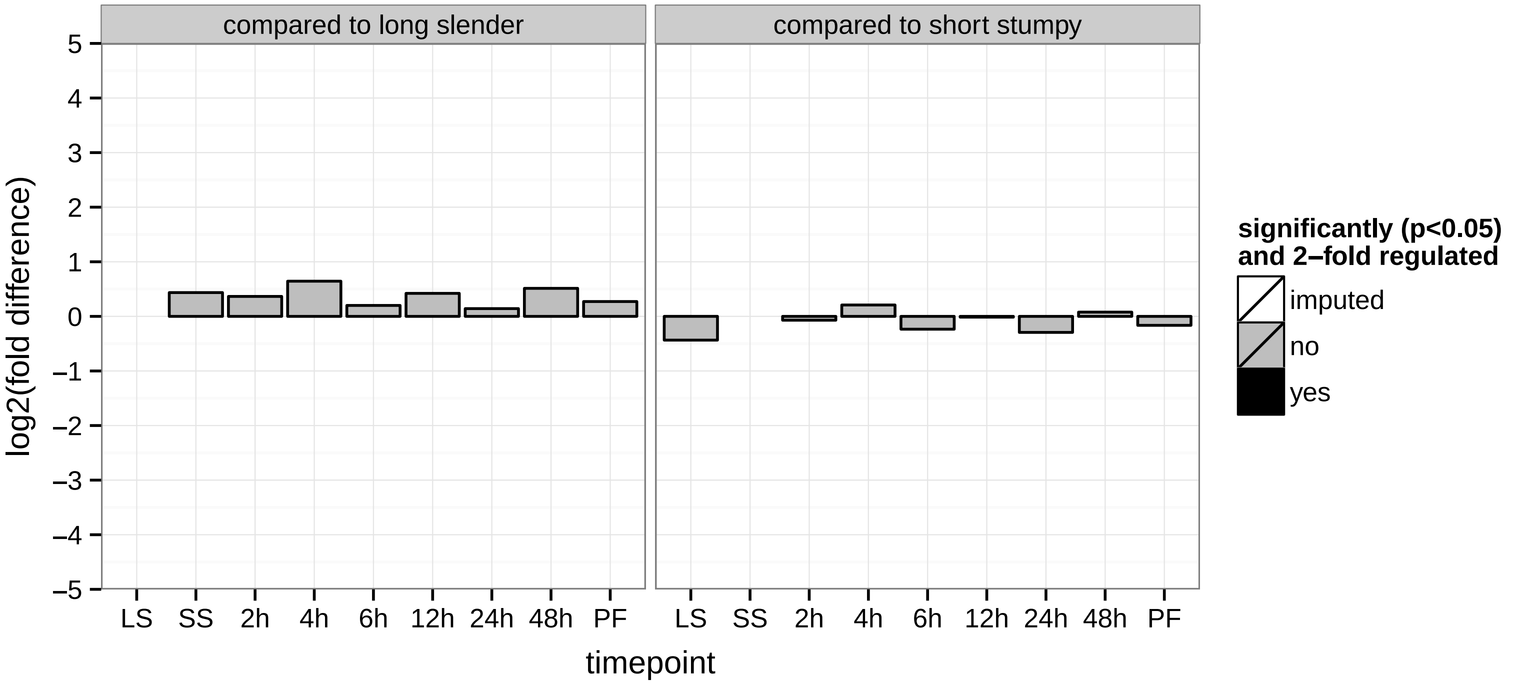

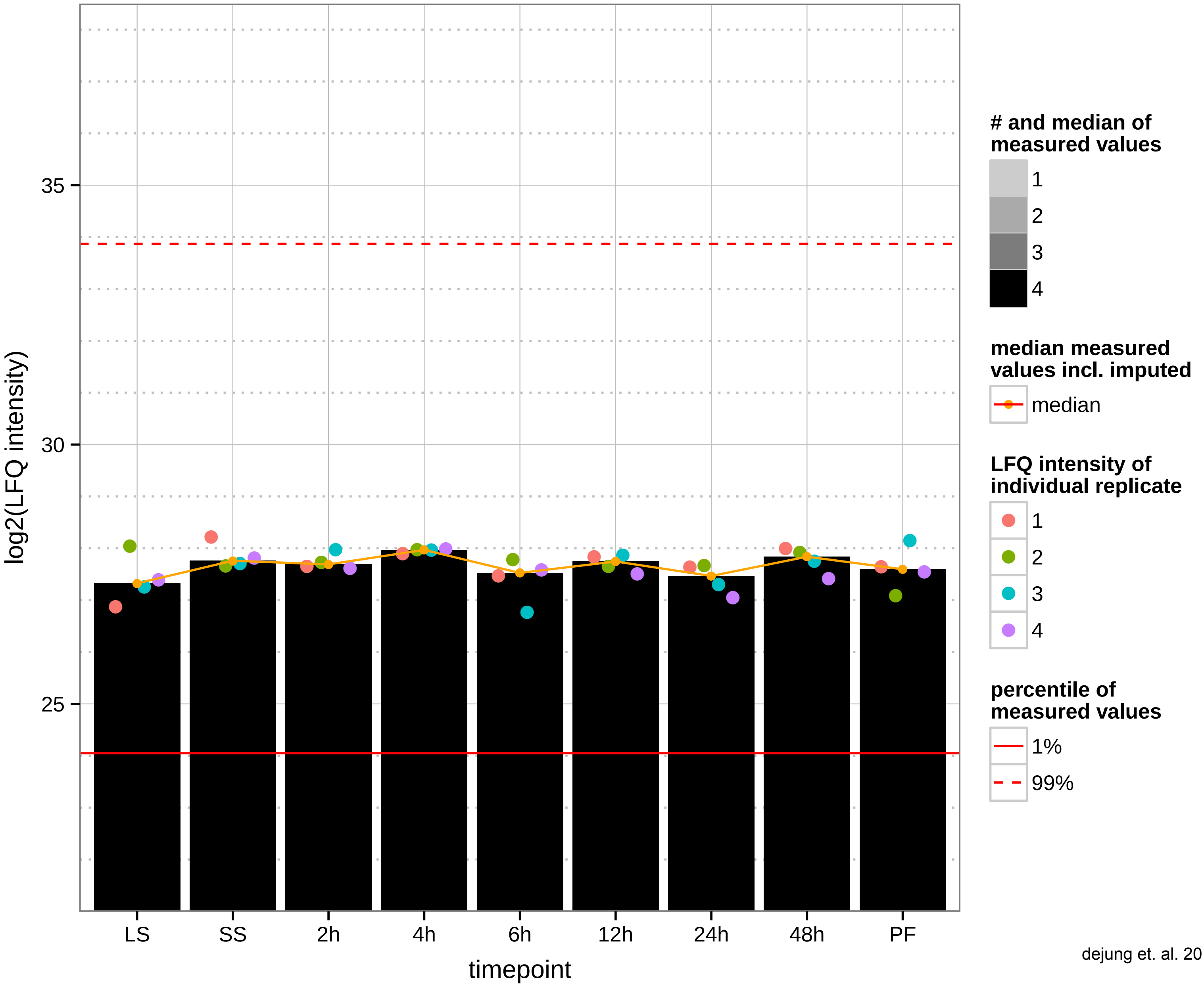

dejung et. al. 2015

GPI transamidase, putative, glycosylphosphatidylinositol (GPI) anchor, putative (TbGPI16)
Tb927.4.1920
AGOF: GPI–anchor transamidase activity
AGOC: GPI–anchor transamidase complex
AGOP: attachment of GPI anchor to protein
PGOF: null
PGOC: null
PGOP: null

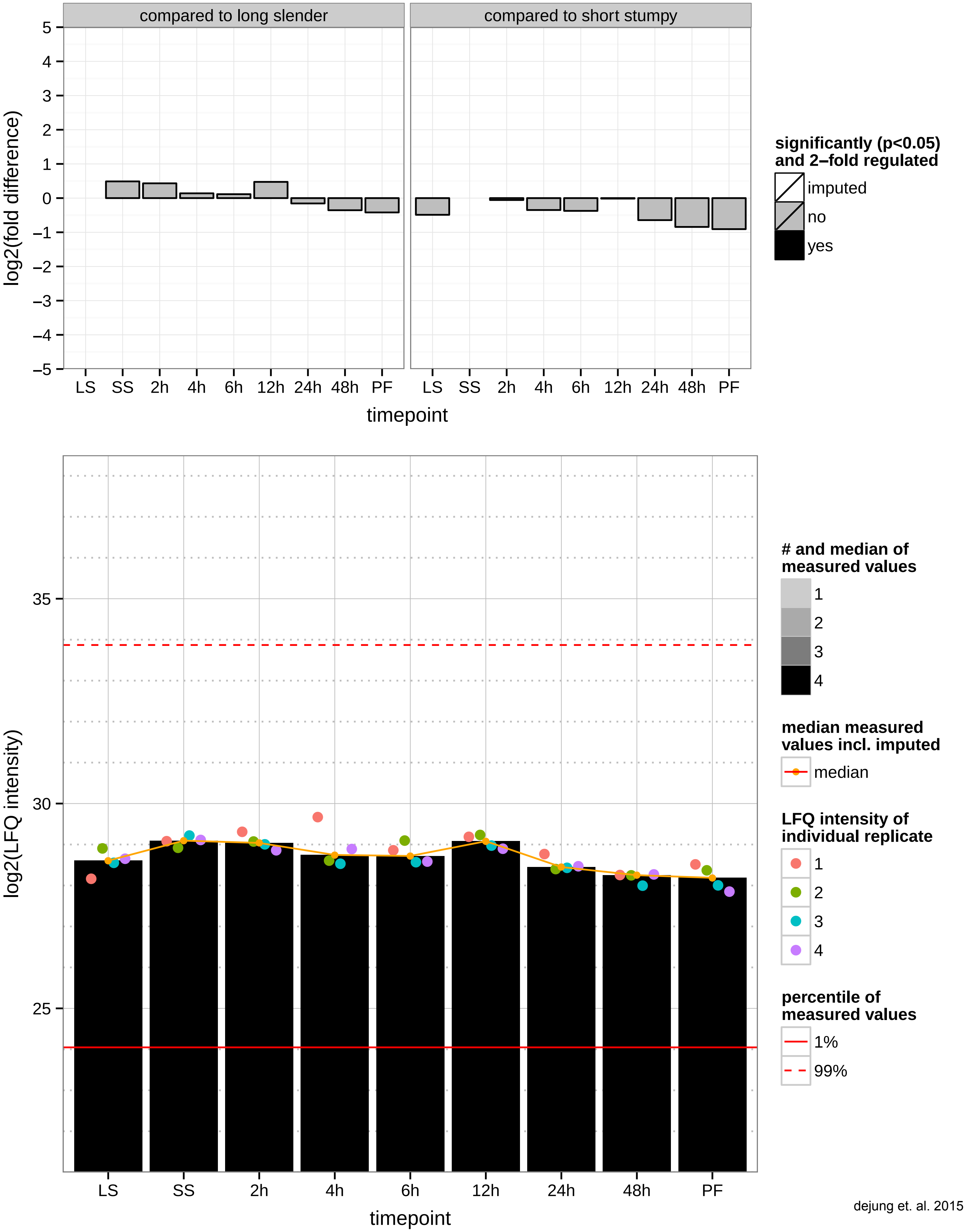

dejung et. al. 2015

hypothetical protein, conserved
Tb927.4.1940
AGOF: null
AGOC: null
AGOP: null
PGOF: protein binding
PGOC: null
PGOP: null

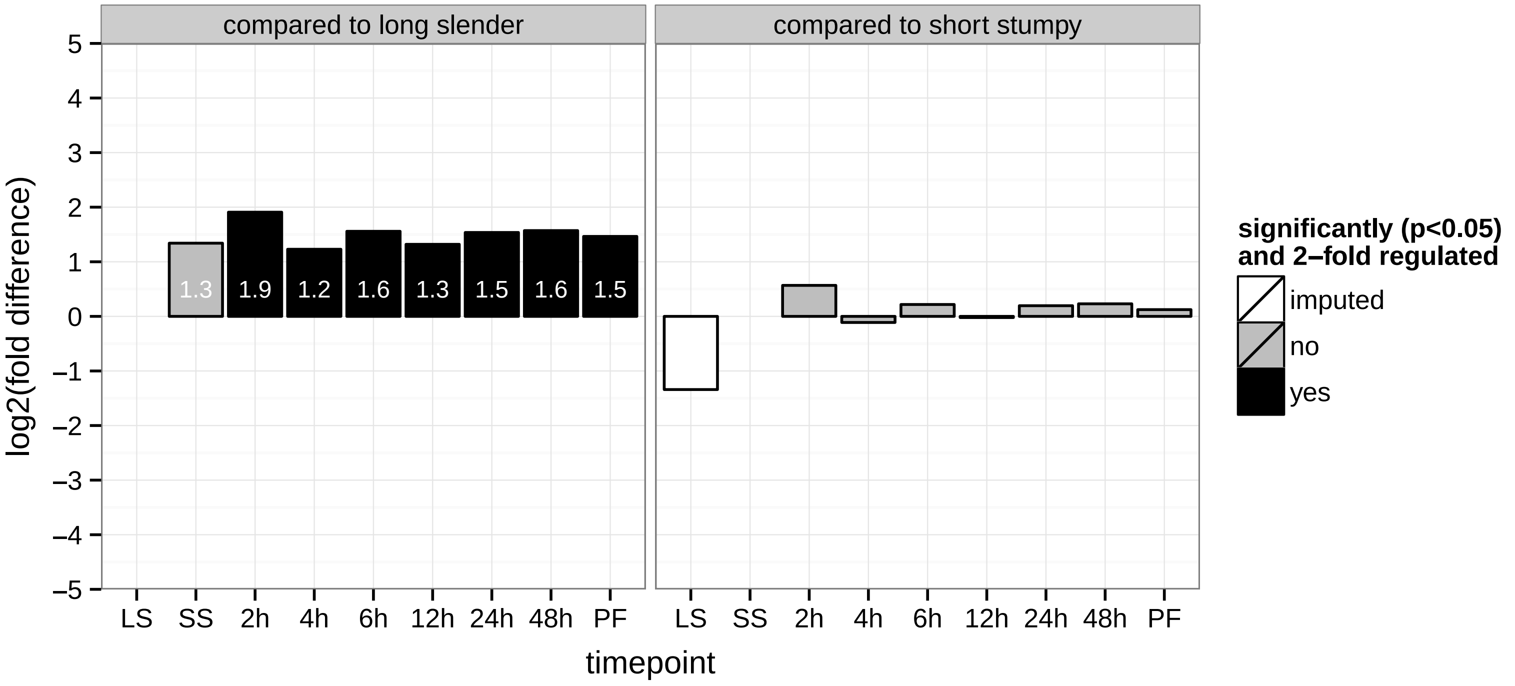

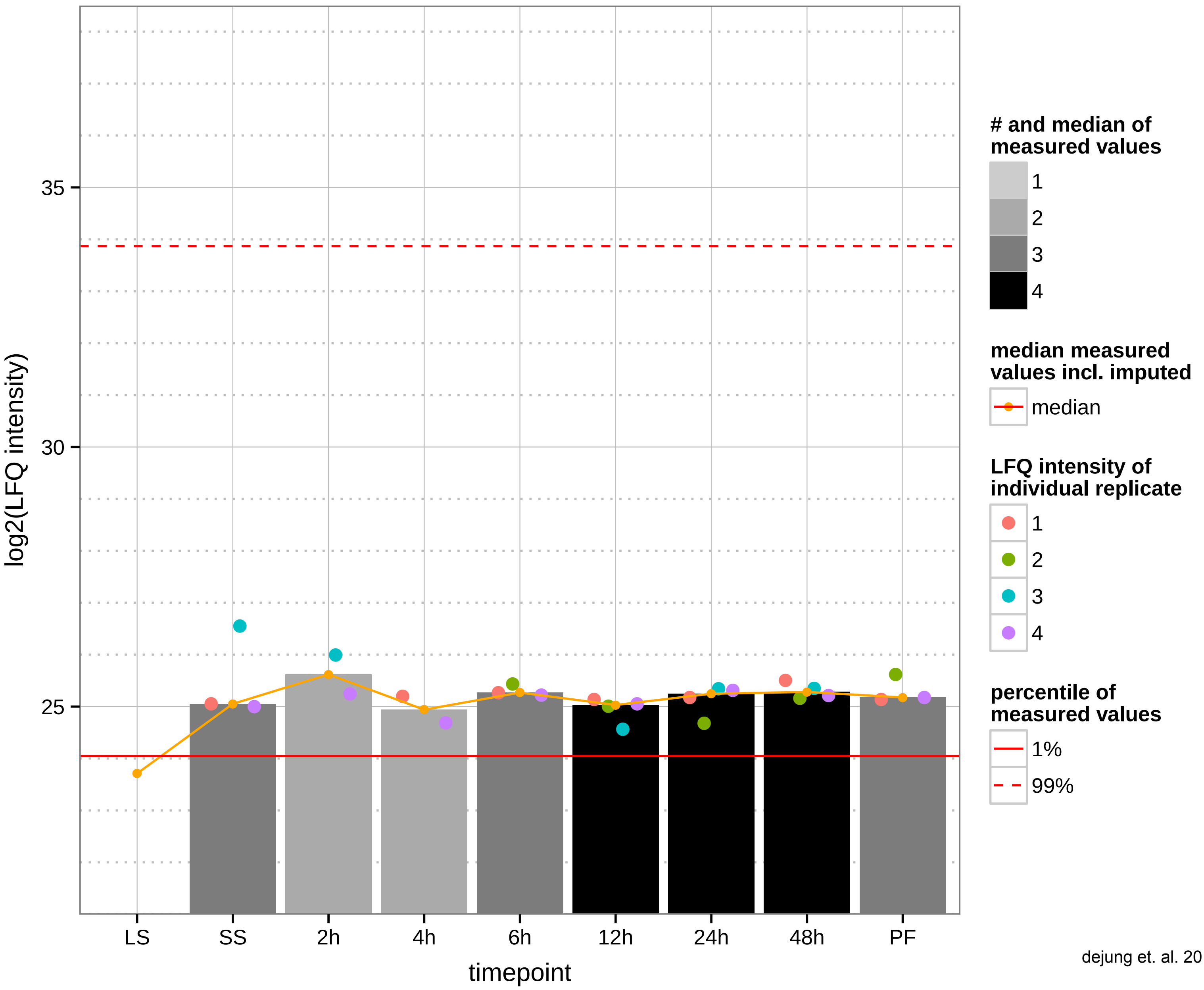

dejung et. al. 2015

NADPH−−cytochrome p450 reductase, putative (CPR)
Tb927.4.1950
AGOF: FMN binding, electron carrier activity, iron ion binding, oxidoreductase activity
AGOC: null
AGOP: null
PGOF: FMN binding, oxidoreductase activity
PGOC: null
PGOP: oxidation−reduction process

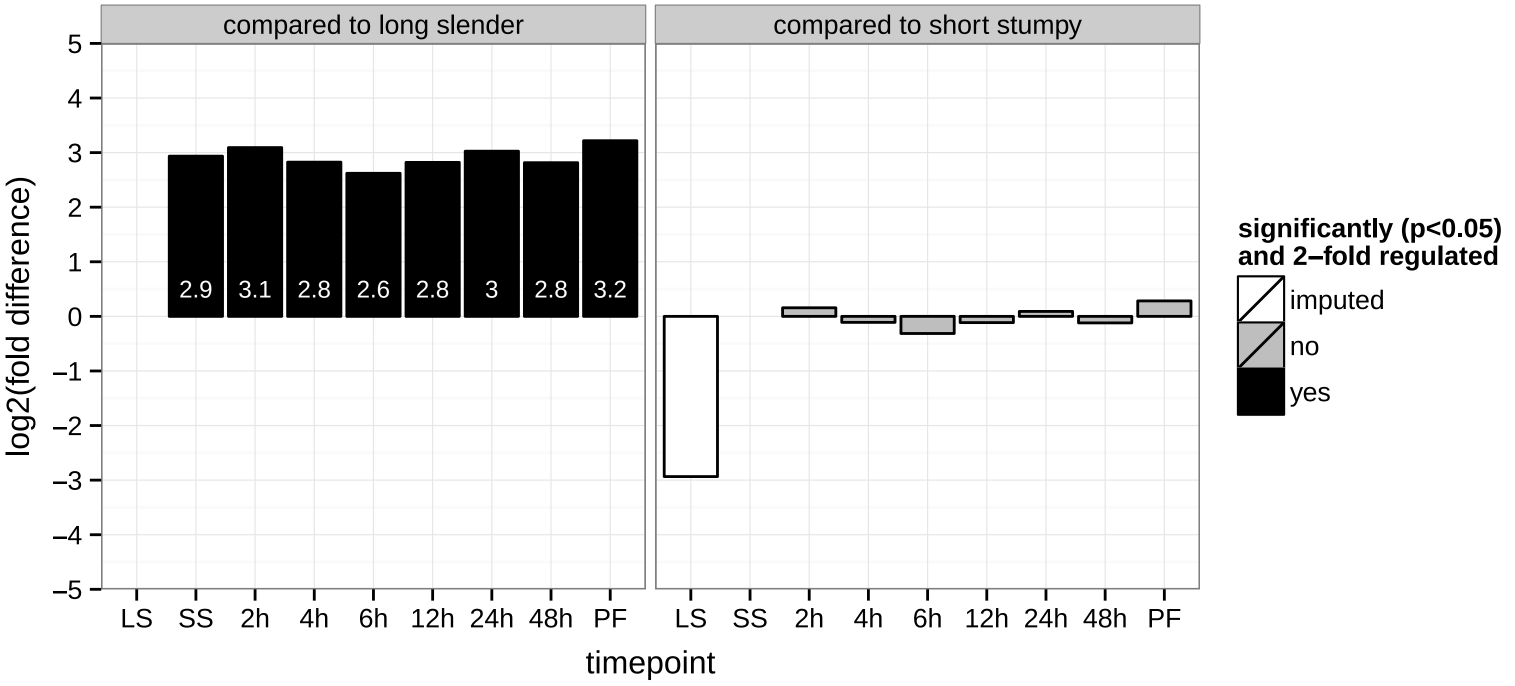

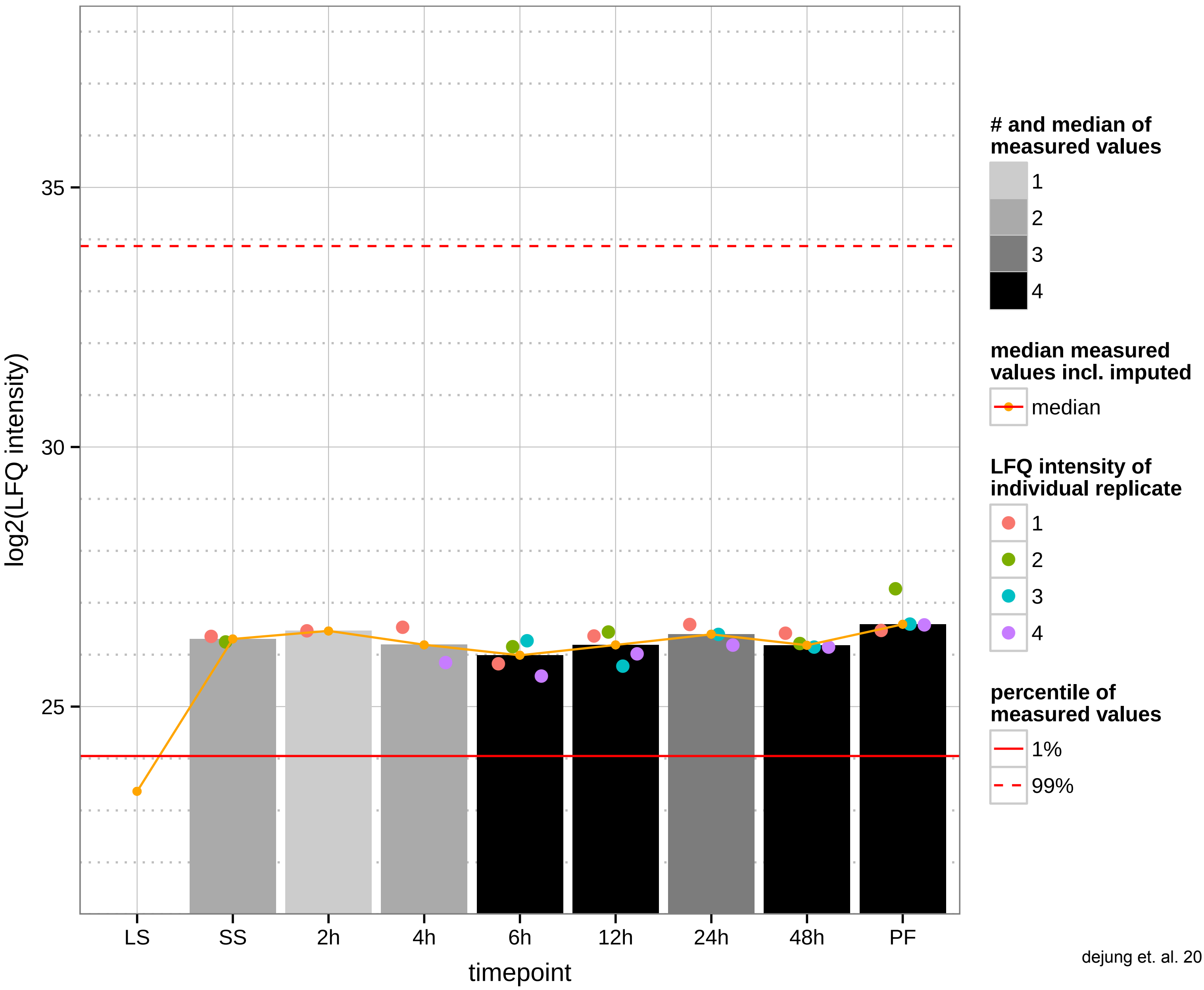

dejung et. al. 2015

hypothetical protein, conserved
Tb927.4.1970
AGOF: null
AGOC: null
AGOP: null
PGOF: protein binding
PGOC: null
PGOP: null

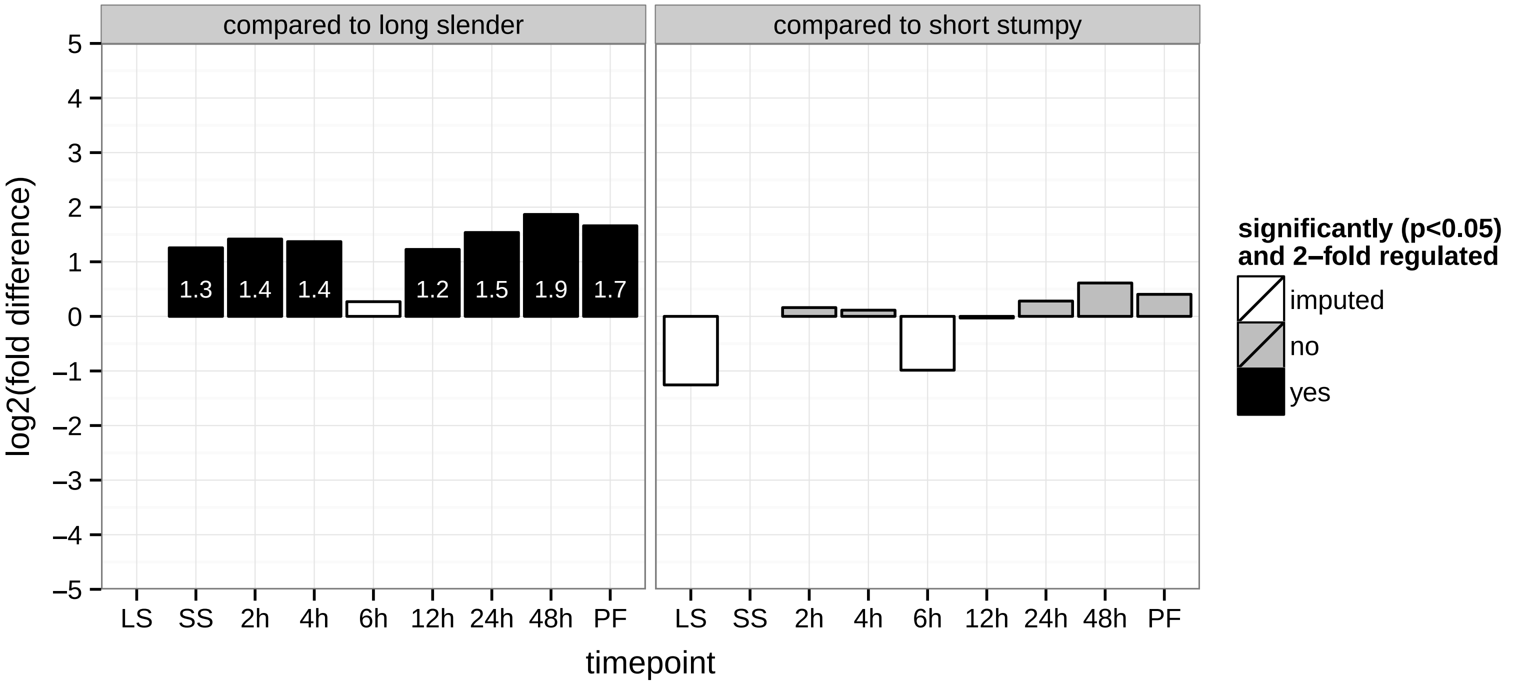

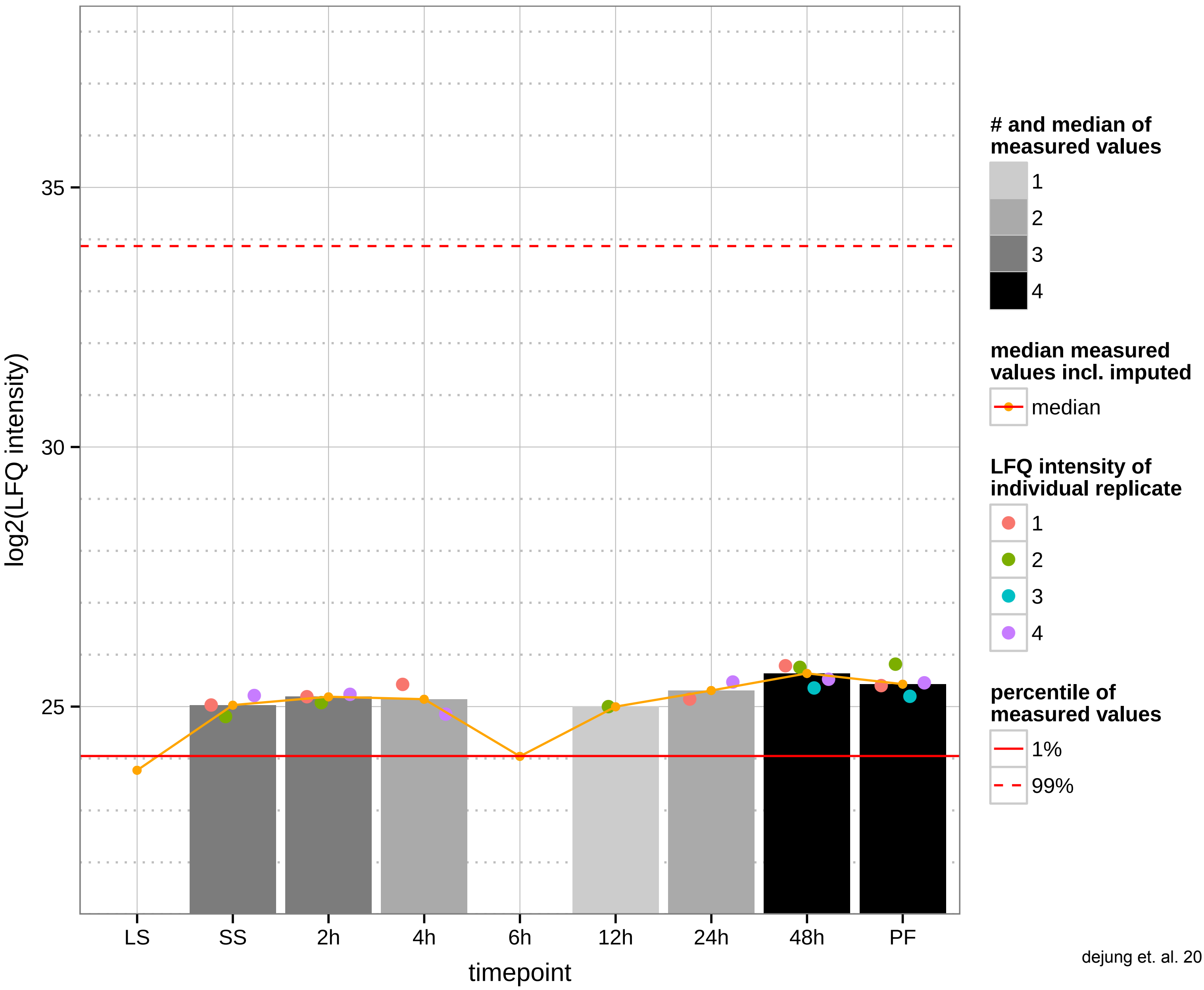

dejung et. al. 2015

ruvB–like DNA helicase, putative, ATP–dependent DNA helicase
Tb927.4.2000
AGOF: ATP binding, DNA helicase activity, nucleoside–triphosphatase activity
AGOC: nucleus
AGOP: null
PGOF: ATP binding, DNA helicase activity, nucleoside–triphosphatase activity, nucleotide binding
PGOC: null
PGOP: null

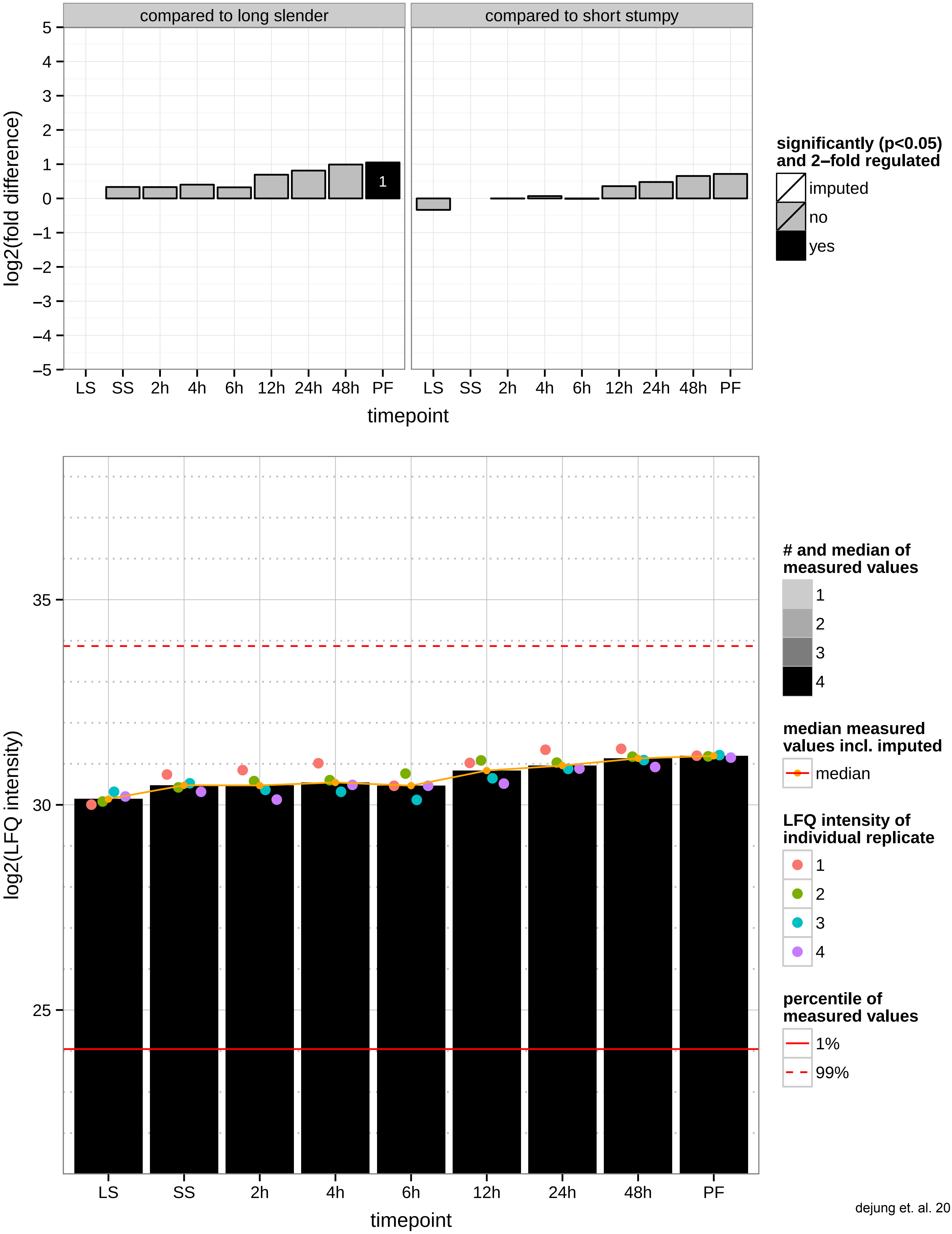

dejung et. al. 2015

mu–adaptin 3, putative, adaptor complex AP–3 medium subunit
Tb927.4.2020
AGOF: clathrin binding
AGOC: clathrin adaptor complex
AGOP: intracellular protein transport, vesicle–mediated transport
PGOF: protein binding
PGOC: clathrin adaptor complex
PGOP: intracellular protein transport, transport, vesicle–mediated transport

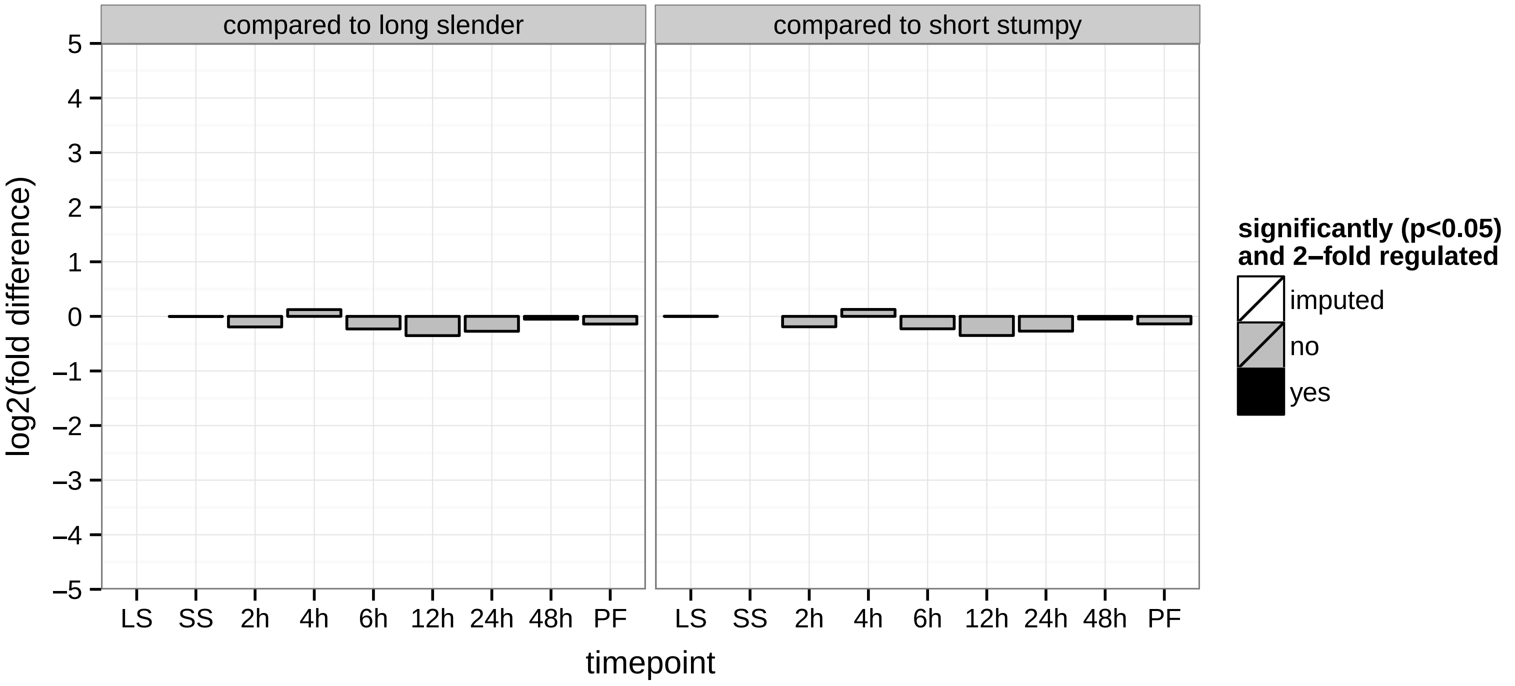

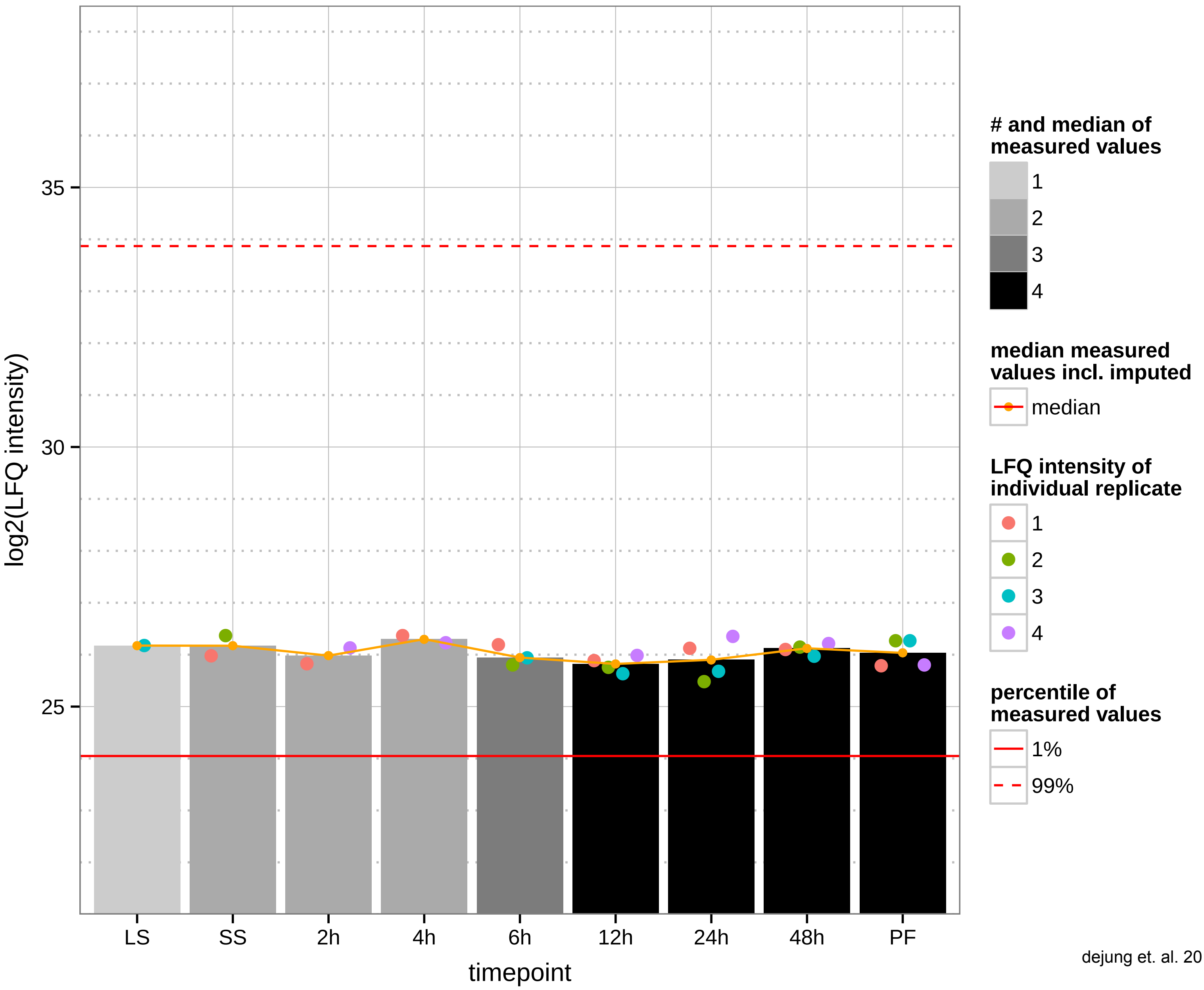

dejung et. al. 2015

ALBA–Domain Protein (ALBA3)
Tb927.4.2040
AGOF: nucleic acid binding
AGOC: cytoplasm, cytoplasmic stress granule
AGOP: null
PGOF: nucleic acid binding
PGOC: null
PGOP: null

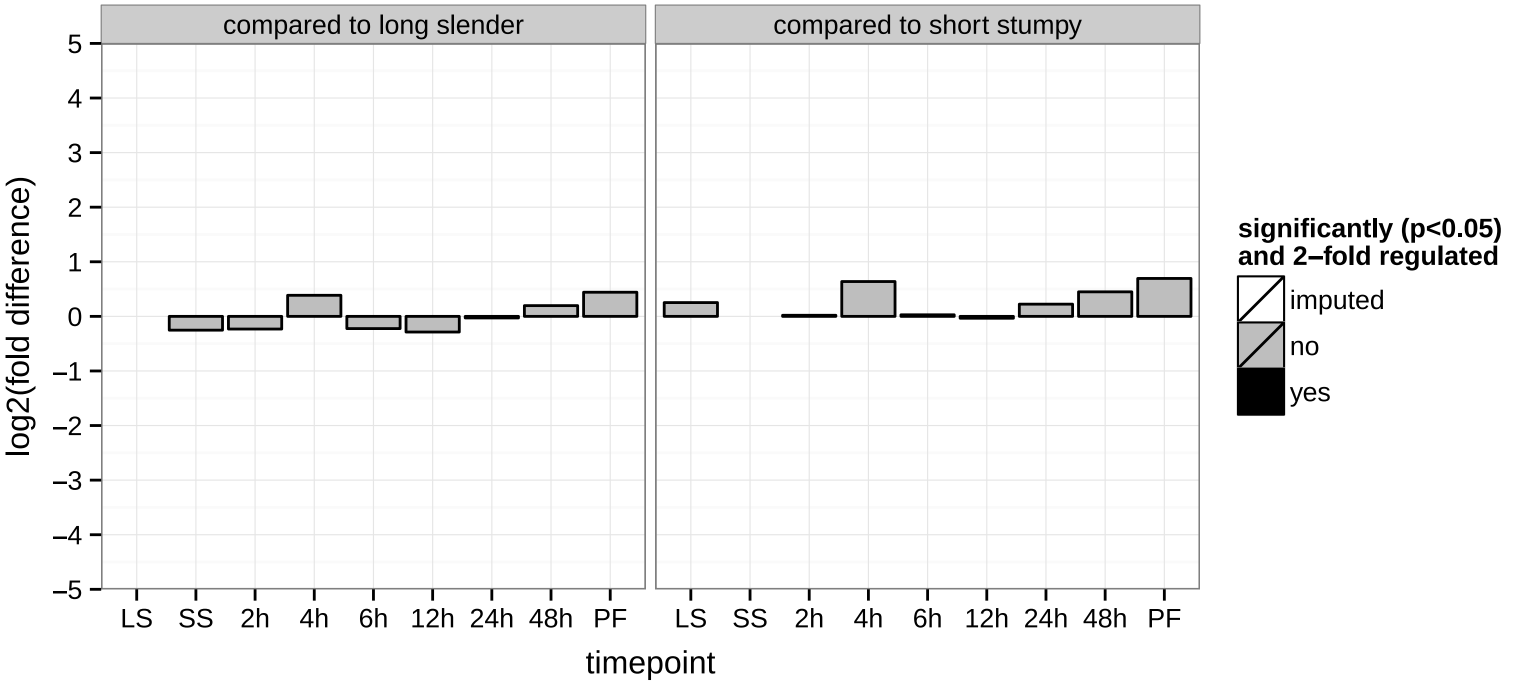

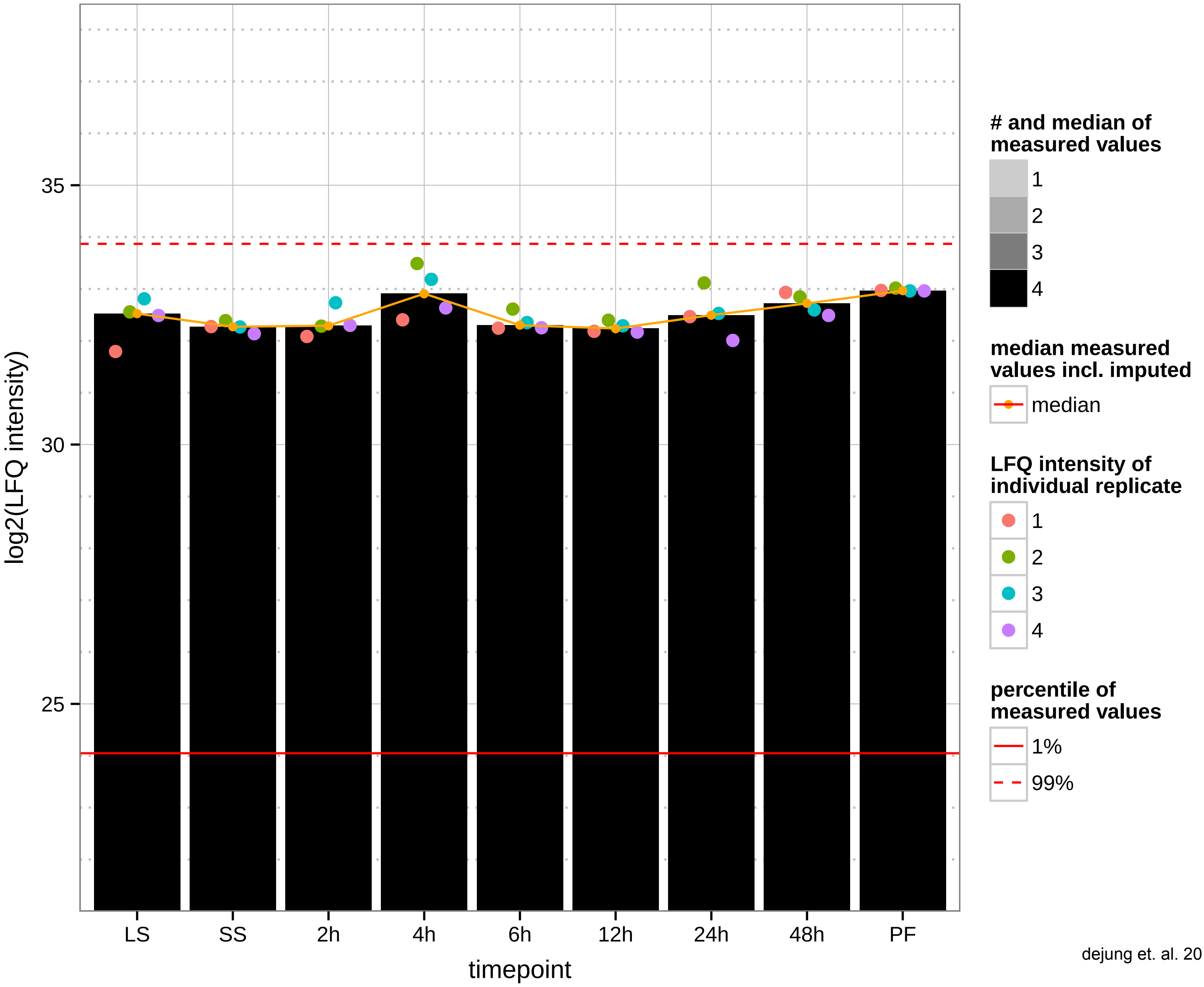

dejung et. al. 2015

hypothetical protein, conserved
Tb927.4.2060
AGOF: null
AGOC: null
AGOP: null
PGOF: null
PGOC: null
PGOP: null

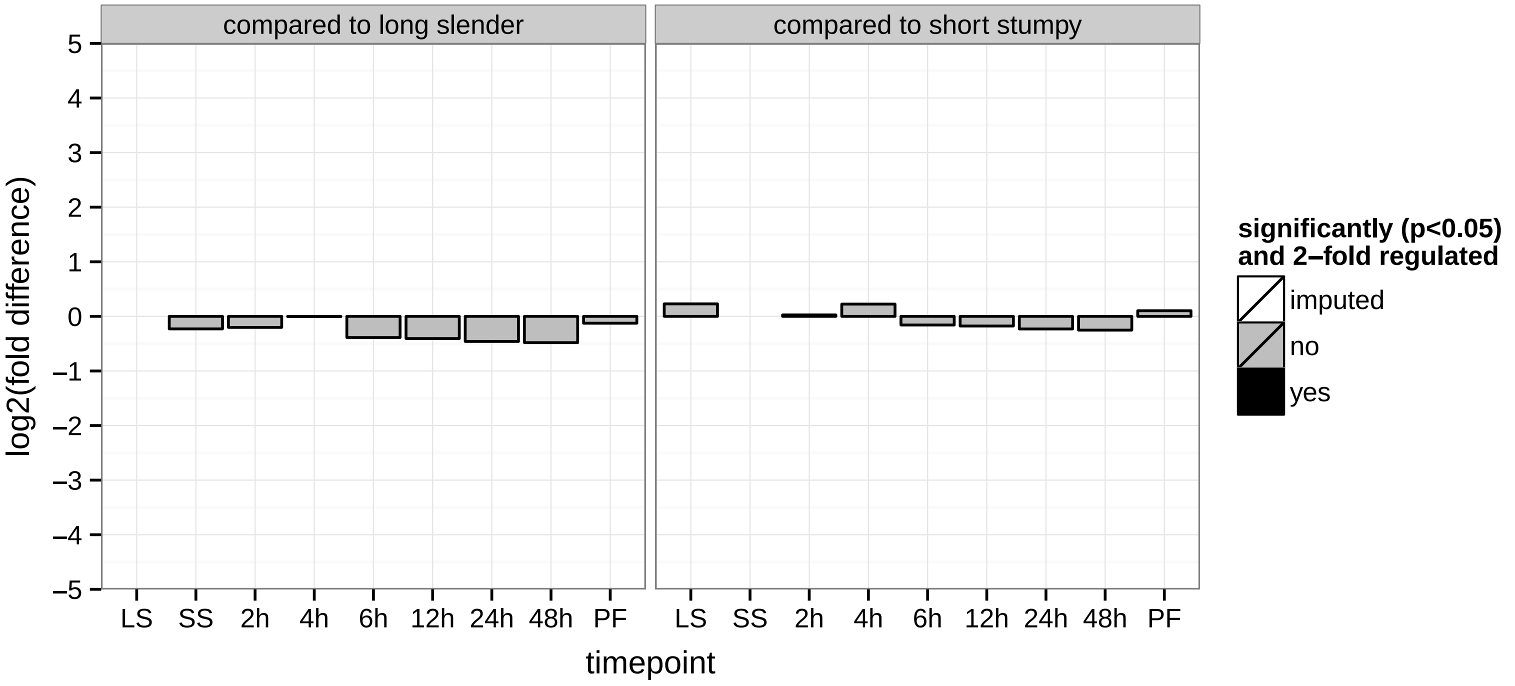

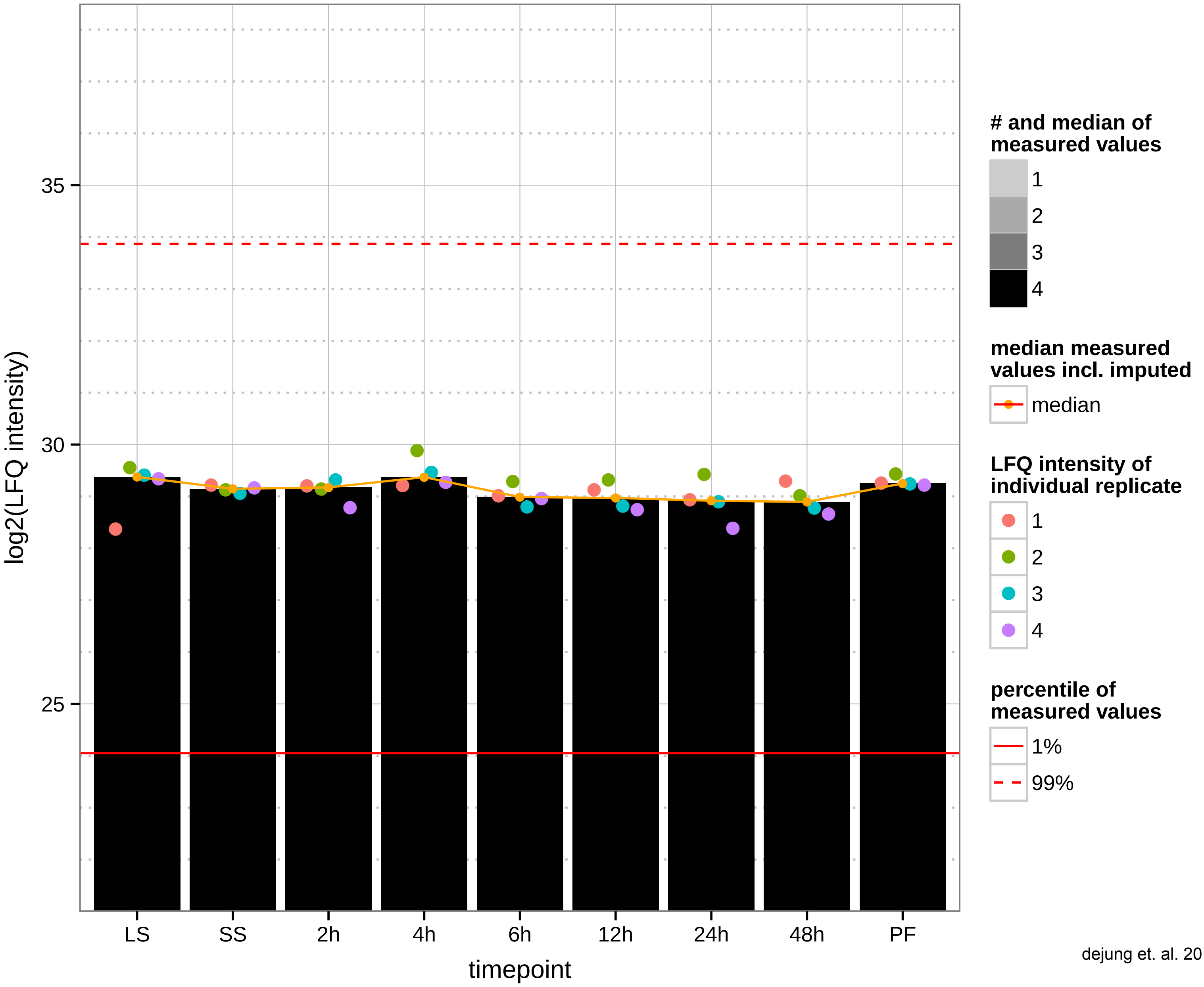

dejung et. al. 2015

C2 domain containing protein (CC2D)
Tb927.4.2080
AGOF: null
AGOC: bacterial–type flagellum basal body, cell body
AGOP: cilium assembly
PGOF: protein binding
PGOC: null
PGOP: null

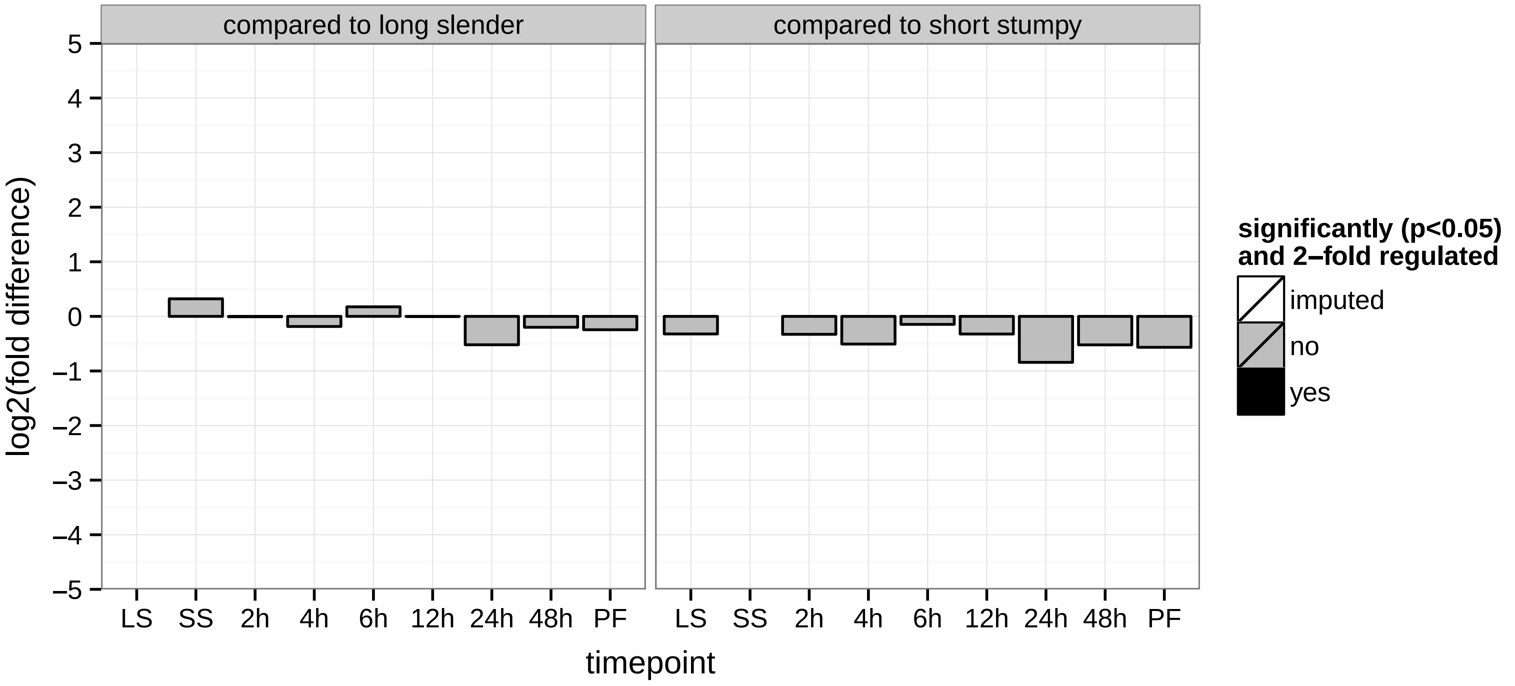

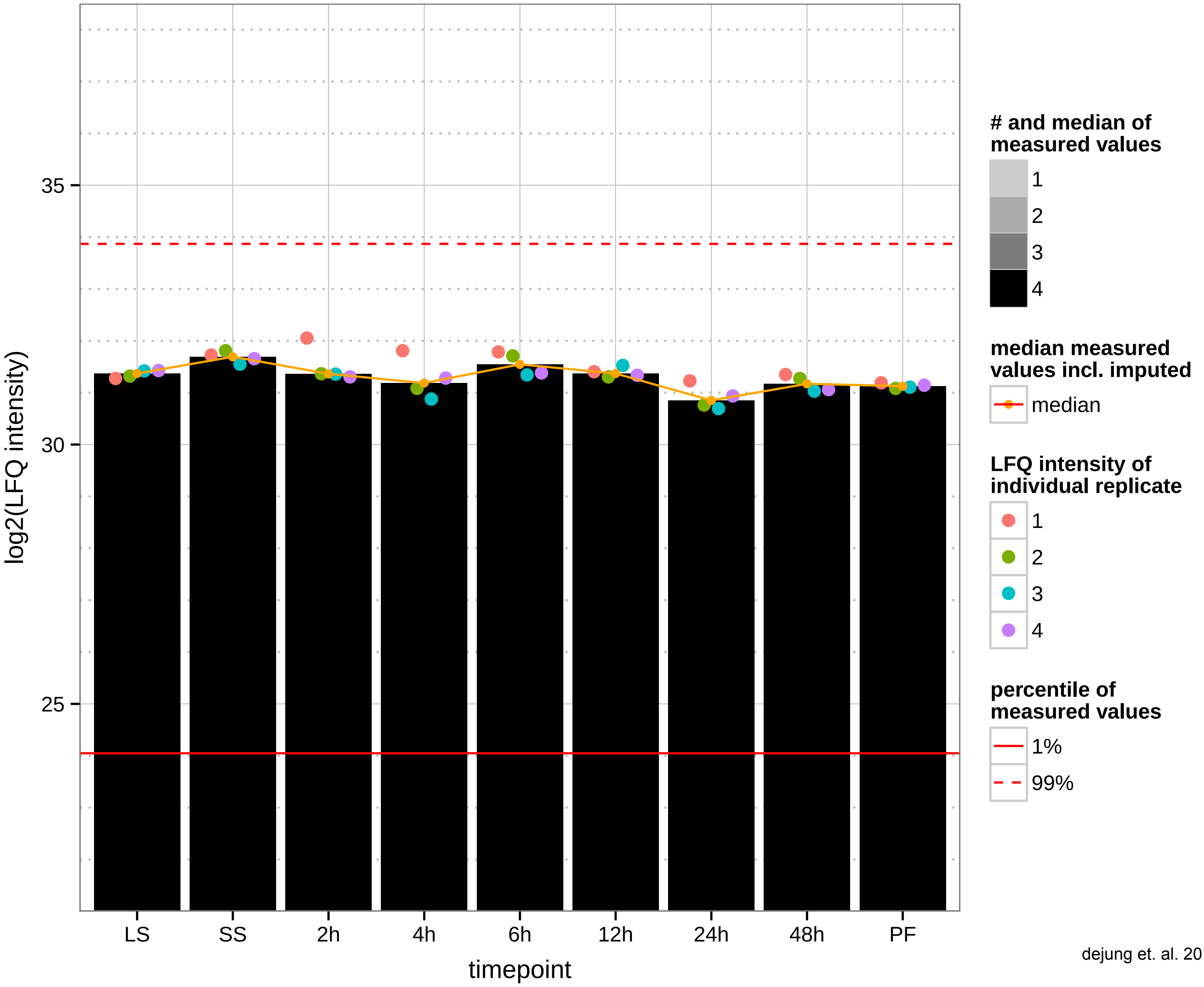

dejung et. al. 2015

protein phosphatase 2C, putative
Tb927.4.2110
AGOF: phosphatase activity
AGOC: null
AGOP: dephosphorylation
PGOF: catalytic activity
PGOC: null
PGOP: null

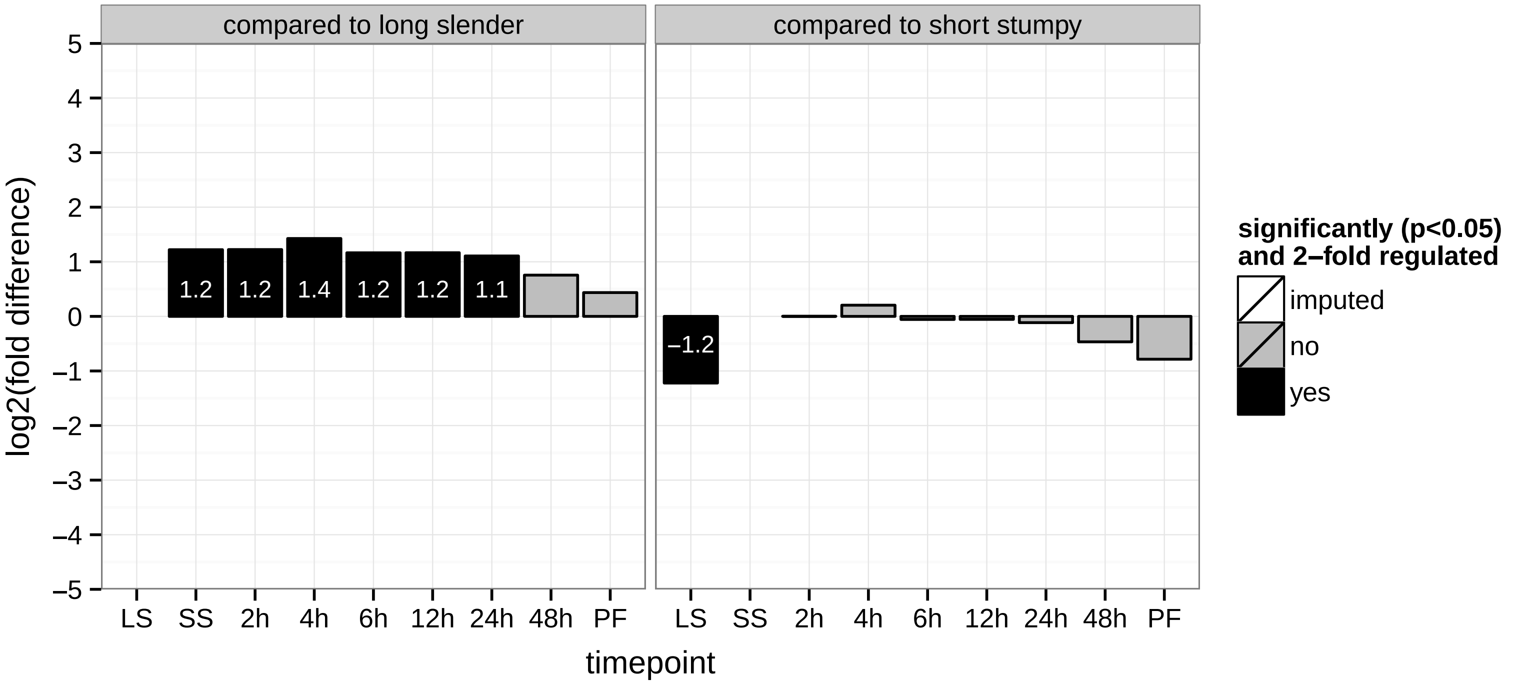

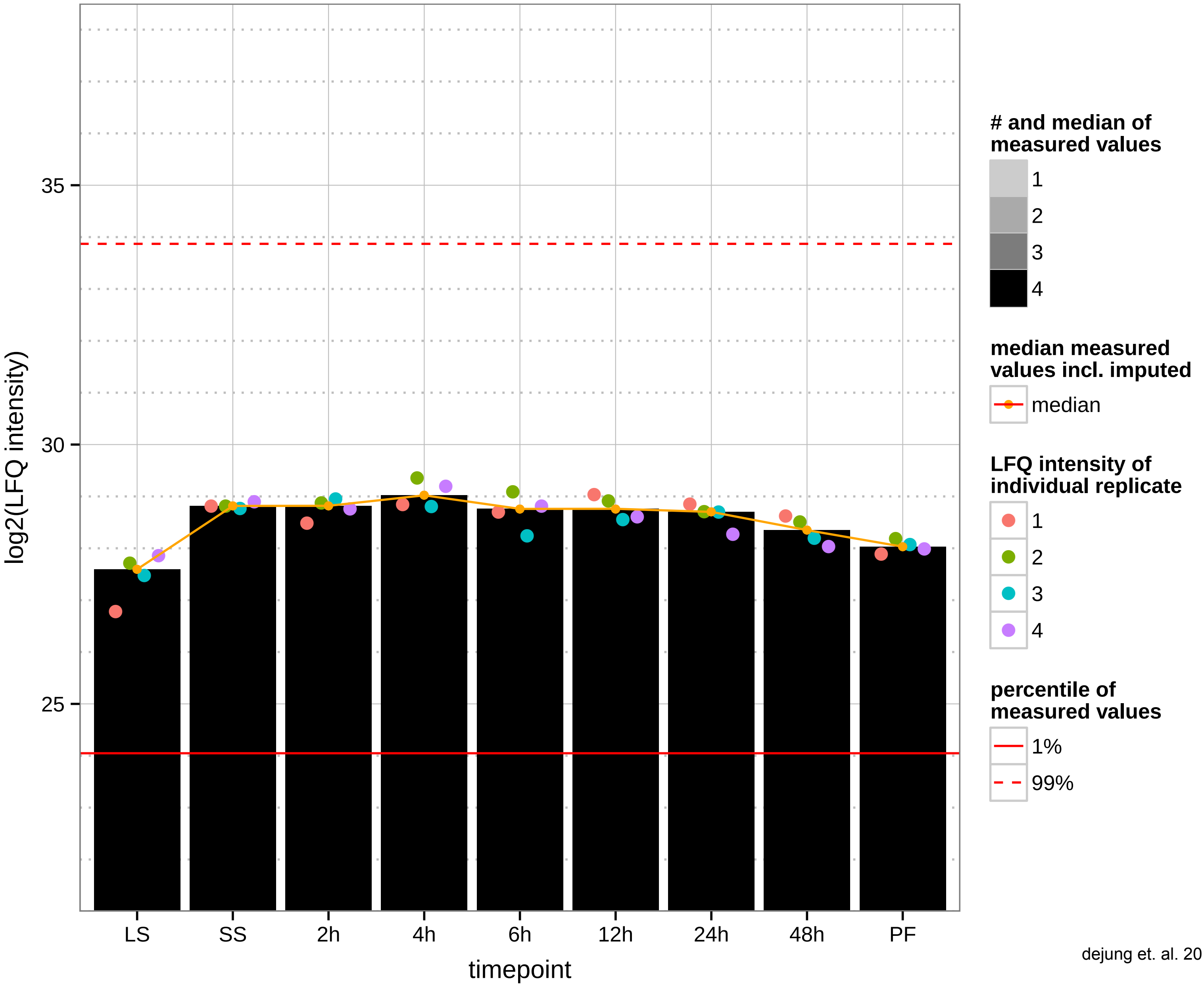

dejung et. al. 2015

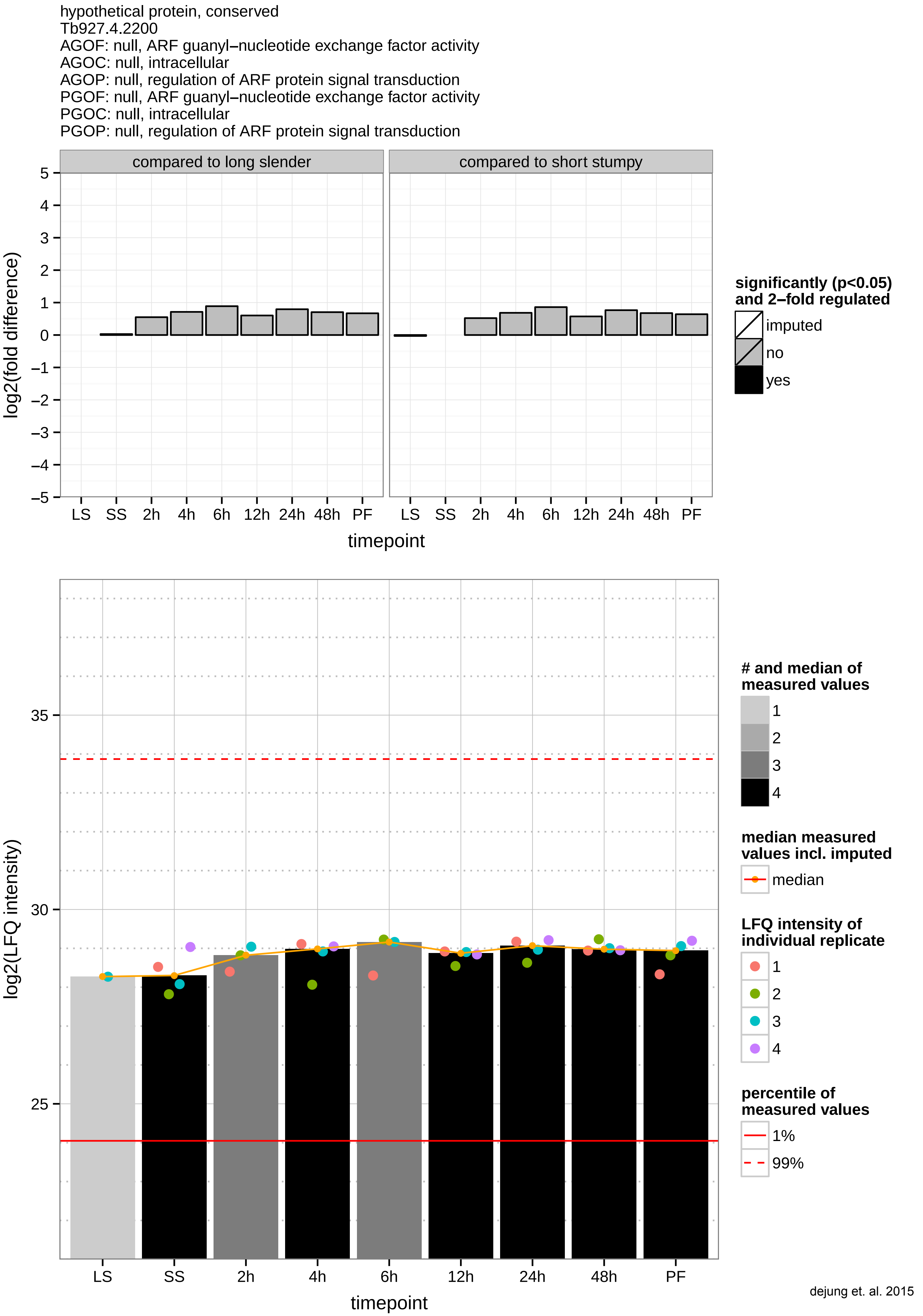
hypothetical protein, conserved
Tb927.4.2200
AGOF: null, ARF guanyl–nucleotide exchange factor activity
AGOC: null, intracellular
AGOP: null, regulation of ARF protein signal transduction
PGOF: null, ARF guanyl–nucleotide exchange factor activity
PGOC: null, intracellular
PGOP: null, regulation of ARF protein signal transduction
compared to long slender
compared to short stumpy
log2(fold difference)
timepoint
LS SS 2h 4h 6h 12h 24h 48h PF
significantly (p<0.05) and 2–fold regulated
imputed
no
yes
log2(LFQ intensity)
# and median of measured values
median measured values incl. imputed
median
LFQ intensity of individual replicate
percentile of measured values
1%
99%
dejung et. al. 2015

hypothetical protein, conserved
Tb927.4.2210
AGOF: null
AGOC: null
AGOP: null
PGOF: null
PGOC: null
PGOP: null

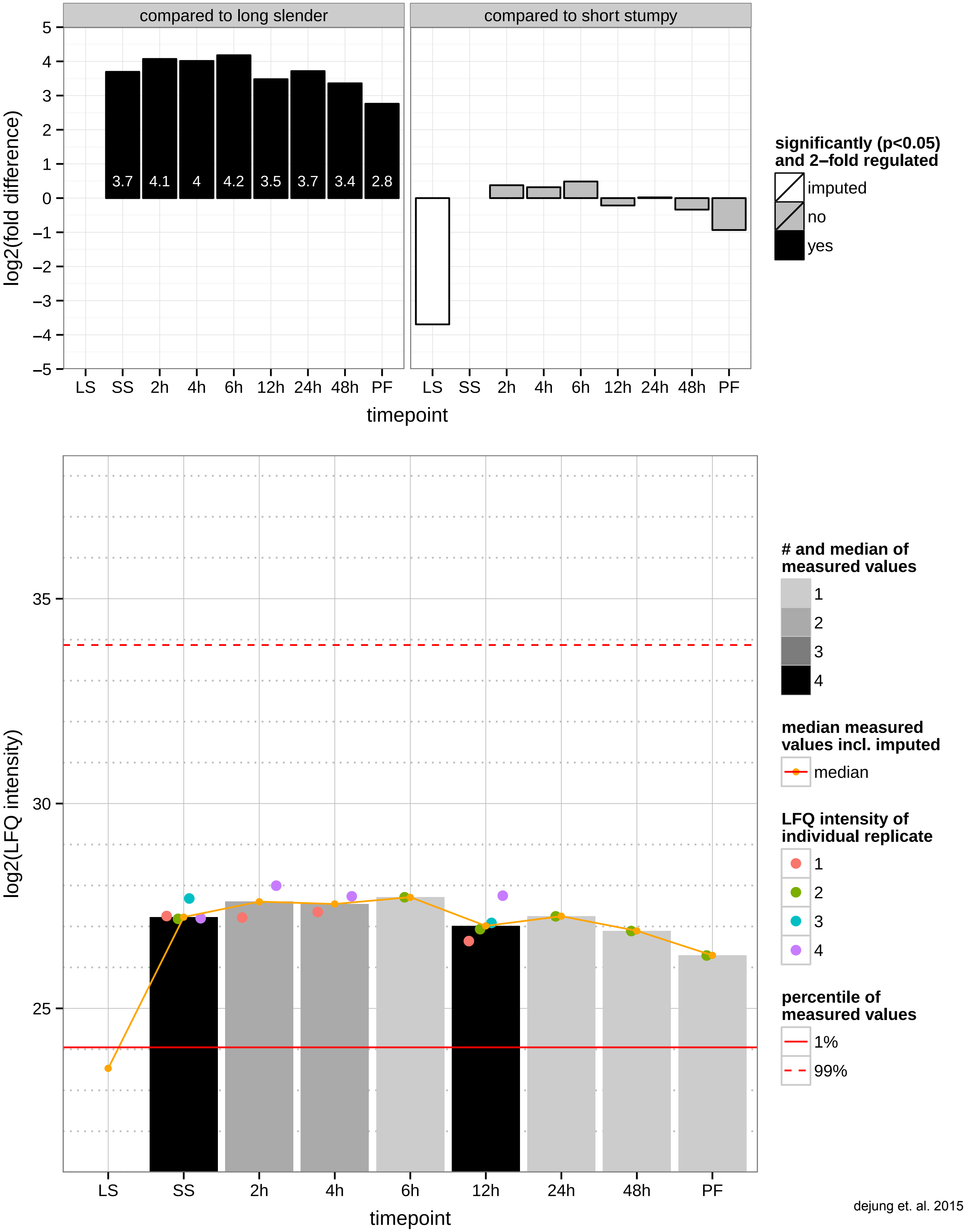

dejung et. al. 2015

dolichyl−P−Man:GDP−Man1GlcNAc2−PP−dolichyl alpha−1, 3−mannosyltransferase, putative
Tb927.4.2230
AGOF: GDP−Man:Man1GlcNAc2−PP−Dol alpha−1, 3−mannosyltransferase activity
AGOC: null
AGOP: biosynthetic process, dolichol−linked oligosaccharide biosynthetic process
PGOF: null
PGOC: null
PGOP: biosynthetic process

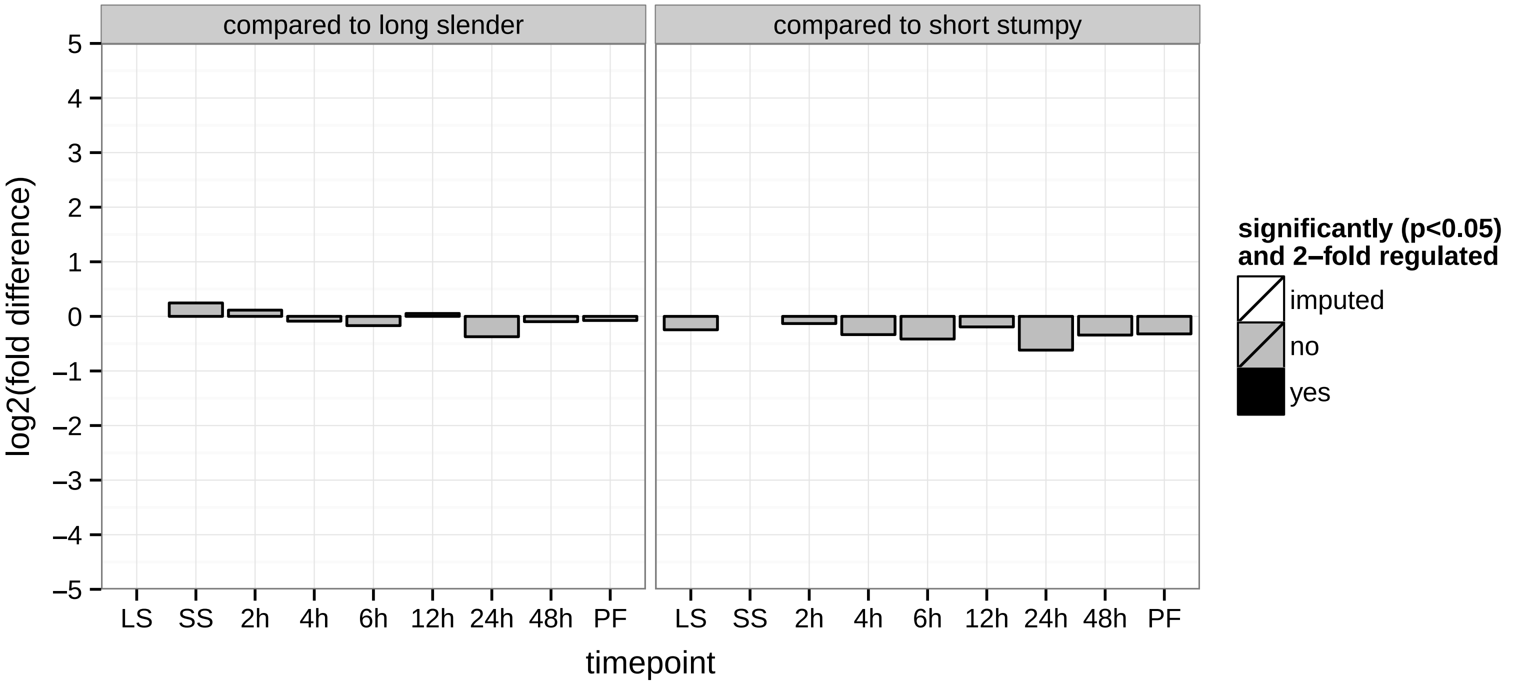

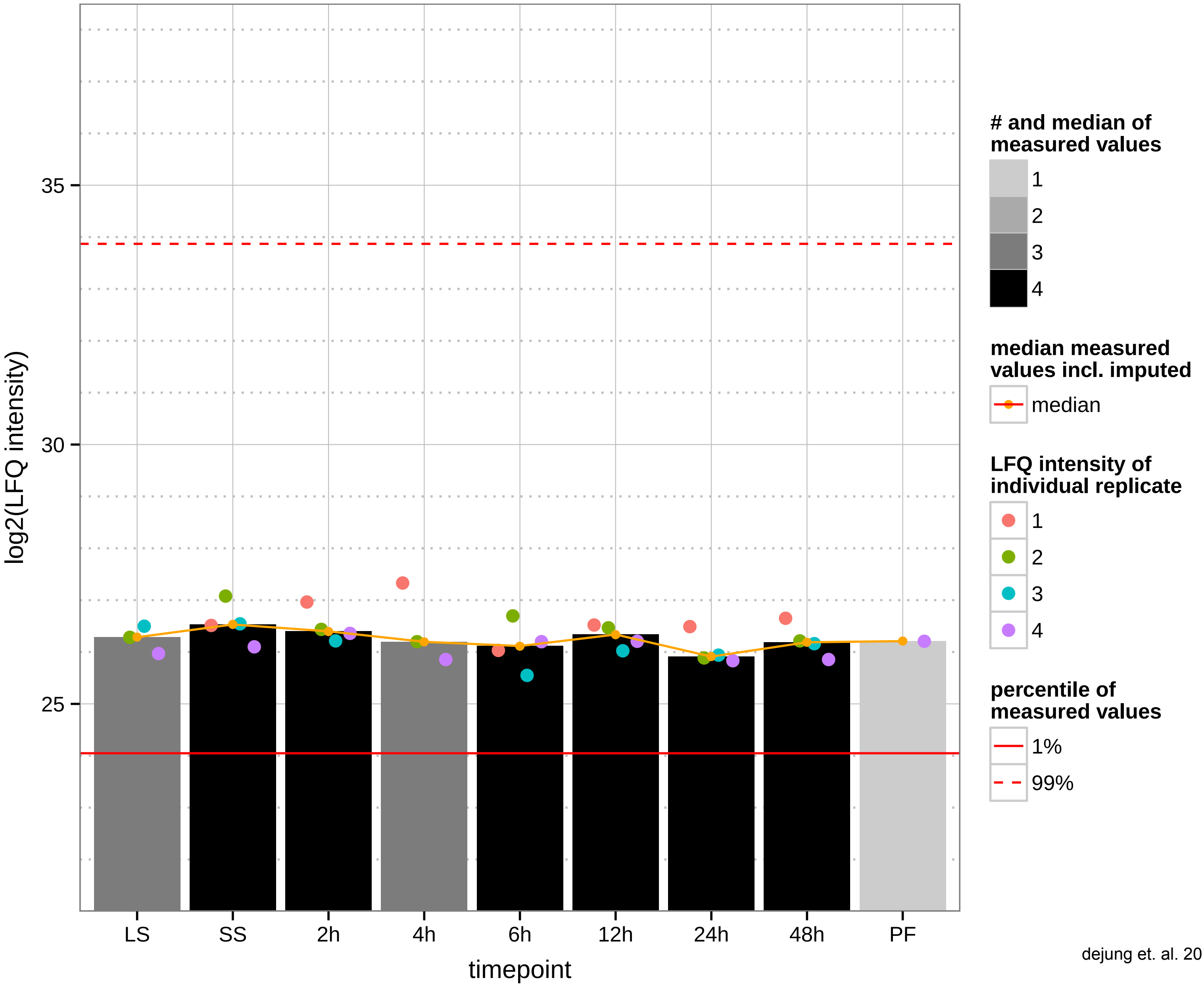

dejung et. al. 2015

hypothetical protein, conserved
Tb927.4.2300
AGOF: null
AGOC: null
AGOP: null
PGOF: null
PGOC: null
PGOP: null

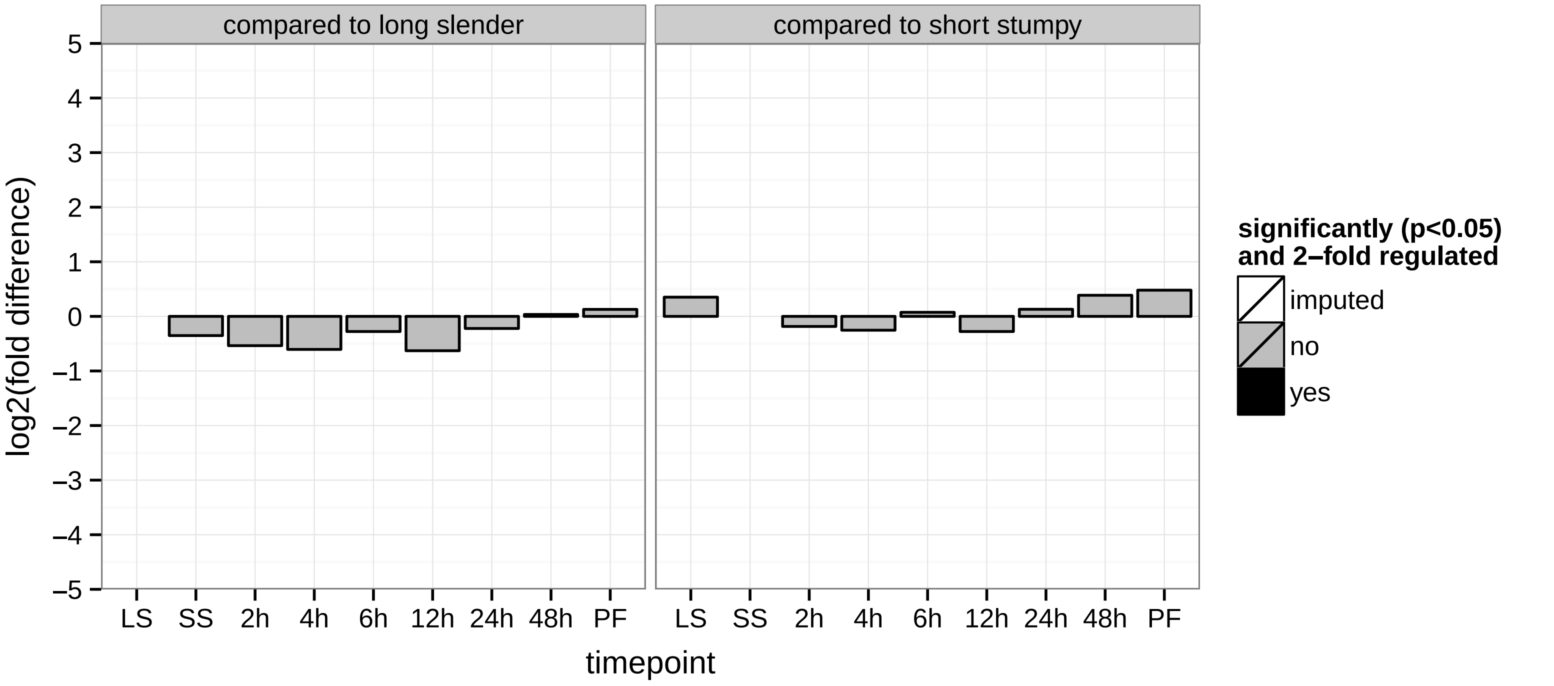

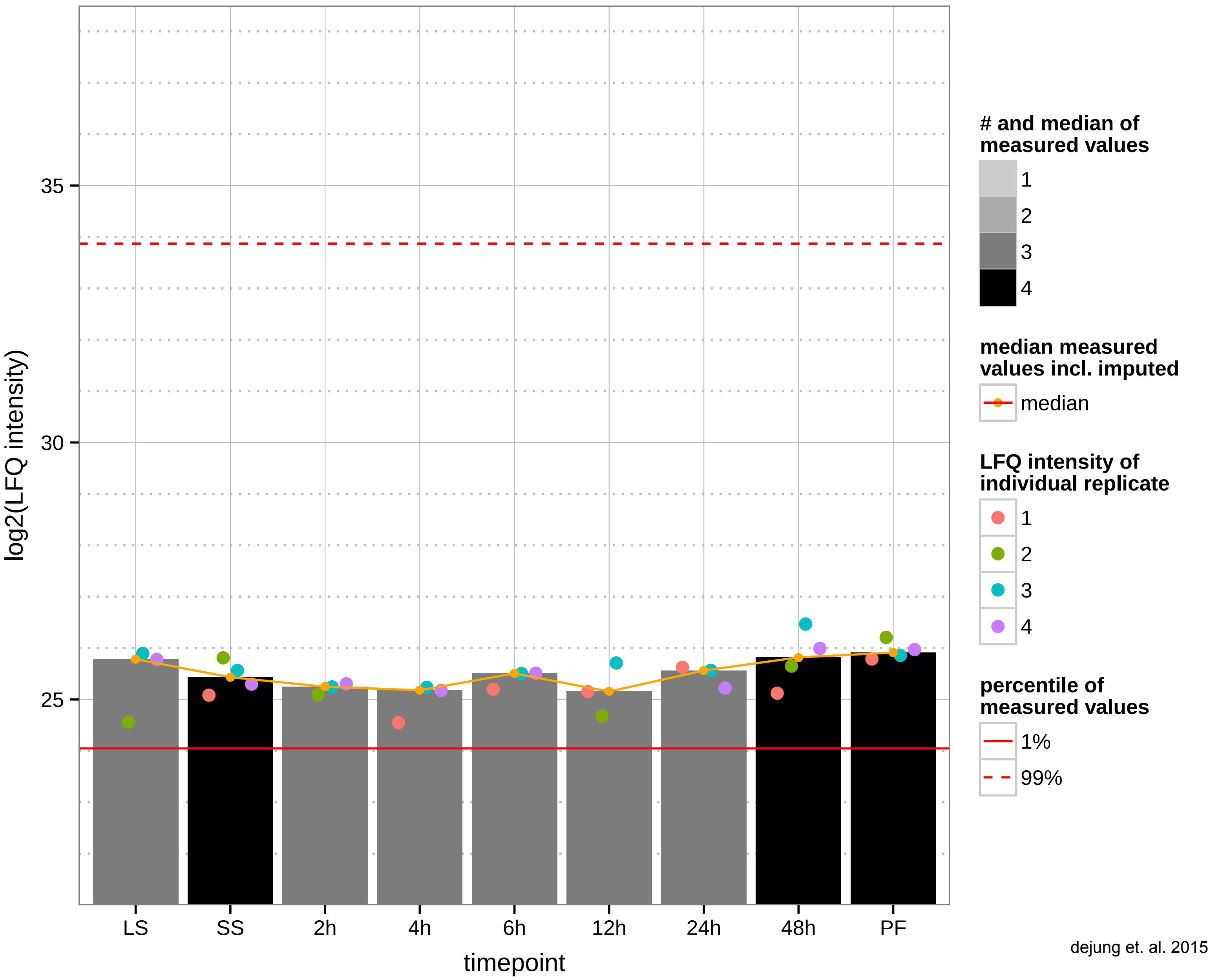

dejung et. al. 2015

asparaginyl–tRNA synthetase, putative
Tb927.4.2310
AGOF: ATP binding, asparagine–tRNA ligase activity, aspartate–tRNA ligase activity, nucleic acid binding
AGOC: cytoplasm, mitochondrion
AGOP: asparaginyl–tRNA aminoacylation, aspartyl–tRNA aminoacylation, translation
PGOF: ATP binding, aminoacyl–tRNA ligase activity, asparagine–tRNA ligase activity, nucleic acid binding, nucleotide binding
PGOC: cytoplasm
PGOP: asparaginyl–tRNA aminoacylation, tRNA aminoacylation for protein translation

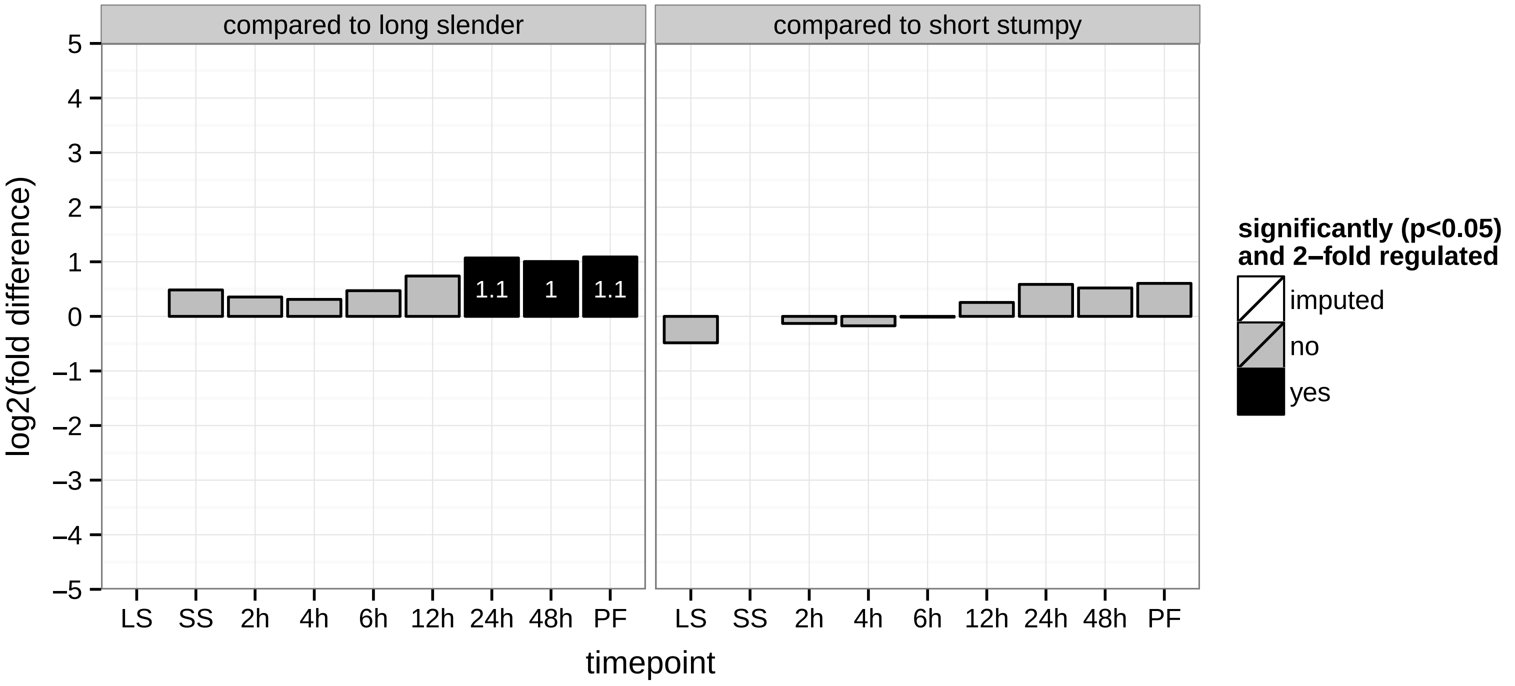

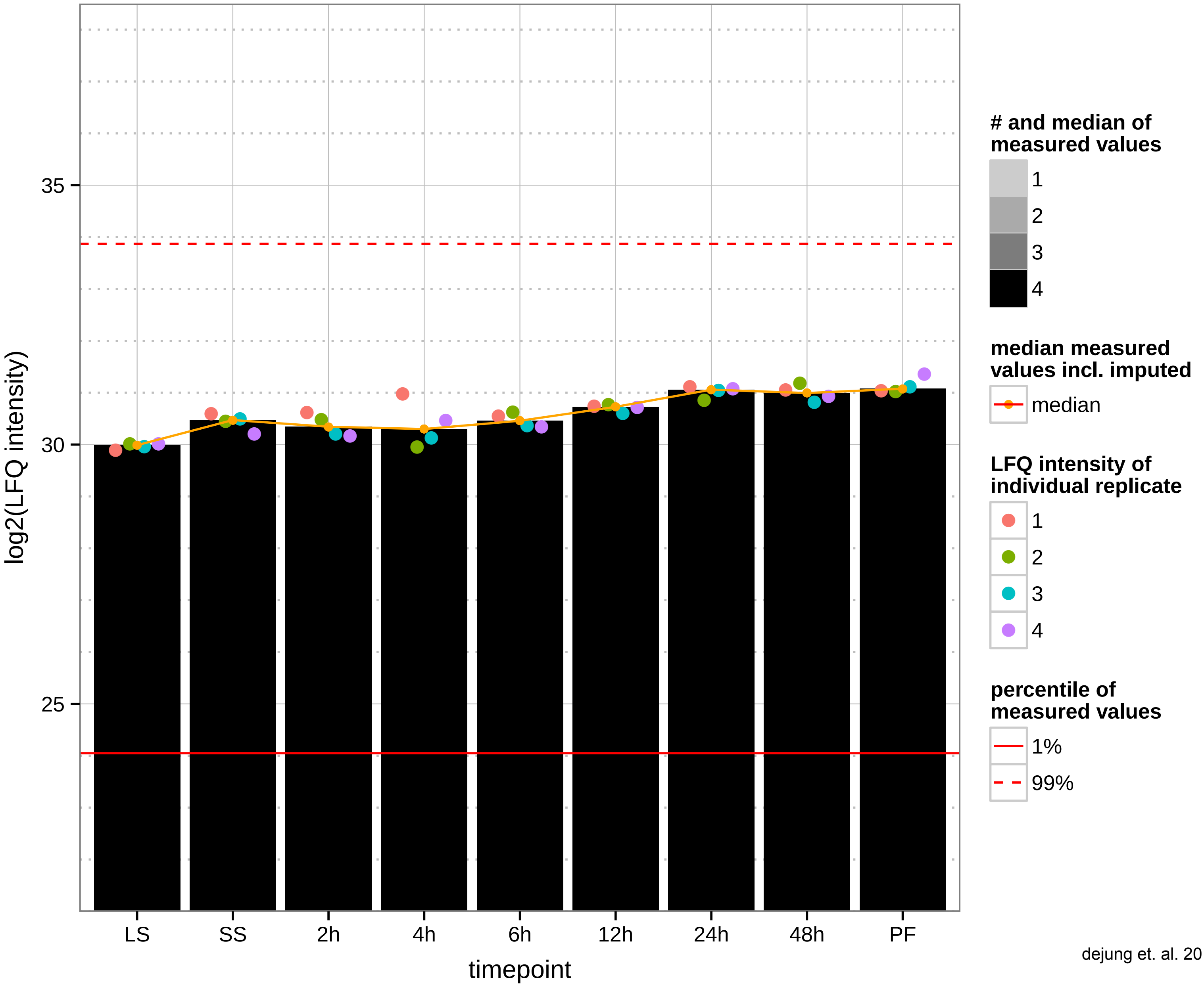

dejung et. al. 2015

hypothetical protein, conserved
Tb927.4.2340
AGOF: null
AGOC: null
AGOP: null
PGOF: null
PGOC: null
PGOP: null

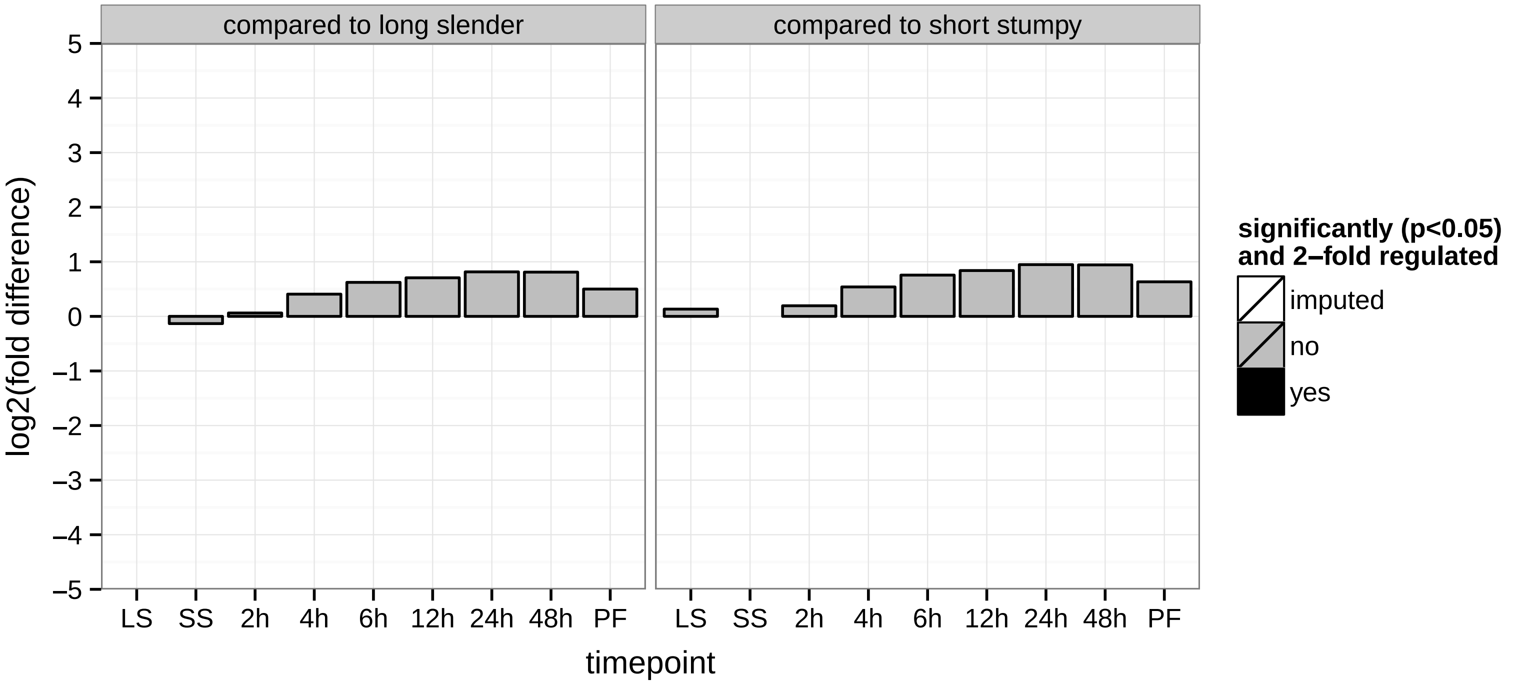

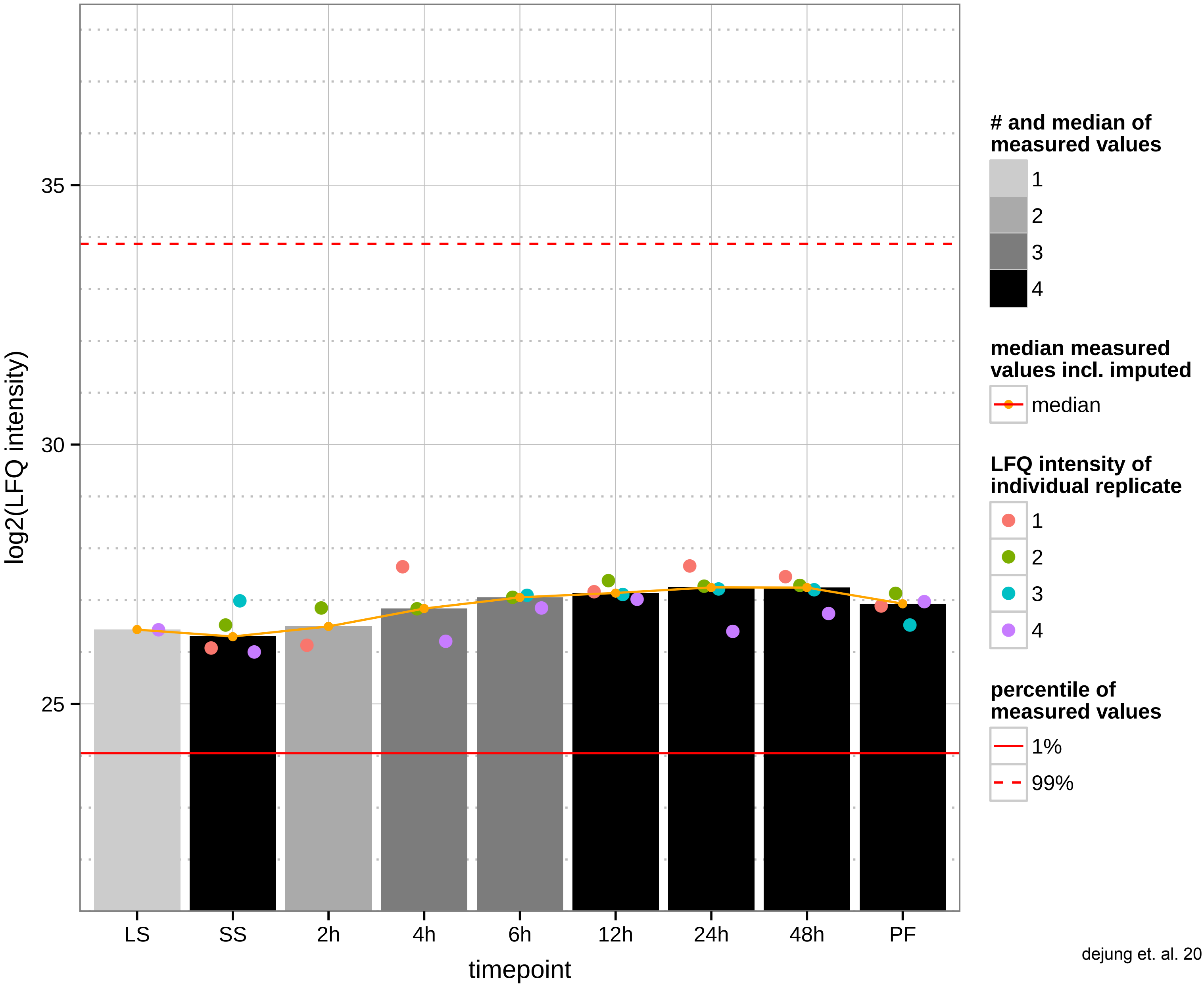

dejung et. al. 2015

hypothetical protein, conserved
Tb927.4.2530
AGOF: null
AGOC: null
AGOP: null
PGOF: null
PGOC: null
PGOP: null

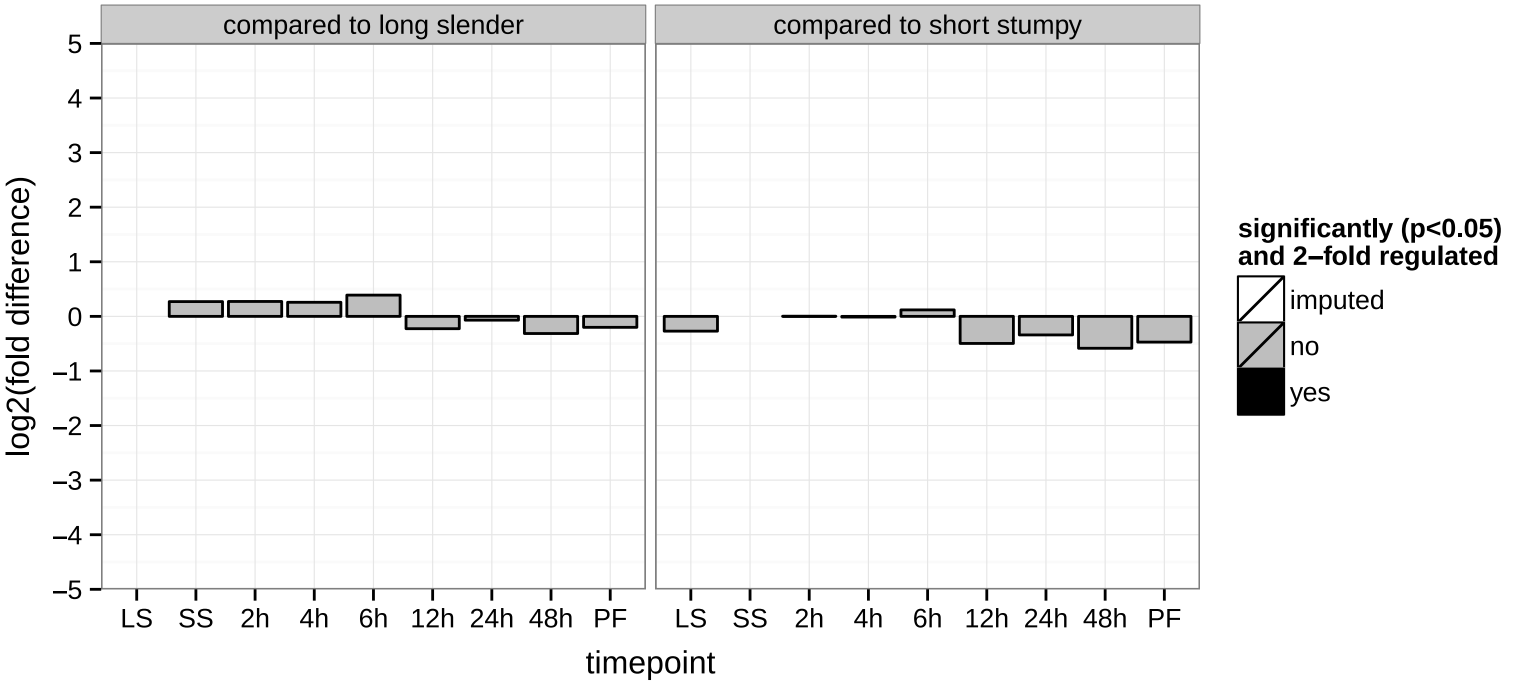

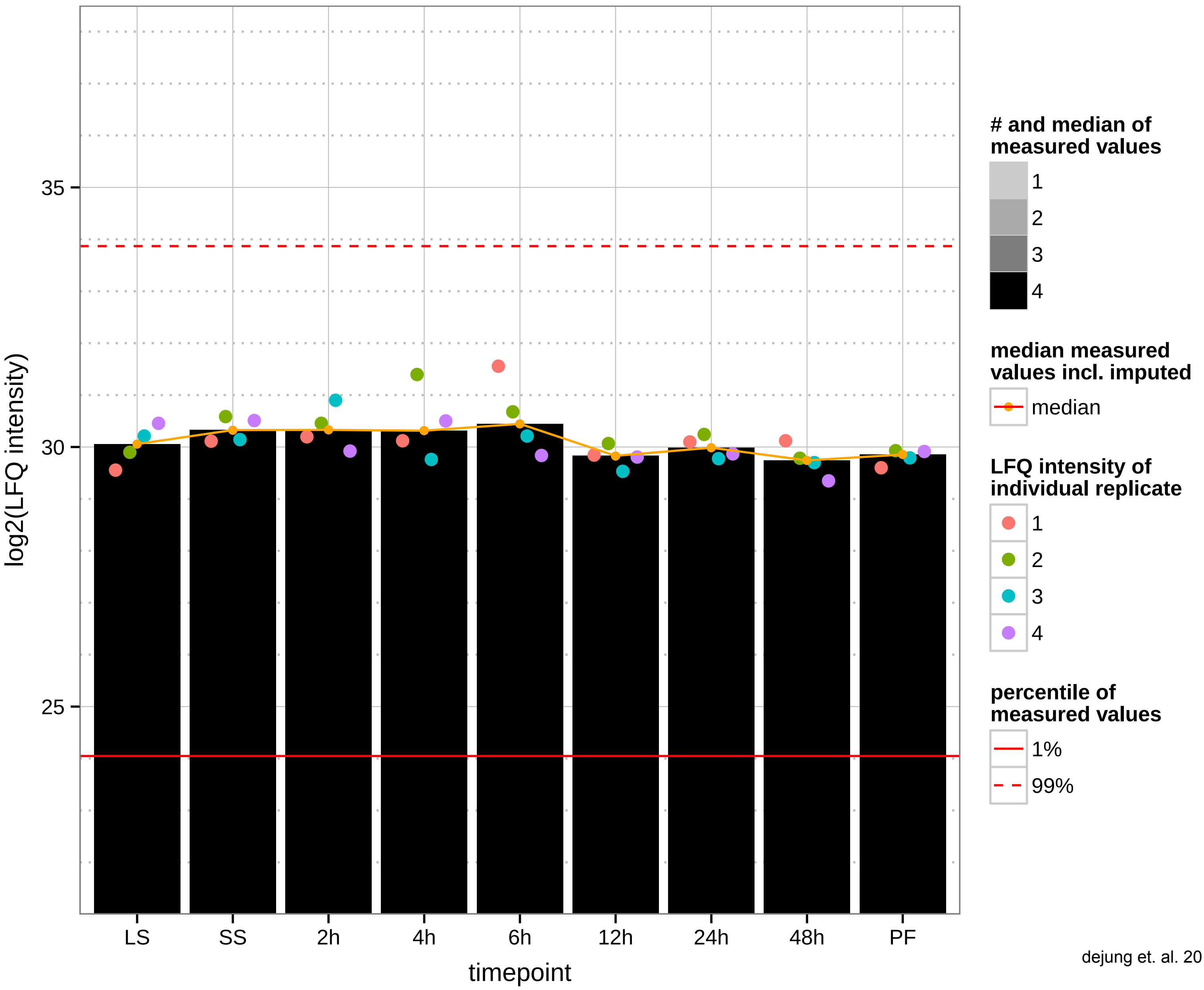

dejung et. al. 2015

hypothetical protein, conserved
Tb927.4.2600
AGOF: null
AGOC: null
AGOP: null
PGOF: null
PGOC: null
PGOP: null

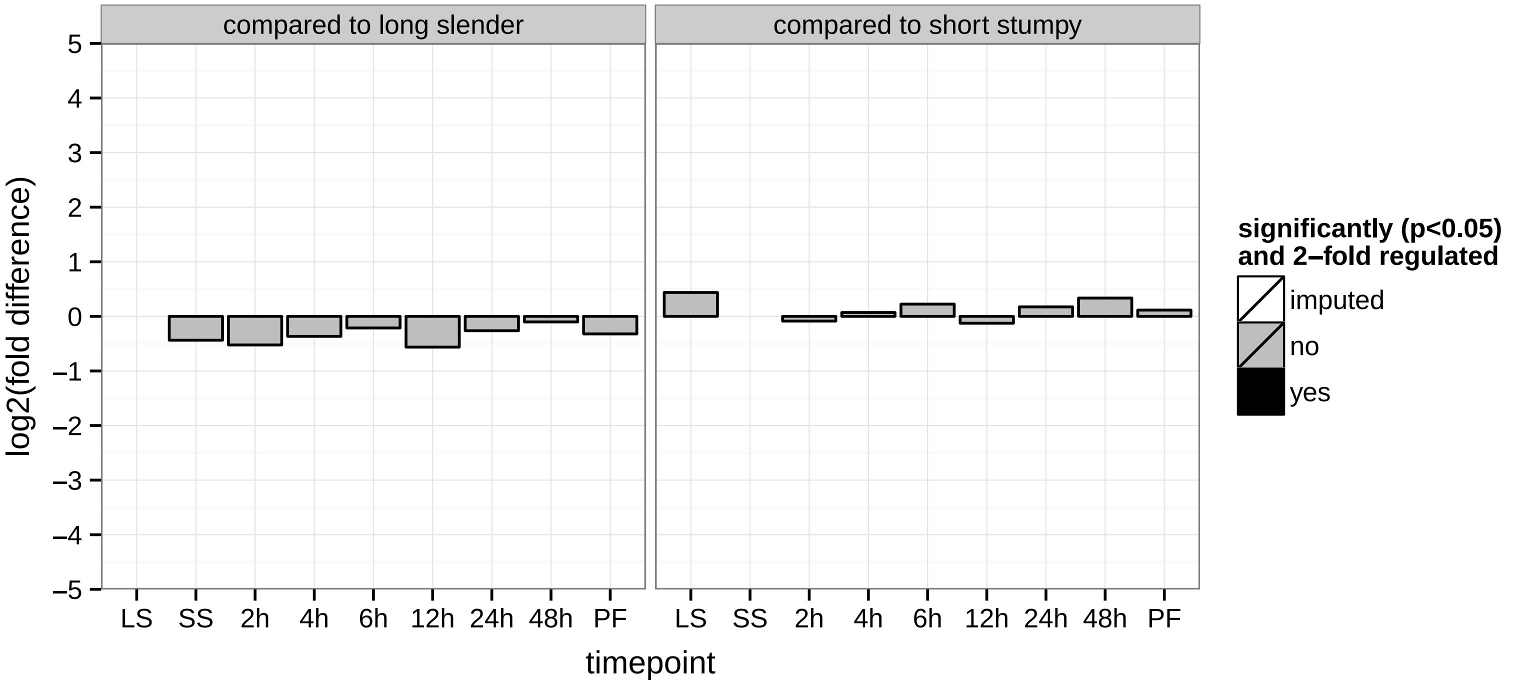

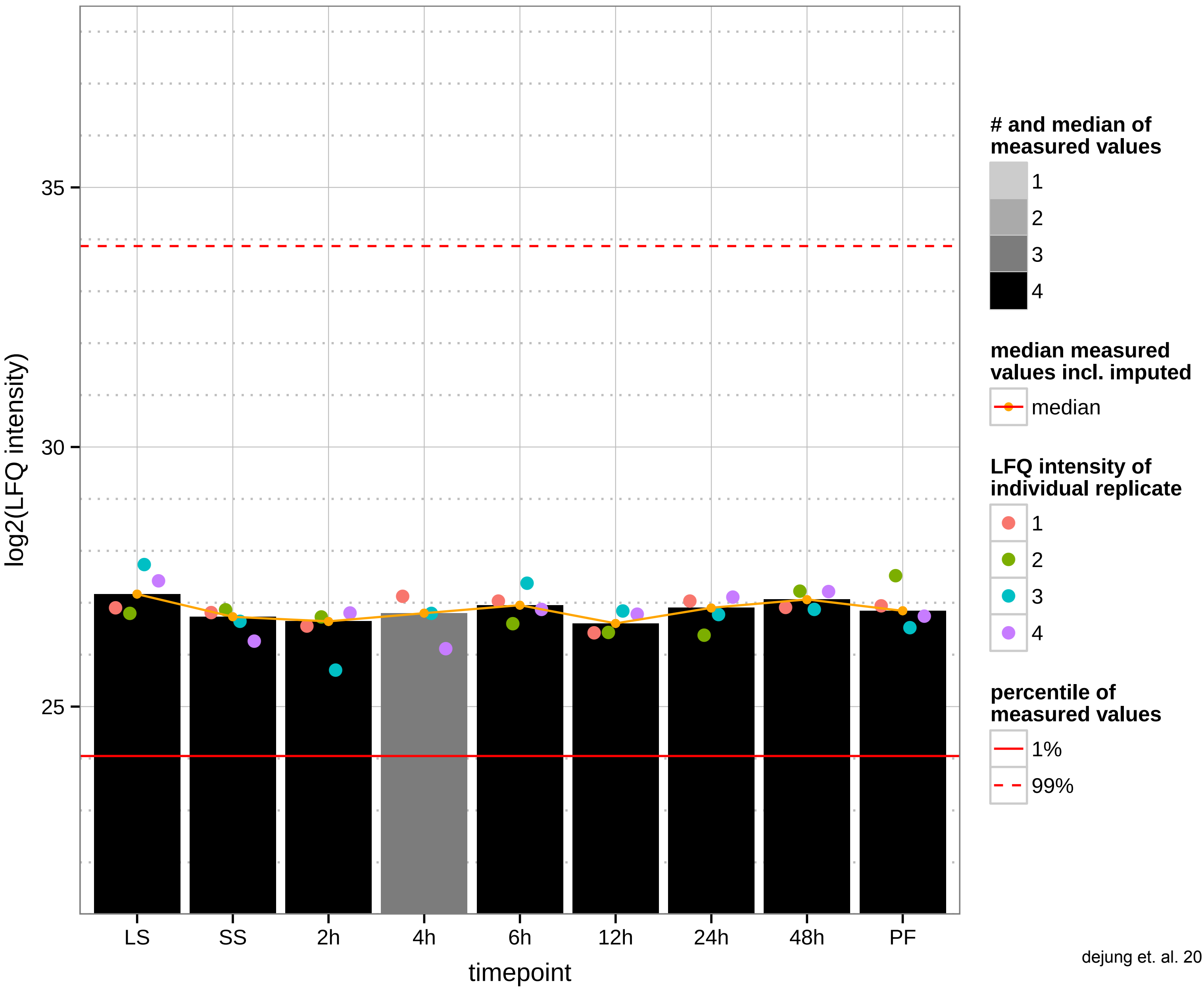

dejung et. al. 2015

hypothetical protein, conserved
Tb927.4.2650
AGOF: null
AGOC: null
AGOP: null
PGOF: null
PGOC: null
PGOP: null

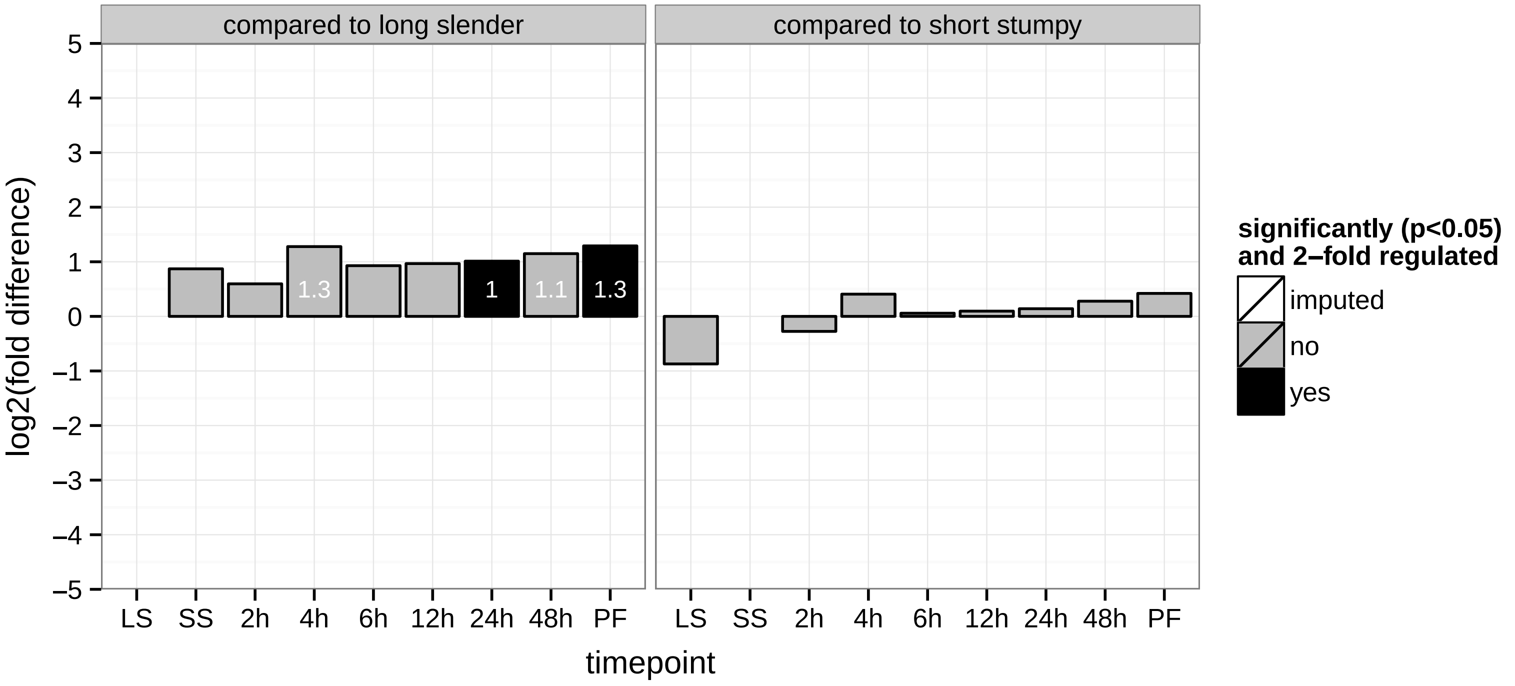

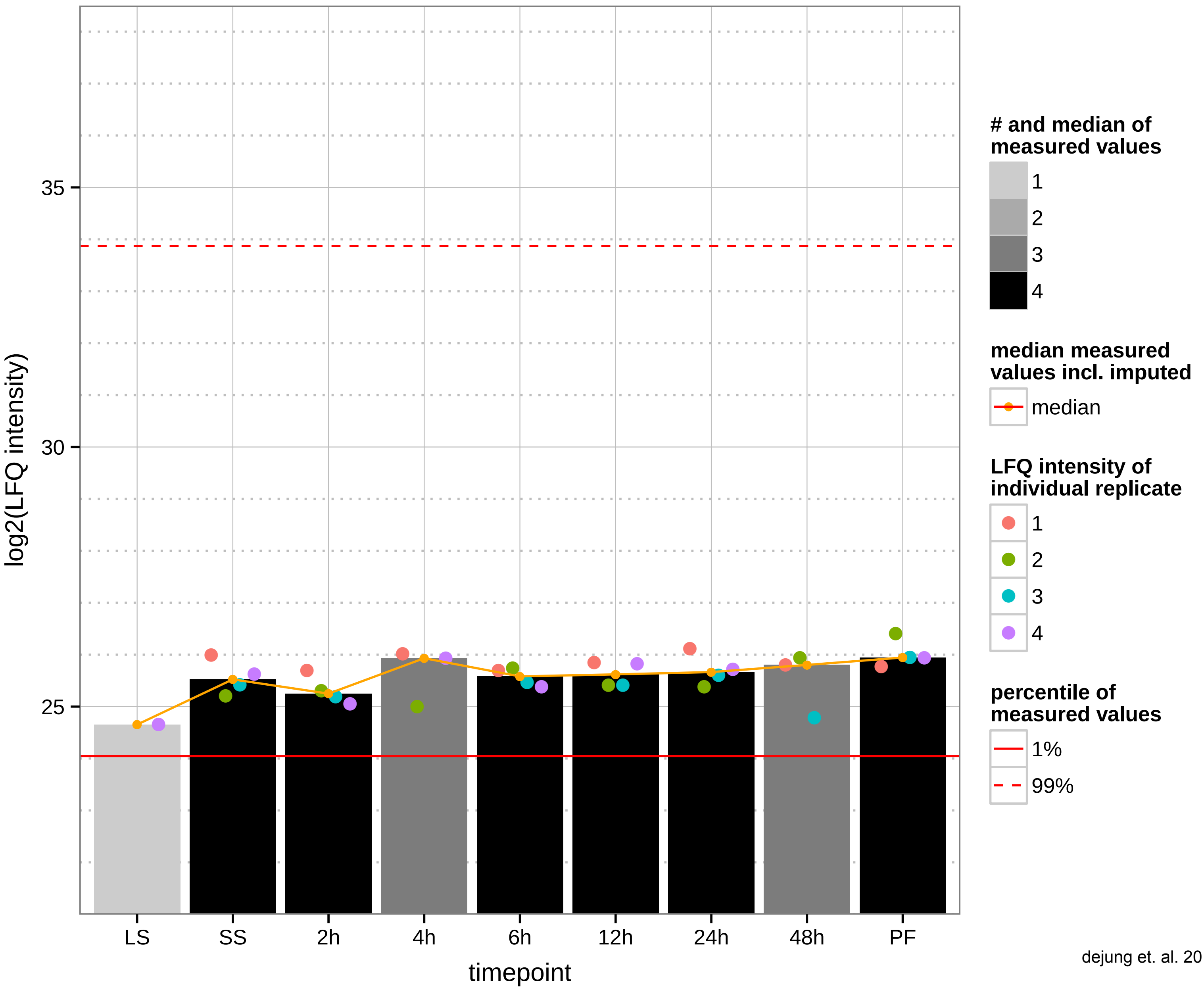

dejung et. al. 2015

hypothetical protein, conserved
Tb927.4.2840
AGOF: null
AGOC: null
AGOP: null
PGOF: null
PGOC: null
PGOP: null

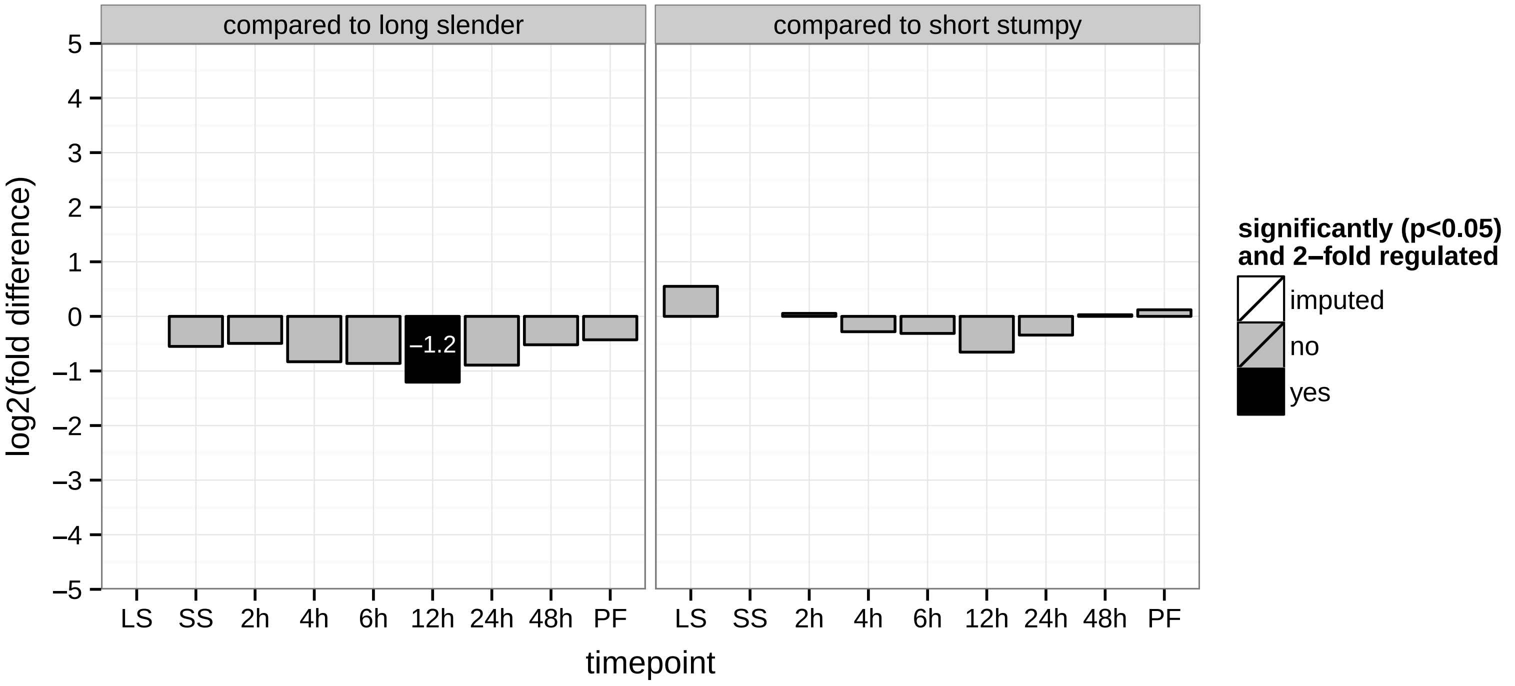

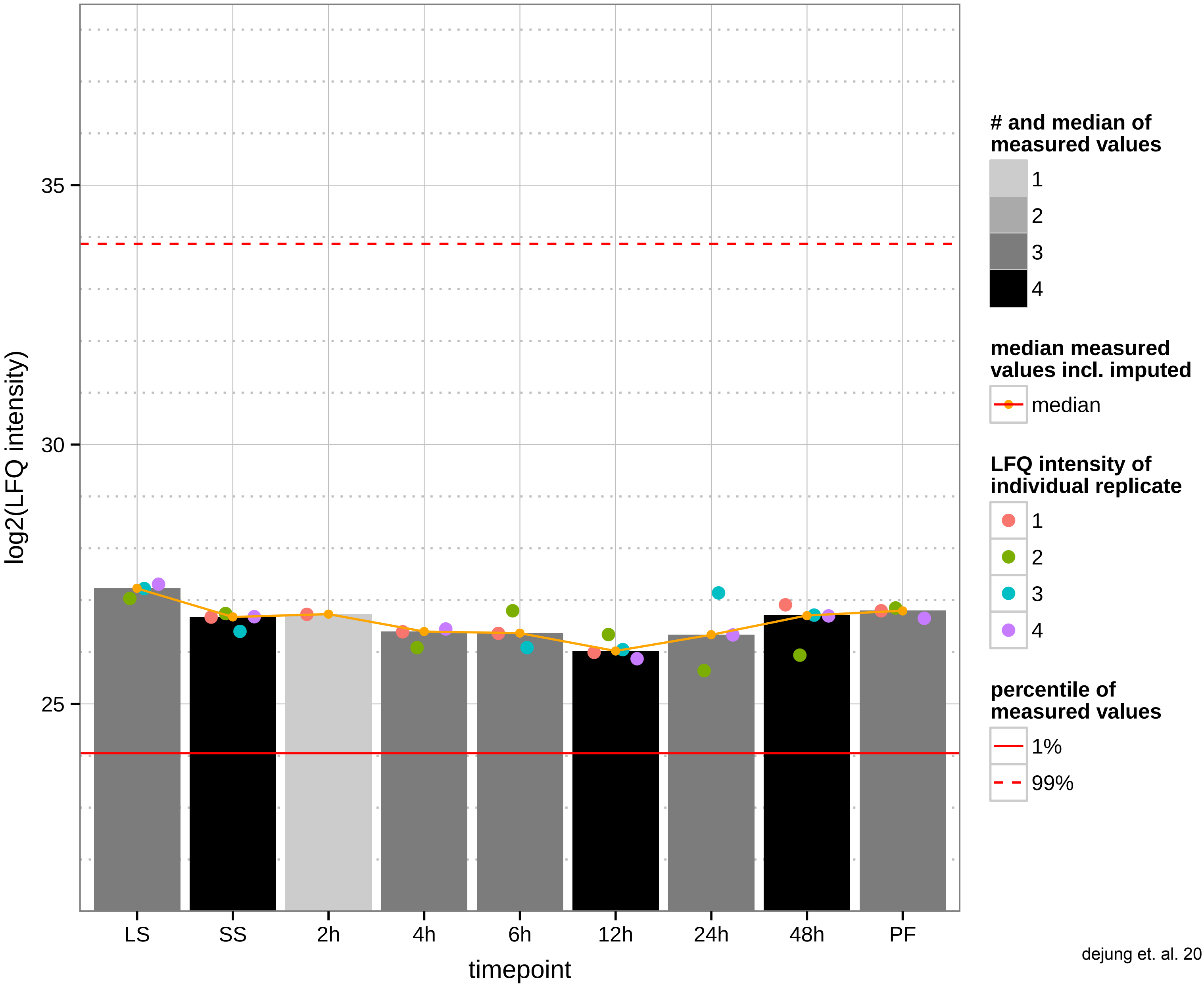

dejung et. al. 2015

hypothetical protein, conserved
Tb927.4.2850
AGOF: null
AGOC: null
AGOP: null
PGOF: null
PGOC: null
PGOP: null

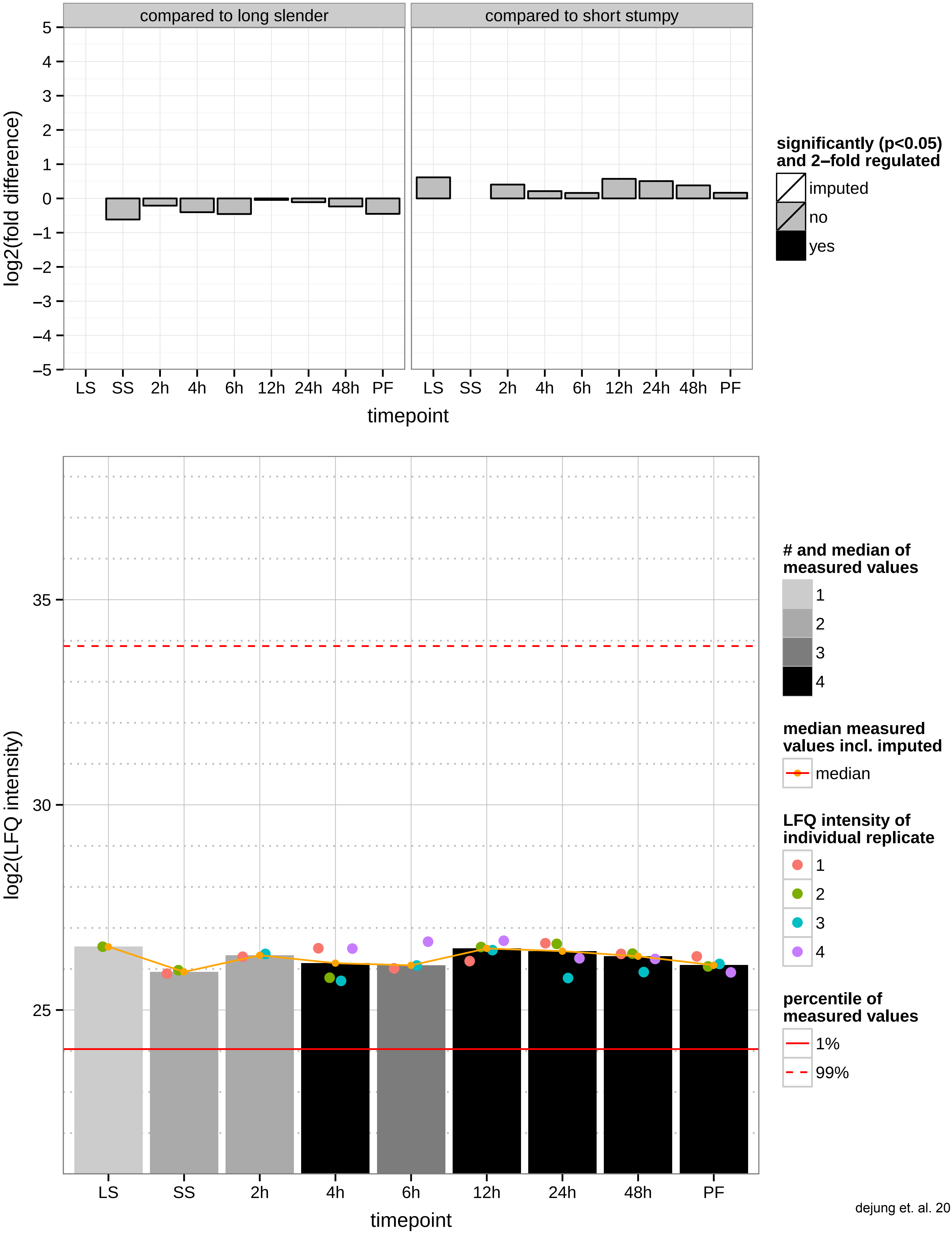

dejung et. al. 2015

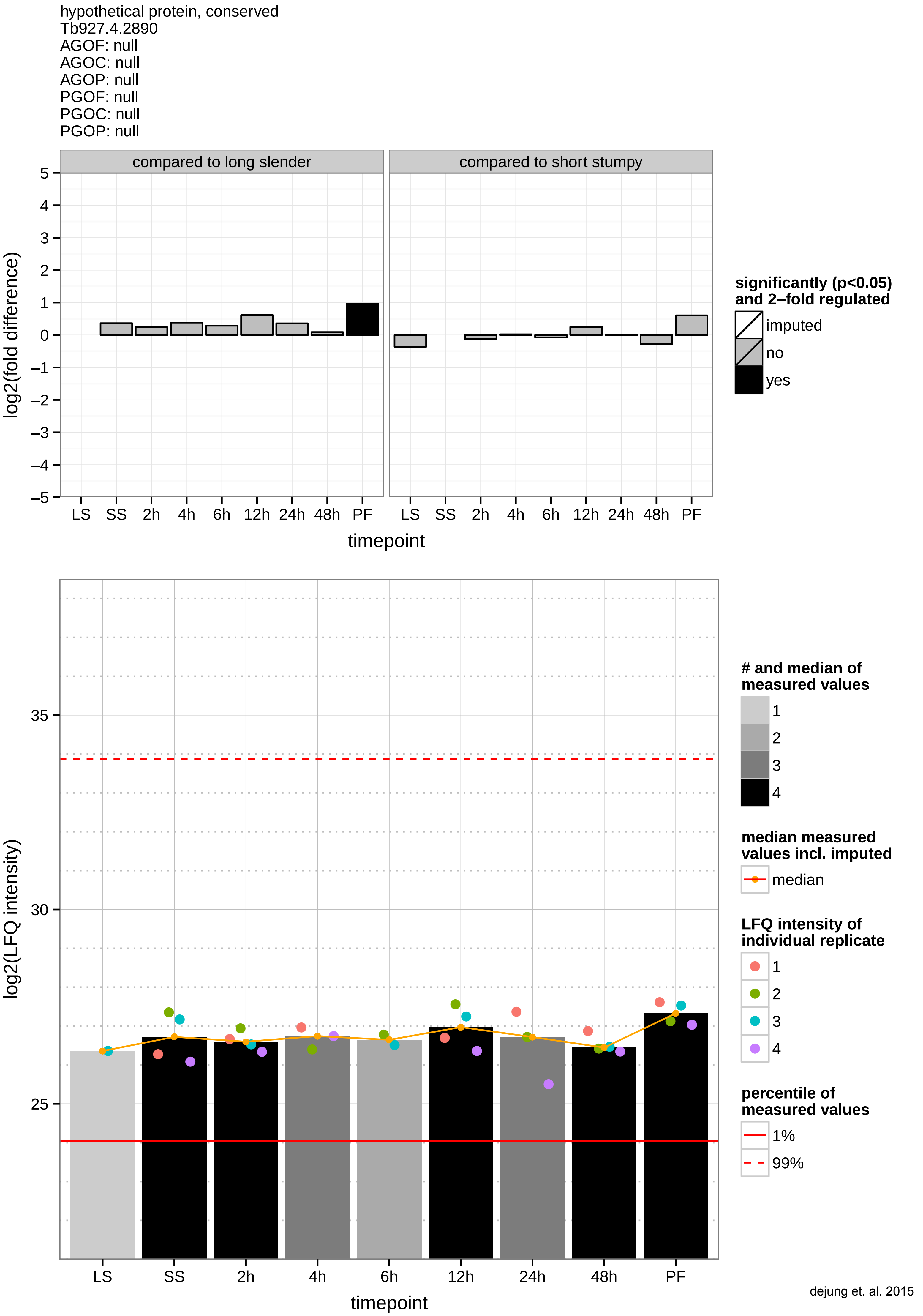
hypothetical protein, conserved
Tb927.4.2890
AGOF: null
AGOC: null
AGOP: null
PGOF: null
PGOC: null
PGOP: null
compared to long slender
compared to short stumpy
log2(fold difference)
timepoint
LS SS 2h 4h 6h 12h 24h 48h PF
significantly (p<0.05) and 2−fold regulated
imputed
no
yes
log2(LFQ intensity)
# and median of measured values
1
2
3
4
median measured values incl. imputed
median
LFQ intensity of individual replicate
percentile of measured values
1%
99%
dejung et. al. 2015

hypothetical protein, conserved
Tb927.4.3060
AGOF: null
AGOC: null
AGOP: null
PGOF: null
PGOC: null
PGOP: null

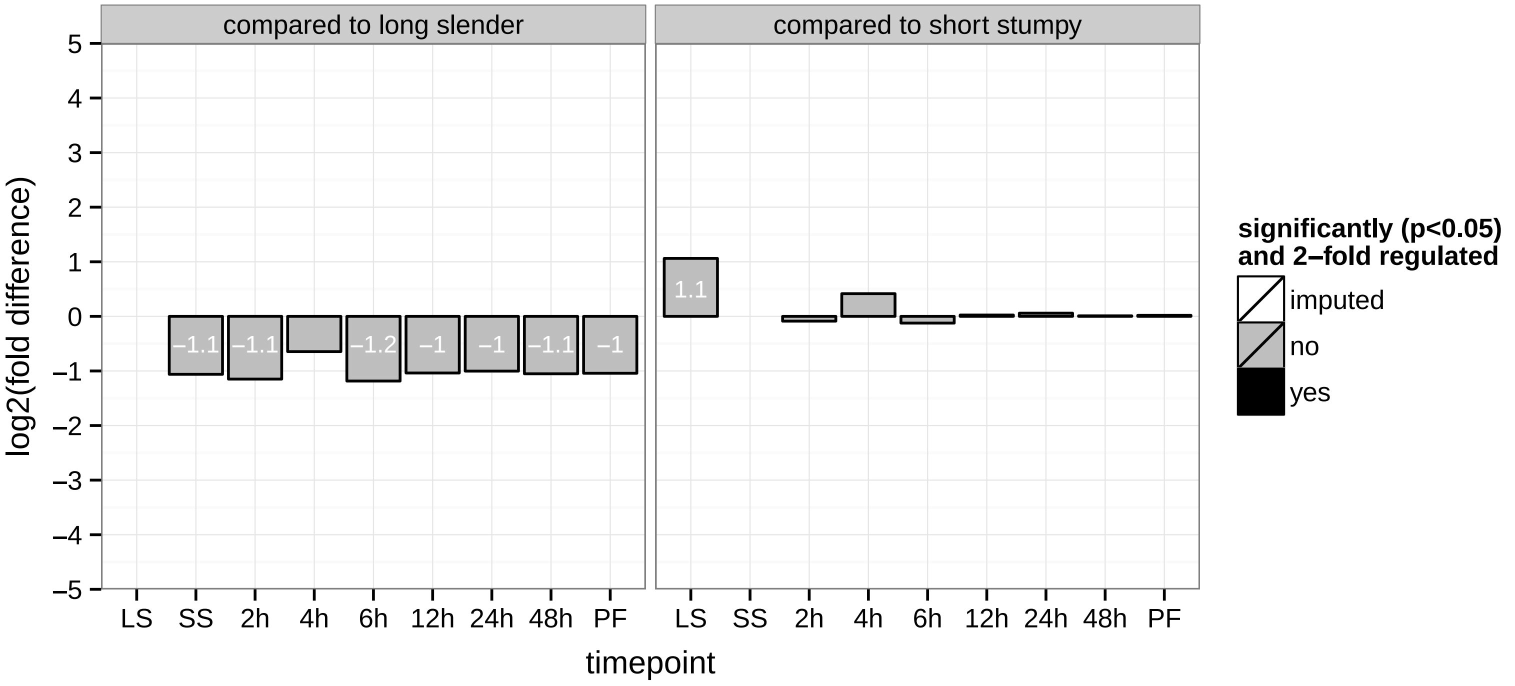

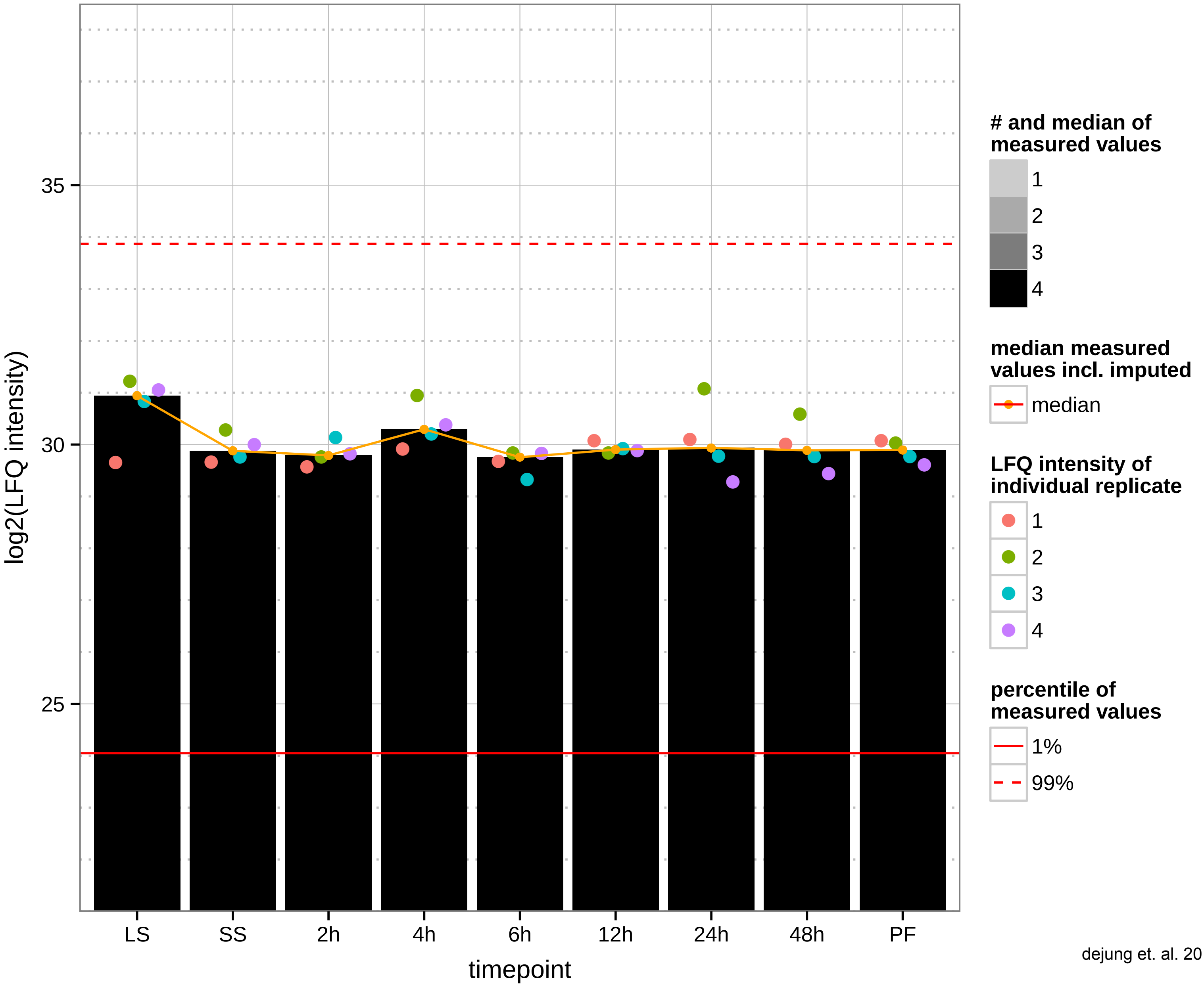

dejung et. al. 2015

hypothetical protein, conserved
Tb927.4.3150
AGOF: null
AGOC: mitochondrion
AGOP: null
PGOF: null
PGOC: null
PGOP: null

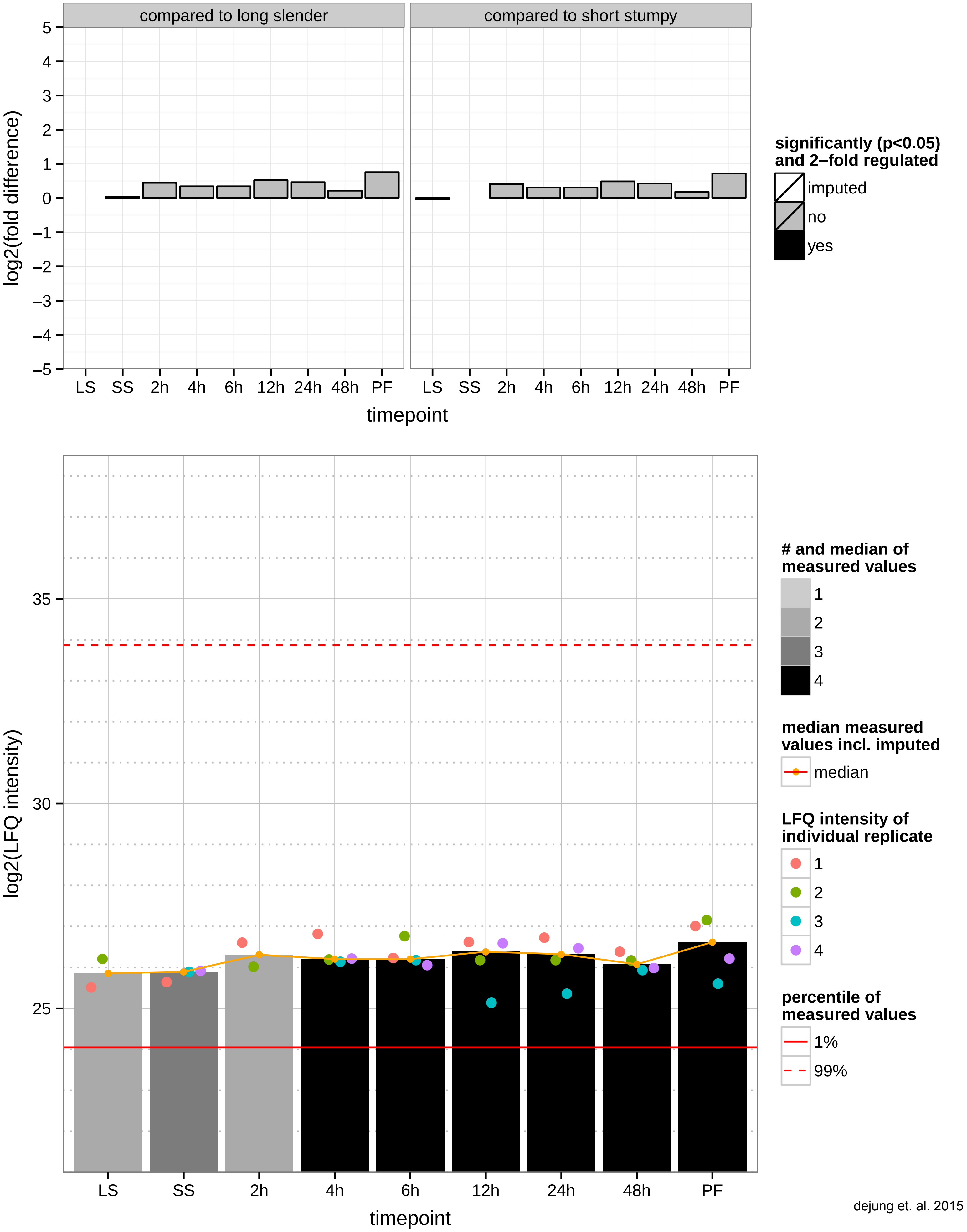

dejung et. al. 2015

dihydroxyacetone phosphate acyltransferase, putative (DAT)
Tb927.4.3160
AGOF: null
AGOC: glycosome
AGOP: ether lipid biosynthetic process
PGOF: transferase activity, transferring acyl groups
PGOC: null
PGOP: metabolic process

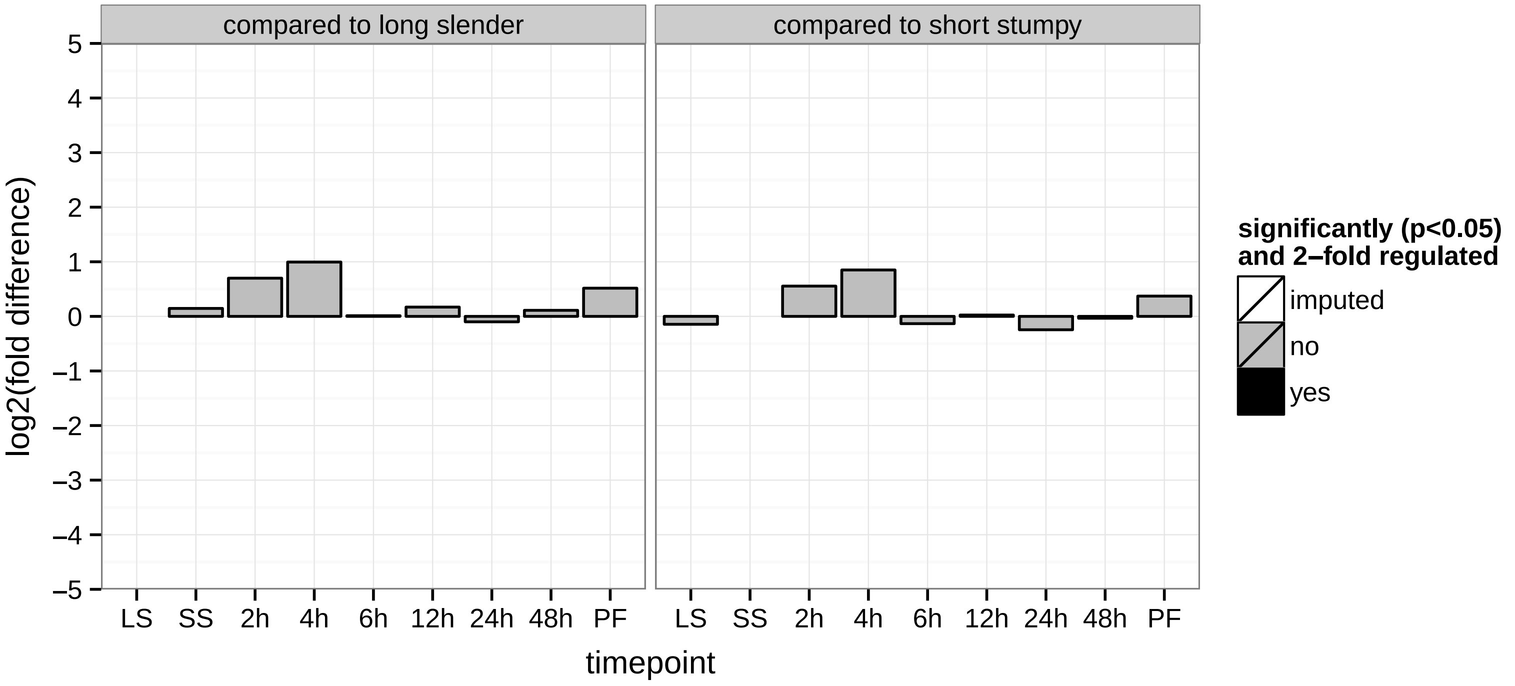

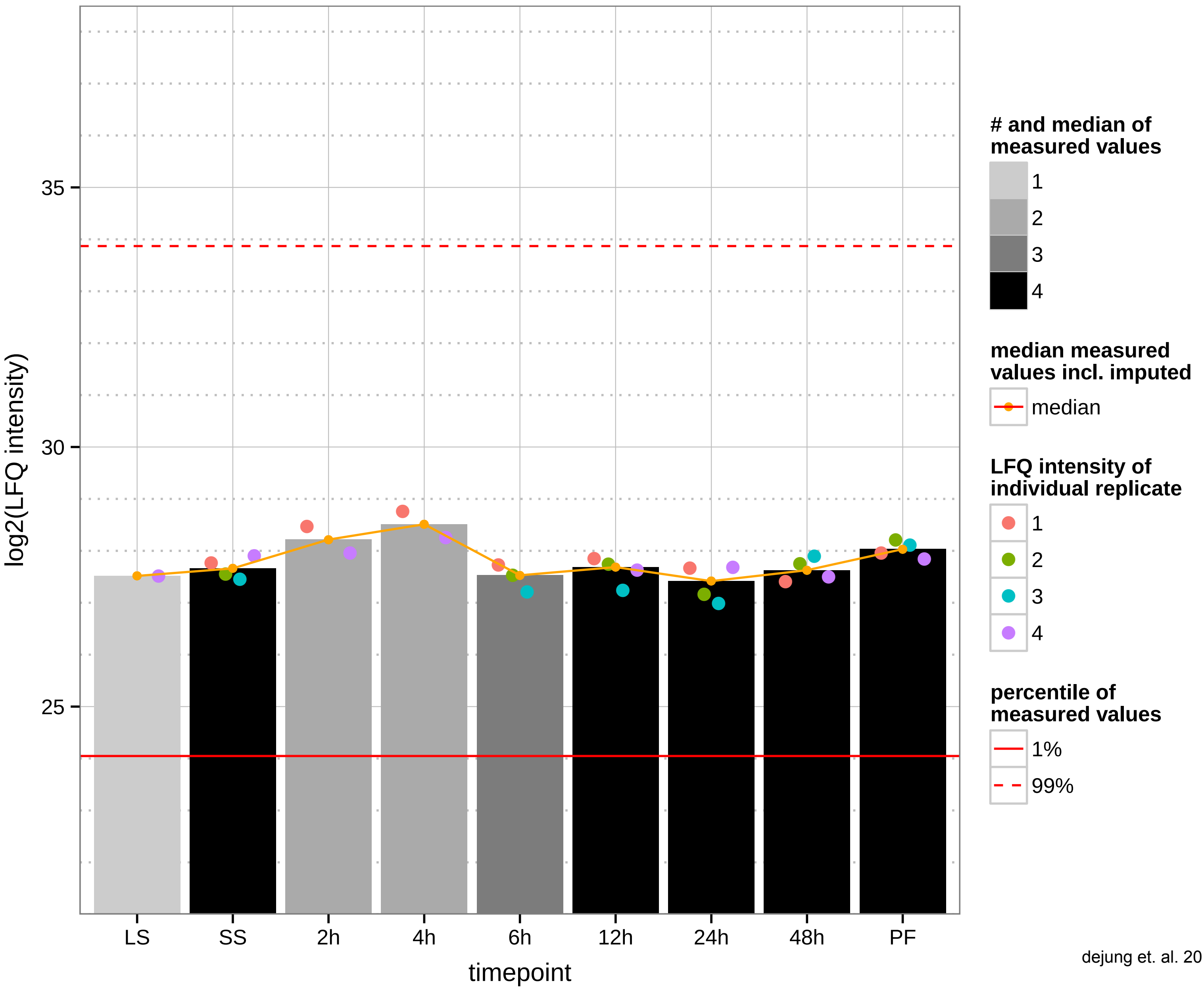

dejung et. al. 2015

hypothetical protein, conserved
Tb927.4.3180
AGOF: null
AGOC: null
AGOP: null
PGOF: protein binding
PGOC: null
PGOP: null

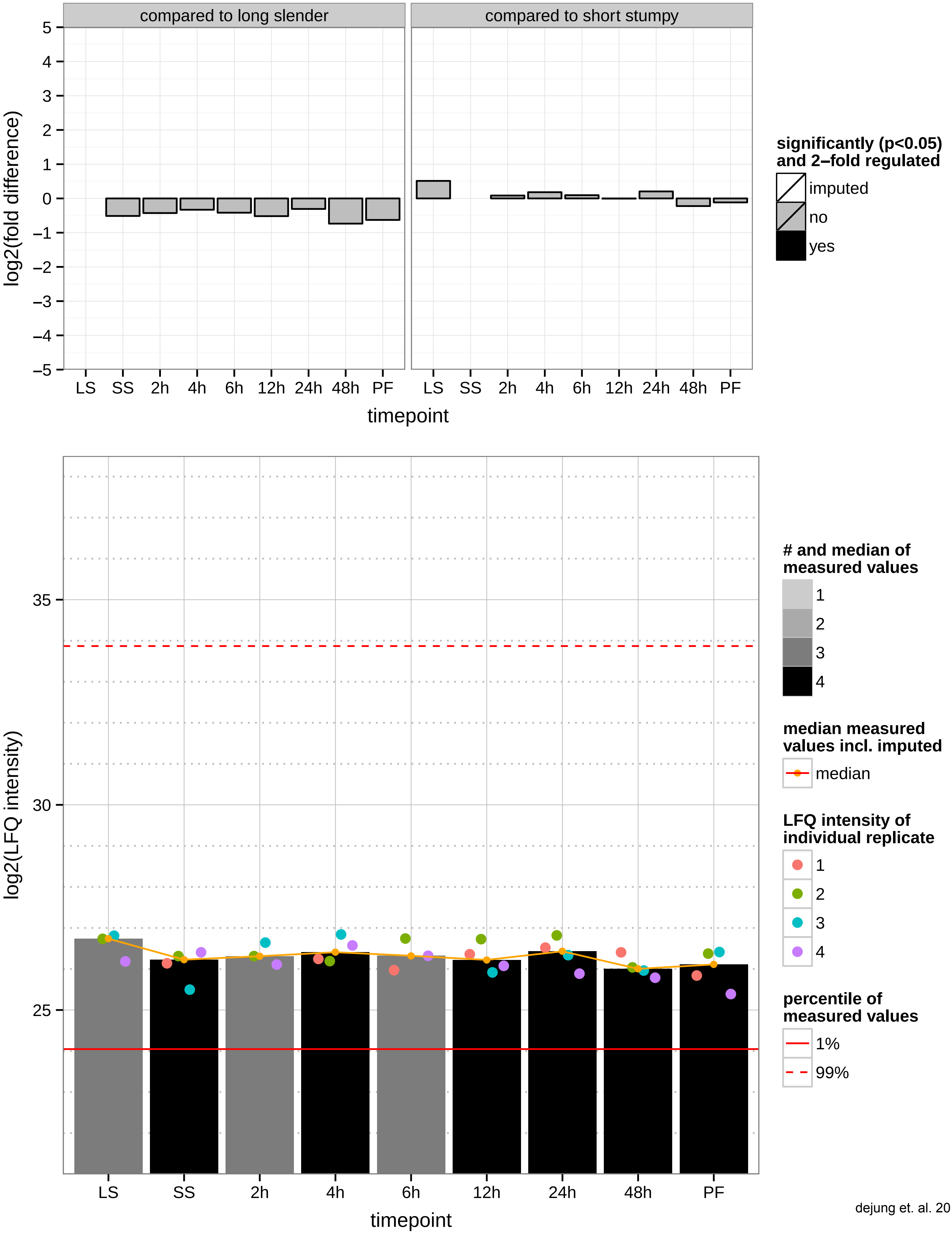

dejung et. al. 2015

hypothetical protein, conserved
Tb927.4.330
AGOF: null
AGOC: mitochondrion
AGOP: null
PGOF: null
PGOC: null
PGOP: null

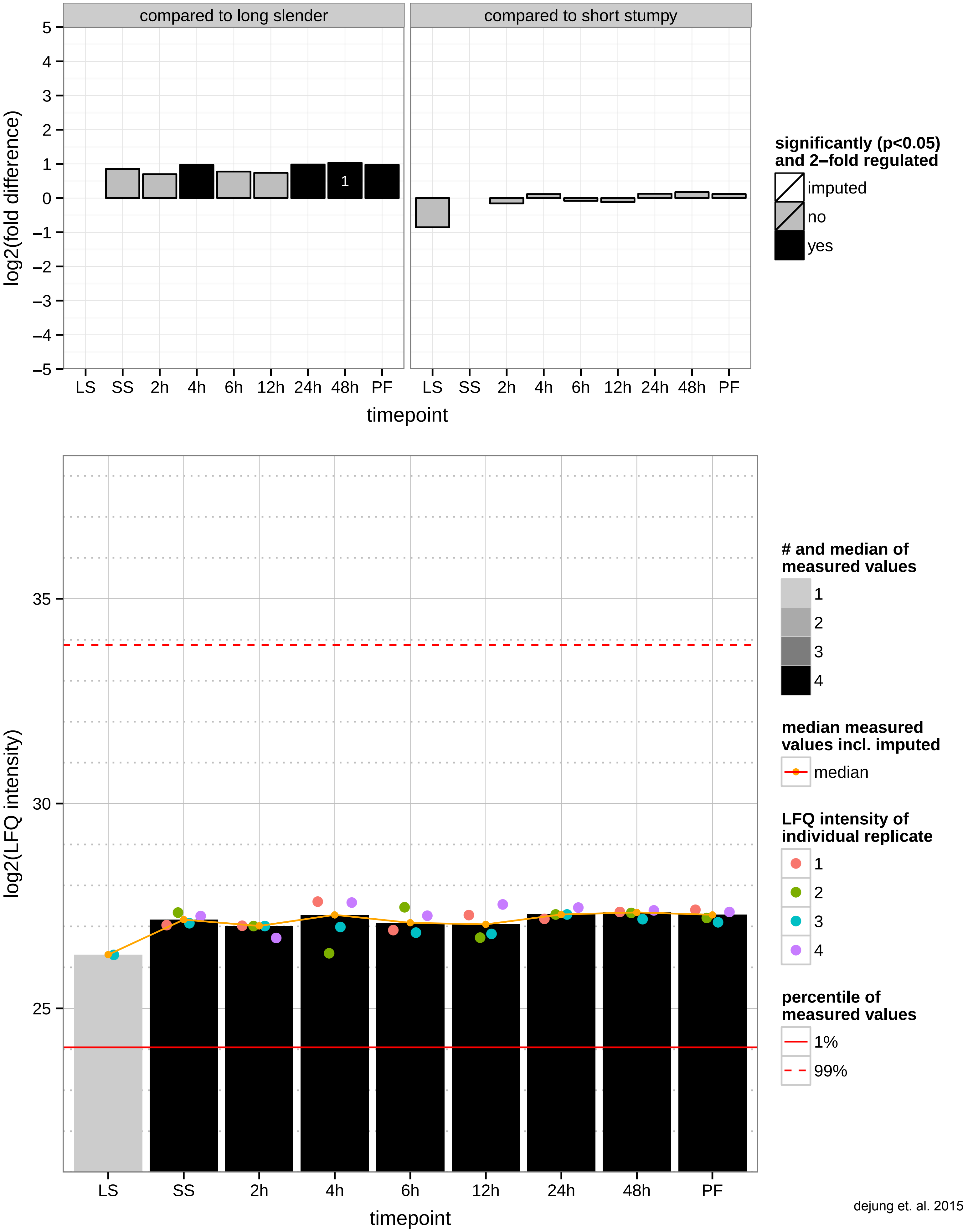

mitochondrial ATP−dependent zinc metallopeptidase, putative, metallo−peptidase, Clan MA(E) Family M41
Tb927.4.3300
AGOF: ATP binding, metalloendopeptidase activity, nucleoside−triphosphatase activity
AGOC: mitochondrial membrane, mitochondrion
AGOP: protein catabolic process, proteolysis
PGOF: ATP binding, metalloendopeptidase activity, nucleoside−triphosphatase activity, nucleotide binding
PGOC: membrane
PGOP: protein catabolic process, proteolysis

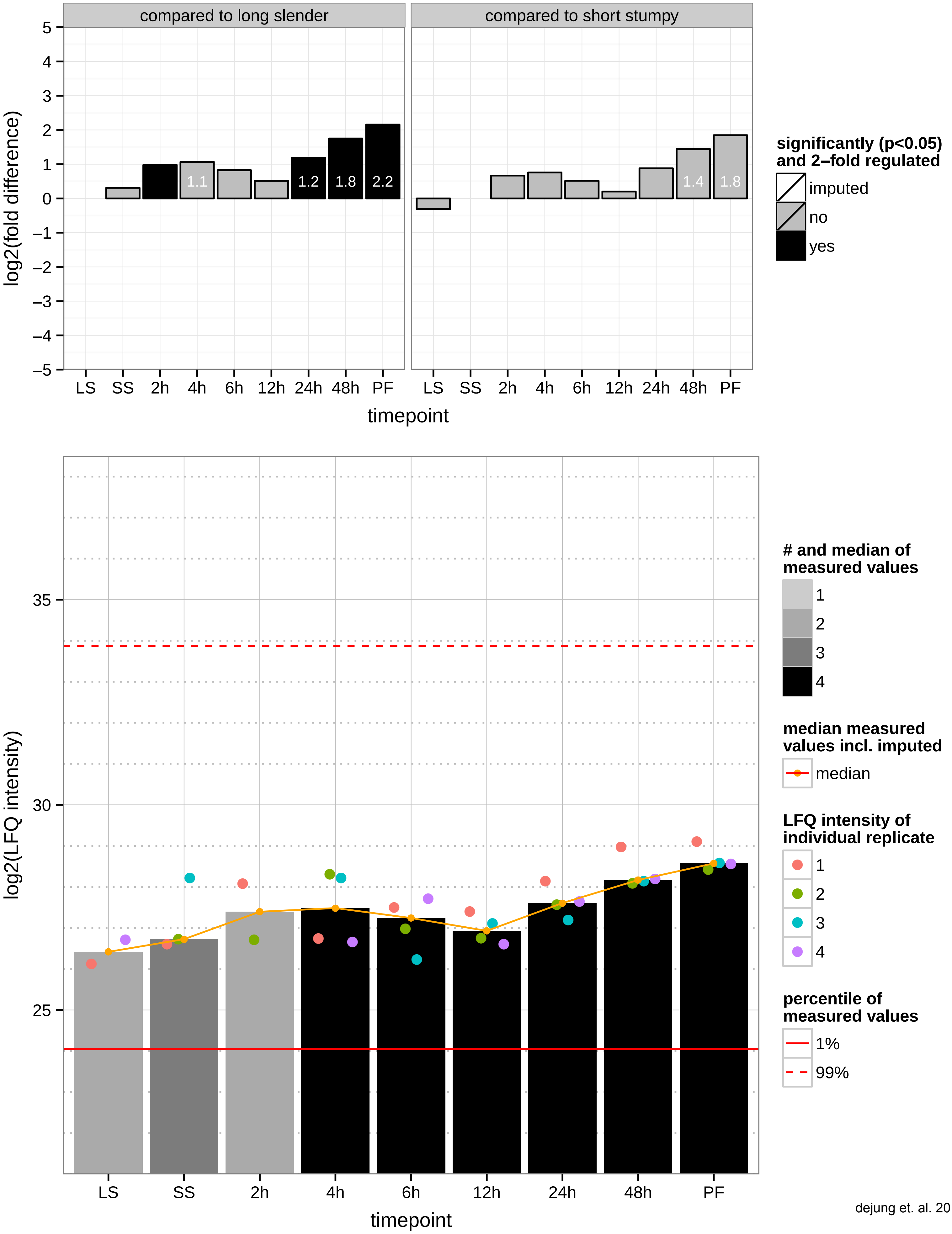

dejung et. al. 2015

selenoprotein, putative
Tb927.4.3410
AGOF: null
AGOC: null
AGOP: null
PGOF: null
PGOC: null
PGOP: null

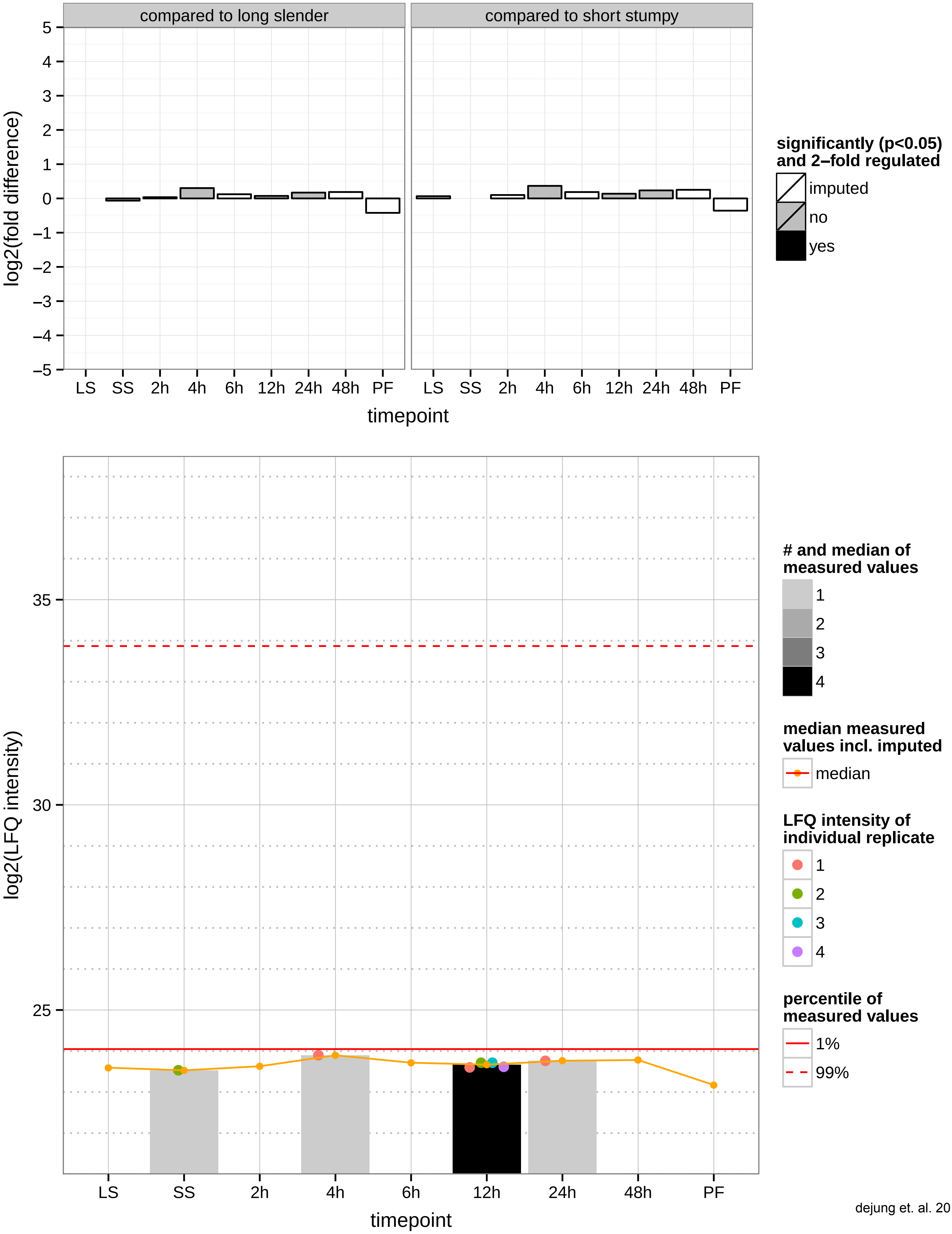

dejung et. al. 2015

60S ribosomal protein L13a, putative
Tb927.5.1610;Tb927.4.3550
AGOF: structural constituent of ribosome
AGOC: intracellular, large ribosomal subunit, ribosome
AGOP: translation
PGOF: structural constituent of ribosome
PGOC: large ribosomal subunit, ribosome
PGOP: translation

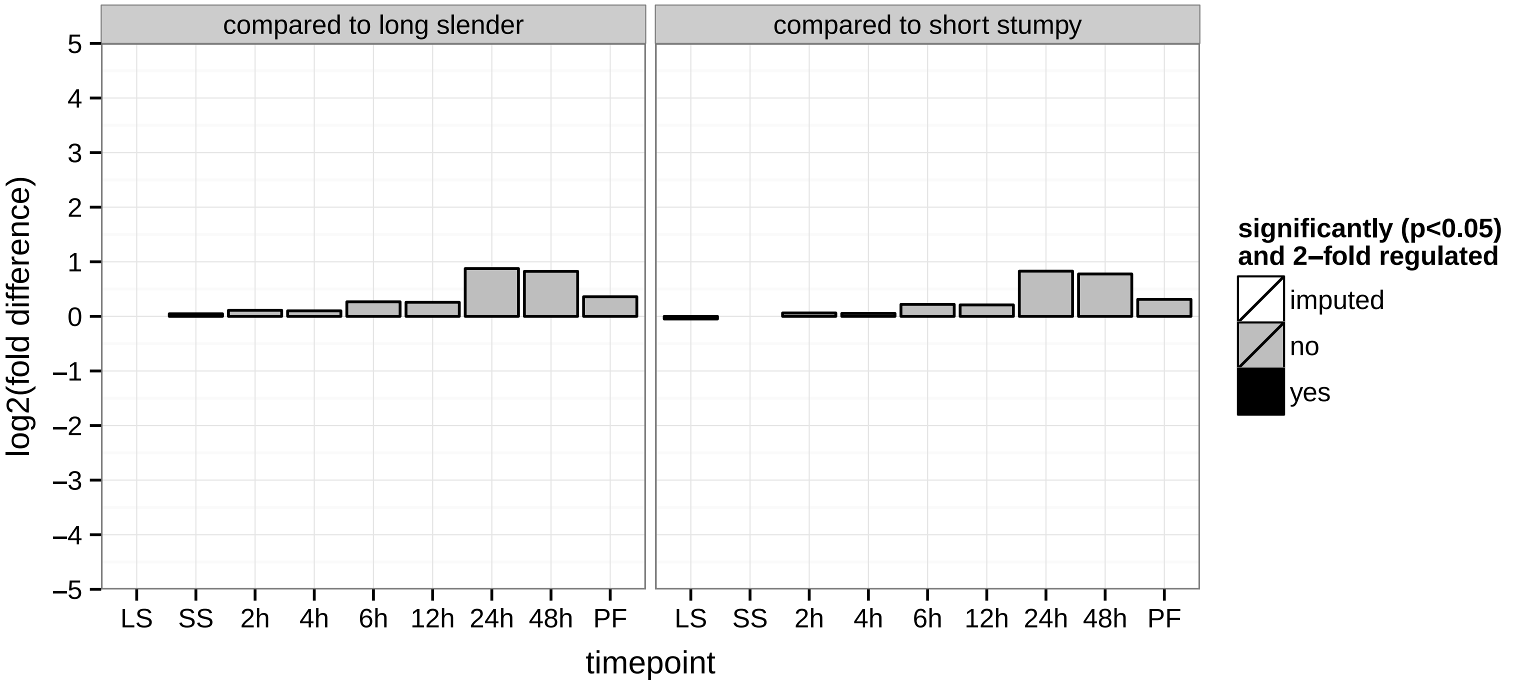

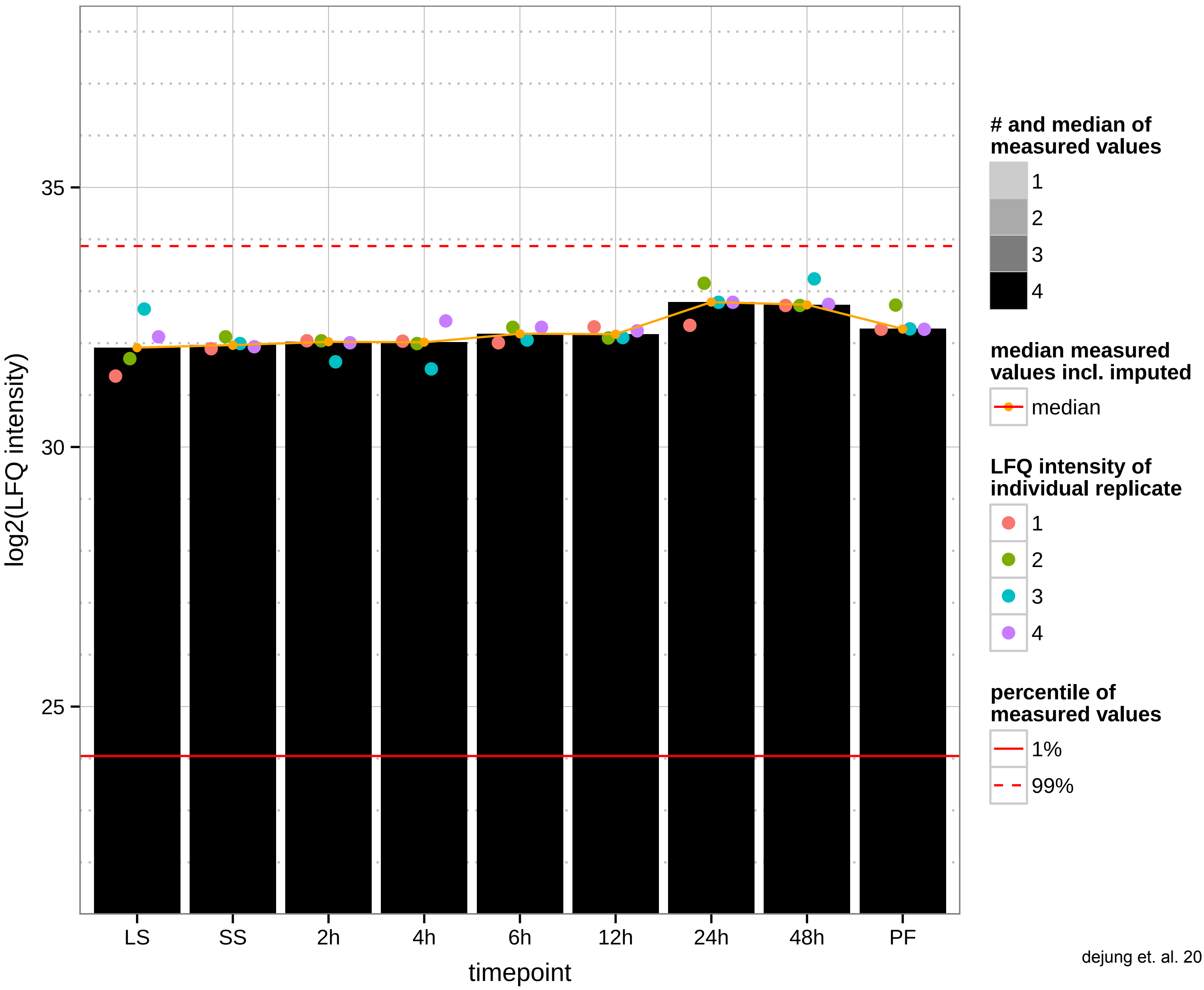

dejung et. al. 2015

protein phoshatase 1, putative, protein phosphatase 1
Tb927.4.3560;Tb927.4.3610
AGOF: hydrolase activity, protein serine/threonine phosphatase activity
AGOC: null
AGOP: protein dephosphorylation
PGOF: hydrolase activity
PGOC: null
PGOP: null

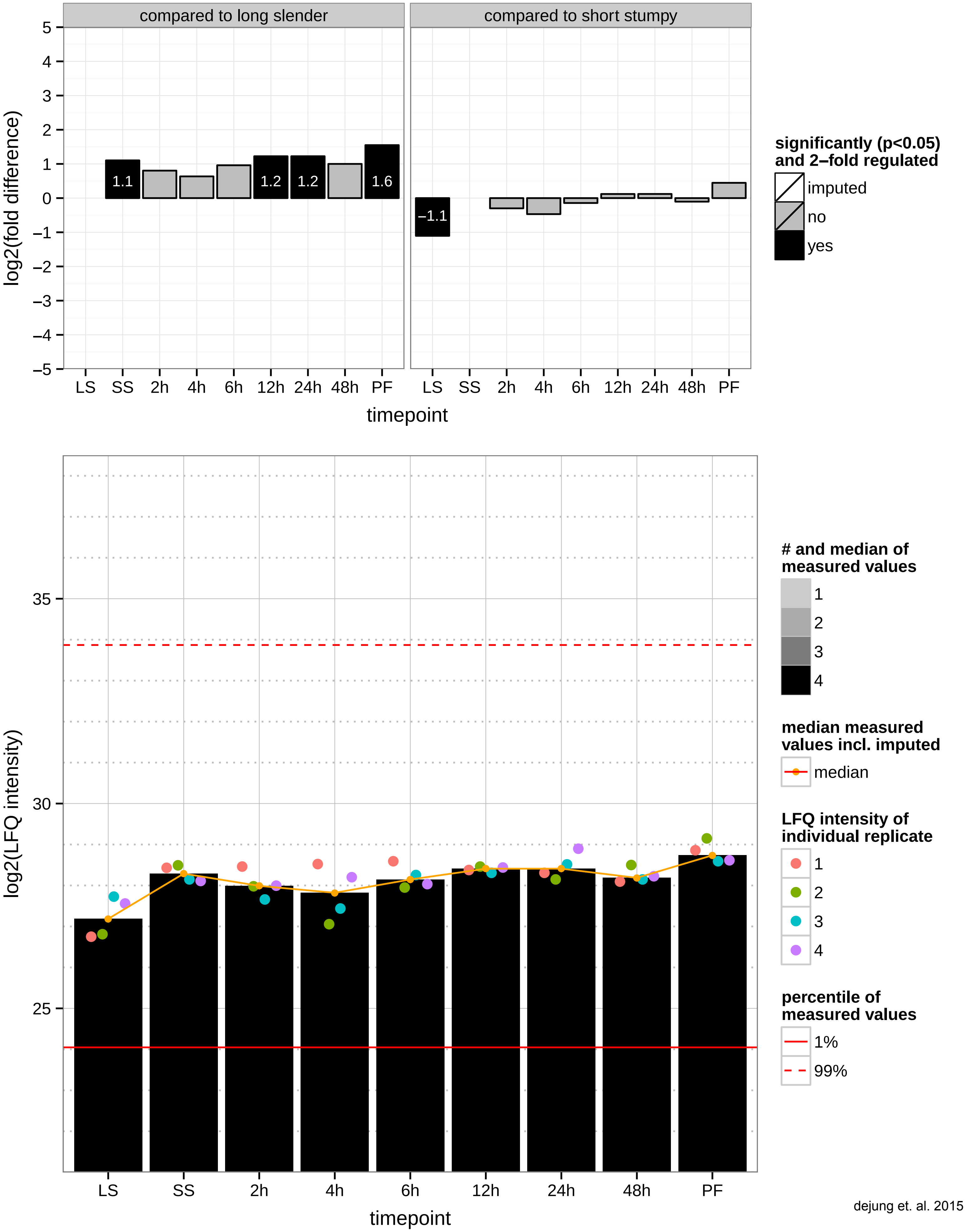

dejung et. al. 2015

1, 2–Dihydroxy–3–keto–5–methylthiopentene dioxygenase, putative
Tb927.4.360
AGOF: dioxygenase activity, metal ion binding
AGOC: cytosol
AGOP: L–methionine salvage from methylthioadenosine
PGOF: acireductone dioxygenase [iron(II)–requiring] activity
PGOC: null
PGOP: oxidation–reduction process

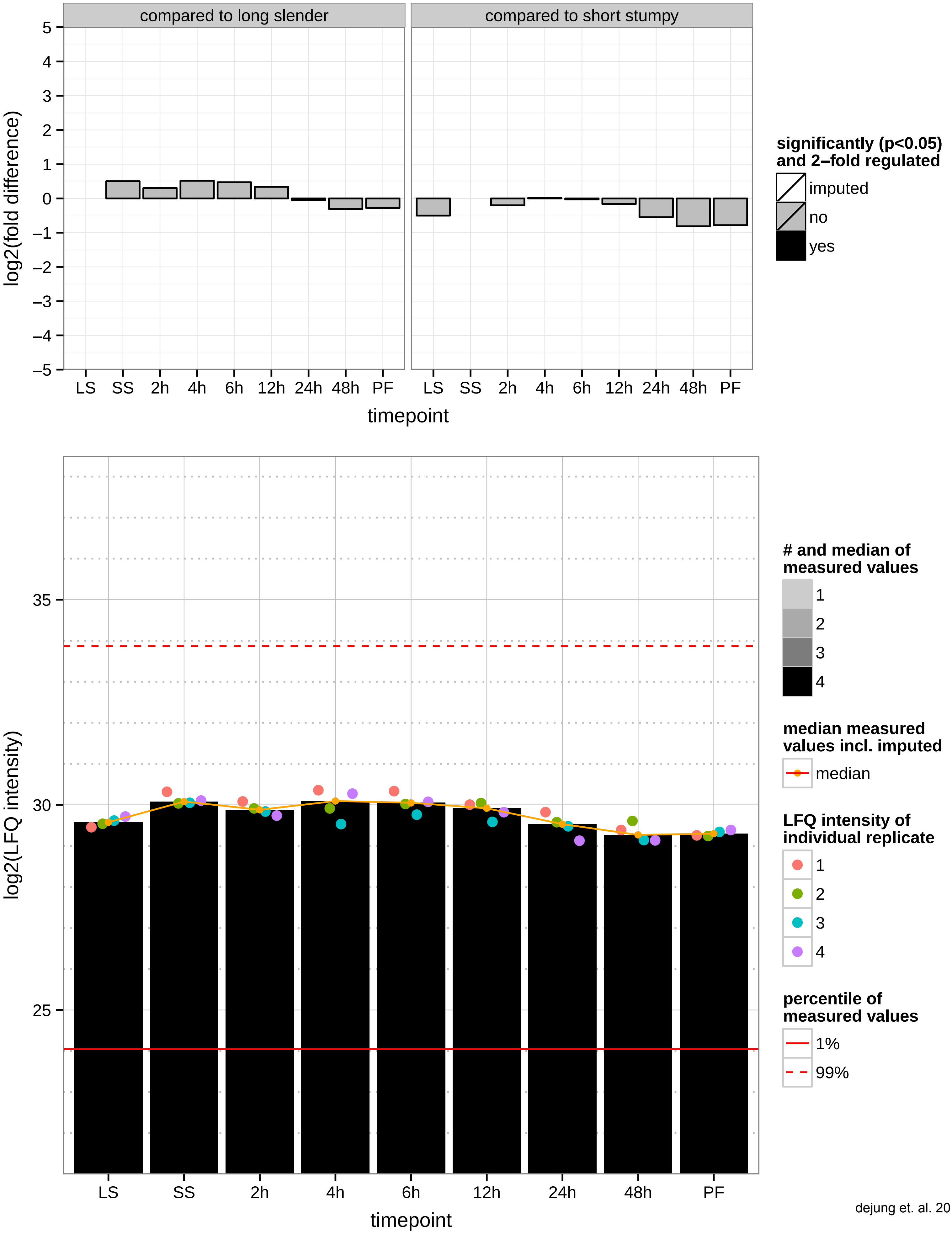

dejung et. al. 2015

serine threonine–protein phosphatase PP1, putative (PP1–6)
Tb927.4.3620
AGOF: hydrolase activity, protein serine/threonine phosphatase activity
AGOC: null
AGOP: modulation of development of symbiont involved in interaction with host, protein dephosphorylation, quorum sensing i
PGOF: hydrolase activity
PGOC: null
PGOP: null

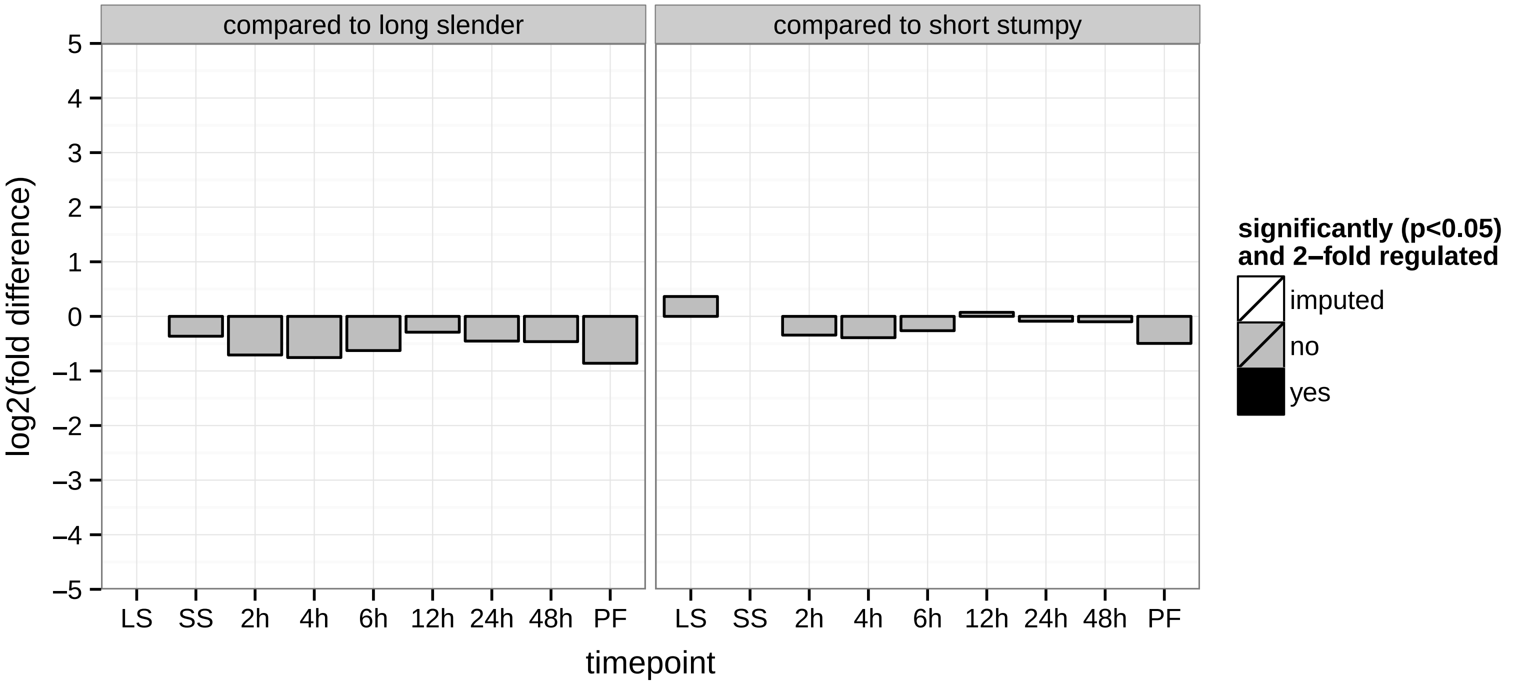

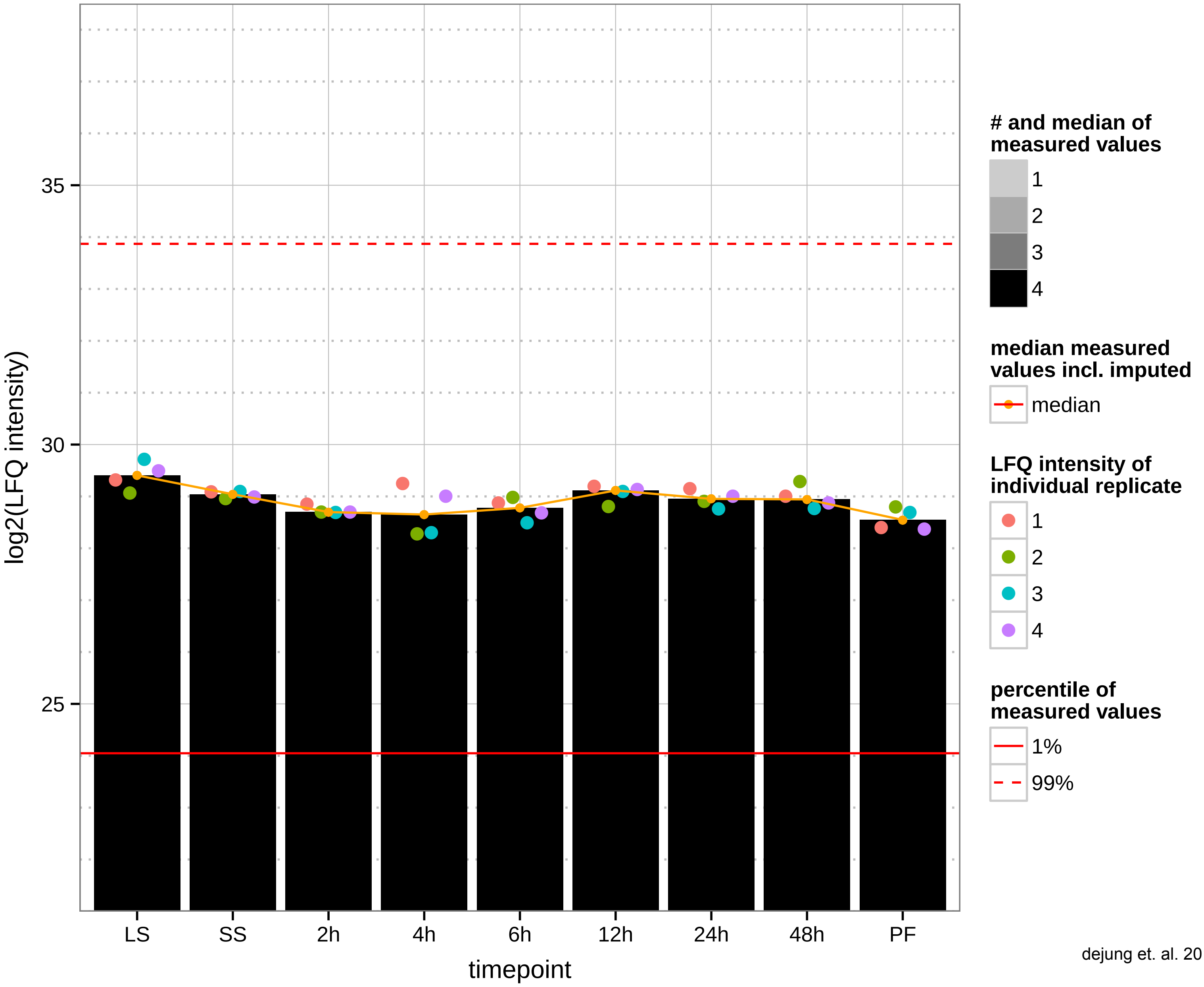

dejung et. al. 2015

hypothetical protein, conserved
Tb927.4.3660
AGOF: null
AGOC: mitochondrion
AGOP: null
PGOF: null
PGOC: null
PGOP: null

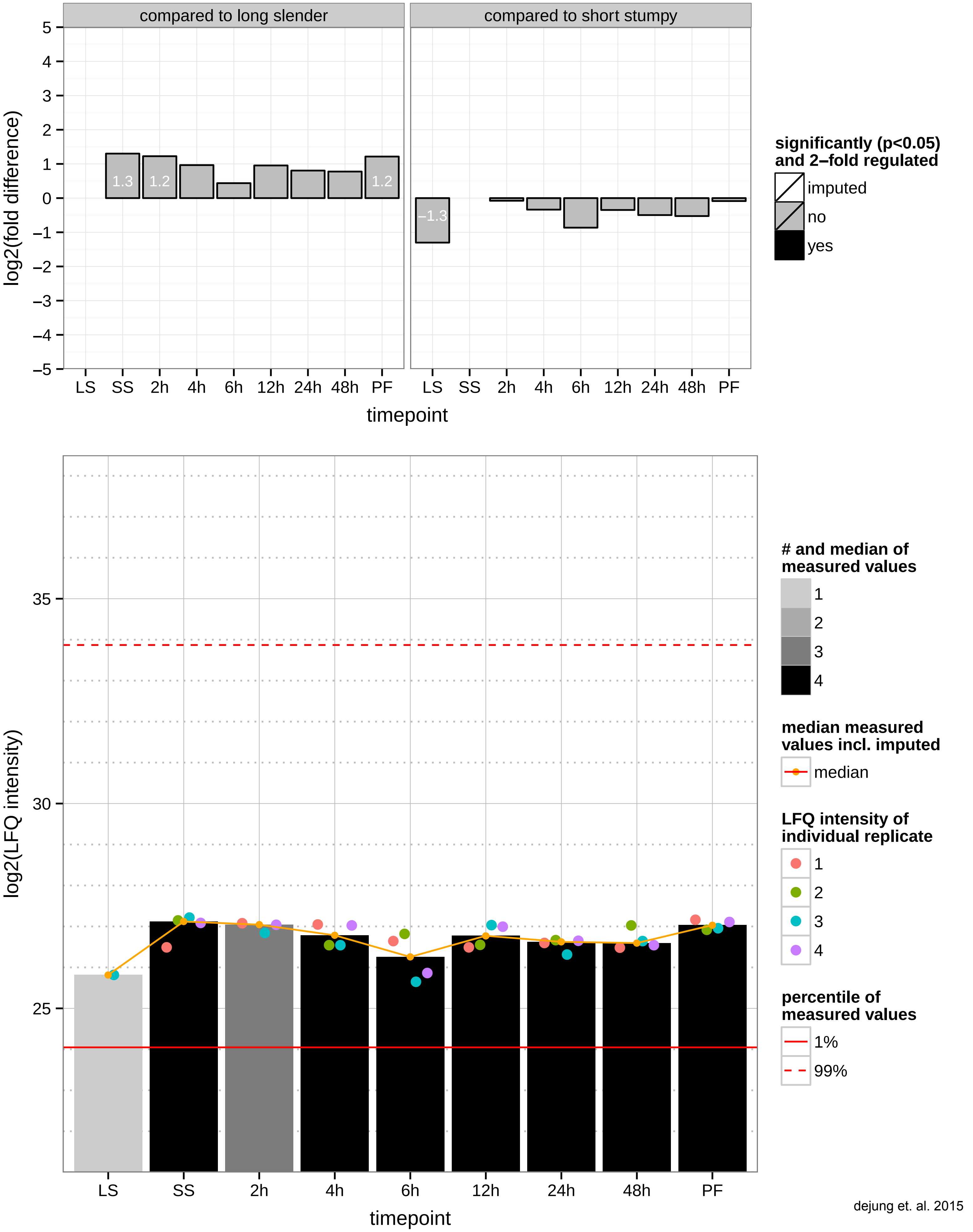

dejung et. al. 2015

flagellar attachment zone protein (FAZ1)
Tb927.4.3740
AGOF: null
AGOC: cilium part, cytoskeletal part
AGOP: cilium assembly, cytokinesis
PGOF: null
PGOC: null
PGOP: null

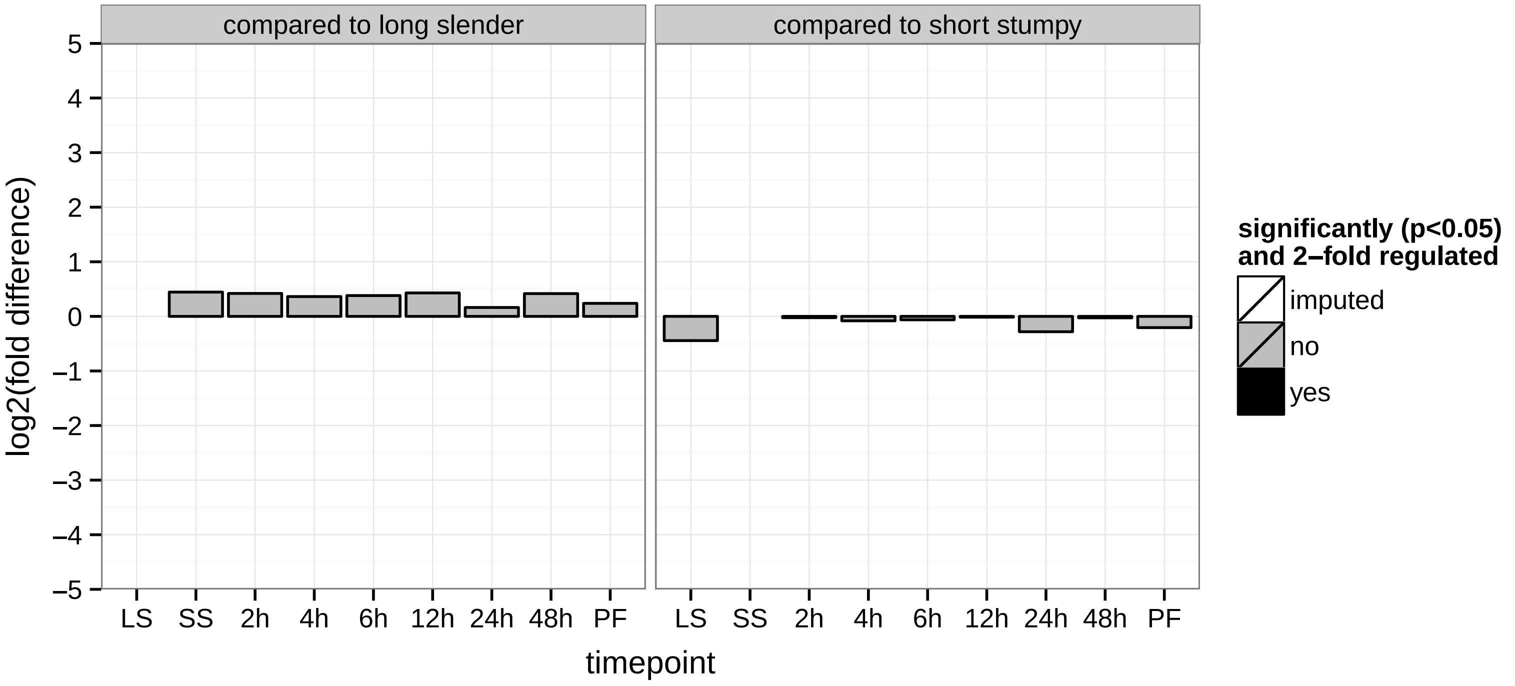

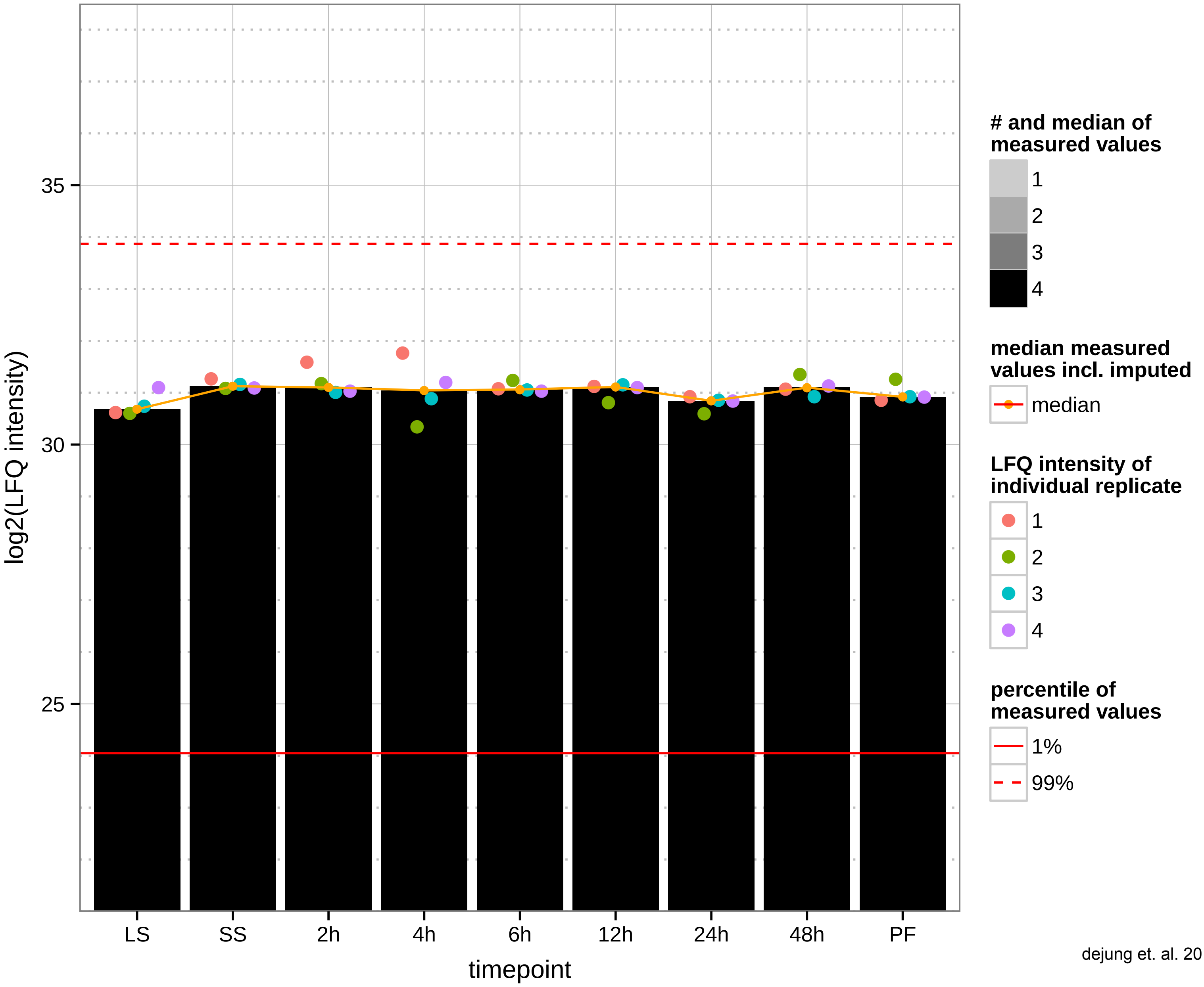

dejung et. al. 2015

ubiquitin carboxyl–terminal hydrolase, putative, cysteine peptidase, Clan CA, family C19
Tb927.4.3790
AGOF: ubiquitin thiolesterase activity
AGOC: null
AGOP: ubiquitin–dependent protein catabolic process
PGOF: protein binding, ubiquitin thiolesterase activity
PGOC: null
PGOP: ubiquitin–dependent protein catabolic process

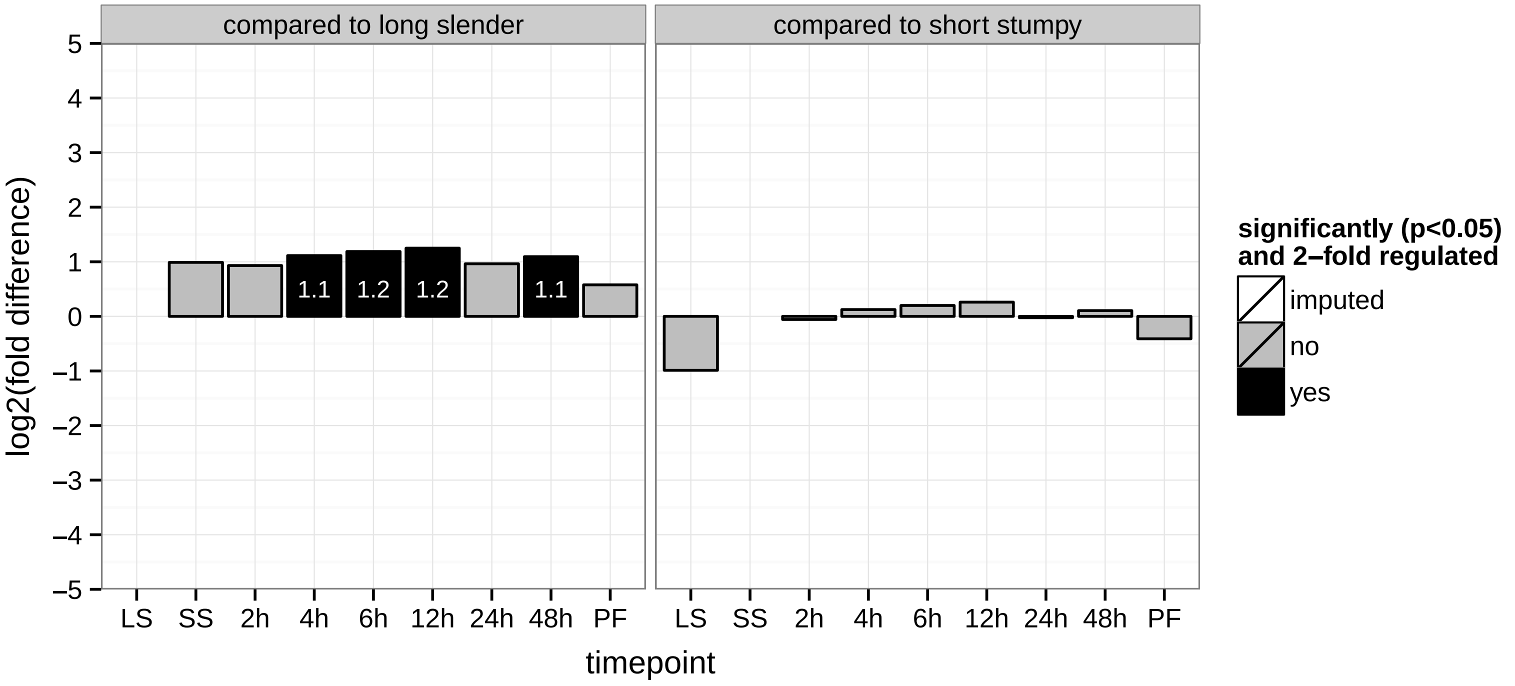

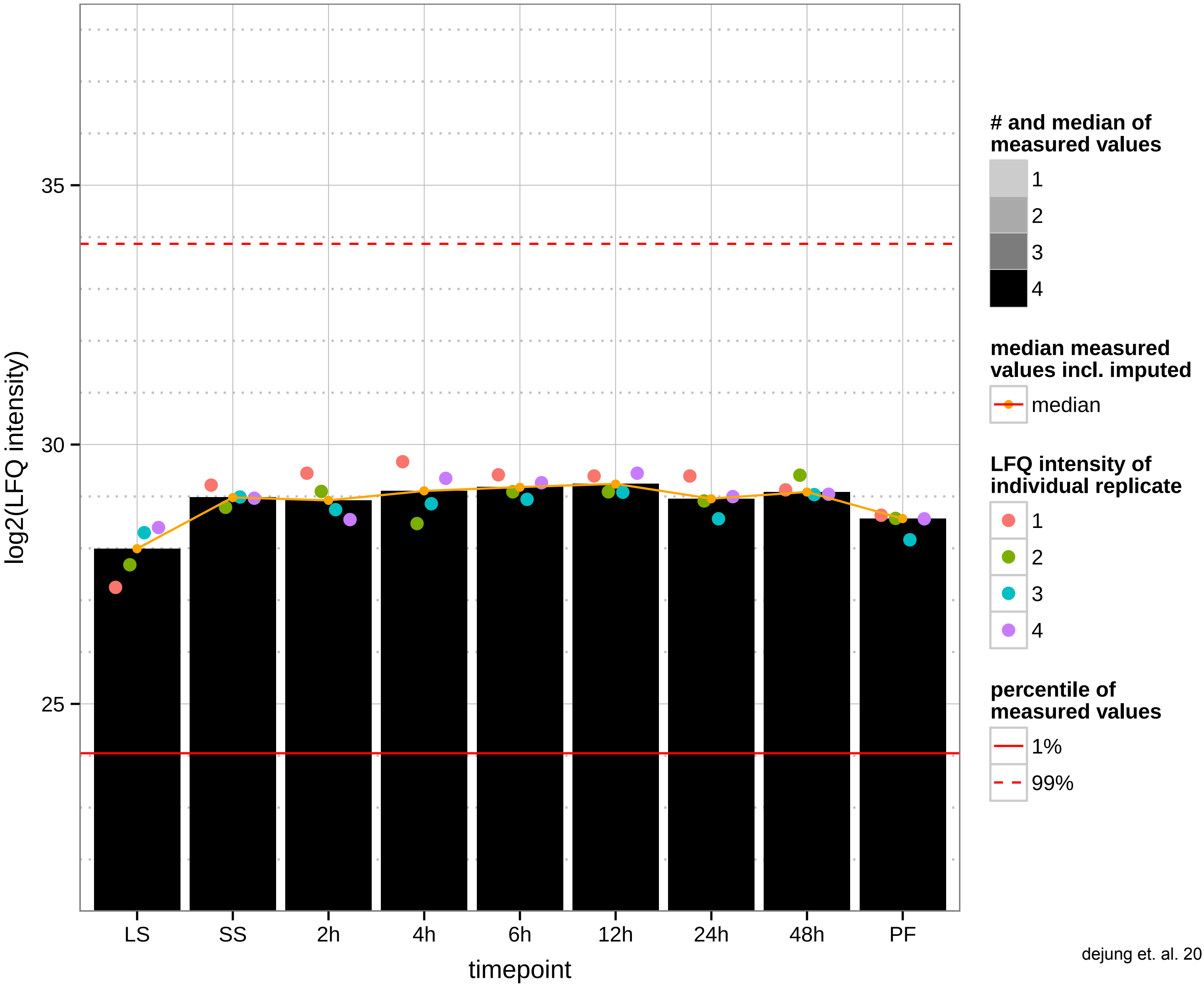

dejung et. al. 2015

hypothetical protein, conserved
Tb927.4.380
AGOF: null
AGOC: null
AGOP: null
PGOF: protein binding
PGOC: null
PGOP: null

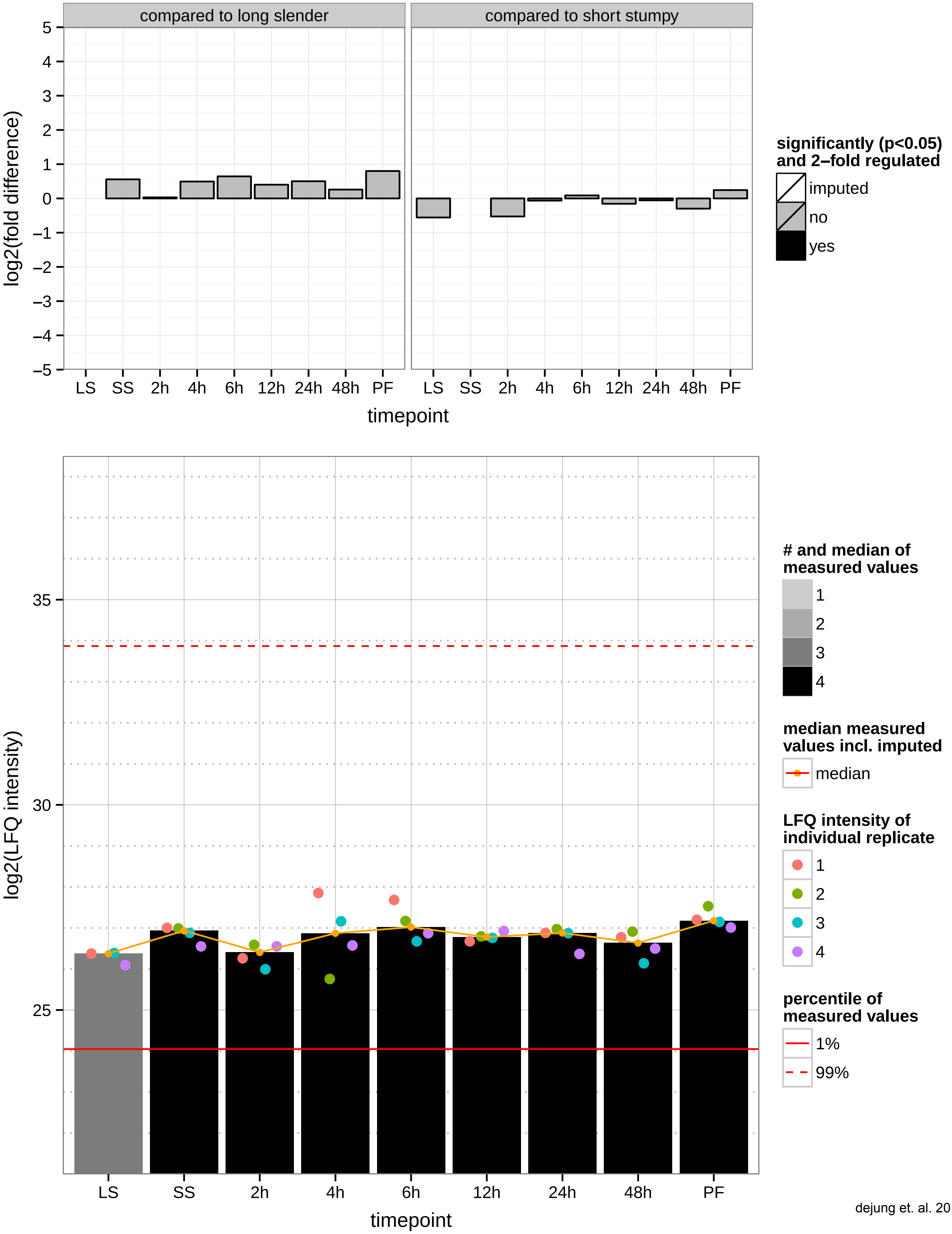

dejung et. al. 2015

DNA–directed RNA polymerase II subunit 2, putative, RNA polymerase subunit, putative (RPB2)
Tb927.4.3810
AGOF: DNA binding, DNA–directed RNA polymerase activity, ribonucleoside binding
AGOC: null
AGOP: transcription, DNA–dependent
PGOF: DNA binding, DNA–directed RNA polymerase activity
PGOC: null
PGOP: transcription, DNA–dependent

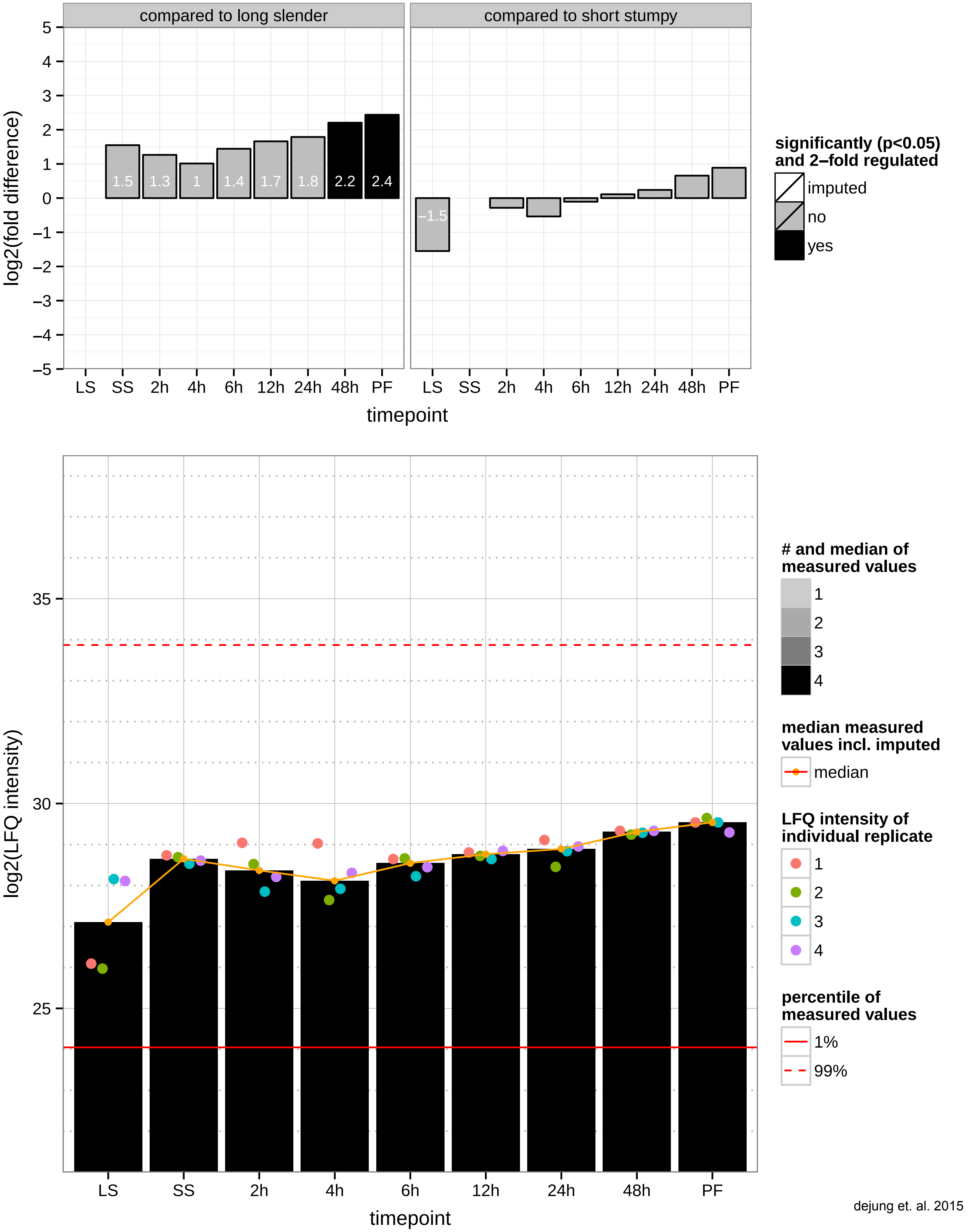

dejung et. al. 2015

nucleolar protein, putative
Tb927.4.3840
AGOF: RNA binding, S–adenosylmethionine–dependent methyltransferase activity
AGOC: null
AGOP: rRNA processing
PGOF: RNA binding, S–adenosylmethionine–dependent methyltransferase activity
PGOC: null
PGOP: rRNA processing

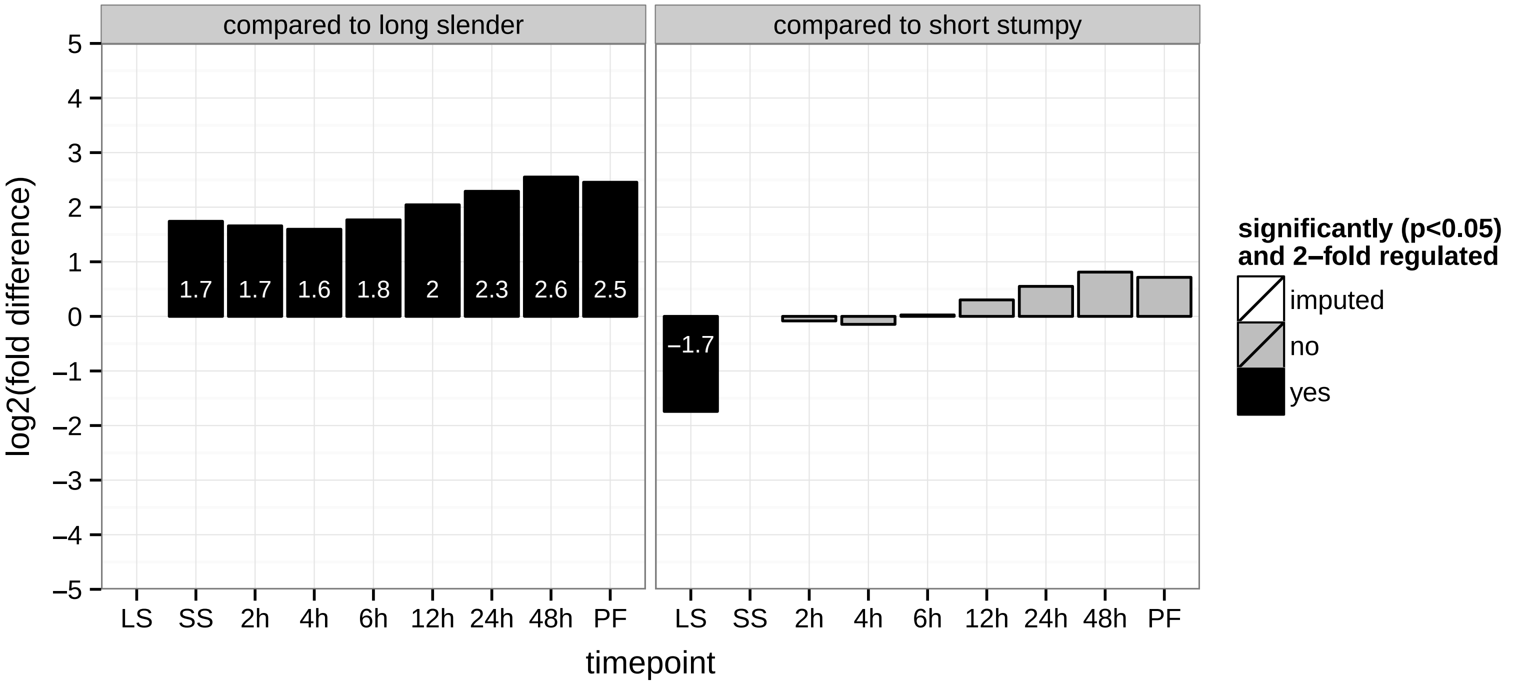

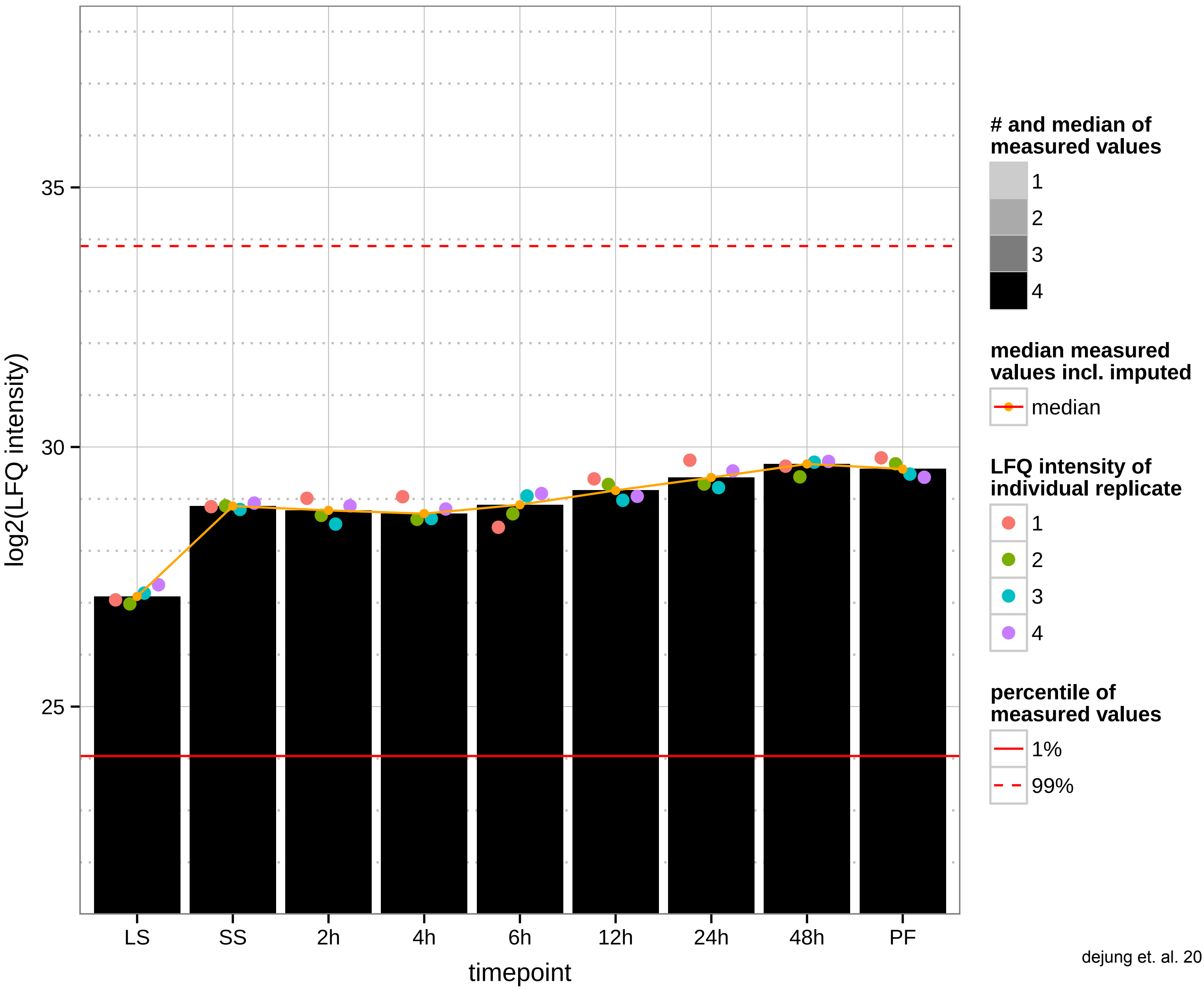

dejung et. al. 2015

hypothetical protein, conserved
Tb927.4.3850
AGOF: null
AGOC: null
AGOP: null
PGOF: protein binding
PGOC: null
PGOP: null

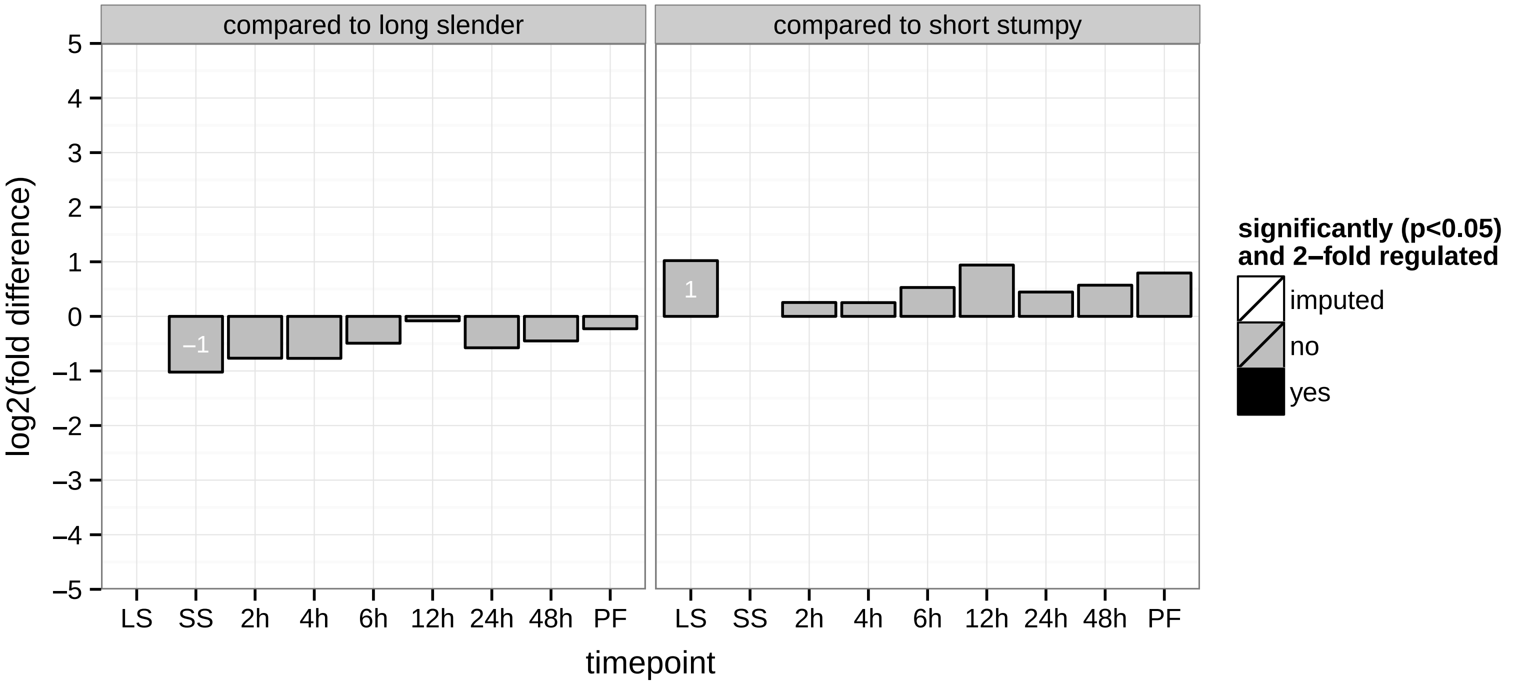

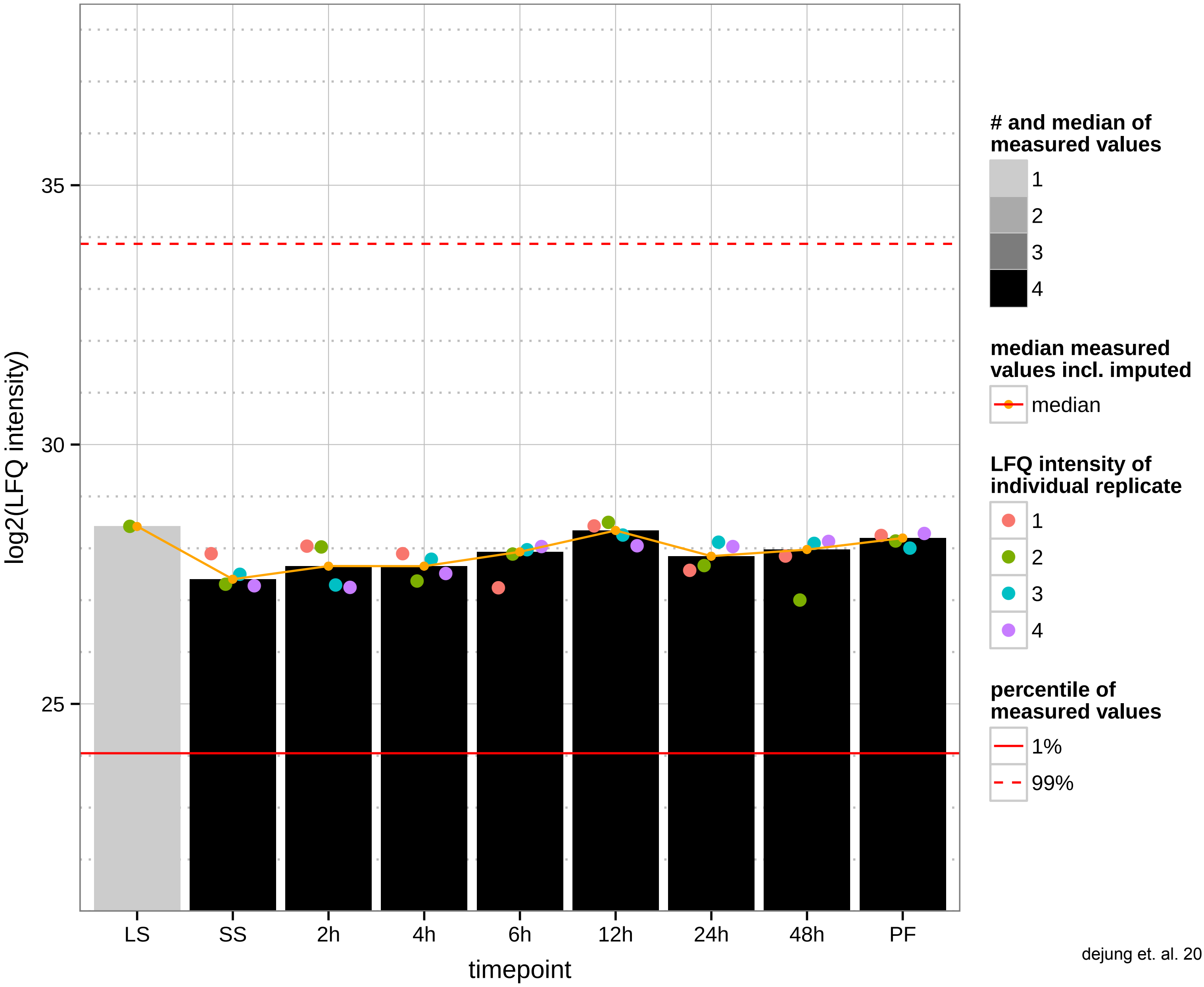

dejung et. al. 2015

hypothetical protein, conserved
Tb927.4.4090
AGOF: null
AGOC: null
AGOP: null
PGOF: null
PGOC: null
PGOP: null

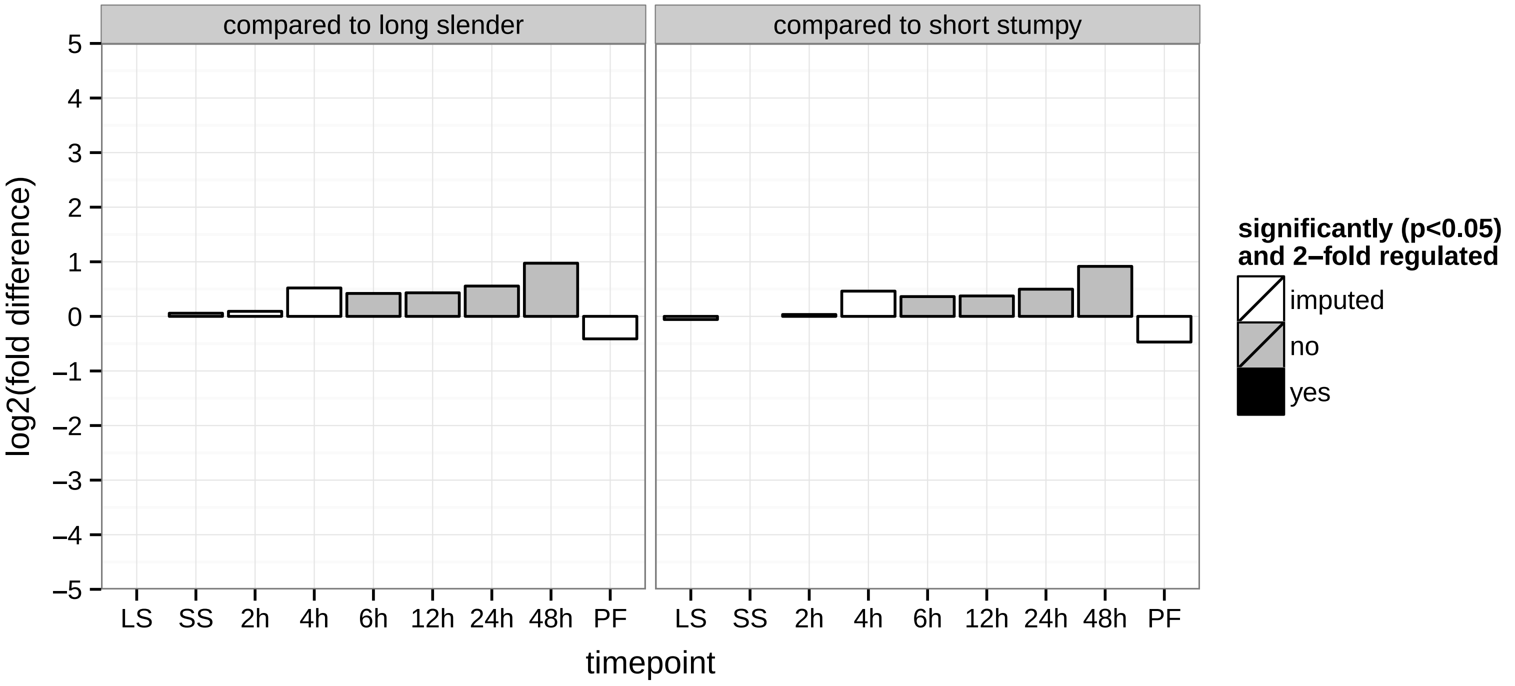

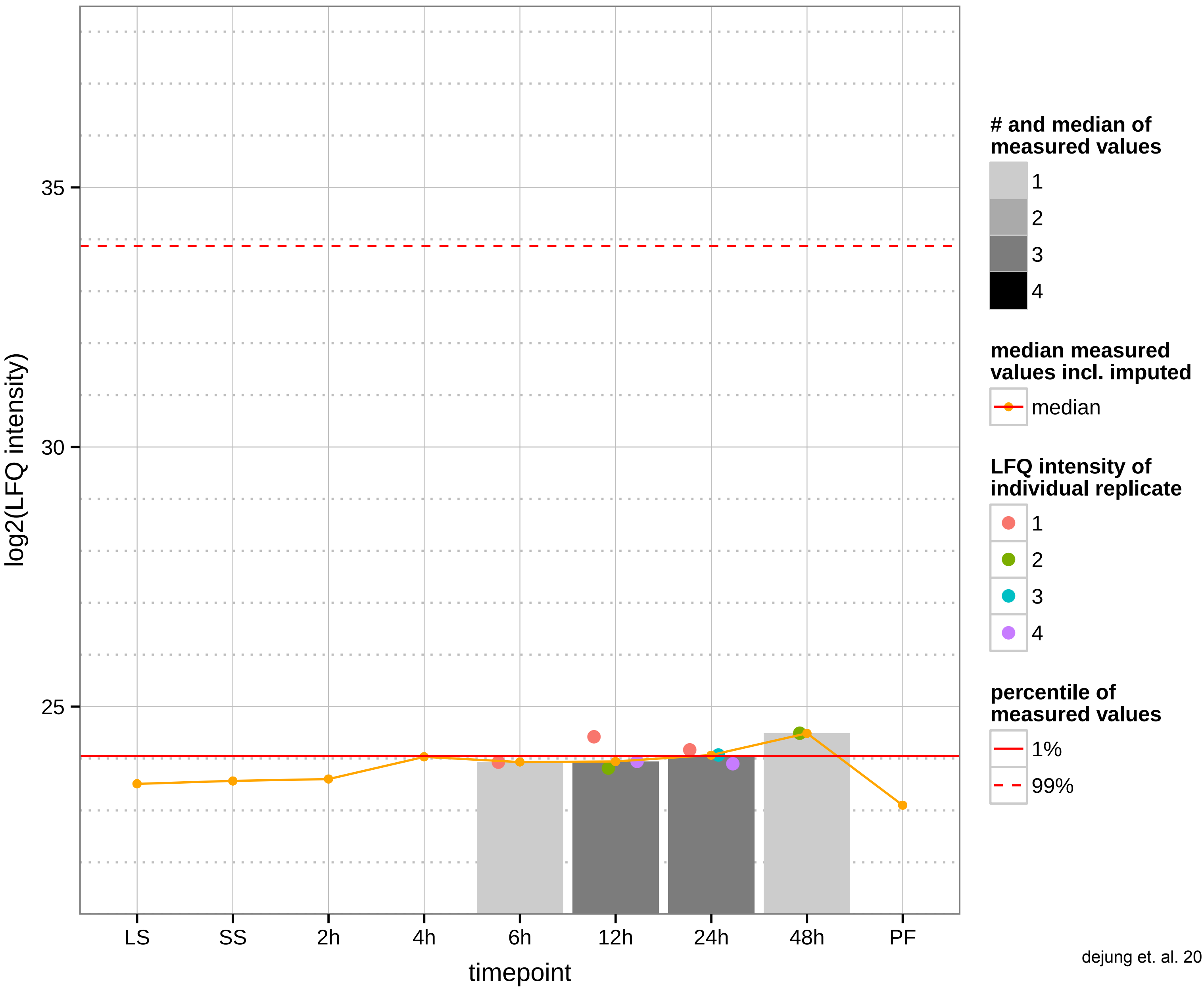

dejung et. al. 2015

CAF 40 (CAF40)
Tb927.4.410
AGOF: null
AGOC: null
AGOP: null
PGOF: binding
PGOC: null
PGOP: null

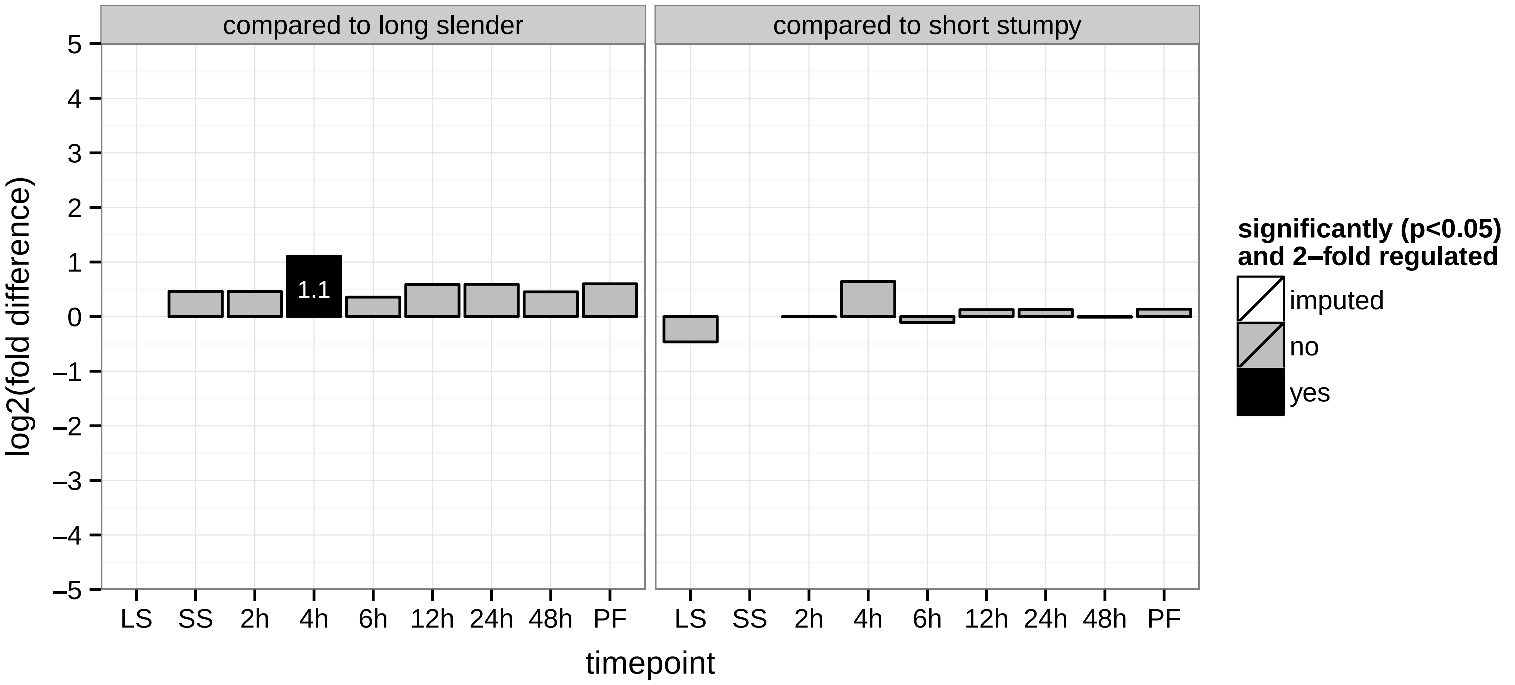

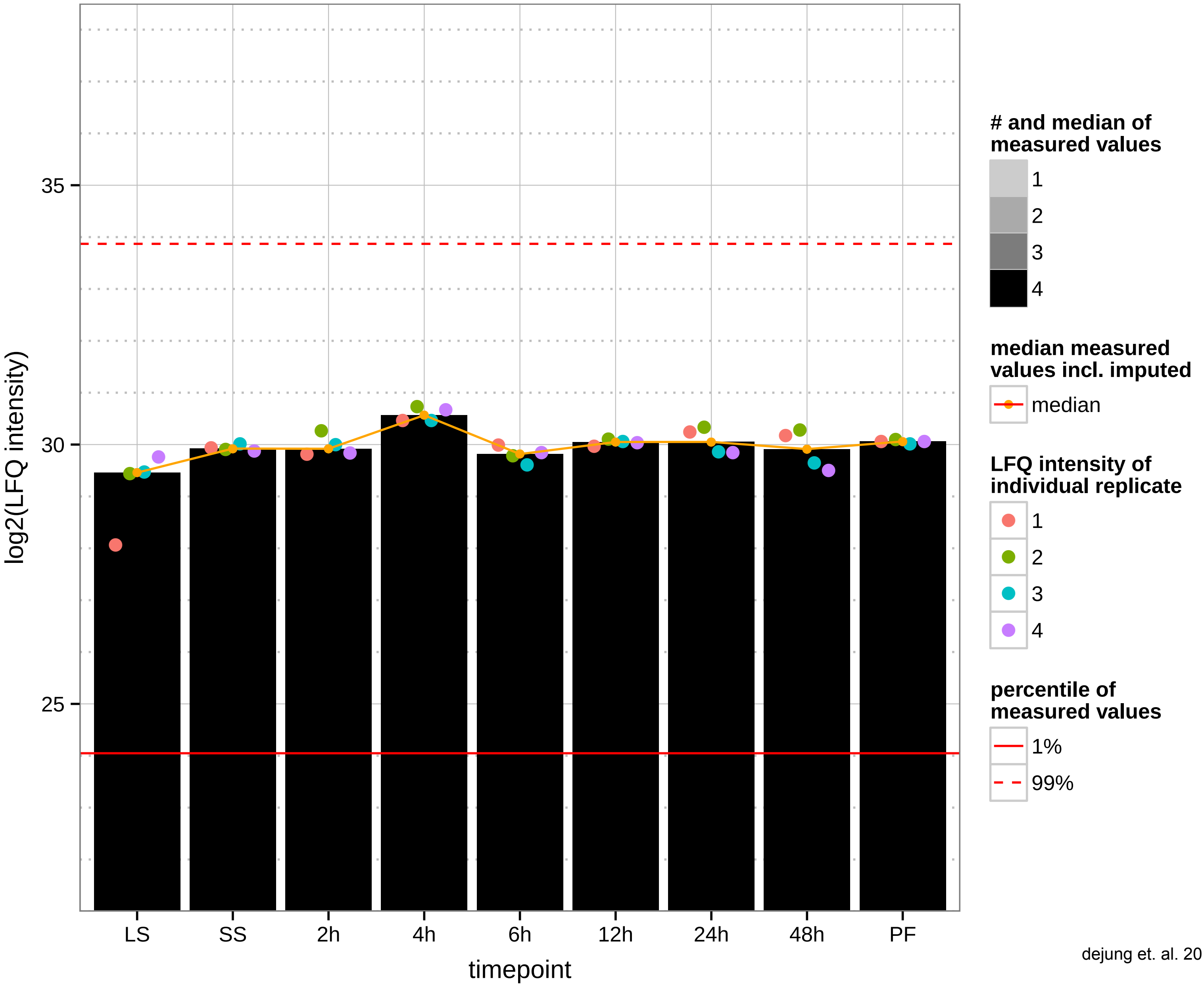

dejung et. al. 2015

hypothetical protein, conserved
Tb927.4.4130
AGOF: null
AGOC: null
AGOP: null
PGOF: null
PGOC: null
PGOP: null

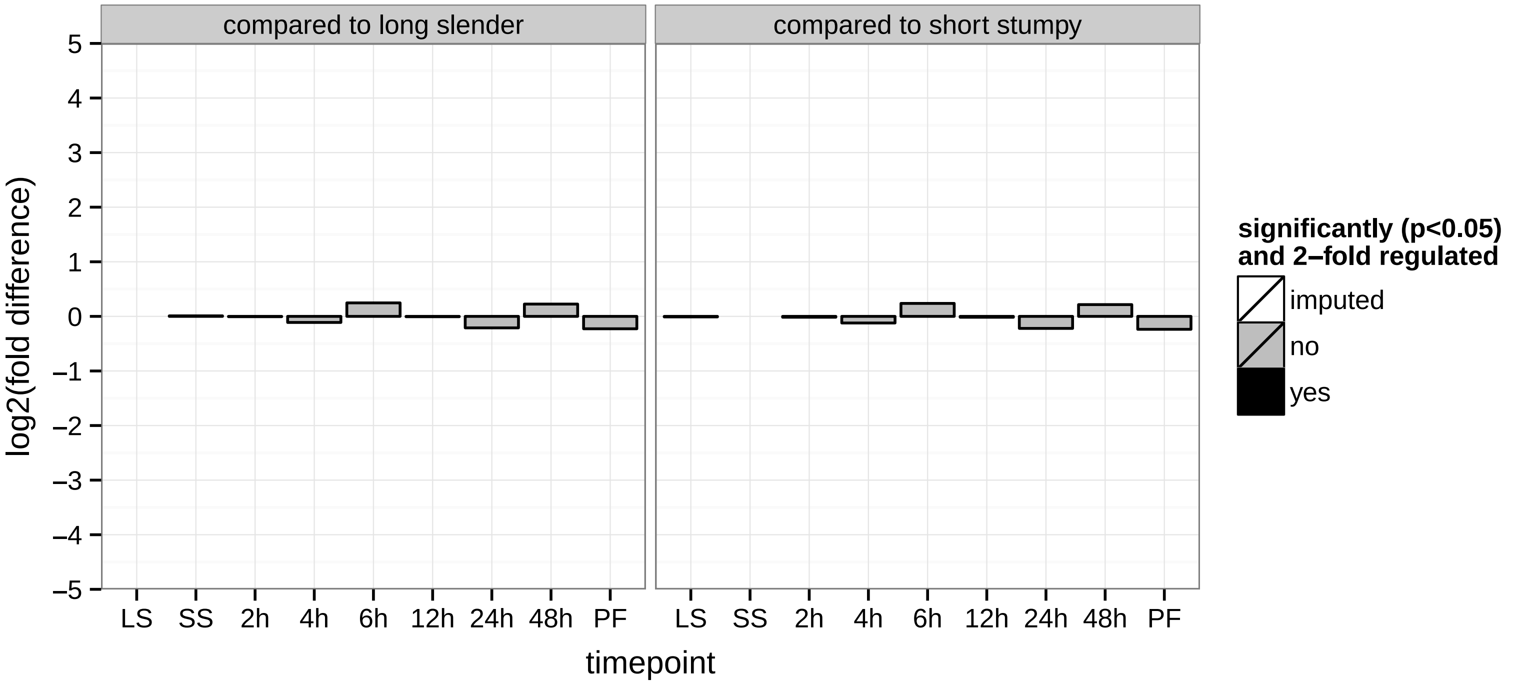

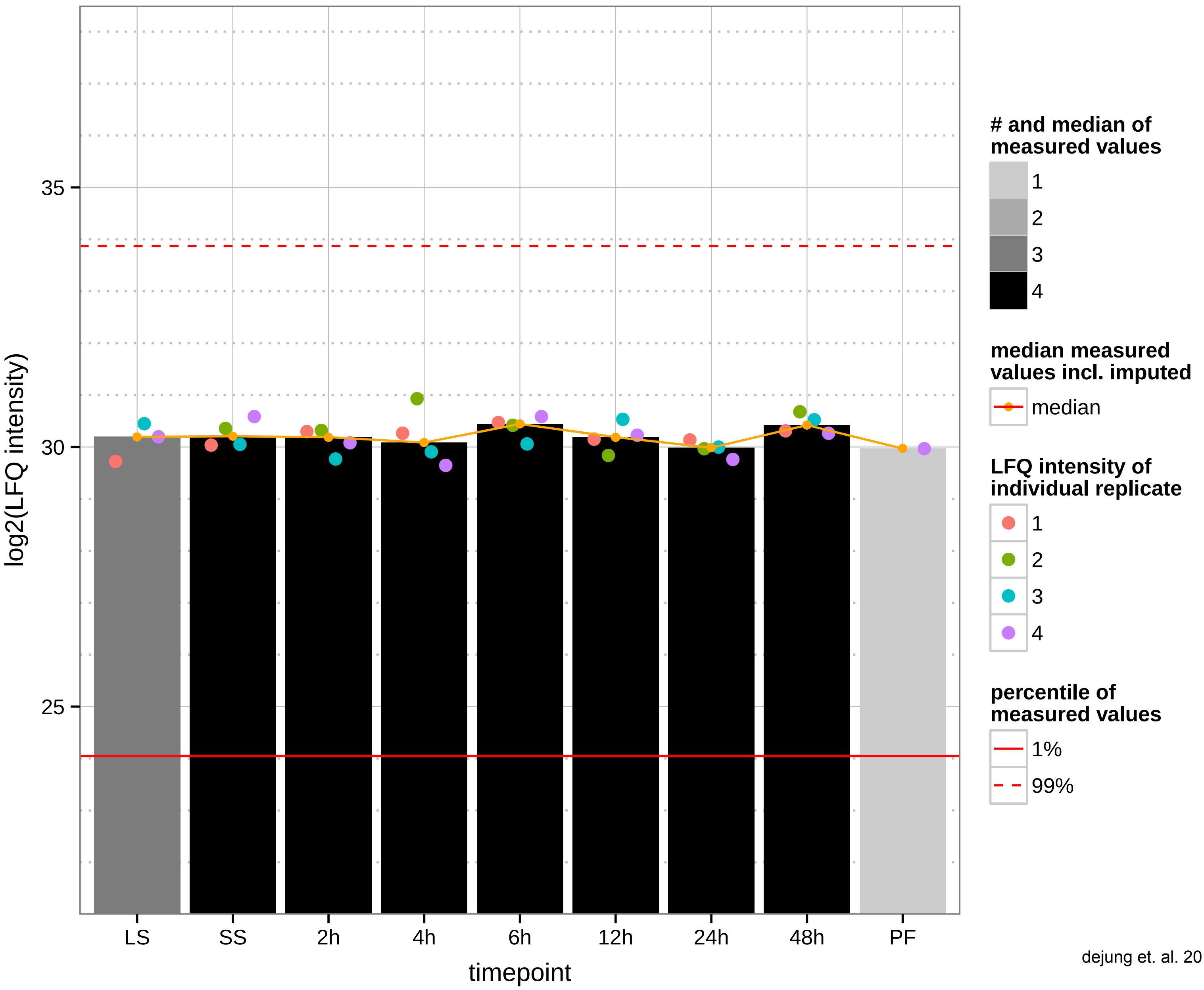

dejung et. al. 2015

Nucleoporin (TbNup64)
Tb927.4.4310
AGOF: null
AGOC: nuclear pore
AGOP: null
PGOF: null
PGOC: null
PGOP: null

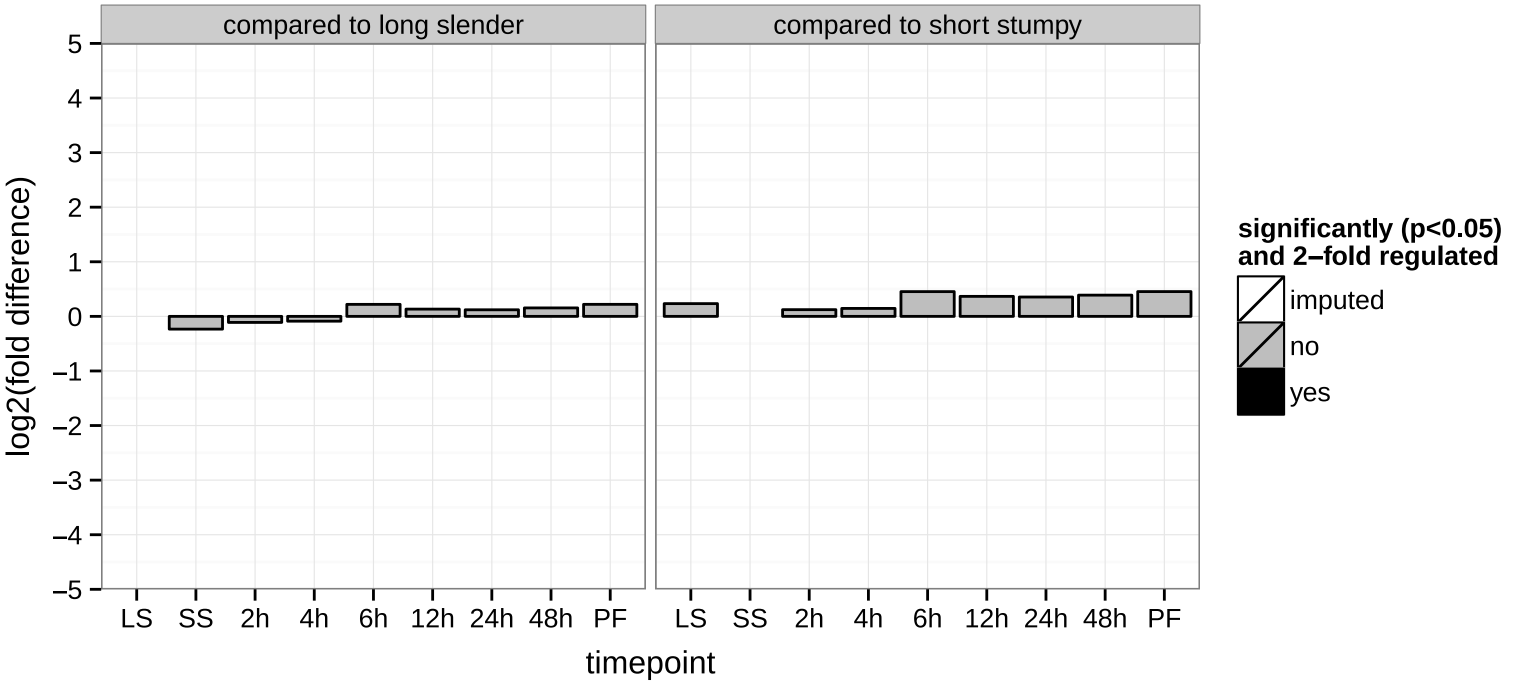

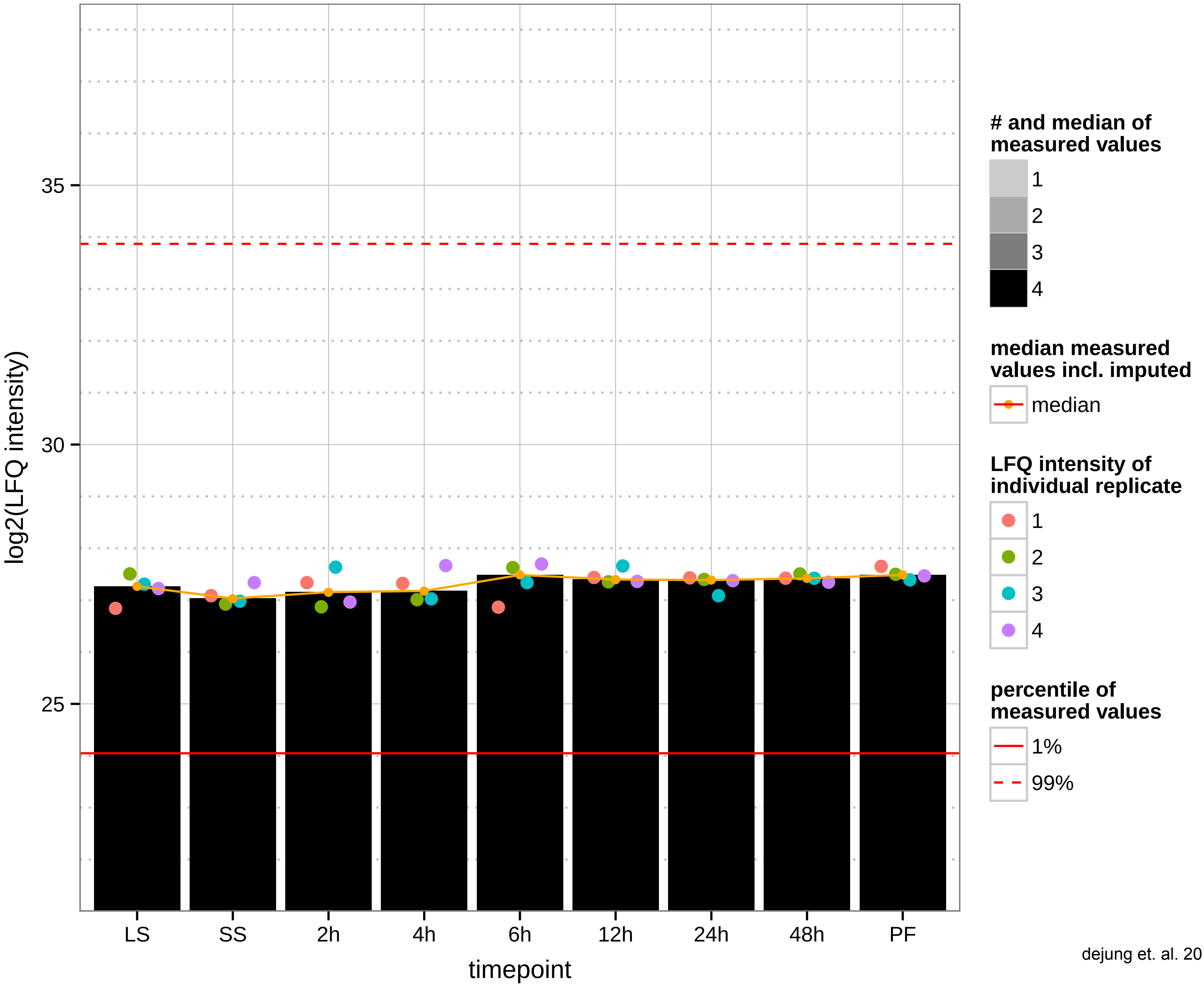

dejung et. al. 2015

divalent cation tolerance protein, putative
Tb927.4.4320
AGOF: null
AGOC: null
AGOP: response to metal ion
PGOF: null
PGOC: null
PGOP: response to metal ion

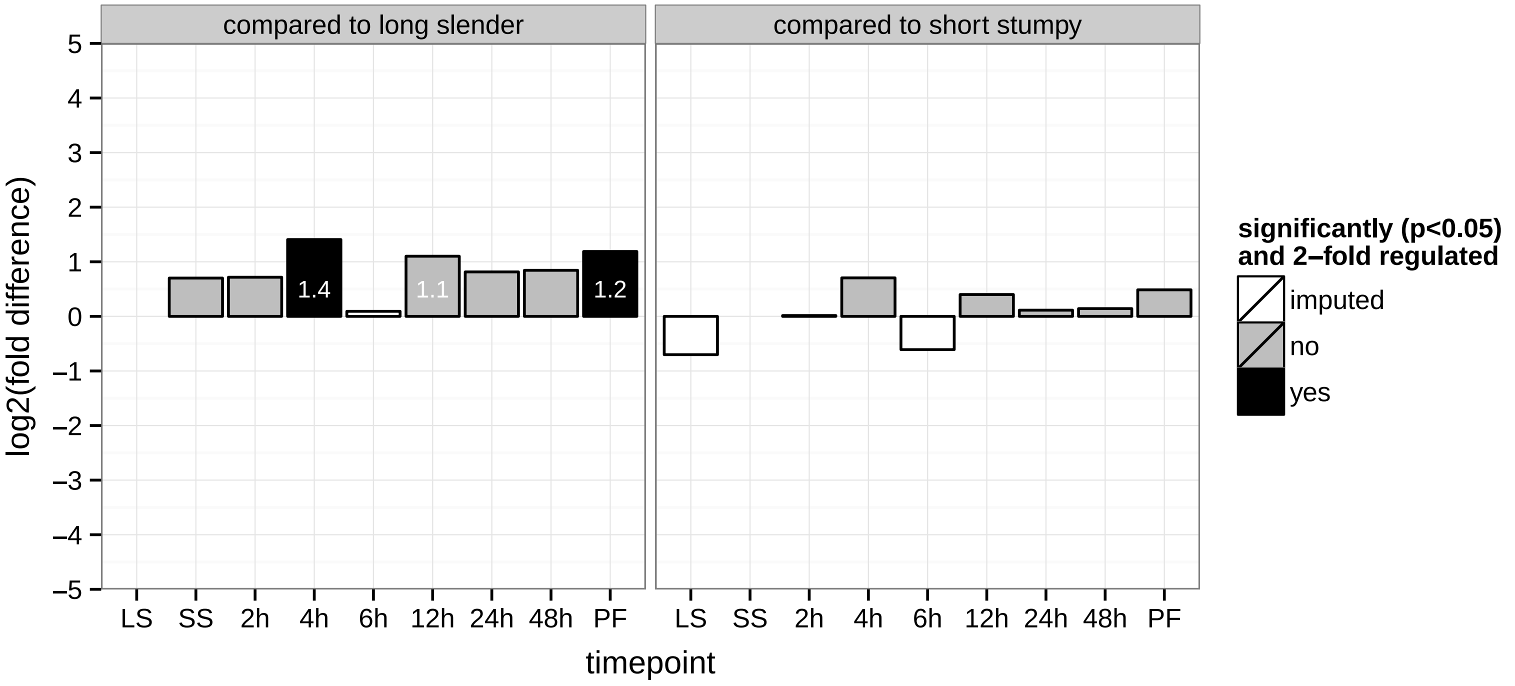

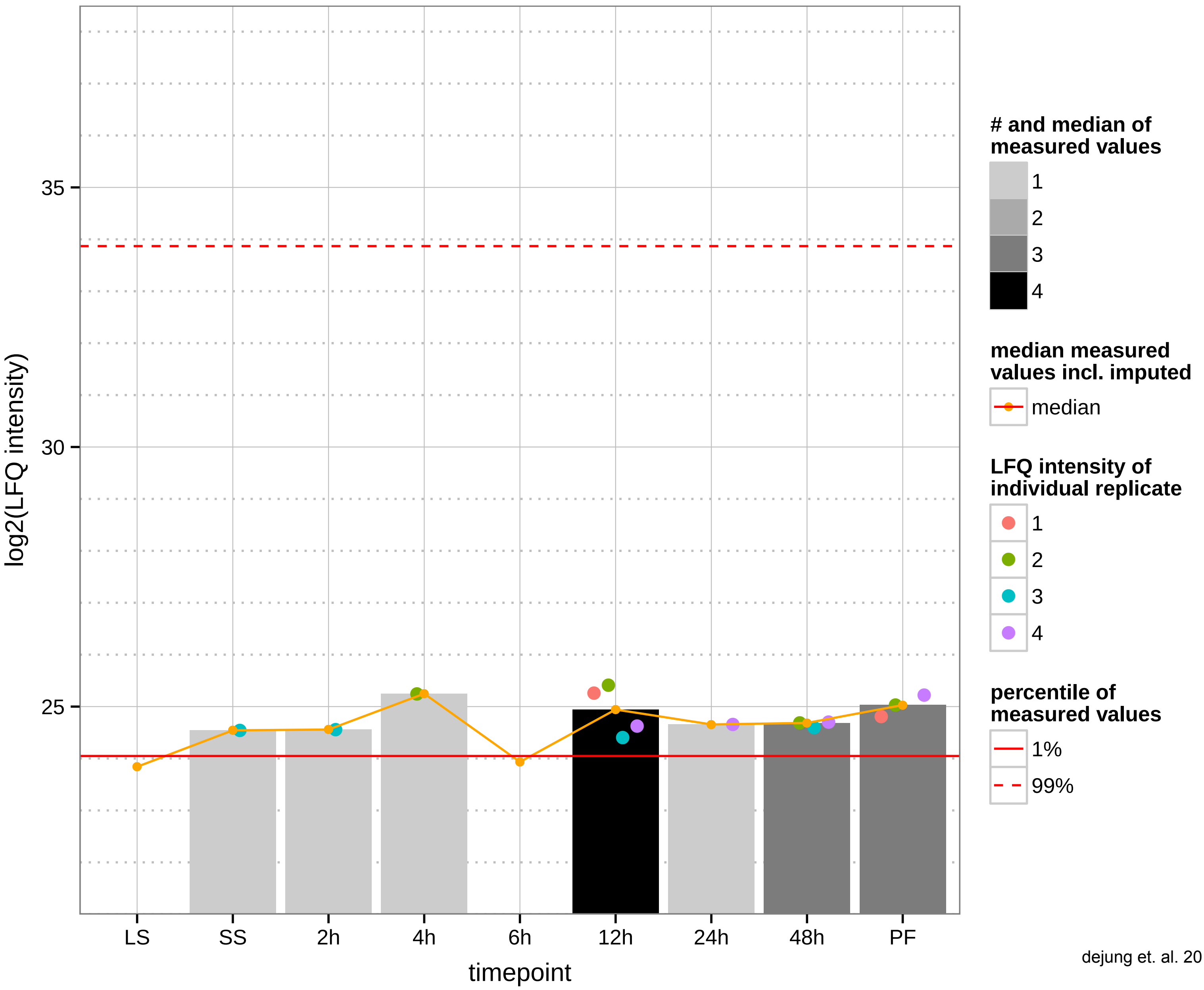

dejung et. al. 2015

monoglyceride lipase, putative
Tb927.4.4360
AGOF: null
AGOC: null
AGOP: null
PGOF: null
PGOC: null
PGOP: null

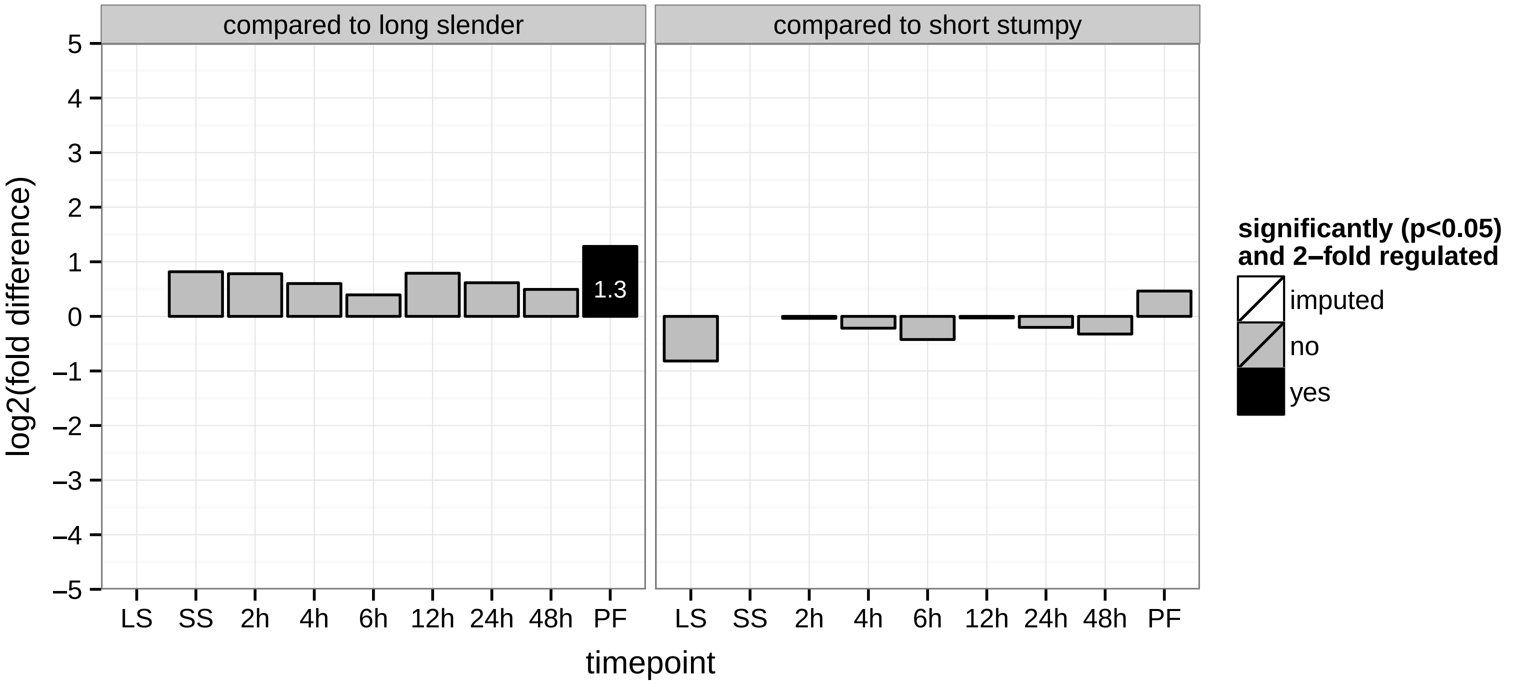

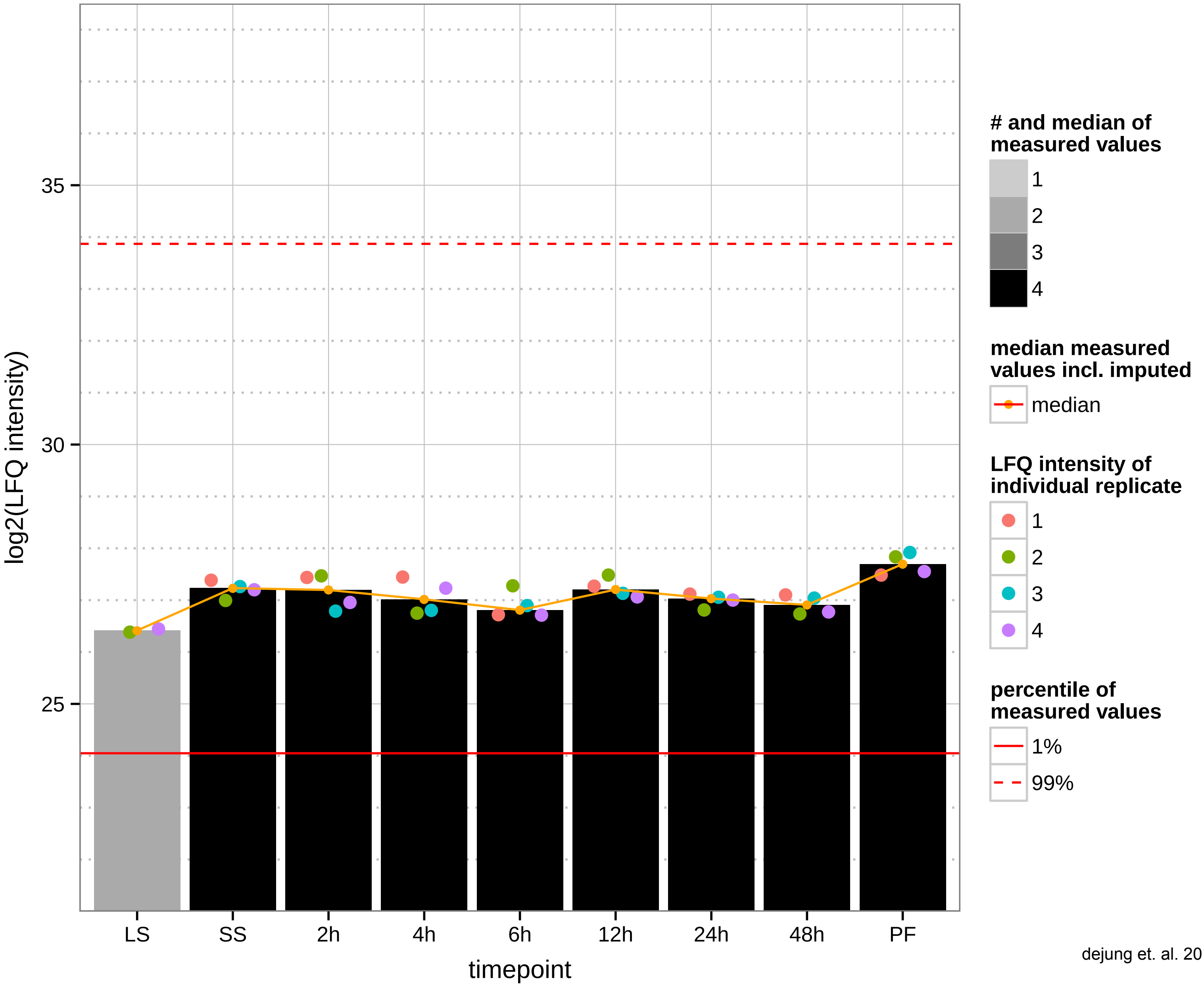

dejung et. al. 2015

vacuolar–type proton translocating pyrophosphatase 1, putative (PPase1), vacuolar–type proton translocating pyrophosphatas
Tb927.8.7980;Tb927.4.4380
AGOF: hydrogen–translocating pyrophosphatase activity, inorganic diphosphatase activity
AGOC: Golgi membrane, acidocalcisome, contractile vacuole, integral to membrane
AGOP: proton transport
PGOF: hydrogen–translocating pyrophosphatase activity, inorganic diphosphatase activity
PGOC: membrane
PGOP: proton transport

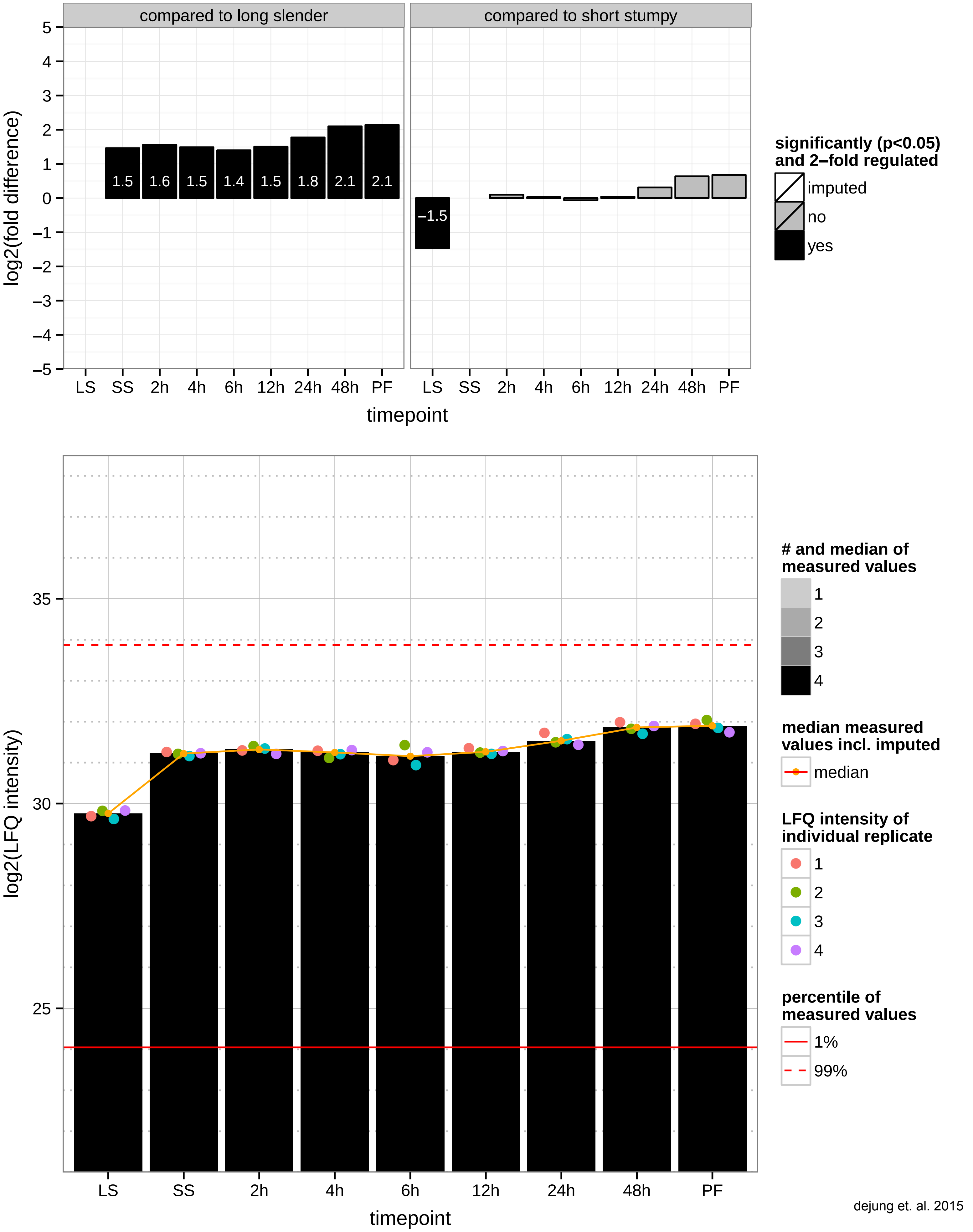

dejung et. al. 2015

multidrug resistance protein E, p−glycoprotein (MRPE)
Tb927.4.4490
AGOF: ATP binding, ATPase activity, coupled to transmembrane movement of substances
AGOC: ATP−binding cassette (ABC) transporter complex, integral to membrane
AGOP: response to drug, transport
PGOF: ATP binding, ATPase activity, coupled to transmembrane movement of substances, nucleoside−triphosphatase activity
PGOC: integral to membrane
PGOP: transmembrane transport, transport

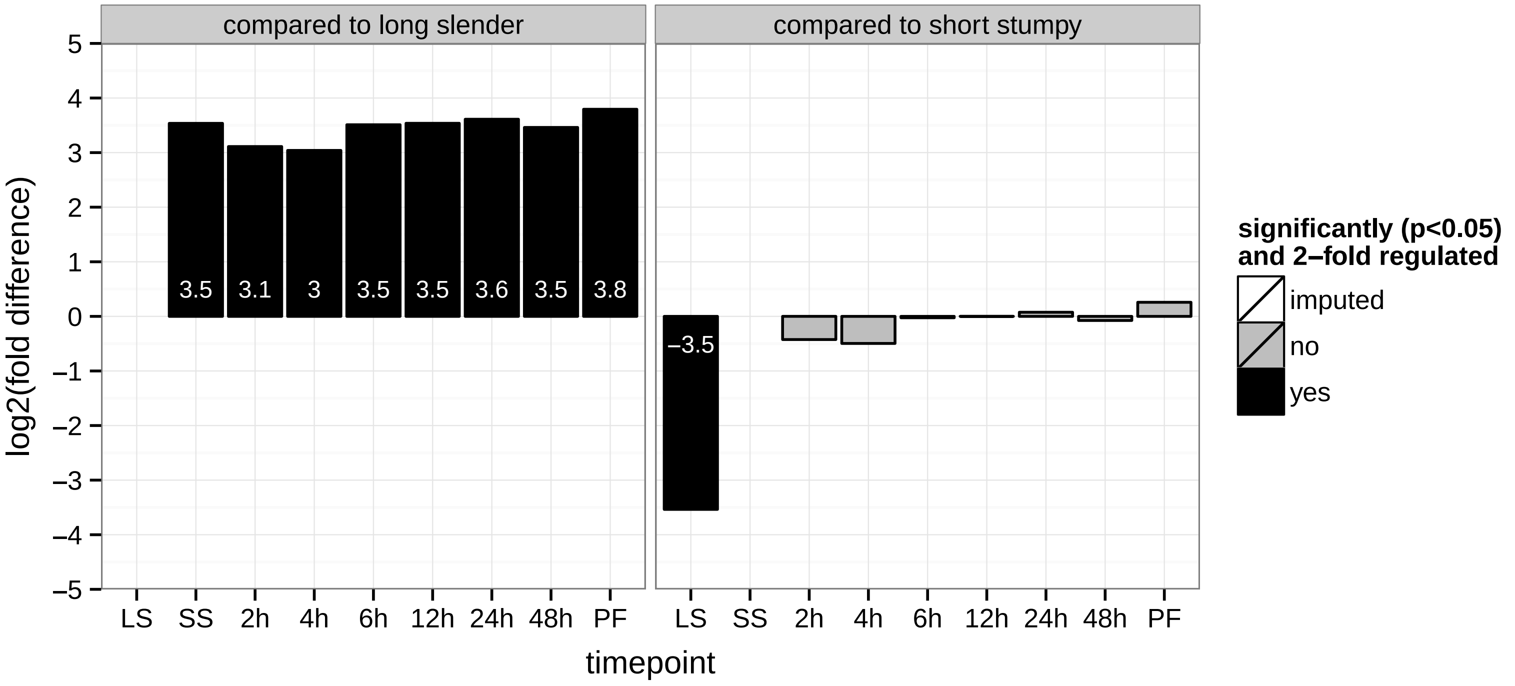

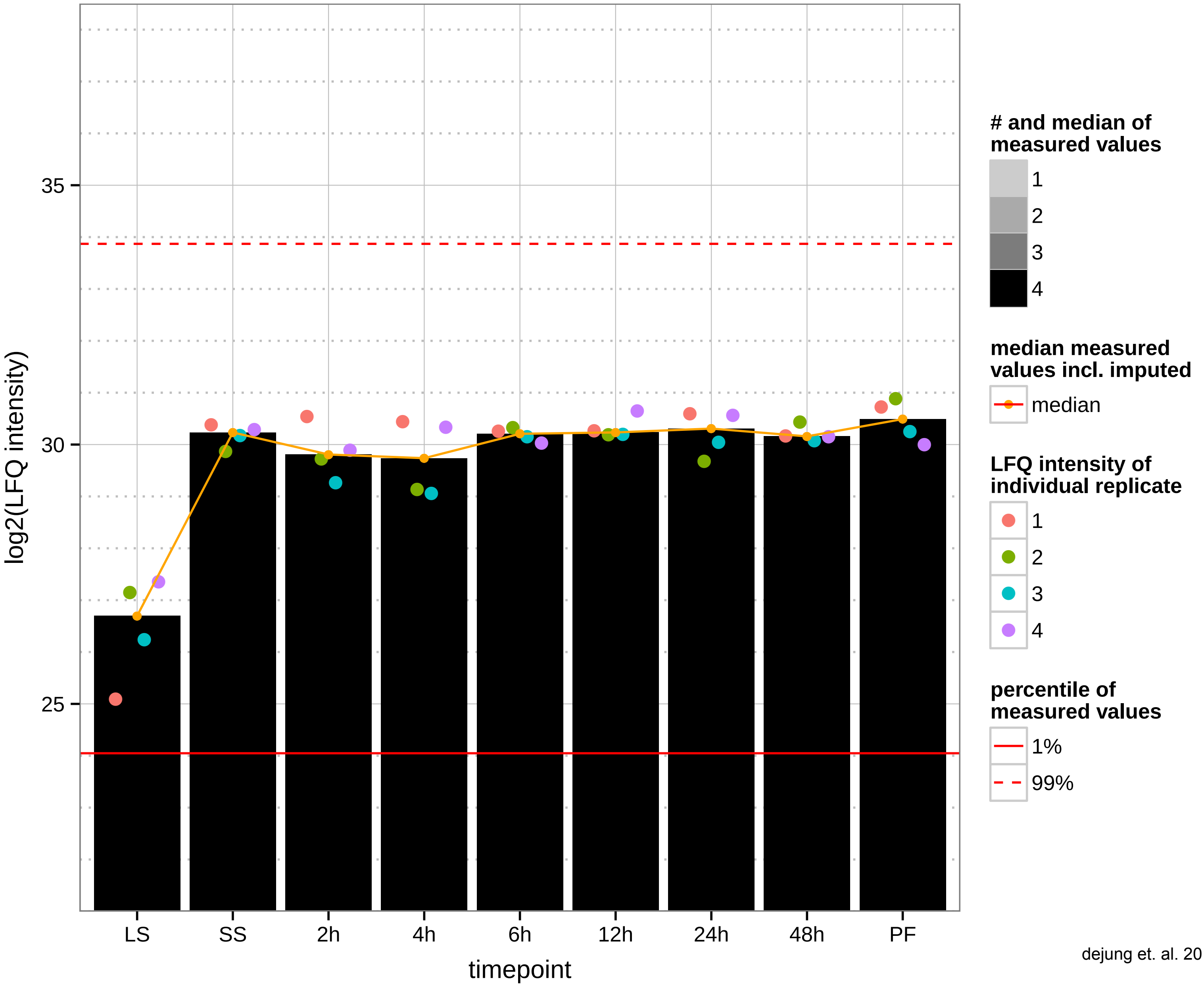

dejung et. al. 2015

coatomer alpha subunit, putative (TbCoatomerAlpha)
Tb927.4.450
AGOF: structural molecule activity, transporter activity
AGOC: COPI vesicle coat
AGOP: ER to Golgi vesicle–mediated transport, intracellular protein transport
PGOF: protein binding, structural molecule activity
PGOC: COPI vesicle coat, membrane coat
PGOP: intracellular protein transport, vesicle–mediated transport

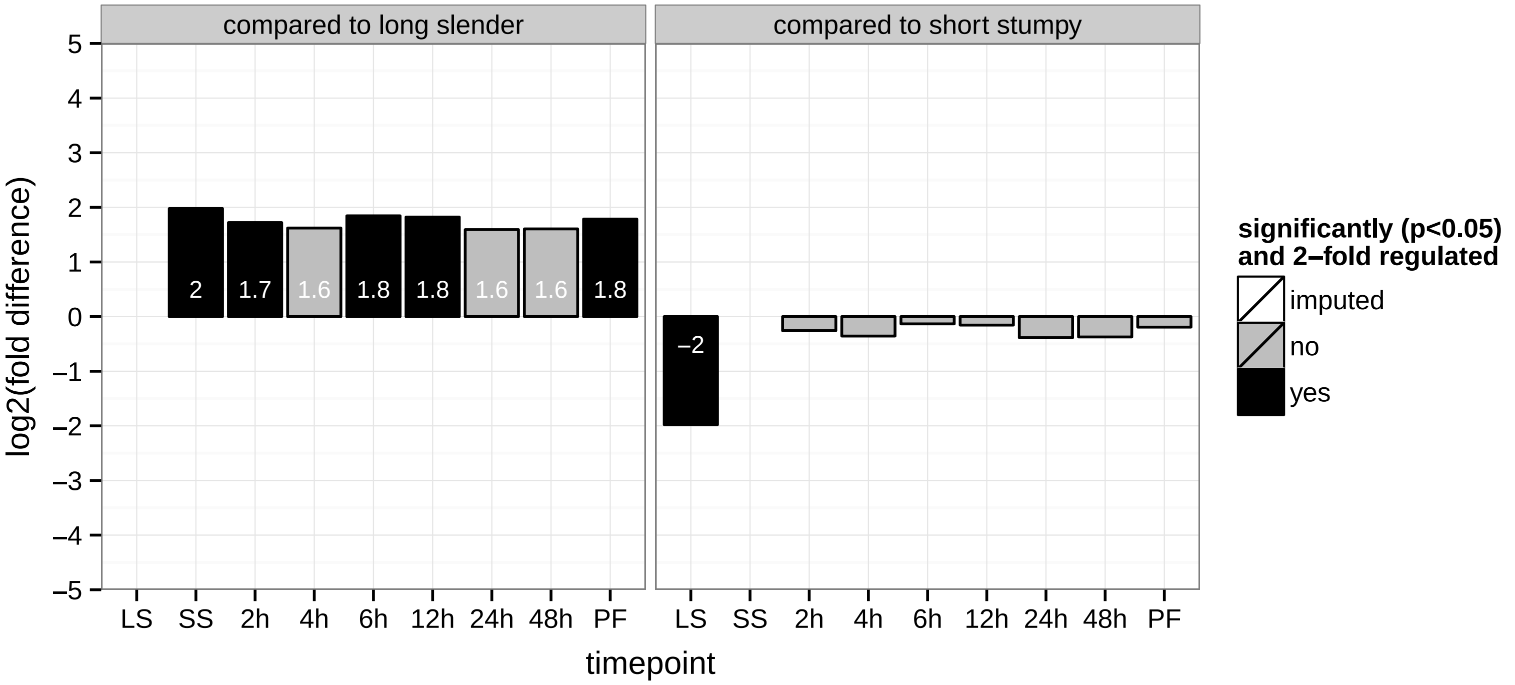

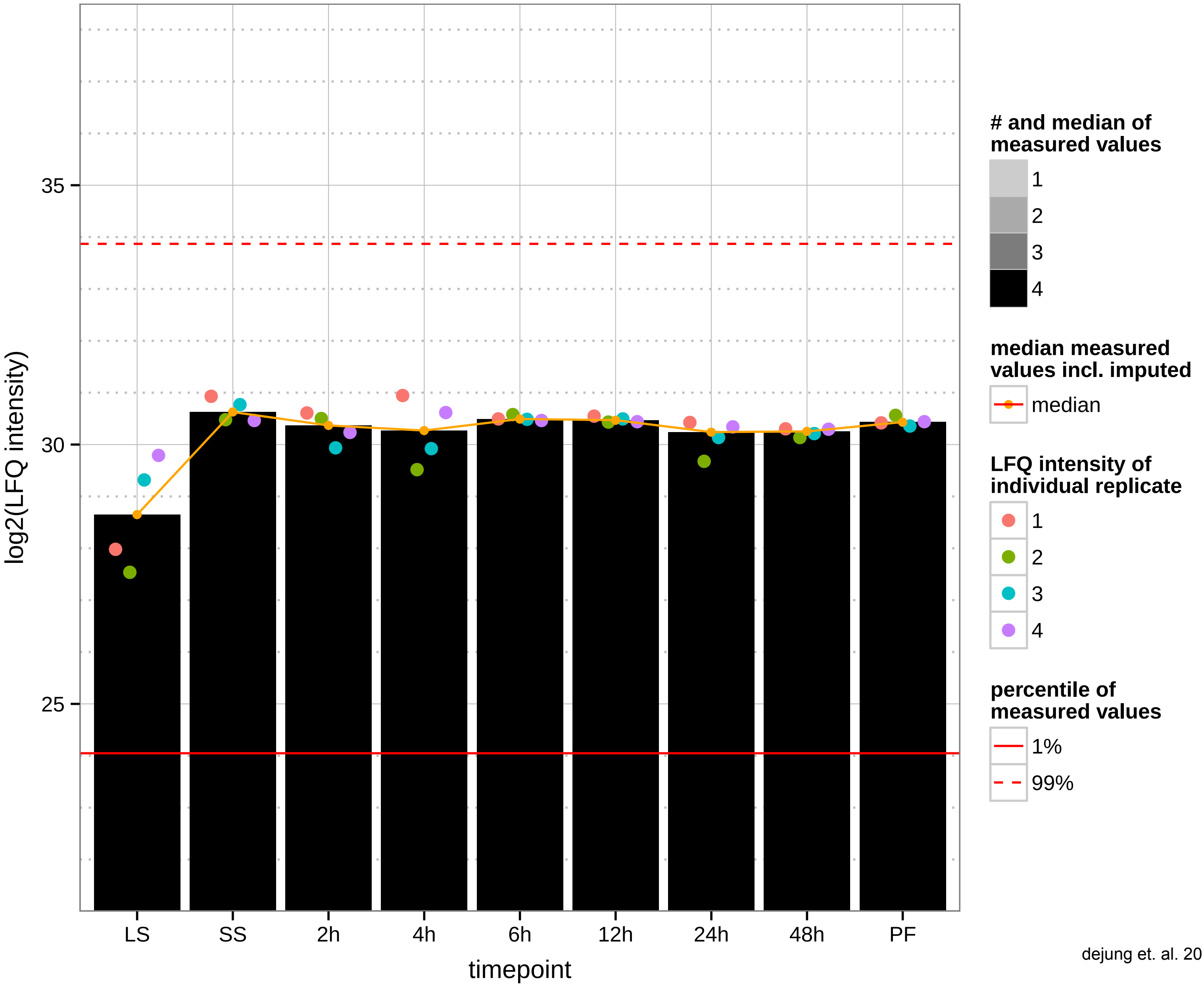

dejung et. al. 2015

hypothetical protein, conserved
Tb927.4.4550
AGOF: null
AGOC: null
AGOP: null
PGOF: null
PGOC: null
PGOP: null

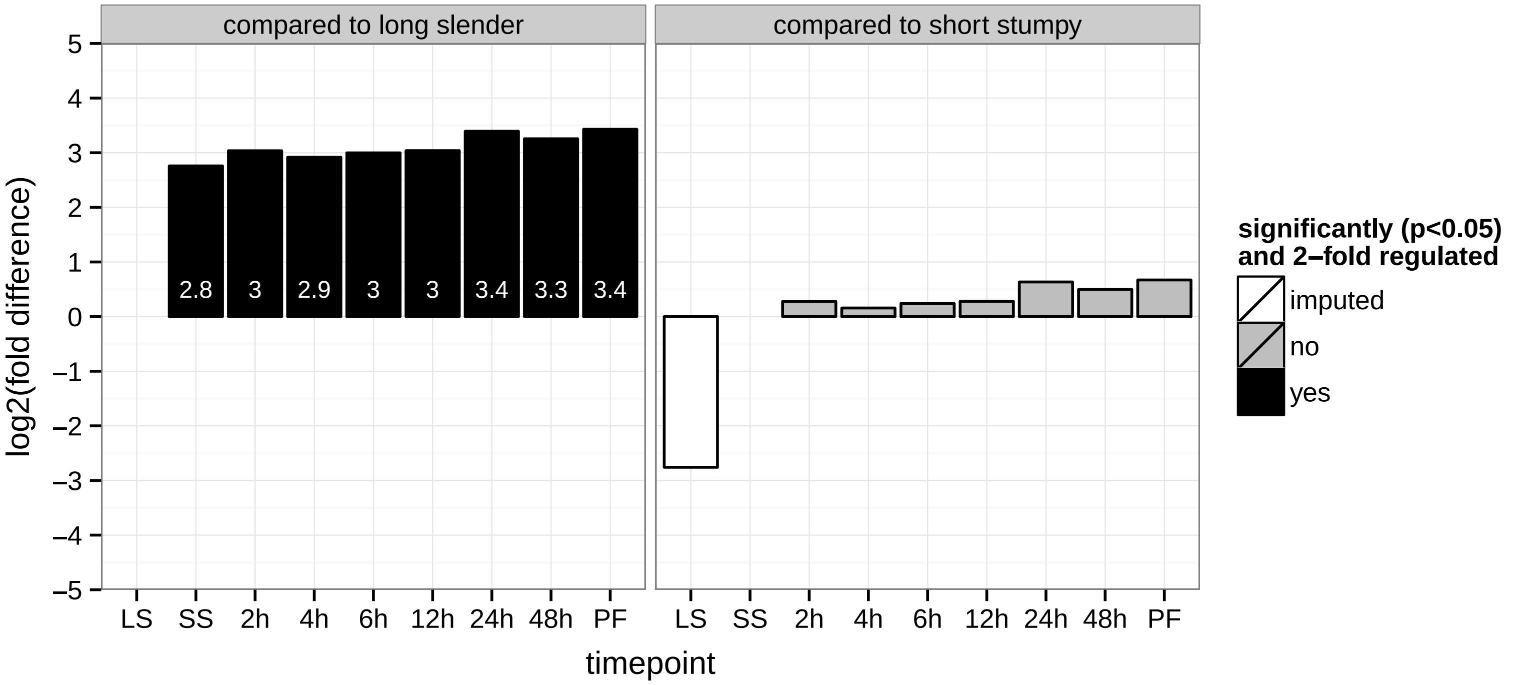

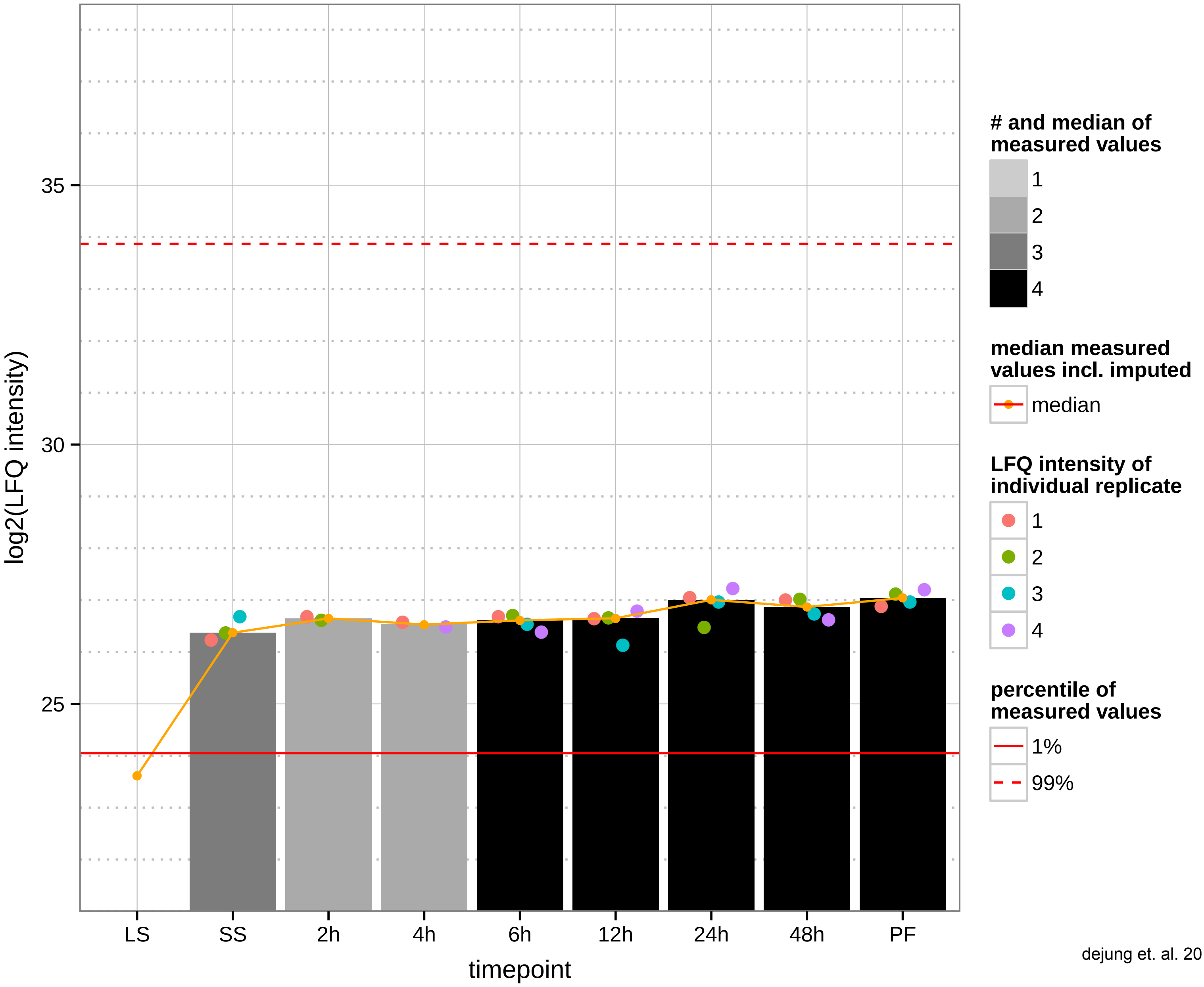

dejung et. al. 2015

diphthine synthase, putative
Tb927.4.4650
AGOF: diphthine synthase activity
AGOC: null
AGOP: peptidyl–diphthamide biosynthetic process from peptidyl–histidine
PGOF: diphthine synthase activity, methyltransferase activity
PGOC: null
PGOP: metabolic process, peptidyl–diphthamide biosynthetic process from peptidyl–histidine

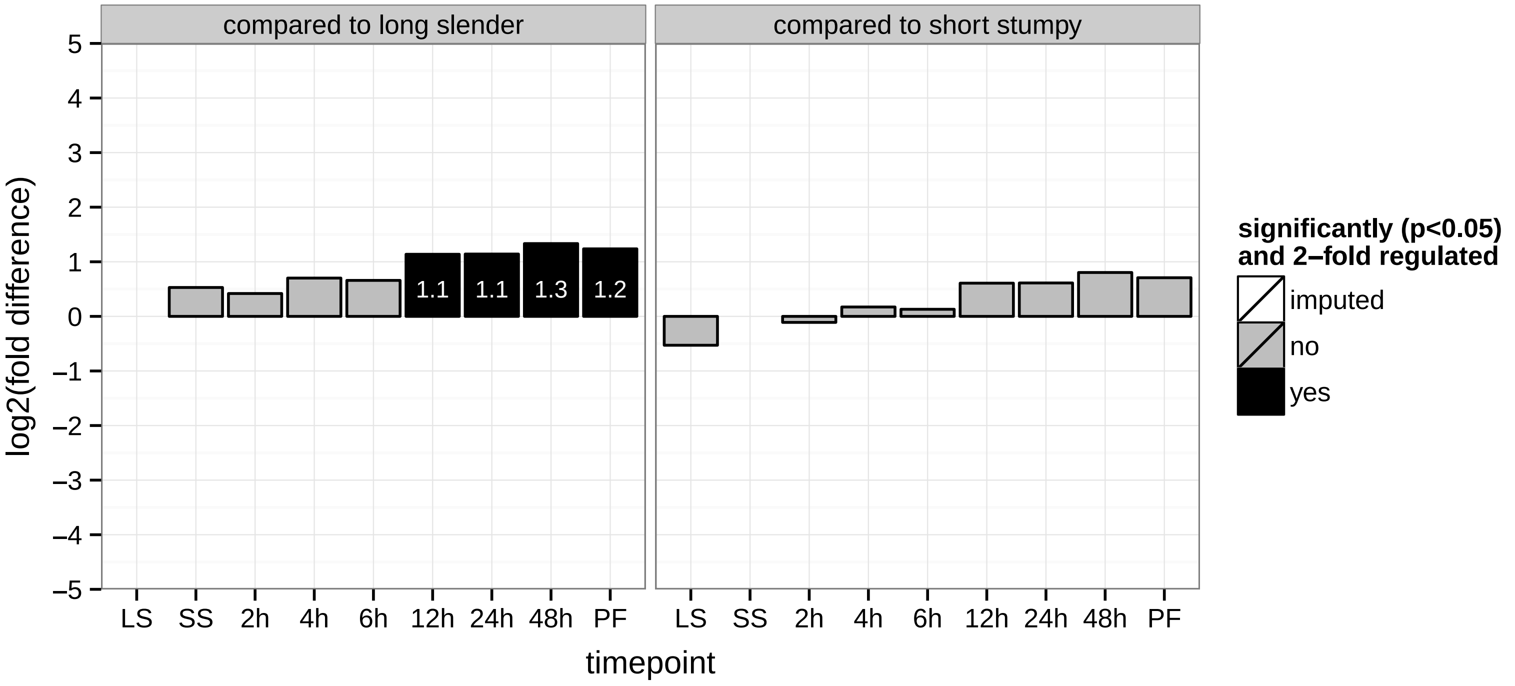

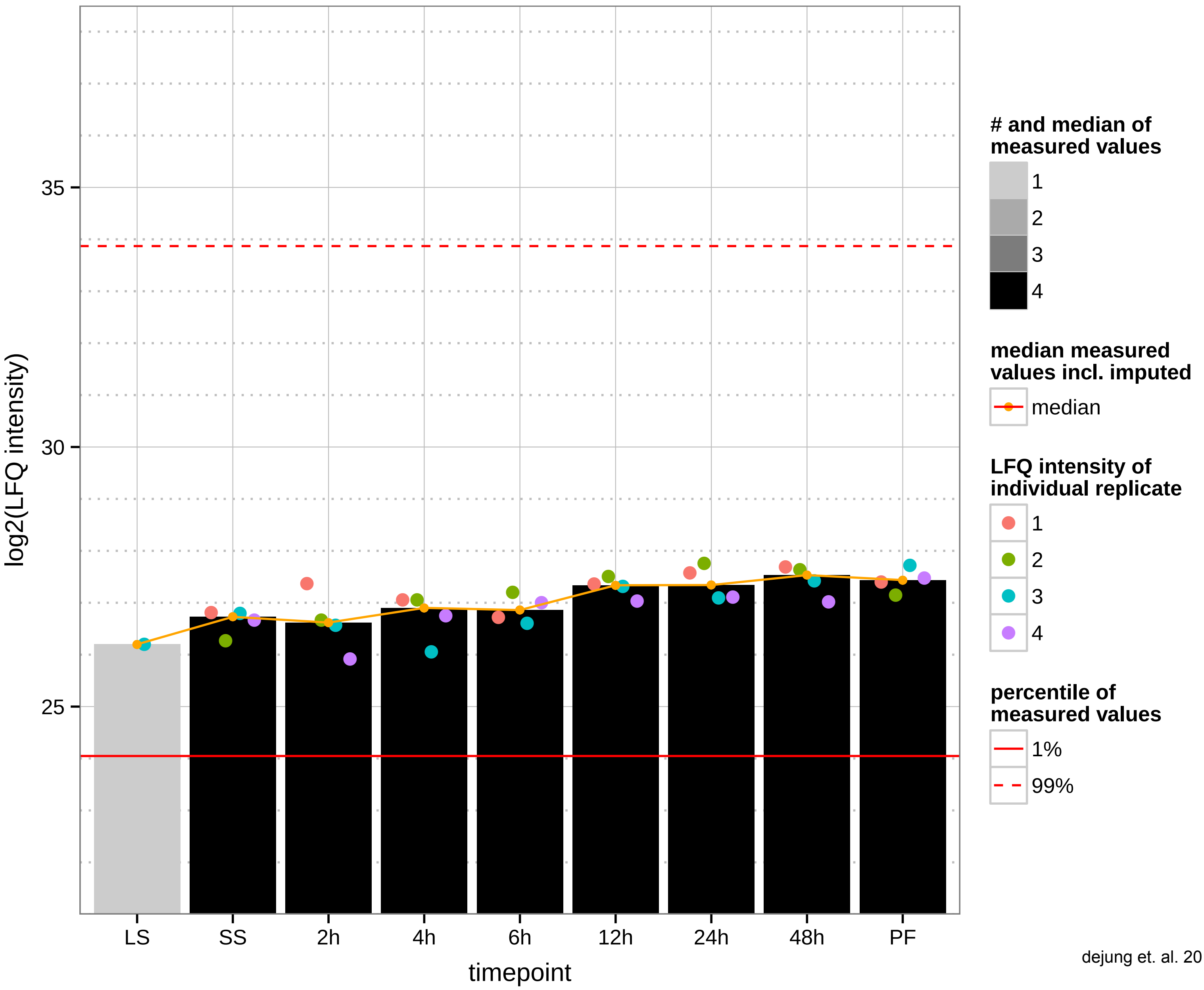

dejung et. al. 2015

hypothetical protein, conserved
Tb927.4.4660
AGOF: null
AGOC: null
AGOP: null
PGOF: null
PGOC: null
PGOP: null

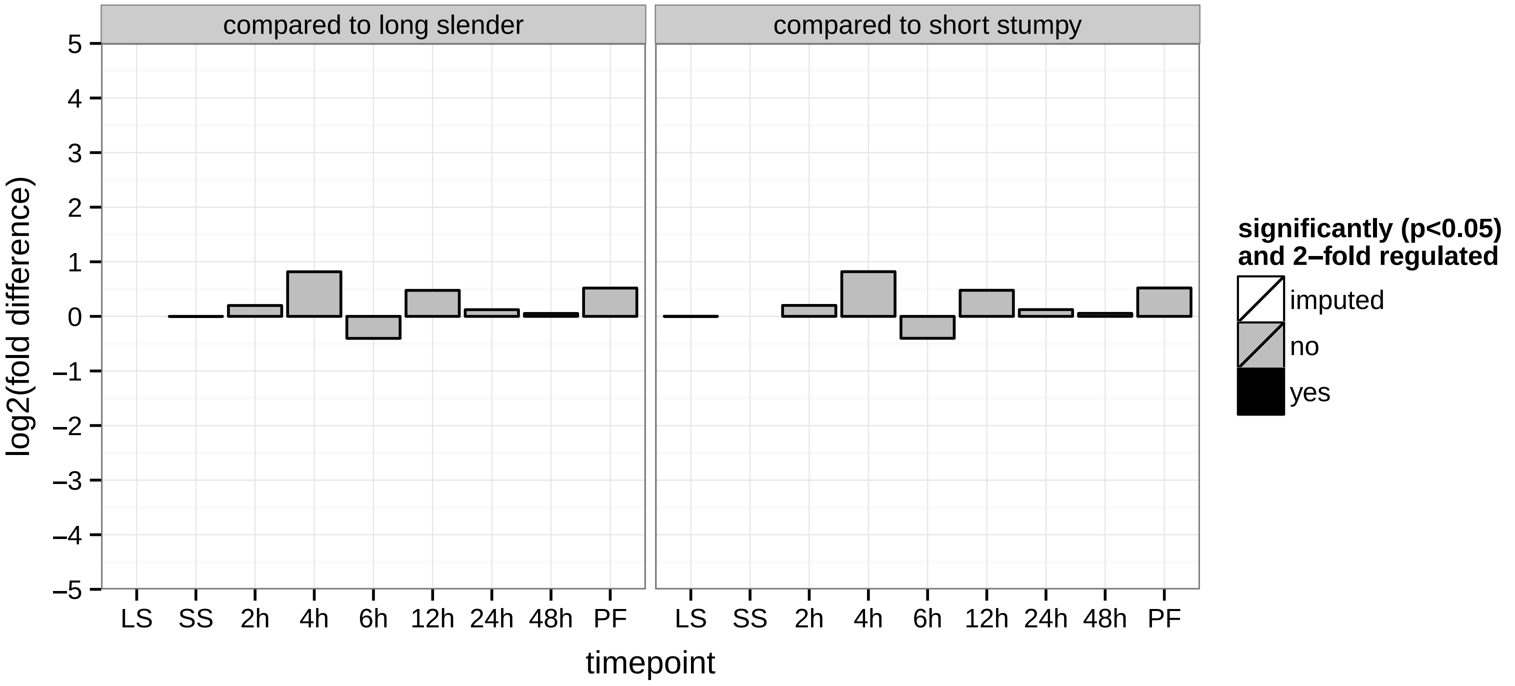

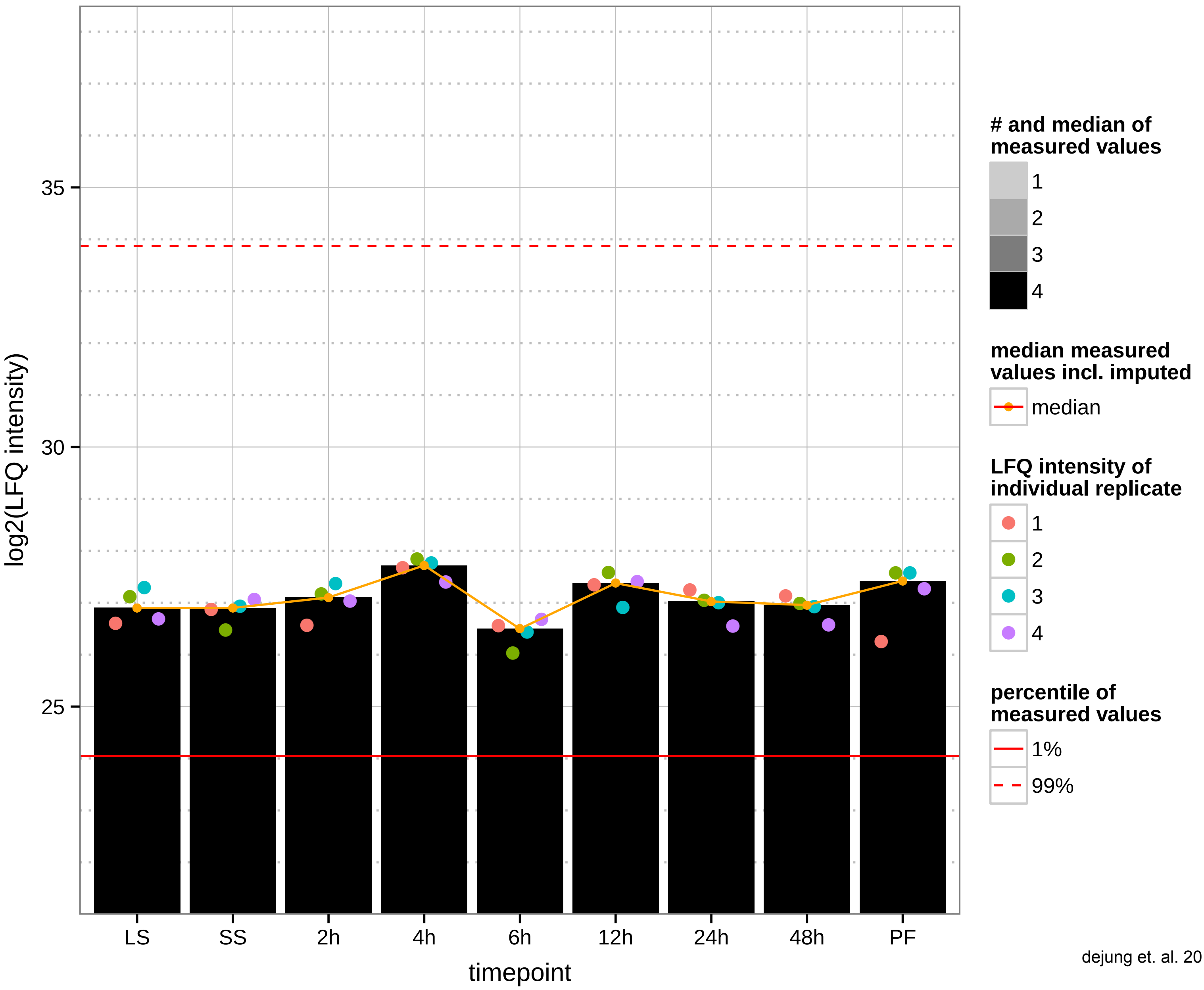

dejung et. al. 2015

hypothetical protein, conserved
Tb927.4.4670
AGOF: zinc ion binding
AGOC: null
AGOP: null
PGOF: zinc ion binding
PGOC: null
PGOP: null

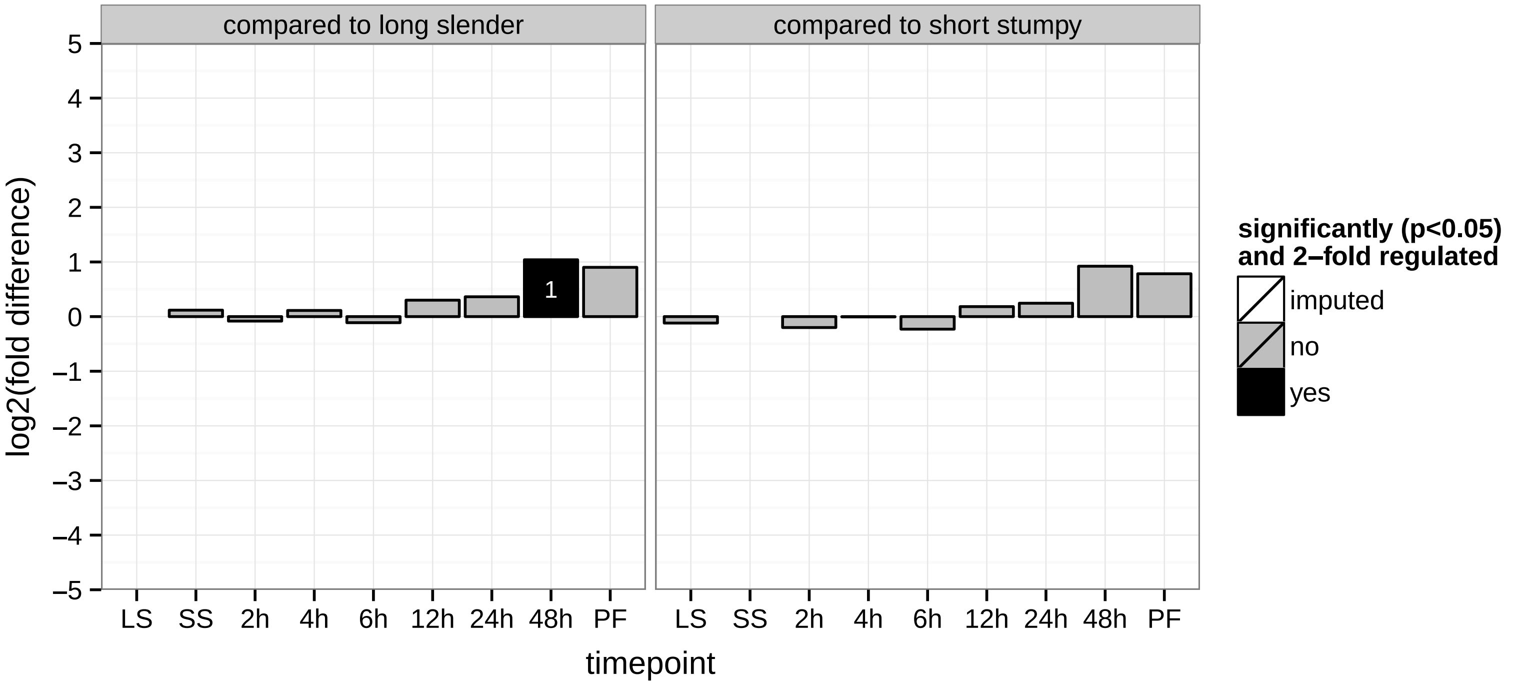

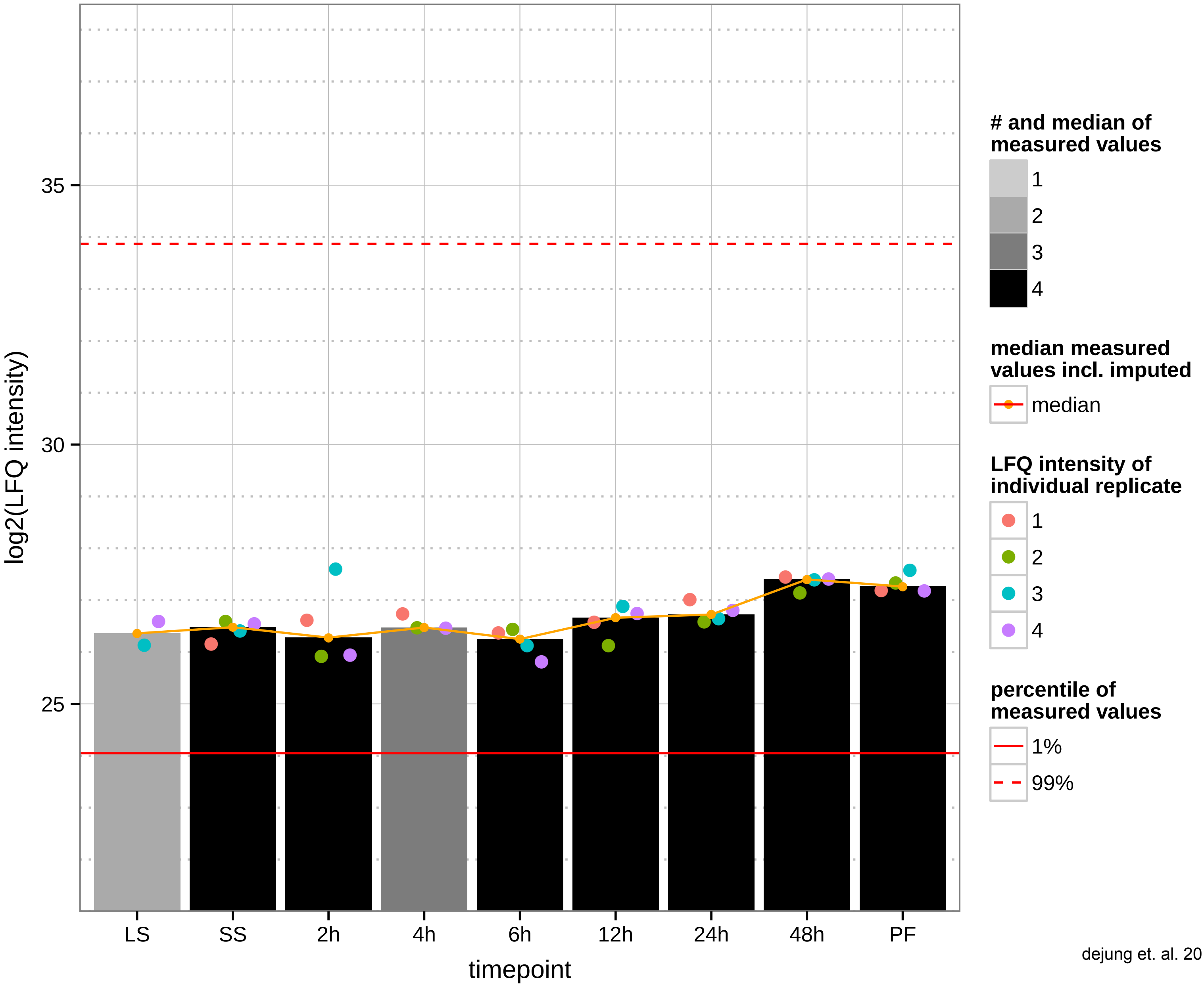

dejung et. al. 2015

hypothetical protein, conserved
Tb927.4.4690
AGOF: null
AGOC: null
AGOP: null
PGOF: null
PGOC: null
PGOP: null

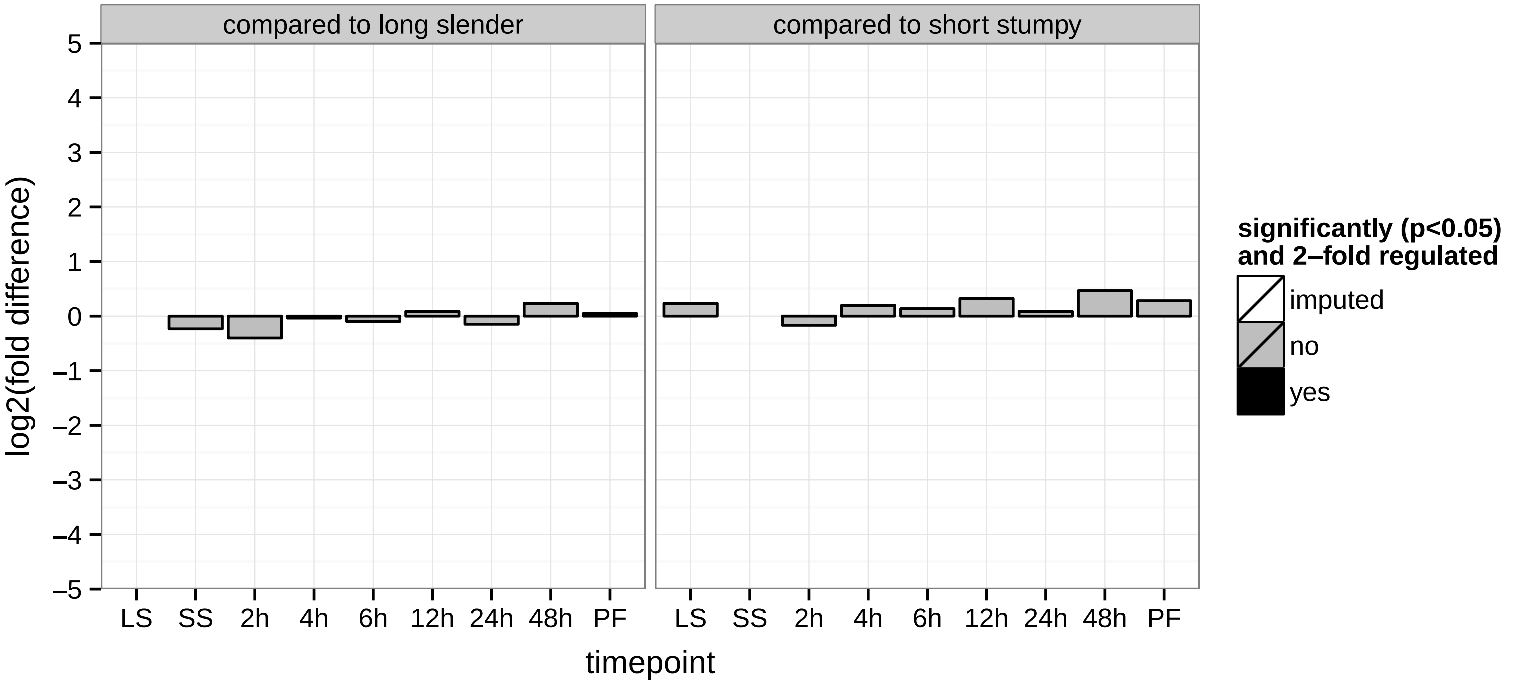

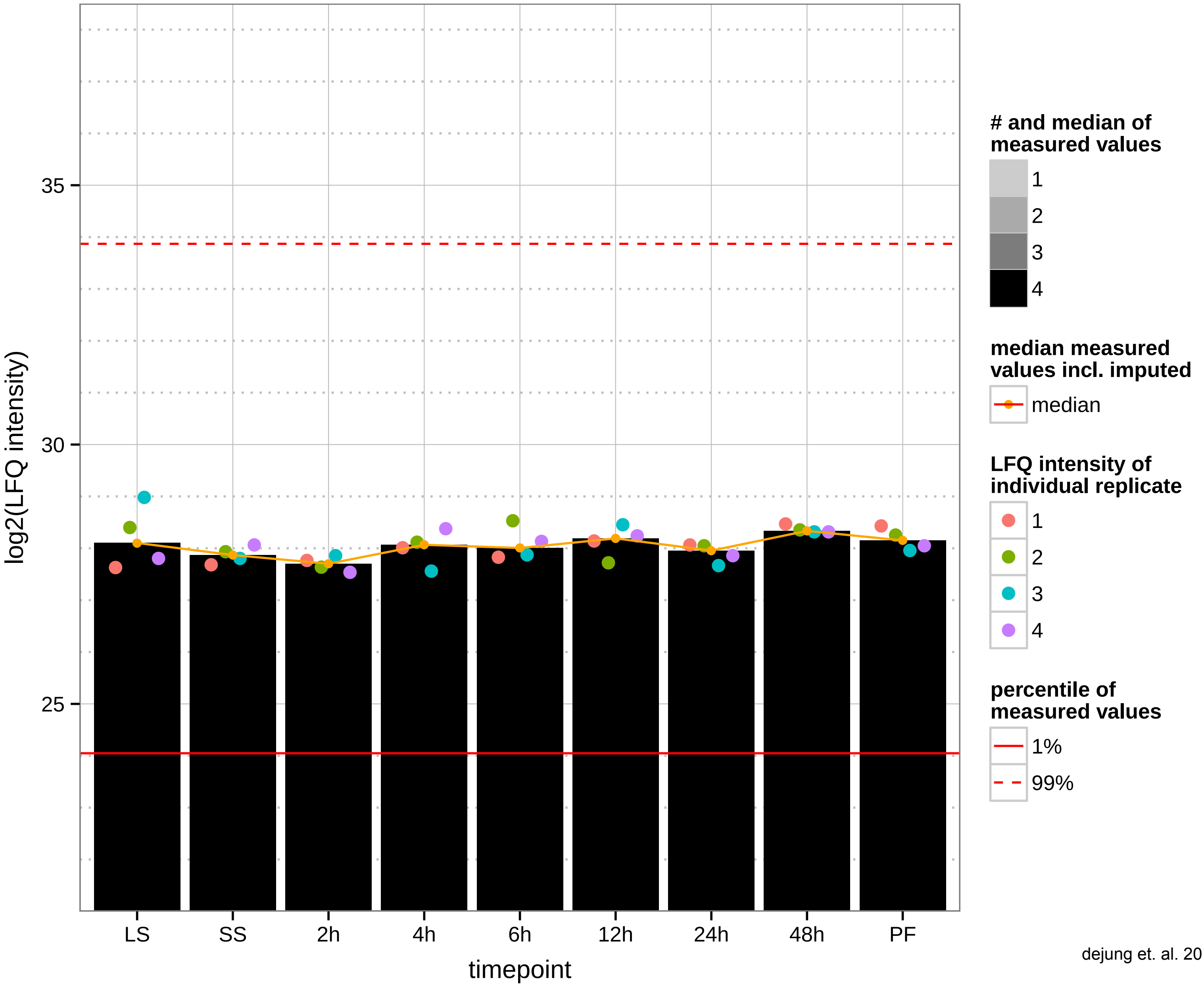

dejung et. al. 2015

snoRNP protein GAR1, putative
Tb927.4.470
AGOF: rRNA binding
AGOC: small nucleolar ribonucleoprotein complex
AGOP: rRNA processing
PGOF: pseudouridine synthase activity, snoRNA binding
PGOC: box H/ACA snoRNP complex
PGOP: ribosome biogenesis, snRNA pseudouridine synthesis

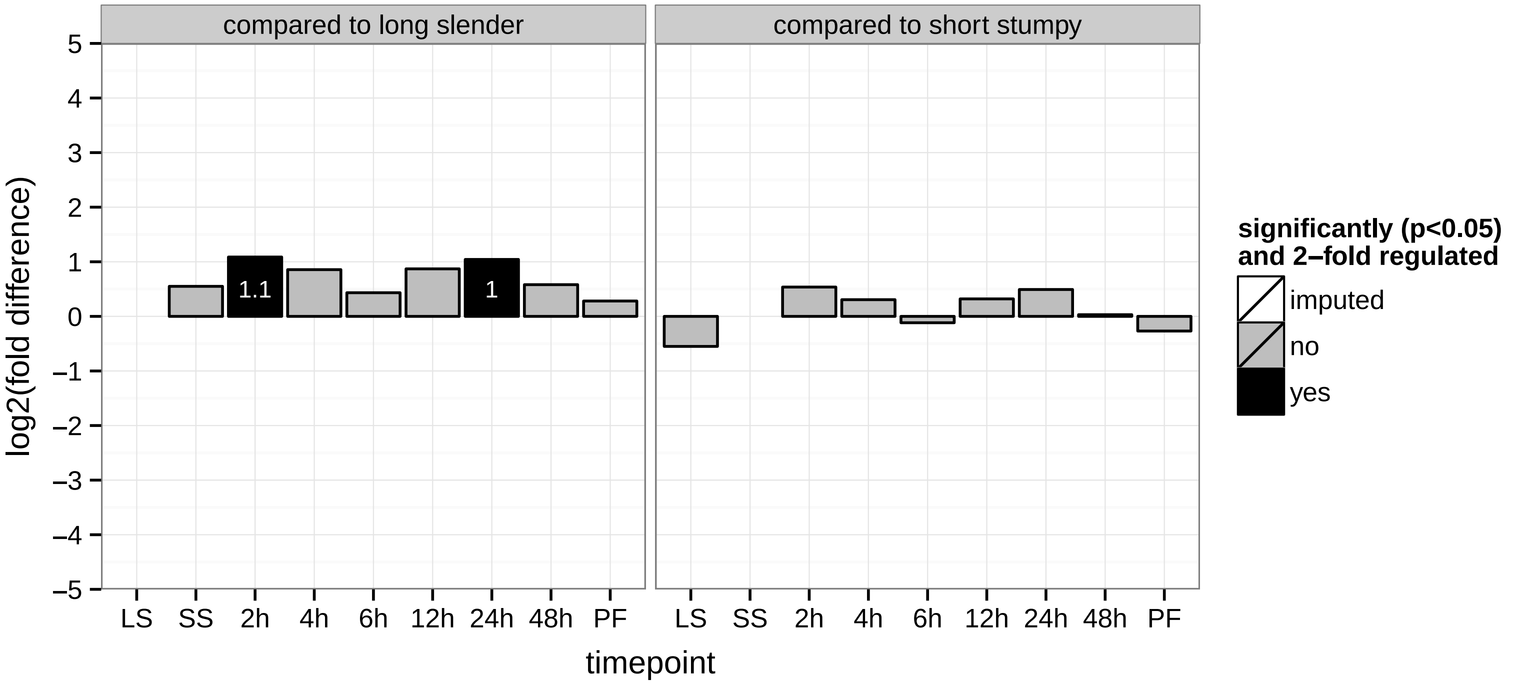

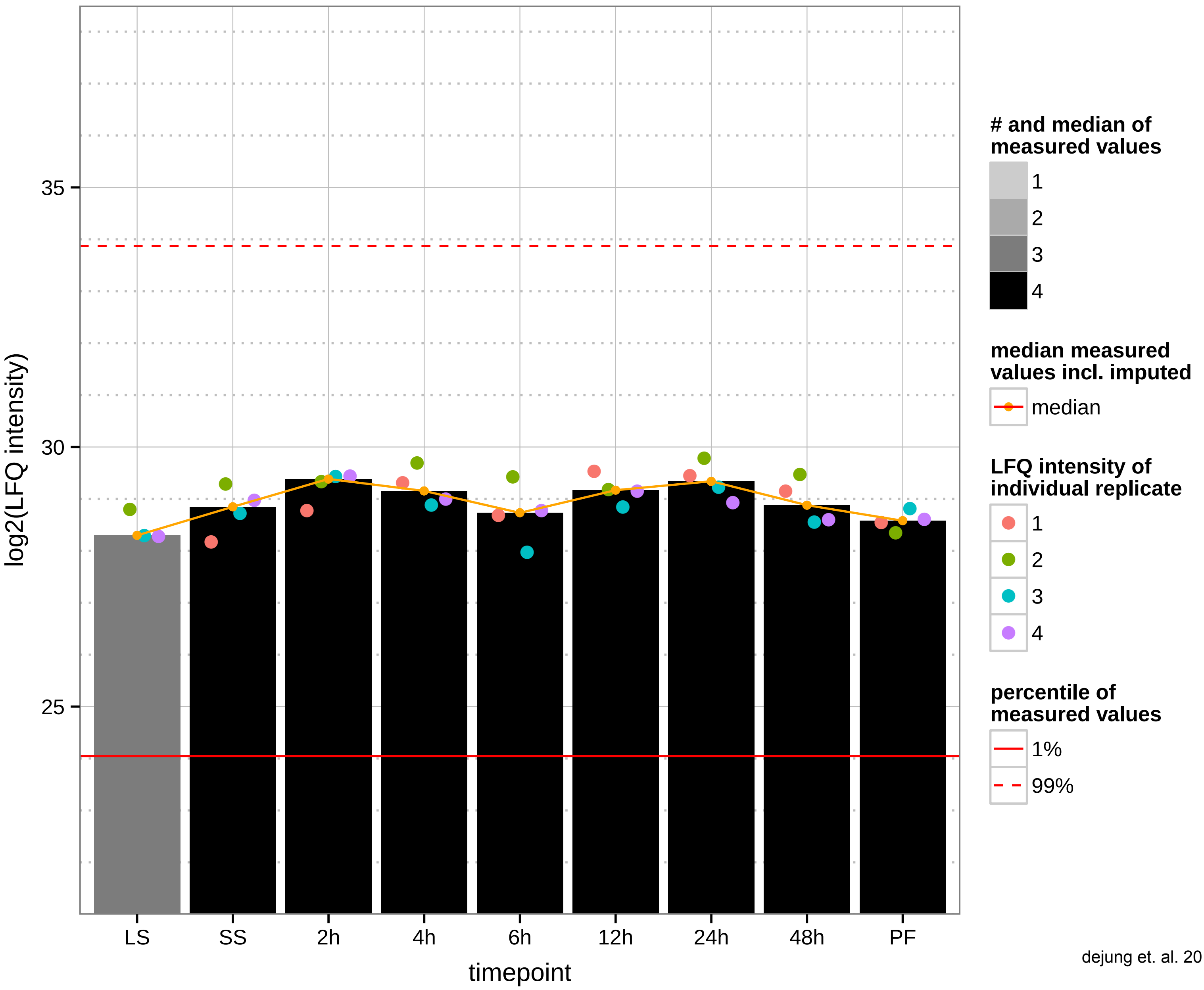

dejung et. al. 2015

hypothetical protein, conserved
Tb927.4.4700
AGOF: null
AGOC: null
AGOP: null
PGOF: null
PGOC: null
PGOP: null

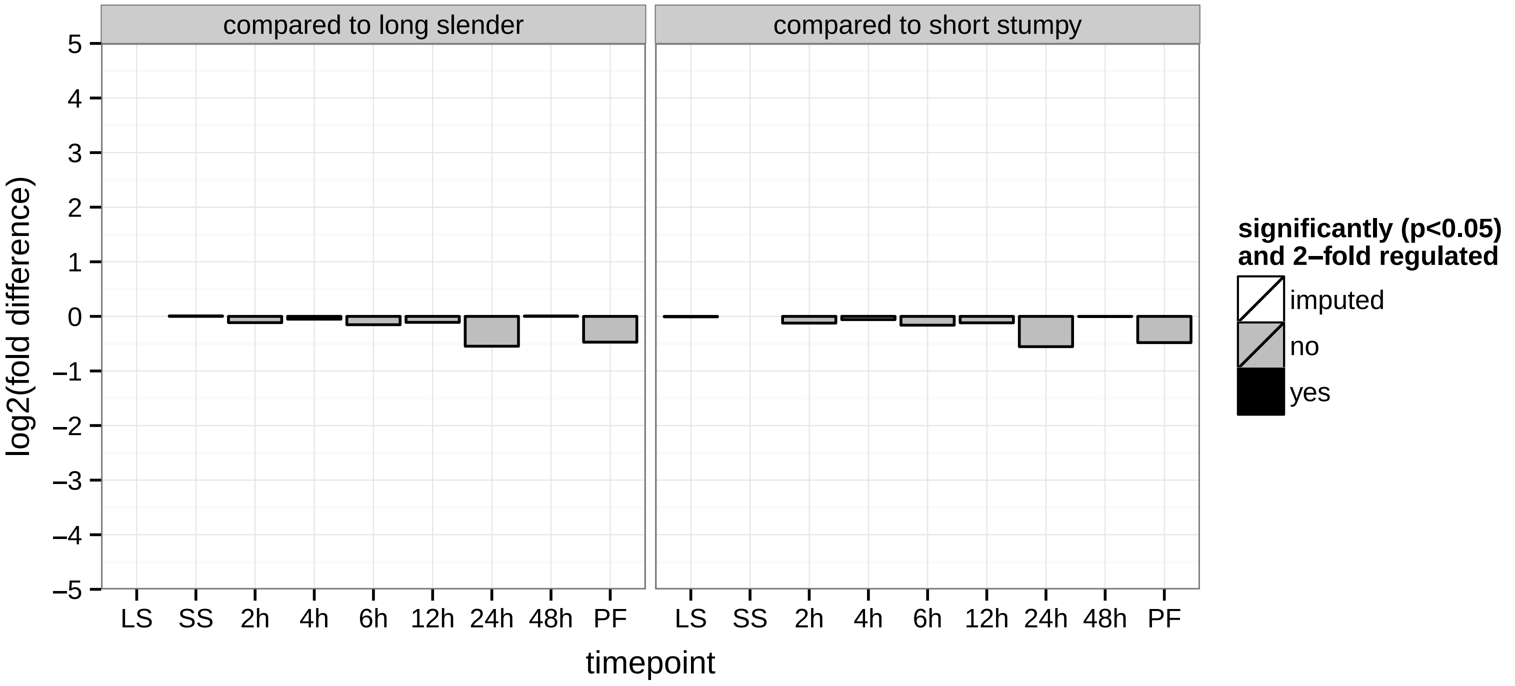

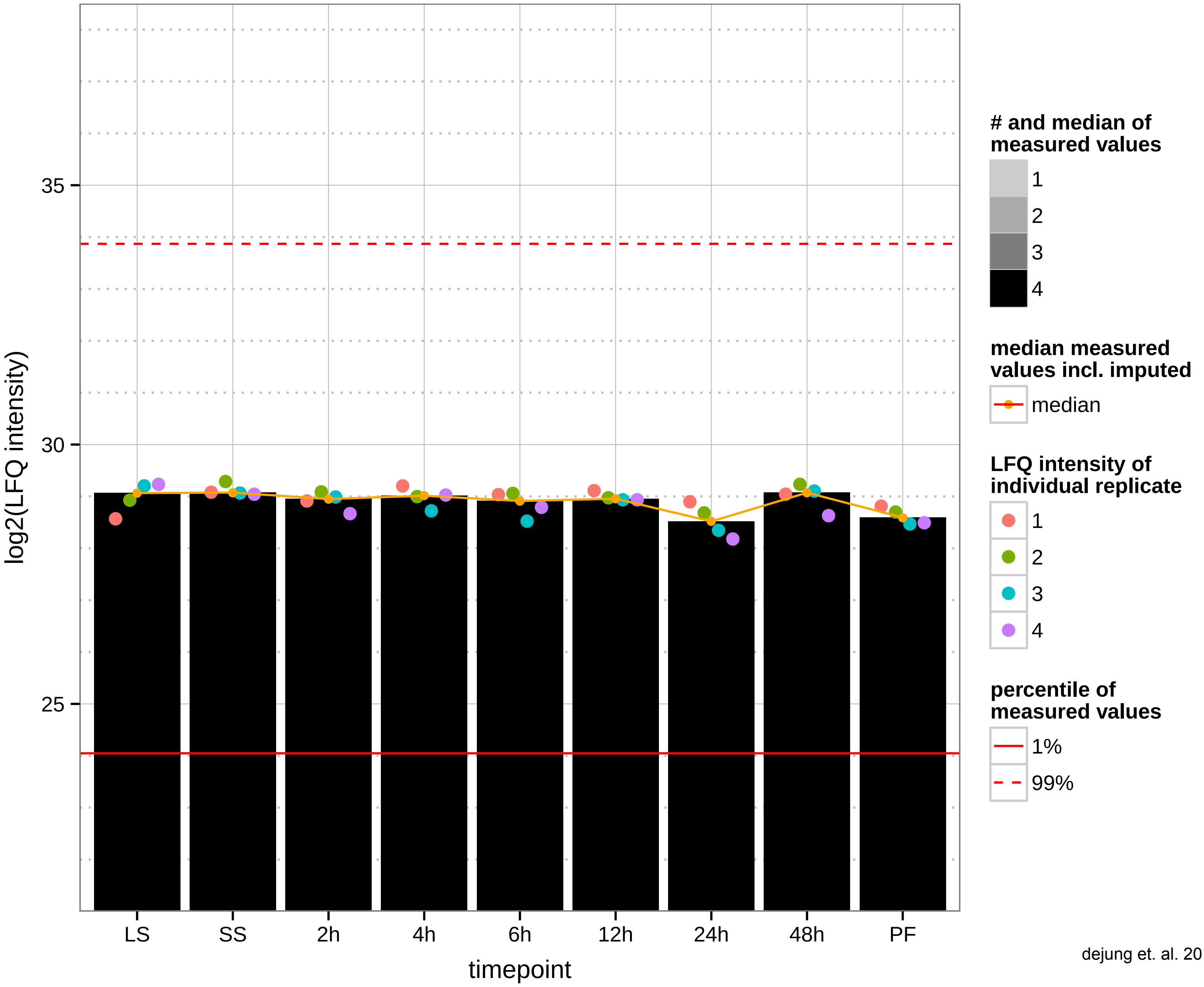

dejung et. al. 2015

hypothetical protein, conserved
Tb927.4.4950;Tb927.8.7470
AGOF: null
AGOC: null
AGOP: null
PGOF: protein binding
PGOC: null
PGOP: null

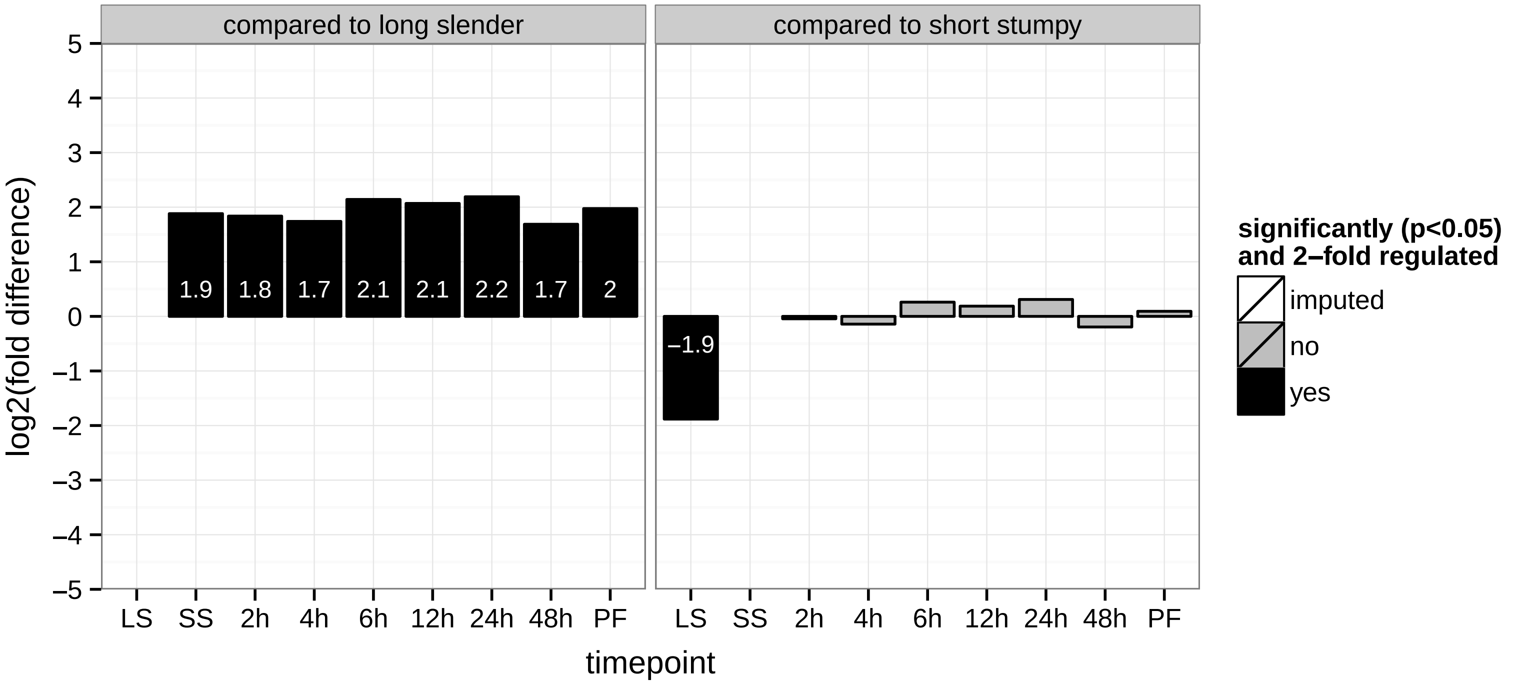

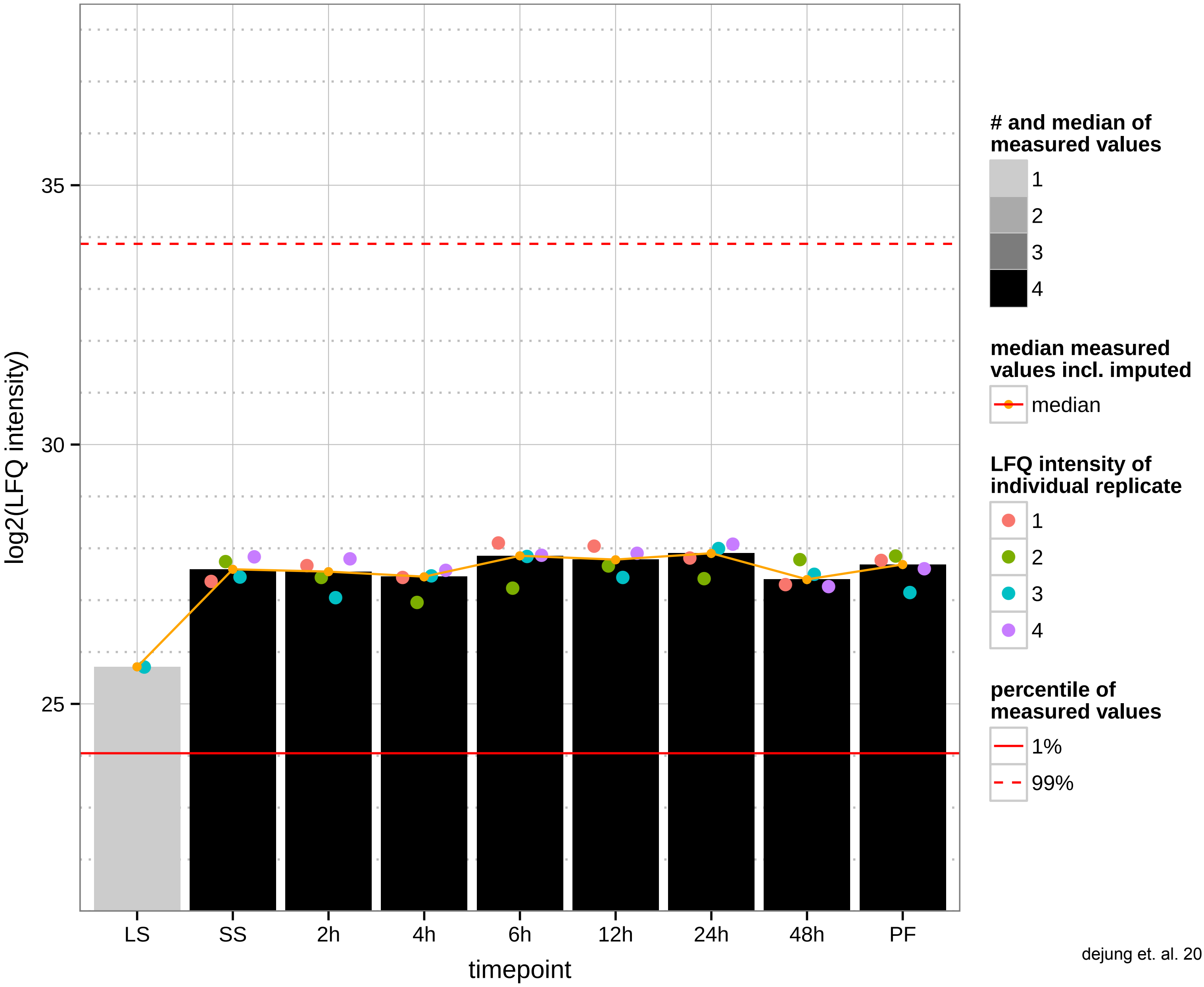

dejung et. al. 2015

hypothetical protein, conserved
Tb927.4.5000;Tb927.8.7420
AGOF: null
AGOC: null
AGOP: null
PGOF: null
PGOC: null
PGOP: null

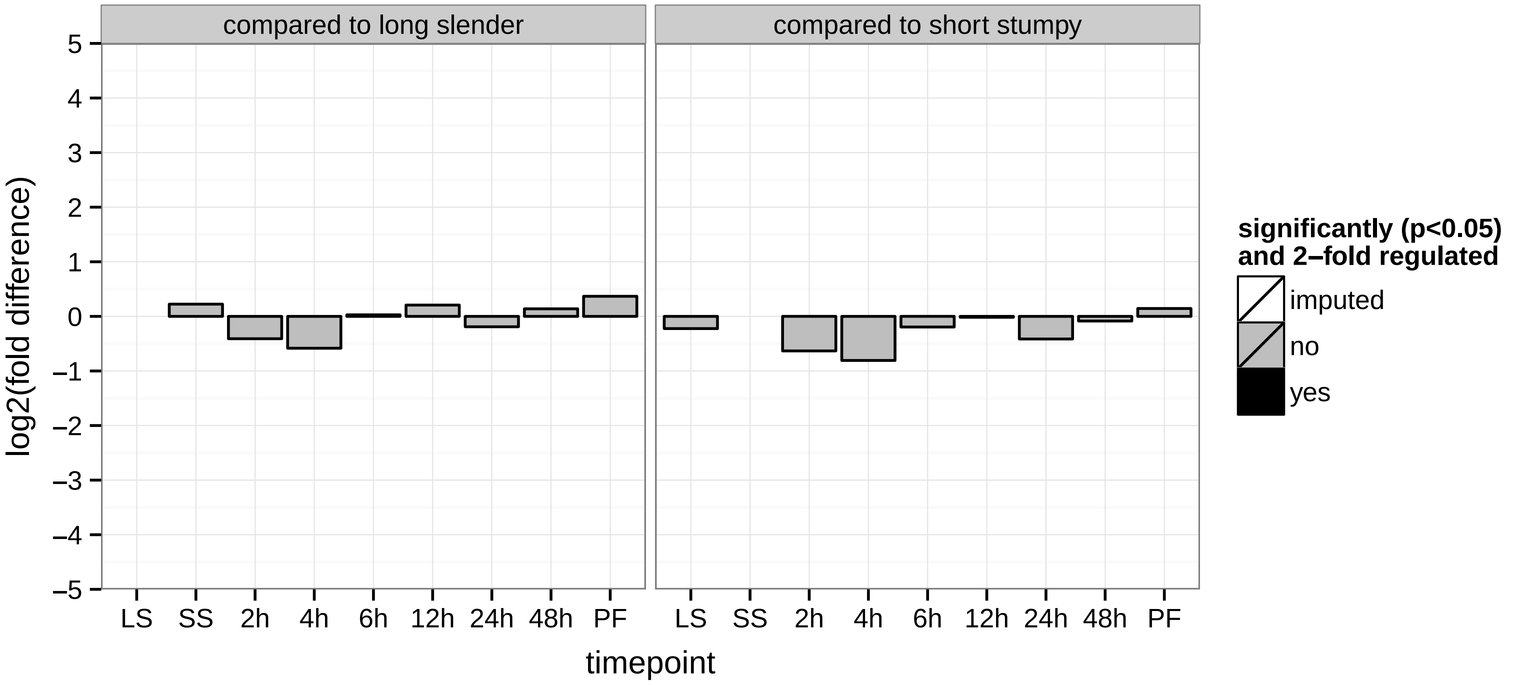

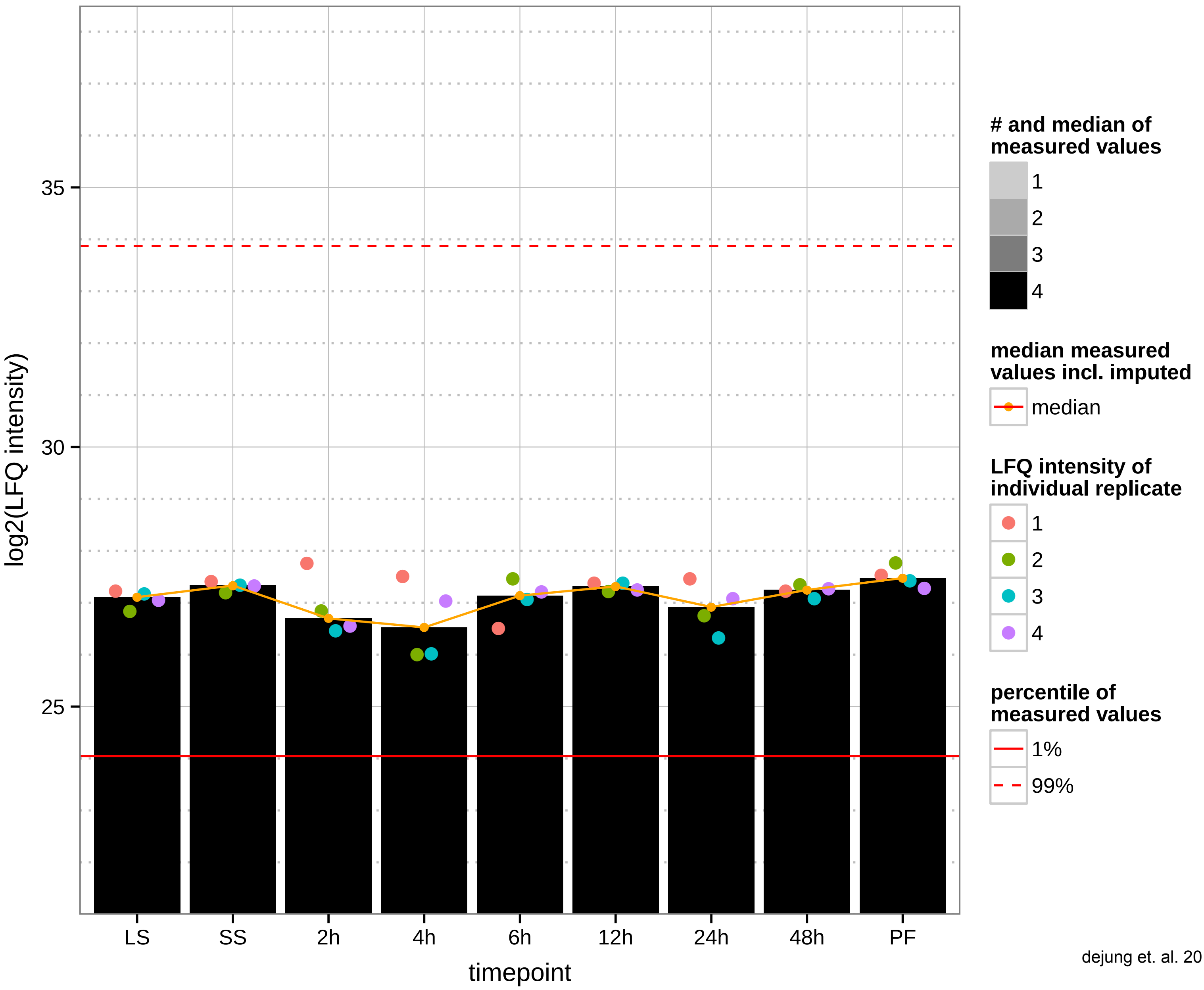

dejung et. al. 2015

dihydrolipoamide dehydrogenase, putative
Tb927.4.5040
AGOF: dihydrolipoyl dehydrogenase activity, flavin adenine dinucleotide binding
AGOC: cytoplasm
AGOP: cell redox homeostasis
PGOF: flavin adenine dinucleotide binding, oxidoreductase activity
PGOC: cytoplasm
PGOP: cell redox homeostasis, oxidation–reduction process

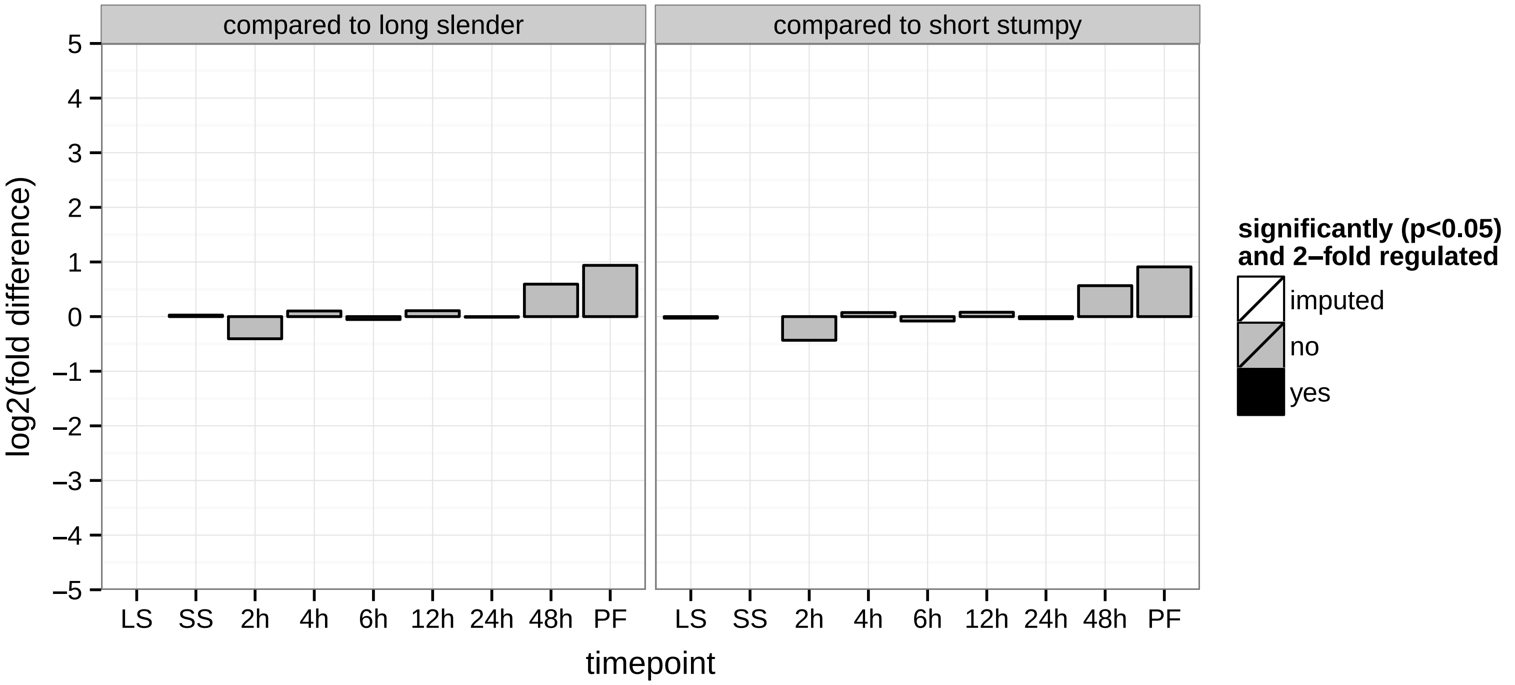

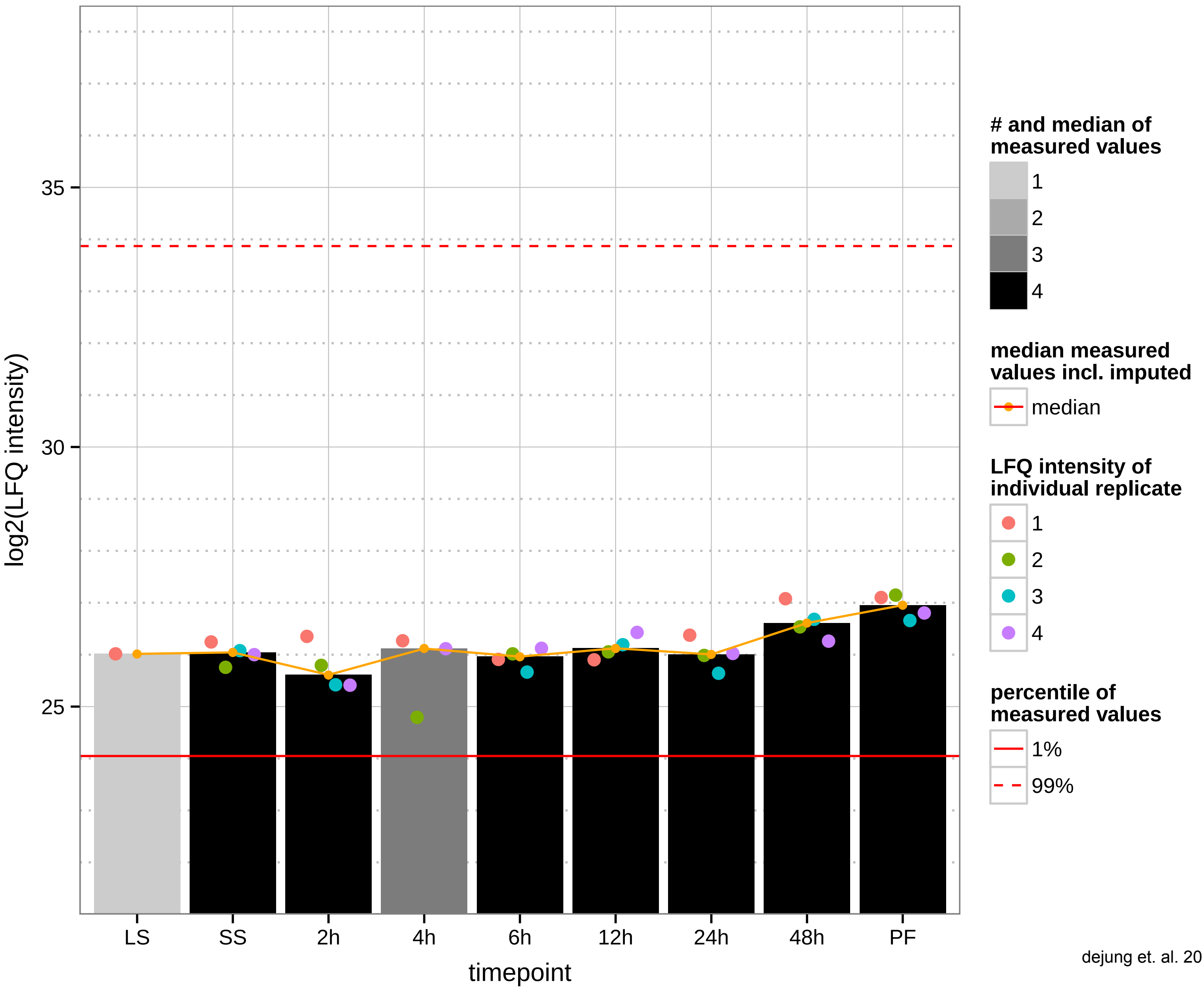

dejung et. al. 2015

hypothetical protein, conserved
Tb927.4.5130
AGOF: RNA binding
AGOC: null
AGOP: null
PGOF: RNA binding
PGOC: null
PGOP: null

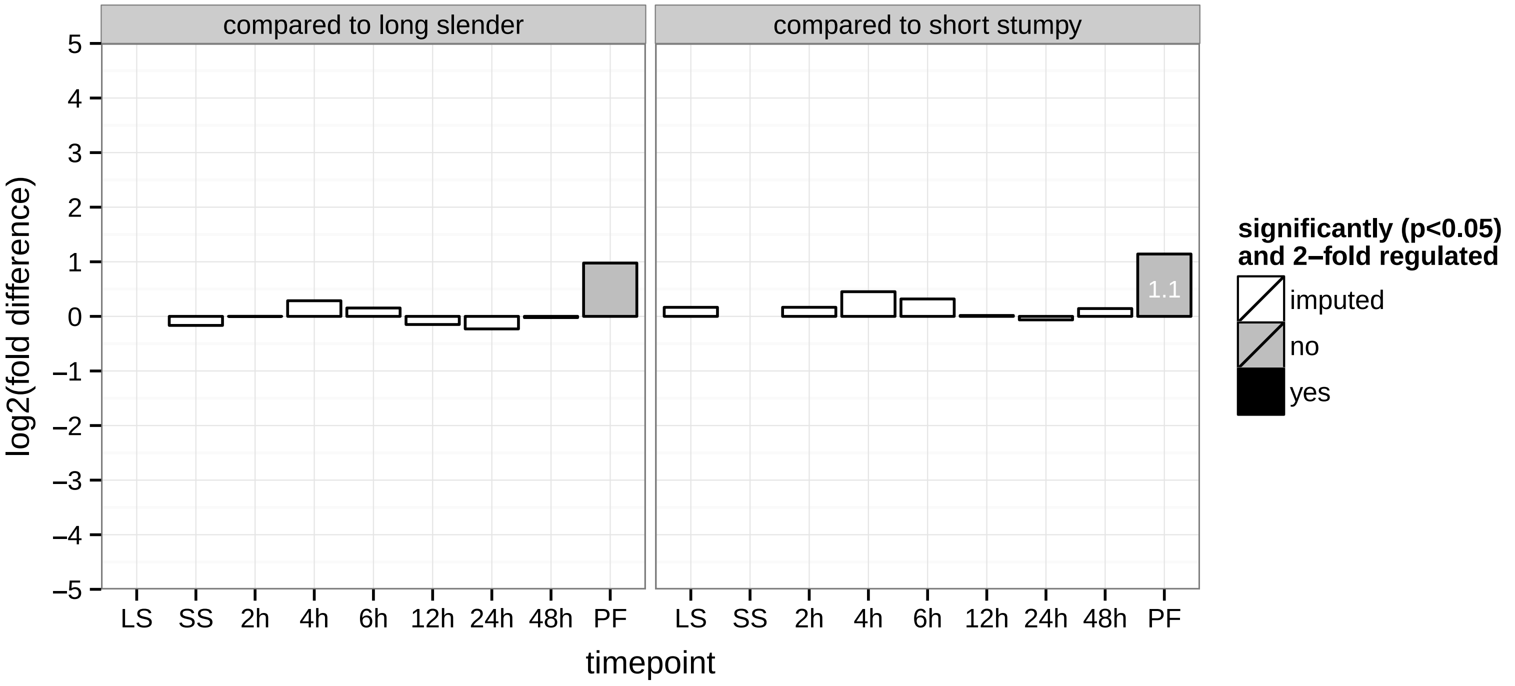

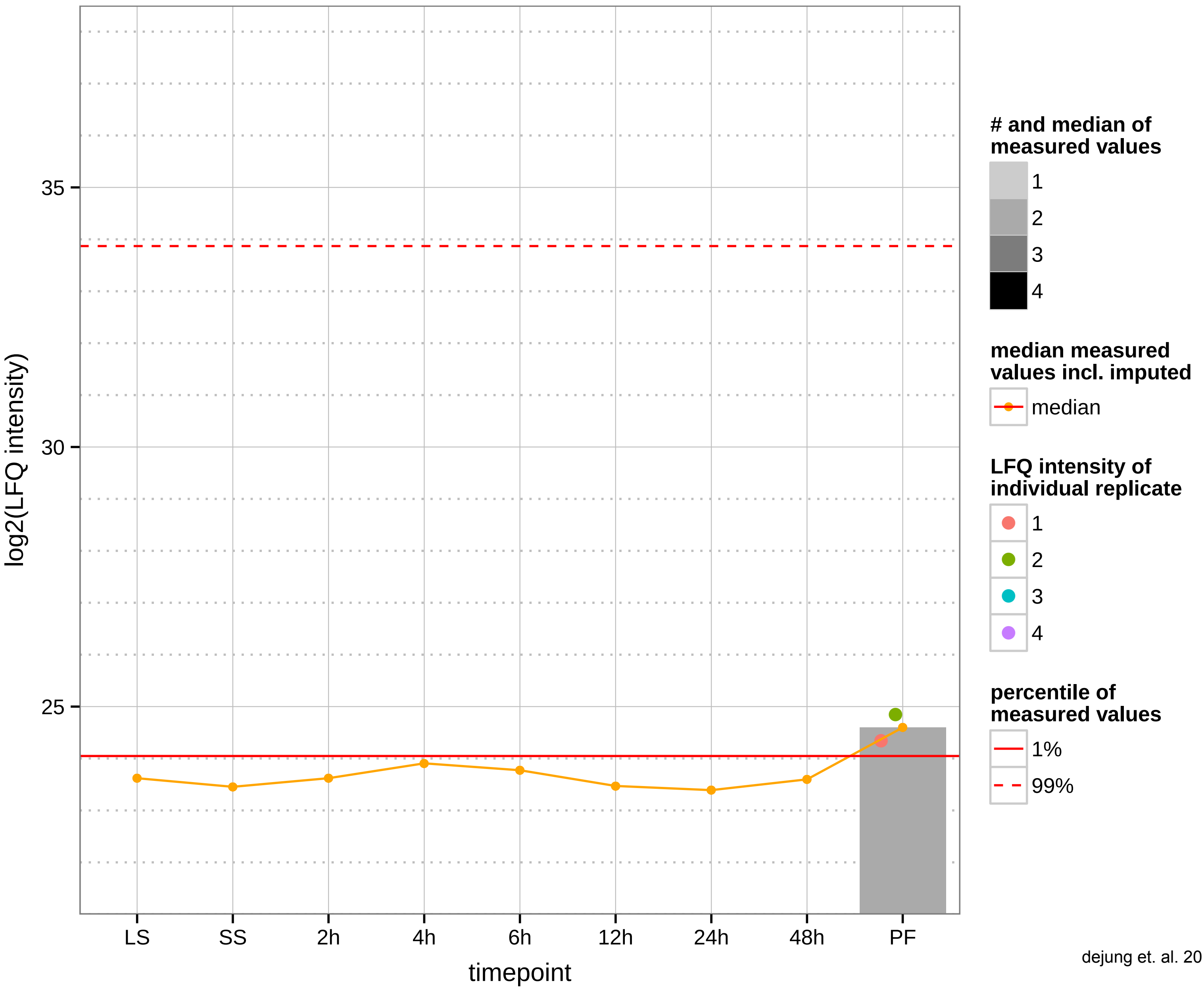

dejung et. al. 2015

hypothetical protein, conserved
Tb927.4.520
AGOF: null
AGOC: null
AGOP: null
PGOF: null
PGOC: null
PGOP: null

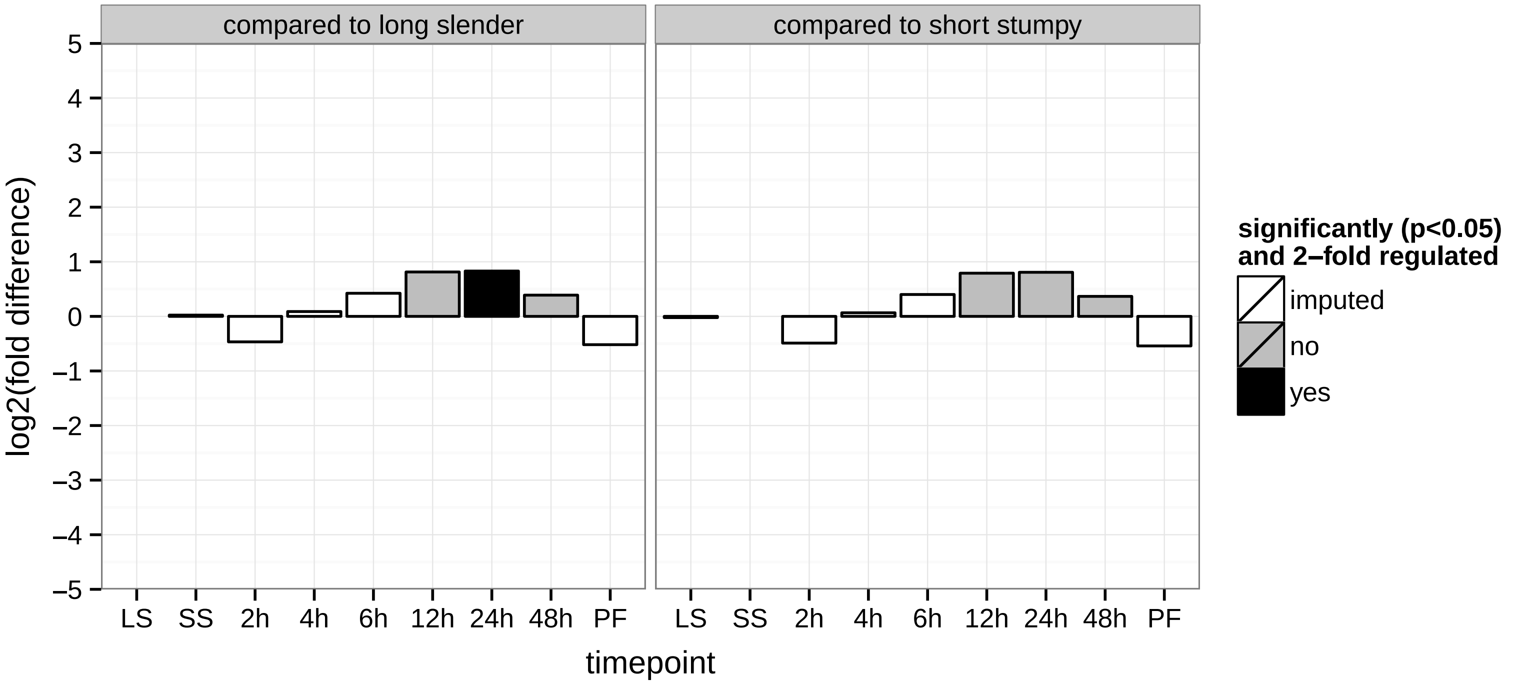

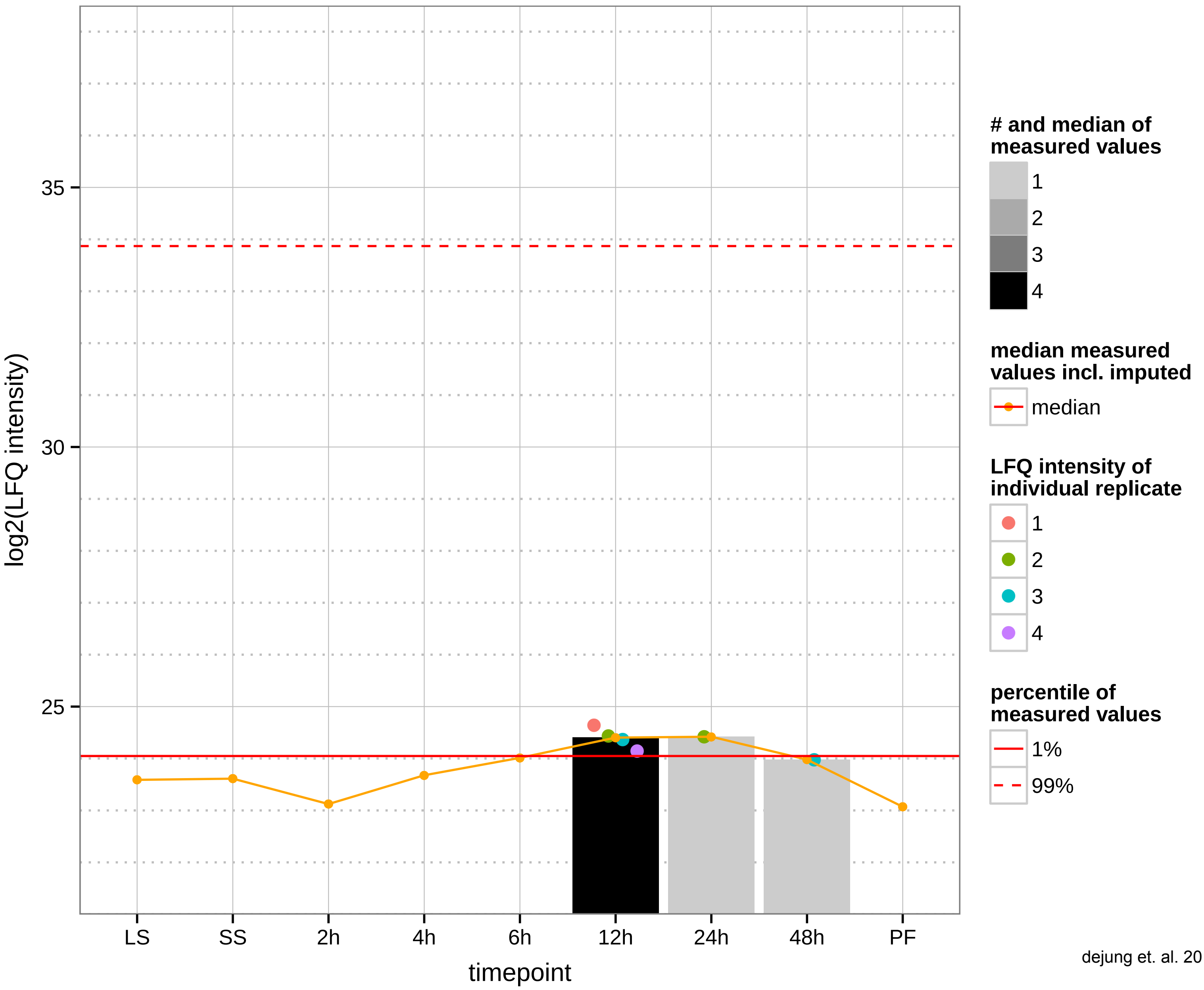

dejung et. al. 2015

nucleoporin (NUP54/57), putative (TbNup62)
Tb927.4.5200
AGOF: null
AGOC: null
AGOP: null
PGOF: null
PGOC: null
PGOP: null

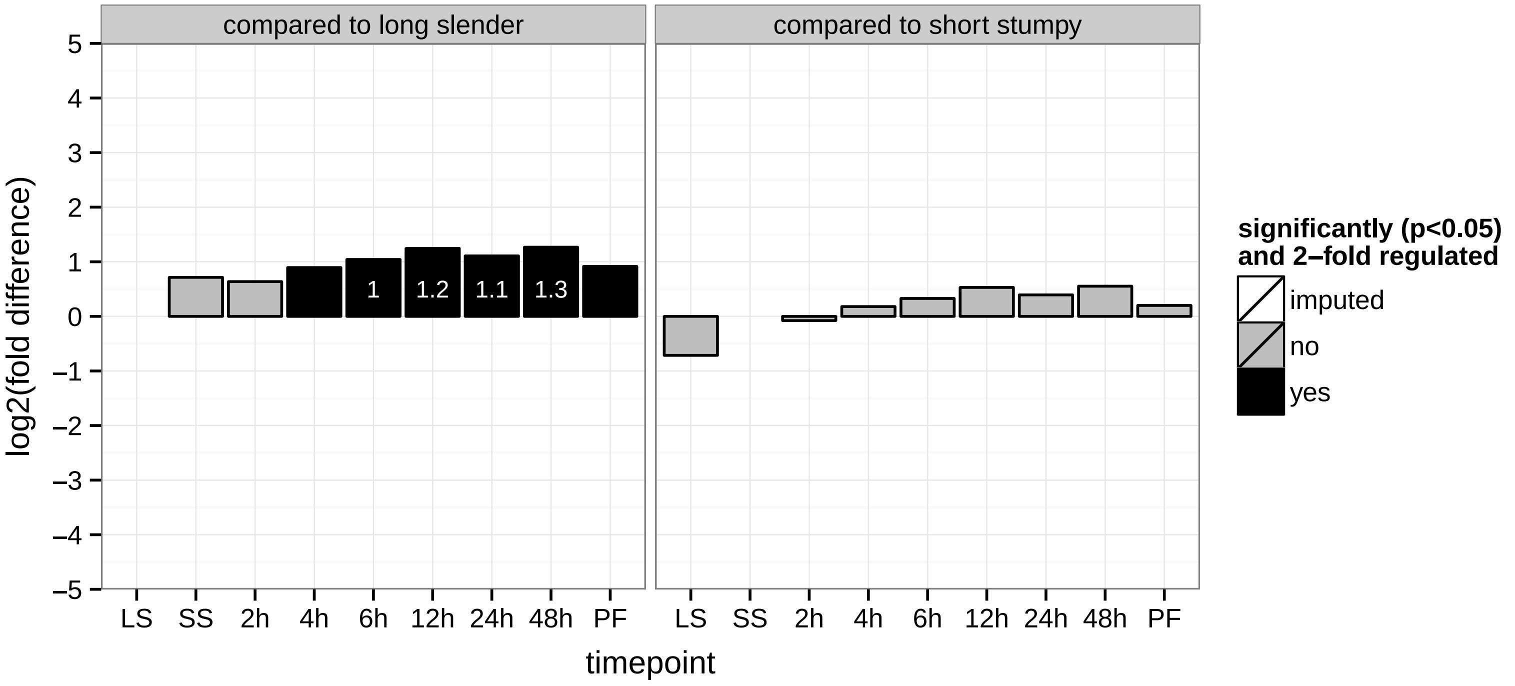

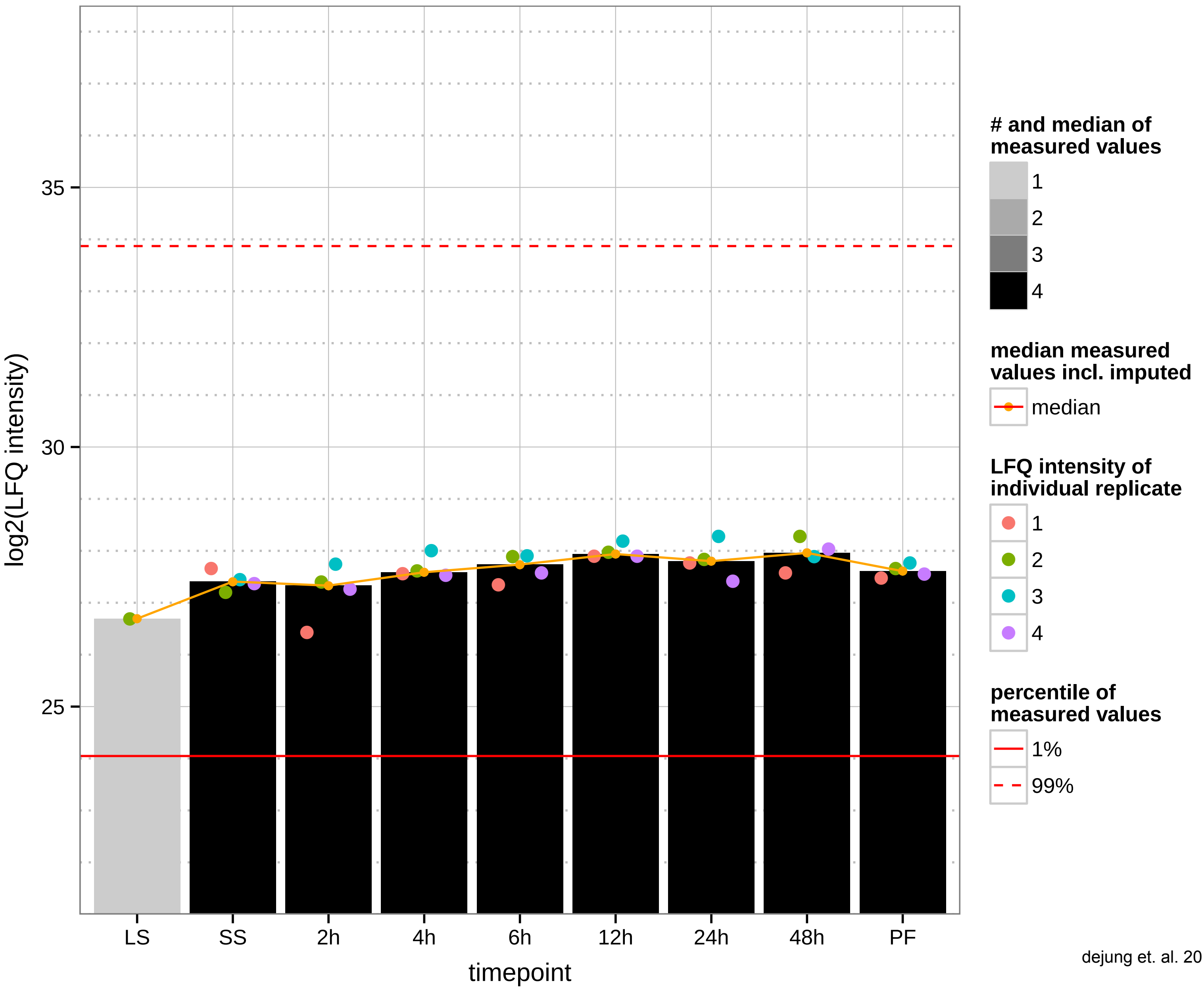

dejung et. al. 2015

hypothetical protein
Tb927.4.5220
AGOF: null
AGOC: null
AGOP: null
PGOF: null
PGOC: null
PGOP: null

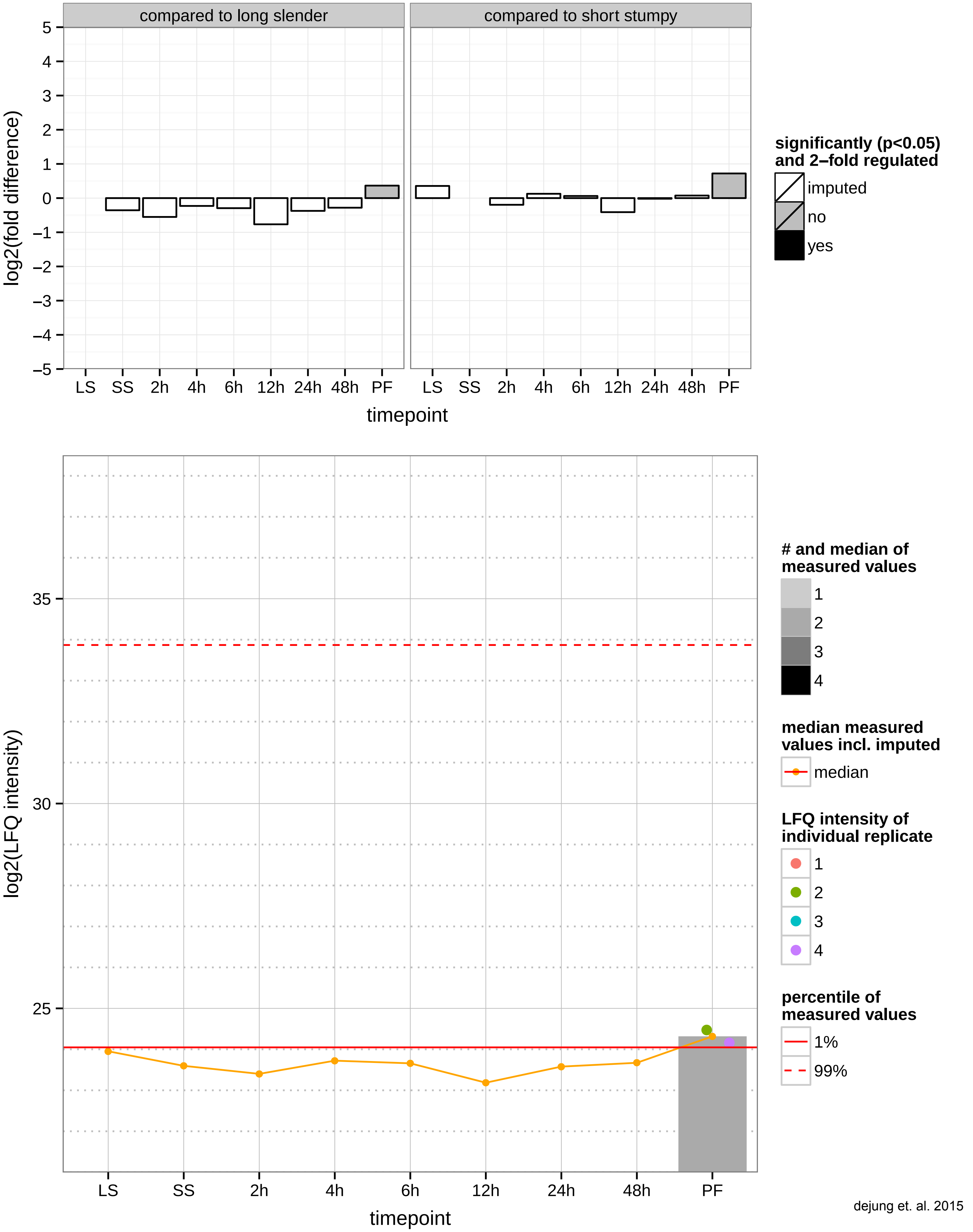

dejung et. al. 2015

hypothetical protein, conserved
Tb927.4.5320
AGOF: null
AGOC: null
AGOP: null
PGOF: null
PGOC: null
PGOP: null

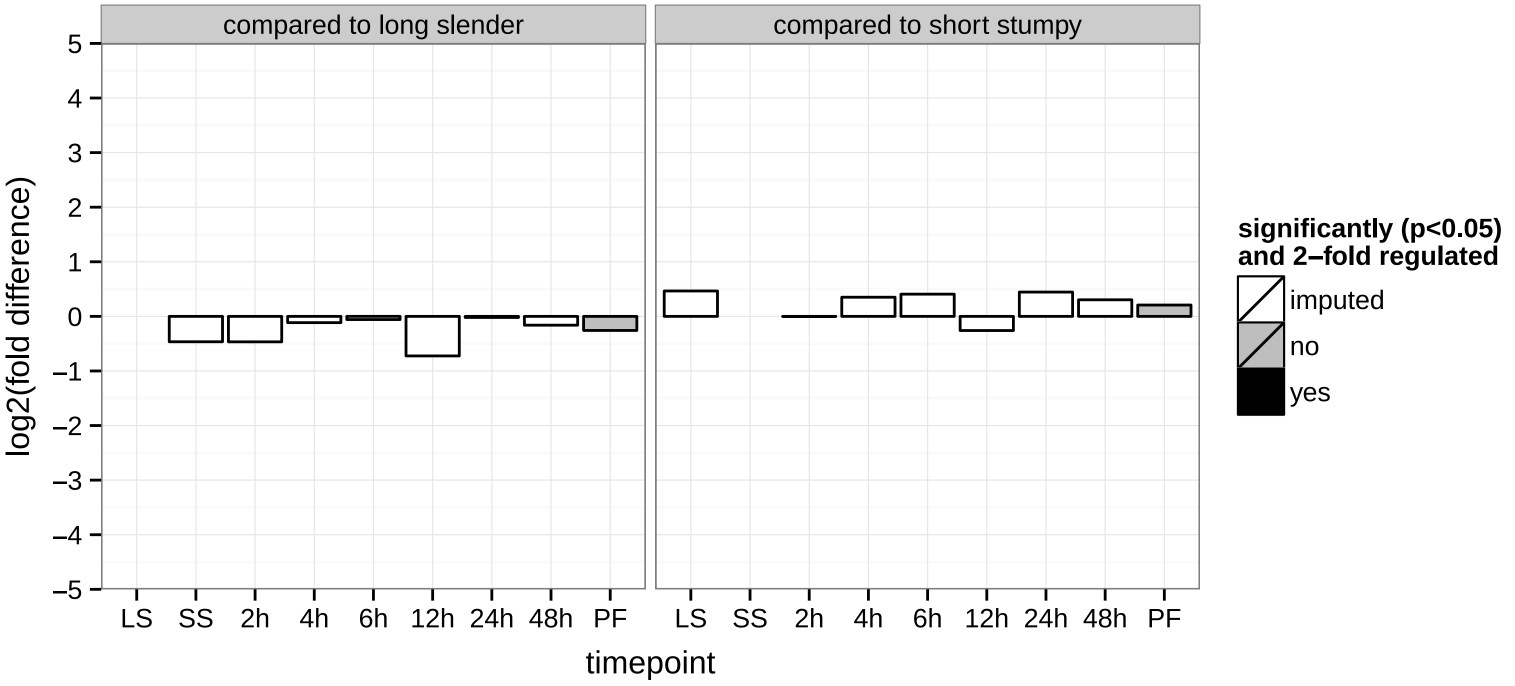

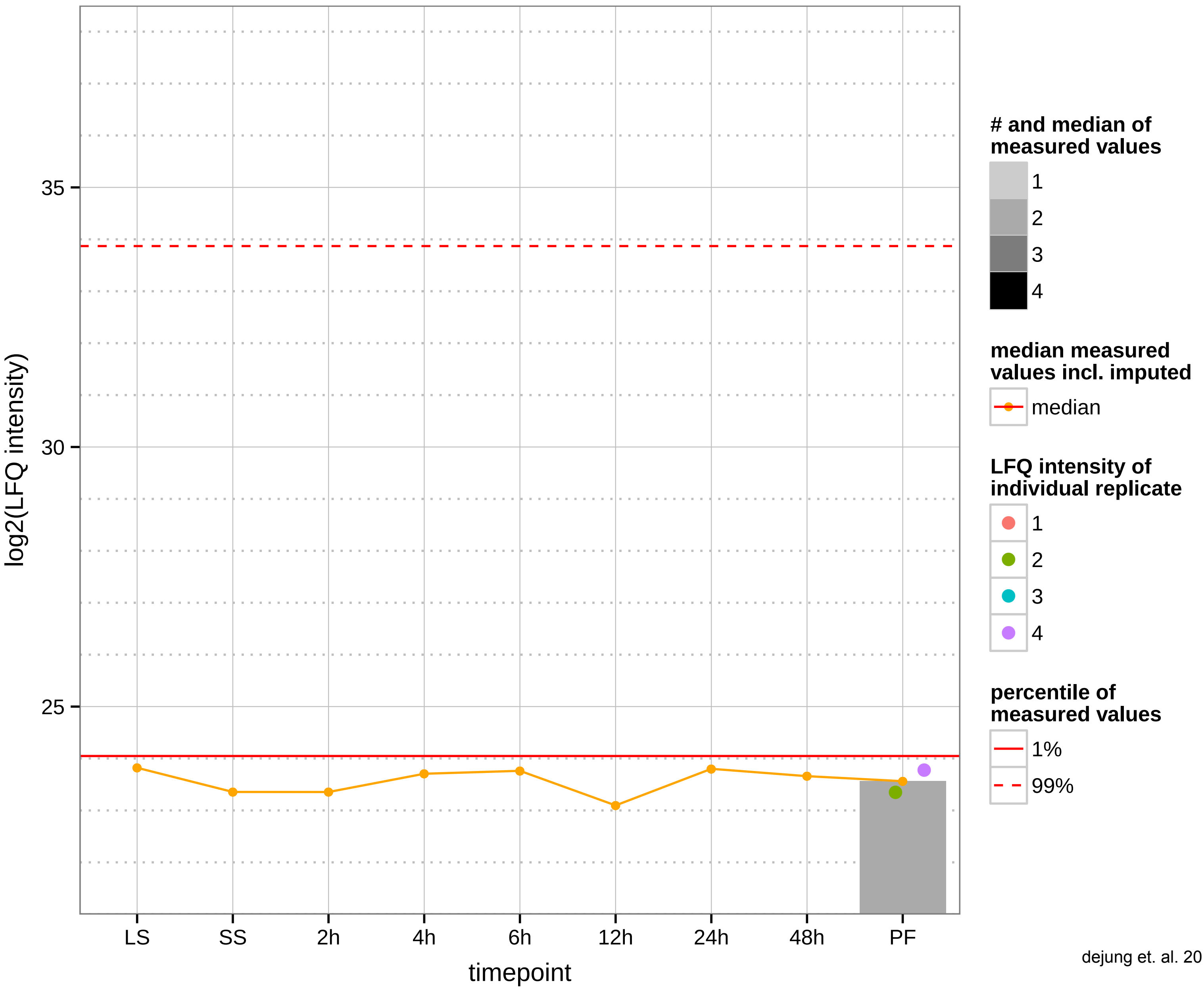

dejung et. al. 2015

hypothetical protein, conserved
Tb927.4.5380;Tb927.8.6940
AGOF: null
AGOC: null
AGOP: null
PGOF: protein binding
PGOC: null
PGOP: null

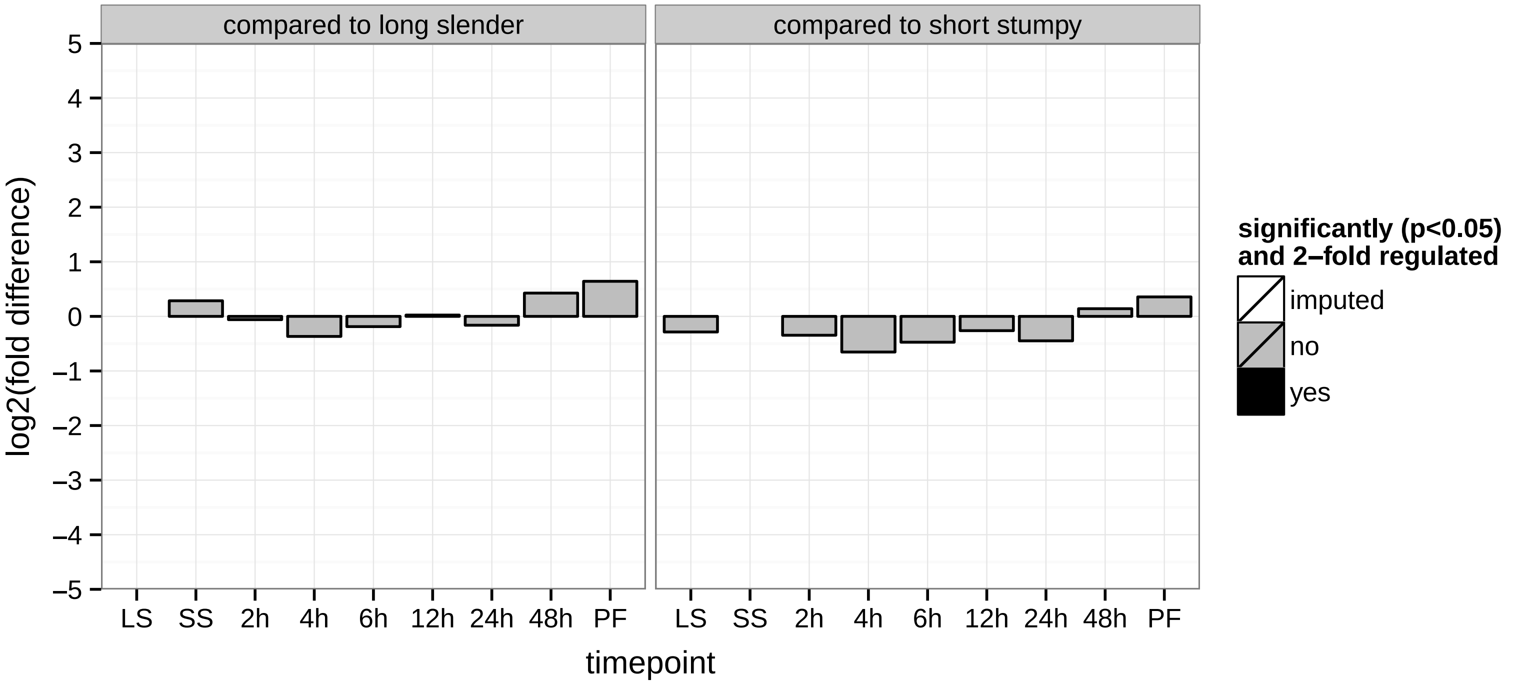

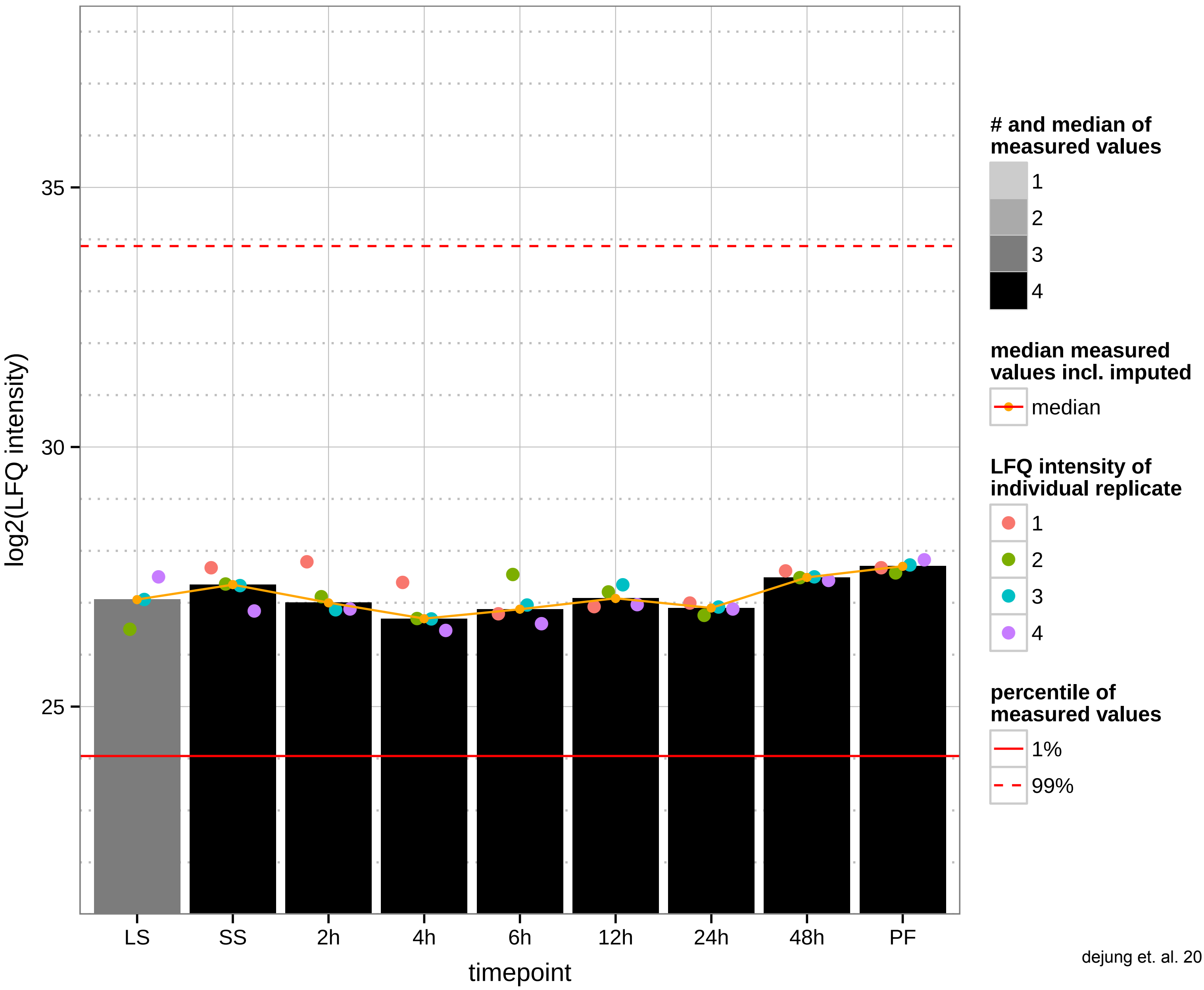

dejung et. al. 2015

hypothetical protein, conserved
Tb927.4.600
AGOF: null
AGOC: mitochondrion
AGOP: null
PGOF: null
PGOC: null
PGOP: null

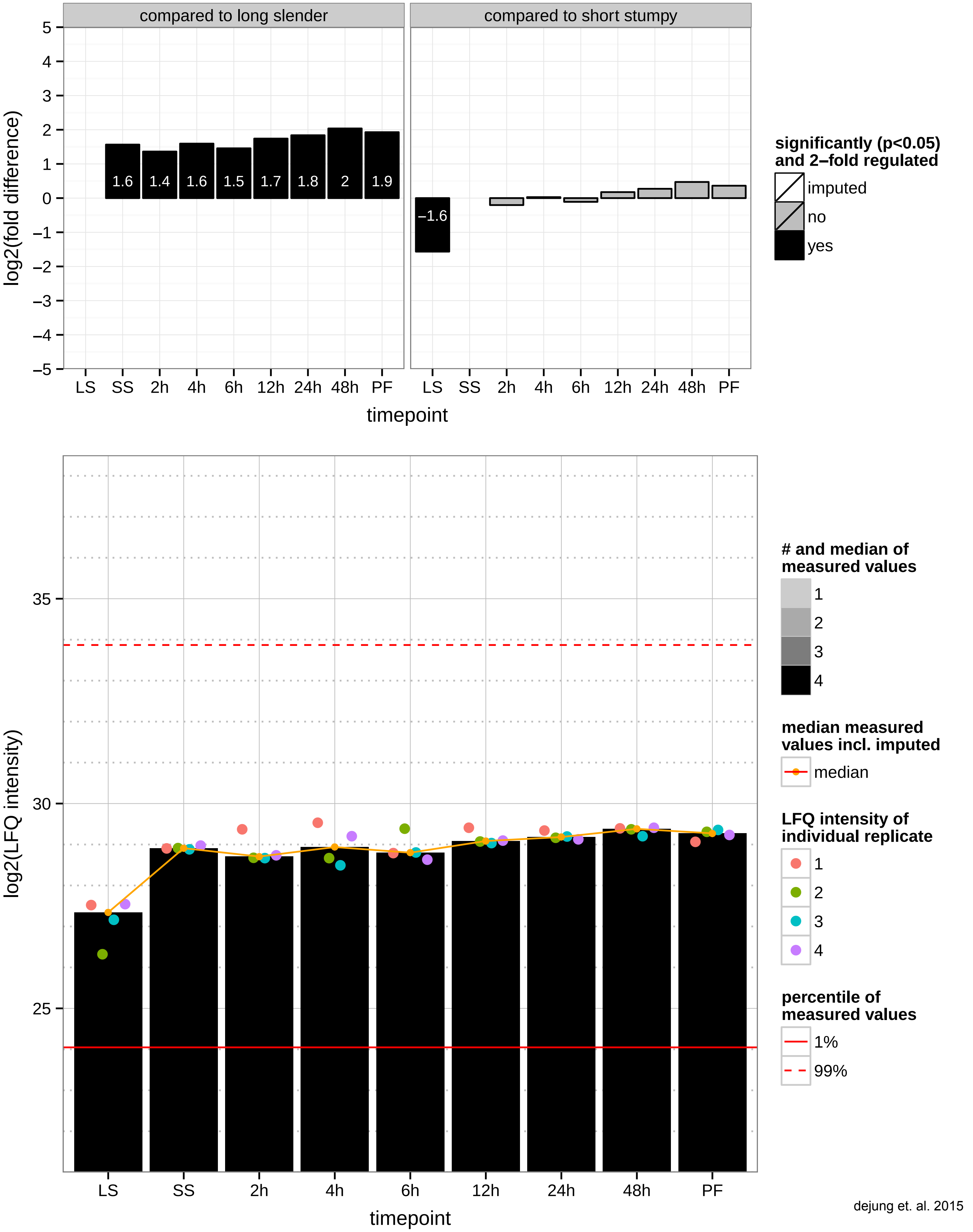

dejung et. al. 2015

hypothetical protein, conserved
Tb927.4.620
AGOF: null
AGOC: null
AGOP: null
PGOF: null
PGOC: null
PGOP: null

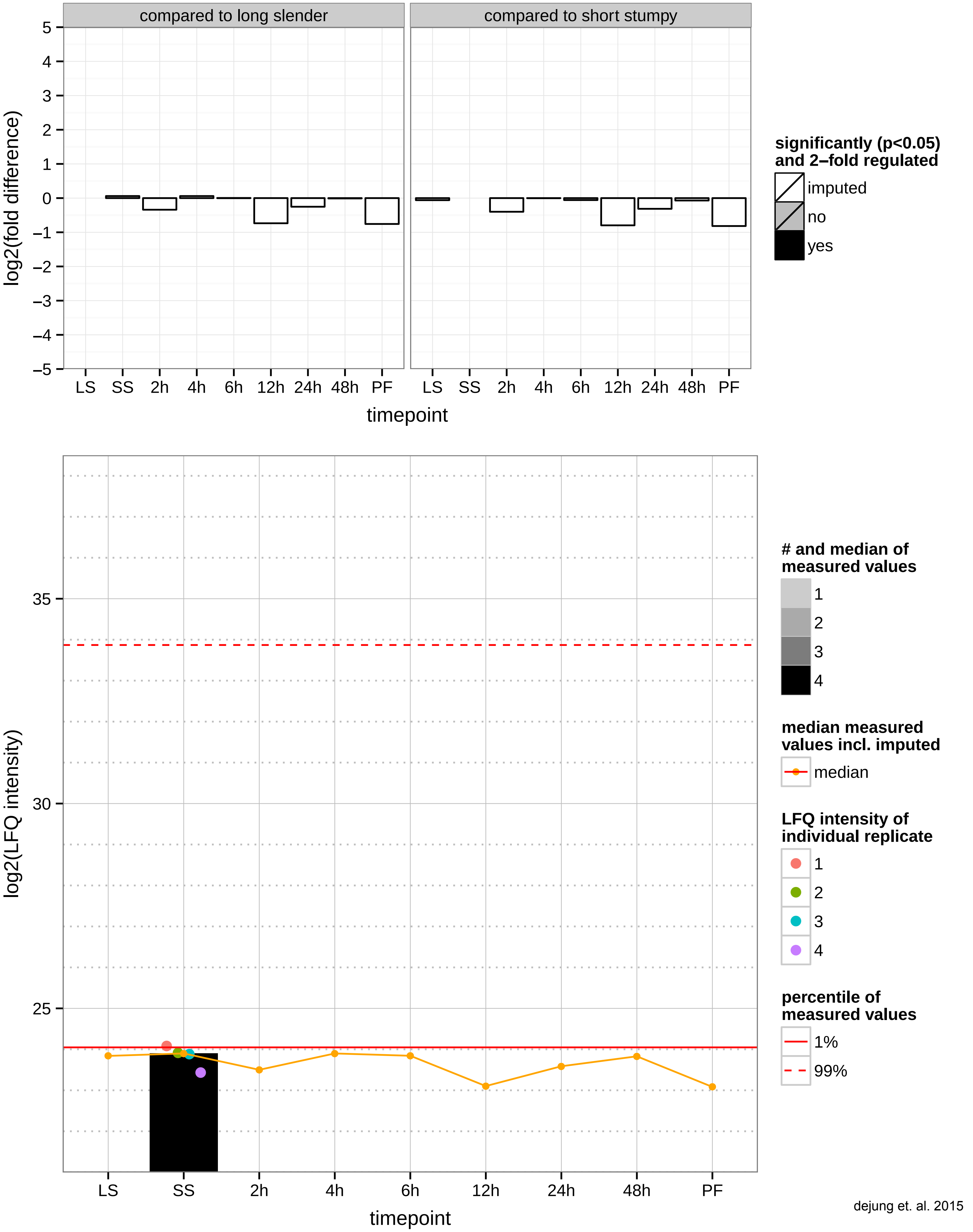

dejung et. al. 2015

hypothetical protein, conserved
Tb927.4.630
AGOF: null
AGOC: mitochondrion
AGOP: null
PGOF: null
PGOC: null
PGOP: null

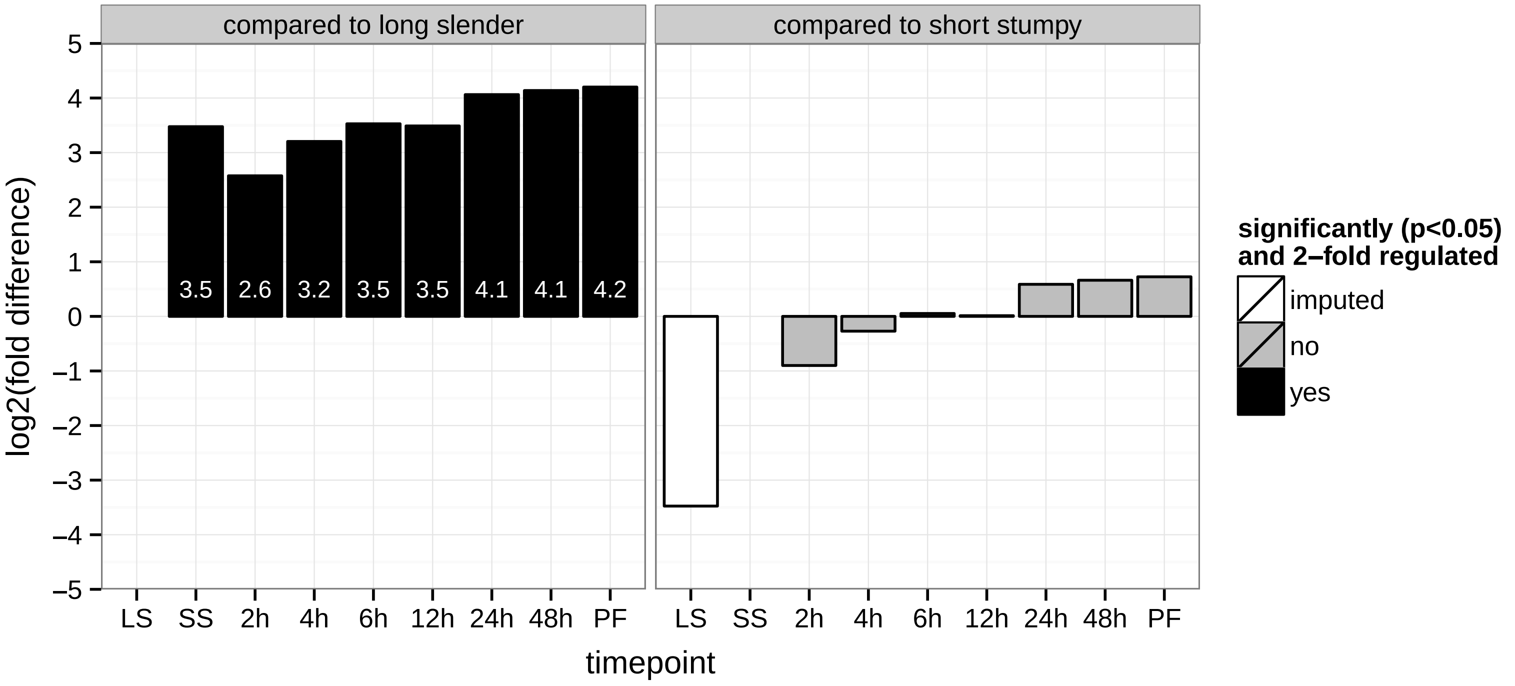

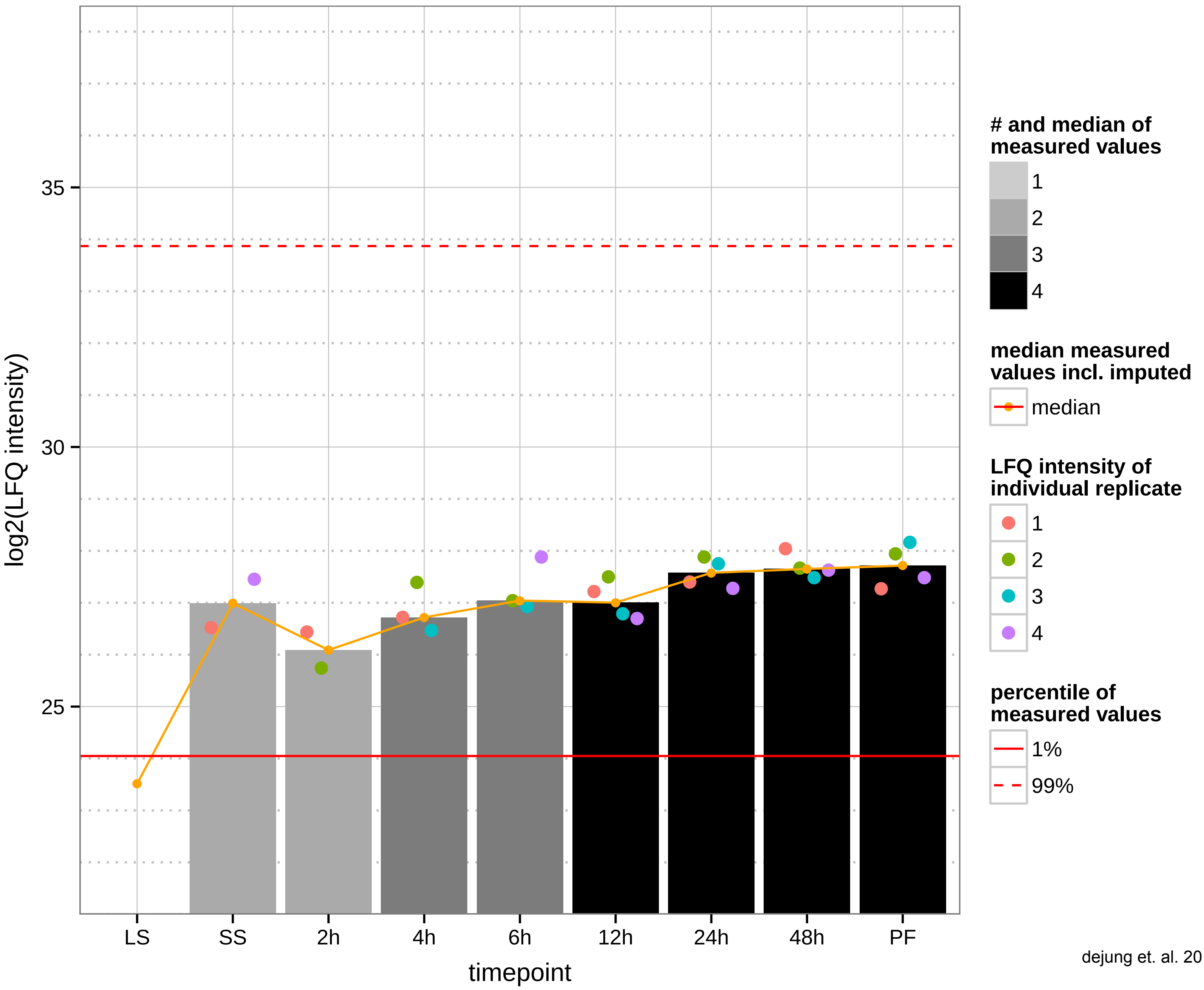

dejung et. al. 2015

hypothetical protein, conserved
Tb927.4.660
AGOF: null
AGOC: null
AGOP: null
PGOF: null
PGOC: null
PGOP: null

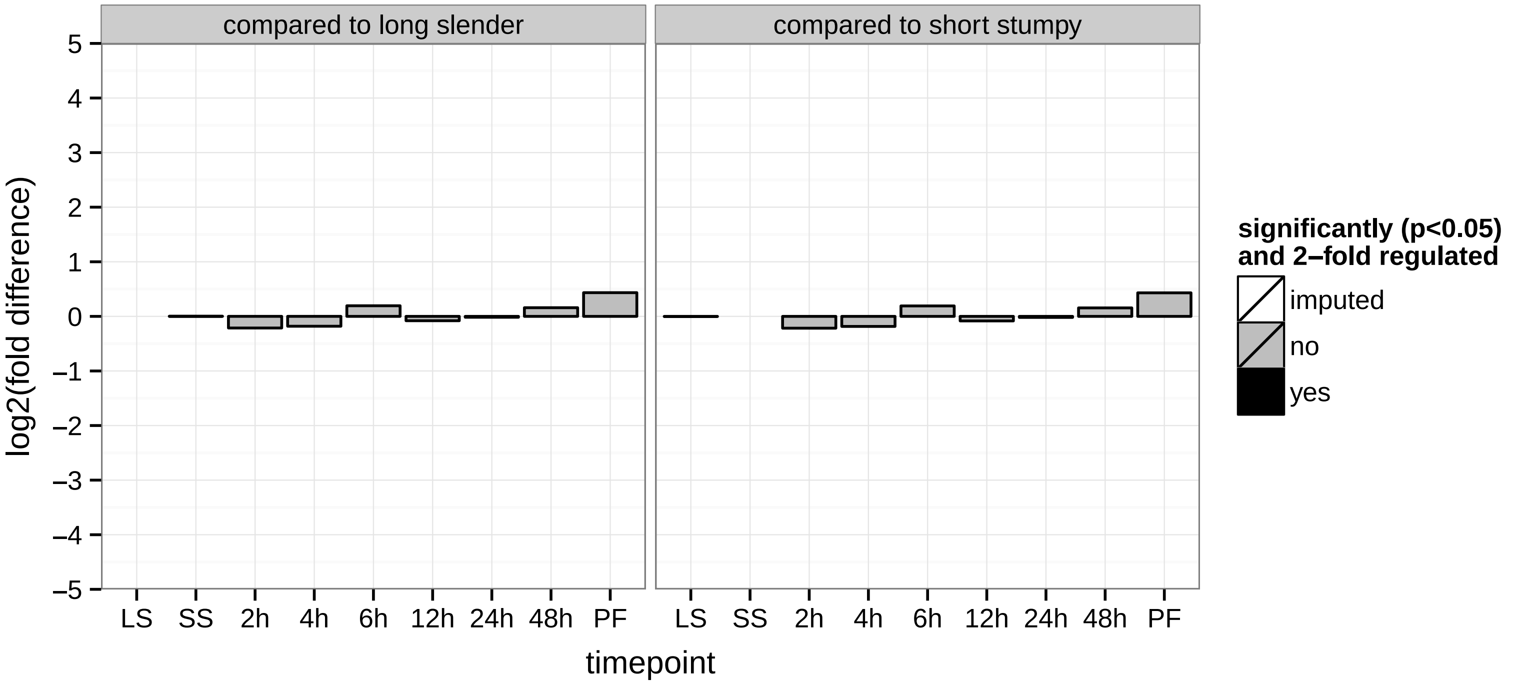

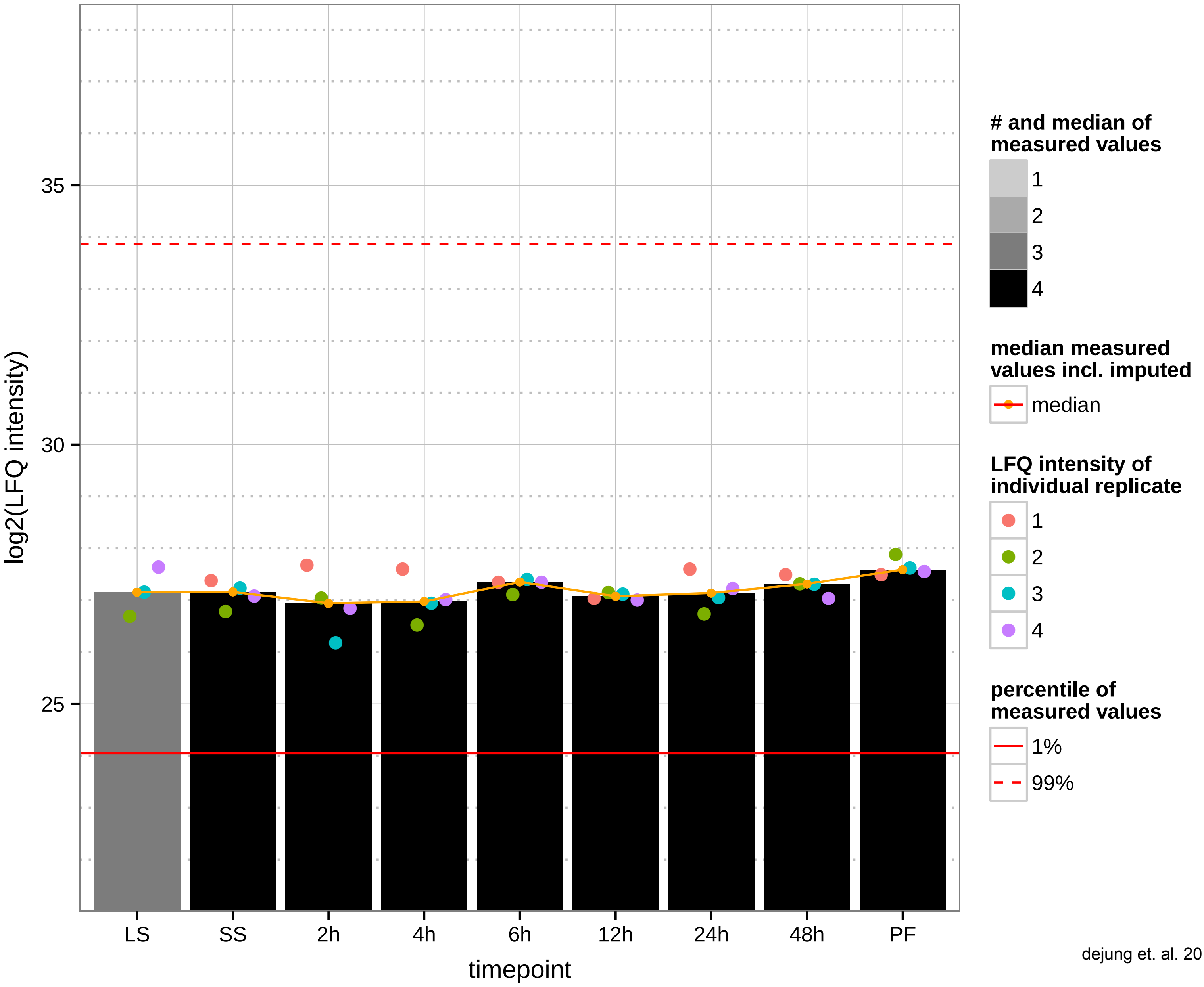

dejung et. al. 2015

50S ribosomal protein L7Ae, putative
Tb927.4.750
AGOF: null
AGOC: null
AGOP: null
PGOF: null
PGOC: null
PGOP: null

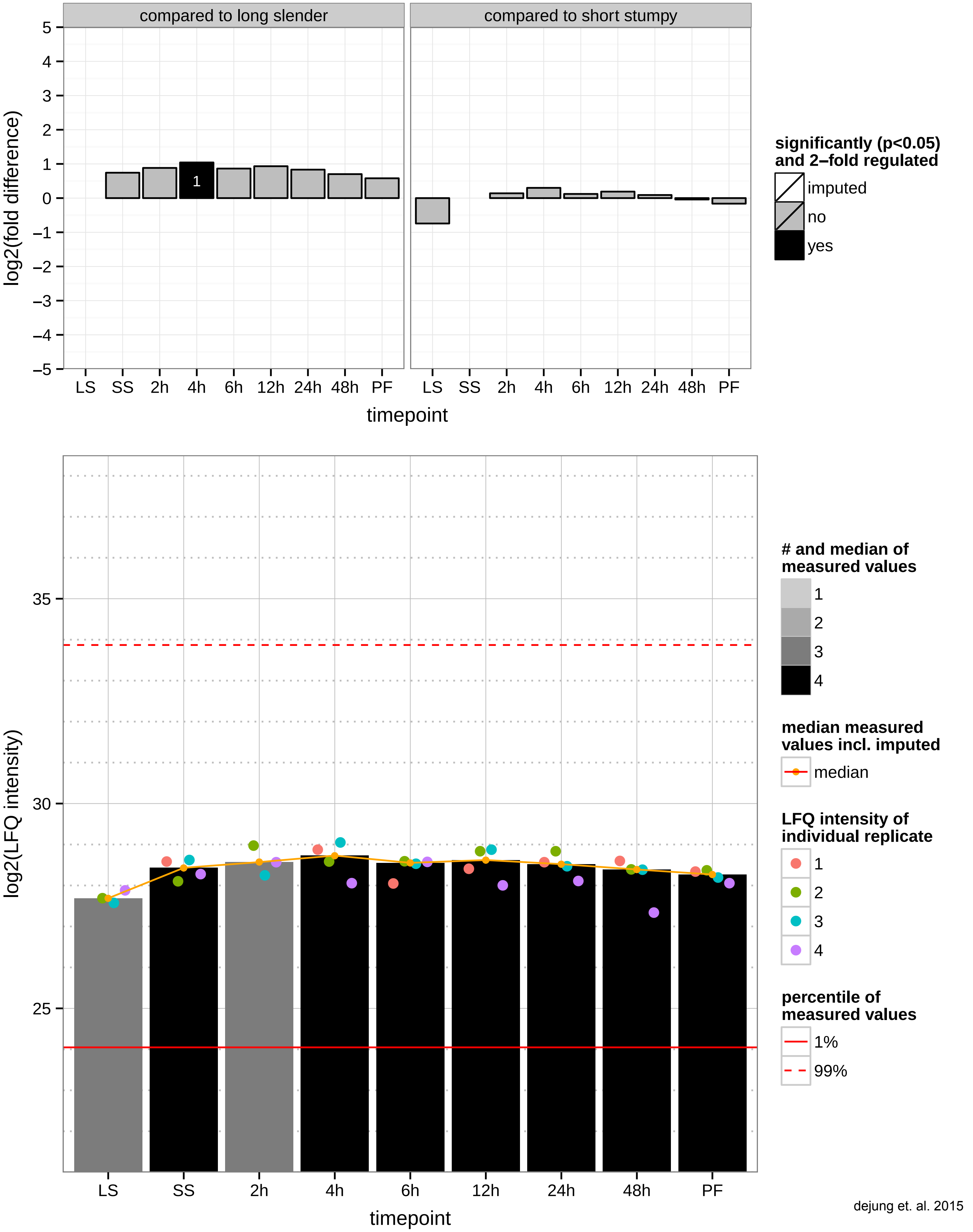

dejung et. al. 2015

hypothetical protein, conserved
Tb927.4.840
AGOF: ATP binding, two−component sensor activity
AGOC: null
AGOP: peptidyl−histidine phosphorylation, signal transduction
PGOF: ATP binding, protein histidine kinase activity, two−component sensor activity
PGOC: null
PGOP: peptidyl−histidine phosphorylation, signal transduction

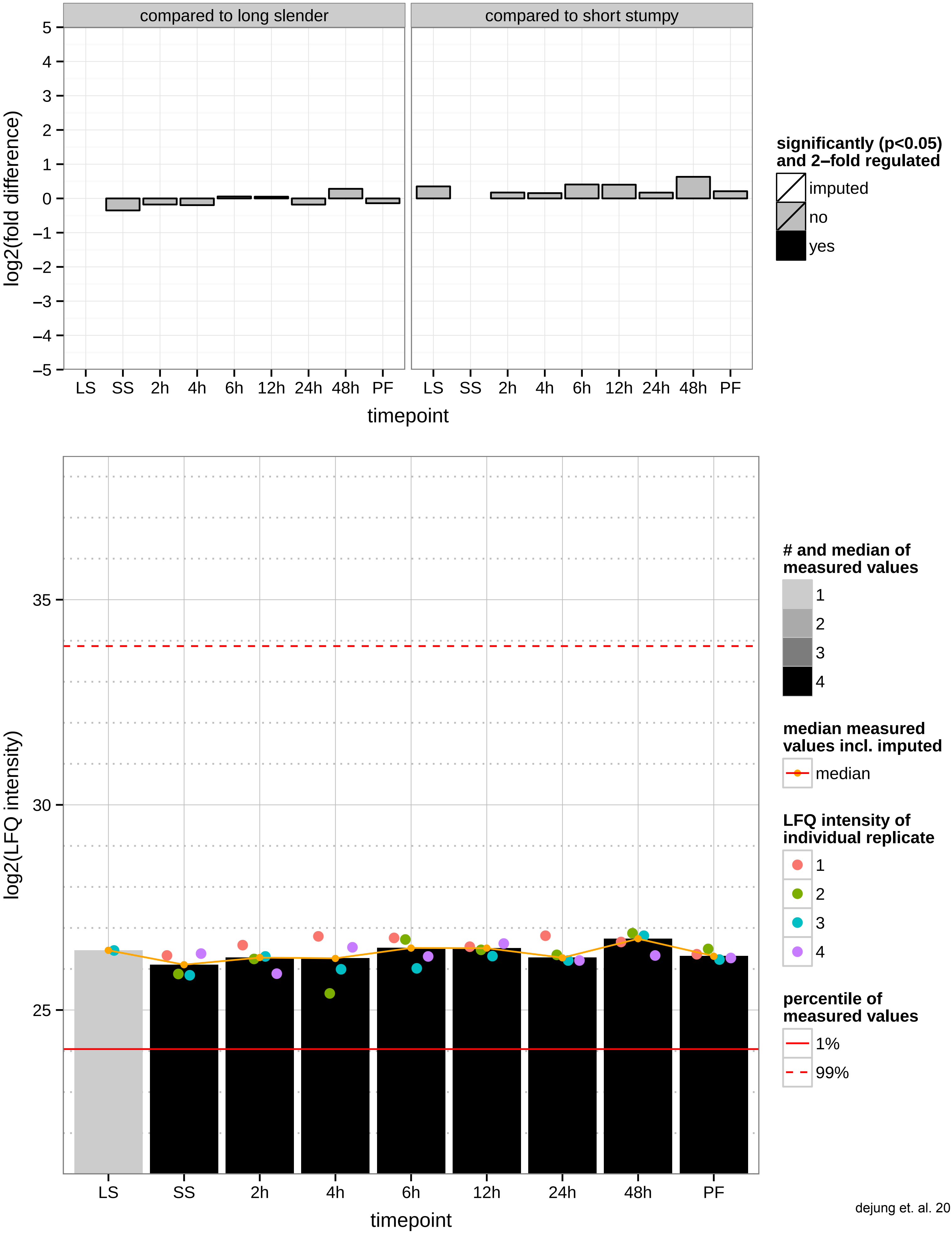

dejung et. al. 2015

hypothetical protein, conserved
Tb927.4.860
AGOF: null
AGOC: null
AGOP: null
PGOF: null
PGOC: null
PGOP: null

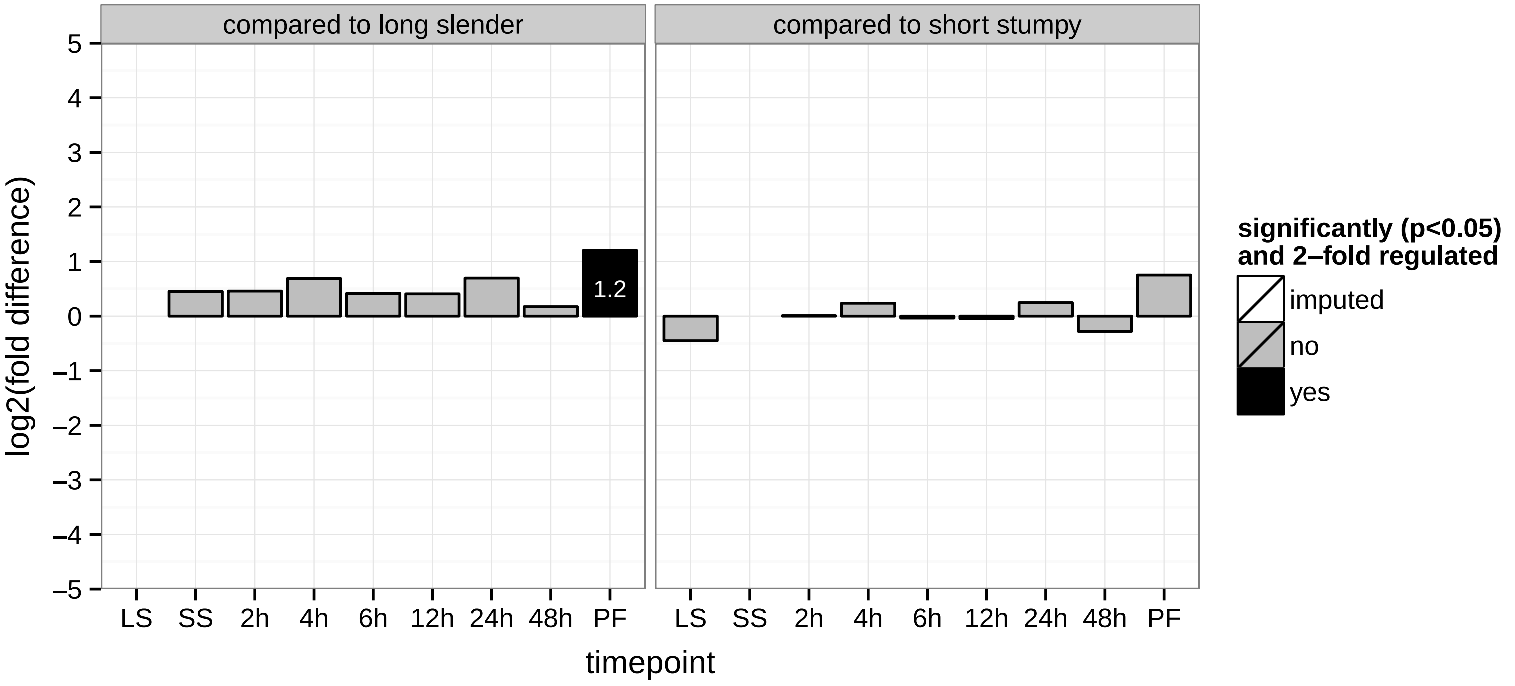

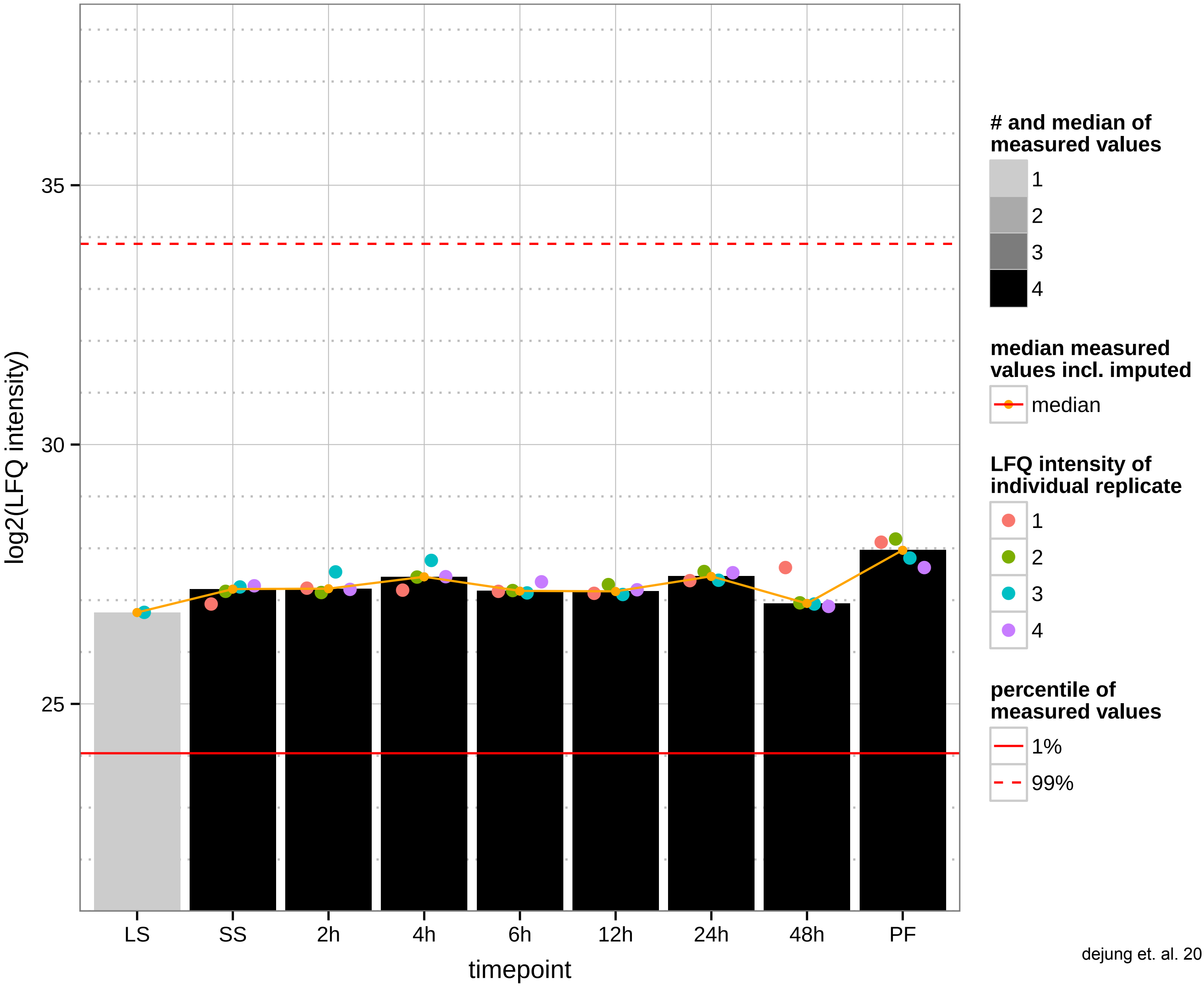

dejung et. al. 2015

dynein heavy chain, putative
Tb927.4.870
AGOF: ATP binding, ATPase activity, microtubule motor activity
AGOC: axonemal dynein complex
AGOP: microtubule–based movement
PGOF: ATP binding, ATPase activity, microtubule motor activity, nucleoside–triphosphatase activity, nucleotide binding
PGOC: dynein complex
PGOP: microtubule–based movement

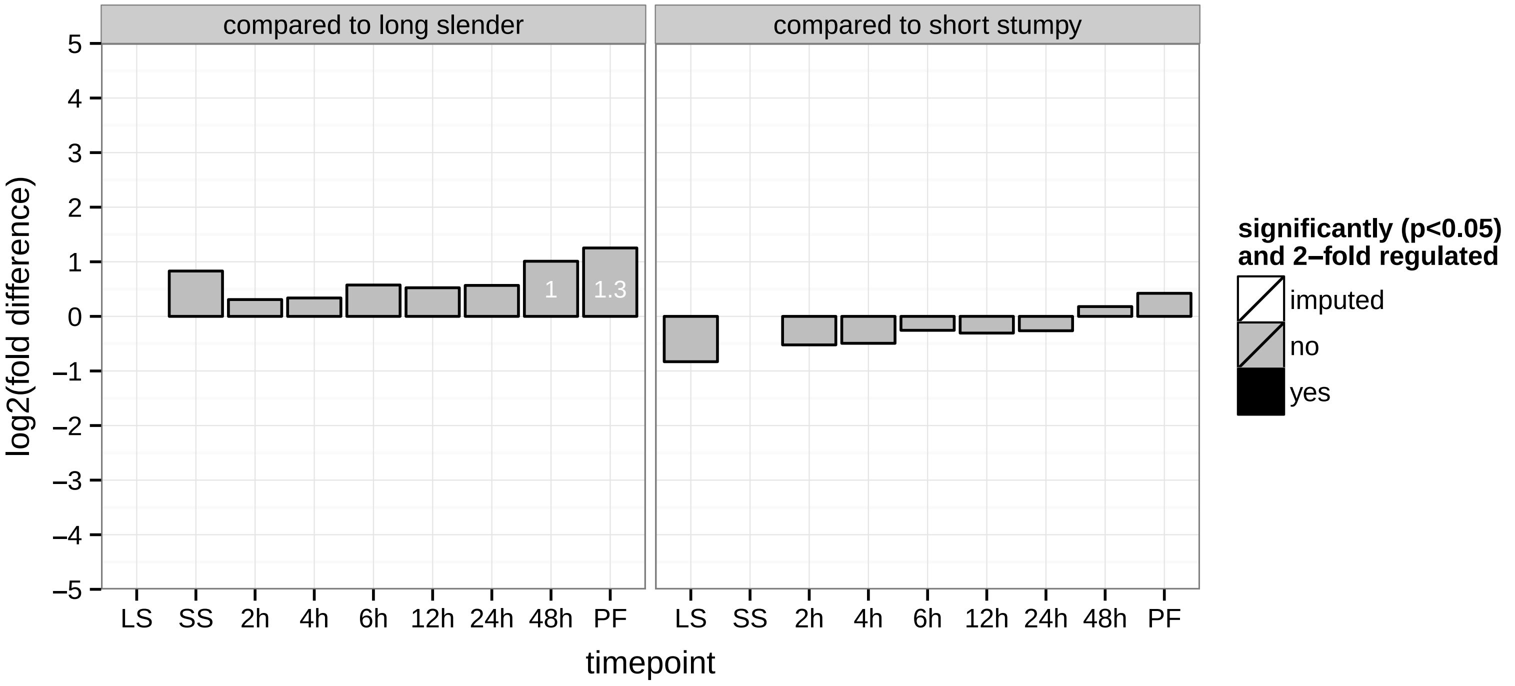

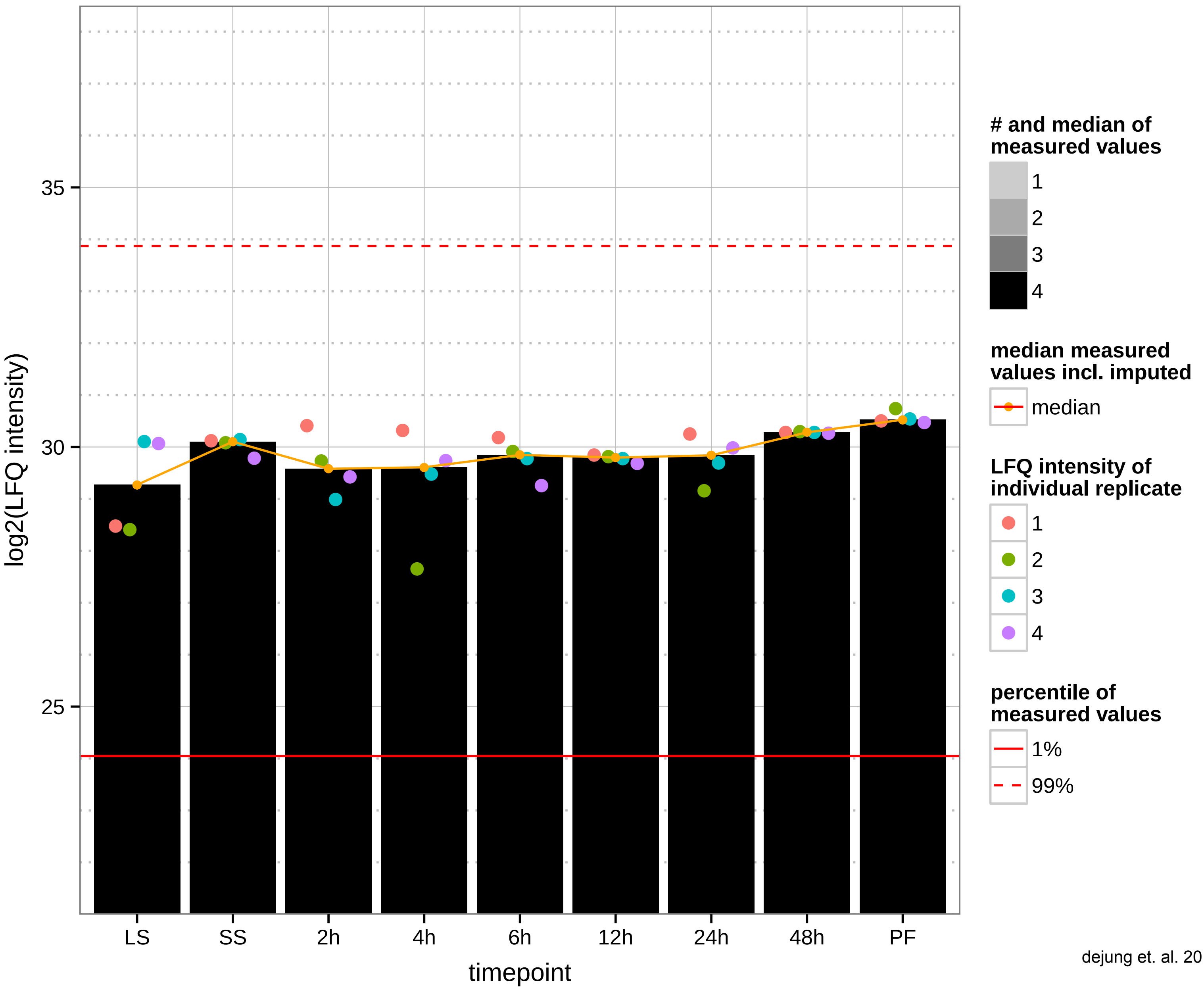

dejung et. al. 2015

small nuclear ribonucleoprotein SmD3, putative (SmD3)
Tb927.4.890
AGOF: null
AGOC: nucleus, small nuclear ribonucleoprotein complex
AGOP: RNA splicing, RNA splicing, via transesterification reactions, mRNA processing
PGOF: null
PGOC: null
PGOP: null

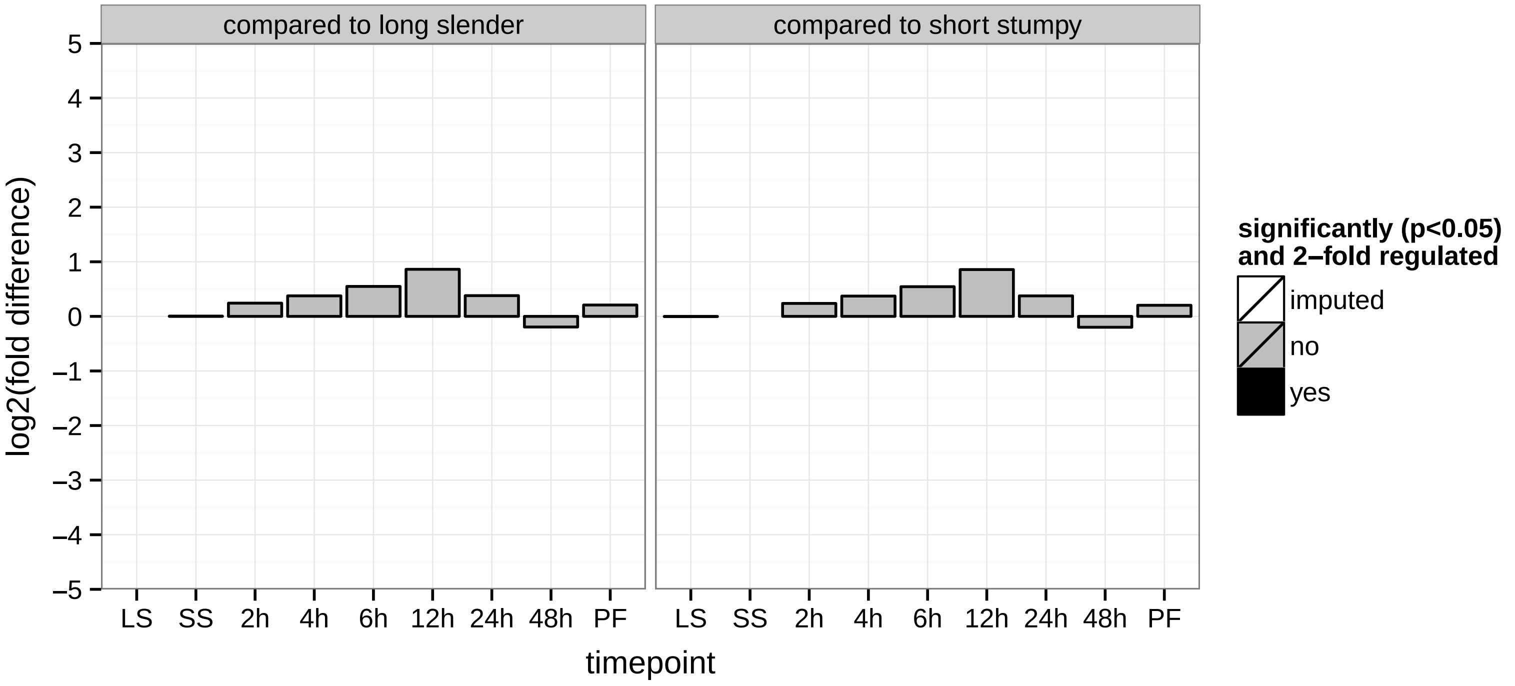

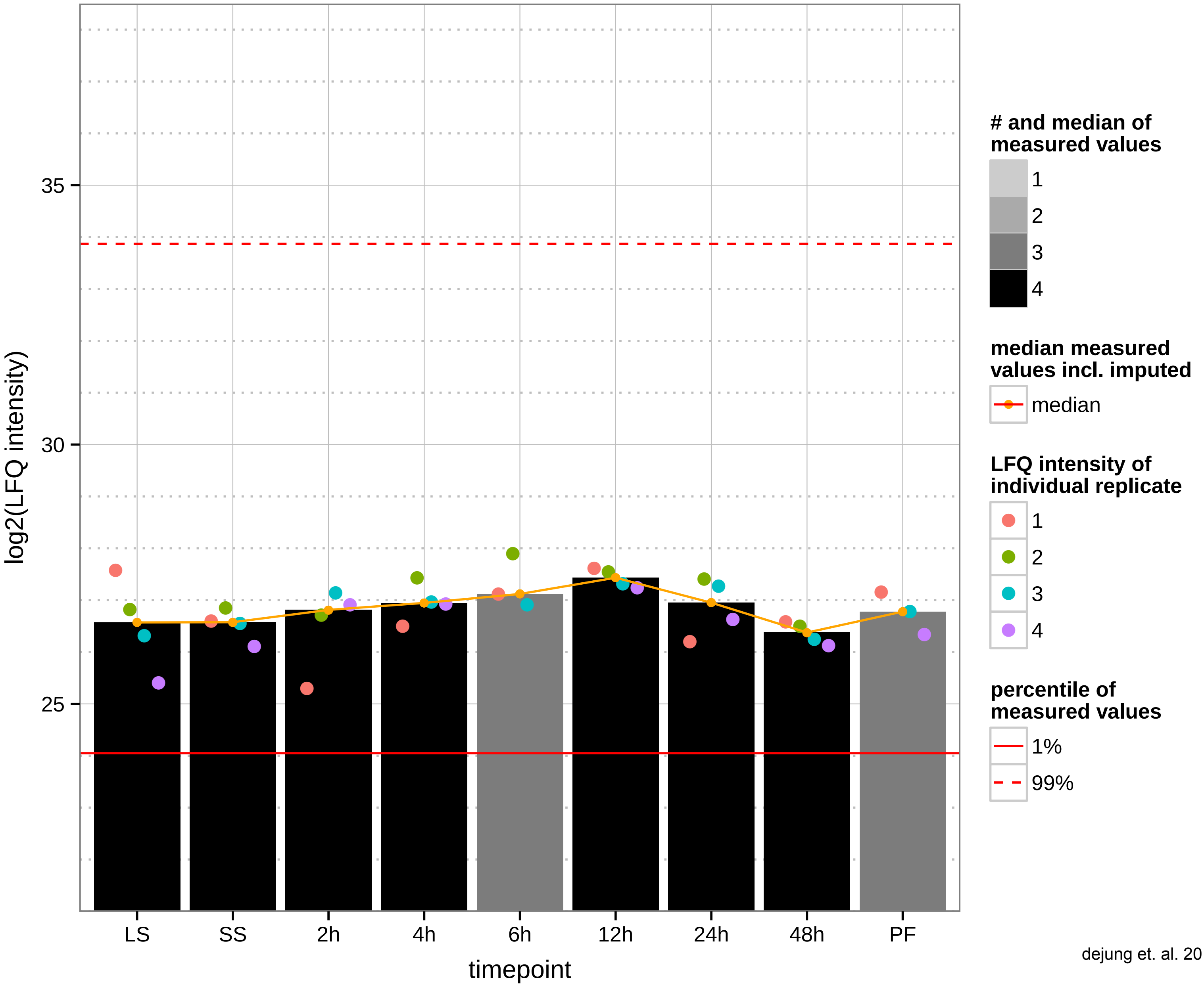

dejung et. al. 2015

hypothetical protein, conserved
Tb927.4.920
AGOF: null
AGOC: null
AGOP: null
PGOF: protein binding
PGOC: null
PGOP: null

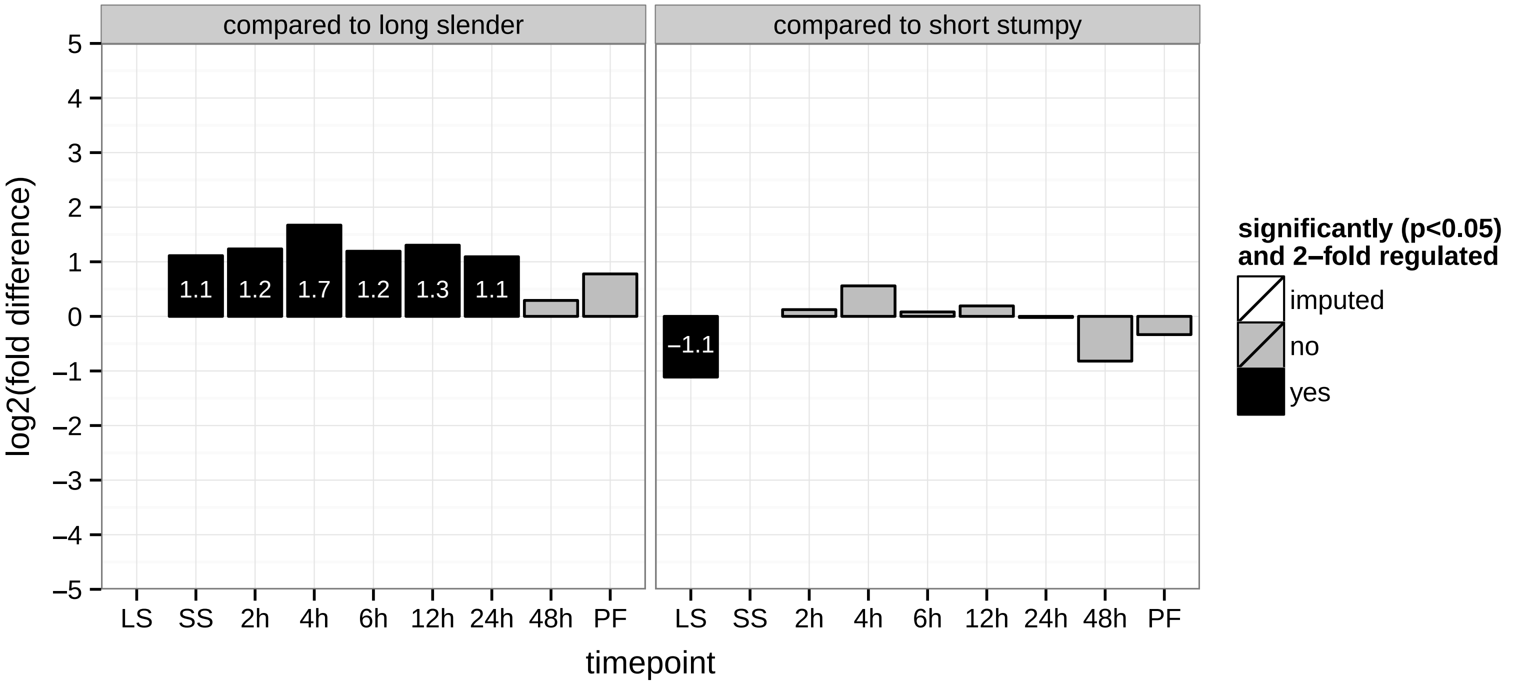

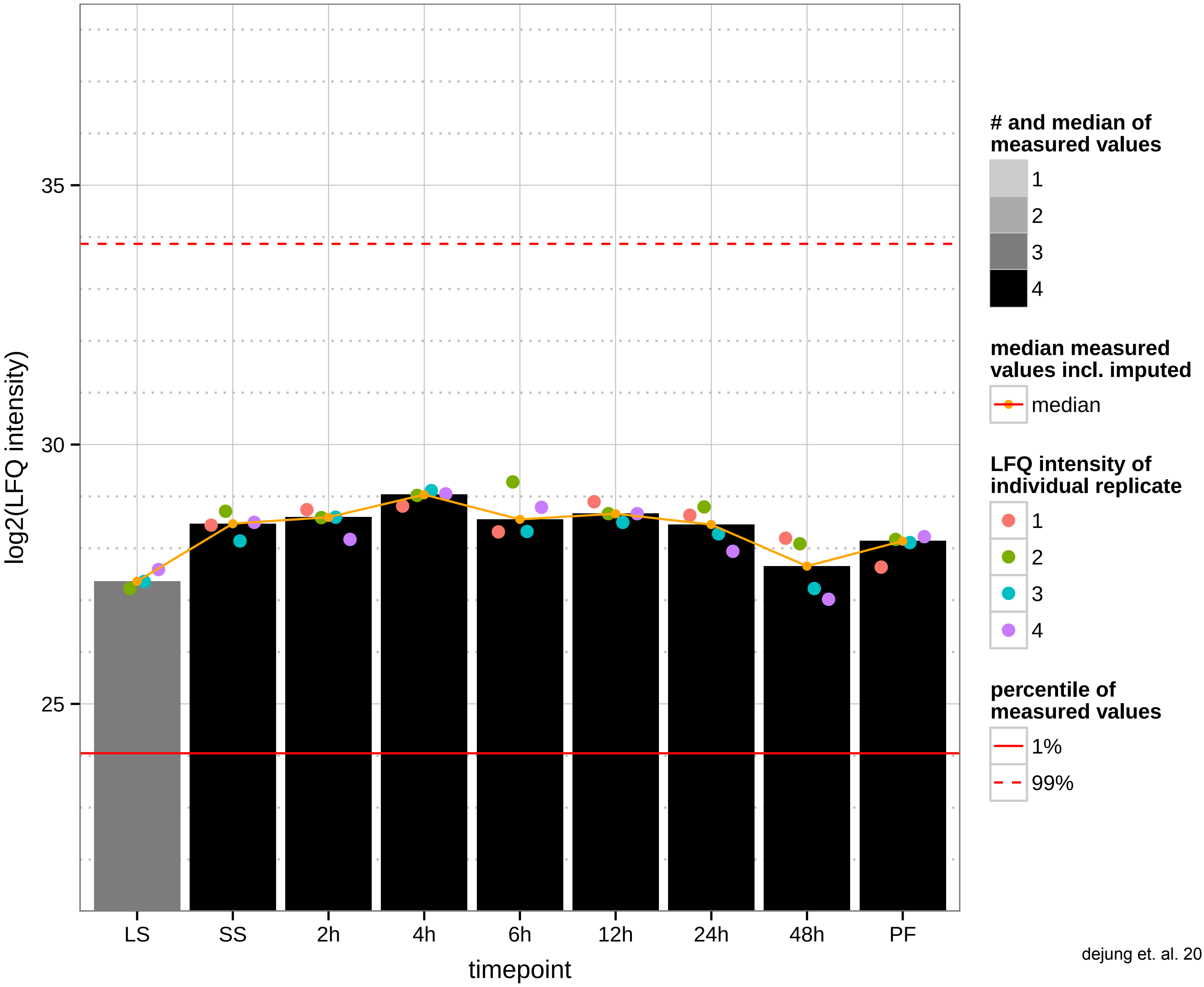

dejung et. al. 2015

hypothetical protein, conserved
Tb927.4.970
AGOF: null
AGOC: null
AGOP: null
PGOF: null
PGOC: null
PGOP: null

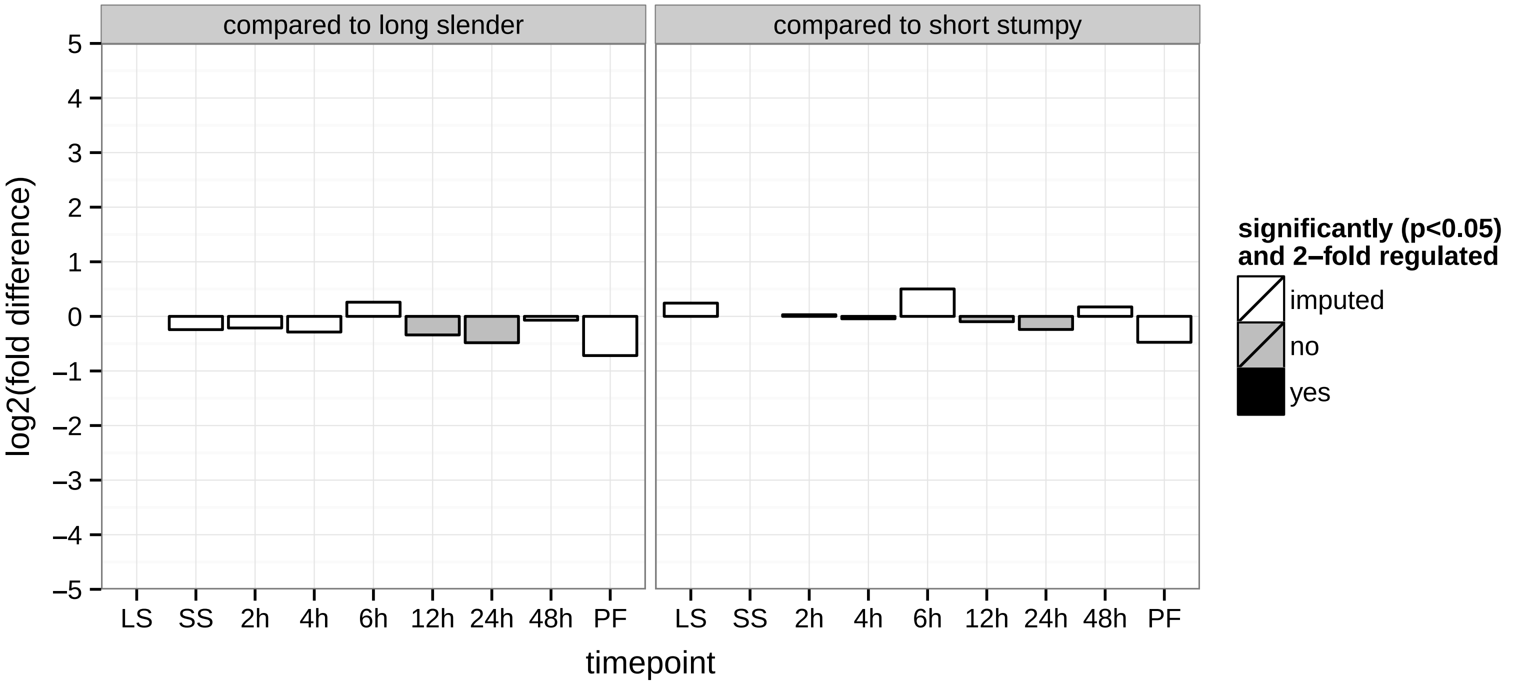

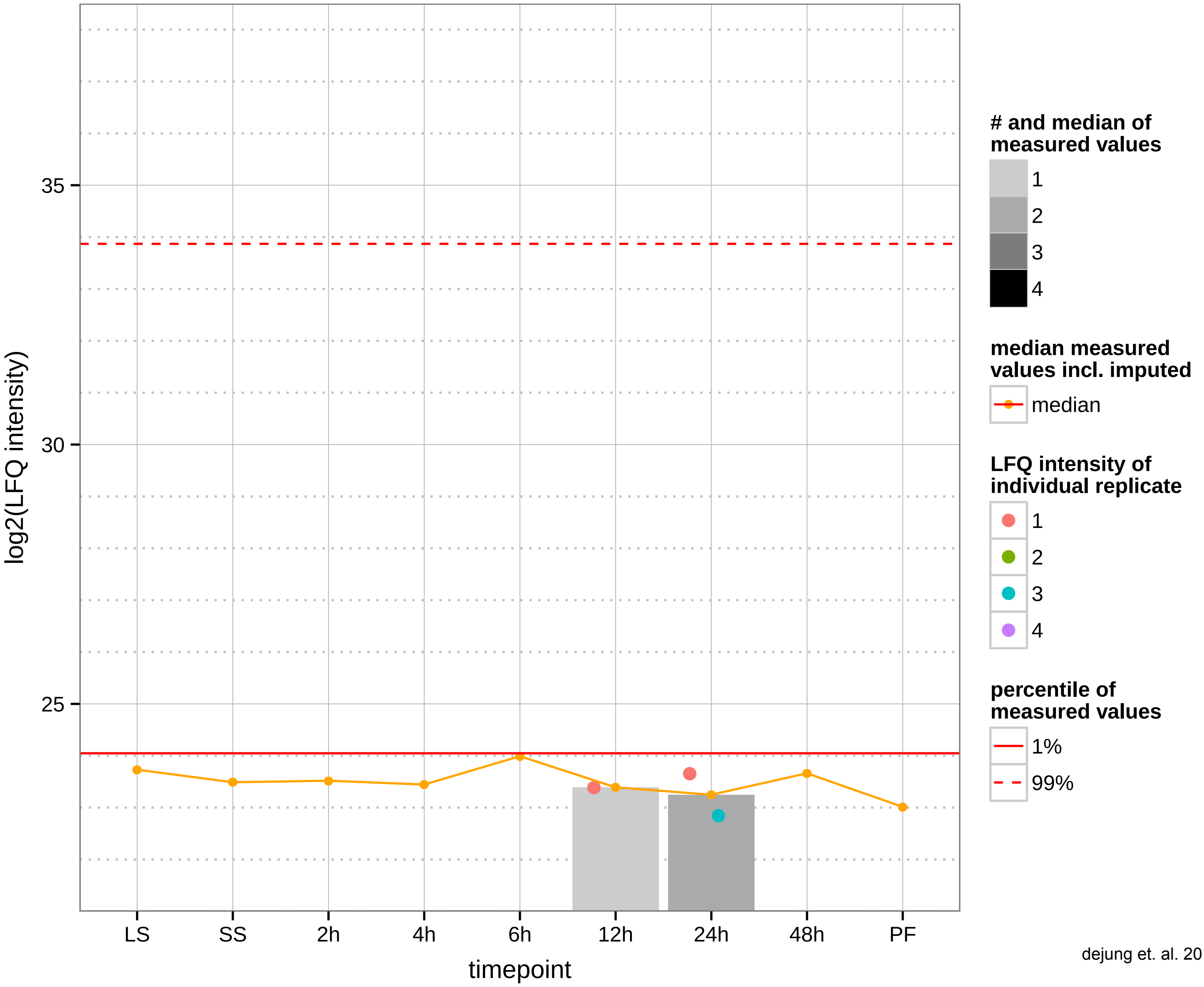

dejung et. al. 2015

ubiquitin−conjugating enzyme E2, putative, ubiquitin carrier protein, ubiquitin−protein ligase
Tb927.5.1000
AGOF: ubiquitin−protein ligase activity
AGOC: null
AGOP: cellular protein modification process, post−translational protein modification, regulation of protein metabolic process
PGOF: acid−amino acid ligase activity
PGOC: null
PGOP: null

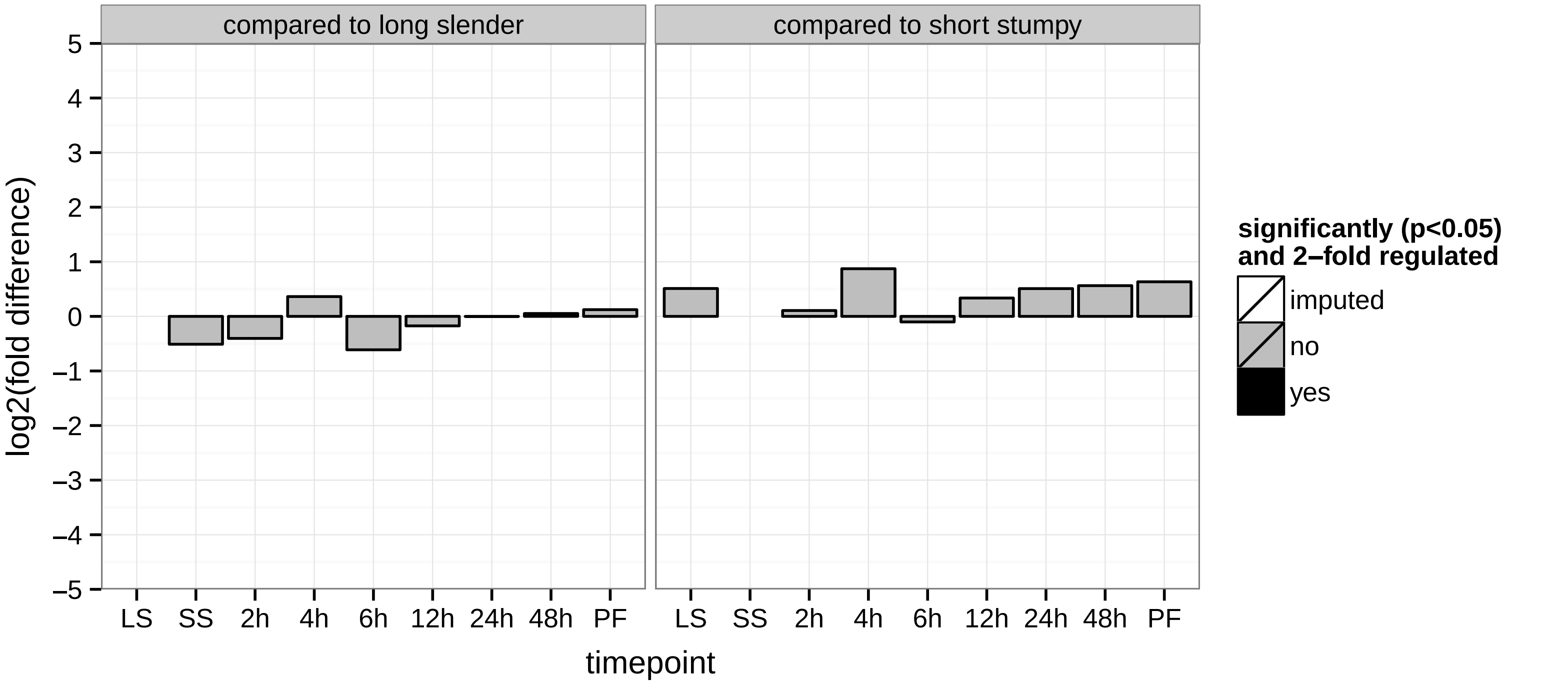

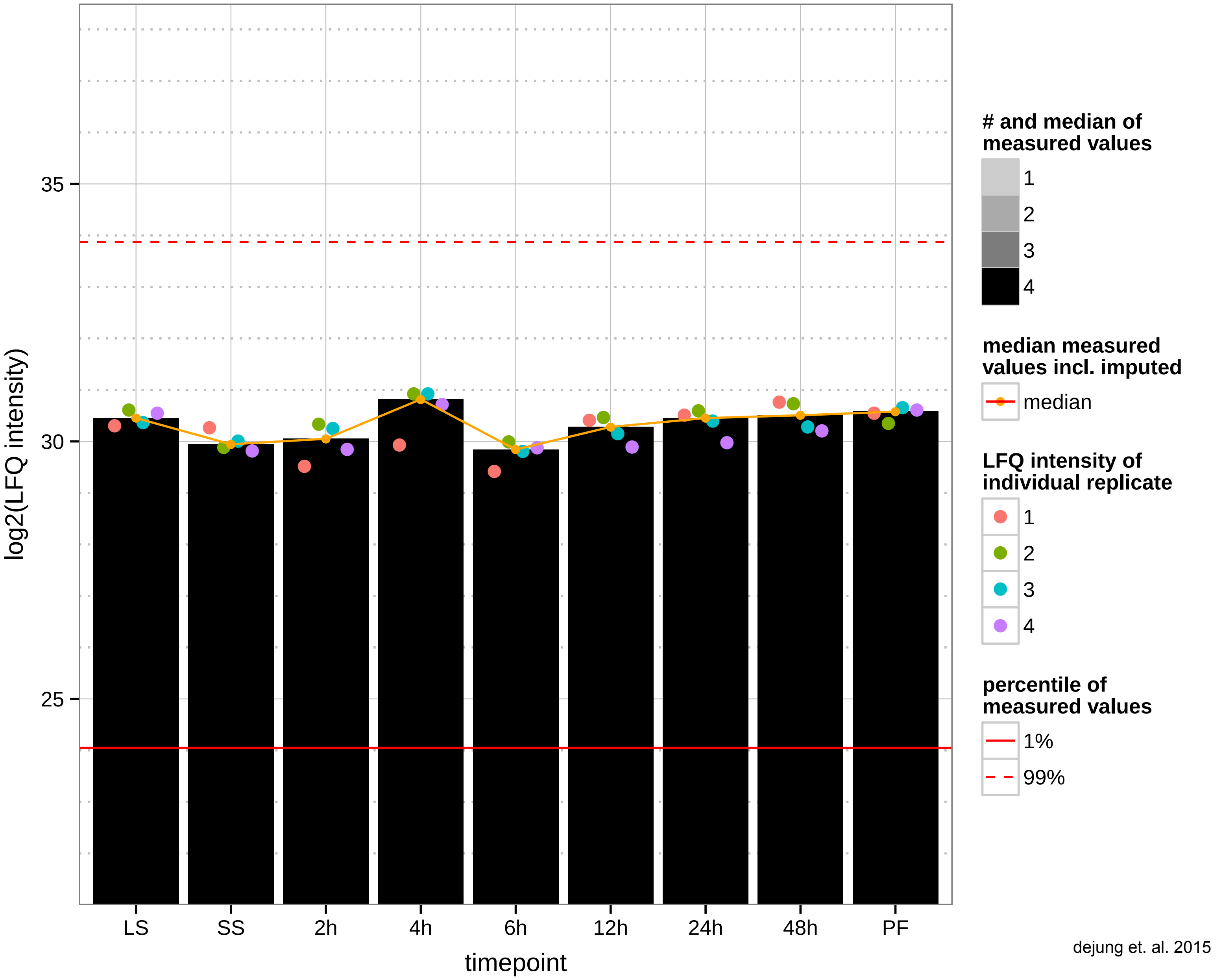

dejung et. al. 2015

hypothetical protein, conserved
Tb927.5.1010
AGOF: null
AGOC: null
AGOP: null
PGOF: null
PGOC: null
PGOP: null

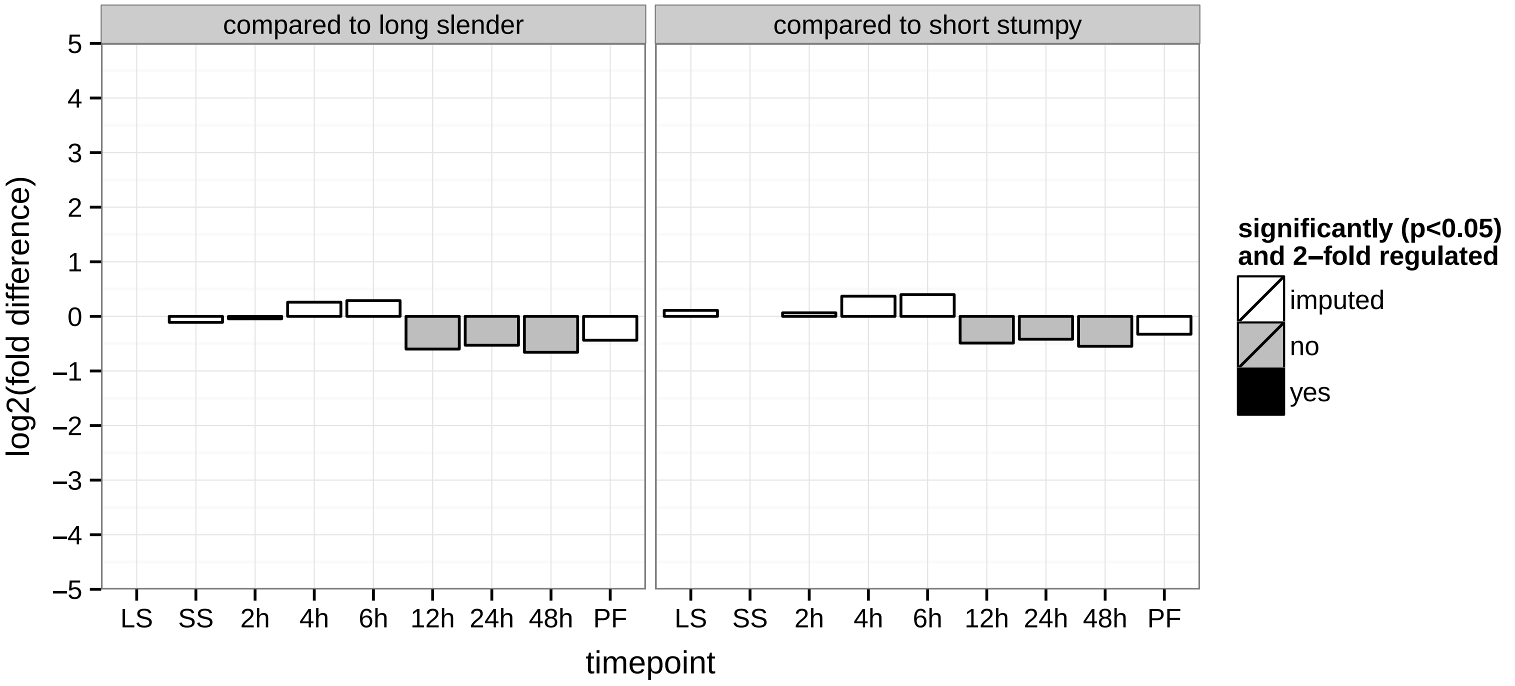

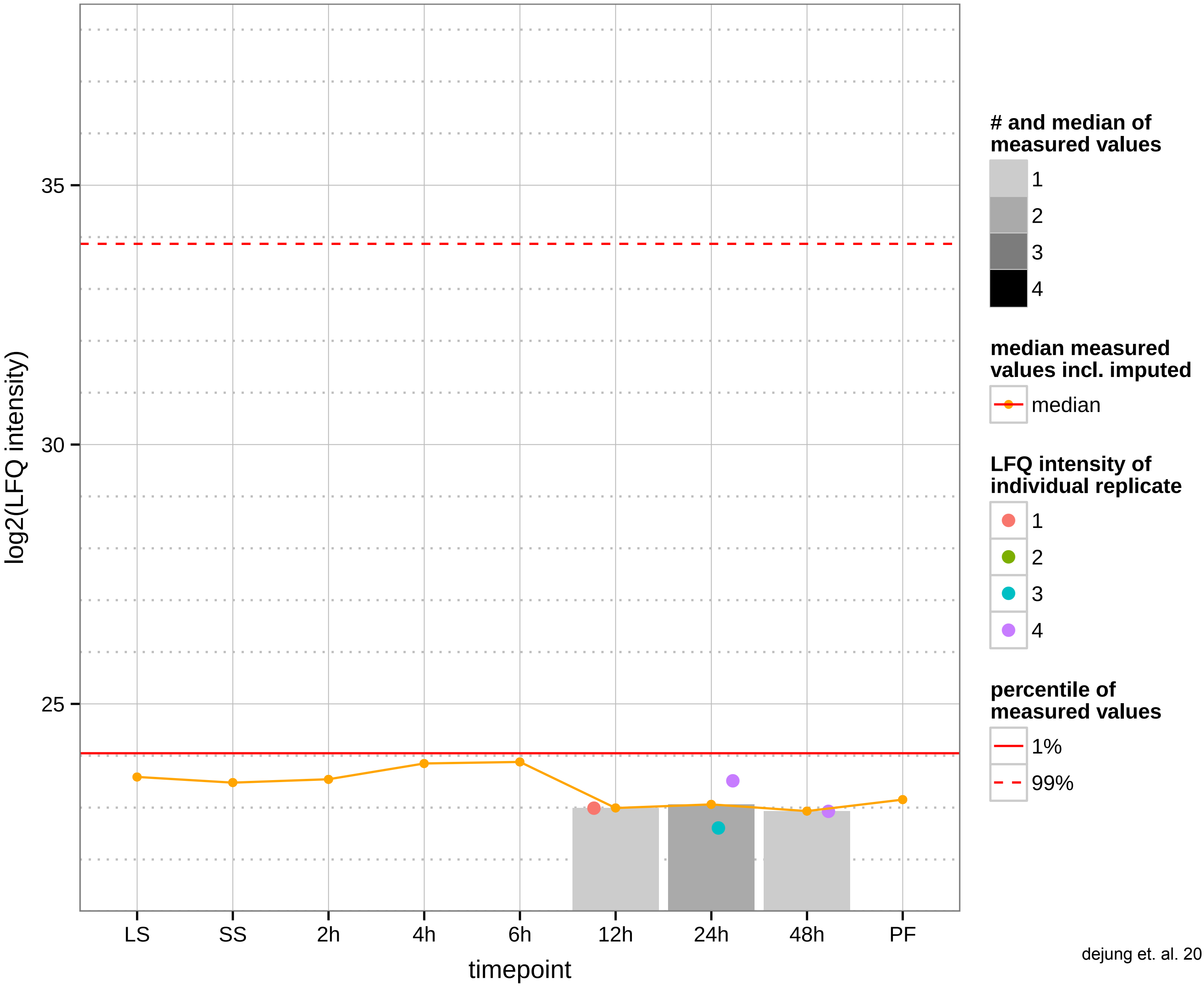

dejung et. al. 2015

RNA–binding protein, putative (RBP22)
Tb927.5.1080
AGOF: RNA binding
AGOC: null
AGOP: RNA processing
PGOF: nucleic acid binding
PGOC: null
PGOP: null

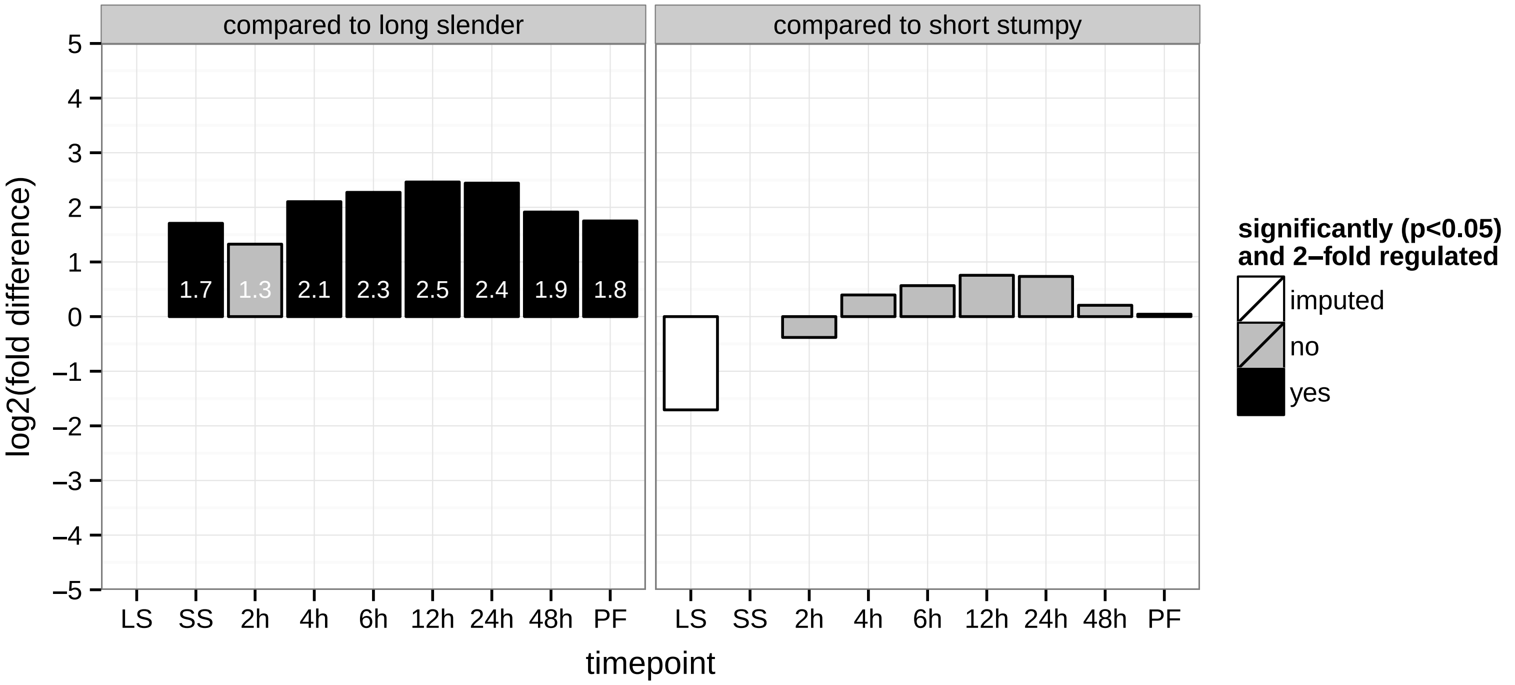

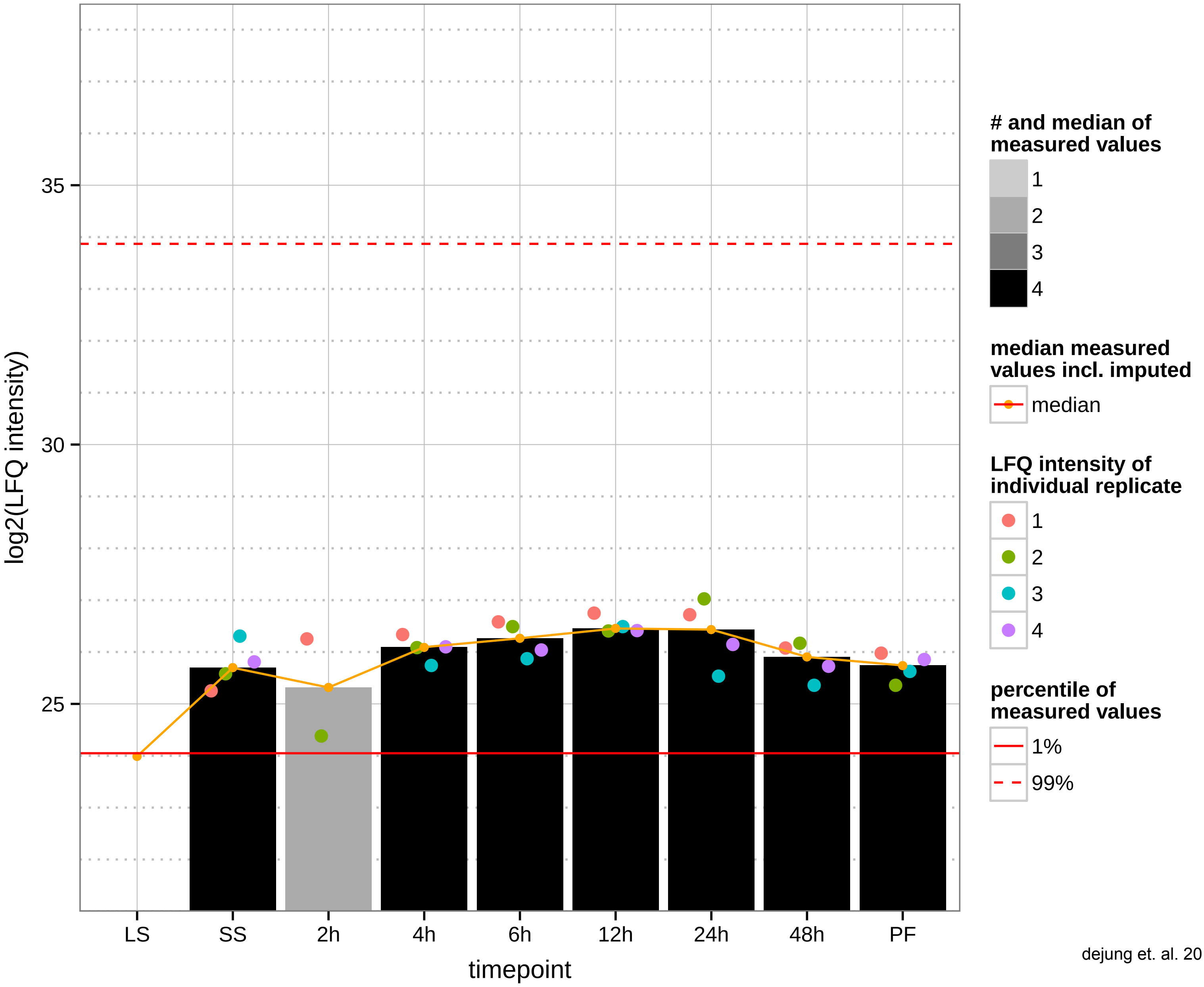

dejung et. al. 2015

hypothetical protein, conserved
Tb927.5.1130
AGOF: null
AGOC: mitochondrion
AGOP: null
PGOF: null
PGOC: null
PGOP: null

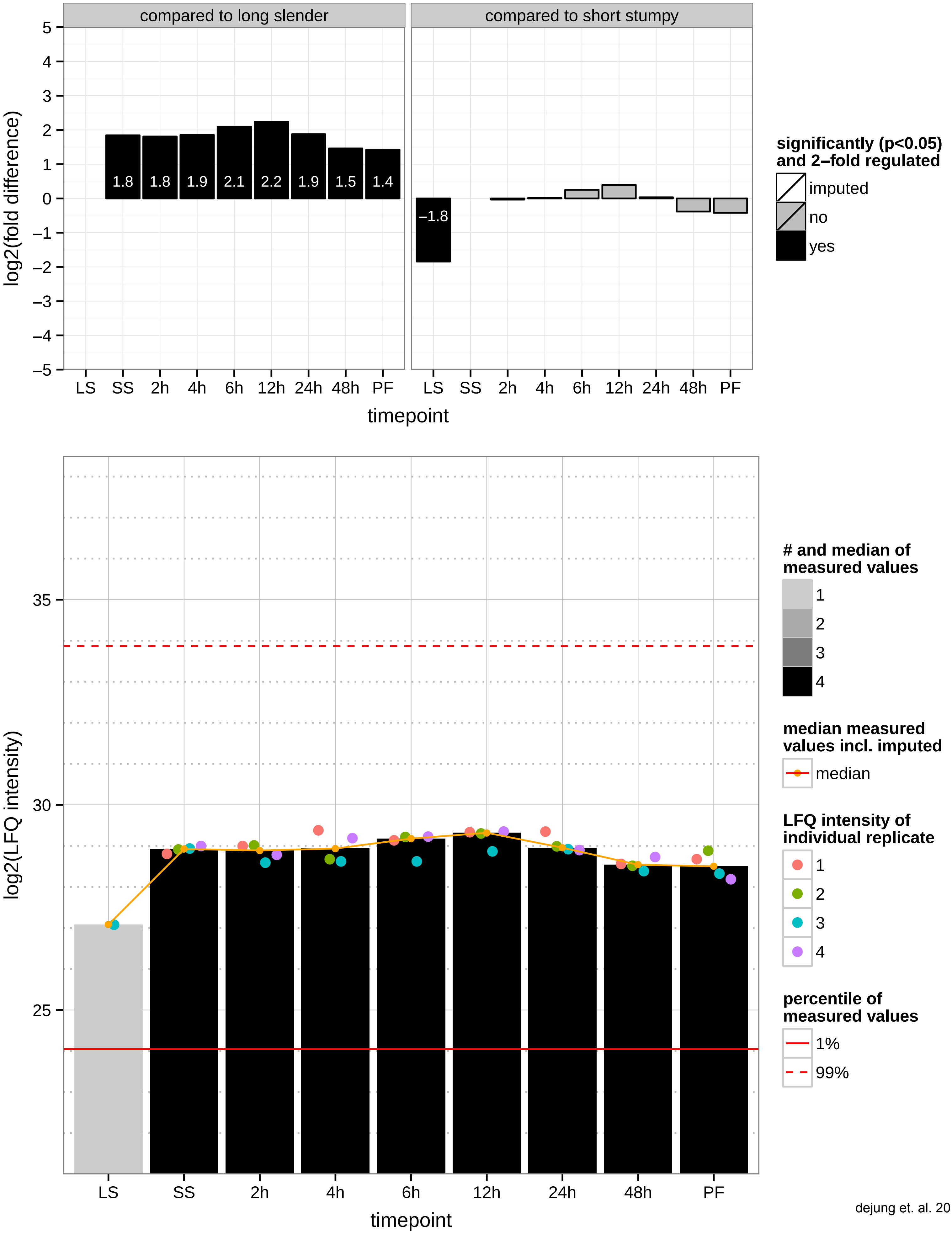

dejung et. al. 2015

exosome component CSL4, Csl4p homologue (CSL4)
Tb927.5.1200
AGOF: null
AGOC: null
AGOP: null
PGOF: null
PGOC: null
PGOP: null

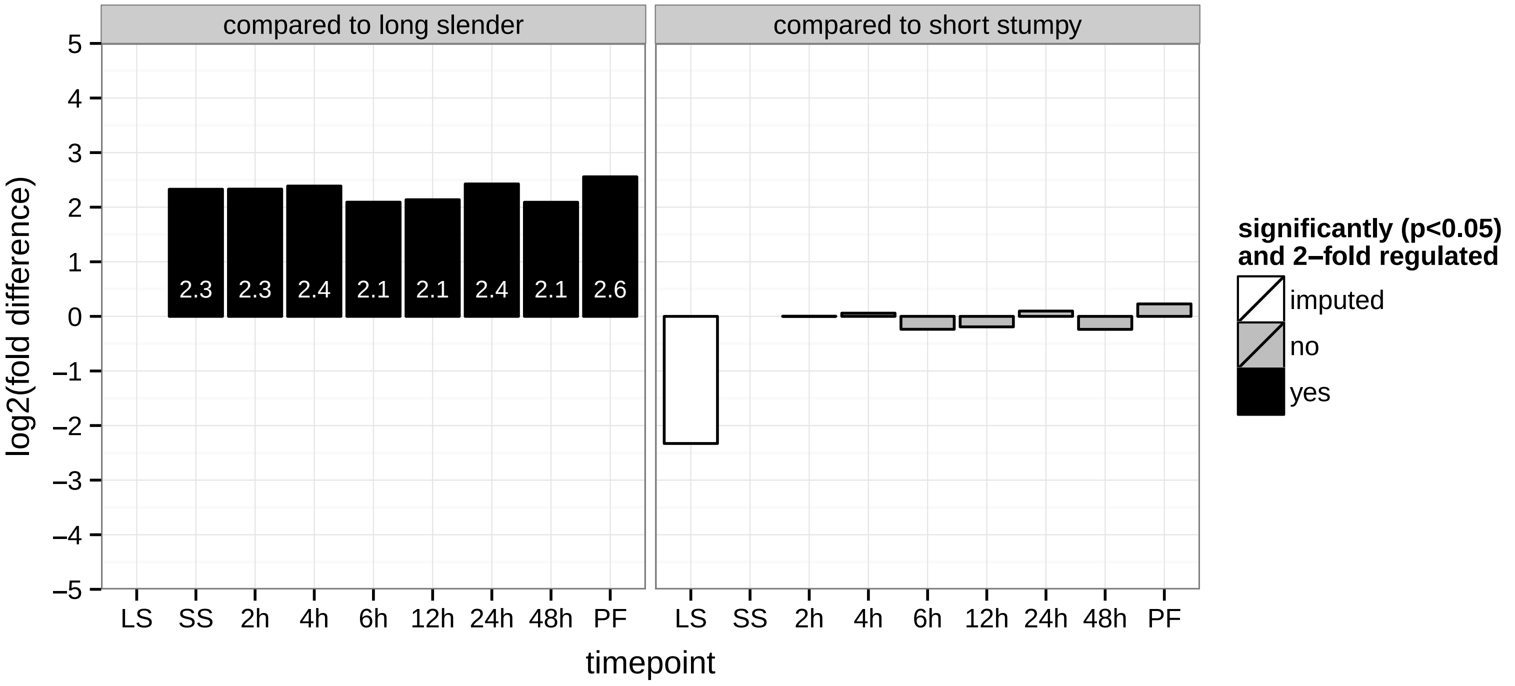

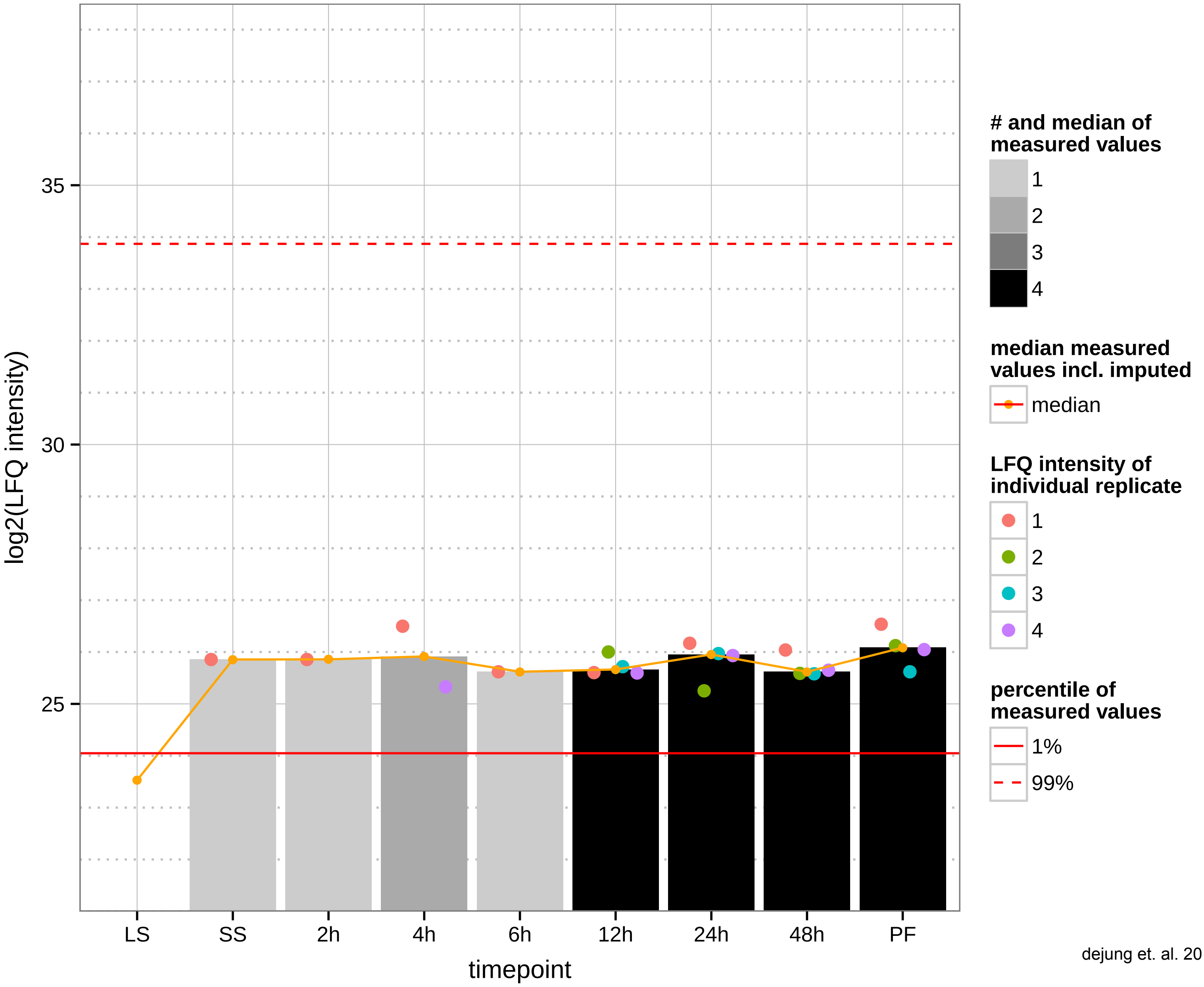

dejung et. al. 2015

hypothetical protein, conserved
Tb927.5.1250
AGOF: null
AGOC: null
AGOP: null
PGOF: protein binding
PGOC: null
PGOP: null

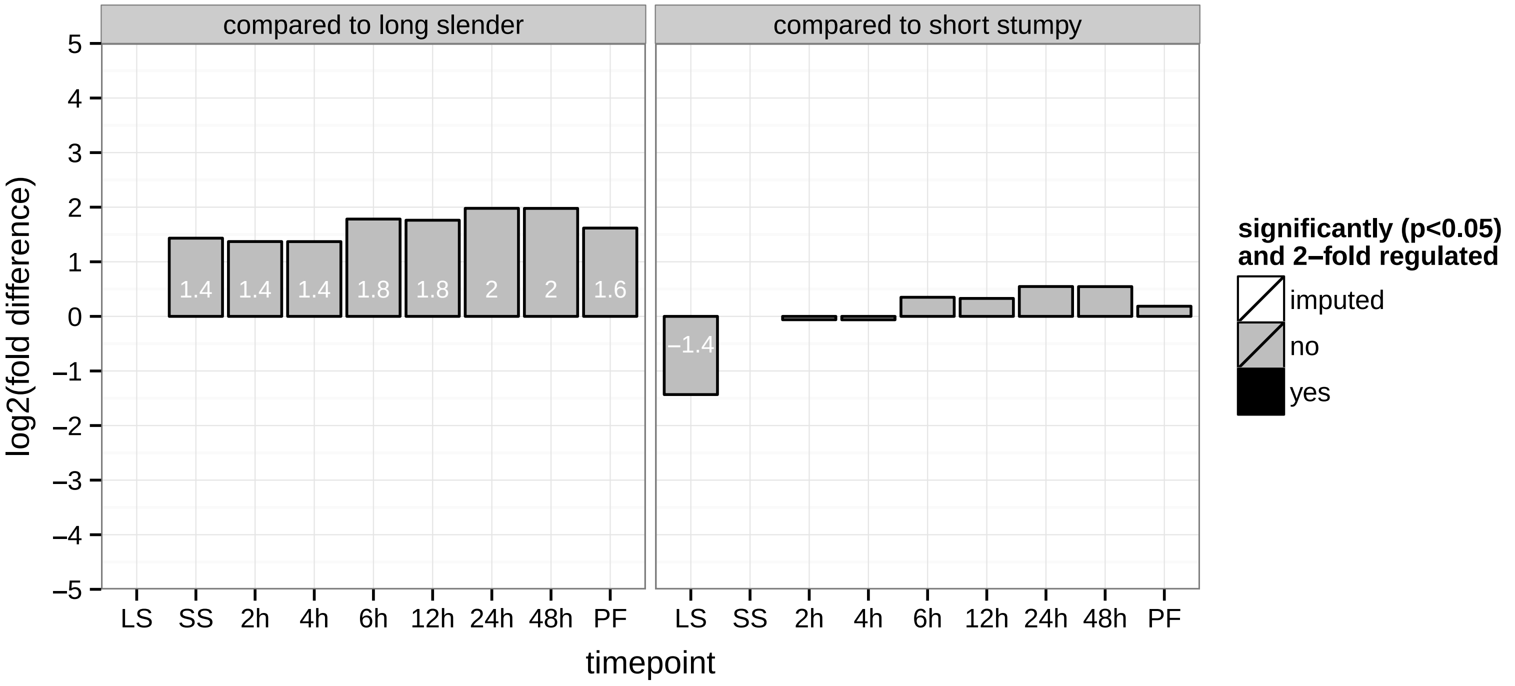

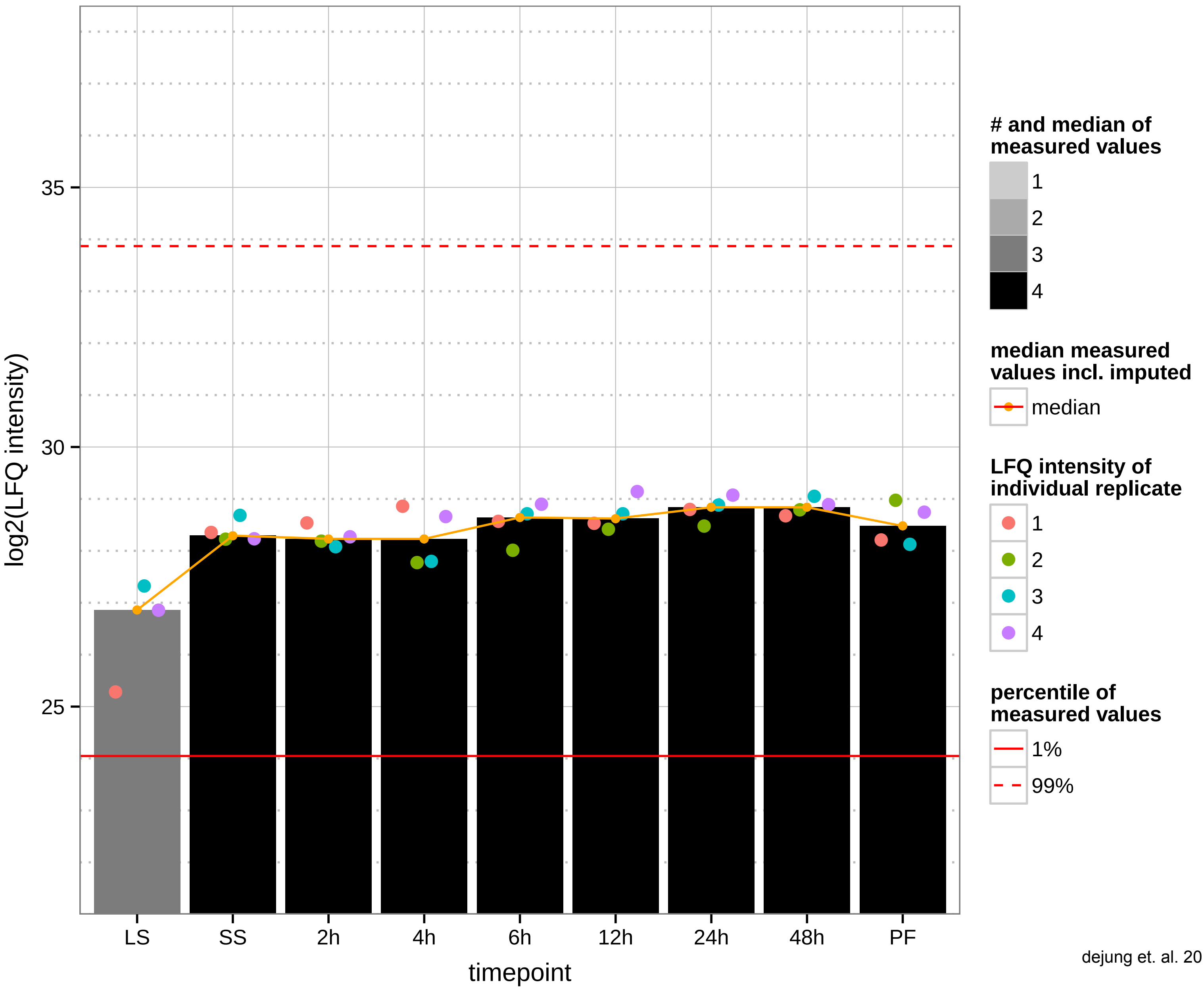

dejung et. al. 2015

hypothetical protein, conserved
Tb927.5.1270
AGOF: null
AGOC: null
AGOP: null
PGOF: null
PGOC: null
PGOP: null

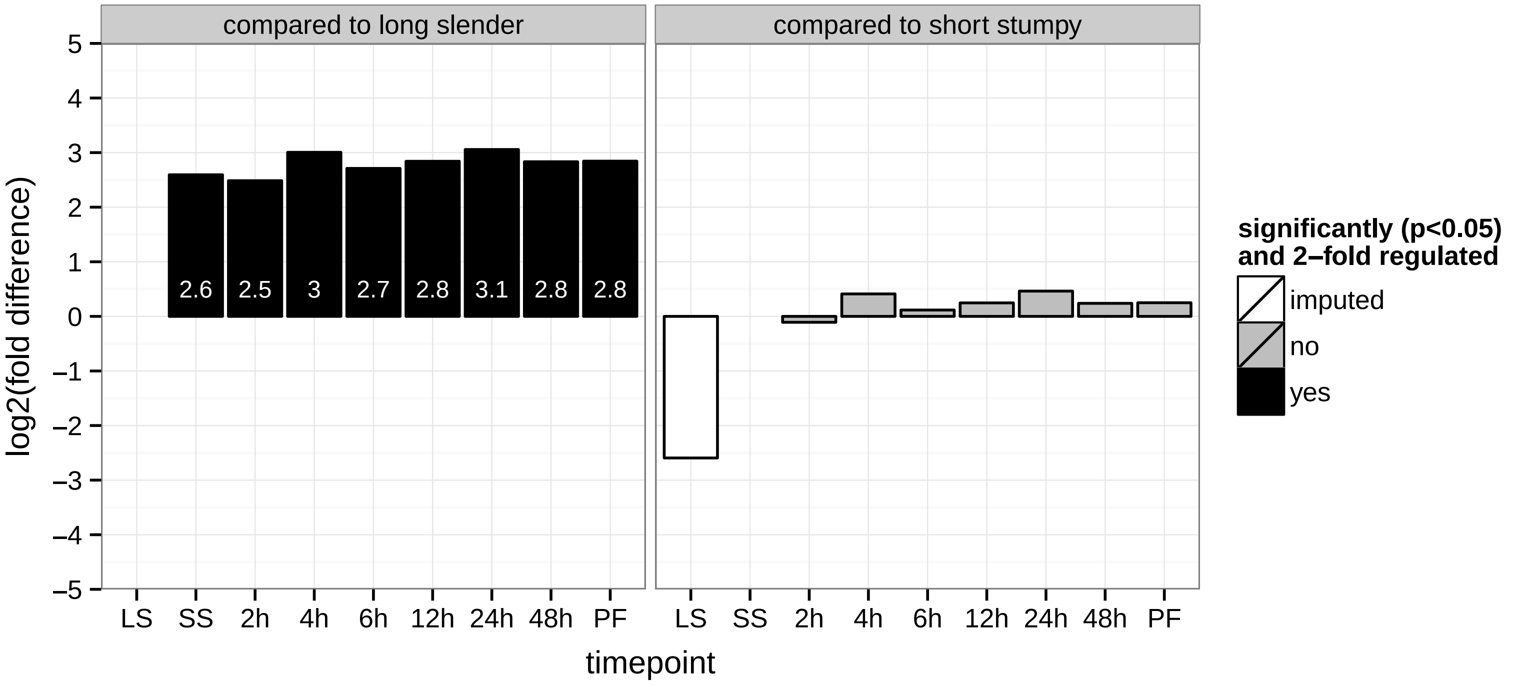

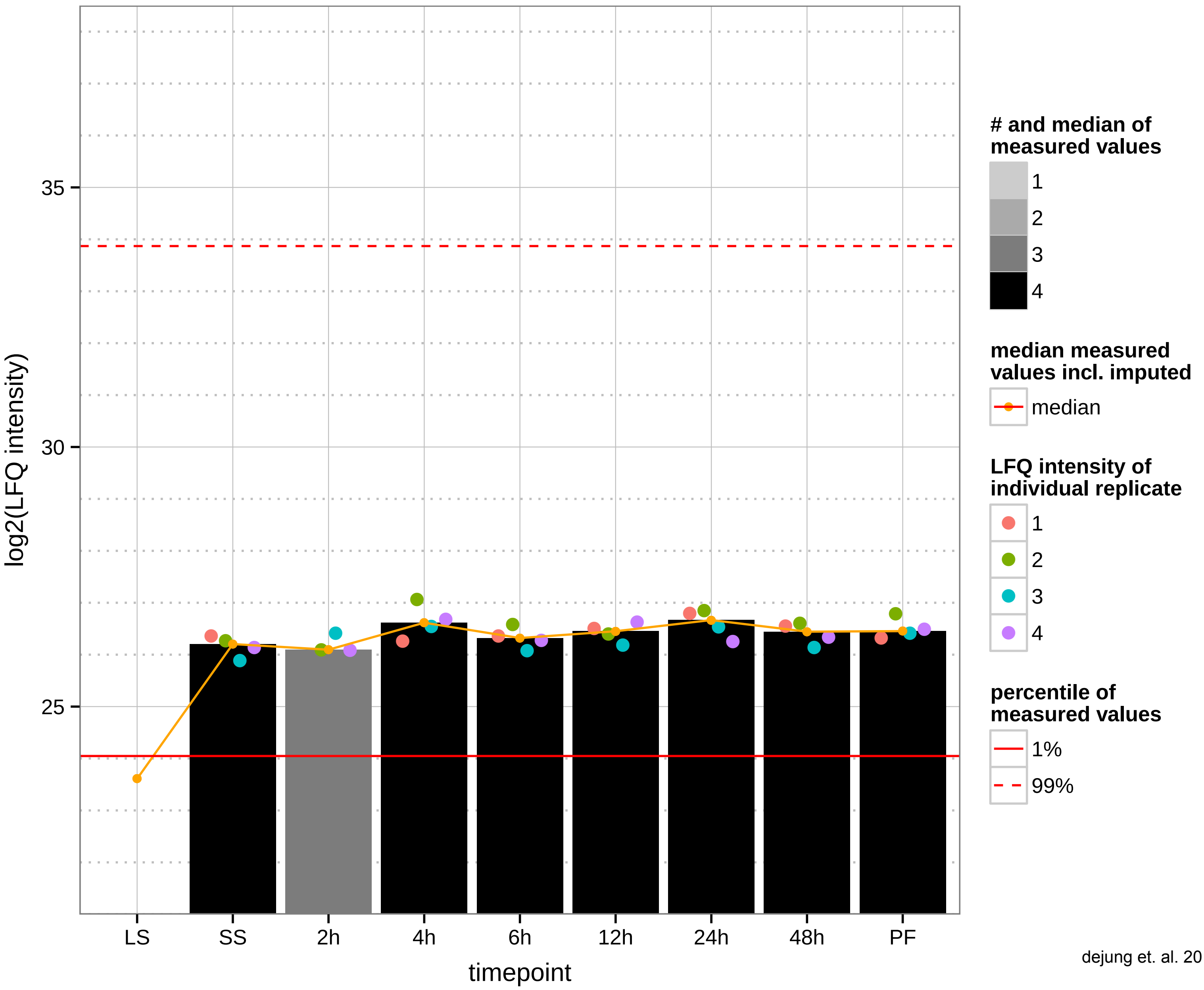

dejung et. al. 2015

nucleoside 2–deoxyribosyltransferase (NDRT)
Tb927.5.1360
AGOF: null
AGOC: null
AGOP: null
PGOF: deoxyribonucleoside 5'–monophosphate N–glycosidase activity, nucleoside deoxyribosyltransferase activity
PGOC: null
PGOP: deoxyribonucleoside monophosphate catabolic process

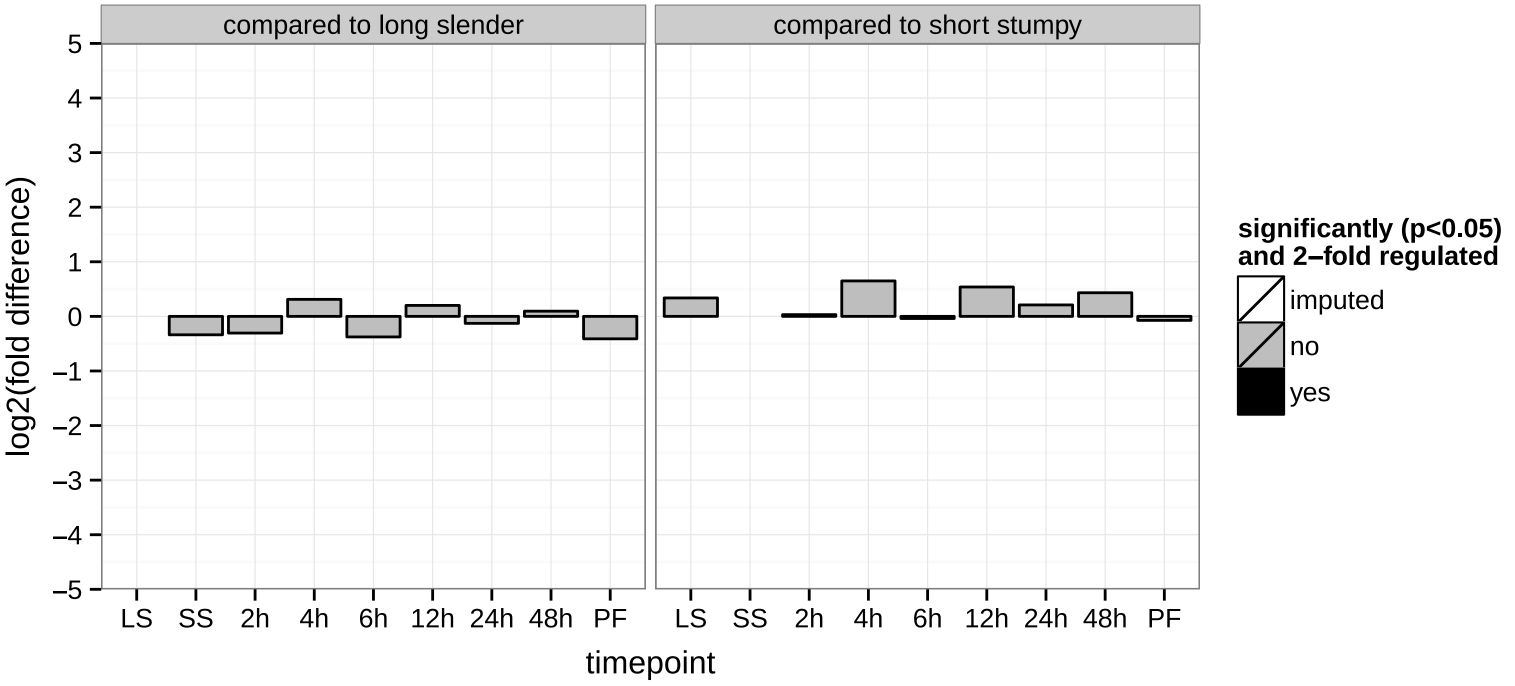

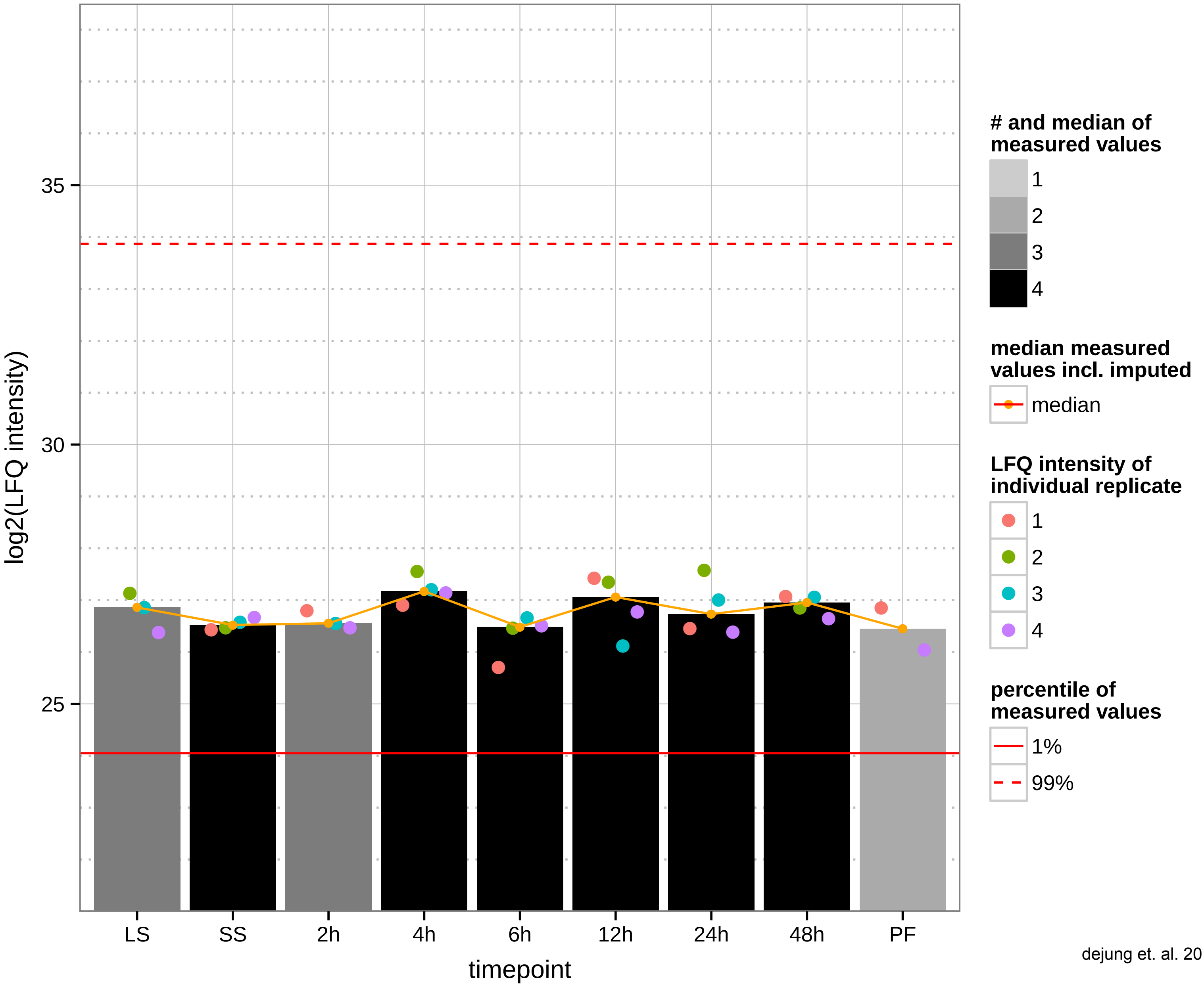

dejung et. al. 2015

NADH−cytochrome b5 reductase, putative (B5R)
Tb927.5.1470
AGOF: electron carrier activity, oxidoreductase activity
AGOC: null
AGOP: null
PGOF: oxidoreductase activity
PGOC: null
PGOP: oxidation−reduction process

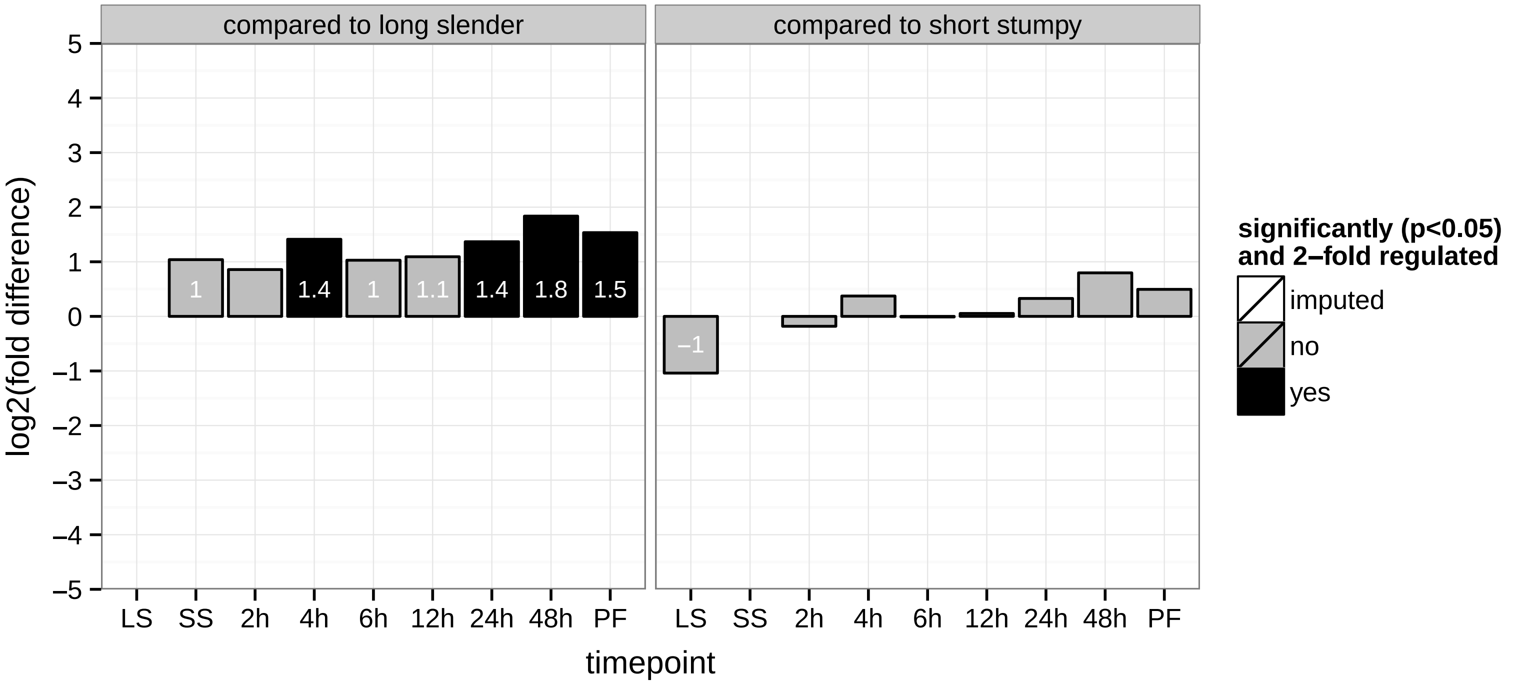

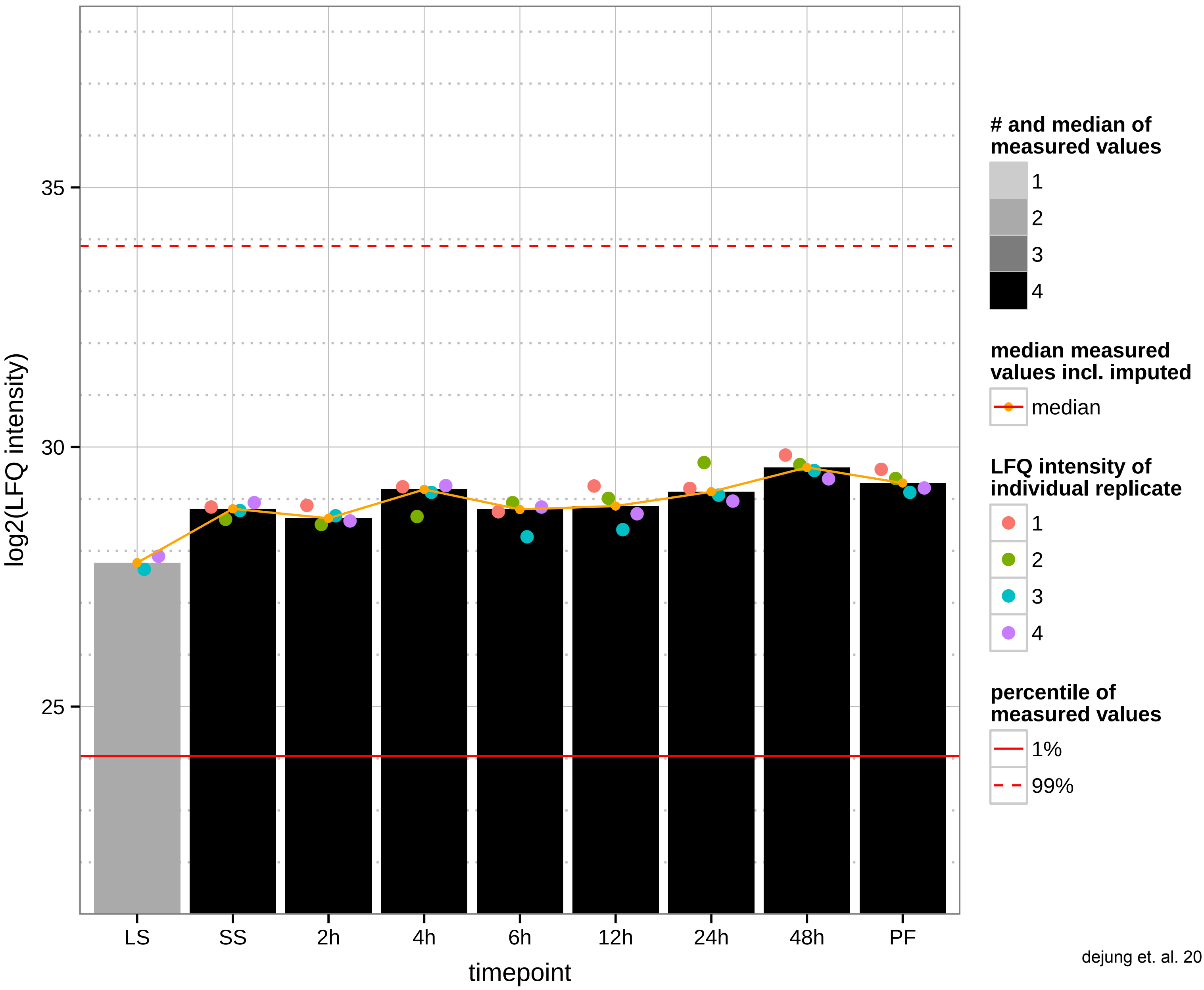

dejung et. al. 2015

hypothetical protein, conserved
Tb927.5.1530
AGOF: N, N−dimethylaniline monooxygenase activity, NADP binding, flavin adenine dinucleotide binding
AGOC: mitochondrion
AGOP: null
PGOF: N, N−dimethylaniline monooxygenase activity, NADP binding, flavin adenine dinucleotide binding
PGOC: null
PGOP: oxidation−reduction process

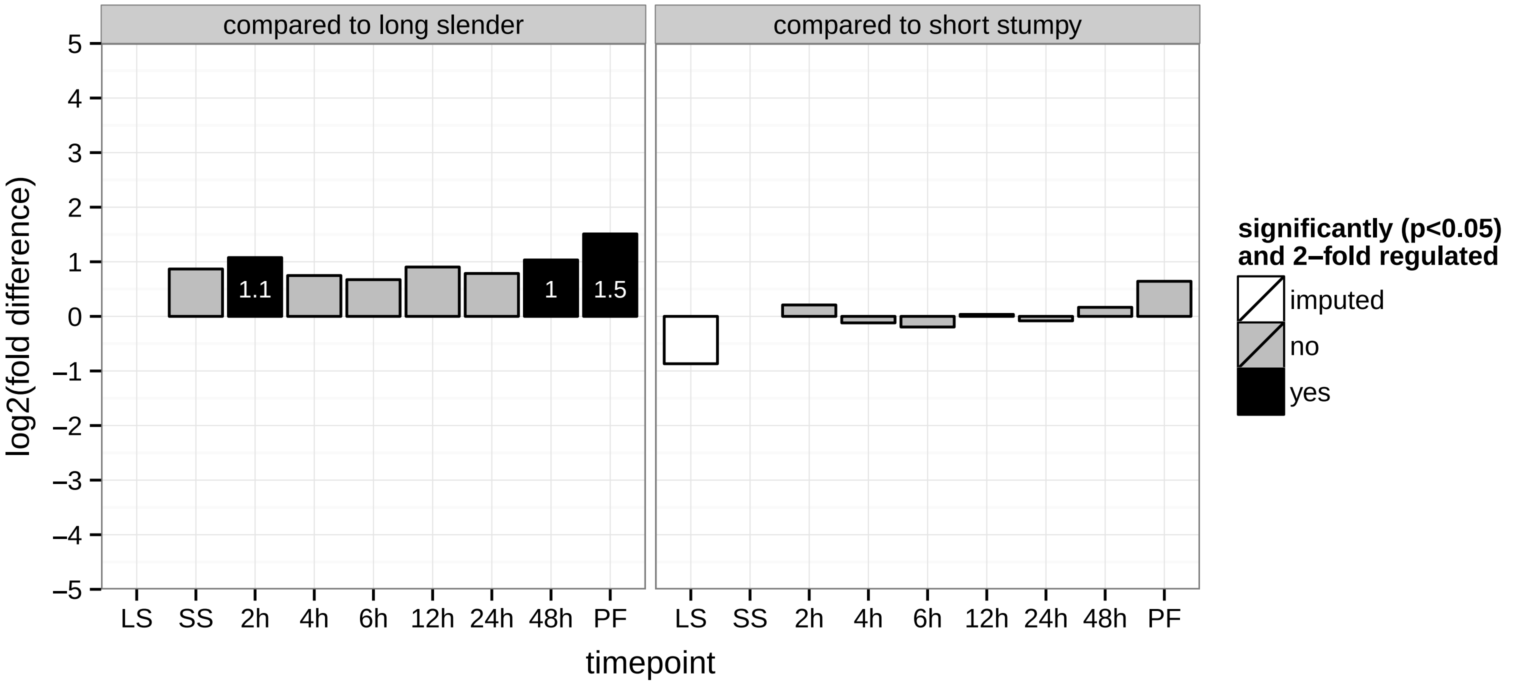

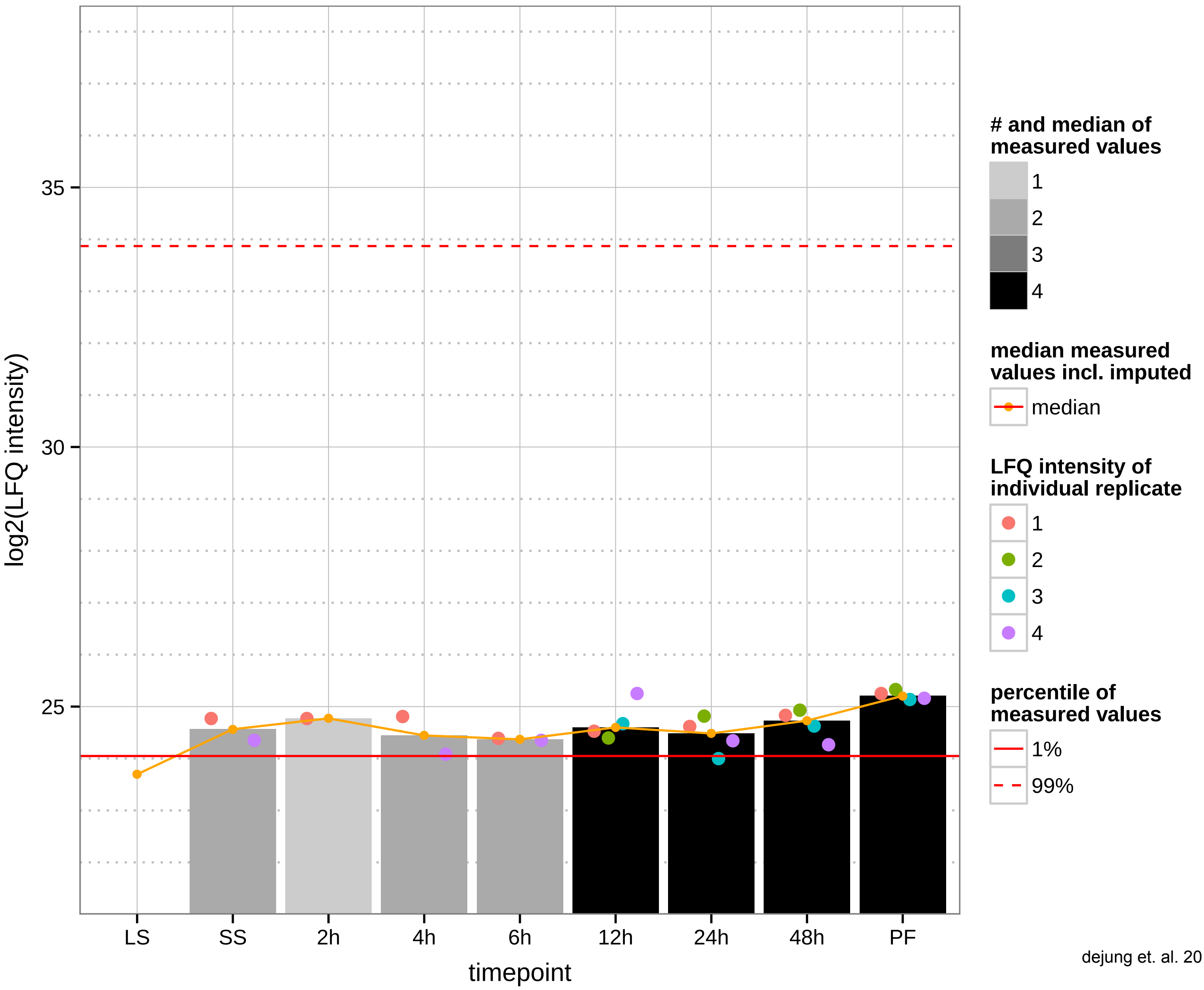

dejung et. al. 2015

hypothetical protein, conserved
Tb927.5.1630
AGOF: heme binding
AGOC: null
AGOP: null
PGOF: heme binding
PGOC: null
PGOP: null

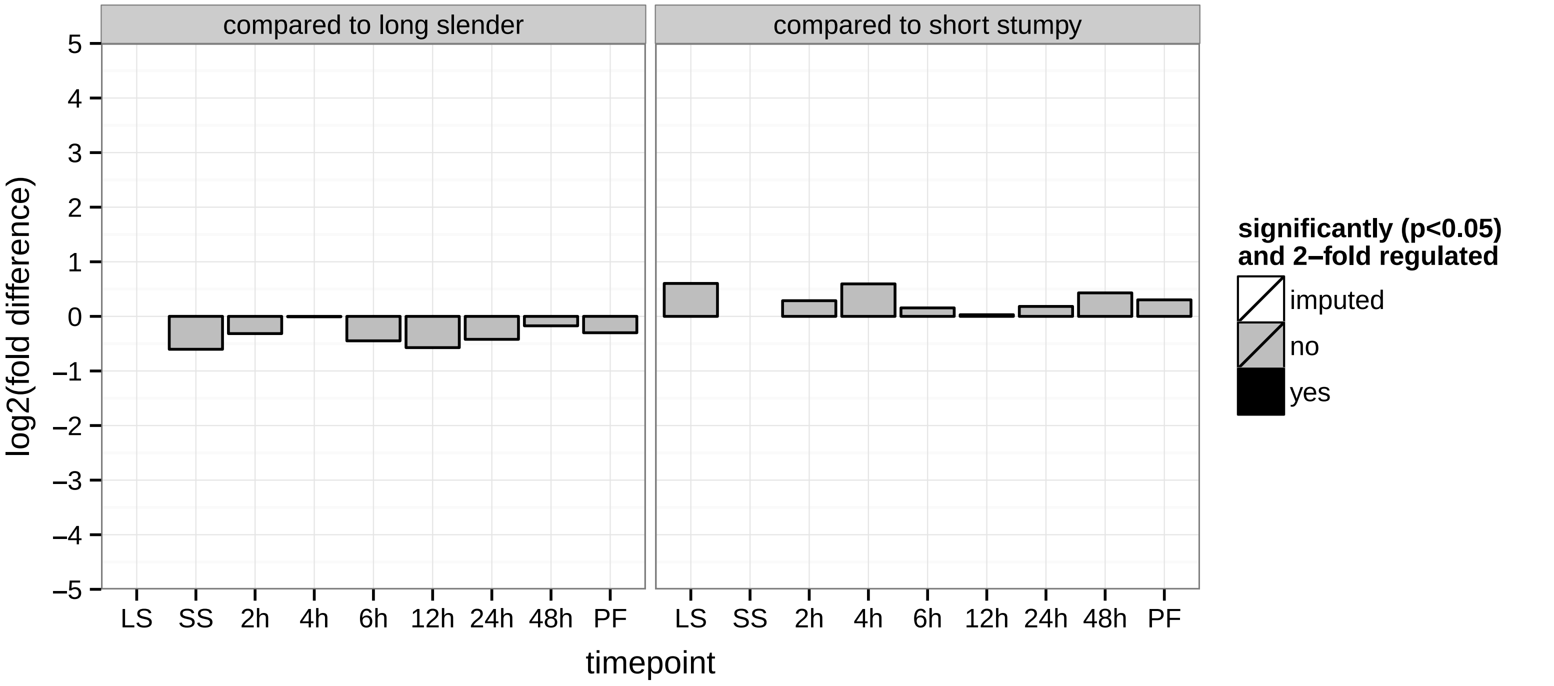

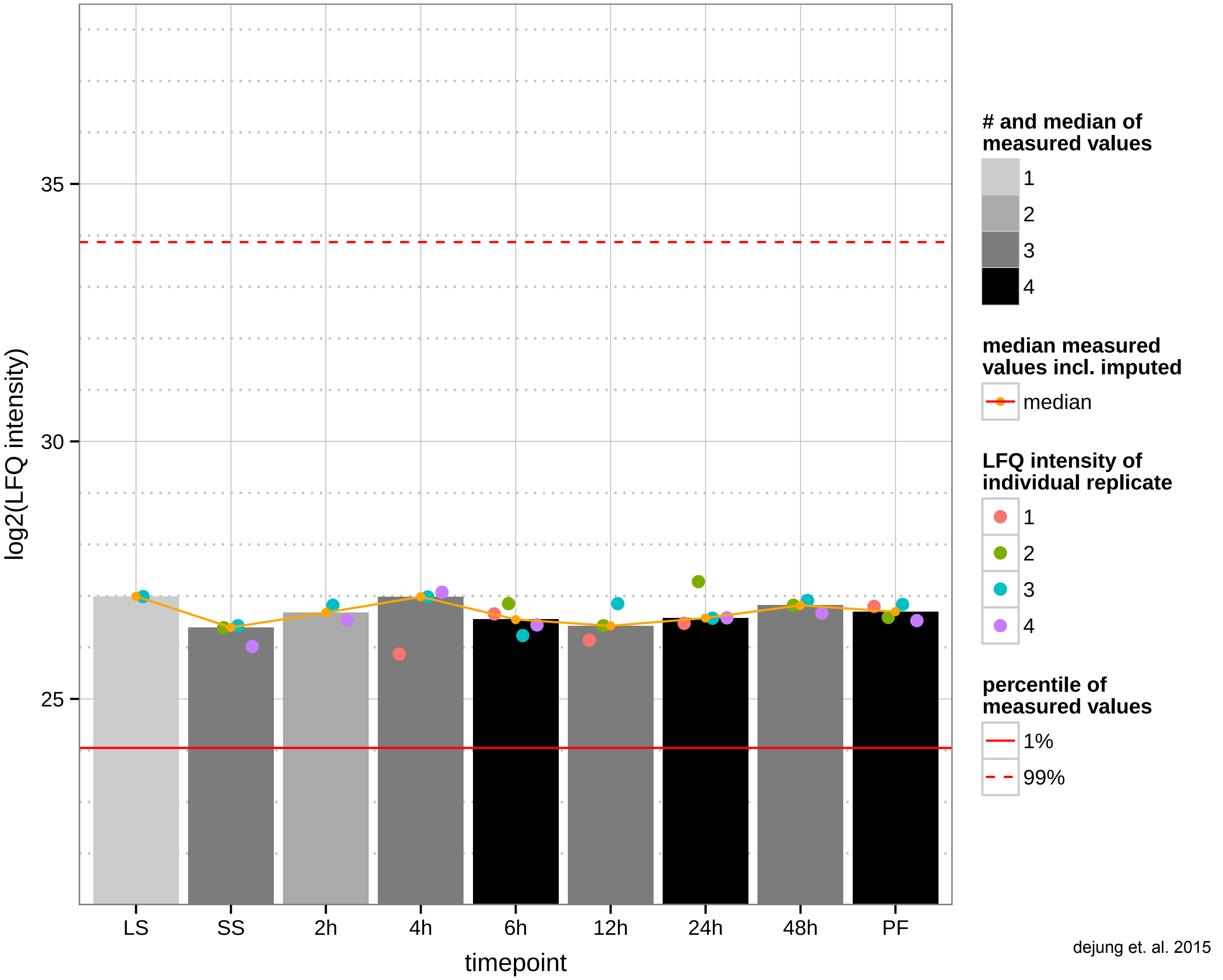

dejung et. al. 2015

hypothetical protein, conserved
Tb927.5.1680
AGOF: null
AGOC: null
AGOP: null
PGOF: null
PGOC: null
PGOP: null

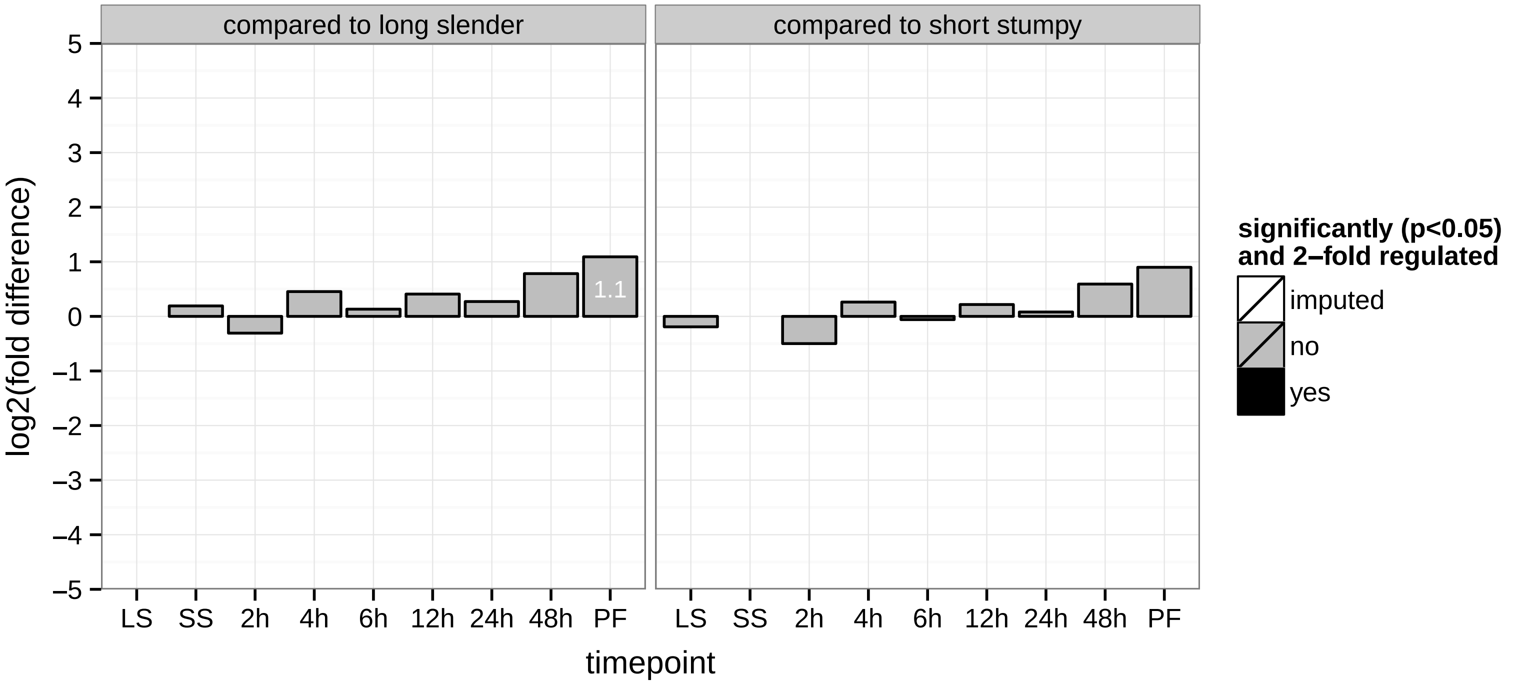

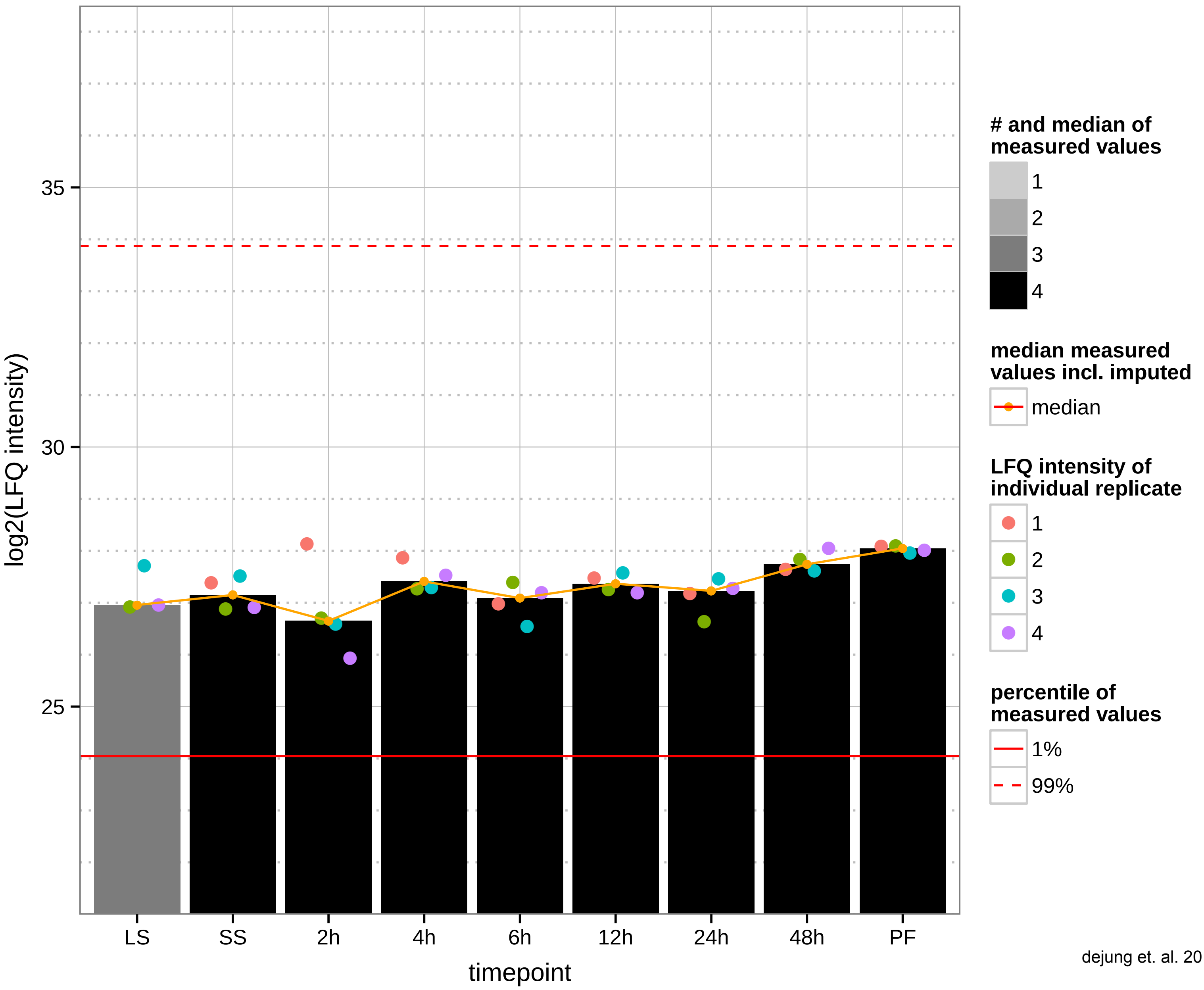

dejung et. al. 2015

hypothetical protein, conserved
Tb927.5.1690
AGOF: null
AGOC: null
AGOP: null
PGOF: null
PGOC: null
PGOP: null

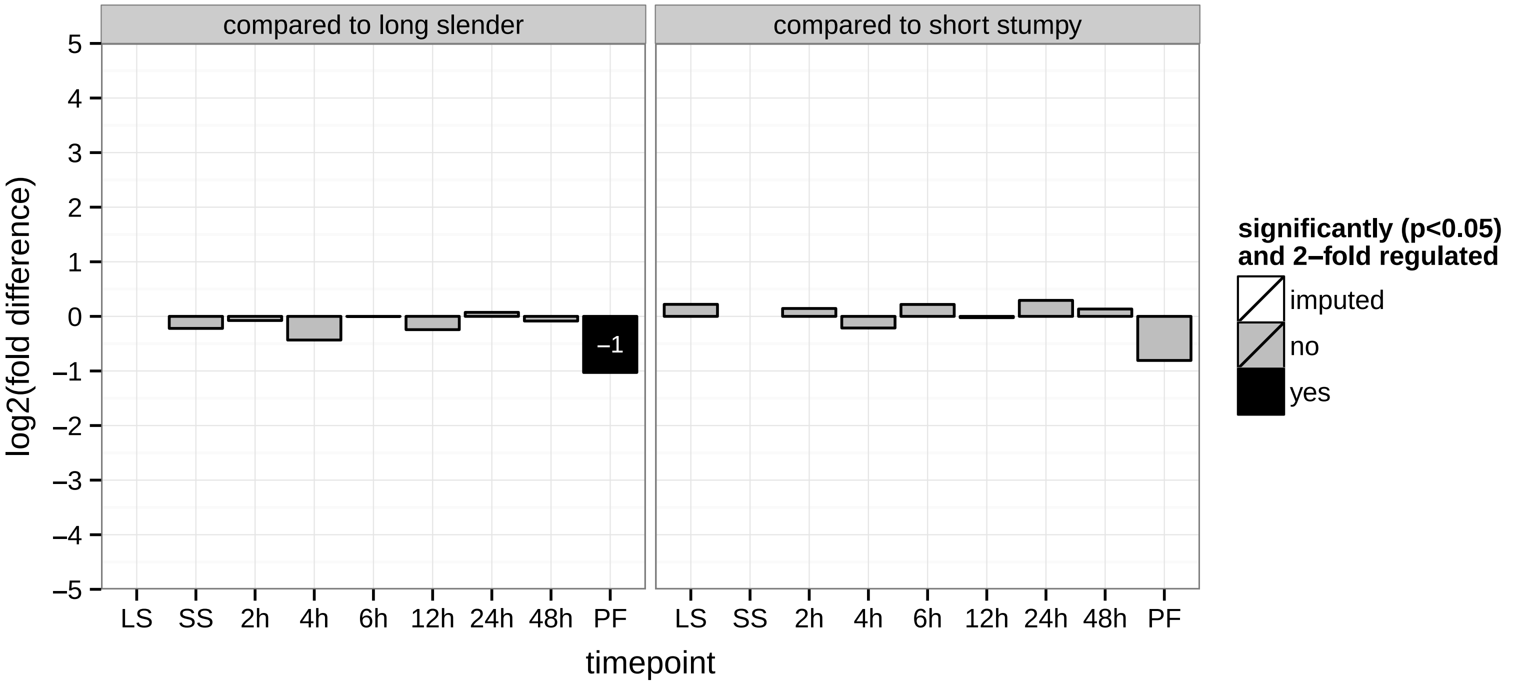

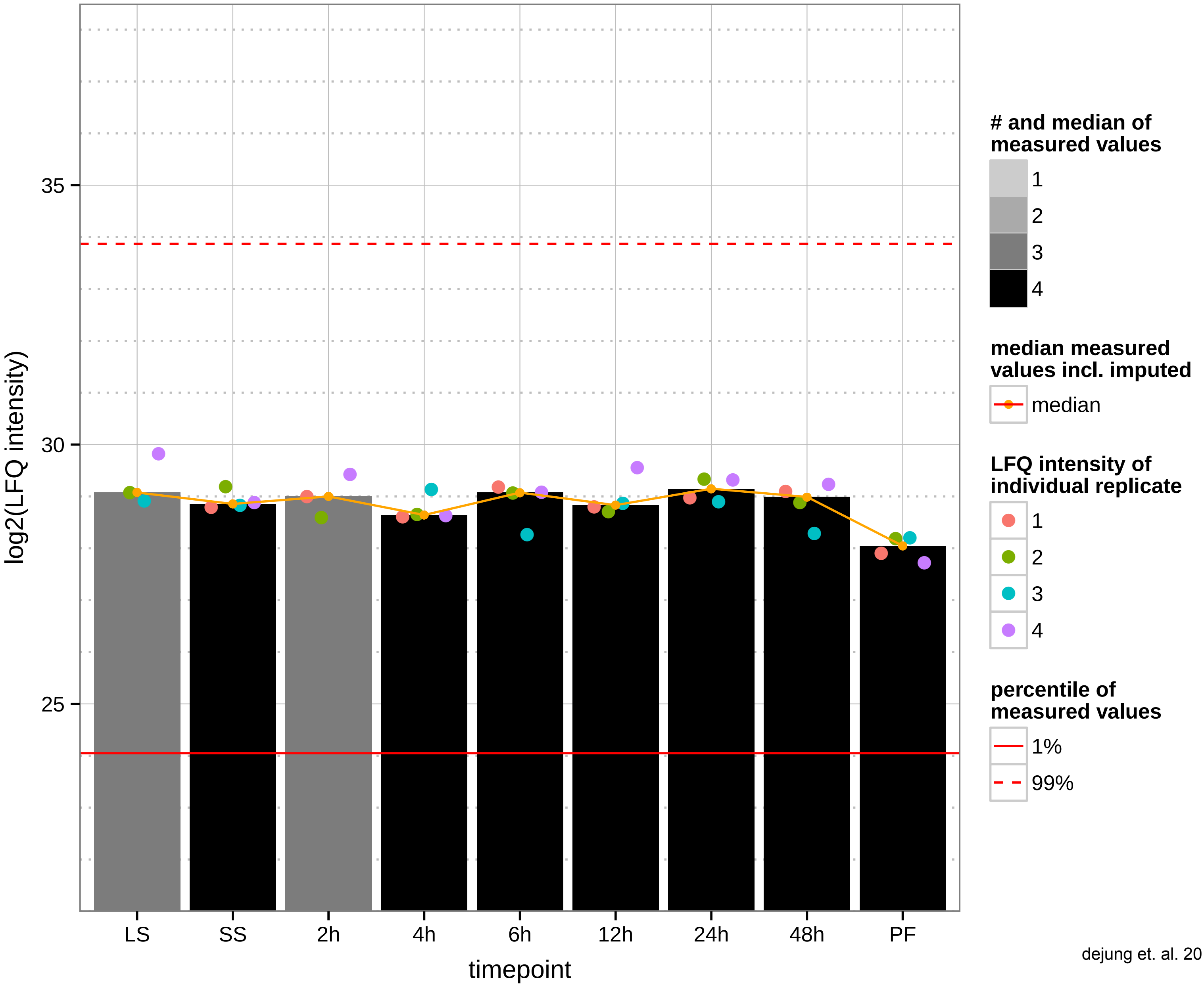

dejung et. al. 2015

replication Factor A 28 kDa subunit, putative
Tb927.5.1700
AGOF: nucleic acid binding
AGOC: null
AGOP: null
PGOF: nucleic acid binding
PGOC: null
PGOP: null

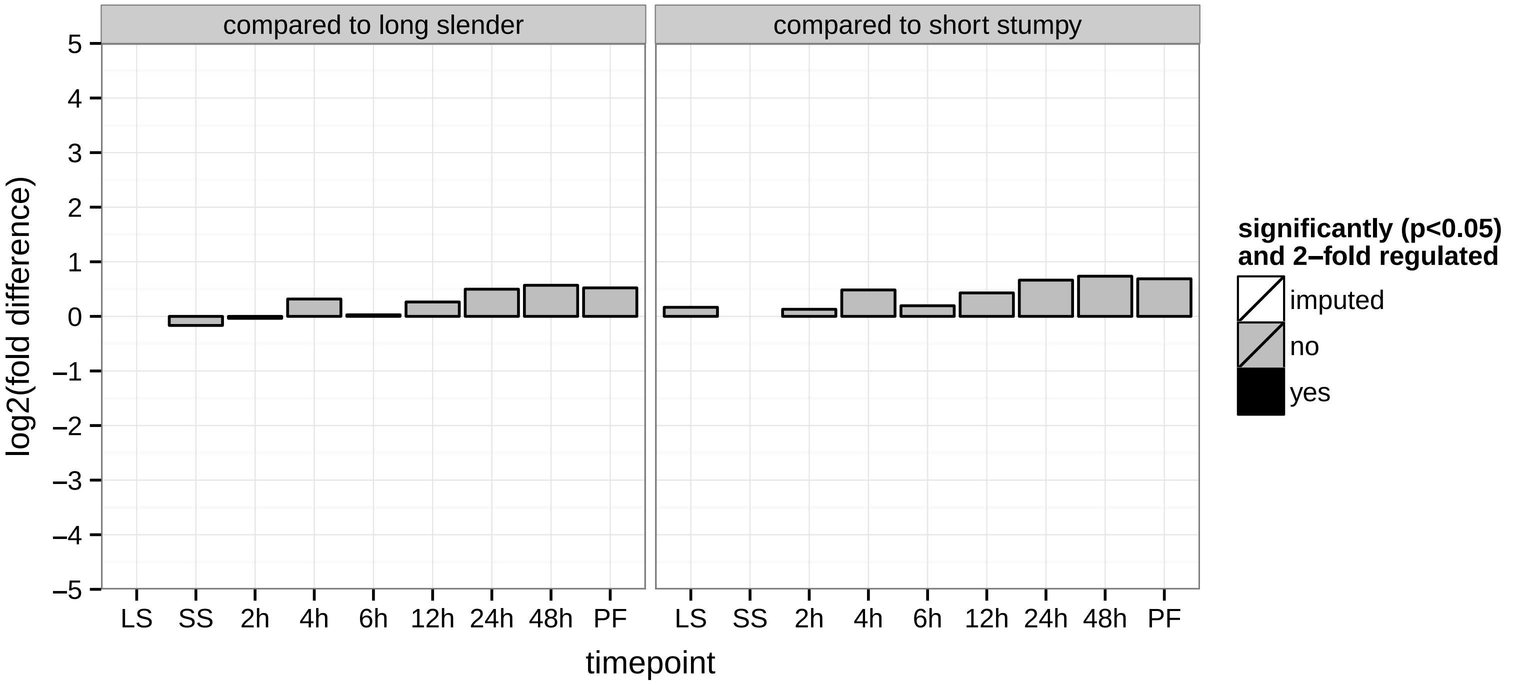

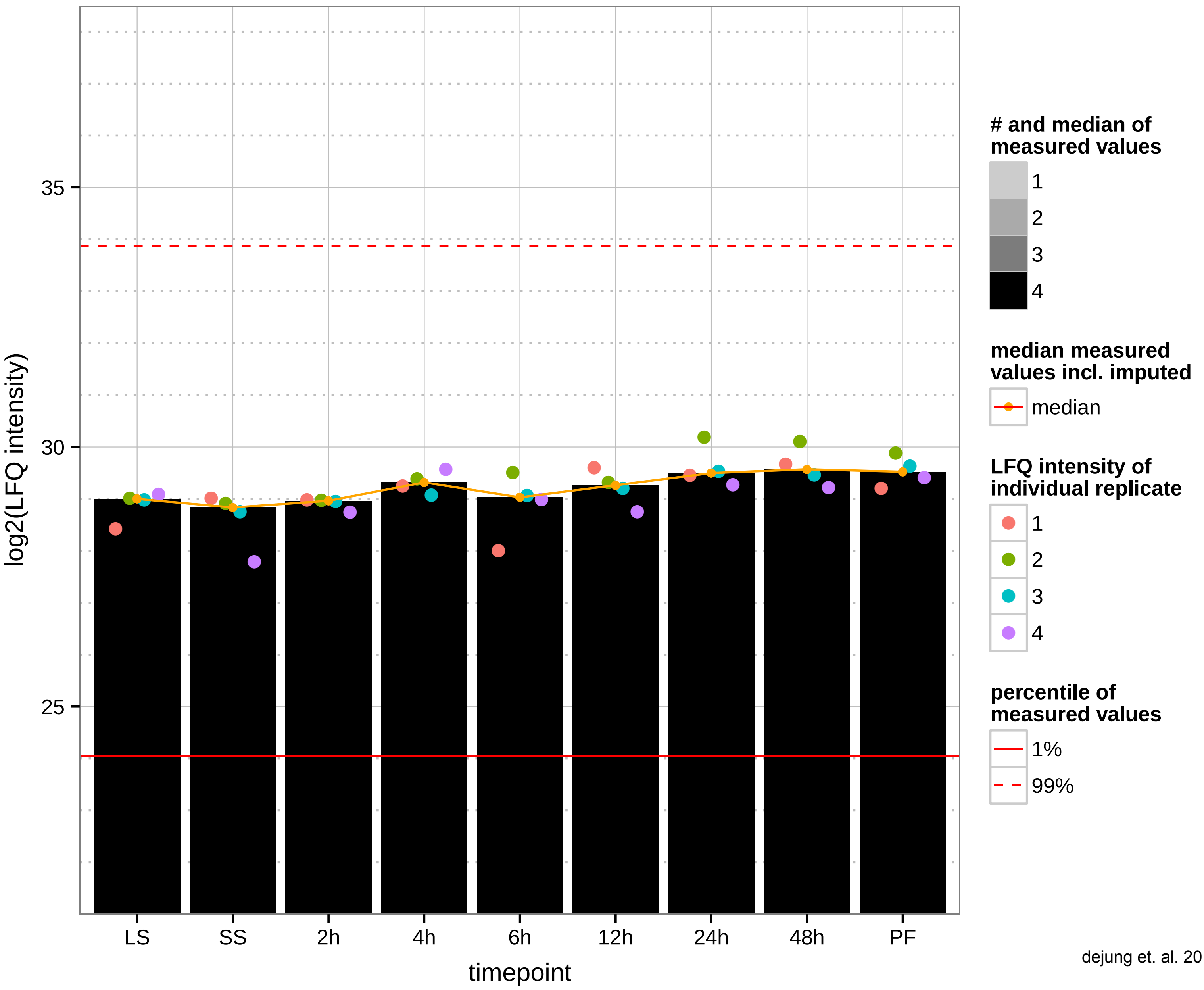

dejung et. al. 2015

ecotin family (I11), putative, inhibitor of serine peptidase (ISP), putative (ISP)
Tb927.5.1730
AGOF: null
AGOC: null
AGOP: null
PGOF: null
PGOC: null
PGOP: null

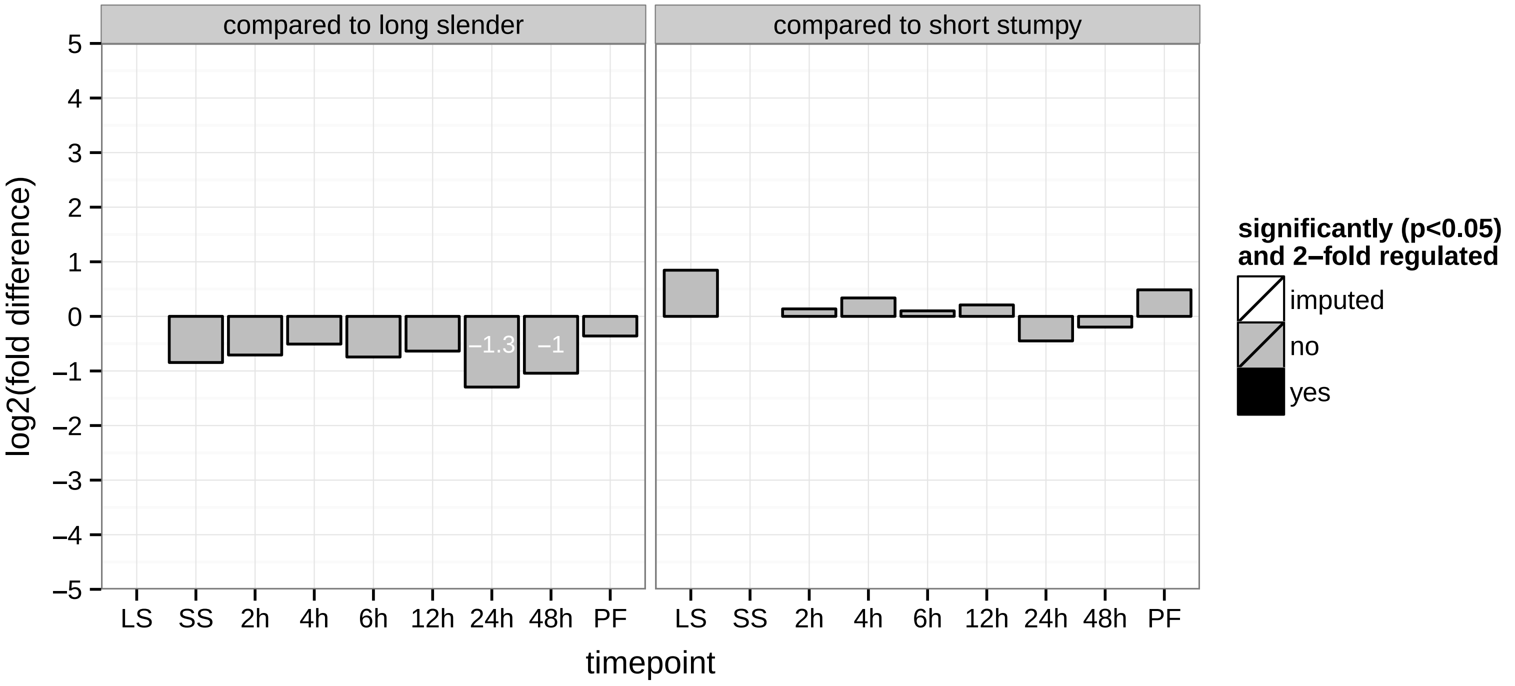

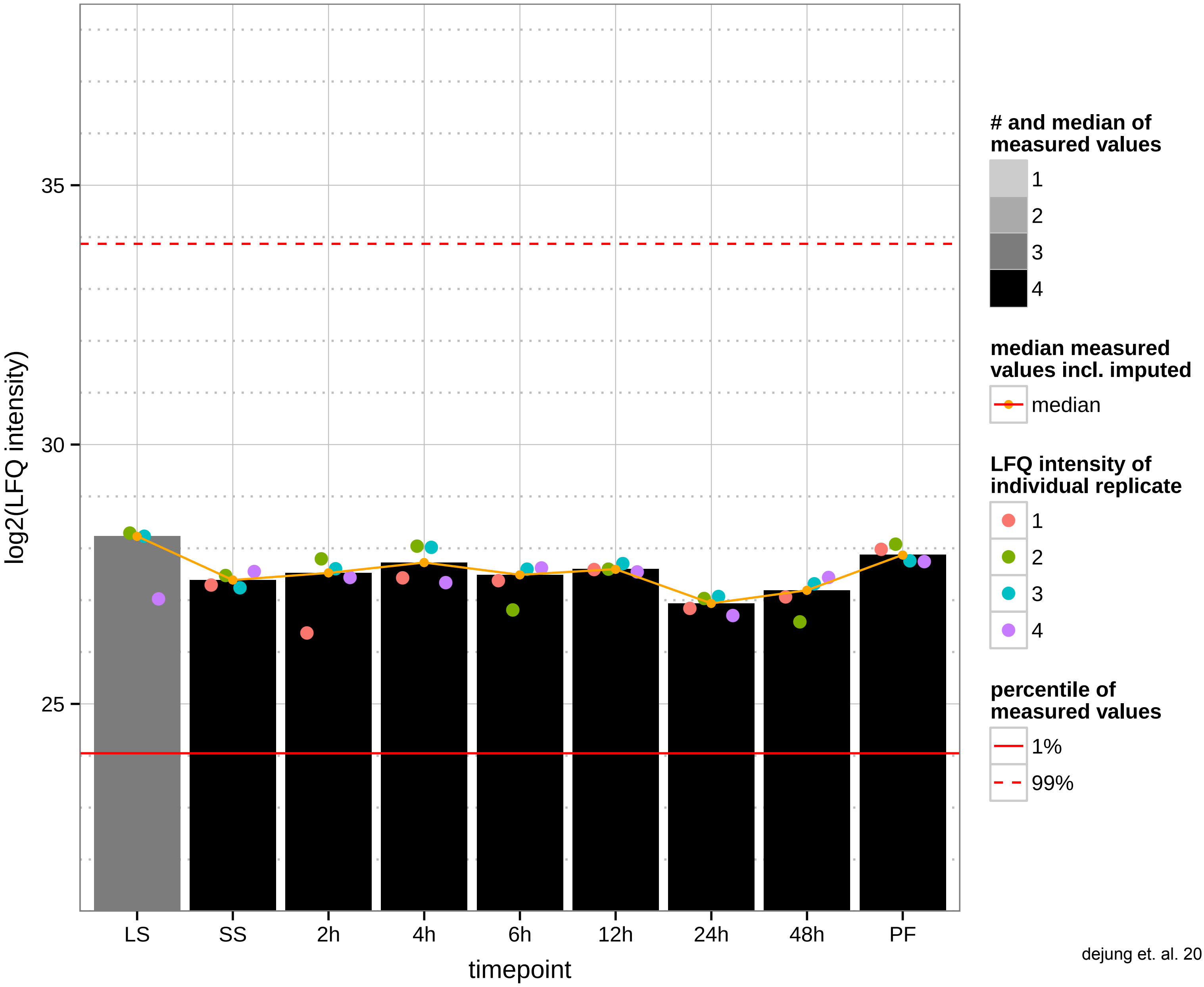

dejung et. al. 2015

lysosomal/endosomal membrane protein p67 (p67), lysosomal/endosomal membrane protein p67, lysosomal membrane glyco
Tb927.5.1810;Tb927.5.1830
AGOF: null
AGOC: null
AGOP: null
PGOF: null
PGOC: null
PGOP: null

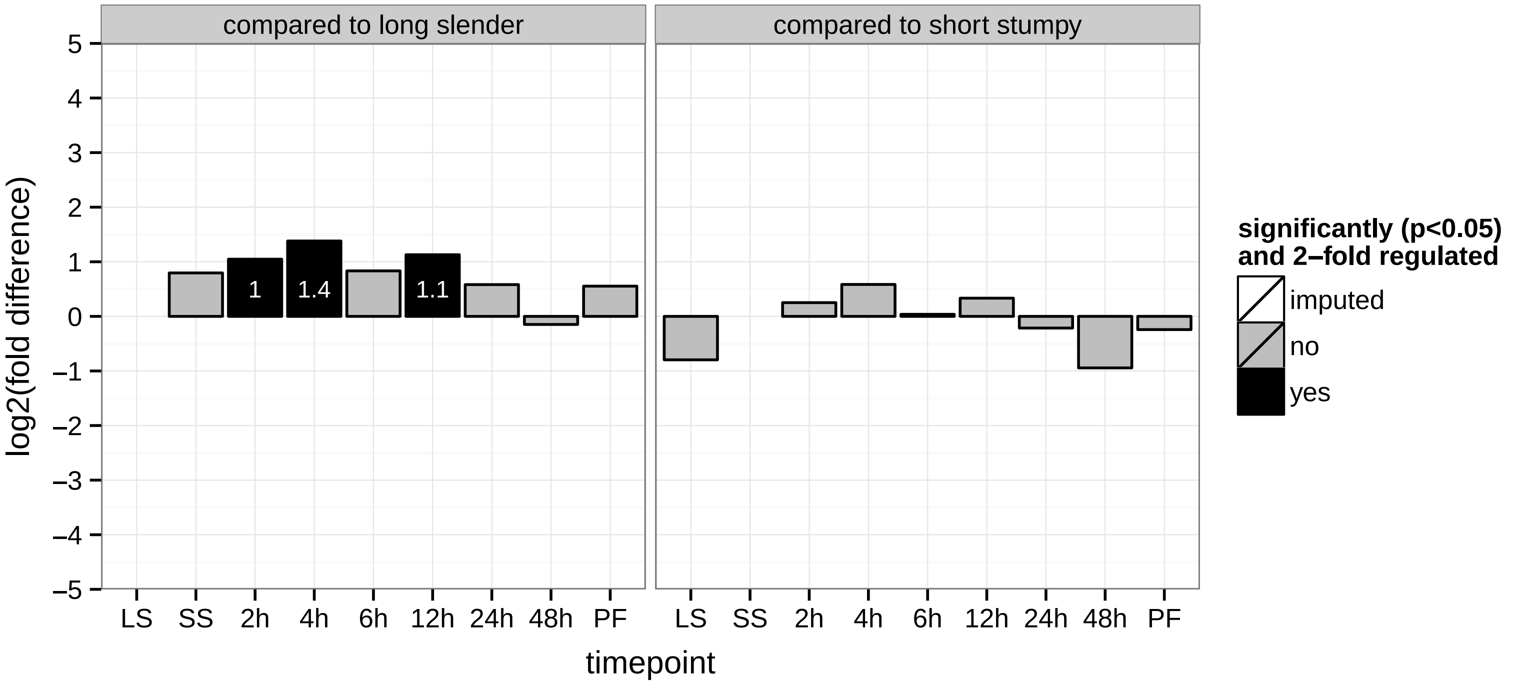

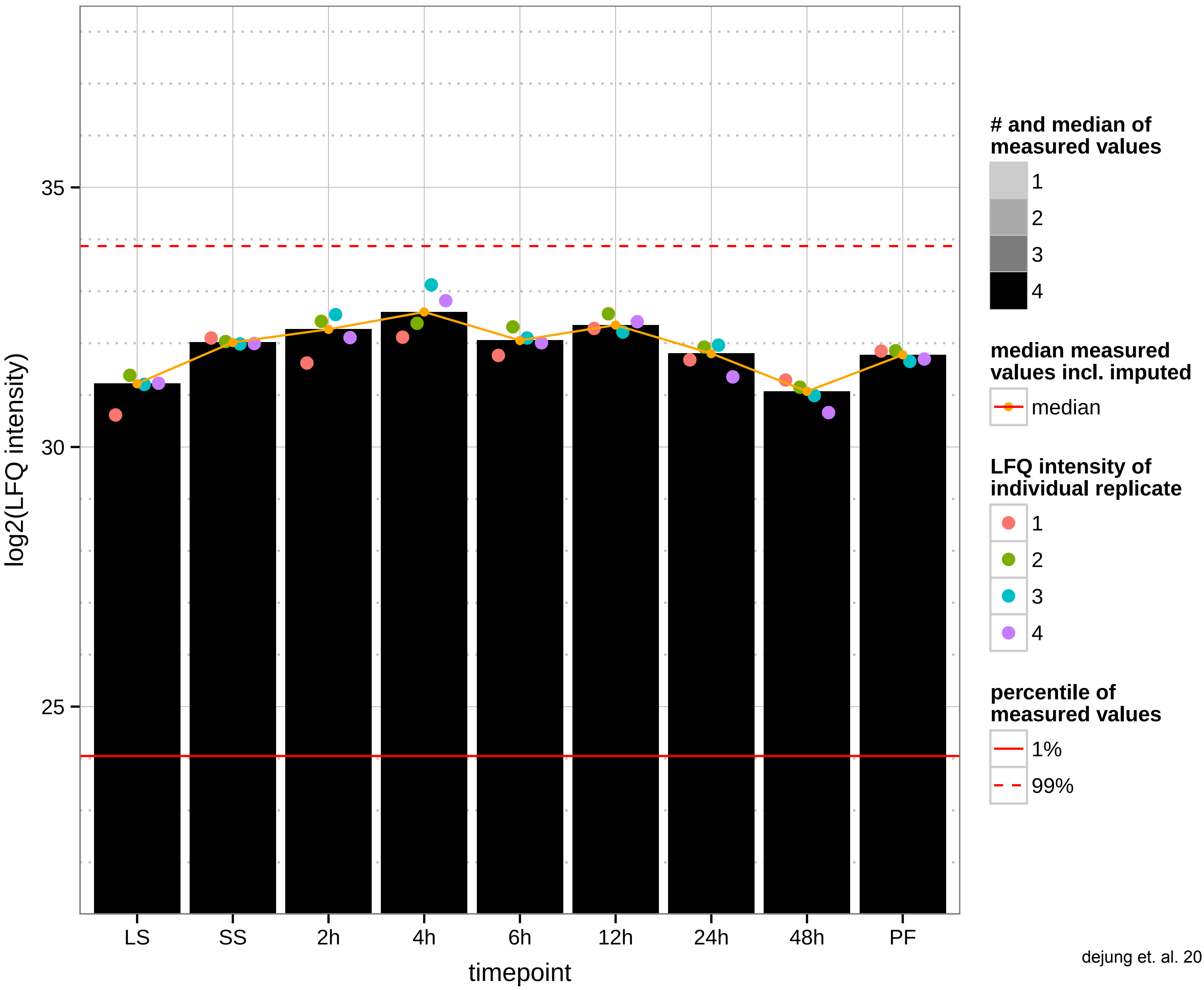

dejung et. al. 2015

ecotin family (I11), putative, inhibitor of serine peptidase (ISP), putative (ISP2)
Tb927.5.1880
AGOF: null
AGOC: null
AGOP: active evasion of host immune response via regulation of host complement system, modulation by symbiont of host ph
PGOF: null
PGOC: null
PGOP: null

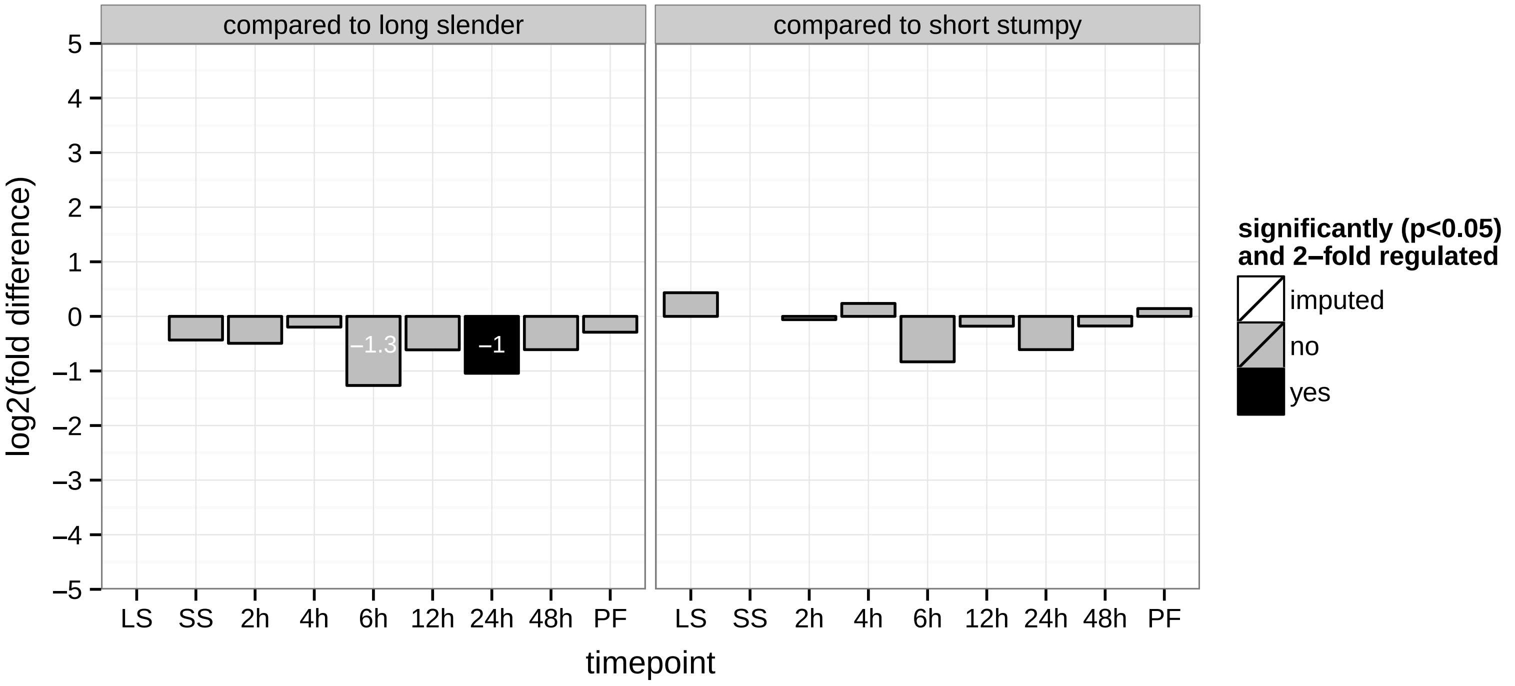

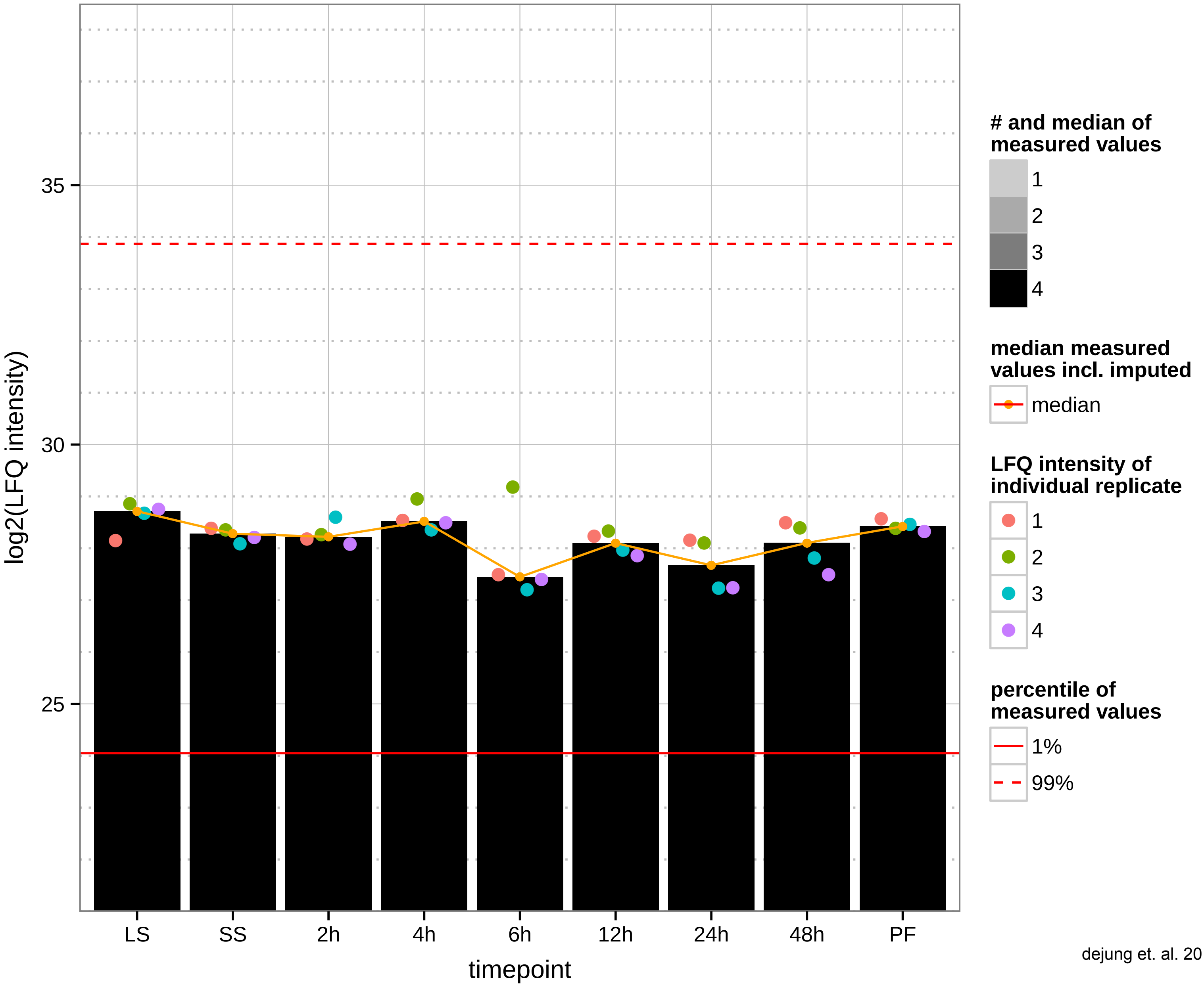

dejung et. al. 2015

hypothetical protein, conserved
Tb927.5.1900
AGOF: null
AGOC: null
AGOP: null
PGOF: null
PGOC: null
PGOP: null

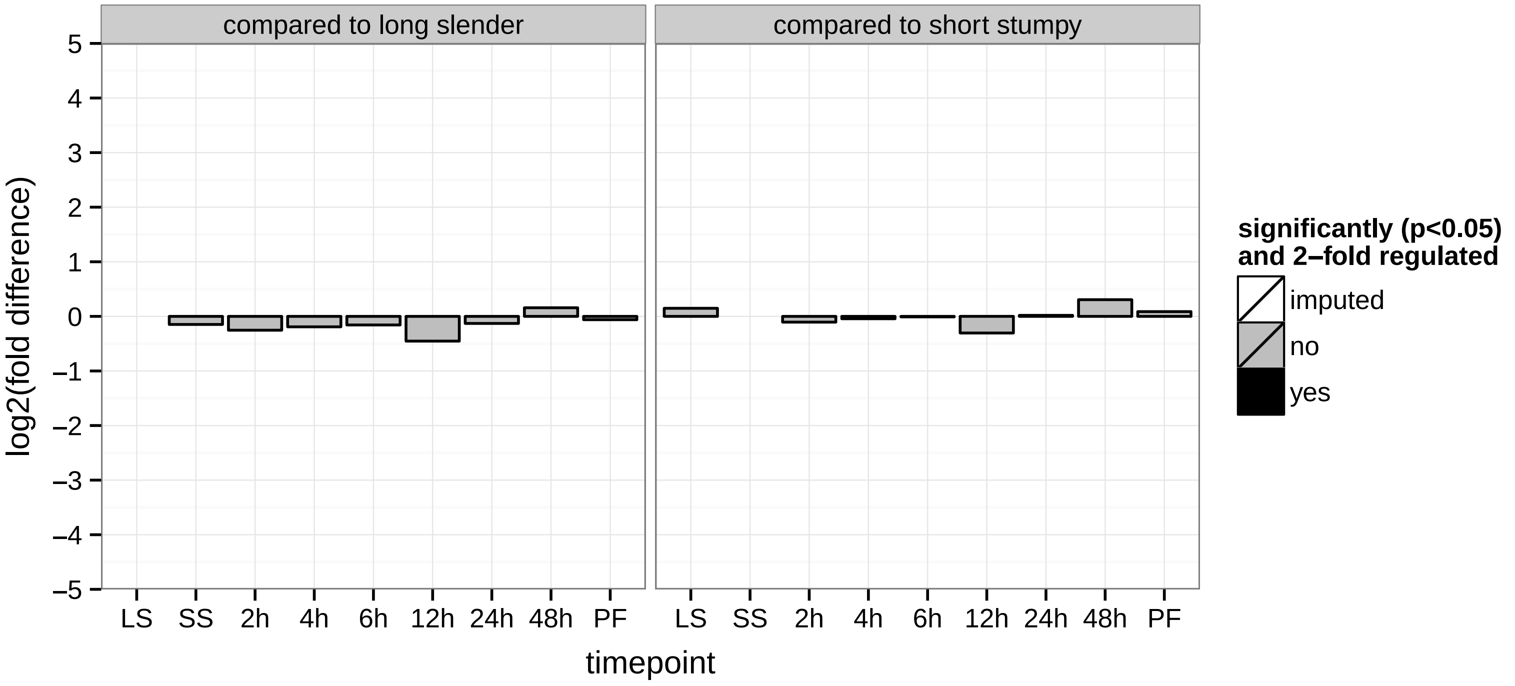

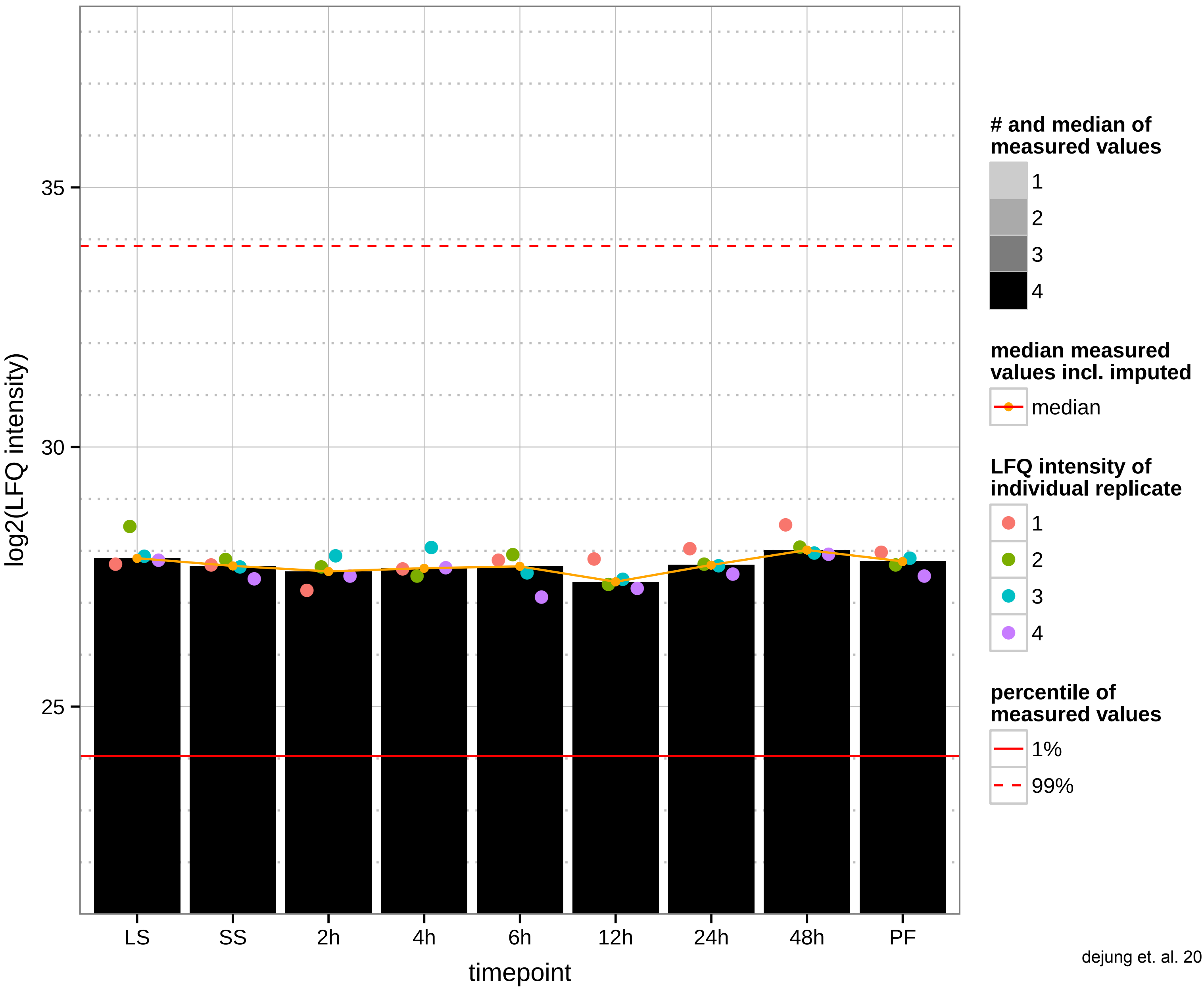

dejung et. al. 2015

hypothetical protein, conserved
Tb927.5.1930
AGOF: peptidase activity
AGOC: integral to membrane, signal peptidase complex
AGOP: signal peptide processing
PGOF: peptidase activity
PGOC: integral to membrane, signal peptidase complex
PGOP: signal peptide processing

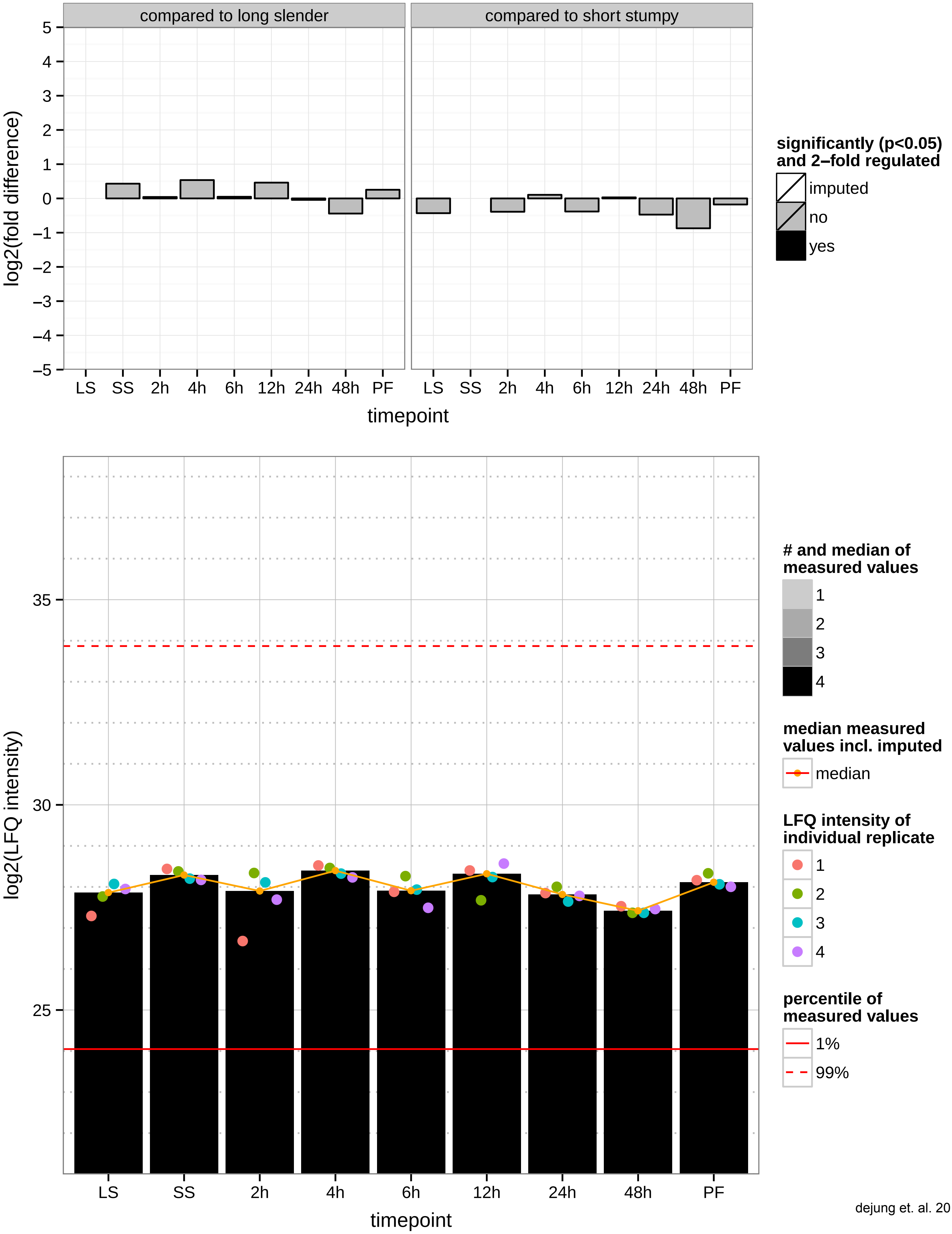

dejung et. al. 2015

hypothetical protein, conserved
Tb927.5.2050
AGOF: null
AGOC: null
AGOP: null
PGOF: null
PGOC: null
PGOP: null

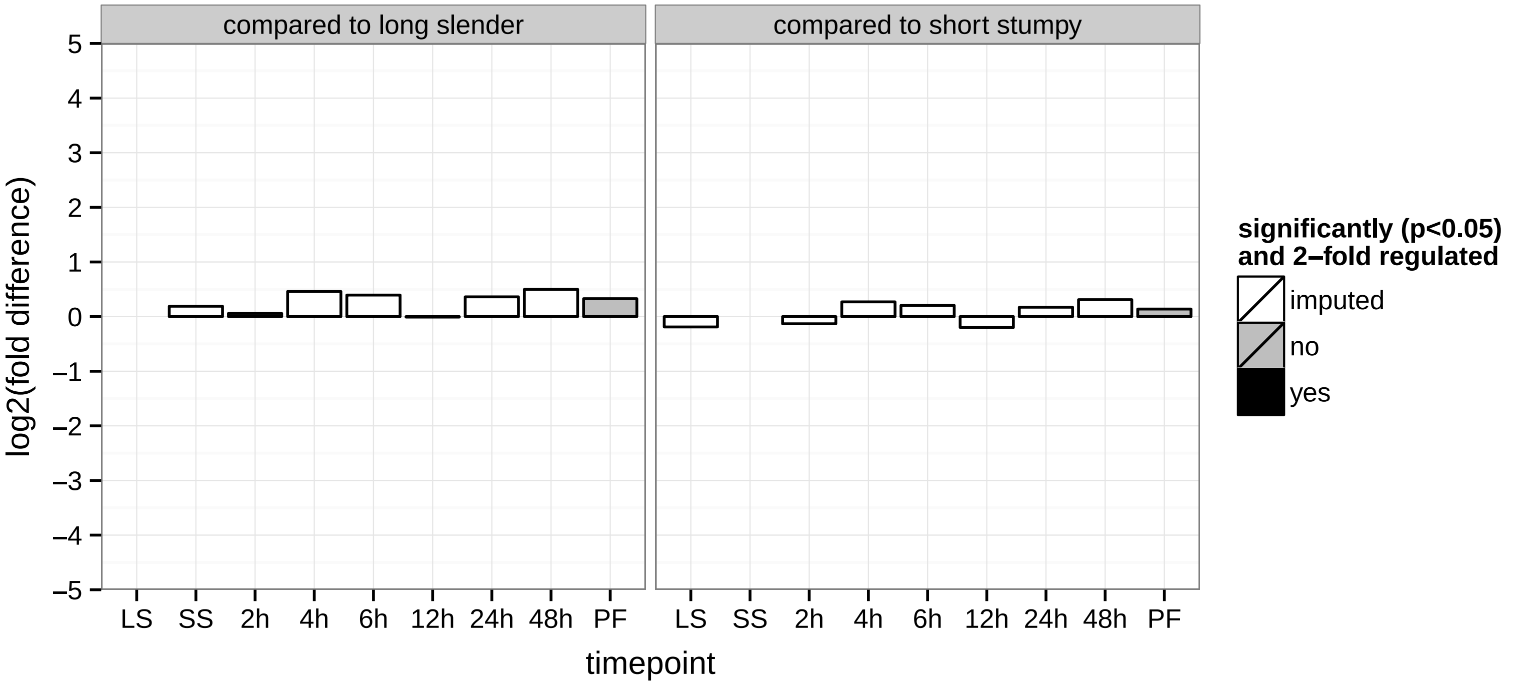

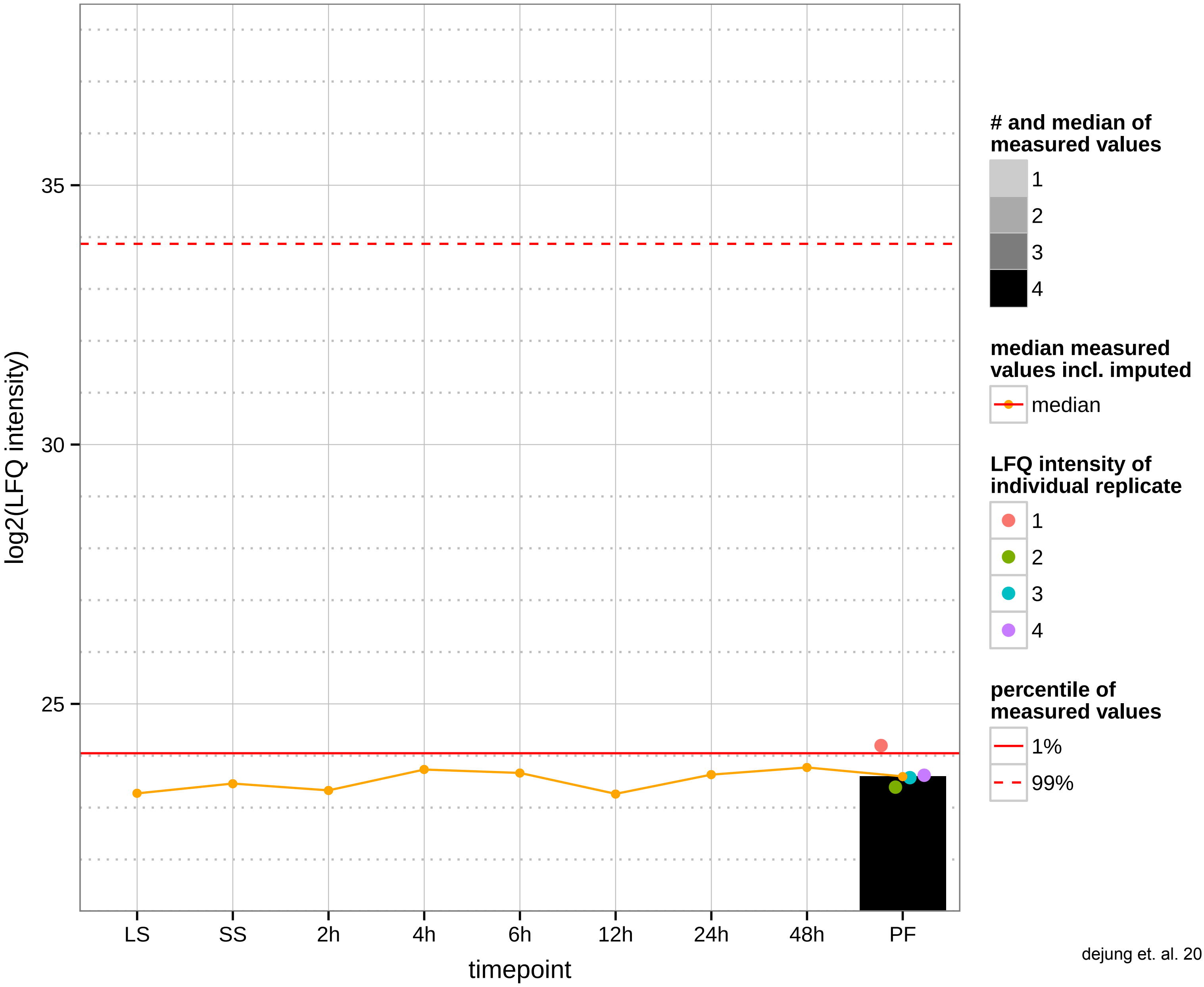

dejung et. al. 2015

guanosine monophosphate reductase, putative
Tb927.5.2080
AGOF: IMP dehydrogenase activity, guanosine deaminase activity
AGOC: null
AGOP: null
PGOF: IMP dehydrogenase activity, catalytic activity, protein binding
PGOC: null
PGOP: oxidation–reduction process

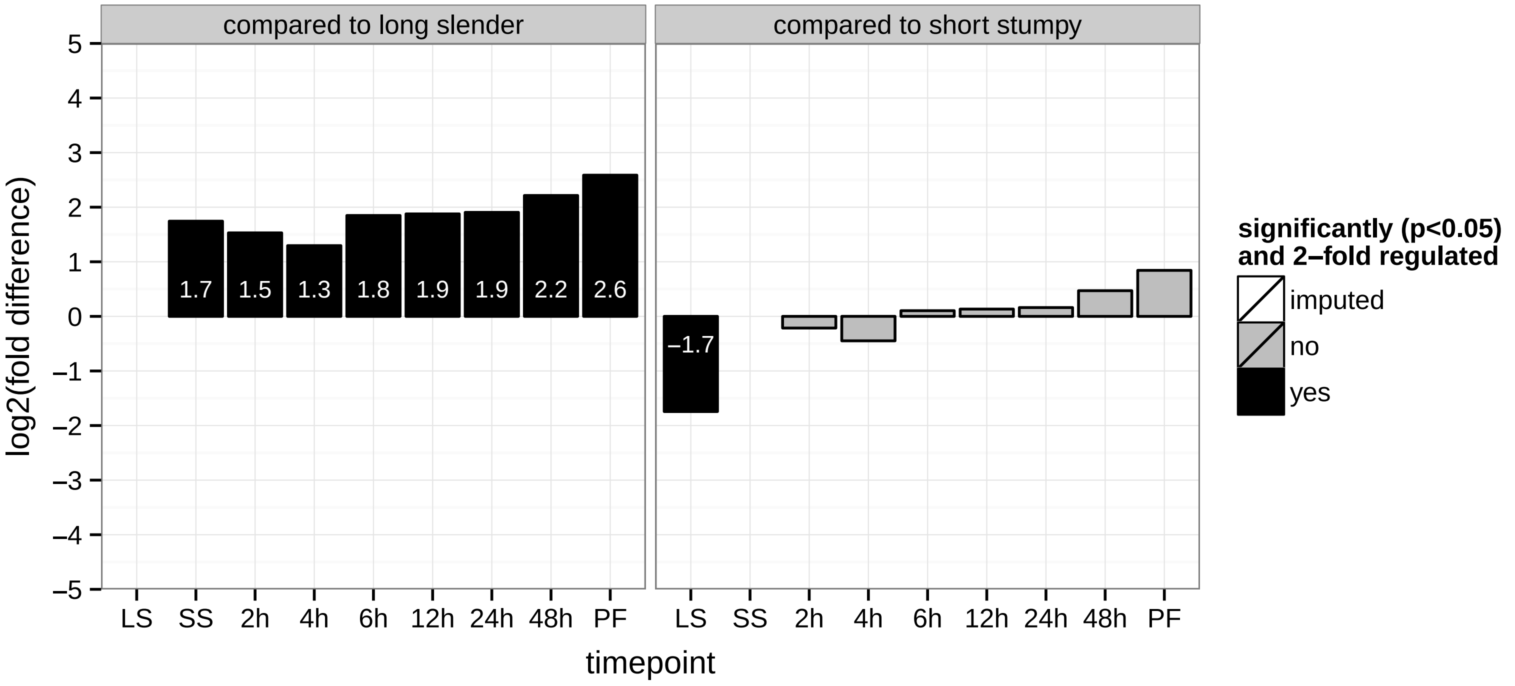

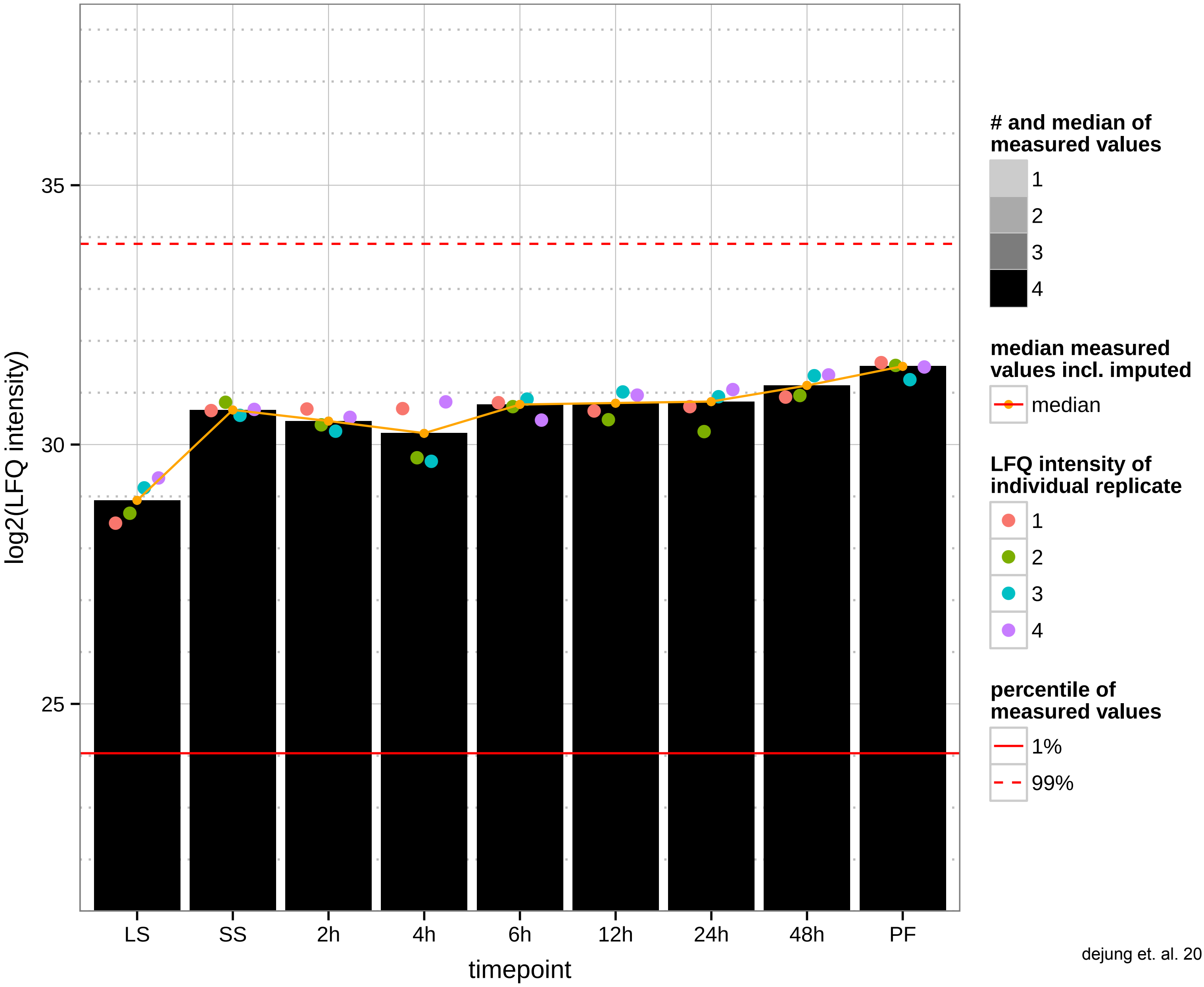

dejung et. al. 2015

RNA–binding protein, putative (RBP30)
Tb927.5.2100
AGOF: RNA binding
AGOC: mitochondrion
AGOP: null
PGOF: nucleic acid binding
PGOC: null
PGOP: null

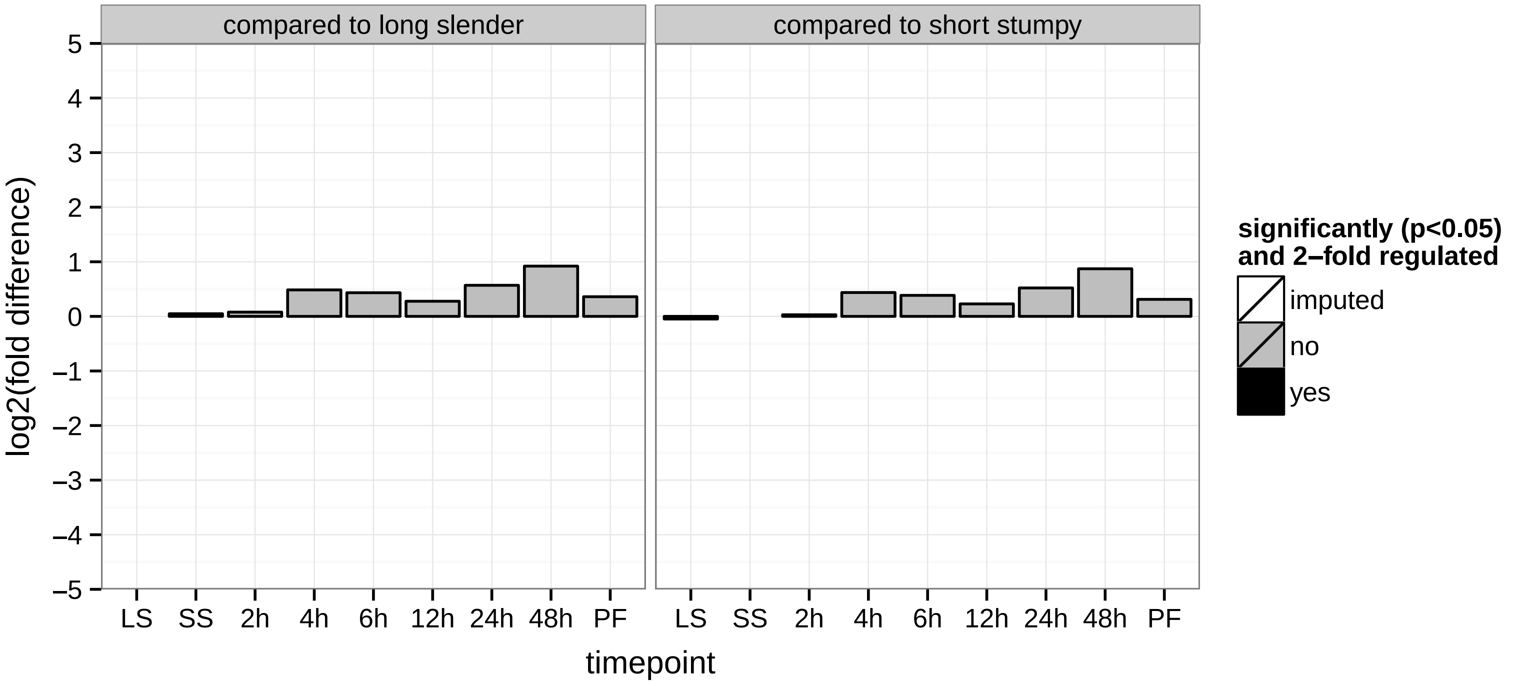

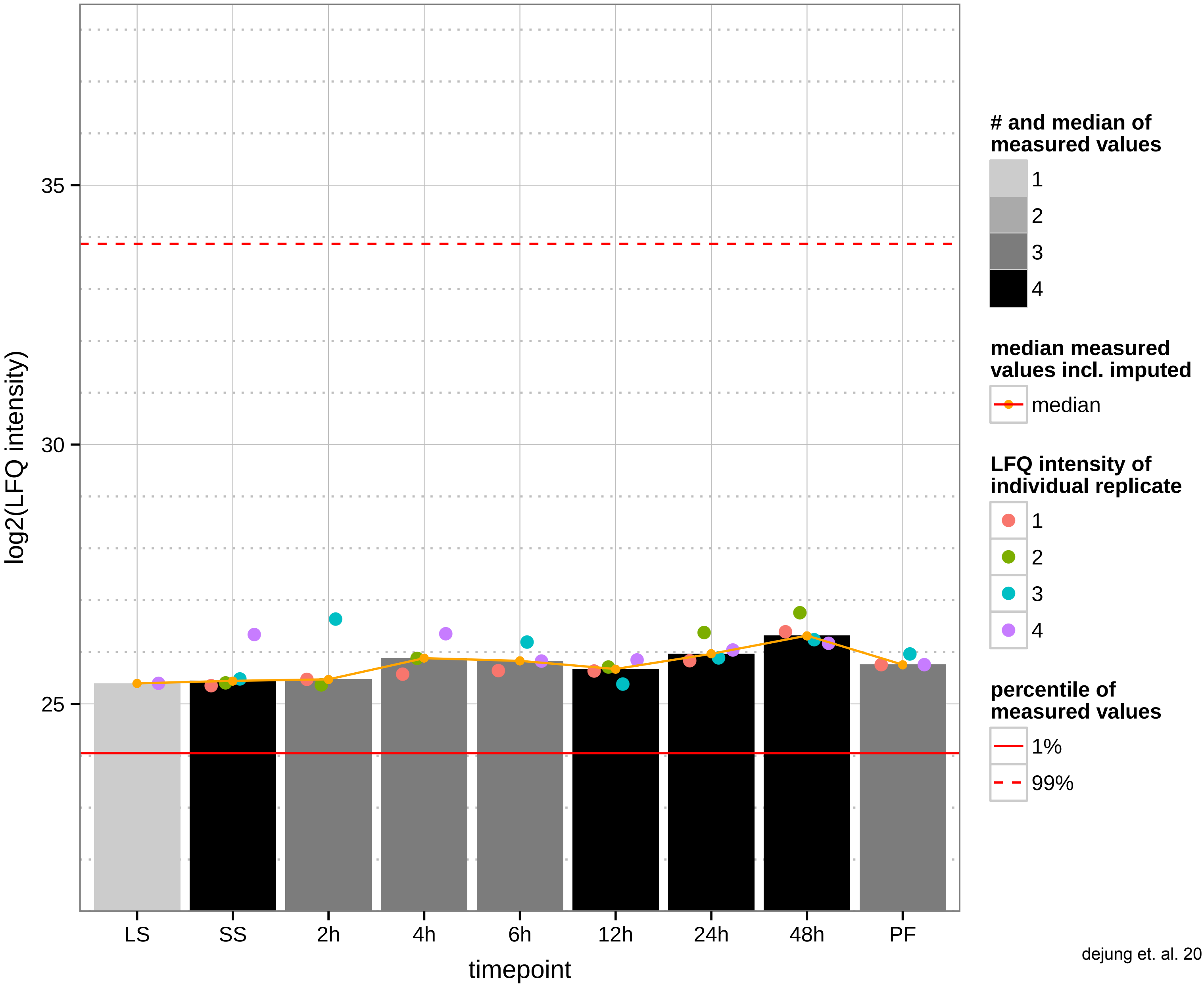

dejung et. al. 2015

hypothetical protein, conserved
Tb927.5.2270
AGOF: null
AGOC: null
AGOP: ciliary or flagellar motility
PGOF: null
PGOC: null
PGOP: null

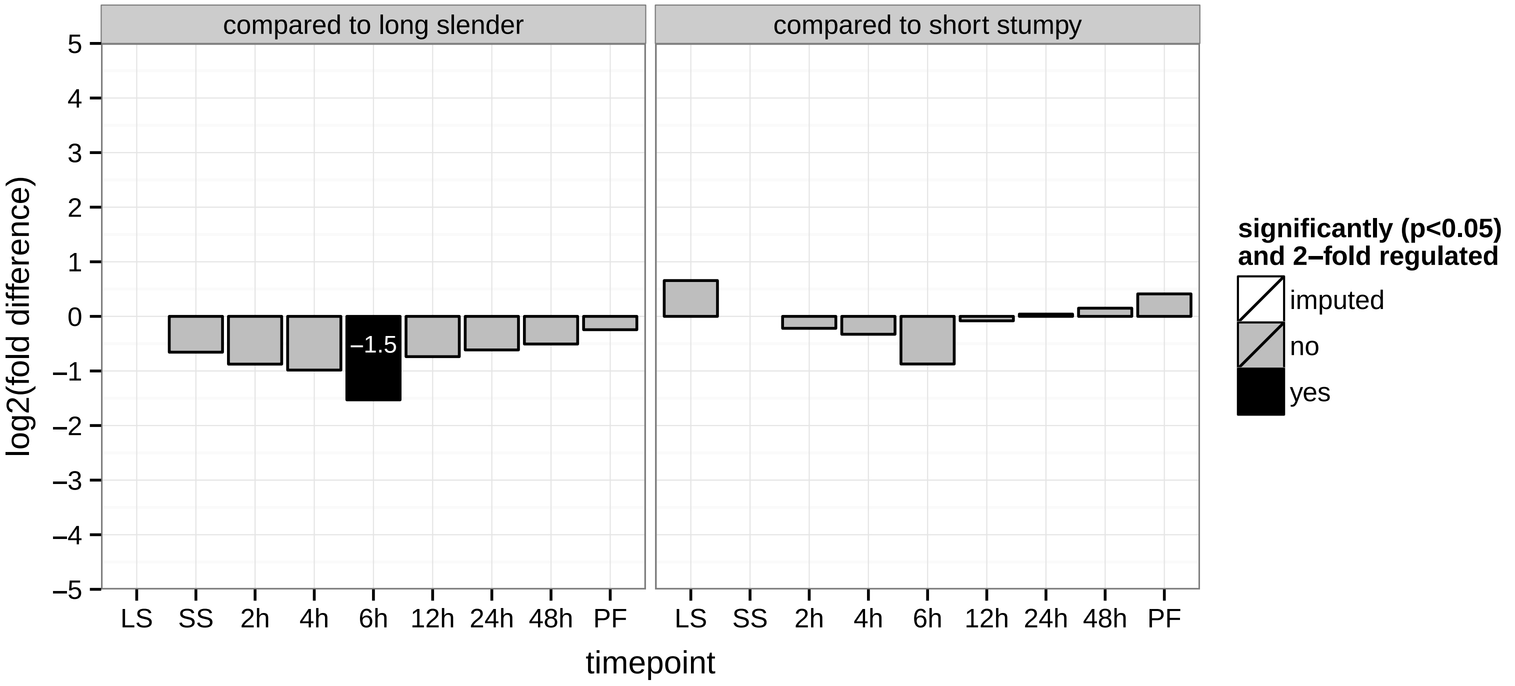

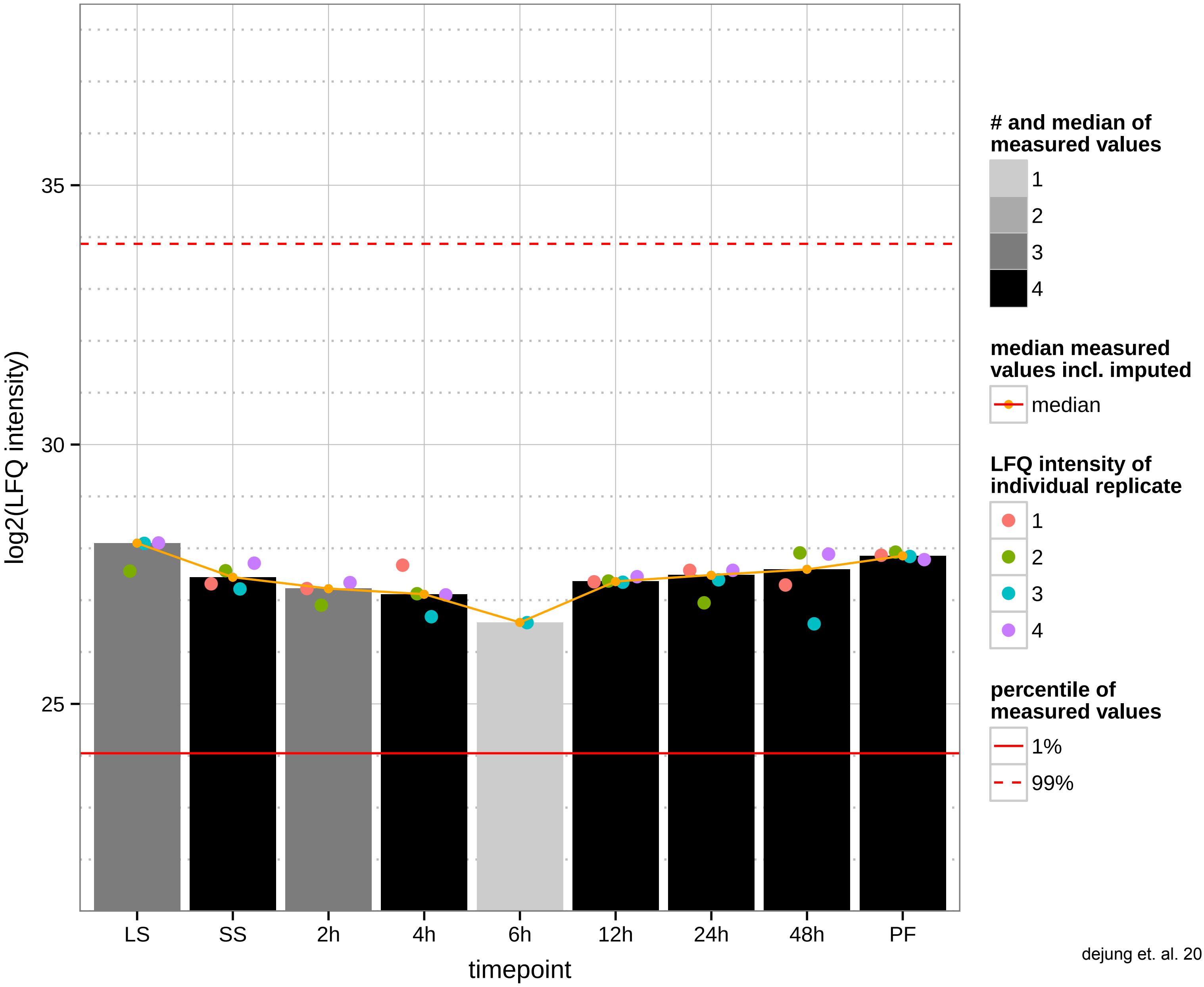

dejung et. al. 2015

hypothetical protein, conserved
Tb927.5.2310
AGOF: null
AGOC: null
AGOP: null
PGOF: null
PGOC: null
PGOP: null

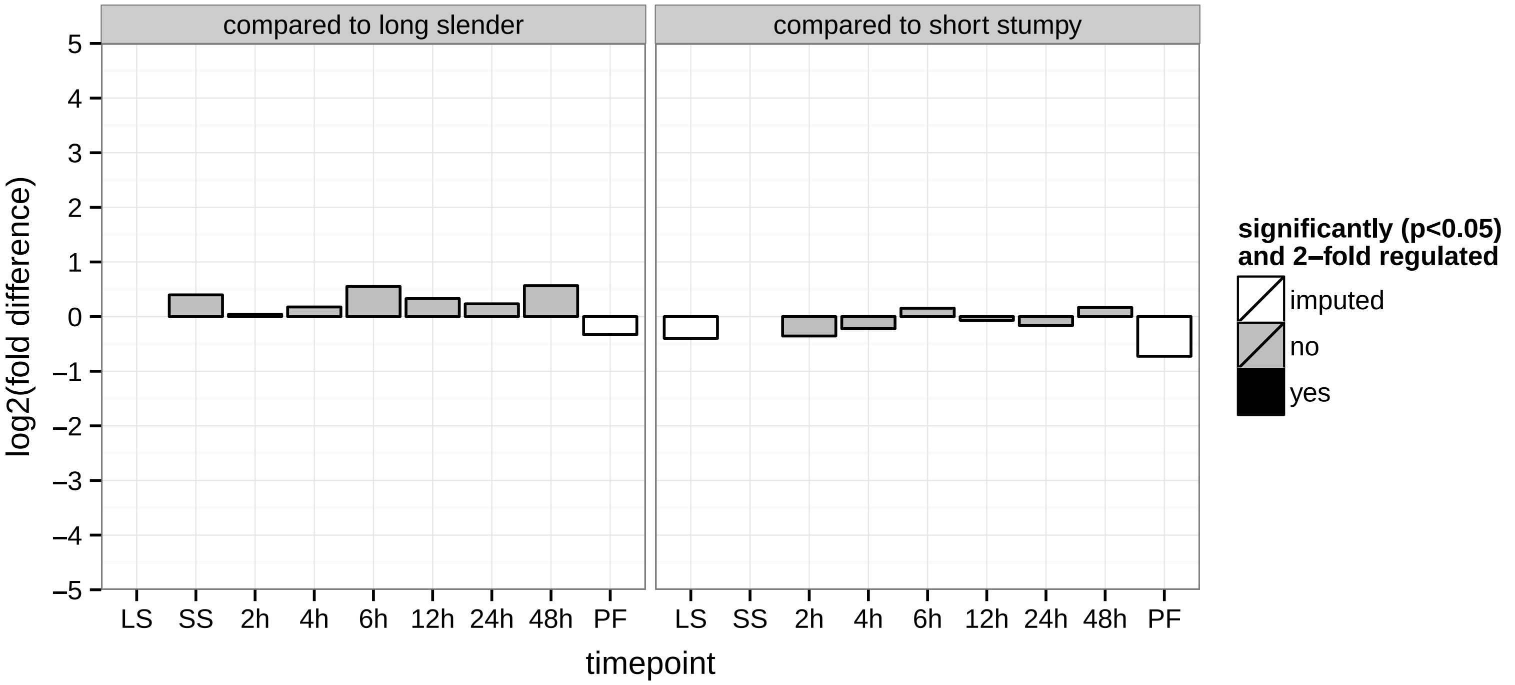

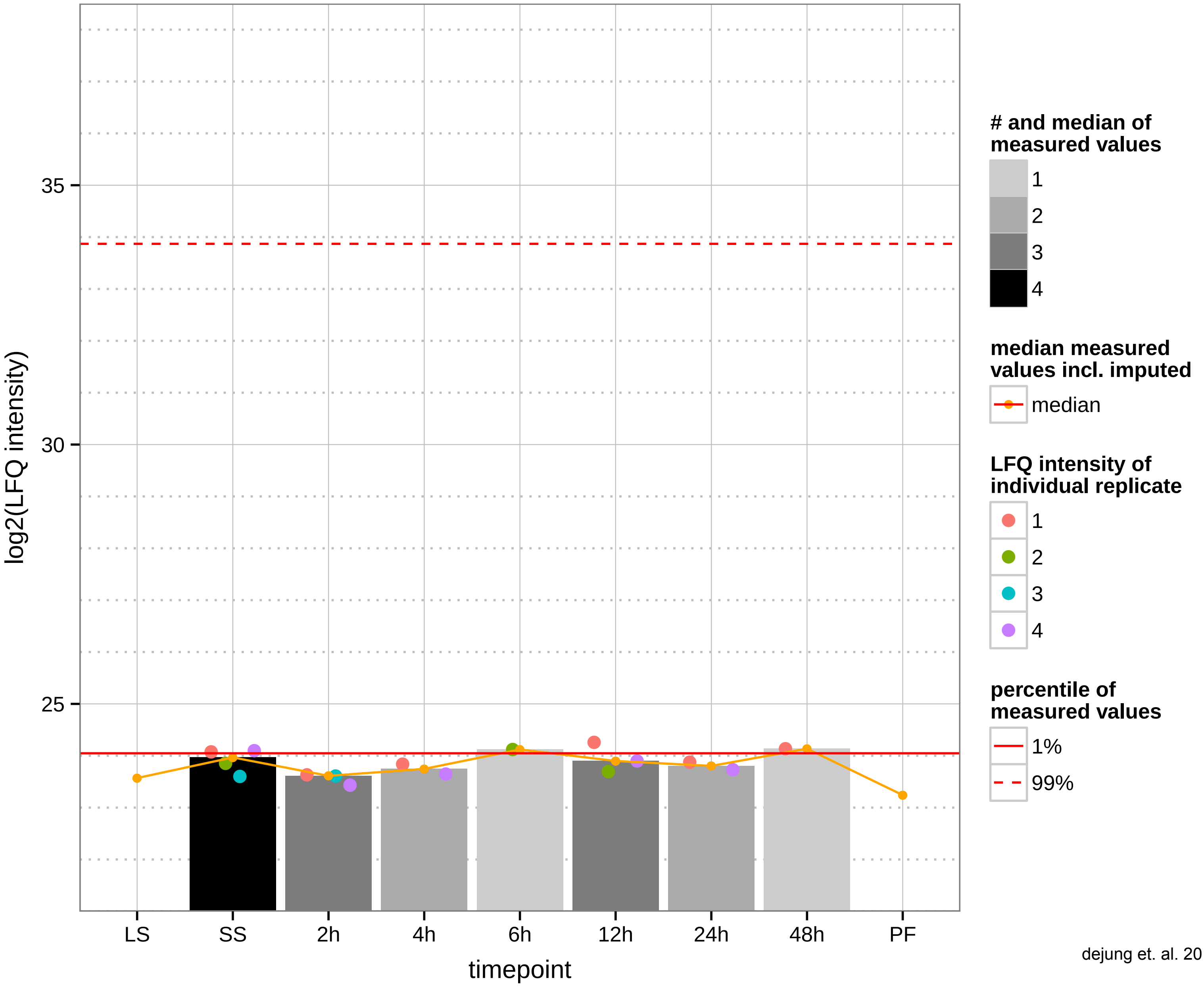

dejung et. al. 2015

hydrolase, alpha/beta fold family, putative
Tb927.5.2380
AGOF: null
AGOC: null
AGOP: null
PGOF: null
PGOC: null
PGOP: null

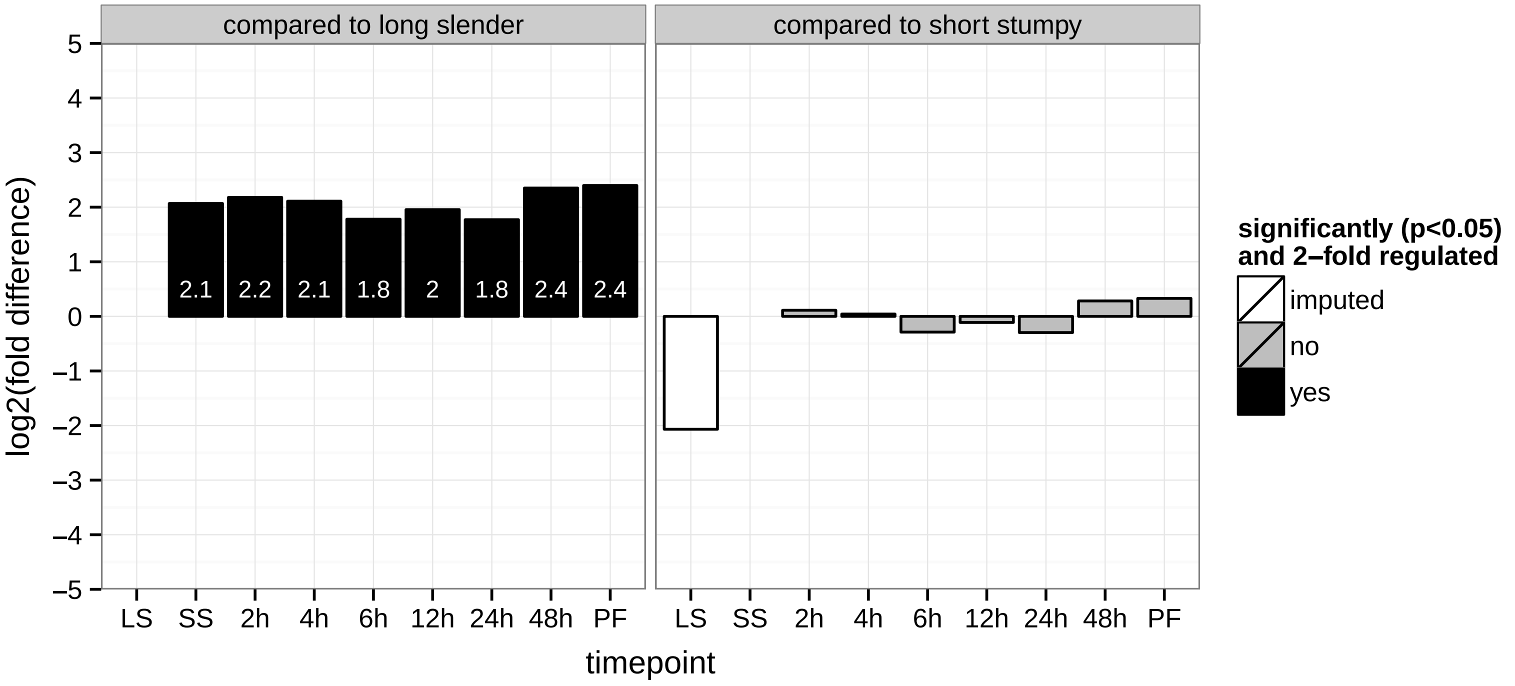

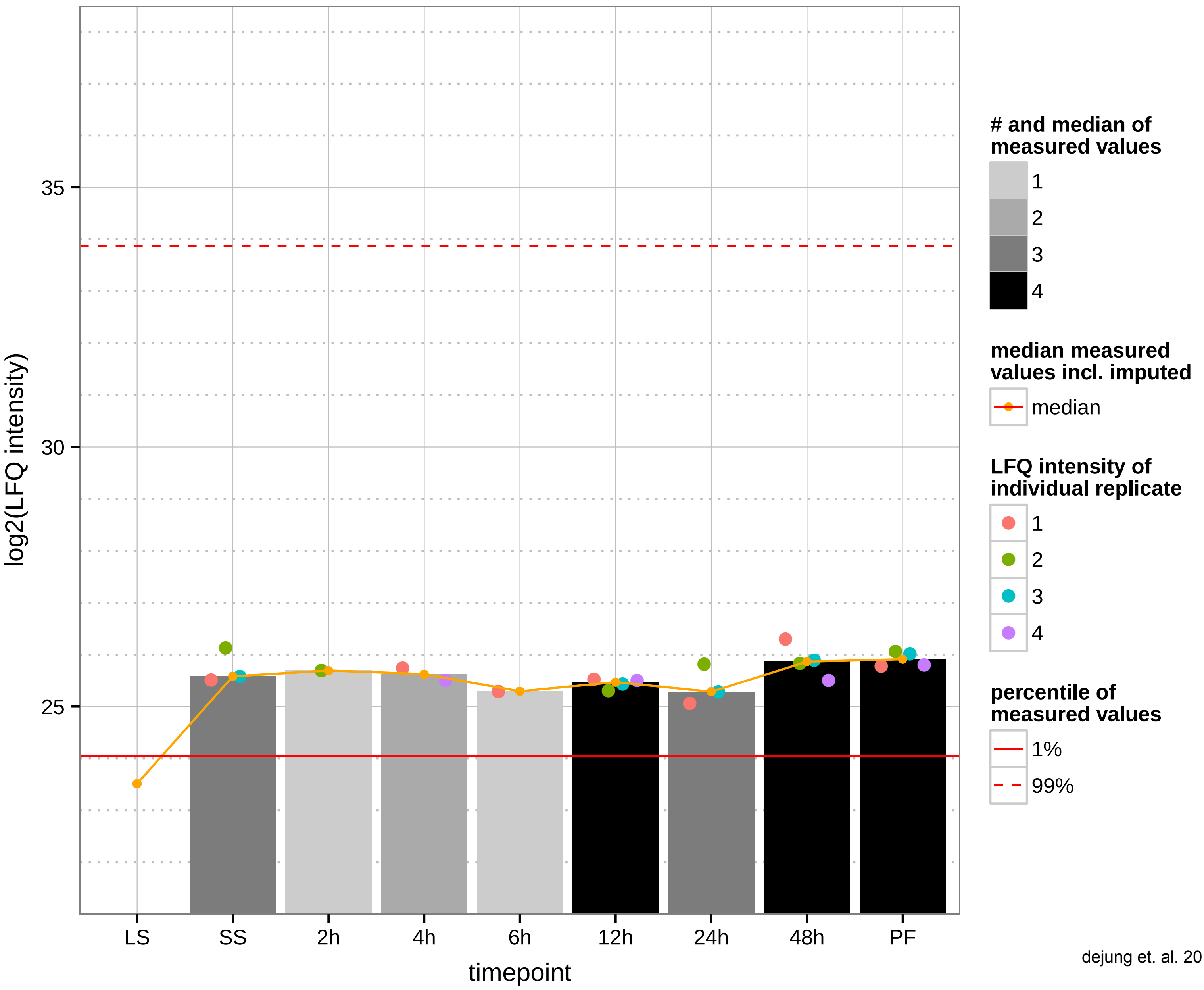

dejung et. al. 2015

kinesin, putative
Tb927.5.2410
AGOF: ATP binding, calcium−dependent cysteine−type endopeptidase activity, microtubule motor activity
AGOC: intracellular
AGOP: microtubule−based movement, proteolysis
PGOF: ATP binding, binding, microtubule motor activity, protein binding
PGOC: null
PGOP: microtubule−based movement

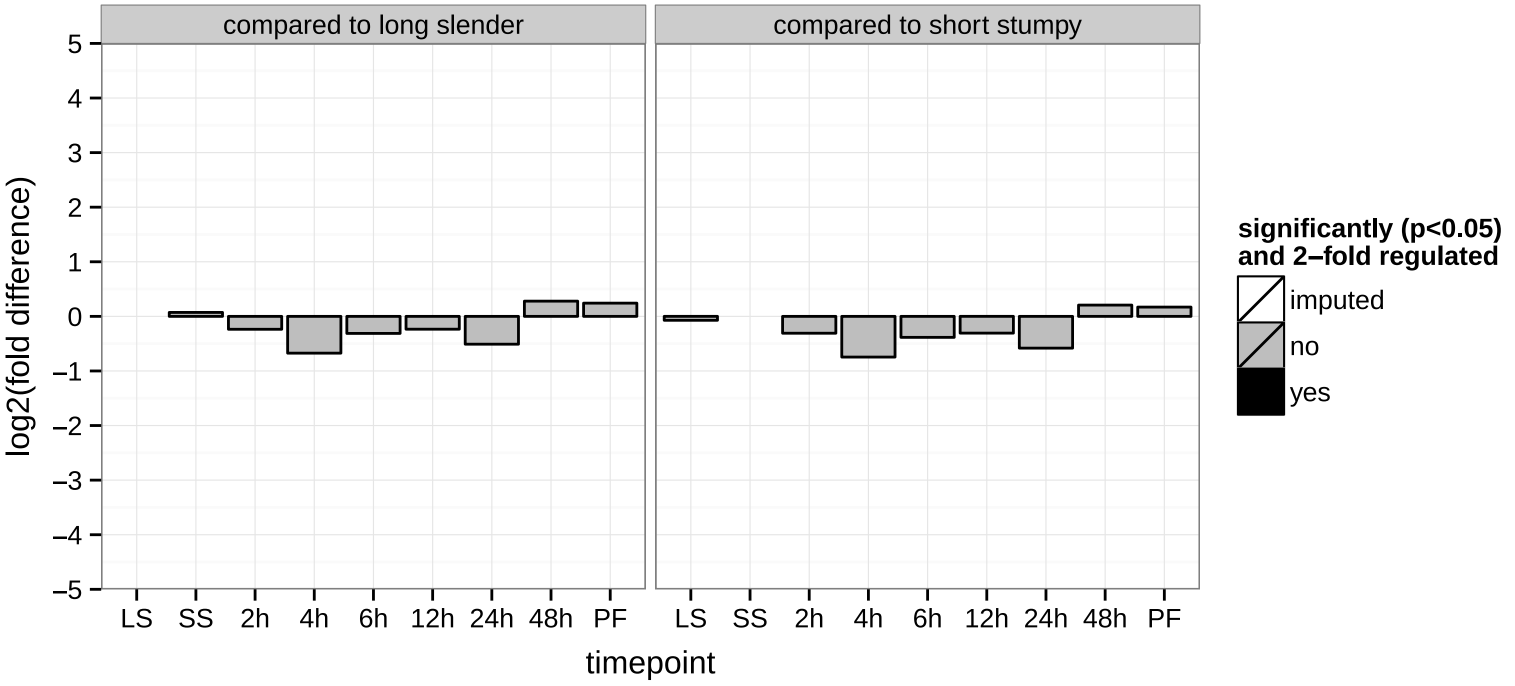

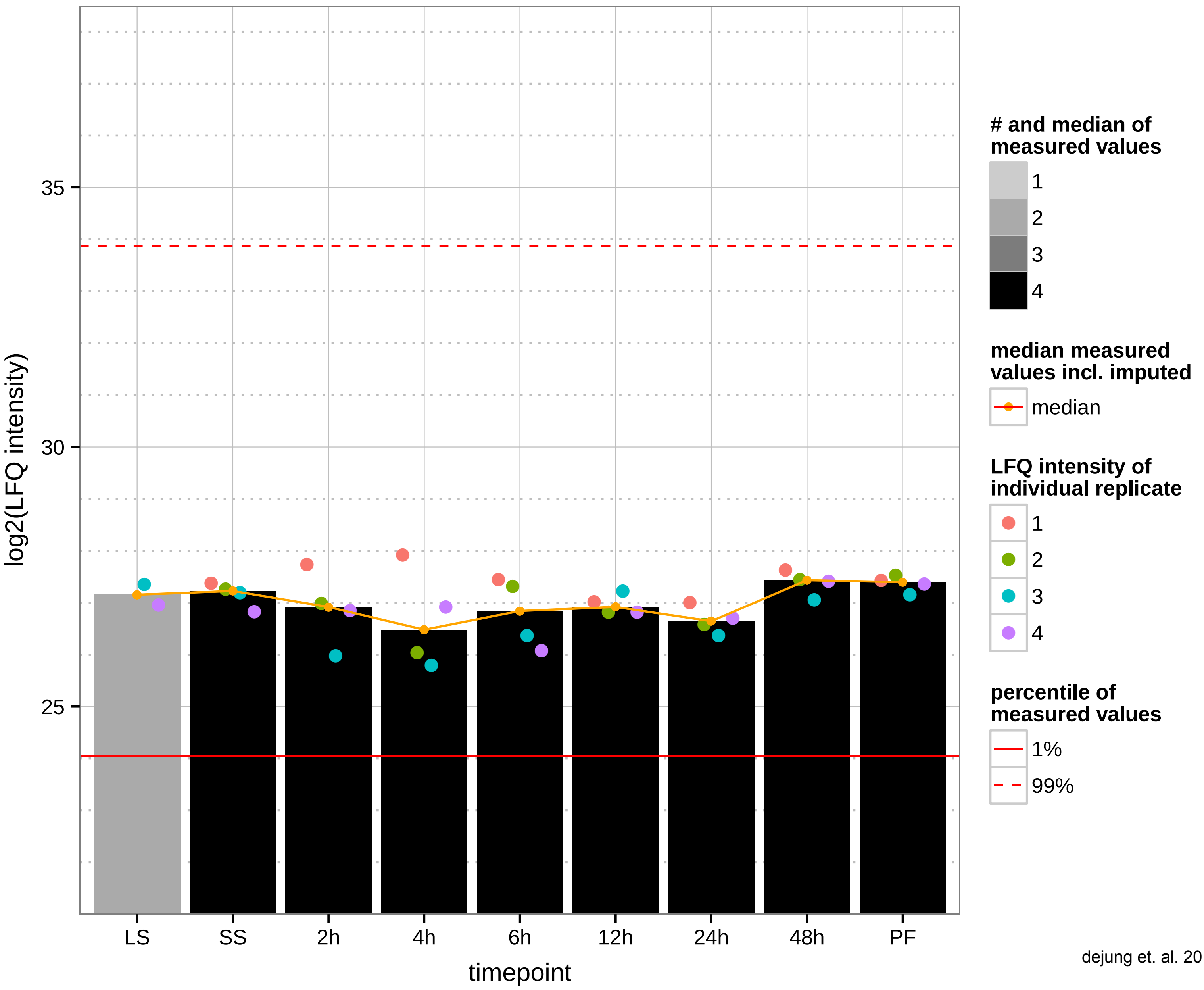

dejung et. al. 2015

hypothetical protein, conserved
Tb927.5.2500
AGOF: null
AGOC: null
AGOP: null
PGOF: null
PGOC: null
PGOP: null

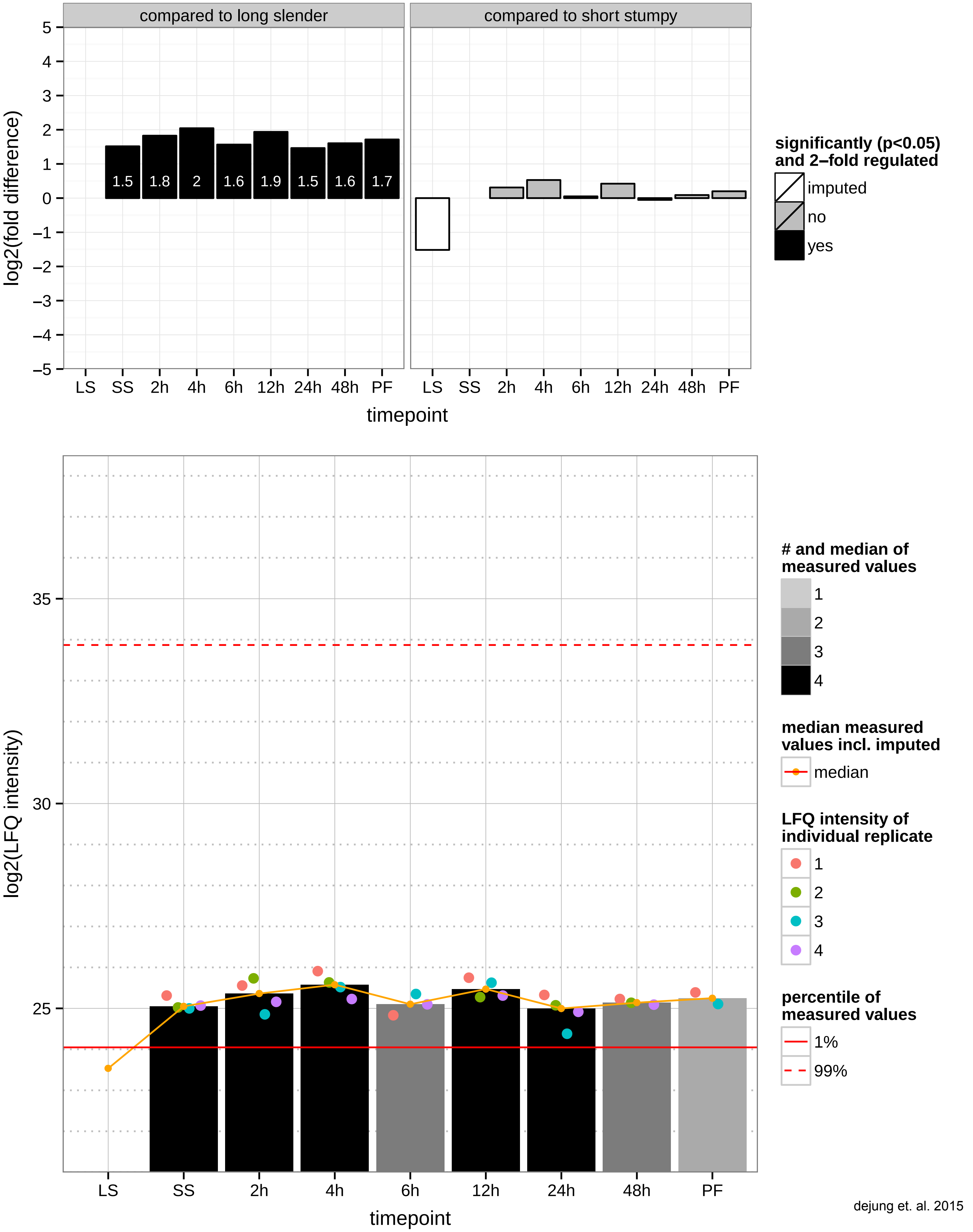

dejung et. al. 2015

translation initiation factor, putative (EIF3B)
Tb927.5.2570
AGOF: RNA binding, translation initiation factor activity
AGOC: null
AGOP: translational initiation
PGOF: null
PGOC: null
PGOP: null

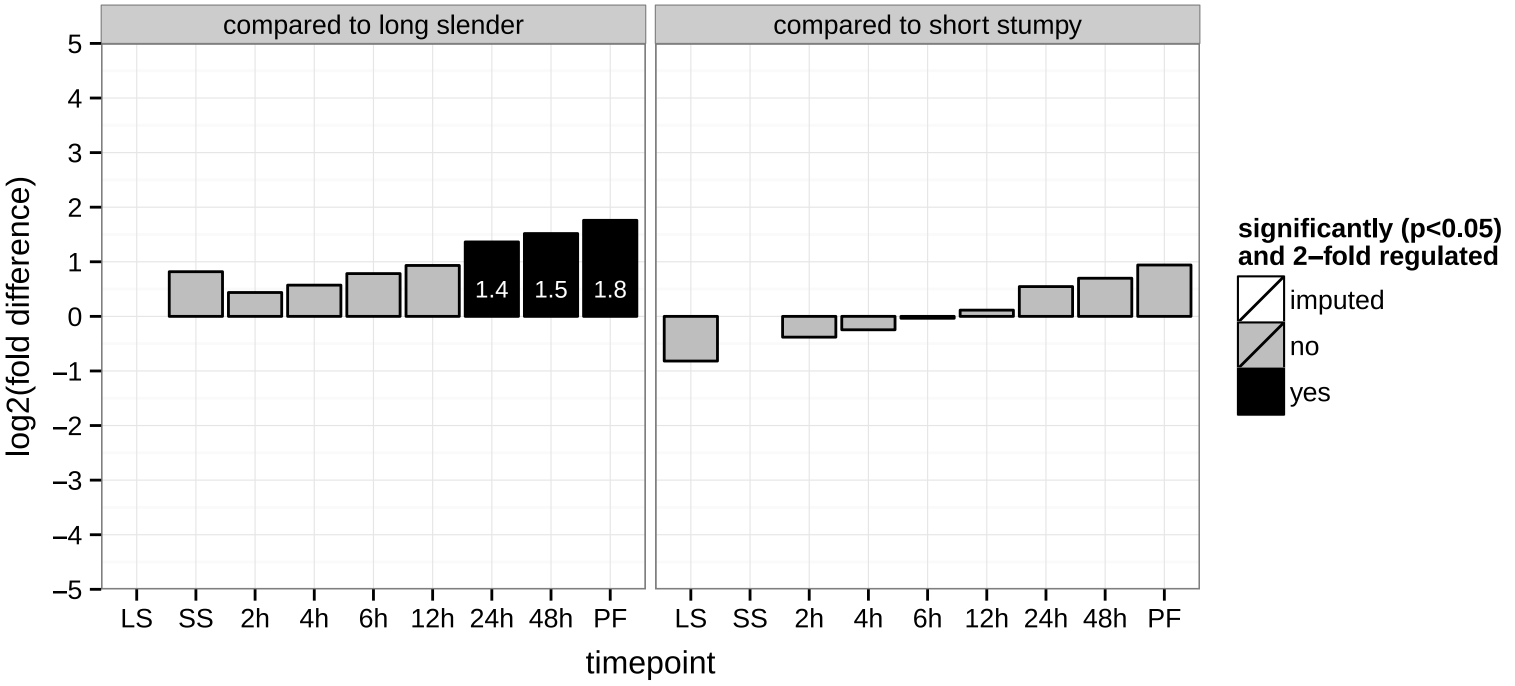

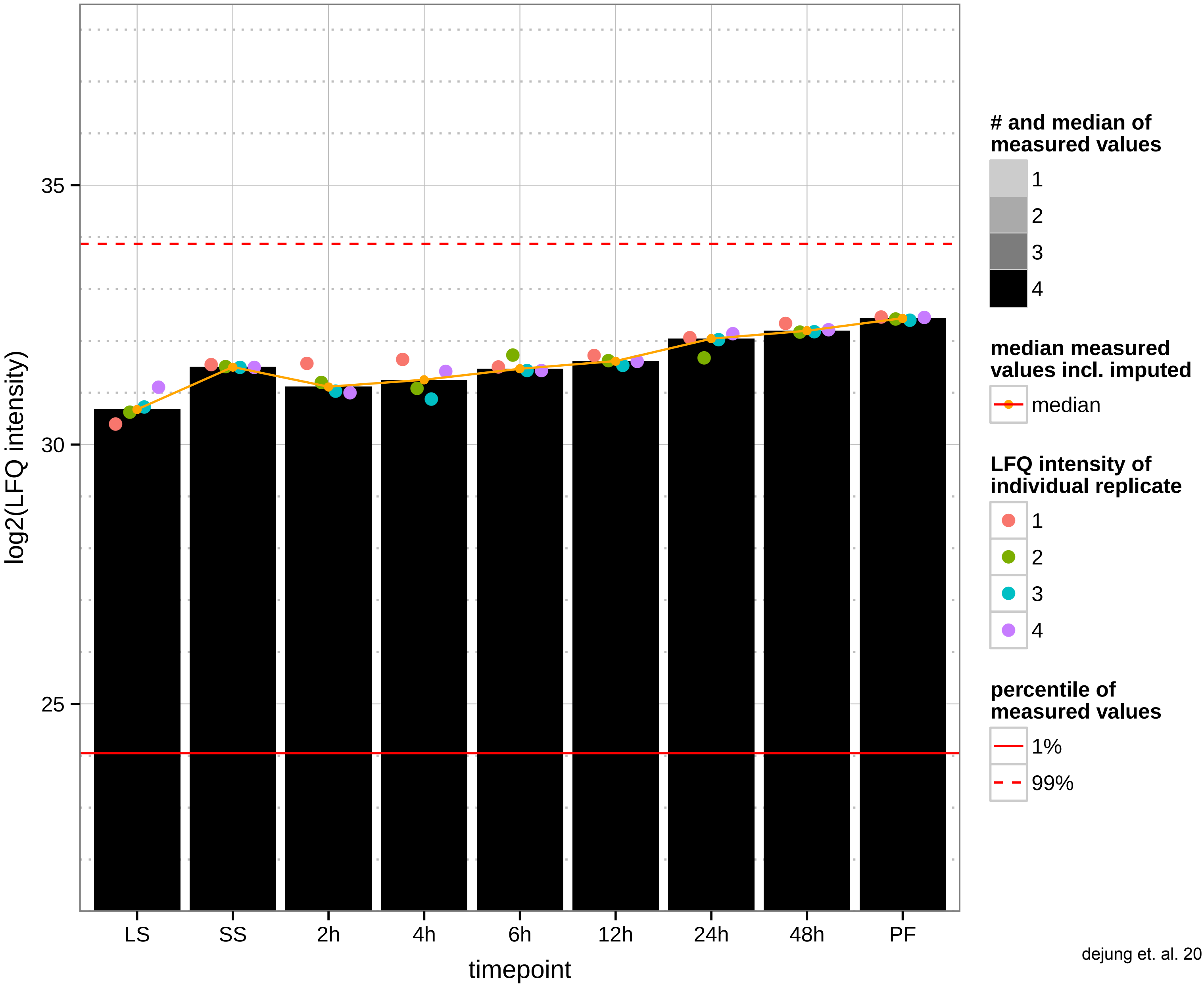

dejung et. al. 2015

hypothetical protein, conserved
Tb927.5.2590
AGOF: null
AGOC: null
AGOP: null
PGOF: null
PGOC: null
PGOP: null

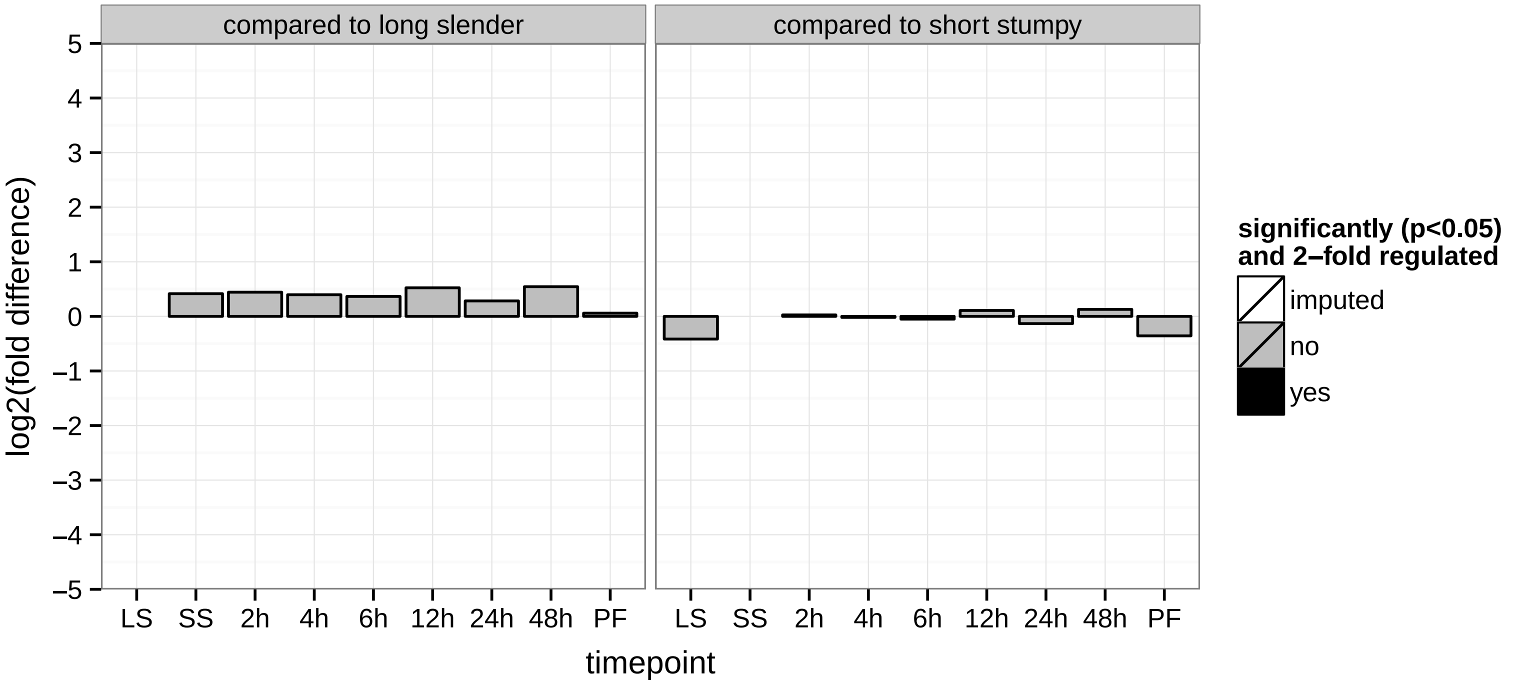

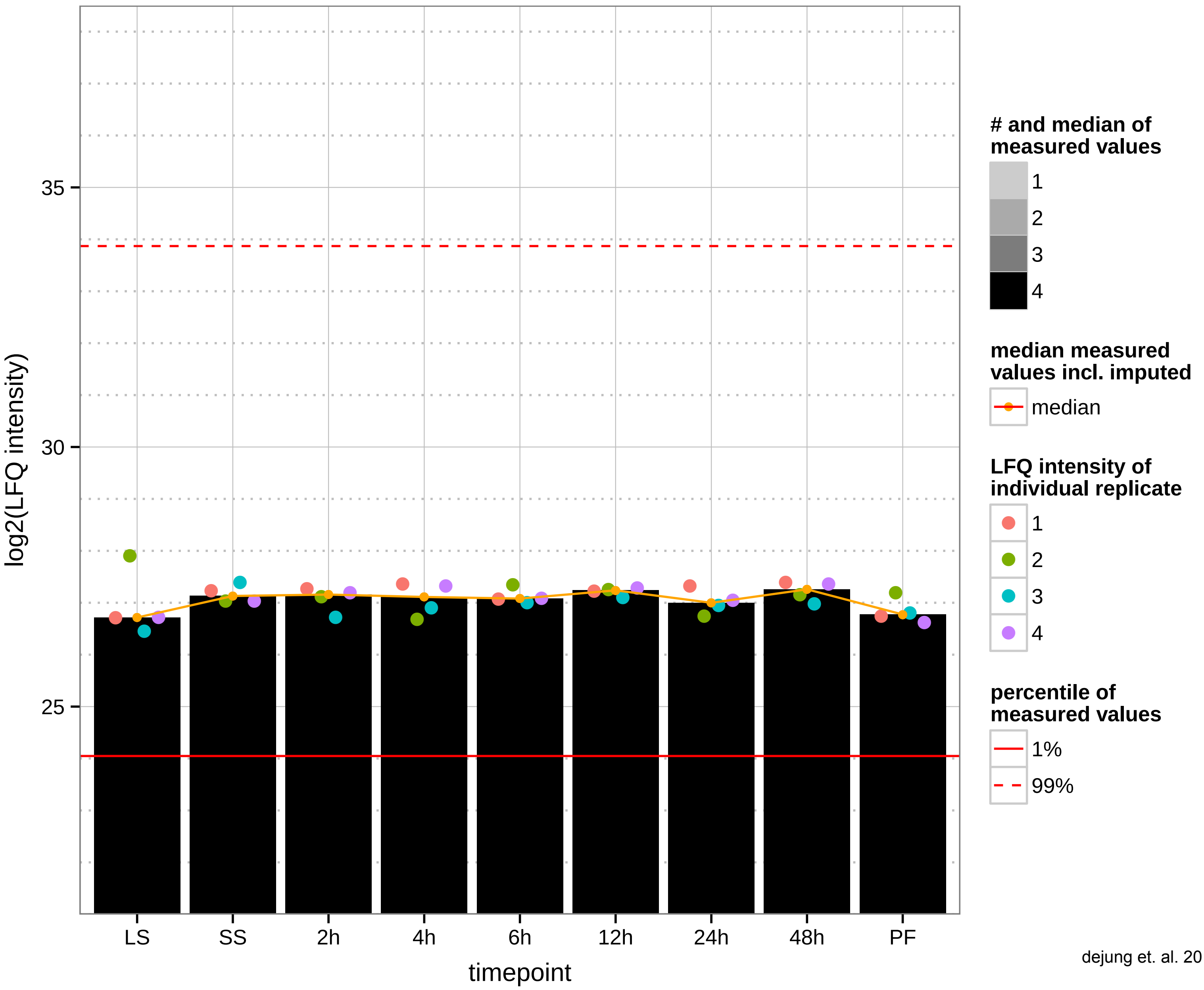

dejung et. al. 2015

hypothetical protein, conserved
Tb927.5.2640
AGOF: nucleotidyltransferase activity
AGOC: null
AGOP: NAD biosynthetic process
PGOF: nucleotidyltransferase activity
PGOC: null
PGOP: biosynthetic process

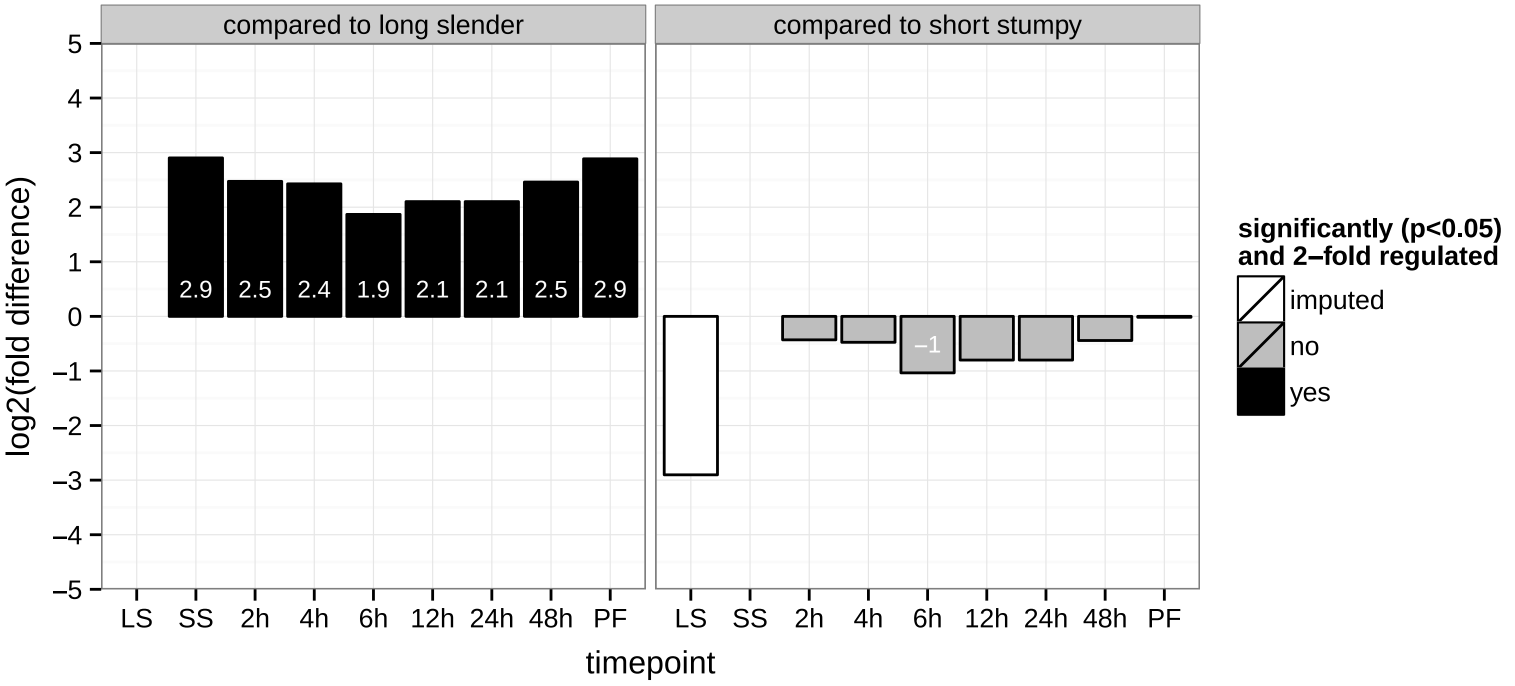

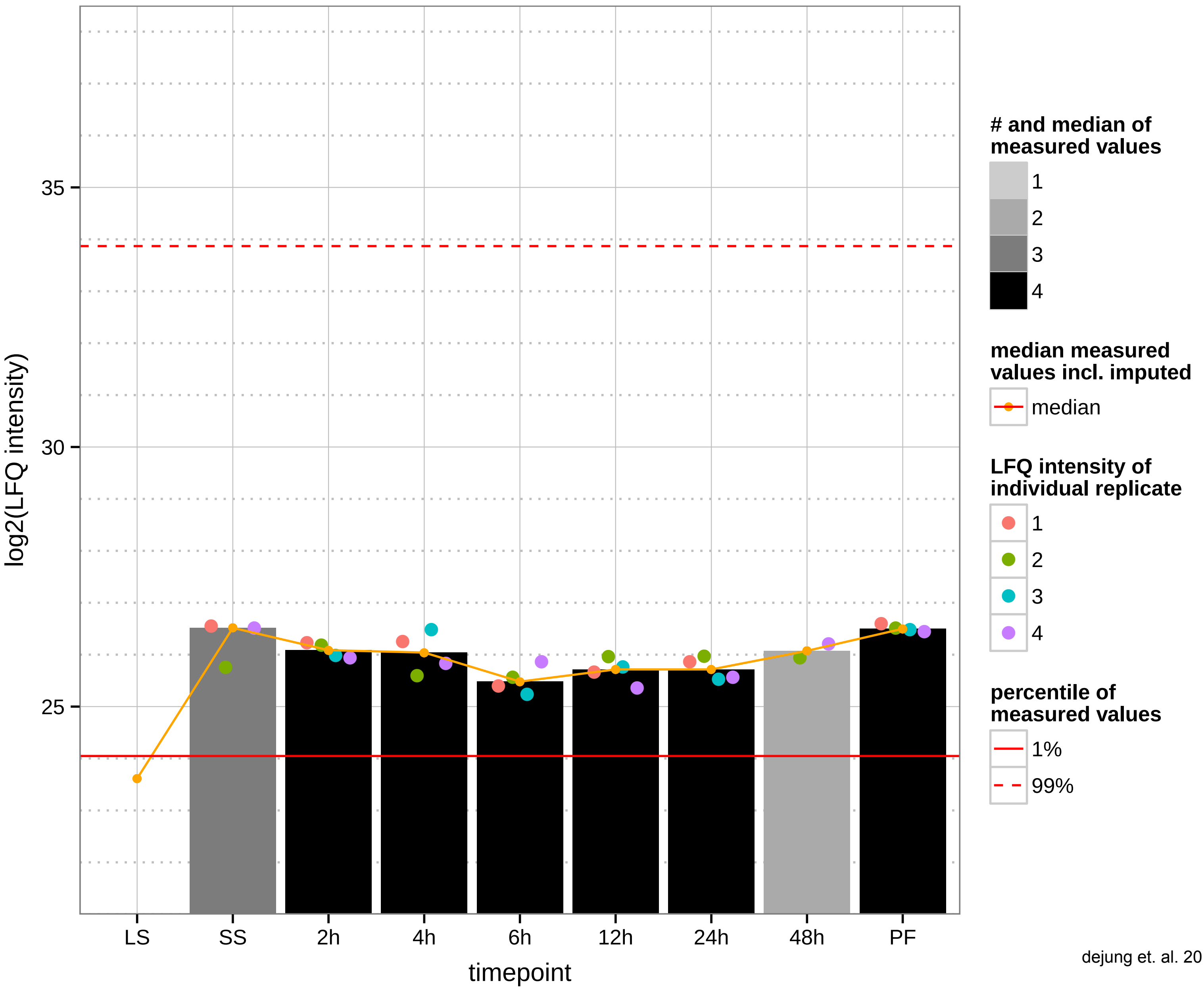

dejung et. al. 2015

hypothetical protein, conserved
Tb927.5.2660
AGOF: null
AGOC: null
AGOP: null
PGOF: binding
PGOC: null
PGOP: null

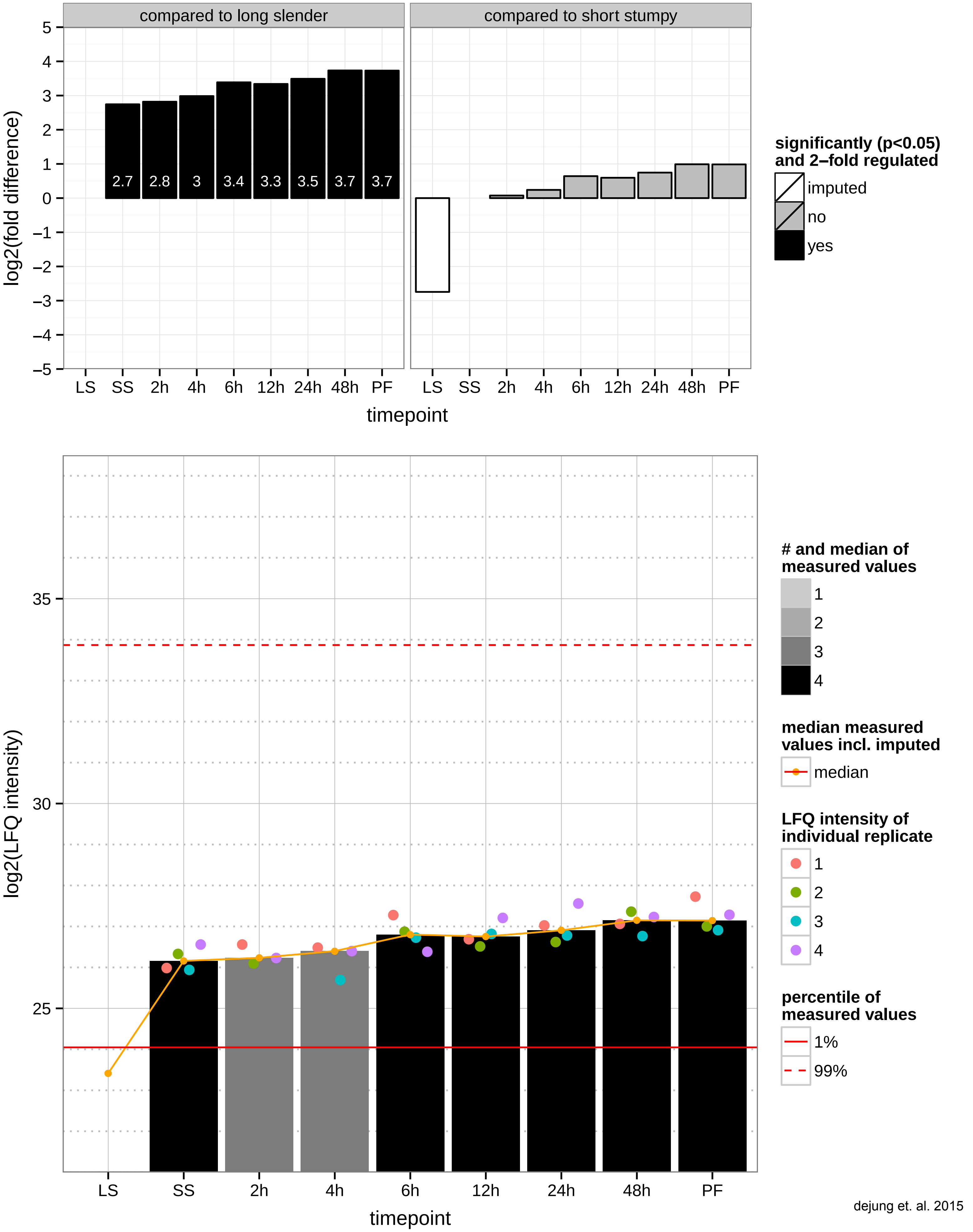

dejung et. al. 2015

otubain, putative, otubain cysteine peptidase, Clan CA, family C65
Tb927.5.2700
AGOF: null
AGOC: null
AGOP: null
PGOF: omega peptidase activity
PGOC: null
PGOP: protein metabolic process

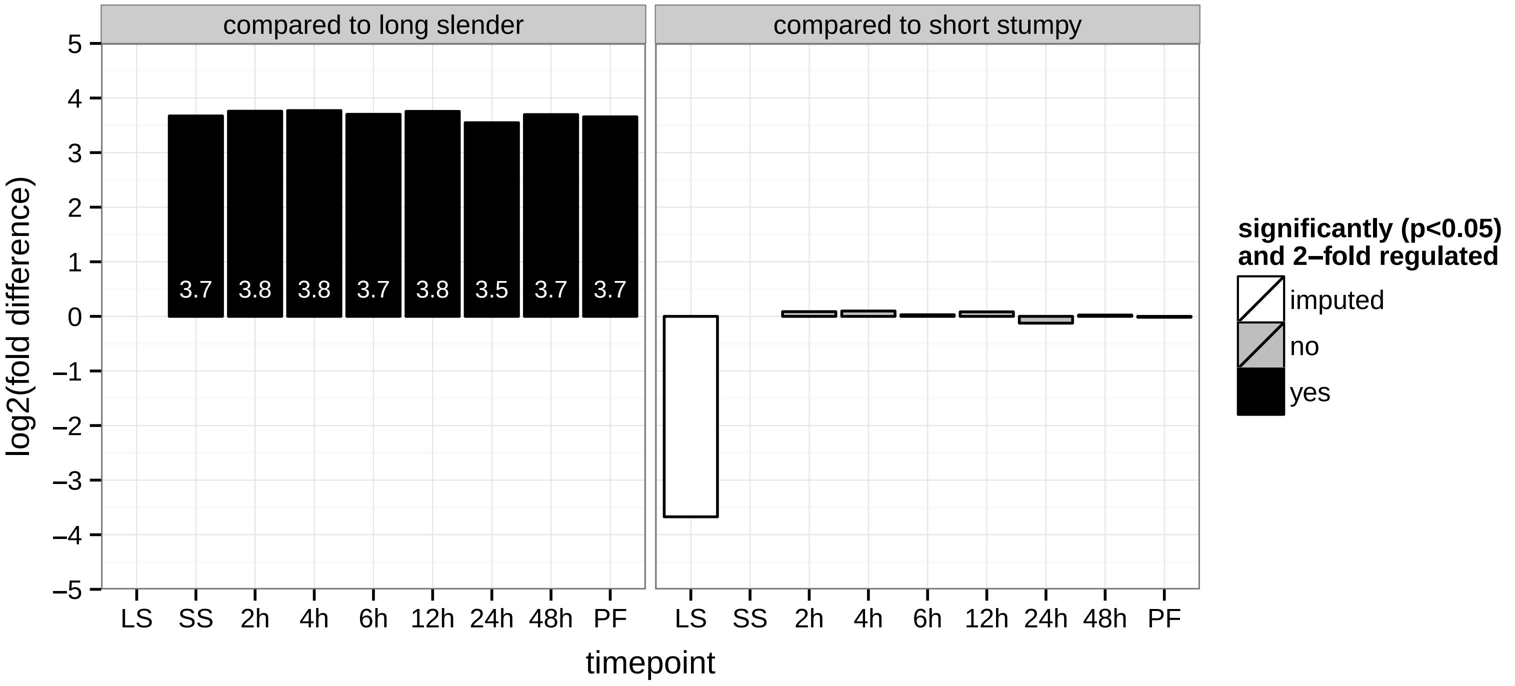

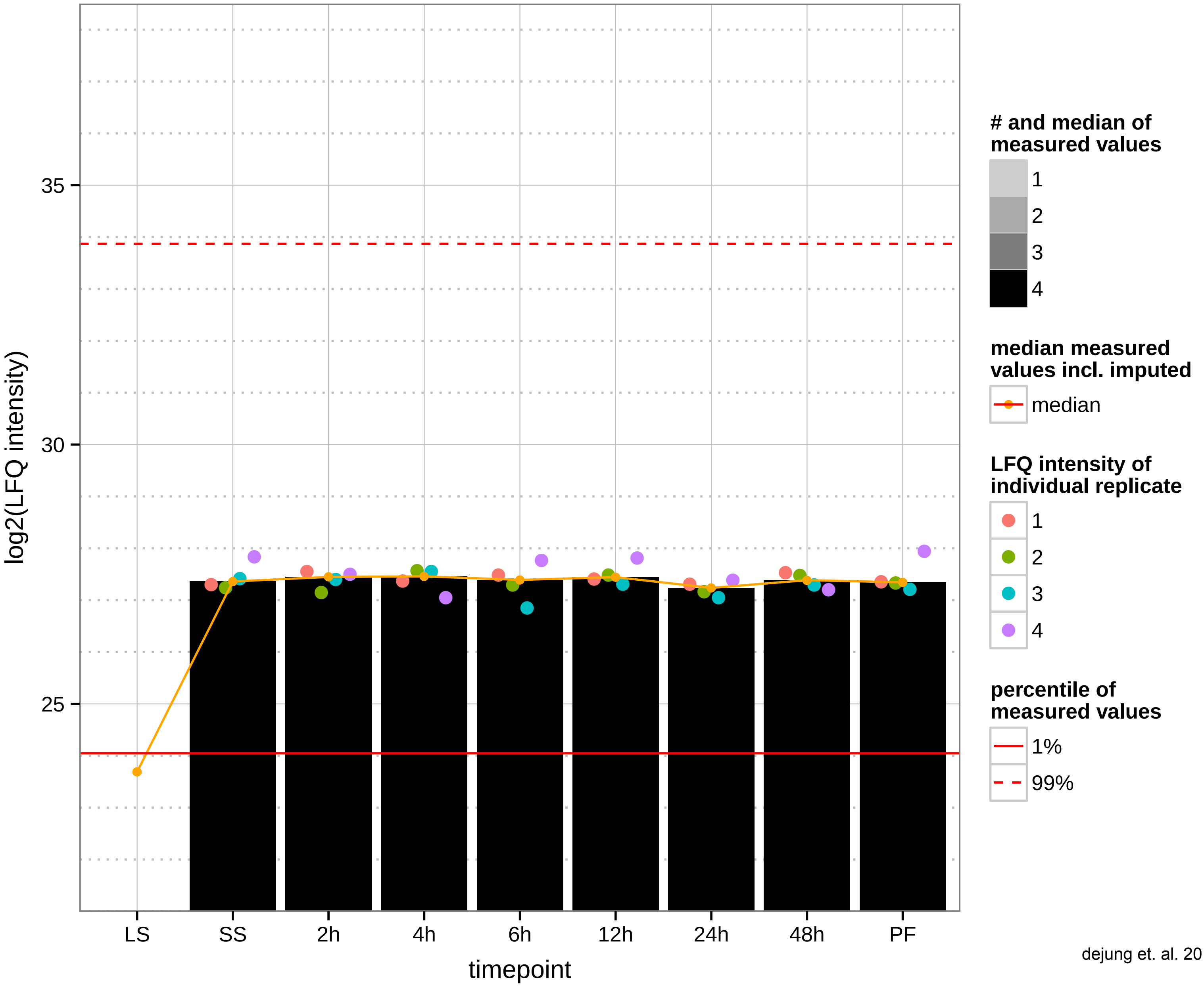

dejung et. al. 2015

hypothetical protein, conserved
Tb927.5.2770
AGOF: null
AGOC: null
AGOP: null
PGOF: protein binding
PGOC: null
PGOP: null

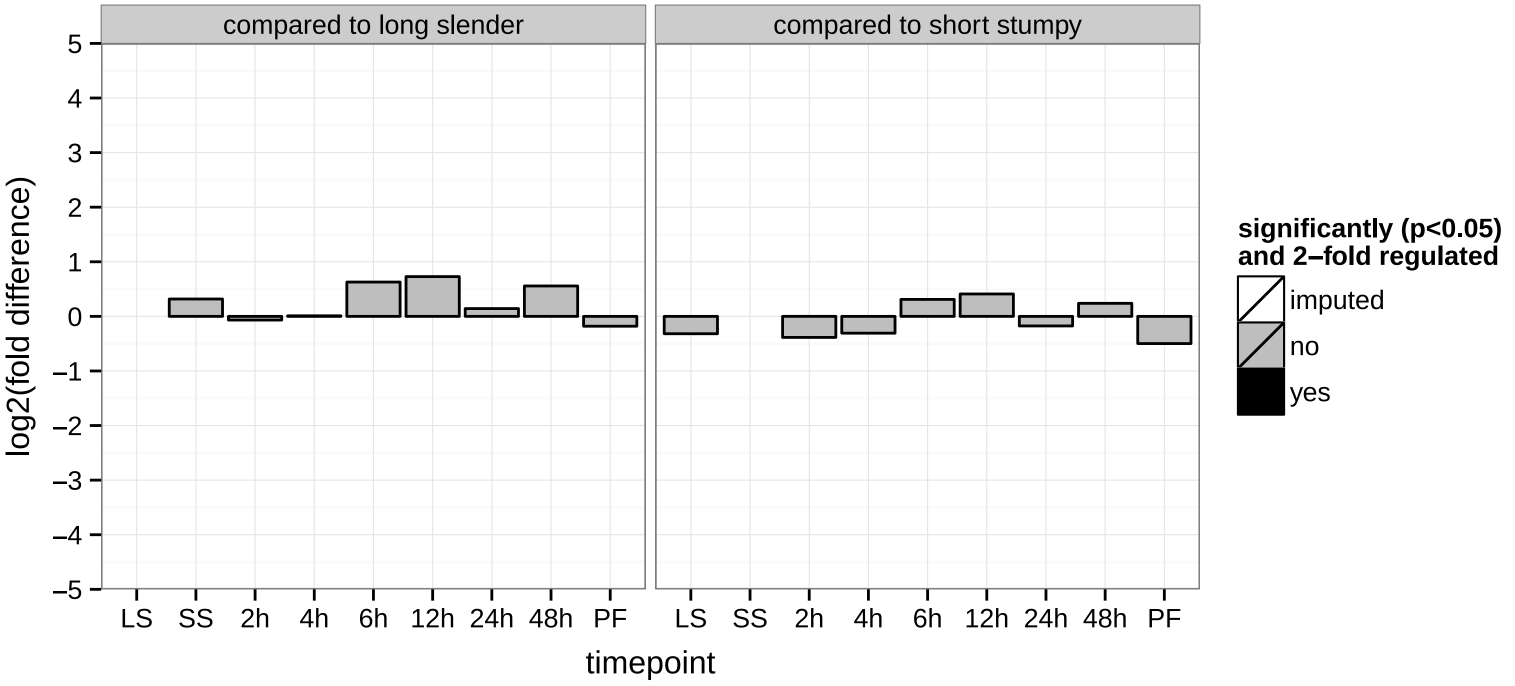

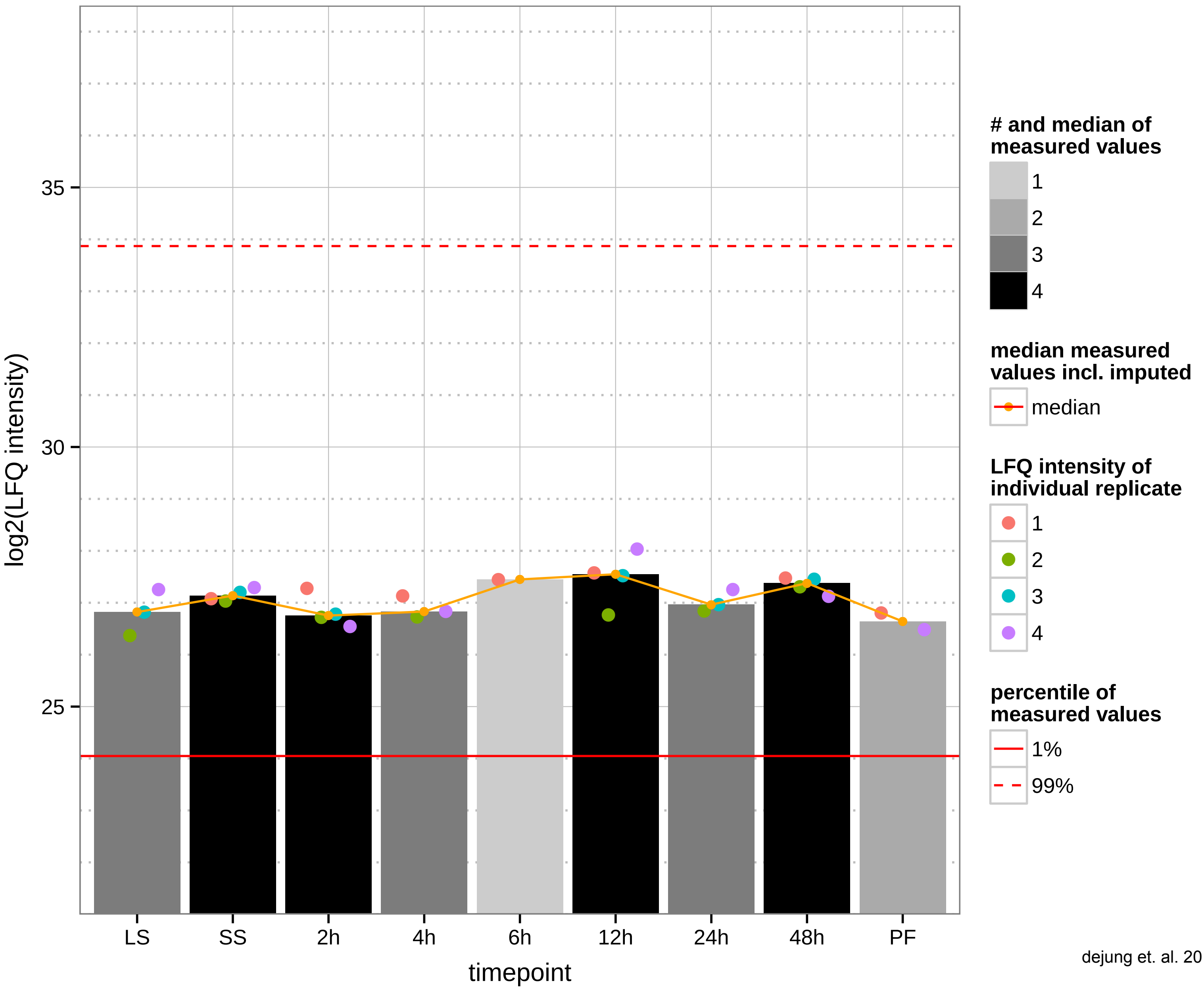

dejung et. al. 2015

radial spoke protein RSP2, putative
Tb927.5.2850
AGOF: null
AGOC: null
AGOP: null
PGOF: null
PGOC: null
PGOP: null

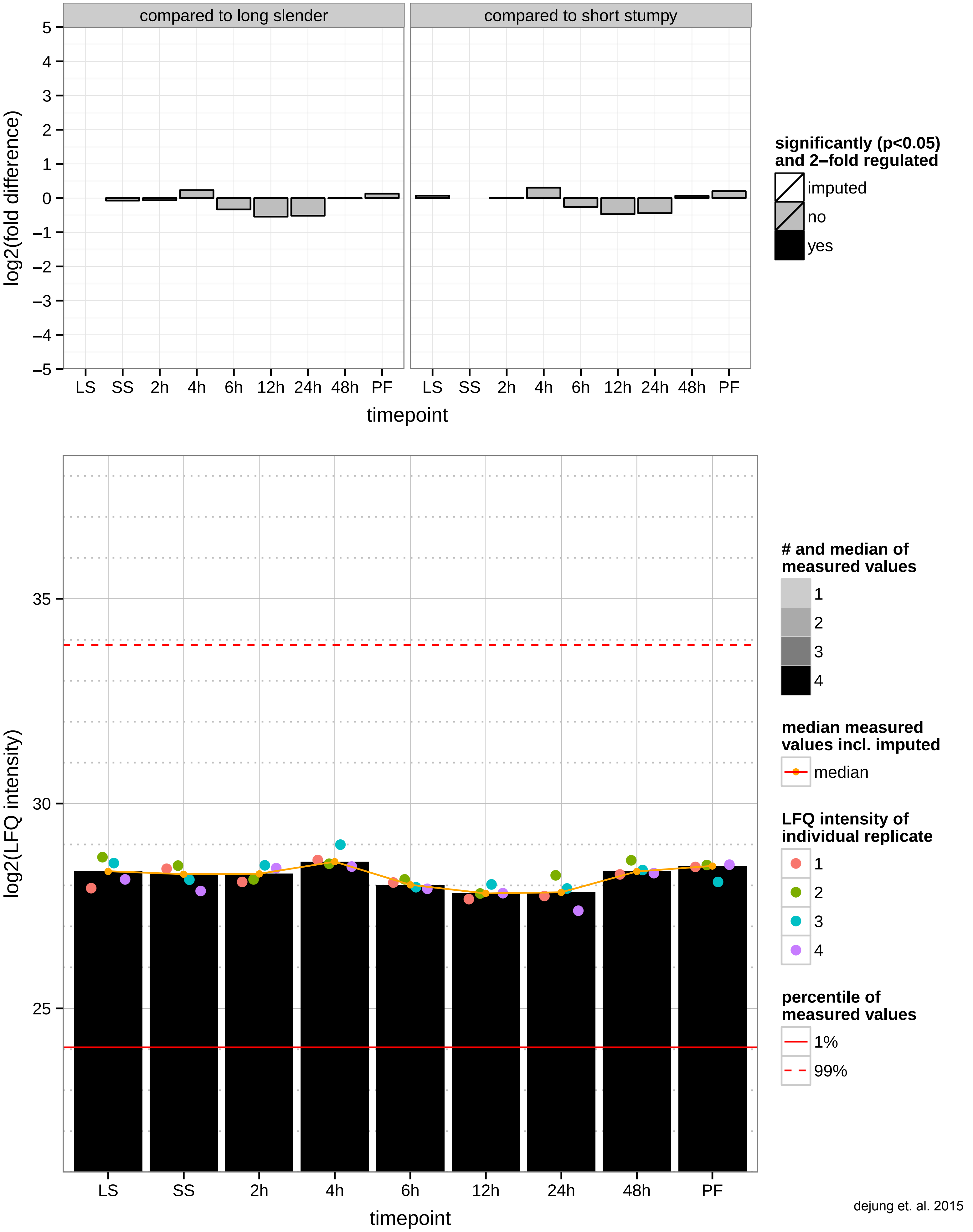

dejung et. al. 2015

stress–induced protein sti1, putative
Tb927.5.2940
AGOF: null
AGOC: null
AGOP: null
PGOF: protein binding
PGOC: null
PGOP: null

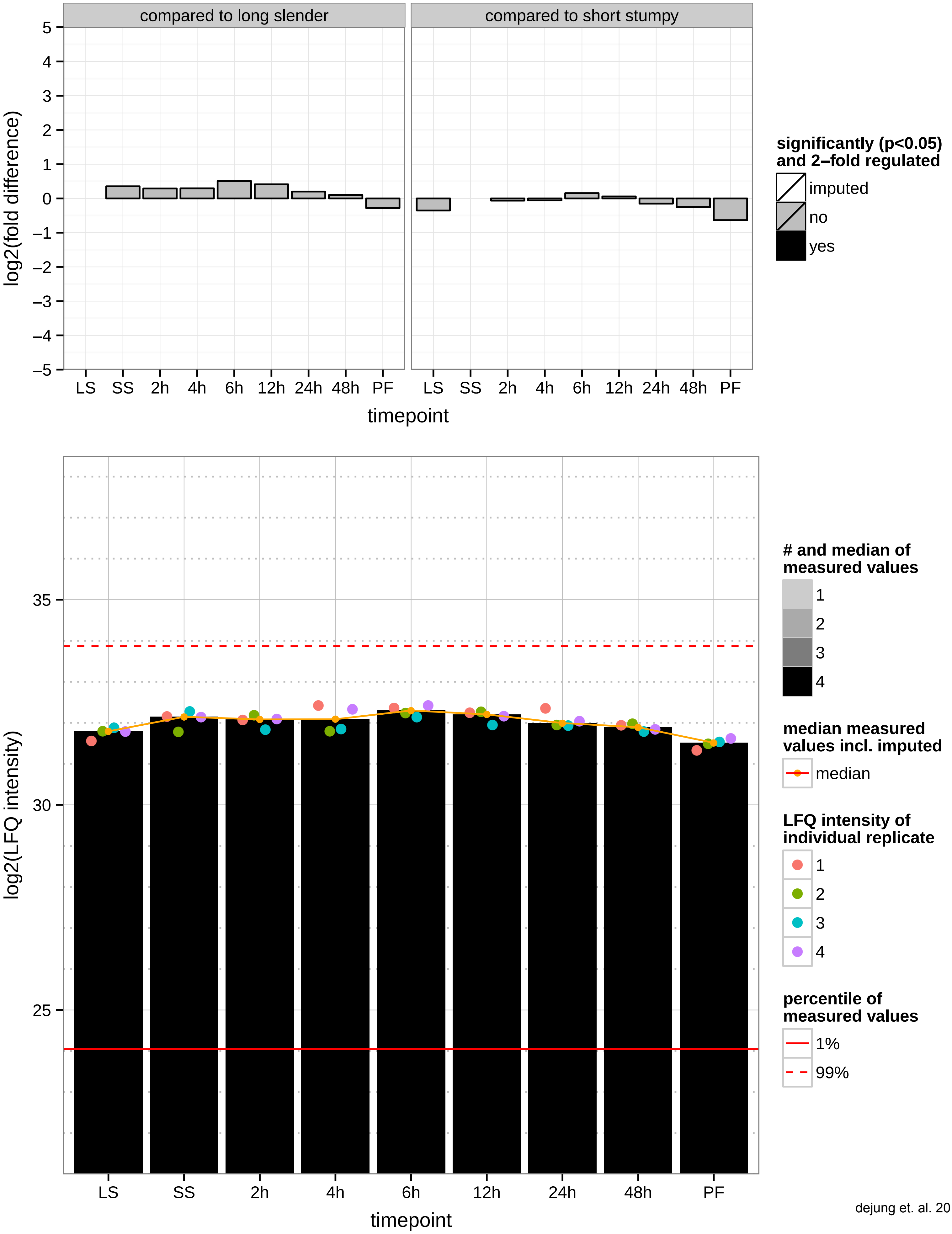

dejung et. al. 2015

hypothetical protein, conserved
Tb927.5.2950
AGOF: null
AGOC: null
AGOP: ciliary or flagellar motility
PGOF: null
PGOC: null
PGOP: null

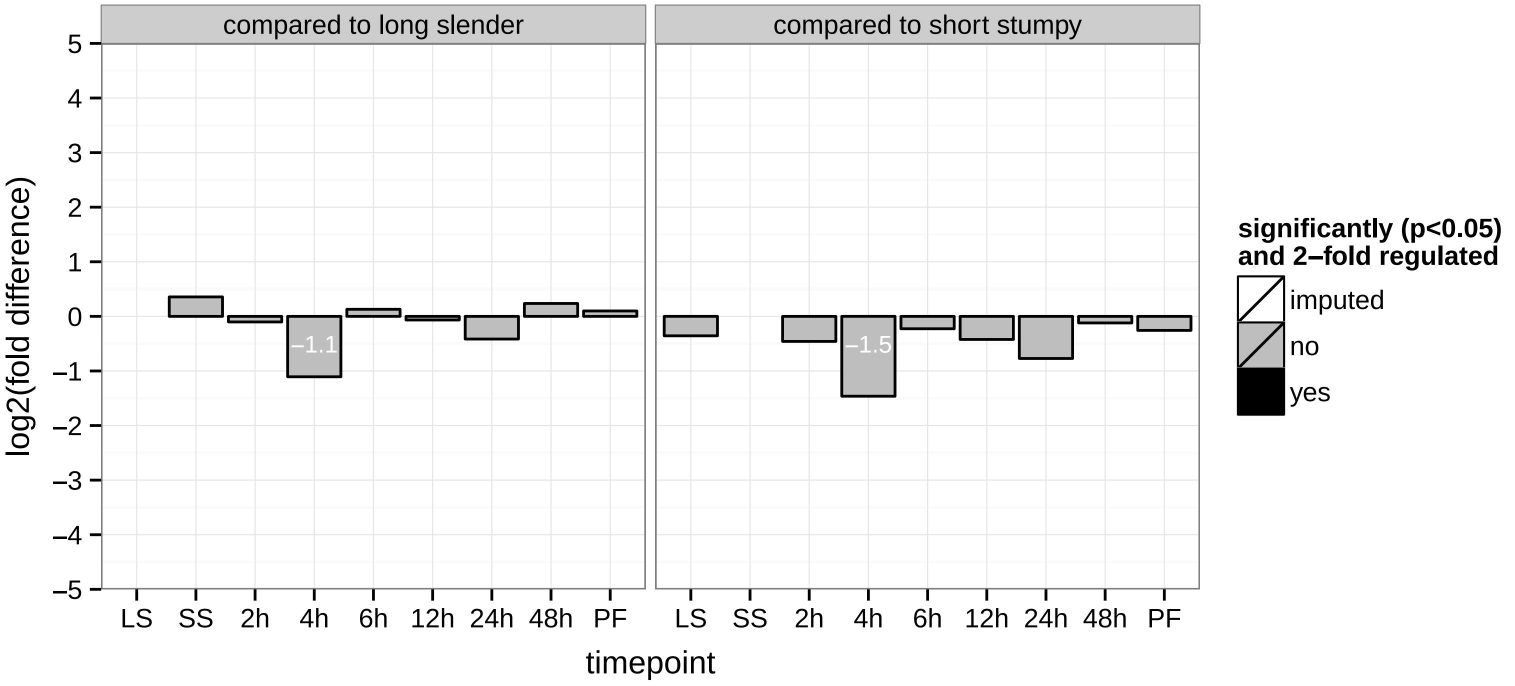

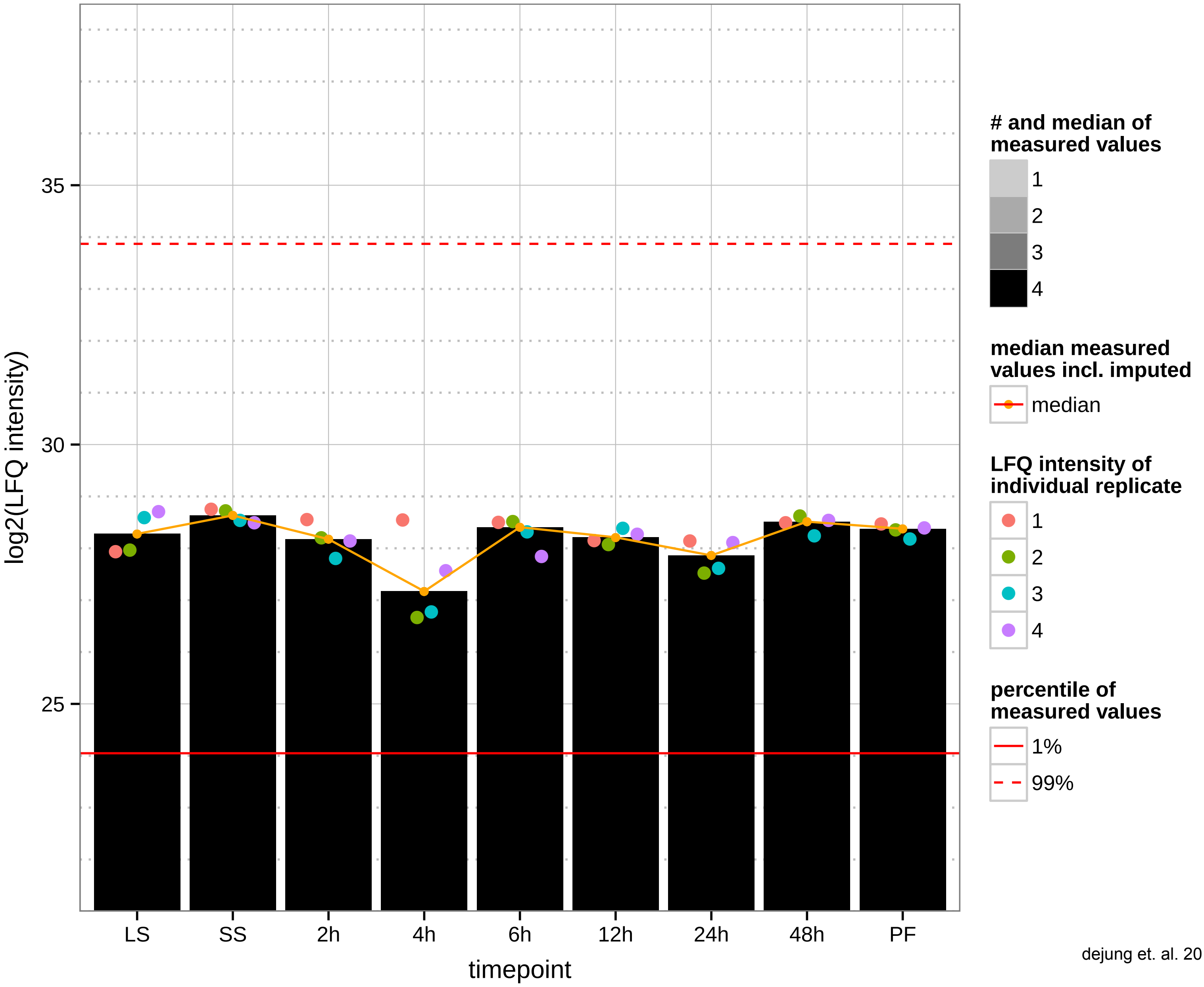

dejung et. al. 2015

phosphoribosylpyrophosphate synthetase, putative (PRS)
Tb927.5.2960
AGOF: magnesium ion binding, ribose phosphate diphosphokinase activity
AGOC: null
AGOP: nucleoside metabolic process, nucleotide biosynthetic process
PGOF: magnesium ion binding, ribose phosphate diphosphokinase activity
PGOC: null
PGOP: nucleotide biosynthetic process

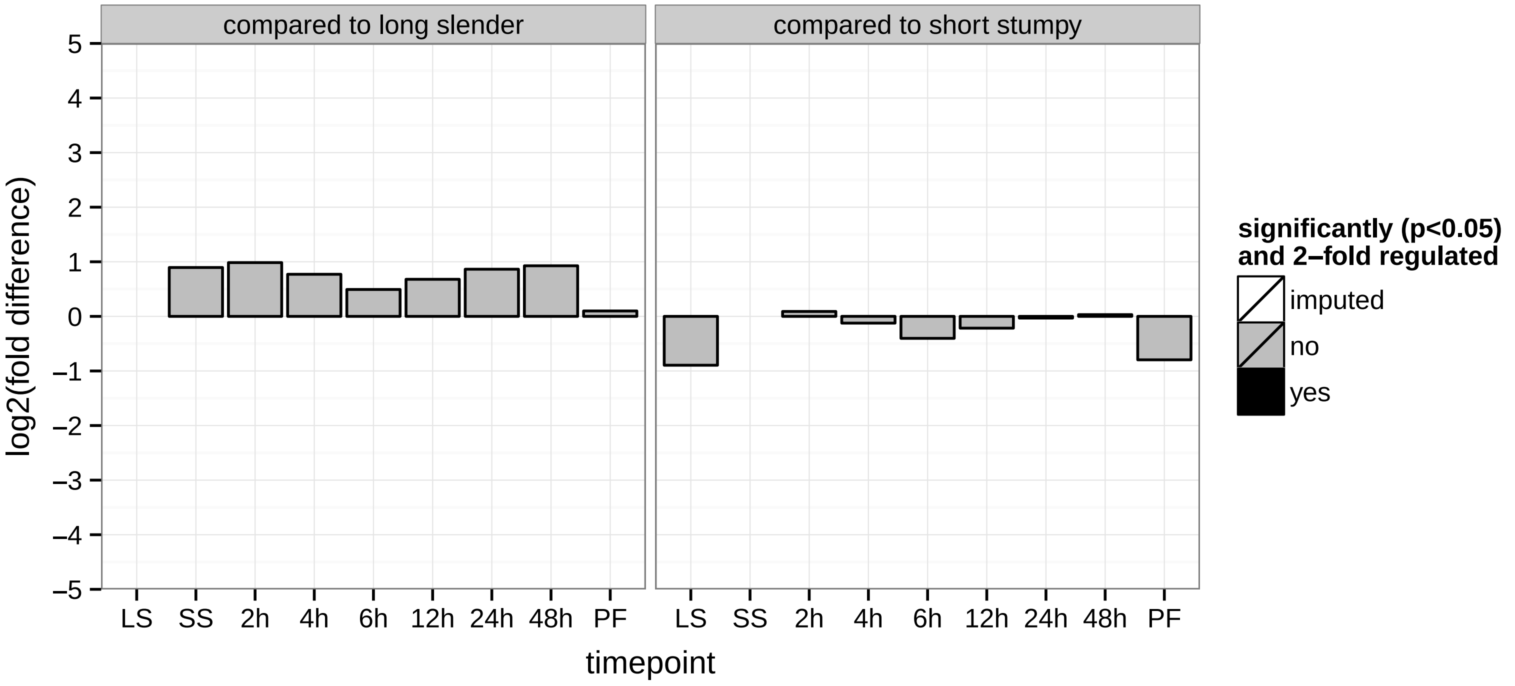

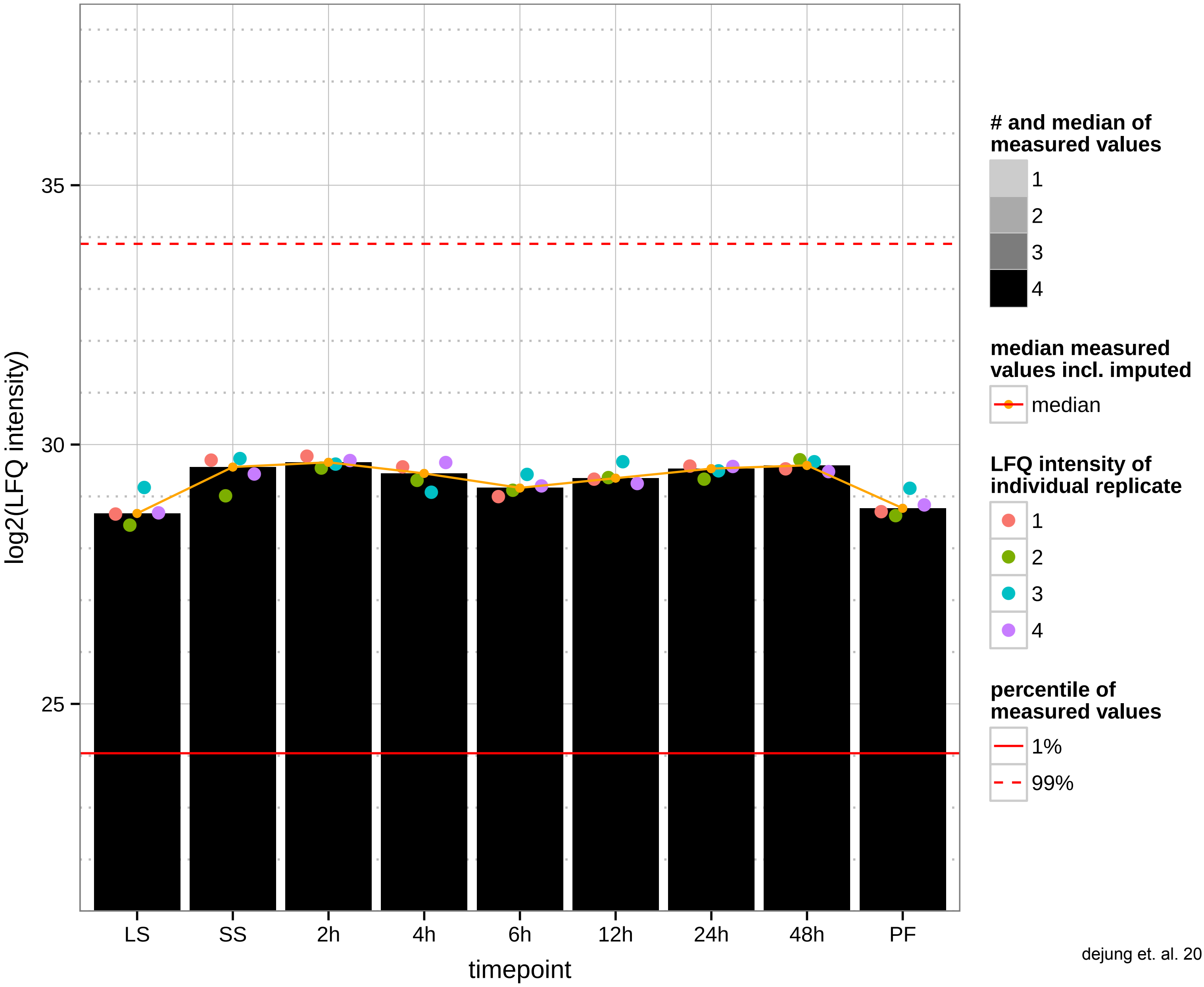

dejung et. al. 2015

hypothetical protein, conserved, MRB1–associated protein, putative, guide RNA binding protein, putative (mrb3010)
Tb927.5.3010;Tb11.v5.0691
AGOF: null, mRNA binding
AGOC: null, mitochondrion
AGOP: null, RNA processing
PGOF: null
PGOC: null
PGOP: null

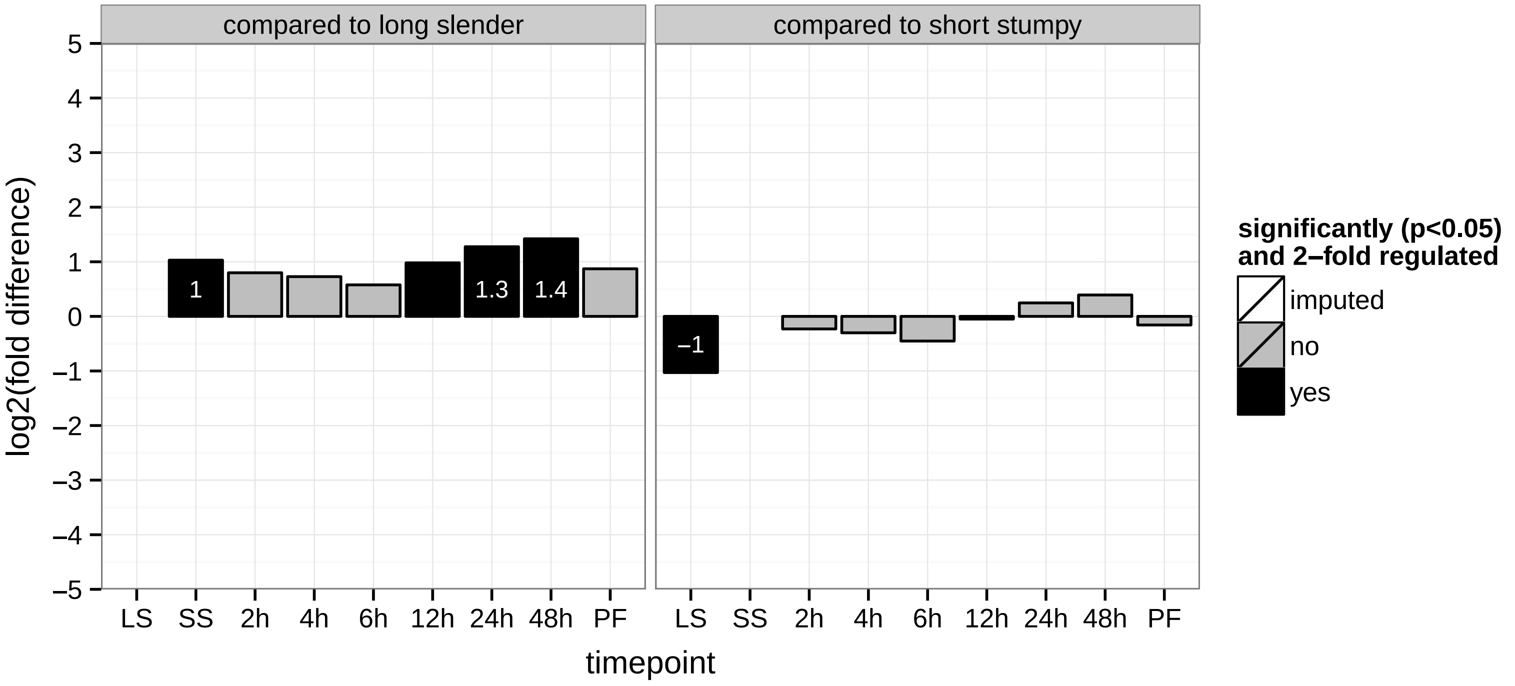

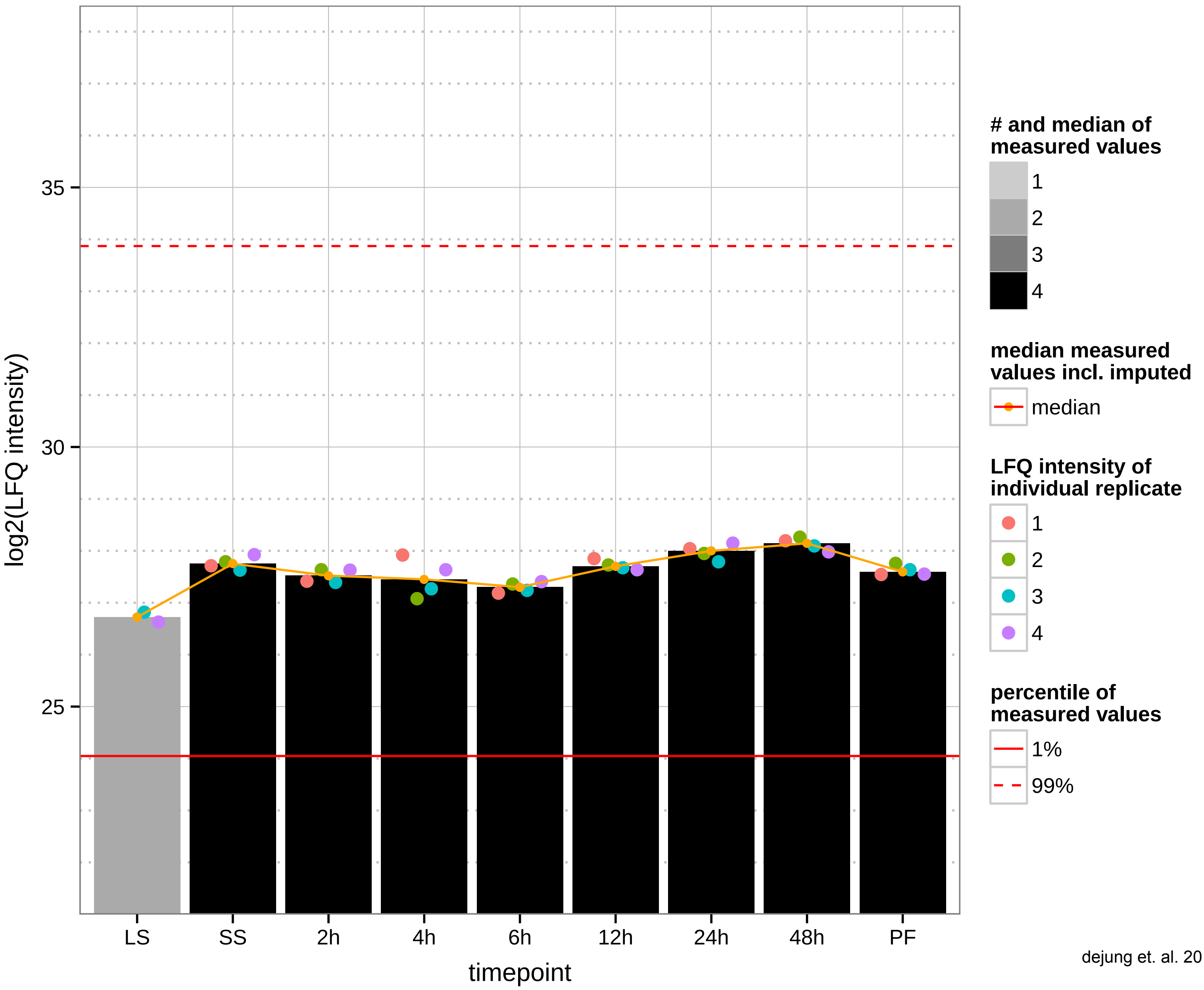

dejung et. al. 2015

intraflagellar transport protein IFT122, putative (IFT122B)
Tb927.5.3030
AGOF: null
AGOC: bacterial–type flagellum basal body
AGOP: intraflagellar transport
PGOF: protein binding
PGOC: null
PGOP: null

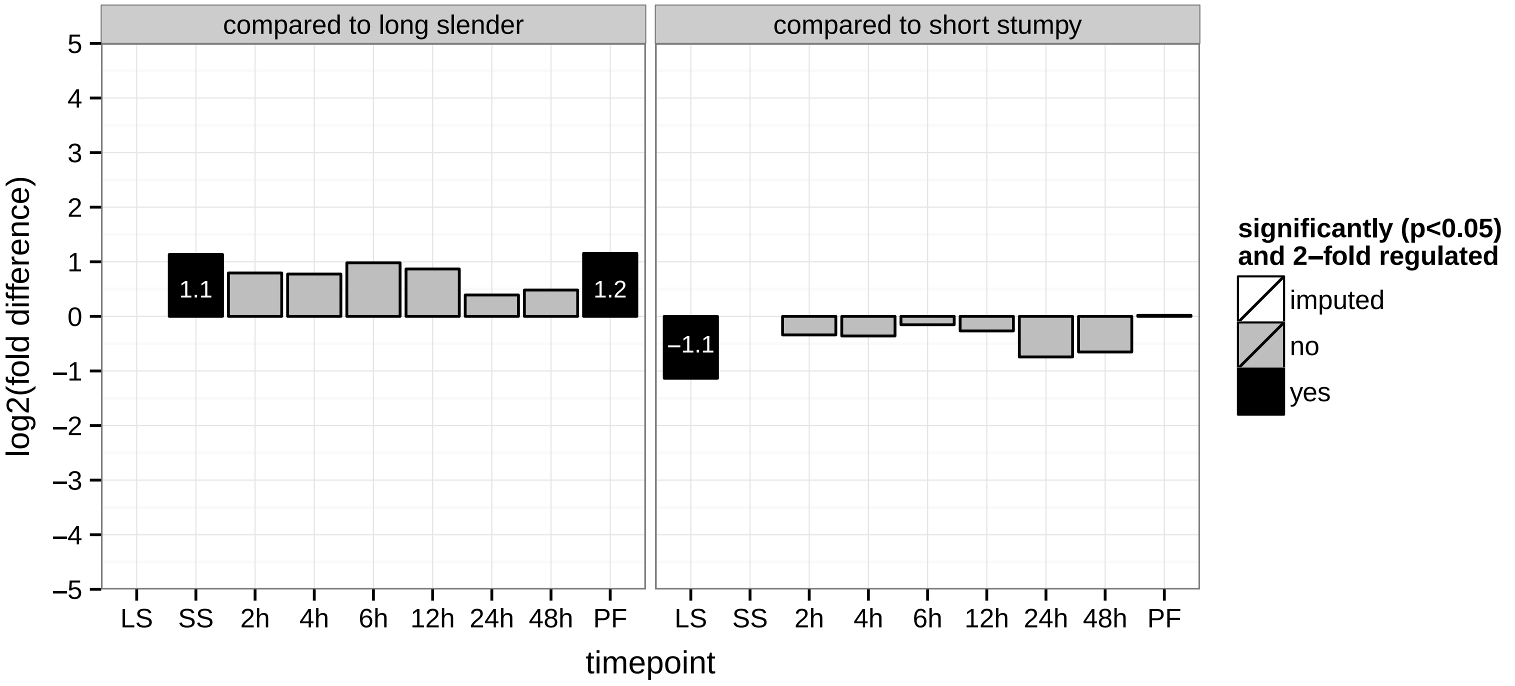

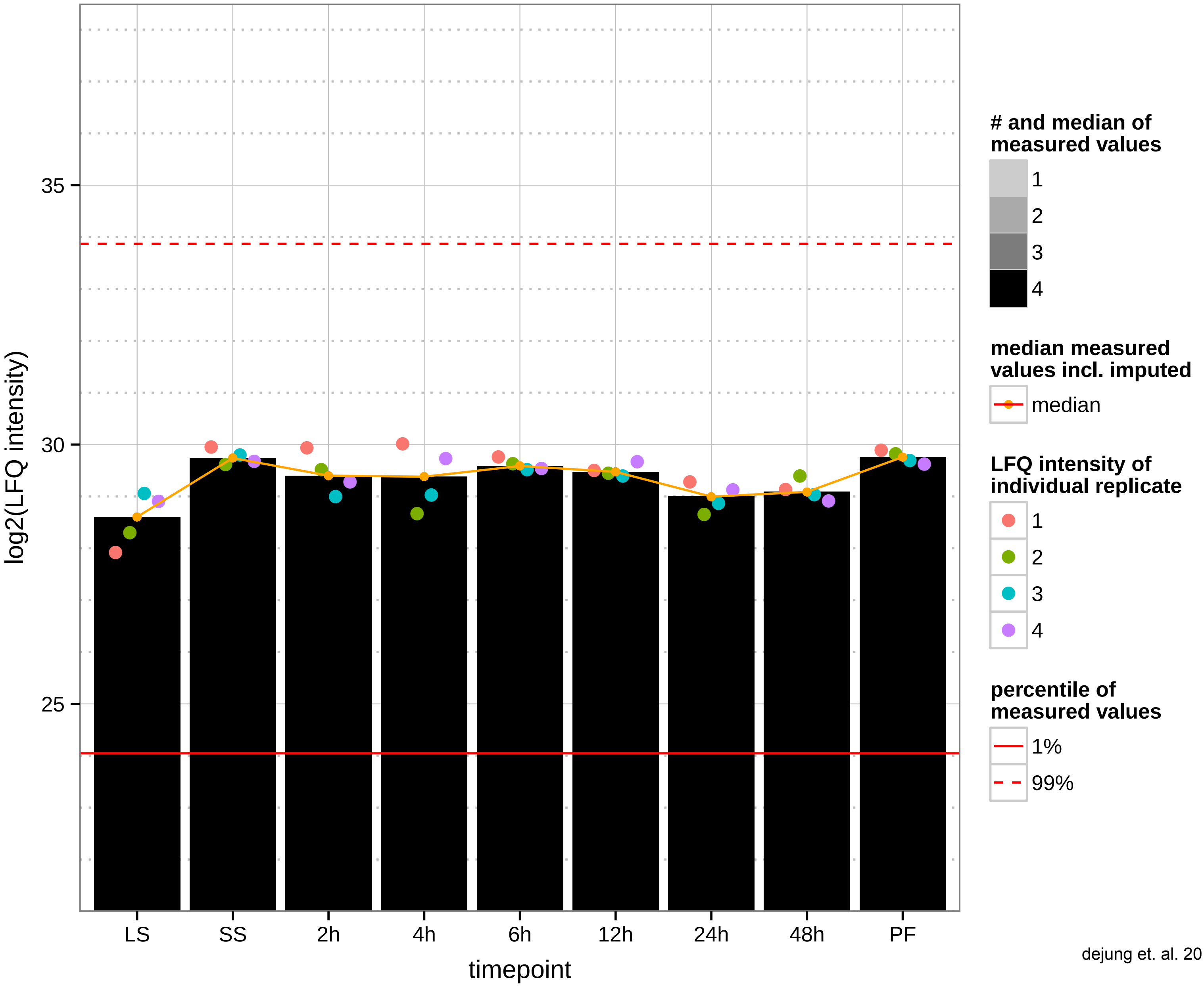

dejung et. al. 2015

ribose–phosphate pyrophosphokinase, putative (PRPS5)
Tb927.5.3170
AGOF: magnesium ion binding, ribose phosphate diphosphokinase activity
AGOC: null
AGOP: nucleotide biosynthetic process
PGOF: null
PGOC: null
PGOP: null

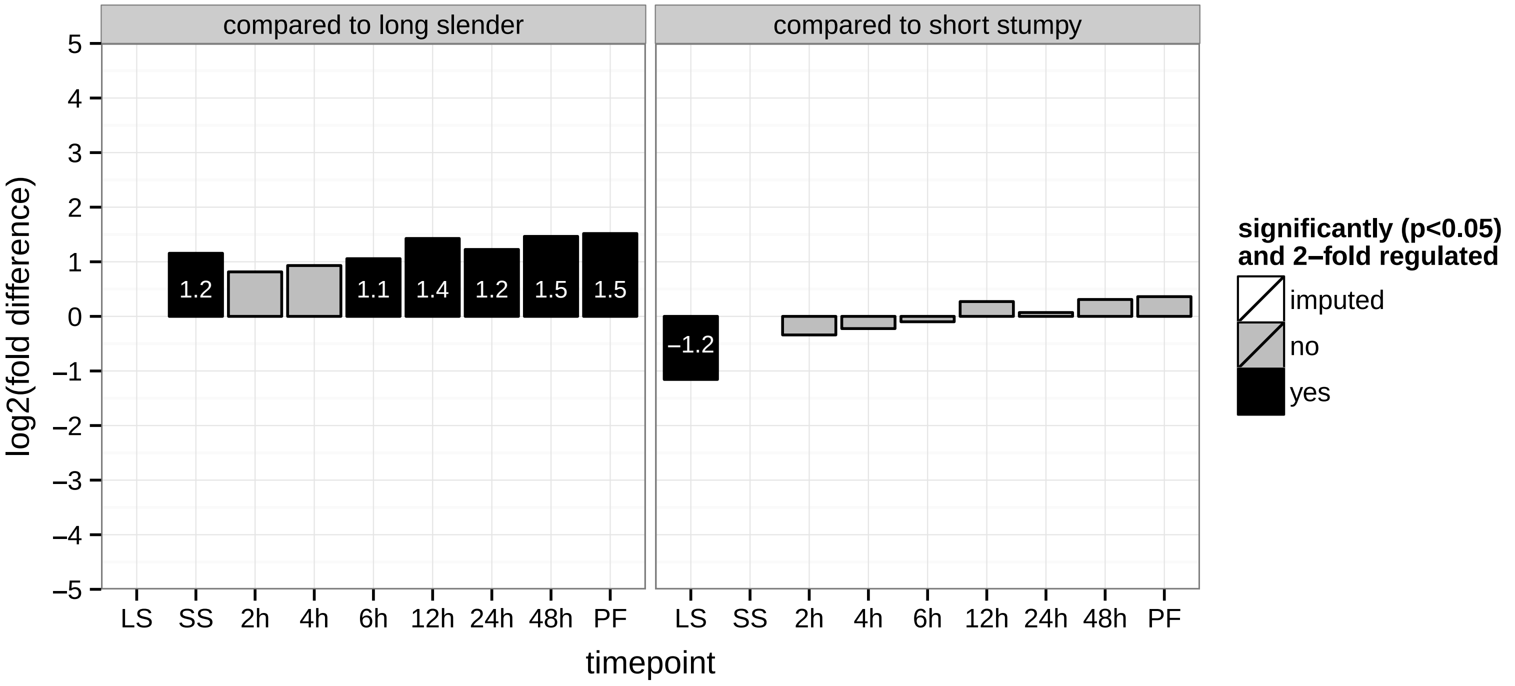

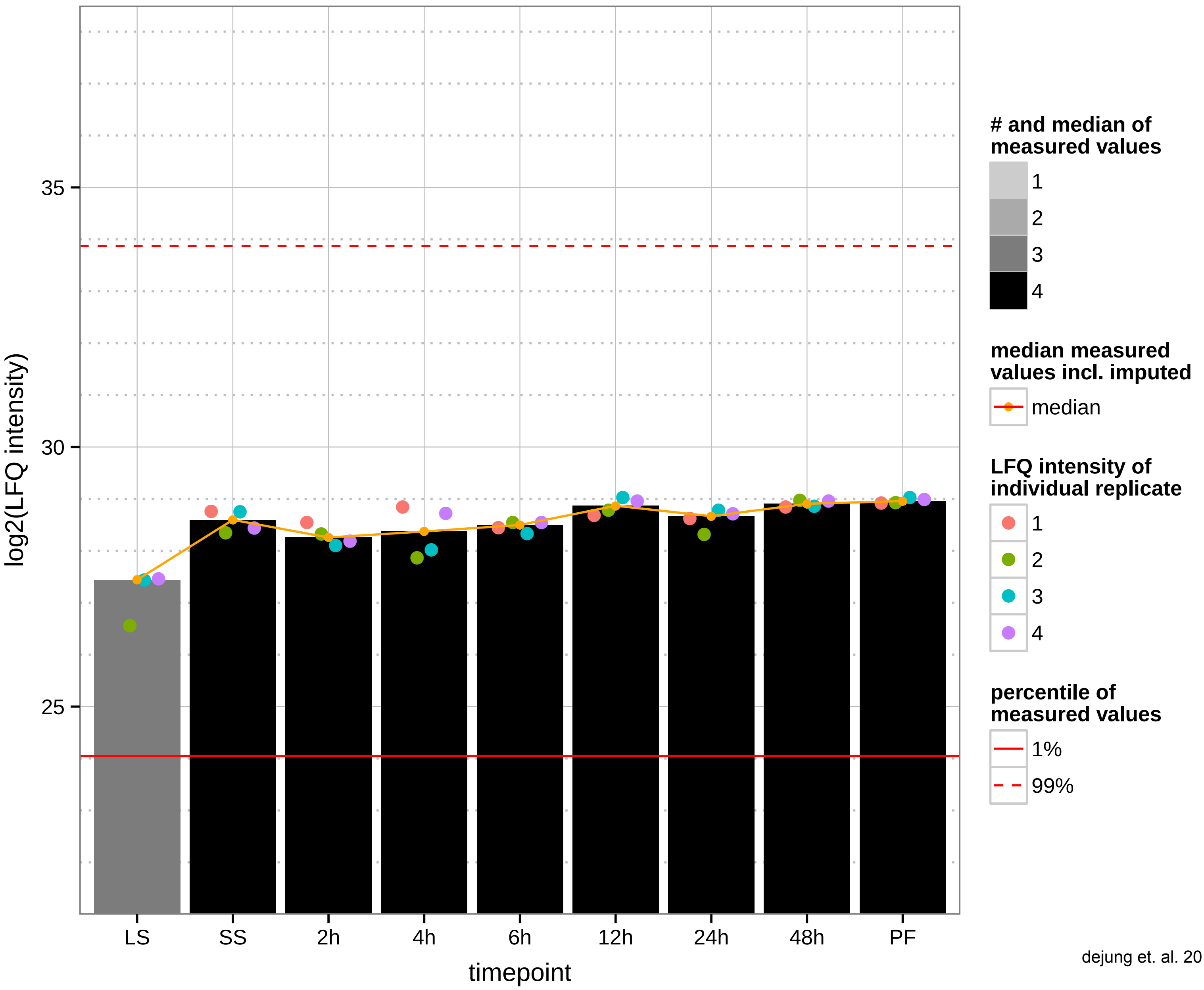

dejung et. al. 2015

small ubiquitin–related modifier (SUMO)
Tb927.5.3210
AGOF: null
AGOC: nucleus
AGOP: null
PGOF: protein binding
PGOC: null
PGOP: null

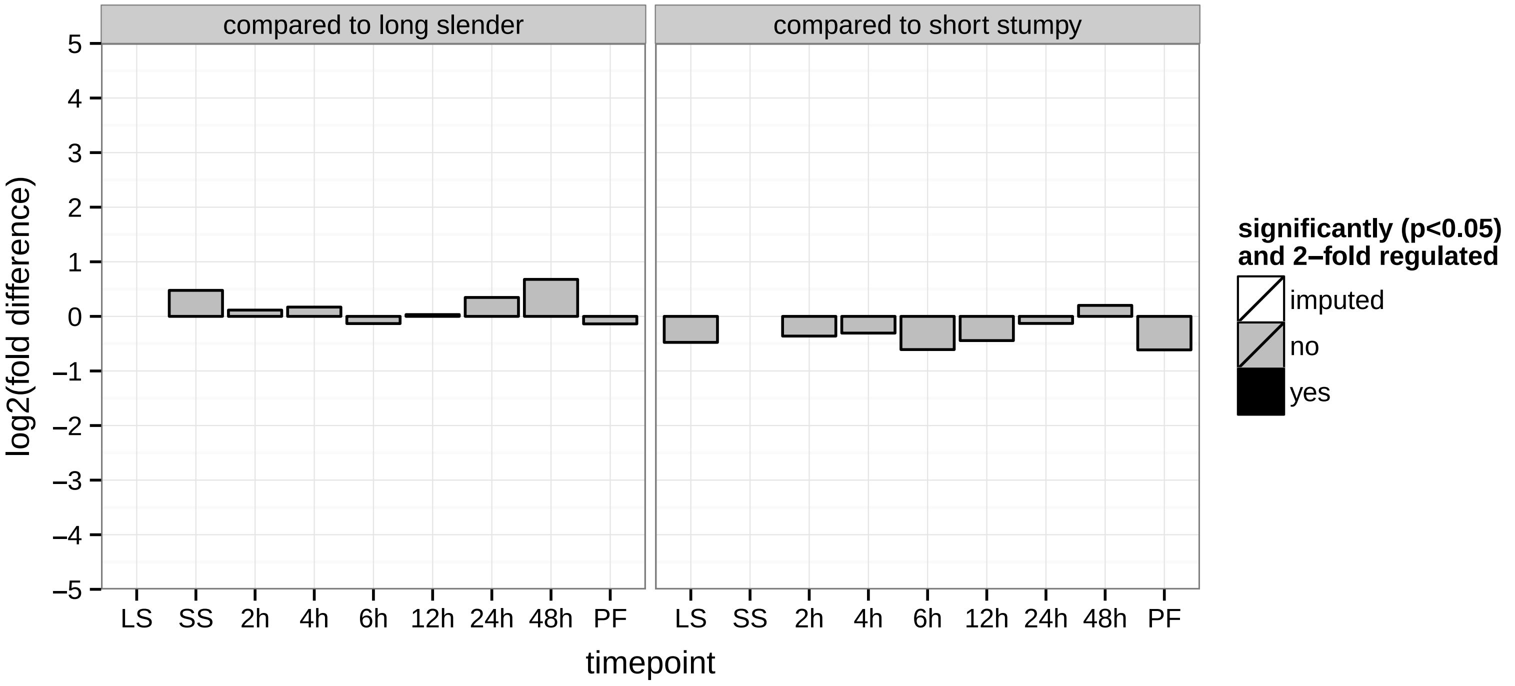

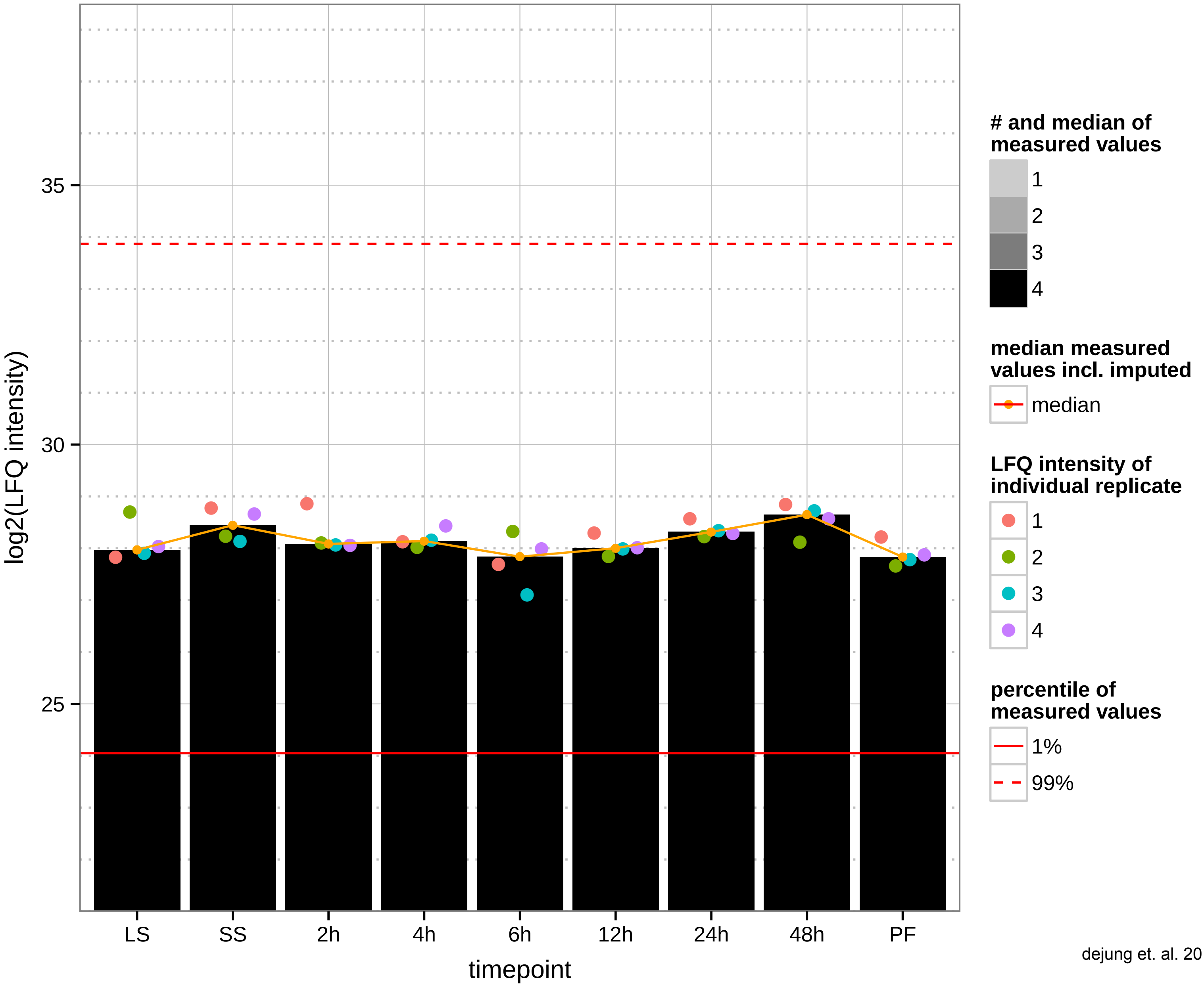

dejung et. al. 2015

hypothetical protein, conserved
Tb927.5.3250
AGOF: null
AGOC: null
AGOP: null
PGOF: protein binding
PGOC: null
PGOP: null

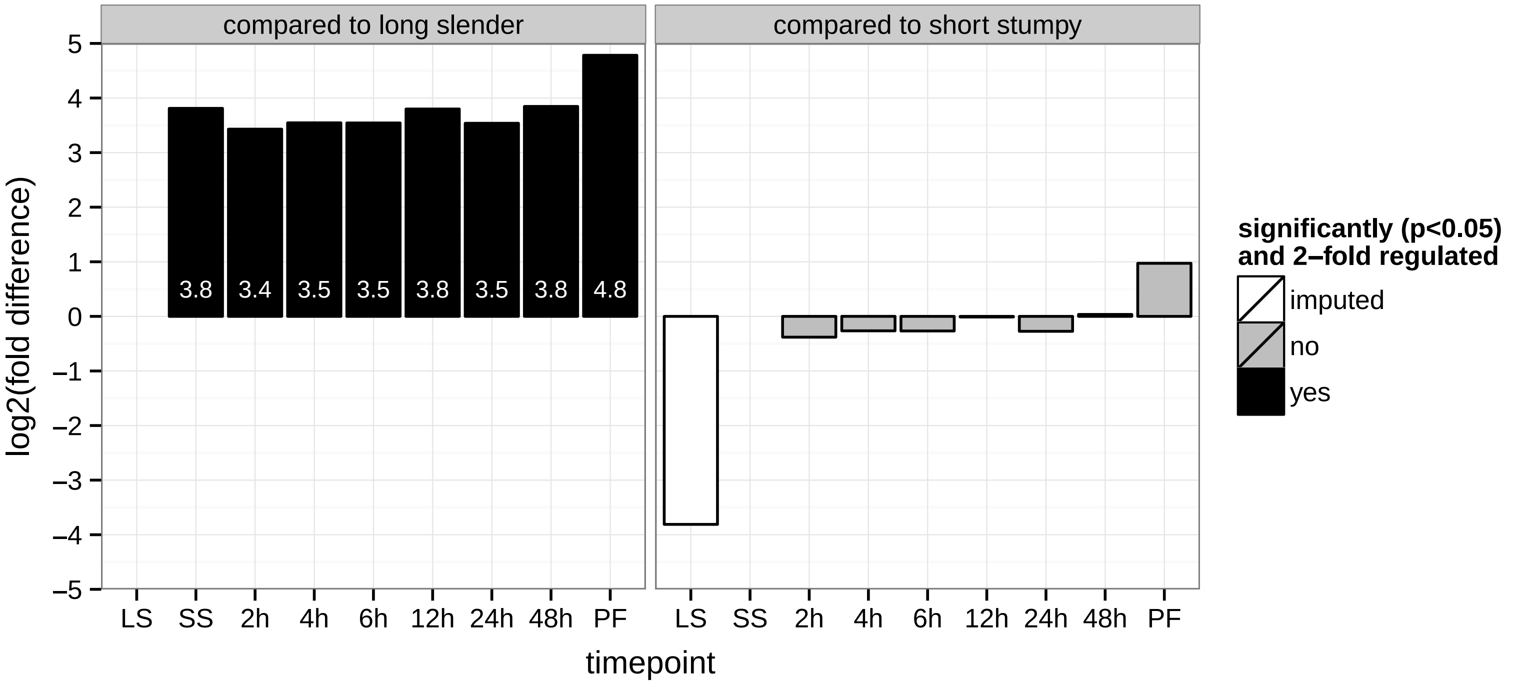

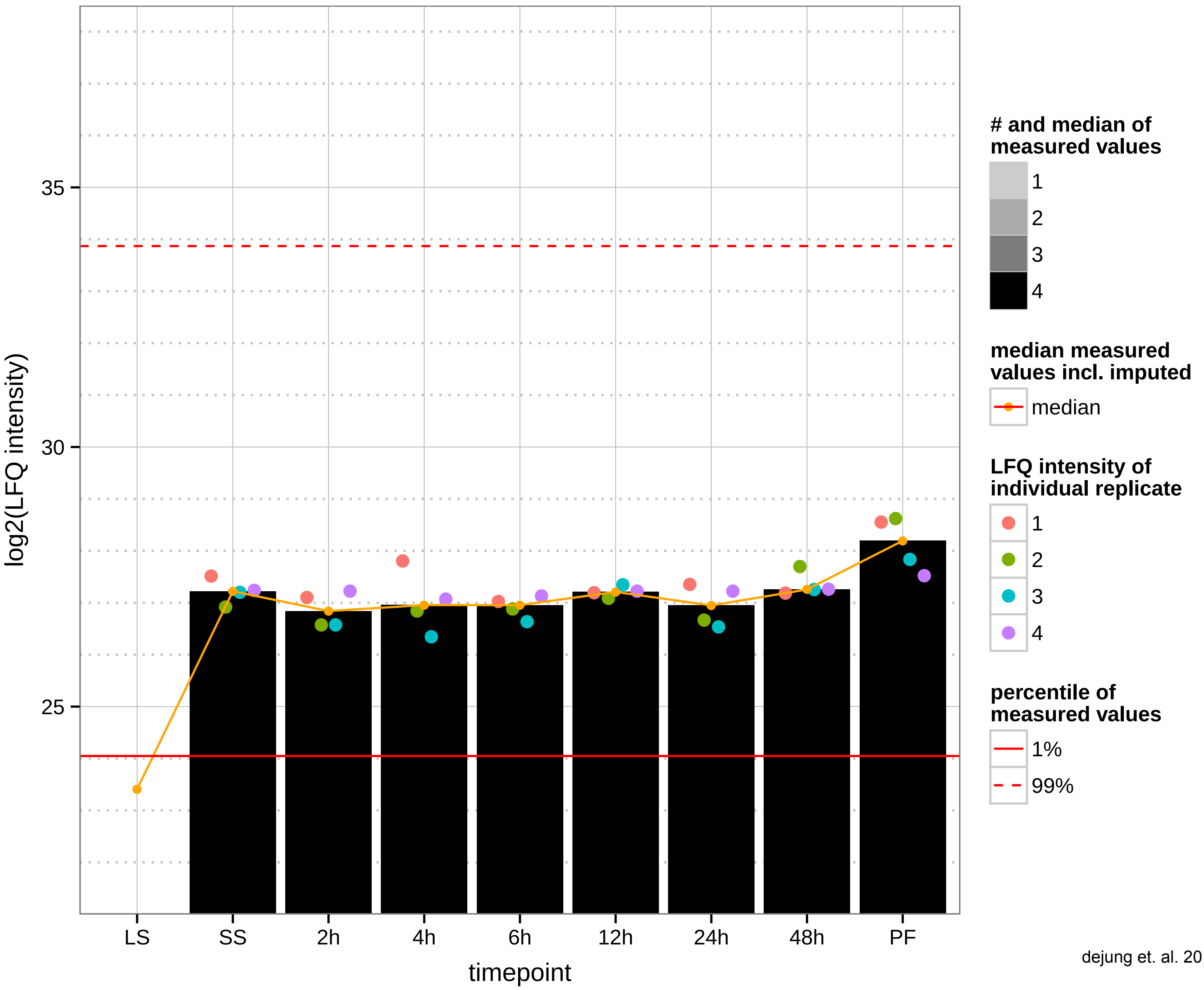

dejung et. al. 2015

hypothetical protein, conserved
Tb927.5.3260
AGOF: null
AGOC: null
AGOP: null
PGOF: protein binding
PGOC: null
PGOP: null

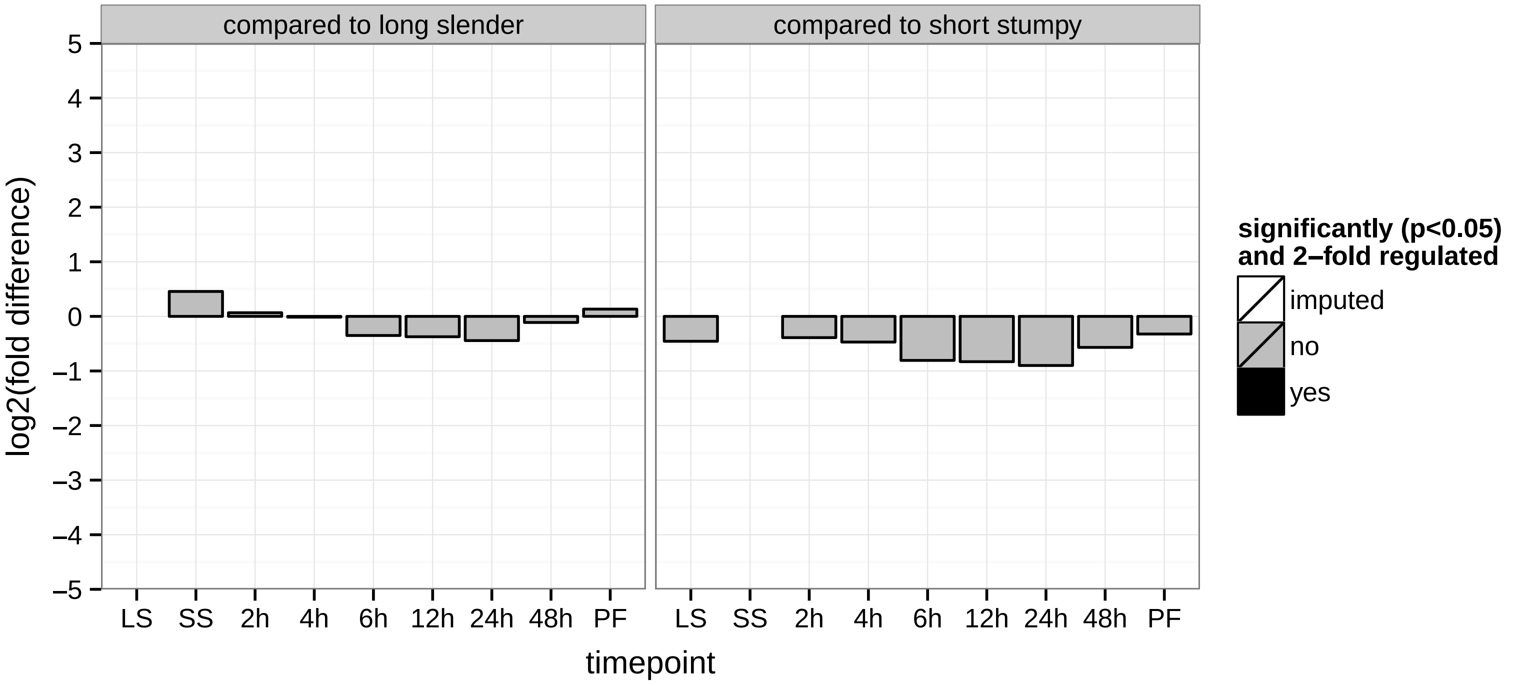

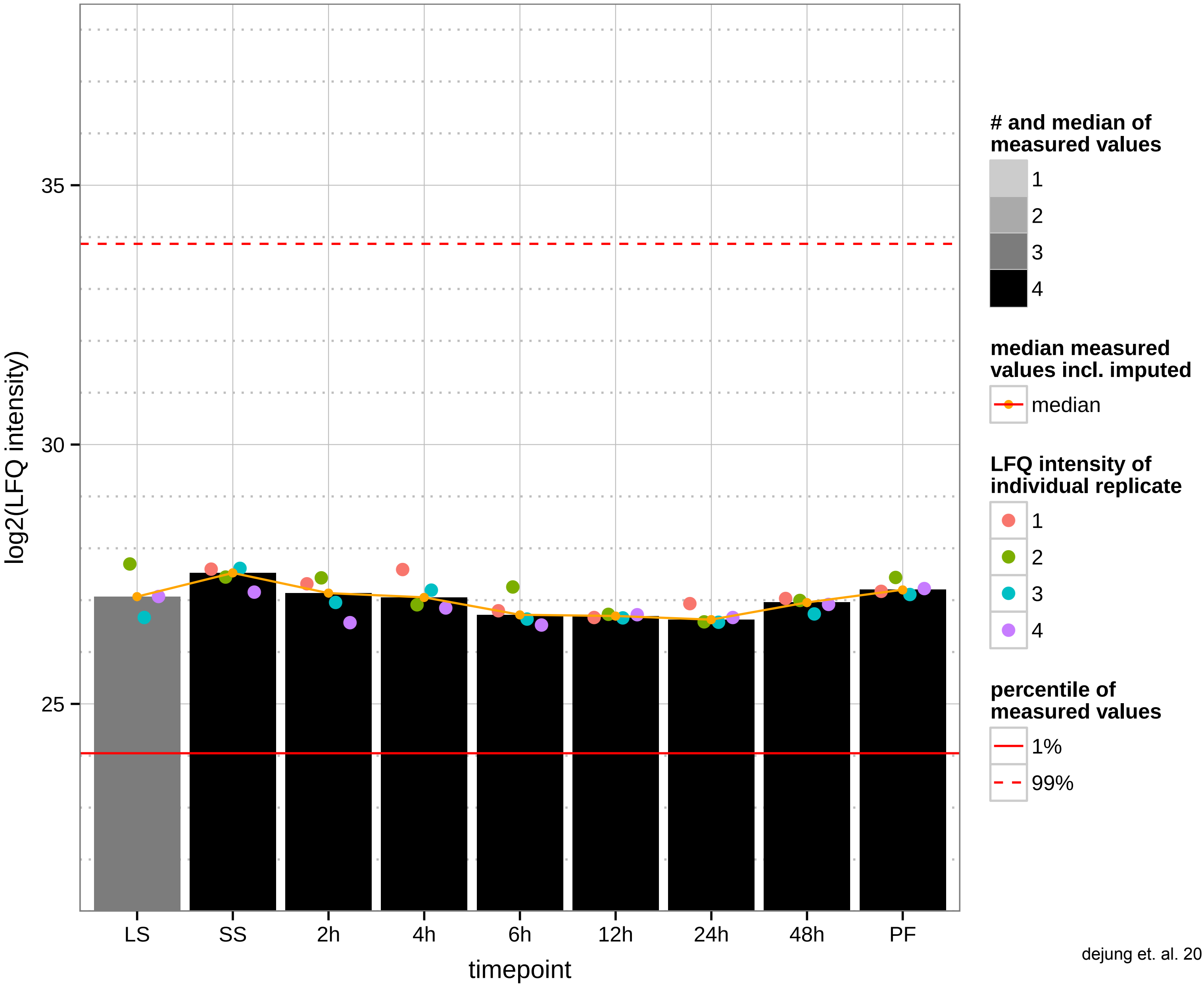

dejung et. al. 2015

iron superoxide dismutase, putative
Tb927.5.3350
AGOF: metal ion binding, superoxide dismutase activity
AGOC: mitochondrion
AGOP: oxidation–reduction process, superoxide metabolic process
PGOF: metal ion binding, superoxide dismutase activity
PGOC: null
PGOP: oxidation–reduction process, superoxide metabolic process

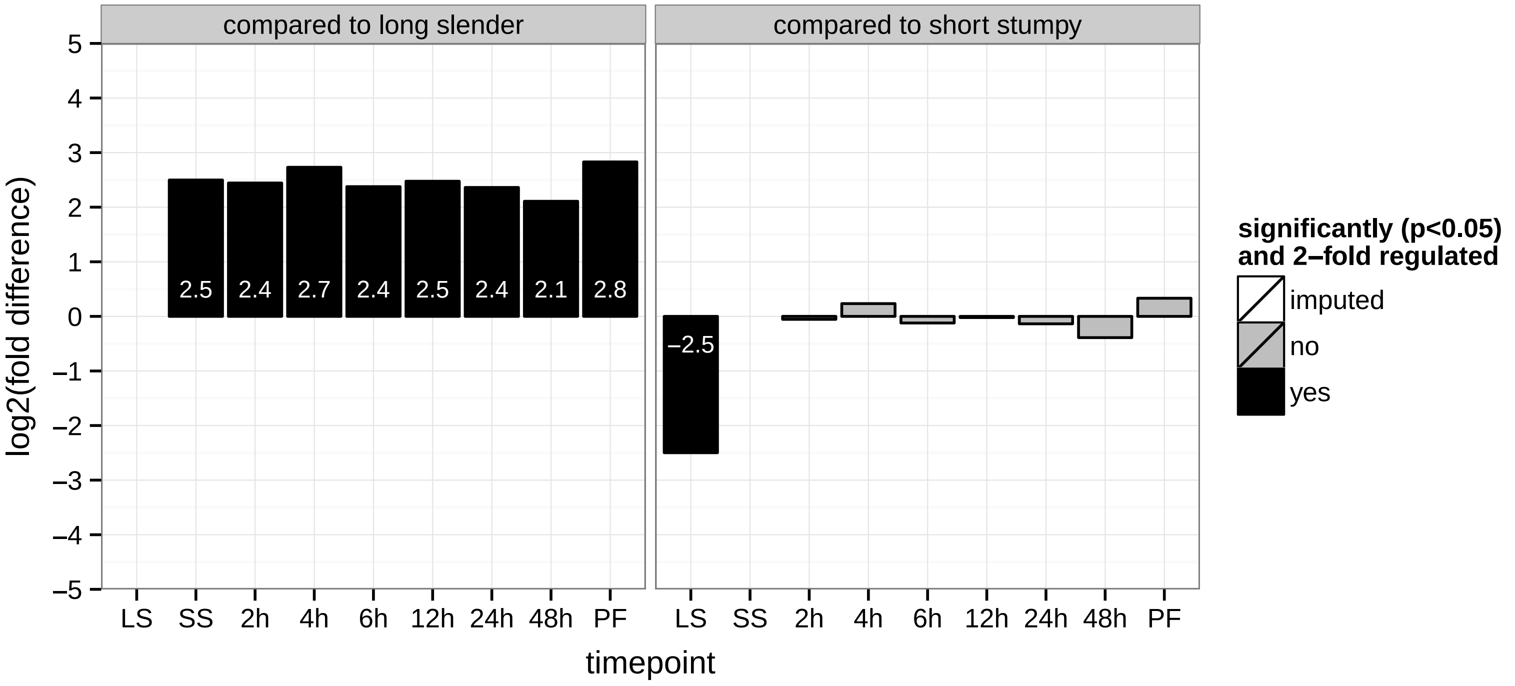

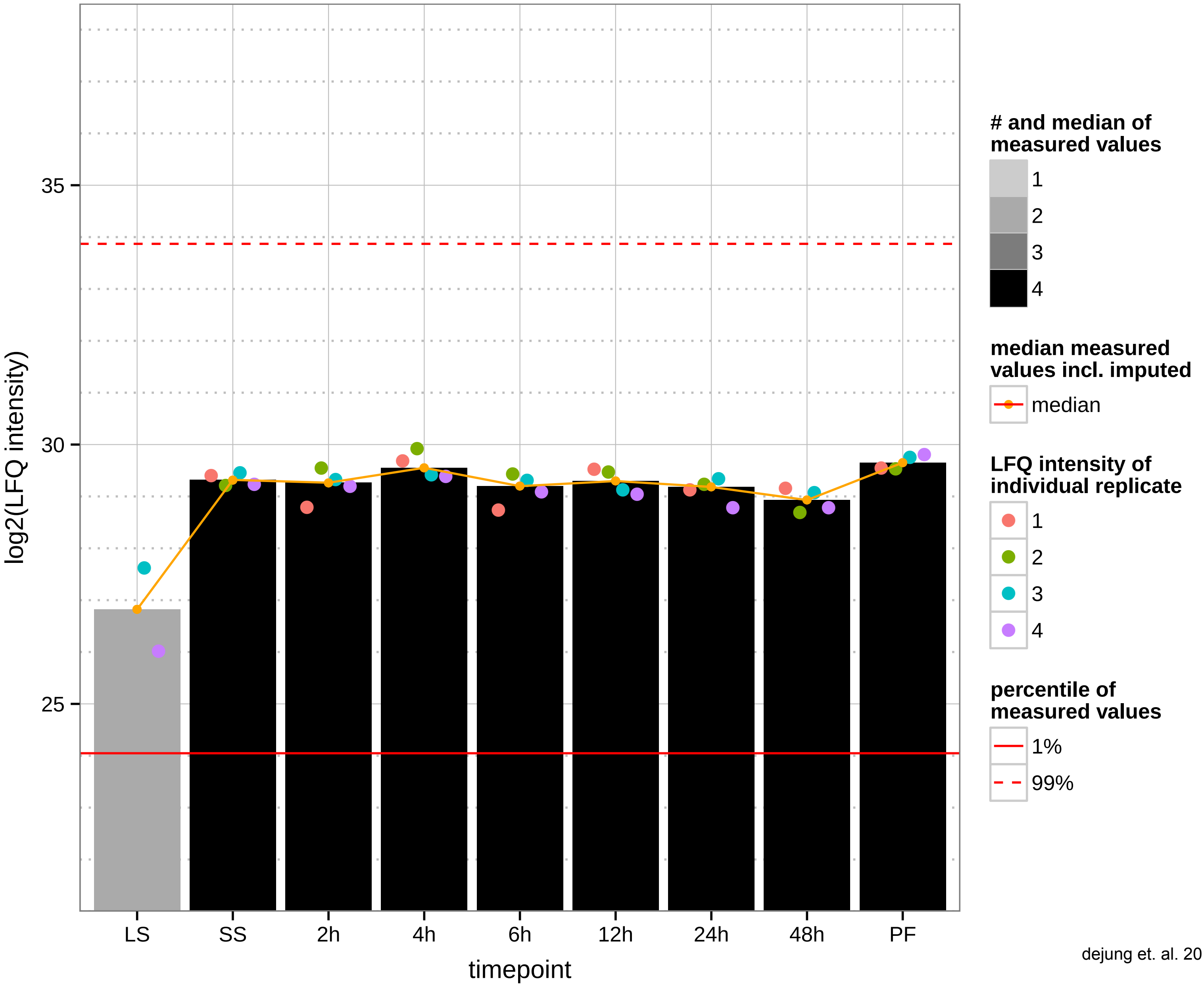

dejung et. al. 2015

50S ribosomal protein L2, putative
Tb927.5.3360
AGOF: structural constituent of ribosome
AGOC: intracellular, mitochondrion, ribosome
AGOP: translation
PGOF: structural constituent of ribosome
PGOC: intracellular, ribosome
PGOP: translation

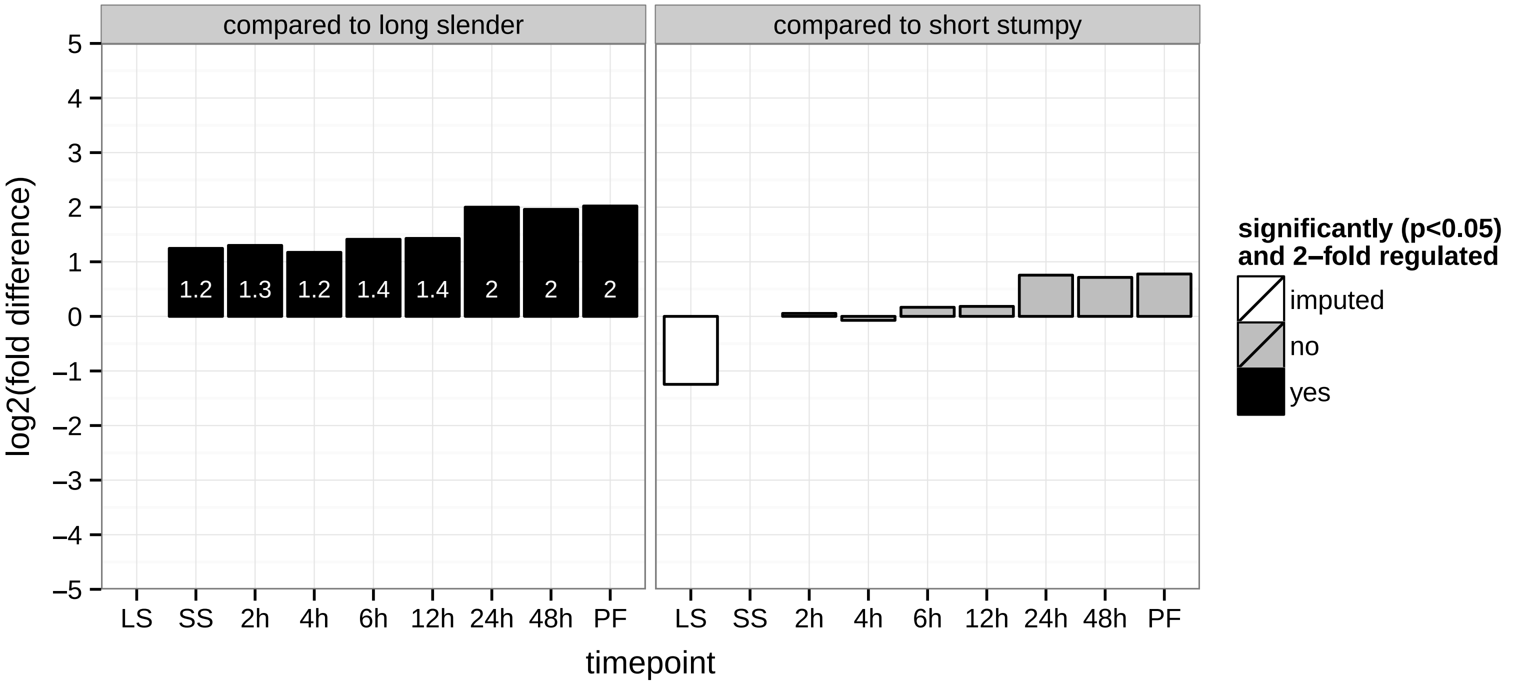

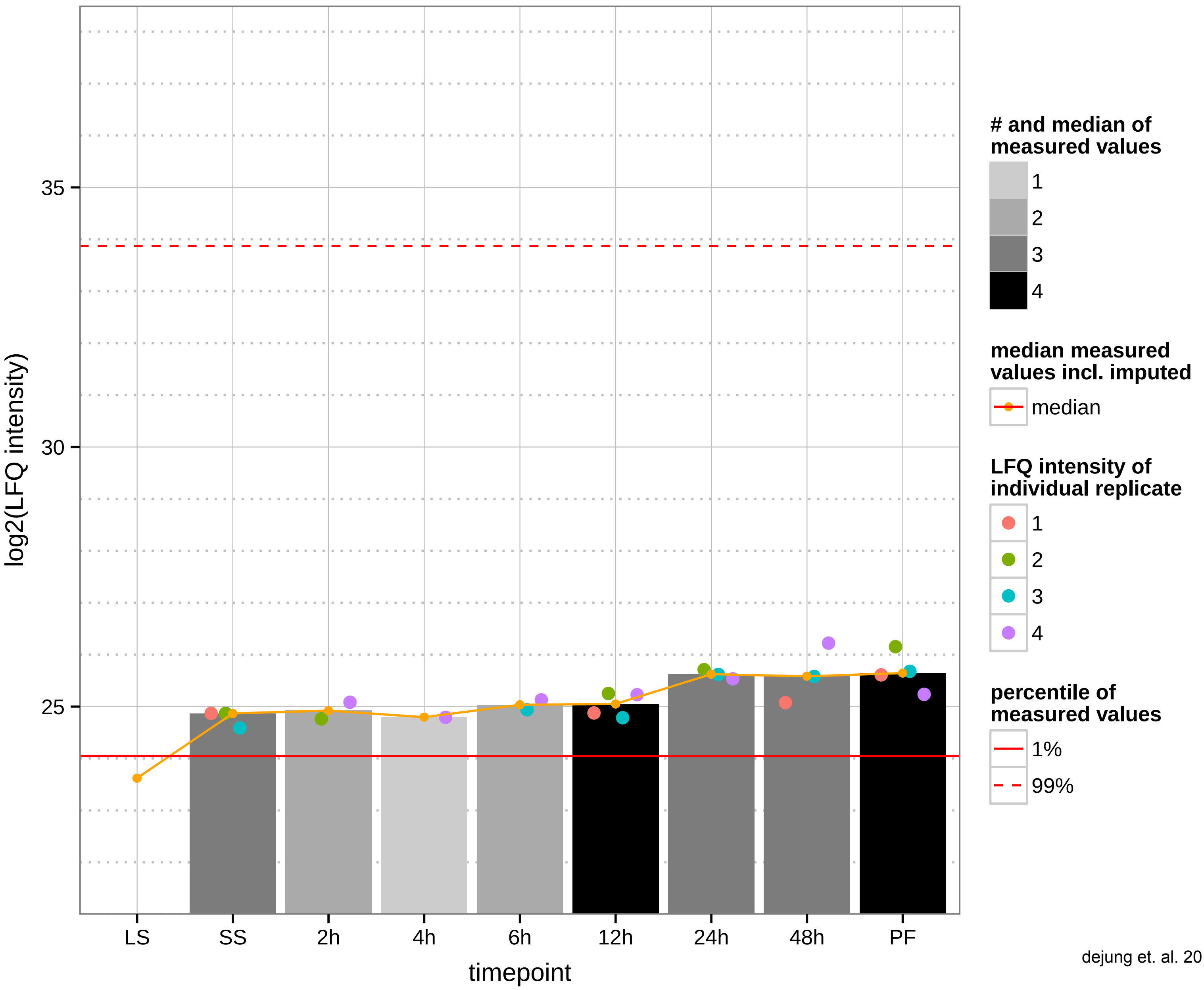

dejung et. al. 2015

calcium−translocating P−type ATPase, calcium pump
Tb927.5.3400
AGOF: ATP binding, calcium−transporting ATPase activity
AGOC: integral to membrane, mitochondrial inner membrane
AGOP: ATP biosynthetic process, calcium ion transport, metabolic process
PGOF: ATP binding, ATPase activity, coupled to transmembrane movement of ions, phosphorylative mechanism, calcium−tran
PGOC: integral to membrane, membrane
PGOP: ATP biosynthetic process, calcium ion transport, cation transport, metabolic process

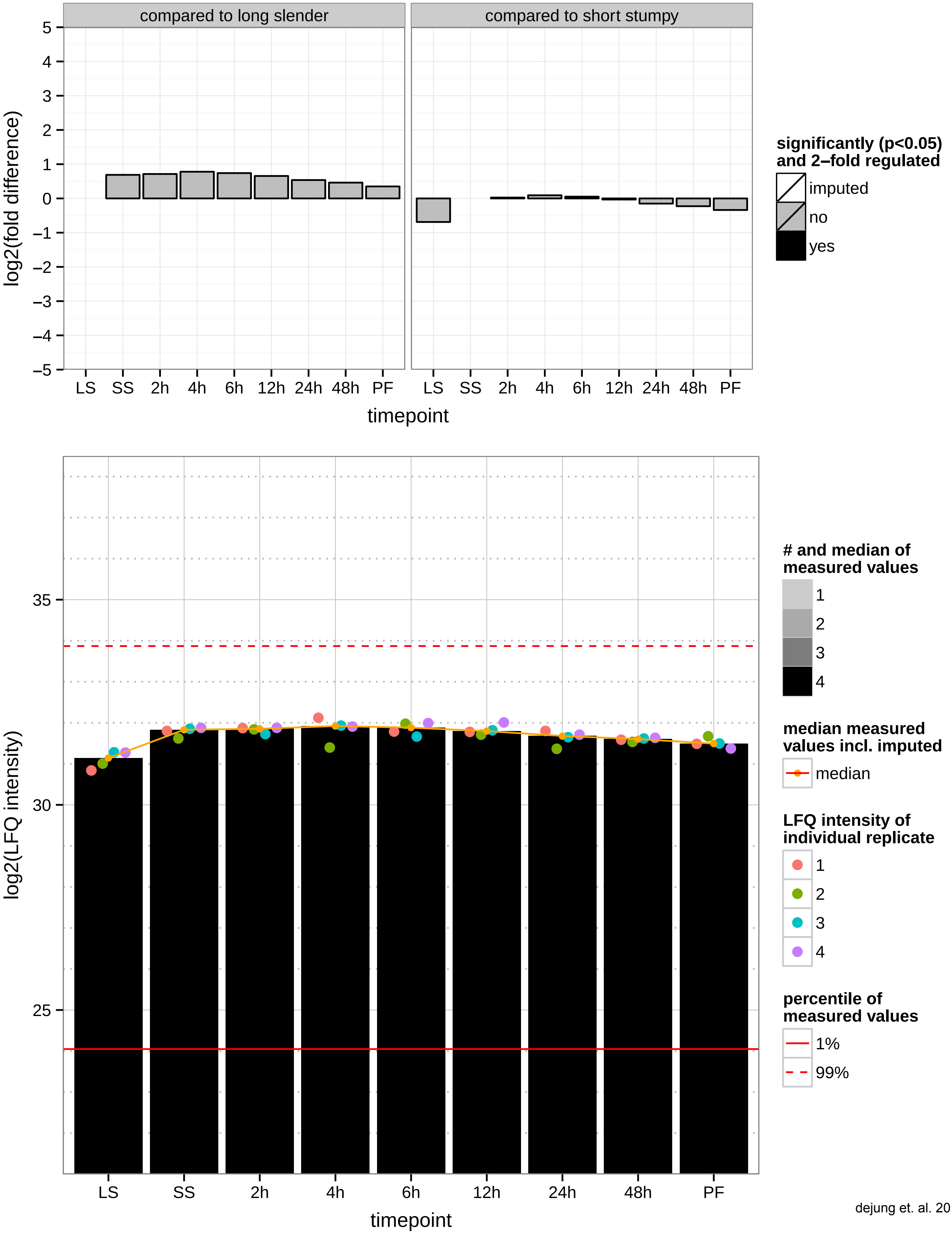

dejung et. al. 2015

ubiquitin–activating enzyme E1, putative
Tb927.5.3430
AGOF: ATP binding, small protein activating enzyme activity
AGOC: null
AGOP: cellular protein modification process
PGOF: ATP binding, catalytic activity, small protein activating enzyme activity
PGOC: null
PGOP: cellular protein modification process

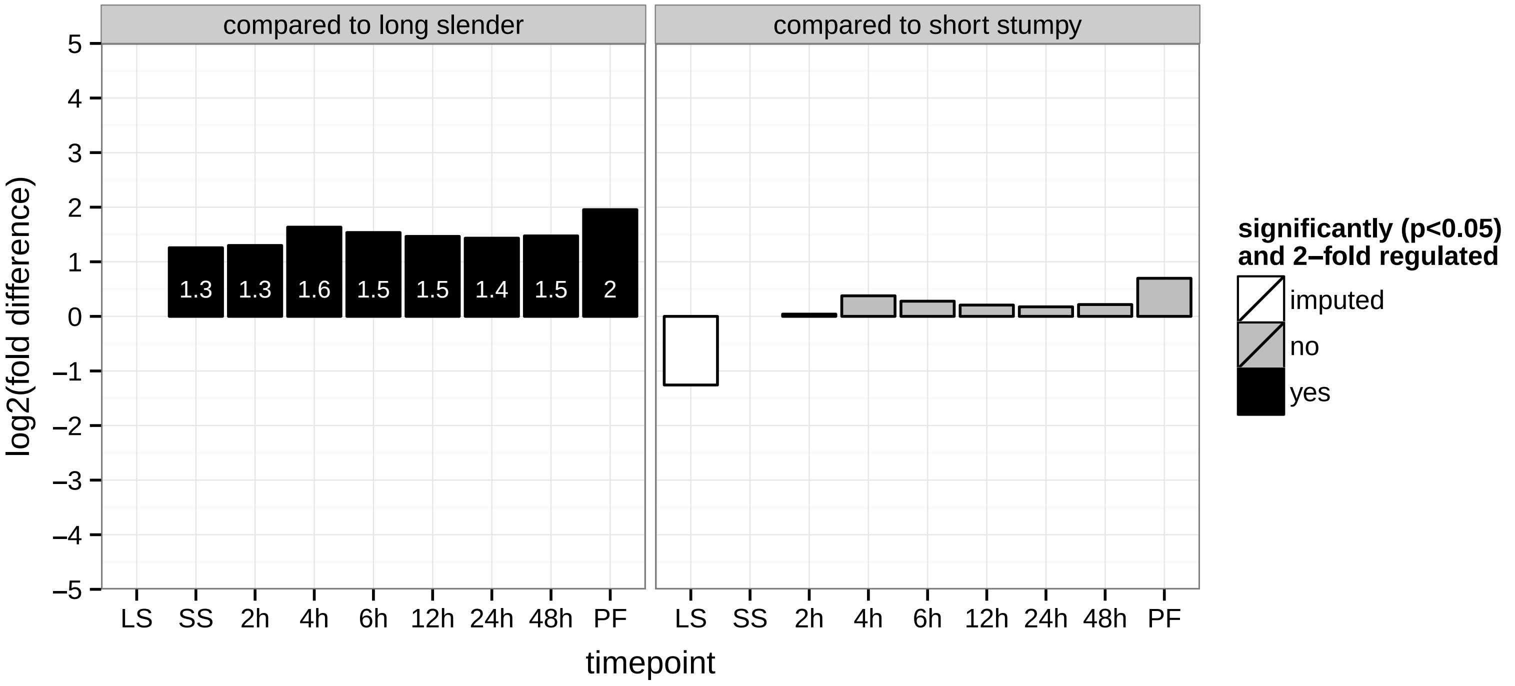

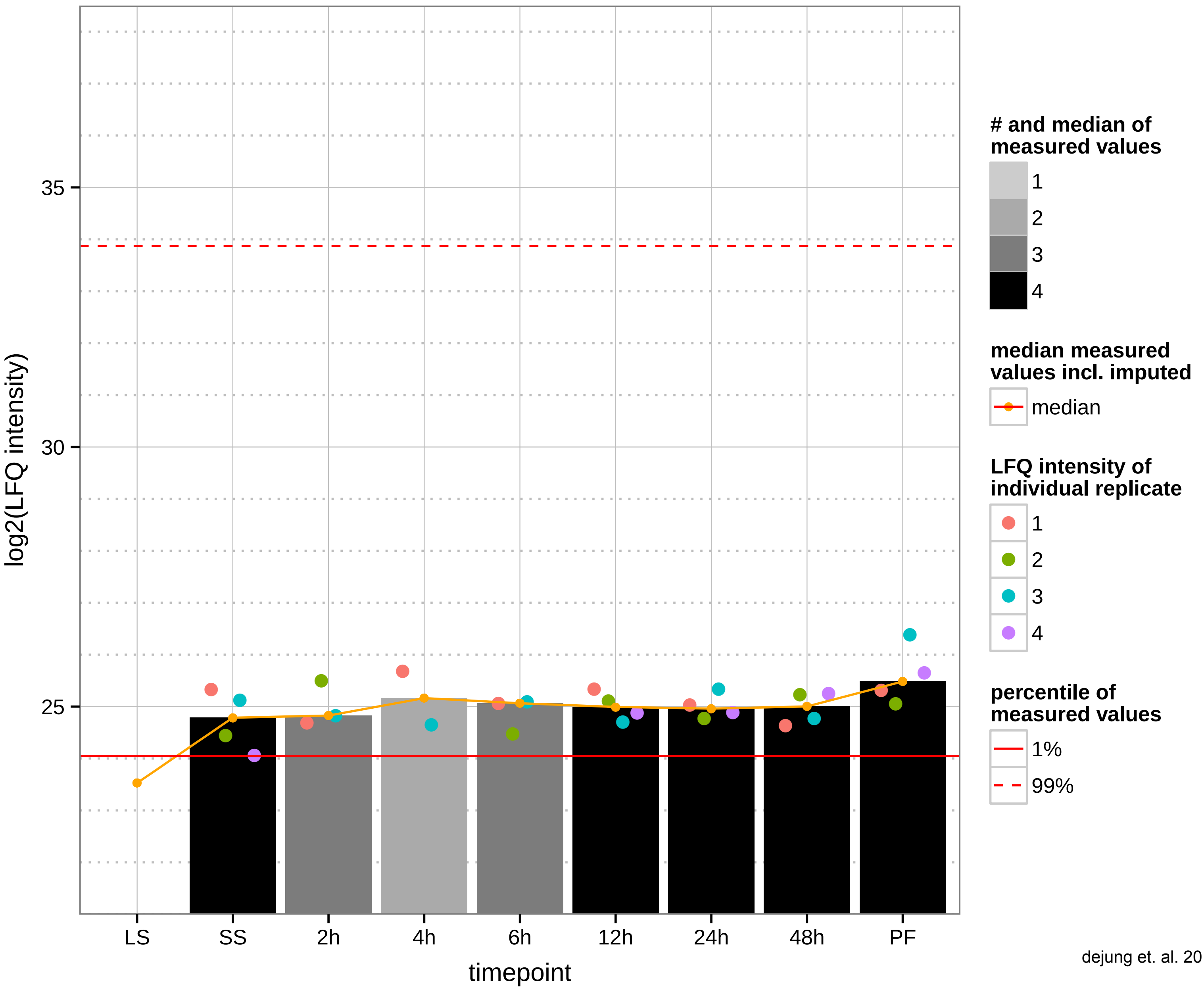

dejung et. al. 2015

hypothetical protein, conserved
Tb927.5.3450
AGOF: null
AGOC: null
AGOP: null
PGOF: null
PGOC: null
PGOP: null

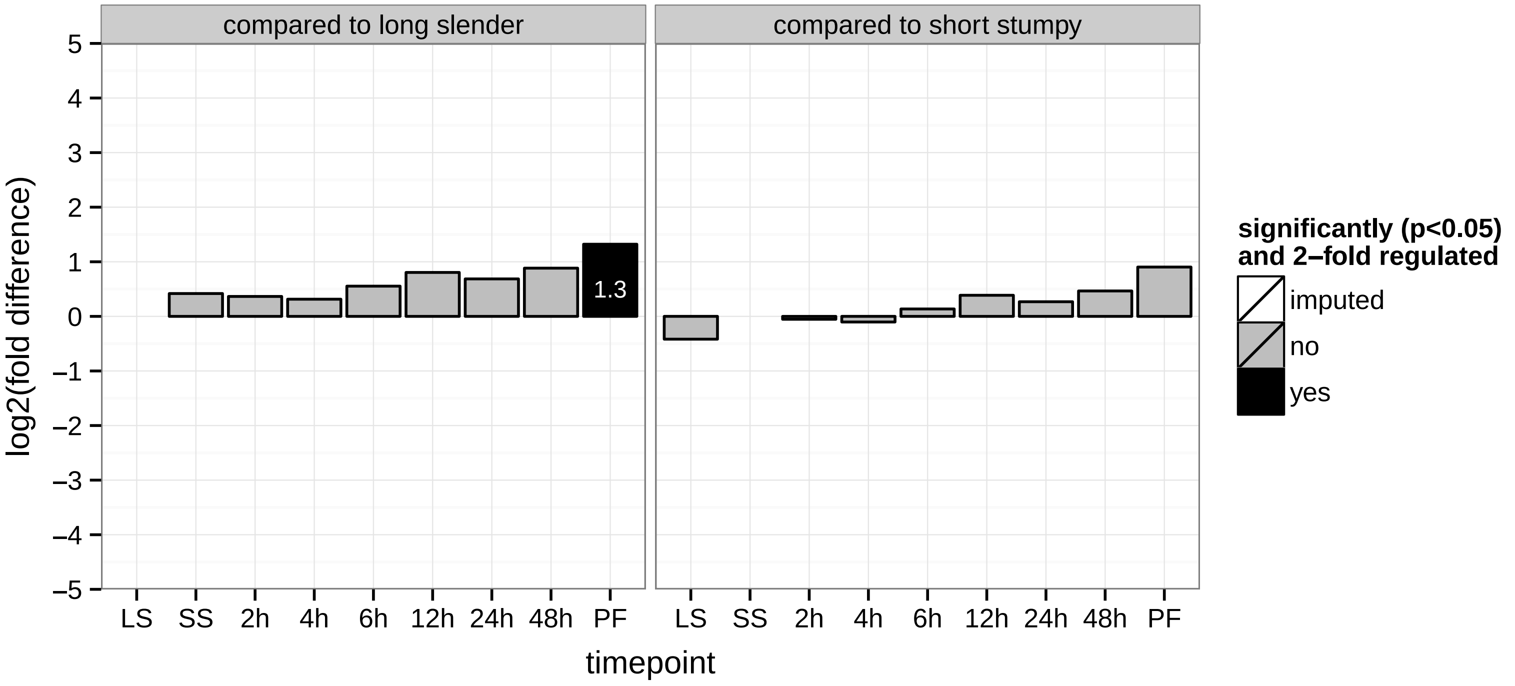

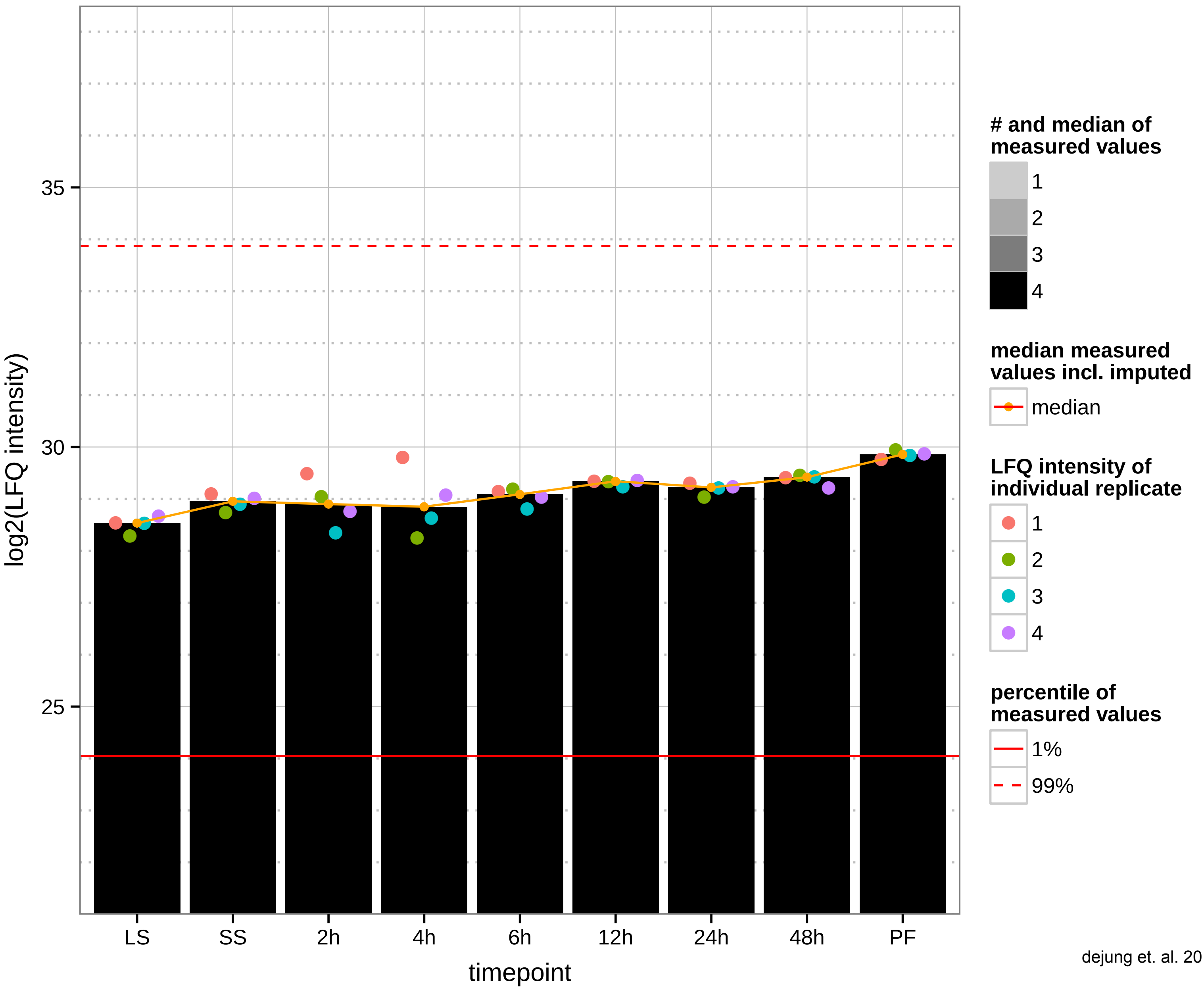

dejung et. al. 2015

structural maintenance of chromosome 3, putative (SMC3)
Tb927.5.3510
AGOF: ATP binding
AGOC: chromosome
AGOP: chromosome organization
PGOF: ATP binding, protein binding
PGOC: chromosome
PGOP: chromosome organization

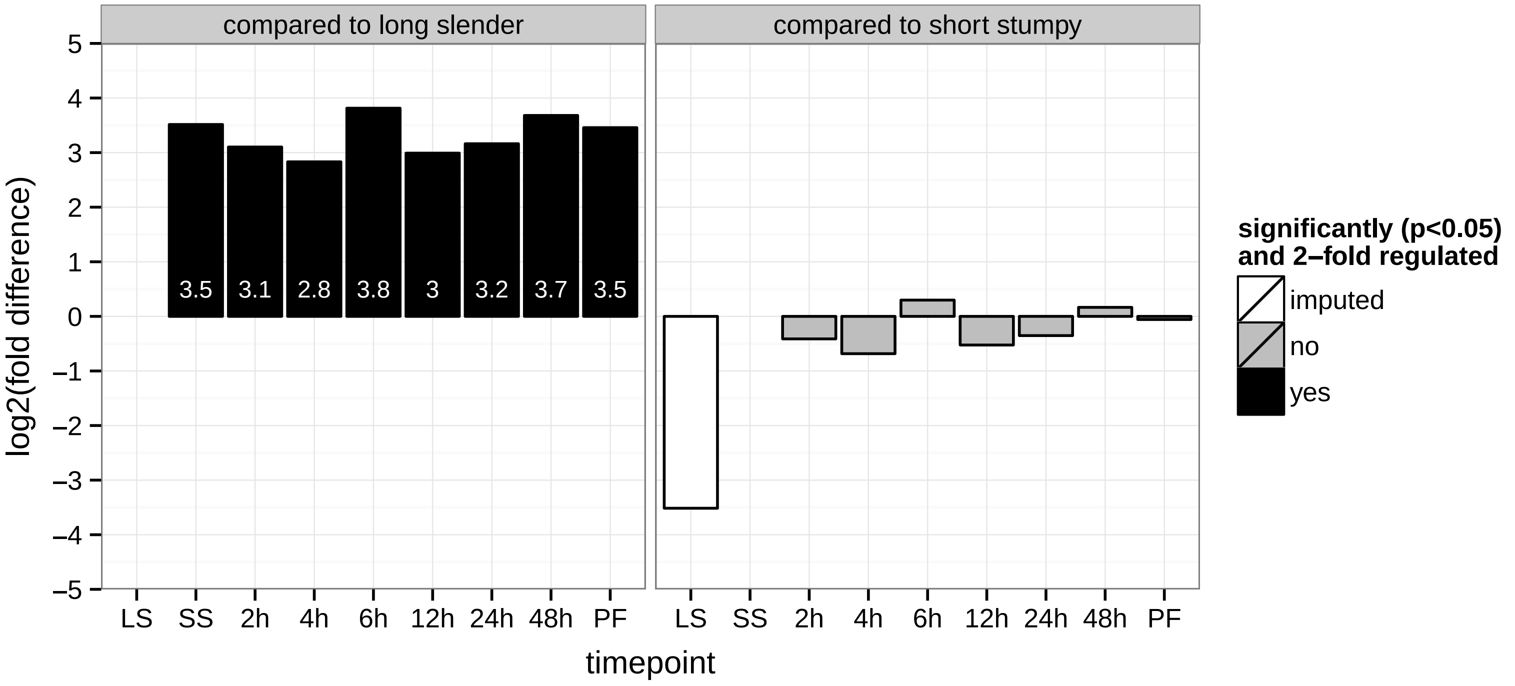

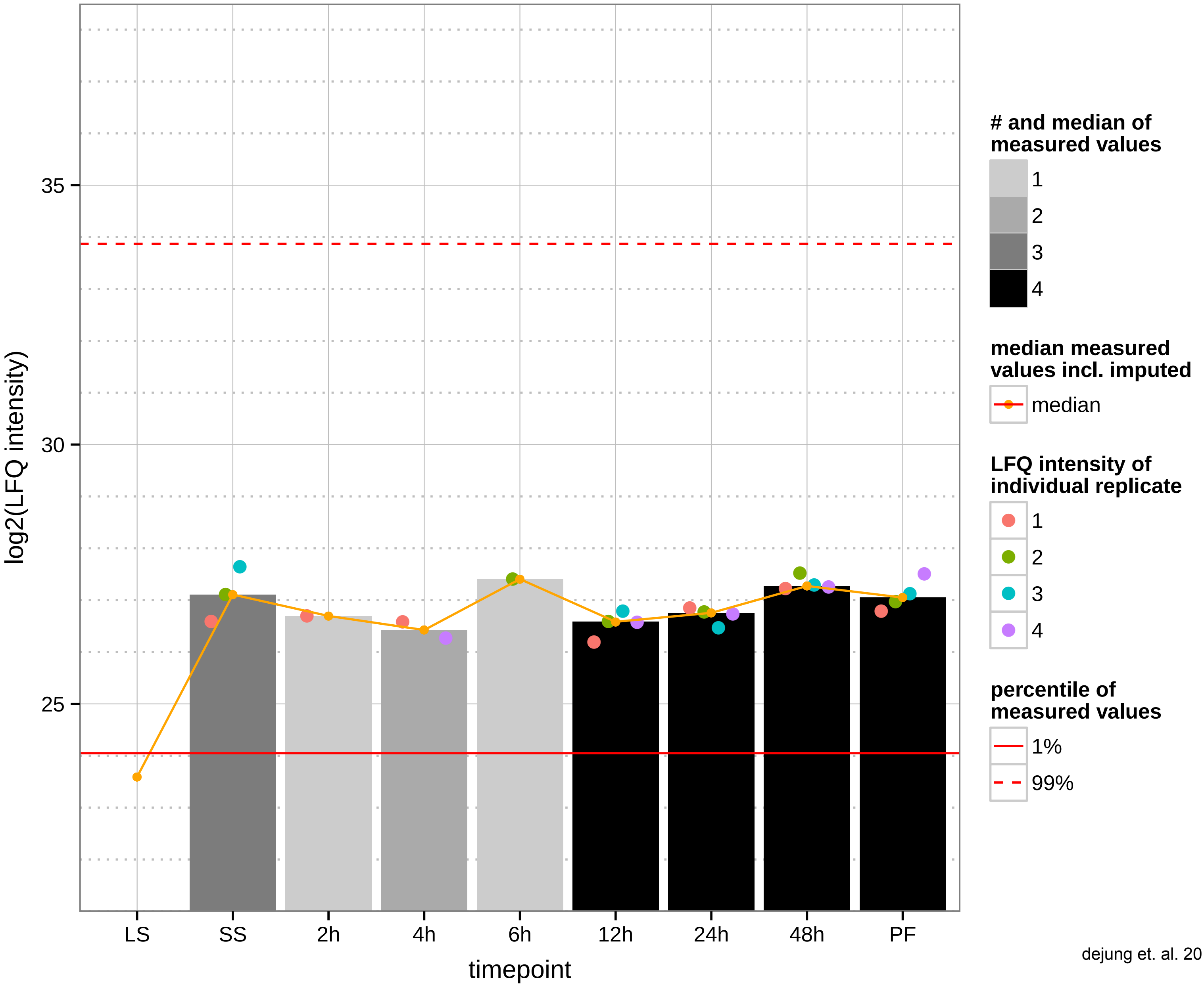

dejung et. al. 2015

adaptor complex protein (AP) 3 delta subunit 1, putative
Tb927.5.3610
AGOF: protein transporter activity
AGOC: Golgi apparatus, membrane coat
AGOP: intracellular protein transport, vesicle–mediated transport
PGOF: binding, protein transporter activity
PGOC: Golgi apparatus, membrane coat
PGOP: intracellular protein transport, protein transport, vesicle–mediated transport

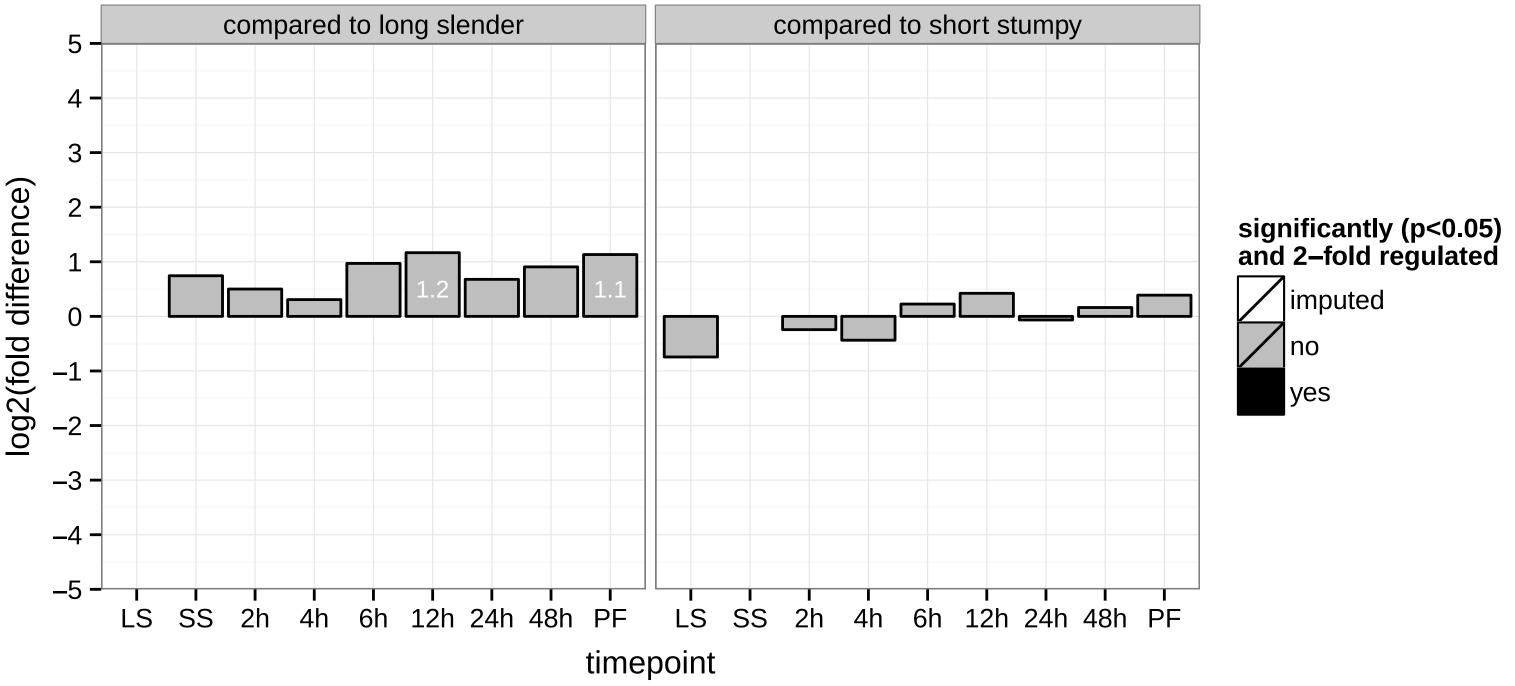

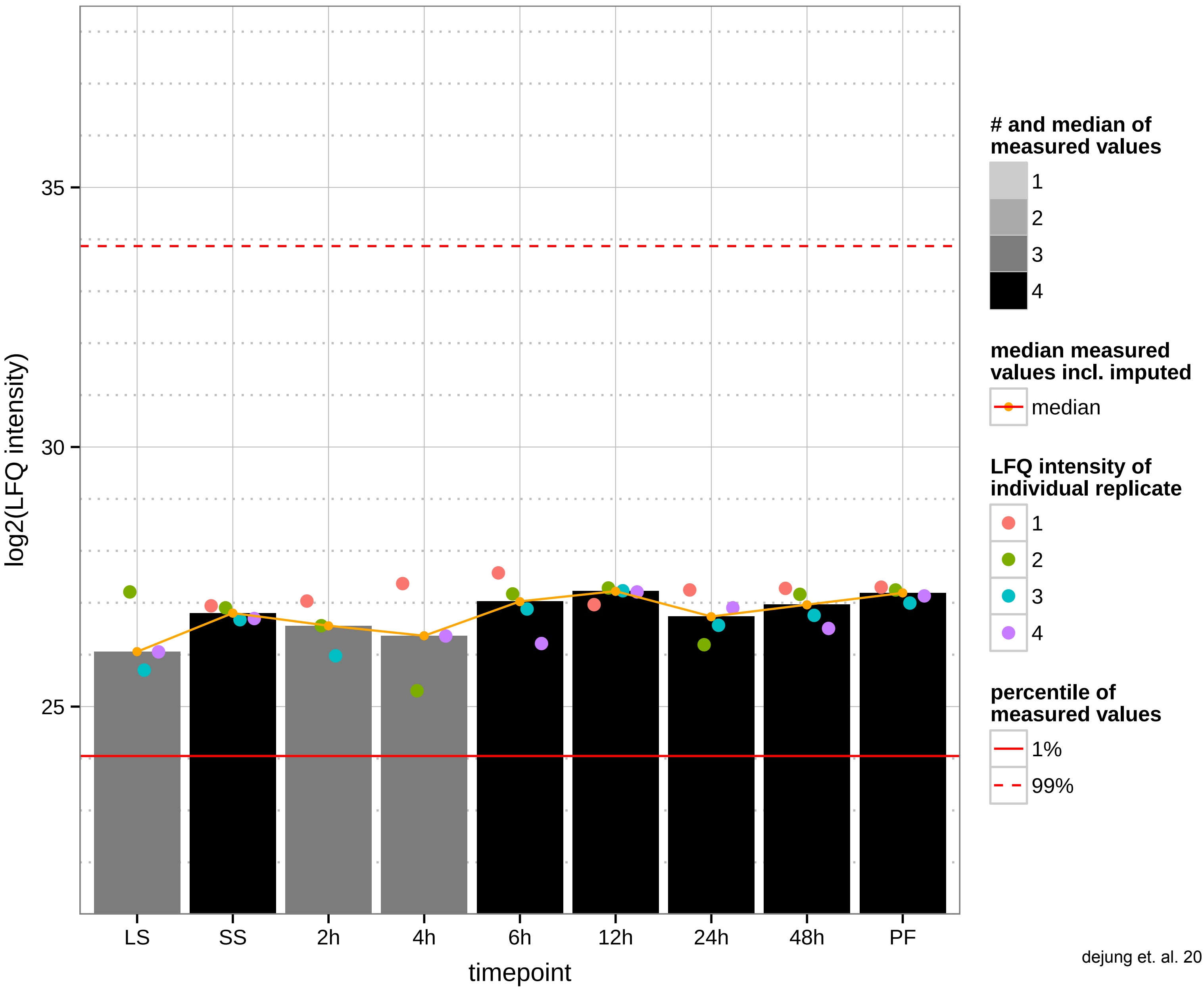

dejung et. al. 2015

hypothetical protein, conserved
Tb927.5.3640
AGOF: null
AGOC: mitochondrion
AGOP: null
PGOF: null
PGOC: null
PGOP: null

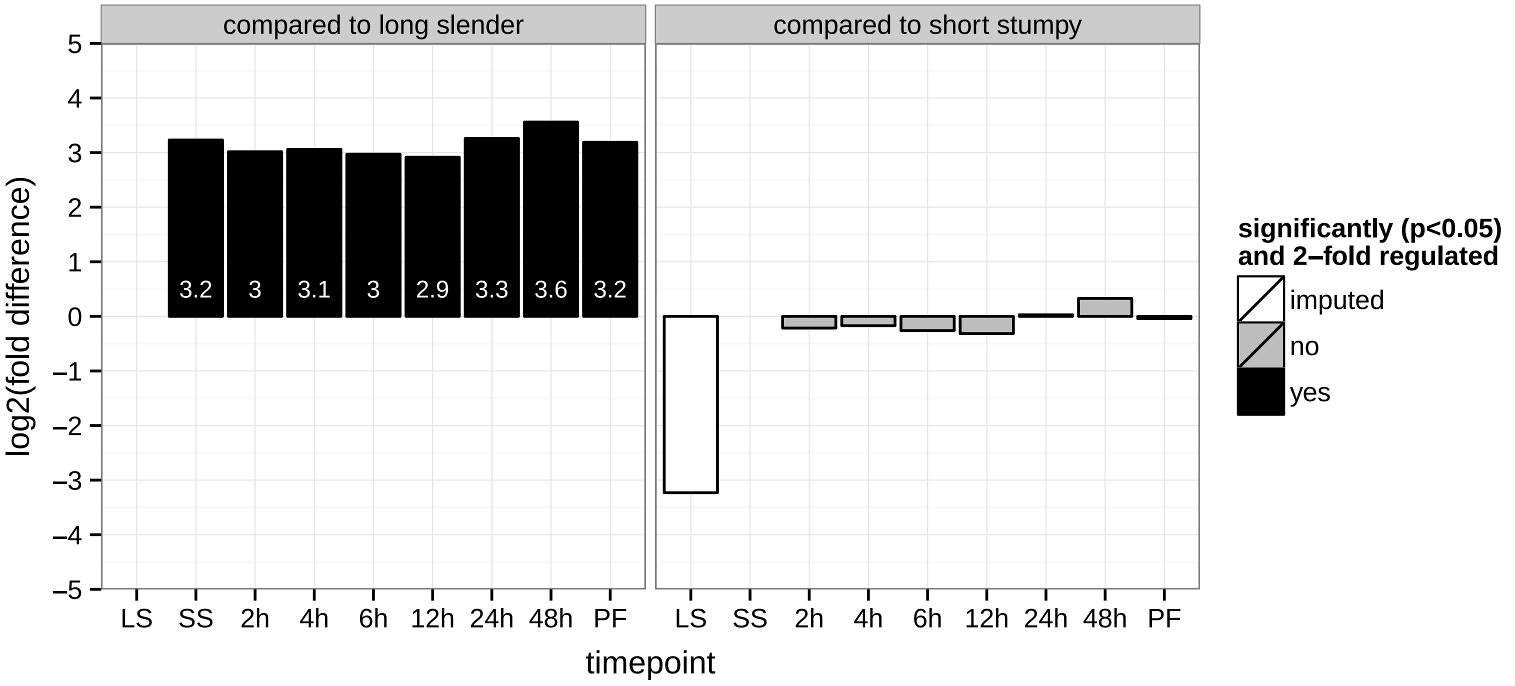

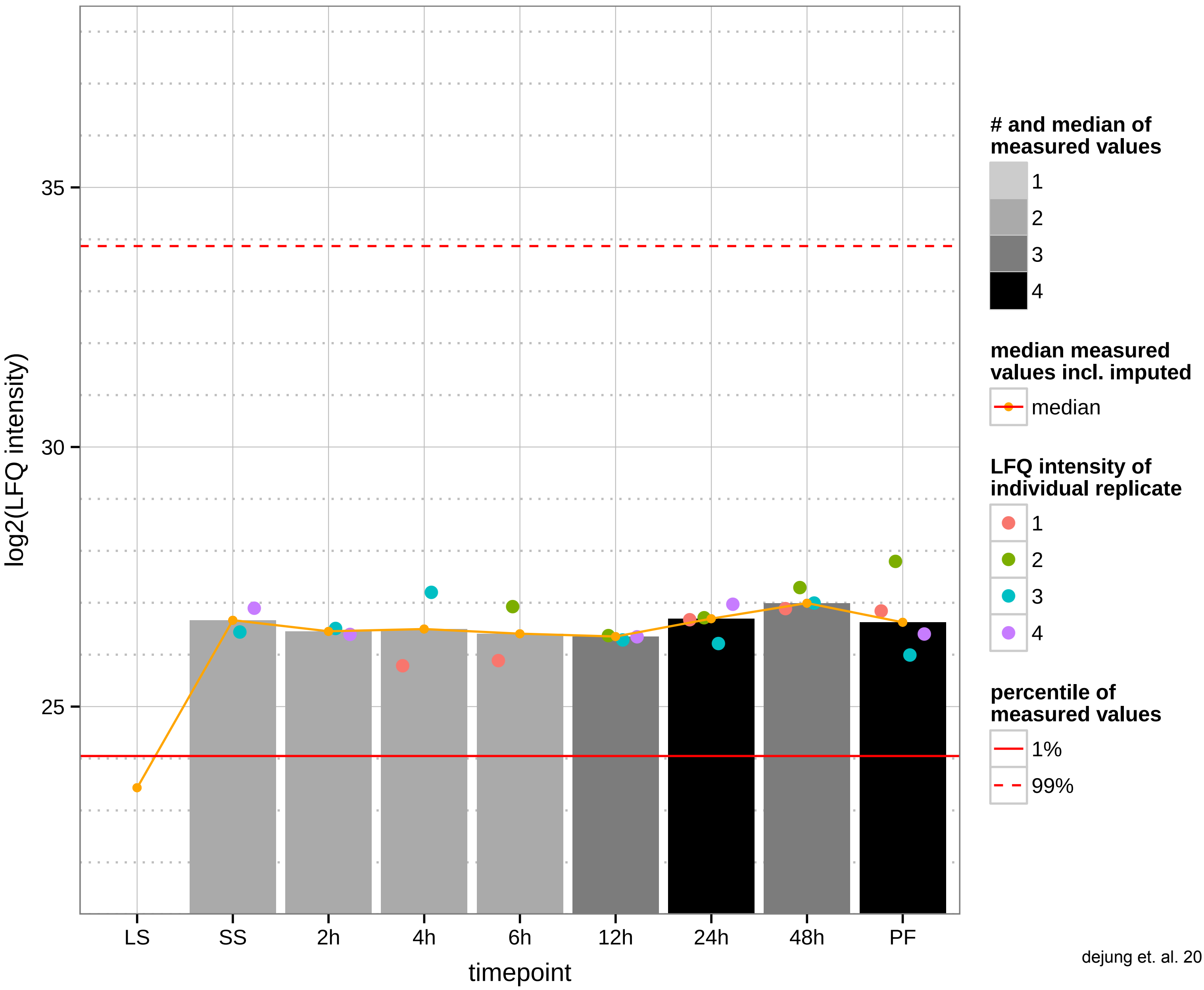

dejung et. al. 2015

hypothetical protein, conserved
Tb927.5.3650
AGOF: zinc ion binding
AGOC: null
AGOP: null
PGOF: null
PGOC: null
PGOP: null

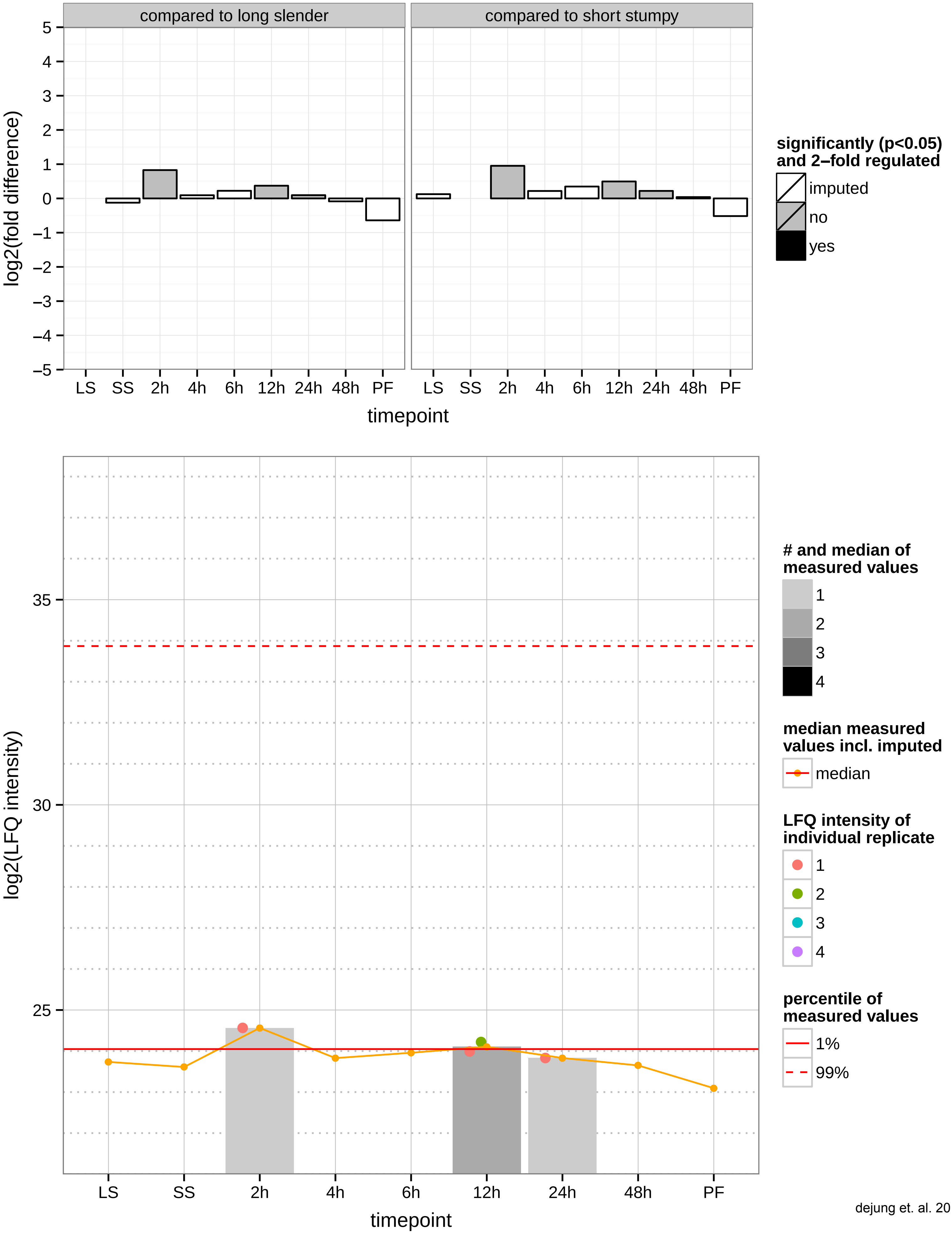

dejung et. al. 2015

essential neutral sphingomyelinase (TbnSMase)
Tb927.5.3710
AGOF: sphingomyelin phosphodiesterase activity
AGOC: endoplasmic reticulum
AGOP: ceramide biosynthetic process, sphingomyelin catabolic process
PGOF: null
PGOC: null
PGOP: null

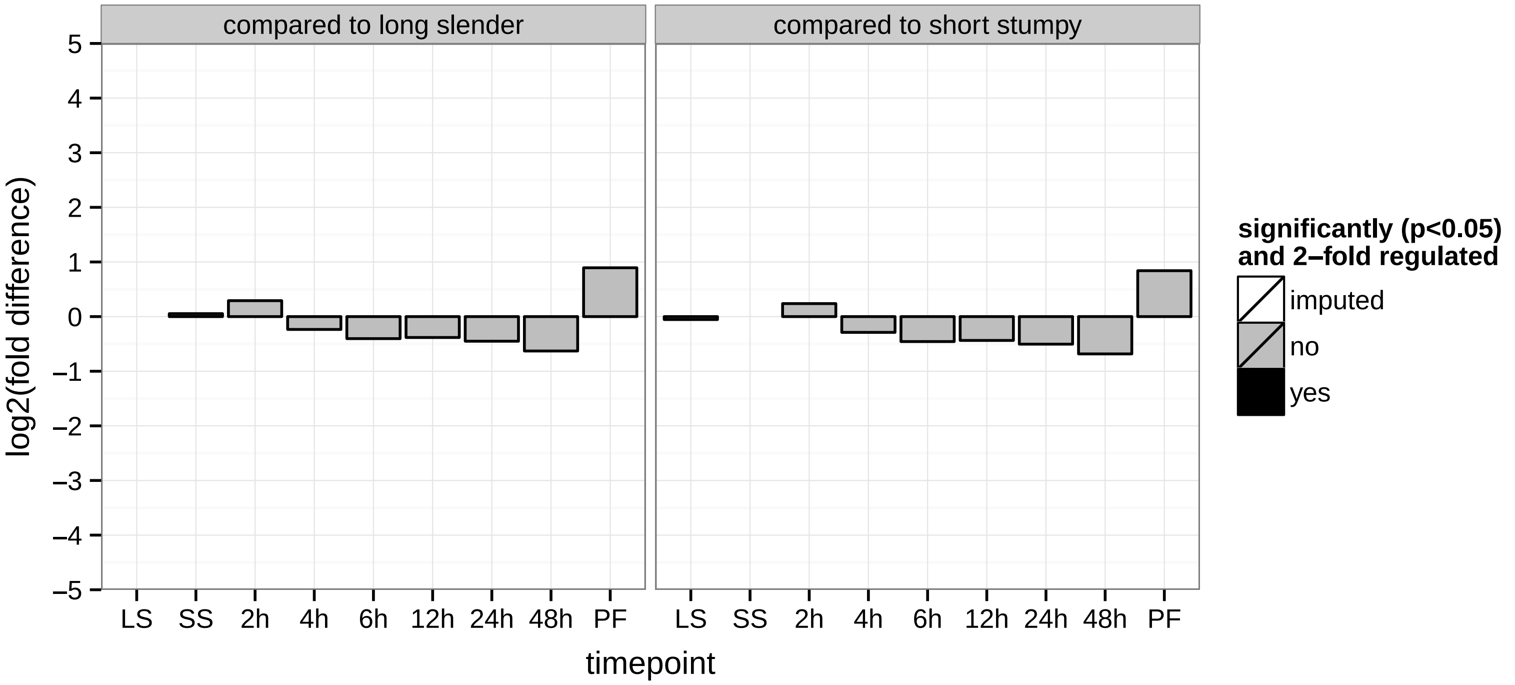

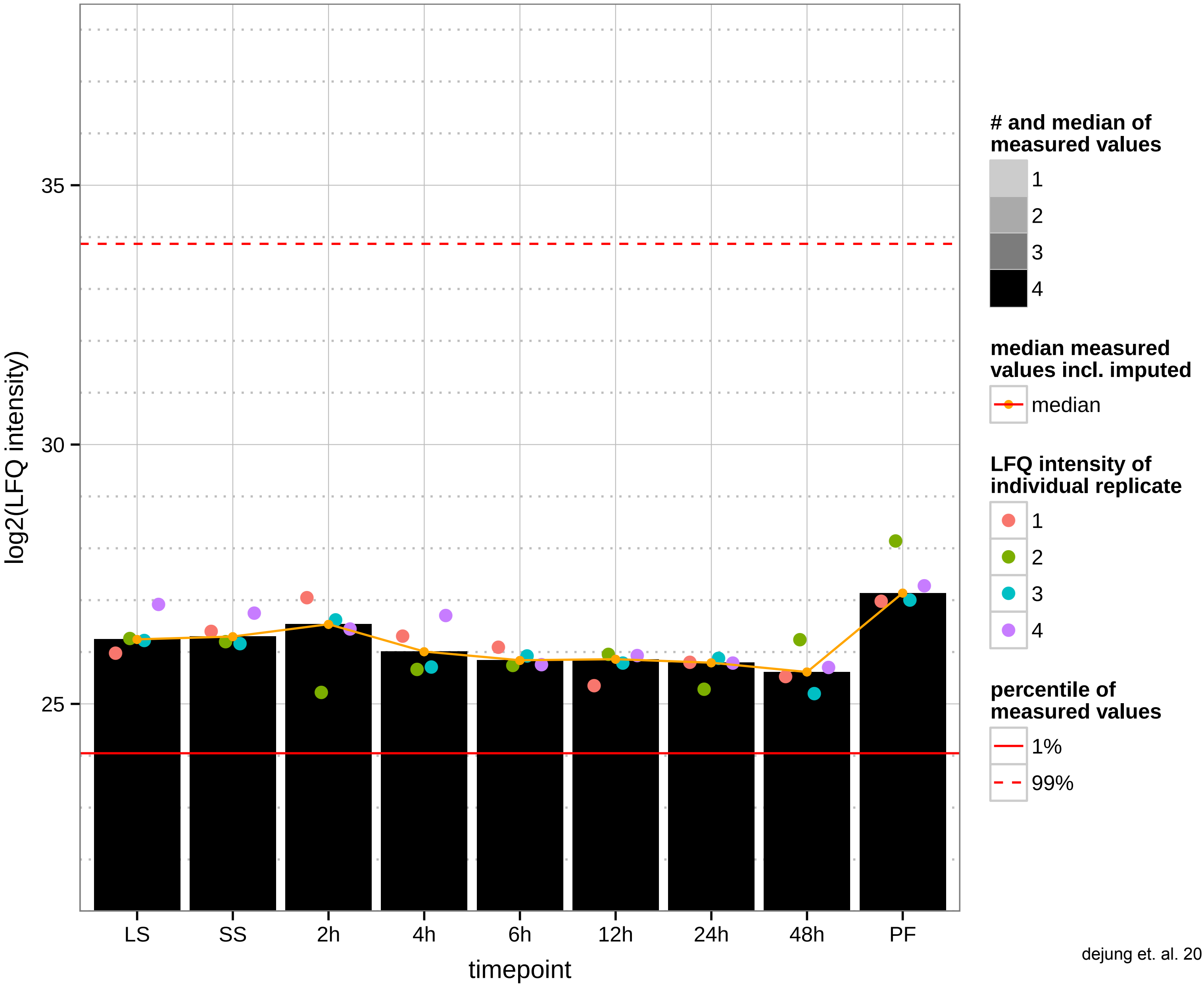

dejung et. al. 2015

orotidine−5−phosphate decarboxylase/orotate phosphoribosyltransferase, putative, OMPDCase−OPRTase
Tb927.5.3810
AGOF: orotate phosphoribosyltransferase activity, orotidine−5'−phosphate decarboxylase activity
AGOC: glycosome
AGOP: 'de novo' pyrimidine nucleobase biosynthetic process, nucleoside metabolic process, pyrimidine ribonucleotide biosynt
PGOF: catalytic activity, orotate phosphoribosyltransferase activity, orotidine−5'−phosphate decarboxylase activity
PGOC: null
PGOP: 'de novo' pyrimidine nucleobase biosynthetic process, metabolic process, nucleoside metabolic process, pyrimidine nu

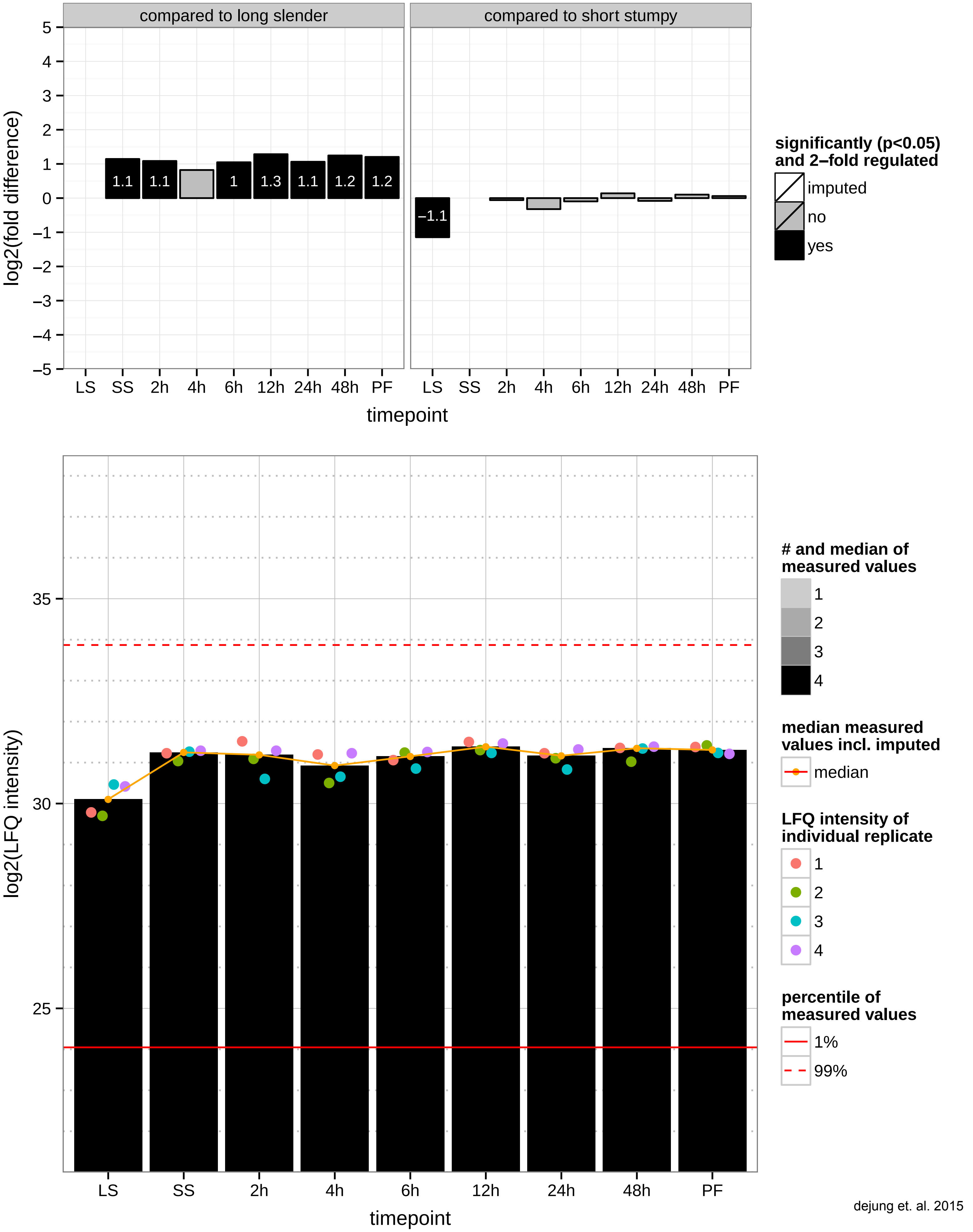

dejung et. al. 2015

aspartate carbamoyltransferase, putative
Tb927.5.3820
AGOF: amino acid binding, aspartate carbamoyltransferase activity
AGOC: null
AGOP: 'de novo' pyrimidine nucleobase biosynthetic process, cellular amino acid metabolic process
PGOF: amino acid binding, aspartate carbamoyltransferase activity, carboxyl– or carbamoyltransferase activity
PGOC: null
PGOP: 'de novo' pyrimidine nucleobase biosynthetic process, cellular amino acid metabolic process

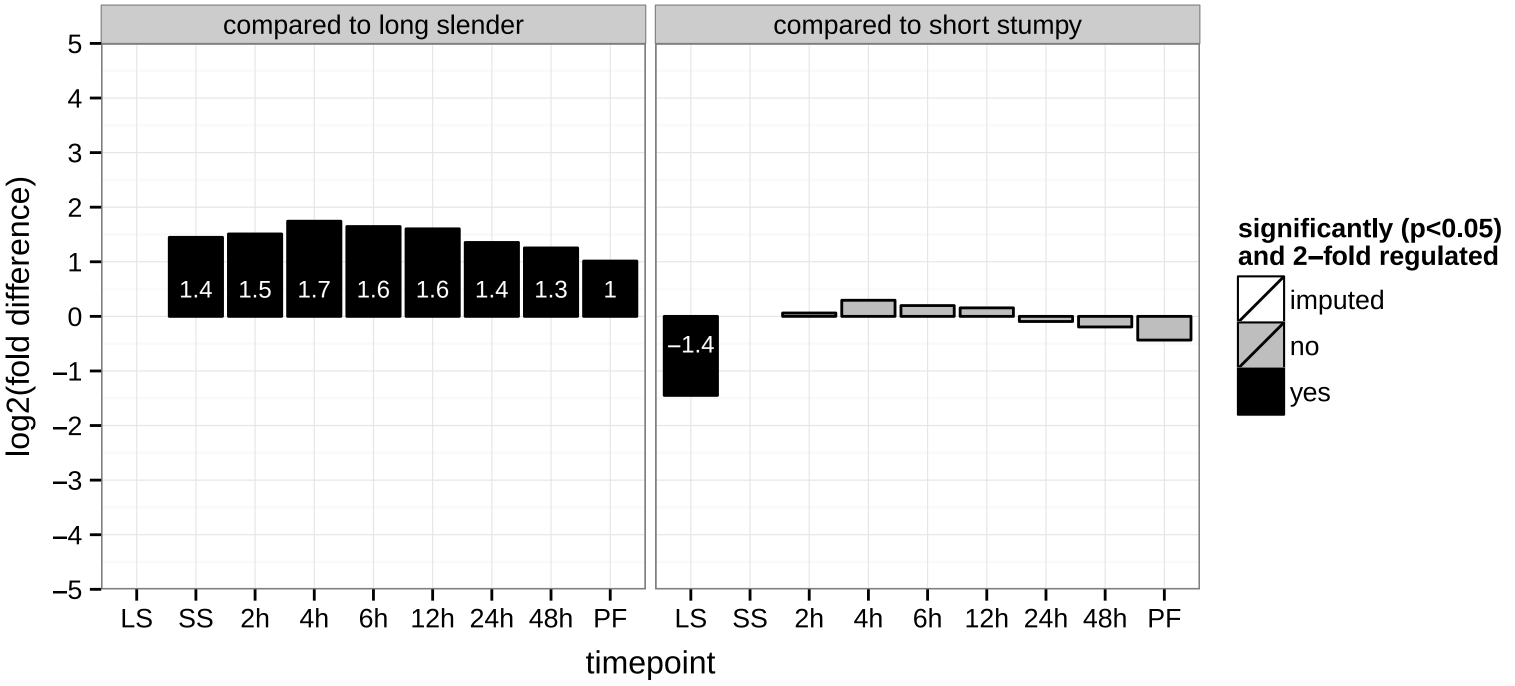

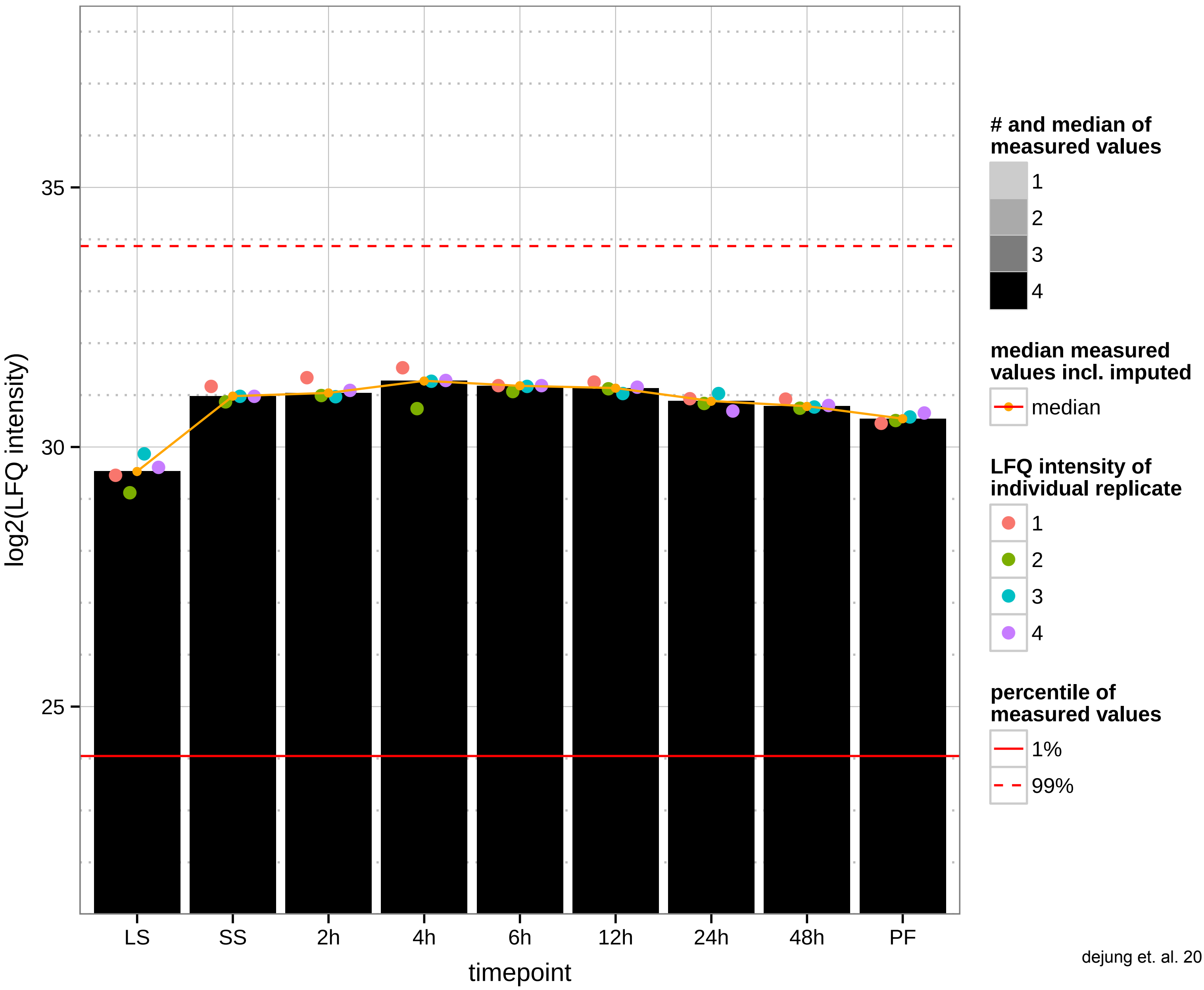

dejung et. al. 2015

dihydroorotate oxidase
Tb927.5.3830
AGOF: FMN binding, dihydroorotate dehydrogenase activity, dihydroorotate oxidase activity
AGOC: cytoplasm, glycosome
AGOP: 'de novo' pyrimidine nucleobase biosynthetic process, UMP biosynthetic process, fumarate metabolic process
PGOF: dihydroorotate dehydrogenase activity, dihydroorotate oxidase activity
PGOC: cytoplasm
PGOP: UMP biosynthetic process, oxidation–reduction process

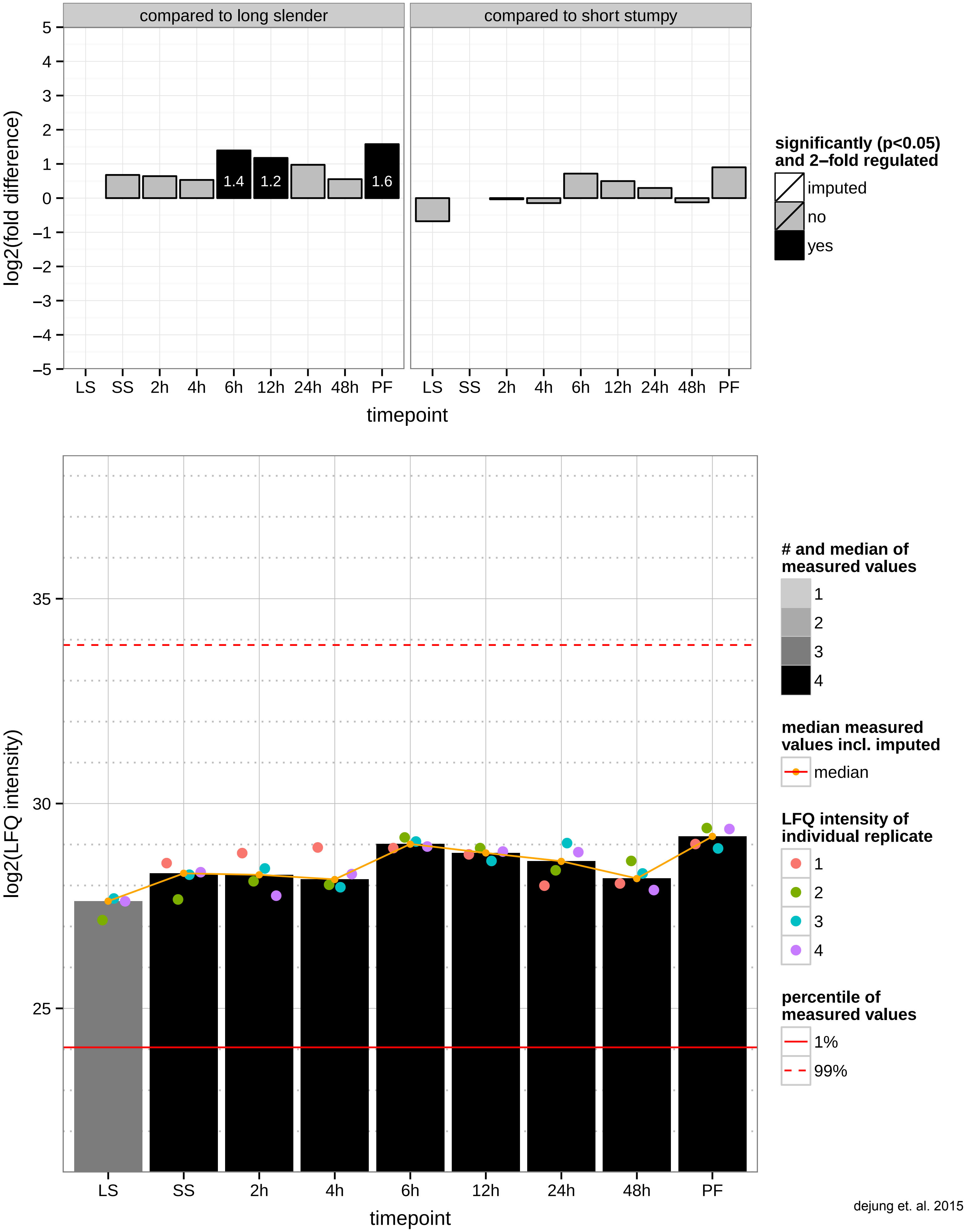

dejung et. al. 2015

hypothetical protein, conserved
Tb927.5.3850
AGOF: null
AGOC: null
AGOP: null
PGOF: exonuclease activity, nucleic acid binding
PGOC: intracellular
PGOP: null

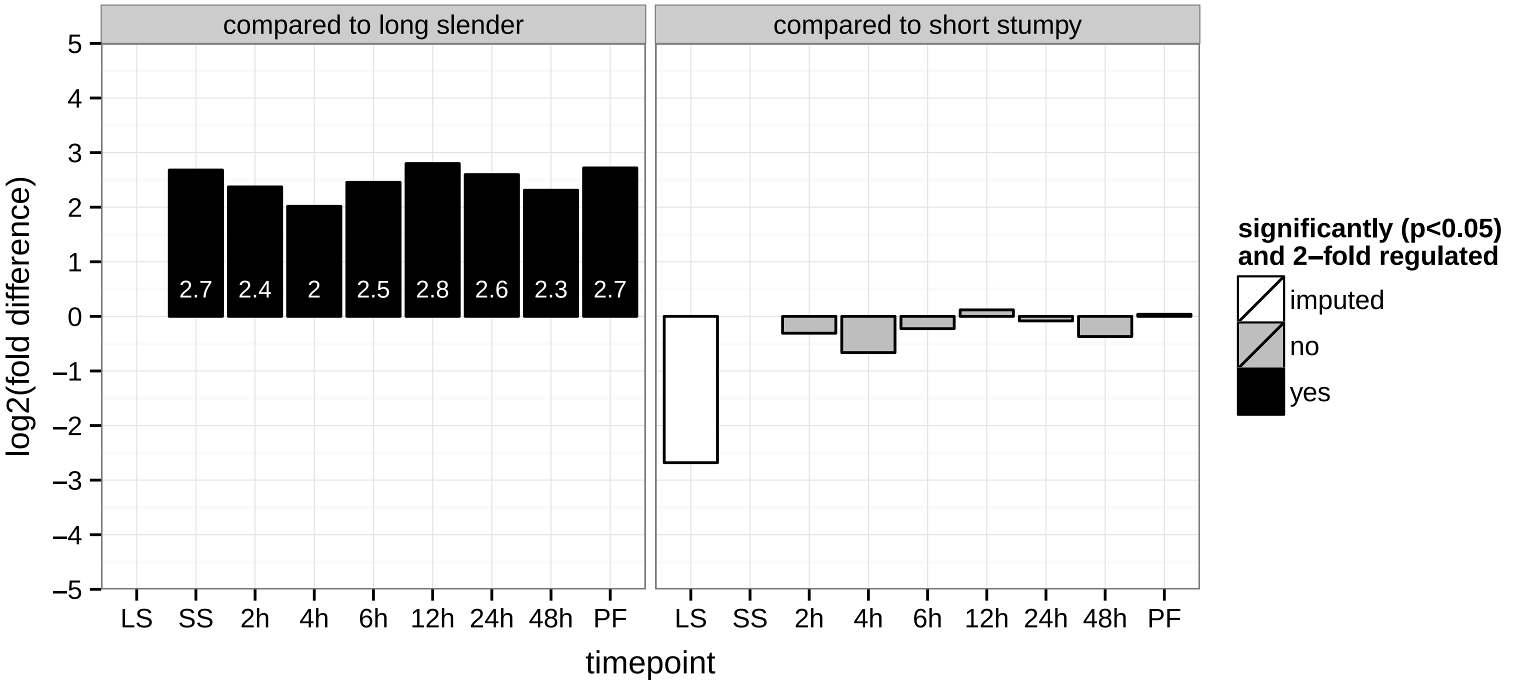

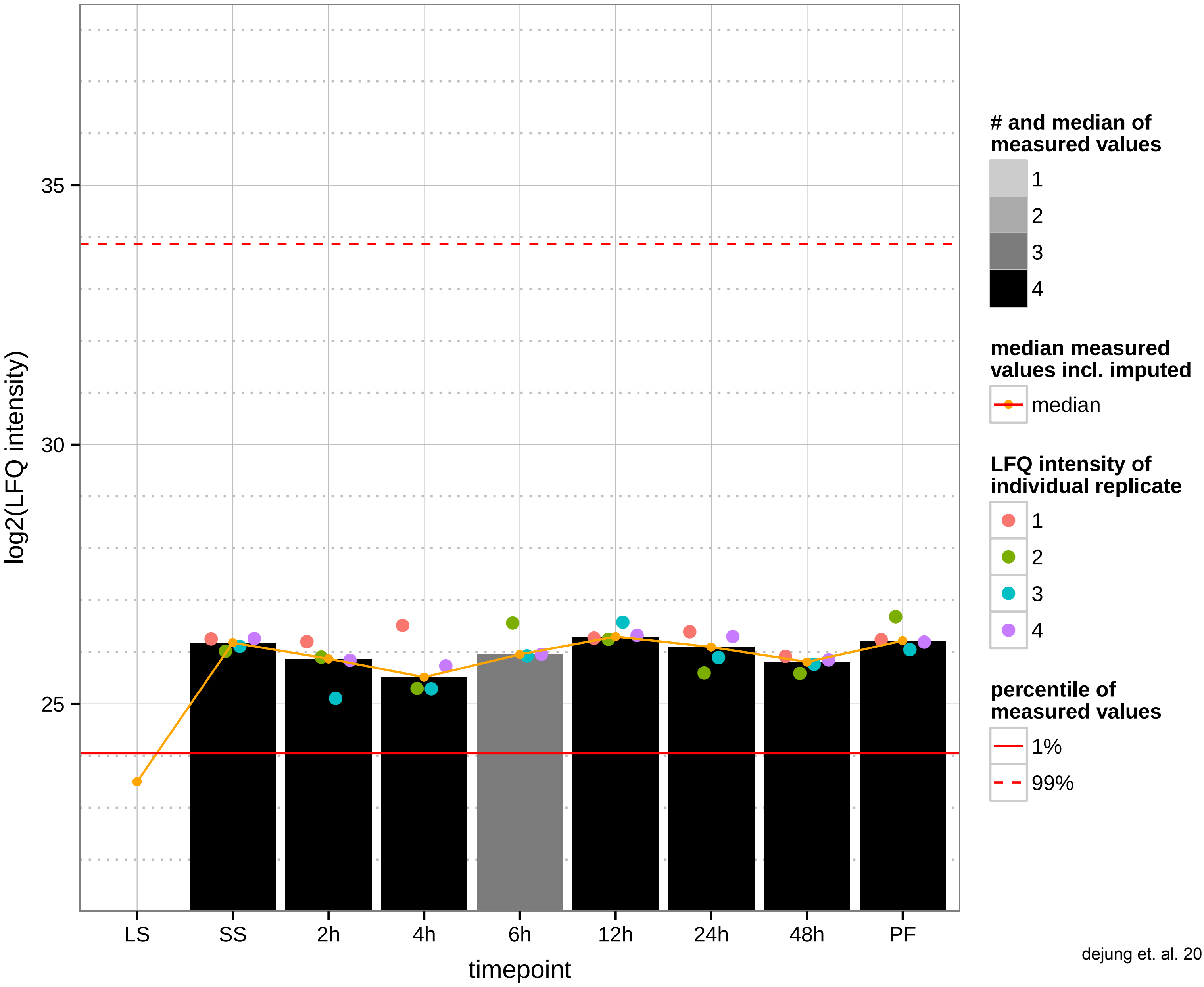

dejung et. al. 2015

hypothetical protein, conserved
Tb927.5.3900
AGOF: null
AGOC: null
AGOP: null
PGOF: null
PGOC: null
PGOP: null

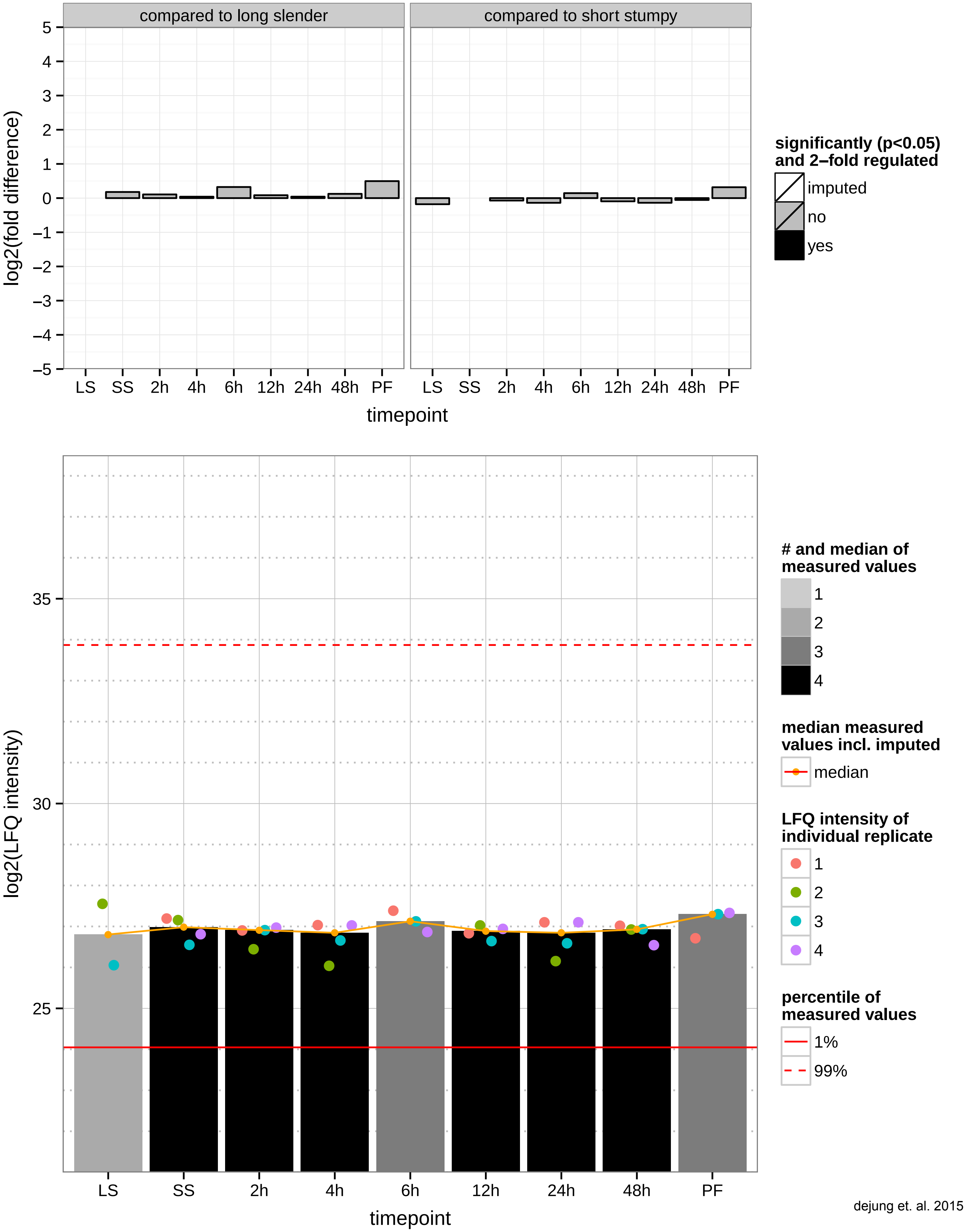

peroxisome assembly protein, putative
Tb927.5.3920
AGOF: ATP binding, ATPase activity
AGOC: null
AGOP: peroxisome organization
PGOF: ATP binding, nucleoside−triphosphatase activity, nucleotide binding
PGOC: null
PGOP: null

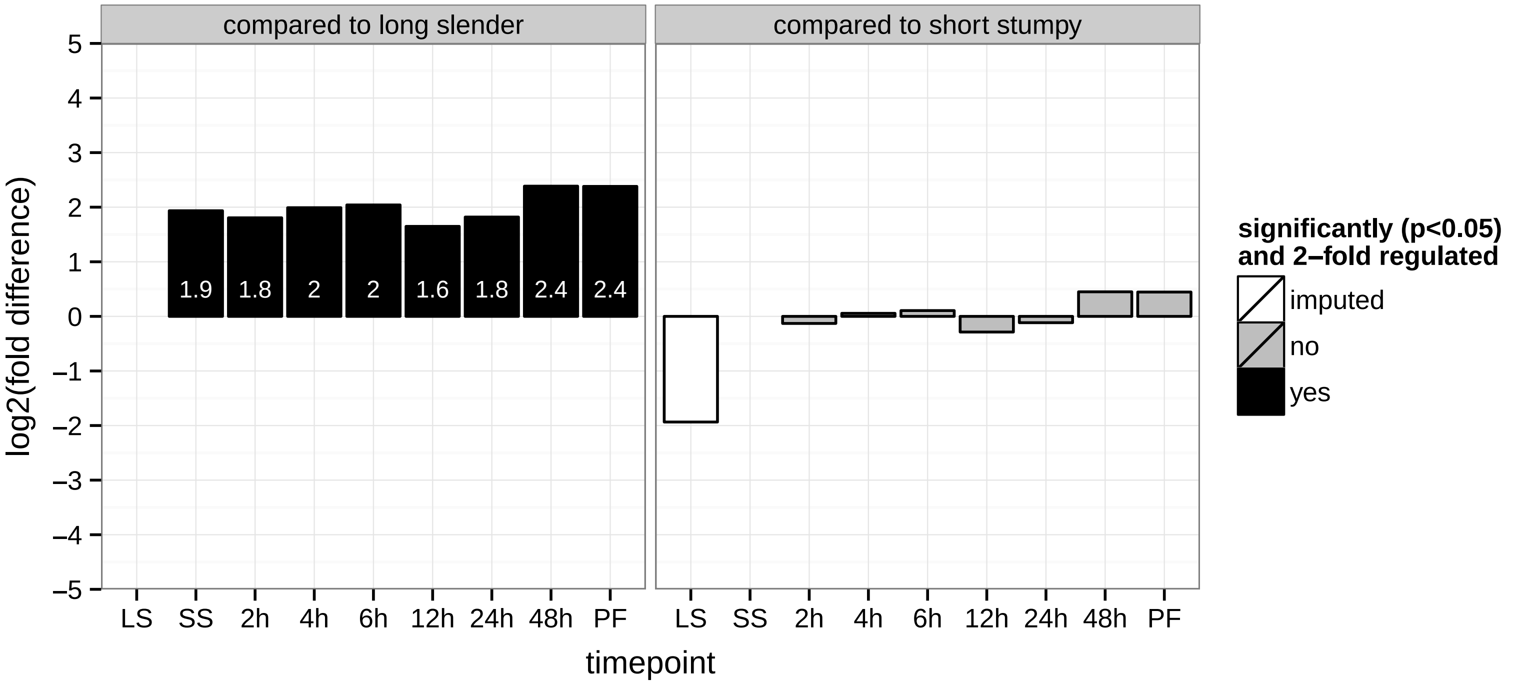

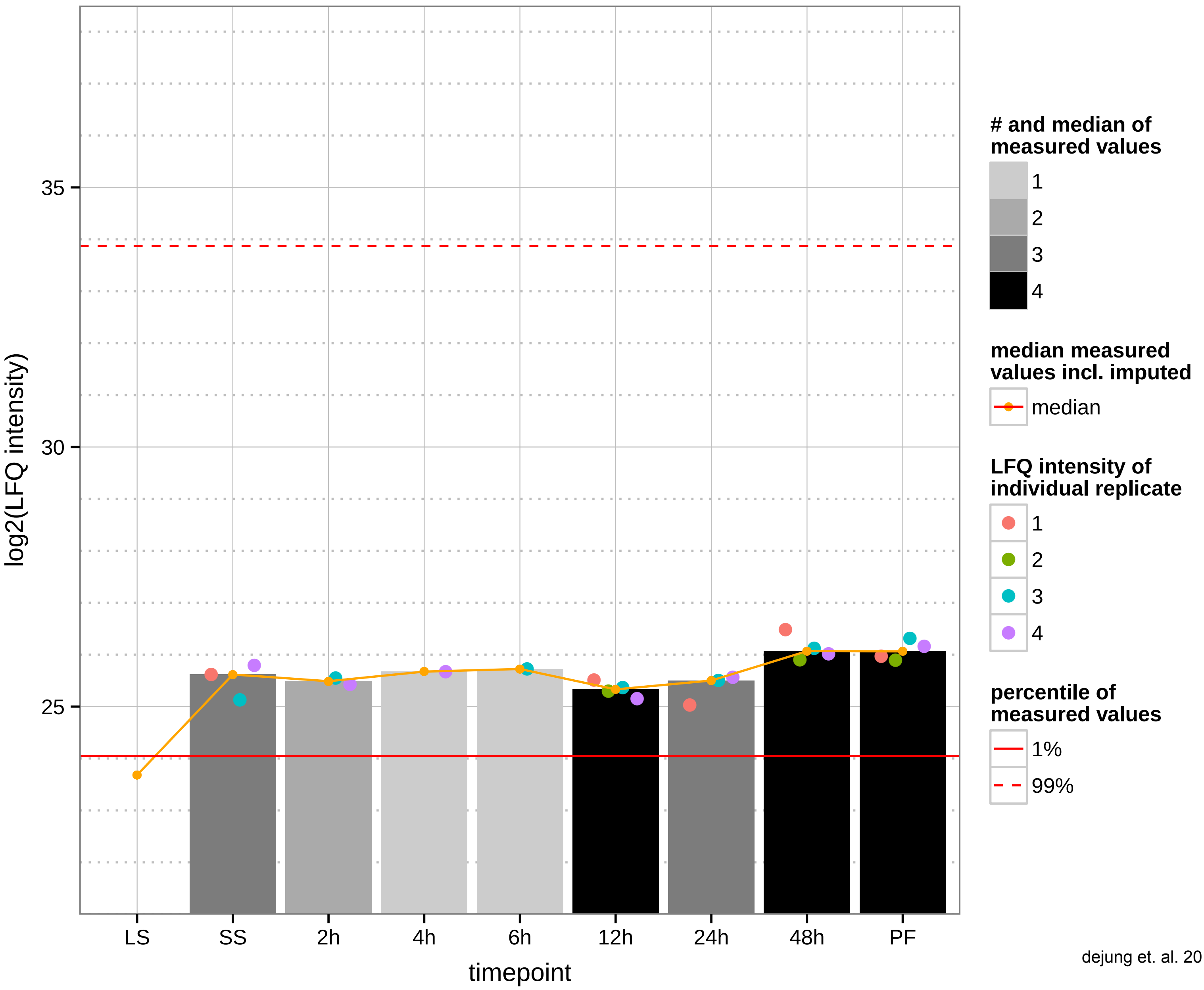

dejung et. al. 2015

helicase, putative
Tb927.5.3940
AGOF: ATP binding, ATP–dependent helicase activity, helicase activity, nucleic acid binding
AGOC: null
AGOP: null
PGOF: ATP binding, ATP–dependent helicase activity, helicase activity, nucleic acid binding
PGOC: null
PGOP: null

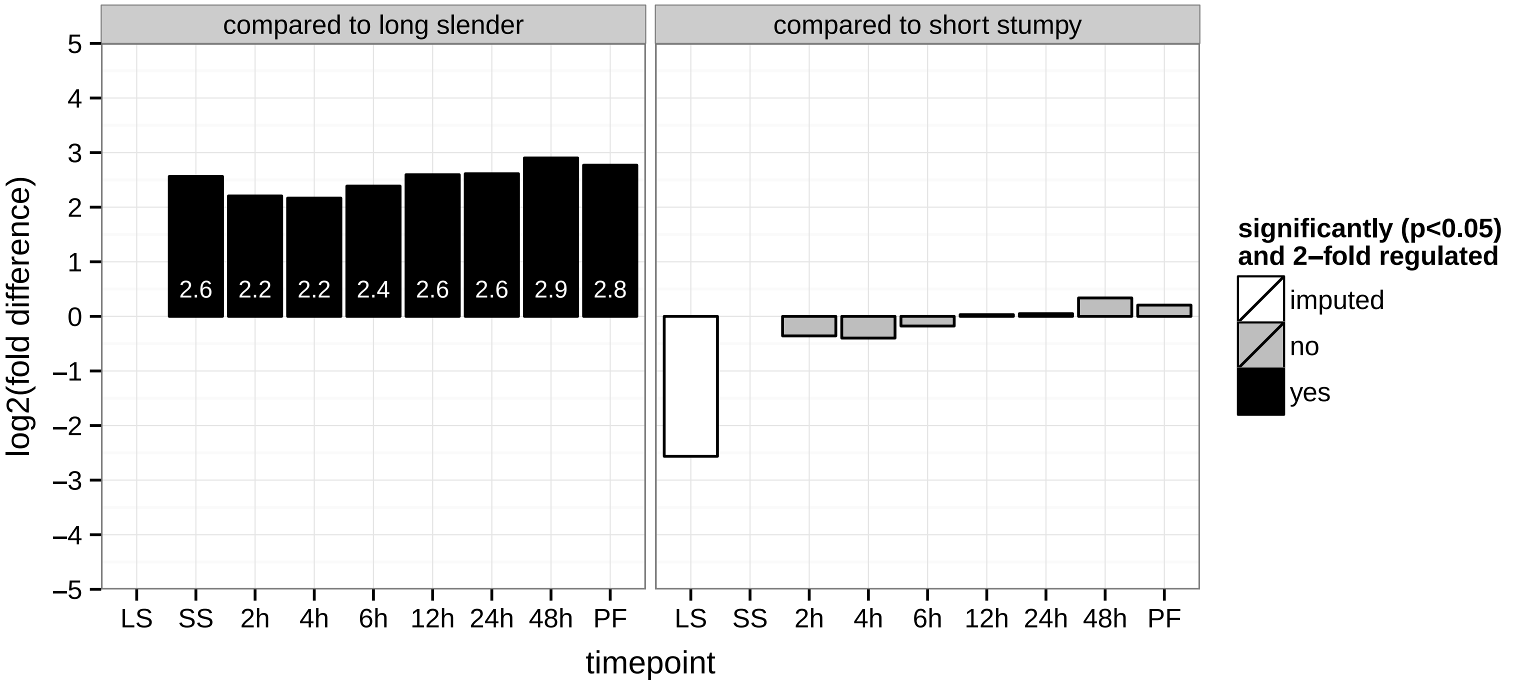

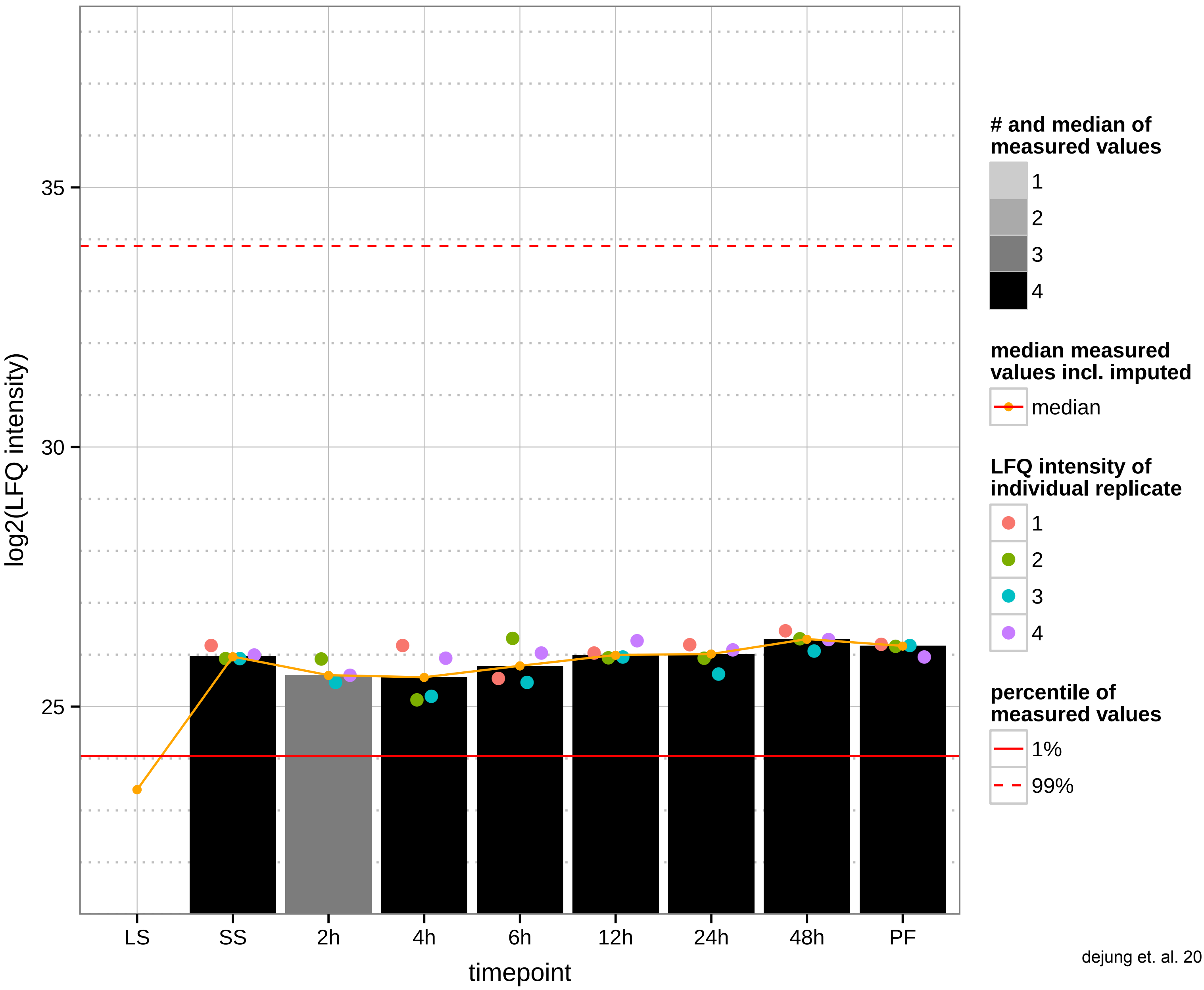

dejung et. al. 2015

adenylate kinase, putative (ADKE)
Tb927.5.3970
AGOF: ATP binding, adenylate kinase activity
AGOC: axoneme
AGOP: nucleobase−containing compound metabolic process, nucleobase−containing small molecule interconversion
PGOF: ATP binding, cAMP−dependent protein kinase regulator activity, nucleobase−containing compound kinase activity
PGOC: null
PGOP: nucleobase−containing compound metabolic process, signal transduction

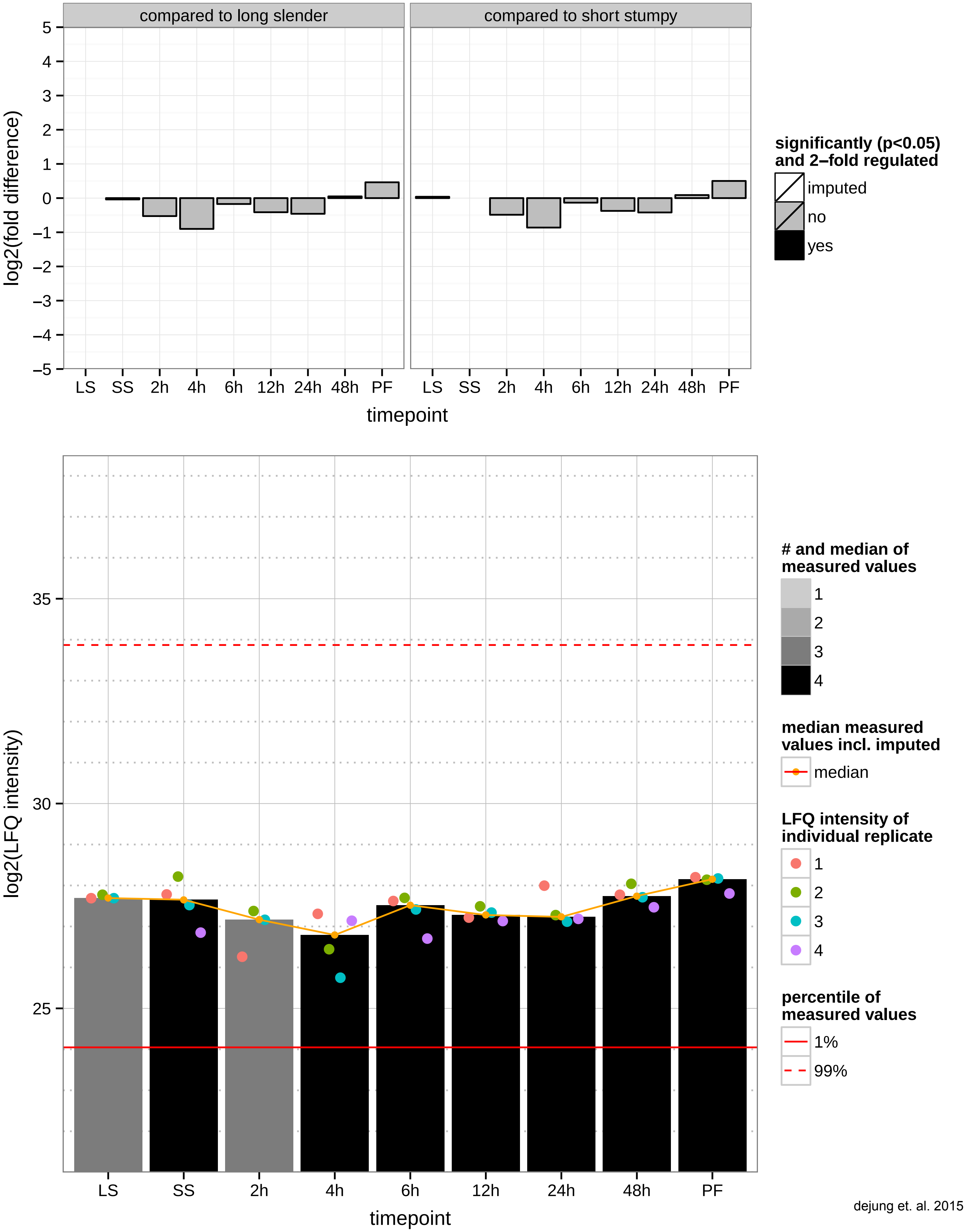

dejung et. al. 2015

hypothetical protein, conserved
Tb927.5.4100
AGOF: null
AGOC: null
AGOP: null
PGOF: protein binding
PGOC: null
PGOP: null

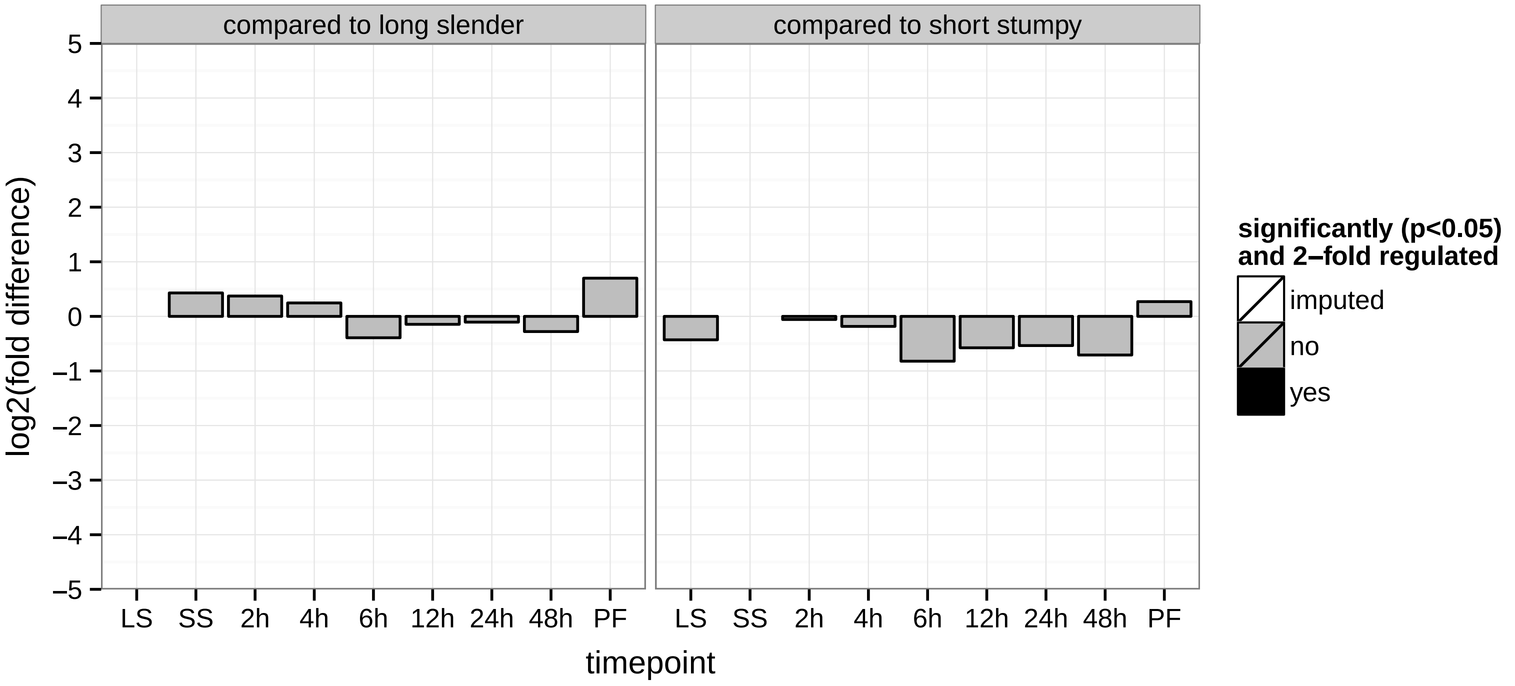

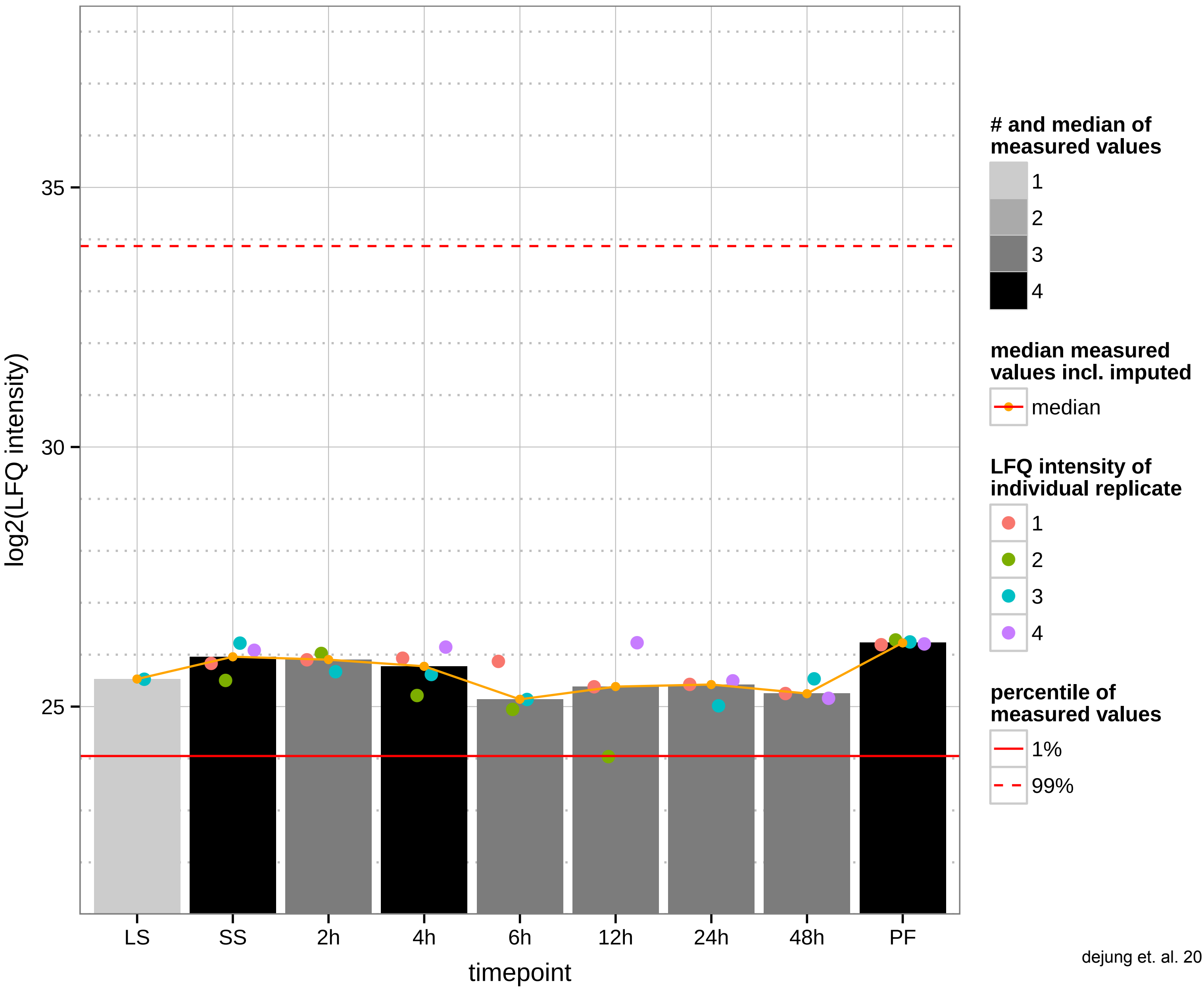

dejung et. al. 2015

hypothetical protein, conserved
Tb927.5.4150
AGOF: null
AGOC: null
AGOP: null
PGOF: null
PGOC: null
PGOP: null

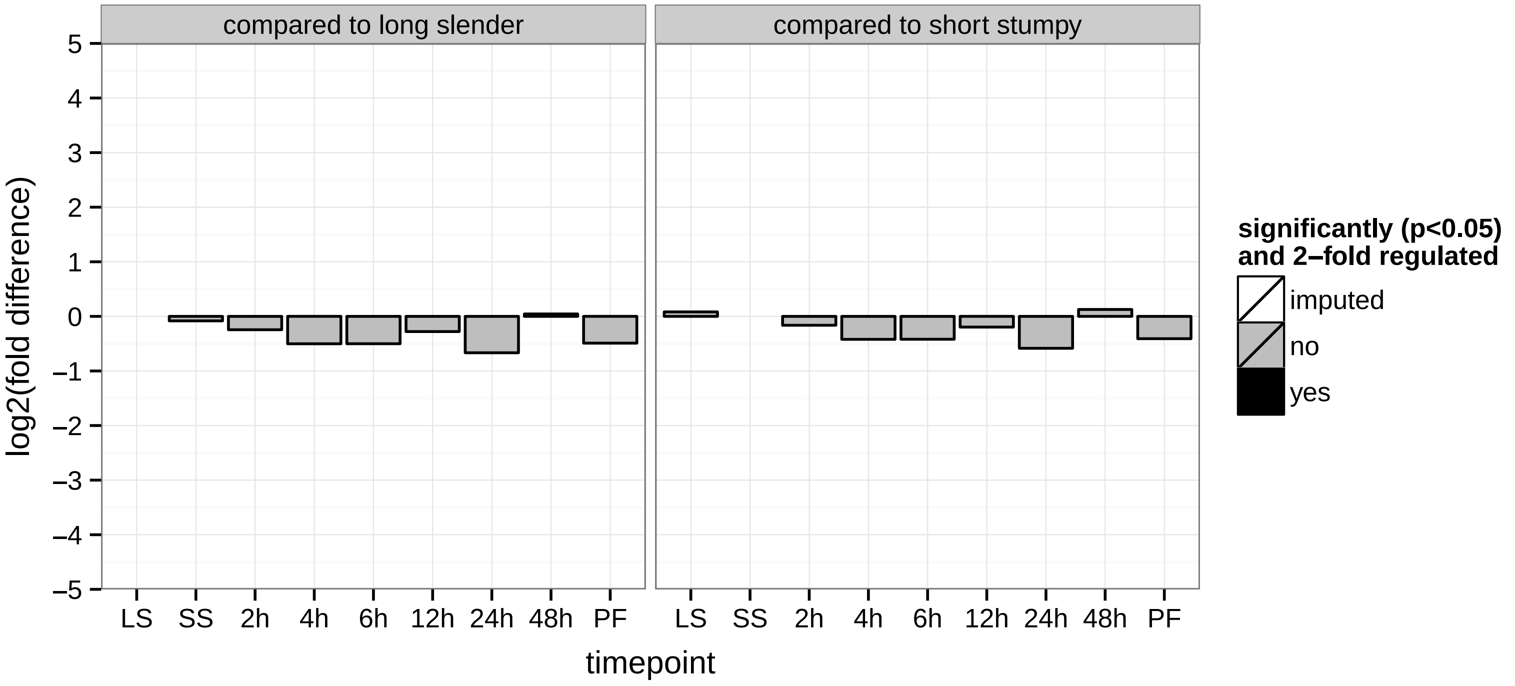

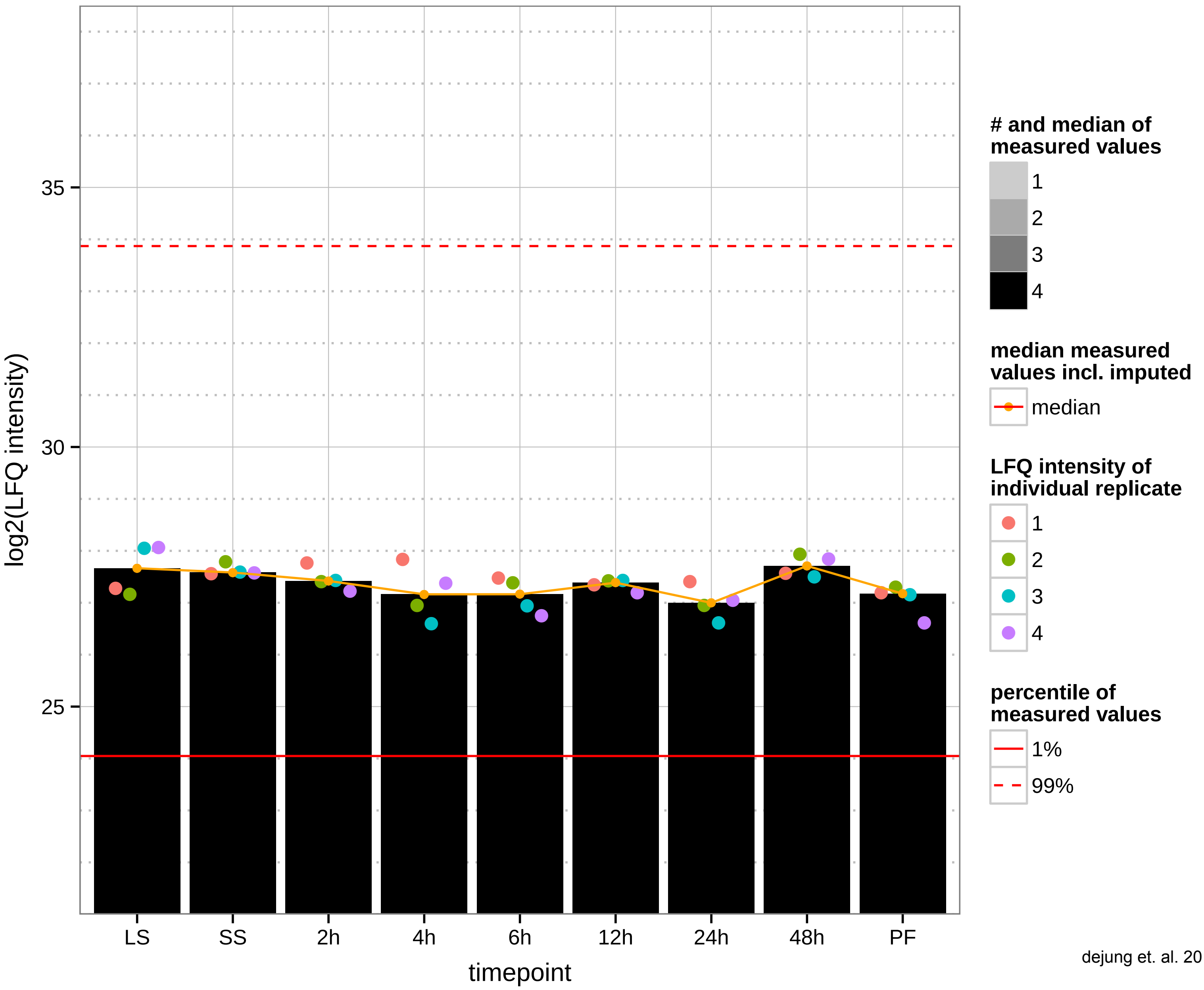

dejung et. al. 2015

histone H4, putative
Tb927.5.4260;Tb927.5.4250;Tb927.5.4240;Tb927.5.4230;Tb927.5.4220;Tb927.5.4210;Tb927.5.4200;Tb927.5.4190;Tb927.5
AGOF: DNA binding
AGOC: nucleosome, nucleus
AGOP: nucleosome assembly
PGOF: null, DNA binding
PGOC: null, nucleosome, nucleus
PGOP: null, nucleosome assembly

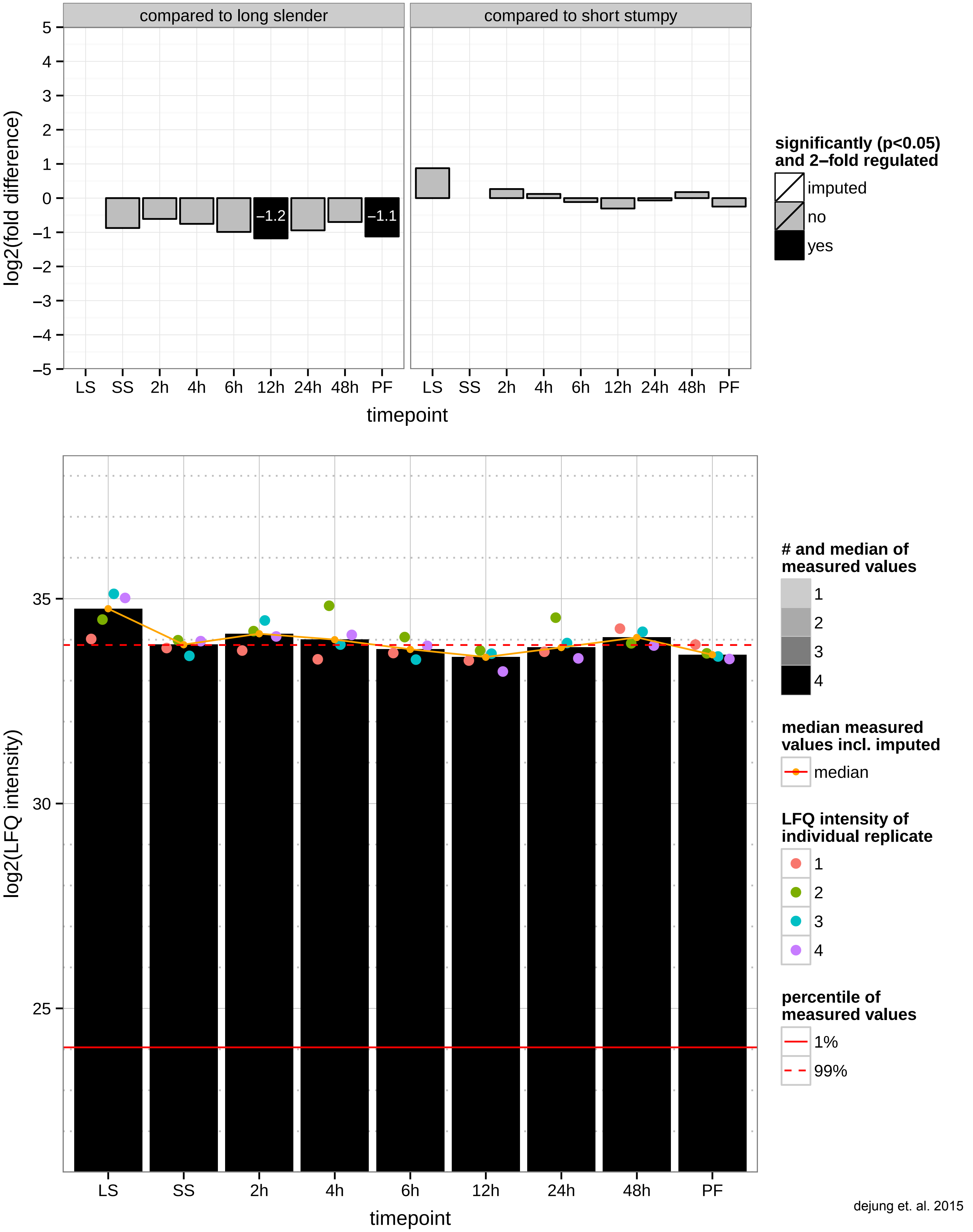

dejung et. al. 2015

ATP−dependent DEAD/H RNA helicase, putative
Tb927.5.4270
AGOF: ATP binding, ATP−dependent helicase activity, helicase activity, nucleic acid binding
AGOC: null
AGOP: nucleobase−containing compound metabolic process
PGOF: ATP binding, ATP−dependent helicase activity, helicase activity, nucleic acid binding
PGOC: null
PGOP: null

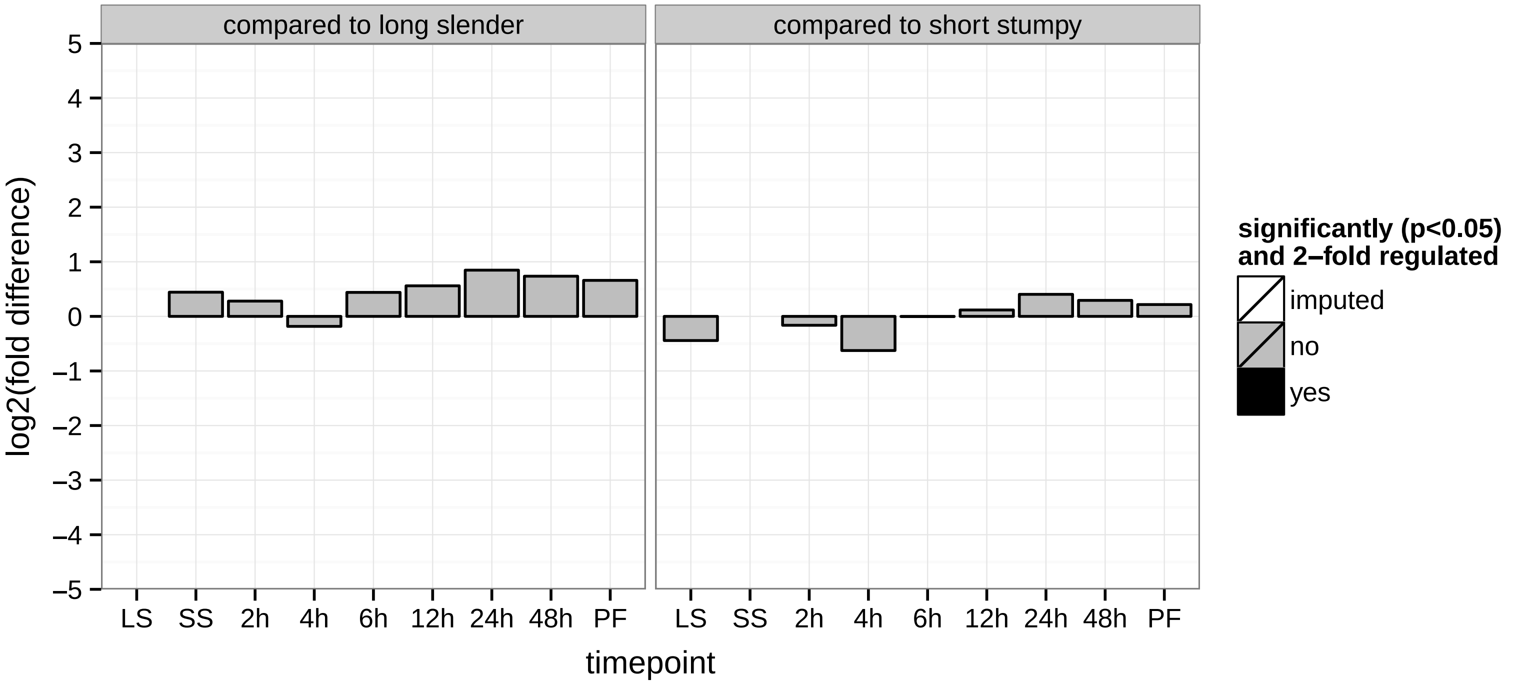

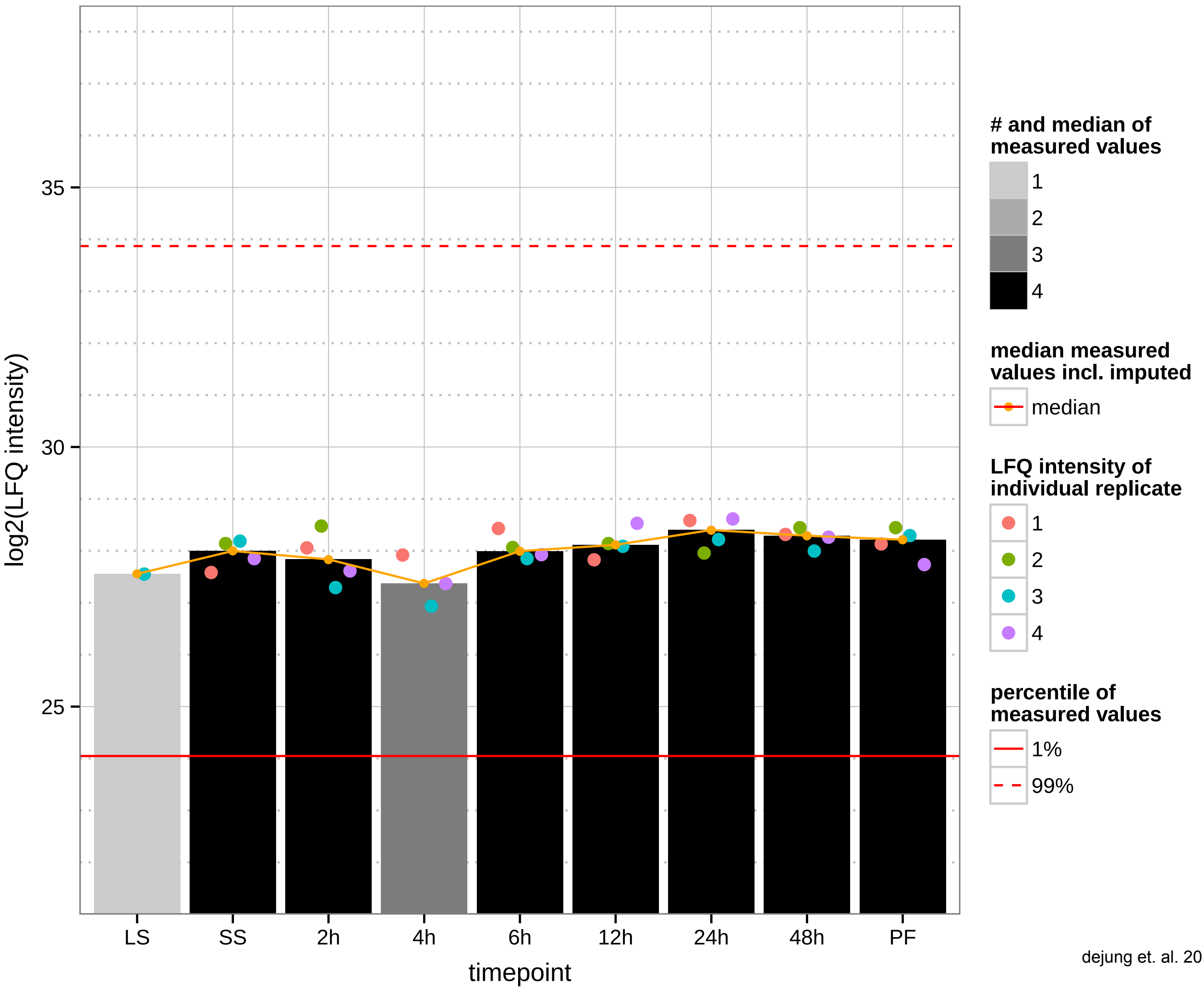

dejung et. al. 2015

NUDIX hydrolase, putative
Tb927.5.4350
AGOF: hydrolase activity
AGOC: null
AGOP: null
PGOF: hydrolase activity
PGOC: null
PGOP: null

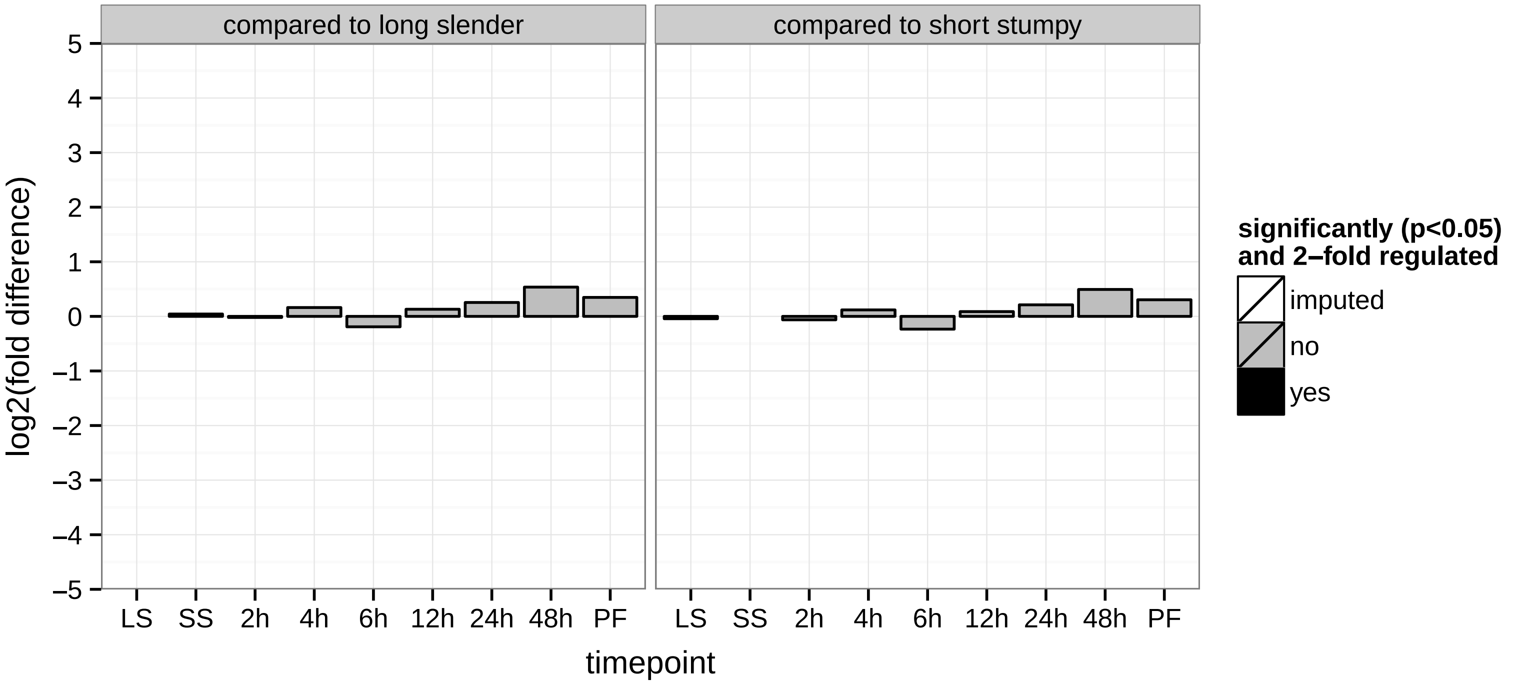

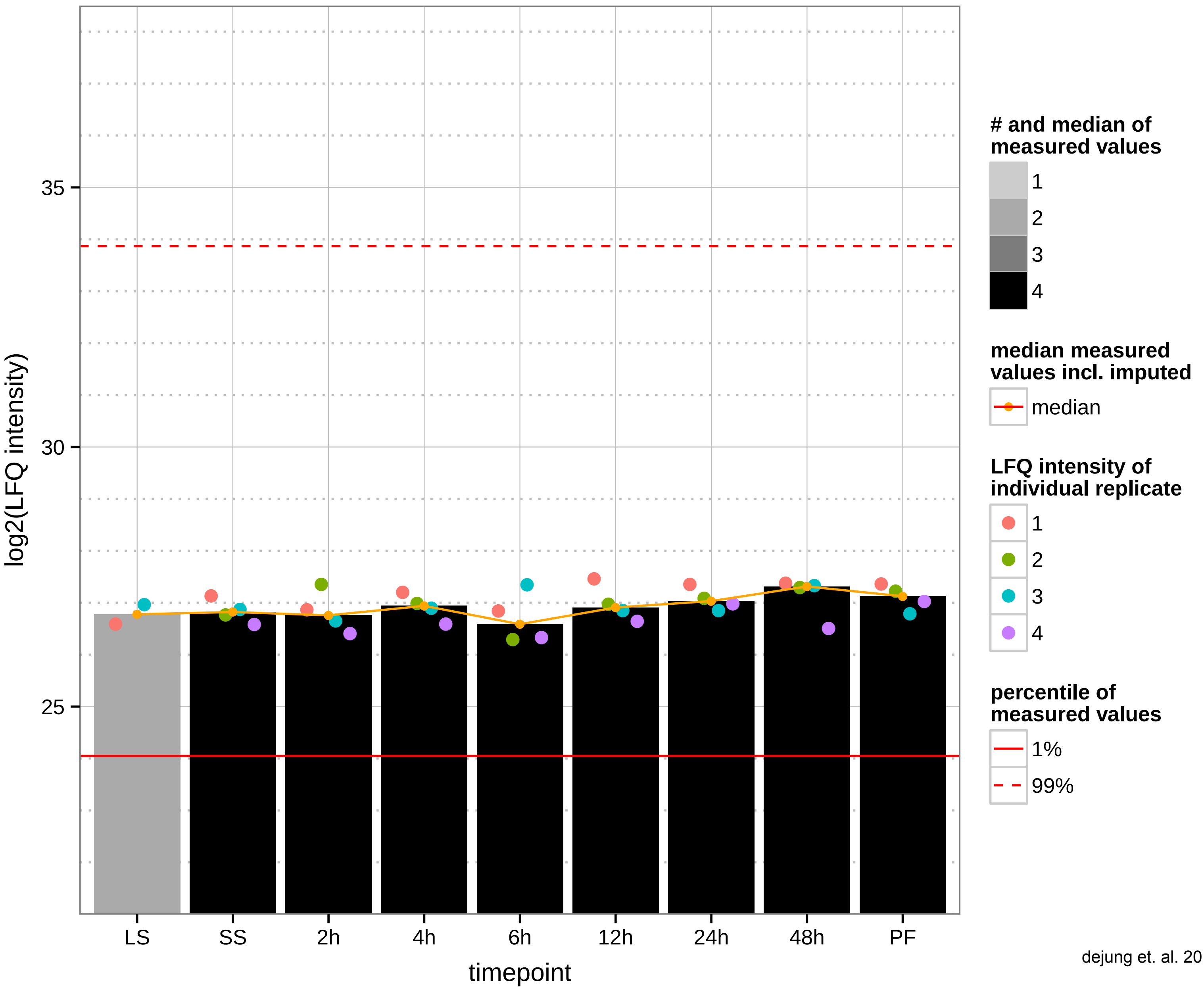

dejung et. al. 2015

Variant Surface Glycoprotein, putative
Tb927.5.5370
AGOF: null
AGOC: null
AGOP: null
PGOF: null
PGOC: null
PGOP: null

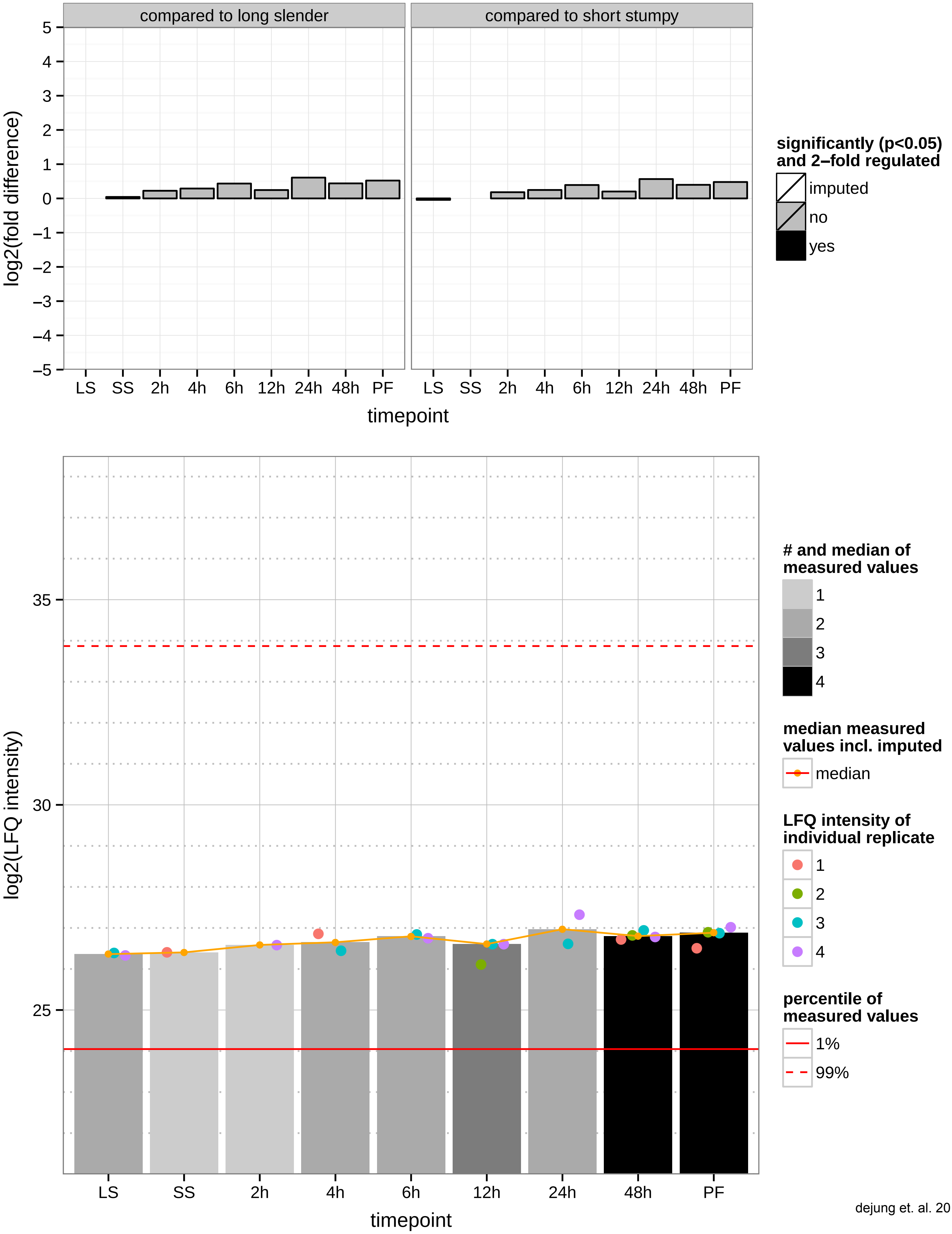

dejung et. al. 2015

hypothetical protein, conserved
Tb927.5.570
AGOF: null
AGOC: null
AGOP: null
PGOF: null
PGOC: null
PGOP: null

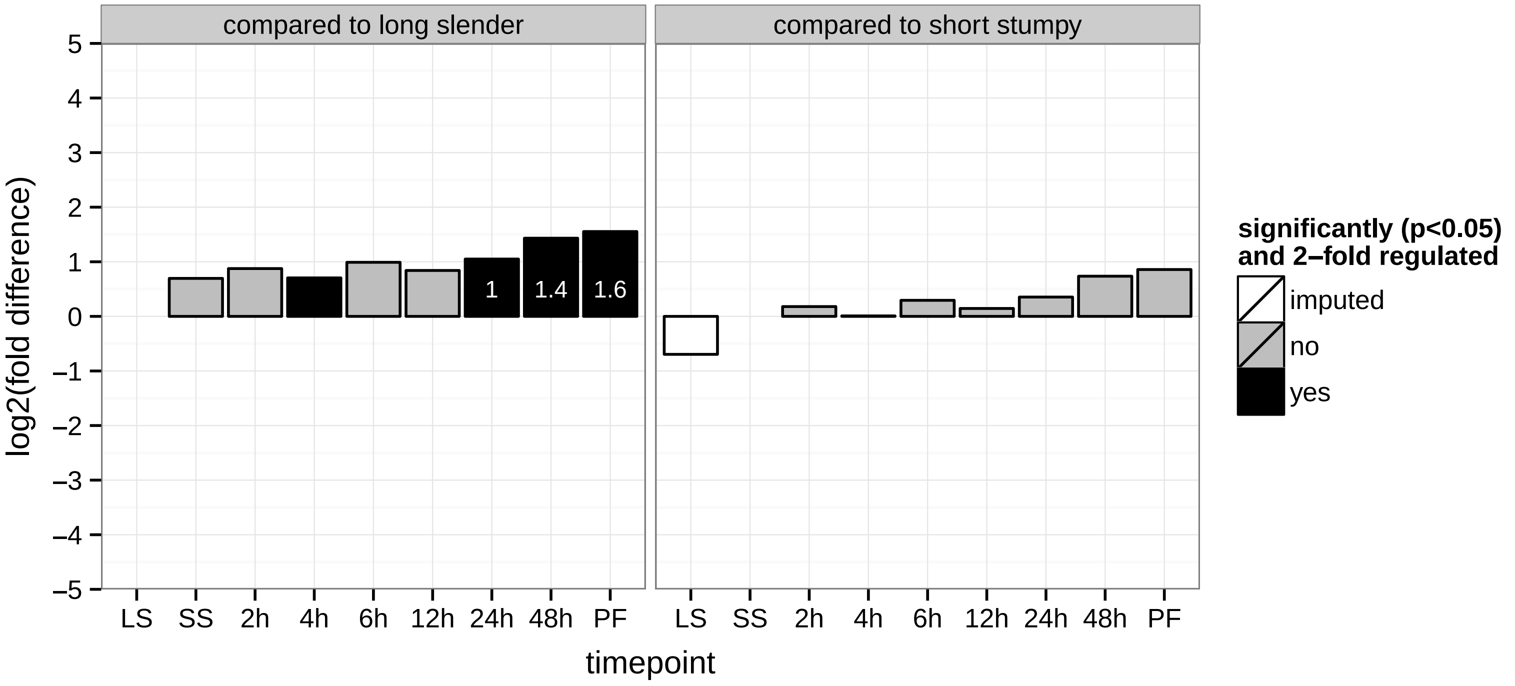

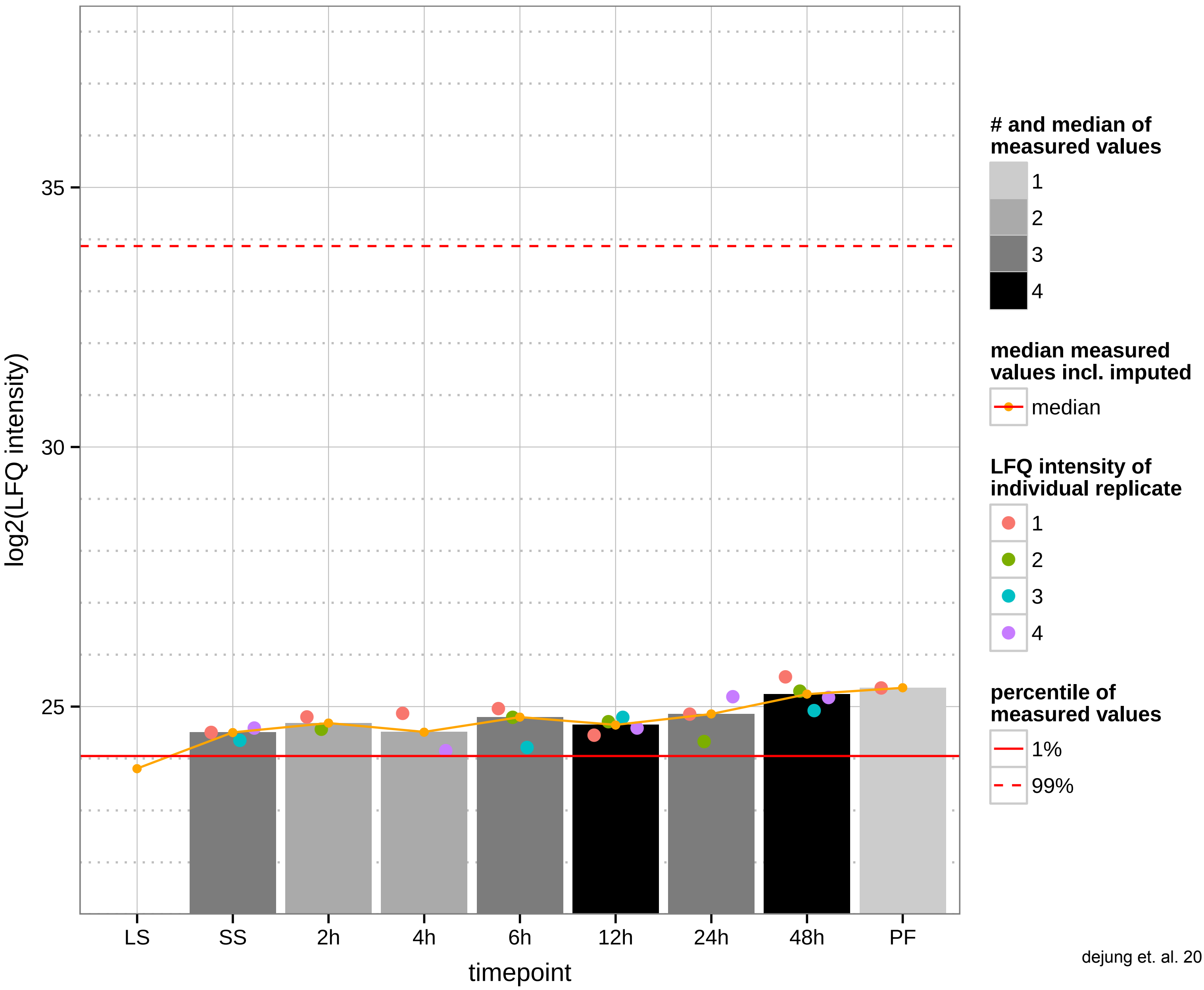

dejung et. al. 2015

acidic phosphatase, putative
Tb927.5.610
AGOF: null
AGOC: null
AGOP: null
PGOF: null
PGOC: null
PGOP: null

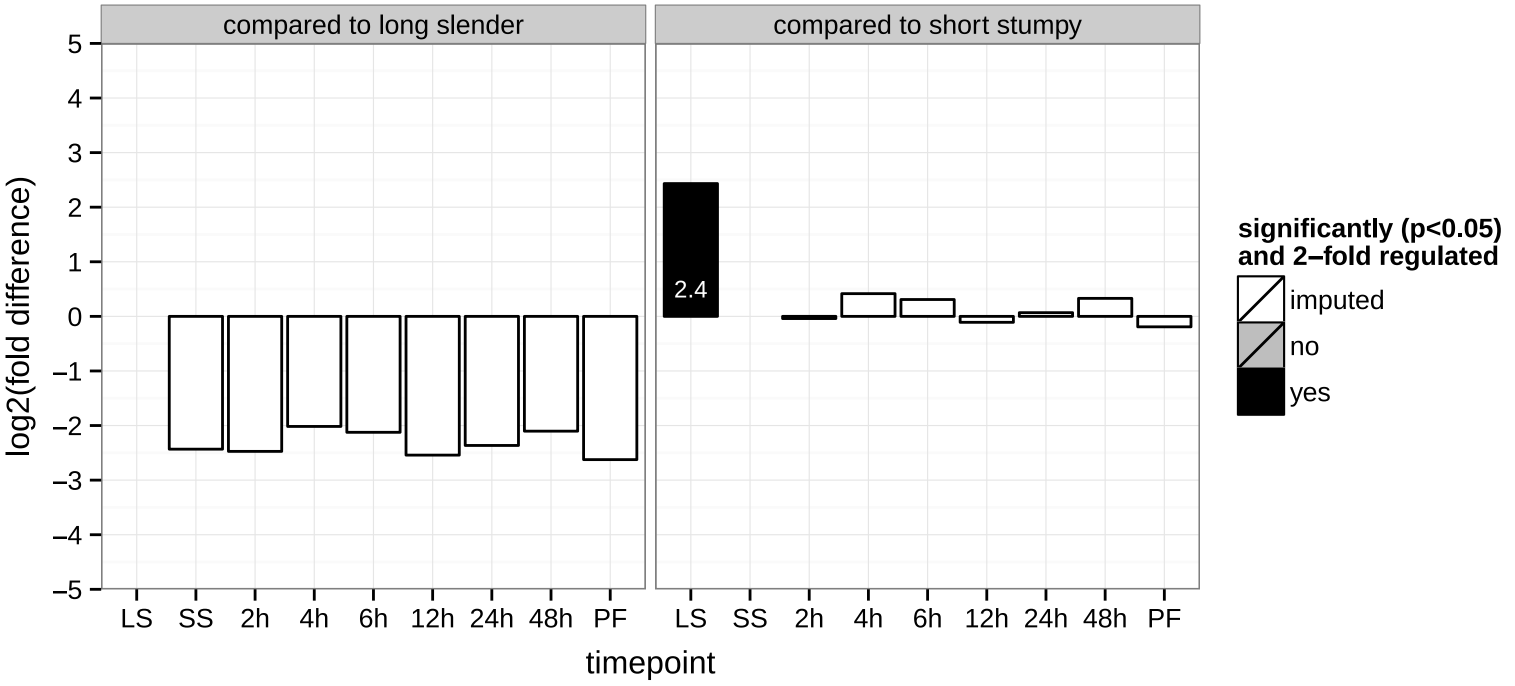

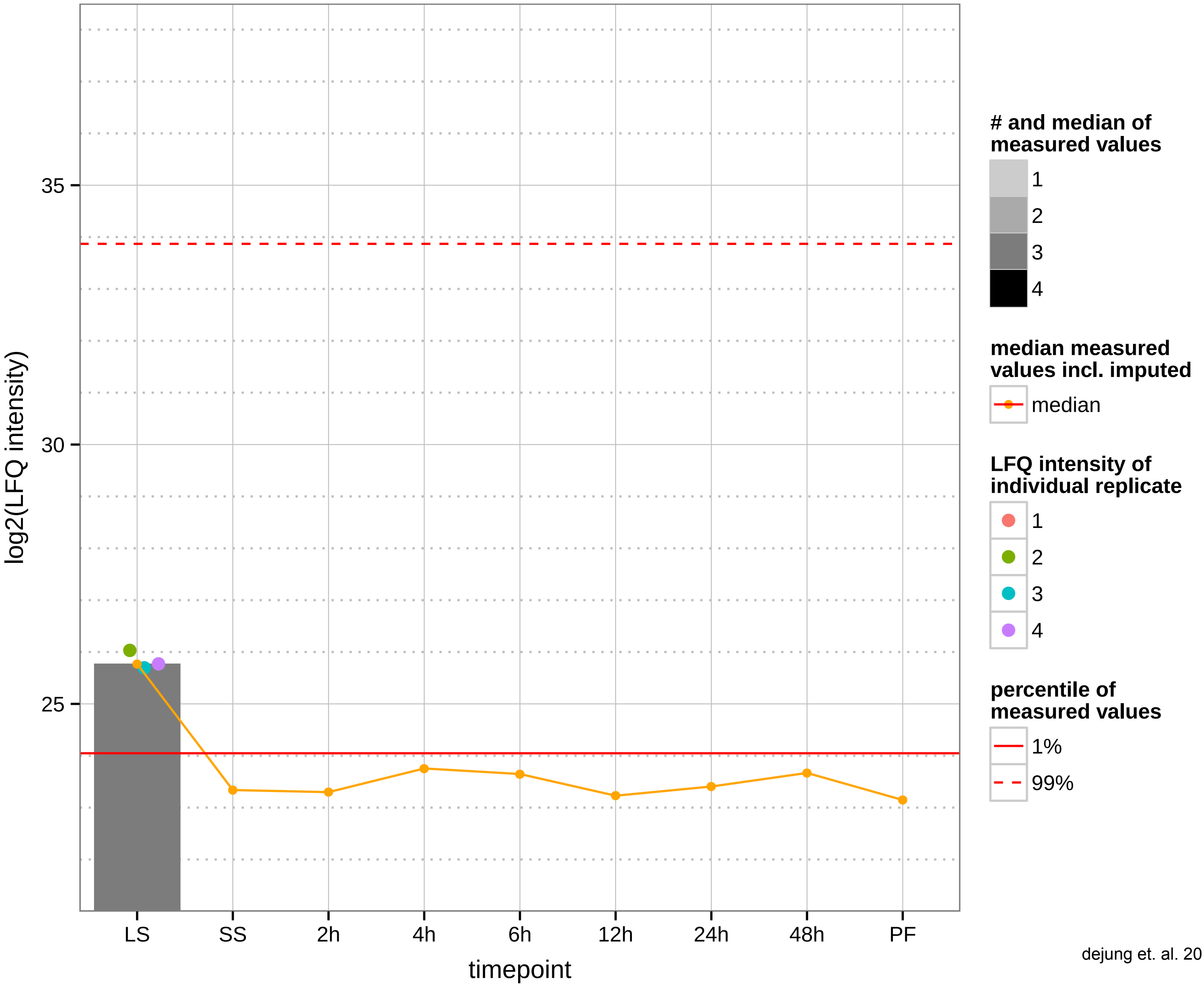

dejung et. al. 2015

hypothetical protein, conserved
Tb927.5.740
AGOF: null
AGOC: mitochondrion
AGOP: null
PGOF: null
PGOC: null
PGOP: null

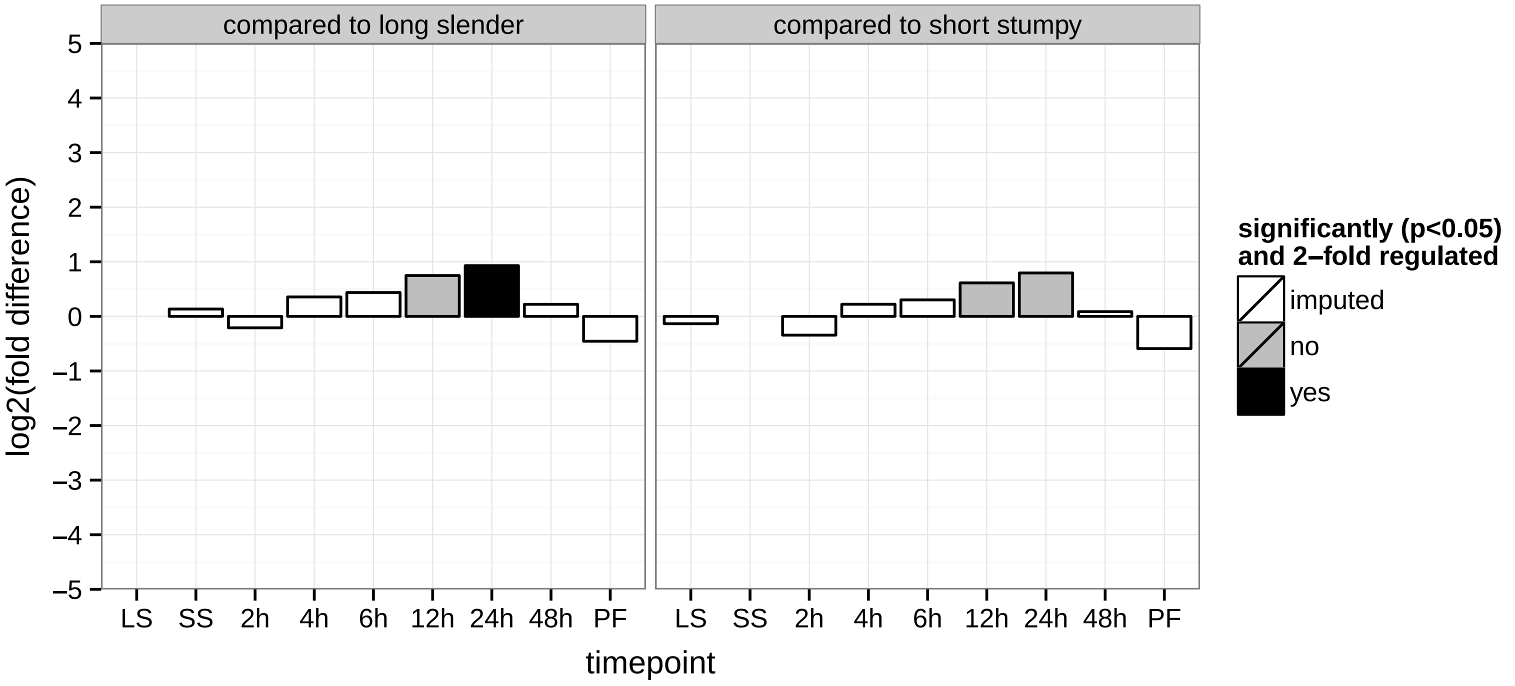

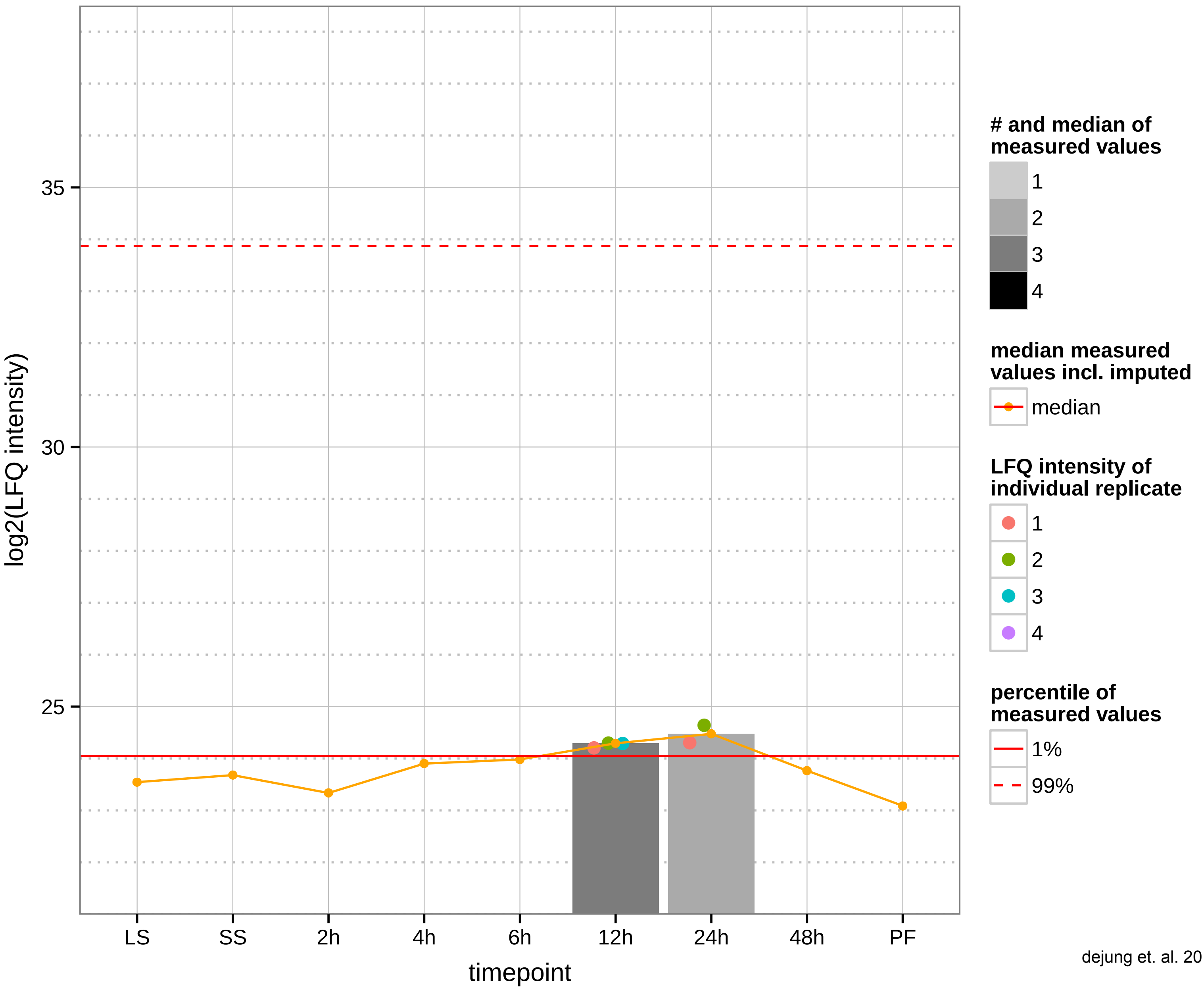

dejung et. al. 2015

hypothetical protein, conserved
Tb927.5.780
AGOF: null
AGOC: null
AGOP: null
PGOF: null
PGOC: null
PGOP: null

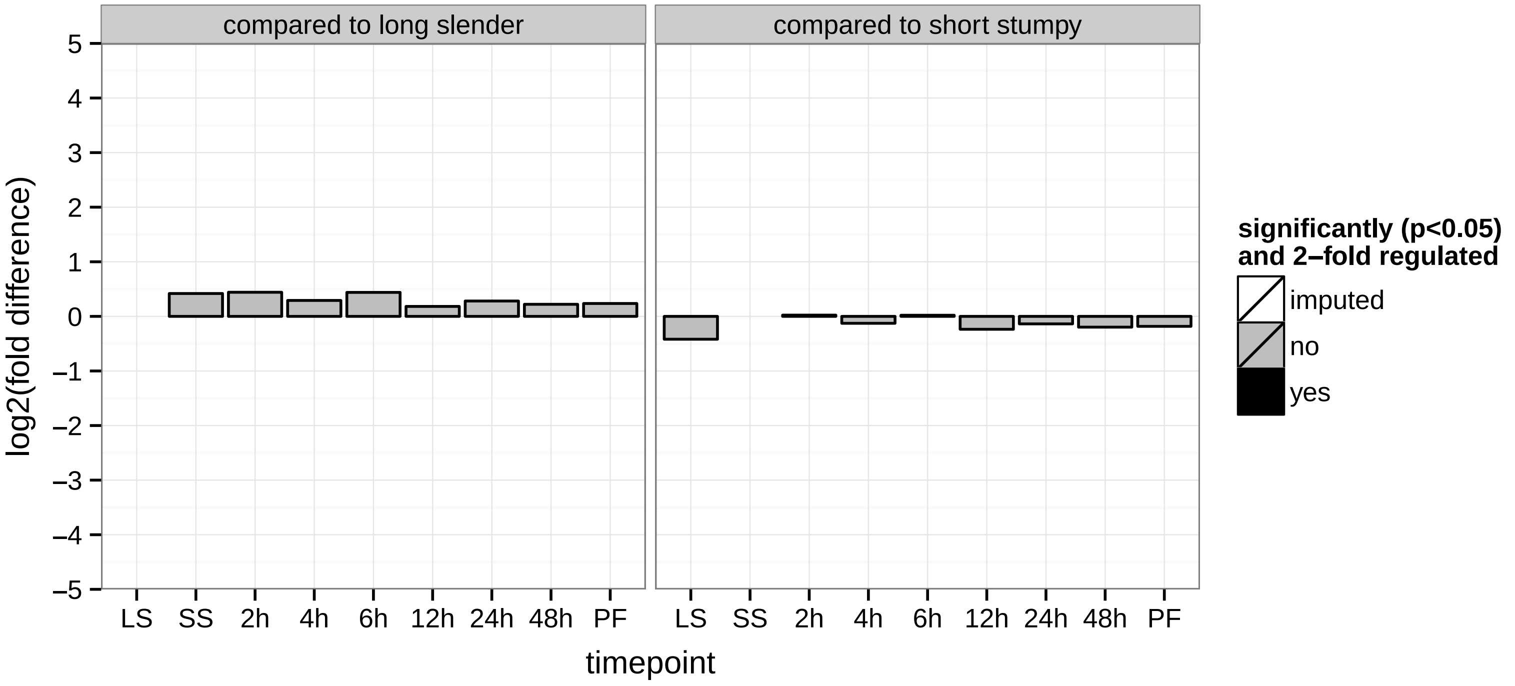

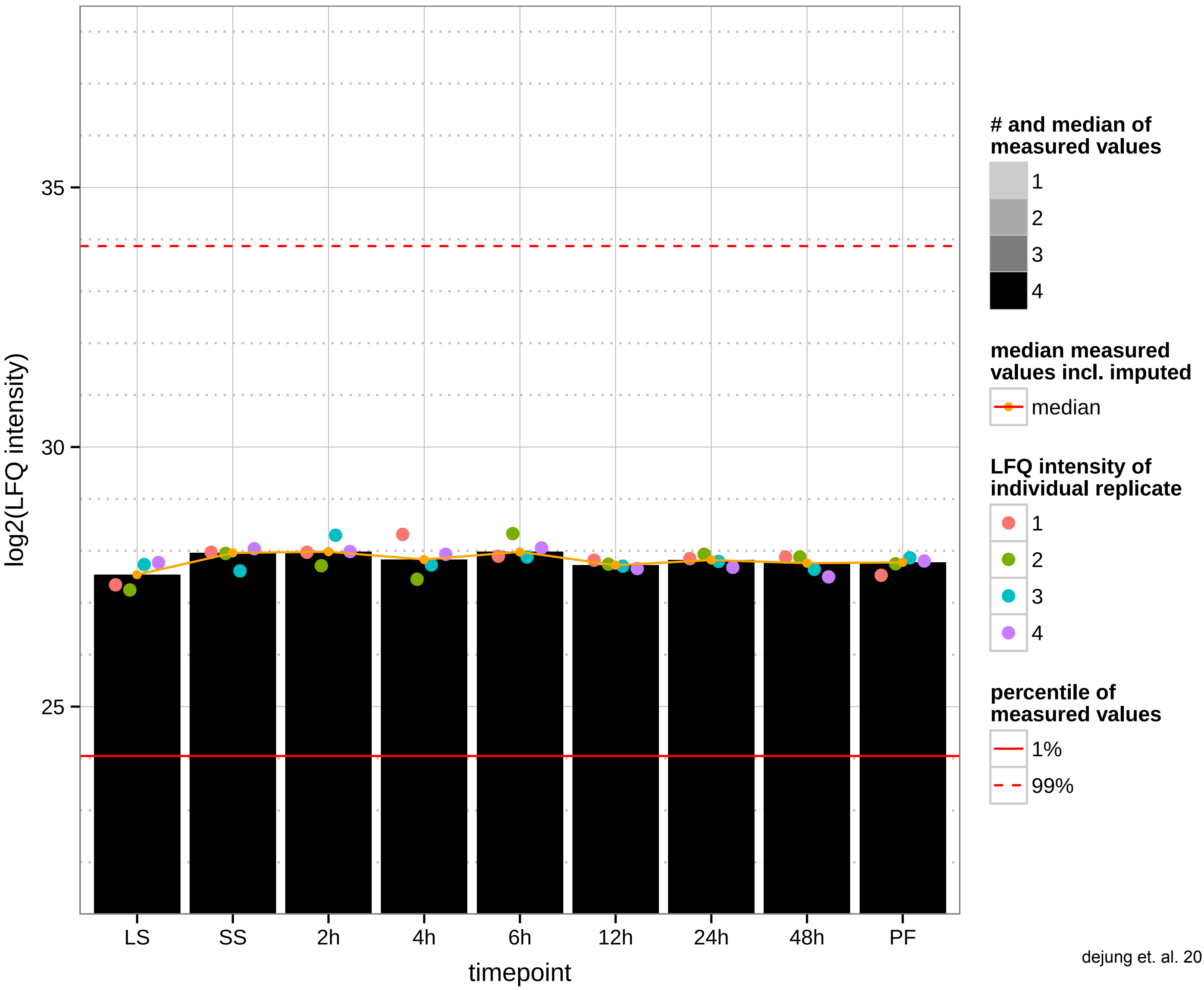

dejung et. al. 2015

casein kinase I isoform 1 (CK1.1), casein kinase I, isoform 2 (CK1.2)
Tb927.5.800
AGOF: ATP binding, protein serine/threonine kinase activity, protein tyrosine kinase activity
AGOC: null
AGOP: protein phosphorylation
PGOF: ATP binding, protein kinase activity, transferase activity, transferring phosphorus–containing groups
PGOC: null
PGOP: protein phosphorylation

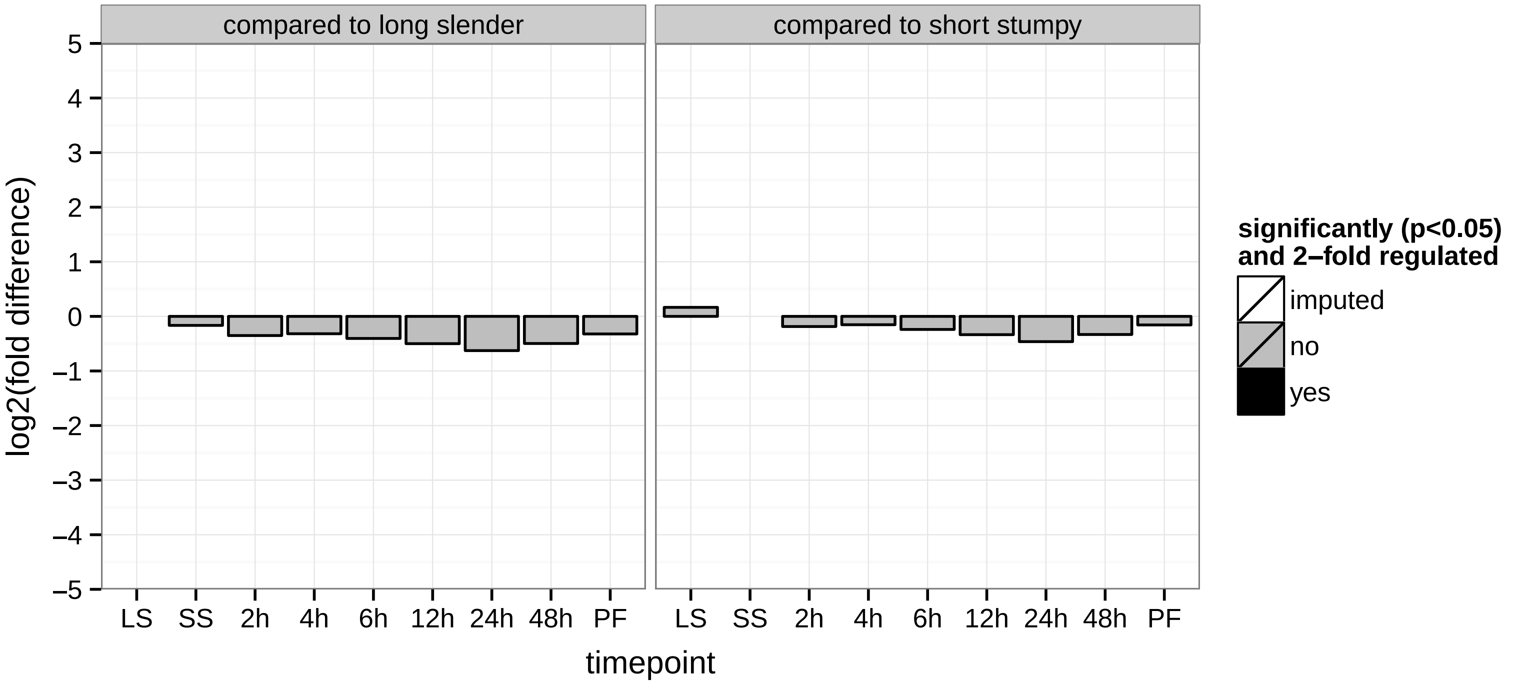

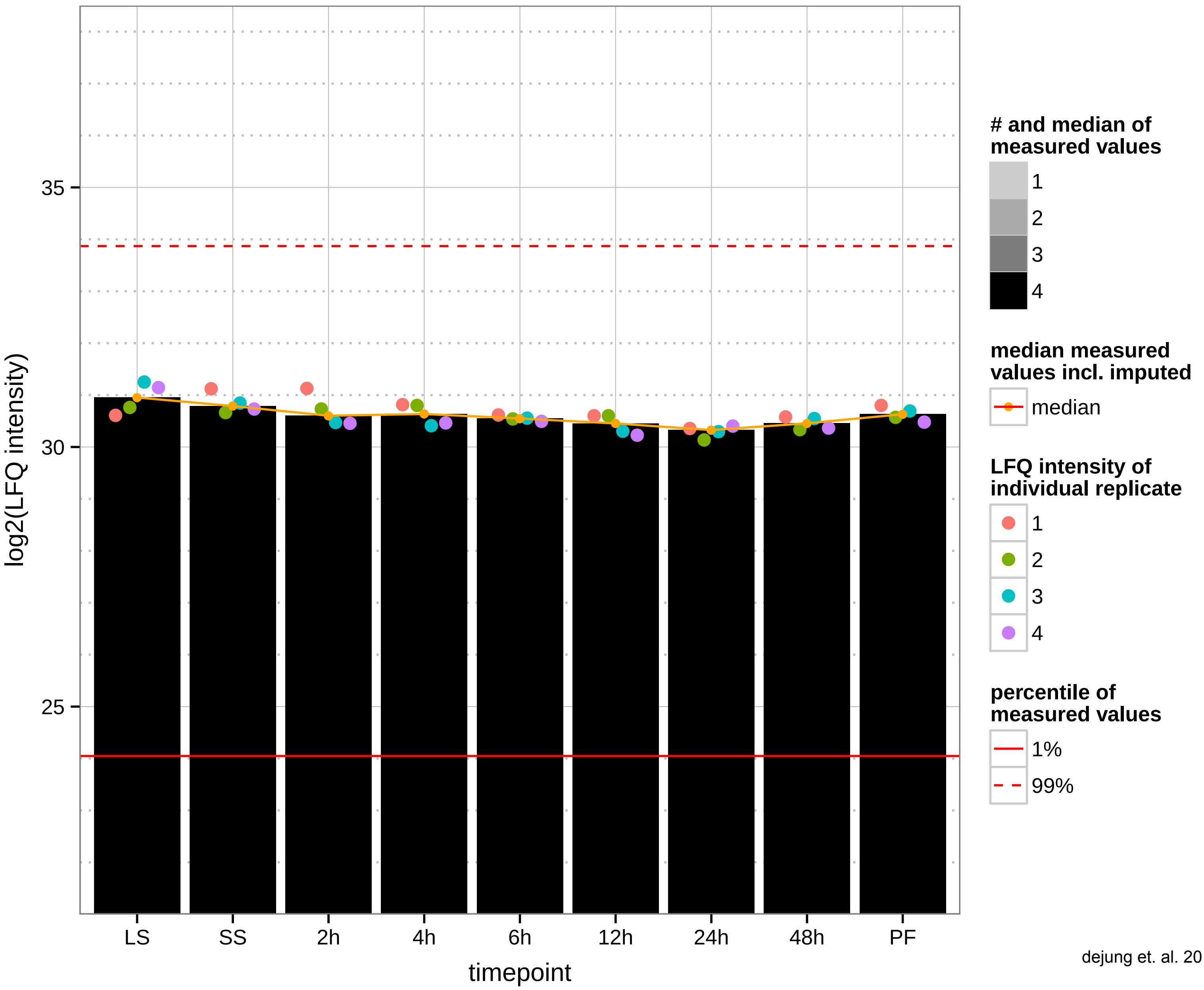

dejung et. al. 2015

hypothetical protein, conserved
Tb927.5.830
AGOF: null
AGOC: null
AGOP: null
PGOF: null
PGOC: null
PGOP: null

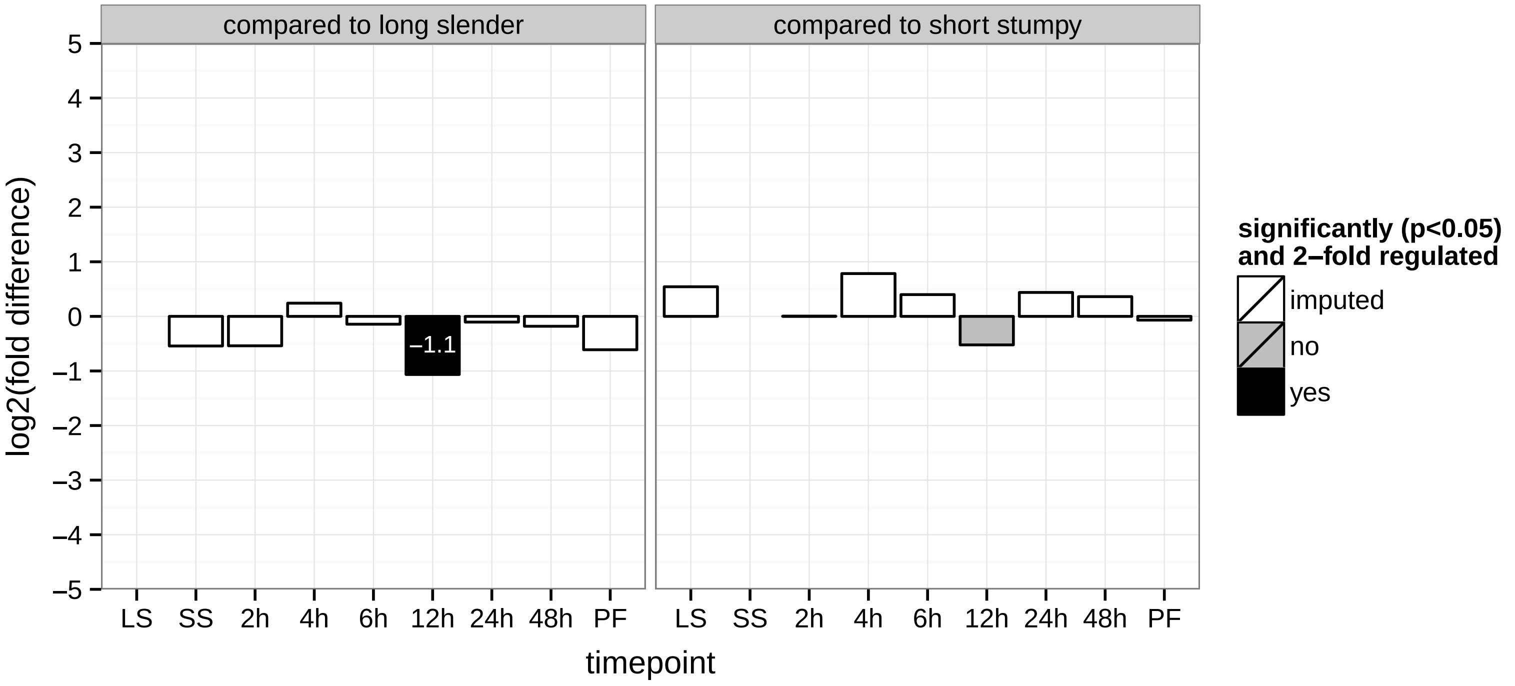

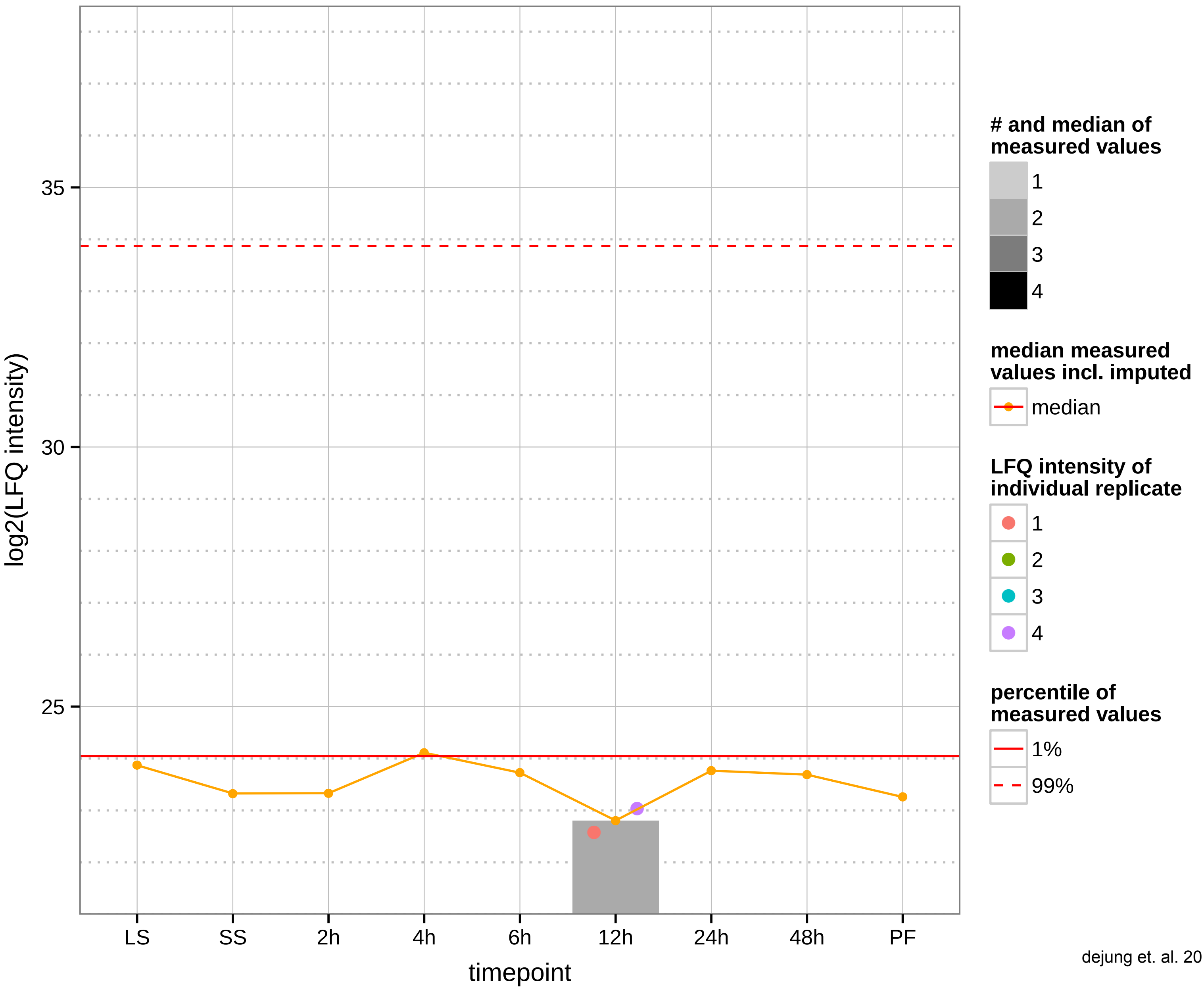

dejung et. al. 2015

oligosaccharyl transferase subunit, putative
Tb927.5.900;Tb11.v5.0559
AGOF: null, oligosaccharyl transferase activity
AGOC: null, membrane
AGOP: null, protein glycosylation
PGOF: oligosaccharyl transferase activity
PGOC: membrane
PGOP: protein glycosylation

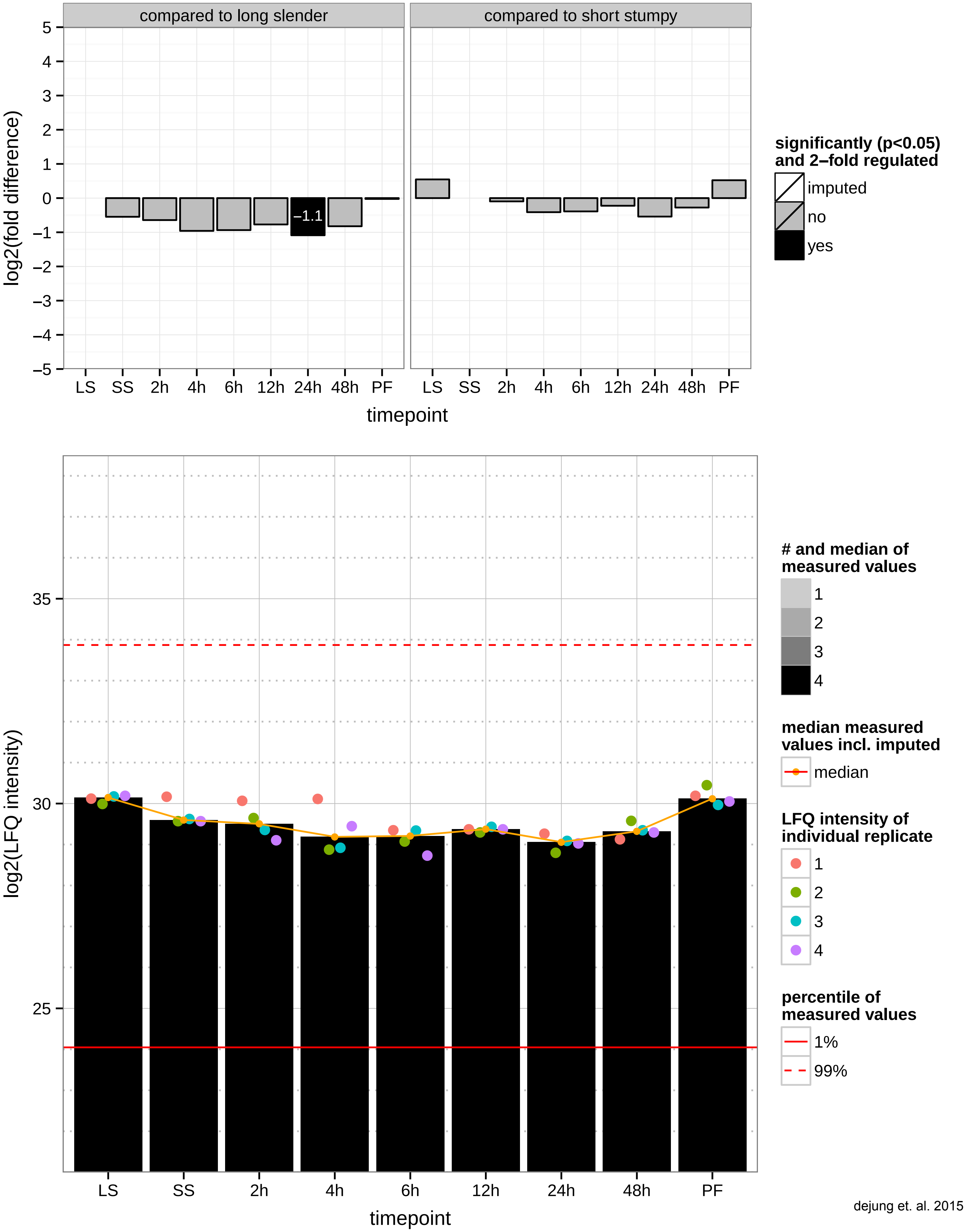

dejung et. al. 2015

hypothetical protein, conserved
Tb927.5.960
AGOF: ATP binding, nucleoside–triphosphatase activity
AGOC: null
AGOP: null
PGOF: ATP binding, nucleoside–triphosphatase activity, nucleotide binding
PGOC: null
PGOP: null

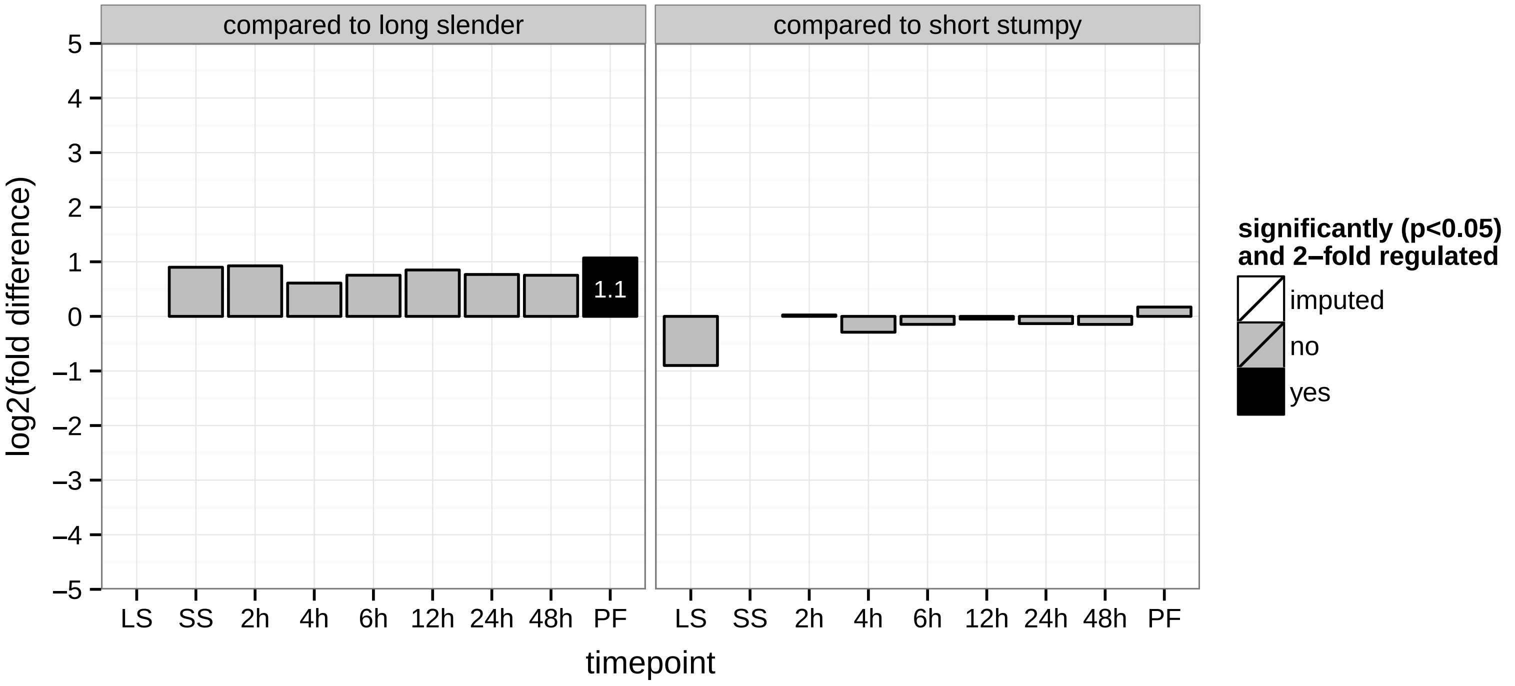

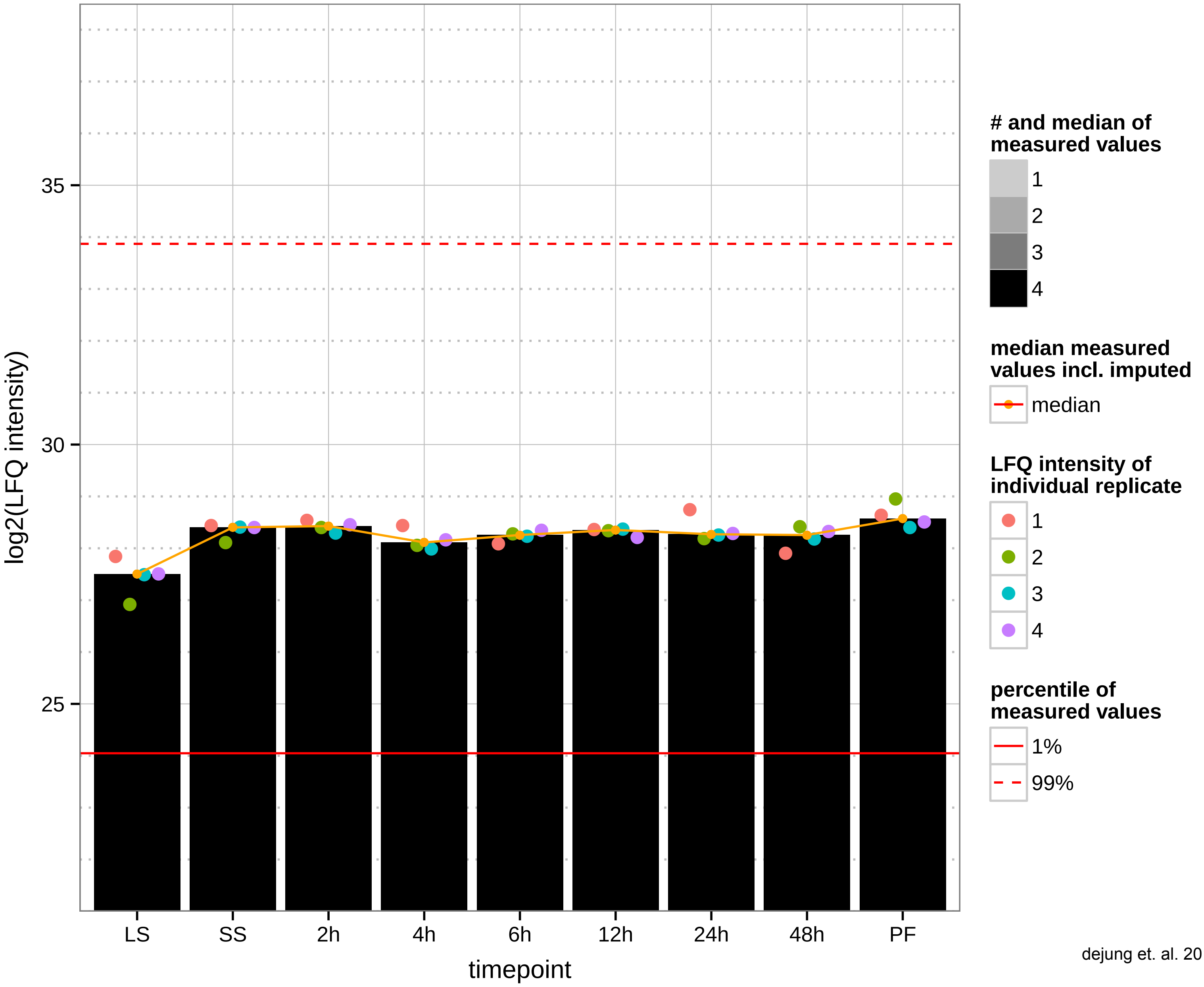

dejung et. al. 2015

glyoxalase II, hydroxyacylglutathione hydrolase, putative (glxII)
Tb927.6.1080
AGOF: hydrolase activity
AGOC: null
AGOP: null
PGOF: hydrolase activity
PGOC: null
PGOP: null

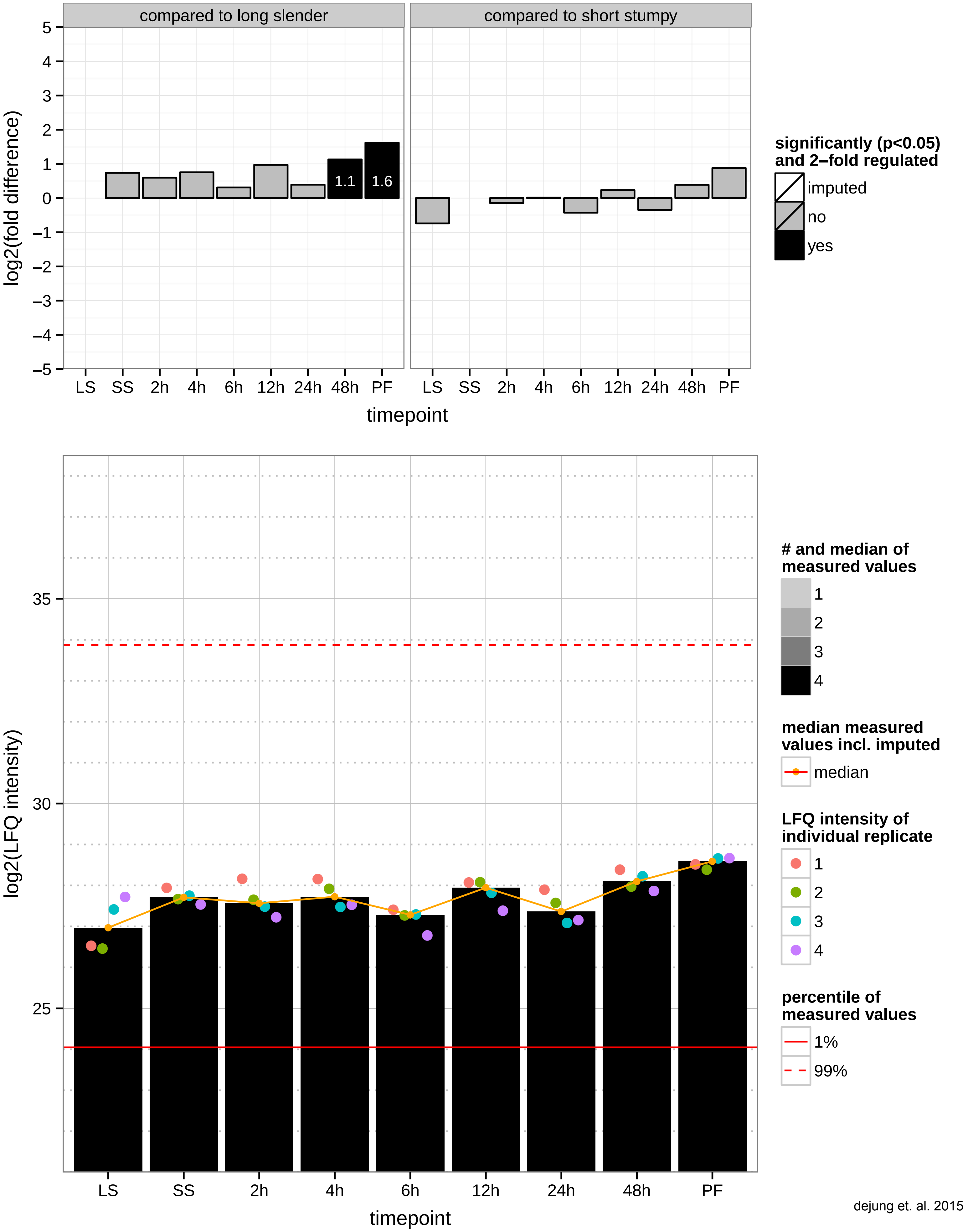

dejung et. al. 2015

proteasome regulatory ATPase subunit 3 (RPT3)
Tb927.6.1090
AGOF: ATP binding, ATPase activity
AGOC: cytoplasm, nucleus, proteasome regulatory particle
AGOP: ubiquitin–dependent protein catabolic process
PGOF: ATP binding, hydrolase activity, nucleoside–triphosphatase activity, nucleotide binding
PGOC: cytoplasm
PGOP: protein catabolic process

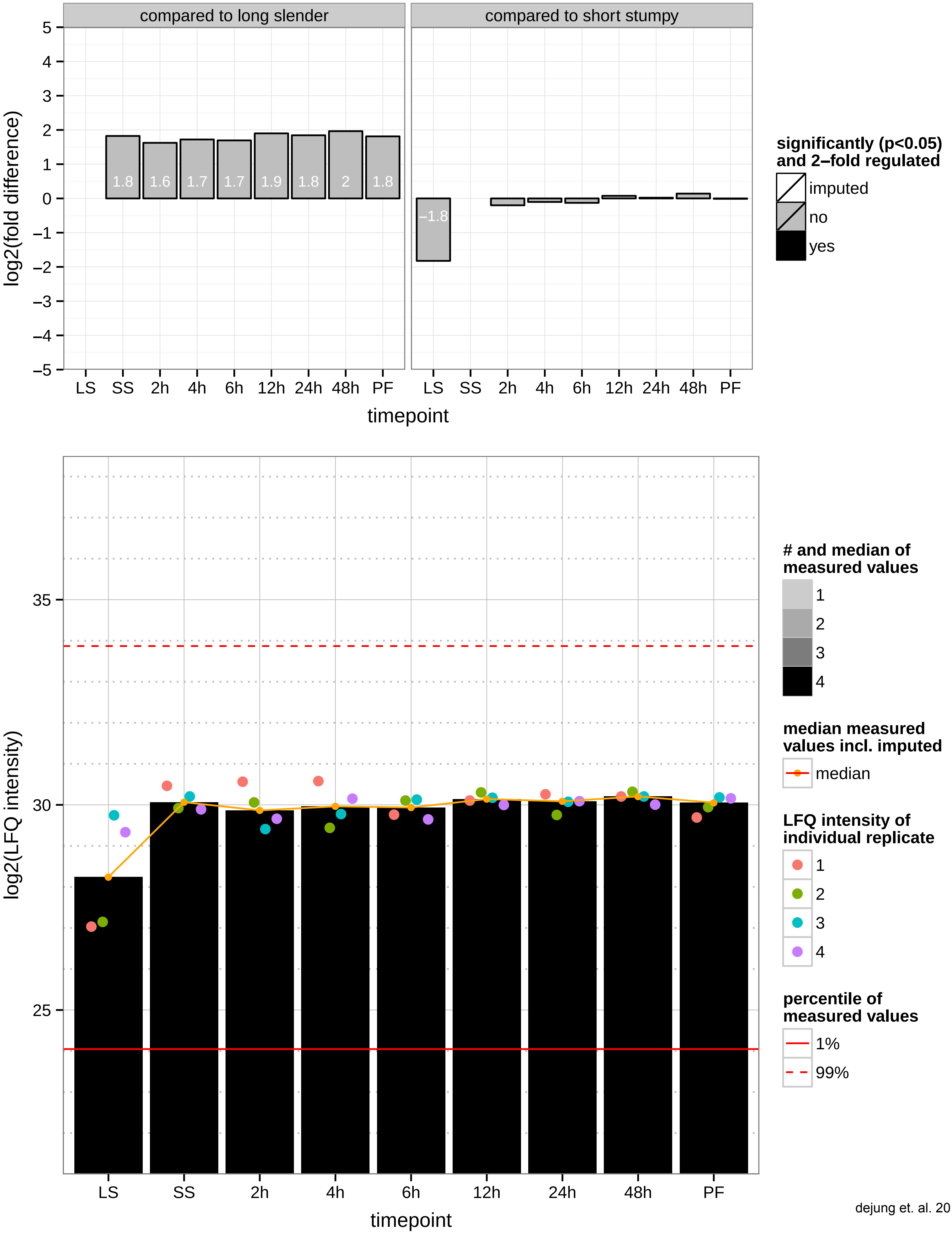

dejung et. al. 2015

dolichyl−P−Man:GDP−Man5GlcNAc2−PP−dolichyl alpha−1, 2−mannosyltransferase, putative, Asparagine−linked glycosylatio
Tb927.6.1140
AGOF: alpha−1, 2−mannosyltransferase activity
AGOC: intrinsic to endoplasmic reticulum membrane
AGOP: GPI anchor biosynthetic process, dolichol−linked oligosaccharide biosynthetic process
PGOF: transferase activity, transferring glycosyl groups
PGOC: intrinsic to endoplasmic reticulum membrane
PGOP: GPI anchor biosynthetic process

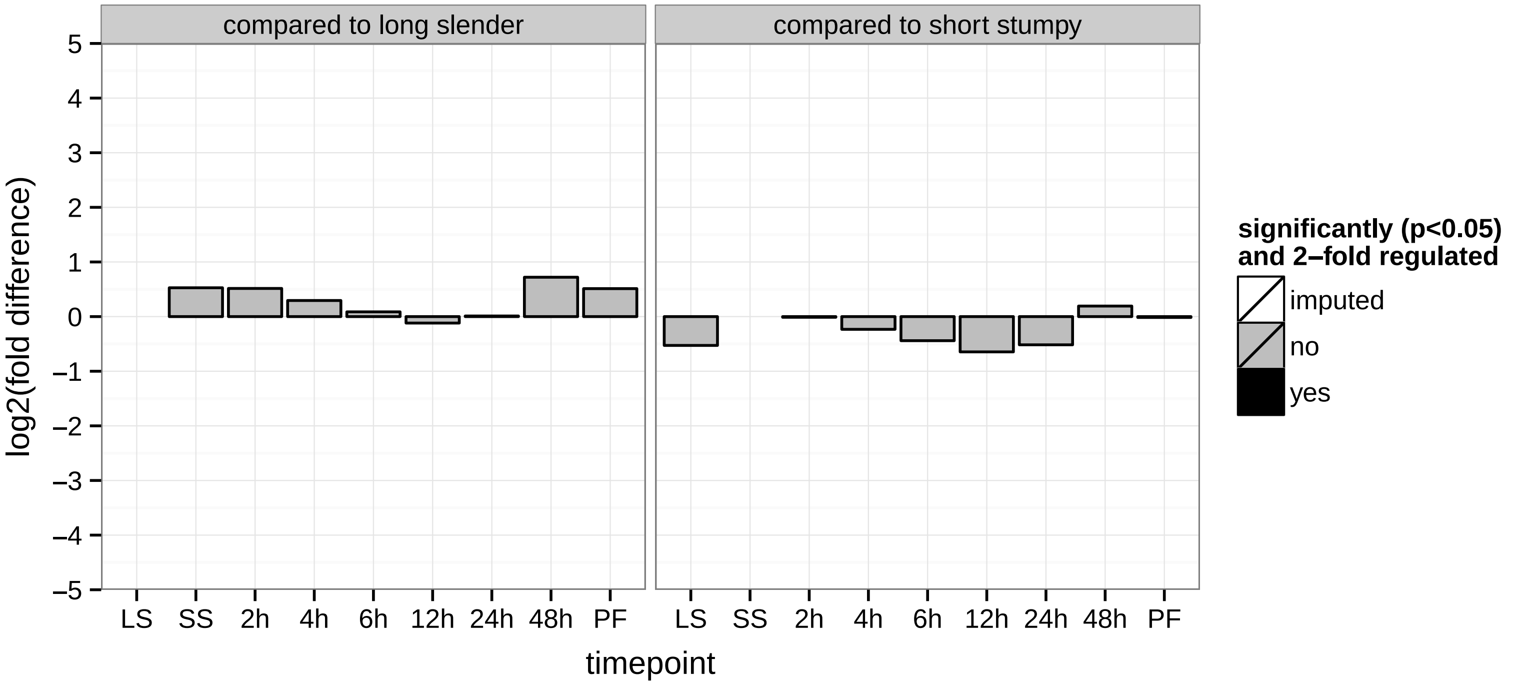

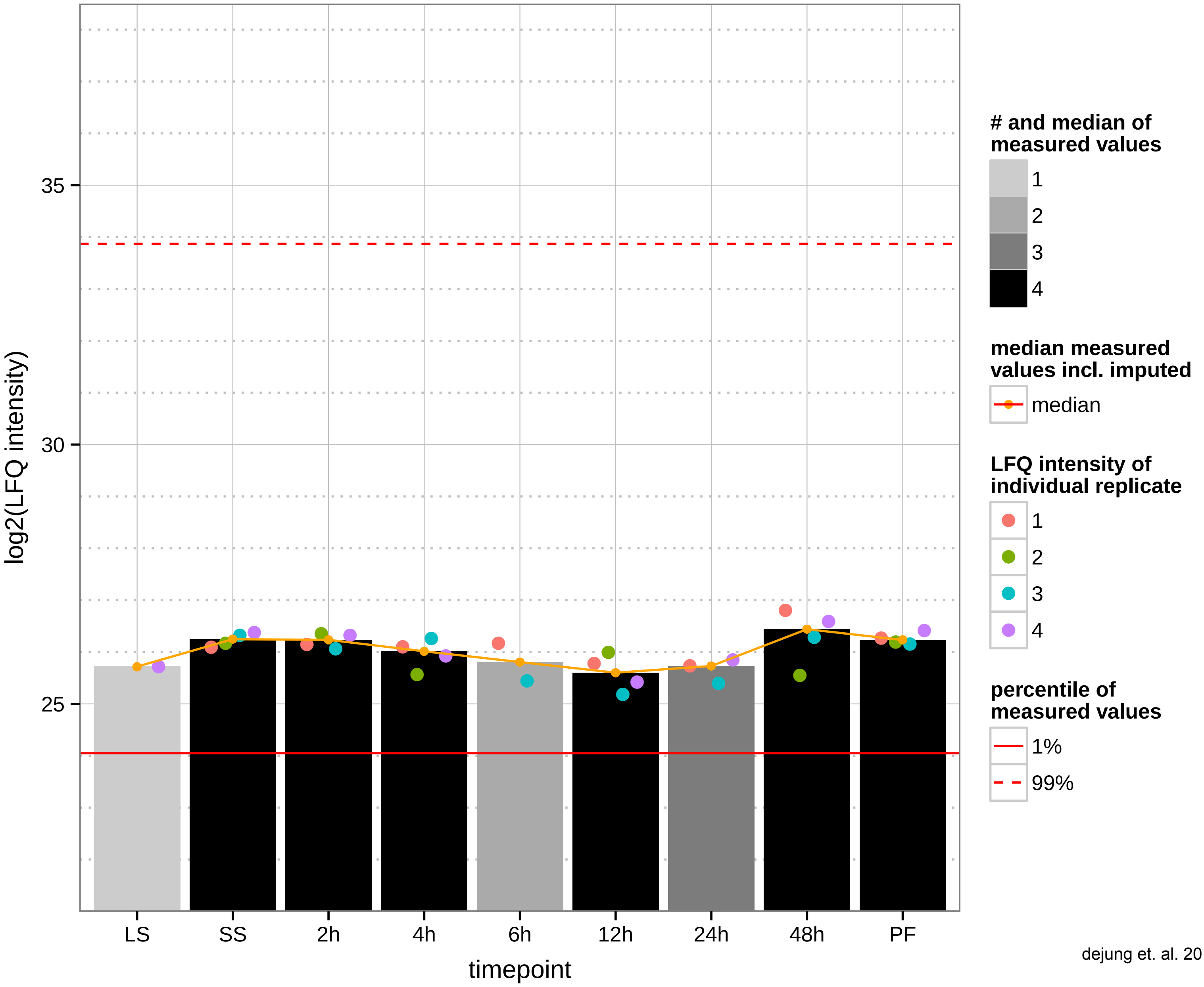

dejung et. al. 2015

hypothetical protein, conserved
Tb927.6.1190
AGOF: DNA binding
AGOC: nucleus
AGOP: DNA–dependent transcription, initiation, histone modification
PGOF: DNA binding
PGOC: nucleus
PGOP: DNA–dependent transcription, initiation, histone modification

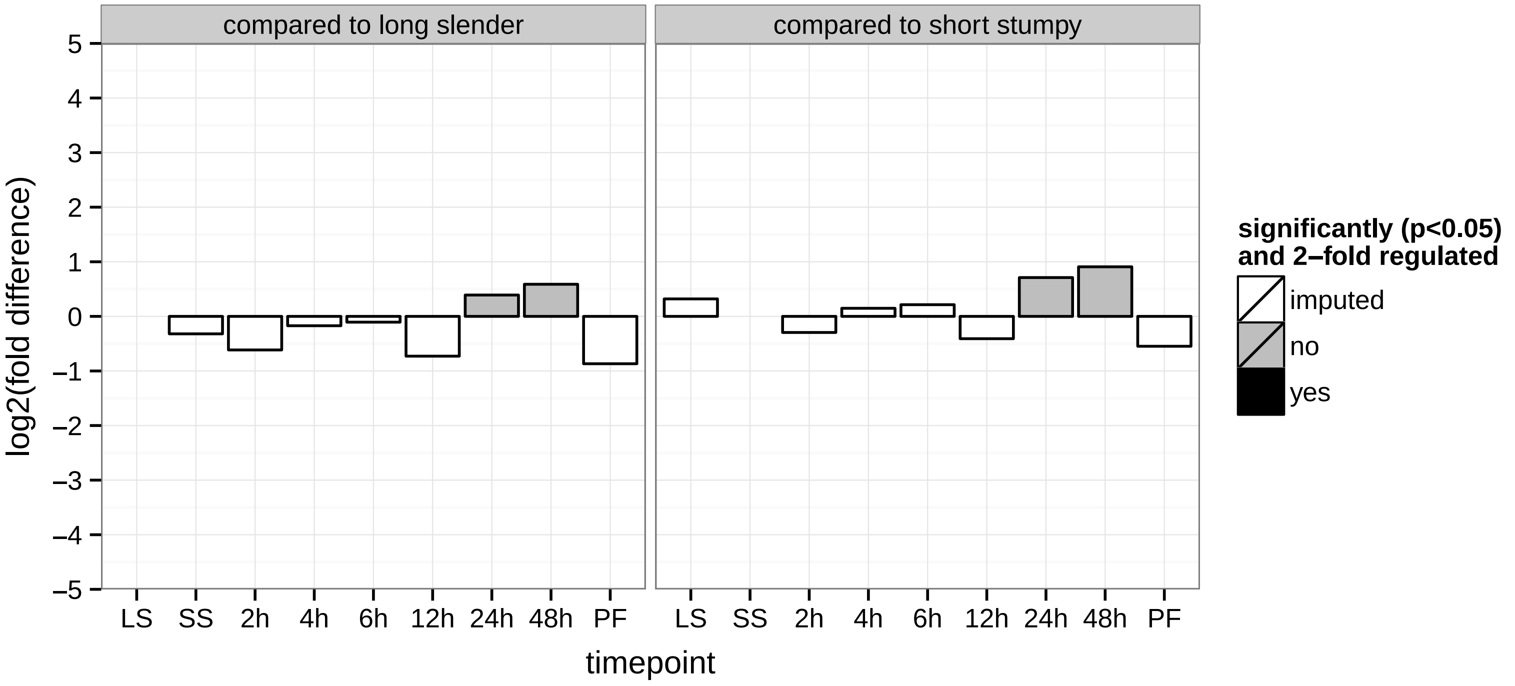

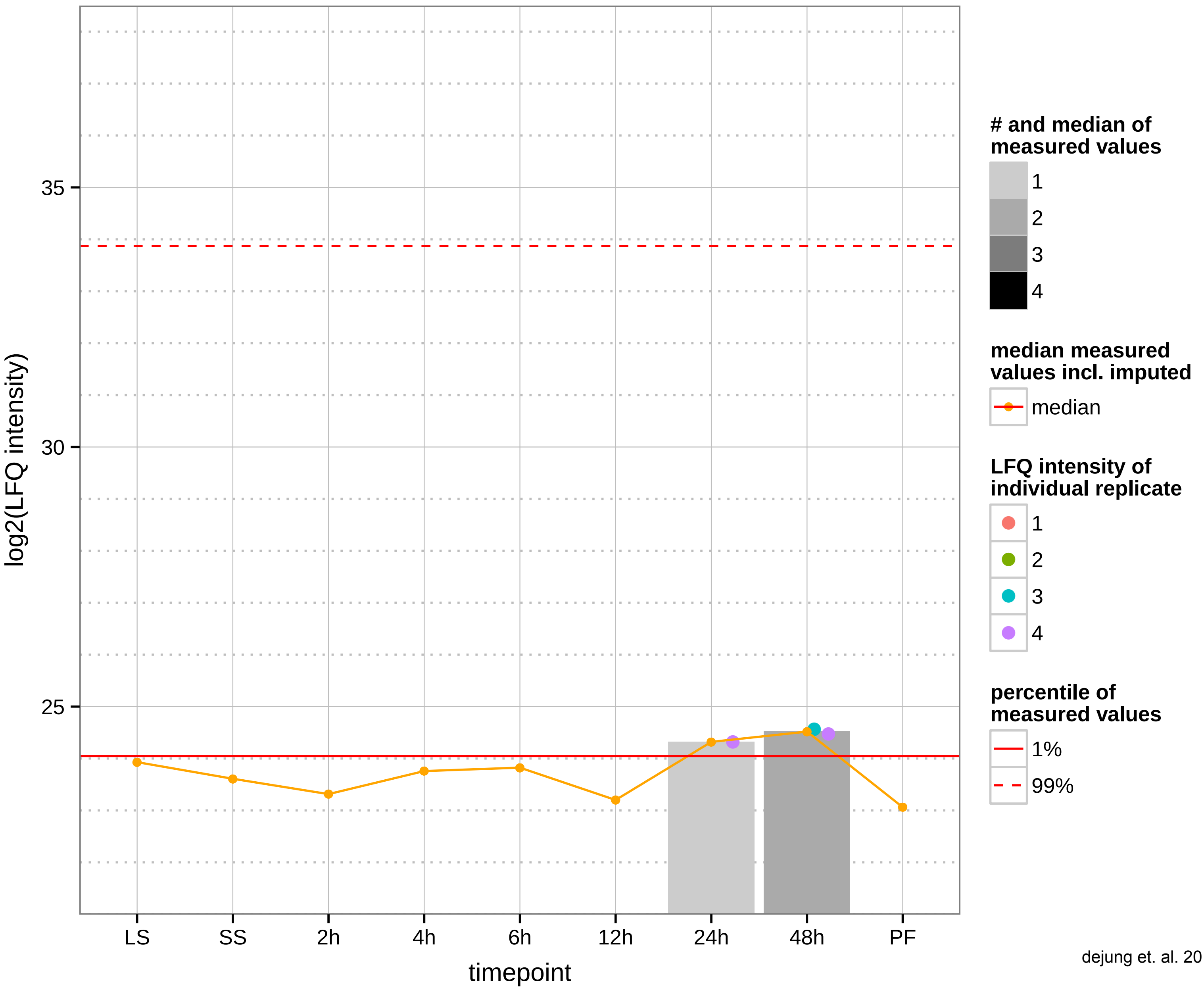

dejung et. al. 2015

translation initiation factor EIF−2b alpha subunit, putative, eIF−2B GDP−GTP exchange factor
Tb927.6.1280
AGOF: null
AGOC: null
AGOP: cellular metabolic process
PGOF: null
PGOC: null
PGOP: cellular metabolic process

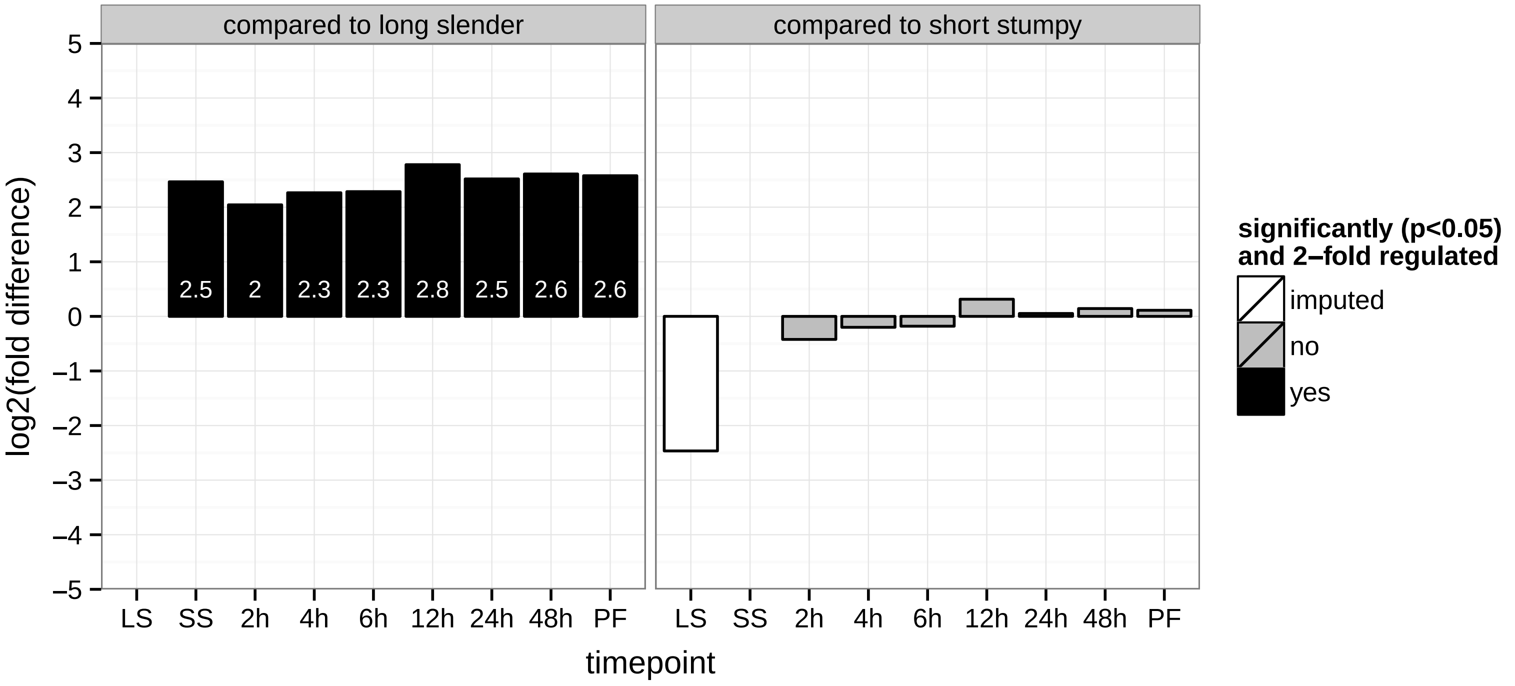

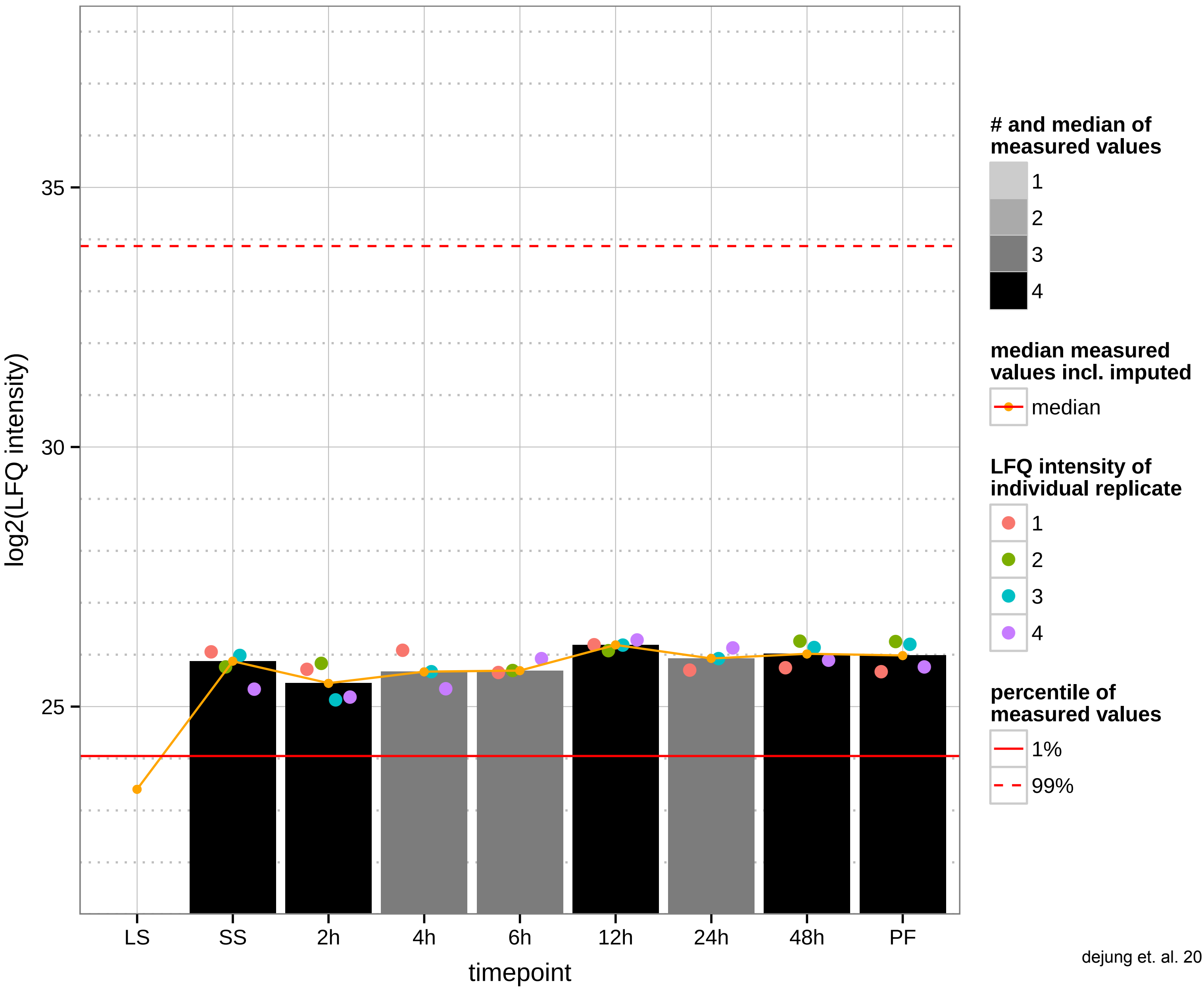

dejung et. al. 2015

2–hydroxy–3–oxopropionate reductase, putative
Tb927.6.1570
AGOF: phosphogluconate dehydrogenase (decarboxylating) activity
AGOC: null
AGOP: pentose–phosphate shunt
PGOF: oxidoreductase activity, phosphogluconate dehydrogenase (decarboxylating) activity
PGOC: null
PGOP: oxidation–reduction process, pentose–phosphate shunt

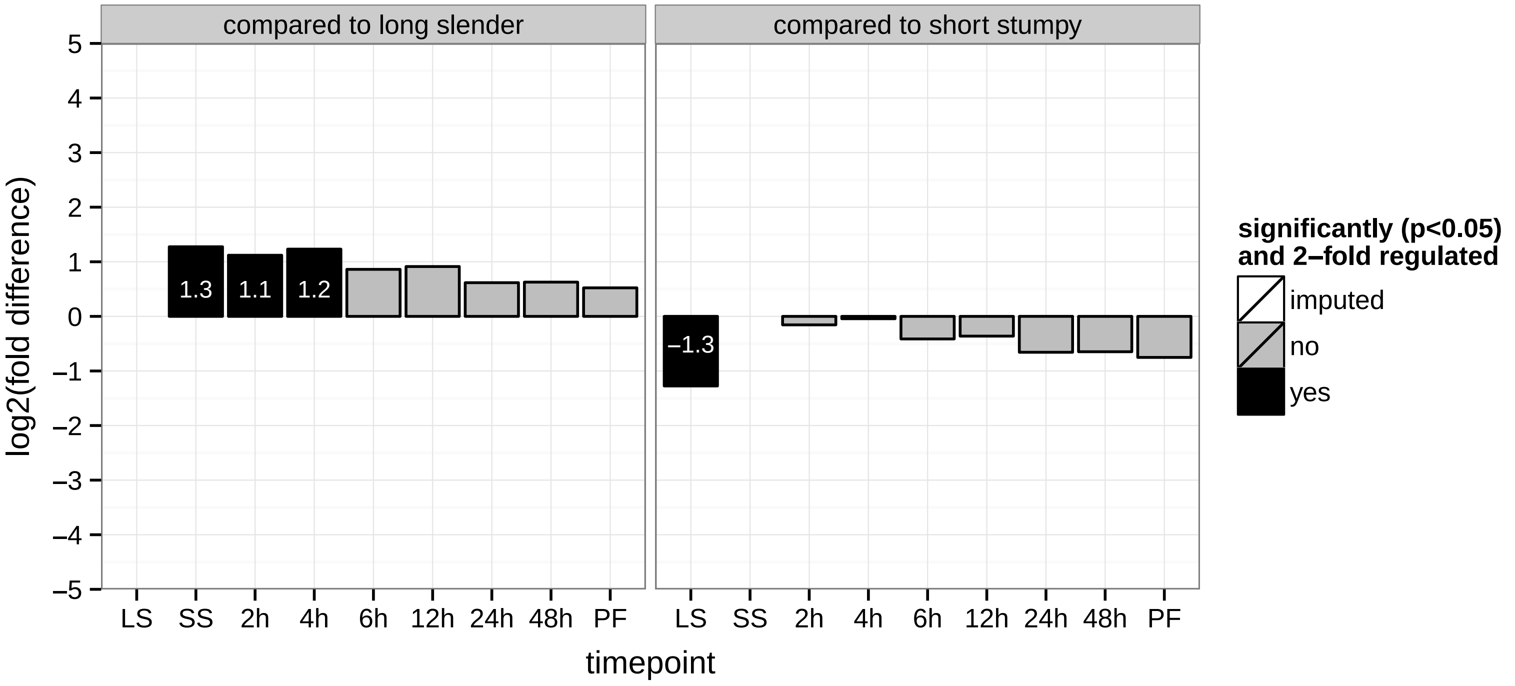

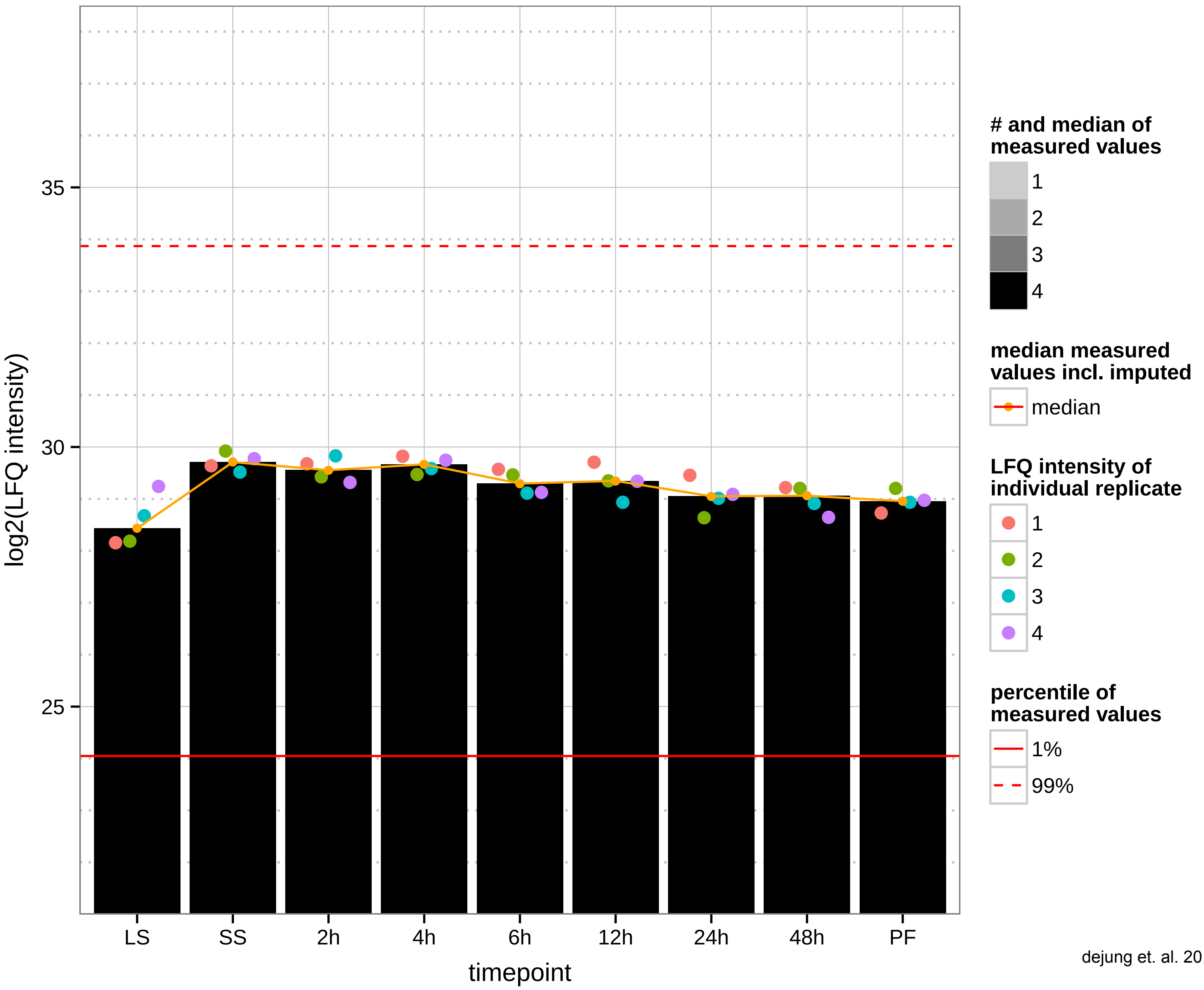

dejung et. al. 2015

hypothetical protein, conserved
Tb927.6.1660
AGOF: null
AGOC: null
AGOP: null
PGOF: null
PGOC: null
PGOP: null

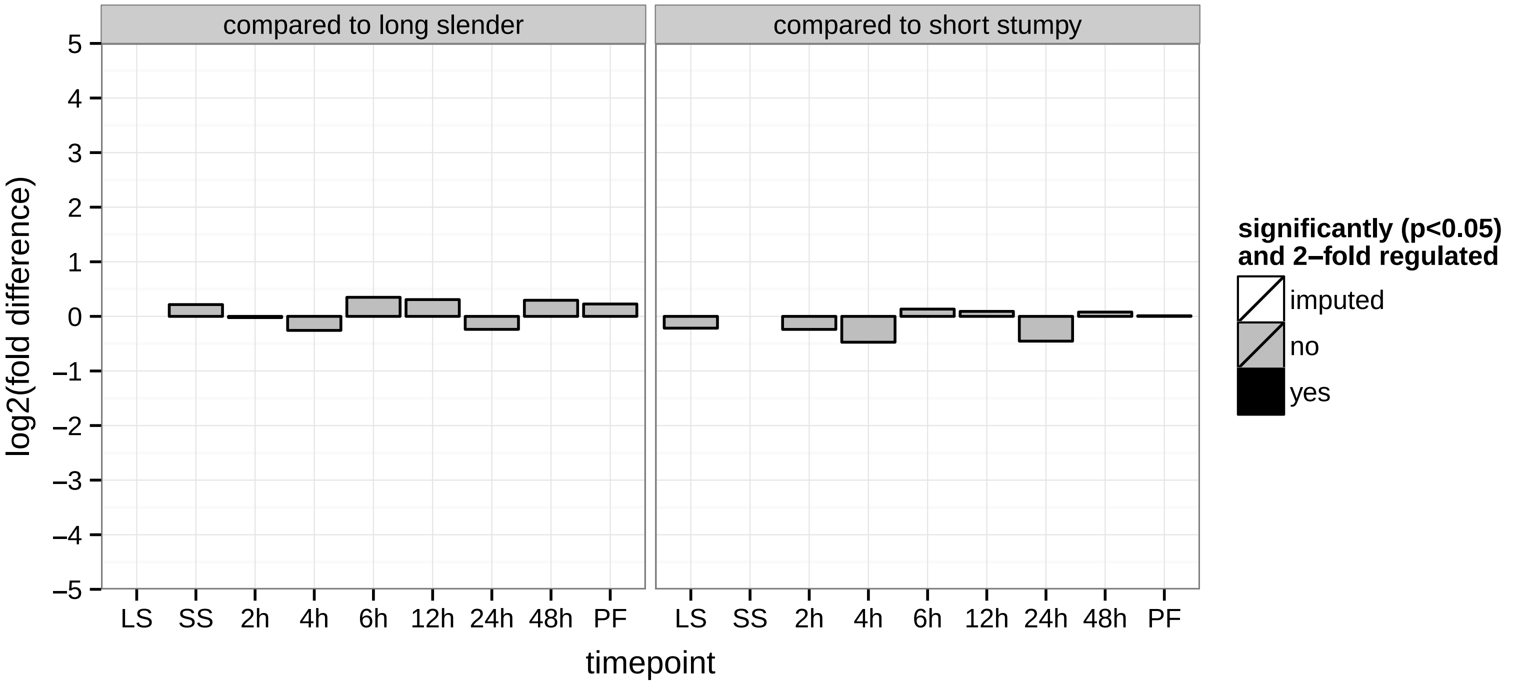

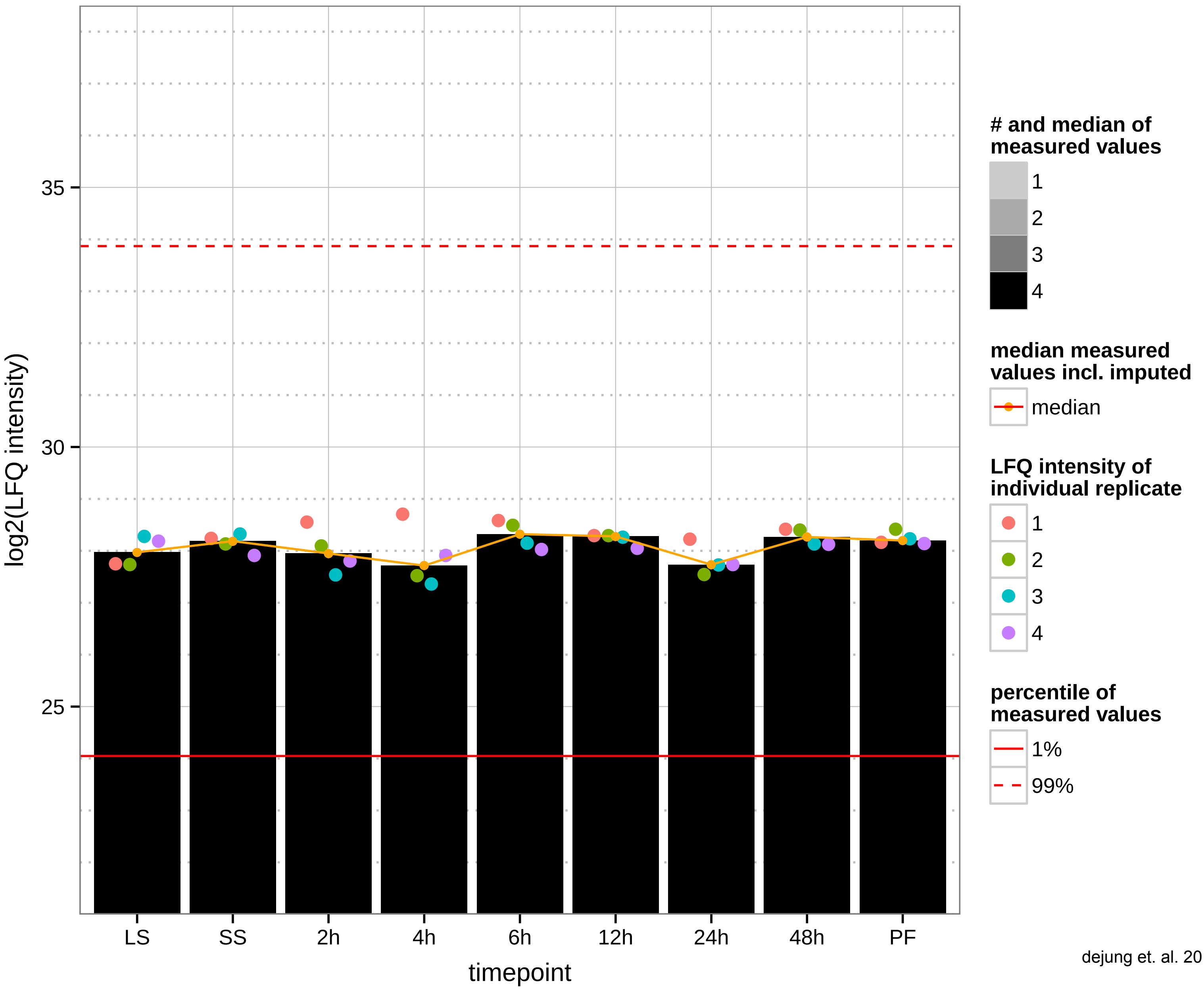

dejung et. al. 2015

aspartyl–tRNA synthetase, putative
Tb927.6.1880
AGOF: ATP binding, aspartate–tRNA ligase activity, nucleic acid binding
AGOC: cytoplasm
AGOP: aspartyl–tRNA aminoacylation, translation
PGOF: ATP binding, aminoacyl–tRNA ligase activity, aspartate–tRNA ligase activity, nucleic acid binding, nucleotide binding
PGOC: cytoplasm
PGOP: aspartyl–tRNA aminoacylation, tRNA aminoacylation for protein translation

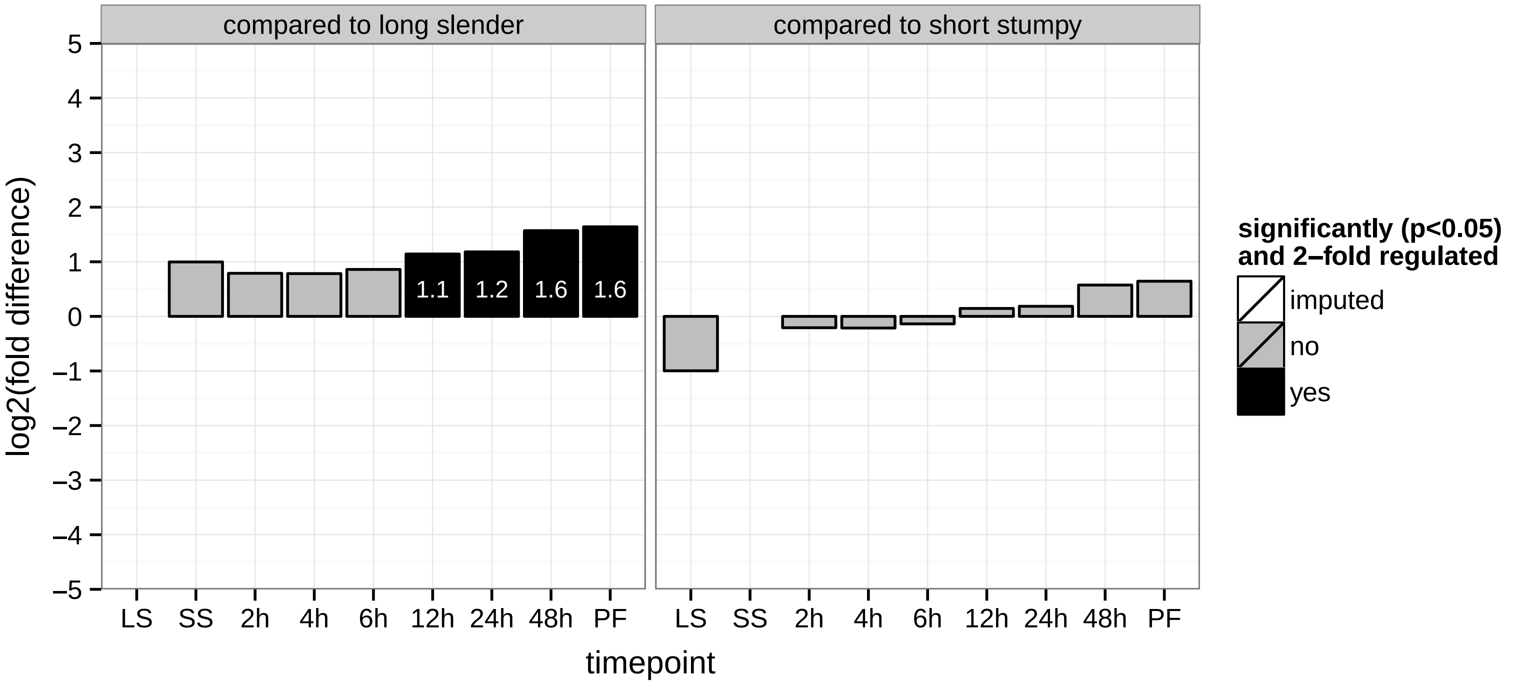

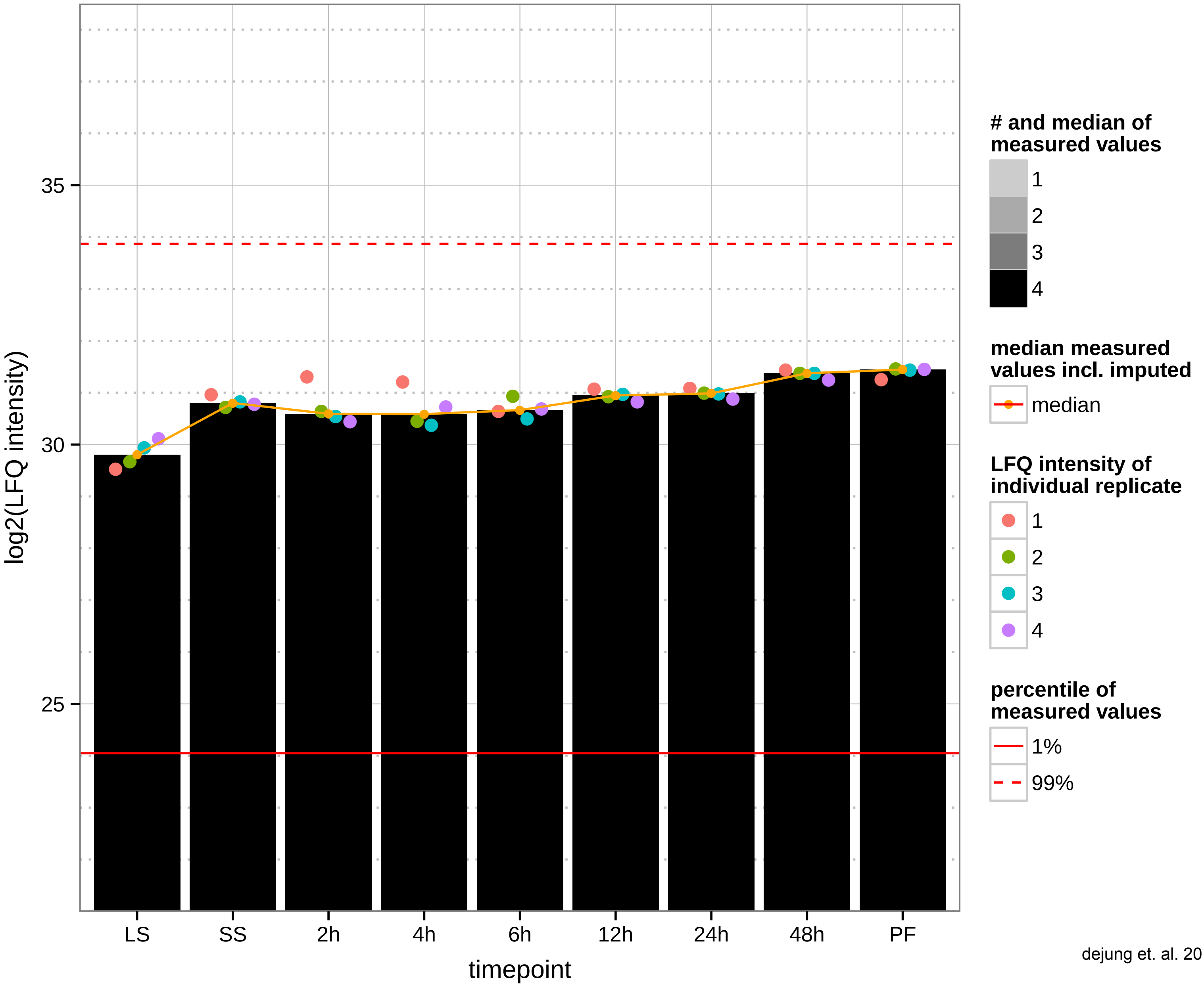

dejung et. al. 2015

Essential nuclear protein 1, putative (enp1)
Tb927.6.1900
AGOF: snoRNA binding
AGOC: nucleolus
AGOP: maturation of SSU−rRNA from tricistronic rRNA transcript (SSU−rRNA, 5.8S rRNA, LSU−rRNA), rRNA processing
PGOF: null
PGOC: null
PGOP: null

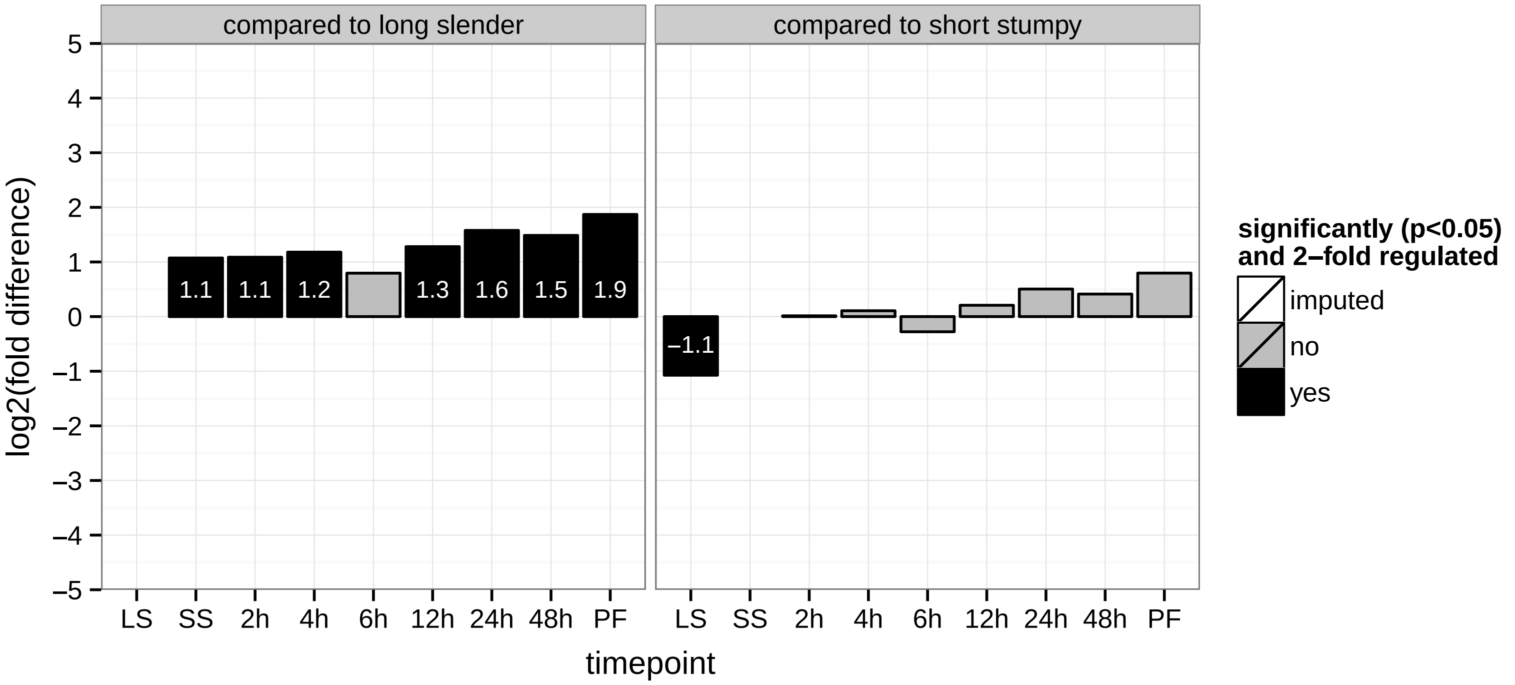

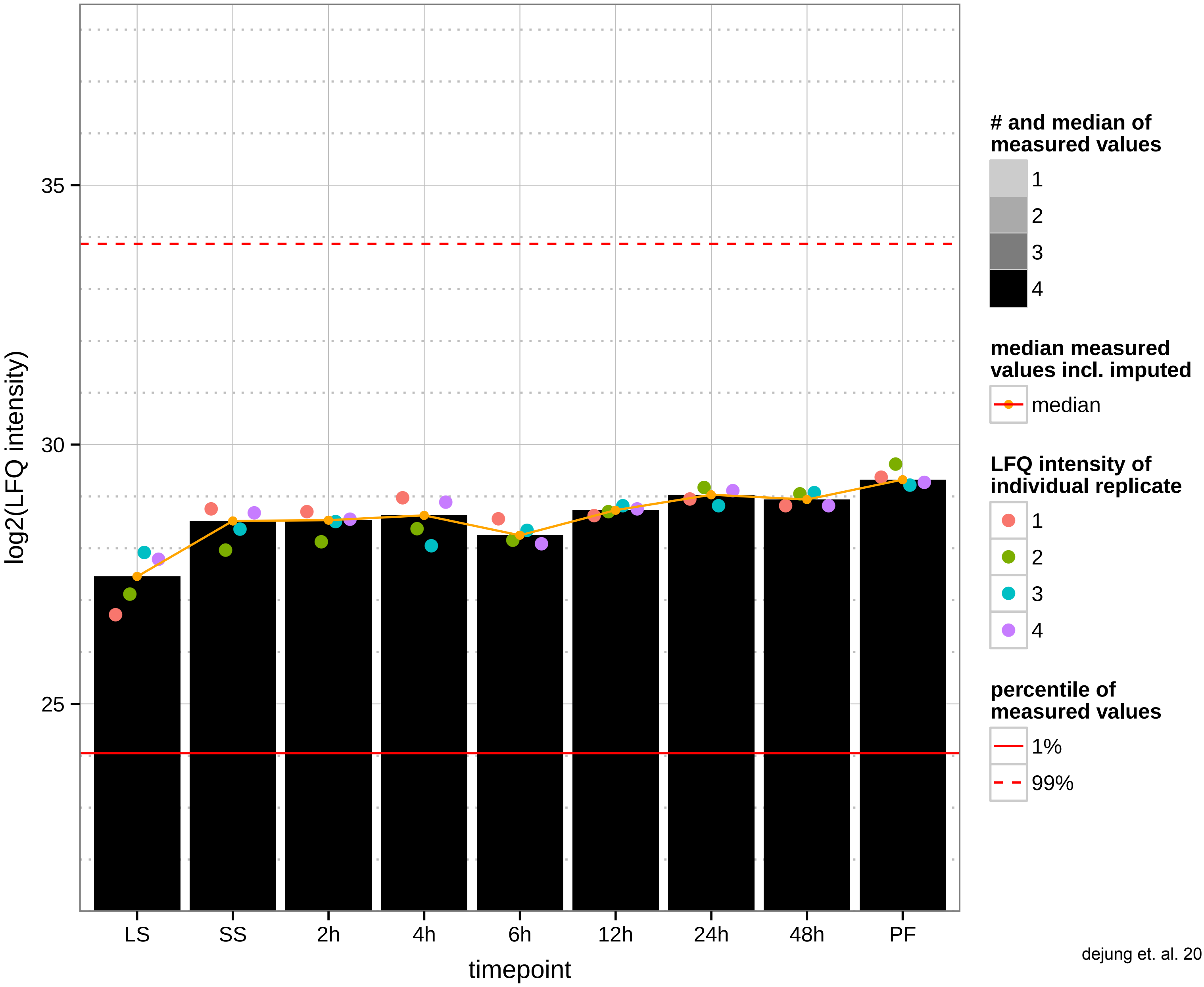

dejung et. al. 2015

hypothetical protein, conserved
Tb927.6.1920
AGOF: null
AGOC: null
AGOP: null
PGOF: null
PGOC: null
PGOP: null

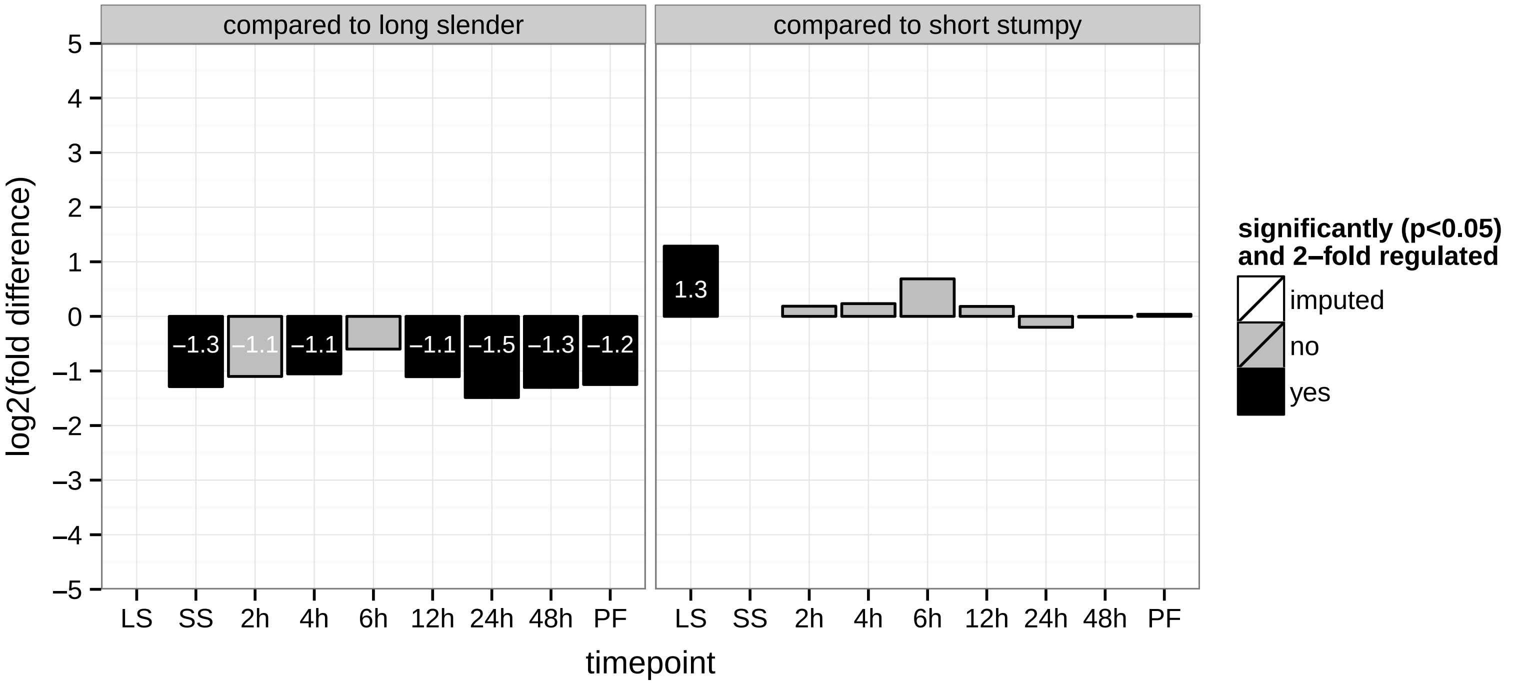

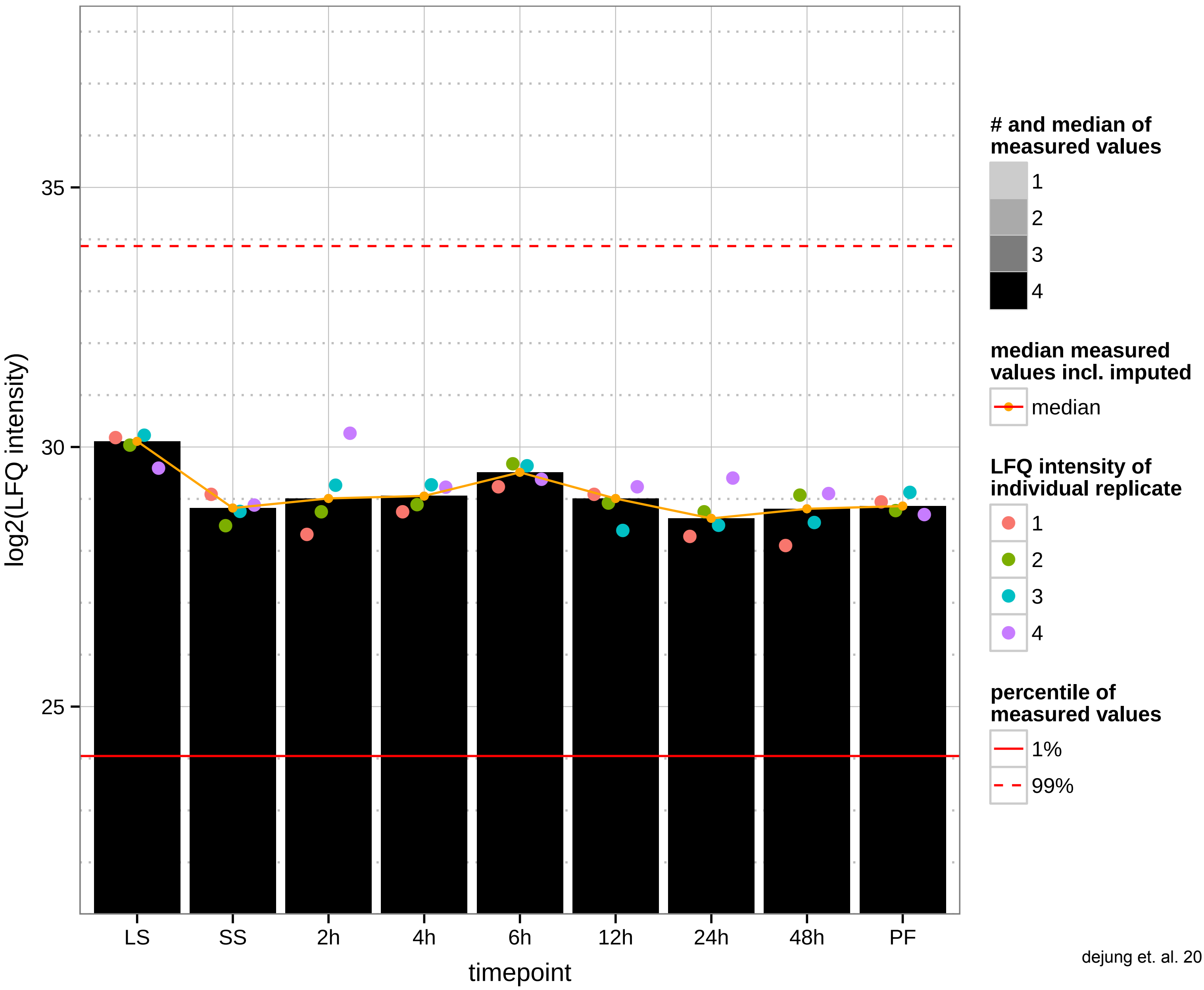

dejung et. al. 2015

hypothetical protein, conserved
Tb927.6.1950
AGOF: null
AGOC: null
AGOP: null
PGOF: null
PGOC: null
PGOP: null

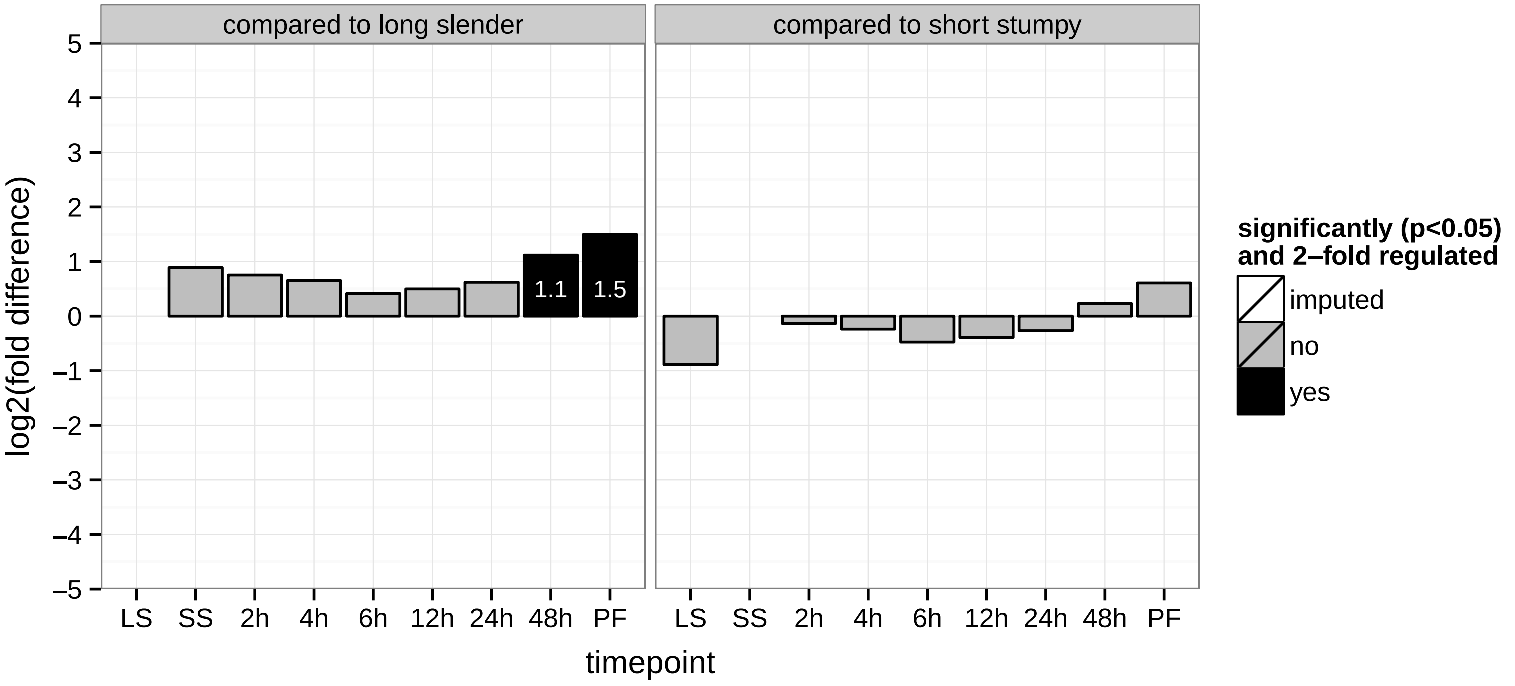

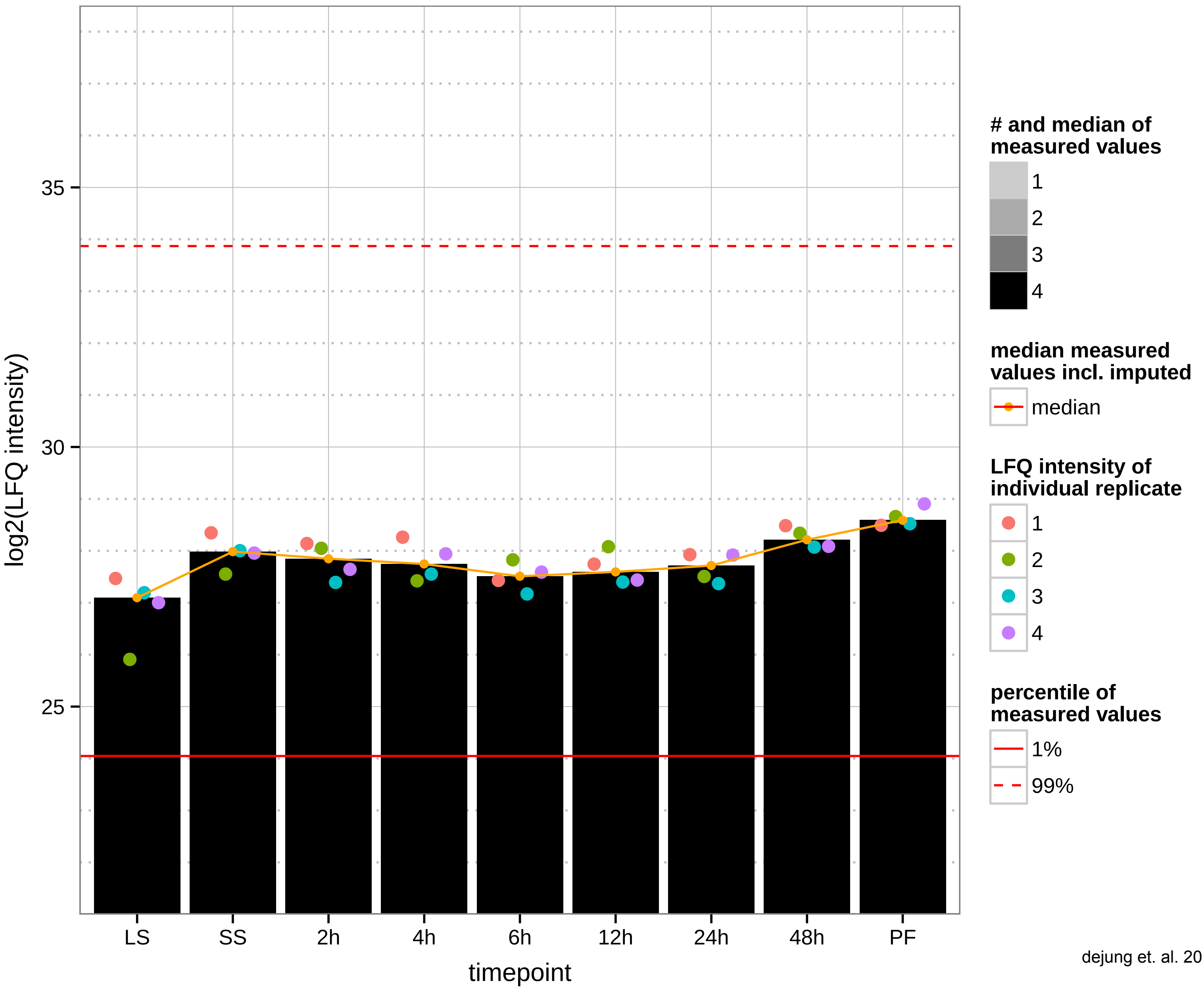

dejung et. al. 2015

NA, hypothetical protein, conserved
Tb927.6.1990
AGOF: NA, null
AGOC: NA, null
AGOP: NA, null
PGOF: NA, null
PGOC: NA, null
PGOP: NA, null

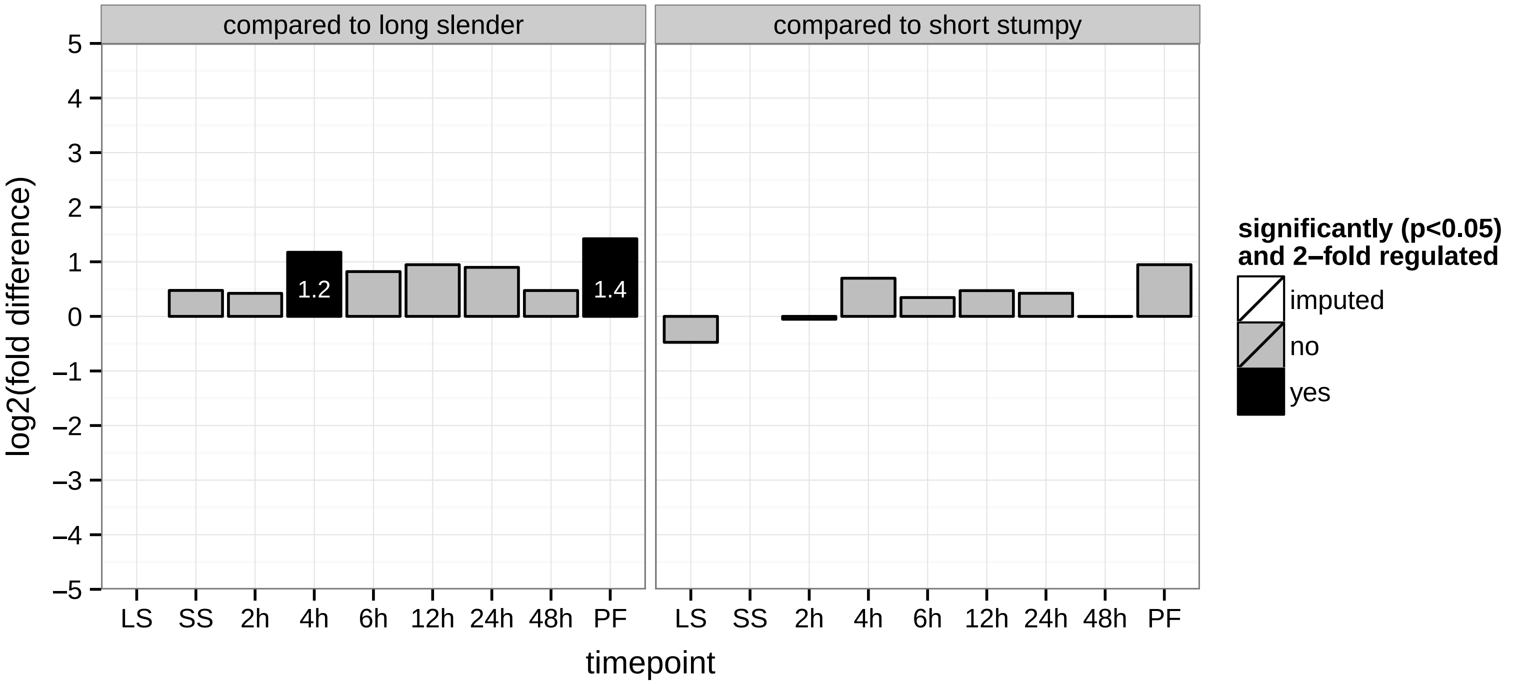

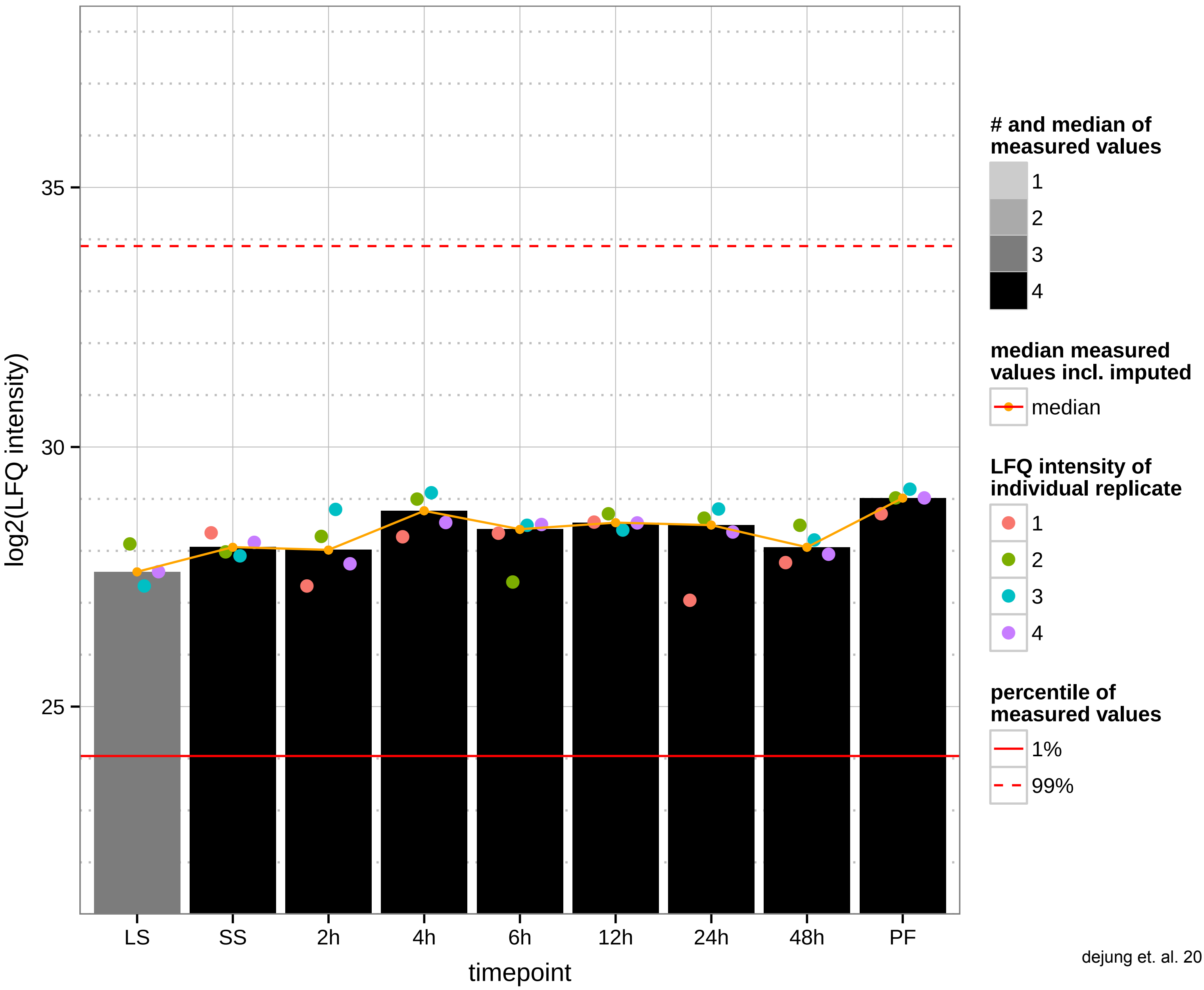

dejung et. al. 2015

spliceosome–associated protein, putative
Tb927.6.2000
AGOF: null
AGOC: nucleus
AGOP: mRNA processing
PGOF: null
PGOC: nucleus
PGOP: null

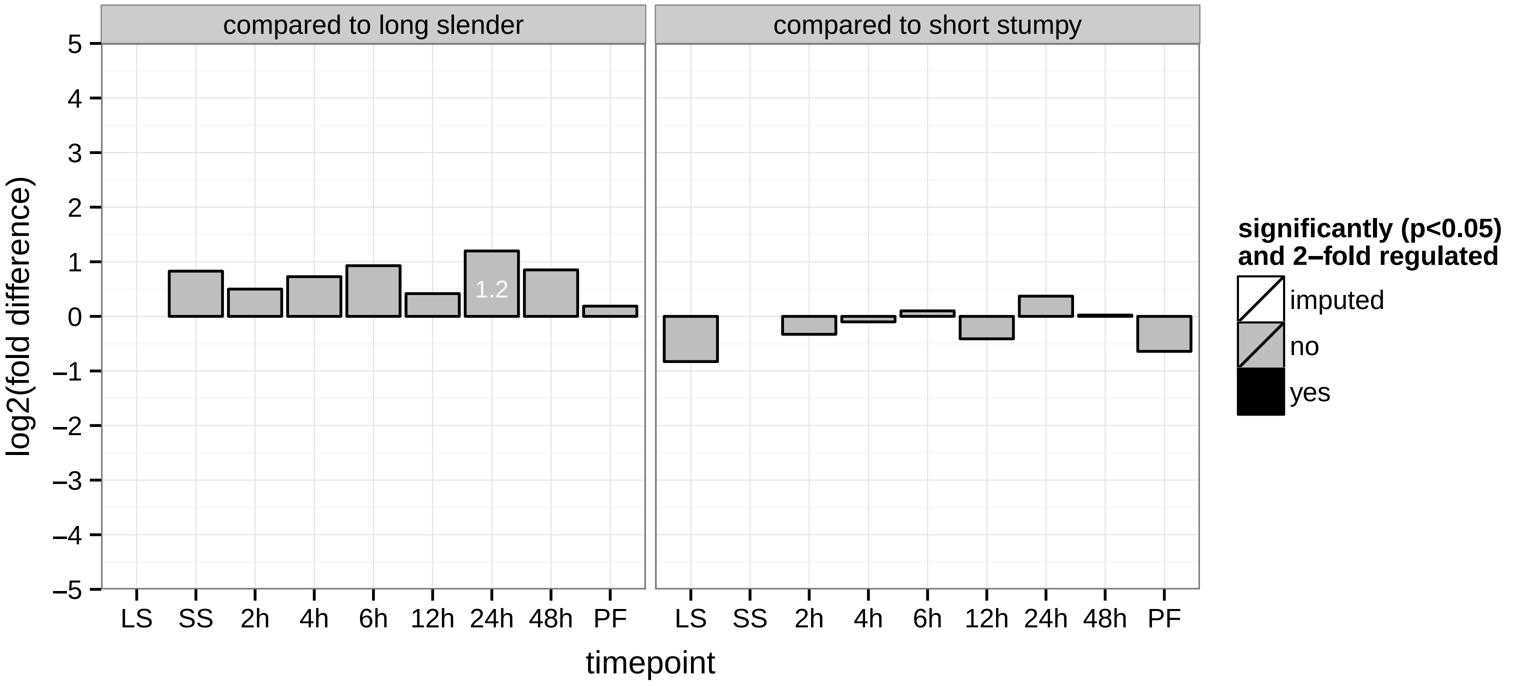

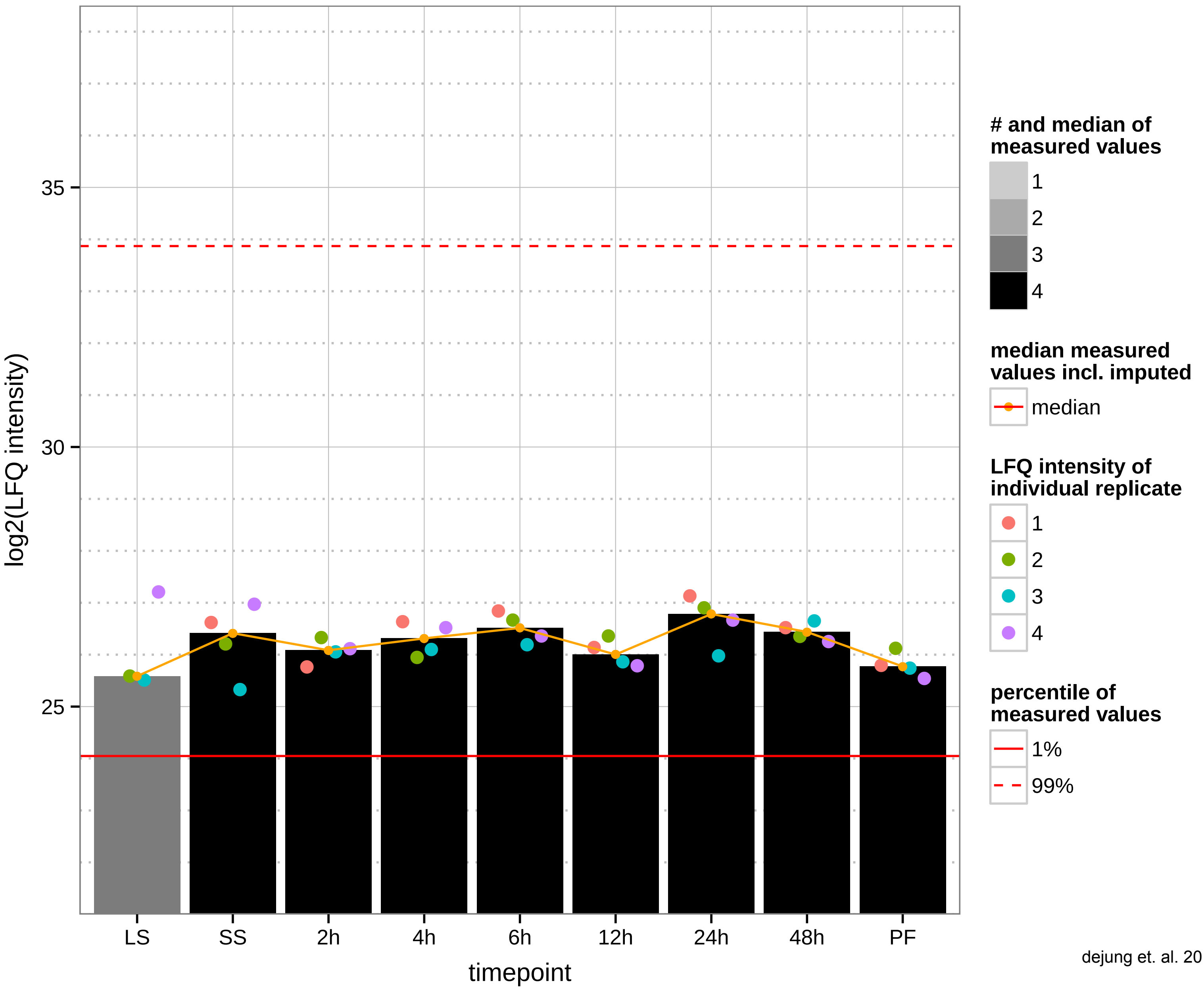

dejung et. al. 2015

ribosome biogenesis regulatory protein (RRS1), putative
Tb927.6.2050
AGOF: null
AGOC: nucleus
AGOP: ribosome biogenesis
PGOF: null
PGOC: nucleus
PGOP: ribosome biogenesis

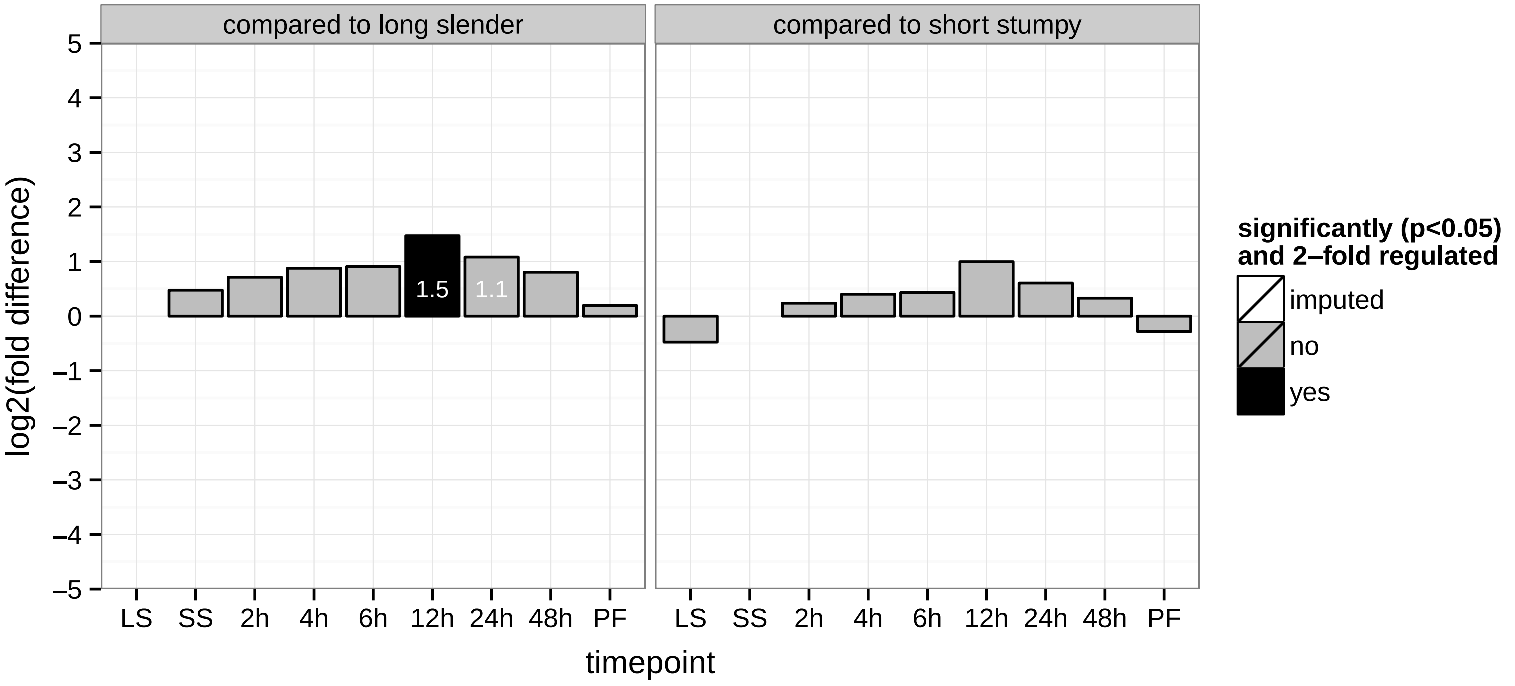

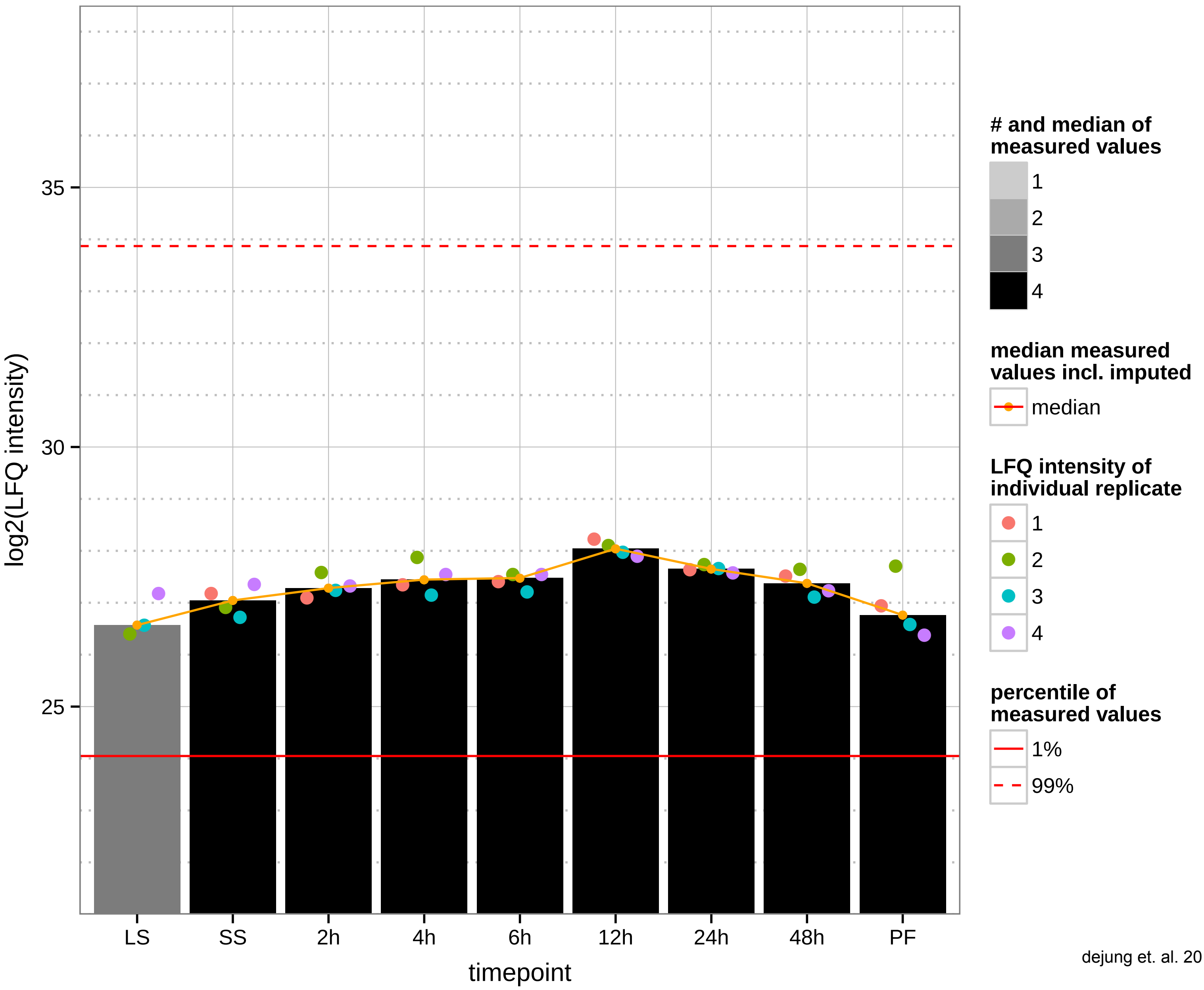

dejung et. al. 2015

histidyl–tRNA synthetase
Tb927.6.2060
AGOF: null
AGOC: cytoplasm
AGOP: translation
PGOF: ATP binding, aminoacyl–tRNA ligase activity, histidine–tRNA ligase activity, nucleotide binding
PGOC: cytoplasm
PGOP: histidyl–tRNA aminoacylation, tRNA aminoacylation for protein translation

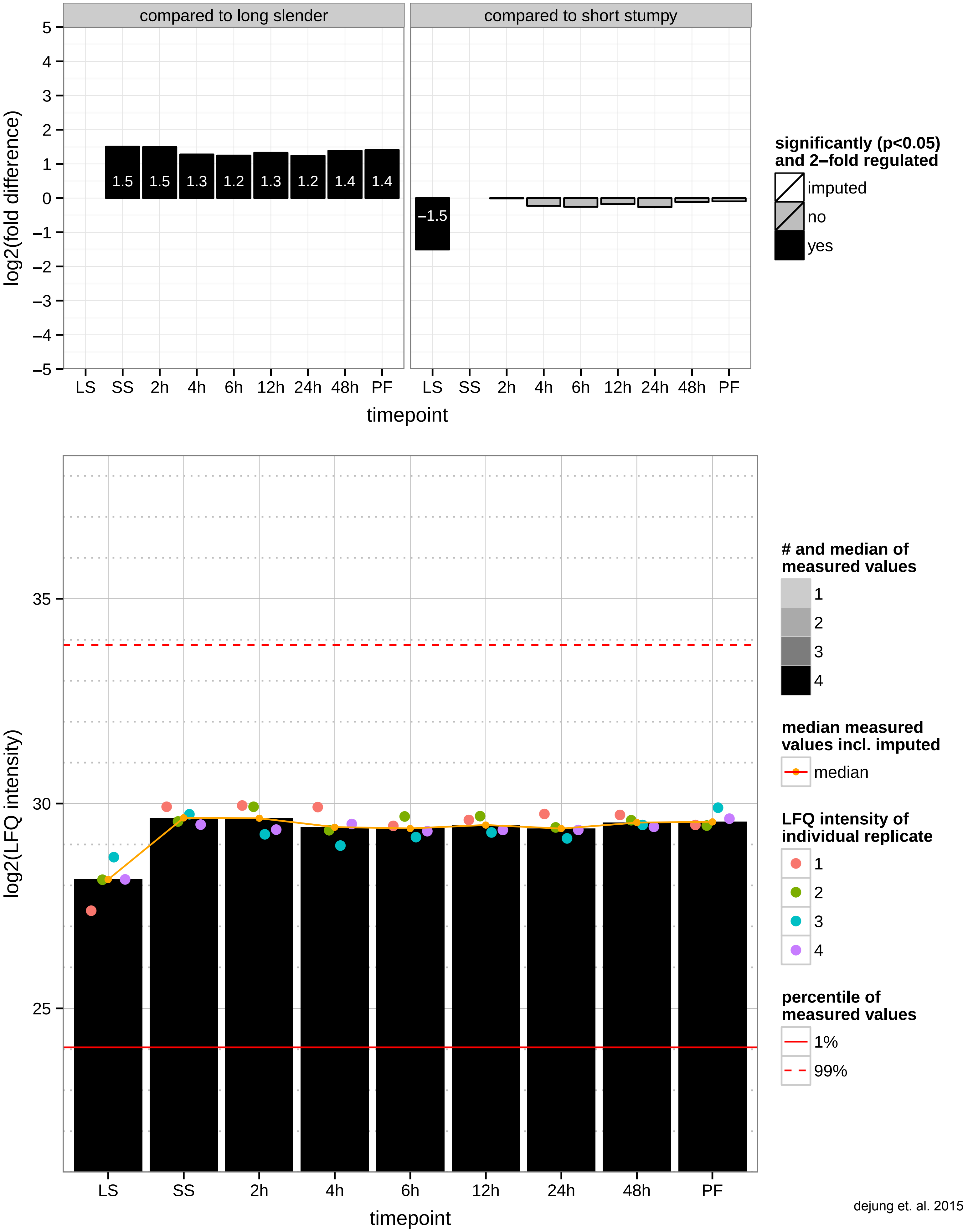

dejung et. al. 2015

hypothetical protein, conserved
Tb927.6.2090
AGOF: phospholipase C activity
AGOC: null
AGOP: intracellular signal transduction, lipid metabolic process
PGOF: phospholipase C activity, phosphoric diester hydrolase activity
PGOC: null
PGOP: intracellular signal transduction, lipid metabolic process

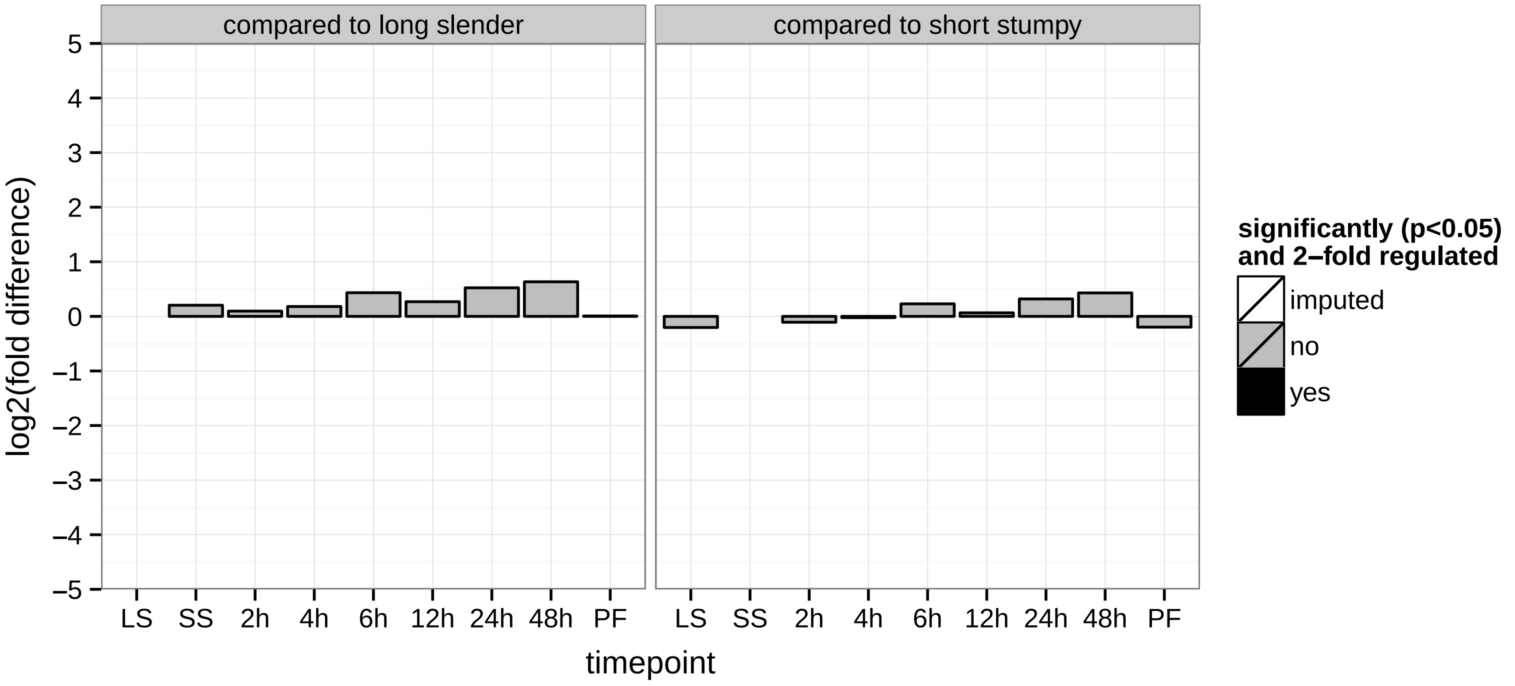

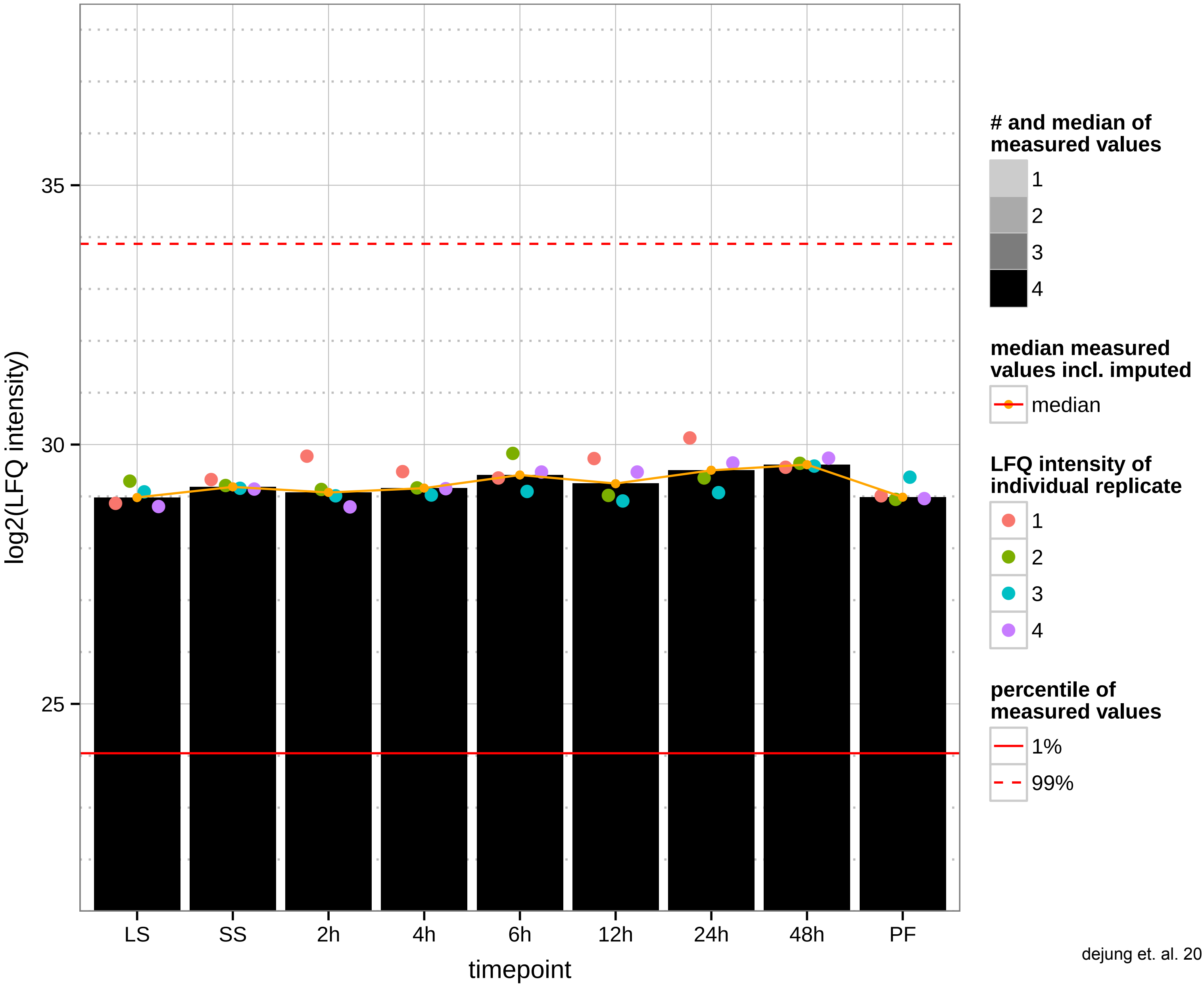

dejung et. al. 2015

nuclear movement protein, putative, NUDC–like protein
Tb927.6.2130
AGOF: null
AGOC: null
AGOP: null
PGOF: null
PGOC: null
PGOP: null

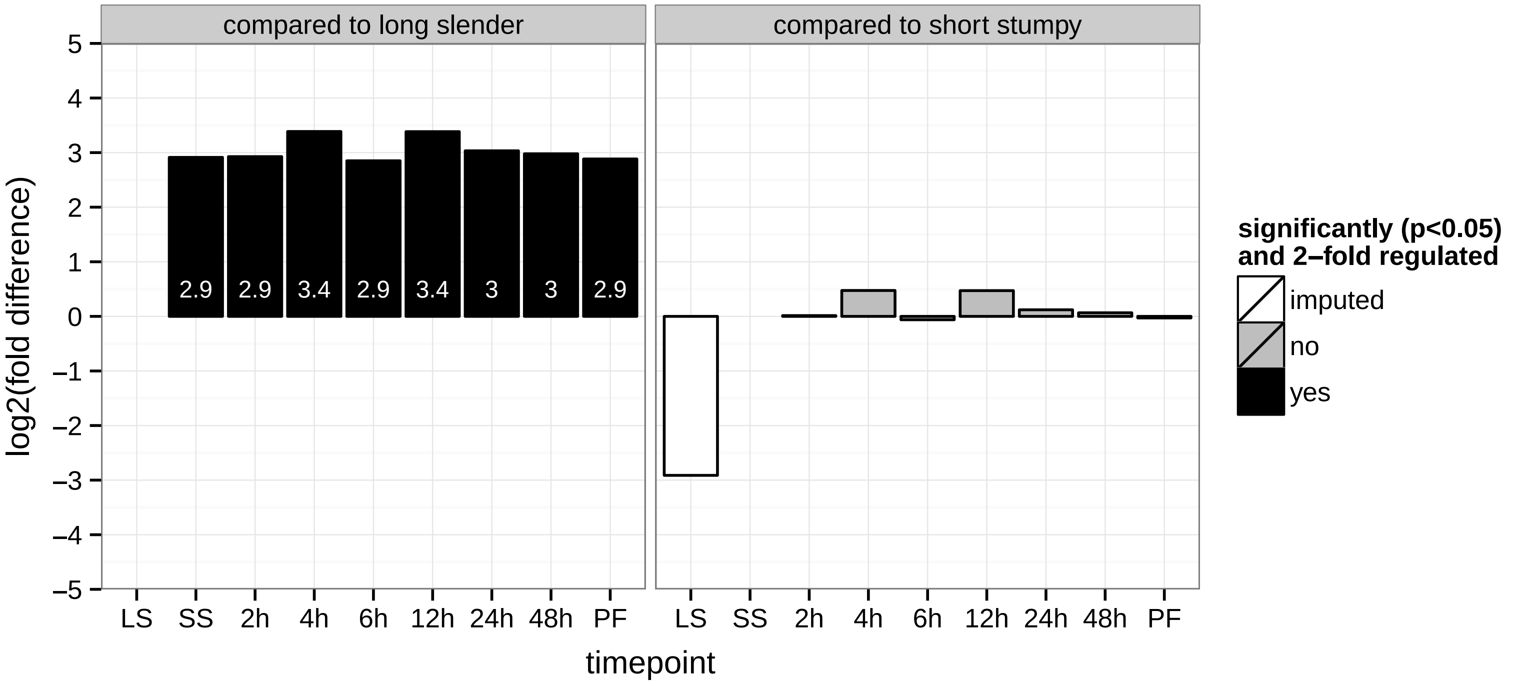

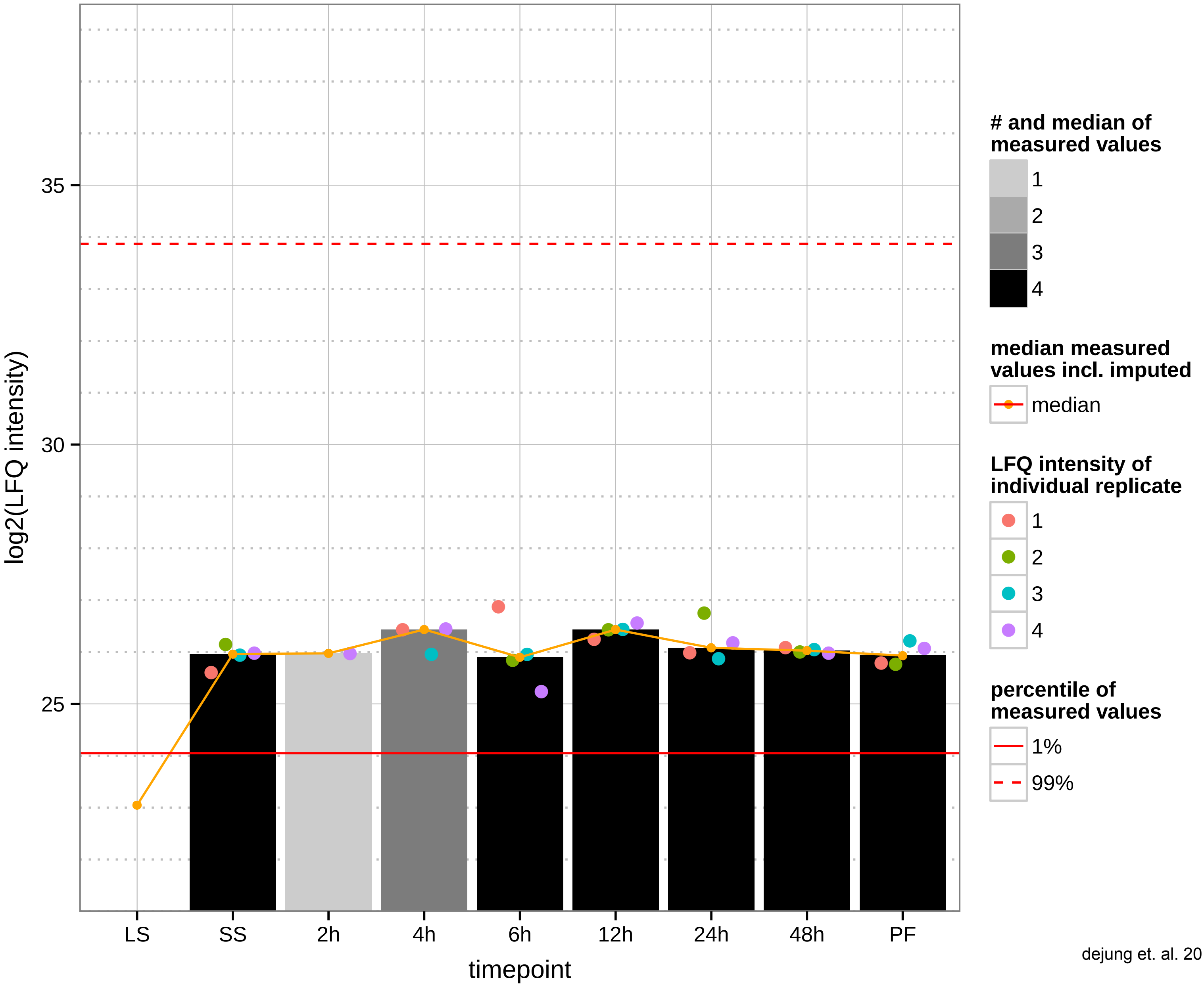

dejung et. al. 2015

hypothetical protein, conserved
Tb927.6.2210
AGOF: null
AGOC: null
AGOP: null
PGOF: binding, protein binding
PGOC: null
PGOP: null

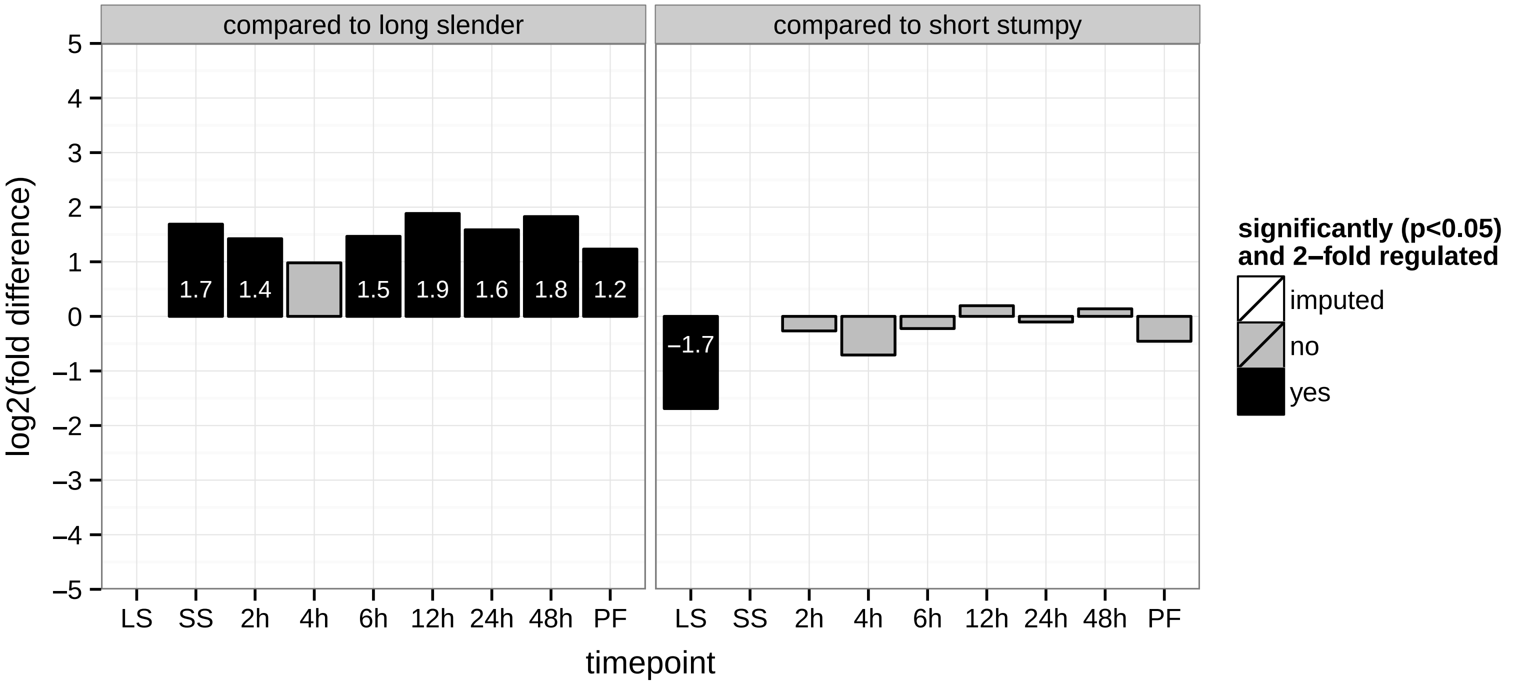

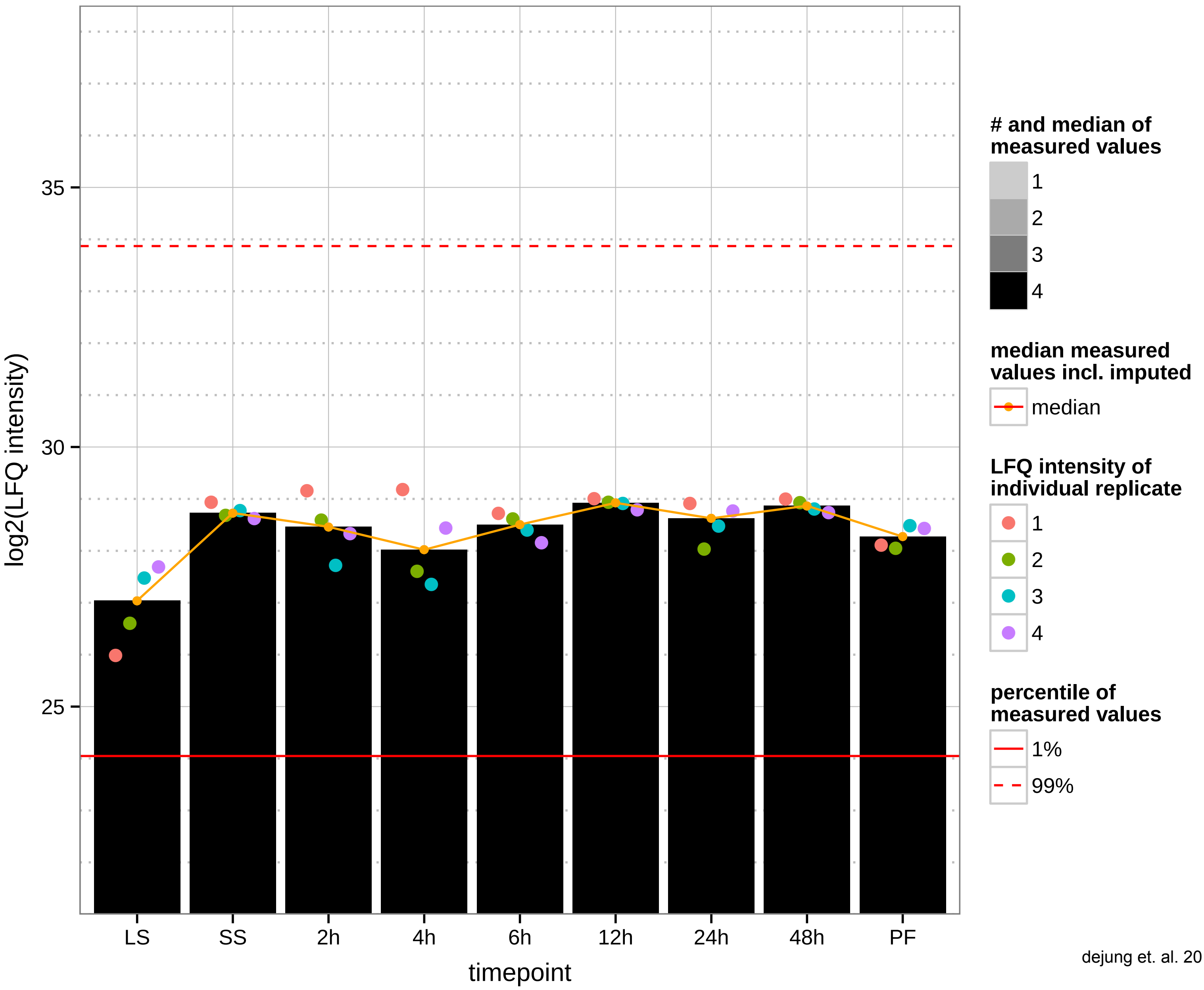

dejung et. al. 2015

RGG protein
Tb927.6.2230
AGOF: RNA binding
AGOC: mitochondrion
AGOP: RNA biosynthetic process
PGOF: null
PGOC: null
PGOP: null

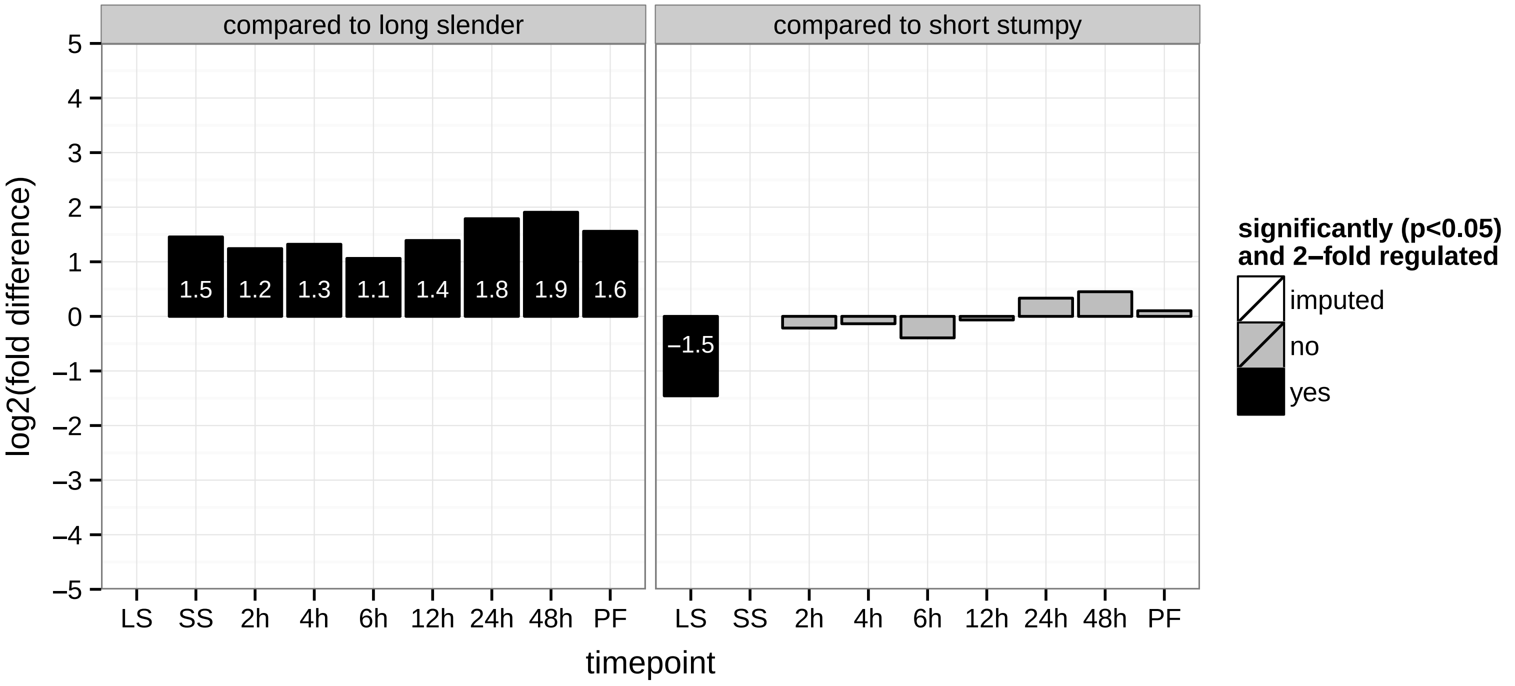

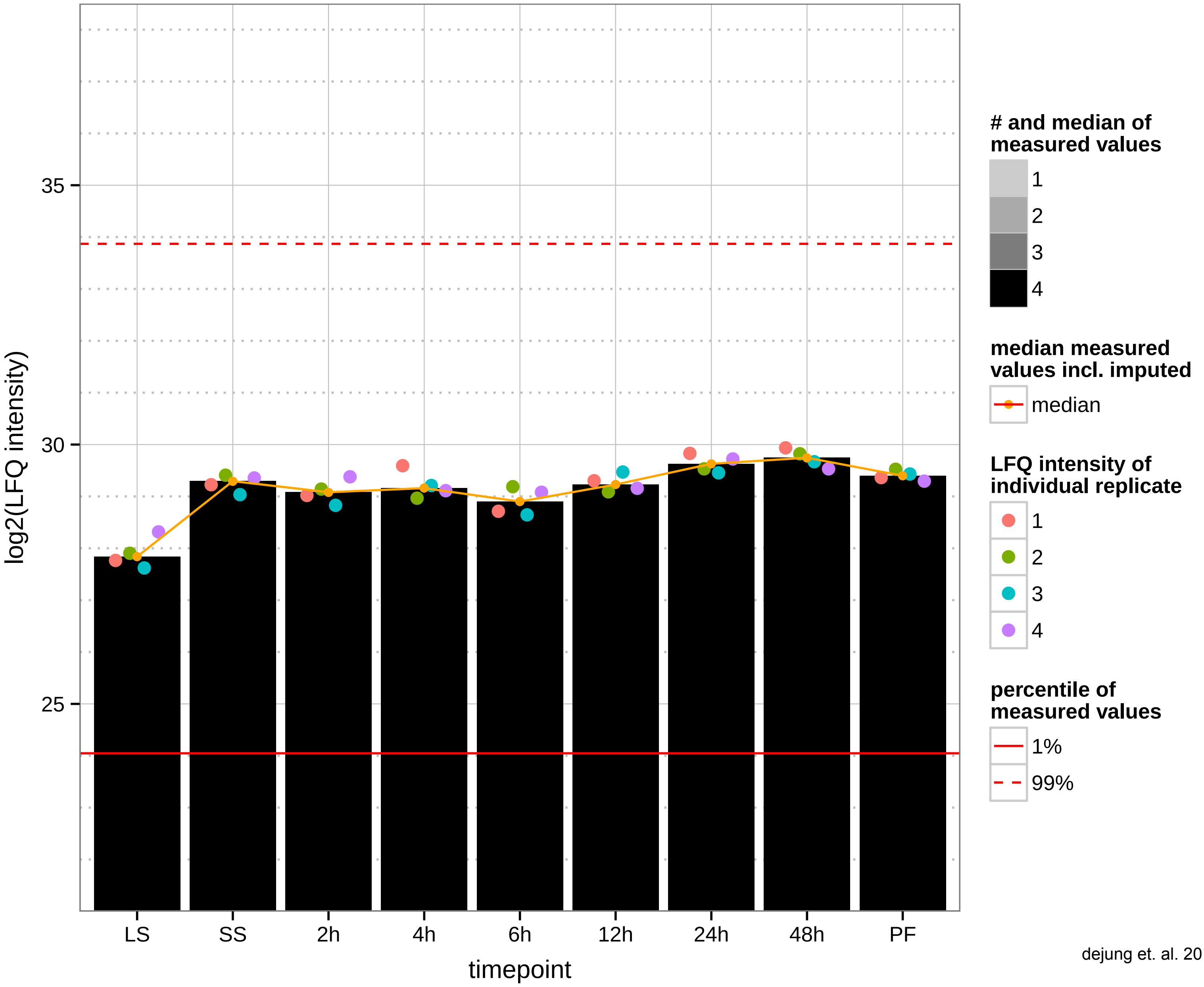

dejung et. al. 2015

hypothetical protein, conserved
Tb927.6.2350;Tb927.6.2290
AGOF: null
AGOC: mitochondrion, null
AGOP: null
PGOF: GTP binding, null
PGOC: null
PGOP: null

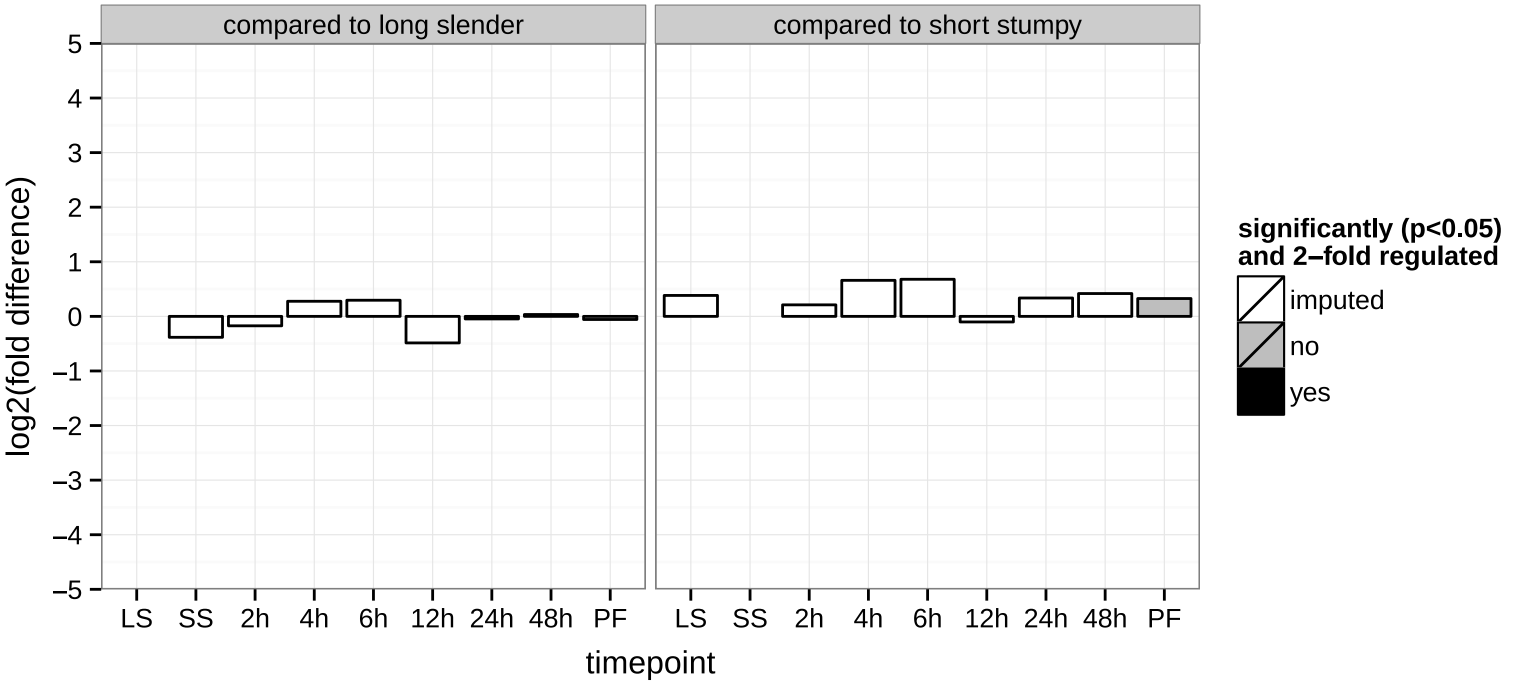

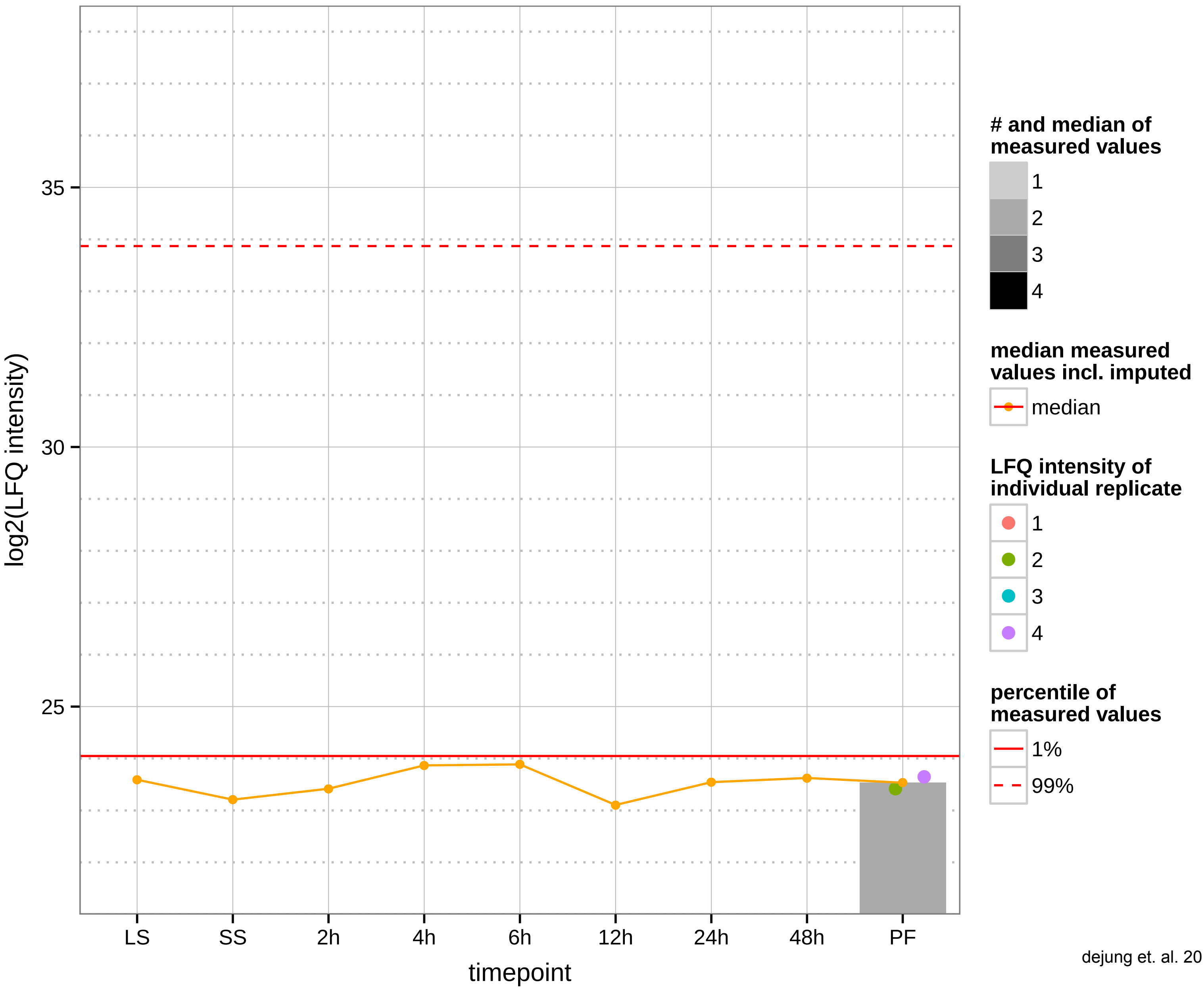

dejung et. al. 2015

adenosine kinase, putative
Tb927.6.2360;Tb927.6.2300
AGOF: adenosine kinase activity
AGOC: null
AGOP: purine ribonucleoside salvage, purine ribonucleotide biosynthetic process
PGOF: null
PGOC: null
PGOP: null

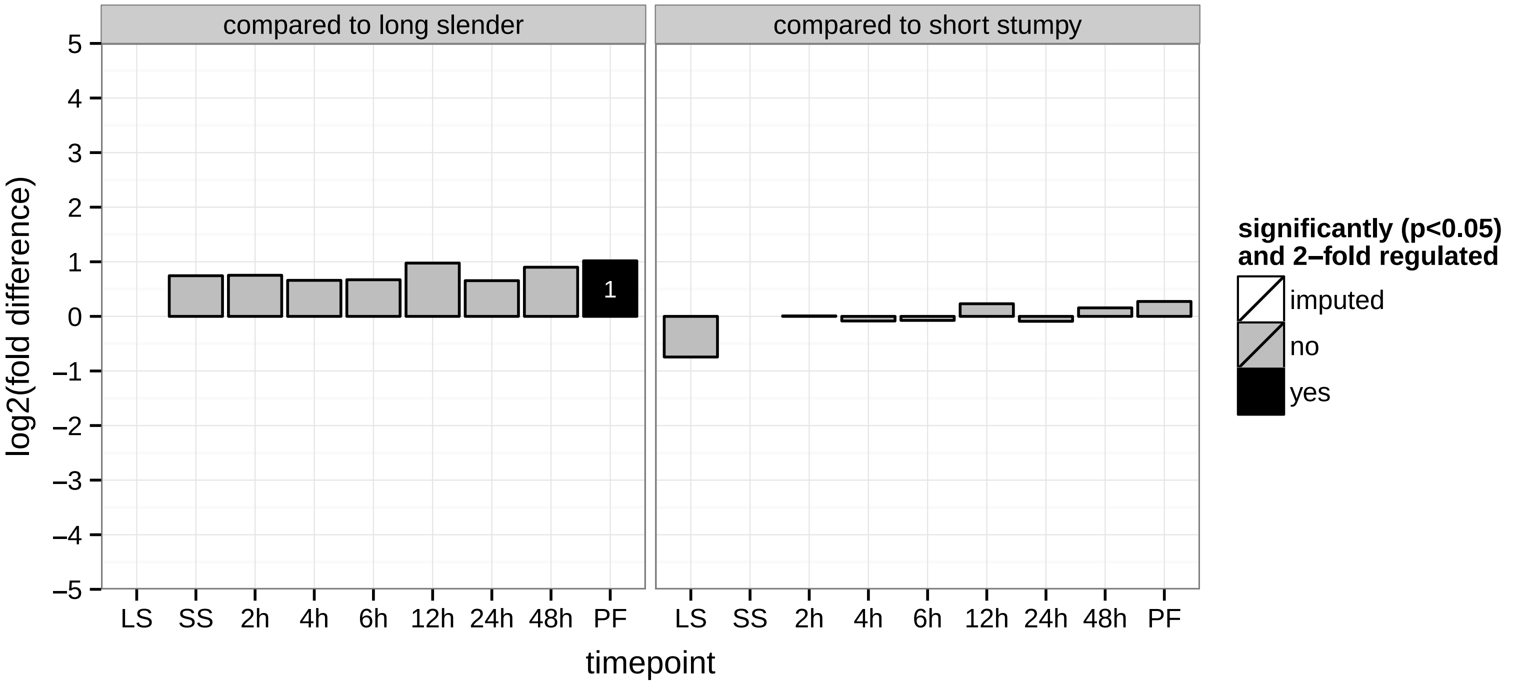

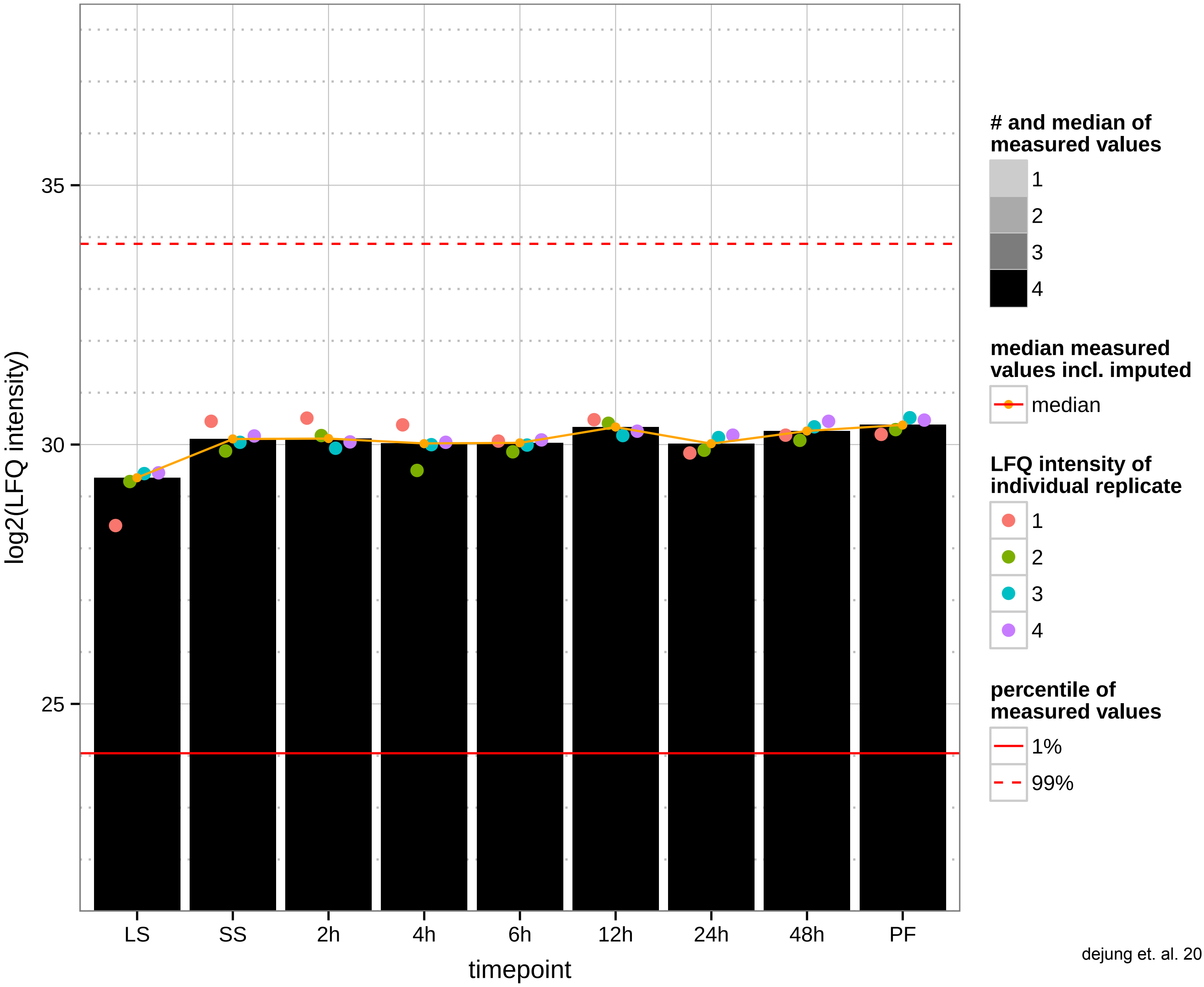

dejung et. al. 2015

ubiquitin–protein ligase, putative
Tb927.6.2370
AGOF: acid–amino acid ligase activity
AGOC: intracellular
AGOP: cellular protein modification process
PGOF: acid–amino acid ligase activity, protein binding
PGOC: intracellular
PGOP: cellular protein modification process

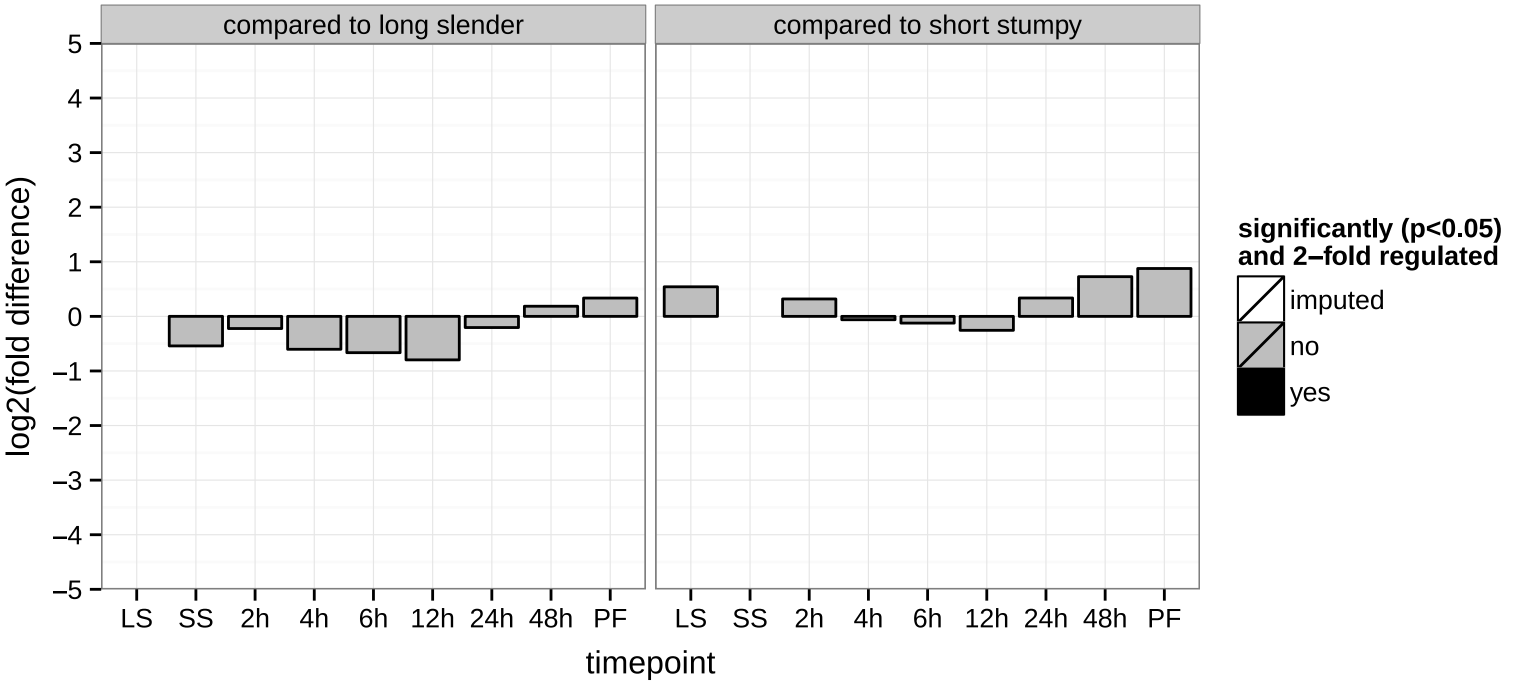

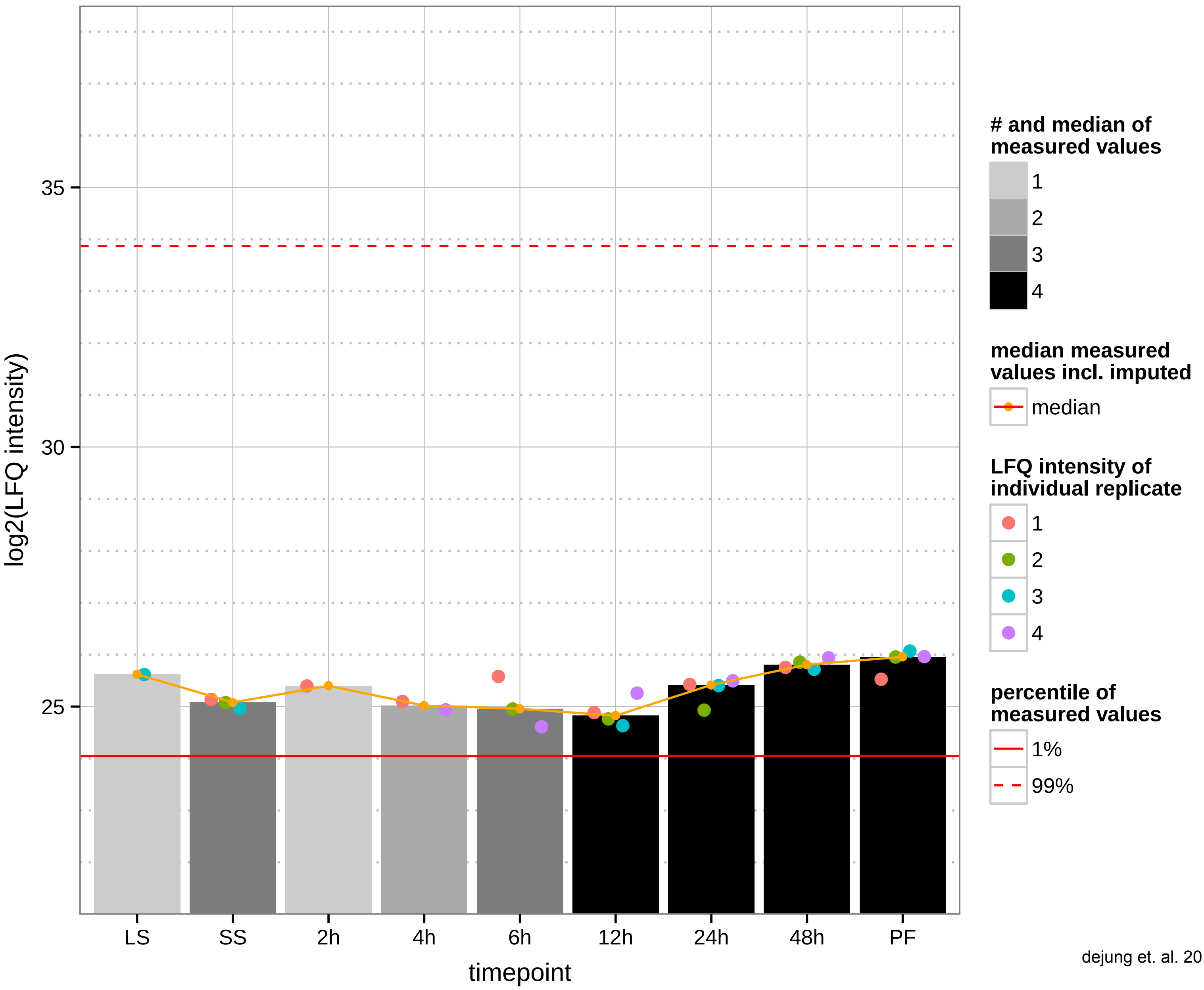

dejung et. al. 2015

hypothetical protein, conserved
Tb927.6.2400
AGOF: null
AGOC: microtubule organizing center, spindle pole
AGOP: microtubule cytoskeleton organization
PGOF: null
PGOC: microtubule organizing center, spindle pole
PGOP: microtubule cytoskeleton organization

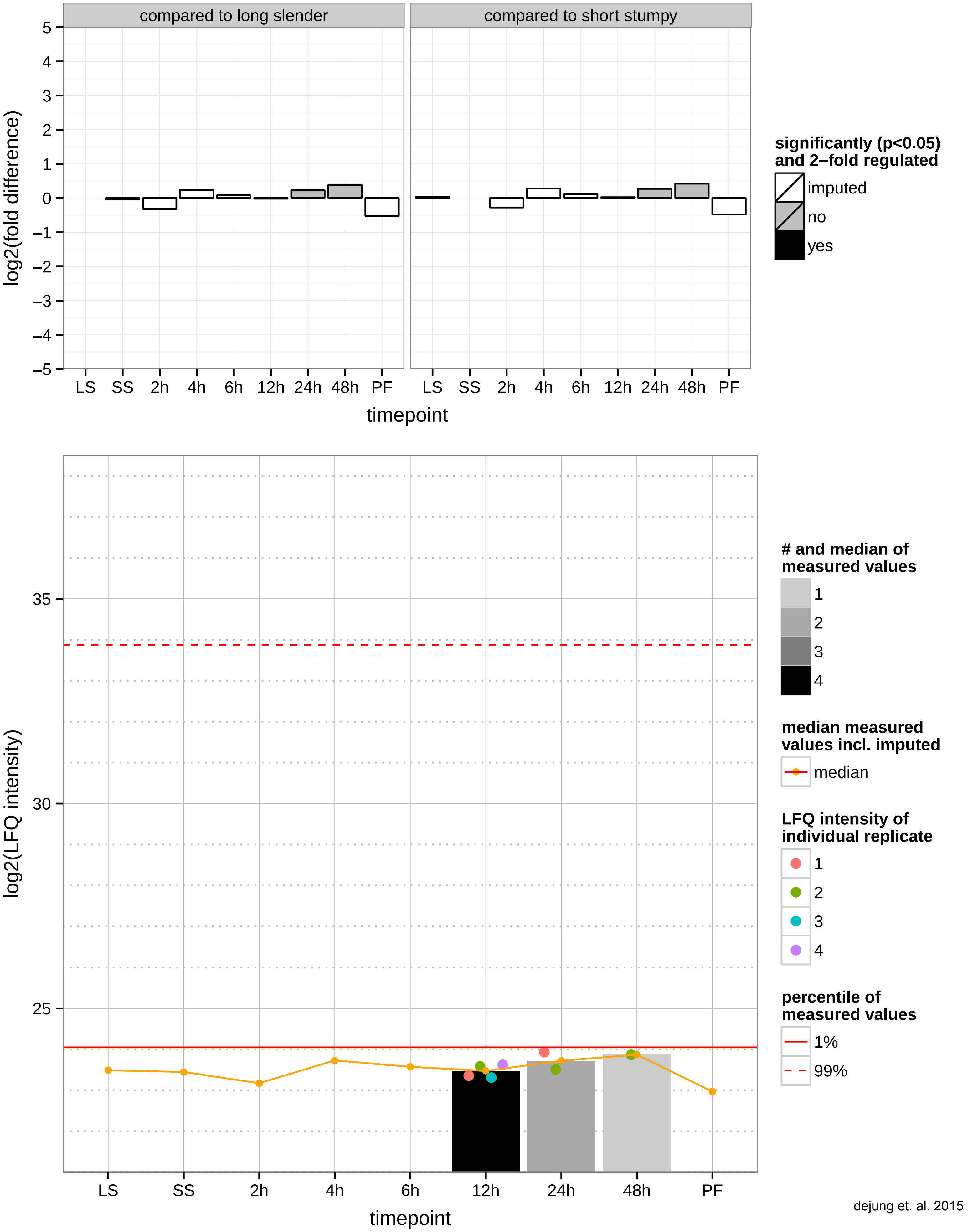

dejung et. al. 2015

p22 protein precursor
Tb927.6.2420
AGOF: protein binding
AGOC: mitochondrial matrix, mitochondrion
AGOP: RNA modification
PGOF: null
PGOC: mitochondrial matrix
PGOP: null

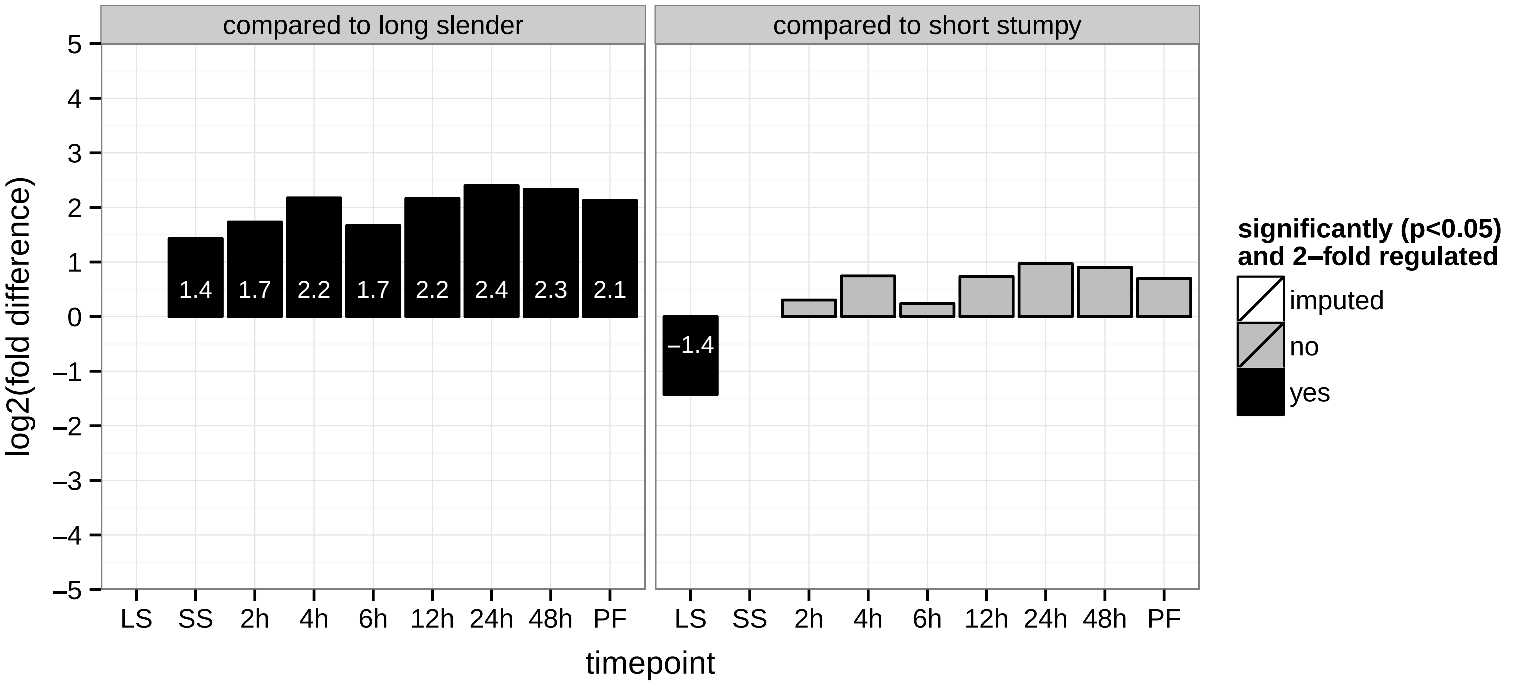

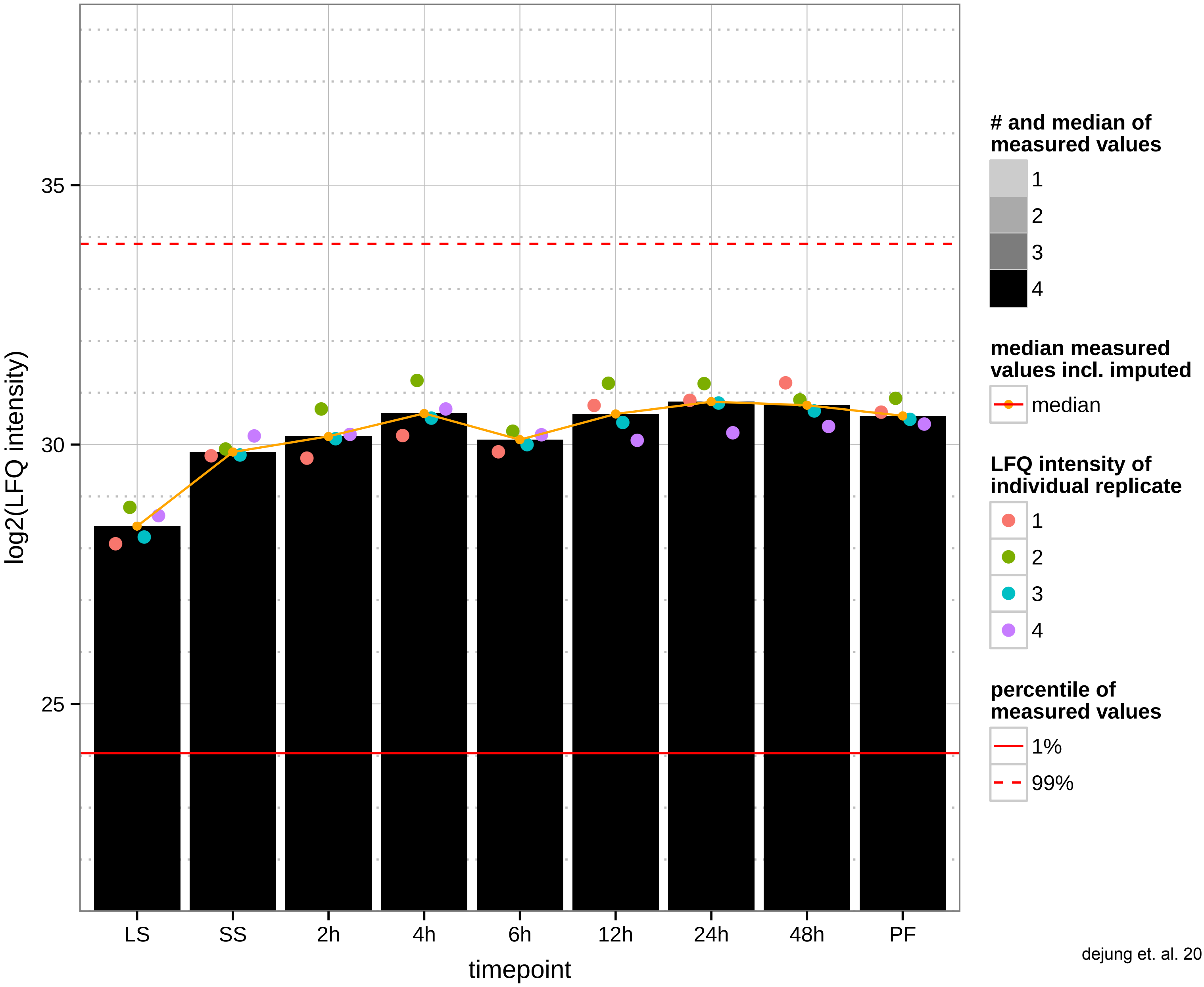

dejung et. al. 2015

hypothetical protein, conserved
Tb927.6.2440
AGOF: null
AGOC: null
AGOP: null
PGOF: binding
PGOC: null
PGOP: null

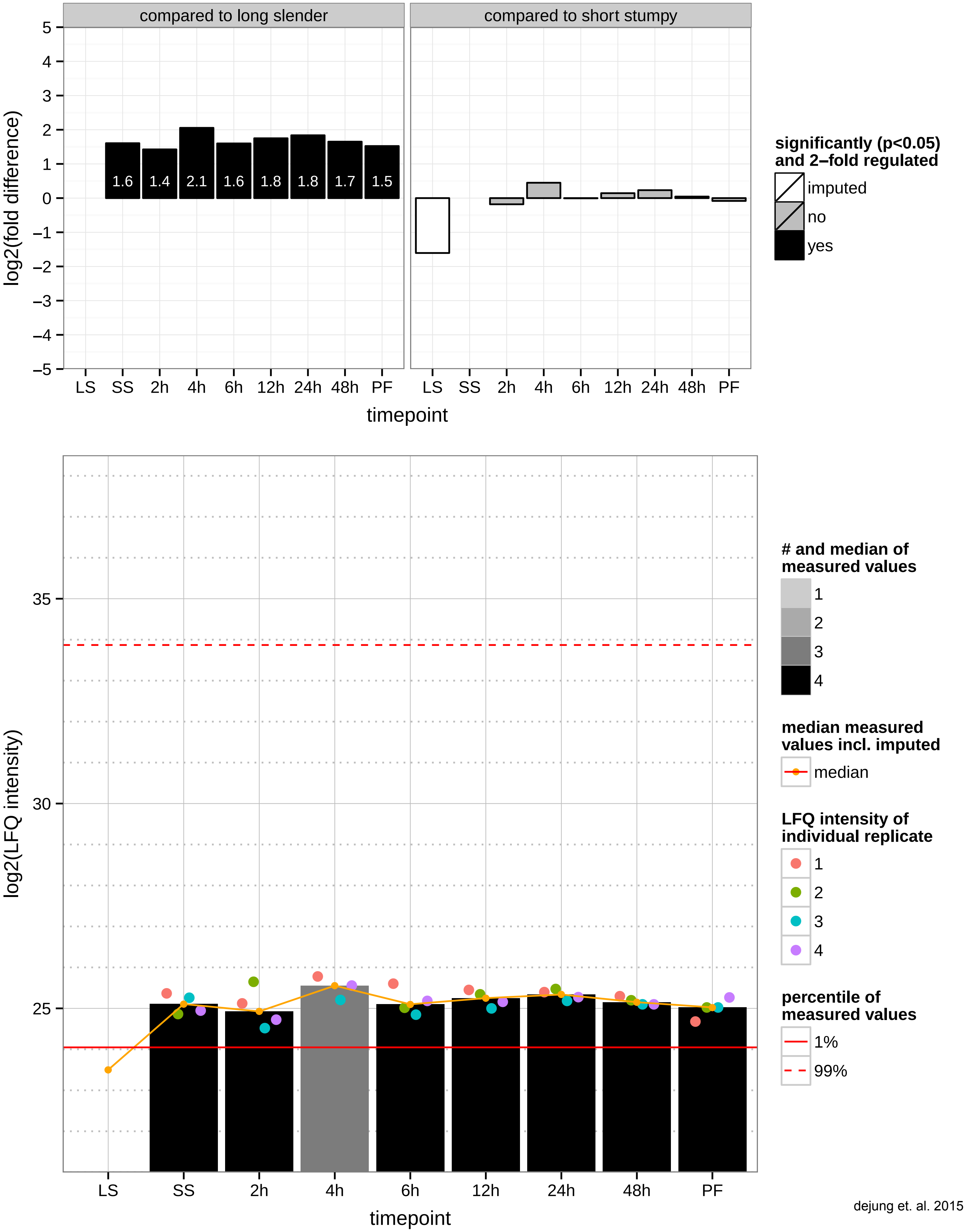

dejung et. al. 2015

ubiquitin fusion degradation protein 2, putative, ubiquitin conjugation factor E4 B
Tb927.6.2460
AGOF: ubiquitin–ubiquitin ligase activity
AGOC: ubiquitin ligase complex
AGOP: cell redox homeostasis, protein ubiquitination, ubiquitin–dependent protein catabolic process
PGOF: ubiquitin–ubiquitin ligase activity
PGOC: ubiquitin ligase complex
PGOP: cell redox homeostasis, protein ubiquitination, ubiquitin–dependent protein catabolic process

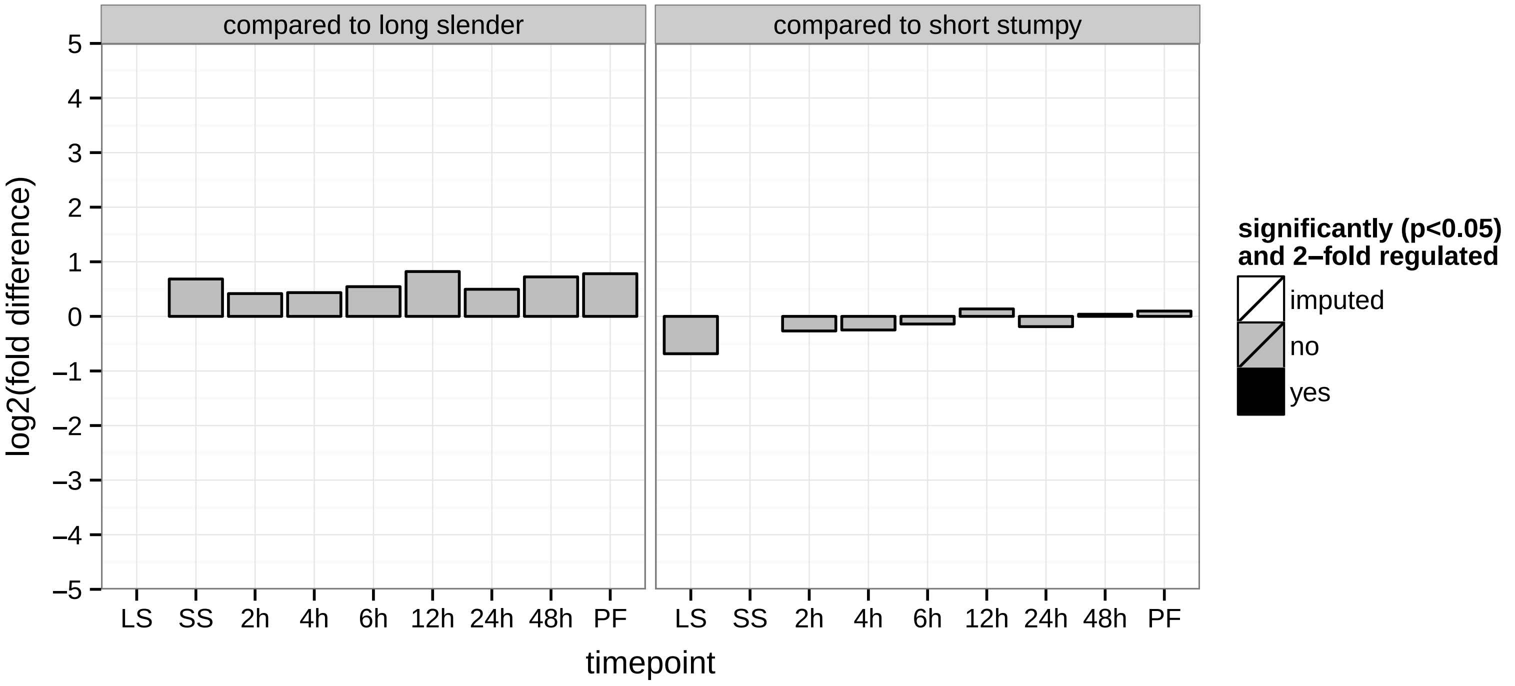

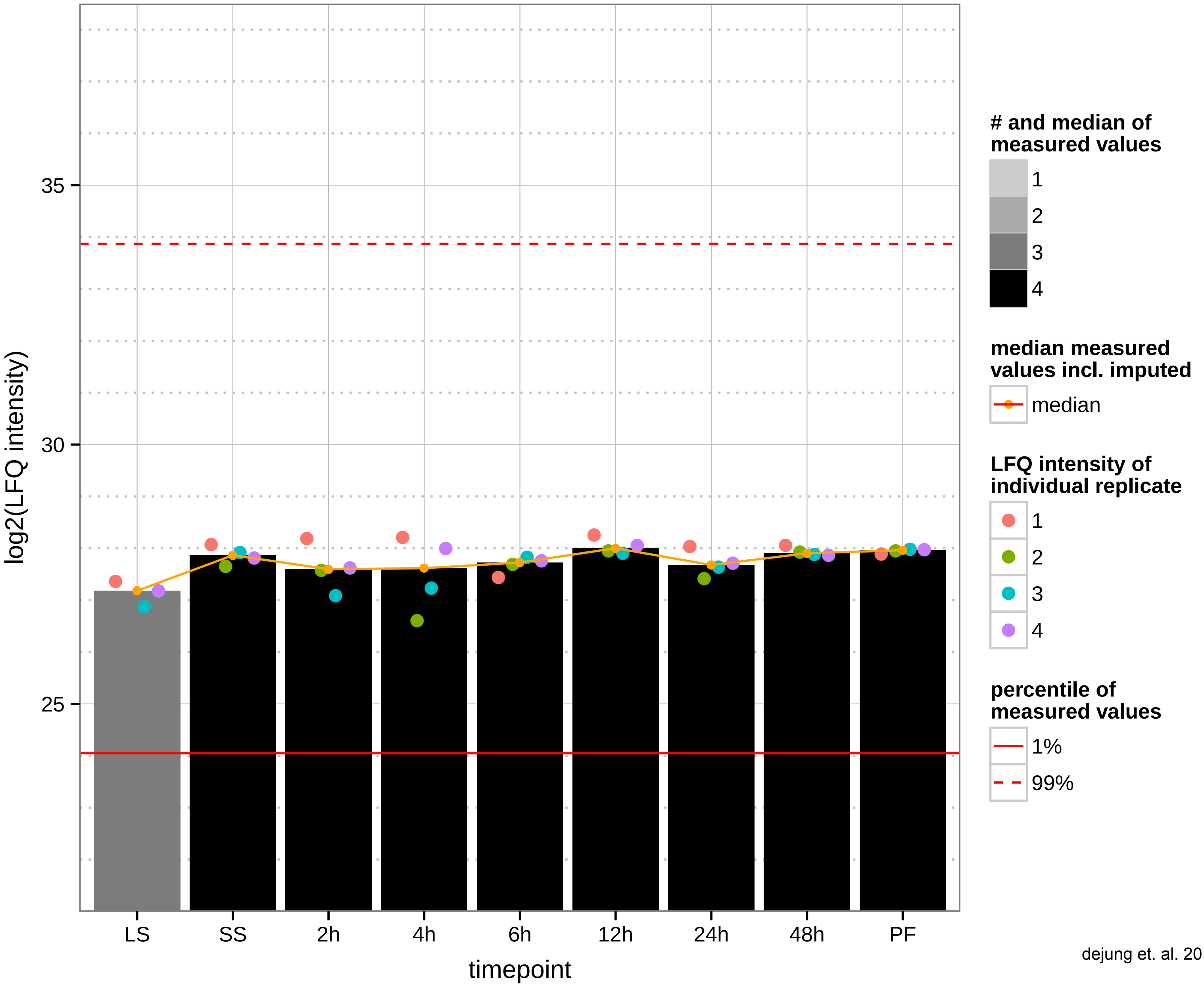

dejung et. al. 2015

hypothetical protein, conserved
Tb927.6.2520
AGOF: null
AGOC: null
AGOP: null
PGOF: null
PGOC: null
PGOP: null

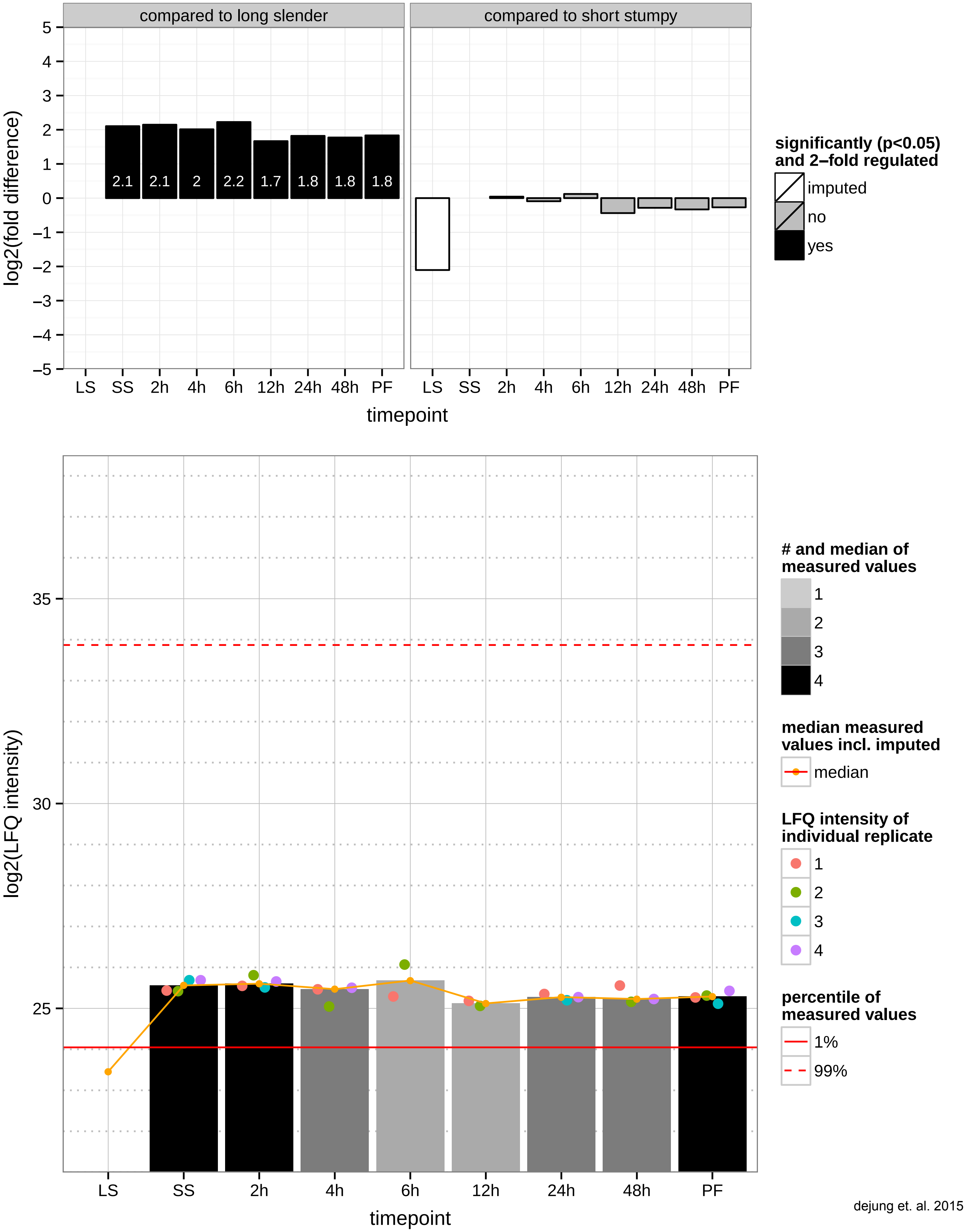

dejung et. al. 2015

RNA–binding protein, putative (TRRM2)
Tb927.6.2550
AGOF: RNA binding
AGOC: null
AGOP: RNA processing
PGOF: nucleic acid binding
PGOC: null
PGOP: null

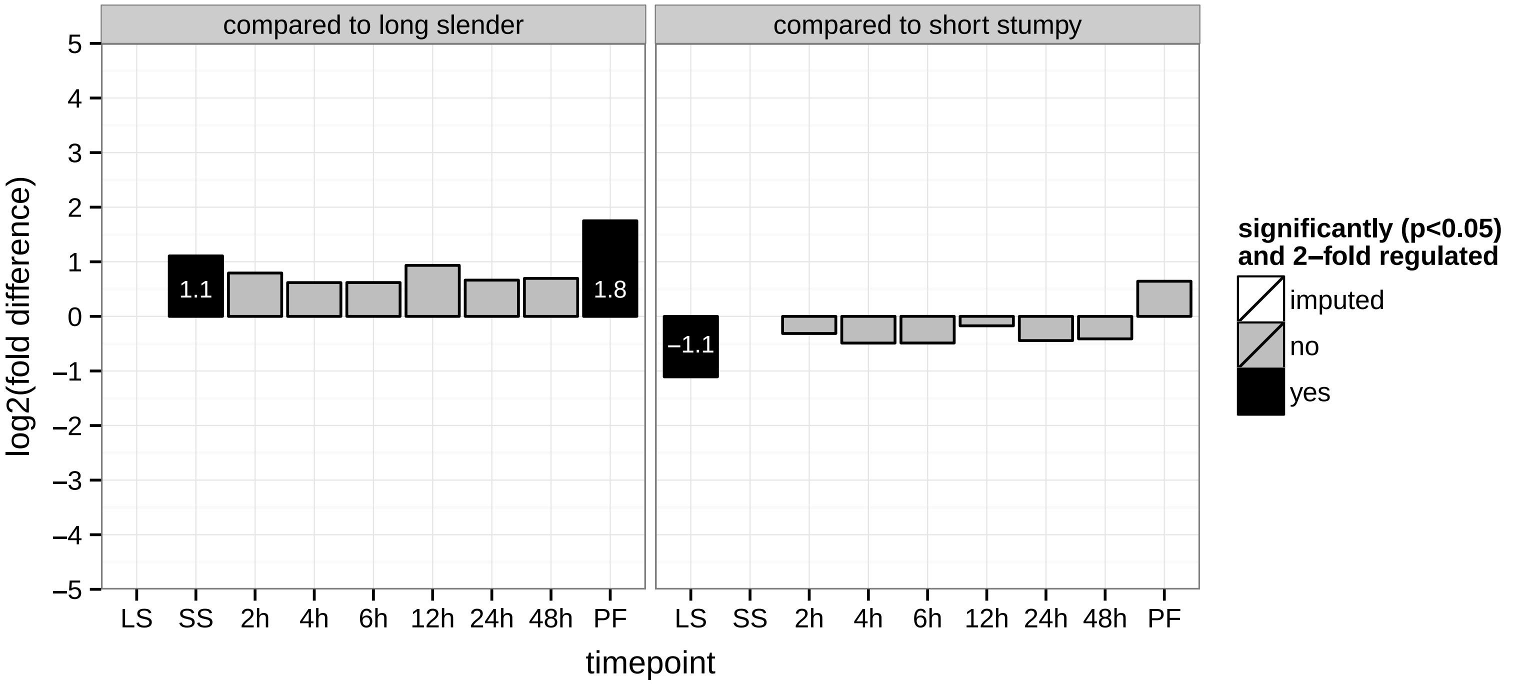

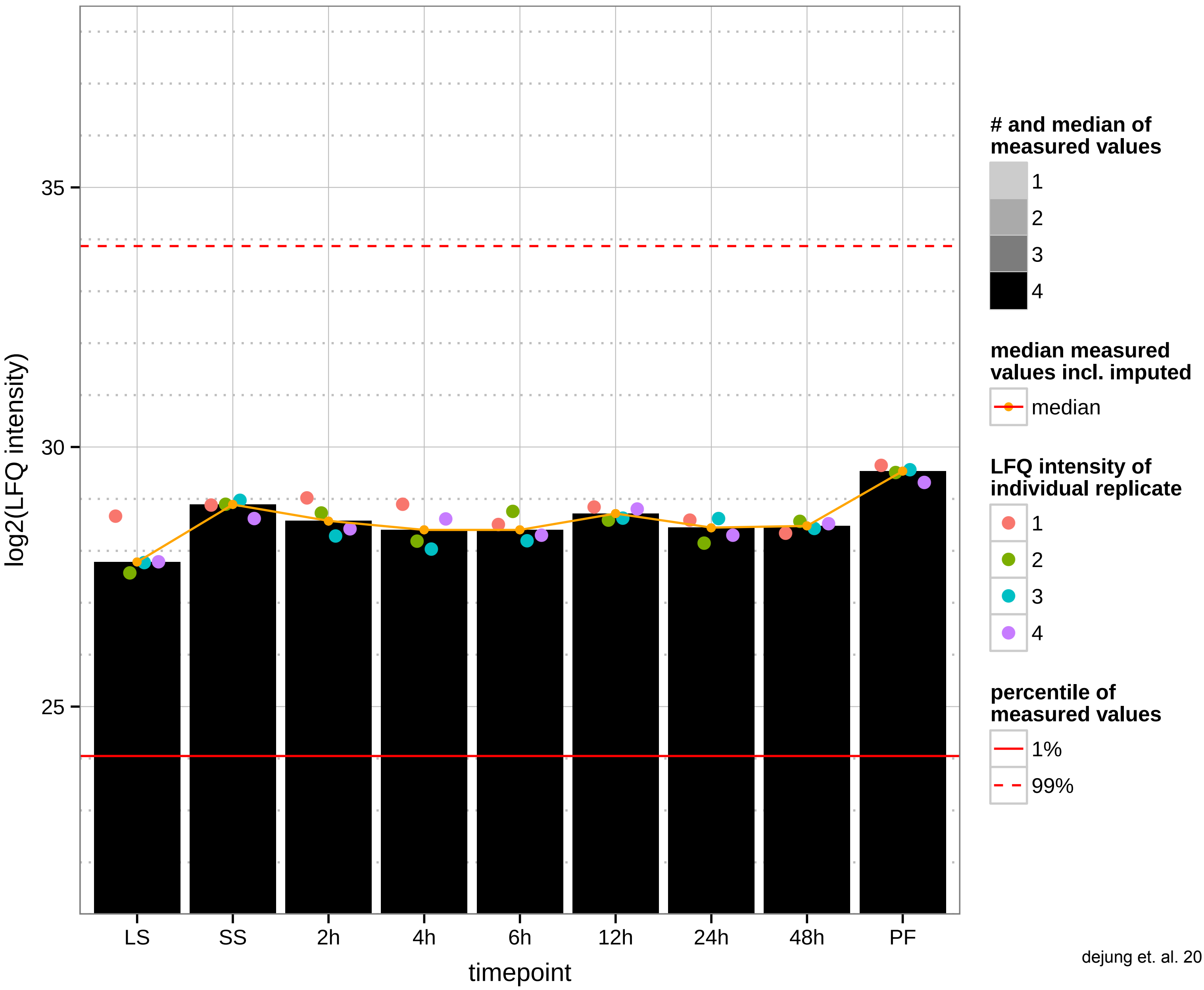

dejung et. al. 2015

importin alpha subunit, putative (TbKap60)
Tb927.6.2640
AGOF: null
AGOC: null
AGOP: null
PGOF: binding, protein binding, protein transporter activity
PGOC: cytoplasm, nucleus
PGOP: protein import into nucleus

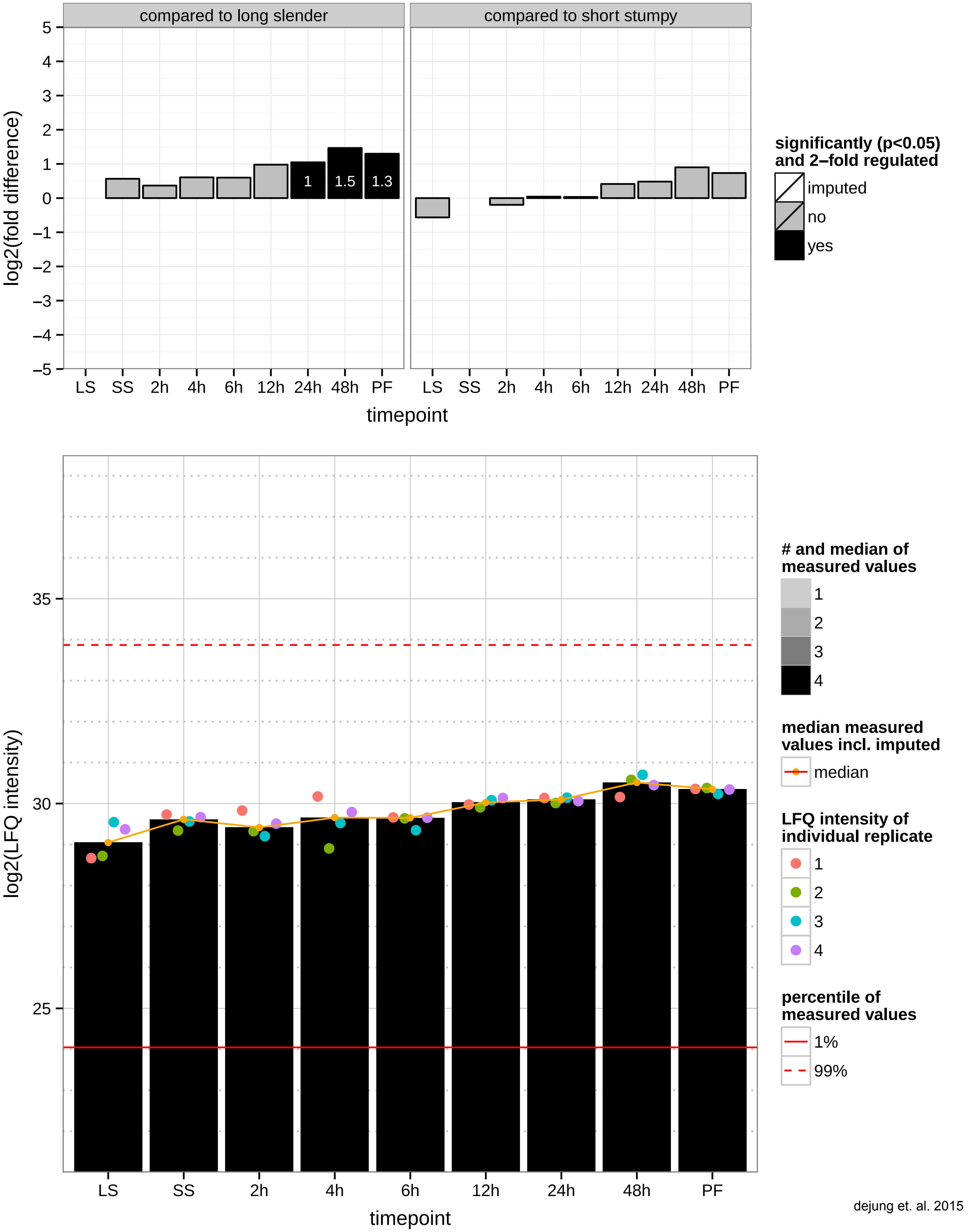

dejung et. al. 2015

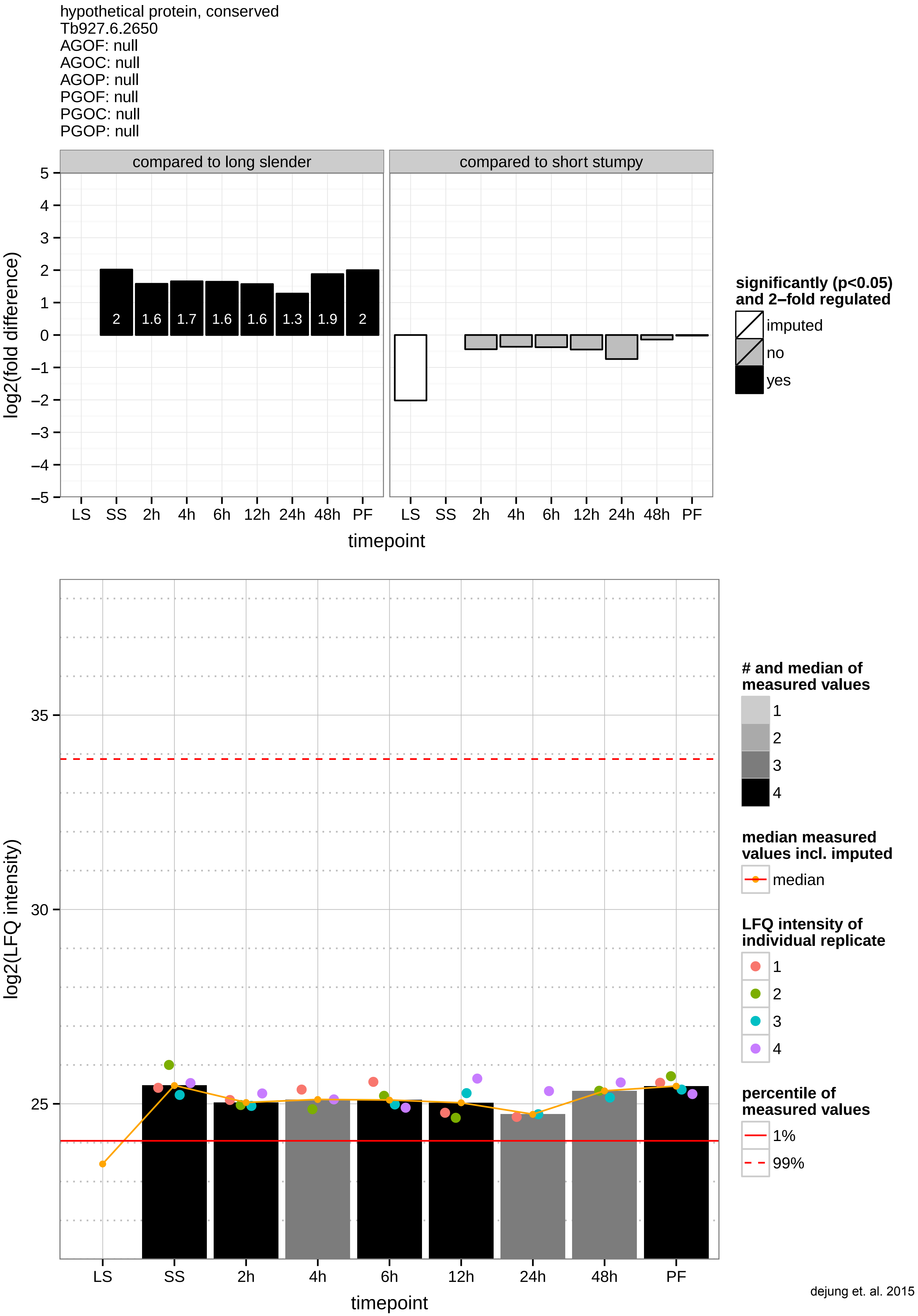
hypothetical protein, conserved
Tb927.6.2650
AGOF: null
AGOC: null
AGOP: null
PGOF: null
PGOC: null
PGOP: null
compared to long slender
compared to short stumpy
log2(fold difference)
2
1.6
1.7
1.6
1.6
1.3
1.9
2
LS
SS
2h
4h
6h
12h
24h
48h
PF
timepoint
significantly (p<0.05) and 2–fold regulated
imputed
no
yes
log2(LFQ intensity)
# and median of measured values
median measured values incl. imputed
median
LFQ intensity of individual replicate
percentile of measured values
1%
99%
dejung et. al. 2015

ubiquitin carboxyl–terminal hydrolase, putative, cysteine peptidase, Clan CA, family C19
Tb927.6.2690
AGOF: ubiquitin thiolesterase activity
AGOC: null
AGOP: ubiquitin–dependent protein catabolic process
PGOF: ubiquitin thiolesterase activity
PGOC: null
PGOP: ubiquitin–dependent protein catabolic process

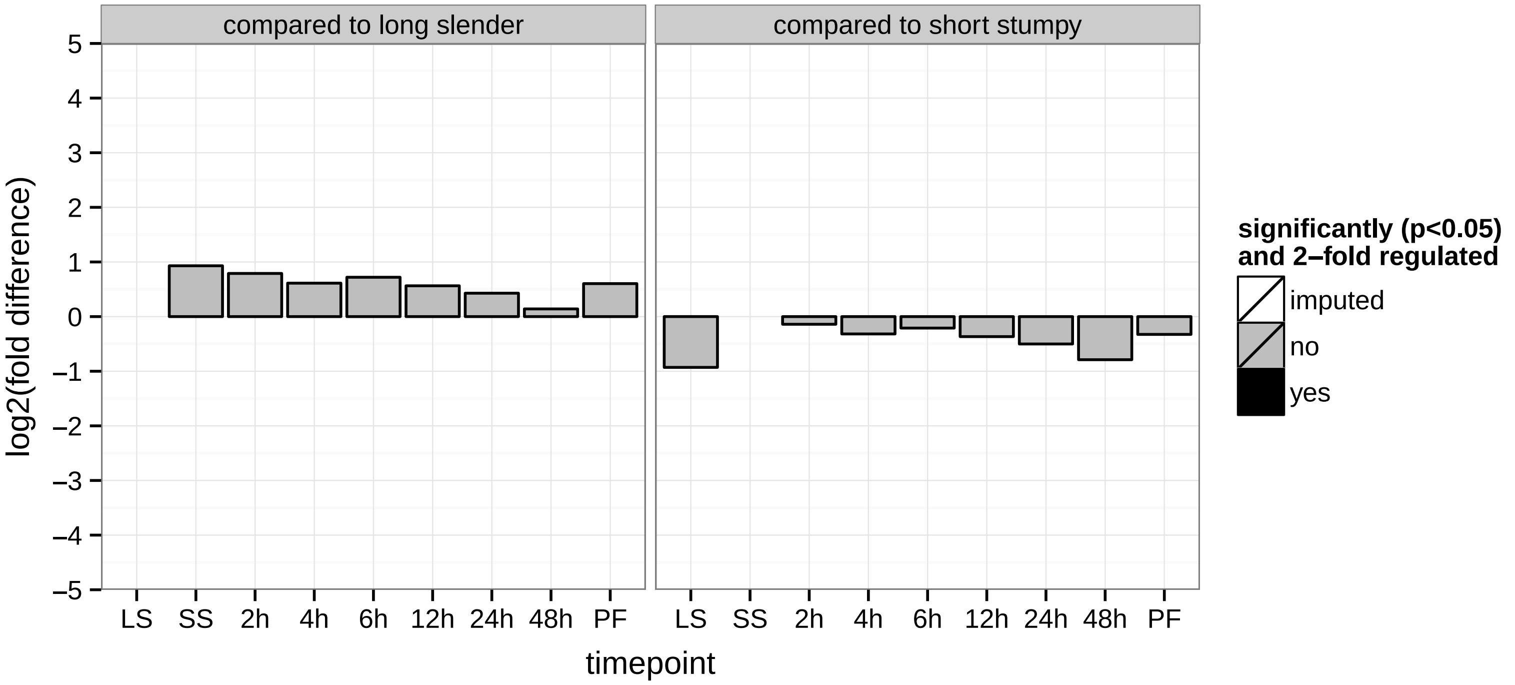

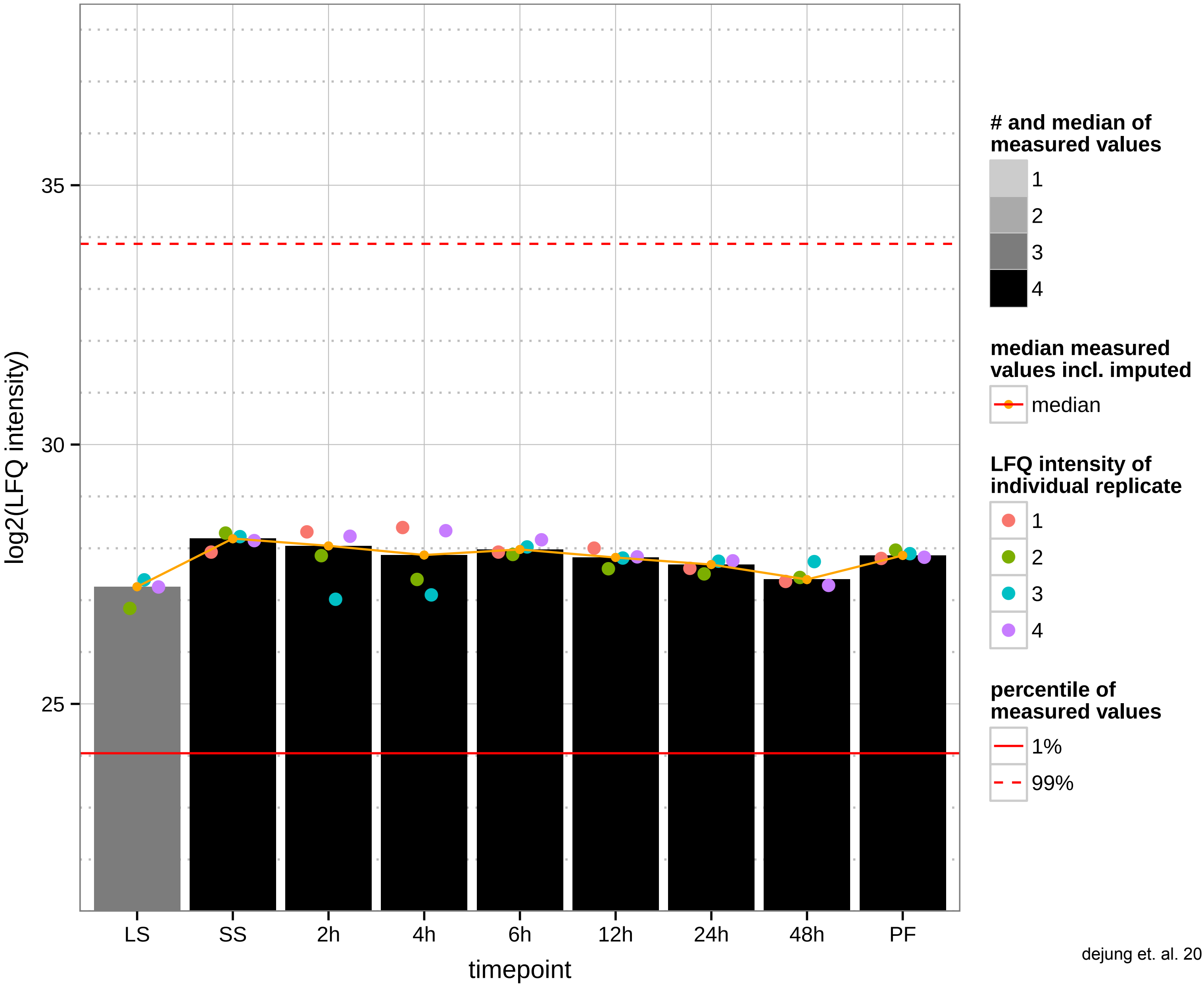

dejung et. al. 2015

small nuclear ribonucleoprotein Sm–E (Sm–E)
Tb927.6.2700
AGOF: null
AGOC: nucleus, small nucleolar ribonucleoprotein complex
AGOP: RNA splicing, RNA splicing, via transesterification reactions, nuclear mRNA trans splicing, SL addition
PGOF: null
PGOC: null
PGOP: null

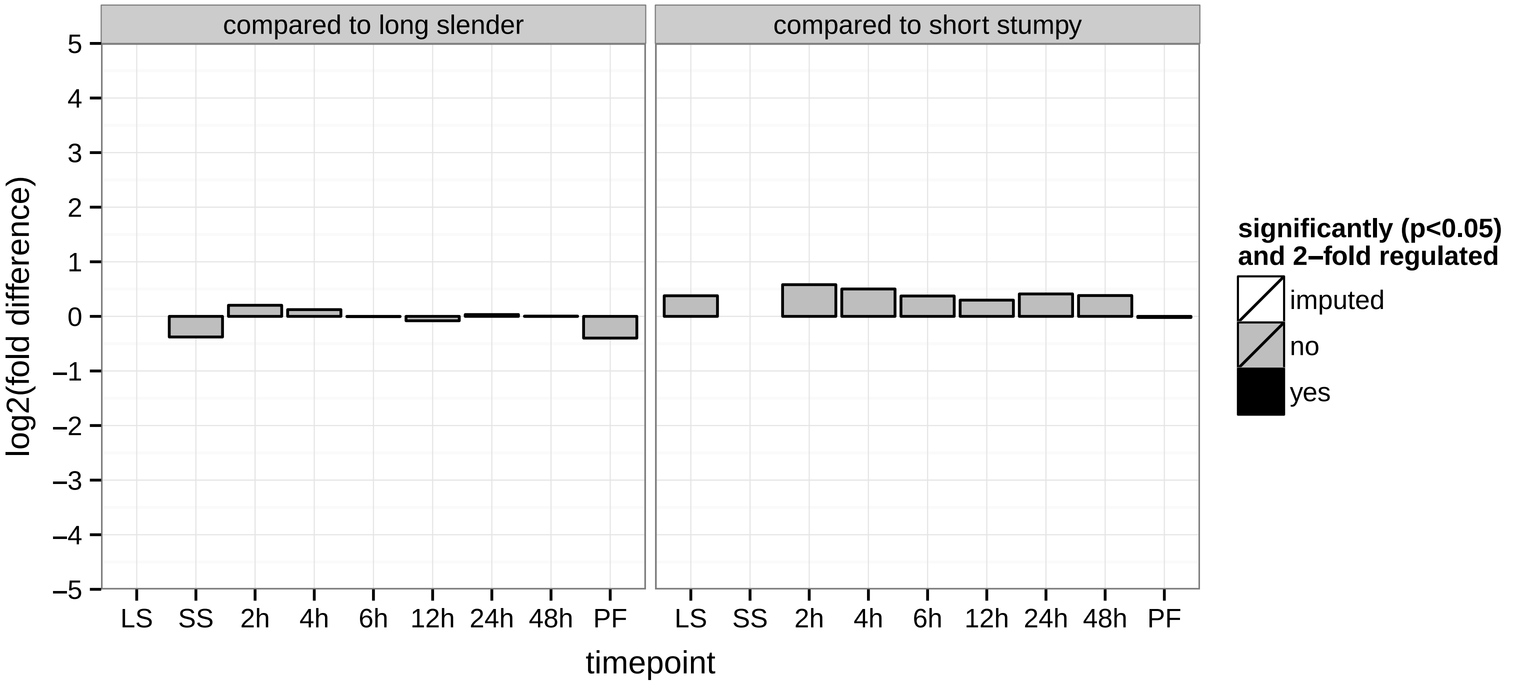

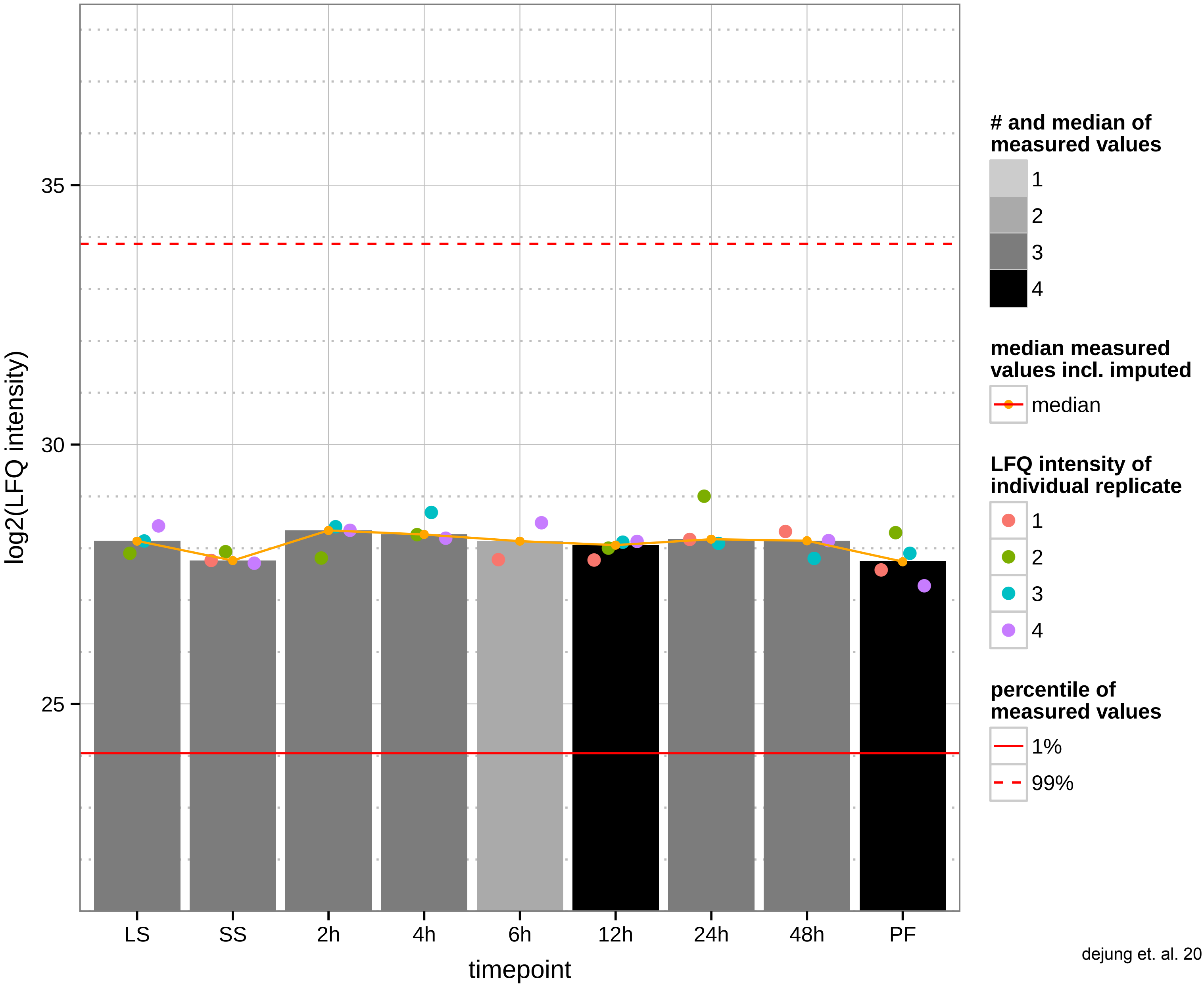

dejung et. al. 2015

calcium–binding protein, putative
Tb927.6.2720
AGOF: null
AGOC: null
AGOP: null
PGOF: null
PGOC: null
PGOP: null

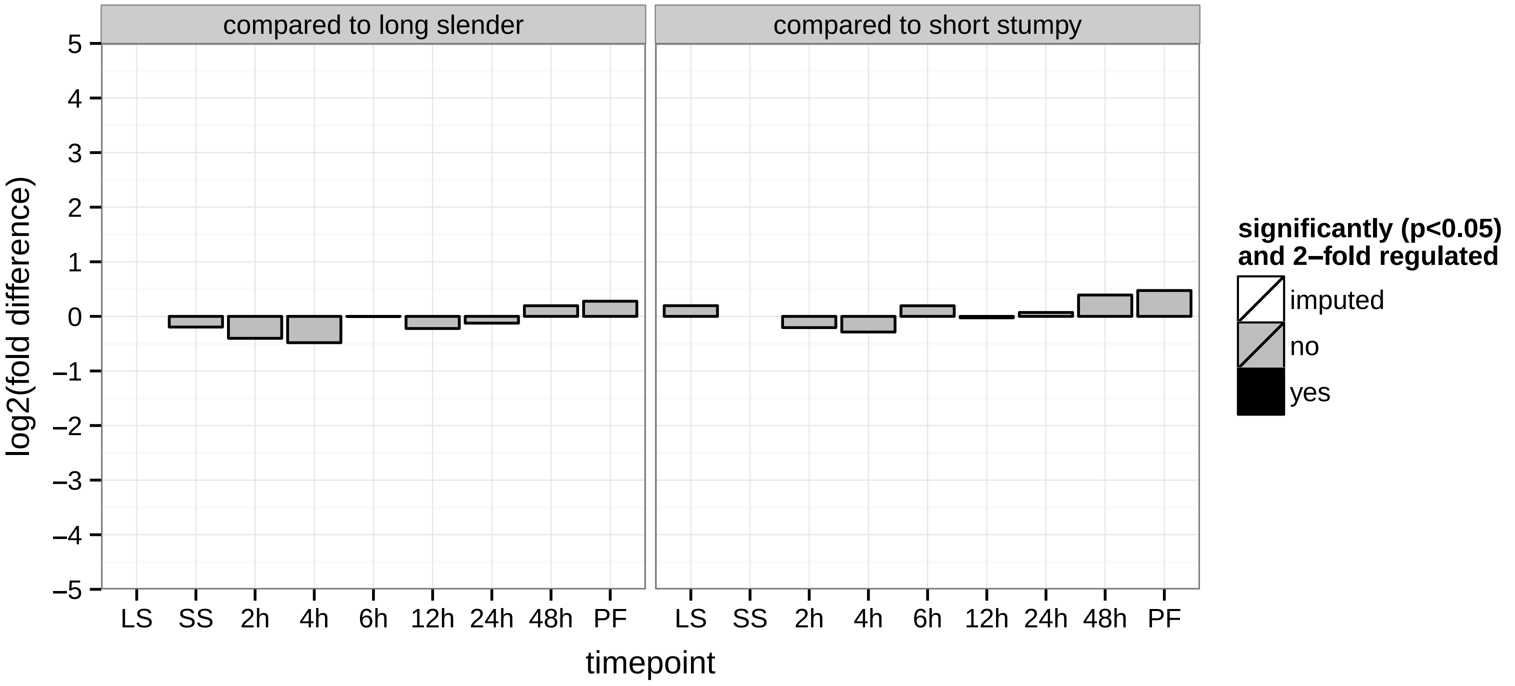

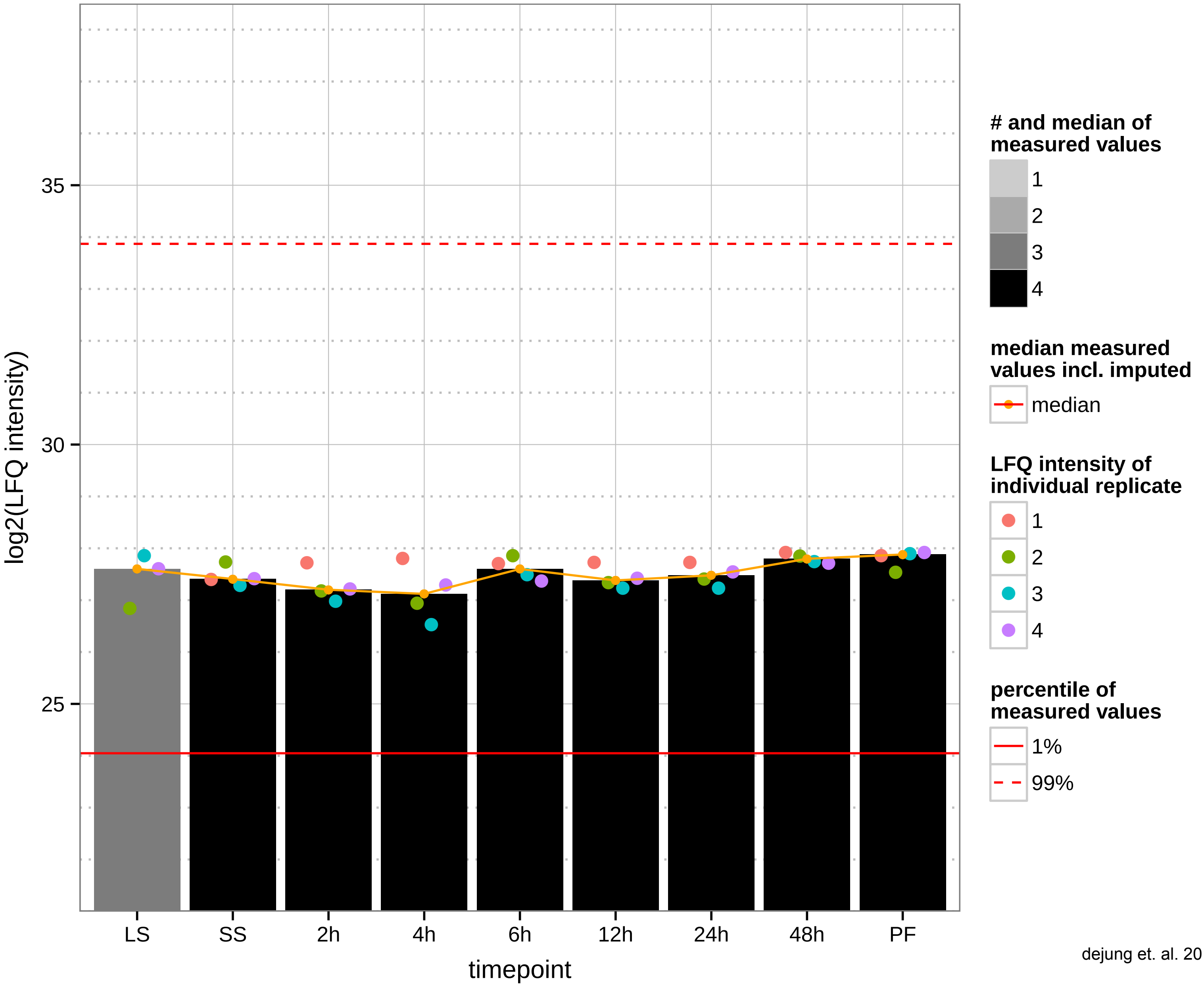

dejung et. al. 2015

pyridoxal kinase (pdxK)
Tb927.6.2740
AGOF: pyridoxal kinase activity
AGOC: null
AGOP: pyridoxine biosynthetic process
PGOF: pyridoxal kinase activity
PGOC: null
PGOP: pyridoxal 5'−phosphate salvage

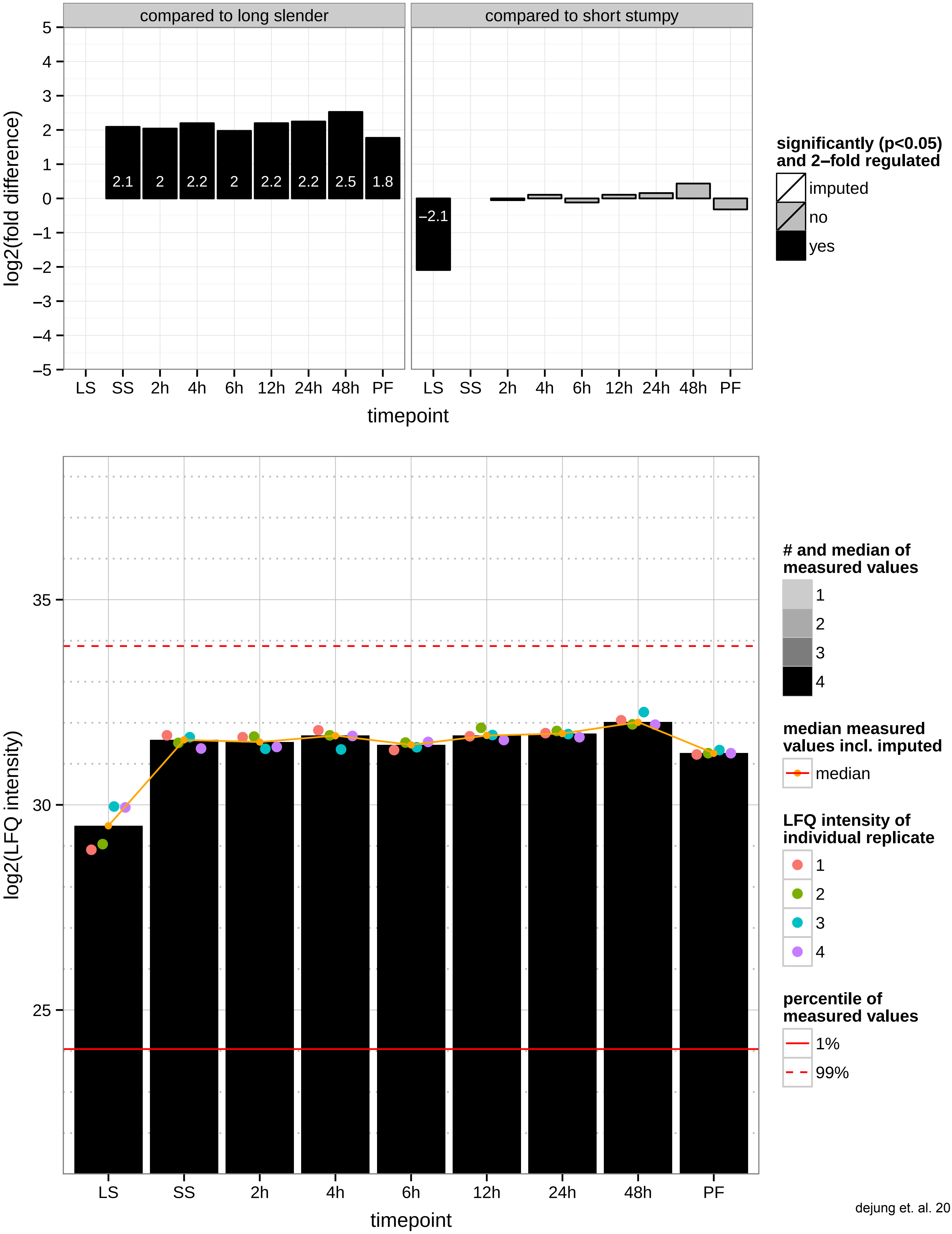

dejung et. al. 2015

U3 small nuclear ribonucleoprotein (snRNP), putative
Tb927.6.2780
AGOF: null
AGOC: null
AGOP: null
PGOF: null
PGOC: null
PGOP: null

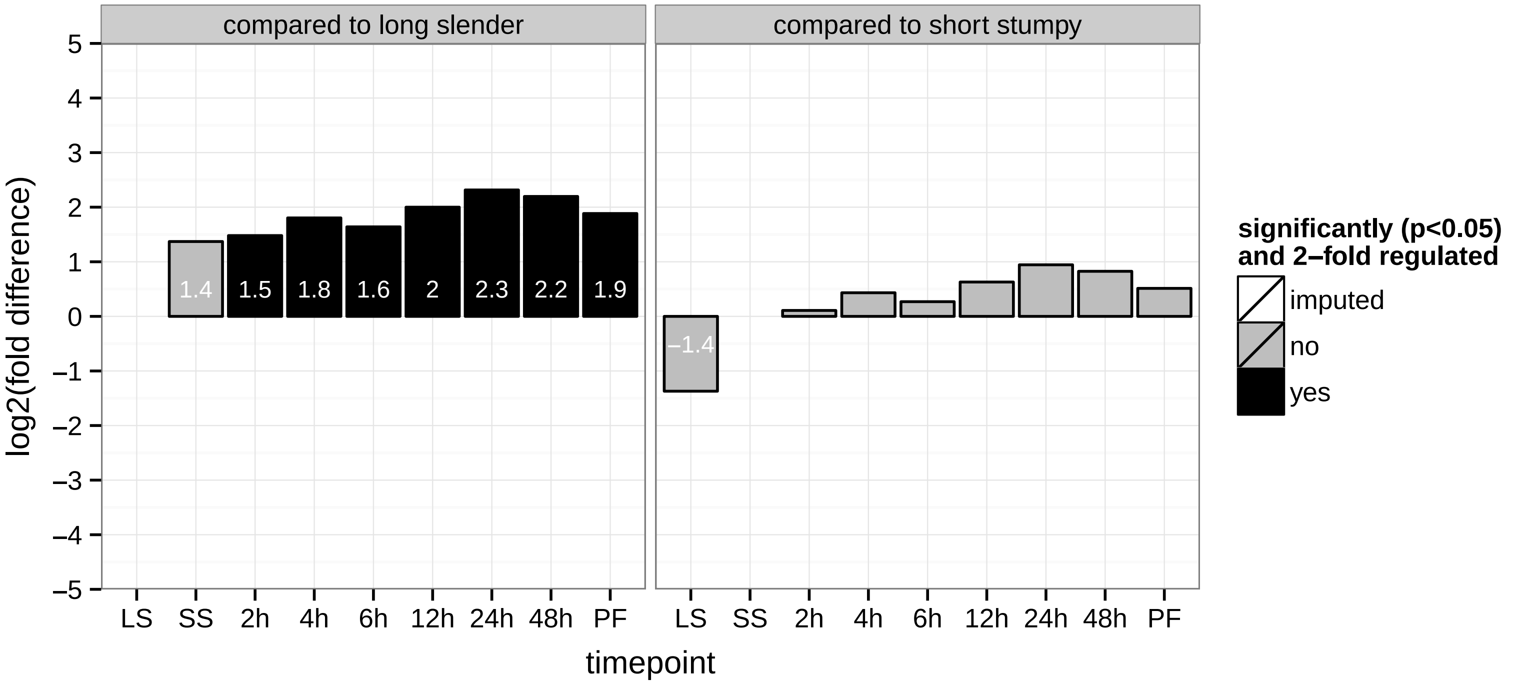

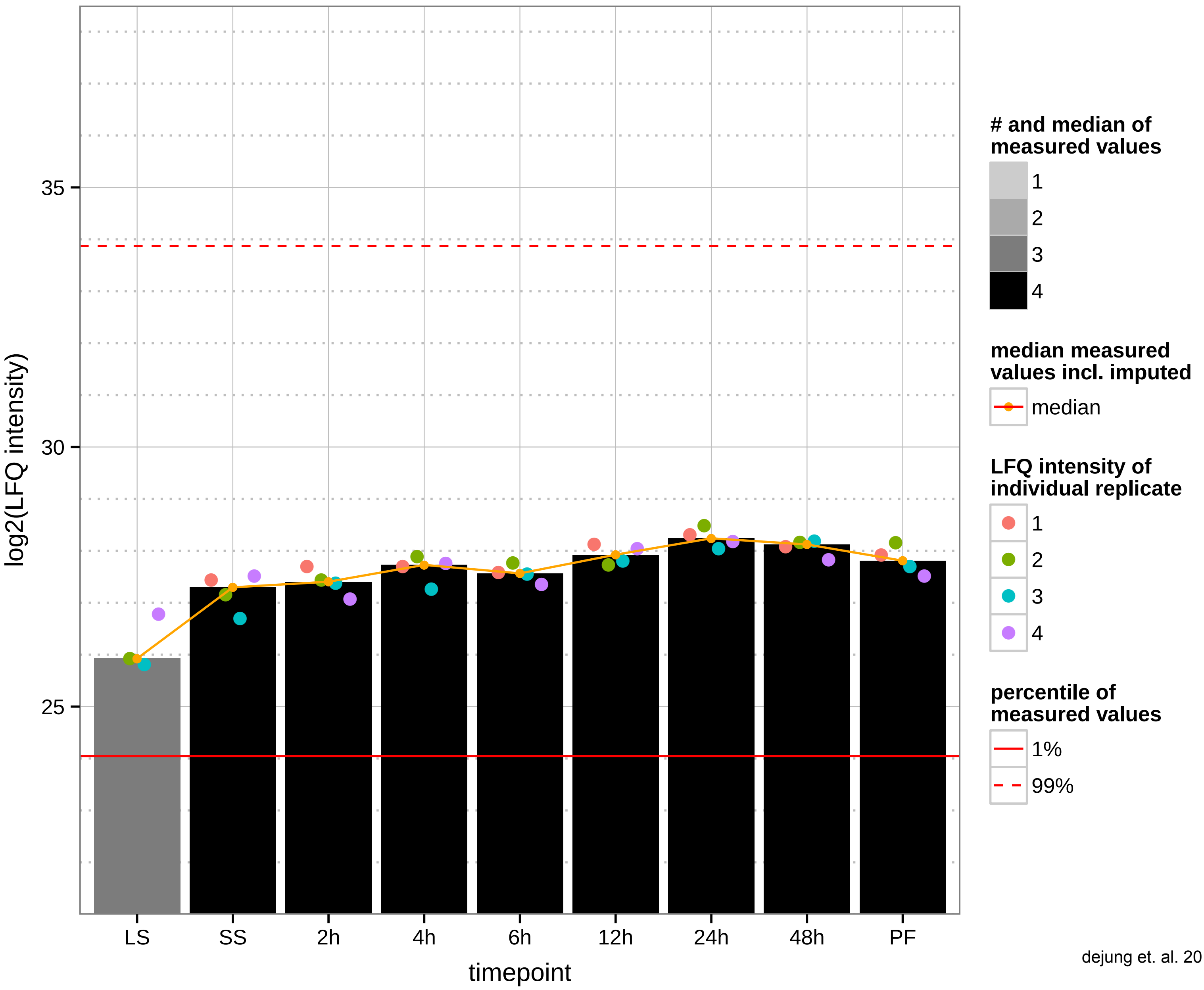

dejung et. al. 2015

NA, L–threonine 3–dehydrogenase, putative
Tb927.6.2790
AGOF: NA, catalytic activity, coenzyme binding
AGOC: NA, mitochondrion
AGOP: NA, cellular metabolic process
PGOF: NA, catalytic activity, coenzyme binding
PGOC: NA, null
PGOP: NA, cellular metabolic process

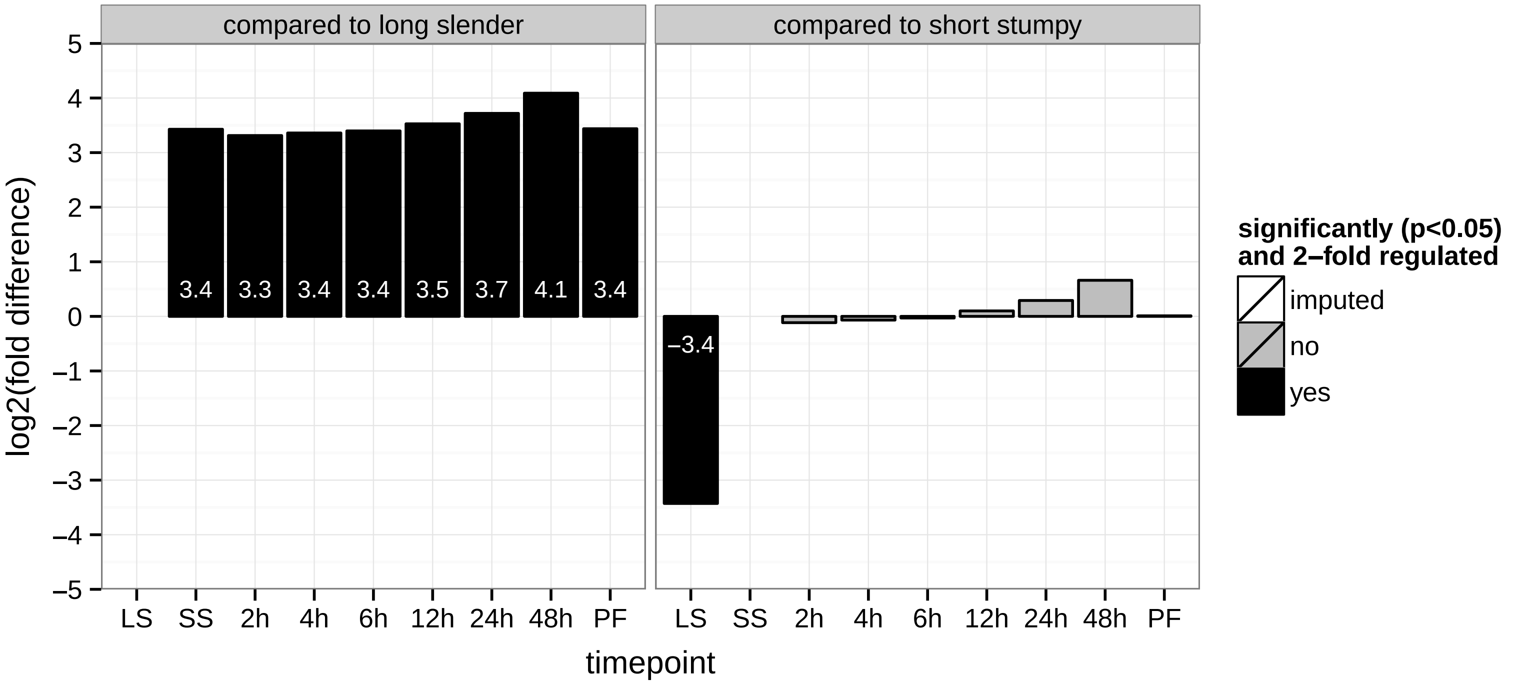

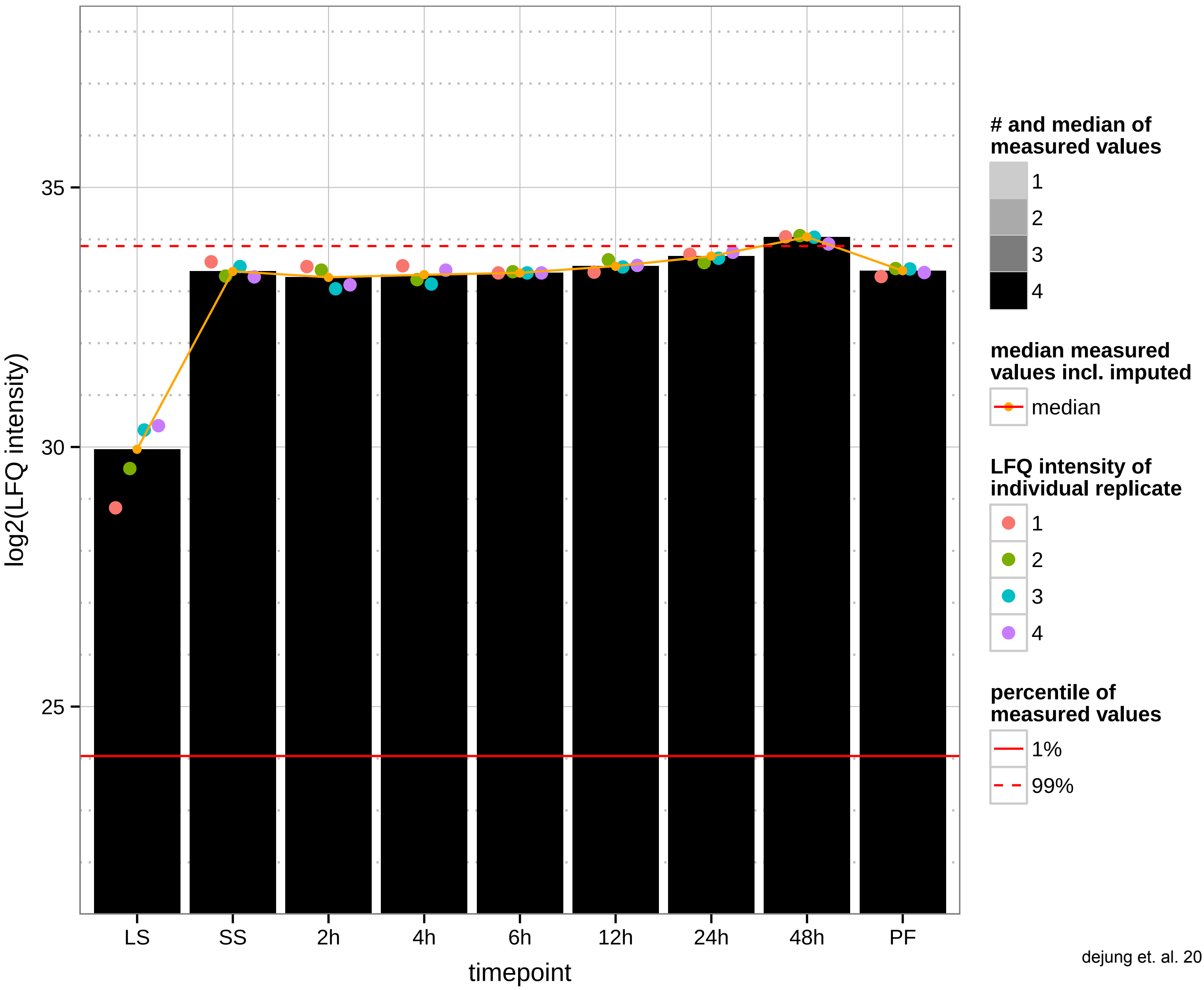

dejung et. al. 2015

hypothetical protein, conserved
Tb927.6.2840
AGOF: ATP binding, protein serine/threonine kinase activity
AGOC: null
AGOP: growth, protein phosphorylation
PGOF: ATP binding, catalytic activity, protein serine/threonine kinase activity, transferase activity, transferring phosphorus–con
PGOC: null
PGOP: protein phosphorylation

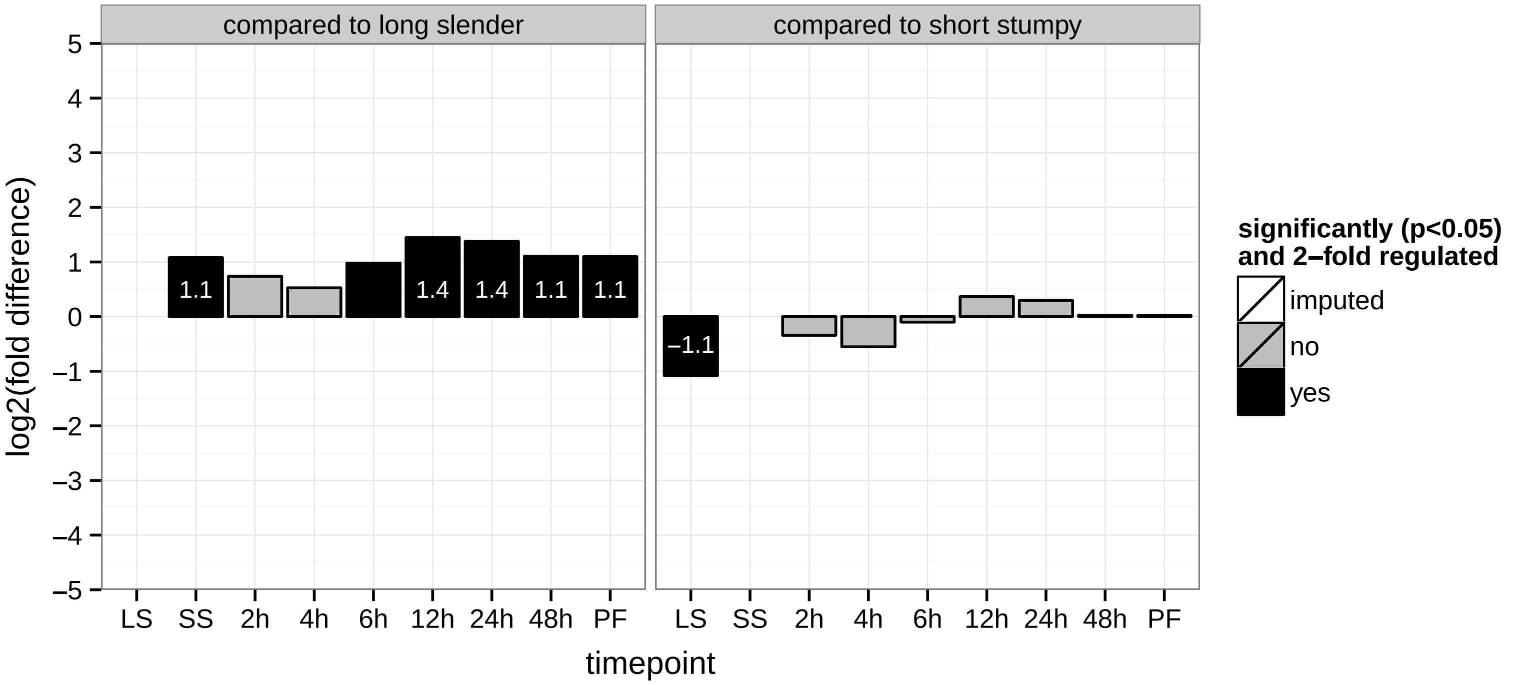

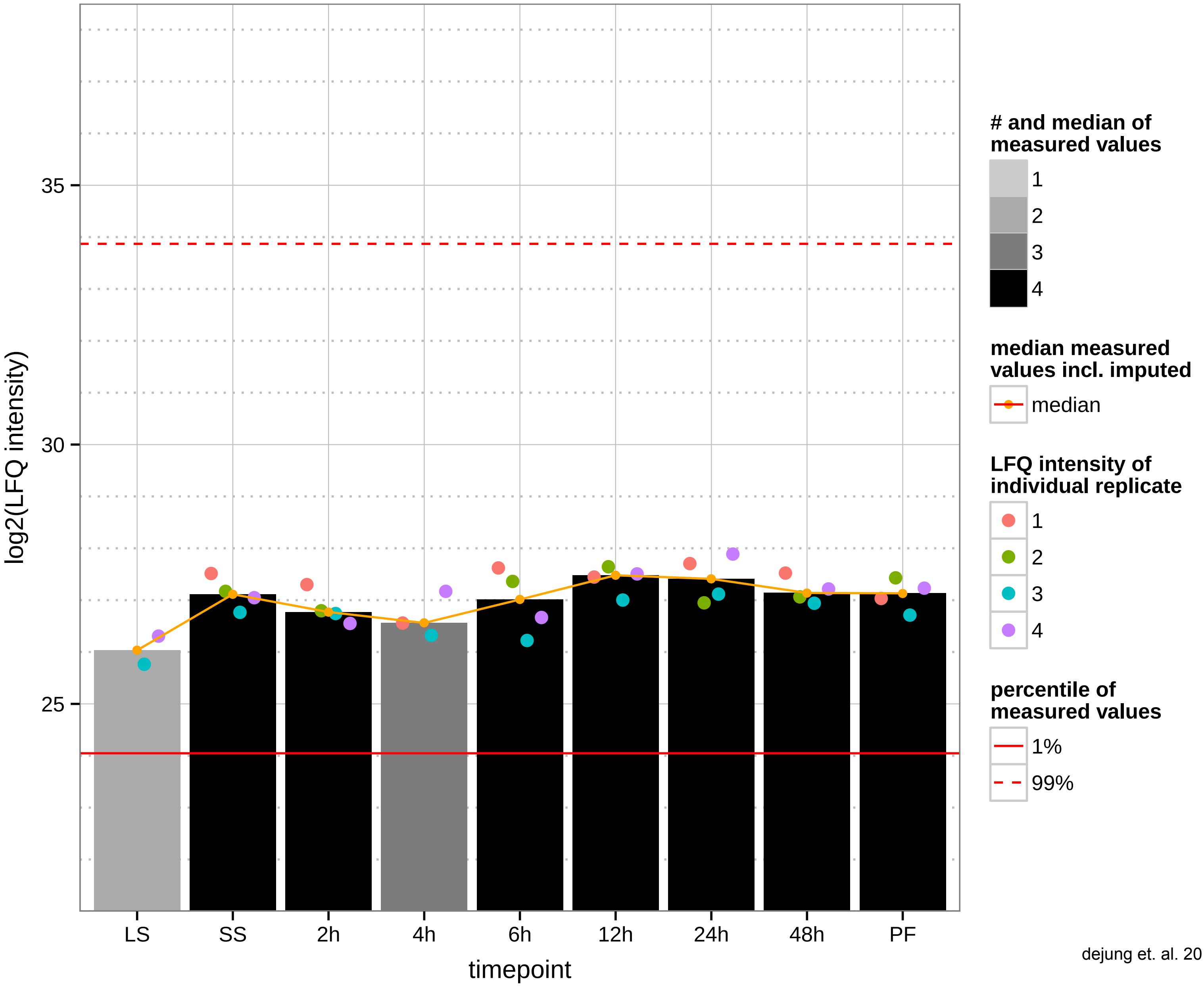

dejung et. al. 2015

hypothetical protein, conserved
Tb927.6.2860
AGOF: null
AGOC: null
AGOP: null
PGOF: protein binding
PGOC: null
PGOP: null

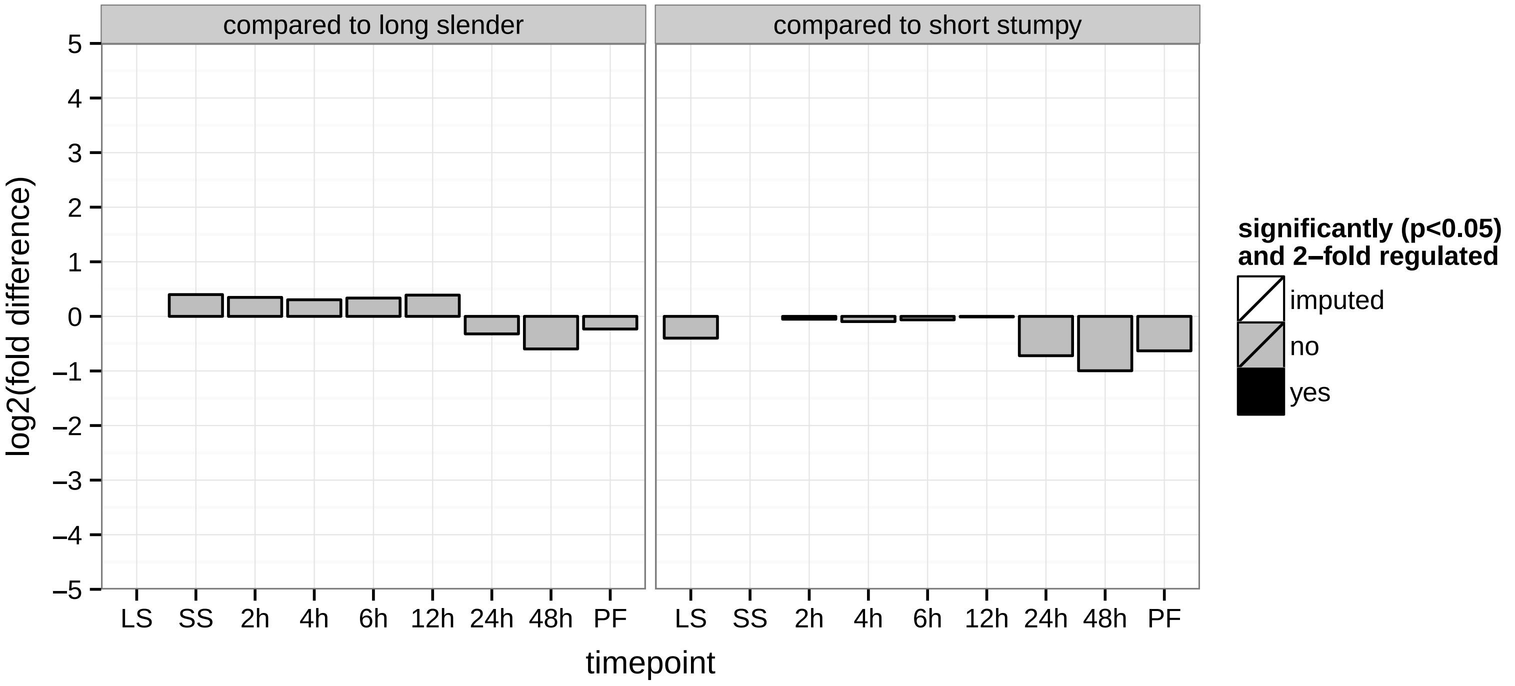

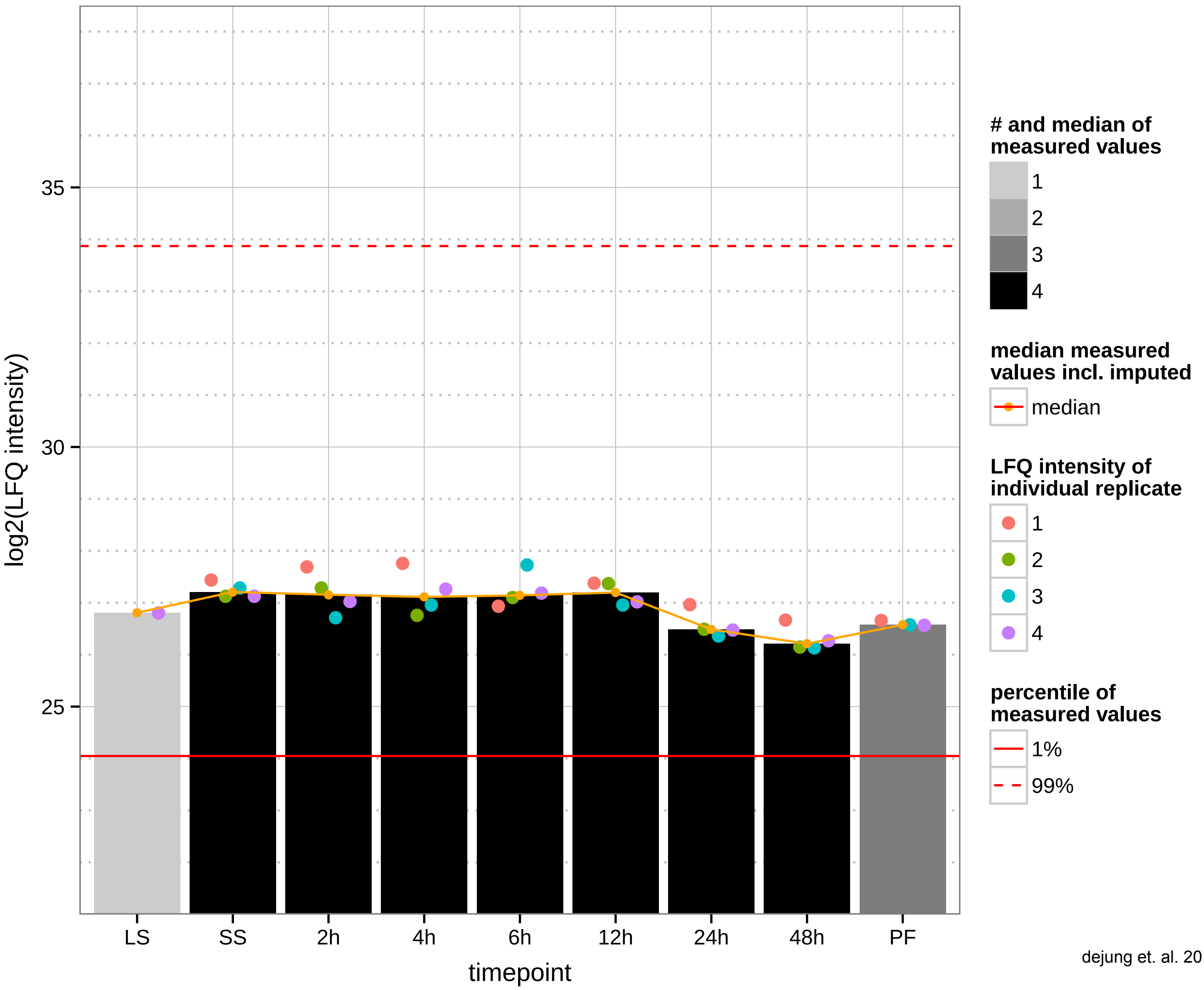

dejung et. al. 2015

phosphopantothenoylcysteine decarboxylase, putative (PPCDC)
Tb927.6.2940
AGOF: phosphopantothenoylcysteine decarboxylase activity
AGOC: null
AGOP: coenzyme A biosynthetic process
PGOF: catalytic activity
PGOC: null
PGOP: null

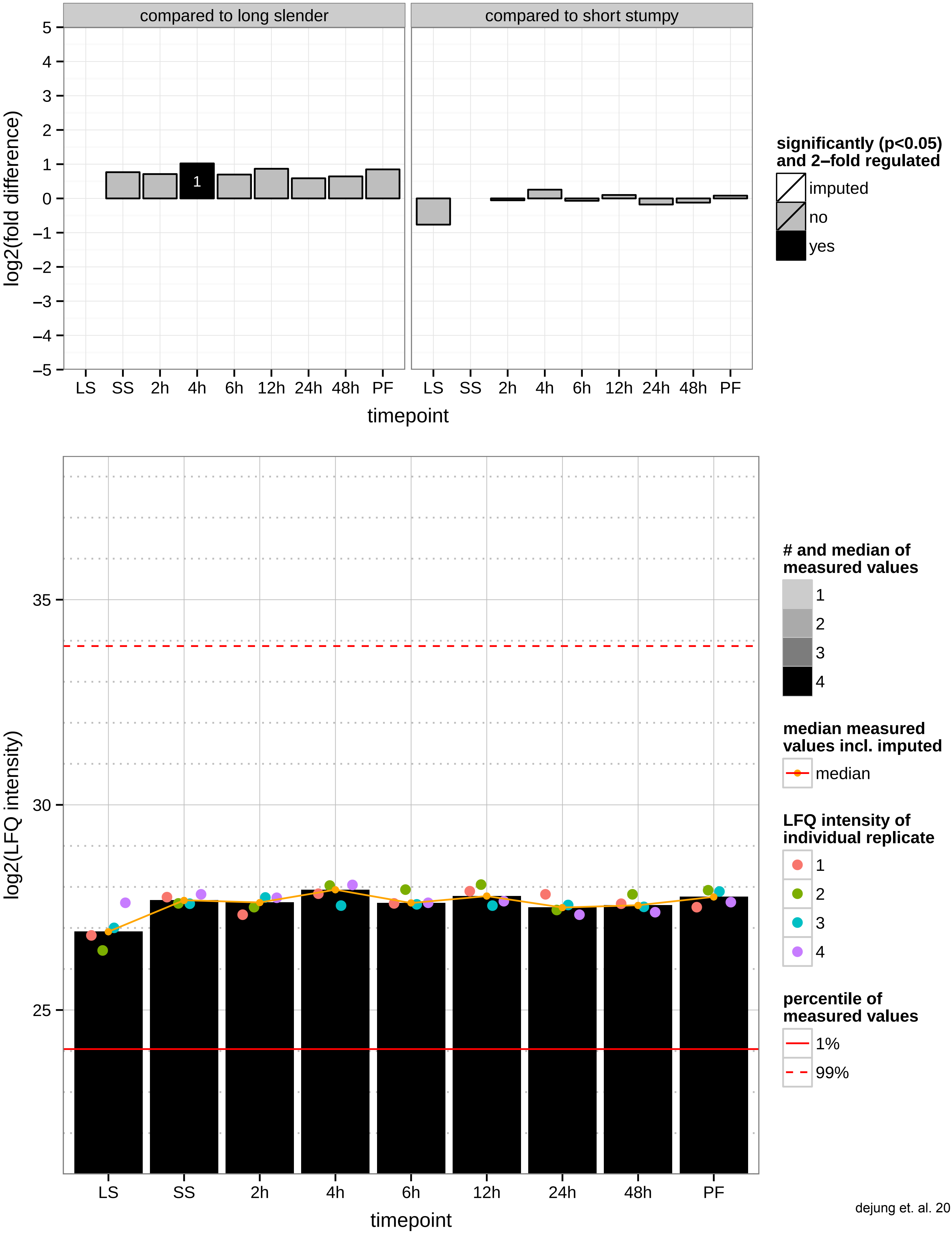

dejung et. al. 2015

hypothetical protein, conserved
Tb927.6.3020
AGOF: null
AGOC: null
AGOP: null
PGOF: null
PGOC: null
PGOP: null

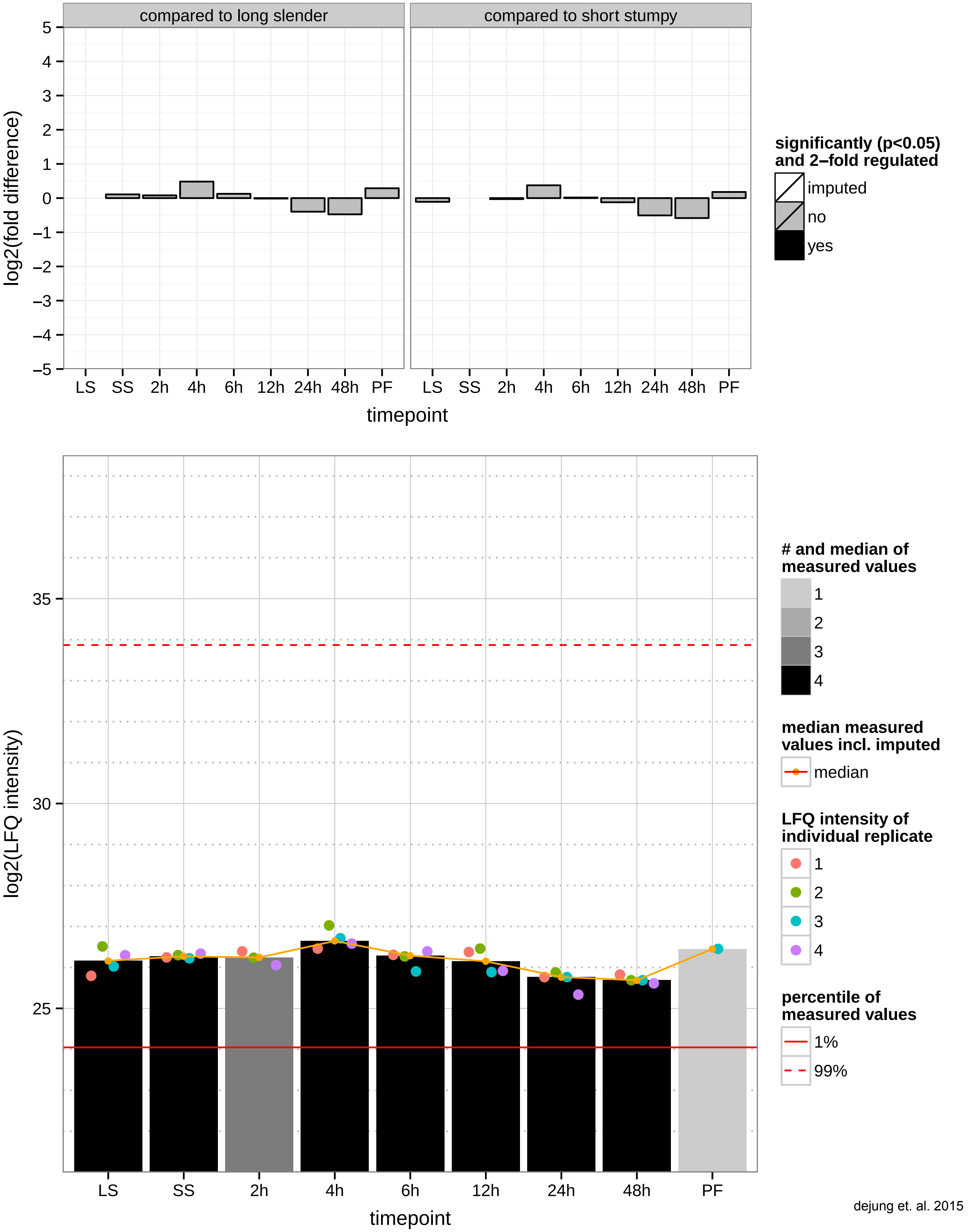

dejung et. al. 2015

small GTP–binding protein Rab28, putative, Ras– related protein Rab–26 (Rab26)
Tb927.6.3040
AGOF: GTP binding, GTPase activity
AGOC: intracellular
AGOP: intracellular protein transport, nucleocytoplasmic transport, small GTPase mediated signal transduction
PGOF: GTP binding, GTPase activity
PGOC: intracellular, membrane
PGOP: GTP catabolic process, intracellular protein transport, nucleocytoplasmic transport, protein transport, signal transducti

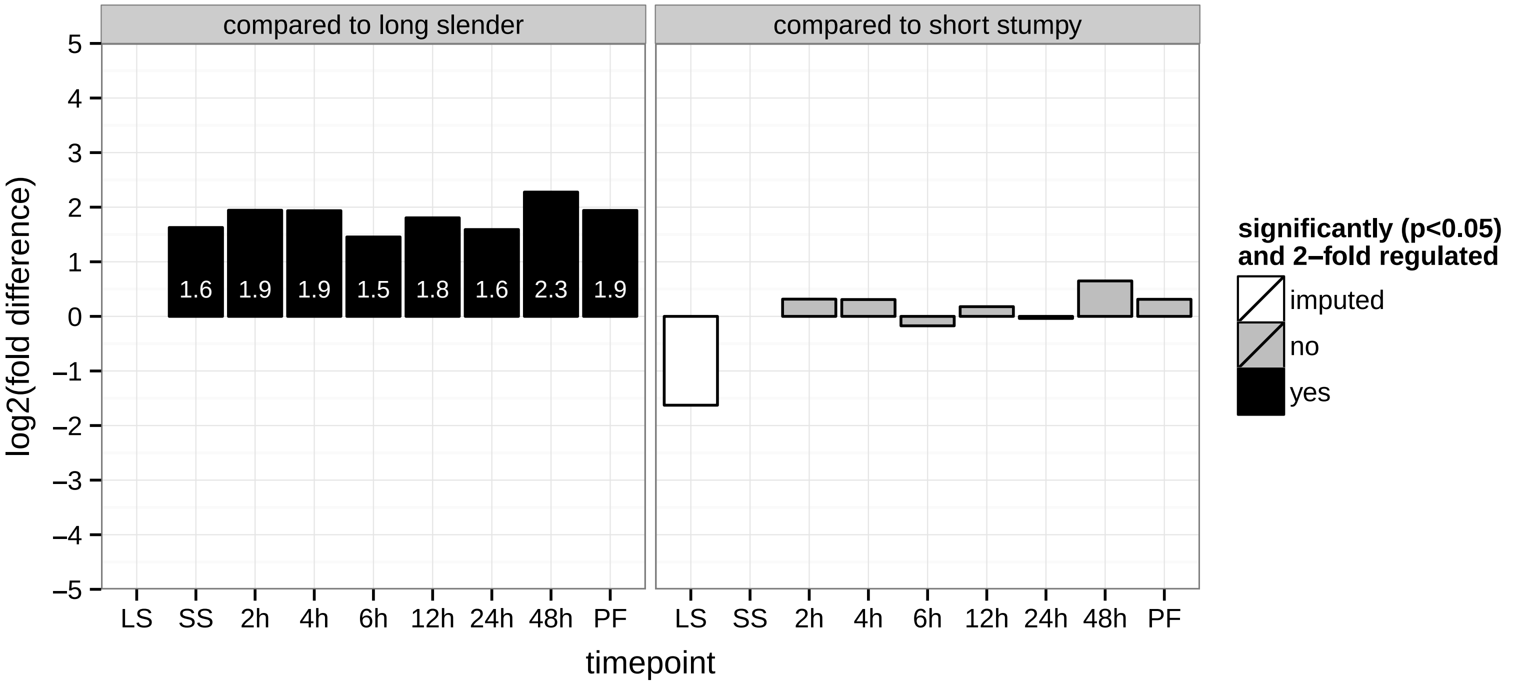

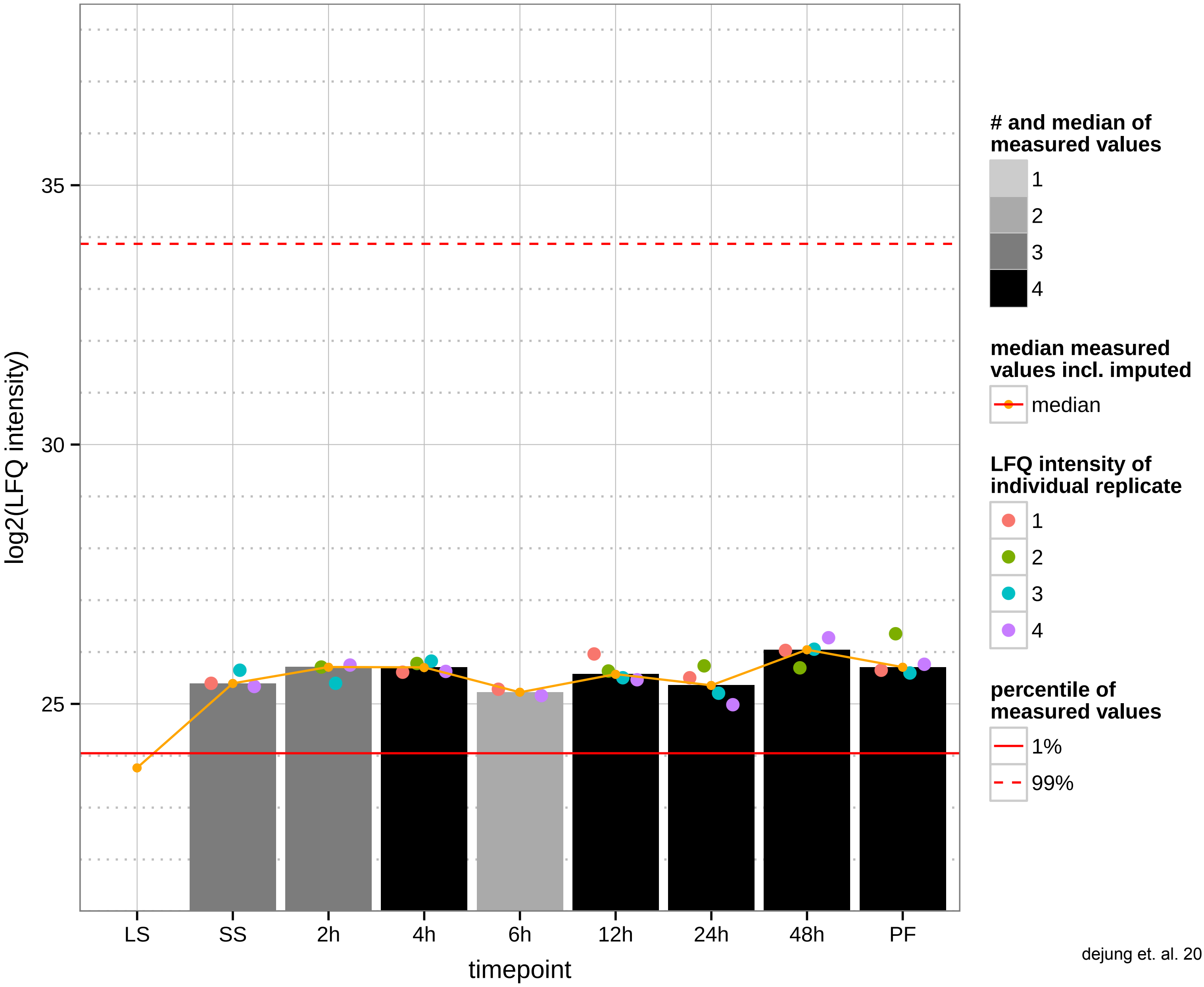

dejung et. al. 2015

aldehyde dehydrogenase family, putative
Tb927.6.3050
AGOF: aldehyde dehydrogenase [NAD(P)+] activity
AGOC: null
AGOP: cellular aldehyde metabolic process, oxidation–reduction process
PGOF: aldehyde dehydrogenase [NAD(P)+] activity, oxidoreductase activity
PGOC: null
PGOP: cellular aldehyde metabolic process, metabolic process, oxidation–reduction process

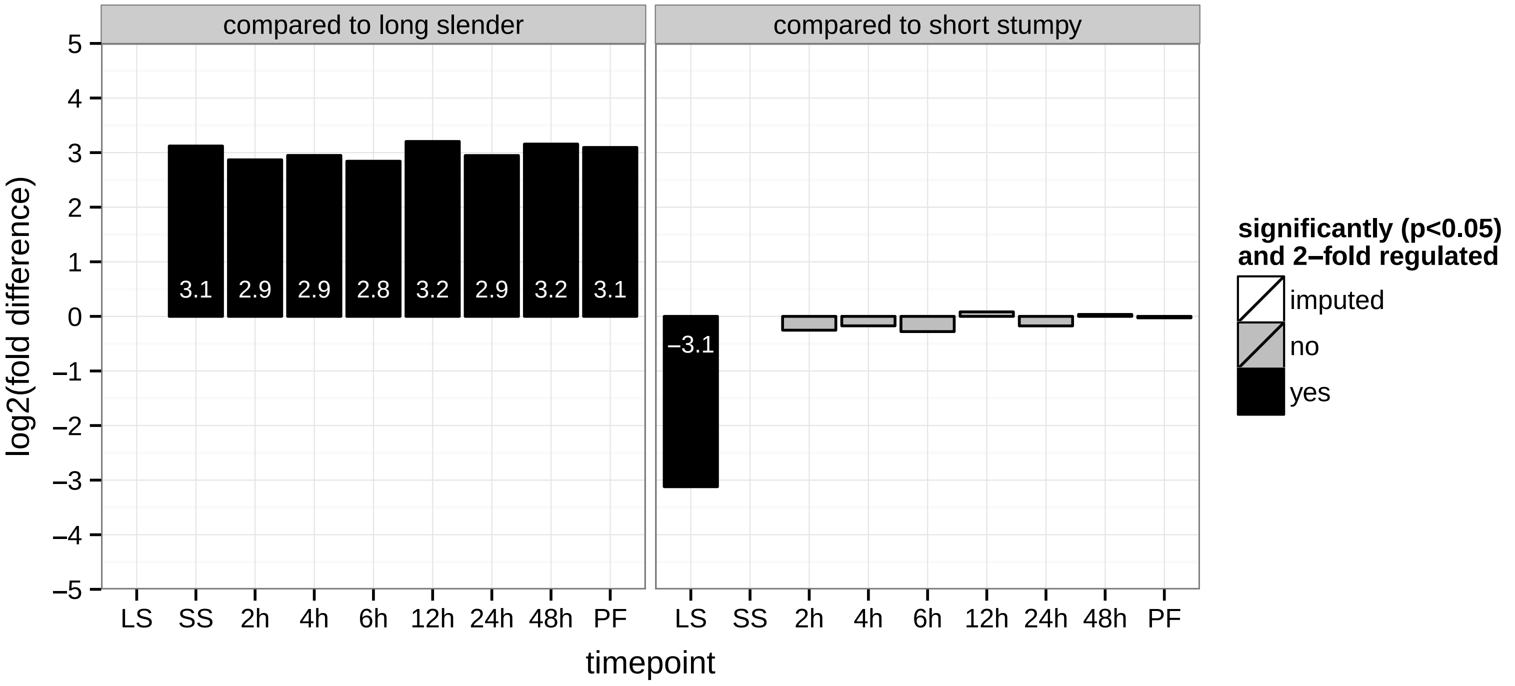

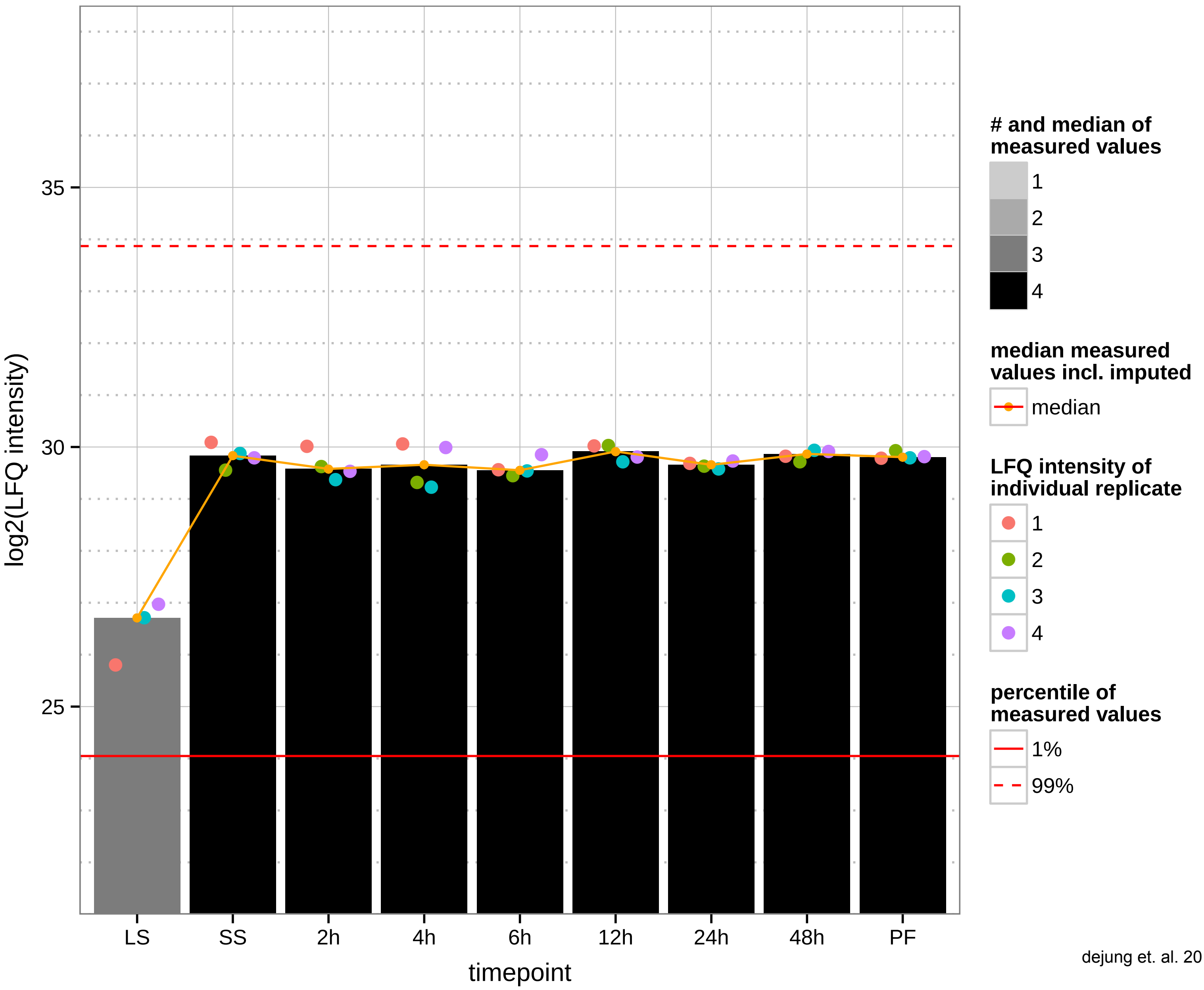

dejung et. al. 2015

cytoskeleton–associated protein, putative
Tb927.6.3090
AGOF: null
AGOC: null
AGOP: null
PGOF: binding
PGOC: null
PGOP: null

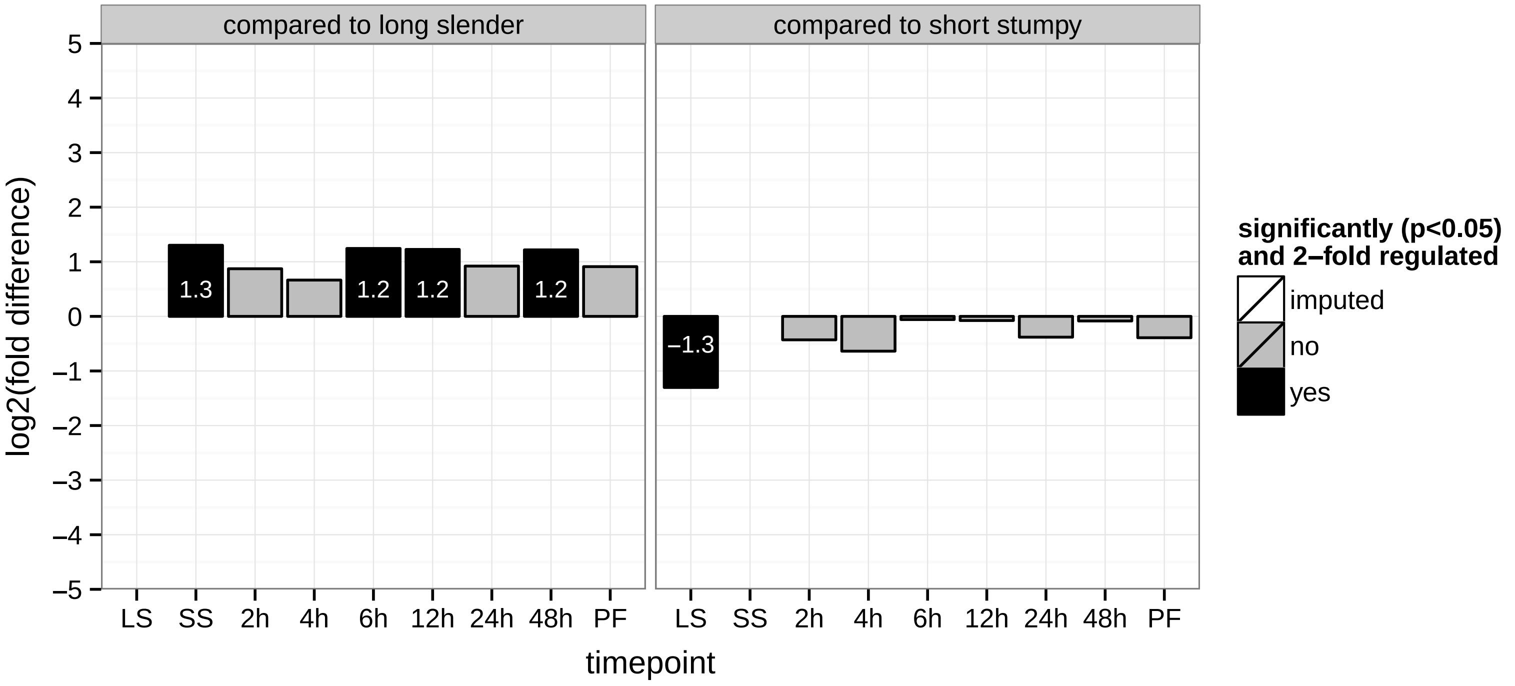

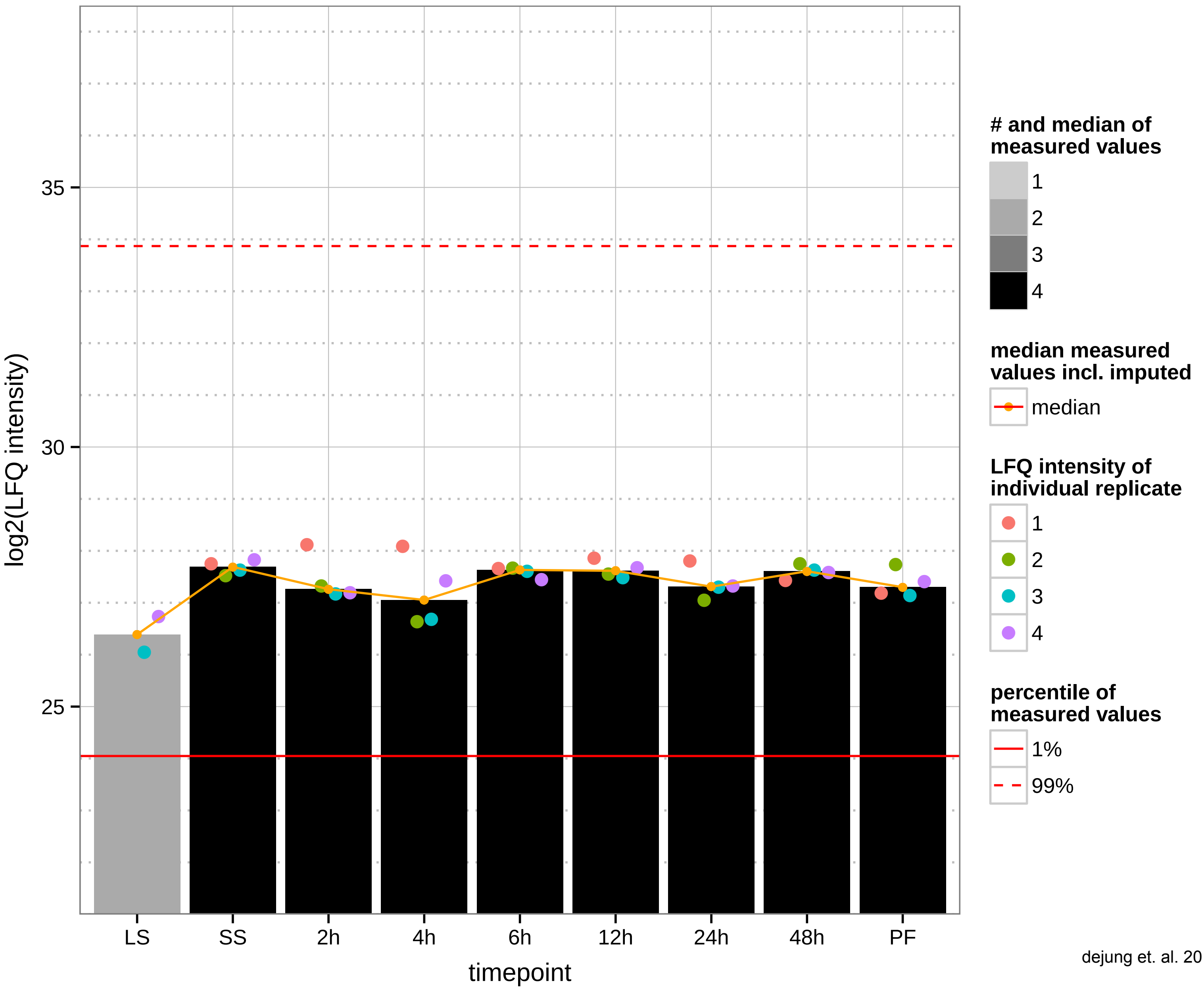

dejung et. al. 2015

hypothetical protein, conserved
Tb927.6.3100
AGOF: null
AGOC: null
AGOP: null
PGOF: null
PGOC: null
PGOP: intraflagellar transport

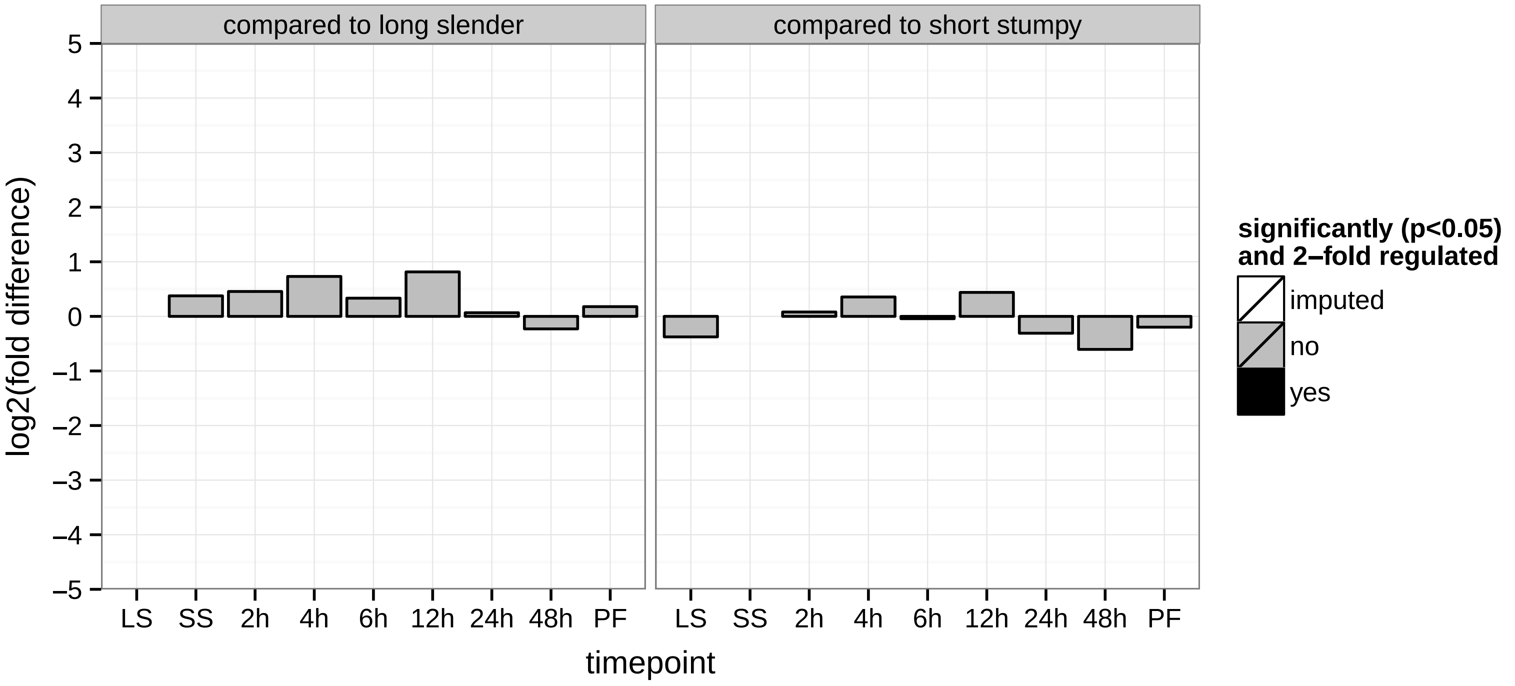

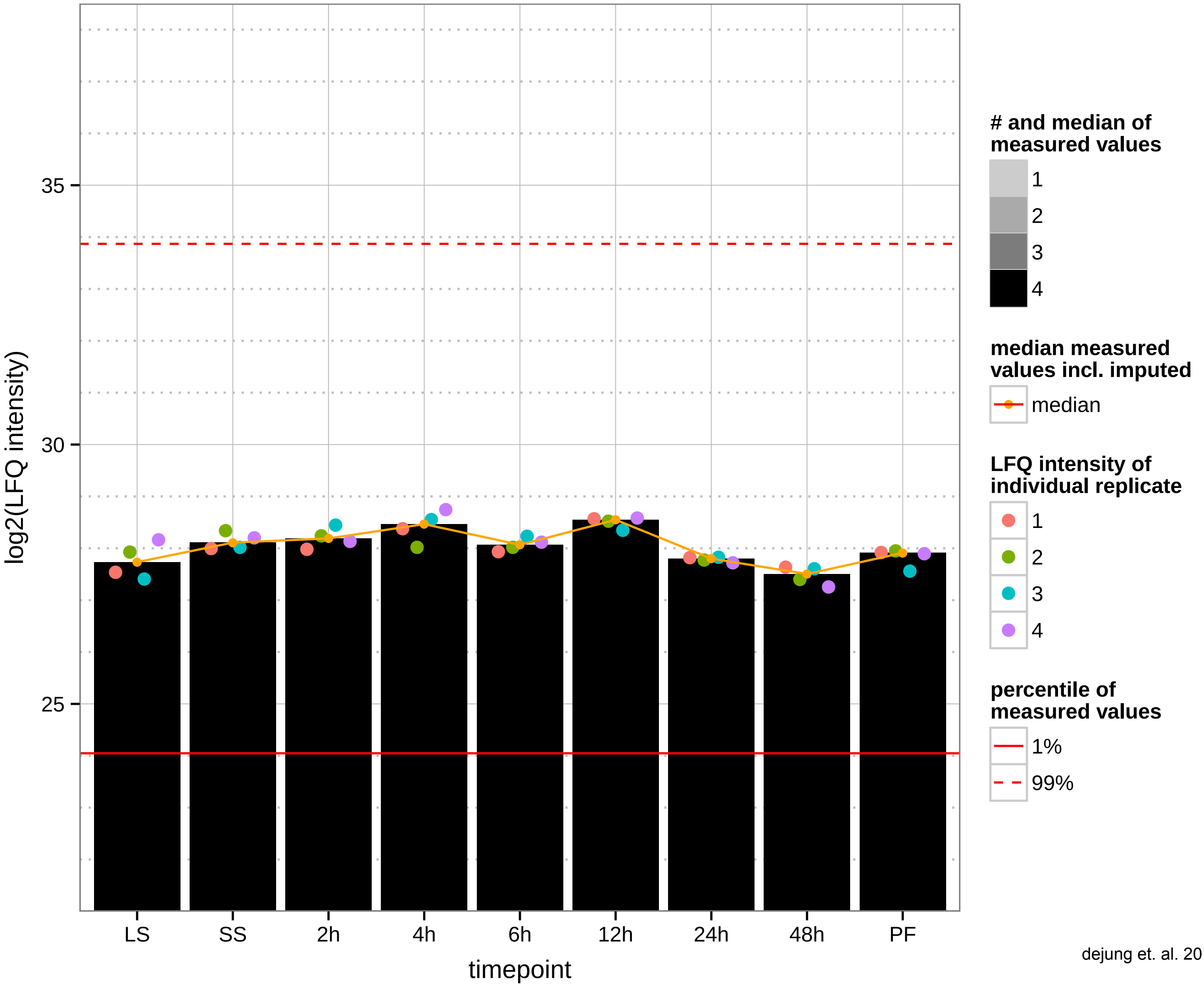

dejung et. al. 2015

queuine tRNA–ribosyltransferase, putative
Tb927.6.3130
AGOF: queuine tRNA–ribosyltransferase activity
AGOC: null
AGOP: queuosine biosynthetic process
PGOF: queuine tRNA–ribosyltransferase activity
PGOC: null
PGOP: queuosine biosynthetic process, tRNA modification

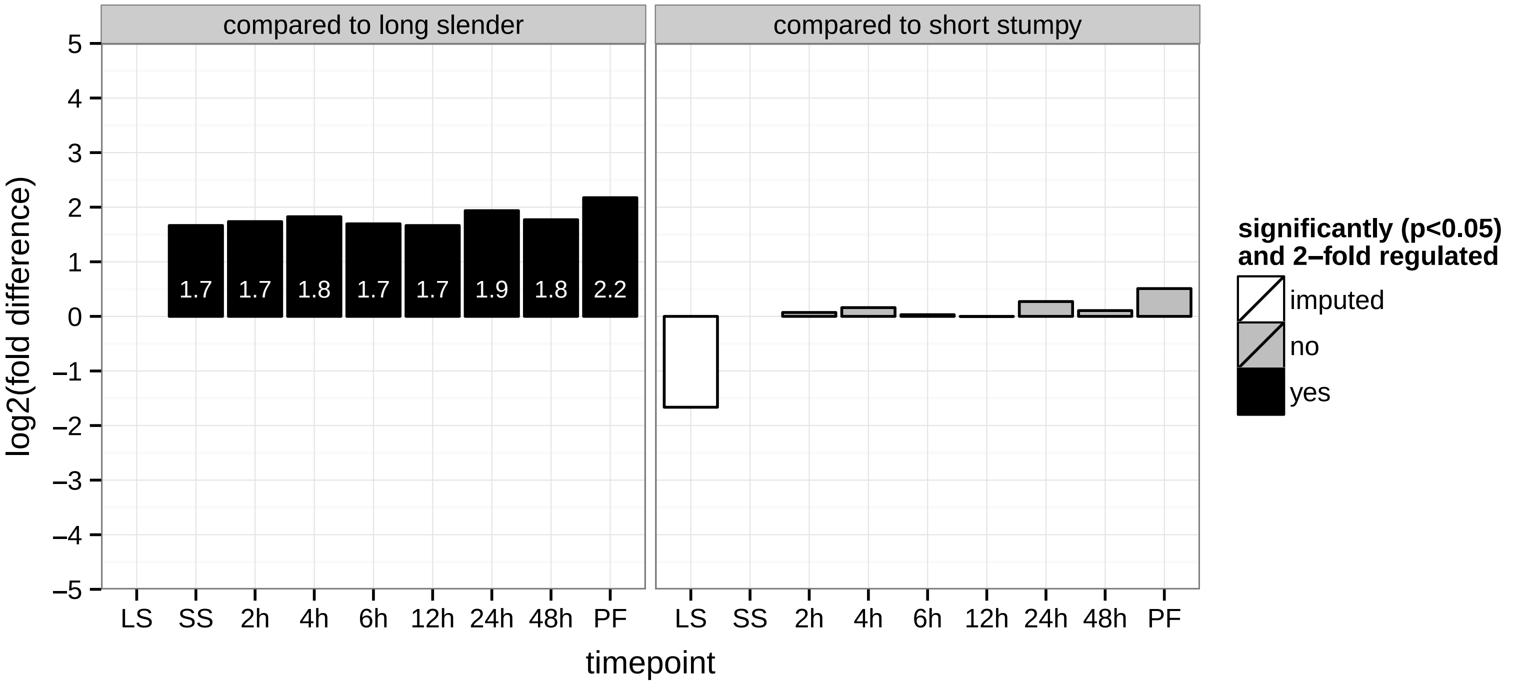

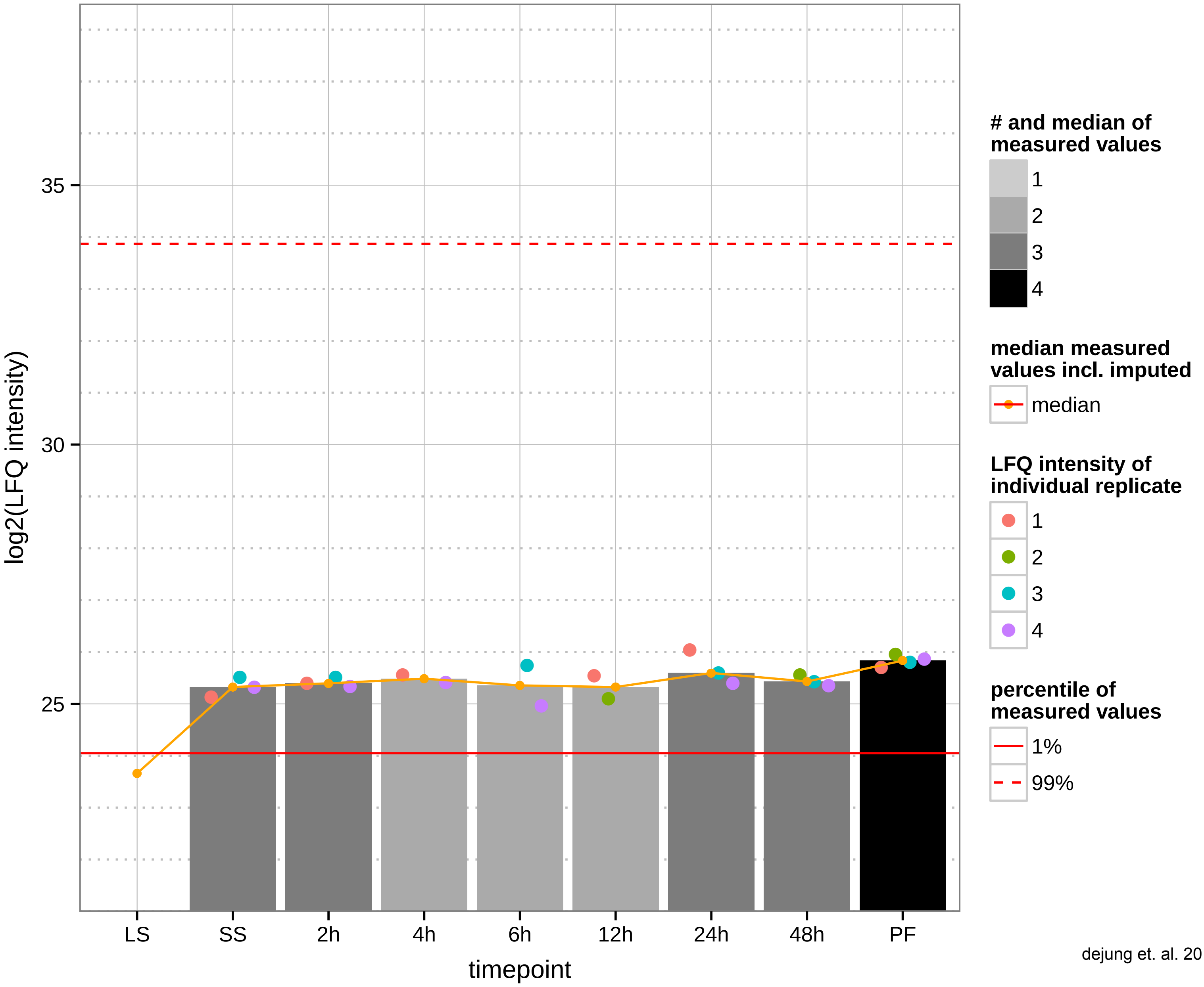

dejung et. al. 2015

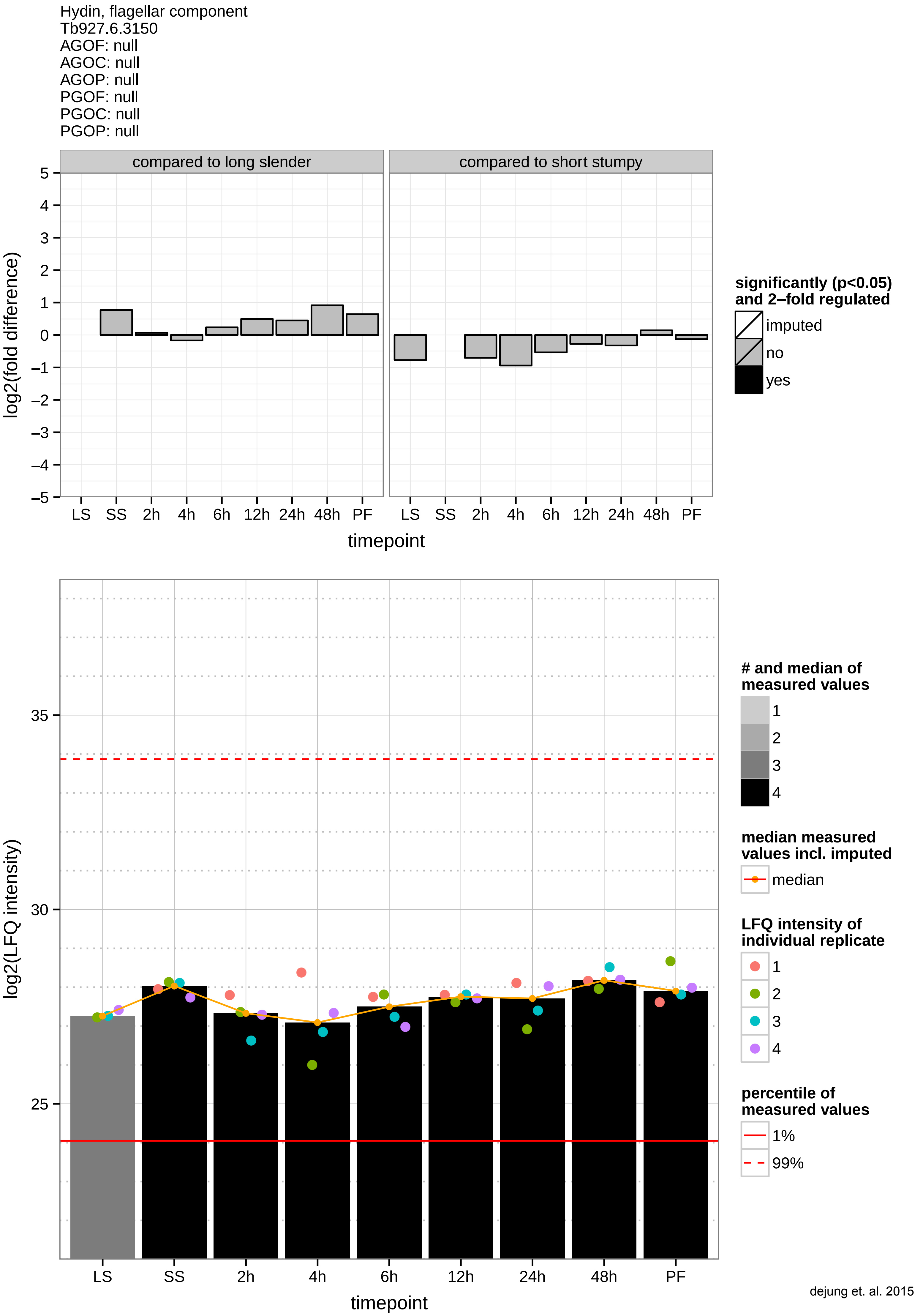

Hydin, flagellar component
Tb927.6.3150
AGOF: null
AGOC: null
AGOP: null
PGOF: null
PGOC: null
PGOP: null
compared to long slender
compared to short stumpy
log2(fold difference)
timepoint
LS
SS
2h
4h
6h
12h
24h
48h
PF
significantly (p<0.05) and 2–fold regulated
imputed
no
yes
log2(LFQ intensity)
# and median of measured values
1
2
3
4
median measured values incl. imputed
median
LFQ intensity of individual replicate
percentile of measured values
1%
99%
dejung et. al. 2015

splicing factor 3a, putative
Tb927.6.3160
AGOF: nucleic acid binding, zinc ion binding
AGOC: nucleus
AGOP: null
PGOF: nucleic acid binding, zinc ion binding
PGOC: nucleus
PGOP: null

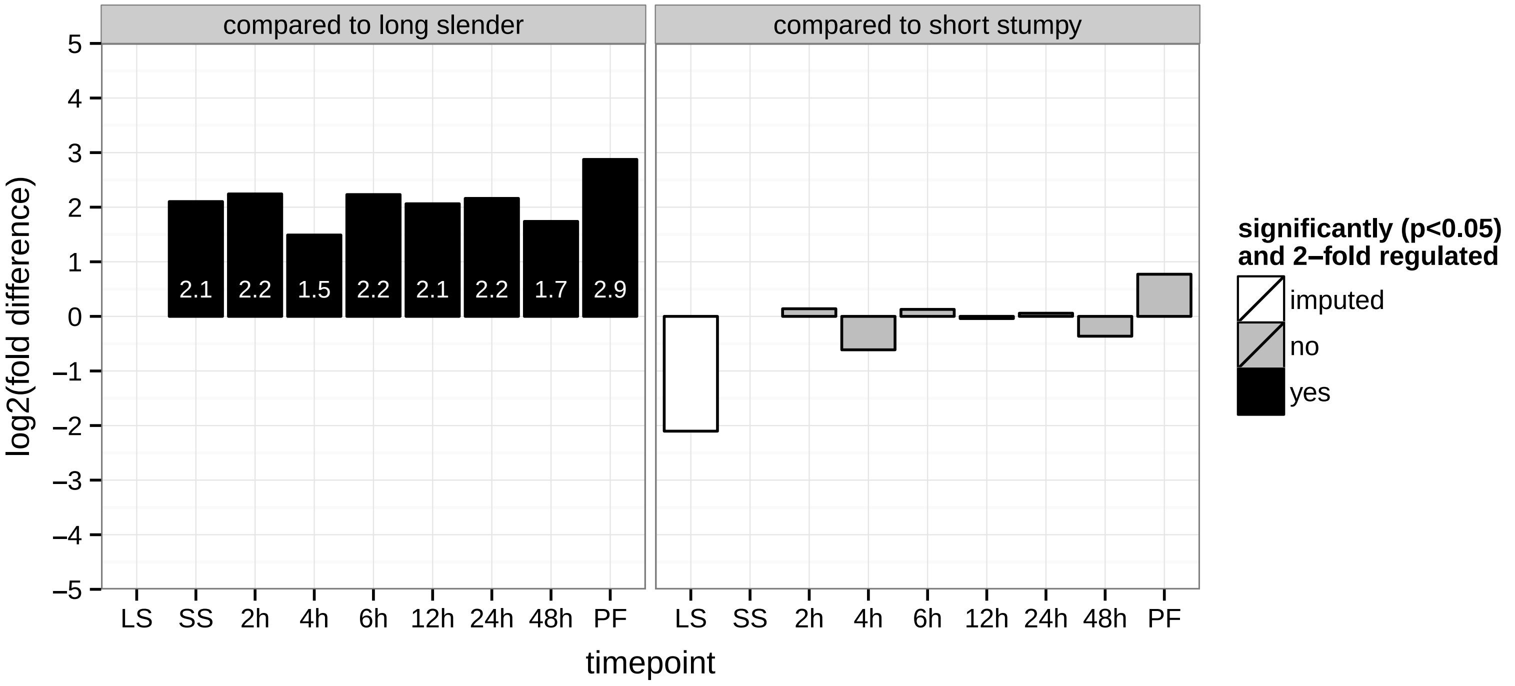

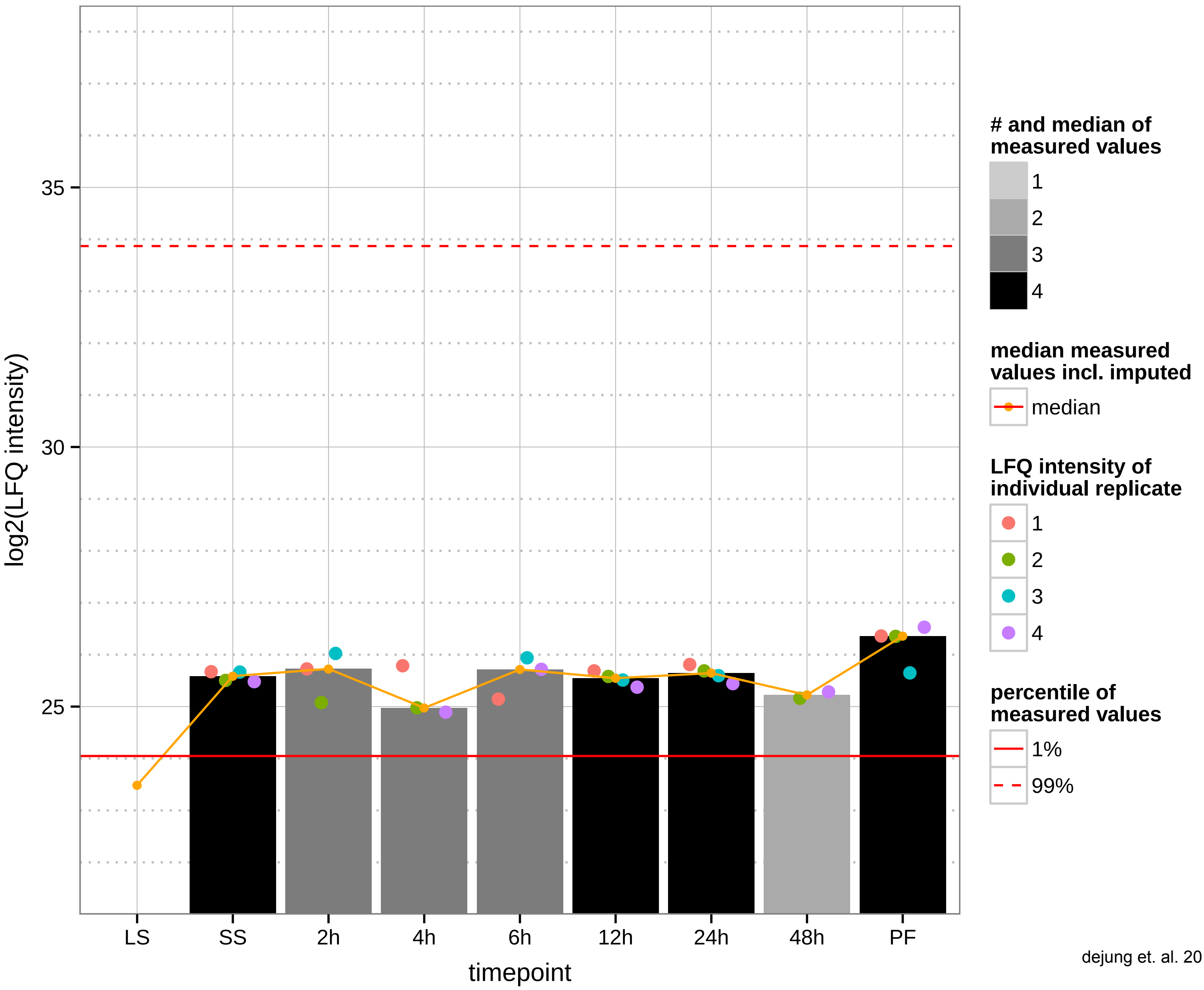

dejung et. al. 2015

hypothetical protein, conserved
Tb927.6.3210
AGOF: null
AGOC: null
AGOP: null
PGOF: null
PGOC: null
PGOP: null

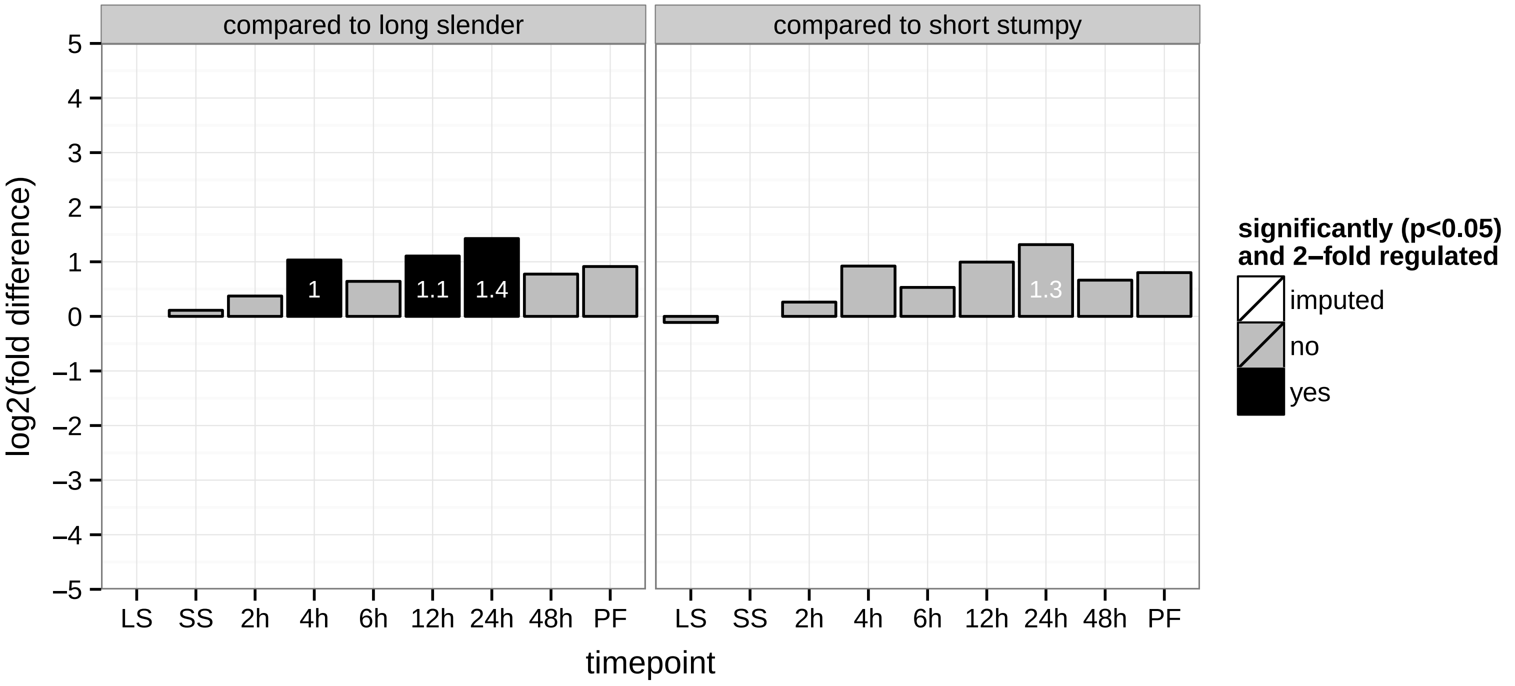

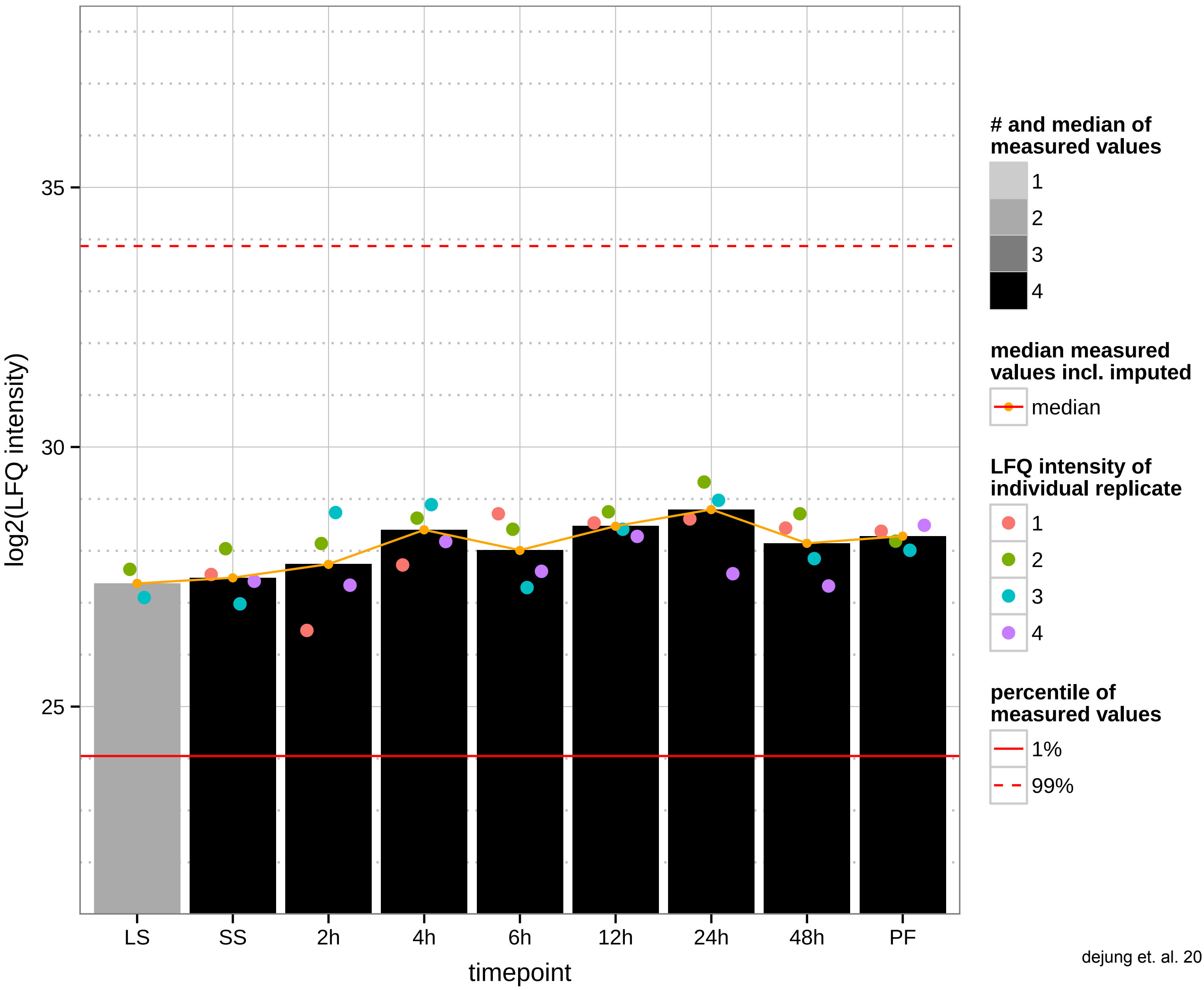

dejung et. al. 2015

intraflagellar transport protein 20, putative (IFT20)
Tb927.6.3290
AGOF: null
AGOC: bacterial–type flagellum basal body
AGOP: null
PGOF: null
PGOC: null
PGOP: null

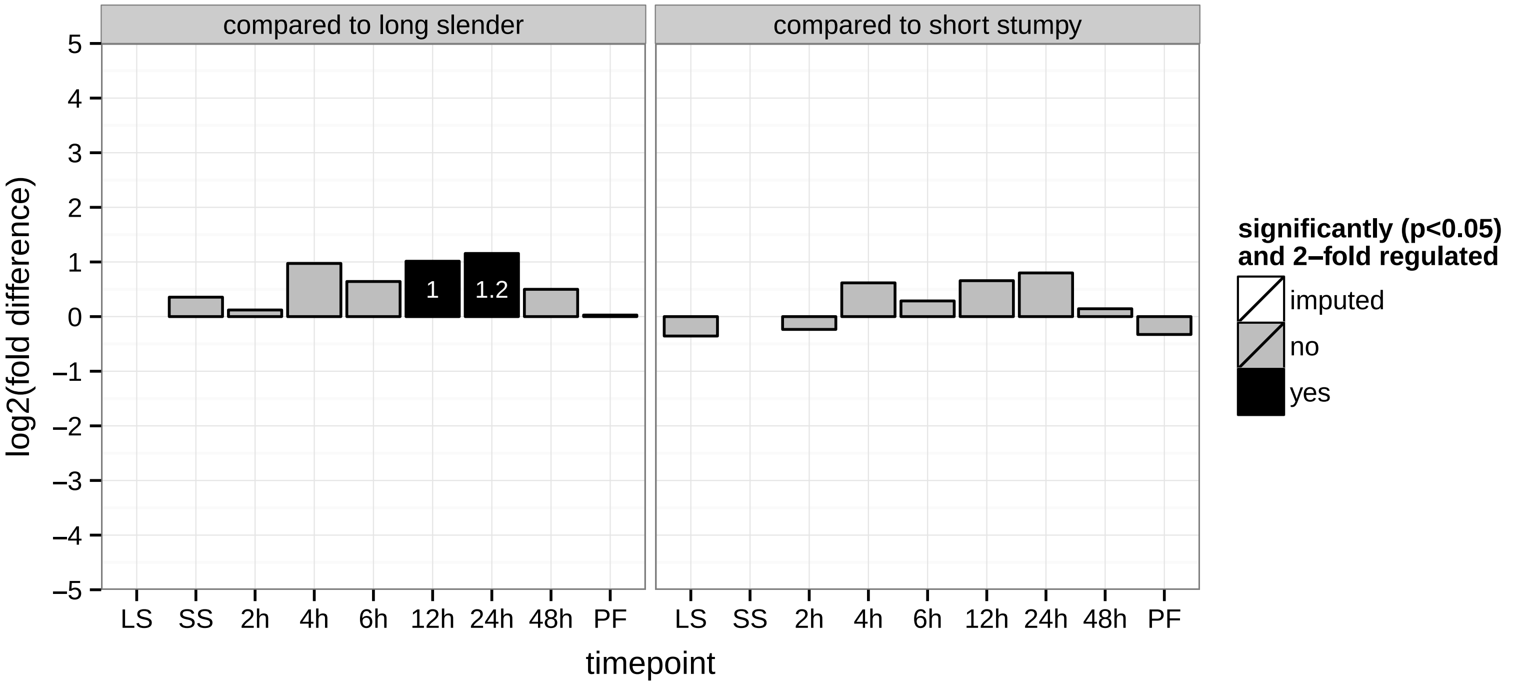

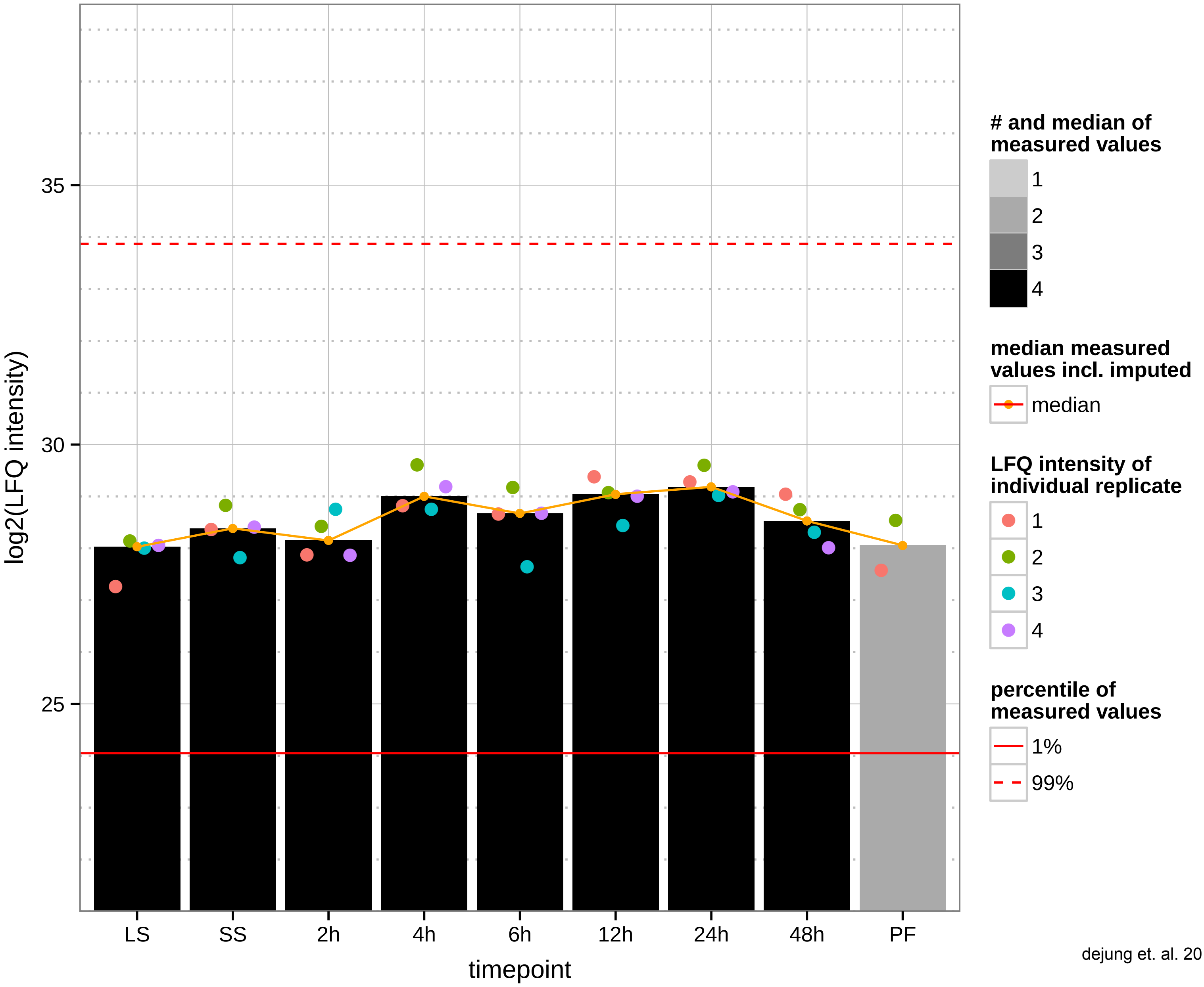

dejung et. al. 2015

calpain–like cysteine peptidase, putative, cysteine peptidase, Clan CA, family C2
Tb927.6.3310
AGOF: calcium–dependent cysteine–type endopeptidase activity
AGOC: intracellular
AGOP: proteolysis
PGOF: calcium–dependent cysteine–type endopeptidase activity
PGOC: intracellular
PGOP: proteolysis

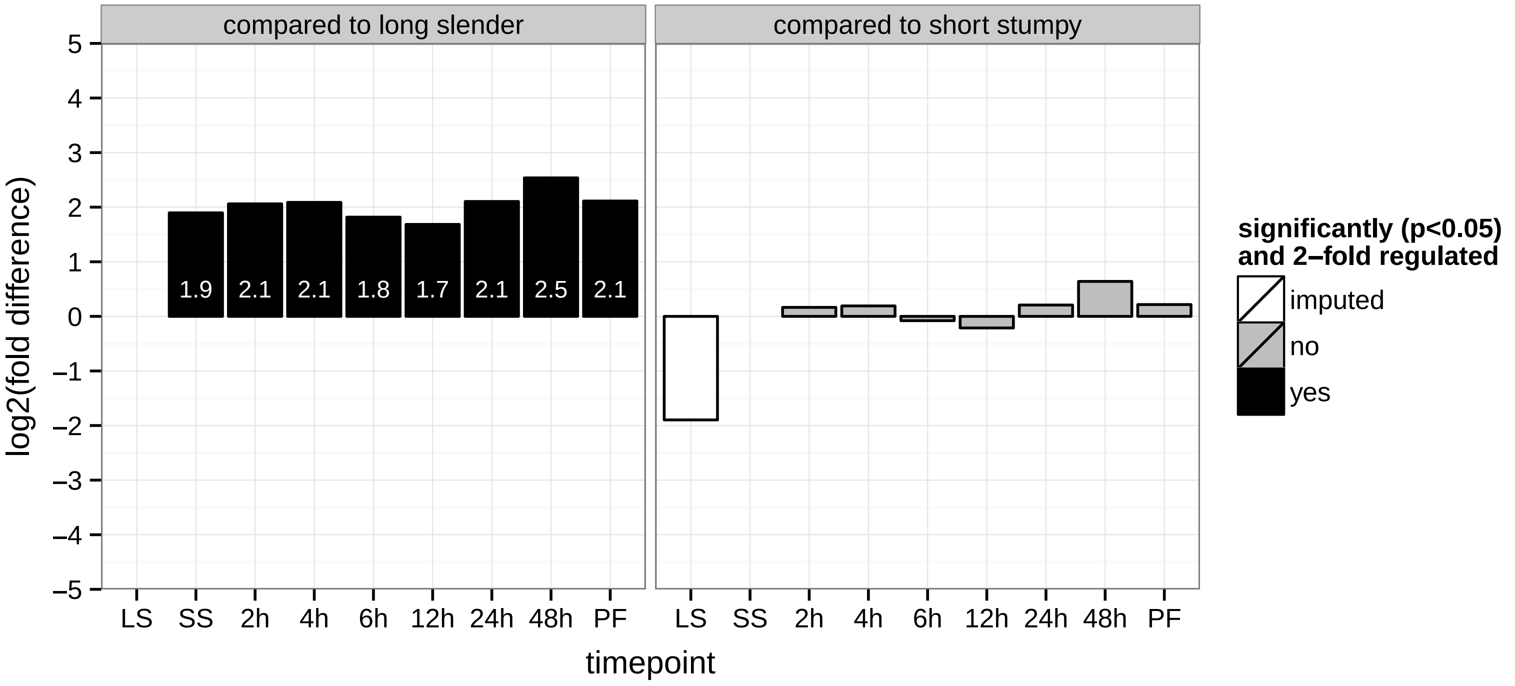

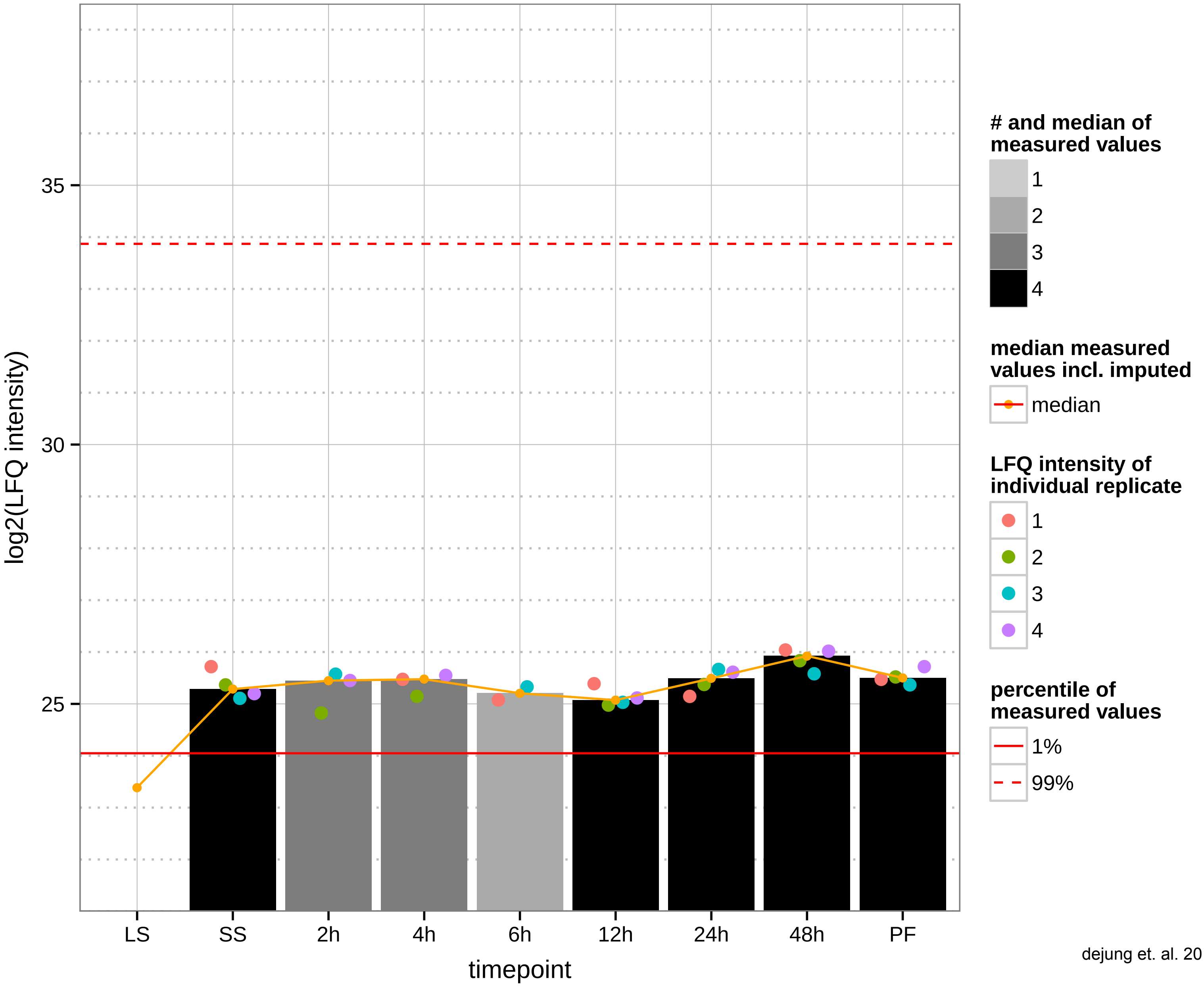

dejung et. al. 2015

hypothetical protein, conserved
Tb927.6.3340
AGOF: null
AGOC: null
AGOP: null
PGOF: null
PGOC: null
PGOP: null

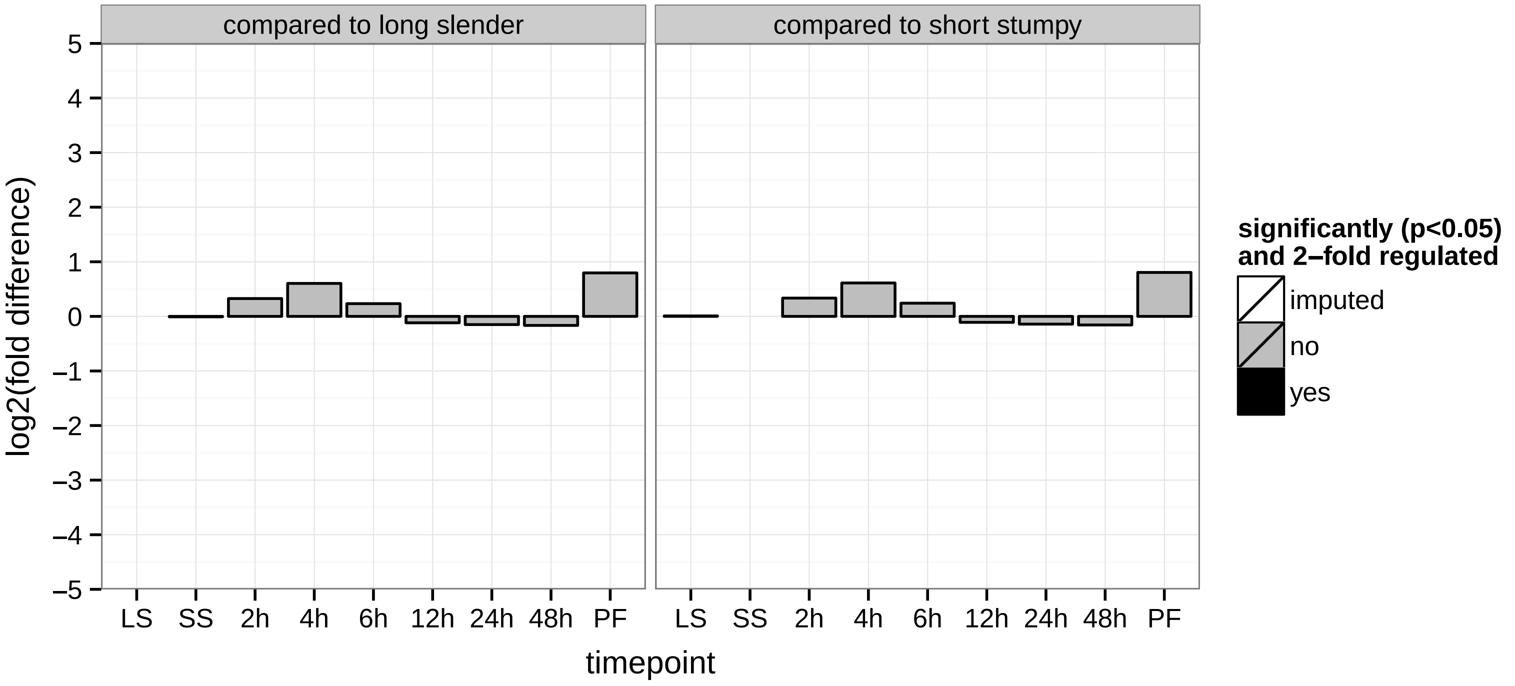

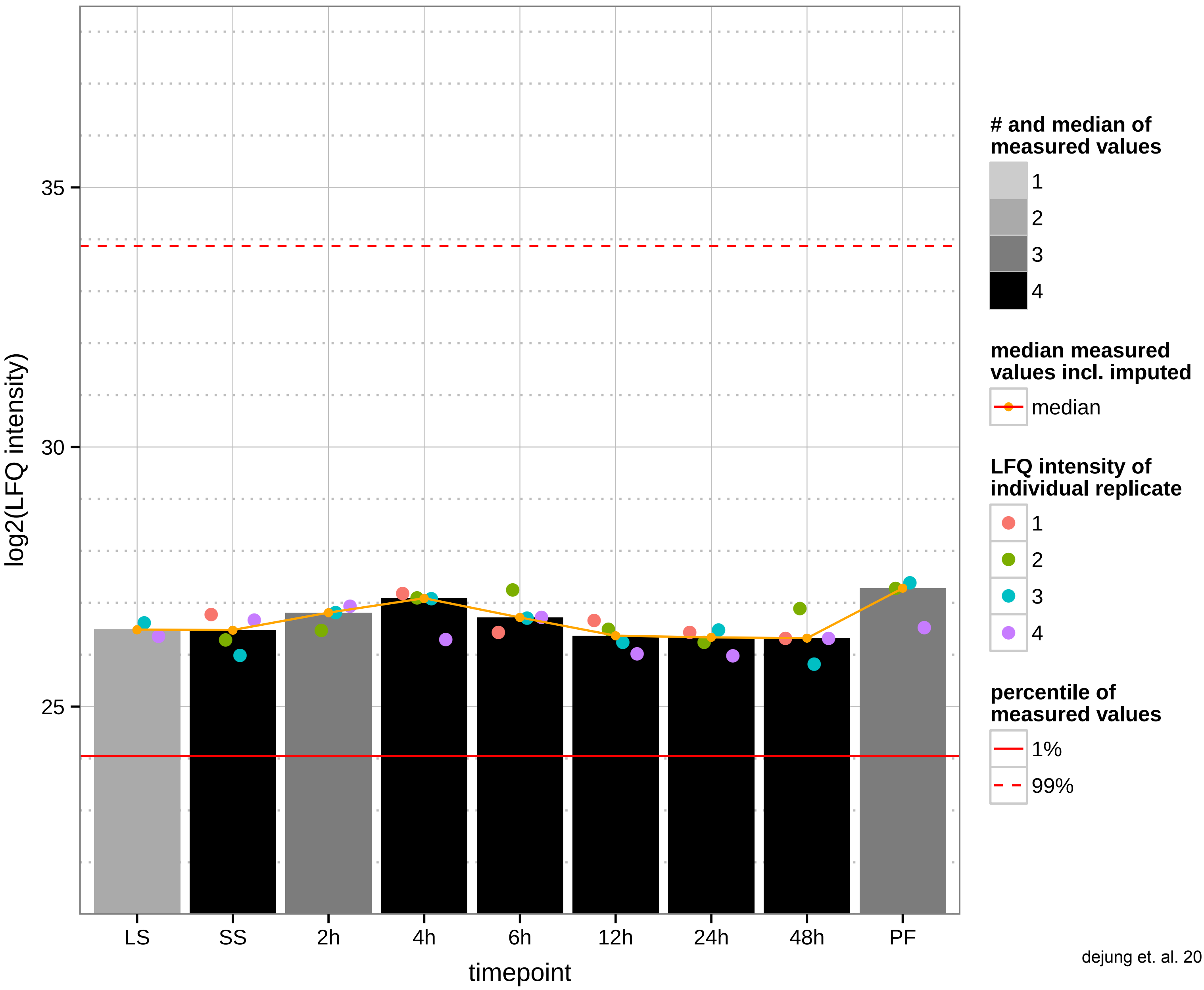

dejung et. al. 2015

hypothetical protein, conserved
Tb927.6.3520;Tb11.v5.0634
AGOF: null
AGOC: null
AGOP: null
PGOF: null
PGOC: null
PGOP: null

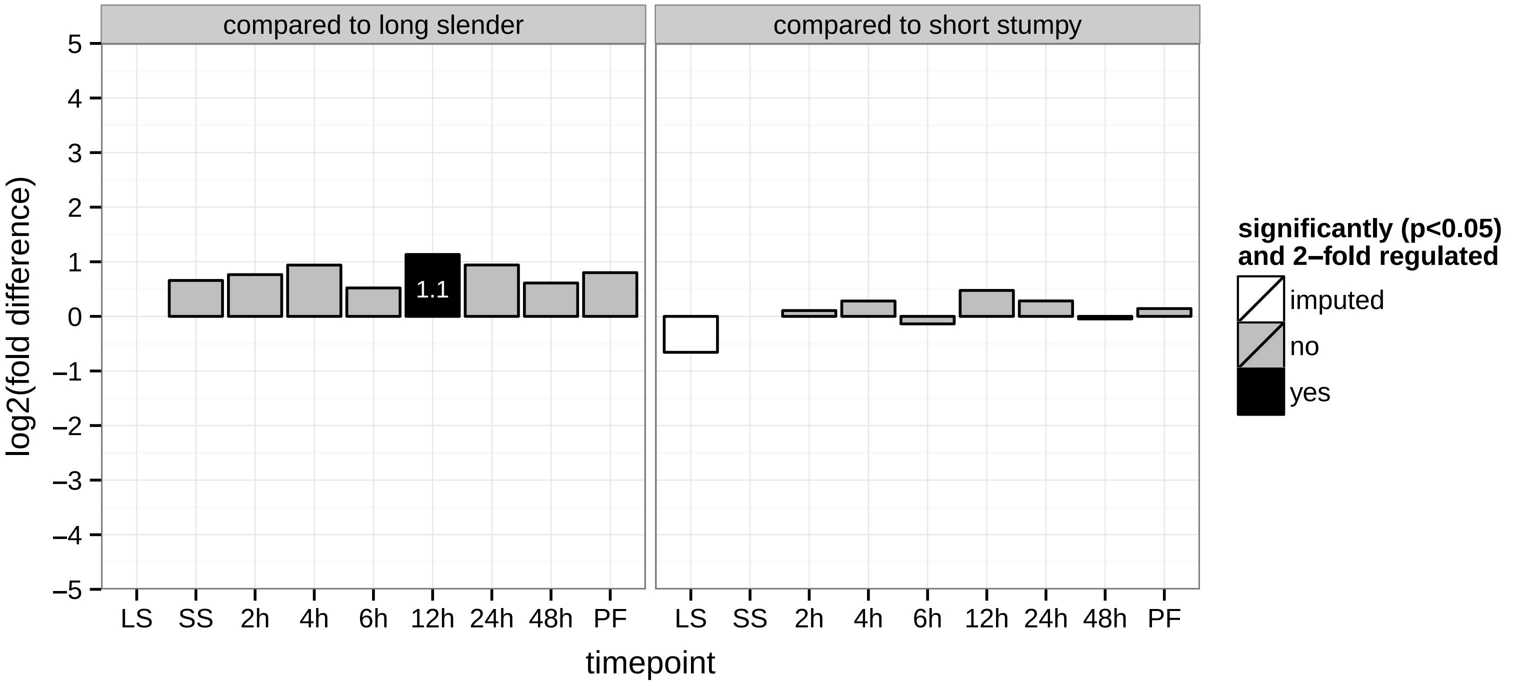

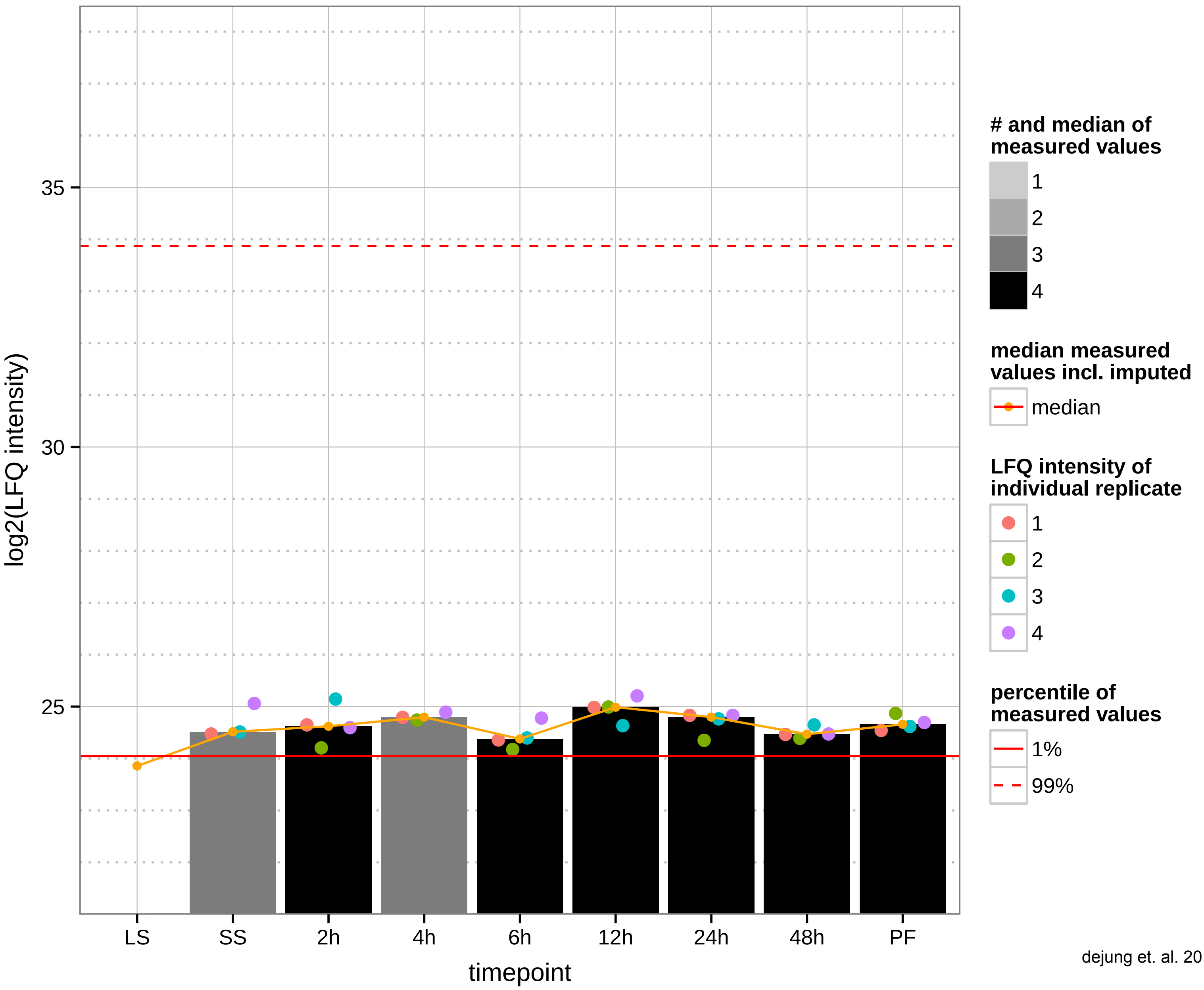

dejung et. al. 2015

ADP–ribosylation factor, putative
Tb927.6.3650
AGOF: GTP binding
AGOC: intracellular
AGOP: intracellular protein transport, small GTPase mediated signal transduction
PGOF: GTP binding
PGOC: intracellular
PGOP: intracellular protein transport, small GTPase mediated signal transduction

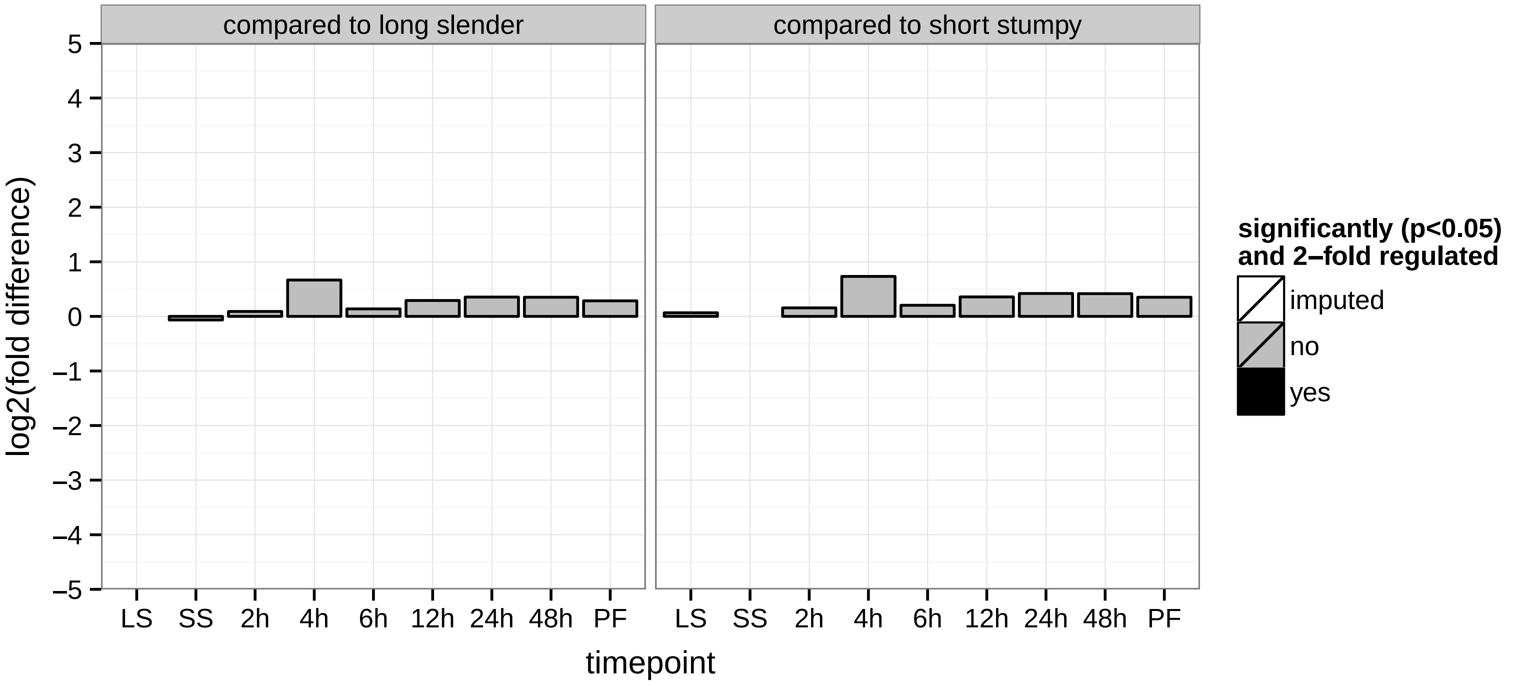

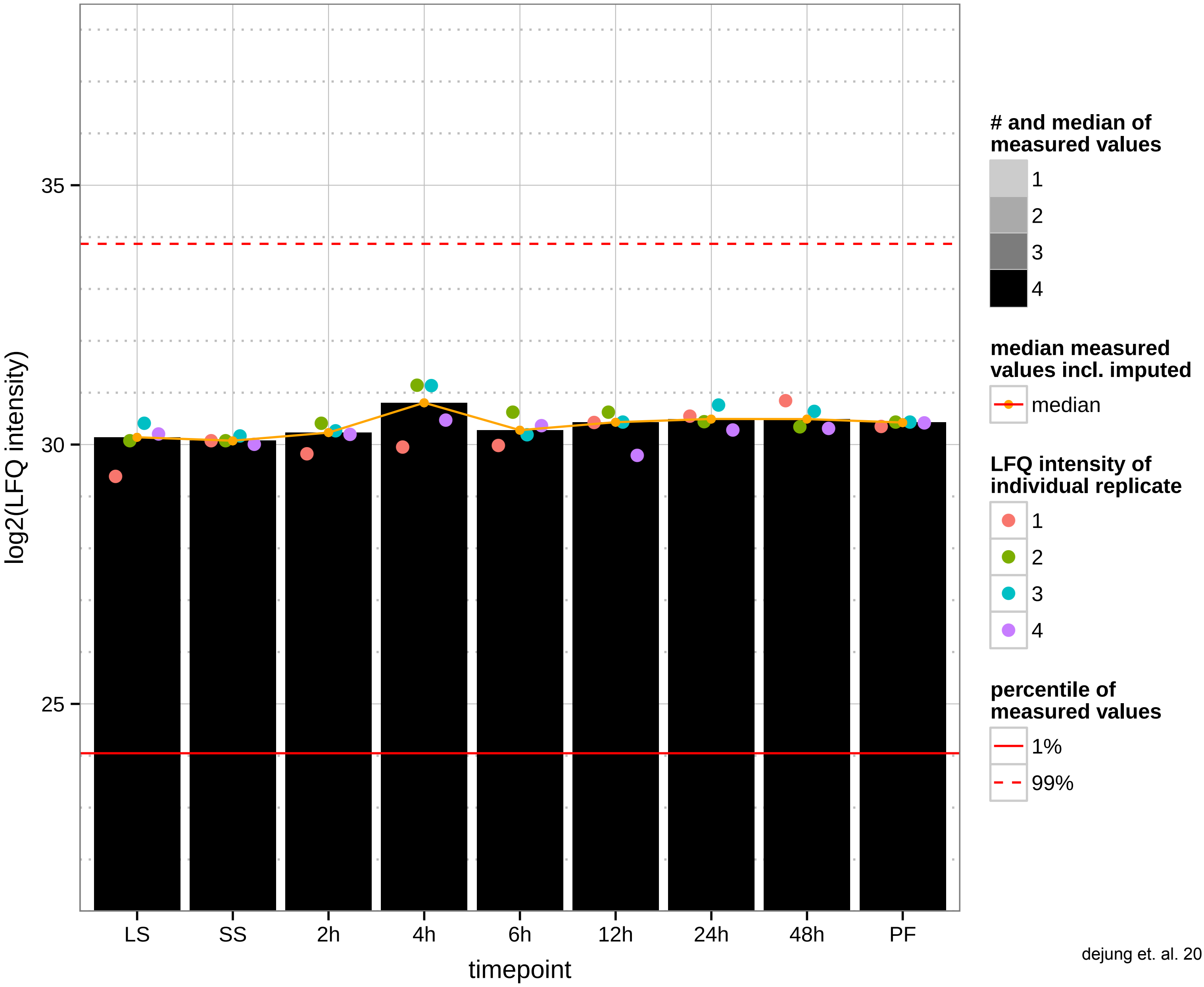

dejung et. al. 2015

paraflagellar rod component, putative (PFC8)
Tb927.6.3670
AGOF: null
AGOC: null
AGOP: null
PGOF: null
PGOC: null
PGOP: null

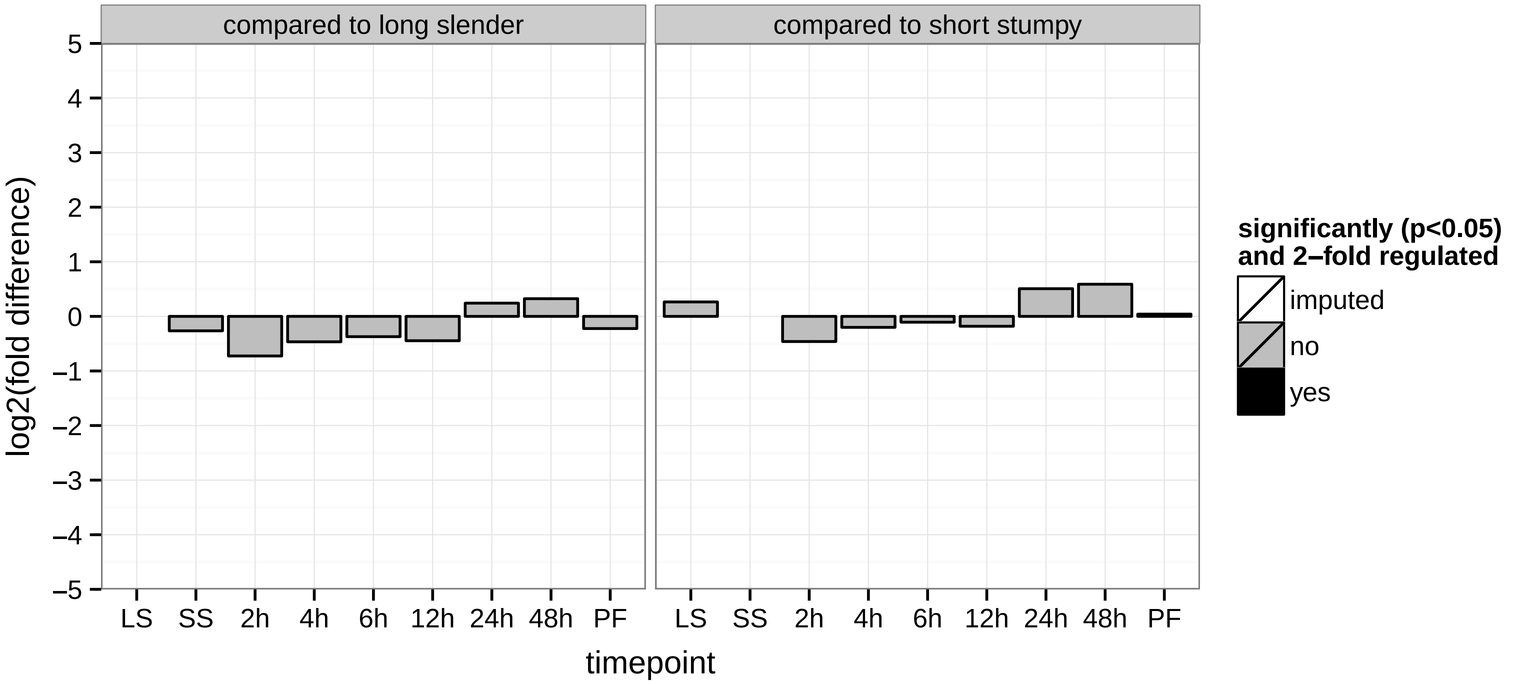

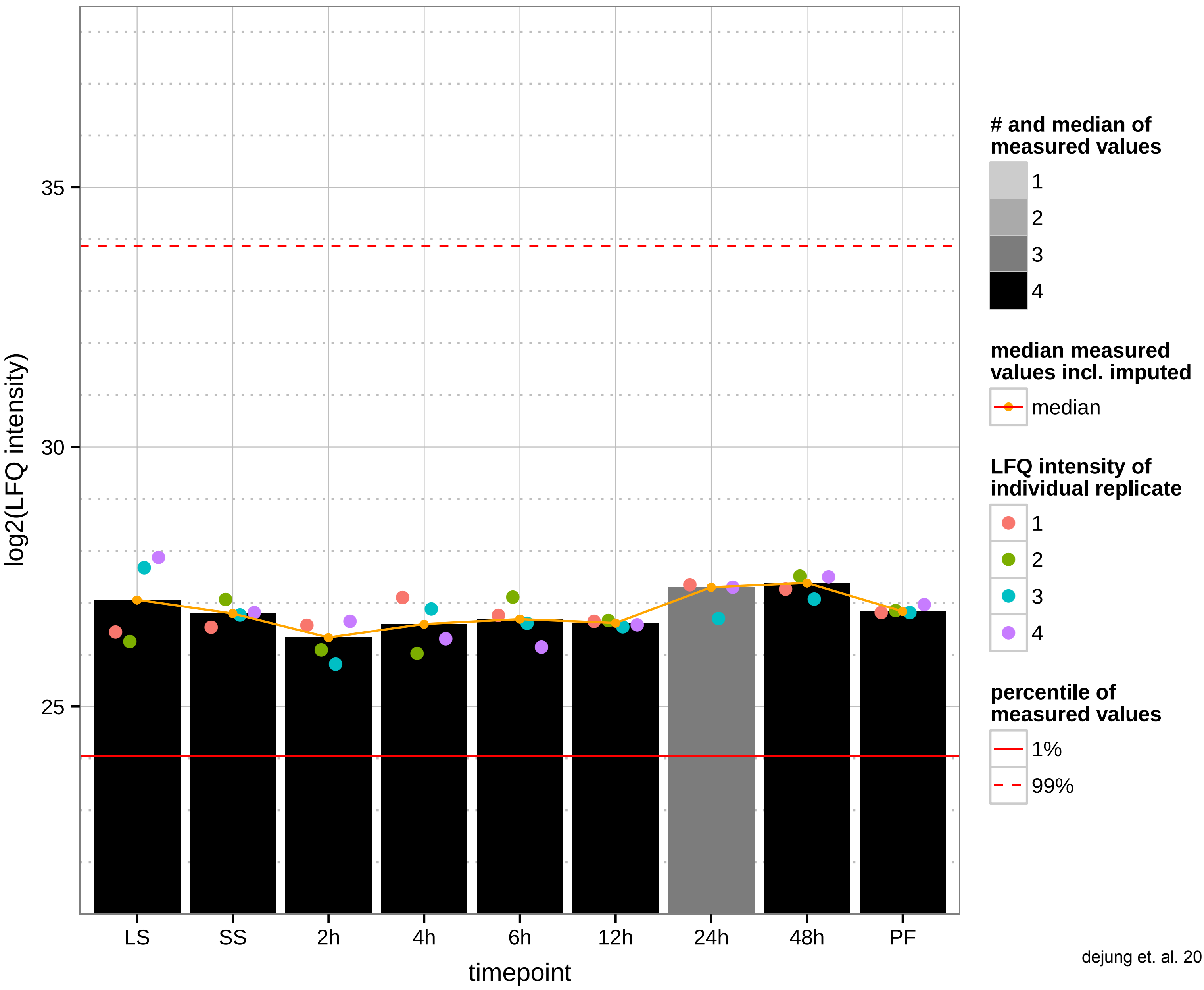

dejung et. al. 2015

hypothetical protein, conserved
Tb927.6.3680
AGOF: null
AGOC: null
AGOP: null
PGOF: null
PGOC: null
PGOP: null

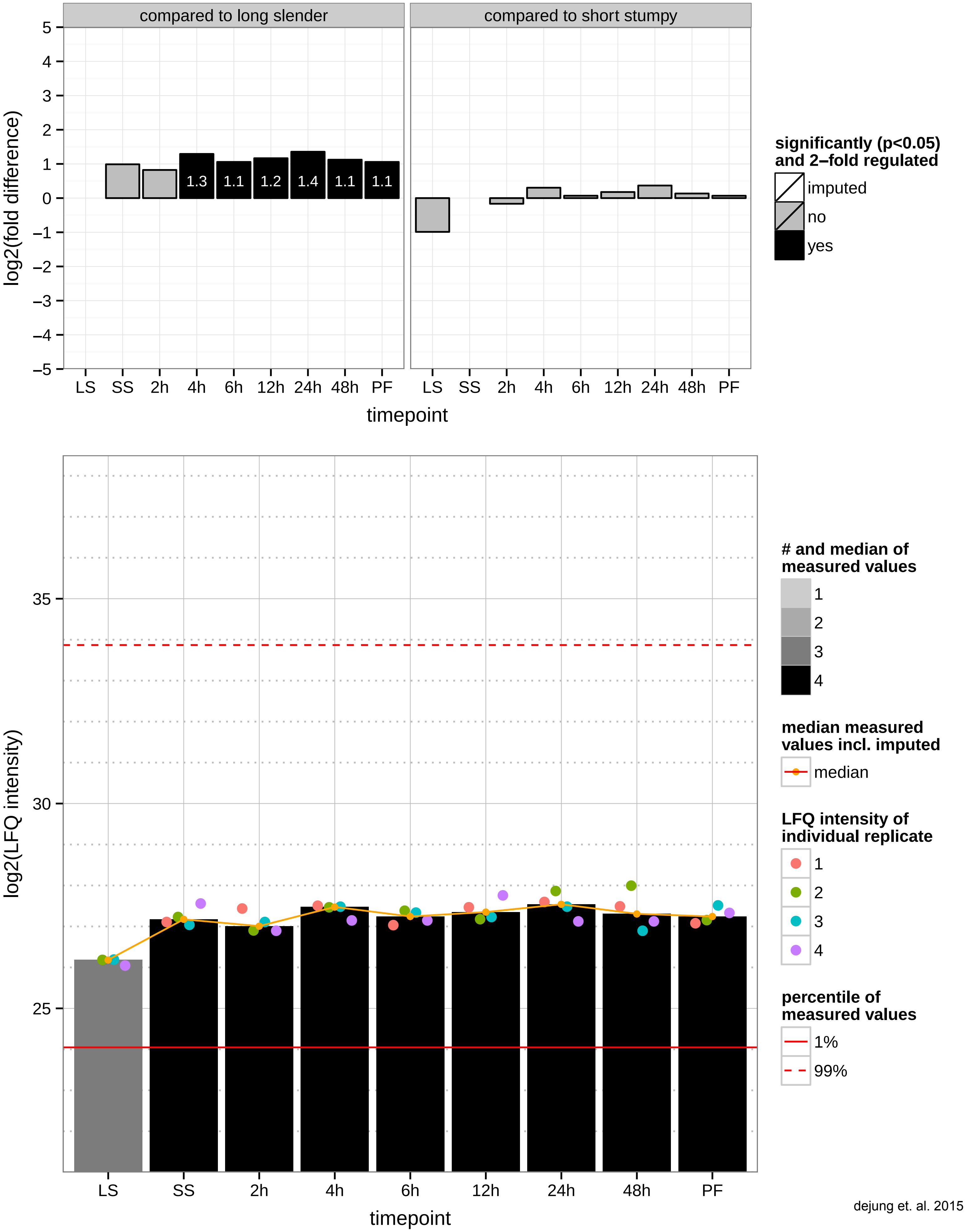

dejung et. al. 2015

pre–mRNA cleavage complex II Clp1–like, conserved
Tb927.6.3690
AGOF: ATP–dependent polyribonucleotide 5'–hydroxyl–kinase activity
AGOC: tRNA–intron endonuclease complex
AGOP: tRNA splicing, via endonucleolytic cleavage and ligation, targeting of mRNA for destruction involved in RNA interferen
PGOF: null
PGOC: null
PGOP: null

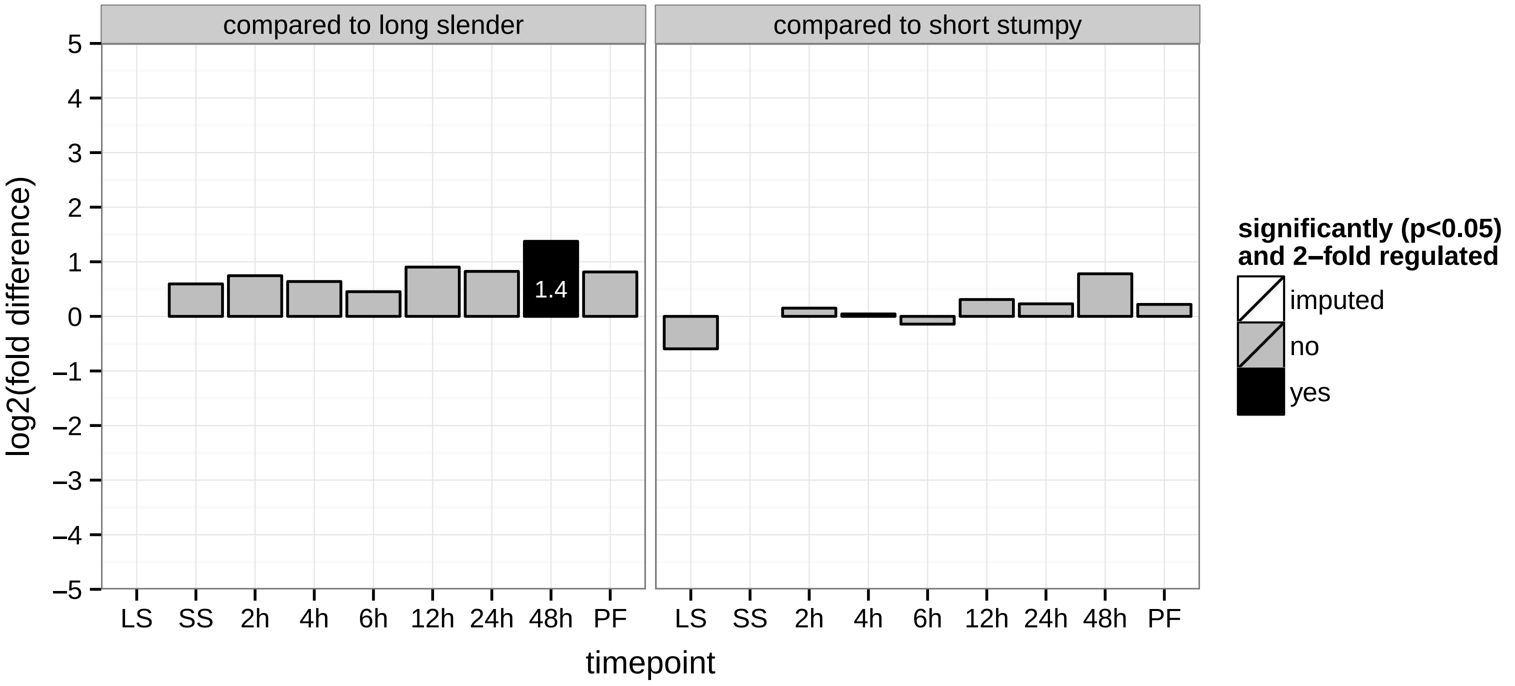

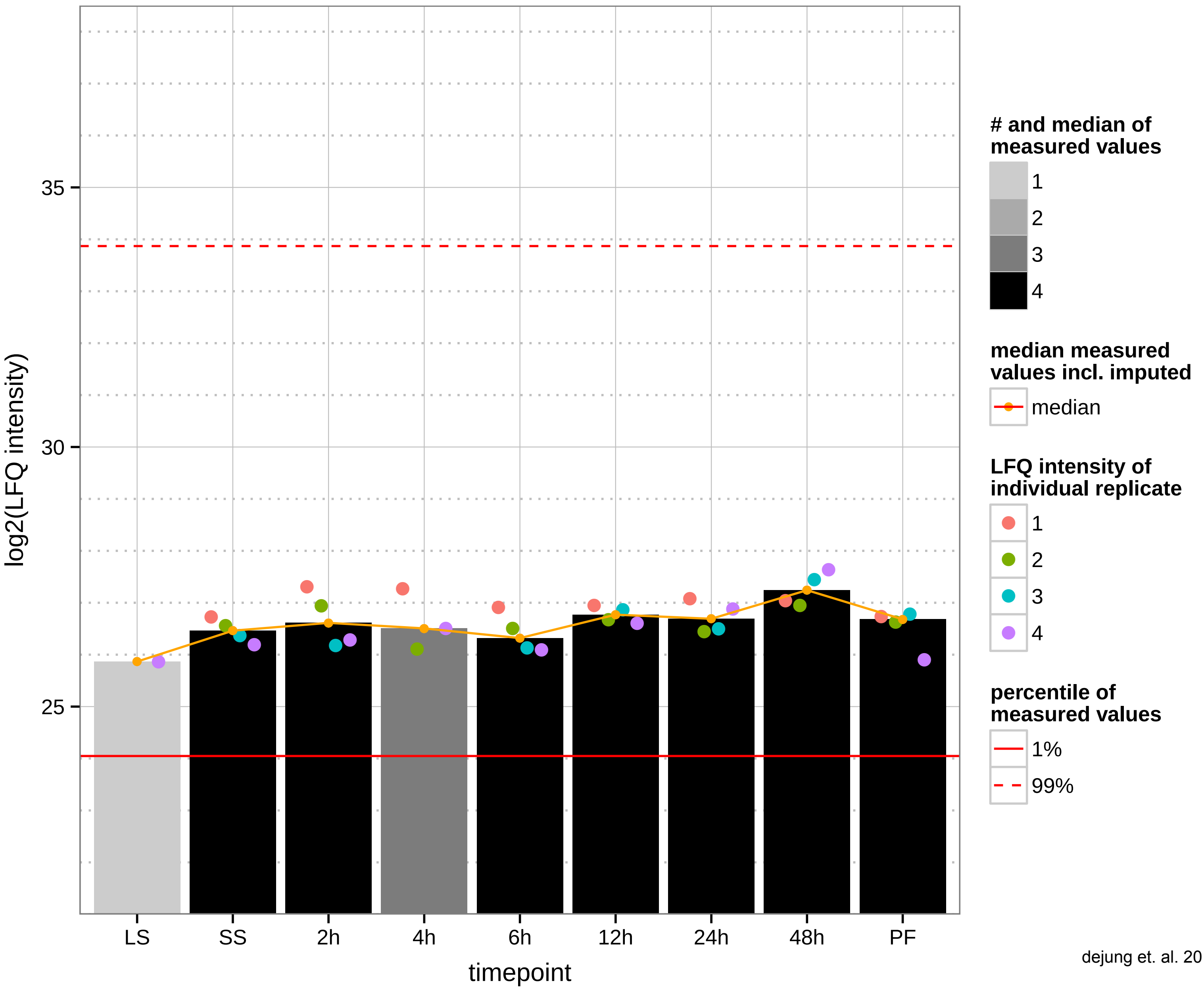

dejung et. al. 2015

heat shock 70 kDa protein, mitochondrial precursor, putative
Tb927.6.3800;Tb927.6.3750;Tb927.6.3740
AGOF: ATP binding, unfolded protein binding
AGOC: mitochondrion, null
AGOP: protein folding
PGOF: ATP binding, unfolded protein binding
PGOC: null
PGOP: protein folding

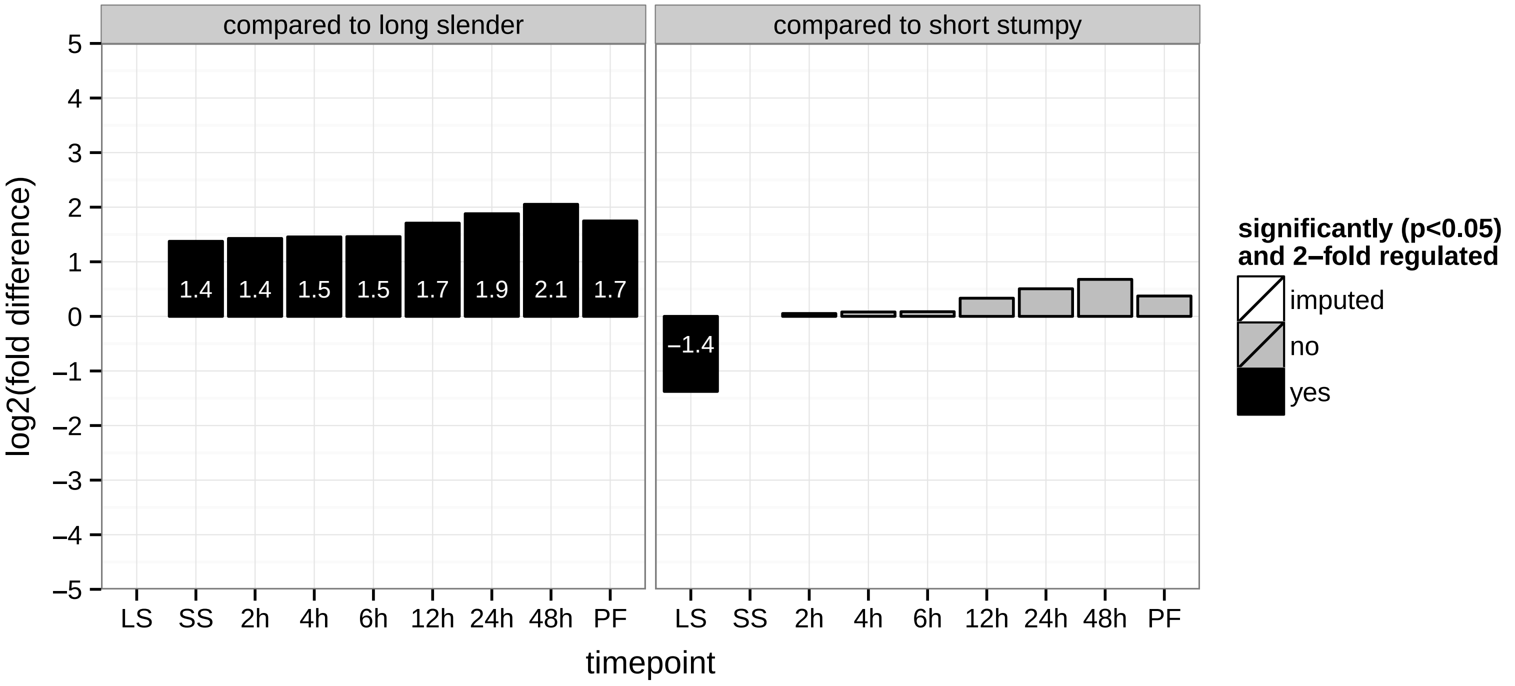

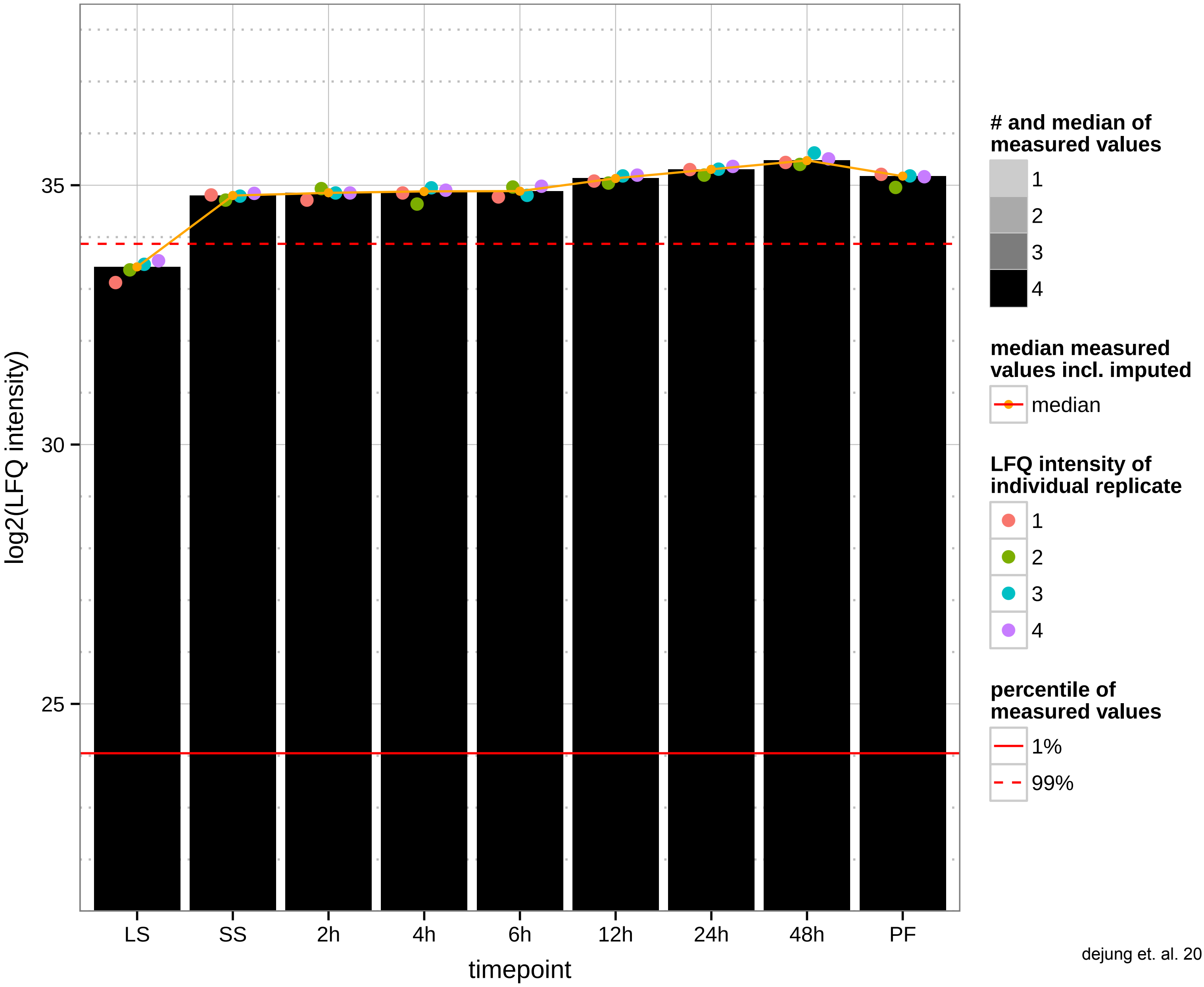

dejung et. al. 2015

hypothetical protein, conserved
Tb927.6.3810
AGOF: null
AGOC: null
AGOP: null
PGOF: null
PGOC: null
PGOP: null

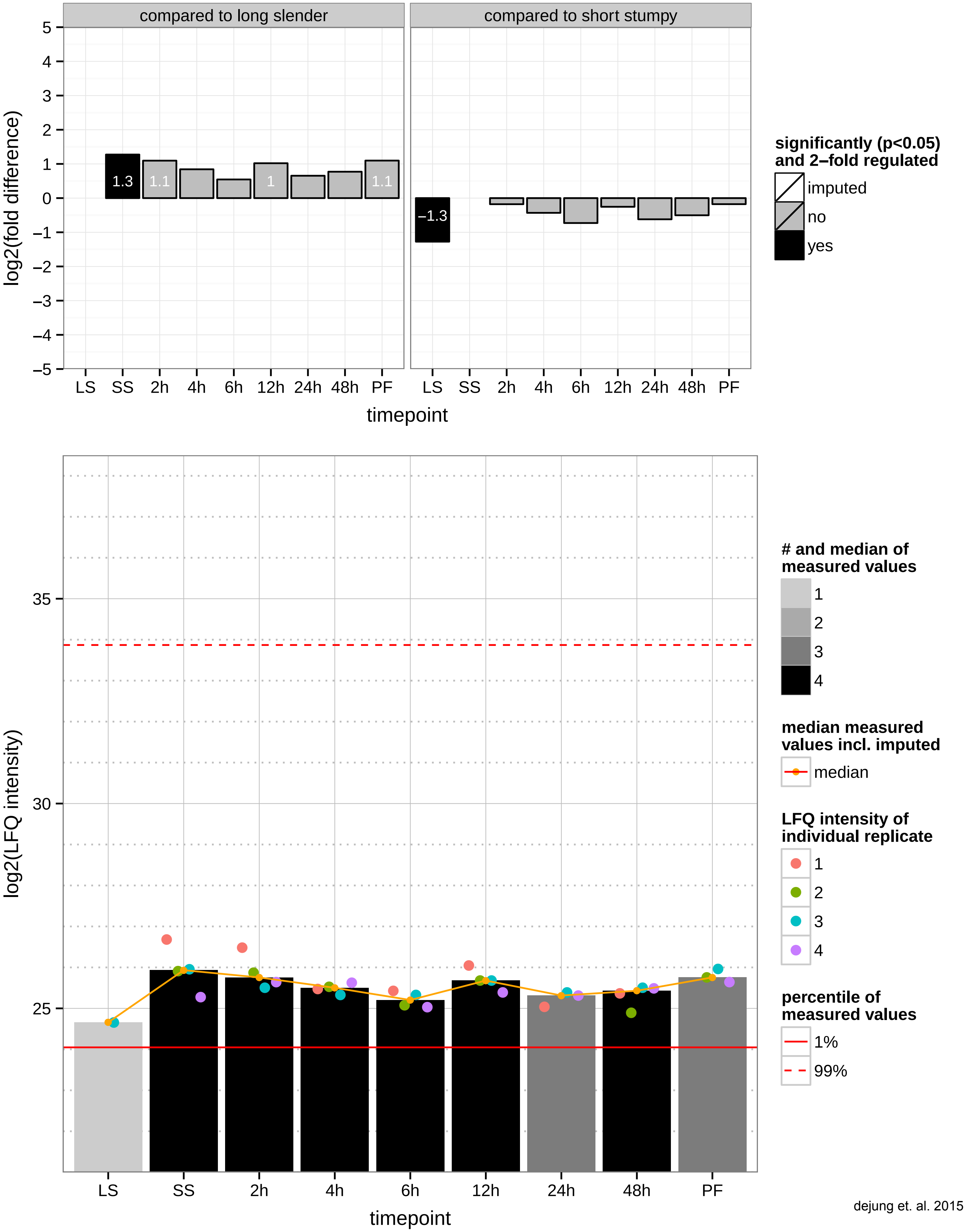

dejung et. al. 2015

reticulon domain protein
Tb927.6.3840
AGOF: null
AGOC: endoplasmic reticulum
AGOP: null
PGOF: null
PGOC: endoplasmic reticulum
PGOP: null

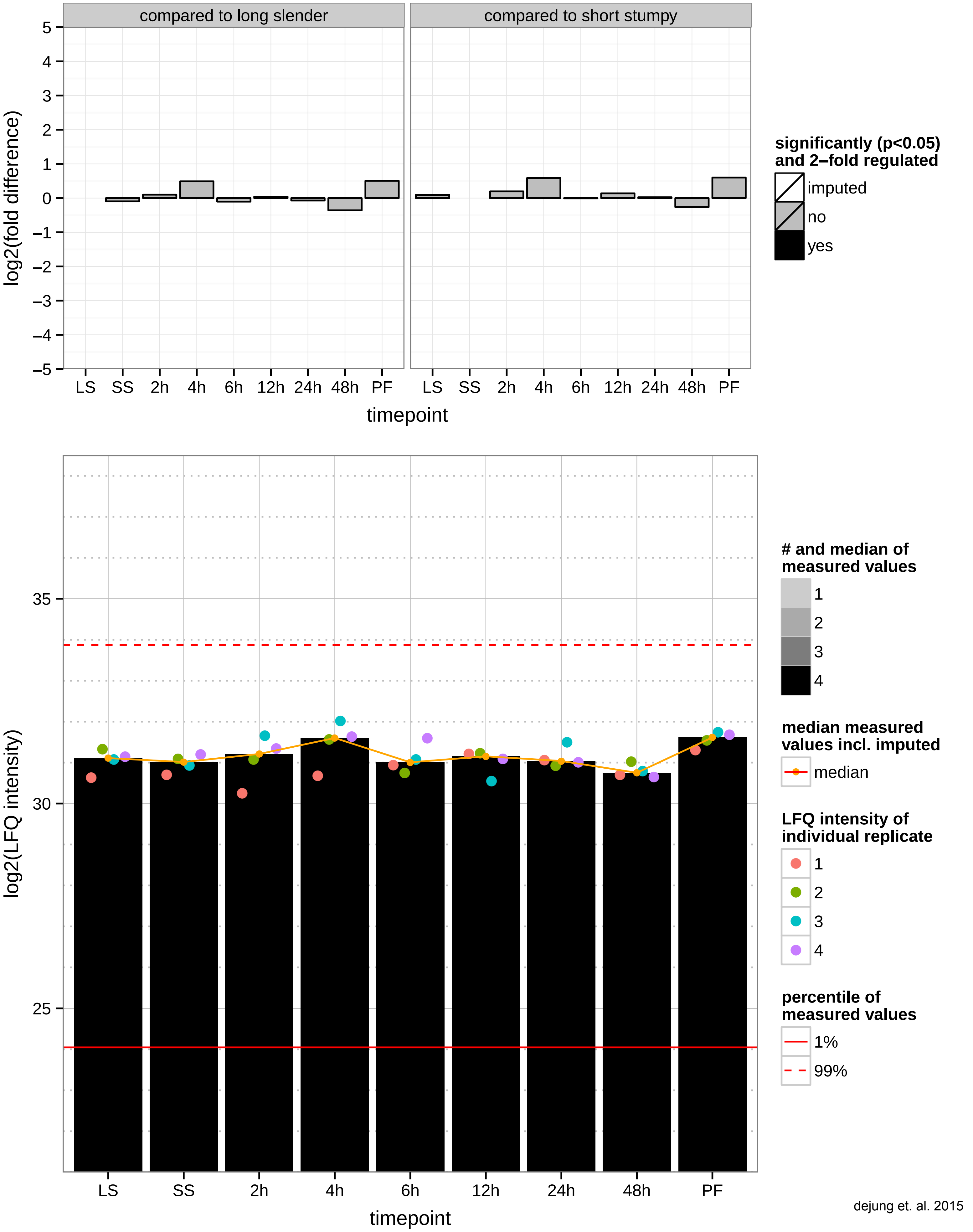

dejung et. al. 2015

RNA–binding protein, putative (SGN1)
Tb927.6.3870
AGOF: RNA binding
AGOC: null
AGOP: RNA processing
PGOF: nucleic acid binding
PGOC: null
PGOP: null

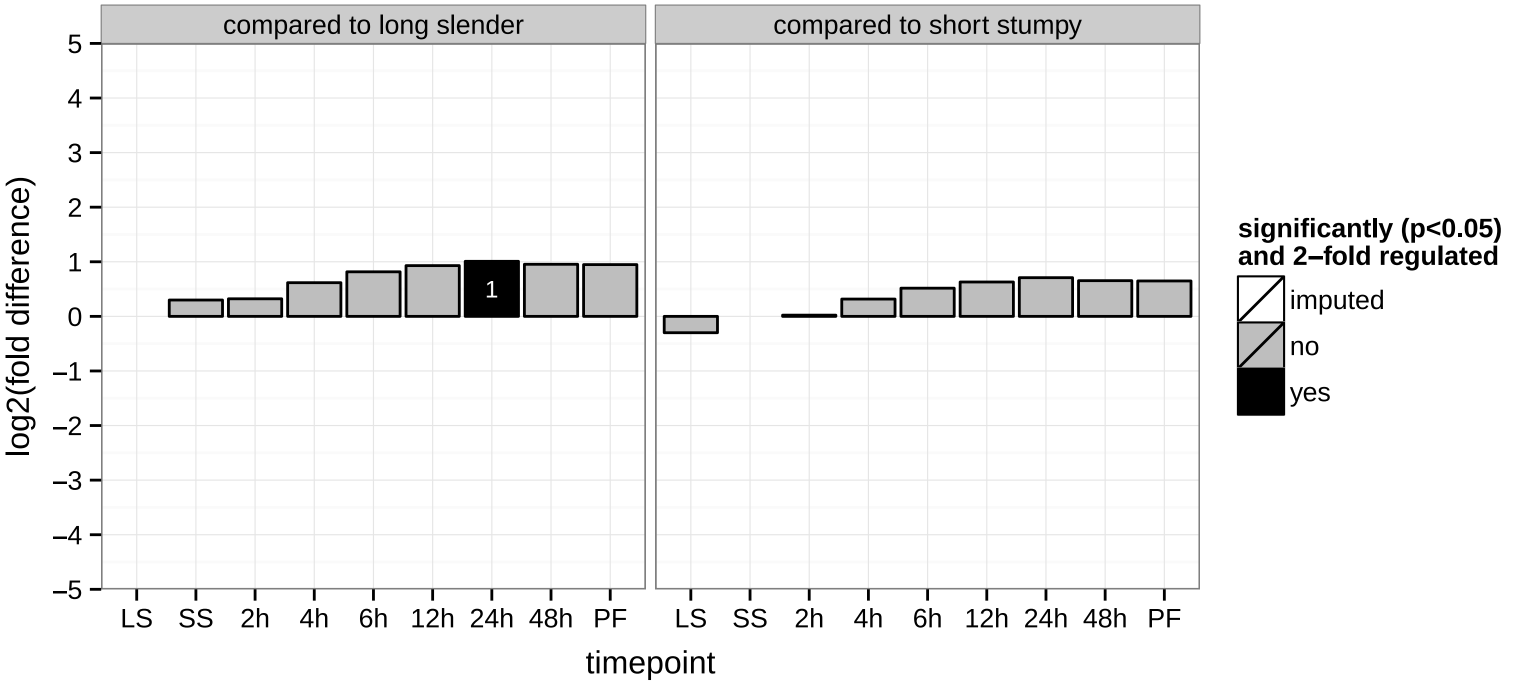

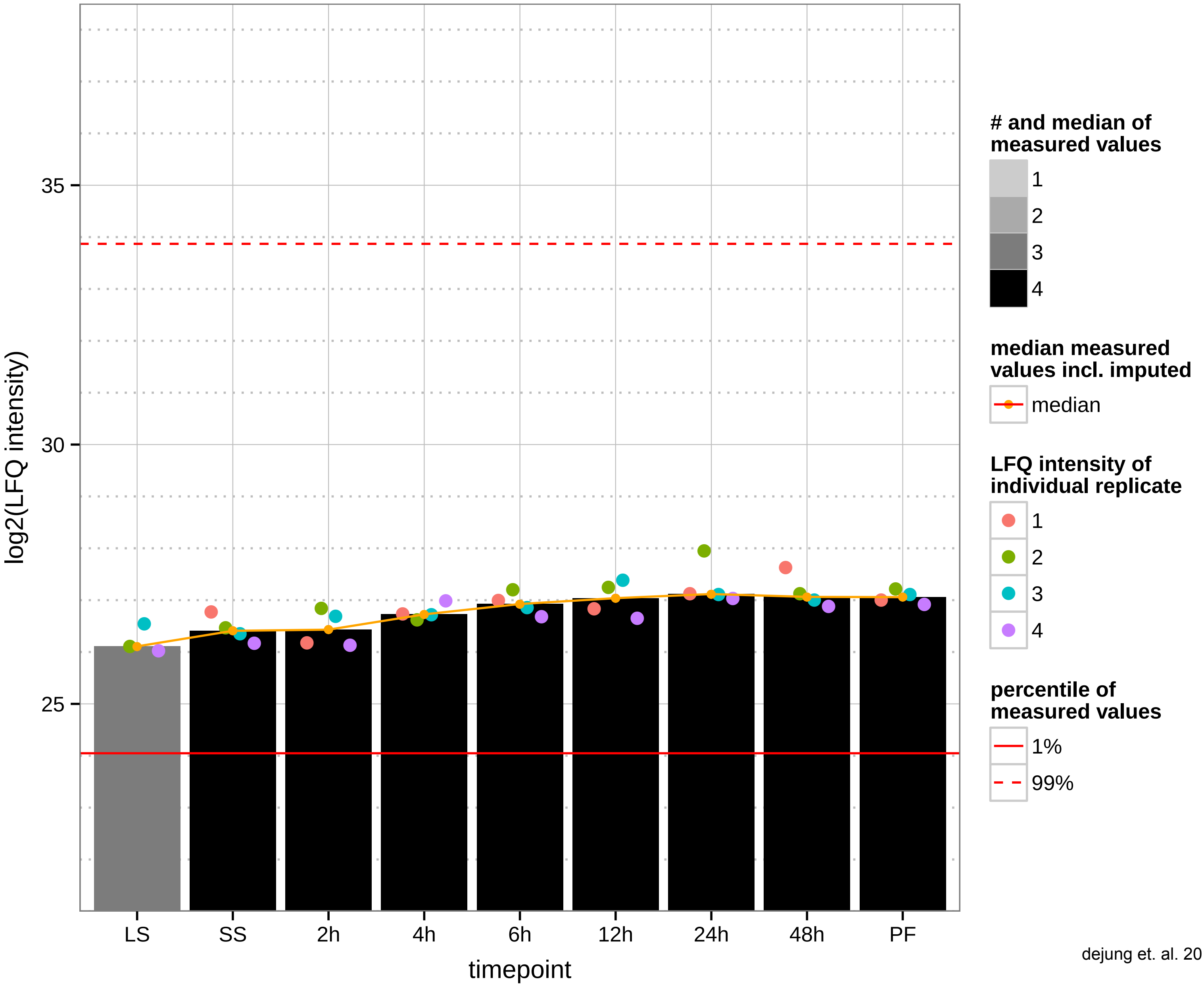

dejung et. al. 2015

replication factor C, subunit 2, putative
Tb927.6.3890
AGOF: ATP binding, DNA binding, DNA clamp loader activity
AGOC: DNA replication factor C complex
AGOP: DNA replication
PGOF: ATP binding, DNA binding, nucleoside−triphosphatase activity, nucleotide binding
PGOC: null
PGOP: DNA replication

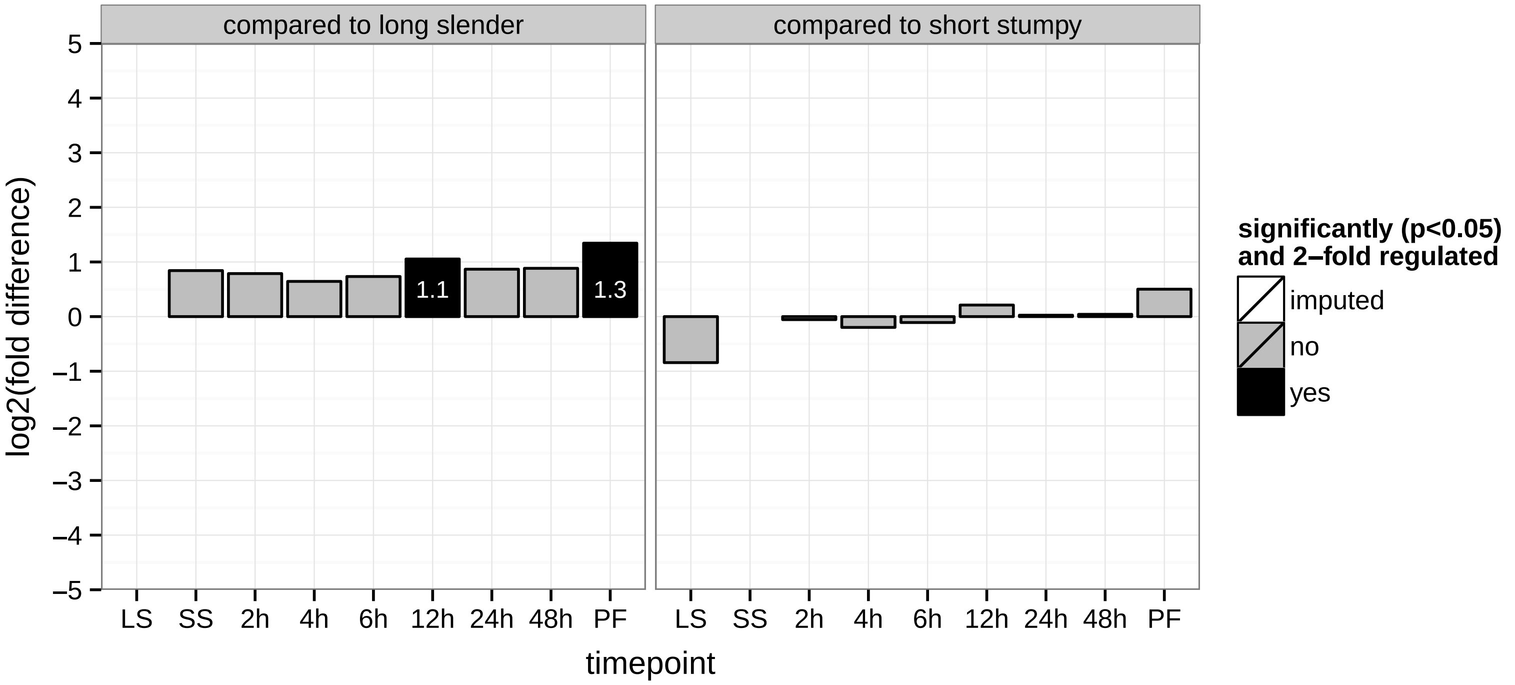

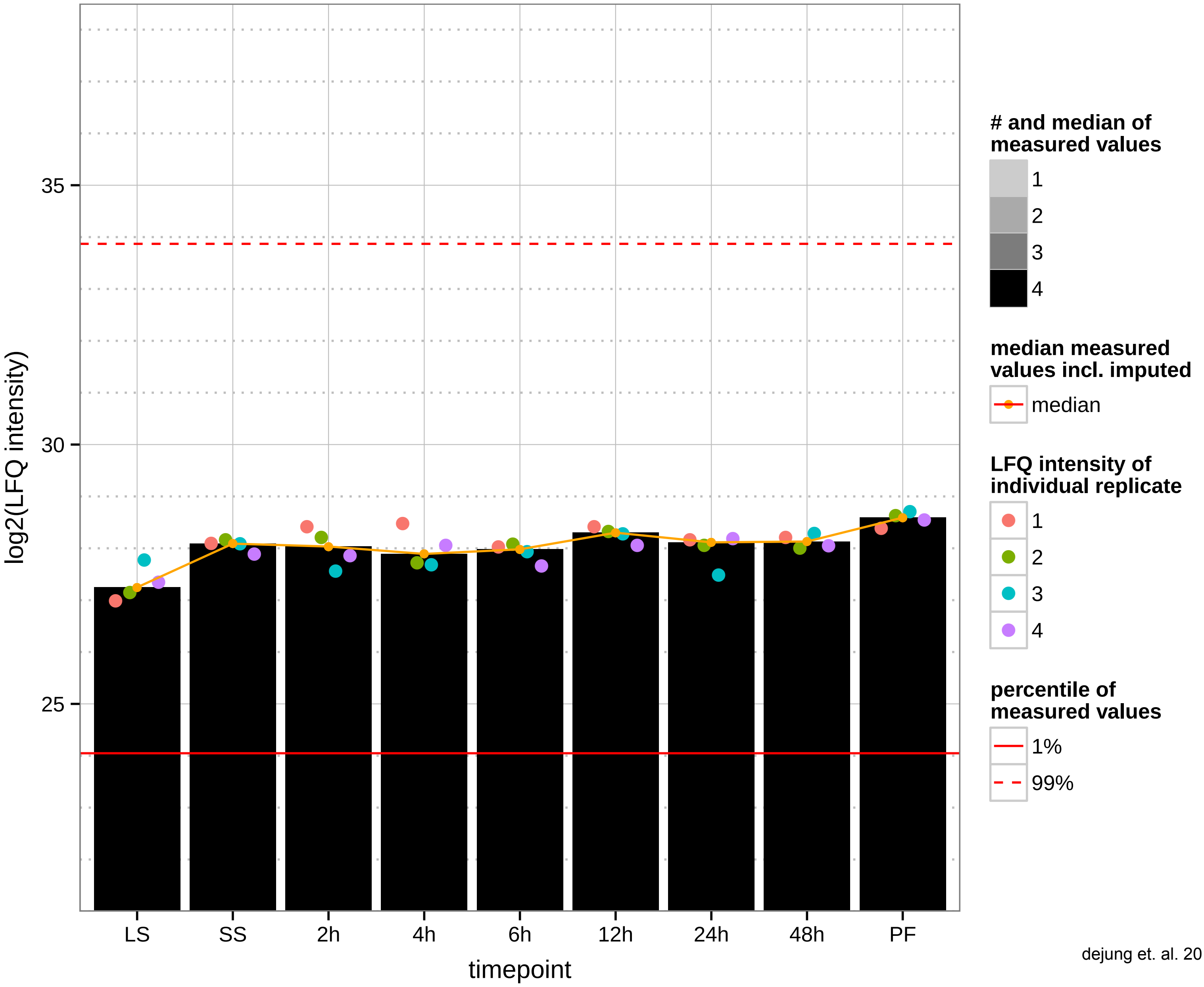

dejung et. al. 2015

small glutamine−rich tetratricopeptide repeat protein, putative, putative (SGT)
Tb927.6.4000
AGOF: chaperone binding
AGOC: cytosol
AGOP: null
PGOF: protein binding
PGOC: null
PGOP: null

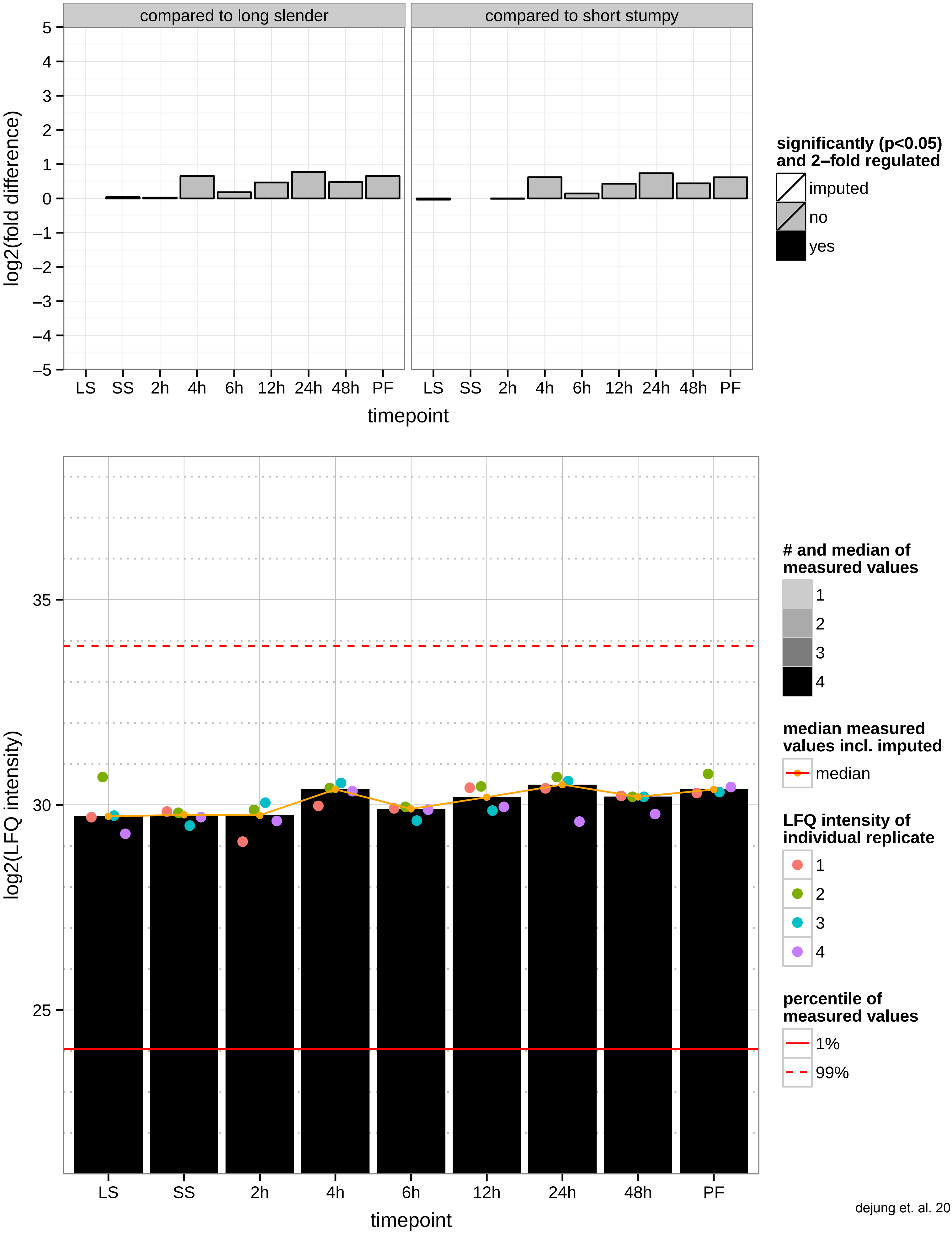

dejung et. al. 2015

hypothetical protein, conserved
Tb927.6.4020
AGOF: null
AGOC: null
AGOP: null
PGOF: null
PGOC: null
PGOP: null

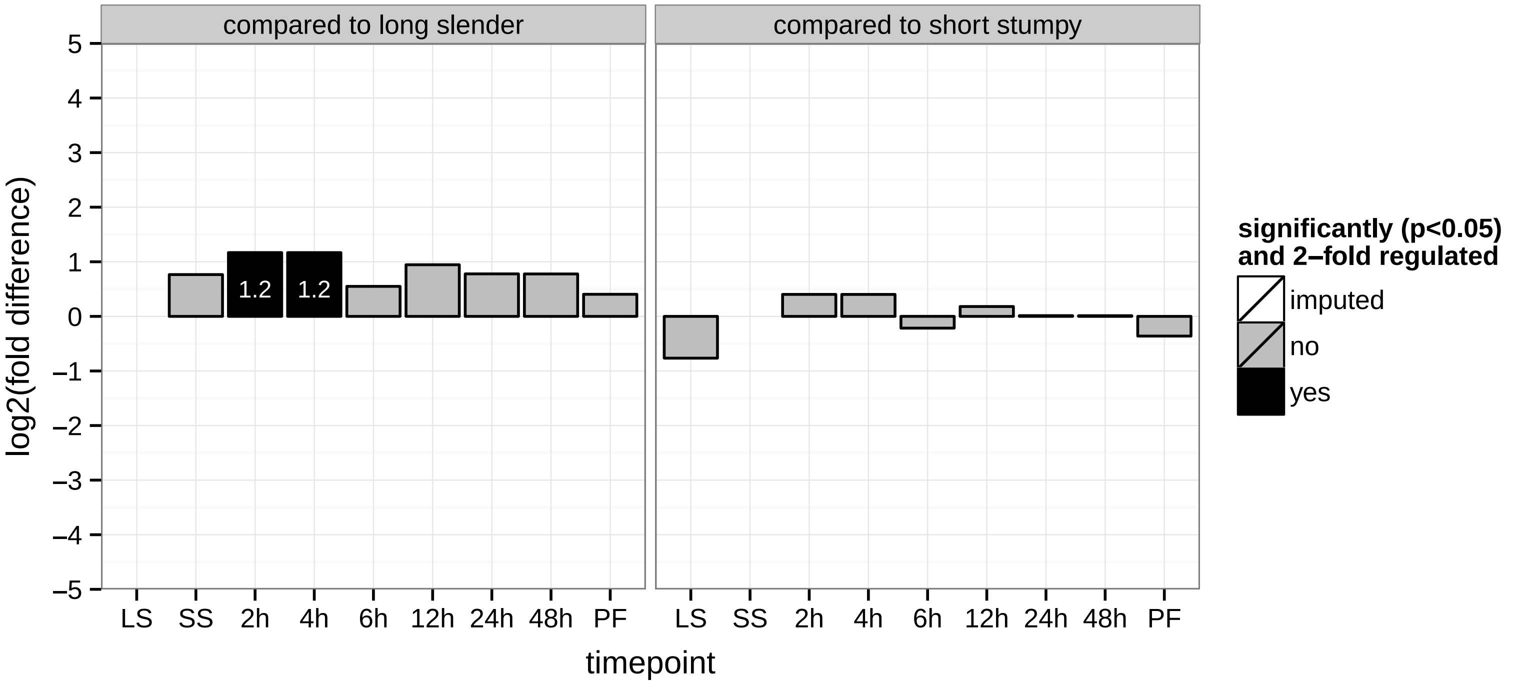

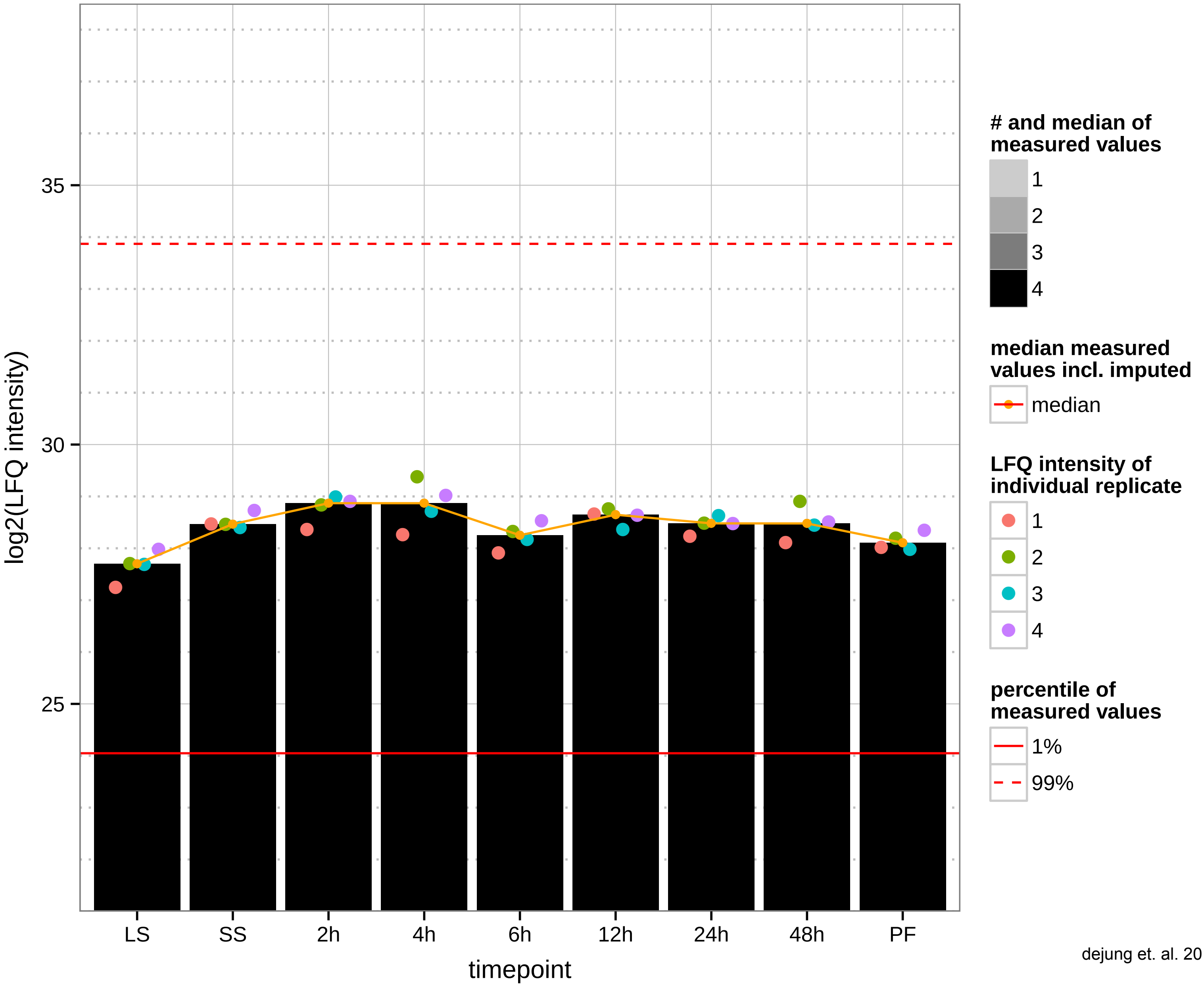

dejung et. al. 2015

chaperonin HSP60, mitochondrial precursor, putative (HSP60)
Tb927.6.4090
AGOF: ATP binding
AGOC: null
AGOP: cellular protein metabolic process
PGOF: ATP binding
PGOC: null
PGOP: cellular protein metabolic process

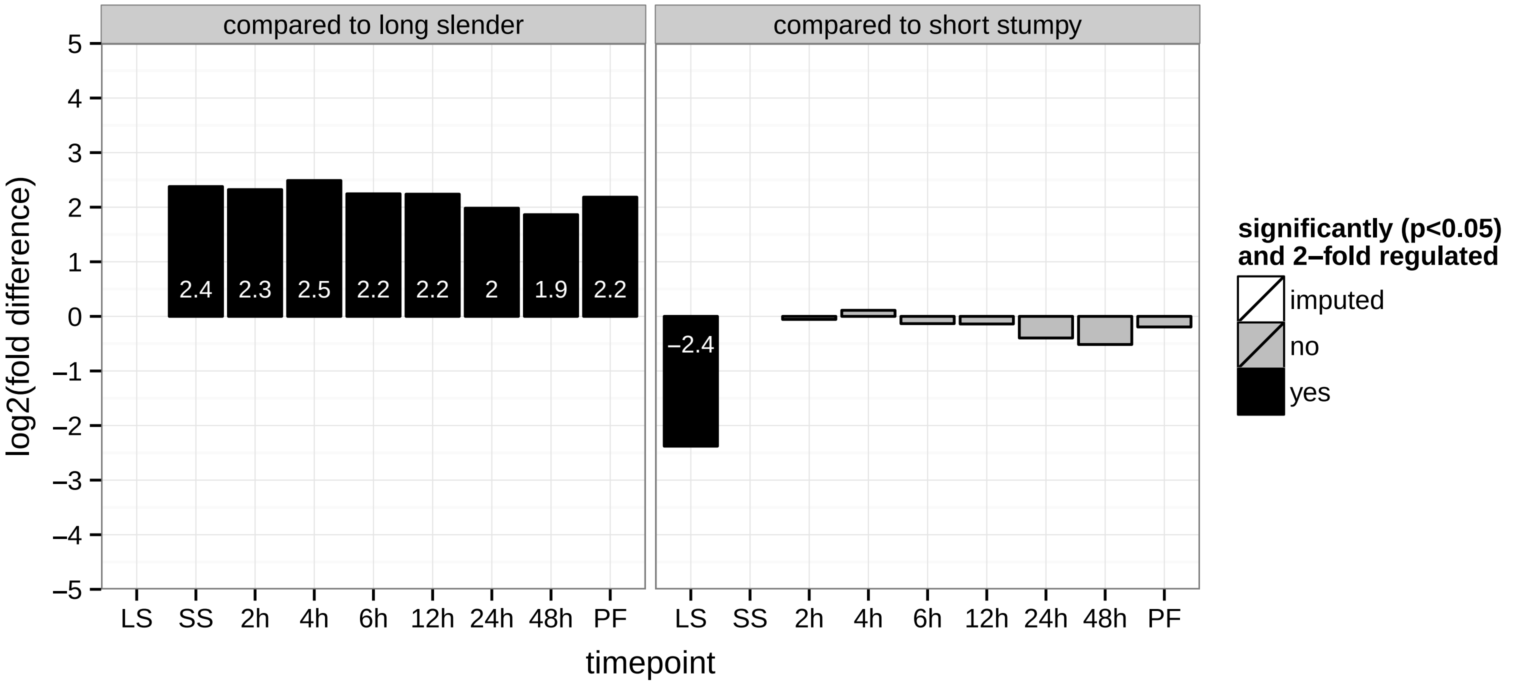

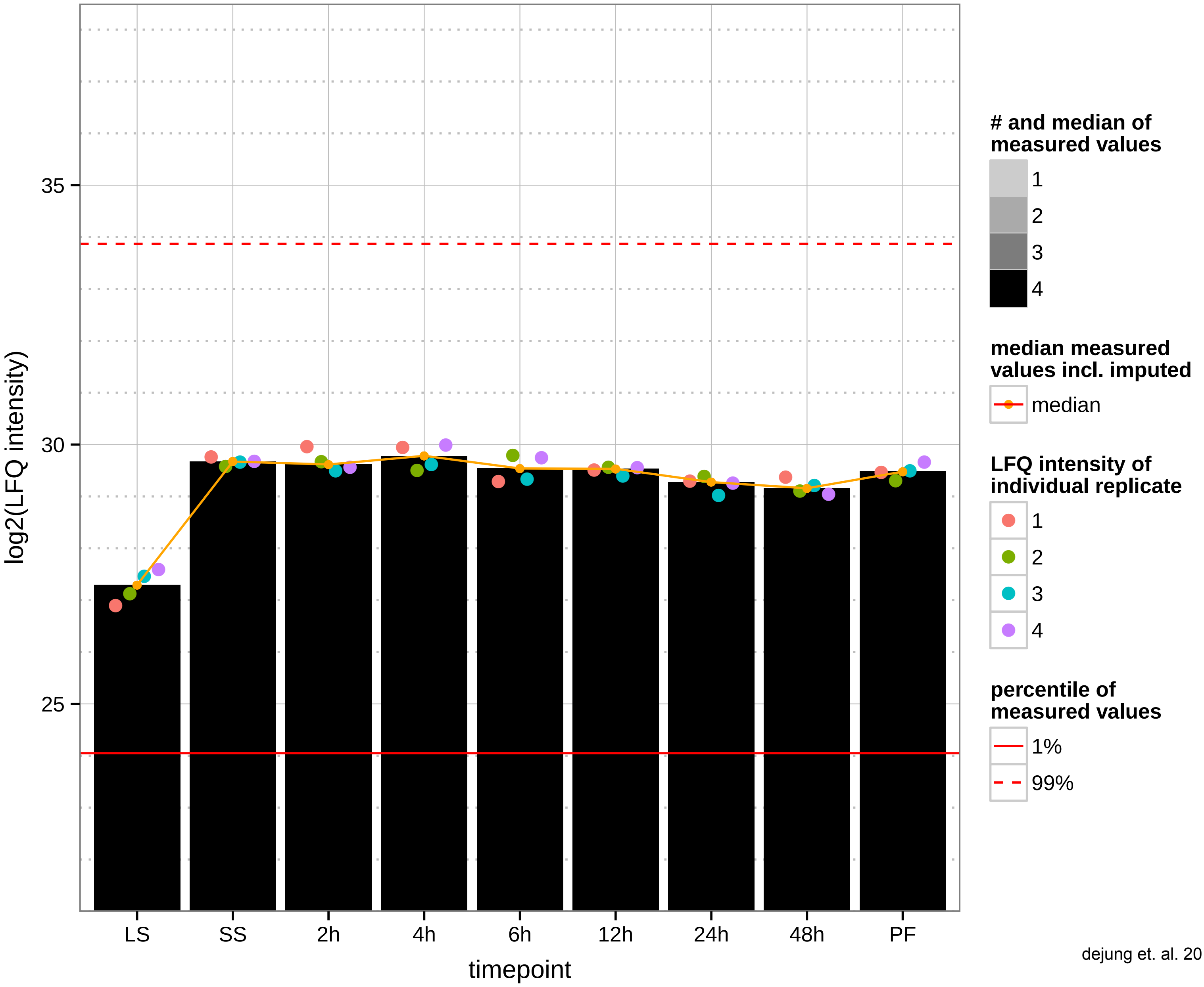

dejung et. al. 2015

GTPase activating protein, putative
Tb927.6.4120
AGOF: Rab GTPase activator activity
AGOC: intracellular
AGOP: regulation of Rab GTPase activity
PGOF: Rab GTPase activator activity
PGOC: intracellular
PGOP: regulation of Rab GTPase activity

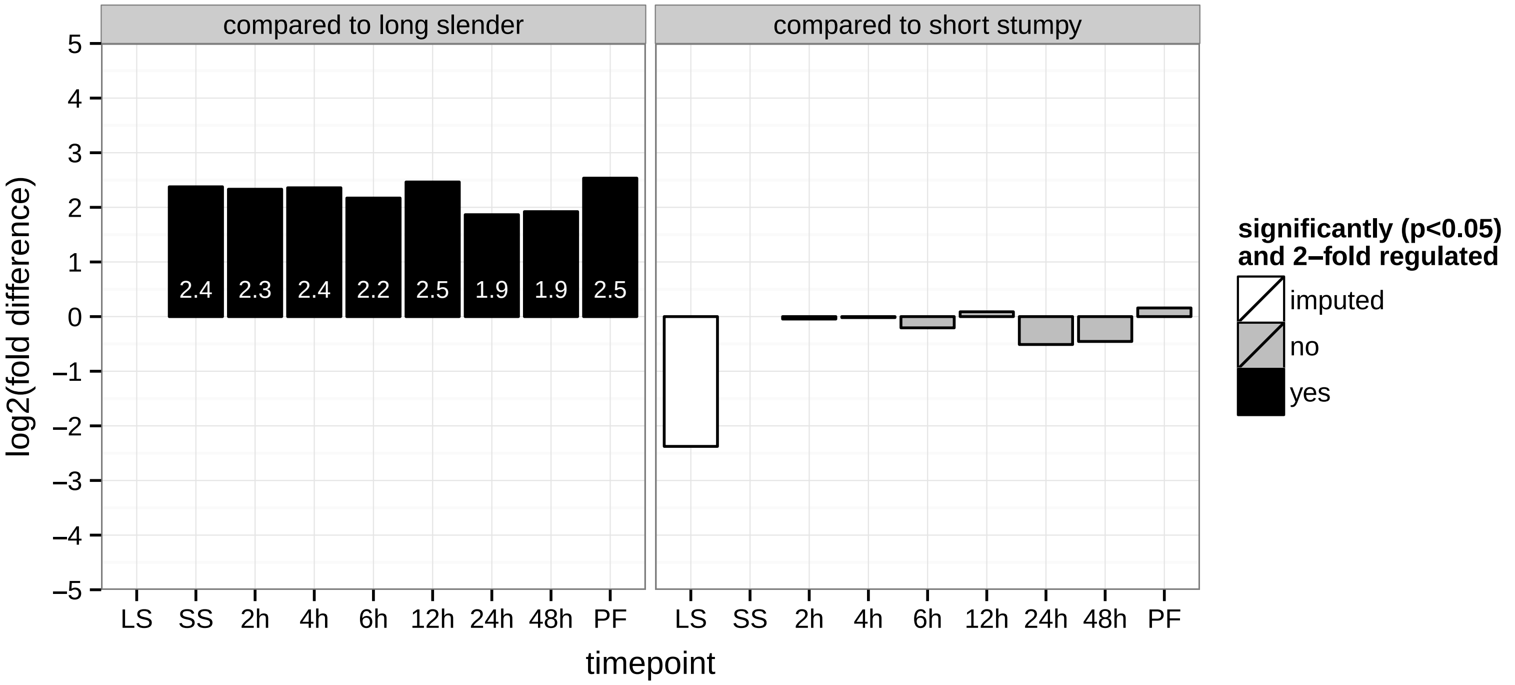

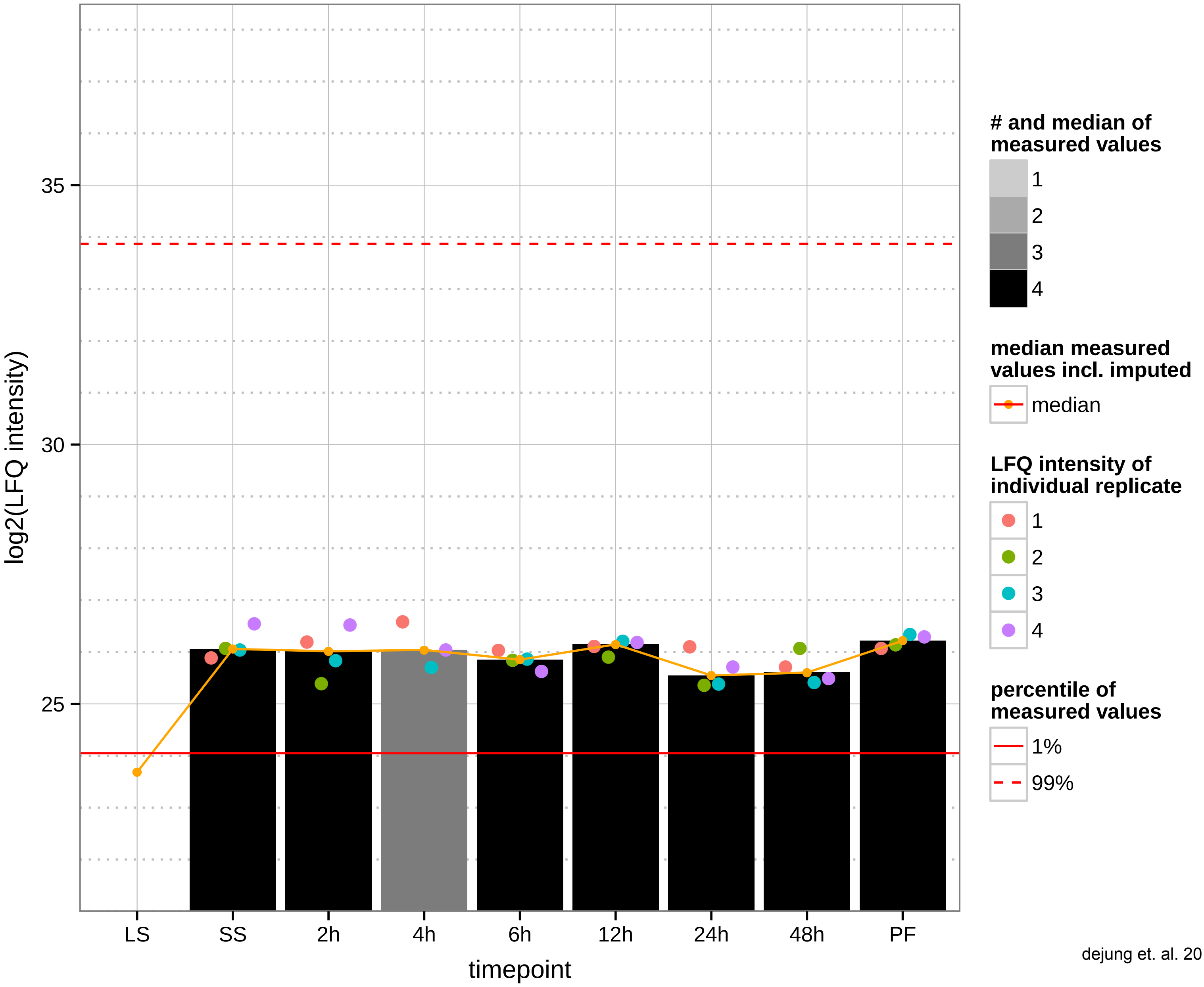

dejung et. al. 2015

paraflagellar rod component, putative (PFC4)
Tb927.6.4140
AGOF: null
AGOC: null
AGOP: null
PGOF: null
PGOC: null
PGOP: null

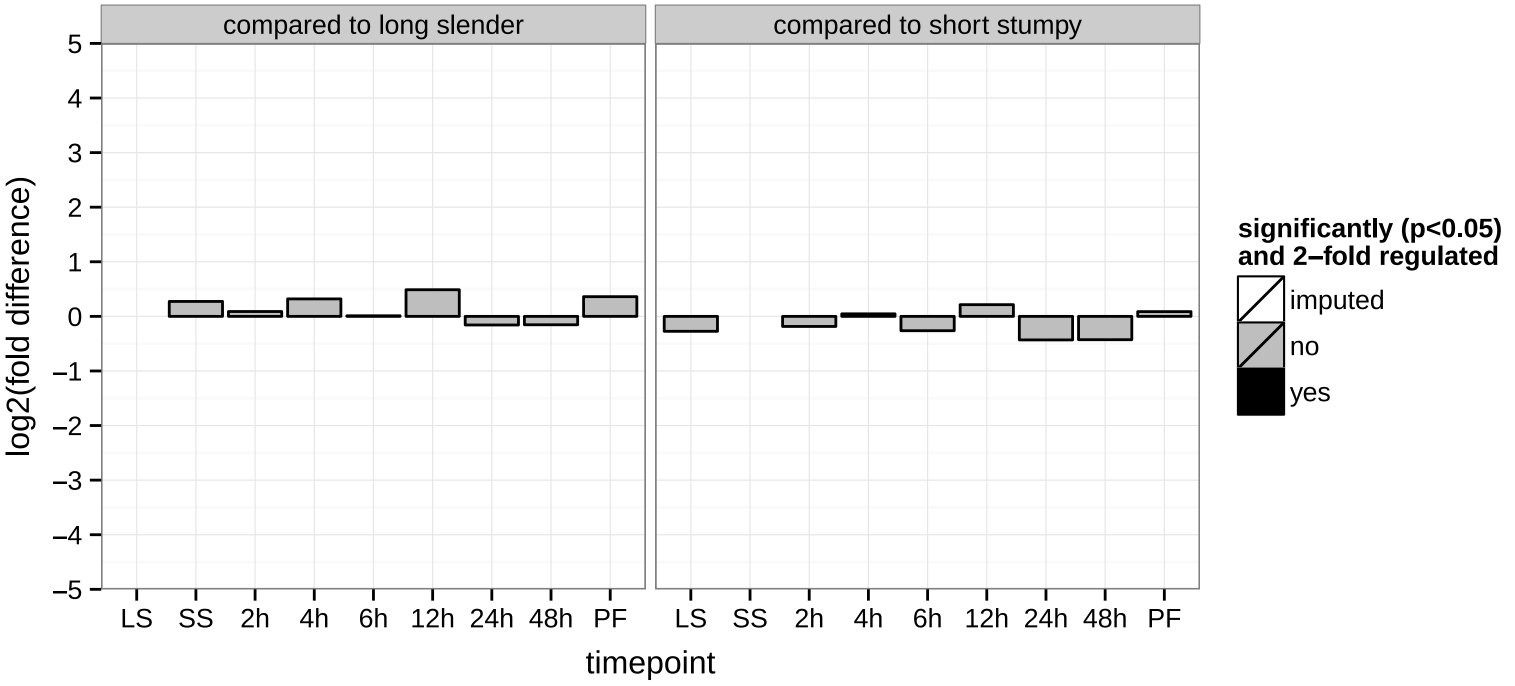

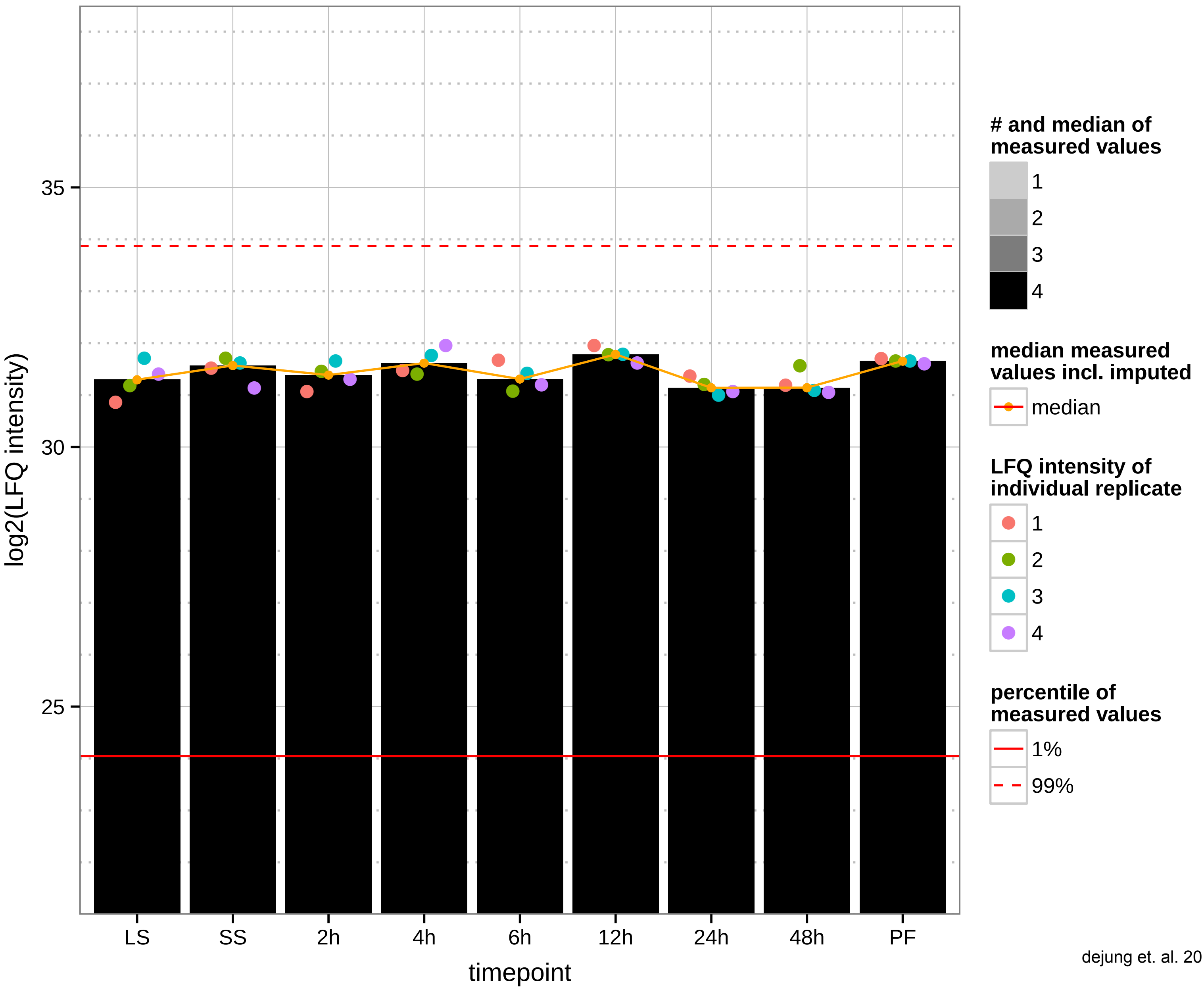

dejung et. al. 2015

aldehyde dehydrogenase, putative (ALDH)
Tb927.6.4210
AGOF: oxidoreductase activity
AGOC: mitochondrial inner membrane
AGOP: metabolic process
PGOF: oxidoreductase activity
PGOC: null
PGOP: metabolic process, oxidation–reduction process

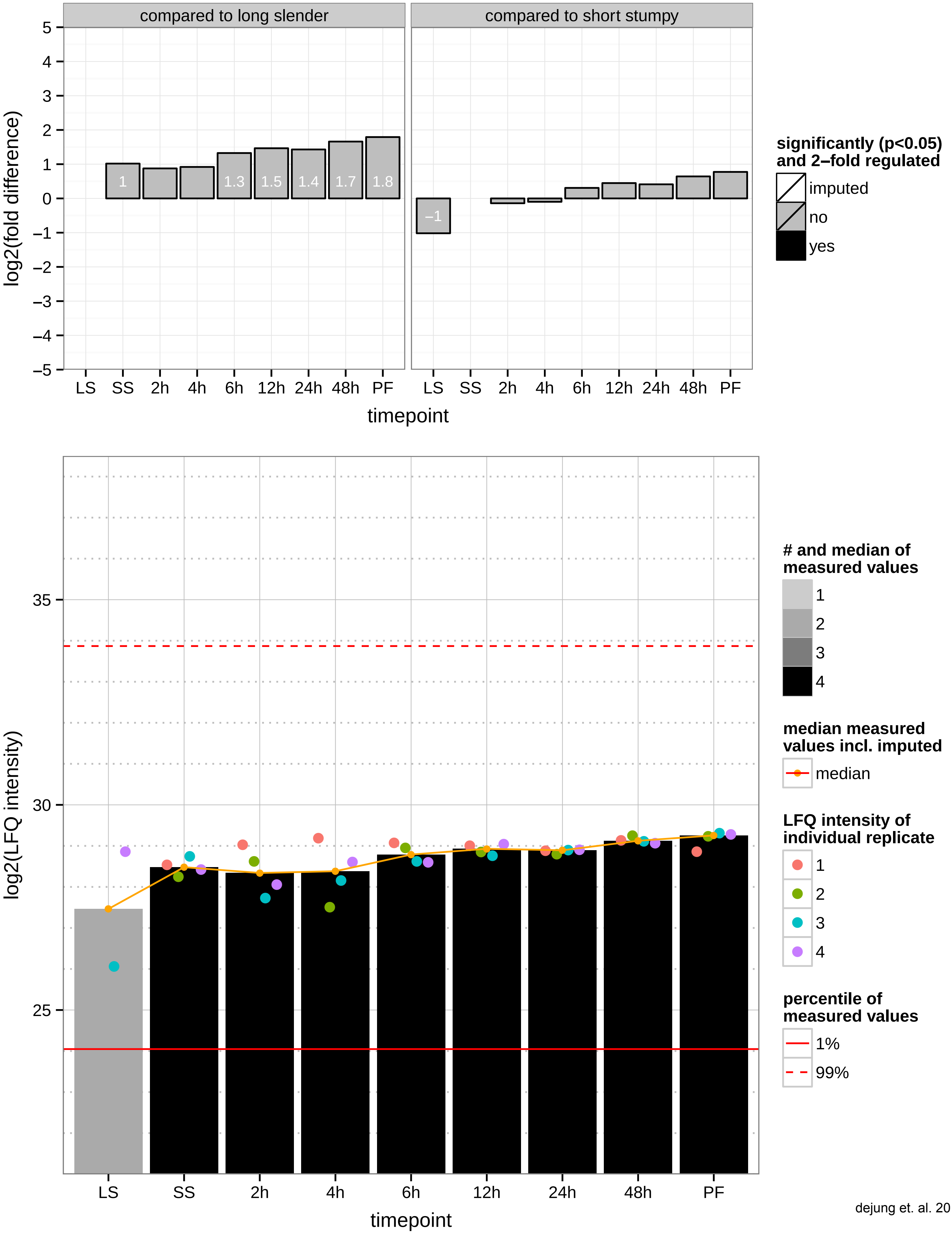

dejung et. al. 2015

hypothetical protein, conserved
Tb927.6.4260
AGOF: null
AGOC: null
AGOP: null
PGOF: null
PGOC: null
PGOP: null

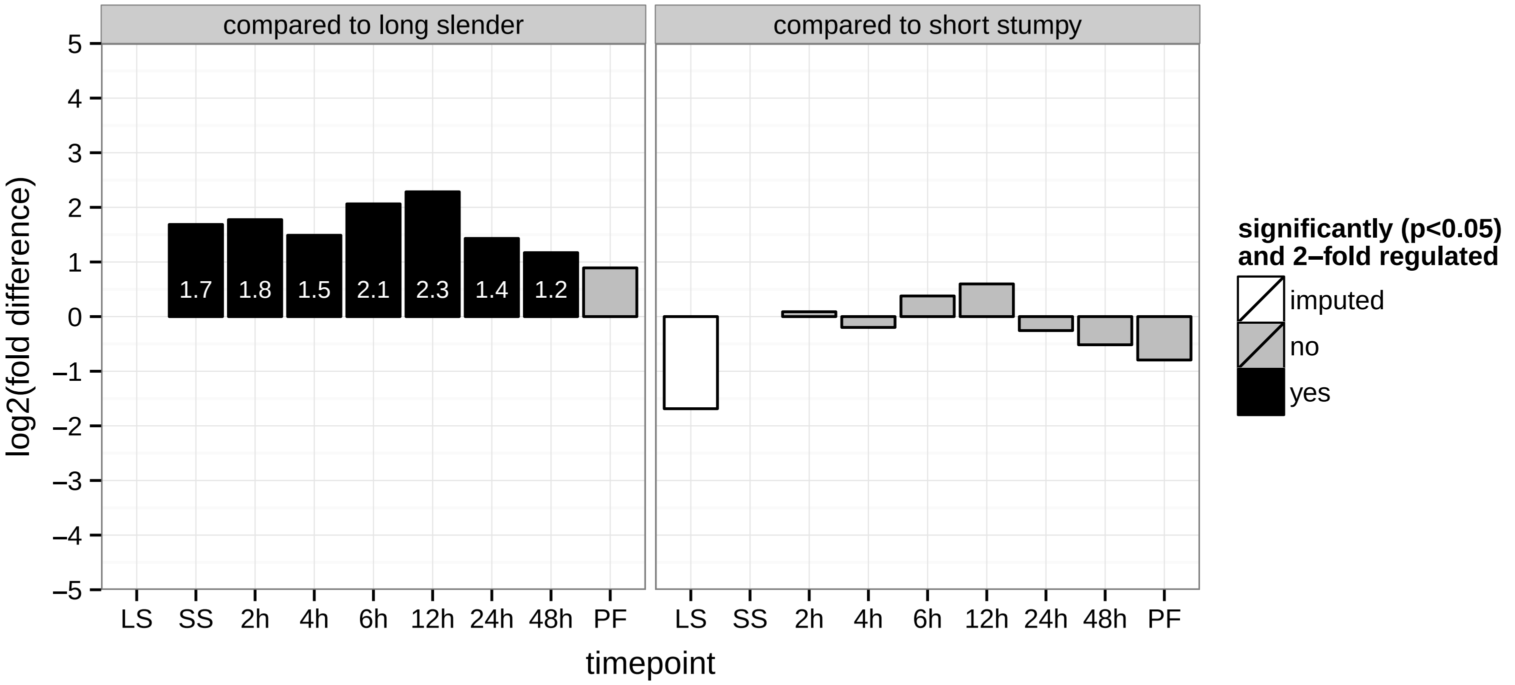

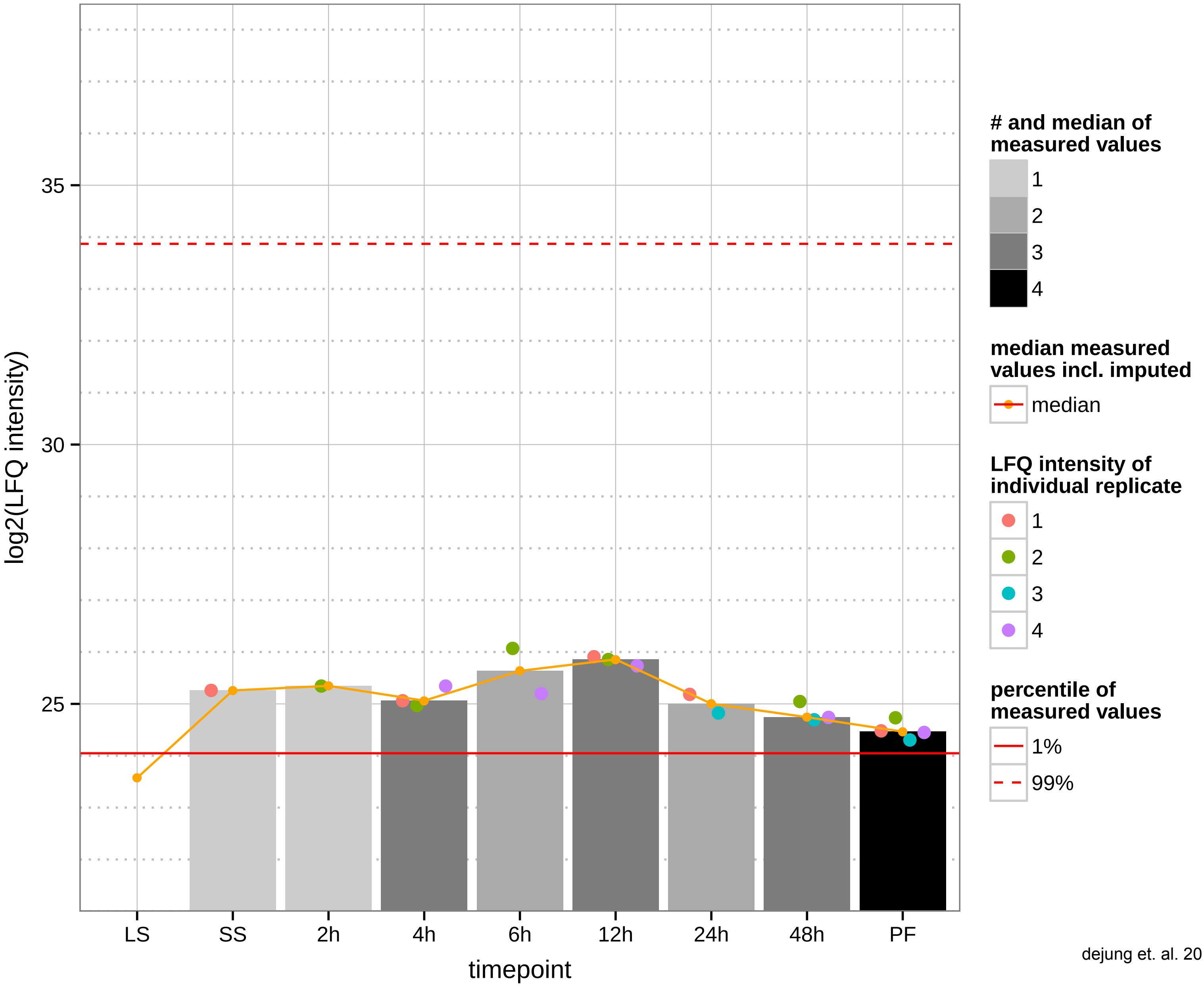

dejung et. al. 2015

hypothetical protein, conserved
Tb927.6.4320
AGOF: null
AGOC: null
AGOP: null
PGOF: null
PGOC: null
PGOP: null

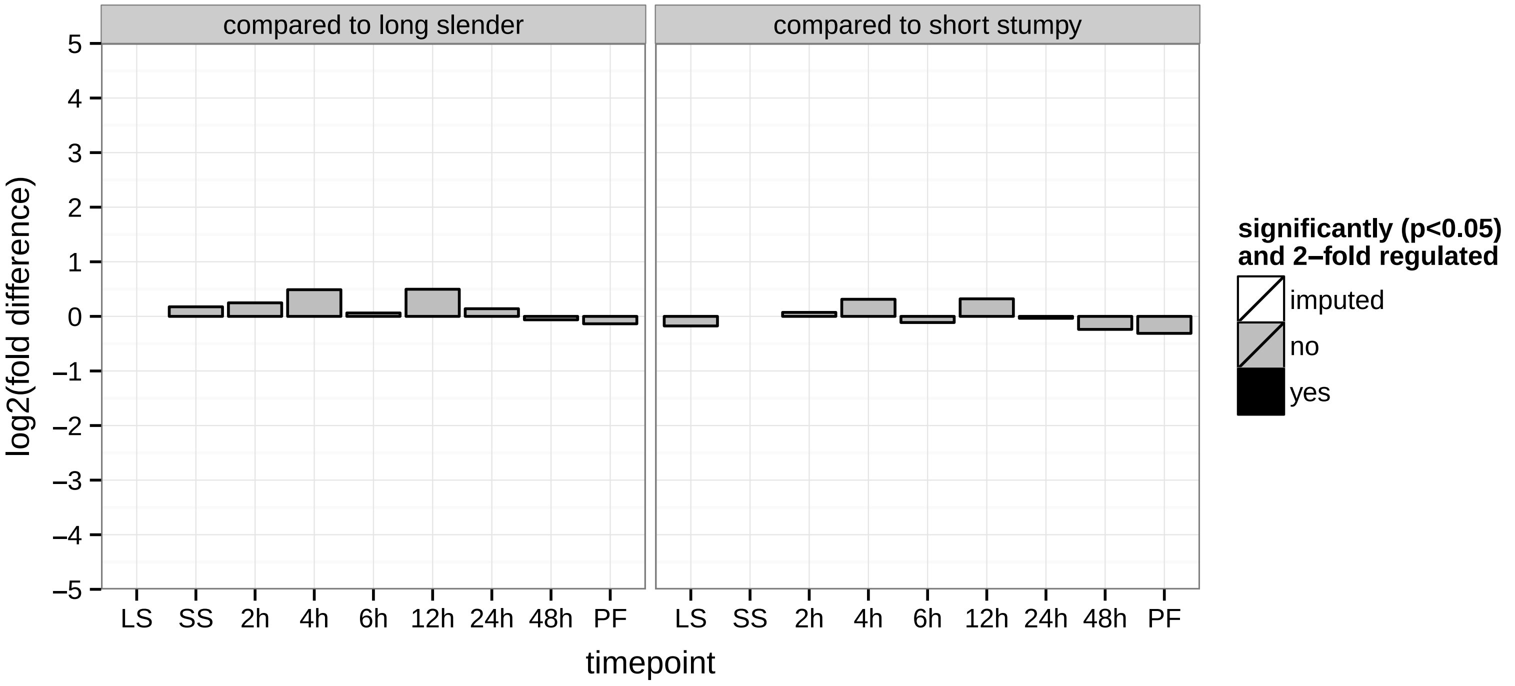

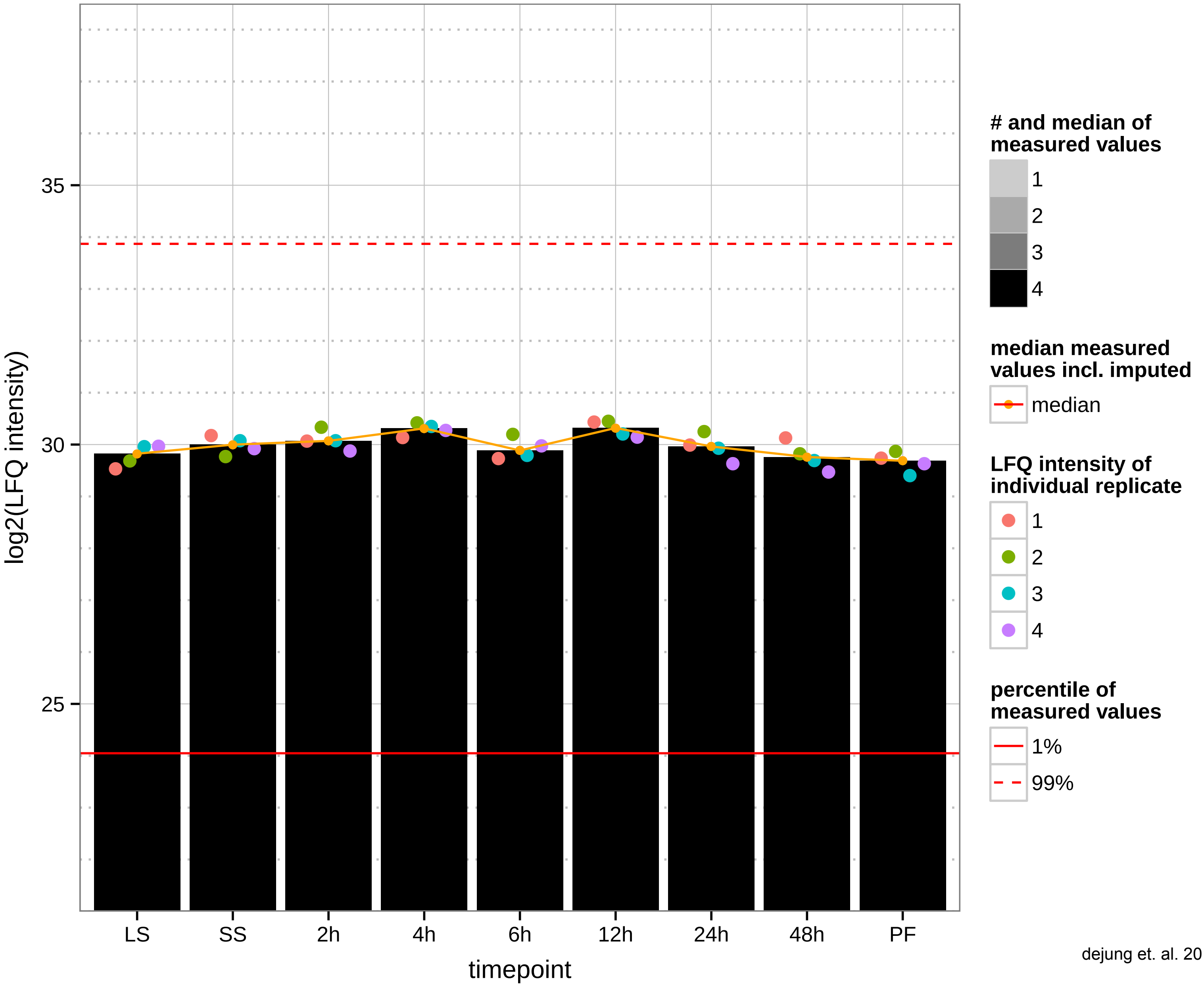

dejung et. al. 2015

U6 snRNA–associated Sm–like protein LSm5p (TbLSm5)
Tb927.6.4340
AGOF: U6 snRNA 3'–end binding
AGOC: nucleus
AGOP: nuclear mRNA splicing, via spliceosome
PGOF: null
PGOC: null
PGOP: null

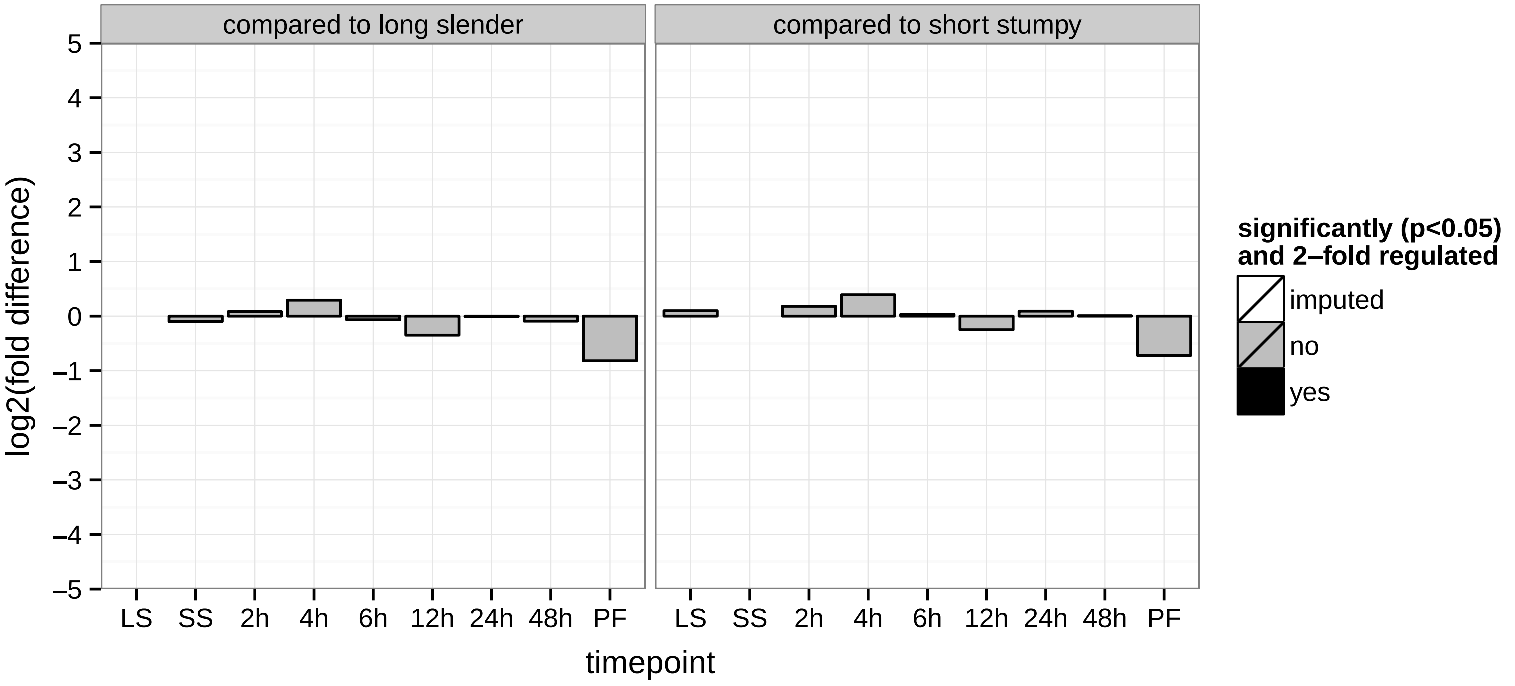

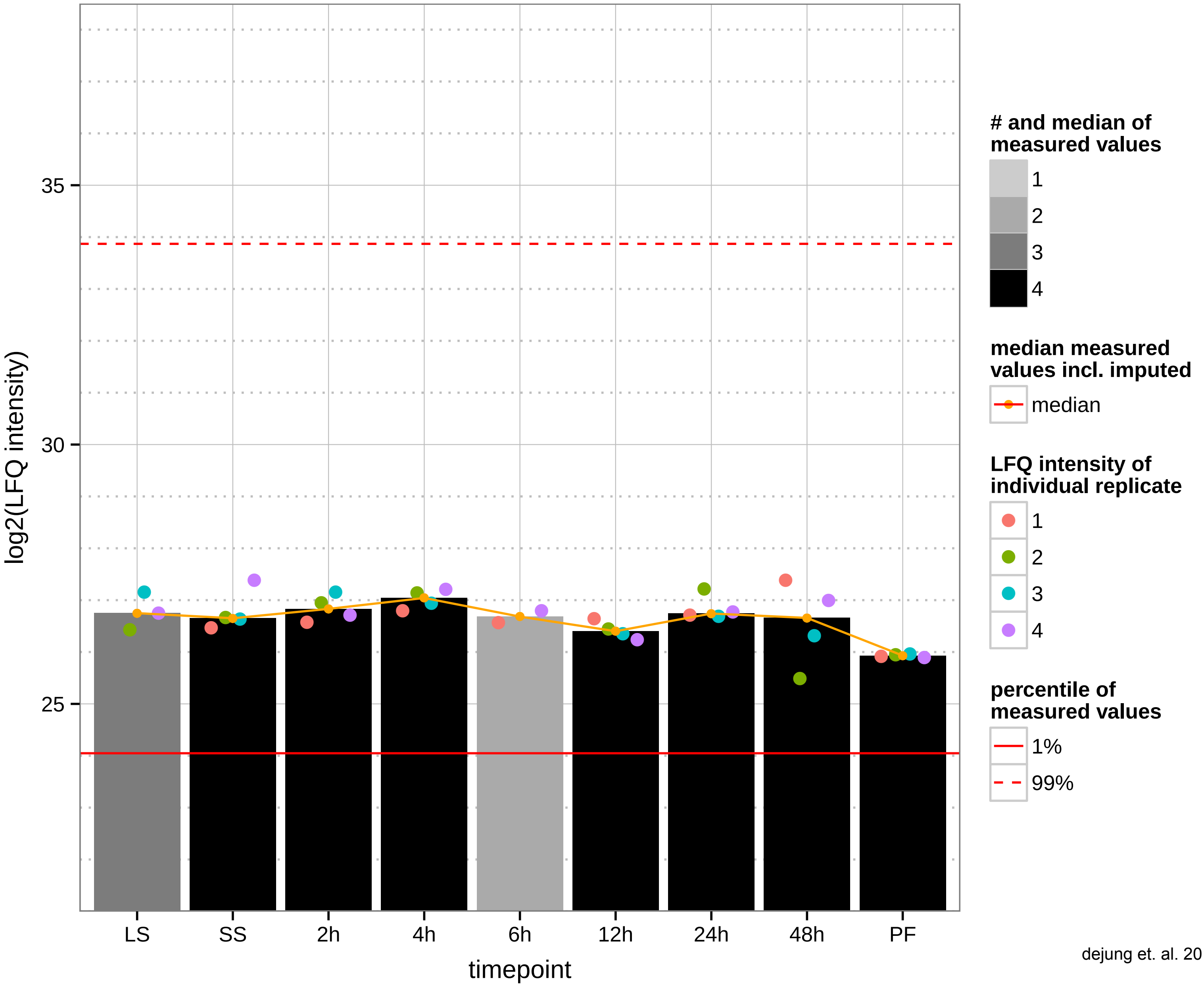

dejung et. al. 2015

ribosomal RNA assembly protein, putative
Tb927.6.4350
AGOF: null
AGOC: null
AGOP: null
PGOF: null
PGOC: null
PGOP: null

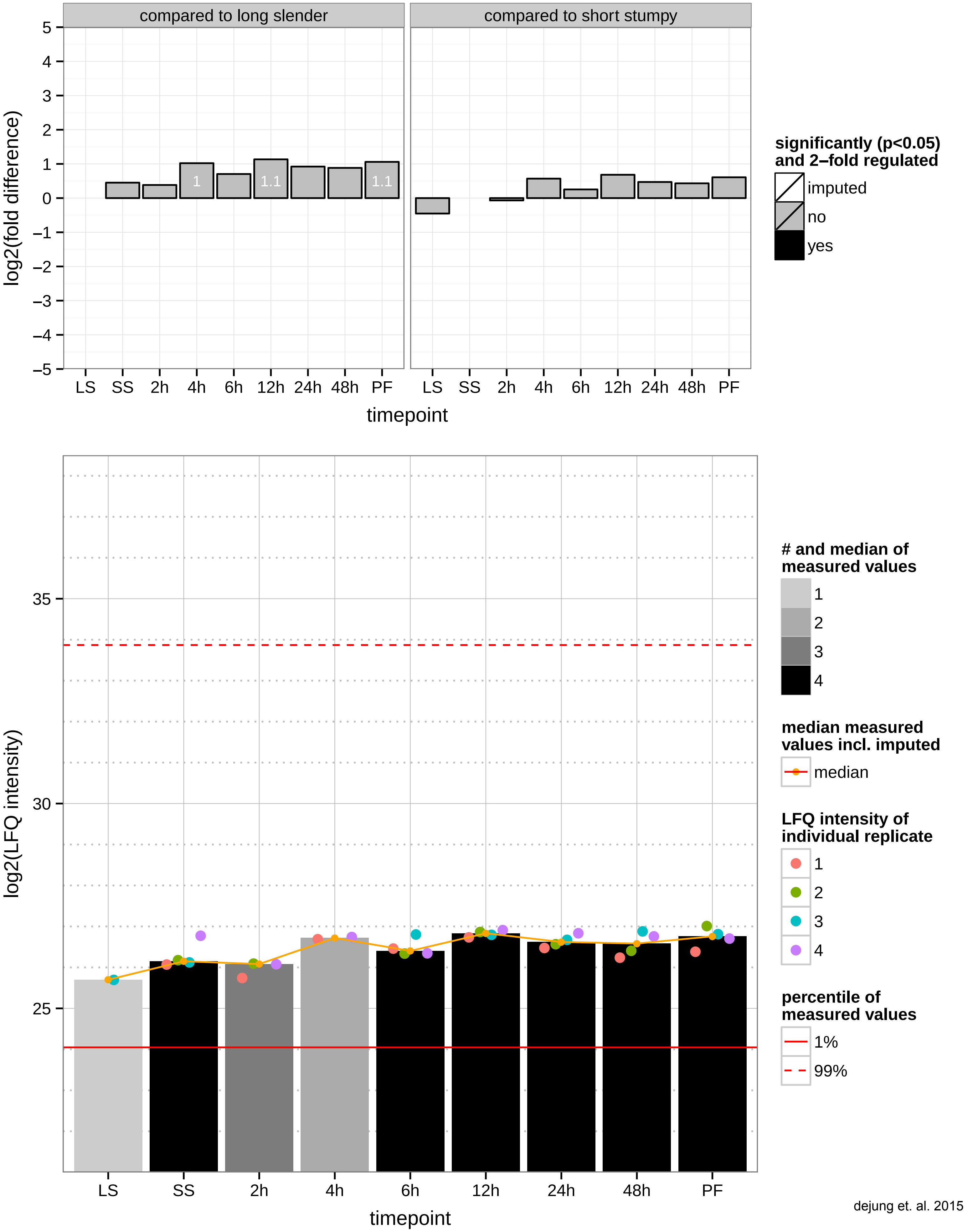

dejung et. al. 2015

eukaryotic translation initiation factor 3 subunit 7–like protein
Tb927.6.4370
AGOF: translation initiation factor activity
AGOC: null
AGOP: translational initiation
PGOF: translation initiation factor activity
PGOC: null
PGOP: translational initiation

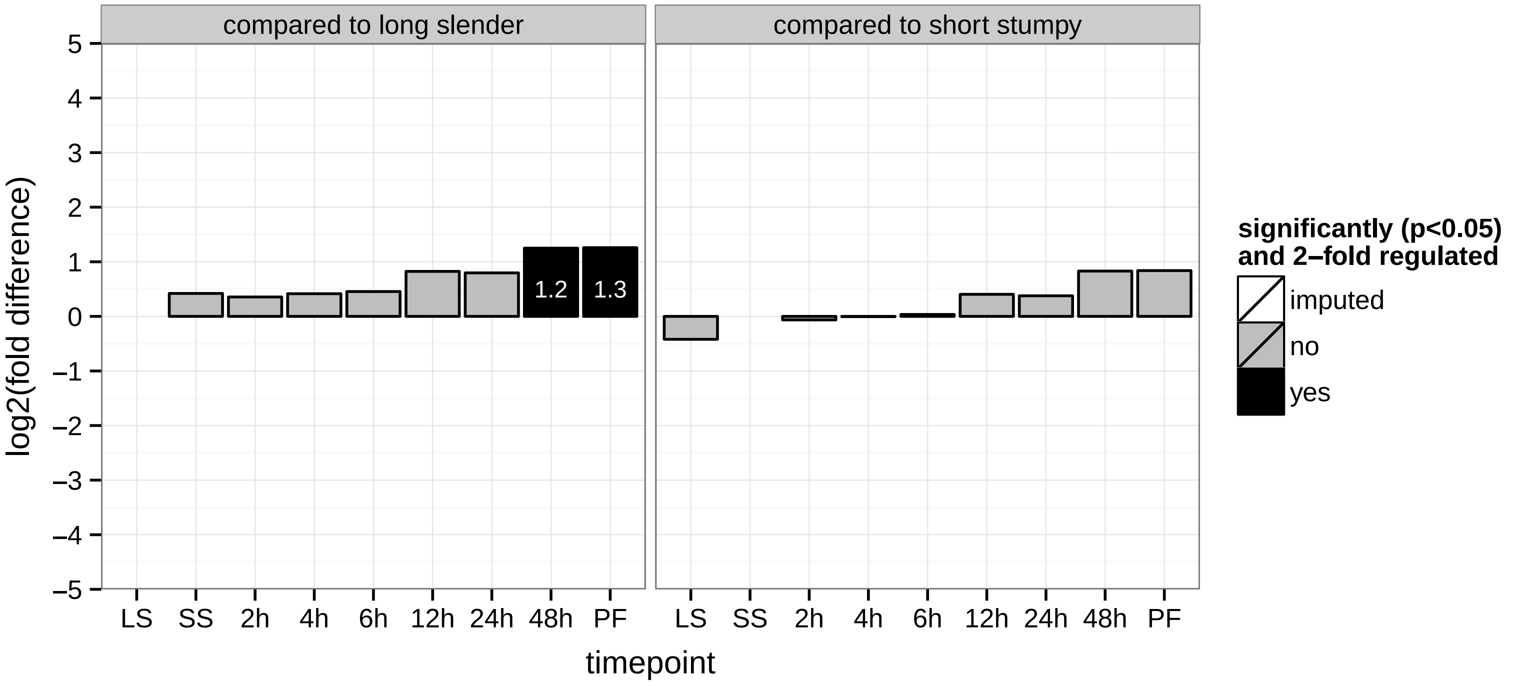

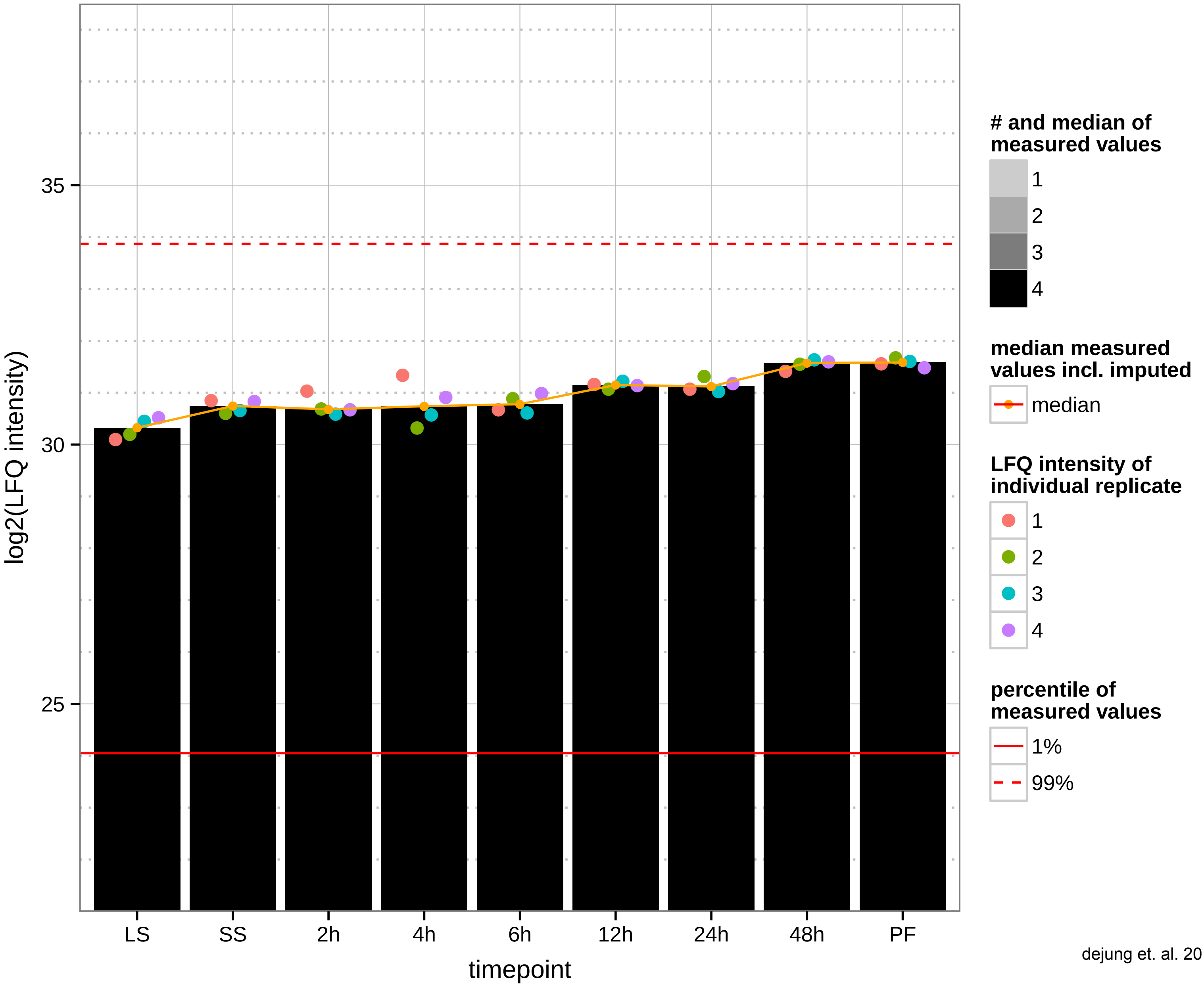

dejung et. al. 2015

S–adenosylmethionine decarboxylase proenzyme, putative, S–adenosylmethionine decarboxylase (AdoMetDC)
Tb927.6.4460;Tb927.6.4410;Tb11.v5.0903
AGOF: null, adenosylmethionine decarboxylase activity
AGOC: null
AGOP: null, spermidine biosynthetic process, spermine biosynthetic process
PGOF: adenosylmethionine decarboxylase activity
PGOC: null
PGOP: spermidine biosynthetic process, spermine biosynthetic process

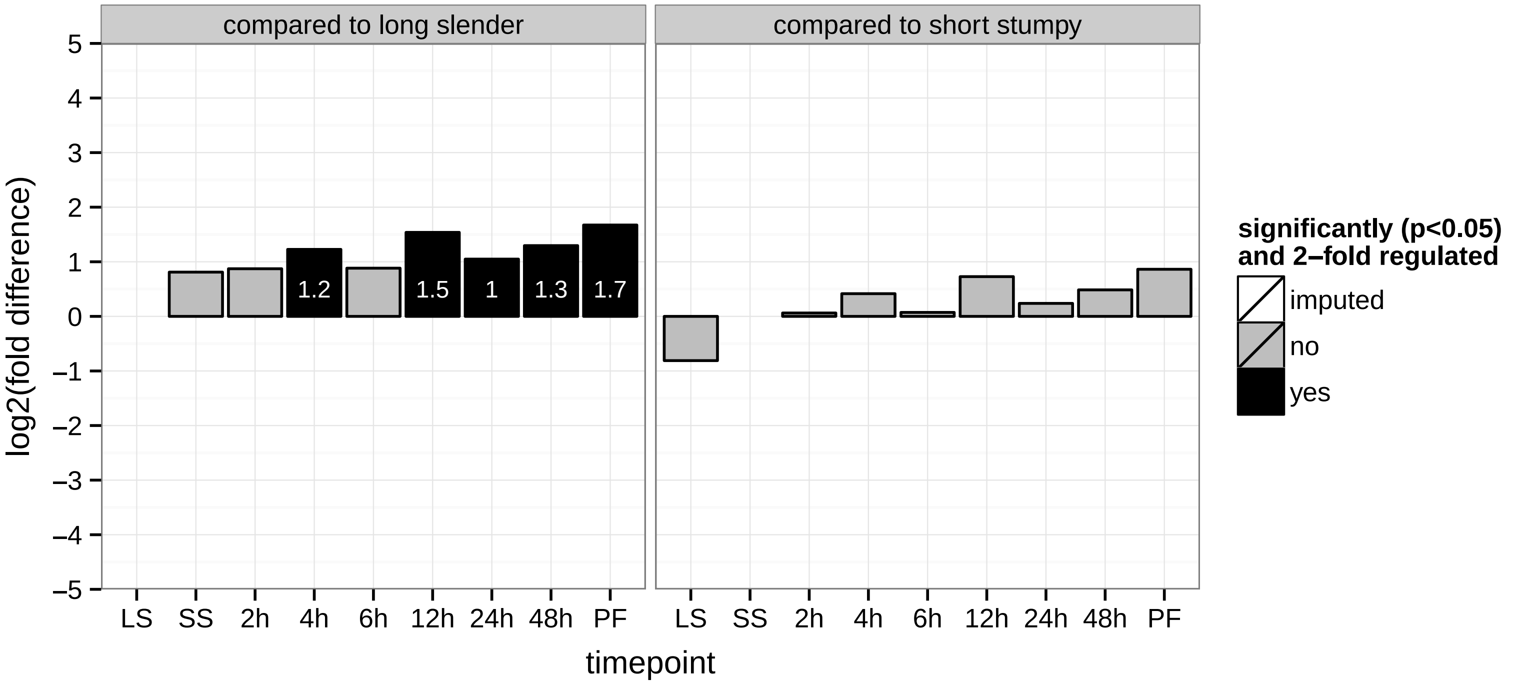

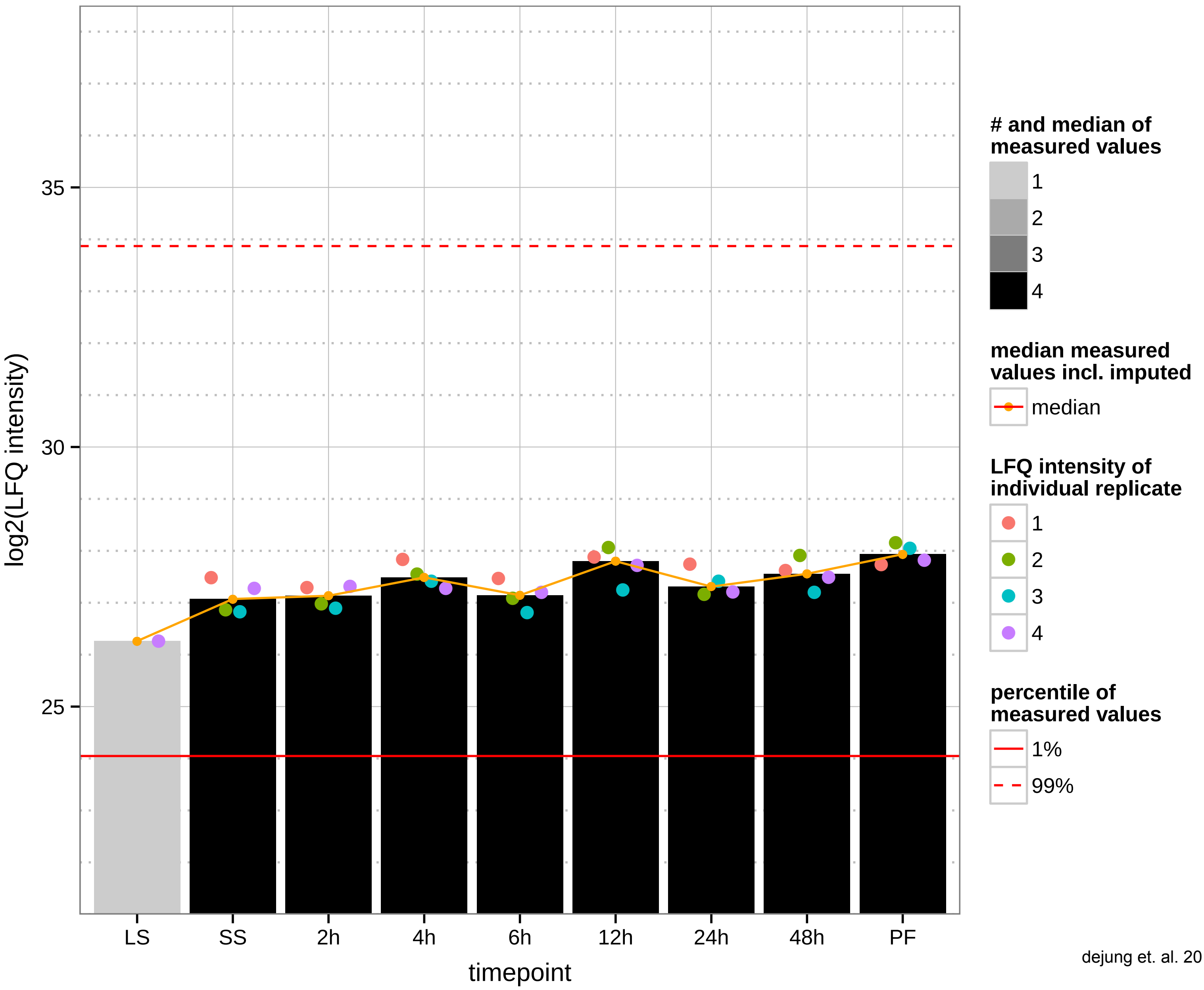

dejung et. al. 2015

hypothetical protein, conserved
Tb927.6.4500
AGOF: null
AGOC: extrachromosomal circular DNA
AGOP: null
PGOF: null
PGOC: endoplasmic reticulum, extrachromosomal circular DNA, integral to membrane
PGOP: null

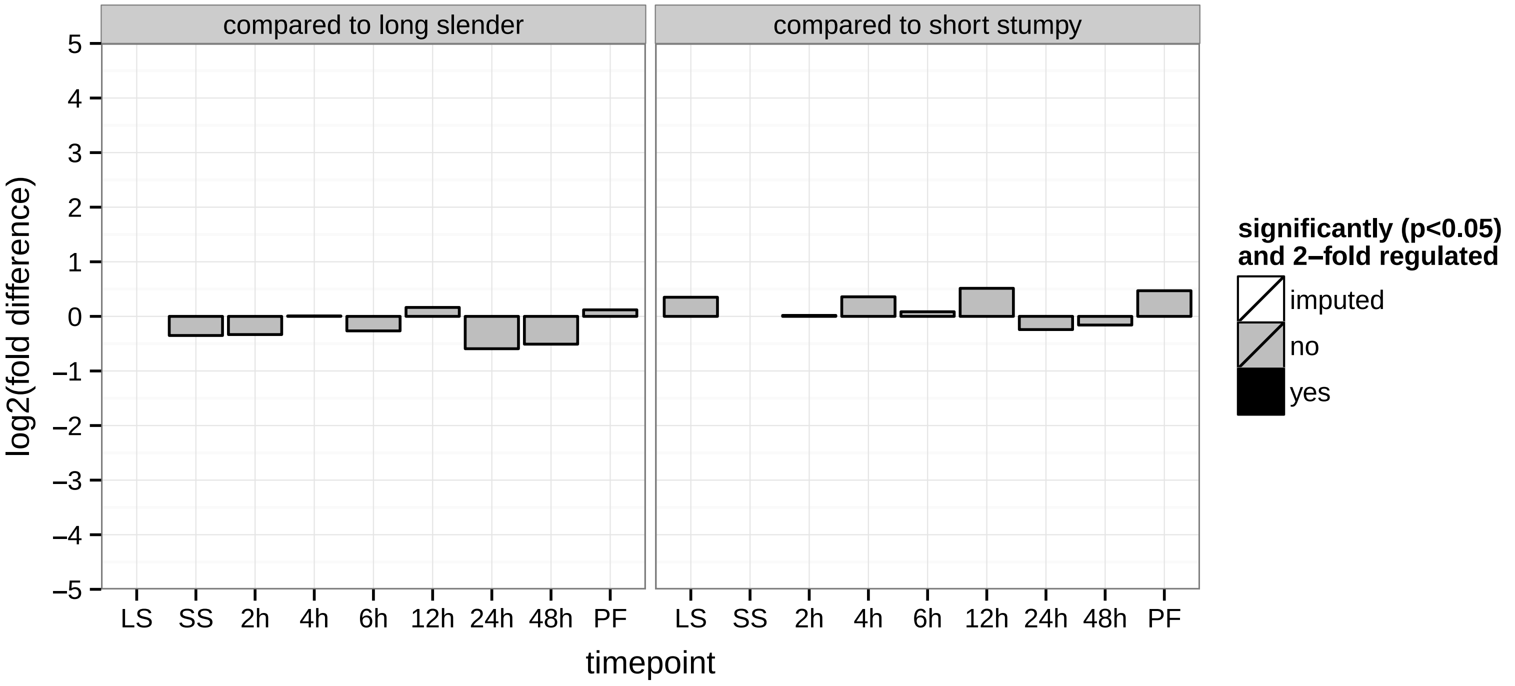

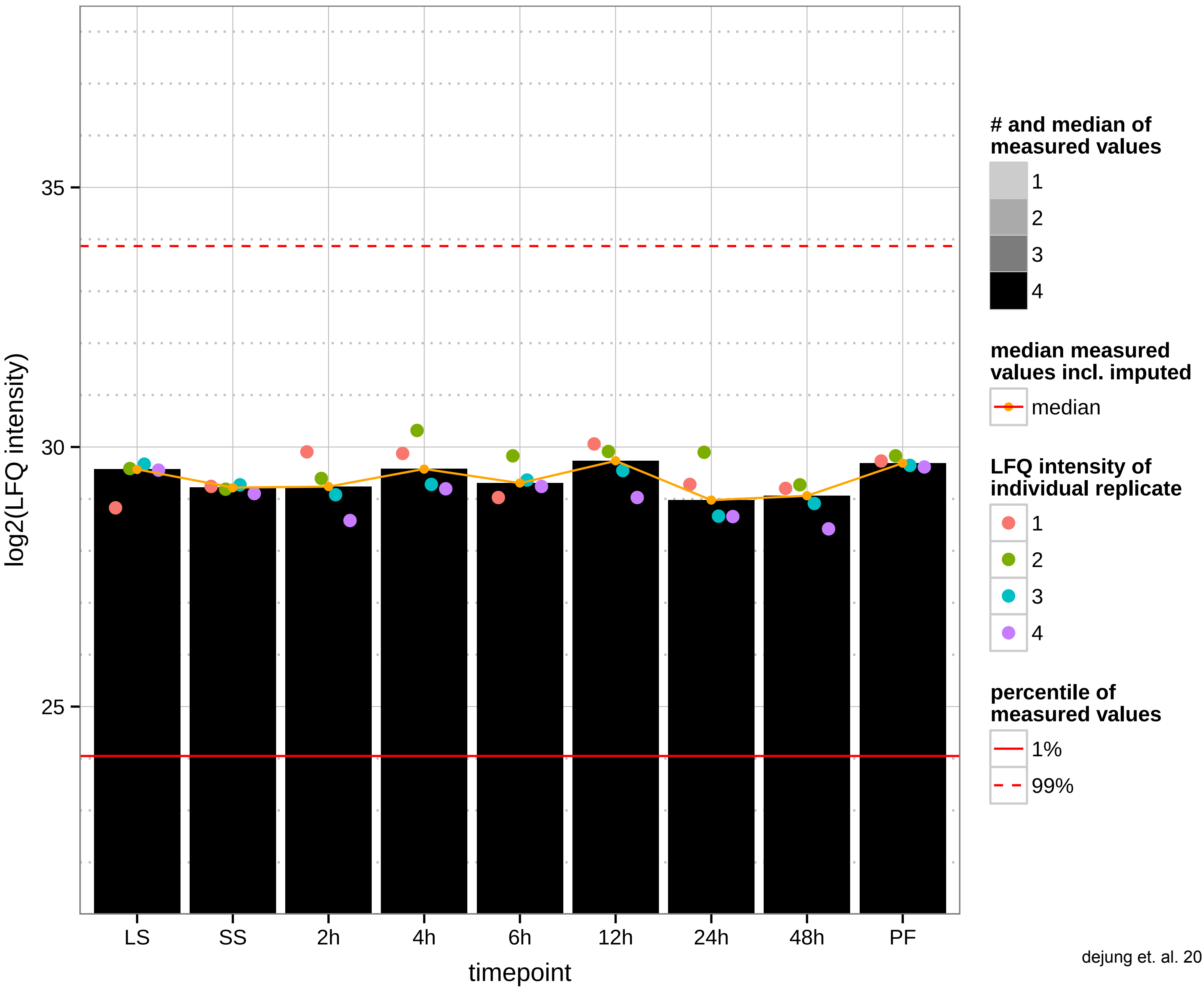

dejung et. al. 2015

hypothetical protein, conserved
Tb927.6.4520
AGOF: null
AGOC: null
AGOP: null
PGOF: null
PGOC: null
PGOP: null

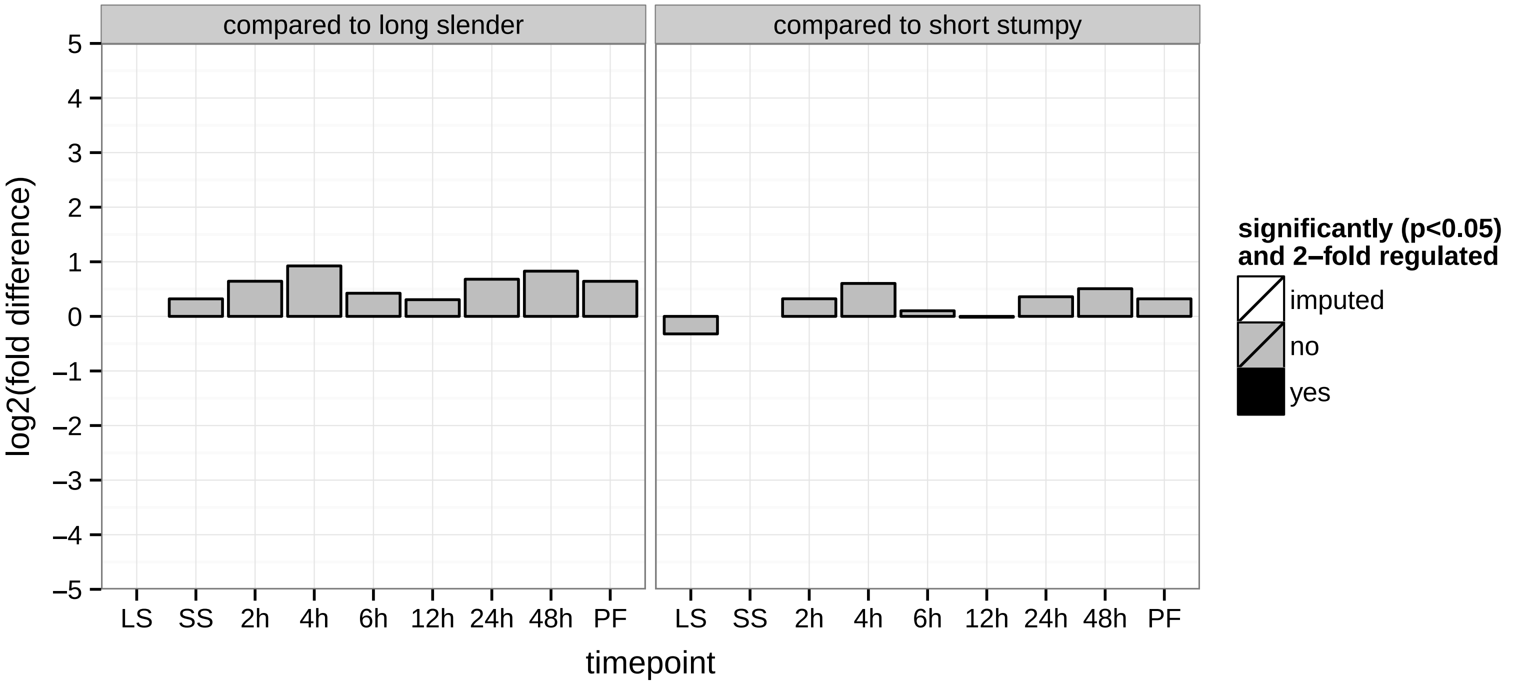

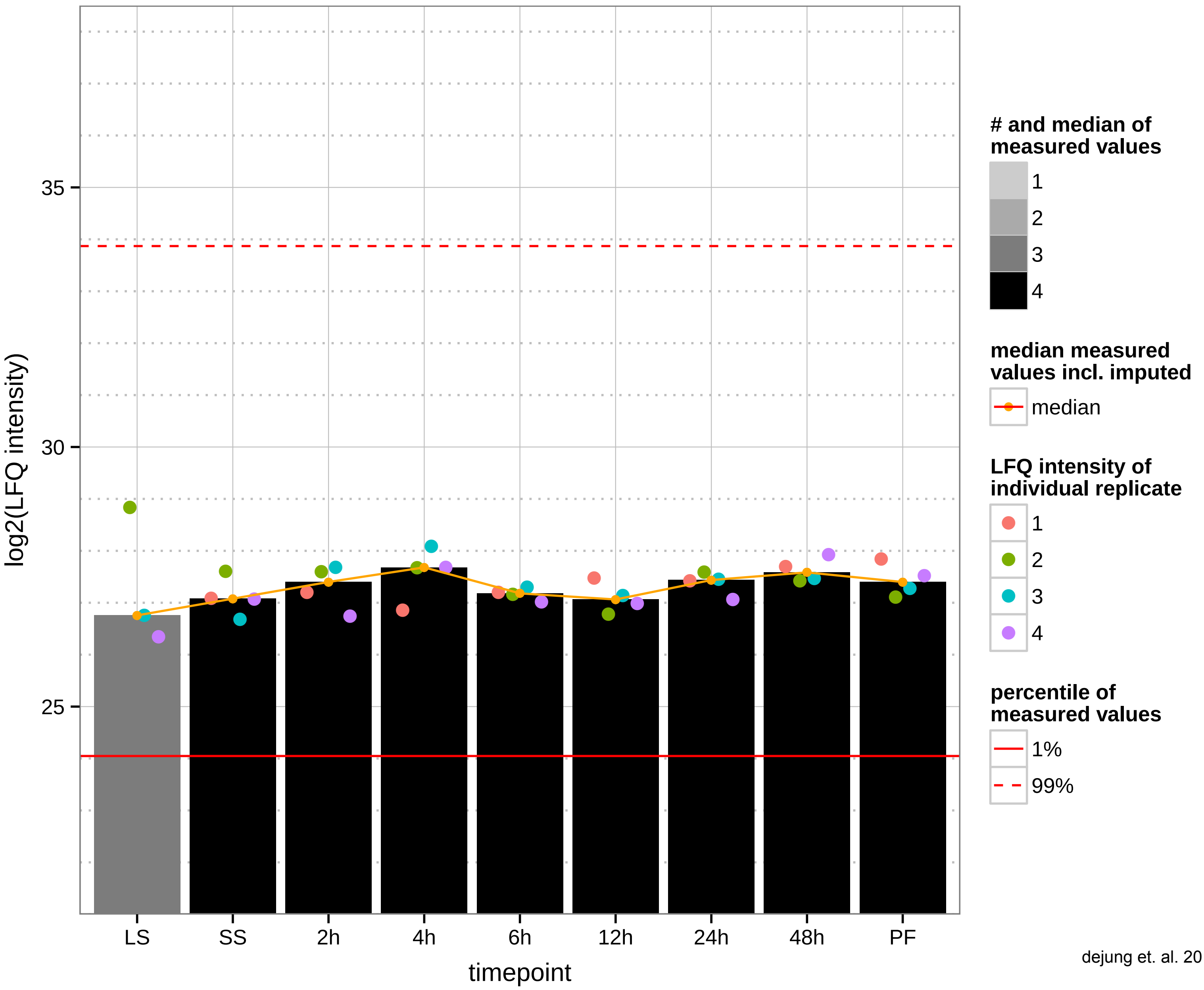

dejung et. al. 2015

glutamyl–tRNA synthetase, putative
Tb927.6.4590;Tb11.v5.0810
AGOF: null, ATP binding, glutamate–tRNA ligase activity
AGOC: null, cytoplasm
AGOP: null, glutamyl–tRNA aminoacylation, translation
PGOF: ATP binding, aminoacyl–tRNA ligase activity, ligase activity, forming aminoacyl–tRNA and related compounds, nucleot
PGOC: cytoplasm
PGOP: tRNA aminoacylation, tRNA aminoacylation for protein translation, translation, glutamyl–tRNA aminoacylation, tRNA a

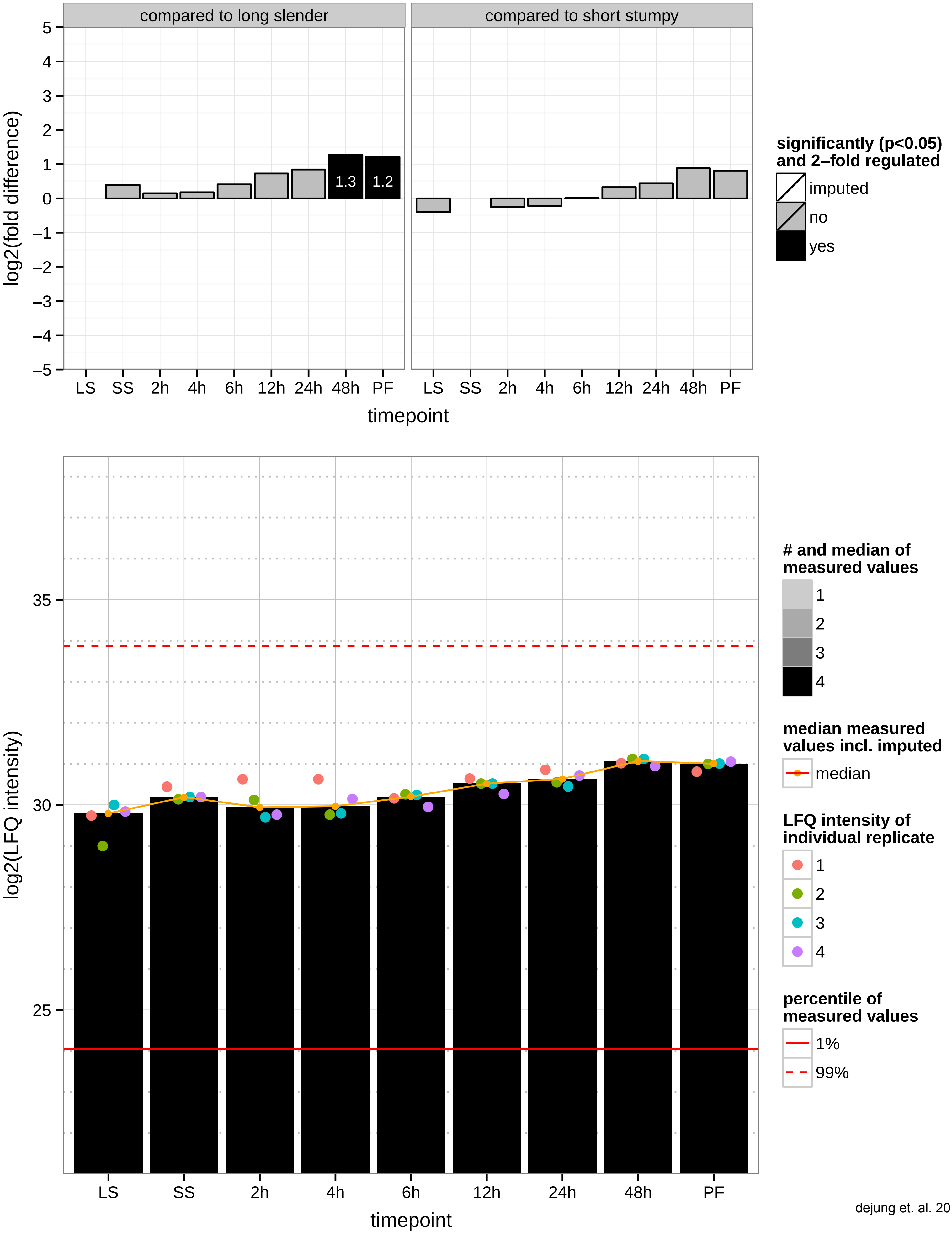

dejung et. al. 2015

hypothetical protein, conserved
Tb927.6.4610
AGOF: null
AGOC: null
AGOP: null
PGOF: protein binding
PGOC: null
PGOP: null

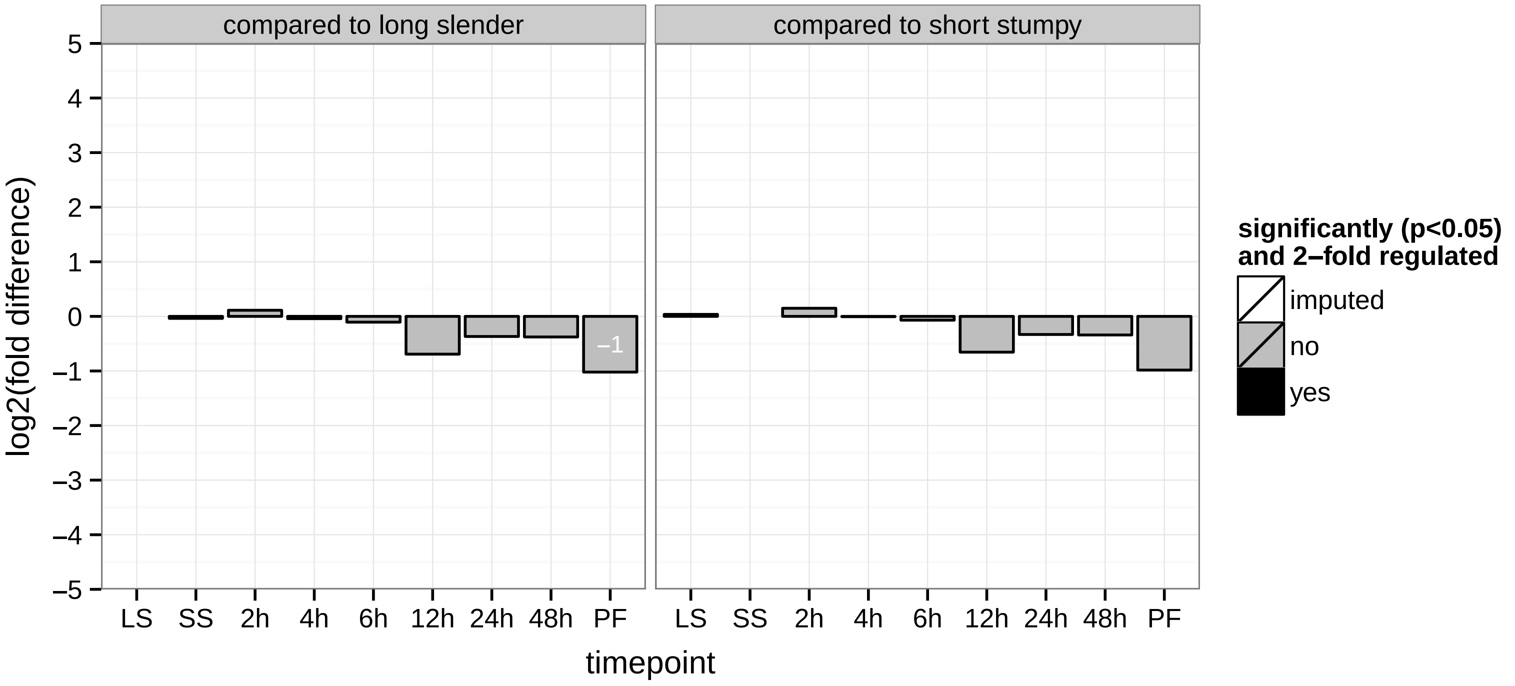

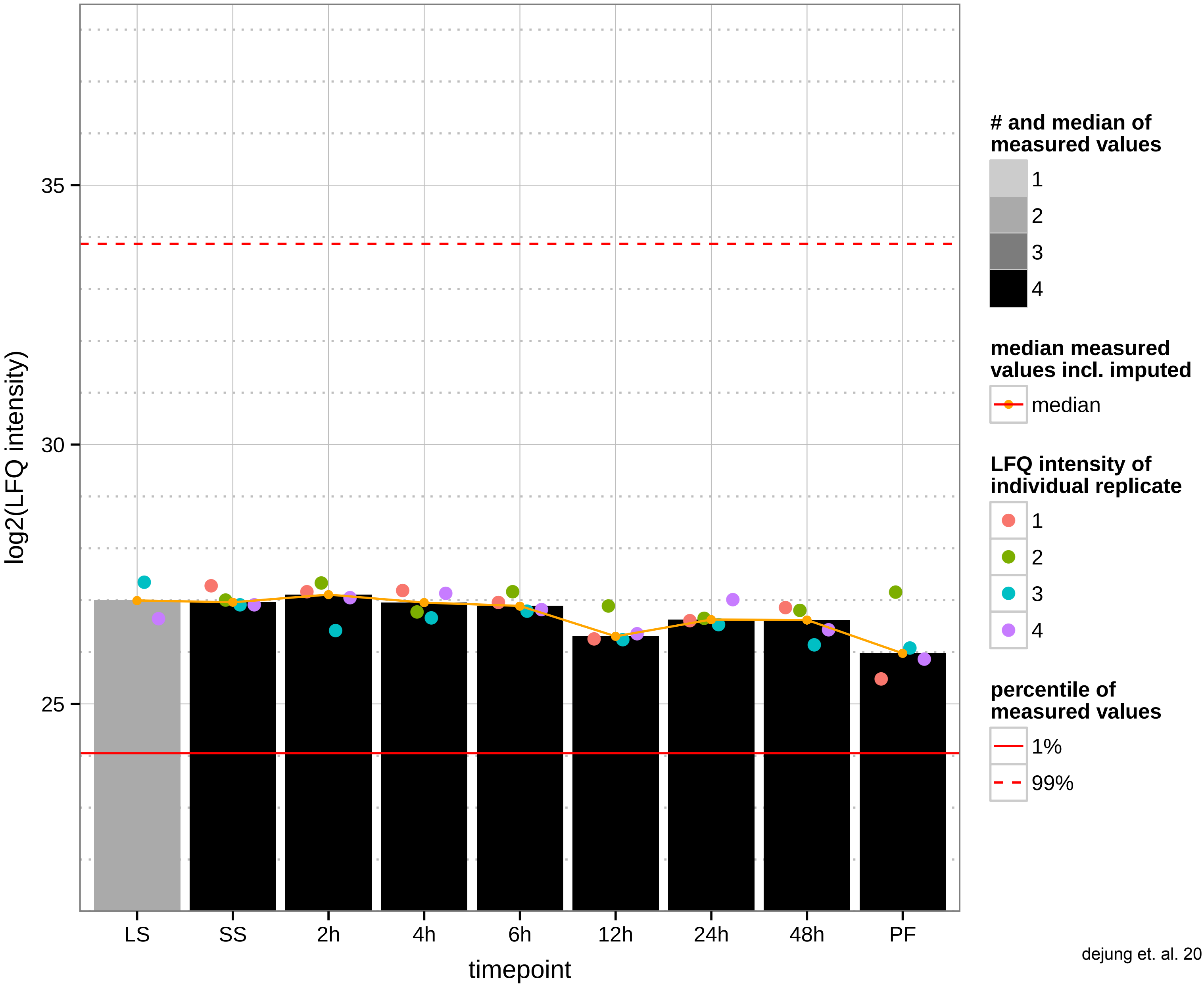

dejung et. al. 2015

hypothetical protein, conserved
Tb927.6.4640
AGOF: null
AGOC: null
AGOP: null
PGOF: null
PGOC: null
PGOP: null

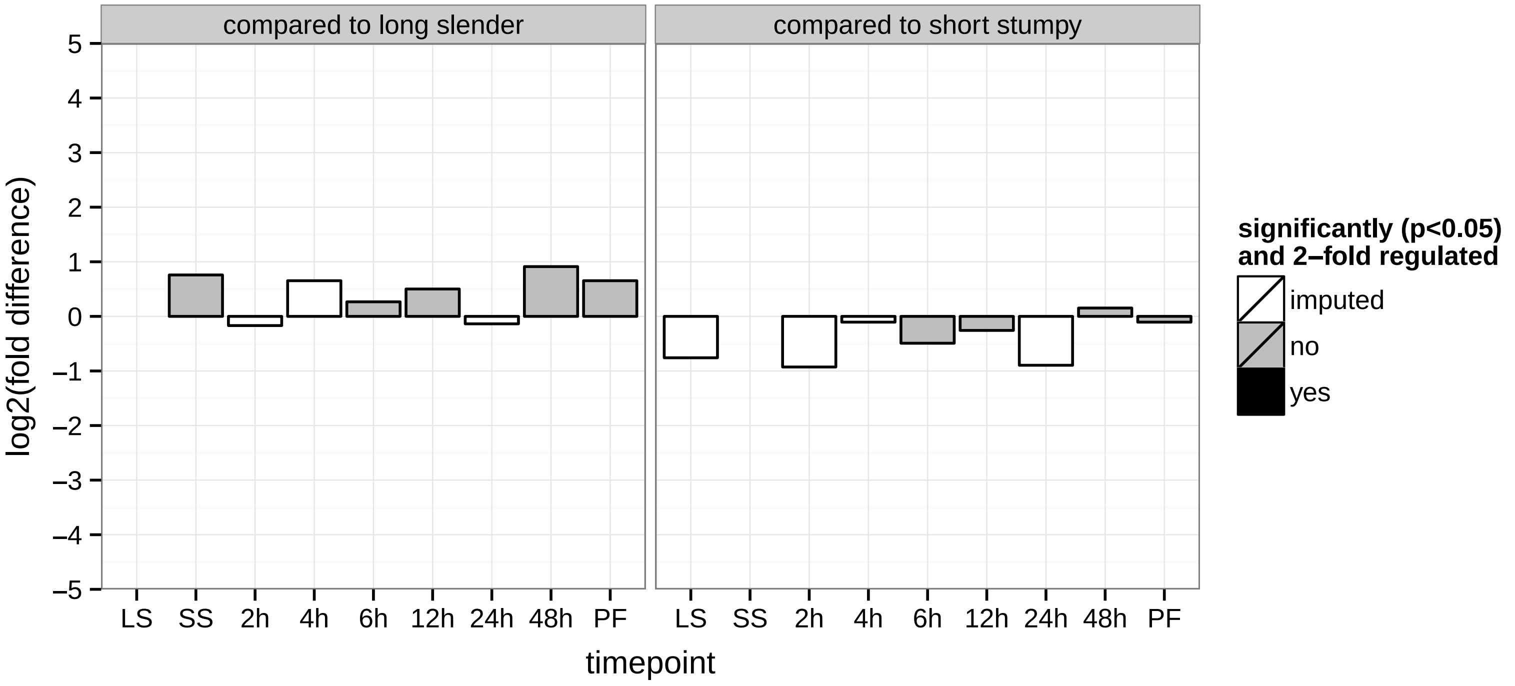

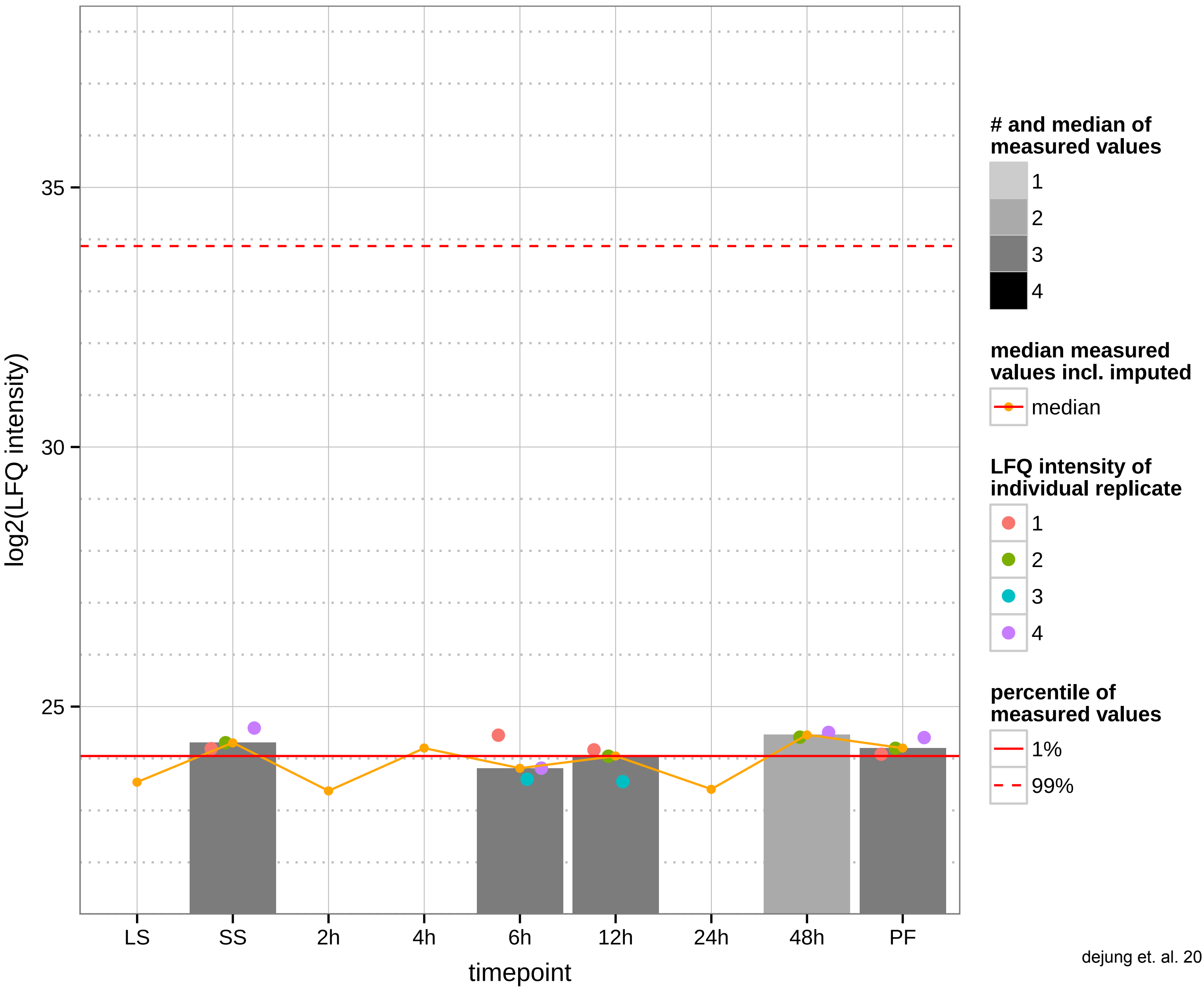

dejung et. al. 2015

MORN repeat–containing protein, CMRP, flagellar component (TbMORN1)
Tb927.6.4670
AGOF: null
AGOC: null
AGOP: null
PGOF: null
PGOC: null
PGOP: null

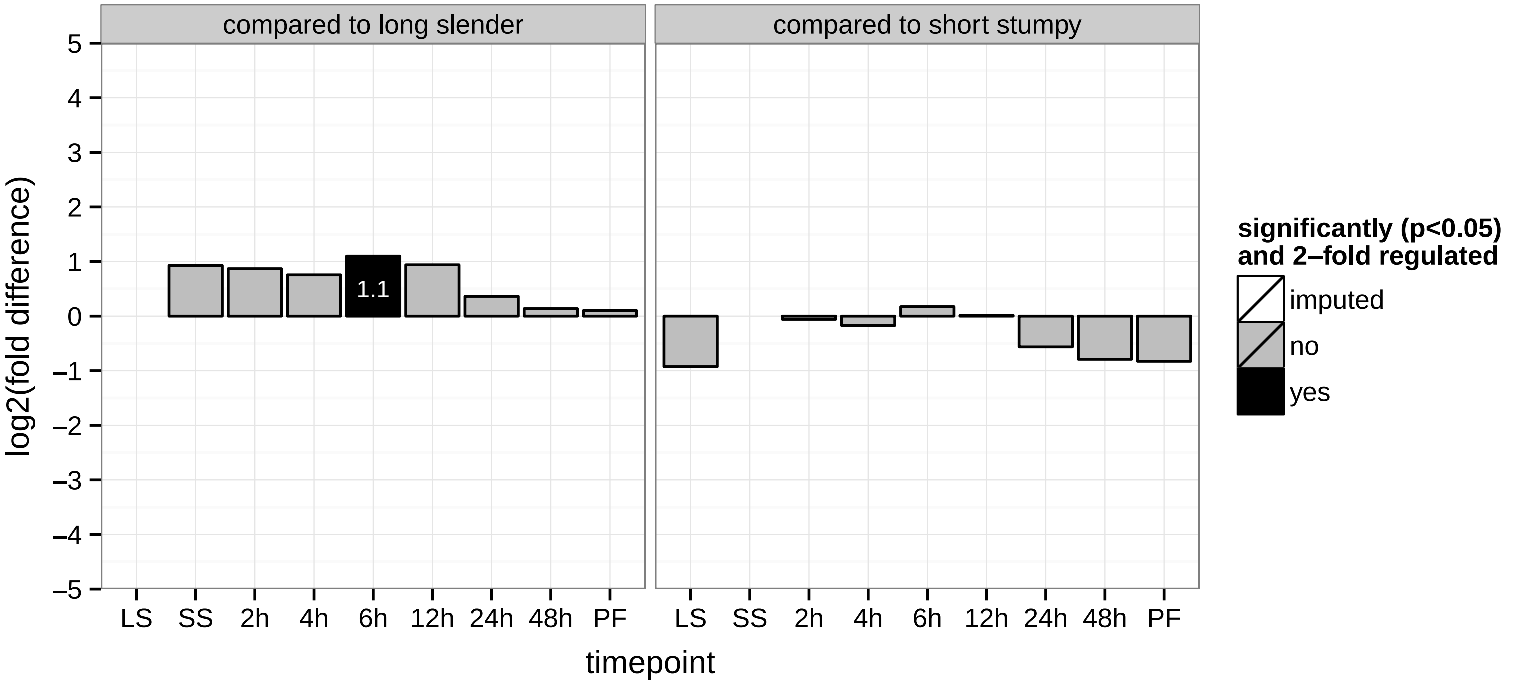

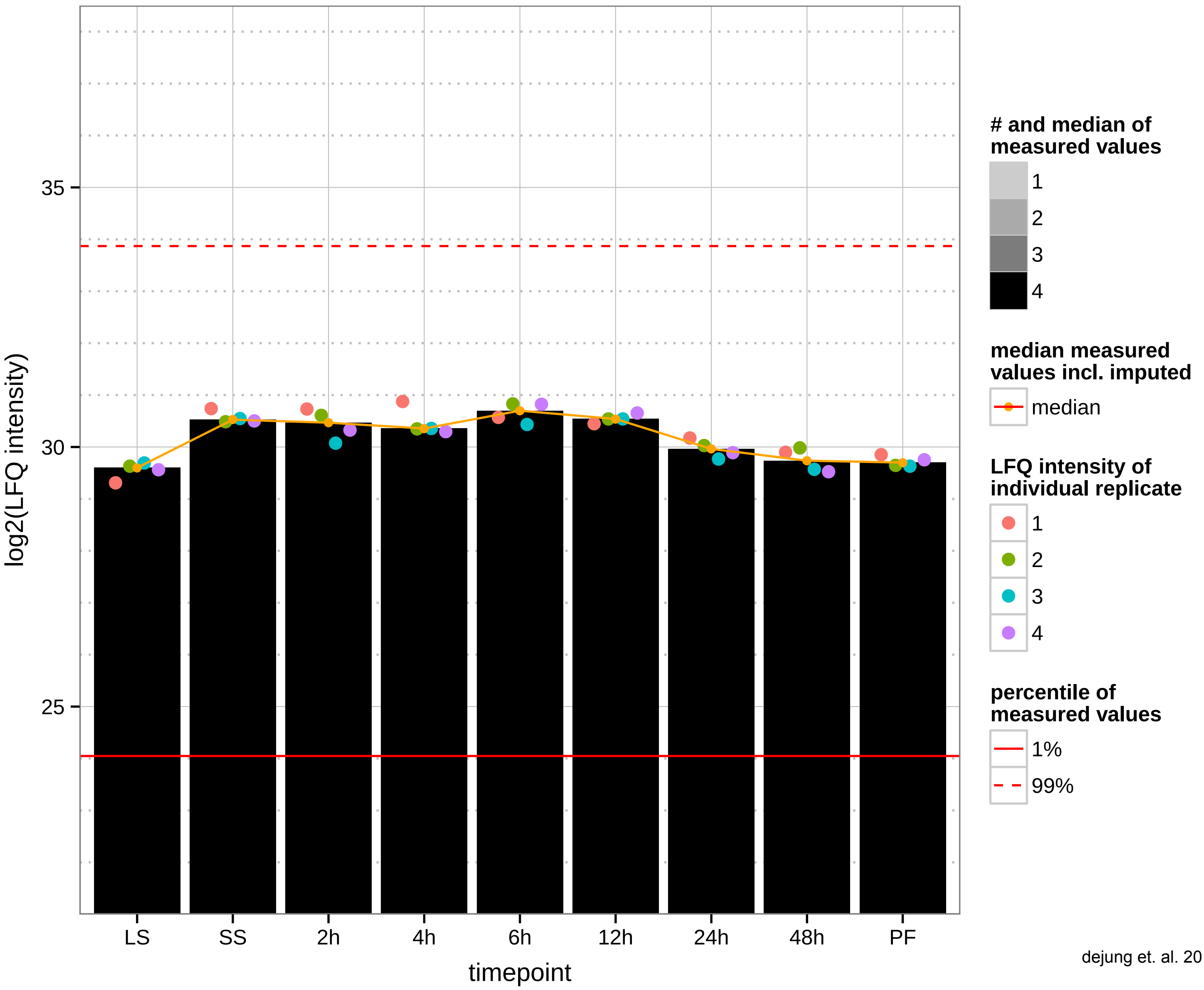

dejung et. al. 2015

hypothetical protein, conserved
Tb927.6.4680
AGOF: null
AGOC: null
AGOP: null
PGOF: null
PGOC: null
PGOP: null

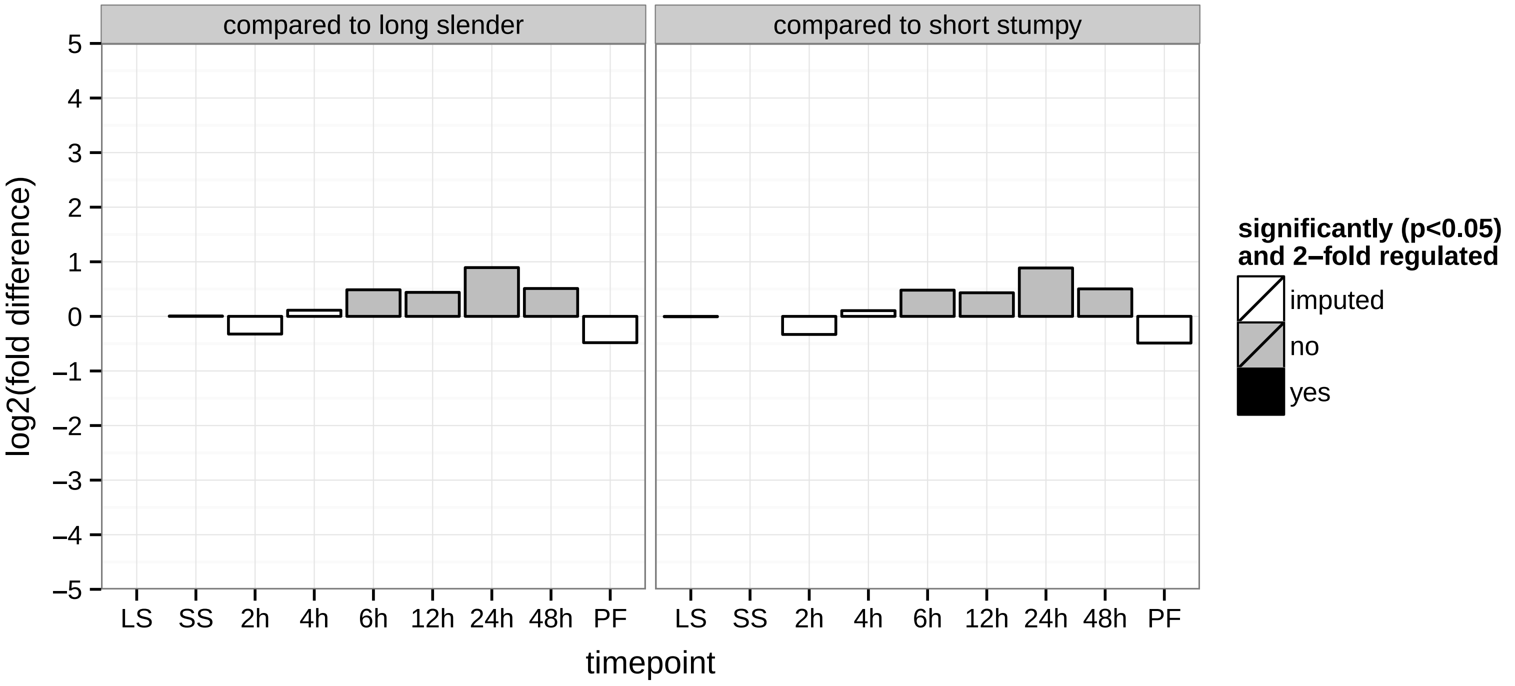

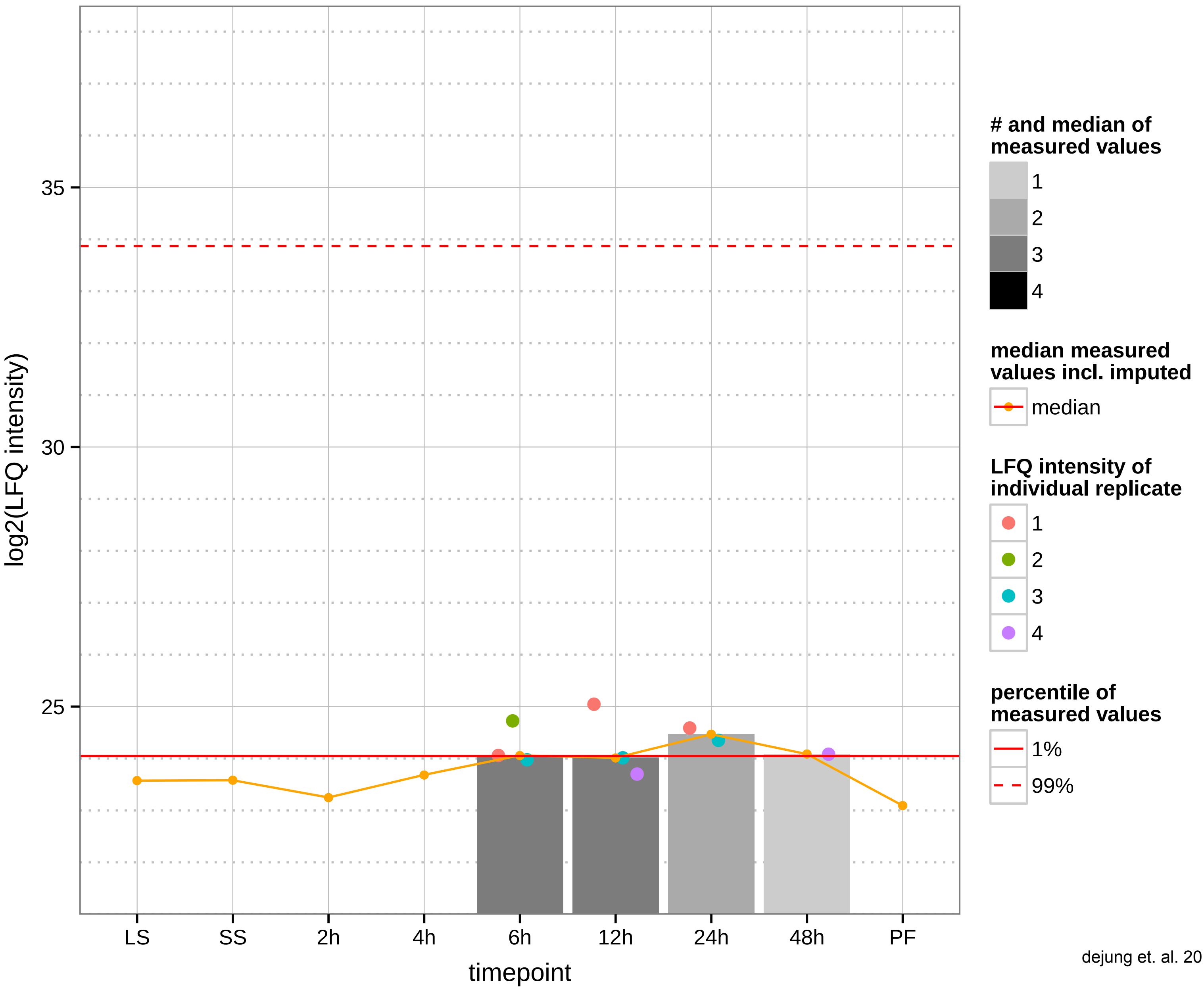

dejung et. al. 2015

calmodulin, putative
Tb927.6.4710
AGOF: calcium ion binding
AGOC: null
AGOP: null
PGOF: calcium ion binding
PGOC: null
PGOP: null

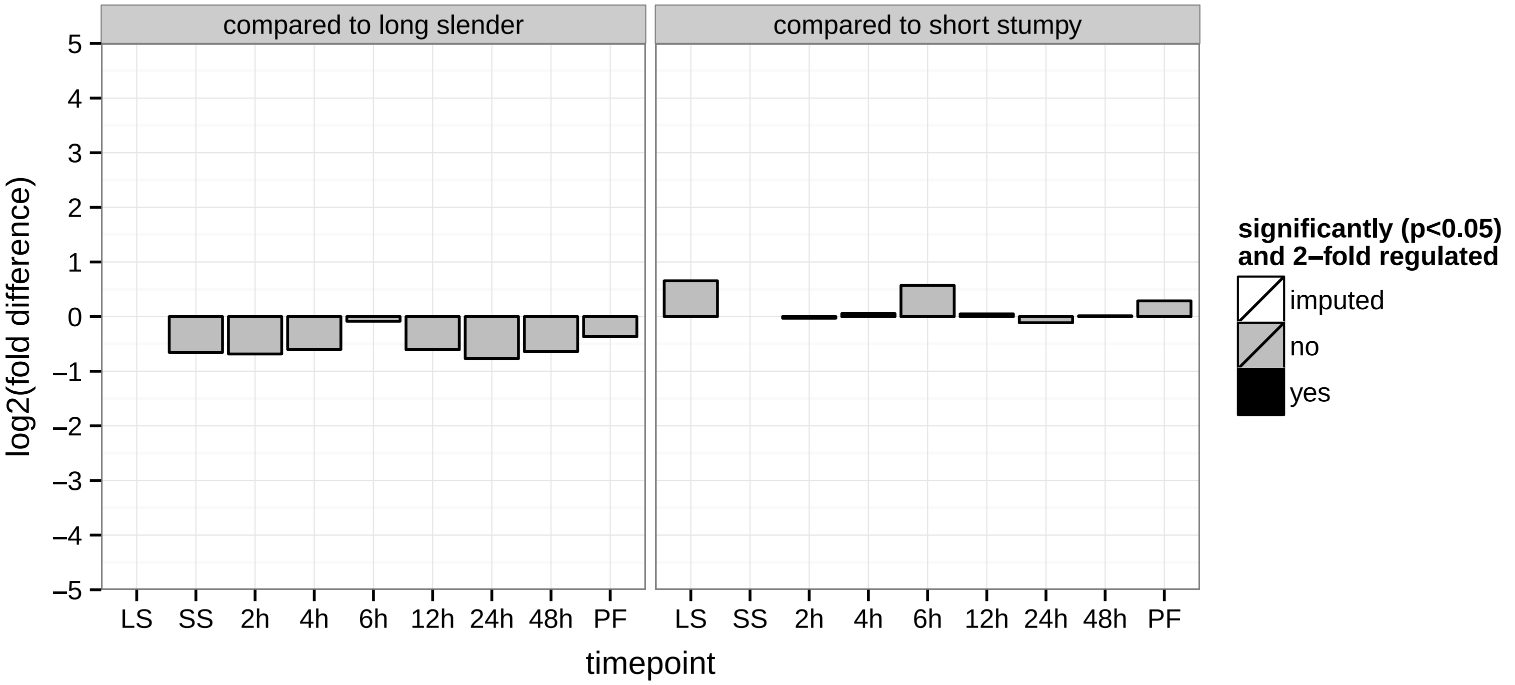

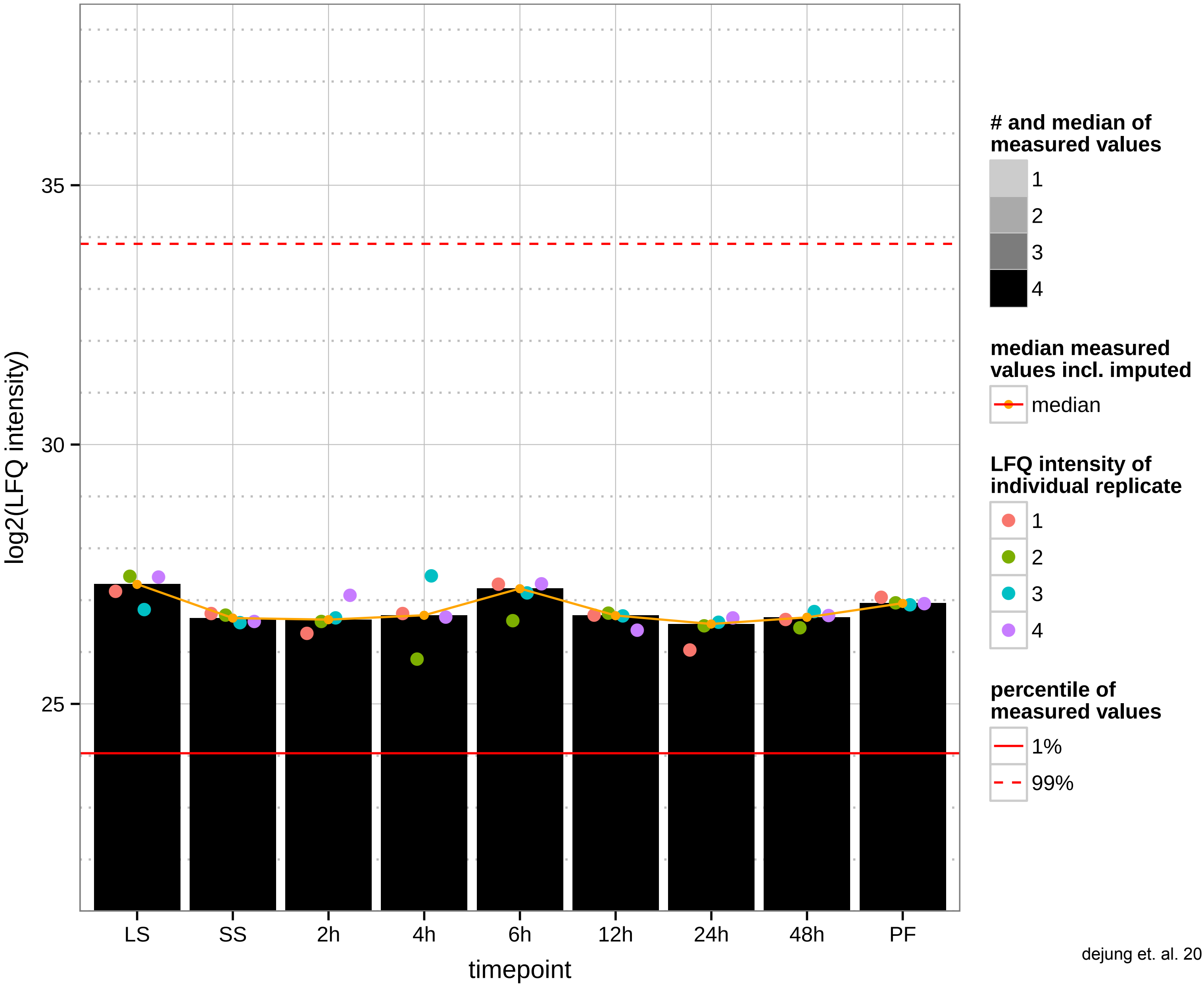

dejung et. al. 2015

importin–alpha re–exporter protein, putative, cellular apoptosis susceptibility protein
Tb927.6.4740
AGOF: importin–alpha export receptor activity, protein transporter activity
AGOC: cytoplasm, nuclear pore, nucleus
AGOP: cell proliferation, intracellular protein transport, protein import into nucleus, docking
PGOF: binding, protein binding, protein transporter activity
PGOC: null
PGOP: intracellular protein transport

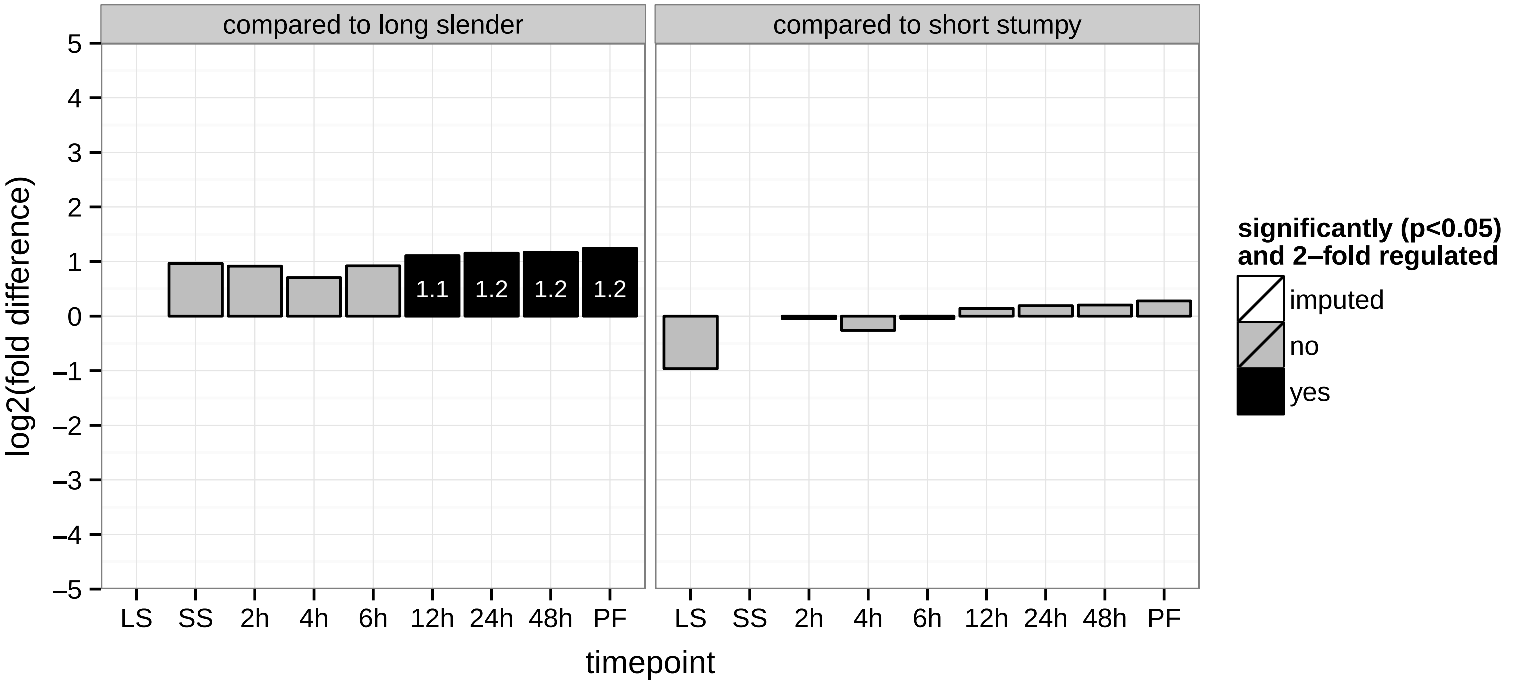

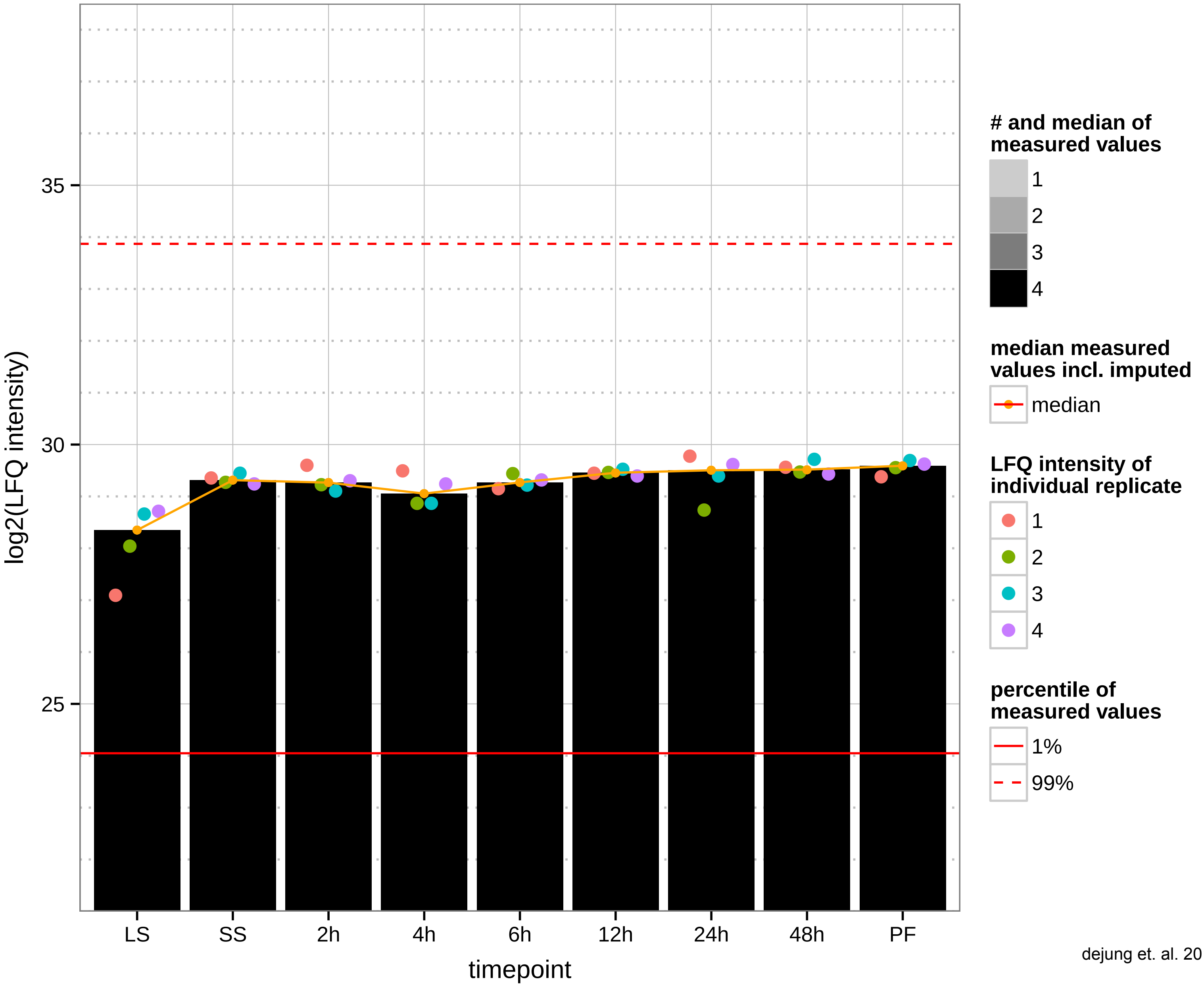

dejung et. al. 2015

hypothetical protein, conserved
Tb927.6.4760
AGOF: null
AGOC: null
AGOP: null
PGOF: null
PGOC: null
PGOP: null

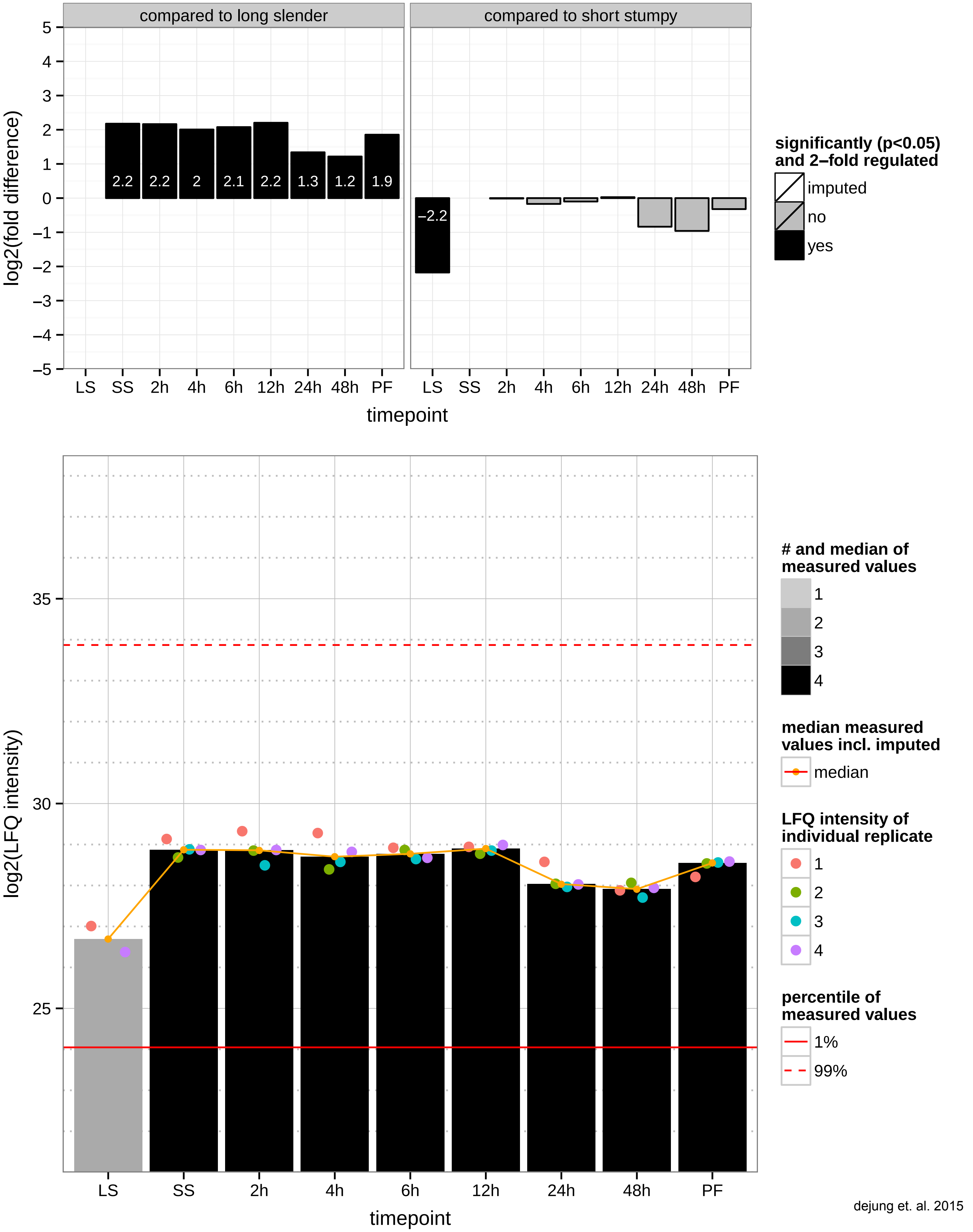

dejung et. al. 2015

protein mkt1, putative (MKT1)
Tb927.6.4770
AGOF: null
AGOC: polysome
AGOP: response to DNA damage stimulus
PGOF: null
PGOC: null
PGOP: null

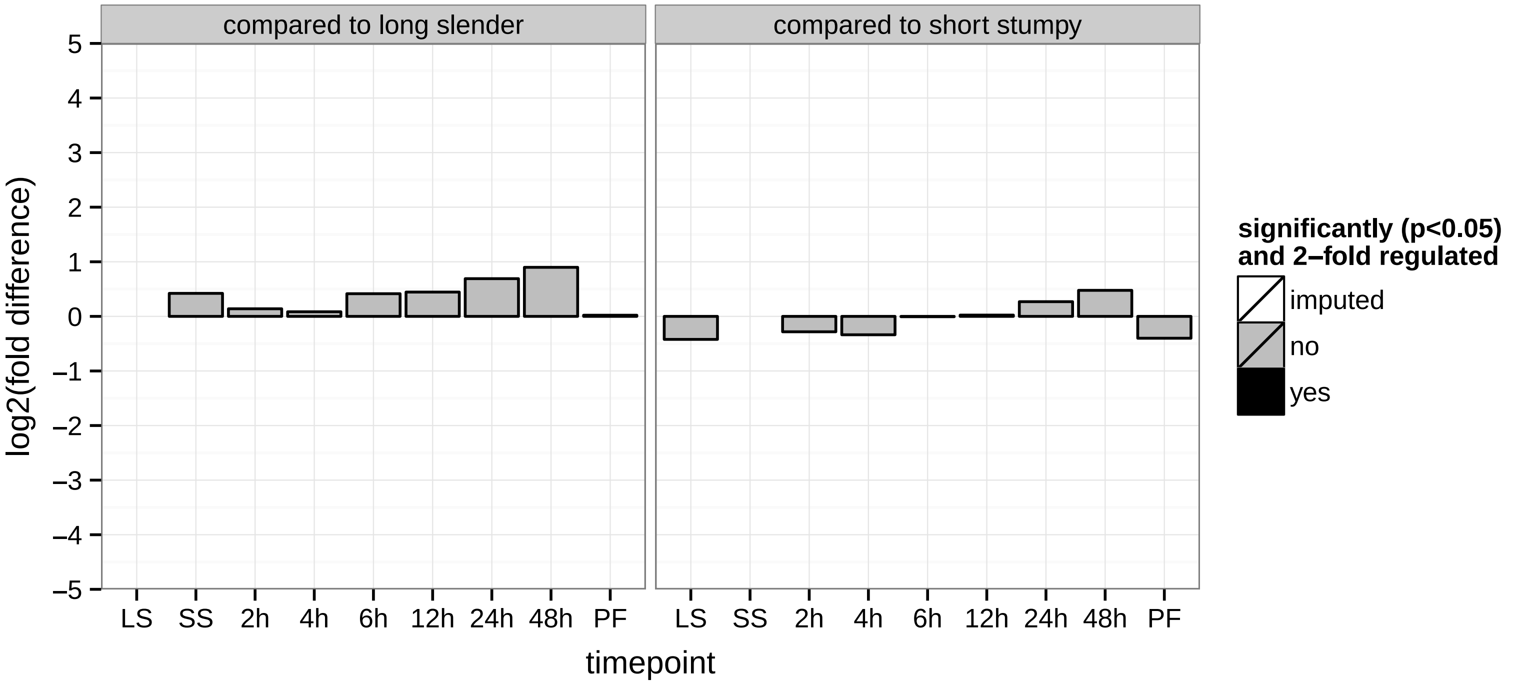

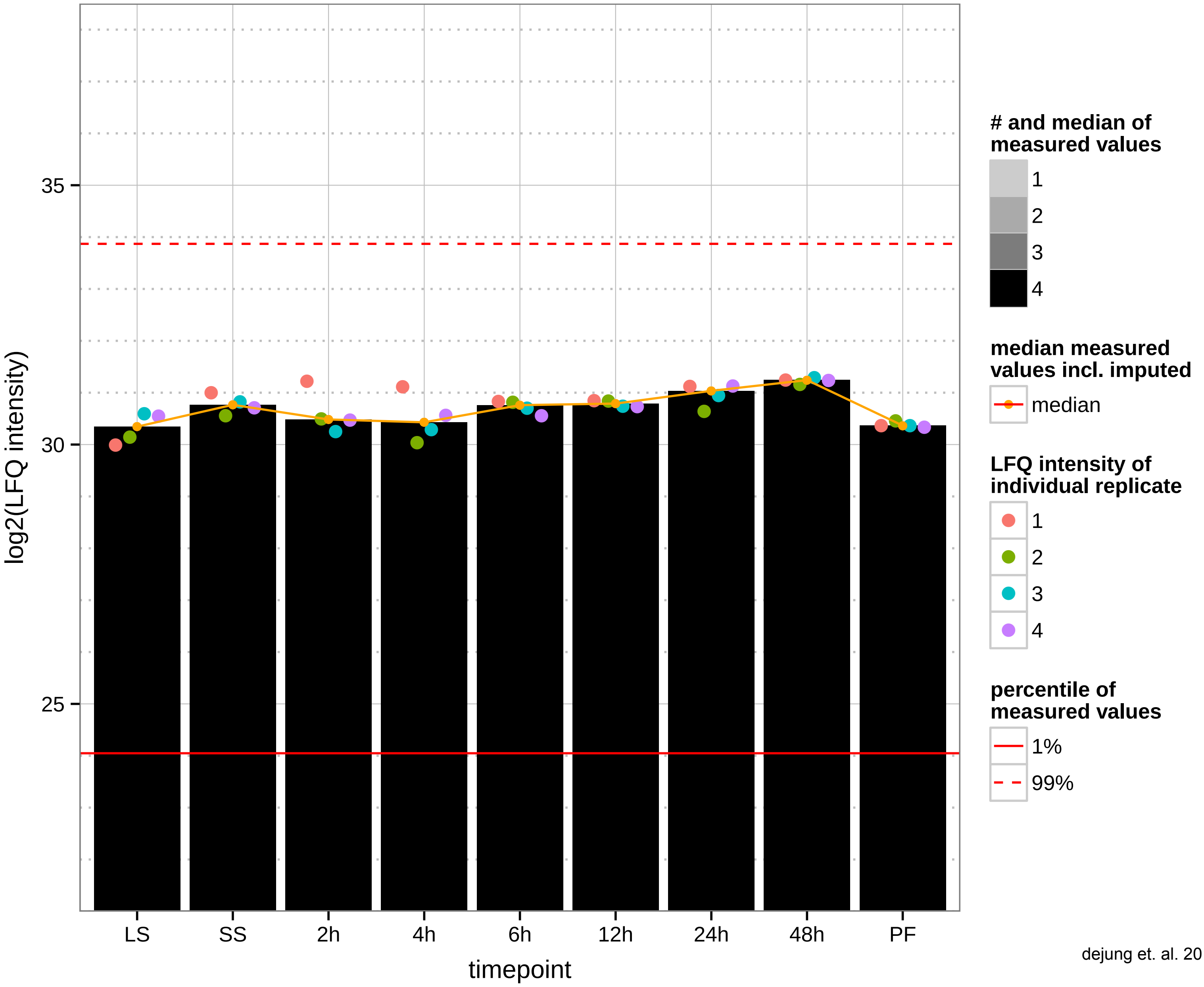

dejung et. al. 2015

DNA ligase I, putative
Tb927.6.4780
AGOF: ATP binding, DNA binding, DNA ligase (ATP) activity
AGOC: null
AGOP: DNA recombination, DNA repair, DNA replication
PGOF: ATP binding, DNA binding, DNA ligase (ATP) activity
PGOC: null
PGOP: DNA recombination, DNA repair, DNA replication

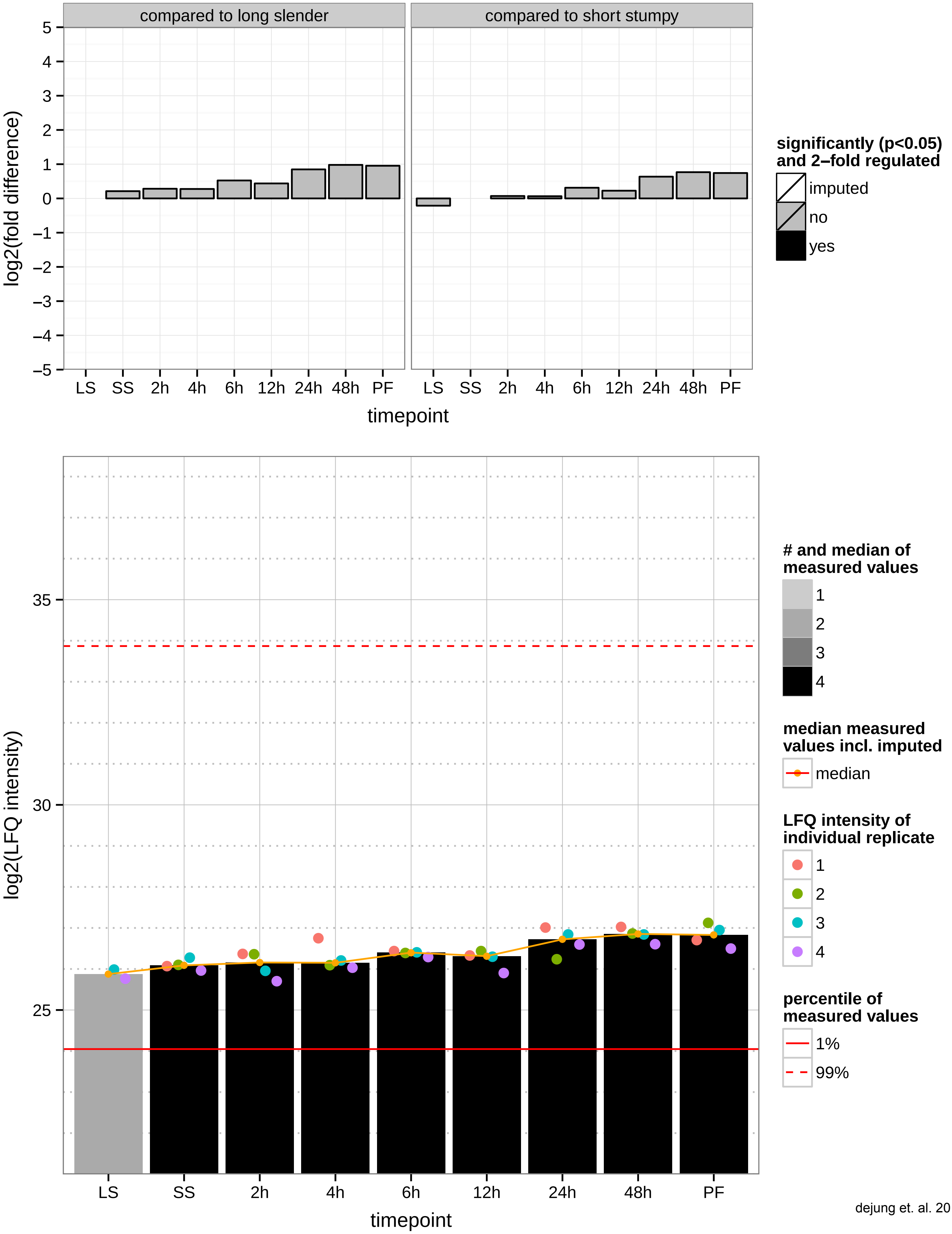

dejung et. al. 2015

mago nashi–like protein, putative
Tb927.6.4950
AGOF: null
AGOC: nucleus
AGOP: null
PGOF: null
PGOC: nucleus
PGOP: null

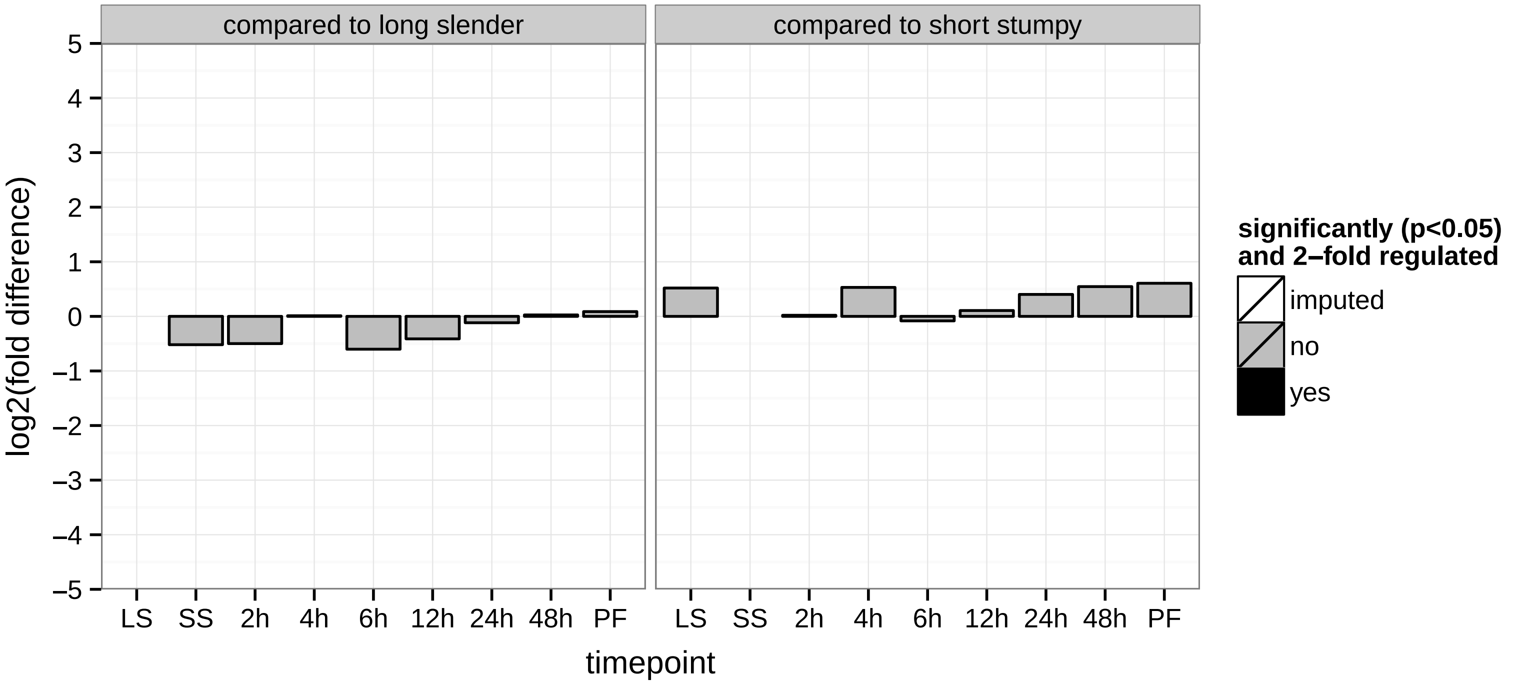

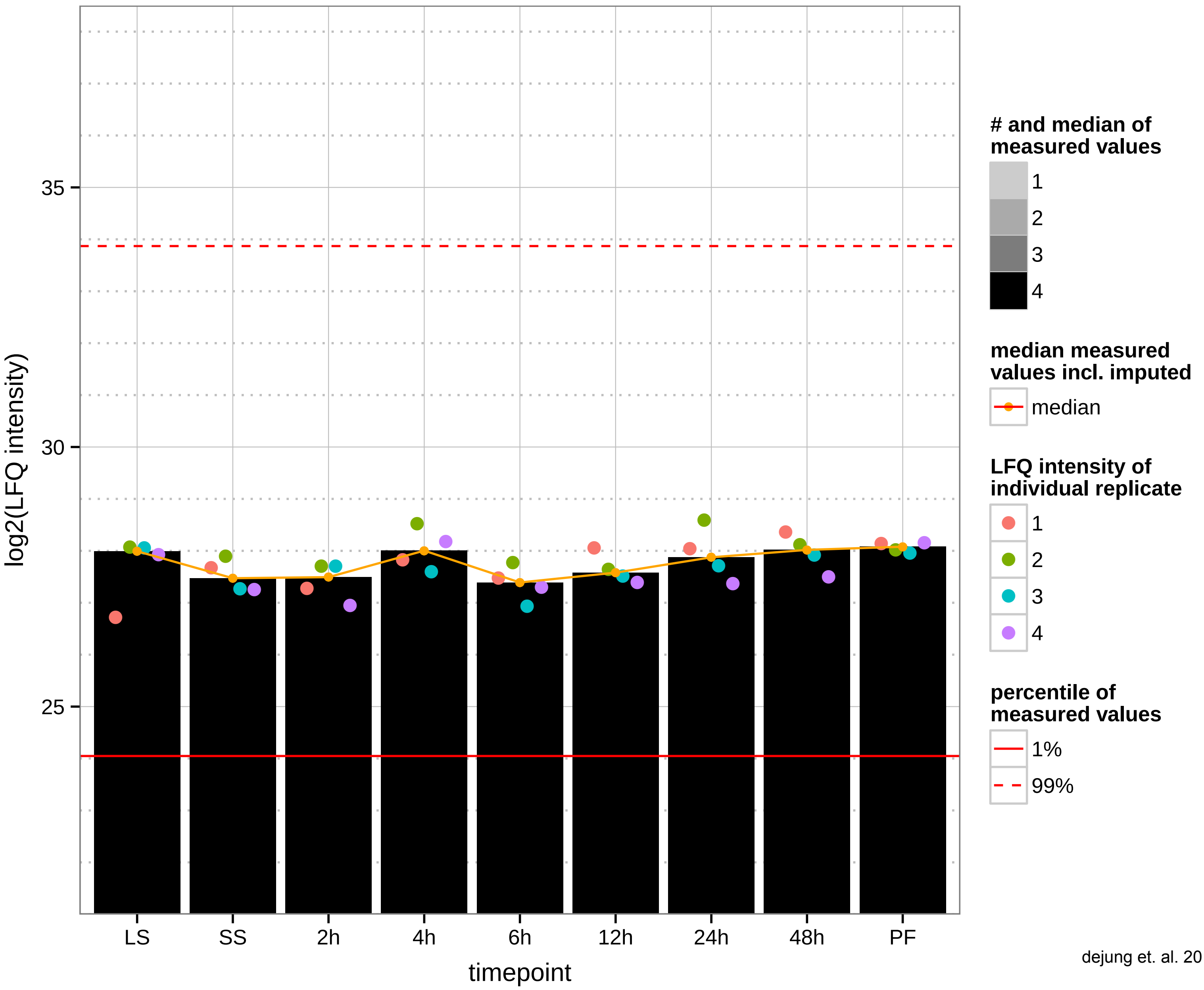

dejung et. al. 2015

hypothetical protein, conserved
Tb927.6.5000
AGOF: null
AGOC: null
AGOP: null
PGOF: null
PGOC: null
PGOP: null

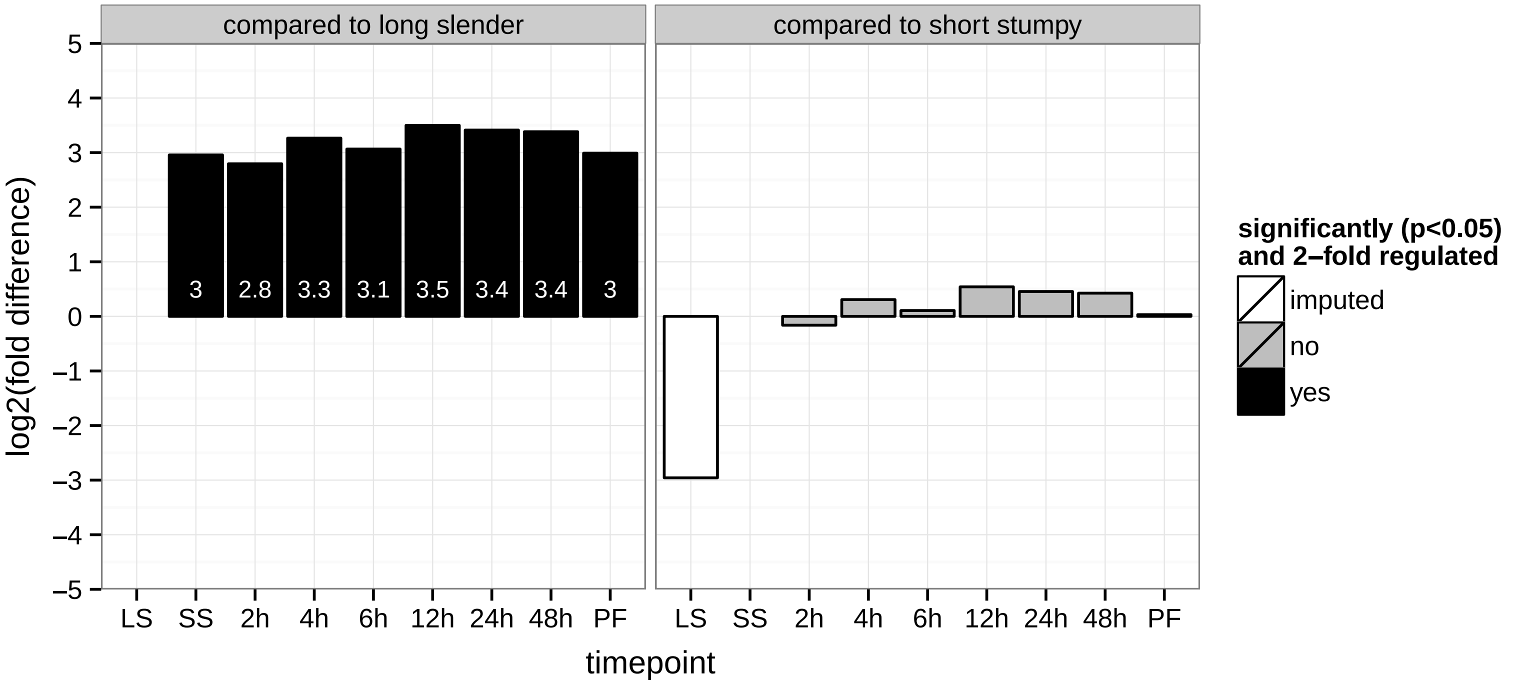

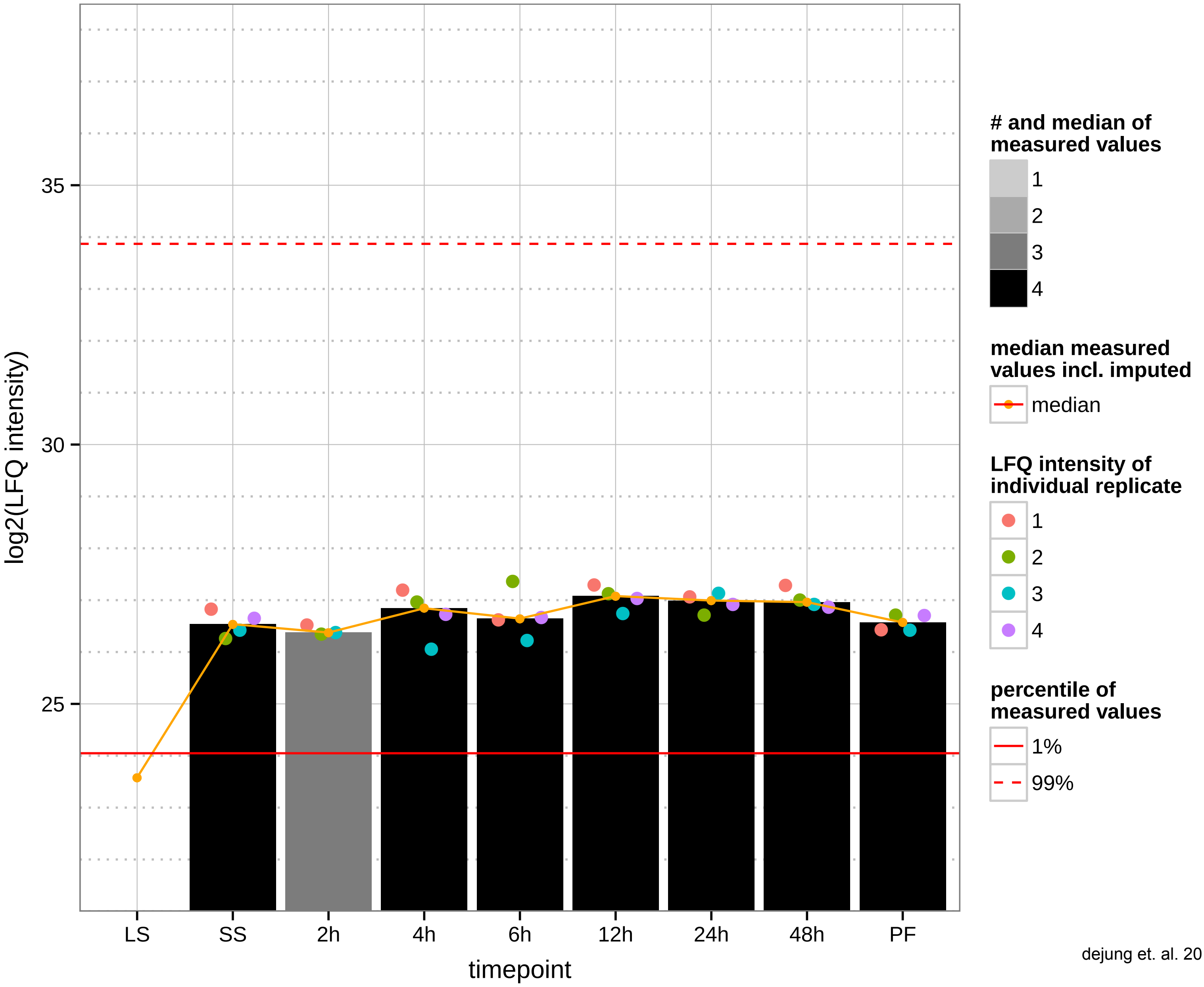

dejung et. al. 2015

leucine–rich repeat protein (LRRP), putative
Tb927.6.5030
AGOF: null
AGOC: null
AGOP: ciliary or flagellar motility
PGOF: protein binding
PGOC: null
PGOP: null

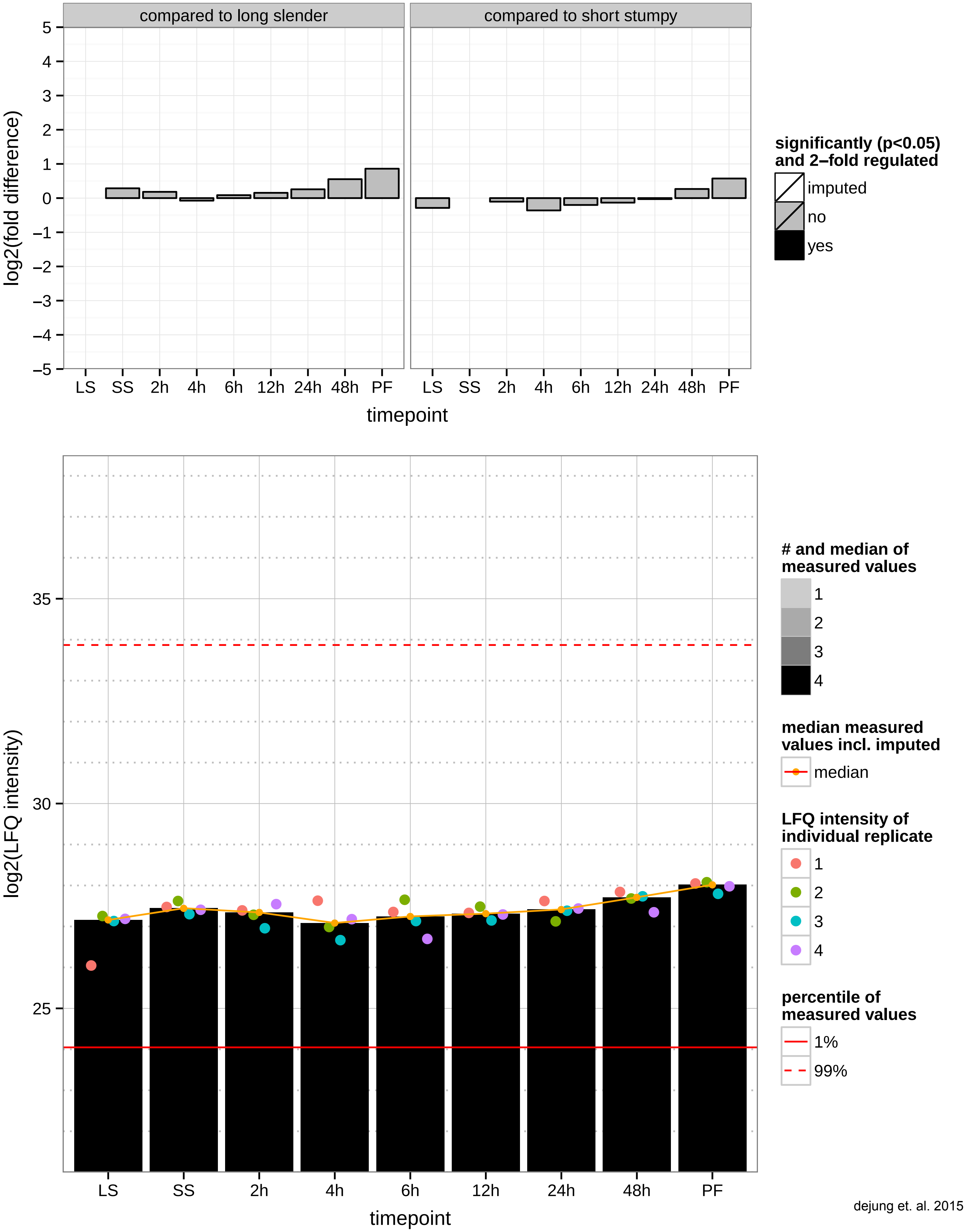

dejung et. al. 2015

V–type ATPase, C subunit, putative
Tb927.6.5050
AGOF: hydrogen ion transmembrane transporter activity
AGOC: proton–transporting V–type ATPase, V0 domain
AGOP: ATP synthesis coupled proton transport
PGOF: hydrogen ion transmembrane transporter activity
PGOC: proton–transporting two–sector ATPase complex, proton–transporting domain
PGOP: ATP hydrolysis coupled proton transport

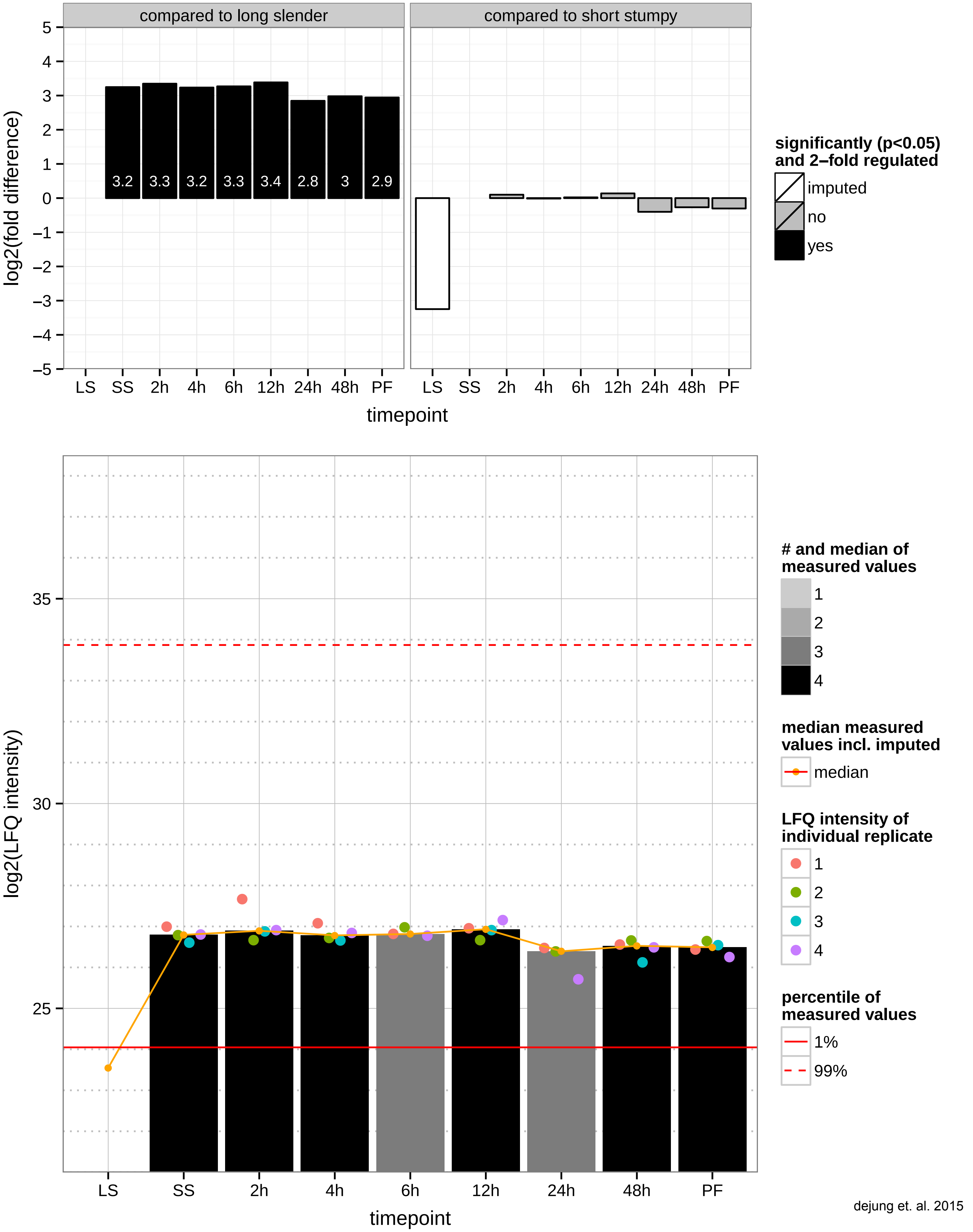

dejung et. al. 2015

NA, hypothetical protein, conserved
Tb927.6.5070
AGOF: NA, null
AGOC: NA, null
AGOP: NA, null
PGOF: NA, null
PGOC: NA, null
PGOP: NA, null

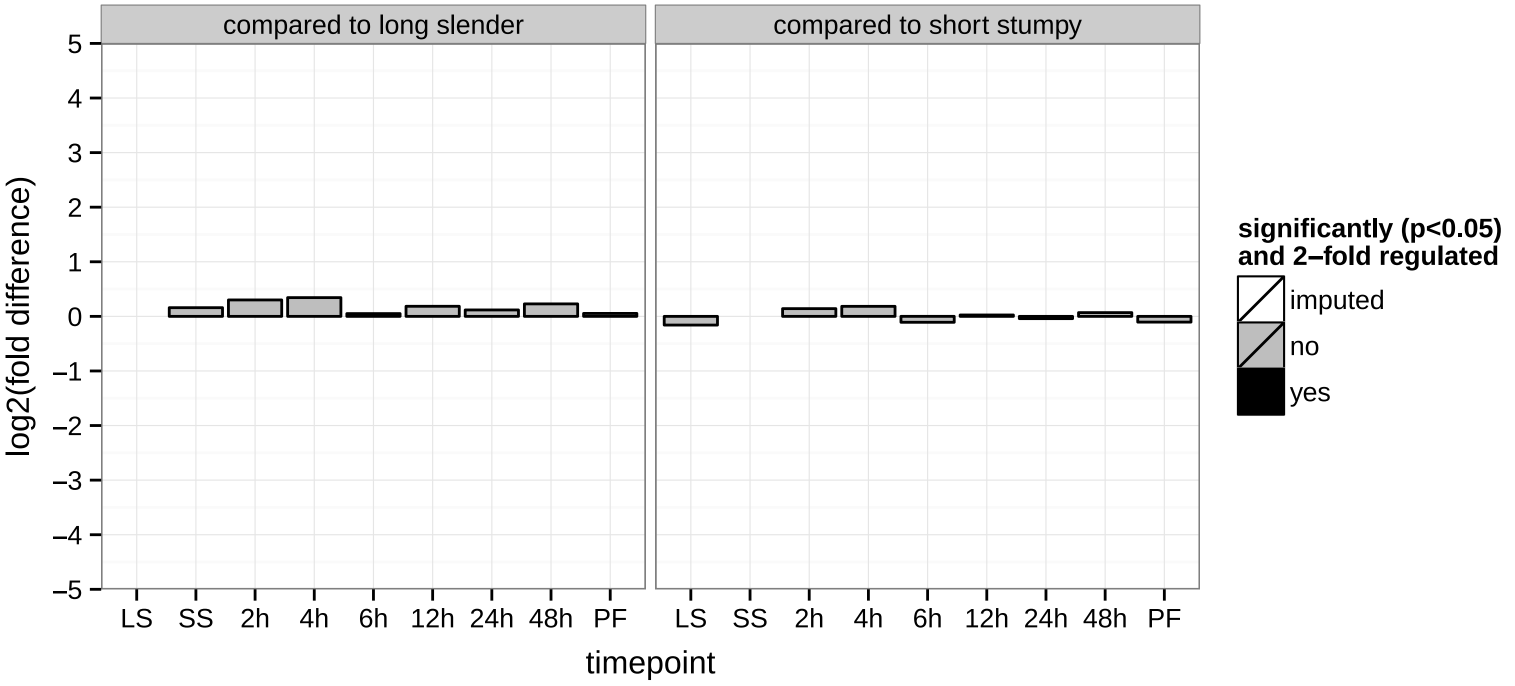

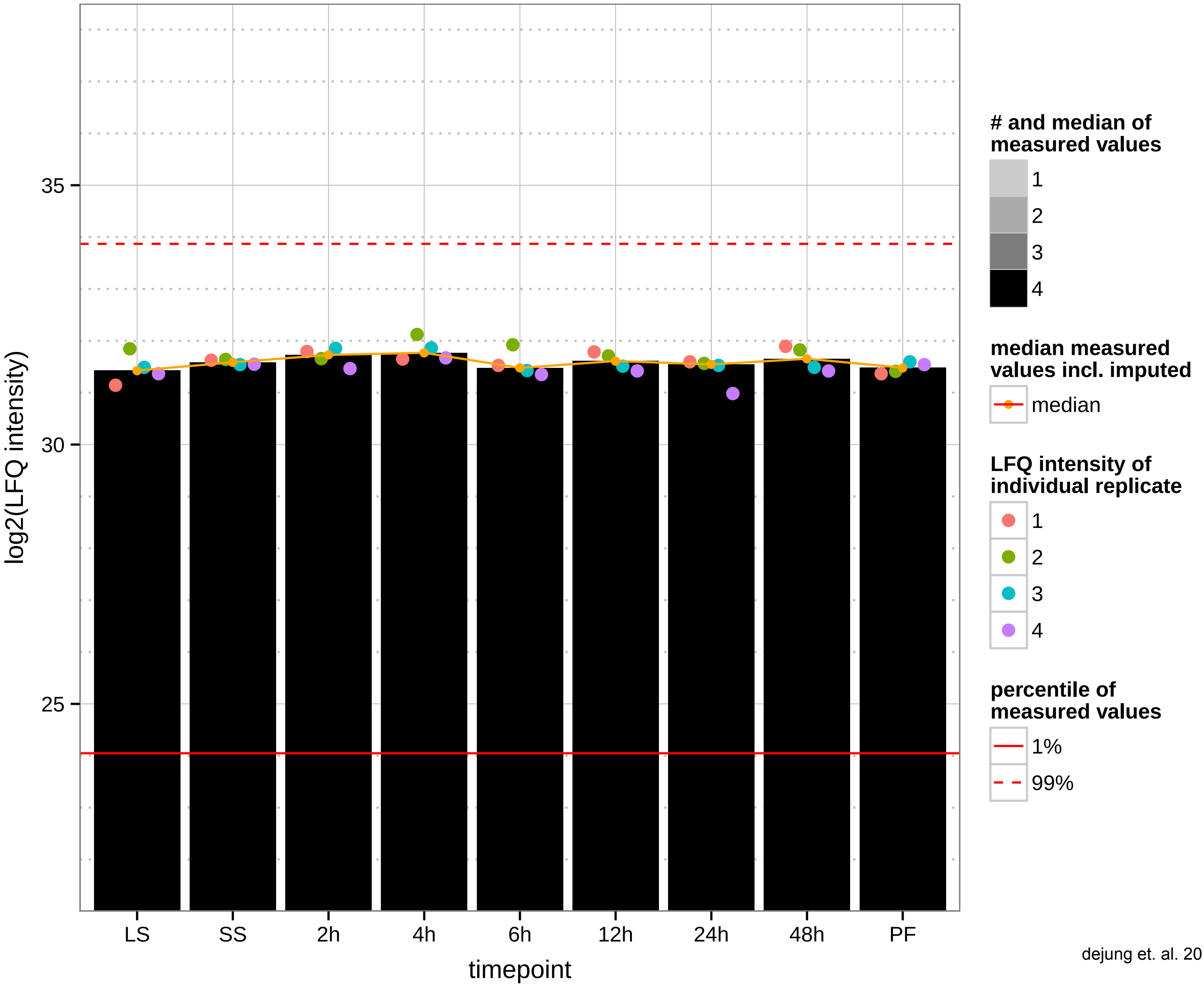

dejung et. al. 2015

hypothetical protein, conserved
Tb927.6.5080
AGOF: null
AGOC: null
AGOP: null
PGOF: null
PGOC: null
PGOP: null

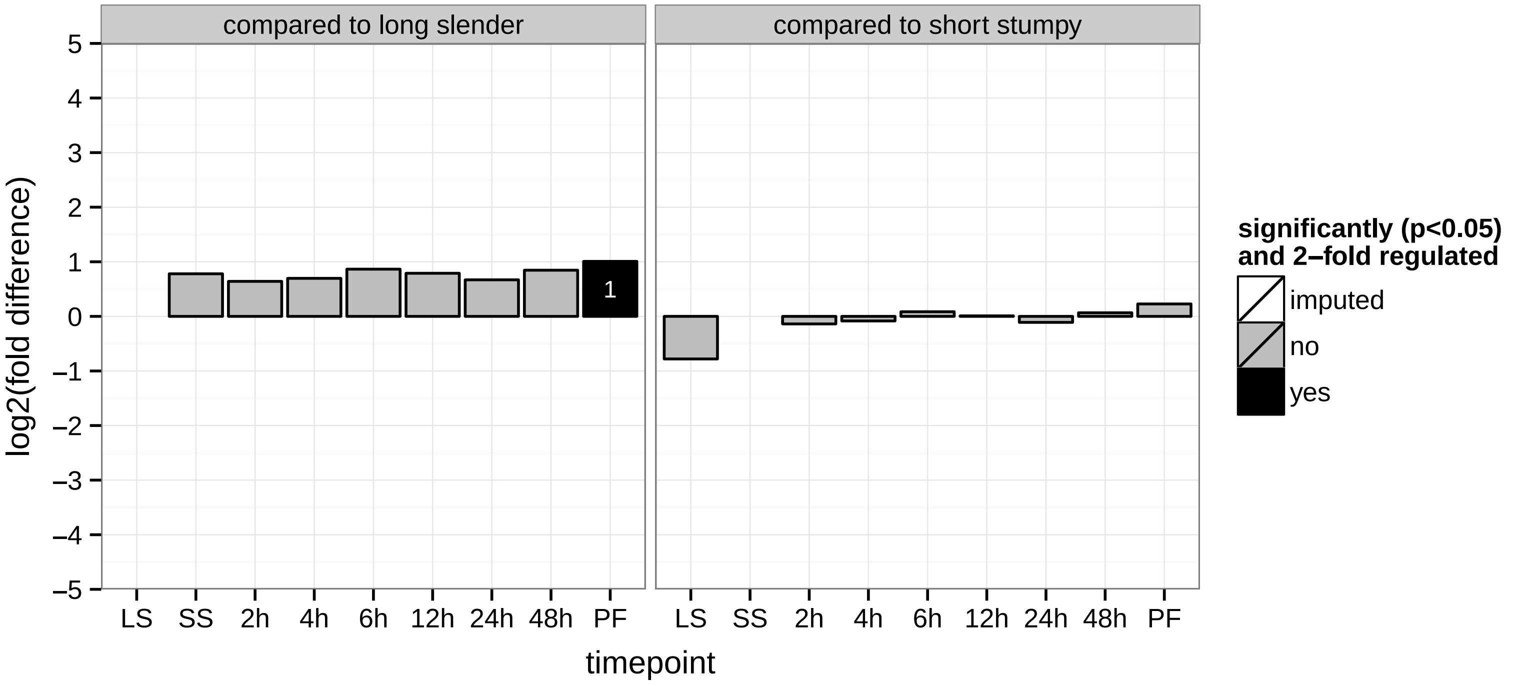

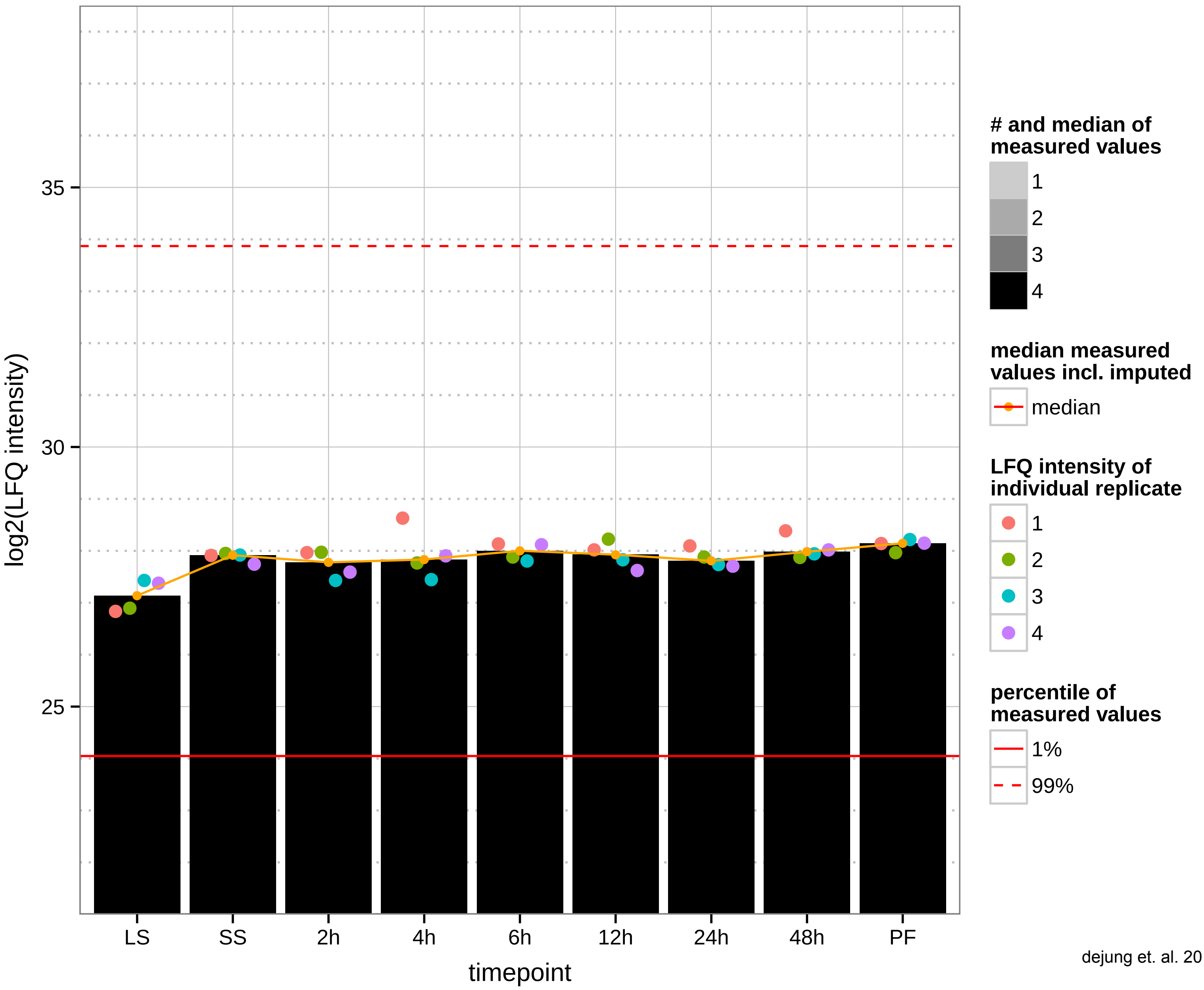

dejung et. al. 2015

damage−specific DNA binding protein, putative
Tb927.6.5110
AGOF: nucleic acid binding
AGOC: nucleus
AGOP: null
PGOF: nucleic acid binding
PGOC: nucleus
PGOP: null

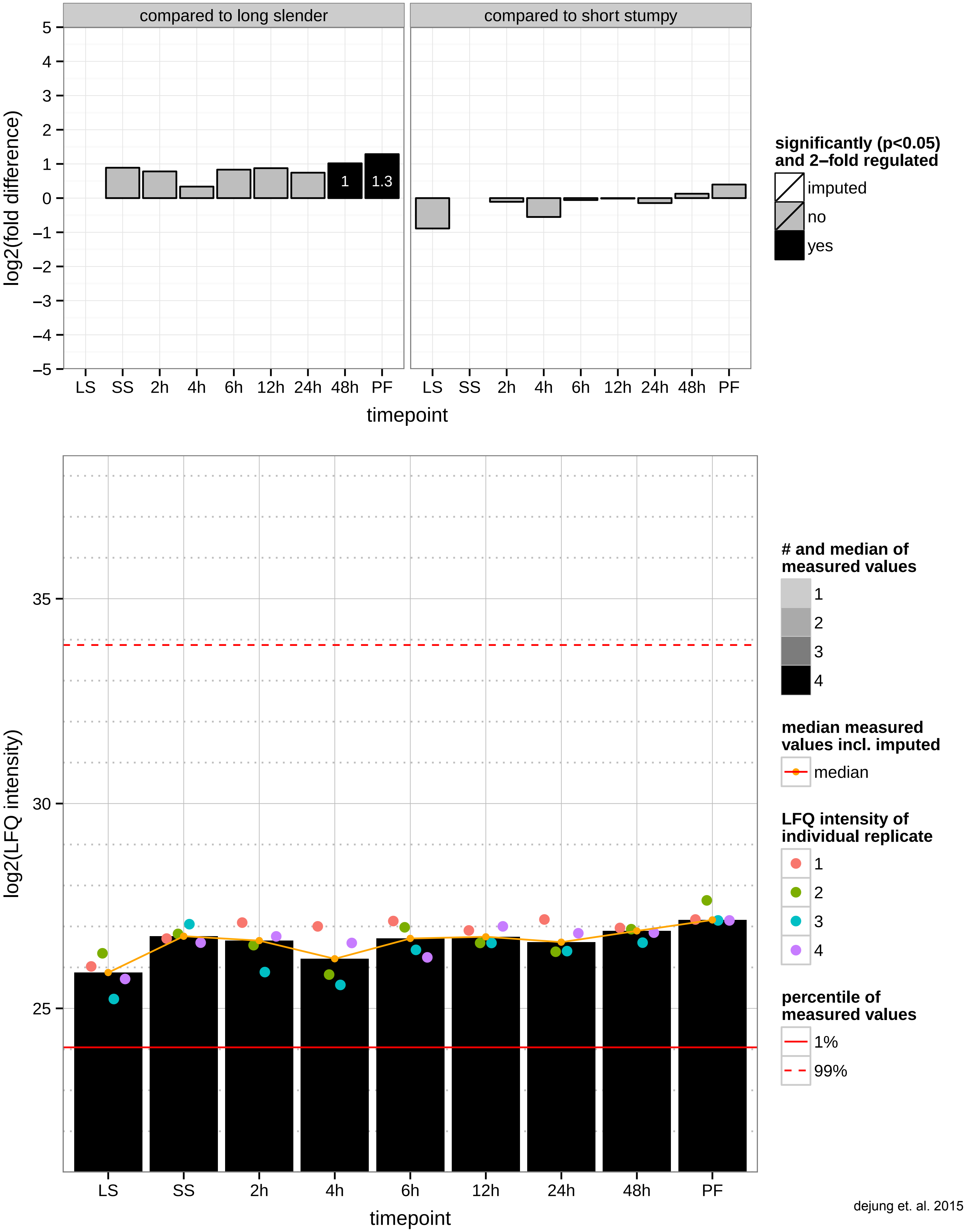

dejung et. al. 2015

60S acidic ribosomal protein P2, putative
Tb927.6.5130;Tb927.6.5120
AGOF: structural constituent of ribosome
AGOC: intracellular, ribosome
AGOP: translation, translational elongation
PGOF: structural constituent of ribosome, null
PGOC: intracellular, ribosome, null
PGOP: translational elongation, null

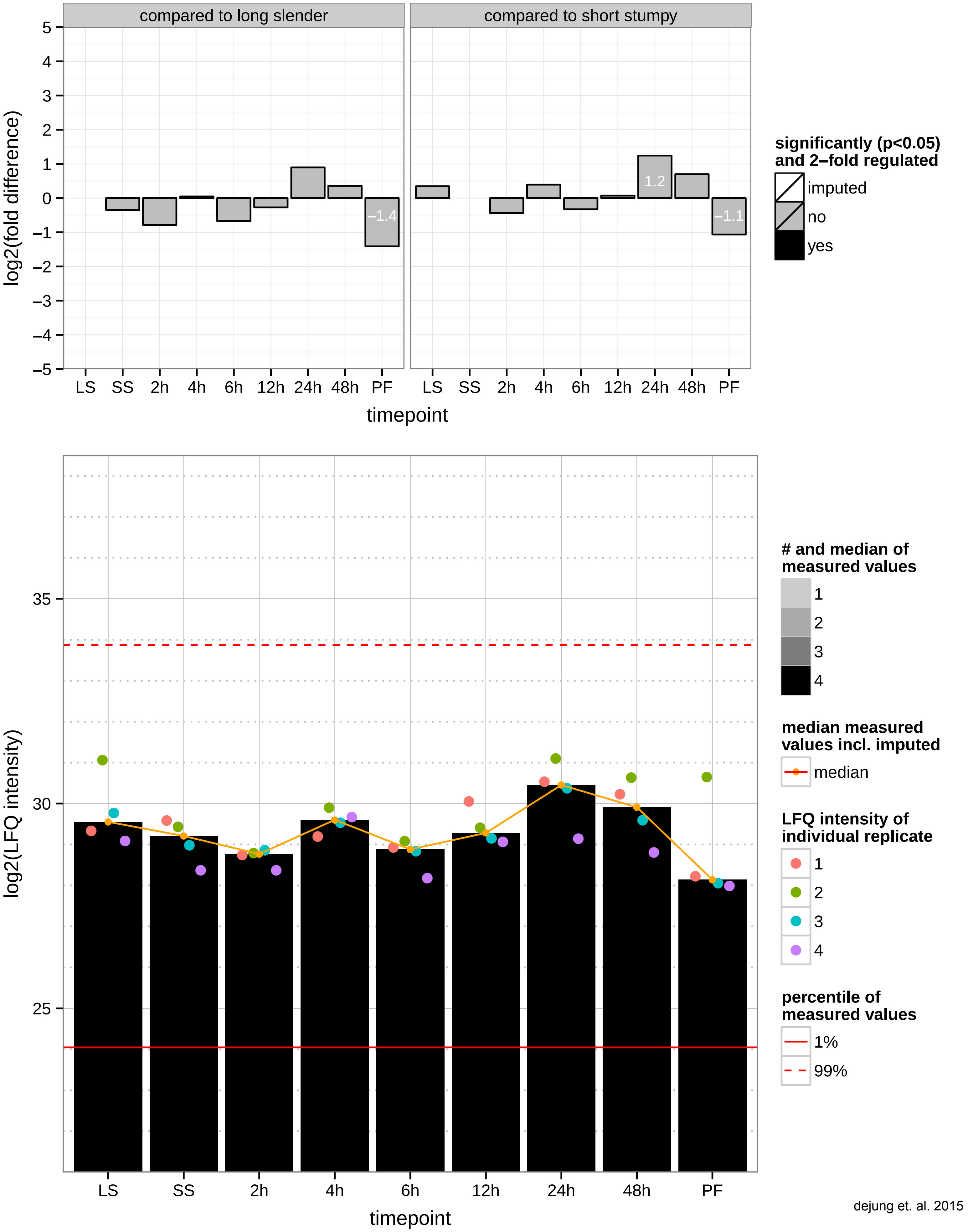

dejung et. al. 2015

ribosomal P protein AGP2beta–1, putative
Tb927.6.5140
AGOF: null
AGOC: null
AGOP: null
PGOF: null
PGOC: null
PGOP: null

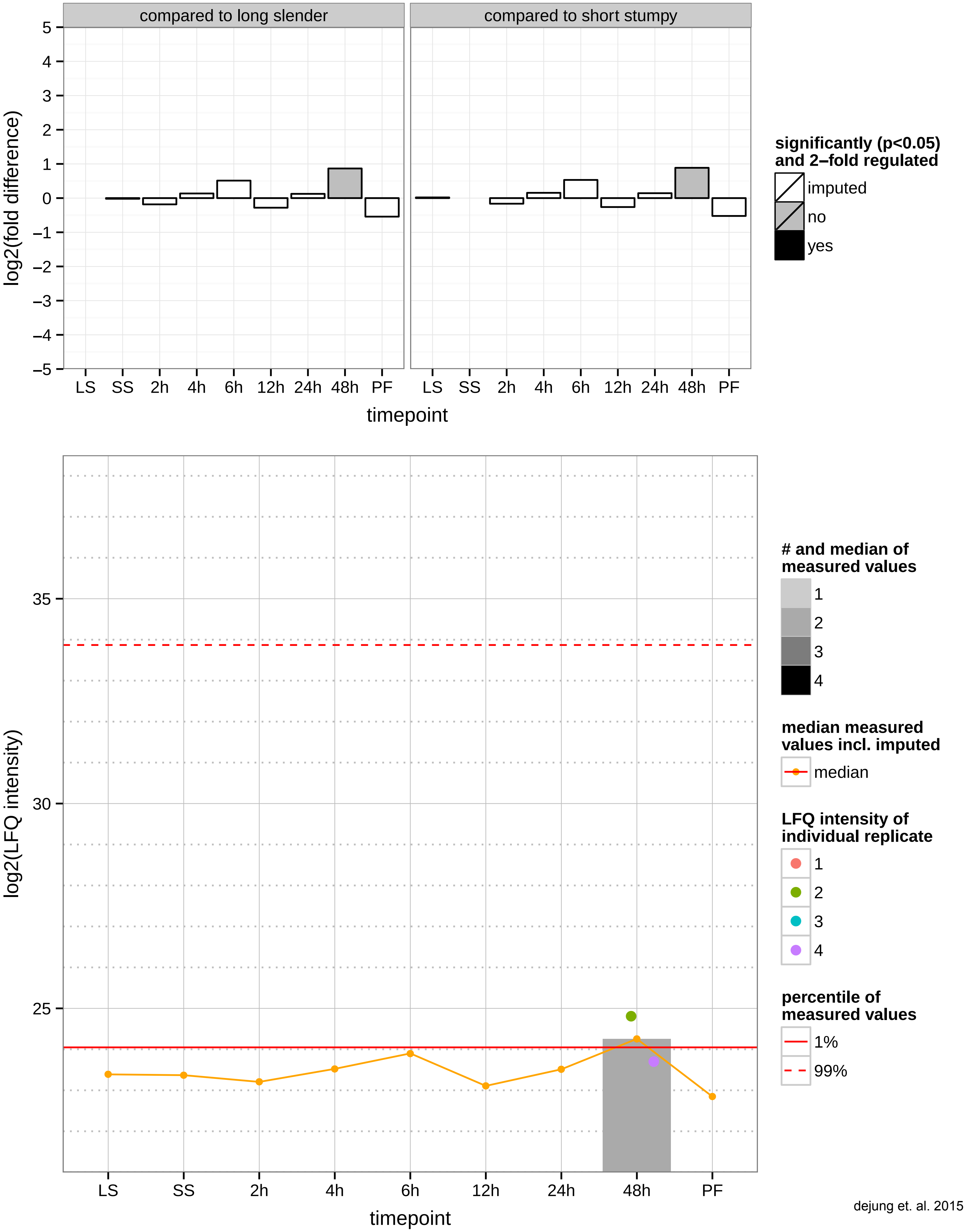

dejung et. al. 2015

hypothetical protein, conserved
Tb927.6.610
AGOF: null
AGOC: mitochondrion
AGOP: null
PGOF: null
PGOC: null
PGOP: null

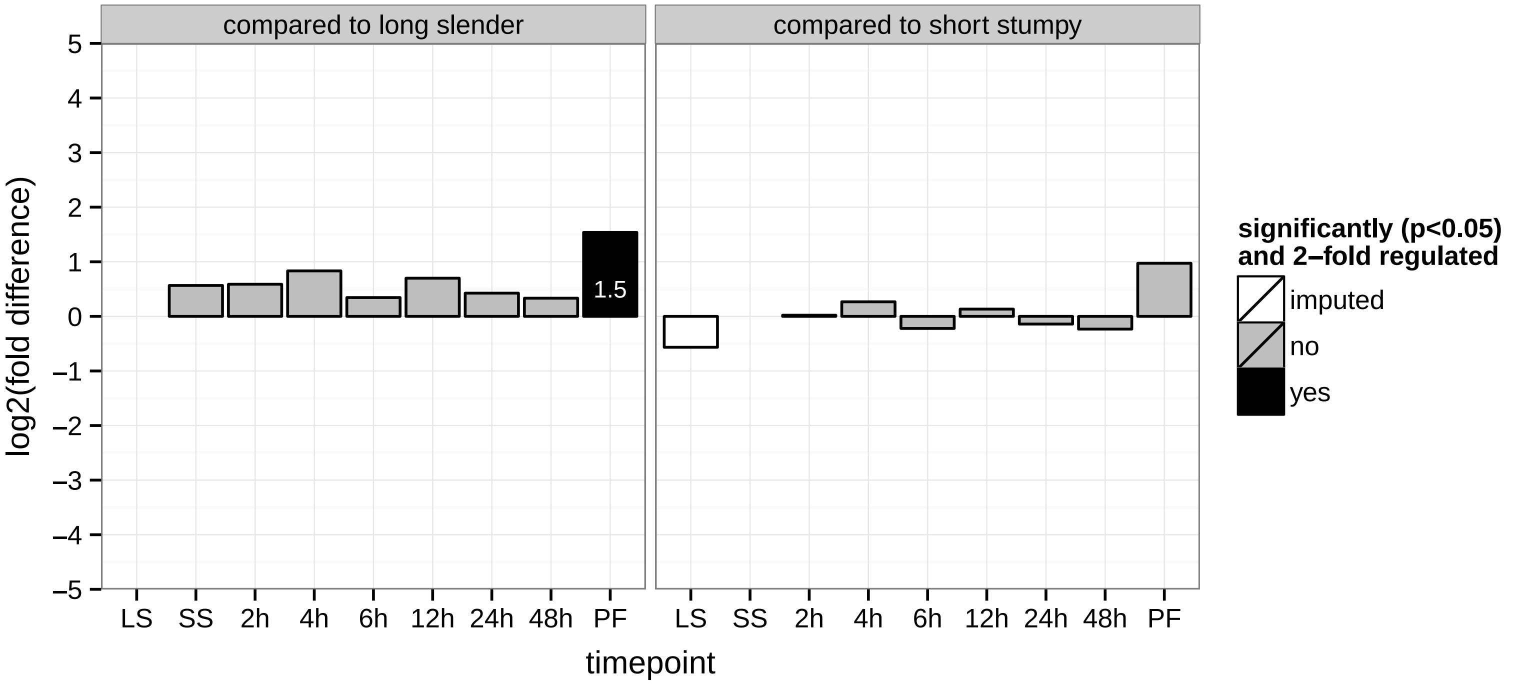

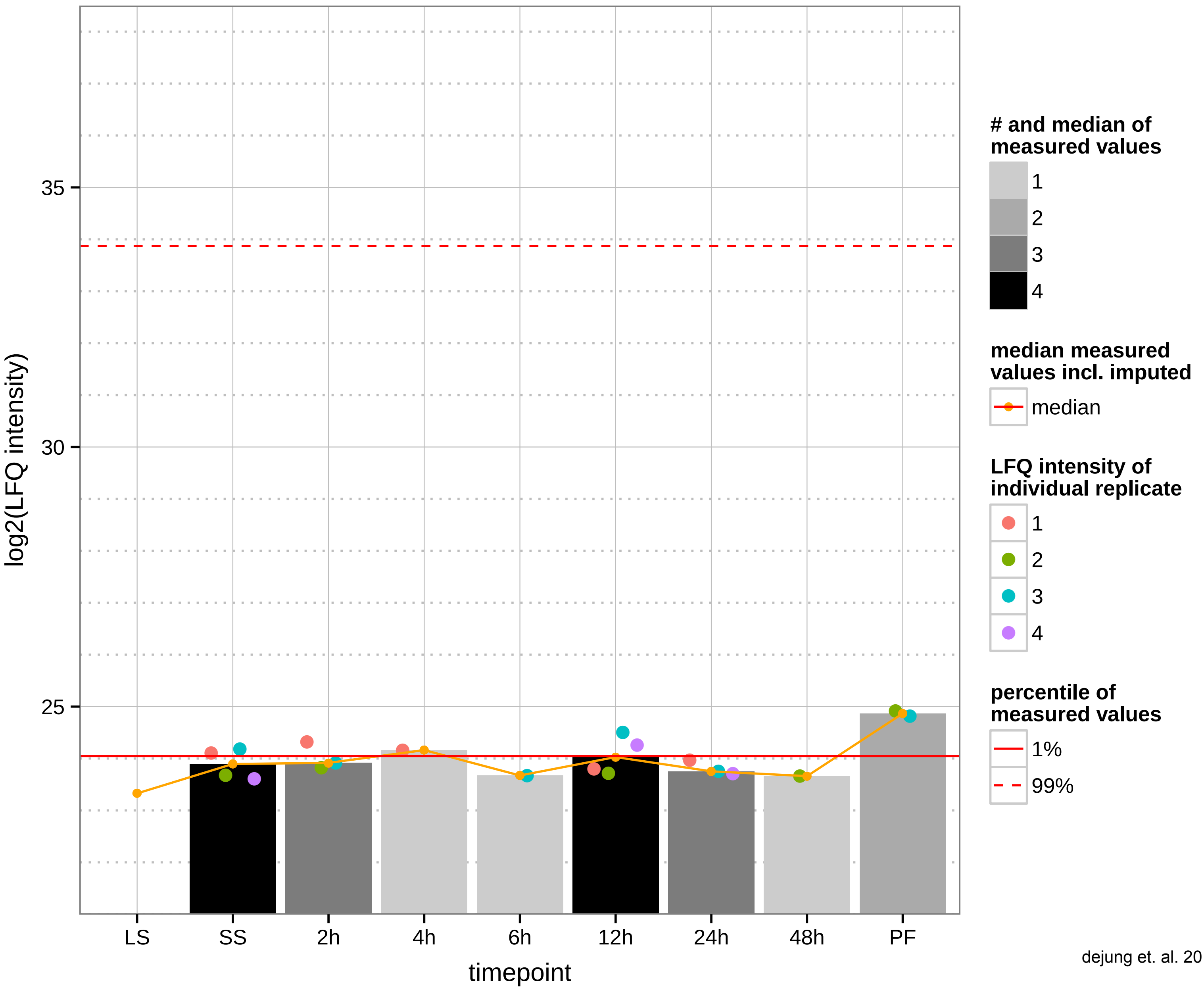

dejung et. al. 2015

hypothetical protein, conserved
Tb927.6.620
AGOF: null
AGOC: null
AGOP: null
PGOF: null
PGOC: null
PGOP: null

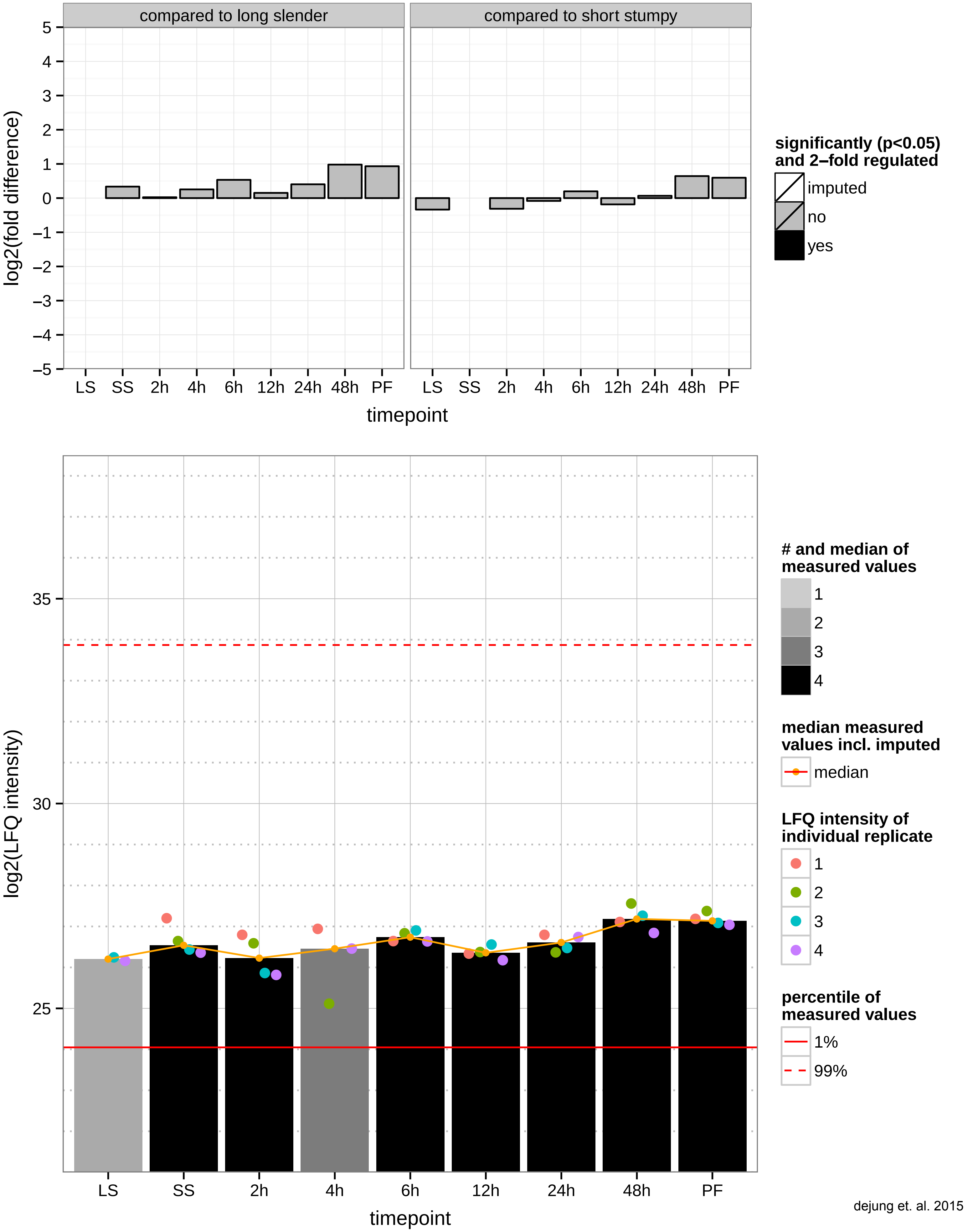

dejung et. al. 2015

kinetoplastid−specific phospho−protein phosphatase, putative
Tb927.6.640
AGOF: protein serine/threonine phosphatase activity
AGOC: null
AGOP: protein dephosphorylation
PGOF: hydrolase activity
PGOC: null
PGOP: null

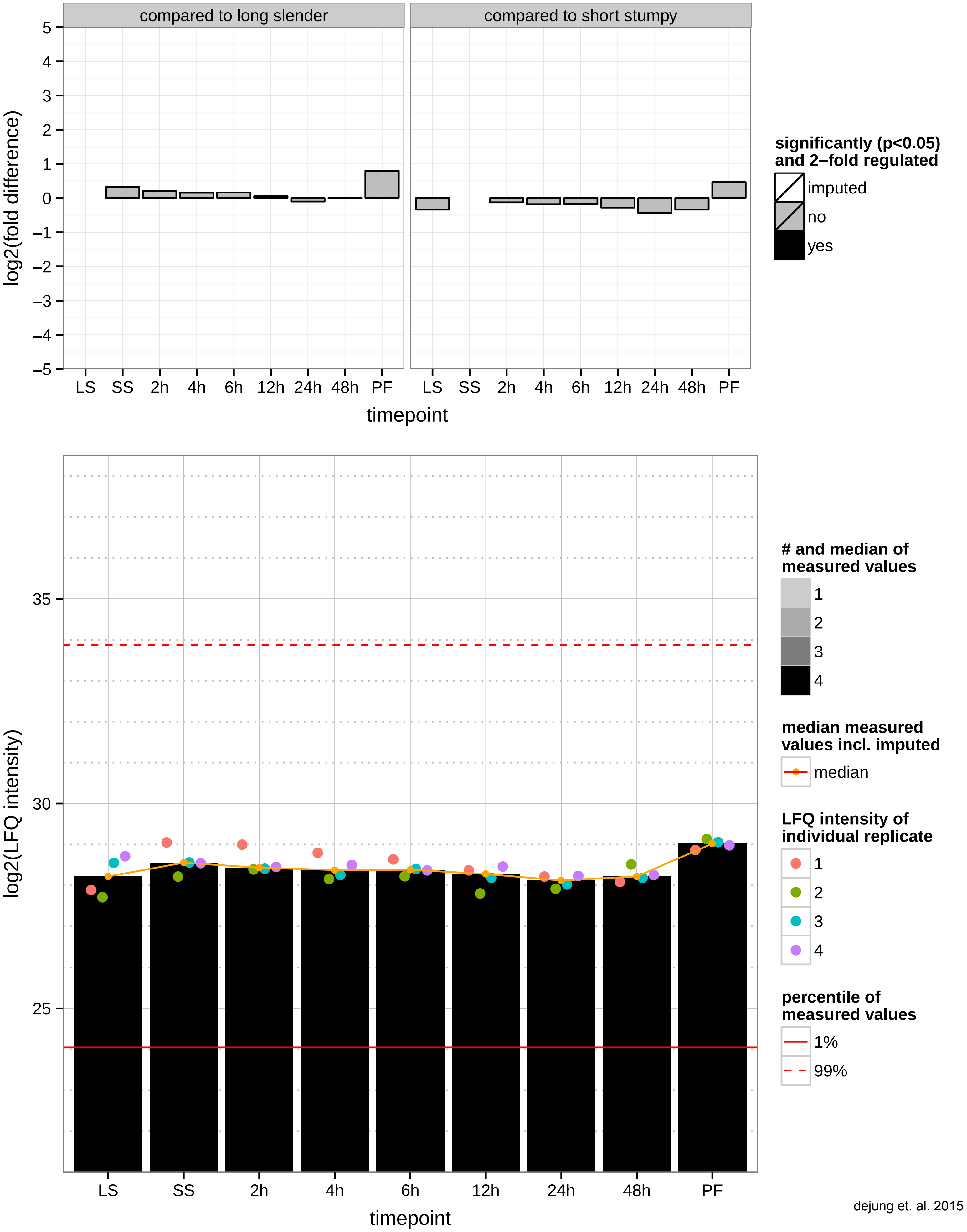

dejung et. al. 2015

ribosomal RNA processing protein 45, exosome complex exonuclease (RRP45)
Tb927.6.670
AGOF: 3'−5'−exoribonuclease activity, RNA binding
AGOC: null
AGOP: RNA processing
PGOF: null
PGOC: null
PGOP: null

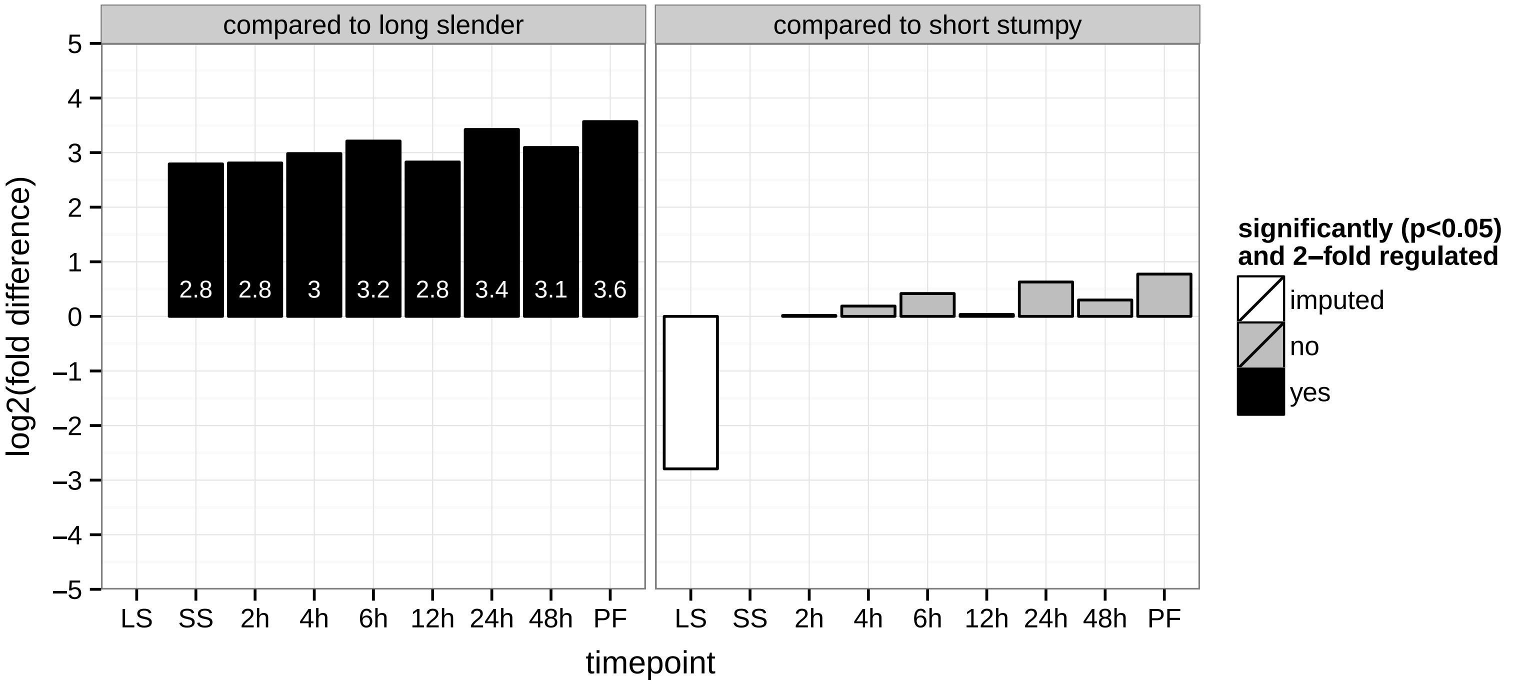

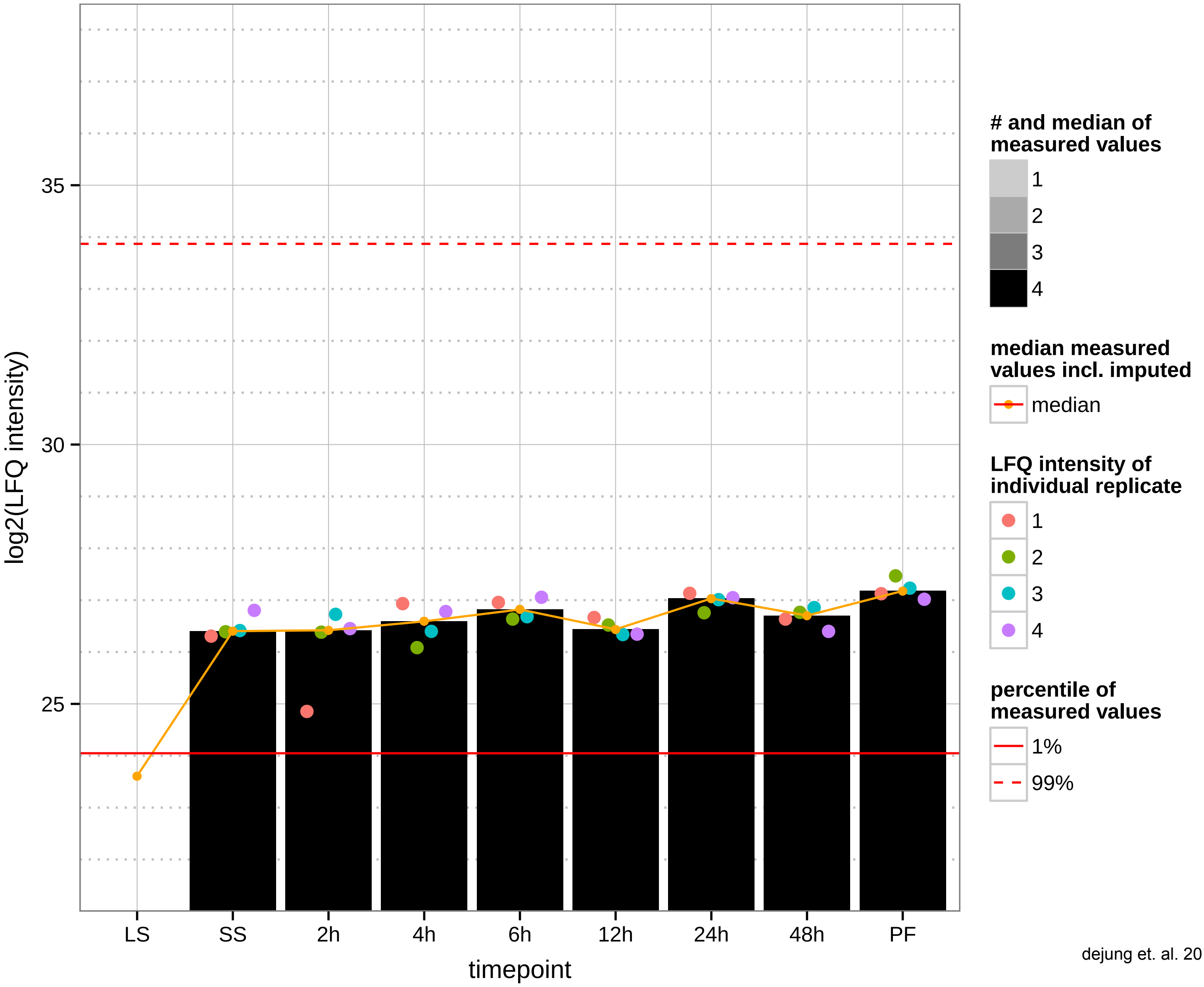

dejung et. al. 2015

alanyl–tRNA synthetase, putative
Tb927.6.700
AGOF: ATP binding, alanine–tRNA ligase activity, nucleic acid binding
AGOC: cytoplasm
AGOP: alanyl–tRNA aminoacylation, translation
PGOF: ATP binding, alanine–tRNA ligase activity, ligase activity, forming aminoacyl–tRNA and related compounds, nucleic ac
PGOC: cytoplasm
PGOP: alanyl–tRNA aminoacylation, tRNA aminoacylation

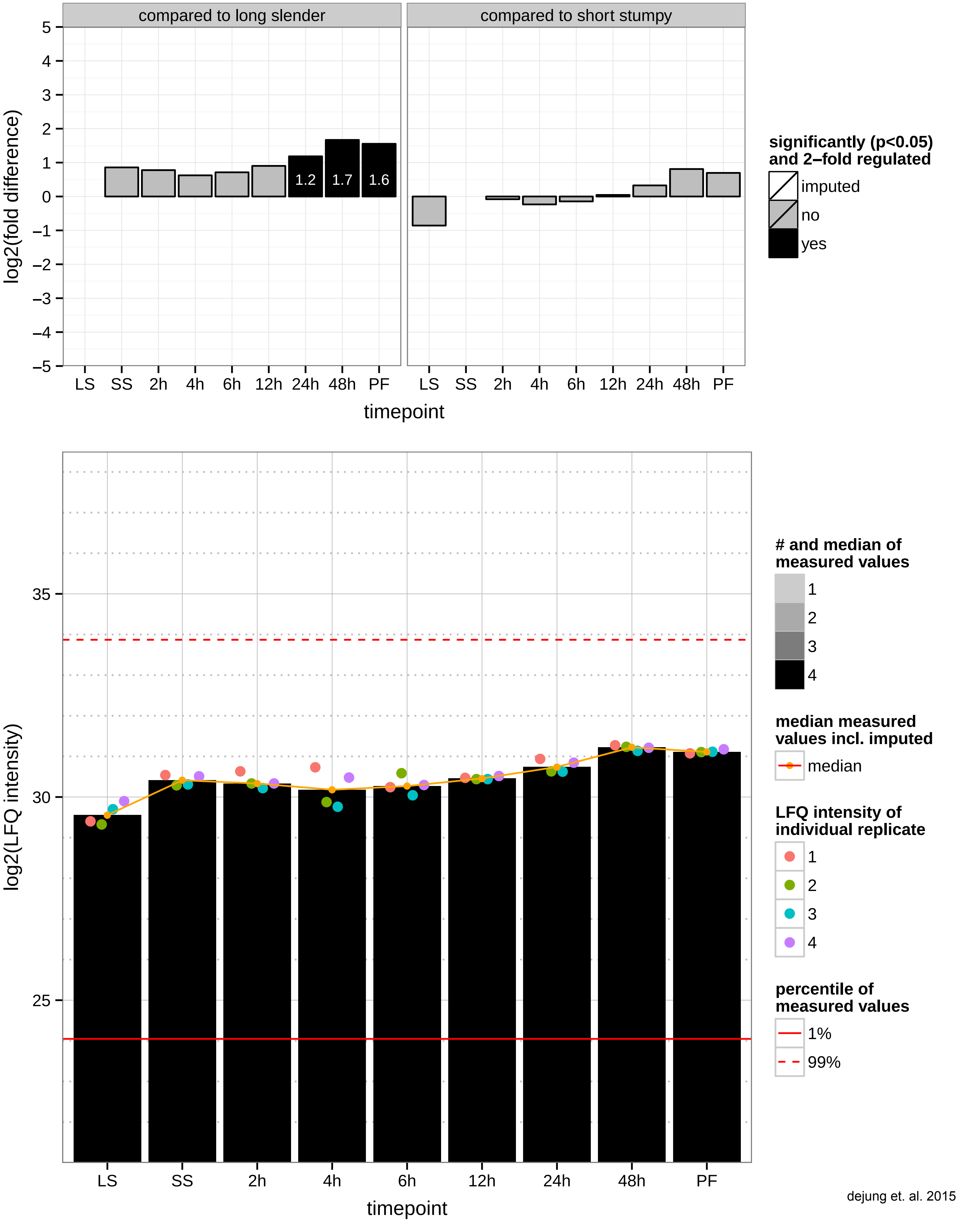

dejung et. al. 2015

60S ribosomal protein L14, putative, 40S ribosomal protein L14
Tb927.6.720;Tb927.11.11230
AGOF: structural constituent of ribosome
AGOC: intracellular, ribosome
AGOP: translation
PGOF: structural constituent of ribosome
PGOC: intracellular, ribosome
PGOP: translation

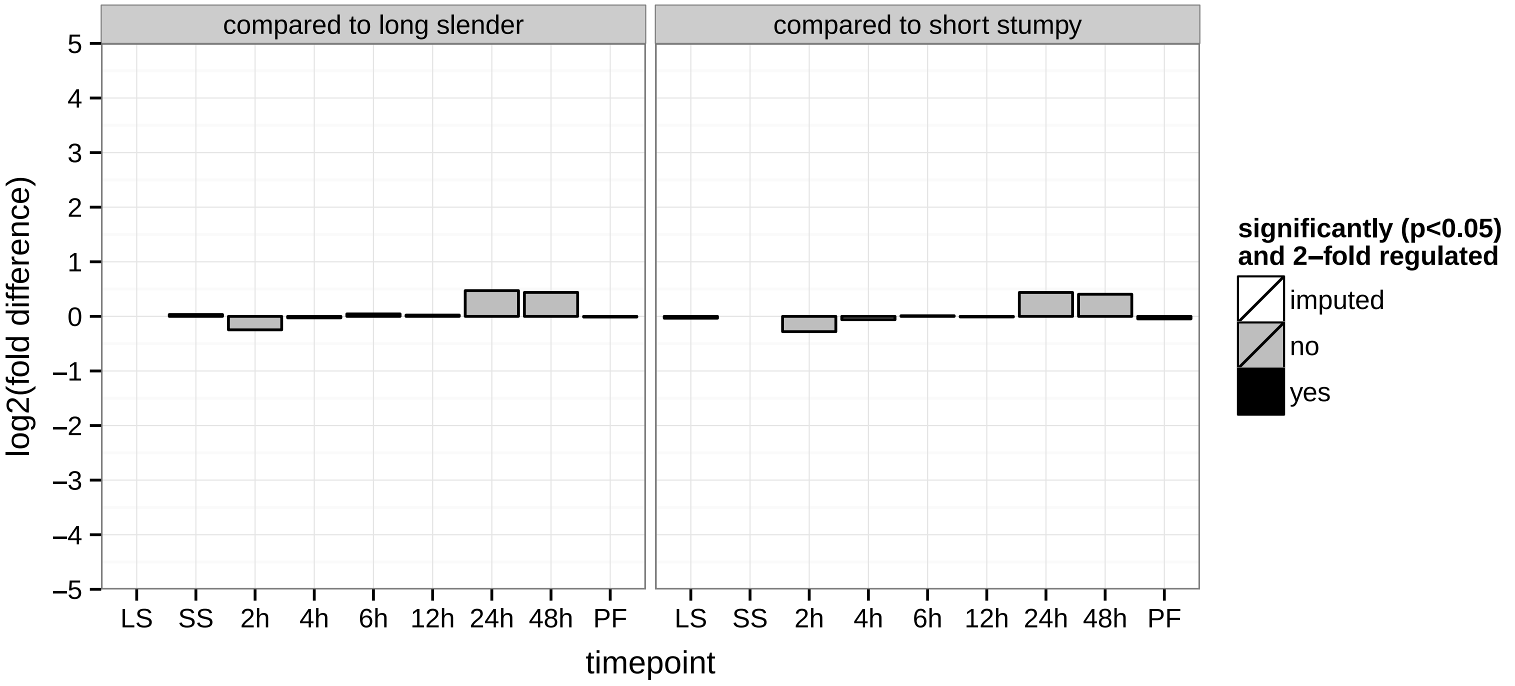

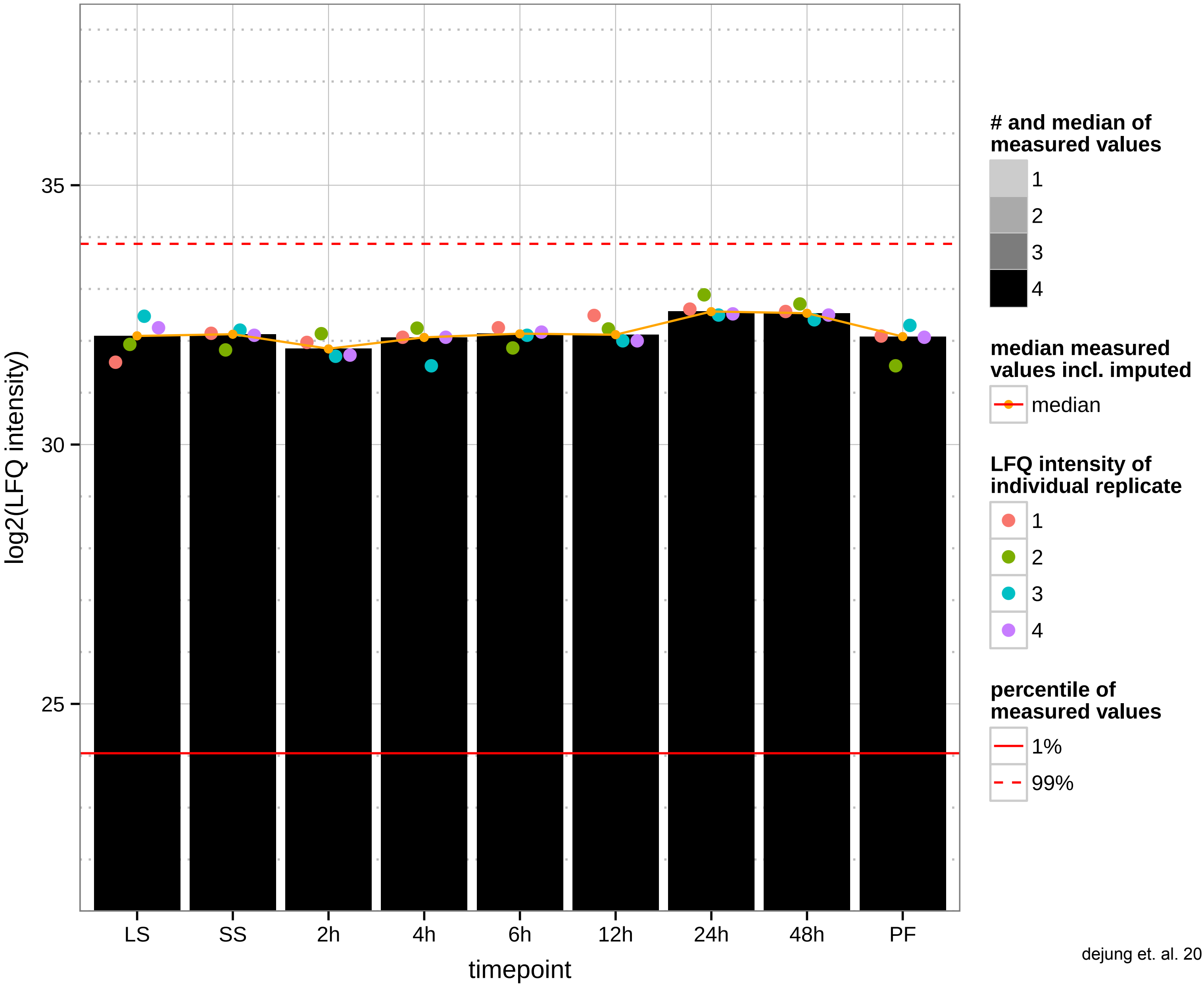

dejung et. al. 2015

ATP–dependent DEAH–box RNA helicase, putative
Tb927.6.740
AGOF: ATP binding, ATP–dependent helicase activity, helicase activity, nucleic acid binding
AGOC: null
AGOP: null
PGOF: ATP binding, ATP–dependent helicase activity, helicase activity, nucleic acid binding
PGOC: null
PGOP: null

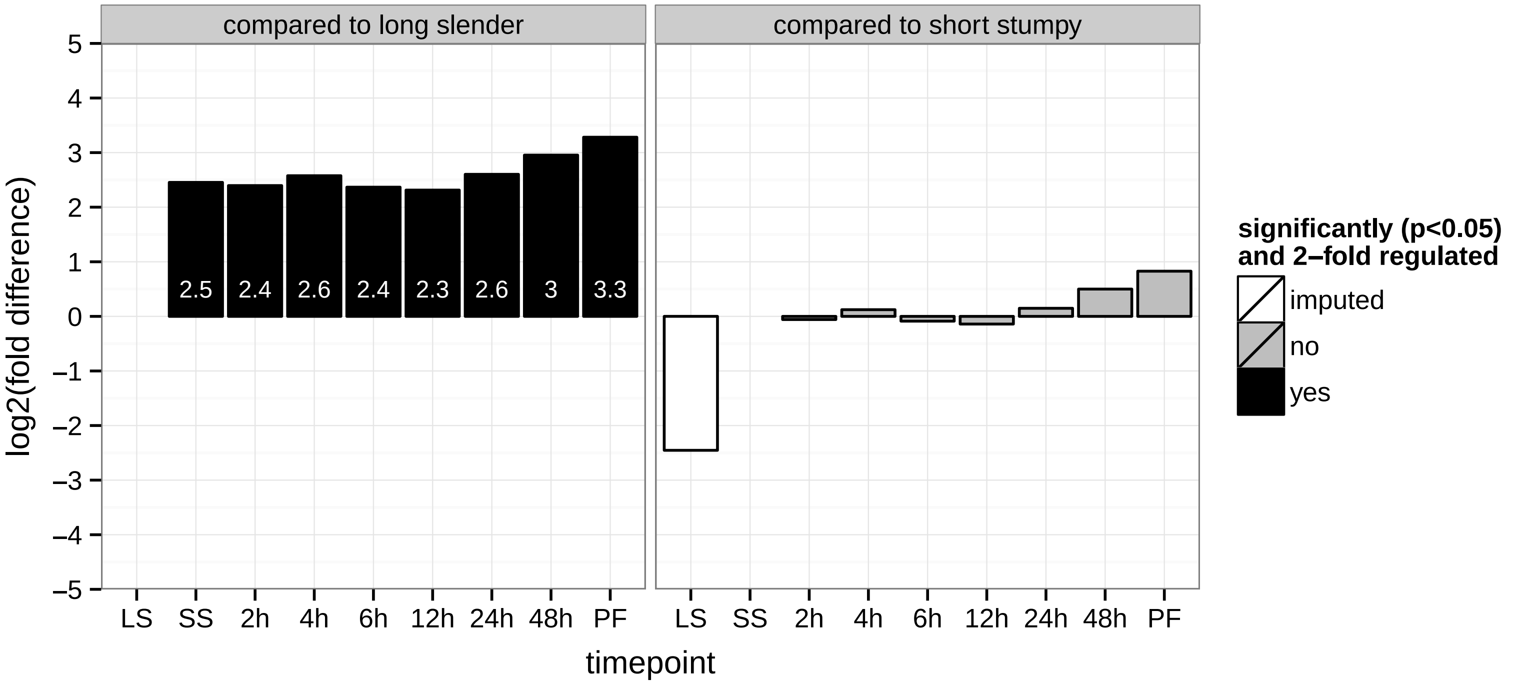

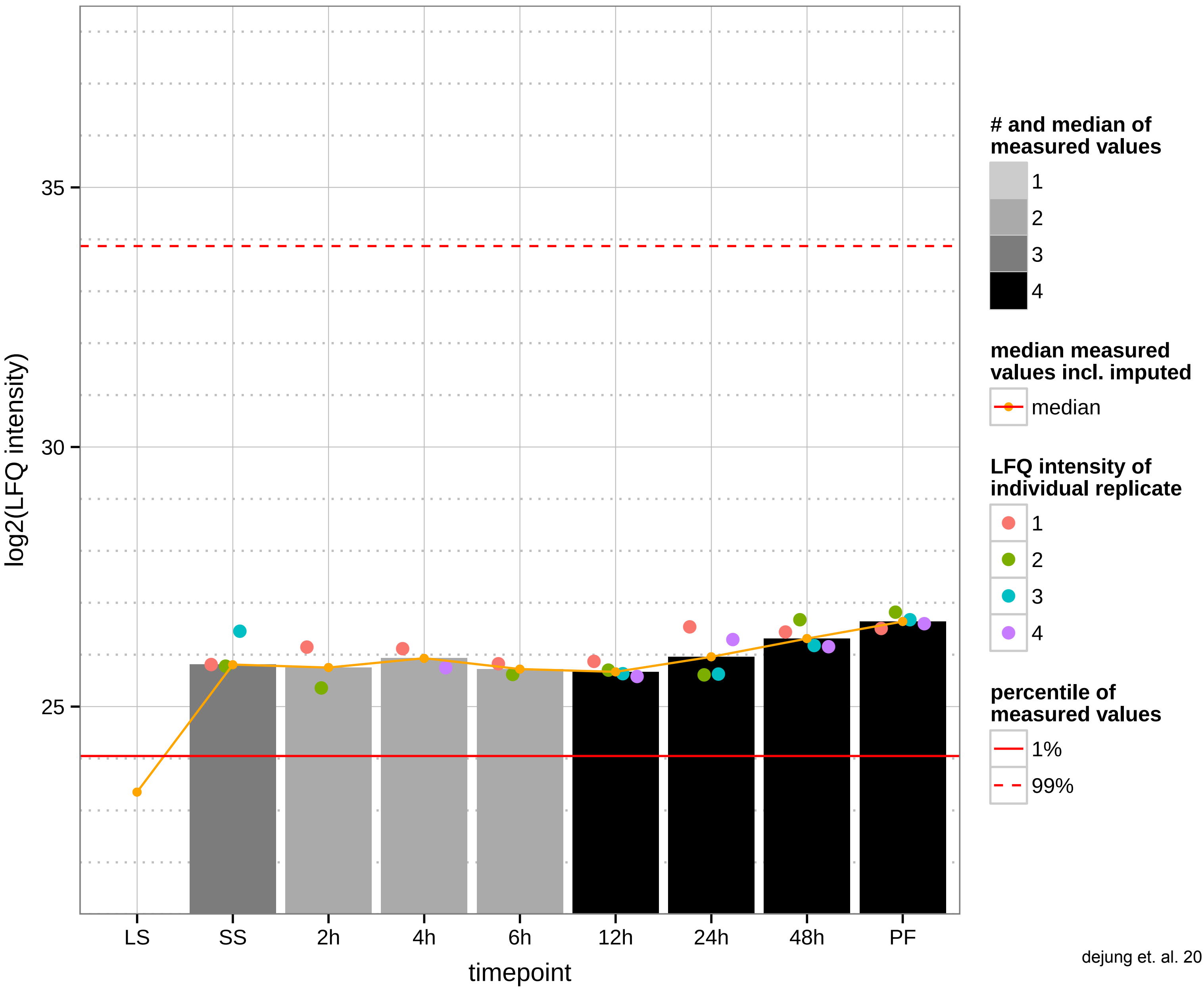

dejung et. al. 2015

kinetoplastid−specific phospho−protein phosphatase, putative
Tb927.6.750
AGOF: protein serine/threonine phosphatase activity
AGOC: null
AGOP: protein dephosphorylation
PGOF: hydrolase activity
PGOC: null
PGOP: null

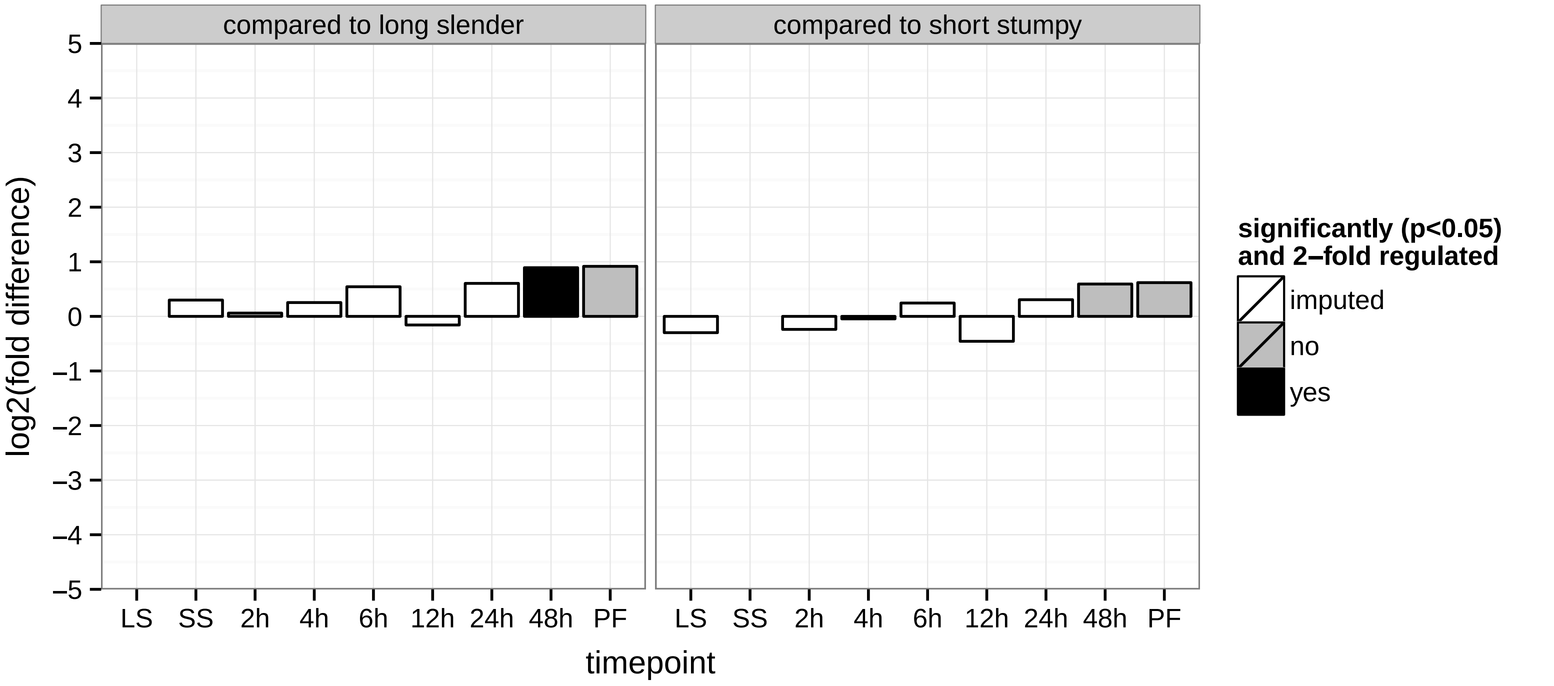

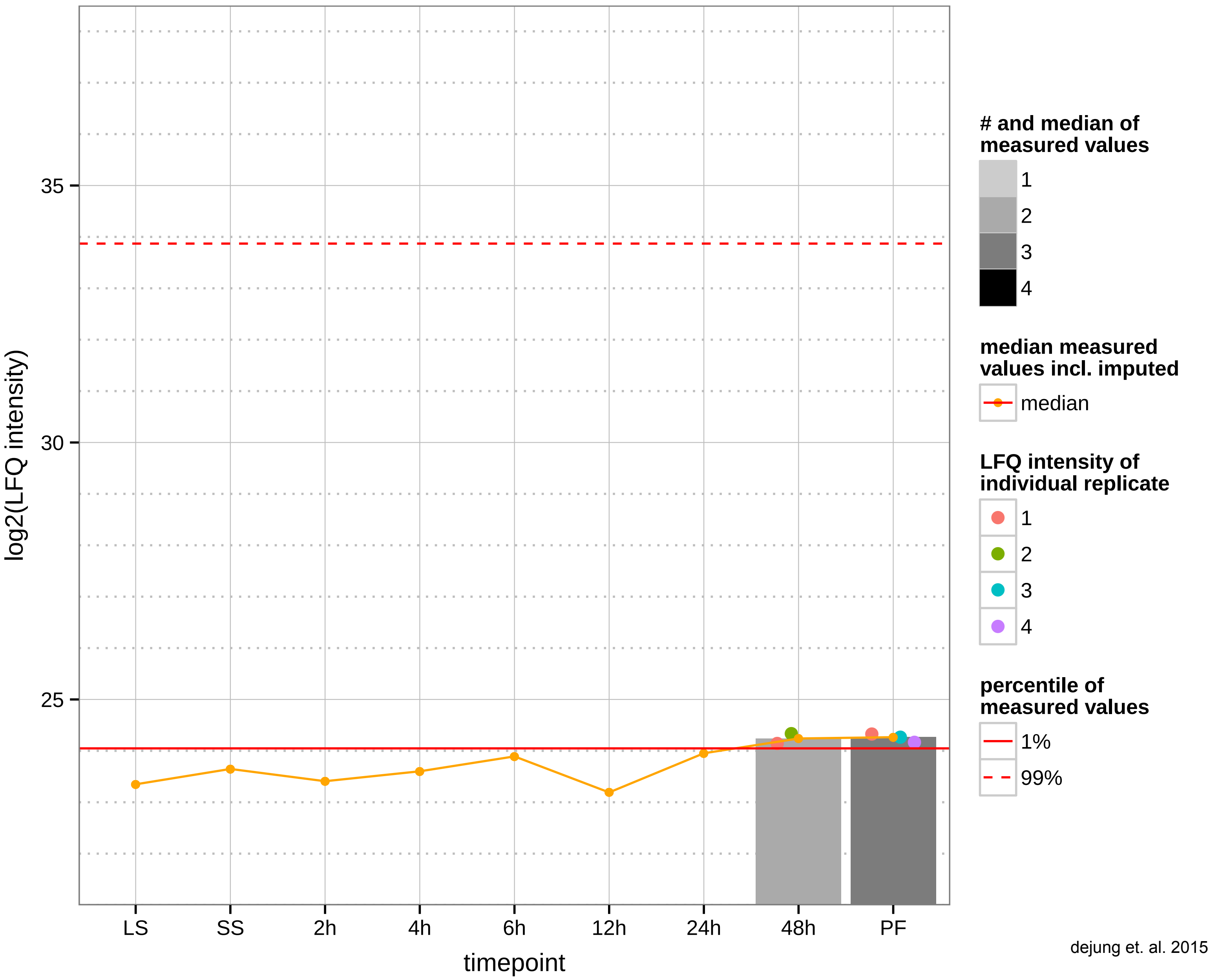

dejung et. al. 2015

hypothetical protein, conserved, ccr4–not transcription complex subunit 2 (NOT2)
Tb927.6.850;Tb11.v5.0689
AGOF: null, RNA polymerase II transcription cofactor activity
AGOC: null, nucleus
AGOP: null, negative regulation of transcription from RNA polymerase II promoter
PGOF: null
PGOC: nucleus
PGOP: regulation of transcription, DNA–dependent

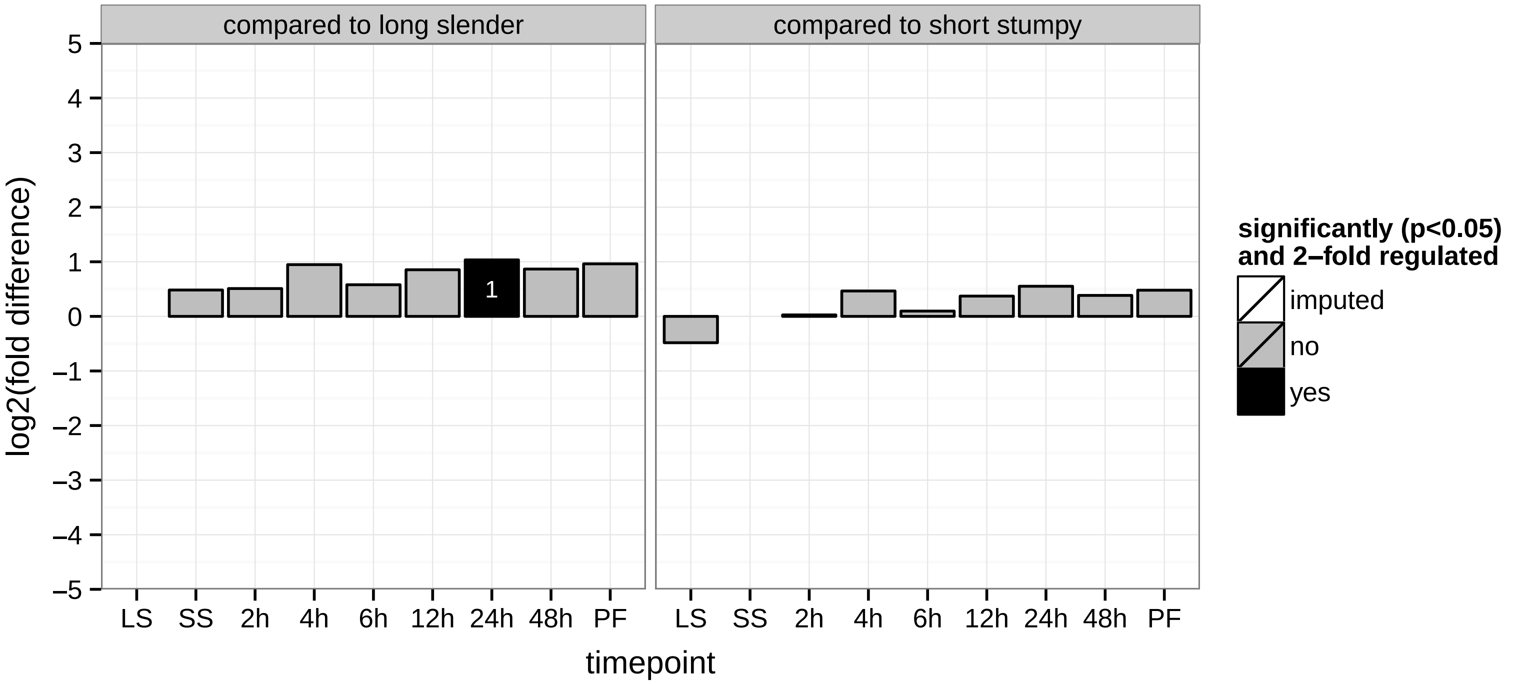

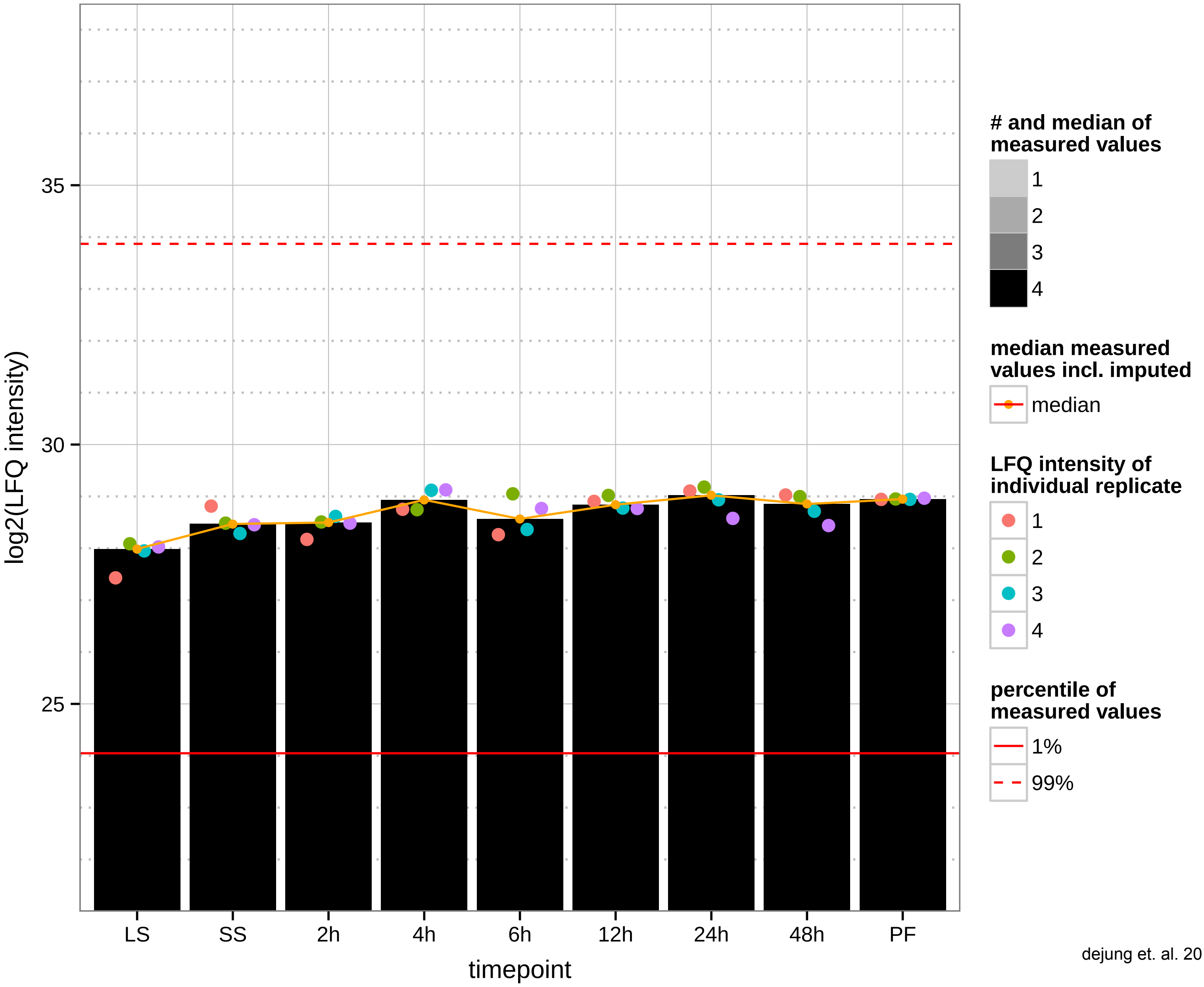

dejung et. al. 2015

hypothetical protein, conserved
Tb927.7.1010
AGOF: null
AGOC: mitochondrion
AGOP: null
PGOF: null
PGOC: null
PGOP: null

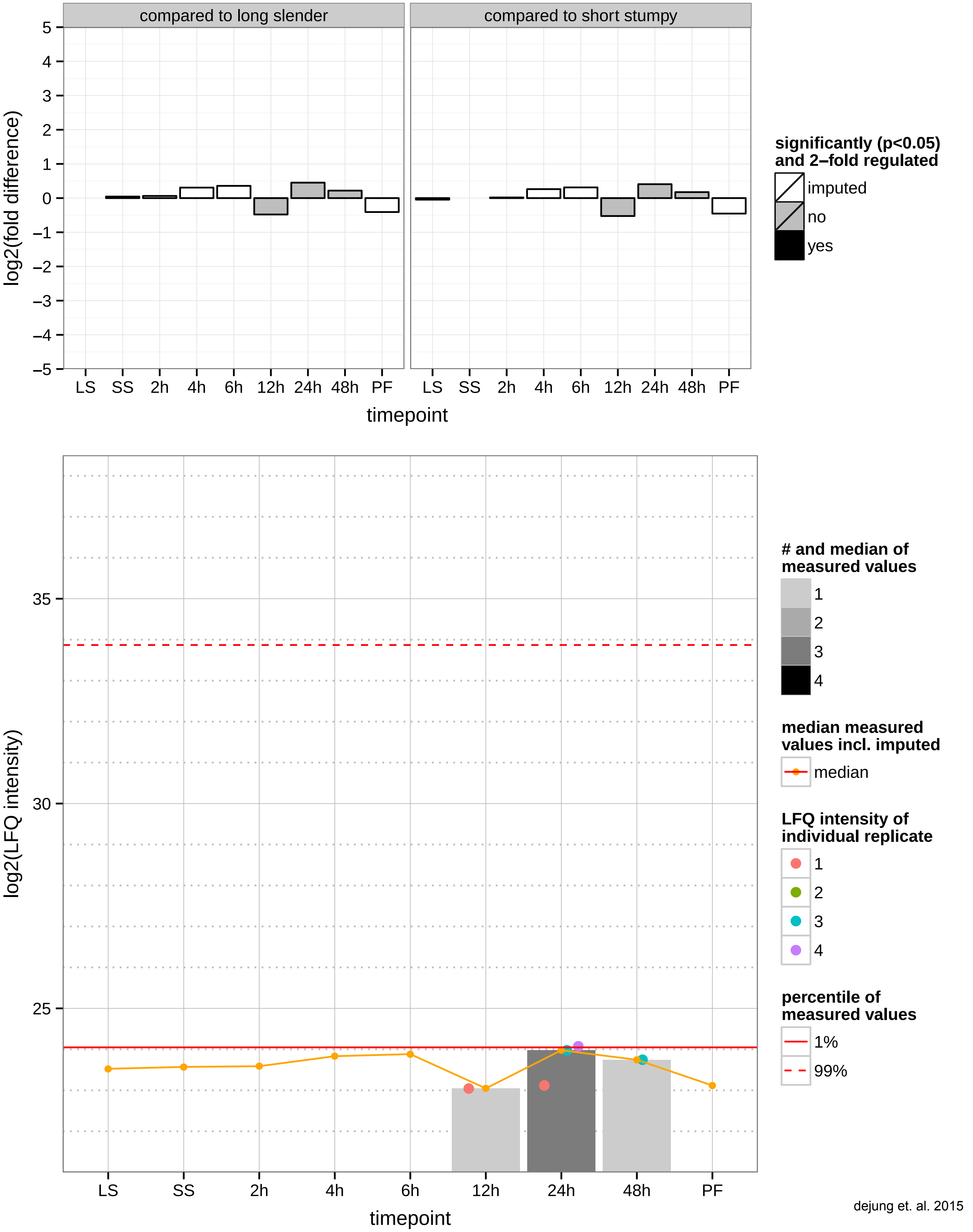

dejung et. al. 2015

40S ribosomal protein S16, putative
Tb927.7.1050;Tb927.7.1040
AGOF: structural constituent of ribosome
AGOC: intracellular, ribosome
AGOP: translation
PGOF: structural constituent of ribosome, null
PGOC: intracellular, ribosome, null
PGOP: translation, null

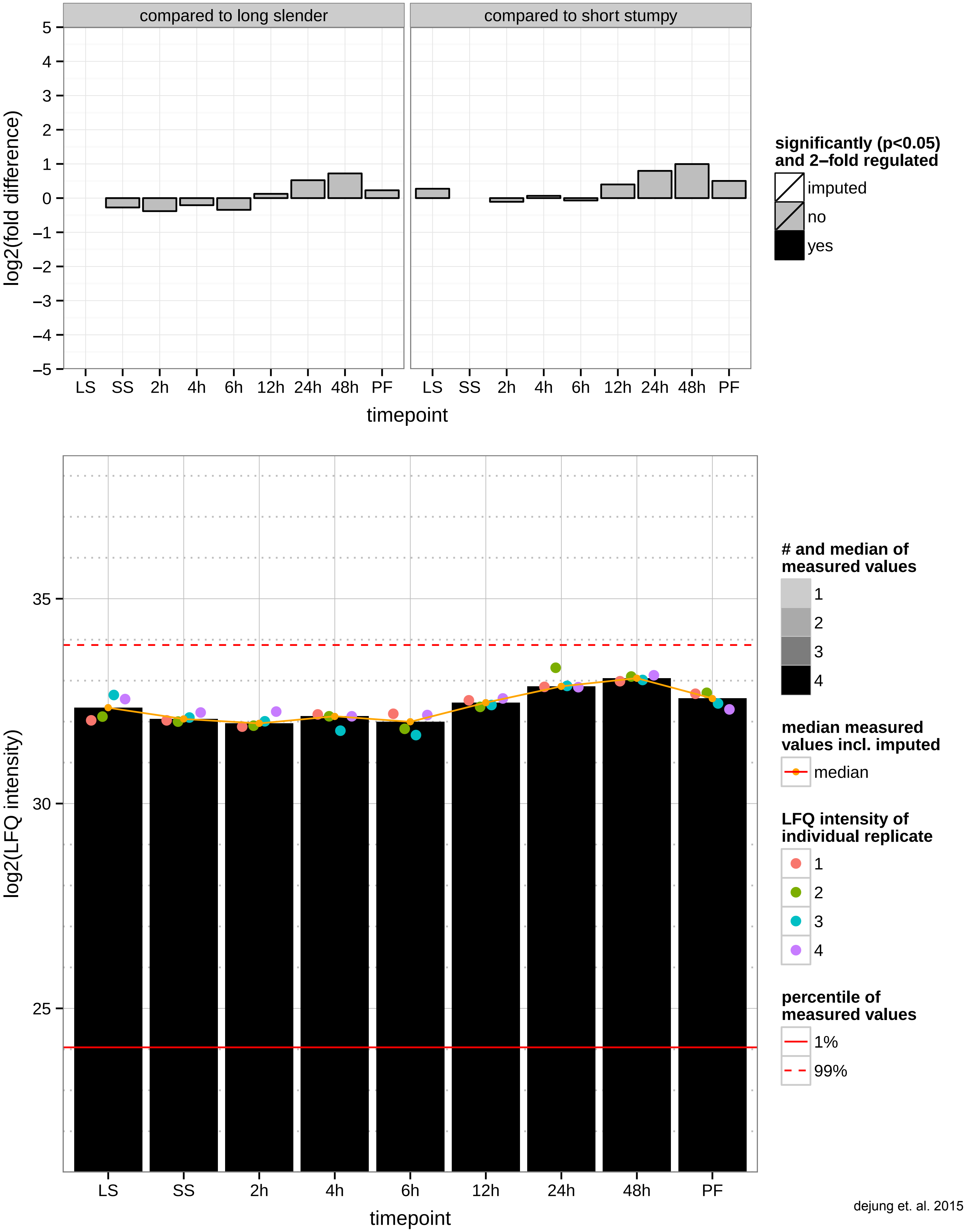

dejung et. al. 2015

hypothetical protein, conserved
Tb927.7.1060
AGOF: null
AGOC: null
AGOP: null
PGOF: null
PGOC: nucleus
PGOP: null

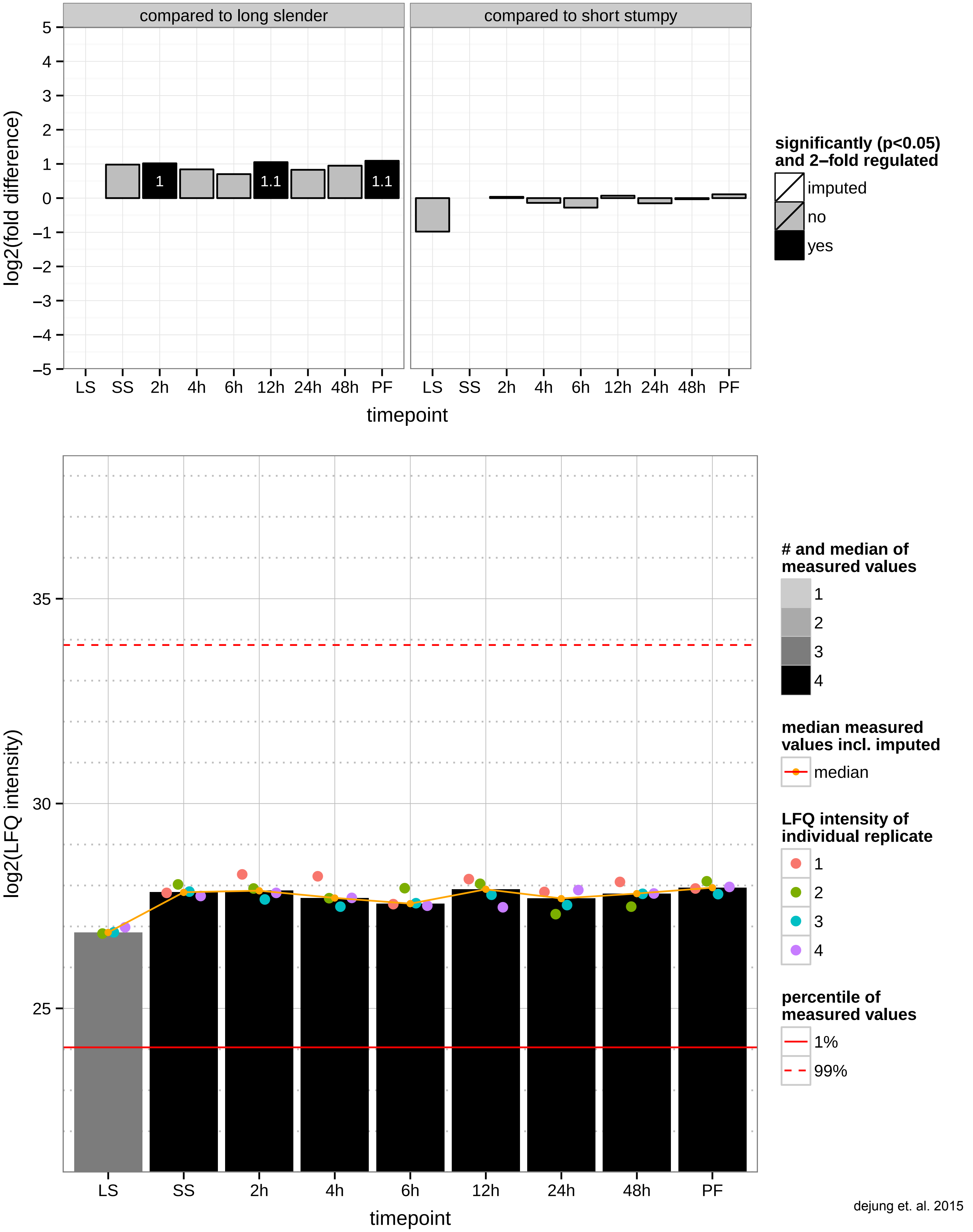

dejung et. al. 2015

hypothetical protein, conserved
Tb927.7.1080
AGOF: null
AGOC: mitochondrion
AGOP: null
PGOF: null
PGOC: null
PGOP: null

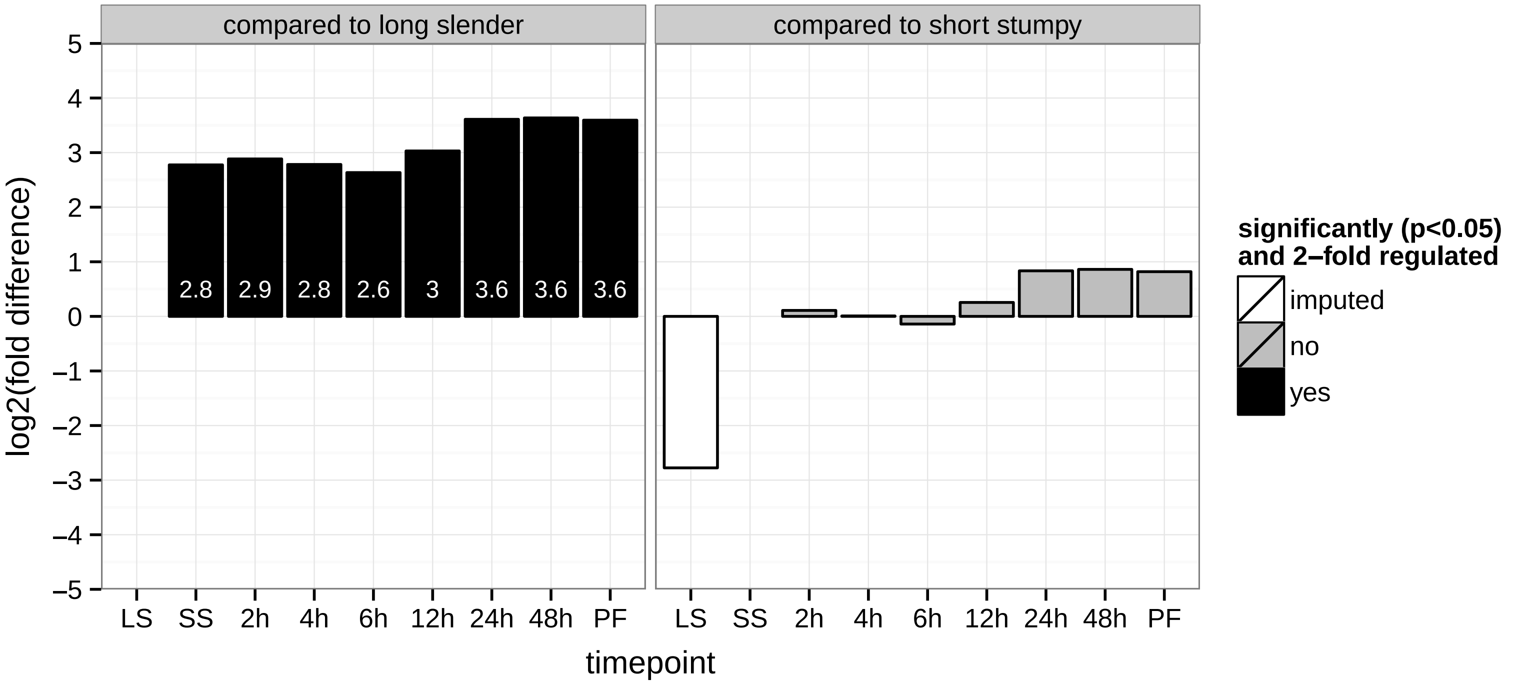

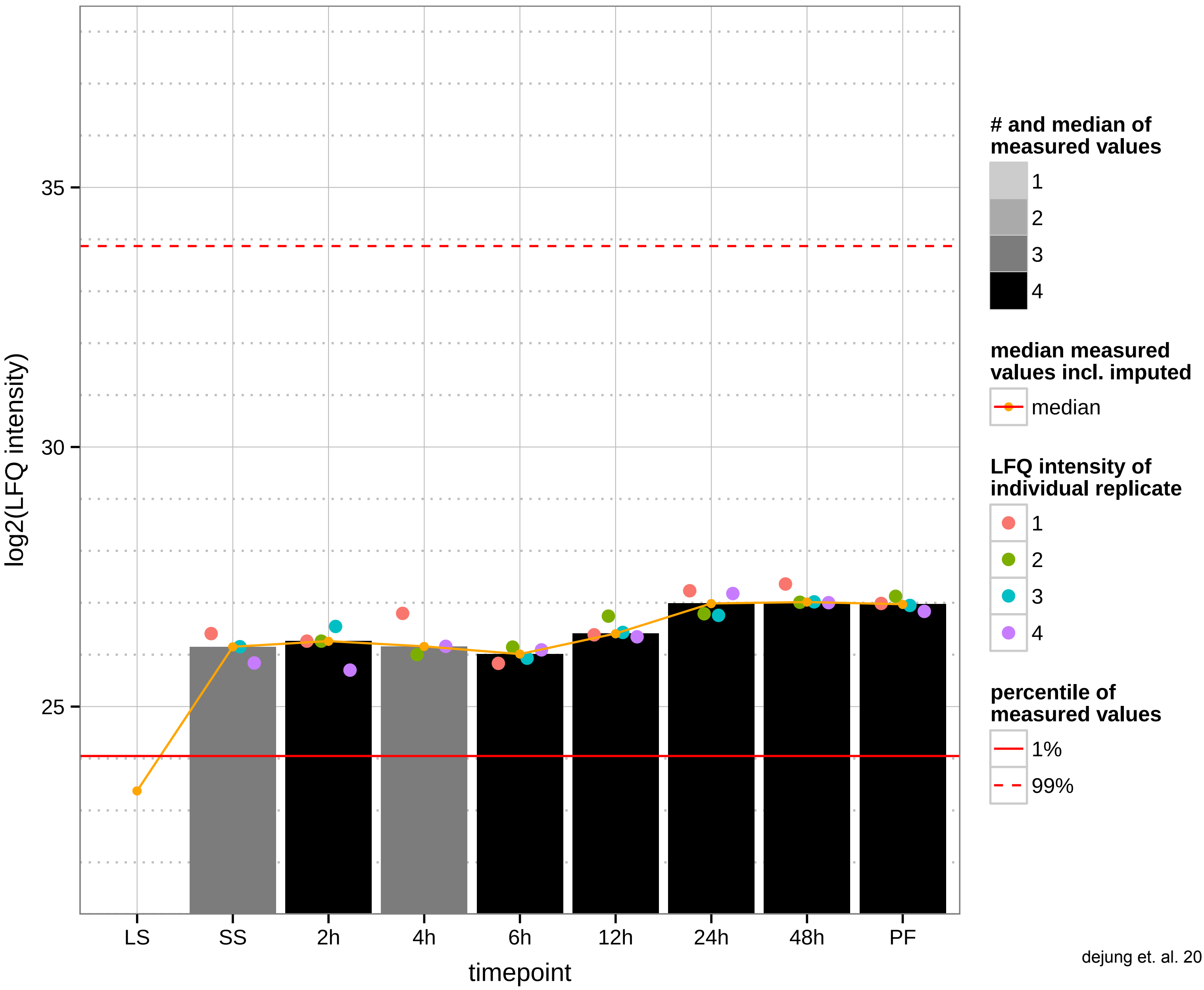

dejung et. al. 2015

hypothetical protein, conserved
Tb927.7.1100
AGOF: null
AGOC: null
AGOP: null
PGOF: null
PGOC: null
PGOP: null

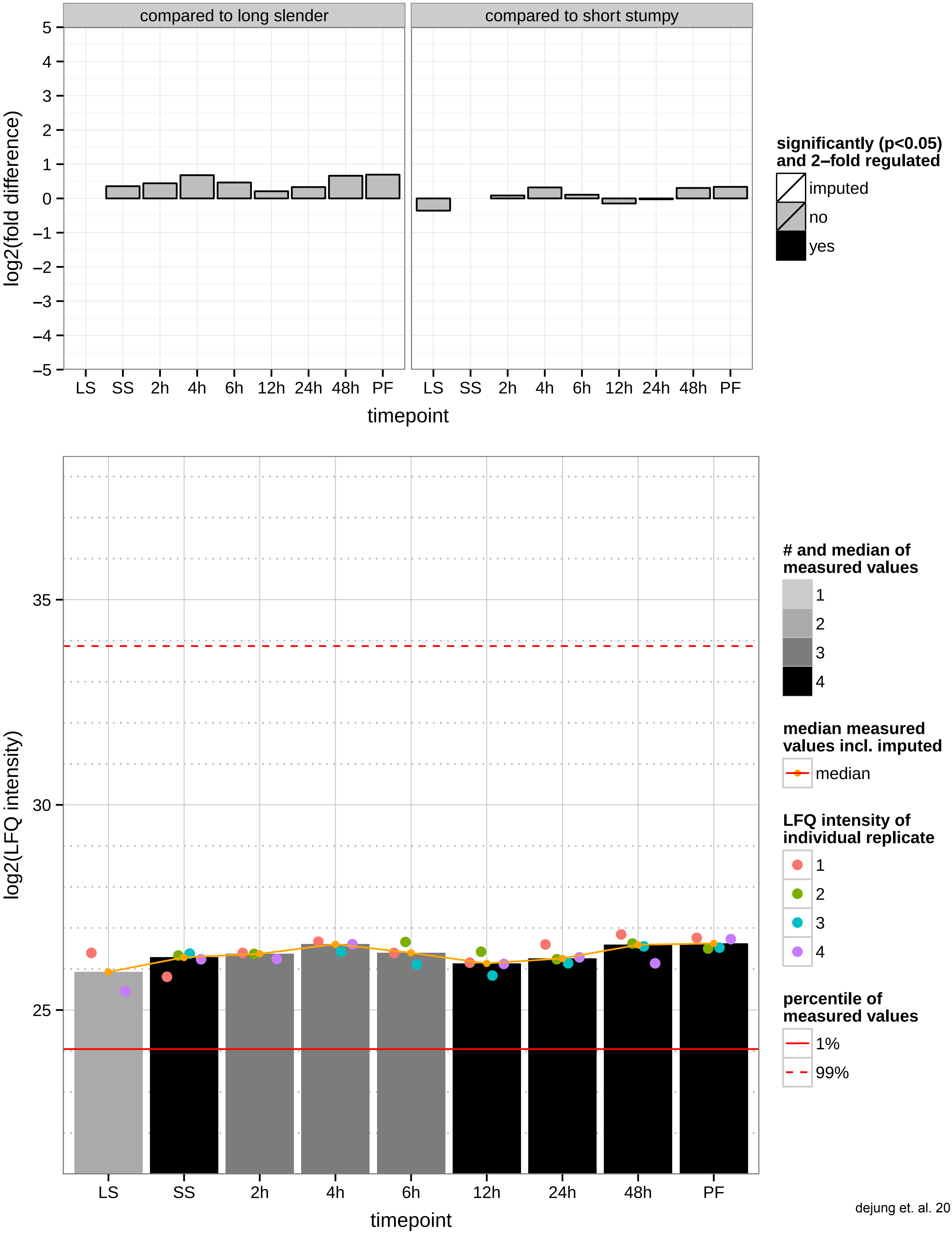

dejung et. al. 2015

trypanothione/tryparedoxin dependent peroxidase 2, glutathione peroxidase−like 2 (TDPX2)
Tb927.7.1130
AGOF: glutathione peroxidase activity
AGOC: null
AGOP: response to oxidative stress
PGOF: glutathione peroxidase activity
PGOC: null
PGOP: oxidation−reduction process, response to oxidative stress

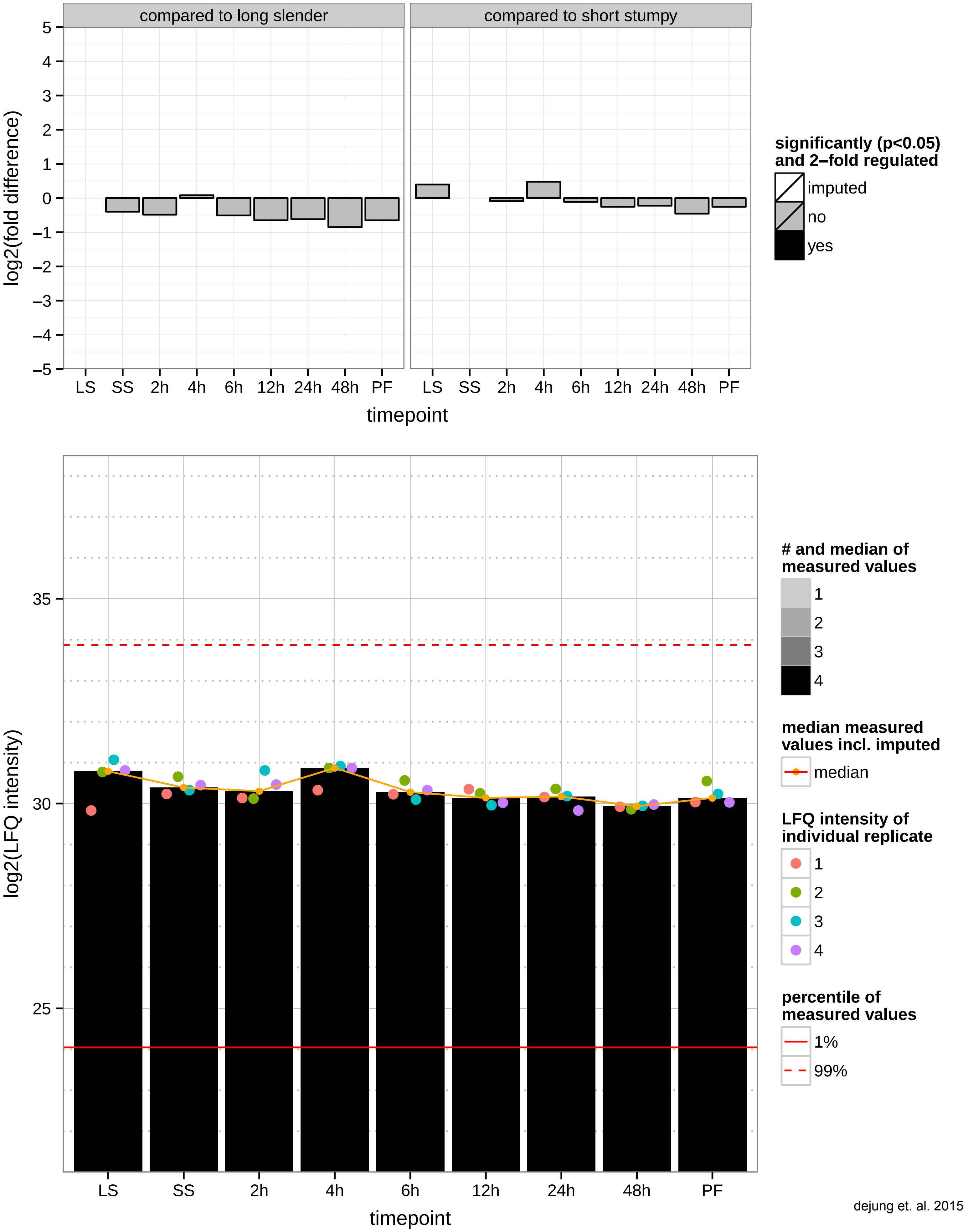

dejung et. al. 2015

regulator of chromosome condensation, putative
Tb927.7.1210
AGOF: null
AGOC: null
AGOP: null
PGOF: null
PGOC: null
PGOP: null

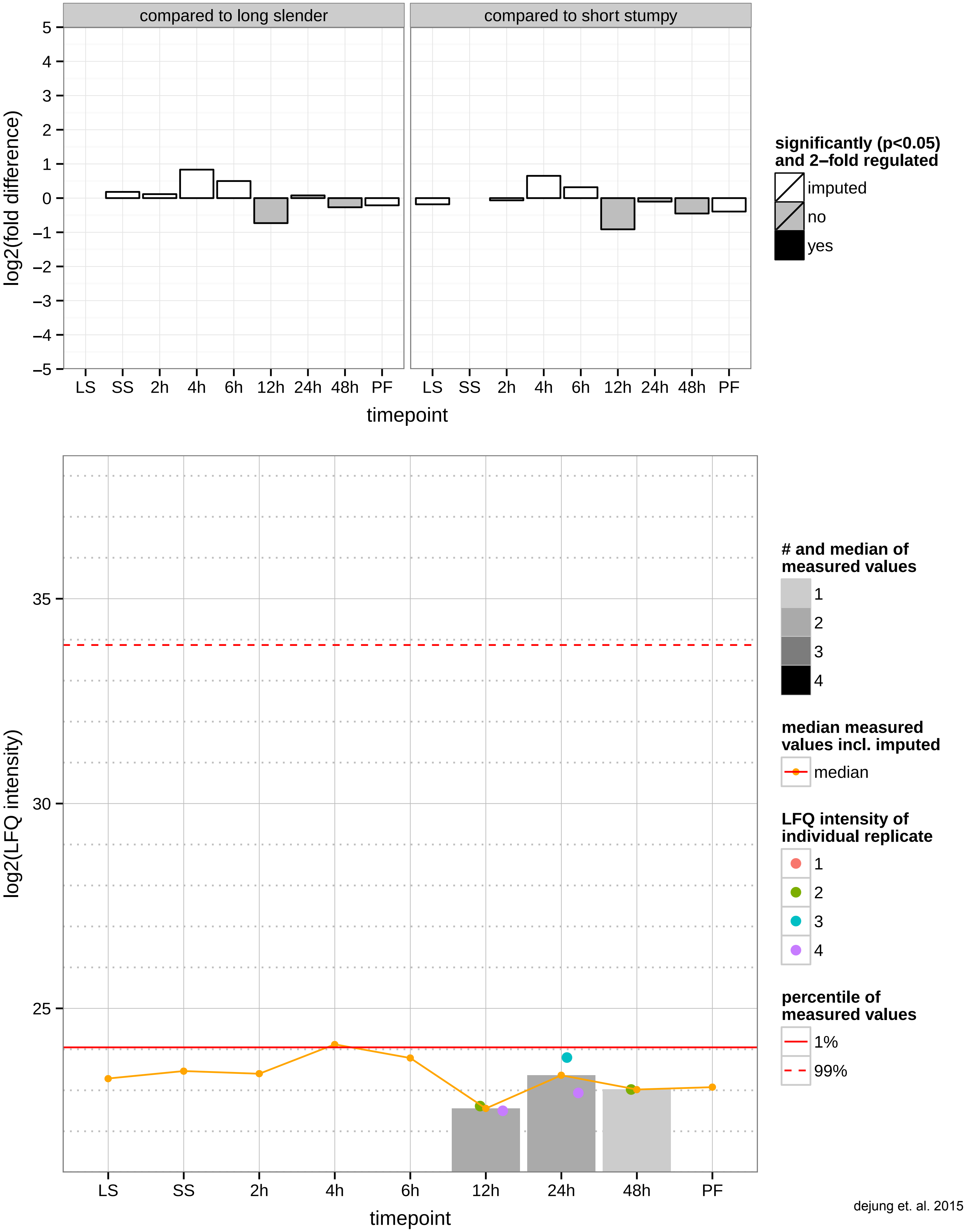

dejung et. al. 2015

sphingosine kinase A, B, putative
Tb927.7.1240
AGOF: diacylglycerol kinase activity, sphinganine kinase activity
AGOC: cytosol
AGOP: protein kinase C–activating G–protein coupled receptor signaling pathway, sphinganine metabolic process
PGOF: NAD+ kinase activity, diacylglycerol kinase activity
PGOC: null
PGOP: metabolic process, protein kinase C–activating G–protein coupled receptor signaling pathway

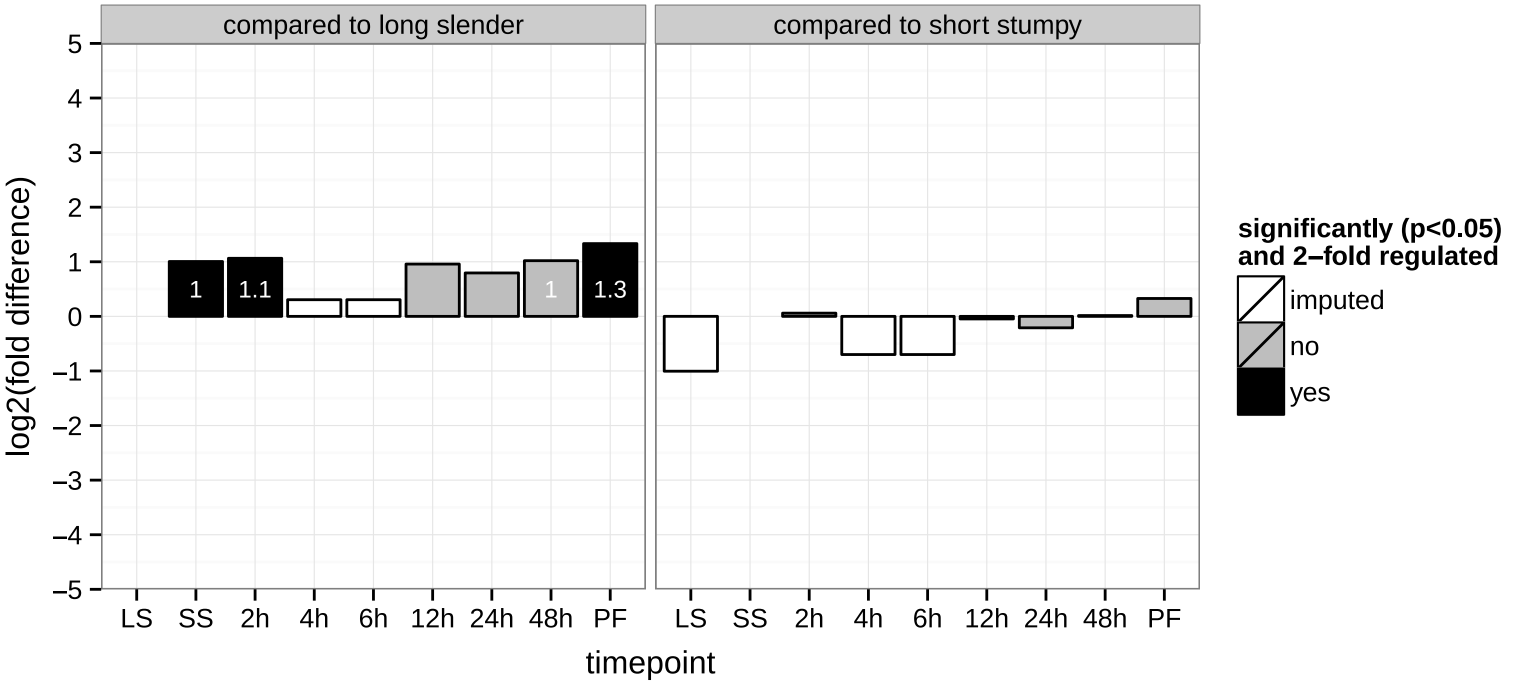

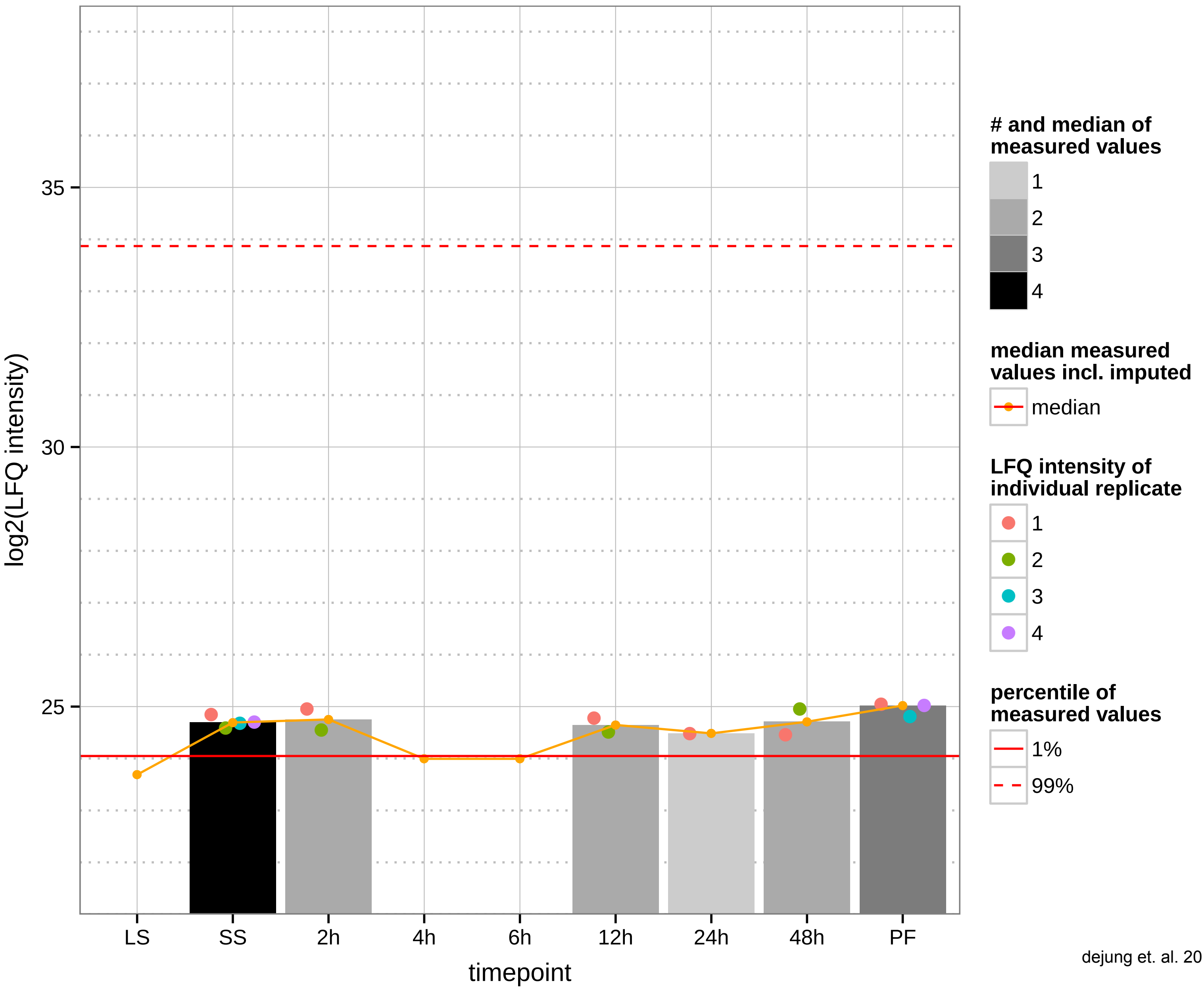

dejung et. al. 2015

hypothetical protein, conserved
Tb927.7.1290
AGOF: null
AGOC: null
AGOP: null
PGOF: carbohydrate binding
PGOC: null
PGOP: null

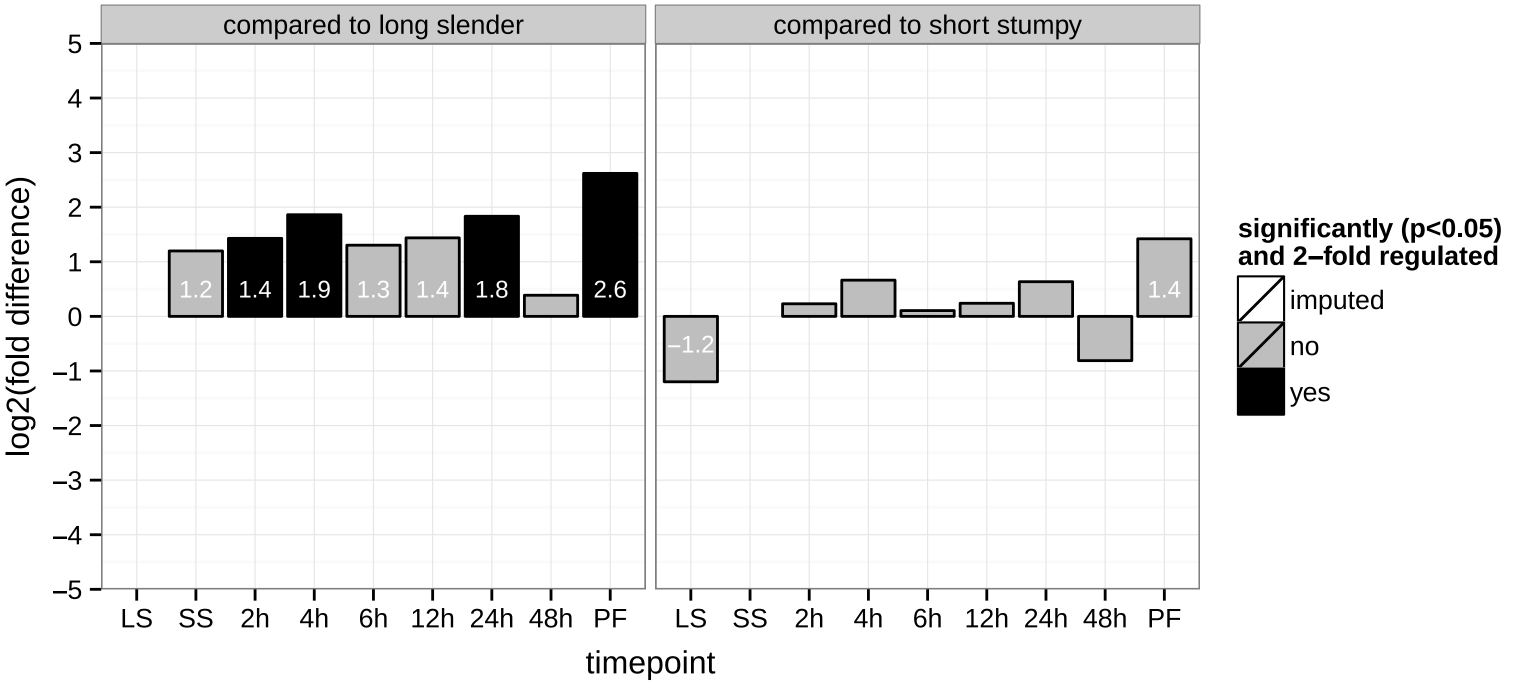

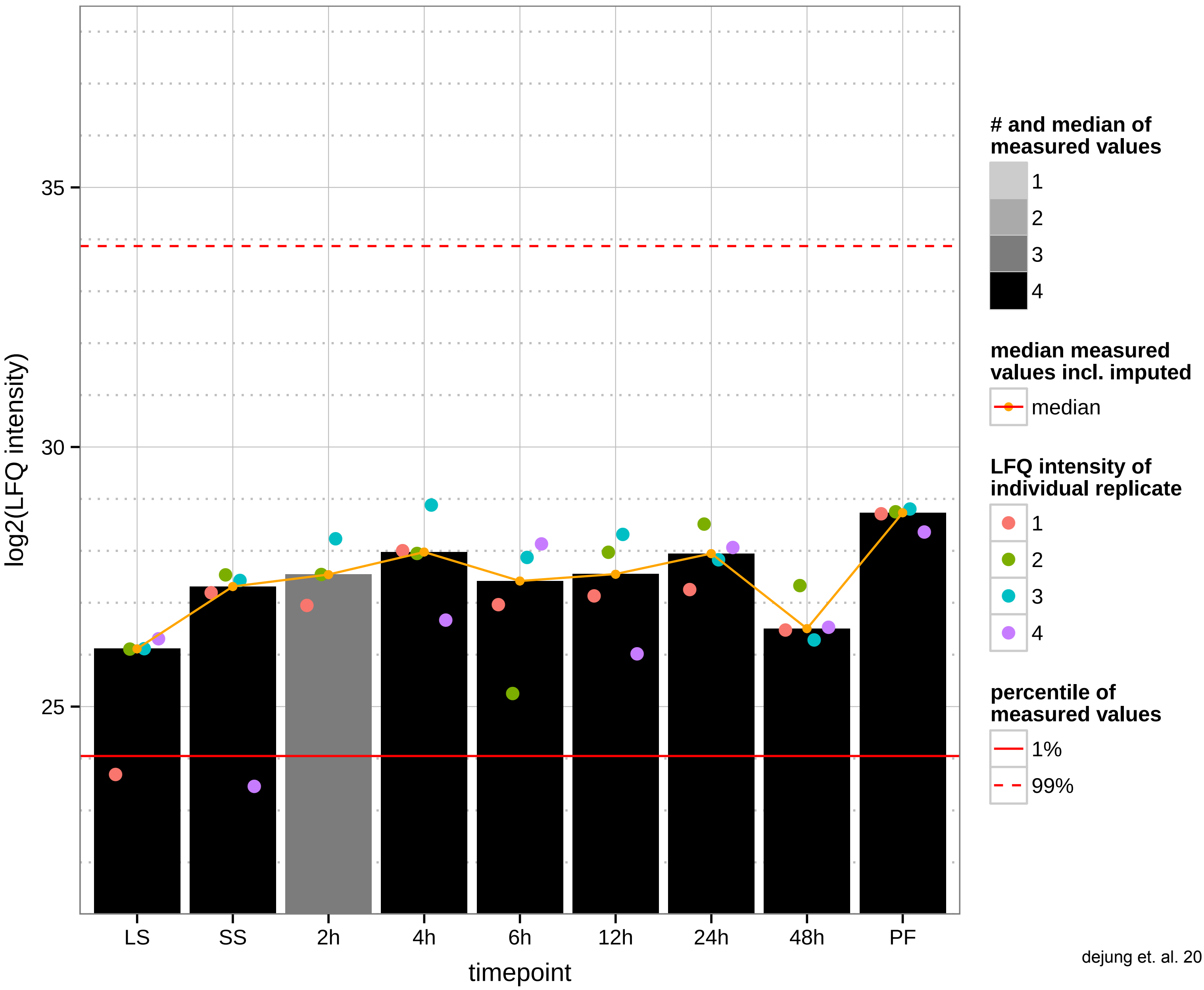

dejung et. al. 2015

protein disulfide isomerase, putative
Tb927.7.1300
AGOF: isomerase activity
AGOC: endoplasmic reticulum
AGOP: cell redox homeostasis
PGOF: isomerase activity
PGOC: endoplasmic reticulum
PGOP: cell redox homeostasis

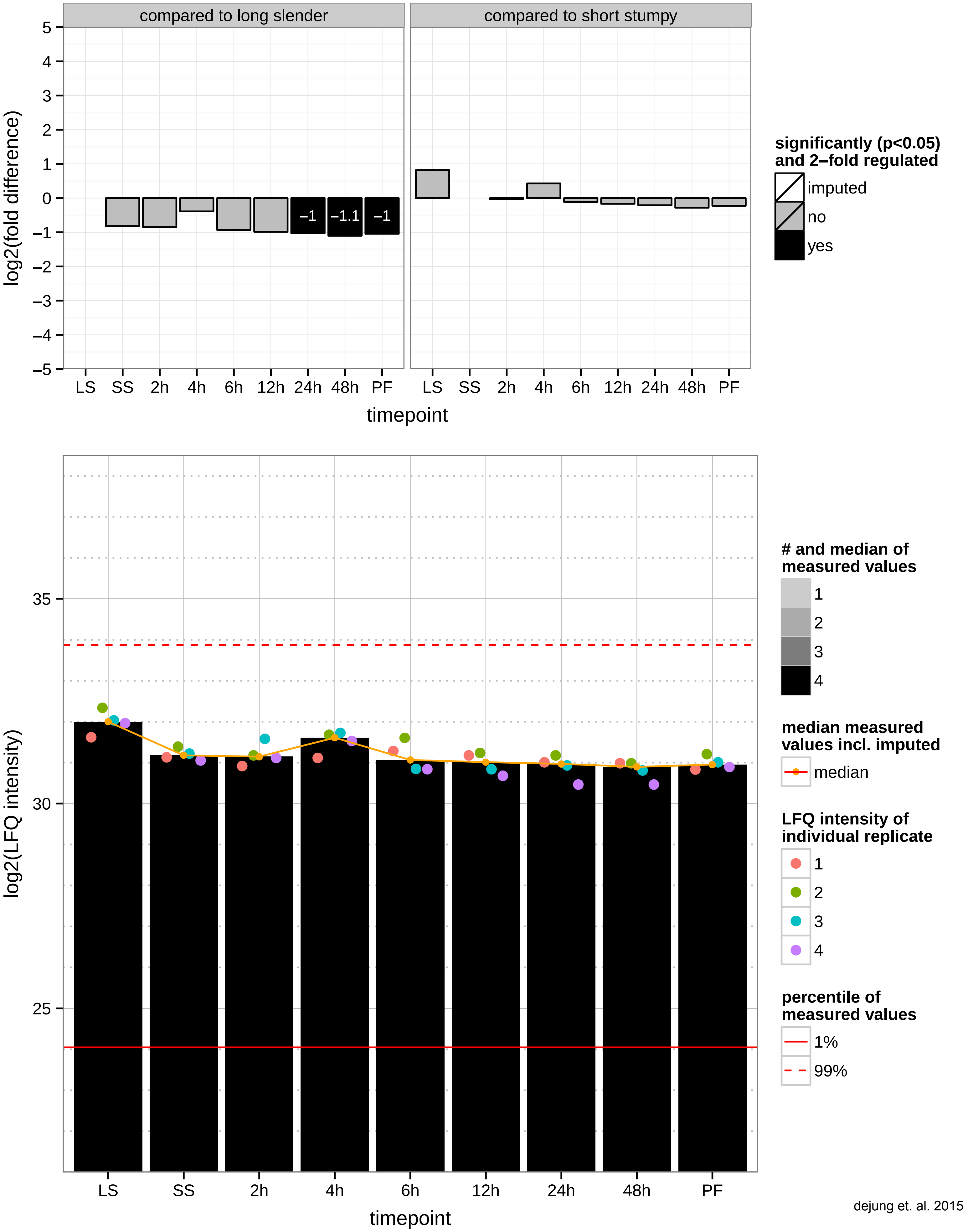

dejung et. al. 2015

hypothetical protein, conserved
Tb927.7.1310
AGOF: null
AGOC: null
AGOP: null
PGOF: null
PGOC: null
PGOP: null

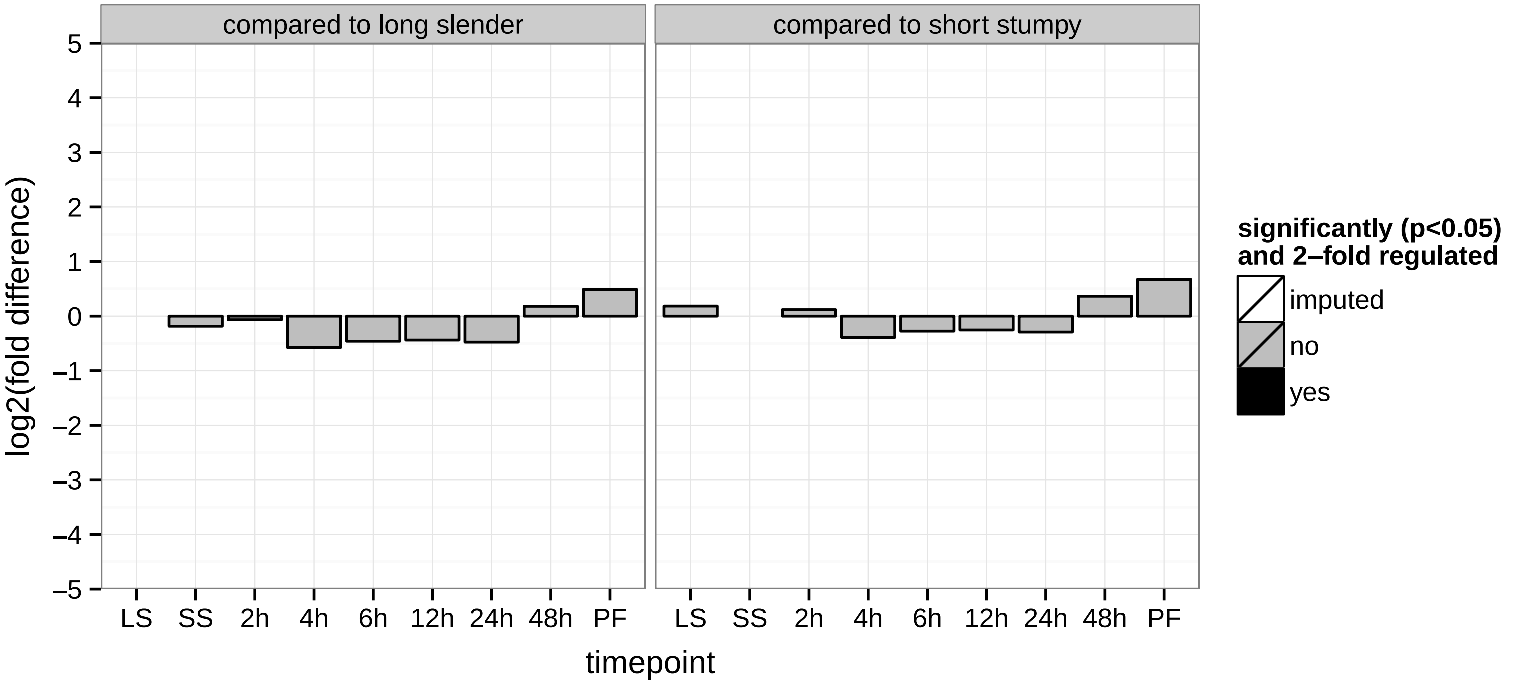

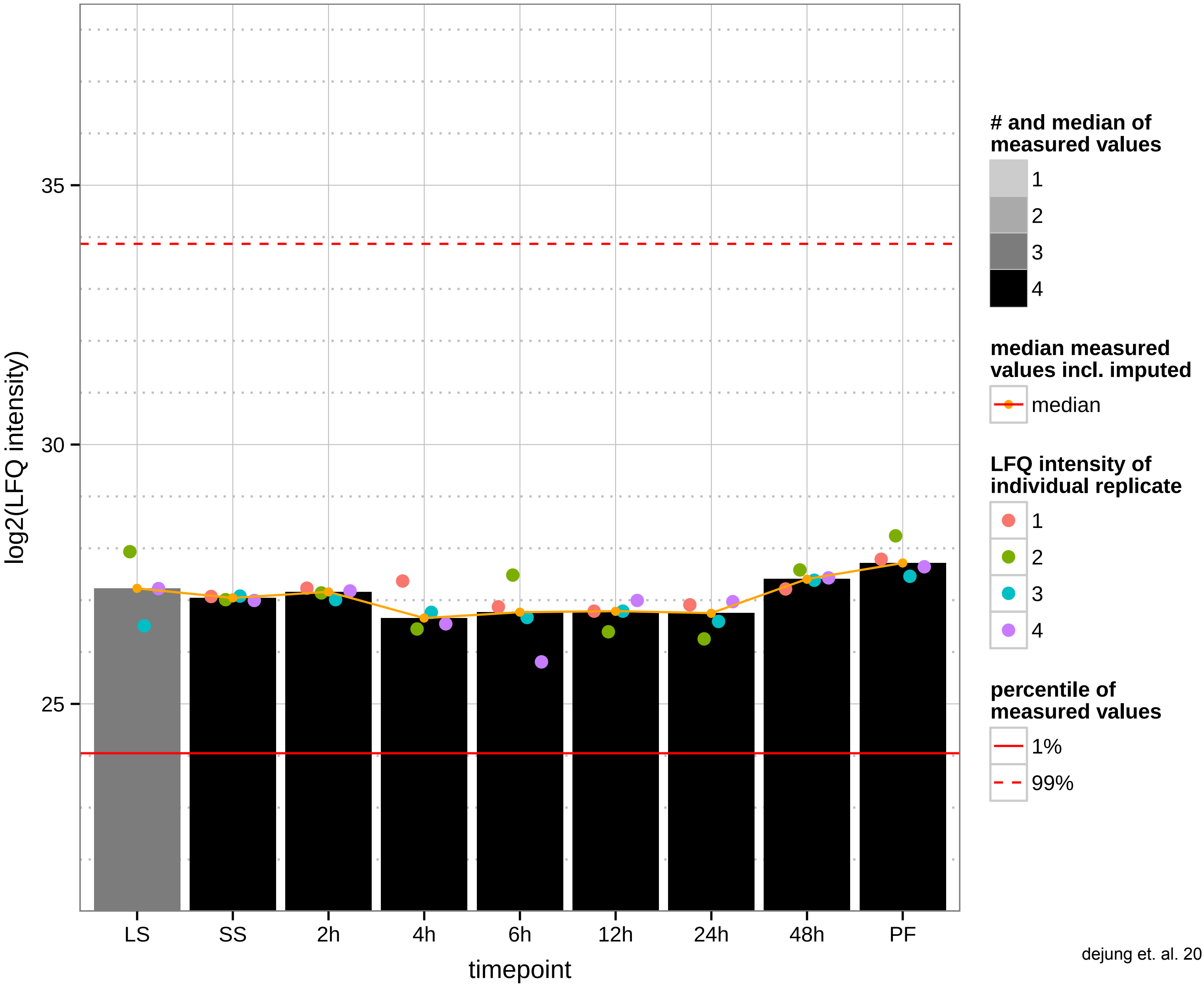

dejung et. al. 2015

10 kDa heat shock protein, putative (HSP10)
Tb927.7.1340;Tb927.7.1320
AGOF: ATP binding, unfolded protein binding
AGOC: mitochondrial matrix, mitochondrial membrane, mitochondrion
AGOP: protein folding
PGOF: null
PGOC: cytoplasm
PGOP: protein folding

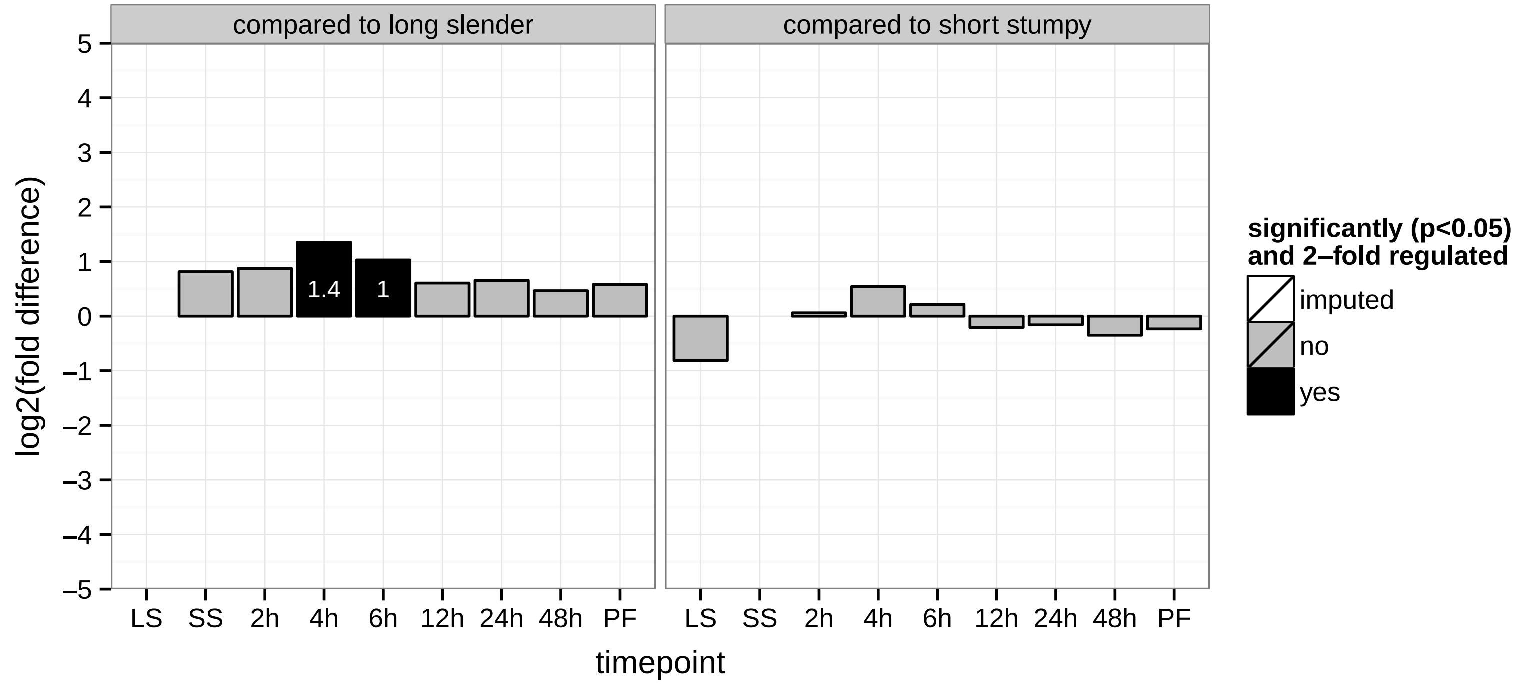

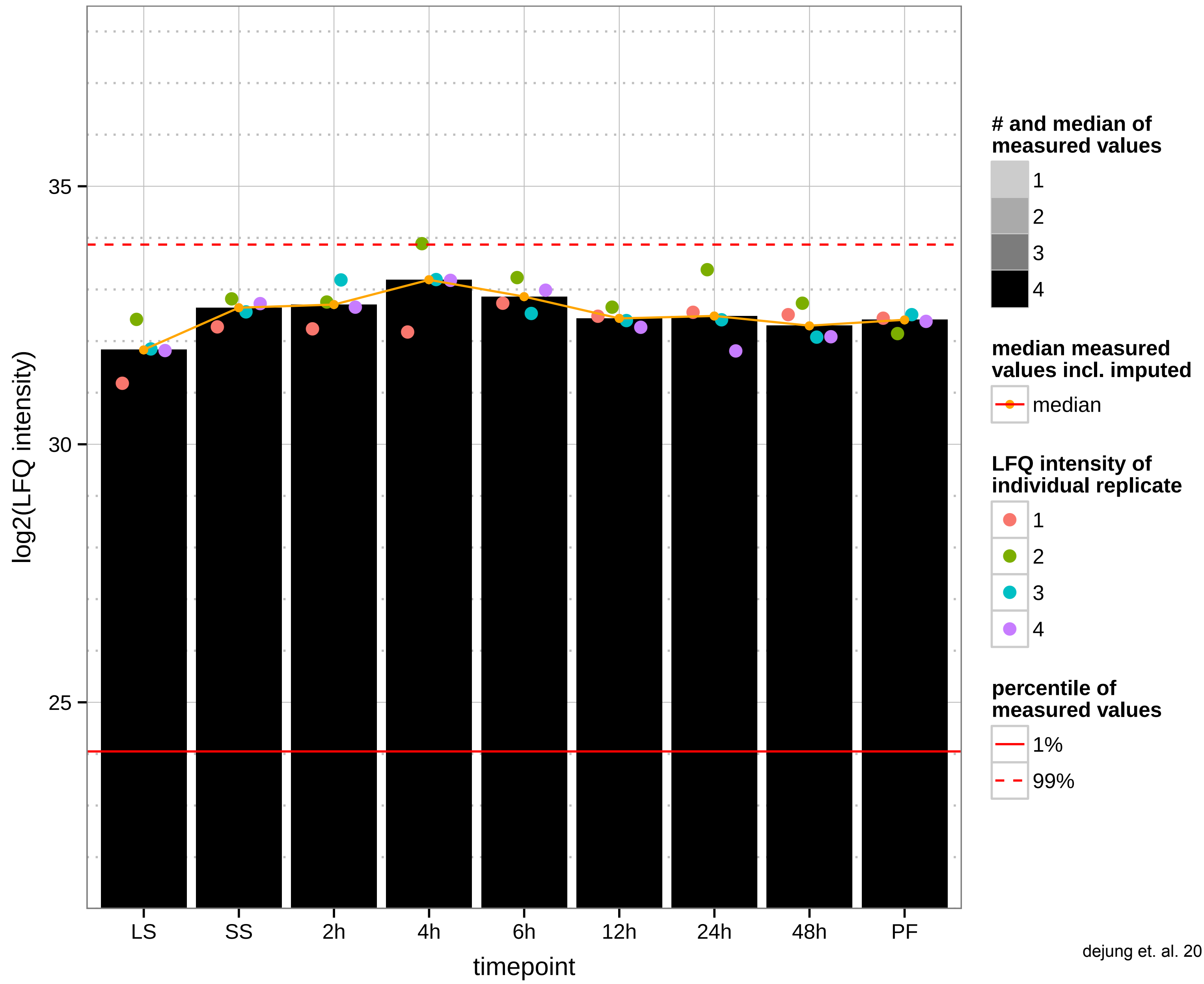

dejung et. al. 2015

hypothetical protein, conserved
Tb927.7.1350
AGOF: null
AGOC: mitochondrion
AGOP: null
PGOF: null
PGOC: null
PGOP: null

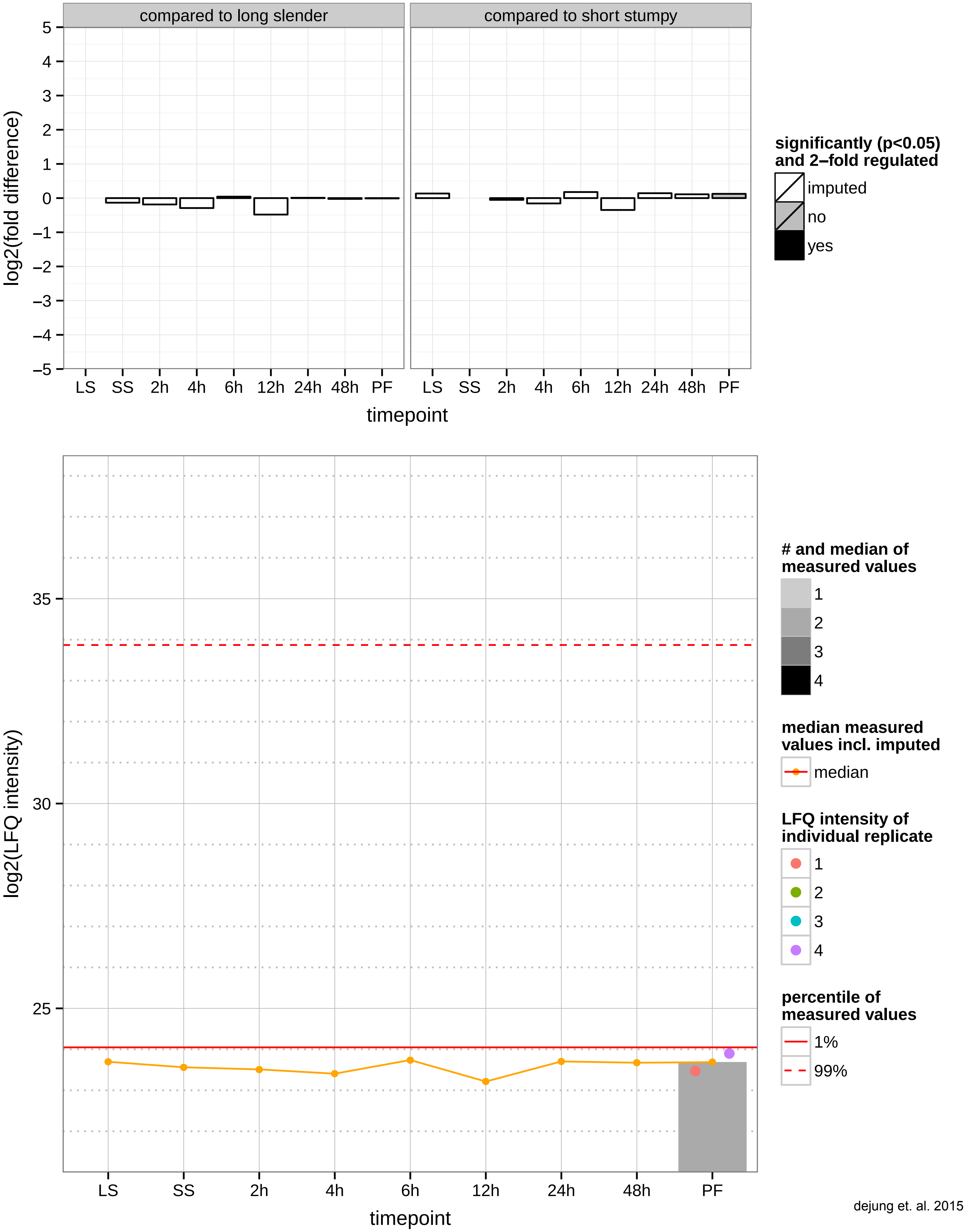

dejung et. al. 2015

hypothetical protein, conserved
Tb927.7.1380
AGOF: null
AGOC: null
AGOP: null
PGOF: null
PGOC: null
PGOP: null

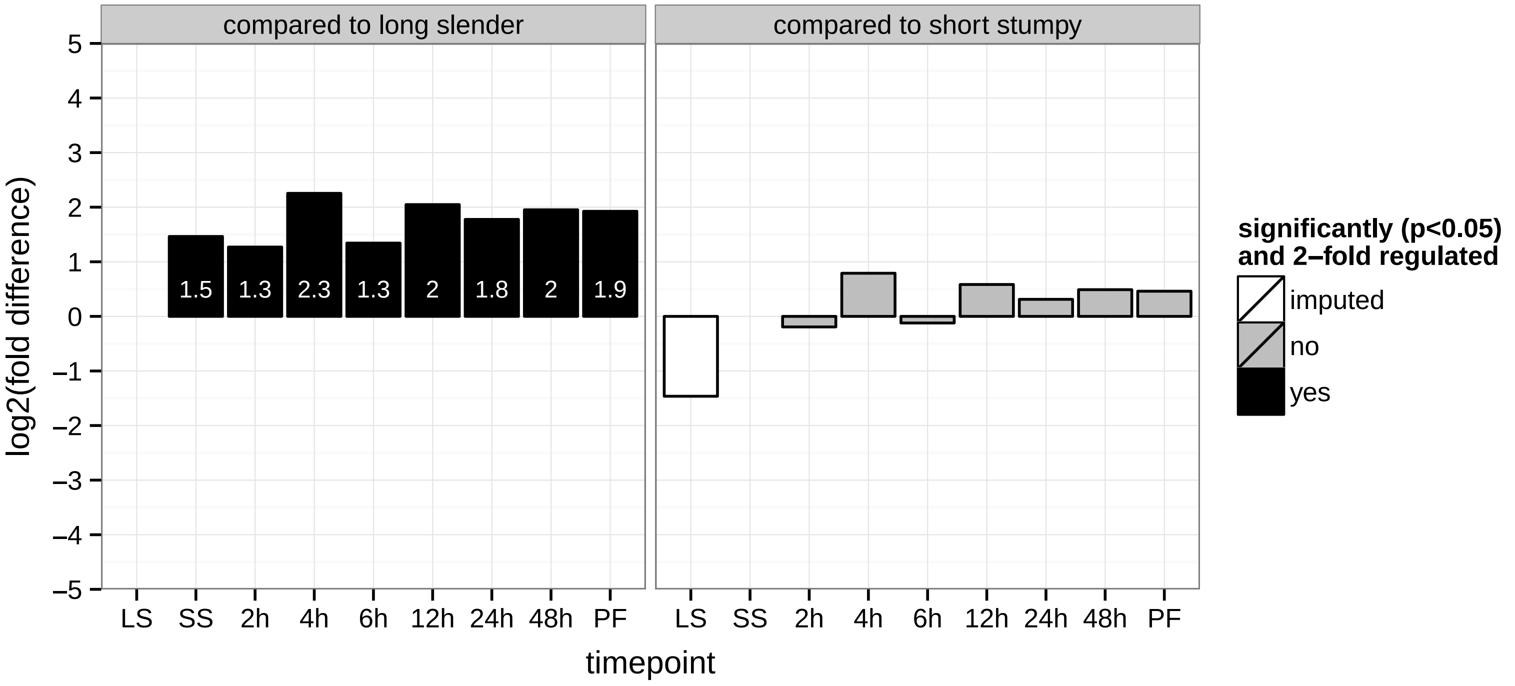

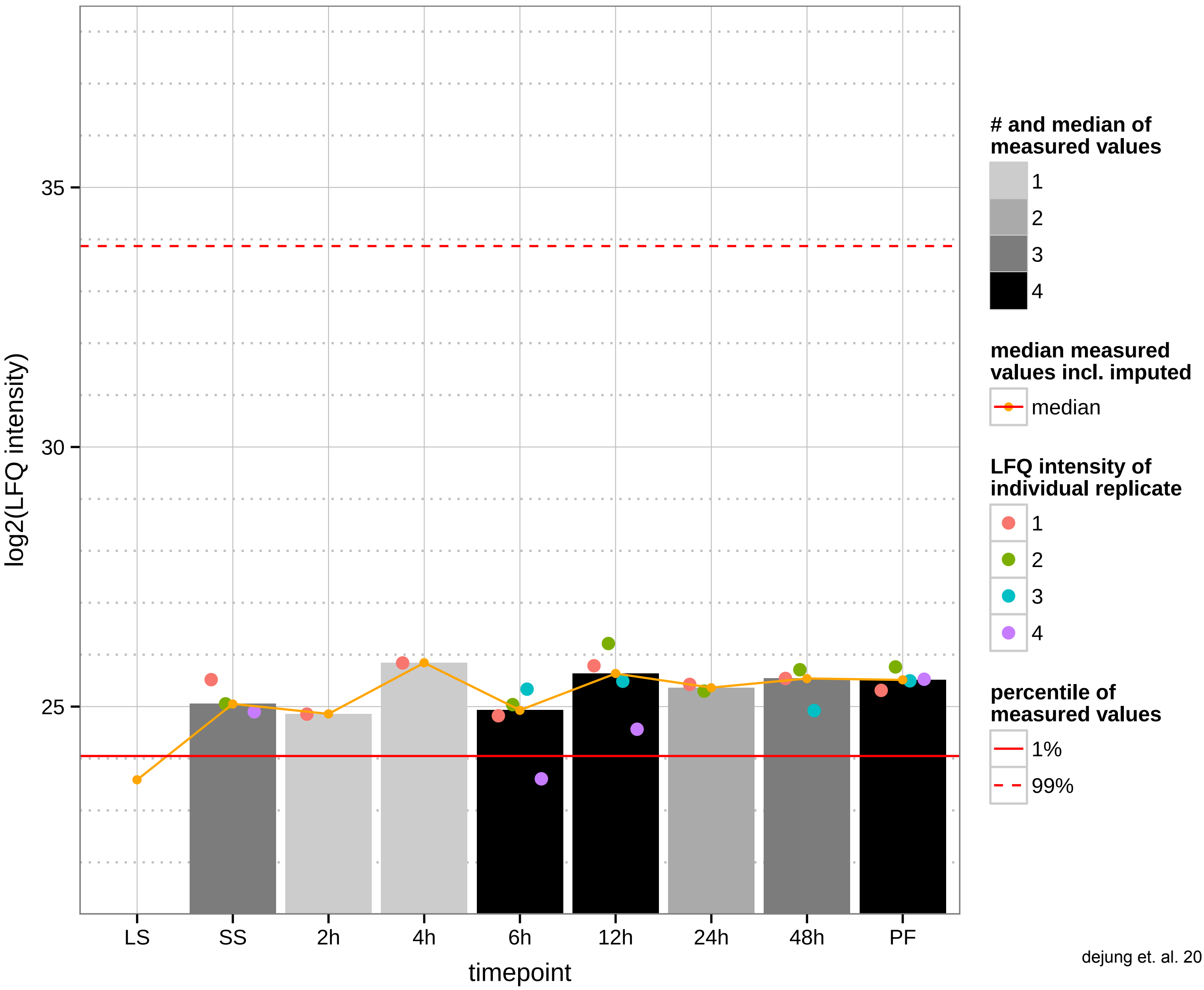

dejung et. al. 2015

leucine–rich repeat protein (LRRP), putative
Tb927.7.1430
AGOF: null
AGOC: null
AGOP: null
PGOF: null
PGOC: null
PGOP: null

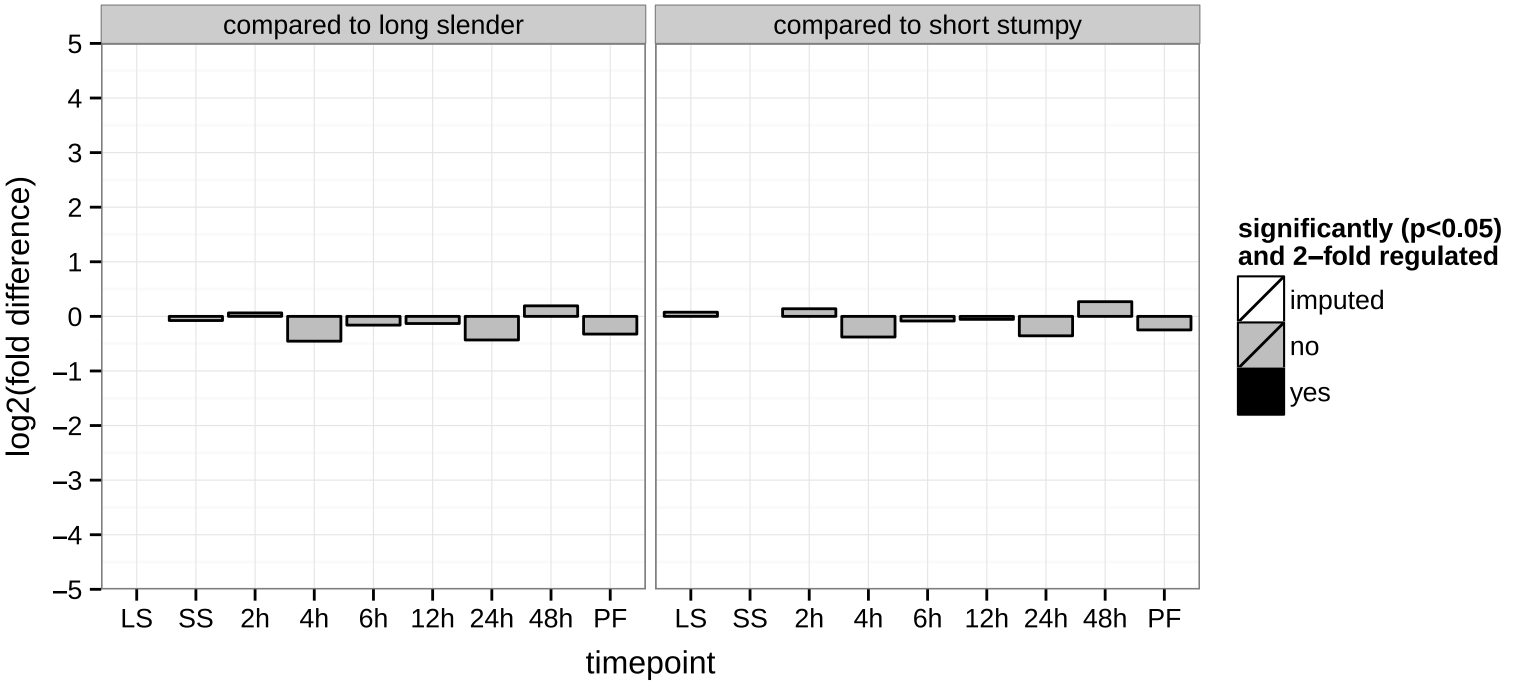

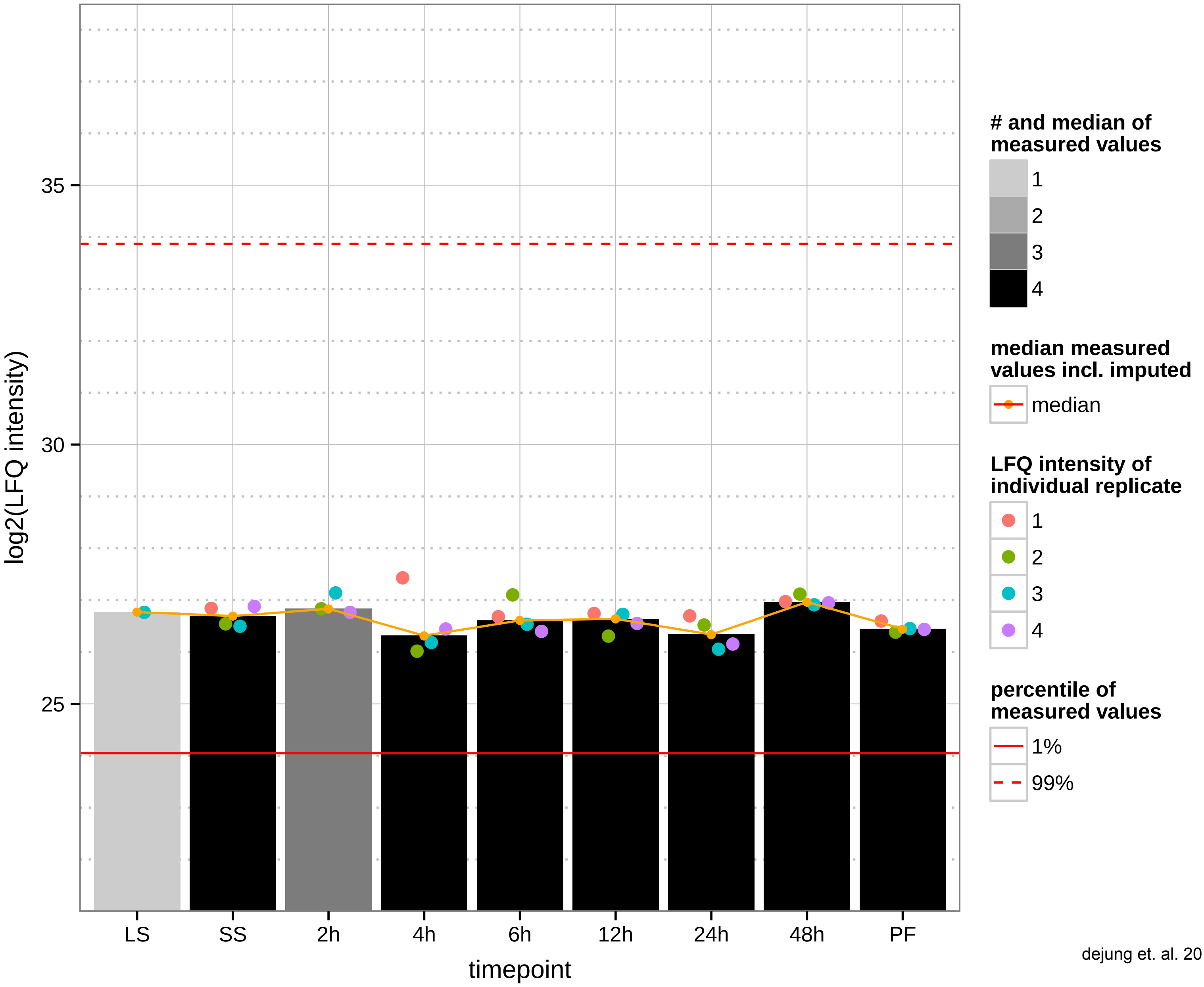

dejung et. al. 2015

nucleotide binding protein, putative (NBP 2)
Tb927.7.1500
AGOF: nucleotide binding
AGOC: cytosol
AGOP: null
PGOF: null
PGOC: null
PGOP: null

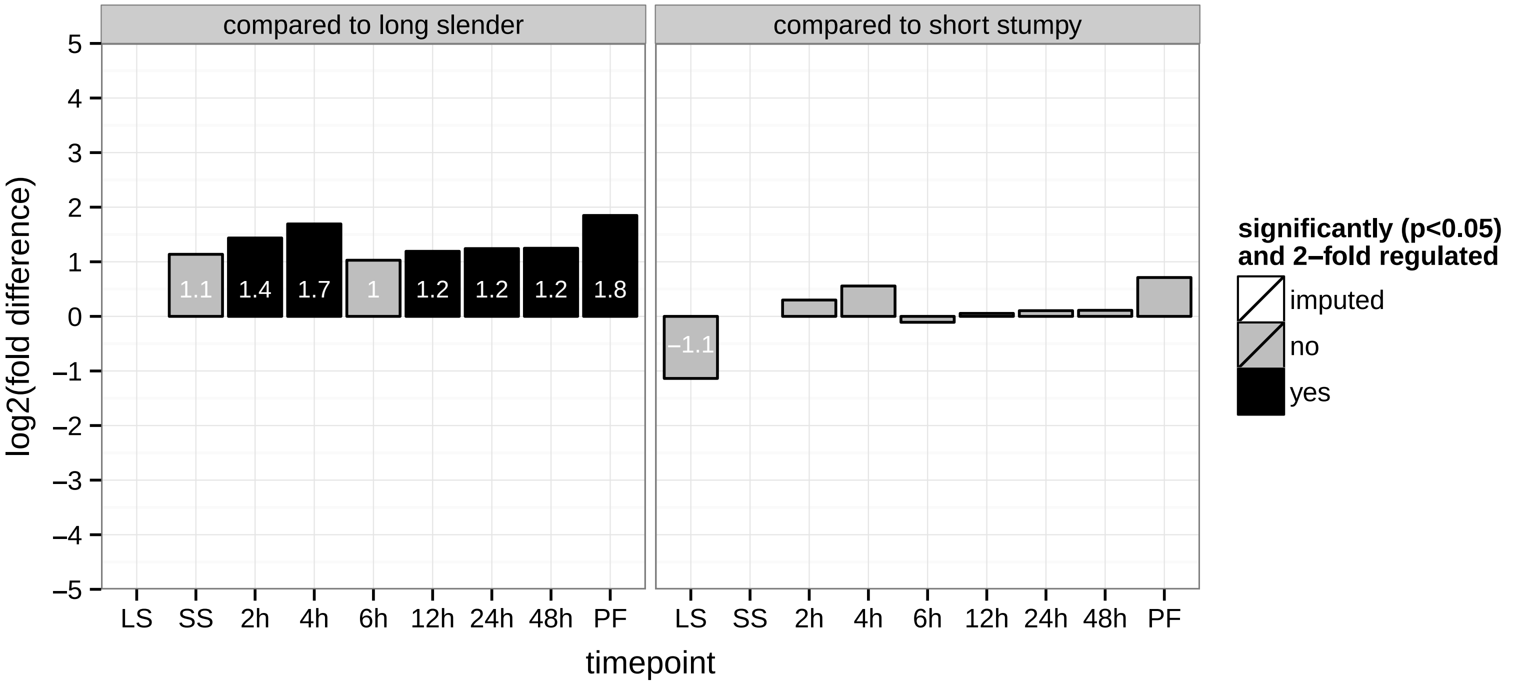

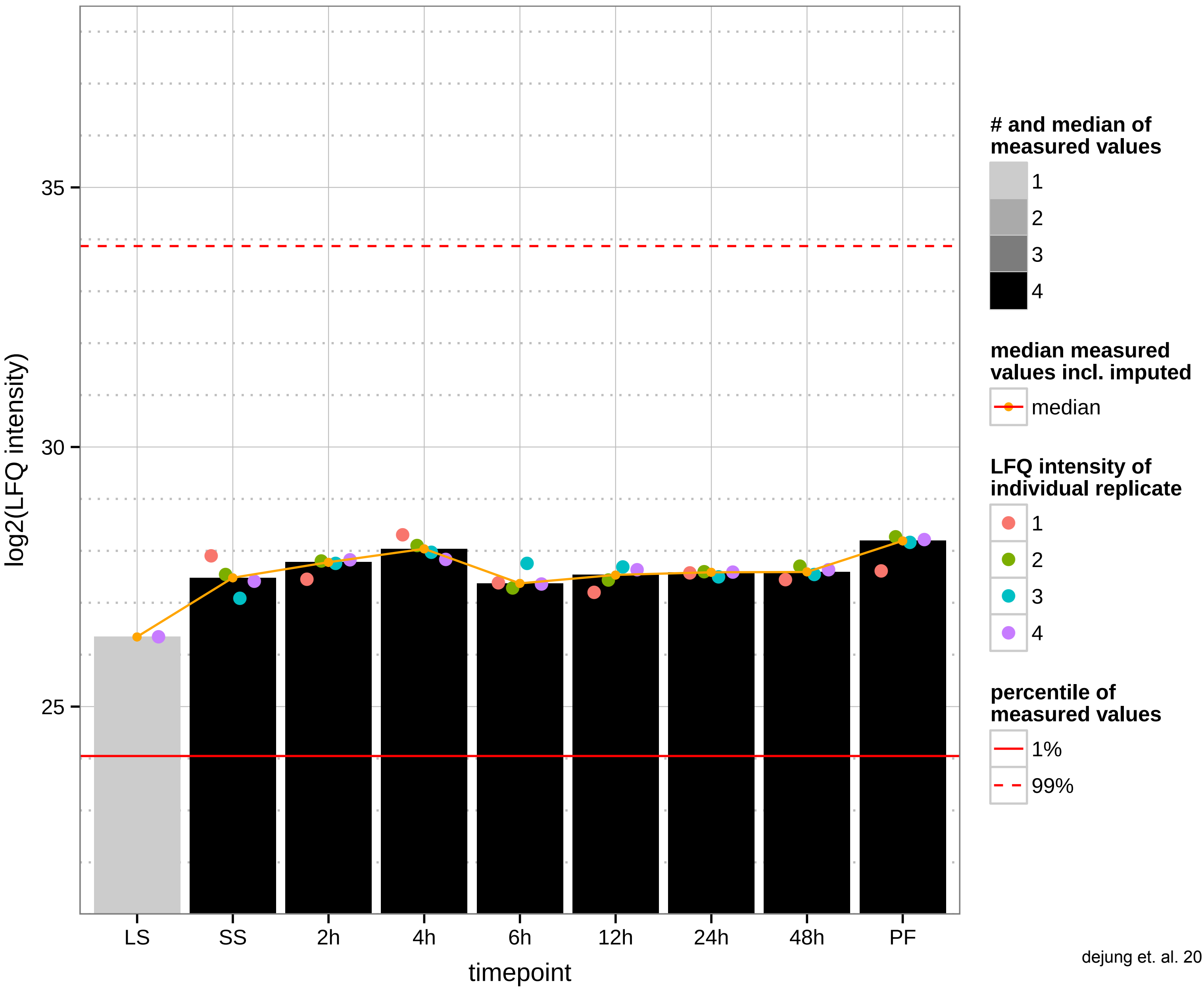

dejung et. al. 2015

pseudouridylate synthase I, putative
Tb927.7.1510
AGOF: RNA binding, pseudouridine synthase activity
AGOC: null
AGOP: pseudouridine synthesis
PGOF: RNA binding, pseudouridine synthase activity
PGOC: null
PGOP: RNA modification, pseudouridine synthesis

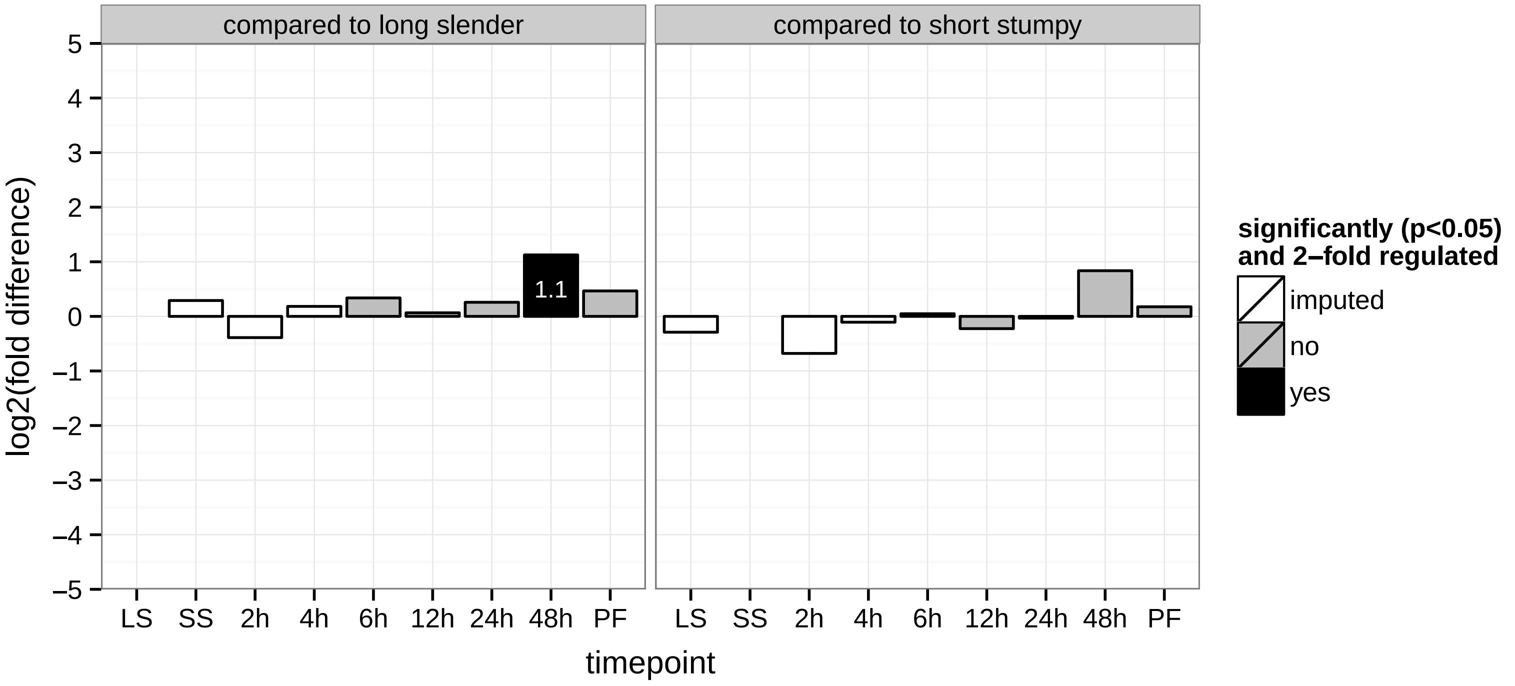

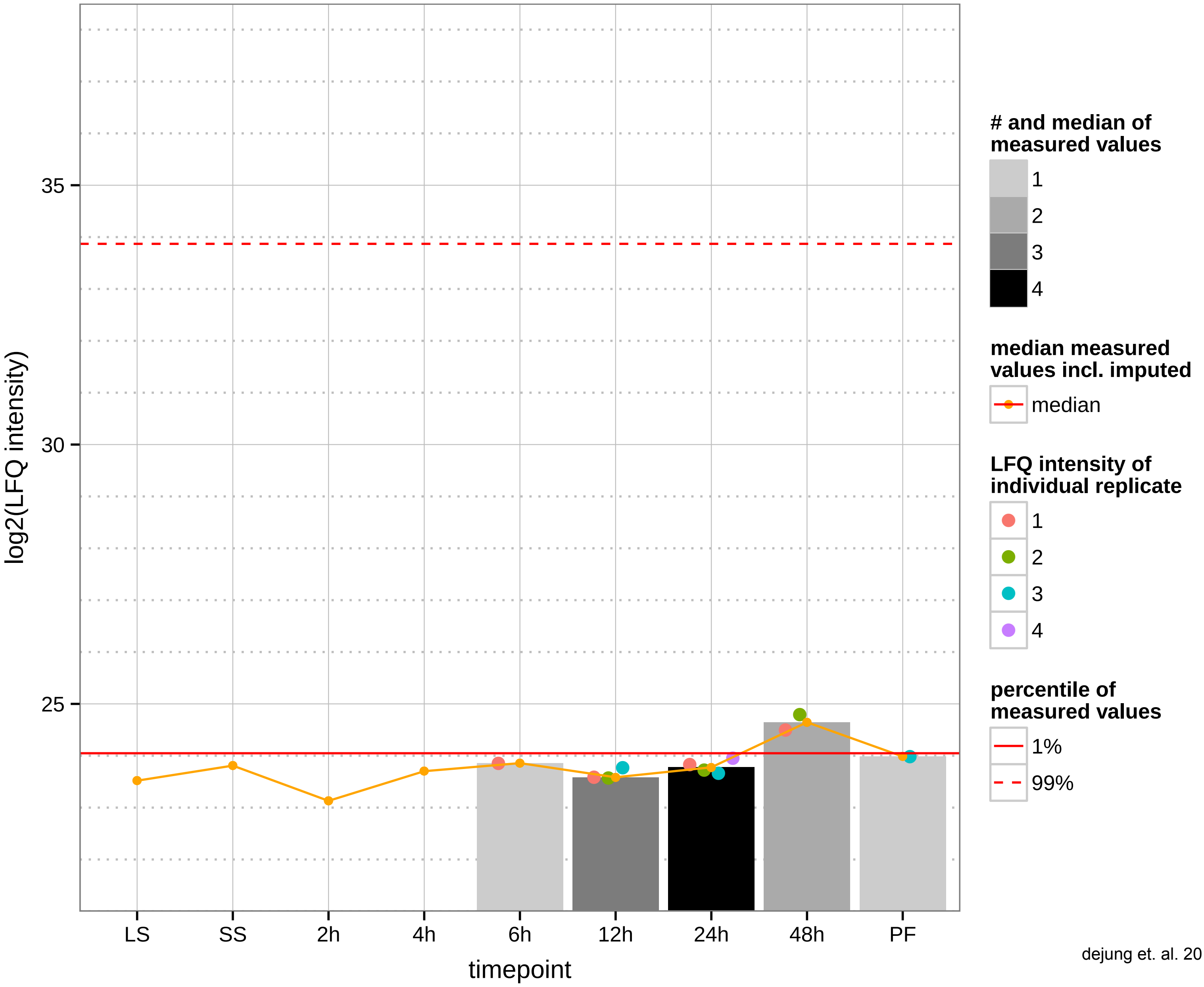

dejung et. al. 2015

hypothetical protein, conserved
Tb927.7.1630
AGOF: null
AGOC: null
AGOP: null
PGOF: null
PGOC: null
PGOP: null

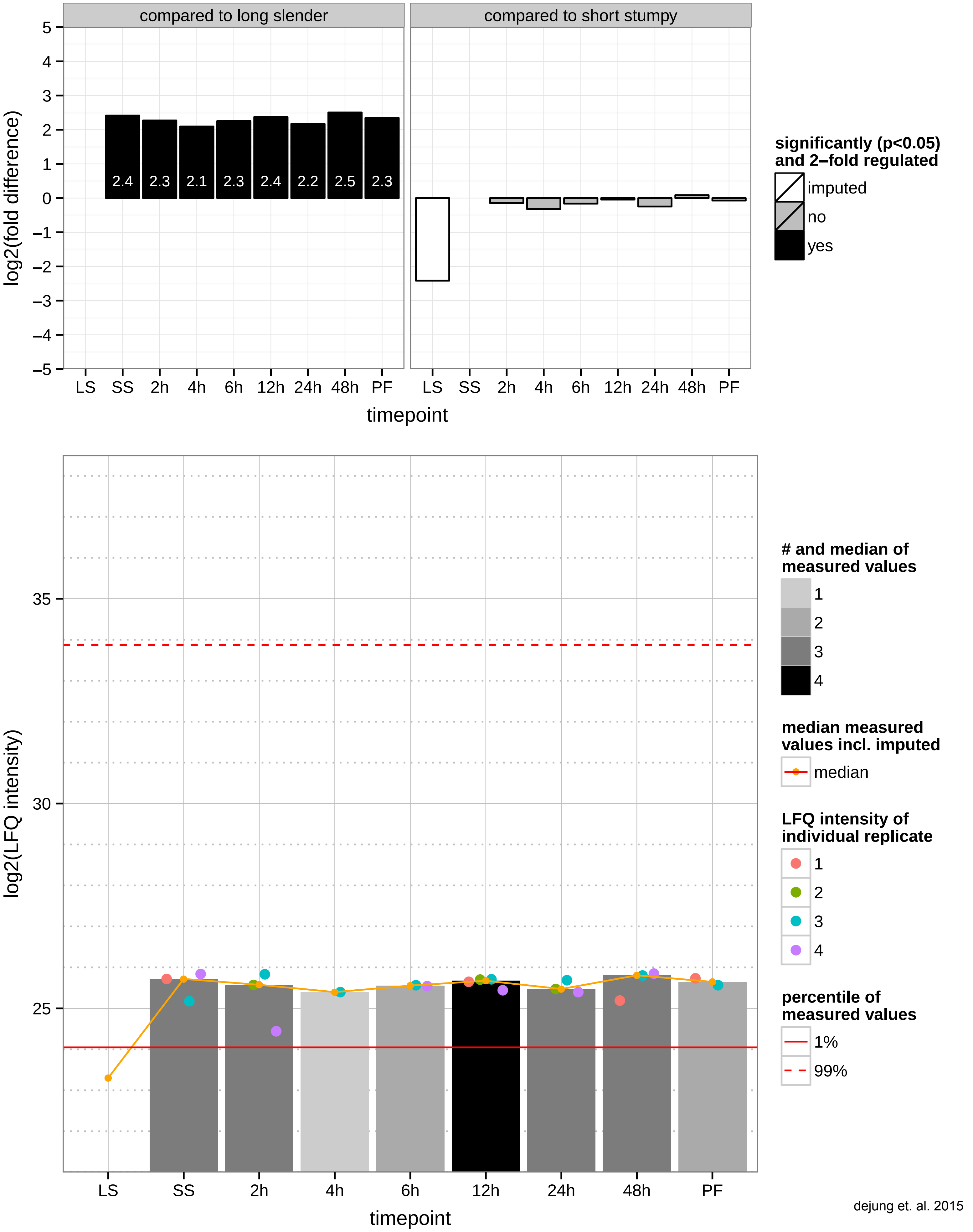

dejung et. al. 2015

hypothetical protein, conserved
Tb927.7.1650
AGOF: null
AGOC: null
AGOP: null
PGOF: null
PGOC: null
PGOP: null

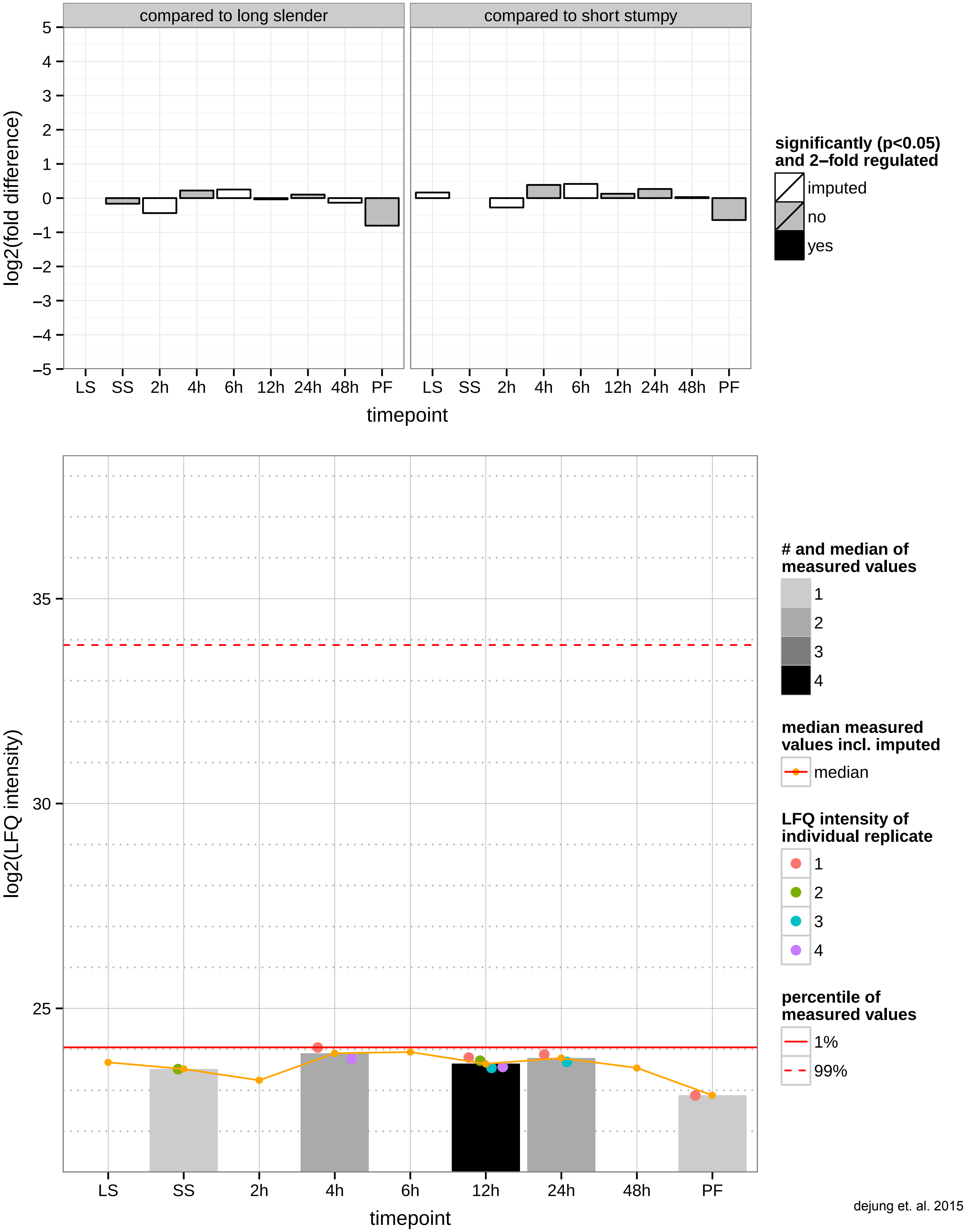

dejung et. al. 2015

hypothetical protein, conserved
Tb927.7.1770
AGOF: null
AGOC: null
AGOP: null
PGOF: null
PGOC: null
PGOP: null

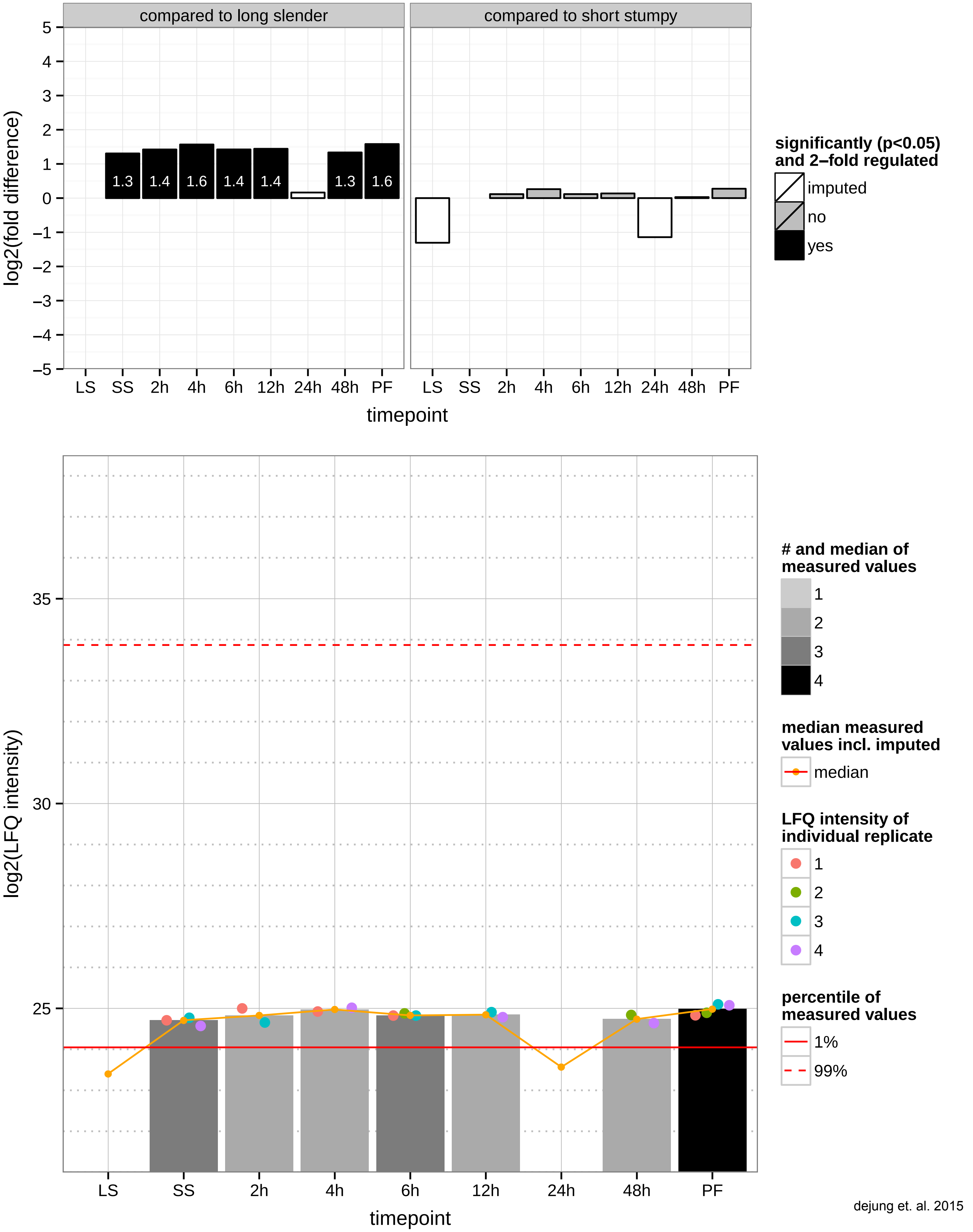

dejung et. al. 2015

Adenine phosphoribosyltransferase, putative
Tb927.7.1790
AGOF: adenine phosphoribosyltransferase activity
AGOC: null
AGOP: purine ribonucleoside salvage
PGOF: null
PGOC: null
PGOP: nucleoside metabolic process

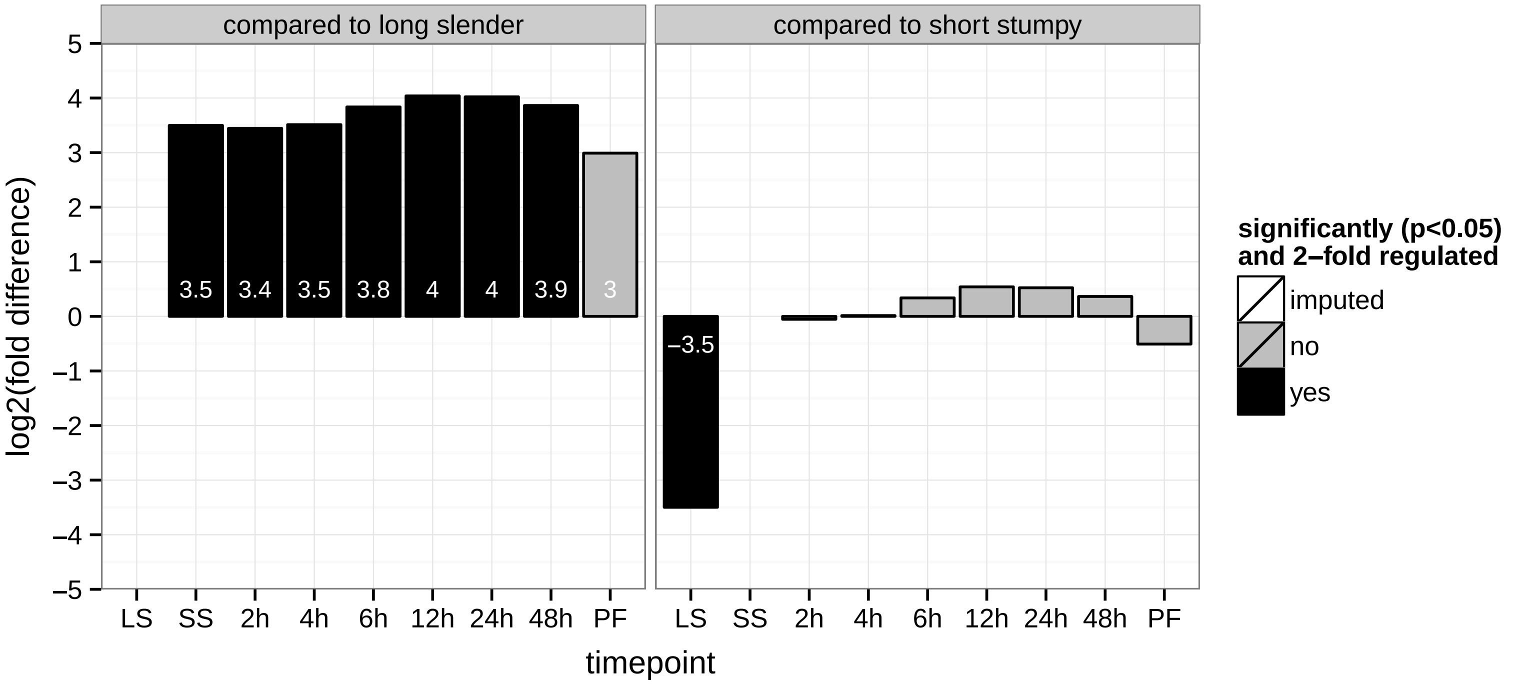

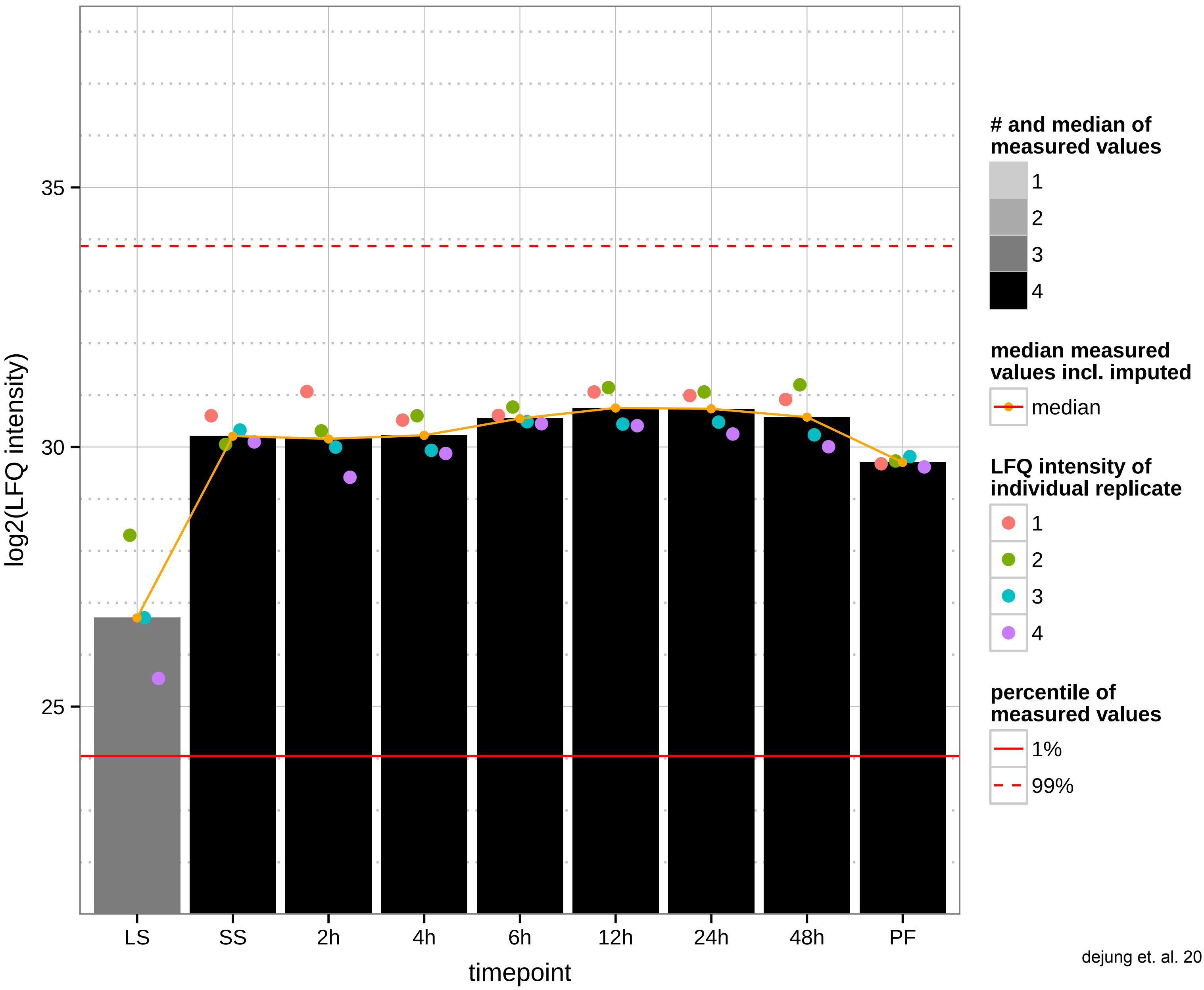

dejung et. al. 2015

paraflagellar rod component, putative (PFC5)
Tb927.7.1920
AGOF: null
AGOC: null
AGOP: null
PGOF: null
PGOC: null
PGOP: null

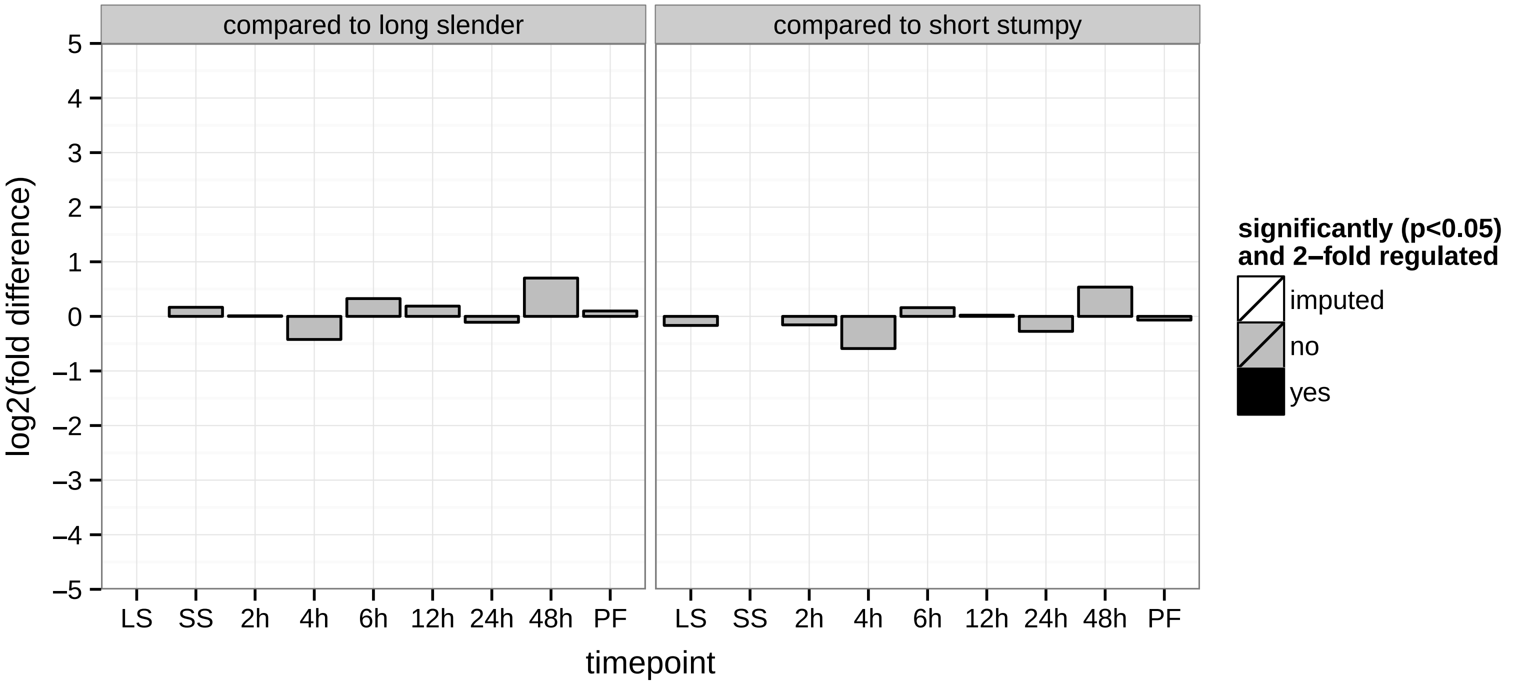

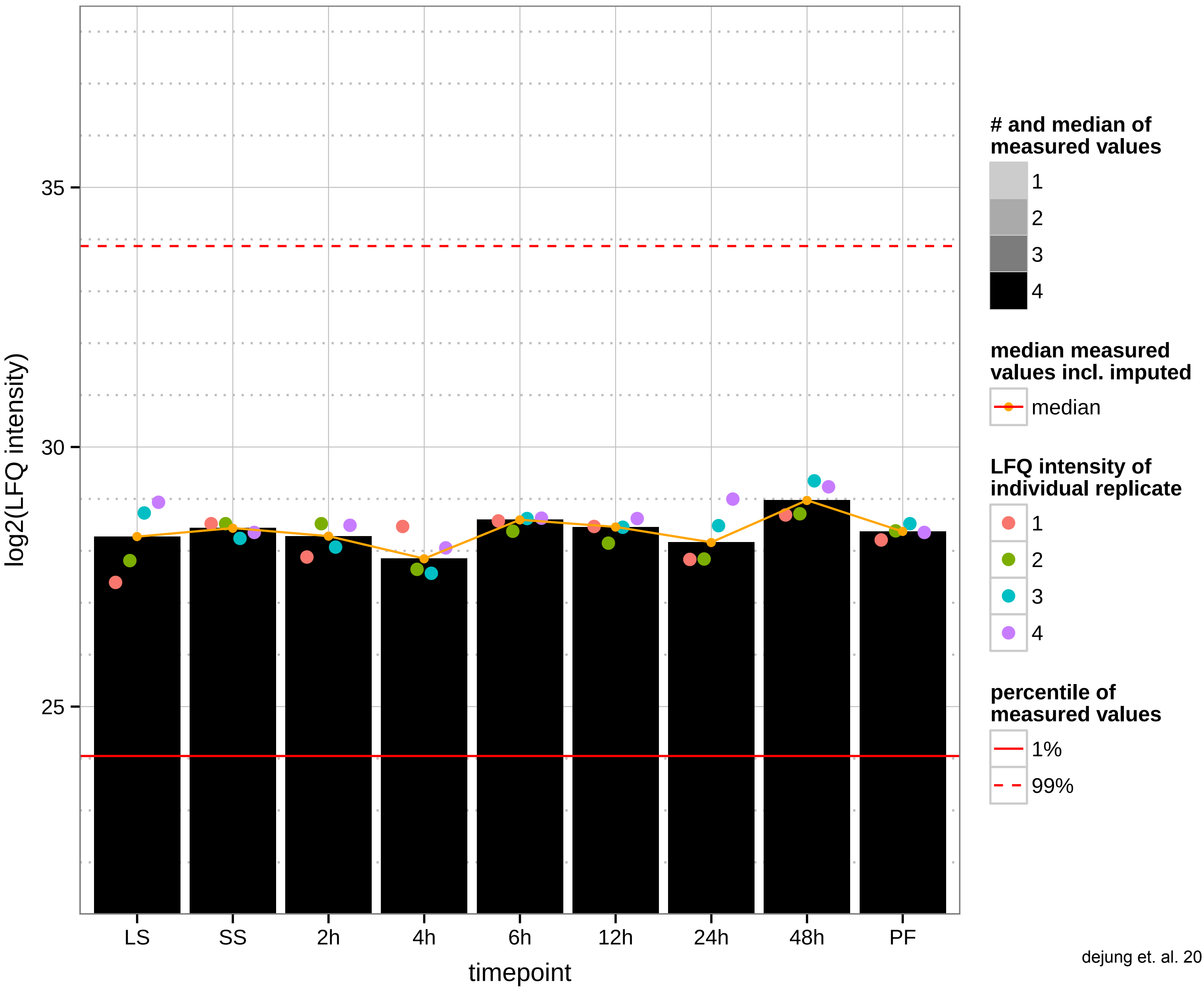

dejung et. al. 2015

heat shock protein DNAJ, putative
Tb927.7.2070
AGOF: heat shock protein binding, unfolded protein binding
AGOC: null
AGOP: protein folding
PGOF: heat shock protein binding, unfolded protein binding
PGOC: null
PGOP: protein folding

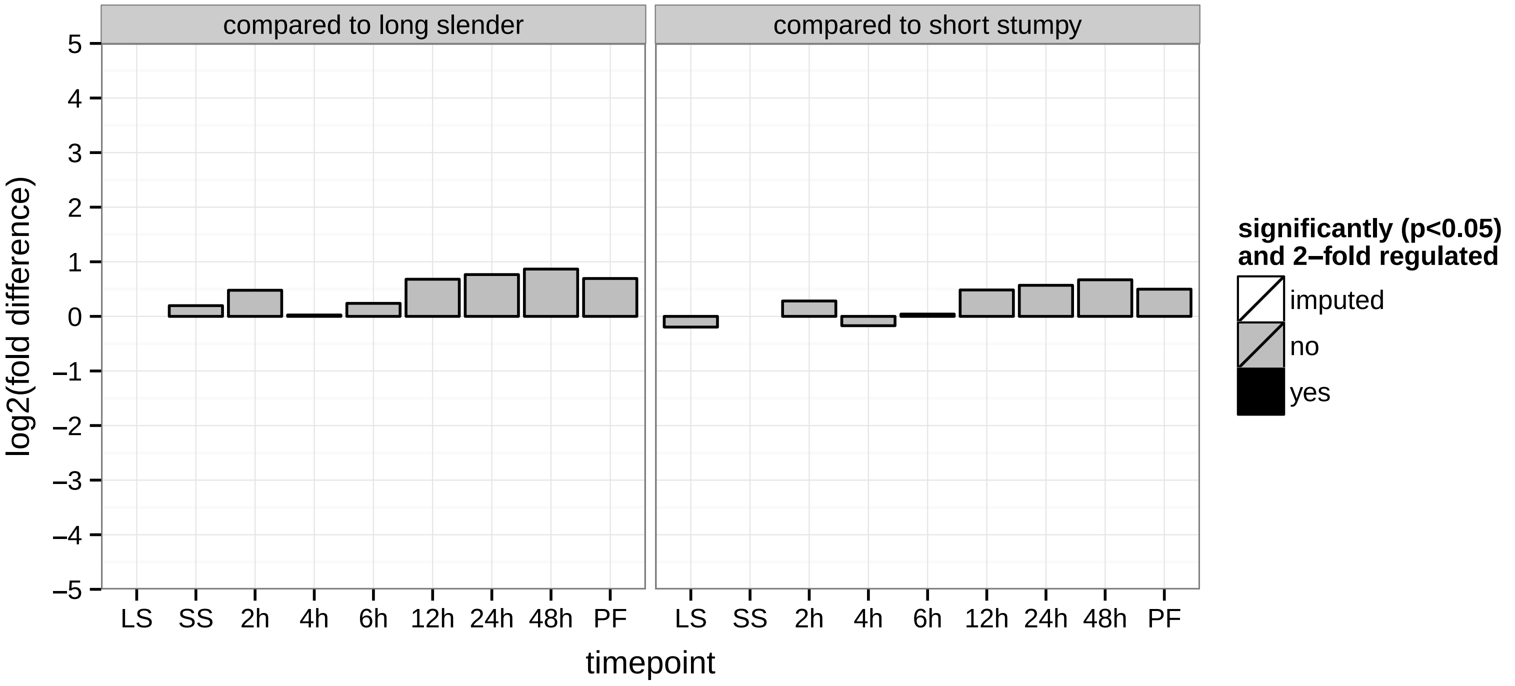

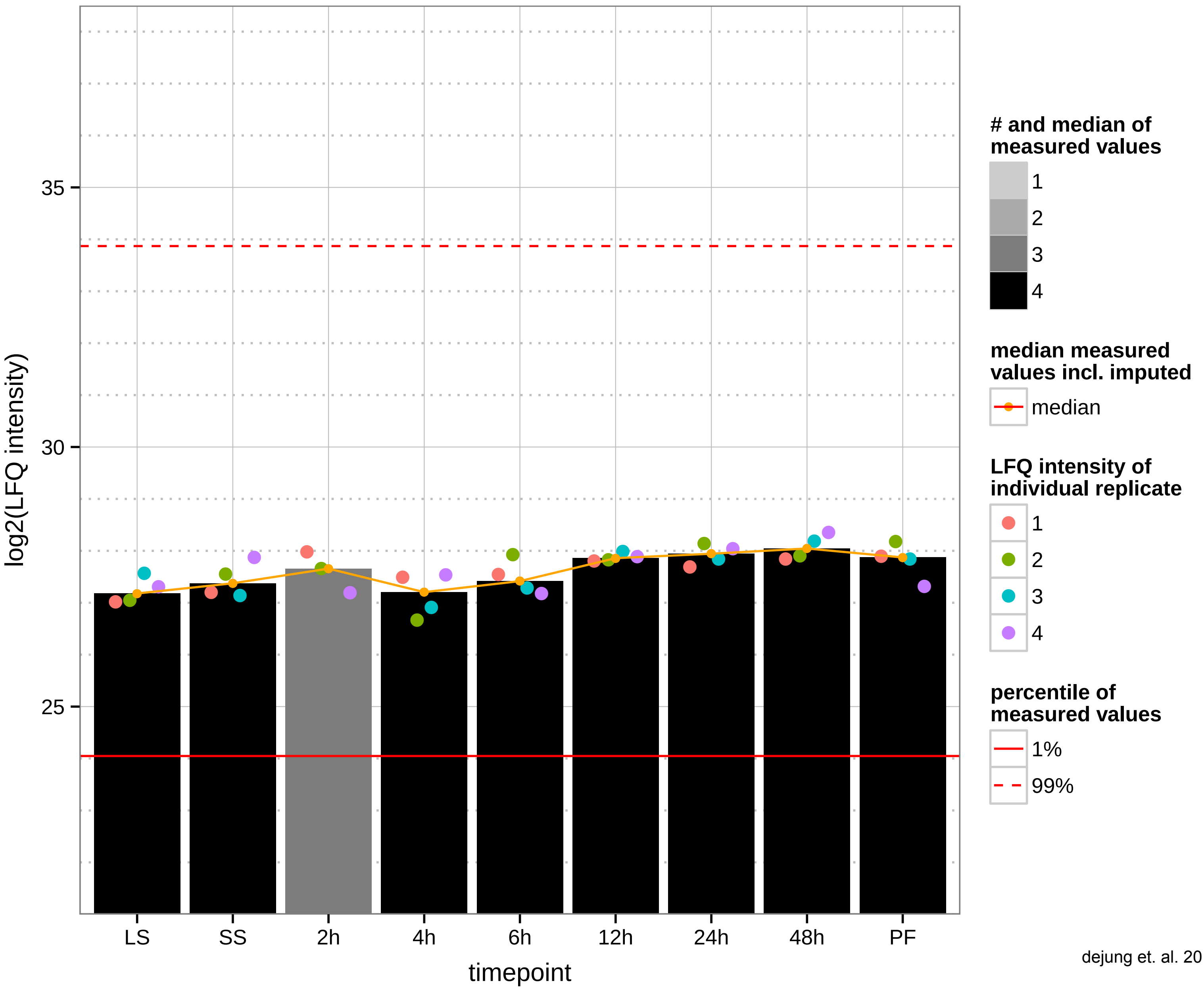

dejung et. al. 2015

methyltransferase, putative, mRNA cap methyltransferase−like protein
Tb927.7.2080
AGOF: null
AGOC: null
AGOP: 7−methylguanosine mRNA capping
PGOF: mRNA guanylyltransferase activity
PGOC: null
PGOP: 7−methylguanosine mRNA capping

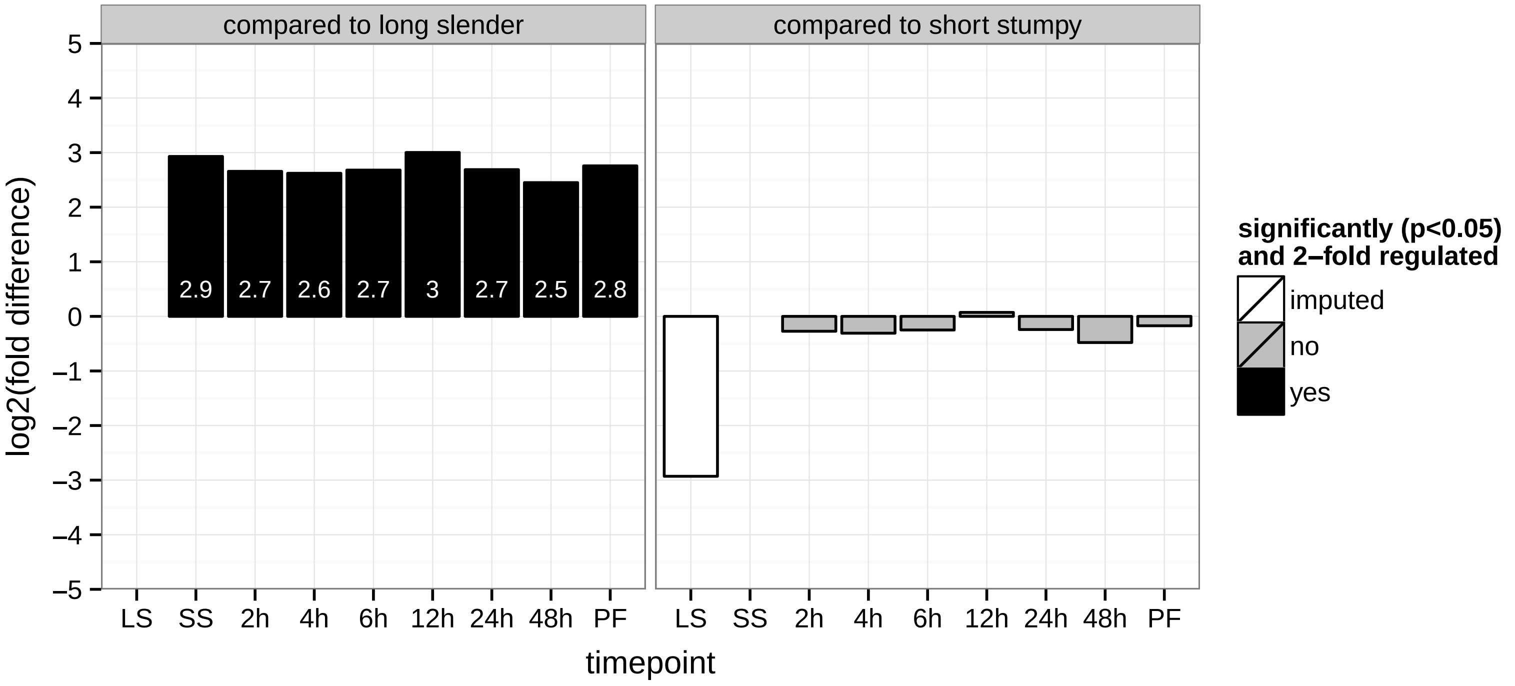

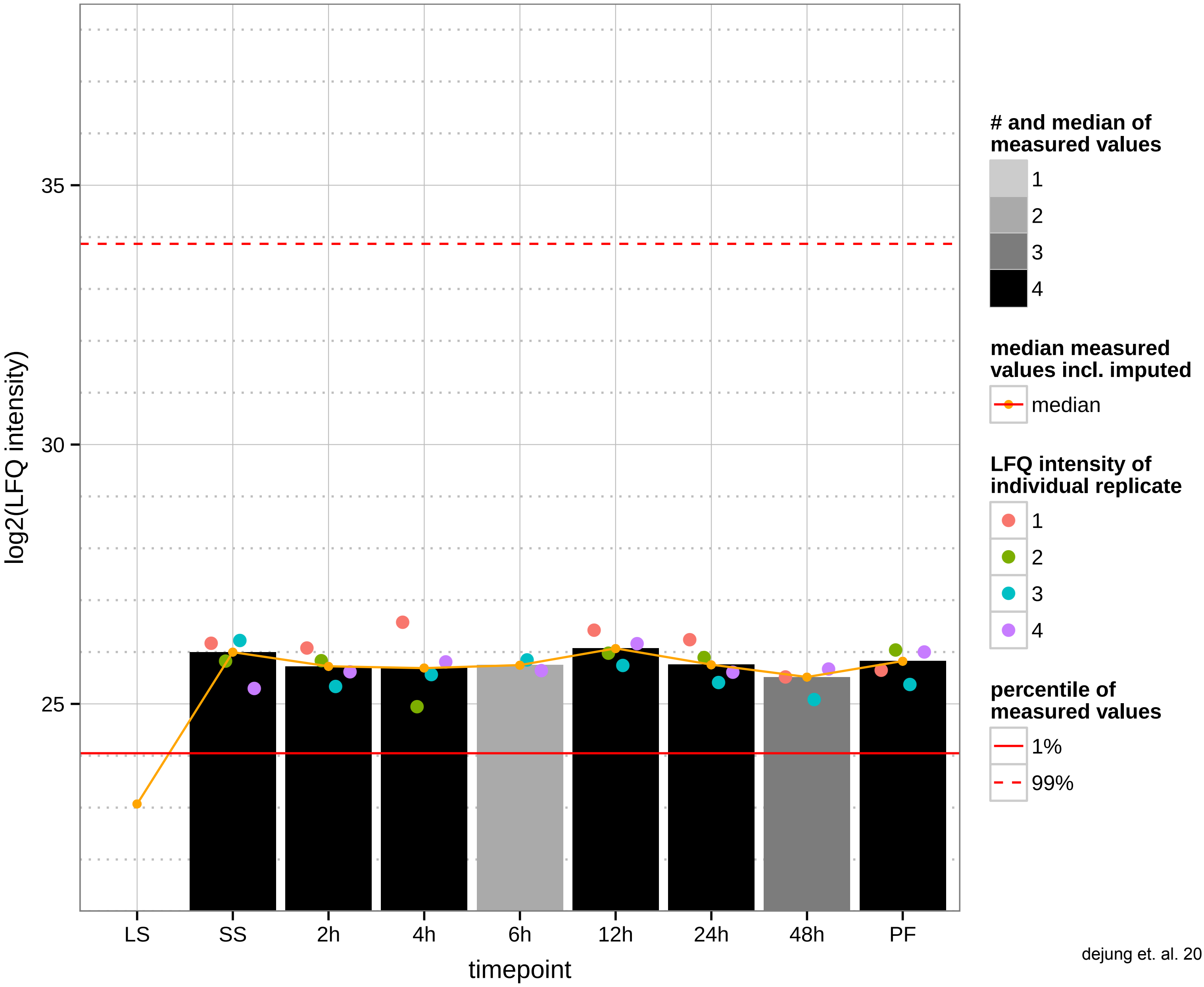

dejung et. al. 2015

proline dehydrogenase
Tb927.7.210
AGOF: proline dehydrogenase activity
AGOC: null
AGOP: glutamate biosynthetic process, proline catabolic process
PGOF: proline dehydrogenase activity
PGOC: null
PGOP: glutamate biosynthetic process, oxidation–reduction process, proline catabolic process

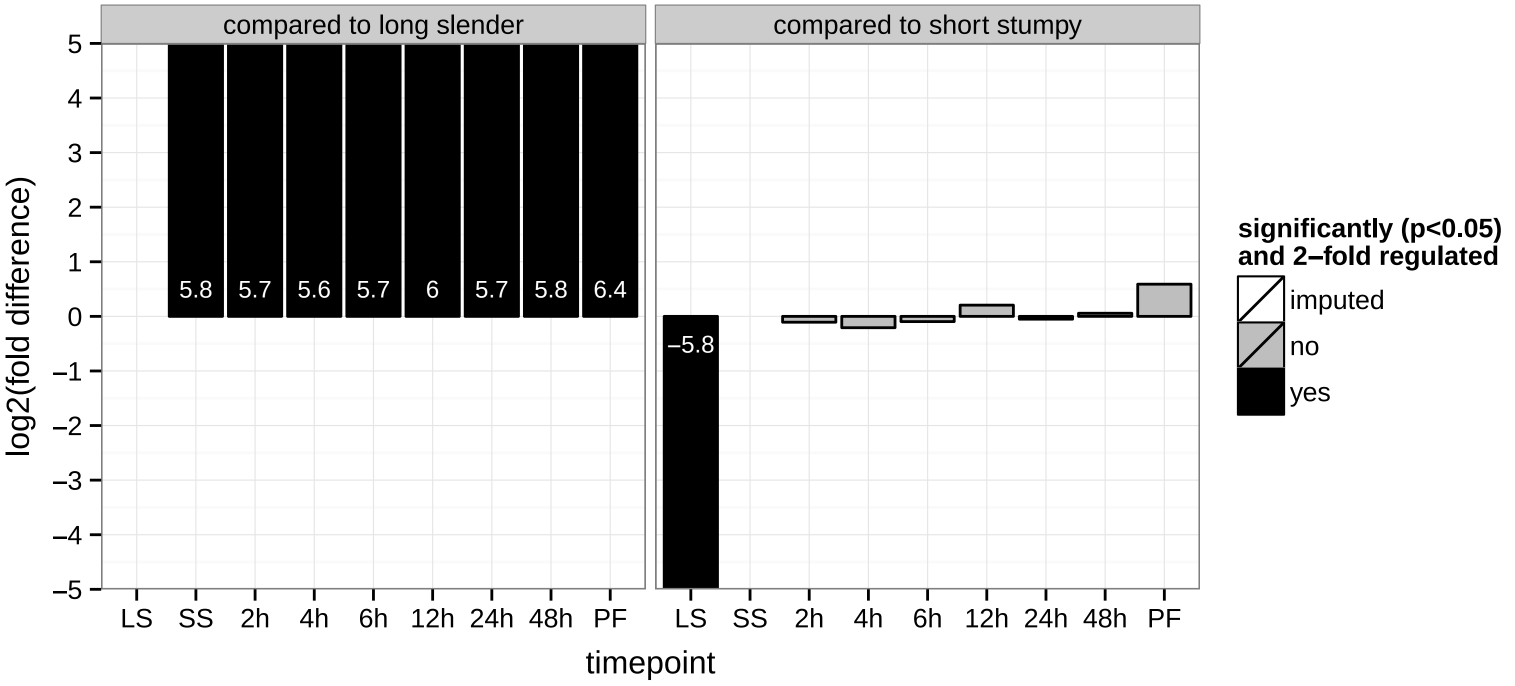

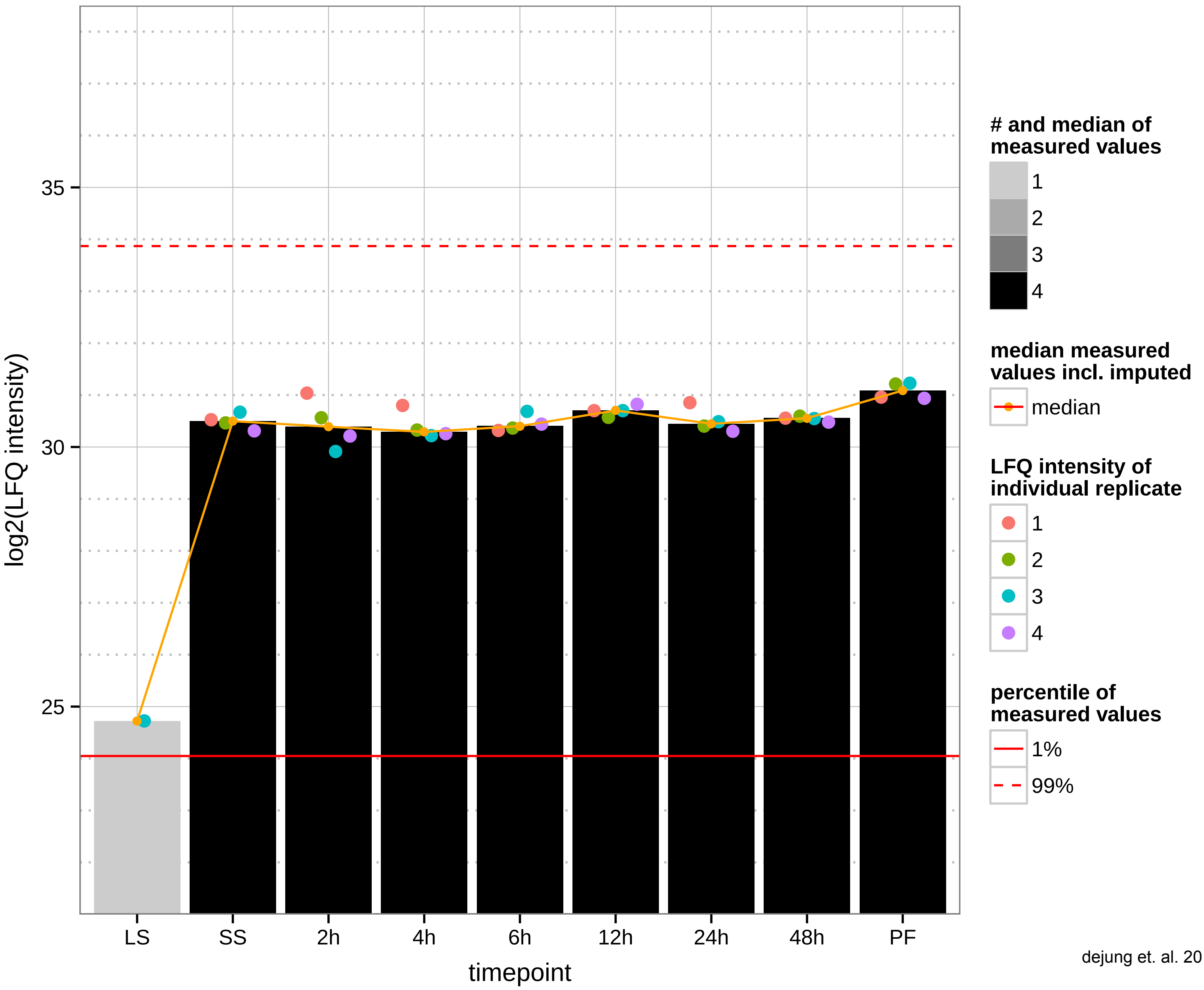

dejung et. al. 2015

GMP synthase, putative, glutamine amidotransferase
Tb927.7.2100
AGOF: ATP binding, GMP synthase (glutamine−hydrolyzing) activity
AGOC: null
AGOP: GMP biosynthetic process, biosynthetic process, glutamine metabolic process, purine ribonucleotide biosynthetic proc
PGOF: ATP binding, GMP synthase (glutamine−hydrolyzing) activity, pyrophosphatase activity
PGOC: null
PGOP: GMP biosynthetic process, purine nucleotide biosynthetic process

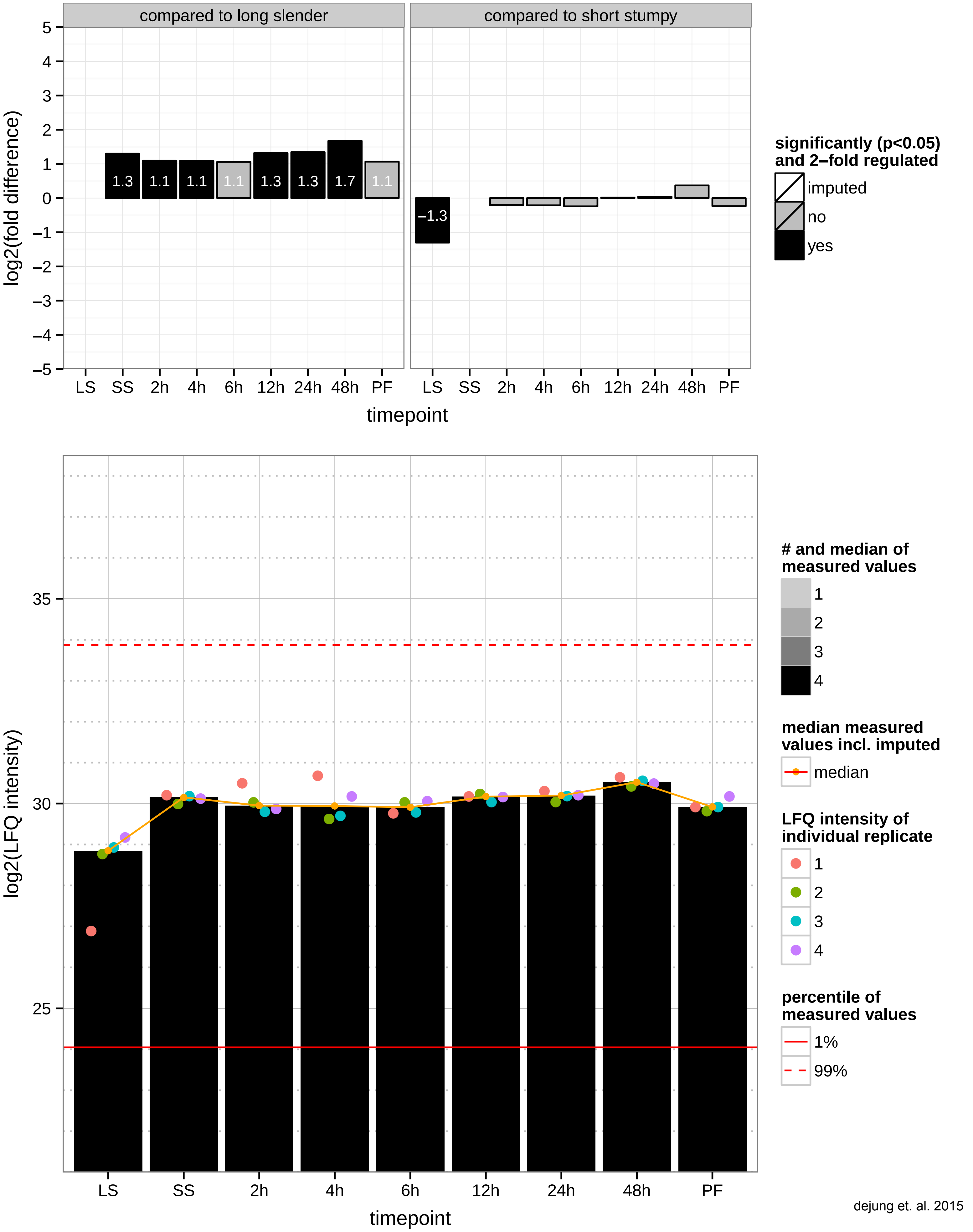

dejung et. al. 2015

hypothetical protein, conserved
Tb927.7.2120
AGOF: null
AGOC: null
AGOP: null
PGOF: null
PGOC: null
PGOP: null

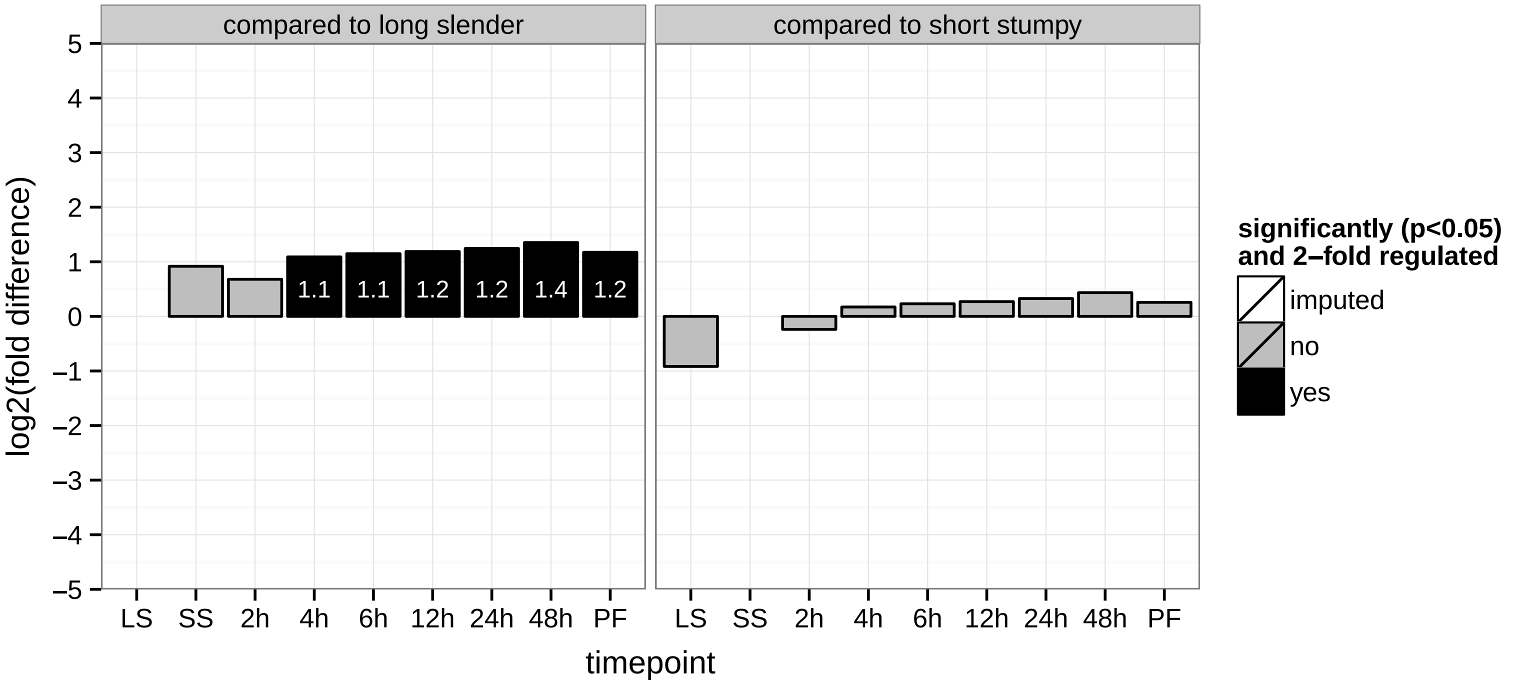

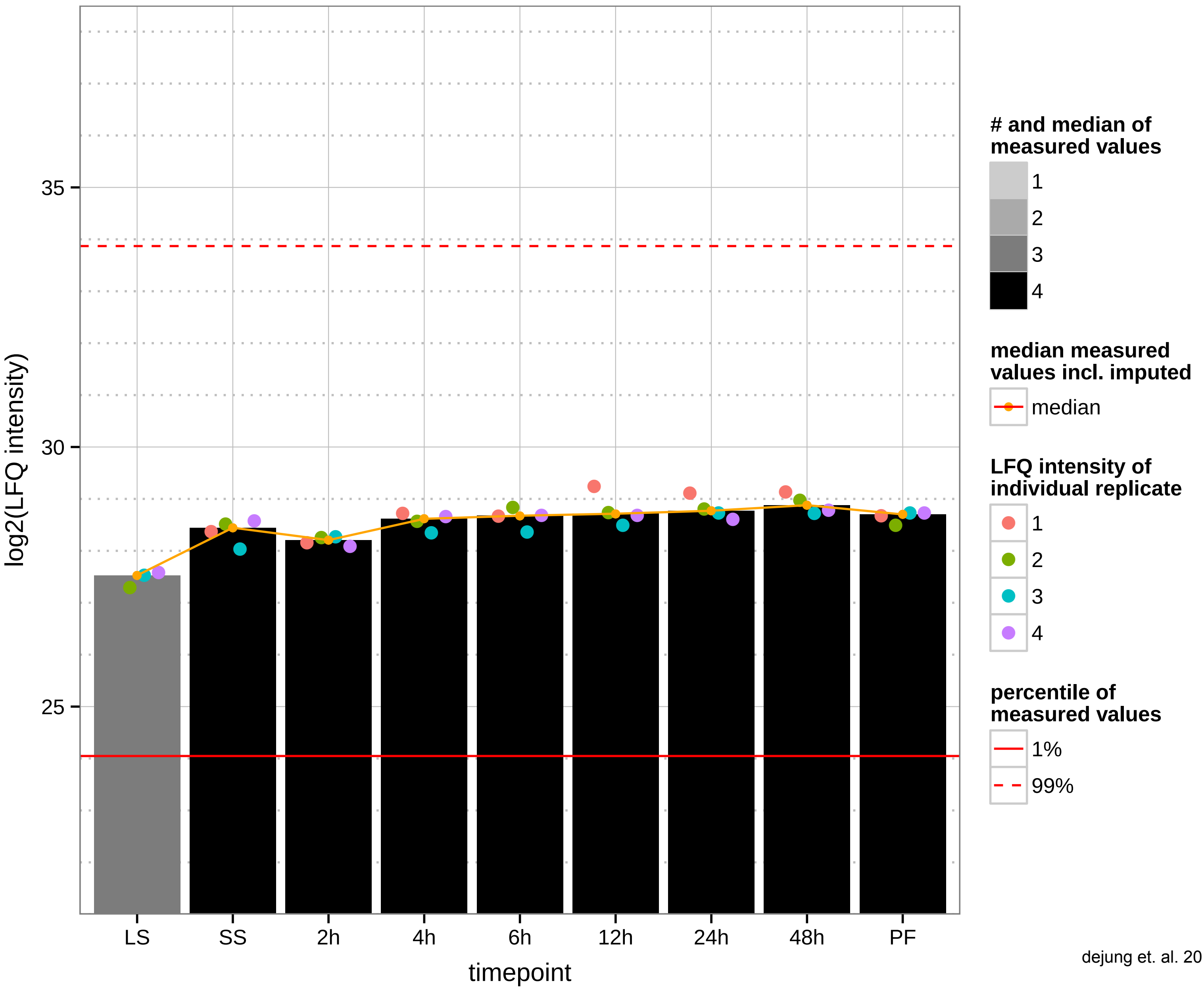

dejung et. al. 2015

hypothetical protein, conserved
Tb927.7.2130
AGOF: null
AGOC: null
AGOP: null
PGOF: null
PGOC: null
PGOP: null

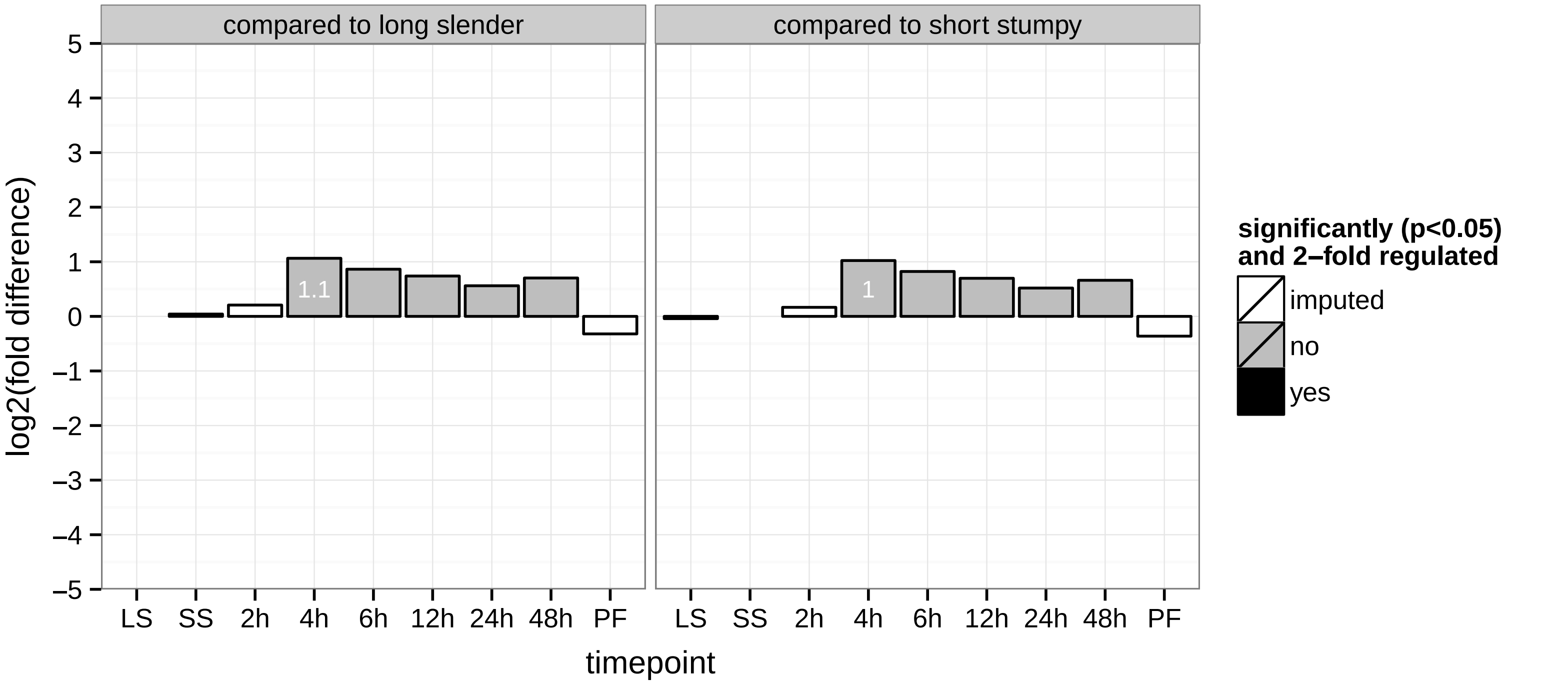

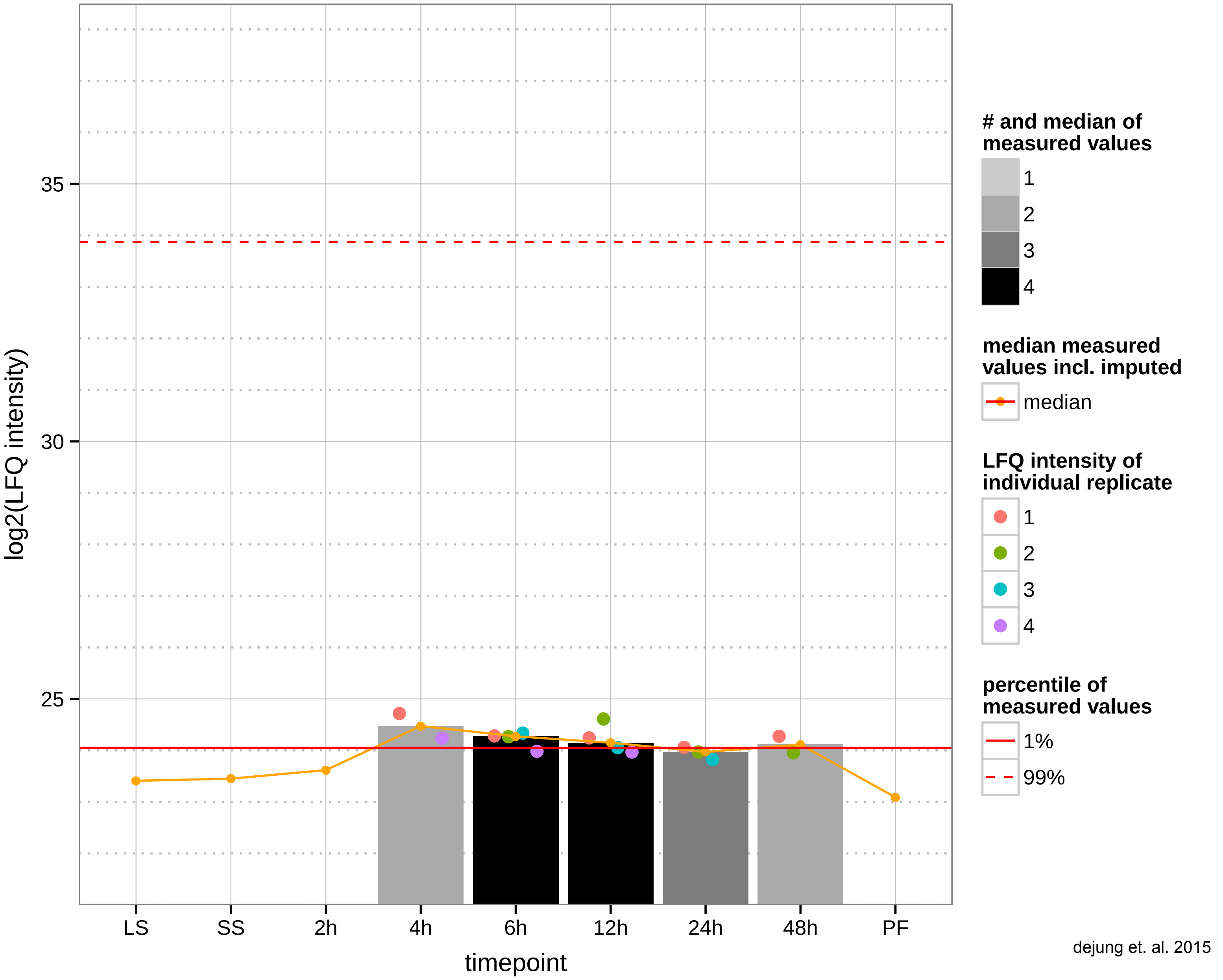

dejung et. al. 2015

hypothetical protein, conserved
Tb927.7.2190
AGOF: null
AGOC: null
AGOP: null
PGOF: null
PGOC: endoplasmic reticulum
PGOP: null

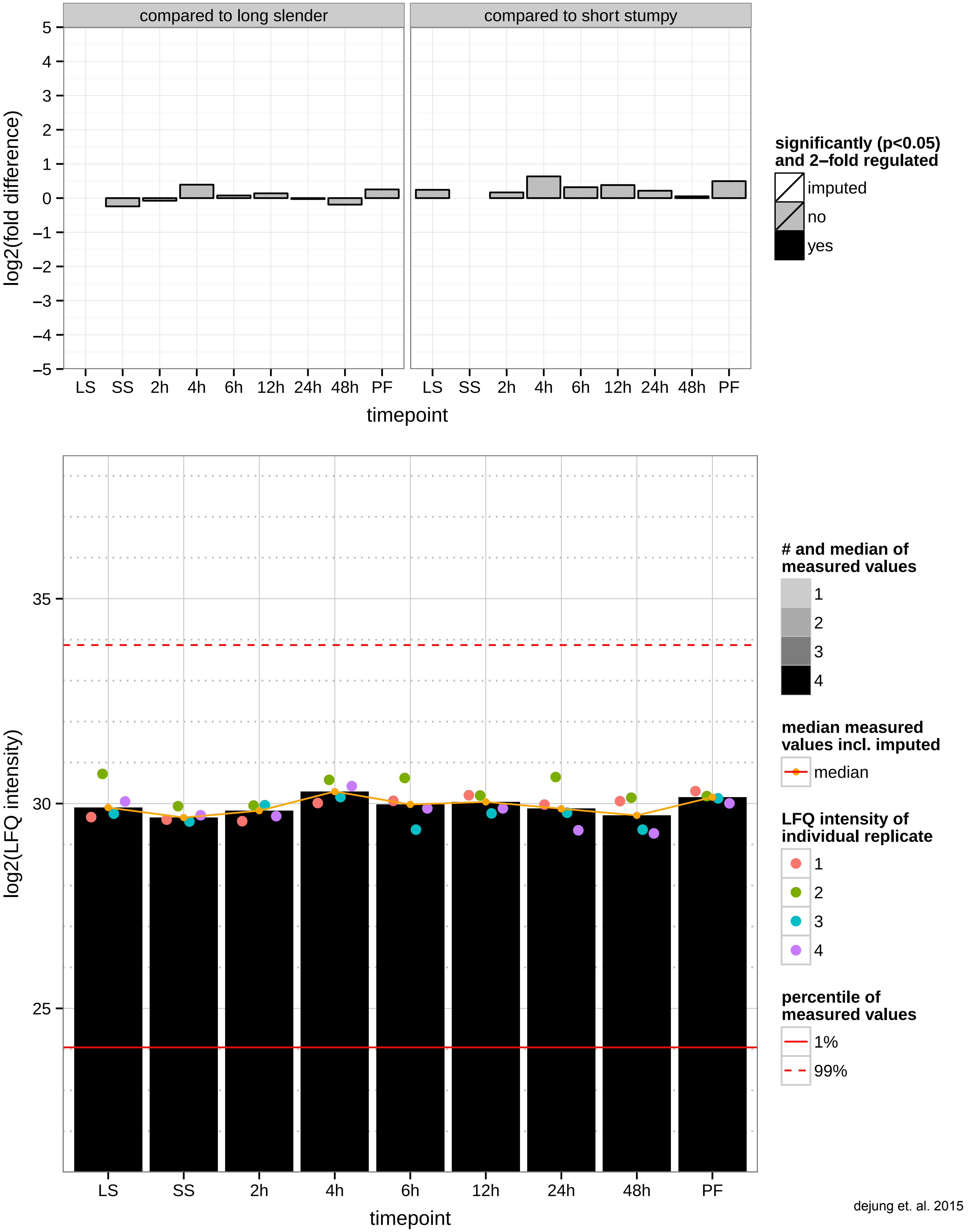

dejung et. al. 2015

CDP–DAG synthase (CDS)
Tb927.7.220
AGOF: phosphatidate cytidylyltransferase activity, transferase activity, transferring phosphorus–containing groups
AGOC: endoplasmic reticulum membrane, integral to Golgi membrane
AGOP: CDP–diacylglycerol biosynthetic process, phosphatidylglycerol biosynthetic process, phosphatidylinositol metabolic pr
PGOF: phosphatidate cytidylyltransferase activity, transferase activity, transferring phosphorus–containing groups
PGOC: membrane
PGOP: null

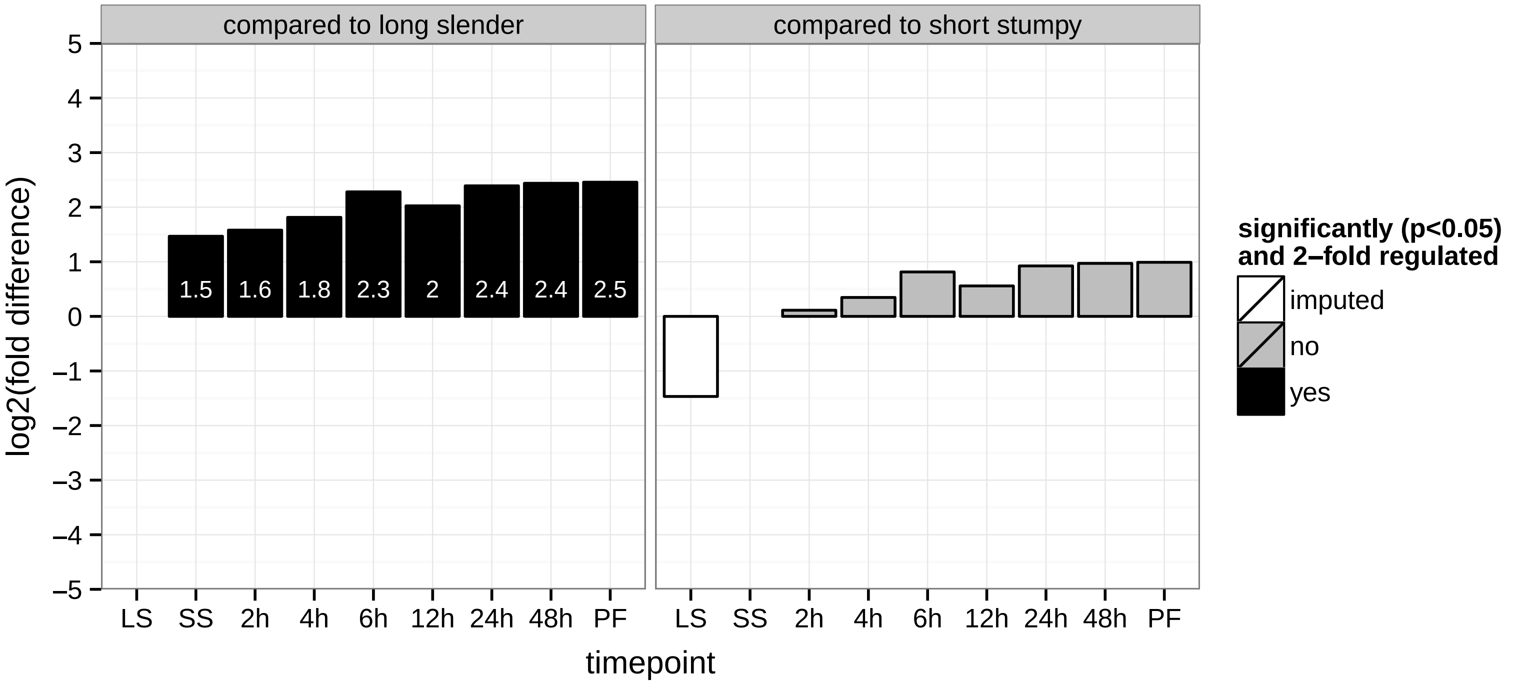

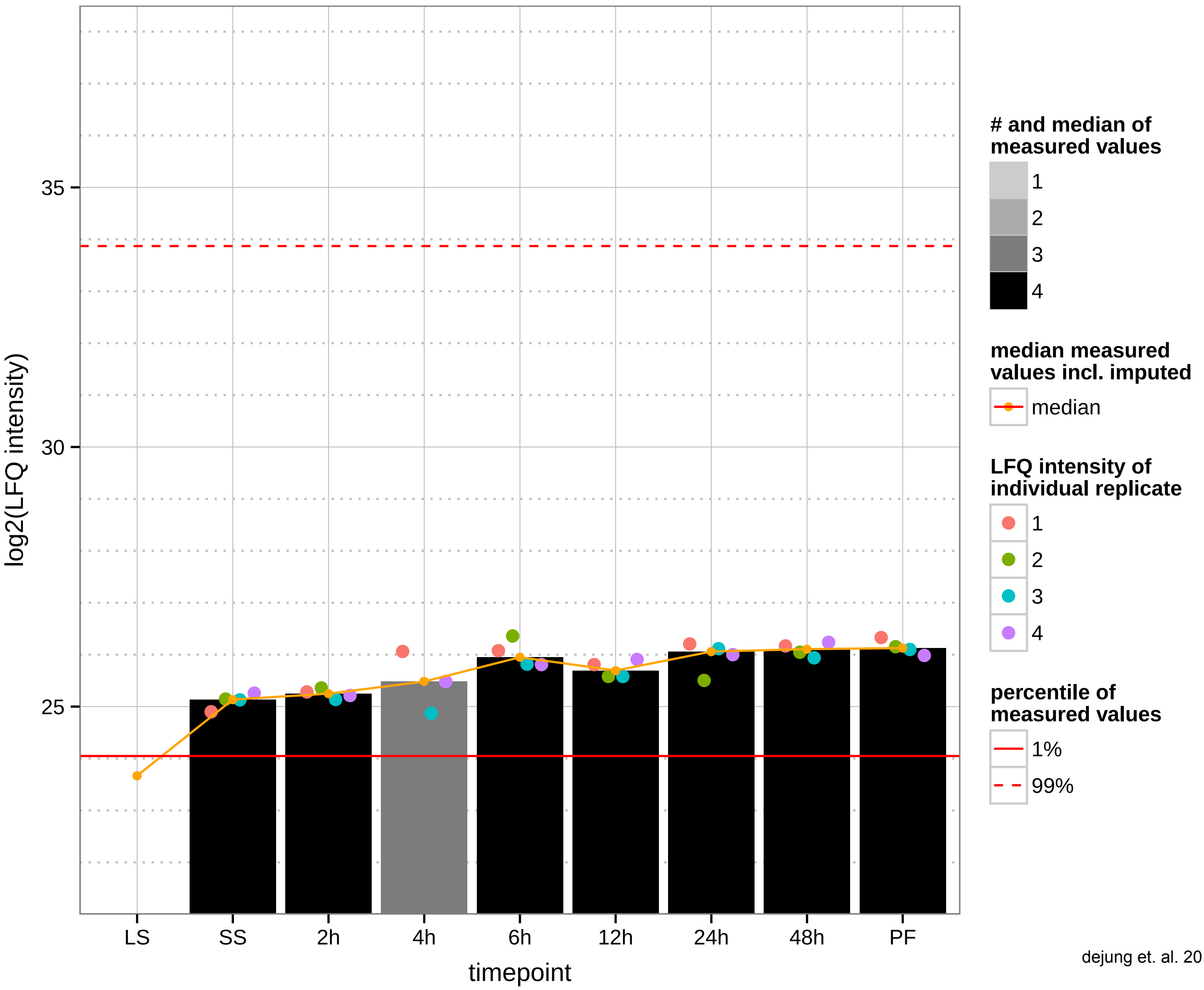

dejung et. al. 2015

hypothetical protein, conserved
Tb927.7.2230
AGOF: null
AGOC: integral to membrane, membrane
AGOP: transport
PGOF: null
PGOC: null
PGOP: null

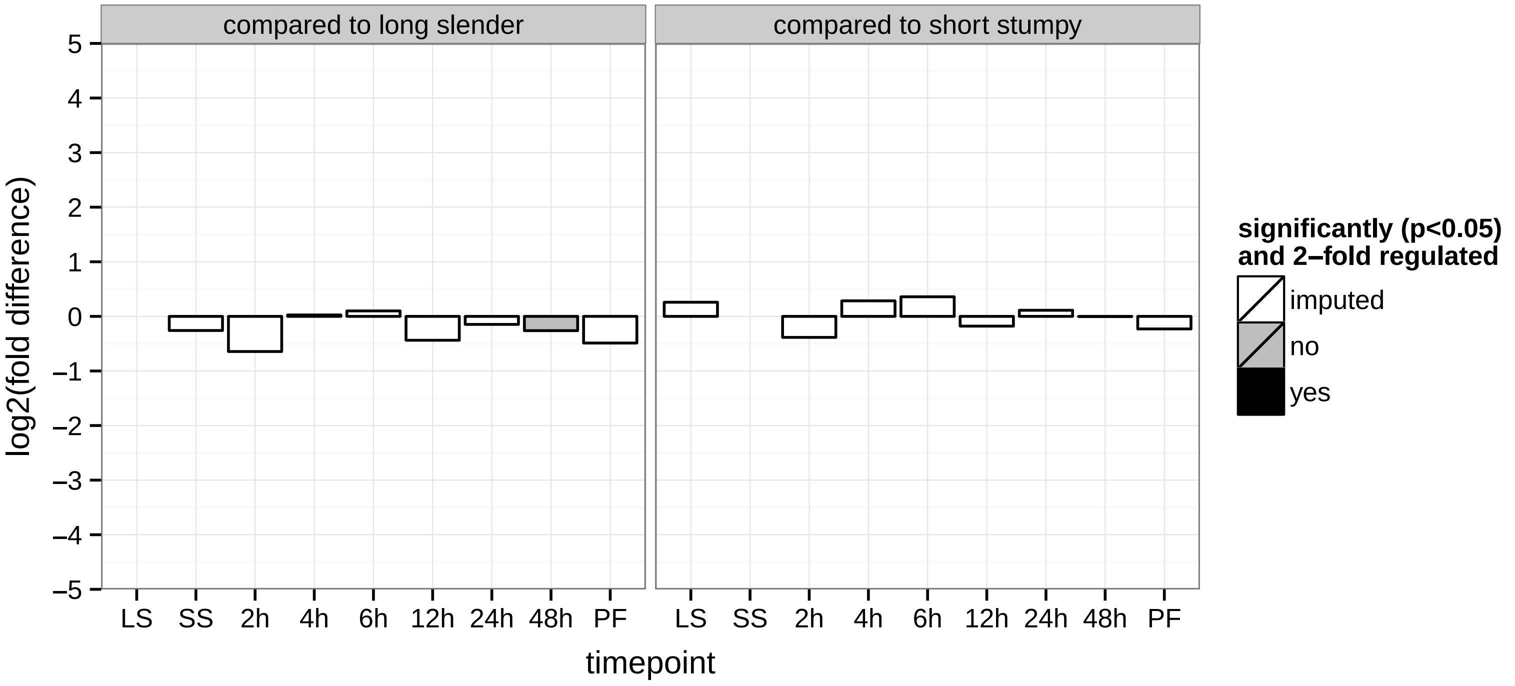

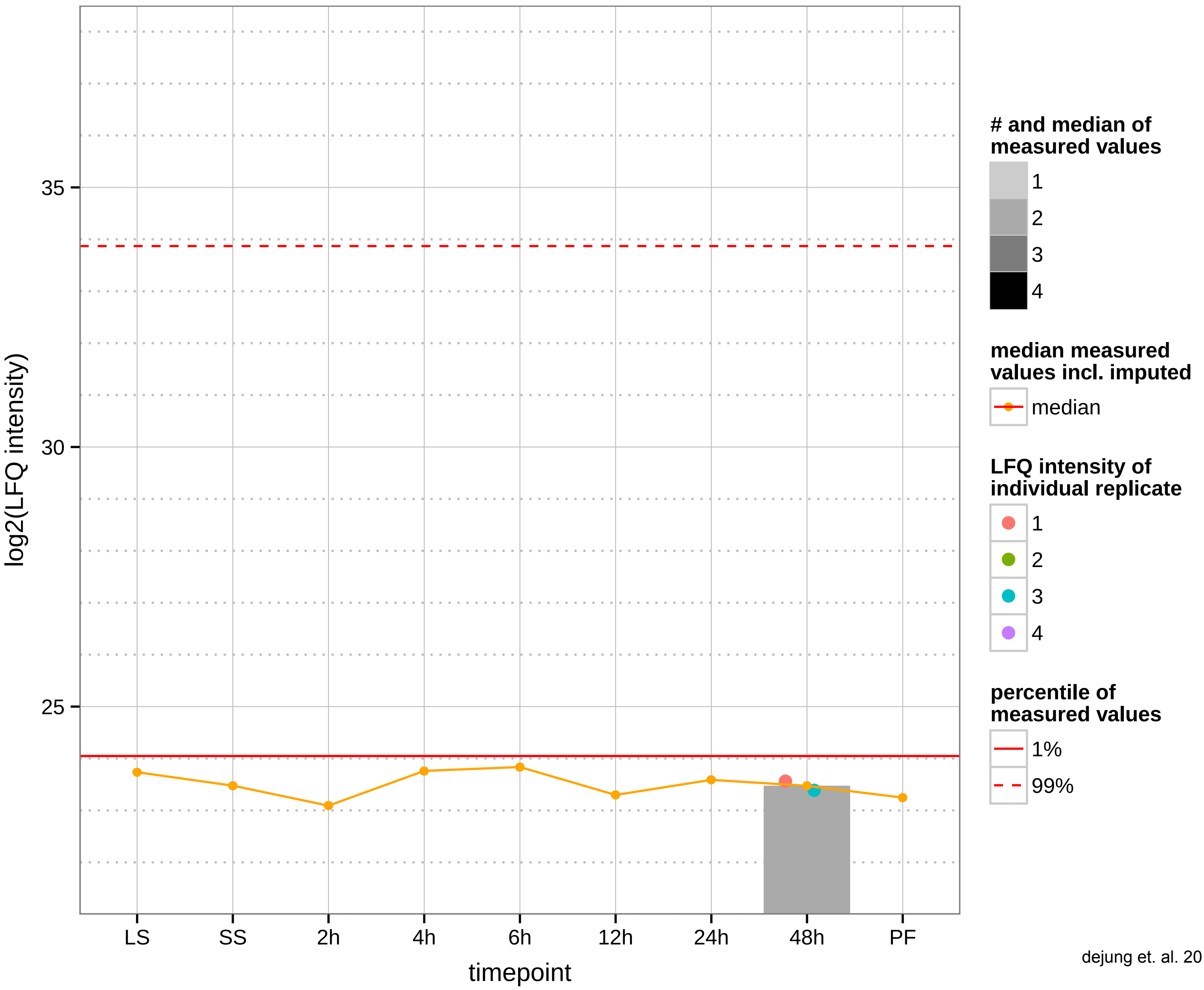

dejung et. al. 2015

hypothetical protein, conserved
Tb927.7.2240
AGOF: null
AGOC: null
AGOP: null
PGOF: binding
PGOC: null
PGOP: null

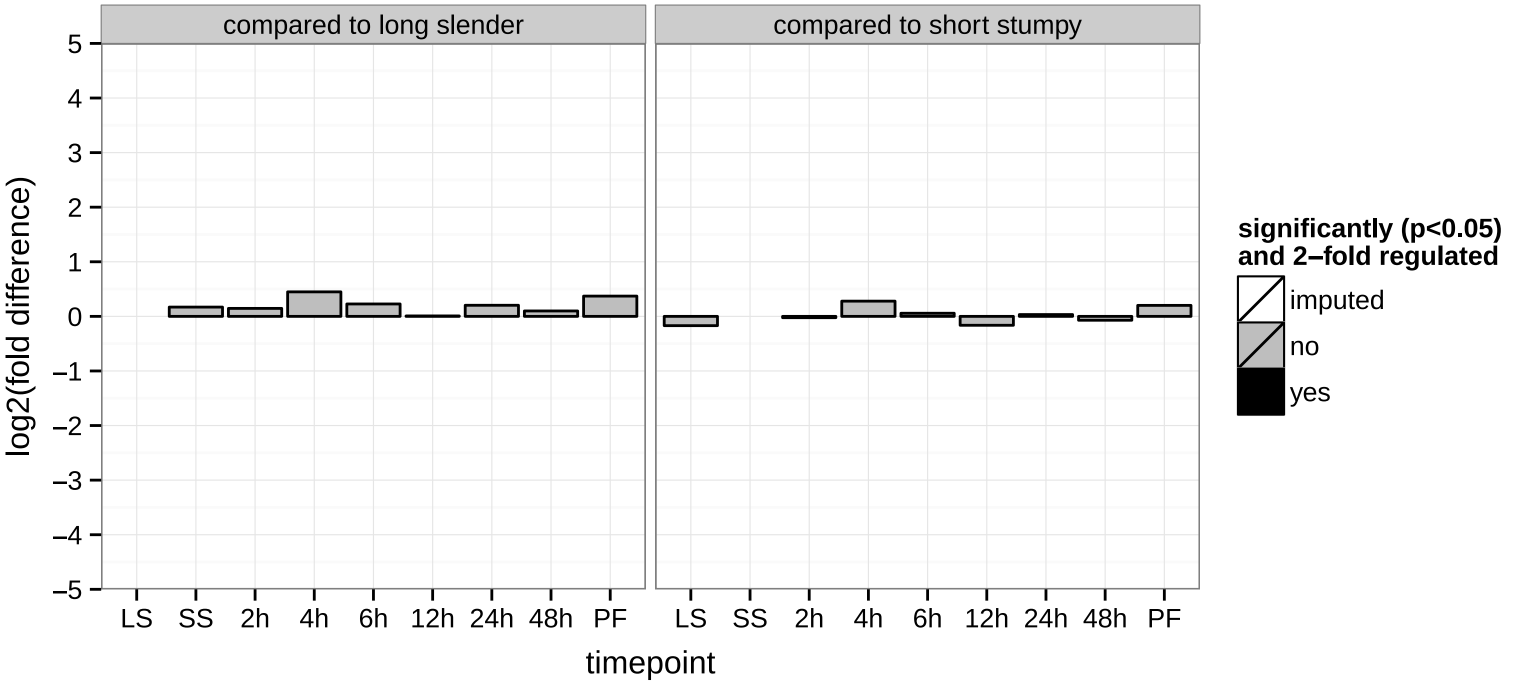

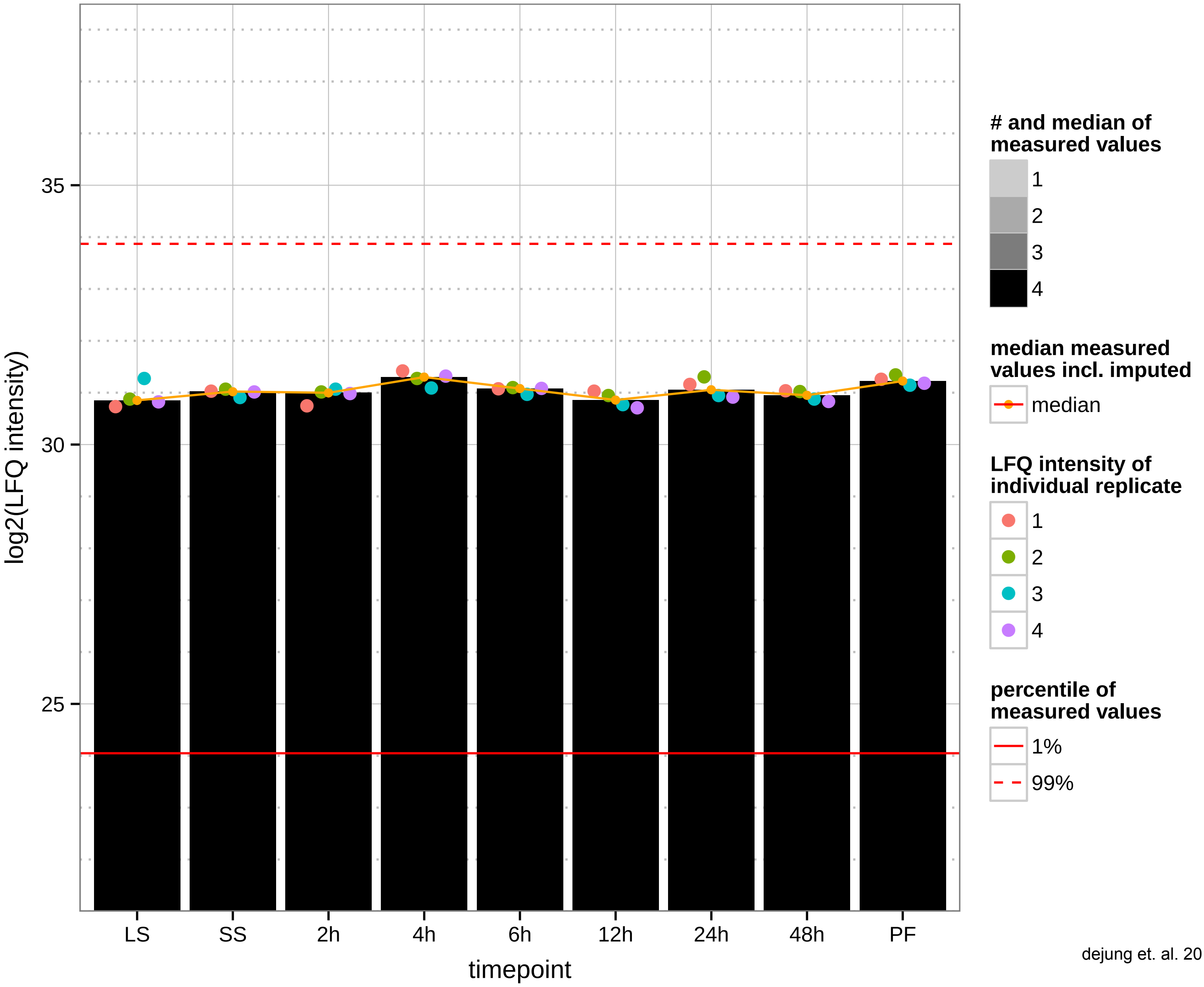

dejung et. al. 2015

hypothetical protein, conserved
Tb927.7.2260
AGOF: null
AGOC: null
AGOP: null
PGOF: null
PGOC: null
PGOP: null

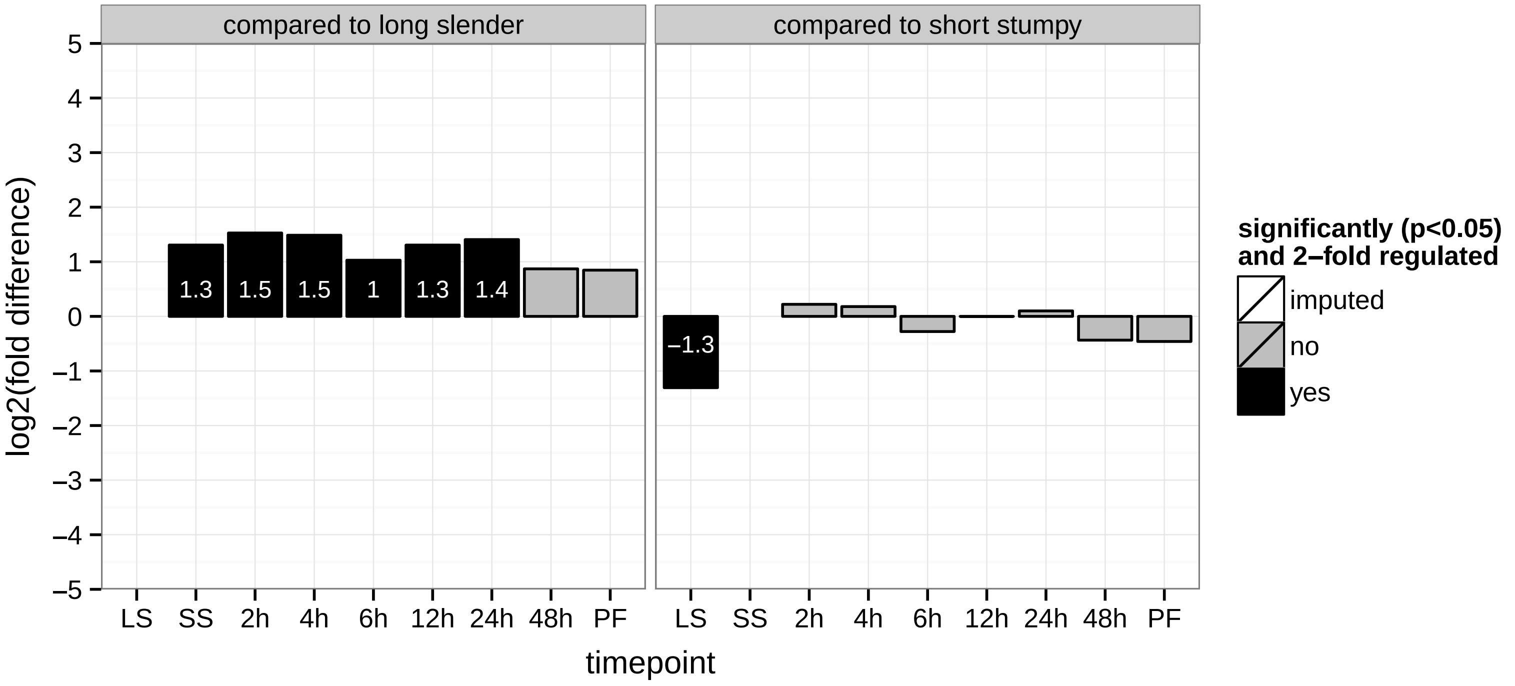

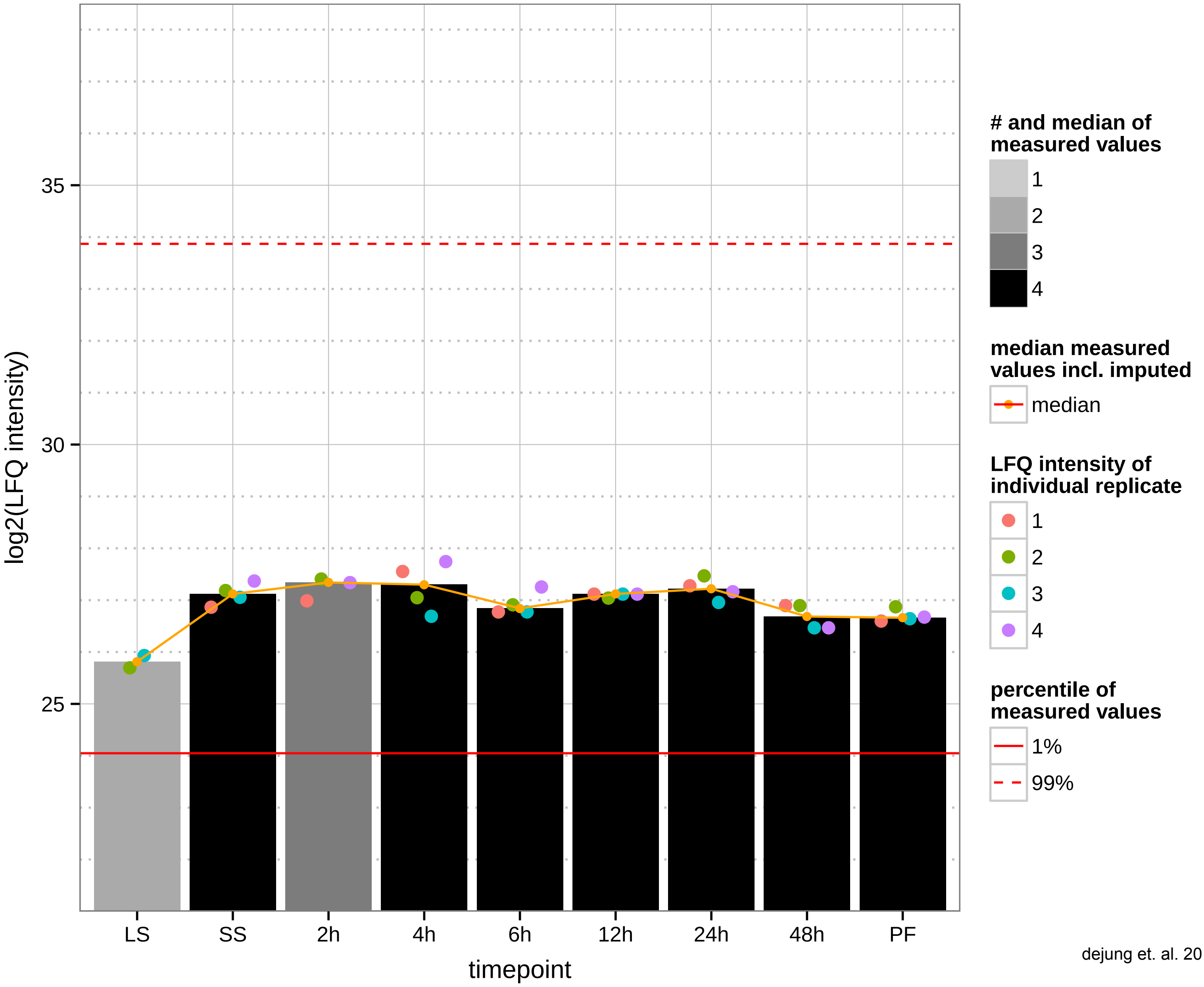

dejung et. al. 2015

Nucleoporin (TbNup132)
Tb927.7.2300
AGOF: null
AGOC: nuclear pore
AGOP: null
PGOF: null
PGOC: null
PGOP: null

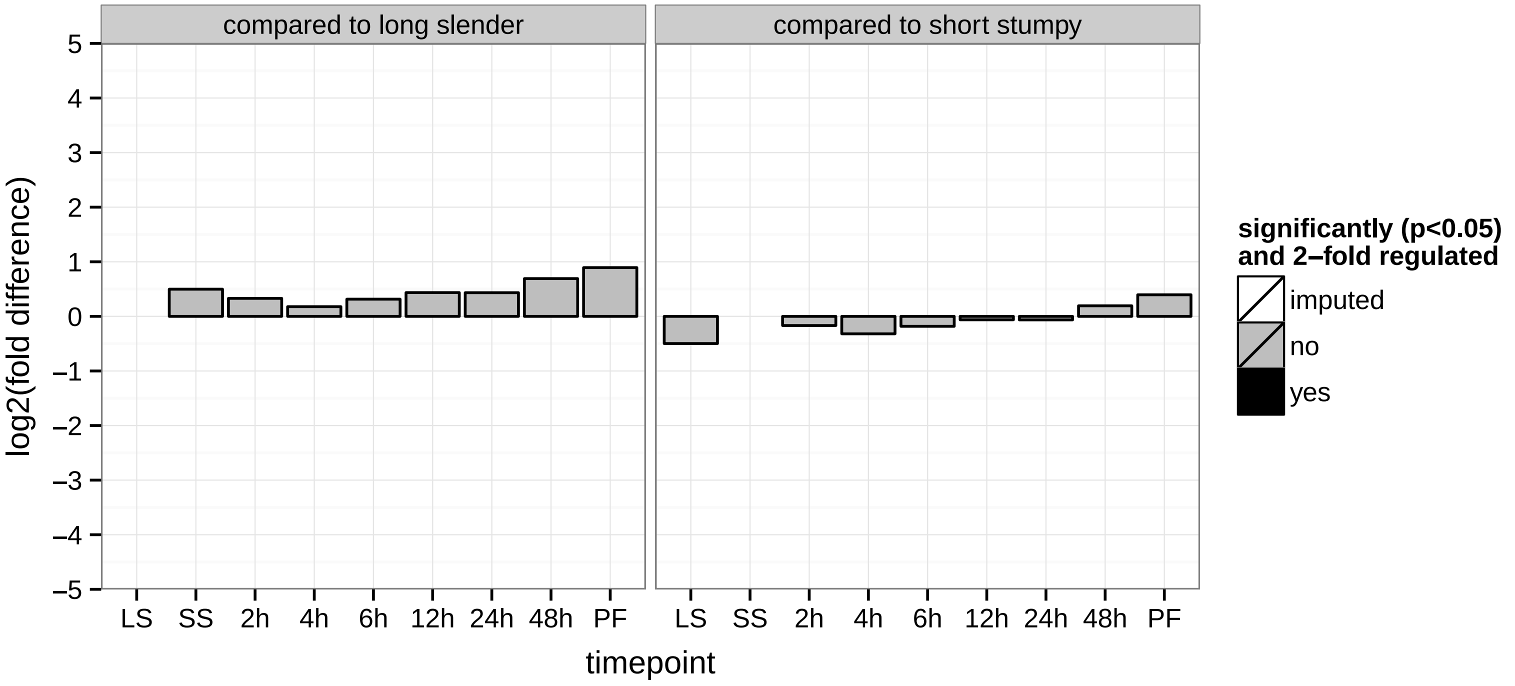

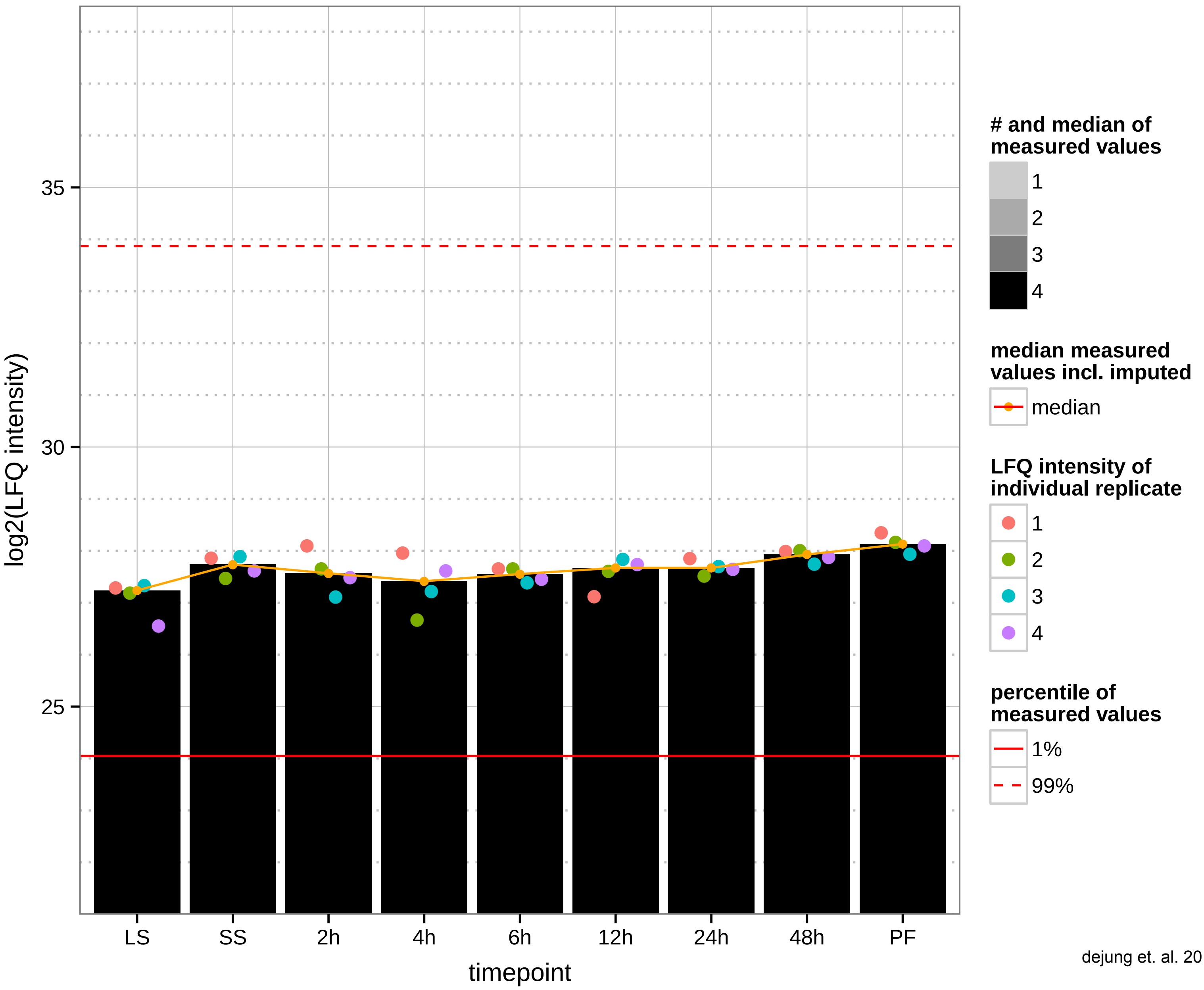

dejung et. al. 2015

pyrroline−5−carboxylate reductase, putative (P5CR)
Tb927.7.2440
AGOF: pyrroline−5−carboxylate reductase activity
AGOC: null
AGOP: proline biosynthetic process
PGOF: oxidoreductase activity, pyrroline−5−carboxylate reductase activity
PGOC: null
PGOP: oxidation−reduction process, proline biosynthetic process

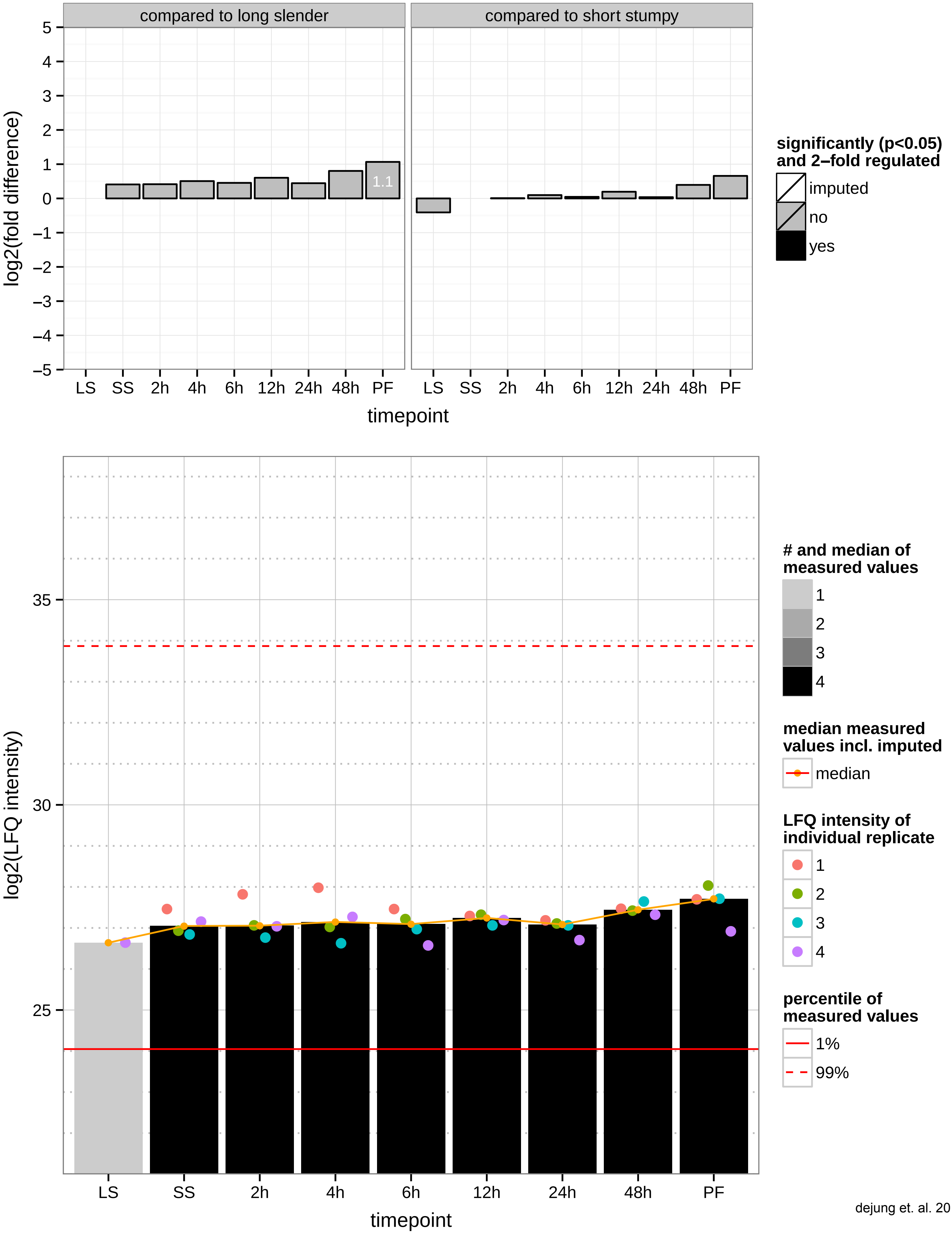

dejung et. al. 2015

hypothetical protein, conserved
Tb927.7.2450
AGOF: null
AGOC: null
AGOP: null
PGOF: null
PGOC: null
PGOP: null

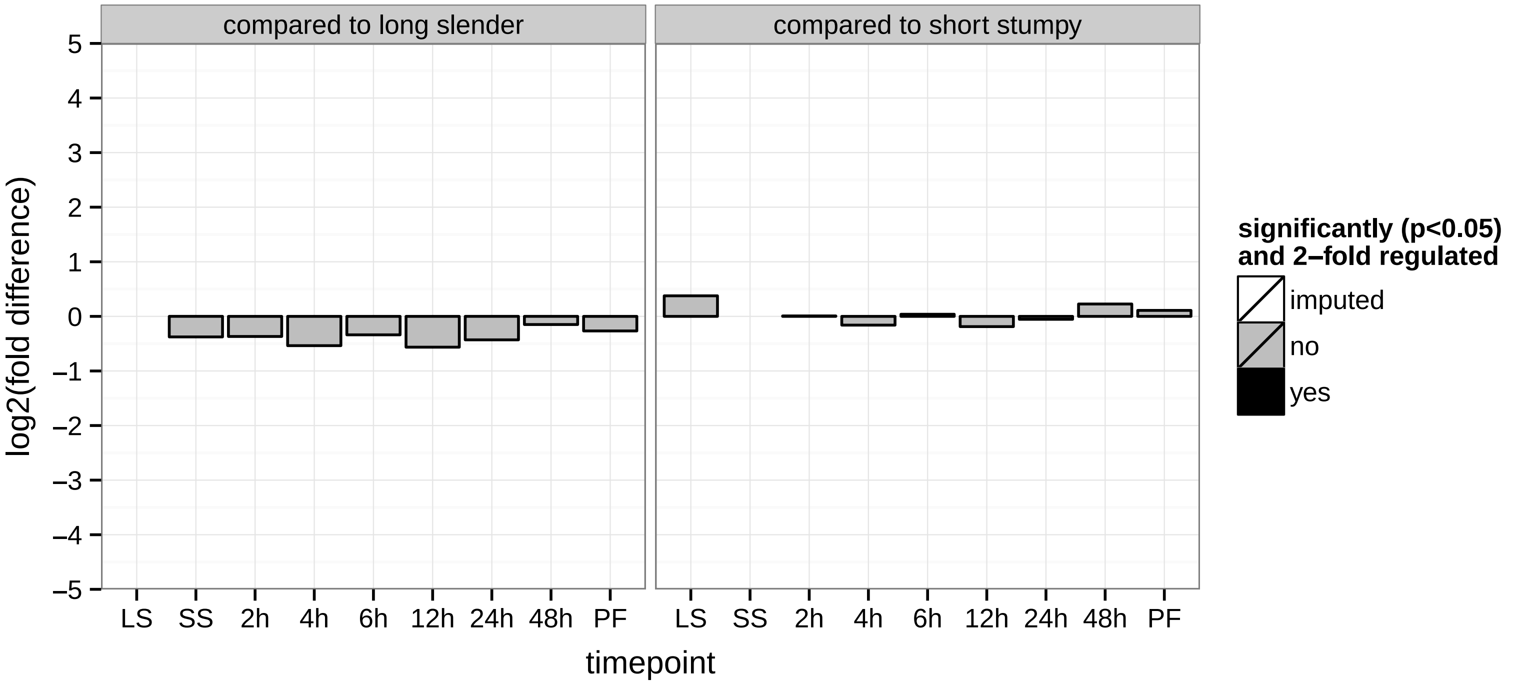

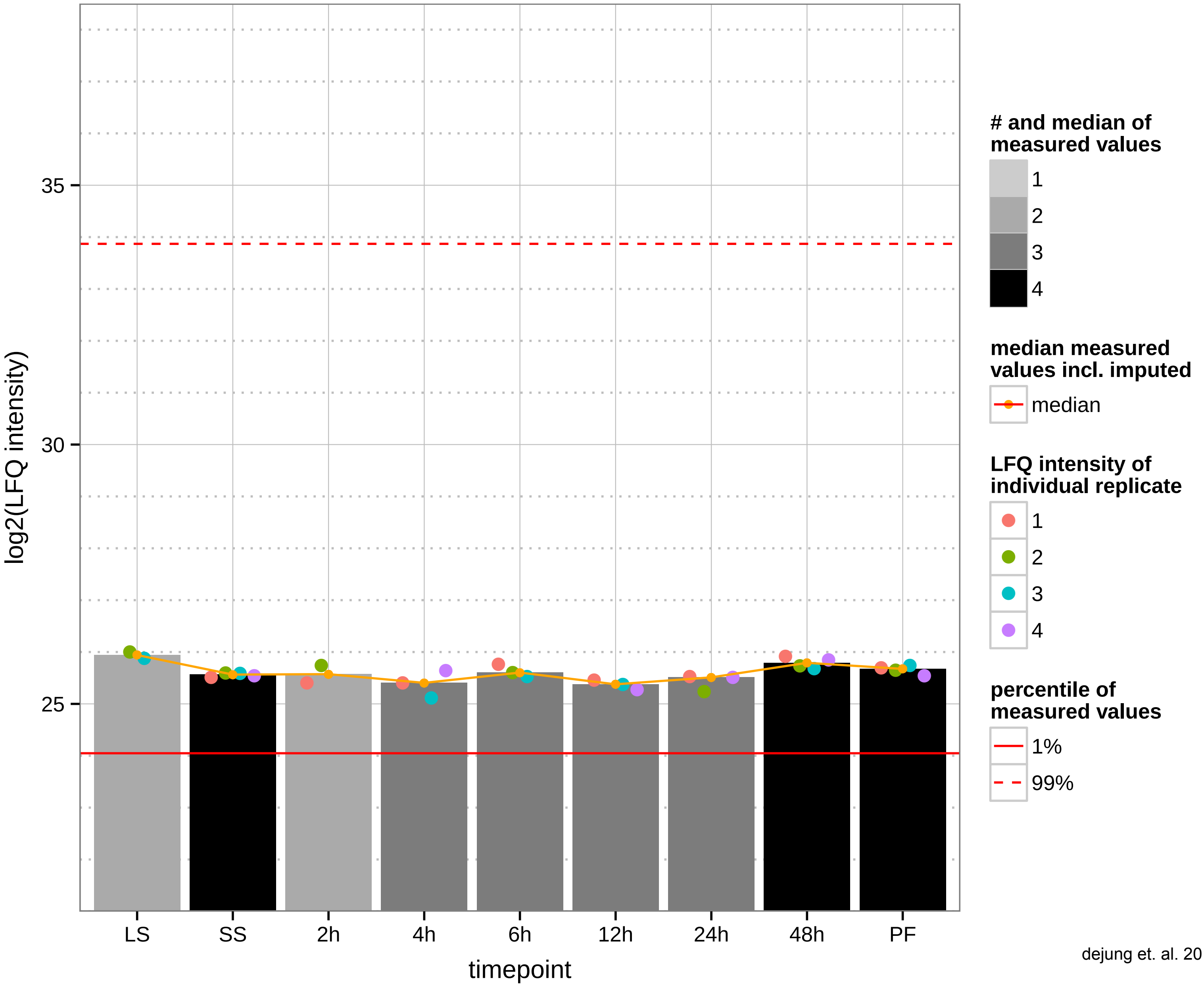

dejung et. al. 2015

proteasome regulatory ATPase subunit 1
Tb927.7.2500
AGOF: ATP binding, nucleoside–triphosphatase activity
AGOC: cytoplasm, nucleus
AGOP: protein catabolic process
PGOF: ATP binding, hydrolase activity, nucleoside–triphosphatase activity, nucleotide binding
PGOC: cytoplasm
PGOP: protein catabolic process

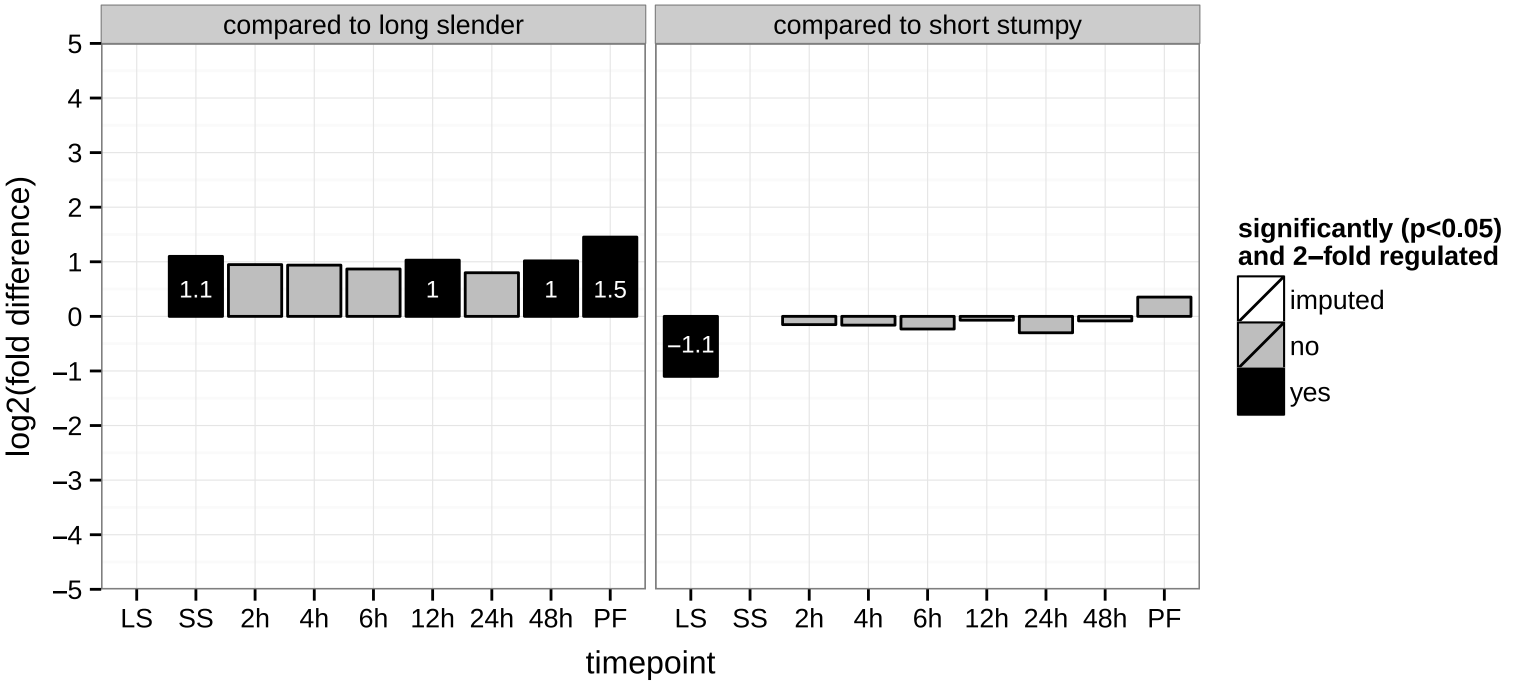

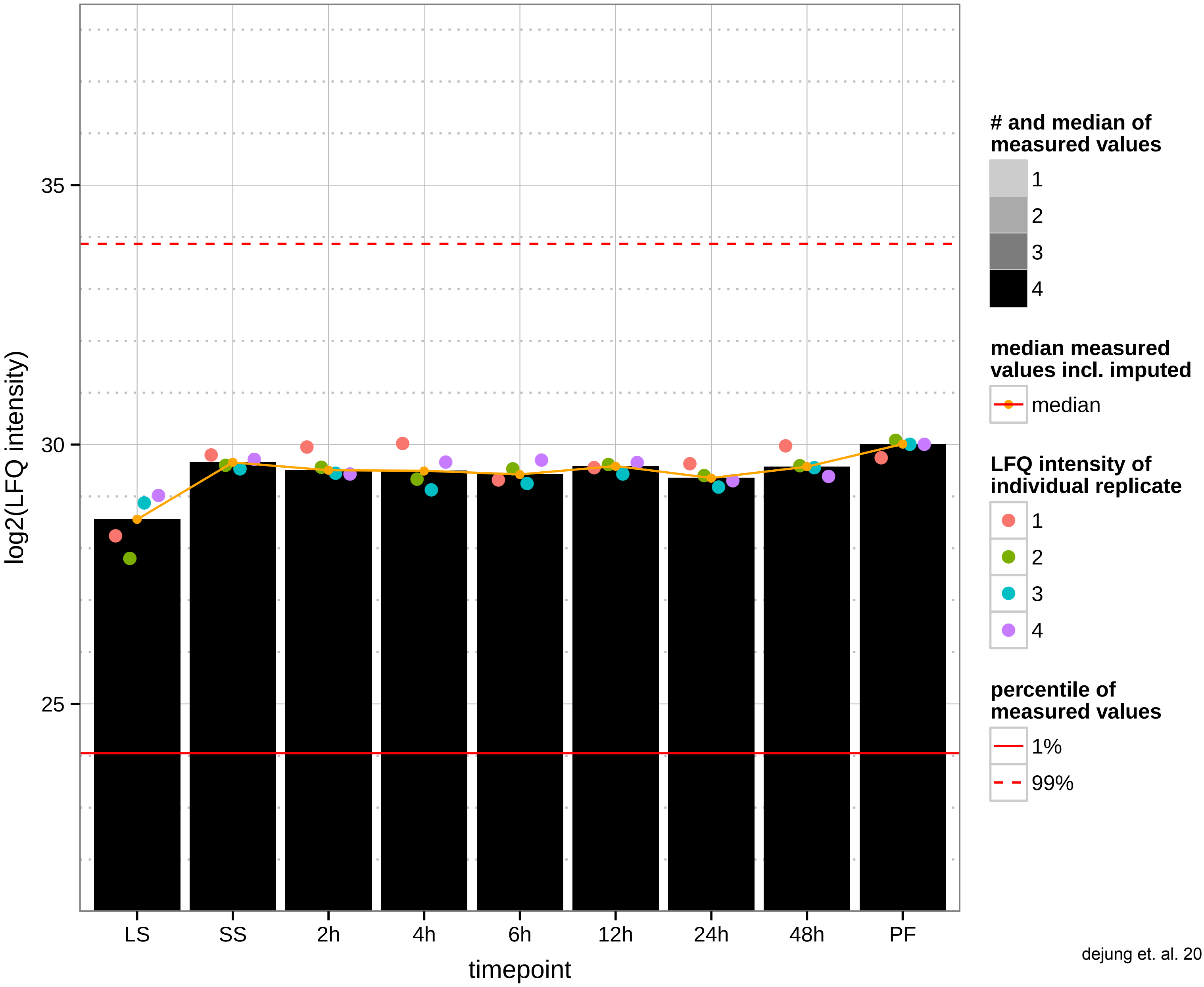

dejung et. al. 2015

proteasome regulatory ATPase subunit 5 (RPT5)
Tb927.7.2550
AGOF: ATP binding, nucleoside−triphosphatase activity
AGOC: cytoplasm, nucleus
AGOP: protein catabolic process
PGOF: ATP binding, hydrolase activity, nucleoside−triphosphatase activity, nucleotide binding
PGOC: cytoplasm
PGOP: protein catabolic process

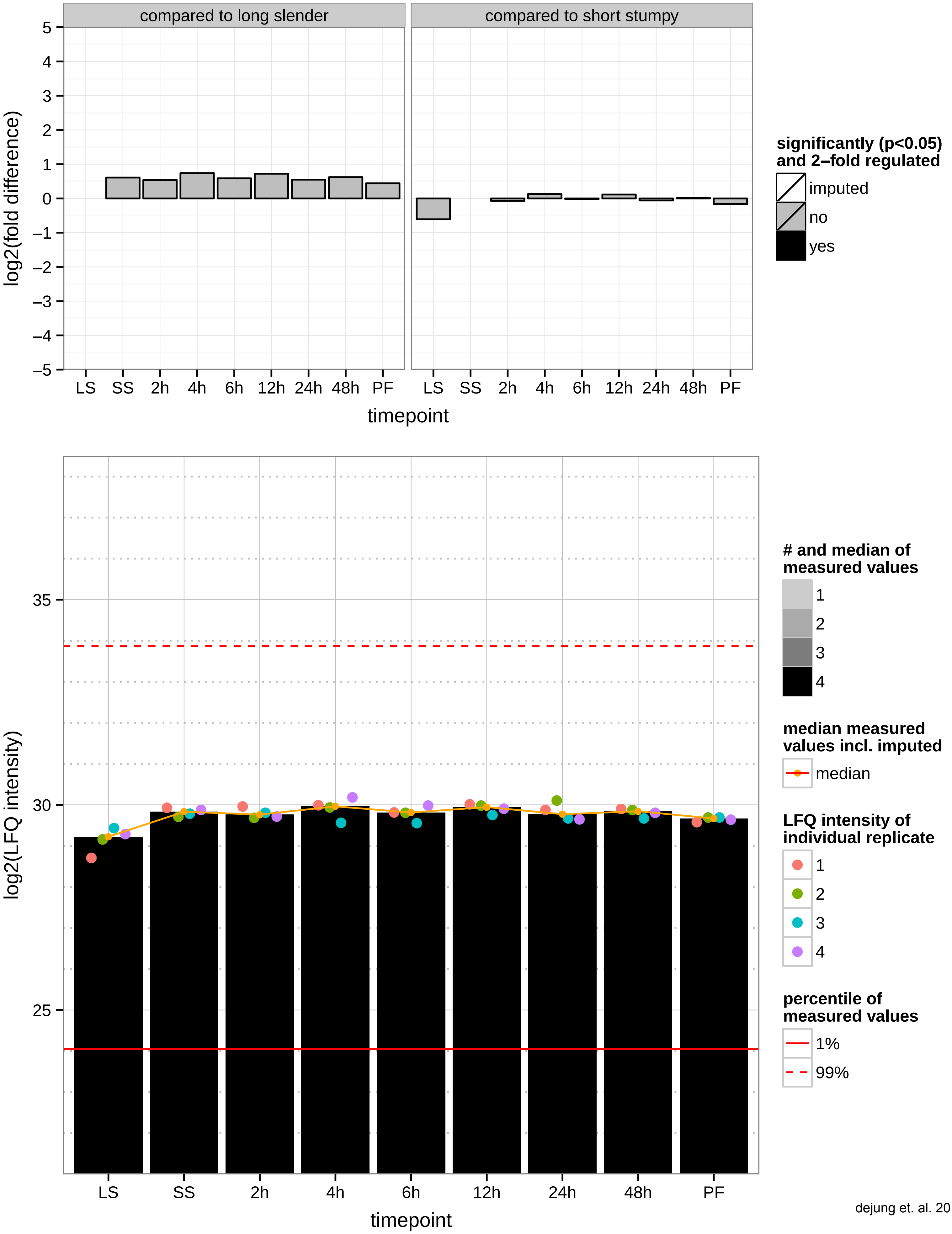

dejung et. al. 2015

ribosome biogenesis protein, putative
Tb927.7.270
AGOF: null
AGOC: null
AGOP: null
PGOF: null
PGOC: null
PGOP: null

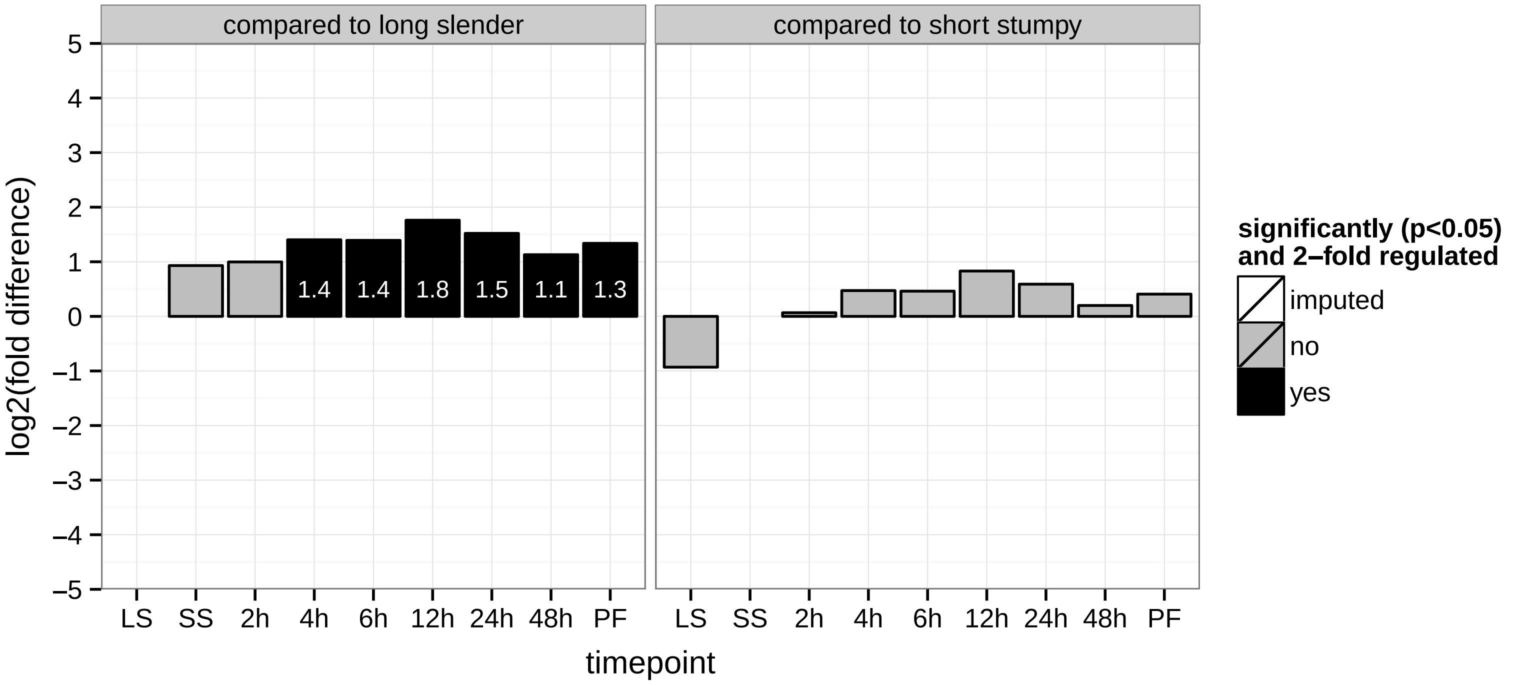

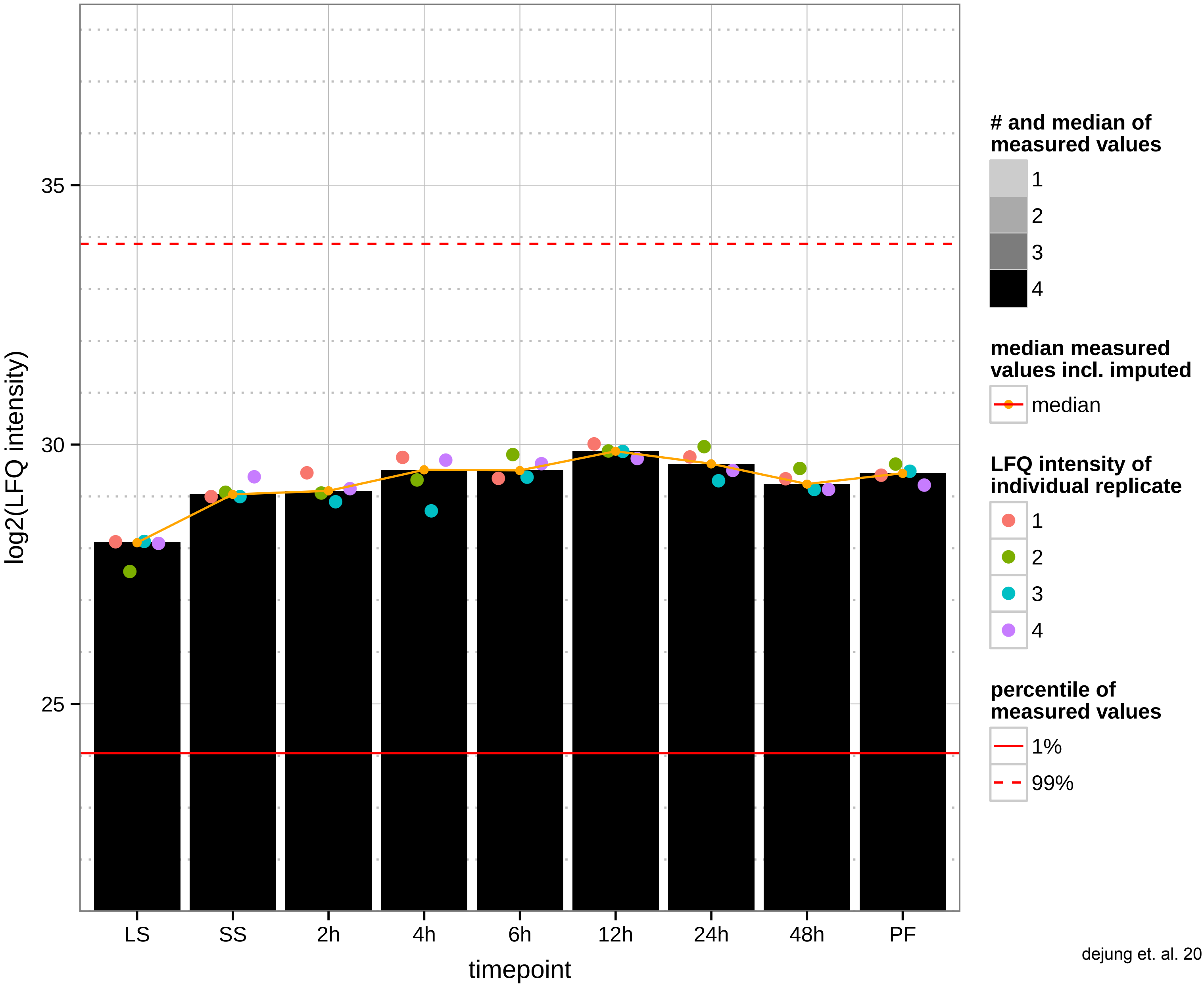

dejung et. al. 2015

hypothetical protein, conserved
Tb927.7.2720
AGOF: RNA binding, translation initiation factor activity
AGOC: null
AGOP: translational initiation
PGOF: RNA binding, translation initiation factor activity
PGOC: null
PGOP: translational initiation

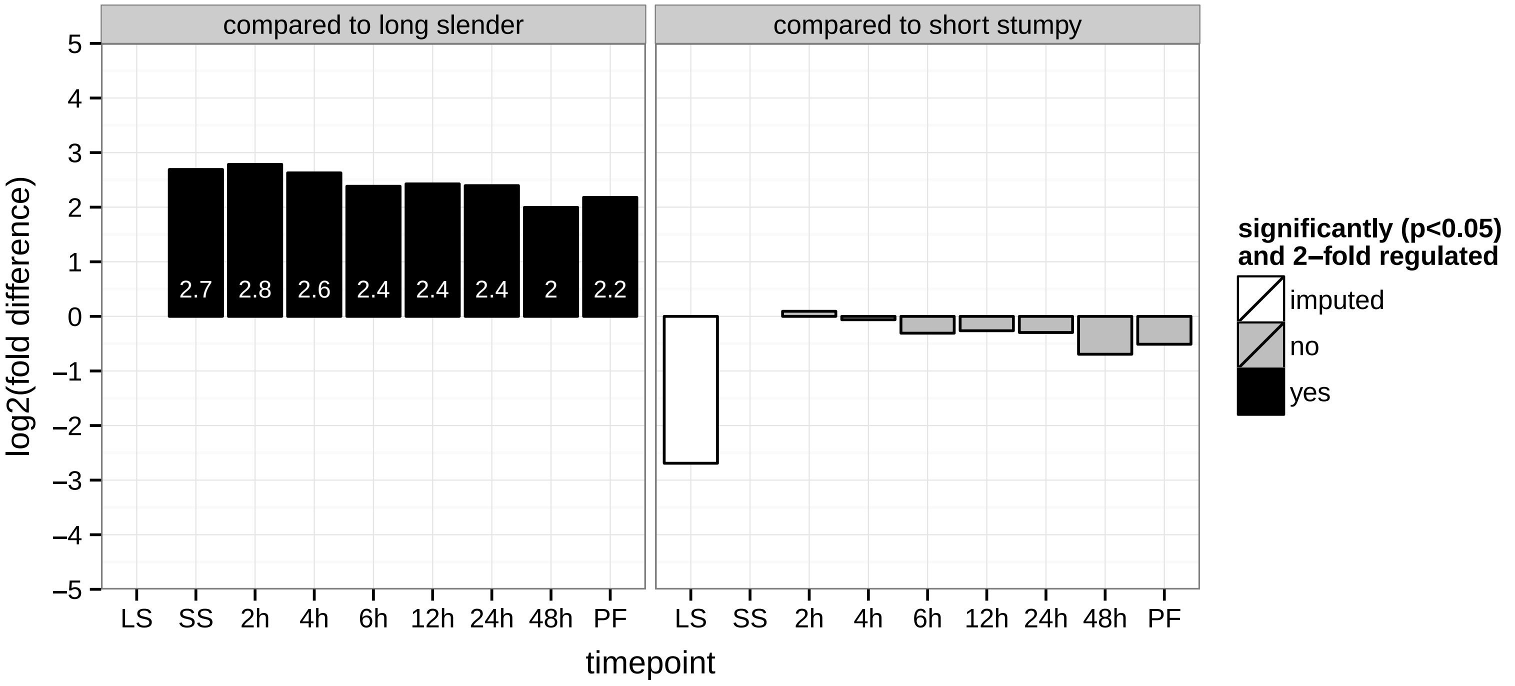

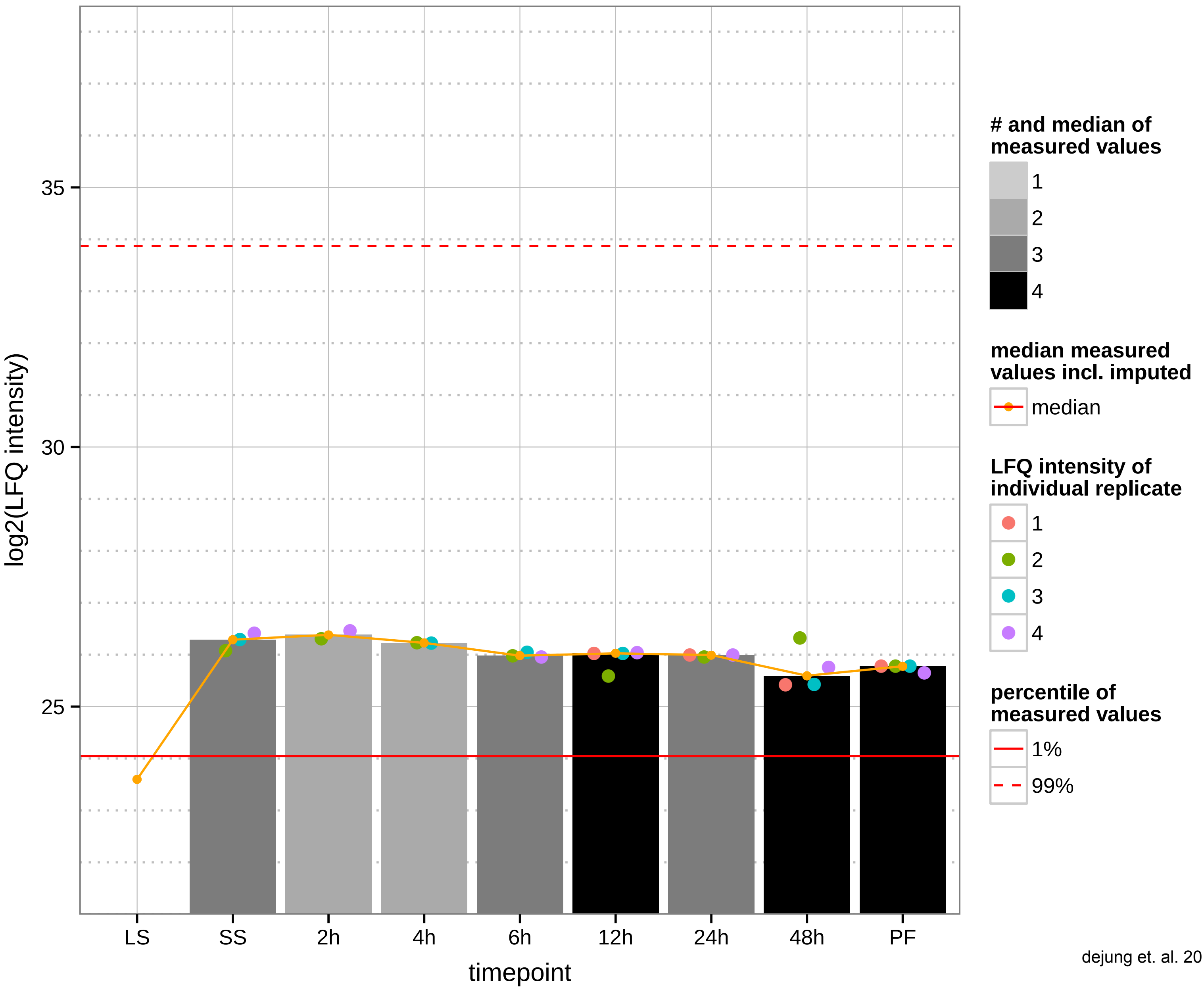

dejung et. al. 2015

leucine–rich repeat protein (LRRP), putative
Tb927.7.2730
AGOF: null
AGOC: null
AGOP: null
PGOF: protein binding
PGOC: null
PGOP: null

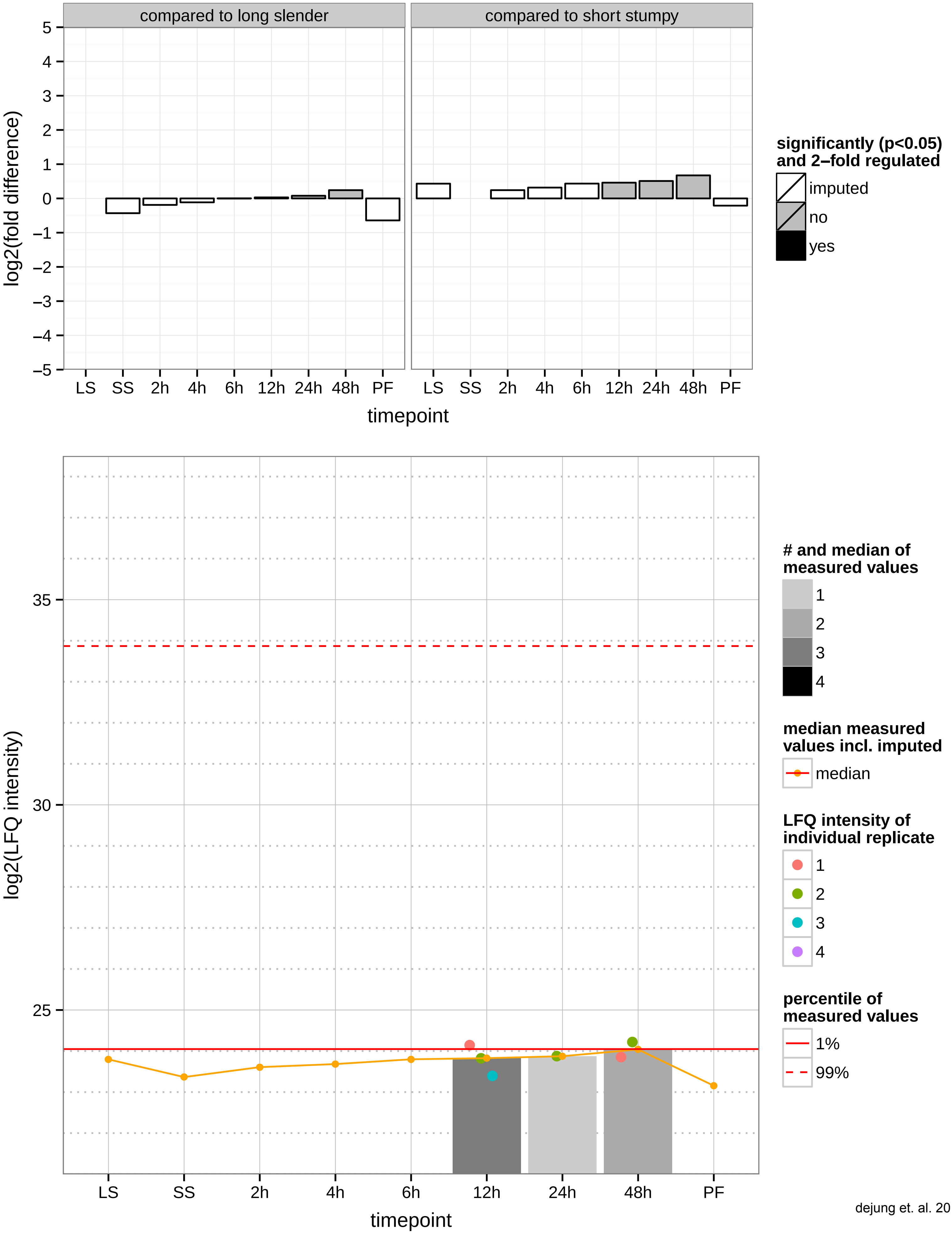

dejung et. al. 2015

protein kinase, putative
Tb927.7.2750
AGOF: ATP binding, protein serine/threonine kinase activity, protein tyrosine kinase activity
AGOC: null
AGOP: growth, protein phosphorylation
PGOF: ATP binding, protein kinase activity, transferase activity, transferring phosphorus–containing groups
PGOC: null
PGOP: protein phosphorylation

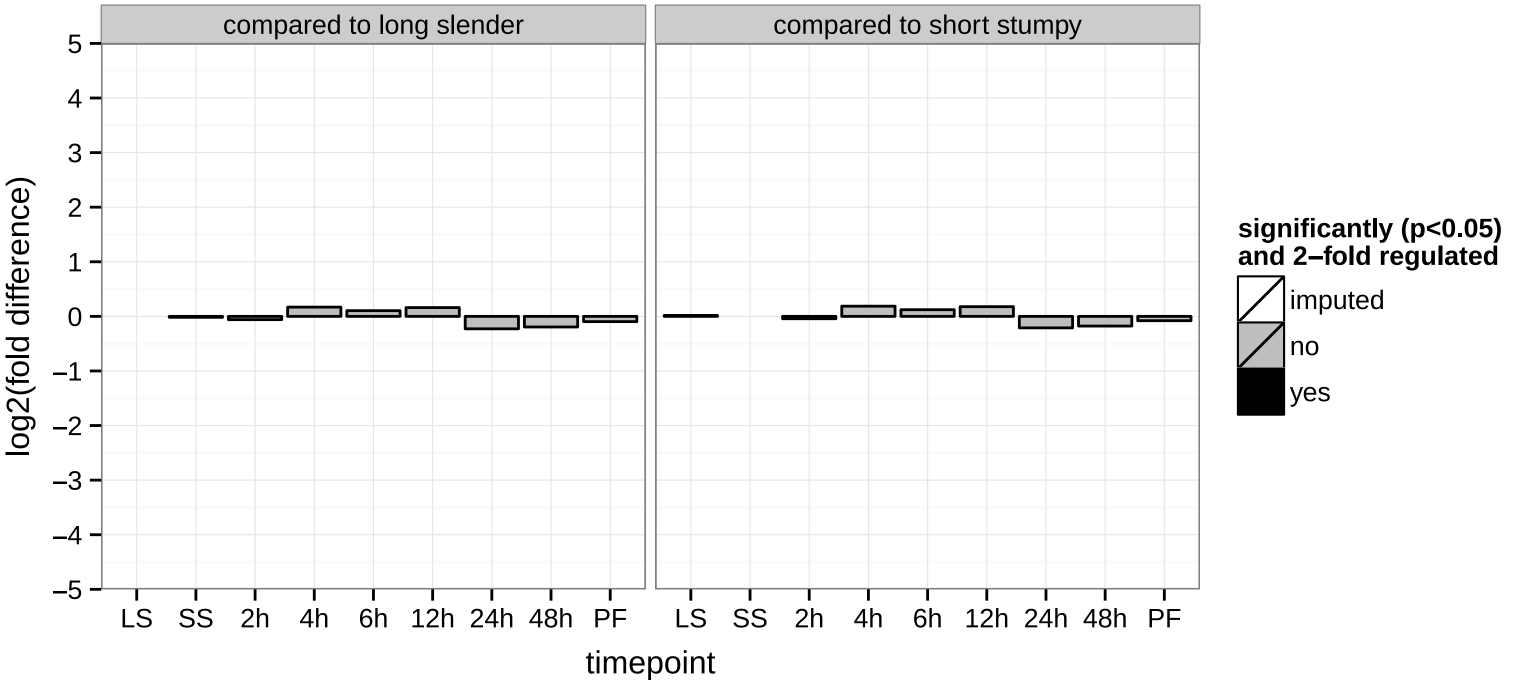

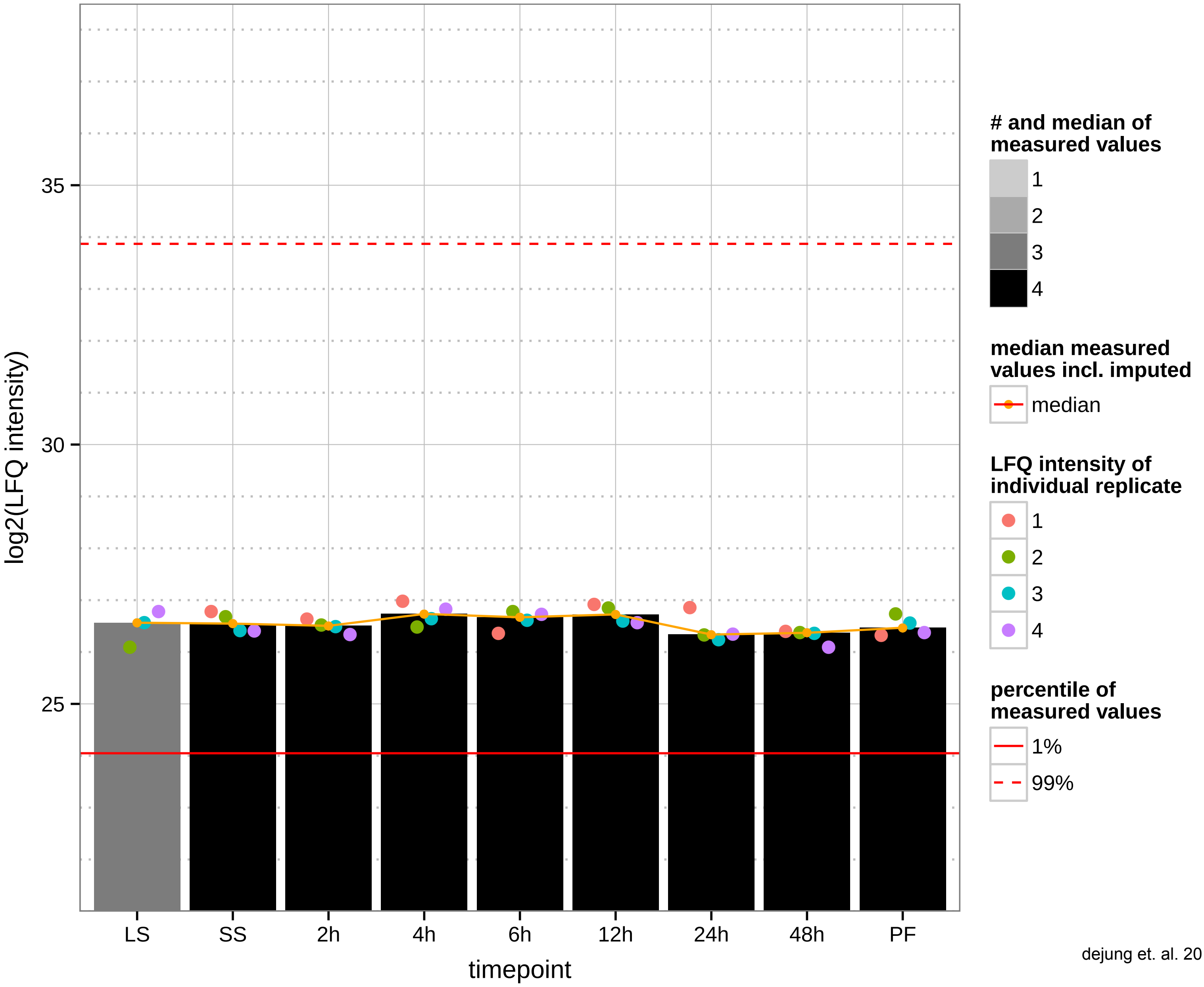

dejung et. al. 2015

hypothetical protein, conserved
Tb927.7.2780
AGOF: null
AGOC: null
AGOP: null
PGOF: null
PGOC: null
PGOP: null

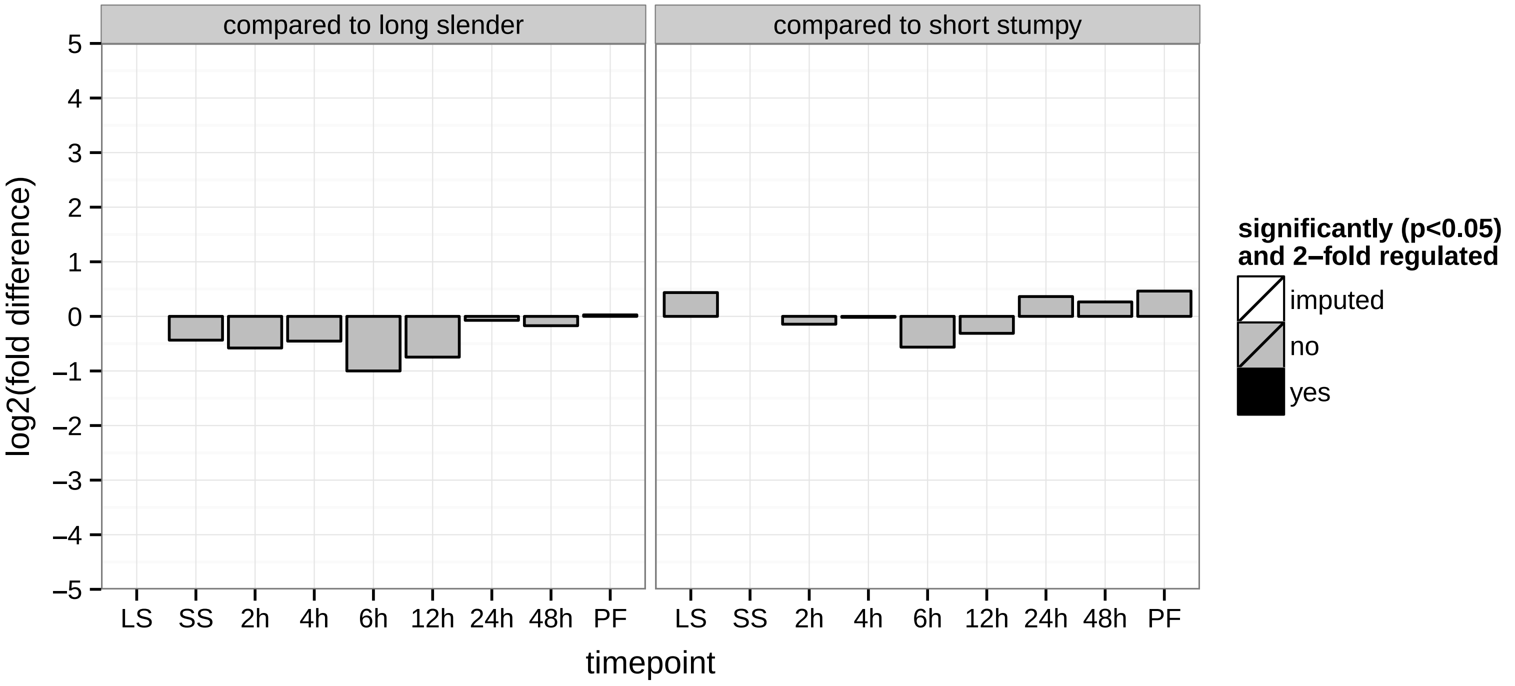

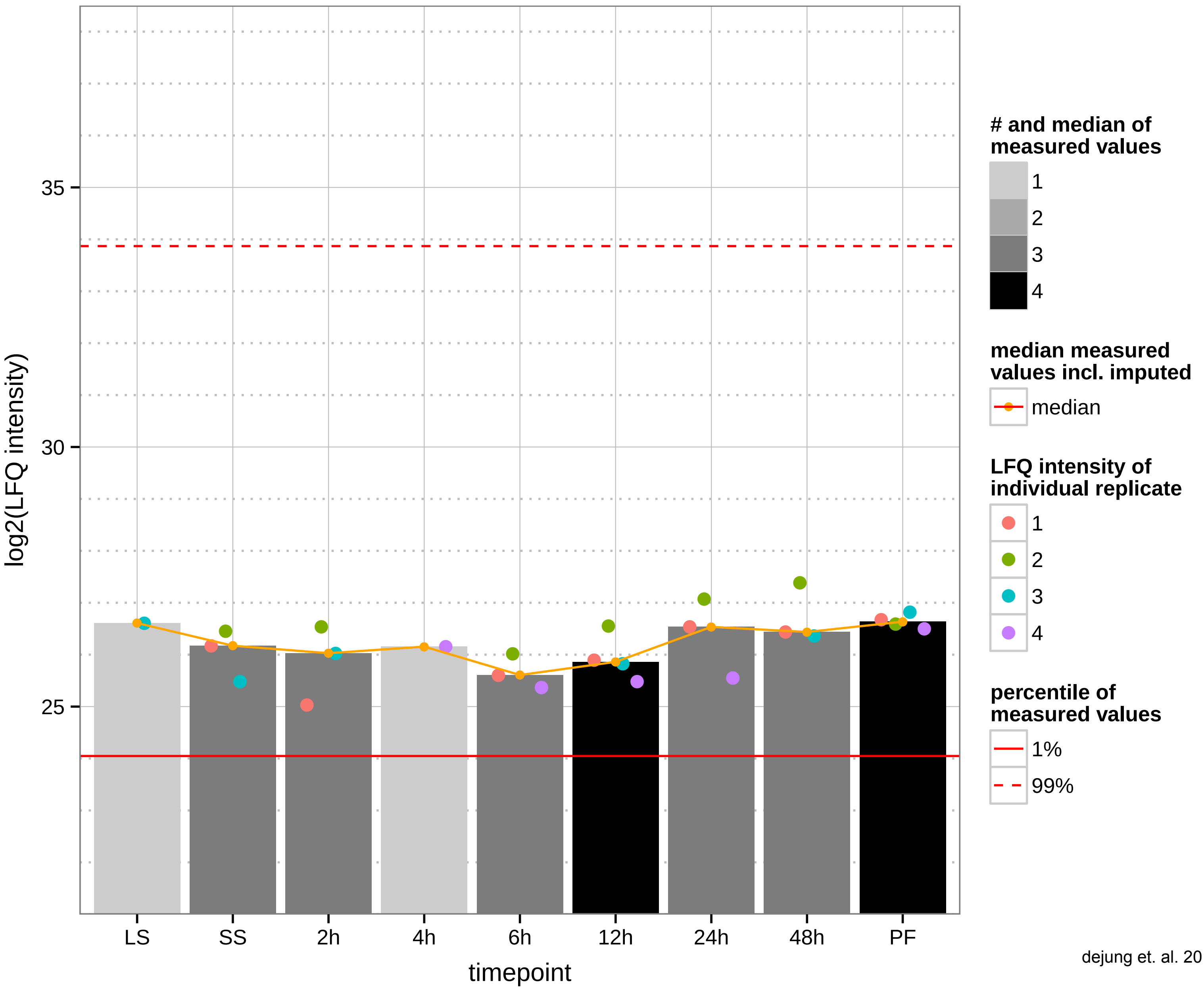

dejung et. al. 2015

hypothetical protein, conserved
Tb927.7.2790
AGOF: null
AGOC: null
AGOP: ciliary or flagellar motility
PGOF: protein binding
PGOC: null
PGOP: null

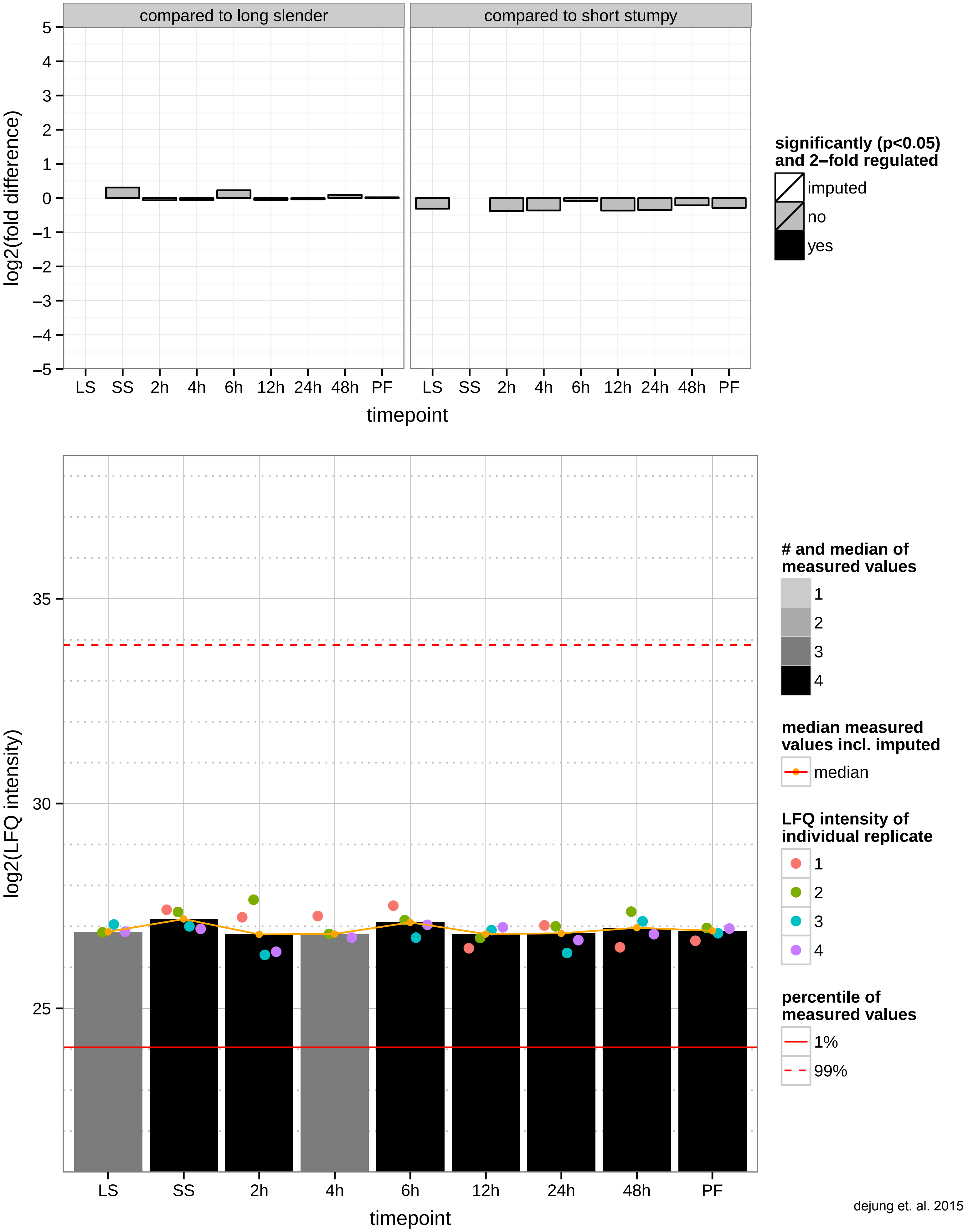

dejung et. al. 2015

histone H2A, putative
Tb927.7.2940;Tb927.7.2930;Tb927.7.2920;Tb927.7.2910;Tb927.7.2900;Tb927.7.2890;Tb927.7.2880;Tb927.7.2870;Tb927.7
AGOF: DNA binding
AGOC: nucleosome, nucleus
AGOP: chromosome organization, nucleosome assembly
PGOF: DNA binding, null
PGOC: nucleosome, nucleus, null
PGOP: nucleosome assembly, null

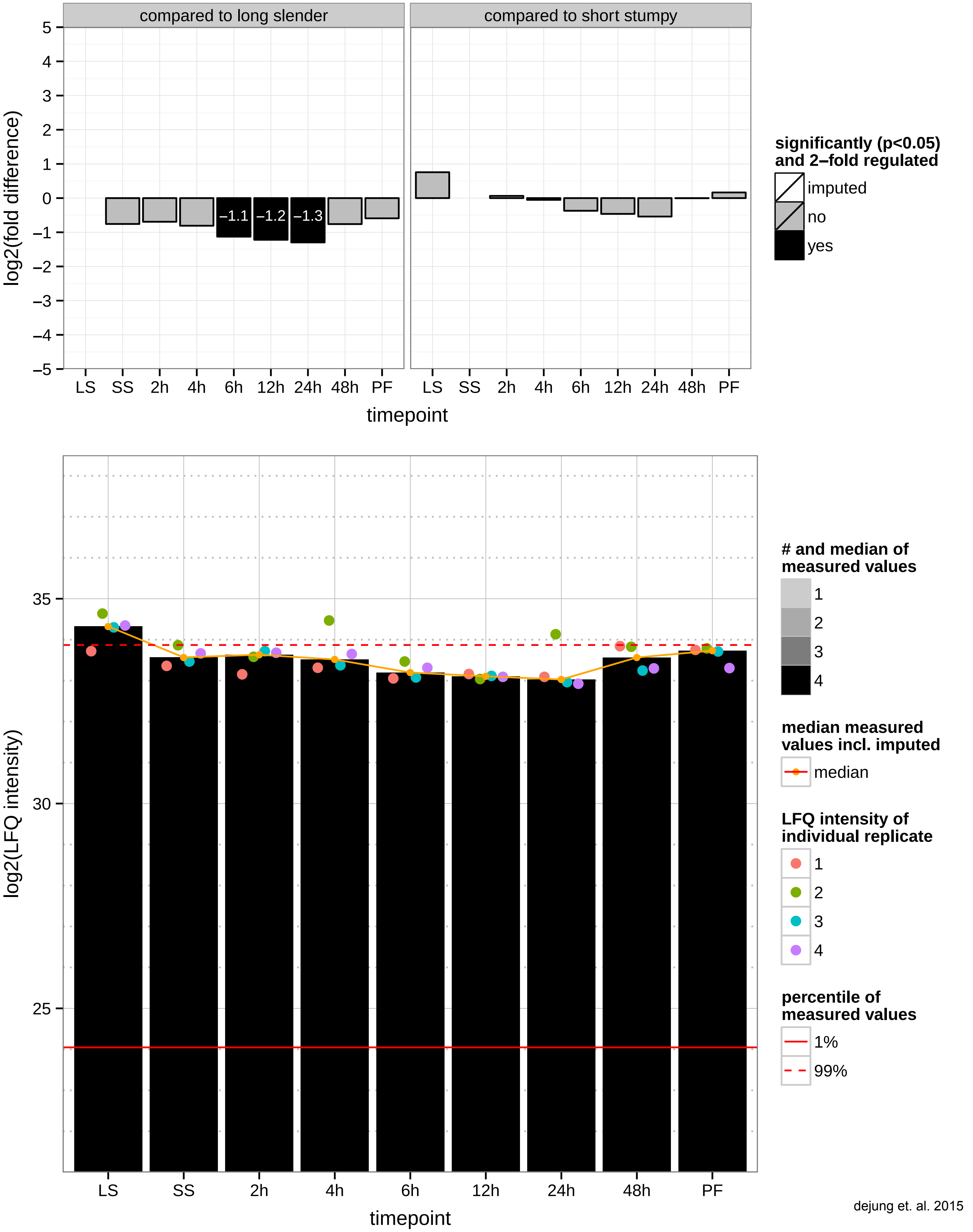

dejung et. al. 2015

hypothetical protein, conserved
Tb927.7.3080
AGOF: null
AGOC: null
AGOP: null
PGOF: null
PGOC: null
PGOP: null

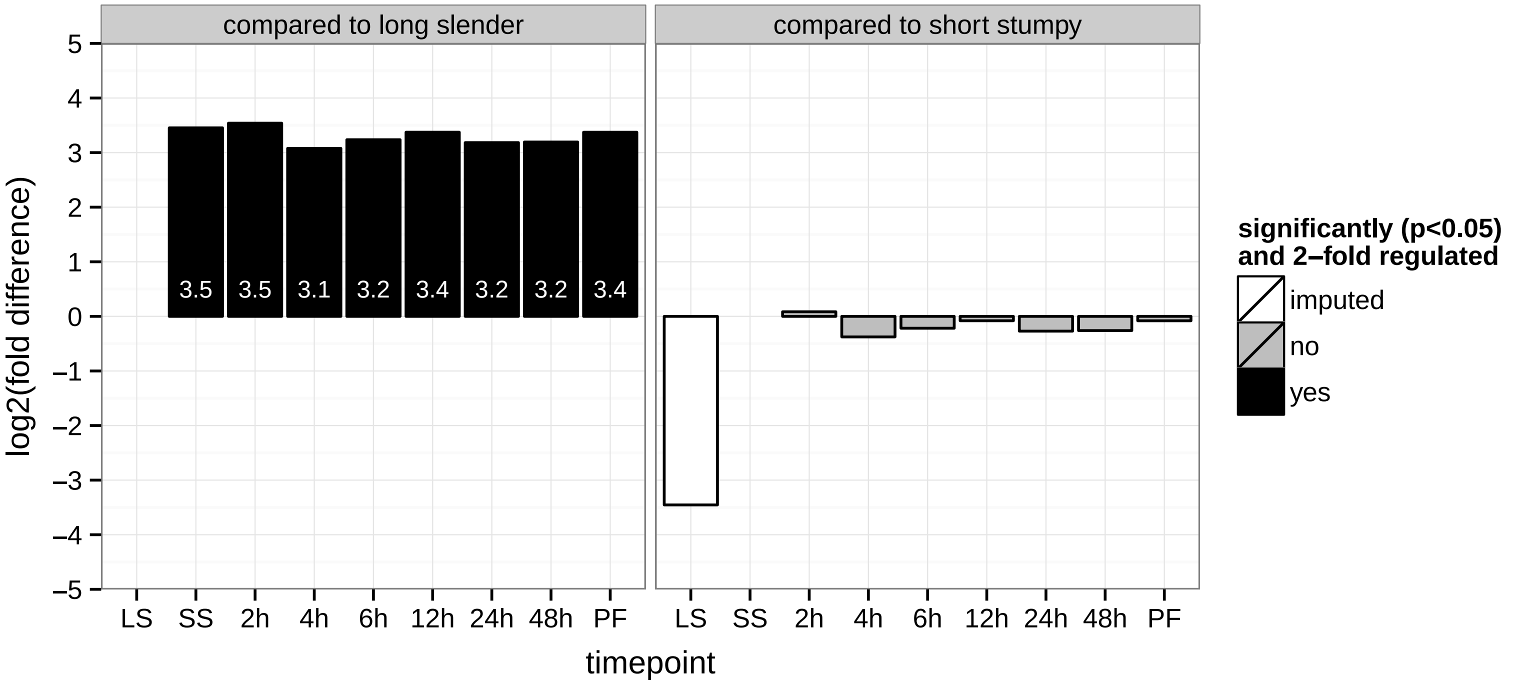

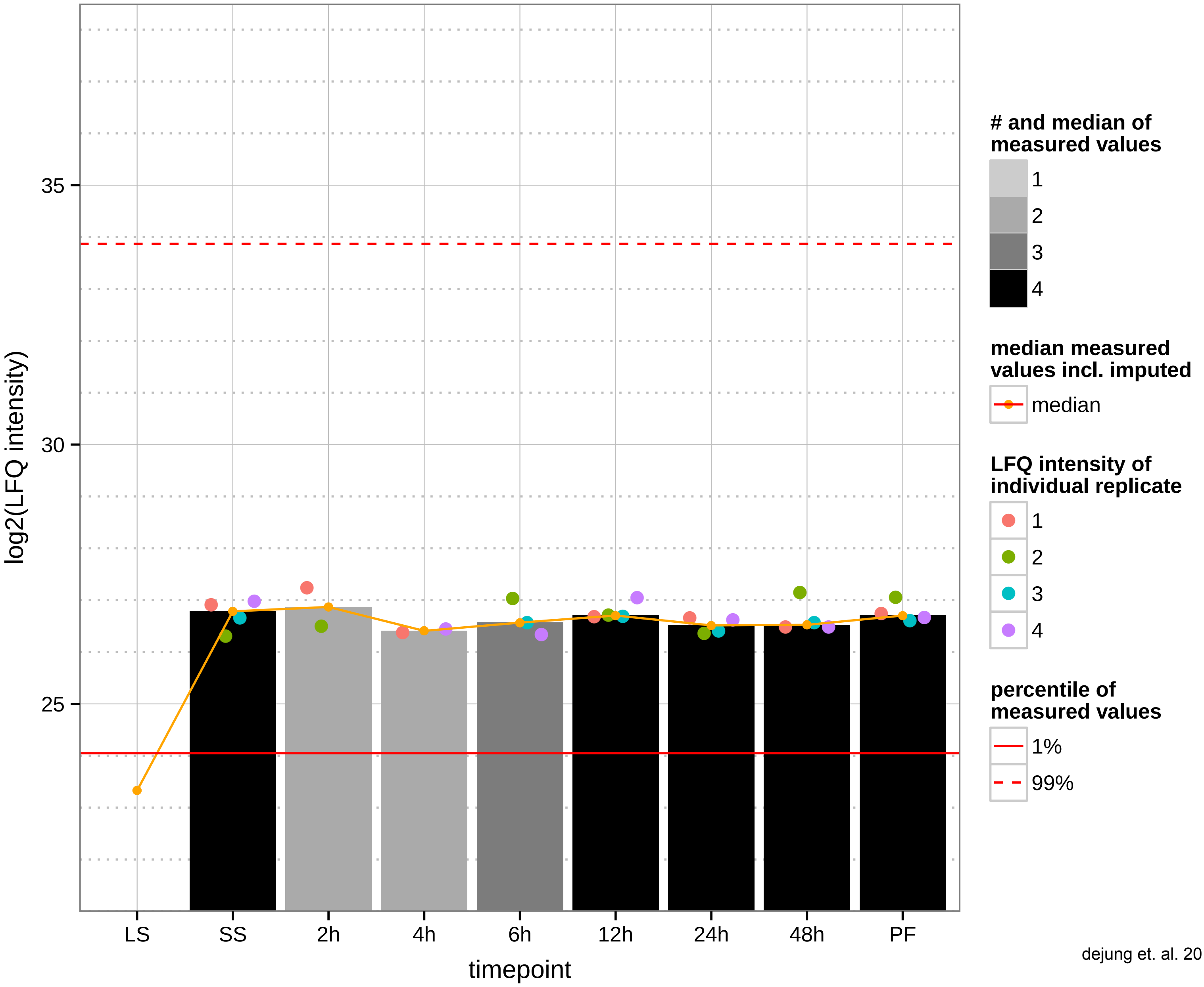

dejung et. al. 2015

hypothetical protein, conserved
Tb927.7.310
AGOF: null
AGOC: null
AGOP: null
PGOF: null
PGOC: null
PGOP: null

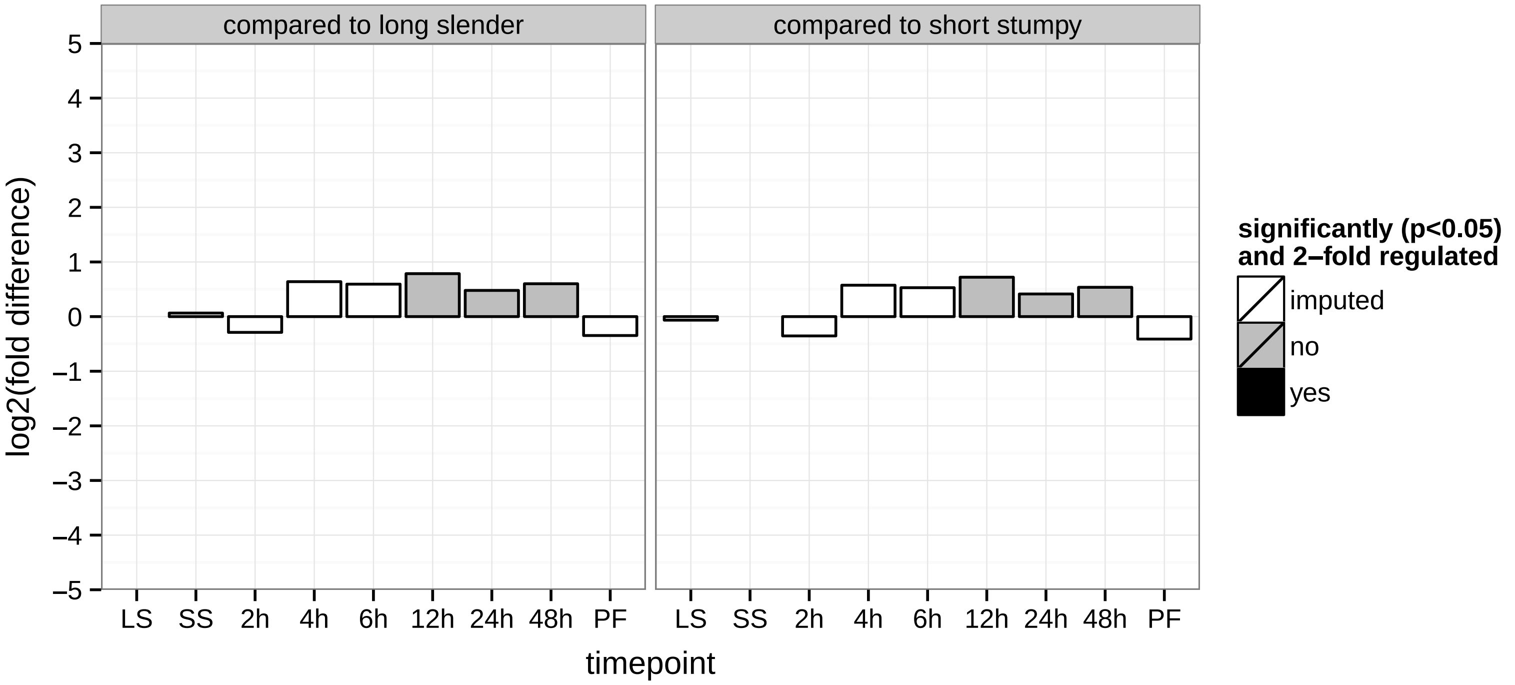

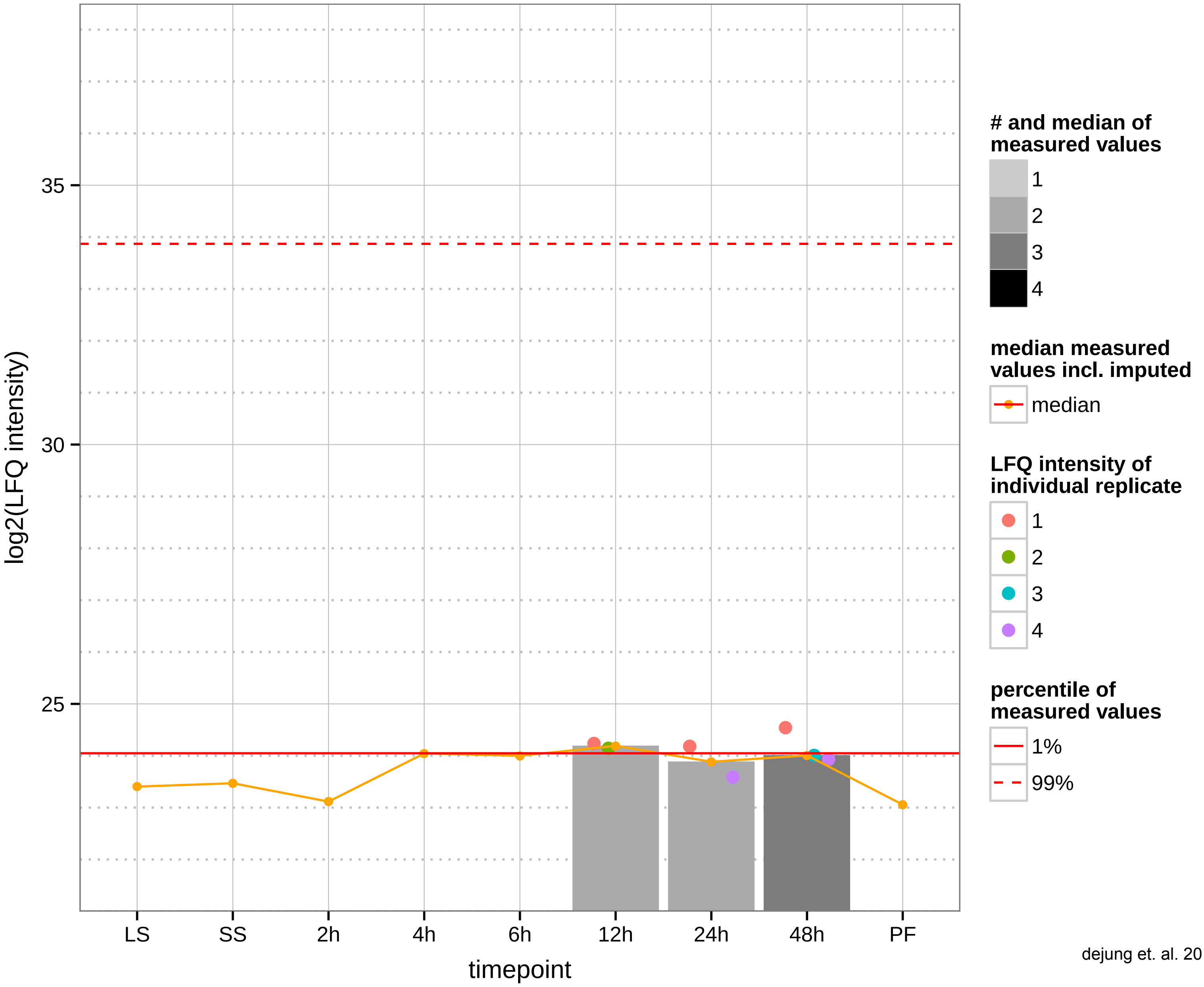

dejung et. al. 2015

small nuclear ribonucleoprotein SmD1, SmD−1 like small nuclear ribonucleoprotein (Sm−D1)
Tb927.7.3120
AGOF: snRNA binding
AGOC: nucleus, small nuclear ribonucleoprotein complex
AGOP: nuclear mRNA trans splicing, SL addition
PGOF: null
PGOC: null
PGOP: null

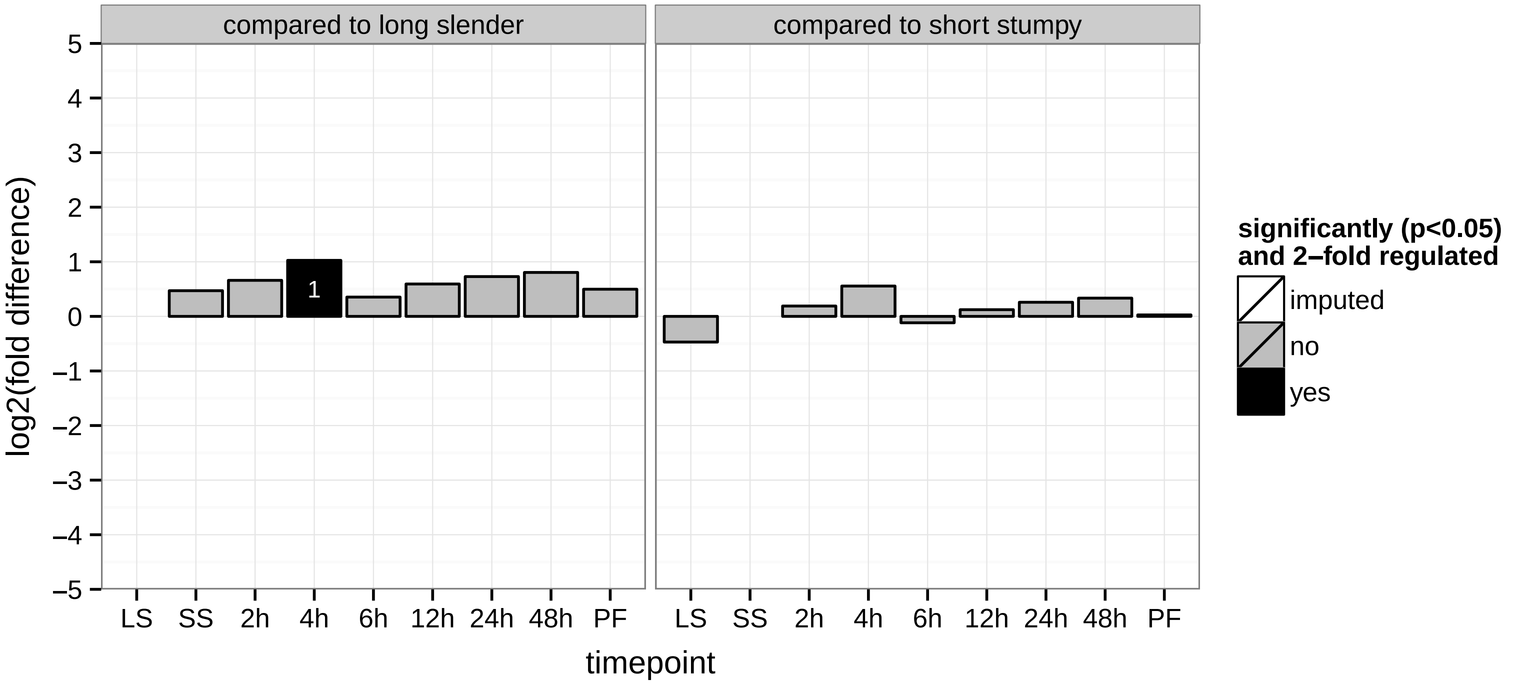

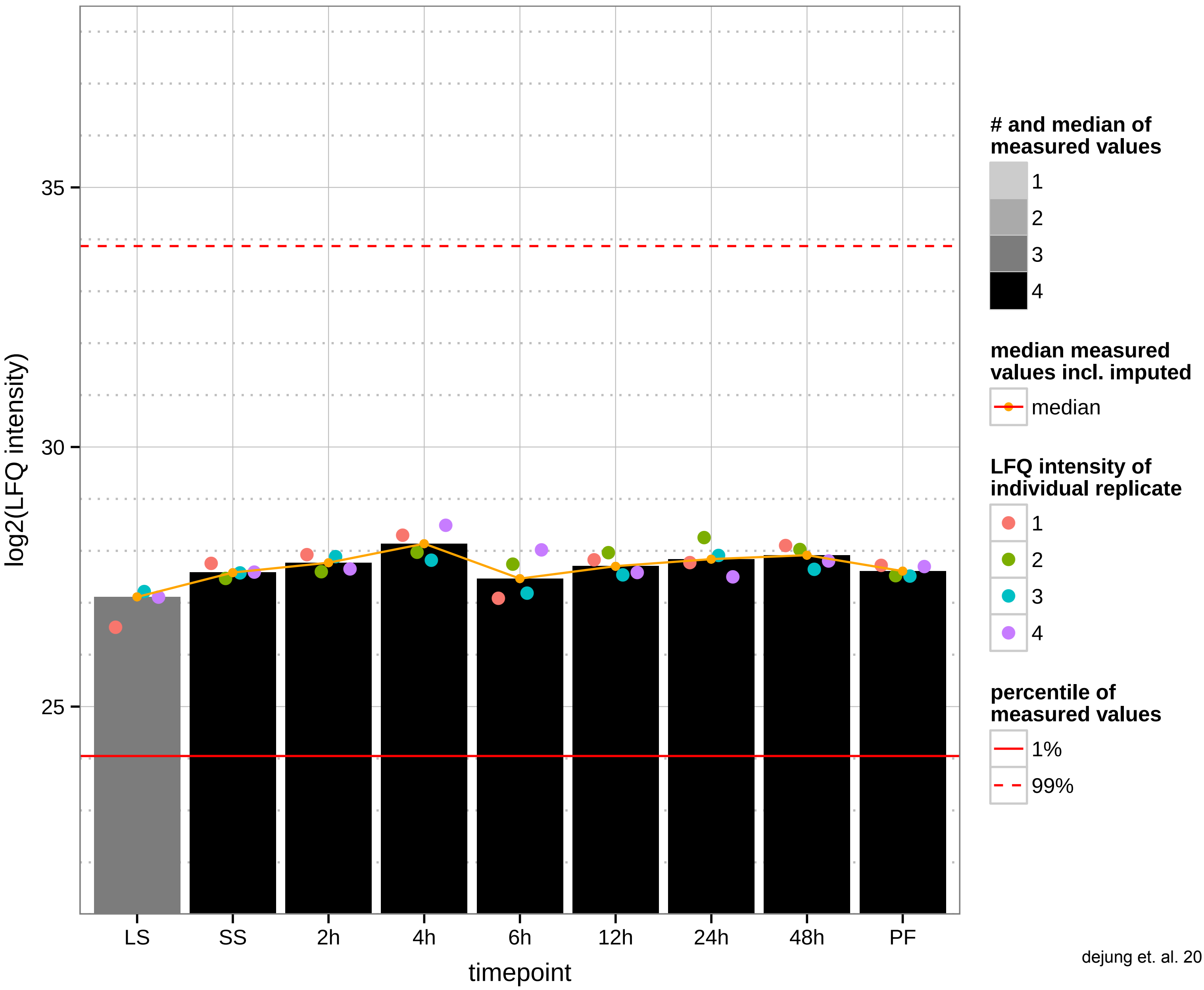

dejung et. al. 2015

hypothetical protein, conserved
Tb927.7.3170
AGOF: null
AGOC: null
AGOP: null
PGOF: null
PGOC: null
PGOP: null

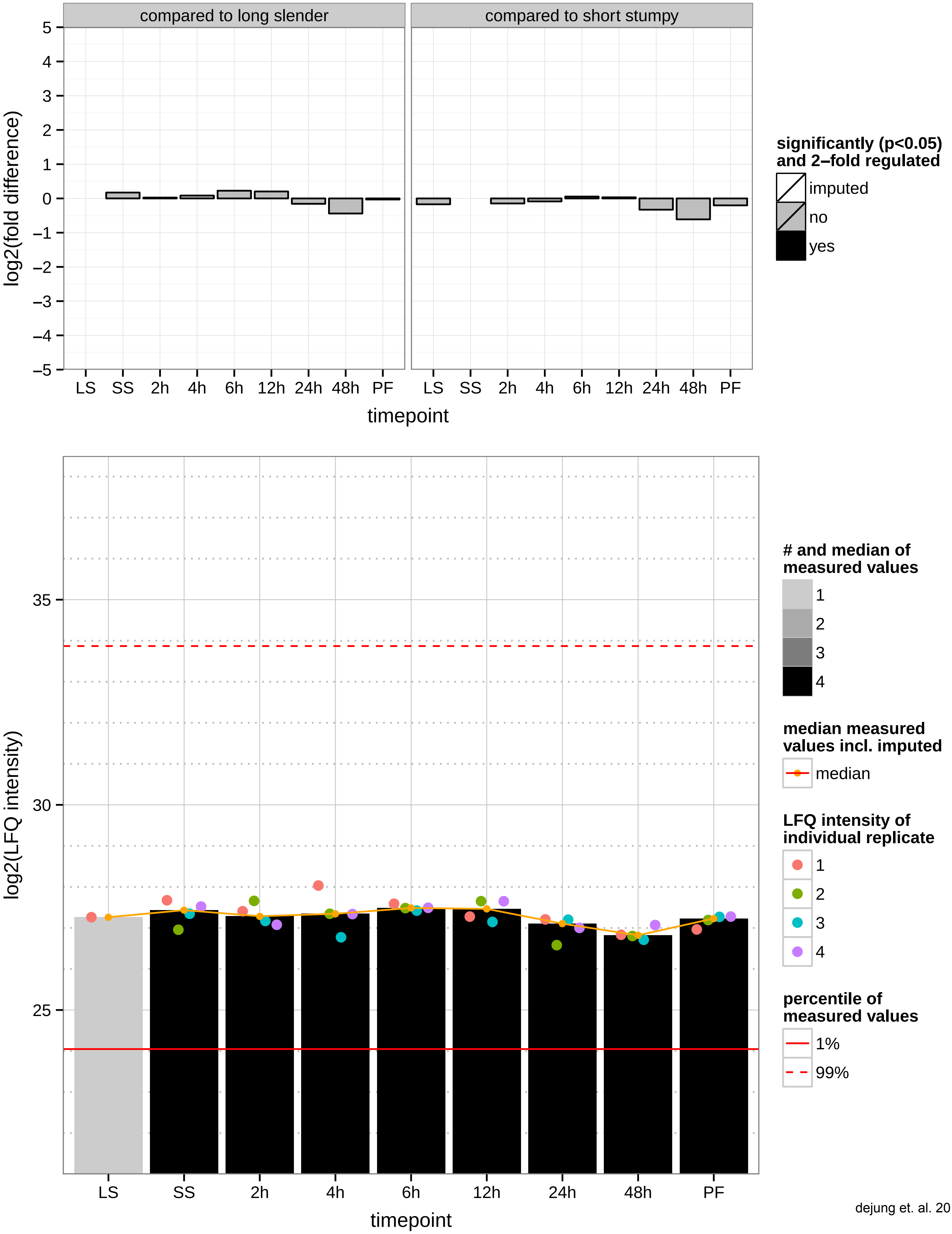

dejung et. al. 2015

hypothetical protein, conserved
Tb927.7.3290
AGOF: null
AGOC: null
AGOP: null
PGOF: protein binding
PGOC: null
PGOP: null

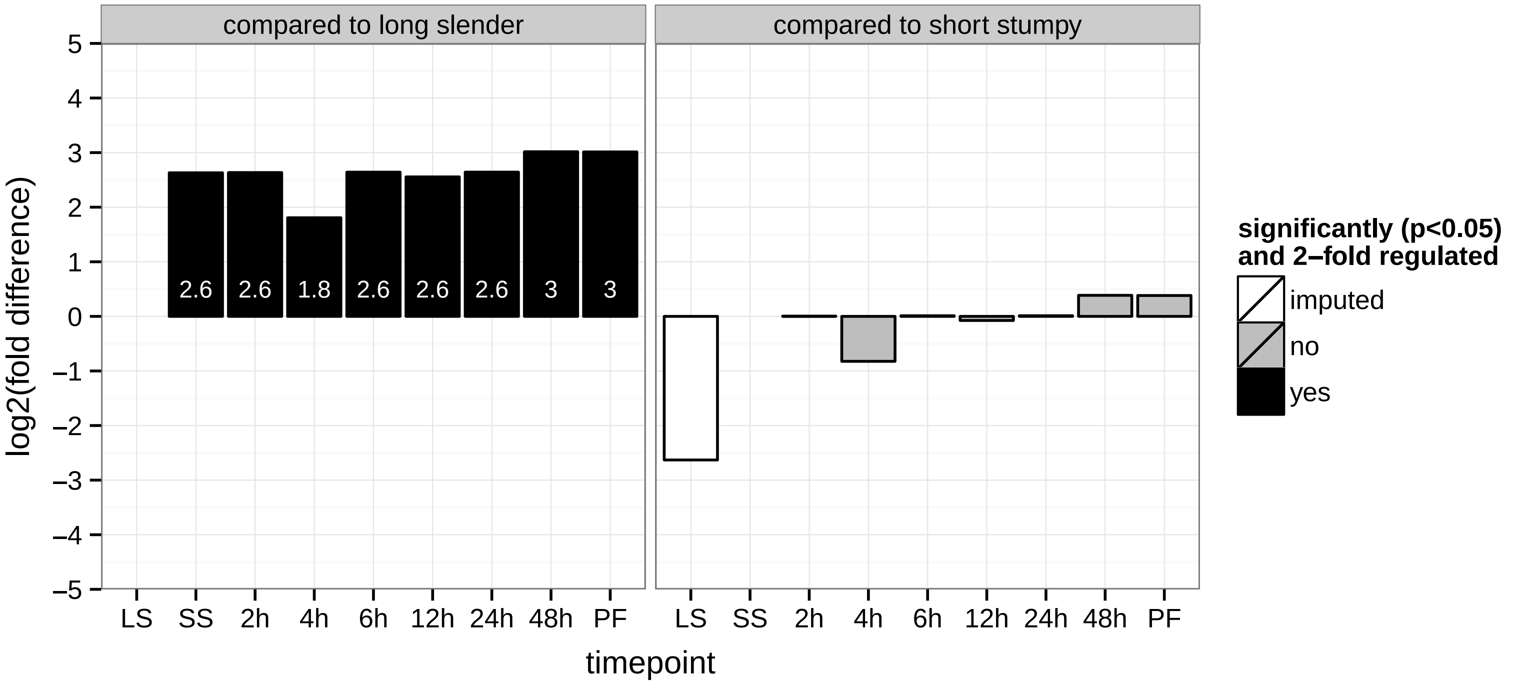

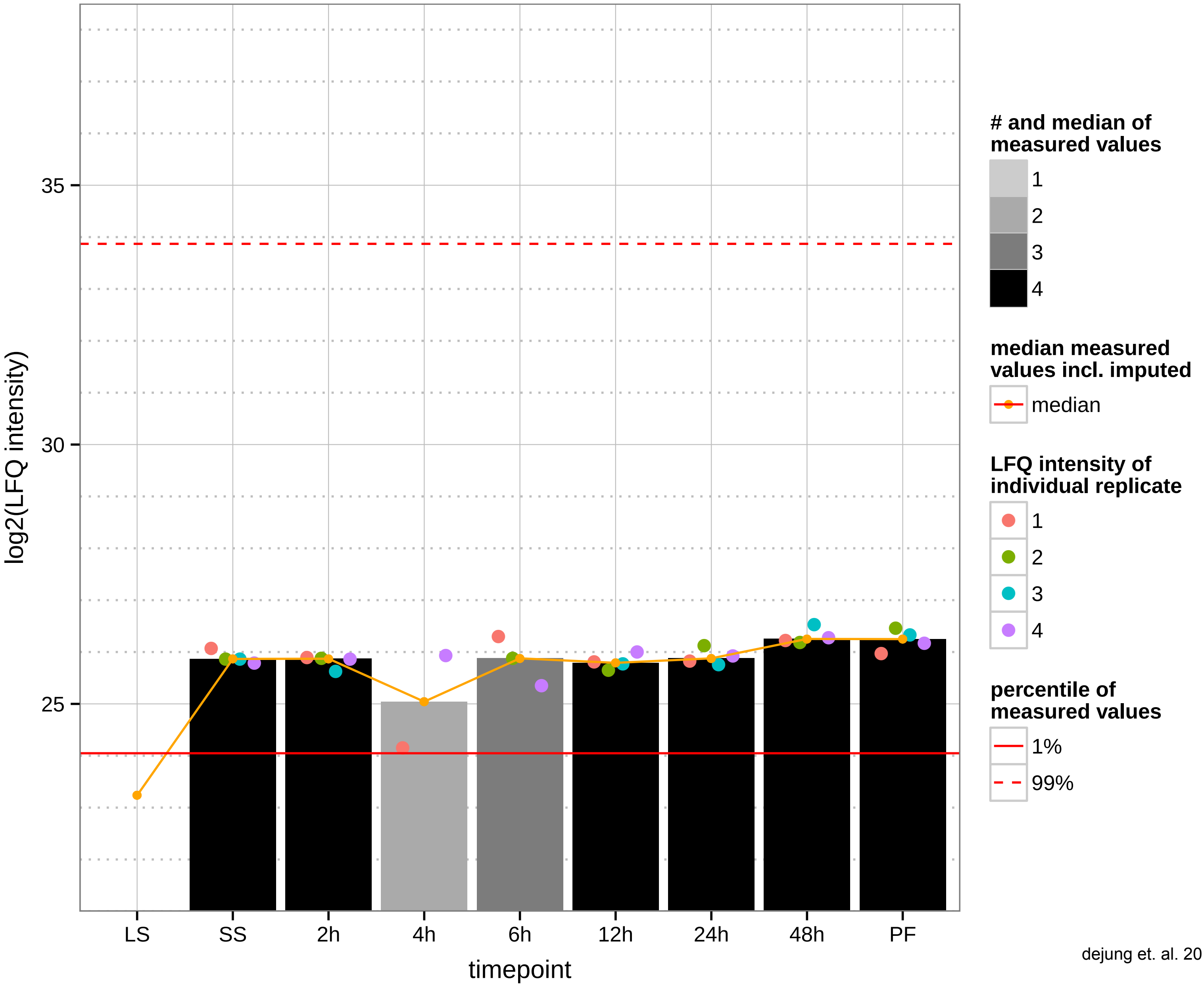

dejung et. al. 2015

hypothetical protein, conserved
Tb927.7.3310
AGOF: null
AGOC: null
AGOP: null
PGOF: null
PGOC: null
PGOP: null

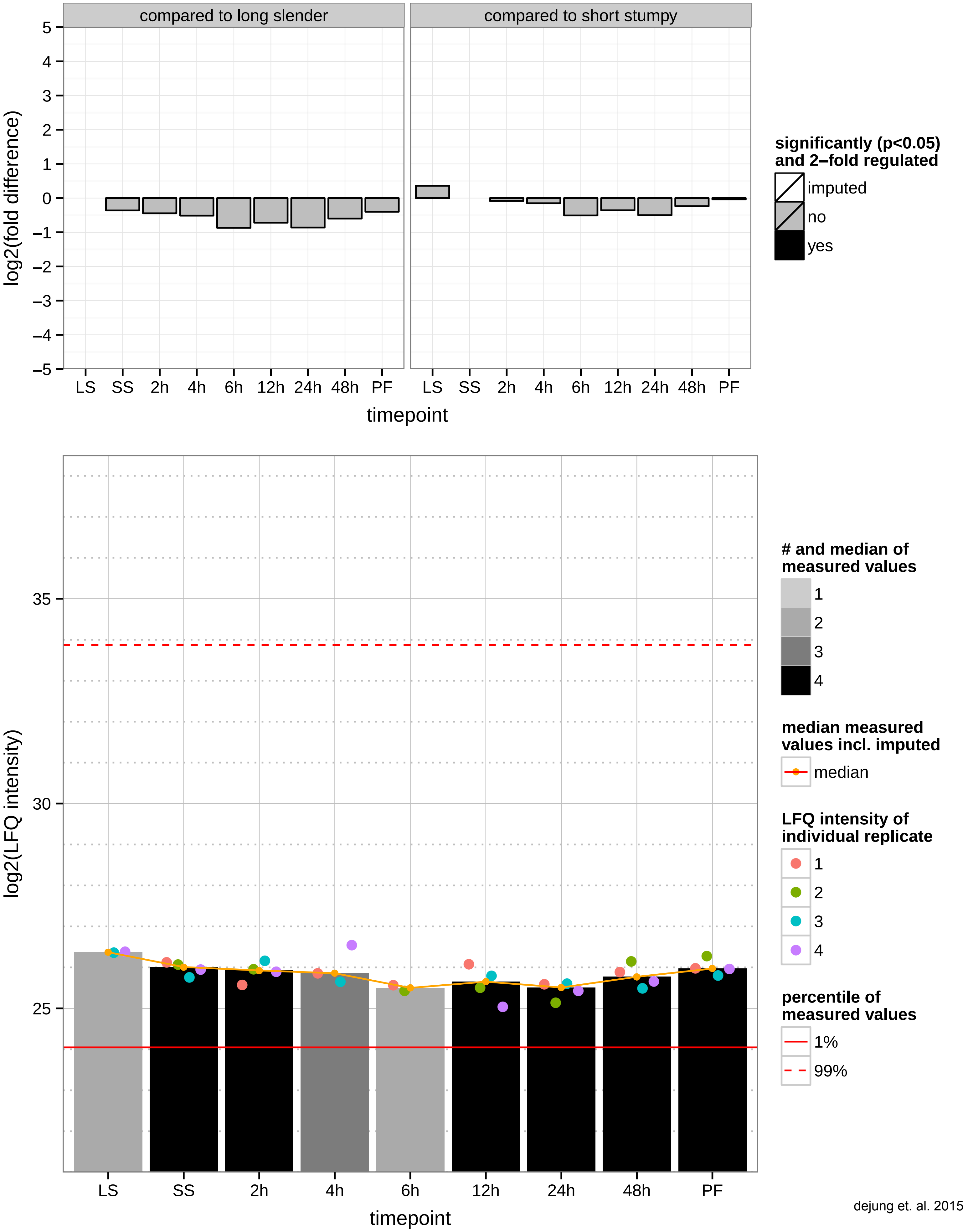

dejung et. al. 2015

hypothetical protein, conserved
Tb927.7.3330
AGOF: null
AGOC: membrane, nucleus
AGOP: regulation of transcription, DNA–dependent, vesicle–mediated transport
PGOF: null
PGOC: null
PGOP: null

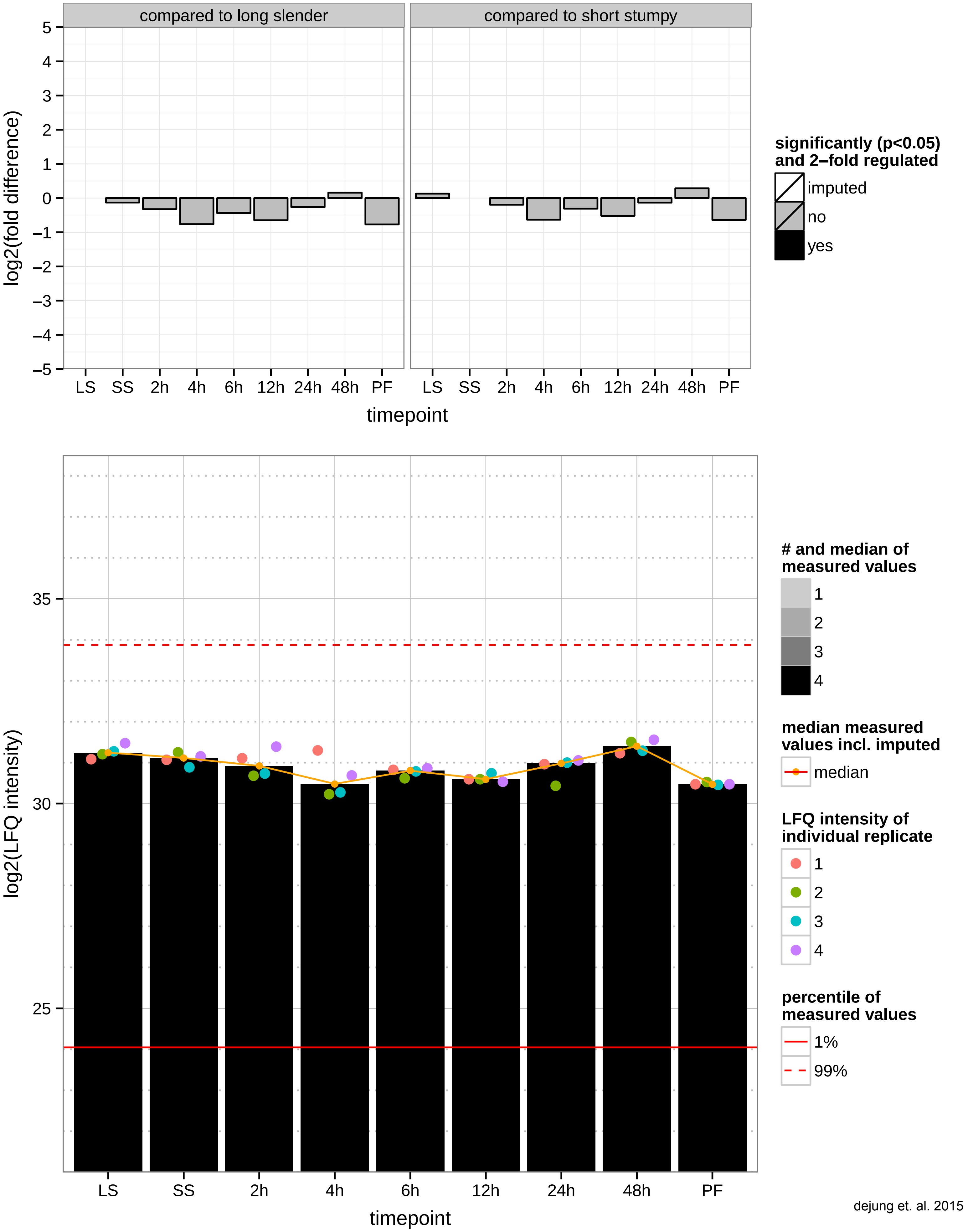

dejung et. al. 2015

hypothetical protein, conserved
Tb927.7.3370
AGOF: null
AGOC: null
AGOP: null
PGOF: null
PGOC: null
PGOP: null

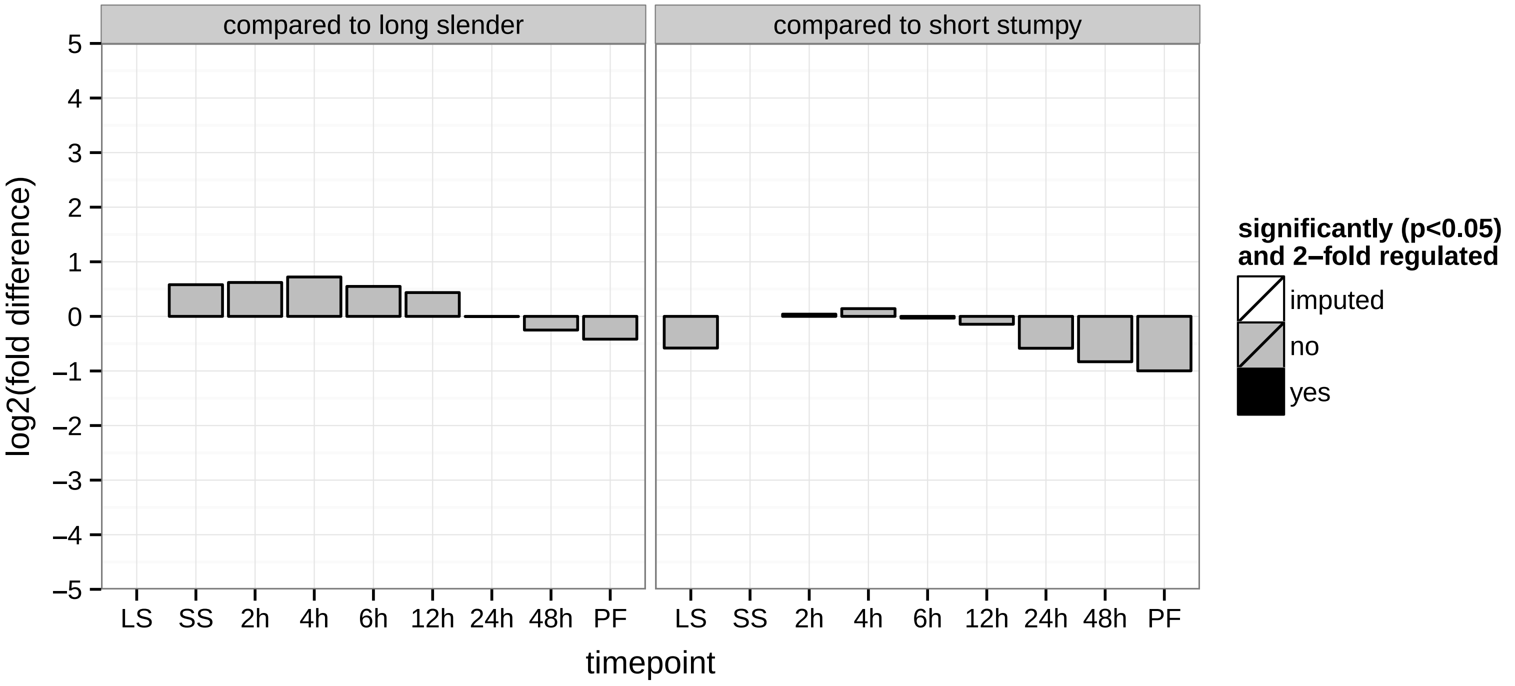

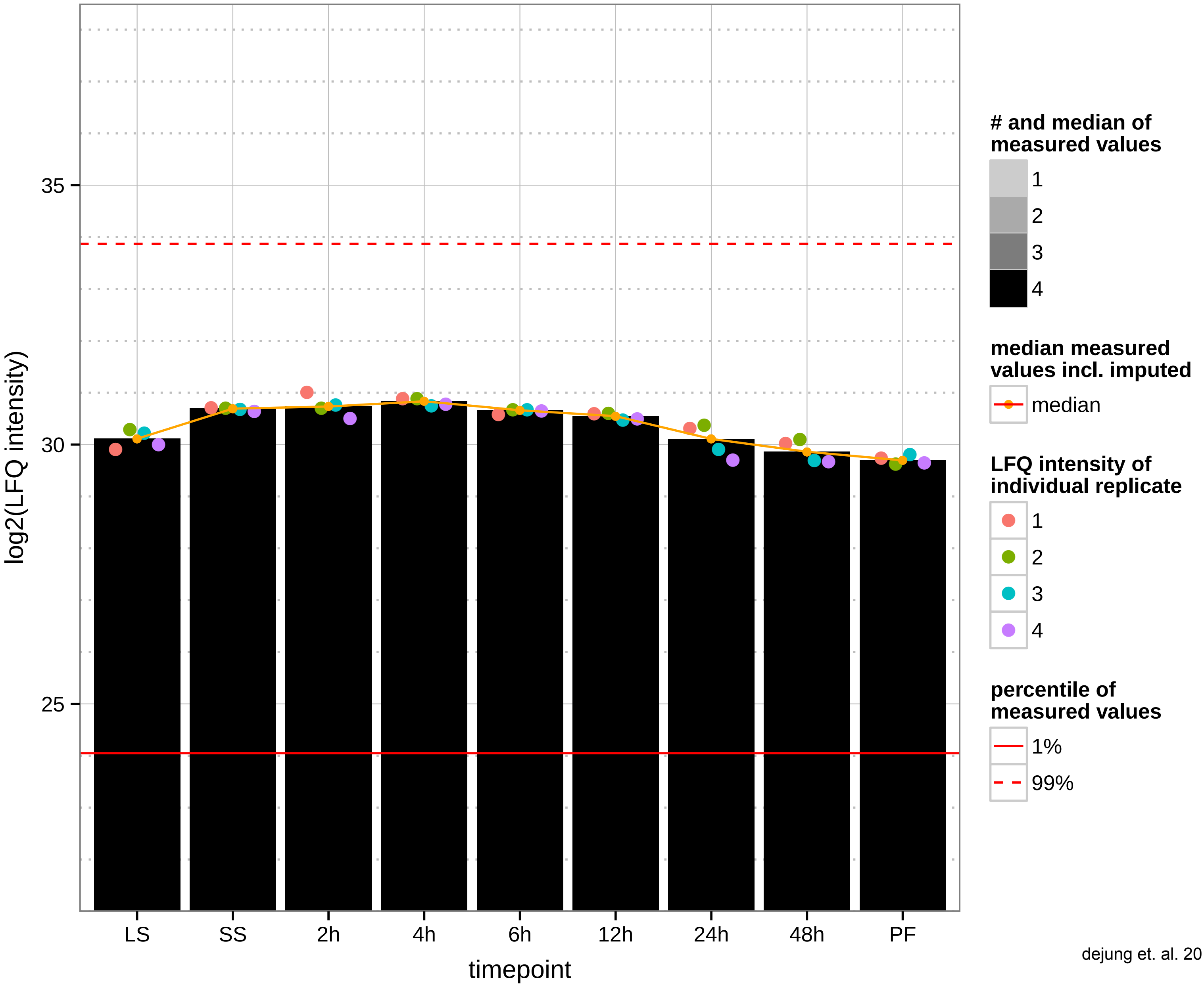

dejung et. al. 2015

I/6 autoantigen
Tb927.7.3440
AGOF: calcium ion binding, structural constituent of cytoskeleton
AGOC: microtubule cytoskeleton
AGOP: null
PGOF: null
PGOC: null
PGOP: null

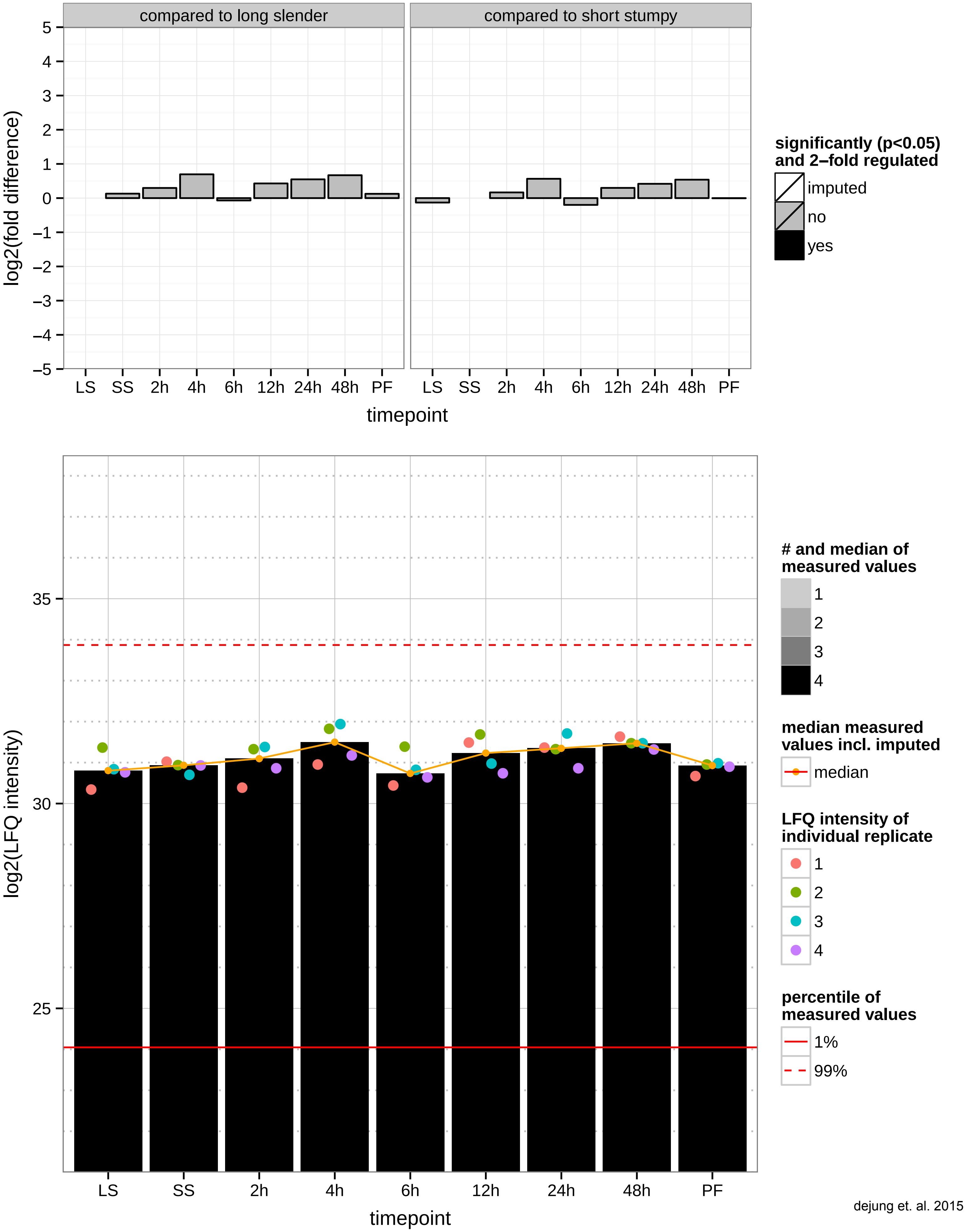

dejung et. al. 2015

mitochondrial pyruvate carrier protein 2, putative
Tb927.7.3520
AGOF: pyruvate transmembrane transporter activity
AGOC: mitochondrial membrane, mitochondrion
AGOP: pyruvate transport
PGOF: null
PGOC: null
PGOP: null

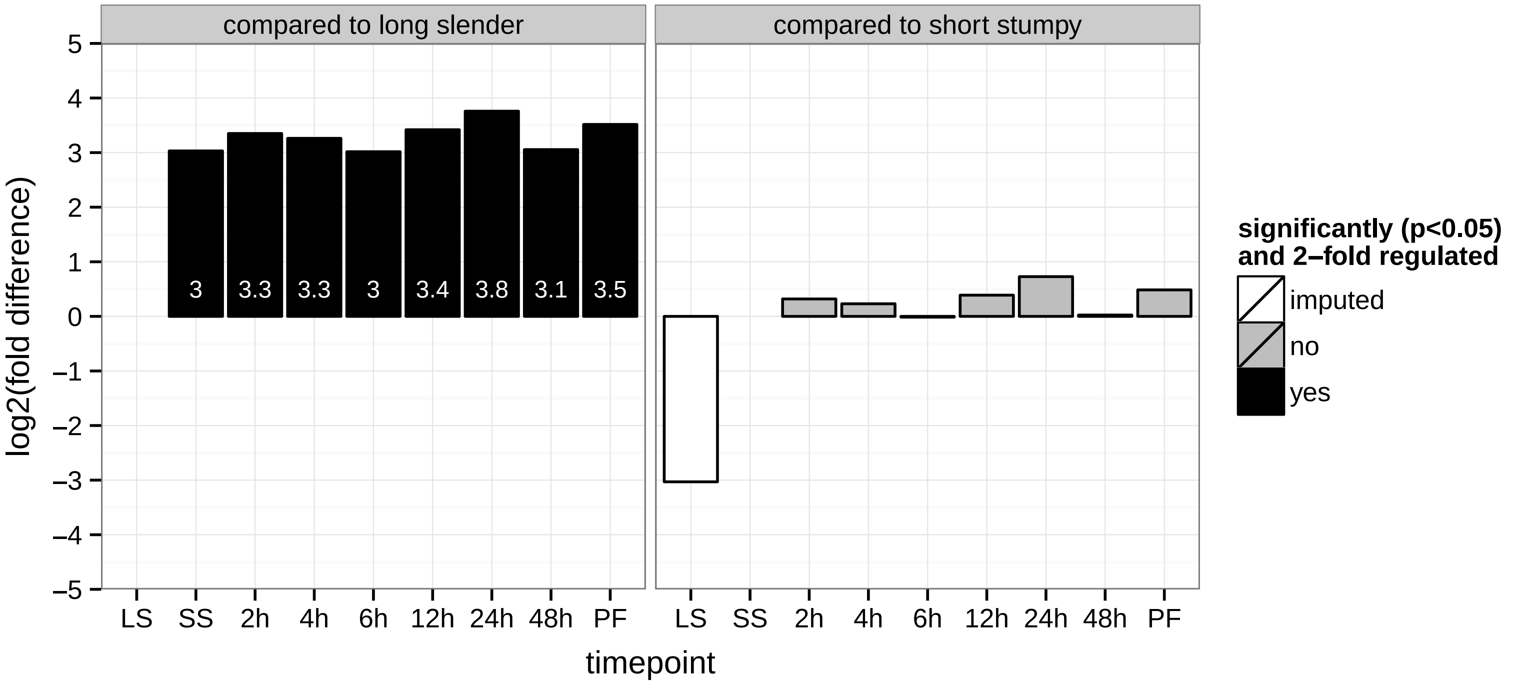

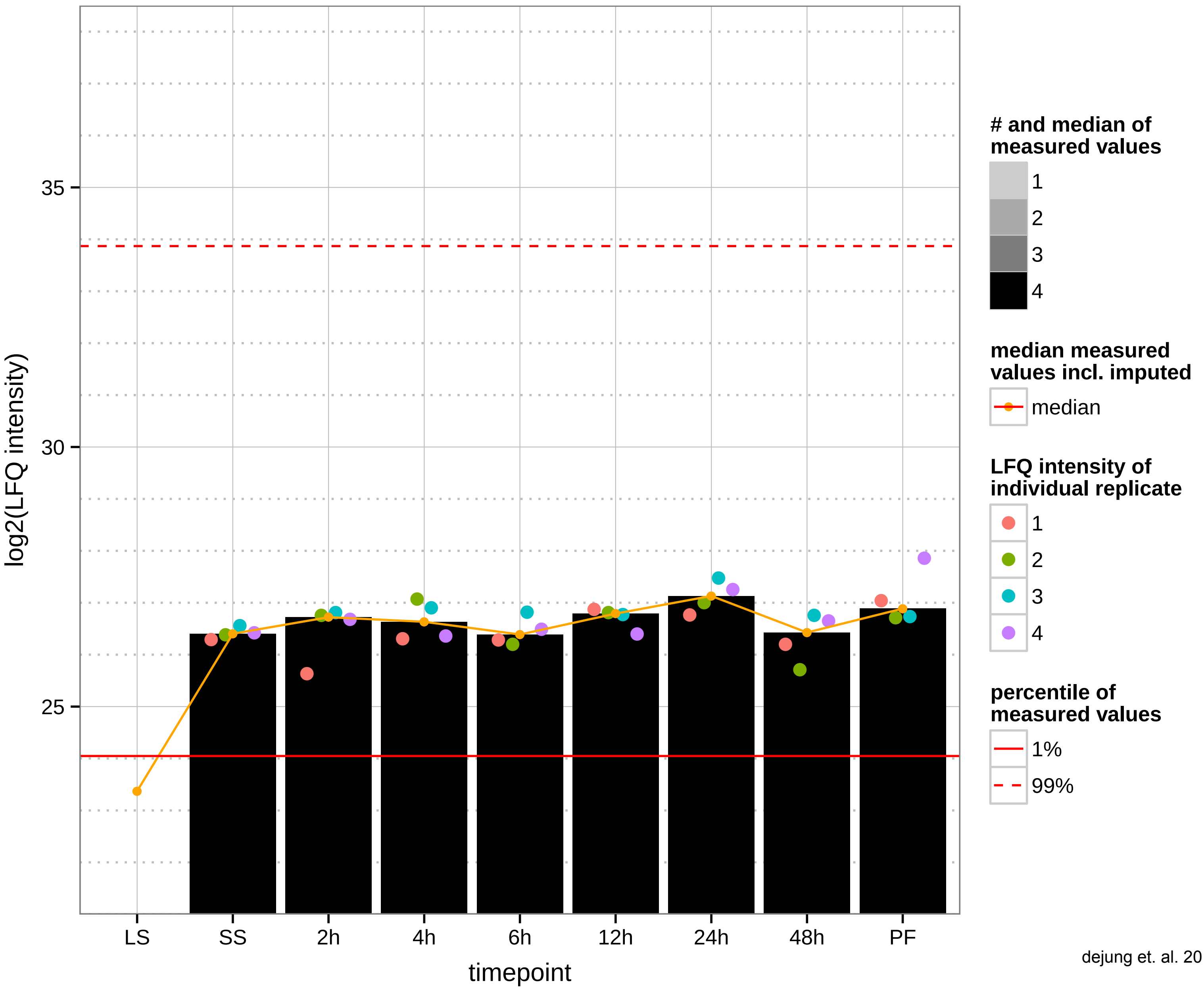

dejung et. al. 2015

hypothetical protein, conserved
Tb927.7.3540
AGOF: null
AGOC: null
AGOP: null
PGOF: null
PGOC: null
PGOP: null

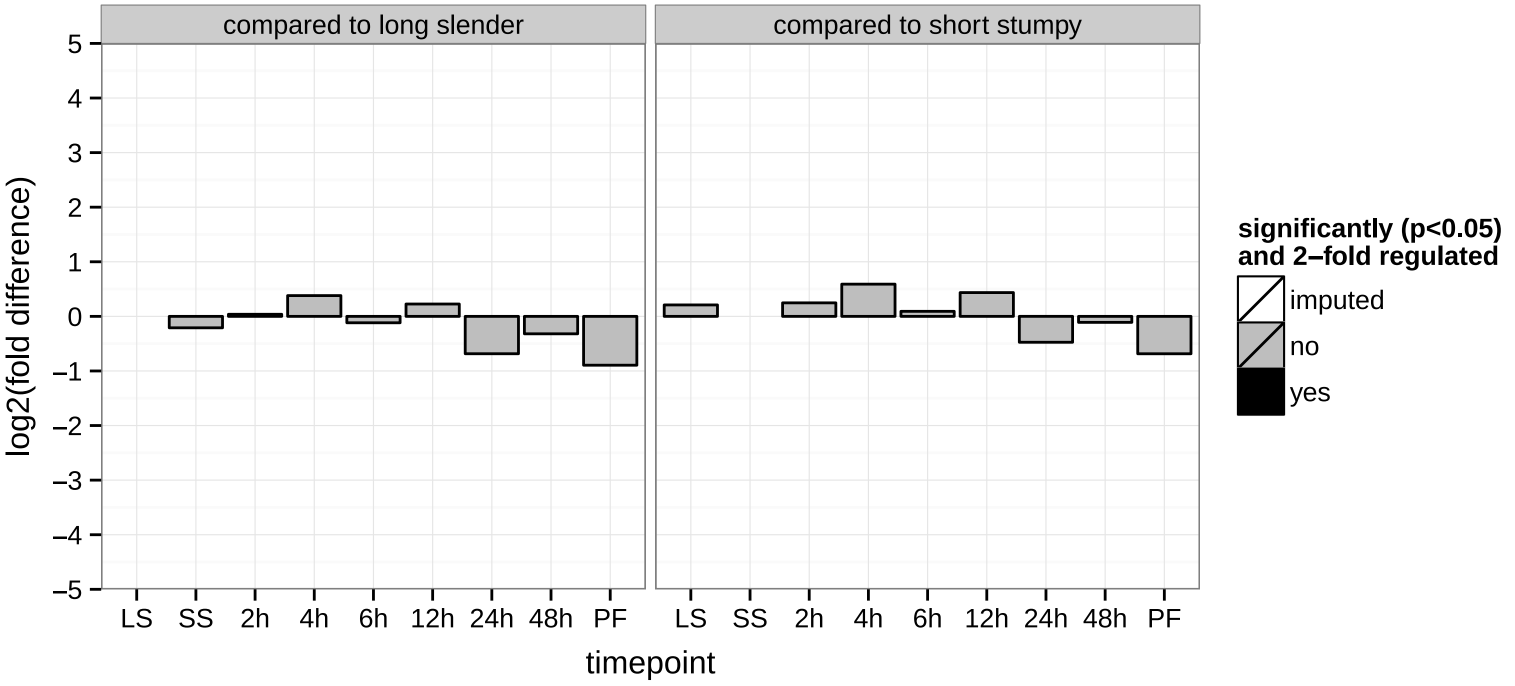

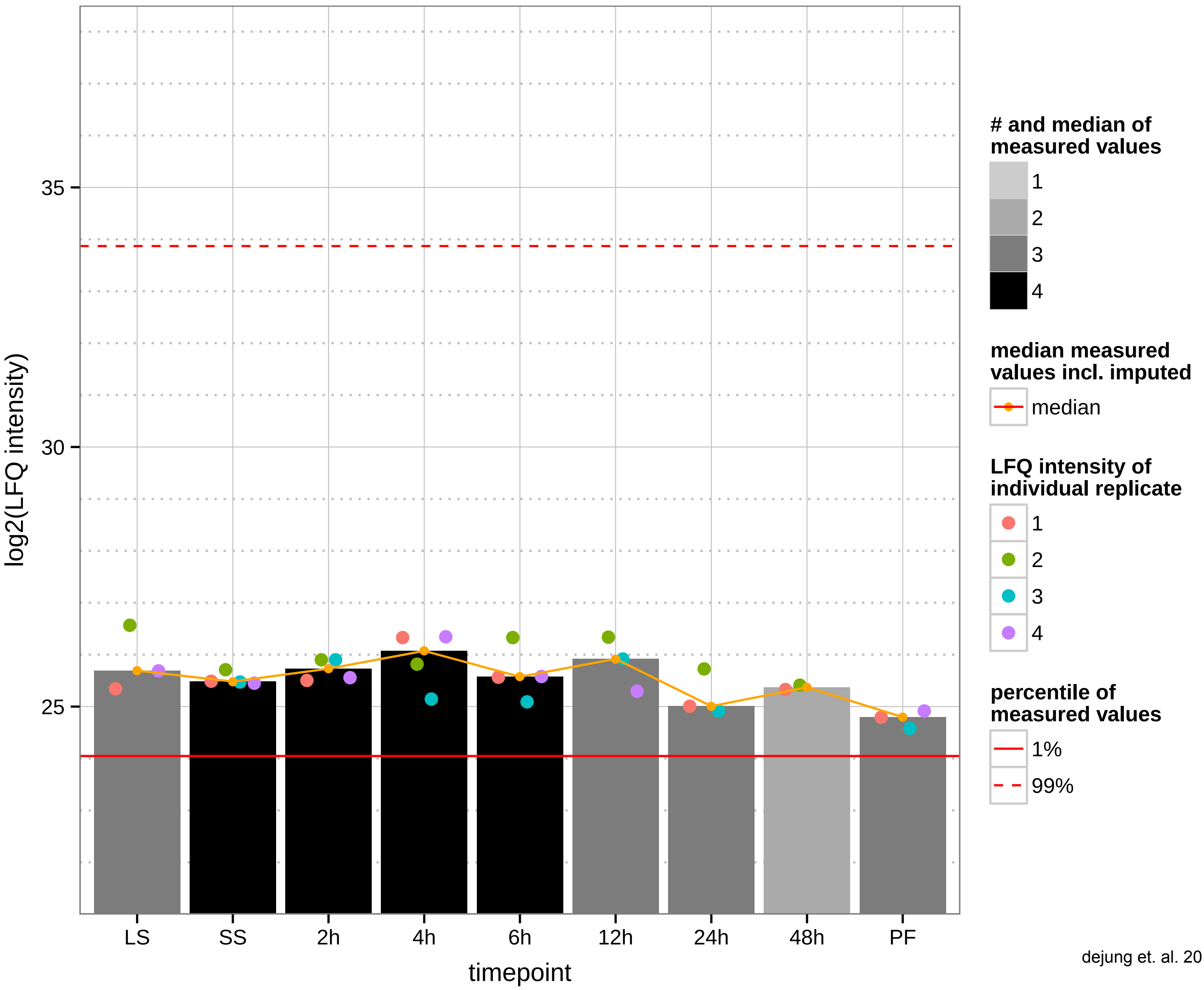

dejung et. al. 2015

hypothetical protein, conserved
Tb927.7.3550
AGOF: null
AGOC: null
AGOP: null
PGOF: protein binding
PGOC: null
PGOP: null

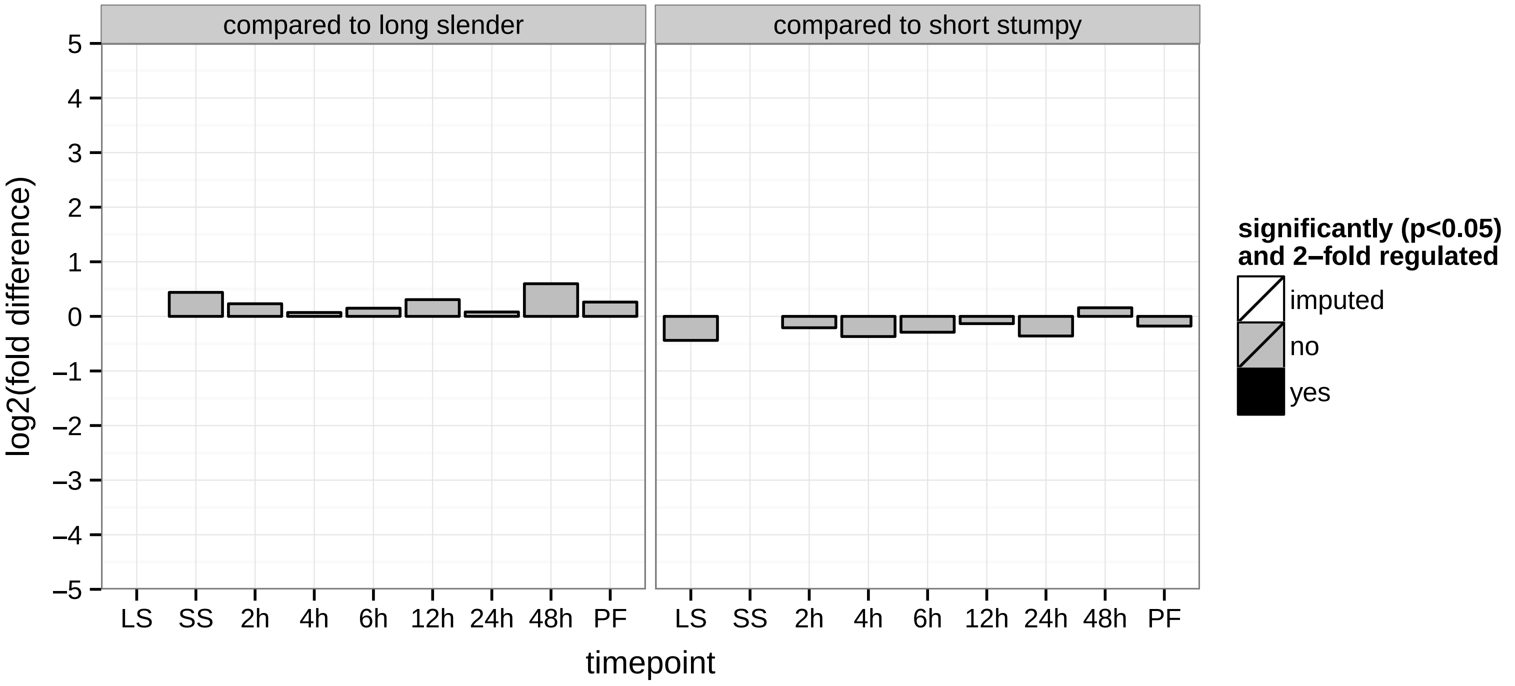

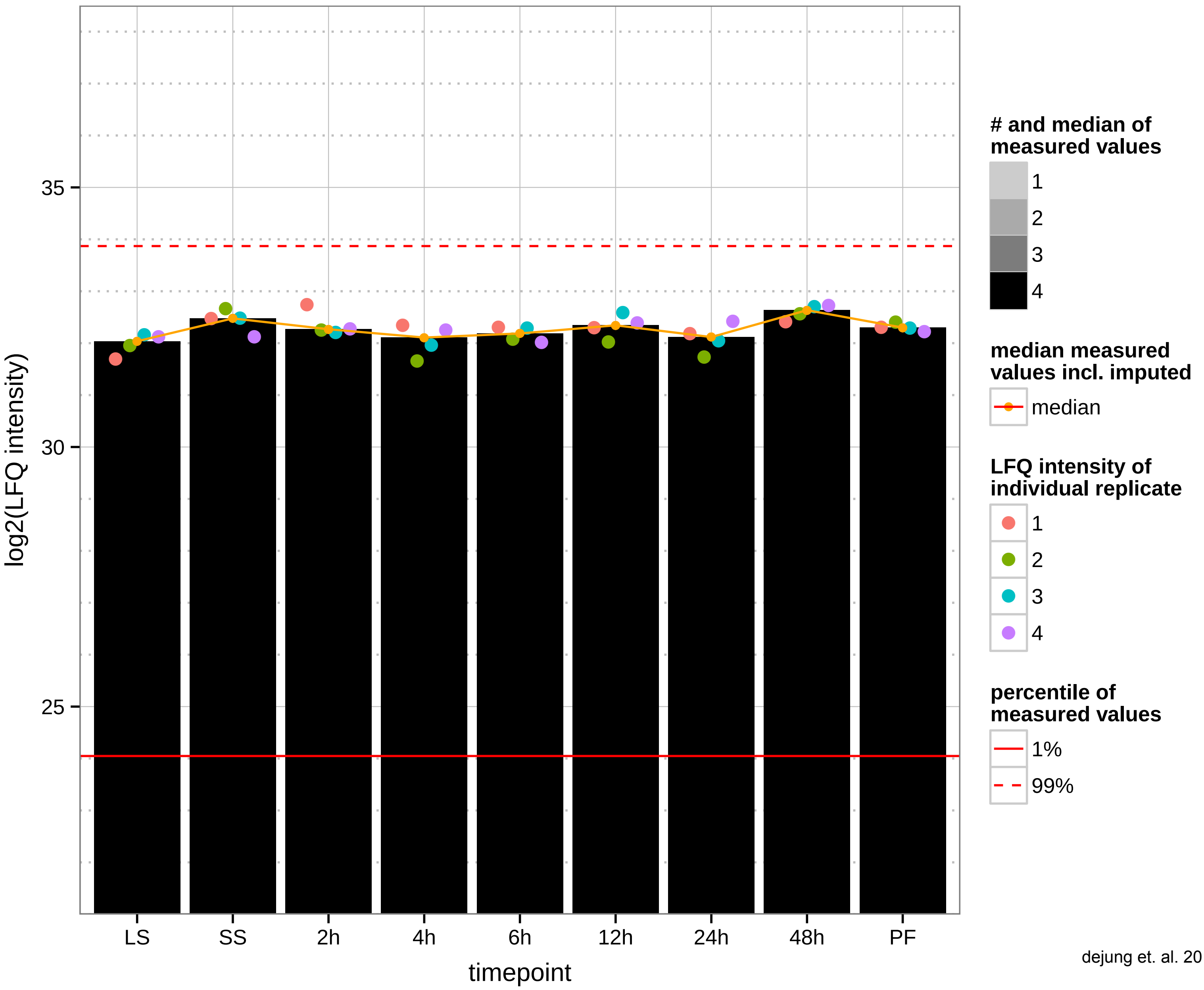

dejung et. al. 2015

hypothetical protein, conserved
Tb927.7.3560
AGOF: null
AGOC: null
AGOP: ciliary or flagellar motility
PGOF: protein binding
PGOC: null
PGOP: null

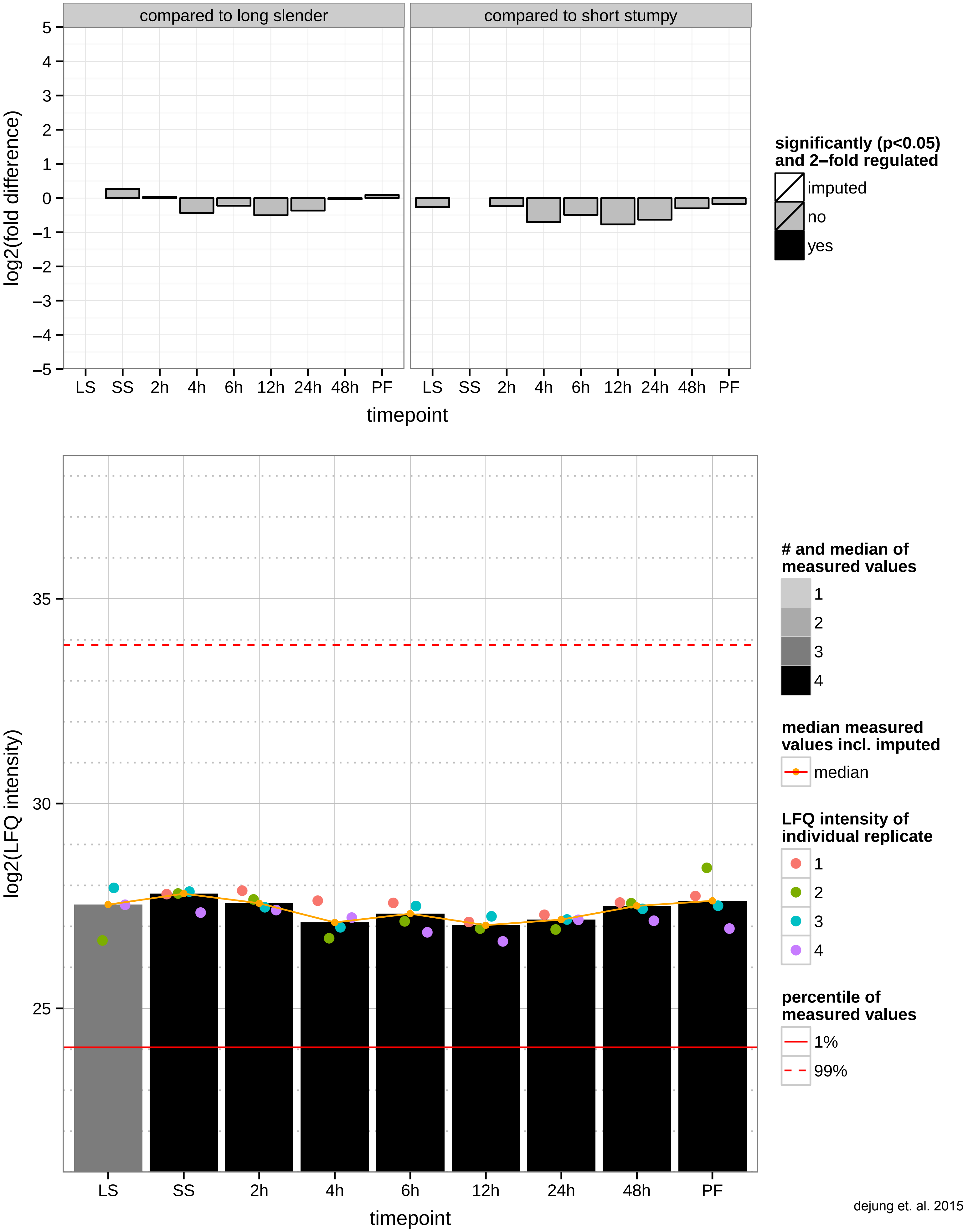

dejung et. al. 2015

tyrosyl–tRNA synthetase, putative
Tb927.7.3620
AGOF: ATP binding, tyrosine–tRNA ligase activity
AGOC: cytoplasm
AGOP: translation, tyrosyl–tRNA aminoacylation
PGOF: ATP binding, aminoacyl–tRNA ligase activity, nucleotide binding
PGOC: cytoplasm
PGOP: tRNA aminoacylation for protein translation

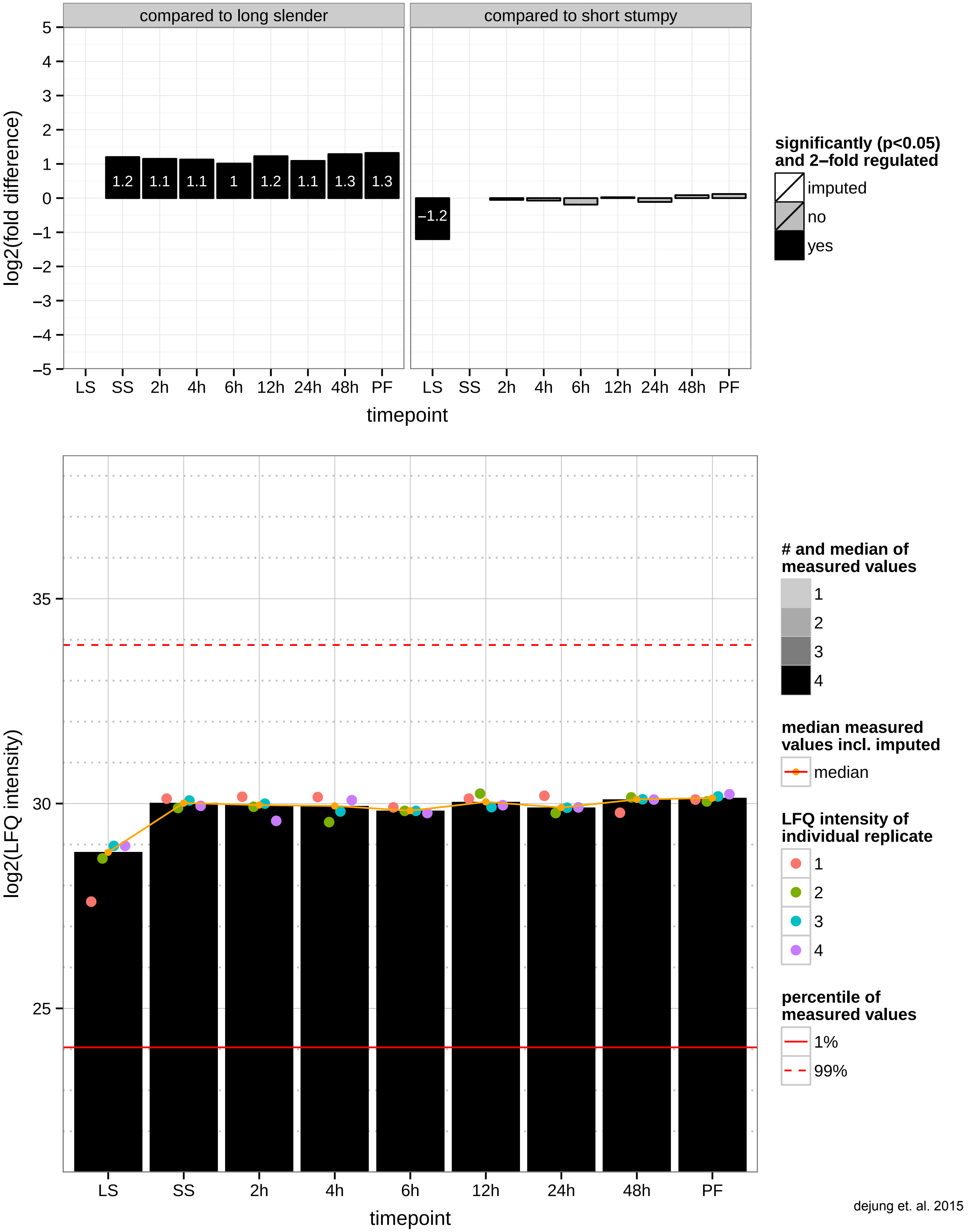

dejung et. al. 2015

hypothetical protein, conserved
Tb927.7.3740
AGOF: null
AGOC: null
AGOP: null
PGOF: null
PGOC: null
PGOP: null

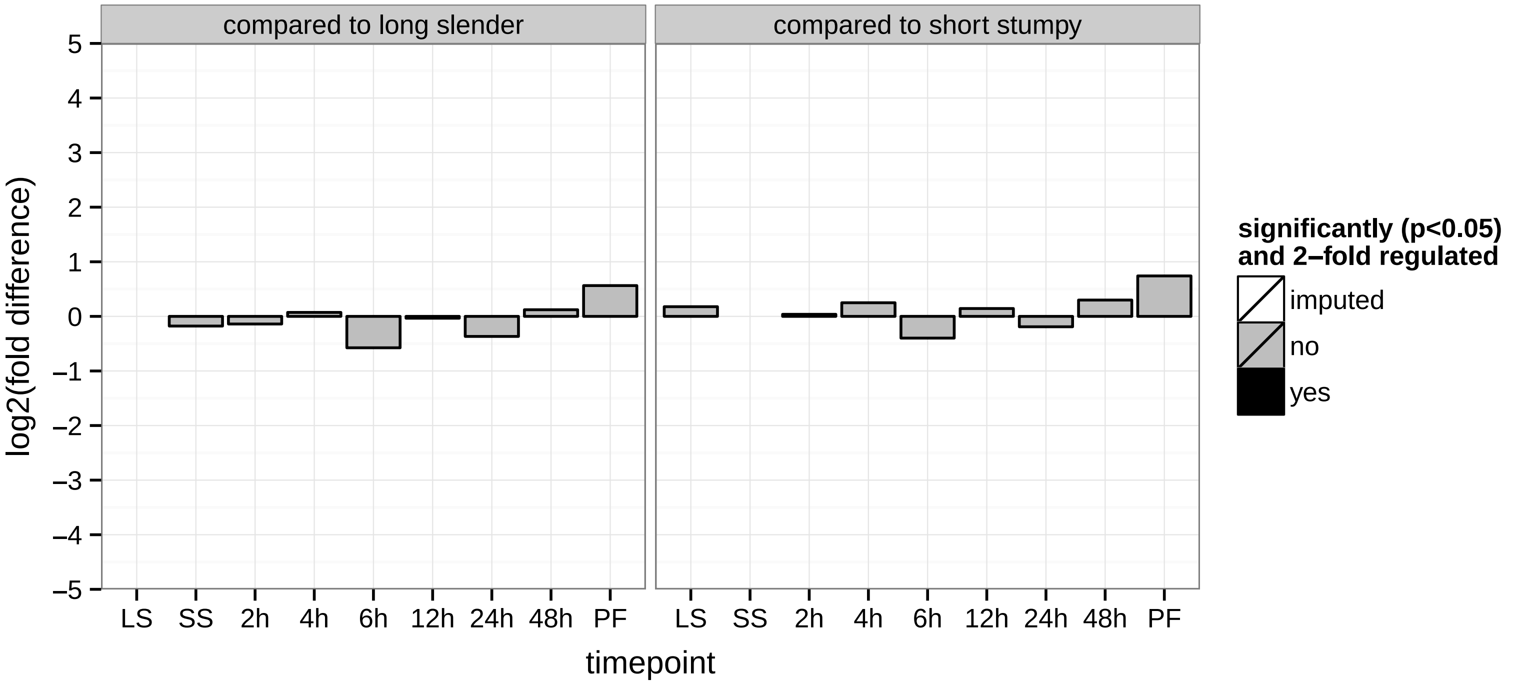

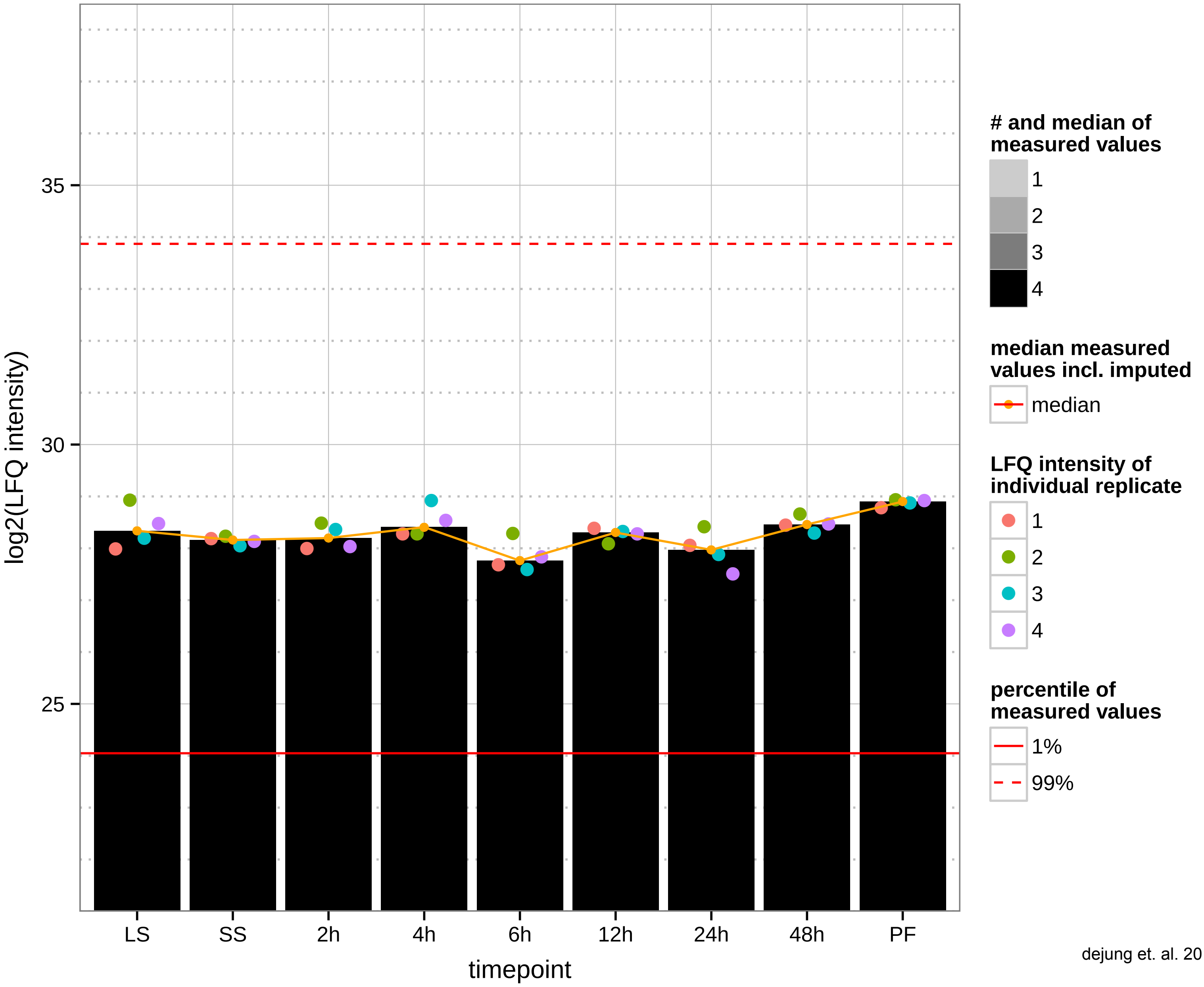

dejung et. al. 2015

TFIIF–stimulated CTD phosphatase, putative
Tb927.7.3750
AGOF: null
AGOC: mitochondrion
AGOP: null
PGOF: protein binding
PGOC: null
PGOP: null

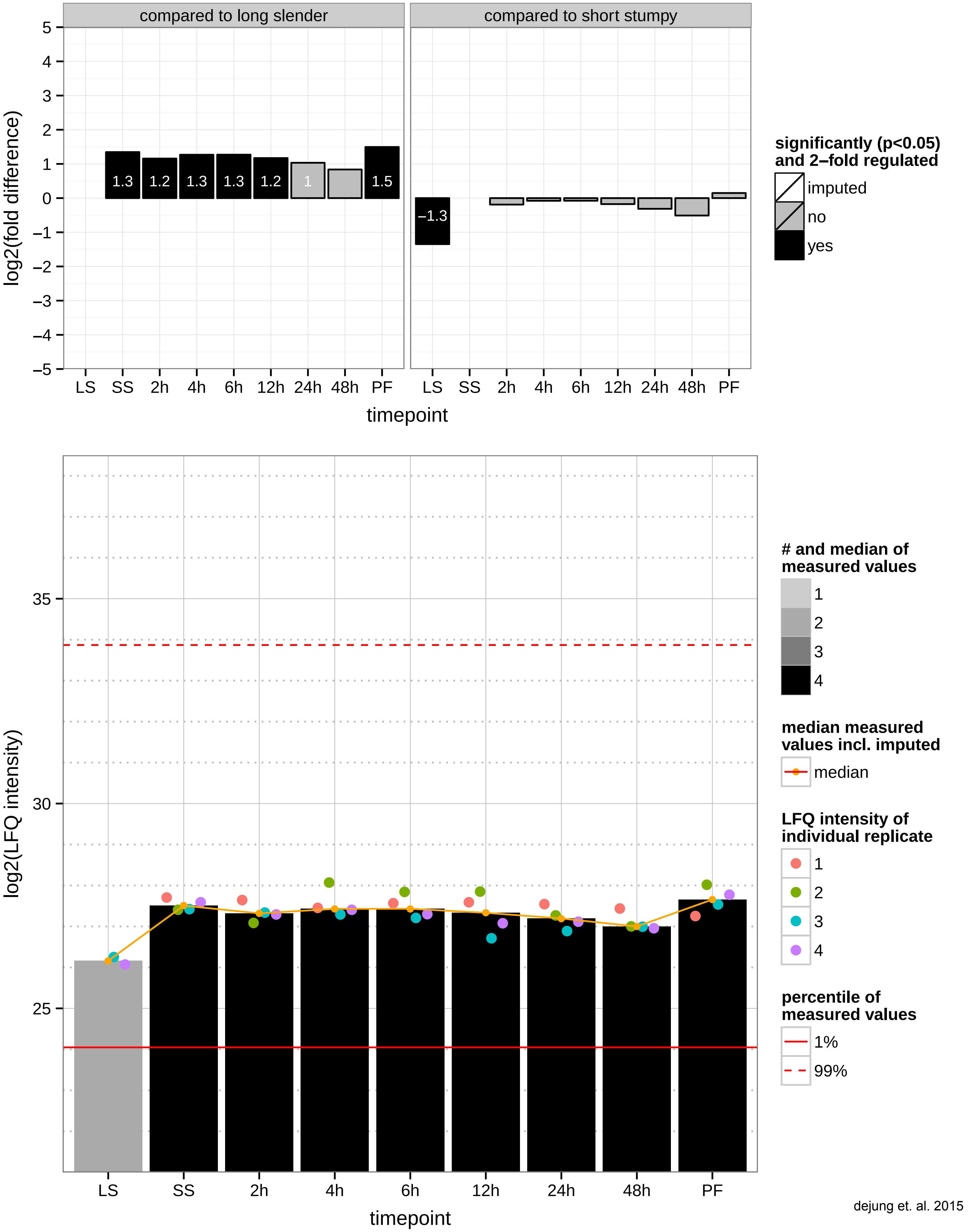

dejung et. al. 2015

phosphatidylserine synthase, putative
Tb927.7.3760
AGOF: CDP−diacylglycerol−serine O−phosphatidyltransferase activity
AGOC: cellular_component
AGOP: phosphatidylserine biosynthetic process
PGOF: null
PGOC: null
PGOP: phosphatidylserine biosynthetic process

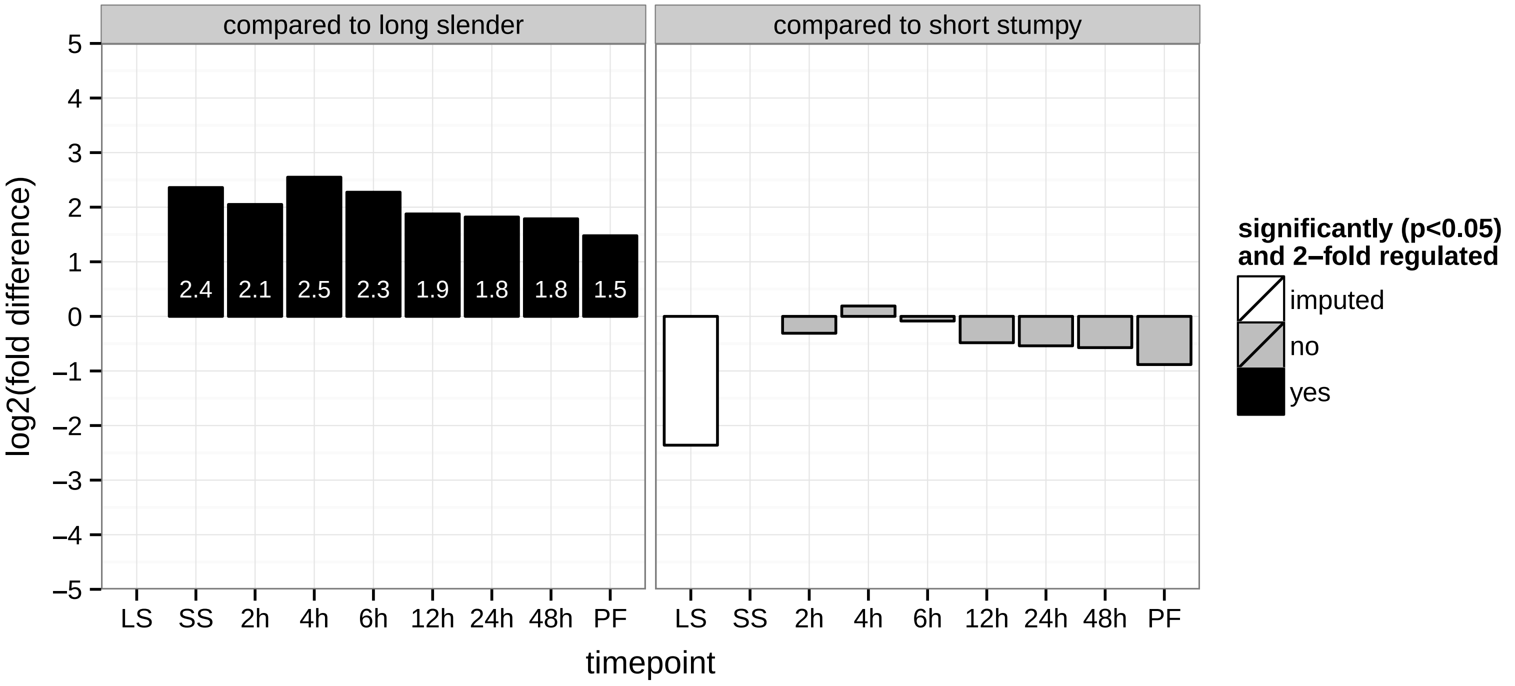

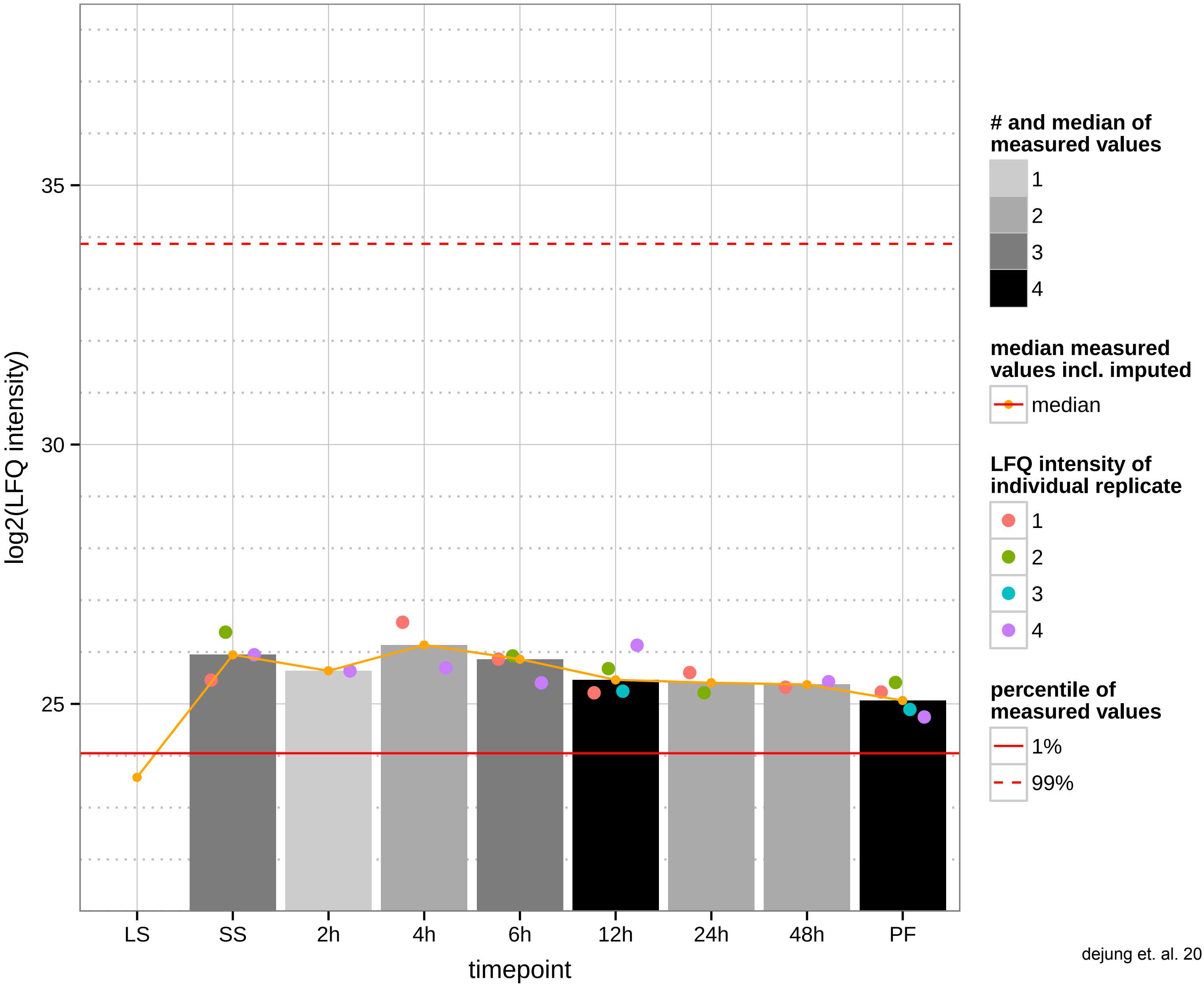

dejung et. al. 2015

hypothetical protein, conserved
Tb927.7.3770
AGOF: null
AGOC: mitochondrion
AGOP: null
PGOF: null
PGOC: null
PGOP: null

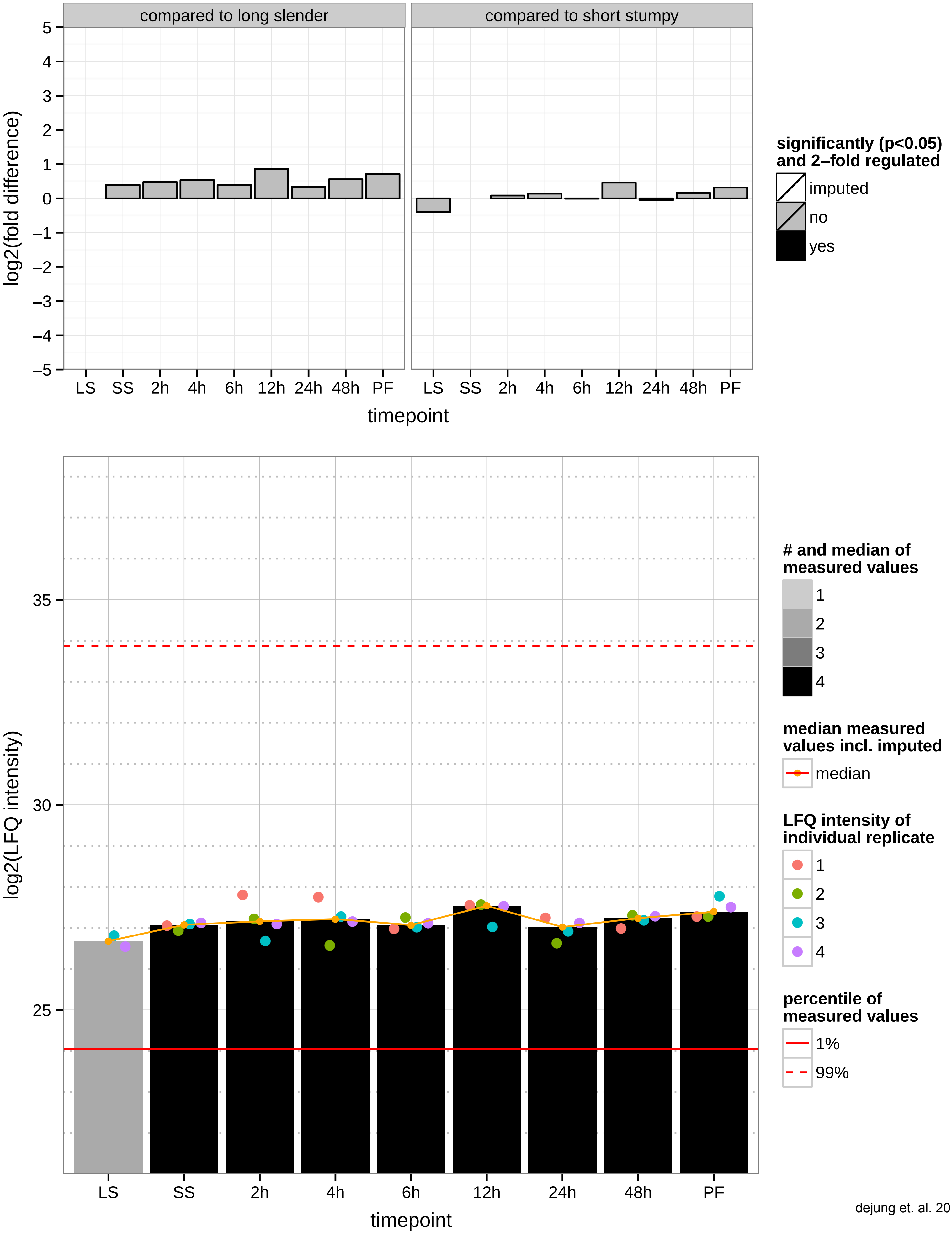

dejung et. al. 2015

hypothetical protein, conserved
Tb927.7.3820
AGOF: null
AGOC: null
AGOP: null
PGOF: protein binding
PGOC: null
PGOP: null

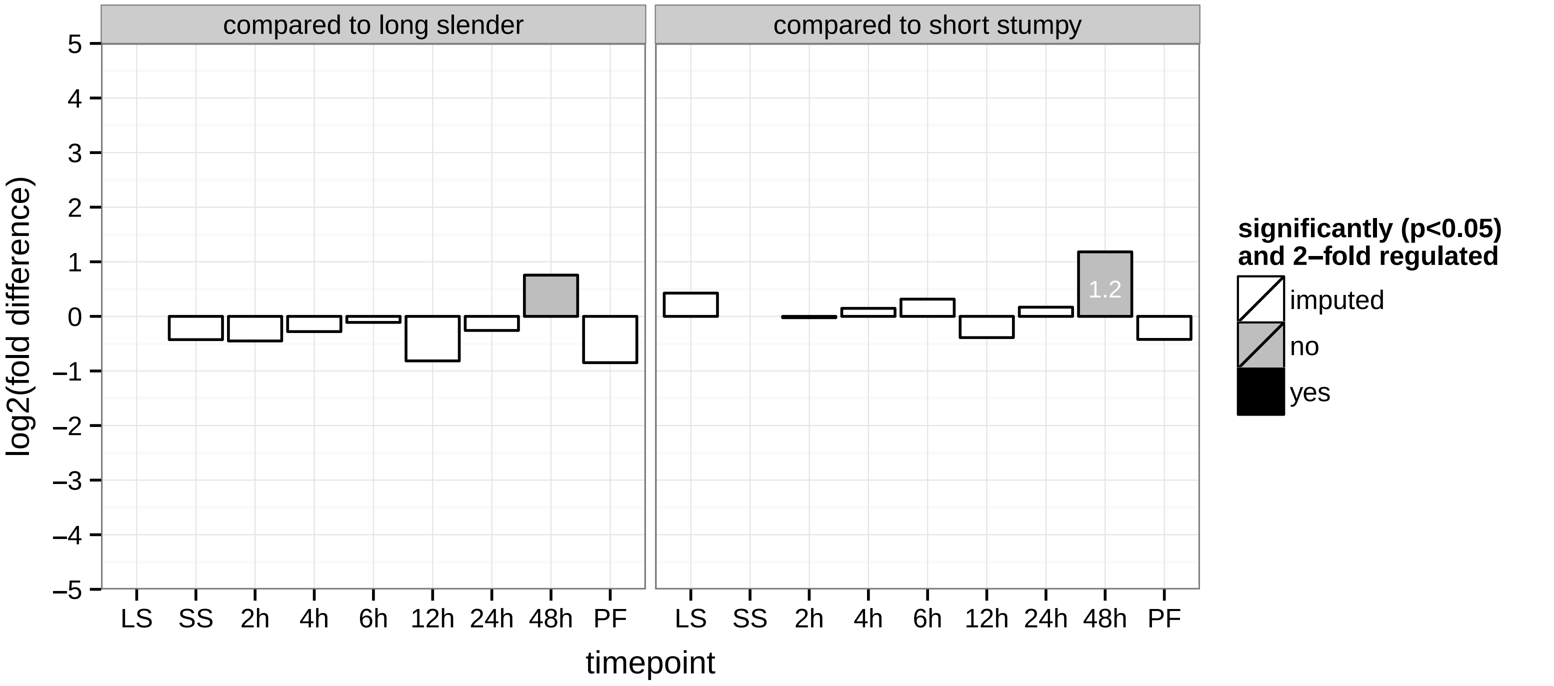

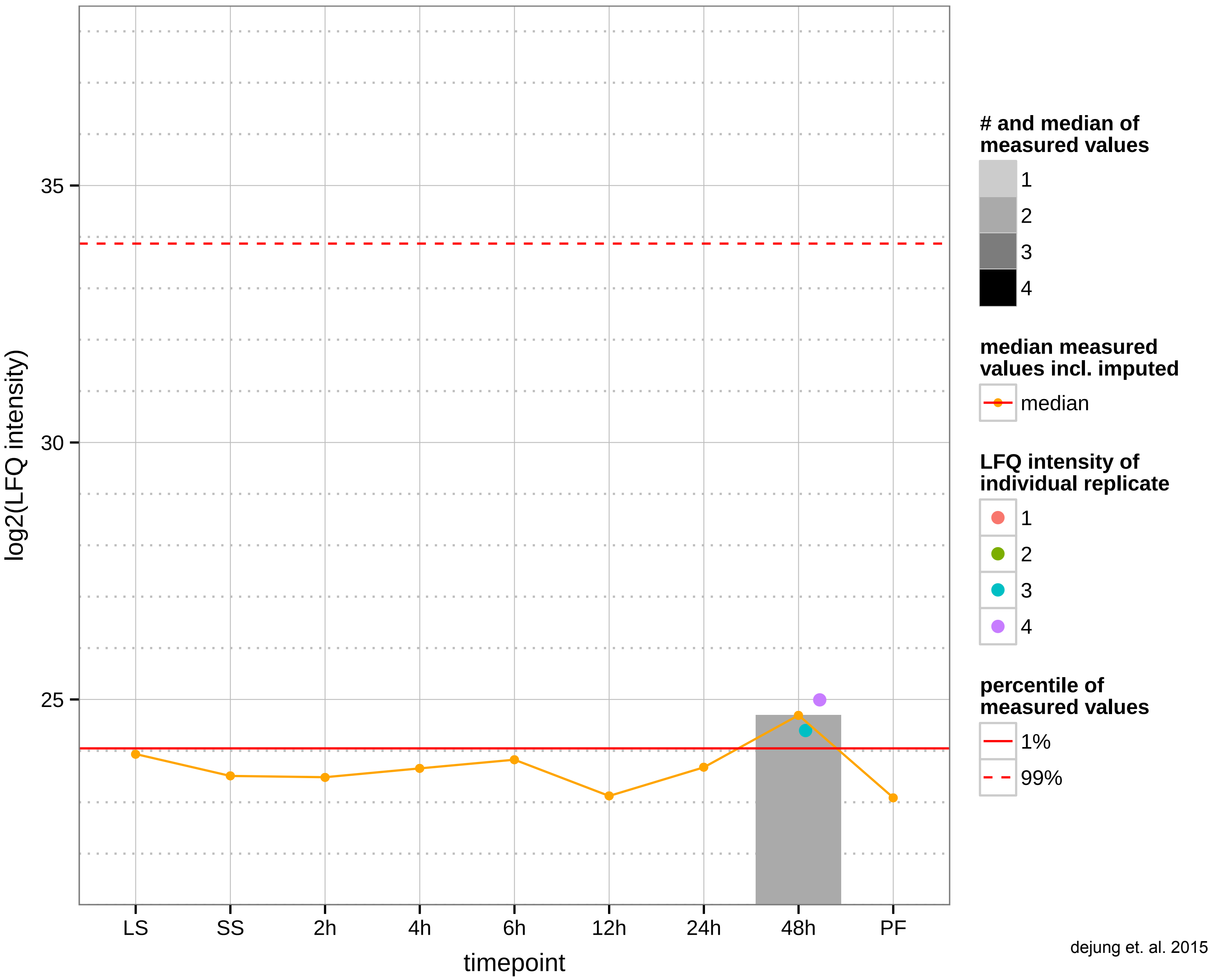

dejung et. al. 2015

protein kinase, putative
Tb927.7.3880
AGOF: ATP binding, protein serine/threonine kinase activity, protein tyrosine kinase activity
AGOC: null
AGOP: protein phosphorylation
PGOF: ATP binding, protein kinase activity, transferase activity, transferring phosphorus–containing groups
PGOC: null
PGOP: protein phosphorylation

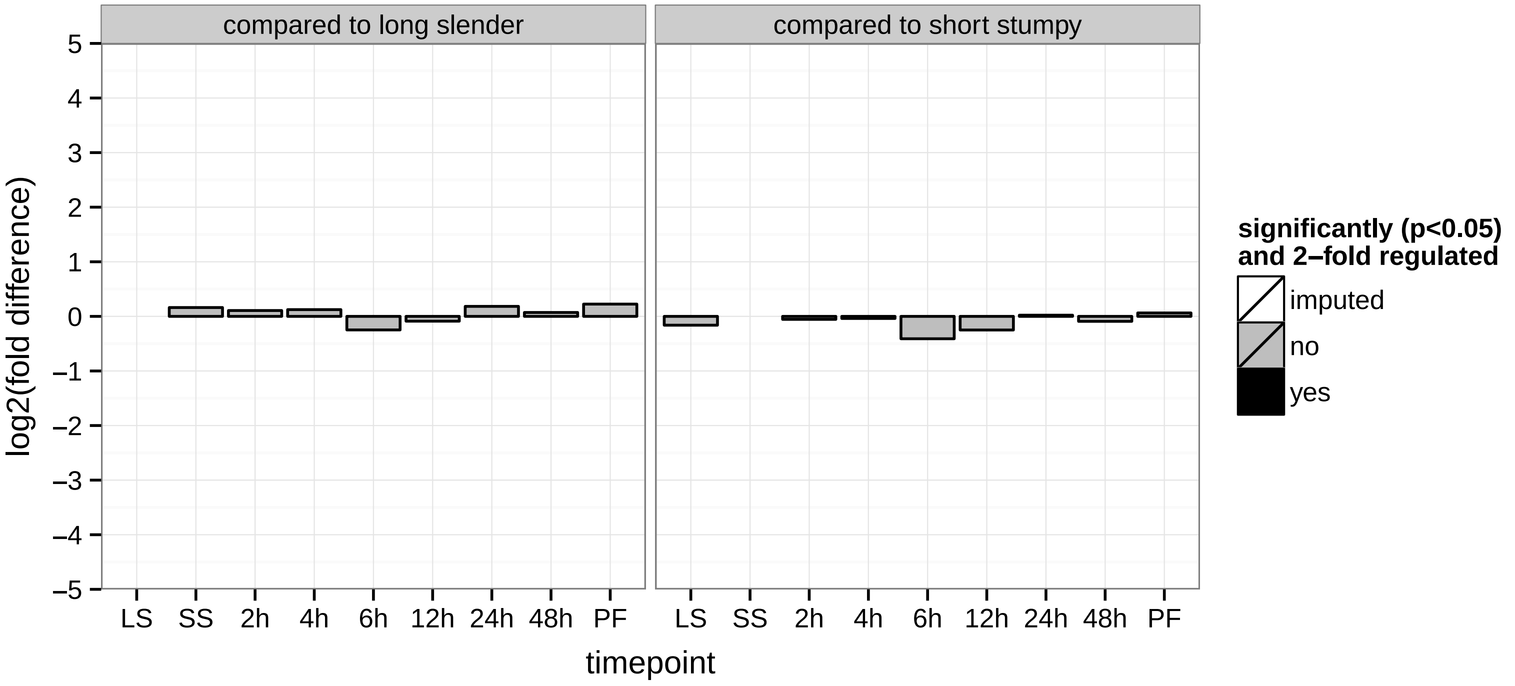

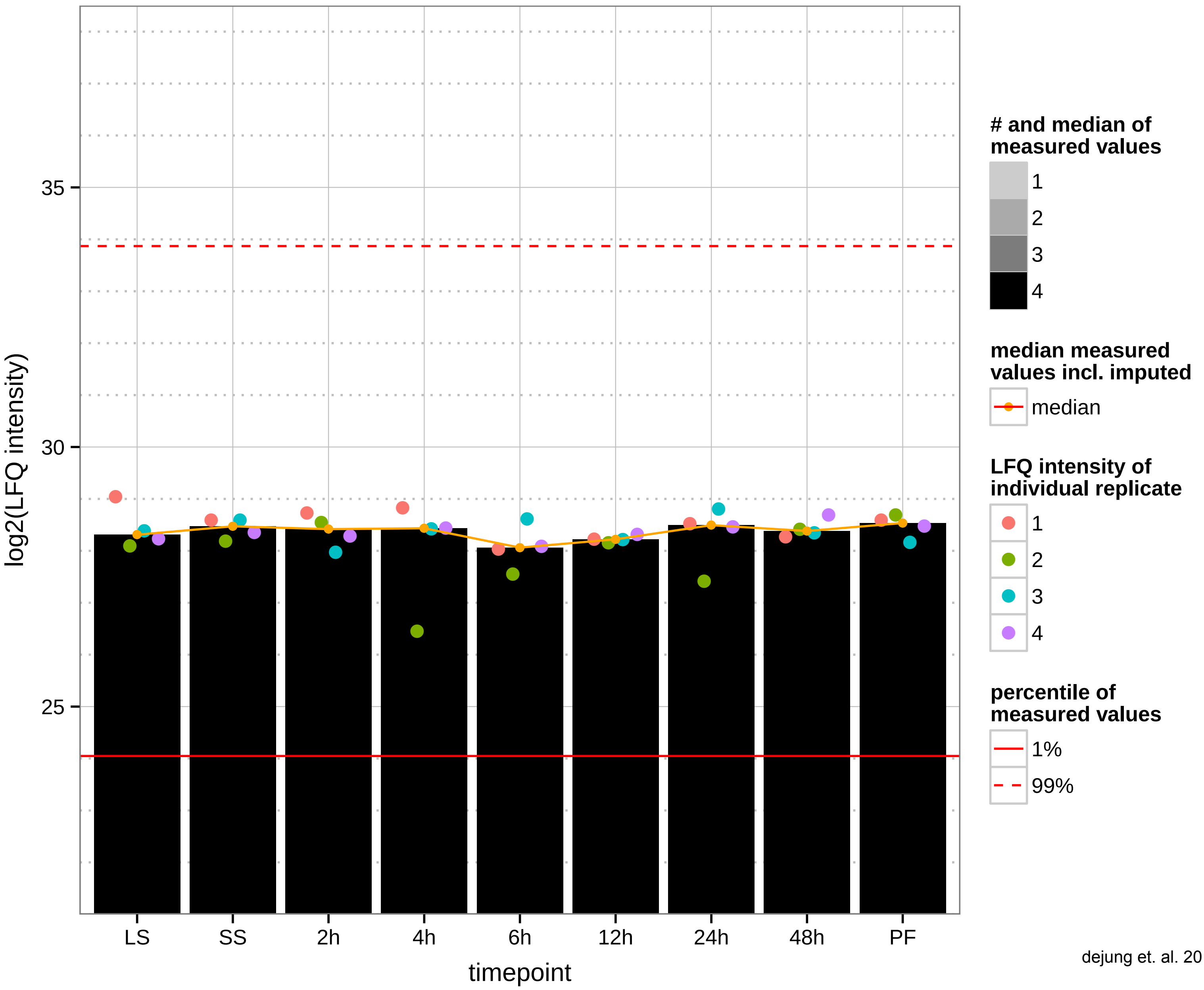

dejung et. al. 2015

hypothetical protein, conserved
Tb927.7.3920
AGOF: null
AGOC: null
AGOP: null
PGOF: null
PGOC: null
PGOP: null

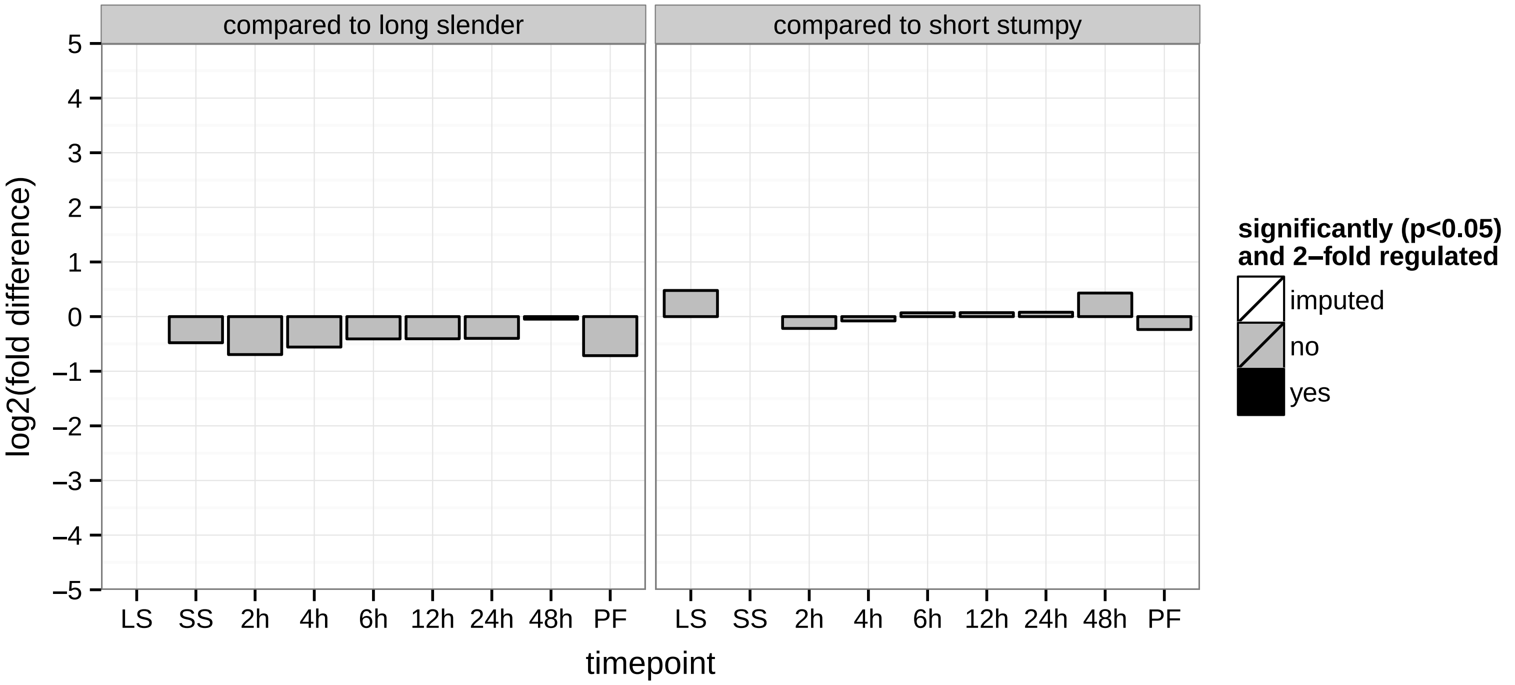

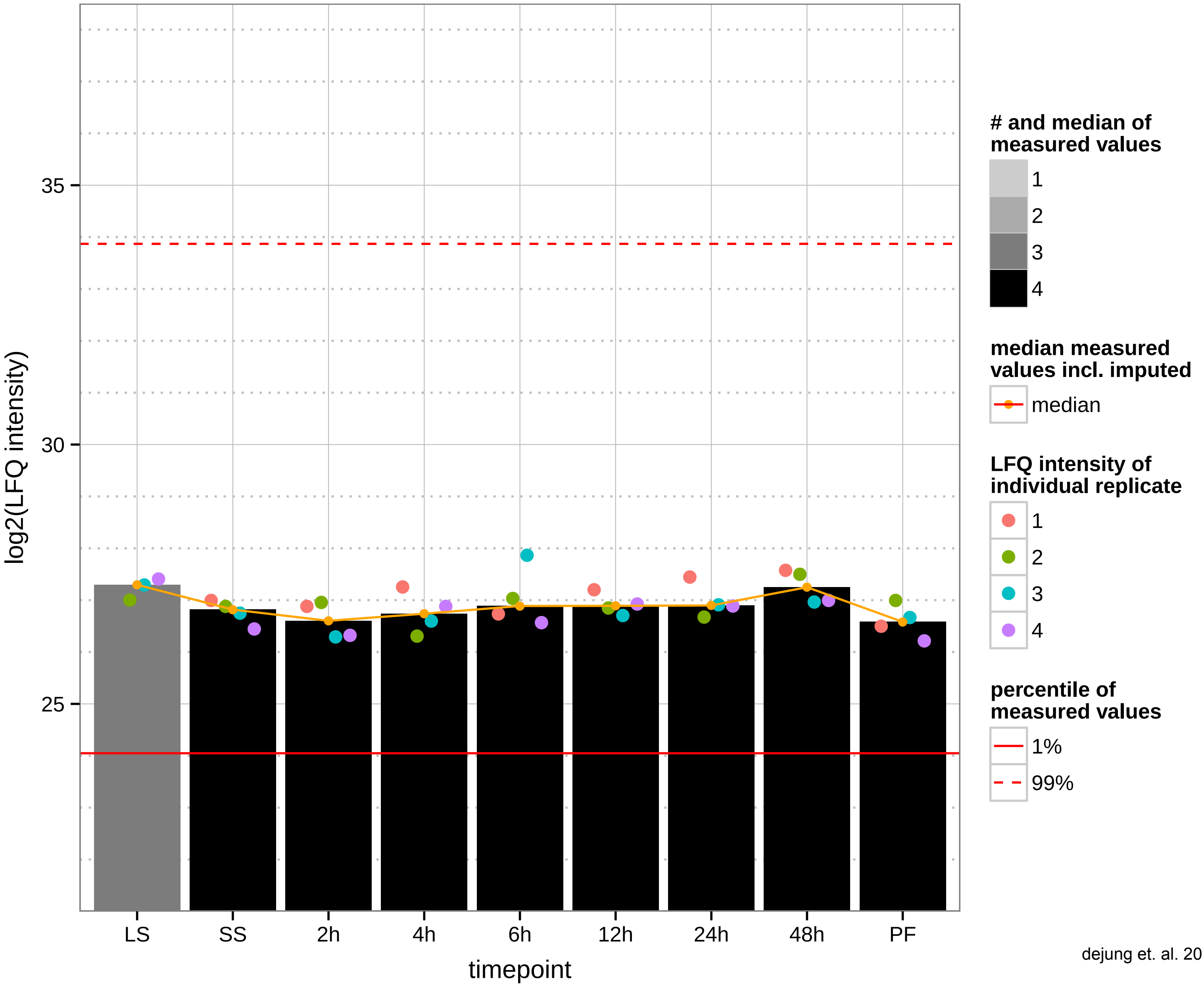

dejung et. al. 2015

immunodominant antigen, putative, tc40 antigen–like
Tb927.7.3980
AGOF: null
AGOC: null
AGOP: null
PGOF: null
PGOC: null
PGOP: null

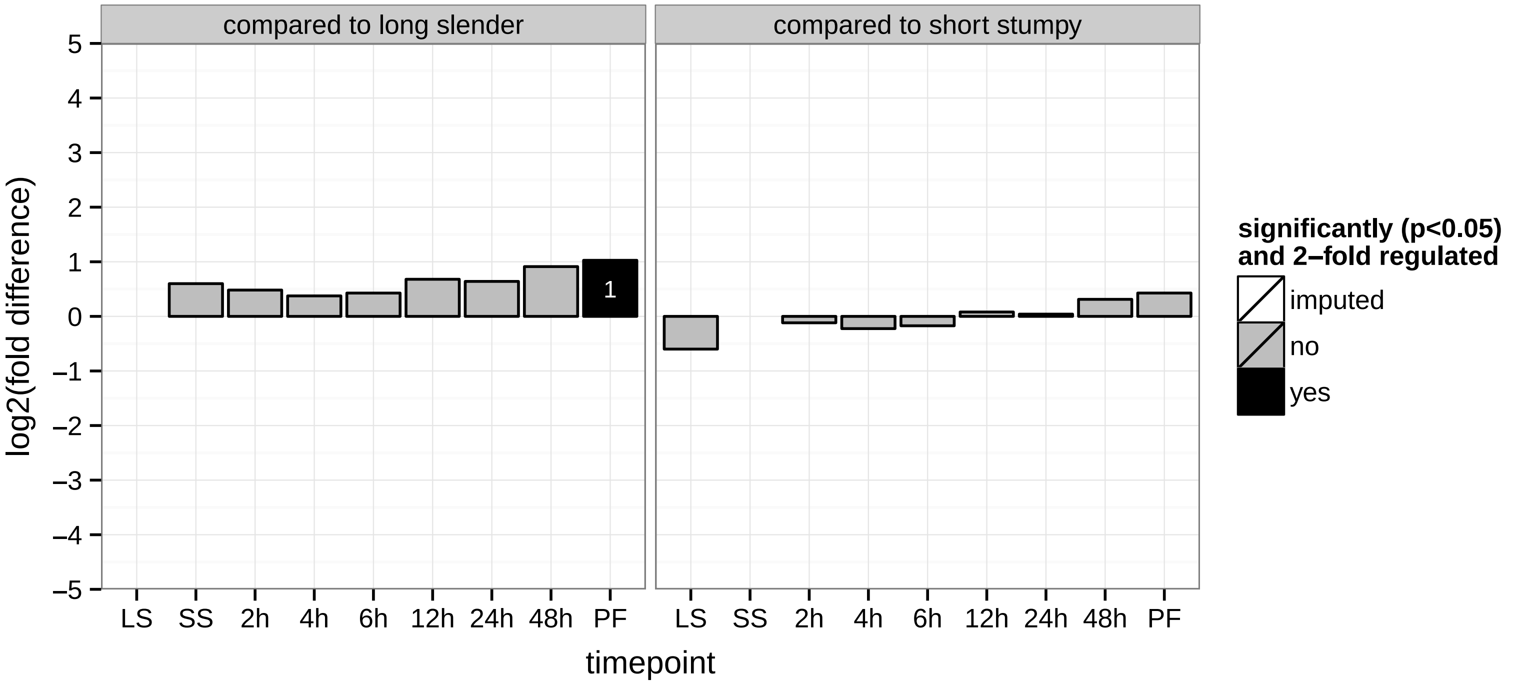

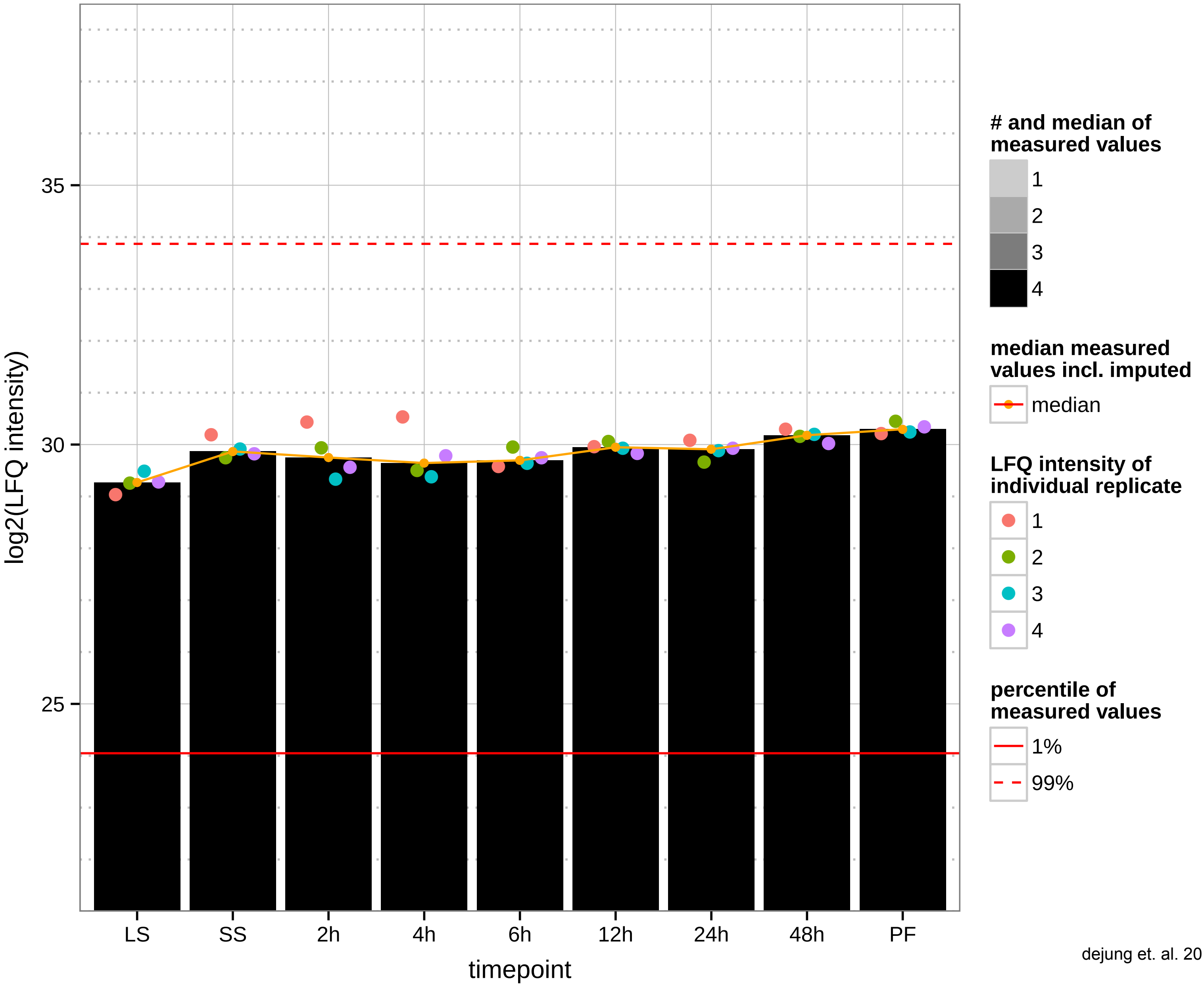

dejung et. al. 2015

glutathione synthetase, putative
Tb927.7.4000
AGOF: ATP binding, glutathione synthase activity
AGOC: null
AGOP: glutathione biosynthetic process
PGOF: ATP binding, glutathione synthase activity
PGOC: null
PGOP: glutathione biosynthetic process

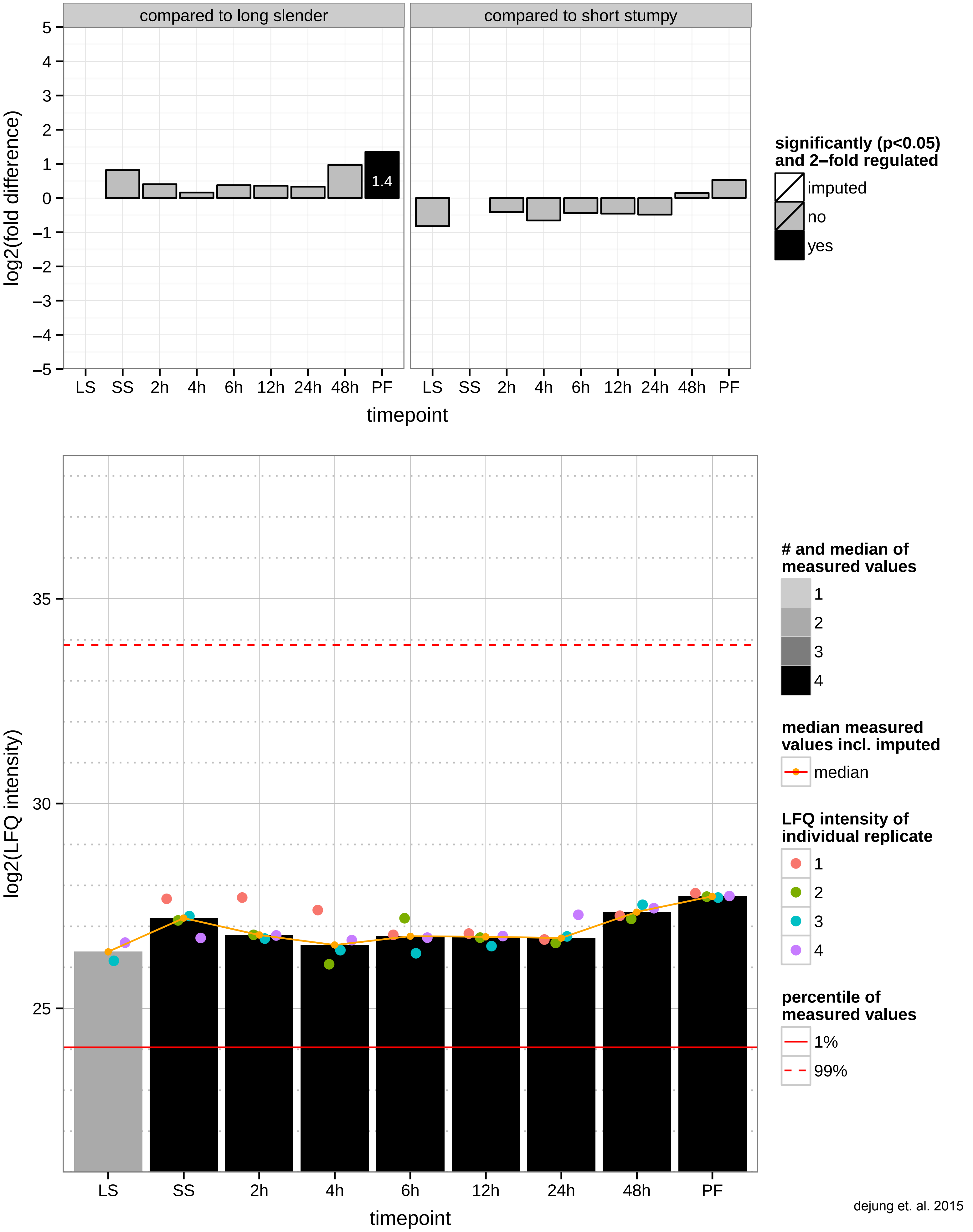

dejung et. al. 2015

protein phosphatase 2C, putative
Tb927.7.4020
AGOF: phosphatase activity
AGOC: null
AGOP: dephosphorylation
PGOF: catalytic activity
PGOC: null
PGOP: null

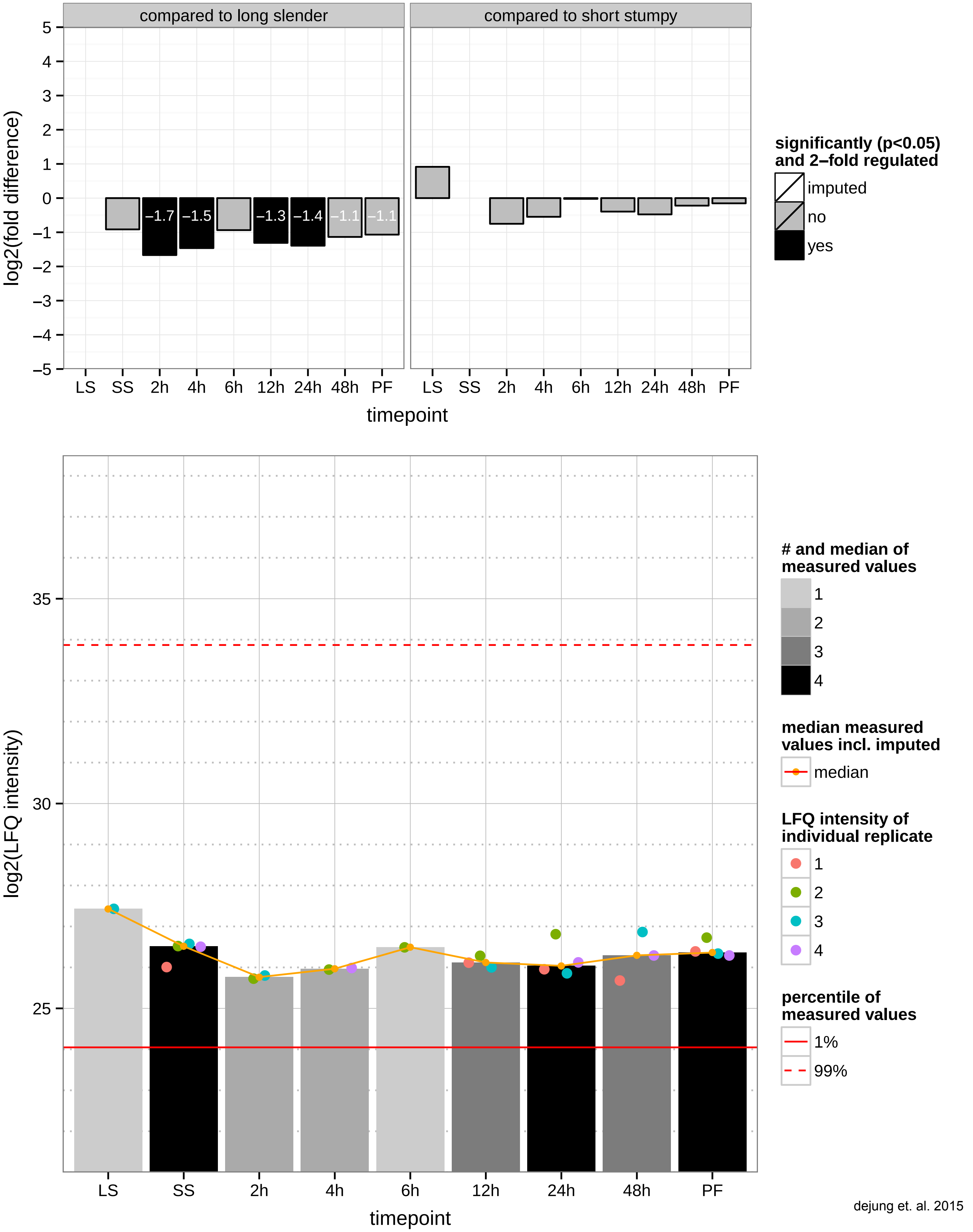

dejung et. al. 2015

fatty acid elongase, putative
Tb927.7.4180
AGOF: fatty acid elongase activity
AGOC: integral to endoplasmic reticulum membrane
AGOP: long−chain fatty acid biosynthetic process
PGOF: null
PGOC: integral to membrane
PGOP: null

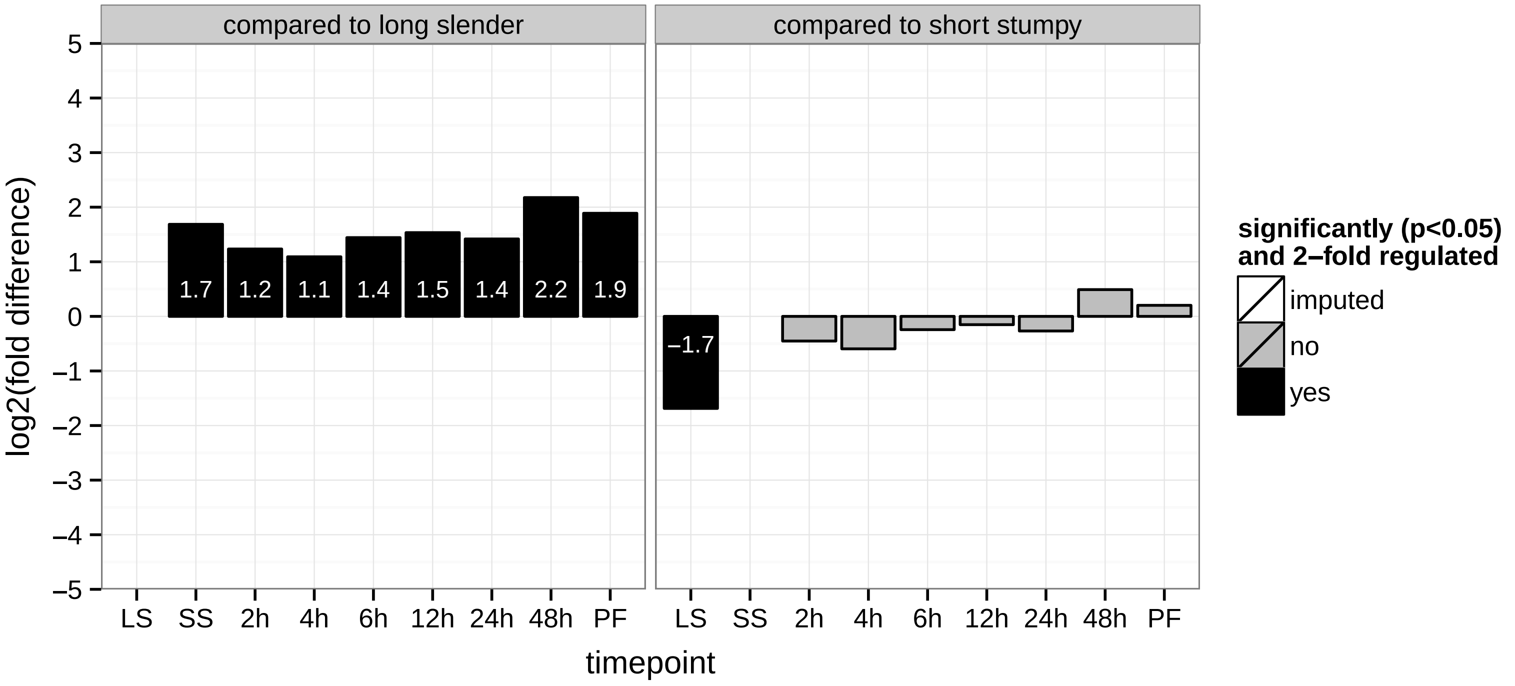

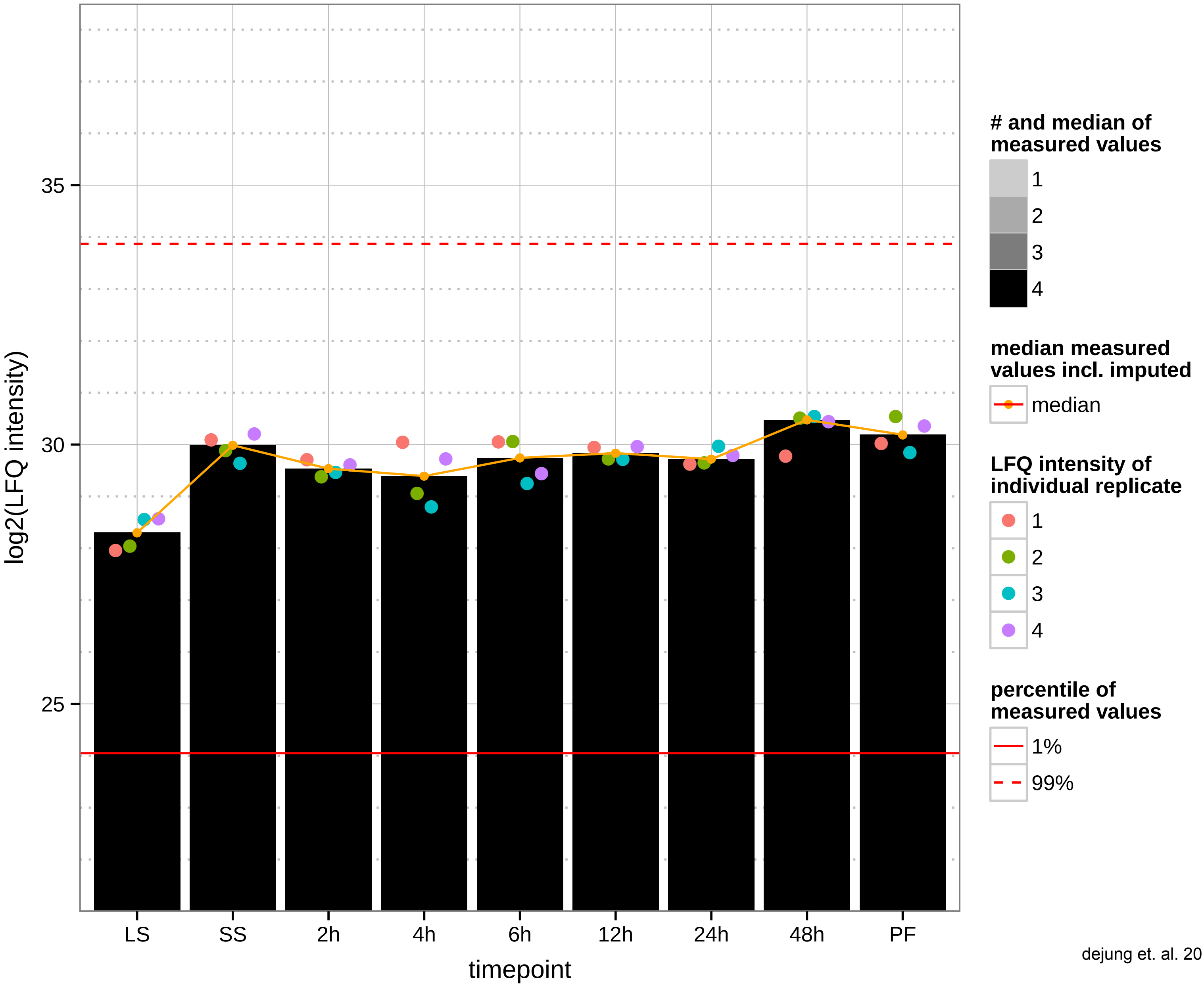

dejung et. al. 2015

hypothetical protein, conserved
Tb927.7.4220
AGOF: null
AGOC: small–subunit processome
AGOP: rRNA processing
PGOF: protein binding
PGOC: null
PGOP: null

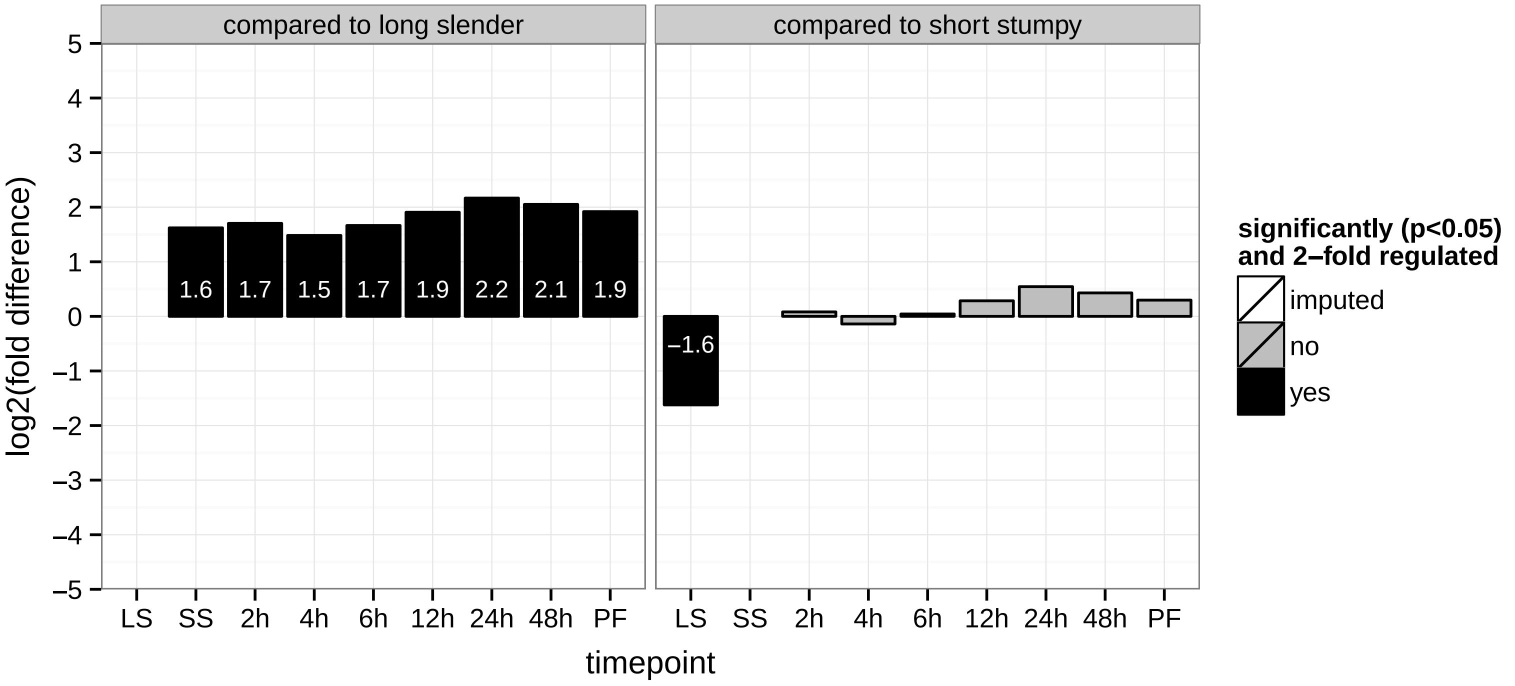

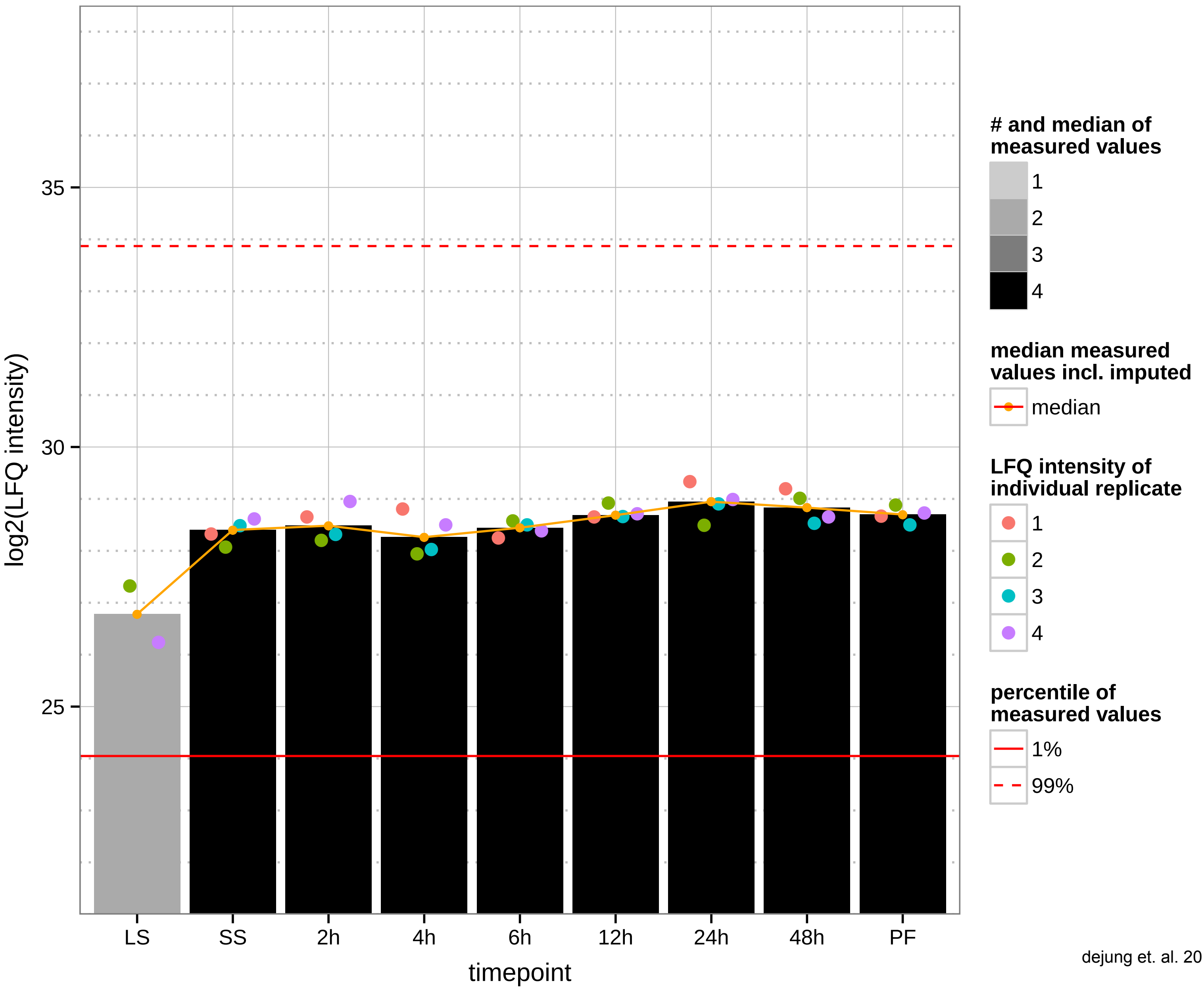

dejung et. al. 2015

hypothetical protein, conserved
Tb927.7.4230
AGOF: null
AGOC: null
AGOP: null
PGOF: null
PGOC: null
PGOP: null

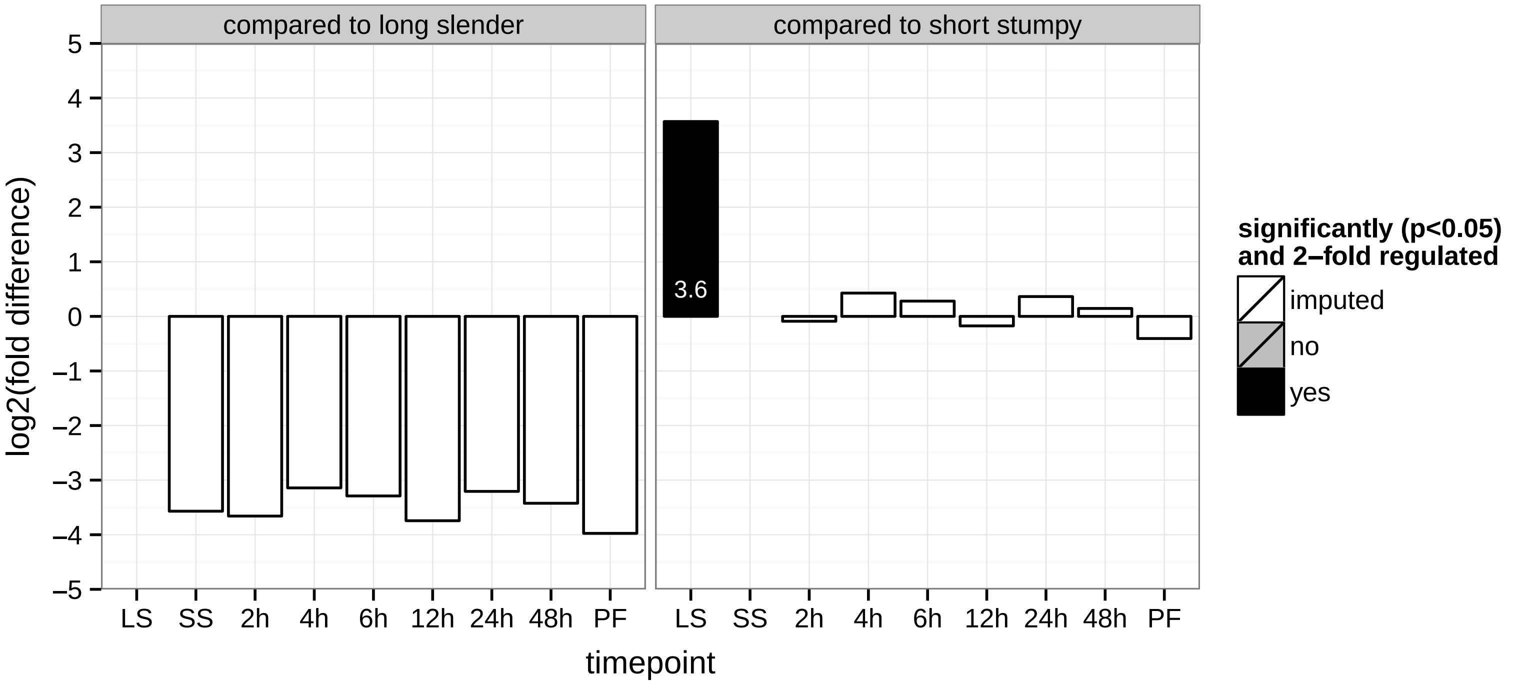

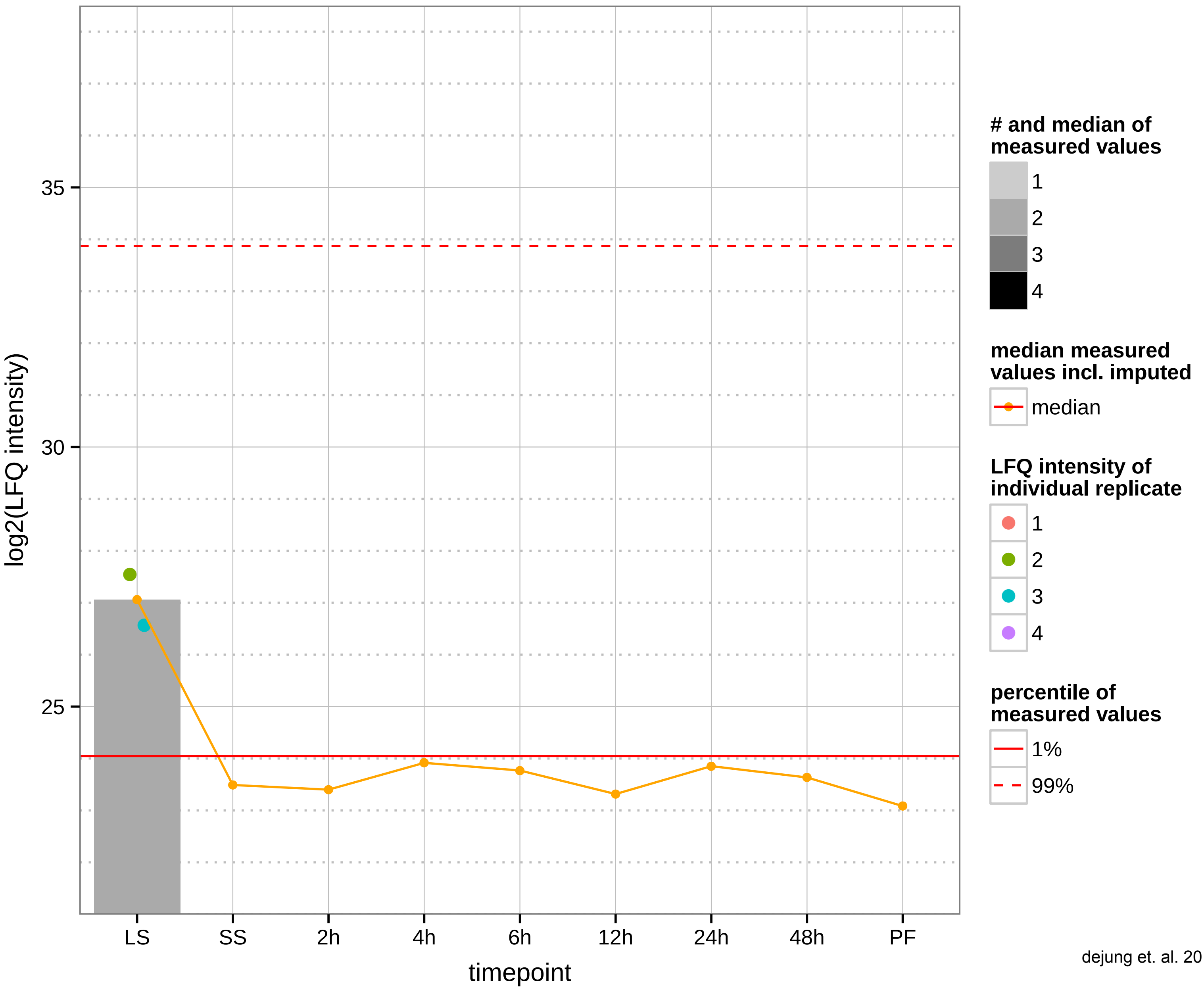

dejung et. al. 2015

hypothetical protein, conserved
Tb927.7.4250;Tb927.7.4240
AGOF: null
AGOC: null
AGOP: null
PGOF: null
PGOC: null
PGOP: null

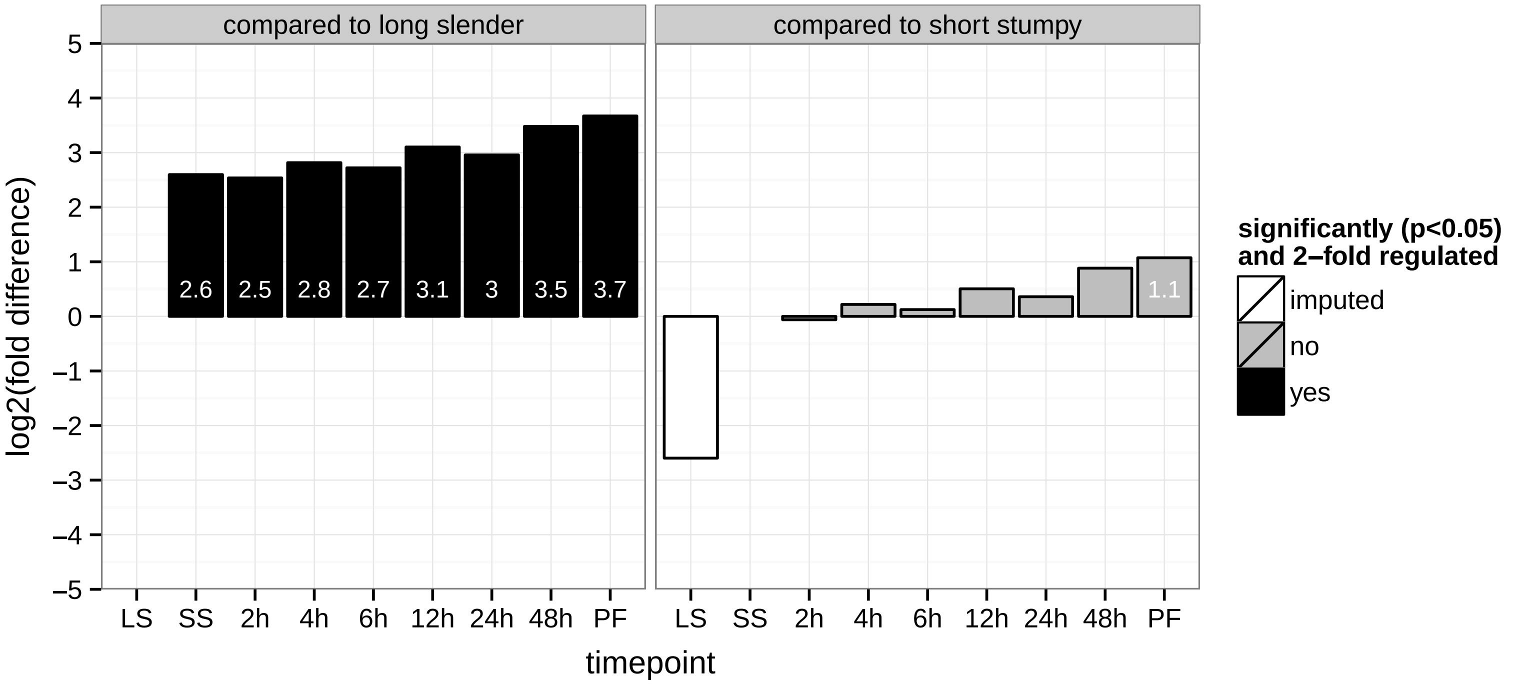

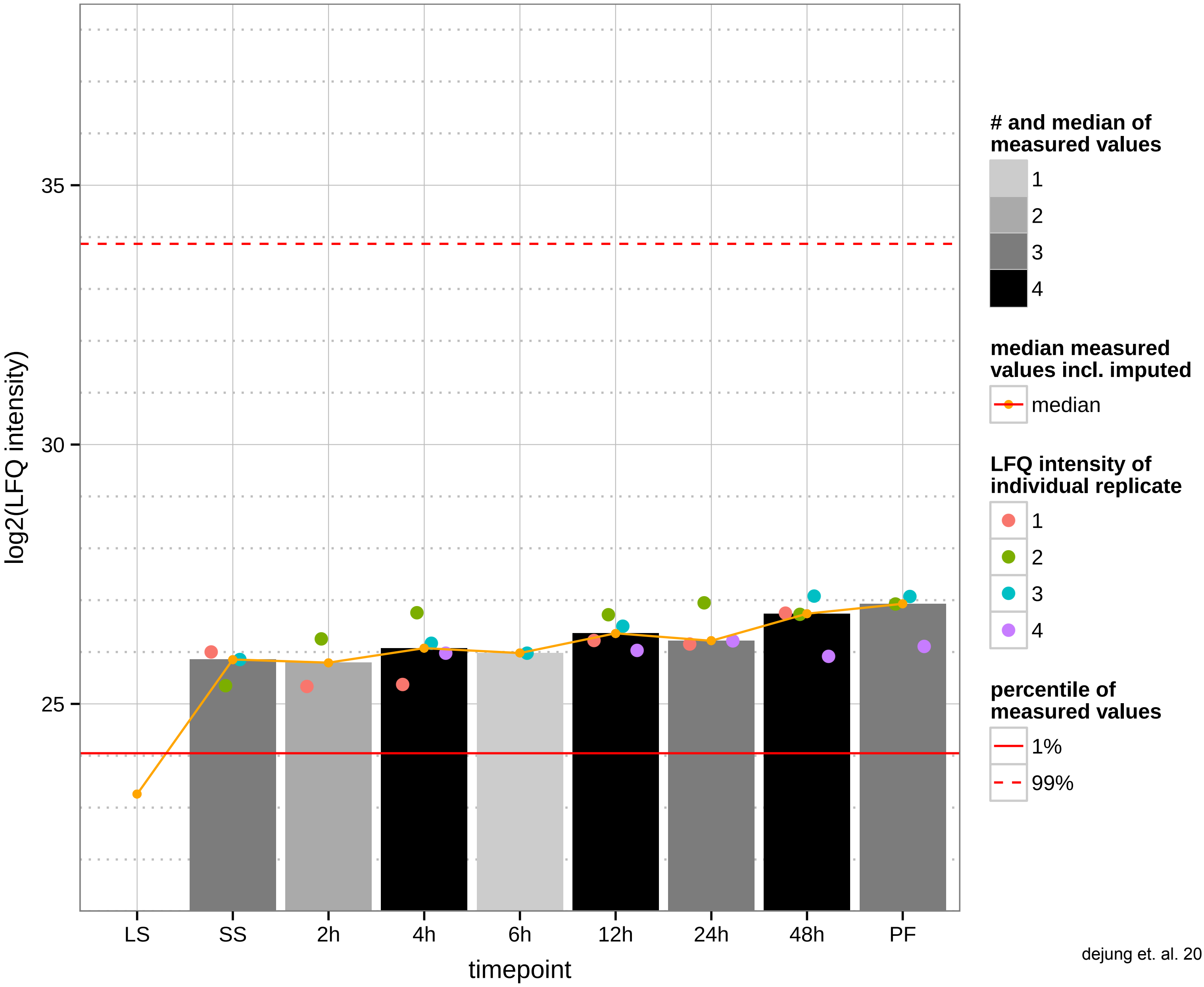

dejung et. al. 2015

hypothetical protein, conserved
Tb927.7.4290
AGOF: null
AGOC: null
AGOP: null
PGOF: null
PGOC: null
PGOP: null

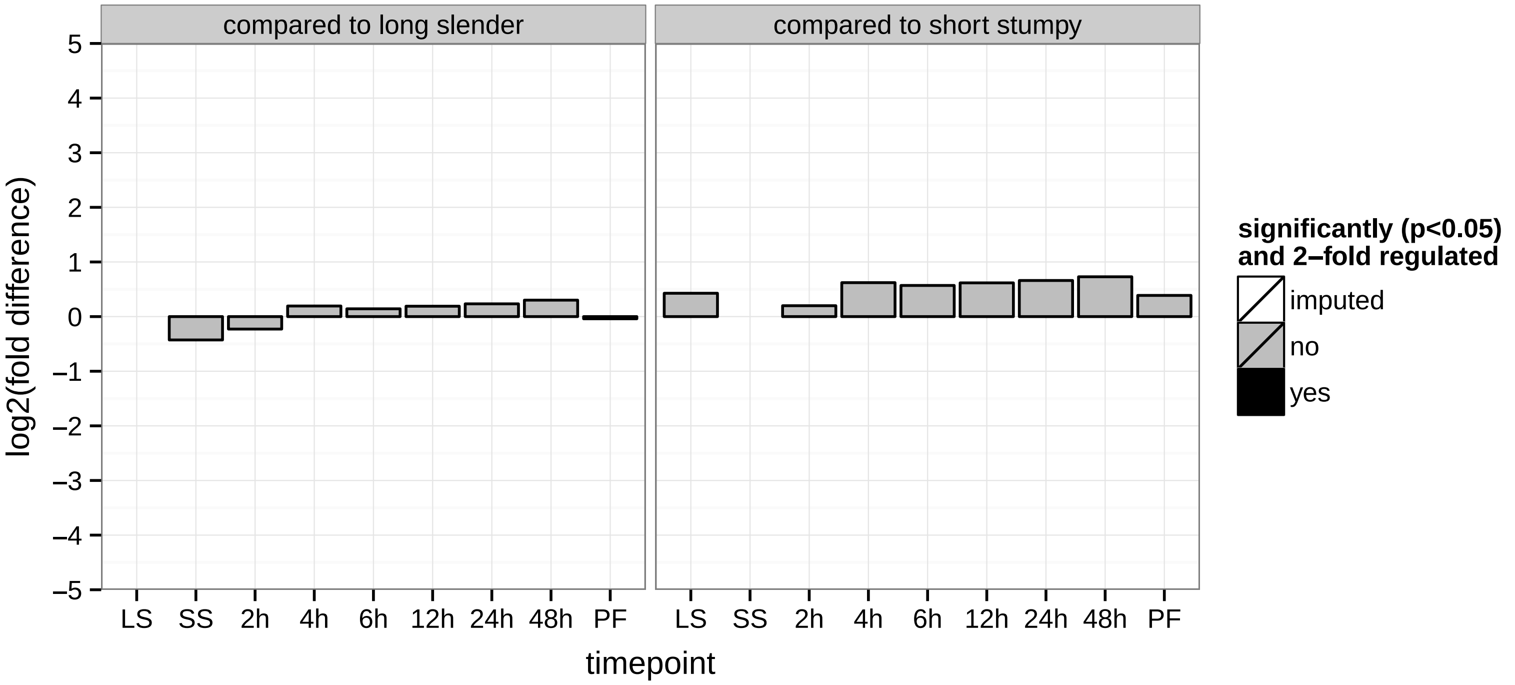

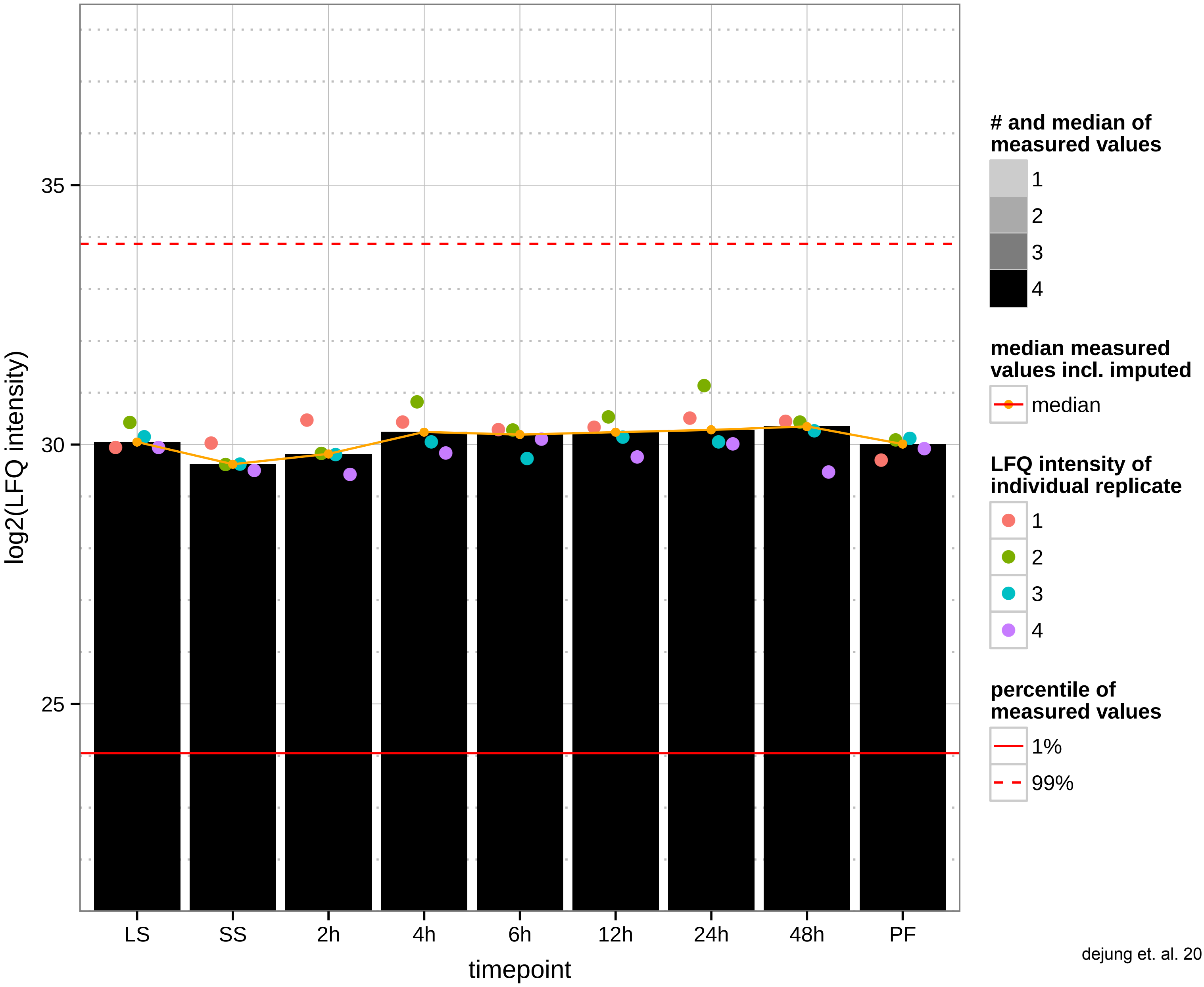

dejung et. al. 2015

hypothetical protein, conserved
Tb927.7.4320
AGOF: polynucleotide 5'−phosphatase activity
AGOC: mRNA cap methyltransferase complex
AGOP: 7−methylguanosine mRNA capping
PGOF: polynucleotide 5'−phosphatase activity, transferase activity
PGOC: null
PGOP: null

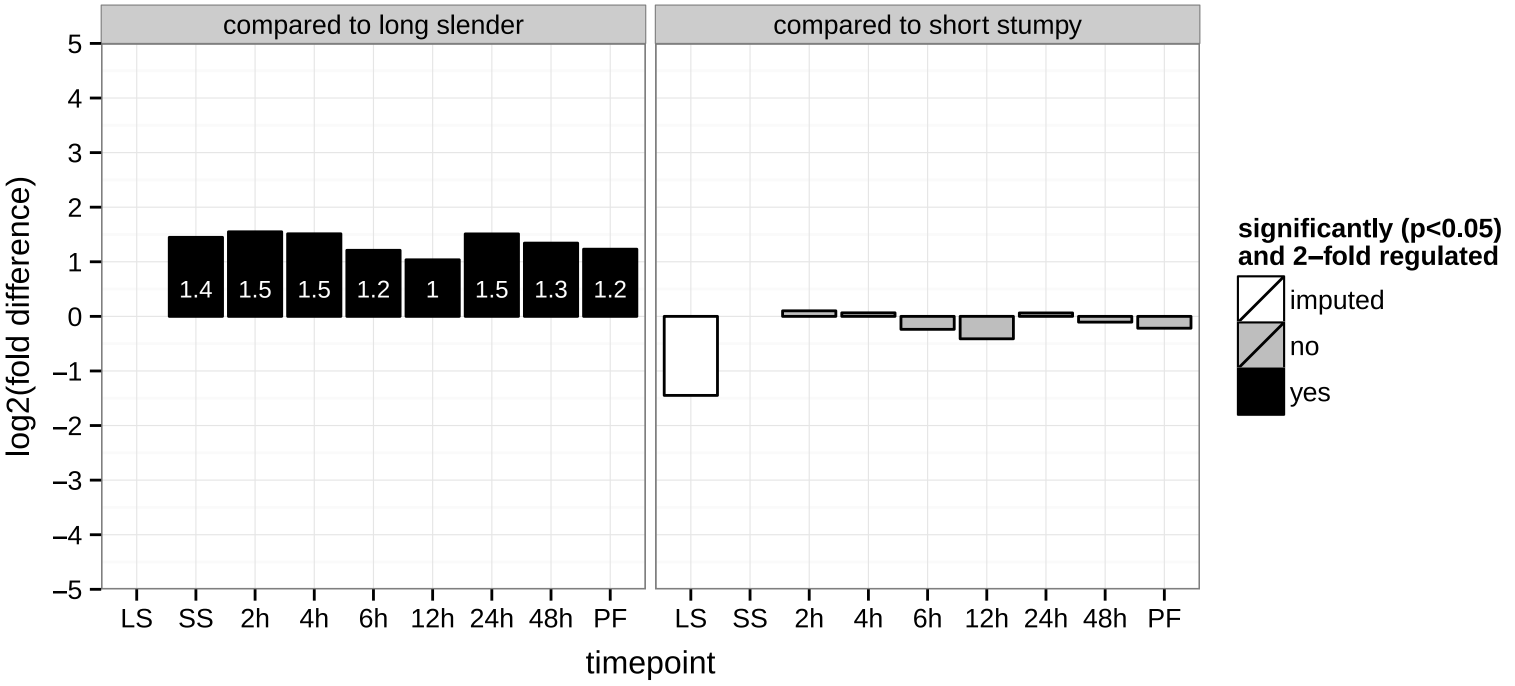

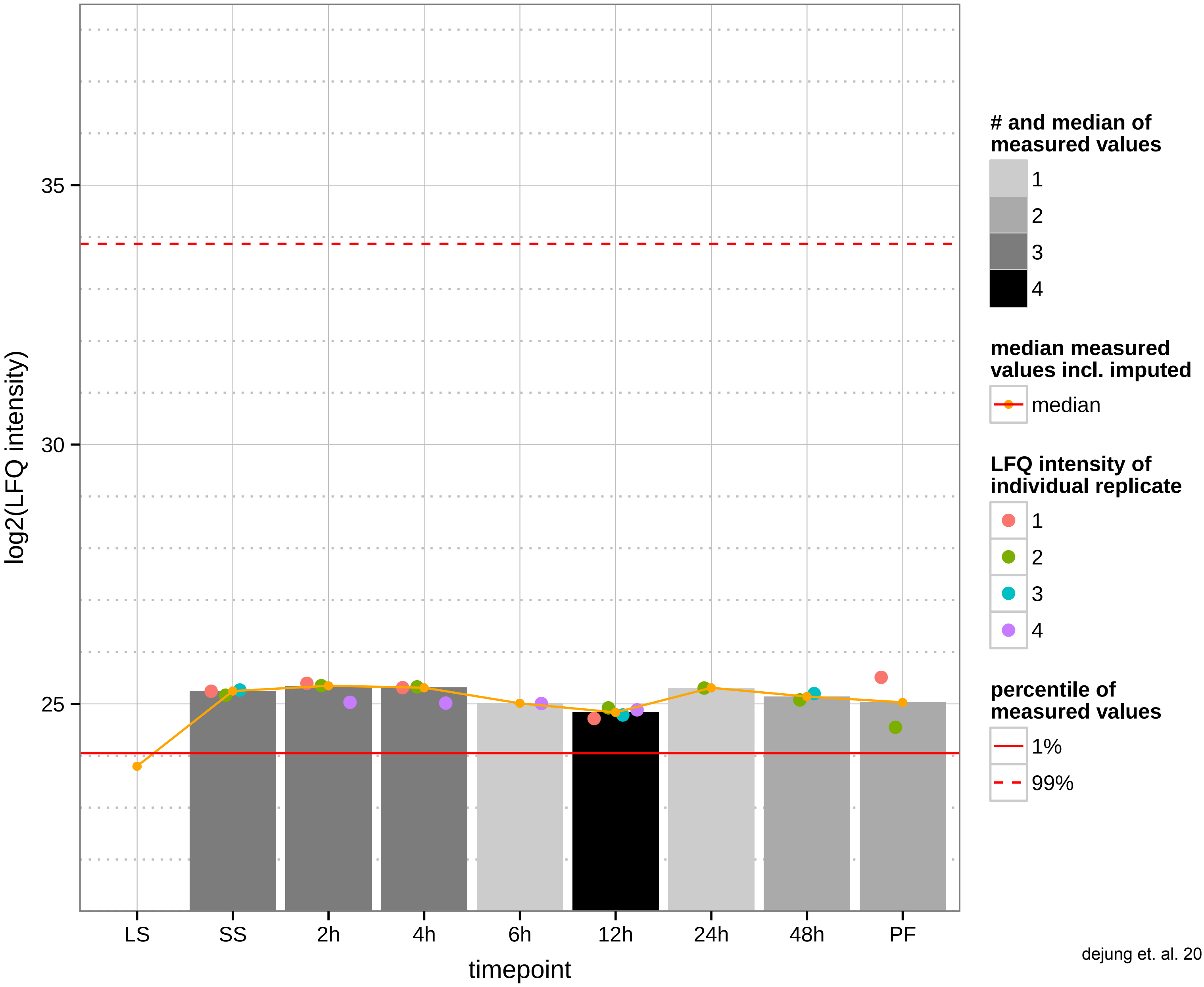

dejung et. al. 2015

hypothetical protein, conserved
Tb927.7.4340
AGOF: null
AGOC: null
AGOP: null
PGOF: null
PGOC: nucleus
PGOP: null

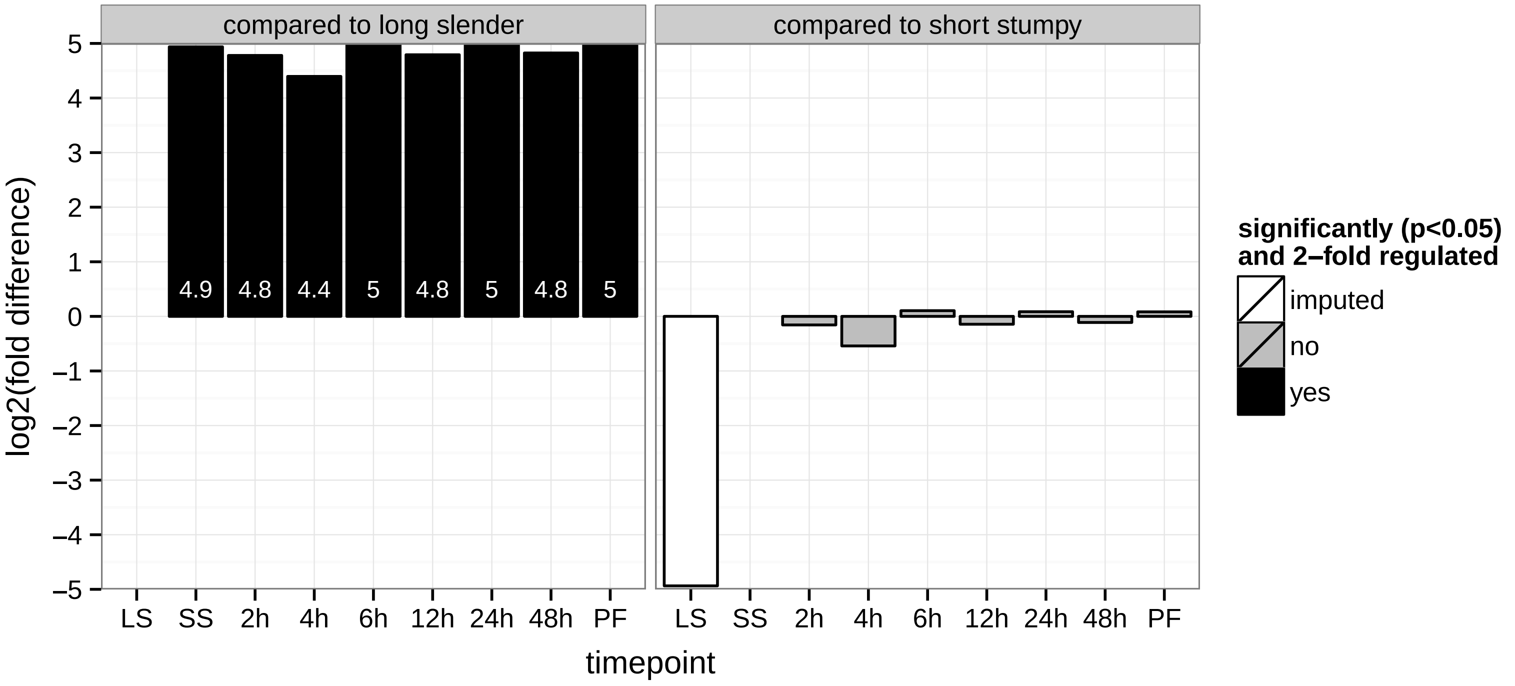

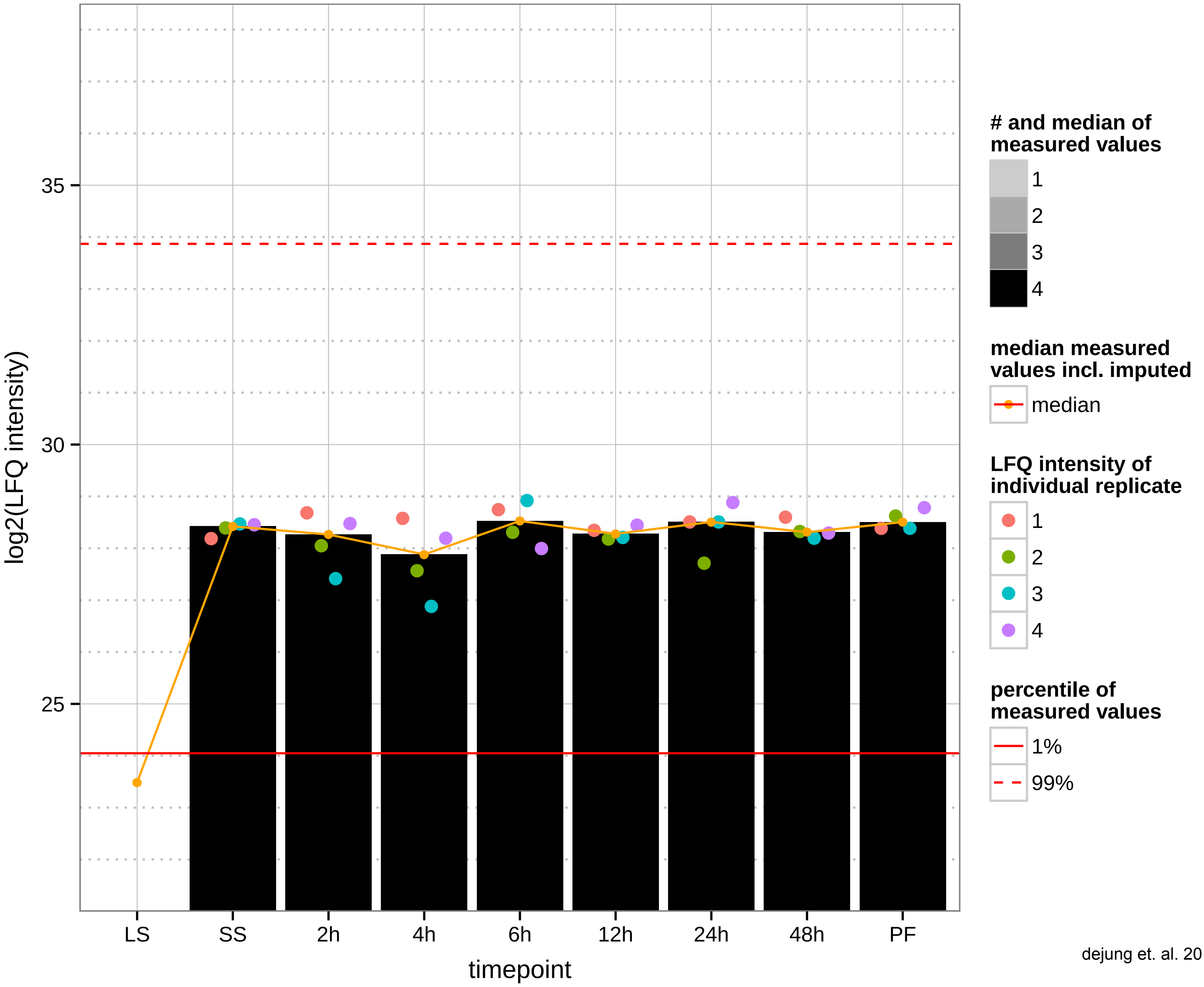

dejung et. al. 2015

threonine synthase, putative
Tb927.7.4390
AGOF: pyridoxal phosphate binding, threonine synthase activity
AGOC: null
AGOP: threonine biosynthetic process
PGOF: catalytic activity, pyridoxal phosphate binding, threonine synthase activity
PGOC: null
PGOP: metabolic process, threonine biosynthetic process

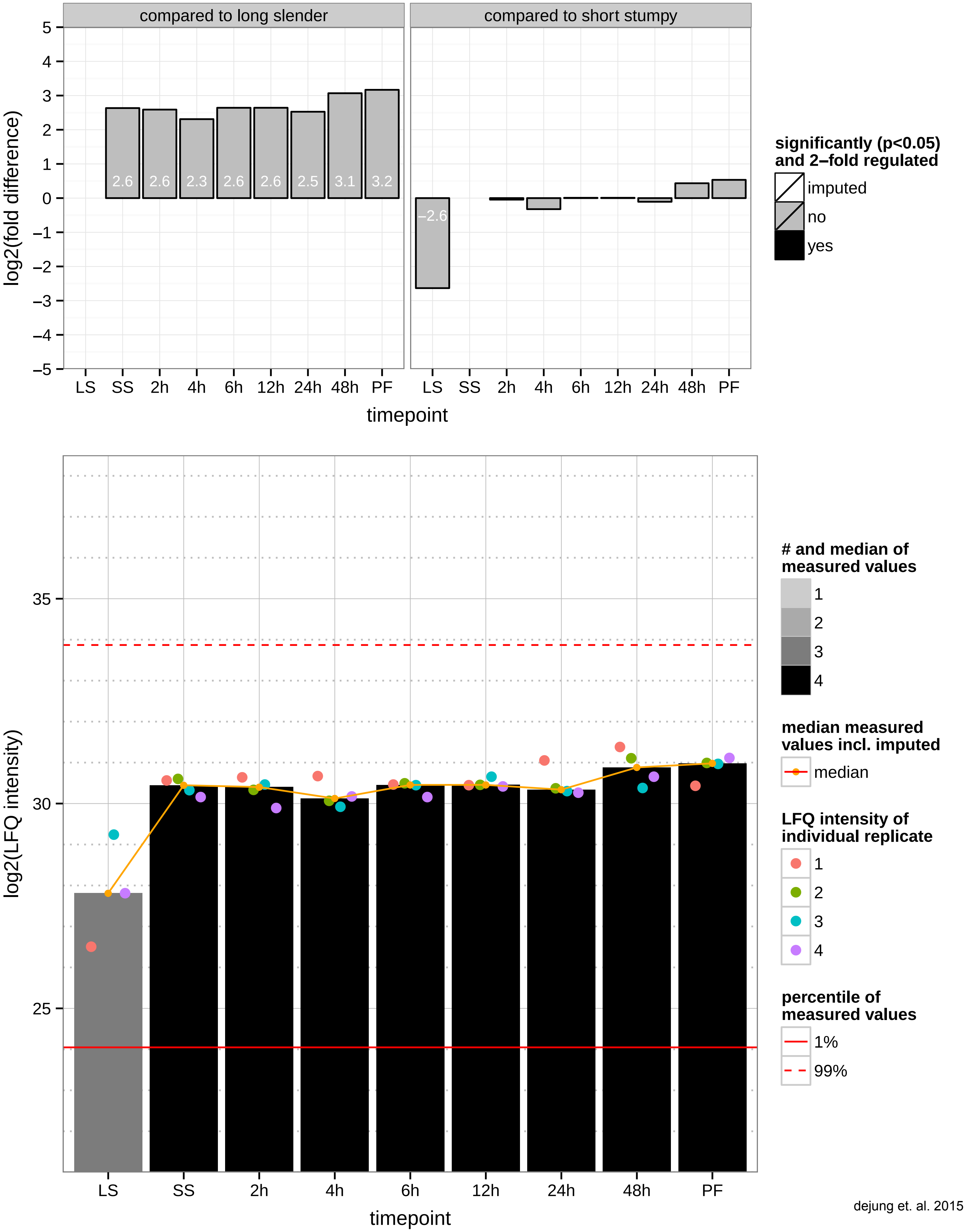

dejung et. al. 2015

proteasome alpha 3 subunit, putative
Tb927.7.4420
AGOF: endopeptidase activity, threonine–type endopeptidase activity
AGOC: proteasome core complex, proteasome core complex, alpha–subunit complex
AGOP: protein metabolic process, ubiquitin–dependent protein catabolic process
PGOF: endopeptidase activity, threonine–type endopeptidase activity
PGOC: proteasome core complex, proteasome core complex, alpha–subunit complex
PGOP: proteolysis involved in cellular protein catabolic process, ubiquitin–dependent protein catabolic process

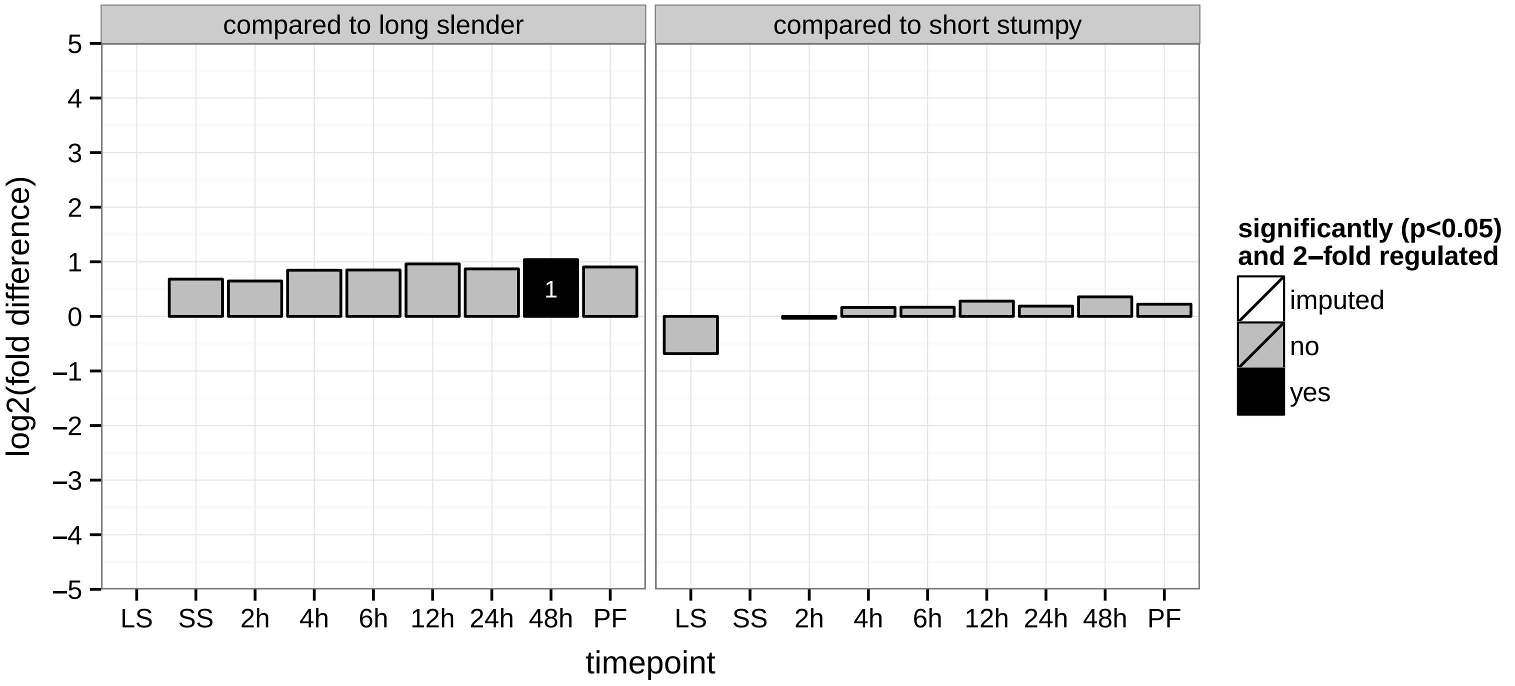

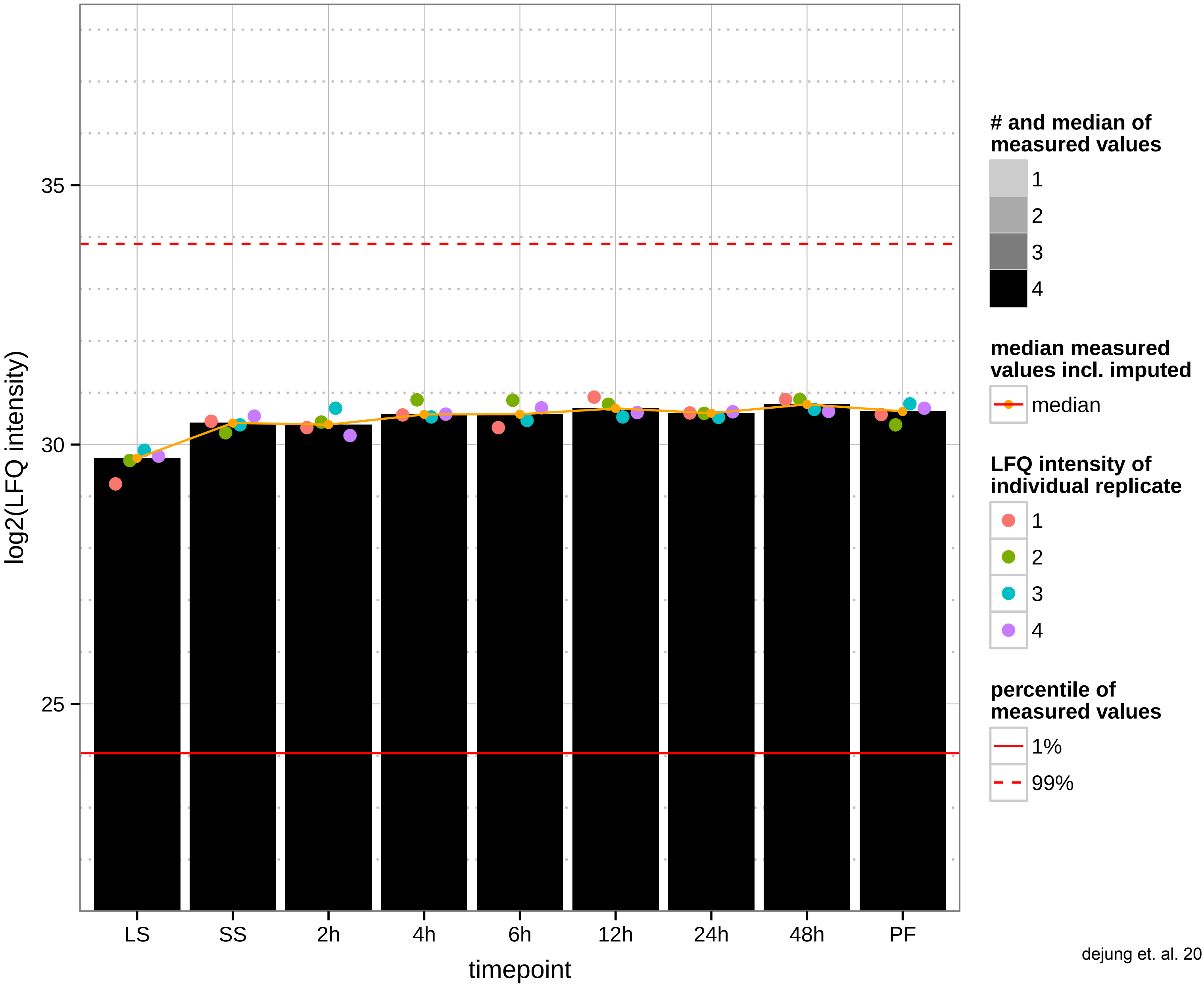

dejung et. al. 2015

hypothetical protein, conserved
Tb927.7.4440
AGOF: catalytic activity, coenzyme binding
AGOC: mitochondrion
AGOP: cellular metabolic process
PGOF: catalytic activity, coenzyme binding
PGOC: null
PGOP: cellular metabolic process

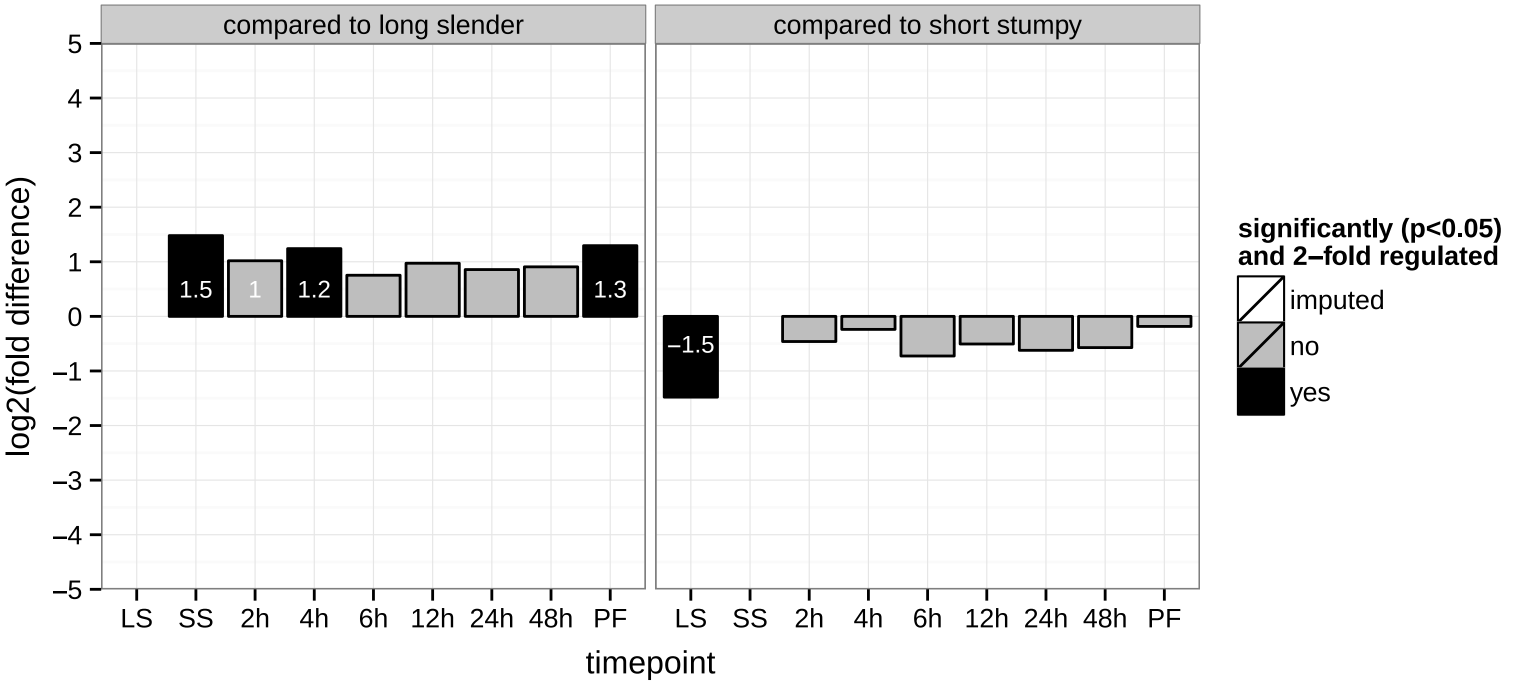

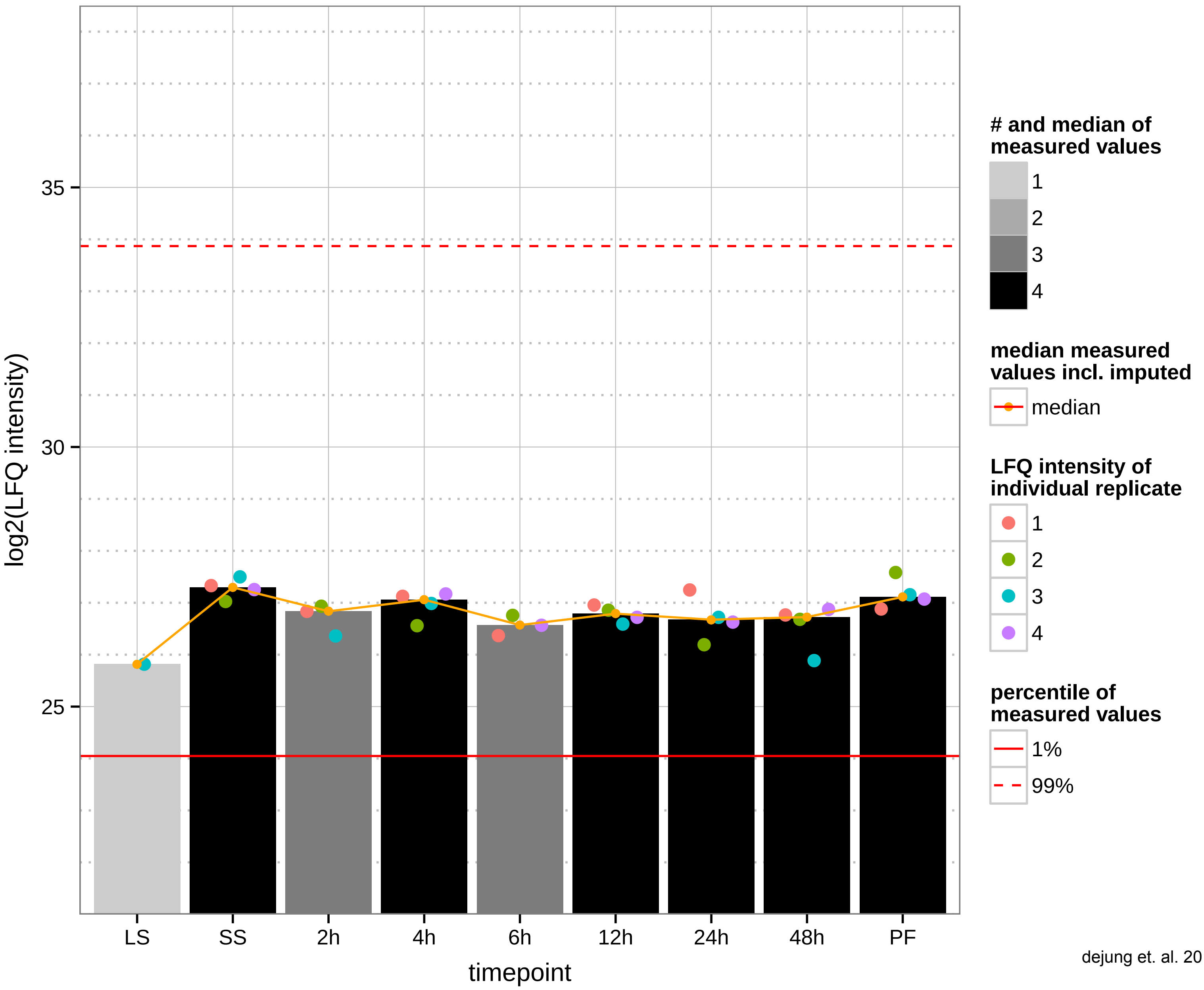

dejung et. al. 2015

hypothetical protein, conserved
Tb927.7.4450
AGOF: null
AGOC: null
AGOP: null
PGOF: null
PGOC: null
PGOP: null

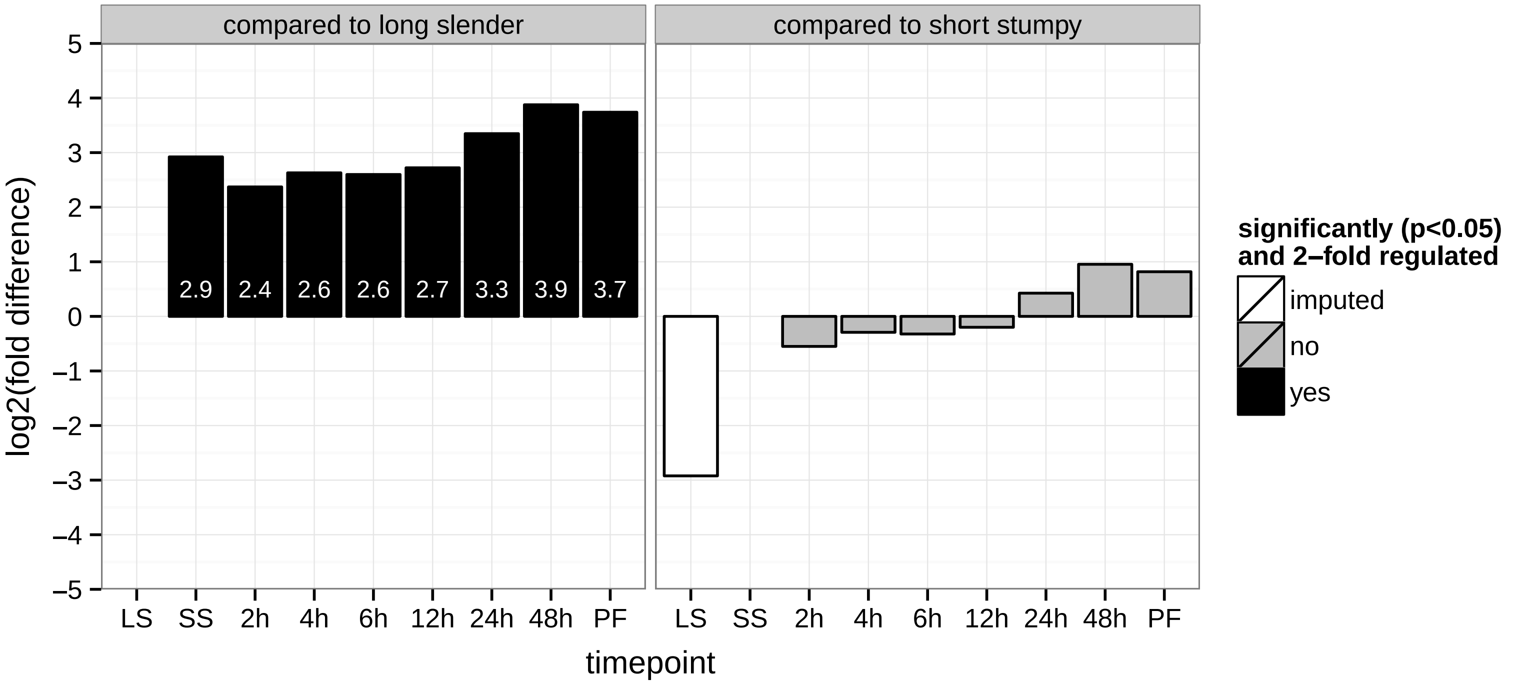

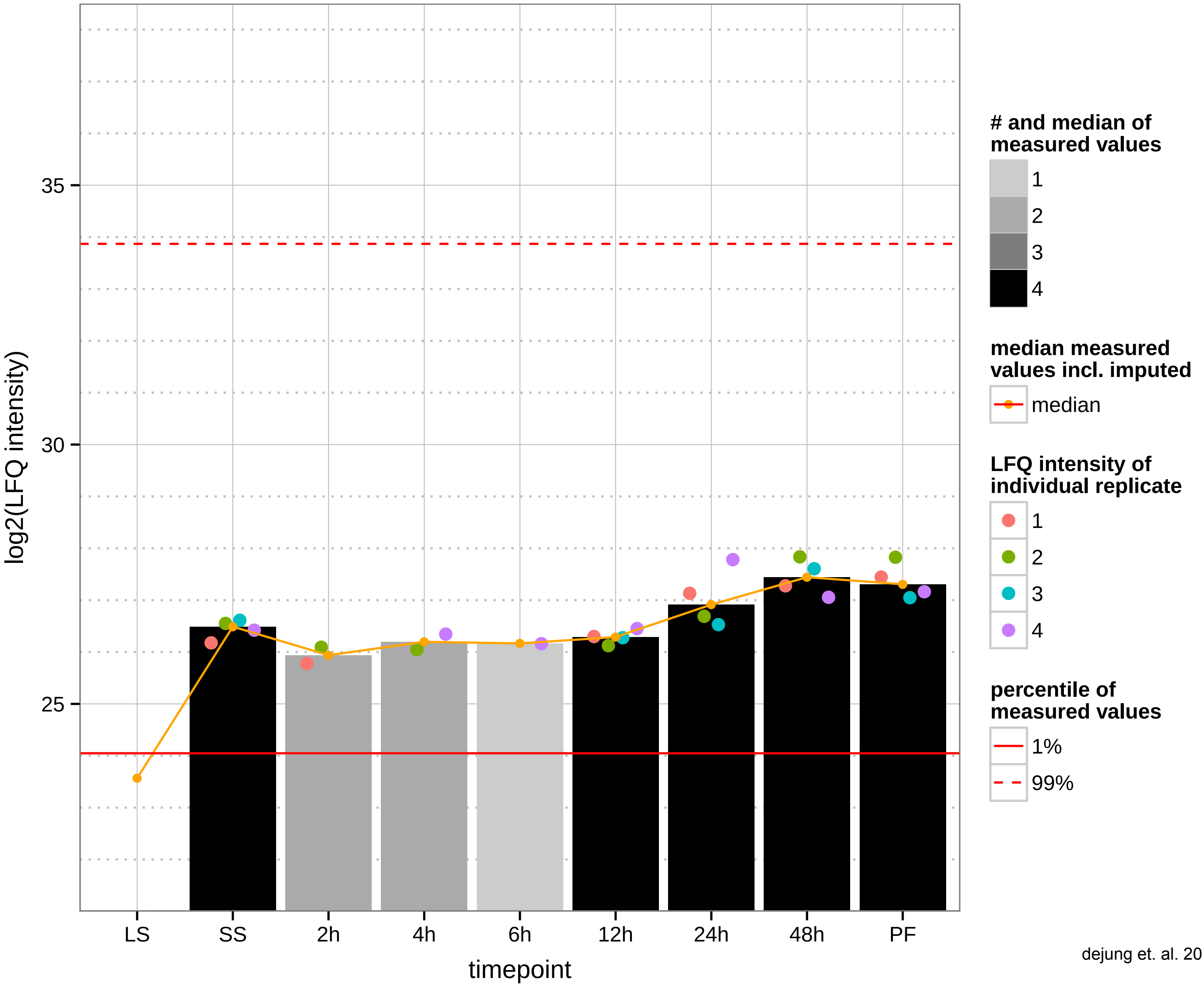

dejung et. al. 2015

hypothetical protein, conserved
Tb927.7.4460
AGOF: null
AGOC: null
AGOP: null
PGOF: null
PGOC: null
PGOP: null

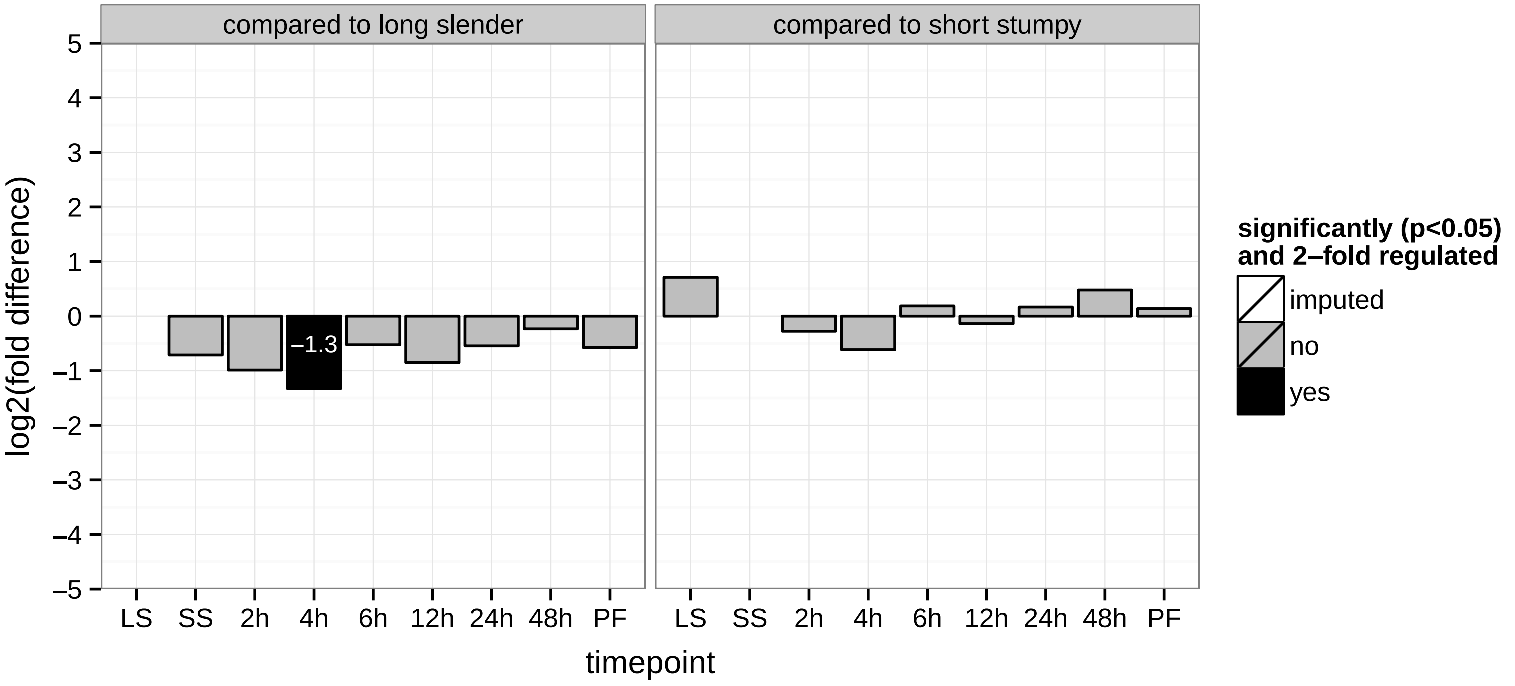

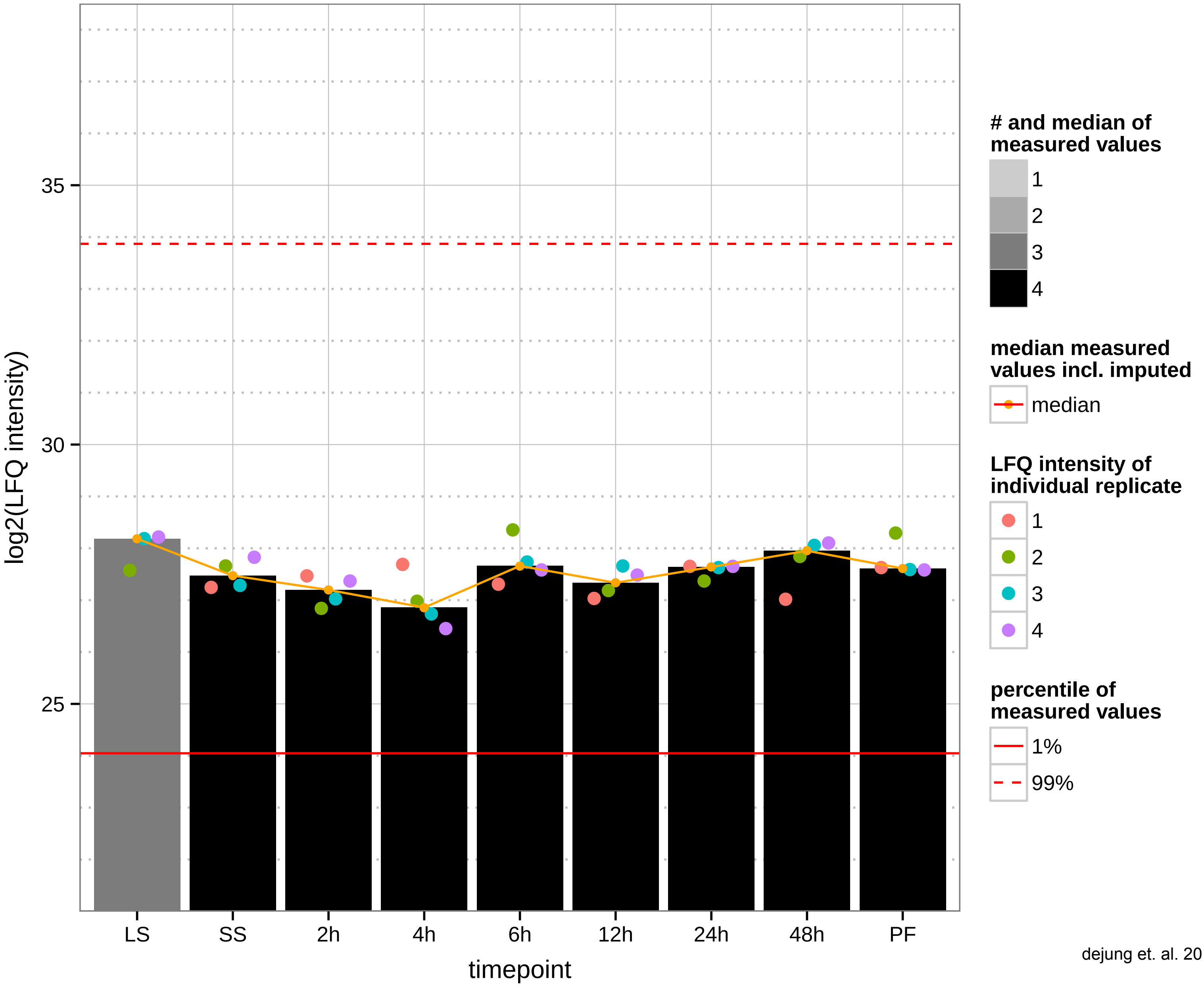

dejung et. al. 2015

hypothetical protein, conserved
Tb927.7.4620
AGOF: null
AGOC: null
AGOP: null
PGOF: null
PGOC: null
PGOP: null

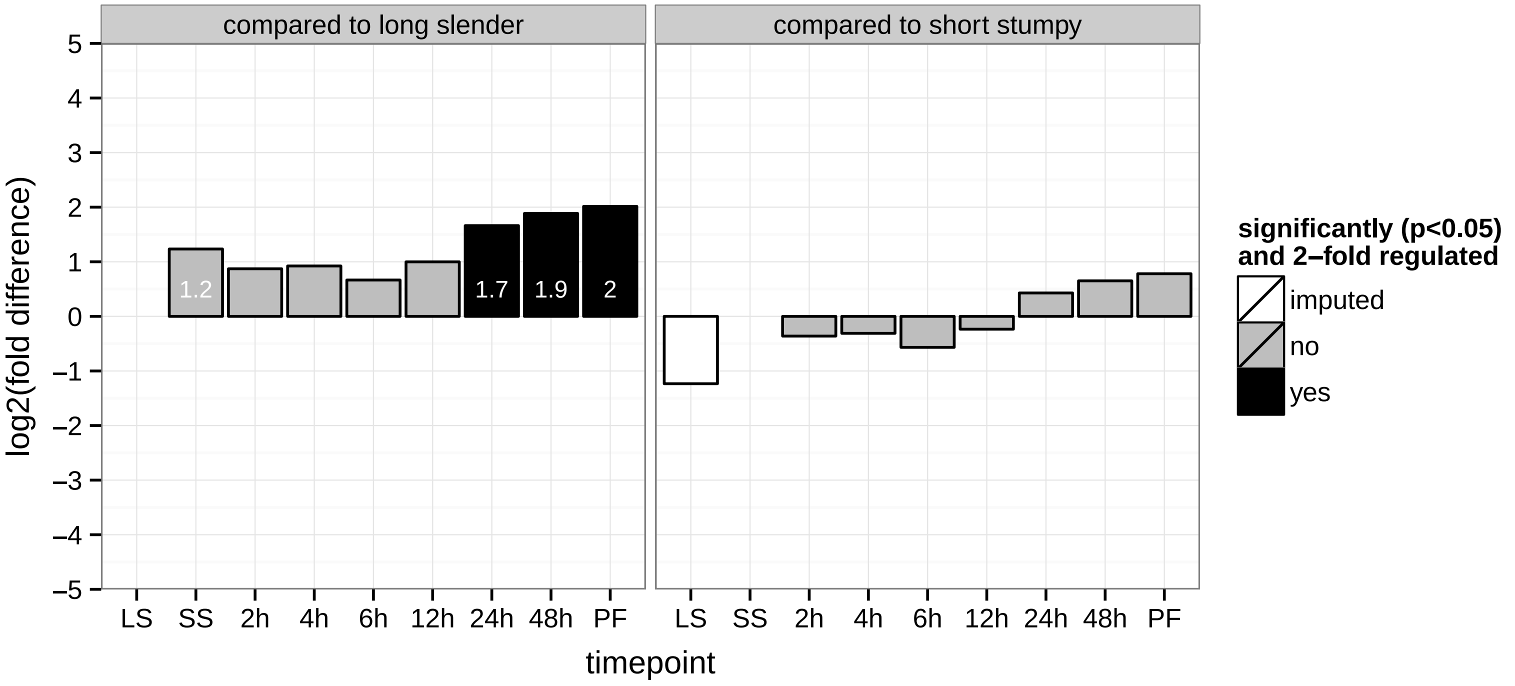

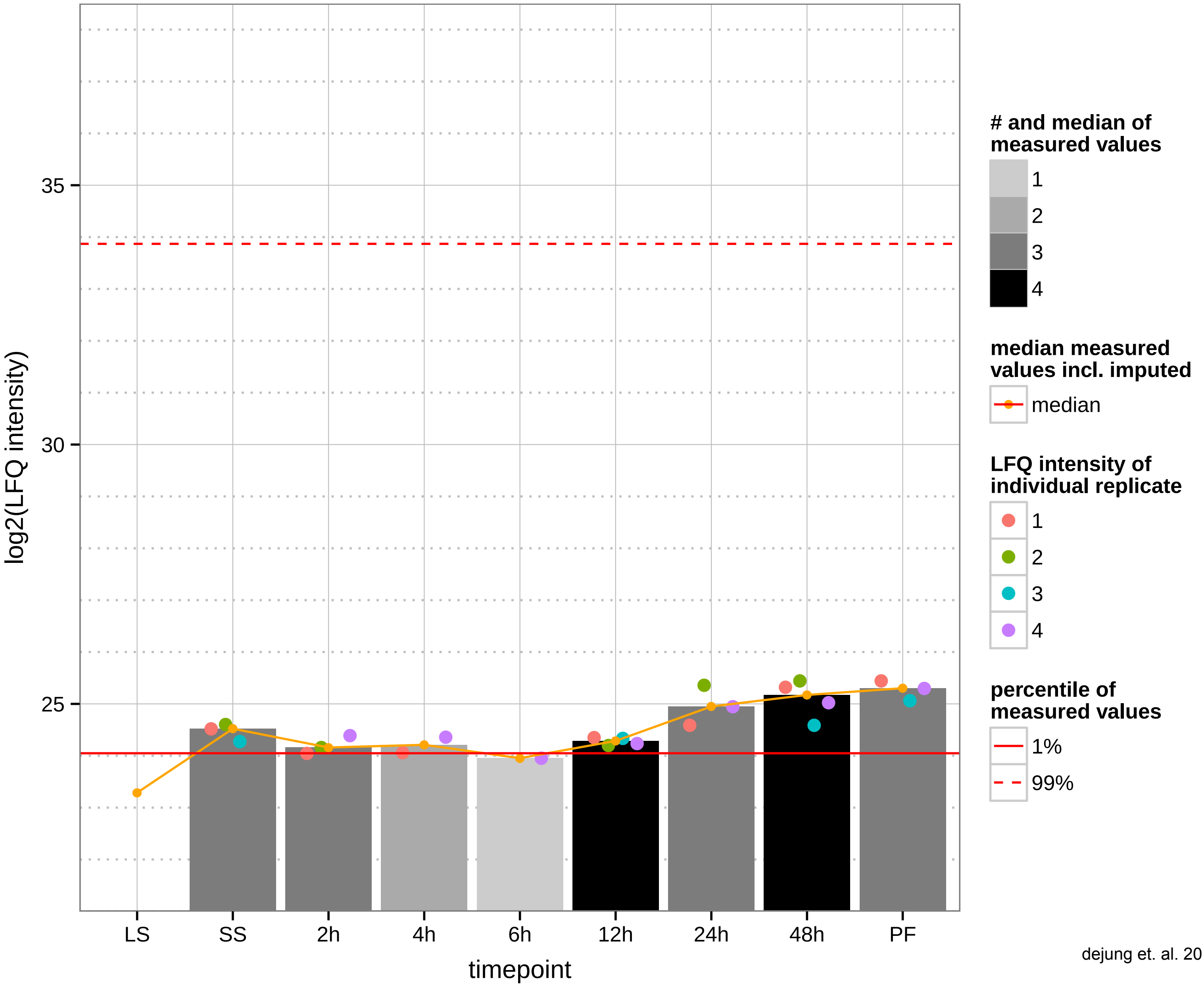

dejung et. al. 2015

hypothetical protein, conserved
Tb927.7.4760;Tb11.v5.0159
AGOF: null
AGOC: null
AGOP: null
PGOF: null
PGOC: null
PGOP: null

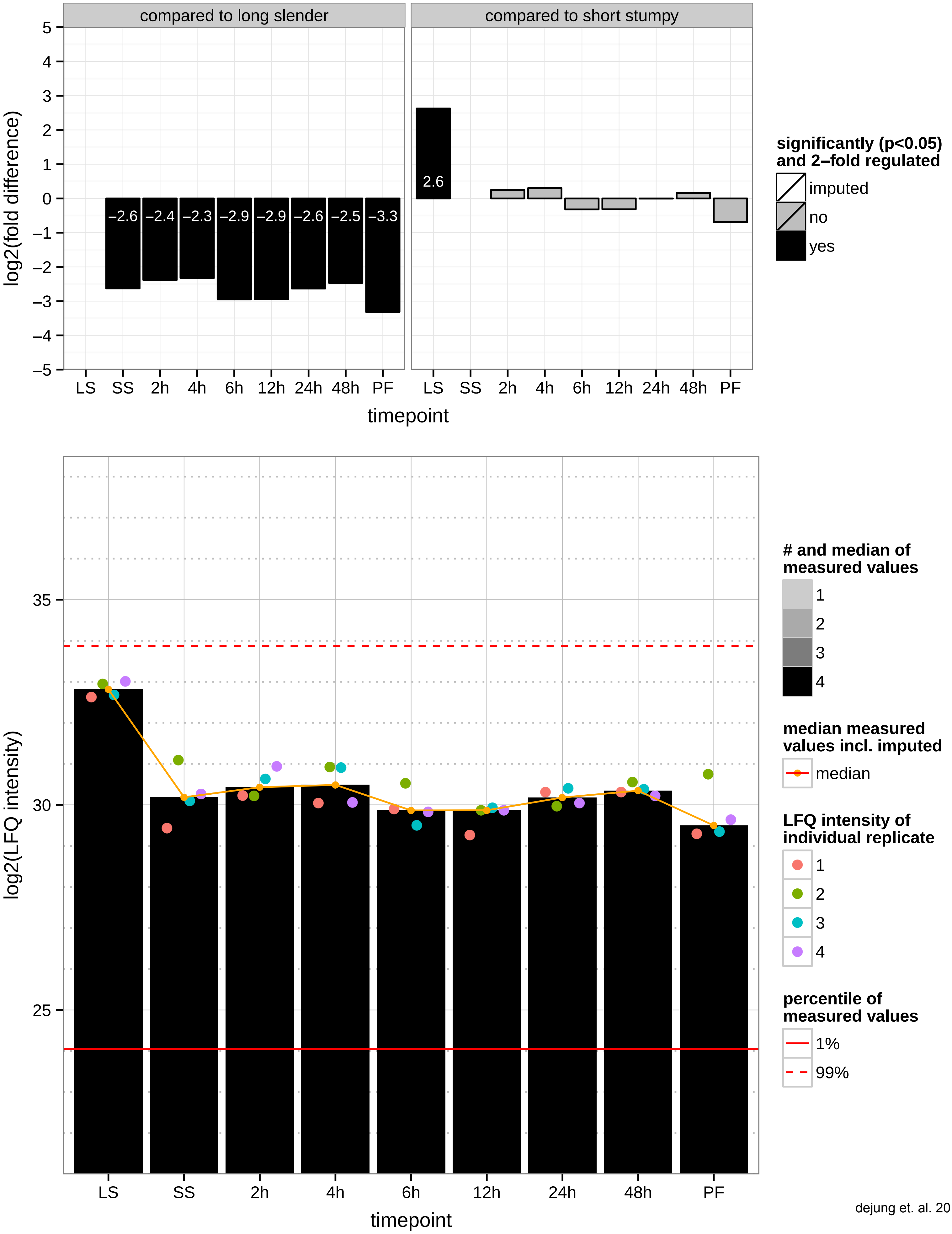

dejung et. al. 2015

hypothetical protein, conserved
Tb927.7.5110
AGOF: null
AGOC: mitochondrion
AGOP: null
PGOF: null
PGOC: null
PGOP: null

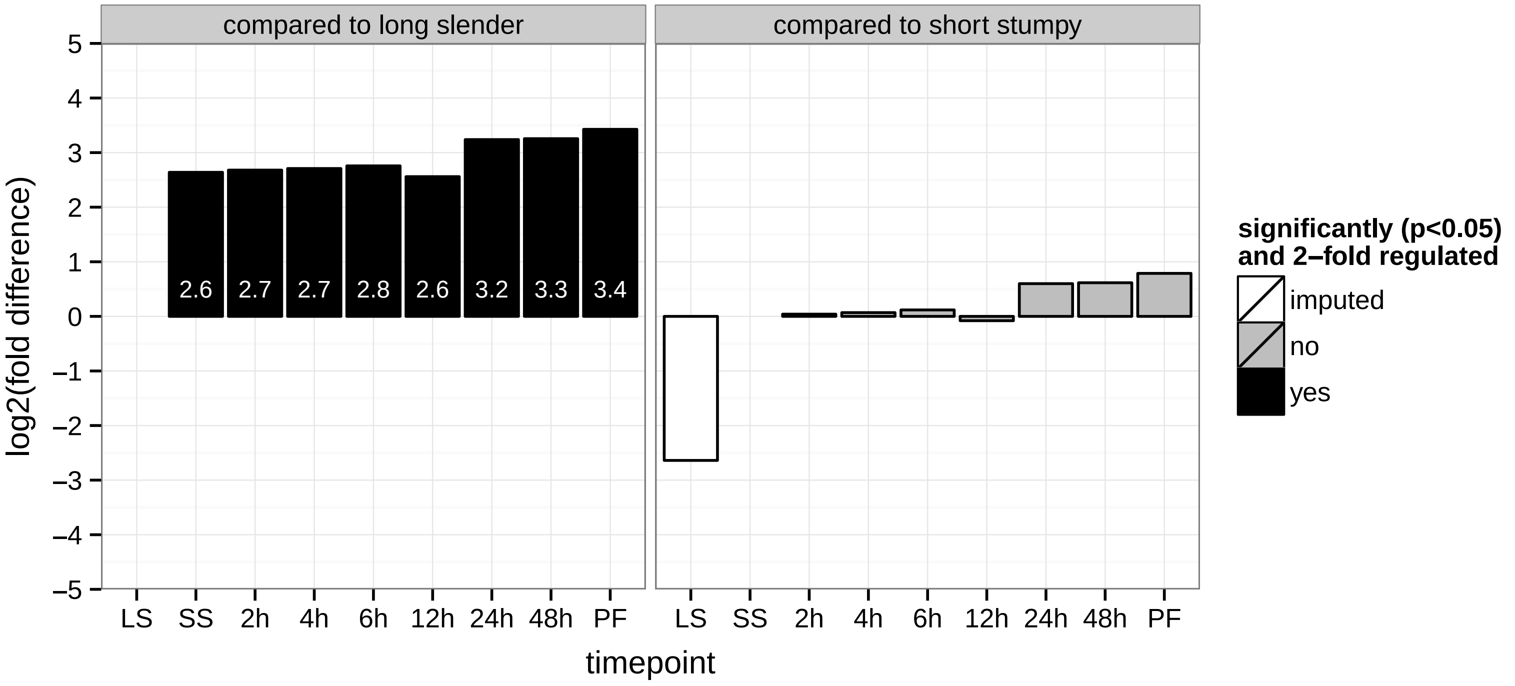

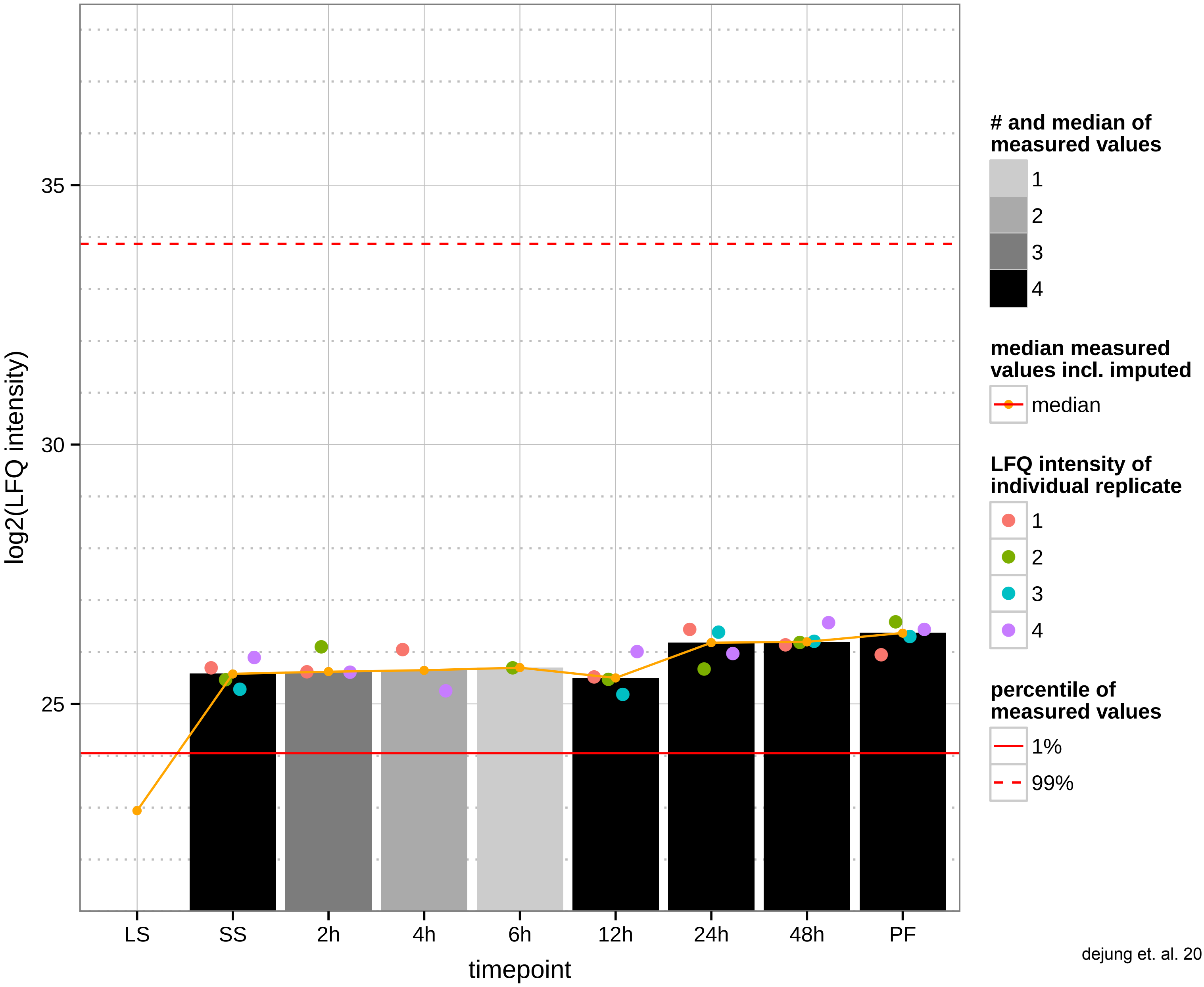

dejung et. al. 2015

hypothetical protein, conserved
Tb927.7.5290
AGOF: null
AGOC: null
AGOP: null
PGOF: null
PGOC: null
PGOP: null

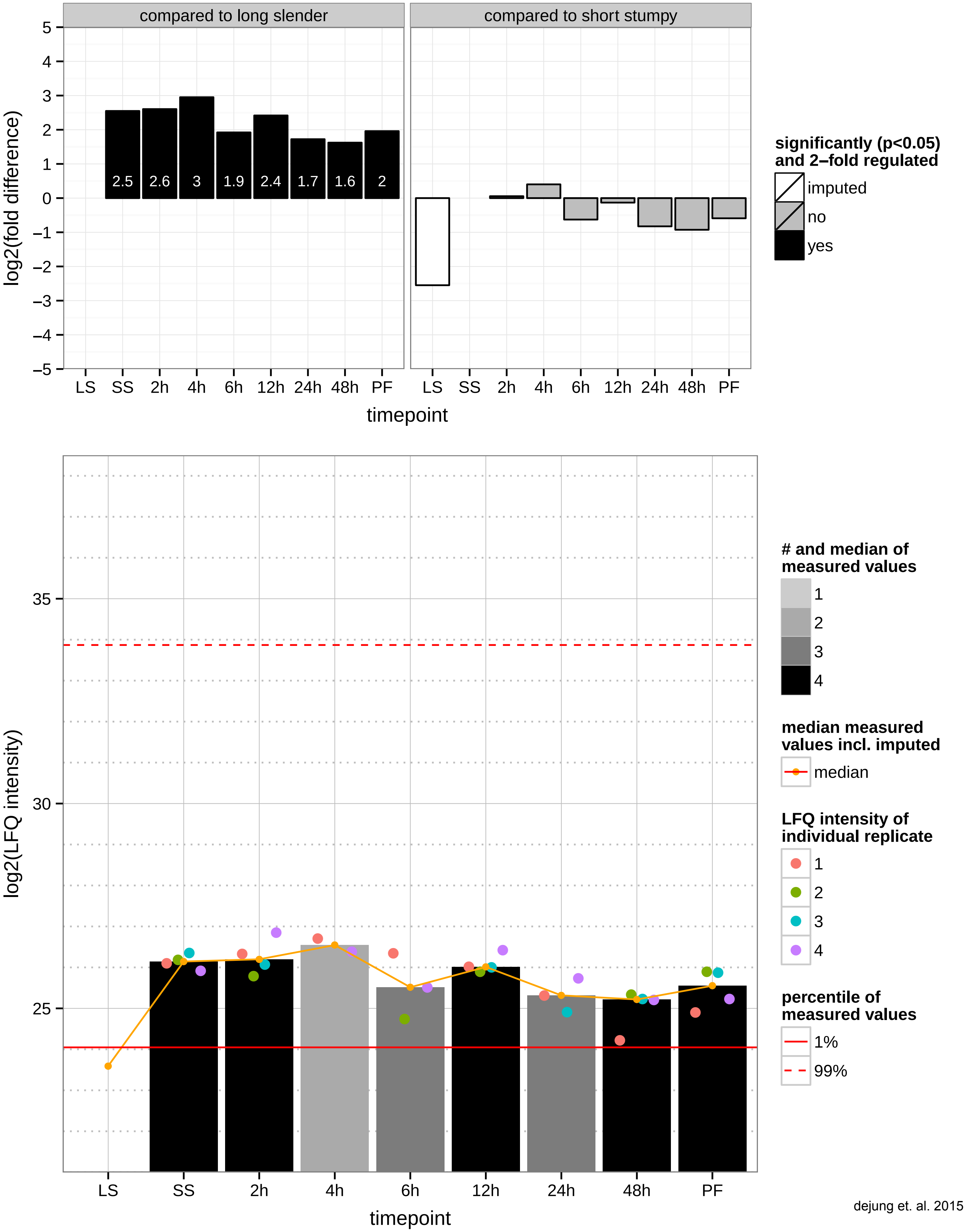

dejung et. al. 2015

hypothetical protein, conserved
Tb927.7.5340
AGOF: null
AGOC: null
AGOP: null
PGOF: null
PGOC: null
PGOP: null

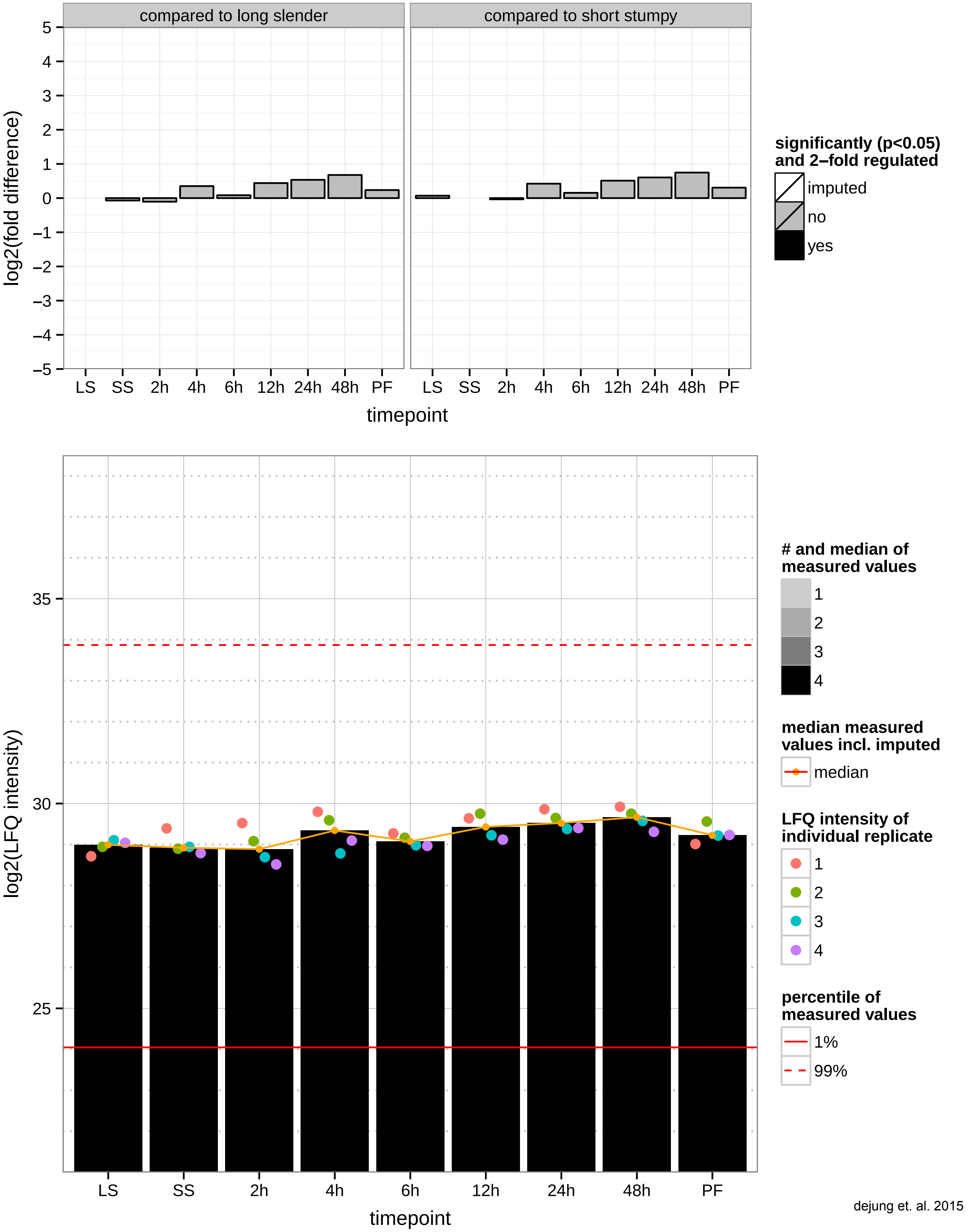

dejung et. al. 2015

hypothetical protein, conserved
Tb927.7.5440
AGOF: null
AGOC: mitochondrion
AGOP: null
PGOF: null
PGOC: null
PGOP: null

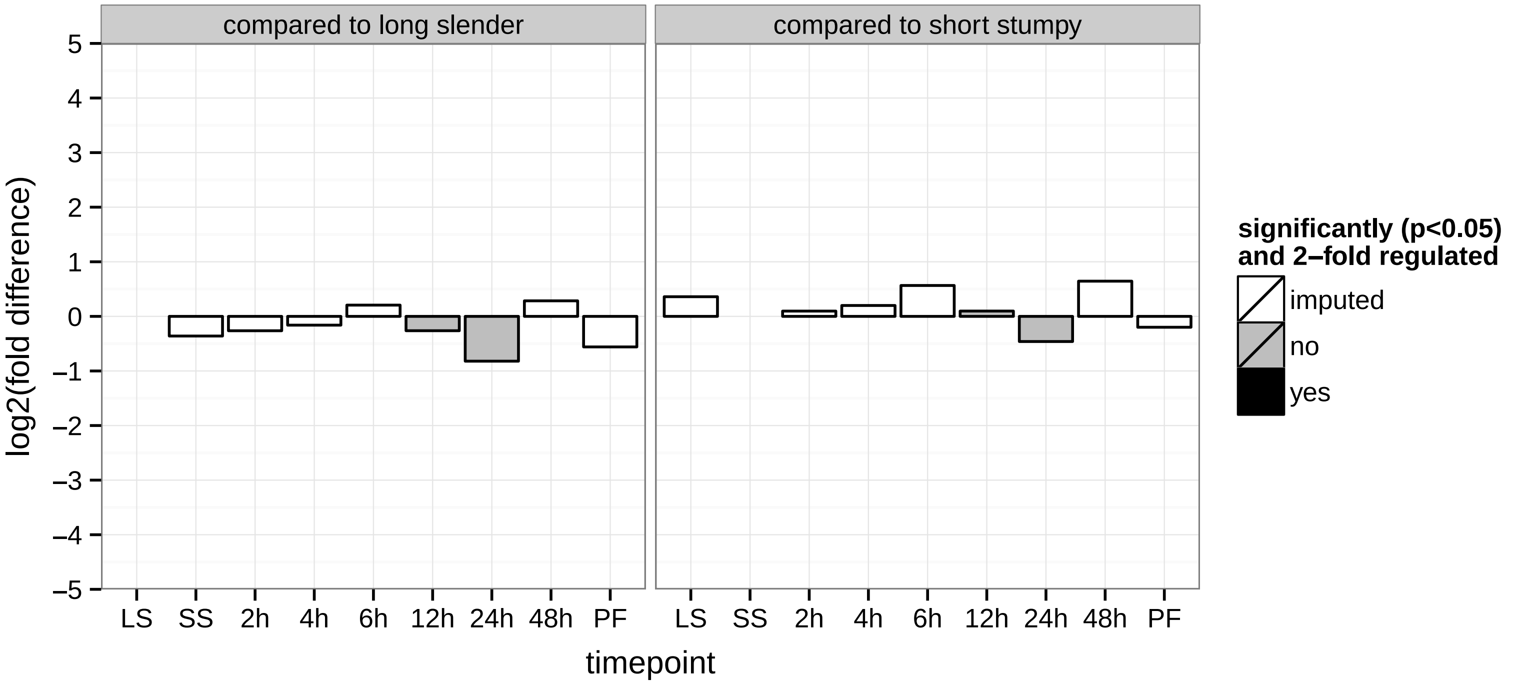

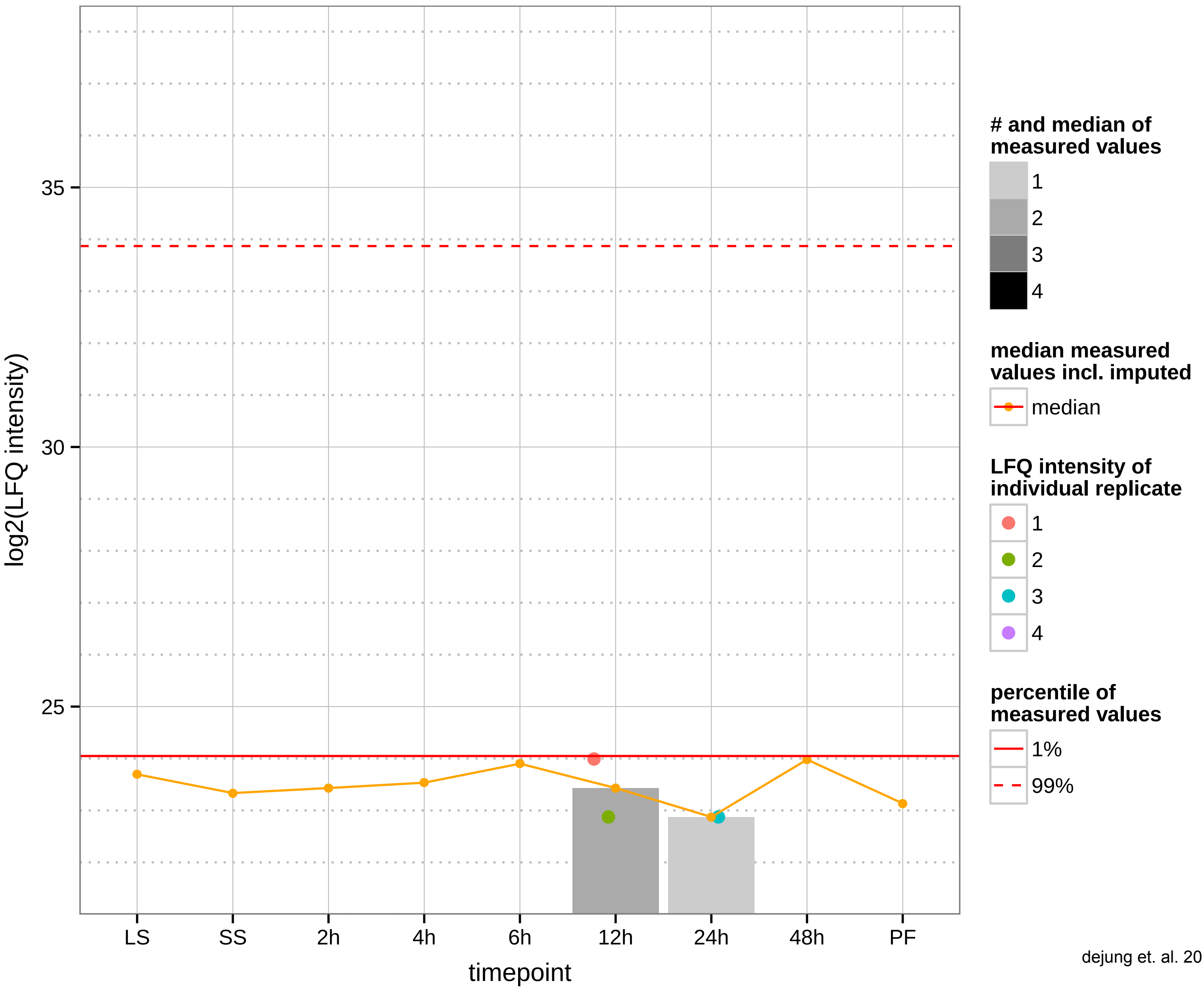

dejung et. al. 2015

arginine N−methyltransferase, type III (PRMT7)
Tb927.7.5490
AGOF: arginine N−methyltransferase activity
AGOC: cytoplasm
AGOP: histone arginine methylation, peptidyl−arginine methylation
PGOF: protein methyltransferase activity
PGOC: cytoplasm
PGOP: protein methylation

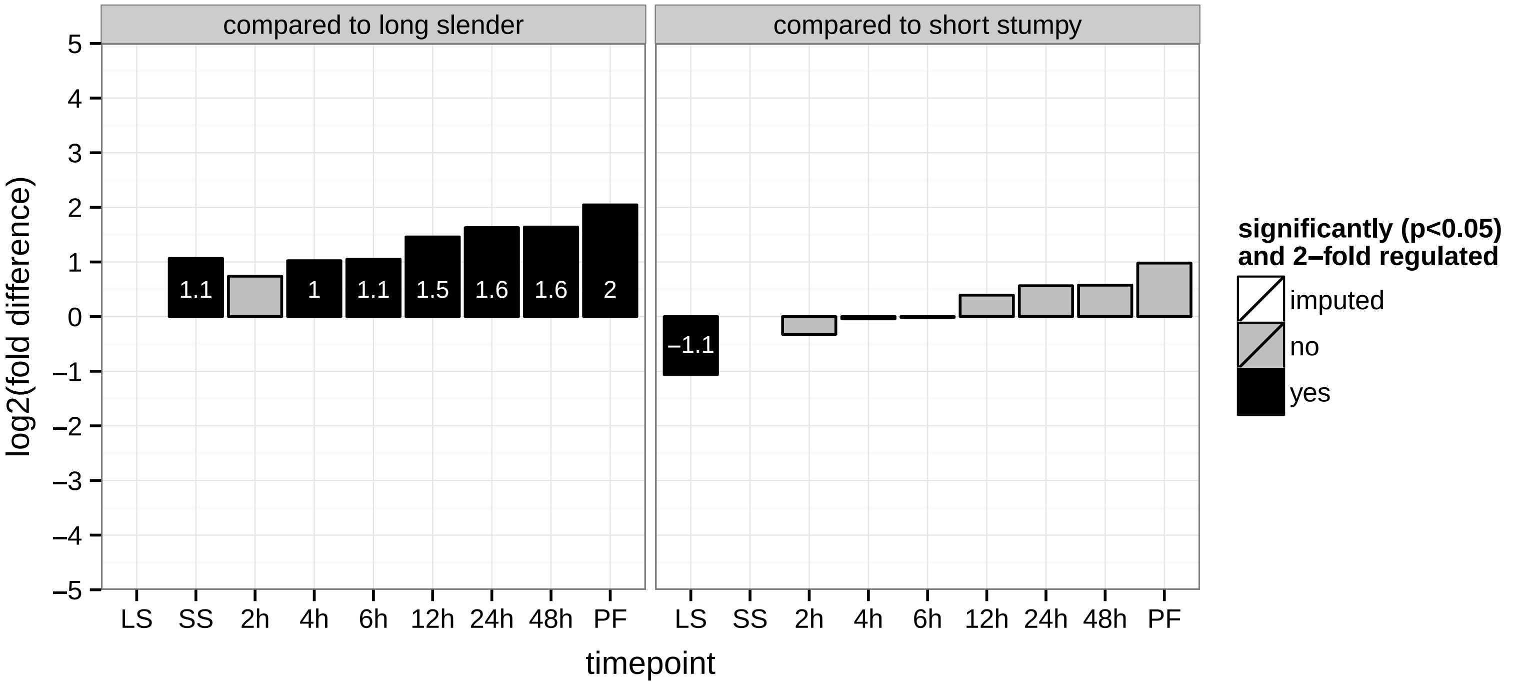

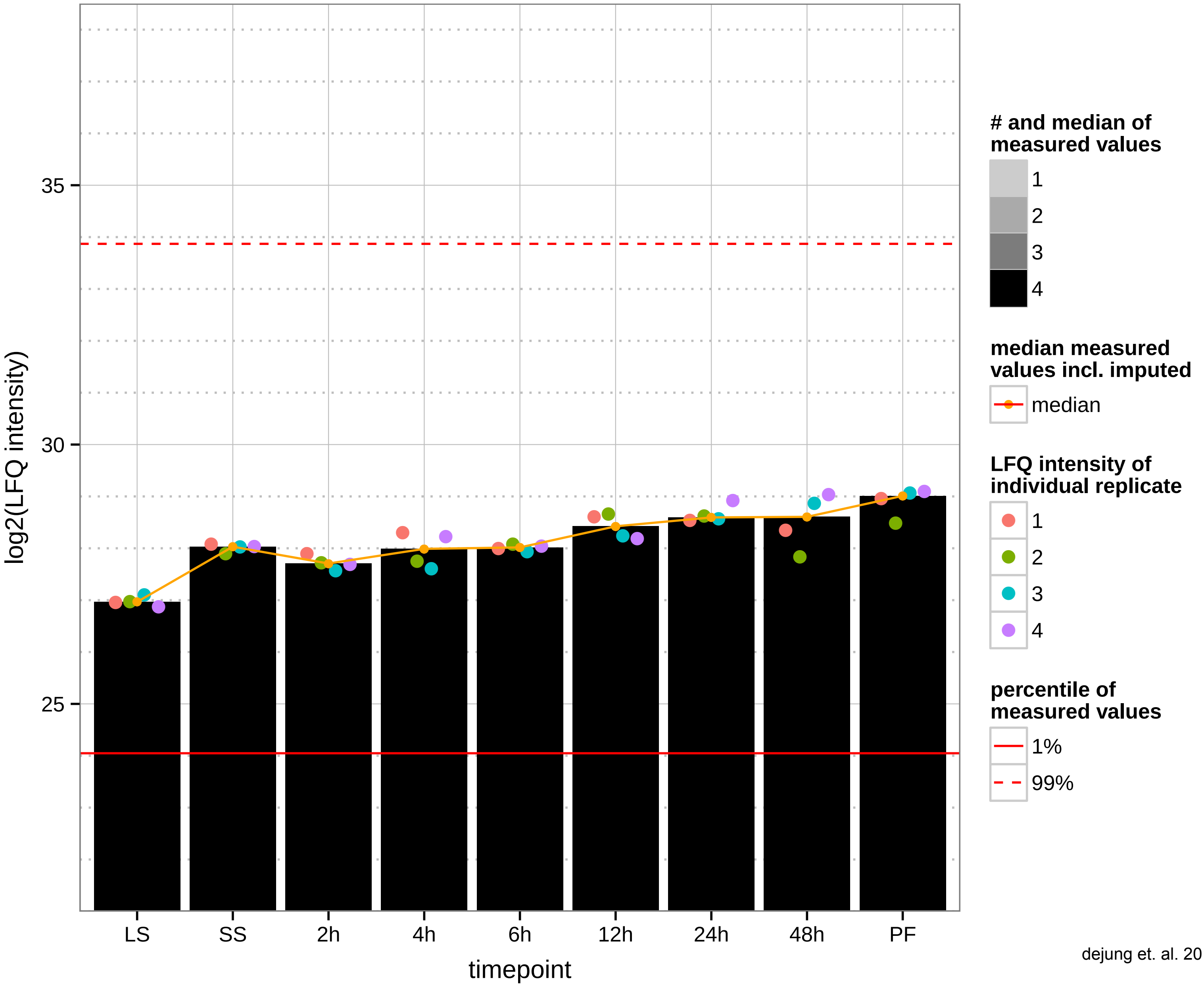

dejung et. al. 2015

hypothetical protein, conserved
Tb927.7.5530
AGOF: null
AGOC: null
AGOP: null
PGOF: null
PGOC: null
PGOP: null

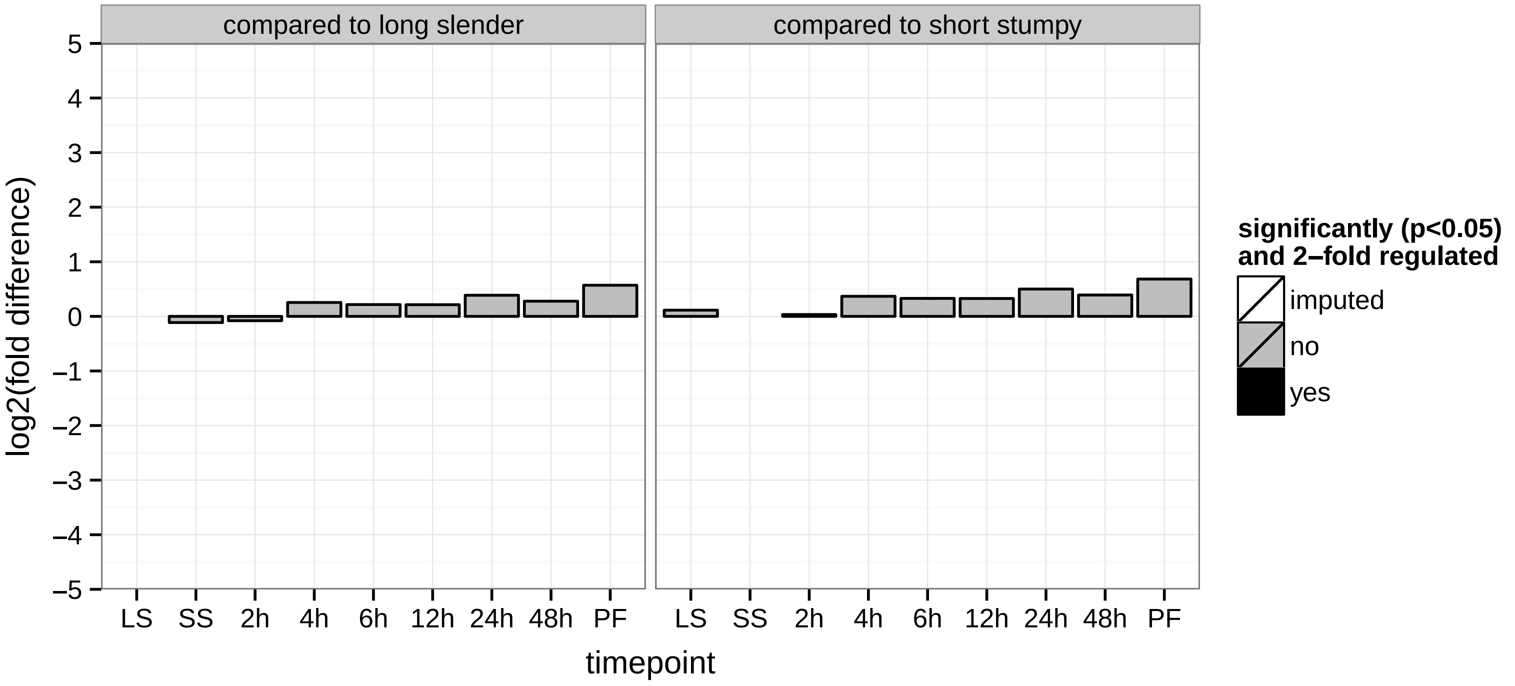

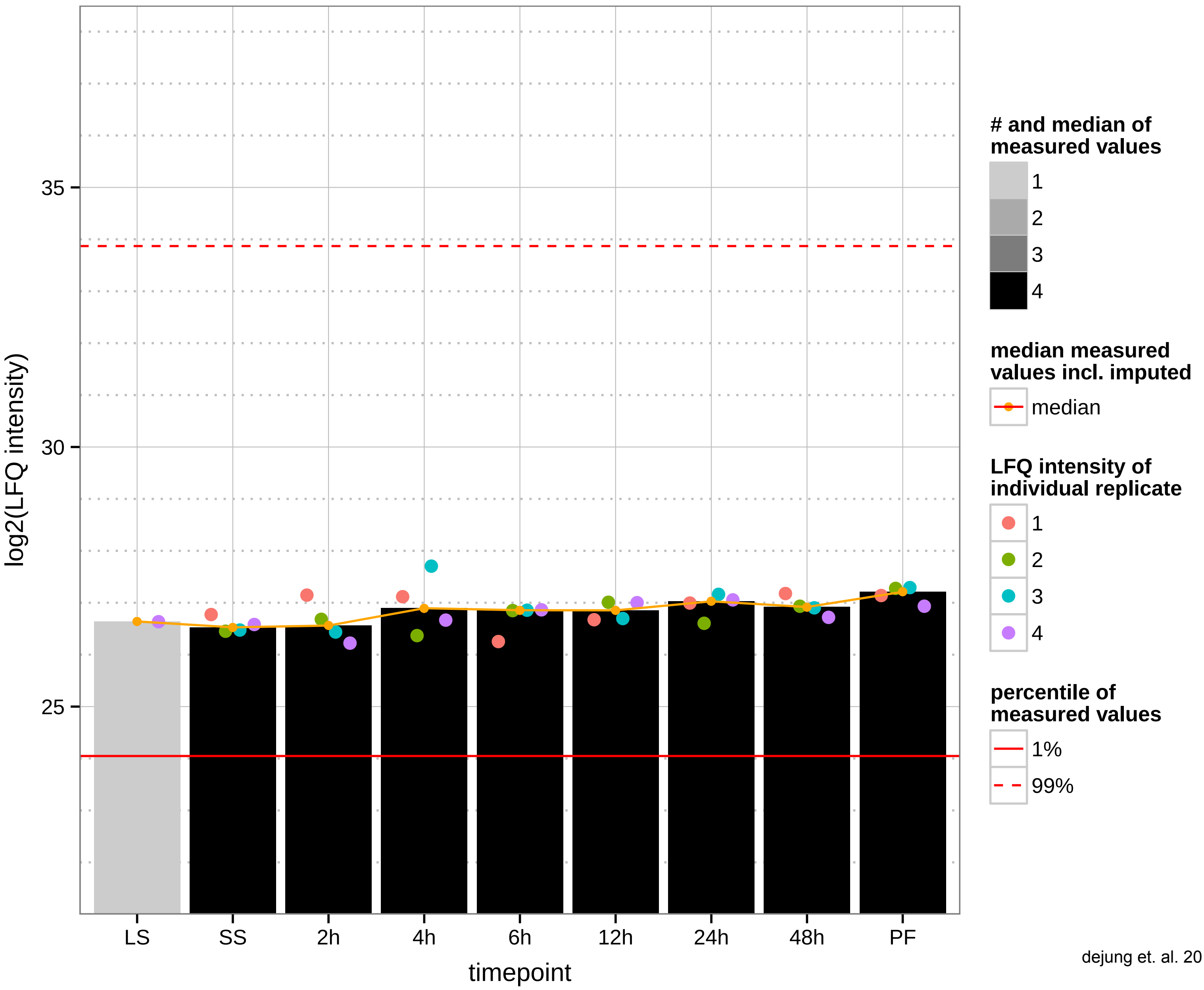

dejung et. al. 2015

glucosamine–fructose–6–phosphate aminotransferase, putative
Tb927.7.5560
AGOF: carbohydrate binding, glutamine–fructose–6–phosphate transaminase (isomerizing) activity
AGOC: cytoplasm
AGOP: carbohydrate biosynthetic process
PGOF: carbohydrate binding, glutamine–fructose–6–phosphate transaminase (isomerizing) activity
PGOC: cytoplasm
PGOP: carbohydrate biosynthetic process, carbohydrate metabolic process, metabolic process

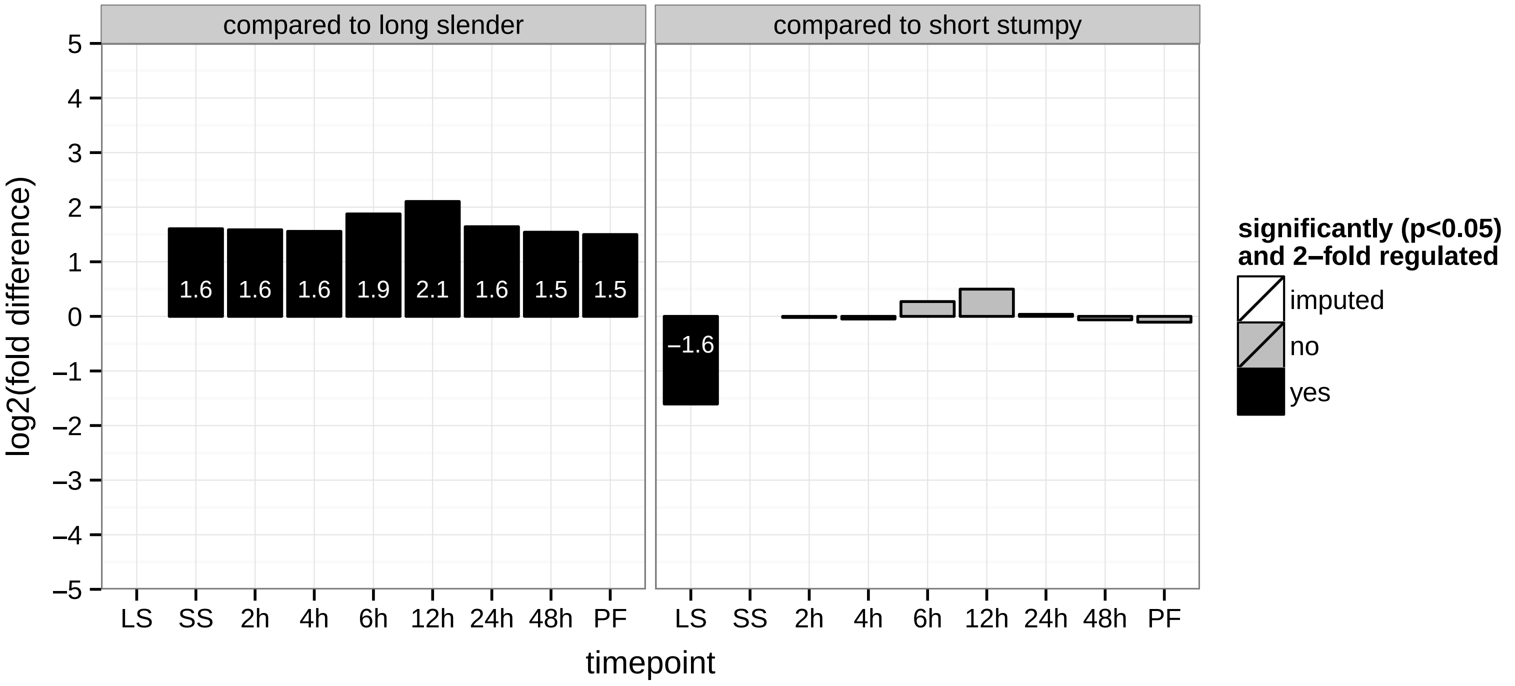

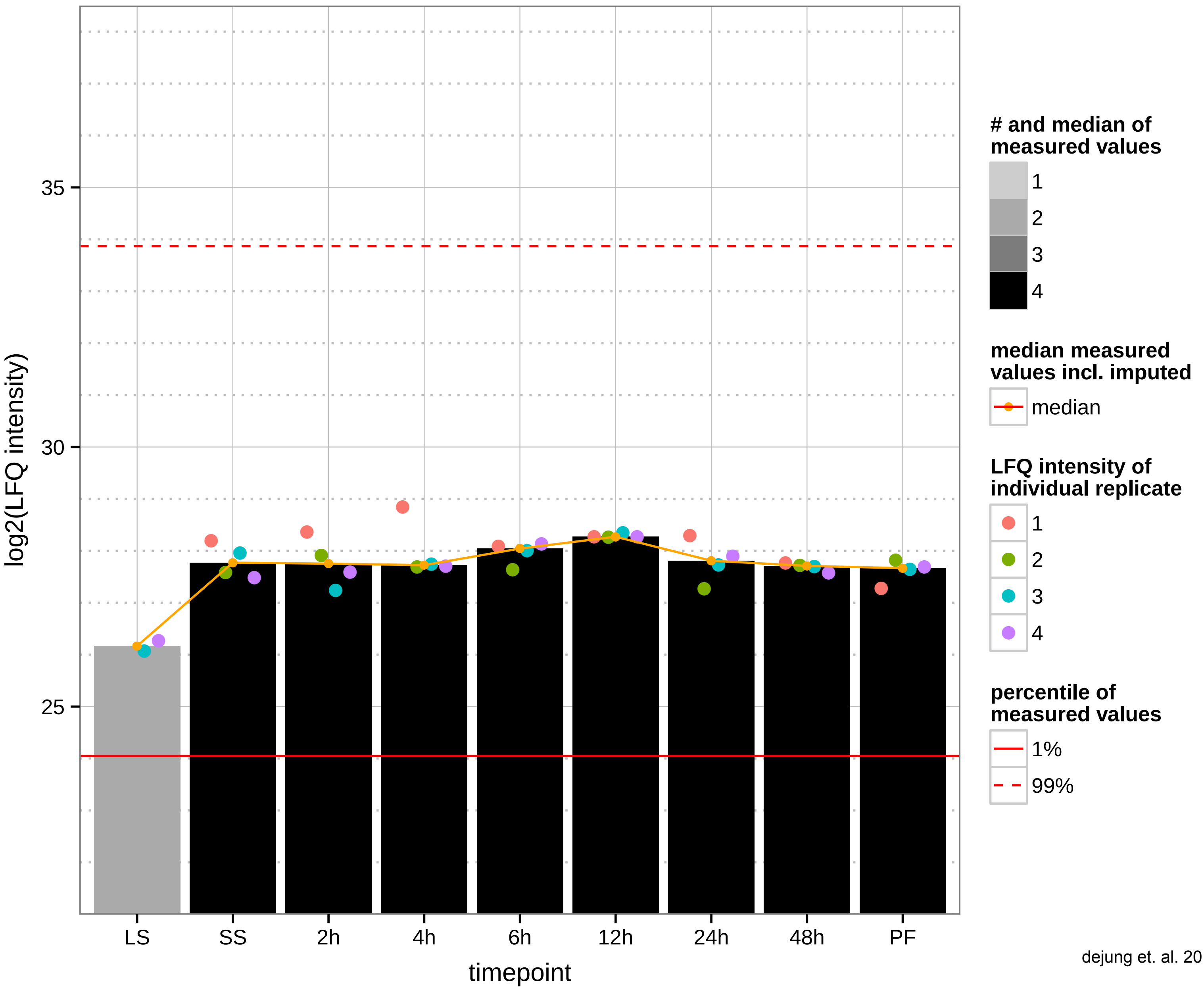

dejung et. al. 2015

cell cycle associated protein MOB1−A (MOB1A), cell cycle associated protein MOB1−B (MOB1B)
Tb927.7.5570;Tb927.7.5580
AGOF: null
AGOC: cytosol, cytoplasm
AGOP: cell division
PGOF: null
PGOC: null
PGOP: null

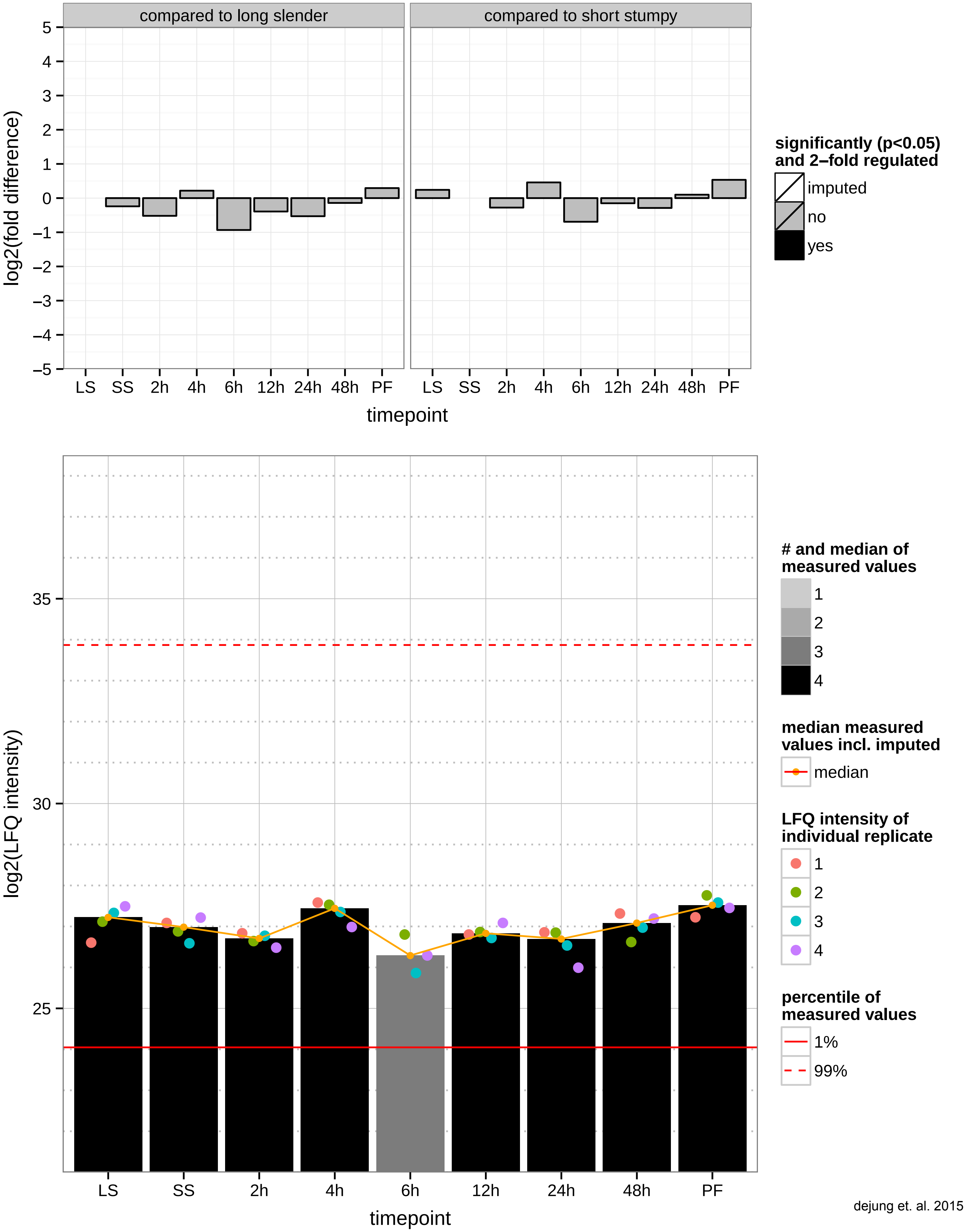

dejung et. al. 2015

hypothetical protein, conserved
Tb927.7.5660
AGOF: null
AGOC: null
AGOP: null
PGOF: null
PGOC: null
PGOP: null

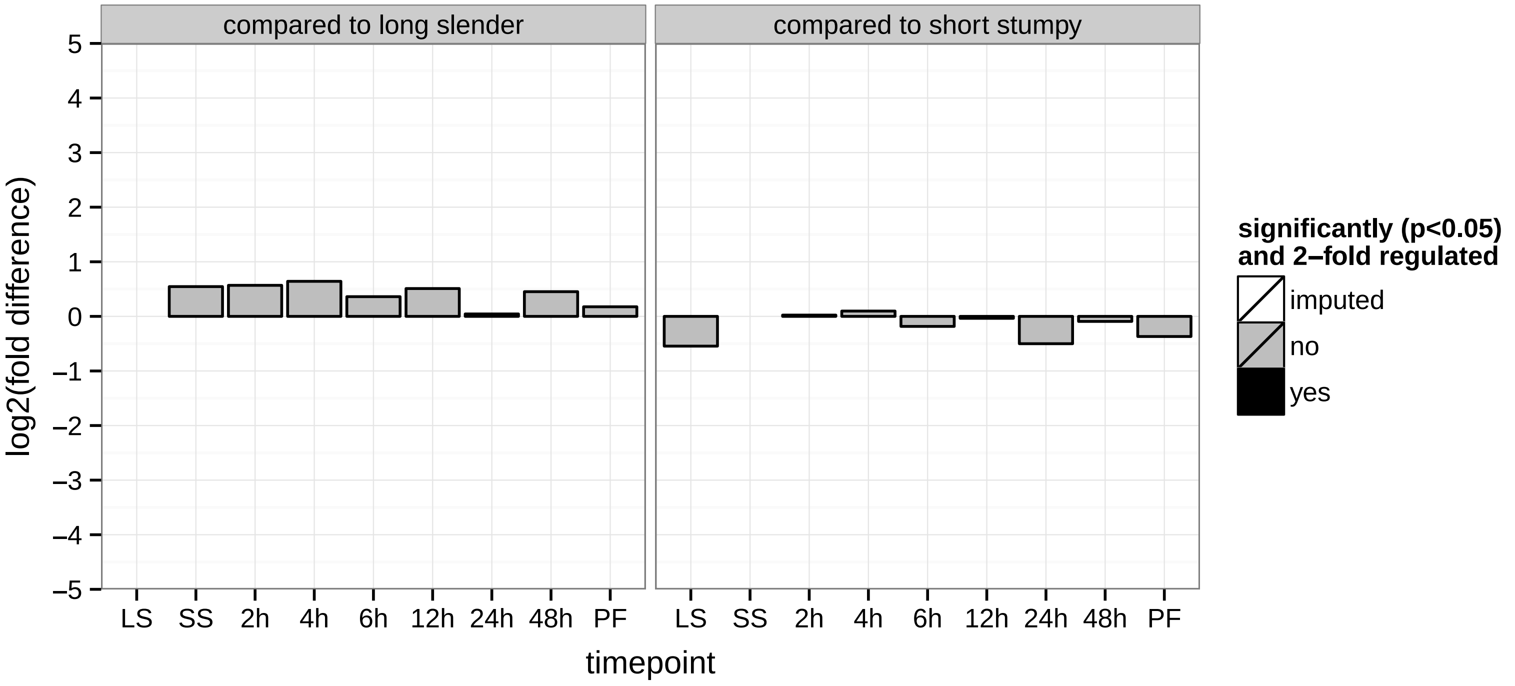

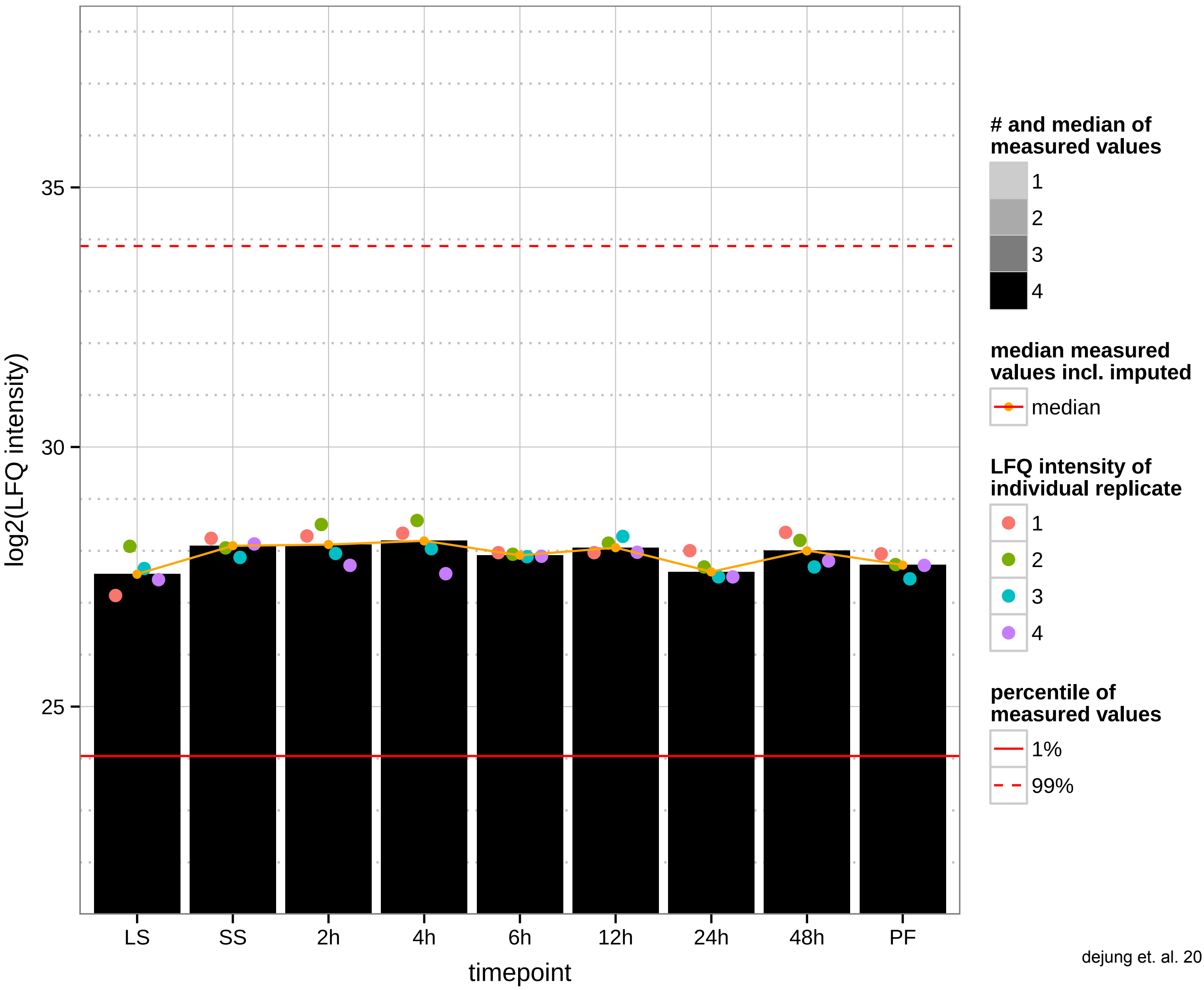

dejung et. al. 2015

hypothetical protein, conserved
Tb927.7.5700
AGOF: null
AGOC: null
AGOP: null
PGOF: null
PGOC: null
PGOP: null

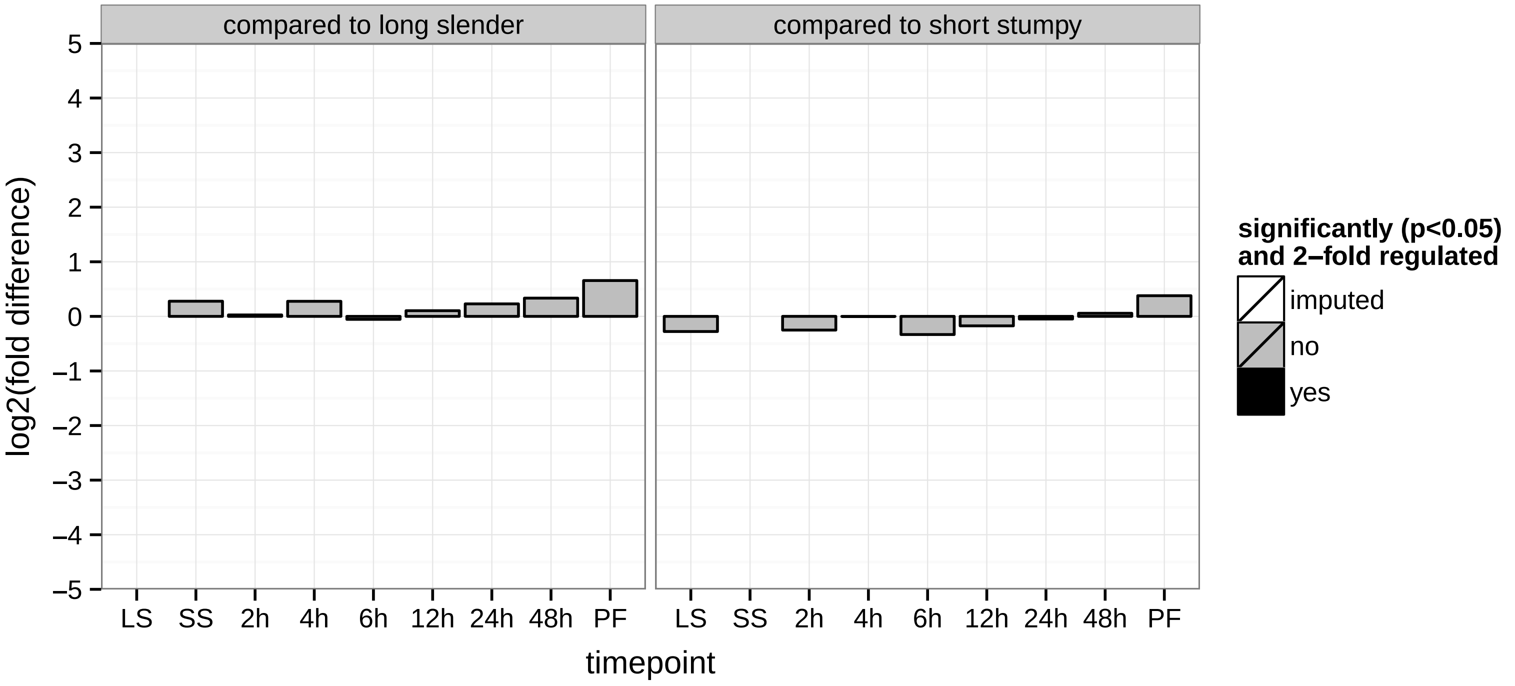

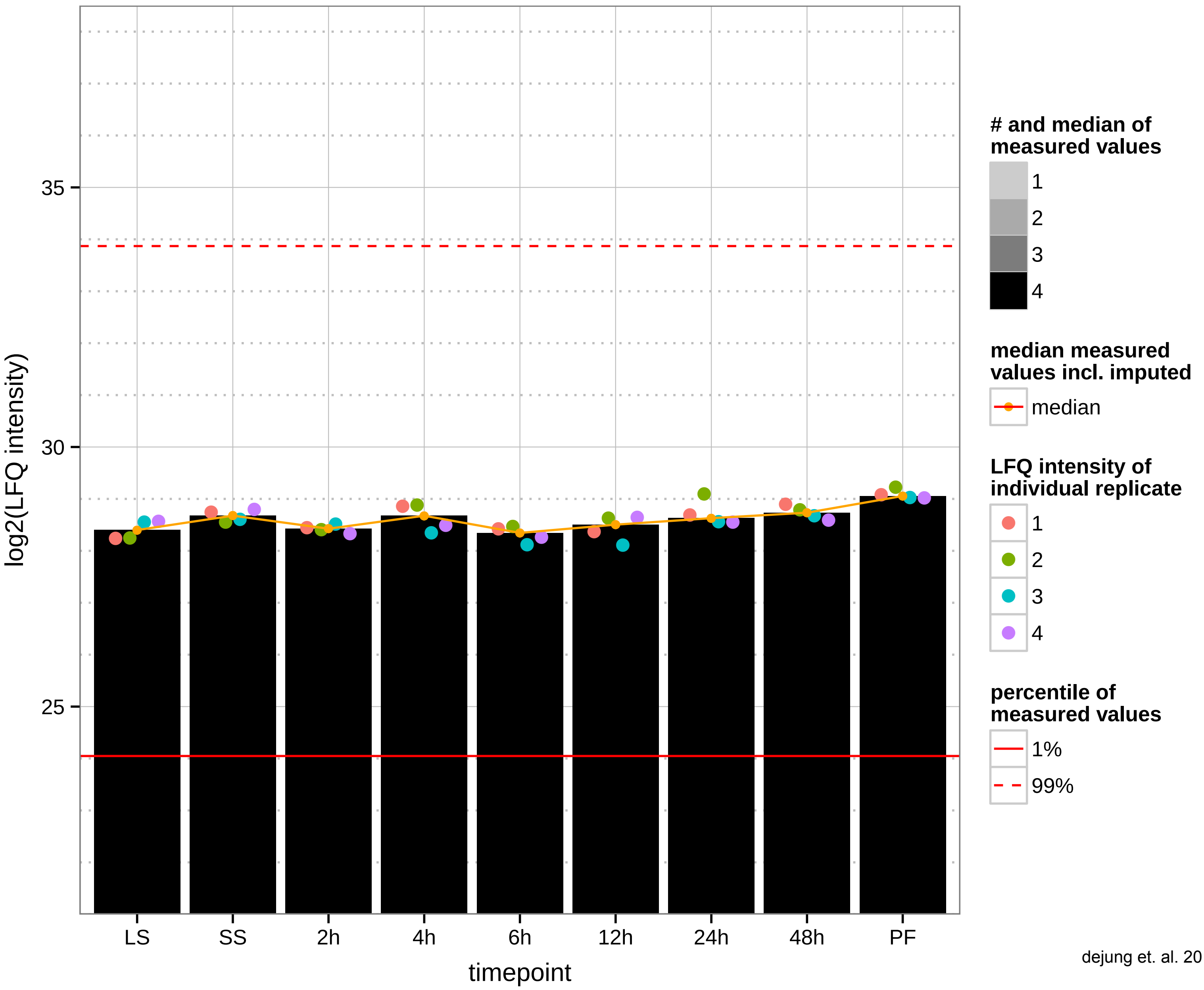

dejung et. al. 2015

hypothetical protein, conserved
Tb927.7.5710
AGOF: null
AGOC: null
AGOP: null
PGOF: null
PGOC: null
PGOP: null

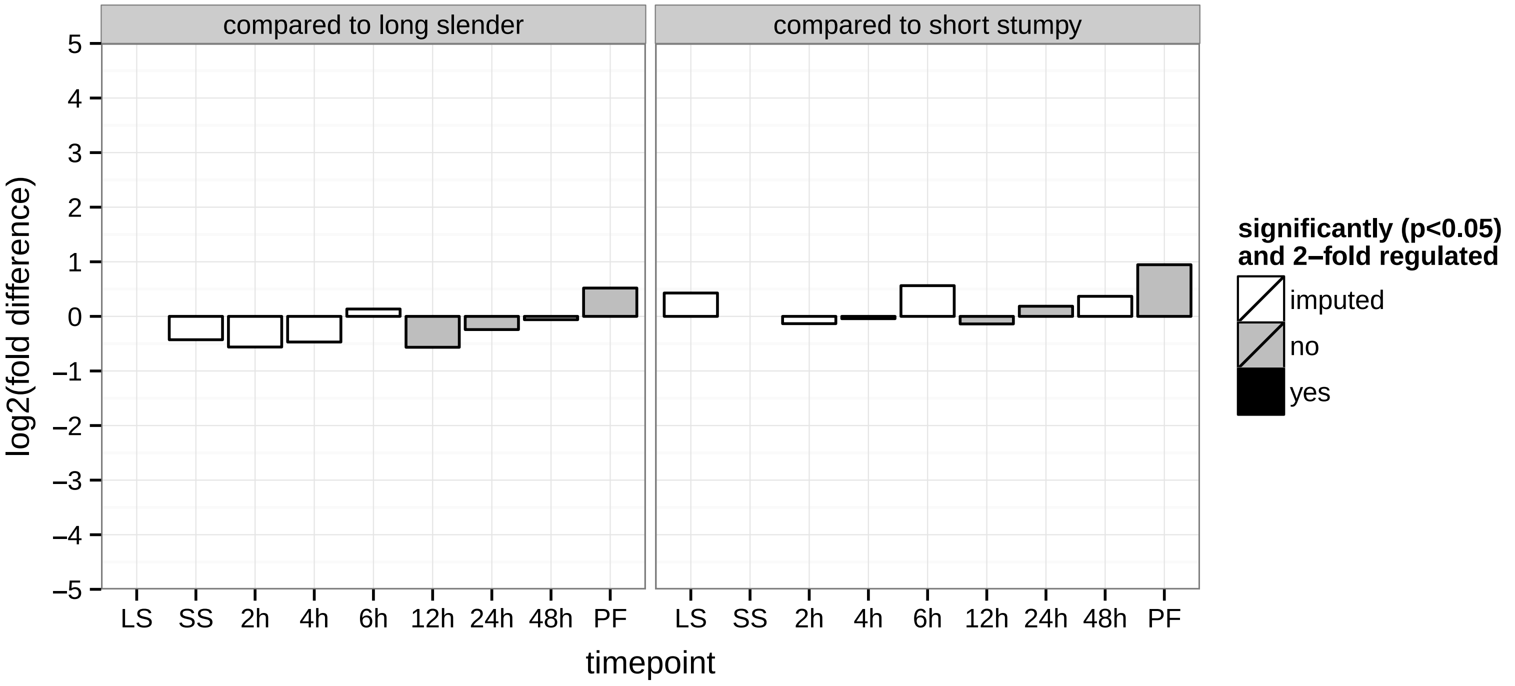

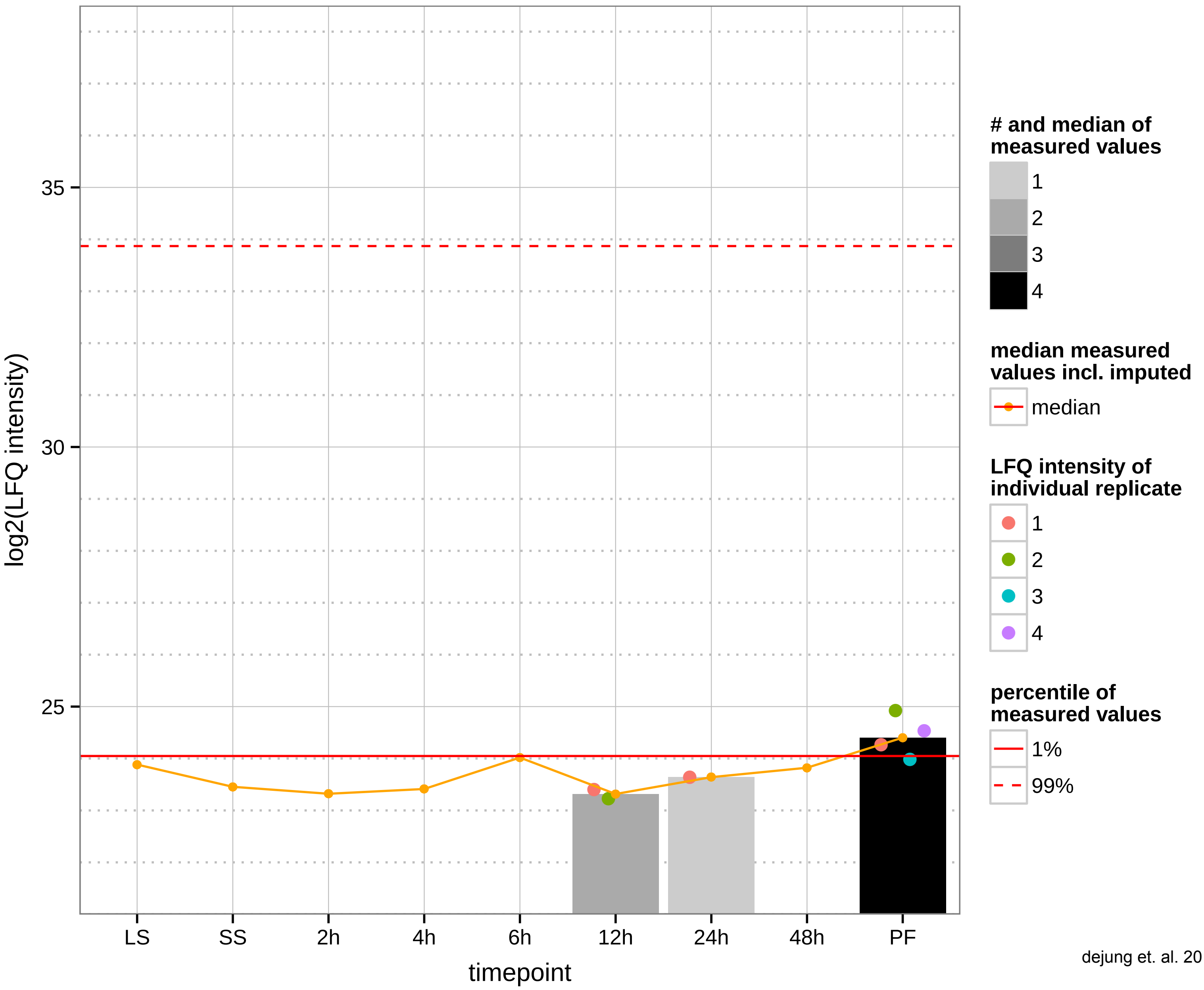

dejung et. al. 2015

nuclear transport factor 2 protein, putative (NFT2)
Tb927.7.5760
AGOF: null
AGOC: intracellular
AGOP: transport
PGOF: null
PGOC: intracellular
PGOP: transport

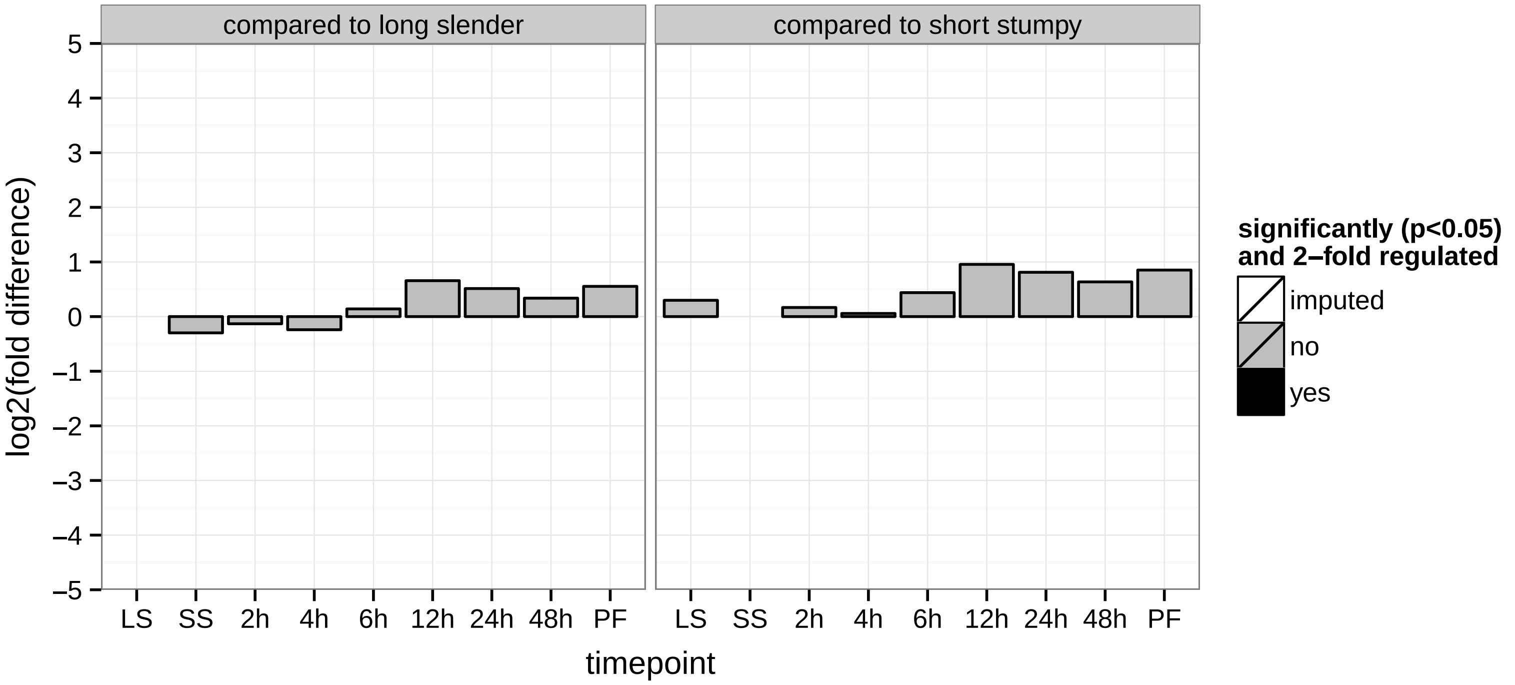

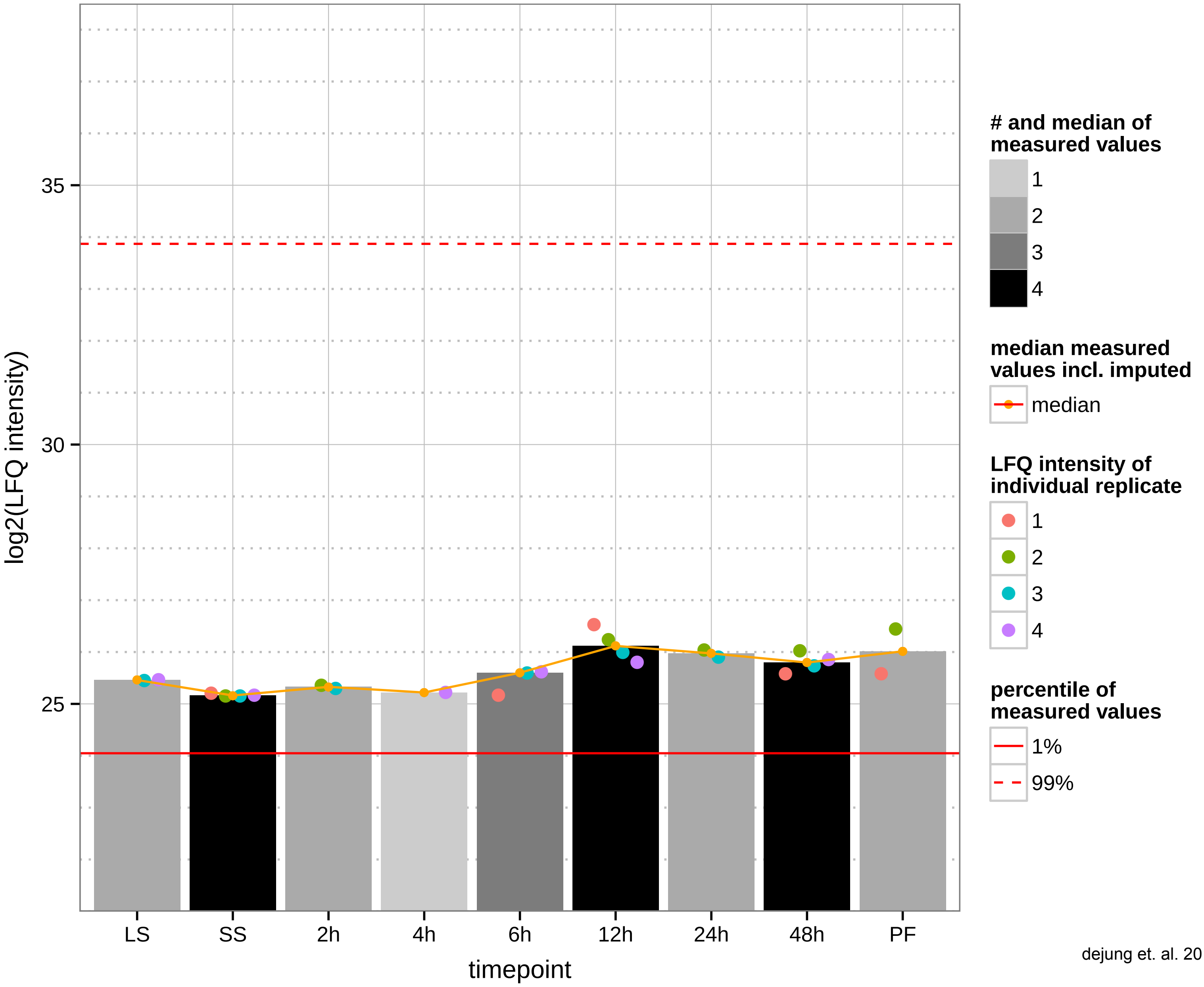

dejung et. al. 2015

hypothetical protein, conserved
Tb927.7.5780
AGOF: null
AGOC: null
AGOP: cell redox homeostasis
PGOF: null
PGOC: null
PGOP: cell redox homeostasis

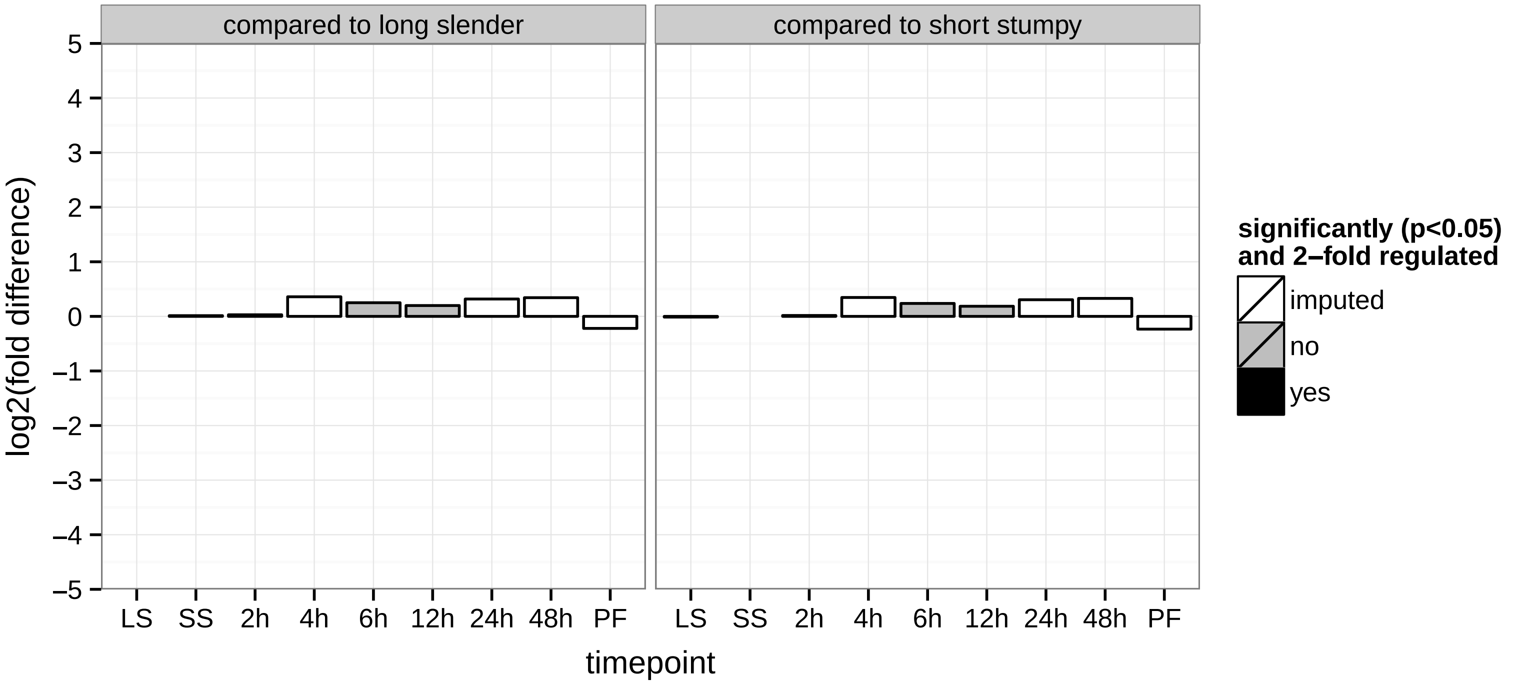

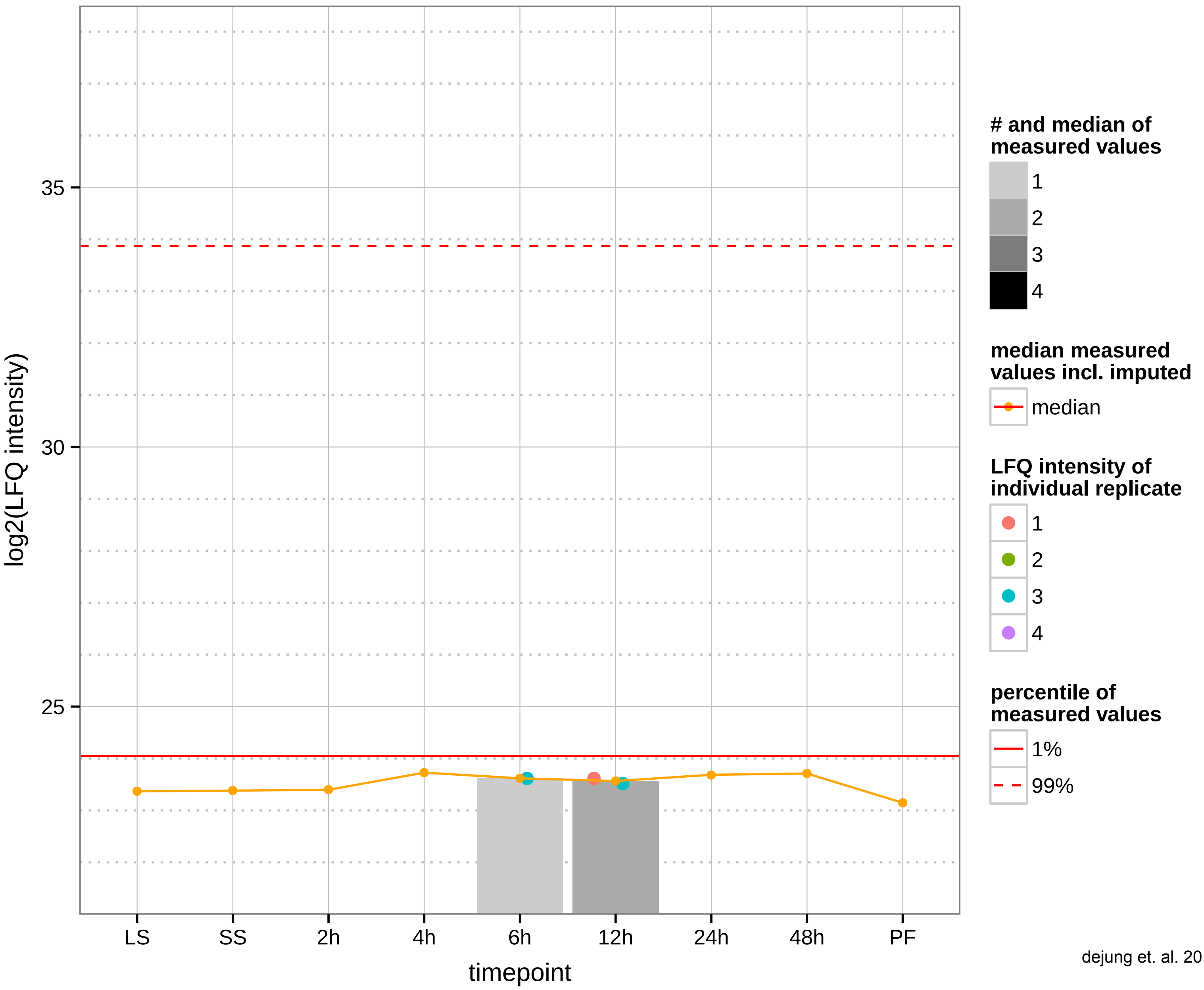

dejung et. al. 2015

GTP–binding protein, putative
Tb927.7.580
AGOF: GTP binding
AGOC: intracellular
AGOP: null
PGOF: GTP binding
PGOC: null
PGOP: null

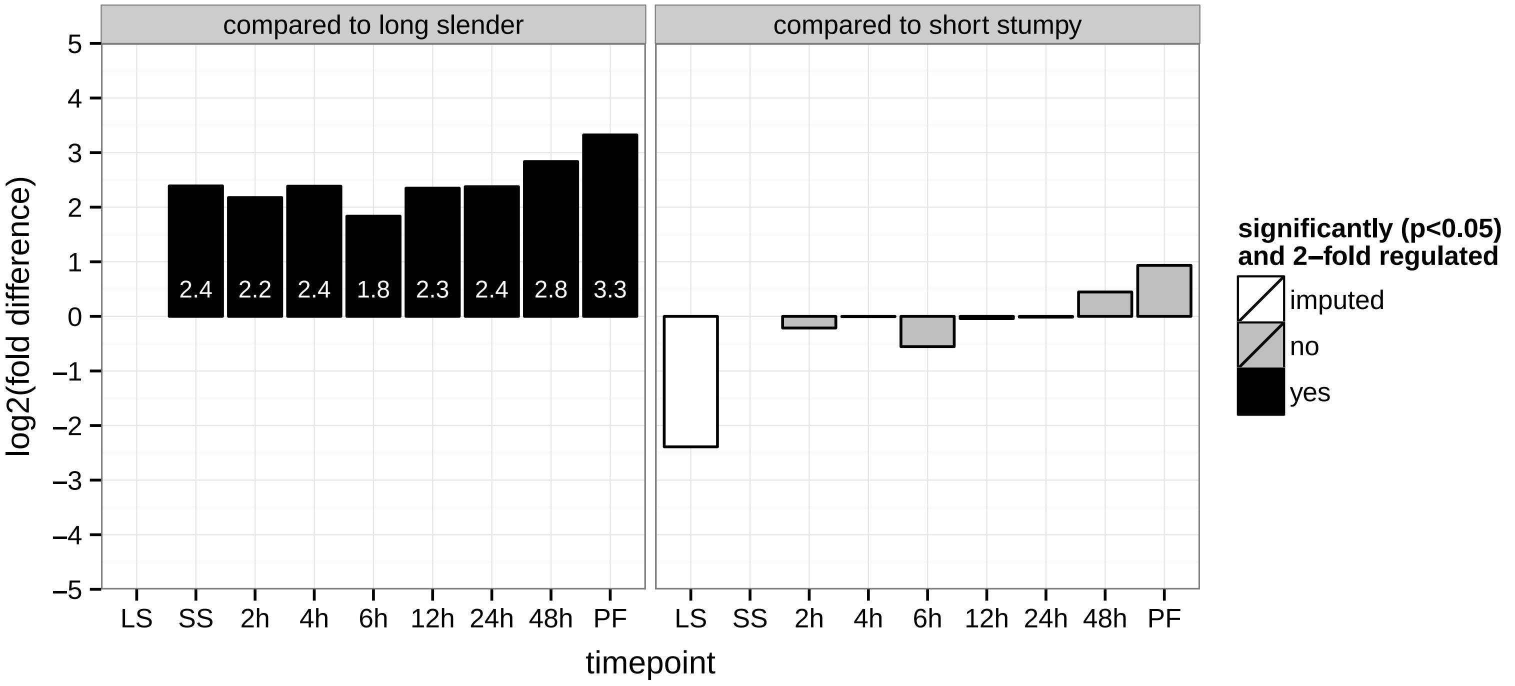

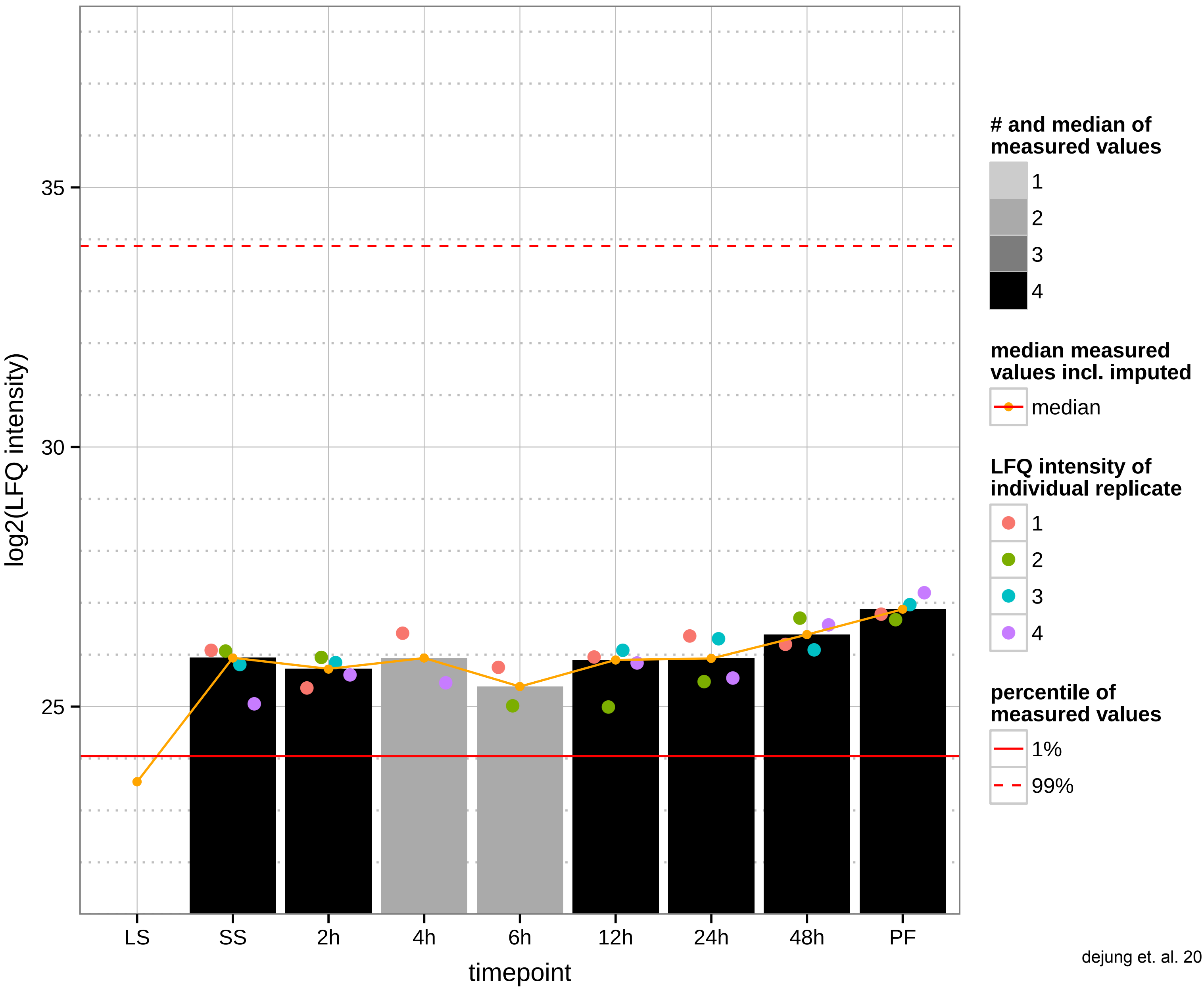

dejung et. al. 2015

Monooxygenase, putative
Tb927.7.5820
AGOF: monooxygenase activity
AGOC: mitochondrial membrane, mitochondrion
AGOP: metabolic process
PGOF: null
PGOC: null
PGOP: null

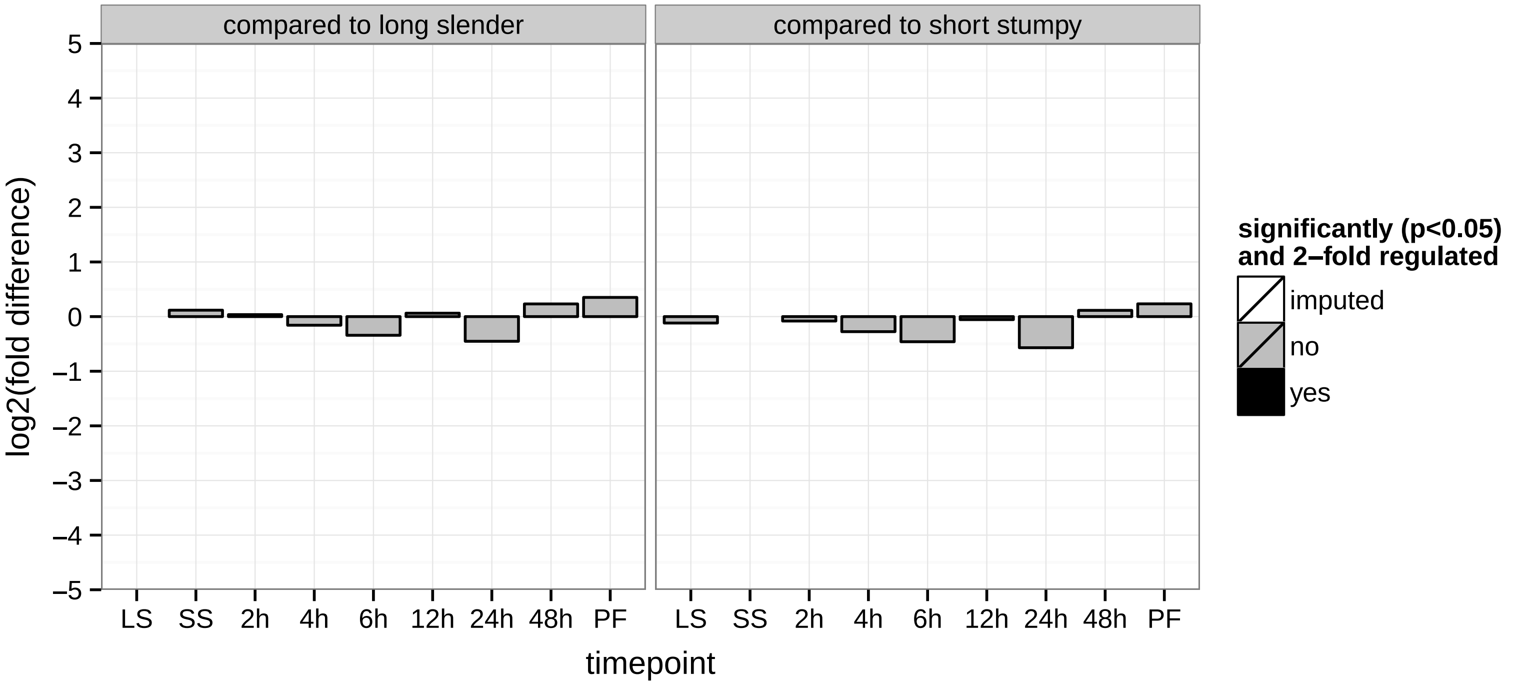

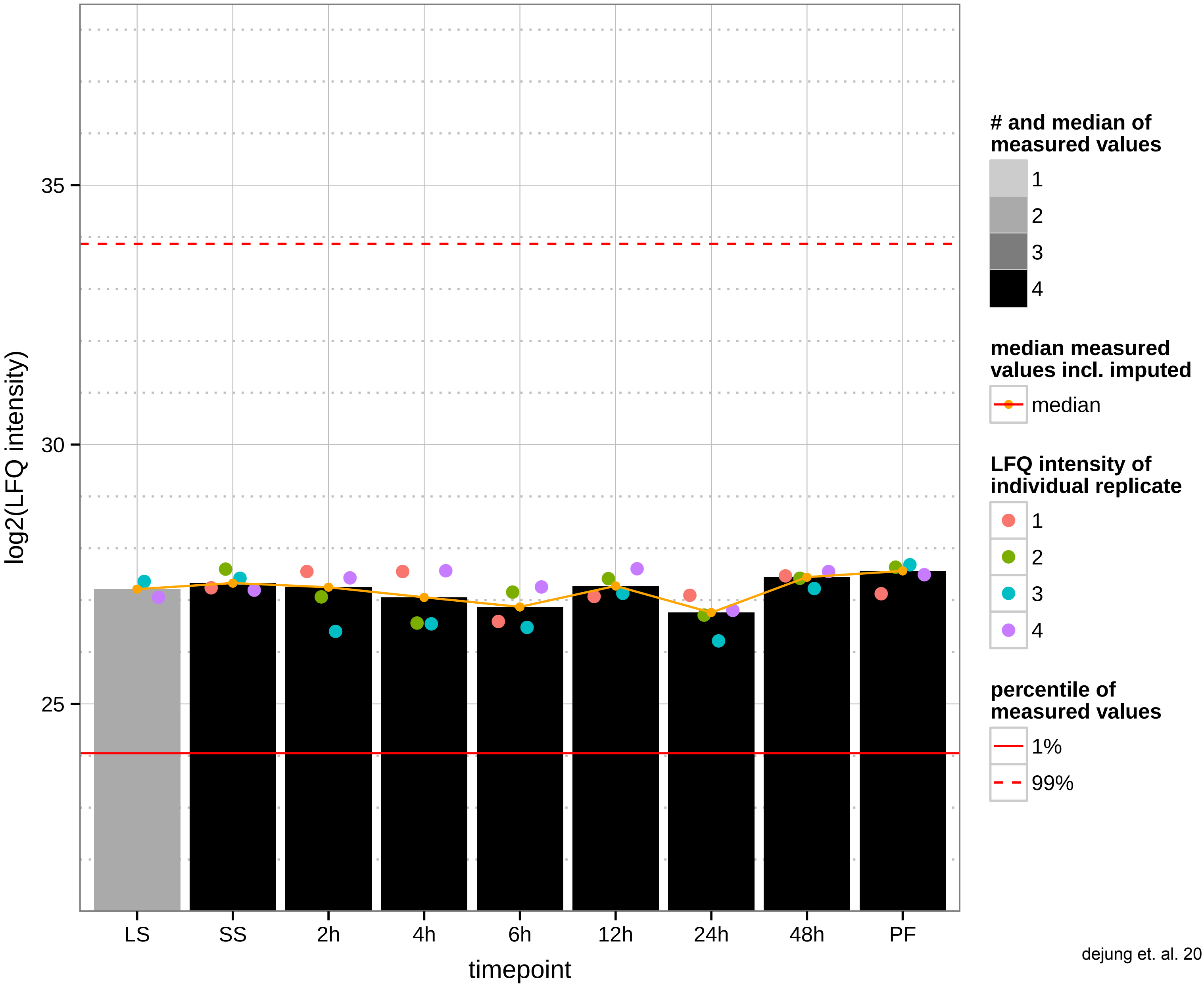

dejung et. al. 2015

hypothetical protein, conserved
Tb927.7.5840
AGOF: null
AGOC: null
AGOP: null
PGOF: null
PGOC: null
PGOP: null

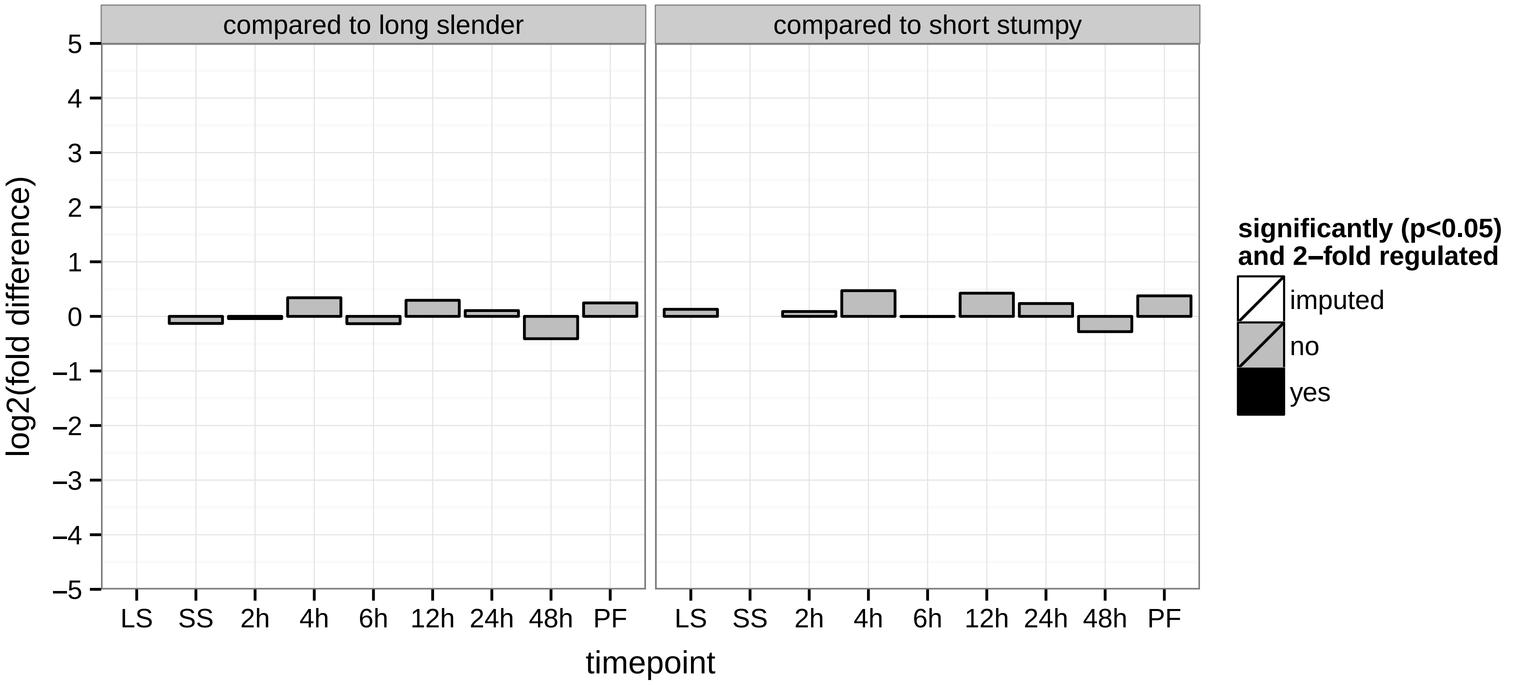

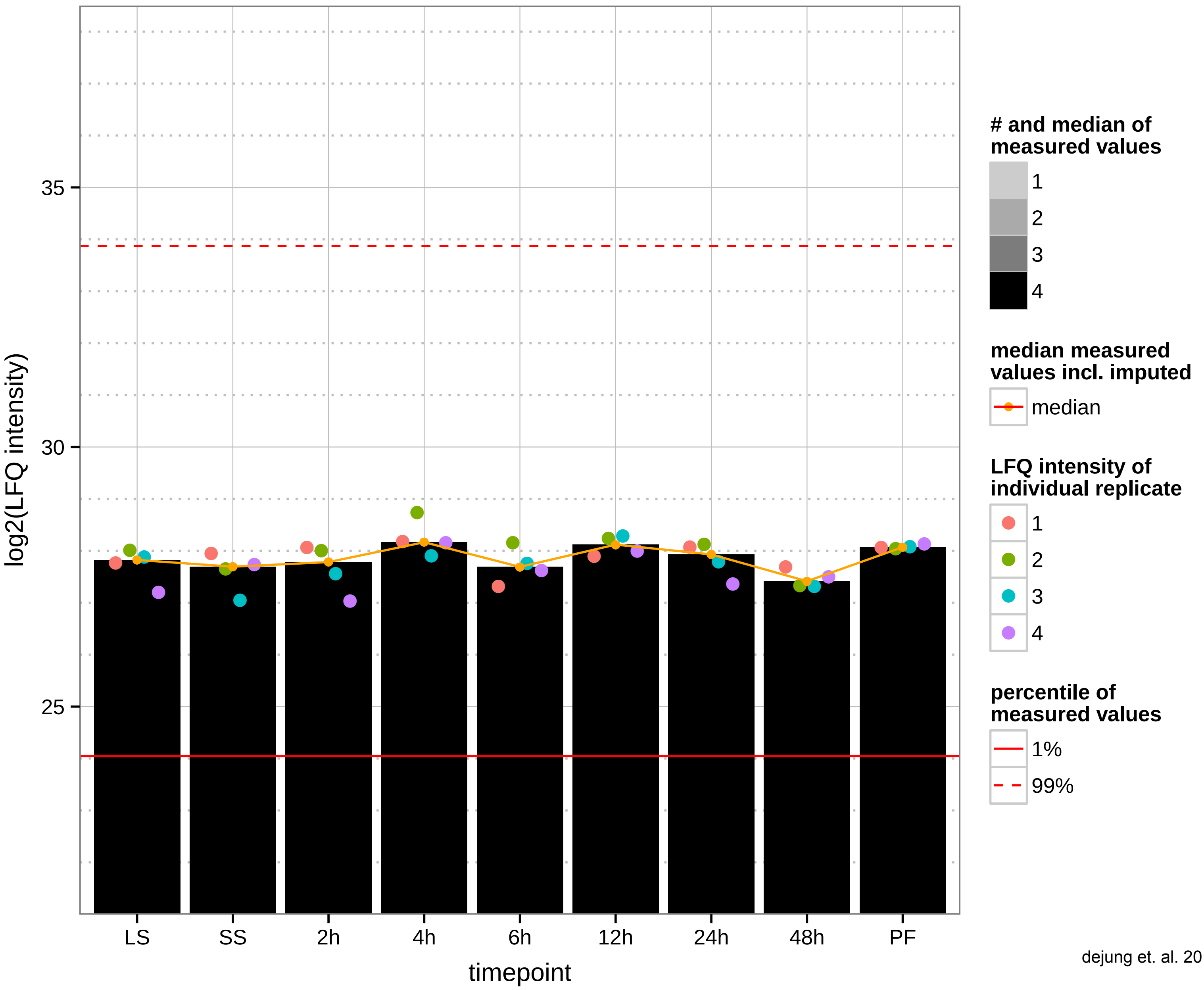

dejung et. al. 2015

mercaptopyruvate sulfurtransferase, putative (MST)
Tb927.7.5920
AGOF: 3–mercaptopyruvate sulfurtransferase activity
AGOC: cytosol
AGOP: cyanide metabolic process, cysteine biosynthetic process via cystathionine
PGOF: null
PGOC: null
PGOP: null

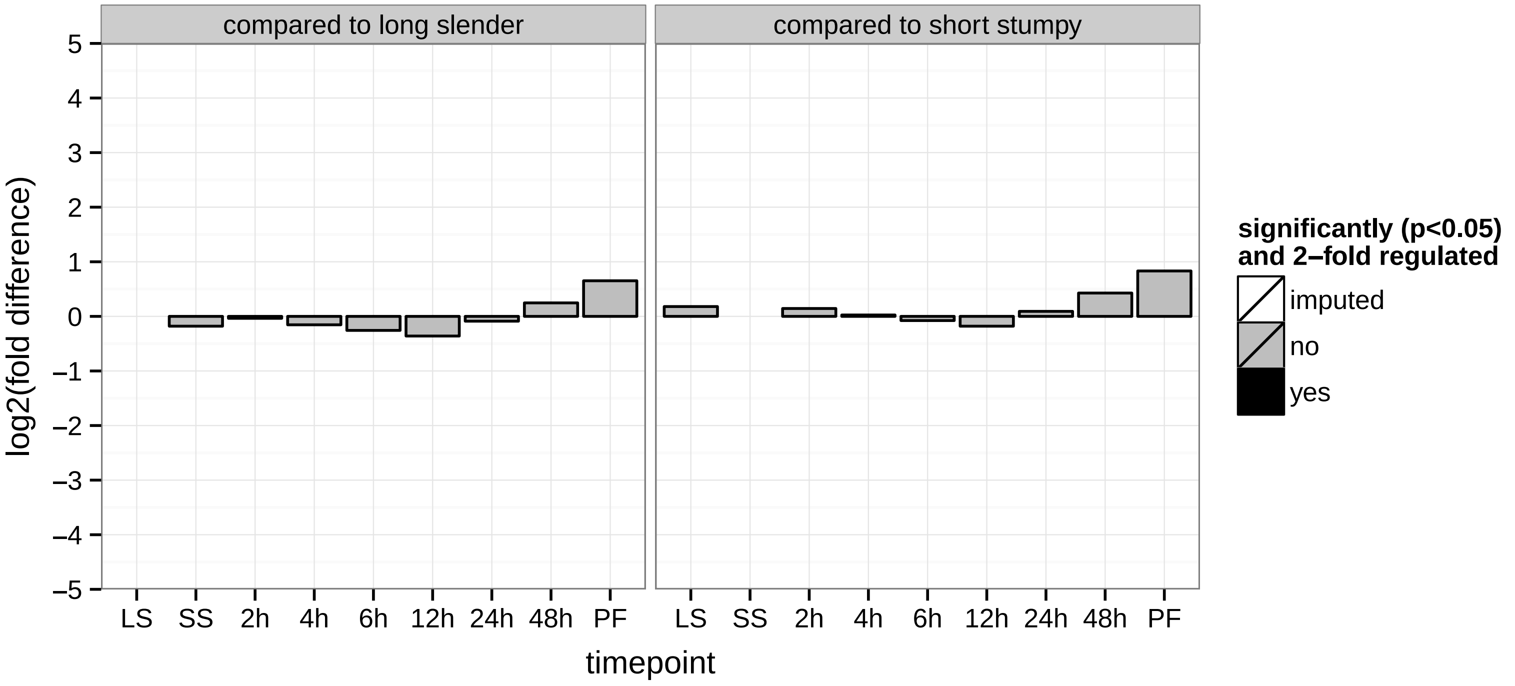

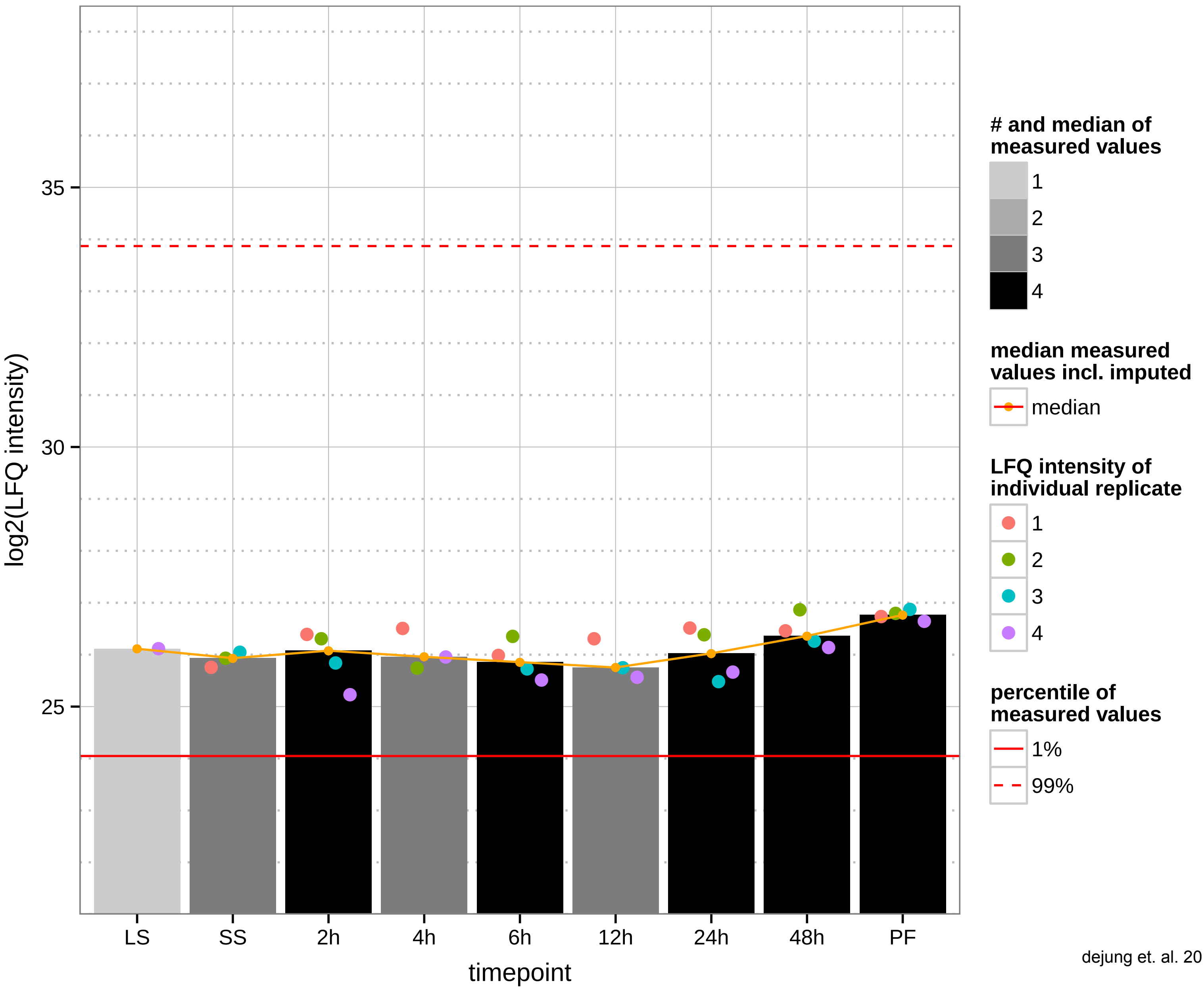

dejung et. al. 2015

hypothetical protein, conserved
Tb927.7.6090
AGOF: null
AGOC: null
AGOP: null
PGOF: null
PGOC: null
PGOP: null

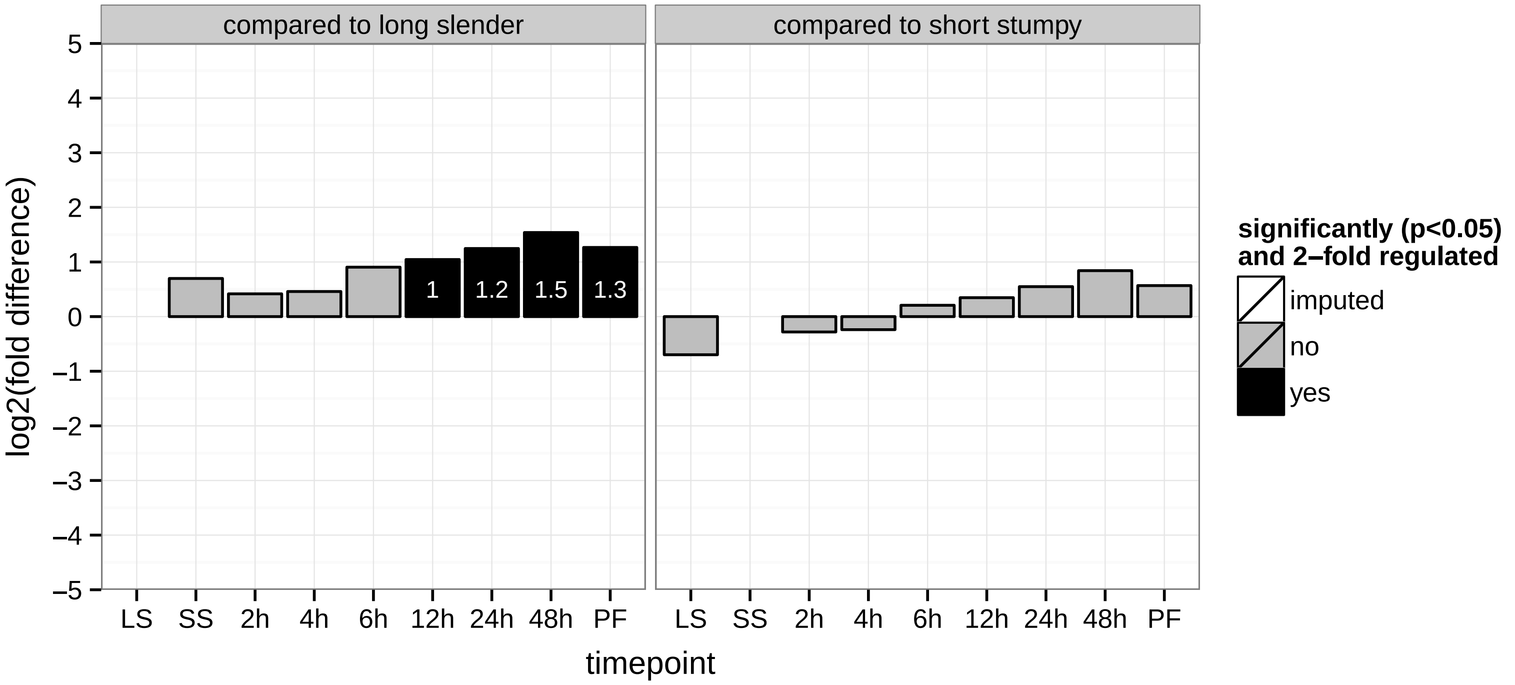

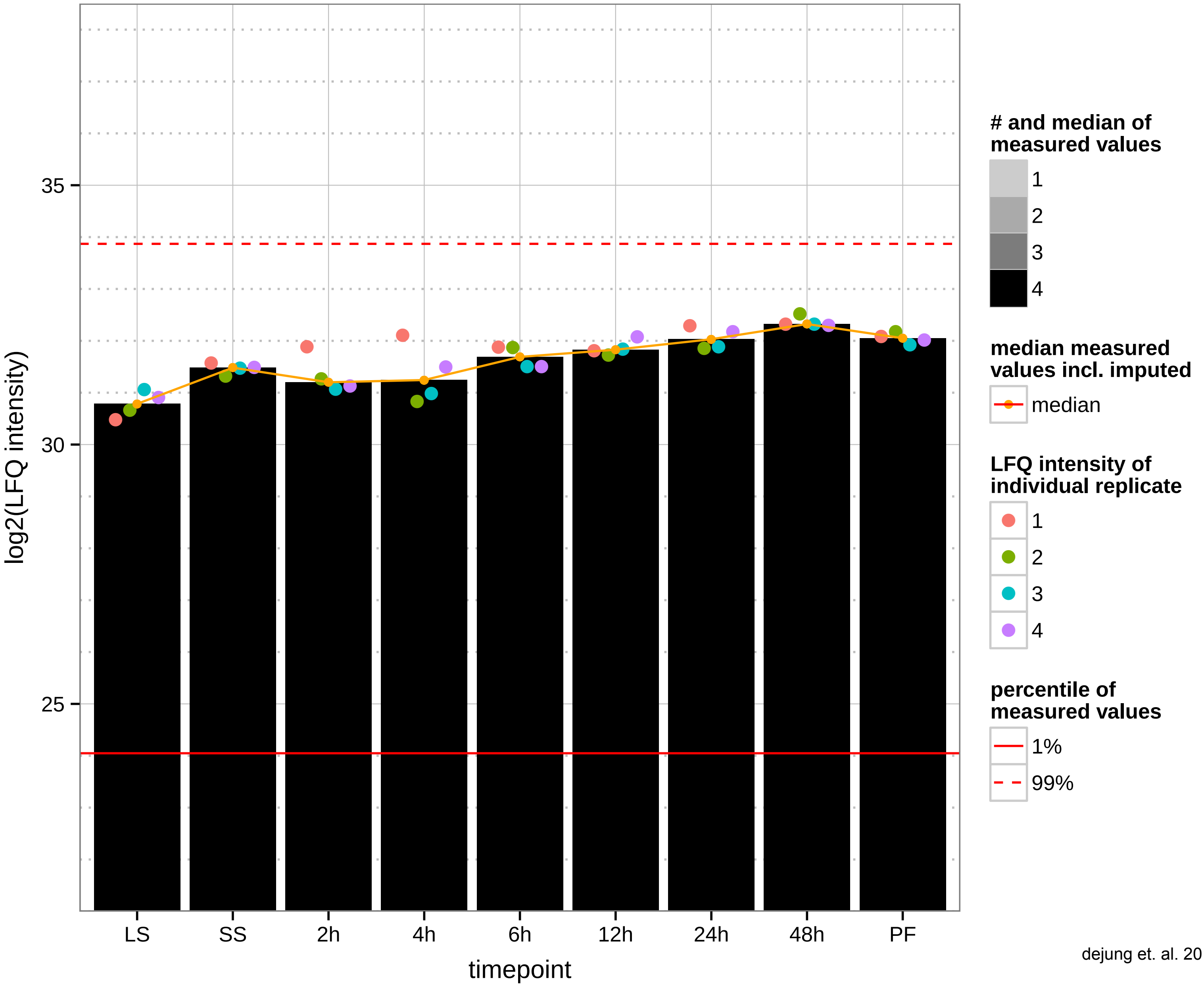

dejung et. al. 2015

ADP–ribosylation factor, putative (ARF3)
Tb927.7.6230
AGOF: GTP binding
AGOC: intracellular
AGOP: intracellular protein transport, small GTPase mediated signal transduction
PGOF: GTP binding
PGOC: intracellular
PGOP: intracellular protein transport, small GTPase mediated signal transduction

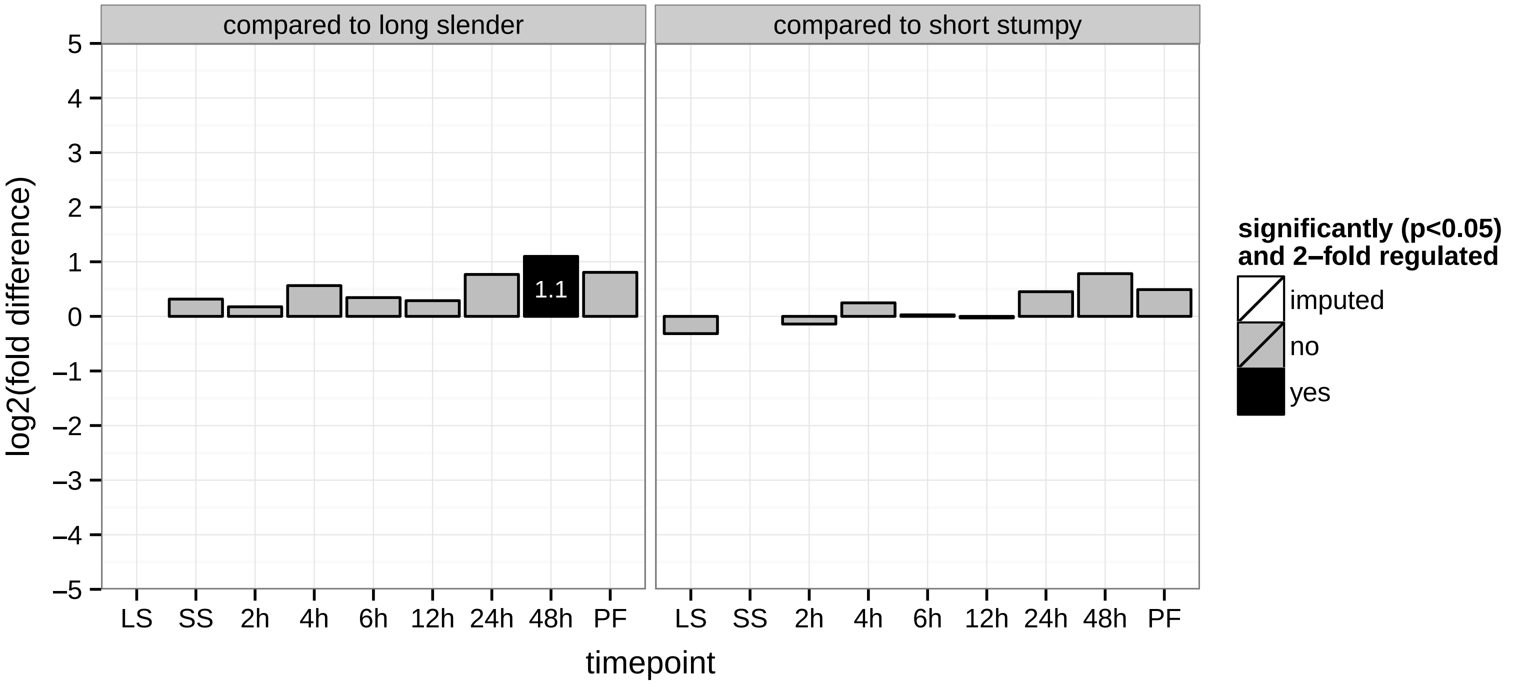

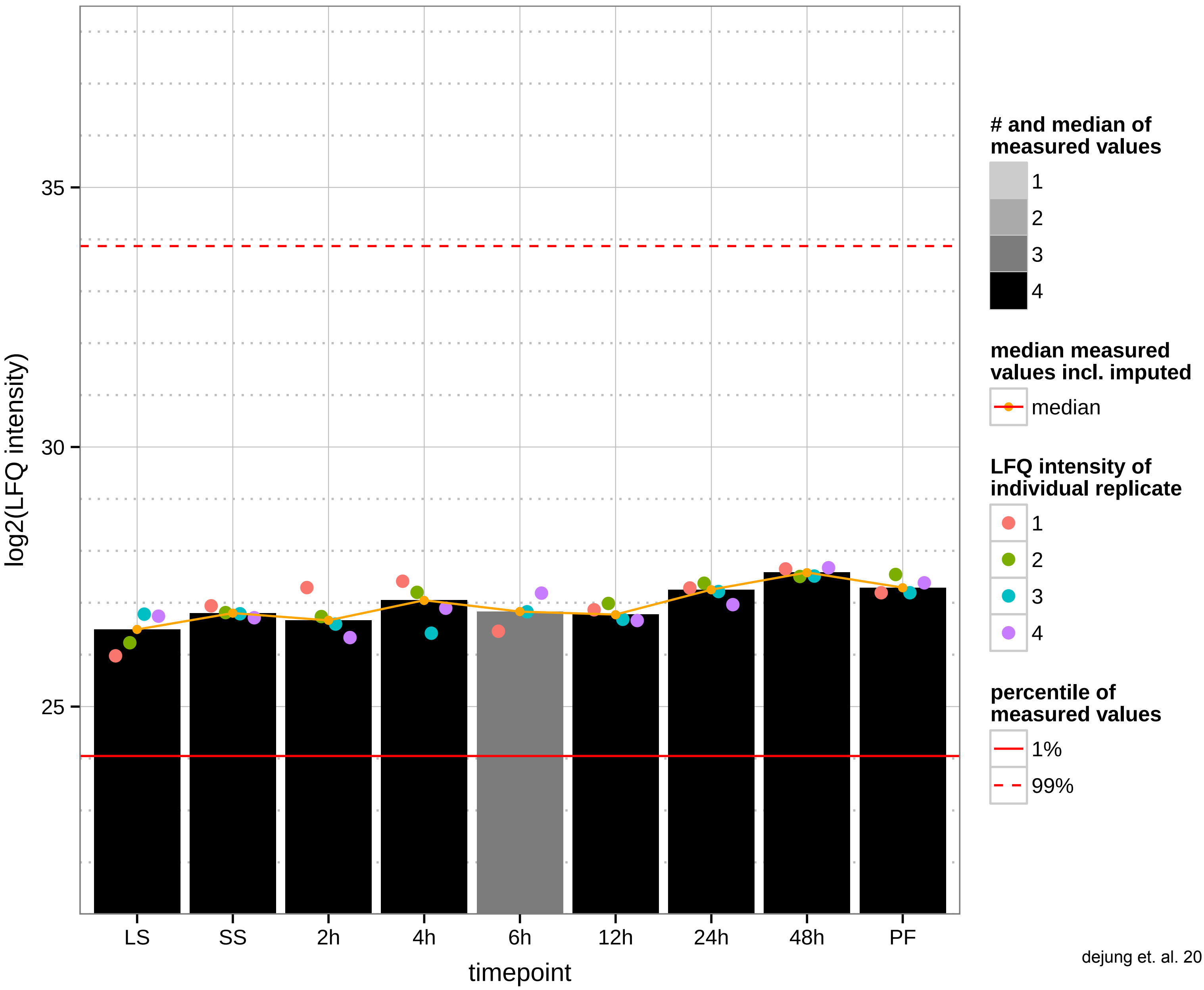

dejung et. al. 2015

hypothetical protein, conserved
Tb927.7.6280
AGOF: null
AGOC: null
AGOP: null
PGOF: null
PGOC: null
PGOP: null

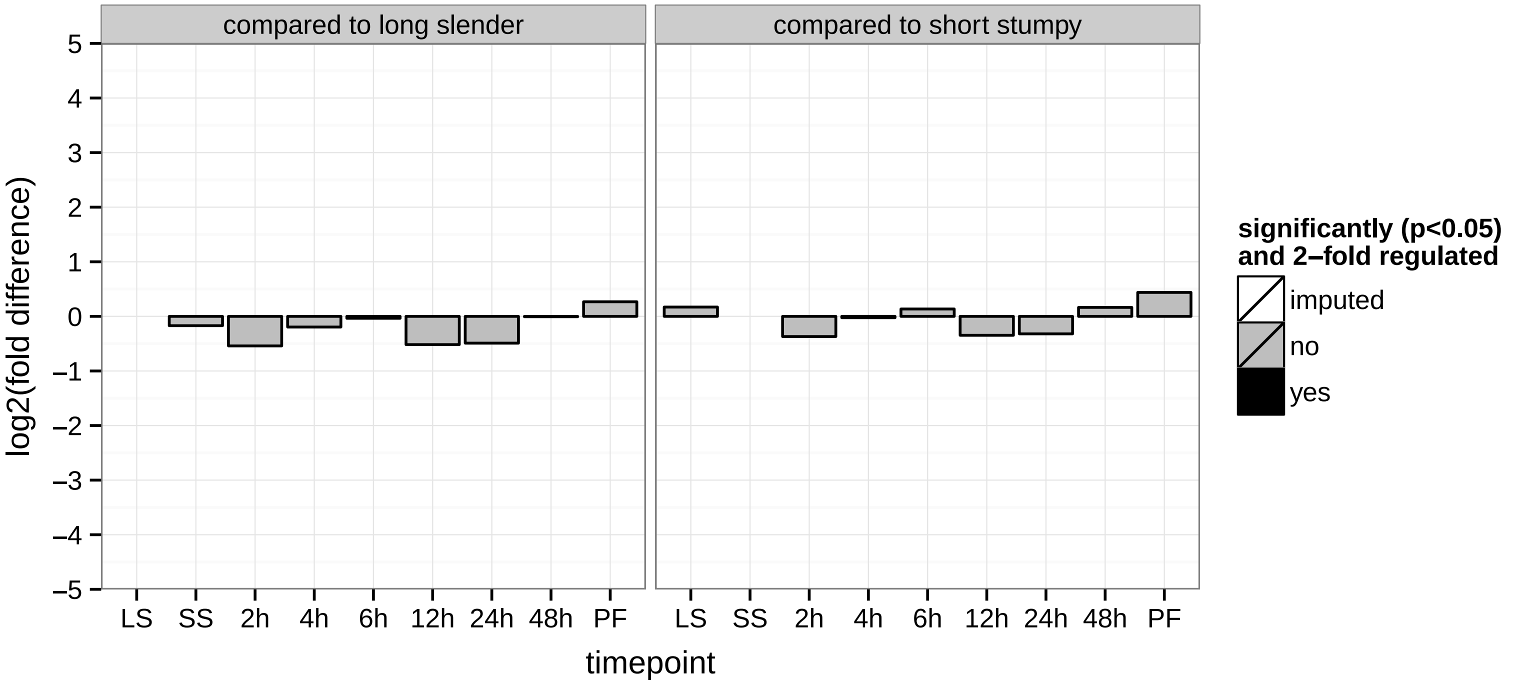

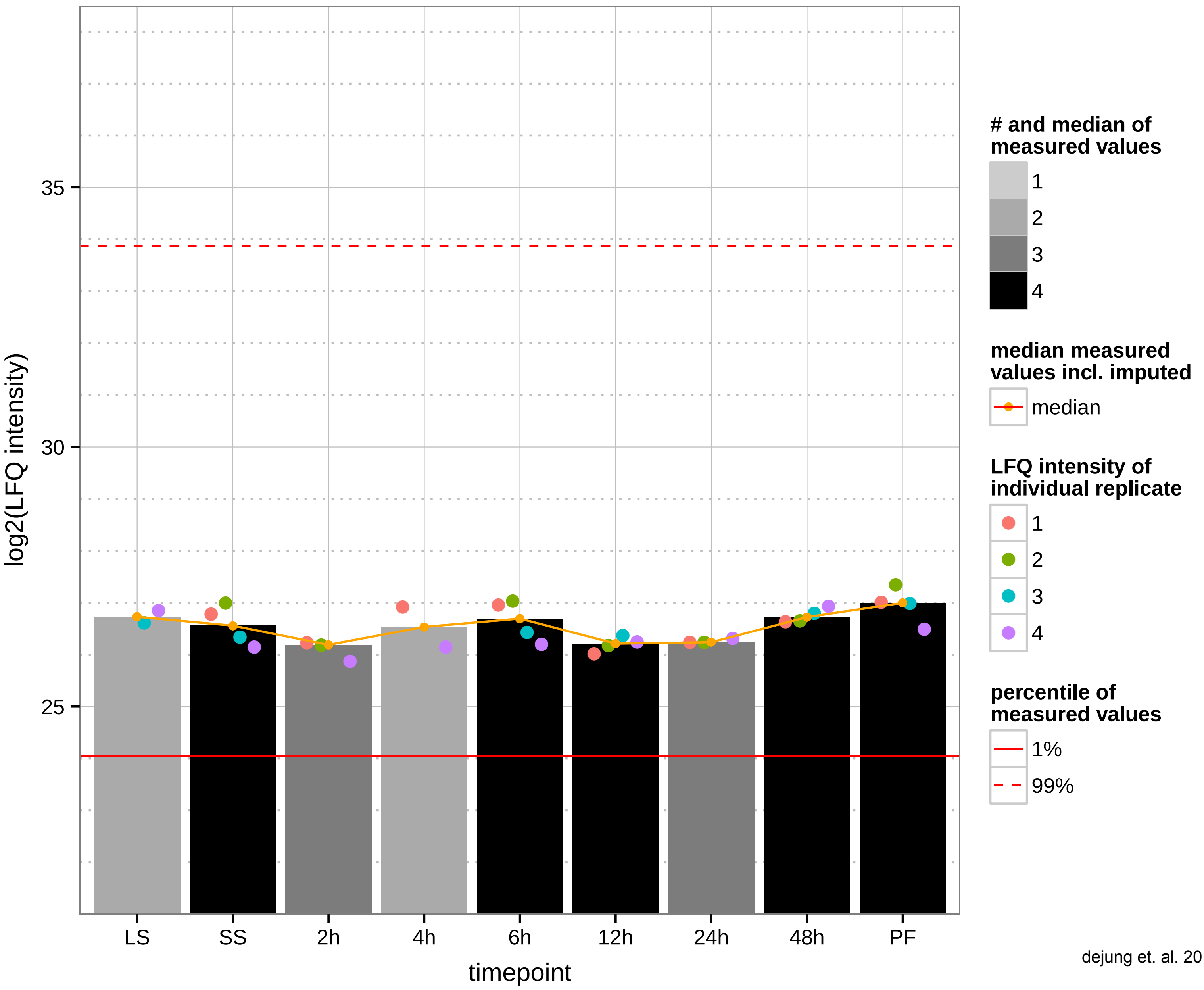

dejung et. al. 2015

kinesin, putative (TbKIF9A)
Tb927.7.6290
AGOF: ATP binding, microtubule motor activity, motor activity
AGOC: axoneme, microtubule associated complex
AGOP: ciliary cell motility, microtubule–based movement
PGOF: ATP binding, microtubule motor activity
PGOC: null
PGOP: microtubule–based movement

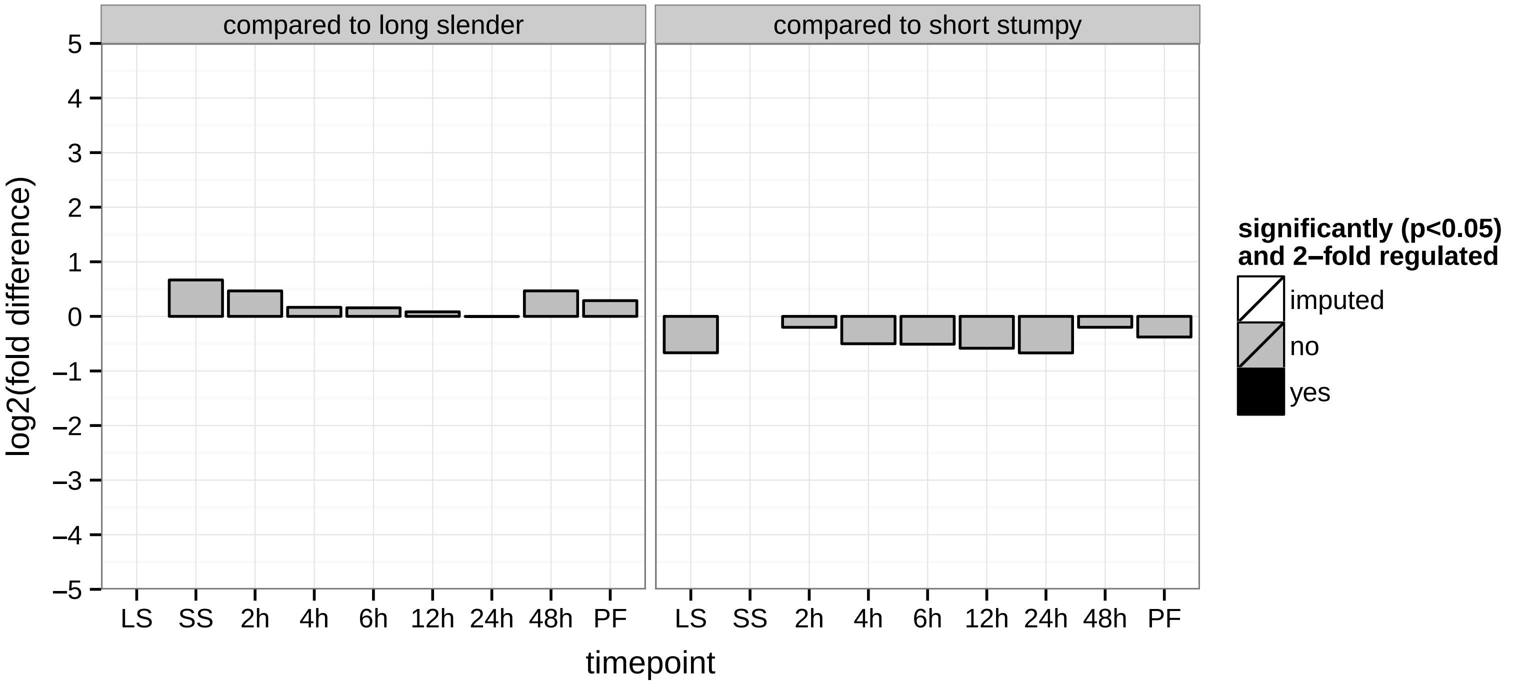

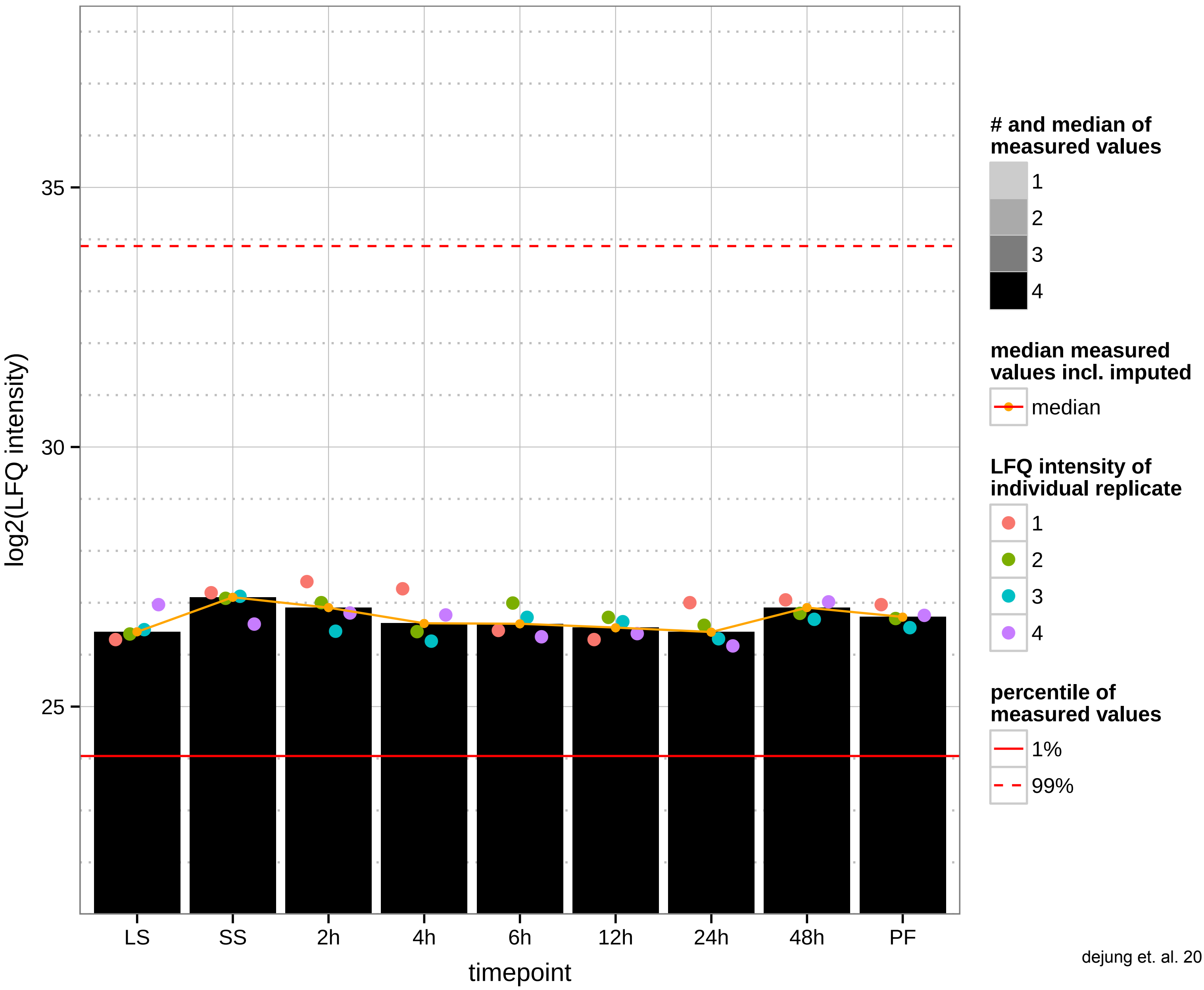

dejung et. al. 2015

histone H2A variant, H2Az (h2aZ)
Tb927.7.6360
AGOF: DNA binding
AGOC: nucleosome, nucleus
AGOP: chromatin organization, chromosome organization, nucleosome assembly, transcription initiation from RNA polymeras
PGOF: DNA binding
PGOC: nucleosome, nucleus
PGOP: nucleosome assembly

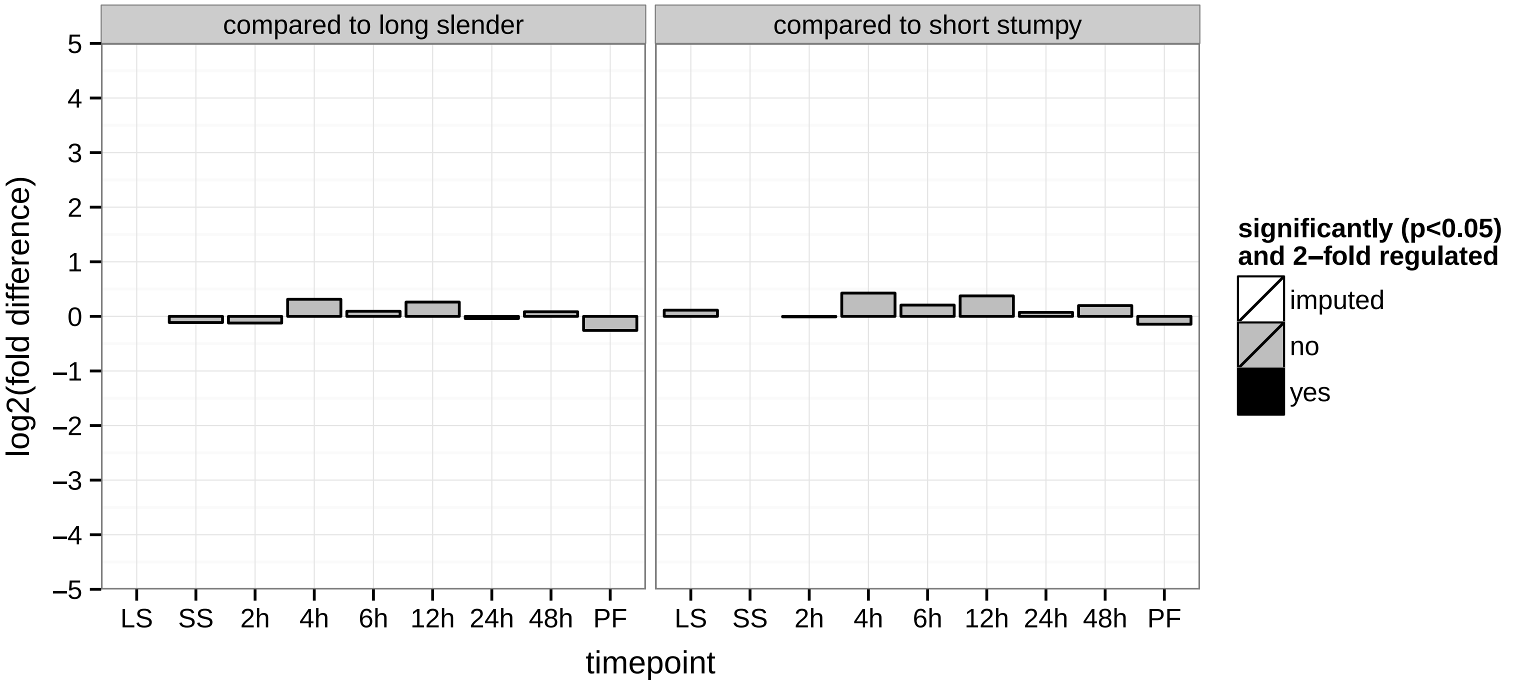

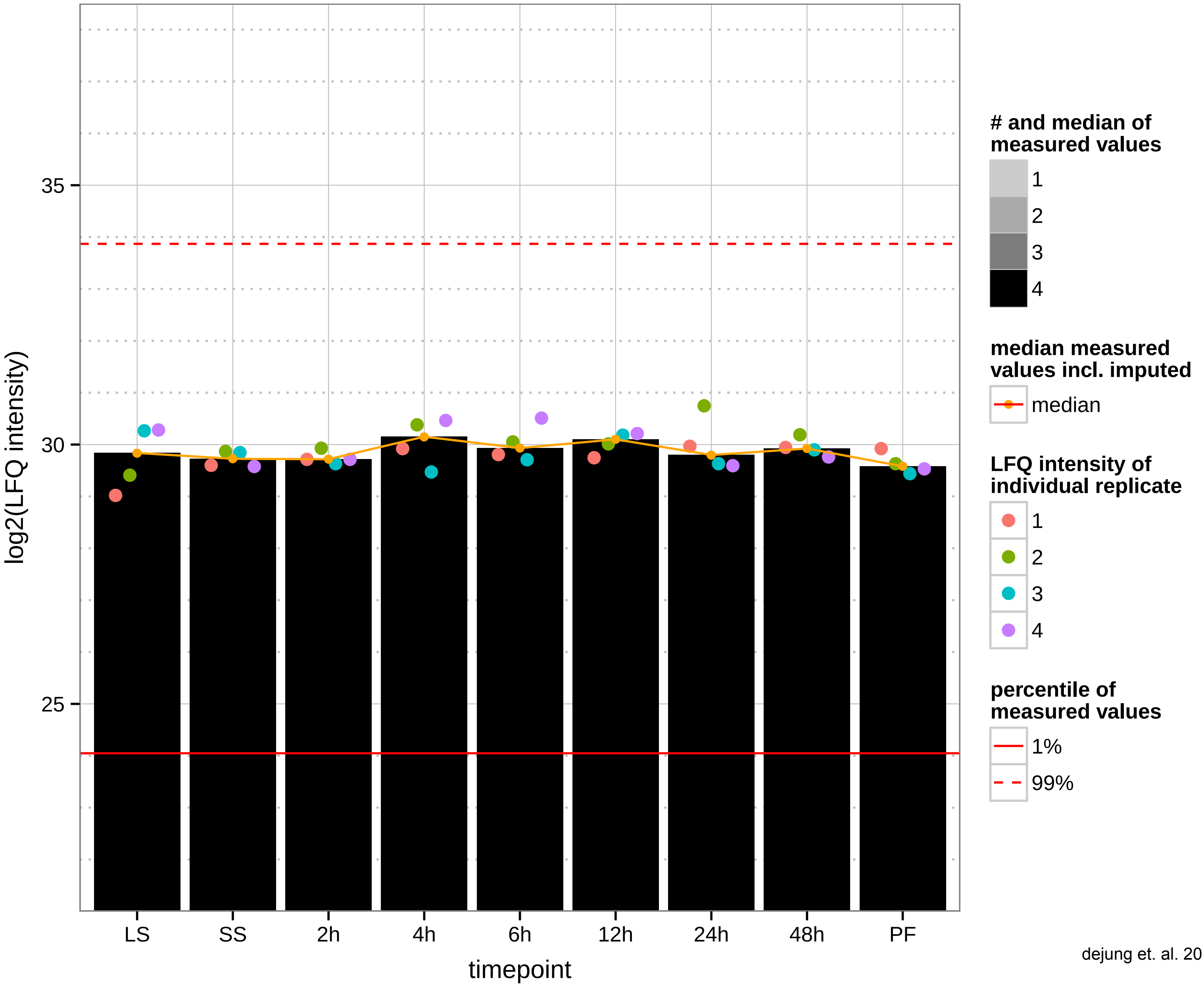

dejung et. al. 2015

hypothetical protein, conserved
Tb927.7.640
AGOF: null
AGOC: mitochondrial membrane, mitochondrion
AGOP: null
PGOF: null
PGOC: null
PGOP: null

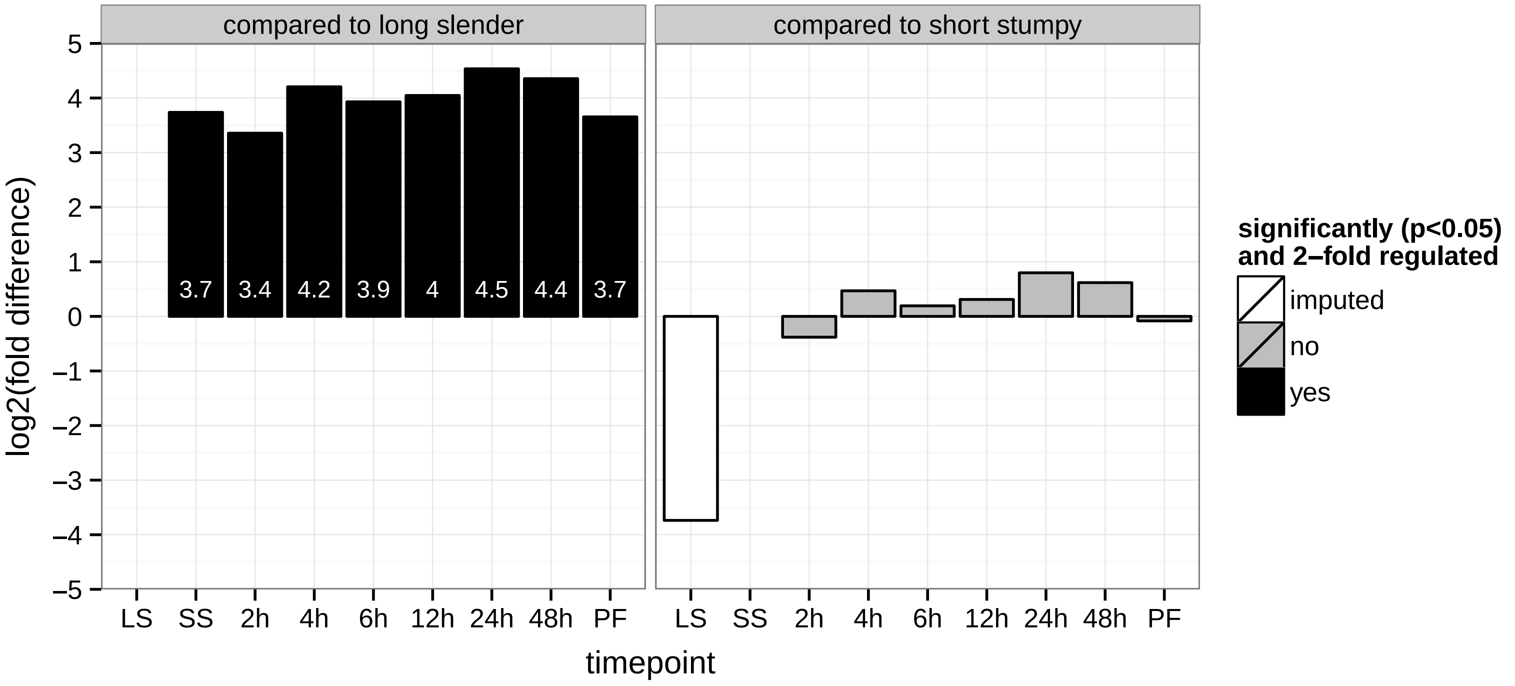

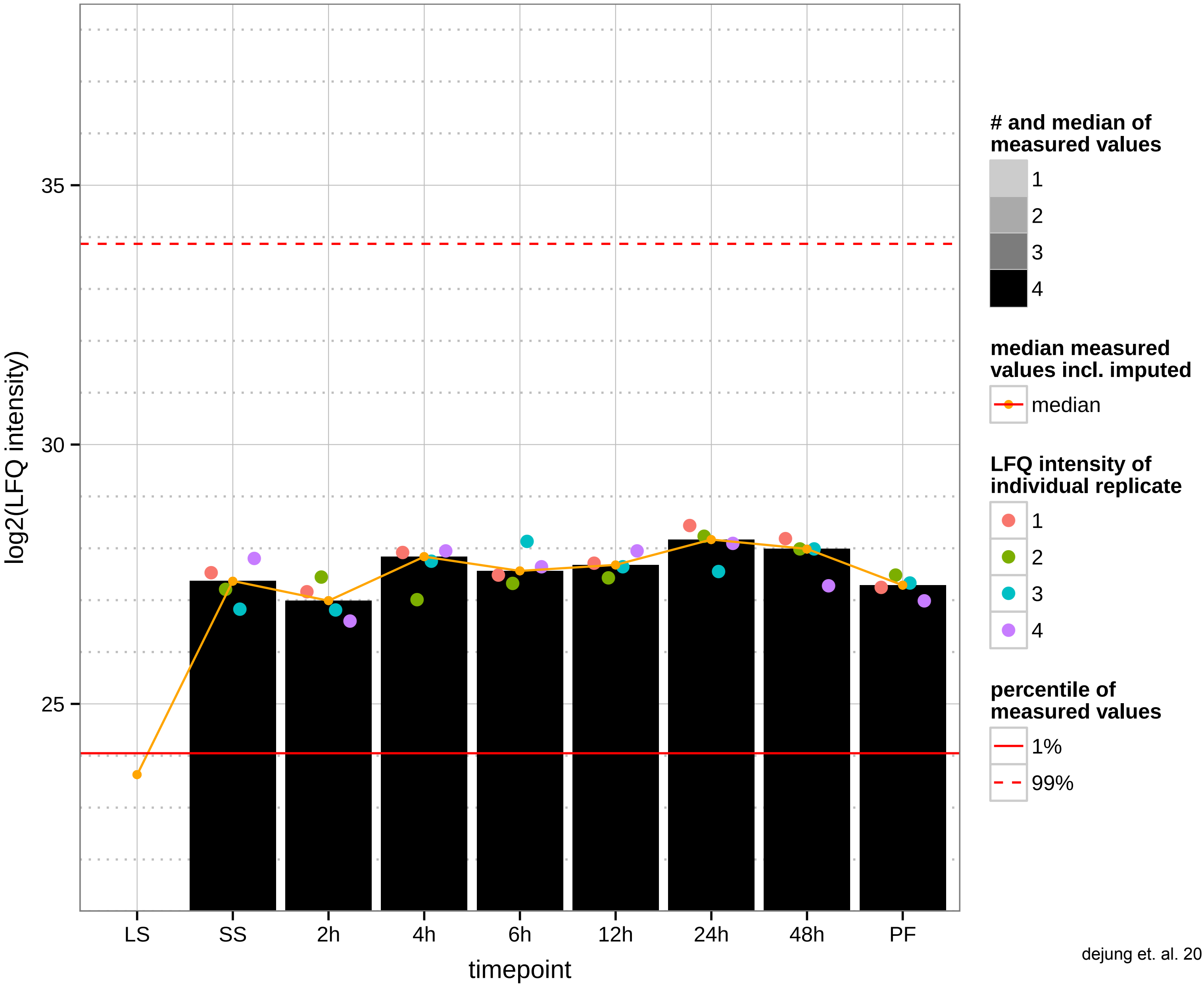

dejung et. al. 2015

hypothetical protein, conserved
Tb927.7.6640
AGOF: null
AGOC: null
AGOP: null
PGOF: null
PGOC: null
PGOP: null

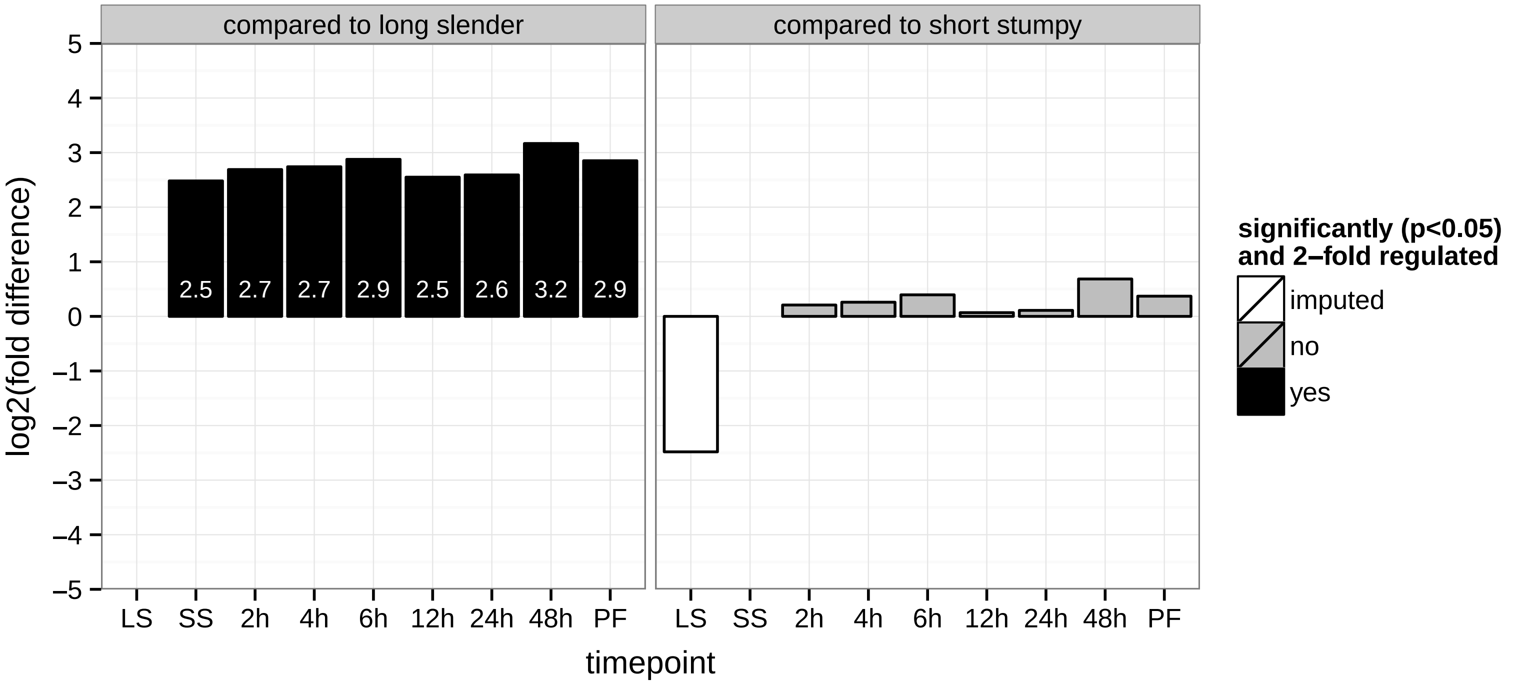

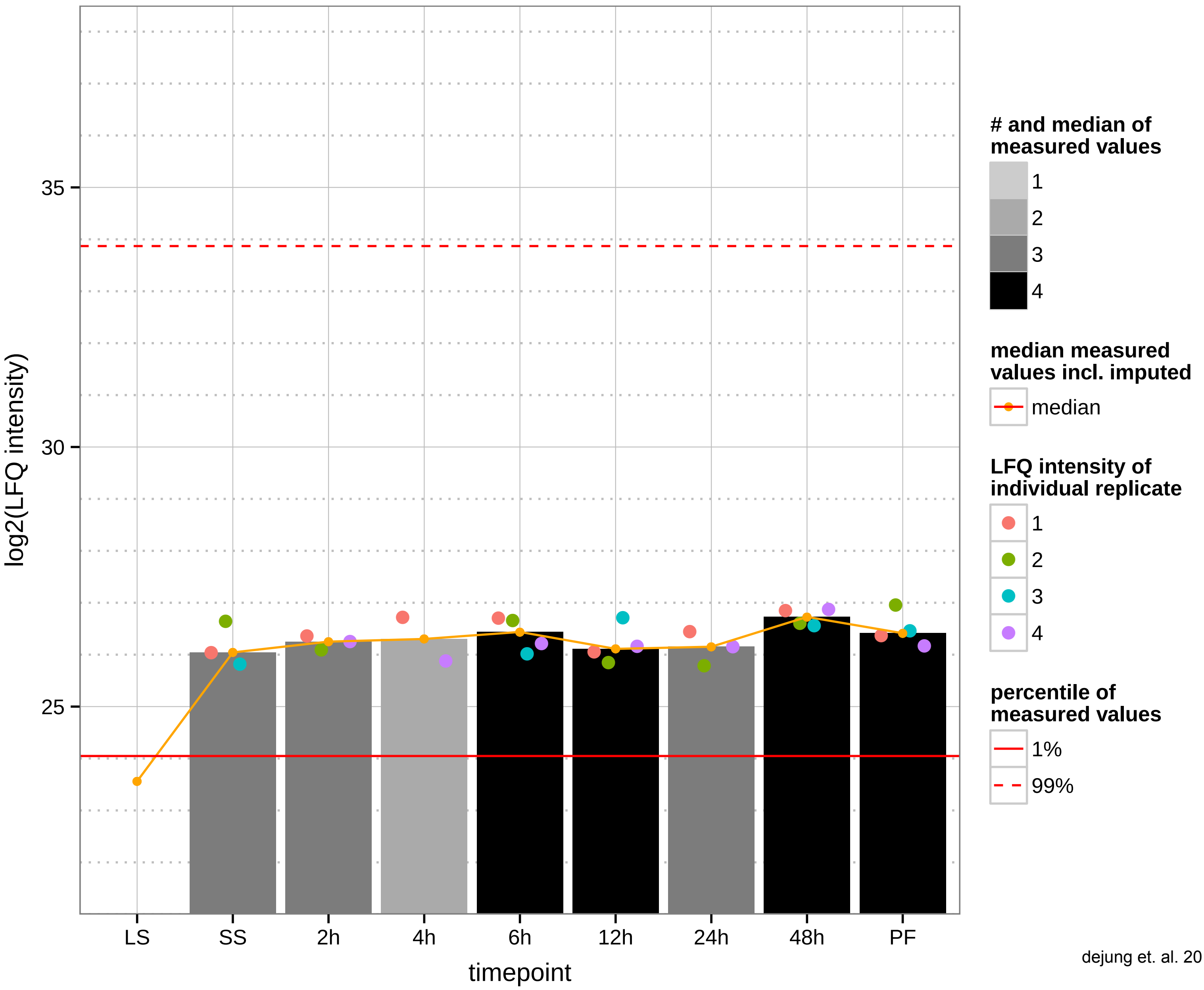

dejung et. al. 2015

protein kinase, putative
Tb927.7.6680
AGOF: ATP binding, protein tyrosine kinase activity
AGOC: null
AGOP: protein phosphorylation
PGOF: ATP binding, protein kinase activity, transferase activity, transferring phosphorus–containing groups
PGOC: null
PGOP: protein phosphorylation

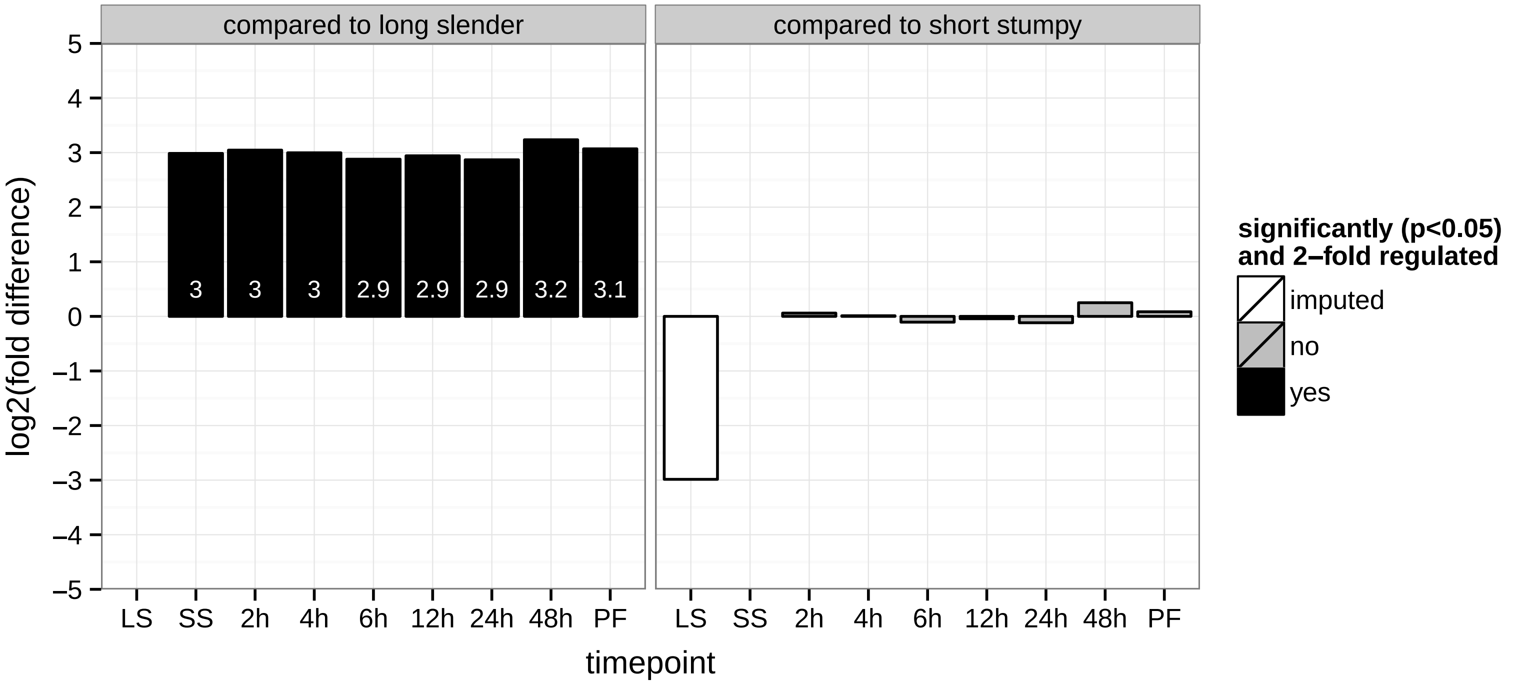

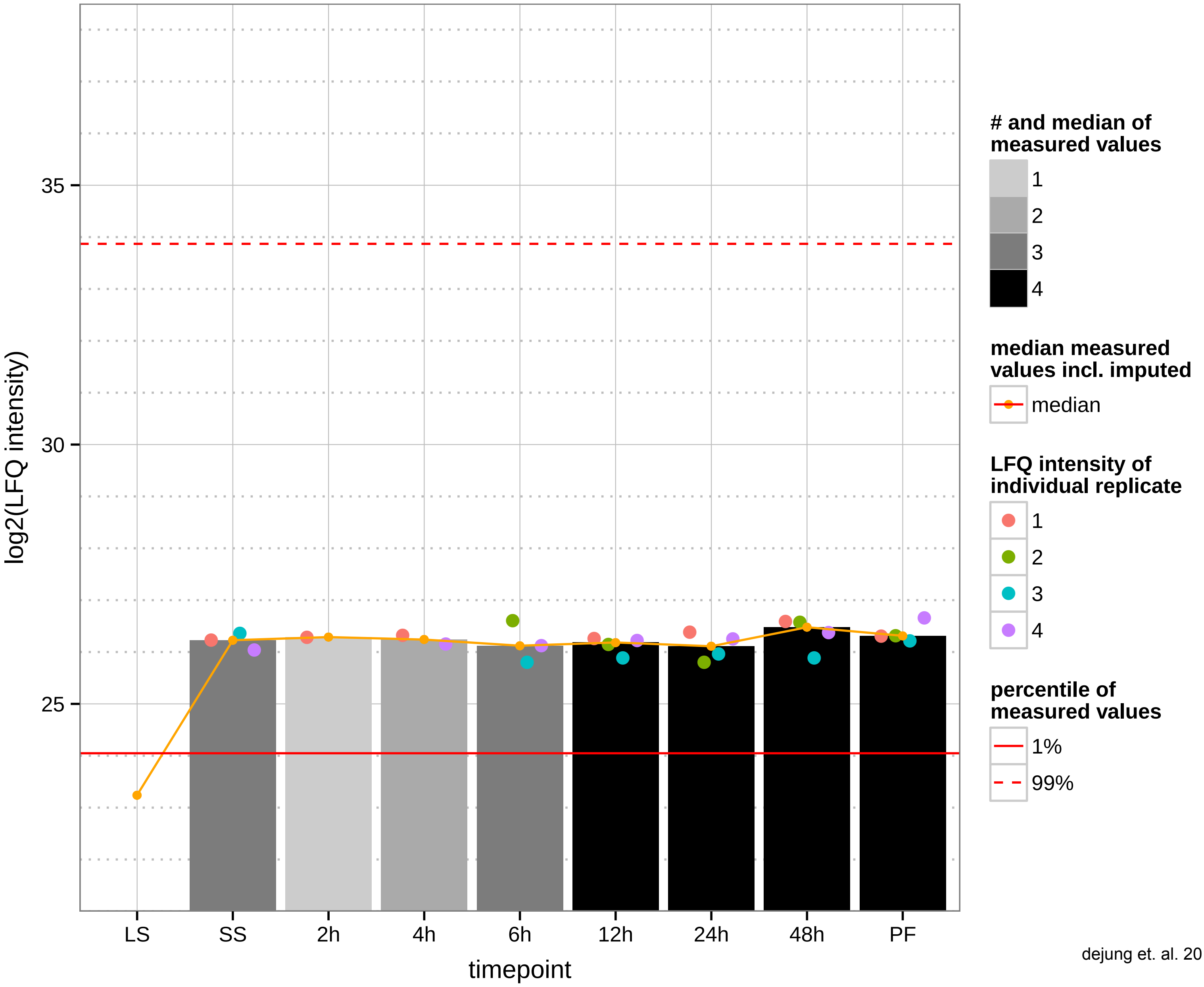

dejung et. al. 2015

hypothetical protein, conserved
Tb927.7.6910
AGOF: null
AGOC: null
AGOP: null
PGOF: null
PGOC: null
PGOP: null

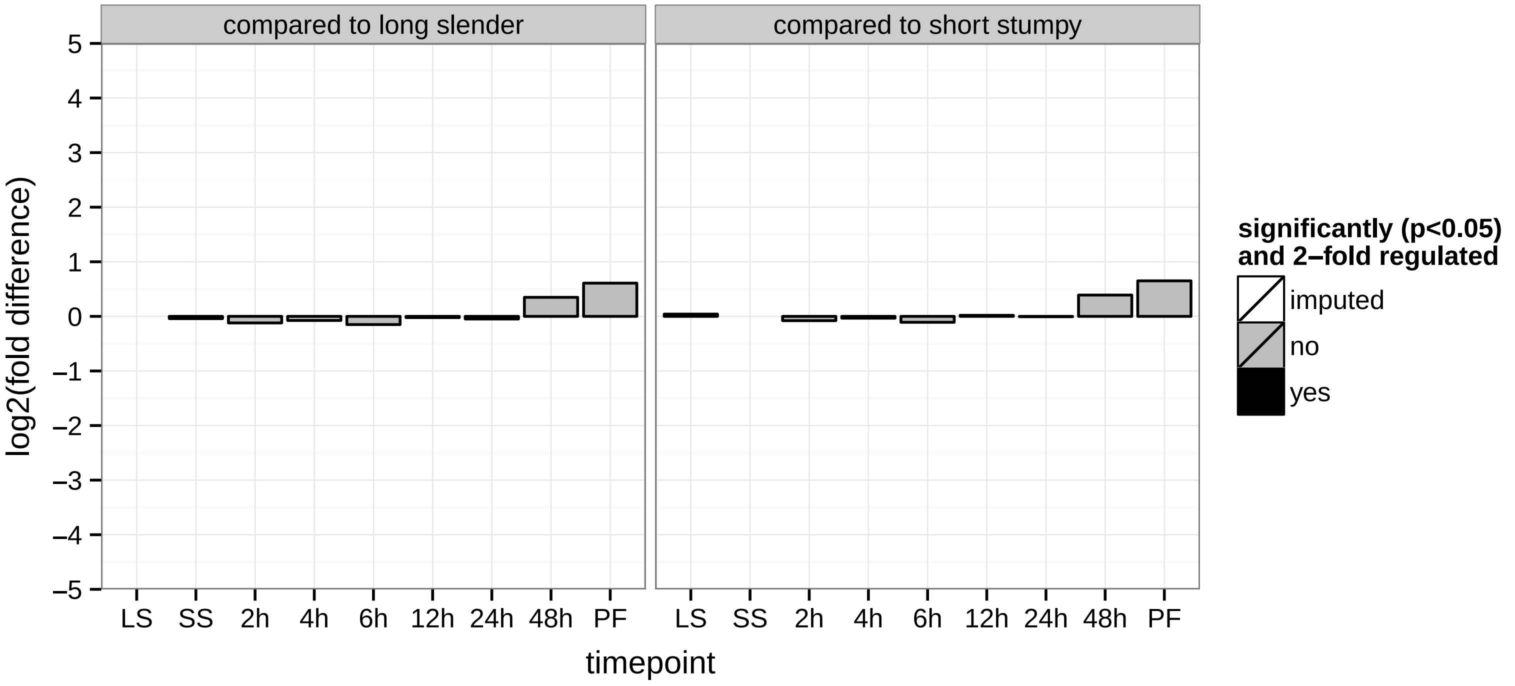

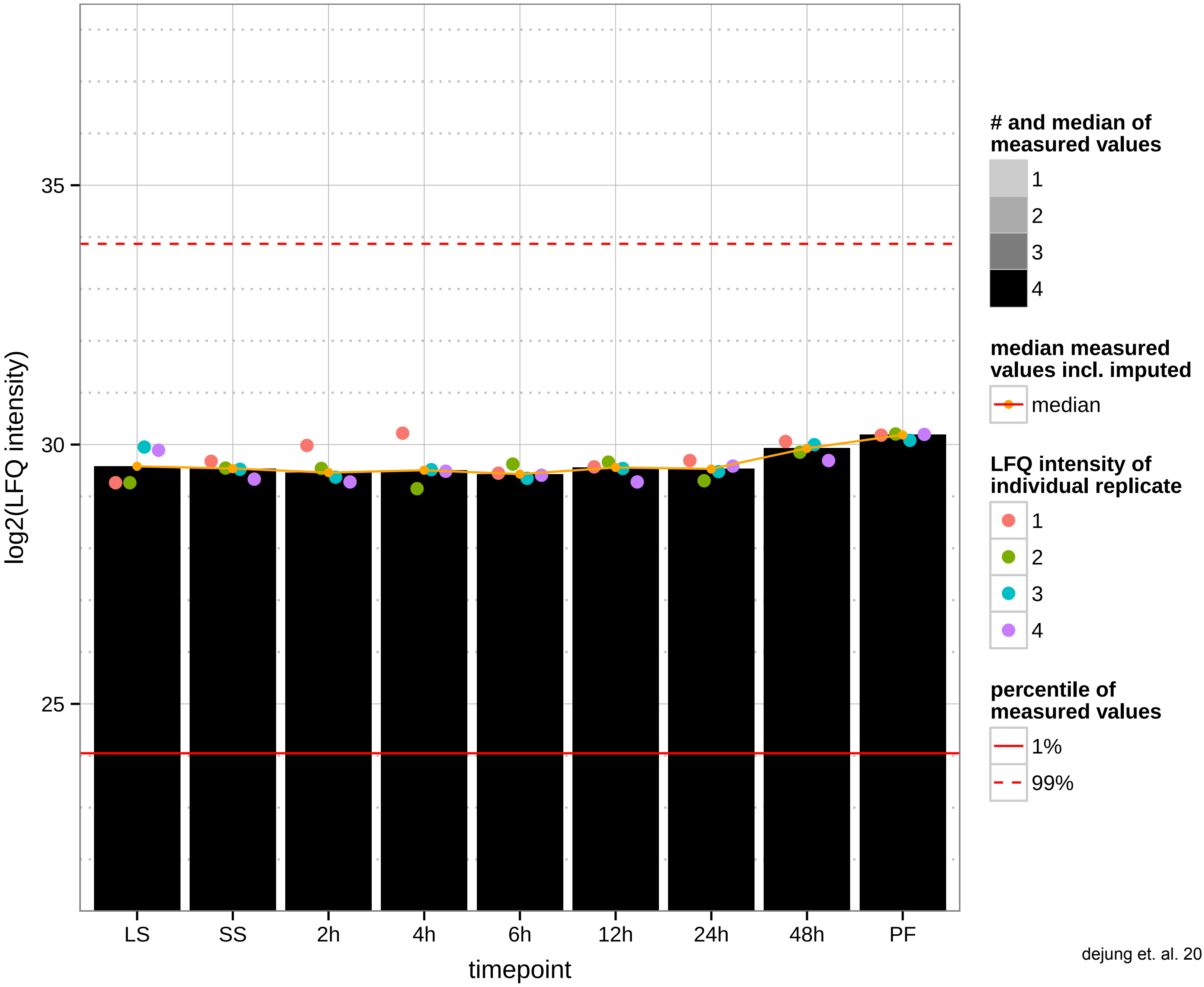

dejung et. al. 2015

ATPase, putative
Tb927.7.6930
AGOF: ATP binding
AGOC: null
AGOP: null
PGOF: ATP binding
PGOC: null
PGOP: null

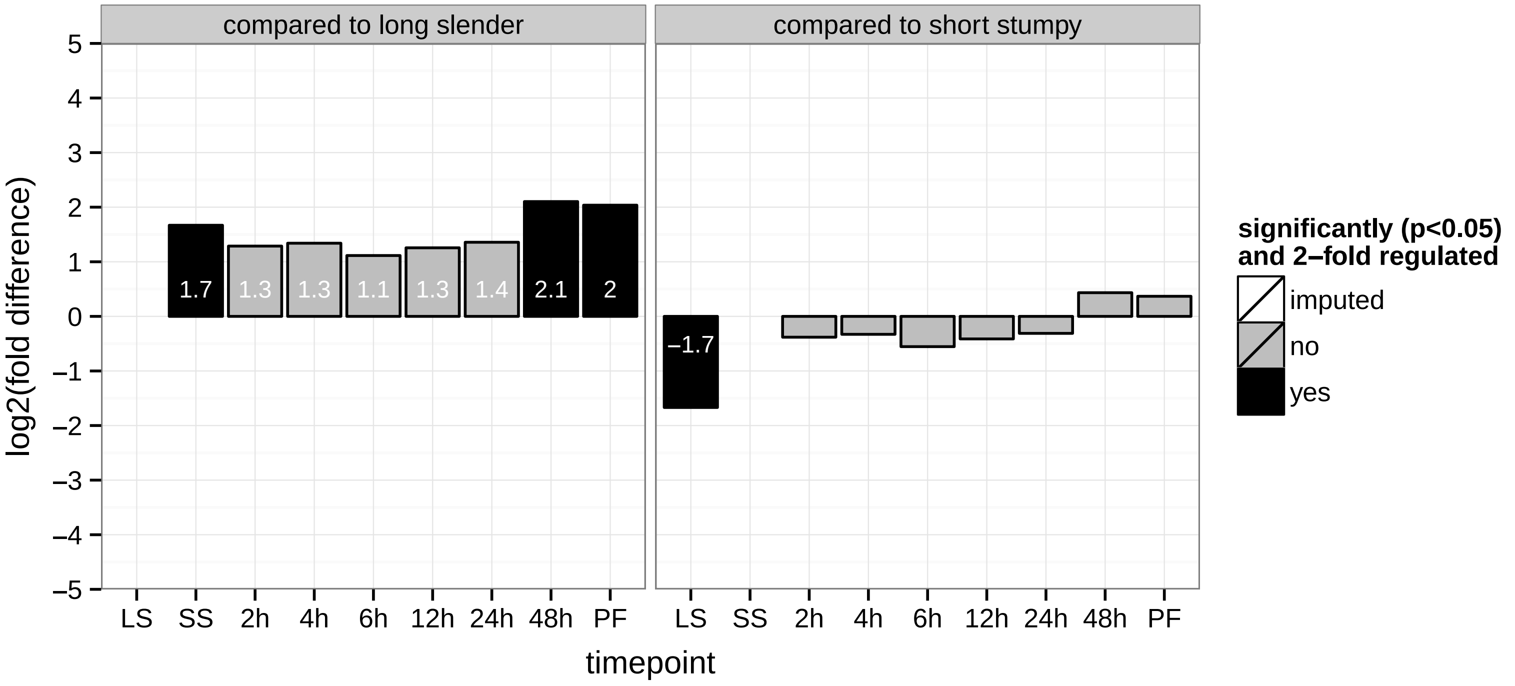

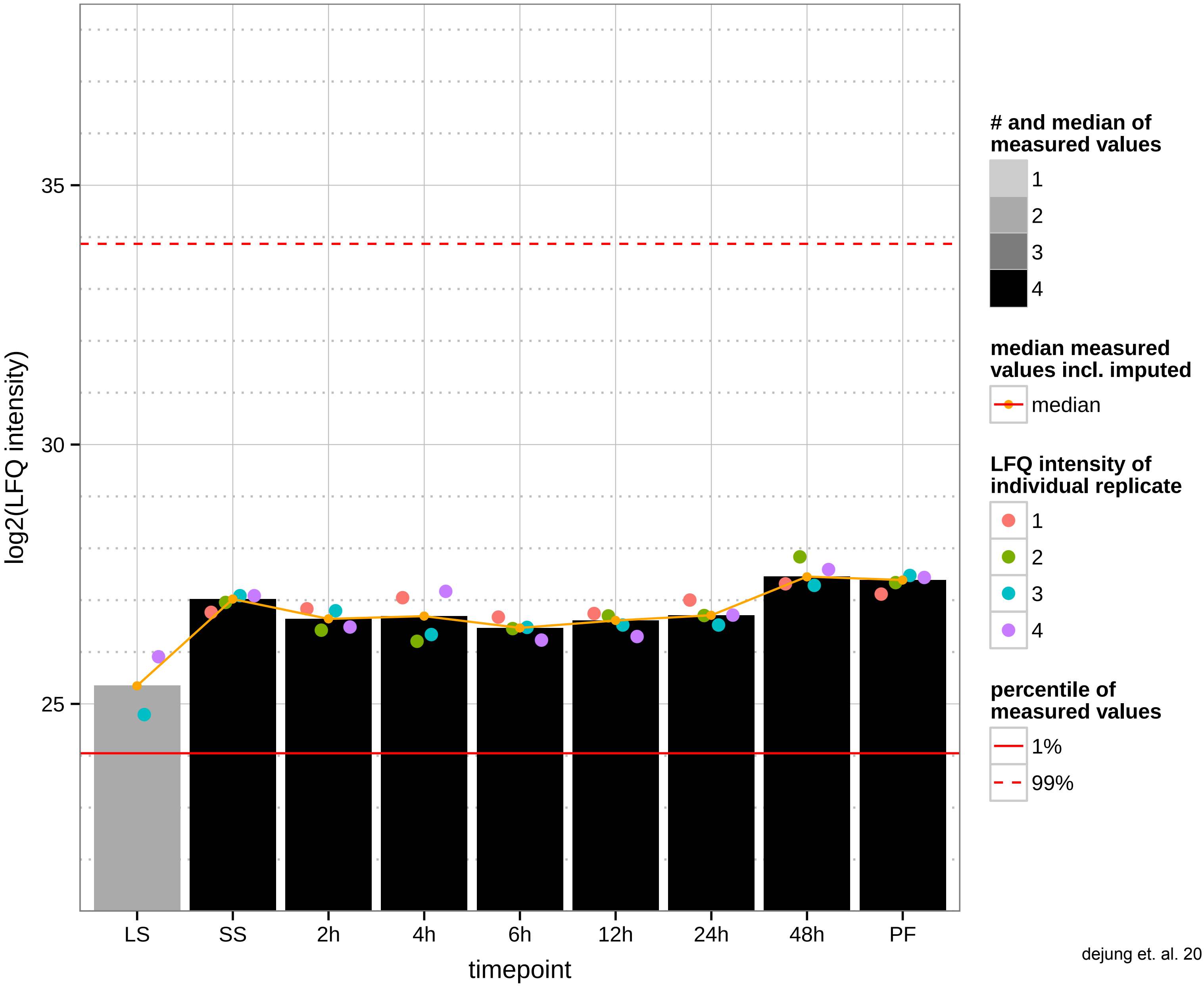

dejung et. al. 2015

hypothetical protein, conserved
Tb927.7.6980
AGOF: null
AGOC: null
AGOP: null
PGOF: nucleic acid binding
PGOC: nucleus
PGOP: null

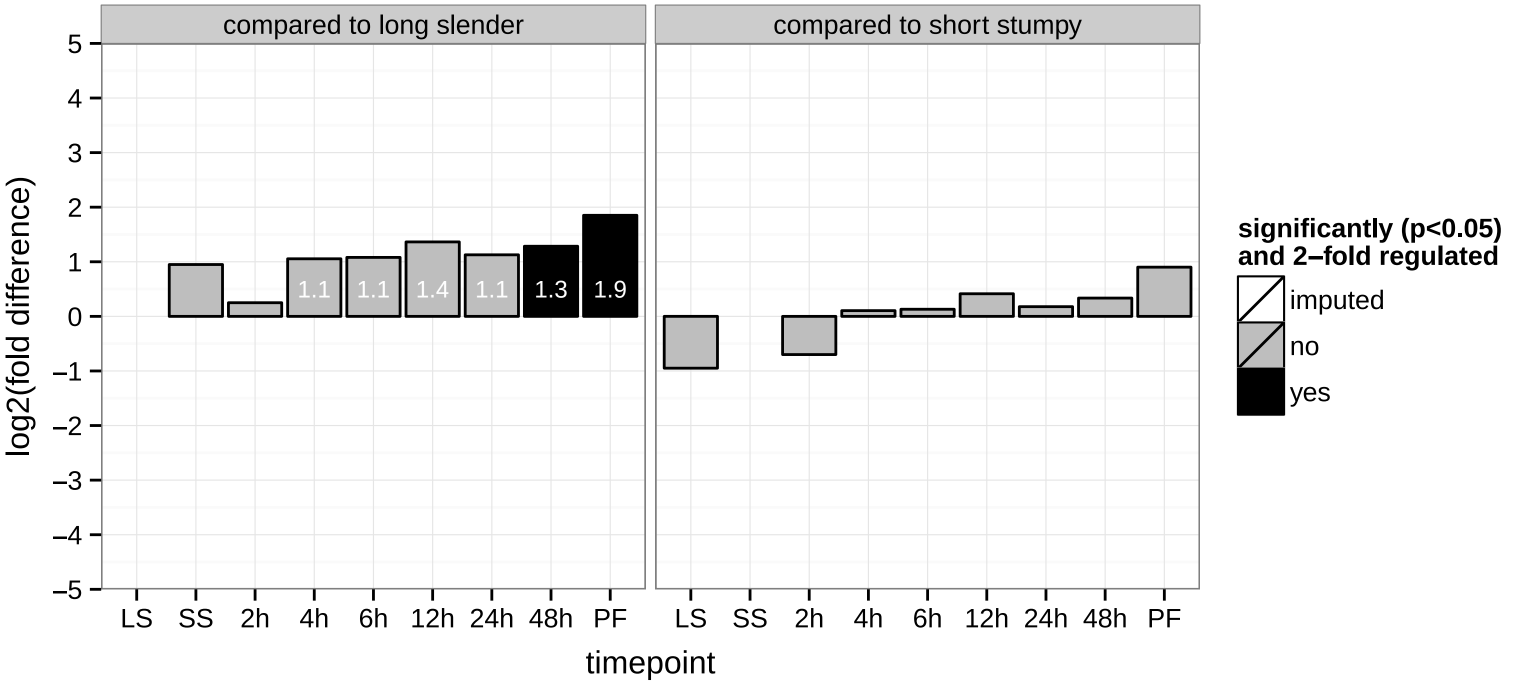

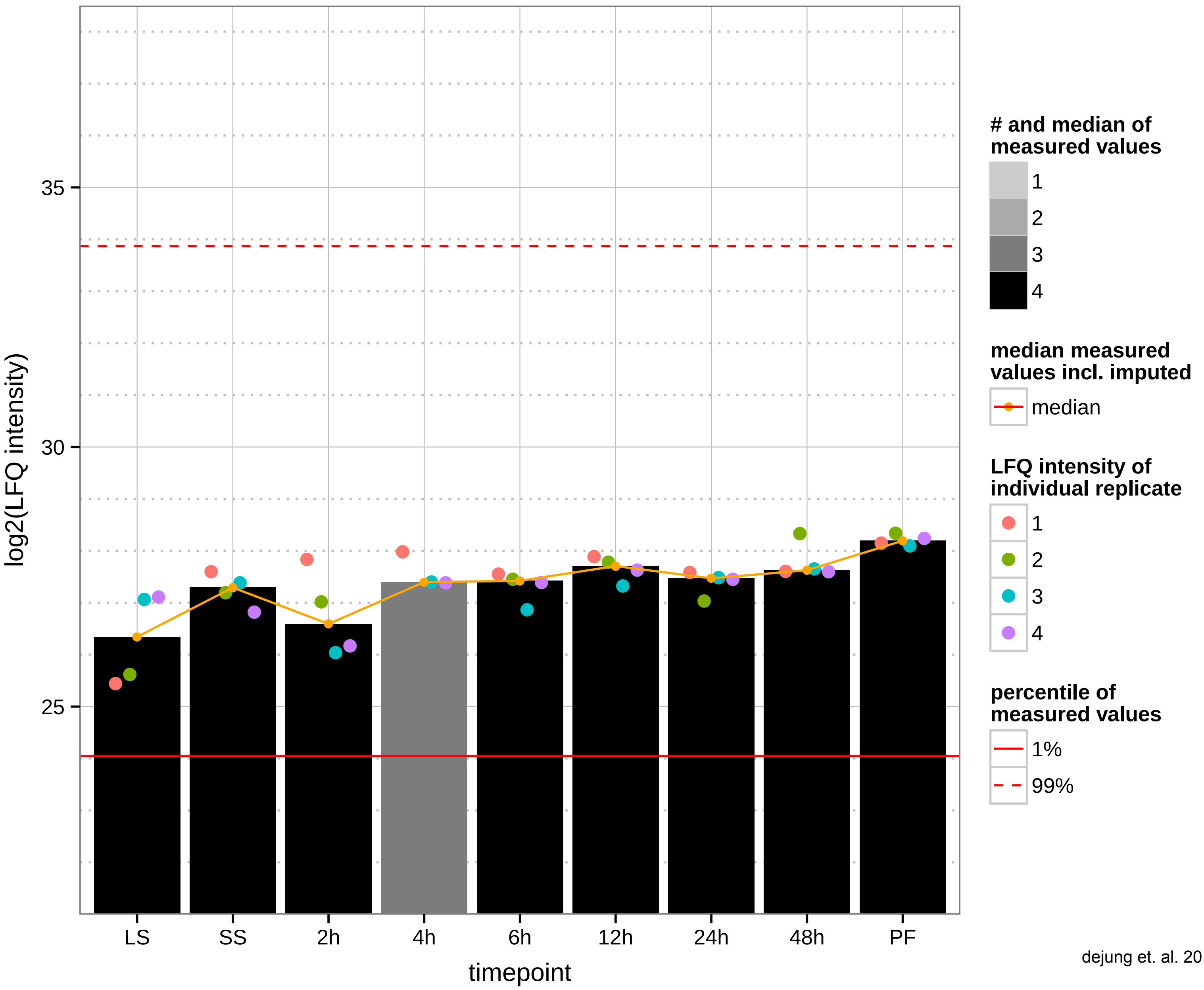

dejung et. al. 2015

hypothetical protein, conserved
Tb927.7.700
AGOF: null
AGOC: null
AGOP: null
PGOF: null
PGOC: null
PGOP: null

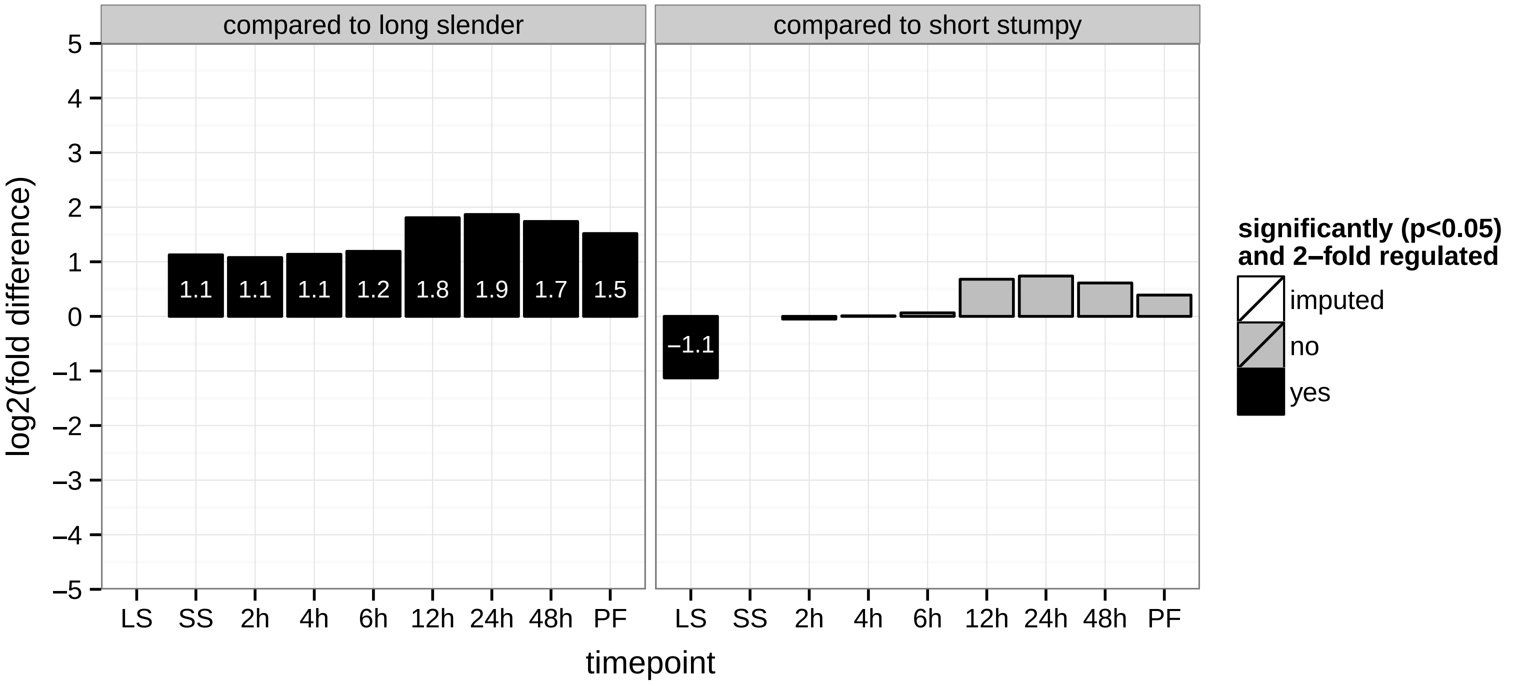

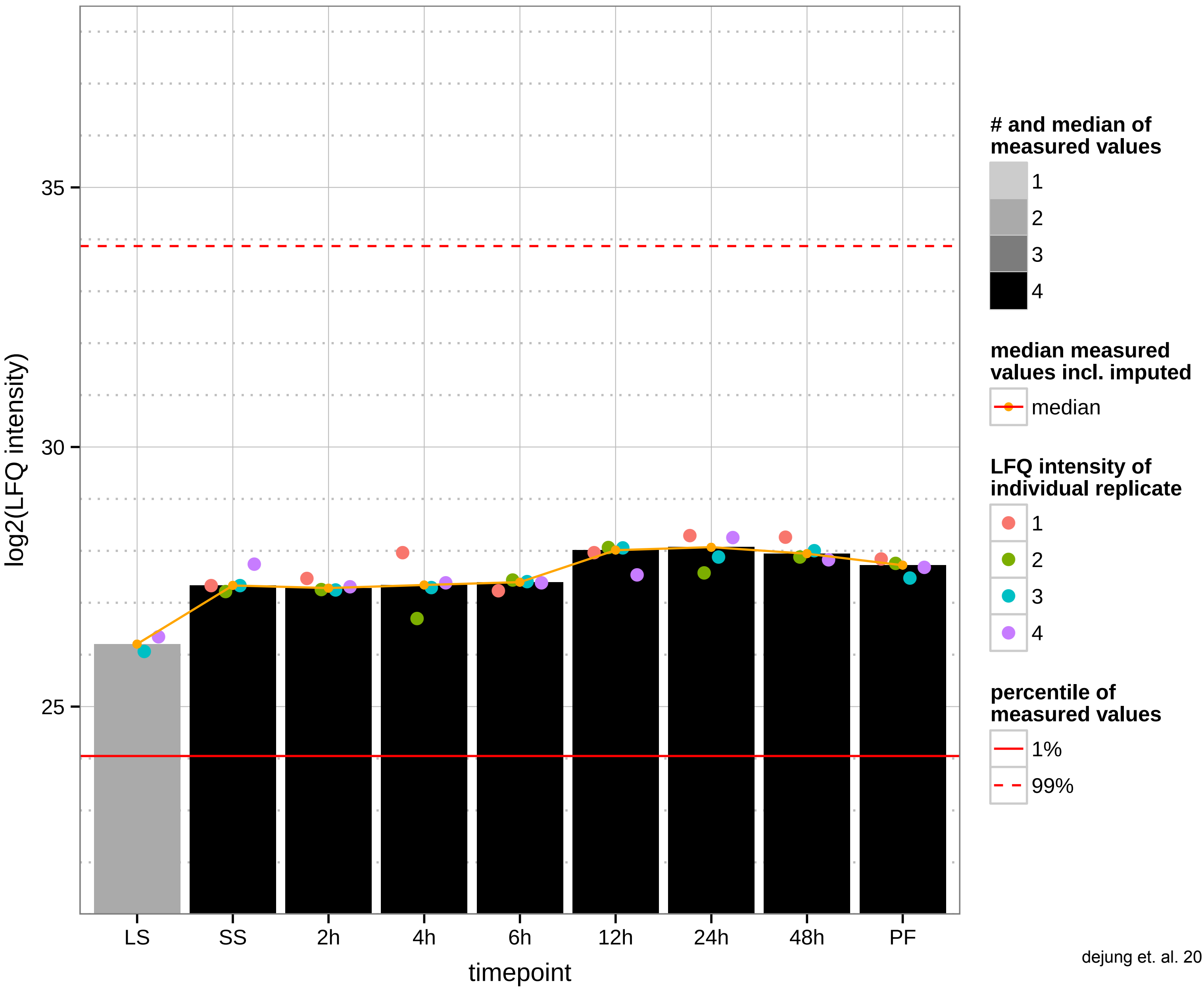

dejung et. al. 2015

methylthioadenosine phosphorylase, putative (MTAP)
Tb927.7.7040
AGOF: transferase activity, transferring pentosyl groups
AGOC: null
AGOP: nucleoside metabolic process
PGOF: catalytic activity, transferase activity, transferring pentosyl groups
PGOC: null
PGOP: nucleoside metabolic process

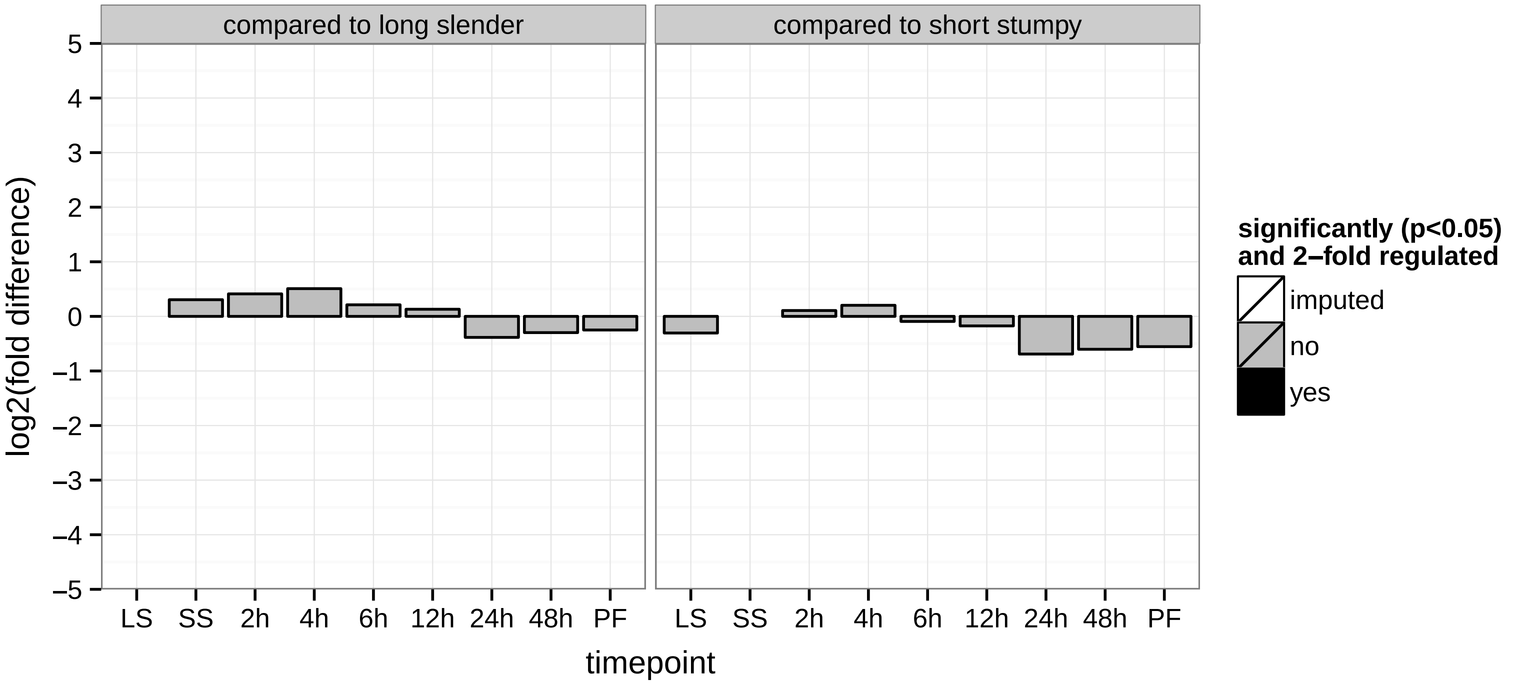

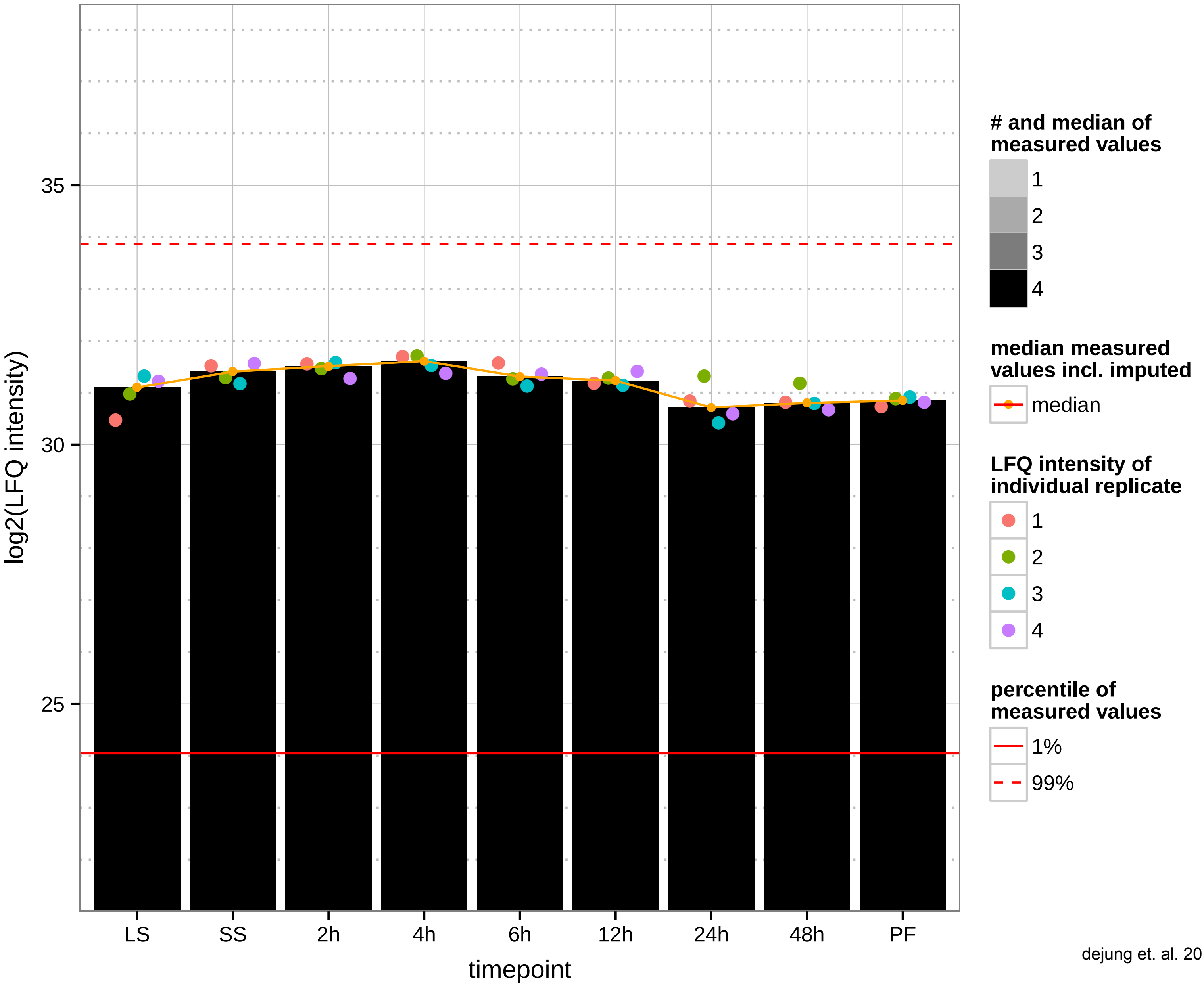

dejung et. al. 2015

mitochondrial glycoprotein–like protein
Tb927.7.7080
AGOF: null
AGOC: mitochondrial matrix, mitochondrion
AGOP: null
PGOF: null
PGOC: mitochondrial matrix
PGOP: null

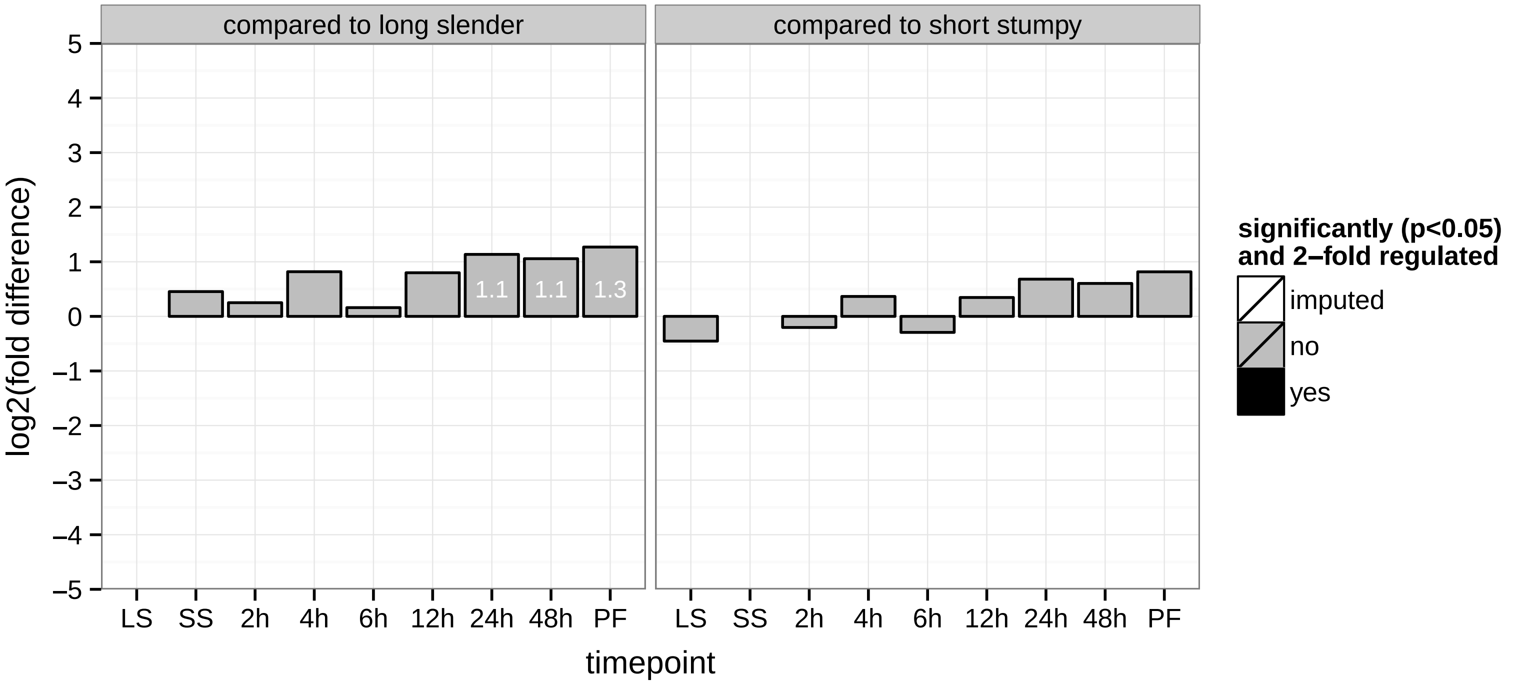

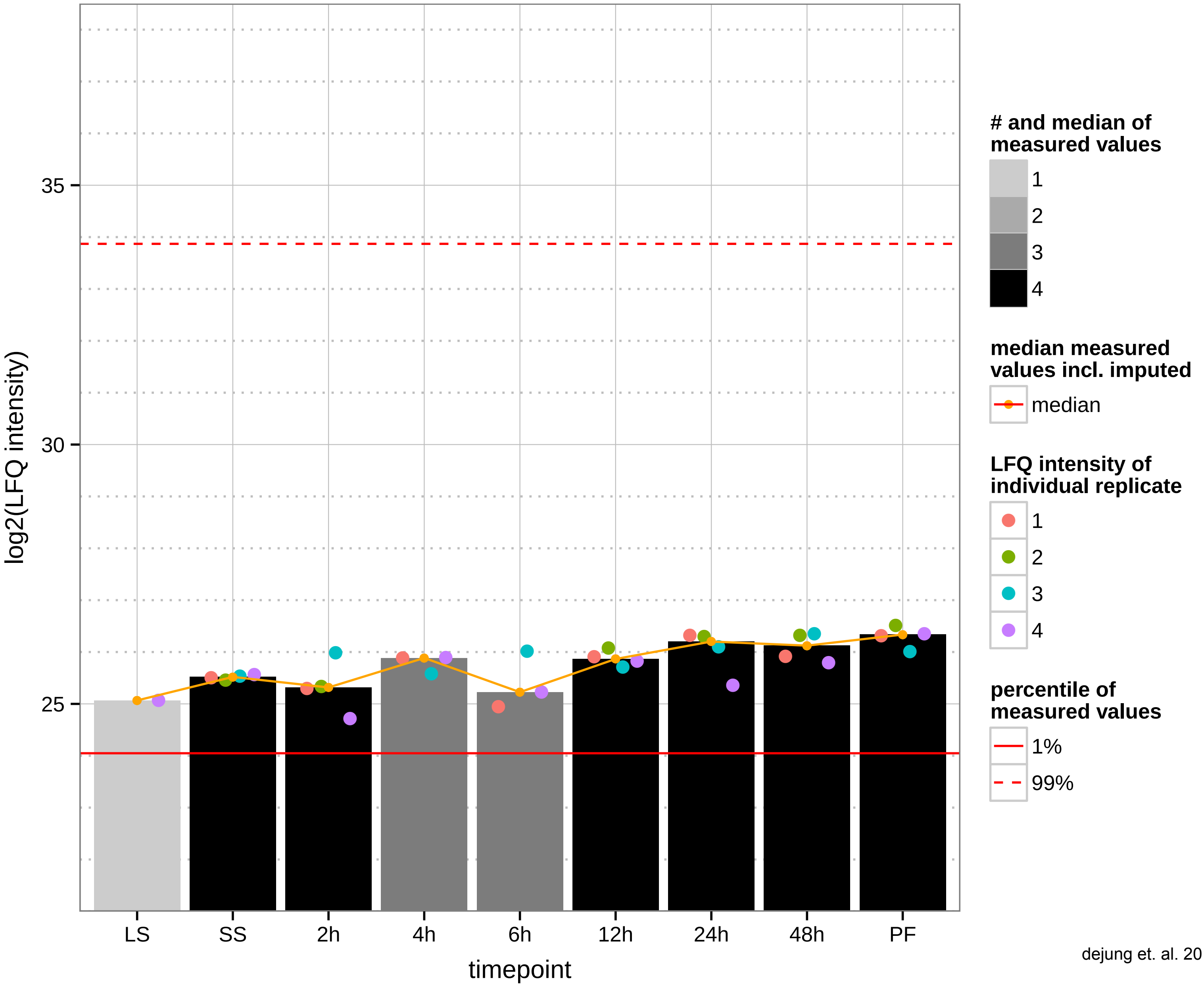

dejung et. al. 2015

heat shock 70 kDa protein, putative (HSP70)
Tb927.7.710
AGOF: ATP binding
AGOC: null
AGOP: response to stress
PGOF: null
PGOC: null
PGOP: null

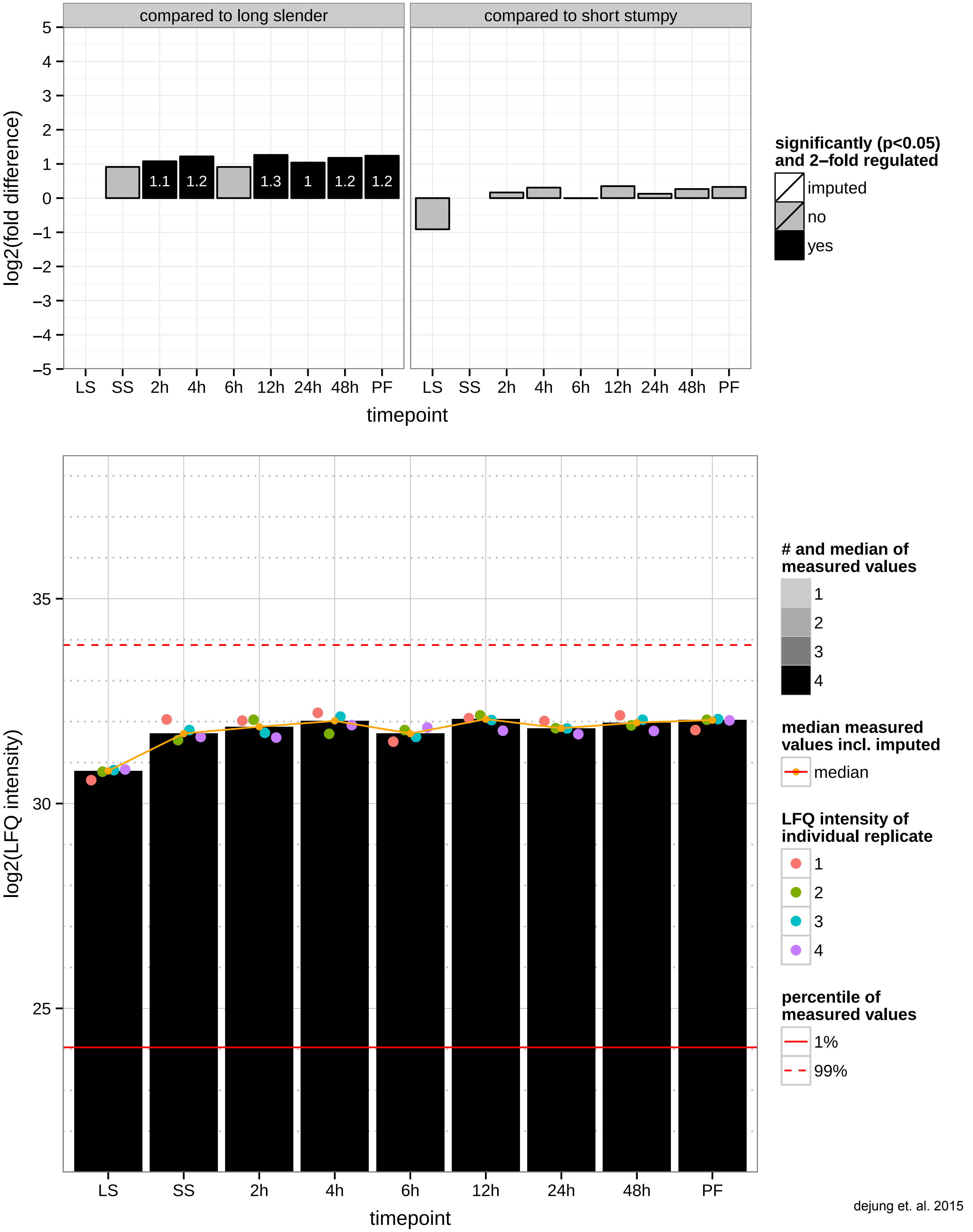

dejung et. al. 2015

hypothetical protein, conserved
Tb927.7.7140
AGOF: null
AGOC: null
AGOP: null
PGOF: null
PGOC: null
PGOP: null

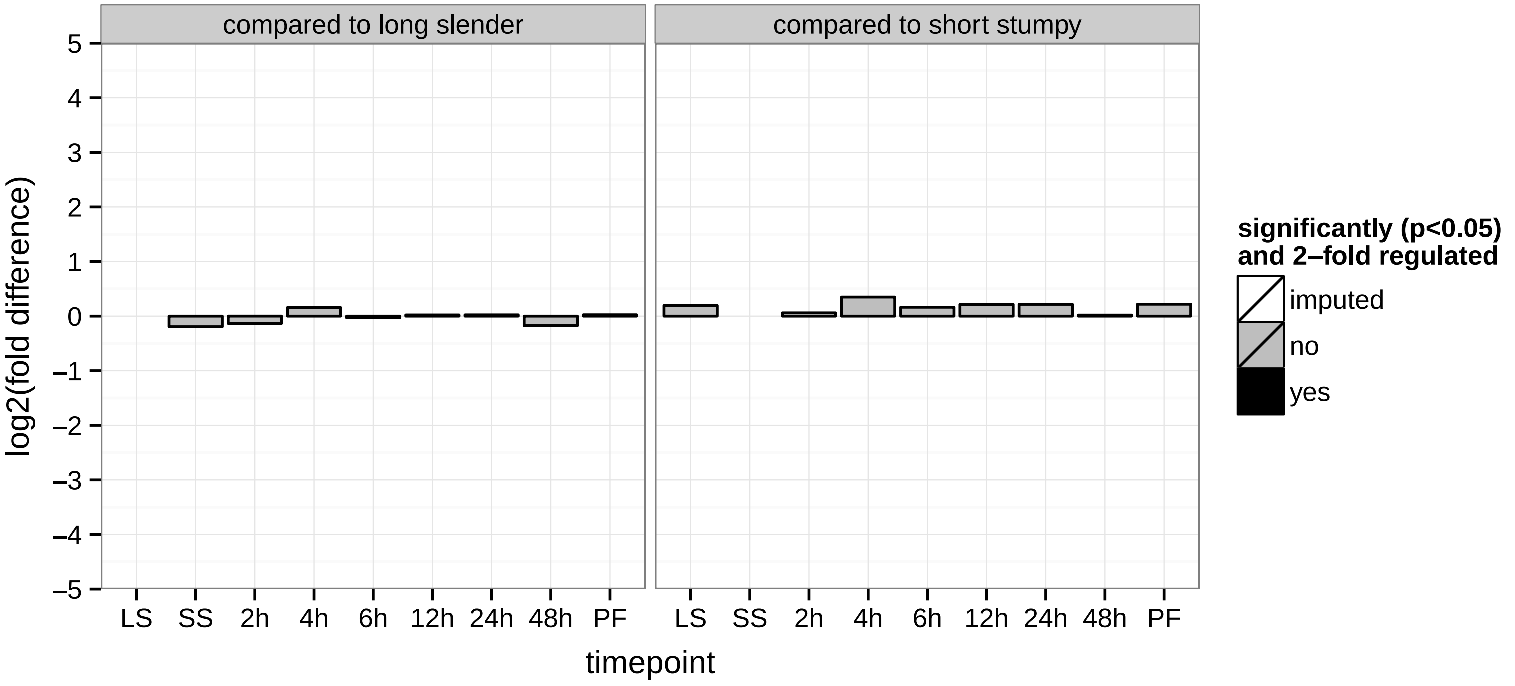

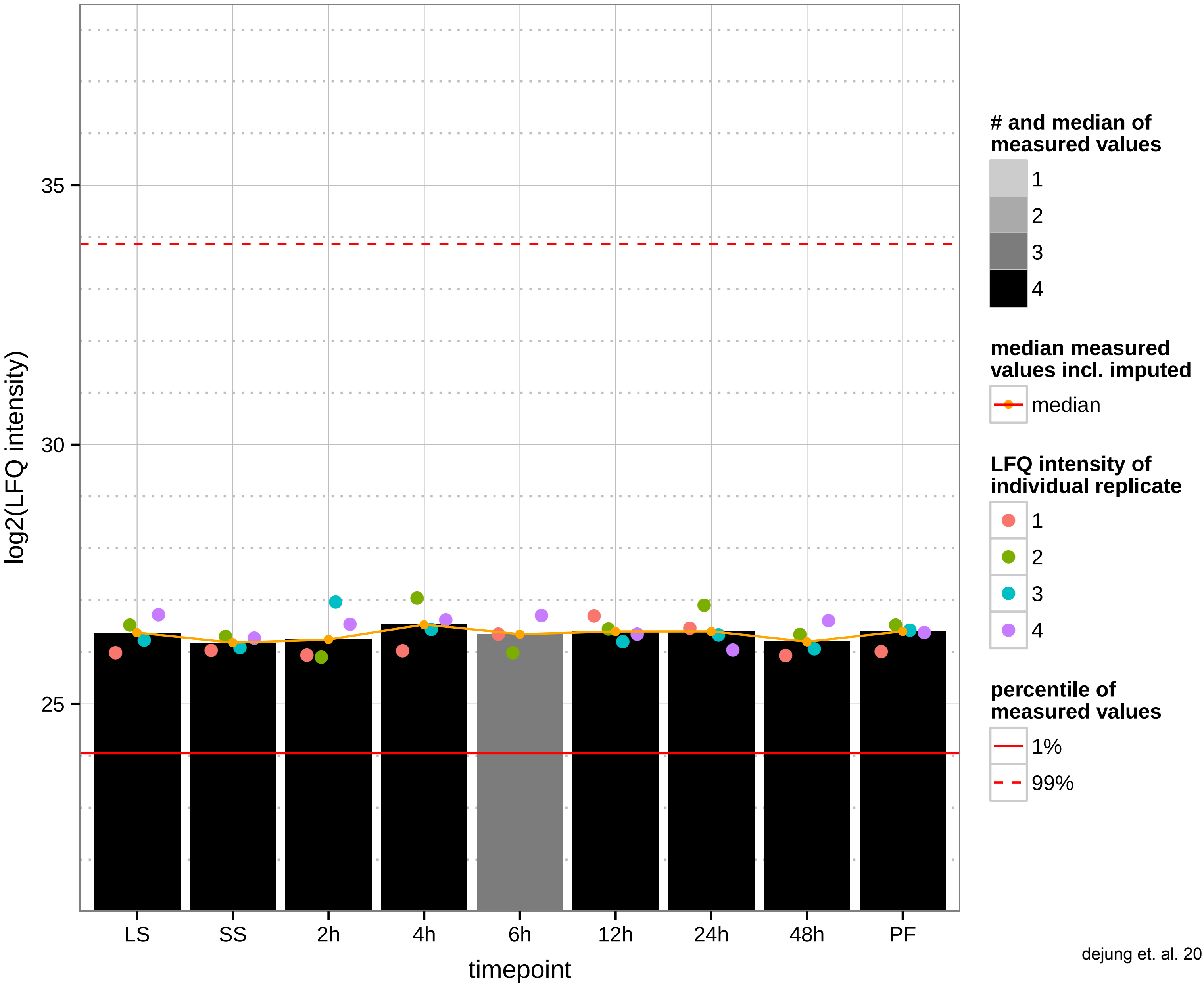

dejung et. al. 2015

hypothetical protein, conserved
Tb927.7.7190
AGOF: null
AGOC: null
AGOP: null
PGOF: null
PGOC: null
PGOP: null

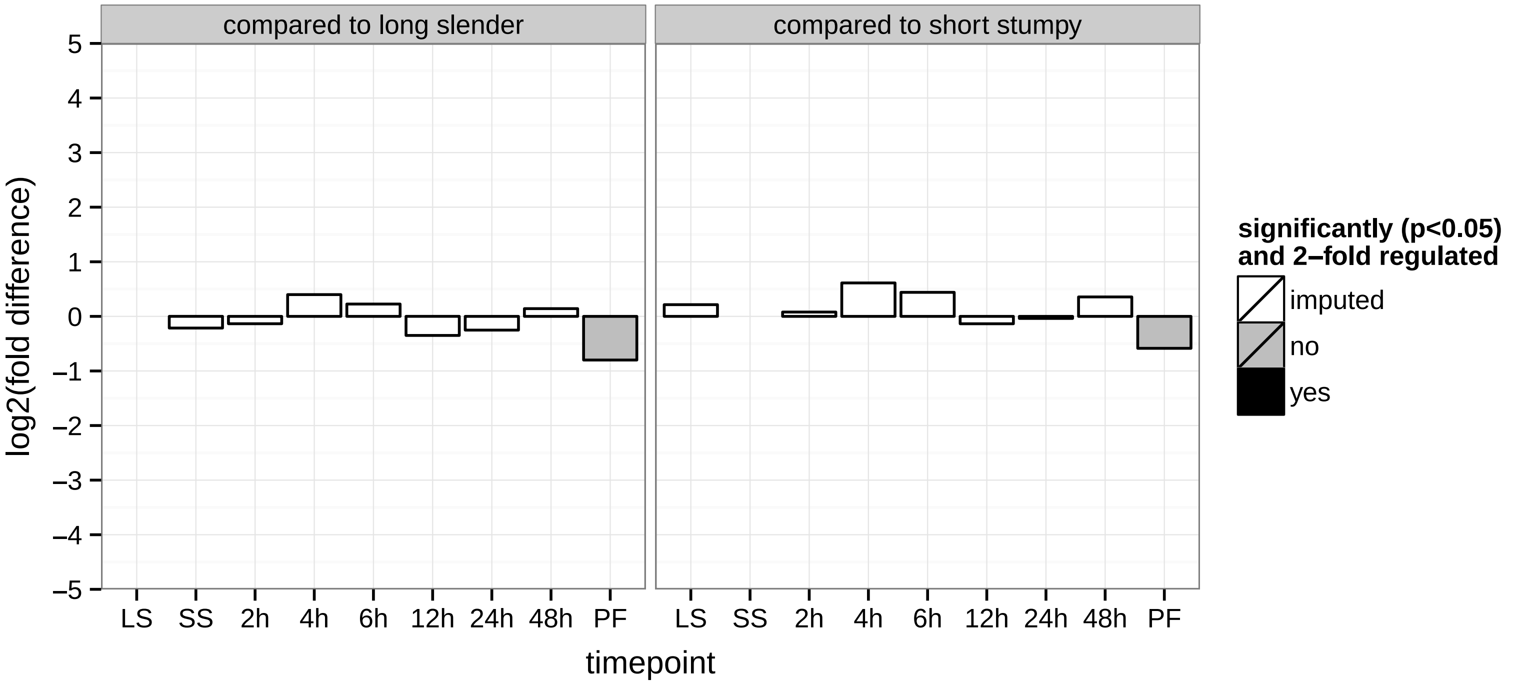

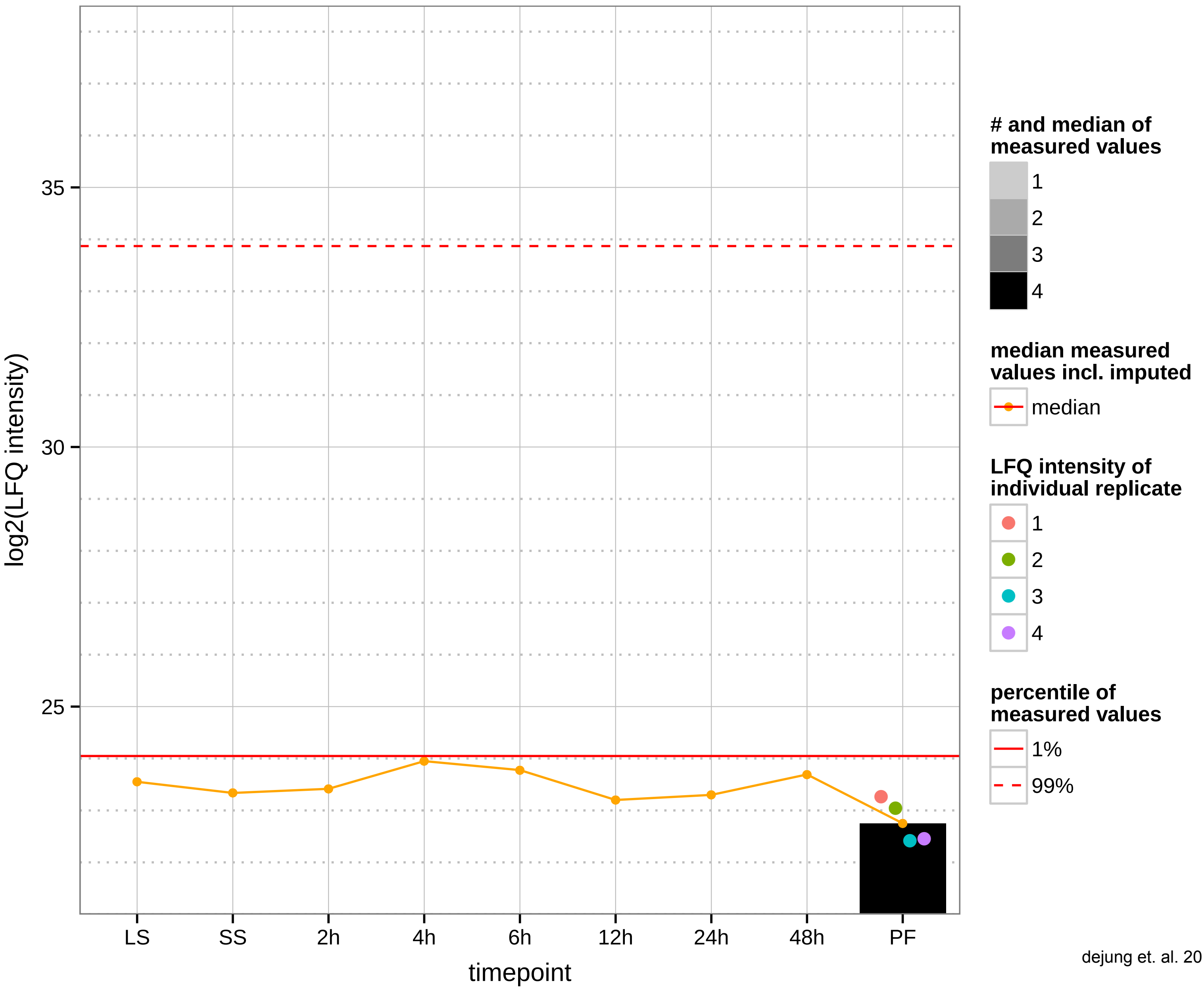

dejung et. al. 2015

leucine−rich repeat protein (LRRP), putative
Tb927.7.7240
AGOF: null
AGOC: null
AGOP: null
PGOF: null
PGOC: null
PGOP: null

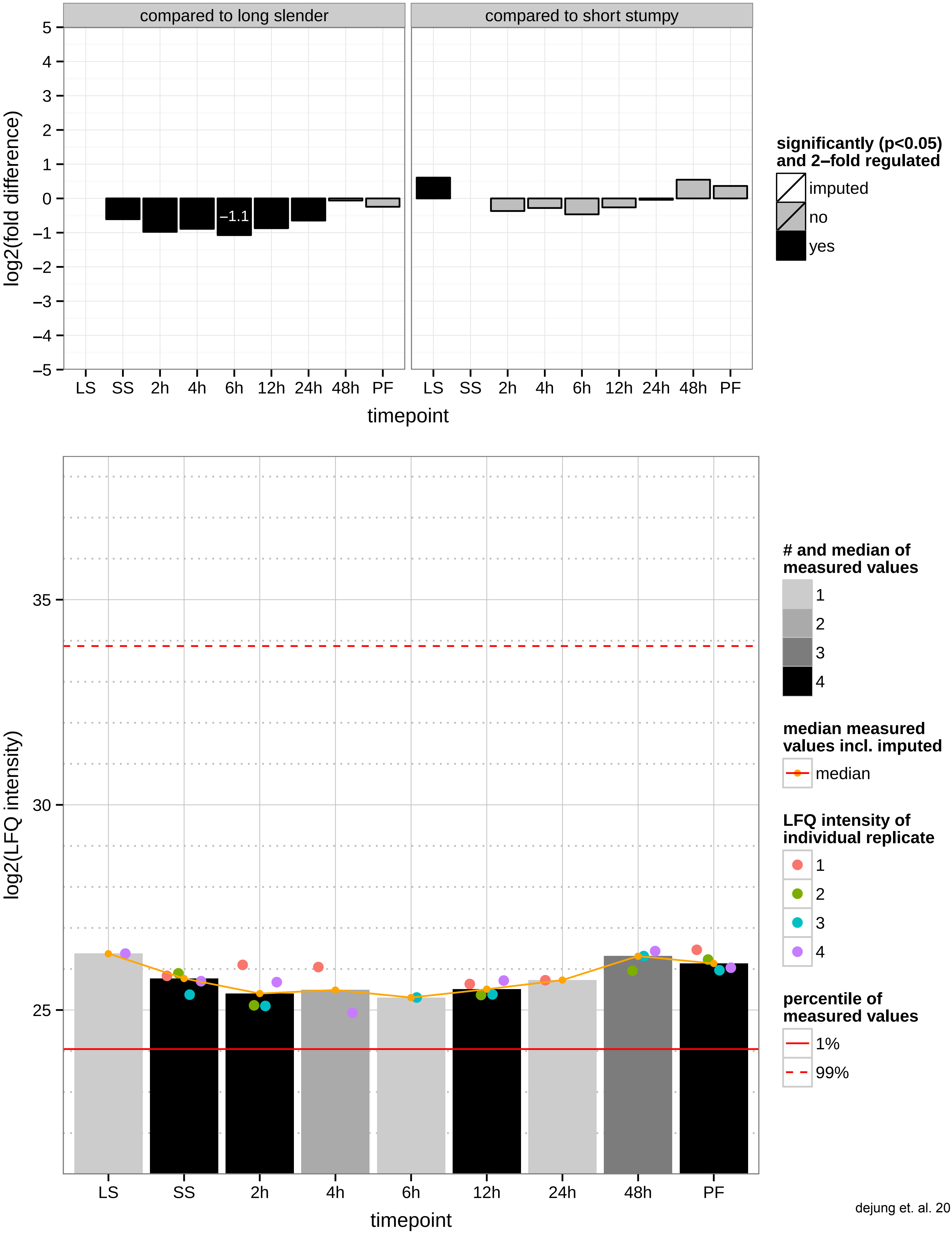

dejung et. al. 2015

hypothetical protein, conserved
Tb927.7.7250
AGOF: null
AGOC: null
AGOP: null
PGOF: protein binding
PGOC: null
PGOP: null

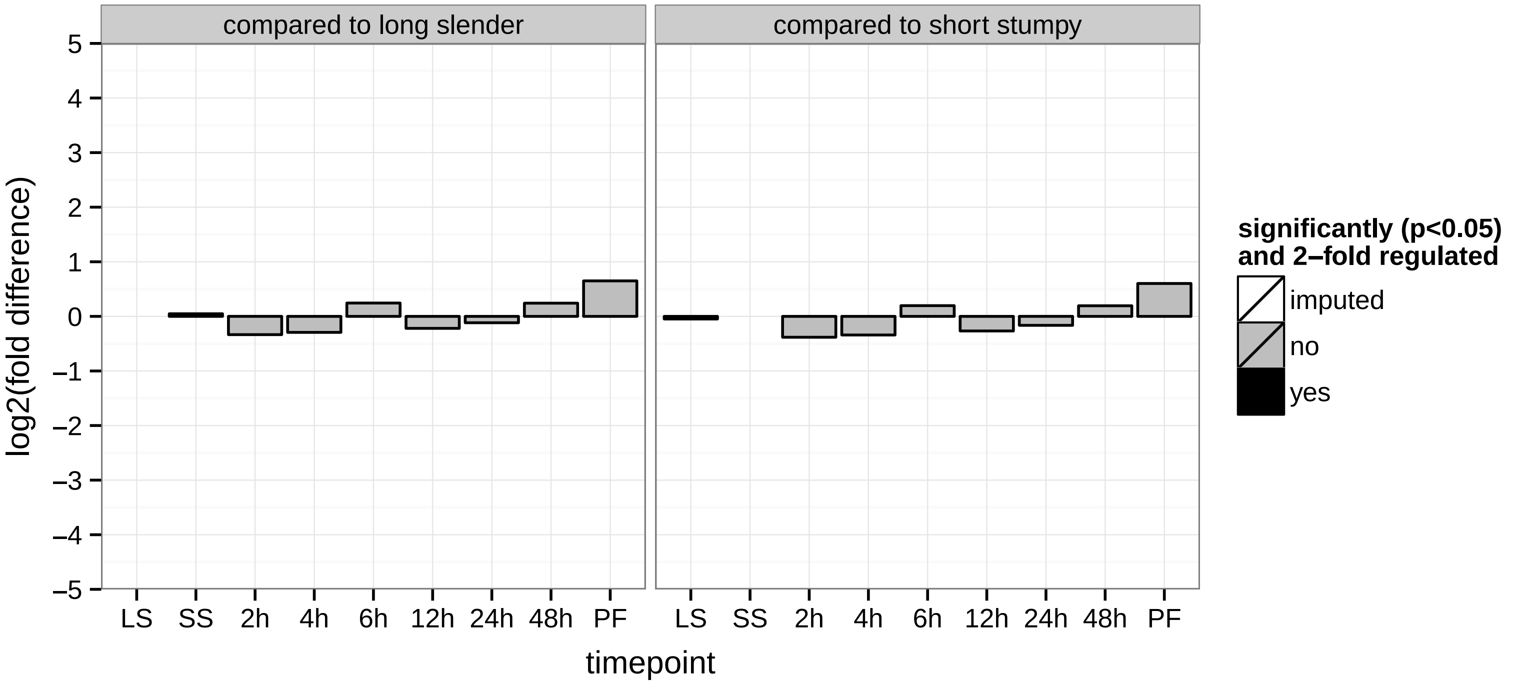

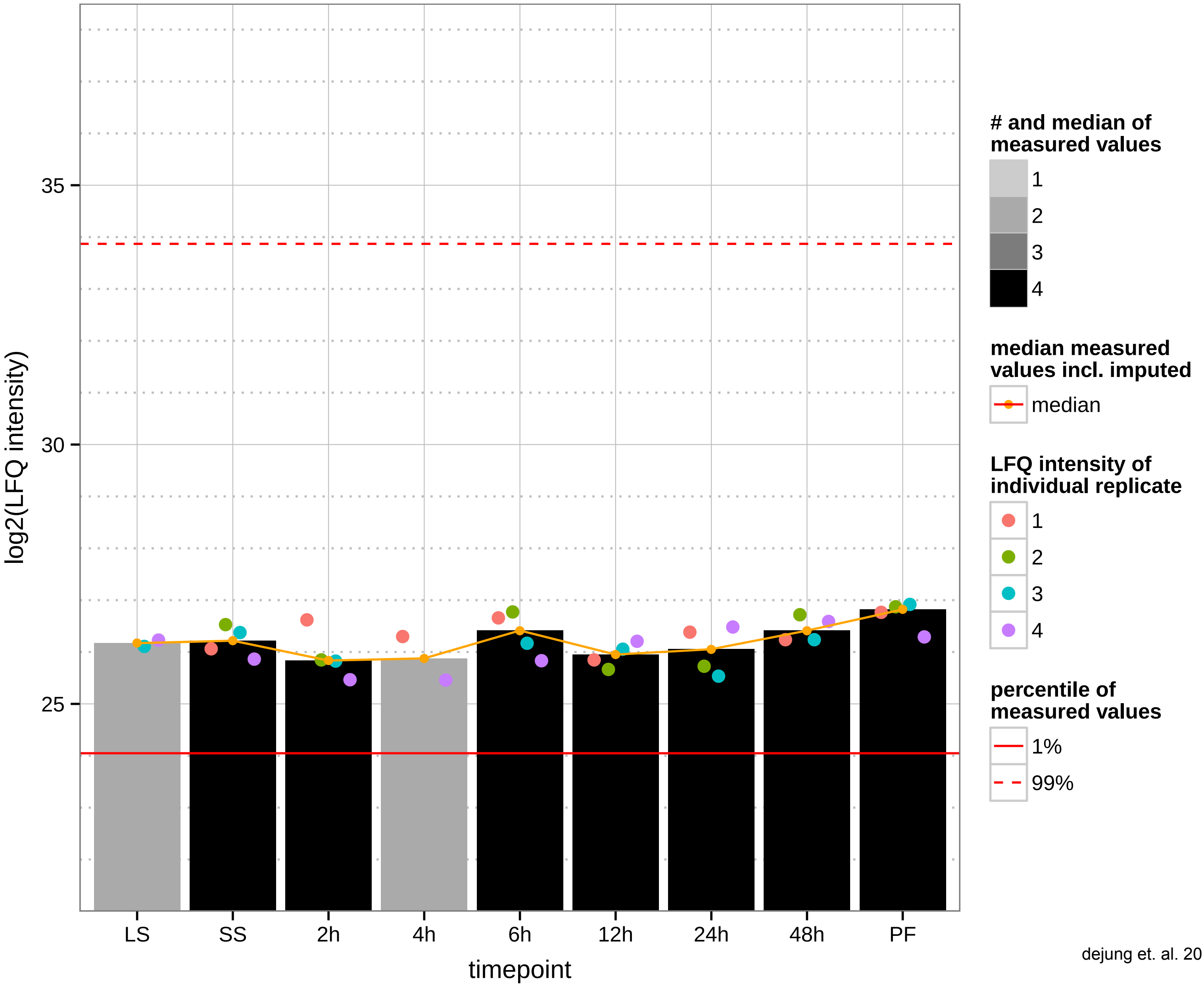

dejung et. al. 2015

hypothetical protein, conserved
Tb927.7.7290
AGOF: null
AGOC: null
AGOP: null
PGOF: null
PGOC: null
PGOP: null

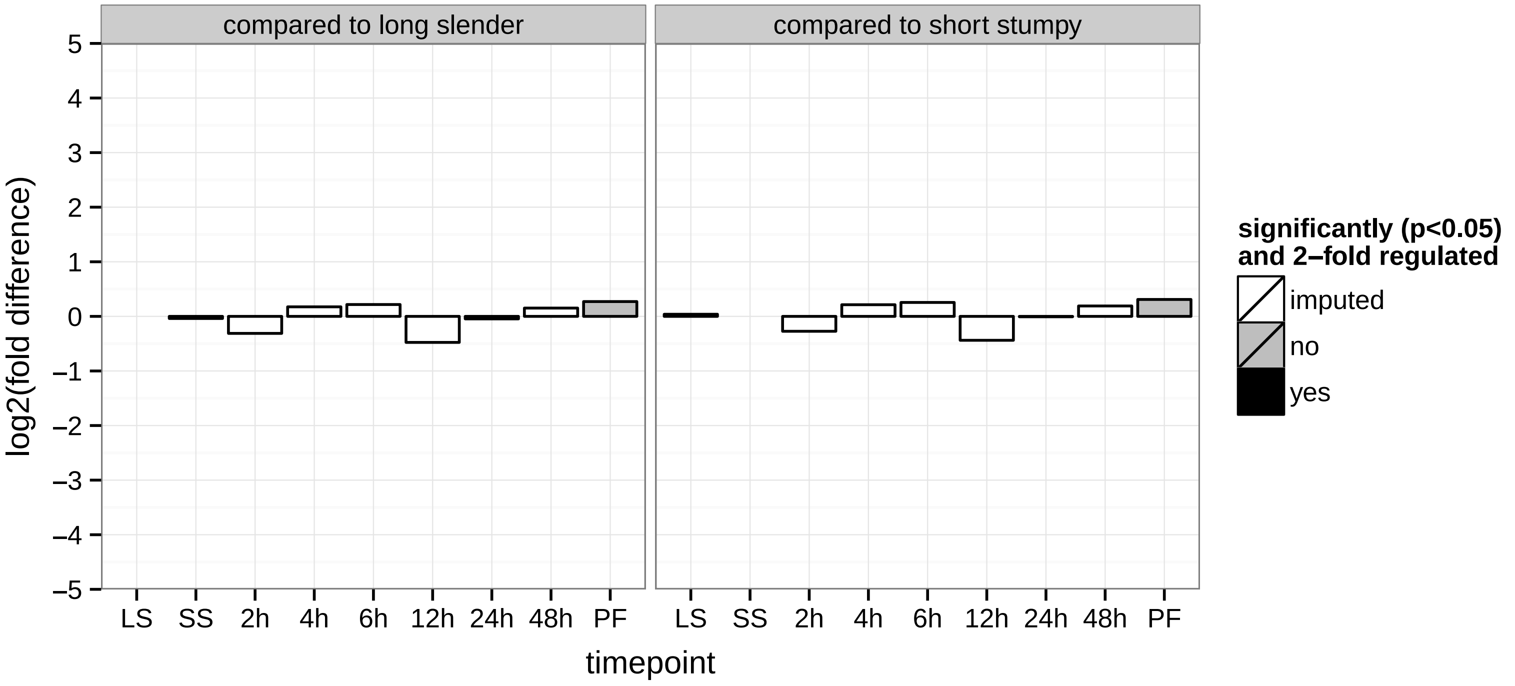

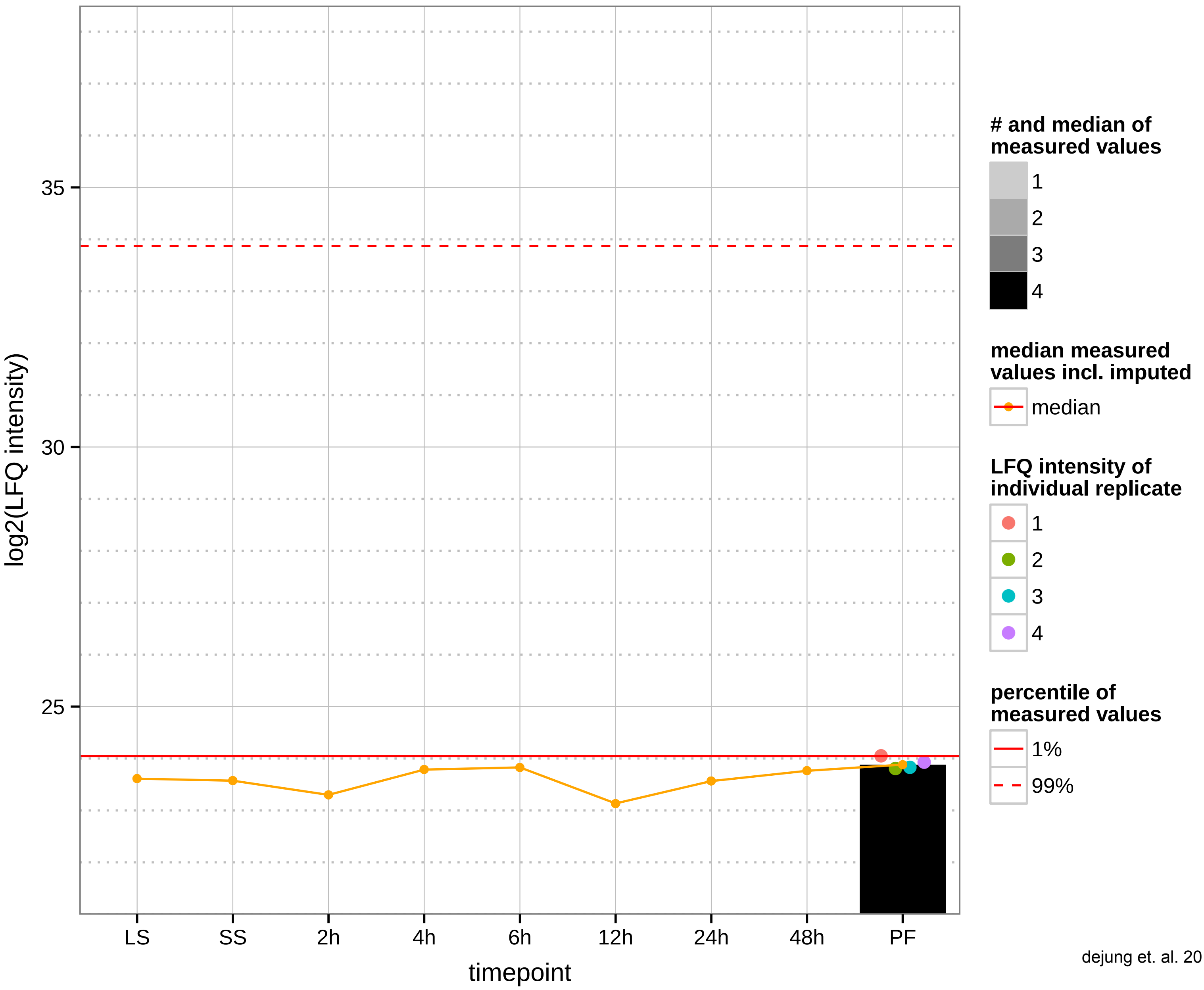

dejung et. al. 2015

ATP−dependent DEAD/H RNA helicase, putative
Tb927.7.7300
AGOF: ATP binding, ATP−dependent helicase activity, helicase activity, nucleic acid binding
AGOC: null
AGOP: nucleobase−containing compound metabolic process
PGOF: ATP binding, ATP−dependent helicase activity, helicase activity, nucleic acid binding
PGOC: null
PGOP: null

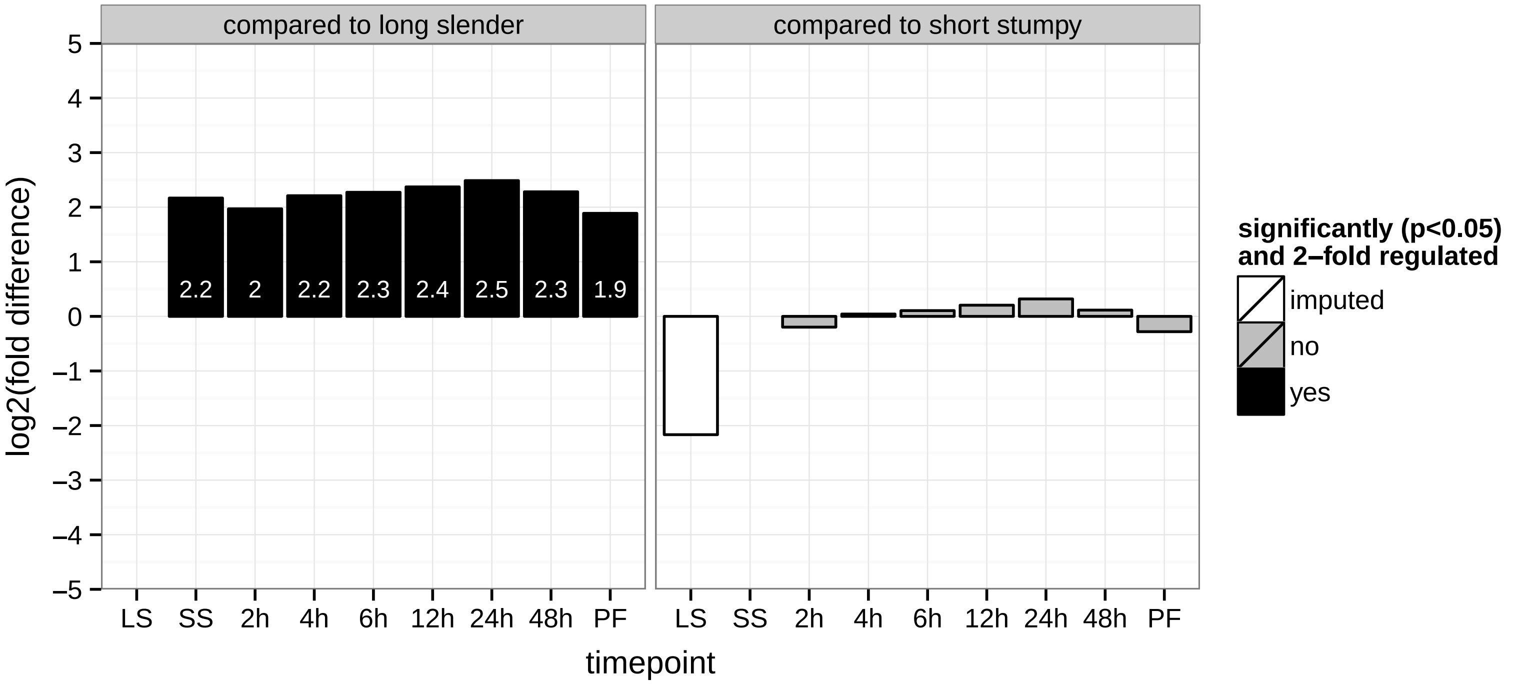

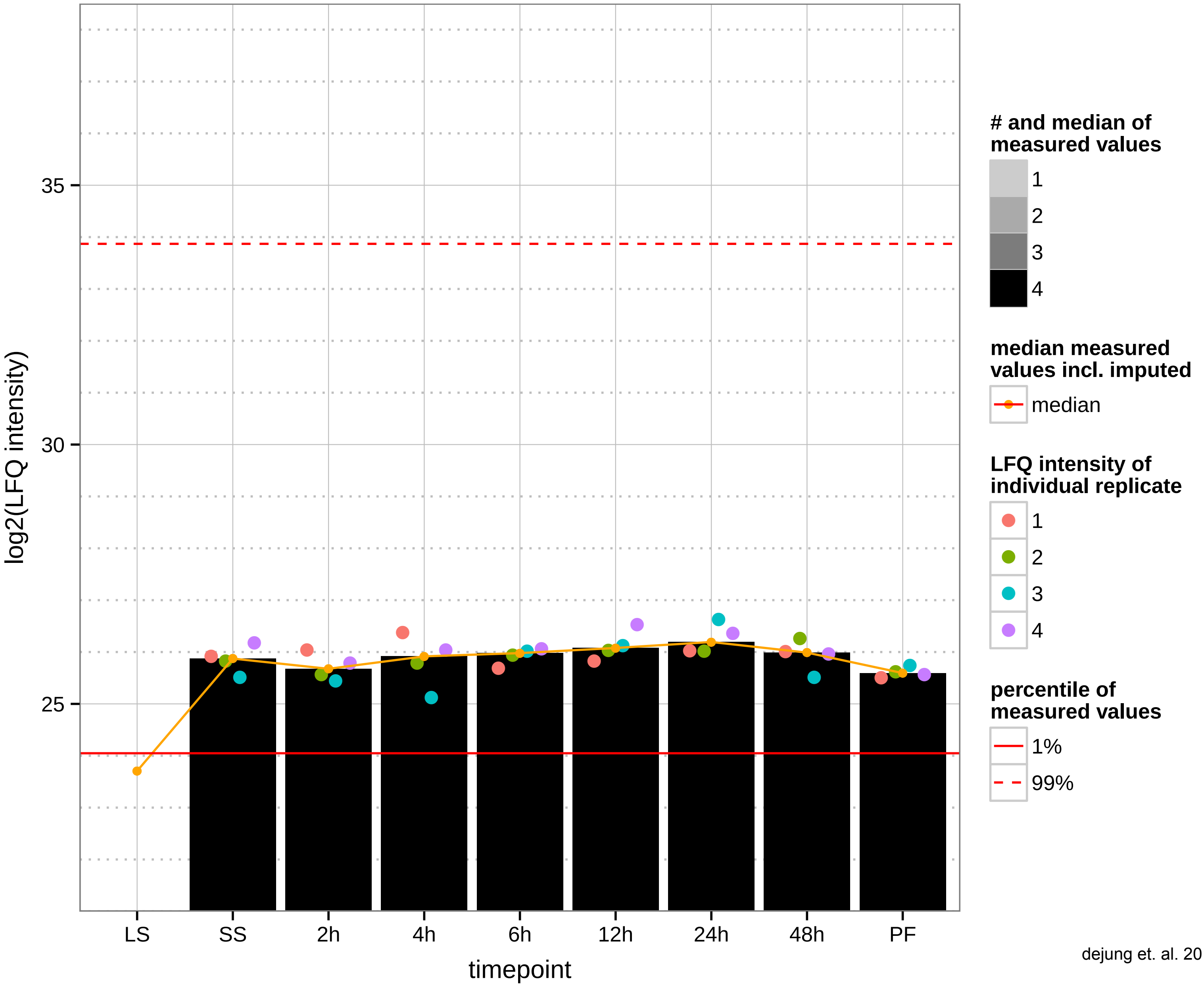

dejung et. al. 2015

hypothetical protein, conserved
Tb927.7.7320
AGOF: null
AGOC: null
AGOP: null
PGOF: null
PGOC: null
PGOP: null

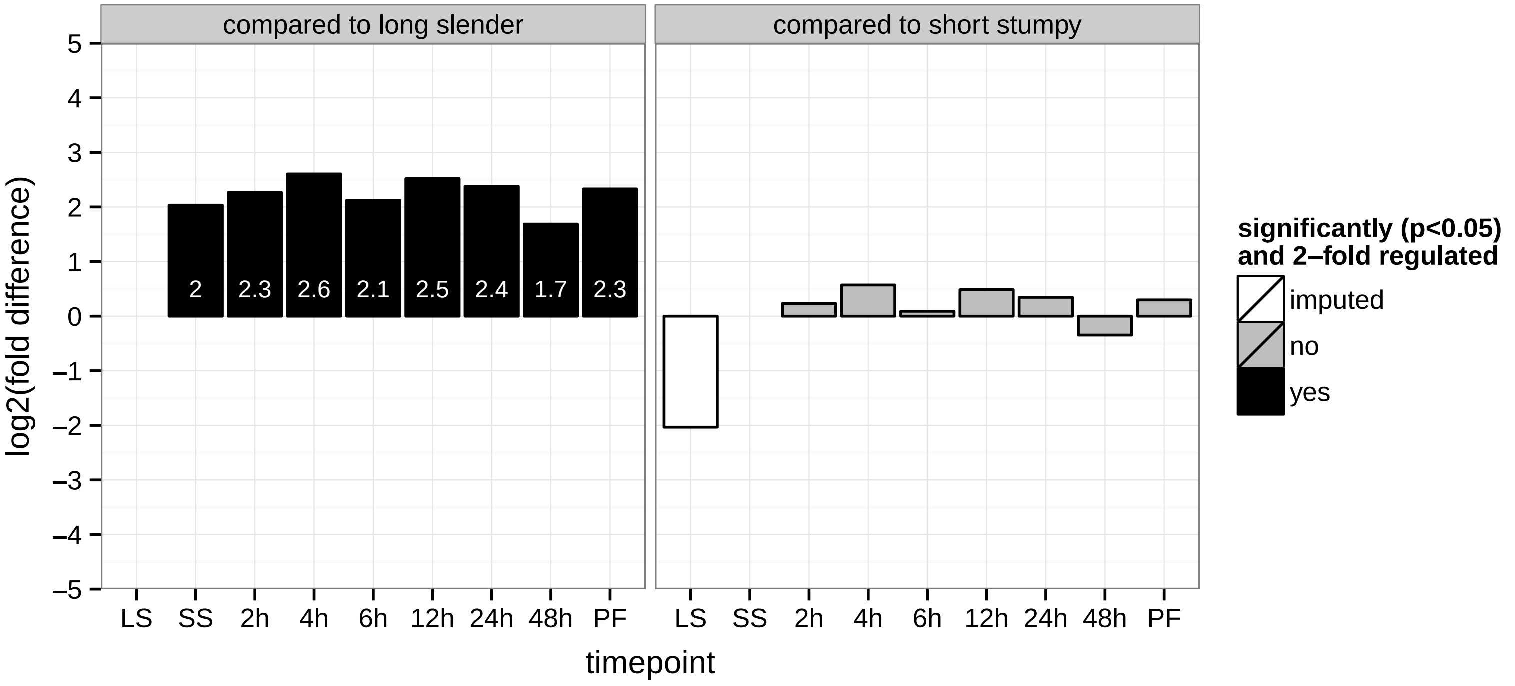

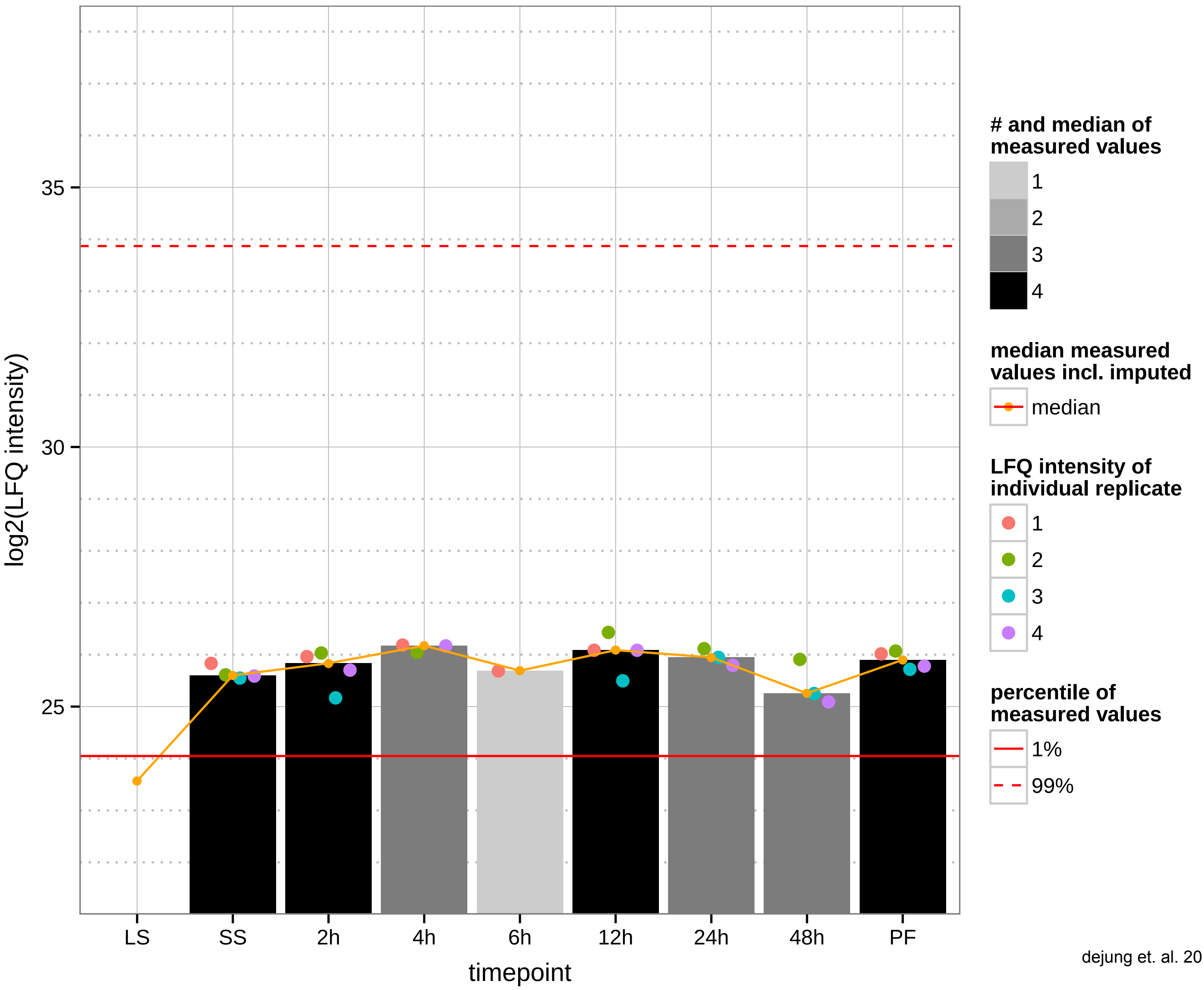

dejung et. al. 2015

hypothetical protein, conserved
Tb927.7.7330
AGOF: null
AGOC: mitochondrion
AGOP: null
PGOF: null
PGOC: null
PGOP: null

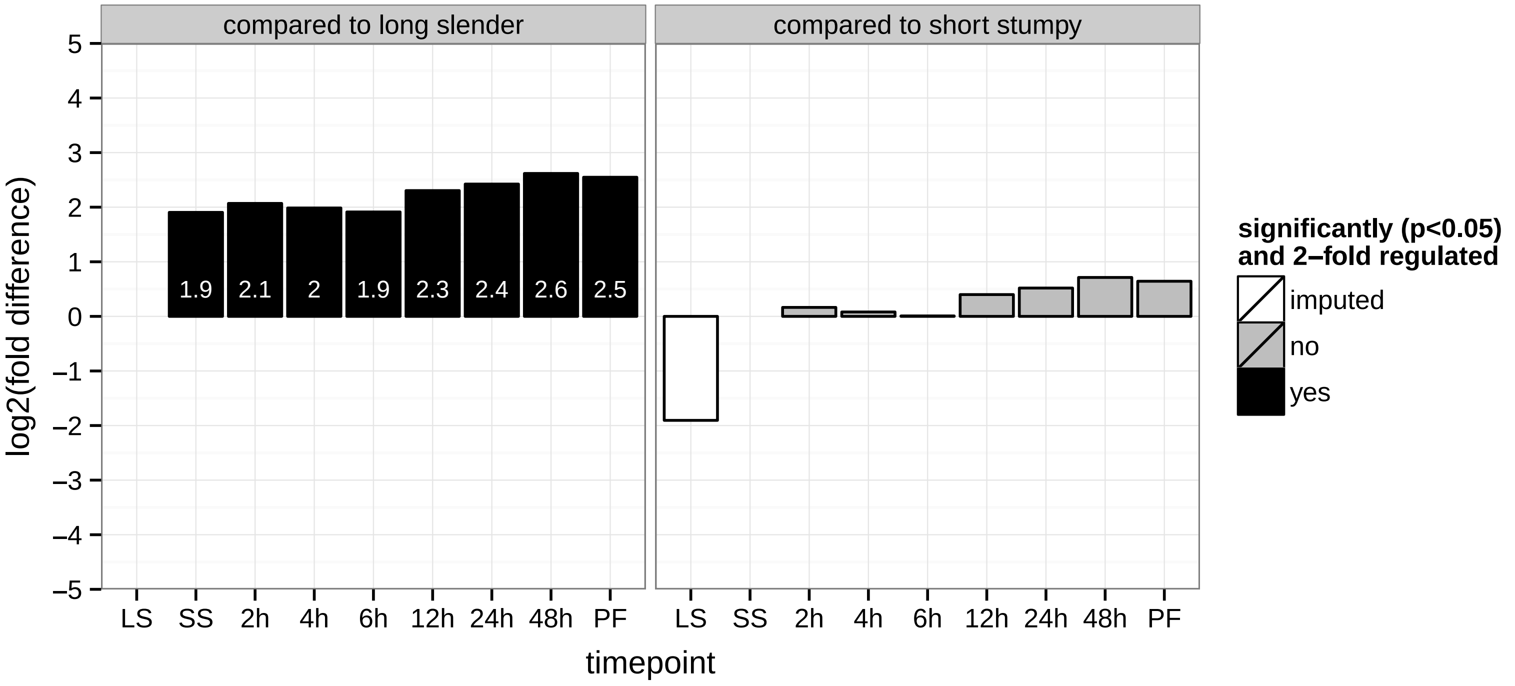

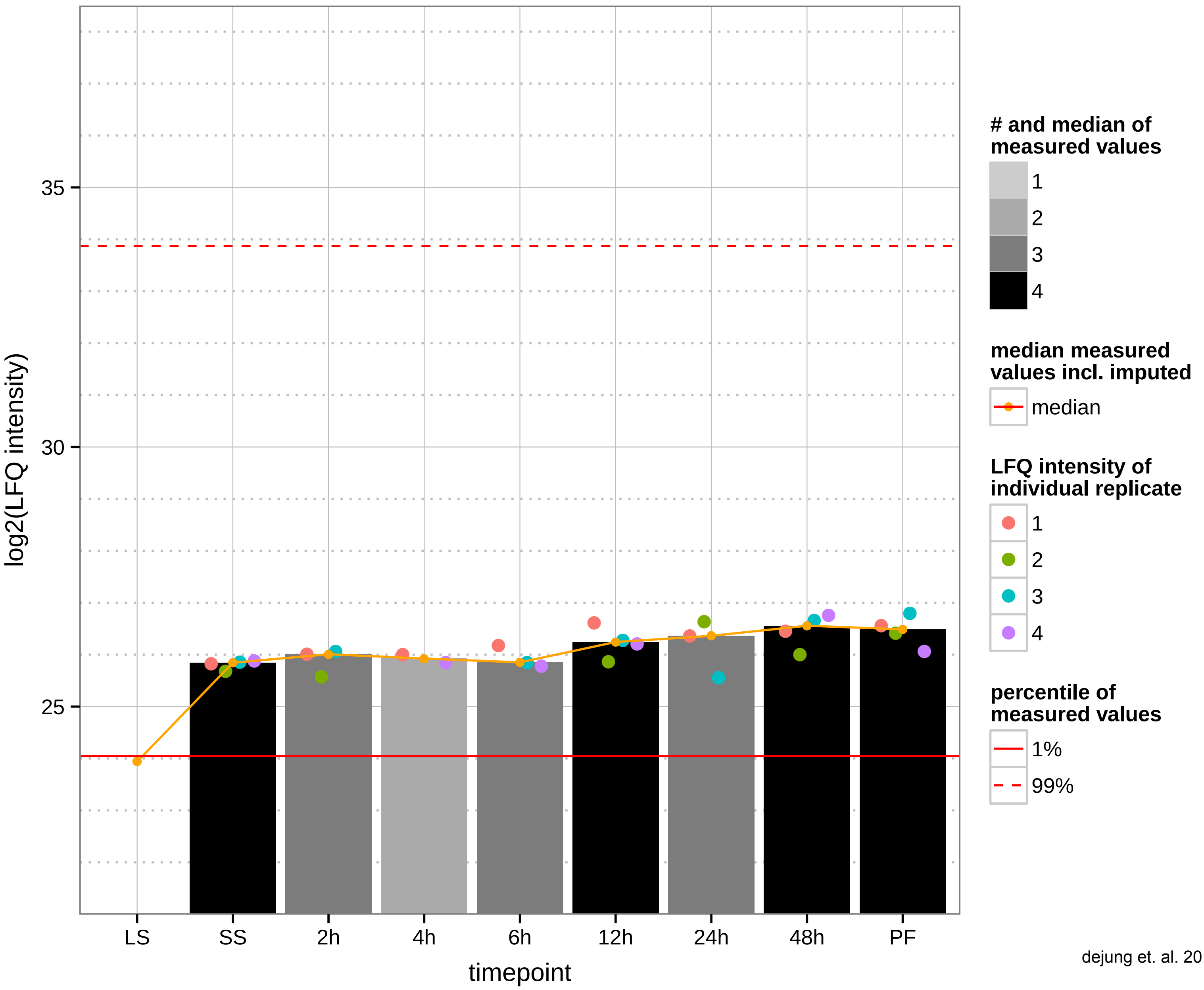

dejung et. al. 2015

chaperone protein DNAj, putative
Tb927.7.740
AGOF: heat shock protein binding, unfolded protein binding
AGOC: integral to membrane, mitochondrion
AGOP: protein folding
PGOF: heat shock protein binding
PGOC: null
PGOP: null

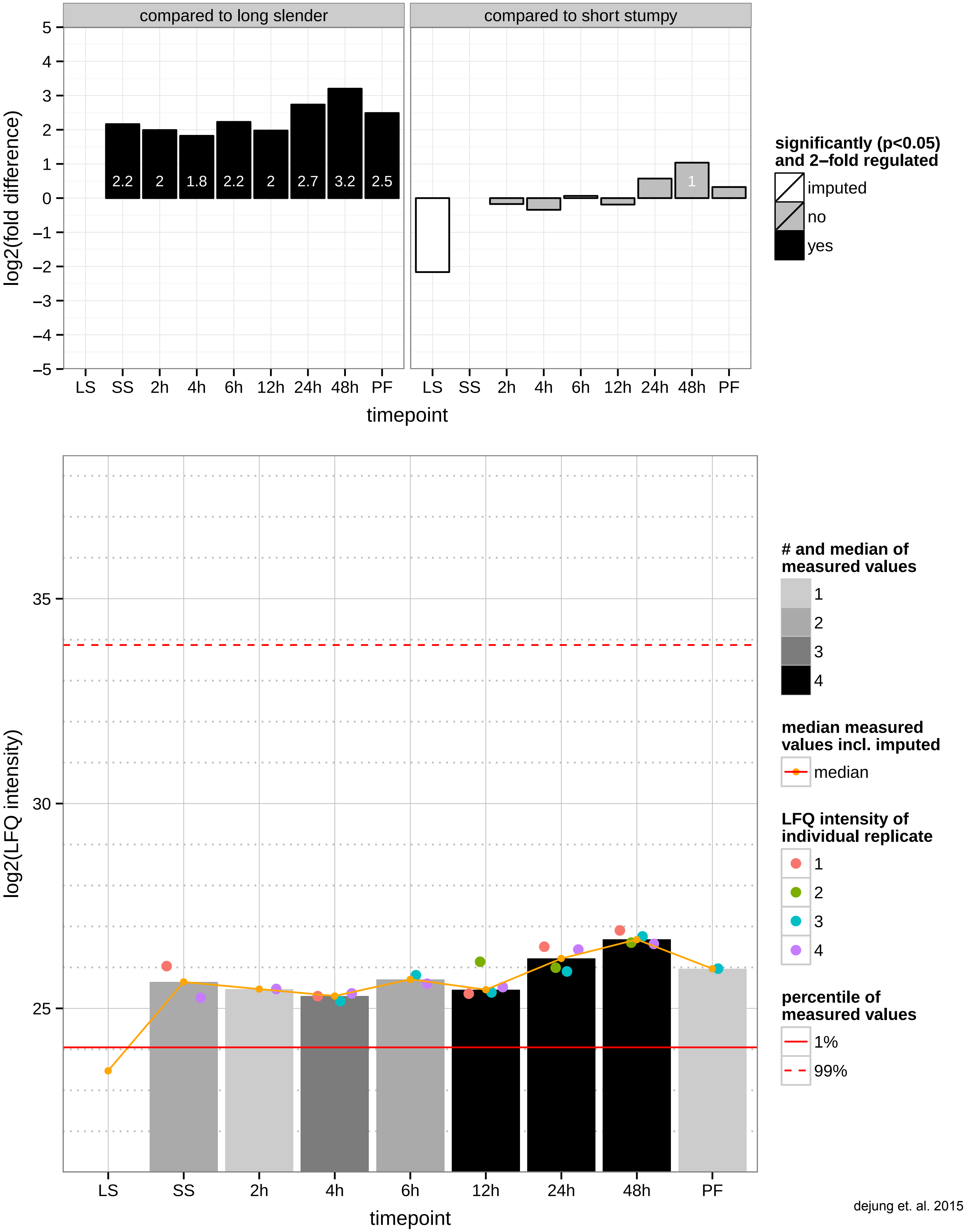

dejung et. al. 2015

oxidoreductase, putative
Tb927.7.7410
AGOF: oxidoreductase activity, zinc ion binding
AGOC: mitochondrion
AGOP: metabolic process, oxidation–reduction process
PGOF: null
PGOC: null
PGOP: null

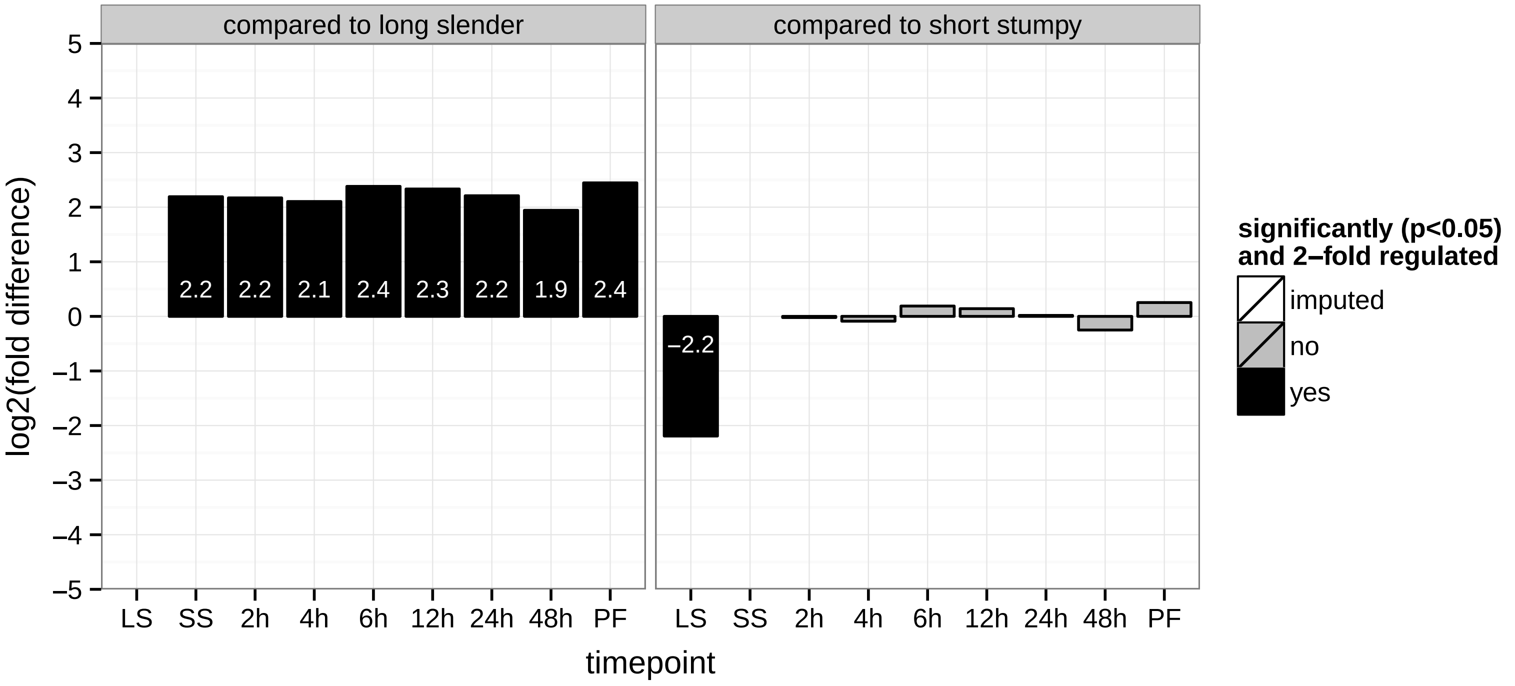

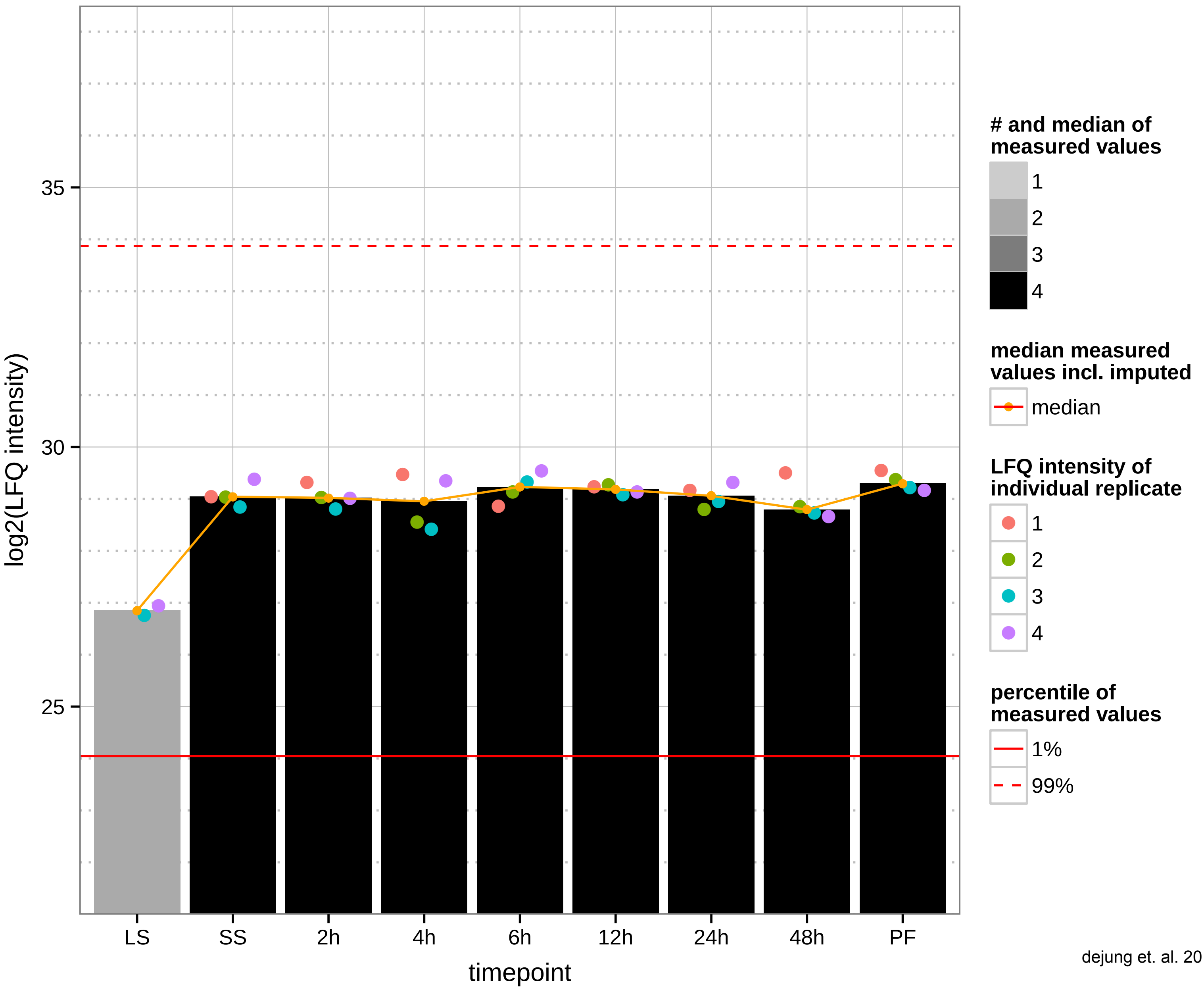

dejung et. al. 2015

GTP–binding protein, putative
Tb927.7.7450
AGOF: GTP binding
AGOC: intracellular
AGOP: null
PGOF: GTP binding
PGOC: null
PGOP: null

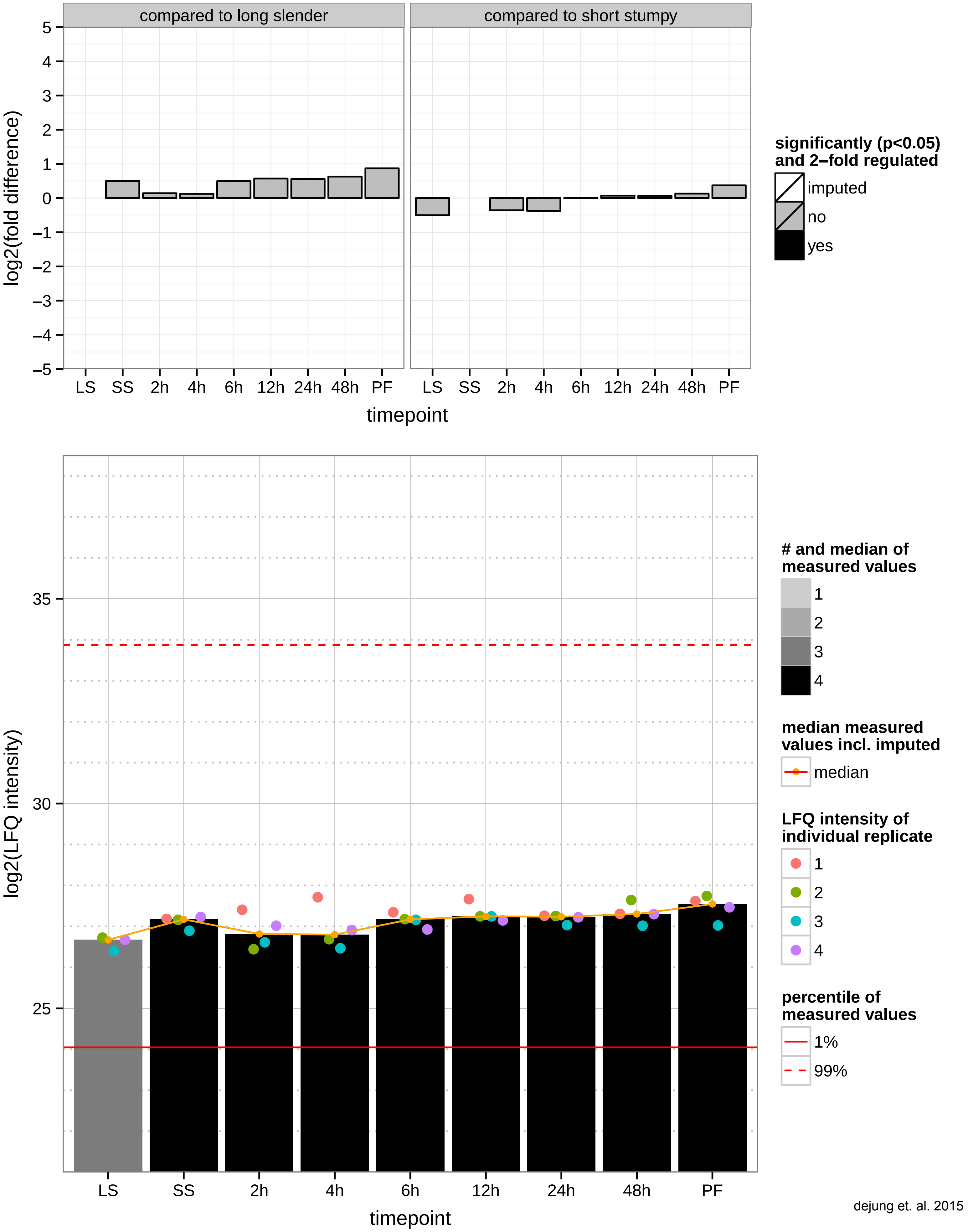

dejung et. al. 2015

hypothetical protein, conserved
Tb927.7.760
AGOF: null
AGOC: null
AGOP: null
PGOF: null
PGOC: null
PGOP: null

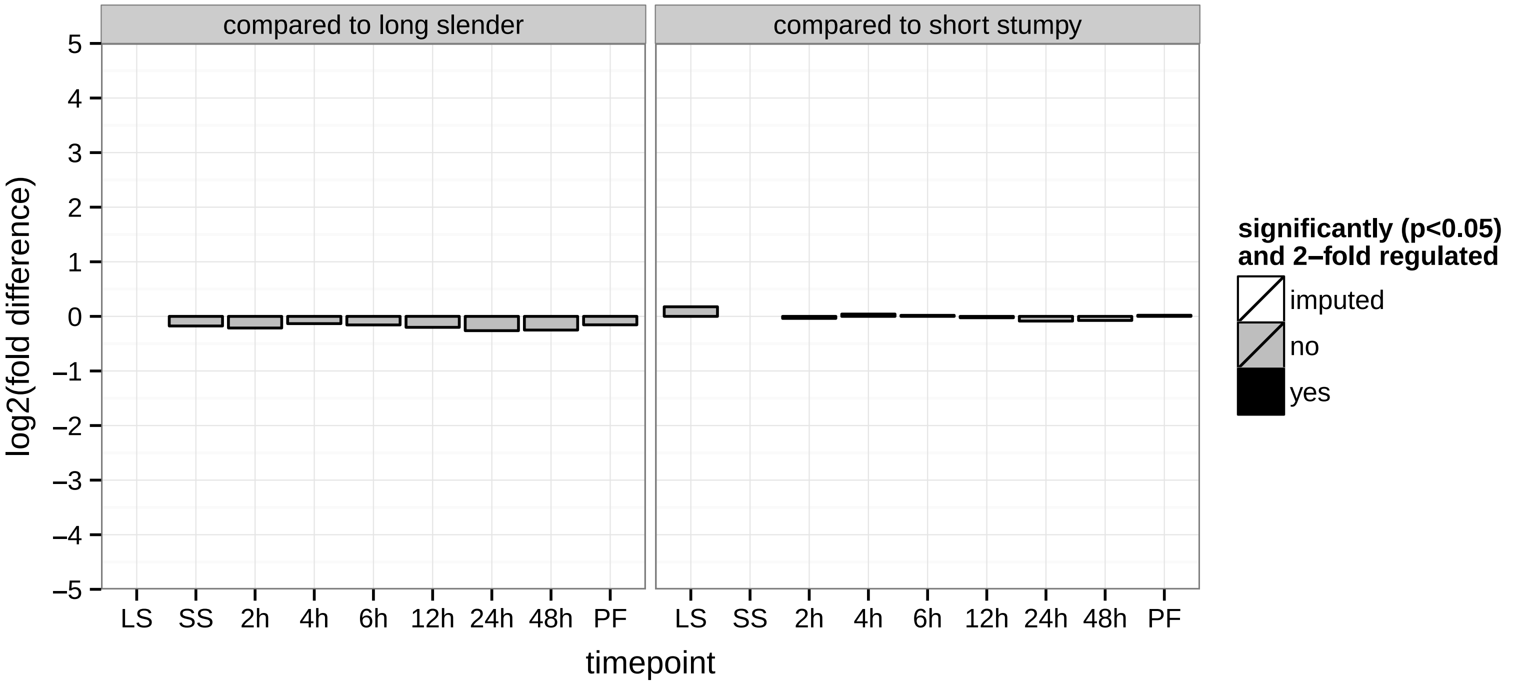

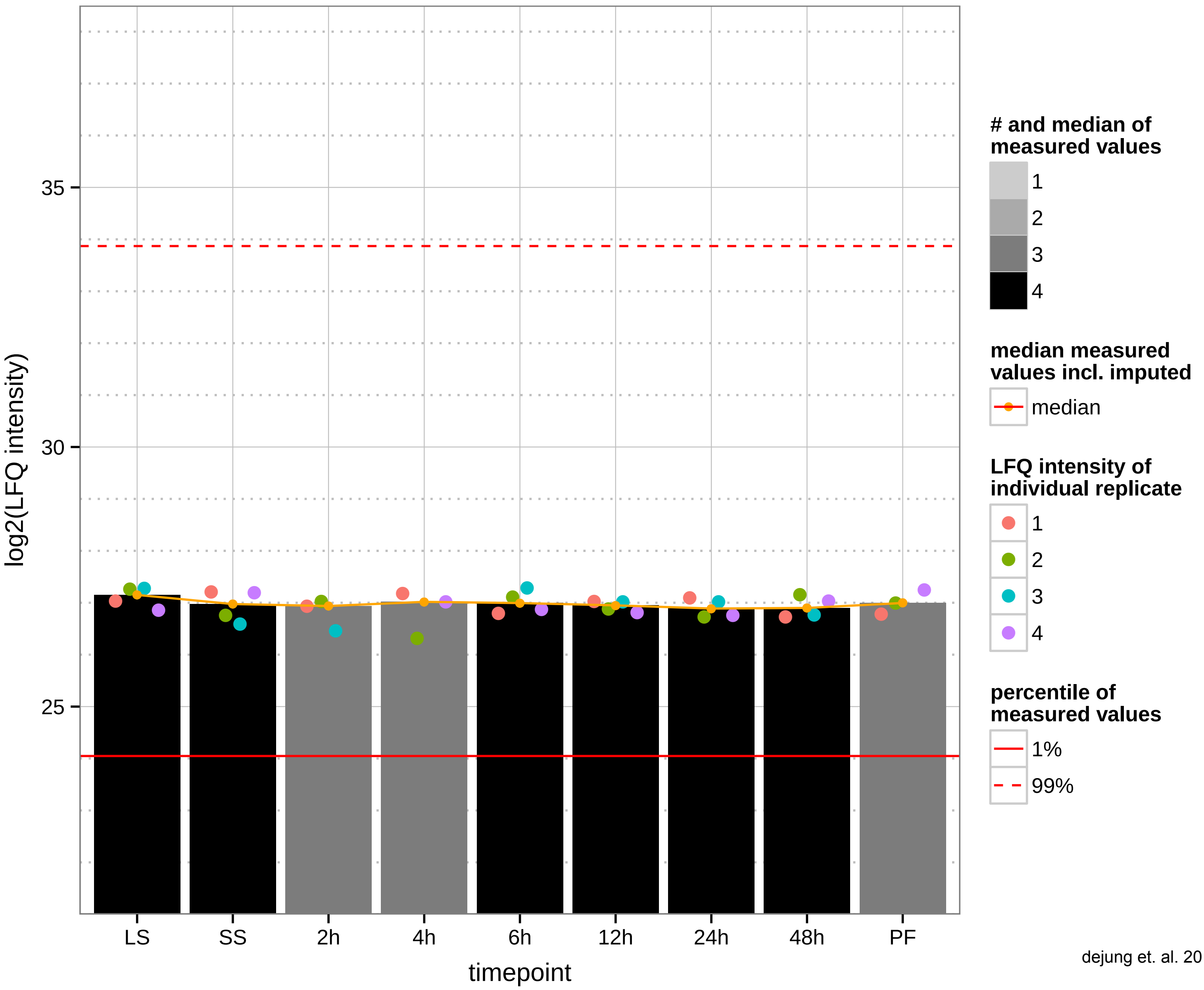

dejung et. al. 2015

RNA–binding protein, putative (RPB25)
Tb927.7.880
AGOF: RNA binding
AGOC: cytosol, nucleus
AGOP: RNA processing
PGOF: nucleic acid binding
PGOC: null
PGOP: null

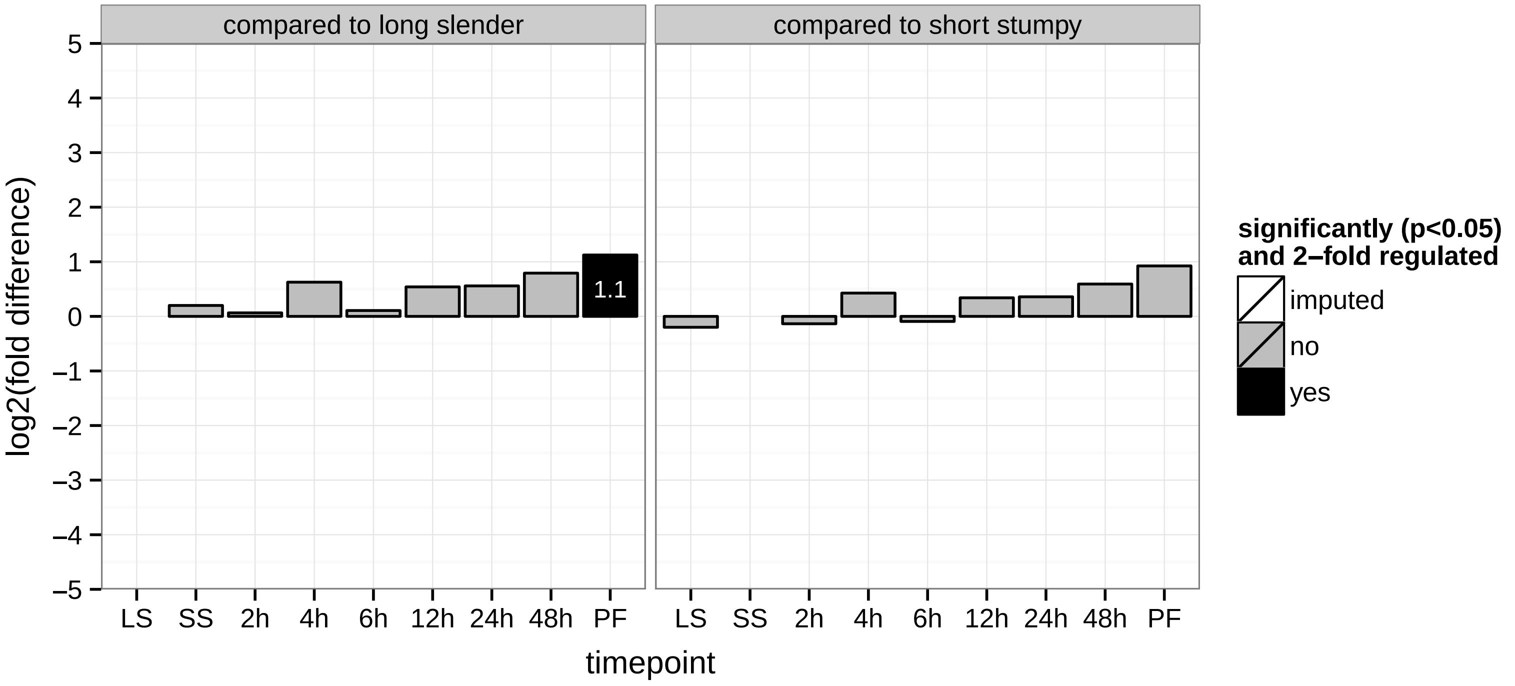

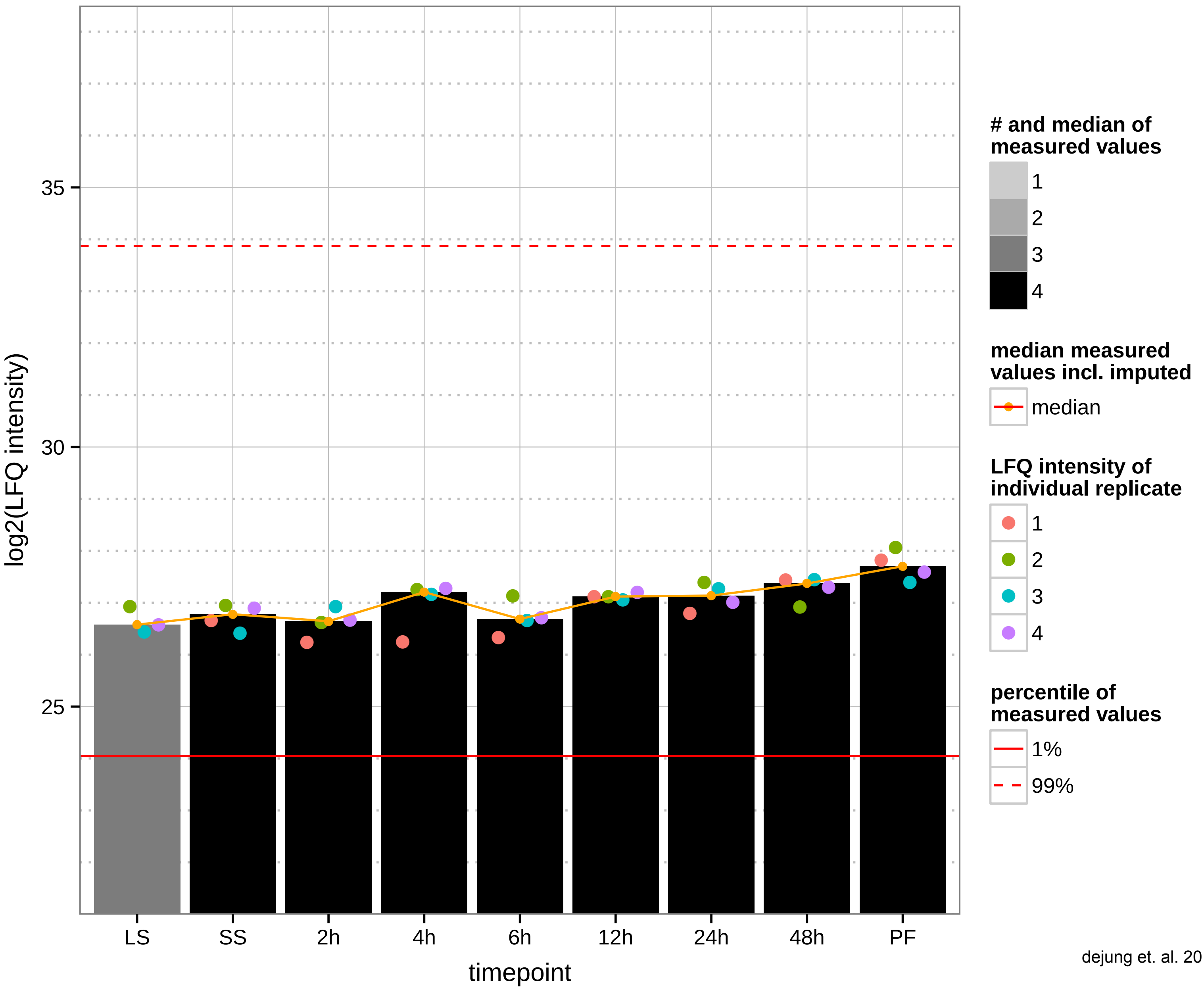

dejung et. al. 2015

dynein heavy chain, putative
Tb927.7.920
AGOF: null, ATP binding, ATPase activity, microtubule motor activity
AGOC: null, dynein complex
AGOP: null, microtubule–based movement
PGOF: null, microtubule motor activity
PGOC: null, dynein complex
PGOP: null, microtubule–based movement

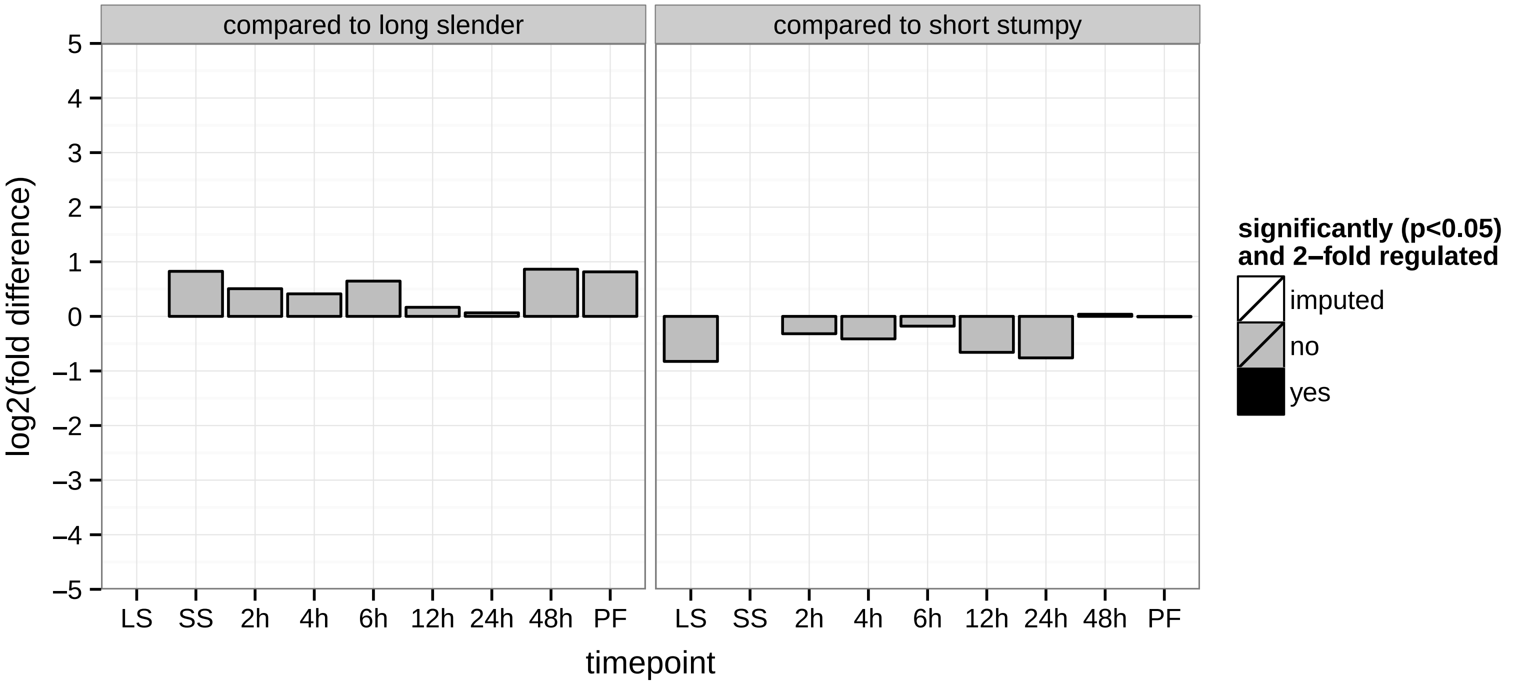

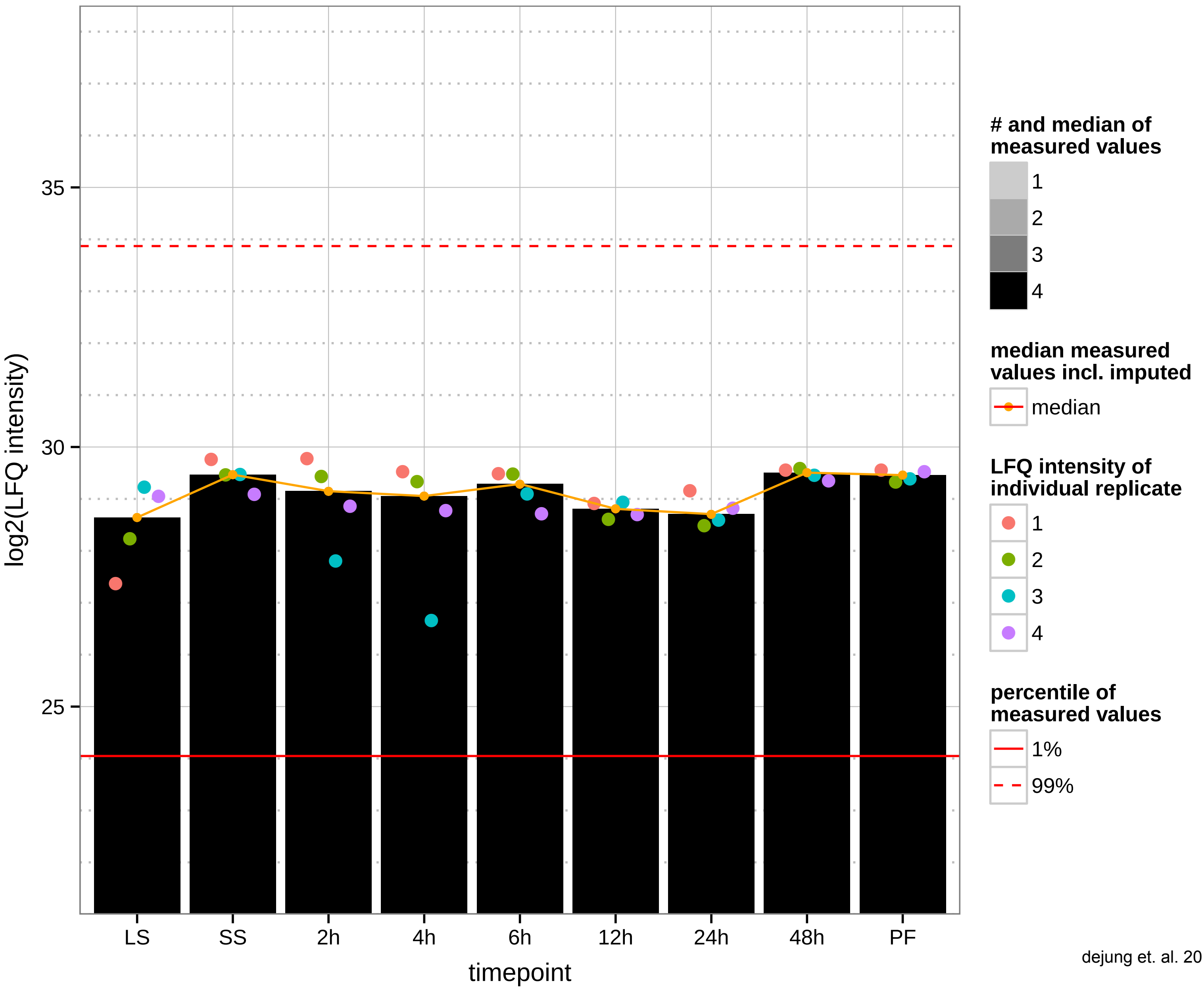

dejung et. al. 2015

protein kinase C substrate protein, heavy chain, putative, glucosidase II beta subunit
Tb927.7.940
AGOF: null
AGOC: null
AGOP: null
PGOF: null
PGOC: null
PGOP: null

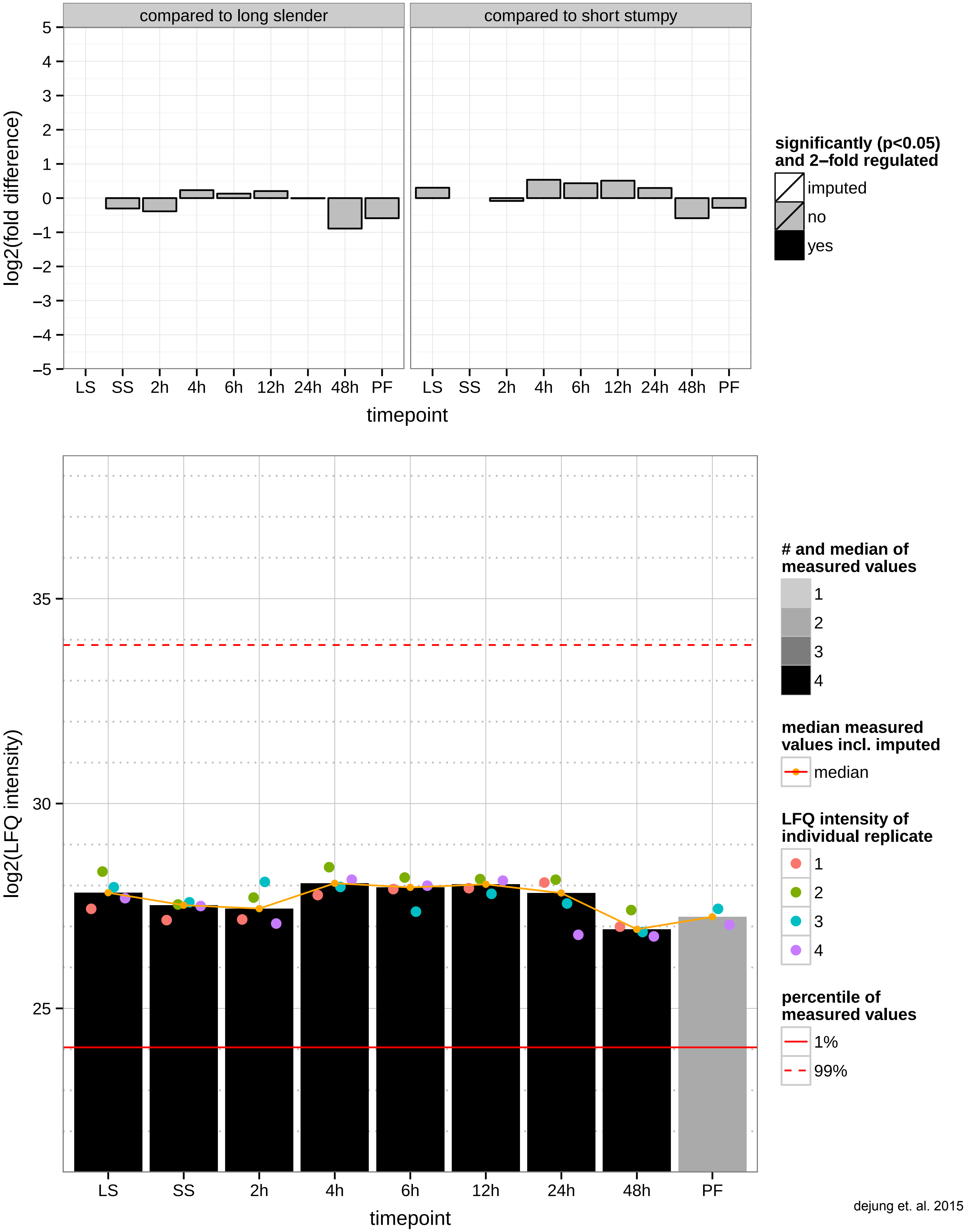

dejung et. al. 2015

NPAPL, nuclear−poly(A)−polymerase−like (NPAPL)
Tb927.8.1090
AGOF: ATP−dependent RNA helicase activity, nucleic acid binding, nucleotidyltransferase activity
AGOC: nucleus
AGOP: null
PGOF: nucleotidyltransferase activity
PGOC: null
PGOP: null

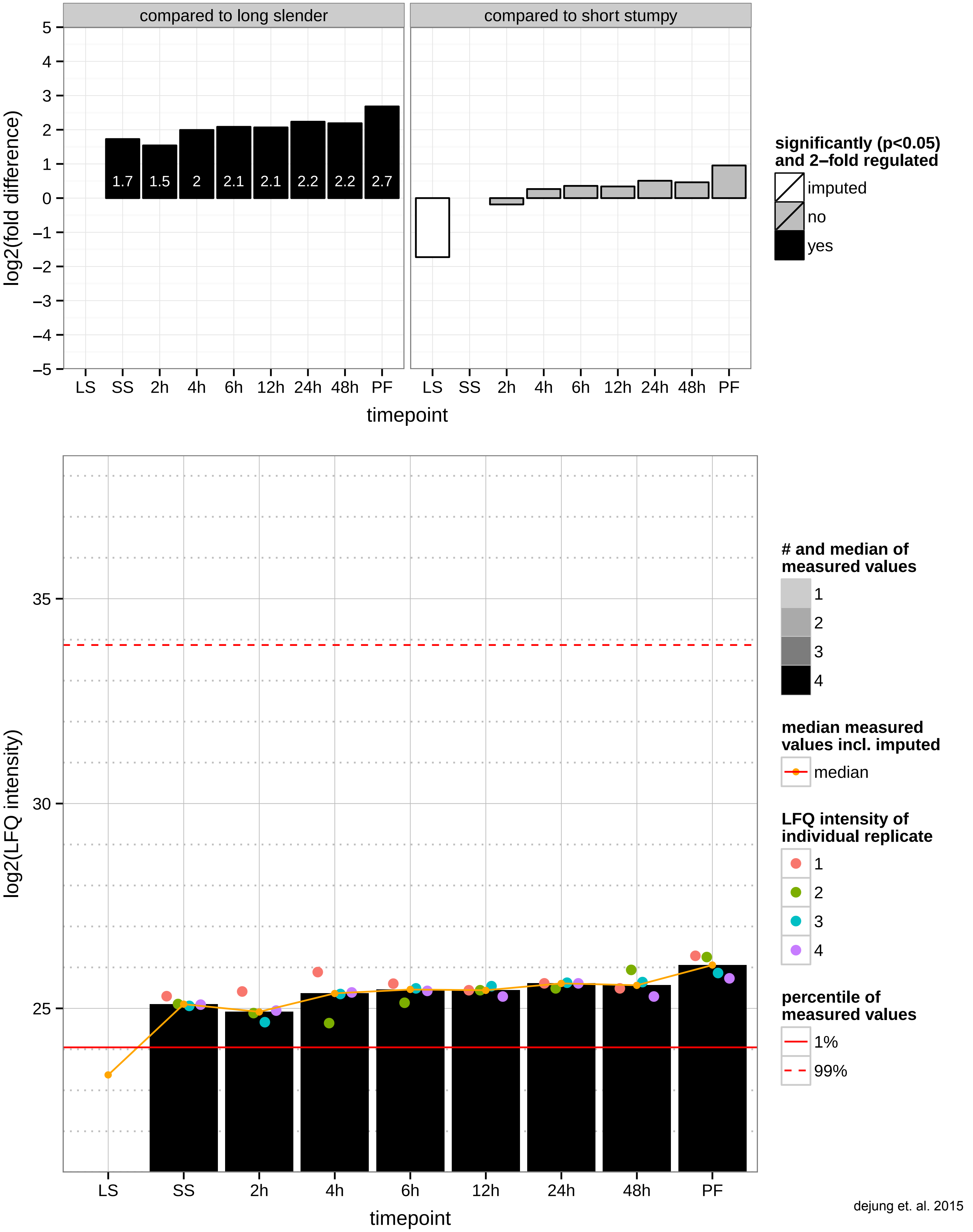

dejung et. al. 2015

hypothetical protein, conserved
Tb927.8.1190;Tb927.8.1170;Tb11.v5.0871
AGOF: null
AGOC: null
AGOP: null
PGOF: null
PGOC: null
PGOP: null

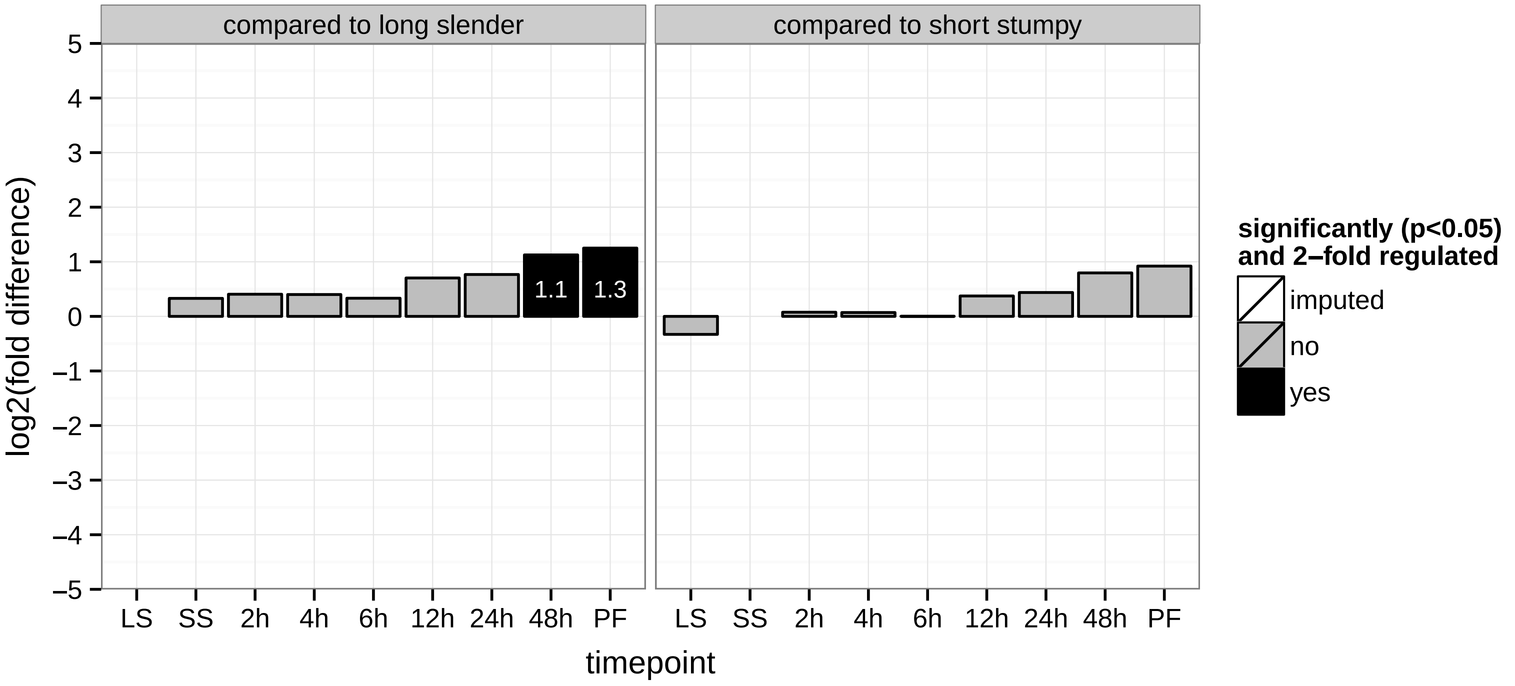

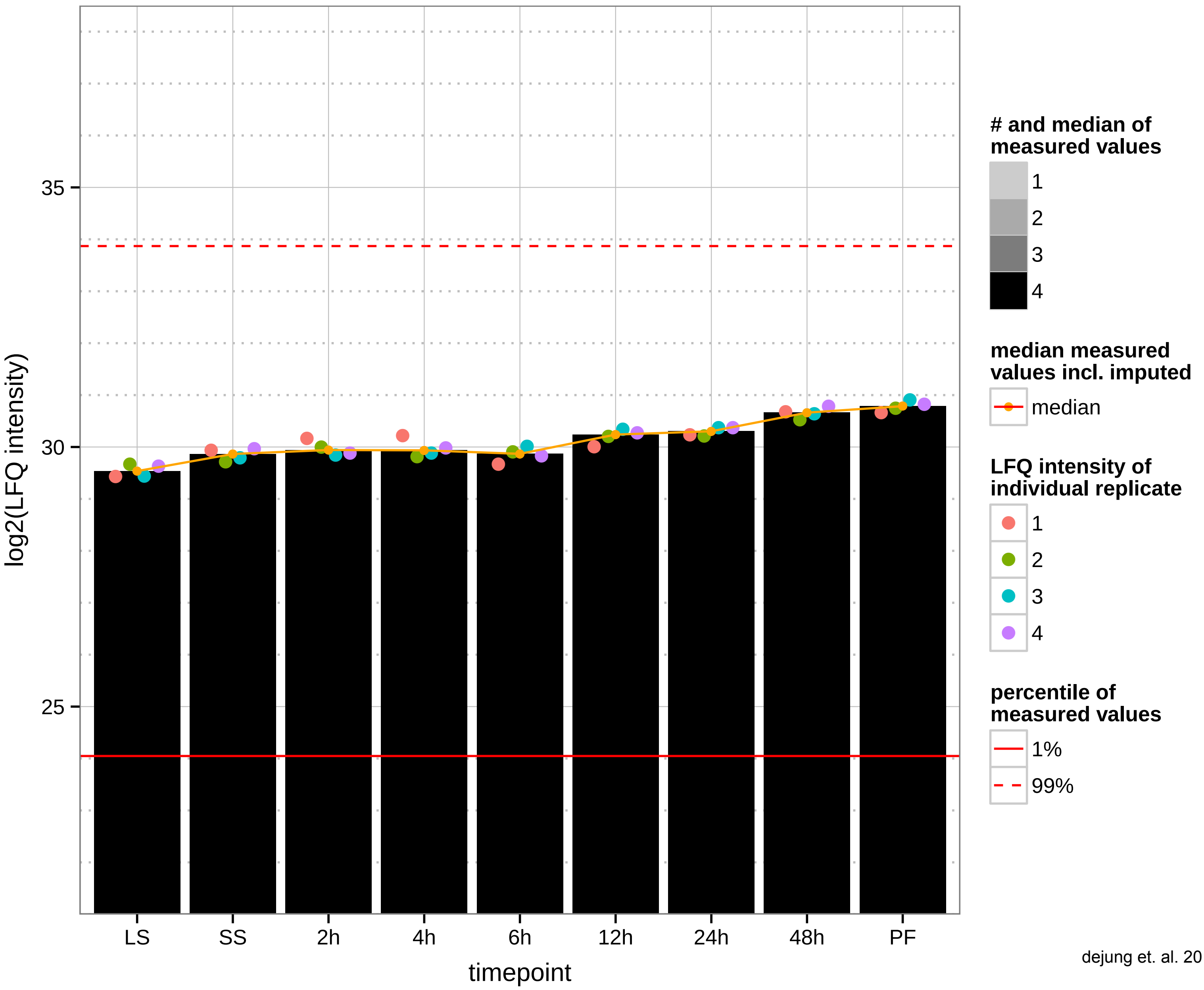

dejung et. al. 2015

vacuolar–type Ca2+–ATPase 2 (TbA2)
Tb927.8.1200
AGOF: ATP binding, calcium–transporting ATPase activity
AGOC: plasma membrane
AGOP: ATP biosynthetic process, calcium ion transport, cell growth, cellular calcium ion homeostasis, metabolic process
PGOF: ATP binding, ATPase activity, coupled to transmembrane movement of ions, phosphorylative mechanism, calcium–tran
PGOC: membrane
PGOP: ATP biosynthetic process, calcium ion transmembrane transport, cation transport, metabolic process

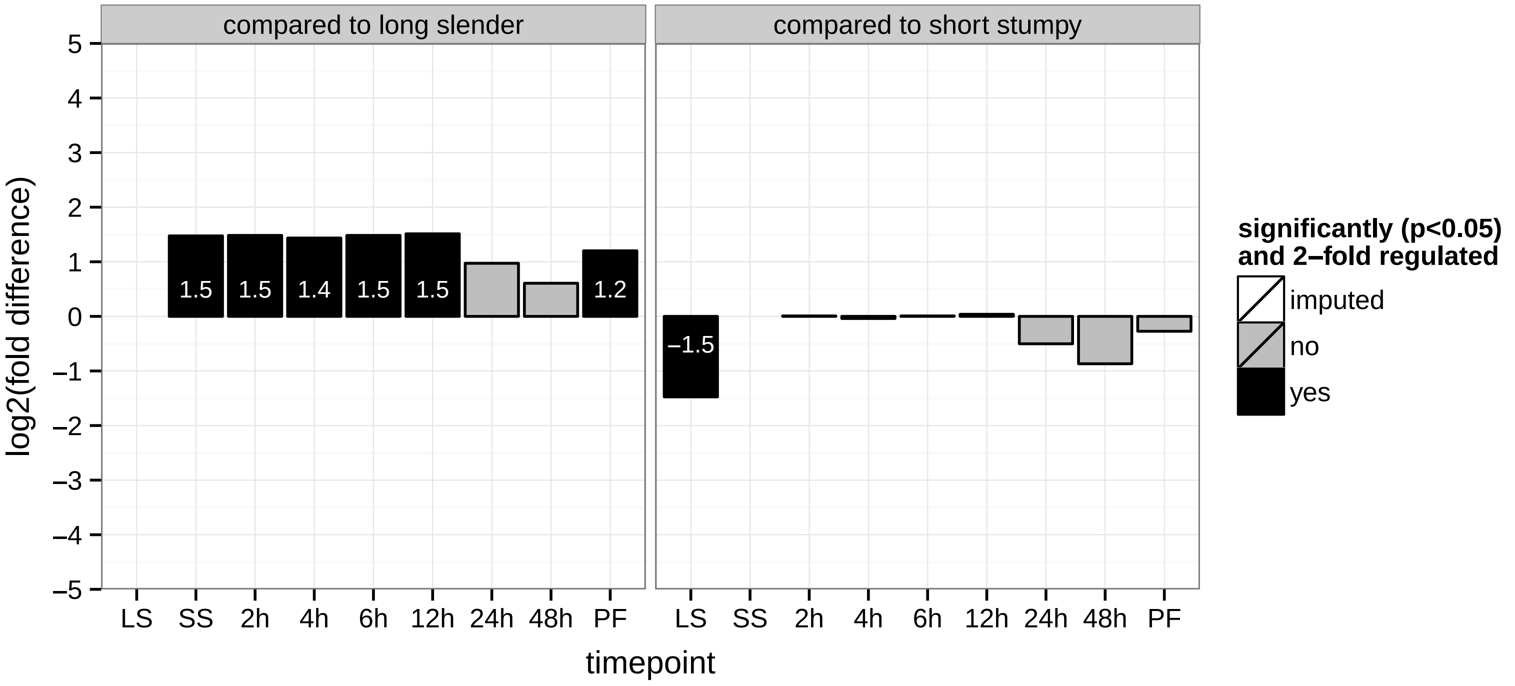

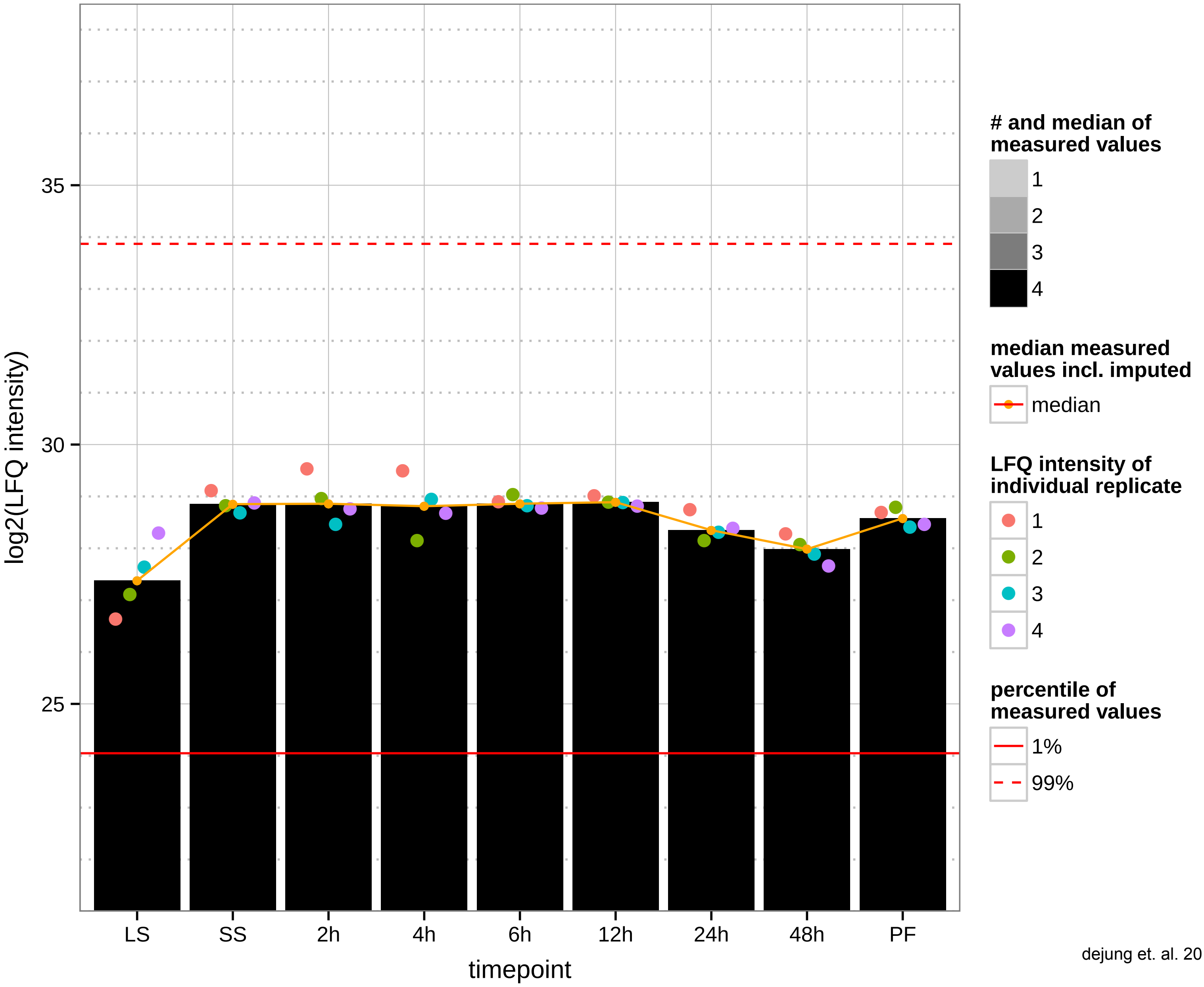

dejung et. al. 2015

hypothetical protein, conserved (pseudogene)
Tb927.8.1220
AGOF: null
AGOC: null
AGOP: null
PGOF: null
PGOC: null
PGOP: null

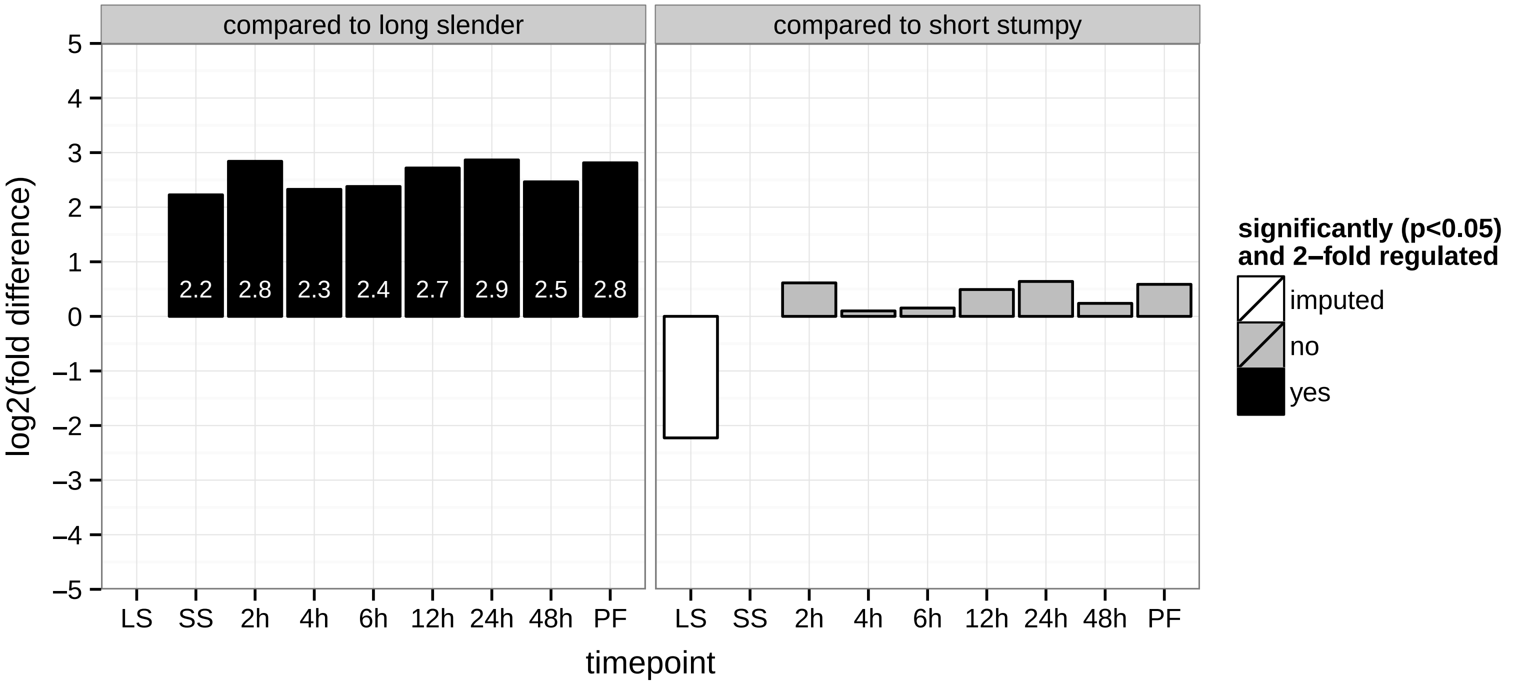

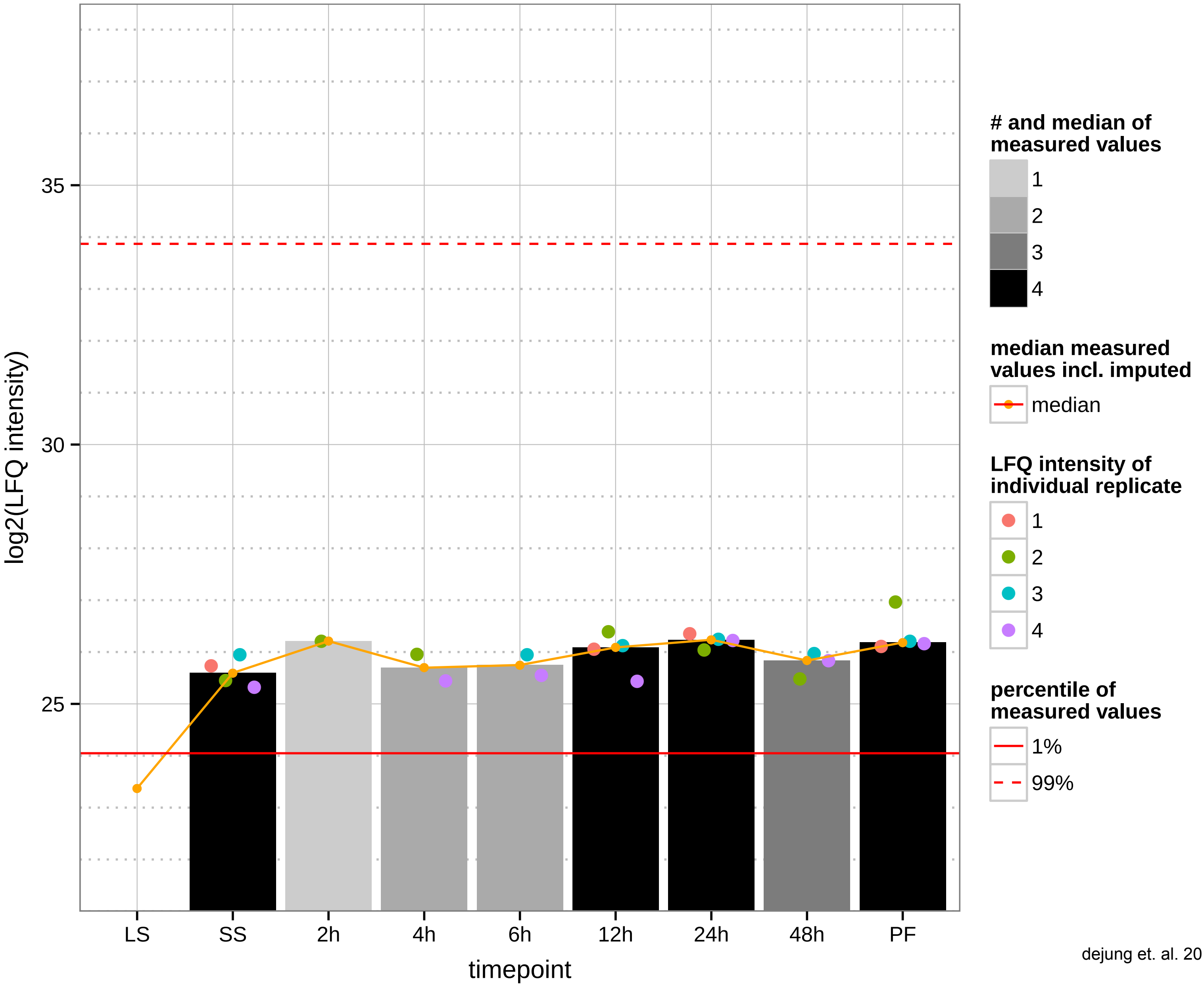

dejung et. al. 2015

hypothetical protein, conserved, pre−rrna−processing protein tsr1 homolog
Tb927.8.1410;Tb11.v5.0588
AGOF: null
AGOC: null, cytoplasm, nucleolus, nucleus
AGOP: null, ribosome biogenesis
PGOF: null
PGOC: nucleus
PGOP: ribosome biogenesis

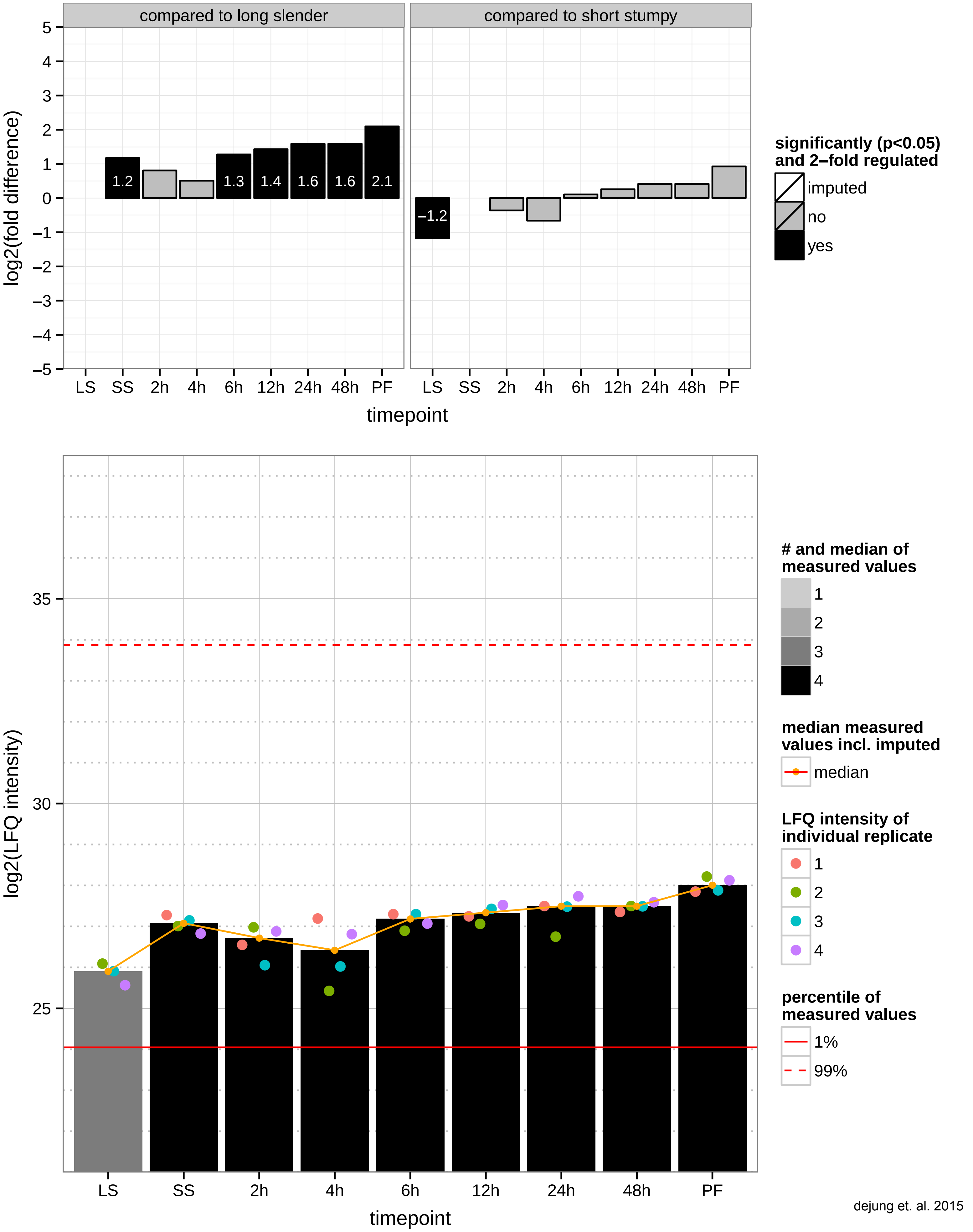

dejung et. al. 2015

acyl–CoA dehydrogenase, mitochondrial precursor, putative
Tb927.8.1420
AGOF: acyl–CoA dehydrogenase activity
AGOC: mitochondrial membrane, mitochondrion
AGOP: metabolic process
PGOF: acyl–CoA dehydrogenase activity, oxidoreductase activity, acting on the CH–CH group of donors
PGOC: null
PGOP: metabolic process, oxidation–reduction process

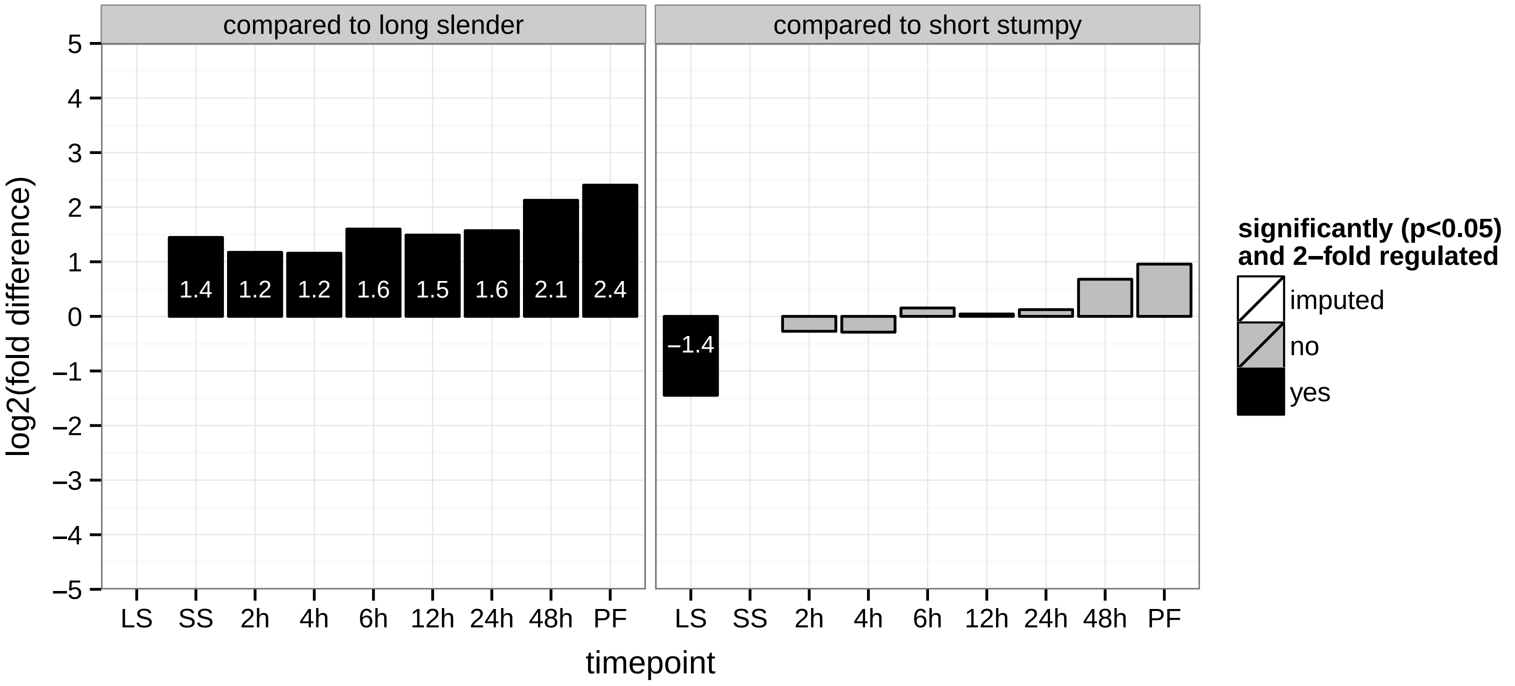

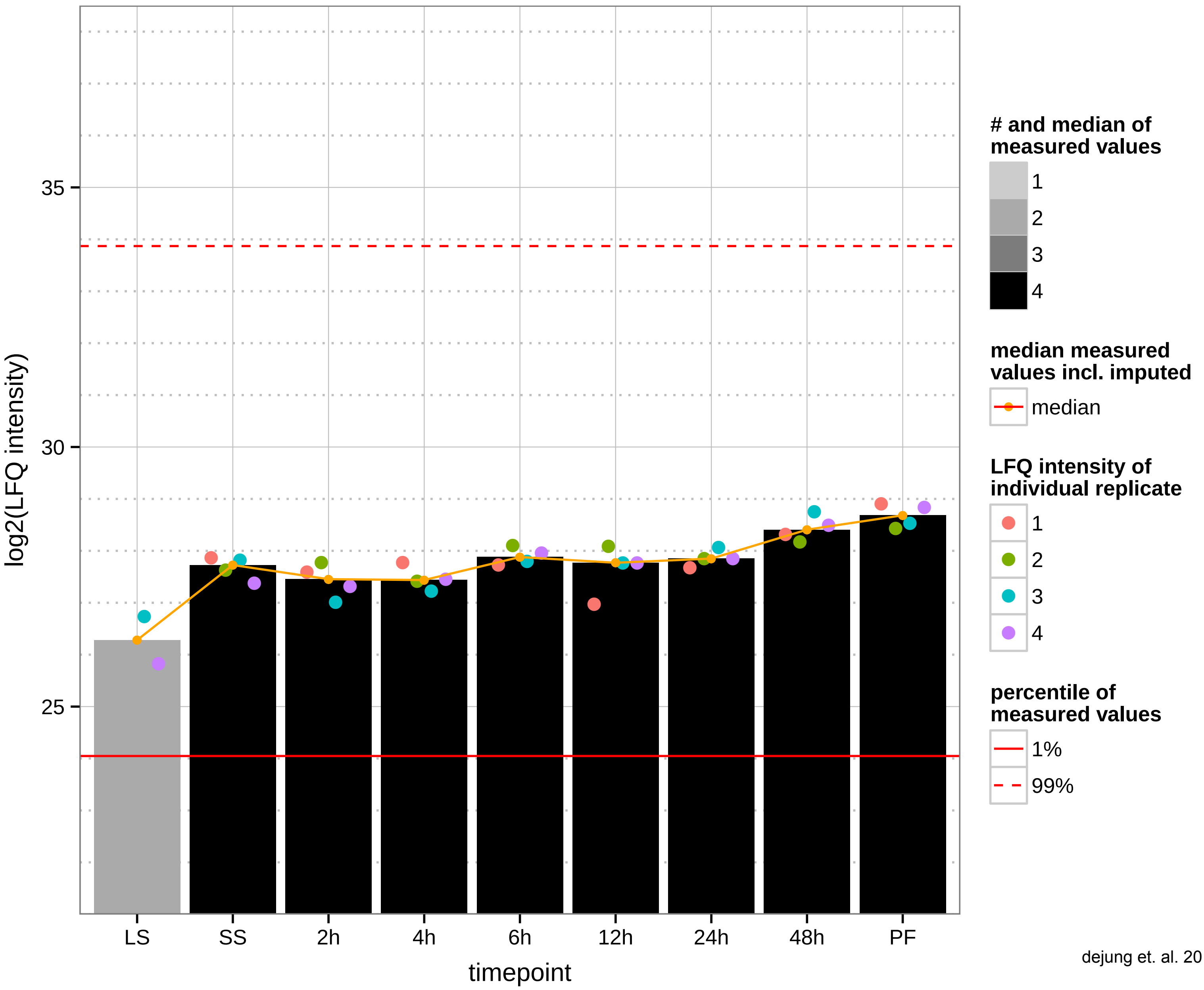

dejung et. al. 2015

maoC–like dehydratase, putative
Tb927.8.1440
AGOF: fatty acid synthase activity, oxidoreductase activity
AGOC: mitochondrion
AGOP: fatty acid biosynthetic process
PGOF: oxidoreductase activity
PGOC: null
PGOP: metabolic process

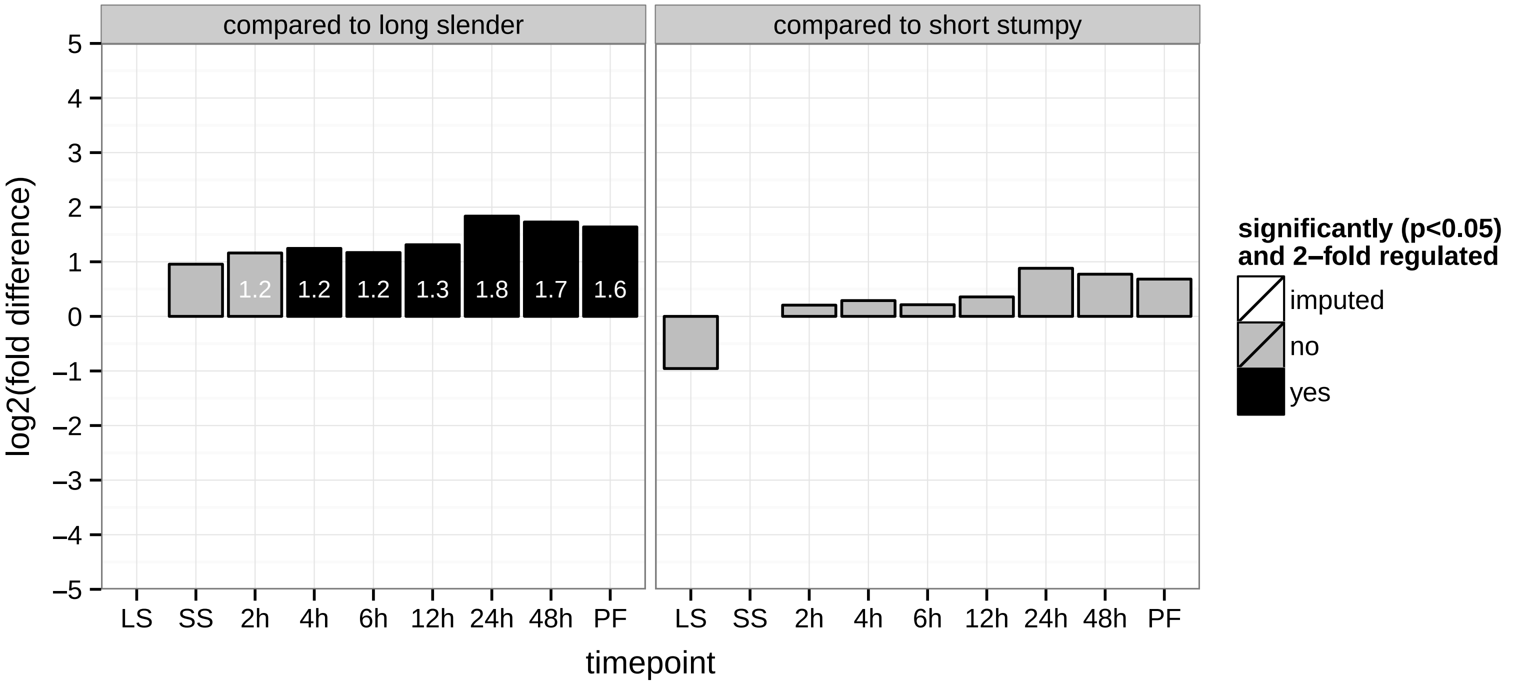

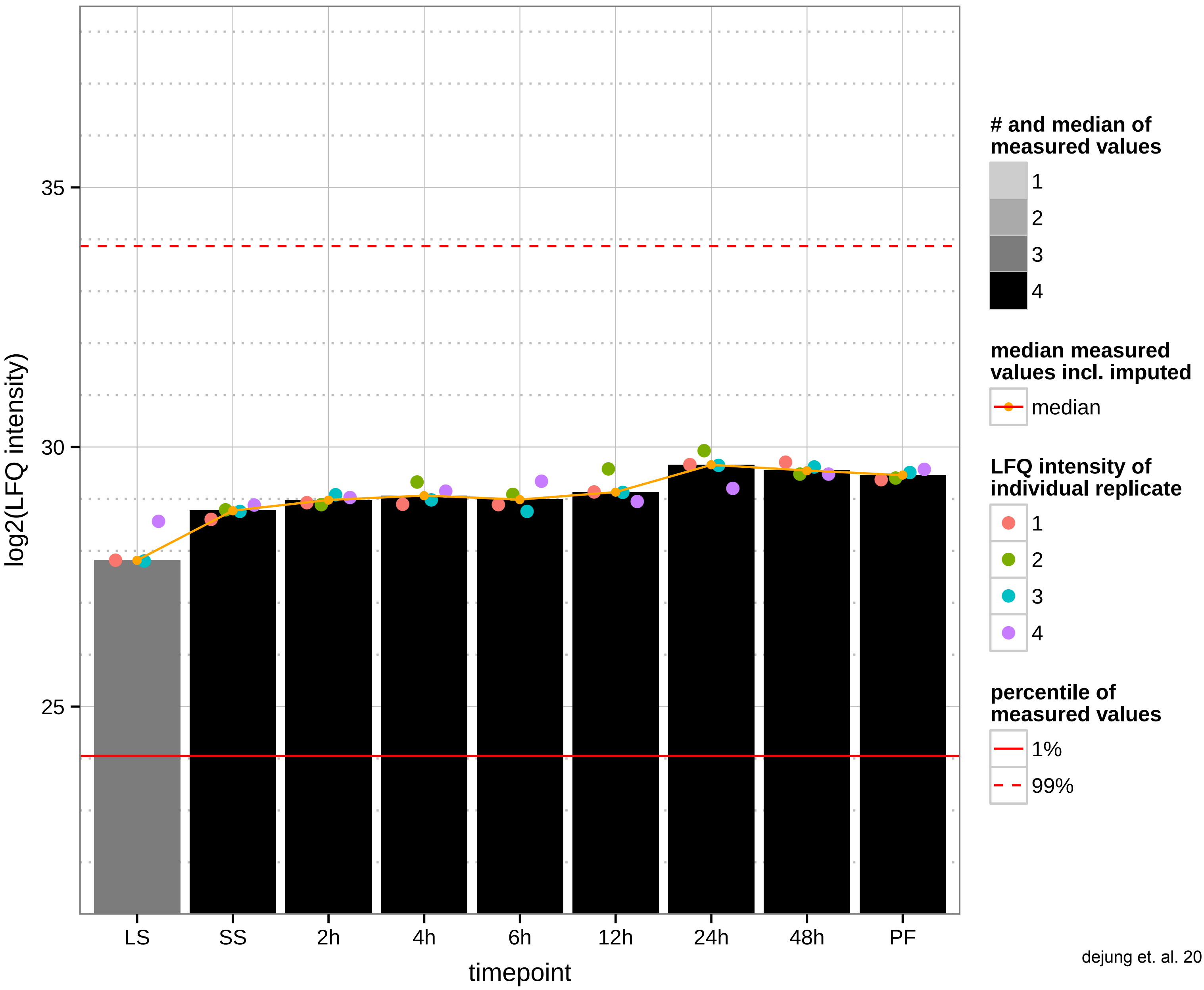

dejung et. al. 2015

hypothetical protein, conserved
Tb927.8.1470
AGOF: null
AGOC: null
AGOP: null
PGOF: null
PGOC: null
PGOP: null

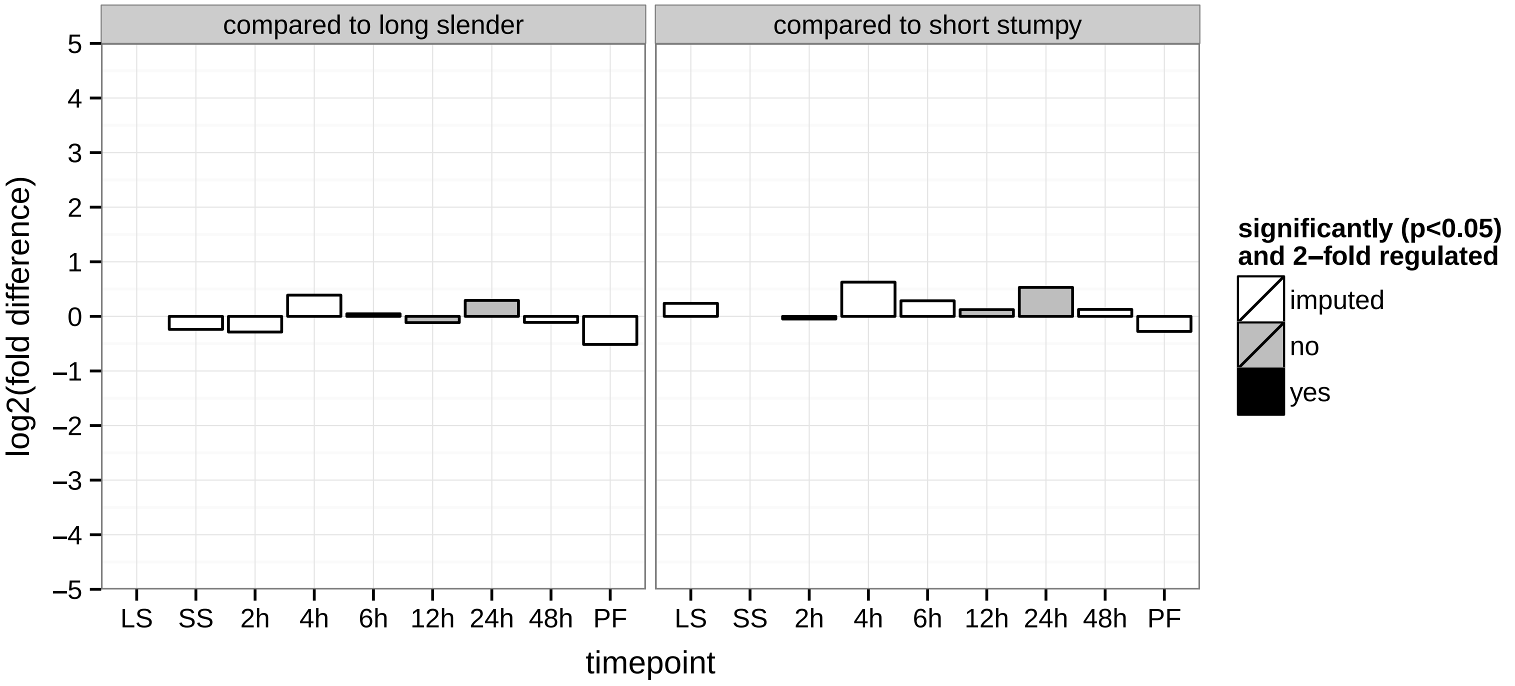

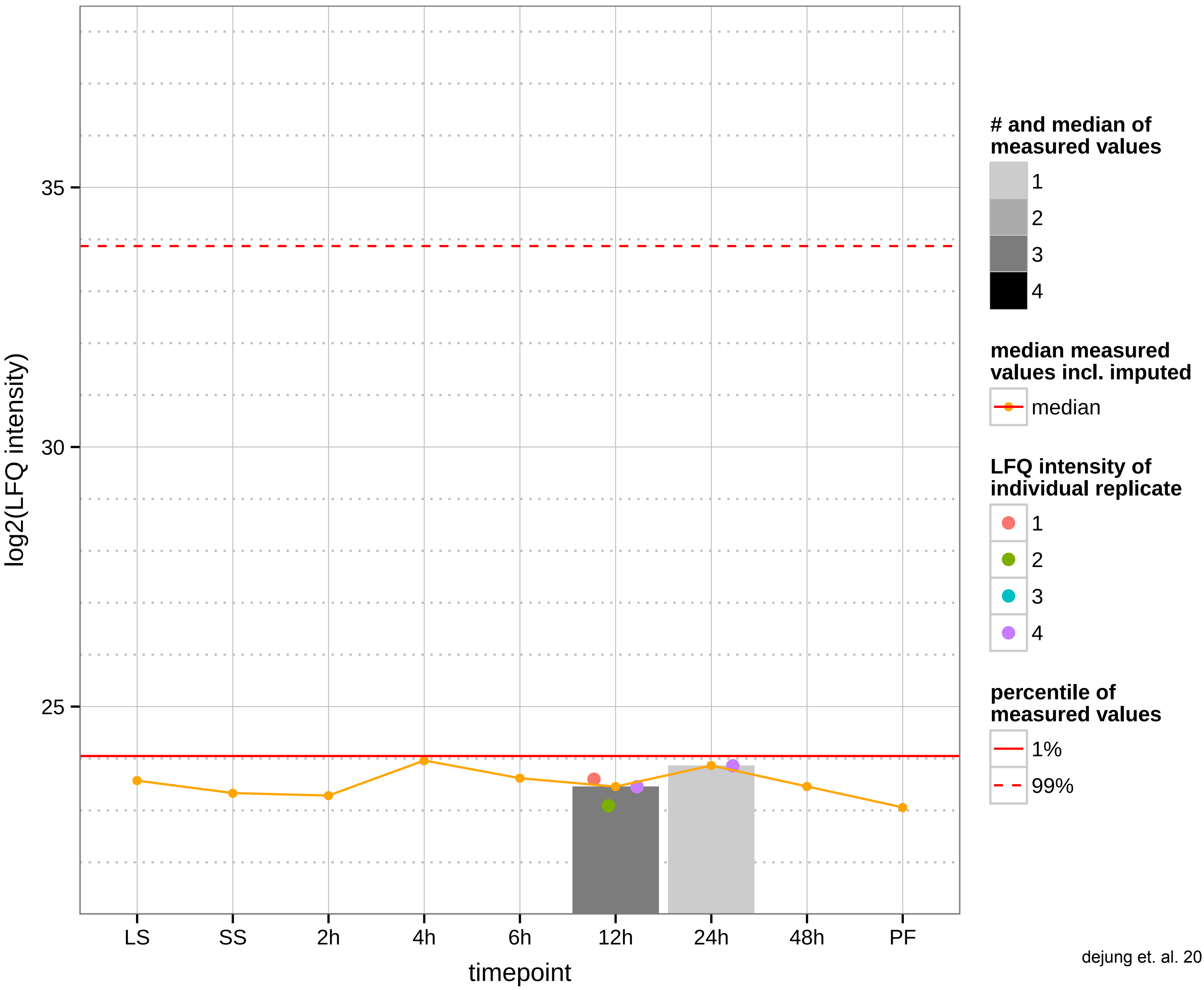

dejung et. al. 2015

hypothetical protein, conserved
Tb927.8.1540
AGOF: null
AGOC: null
AGOP: null
PGOF: null
PGOC: null
PGOP: null

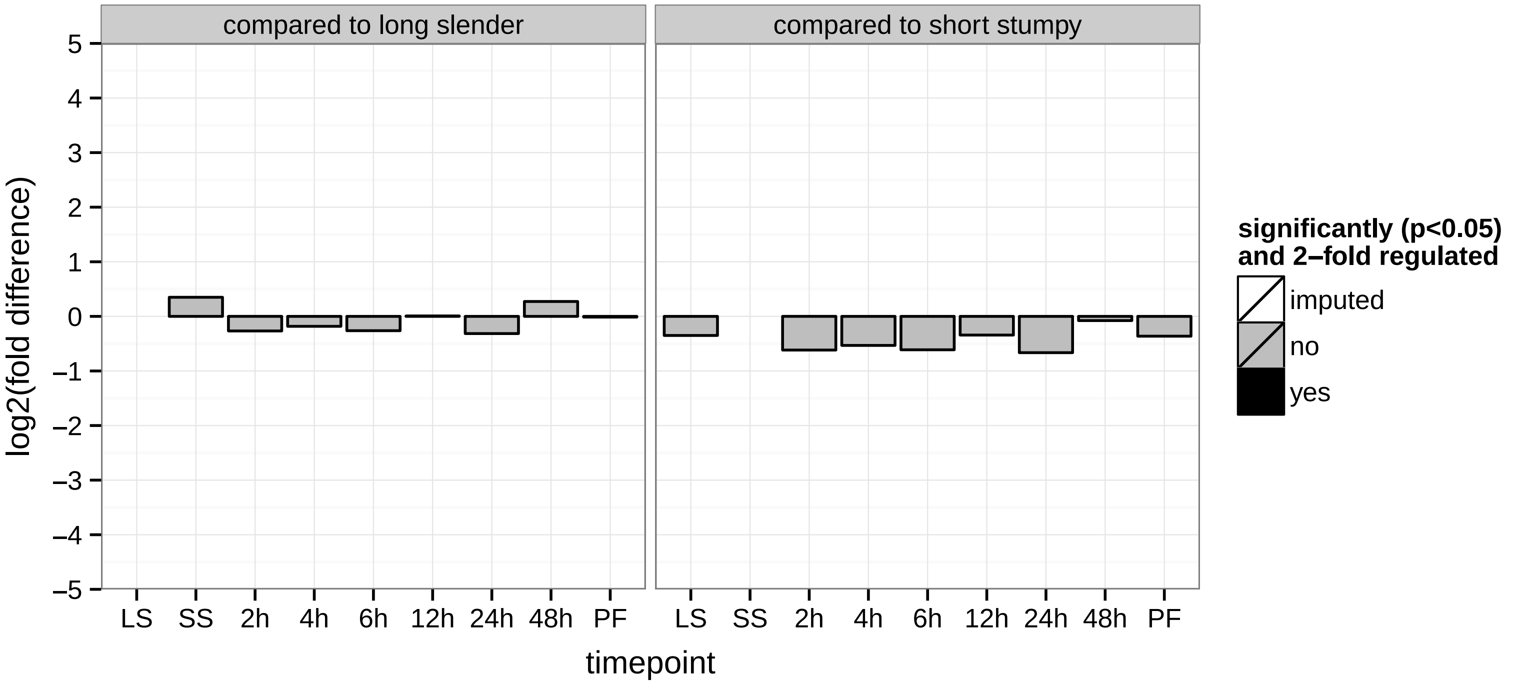

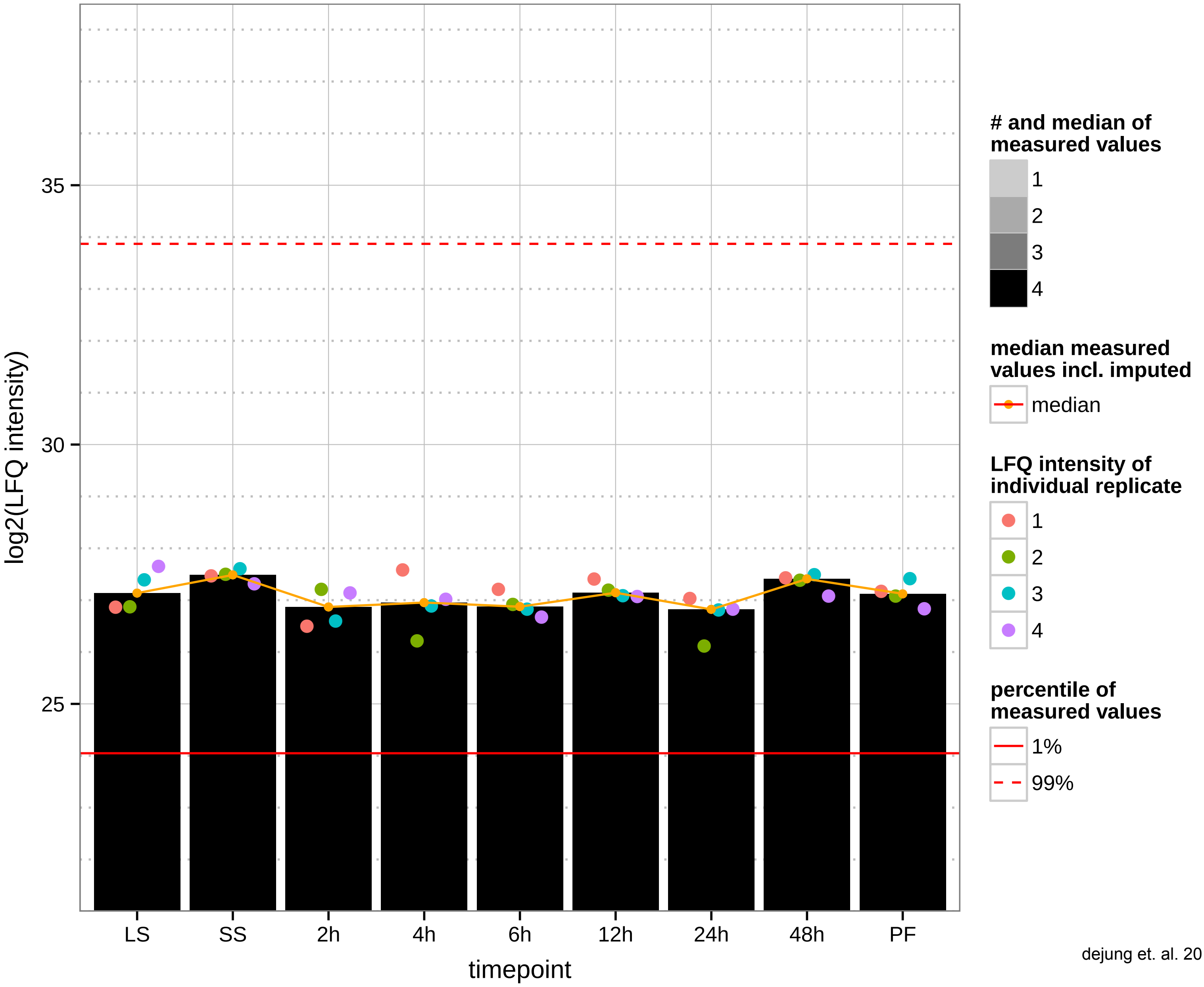

dejung et. al. 2015

paraflagellar rod component, putative (PFC3)
Tb927.8.1550
AGOF: null
AGOC: null
AGOP: null
PGOF: calmodulin binding
PGOC: microtubule–based flagellum
PGOP: null

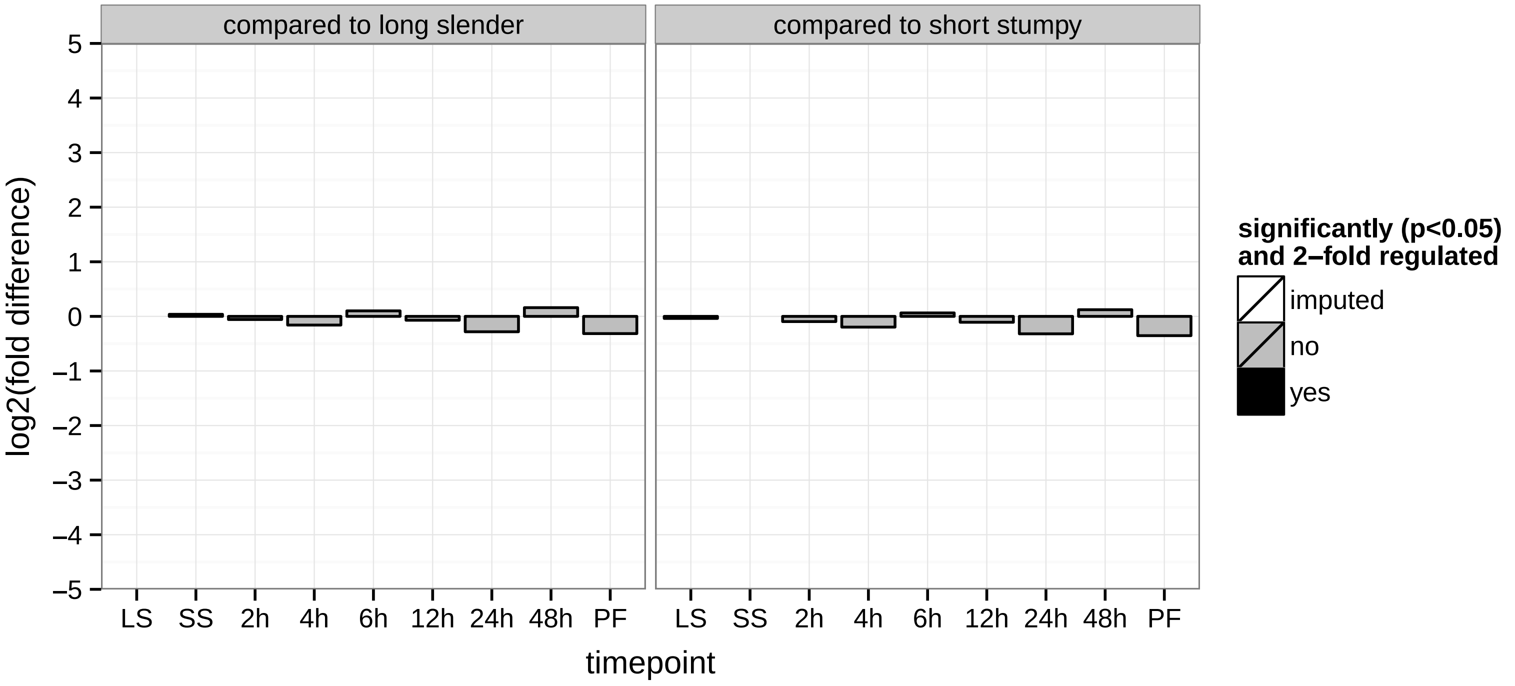

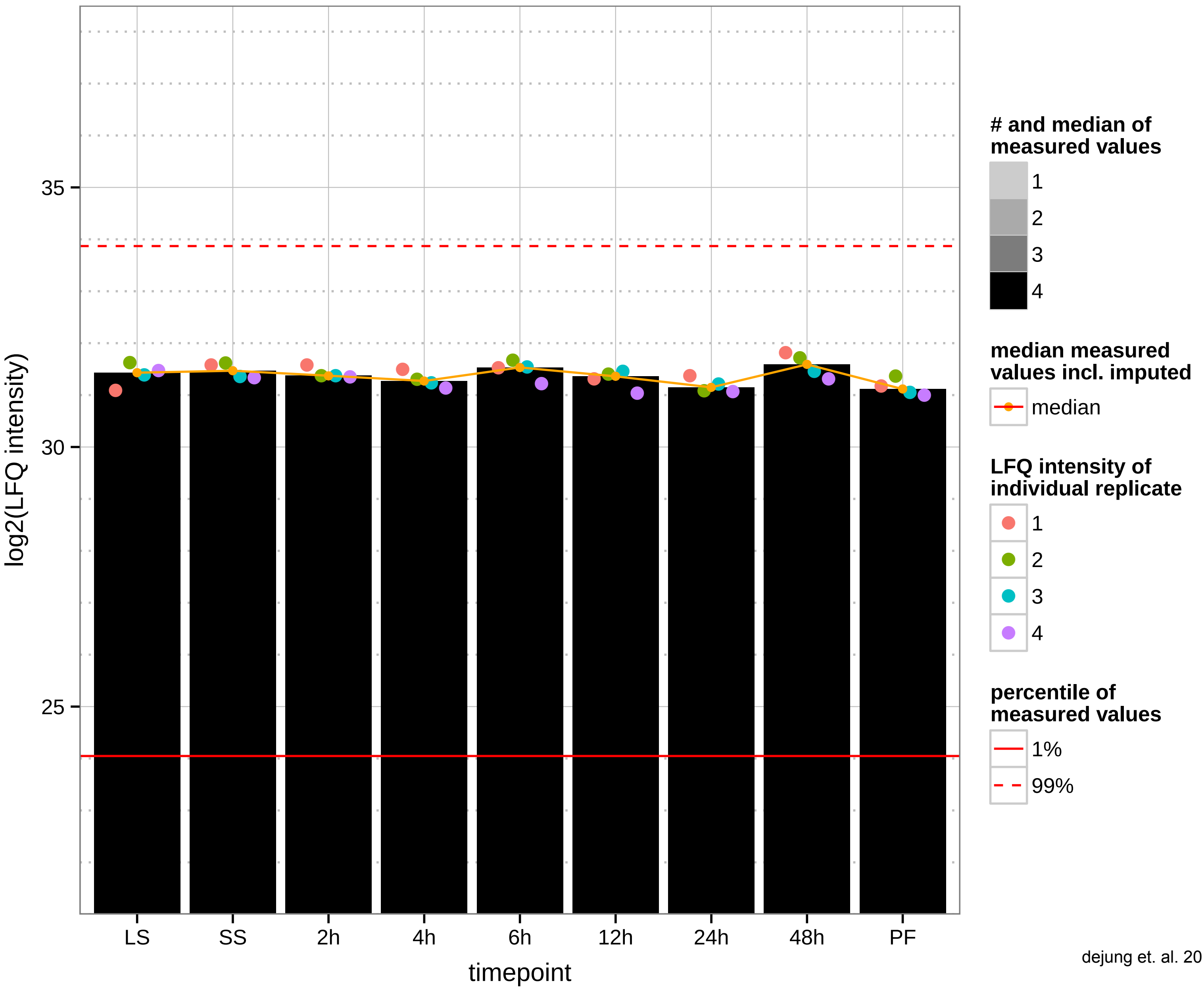

dejung et. al. 2015

lysyl–tRNA synthetase, putative
Tb927.8.1600
AGOF: ATP binding, lysine–tRNA ligase activity, nucleic acid binding
AGOC: cytoplasm
AGOP: lysyl–tRNA aminoacylation, tRNA aminoacylation for protein translation, translation
PGOF: ATP binding, aminoacyl–tRNA ligase activity, lysine–tRNA ligase activity, nucleic acid binding, nucleotide binding
PGOC: cytoplasm
PGOP: lysyl–tRNA aminoacylation, tRNA aminoacylation for protein translation

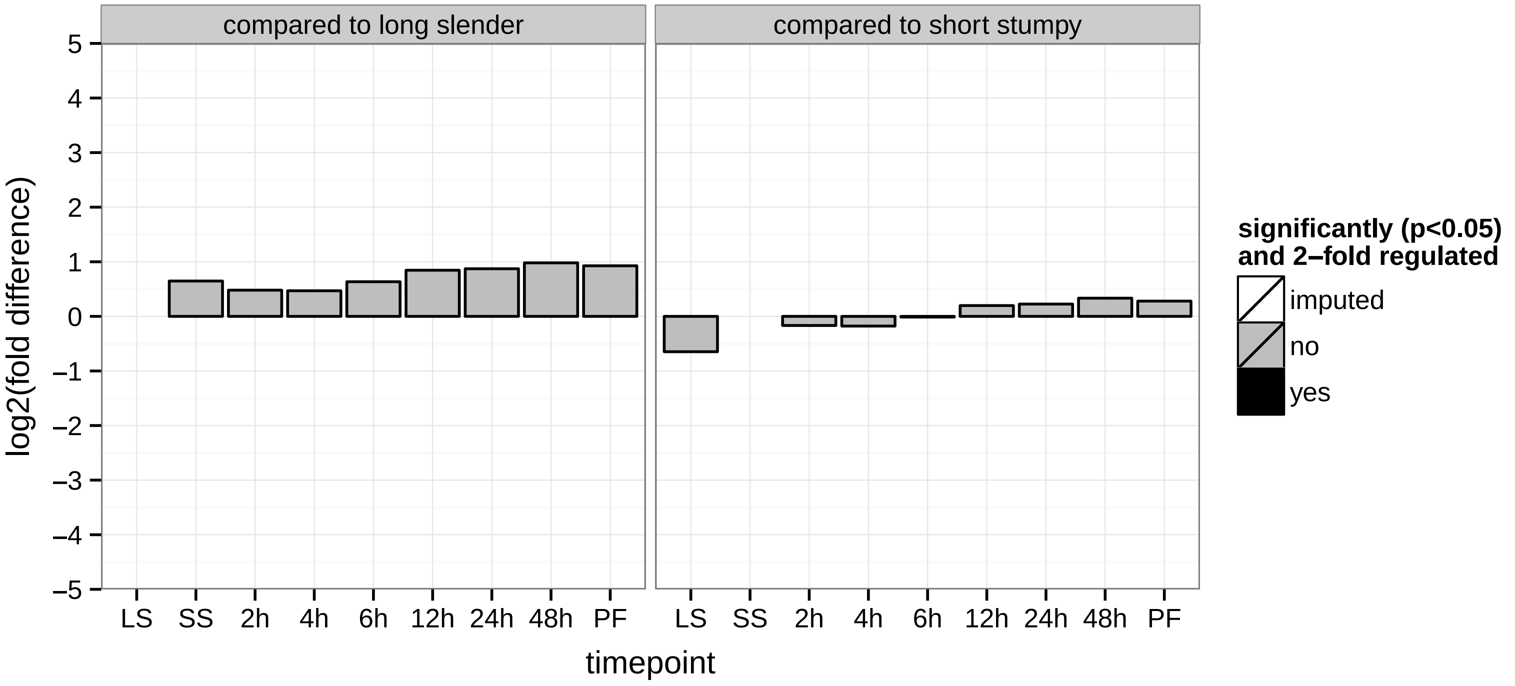

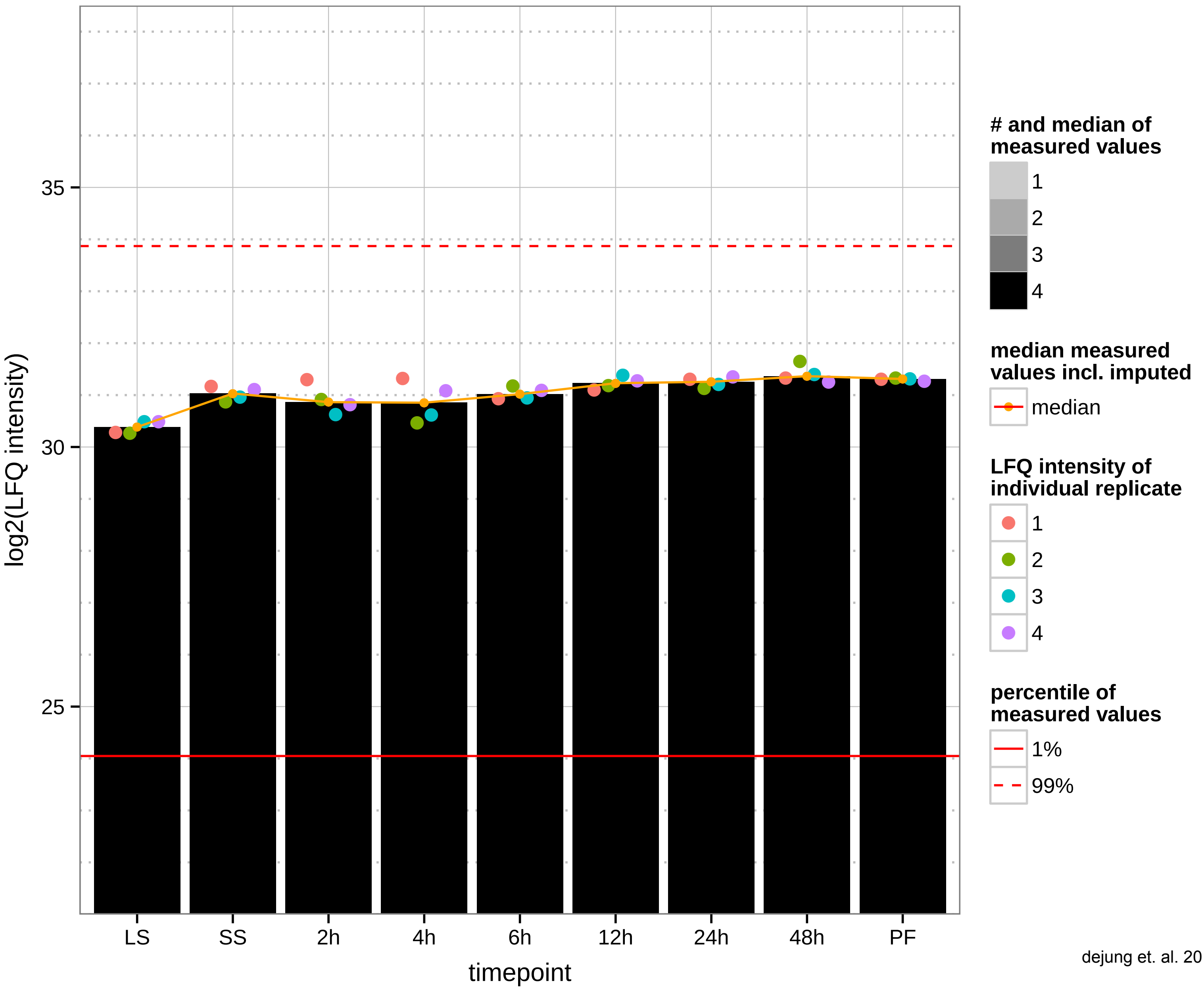

dejung et. al. 2015

protein phosphatase 2A, regulatory subunit B, putative, phosphotyrosyl phosphate activator protein
Tb927.8.1710
AGOF: phosphatase activator activity
AGOC: null
AGOP: null
PGOF: phosphatase activator activity
PGOC: null
PGOP: null

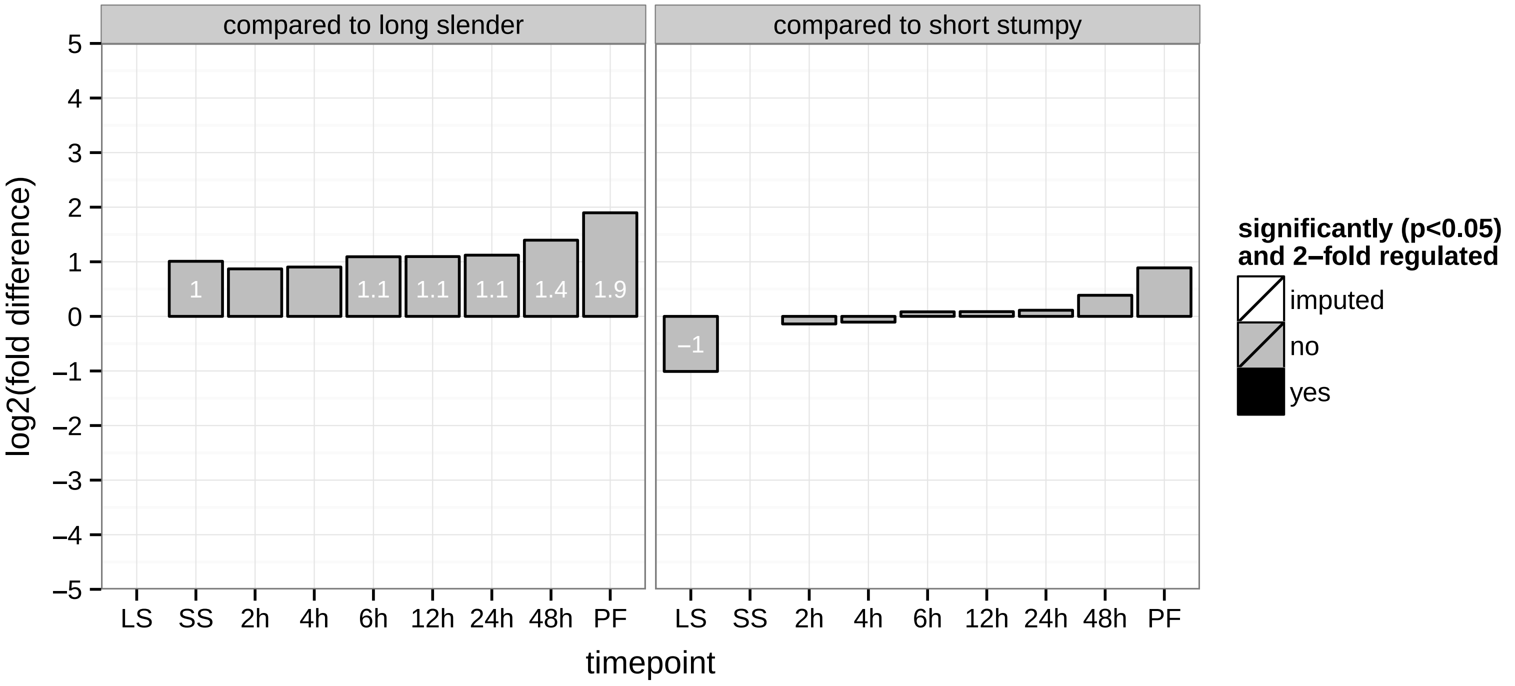

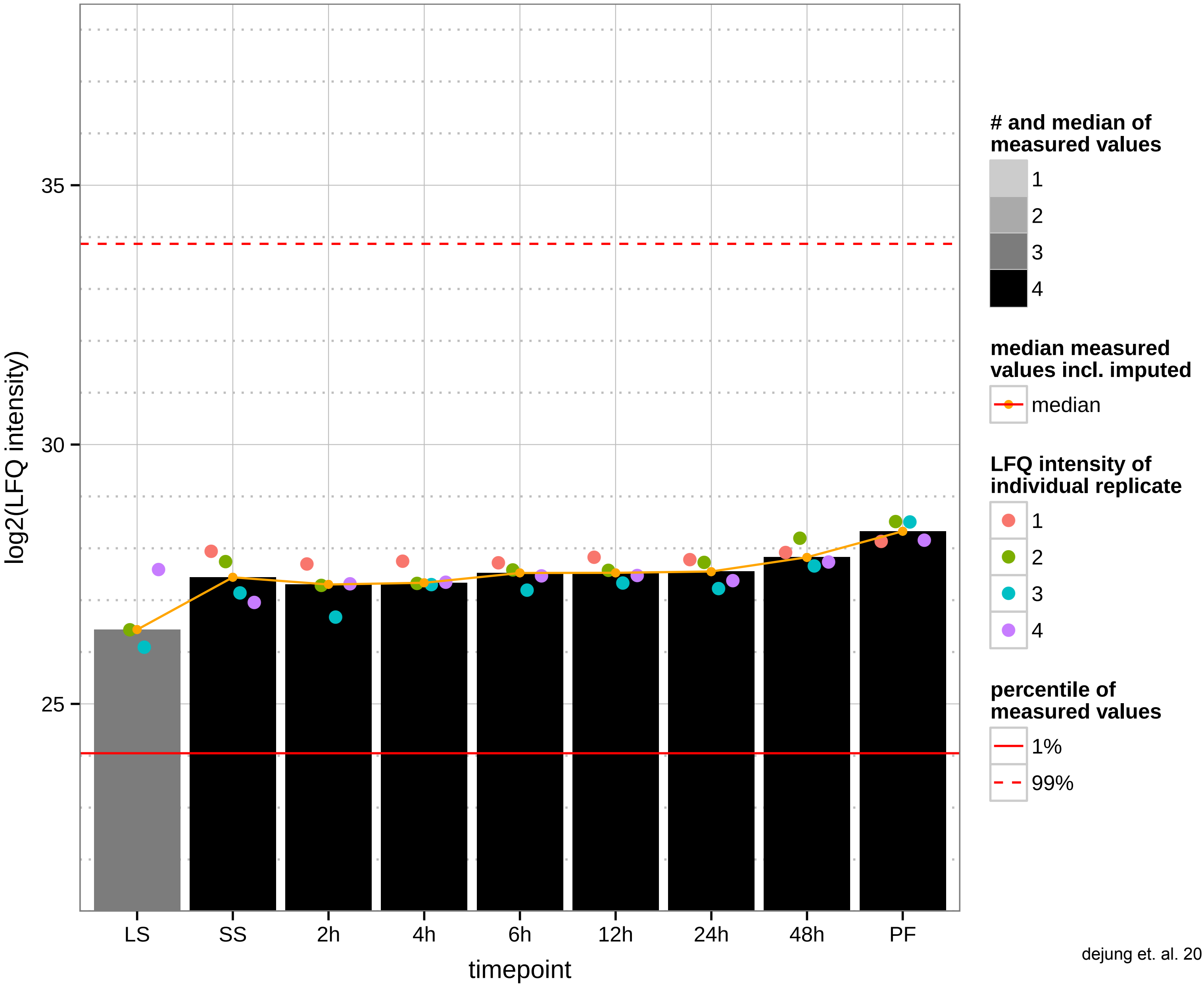

dejung et. al. 2015

hypothetical protein, conserved
Tb927.8.1740
AGOF: null
AGOC: mitochondrion
AGOP: null
PGOF: null
PGOC: null
PGOP: null

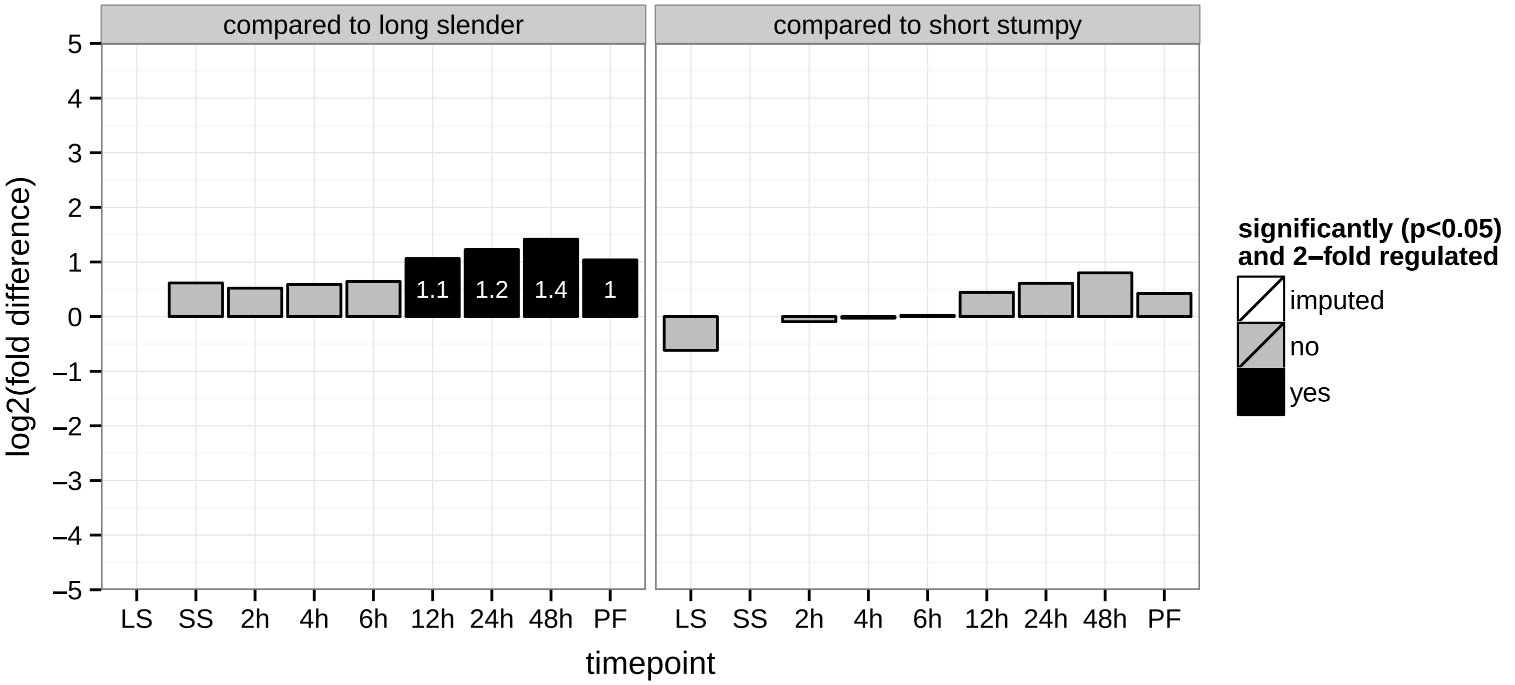

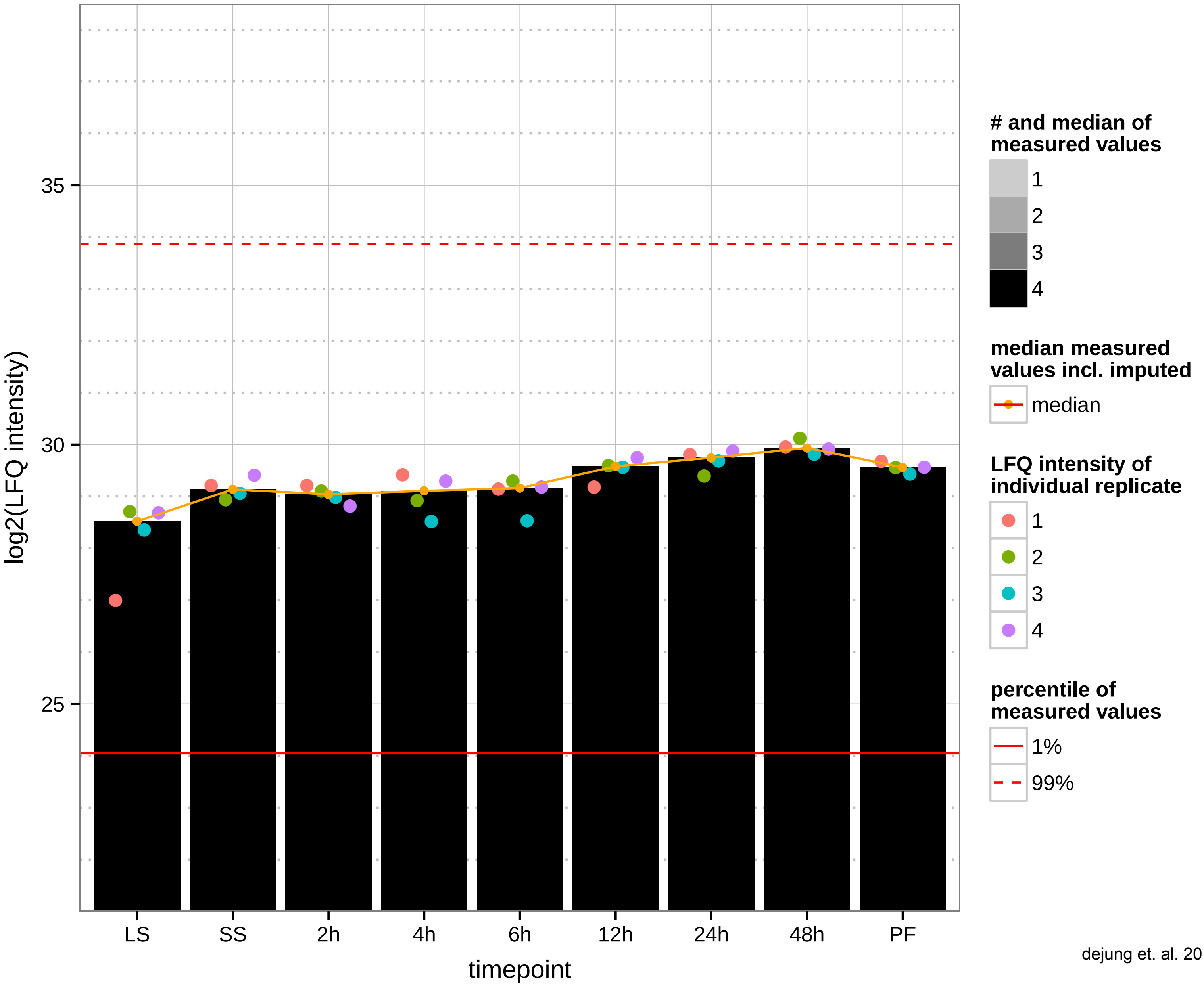

dejung et. al. 2015

protein kinase, putative
Tb927.8.1780
AGOF: ATP binding, protein serine/threonine kinase activity, protein tyrosine kinase activity
AGOC: null
AGOP: protein phosphorylation
PGOF: ATP binding, protein kinase activity, transferase activity, transferring phosphorus–containing groups
PGOC: null
PGOP: protein phosphorylation

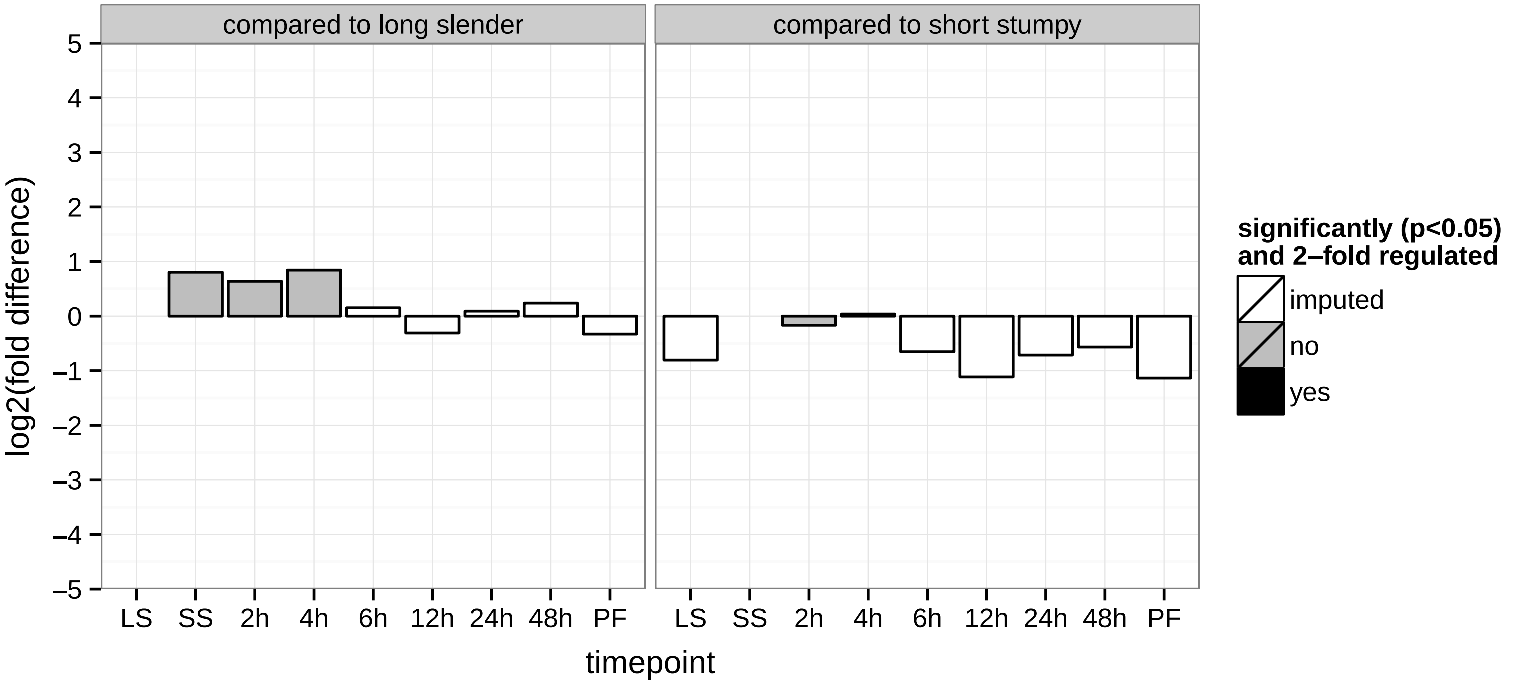

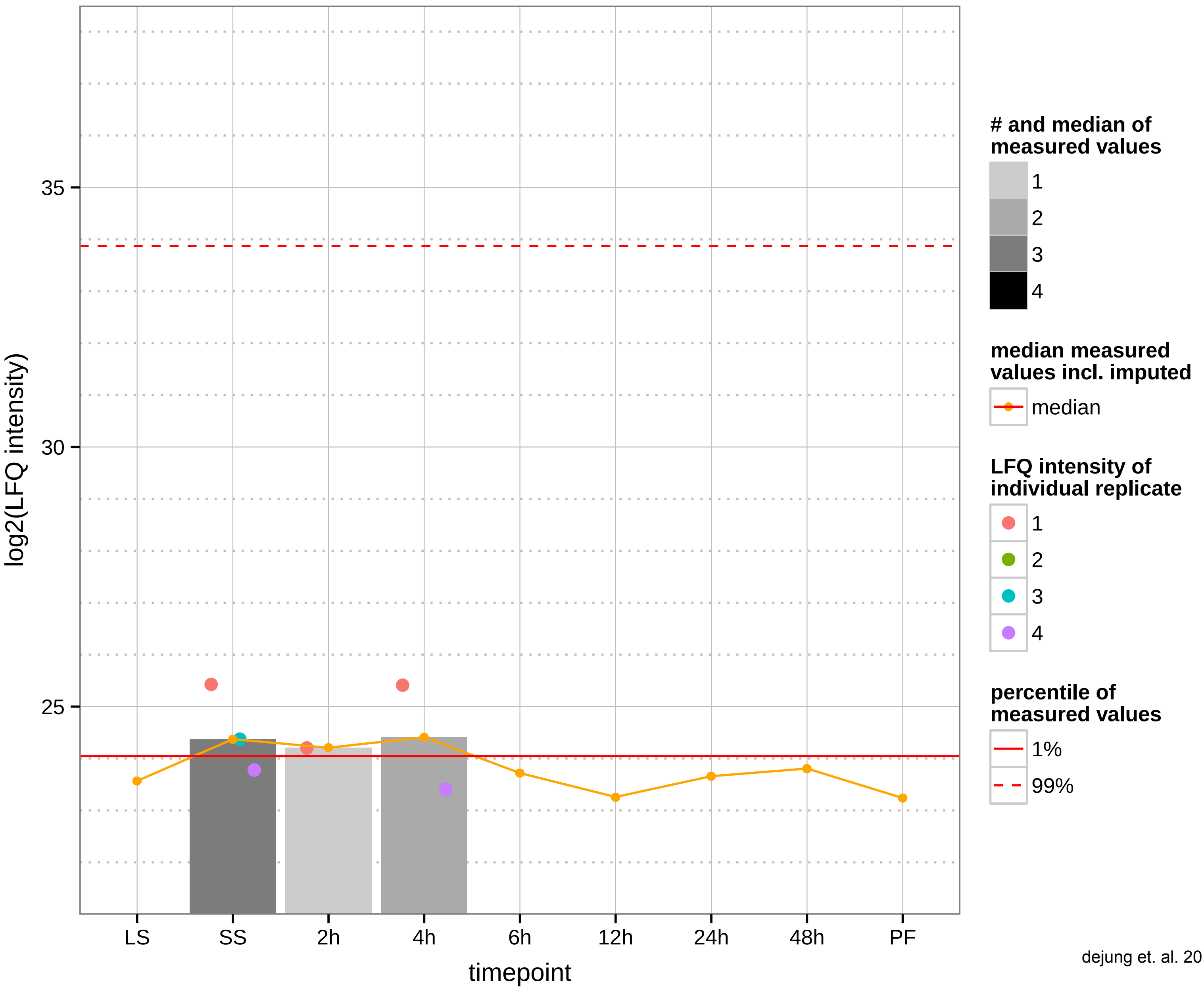

dejung et. al. 2015

hypothetical protein, conserved
Tb927.8.1840
AGOF: ARF guanyl–nucleotide exchange factor activity
AGOC: intracellular
AGOP: regulation of ARF protein signal transduction
PGOF: ARF guanyl–nucleotide exchange factor activity
PGOC: intracellular
PGOP: regulation of ARF protein signal transduction

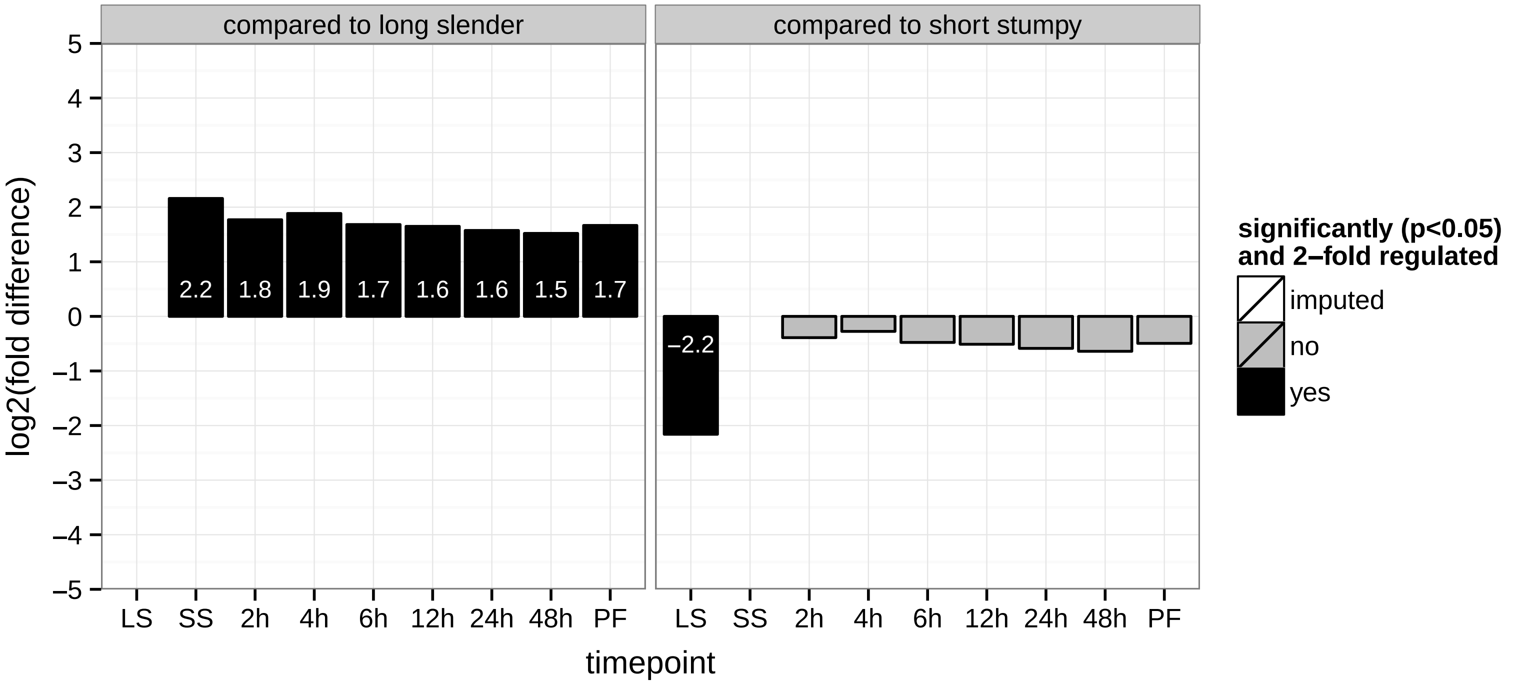

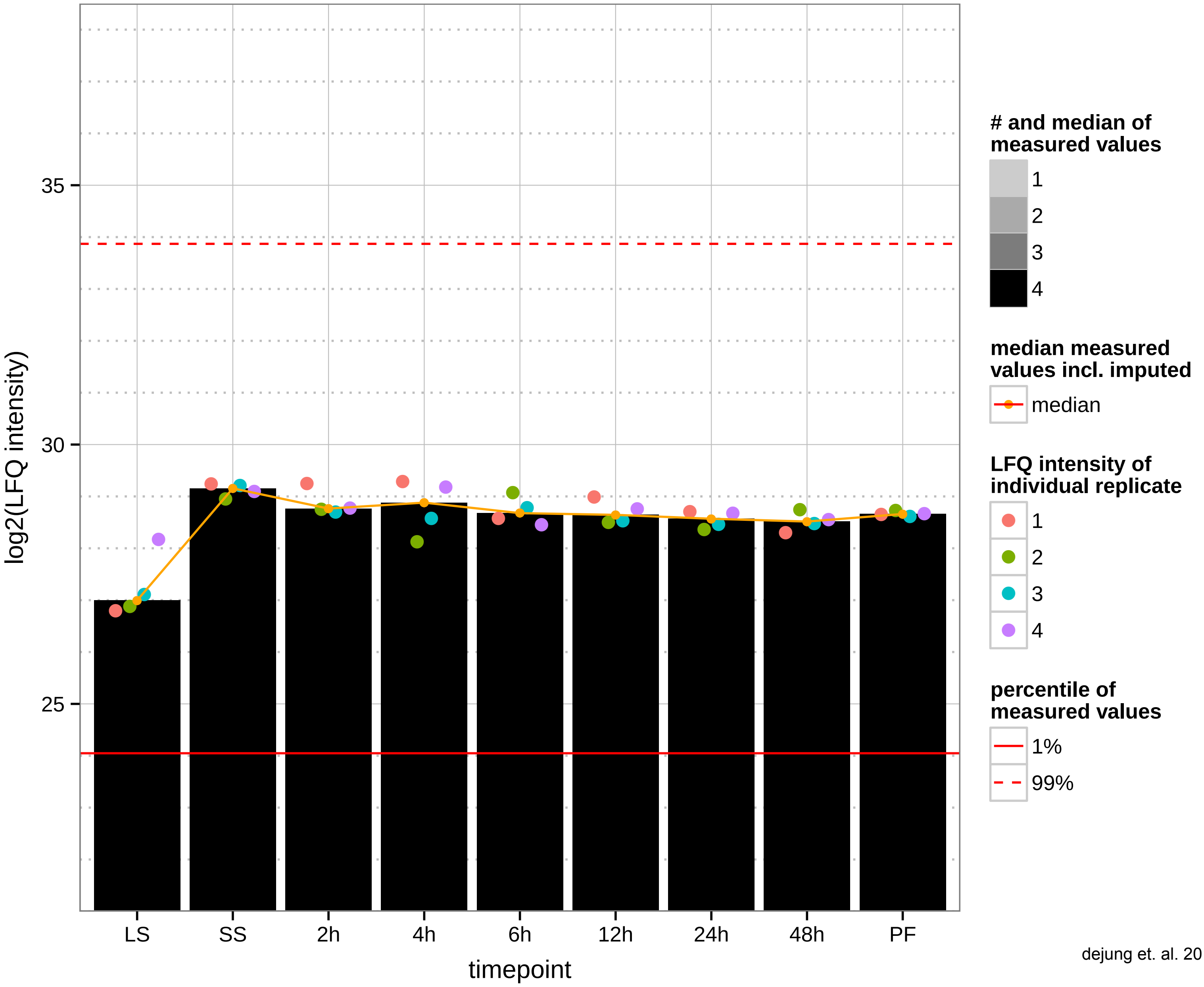

dejung et. al. 2015

hypothetical protein, conserved
Tb927.8.1960
AGOF: null
AGOC: cytoplasm, nucleus
AGOP: null
PGOF: null
PGOC: null
PGOP: null

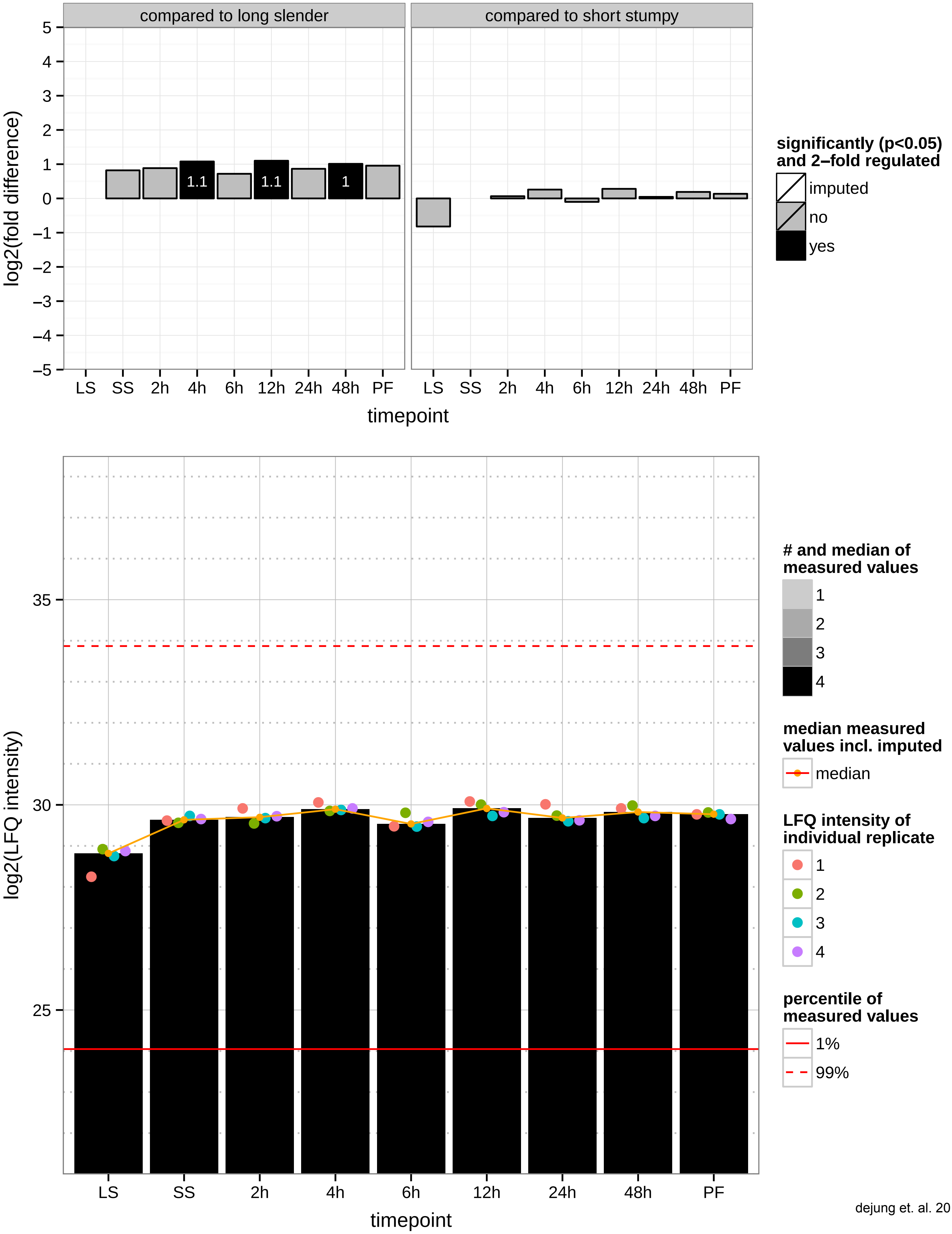

dejung et. al. 2015

hypothetical protein, conserved
Tb927.8.1980
AGOF: null
AGOC: nucleolus
AGOP: rRNA processing
PGOF: protein binding
PGOC: nucleolus
PGOP: rRNA processing

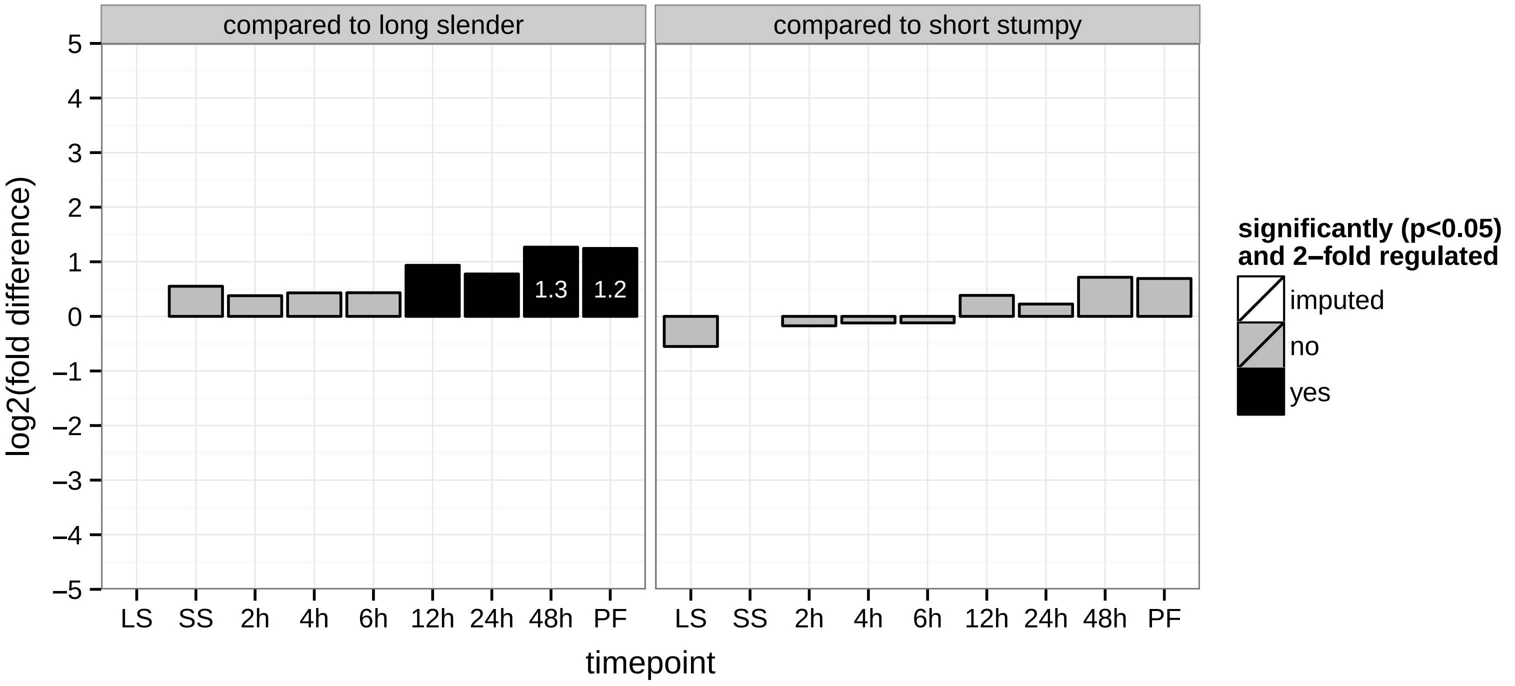

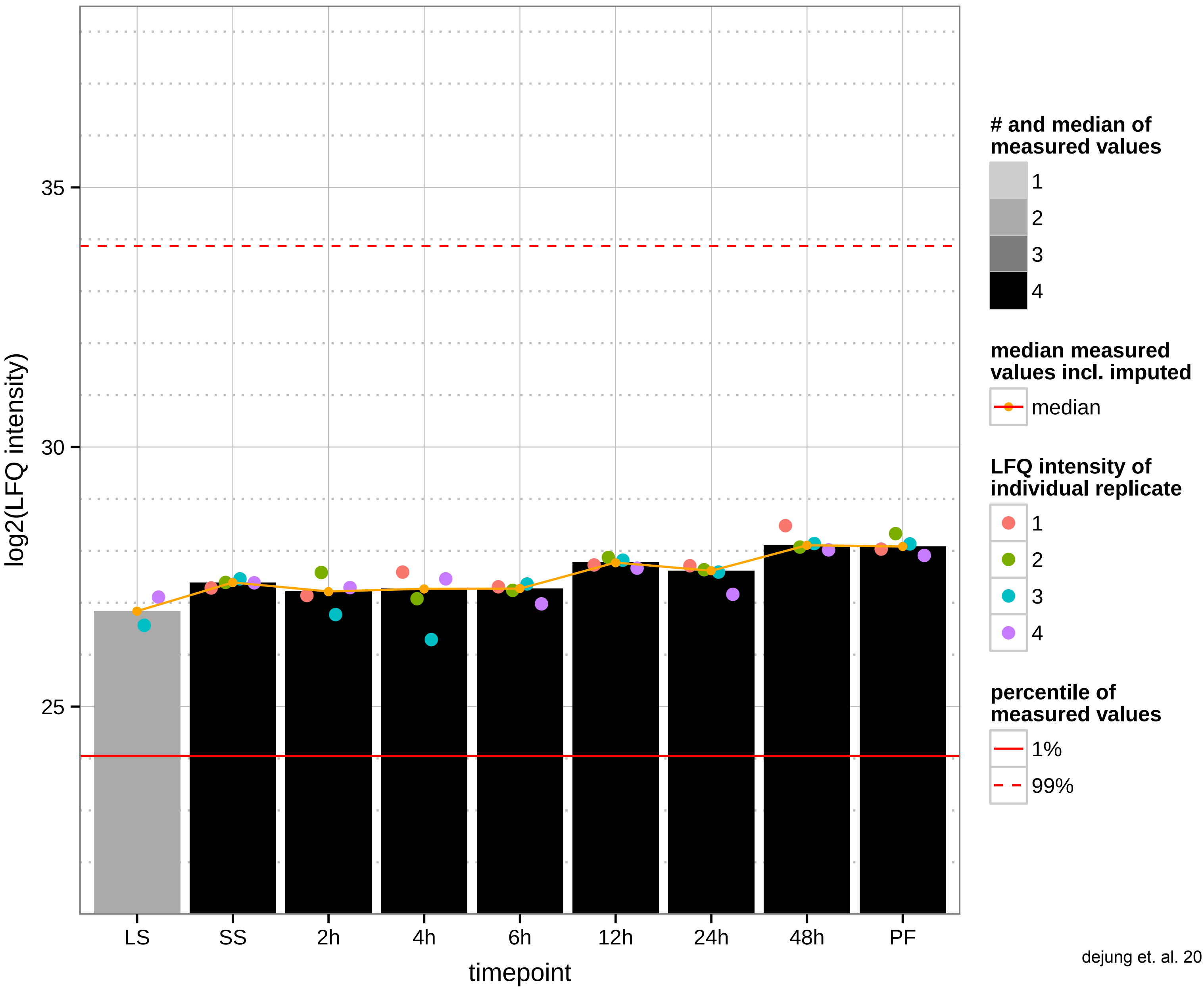

dejung et. al. 2015

peroxidoxin, tryparedoxin peroxidase (TRYP2)
Tb927.8.1990
AGOF: antioxidant activity, trypanothione−disulfide reductase activity
AGOC: mitochondrion
AGOP: cell redox homeostasis, oxidation−reduction process
PGOF: antioxidant activity, oxidoreductase activity, peroxiredoxin activity
PGOC: null
PGOP: oxidation−reduction process

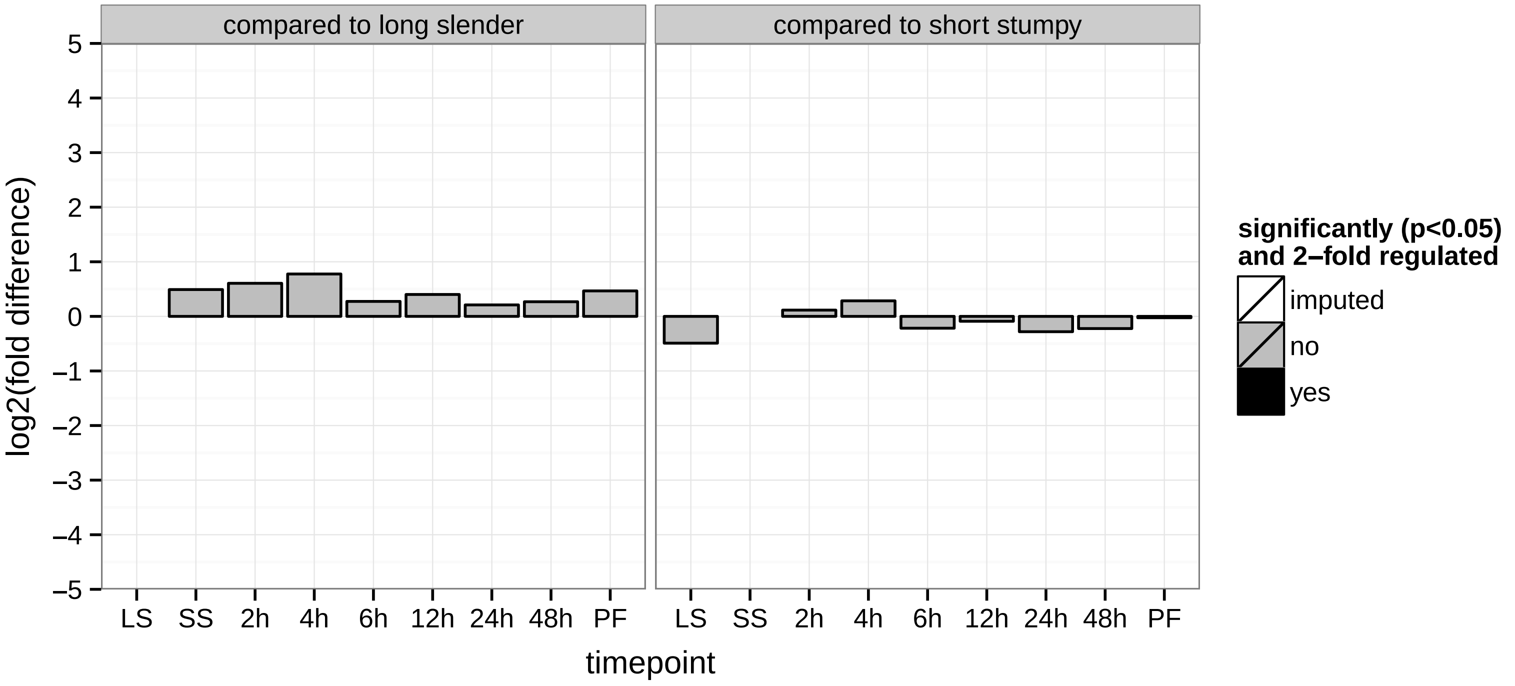

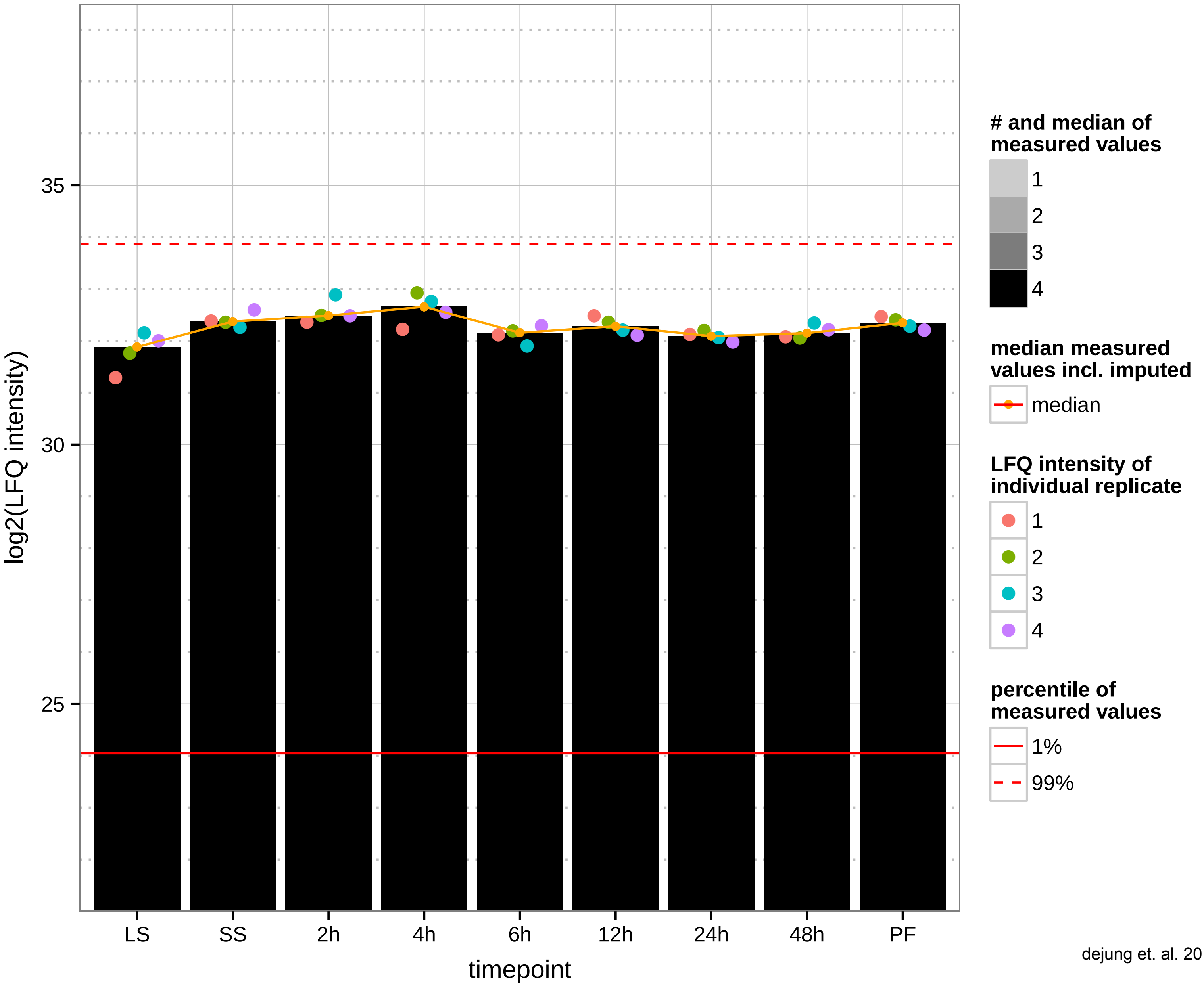

dejung et. al. 2015

agmatinase, putative
Tb927.8.2020
AGOF: arginase activity, metal ion binding
AGOC: null
AGOP: arginine metabolic process
PGOF: hydrolase activity, acting on carbon–nitrogen (but not peptide) bonds, in linear amidines, metal ion binding
PGOC: null
PGOP: null

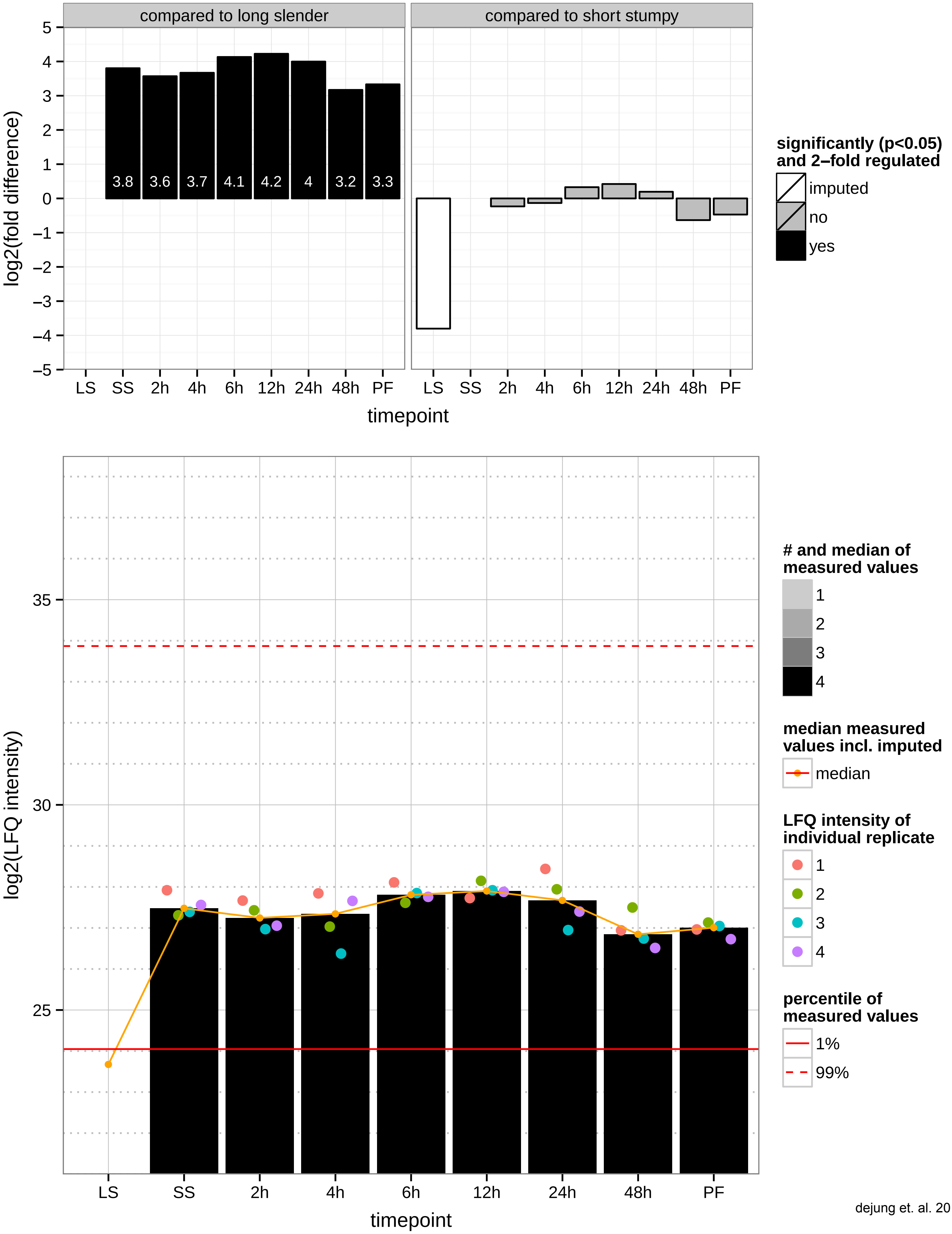

dejung et. al. 2015

GDP–mannose pyrophosphorylase
Tb927.8.2050
AGOF: mannose–1–phosphate guanylyltransferase activity, nucleotidyltransferase activity
AGOC: cytoplasm
AGOP: GDP–mannose biosynthetic process, protein glycosylation
PGOF: nucleotidyltransferase activity
PGOC: null
PGOP: biosynthetic process

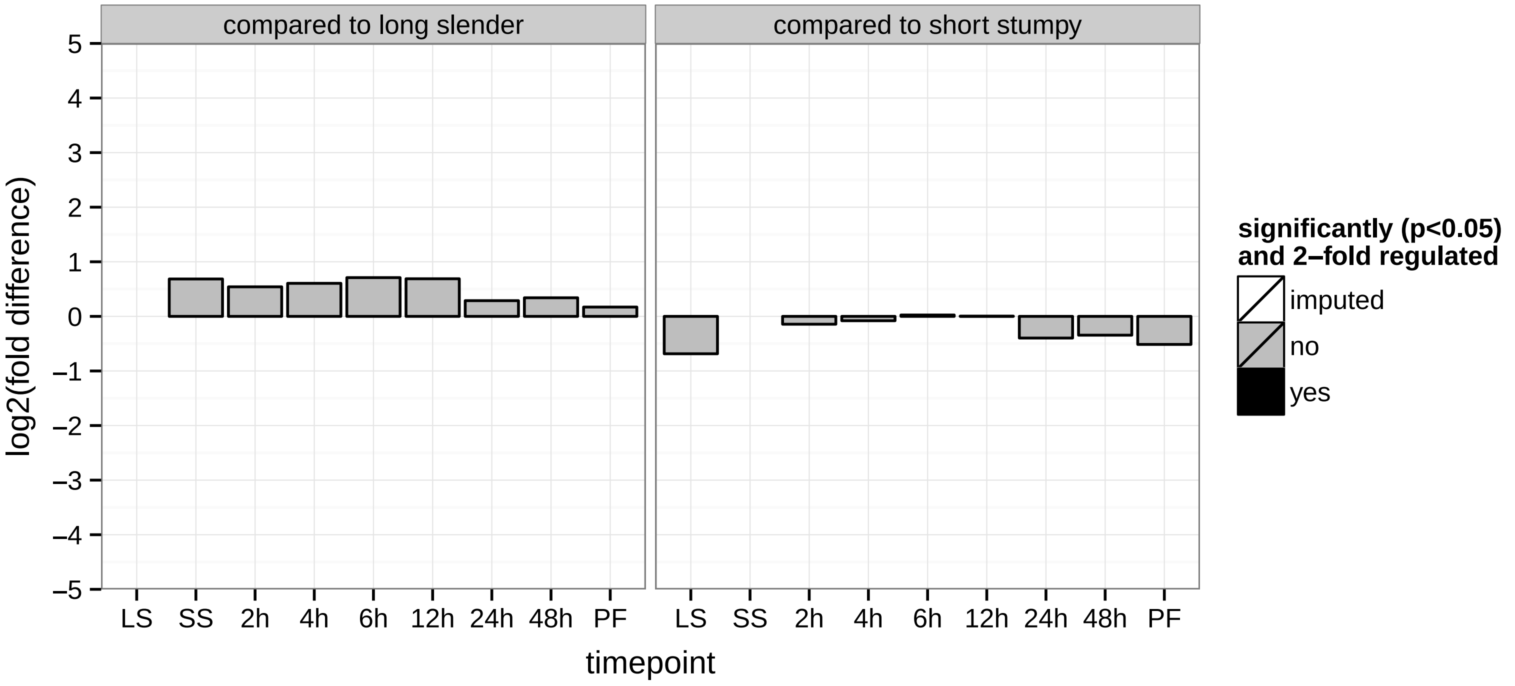

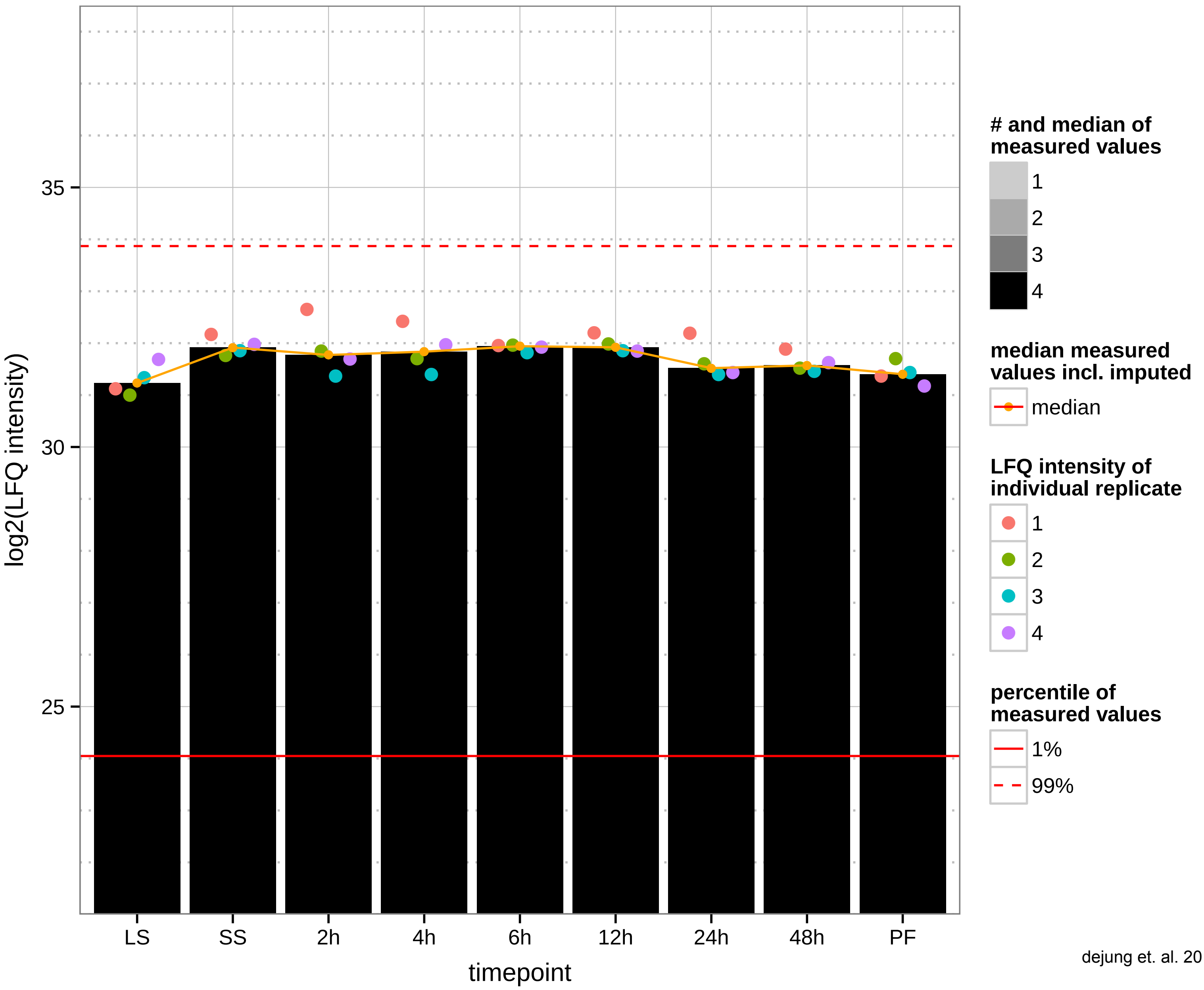

dejung et. al. 2015

hypothetical protein, conserved
Tb927.8.2120
AGOF: null
AGOC: null
AGOP: null
PGOF: null
PGOC: null
PGOP: null

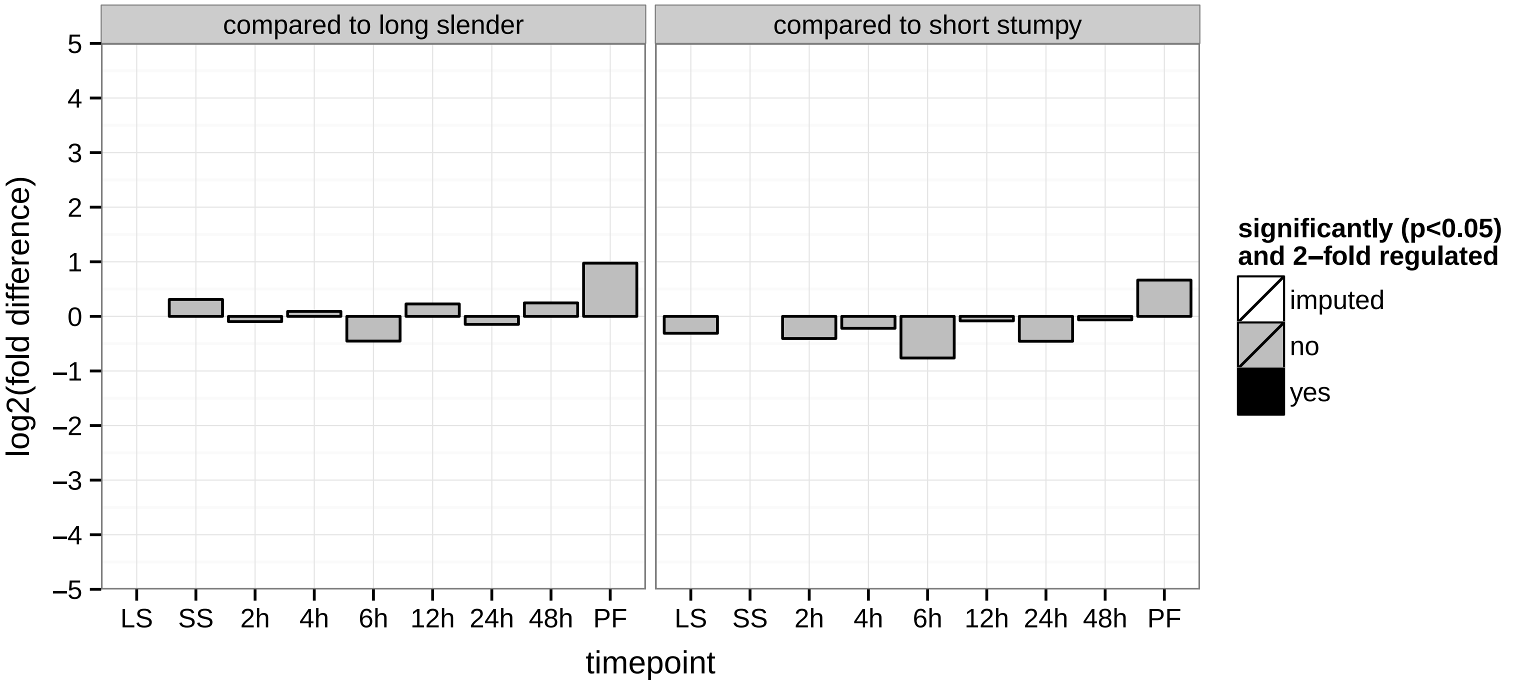

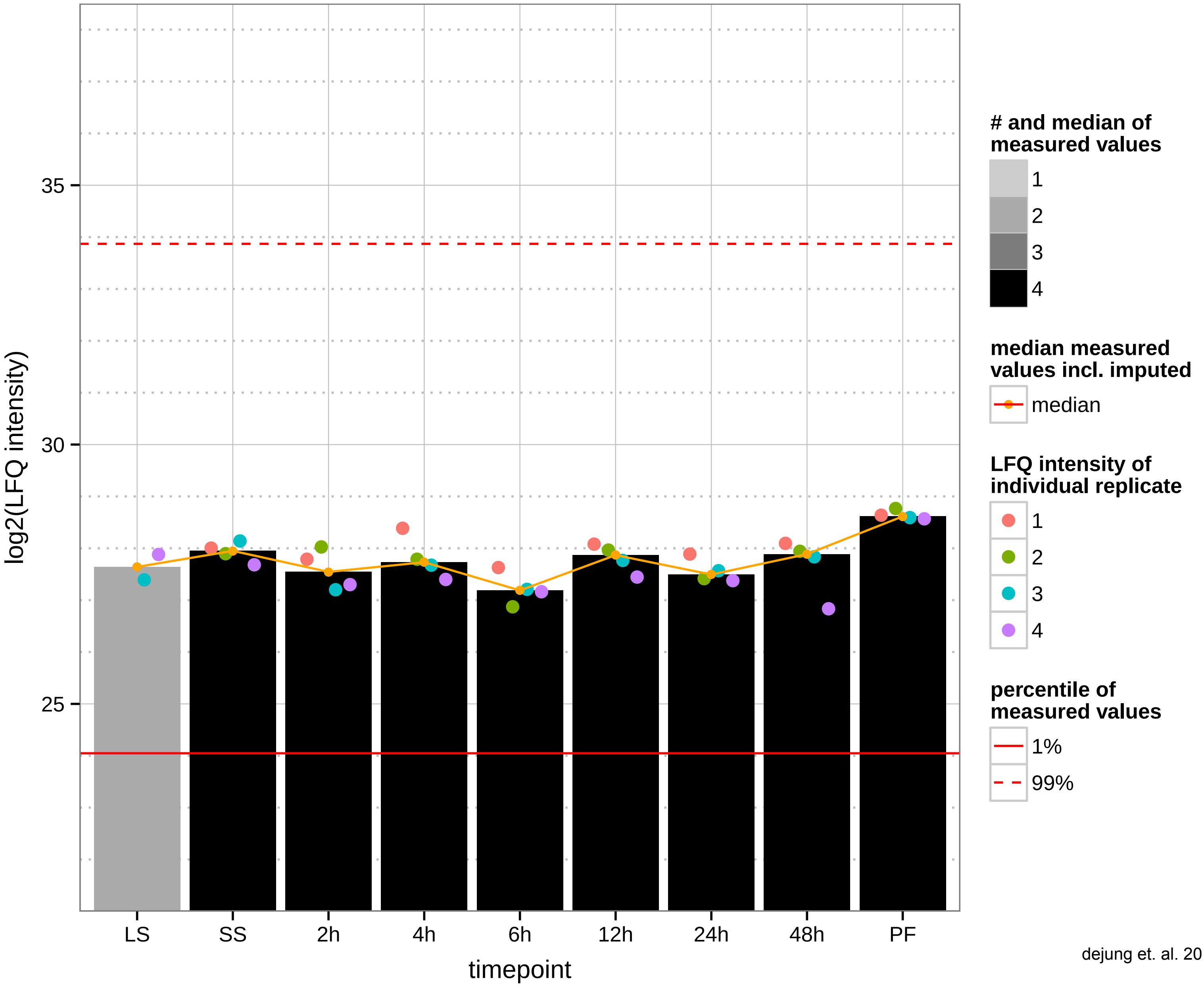

dejung et. al. 2015

terbinafine resistance locus protein (yip1), putative
Tb927.8.2200
AGOF: null
AGOC: Golgi apparatus, integral to membrane
AGOP: response to drug, vesicle−mediated transport
PGOF: null
PGOC: membrane
PGOP: null

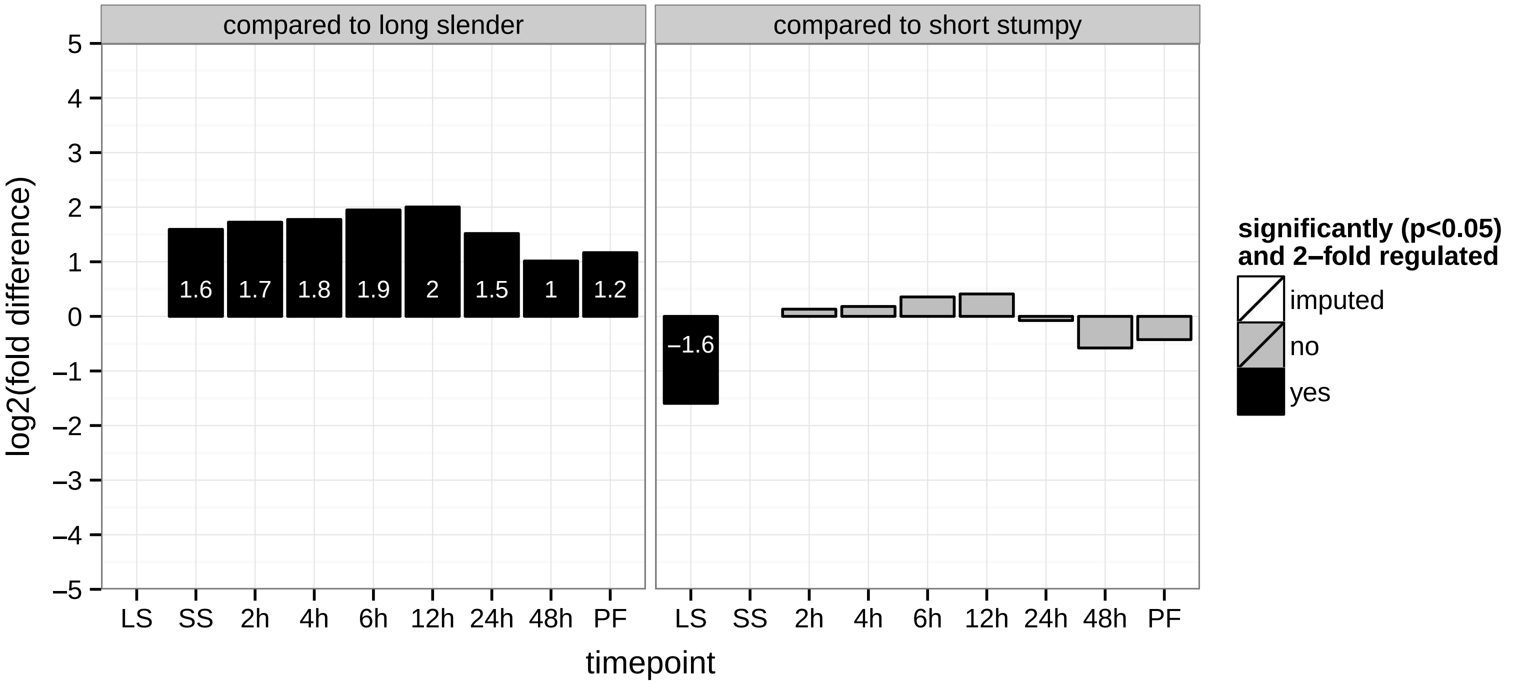

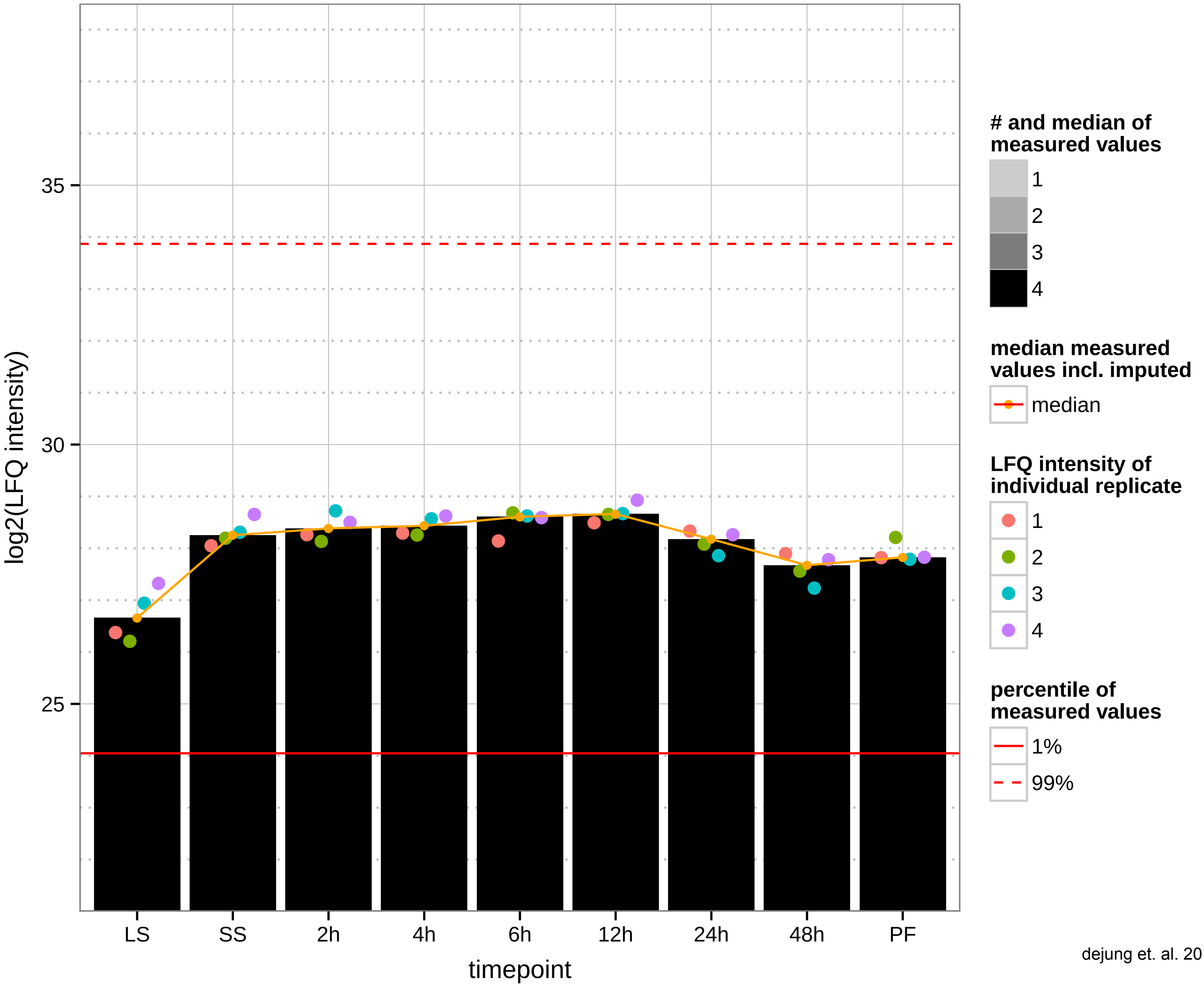

dejung et. al. 2015

pteridine reductase (PTR1)
Tb927.8.2210
AGOF: oxidoreductase activity, pteridine reductase activity
AGOC: cytosol
AGOP: metabolic process
PGOF: null
PGOC: null
PGOP: null

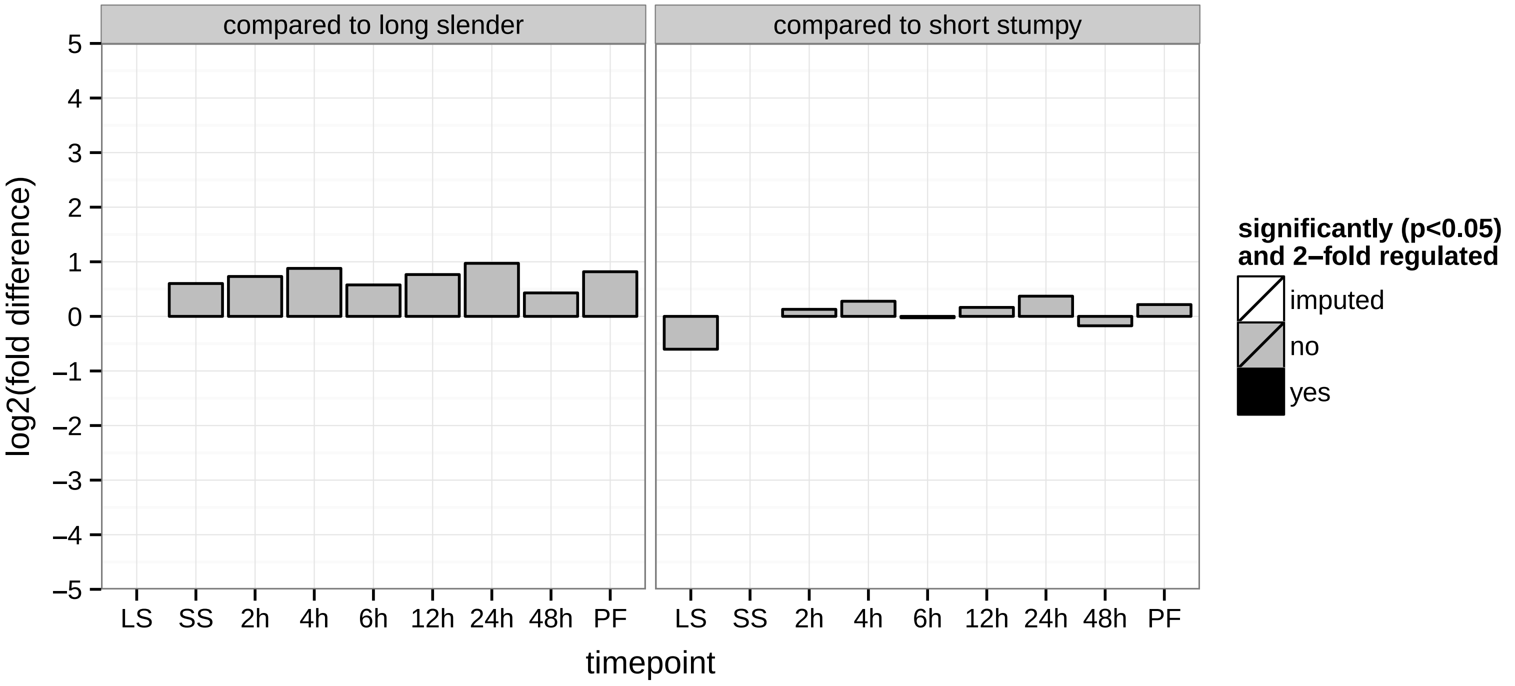

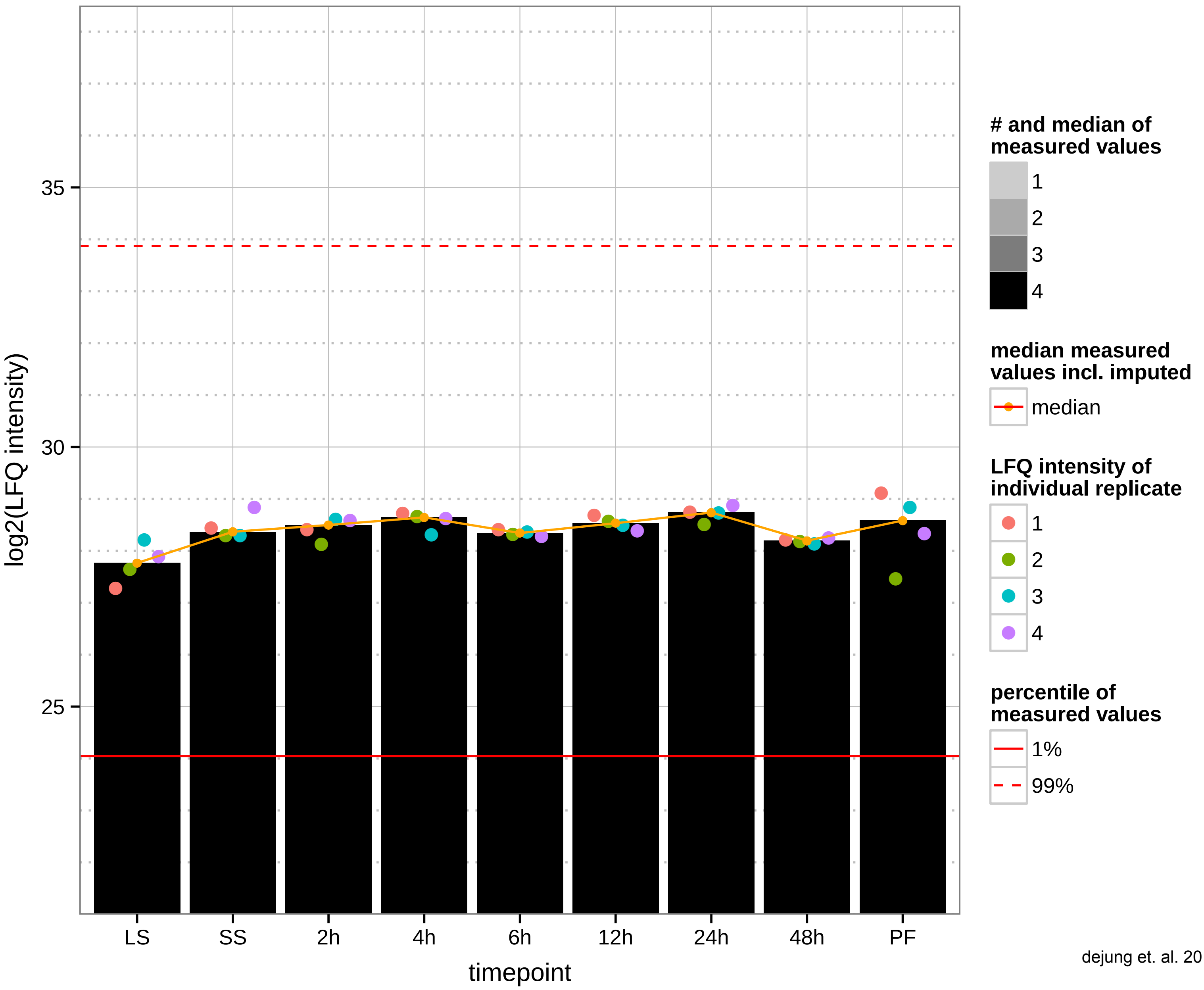

dejung et. al. 2015

hypothetical protein, conserved
Tb927.8.2290
AGOF: null
AGOC: null
AGOP: null
PGOF: null
PGOC: null
PGOP: null

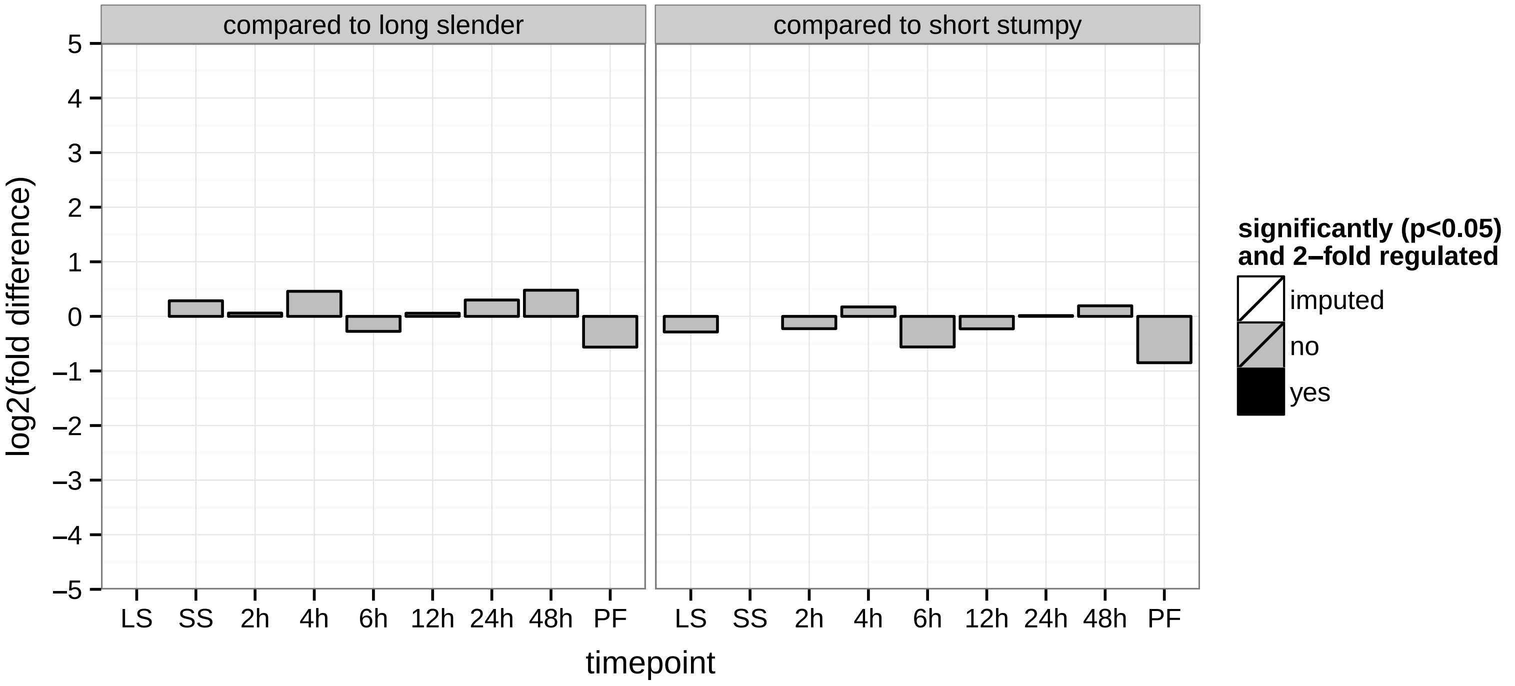

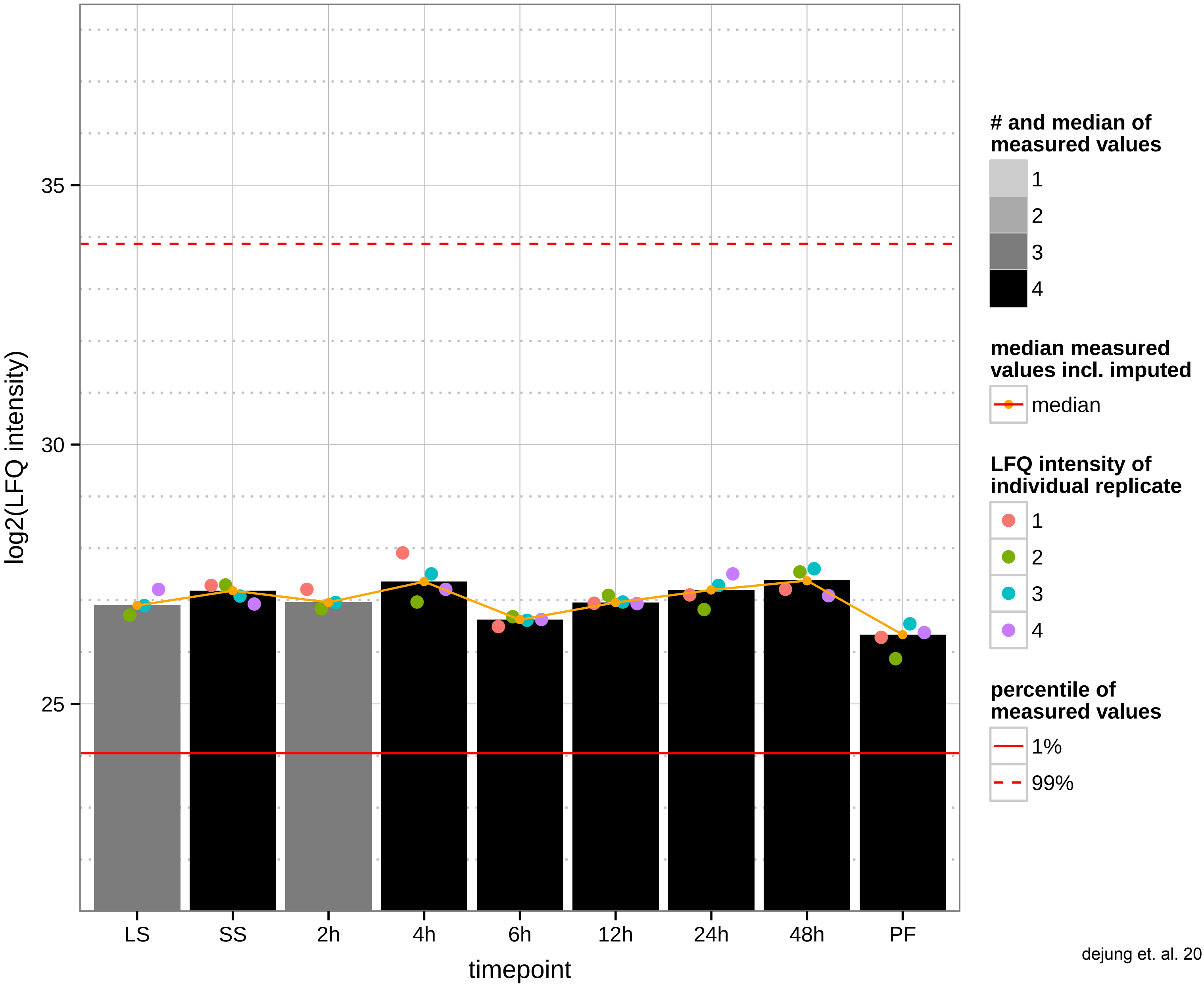

dejung et. al. 2015

(H+)–ATPase G subunit, putative
Tb927.8.2310
AGOF: ATPase activity, hydrolase activity, acting on acid anhydrides, catalyzing transmembrane movement of substances
AGOC: myosin complex, vacuolar proton–transporting V–type ATPase complex
AGOP: proton transport, vacuolar acidification
PGOF: hydrolase activity, acting on acid anhydrides, catalyzing transmembrane movement of substances
PGOC: vacuolar proton–transporting V–type ATPase complex
PGOP: proton transport

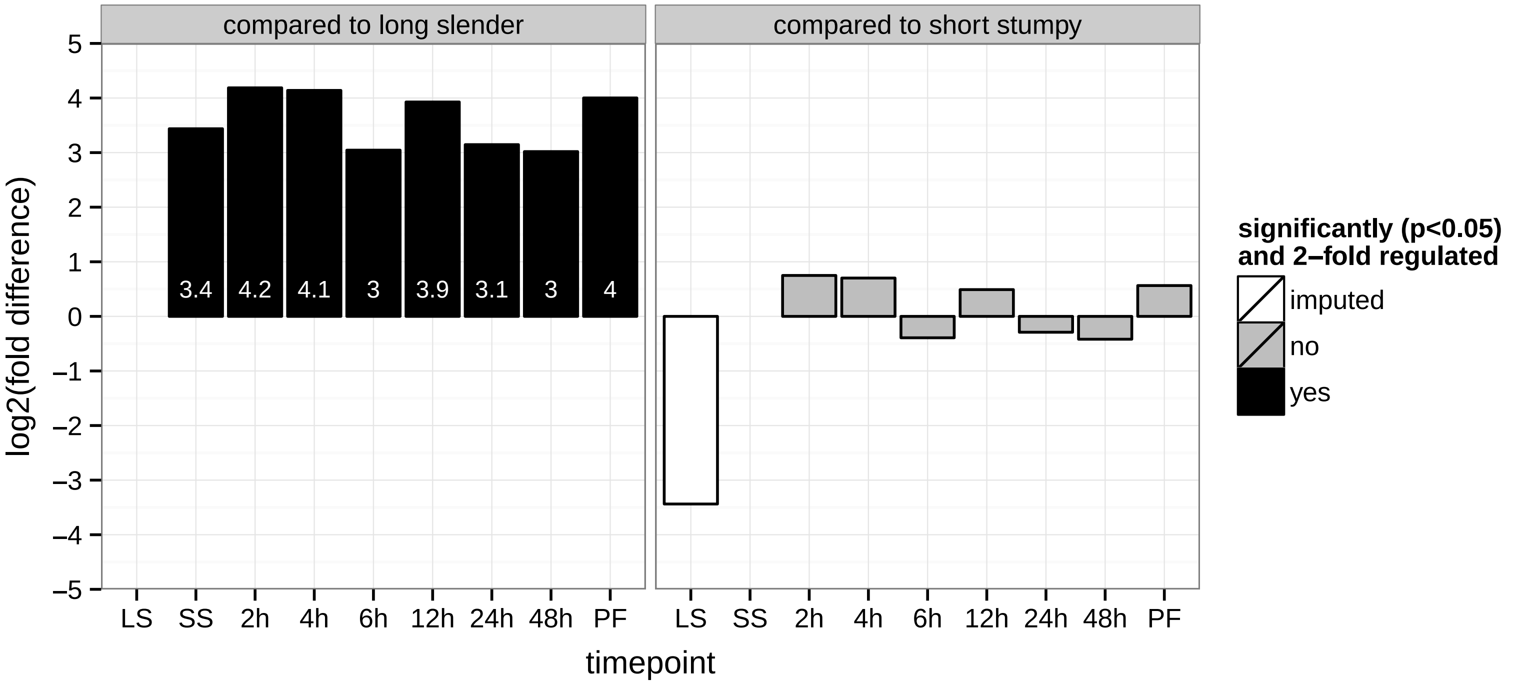

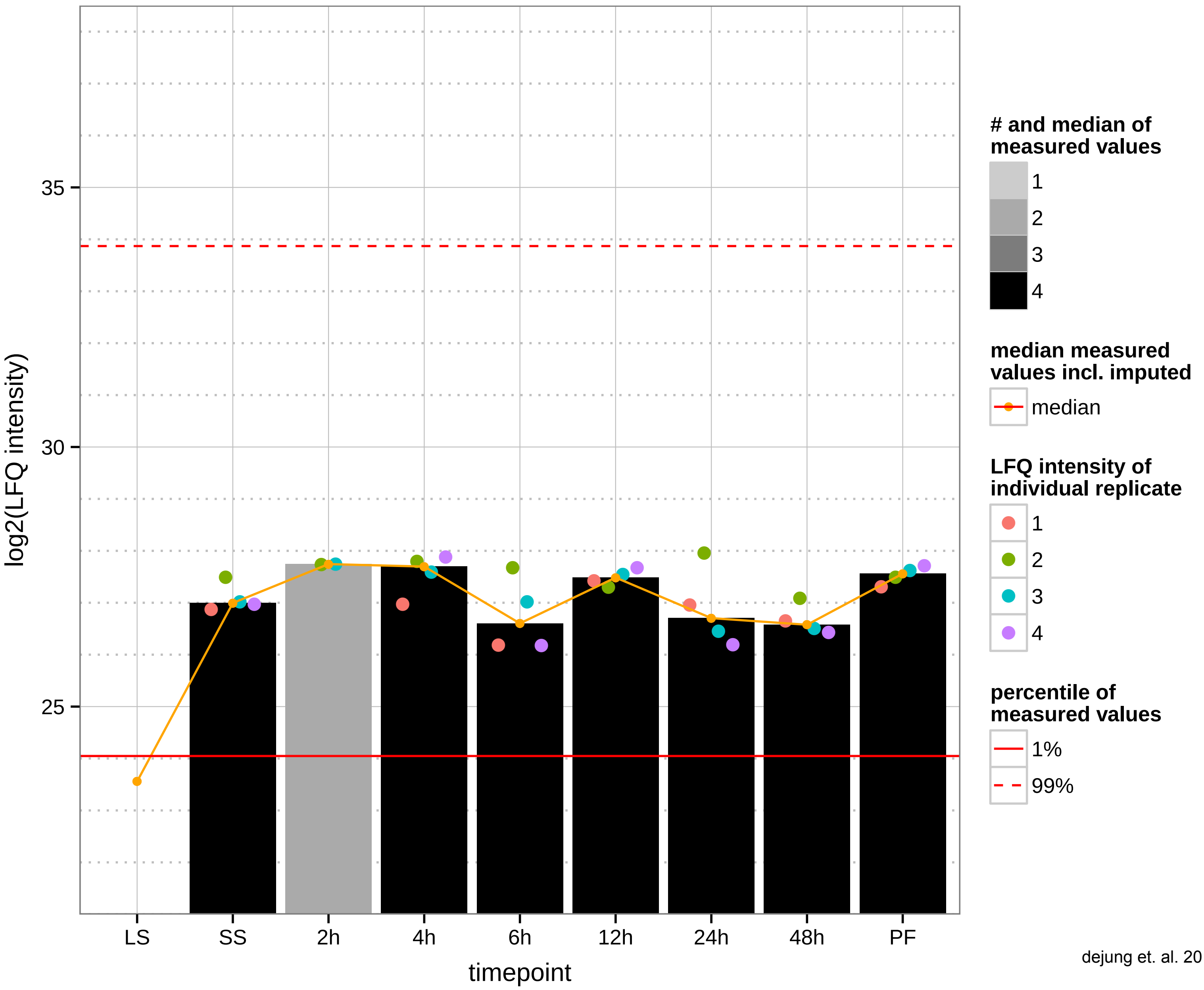

dejung et. al. 2015

ABC transporter, putative
Tb927.8.2380
AGOF: ATP binding, ATPase activity, coupled to transmembrane movement of substances, heme binding, protein homodimeri
AGOC: flagellar pocket, integral to membrane, membrane, vesicle
AGOP: heme transport
PGOF: ATP binding, ATPase activity, nucleoside–triphosphatase activity, nucleotide binding
PGOC: membrane
PGOP: null

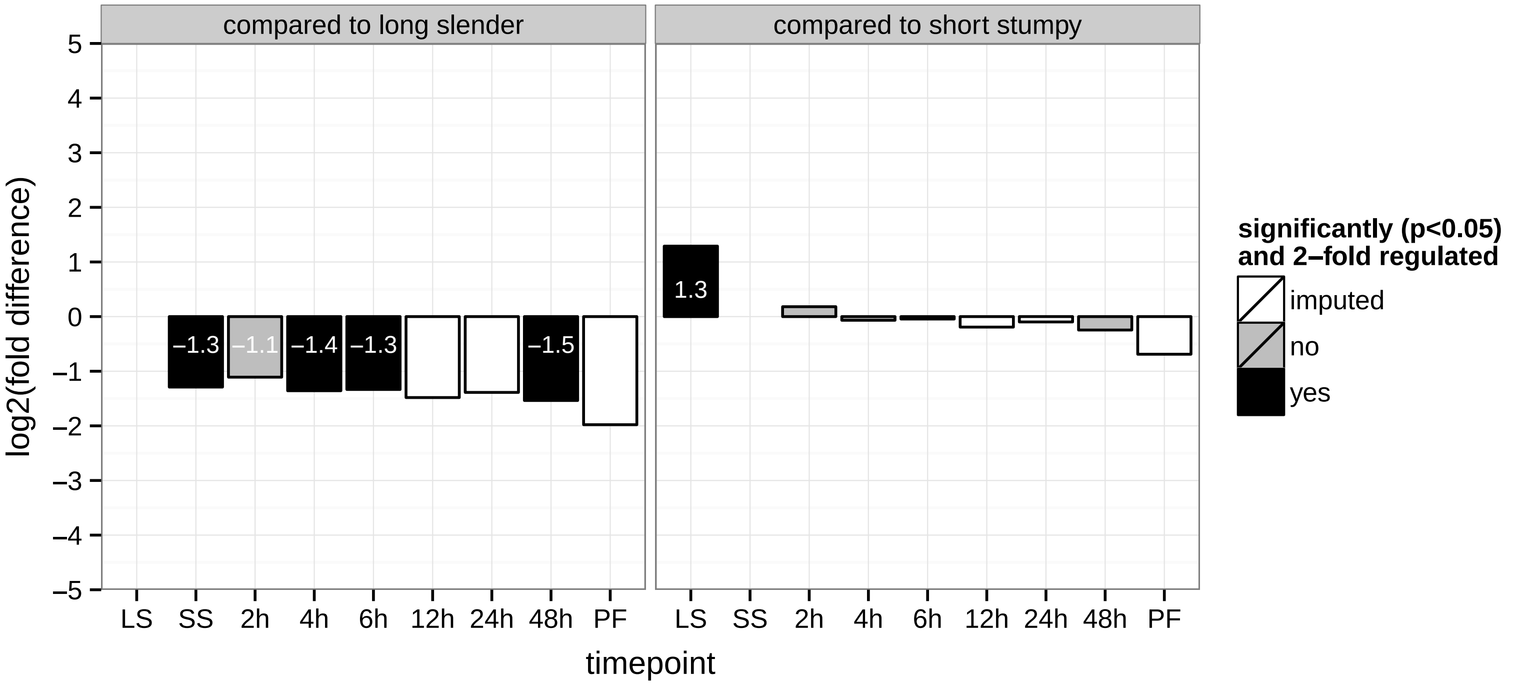

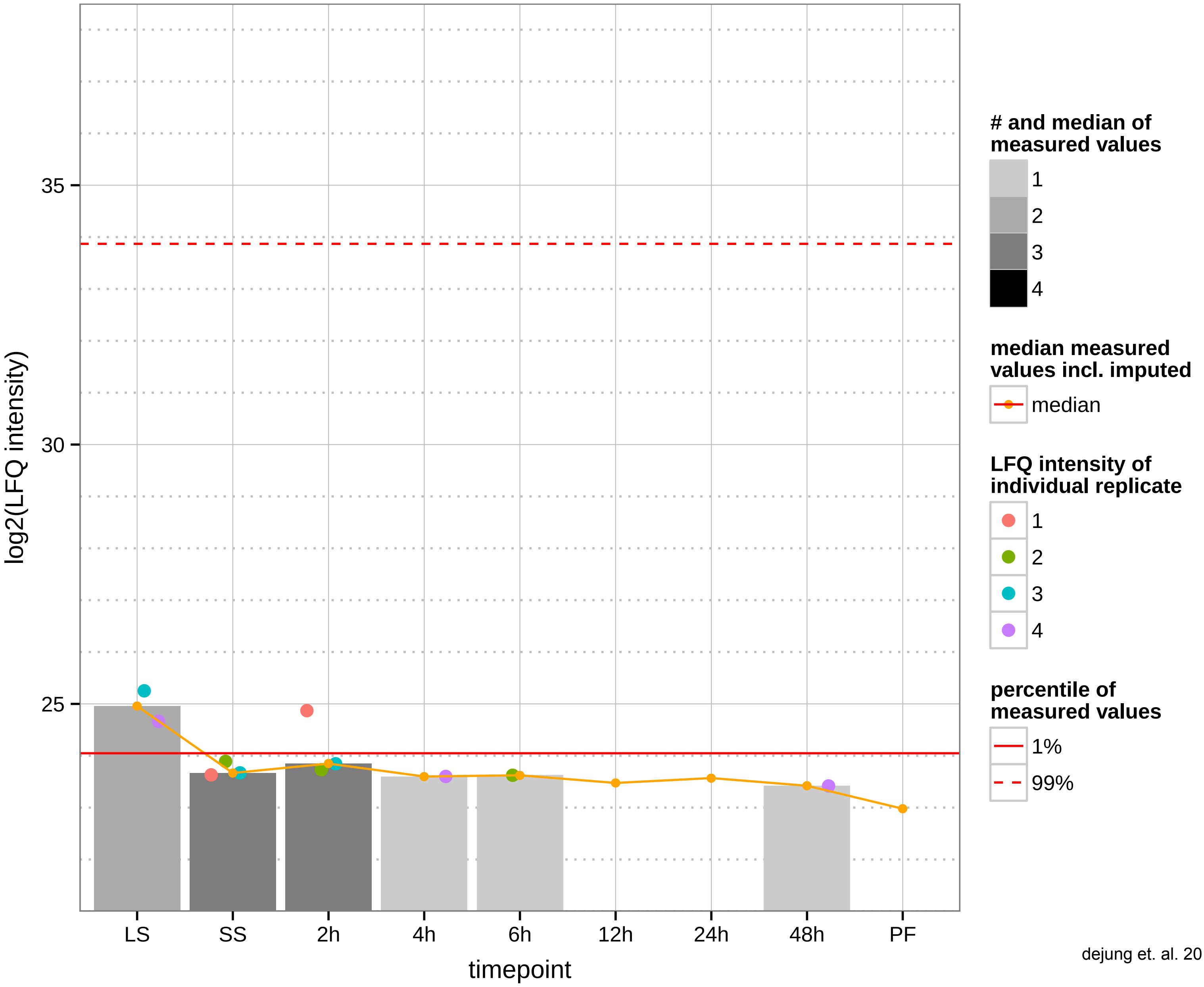

dejung et. al. 2015

hypothetical protein, conserved
Tb927.8.2450
AGOF: null
AGOC: null
AGOP: null
PGOF: protein binding
PGOC: null
PGOP: null

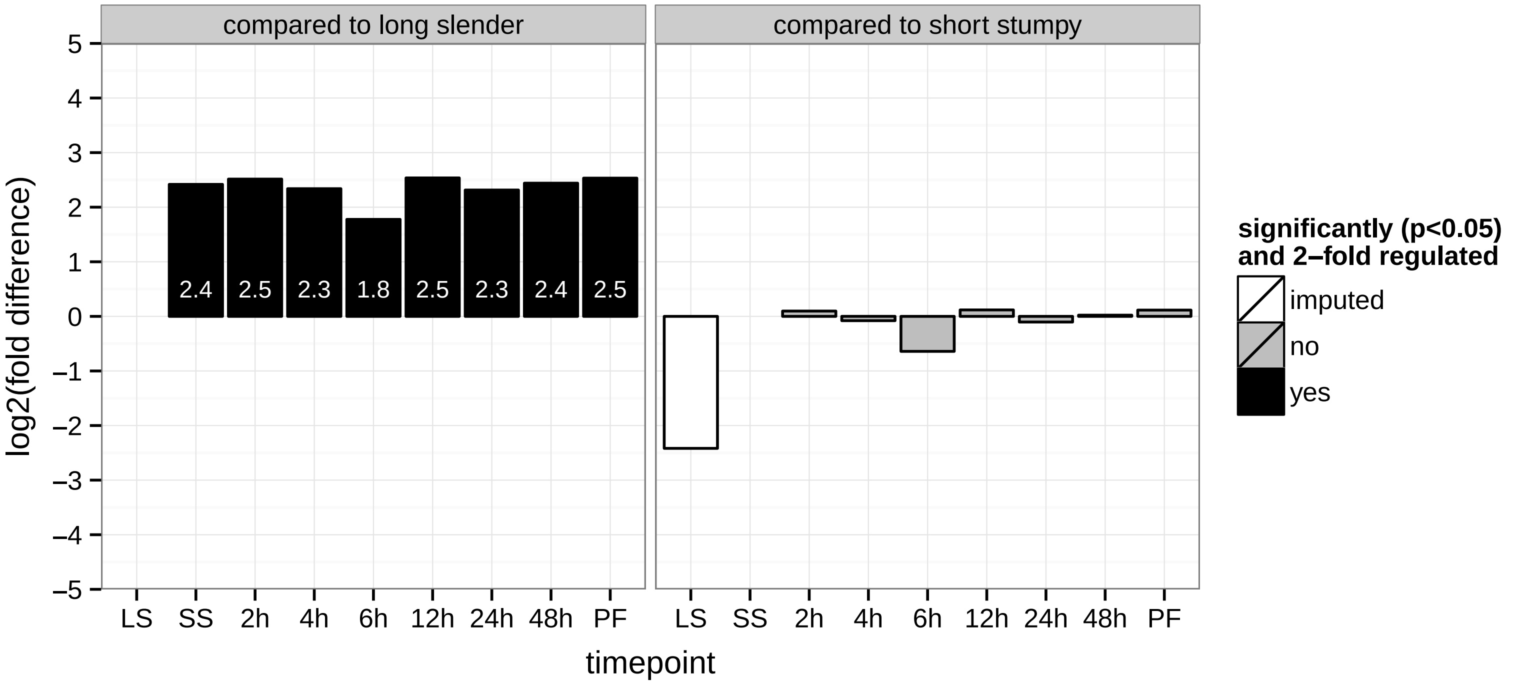

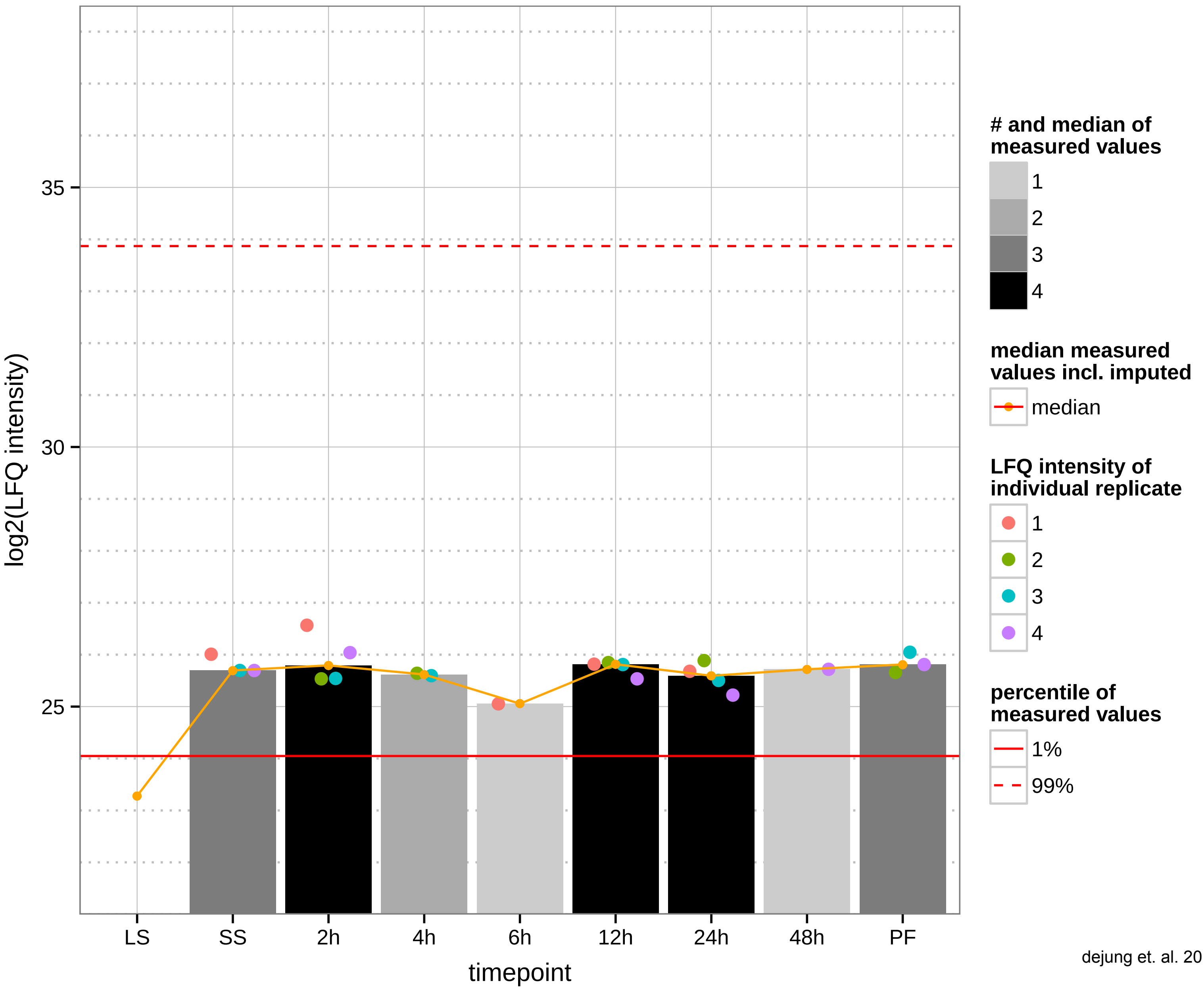

dejung et. al. 2015

hypothetical protein, conserved
Tb927.8.2460
AGOF: null
AGOC: null
AGOP: null
PGOF: null
PGOC: null
PGOP: null

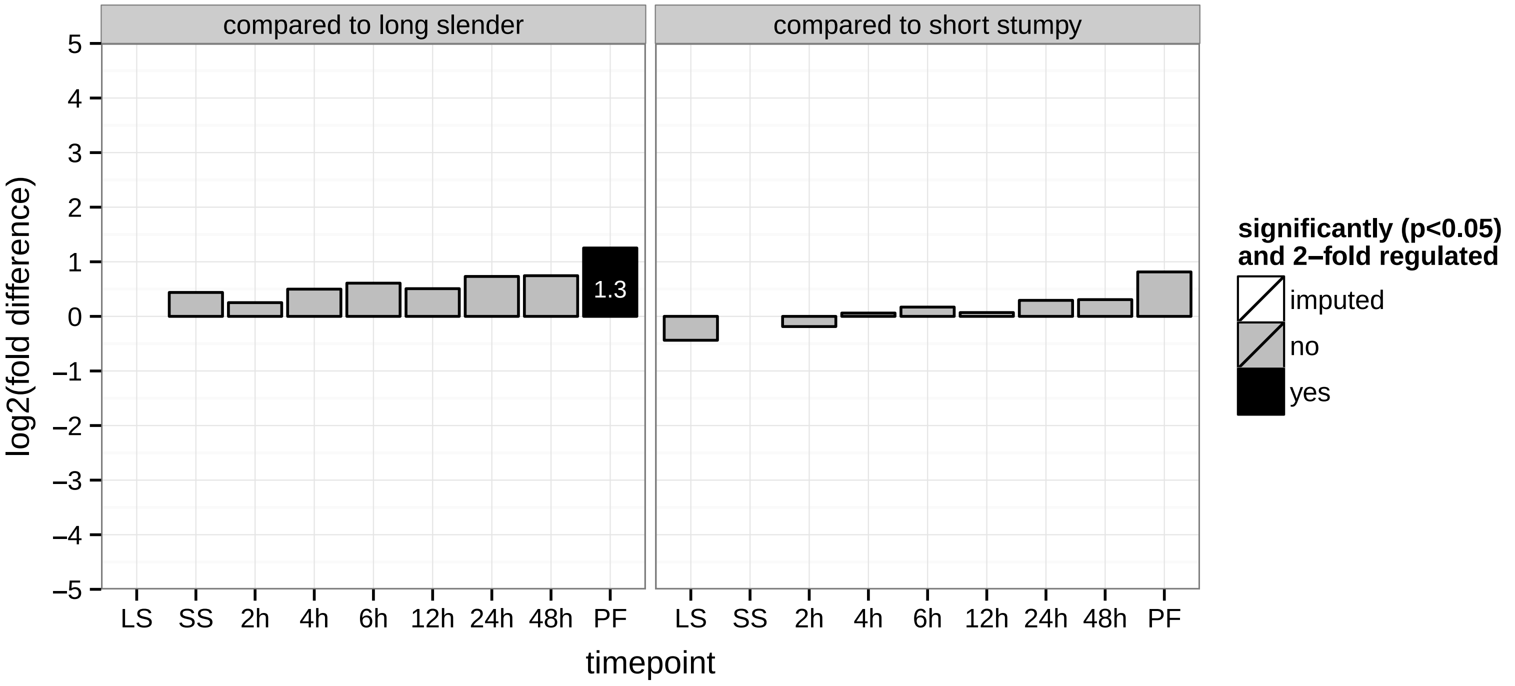

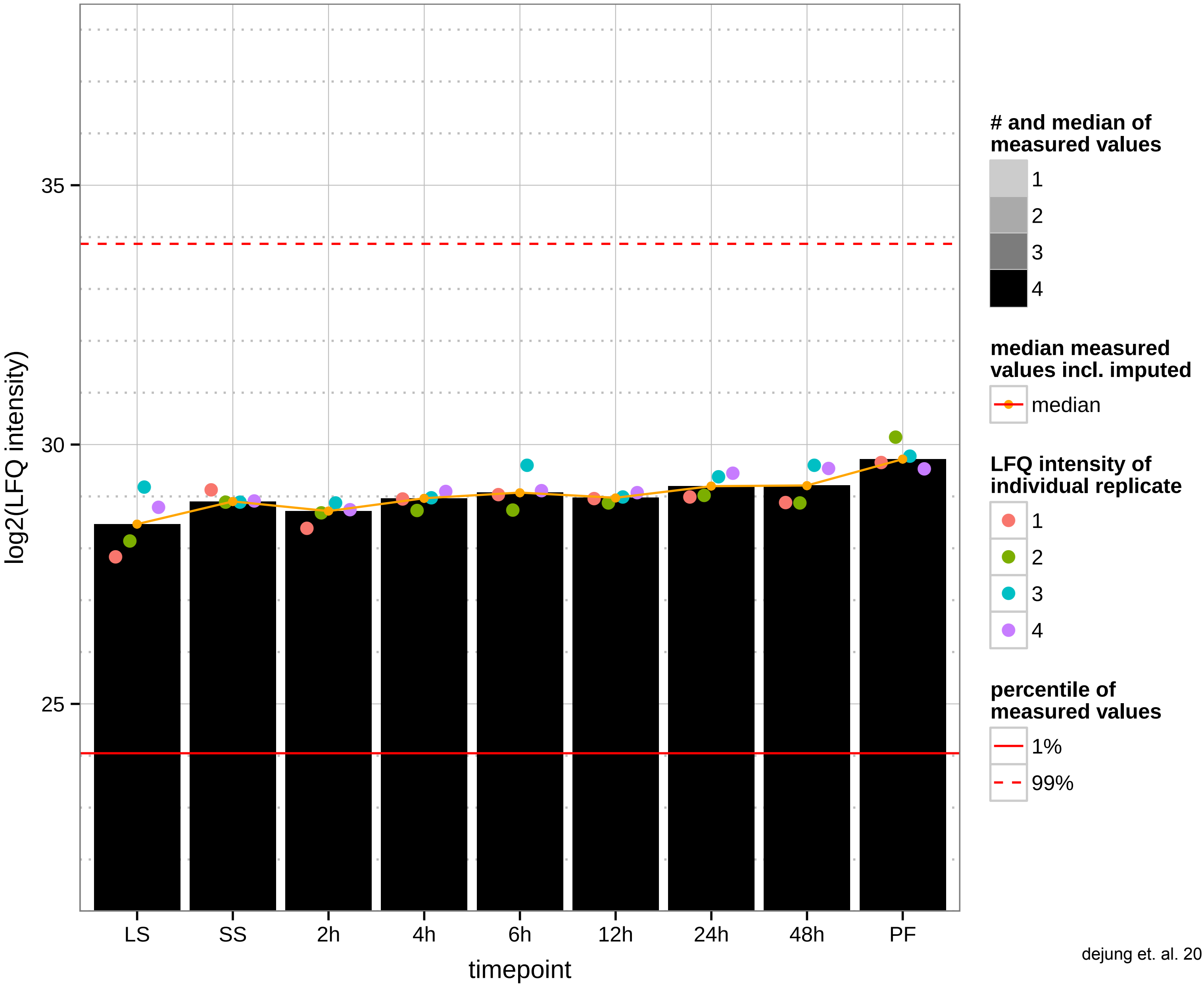

dejung et. al. 2015

hypothetical protein, conserved
Tb927.8.2590
AGOF: null
AGOC: null
AGOP: null
PGOF: null
PGOC: null
PGOP: null

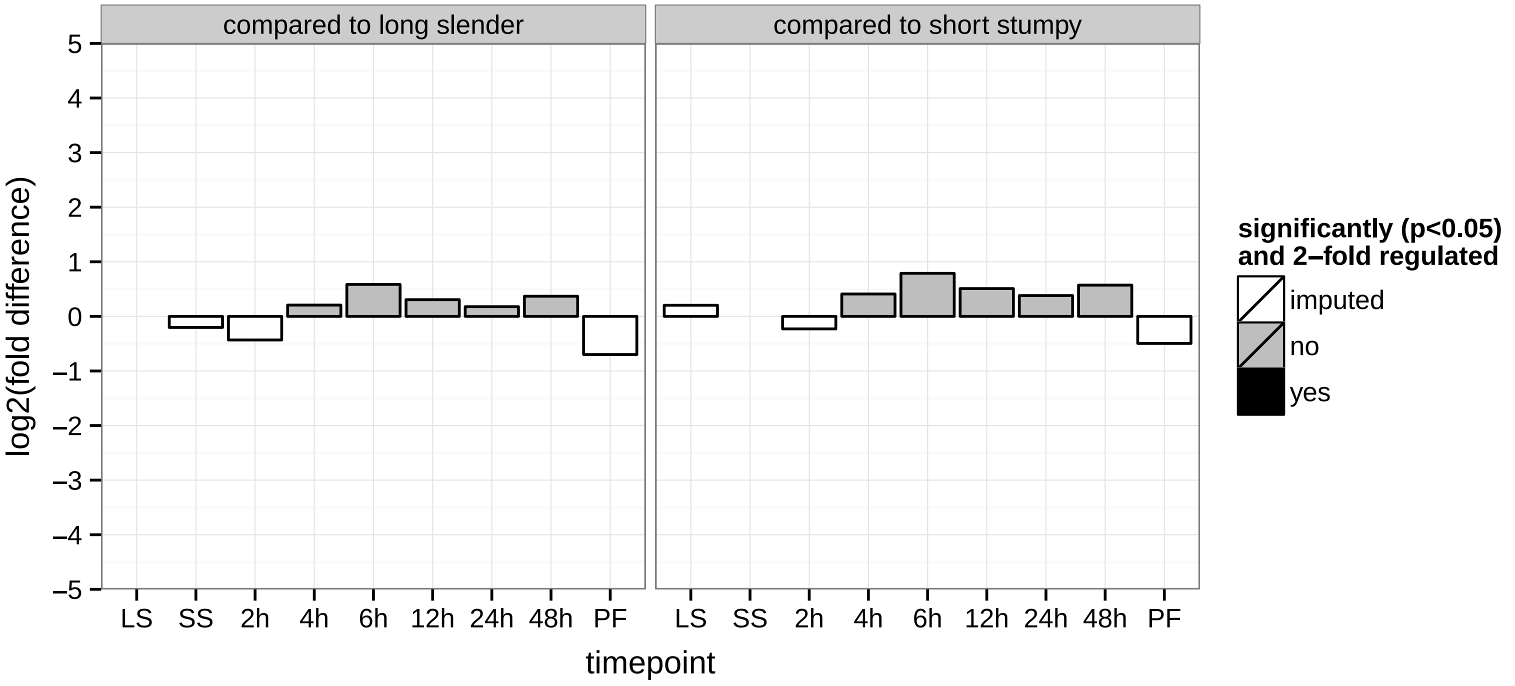

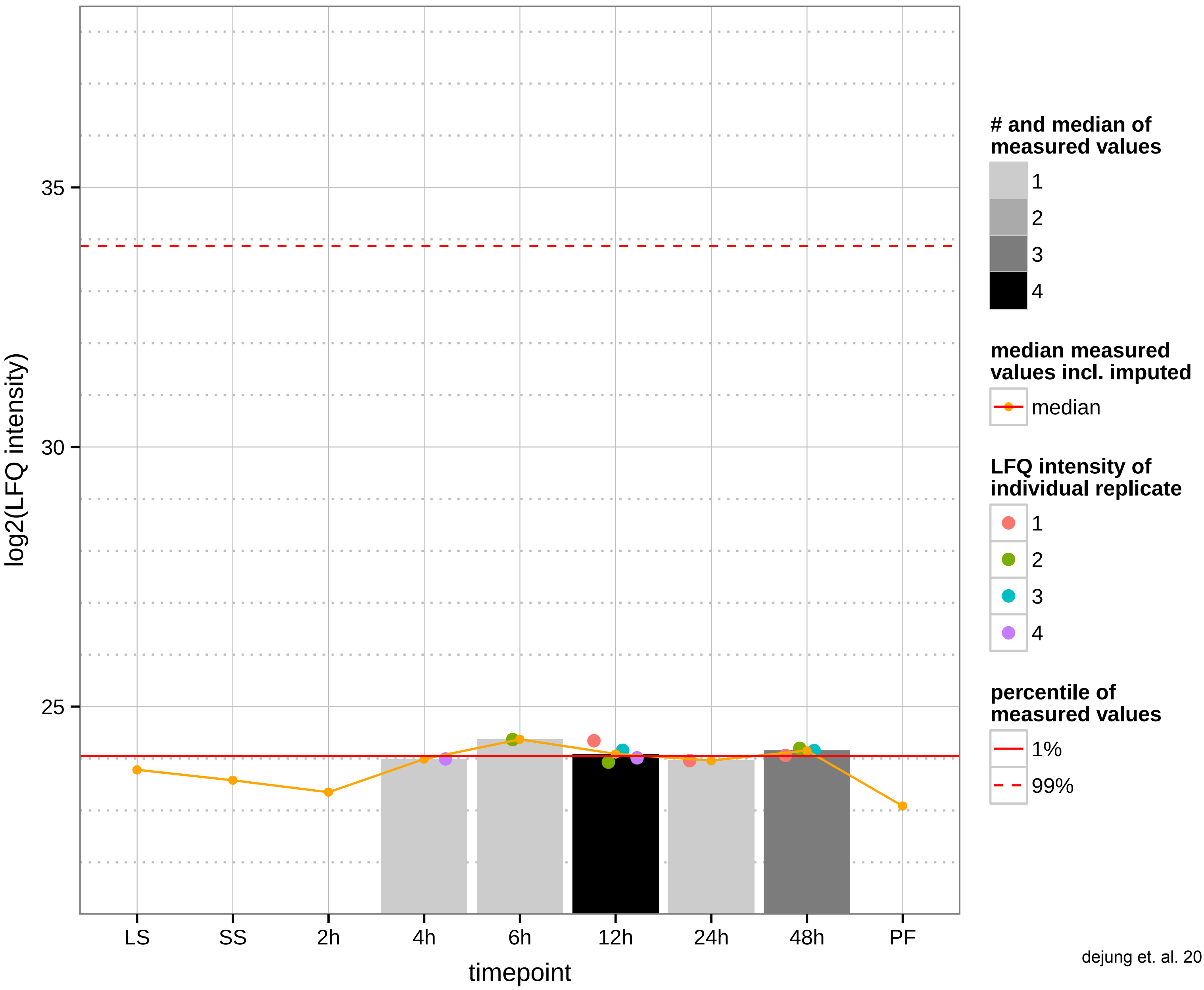

dejung et. al. 2015

hypothetical protein, conserved
Tb927.8.2600
AGOF: null
AGOC: null
AGOP: null
PGOF: protein binding
PGOC: null
PGOP: null

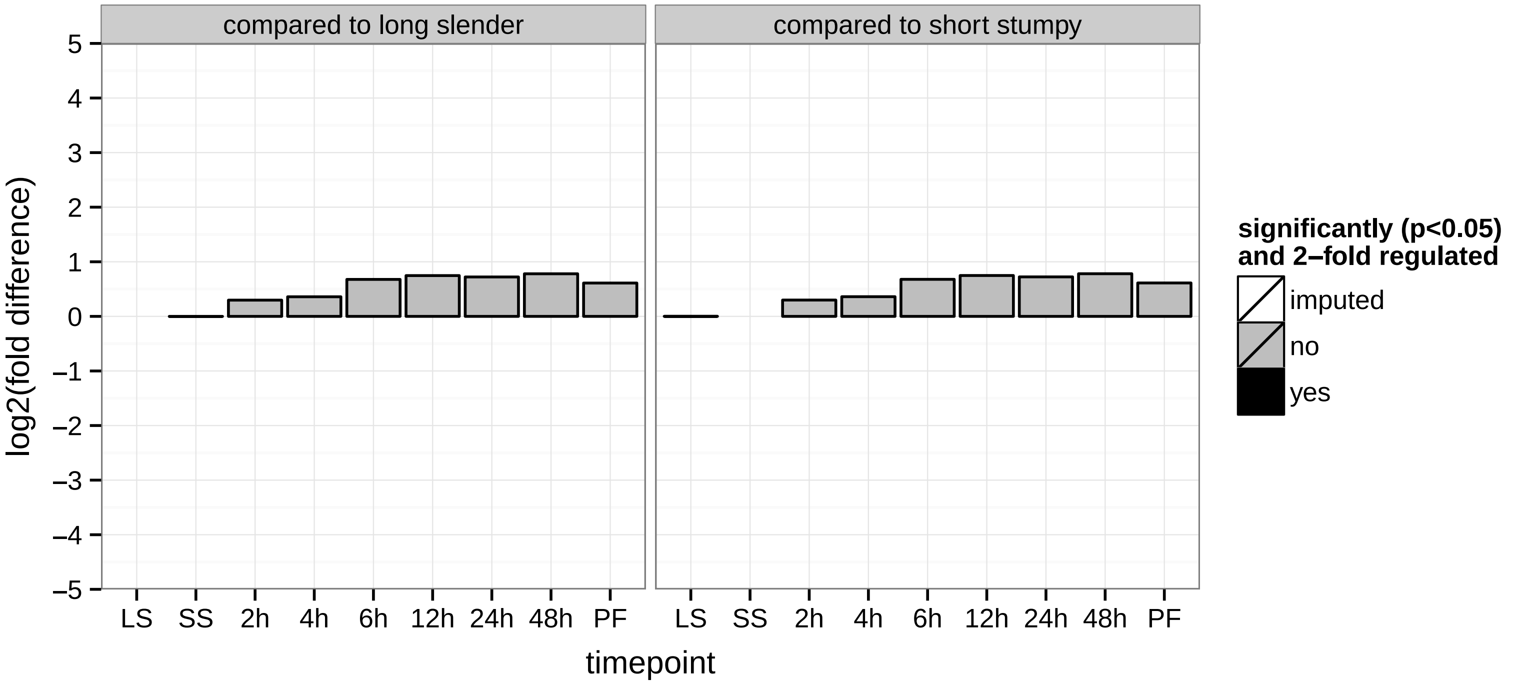

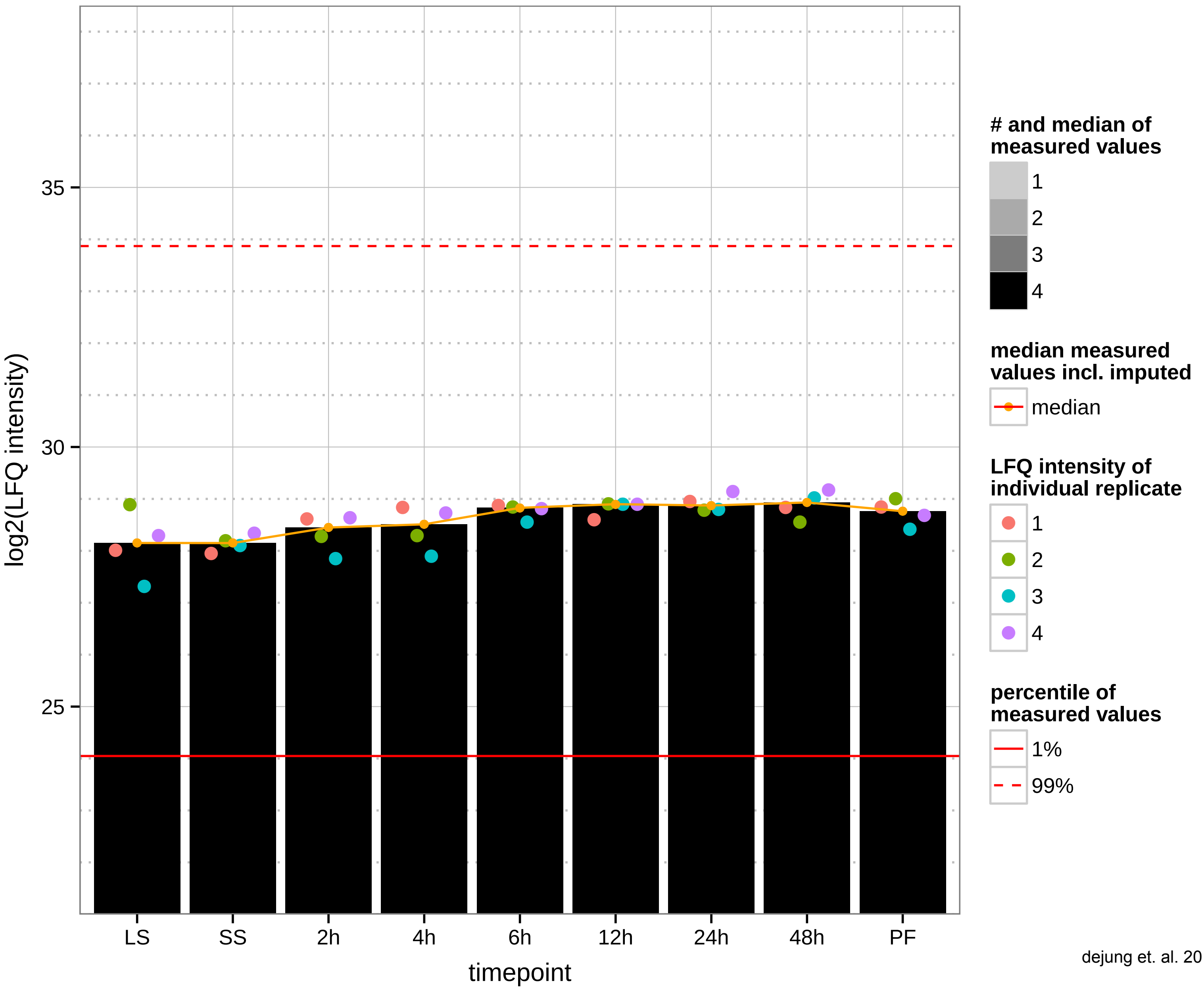

dejung et. al. 2015

hypothetical protein, conserved
Tb927.8.2620
AGOF: null
AGOC: null
AGOP: null
PGOF: null
PGOC: null
PGOP: null

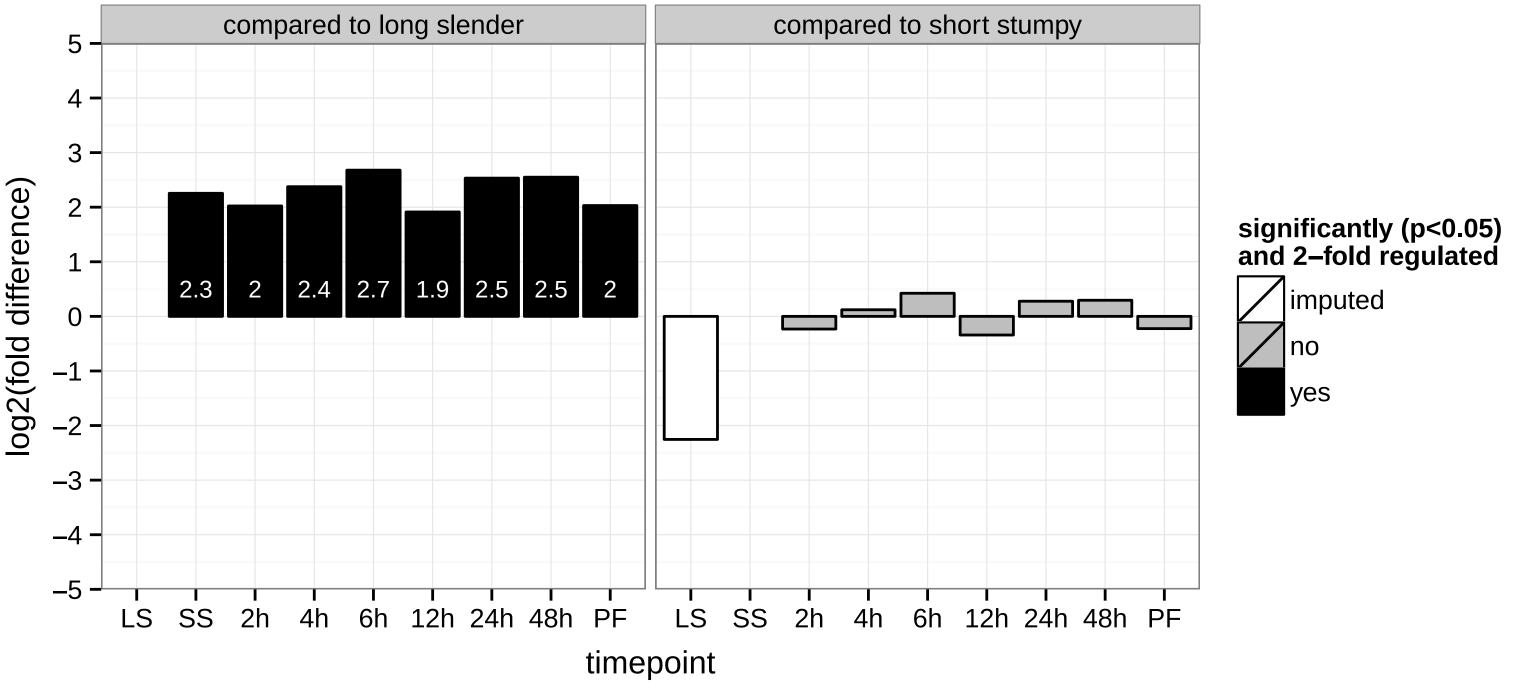

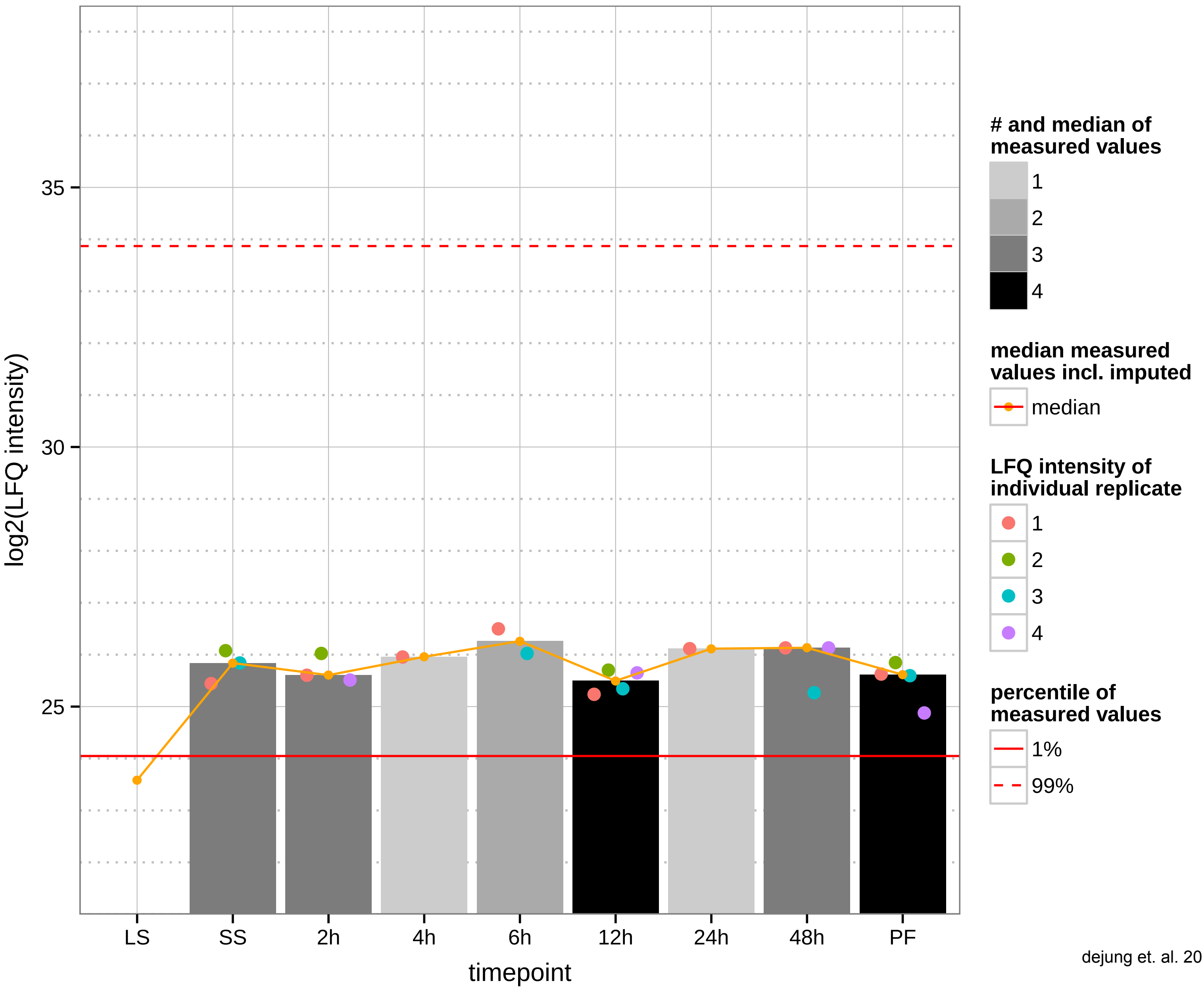

dejung et. al. 2015

ubiquitin–activating enzyme E1, putative (UBA1)
Tb927.8.2640
AGOF: ATP binding, ubiquitin activating enzyme activity, ubiquitin–protein ligase activity
AGOC: null
AGOP: cellular protein modification process
PGOF: ATP binding, catalytic activity, small protein activating enzyme activity
PGOC: null
PGOP: cellular protein modification process

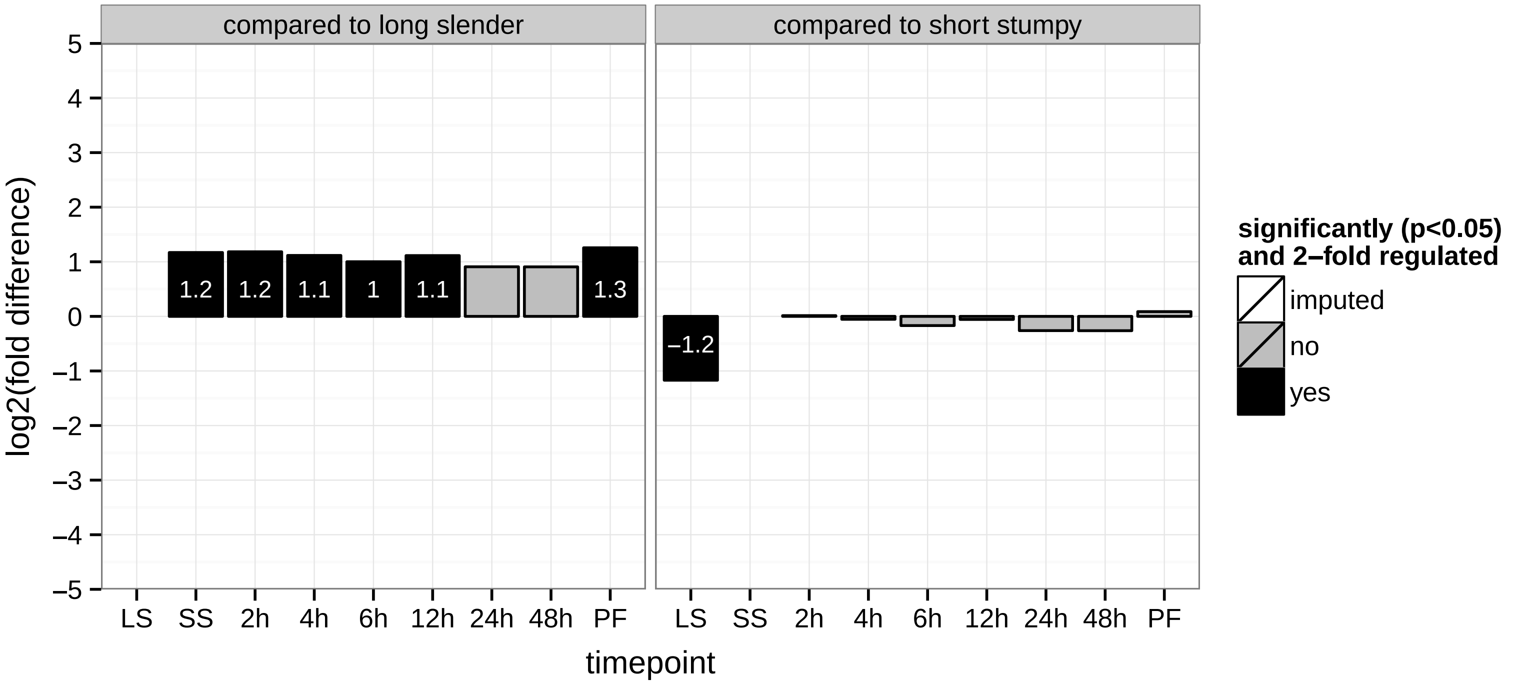

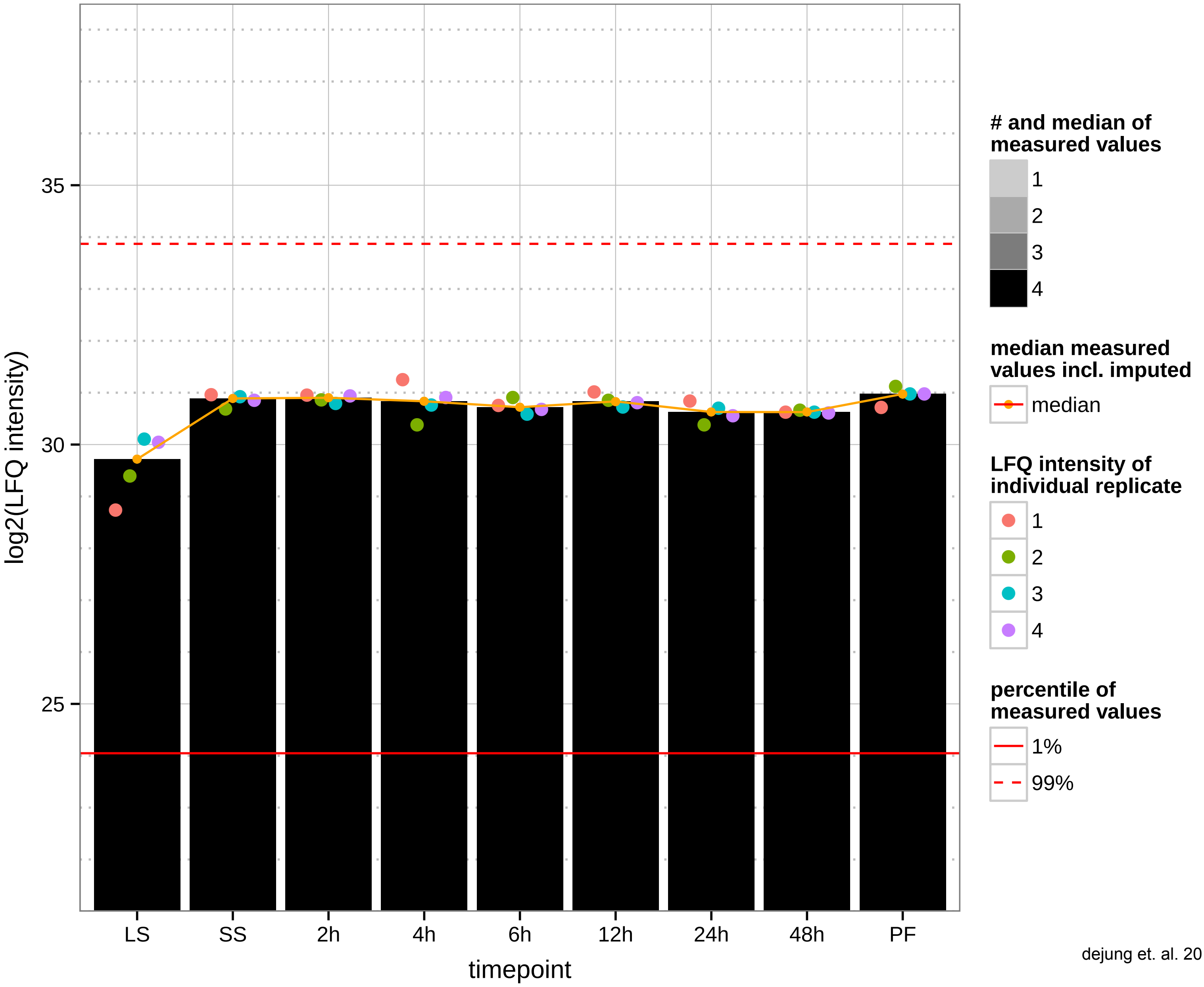

dejung et. al. 2015

hypothetical protein, conserved
Tb927.8.2670
AGOF: null
AGOC: mitochondrion
AGOP: null
PGOF: null
PGOC: null
PGOP: null

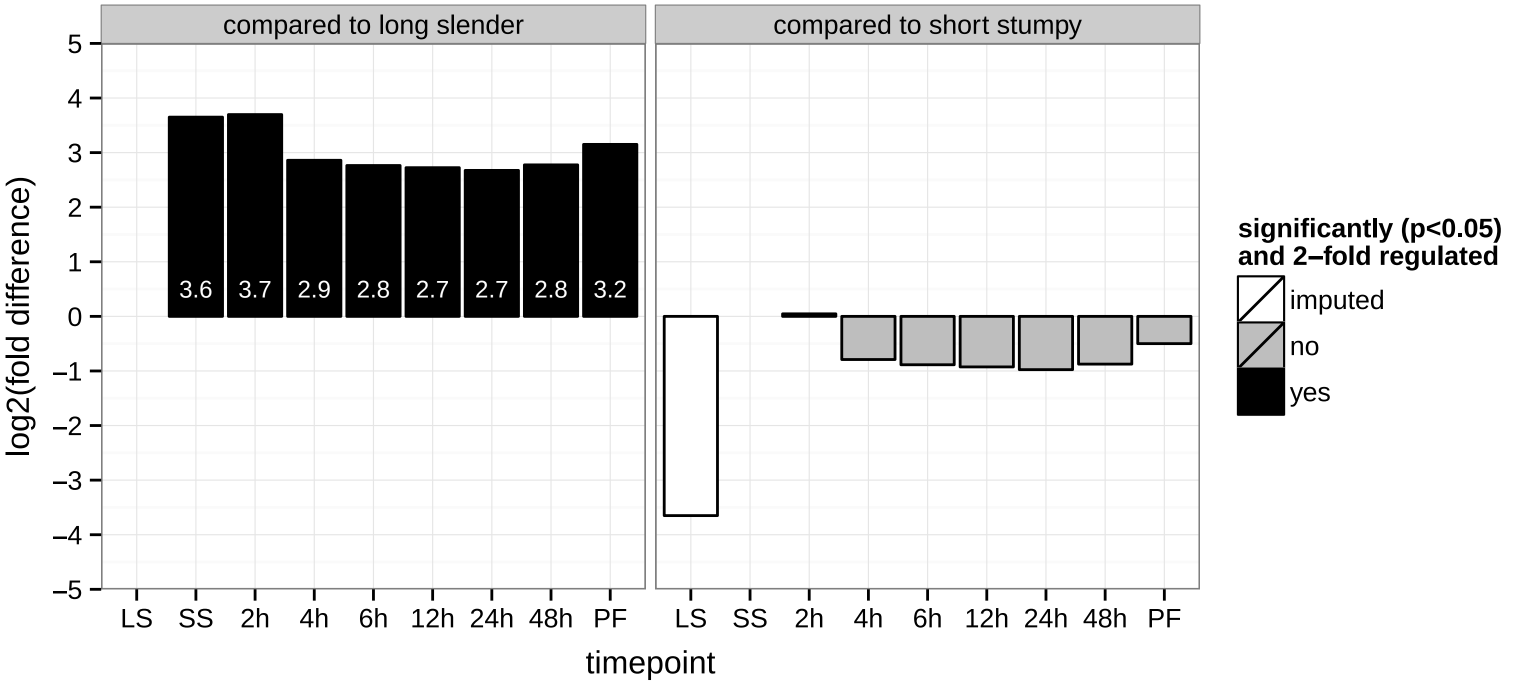

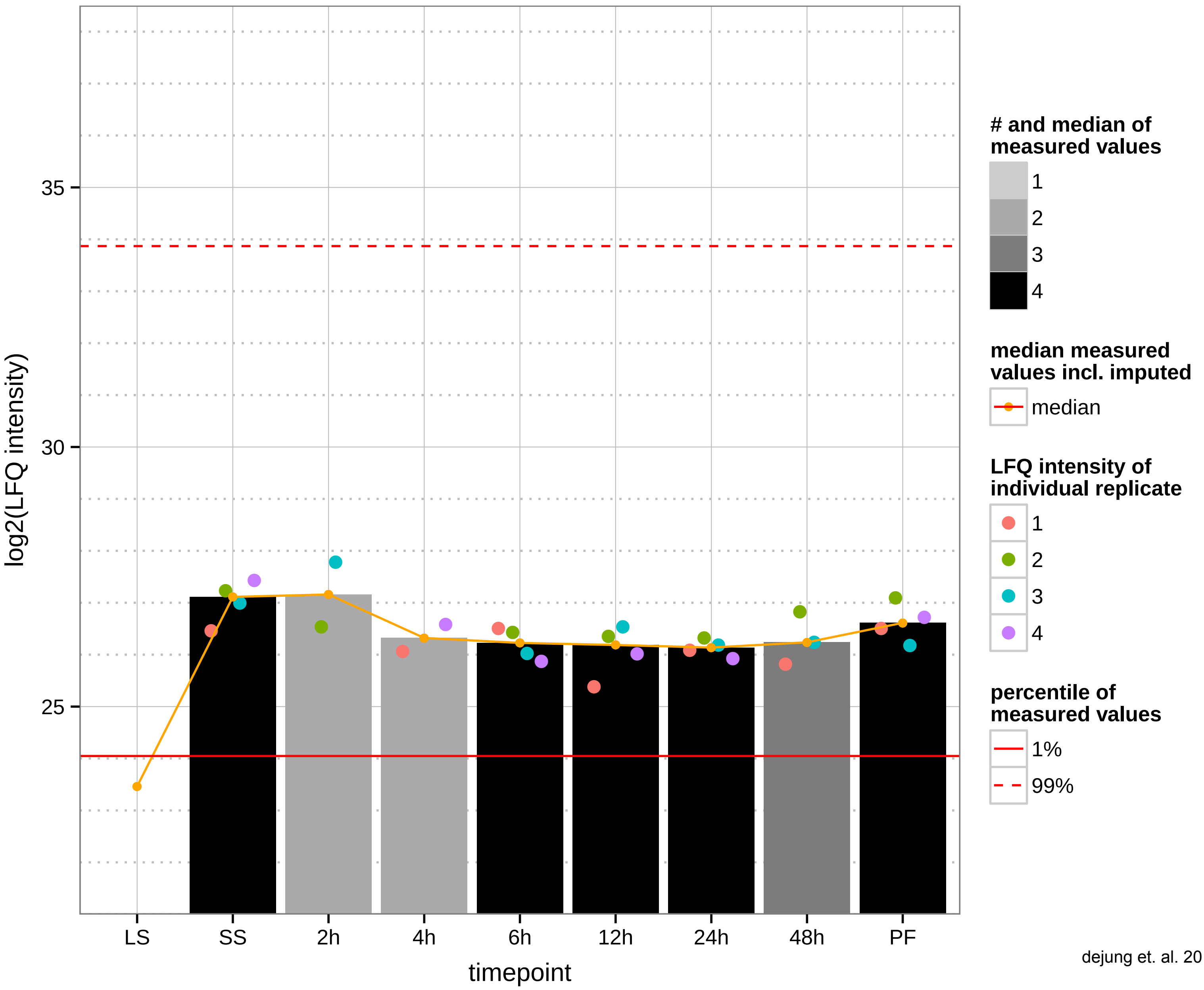

dejung et. al. 2015

RNA–binding protein RBP10, putative (RBP10)
Tb927.8.2780
AGOF: RNA binding
AGOC: null
AGOP: RNA processing
PGOF: nucleic acid binding
PGOC: null
PGOP: null

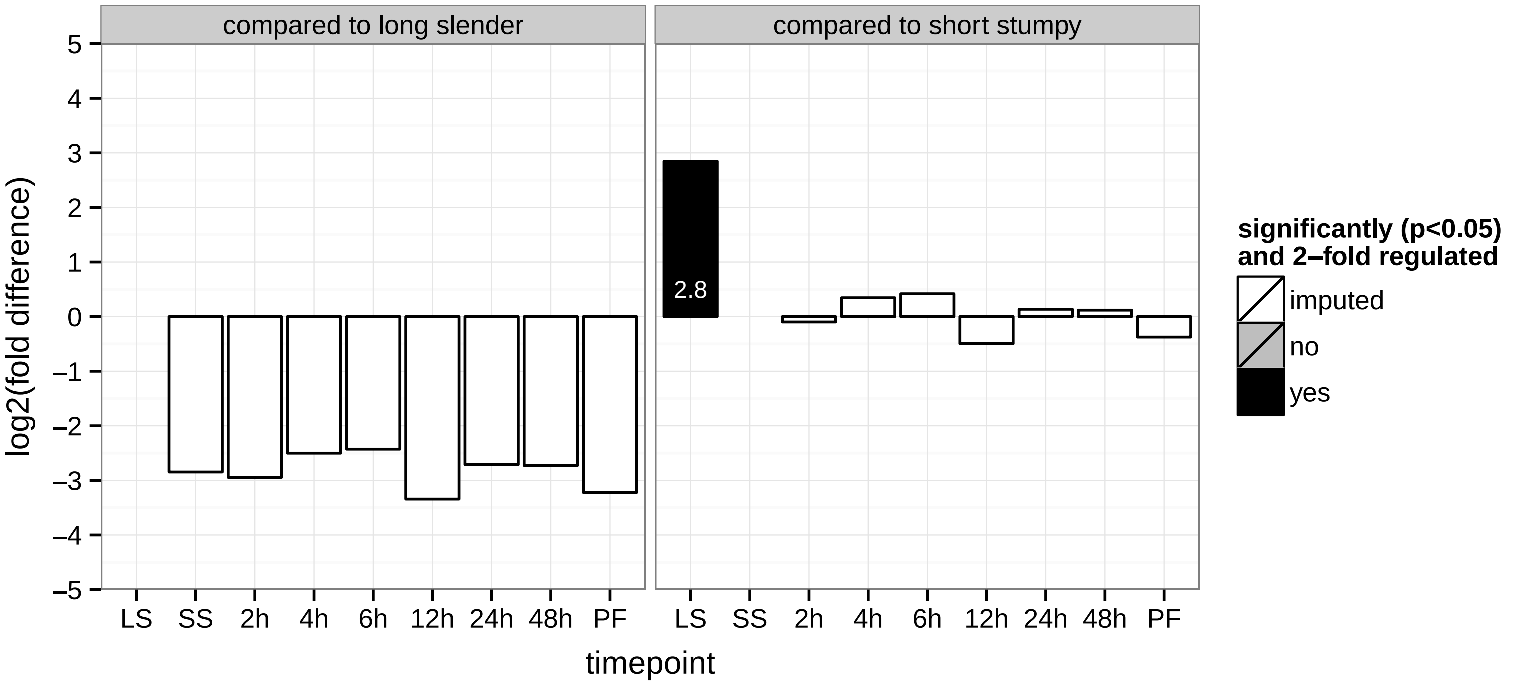

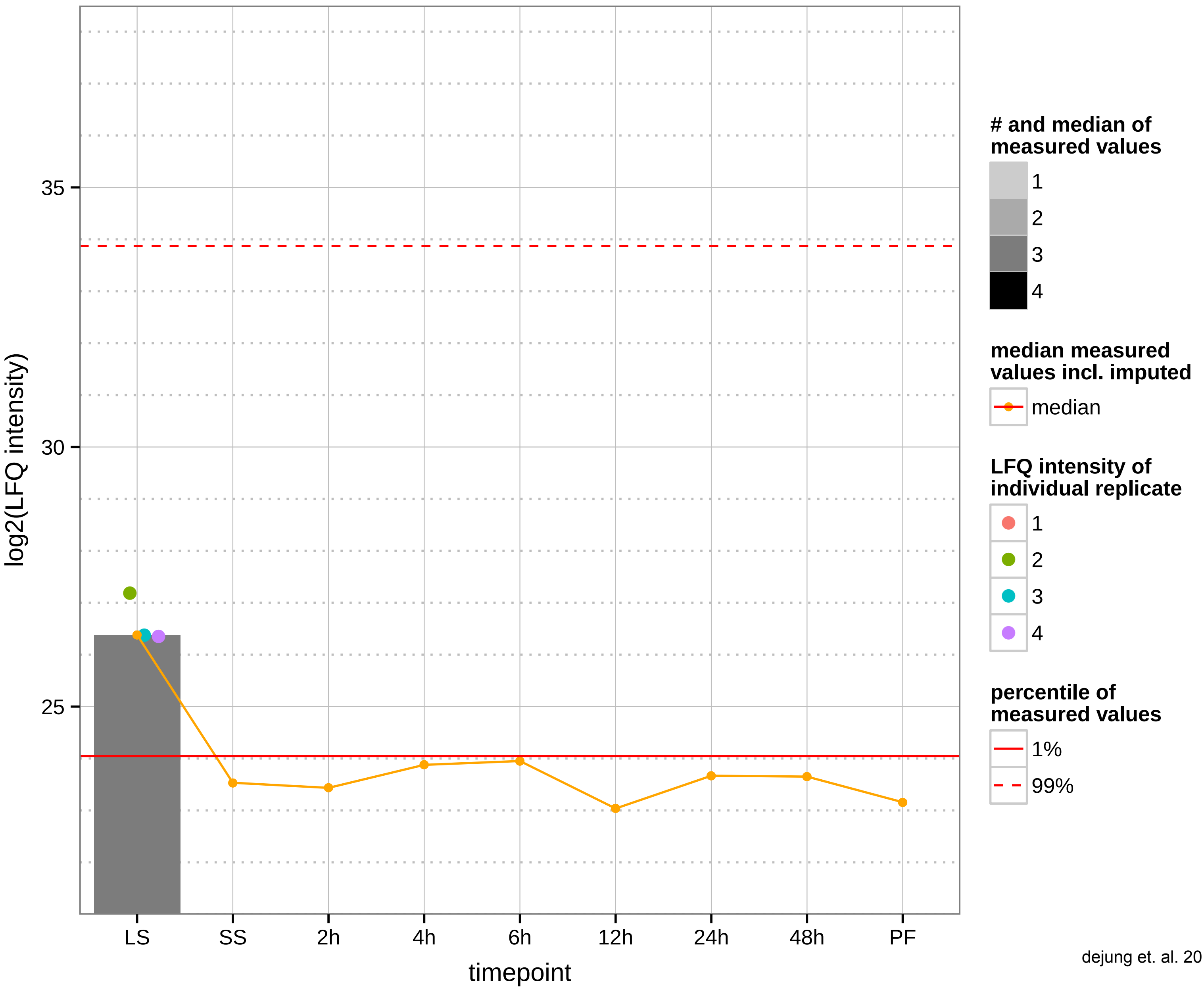

dejung et. al. 2015

hypothetical protein, conserved
Tb927.8.2820
AGOF: null
AGOC: null
AGOP: null
PGOF: null
PGOC: null
PGOP: null

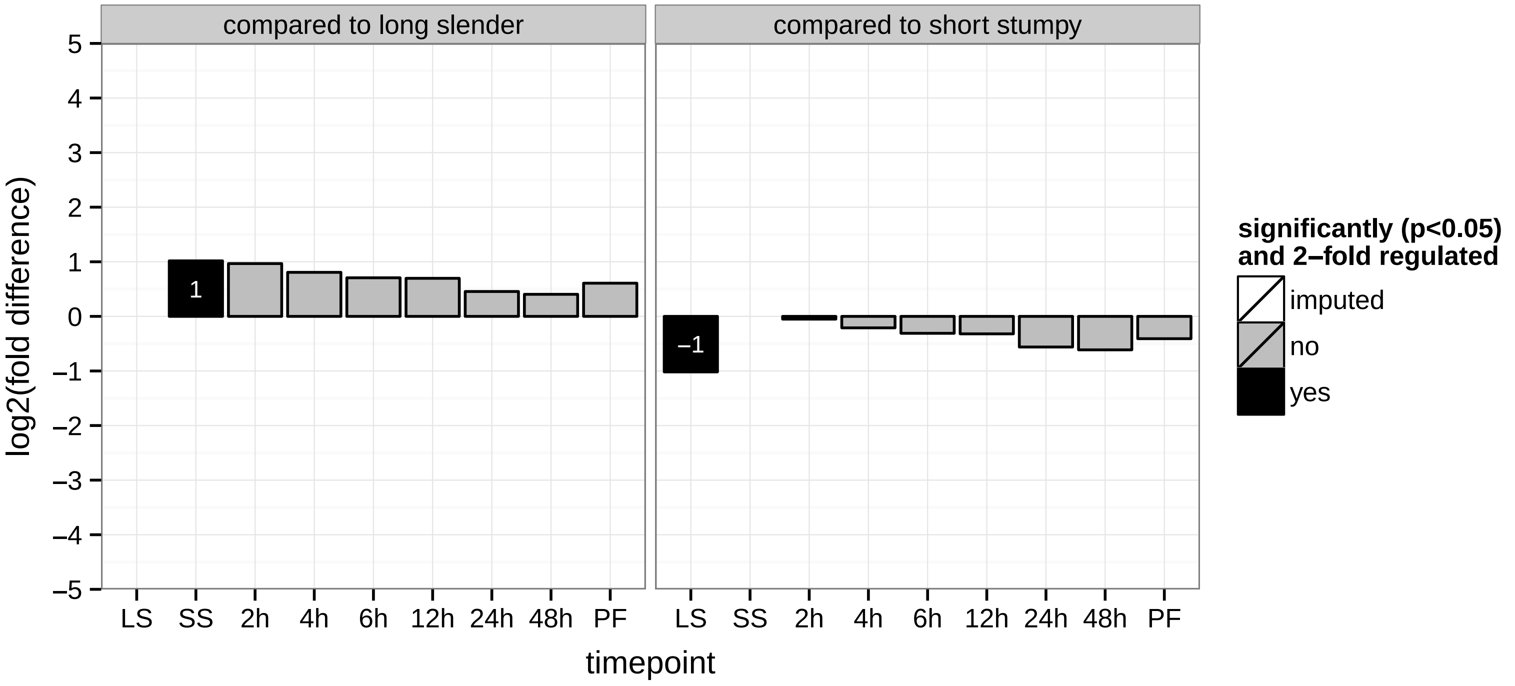

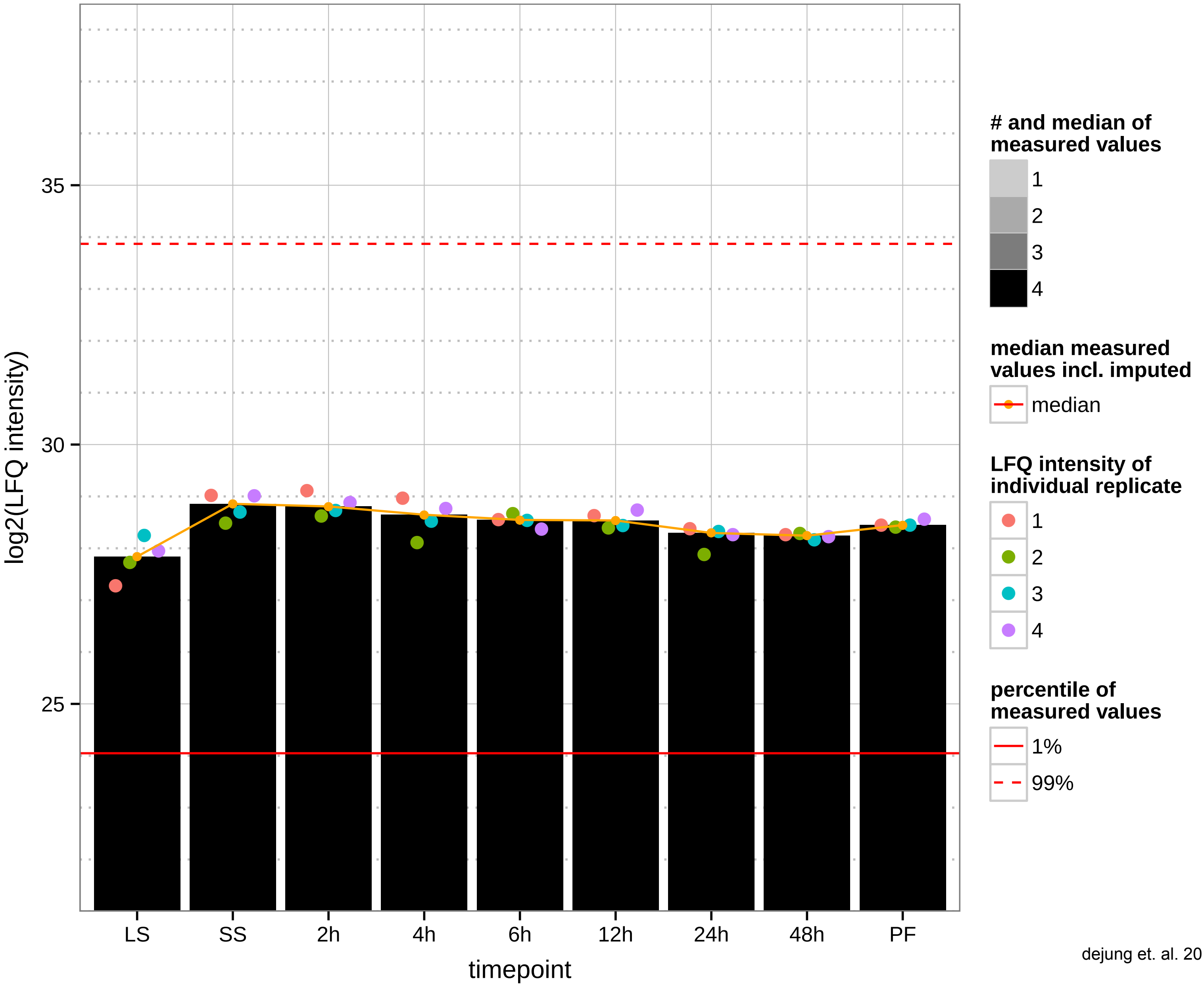

dejung et. al. 2015

Poly(A)–specific ribonuclease PARN–1
Tb927.8.2850
AGOF: nuclease activity, nucleic acid binding
AGOC: cytoplasm, nucleus
AGOP: null
PGOF: nucleic acid binding
PGOC: nucleus
PGOP: null

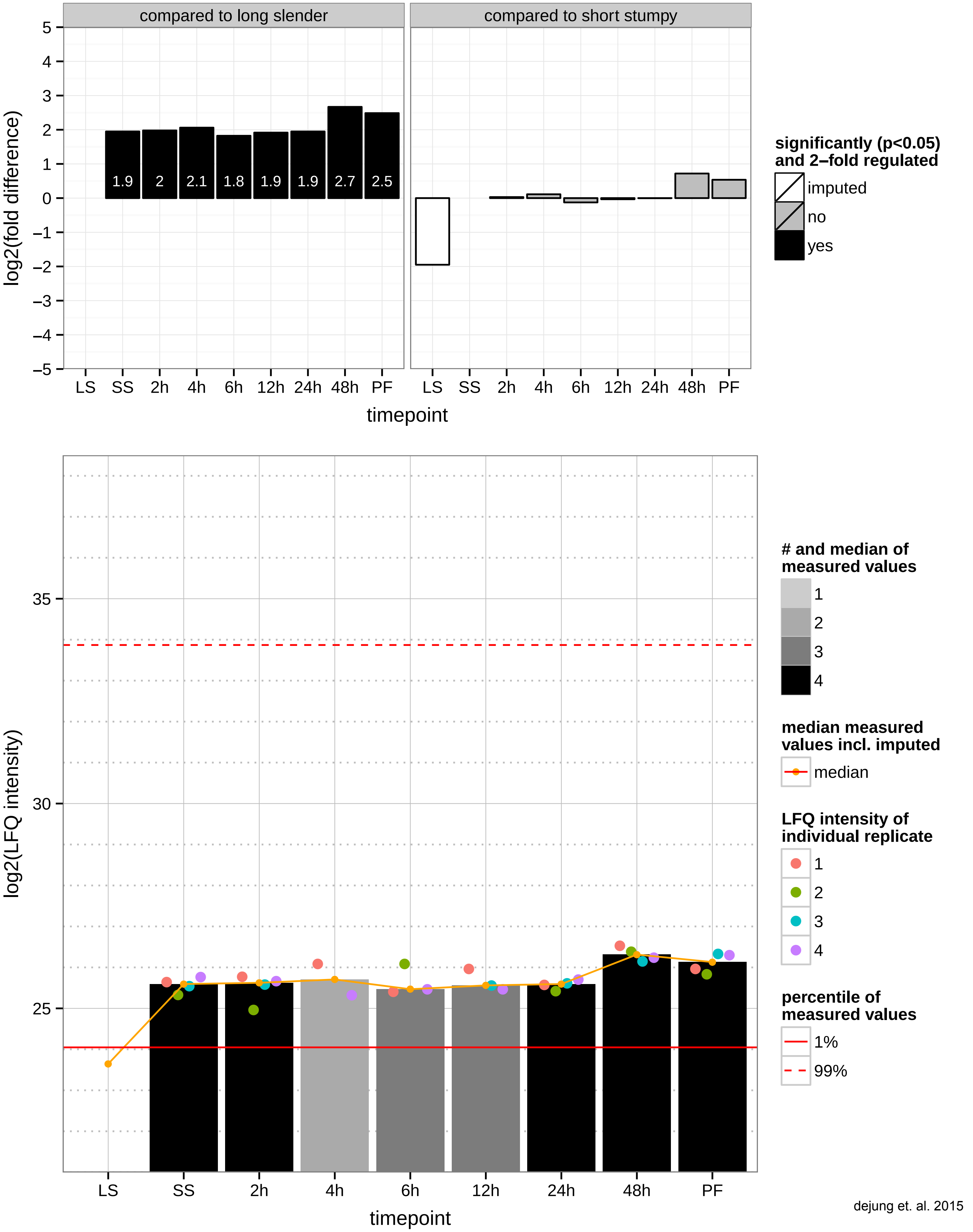

dejung et. al. 2015

hypothetical protein, conserved
Tb927.8.2970
AGOF: null
AGOC: mitochondrion
AGOP: null
PGOF: null
PGOC: null
PGOP: null

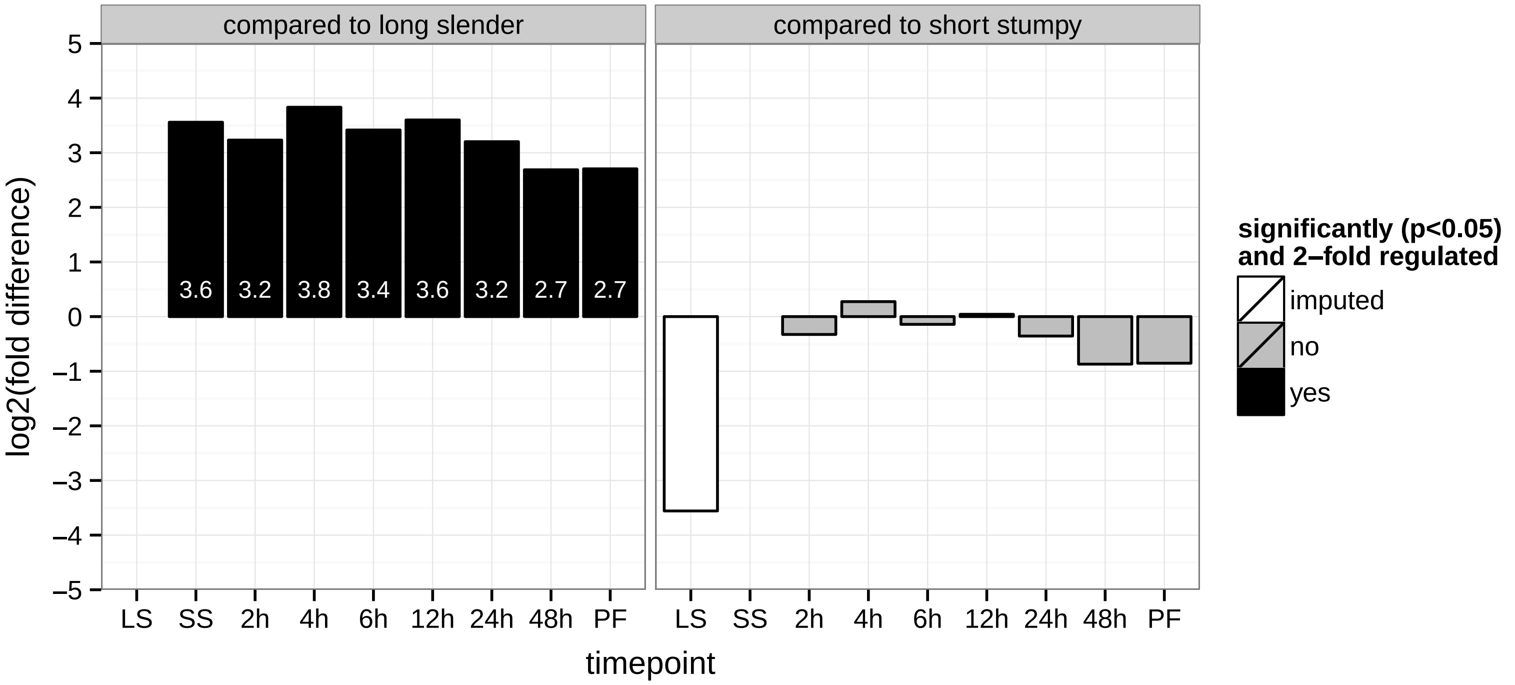

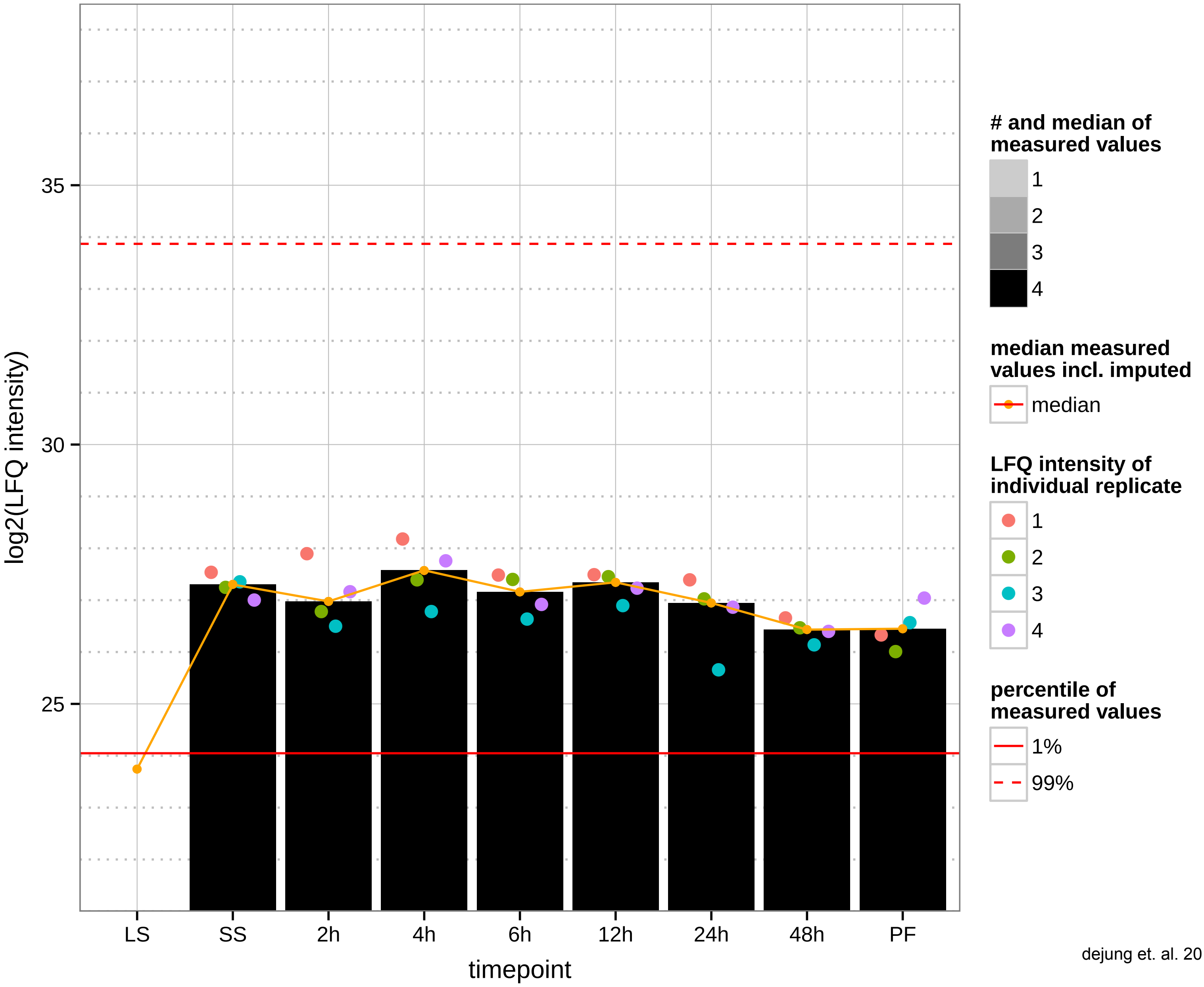

dejung et. al. 2015

hypothetical protein, conserved
Tb927.8.3020
AGOF: null
AGOC: null
AGOP: null
PGOF: null
PGOC: null
PGOP: null

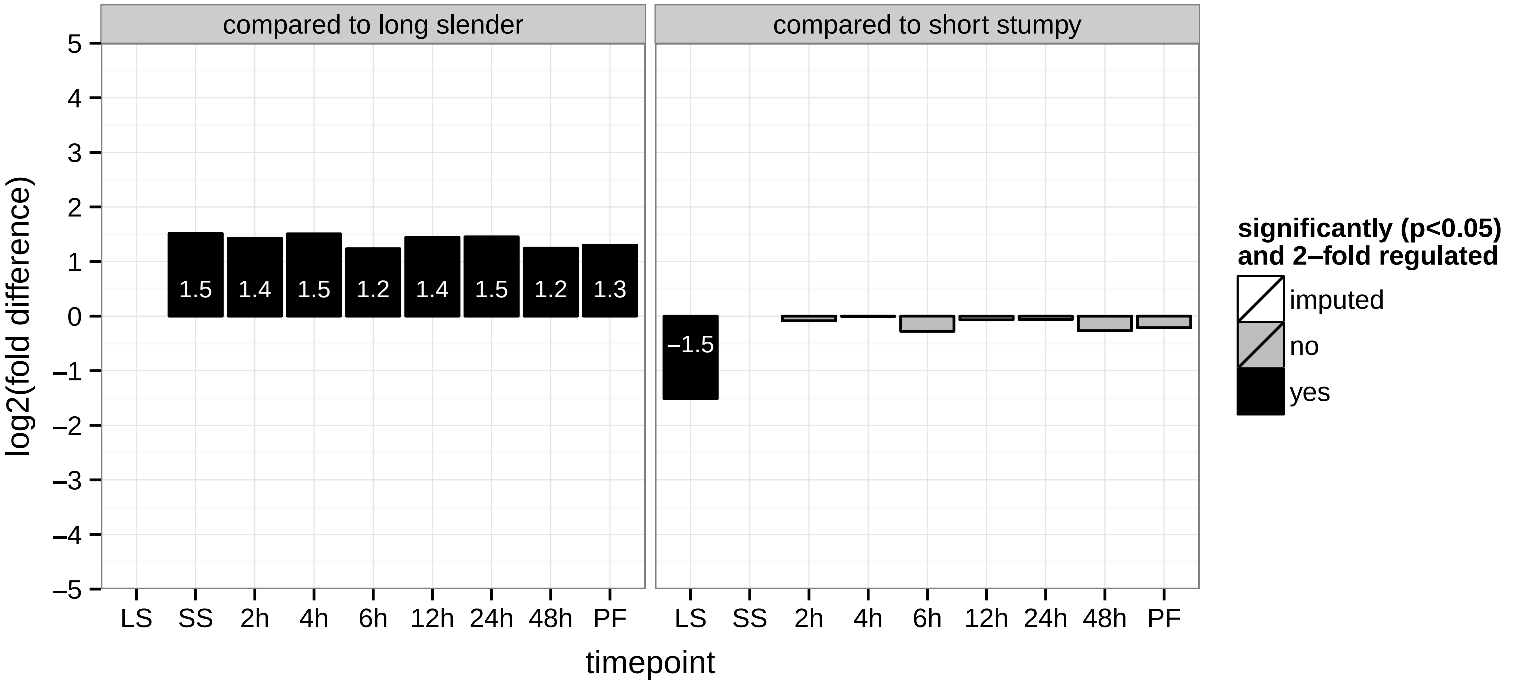

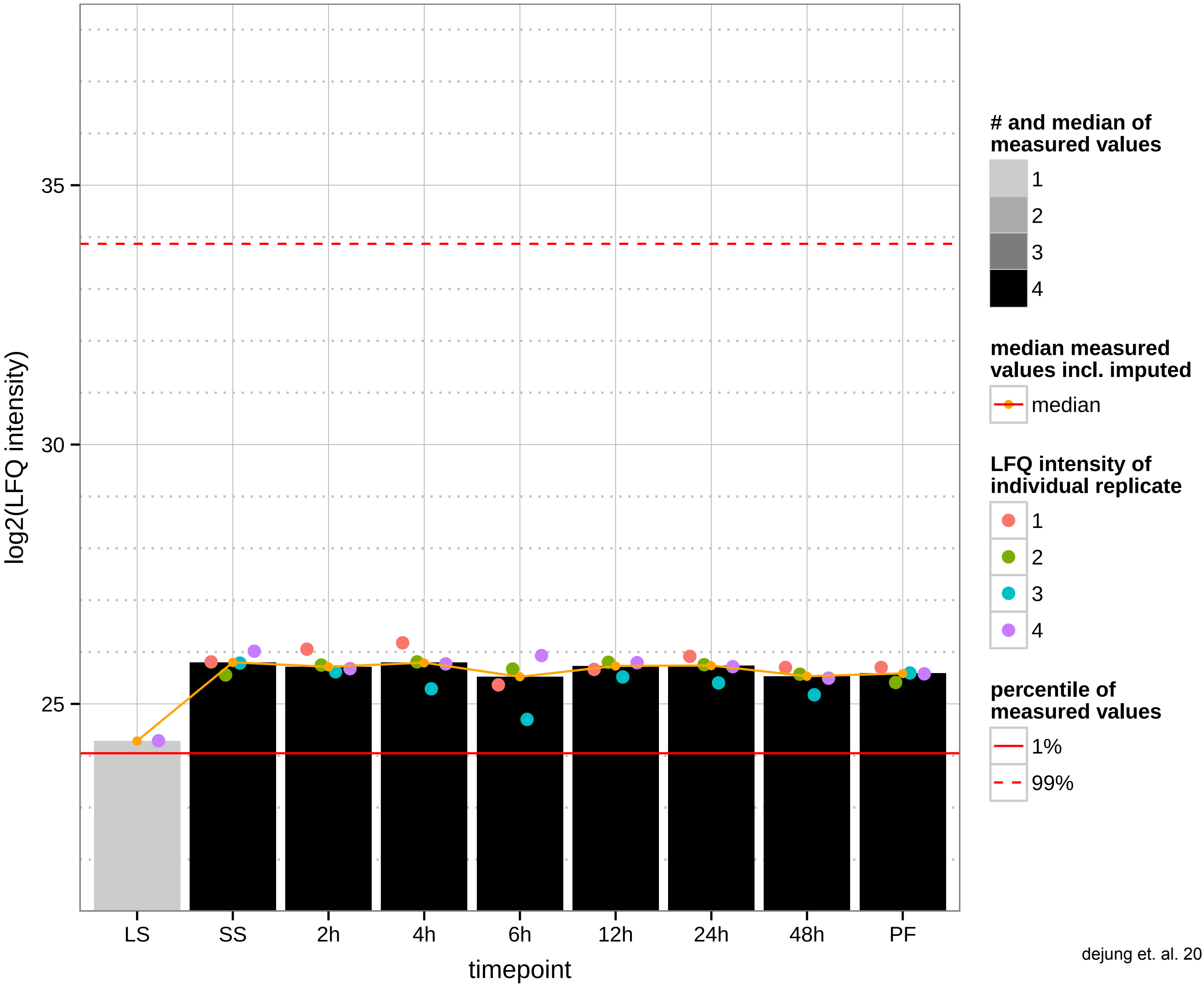

dejung et. al. 2015

cytosolic leucyl aminopeptidase, putative, metallo–peptidase, Clan MF, Family M17
Tb927.8.3060
AGOF: aminopeptidase activity, manganese ion binding, metalloexopeptidase activity
AGOC: cytoplasm, intracellular
AGOP: protein metabolic process, proteolysis
PGOF: aminopeptidase activity
PGOC: intracellular
PGOP: proteolysis

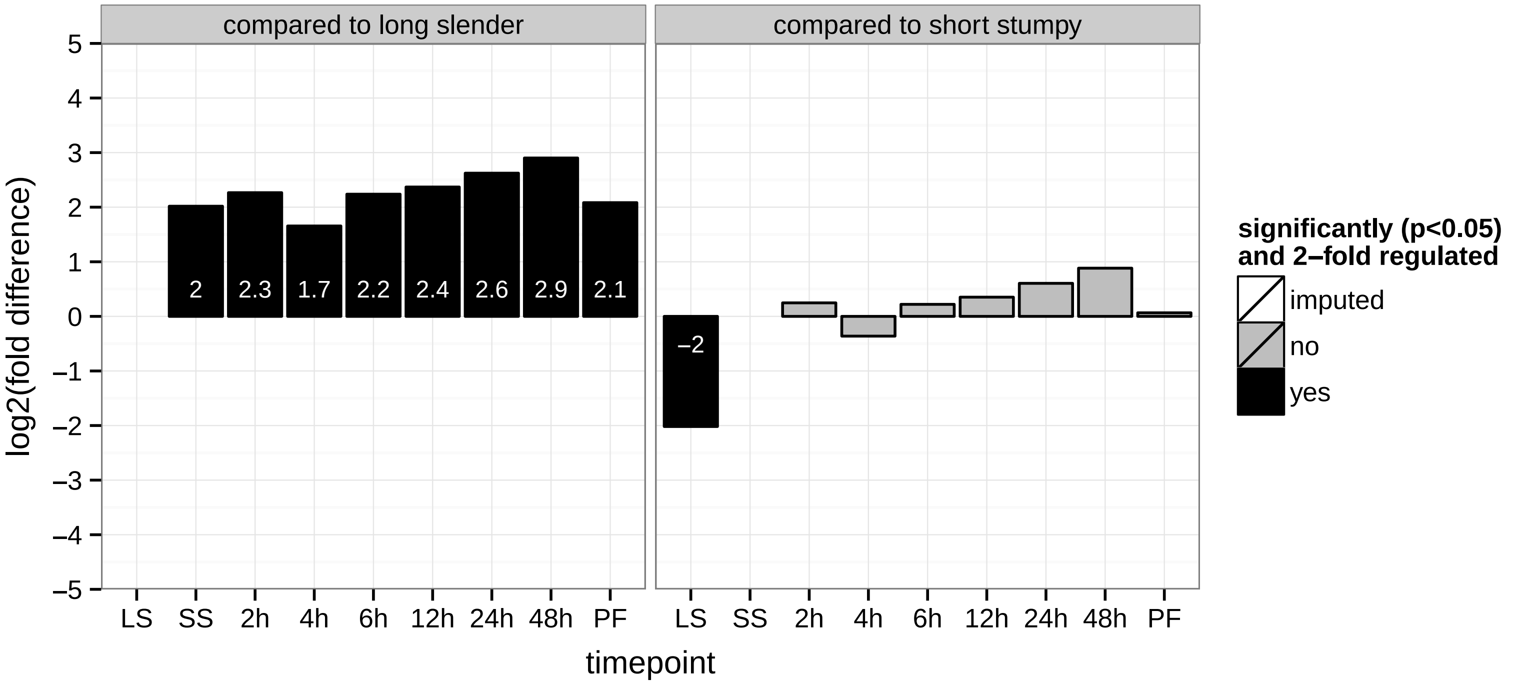

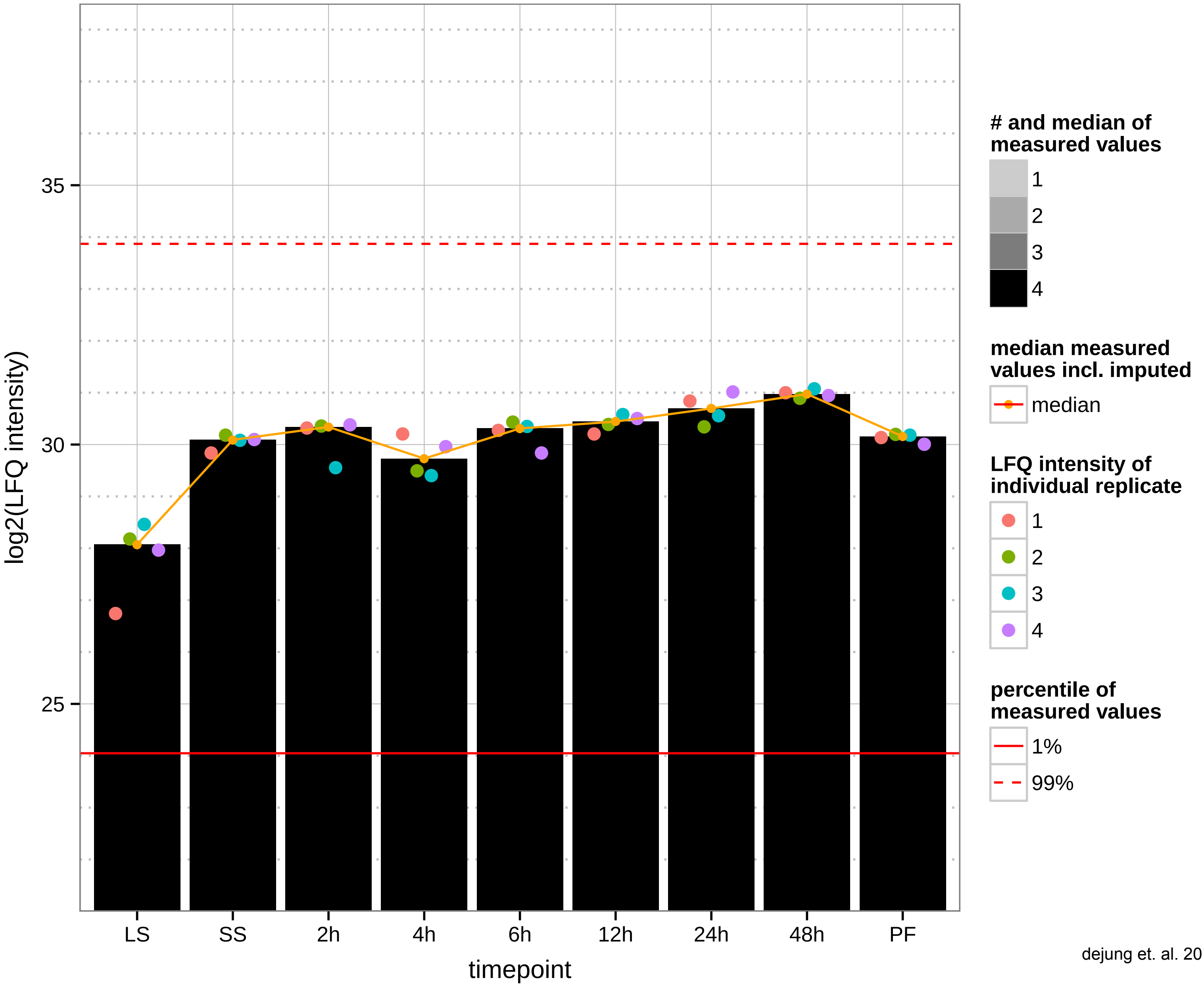

dejung et. al. 2015

T−complex protein 1, gamma subunit, putative (TCP−1−gamma)
Tb927.8.3150
AGOF: ATP binding, unfolded protein binding
AGOC: chaperonin−containing T−complex
AGOP: protein folding, regulation of cell cycle
PGOF: ATP binding, unfolded protein binding
PGOC: null
PGOP: cellular protein metabolic process, protein folding

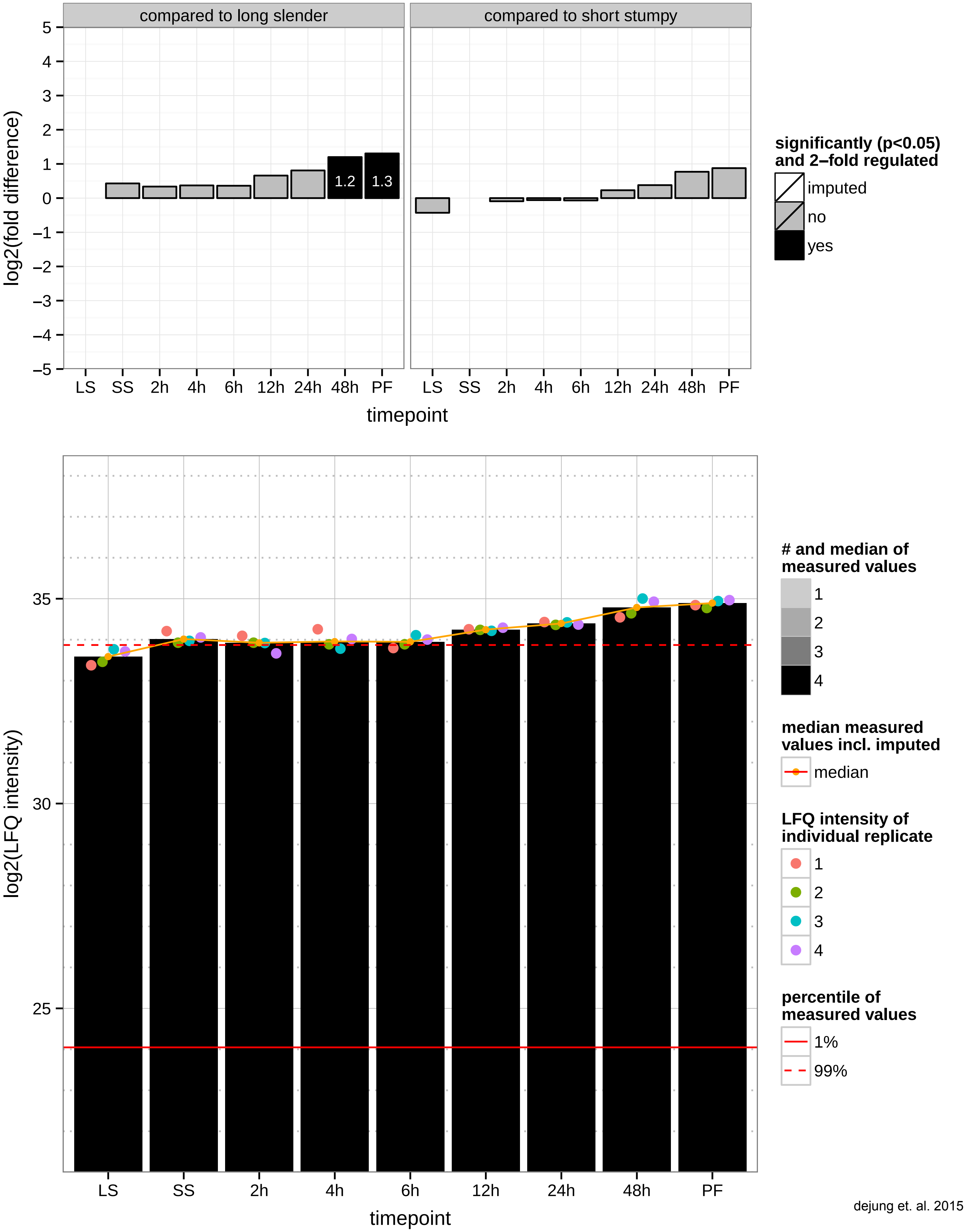

dejung et. al. 2015

hypothetical protein, conserved
Tb927.8.3170
AGOF: null
AGOC: mitochondrion
AGOP: null
PGOF: null
PGOC: null
PGOP: null

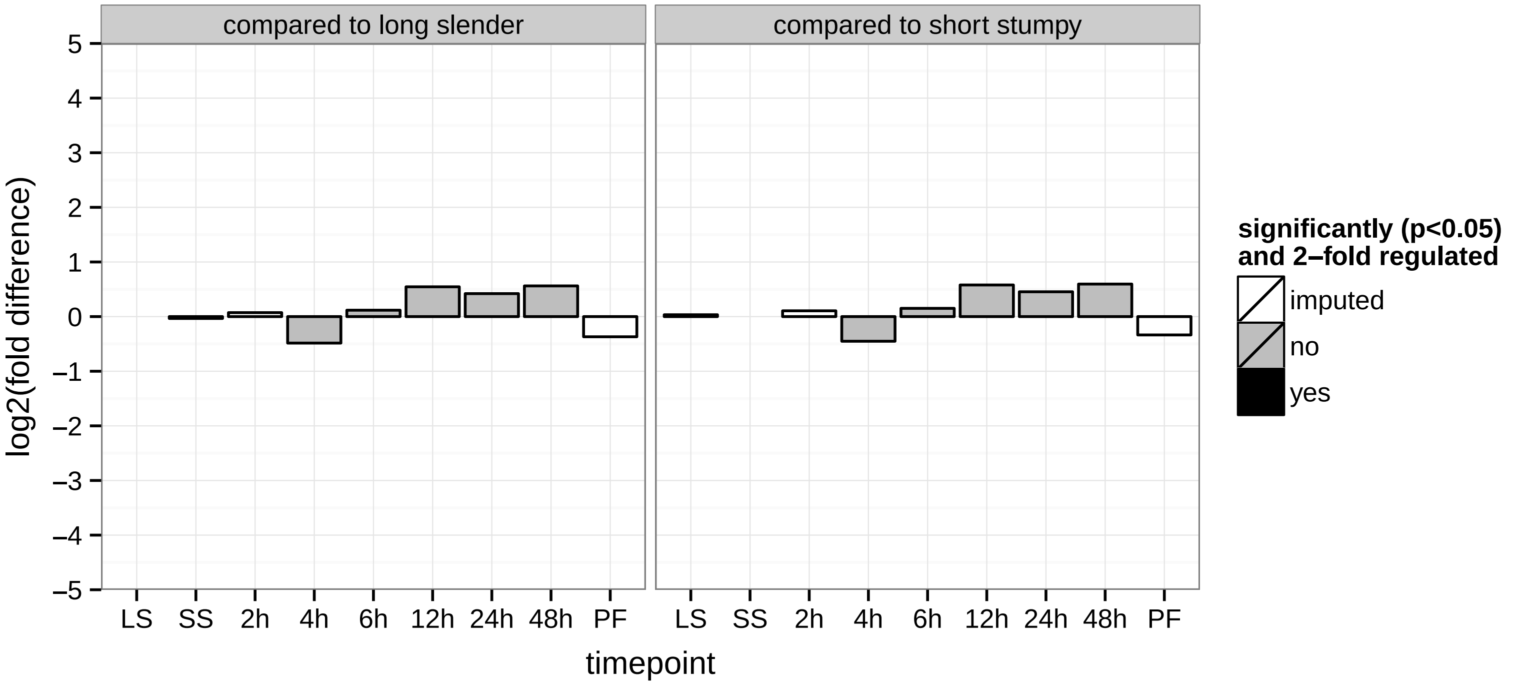

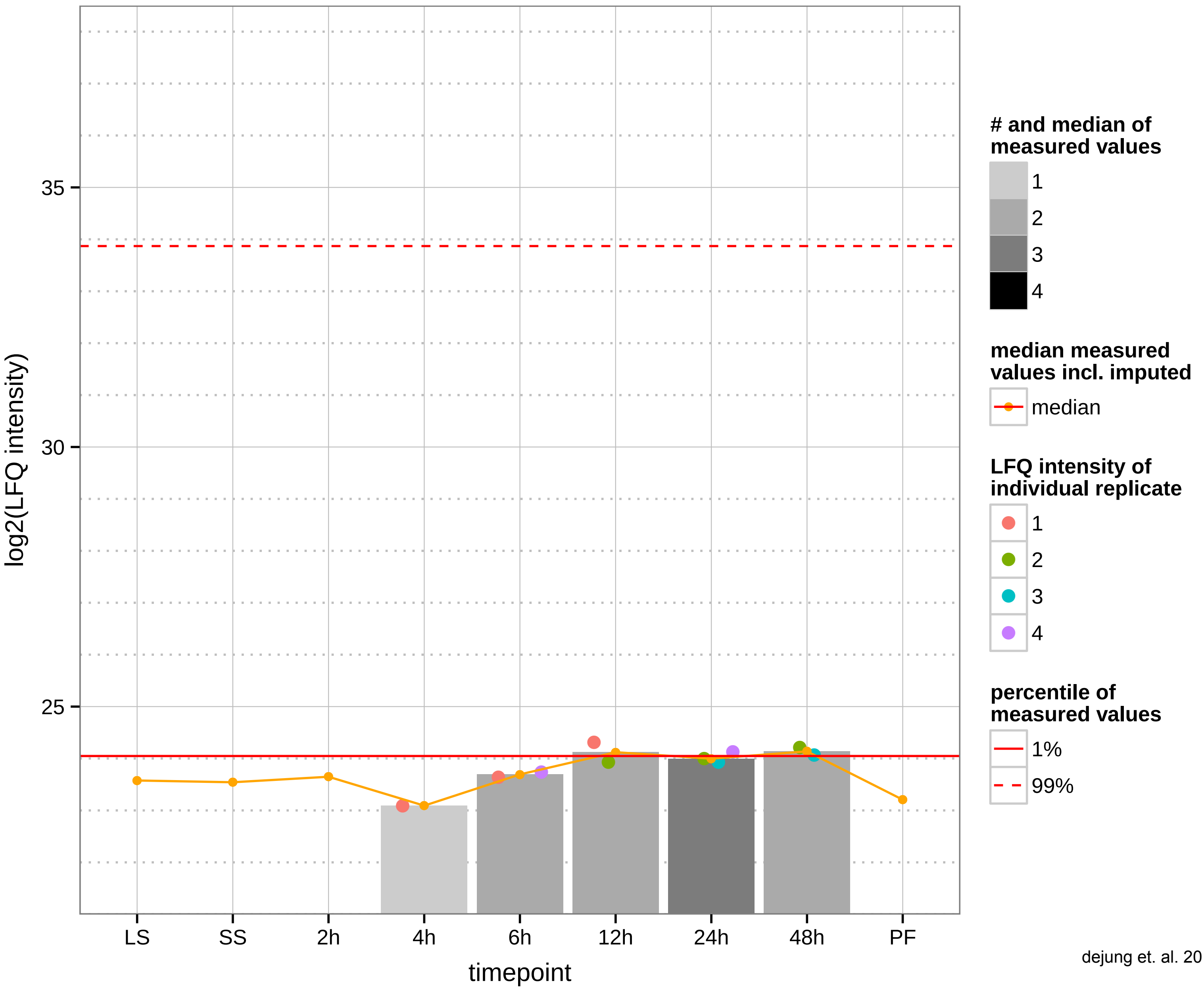

dejung et. al. 2015

hypothetical protein, conserved
Tb927.8.3200
AGOF: null
AGOC: null
AGOP: null
PGOF: binding
PGOC: null
PGOP: null

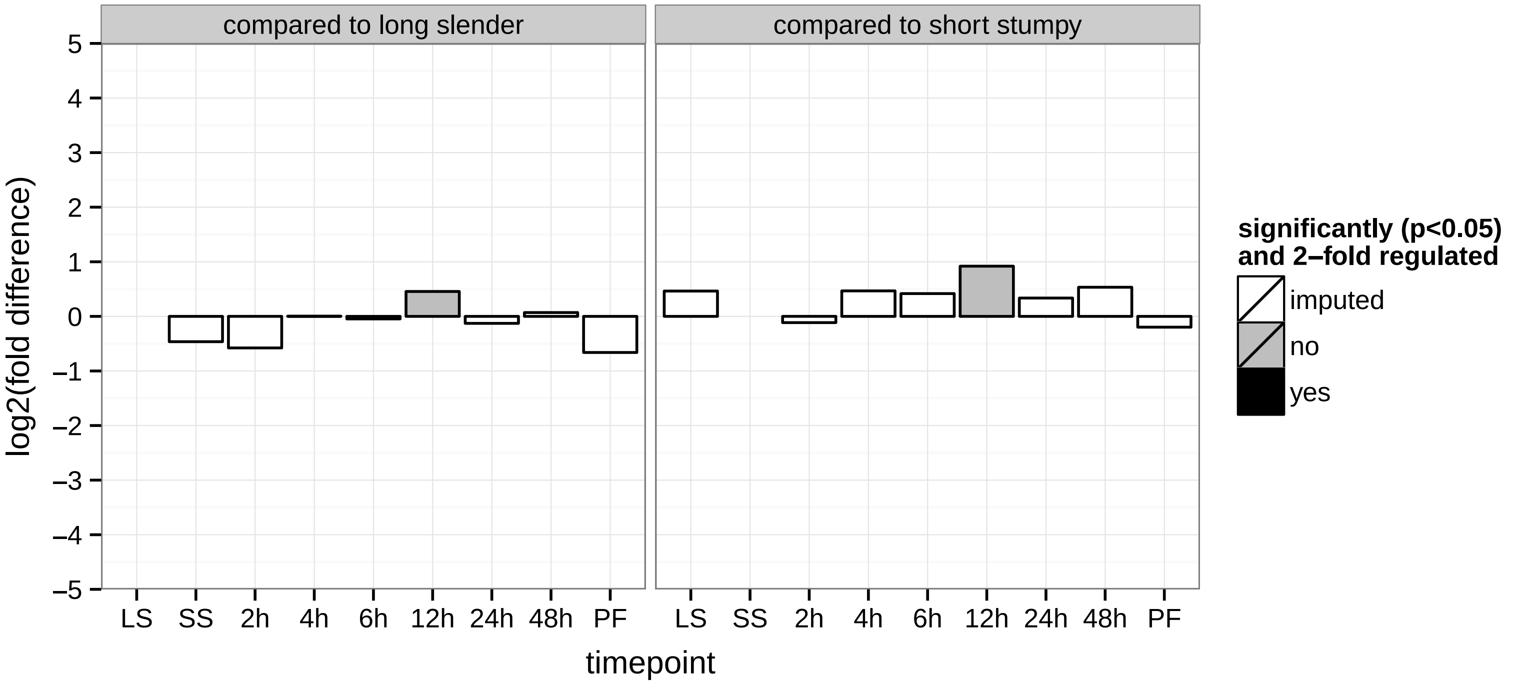

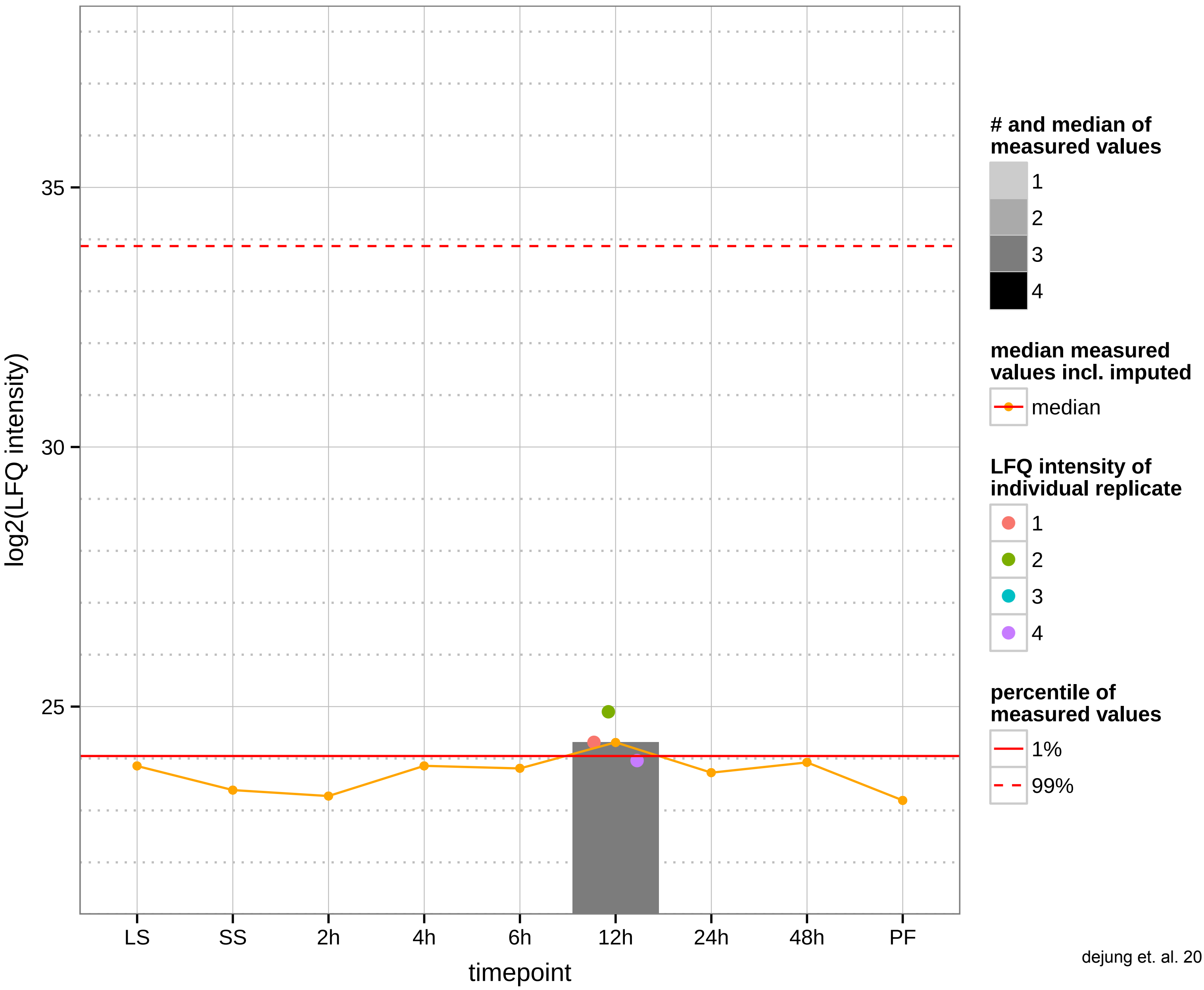

dejung et. al. 2015

dynein heavy chain, putative
Tb927.8.3250
AGOF: ATP binding, ATPase activity, microtubule motor activity
AGOC: dynein complex
AGOP: microtubule–based movement
PGOF: ATP binding, ATPase activity, microtubule motor activity, nucleoside–triphosphatase activity, nucleotide binding
PGOC: dynein complex
PGOP: microtubule–based movement

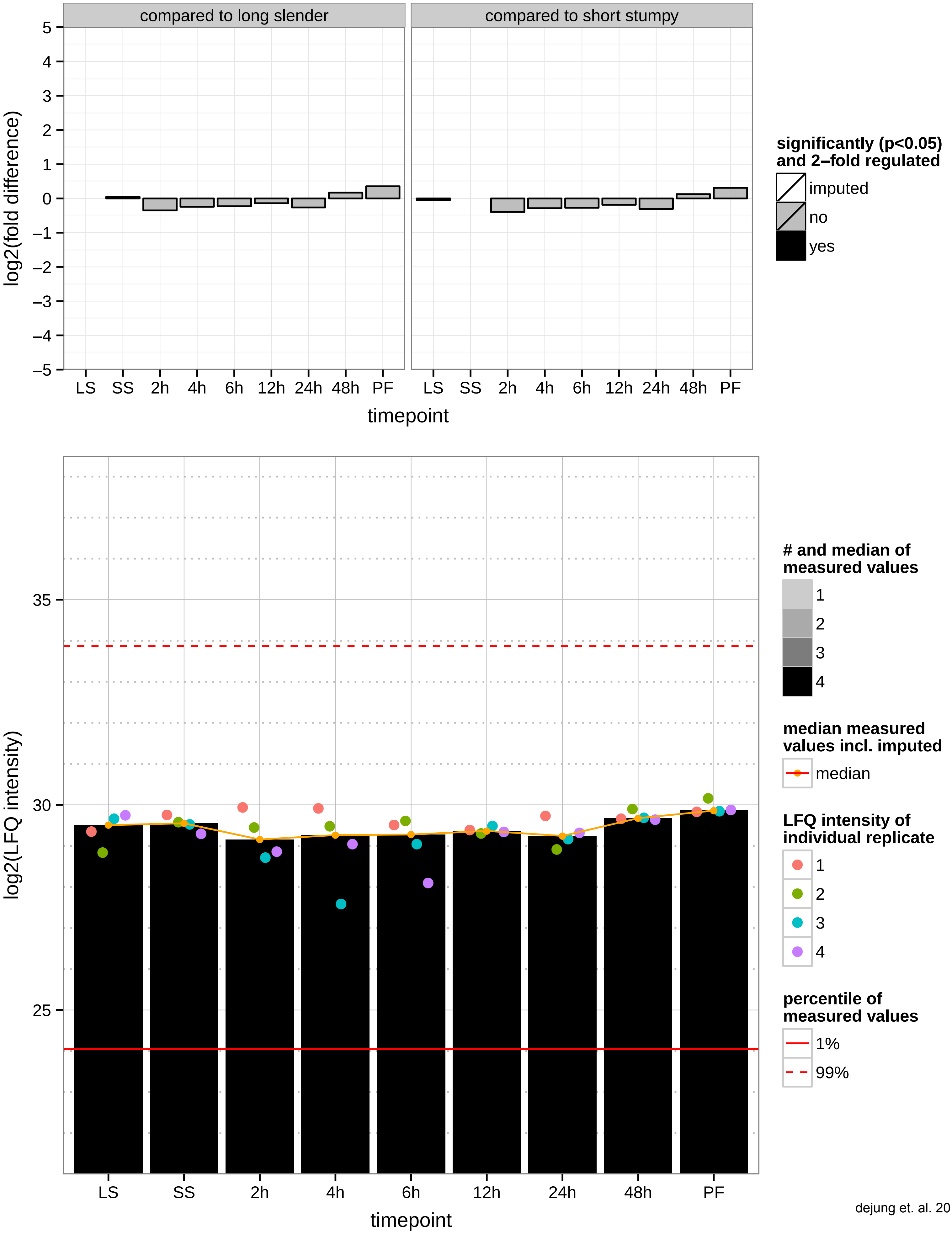

dejung et. al. 2015

hypothetical protein, conserved
Tb927.8.3280
AGOF: null
AGOC: null
AGOP: null
PGOF: null
PGOC: null
PGOP: null

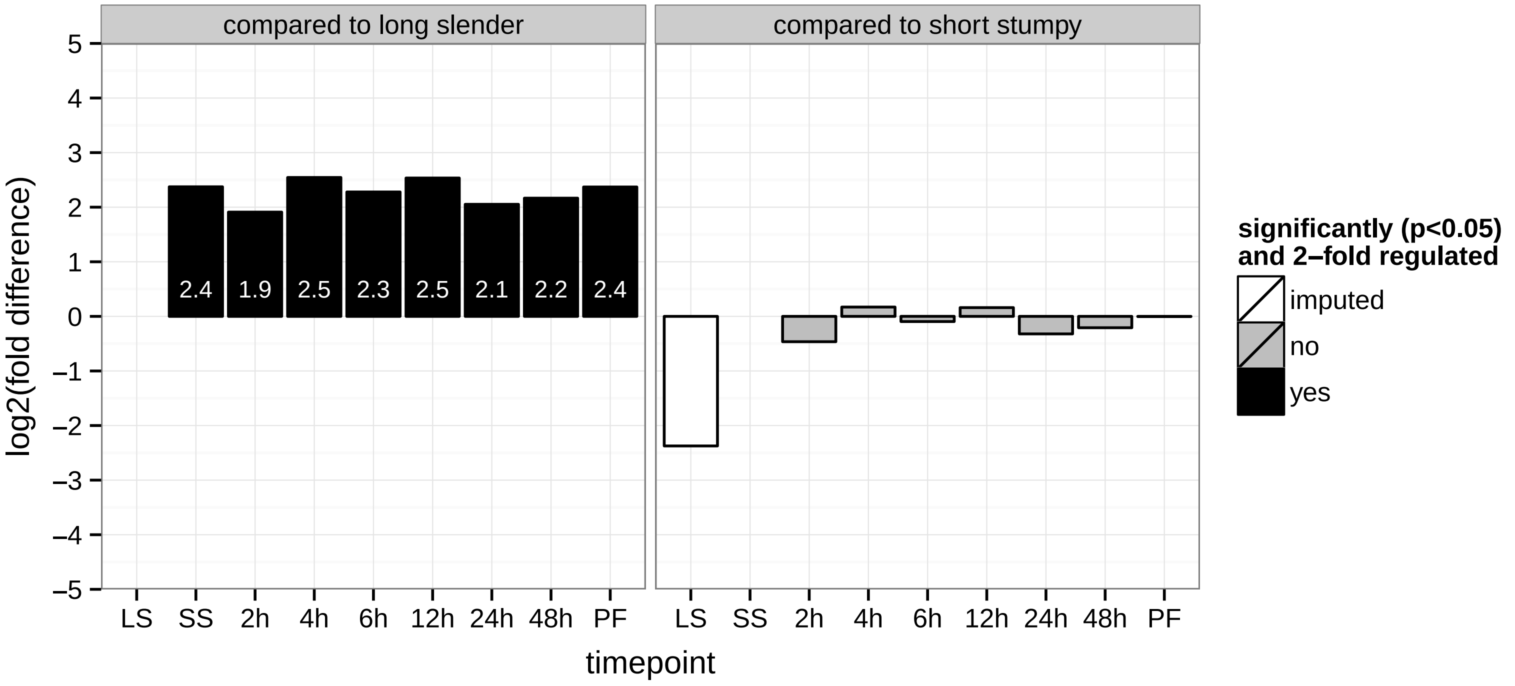

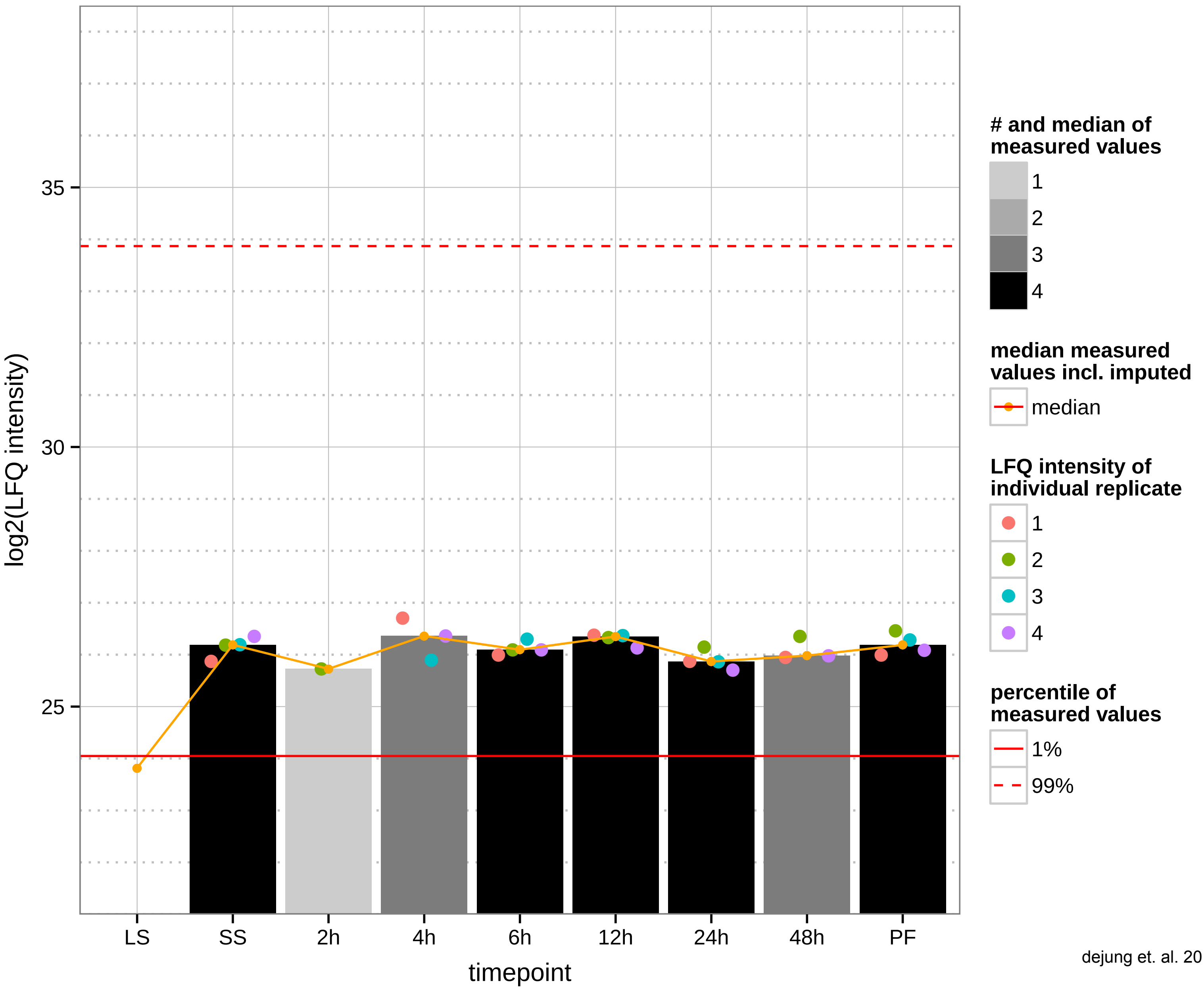

dejung et. al. 2015

DNA polymerase theta (helicase domain only), putative
Tb927.8.3350;Tb11.v5.0792
AGOF: null, ATP binding, ATP−dependent helicase activity, helicase activity, nucleic acid binding
AGOC: null
AGOP: null, nucleobase−containing compound metabolic process
PGOF: ATP binding, ATP−dependent helicase activity, helicase activity, nucleic acid binding
PGOC: null
PGOP: null

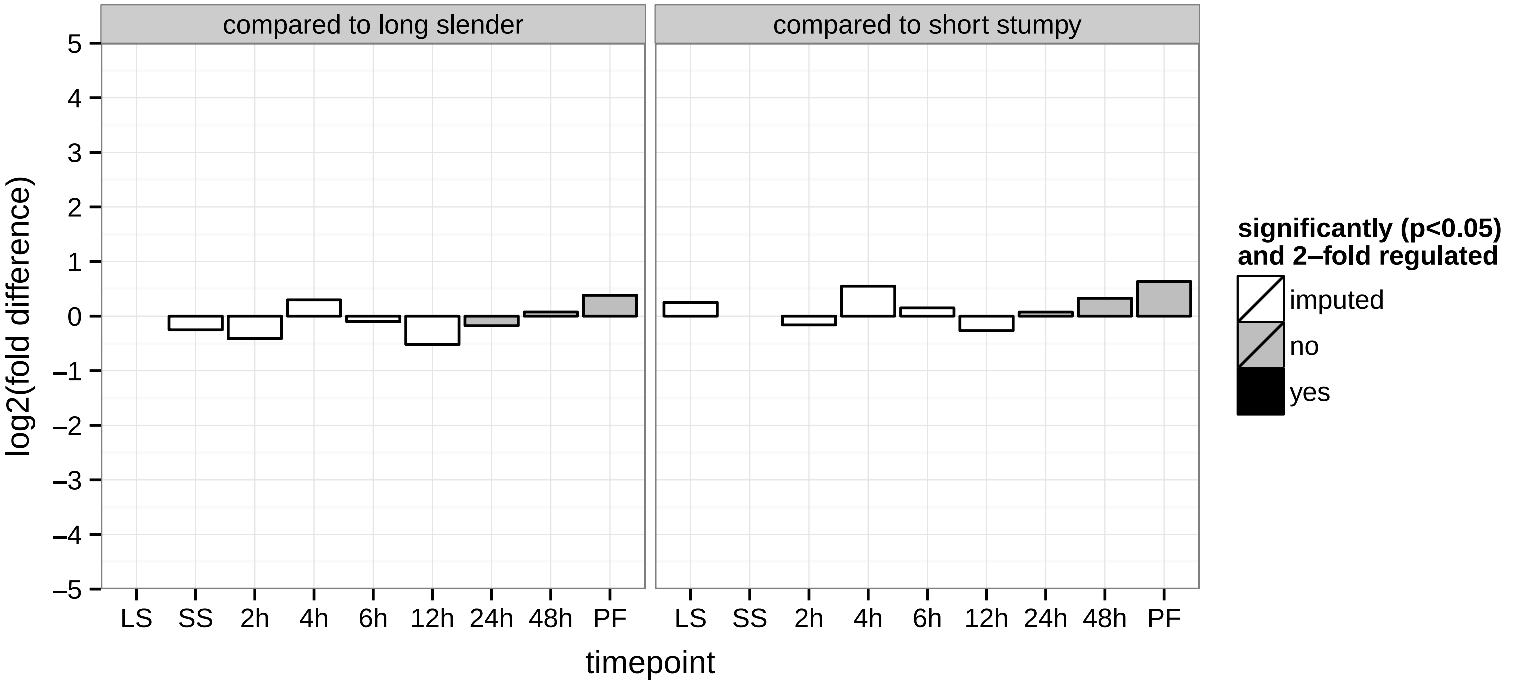

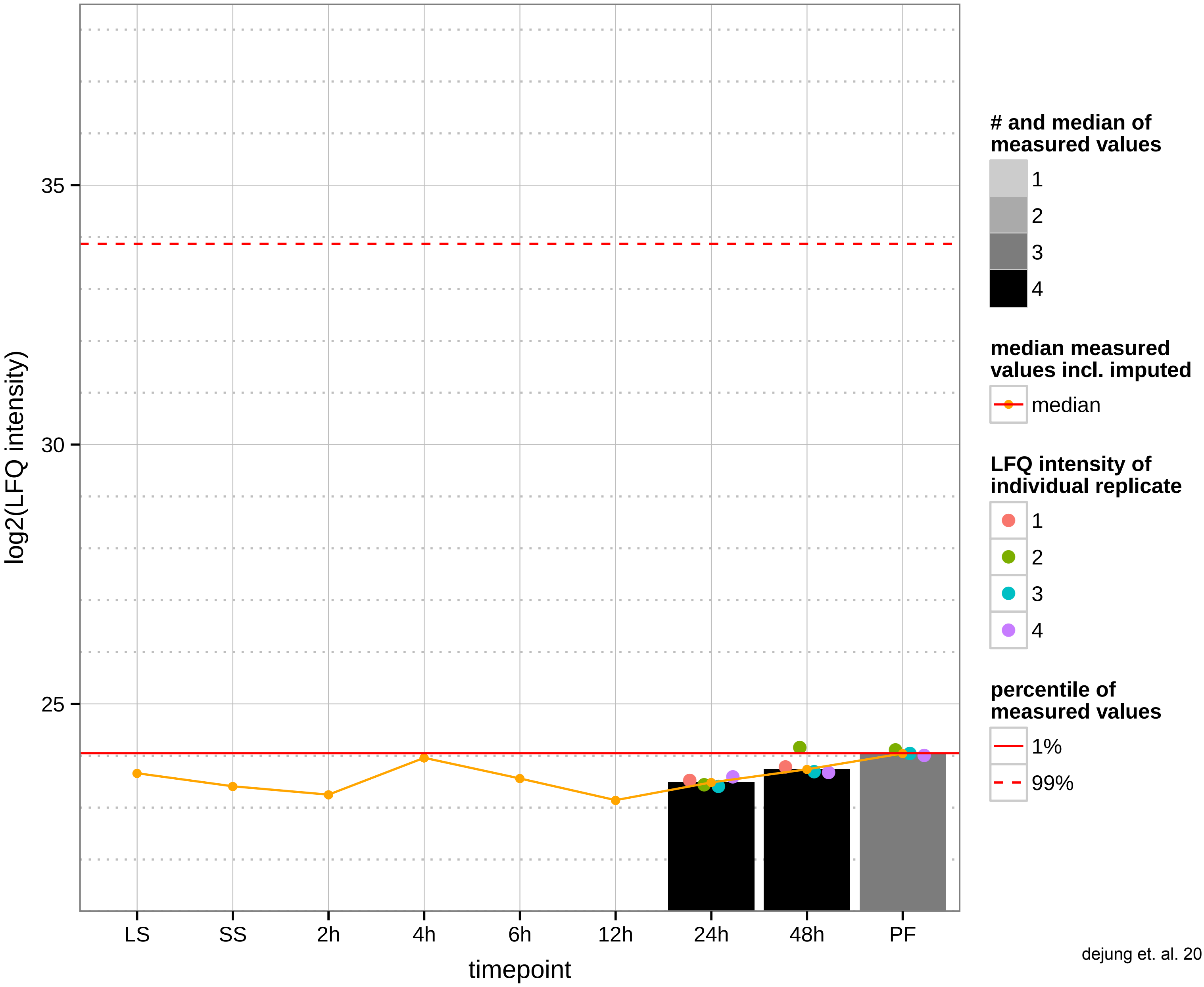

dejung et. al. 2015

hypothetical protein, conserved
Tb927.8.3460
AGOF: zinc ion binding
AGOC: null
AGOP: null
PGOF: protein binding, zinc ion binding
PGOC: null
PGOP: null

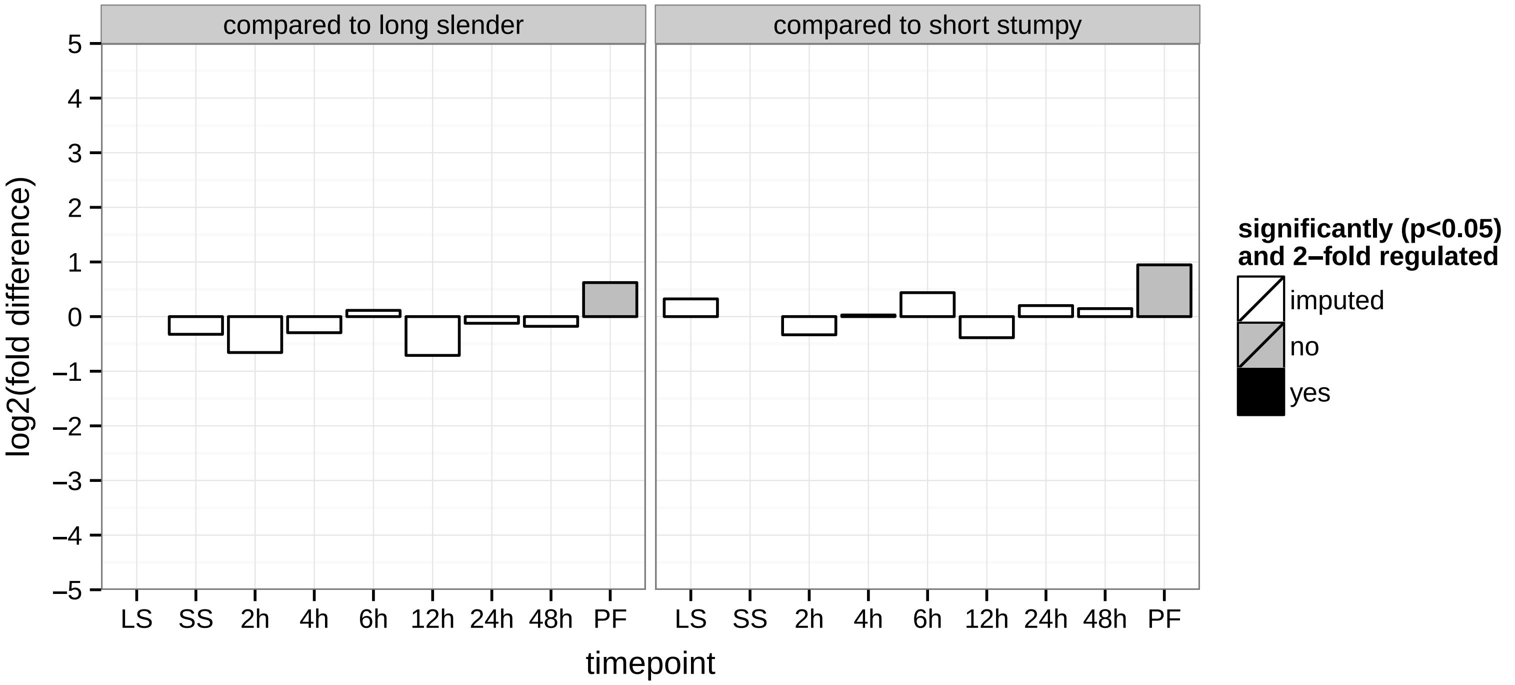

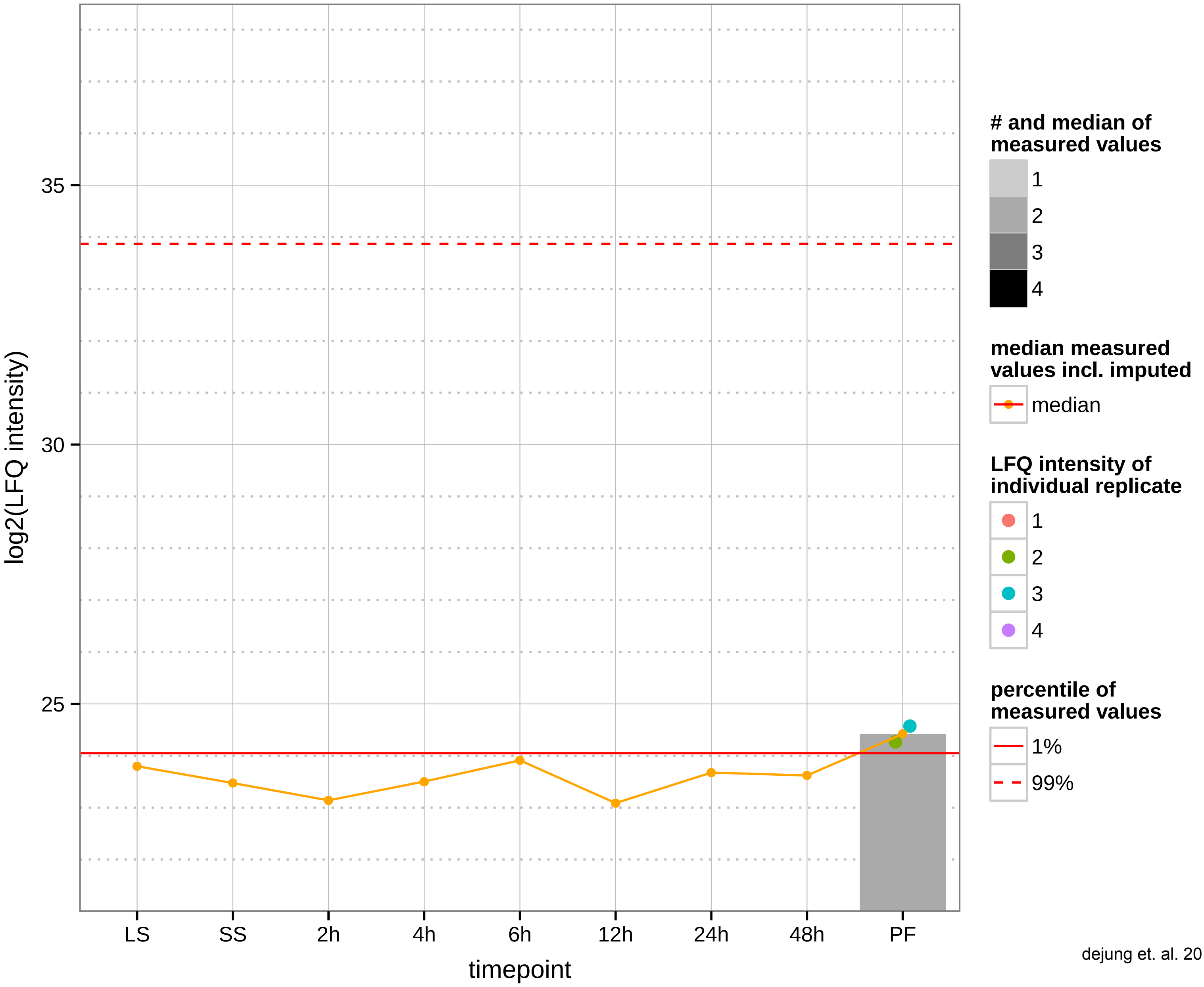

dejung et. al. 2015

hypothetical protein, conserved
Tb927.8.3920;Tb927.8.3500
AGOF: null
AGOC: null
AGOP: null
PGOF: binding
PGOC: null
PGOP: null

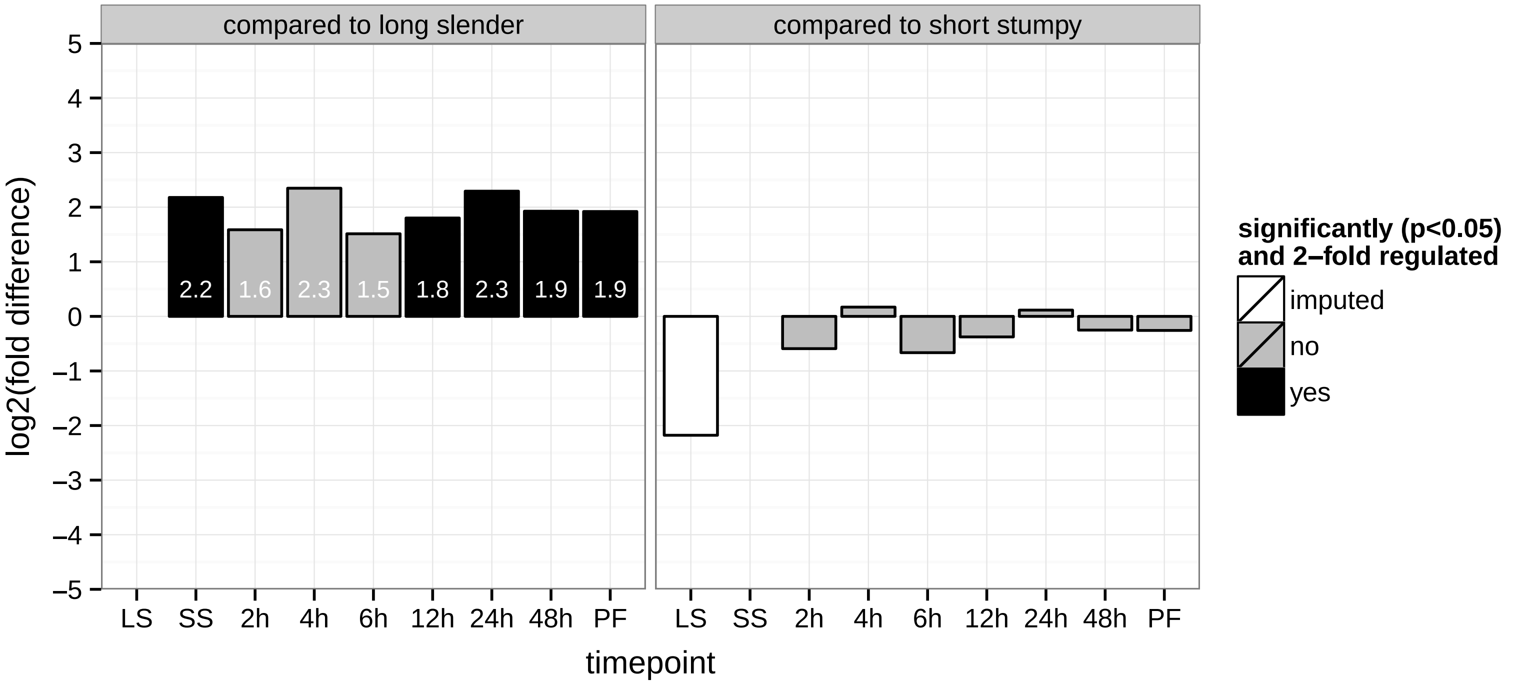

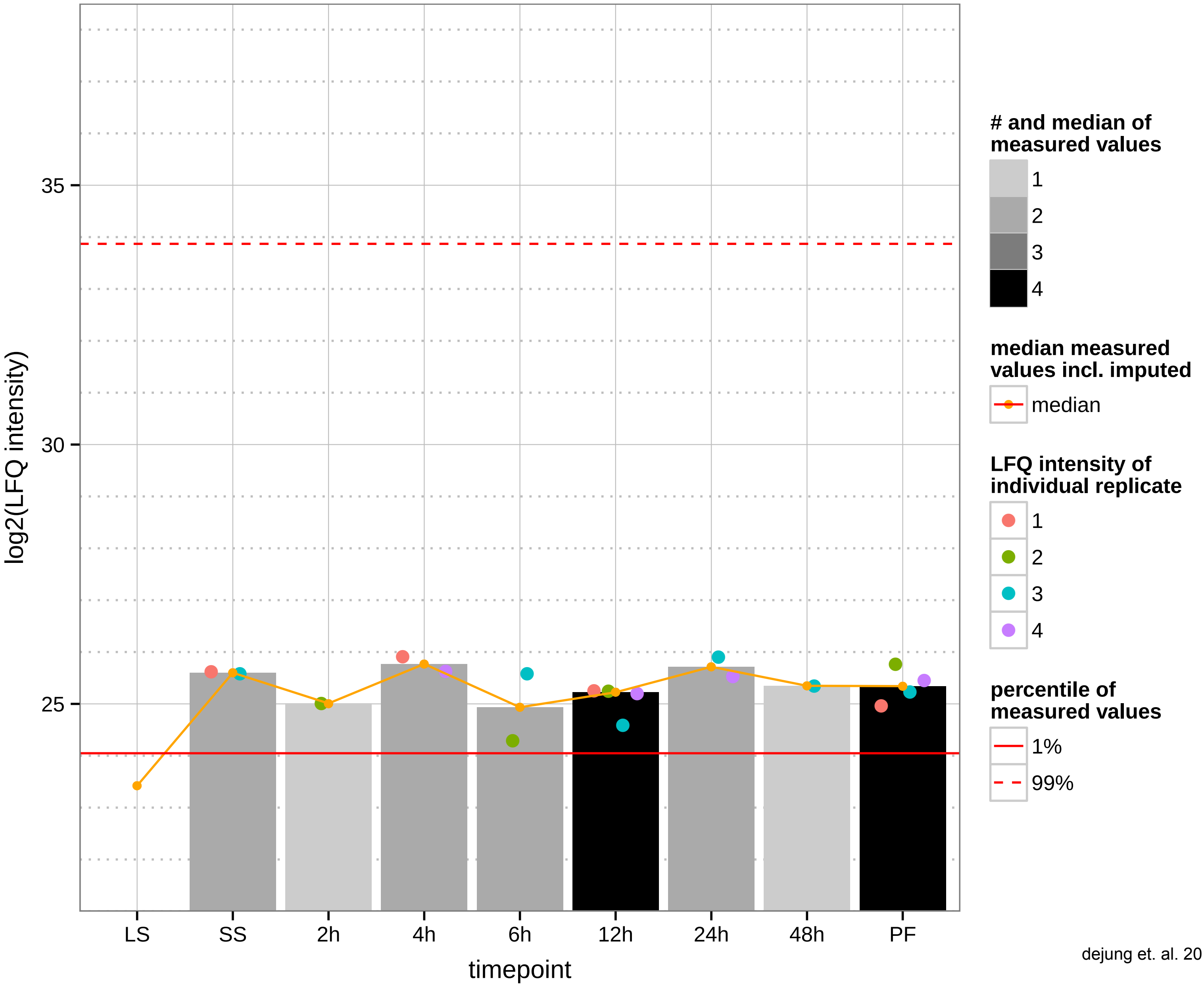

dejung et. al. 2015

glycerol–3–phosphate dehydrogenase [NAD+], glycosomal
Tb927.8.3530
AGOF: NAD binding, glycerol–3–phosphate dehydrogenase [NAD+] activity, protein homodimerization activity
AGOC: cytoplasm, glycerol–3–phosphate dehydrogenase complex, glycosome
AGOP: carbohydrate metabolic process, glycerol–3–phosphate catabolic process, glycerol–3–phosphate metabolic process, c
PGOF: NAD binding, glycerol–3–phosphate dehydrogenase [NAD+] activity, oxidoreductase activity, acting on CH–OH group
PGOC: cytoplasm, glycerol–3–phosphate dehydrogenase complex
PGOP: carbohydrate metabolic process, glycerol–3–phosphate catabolic process, glycerol–3–phosphate metabolic process, c

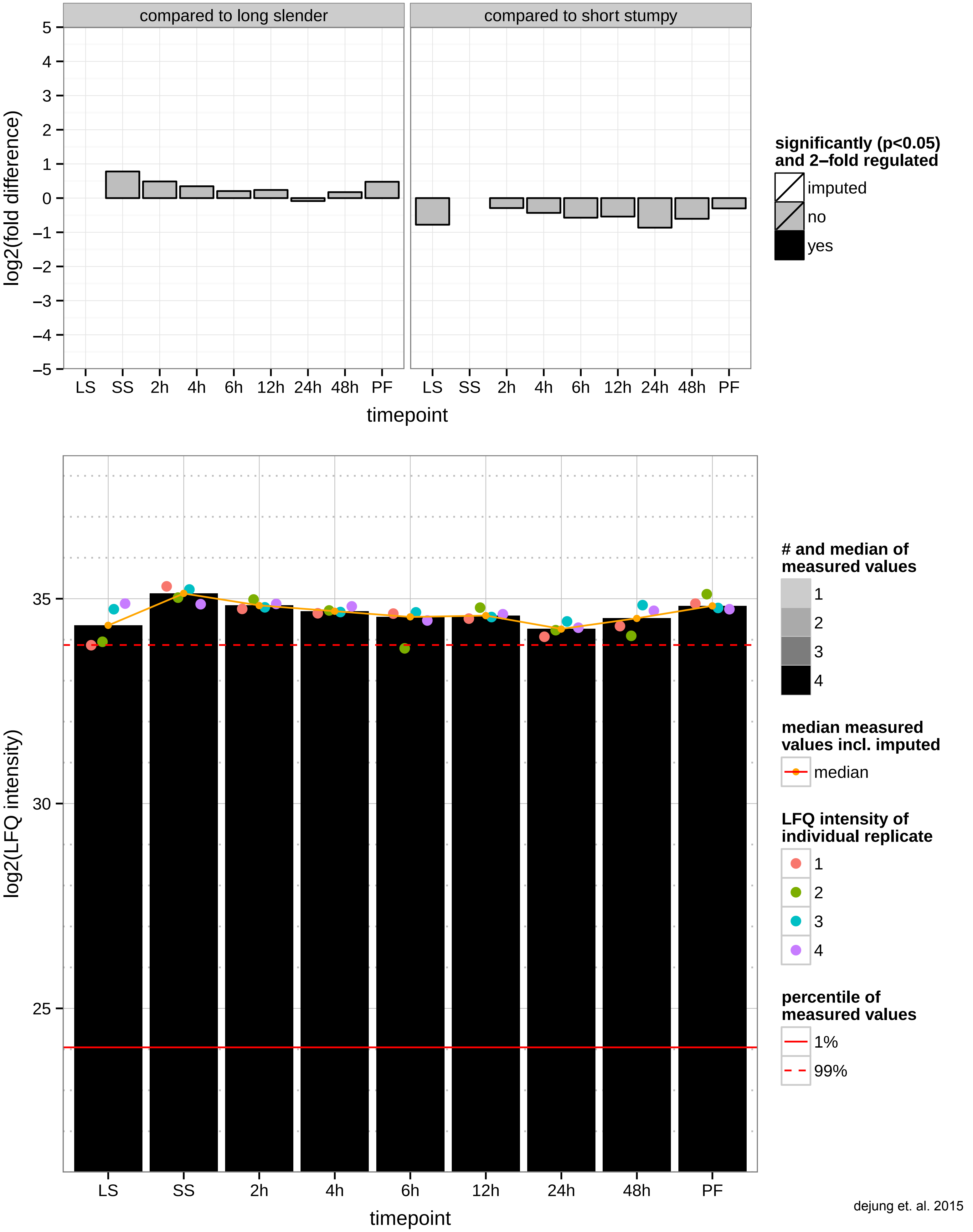

dejung et. al. 2015

protein transport protein SEC23 (SEC23.1)
Tb927.8.3660
AGOF: GTPase activator activity, protein binding, zinc ion binding
AGOC: COPII vesicle coat
AGOP: ER to Golgi vesicle–mediated transport, intracellular protein transport
PGOF: zinc ion binding
PGOC: COPII vesicle coat
PGOP: ER to Golgi vesicle–mediated transport, intracellular protein transport

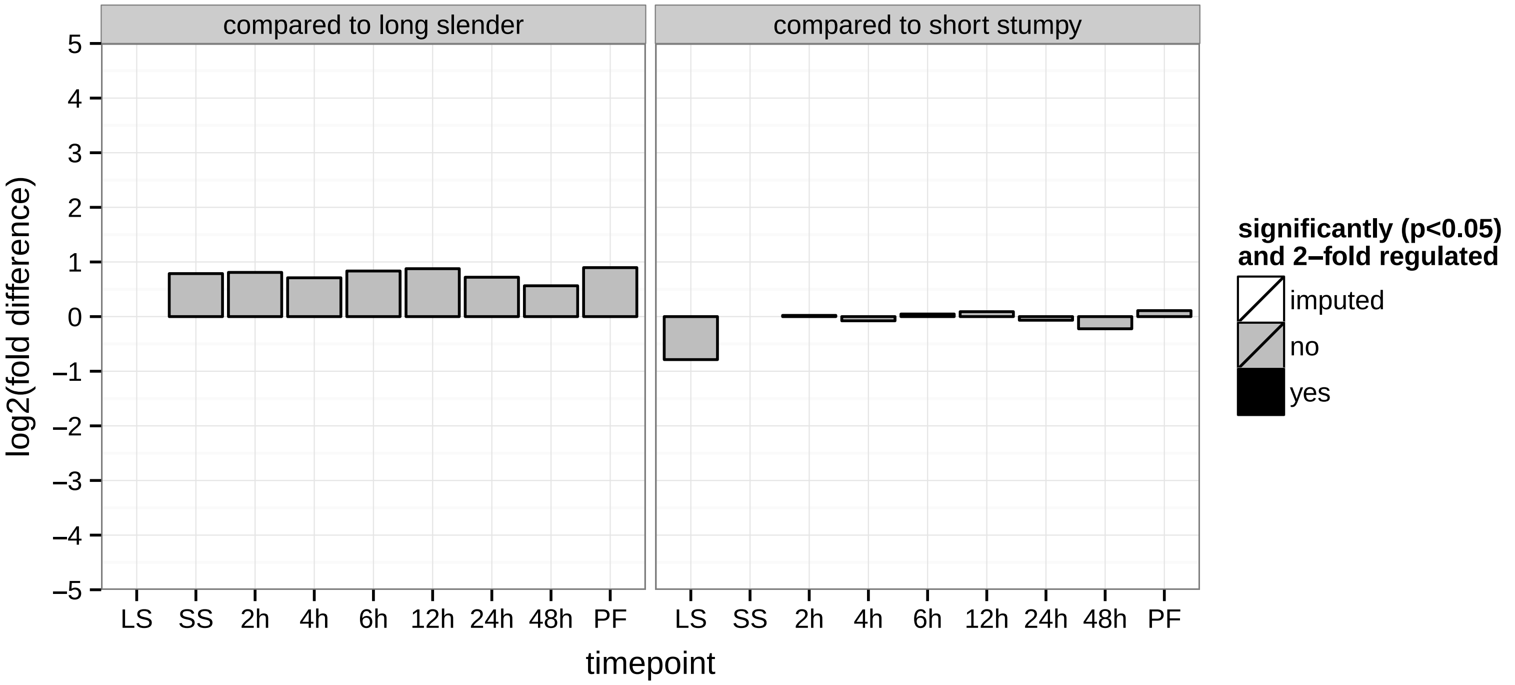

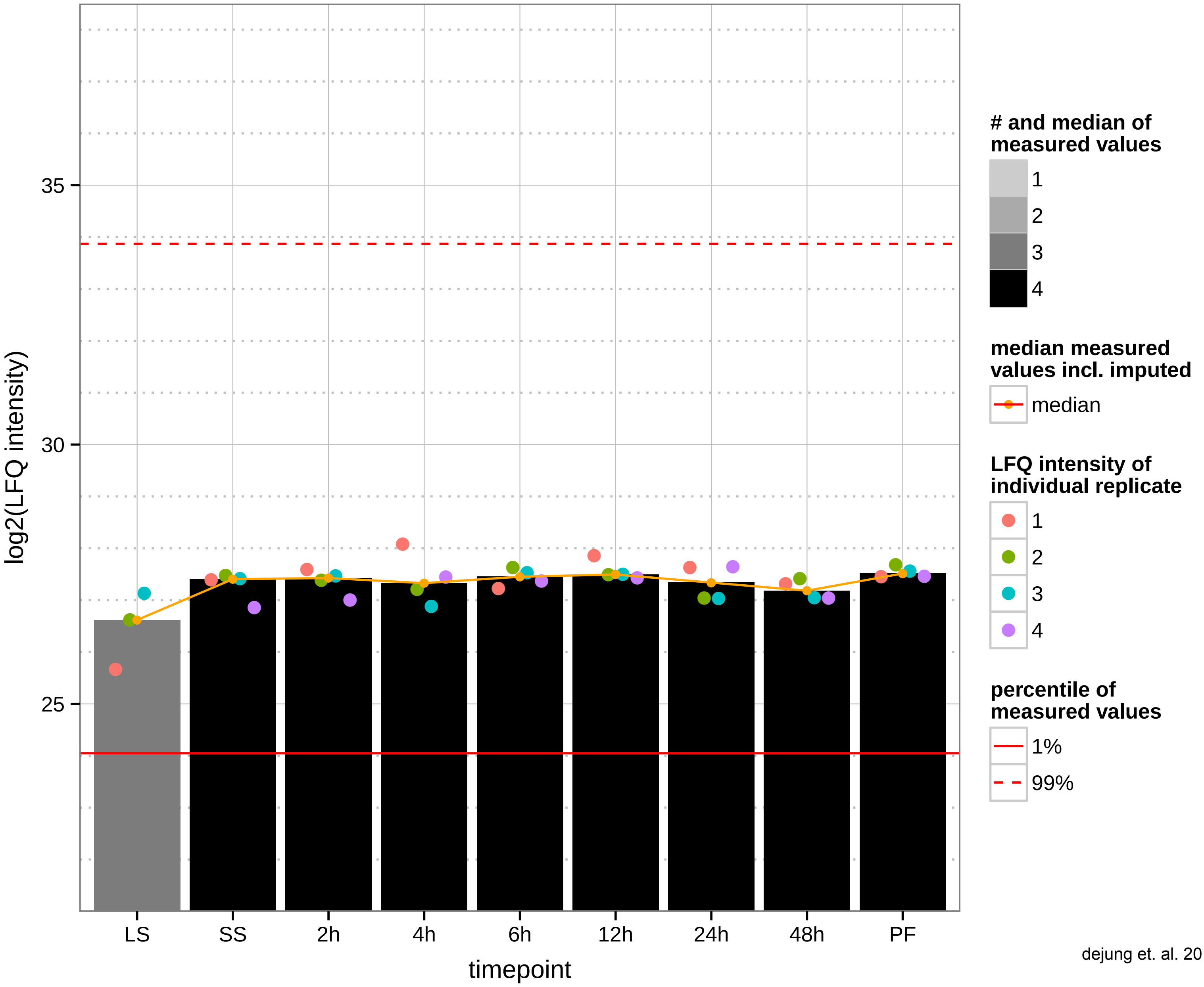

dejung et. al. 2015

isocitrate dehydrogenase [NADP], mitochondrial precursor, putative (IDH)
Tb927.8.3690
AGOF: NAD binding, isocitrate dehydrogenase (NADP+) activity, magnesium ion binding
AGOC: mitochondrion
AGOP: isocitrate metabolic process, oxidation–reduction process, tricarboxylic acid cycle
PGOF: isocitrate dehydrogenase (NADP+) activity, oxidoreductase activity, acting on the CH–OH group of donors, NAD or NA
PGOC: null
PGOP: isocitrate metabolic process, oxidation–reduction process

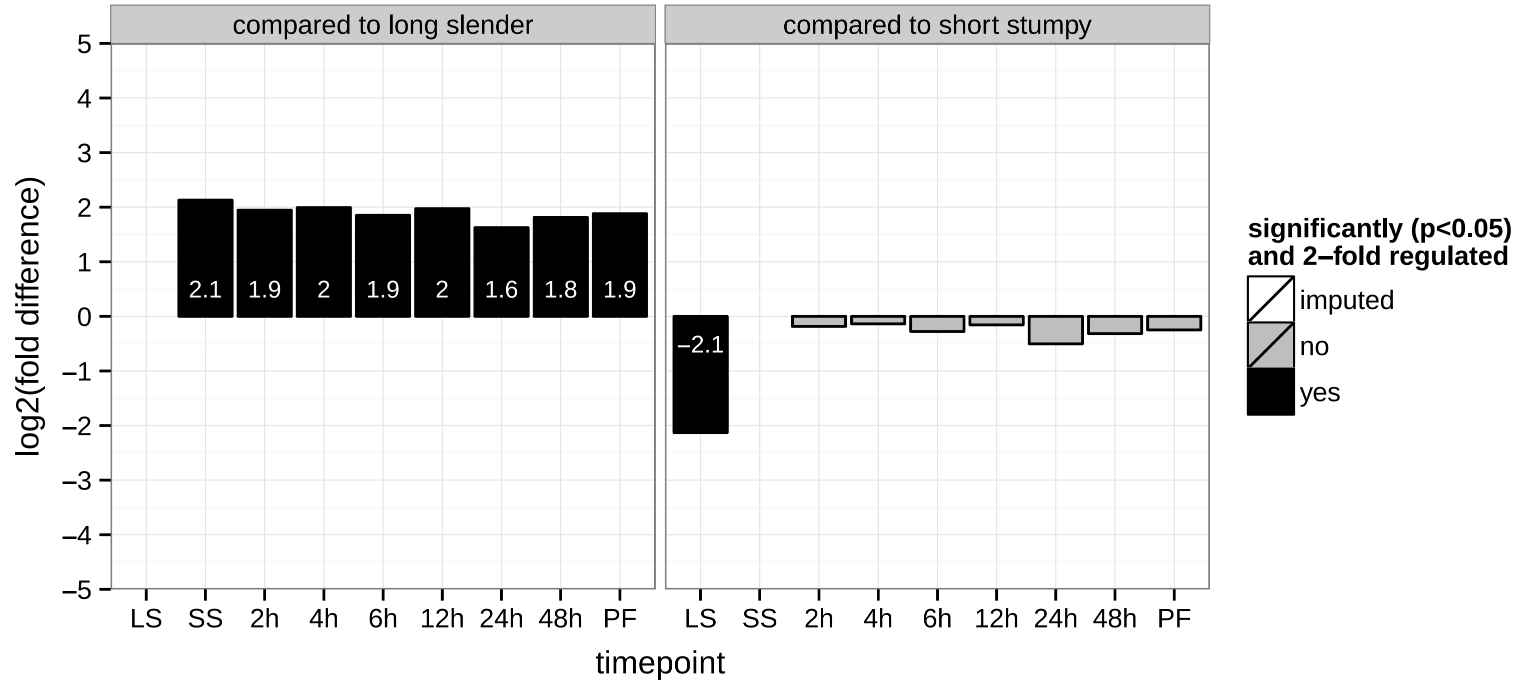

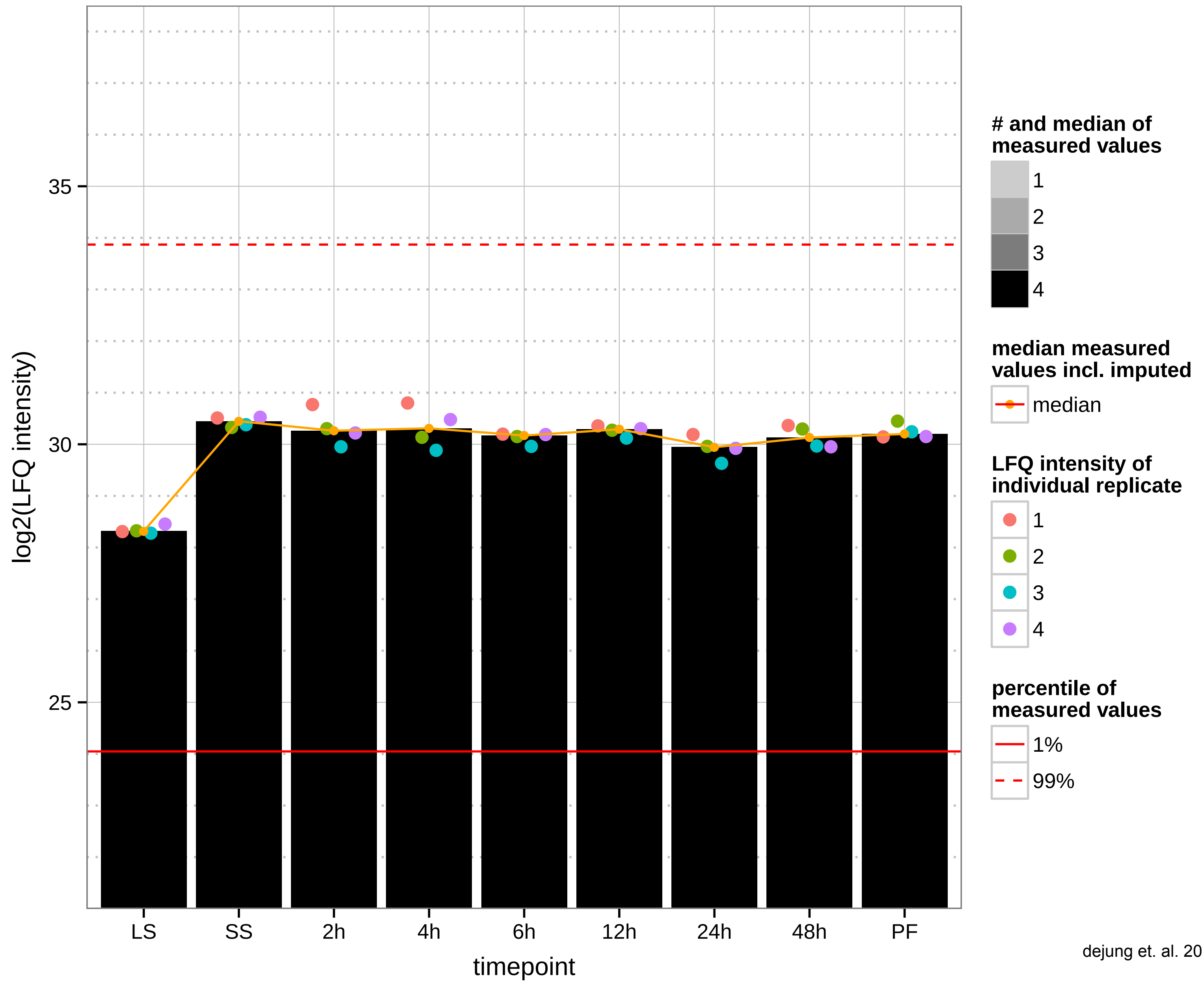

dejung et. al. 2015

Nucleolar protein 56, putative (NOP56)
Tb927.8.3750
AGOF: snoRNA binding
AGOC: box C/D snoRNP complex, small−subunit processome
AGOP: nuclear mRNA splicing, via spliceosome, rRNA processing
PGOF: null
PGOC: null
PGOP: null

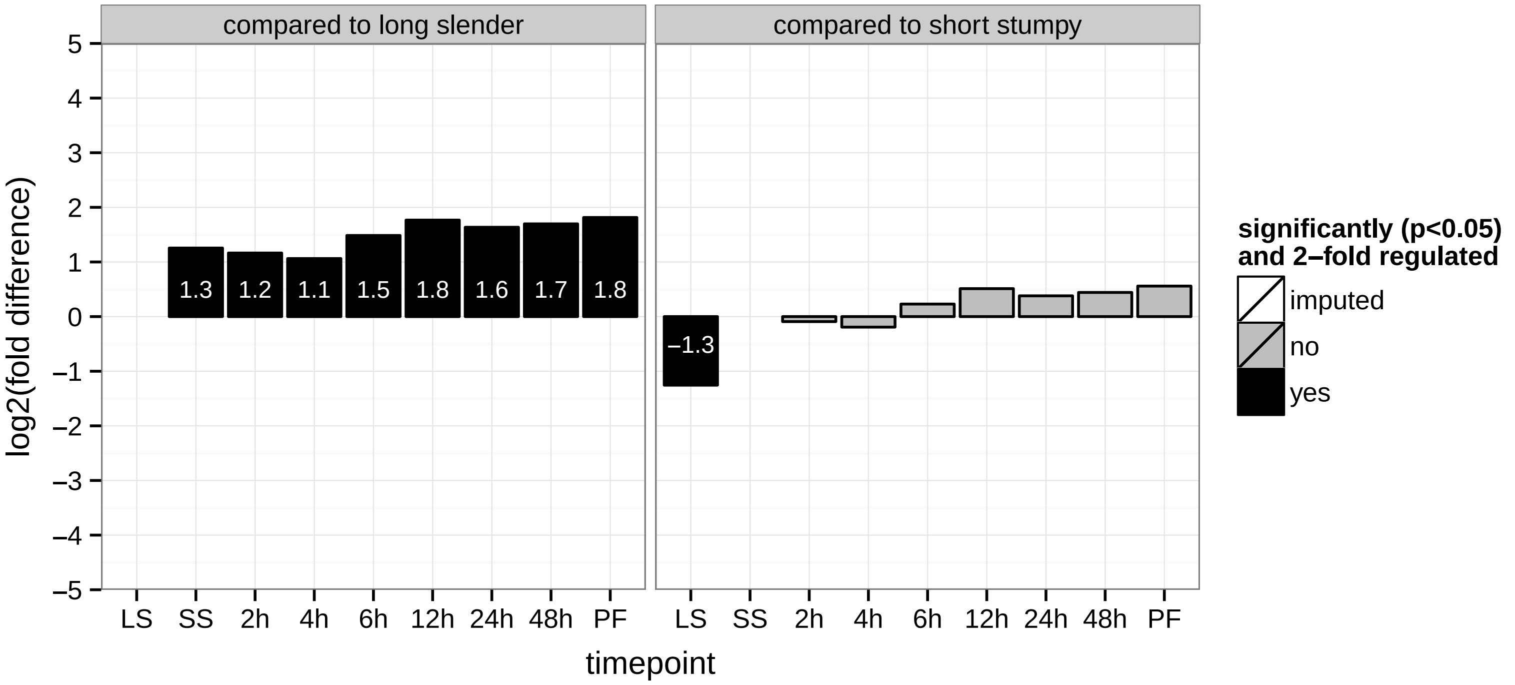

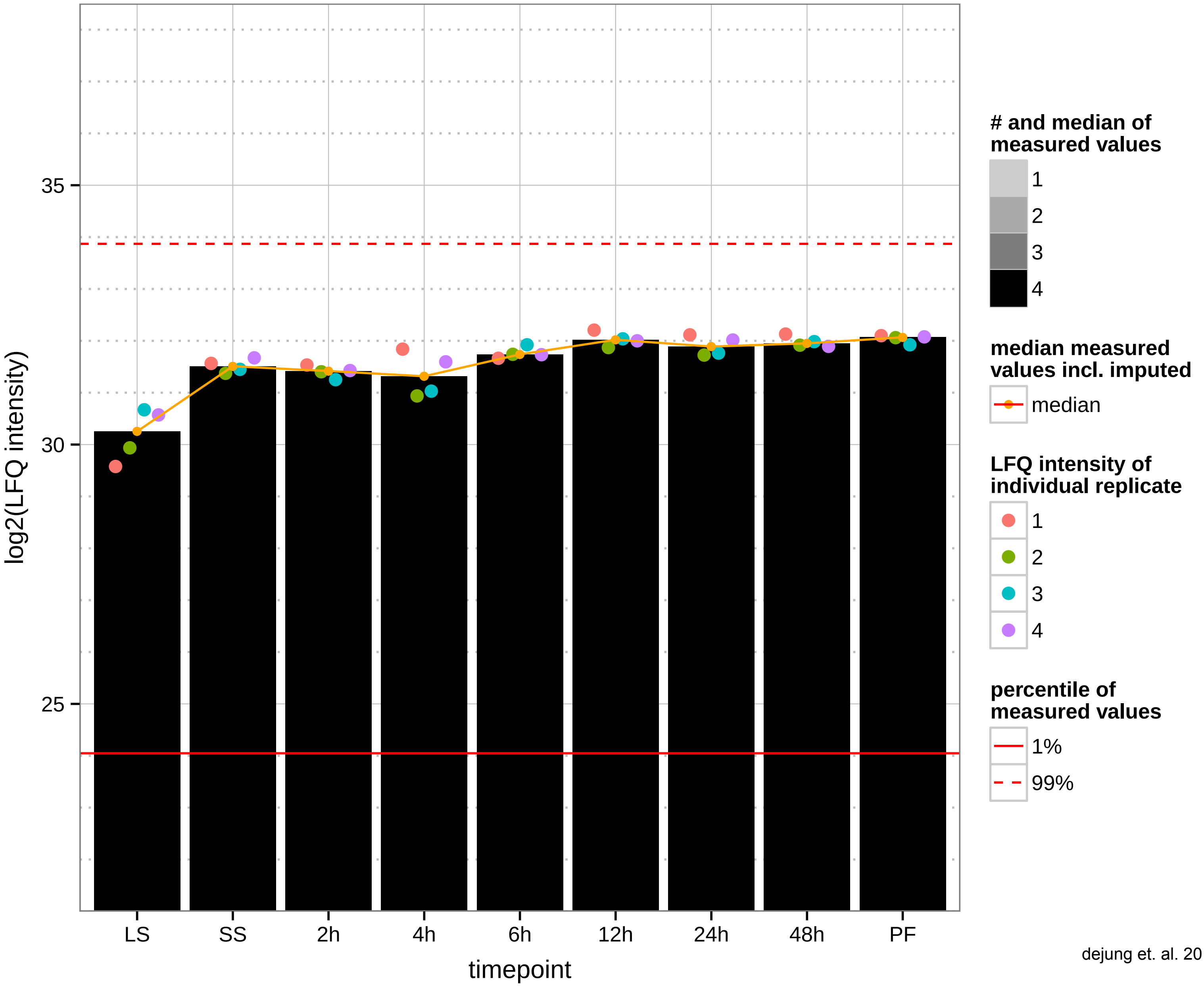

dejung et. al. 2015

hypothetical protein, conserved
Tb927.8.3780
AGOF: null
AGOC: null
AGOP: null
PGOF: null
PGOC: null
PGOP: null

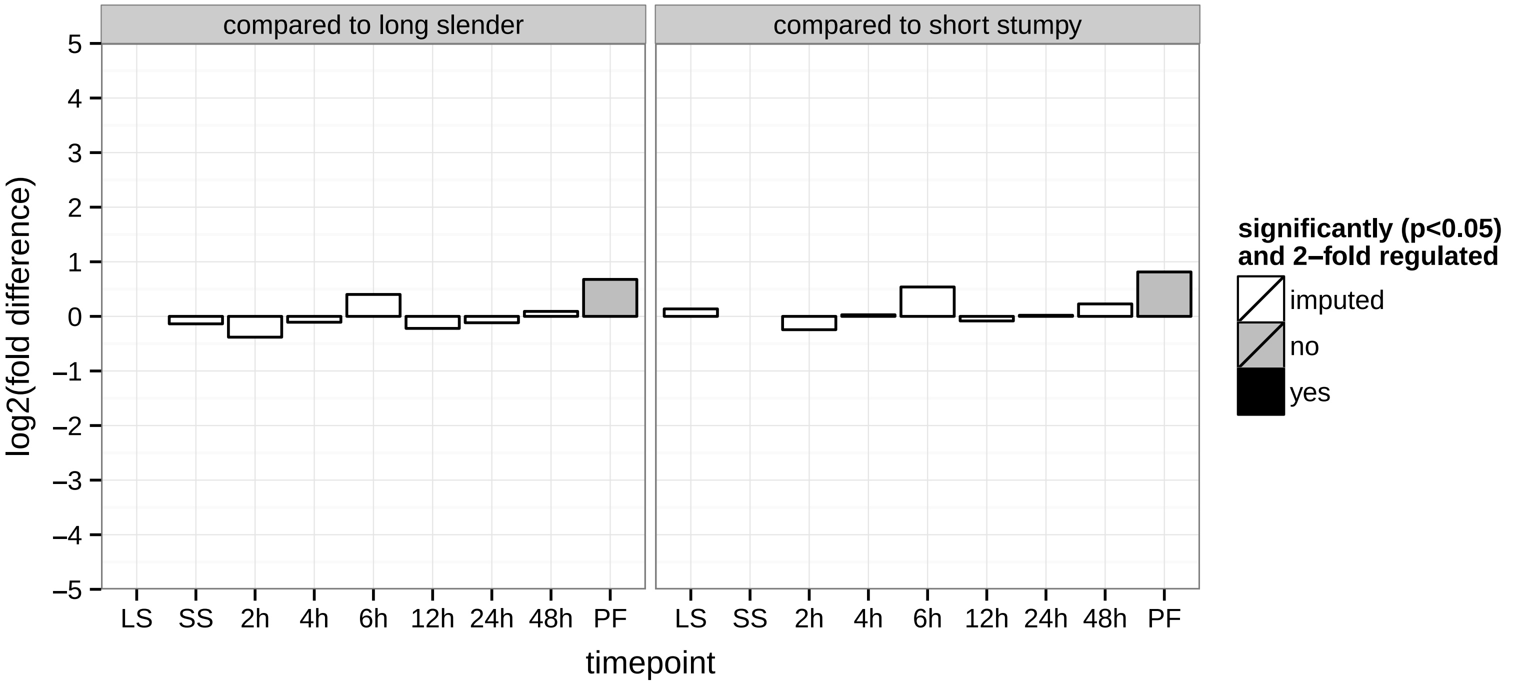

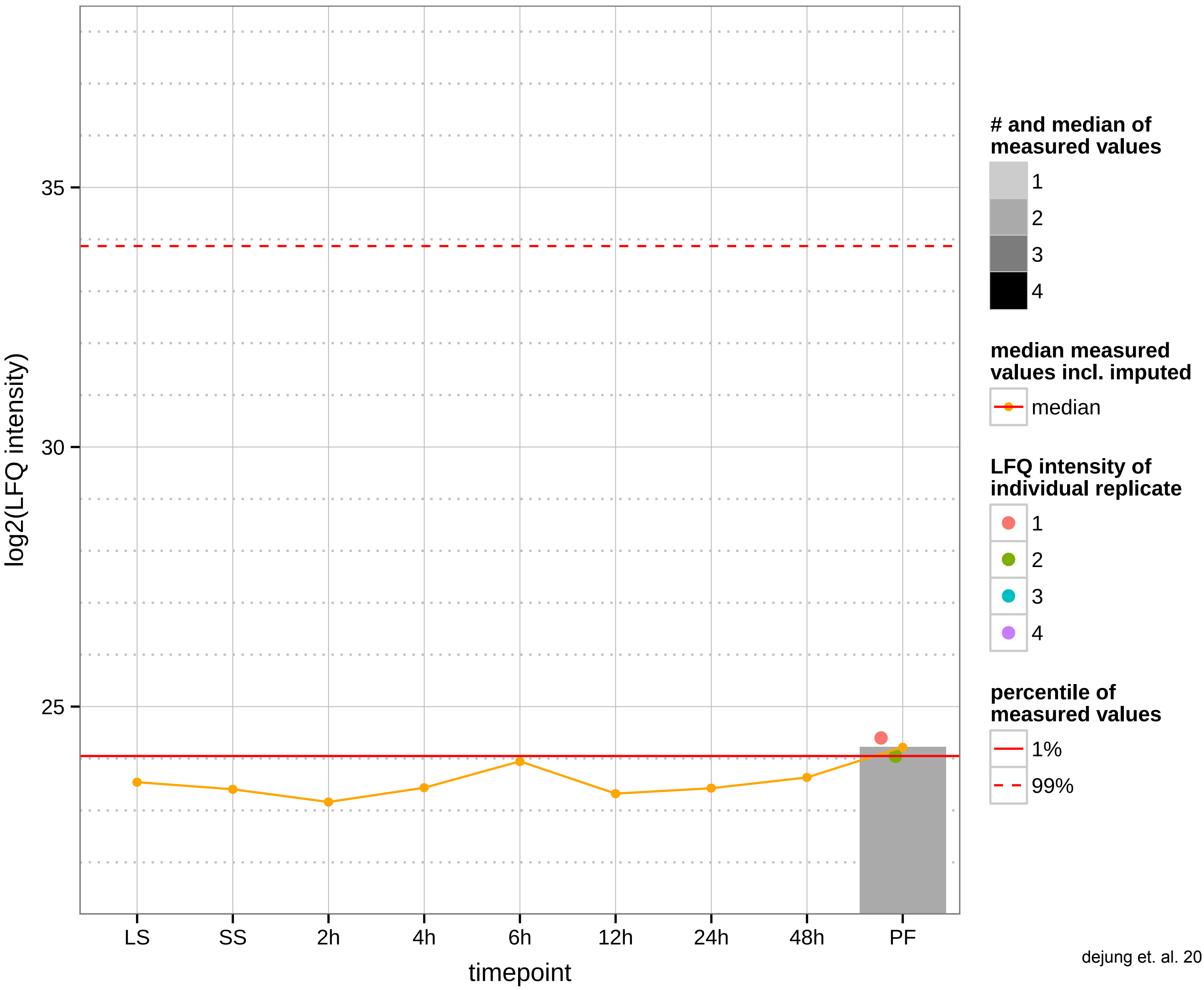

dejung et. al. 2015

paraflagellar rod component, putative (PFC2)
Tb927.8.3790
AGOF: null
AGOC: null
AGOP: null
PGOF: null
PGOC: null
PGOP: null

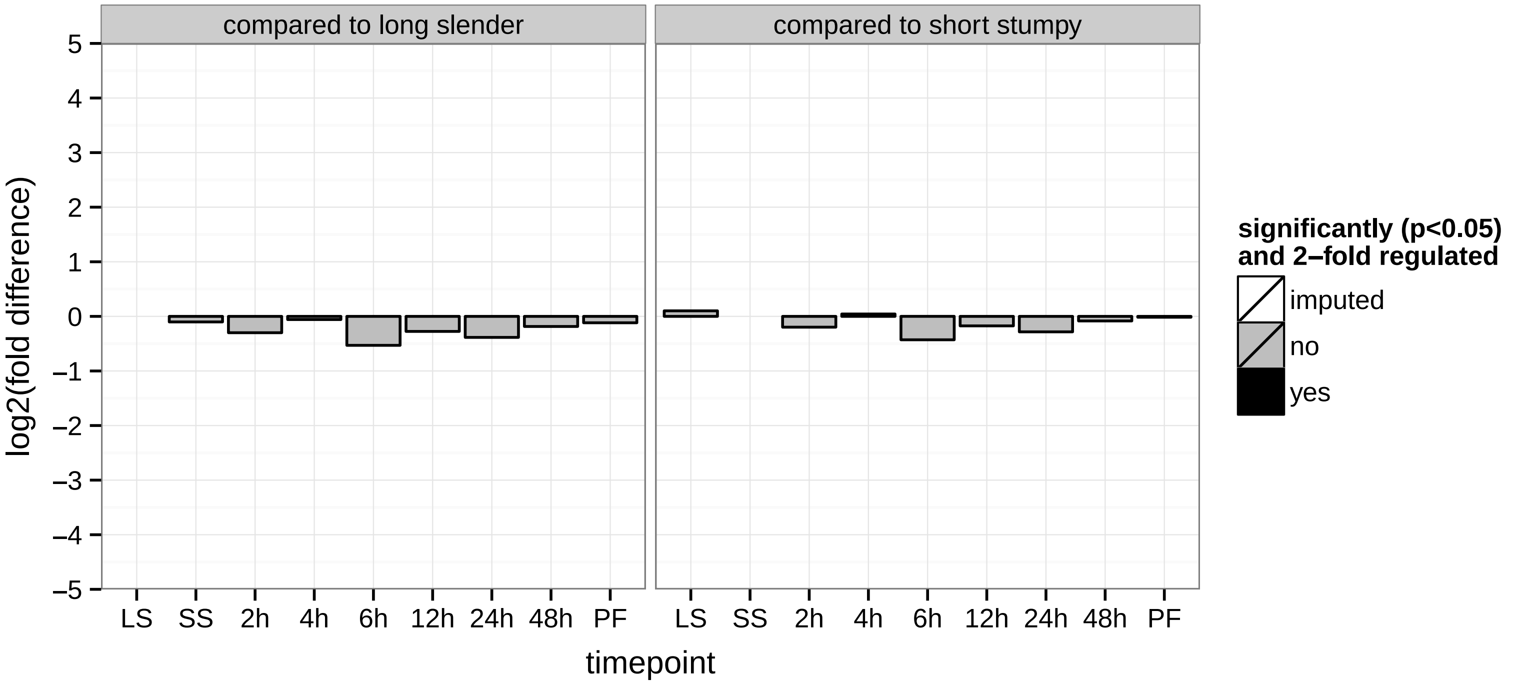

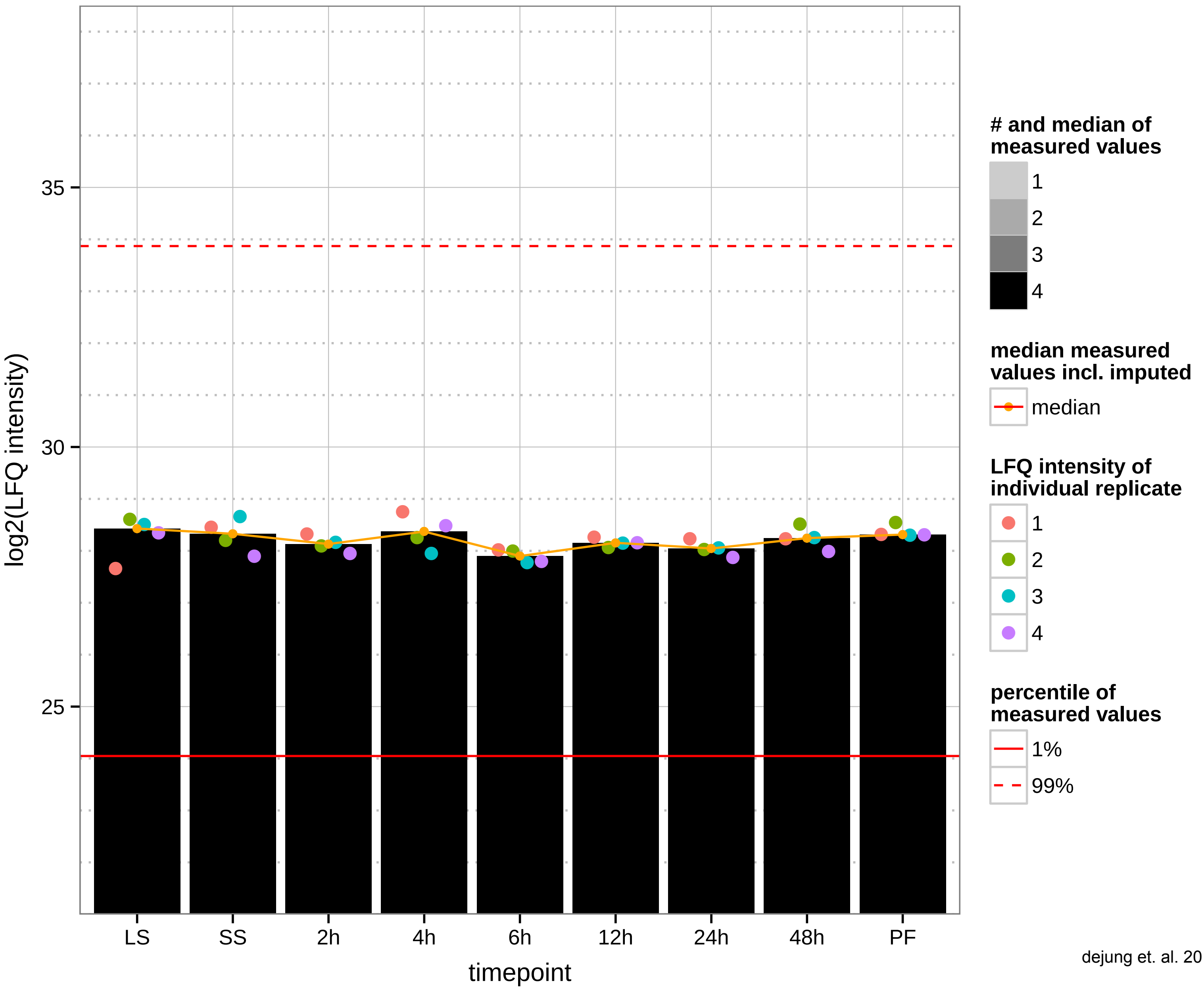

dejung et. al. 2015

variant surface glycoprotein (VSG), putative, hypothetical protein, conserved
Tb927.8.3840
AGOF: null
AGOC: null
AGOP: null
PGOF: null
PGOC: null
PGOP: evasion or tolerance of host immune response, null

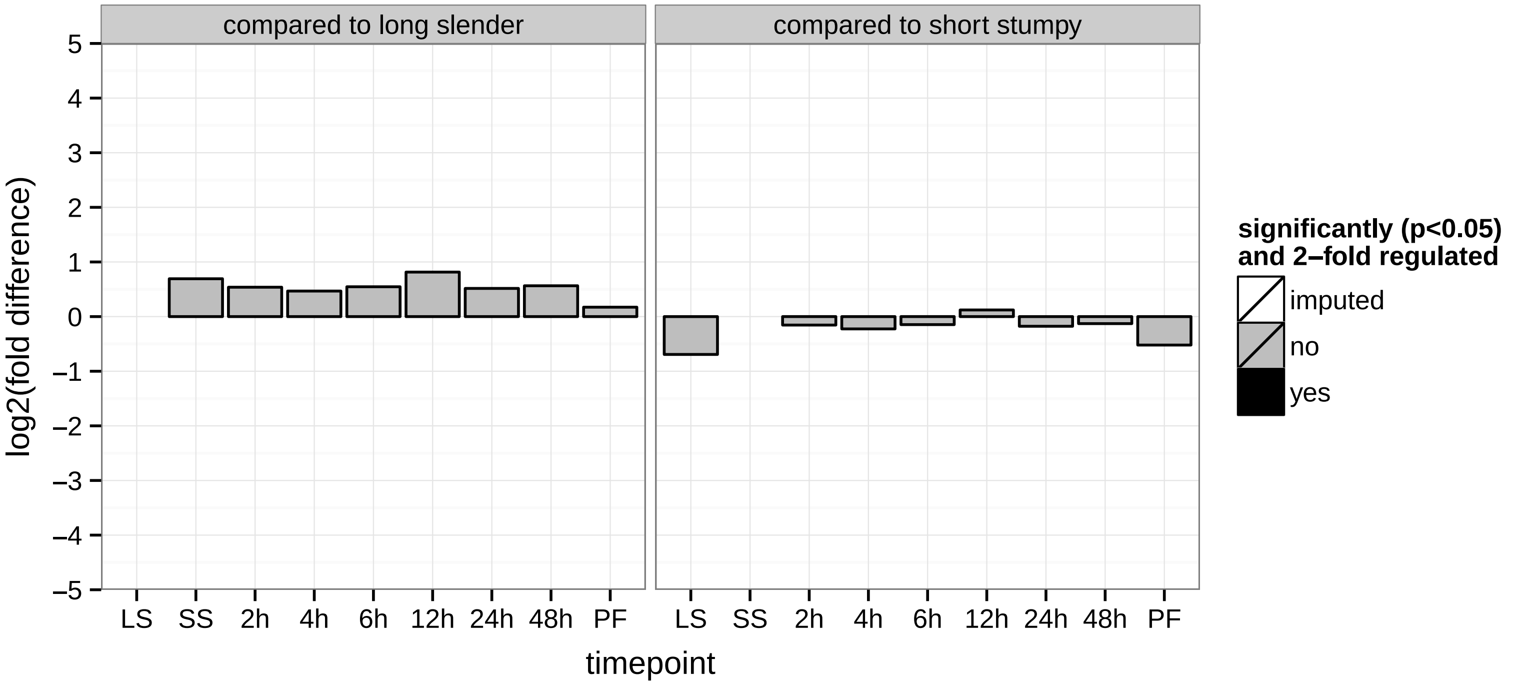

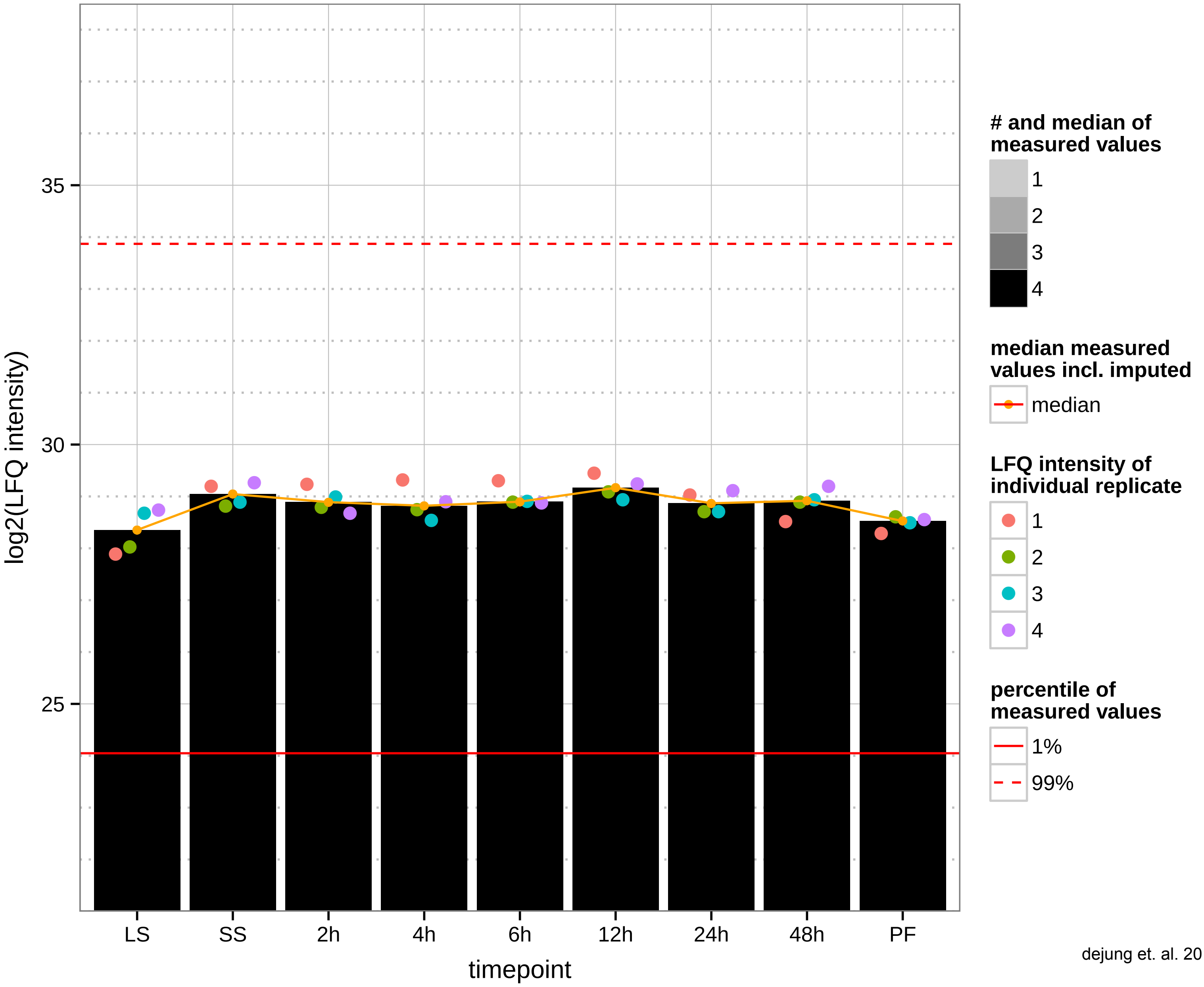

dejung et. al. 2015

hypothetical protein, conserved
Tb927.8.3870;Tb11.v5.0681
AGOF: null, structural constituent of cell wall
AGOC: null
AGOP: null
PGOF: null
PGOC: null
PGOP: null

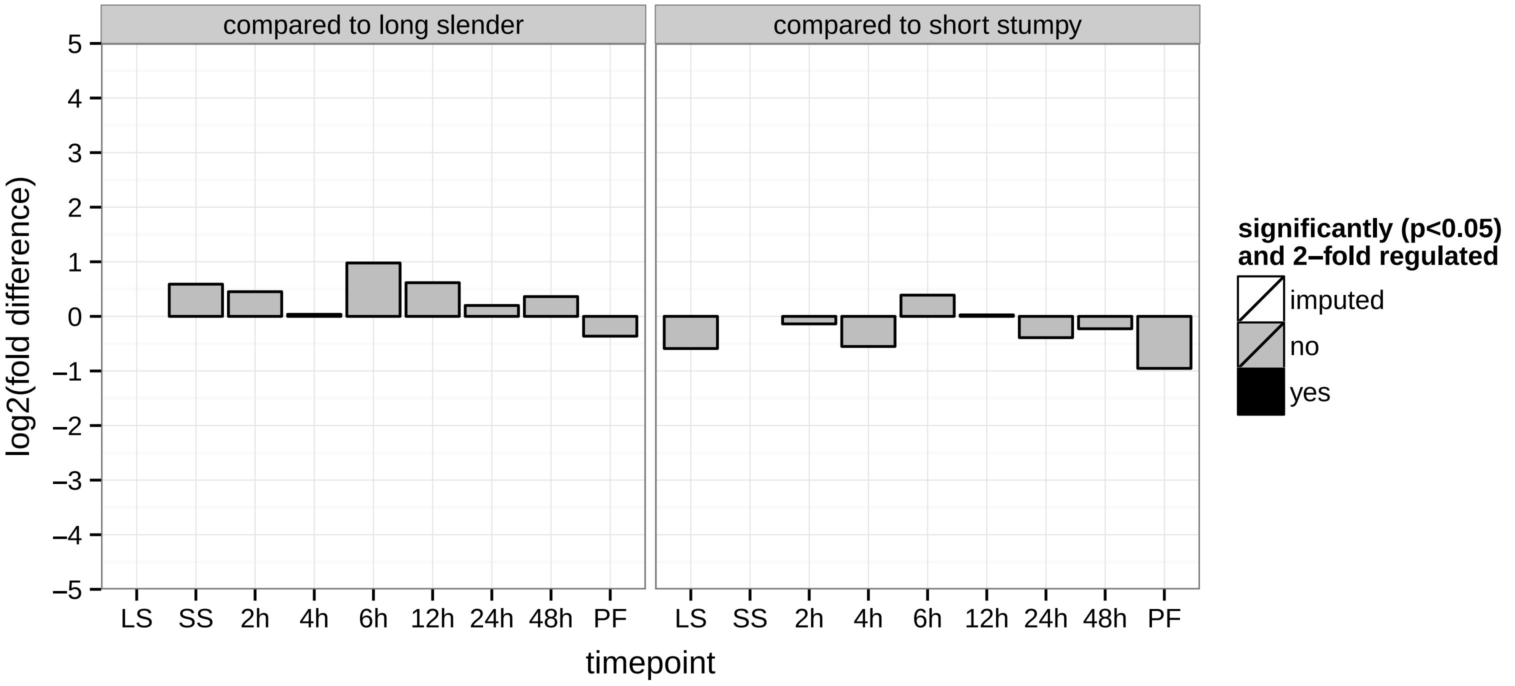

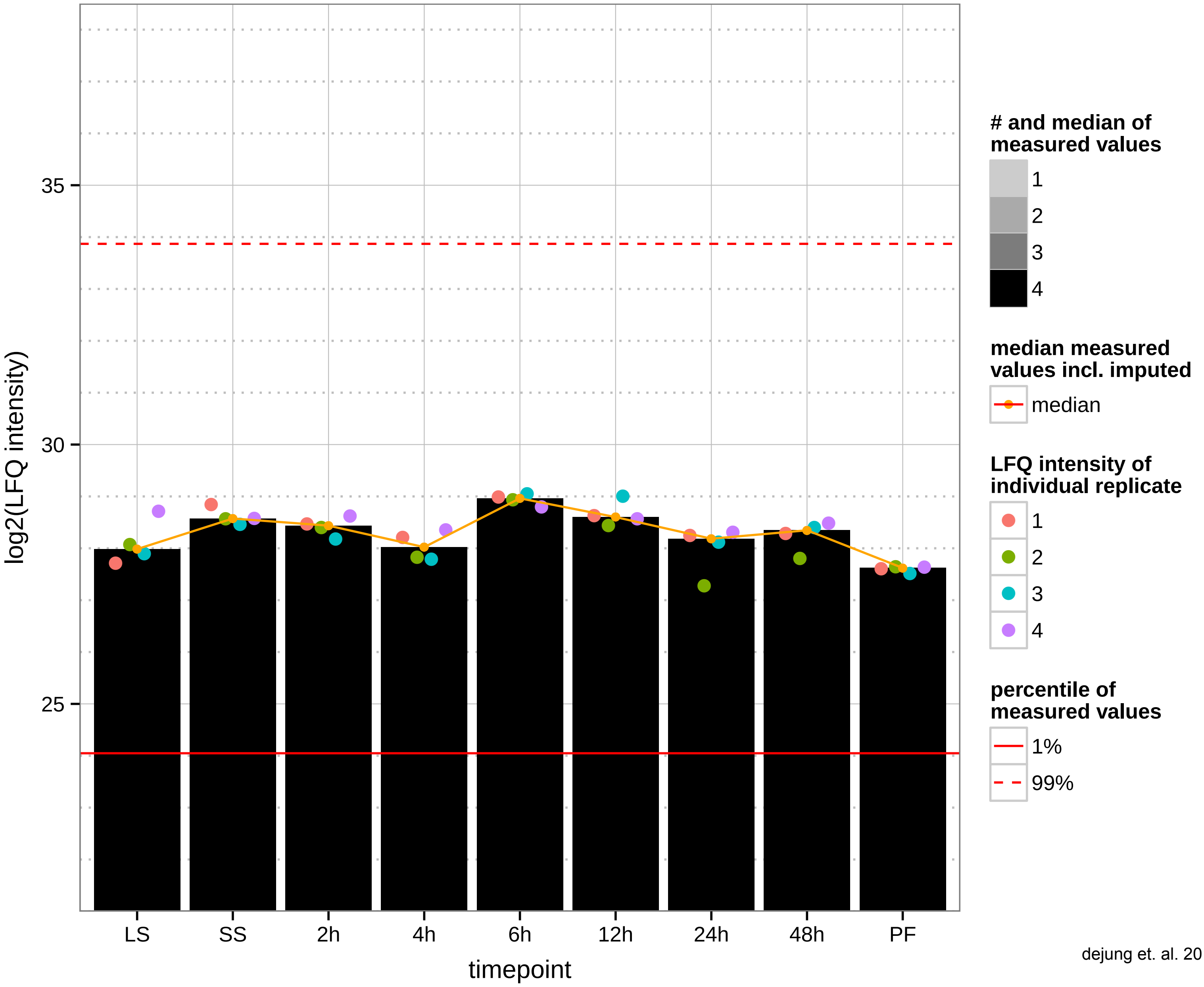

dejung et. al. 2015

hypothetical protein, conserved
Tb927.8.3950
AGOF: null
AGOC: null
AGOP: null
PGOF: null
PGOC: null
PGOP: null

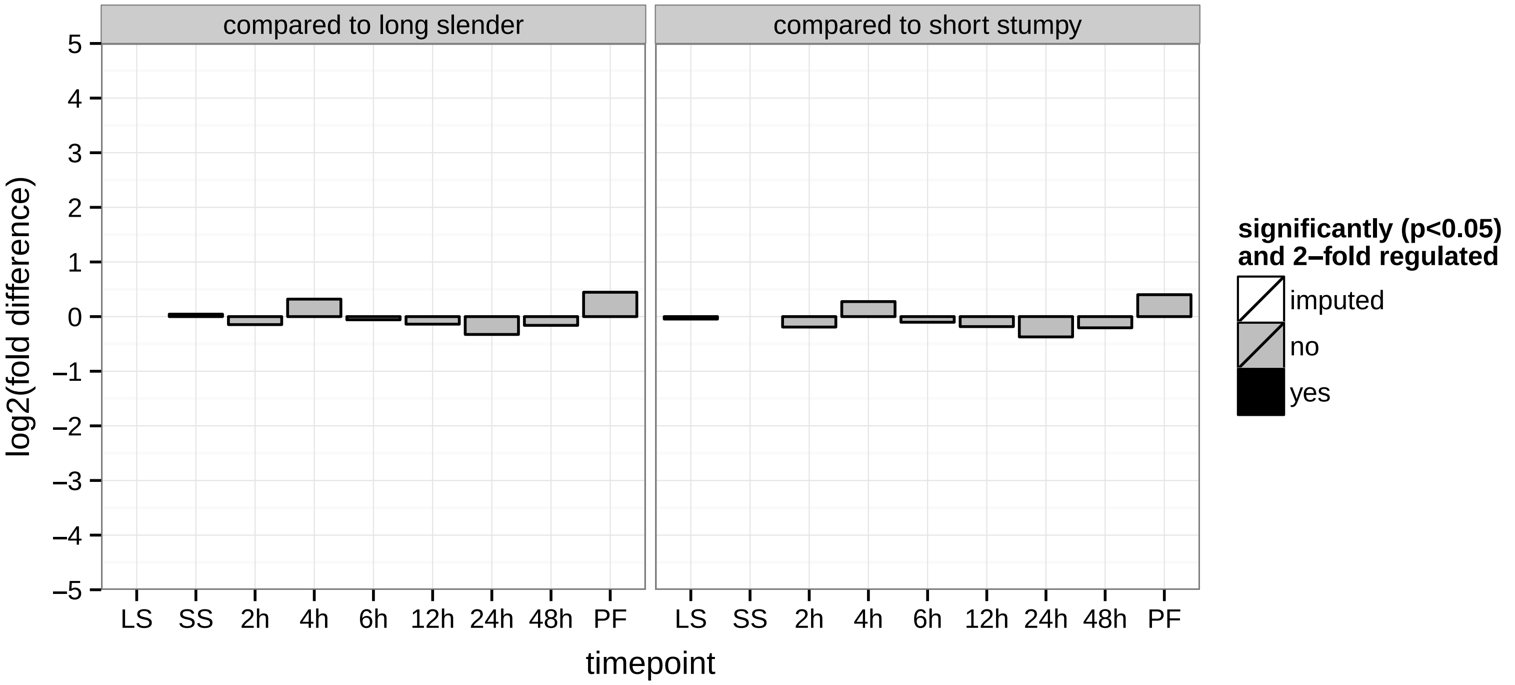

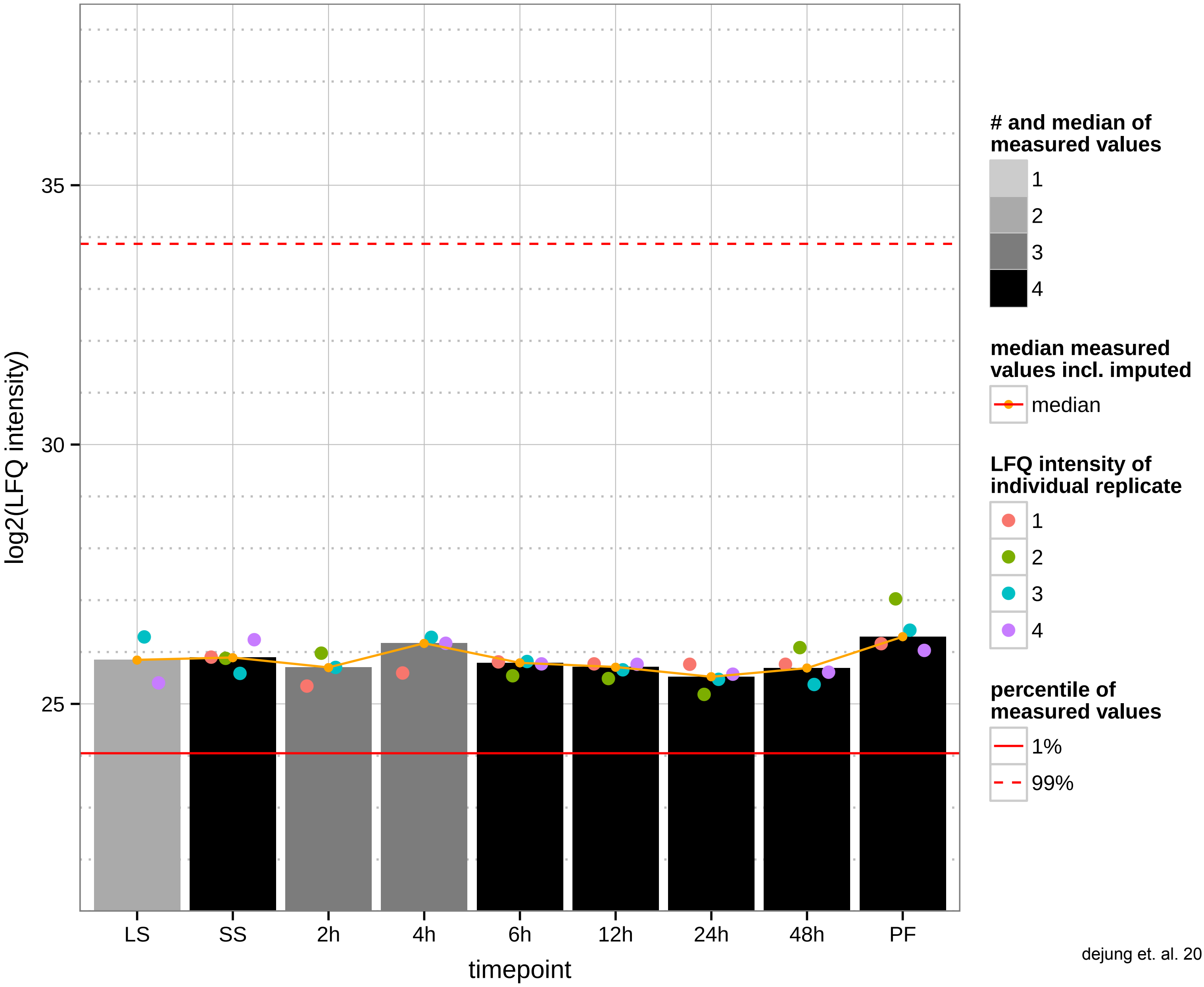

dejung et. al. 2015

oxidoreductase, putative
Tb927.8.3970
AGOF: alcohol dehydrogenase activity, zinc–dependent, oxidoreductase activity, zinc ion binding
AGOC: null
AGOP: metabolic process, oxidation–reduction process
PGOF: oxidoreductase activity, transferase activity, transferring acyl groups other than amino–acyl groups, zinc ion binding
PGOC: null
PGOP: oxidation–reduction process

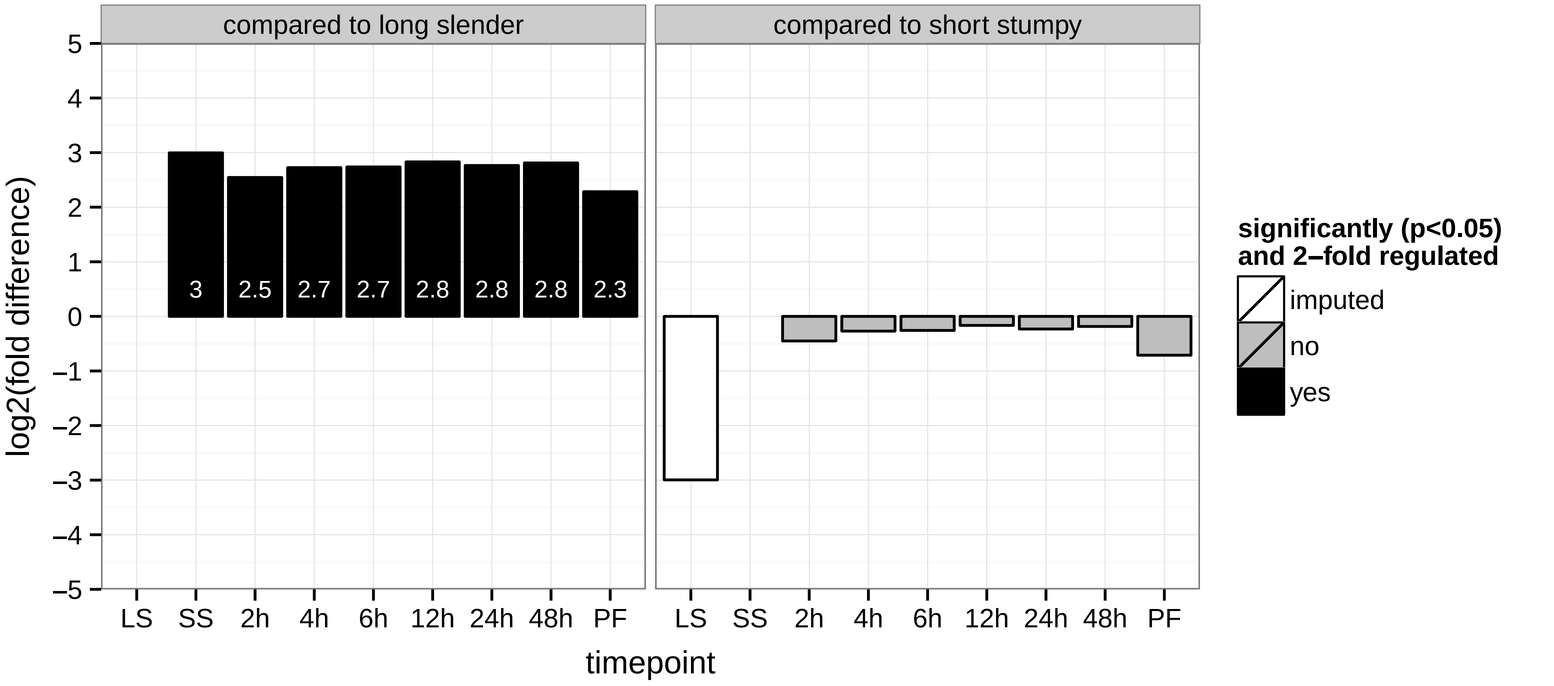

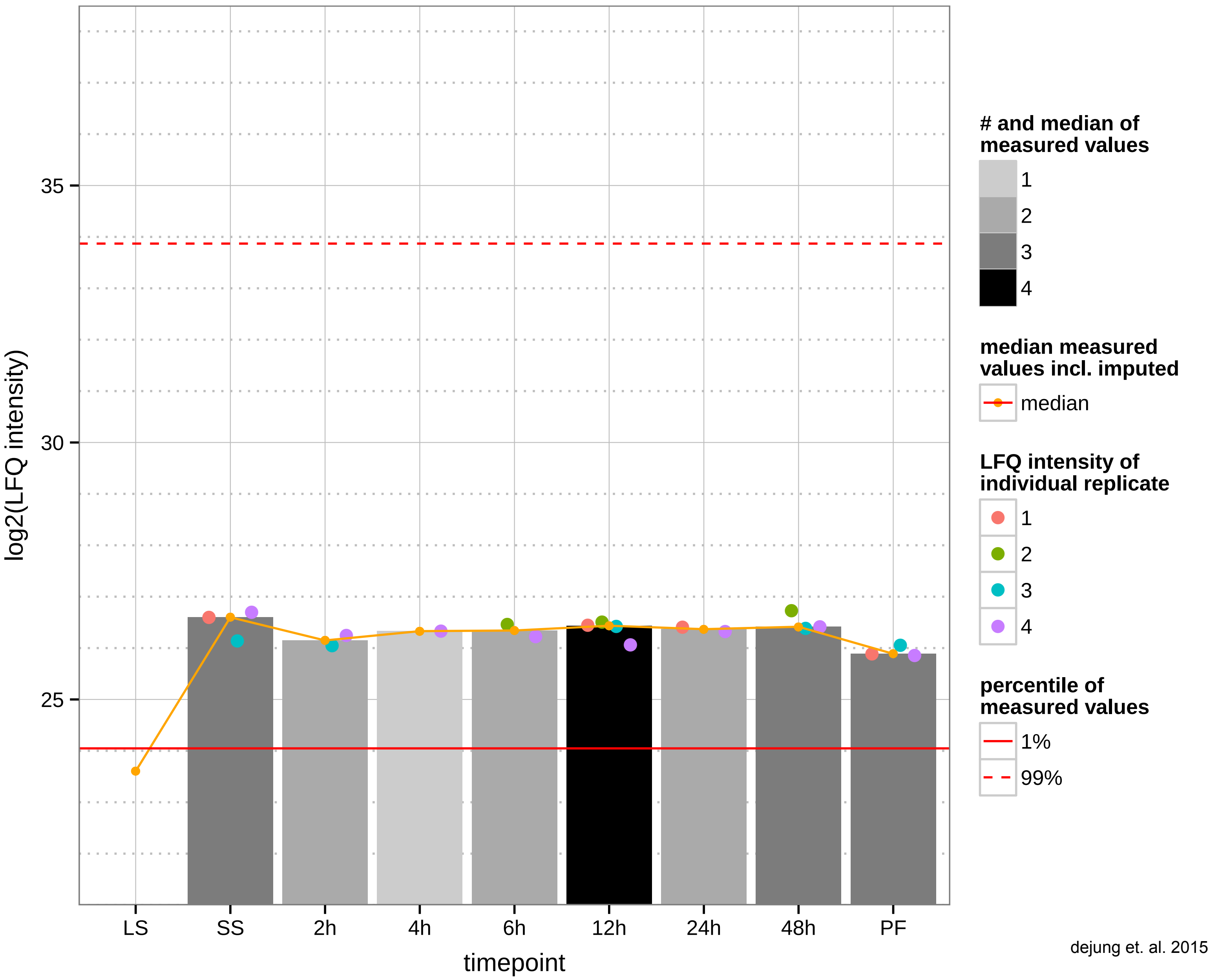

dejung et. al. 2015

class I transcription factor A, subunit 5b (CITFA–5b)
Tb927.8.4130
AGOF: null
AGOC: null
AGOP: null
PGOF: null
PGOC: null
PGOP: null

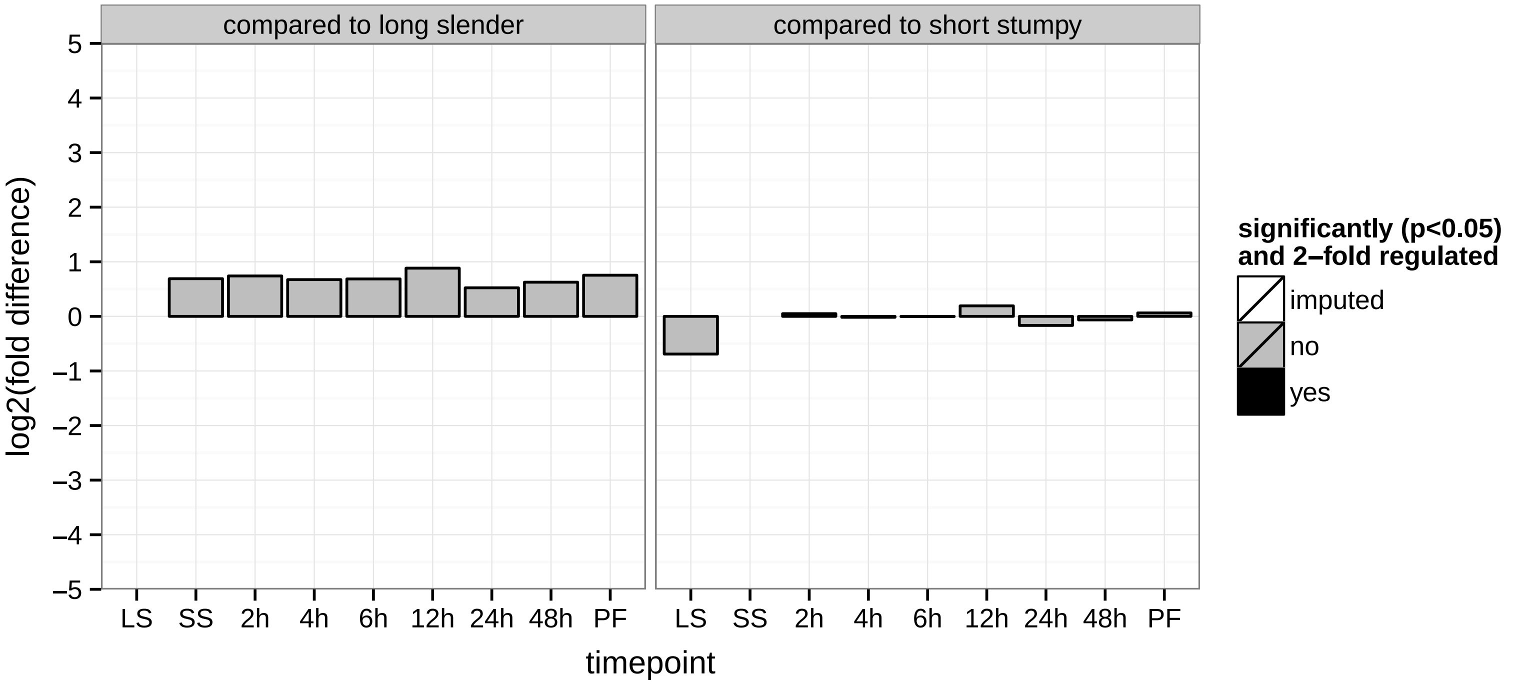

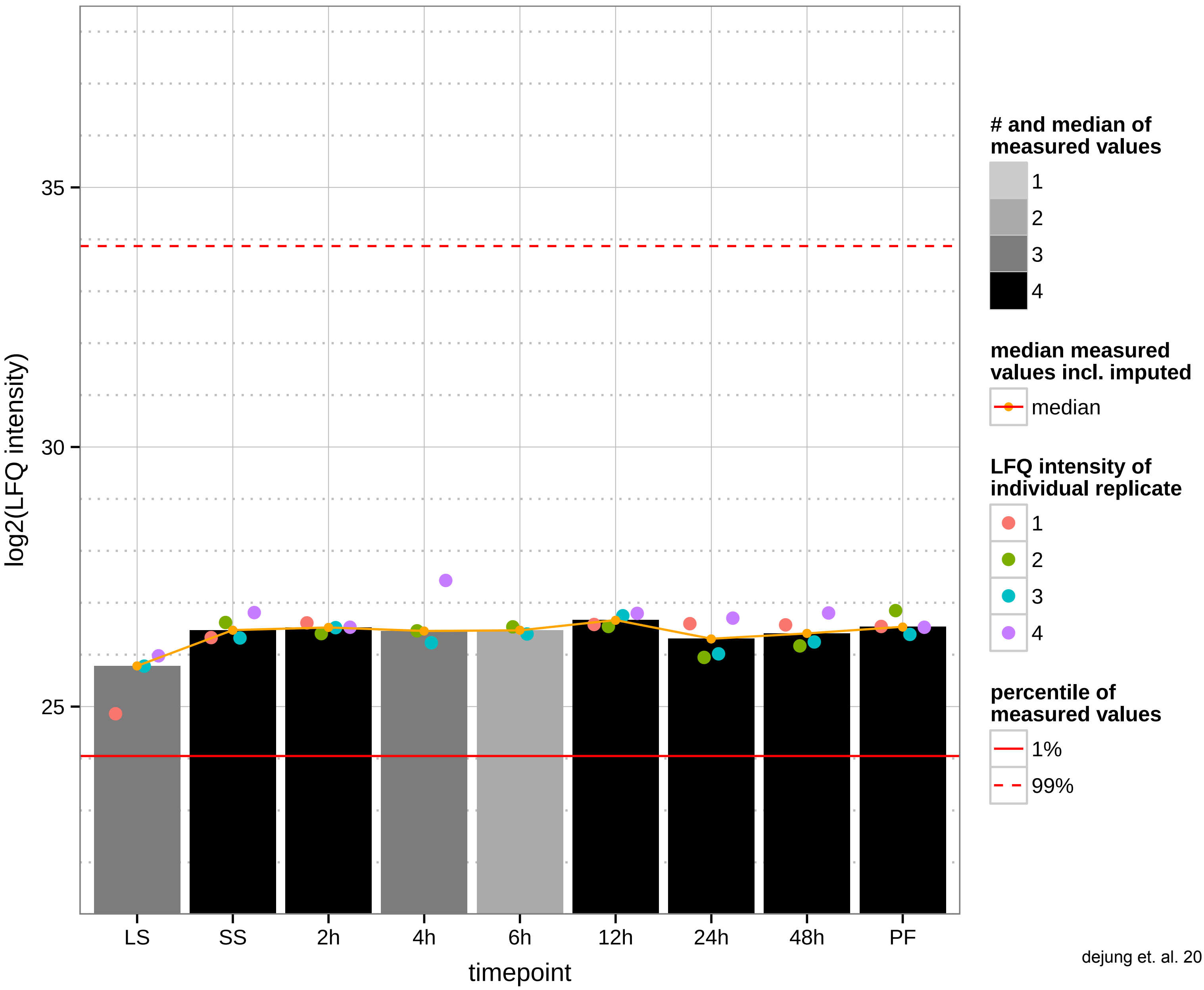

dejung et. al. 2015

hypothetical protein, conserved
Tb927.8.4160
AGOF: null
AGOC: null
AGOP: null
PGOF: binding
PGOC: null
PGOP: null

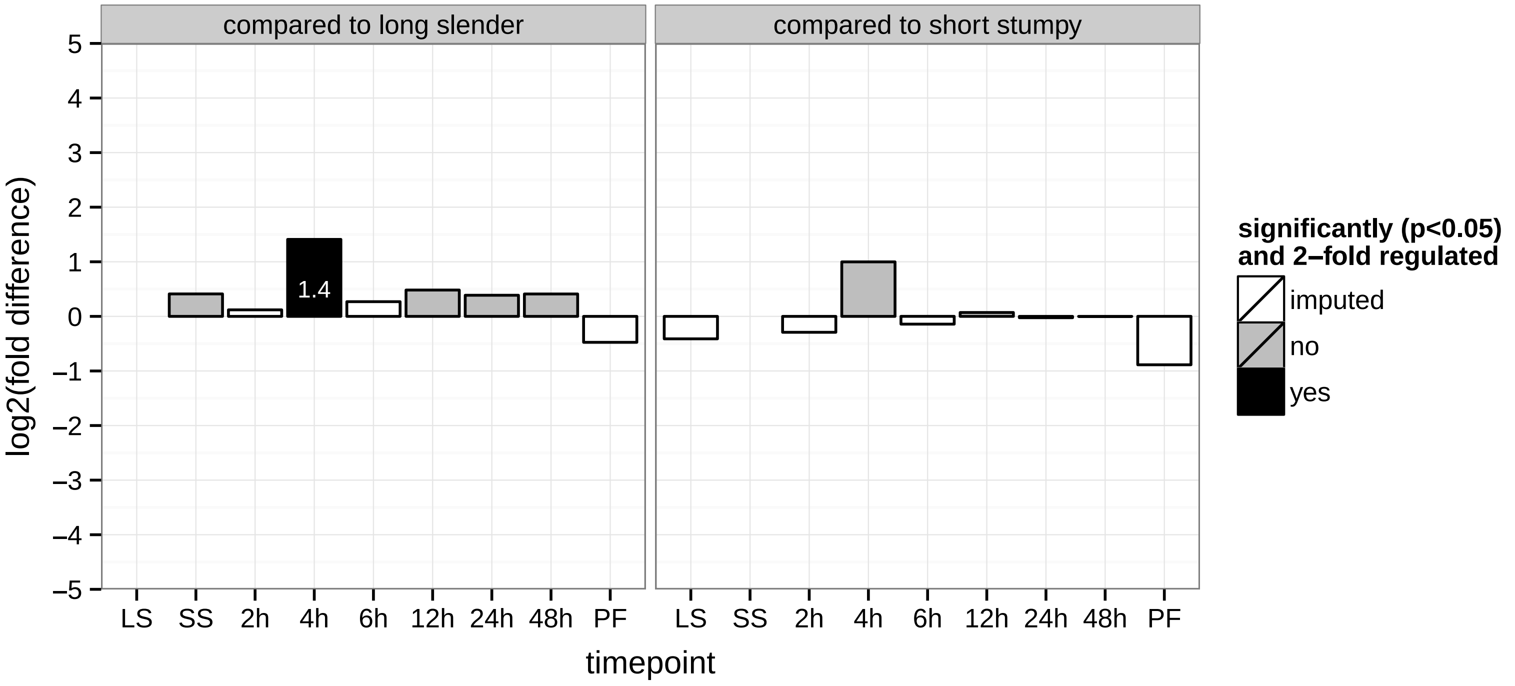

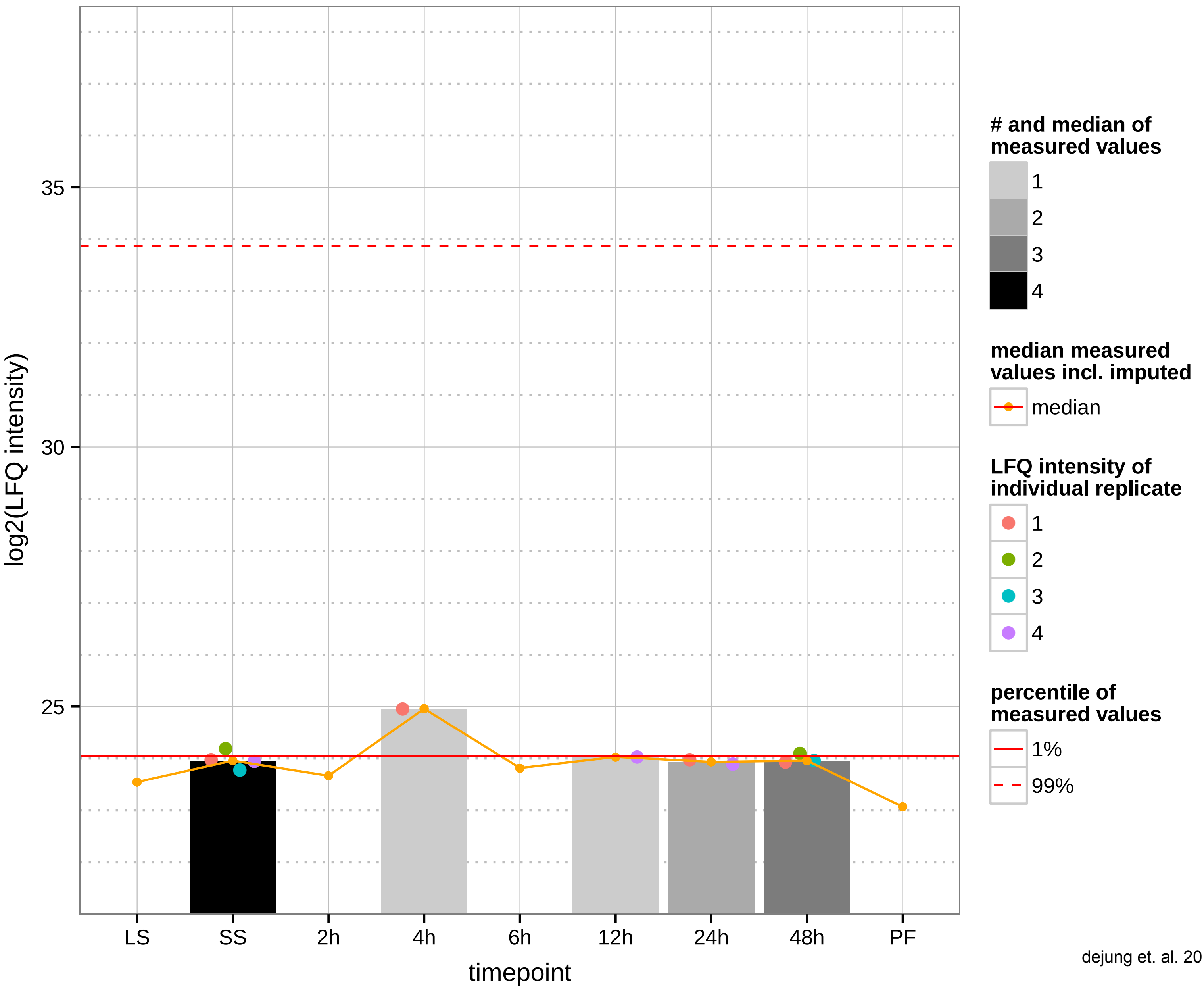

dejung et. al. 2015

RNA–binding protein, putative (MRD1)
Tb927.8.4170
AGOF: RNA binding
AGOC: null
AGOP: RNA processing
PGOF: nucleic acid binding
PGOC: null
PGOP: null

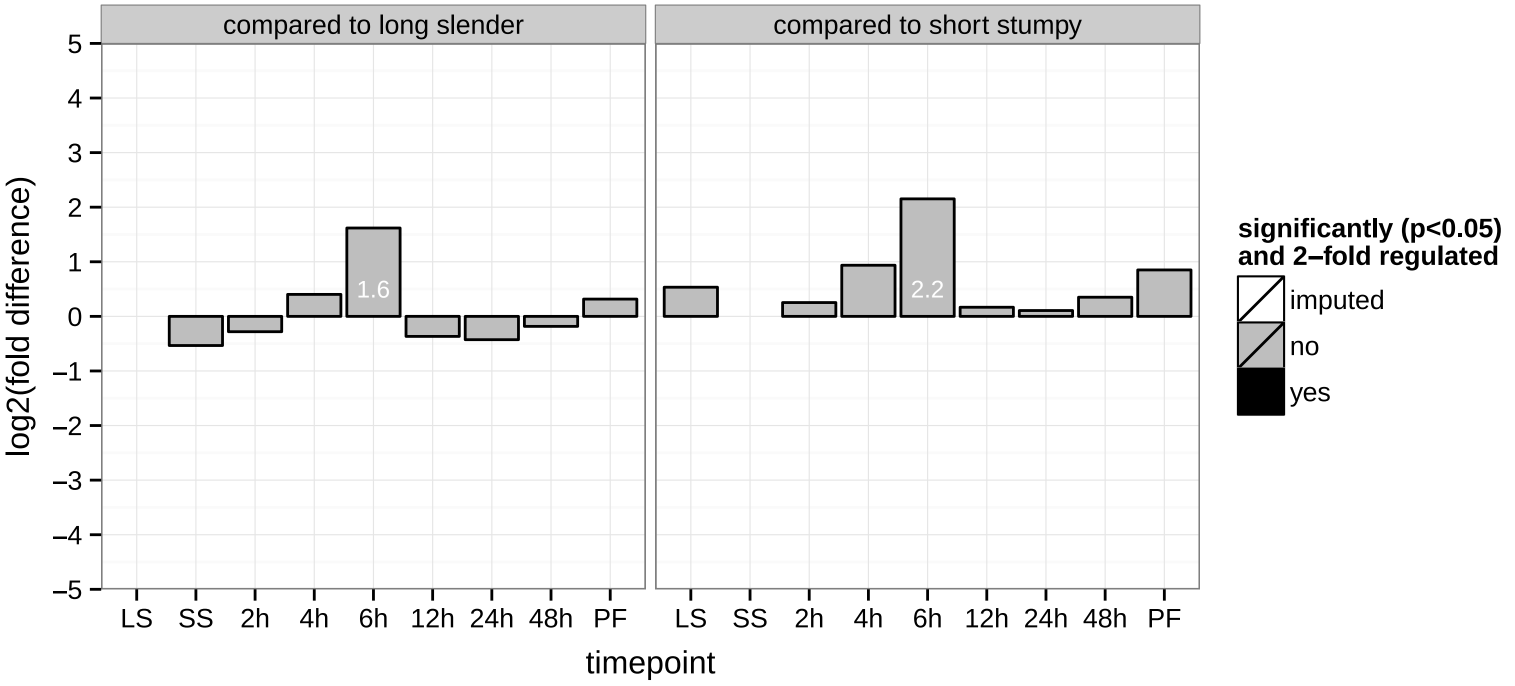

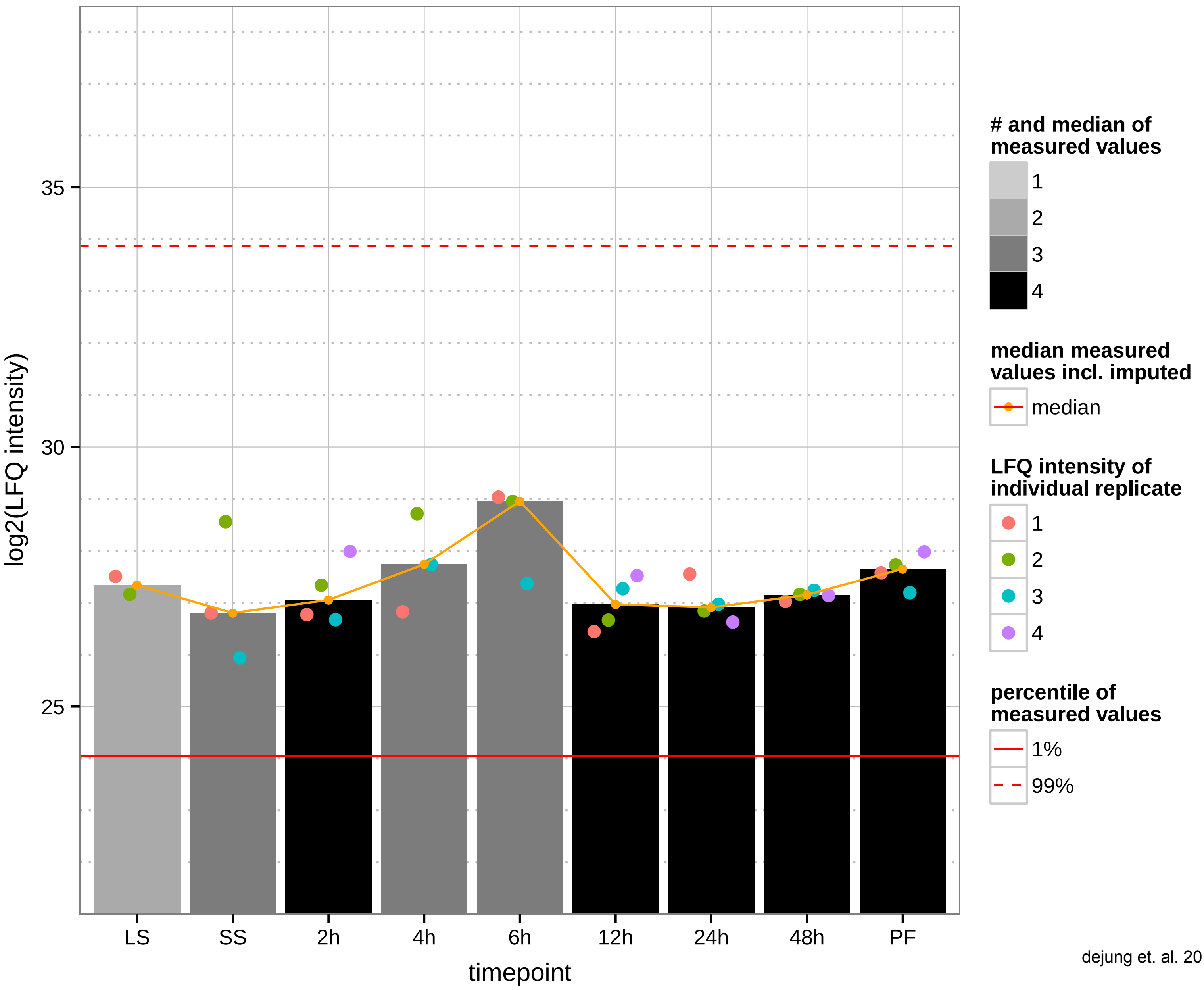

dejung et. al. 2015

hypothetical protein, conserved
Tb927.8.4210
AGOF: null
AGOC: null
AGOP: null
PGOF: protein binding
PGOC: null
PGOP: null

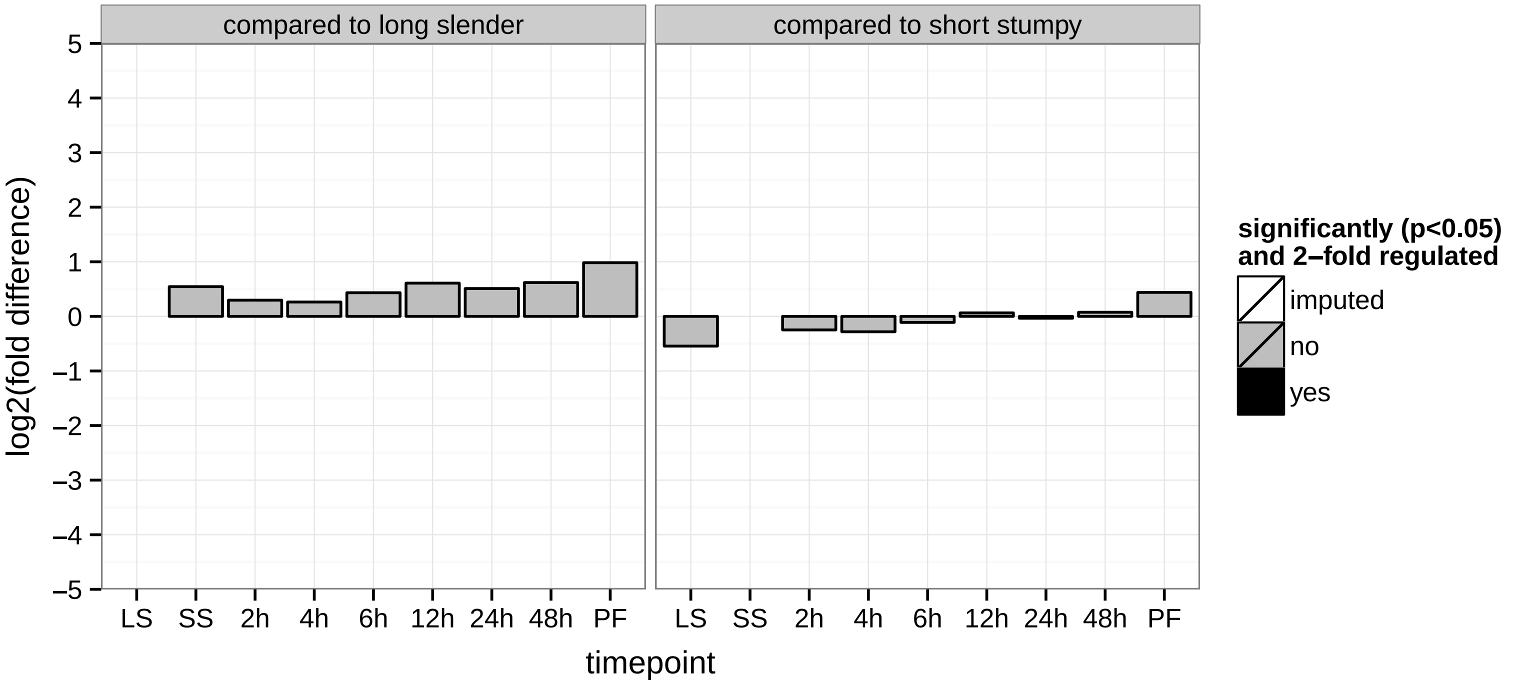

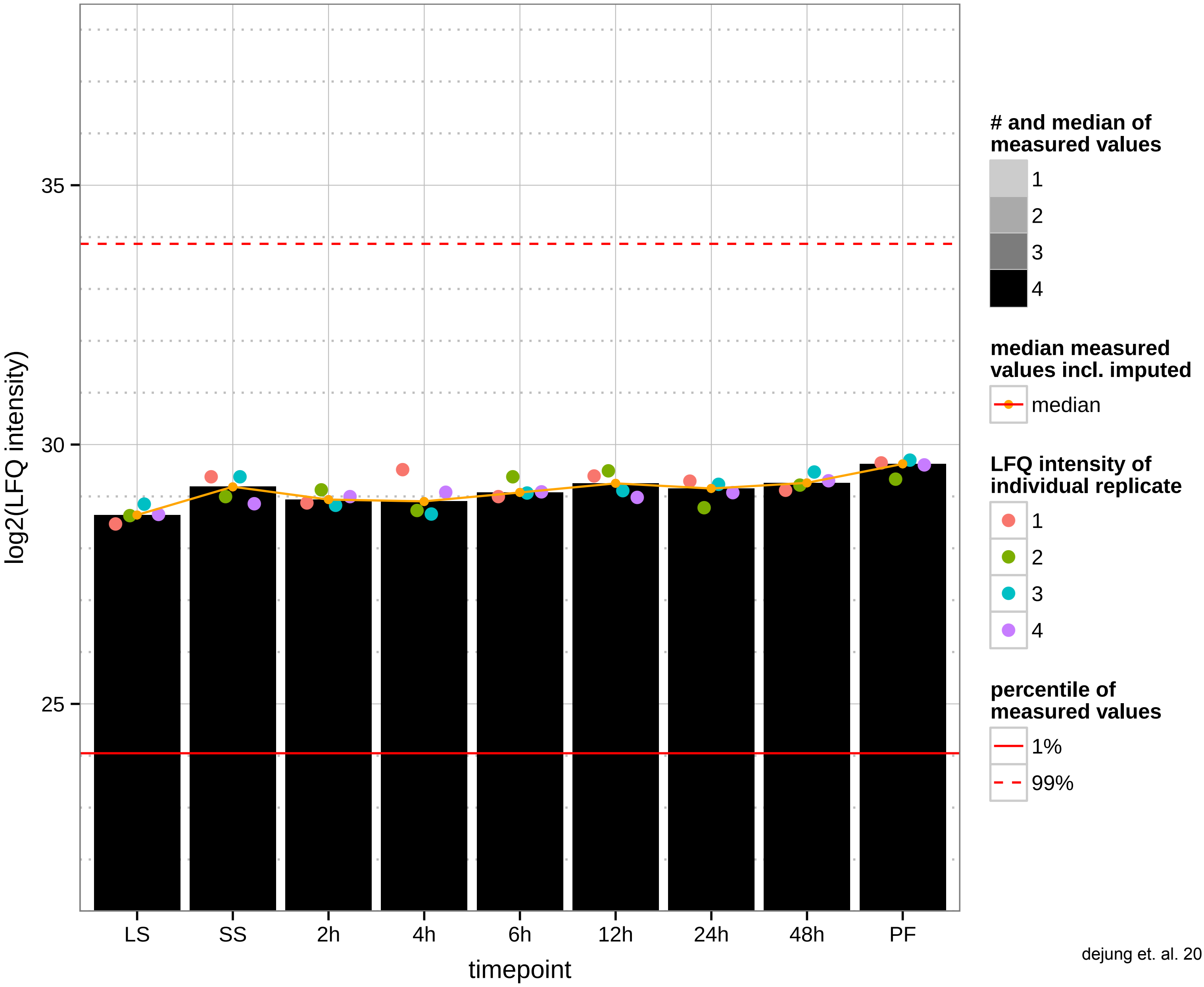

dejung et. al. 2015

hypothetical protein, conserved
Tb927.8.4270
AGOF: deaminase activity
AGOC: null
AGOP: purine ribonucleoside monophosphate biosynthetic process
PGOF: null
PGOC: null
PGOP: null

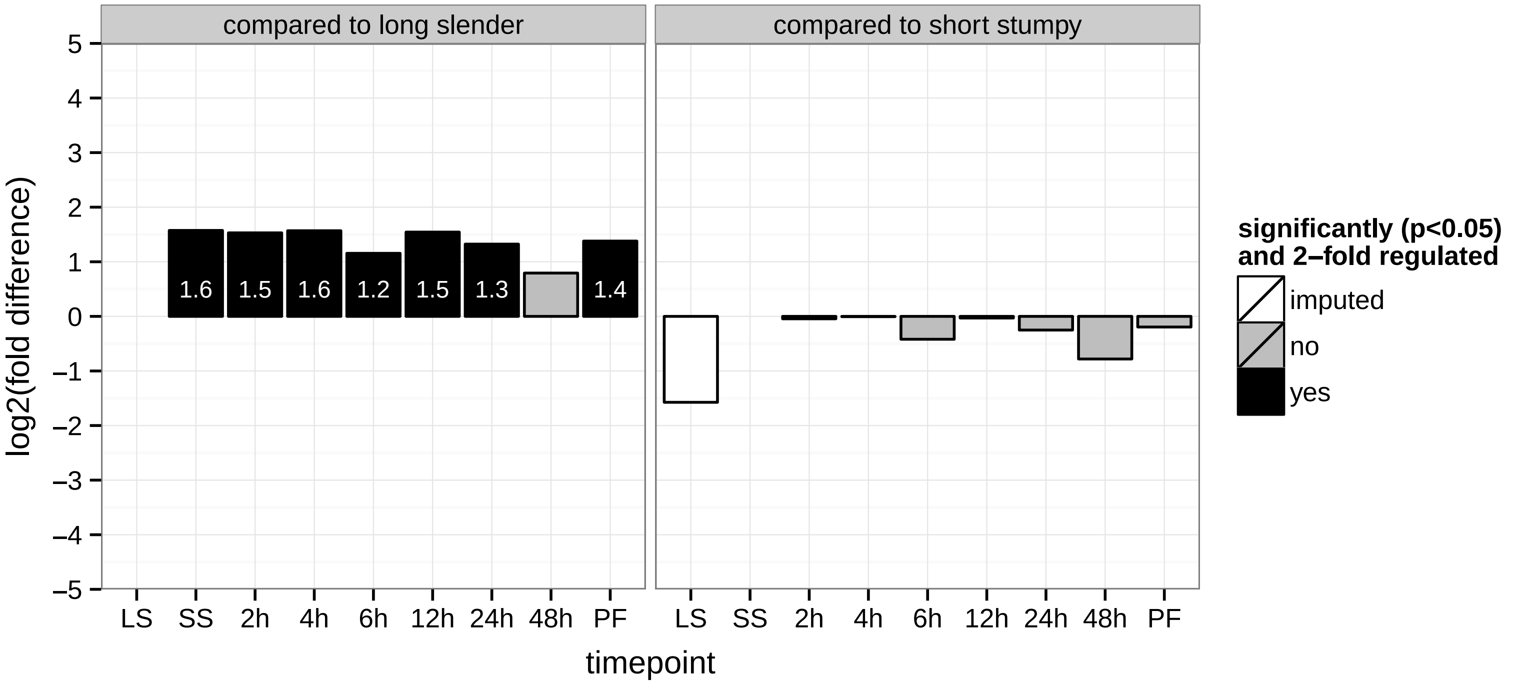

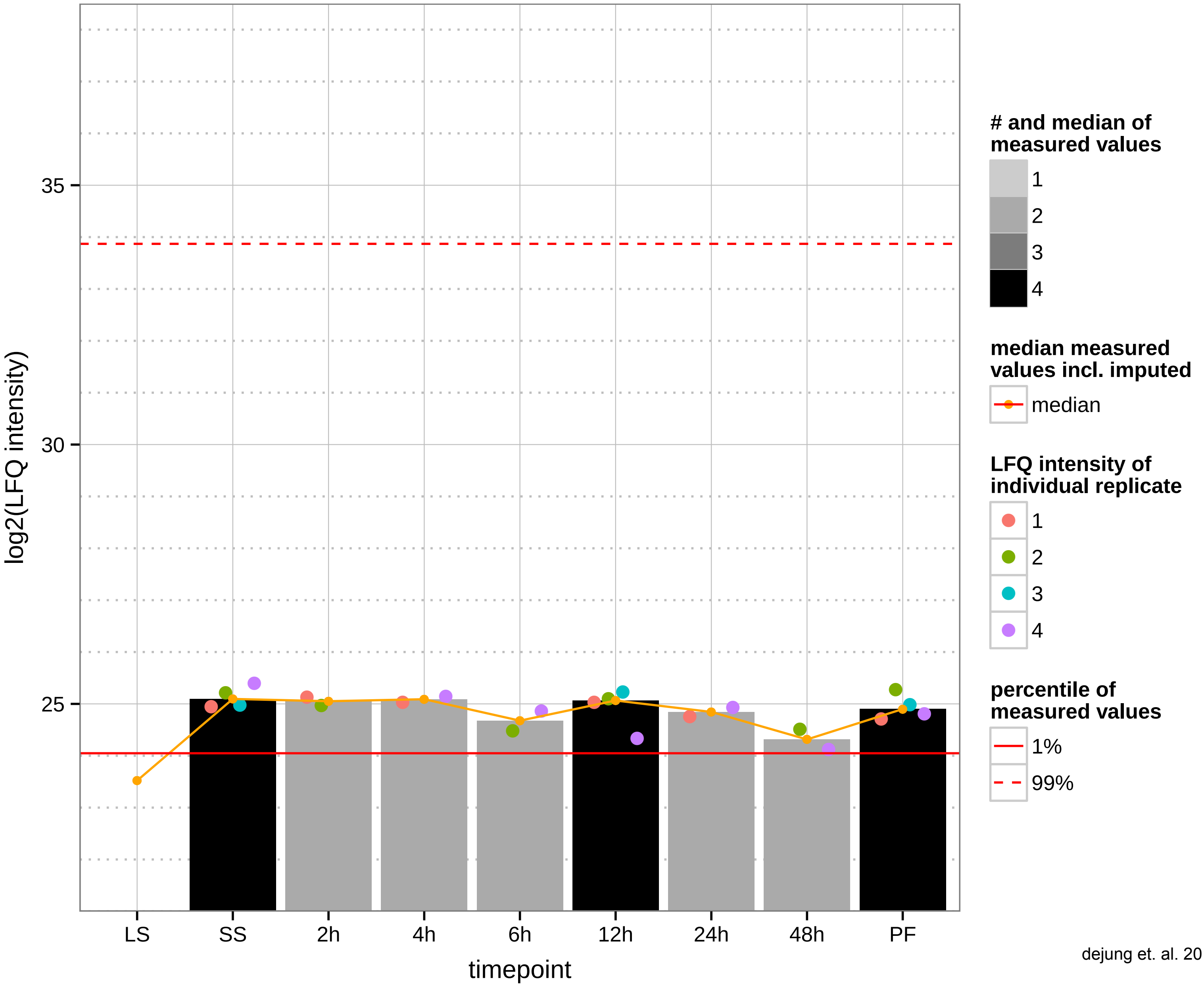

dejung et. al. 2015

nuclear transport factor 2, putative
Tb927.8.4280
AGOF: protein transporter activity
AGOC: intracellular, nucleus
AGOP: protein import into nucleus
PGOF: null
PGOC: intracellular
PGOP: transport

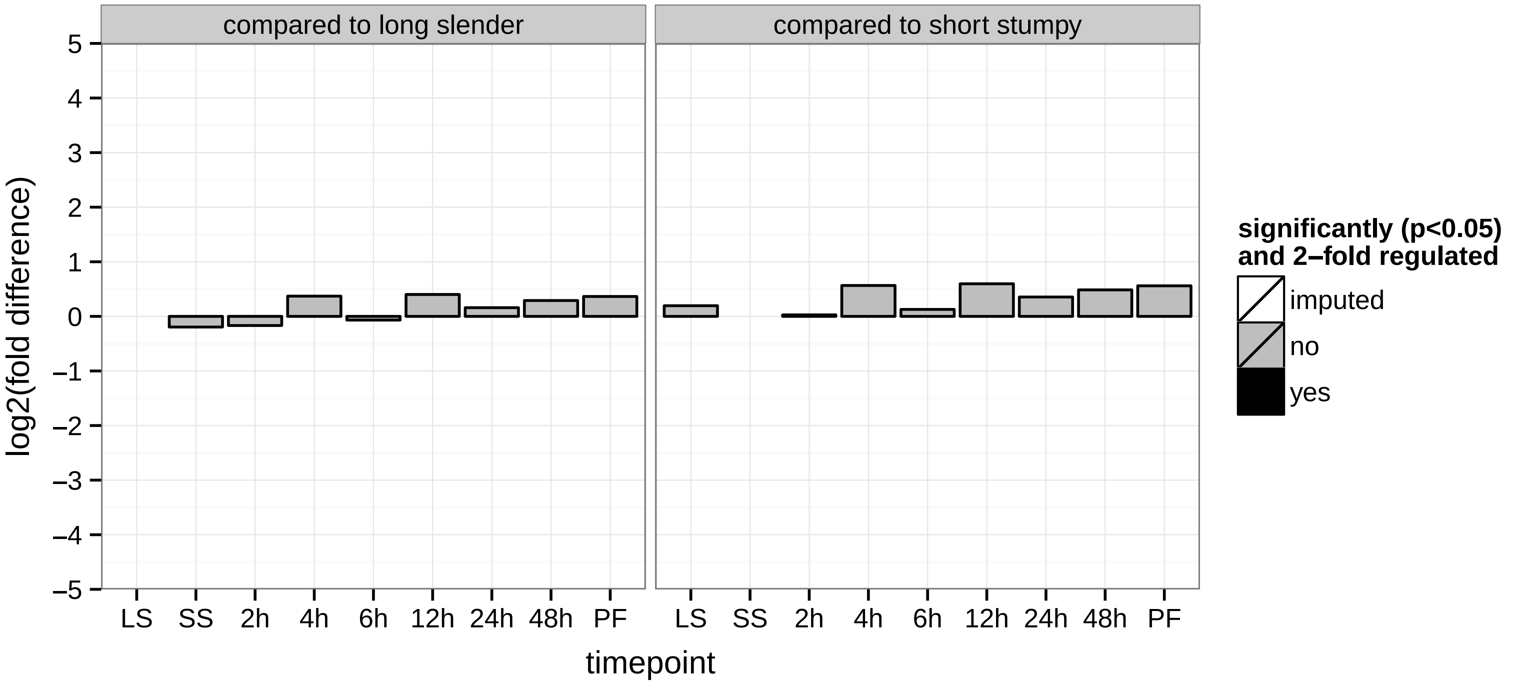

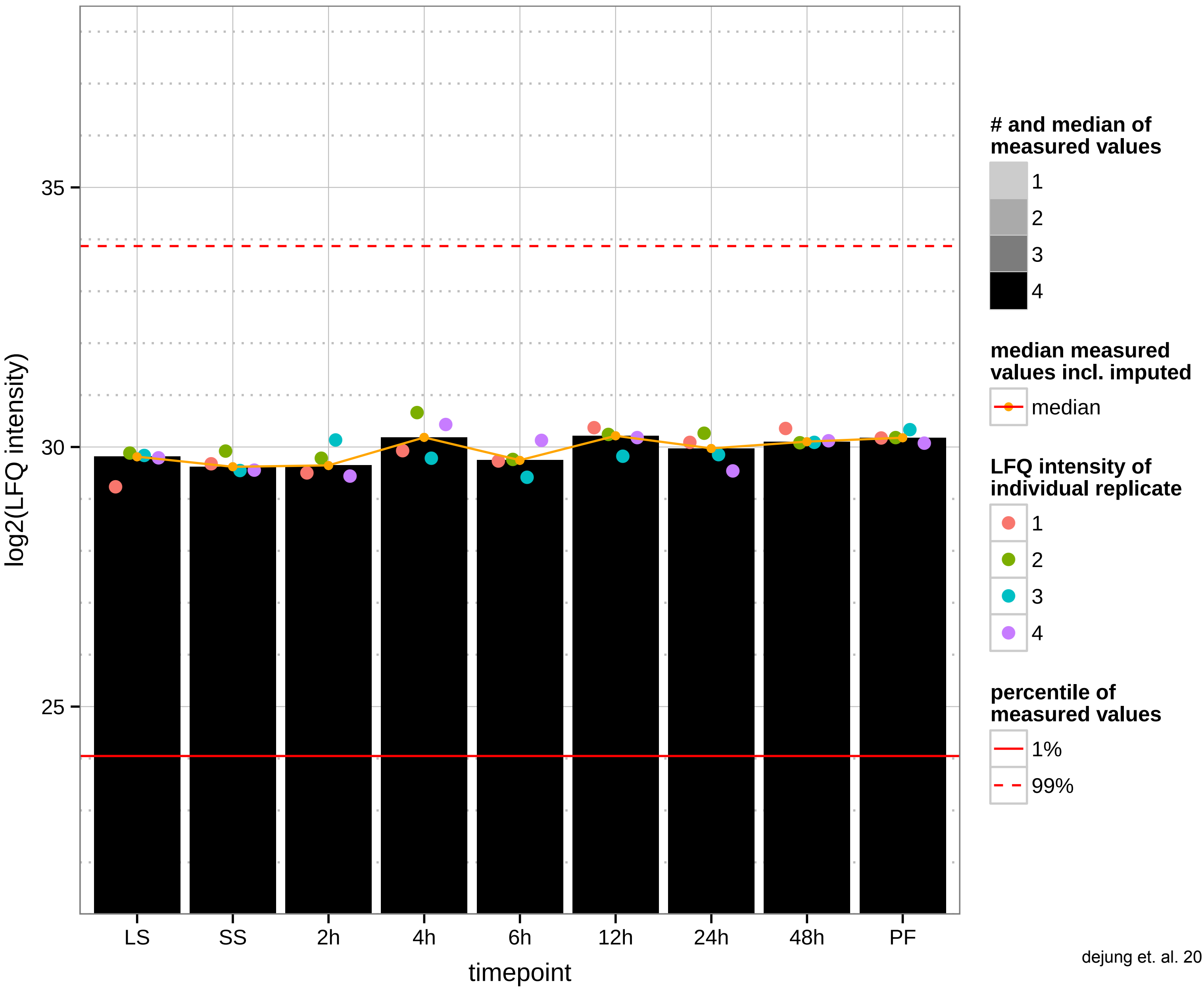

dejung et. al. 2015

hypothetical protein, conserved
Tb927.8.4320
AGOF: null
AGOC: null
AGOP: null
PGOF: protein binding
PGOC: null
PGOP: null

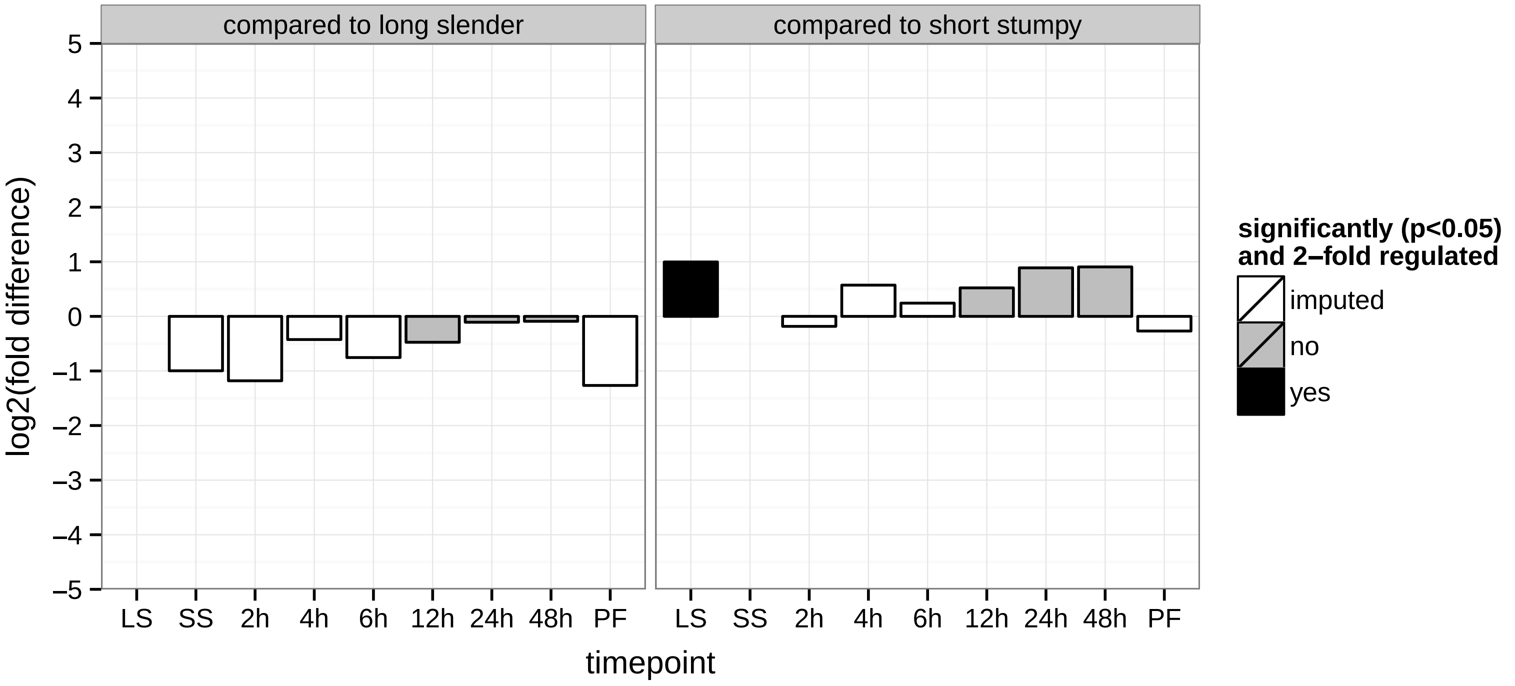

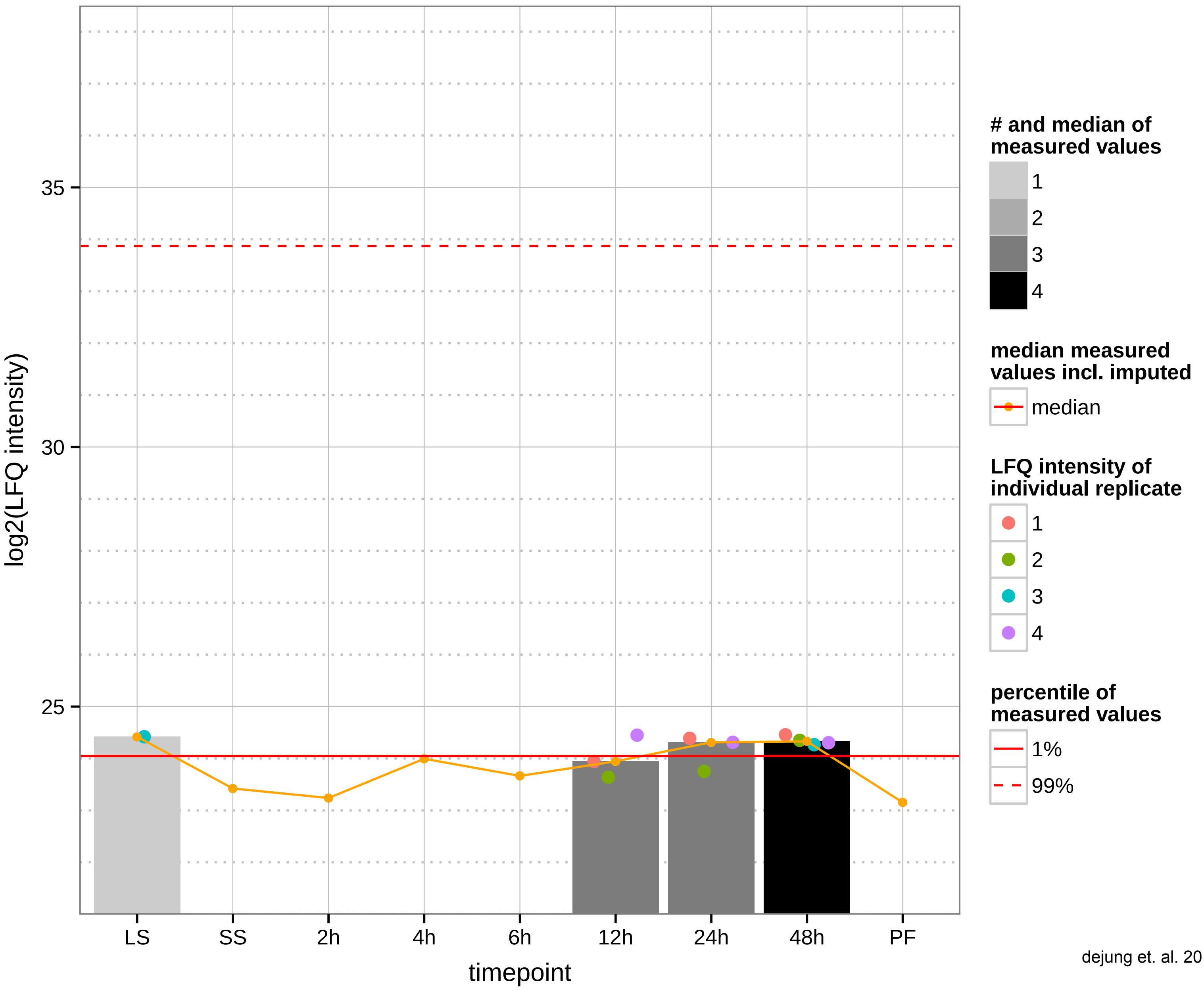

dejung et. al. 2015

hypothetical protein, conserved
Tb927.8.4370
AGOF: null
AGOC: null
AGOP: null
PGOF: null
PGOC: null
PGOP: null

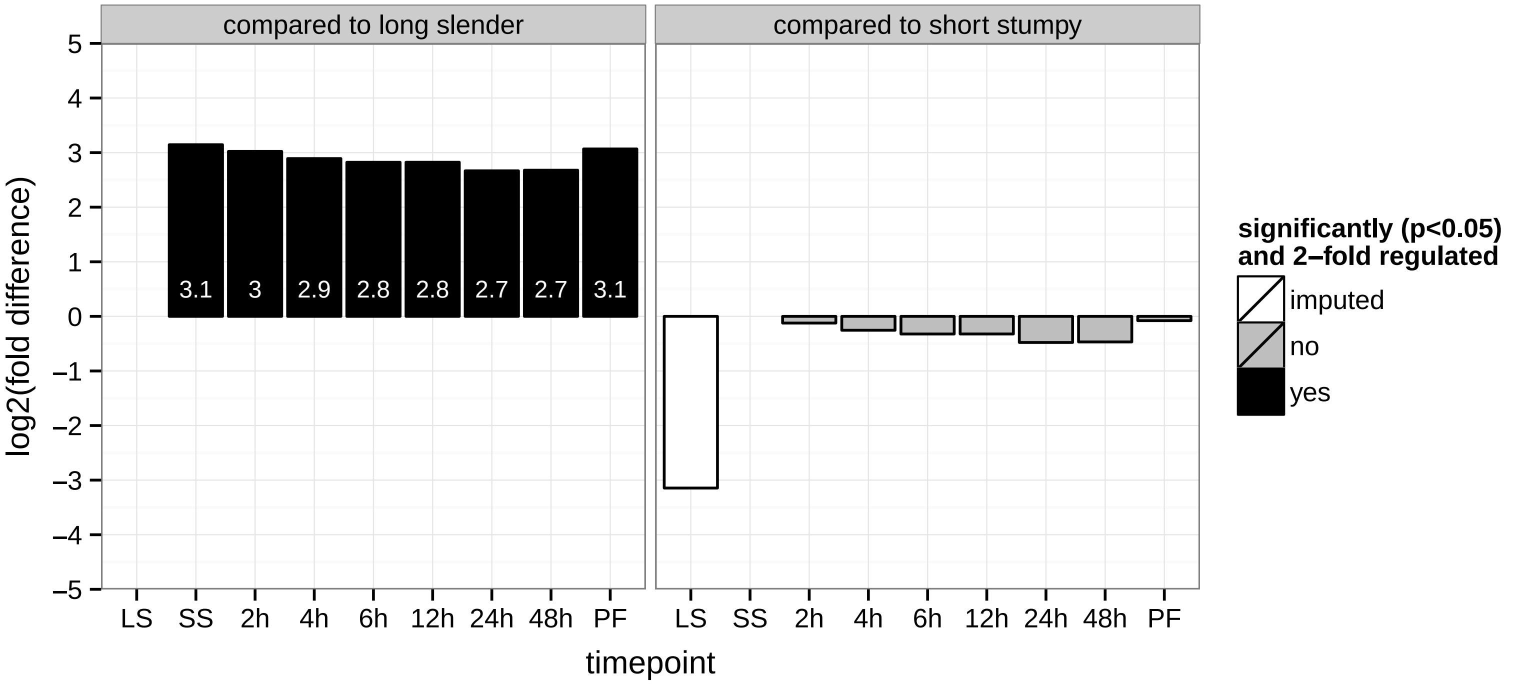

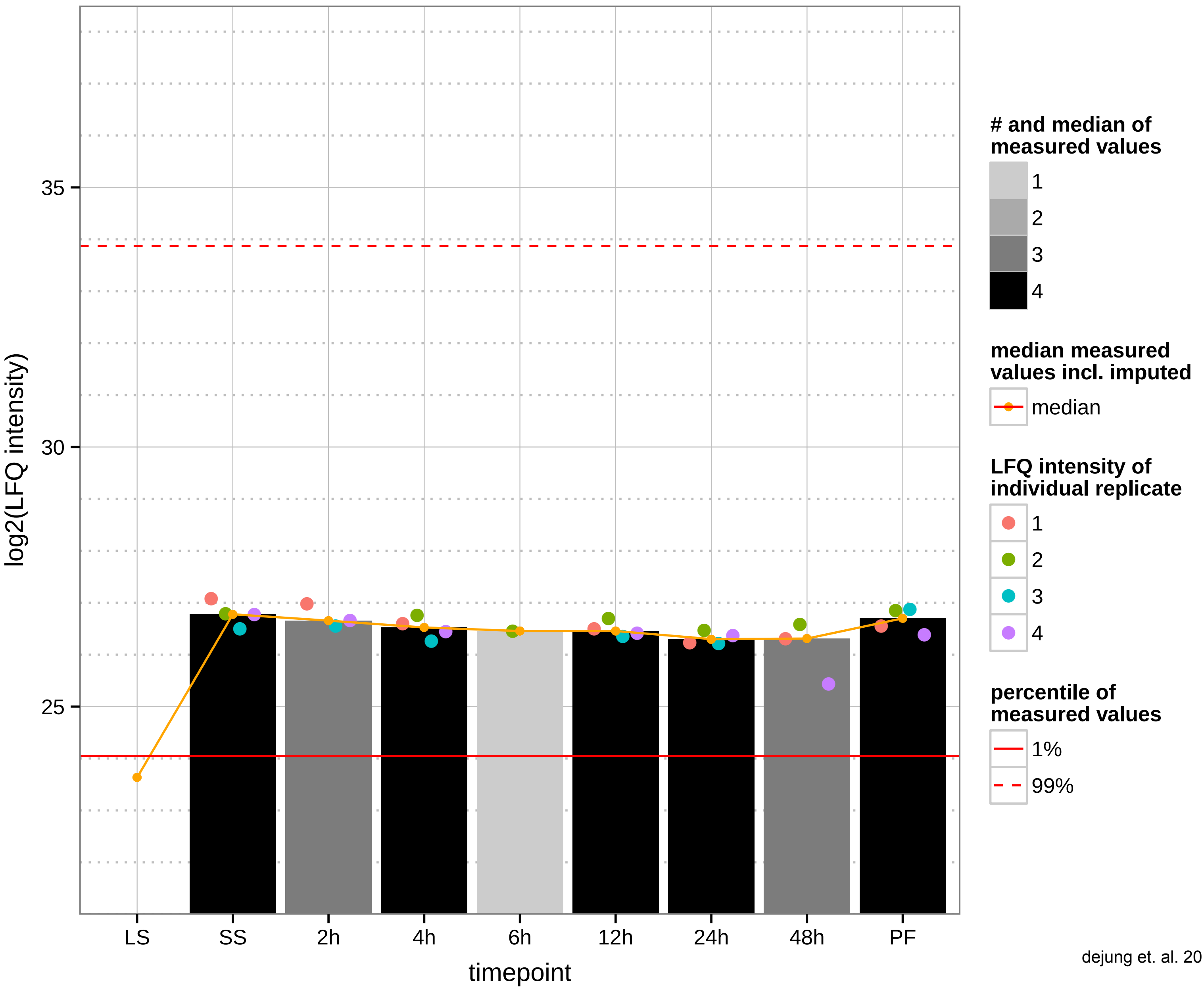

dejung et. al. 2015

hypothetical protein, conserved
Tb927.8.4400
AGOF: null
AGOC: null
AGOP: null
PGOF: null
PGOC: null
PGOP: null

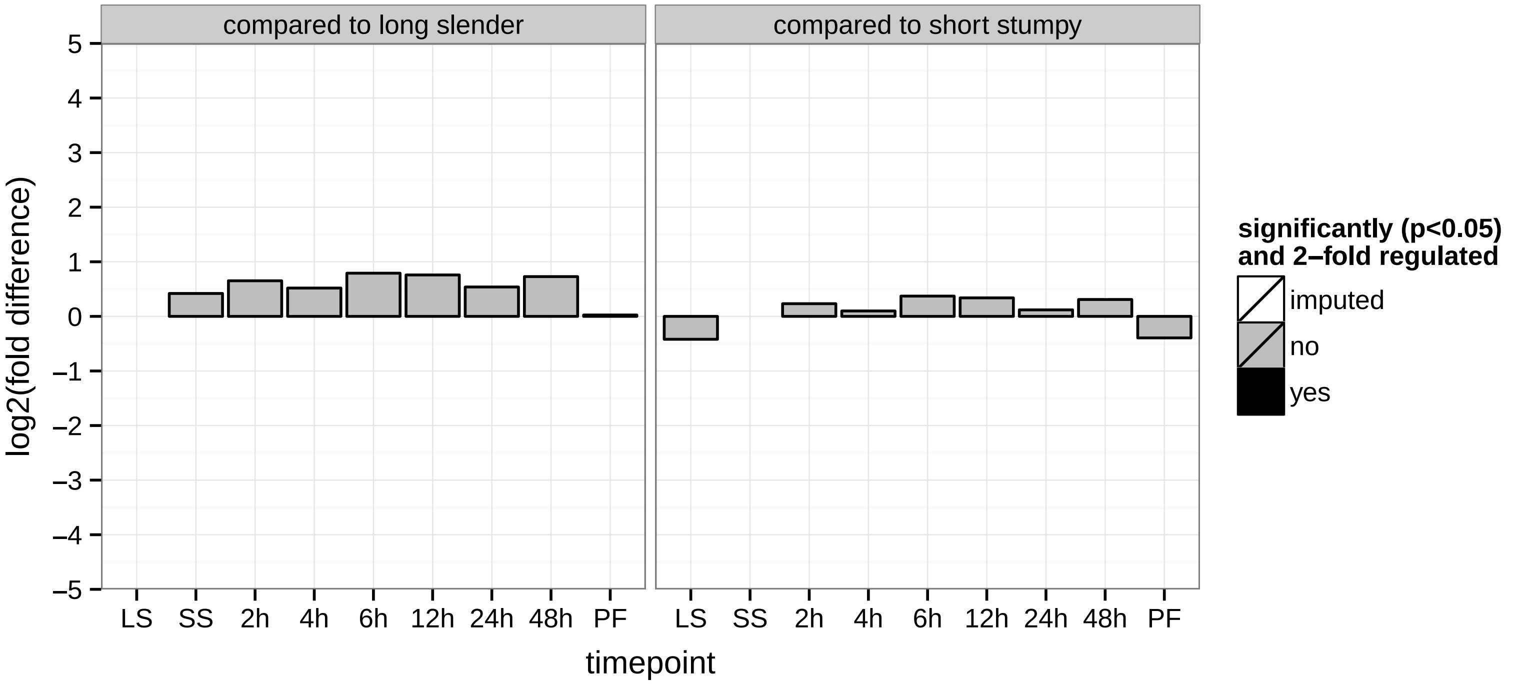

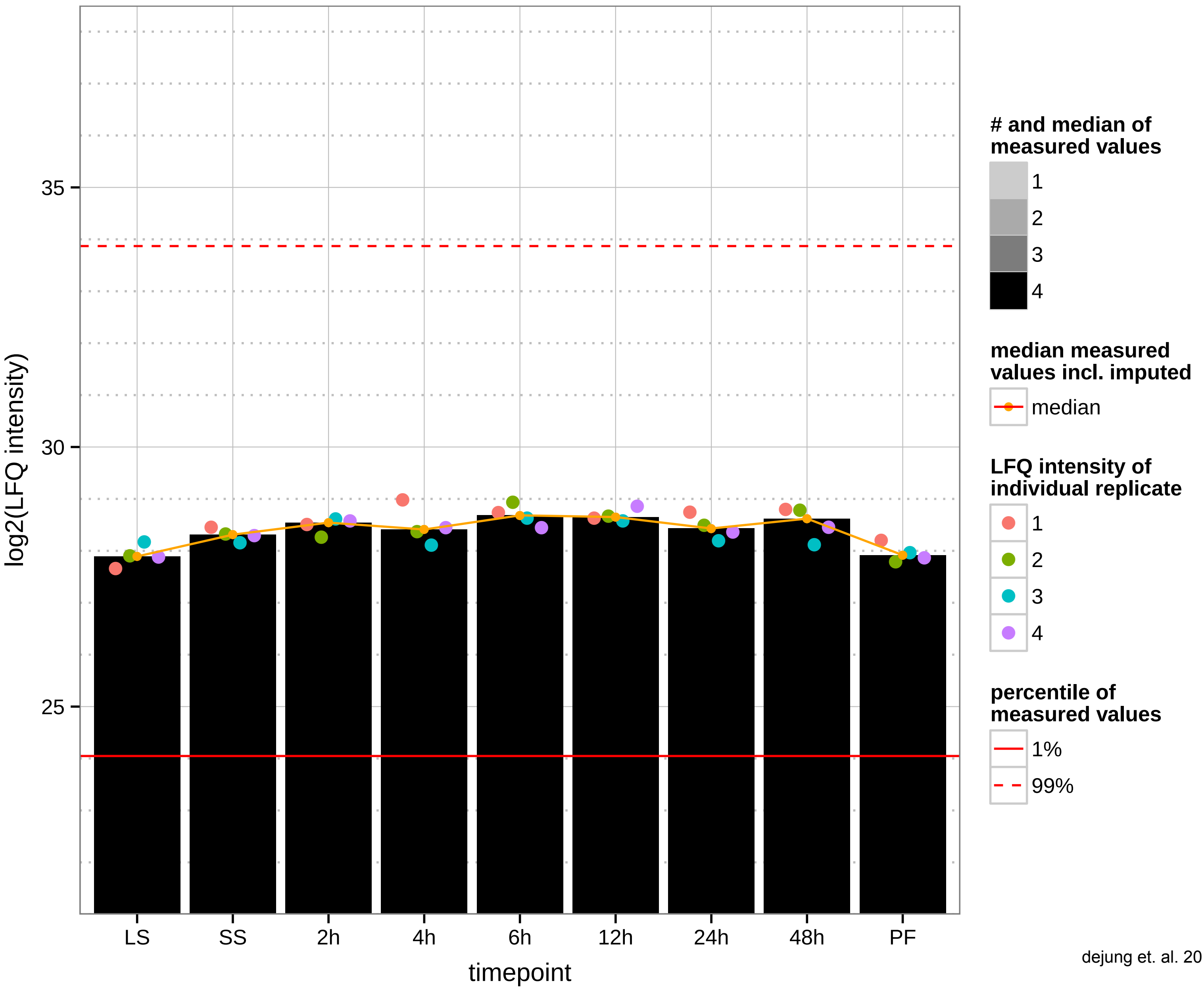

dejung et. al. 2015

hypothetical protein, conserved
Tb927.8.4460
AGOF: null
AGOC: null
AGOP: null
PGOF: null
PGOC: null
PGOP: null

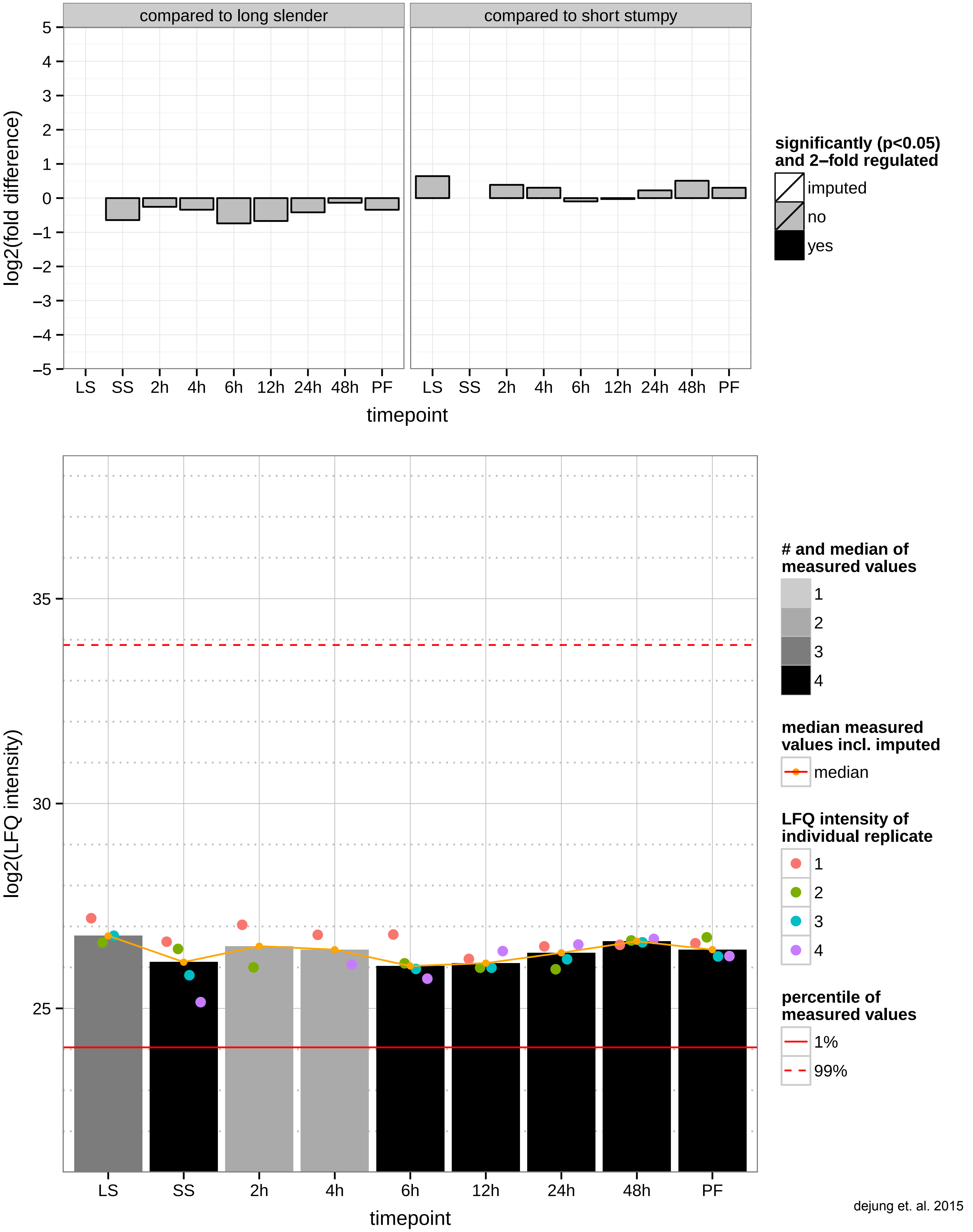

dejung et. al. 2015

hypothetical protein, conserved, Eukaryotic translation initiation factor 4 gamma type 5 (eif4g5)
Tb927.8.4500;Tb11.v5.0763
AGOF: null, RNA binding, protein binding
AGOC: null, eukaryotic translation initiation factor 4F complex
AGOP: null, RNA metabolic process, translation
PGOF: DNA binding, RNA binding, binding, protein binding
PGOC: null
PGOP: null

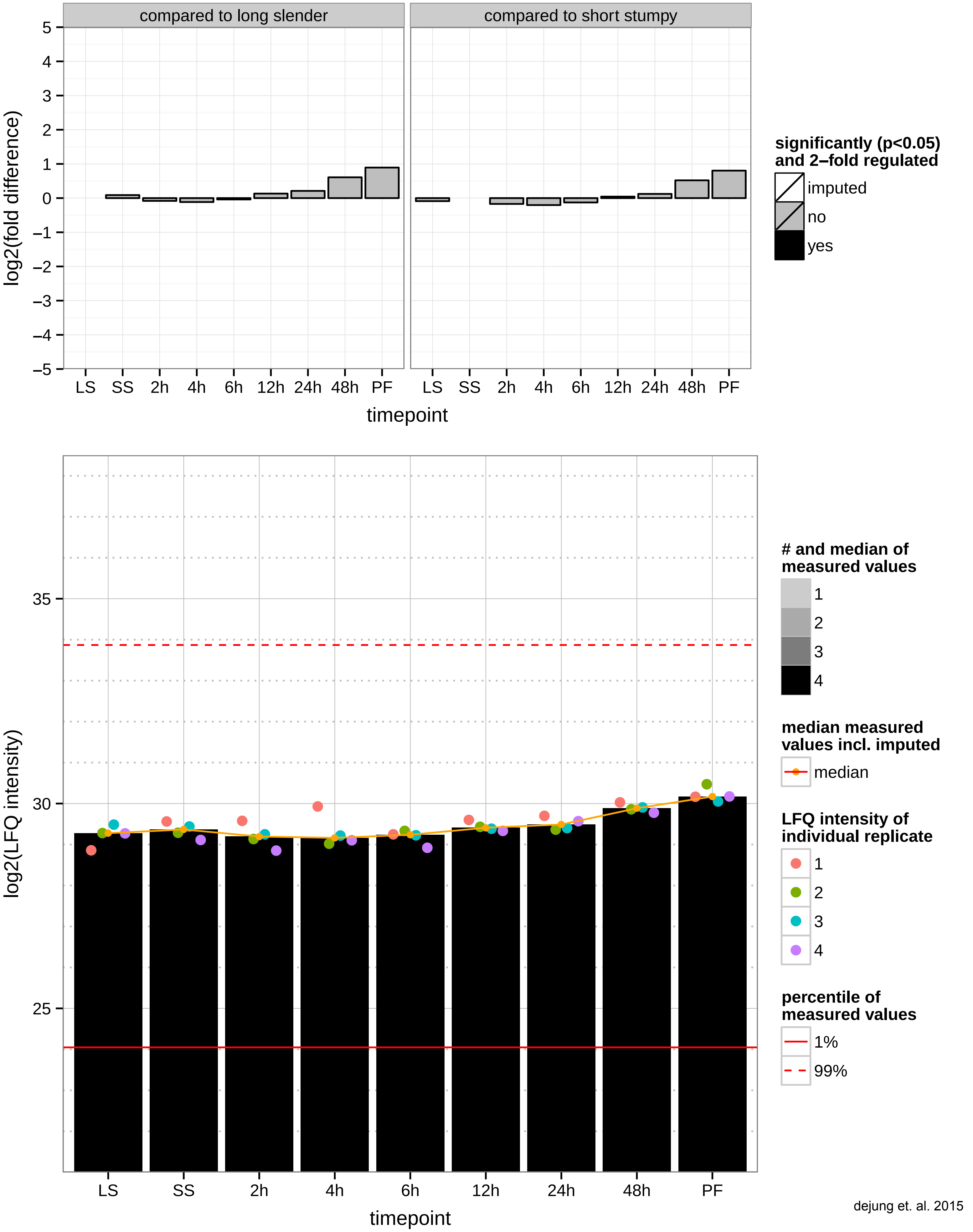

dejung et. al. 2015

PAB1–binding protein, putative (PBP1)
Tb927.8.4540
AGOF: null
AGOC: cytoplasmic stress granule, polysome
AGOP: positive regulation of translation, stress granule assembly
PGOF: null
PGOC: null
PGOP: null

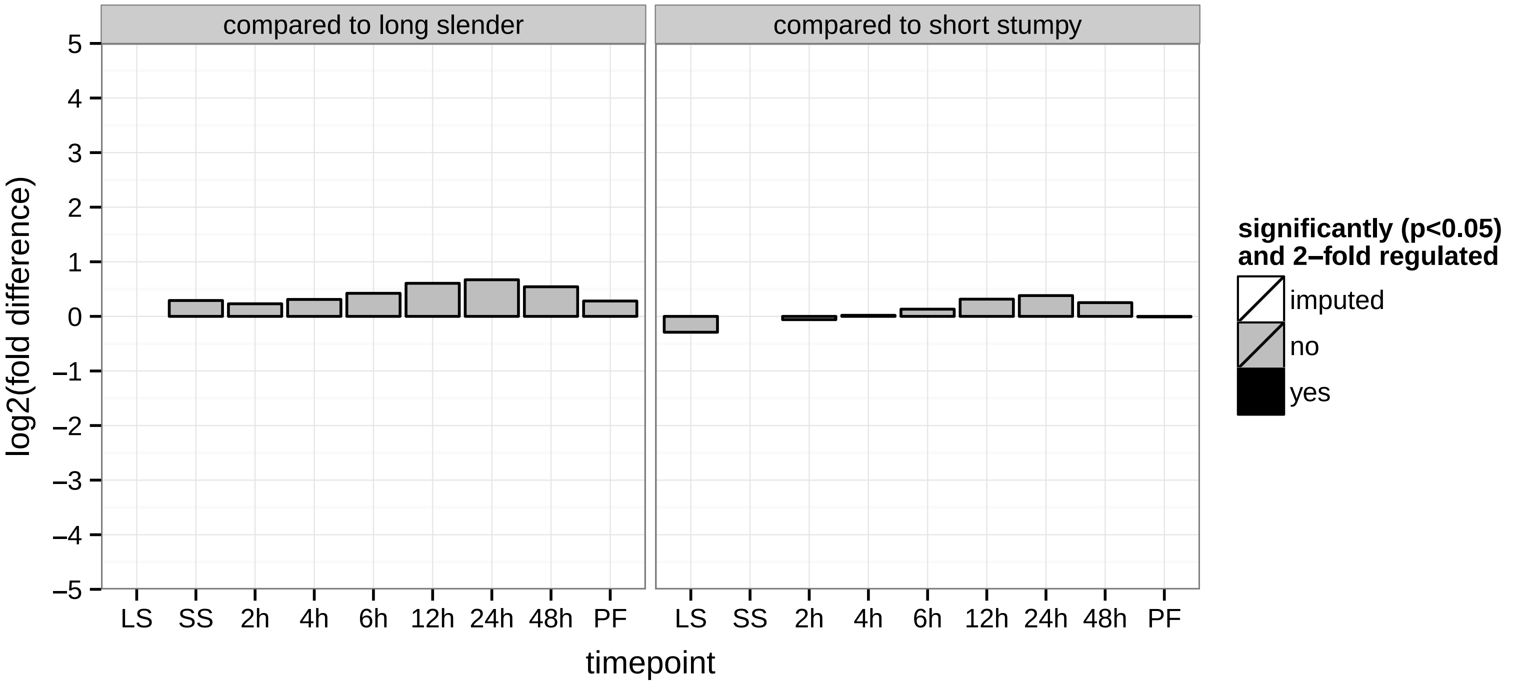

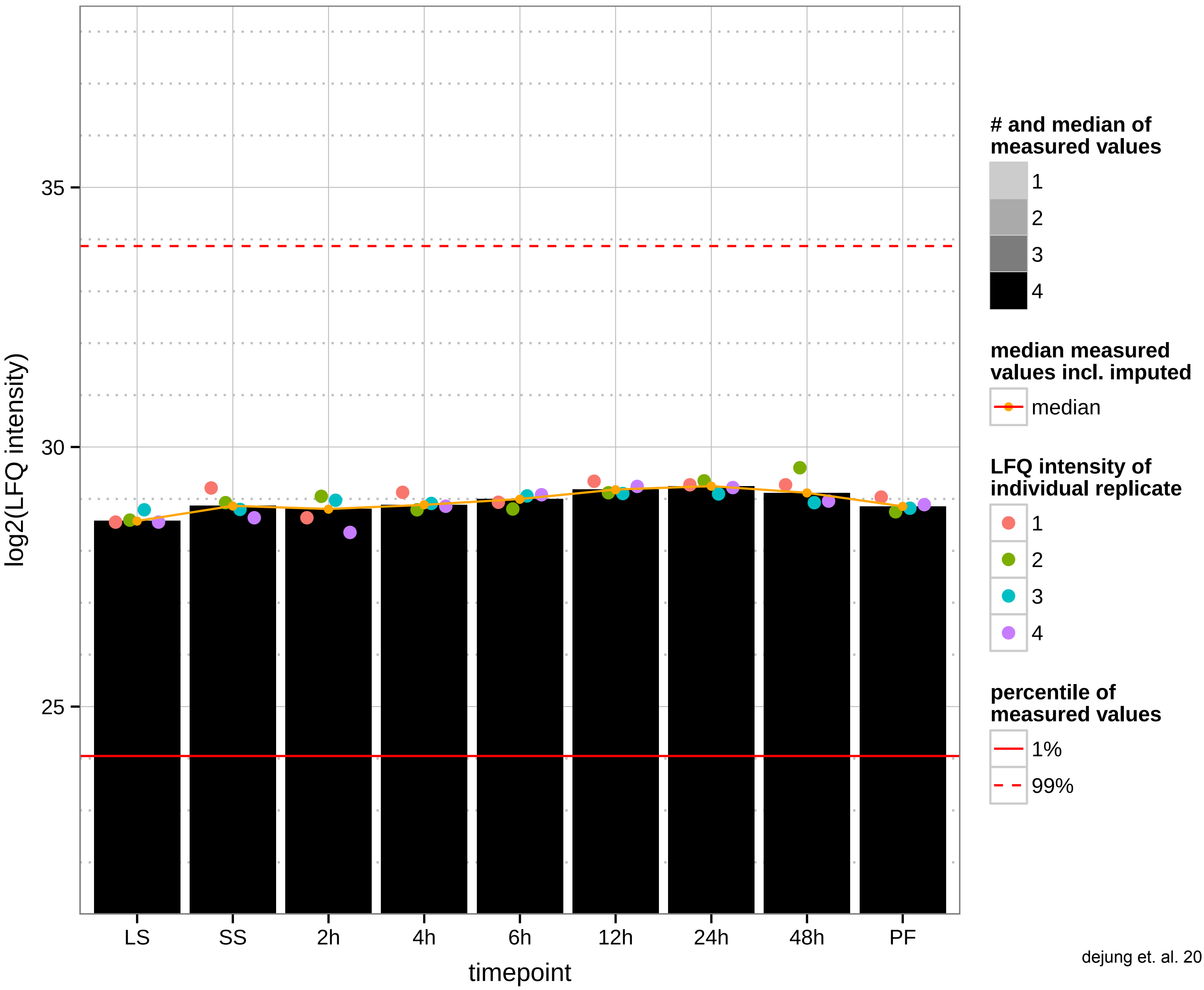

dejung et. al. 2015

hypothetical protein, conserved
Tb927.8.4560
AGOF: null
AGOC: null
AGOP: null
PGOF: protein binding
PGOC: null
PGOP: null

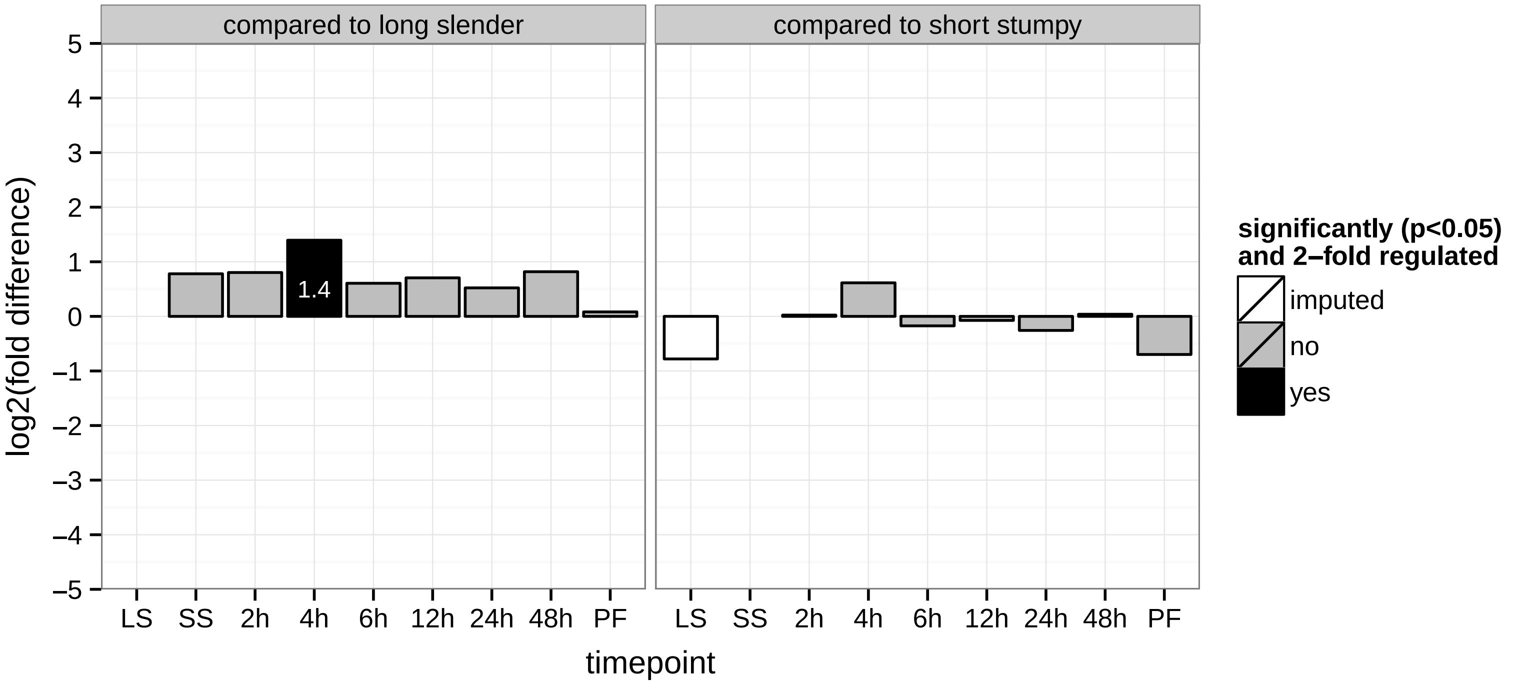

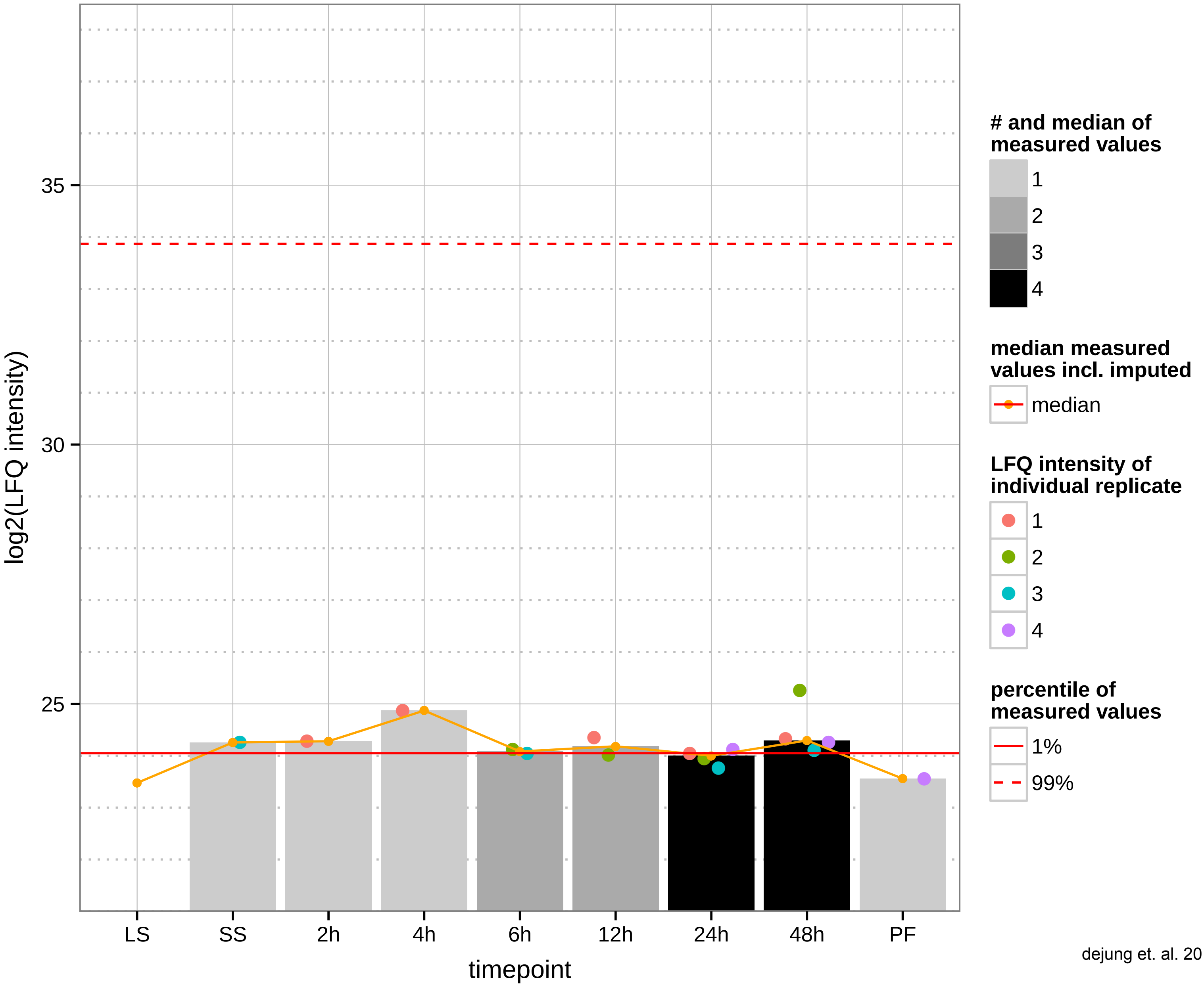

dejung et. al. 2015

hypothetical protein, conserved
Tb927.8.4580
AGOF: null
AGOC: null
AGOP: null
PGOF: null
PGOC: null
PGOP: null

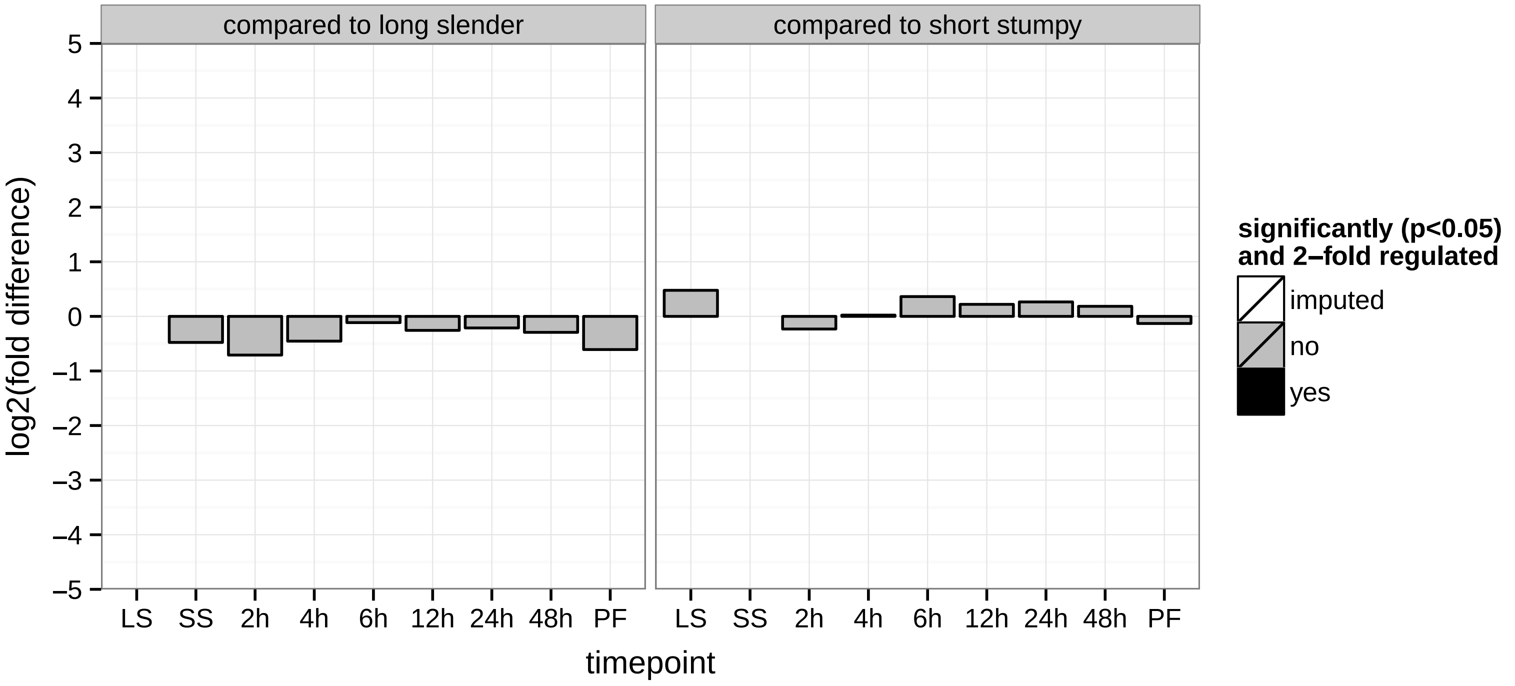

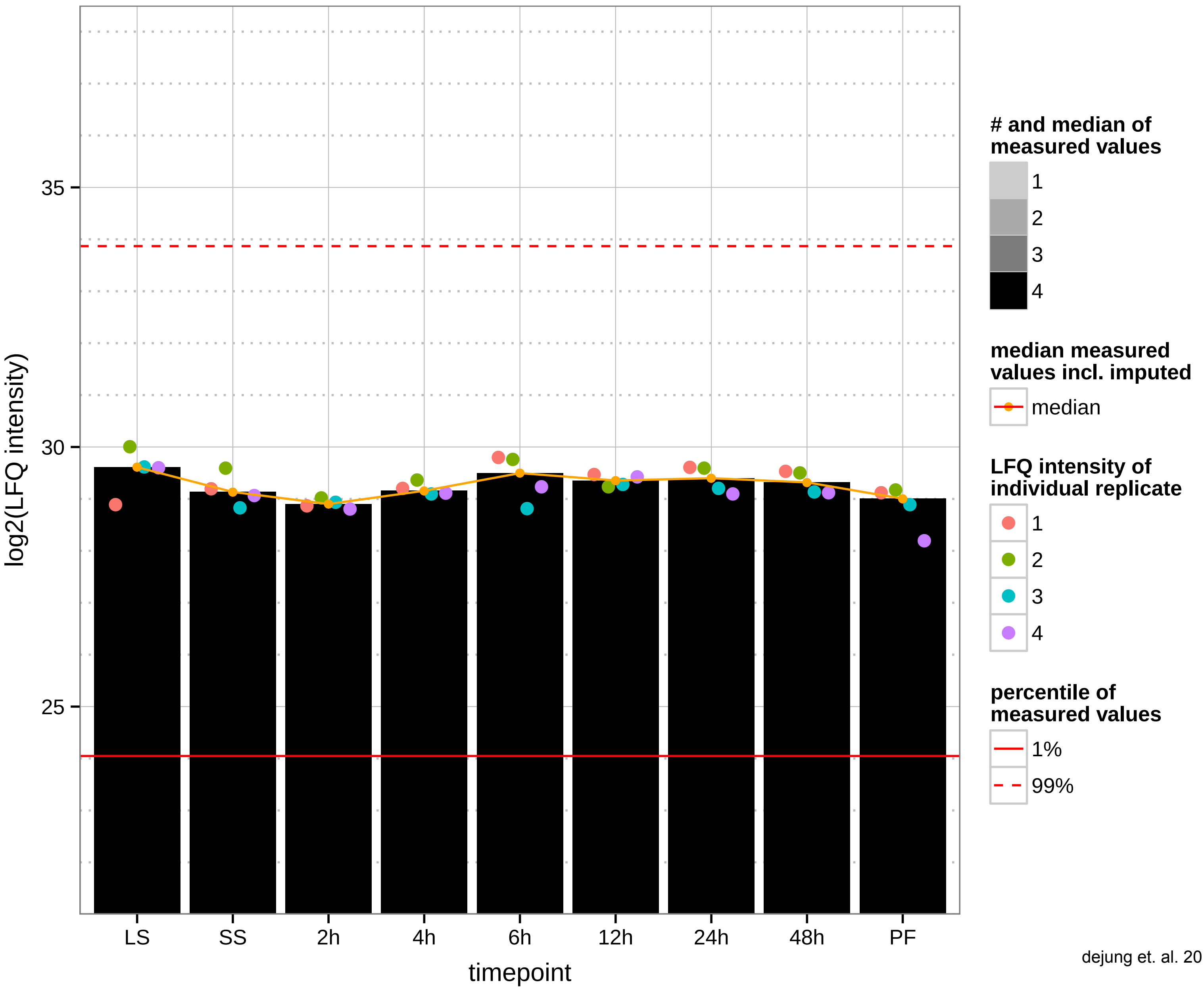

dejung et. al. 2015

Ras−related protein RabX1 (RABX1)
Tb927.8.4610
AGOF: GTP binding, GTPase activity
AGOC: endoplasmic reticulum, integral to membrane, intracellular
AGOP: intracellular protein transport, nucleocytoplasmic transport, small GTPase mediated signal transduction
PGOF: GTP binding, GTPase activity
PGOC: intracellular, membrane
PGOP: GTP catabolic process, intracellular protein transport, nucleocytoplasmic transport, protein transport, signal transducti

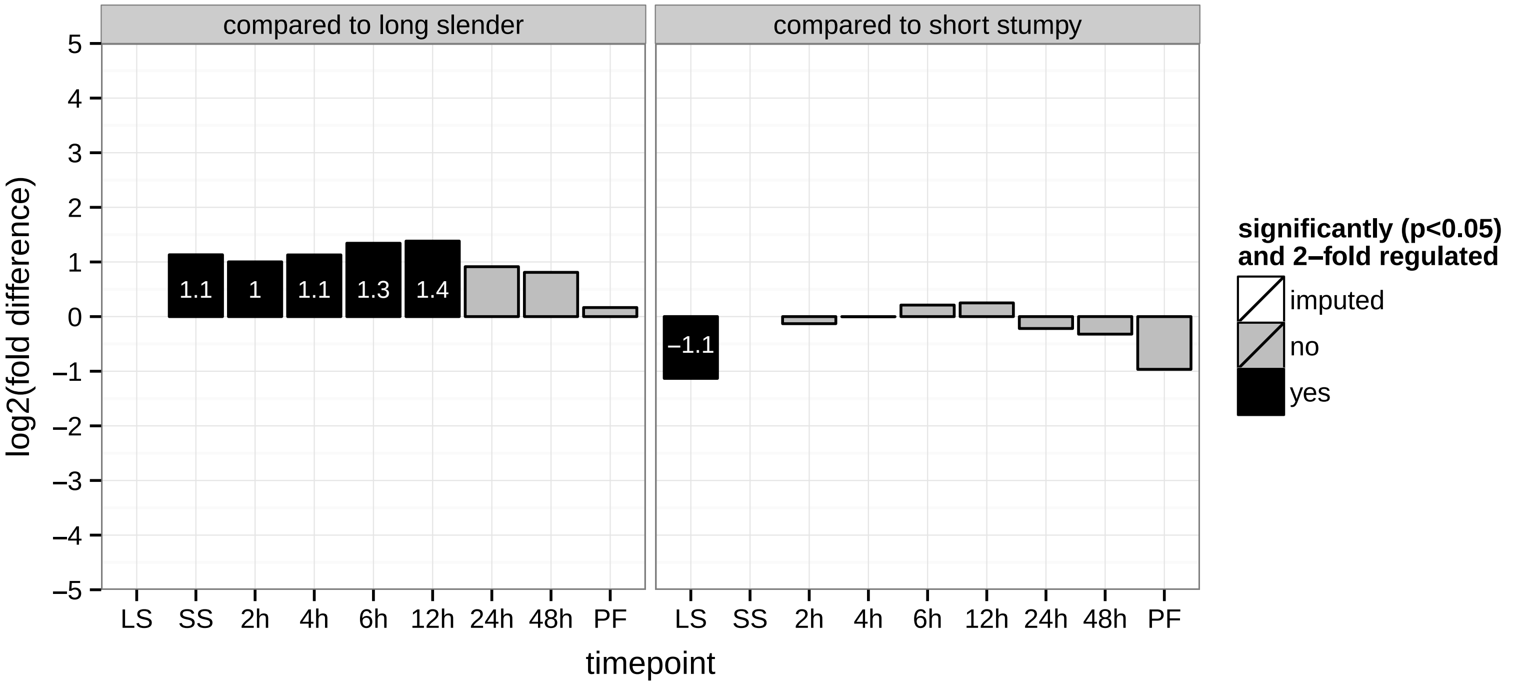

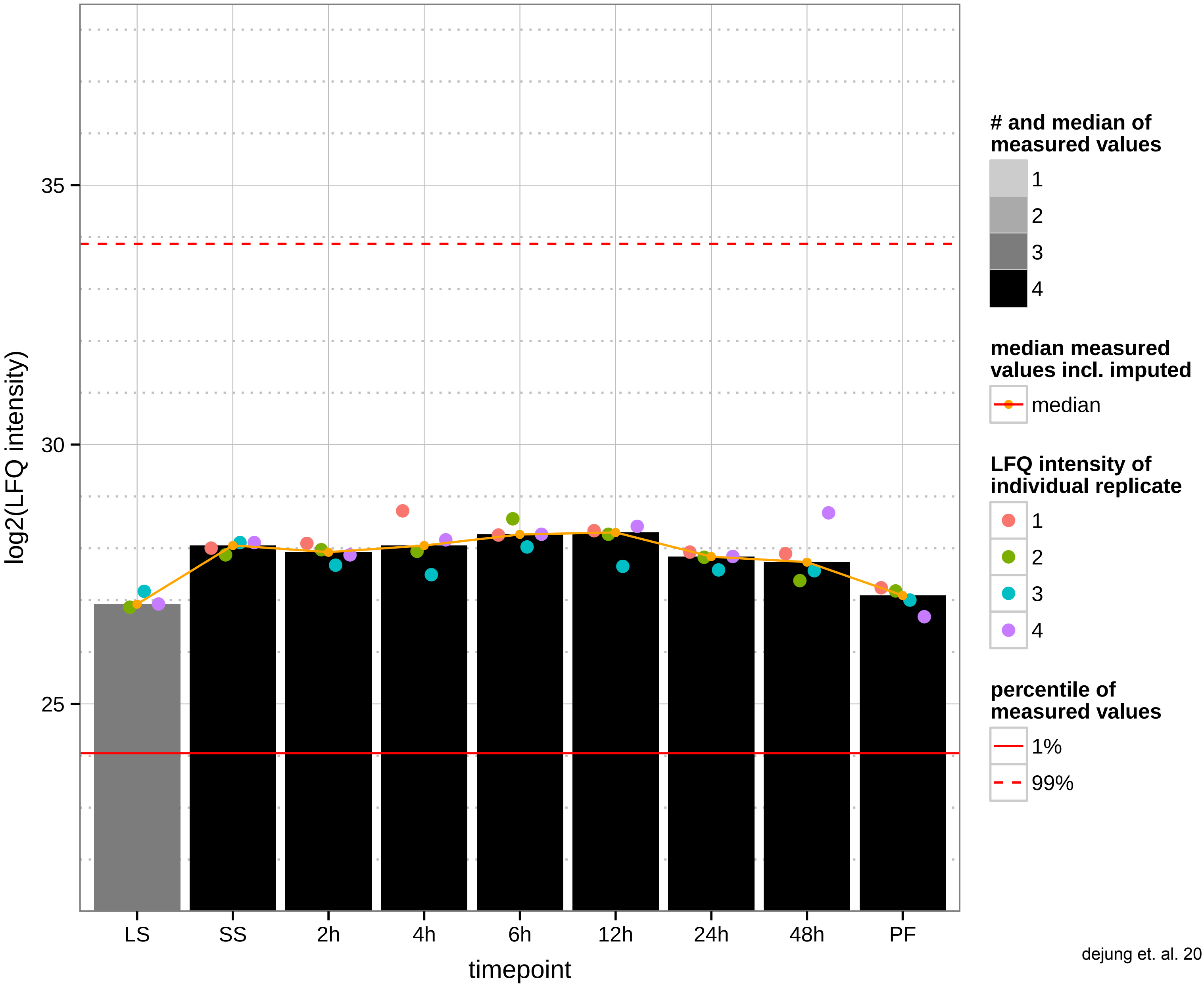

dejung et. al. 2015

flagellar protofilament ribbon protein, putative
Tb927.8.4640
AGOF: null
AGOC: cilium
AGOP: ciliary or flagellar motility, negative regulation of microtubule depolymerization
PGOF: null
PGOC: null
PGOP: null

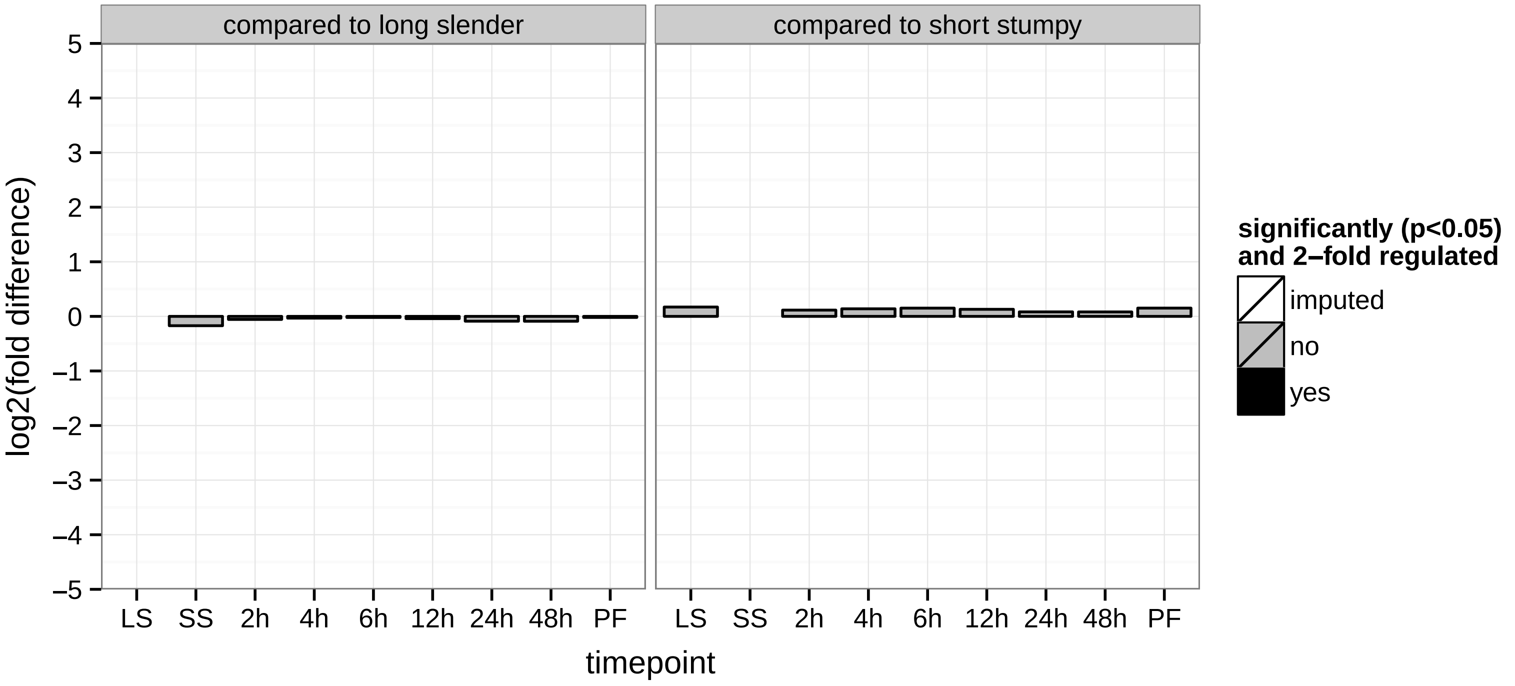

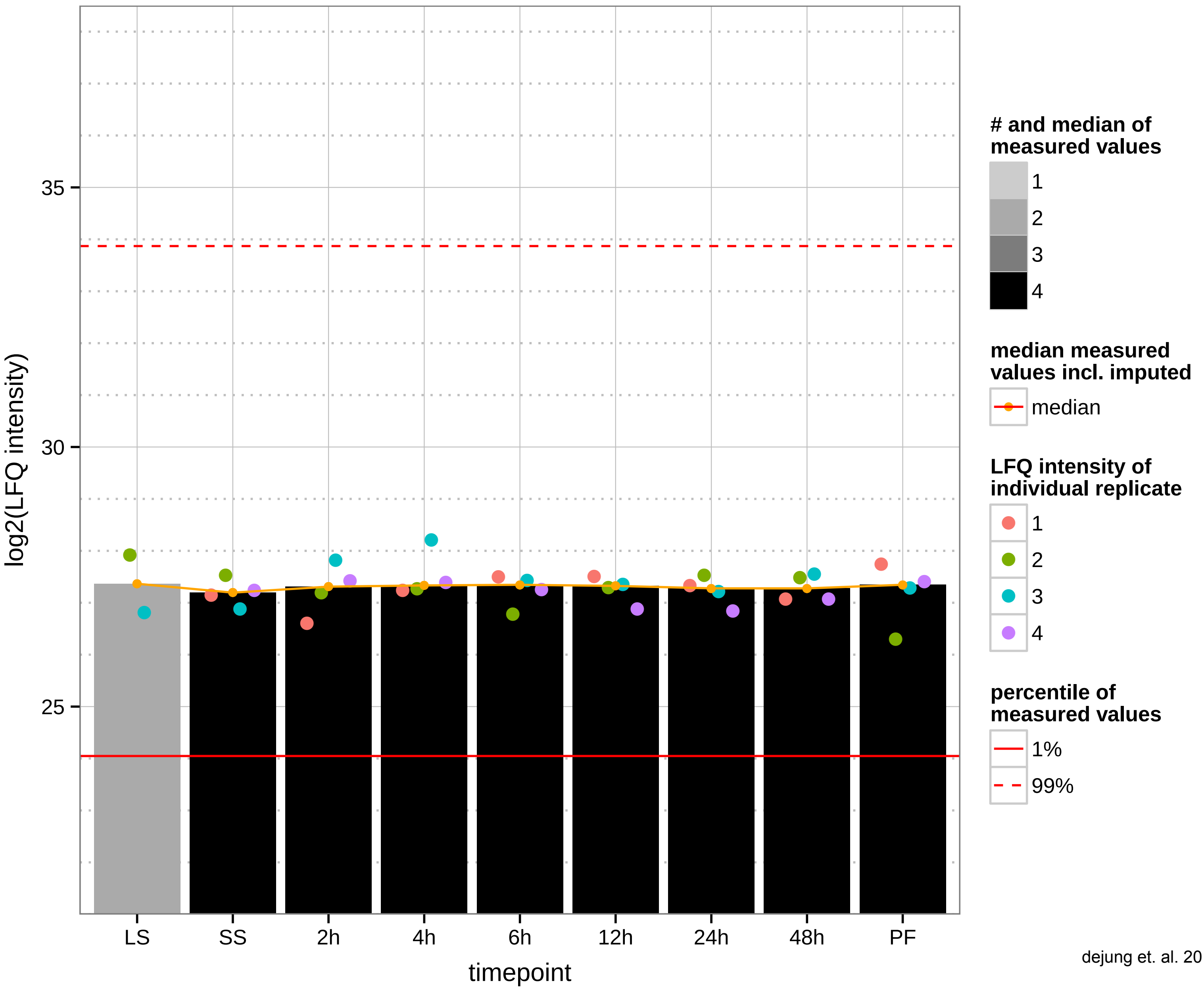

dejung et. al. 2015

small GTP–binding protein Rab18 (TbRAB18)
Tb927.8.4770
AGOF: GTP binding, GTPase activity
AGOC: intracellular
AGOP: protein transport, small GTPase mediated signal transduction
PGOF: GTP binding, GTPase activity
PGOC: intracellular, membrane
PGOP: GTP catabolic process, protein transport, signal transduction, small GTPase mediated signal transduction

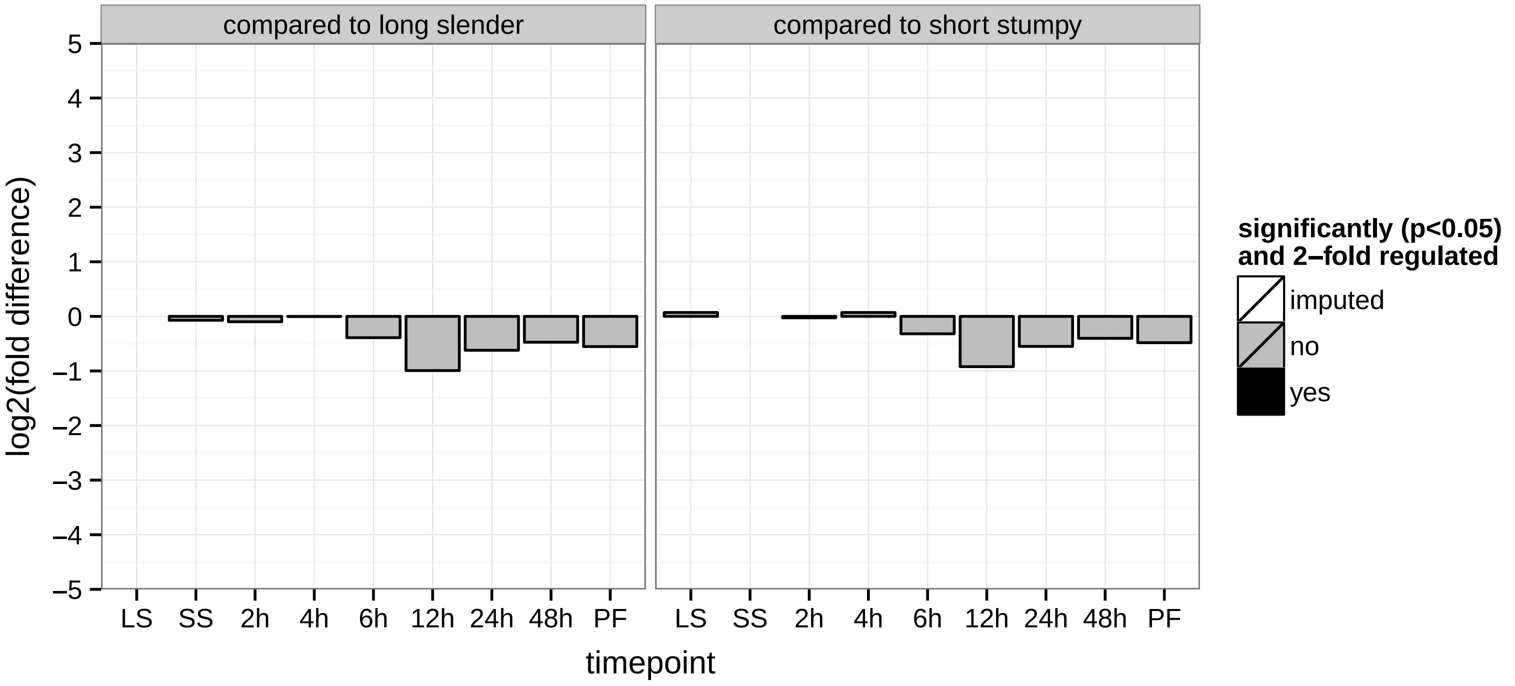

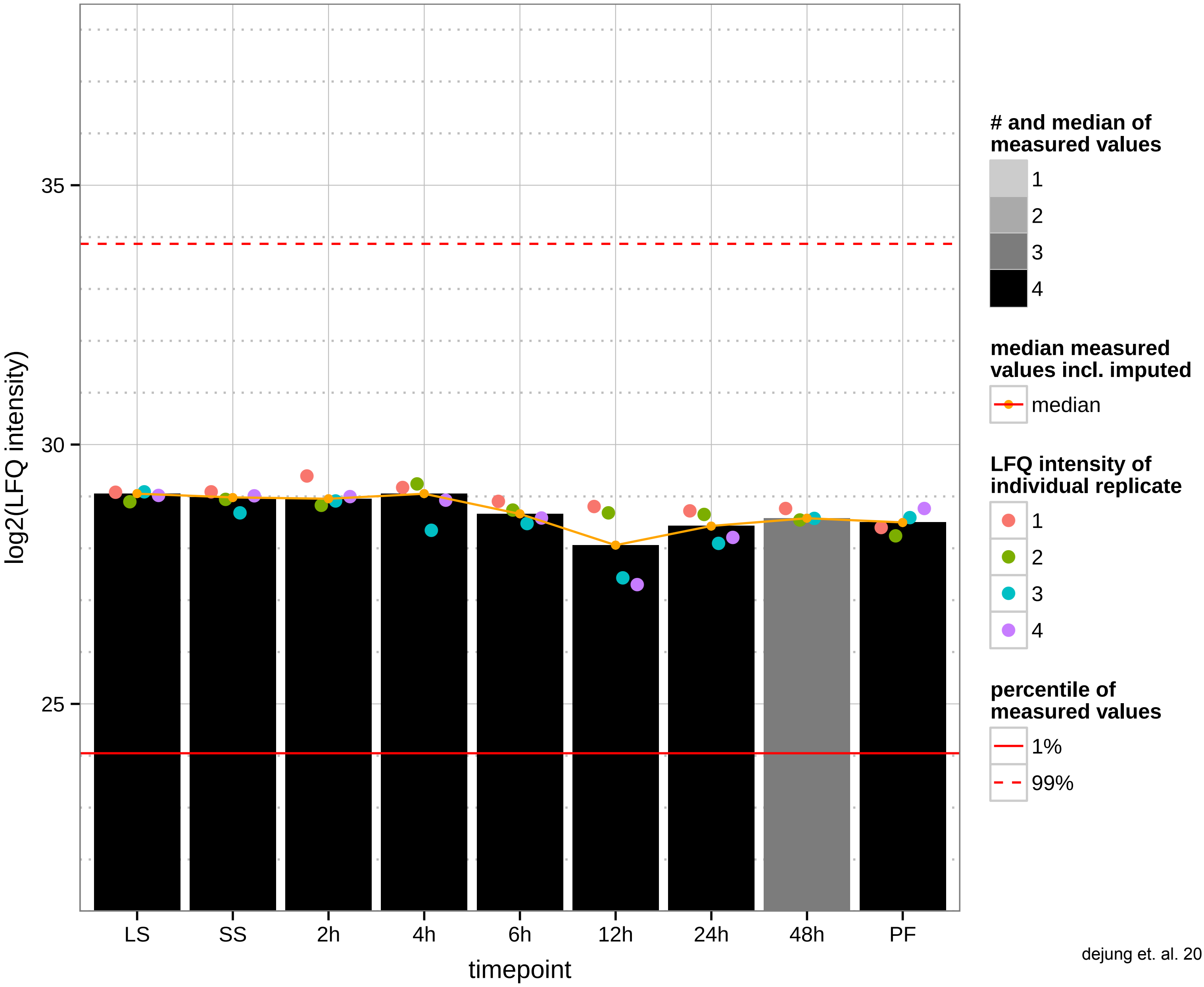

dejung et. al. 2015

hypothetical protein, conserved
Tb927.8.4780
AGOF: null
AGOC: null
AGOP: null
PGOF: null
PGOC: null
PGOP: null

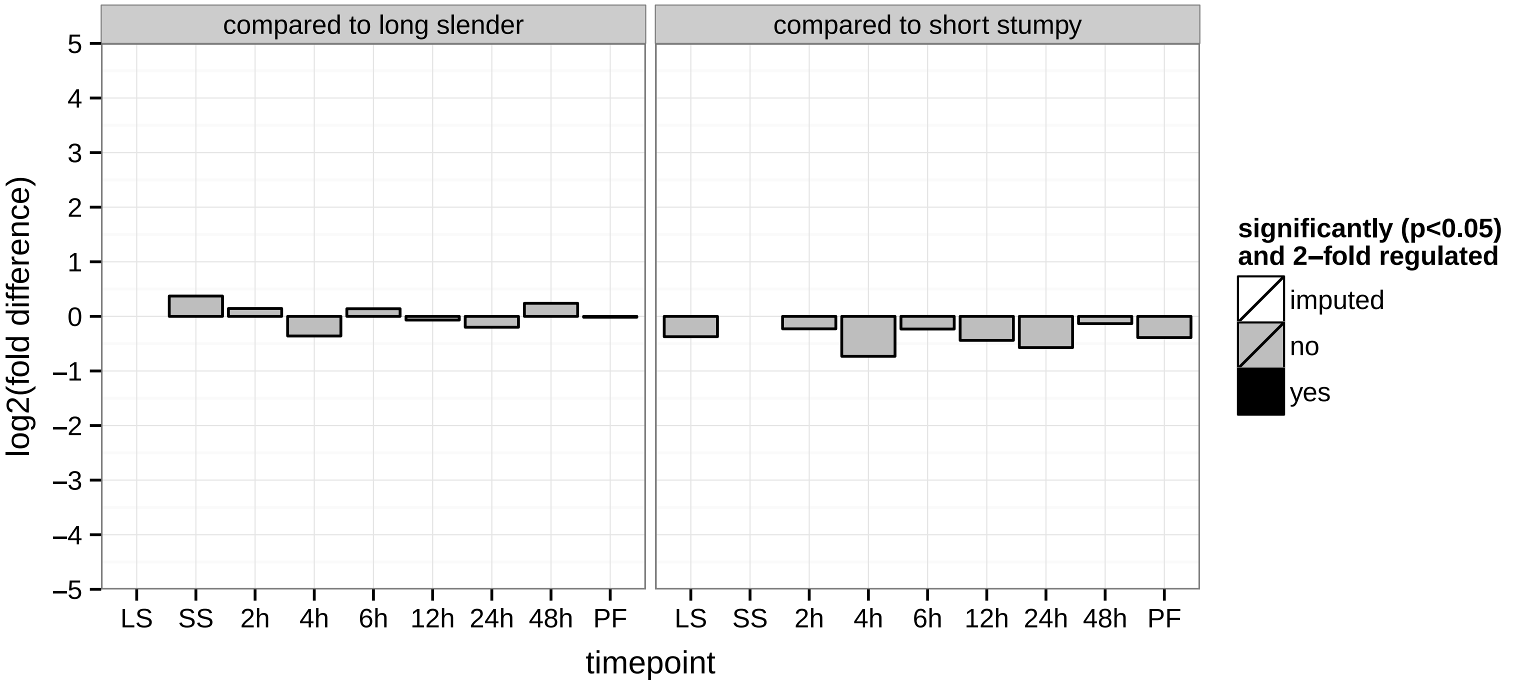

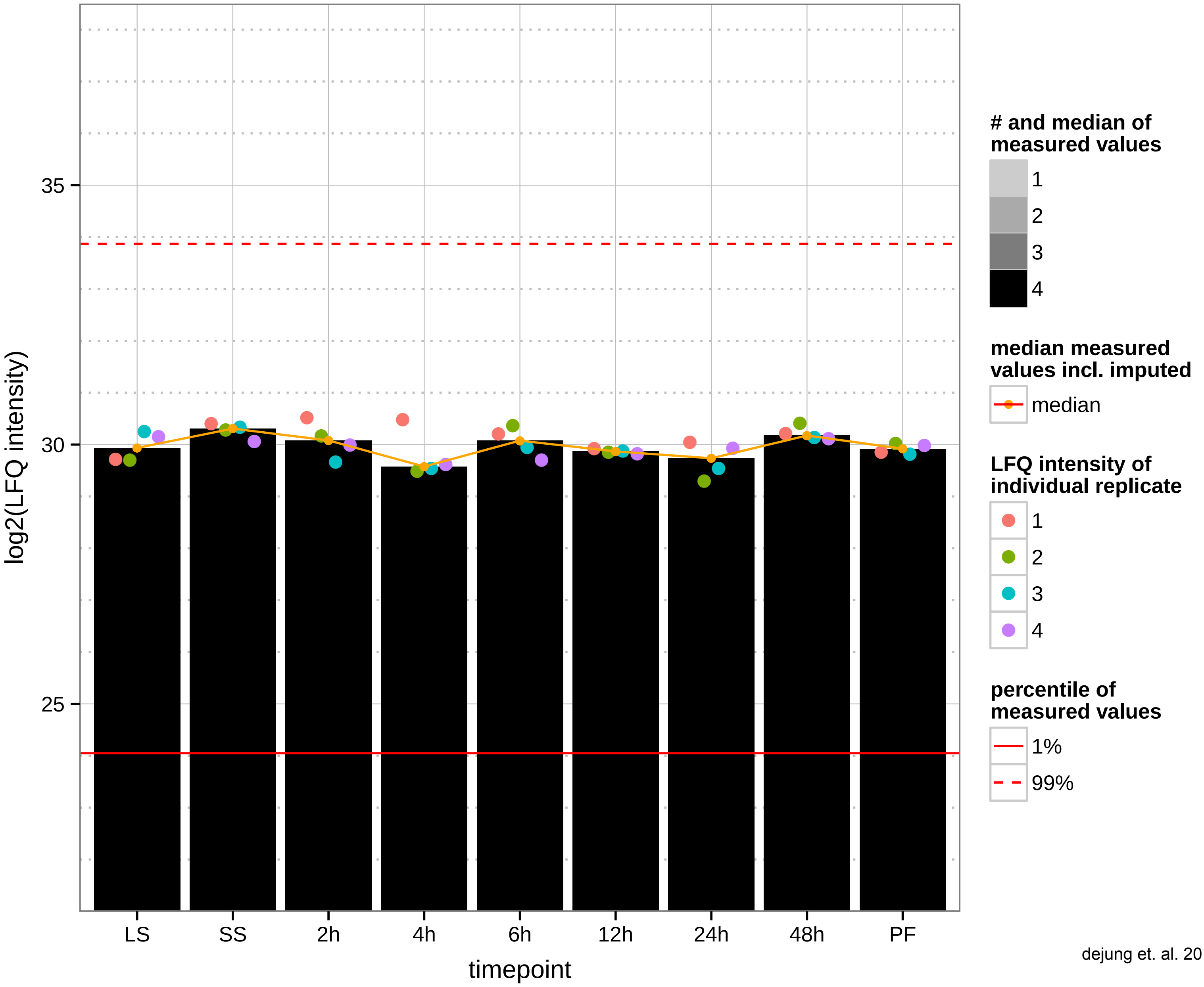

dejung et. al. 2015

kinesin, putative
Tb927.8.4840
AGOF: ATP binding, microtubule motor activity, motor activity
AGOC: kinesin complex, microtubule associated complex
AGOP: microtubule–based movement
PGOF: ATP binding, microtubule motor activity
PGOC: null
PGOP: microtubule–based movement

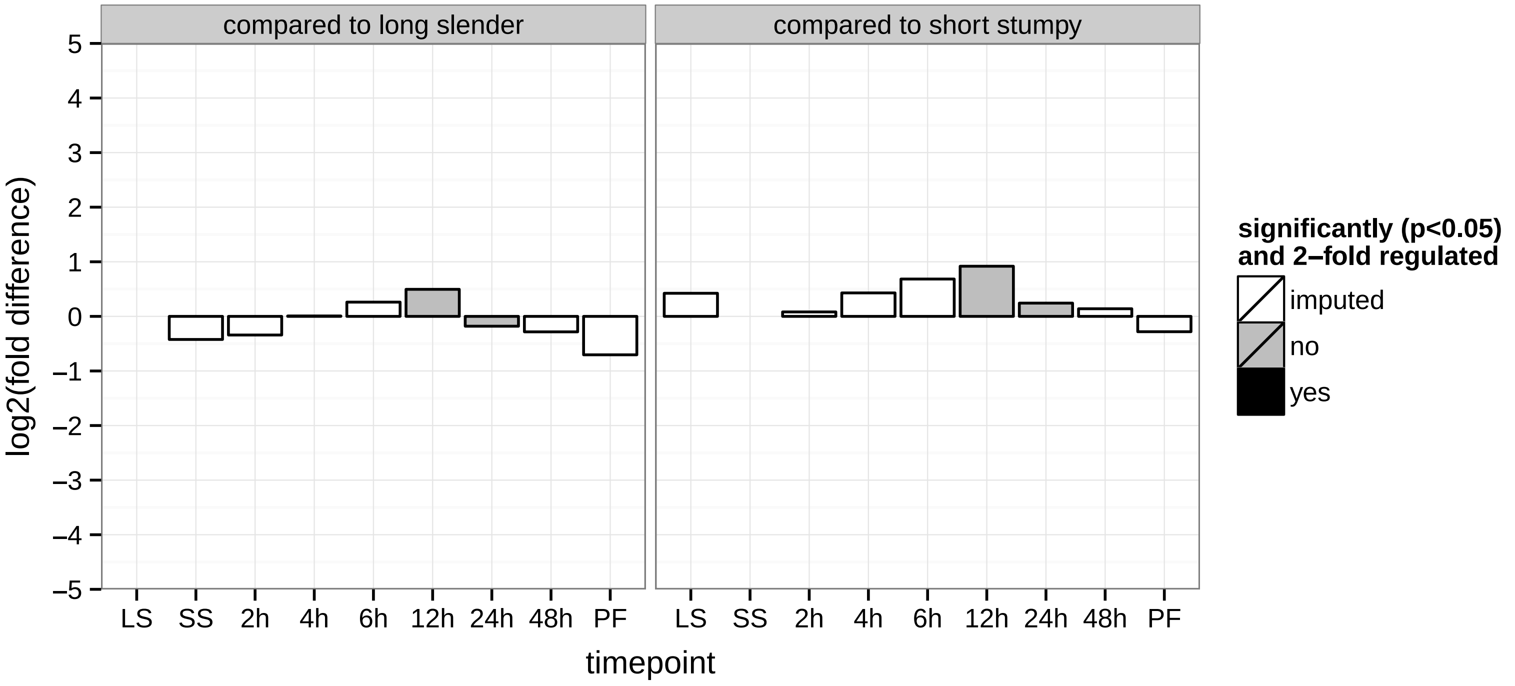

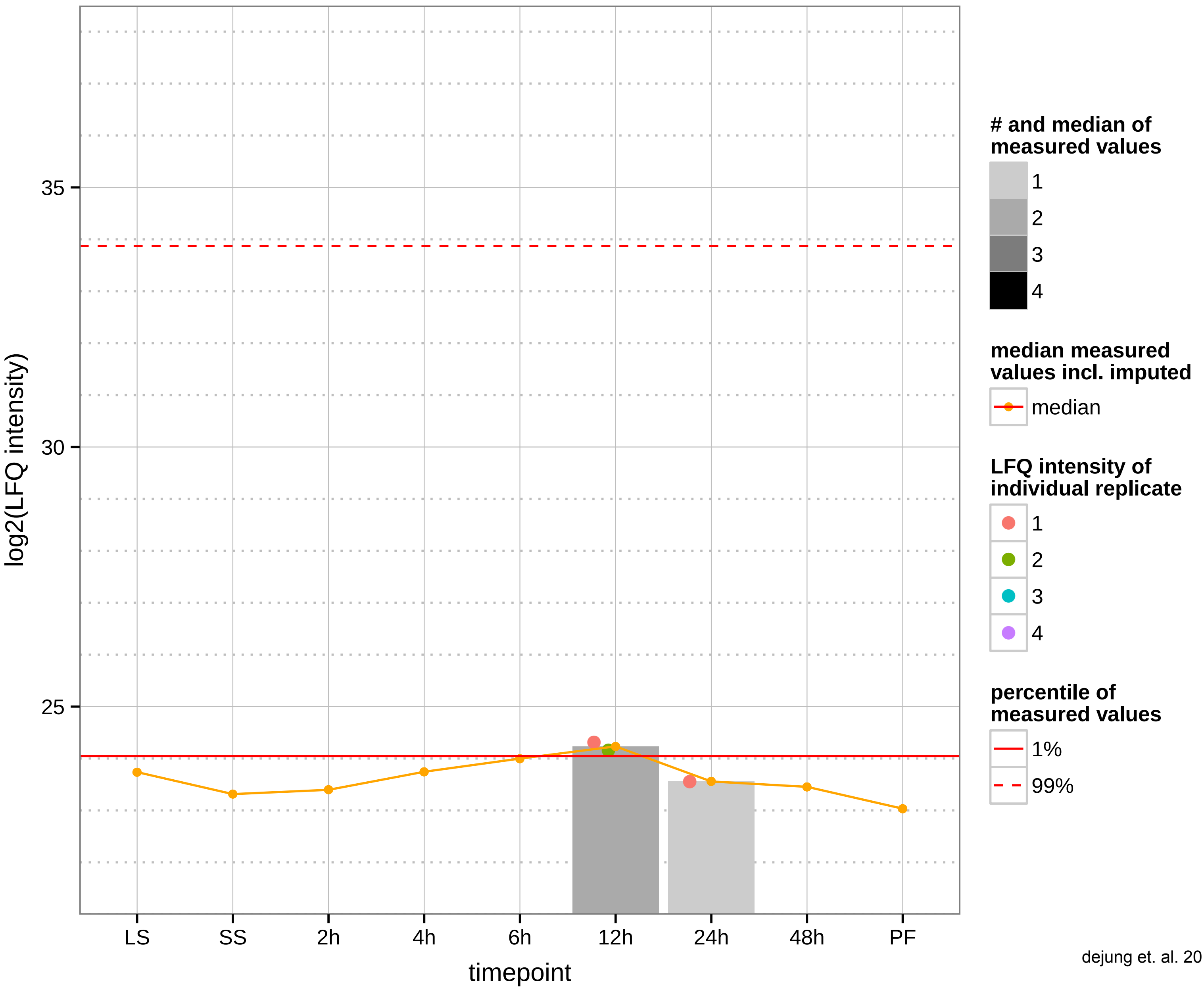

dejung et. al. 2015

DIGIT, flagellar component
Tb927.8.4870
AGOF: null
AGOC: null
AGOP: ciliary or flagellar motility
PGOF: protein binding
PGOC: null
PGOP: null

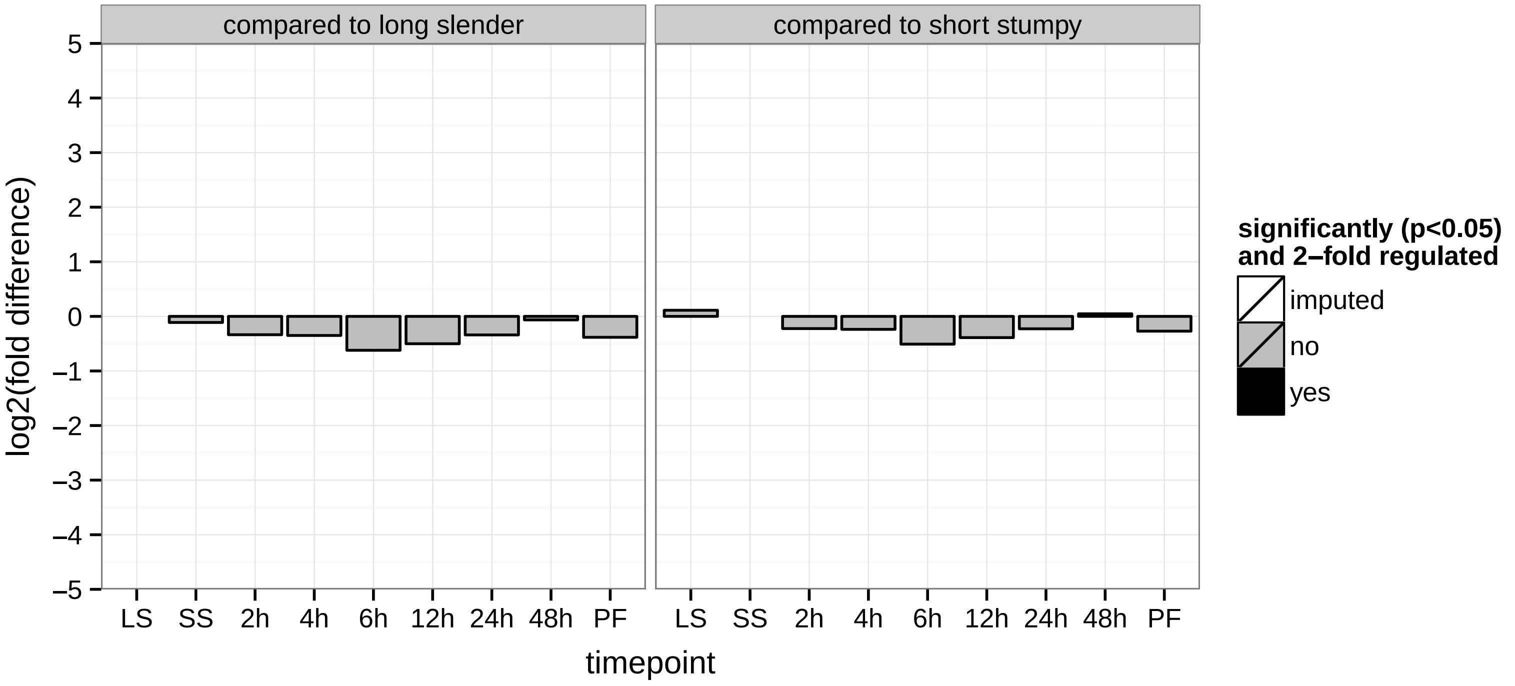

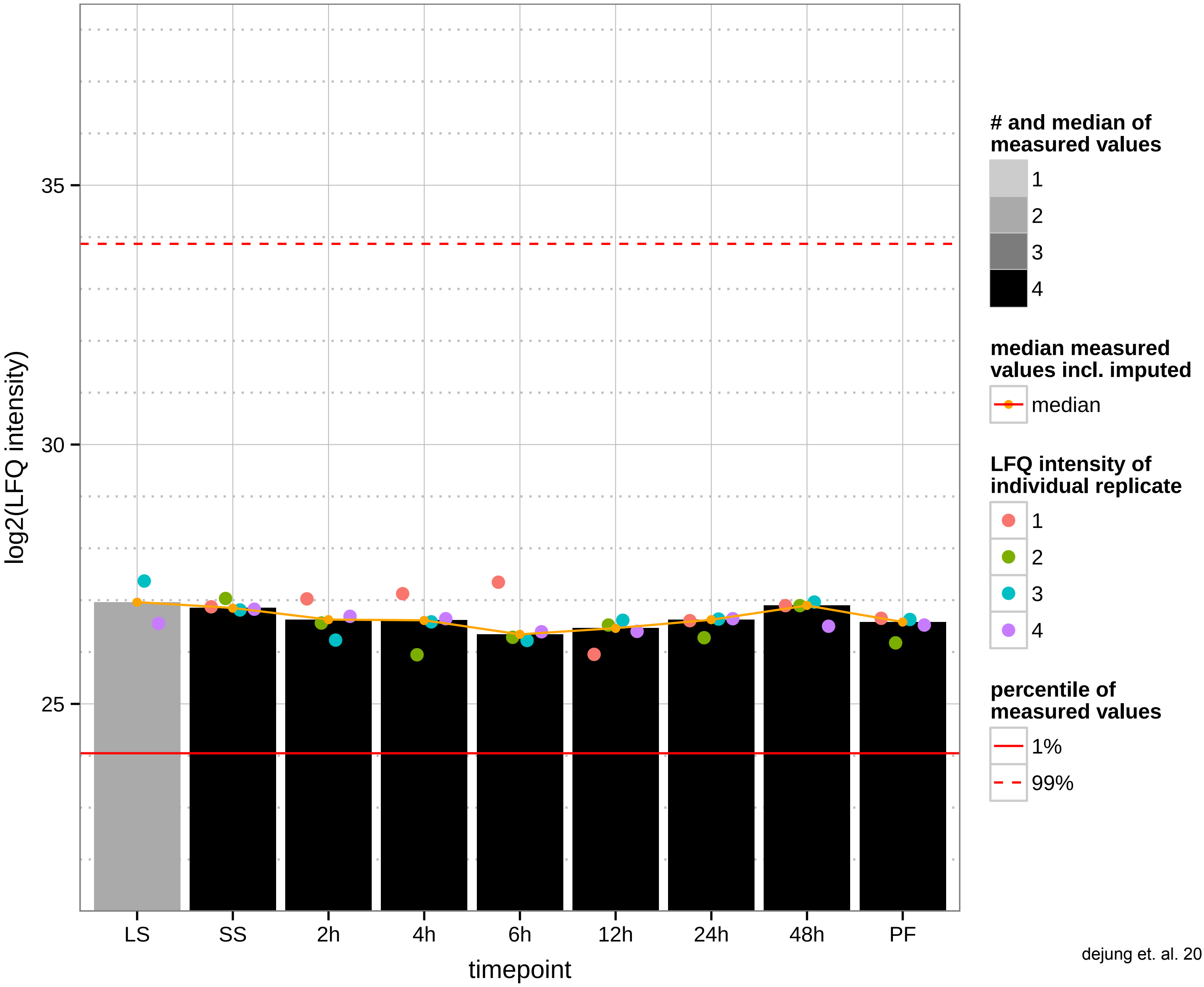

dejung et. al. 2015

hypothetical protein, conserved
Tb927.8.4940
AGOF: null
AGOC: null
AGOP: null
PGOF: null
PGOC: null
PGOP: null

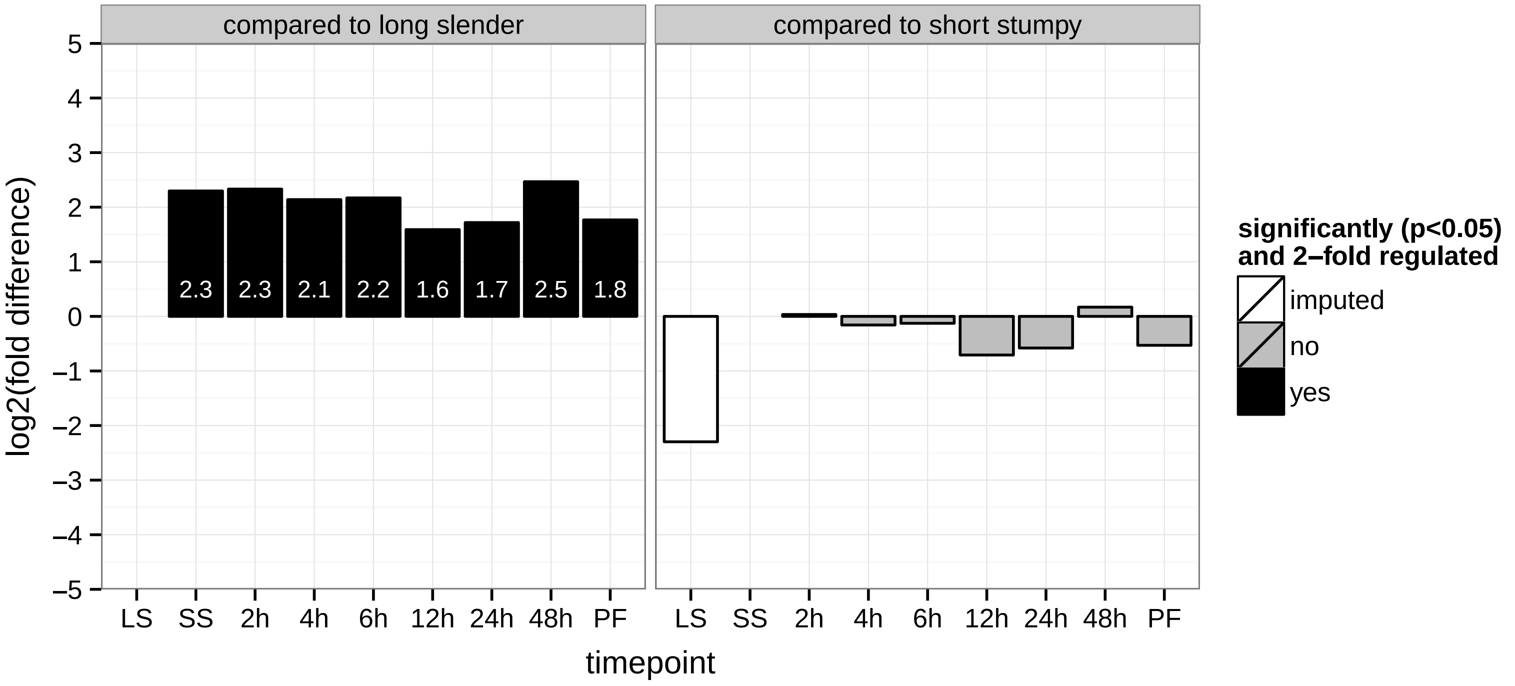

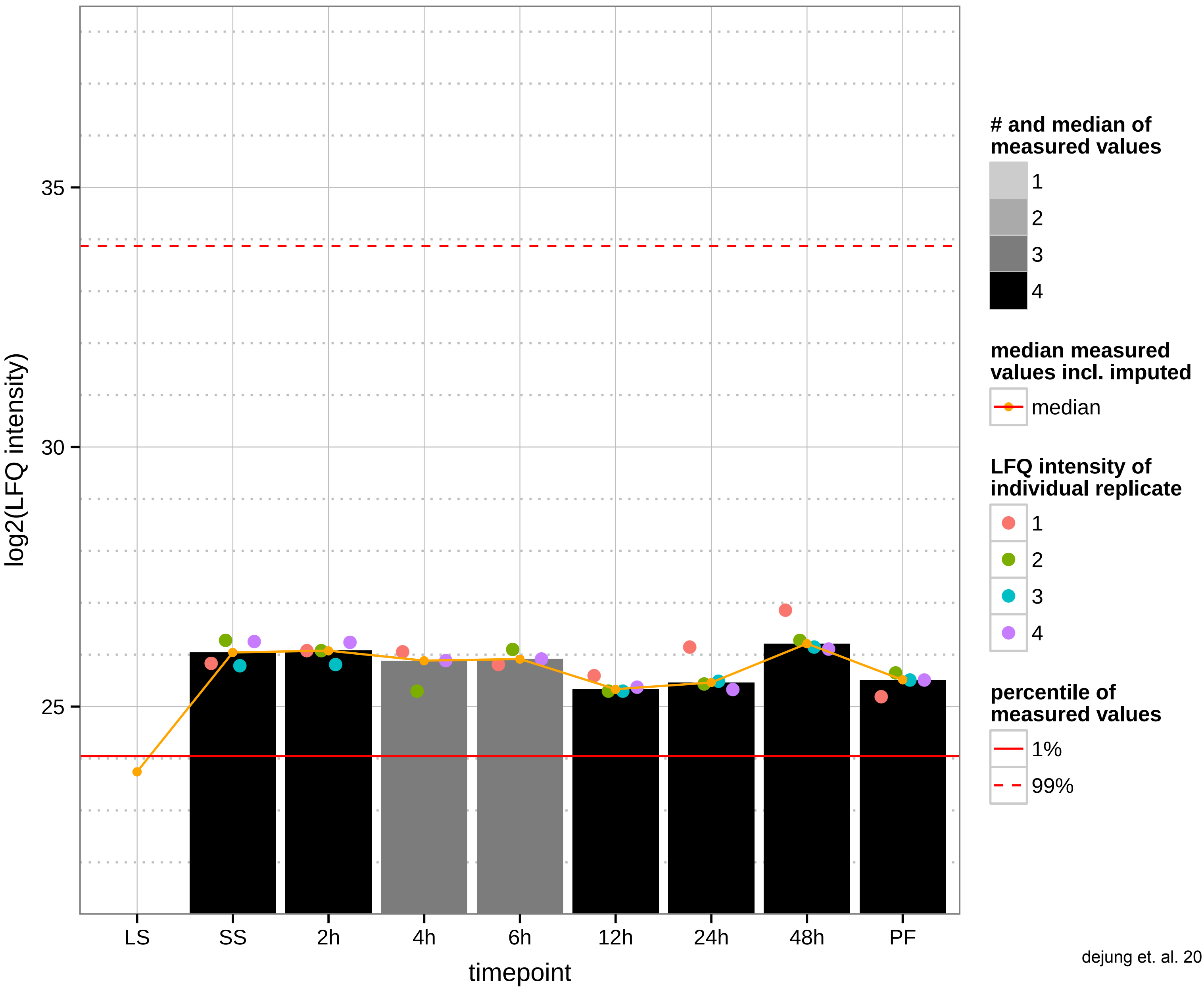

dejung et. al. 2015

hypothetical protein, conserved
Tb927.8.5020
AGOF: null
AGOC: null
AGOP: null
PGOF: protein binding
PGOC: null
PGOP: null

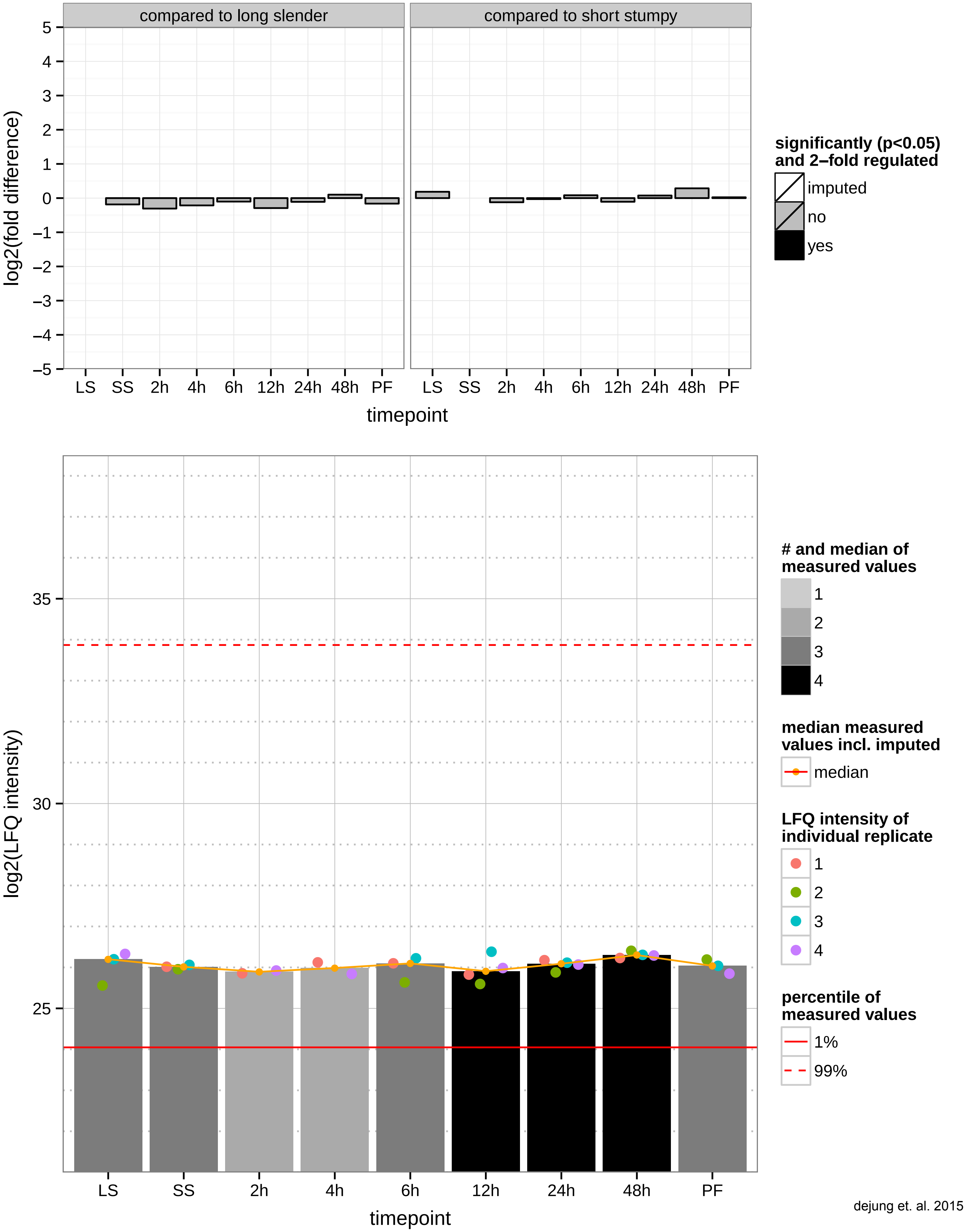

dejung et. al. 2015

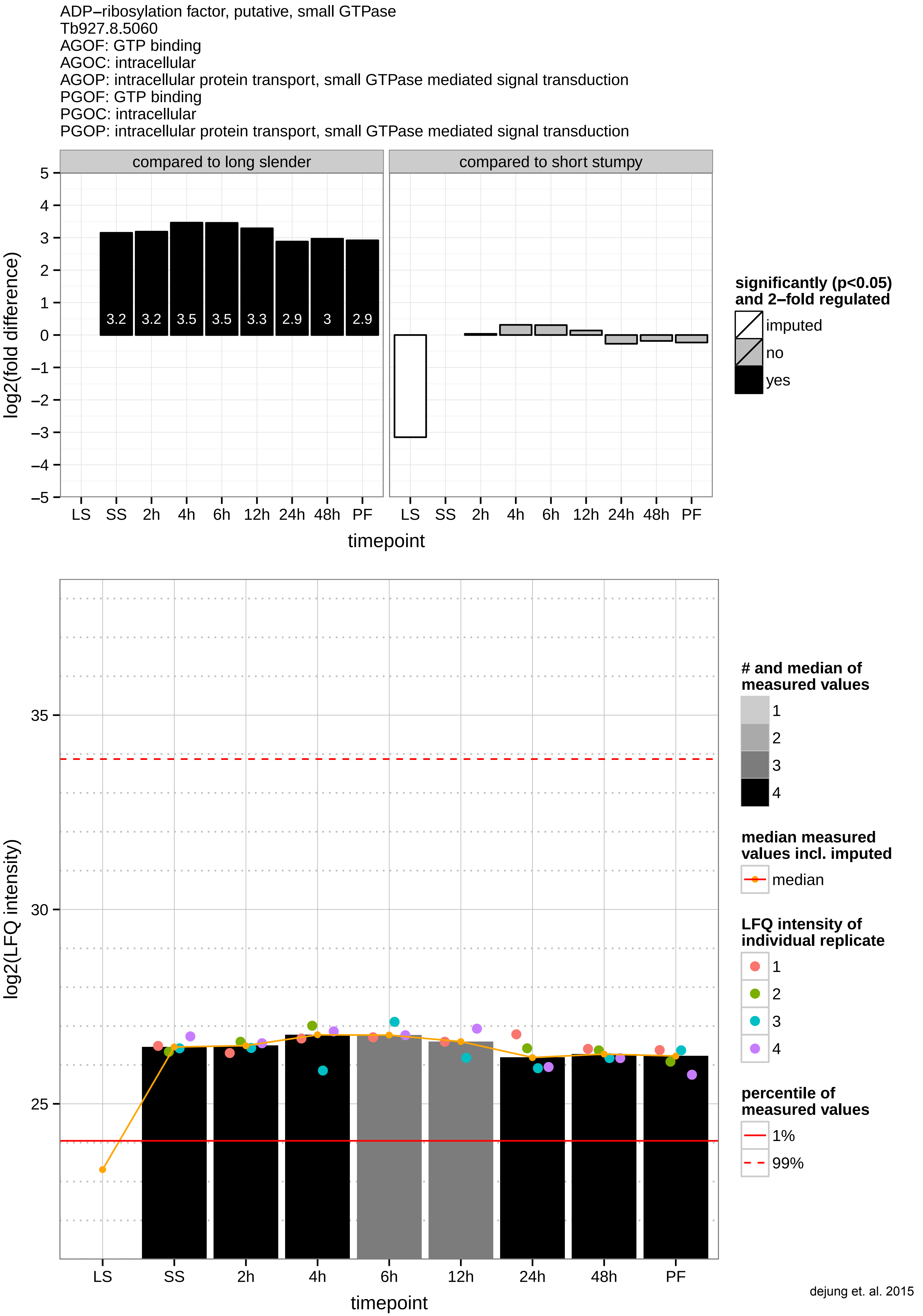
ADP–ribosylation factor, putative, small GTPase
Tb927.8.5060
AGOF: GTP binding
AGOC: intracellular
AGOP: intracellular protein transport, small GTPase mediated signal transduction
PGOF: GTP binding
PGOC: intracellular
PGOP: intracellular protein transport, small GTPase mediated signal transduction
compared to long slender
compared to short stumpy
log2(fold difference)
3.2
3.2
3.5
3.5
3.3
2.9
3
2.9
LS SS 2h 4h 6h 12h 24h 48h PF
timepoint
significantly (p<0.05) and 2–fold regulated
imputed
no
yes
log2(LFQ intensity)
# and median of measured values
1
2
3
4
median measured values incl. imputed
median
LFQ intensity of individual replicate
1
2
3
4
percentile of measured values
1%
99%
dejung et. al. 2015

cyclophilin–type peptidyl–prolyl cis–trans isomerase, putative, PPIase, rotamase, cyclophilin, putative (PPIase)
Tb927.8.5230
AGOF: peptidyl–prolyl cis–trans isomerase activity
AGOC: null
AGOP: protein folding
PGOF: peptidyl–prolyl cis–trans isomerase activity
PGOC: null
PGOP: protein folding

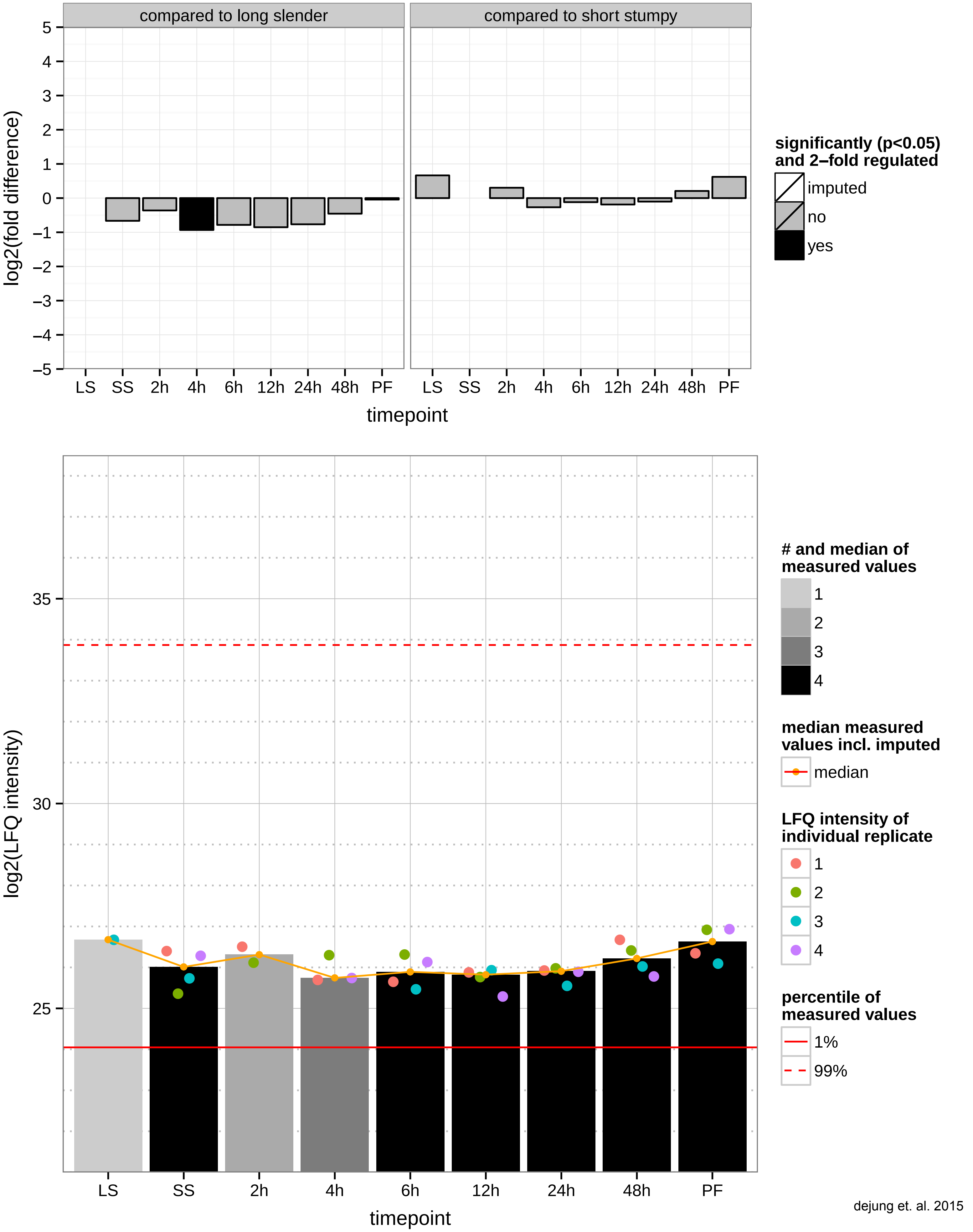

dejung et. al. 2015

coatomer delta subunit, putative, delta–COP, delta–coat protein
Tb927.8.5250
AGOF: null
AGOC: clathrin adaptor complex, cytoplasm
AGOP: intracellular protein transport, vesicle–mediated transport
PGOF: protein binding
PGOC: clathrin adaptor complex
PGOP: intracellular protein transport, transport, vesicle–mediated transport

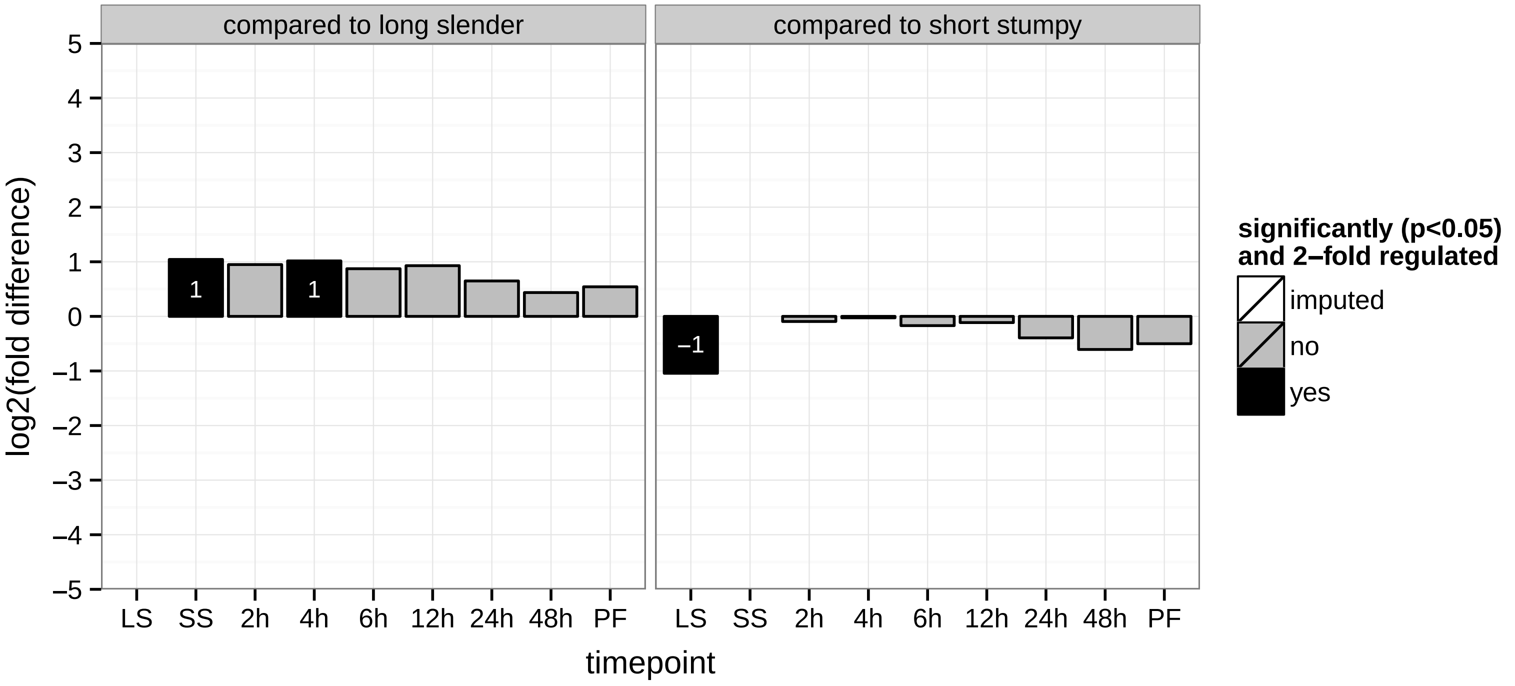

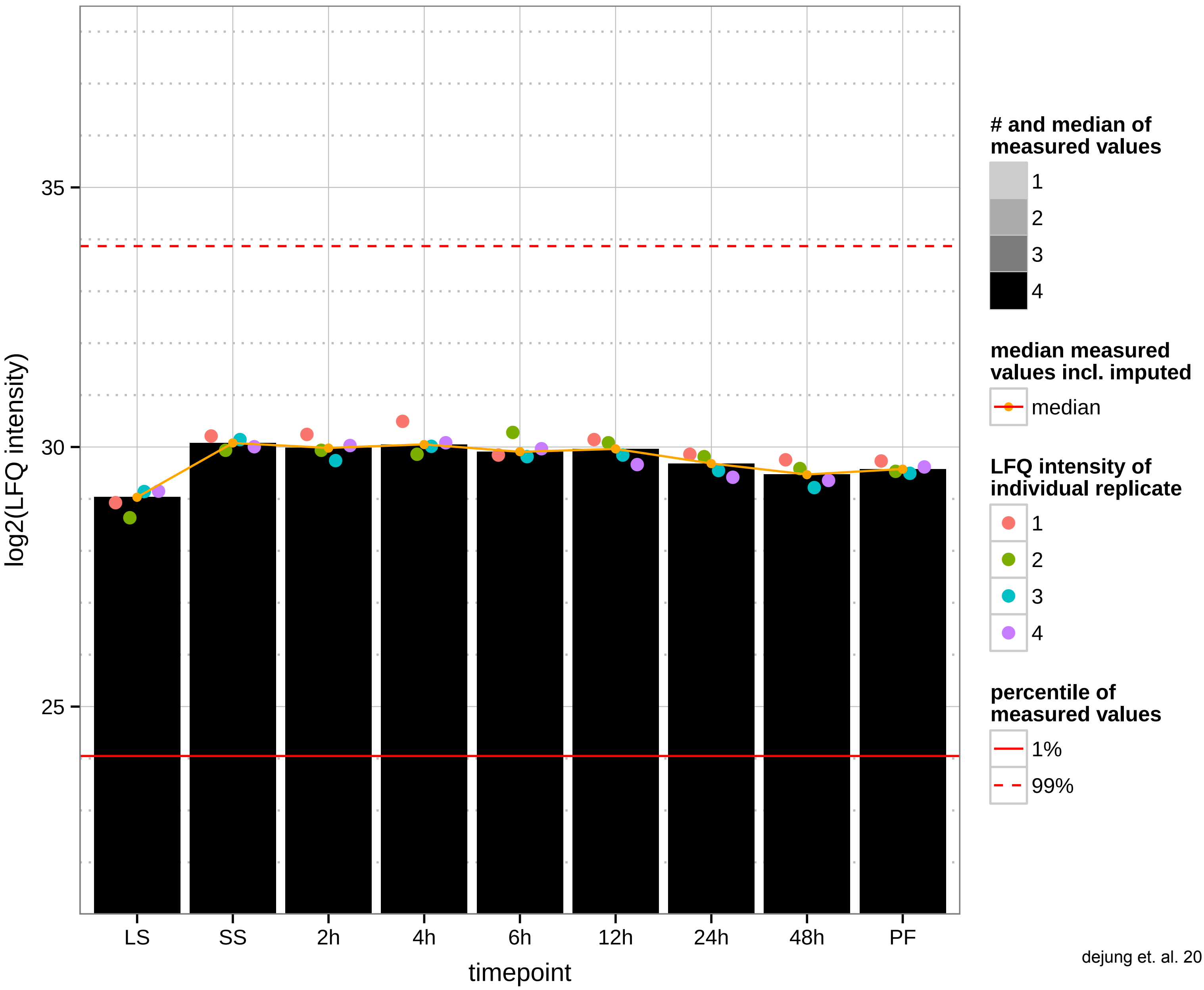

dejung et. al. 2015

hypothetical protein, conserved
Tb927.8.5290
AGOF: null
AGOC: null
AGOP: null
PGOF: protein binding
PGOC: null
PGOP: null

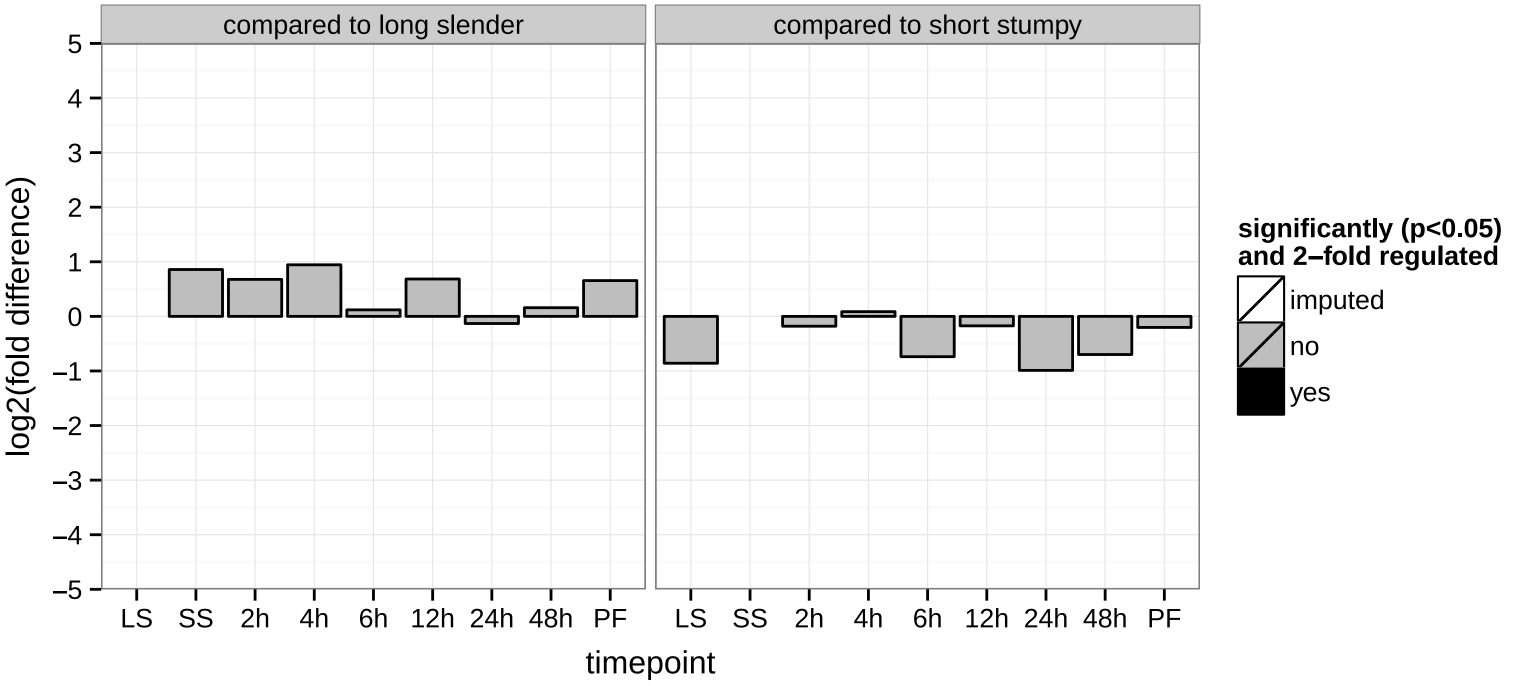

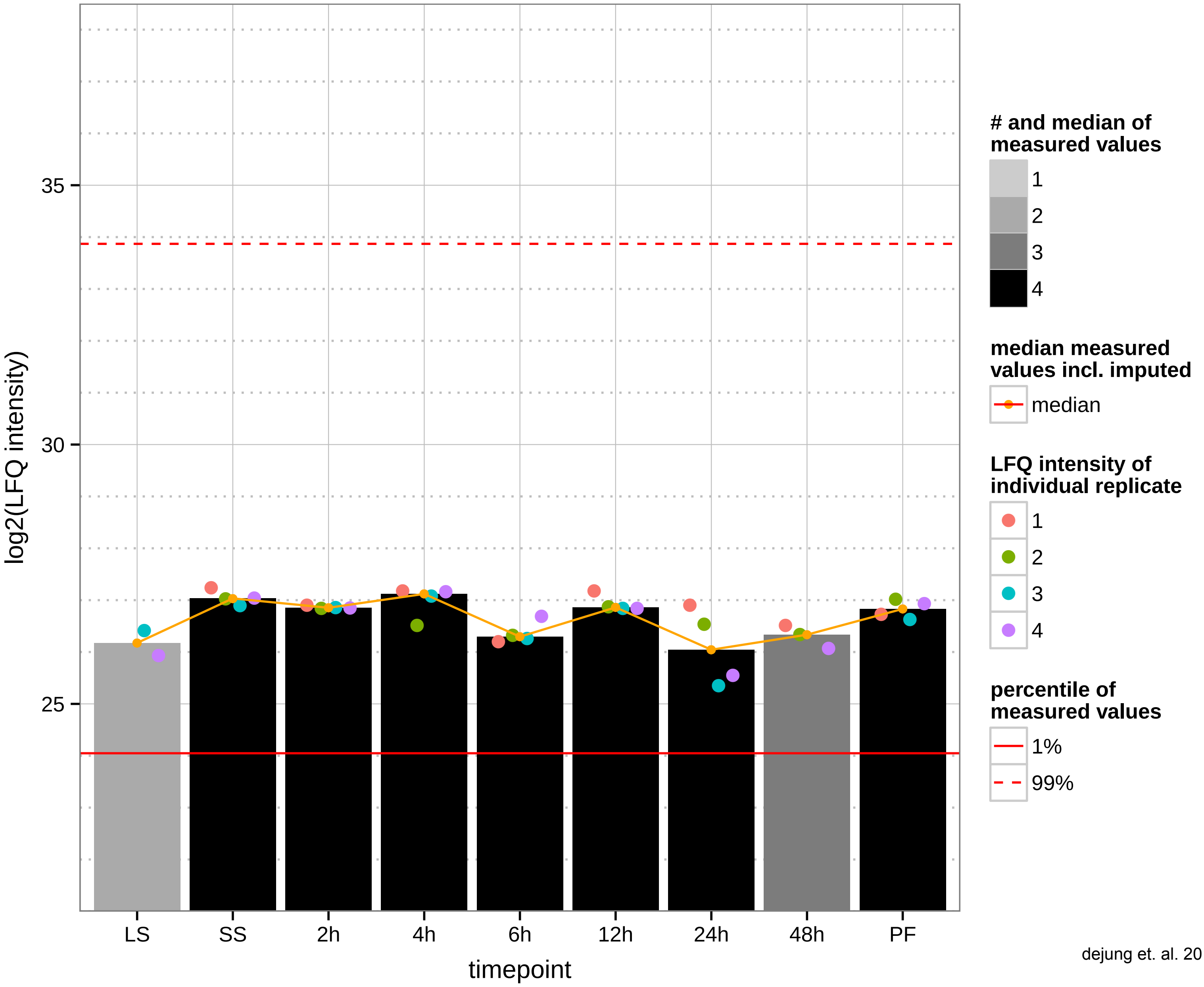

dejung et. al. 2015

hypothetical protein, conserved
Tb927.8.5310
AGOF: null
AGOC: null
AGOP: null
PGOF: null
PGOC: null
PGOP: null

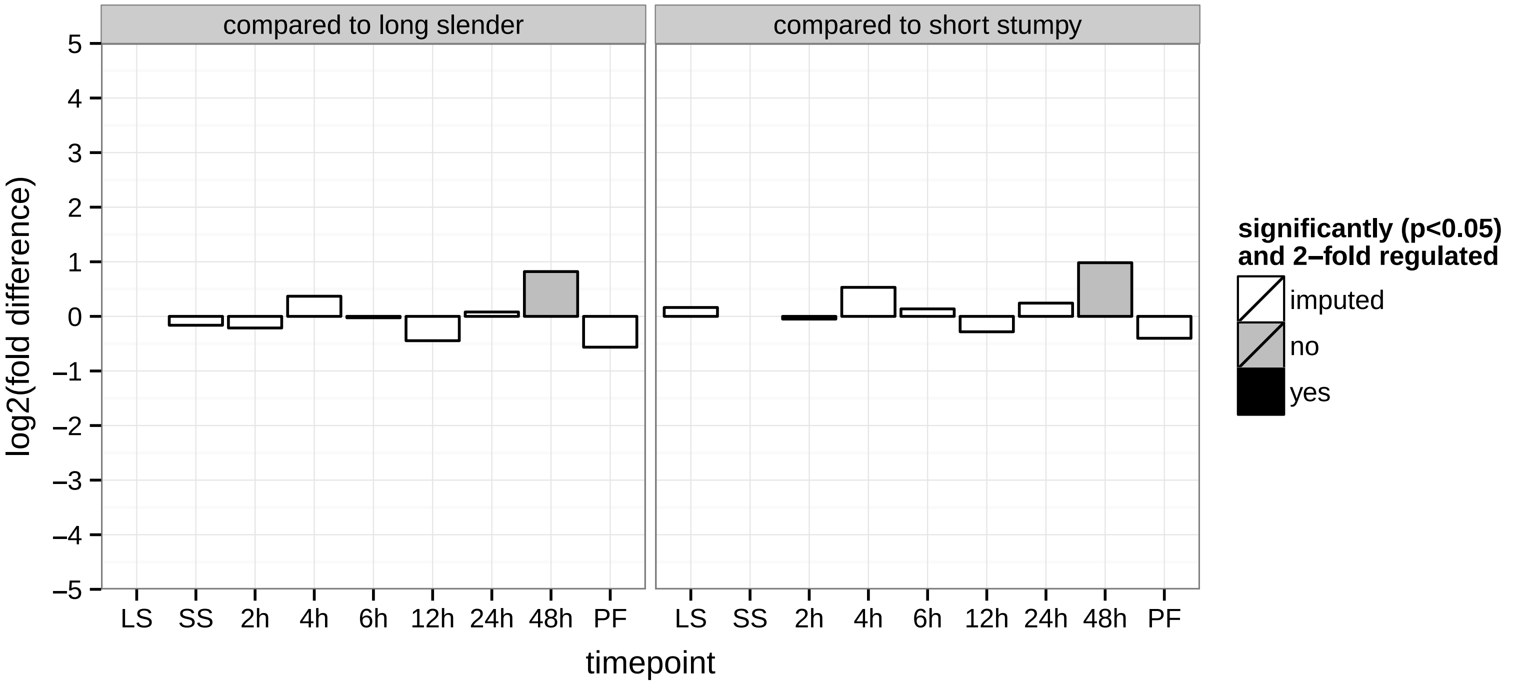

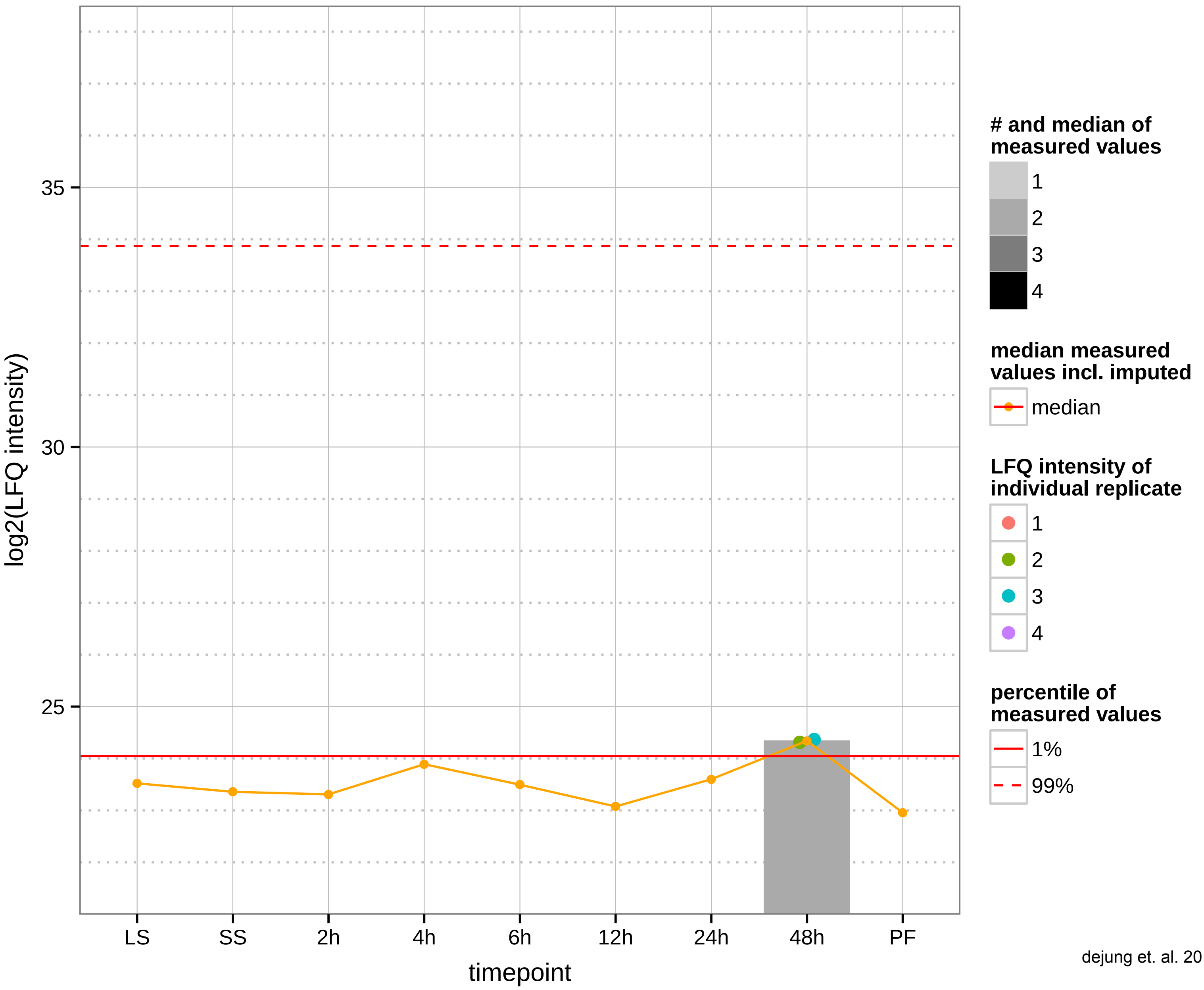

dejung et. al. 2015

tyrosyl/methionyl–tRNA synthetase, putative
Tb927.8.5330
AGOF: tRNA binding
AGOC: null
AGOP: tRNA aminoacylation
PGOF: tRNA binding
PGOC: null
PGOP: null

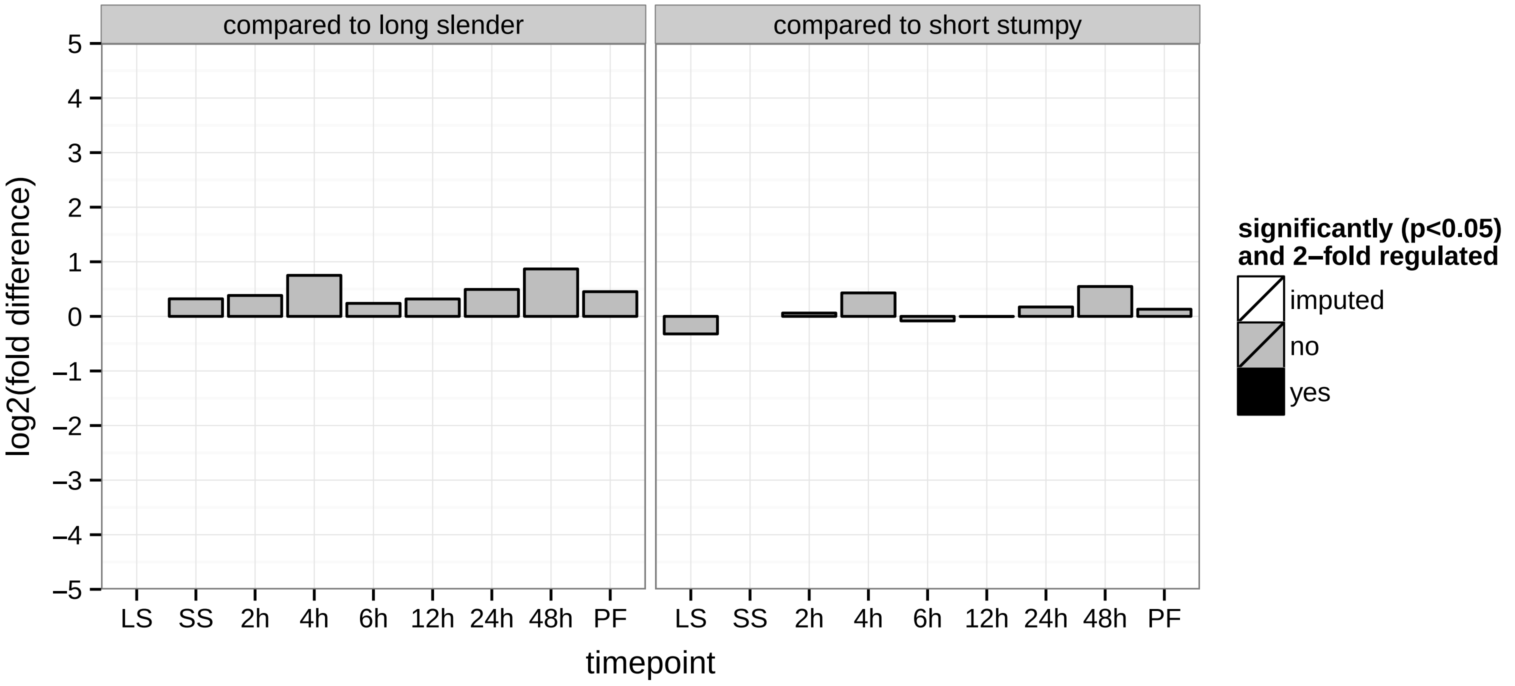

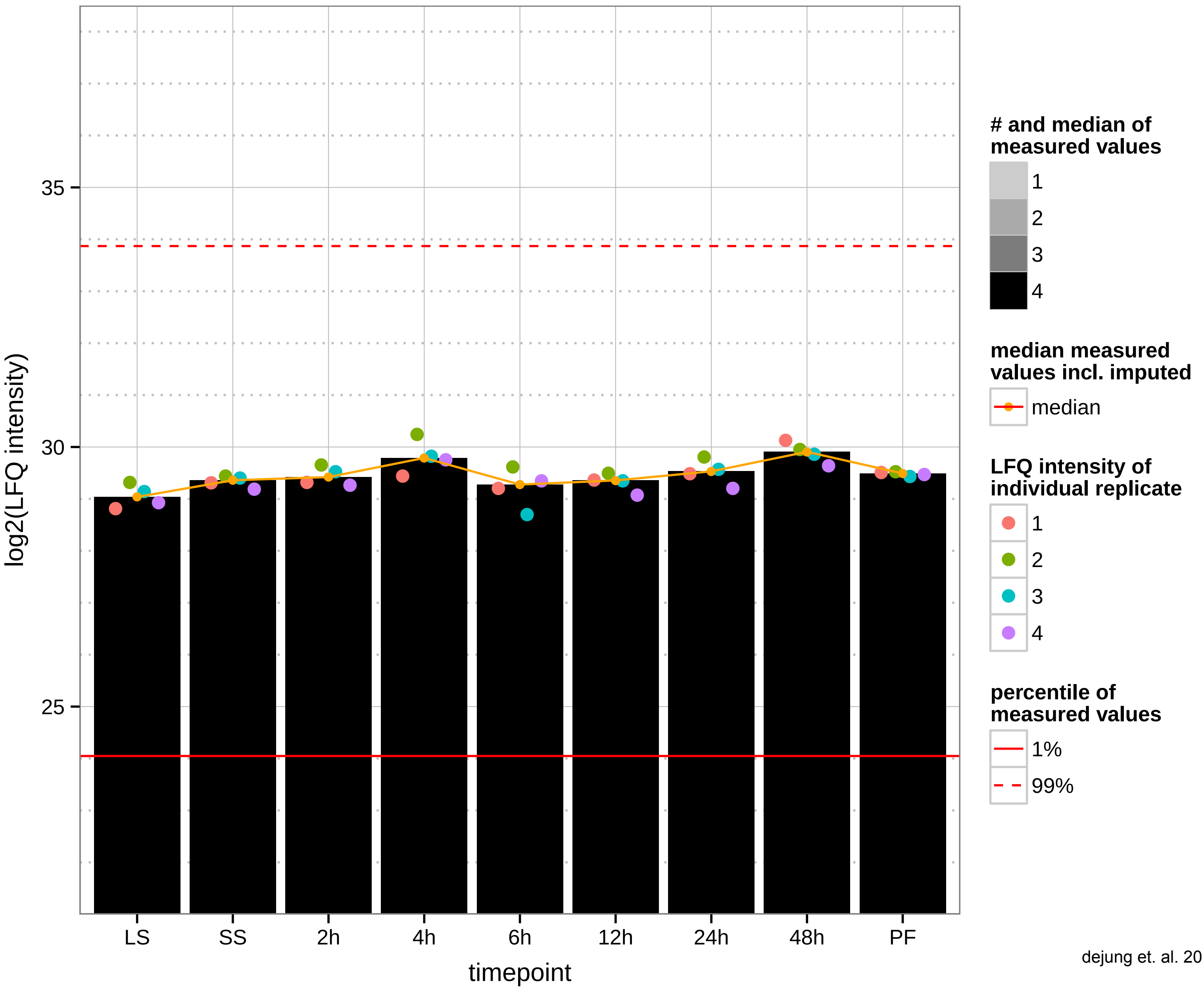

dejung et. al. 2015

ubiquitin fold modifier protein, putative (UFM1)
Tb927.8.5380
AGOF: null
AGOC: mitochondrion
AGOP: protein modification by small protein conjugation
PGOF: null
PGOC: null
PGOP: null

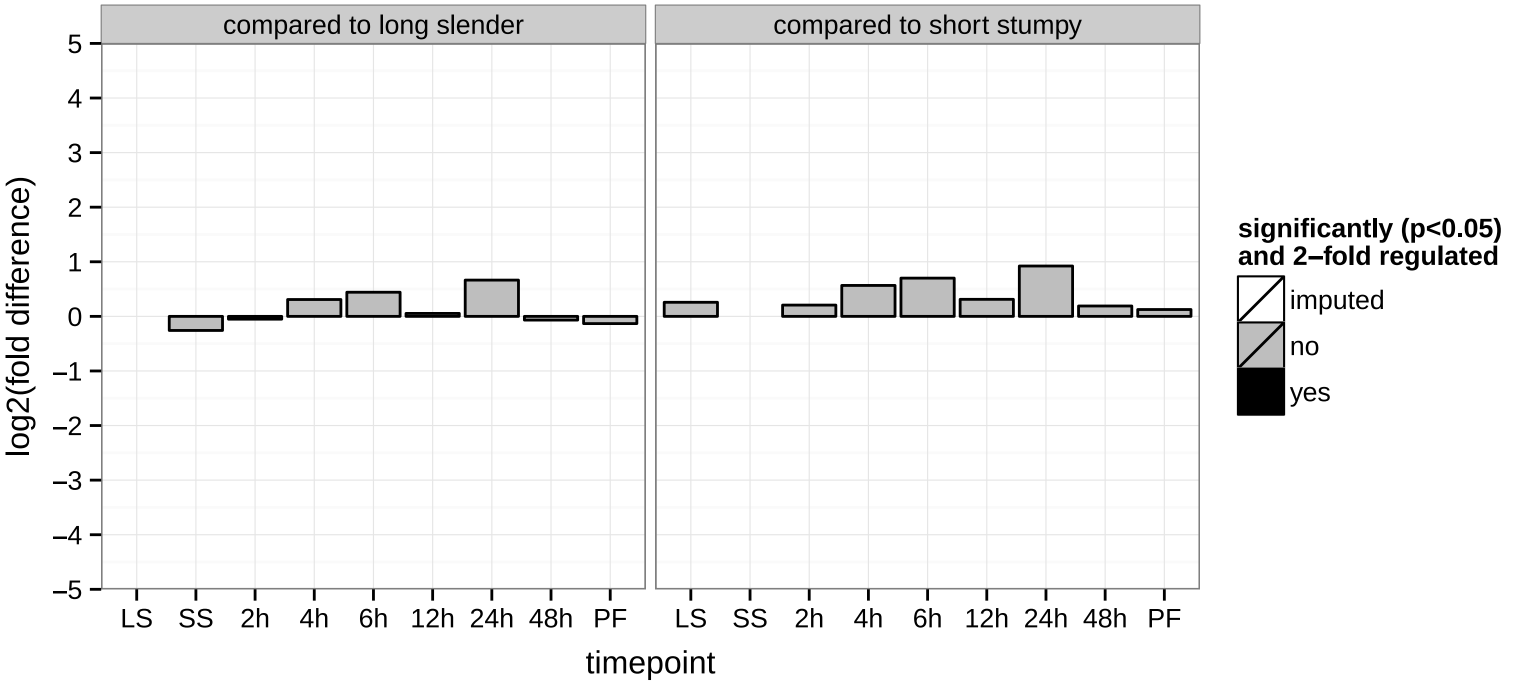

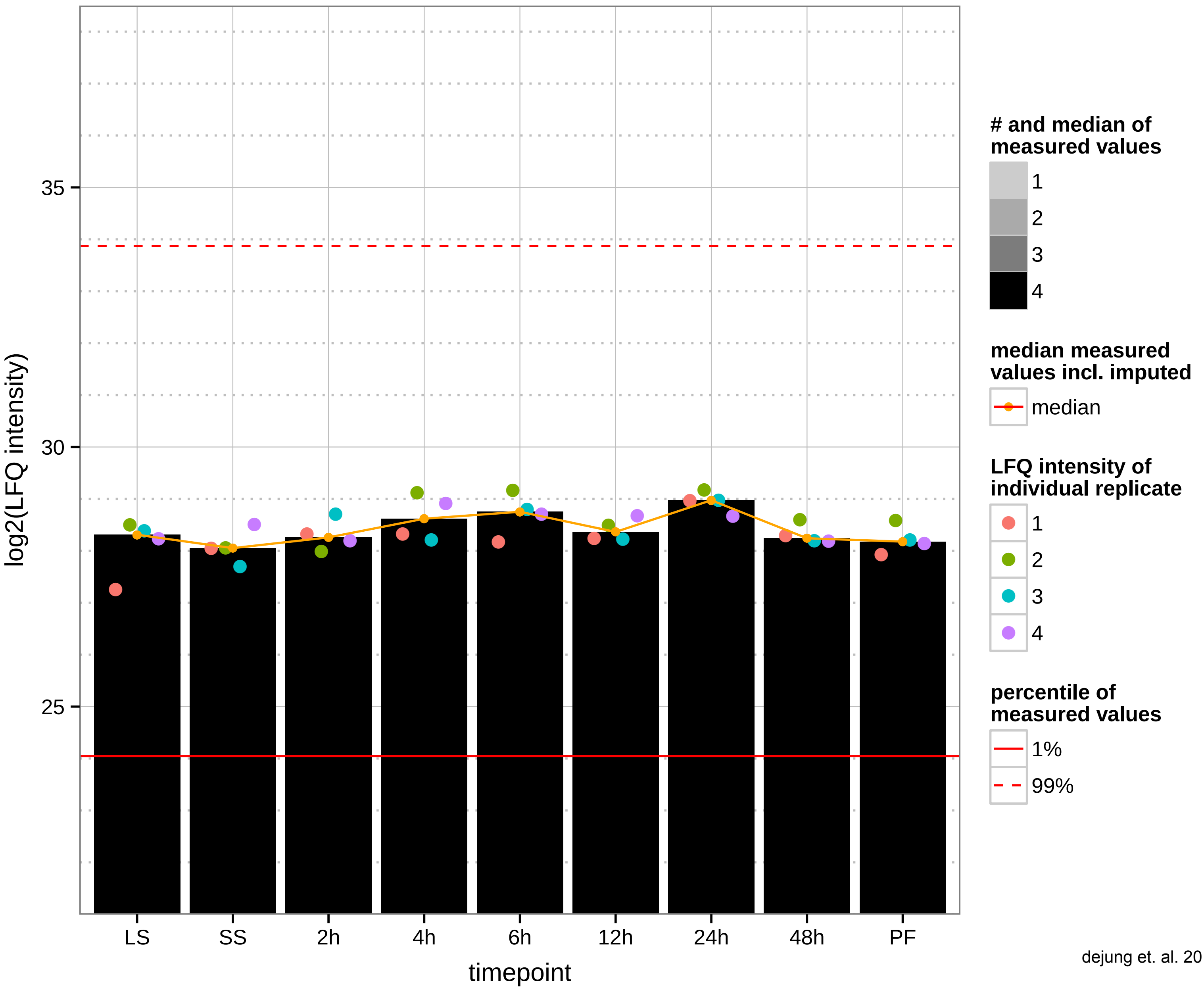

dejung et. al. 2015

flagellar calcium–binding protein, 24 kDa calflagin, 24 kDa calcimedin (Tb–24), 44 kDa calflagin, 44 kDa calcimedin (Tb–44),
Tb927.8.5460;Tb11.v5.0797;Tb927.8.5465;Tb11.v5.0798
AGOF: null, calcium ion binding
AGOC: null, bacterial–type flagellum, cilium, motile cilium
AGOP: null, ciliary or flagellar motility, calcium ion transport, ciliary or flagellar motility
PGOF: calcium ion binding
PGOC: null
PGOP: null

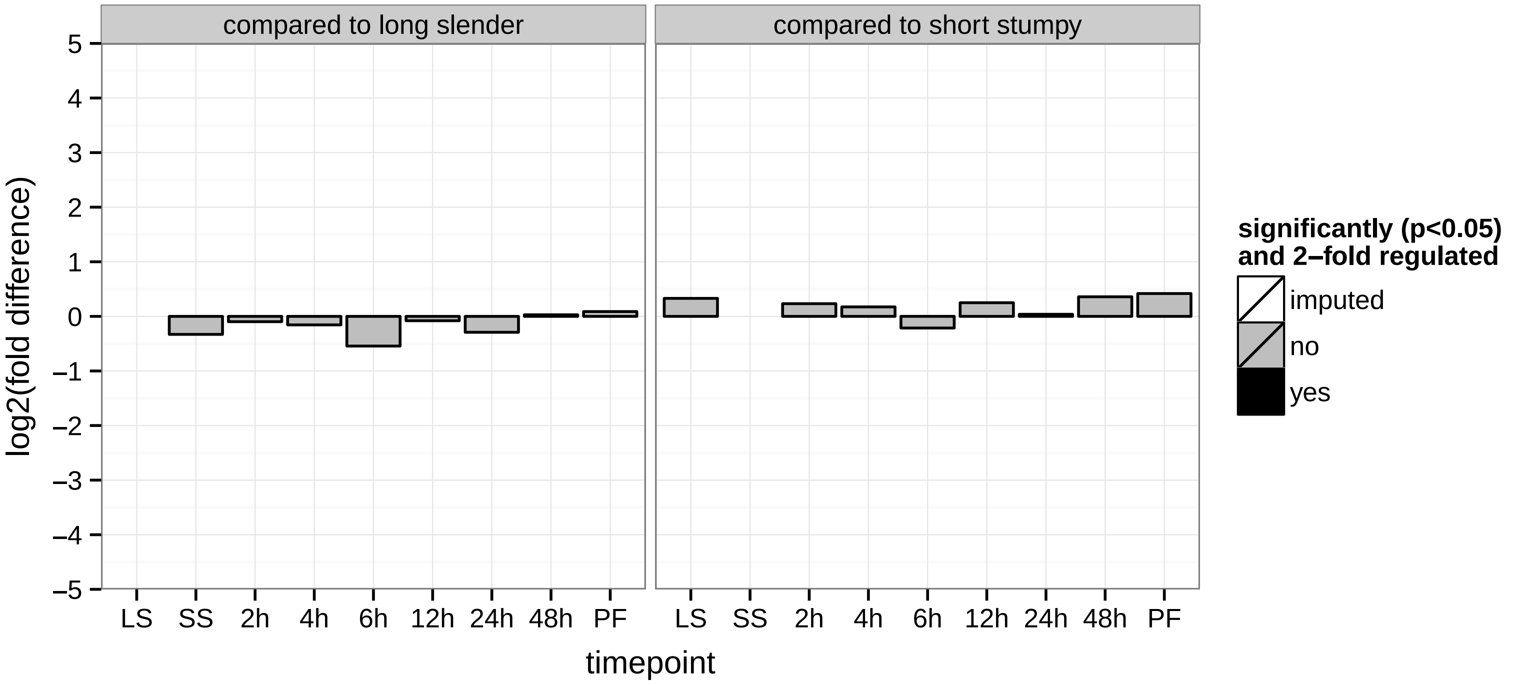

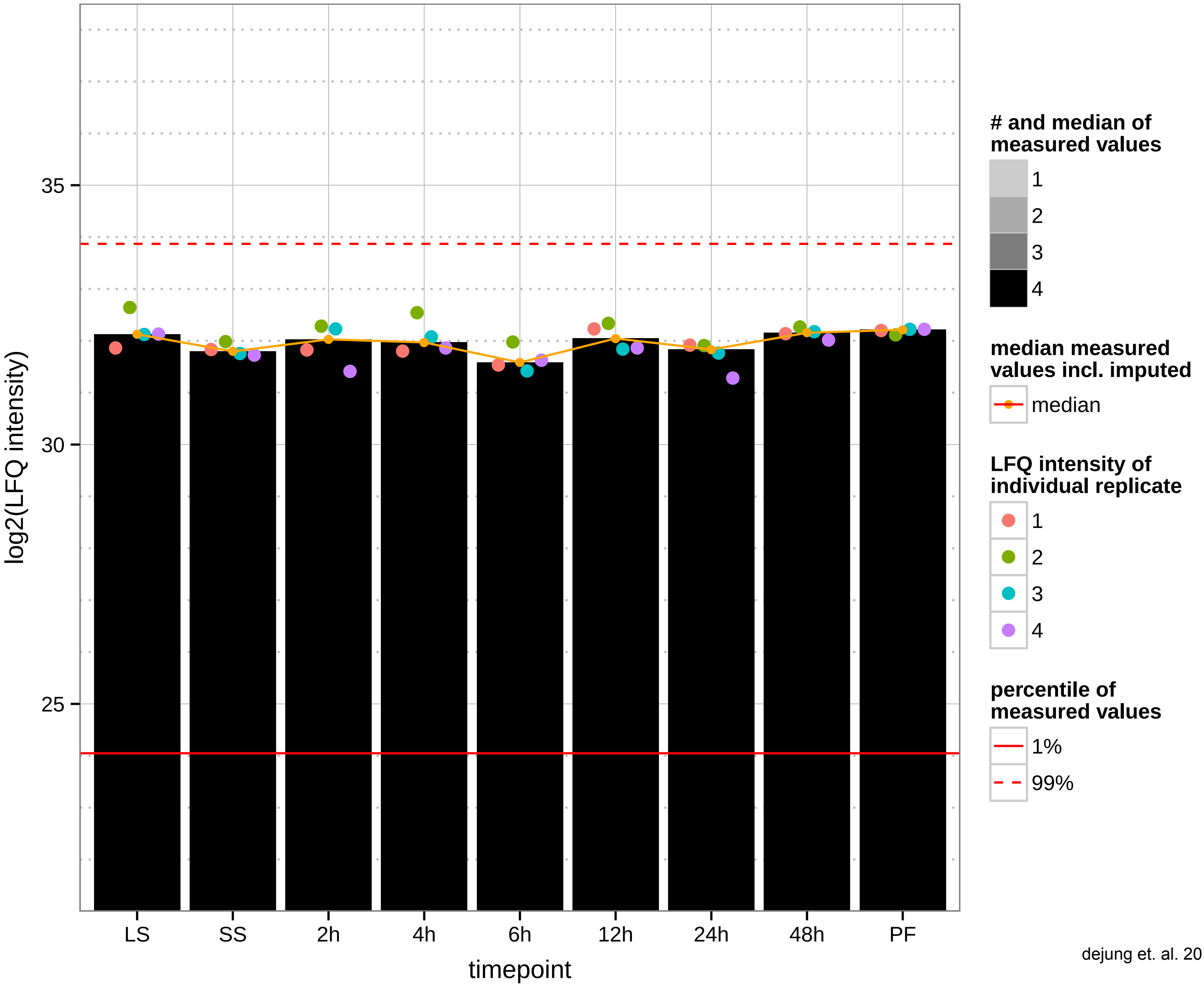

dejung et. al. 2015

apurinic/apyrimidinic endonuclease, putative
Tb927.8.5510
AGOF: nuclease activity
AGOC: null
AGOP: DNA repair
PGOF: nuclease activity
PGOC: null
PGOP: DNA repair

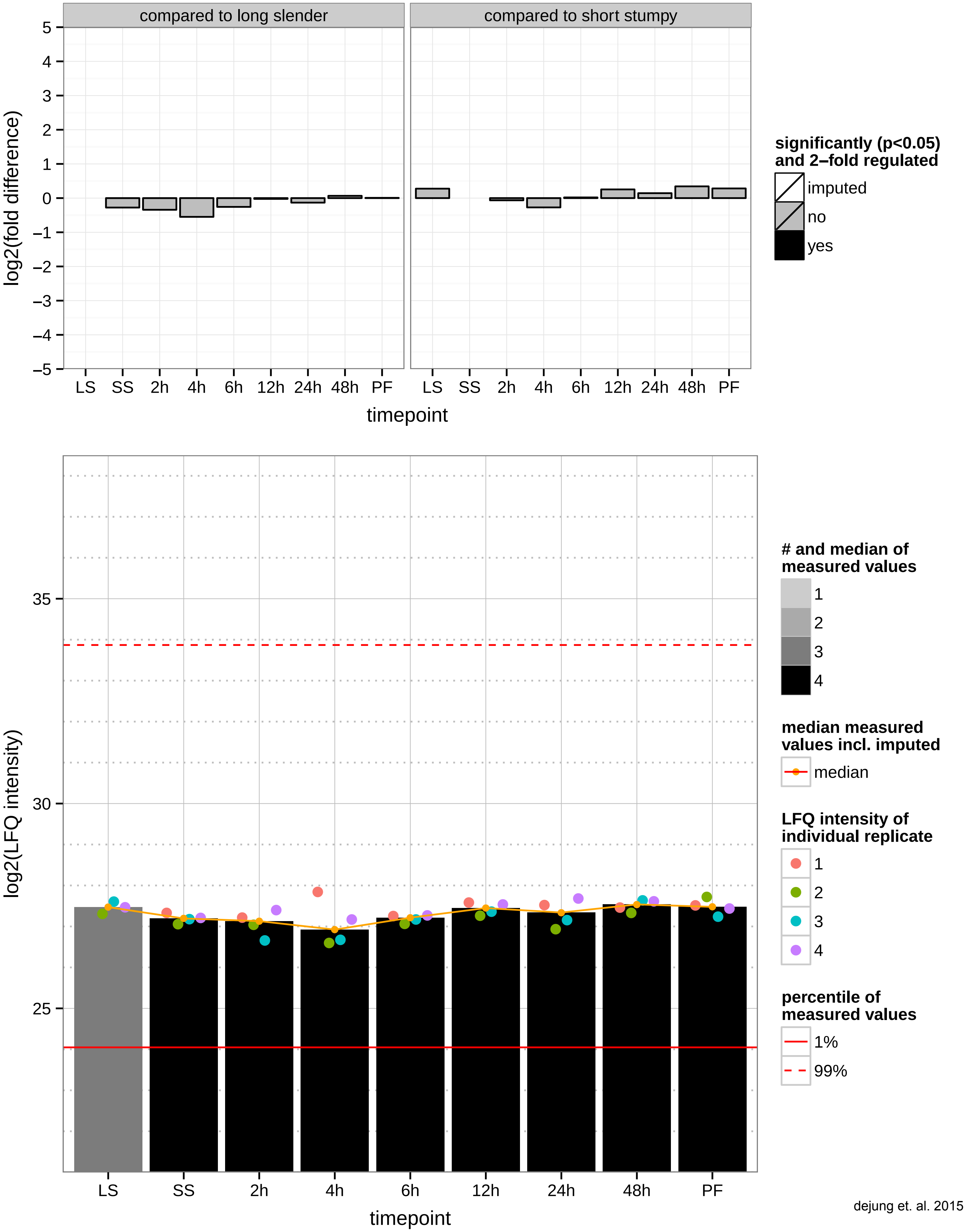

dejung et. al. 2015

hypothetical protein, conserved
Tb927.8.5520
AGOF: null
AGOC: null
AGOP: null
PGOF: null
PGOC: null
PGOP: null

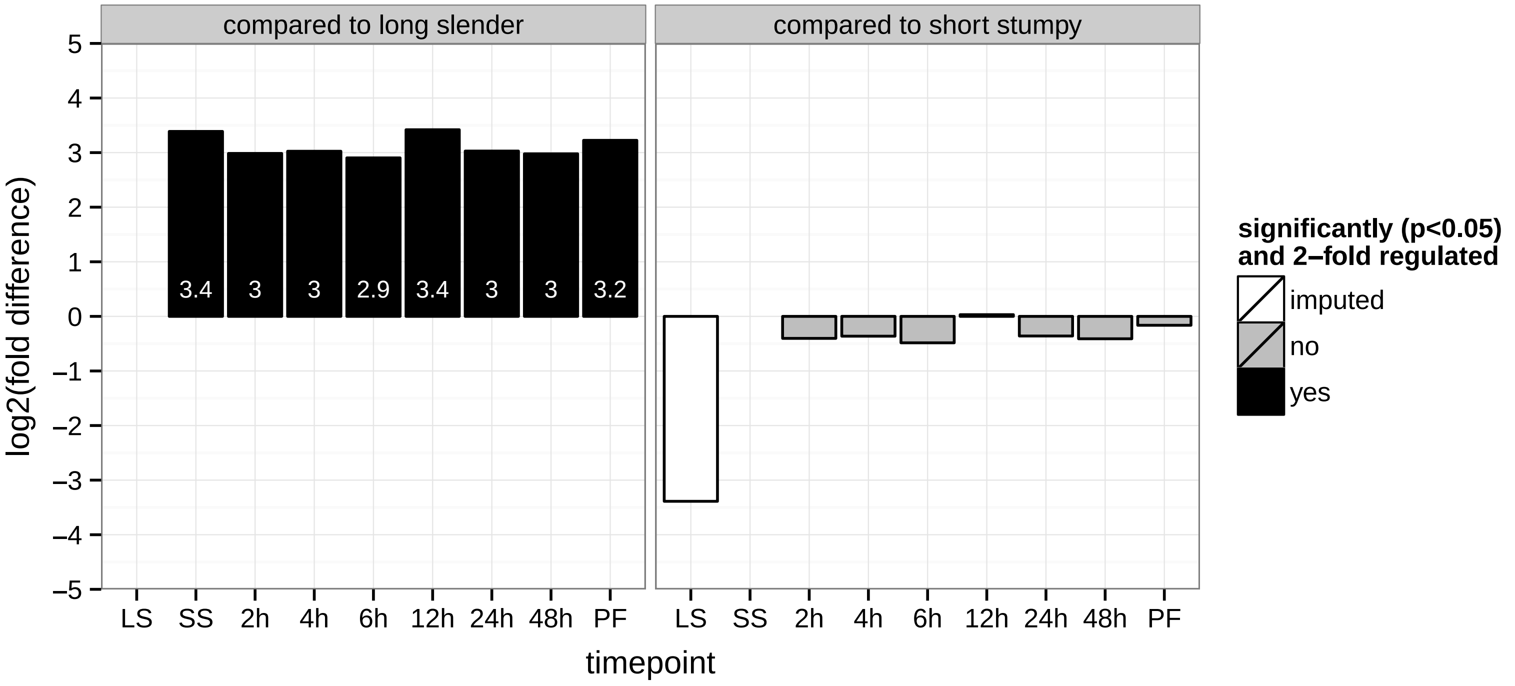

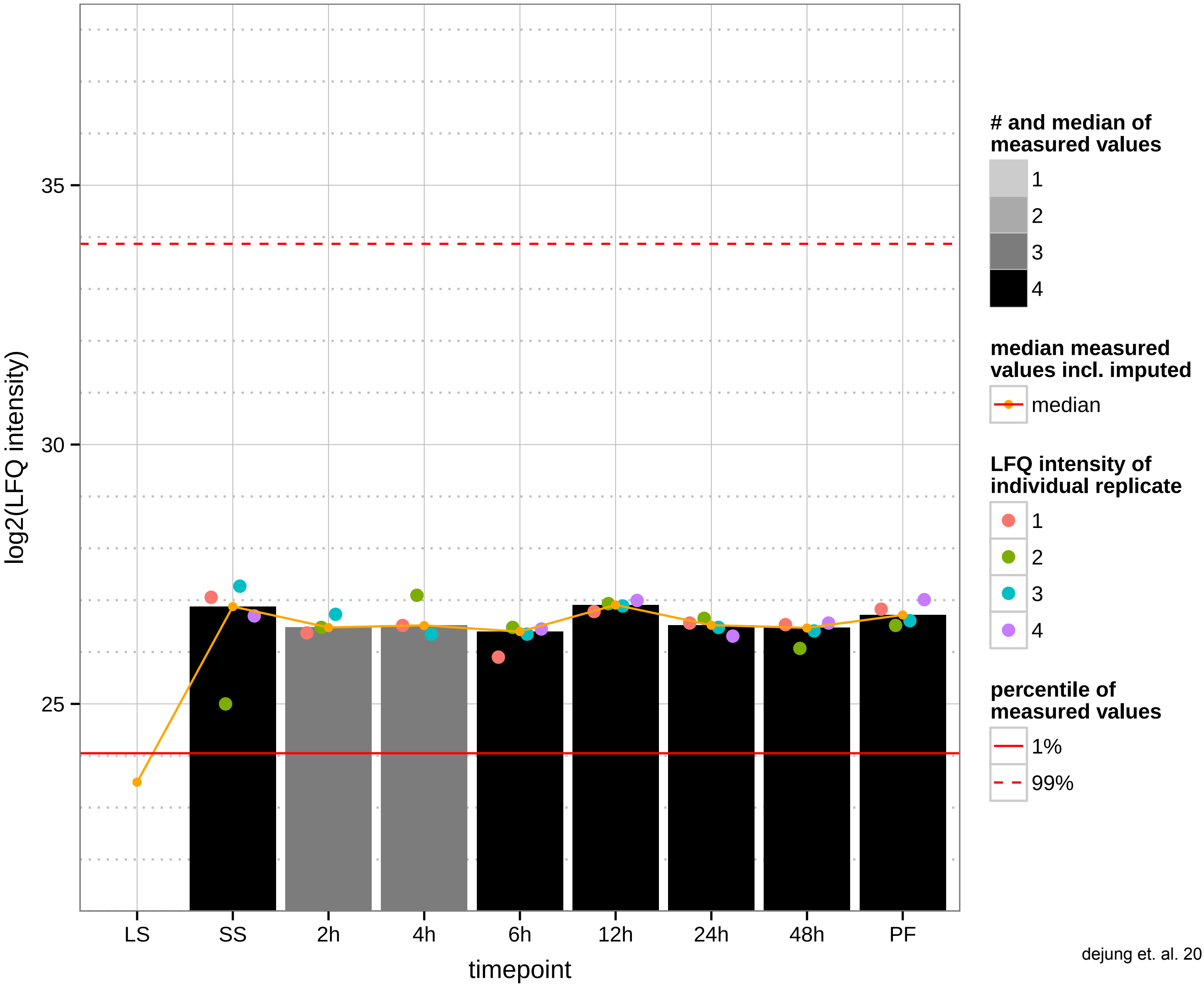

dejung et. al. 2015

hypothetical protein, conserved
Tb927.8.5580
AGOF: null
AGOC: integral to membrane
AGOP: null
PGOF: null
PGOC: integral to membrane
PGOP: null

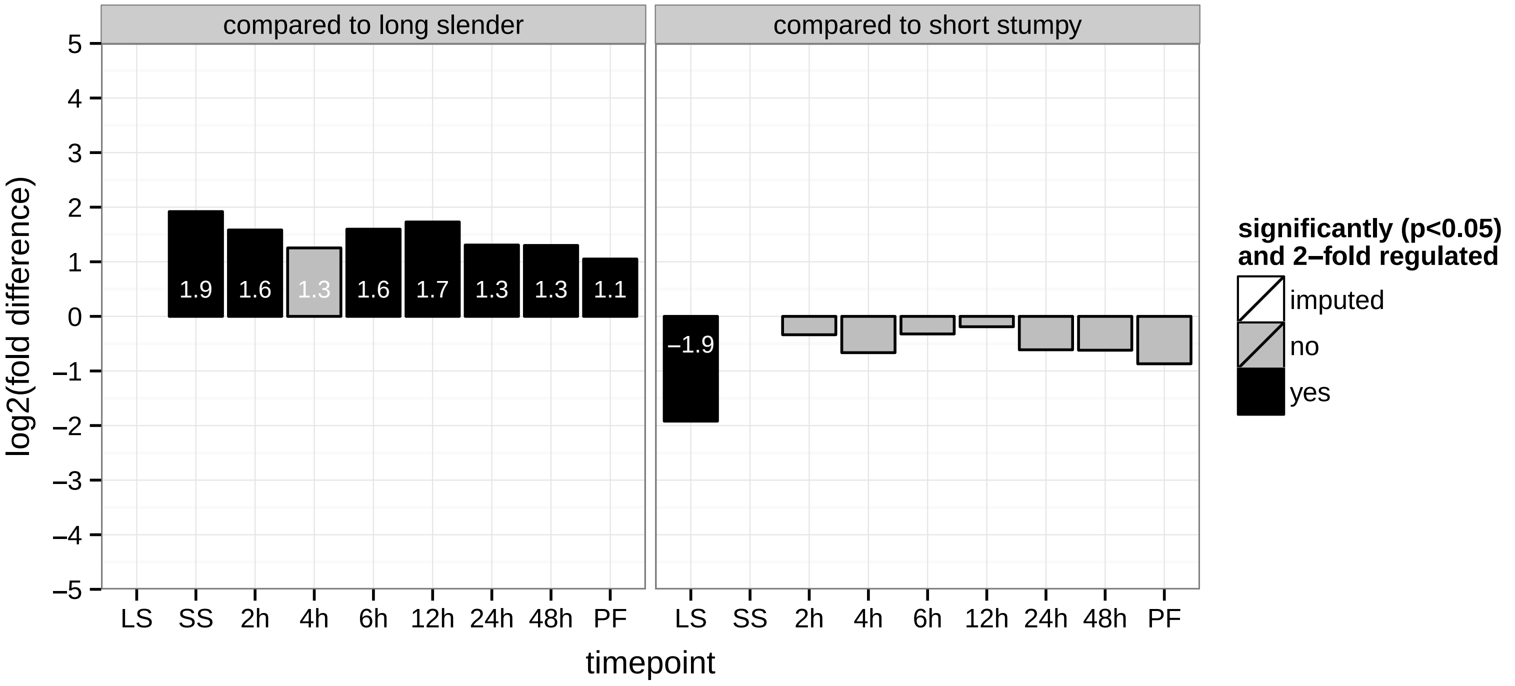

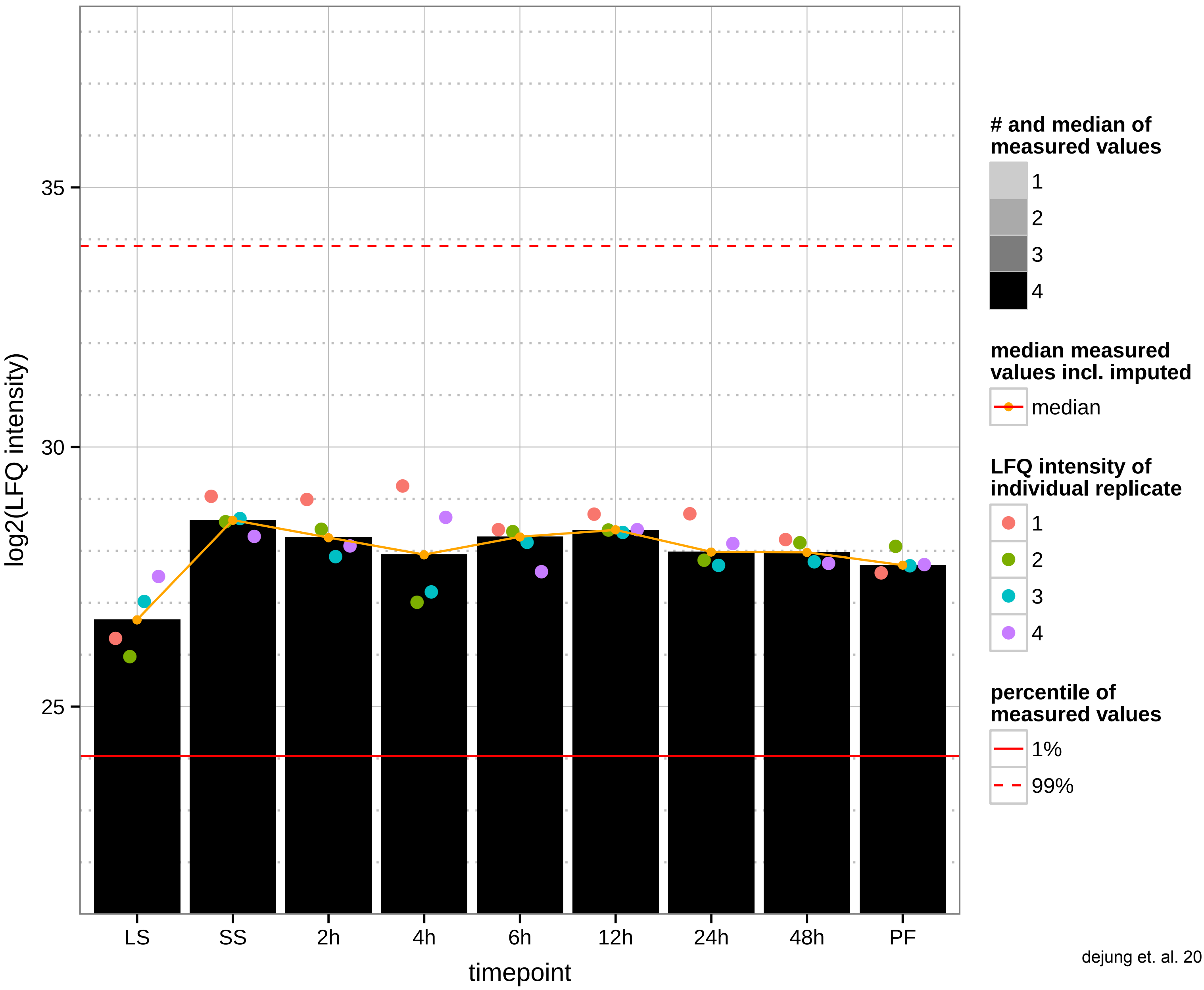

dejung et. al. 2015

transaldolase, putative
Tb927.8.5600
AGOF: sedoheptulose−7−phosphate:D−glyceraldehyde−3−phosphate glyceronetransferase activity
AGOC: cytoplasm
AGOP: pentose−phosphate shunt
PGOF: sedoheptulose−7−phosphate:D−glyceraldehyde−3−phosphate glyceronetransferase activity
PGOC: cytoplasm
PGOP: carbohydrate metabolic process, pentose−phosphate shunt

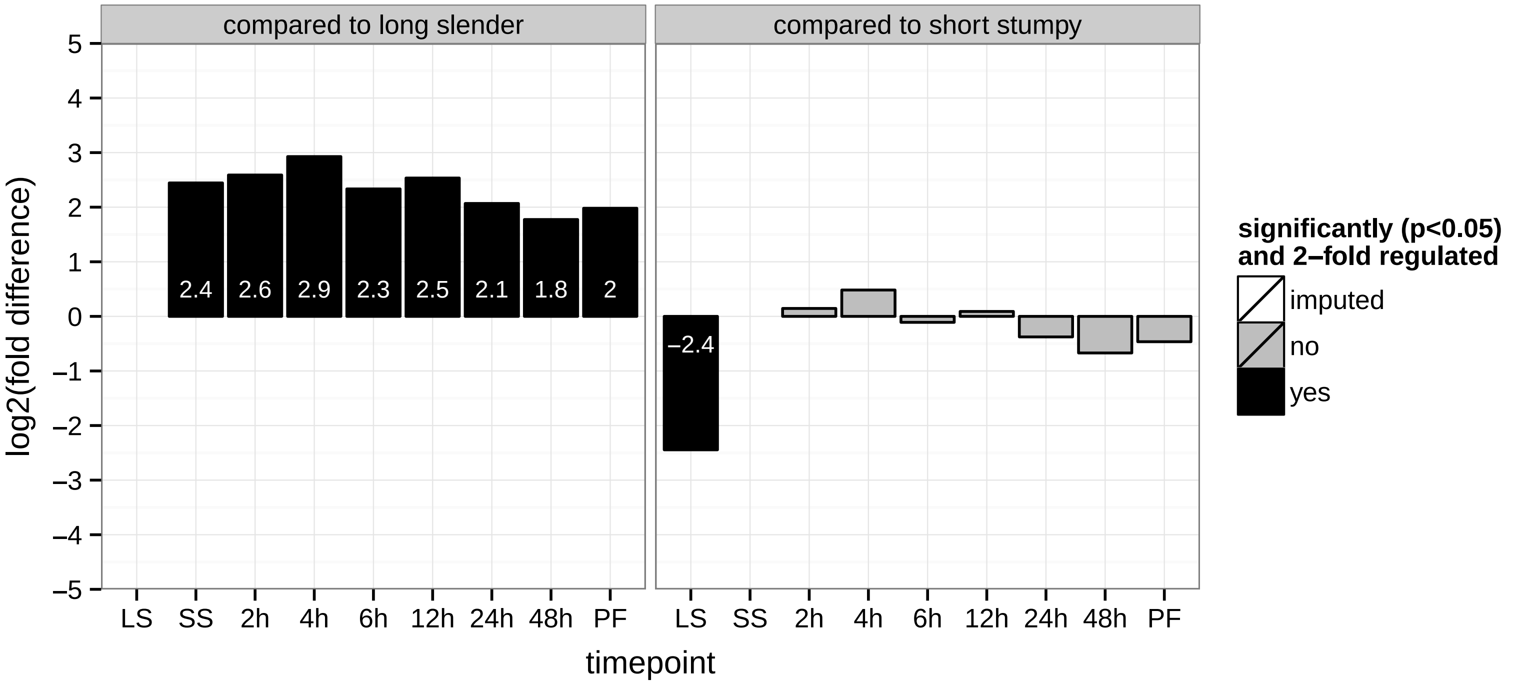

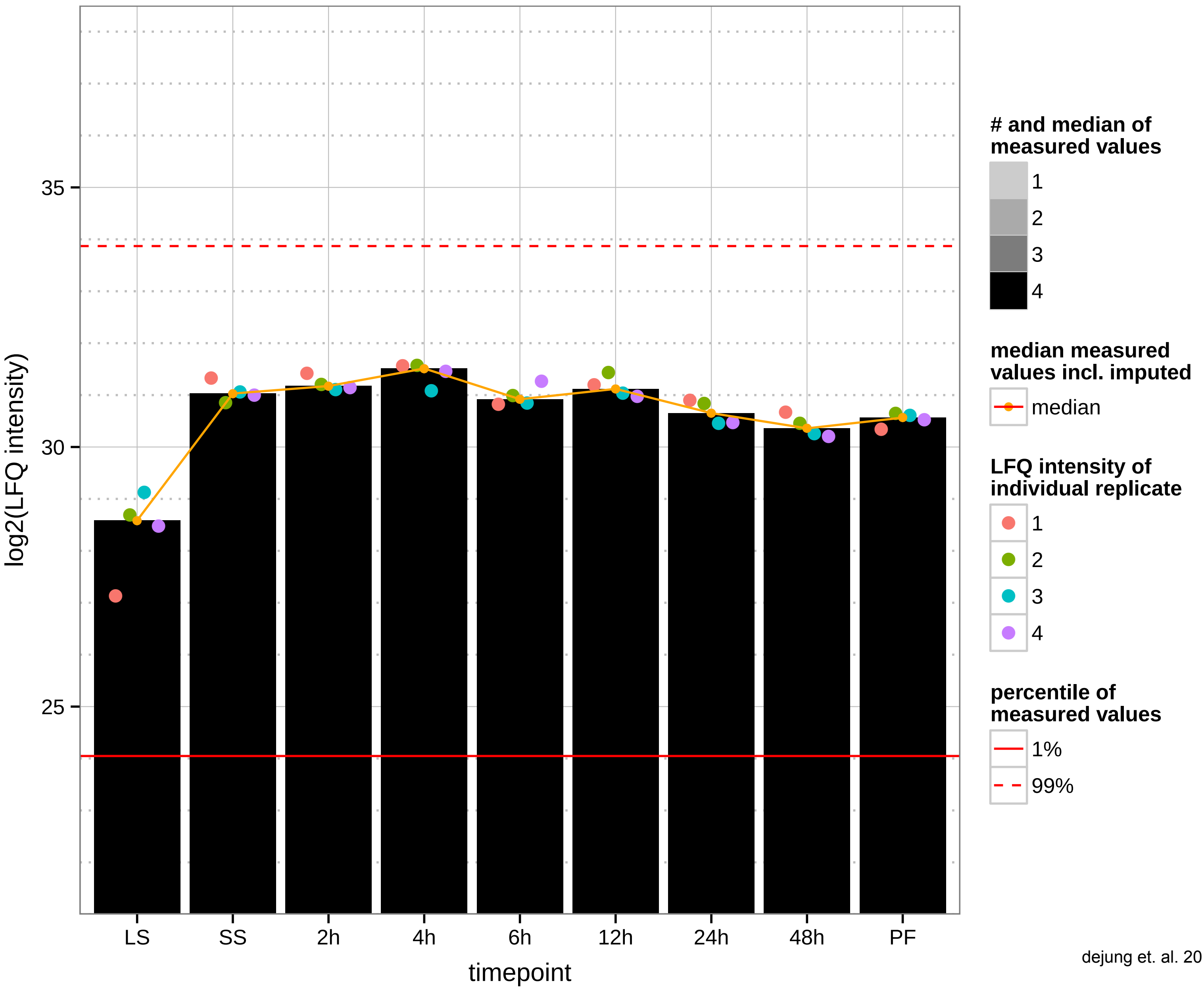

dejung et. al. 2015

dihydroorotase, putative
Tb927.8.5630
AGOF: hydrolase activity, acting on carbon–nitrogen (but not peptide) bonds, in cyclic amides
AGOC: null
AGOP: pyrimidine nucleobase biosynthetic process
PGOF: hydrolase activity, acting on carbon–nitrogen (but not peptide) bonds
PGOC: null
PGOP: null

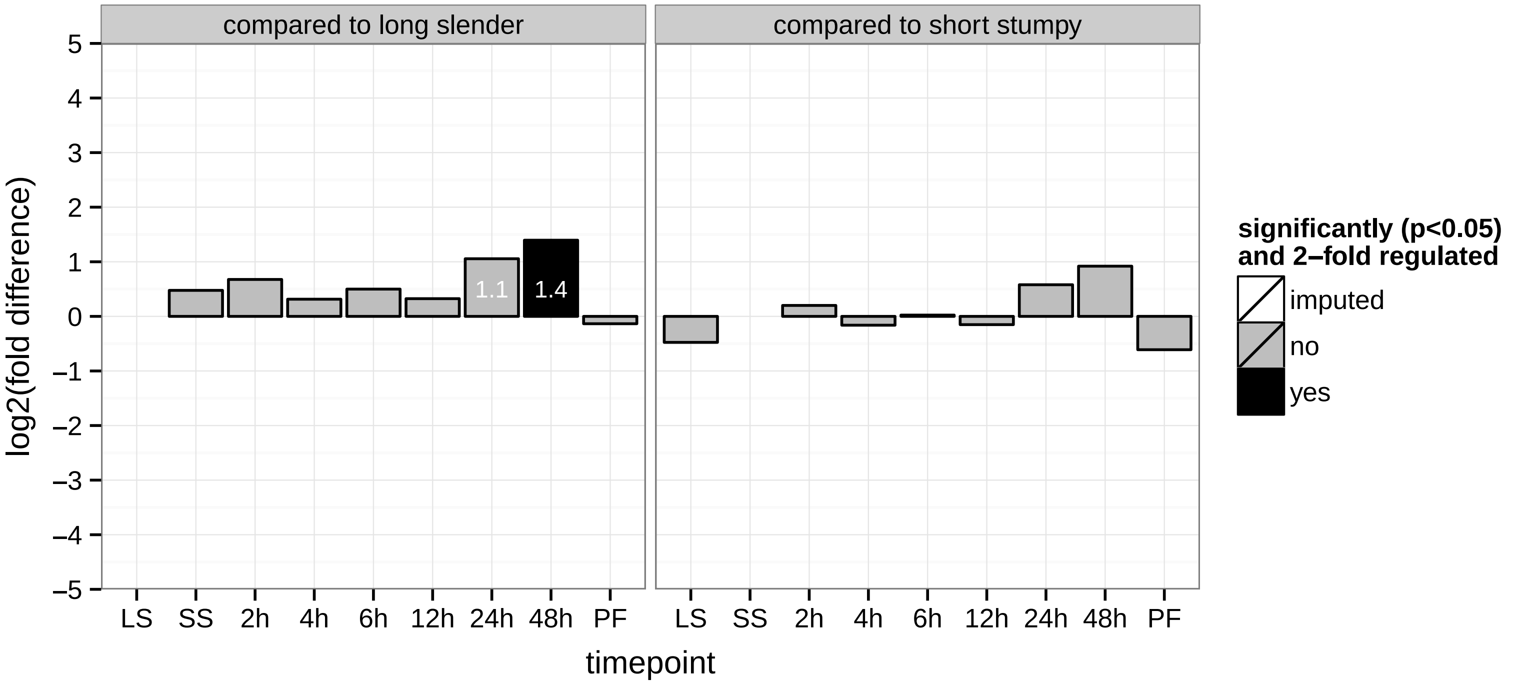

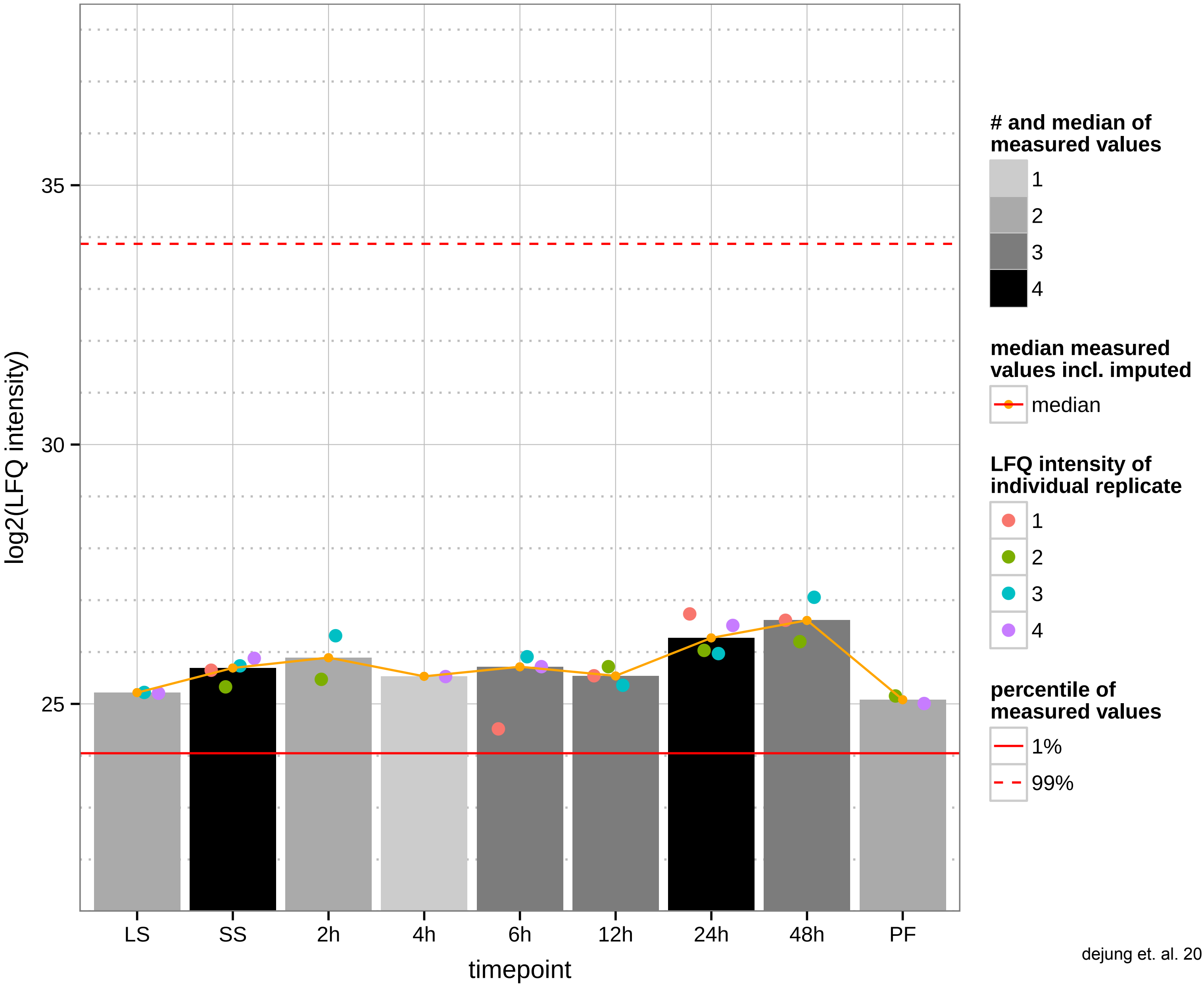

dejung et. al. 2015

proteasome regulatory non–ATP–ase subunit 10
Tb927.8.570
AGOF: null
AGOC: proteasome complex
AGOP: cell growth, ubiquitin–dependent protein catabolic process
PGOF: protein binding
PGOC: null
PGOP: null

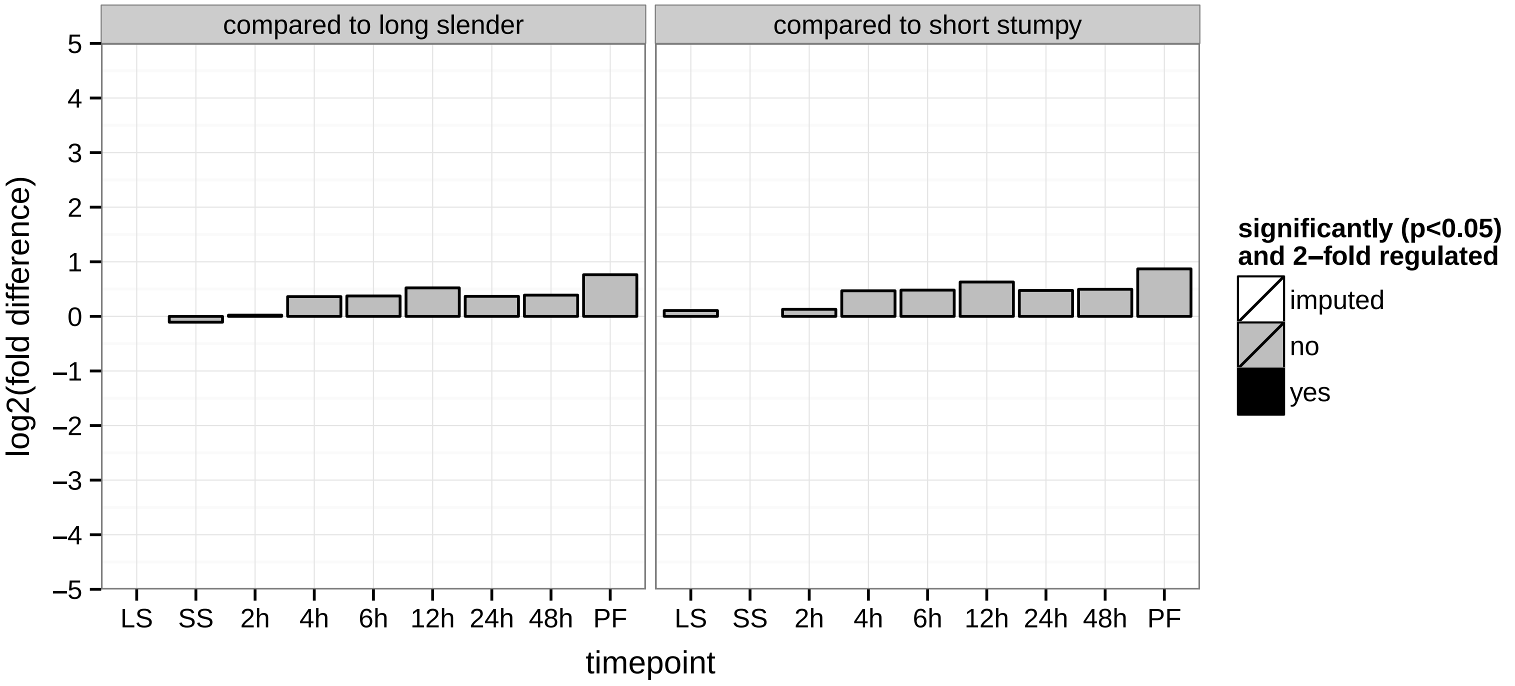

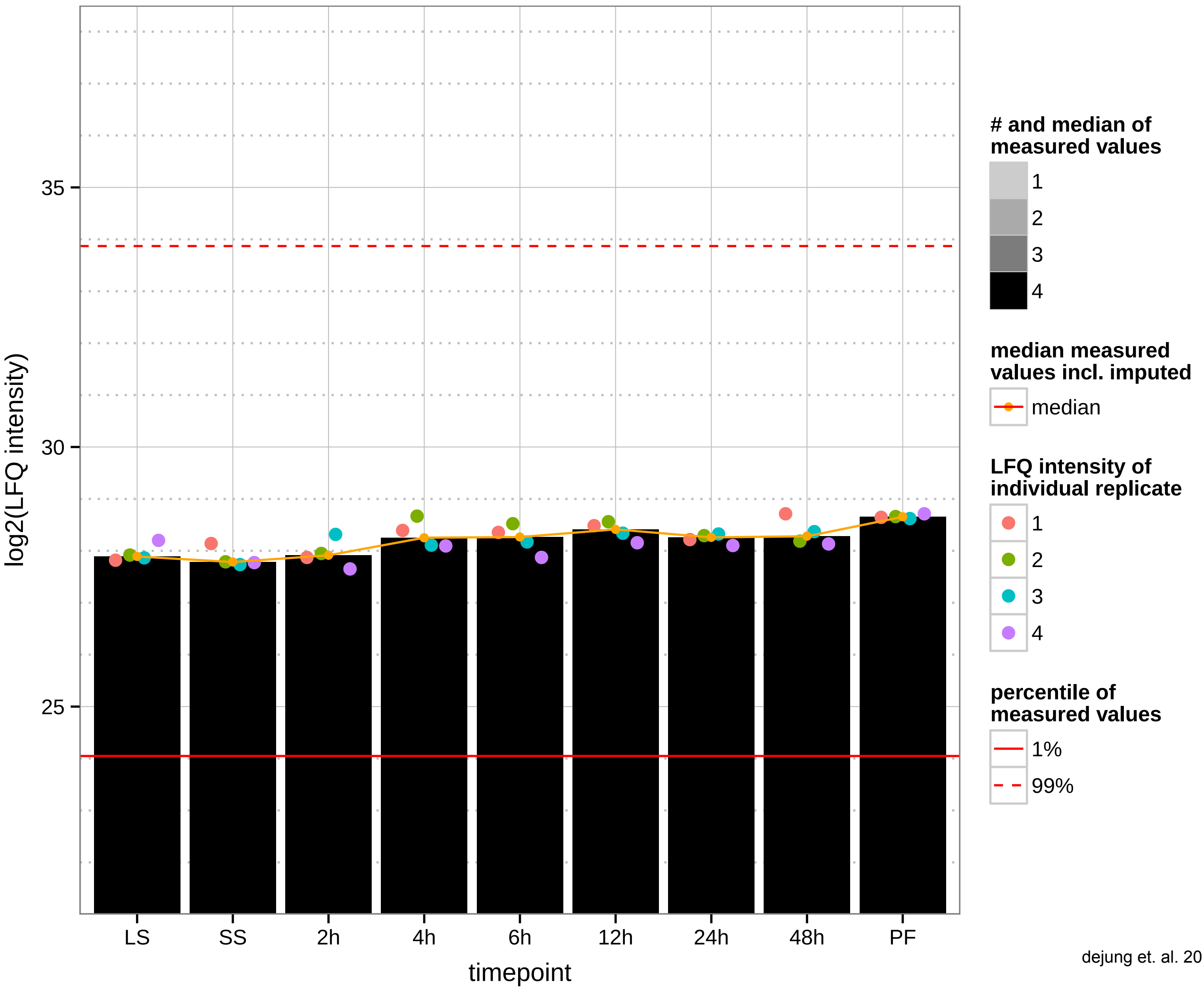

dejung et. al. 2015

protein kinase, putative
Tb927.8.5730
AGOF: ATP binding, protein kinase activity, protein serine/threonine kinase activity, protein tyrosine kinase activity
AGOC: null
AGOP: growth, protein phosphorylation
PGOF: ATP binding, protein kinase activity, transferase activity, transferring phosphorus–containing groups
PGOC: null
PGOP: protein phosphorylation

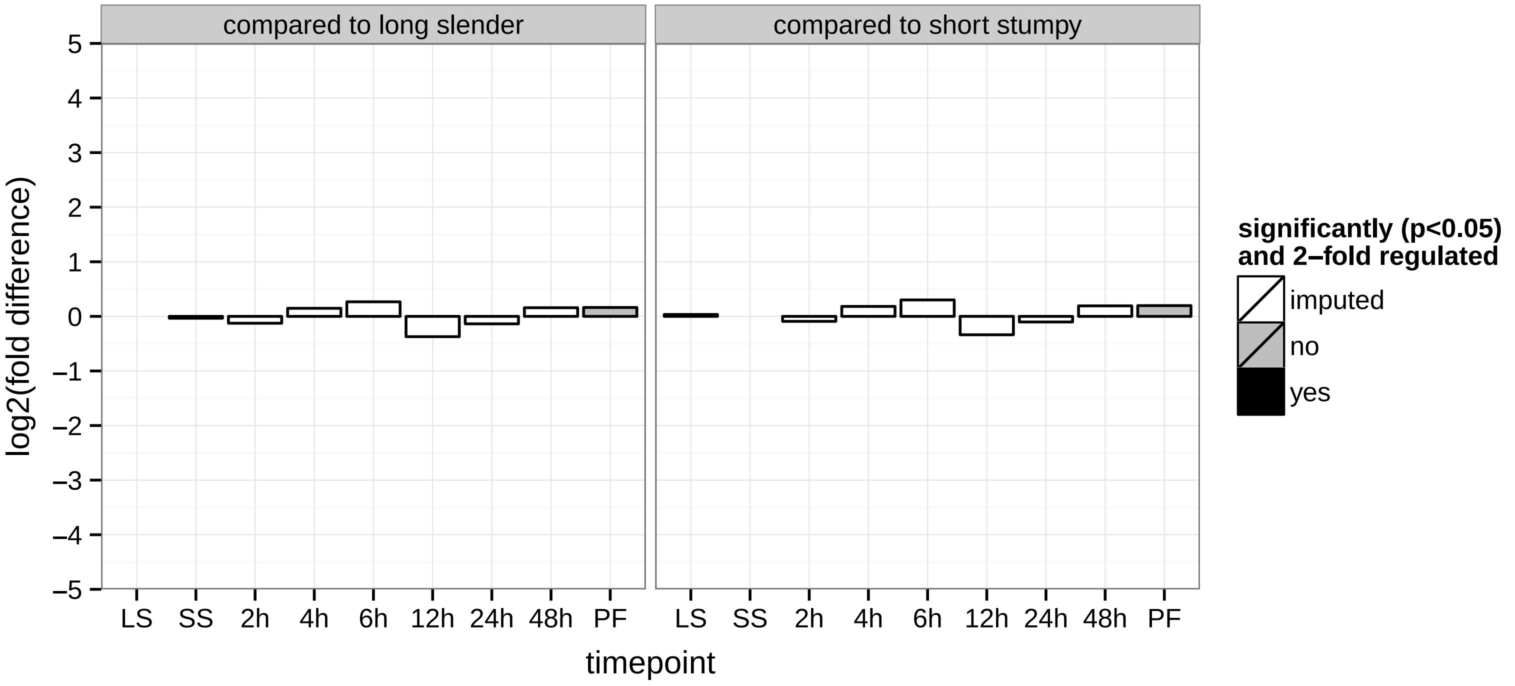

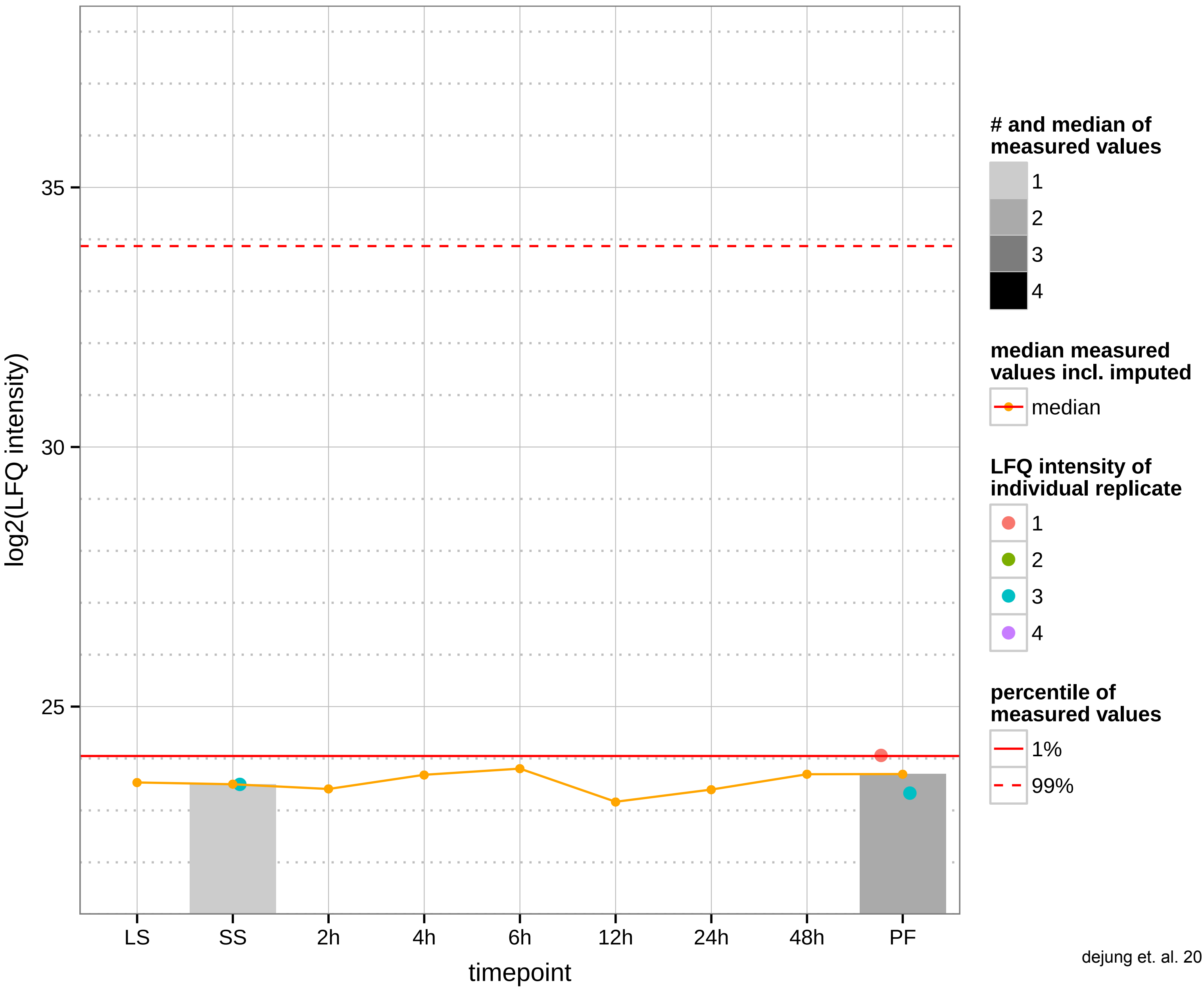

dejung et. al. 2015

hypothetical protein, conserved
Tb927.8.5820
AGOF: null
AGOC: null
AGOP: null
PGOF: null
PGOC: null
PGOP: null

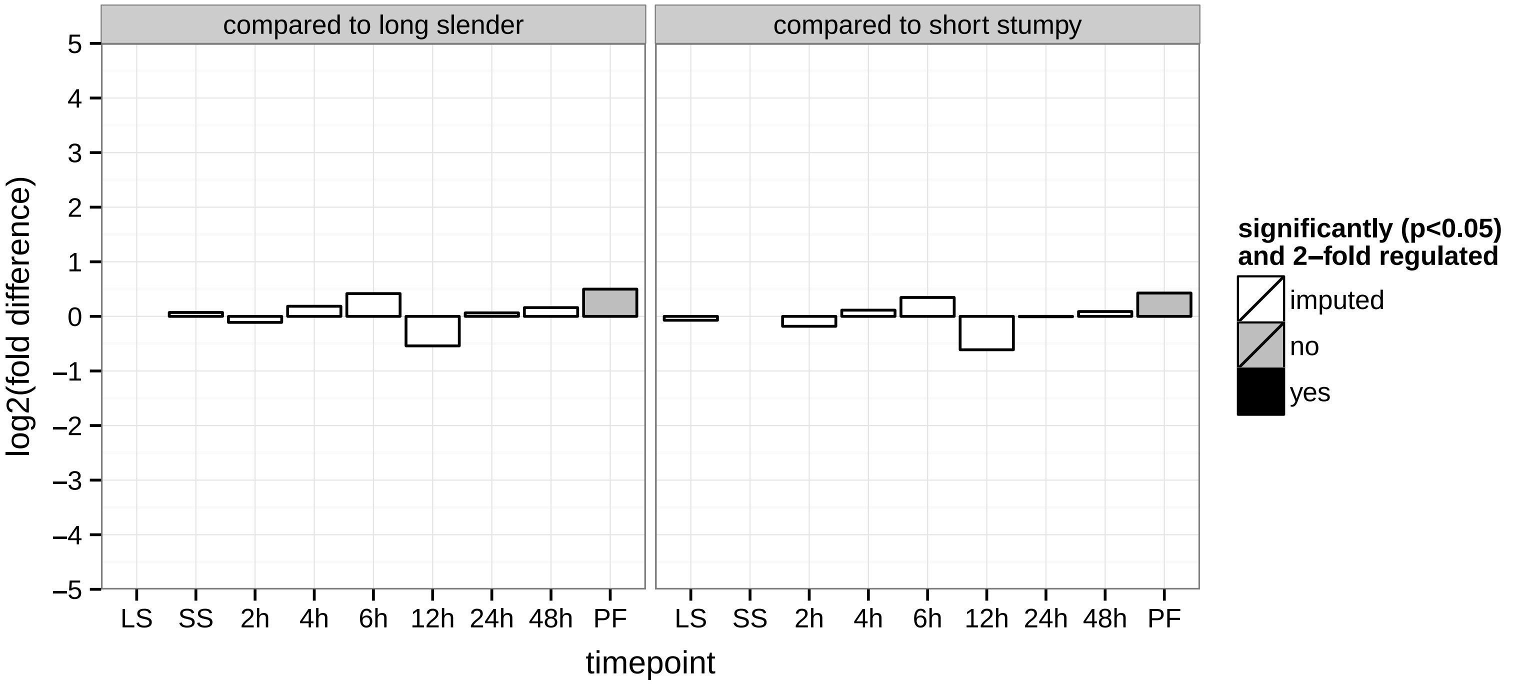

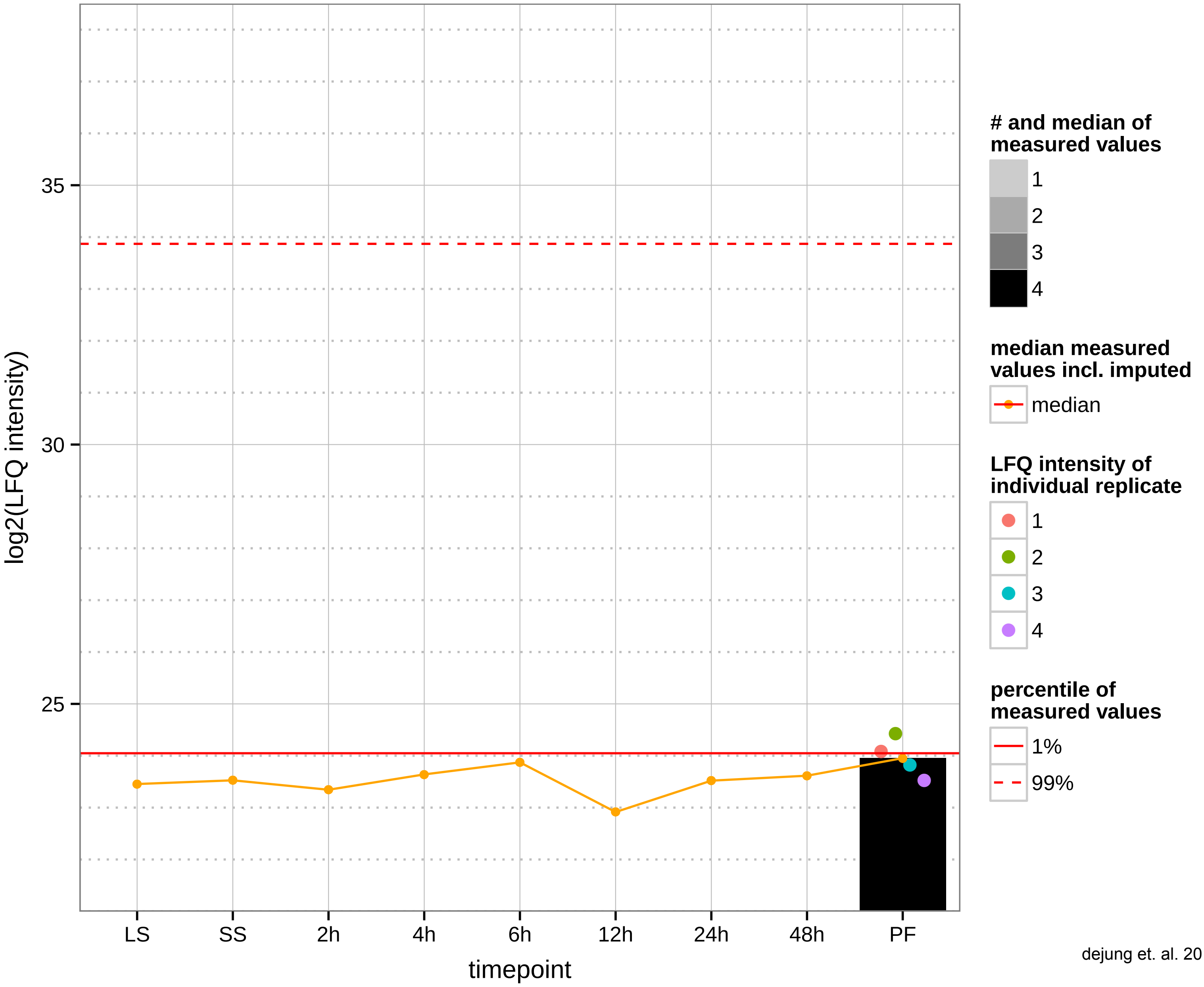

dejung et. al. 2015

hypothetical protein, conserved
Tb927.8.5830
AGOF: null
AGOC: null
AGOP: null
PGOF: null
PGOC: null
PGOP: null

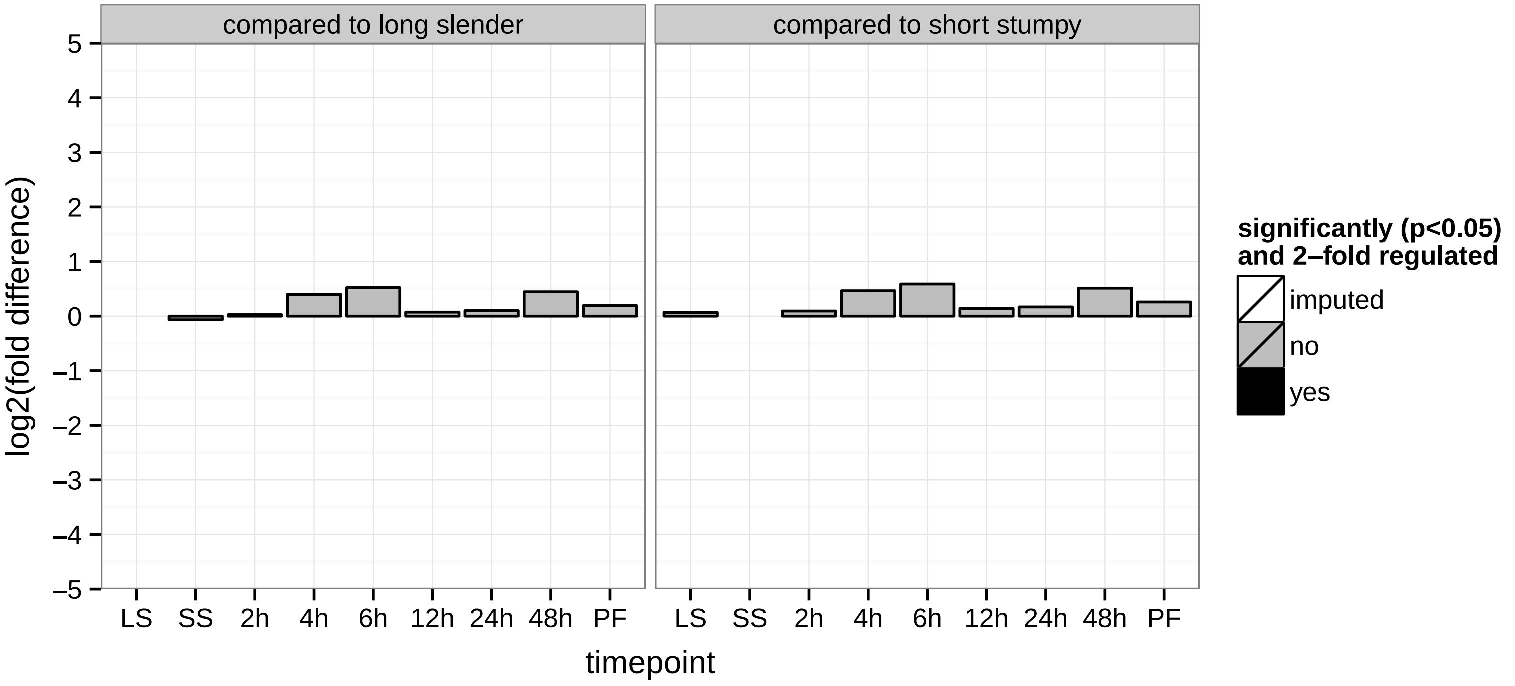

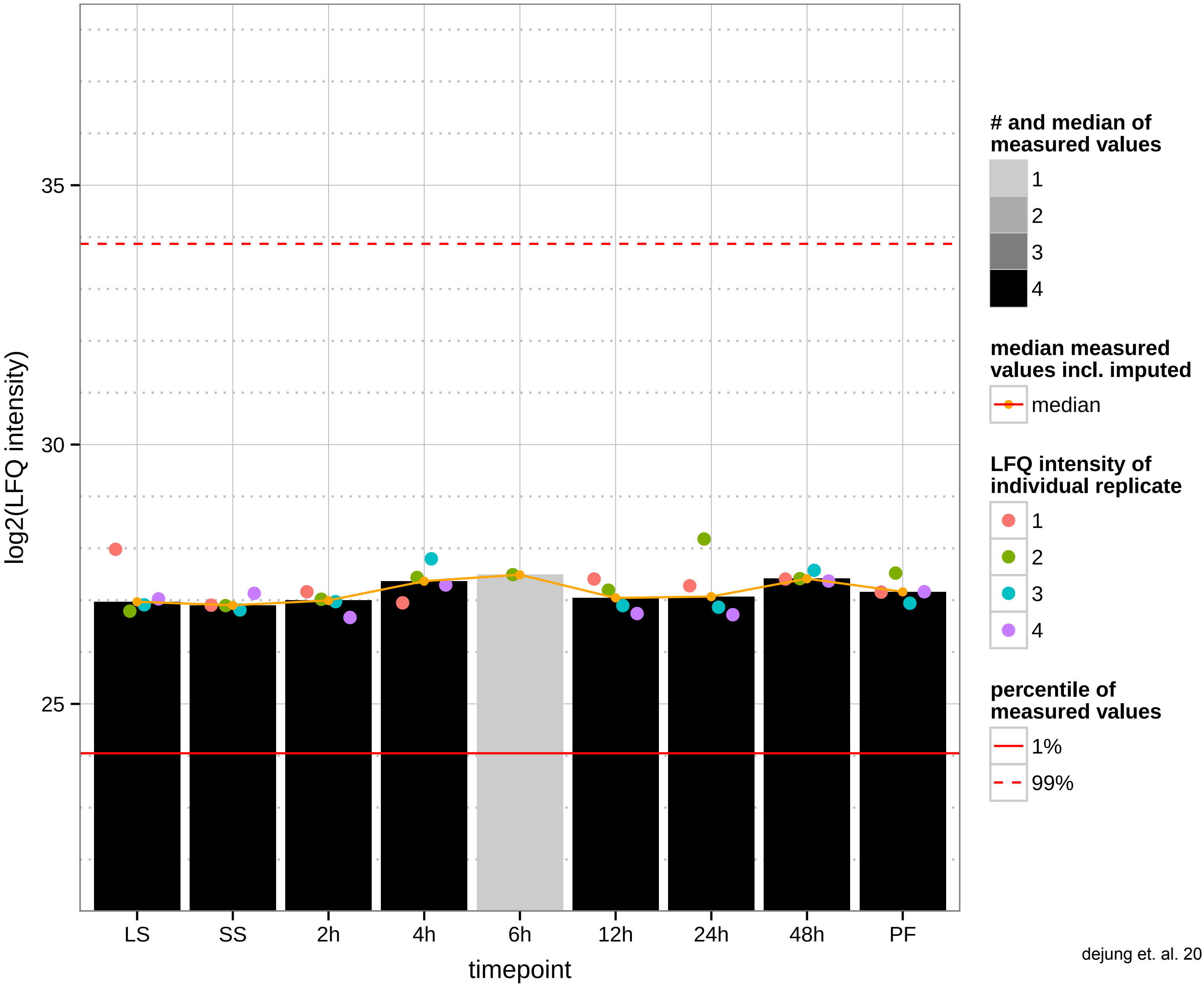

dejung et. al. 2015

hypothetical protein, conserved
Tb927.8.5890
AGOF: null
AGOC: nucleus
AGOP: chromatin assembly or disassembly
PGOF: null
PGOC: nucleus
PGOP: chromatin assembly or disassembly

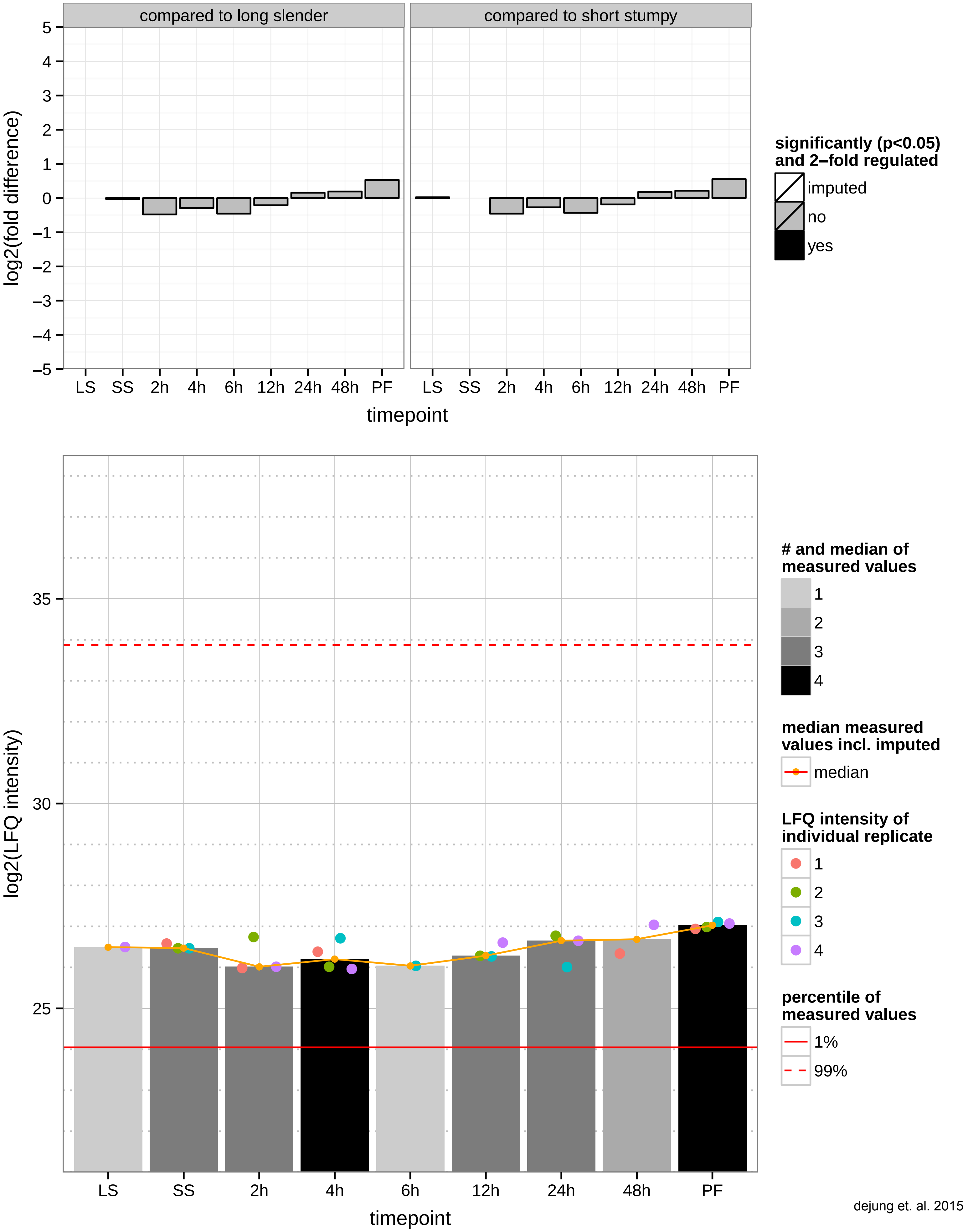

dejung et. al. 2015

carnitine O–palmitoyltransferase, putative
Tb927.8.590
AGOF: carnitine O–acetyltransferase activity
AGOC: mitochondrion
AGOP: generation of precursor metabolites and energy
PGOF: transferase activity, transferring acyl groups
PGOC: null
PGOP: null

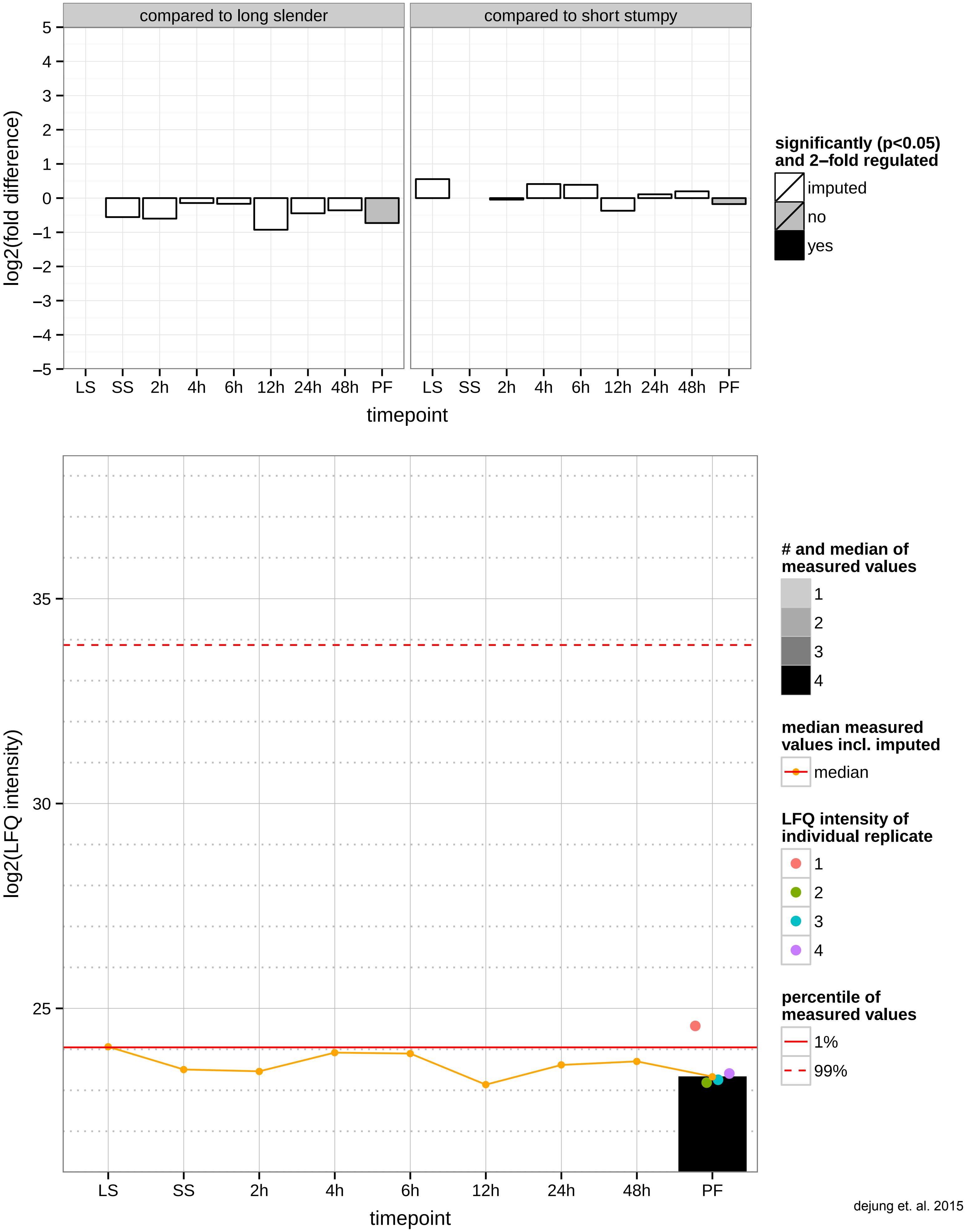

dejung et. al. 2015

hypothetical protein, conserved
Tb927.8.5940
AGOF: null
AGOC: null
AGOP: null
PGOF: null
PGOC: null
PGOP: null

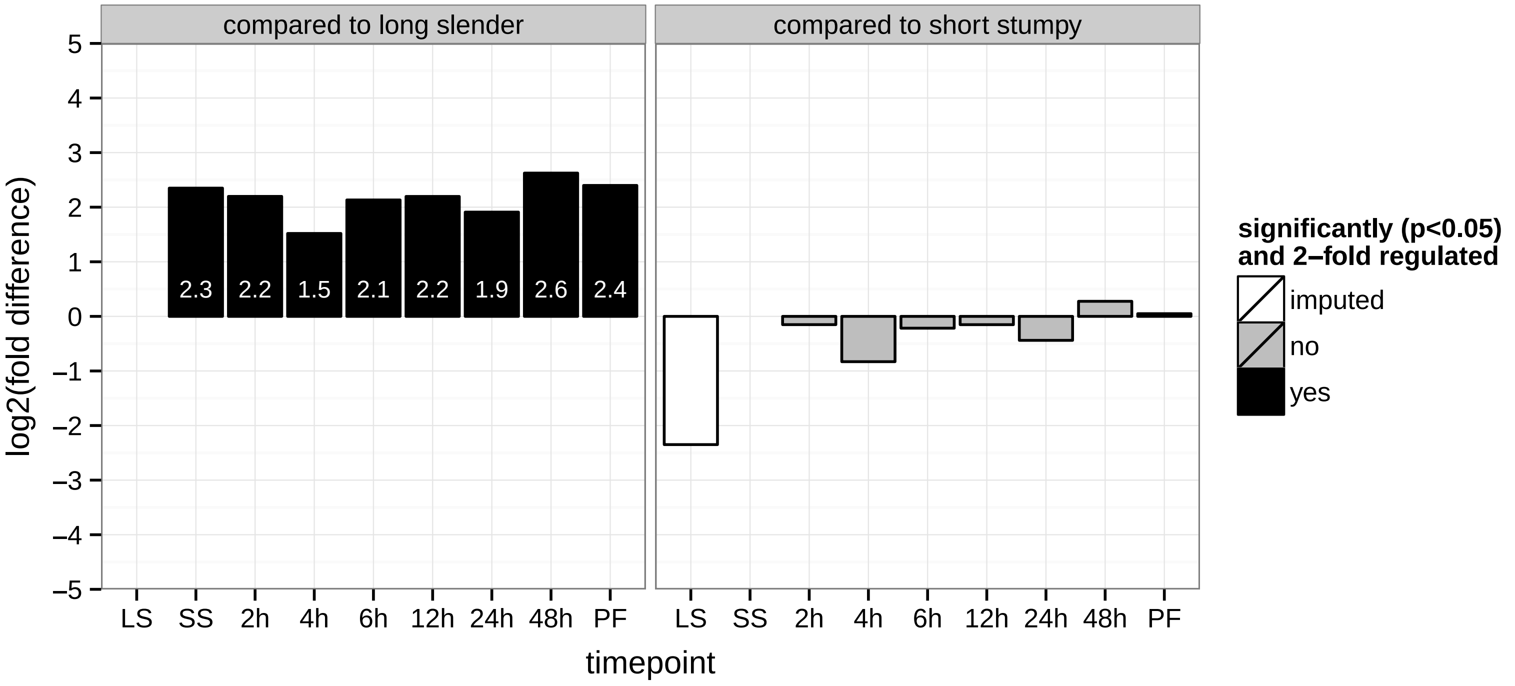

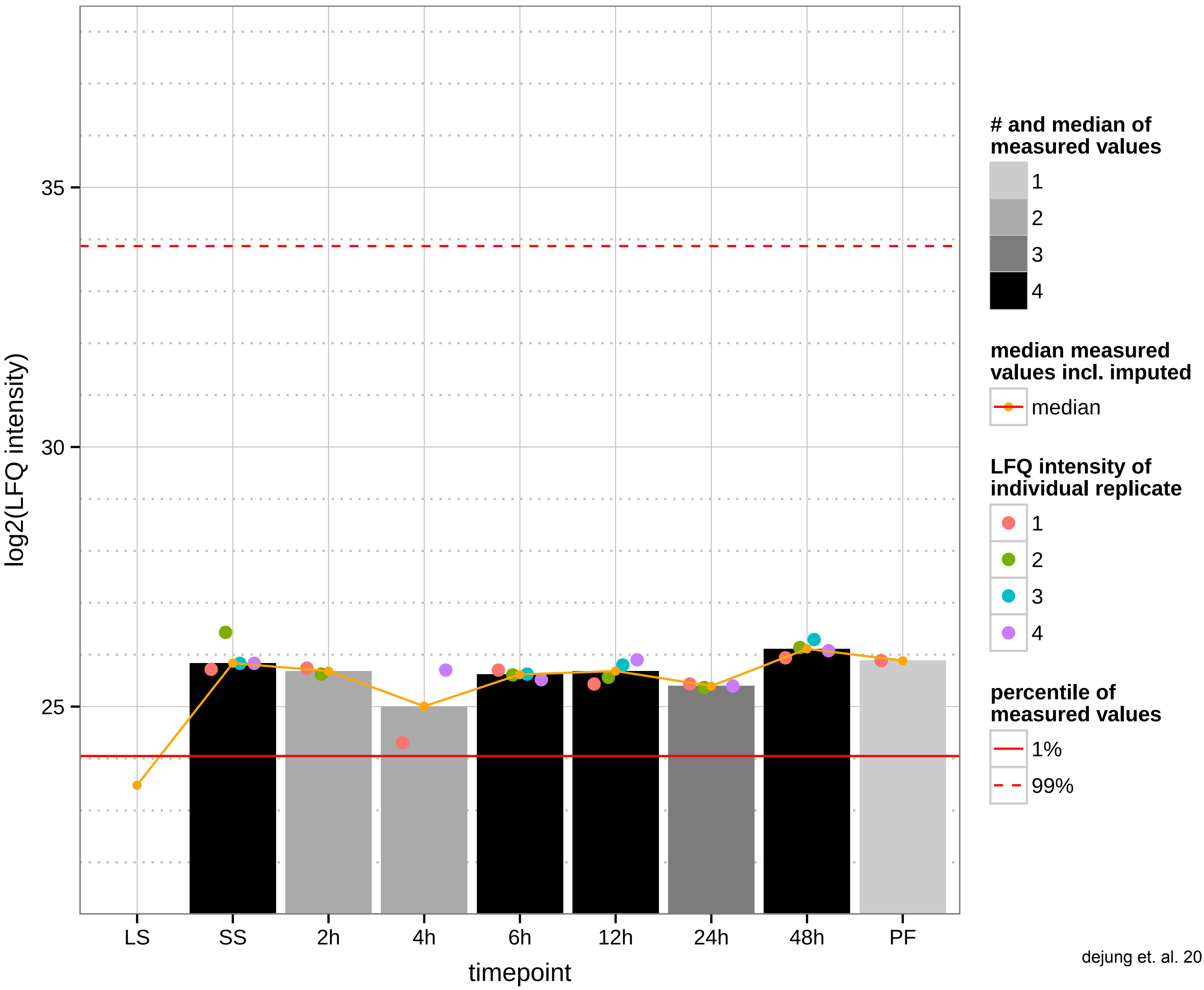

dejung et. al. 2015

60S ribosomal protein L12, putative
Tb927.8.6030;Tb927.9.14000
AGOF: structural constituent of ribosome
AGOC: intracellular, ribosome
AGOP: translation
PGOF: structural constituent of ribosome
PGOC: ribosome
PGOP: translation

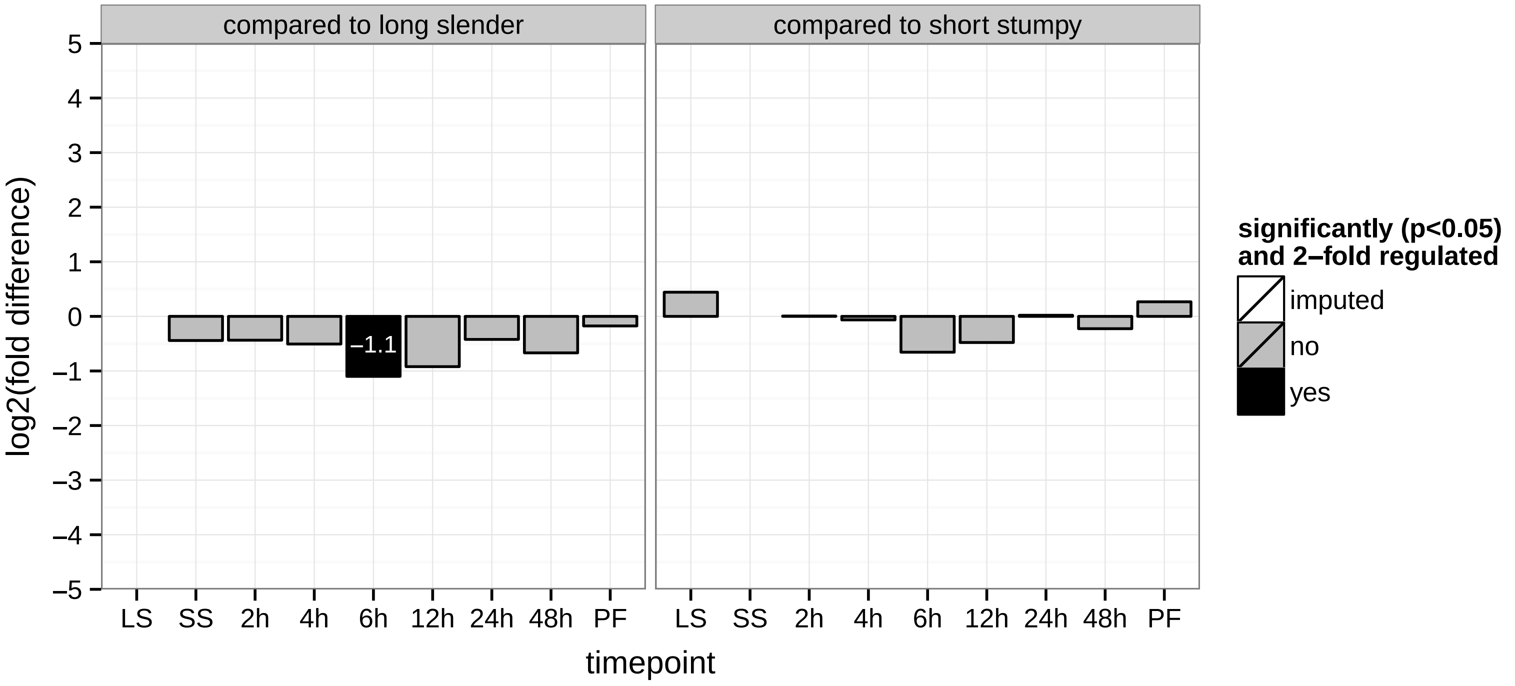

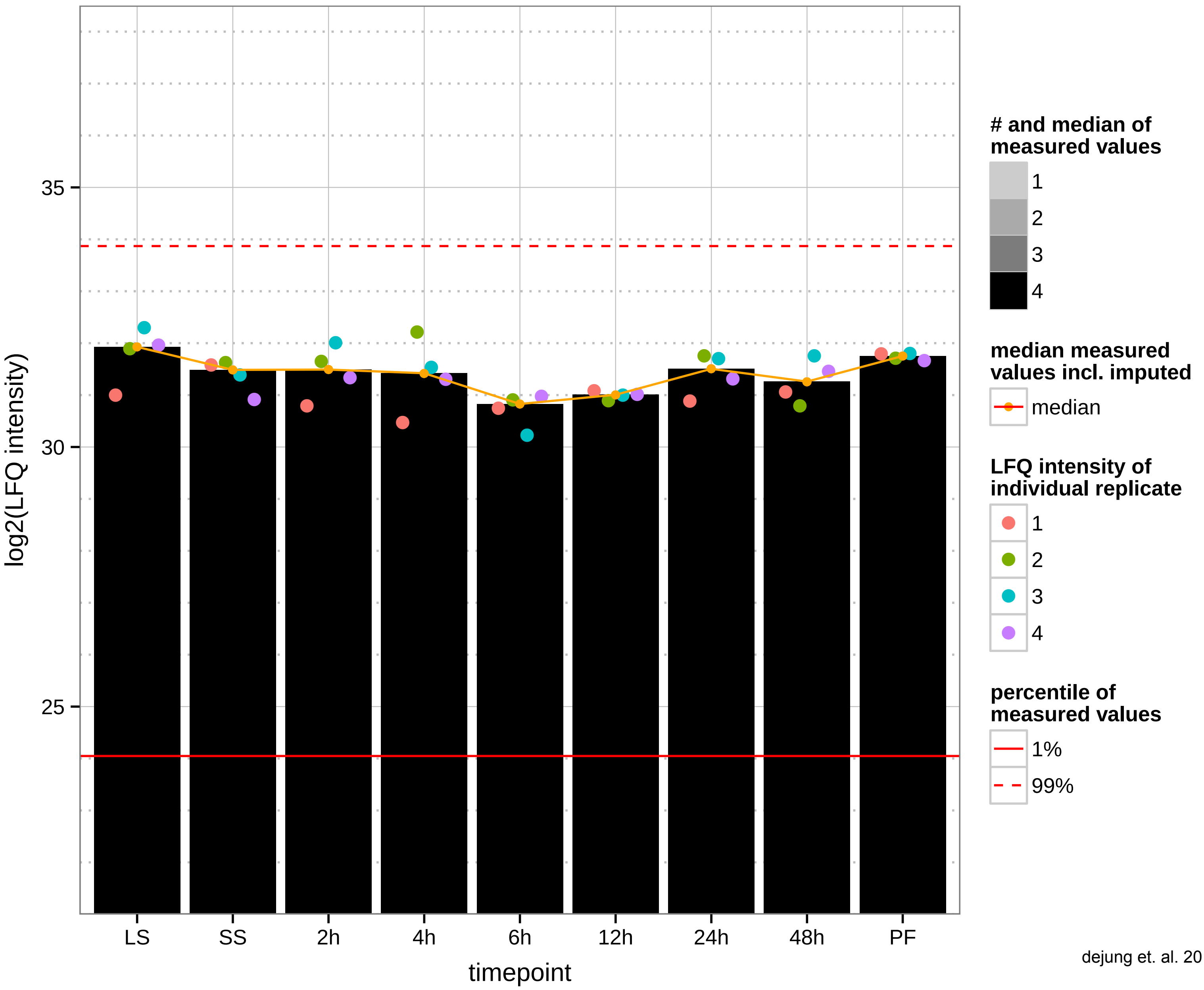

dejung et. al. 2015

2–amino–3–ketobutyrate coenzyme A ligase, putative, glycine acetyltransferase
Tb927.8.6060
AGOF: glycine C–acetyltransferase activity, pyridoxal phosphate binding, transferase activity, transferring nitrogenous groups
AGOC: mitochondrion
AGOP: biosynthetic process, threonine catabolic process
PGOF: glycine C–acetyltransferase activity, pyridoxal phosphate binding, transferase activity
PGOC: null
PGOP: biosynthetic process

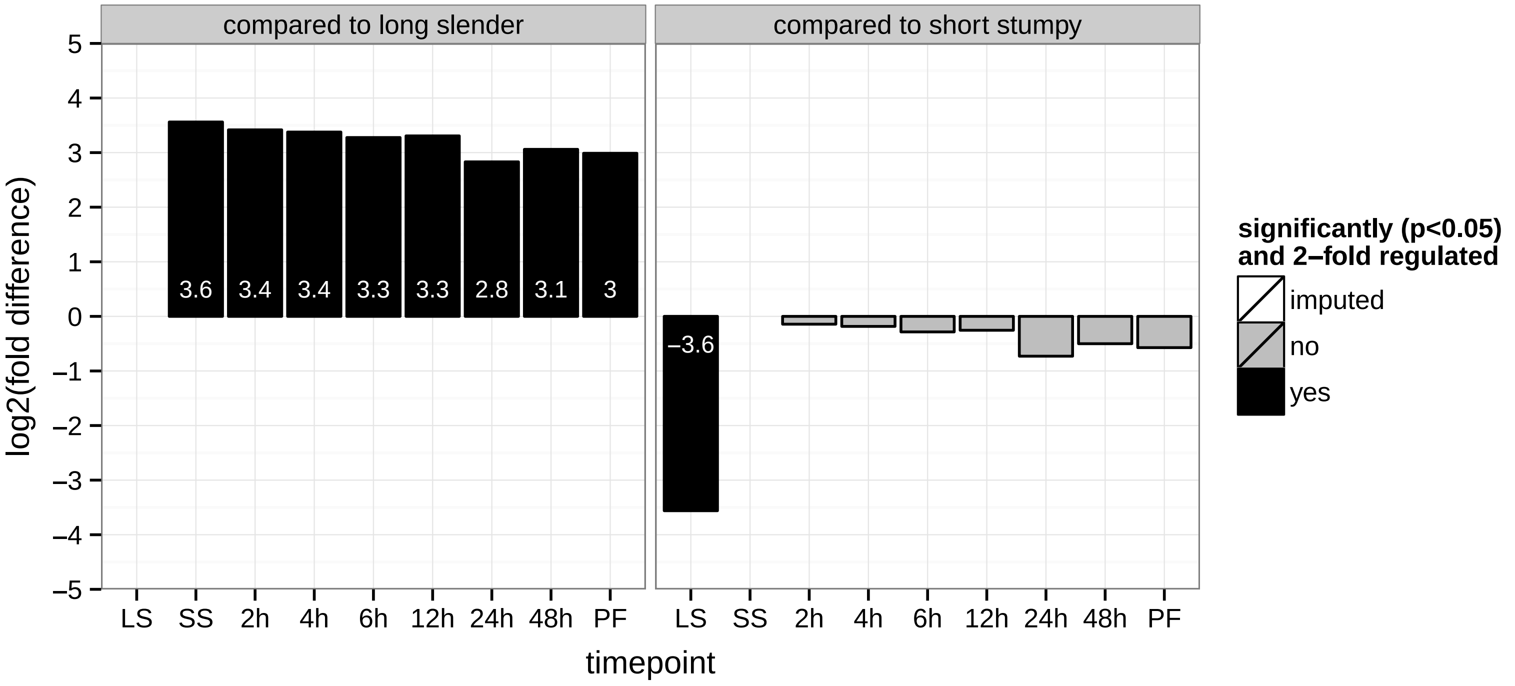

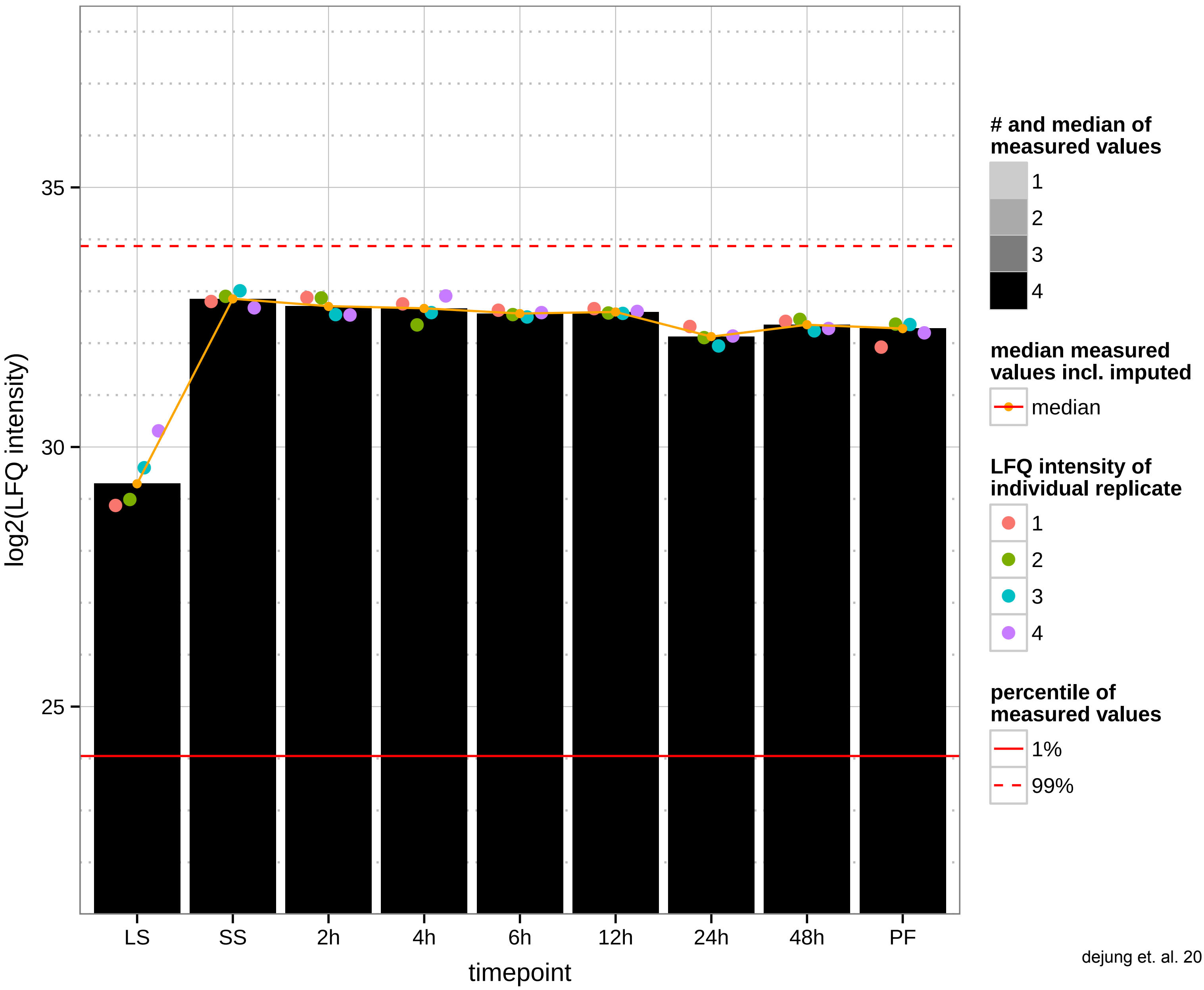

dejung et. al. 2015

40S ribosomal protein S8, putative
Tb927.8.6160;Tb927.8.6150
AGOF: structural constituent of ribosome
AGOC: intracellular, ribosome
AGOP: translation
PGOF: structural constituent of ribosome, null
PGOC: intracellular, ribosome, null
PGOP: translation, null

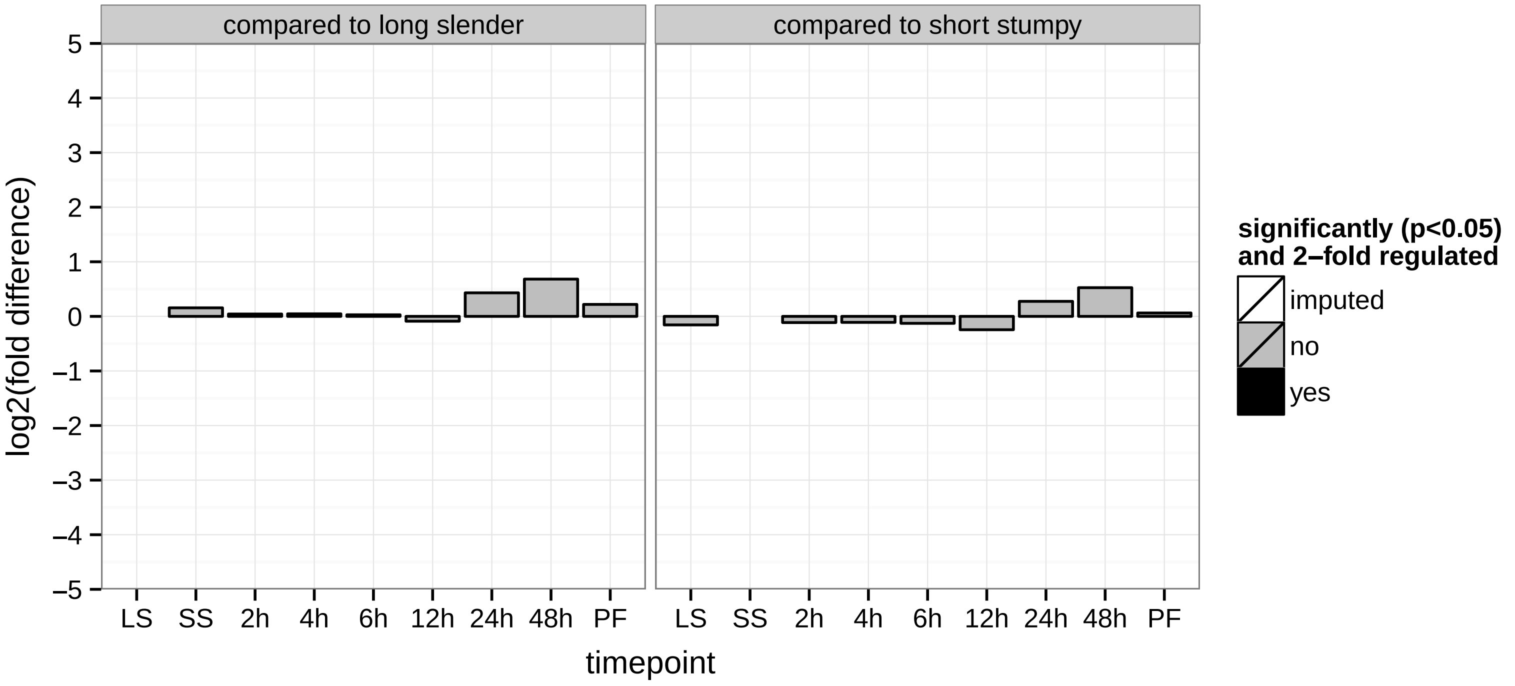

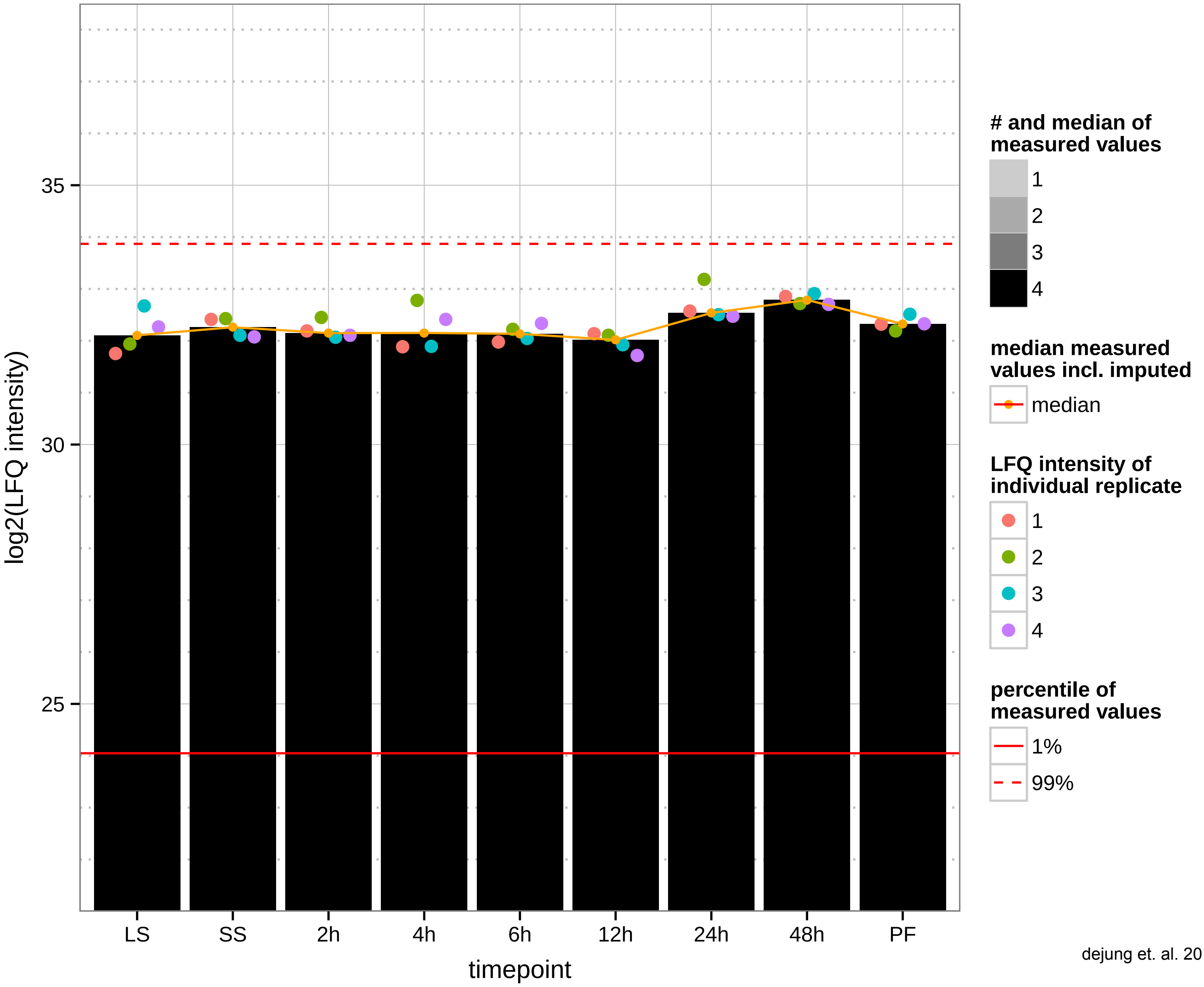

dejung et. al. 2015

hypothetical protein, conserved
Tb927.8.6230
AGOF: null
AGOC: null
AGOP: null
PGOF: null
PGOC: null
PGOP: null

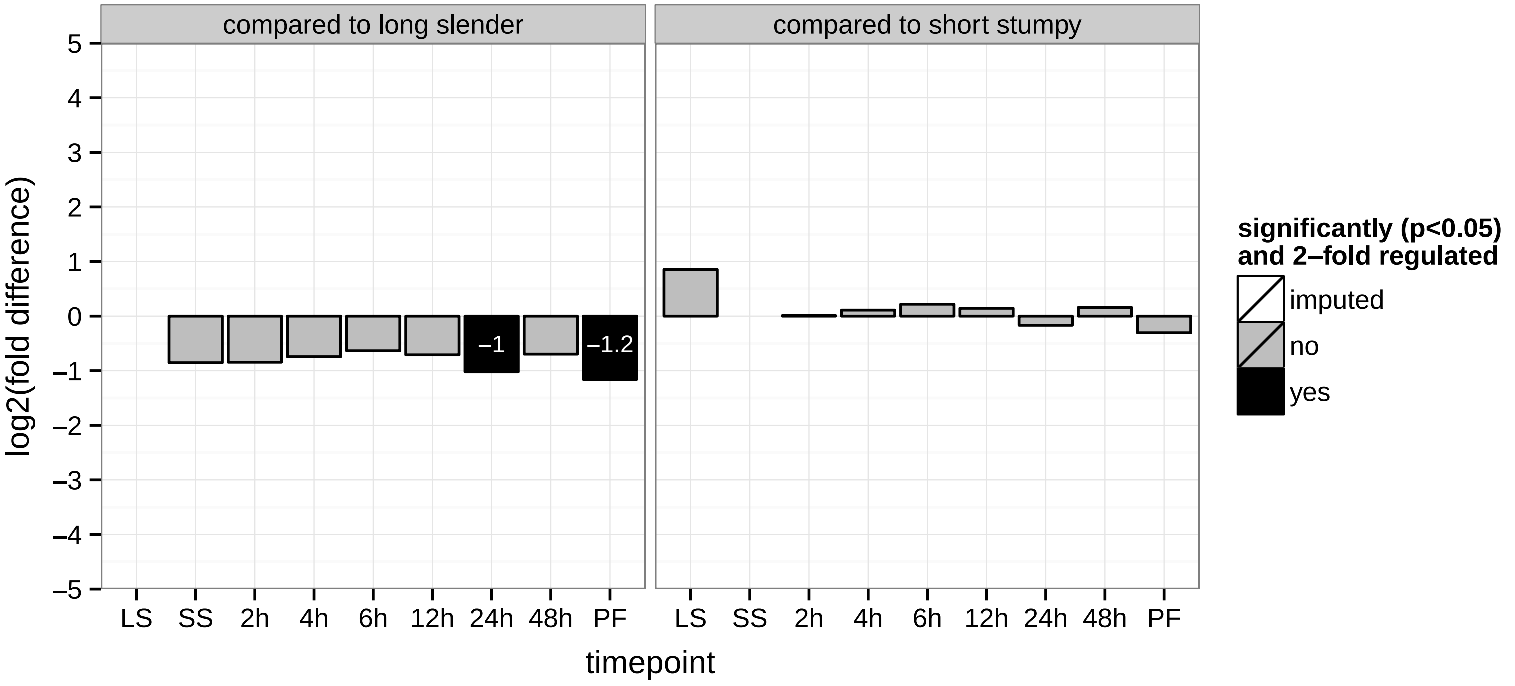

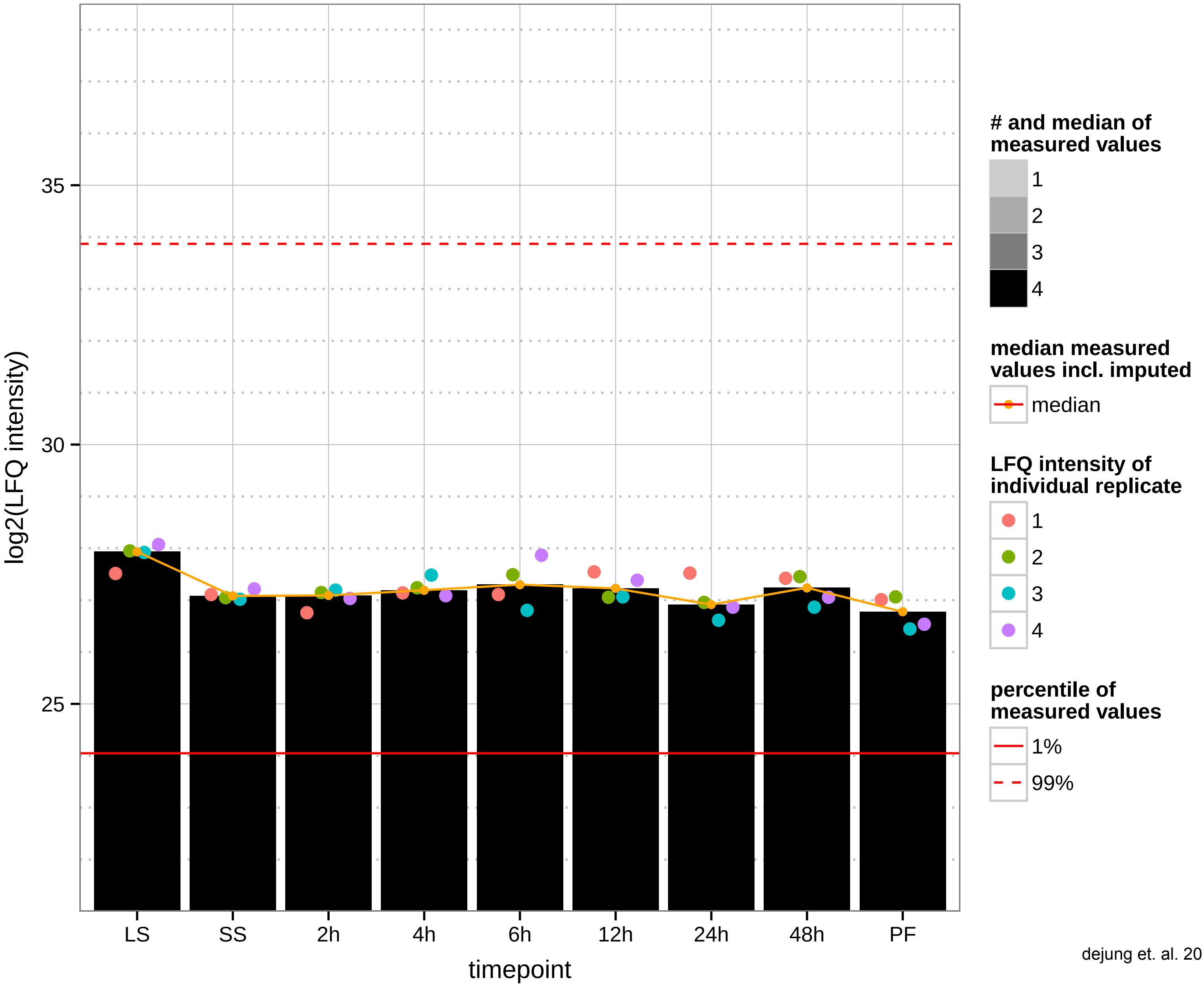

dejung et. al. 2015

STOP axonemal protein
Tb927.8.6240
AGOF: microtubule binding
AGOC: cytoskeleton
AGOP: microtubule anchoring
PGOF: null
PGOC: null
PGOP: null

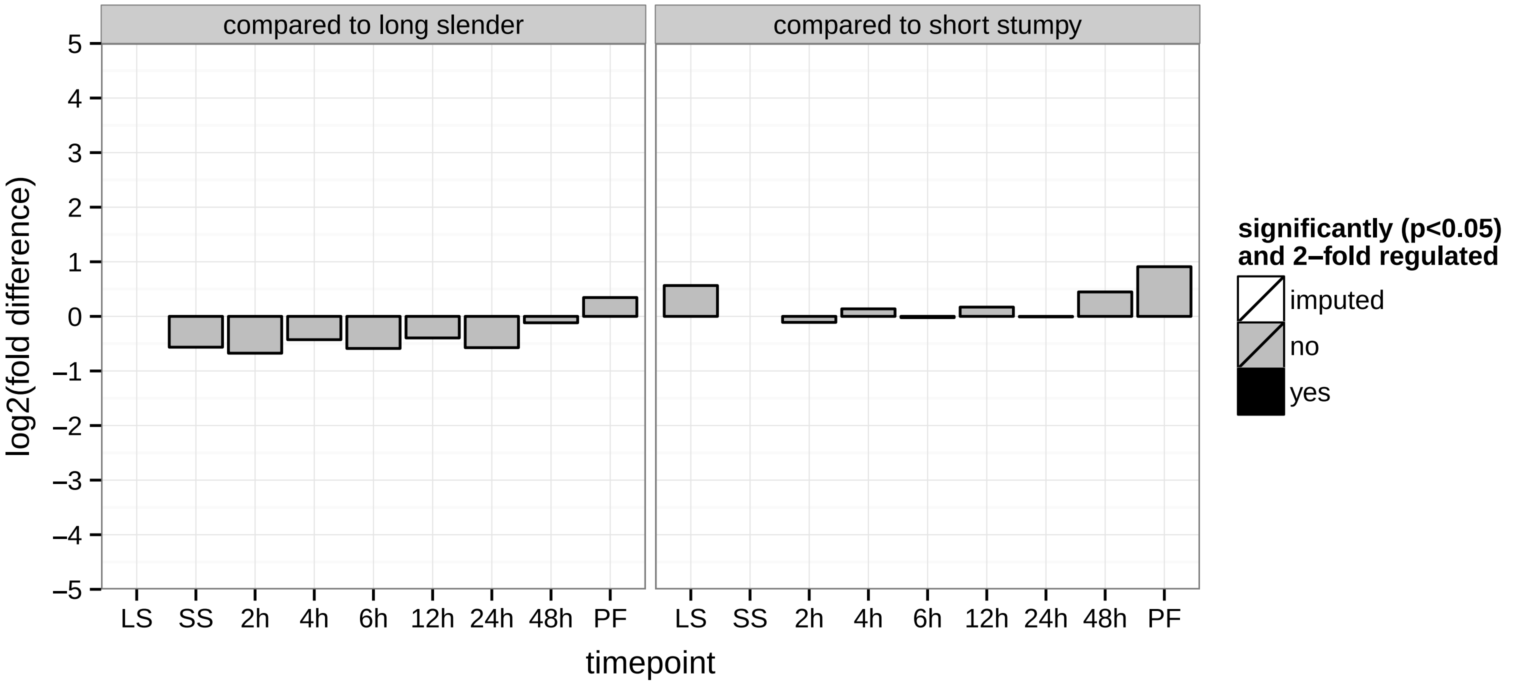

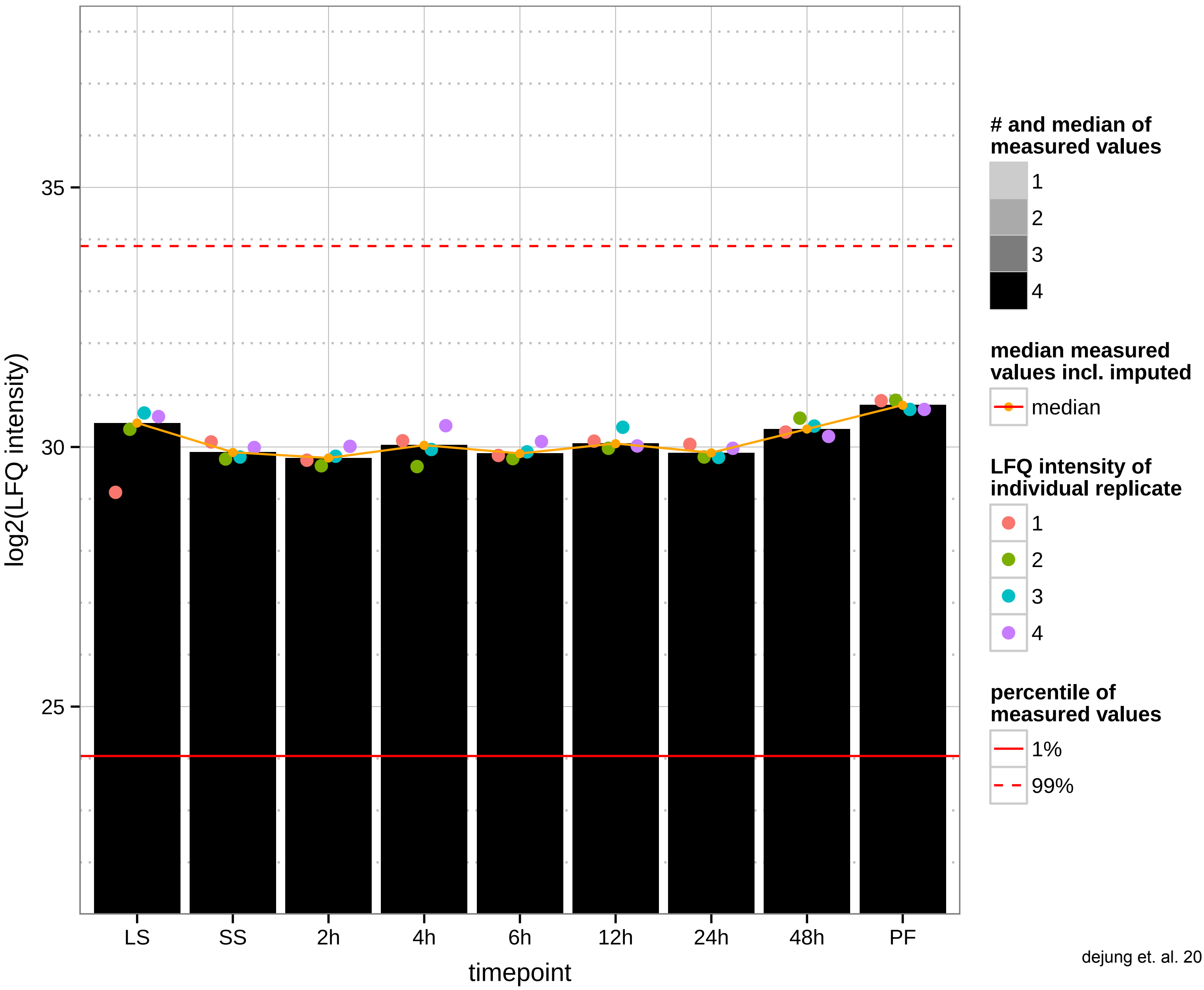

dejung et. al. 2015

hypothetical protein, conserved
Tb927.8.6250
AGOF: null
AGOC: null
AGOP: null
PGOF: null
PGOC: null
PGOP: null

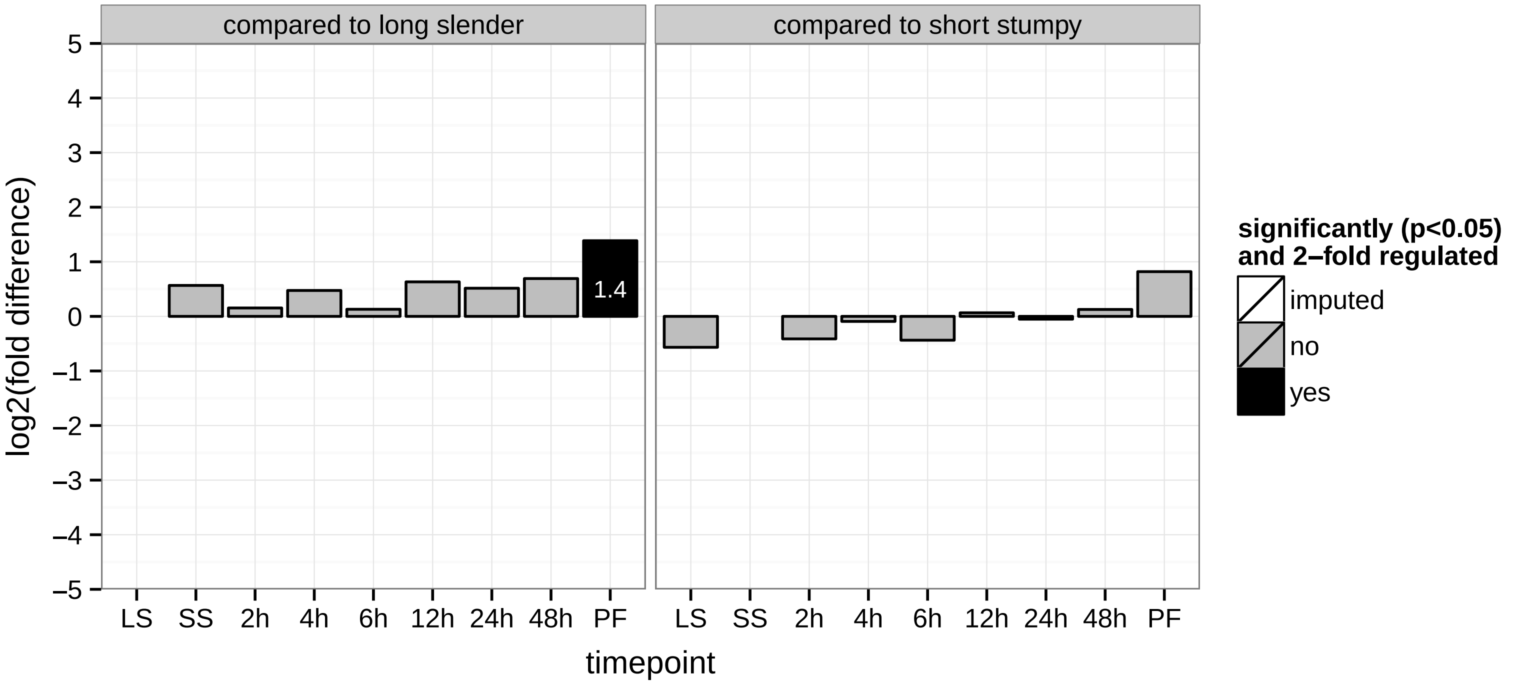

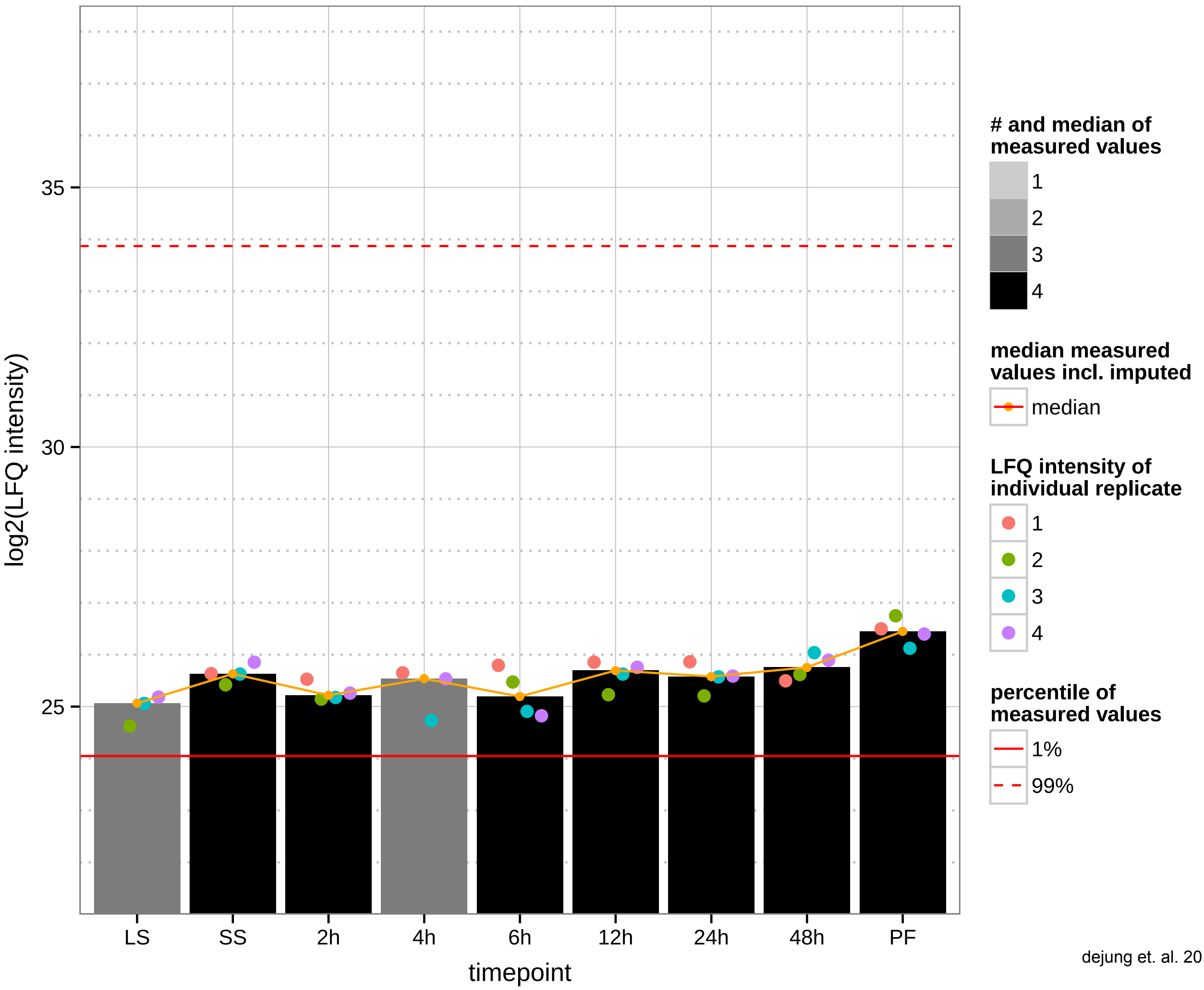

dejung et. al. 2015

hypothetical protein, conserved
Tb927.8.6270
AGOF: null
AGOC: null
AGOP: null
PGOF: null
PGOC: null
PGOP: null

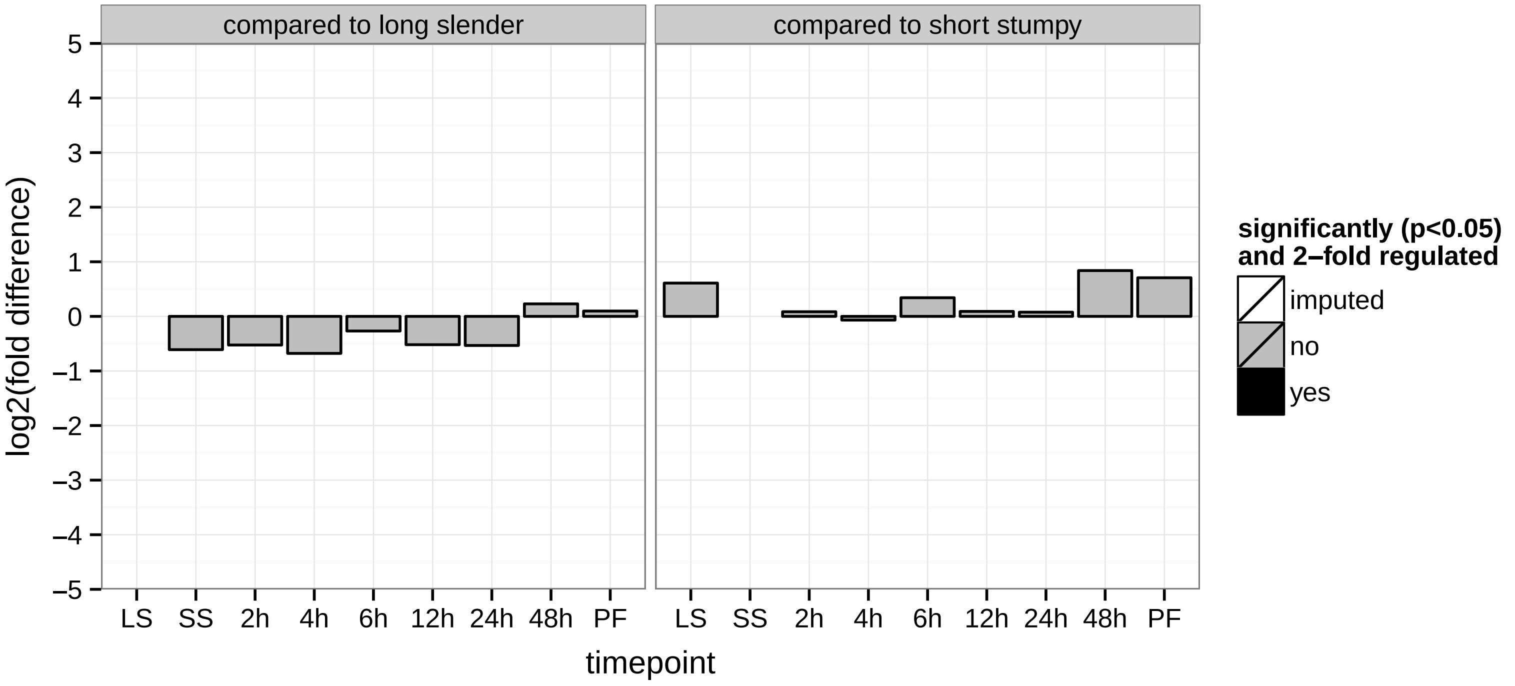

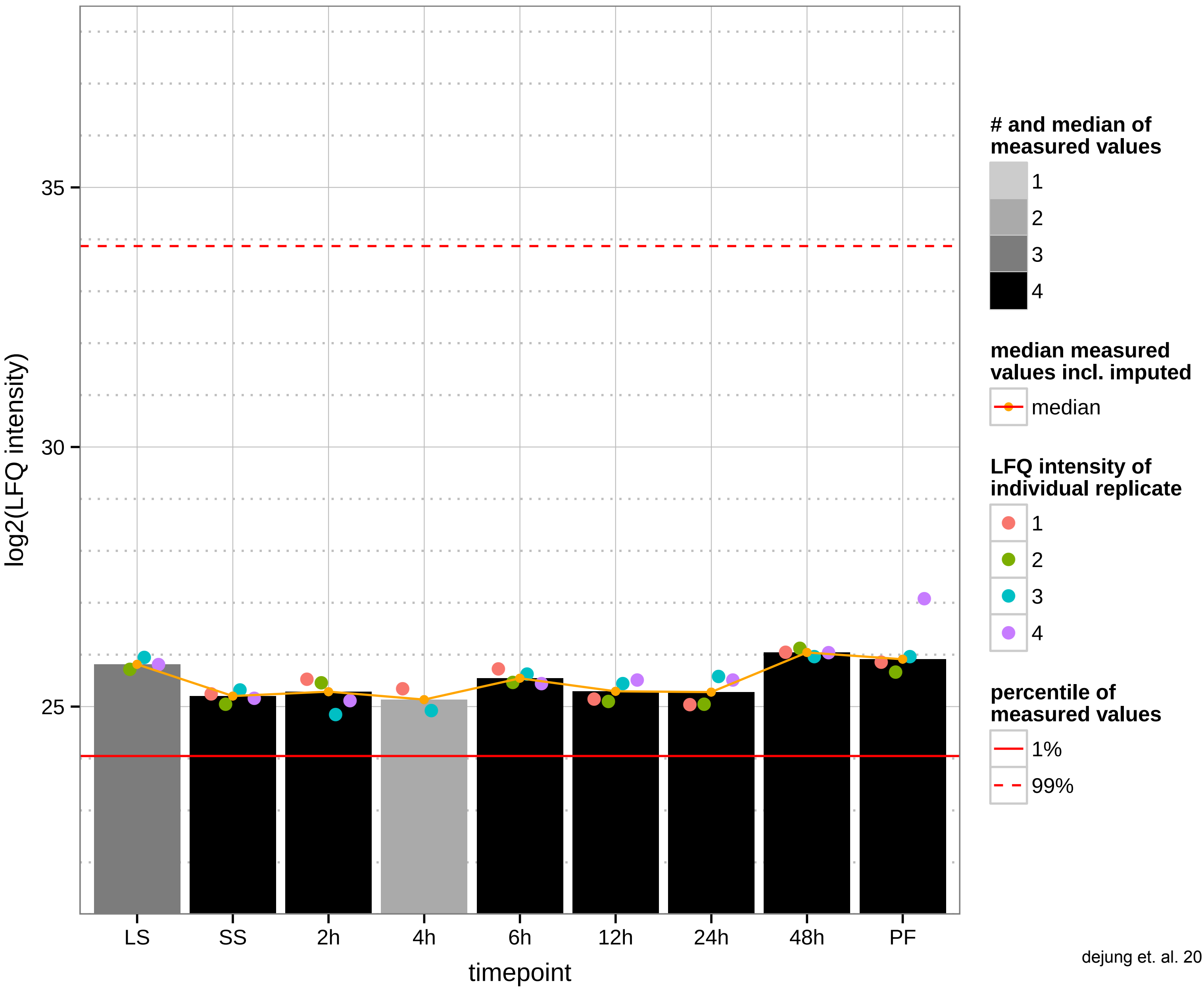

dejung et. al. 2015

hypothetical protein, conserved
Tb927.8.6290
AGOF: ATP binding, inositol tetrakisphosphate 1−kinase activity, magnesium ion binding
AGOC: intracellular
AGOP: inositol trisphosphate metabolic process
PGOF: null
PGOC: null
PGOP: null

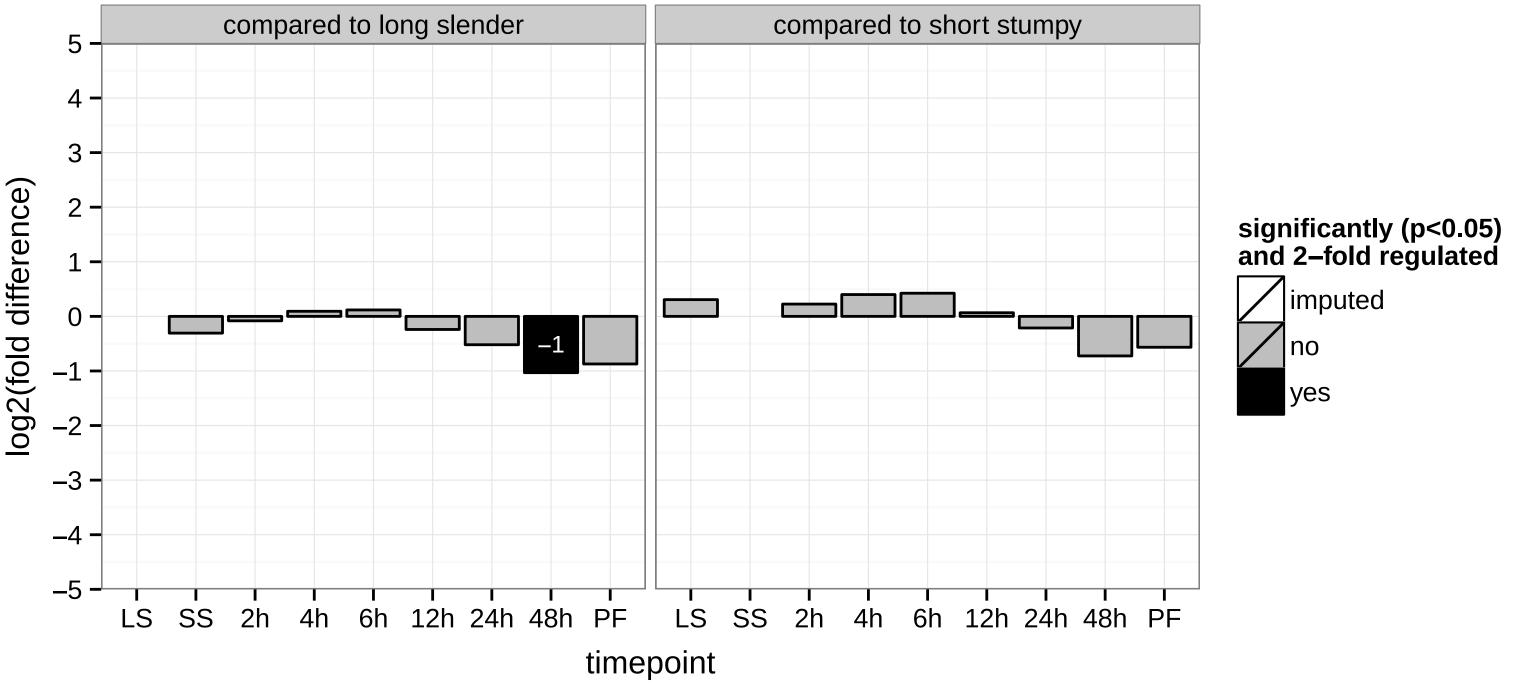

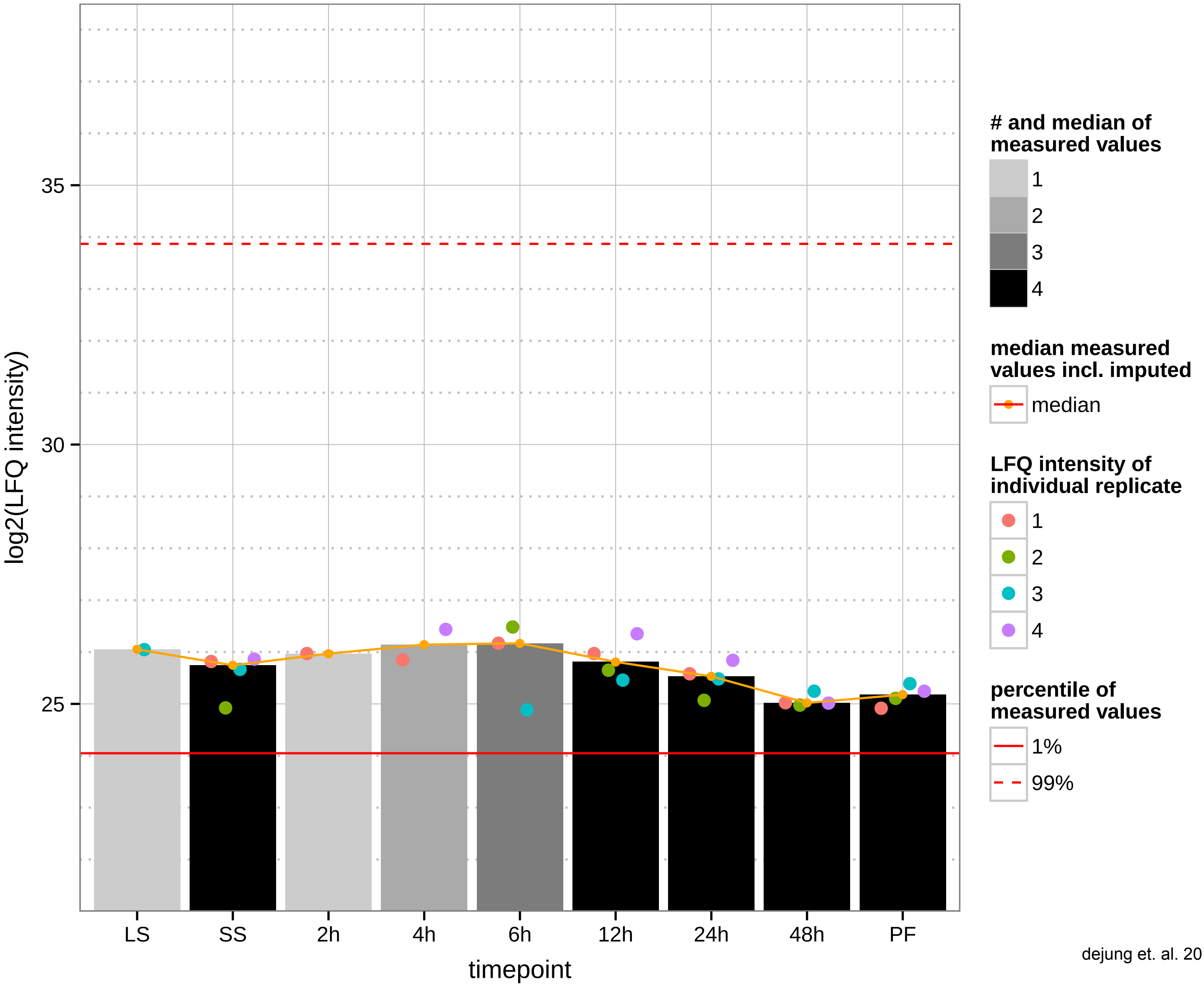

dejung et. al. 2015

hypothetical protein, conserved
Tb927.8.6360
AGOF: zinc ion binding
AGOC: null
AGOP: null
PGOF: protein binding, zinc ion binding
PGOC: null
PGOP: null

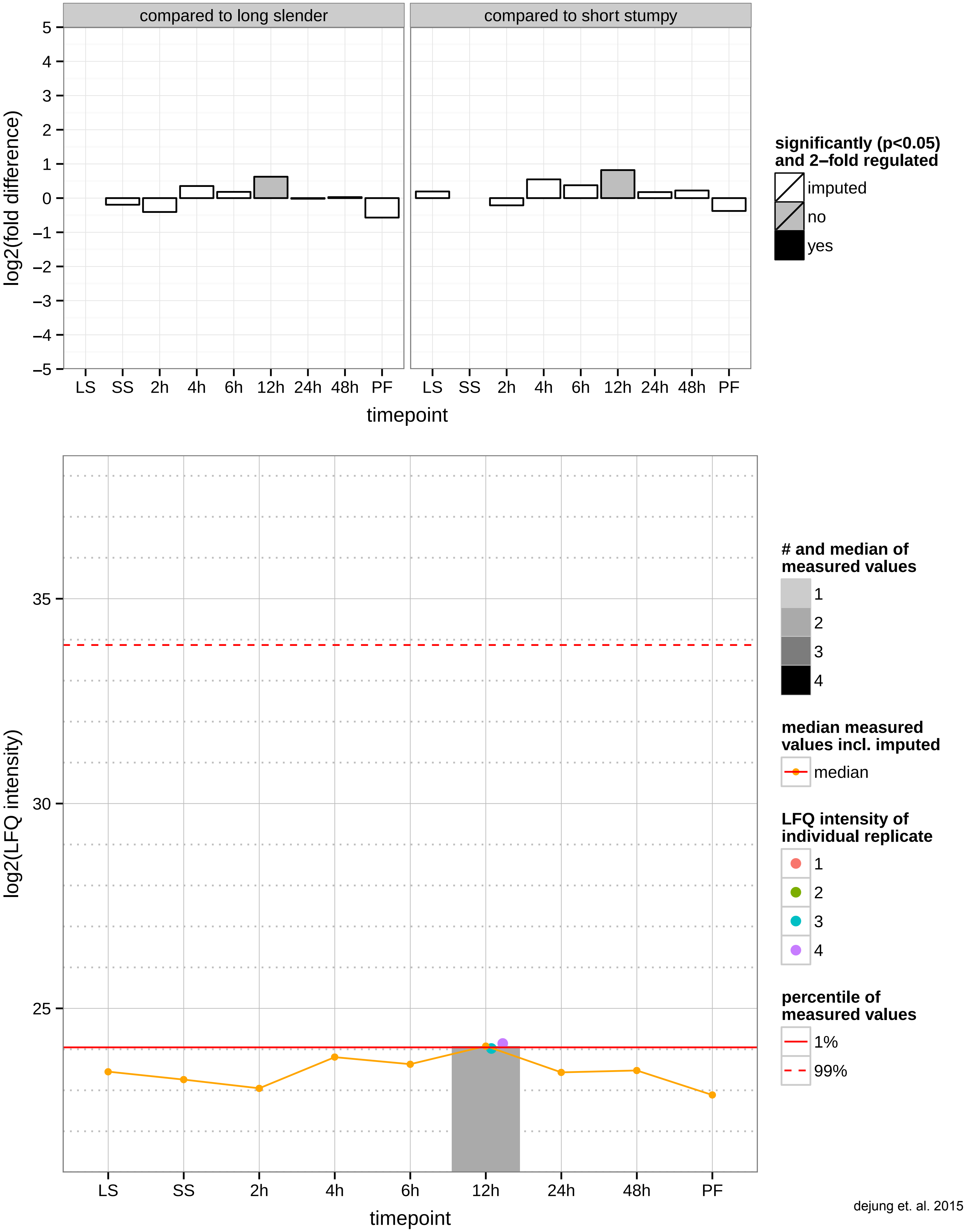

dejung et. al. 2015

lysophospholipase, putative, alpha/beta hydrolase, putative (TbLysoPLA)
Tb927.8.6390
AGOF: hydrolase activity, acting on ester bonds
AGOC: null
AGOP: metabolic process
PGOF: hydrolase activity
PGOC: null
PGOP: null

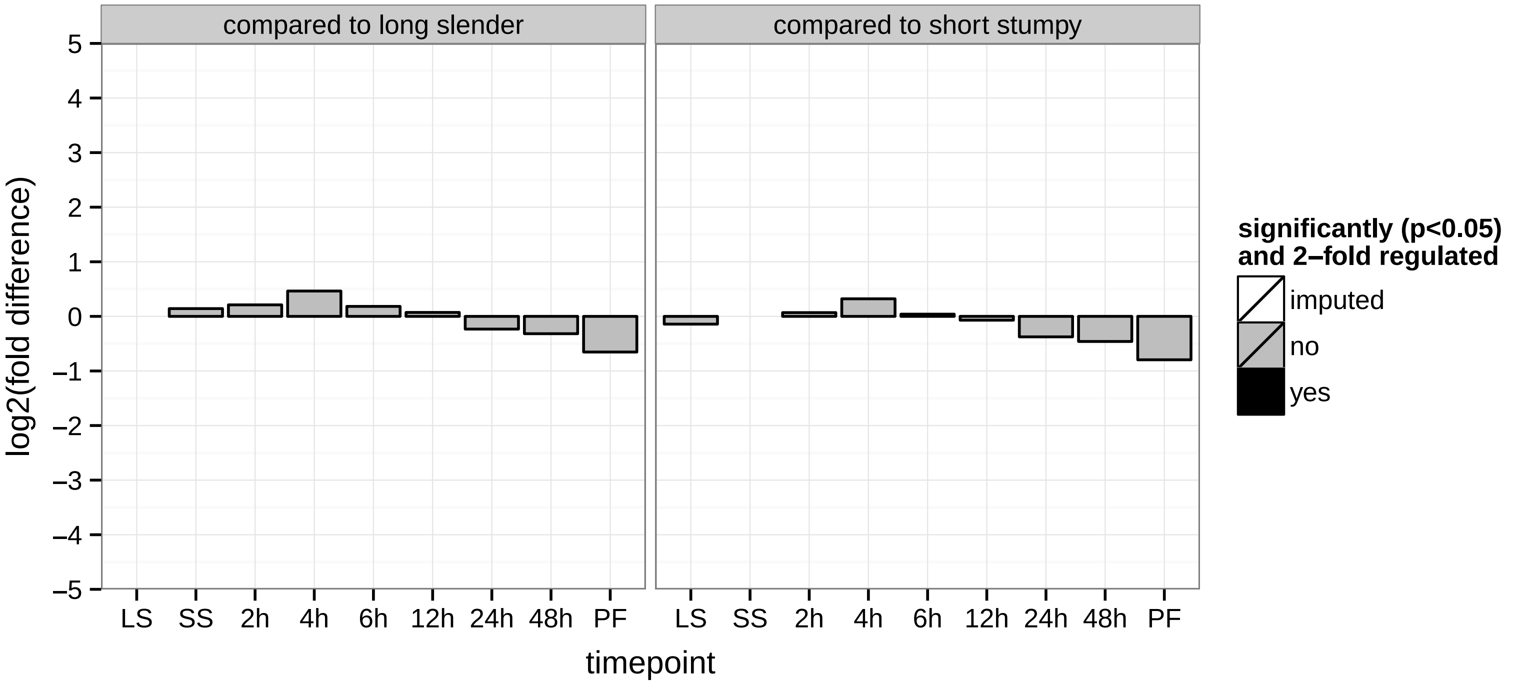

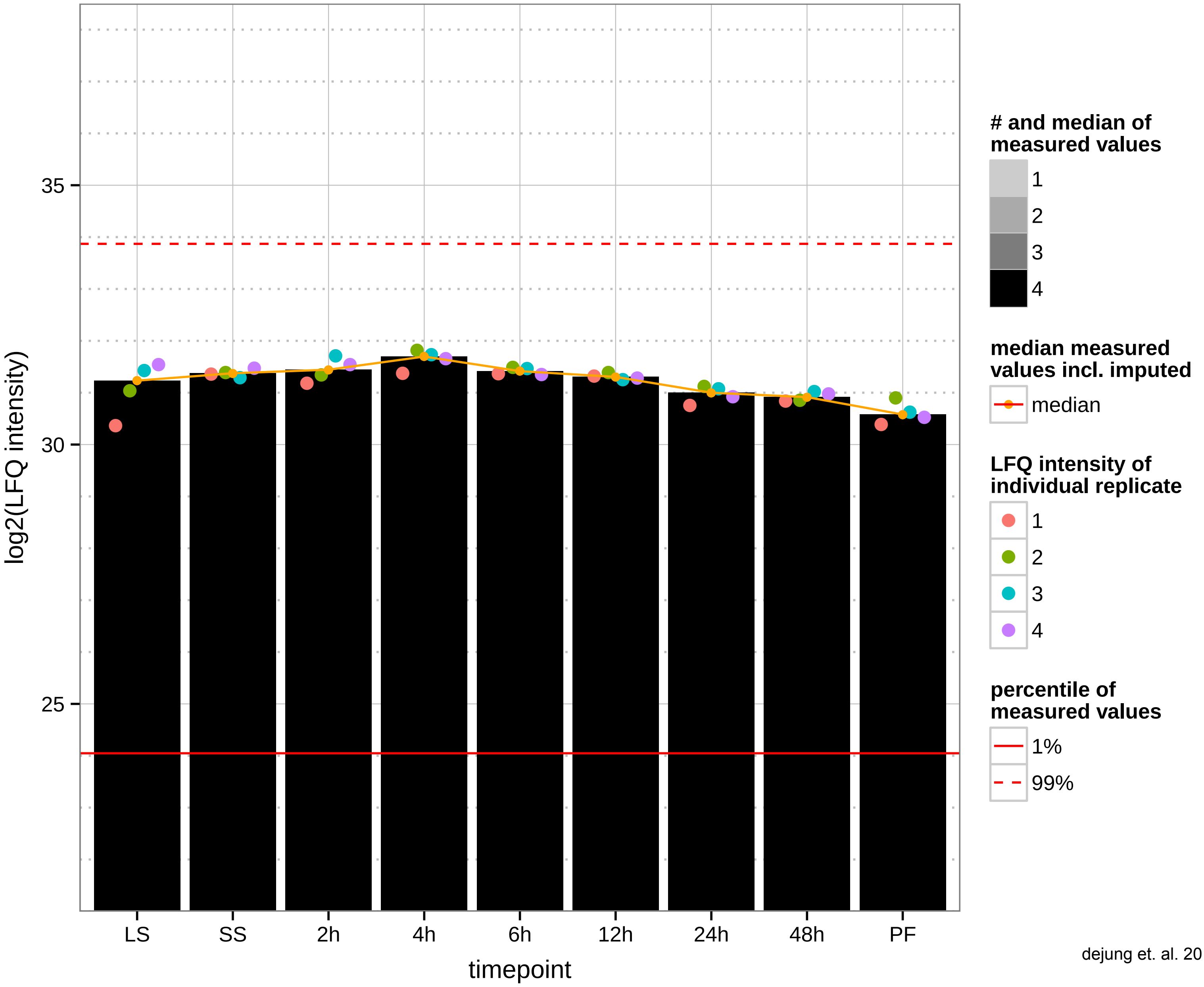

dejung et. al. 2015

hypothetical protein, conserved
Tb927.8.6430
AGOF: null
AGOC: null
AGOP: null
PGOF: null
PGOC: null
PGOP: null

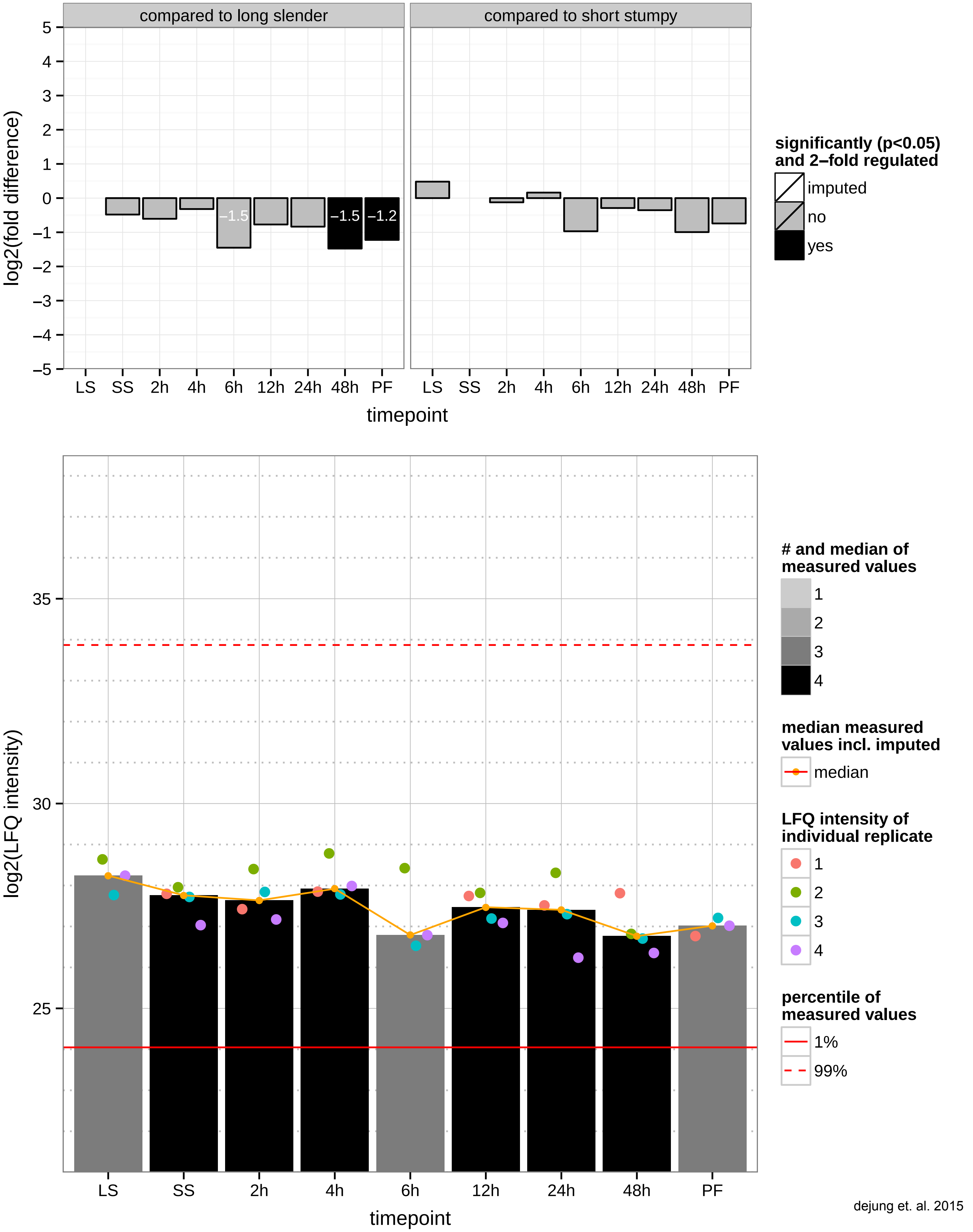

dejung et. al. 2015

RNA–binding protein, putative (RPB20)
Tb927.8.6440
AGOF: RNA binding
AGOC: null
AGOP: RNA processing
PGOF: nucleic acid binding
PGOC: null
PGOP: null

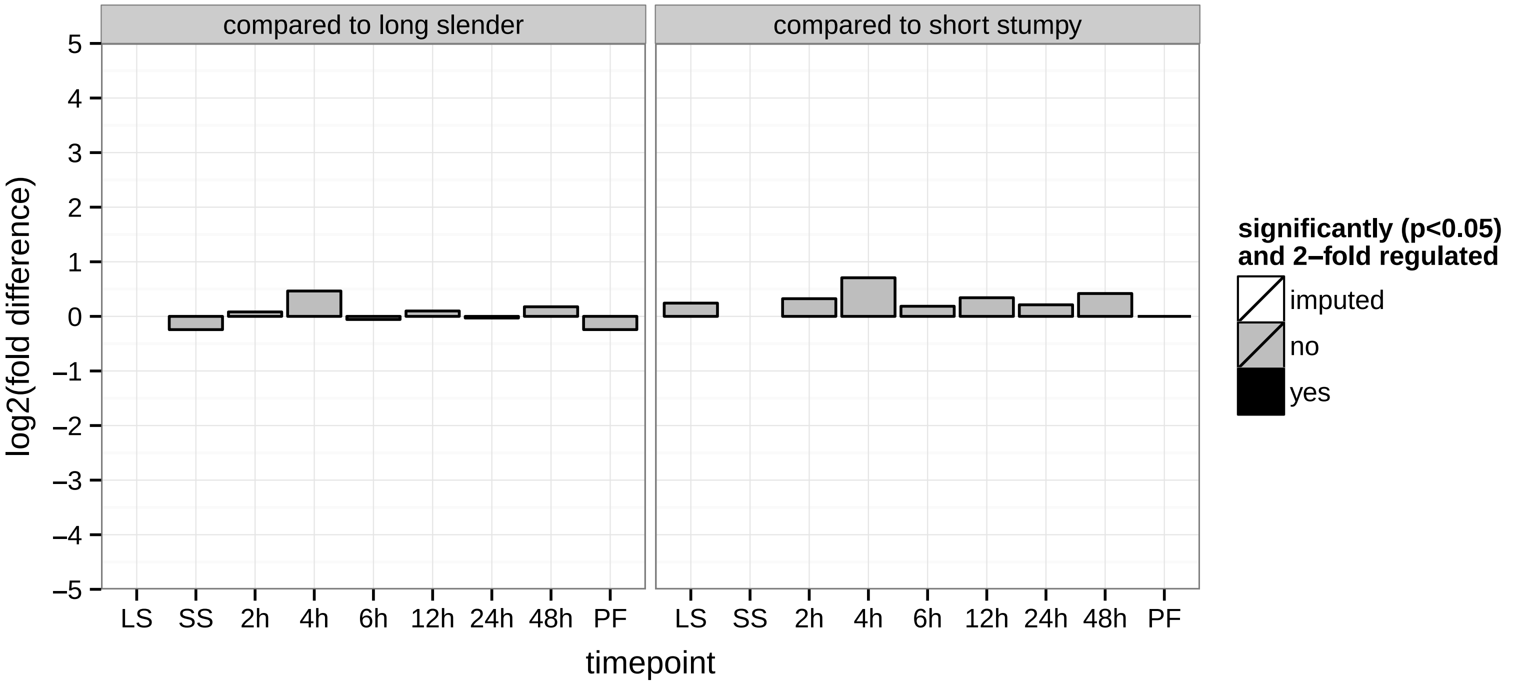

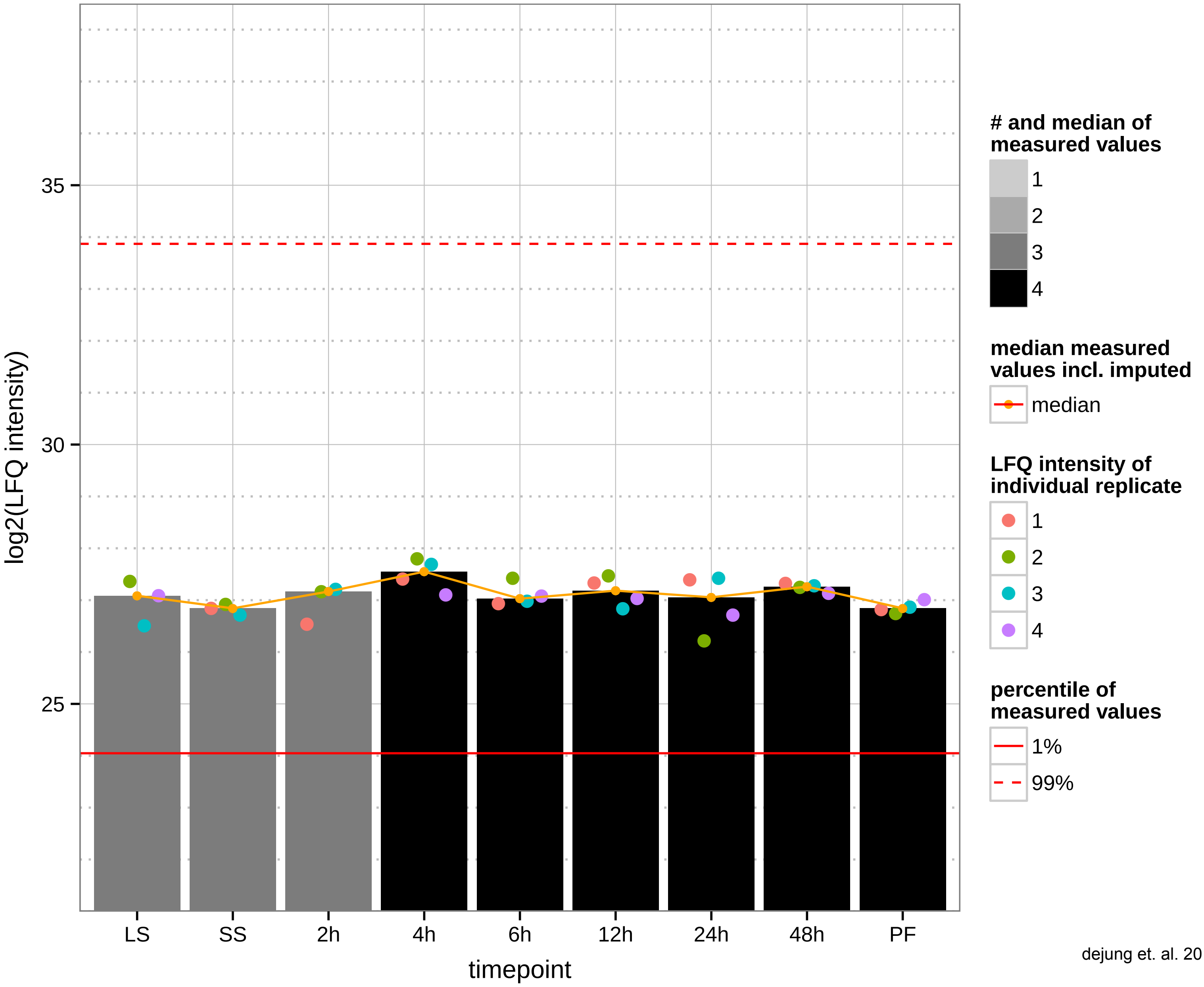

dejung et. al. 2015

hypothetical protein, conserved
Tb927.8.6530
AGOF: null
AGOC: null
AGOP: null
PGOF: protein binding
PGOC: null
PGOP: null

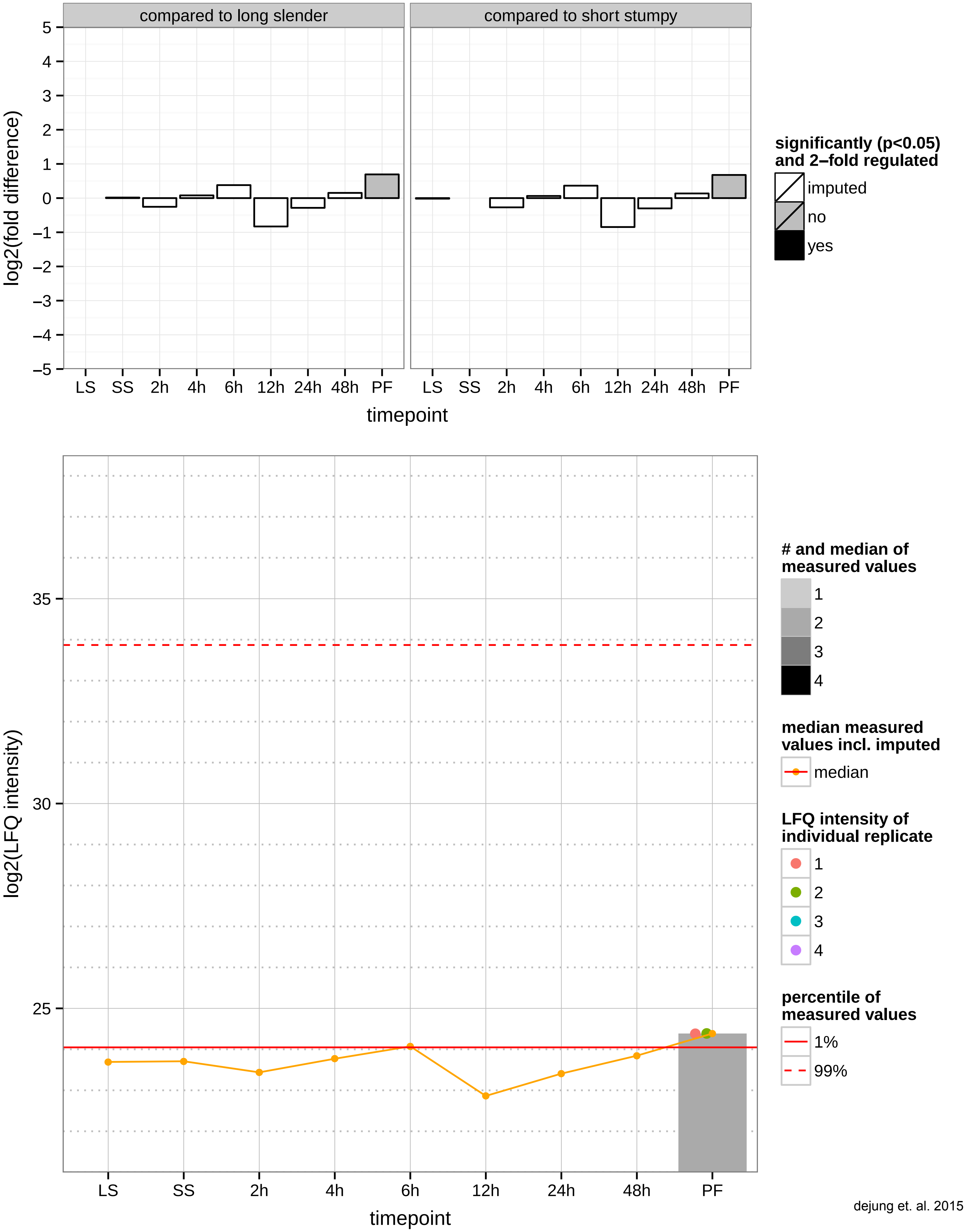

dejung et. al. 2015

hypothetical protein, conserved
Tb927.8.6600
AGOF: null
AGOC: null
AGOP: null
PGOF: null
PGOC: null
PGOP: null

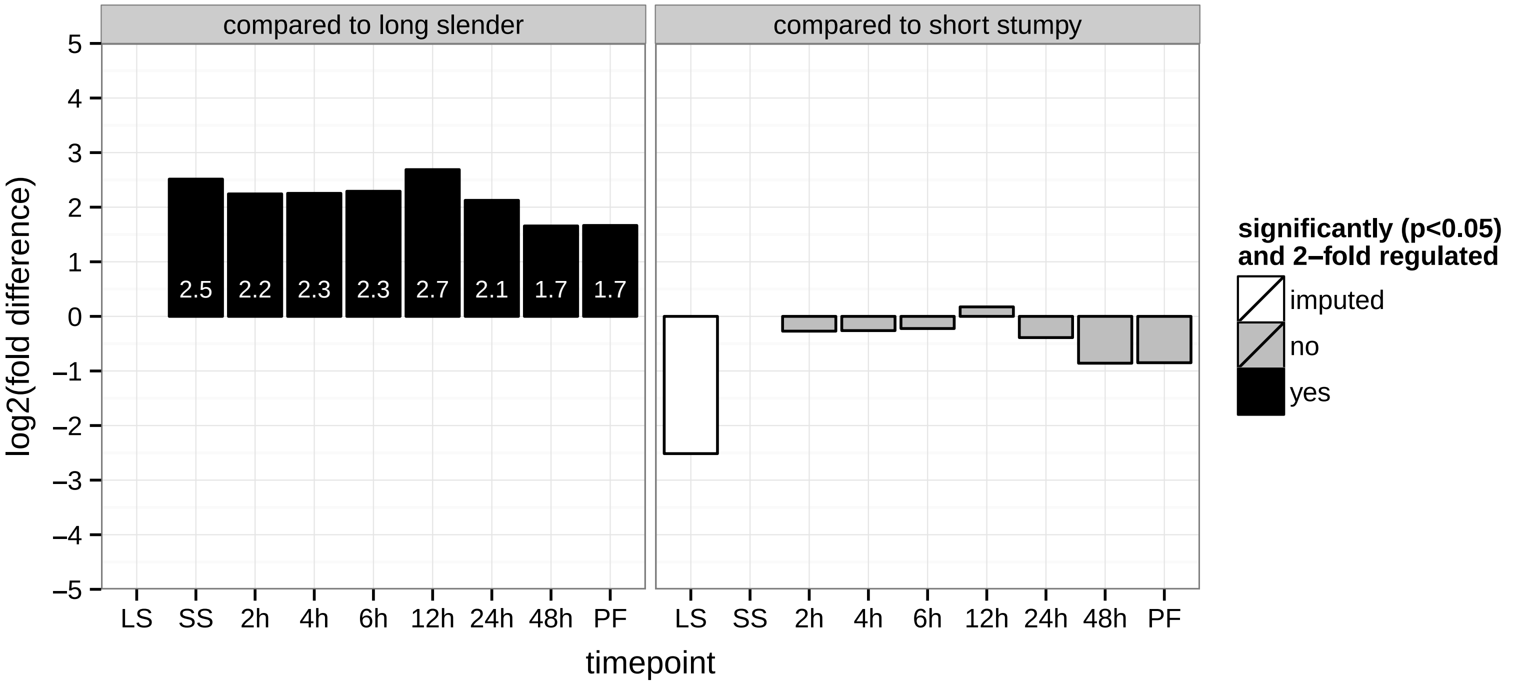

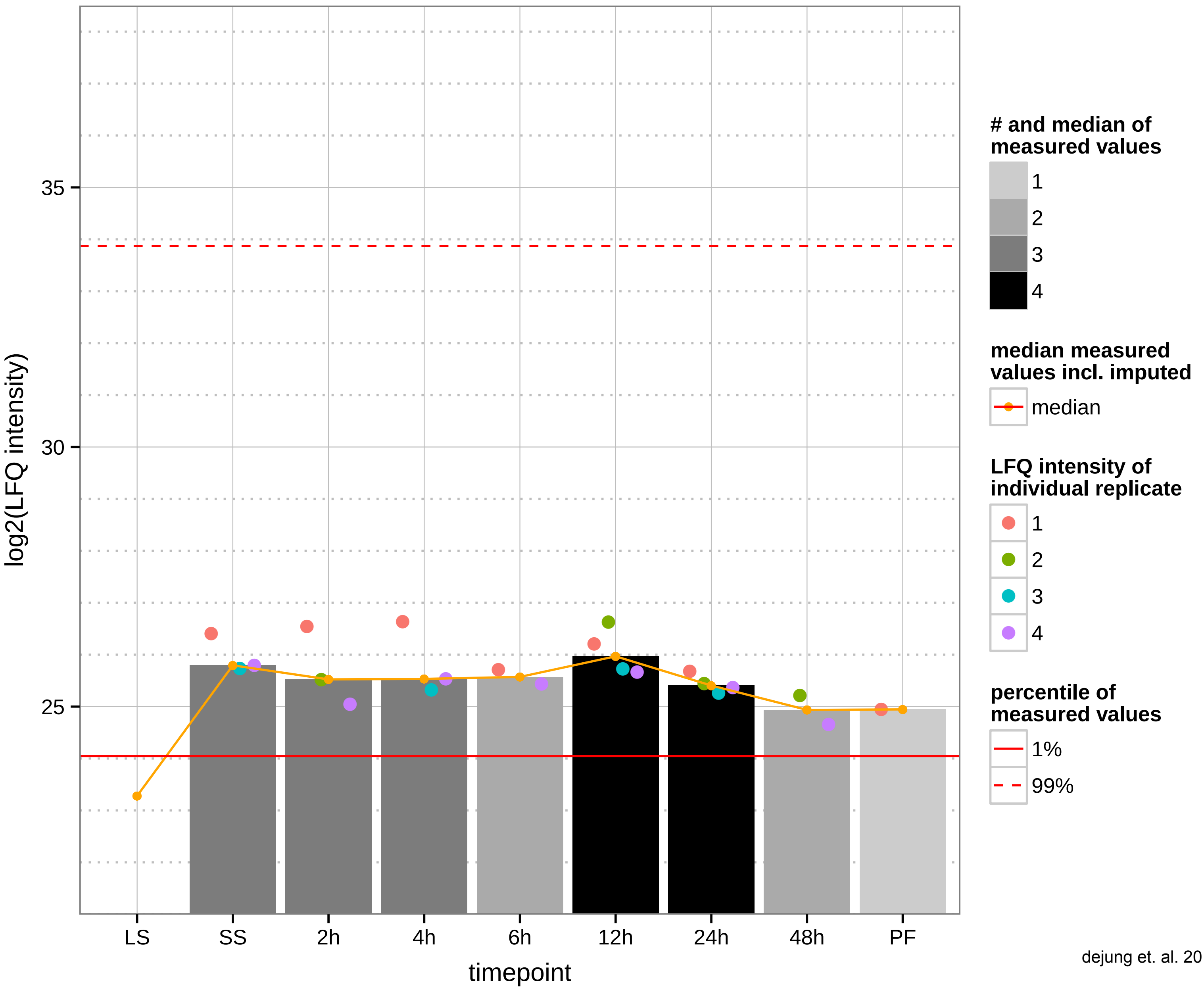

dejung et. al. 2015

paraflagellar rod component, putative (PFC1)
Tb927.8.6660
AGOF: null
AGOC: null
AGOP: null
PGOF: null
PGOC: null
PGOP: null

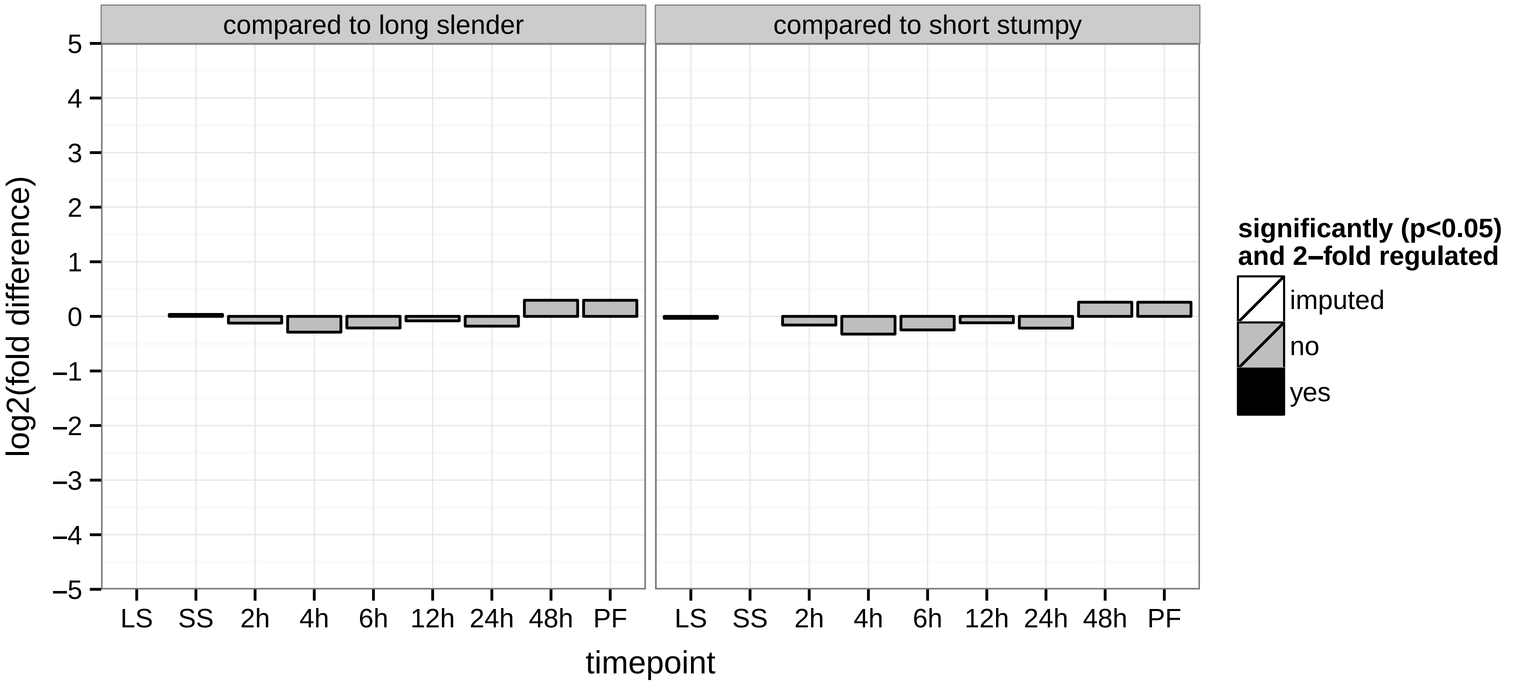

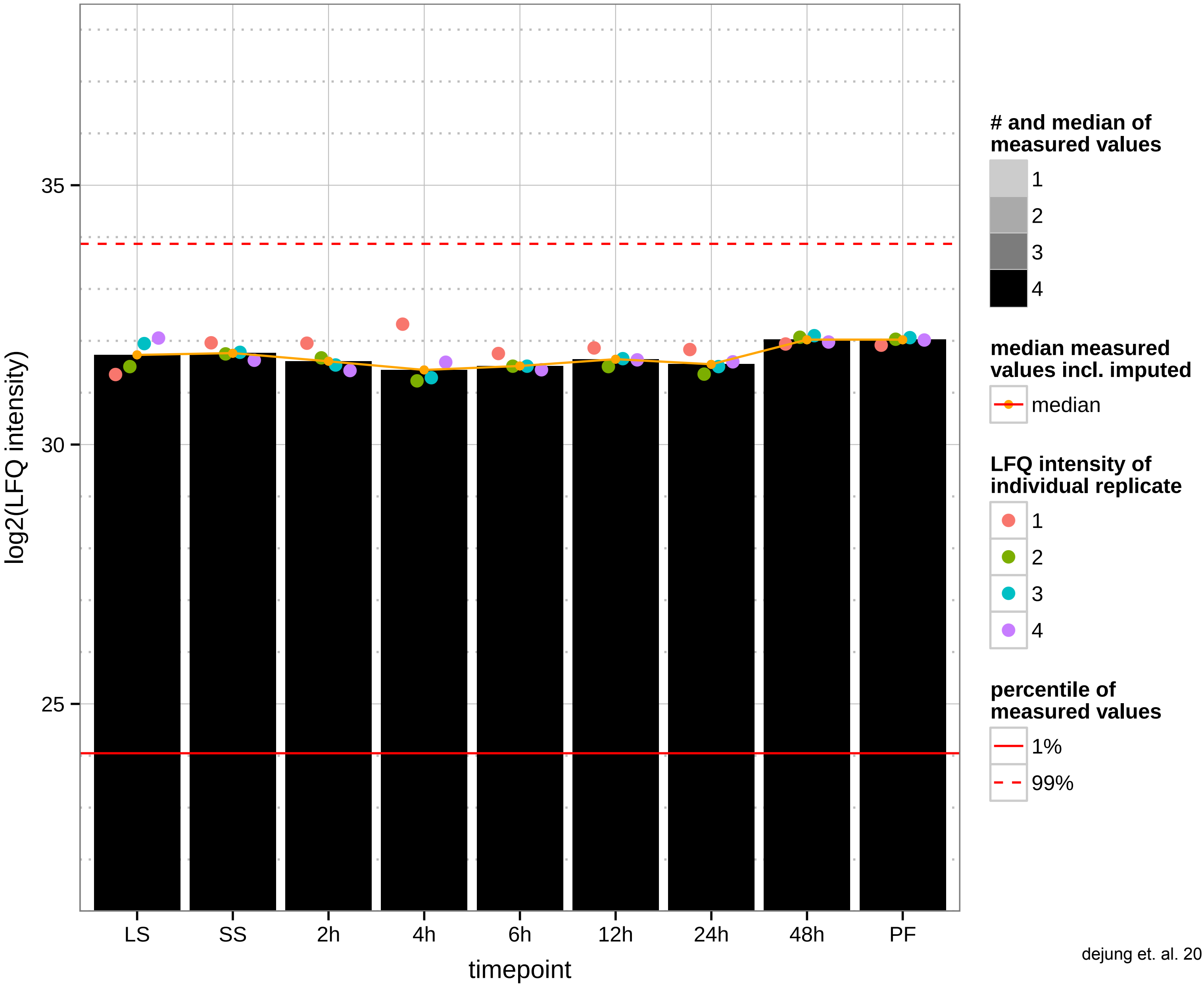

dejung et. al. 2015

hypothetical protein, conserved
Tb927.8.6870
AGOF: null
AGOC: null
AGOP: null
PGOF: protein binding
PGOC: null
PGOP: null

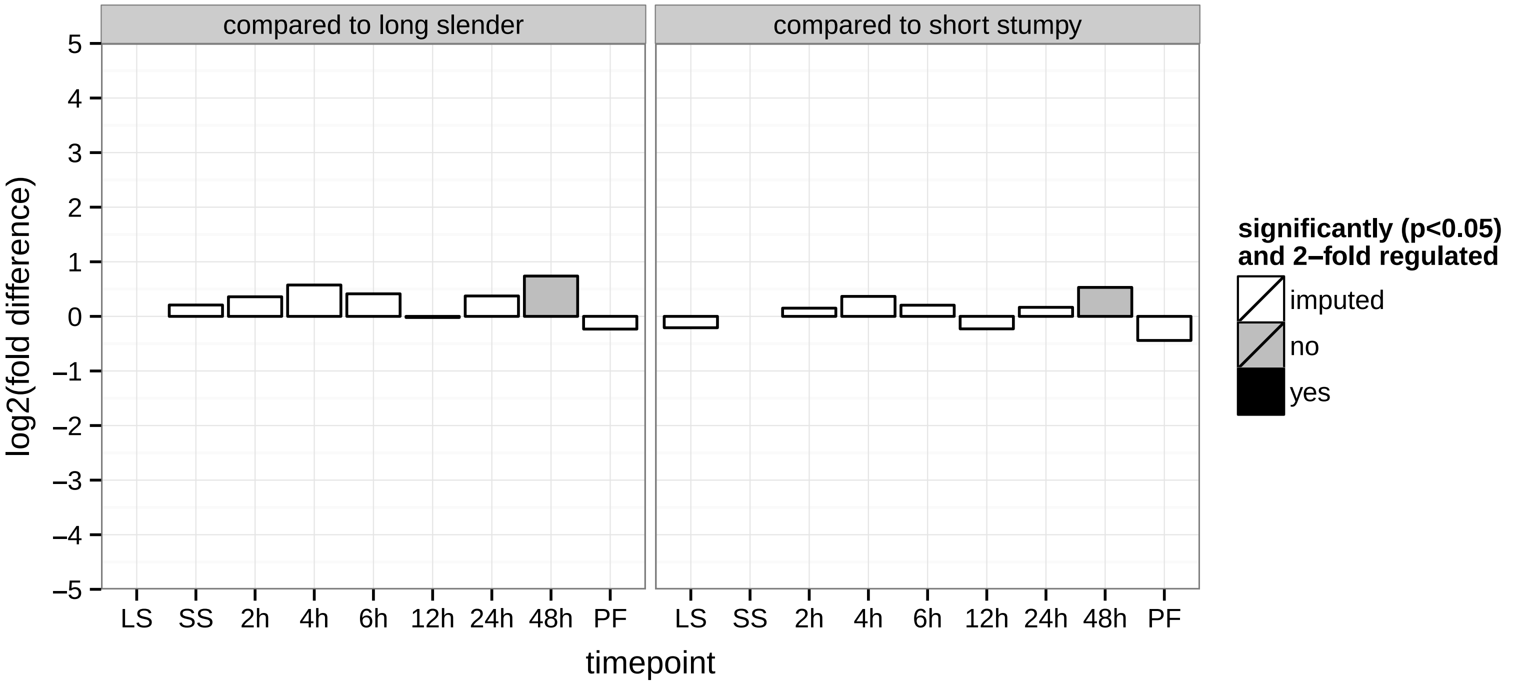

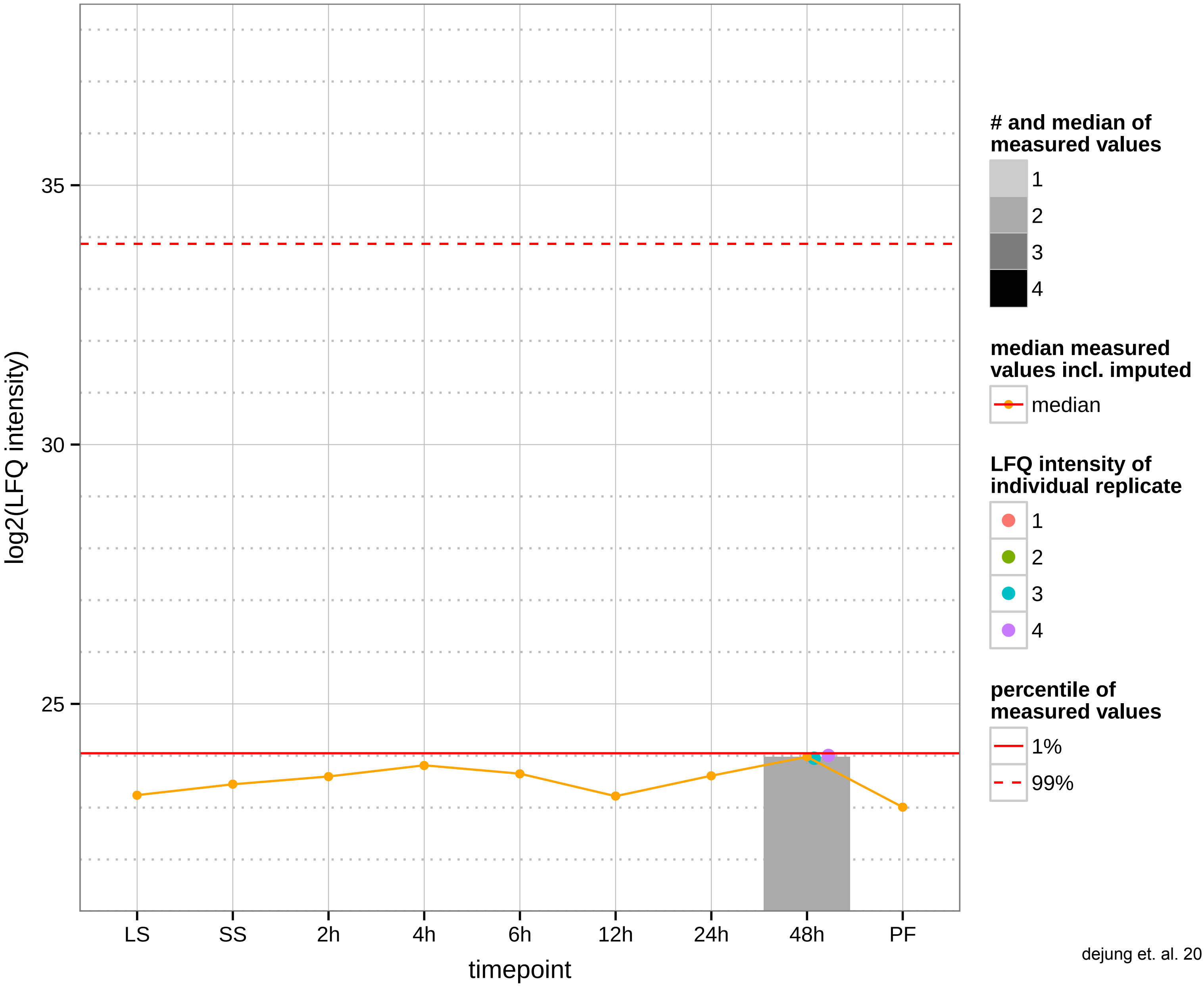

dejung et. al. 2015

peptidyl–prolyl cis–trans isomerase/rotamase, putative, PPIase, parvulin (PIN1)
Tb927.8.690
AGOF: peptidyl–prolyl cis–trans isomerase activity
AGOC: null
AGOP: protein folding
PGOF: isomerase activity
PGOC: null
PGOP: null

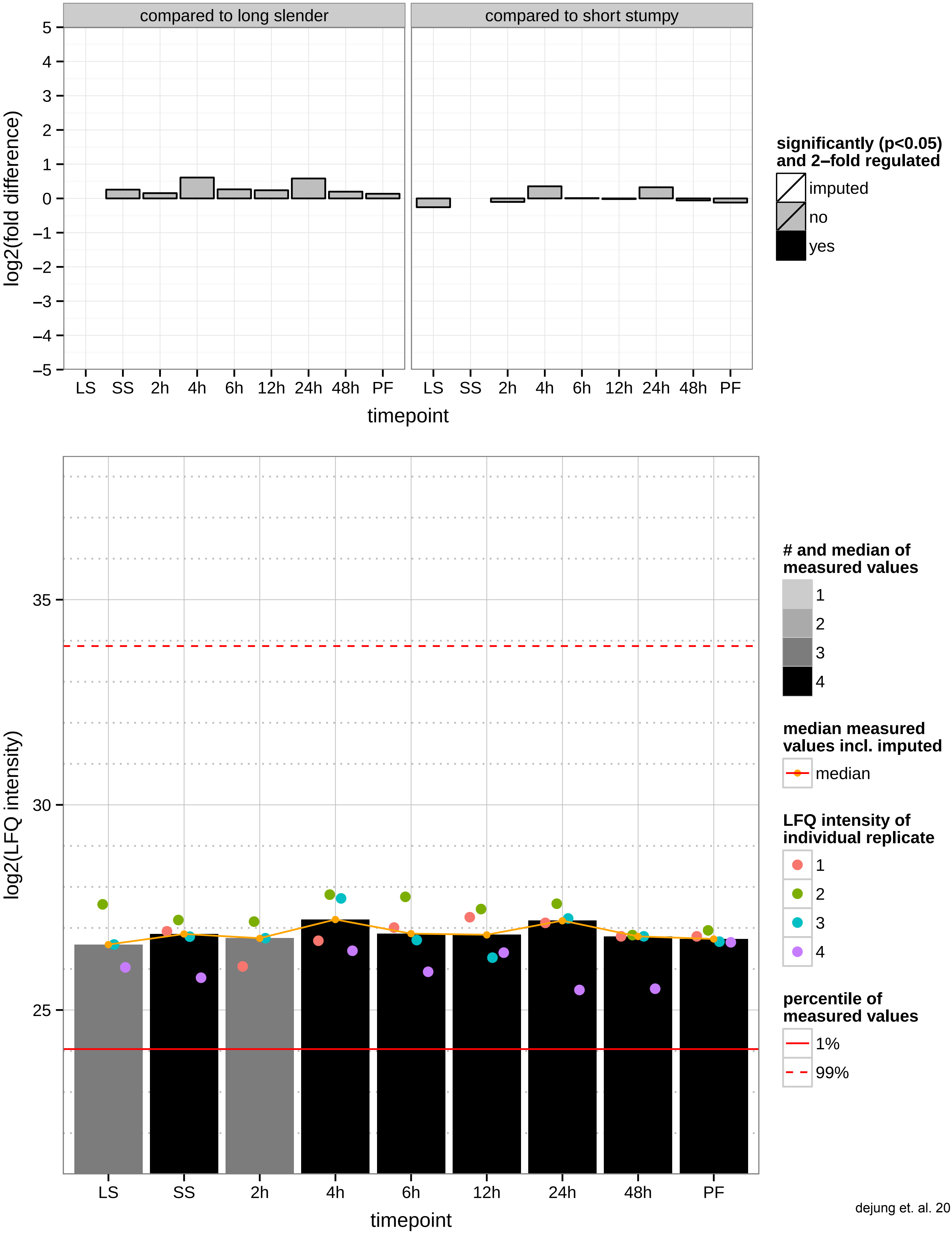

dejung et. al. 2015

hypothetical protein, conserved
Tb927.8.6900
AGOF: null
AGOC: null
AGOP: null
PGOF: null
PGOC: null
PGOP: null

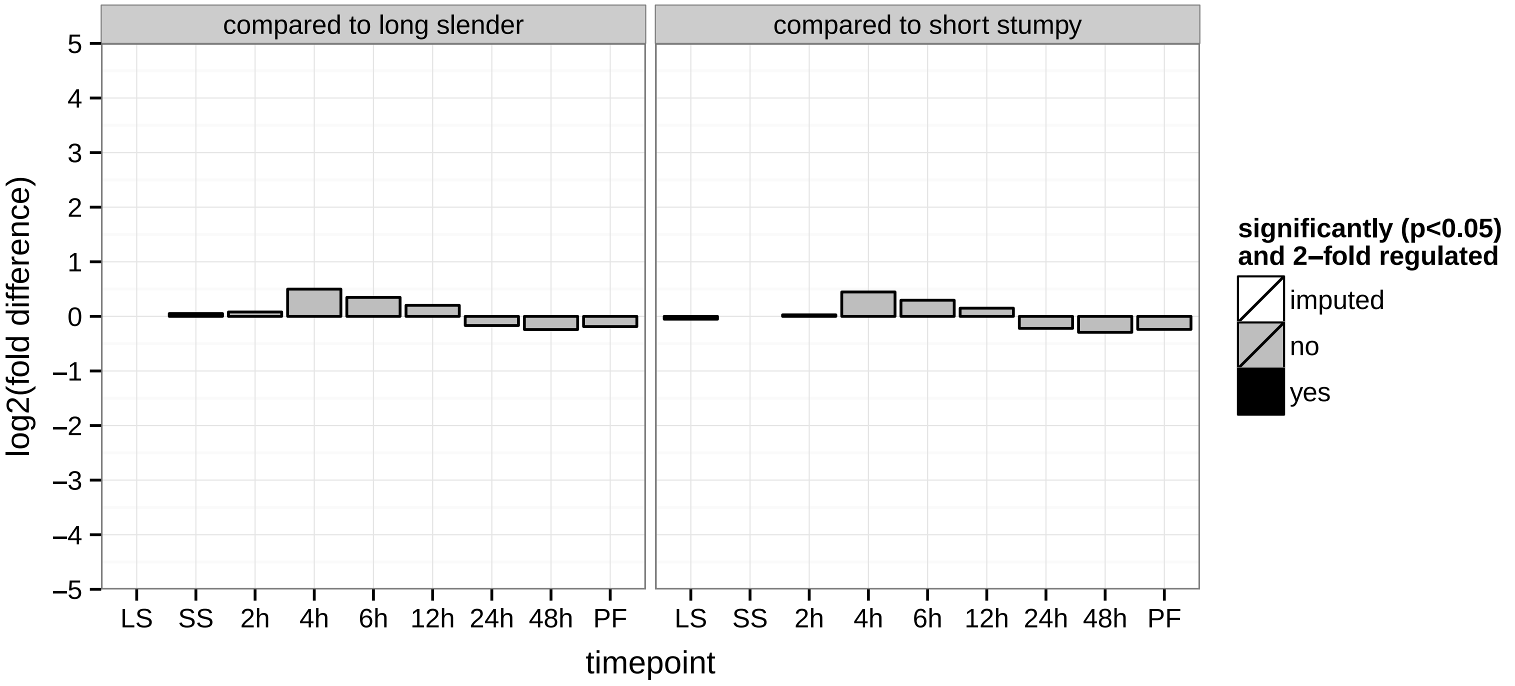

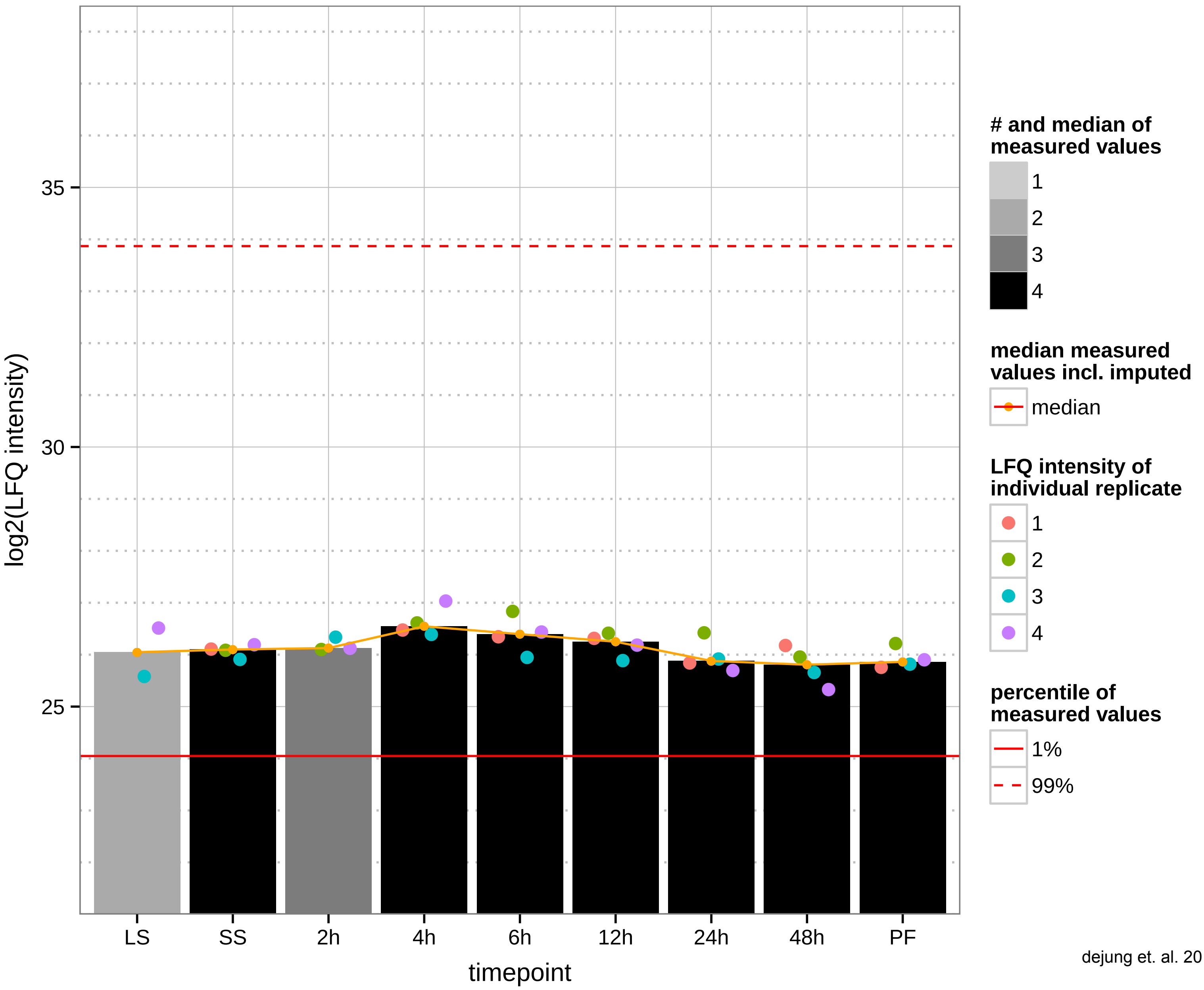

dejung et. al. 2015

cyclophilin, putative
Tb927.8.6910
AGOF: peptidyl–prolyl cis–trans isomerase activity
AGOC: null
AGOP: protein folding
PGOF: peptidyl–prolyl cis–trans isomerase activity
PGOC: null
PGOP: protein folding

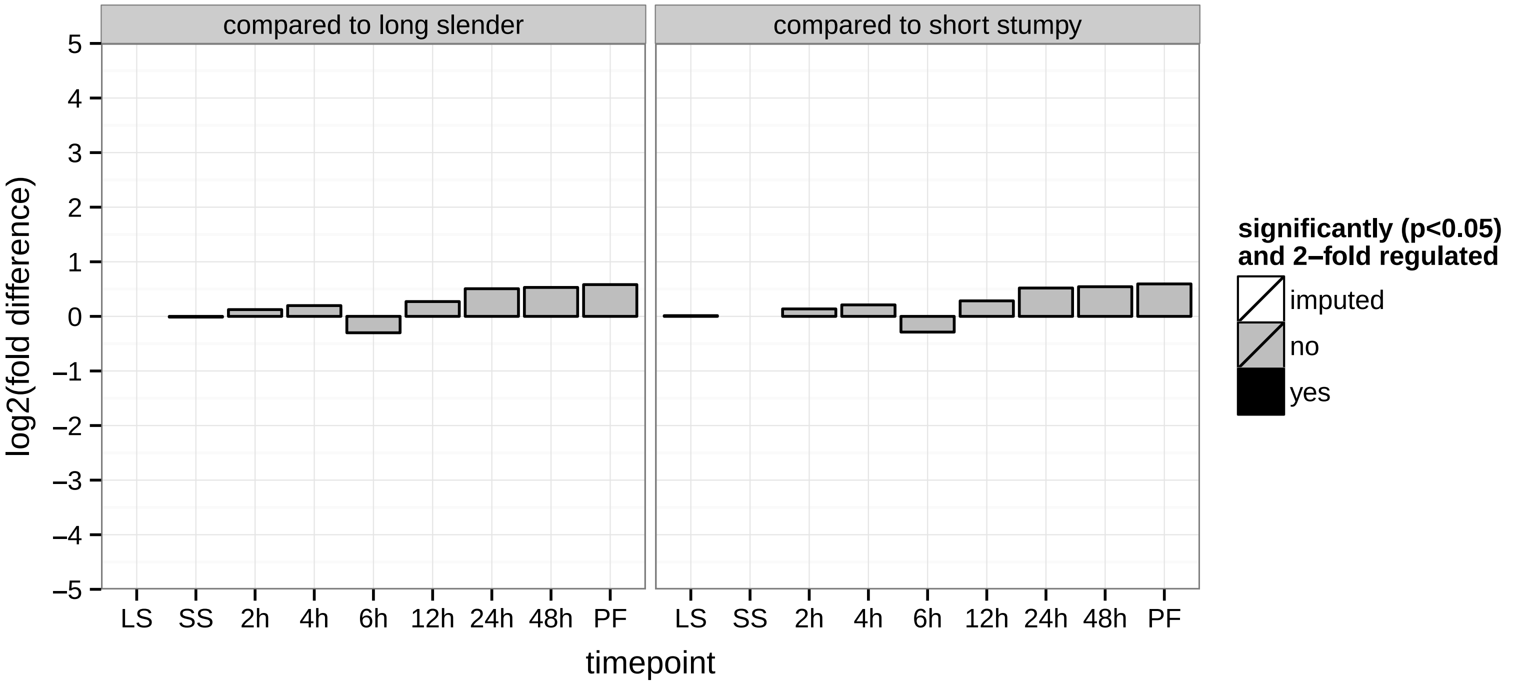

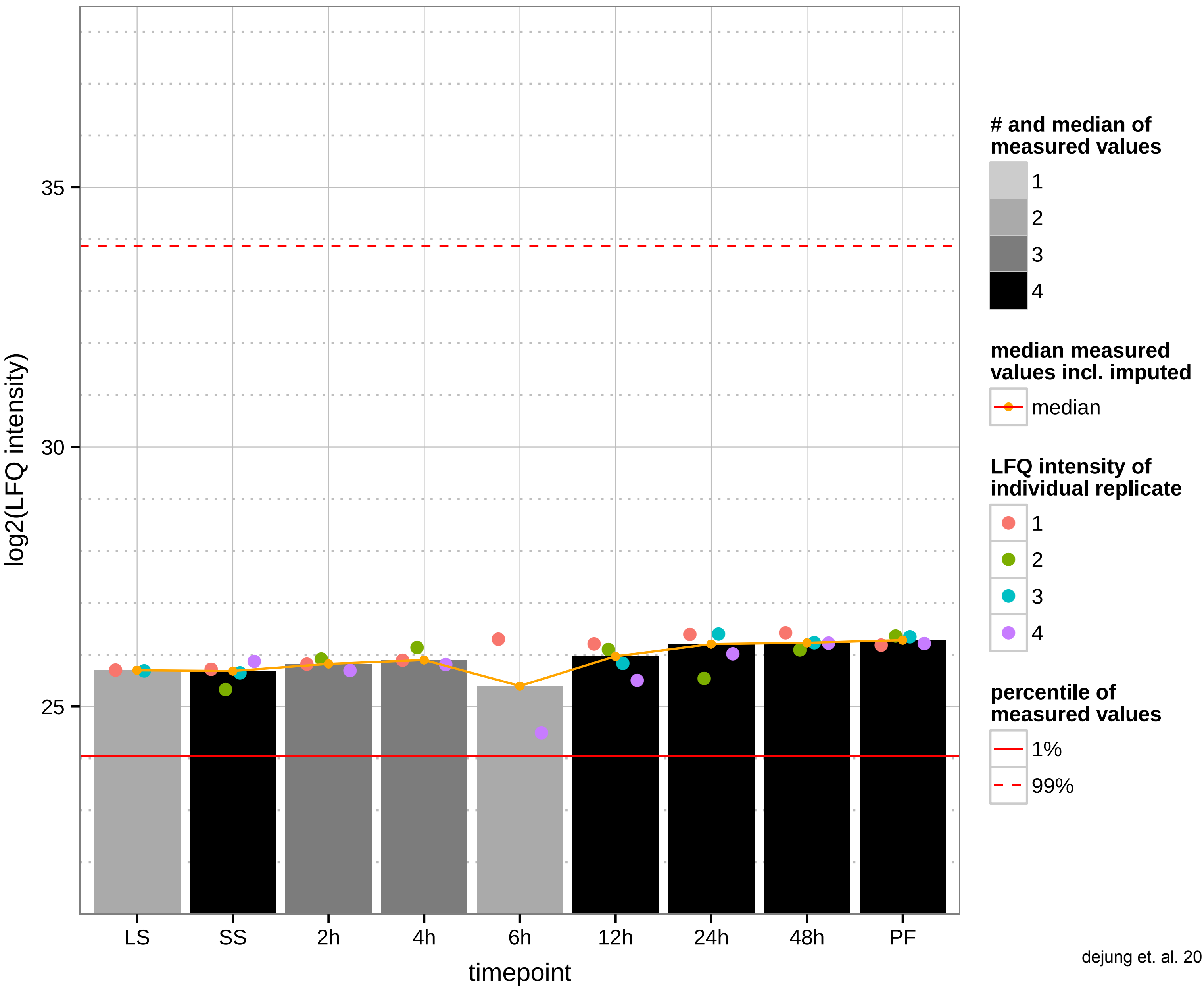

dejung et. al. 2015

hypothetical protein, conserved
Tb927.8.6920
AGOF: null
AGOC: null
AGOP: null
PGOF: null
PGOC: null
PGOP: null

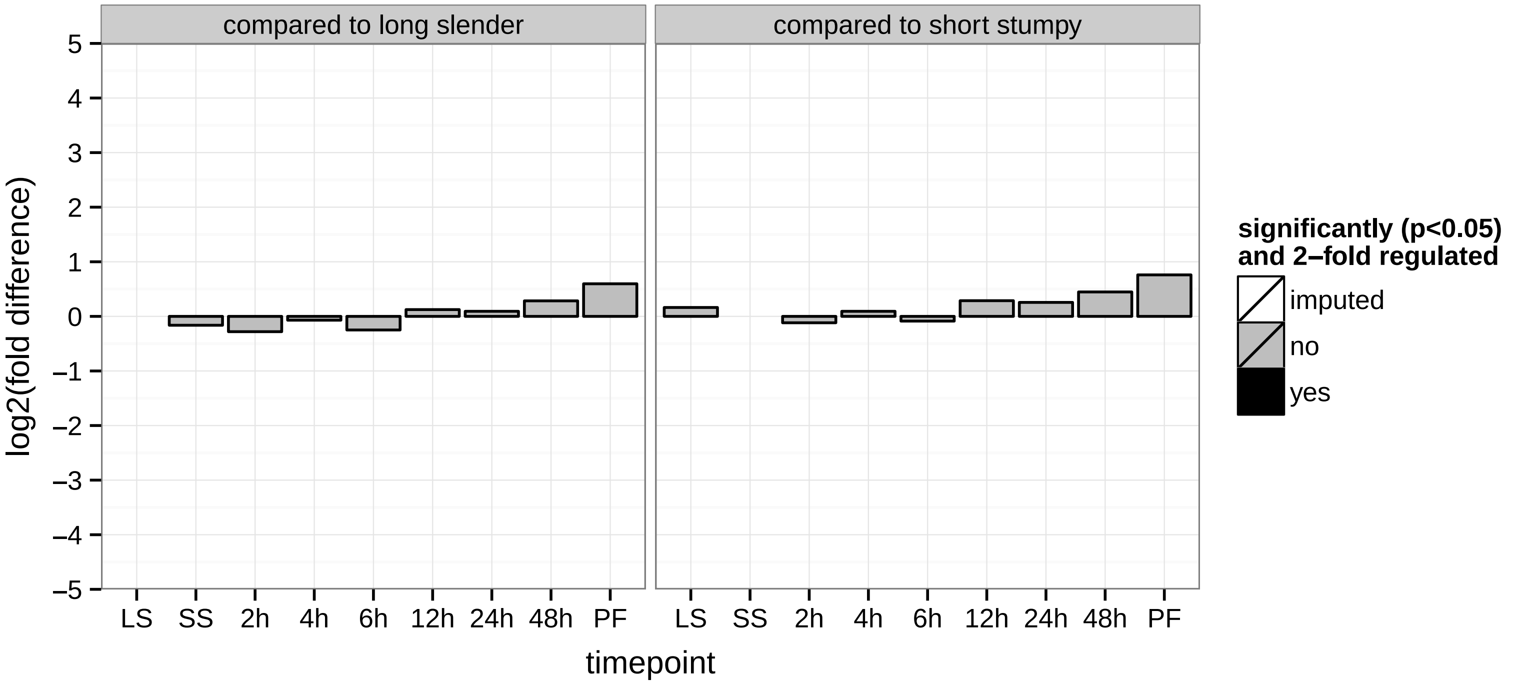

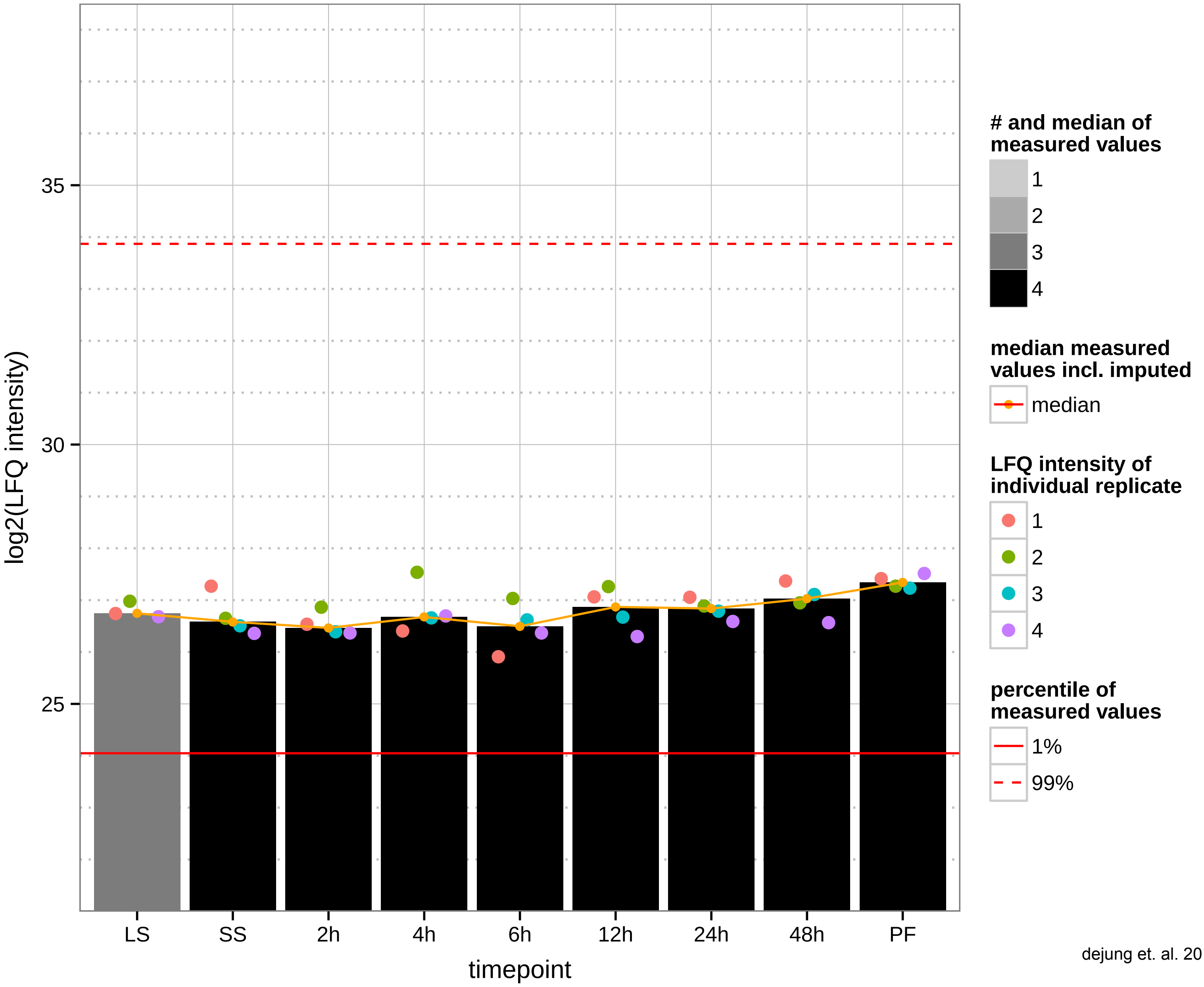

dejung et. al. 2015

dynein light chain 2B, cytoplasmic, putative, predicted dynein modulator, roadblock/LC7 family member
Tb927.8.6950;Tb927.4.5370
AGOF: null, microtubule motor activity
AGOC: null, cytoplasmic dynein complex
AGOP: ciliary or flagellar motility, microtubule–based movement
PGOF: null
PGOC: null
PGOP: null

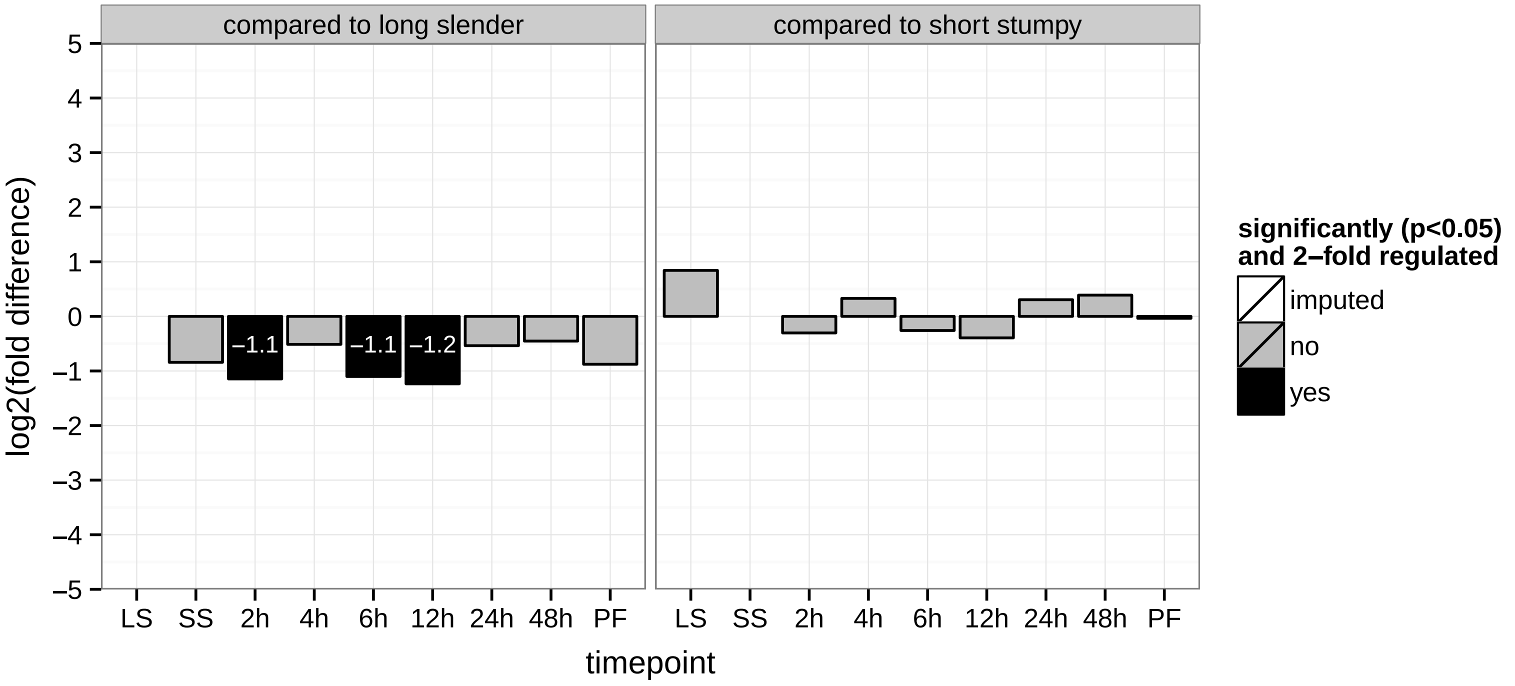

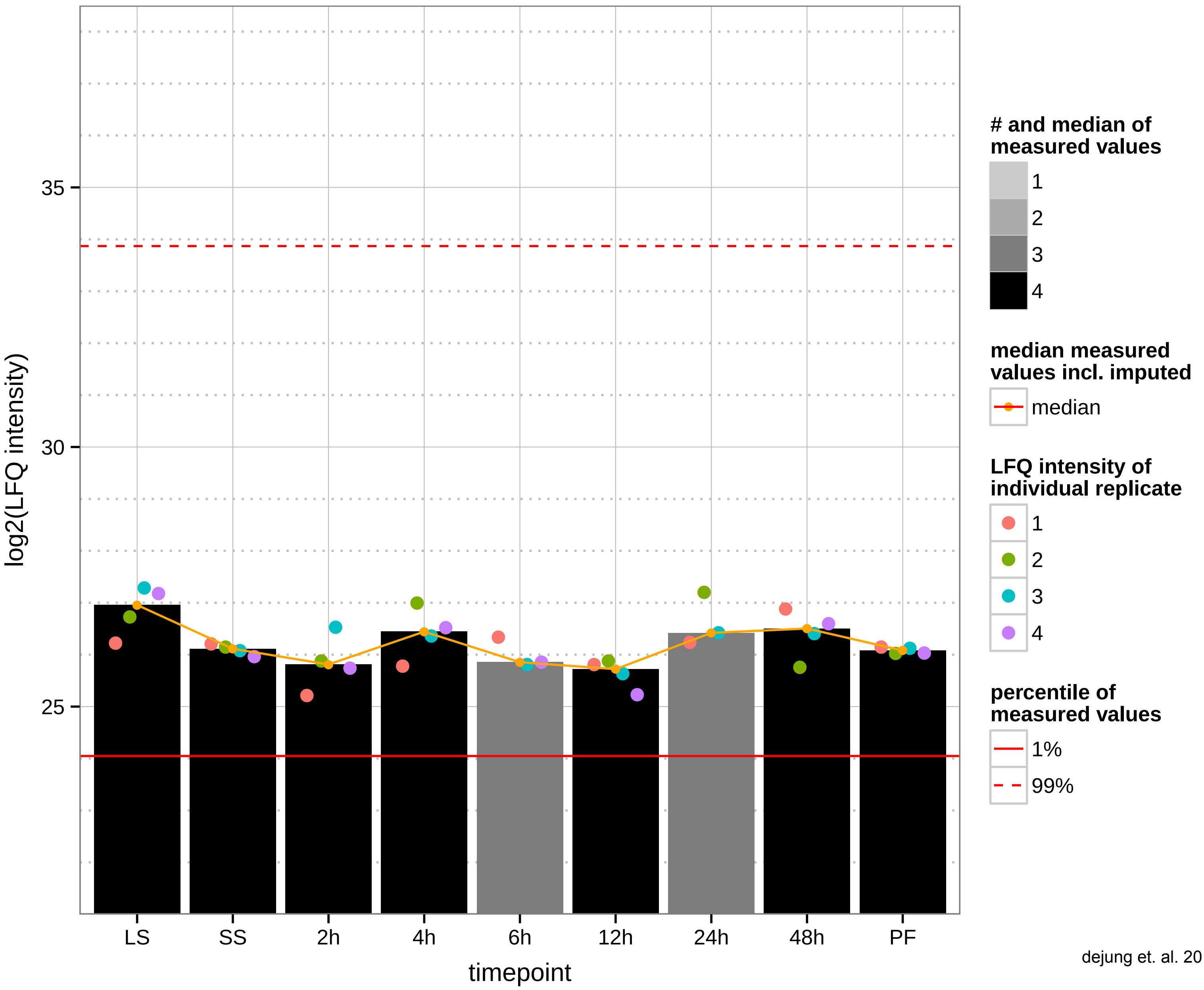

dejung et. al. 2015

hypothetical protein, conserved
Tb927.8.6980
AGOF: null
AGOC: null
AGOP: null
PGOF: null
PGOC: null
PGOP: null

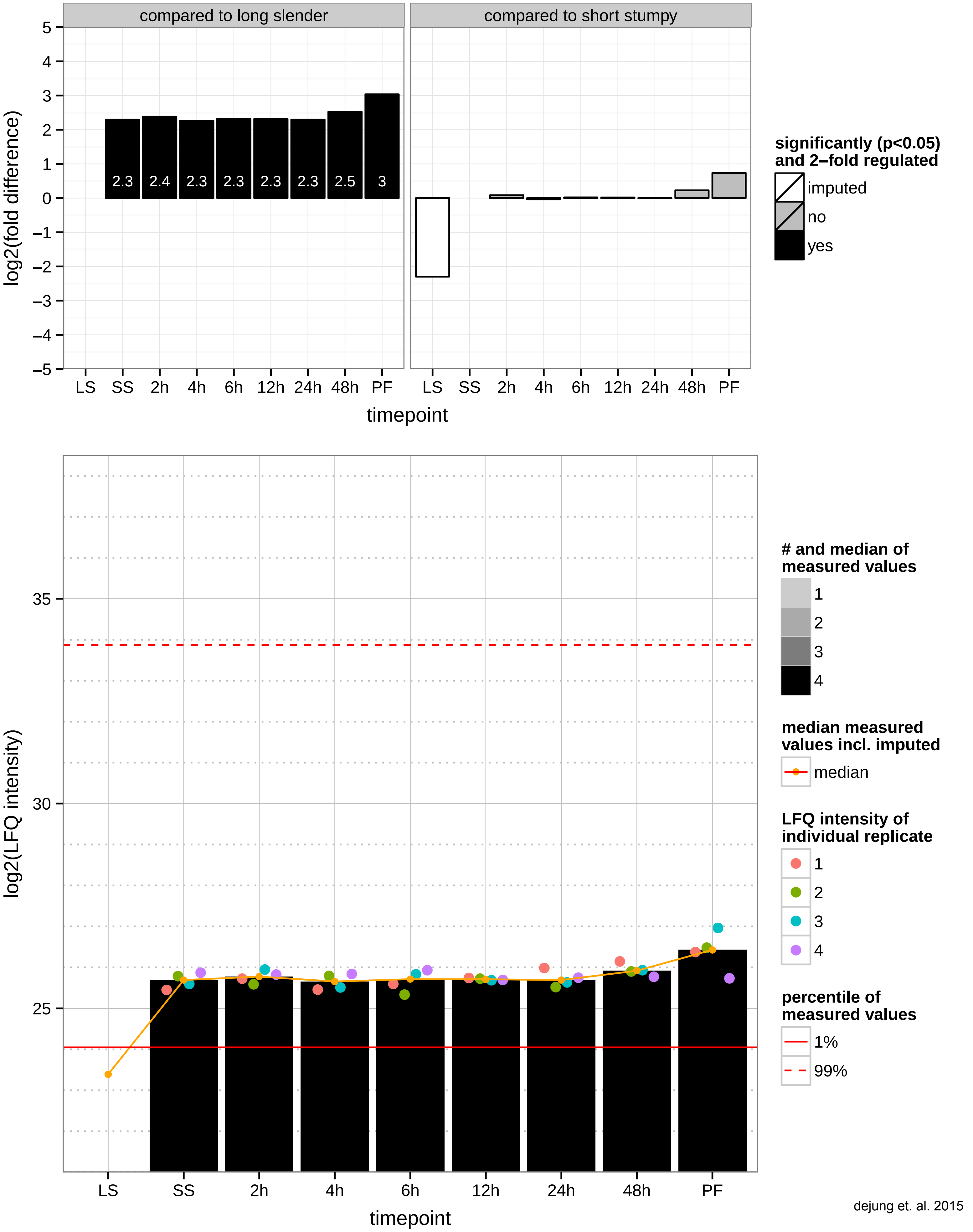

dejung et. al. 2015

peptidase, putative, metallo–peptidase, Clan ME, Family M16
Tb927.8.7020
AGOF: catalytic activity, metalloendopeptidase activity, zinc ion binding
AGOC: null
AGOP: proteolysis
PGOF: catalytic activity, metal ion binding, metalloendopeptidase activity, zinc ion binding
PGOC: null
PGOP: proteolysis

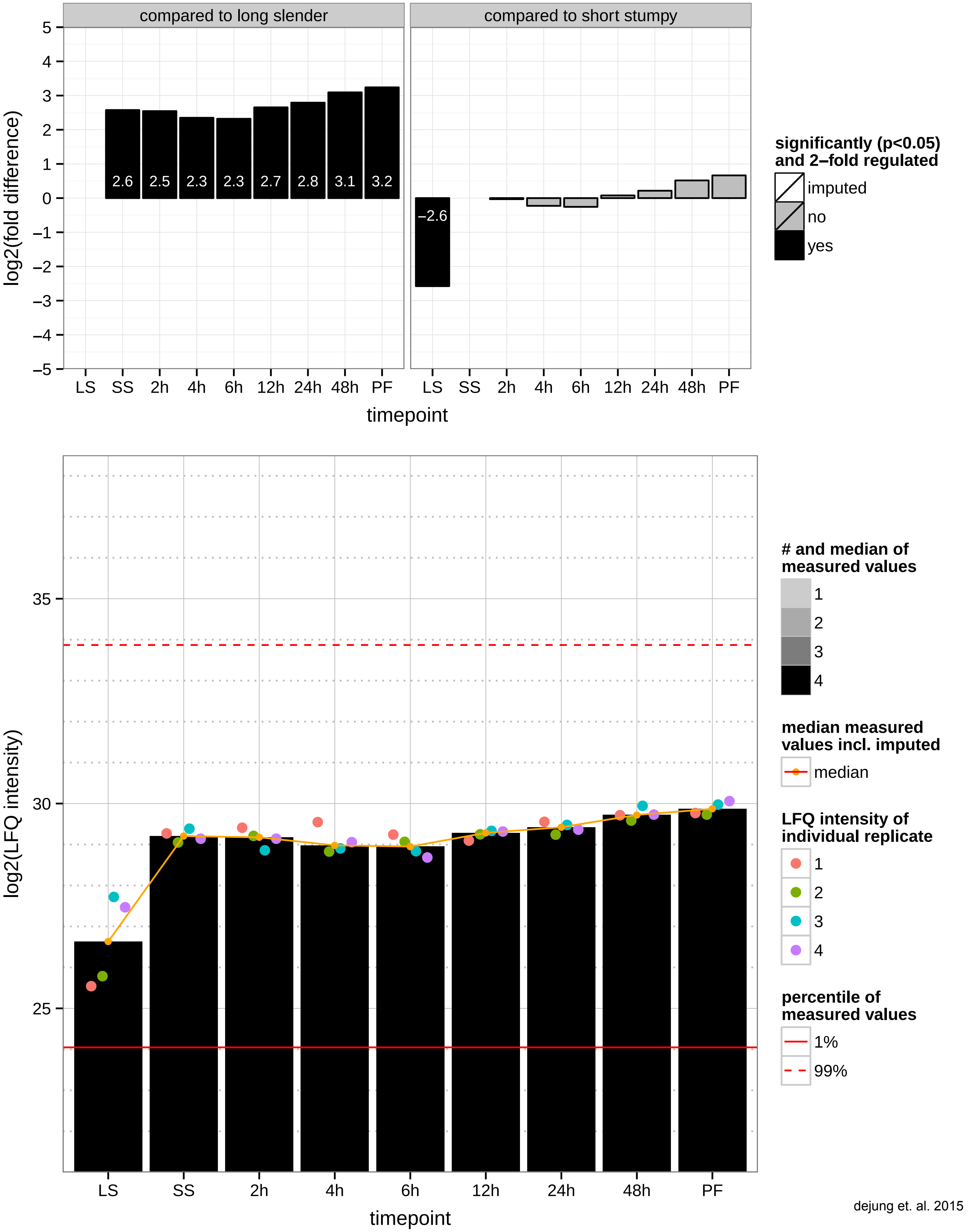

dejung et. al. 2015

acetyl–CoA carboxylase
Tb927.8.7100
AGOF: ATP binding, acetyl–CoA carboxylase activity, biotin binding, biotin carboxylase activity
AGOC: biotin carboxylase complex, cytoplasm, mitochondrion
AGOP: fatty acid biosynthetic process, fatty acid elongation, quorum sensing
PGOF: ATP binding, acetyl–CoA carboxylase activity, biotin carboxylase activity, catalytic activity, ligase activity, metal ion binc
PGOC: null
PGOP: fatty acid biosynthetic process, metabolic process

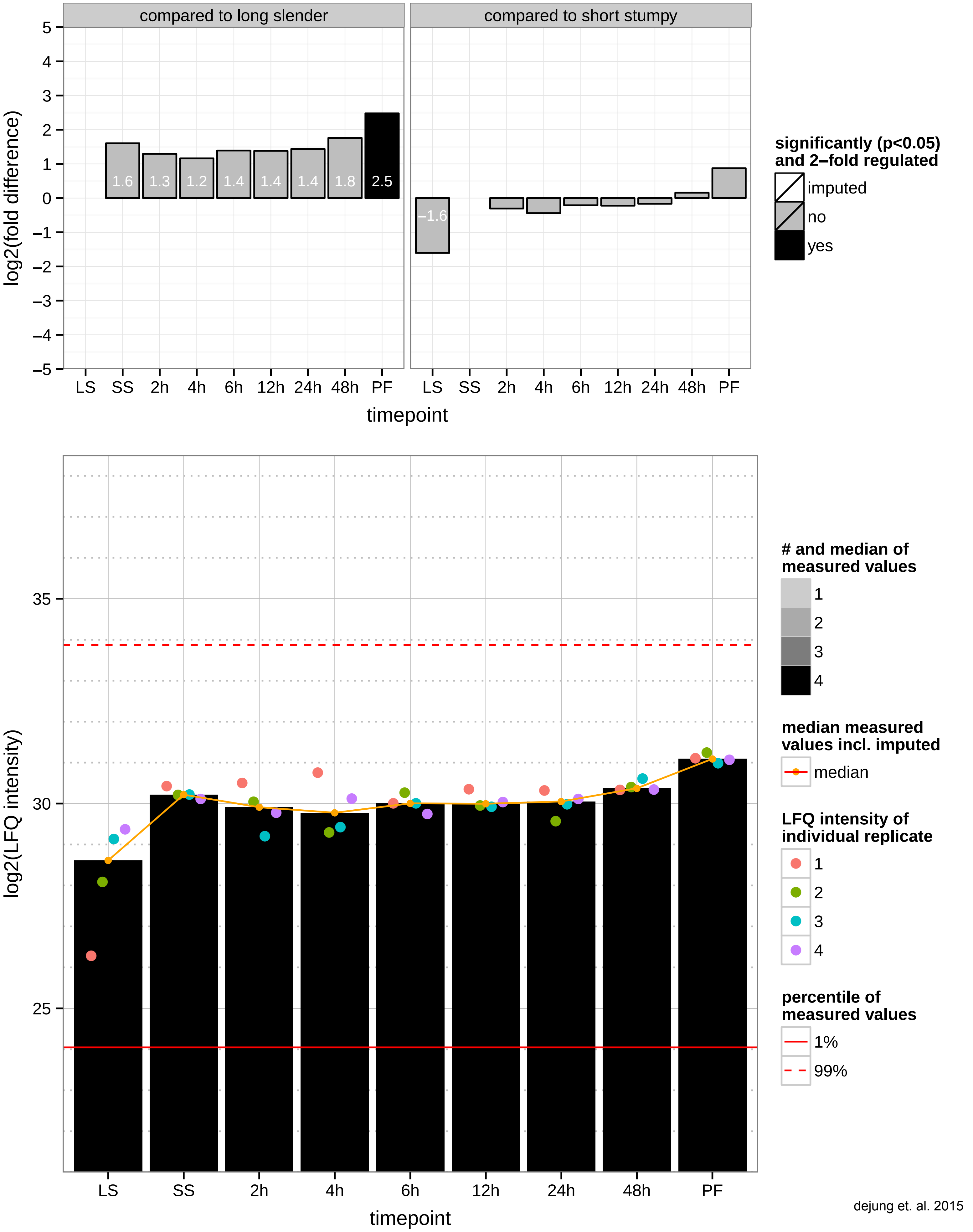

dejung et. al. 2015

inositol polyphosphate 1–phosphatase, putative
Tb927.8.7170
AGOF: null
AGOC: mitochondrion
AGOP: phosphatidylinositol phosphorylation
PGOF: null
PGOC: null
PGOP: phosphatidylinositol phosphorylation

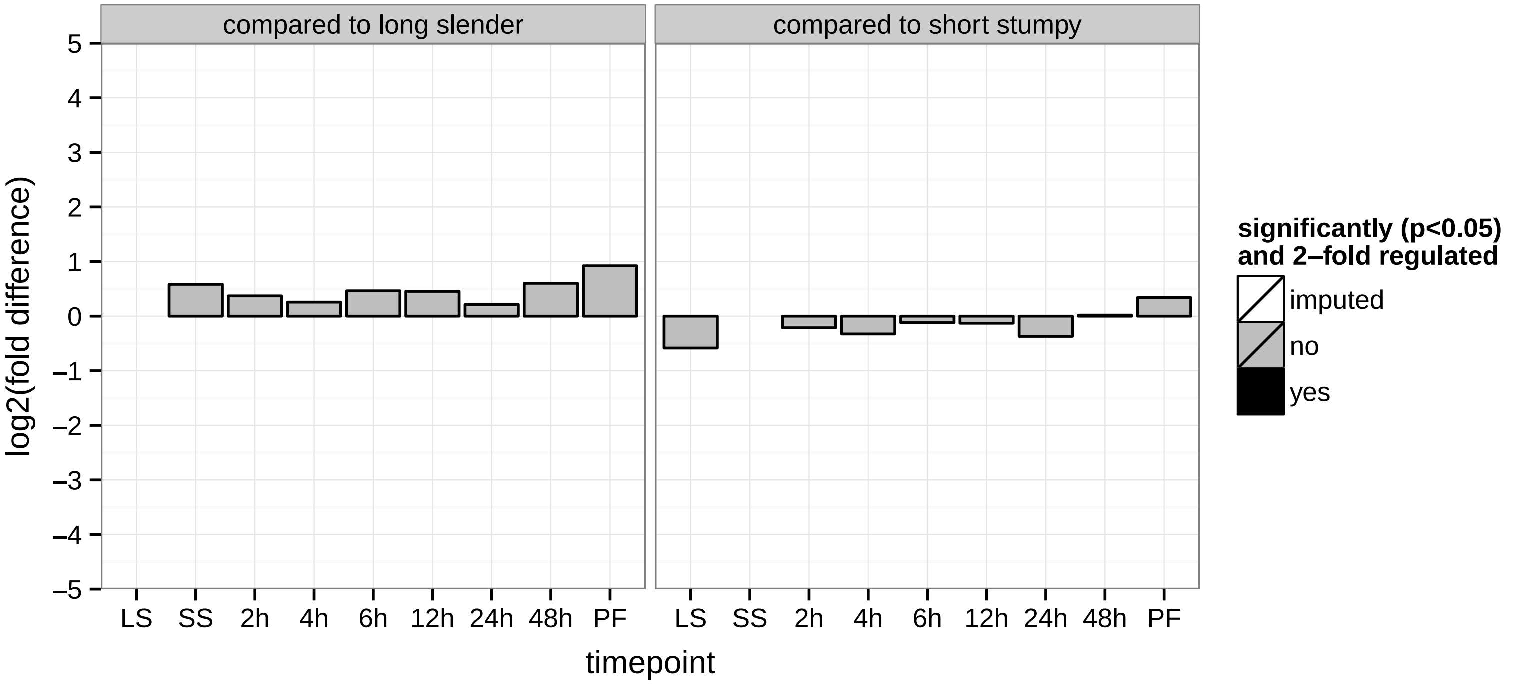

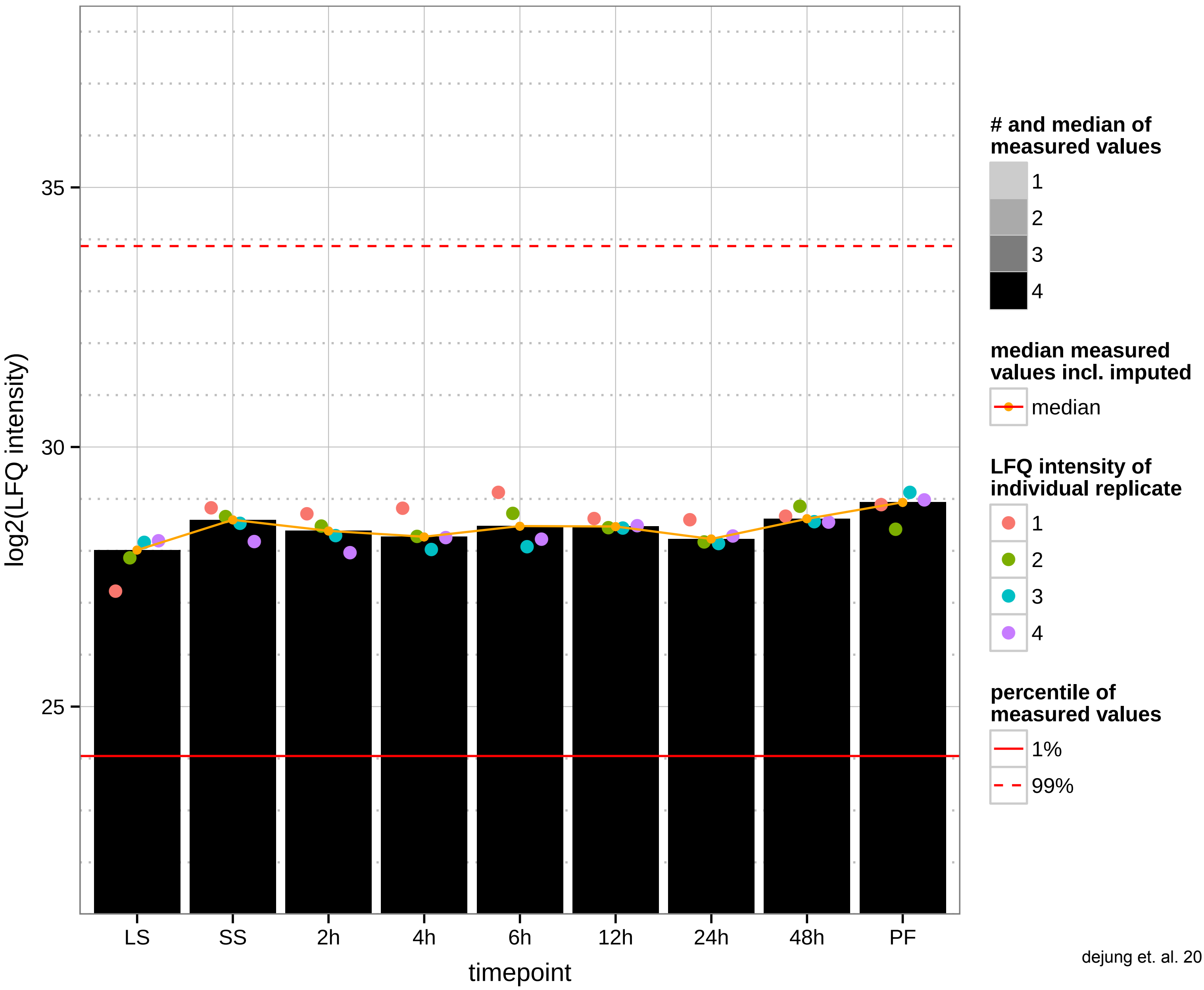

dejung et. al. 2015

nucleolar RNA–binding protein, putative
Tb927.8.730
AGOF: RNA binding
AGOC: nucleolus
AGOP: RNA metabolic process
PGOF: null
PGOC: null
PGOP: null

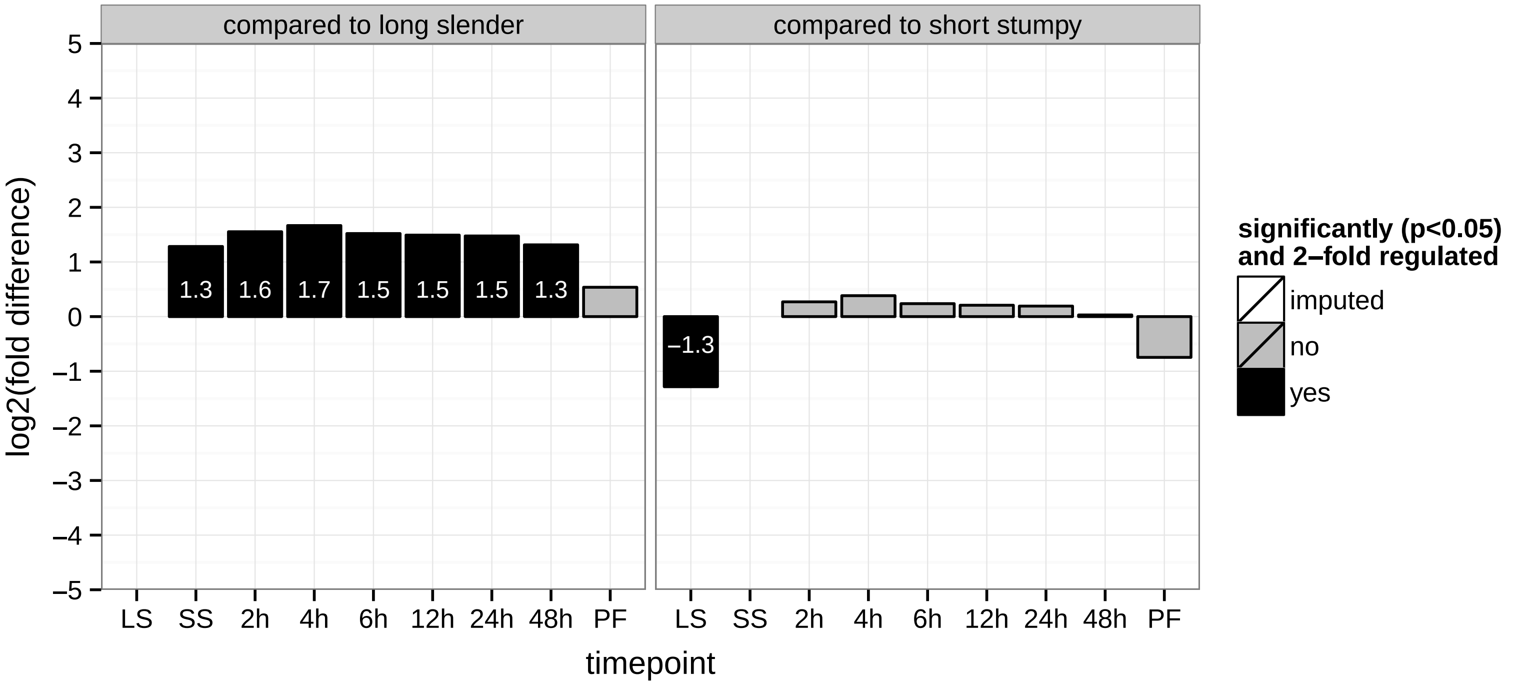

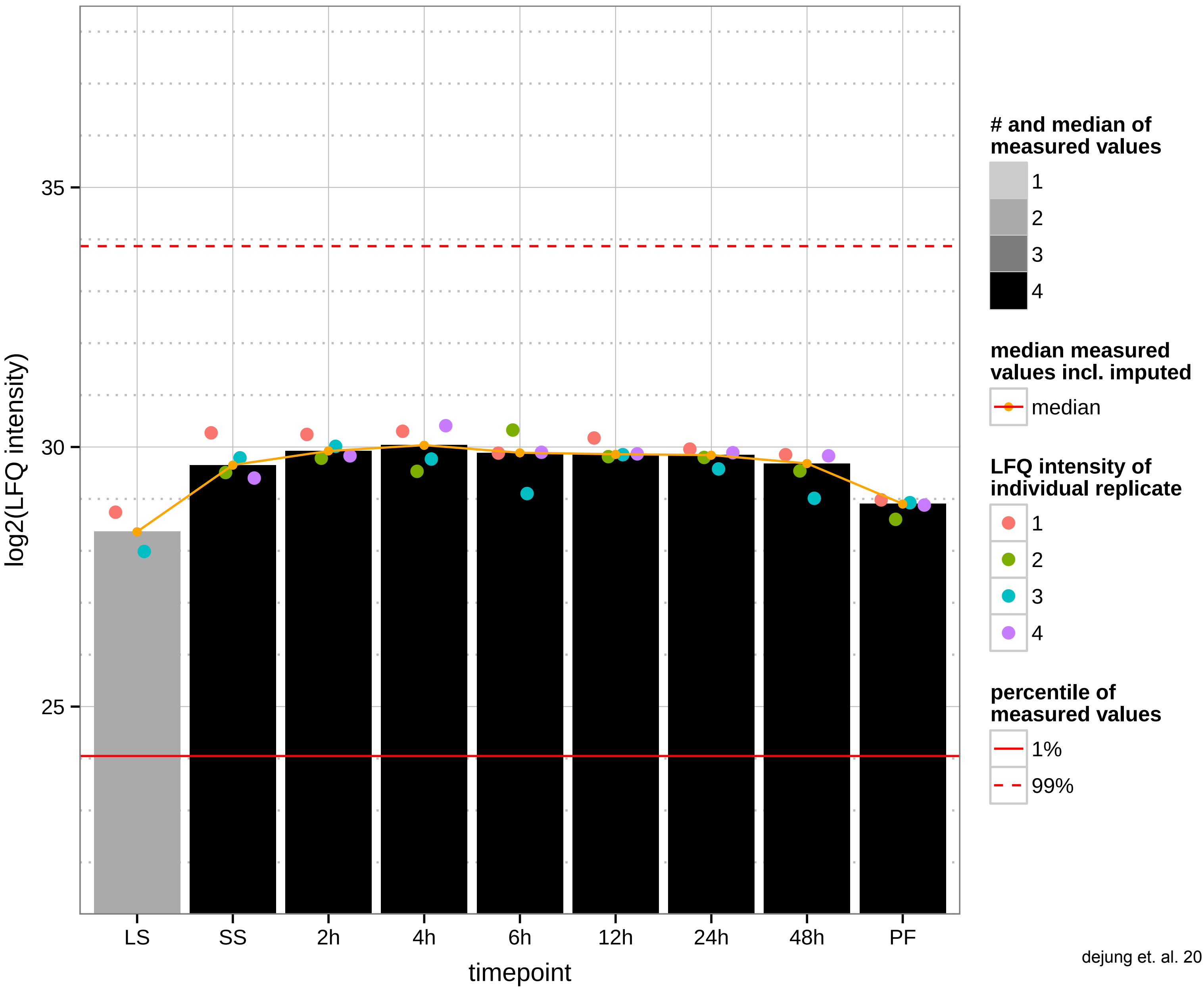

dejung et. al. 2015

RNA polymerase IIA largest subunit, putative, RNA polymerase IIA largest subunit (RPB1)
Tb927.8.7400;Tb927.4.5020;Tb11.v5.0504
AGOF: null, DNA binding, DNA–directed RNA polymerase activity
AGOC: null, nucleus
AGOP: null, transcription, DNA–dependent
PGOF: DNA binding, DNA–directed RNA polymerase activity
PGOC: null
PGOP: transcription, DNA–dependent

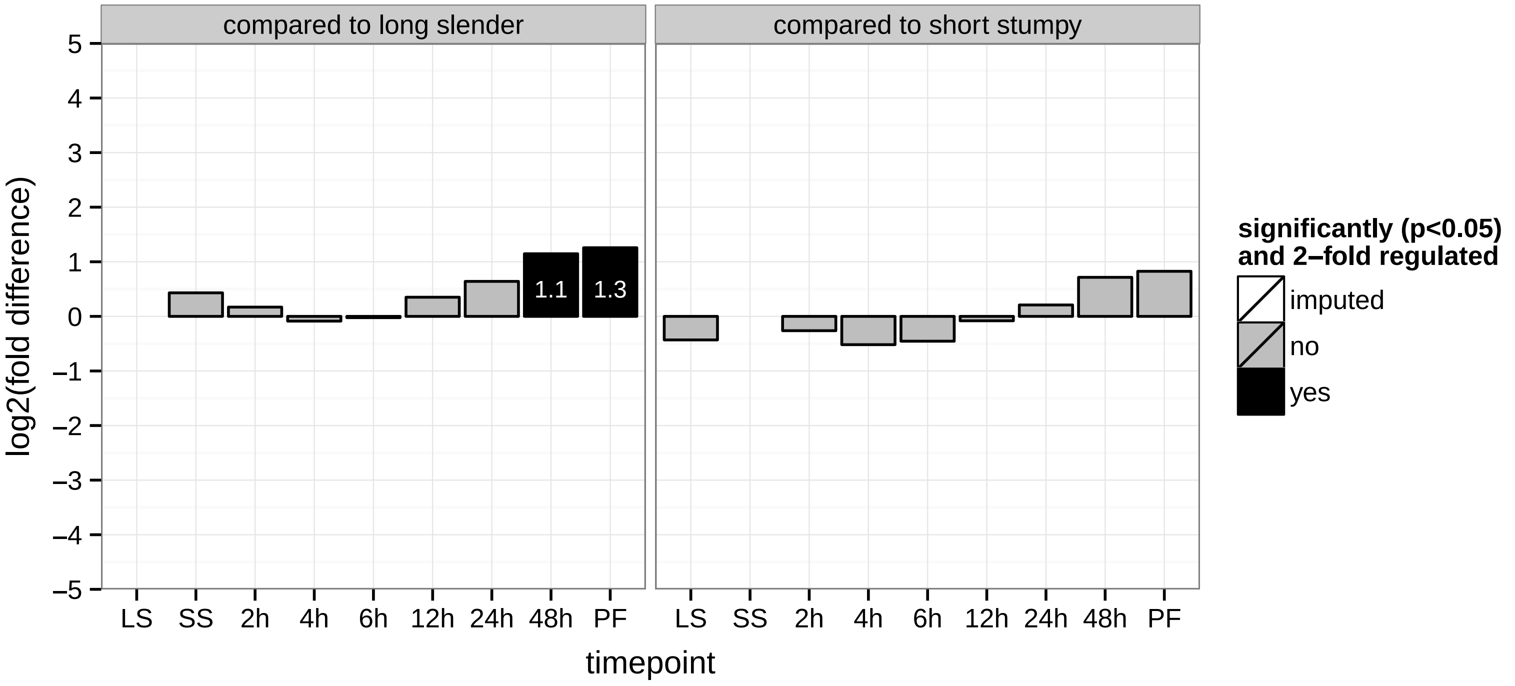

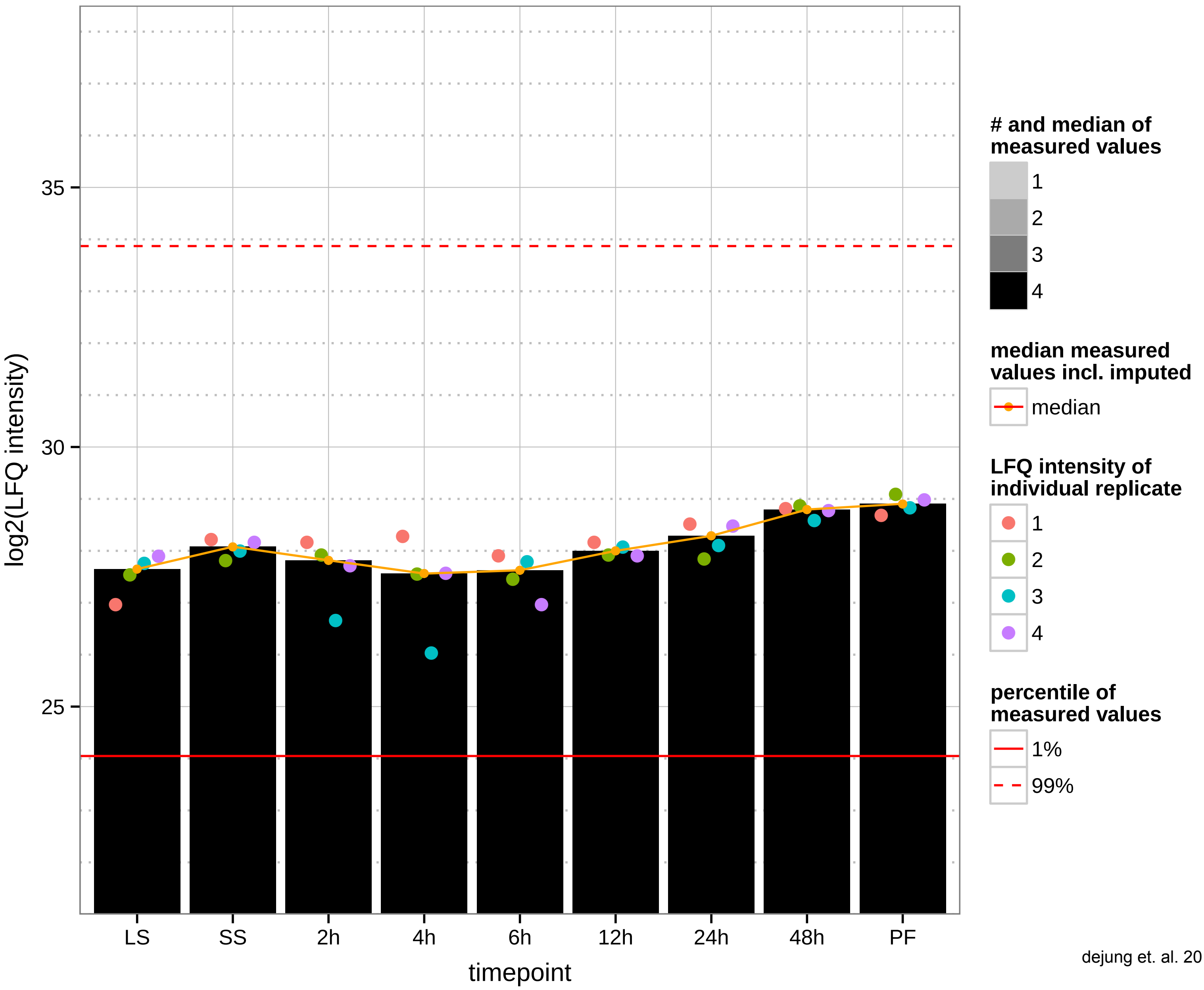

dejung et. al. 2015

calreticulin, putative
Tb927.8.7410
AGOF: calcium ion binding, carbohydrate binding, glucosidase activity, transferase activity, transferring glycosyl groups, unfolc
AGOC: endoplasmic reticulum, integral to membrane
AGOP: protein folding
PGOF: calcium ion binding, unfolded protein binding
PGOC: endoplasmic reticulum
PGOP: protein folding

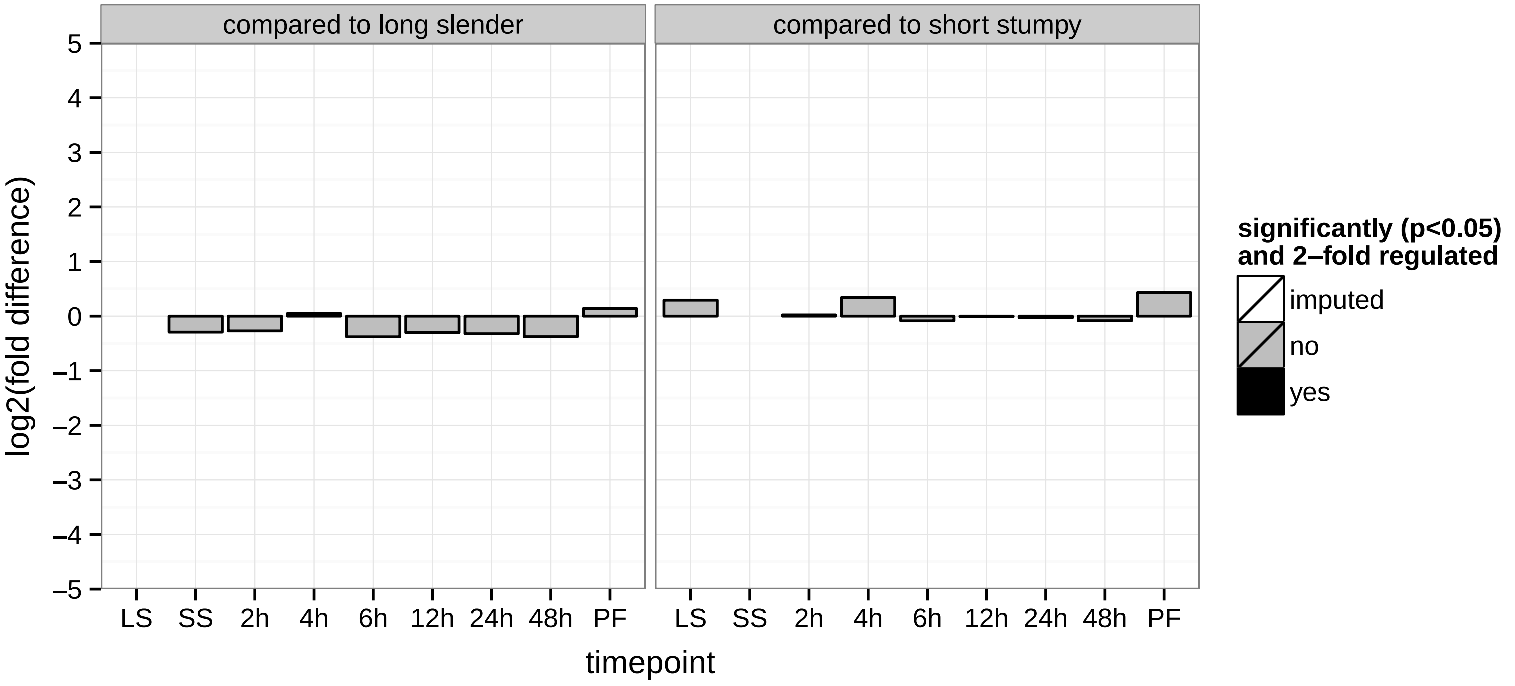

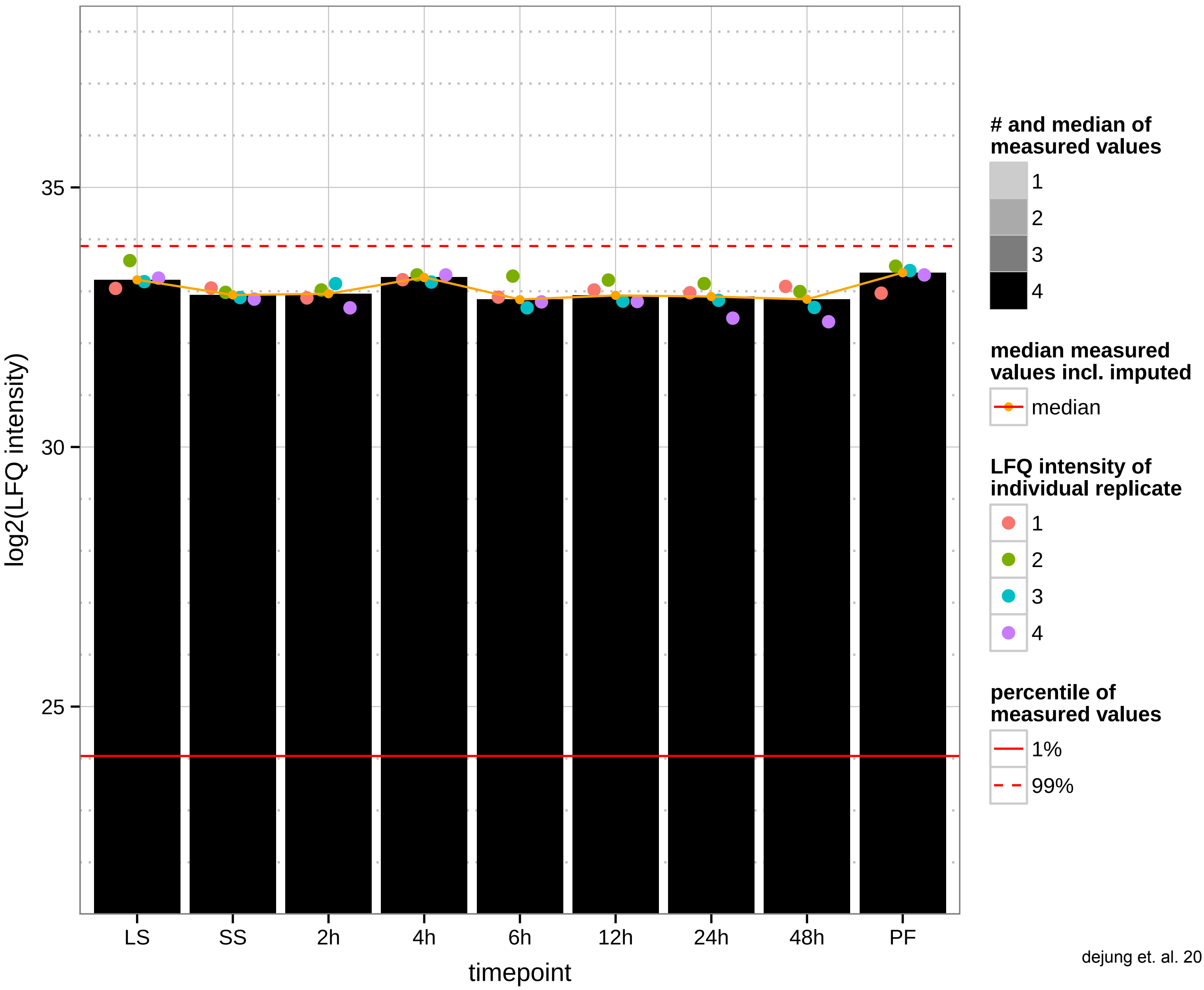

dejung et. al. 2015

hypothetical protein, conserved
Tb927.8.7490
AGOF: null
AGOC: null
AGOP: null
PGOF: null
PGOC: null
PGOP: null

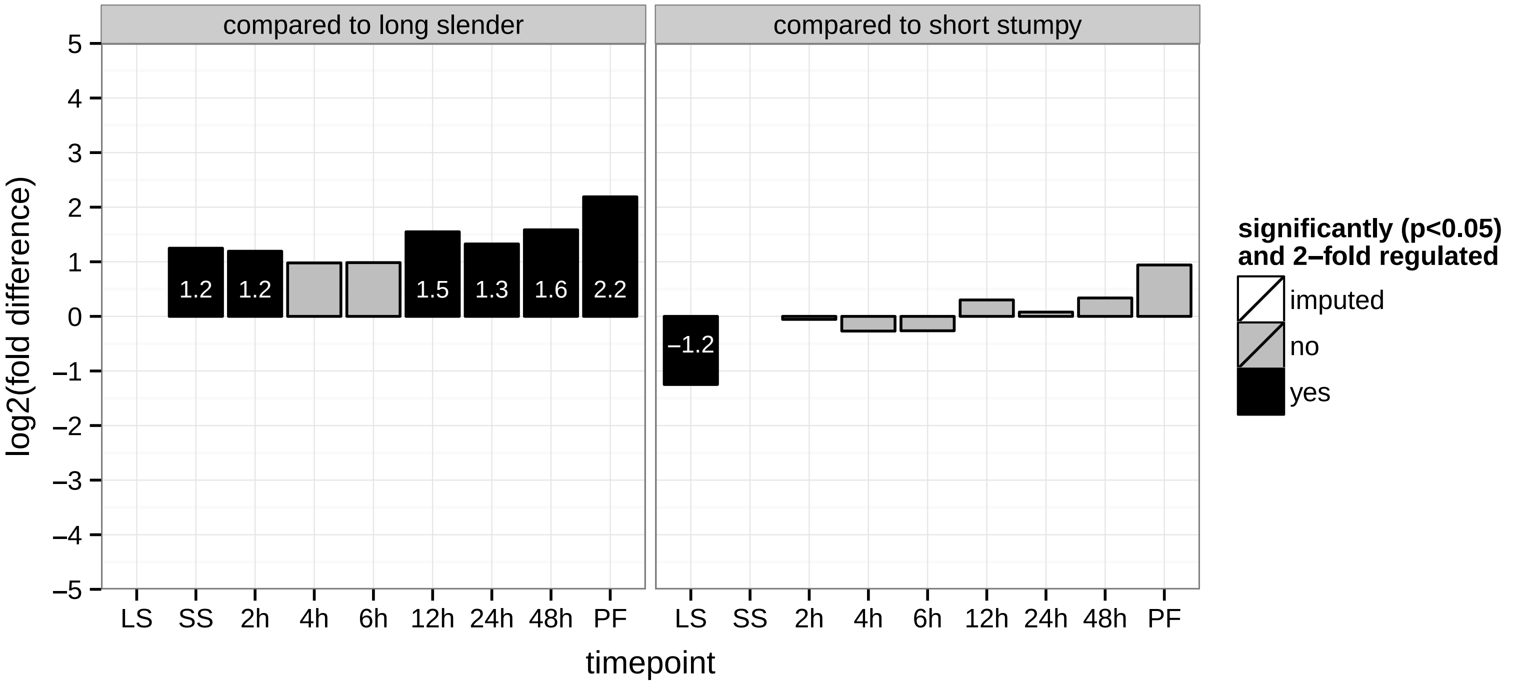

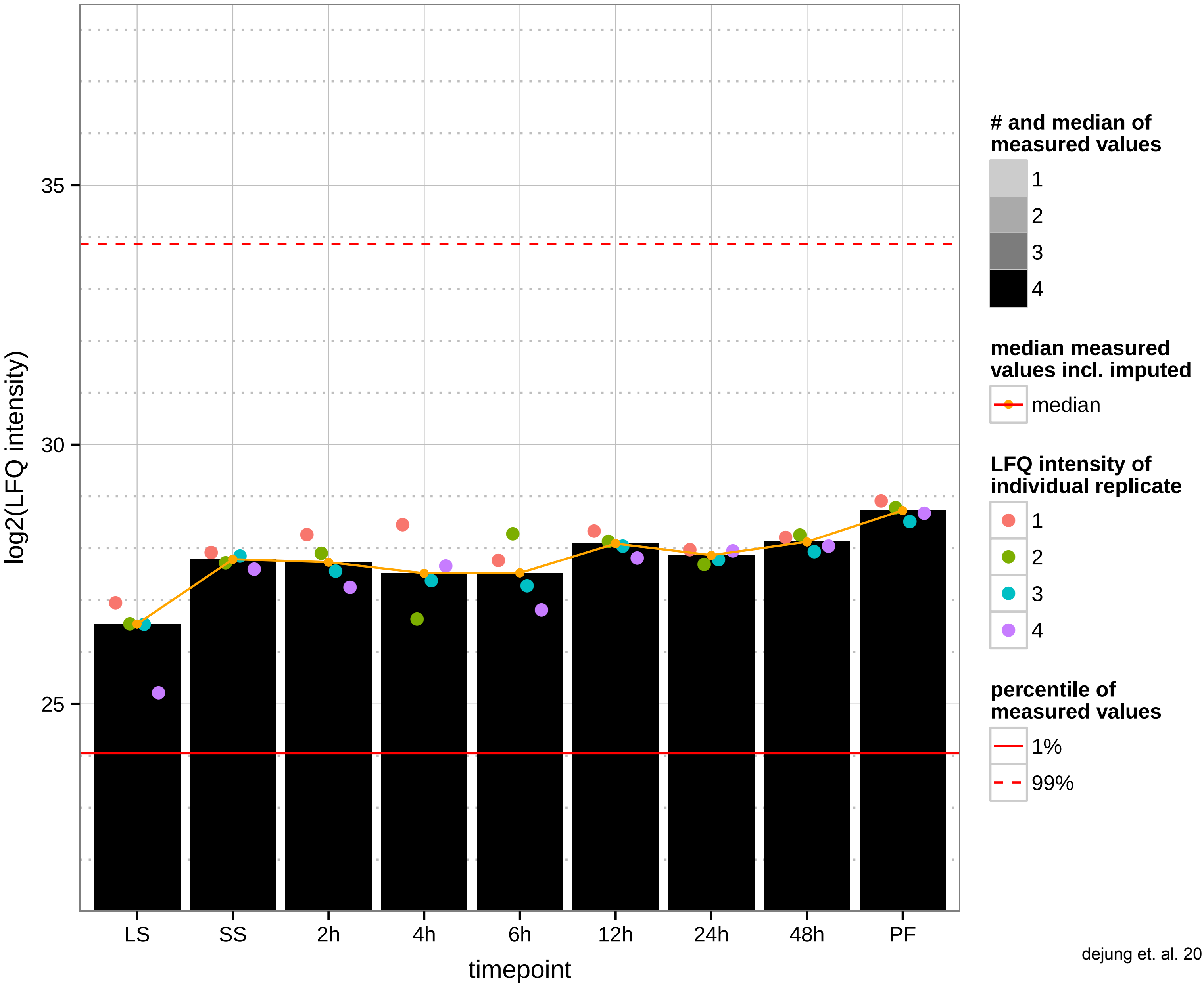

dejung et. al. 2015

nucleolar RNA–binding protein, putative
Tb927.8.750
AGOF: RNA binding
AGOC: nucleolus
AGOP: RNA metabolic process
PGOF: null
PGOC: null
PGOP: null

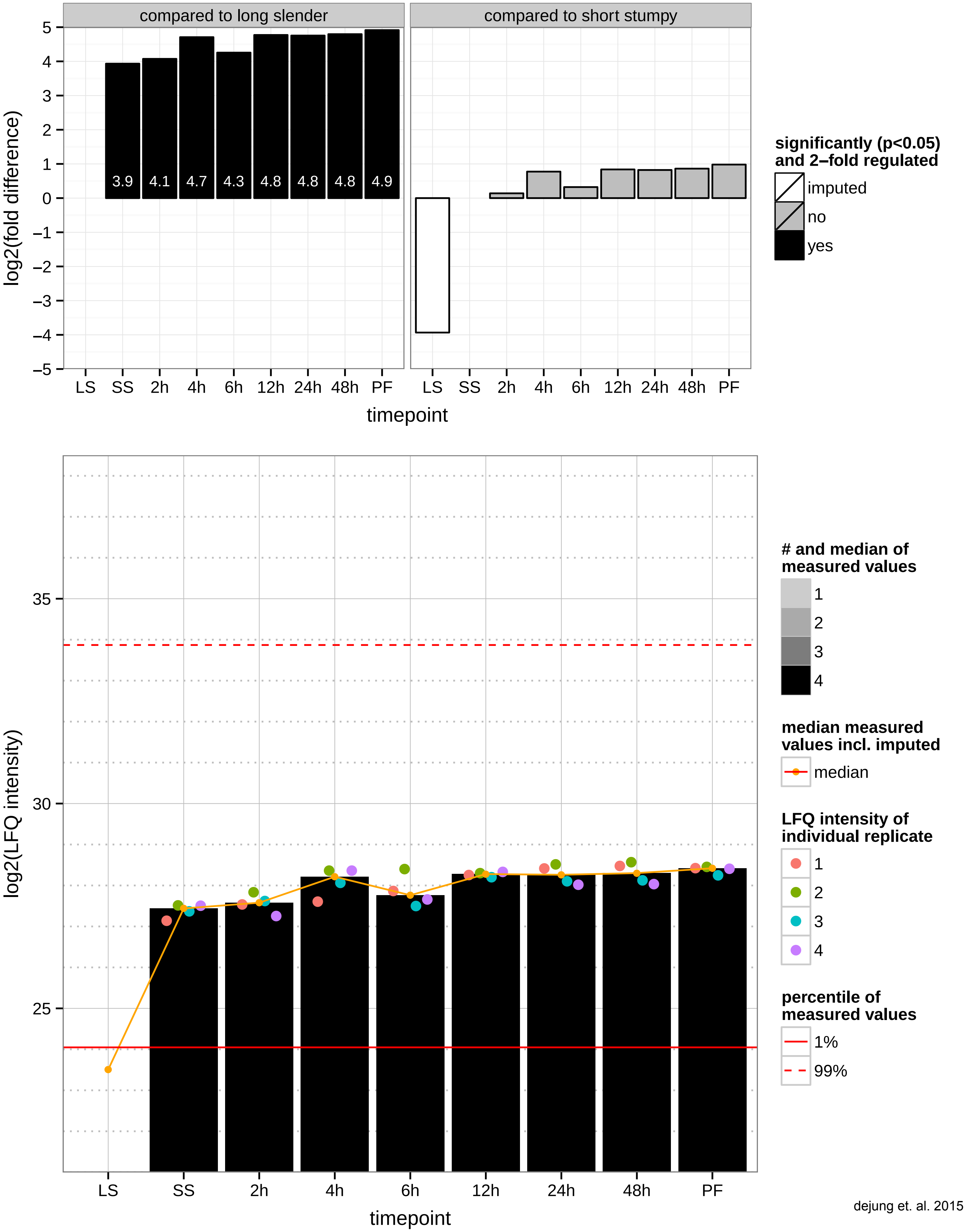

dejung et. al. 2015

p–nitrophenylphosphatase, putative
Tb927.8.7510
AGOF: phosphatase activity
AGOC: null
AGOP: metabolic process
PGOF: null
PGOC: null
PGOP: null

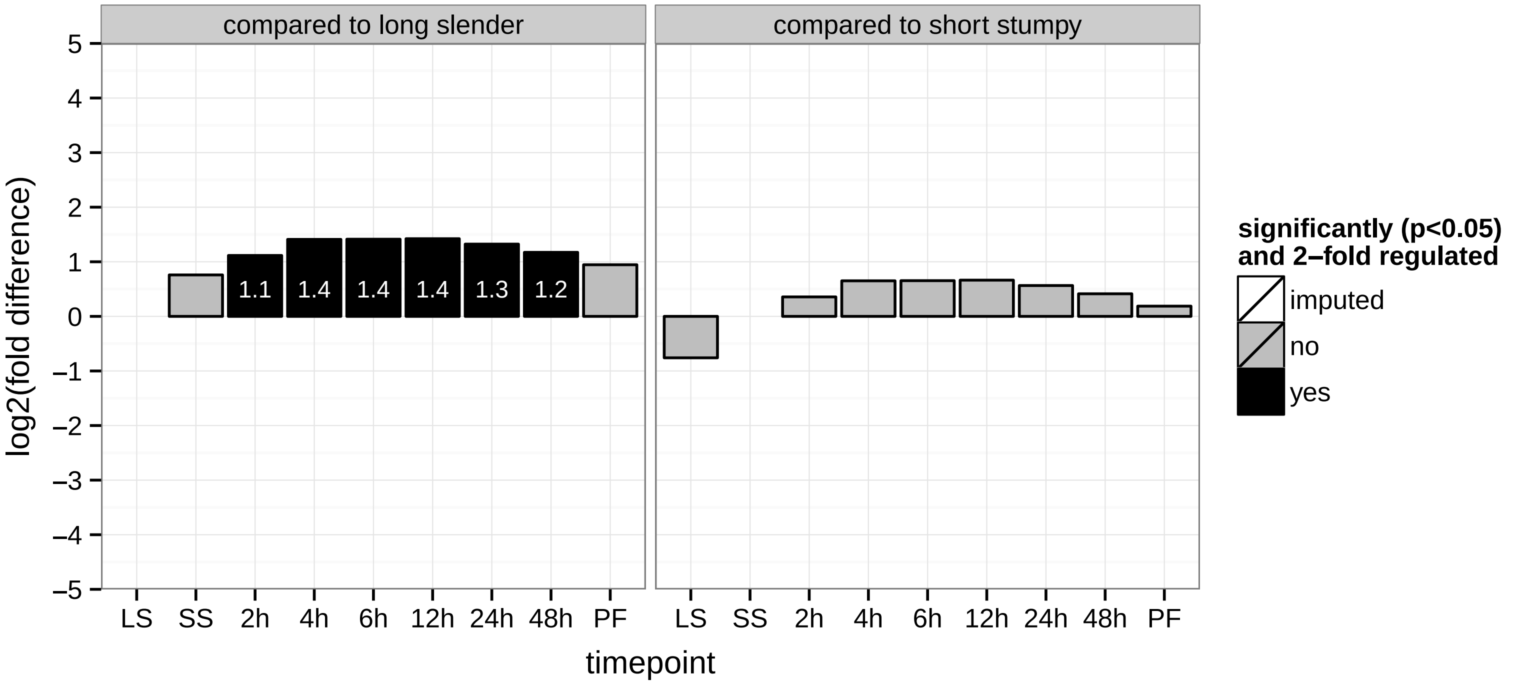

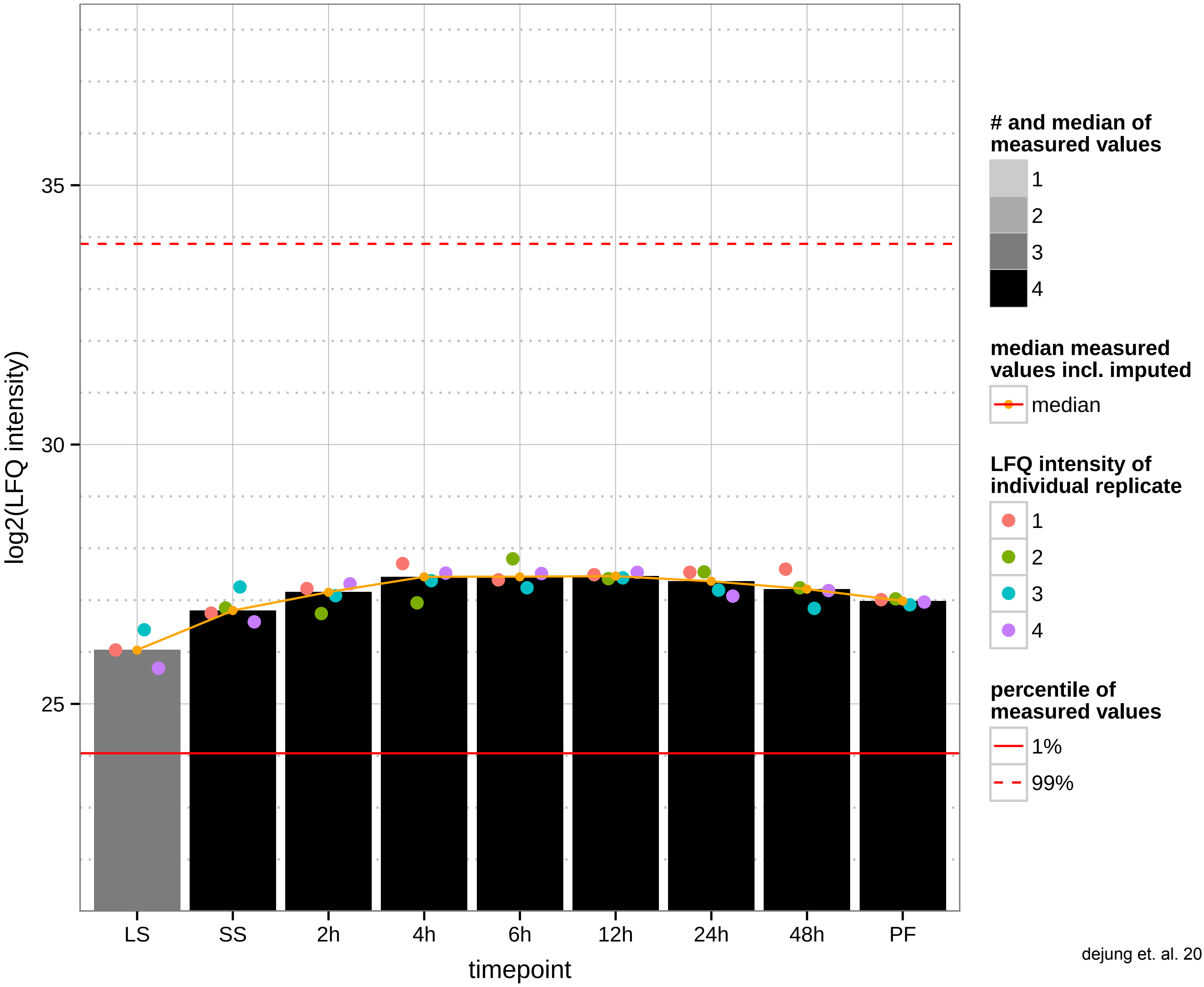

dejung et. al. 2015

nucleolar RNA–binding protein, truncated, nucleolar RNA–binding protein (Nopp44/46)
Tb927.8.760;Tb927.8.740
AGOF: RNA binding, nucleic acid binding, protein binding, protein serine/threonine/tyrosine kinase activity
AGOC: nucleolus
AGOP: null, RNA metabolic process
PGOF: null
PGOC: null
PGOP: null

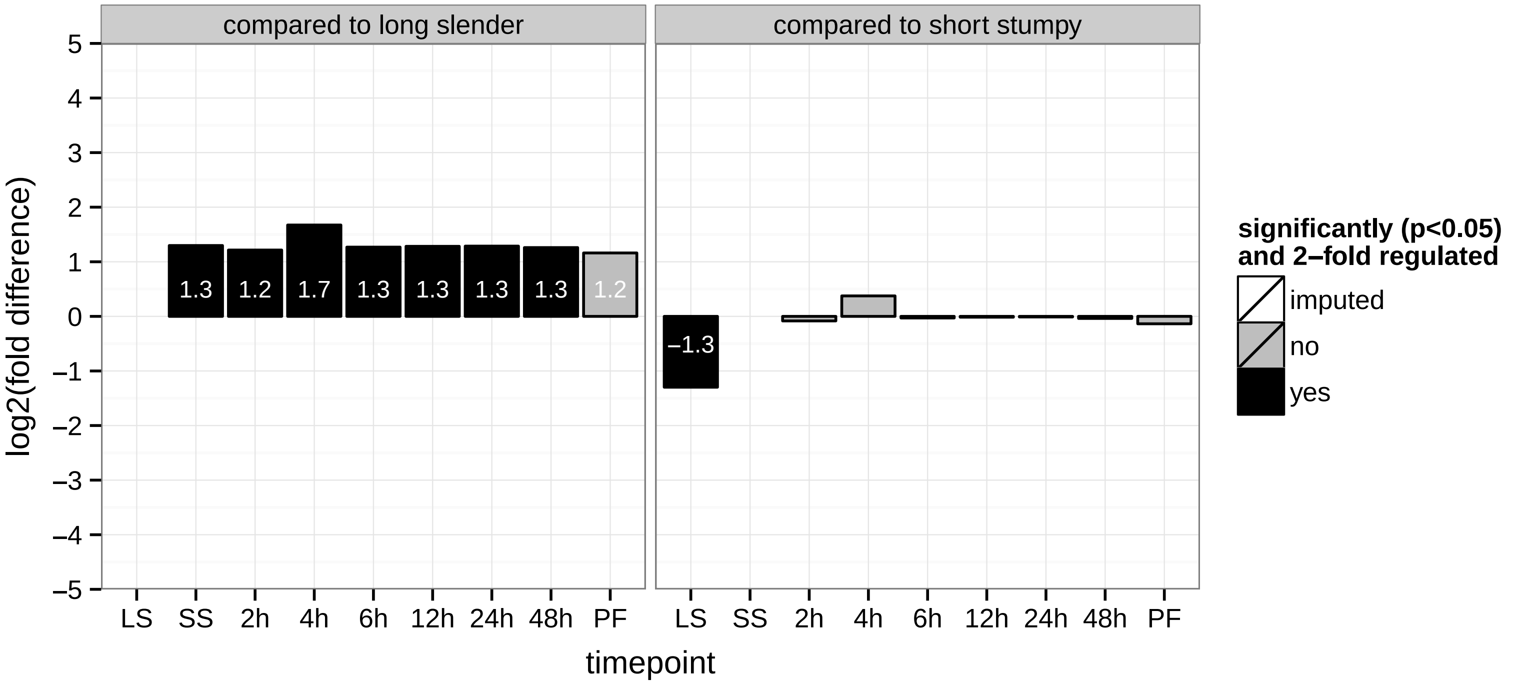

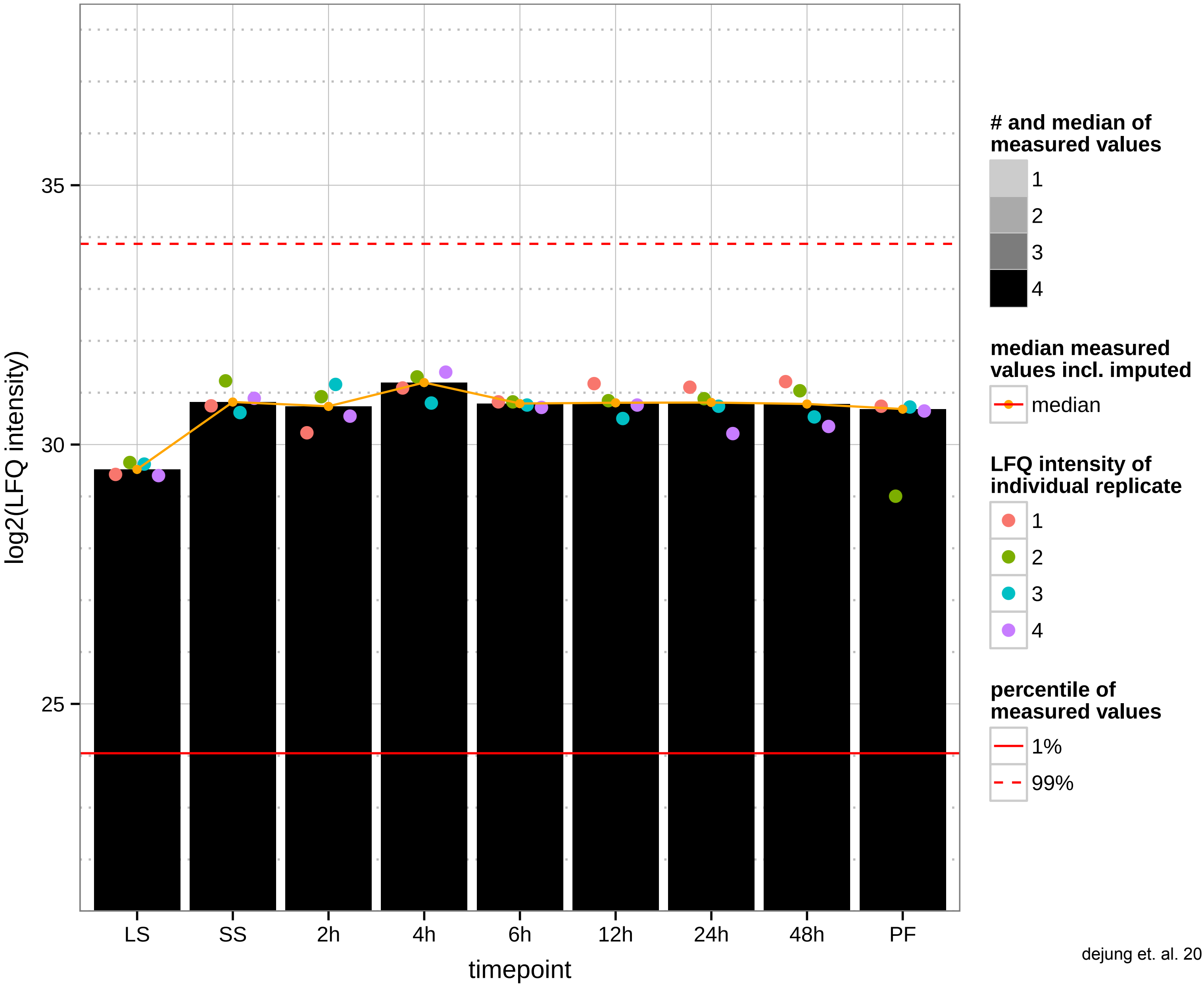

dejung et. al. 2015

hypothetical protein, conserved
Tb927.8.7950
AGOF: null
AGOC: null
AGOP: null
PGOF: protein binding
PGOC: null
PGOP: null

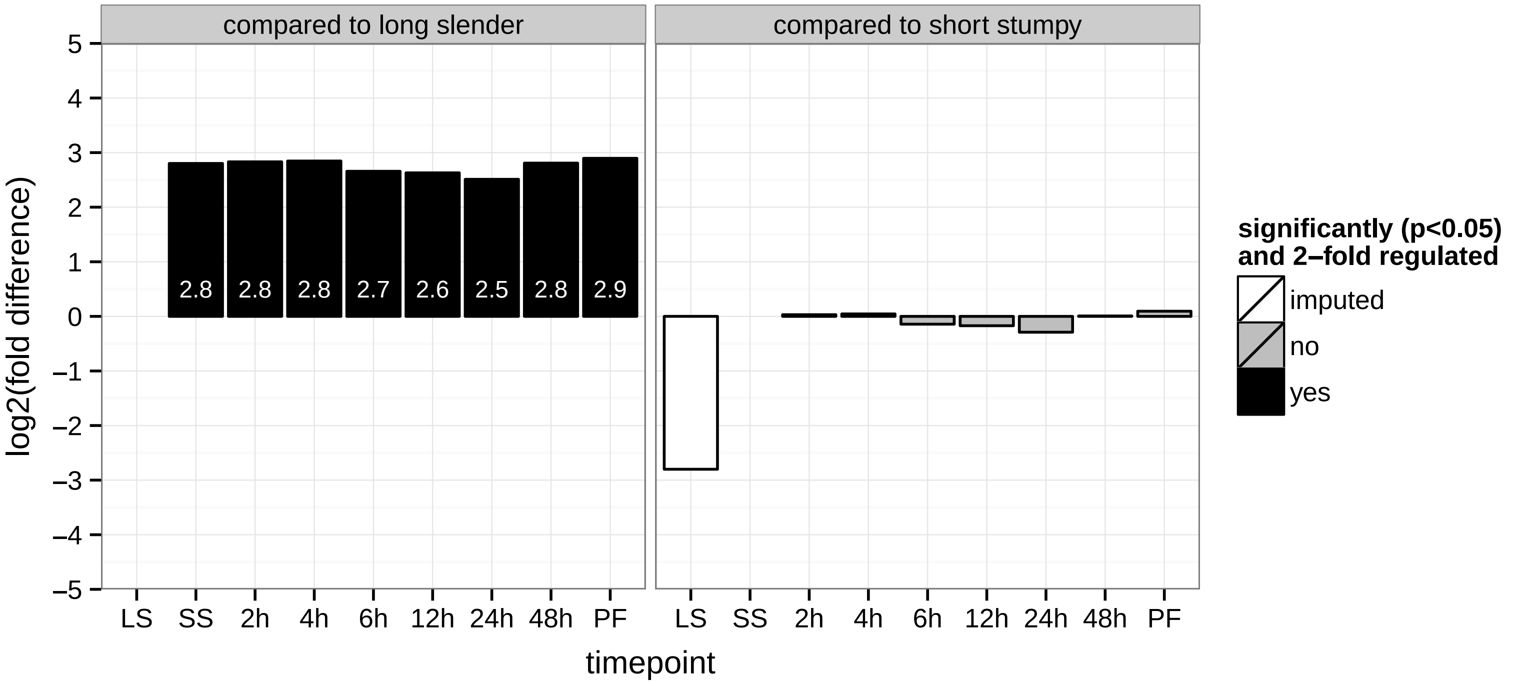

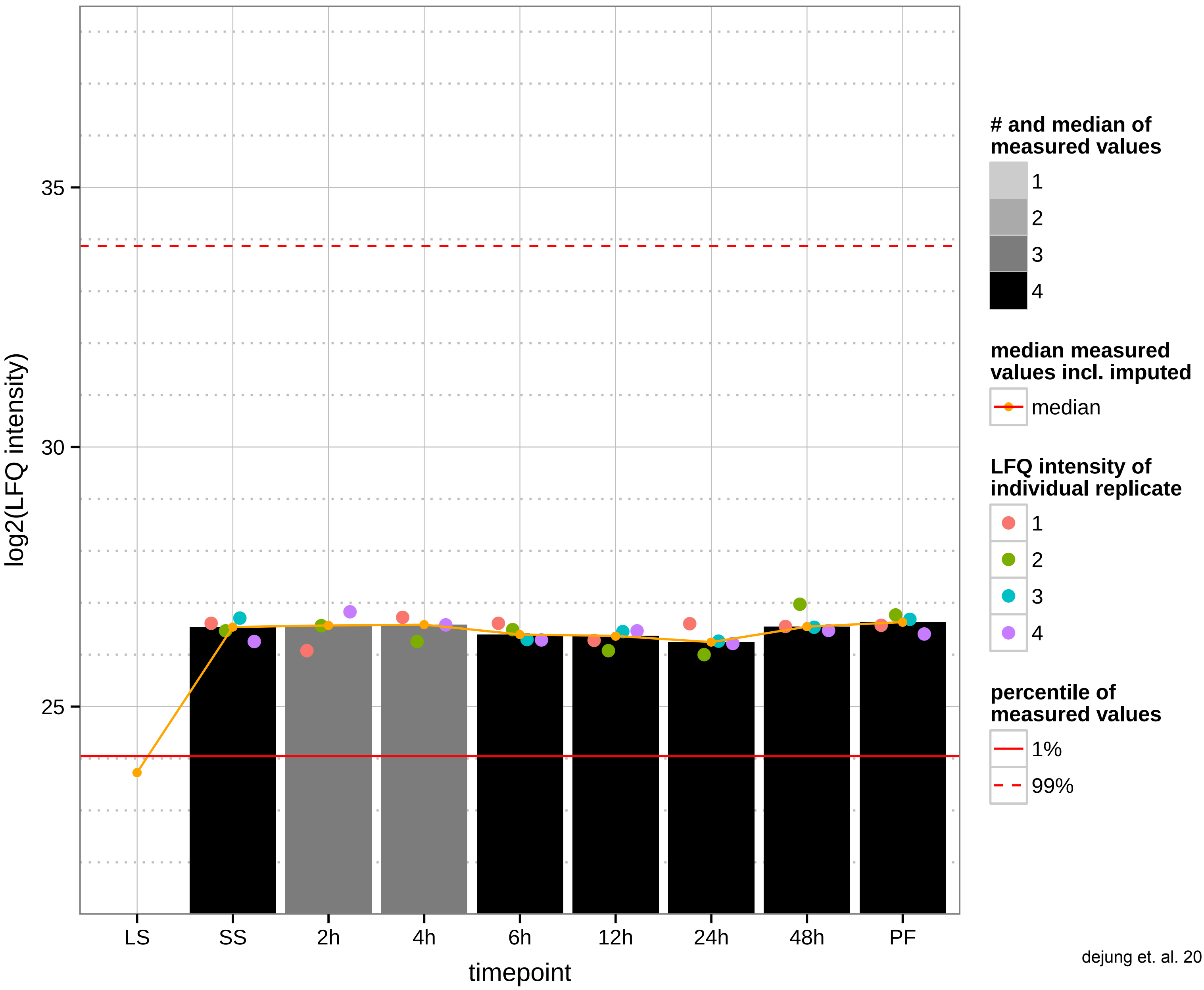

dejung et. al. 2015

monoglyceride lipase, putative
Tb927.8.8020
AGOF: hydrolase activity
AGOC: null
AGOP: null
PGOF: null
PGOC: null
PGOP: null

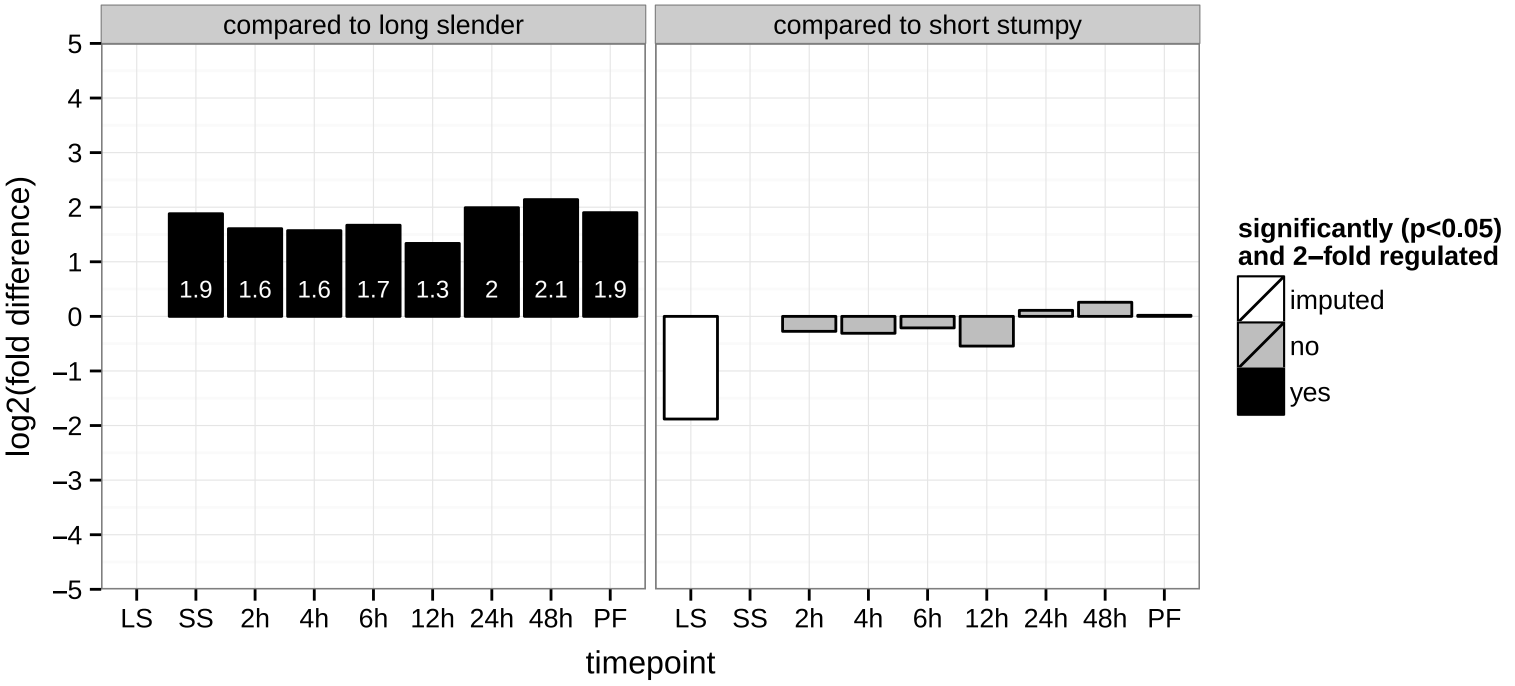

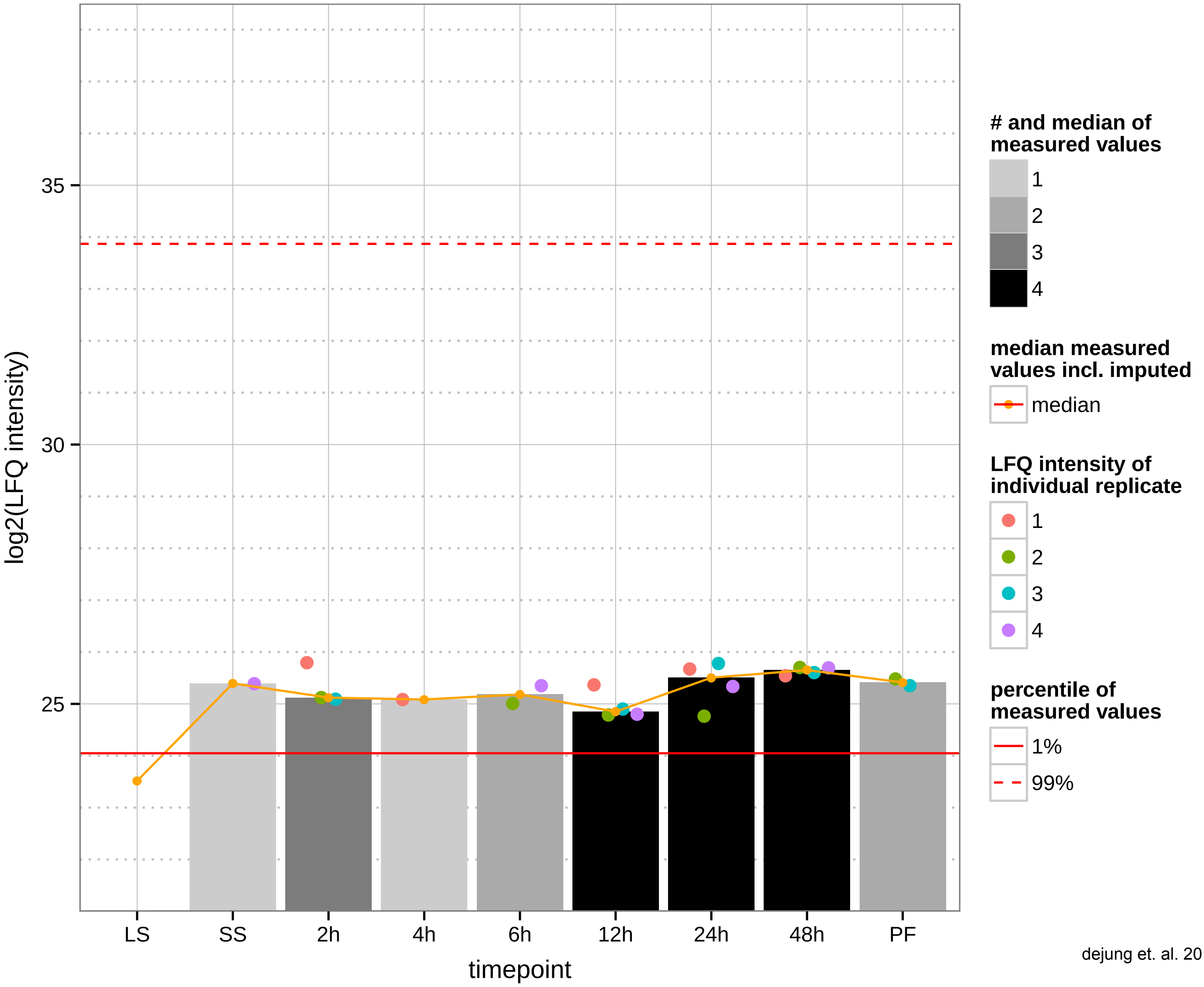

dejung et. al. 2015

radial spoke protein RSP9, putative
Tb927.8.810
AGOF: null
AGOC: null
AGOP: null
PGOF: null
PGOC: null
PGOP: null

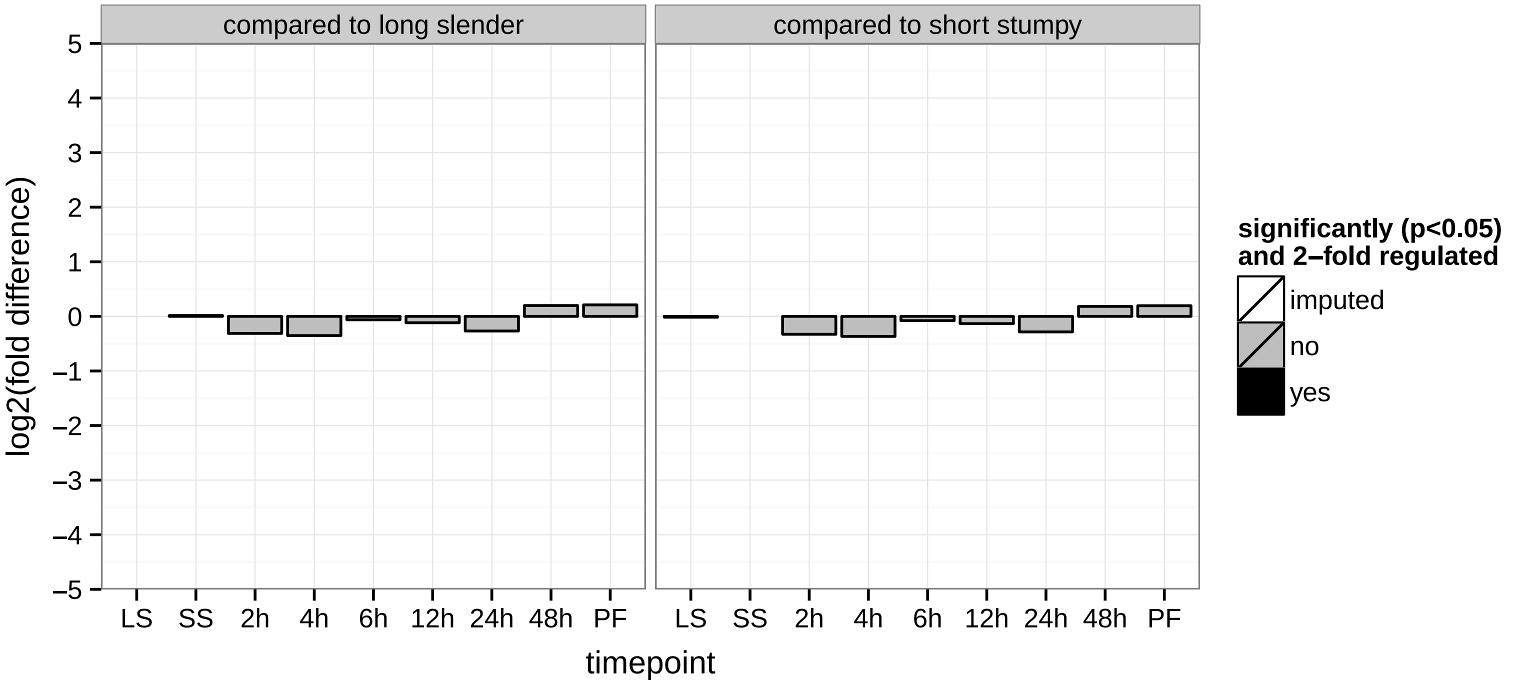

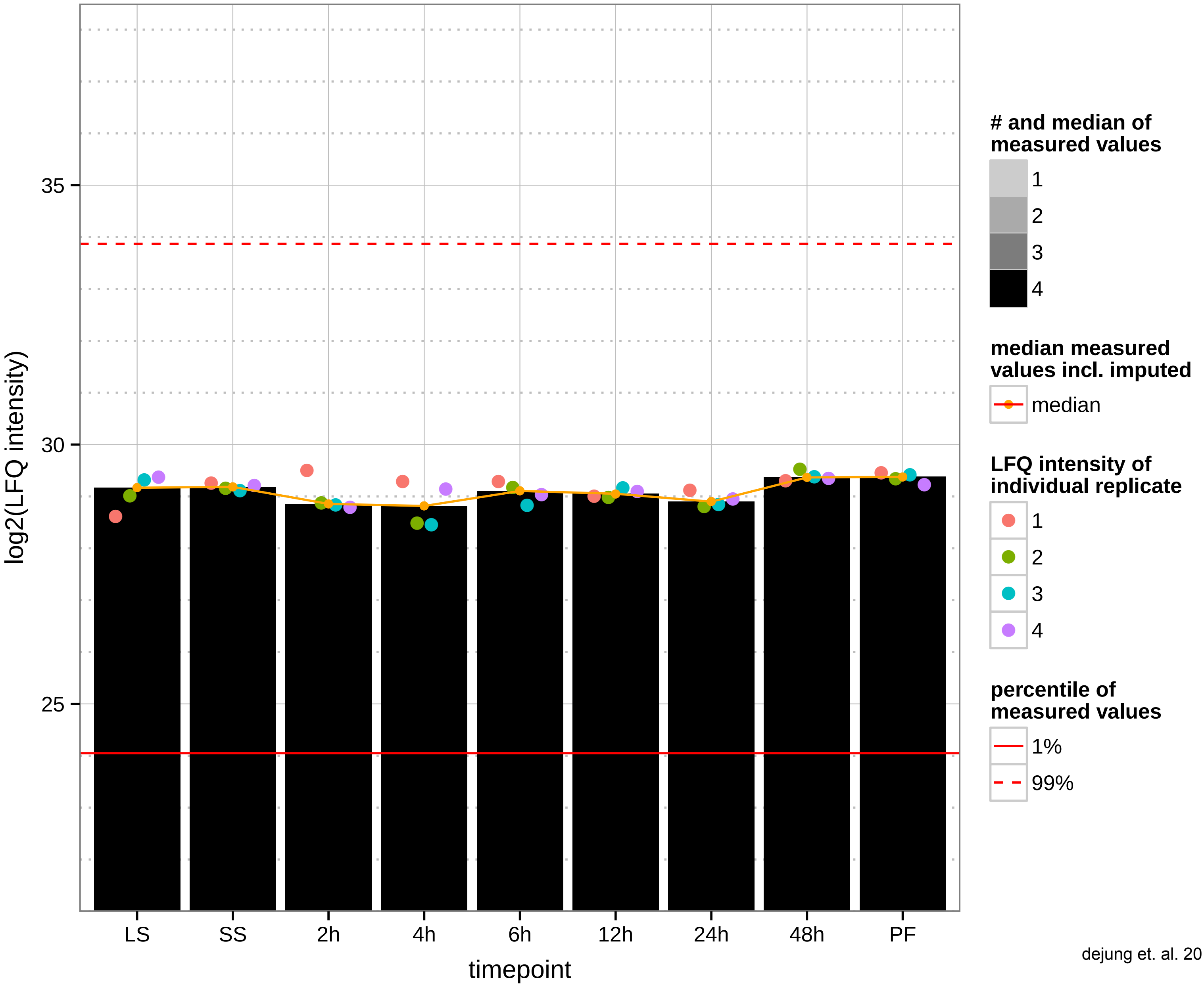

dejung et. al. 2015

mitochondrial RNA binding protein 1, putative (MRB8170)
Tb927.8.8170
AGOF: RNA binding
AGOC: mitochondrion
AGOP: RNA biosynthetic process
PGOF: null
PGOC: null
PGOP: null

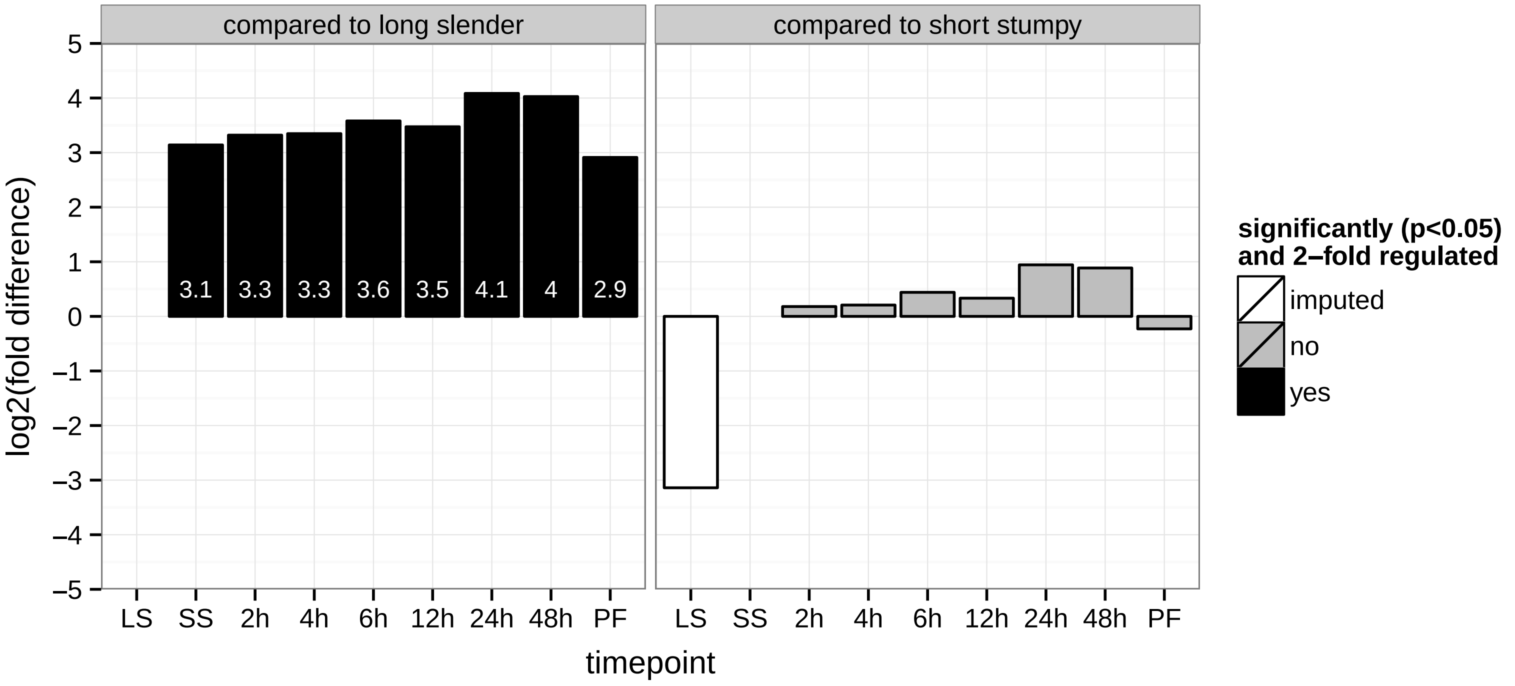

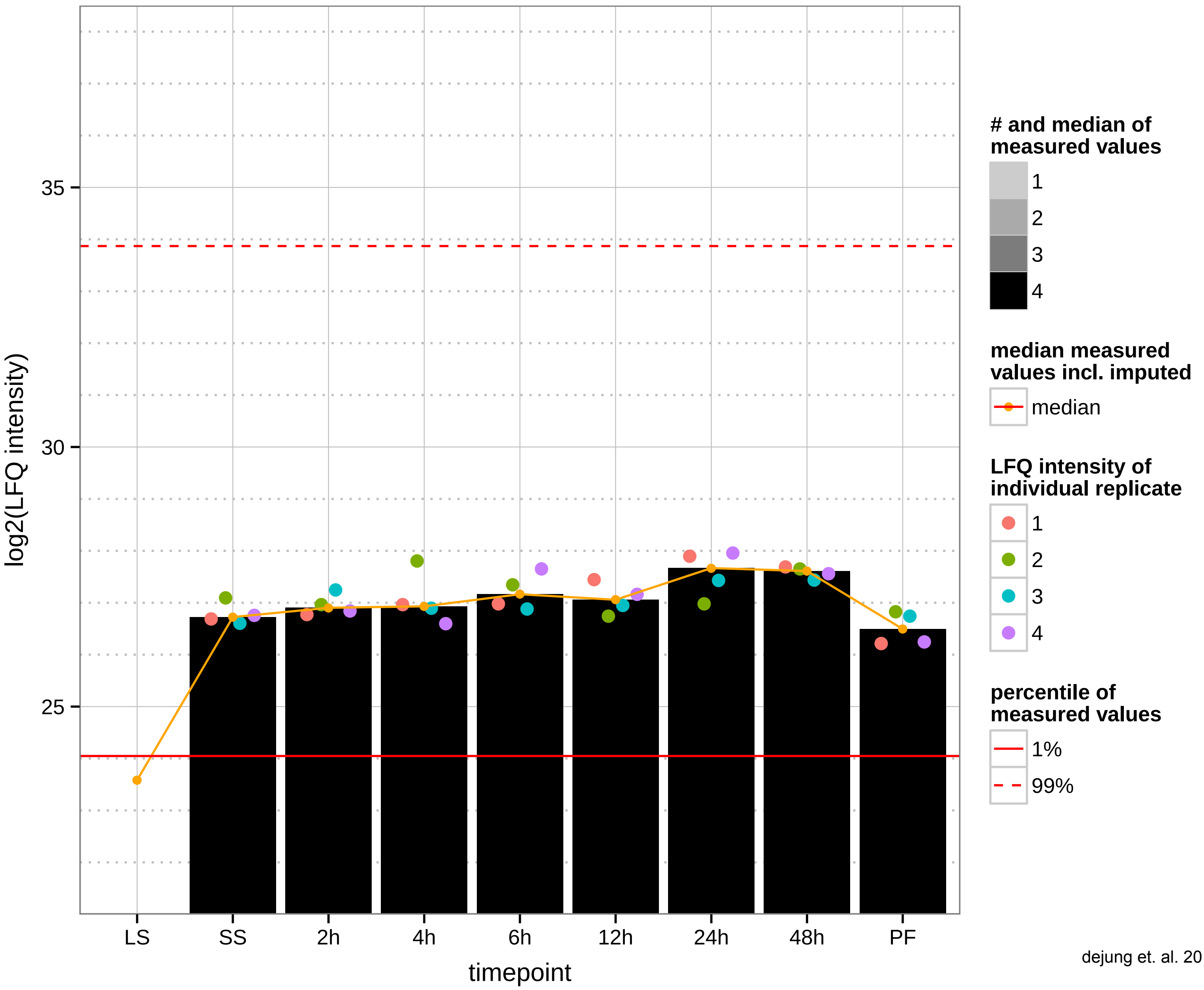

dejung et. al. 2015

amino acid transporter, putative
Tb927.8.8250;Tb927.8.8260
AGOF: amine transmembrane transporter activity
AGOC: integral to membrane
AGOP: amino acid transport
PGOF: null
PGOC: null
PGOP: null

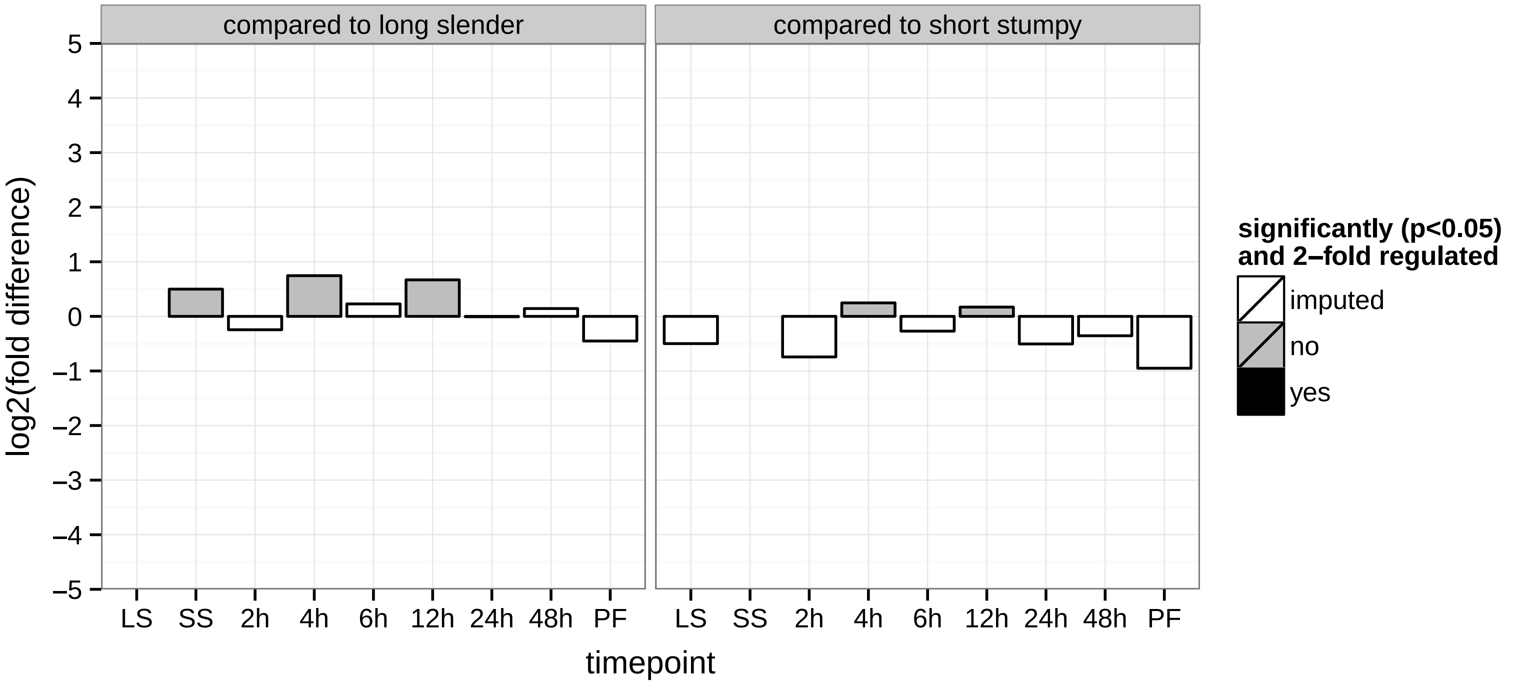

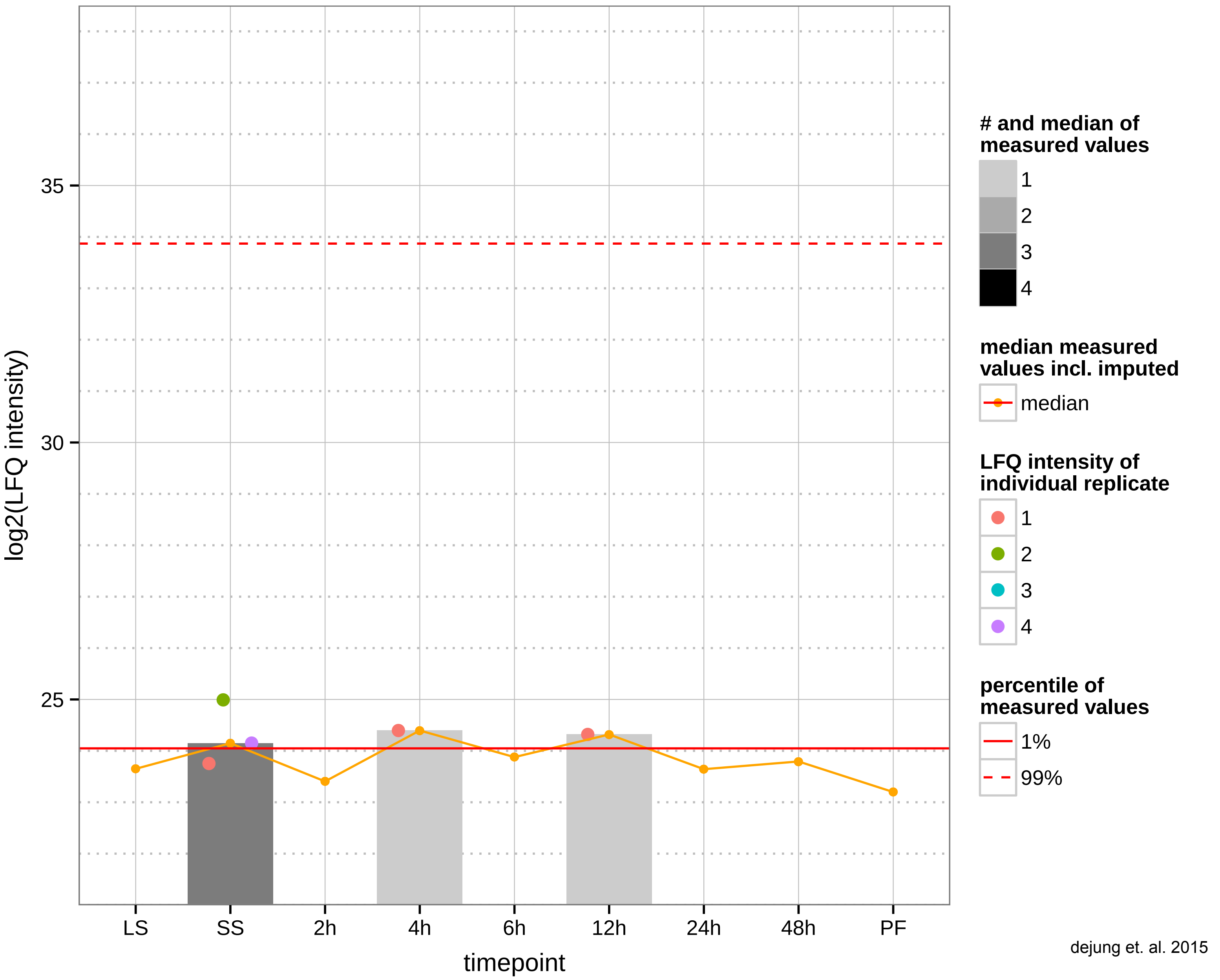

dejung et. al. 2015

ubiquitin–conjugating enzyme E2, putative, ubiquitin–protein ligase, ubiquitin carrier protein, putative (PEX4)
Tb927.8.920
AGOF: ubiquitin–protein ligase activity
AGOC: null
AGOP: cellular protein modification process, post–translational protein modification, regulation of protein metabolic process
PGOF: acid–amino acid ligase activity
PGOC: null
PGOP: null

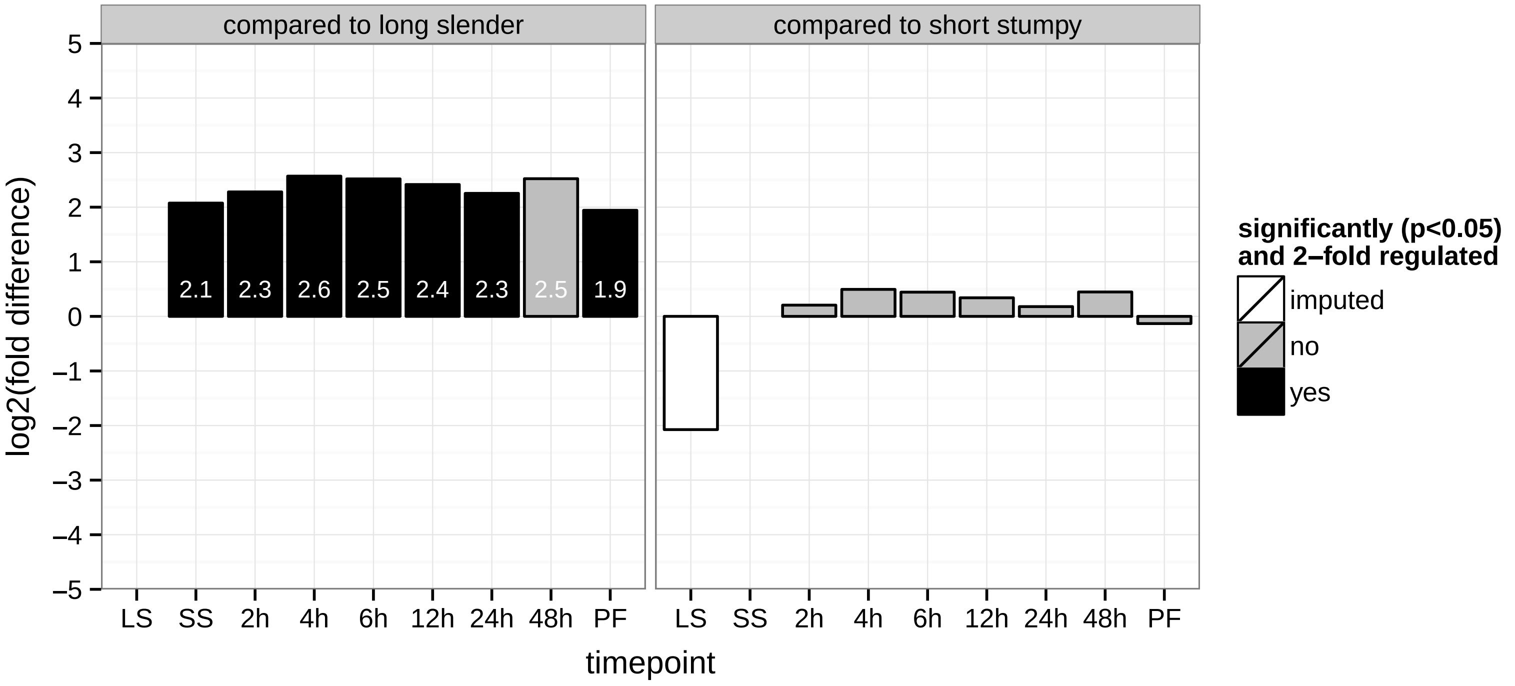

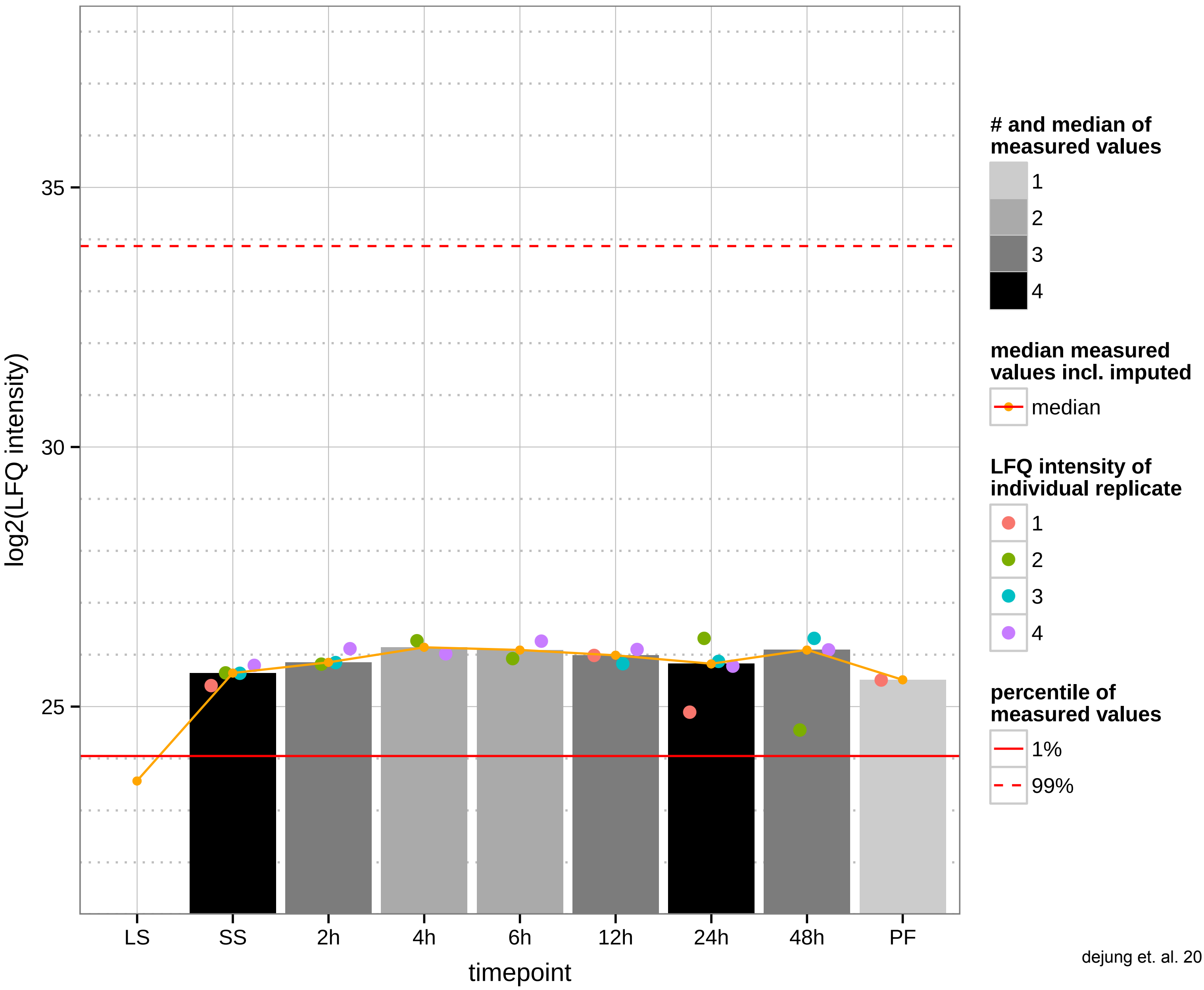

dejung et. al. 2015

phosphoacetylglucosamine mutase, putative, acetylglucosaminephosphomutase, N−acetylglucosamine−phosphate mutase
Tb927.8.980
AGOF: phosphoacetylglucosamine mutase activity
AGOC: null
AGOP: carbohydrate metabolic process, glucosamine metabolic process
PGOF: intramolecular transferase activity, phosphotransferases, phosphoacetylglucosamine mutase activity
PGOC: null
PGOP: carbohydrate metabolic process

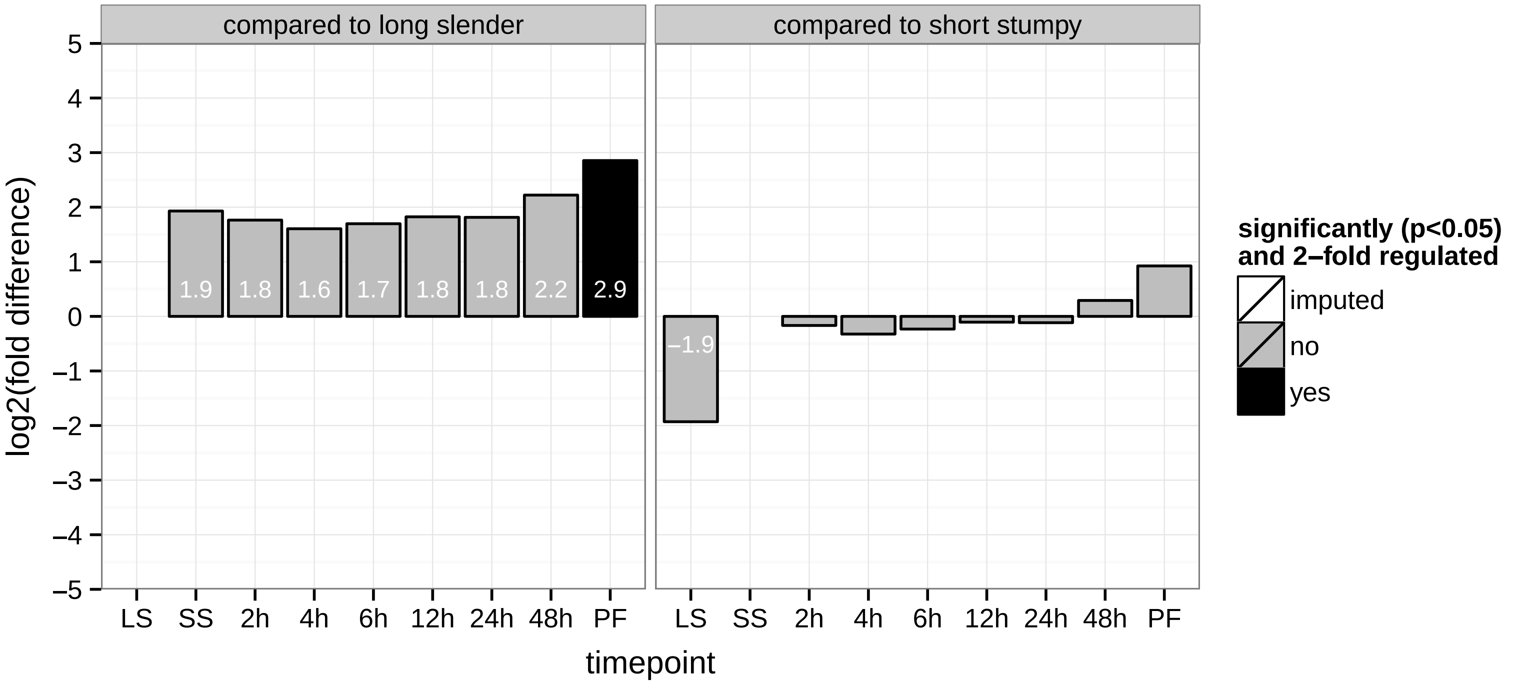

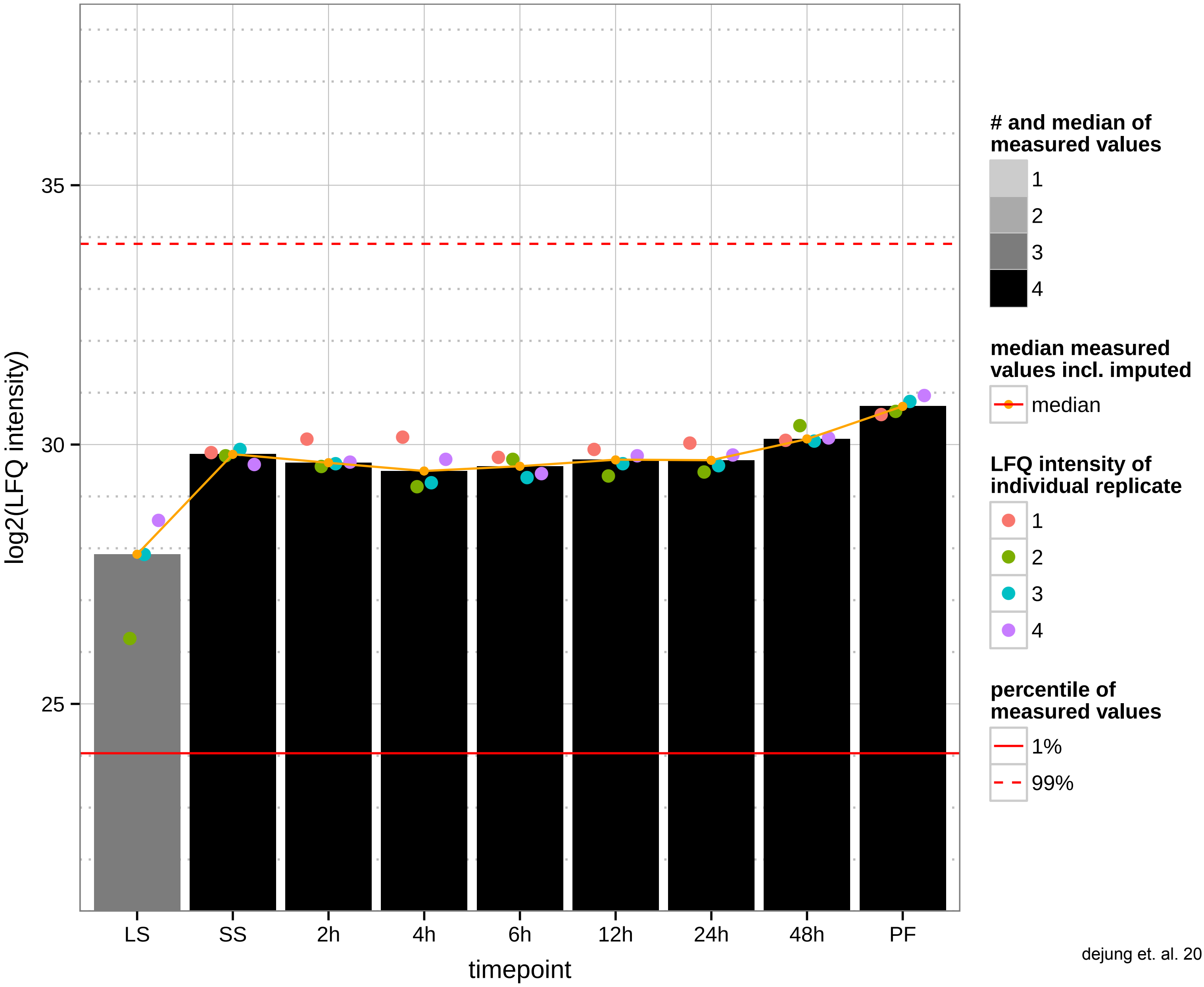

dejung et. al. 2015

RNA–binding protein 33 (RNA–binding motif protein 33) (RBP33)
Tb927.8.990
AGOF: RNA binding, mRNA binding
AGOC: null
AGOP: RNA processing
PGOF: nucleic acid binding
PGOC: null
PGOP: null

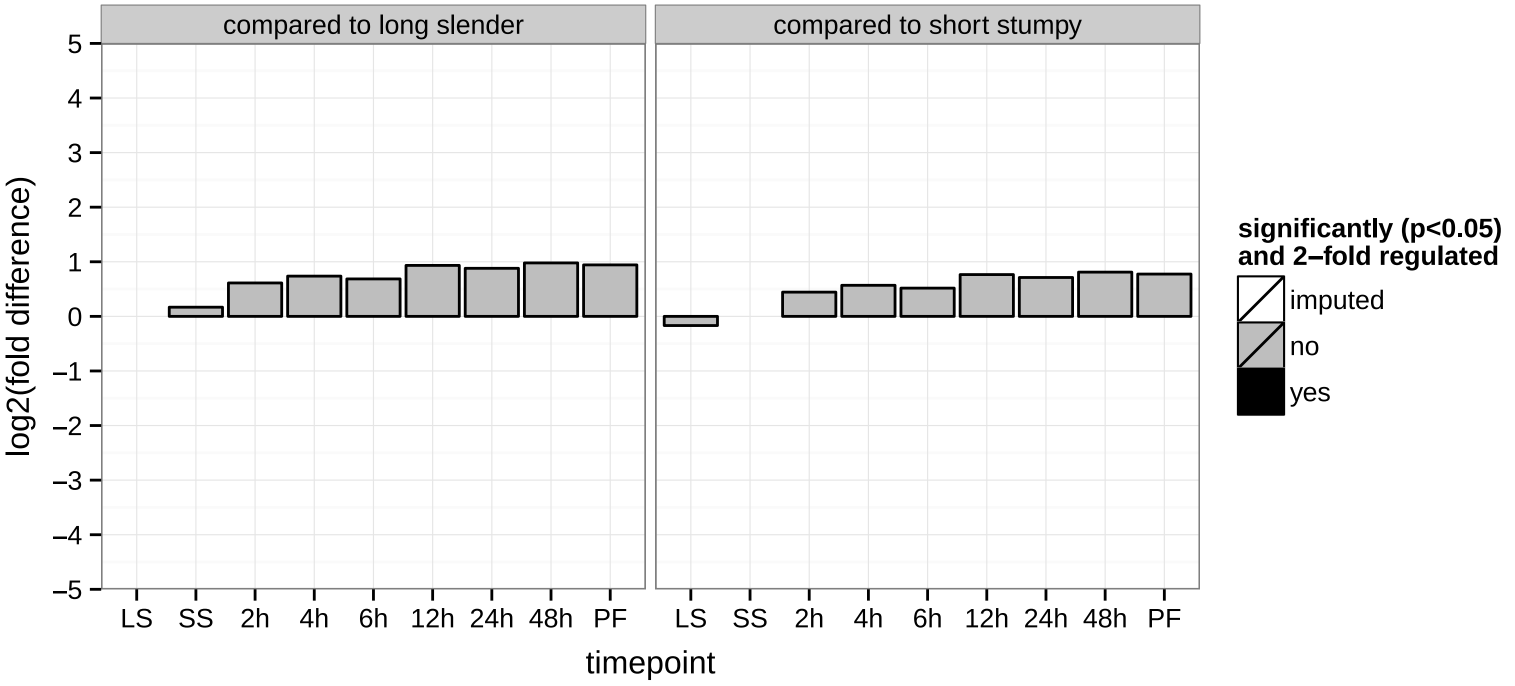

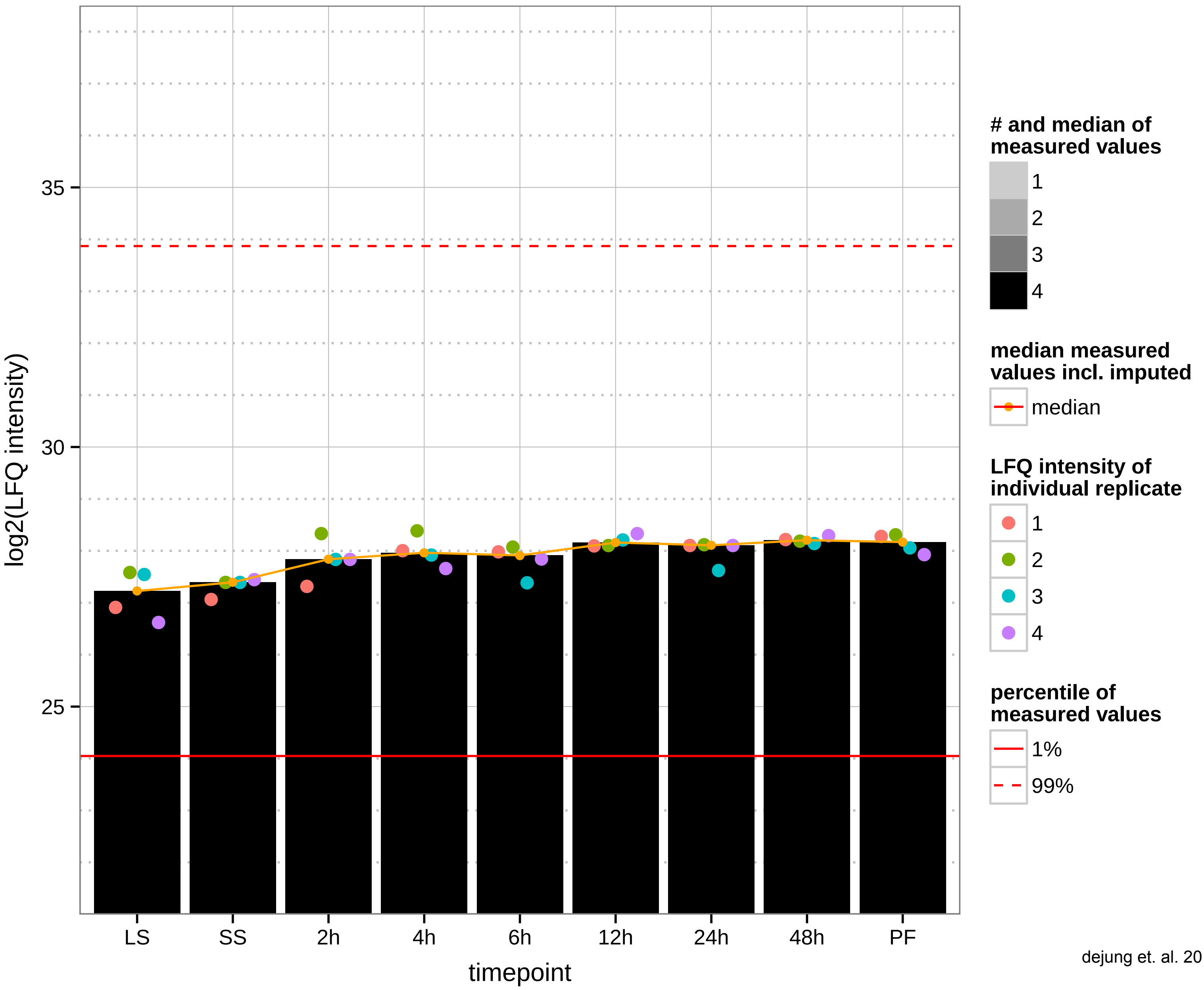

dejung et. al. 2015

chaperone protein DNAj, putative
Tb927.9.10010
AGOF: heat shock protein binding, unfolded protein binding
AGOC: integral to membrane, mitochondrial inner membrane
AGOP: protein folding
PGOF: heat shock protein binding
PGOC: null
PGOP: null

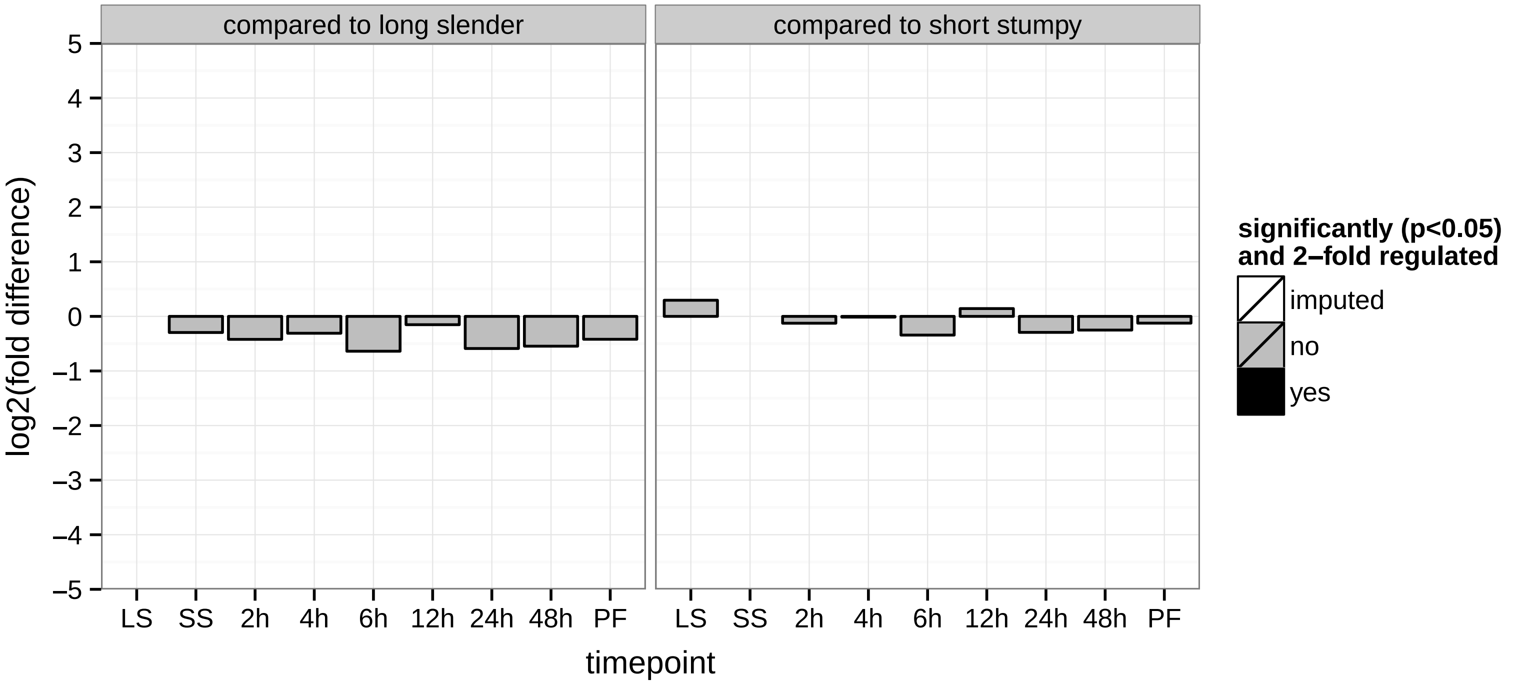

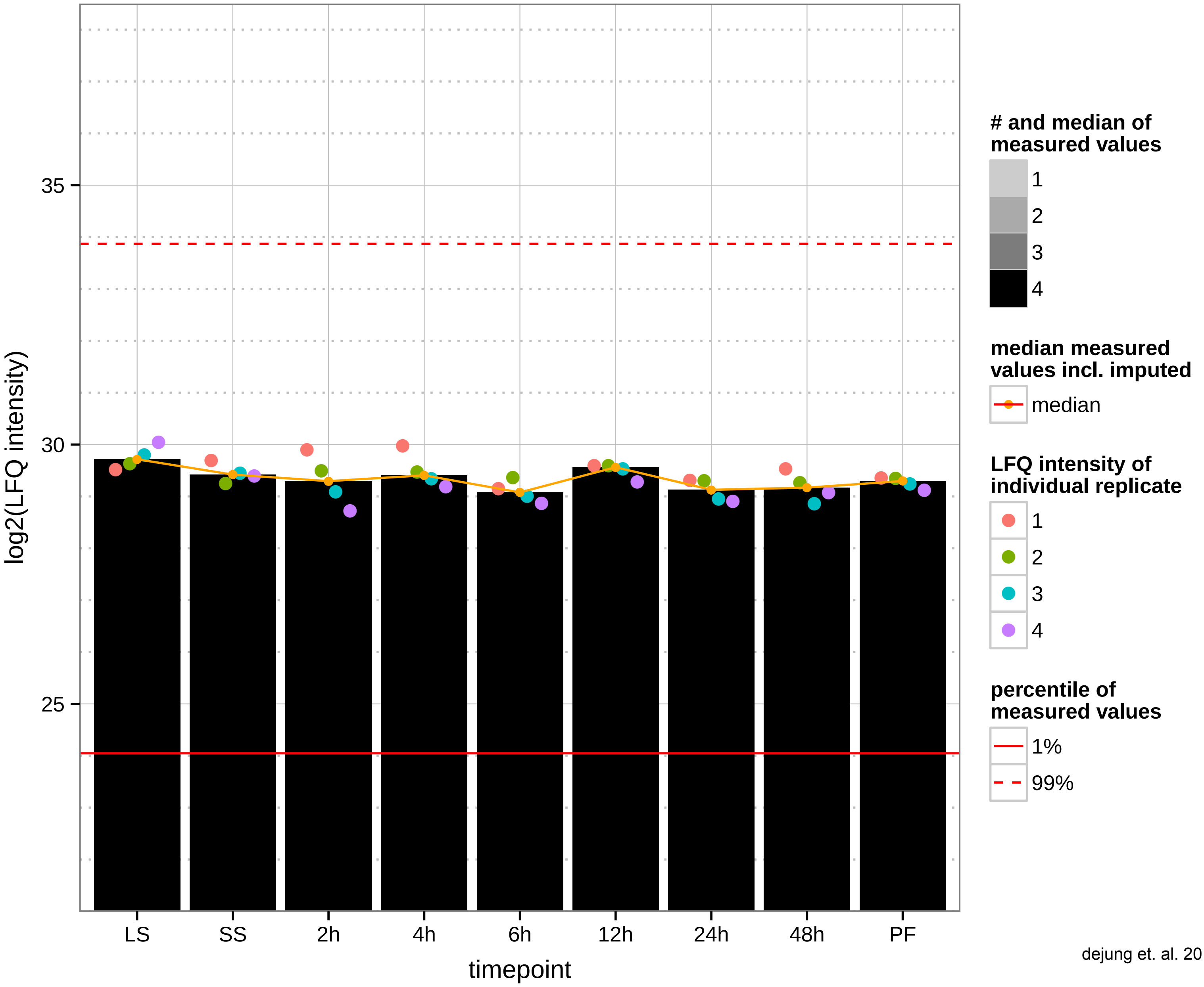

dejung et. al. 2015

hypothetical protein, conserved
Tb927.9.10070
AGOF: null
AGOC: mitochondrion
AGOP: null
PGOF: null
PGOC: null
PGOP: null

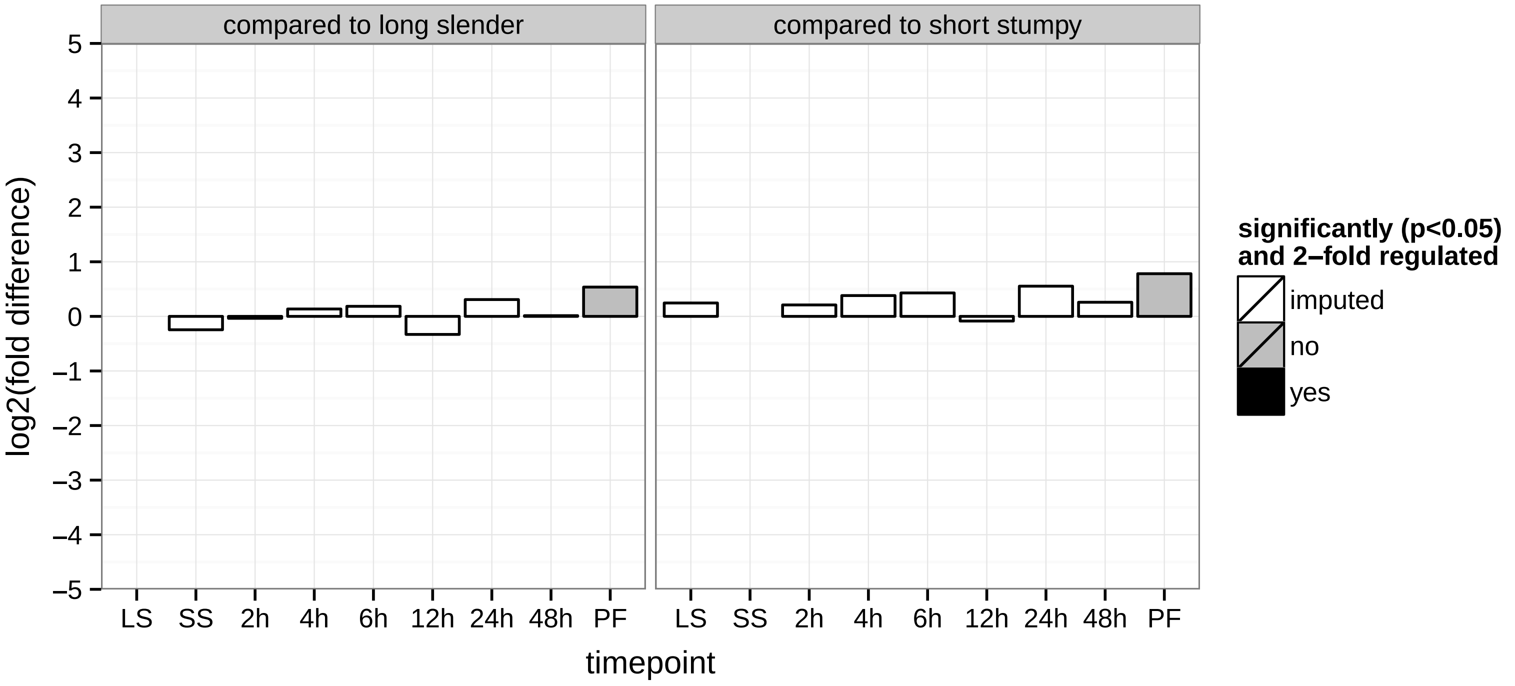

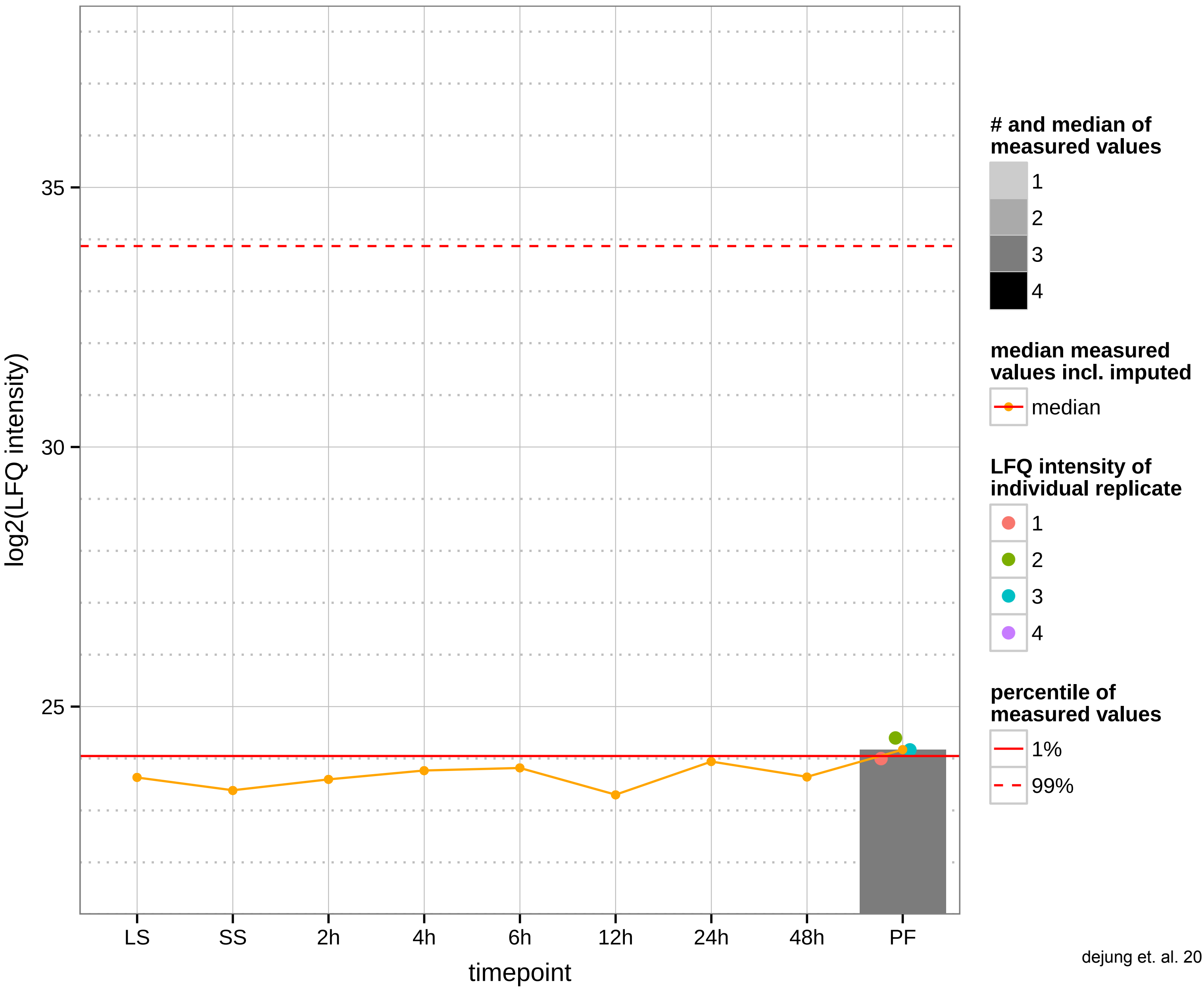

dejung et. al. 2015

phosphatidylserine decarboxylase, putative
Tb927.9.10080
AGOF: phosphatidylserine decarboxylase activity
AGOC: mitochondrion
AGOP: phosphatidylserine catabolic process, phospholipid biosynthetic process
PGOF: phosphatidylserine decarboxylase activity
PGOC: null
PGOP: phospholipid biosynthetic process

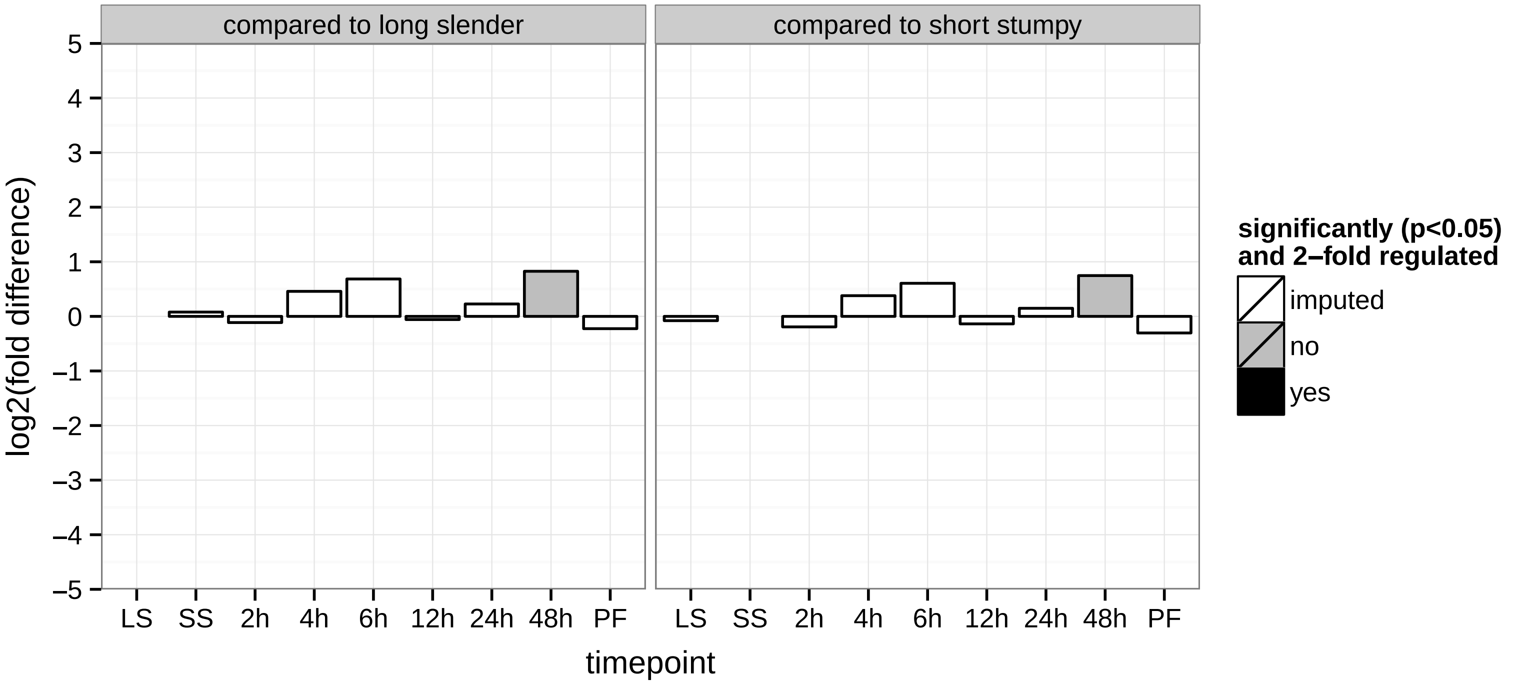

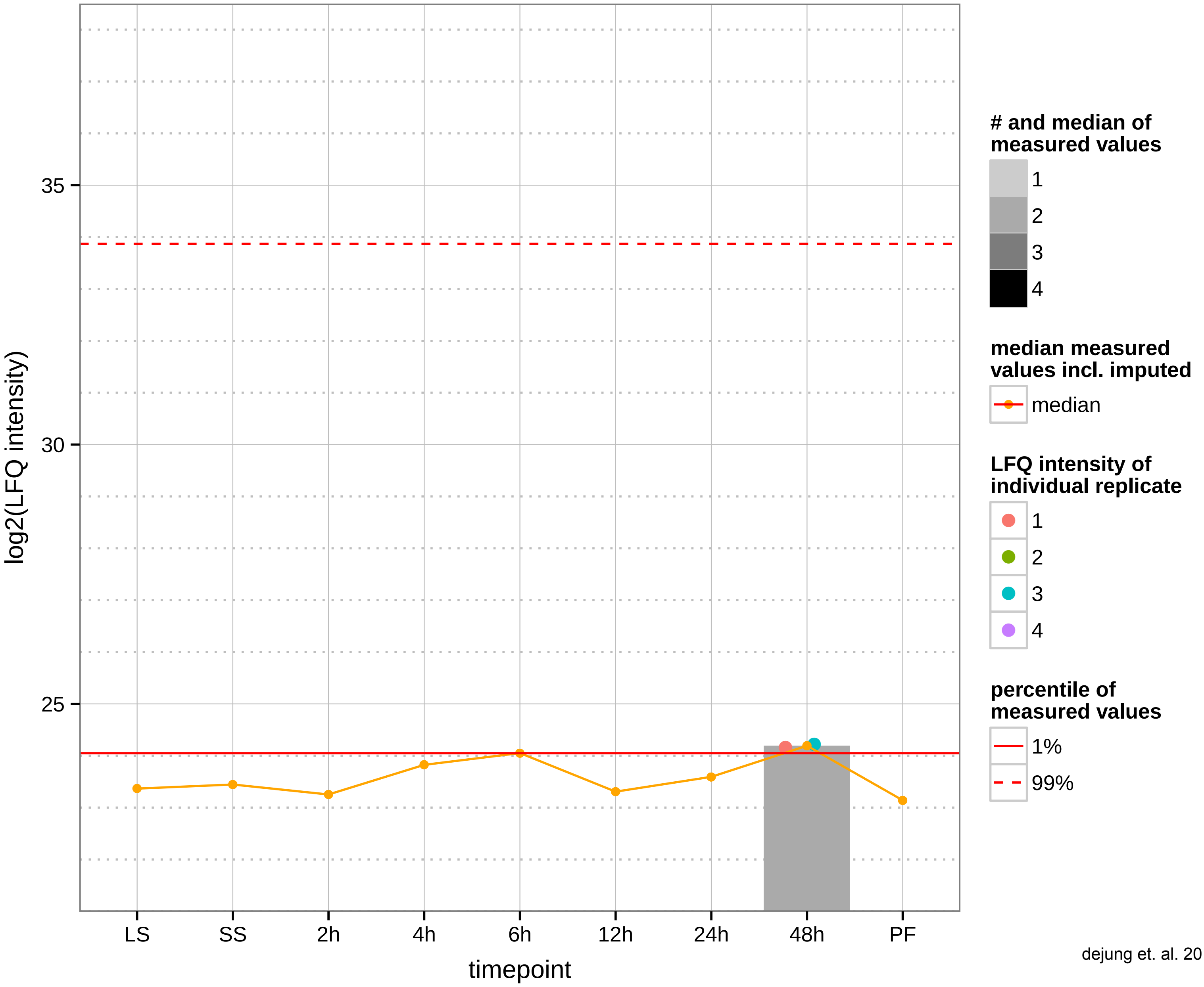

dejung et. al. 2015

hypothetical protein, conserved
Tb927.9.10100
AGOF: null
AGOC: null
AGOP: null
PGOF: protein binding
PGOC: null
PGOP: null

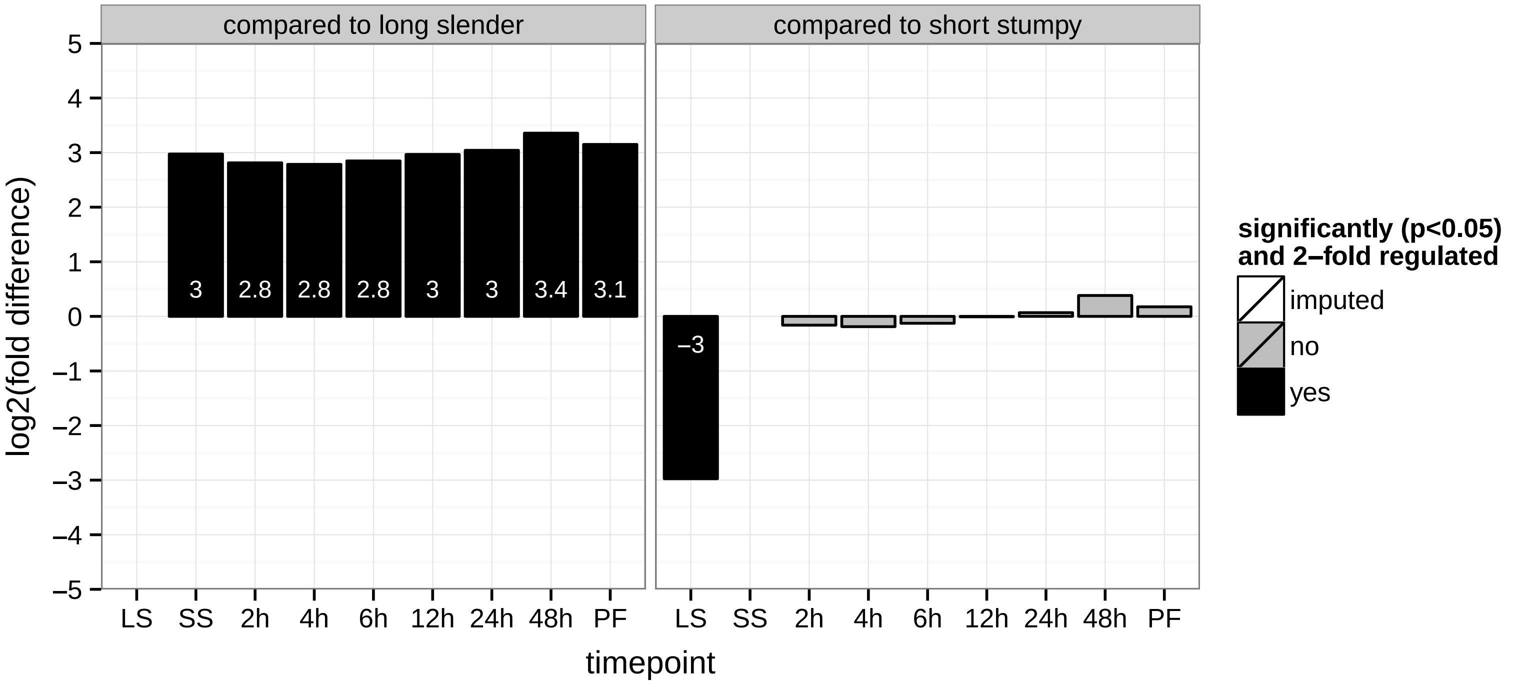

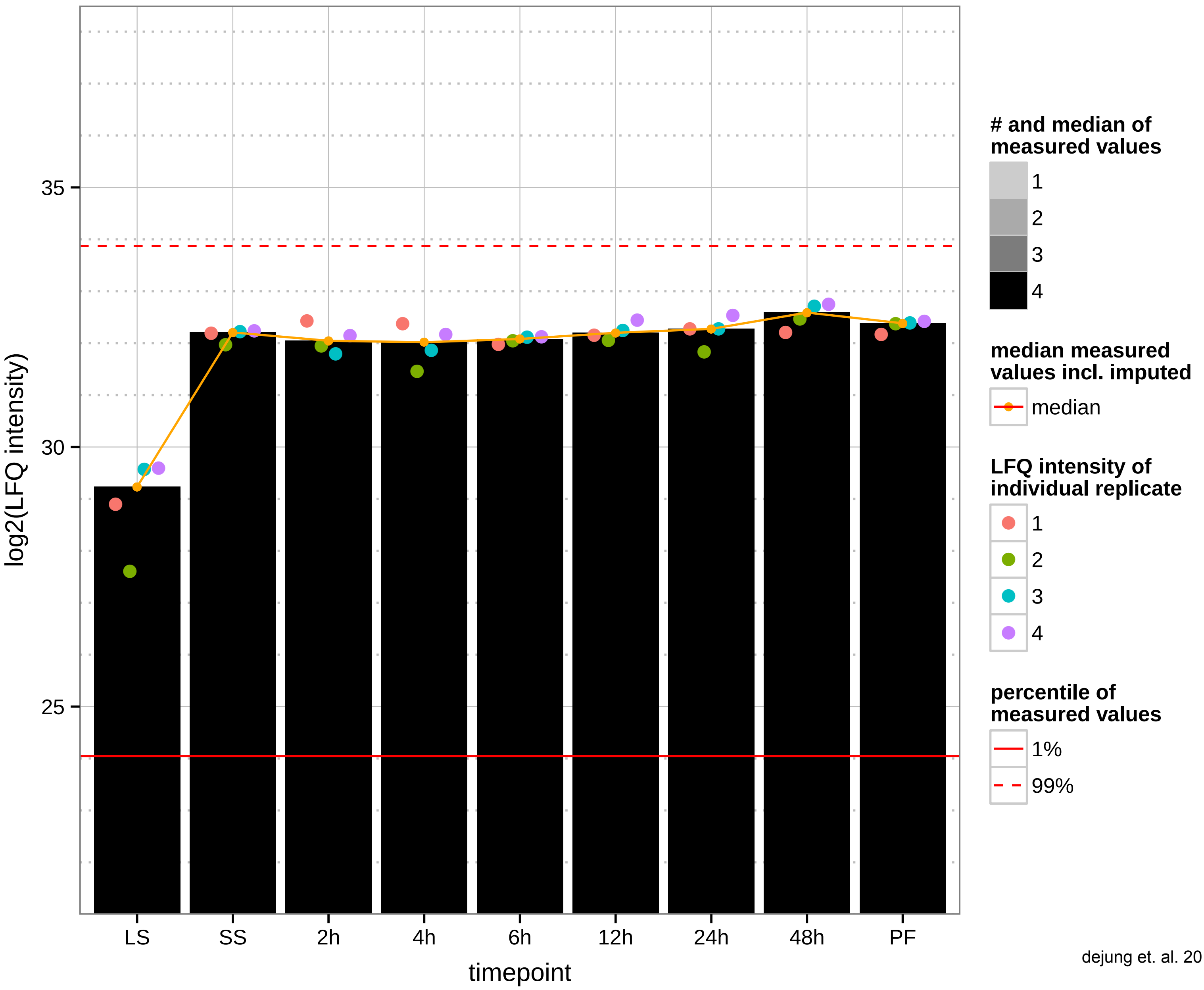

dejung et. al. 2015

hypothetical protein, conserved
Tb927.9.10160
AGOF: null
AGOC: null
AGOP: null
PGOF: null
PGOC: null
PGOP: null

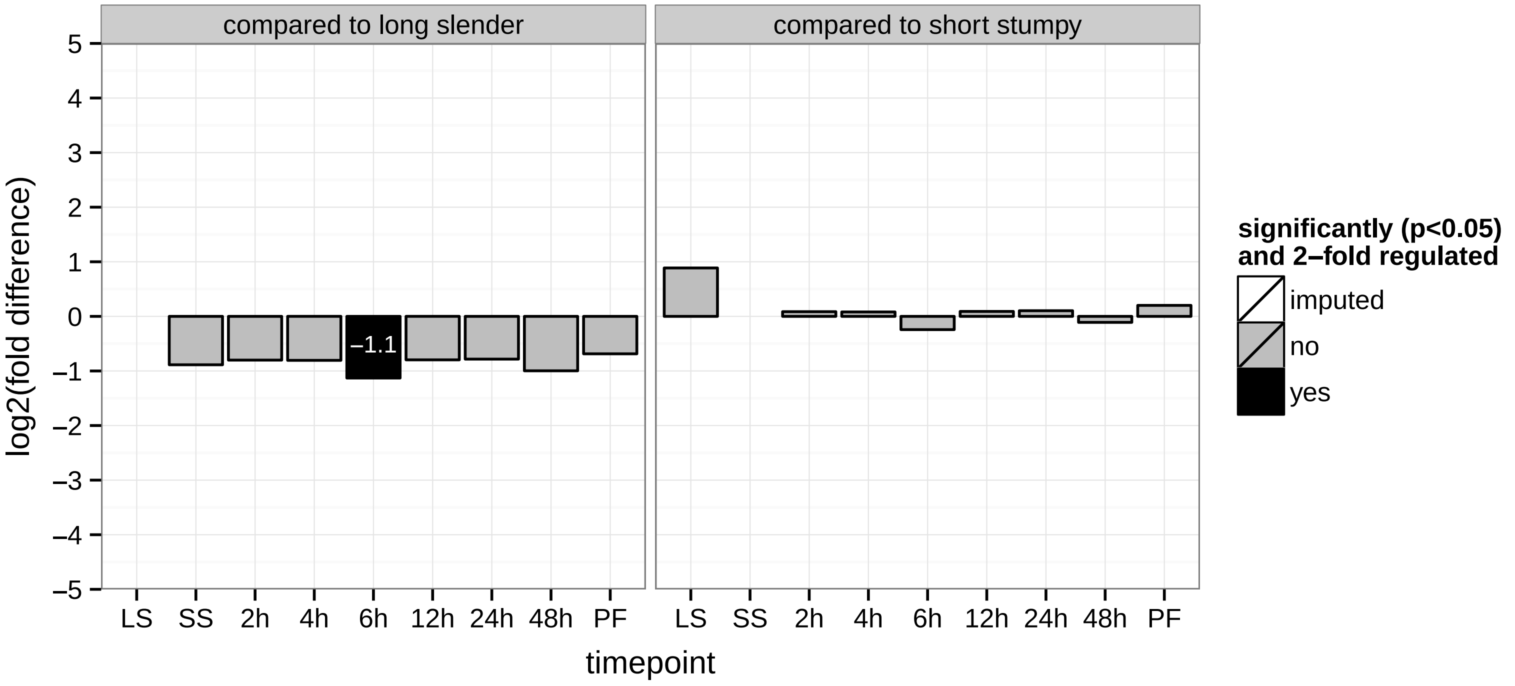

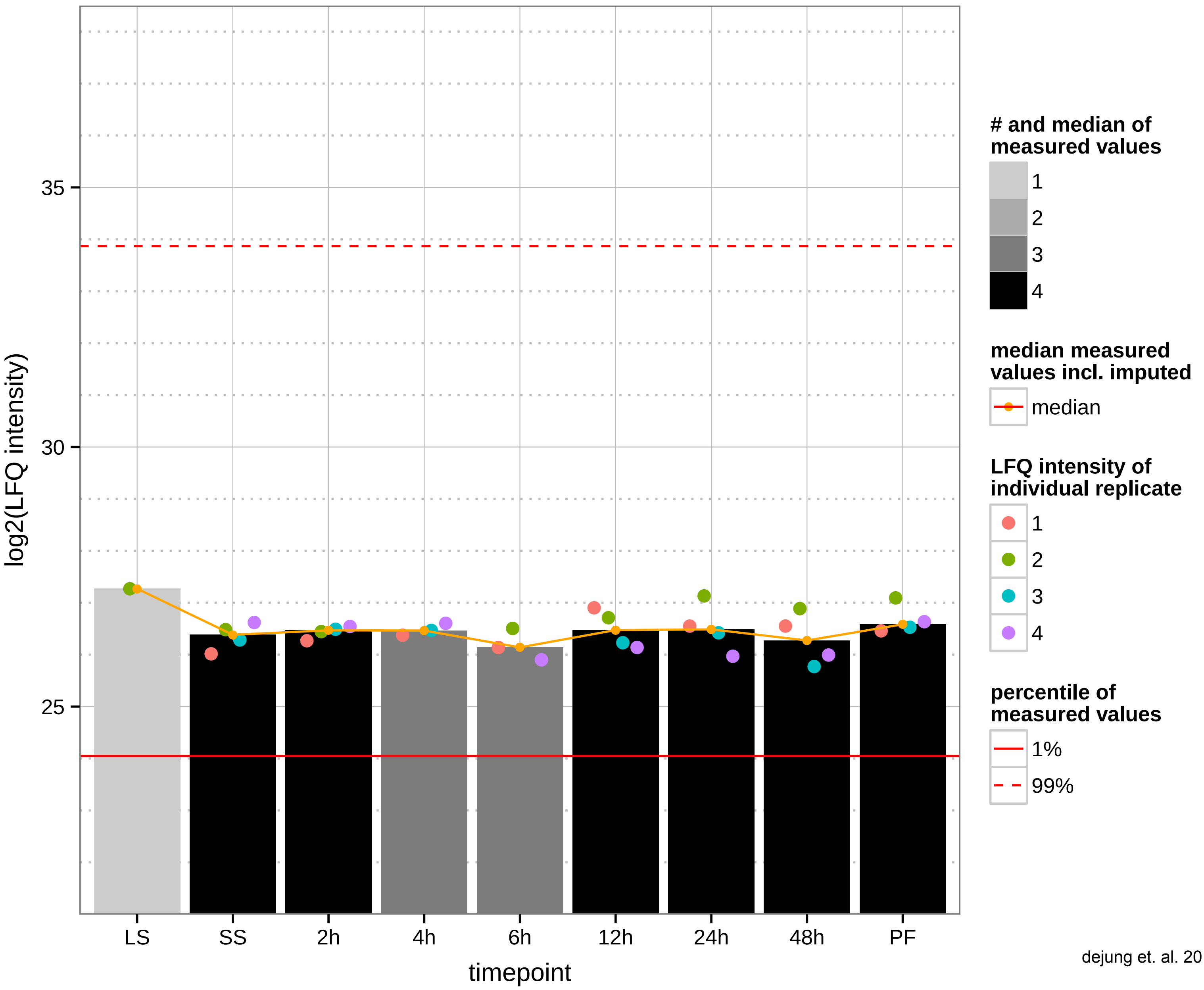

dejung et. al. 2015

hypothetical protein, conserved
Tb927.9.10230
AGOF: null
AGOC: null
AGOP: null
PGOF: null
PGOC: null
PGOP: null

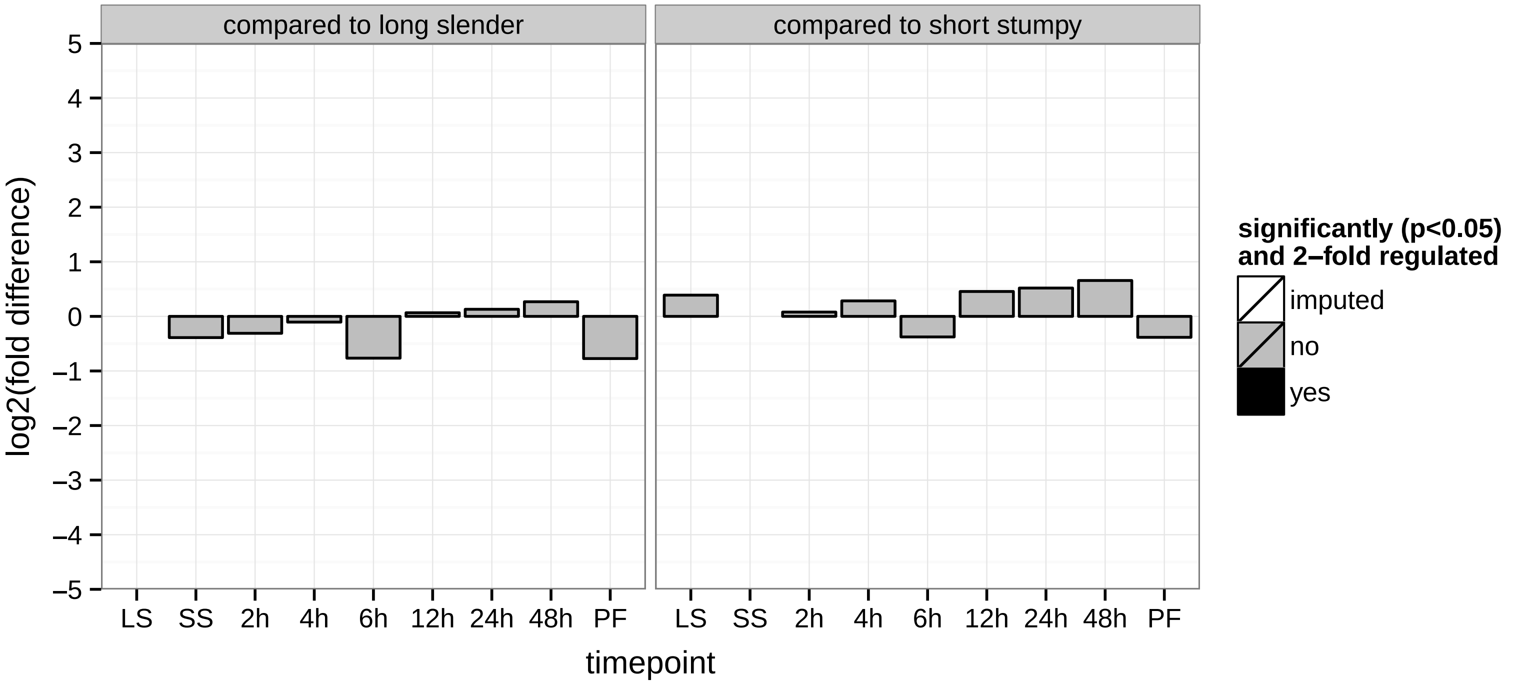

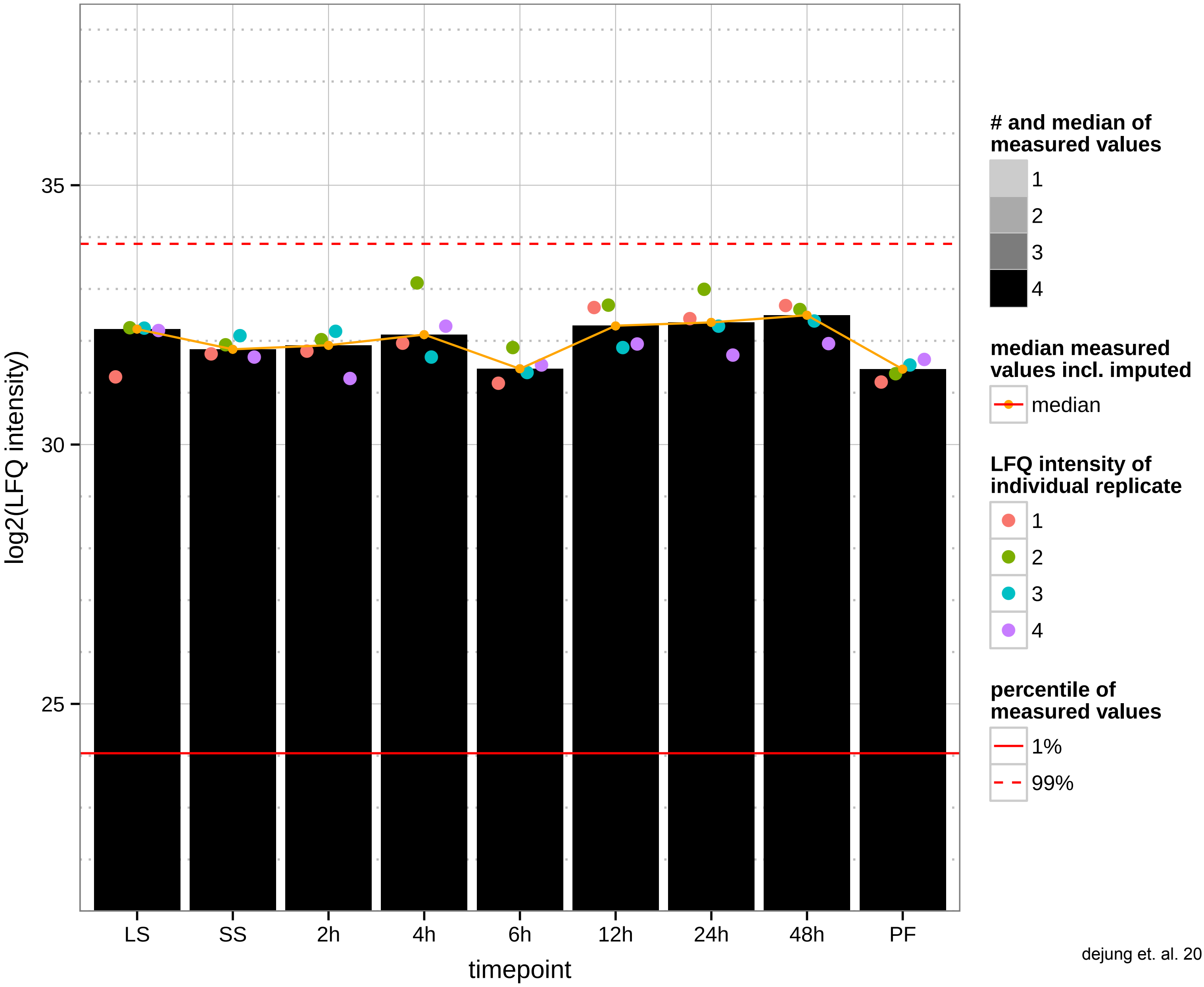

dejung et. al. 2015

hypothetical protein, conserved
Tb927.9.10400
AGOF: null
AGOC: null
AGOP: null
PGOF: protein binding
PGOC: null
PGOP: null

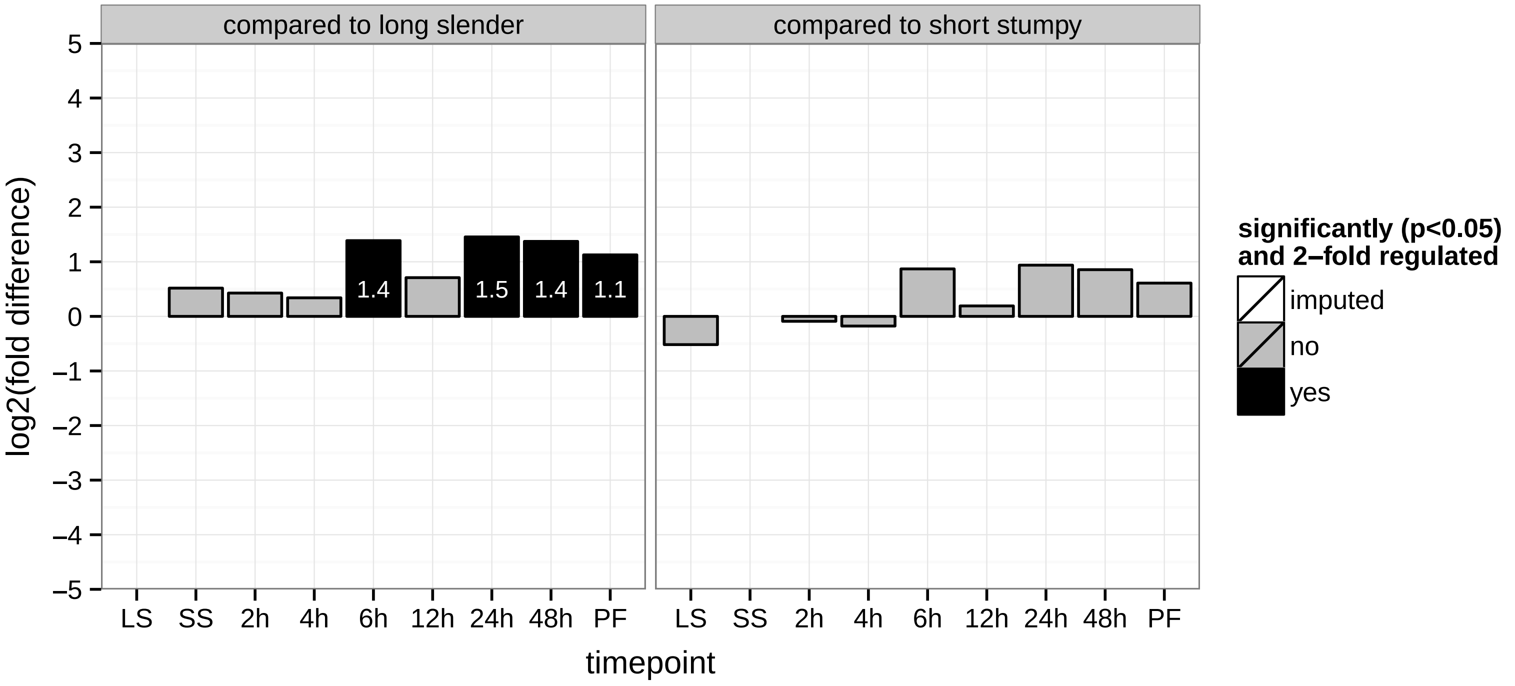

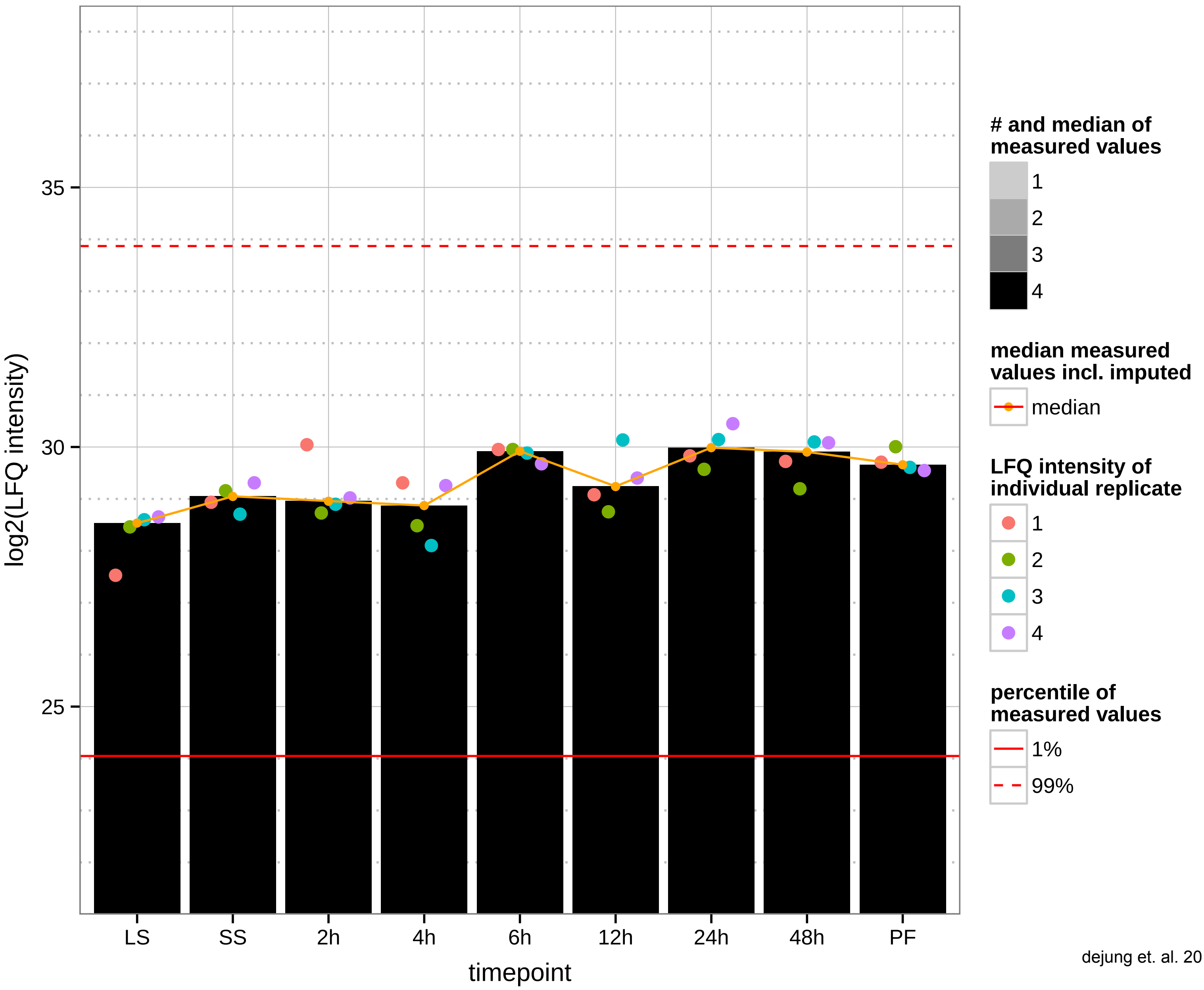

dejung et. al. 2015

hypothetical protein, conserved
Tb927.9.10500
AGOF: hydrolase activity
AGOC: mitochondrion
AGOP: metabolic process
PGOF: hydrolase activity
PGOC: null
PGOP: metabolic process

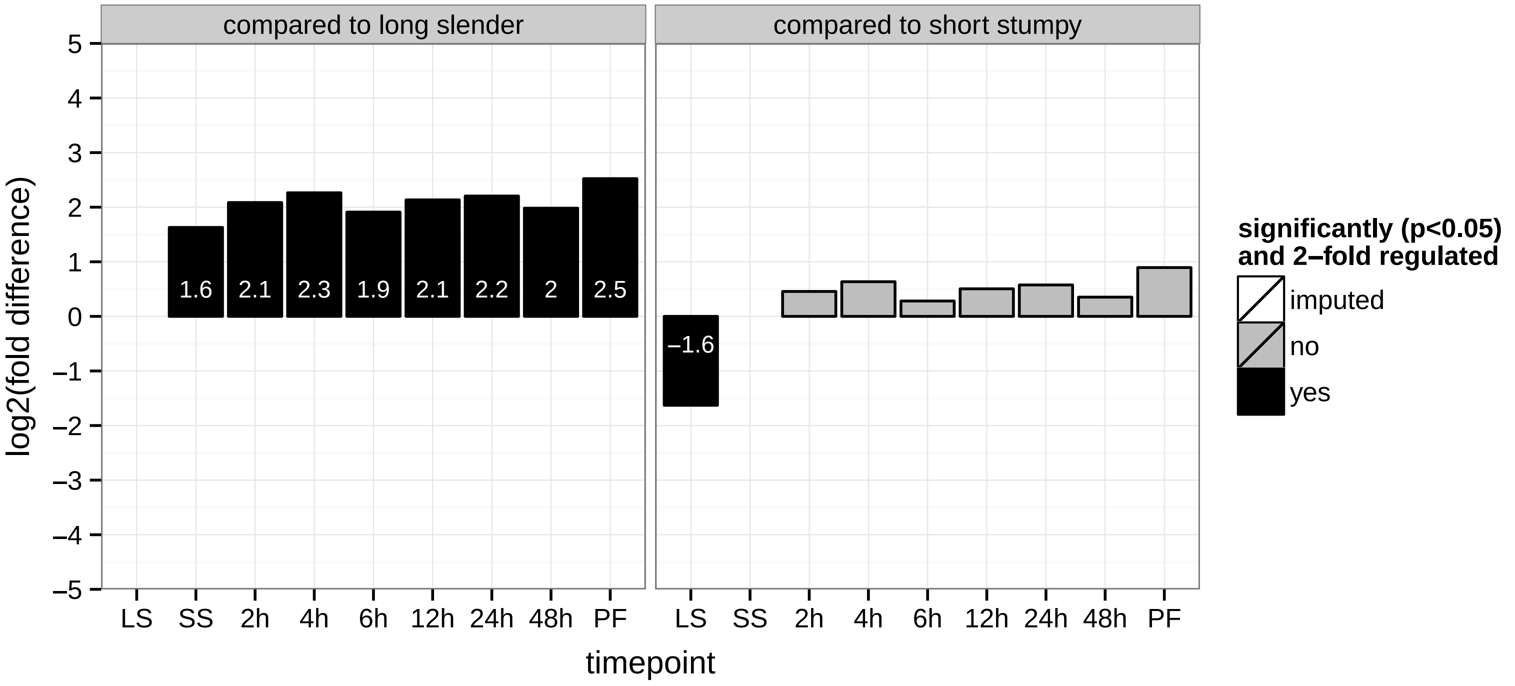

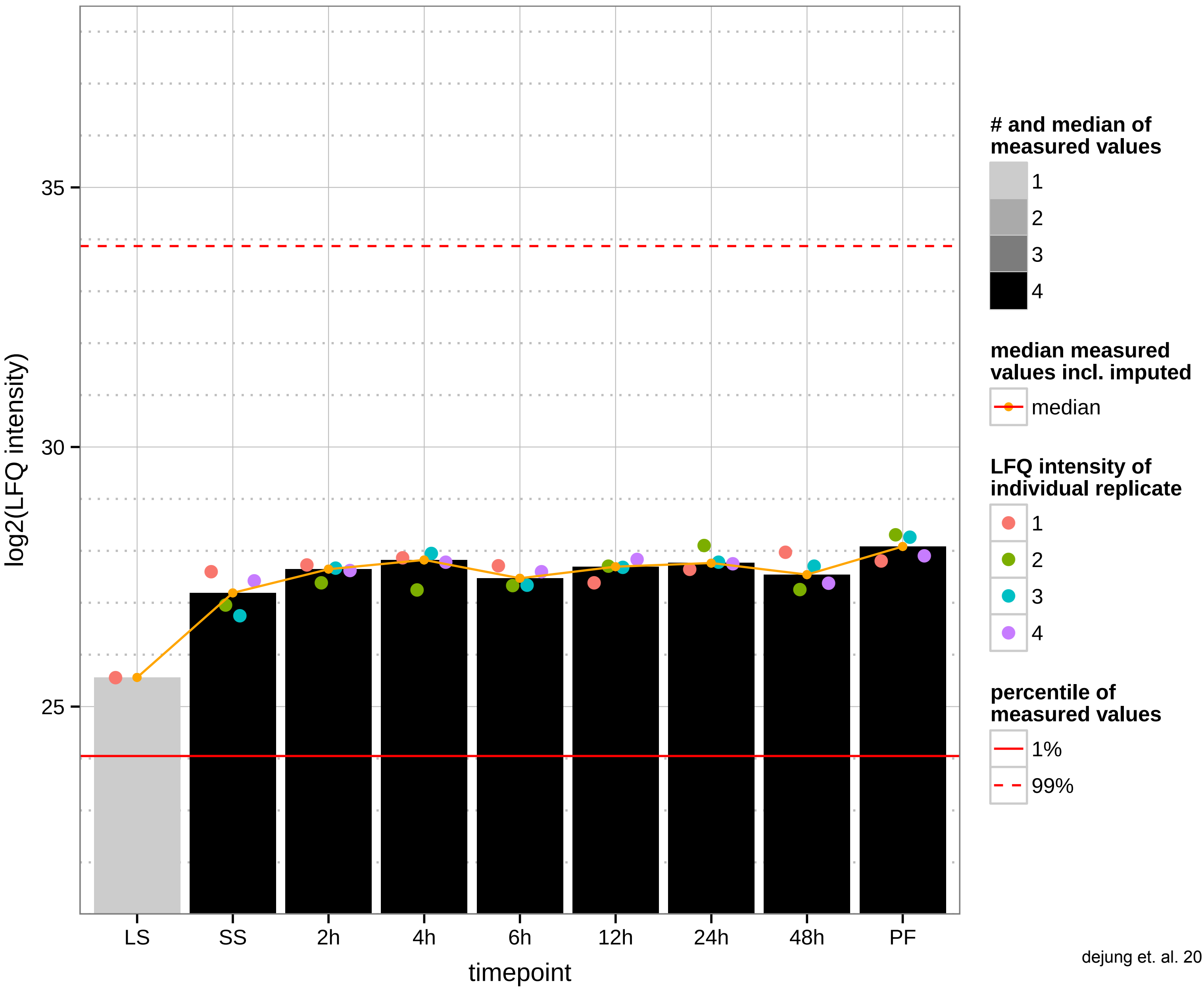

dejung et. al. 2015

hypothetical protein, conserved
Tb927.9.10530
AGOF: null
AGOC: null
AGOP: null
PGOF: null
PGOC: null
PGOP: null

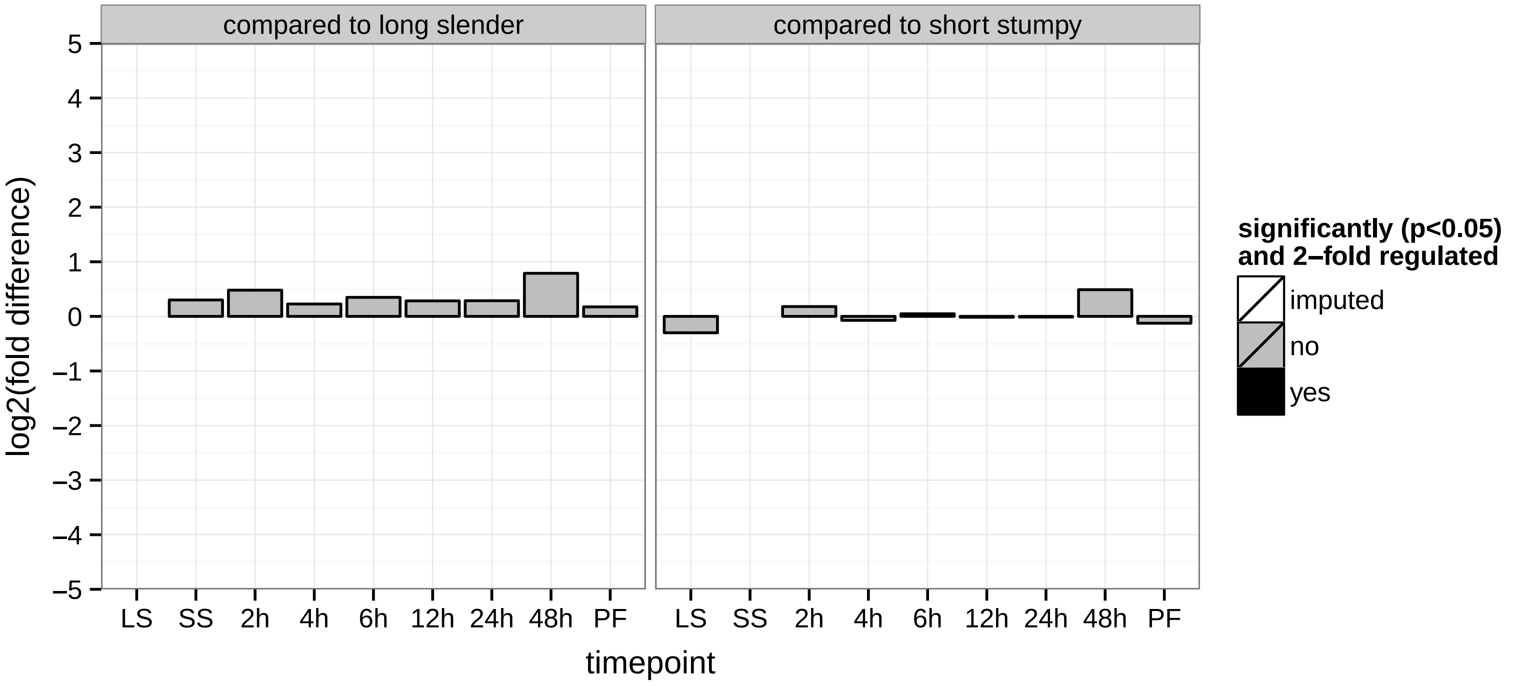

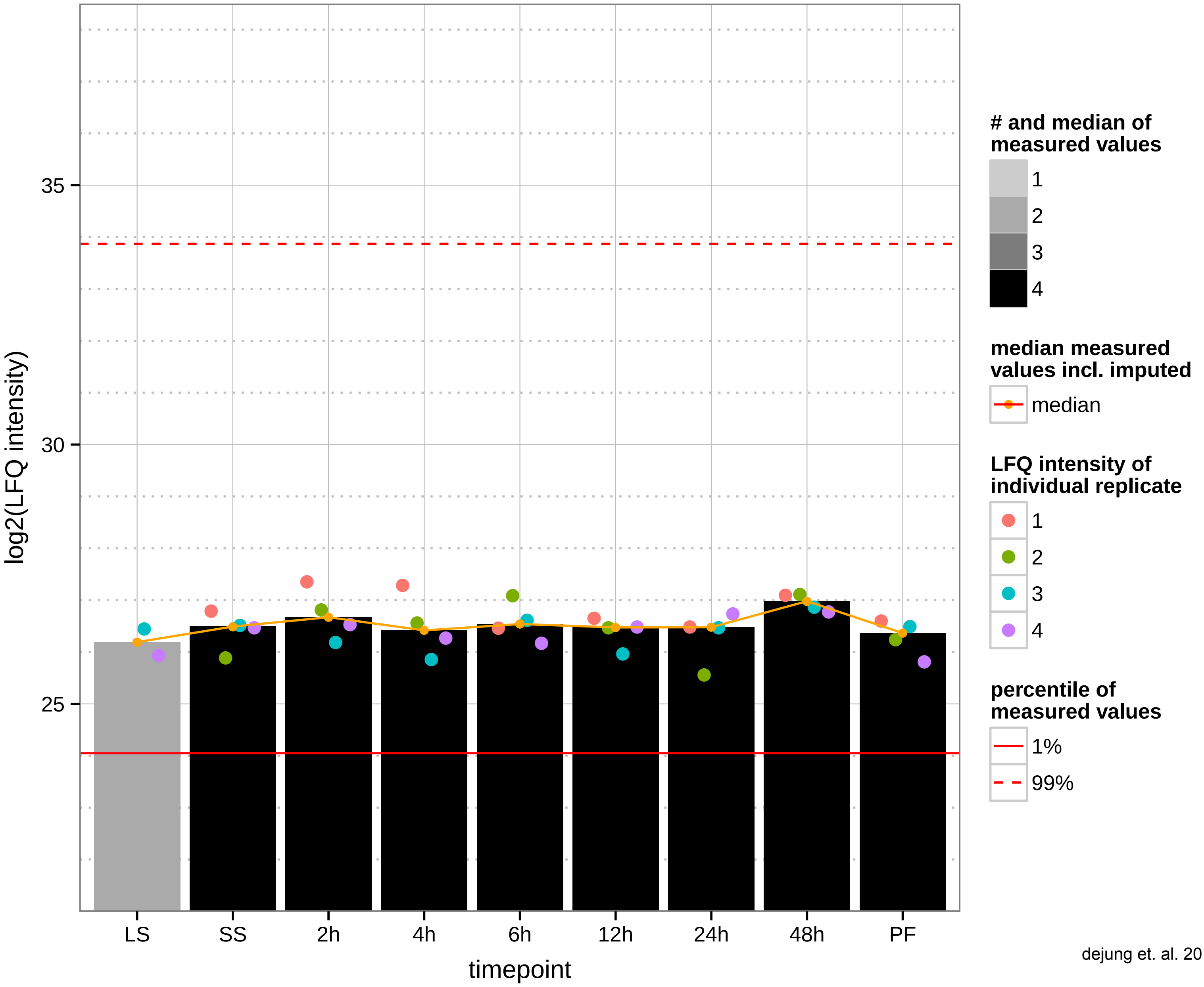

dejung et. al. 2015

predicted zinc finger protein
Tb927.9.10570
AGOF: zinc ion binding
AGOC: null
AGOP: null
PGOF: metal ion binding
PGOC: null
PGOP: null

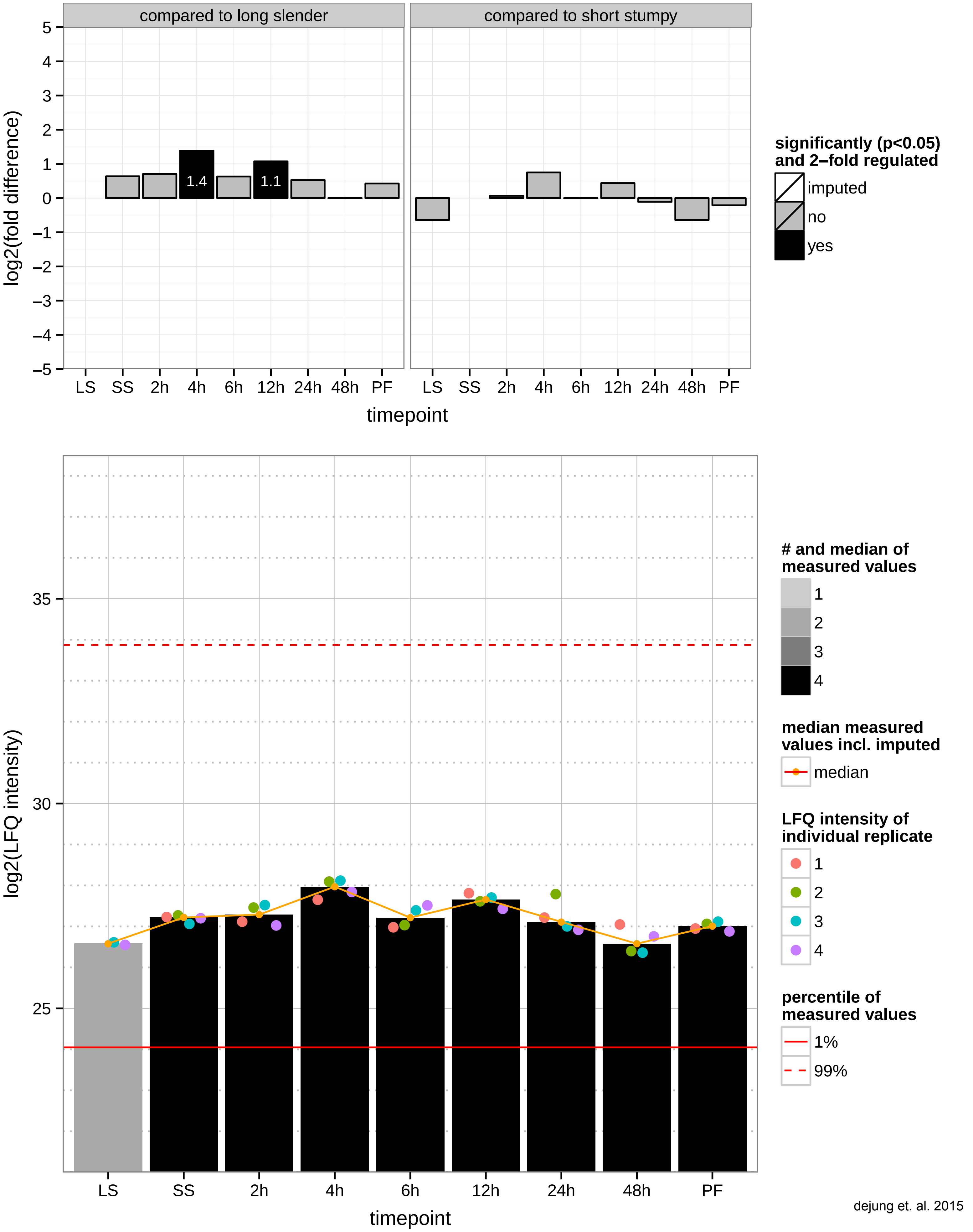

dejung et. al. 2015

3–demethylubiquinone–9 3–methyltransferase, putative
Tb927.9.10580
AGOF: 2–polyprenyl–6–methoxy–1, 4–benzoquinone methyltransferase activity, hexaprenyldihydroxybenzoate methyltransfera
AGOC: mitochondrial membrane, mitochondrion
AGOP: ubiquinone biosynthetic process, ubiquinone metabolic process
PGOF: 2–polyprenyl–6–methoxy–1, 4–benzoquinone methyltransferase activity
PGOC: null
PGOP: ubiquinone biosynthetic process

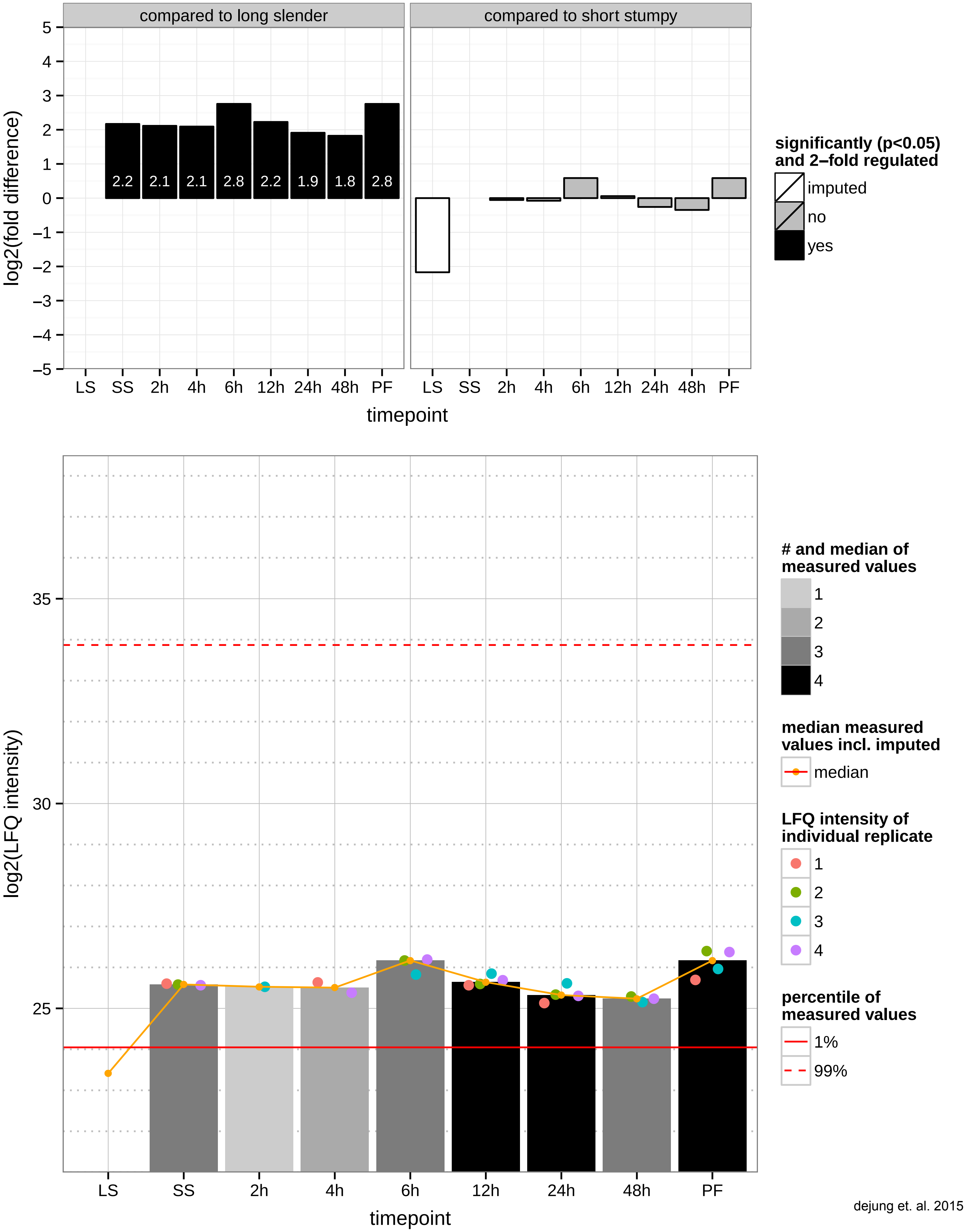

dejung et. al. 2015

hypothetical protein, conserved
Tb927.9.10680
AGOF: null
AGOC: null
AGOP: null
PGOF: DNA binding, DNA–directed RNA polymerase activity
PGOC: DNA–directed RNA polymerase III complex
PGOP: transcription from RNA polymerase III promoter

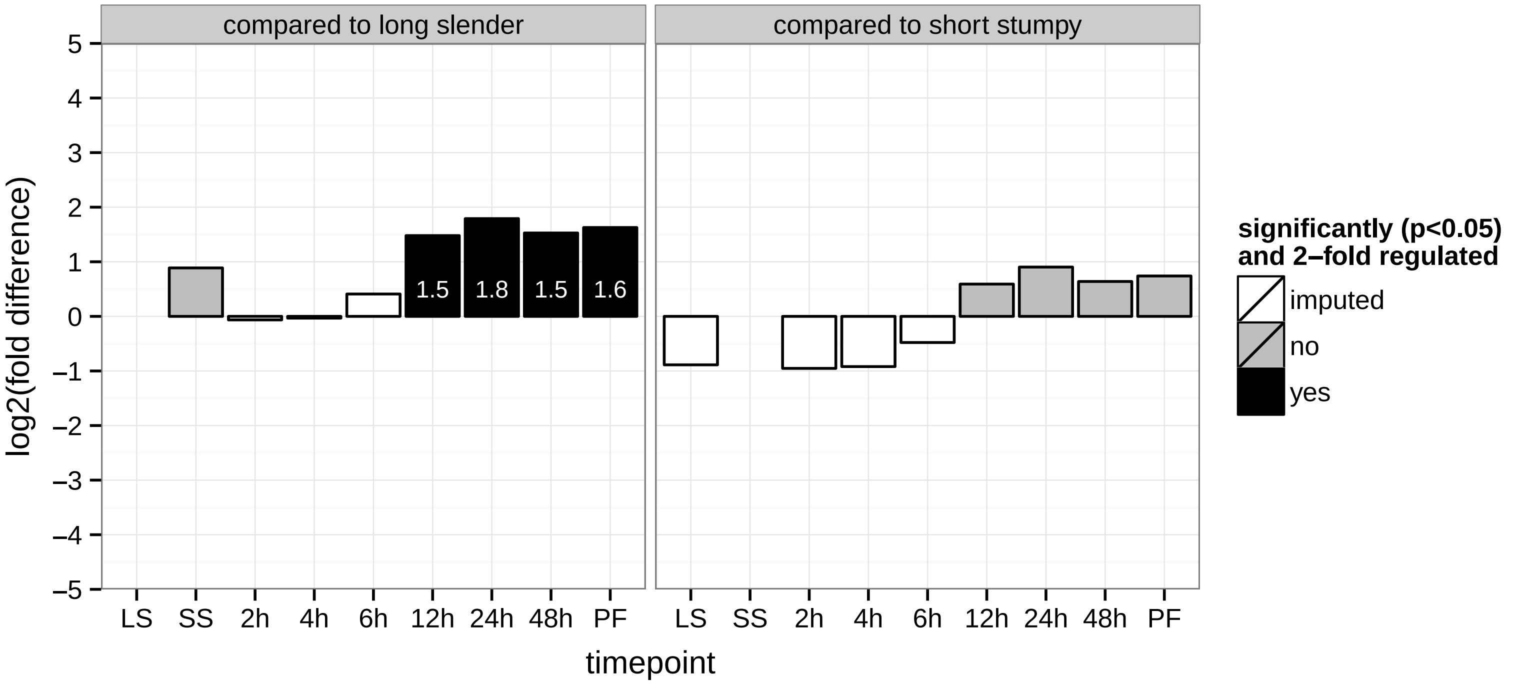

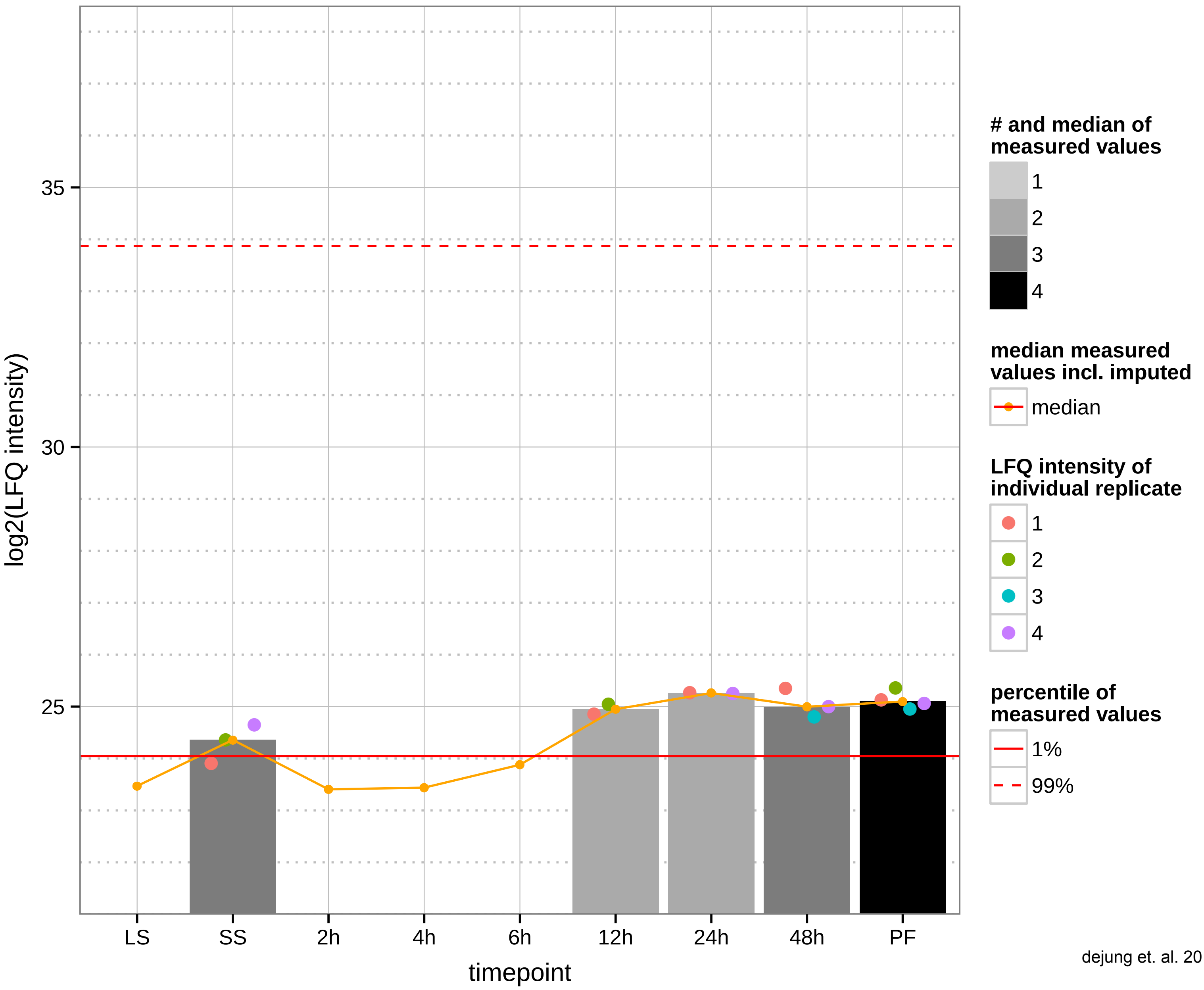

dejung et. al. 2015

hypothetical protein, conserved
Tb927.9.10690
AGOF: null
AGOC: null
AGOP: null
PGOF: null
PGOC: null
PGOP: null

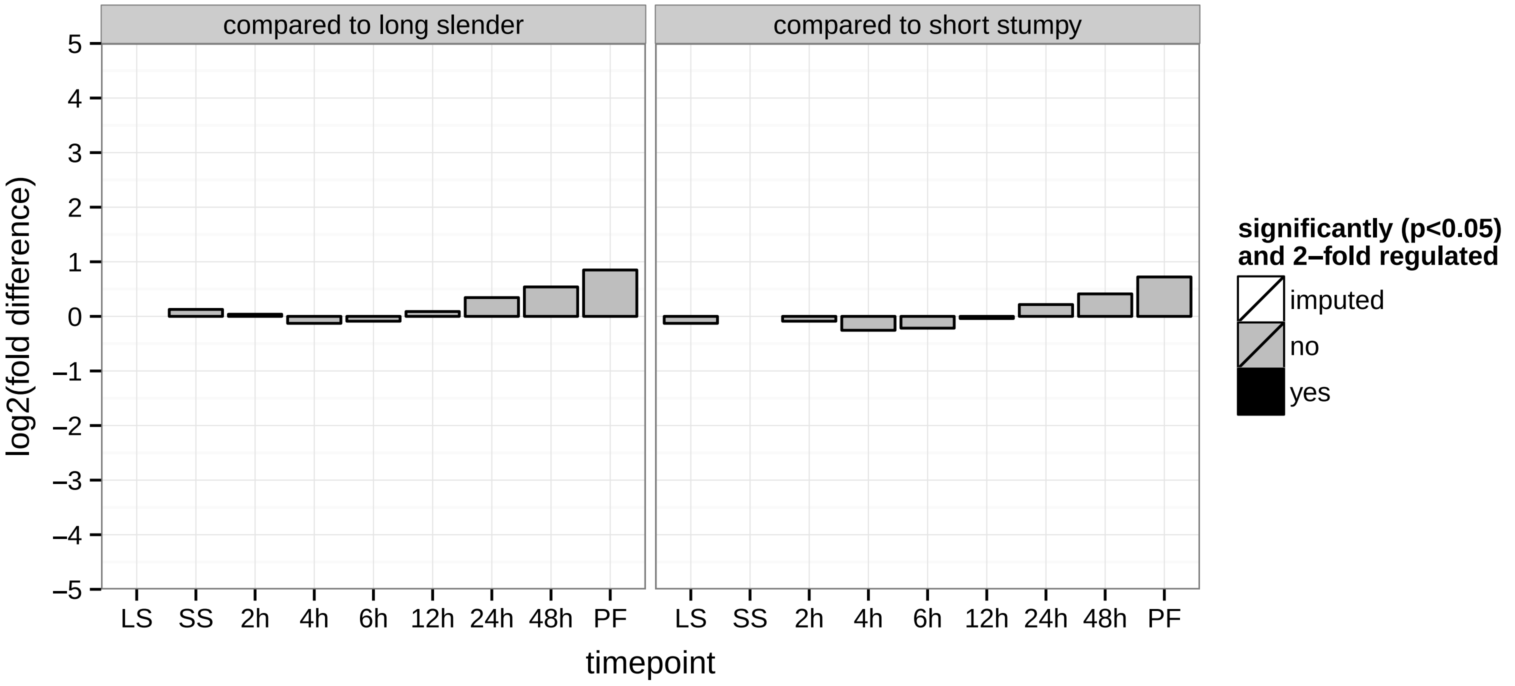

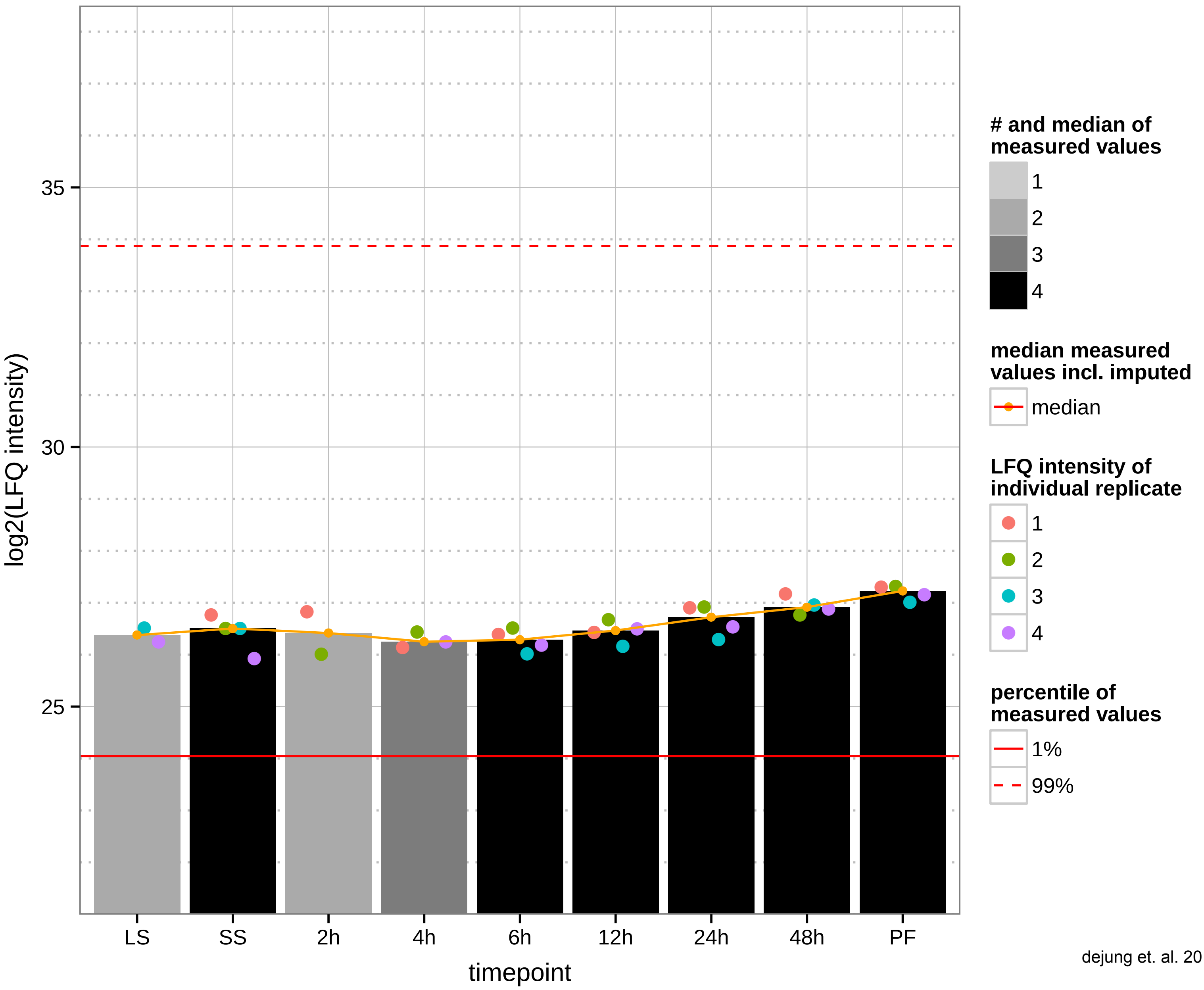

dejung et. al. 2015

hypothetical protein, conserved
Tb927.9.10890
AGOF: cAMP−dependent protein kinase regulator activity
AGOC: null
AGOP: signal transduction
PGOF: cAMP−dependent protein kinase regulator activity
PGOC: null
PGOP: signal transduction

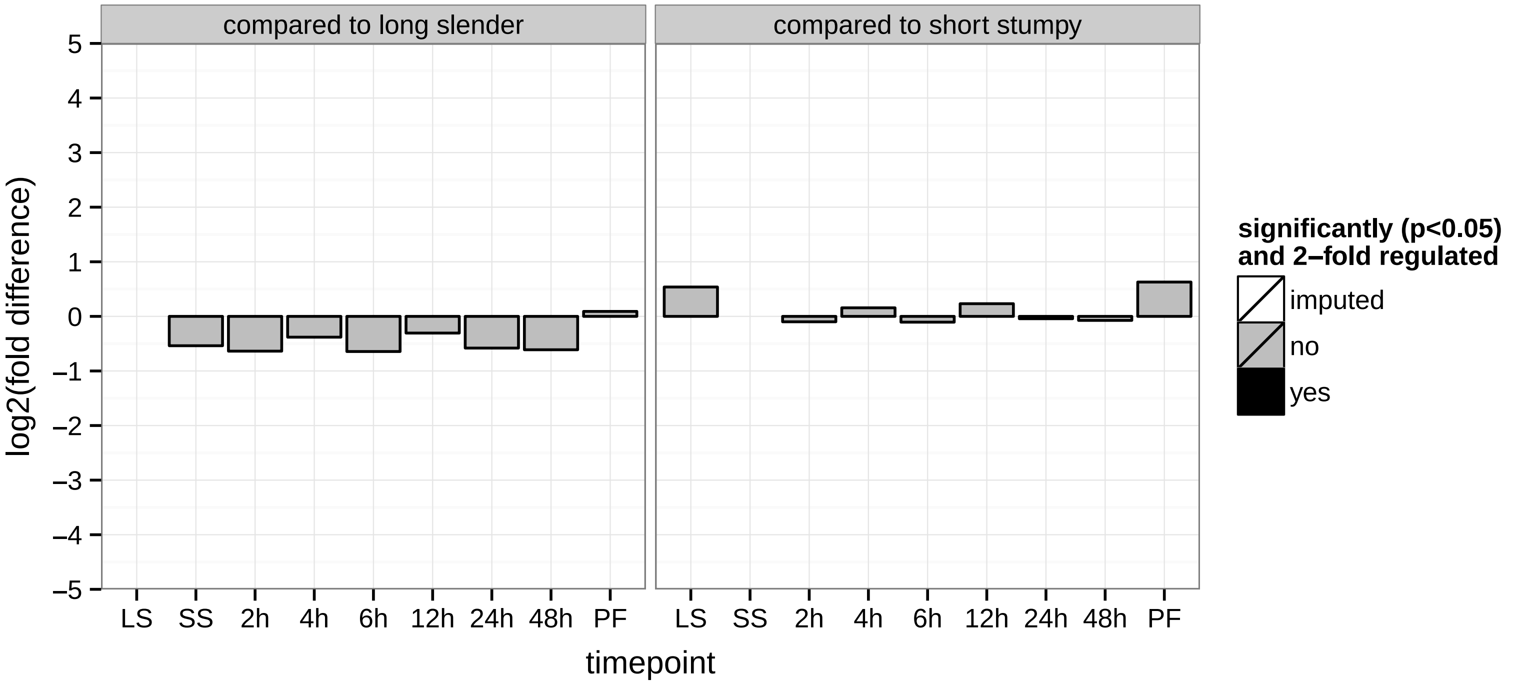

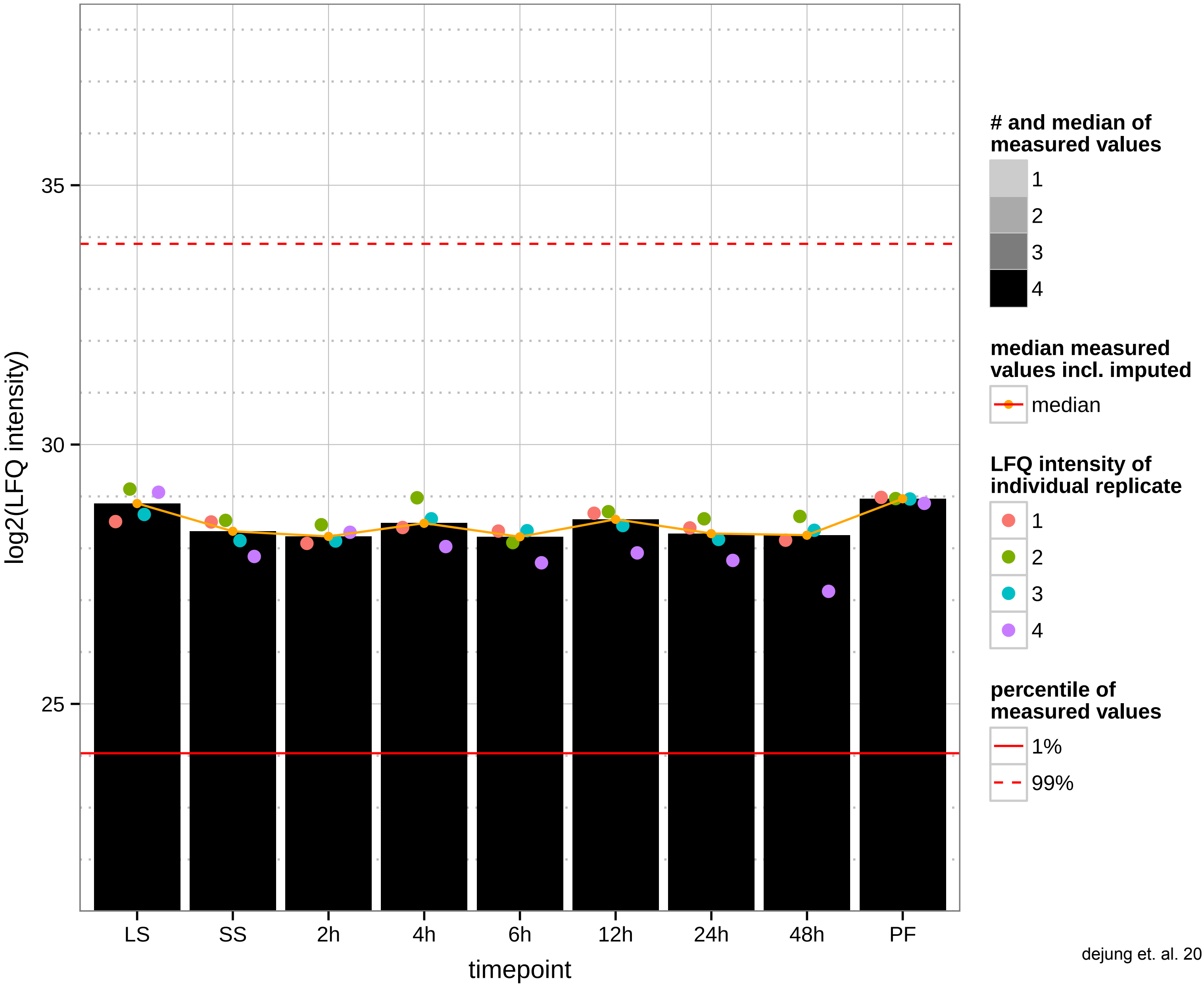

dejung et. al. 2015

ATP−dependent DEAD/H RNA helicase, putative
Tb927.9.10960
AGOF: ATP binding, ATP−dependent helicase activity, helicase activity, nucleic acid binding
AGOC: null
AGOP: nucleobase−containing compound metabolic process
PGOF: ATP binding, ATP−dependent helicase activity, helicase activity, nucleic acid binding
PGOC: null
PGOP: null

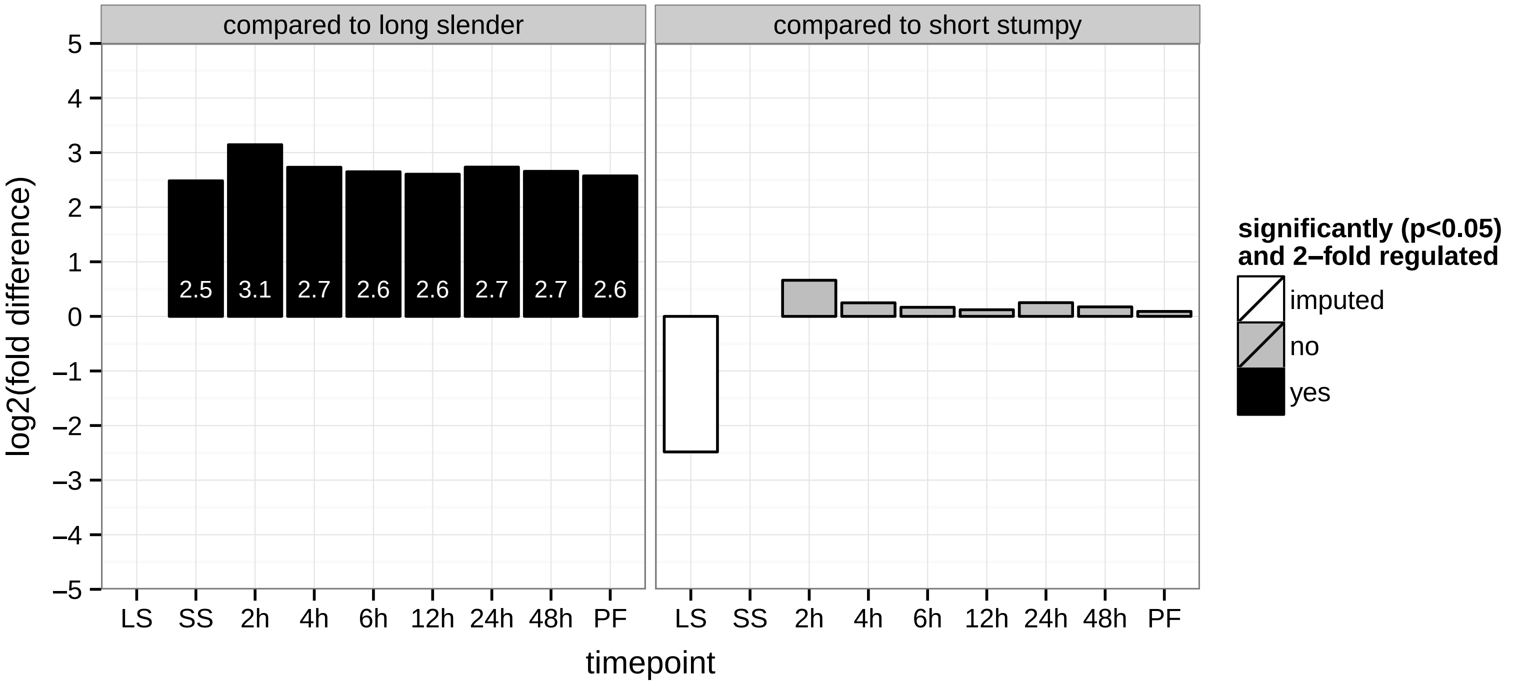

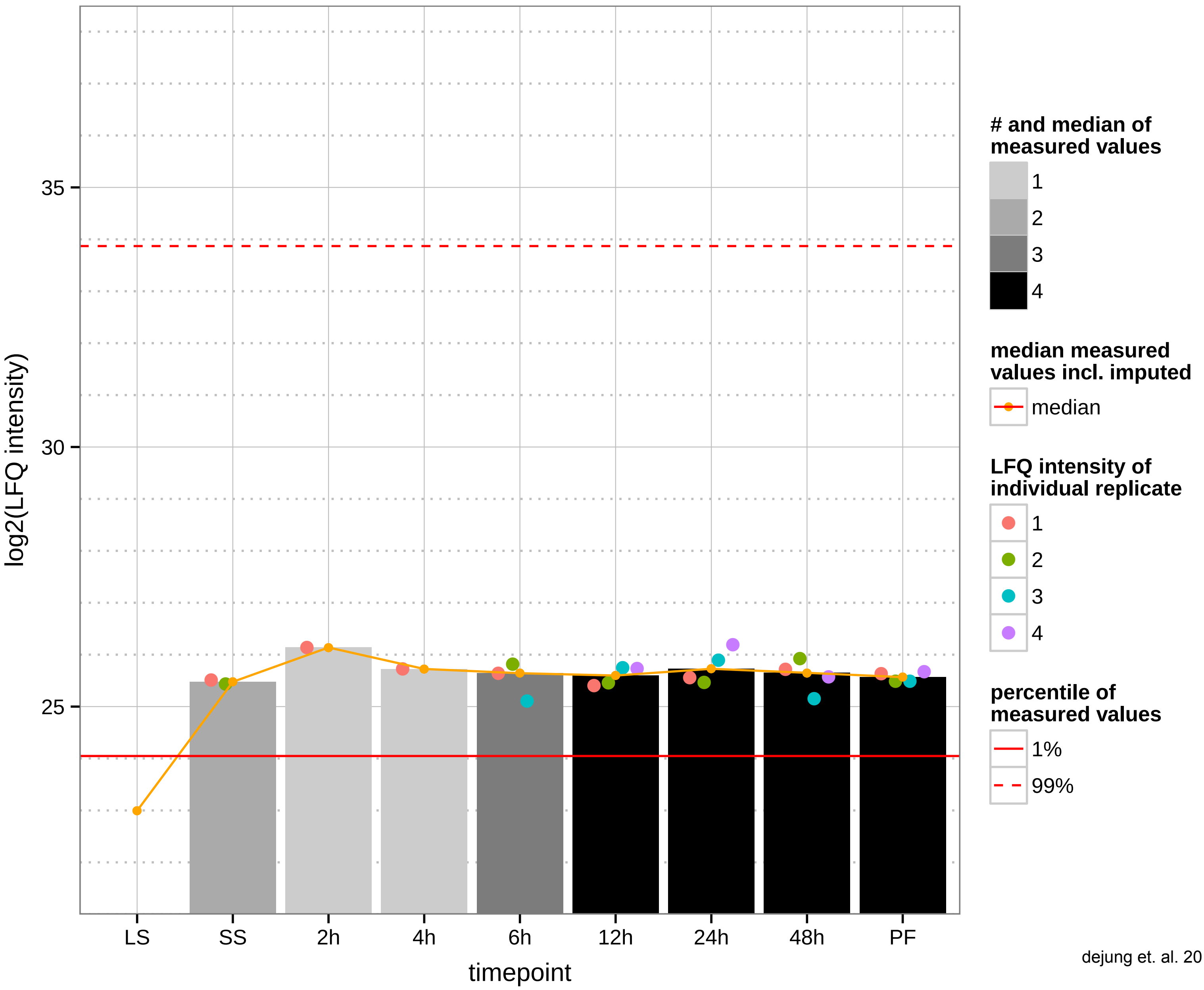

dejung et. al. 2015

mitochondrial carrier protein (MCP3)
Tb927.9.11040
AGOF: null
AGOC: mitochondrial inner membrane, mitochondrial part
AGOP: transport
PGOF: null
PGOC: null
PGOP: null

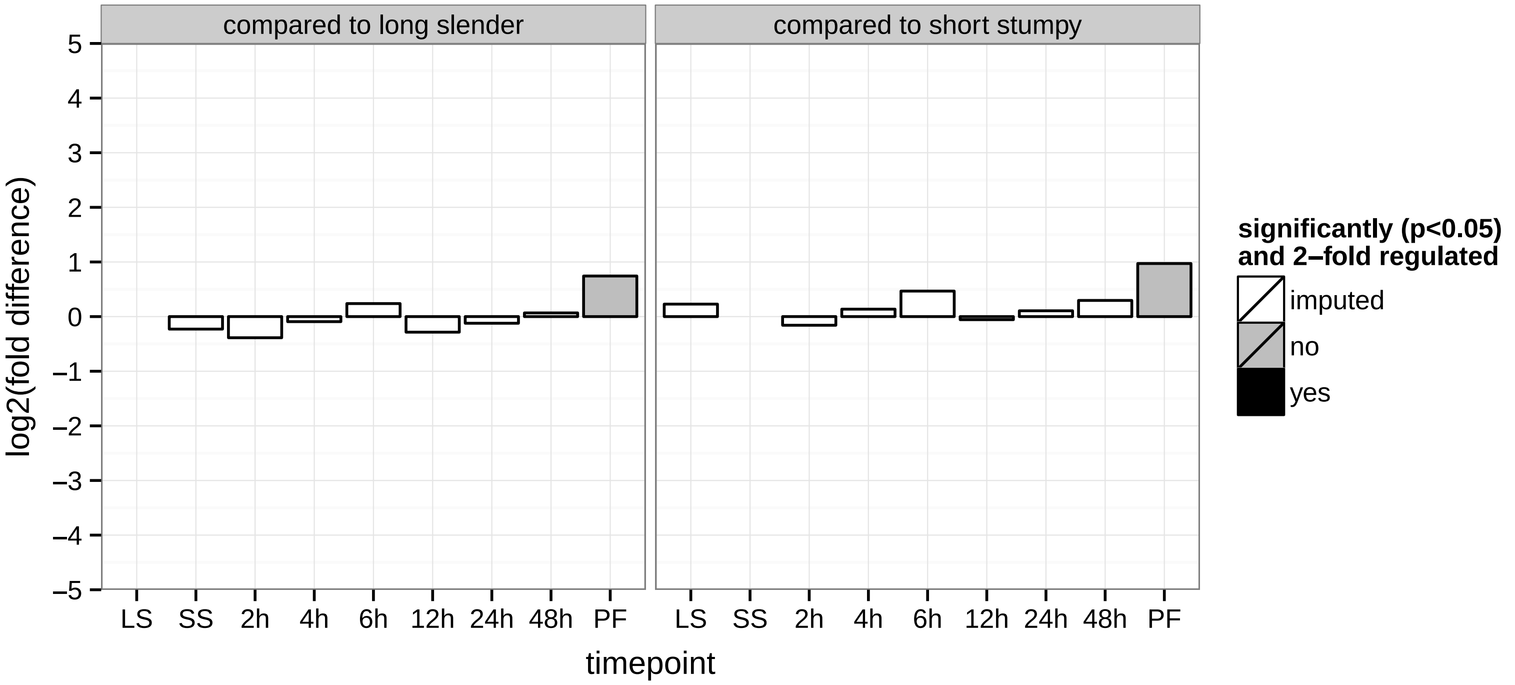

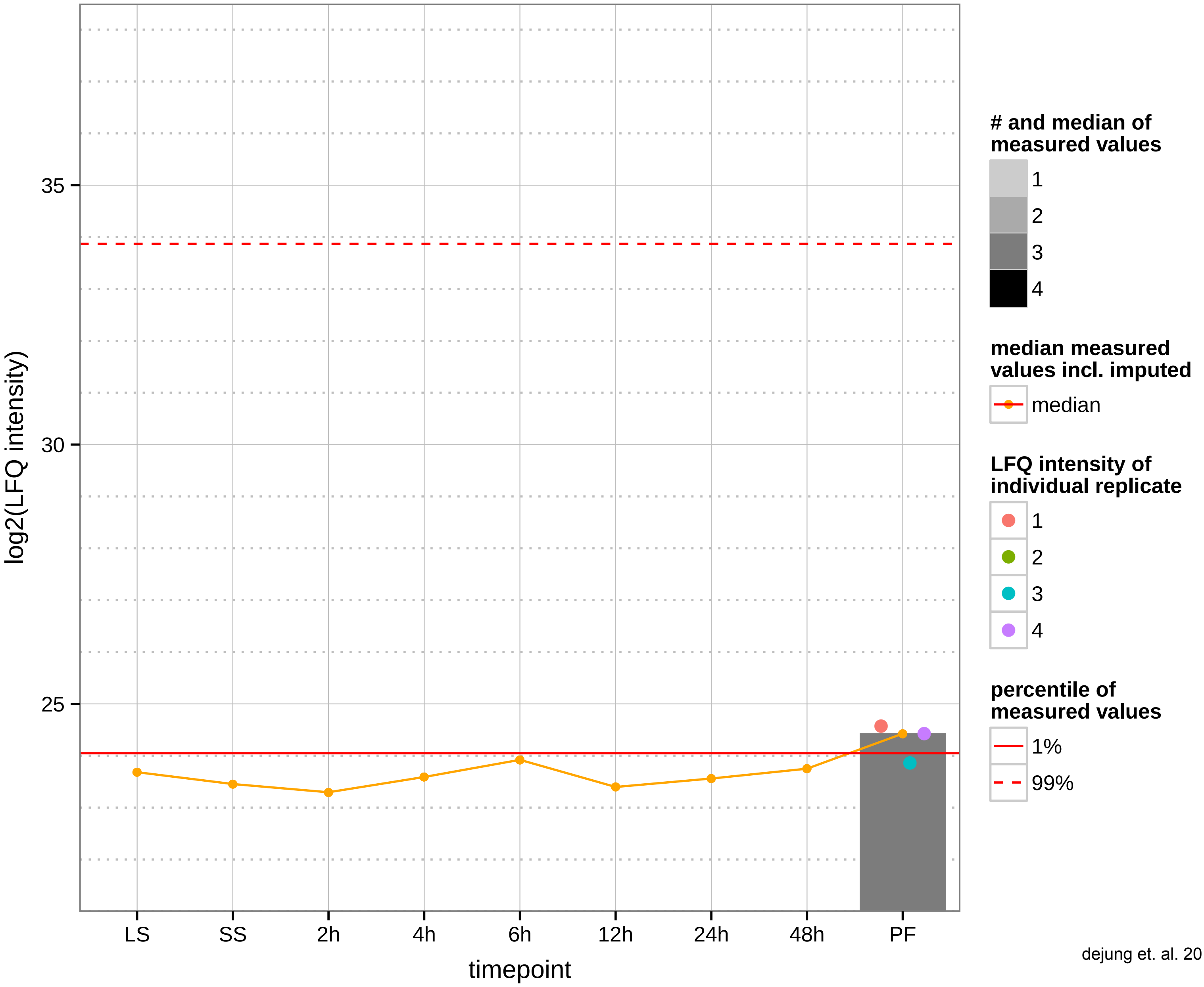

dejung et. al. 2015

PRP8 protein homologue, U5 snRNA–associated splicing factor
Tb927.9.11110
AGOF: null
AGOC: spliceosomal complex
AGOP: nuclear mRNA splicing, via spliceosome
PGOF: nucleic acid binding, protein binding
PGOC: spliceosomal complex
PGOP: nuclear mRNA splicing, via spliceosome

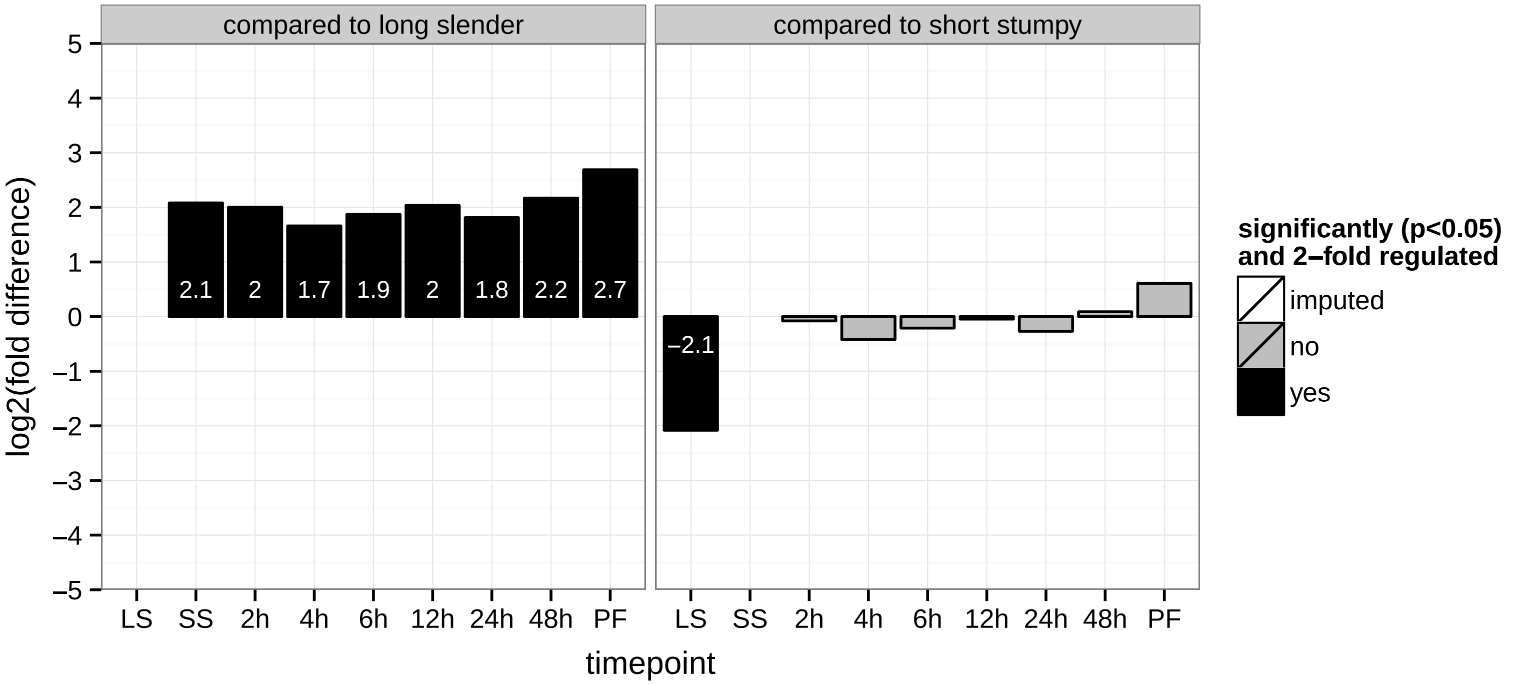

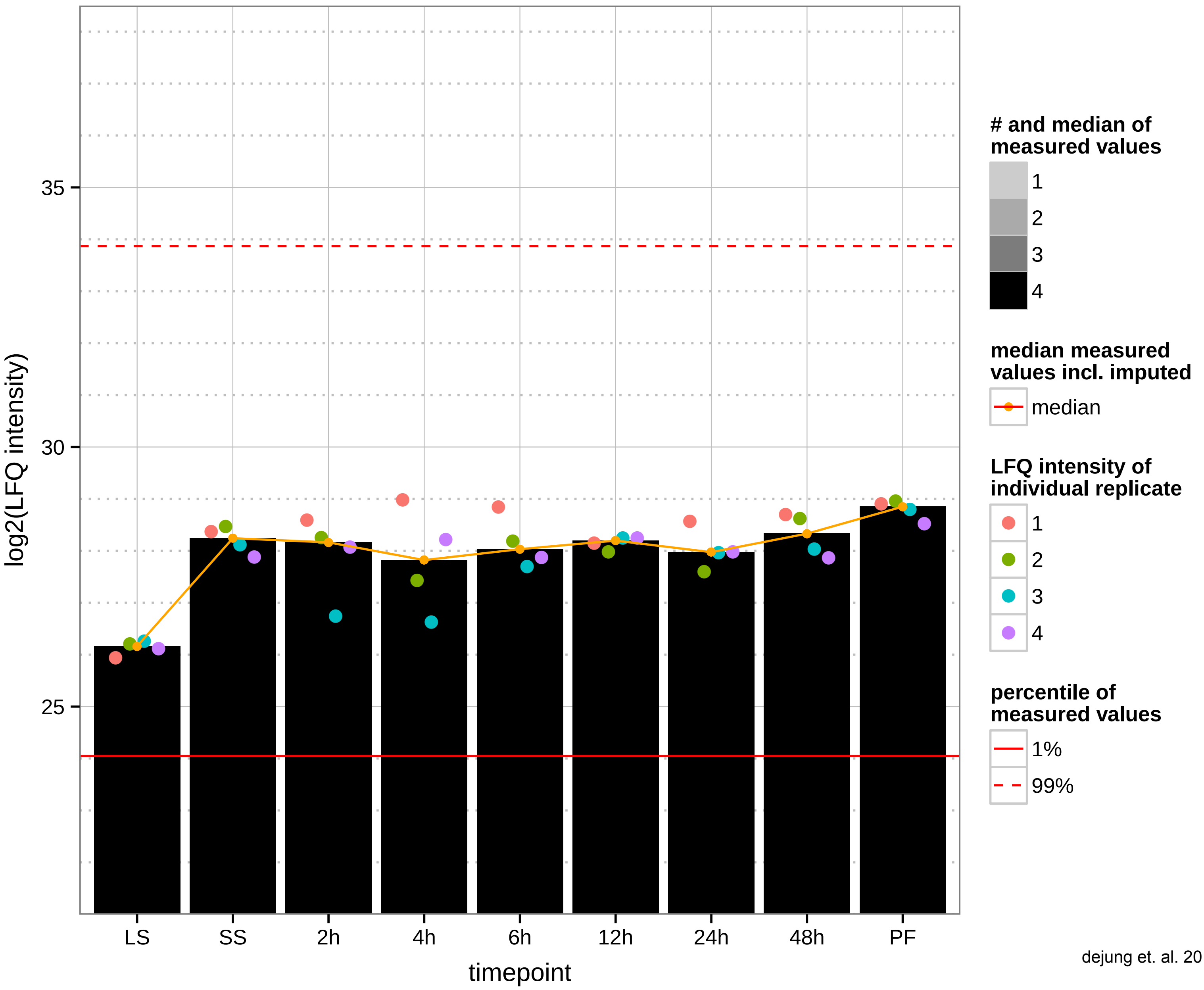

dejung et. al. 2015

hypothetical protein, conserved
Tb927.9.11140
AGOF: null
AGOC: null
AGOP: null
PGOF: null
PGOC: null
PGOP: null

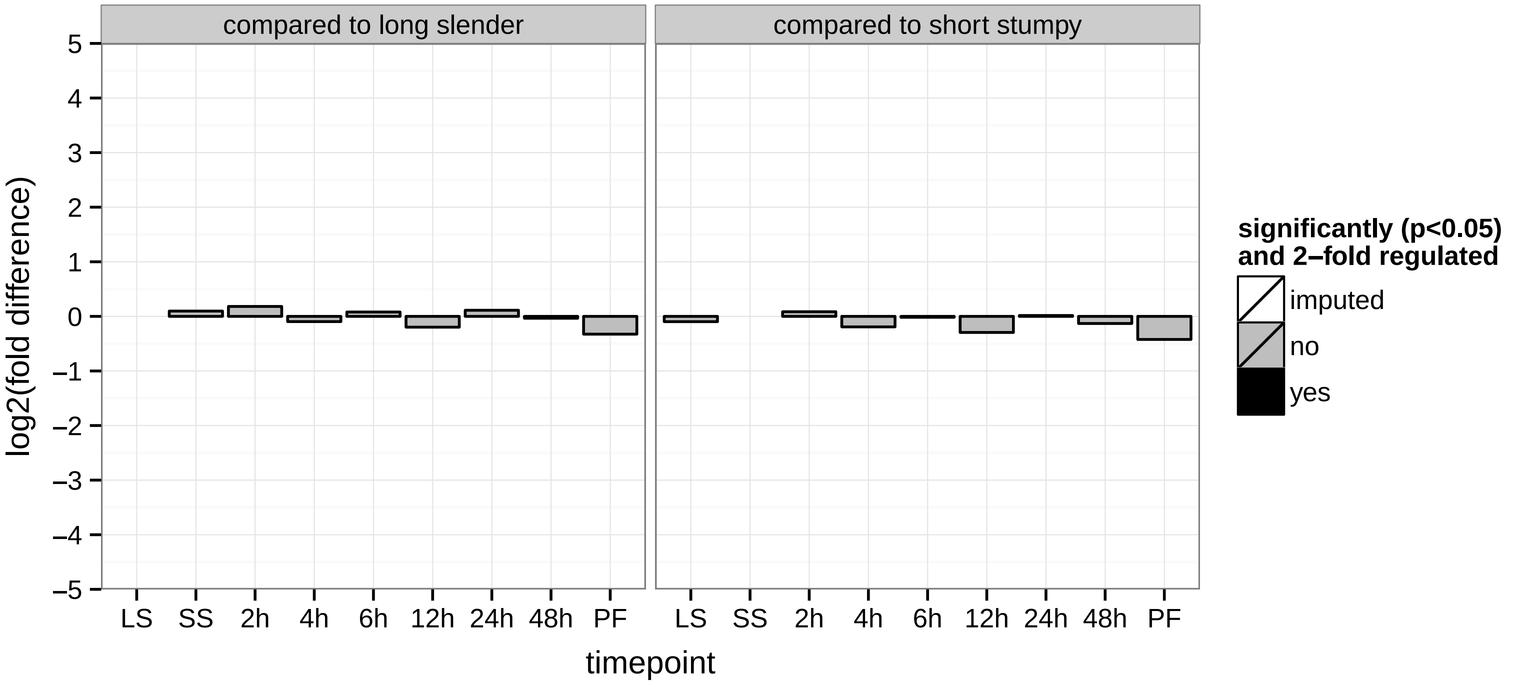

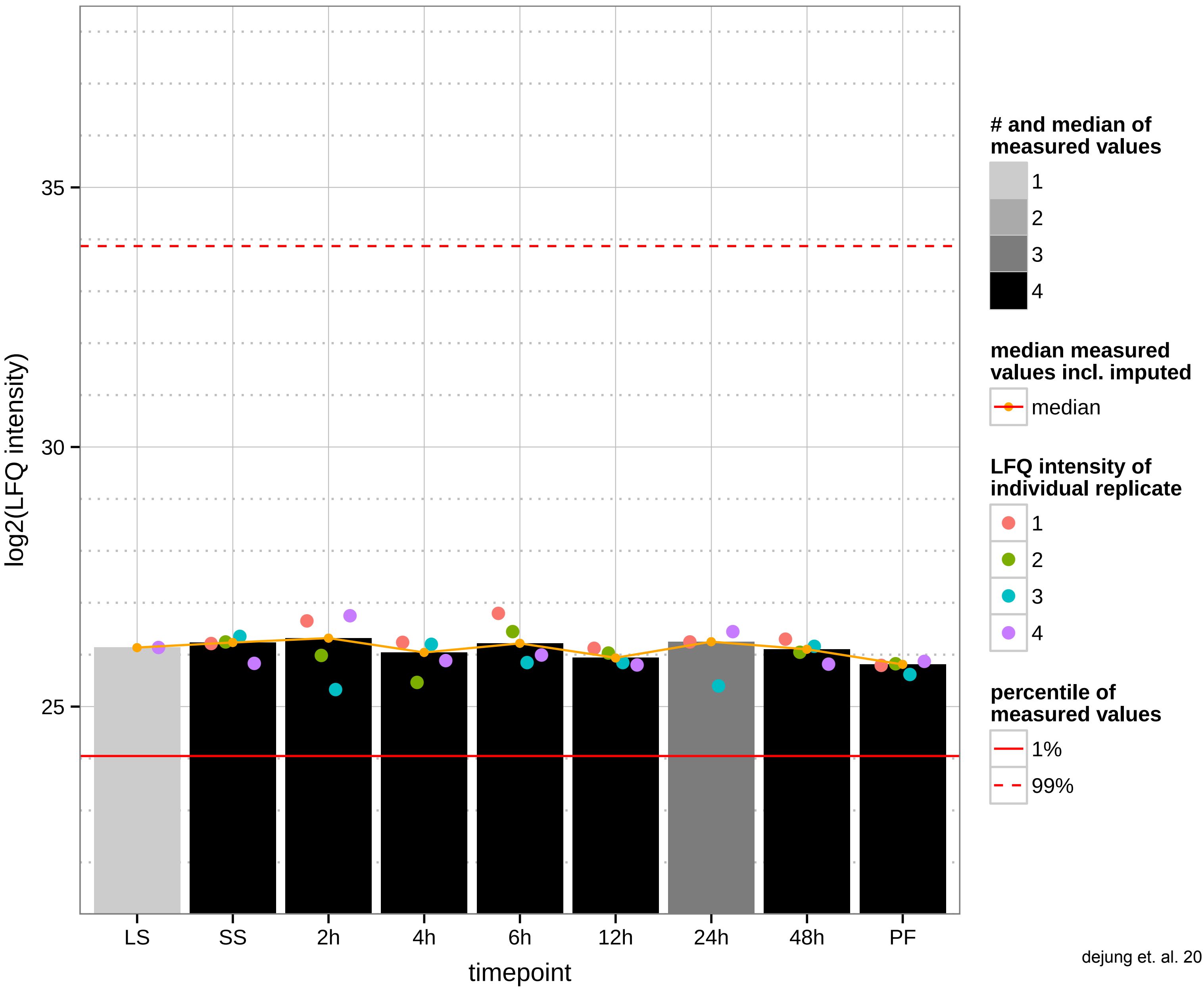

dejung et. al. 2015

hypothetical protein, conserved
Tb927.9.11150
AGOF: null
AGOC: null
AGOP: null
PGOF: null
PGOC: null
PGOP: null

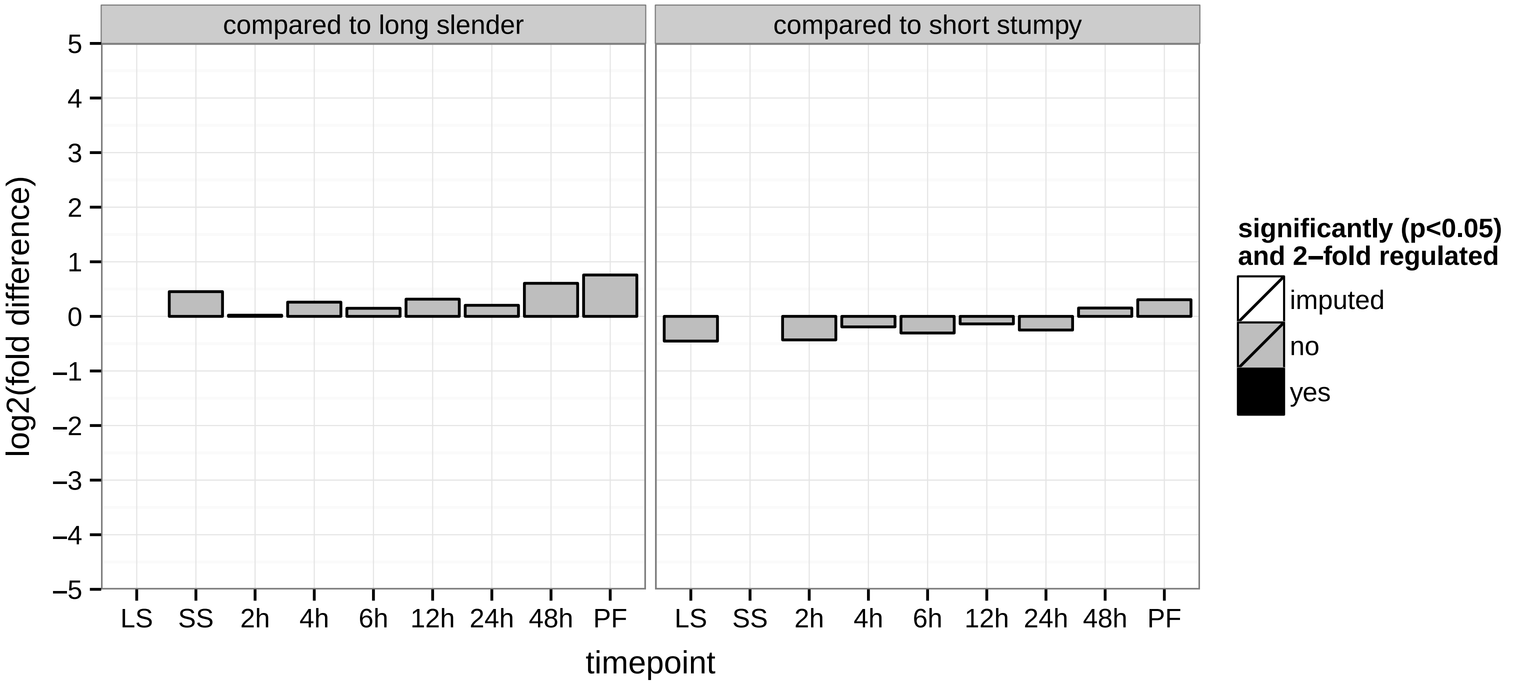

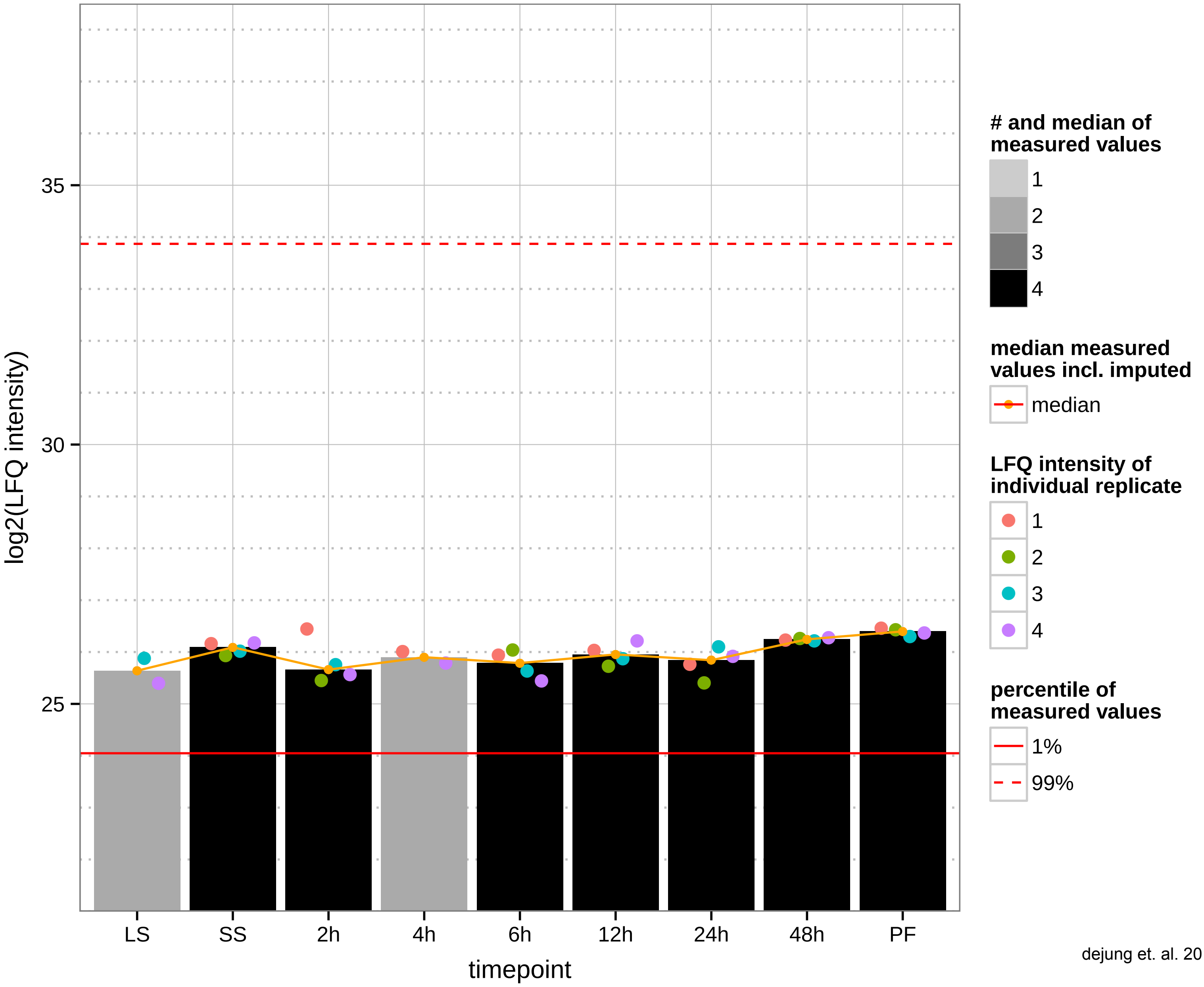

dejung et. al. 2015

predicted WD40 repeat protein
Tb927.9.11250
AGOF: null
AGOC: null
AGOP: null
PGOF: protein binding
PGOC: null
PGOP: null

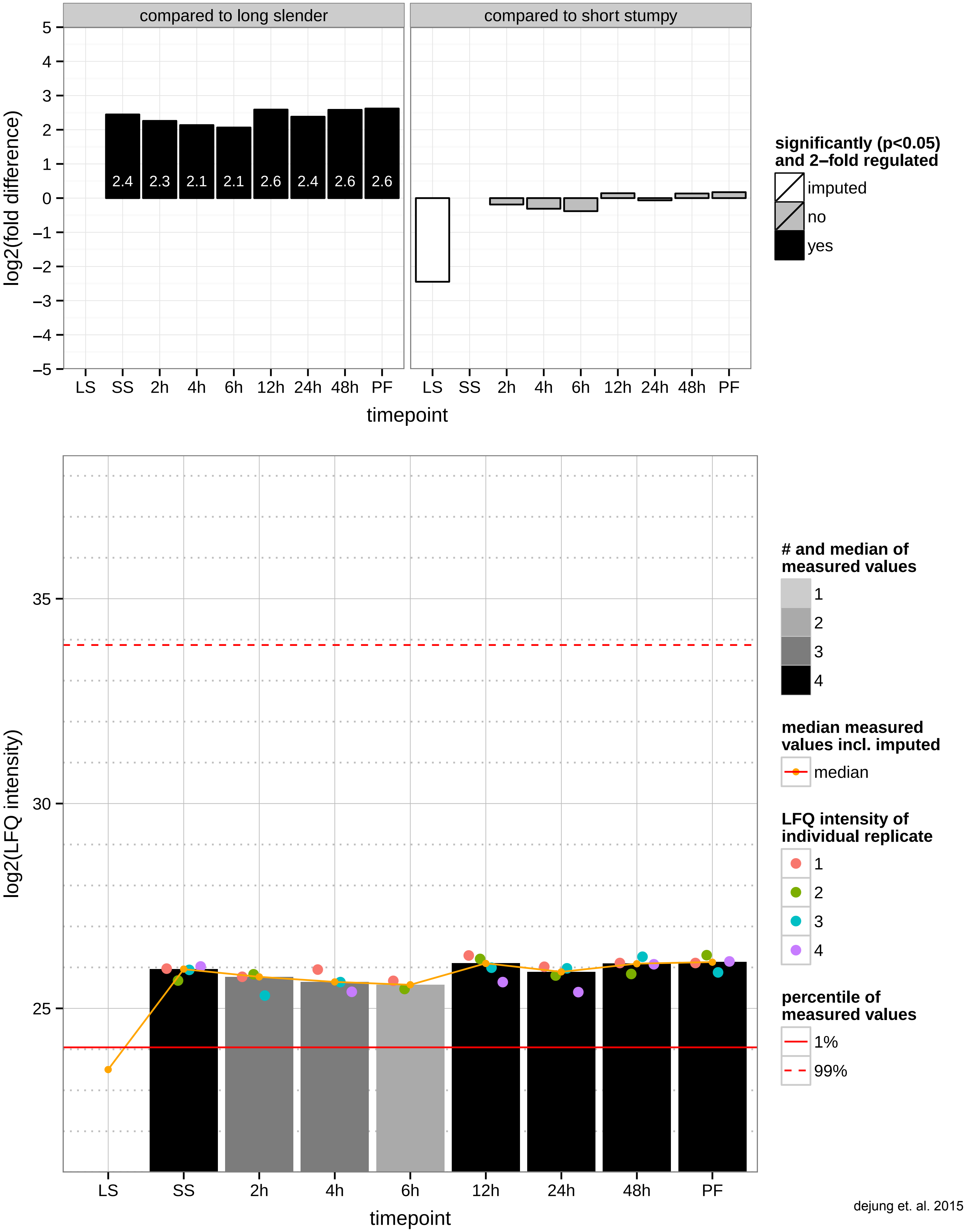

dejung et. al. 2015

nucleoside diphosphate kinase, putative
Tb927.9.11260
AGOF: ATP binding, nucleoside diphosphate kinase activity
AGOC: cilium
AGOP: CTP biosynthetic process, GTP biosynthetic process, UTP biosynthetic process, ciliary or flagellar motility
PGOF: ATP binding, nucleoside diphosphate kinase activity
PGOC: null
PGOP: CTP biosynthetic process, GTP biosynthetic process, UTP biosynthetic process, nucleoside diphosphate phosphoryla

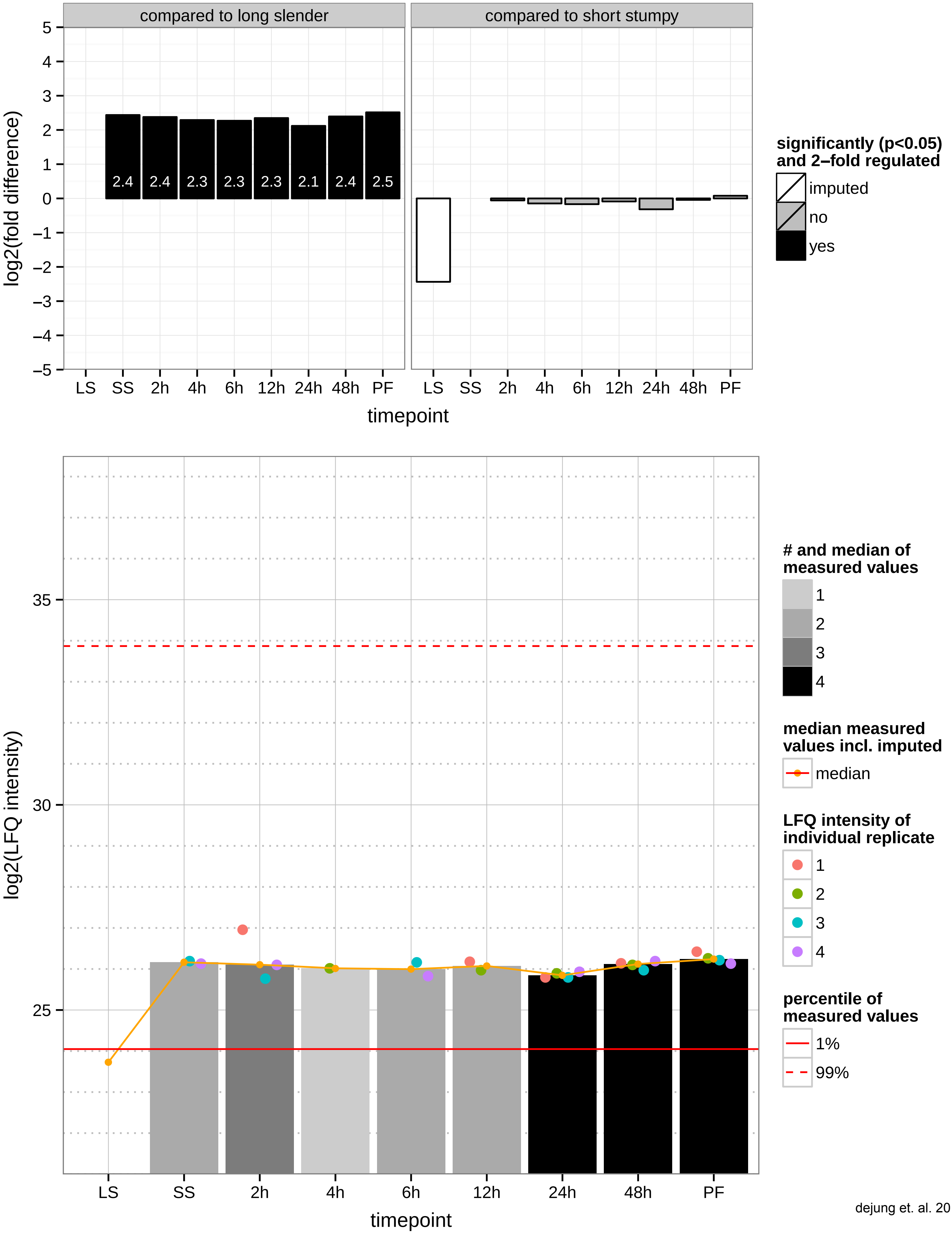

dejung et. al. 2015

unspecified product
Tb927.9.11310
AGOF: null
AGOC: null
AGOP: null
PGOF: threonine−type endopeptidase activity
PGOC: proteasome core complex
PGOP: proteolysis involved in cellular protein catabolic process

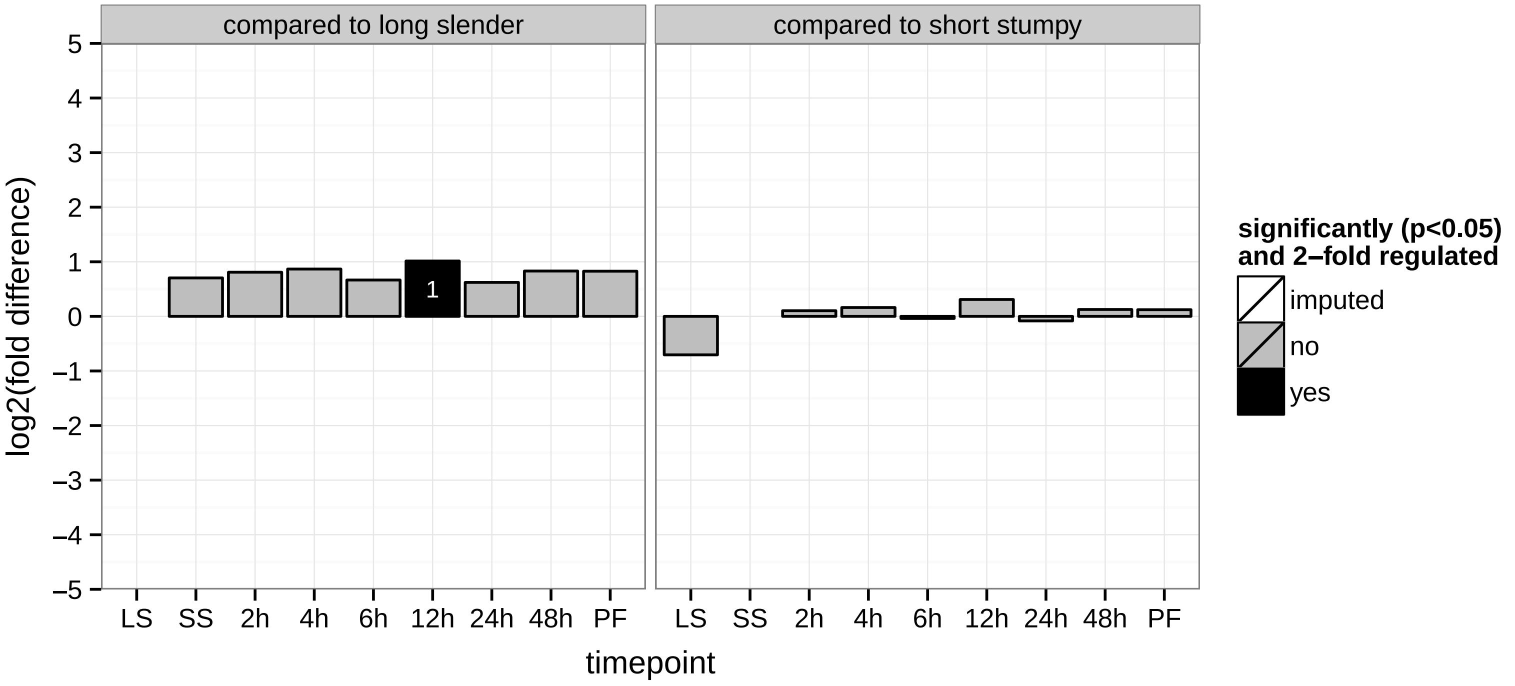

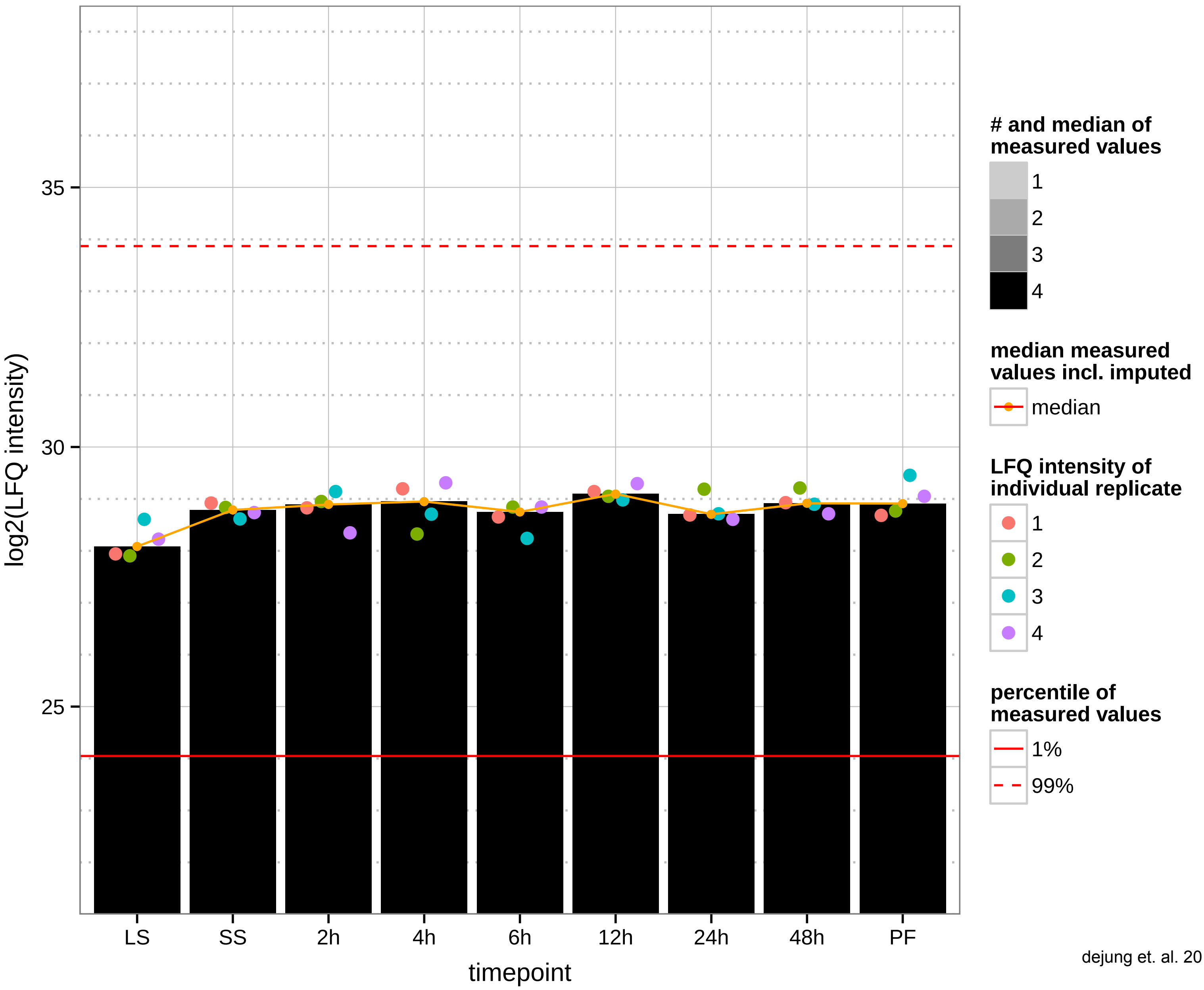

dejung et. al. 2015

unspecified product
Tb927.9.11410;Tb927.9.11380
AGOF: structural constituent of ribosome
AGOC: intracellular, ribosome
AGOP: translation
PGOF: null, structural constituent of ribosome
PGOC: null, ribosome
PGOP: null, translation

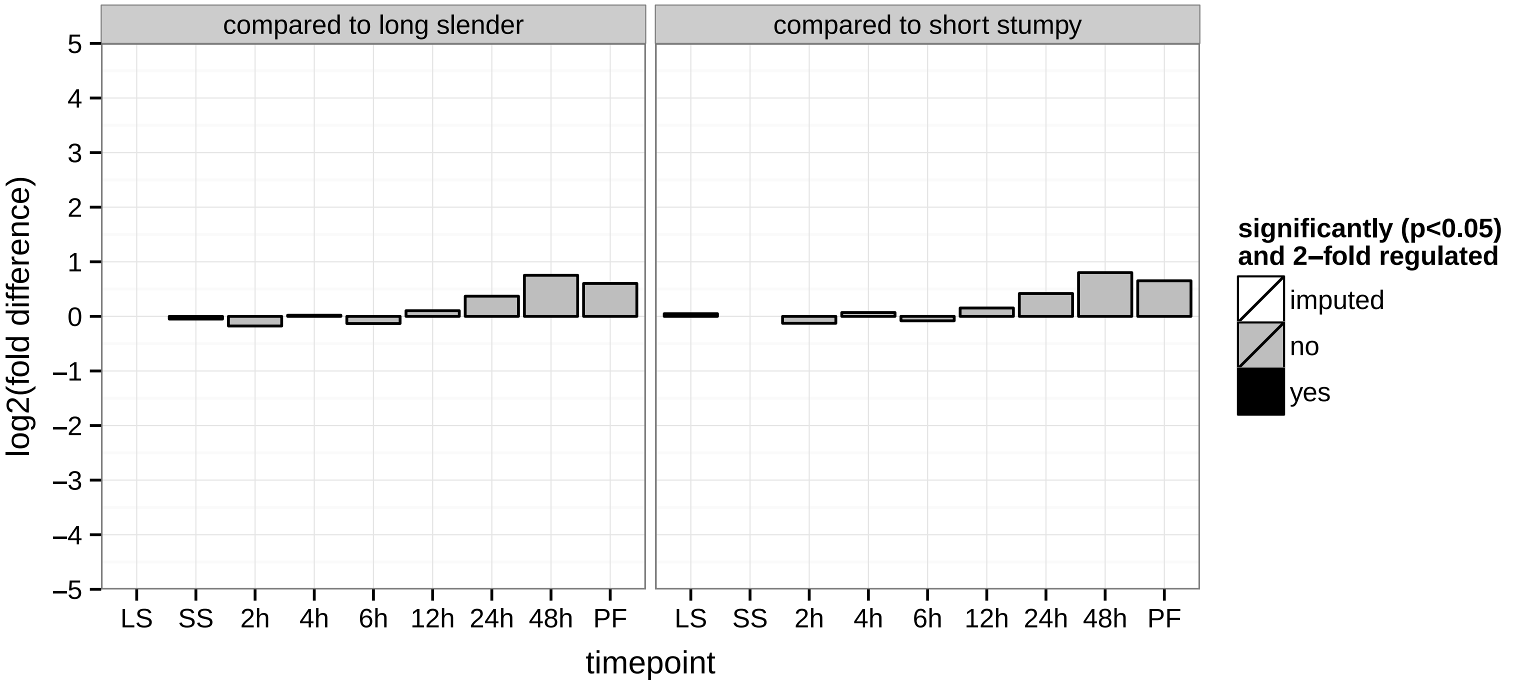

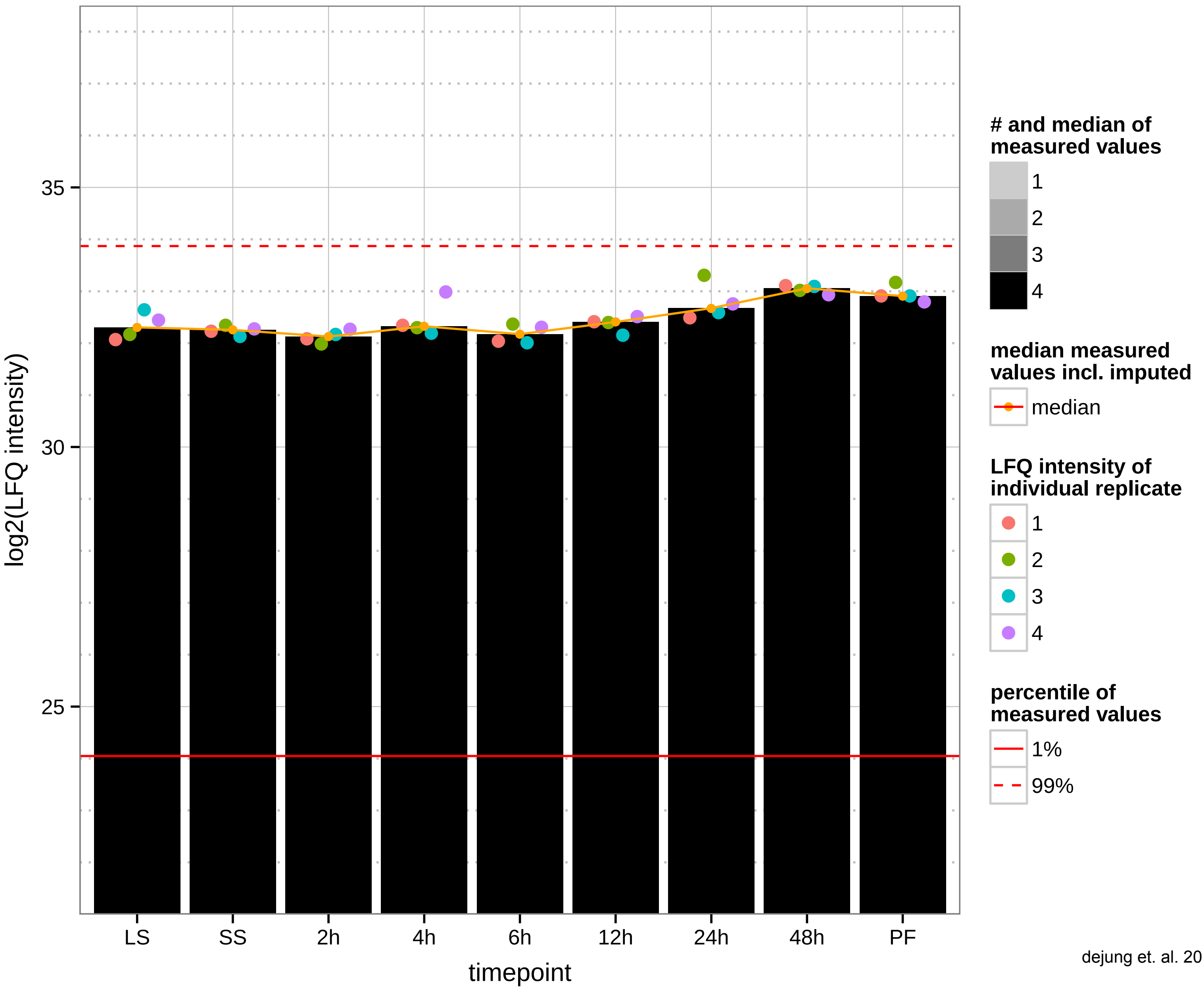

dejung et. al. 2015

Gim5A protein, glycosomal membrane protein (gim5A)
Tb927.9.11580
AGOF: null
AGOC: glycosome membrane
AGOP: null
PGOF: null
PGOC: integral to peroxisomal membrane
PGOP: peroxisome fission

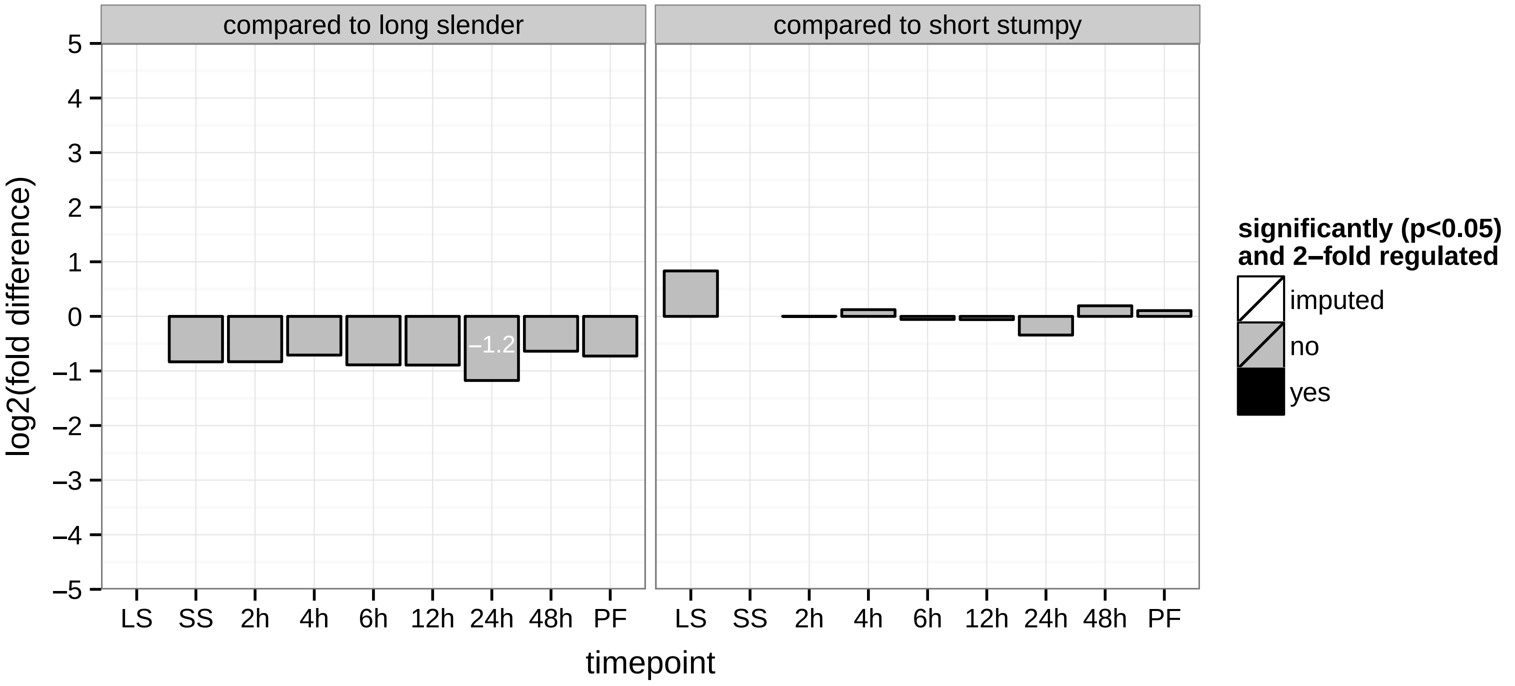

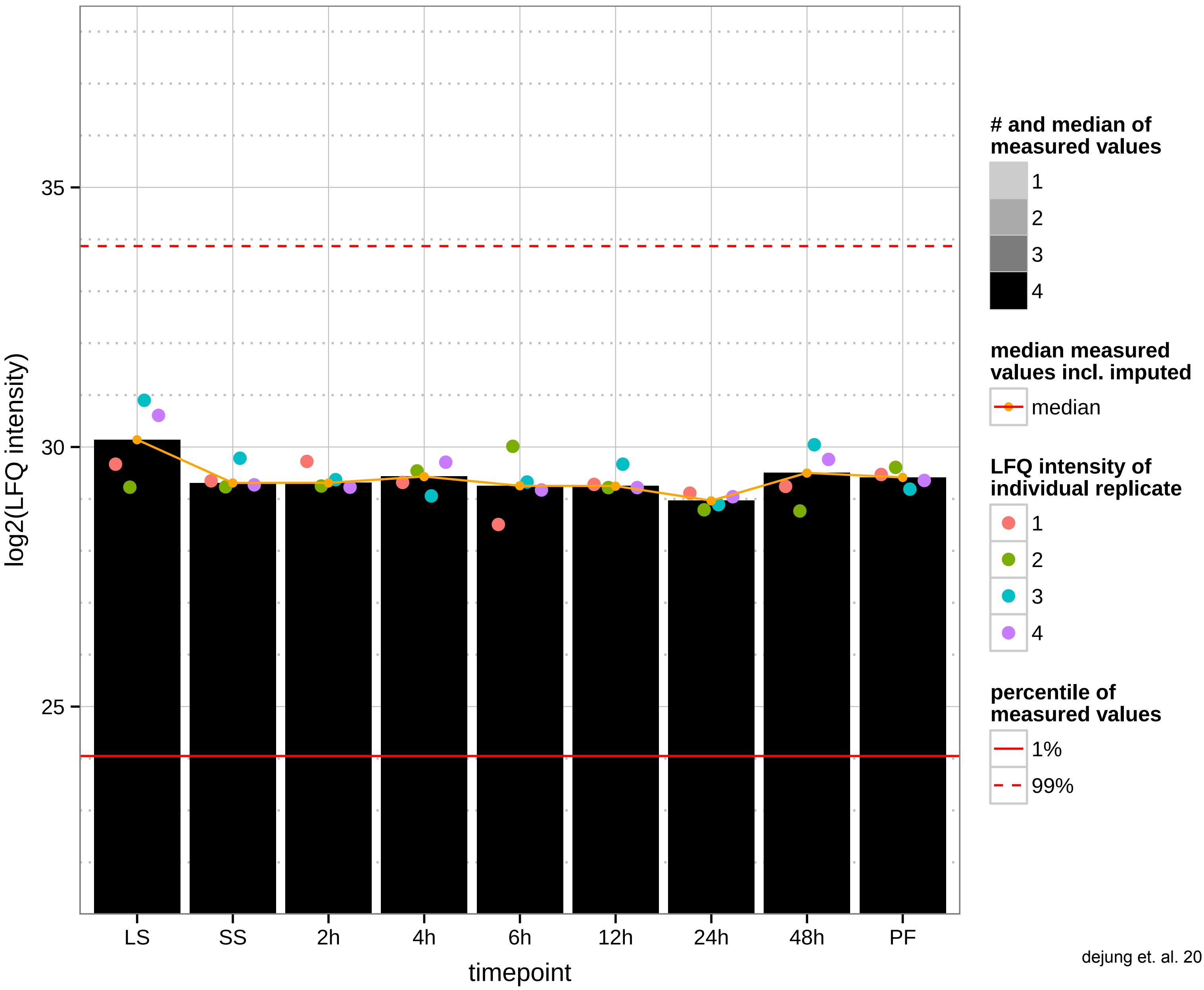

dejung et. al. 2015

unspecified product (gim5B)
Tb927.9.11600
AGOF: null
AGOC: glycosome membrane
AGOP: null
PGOF: null
PGOC: integral to peroxisomal membrane
PGOP: peroxisome fission

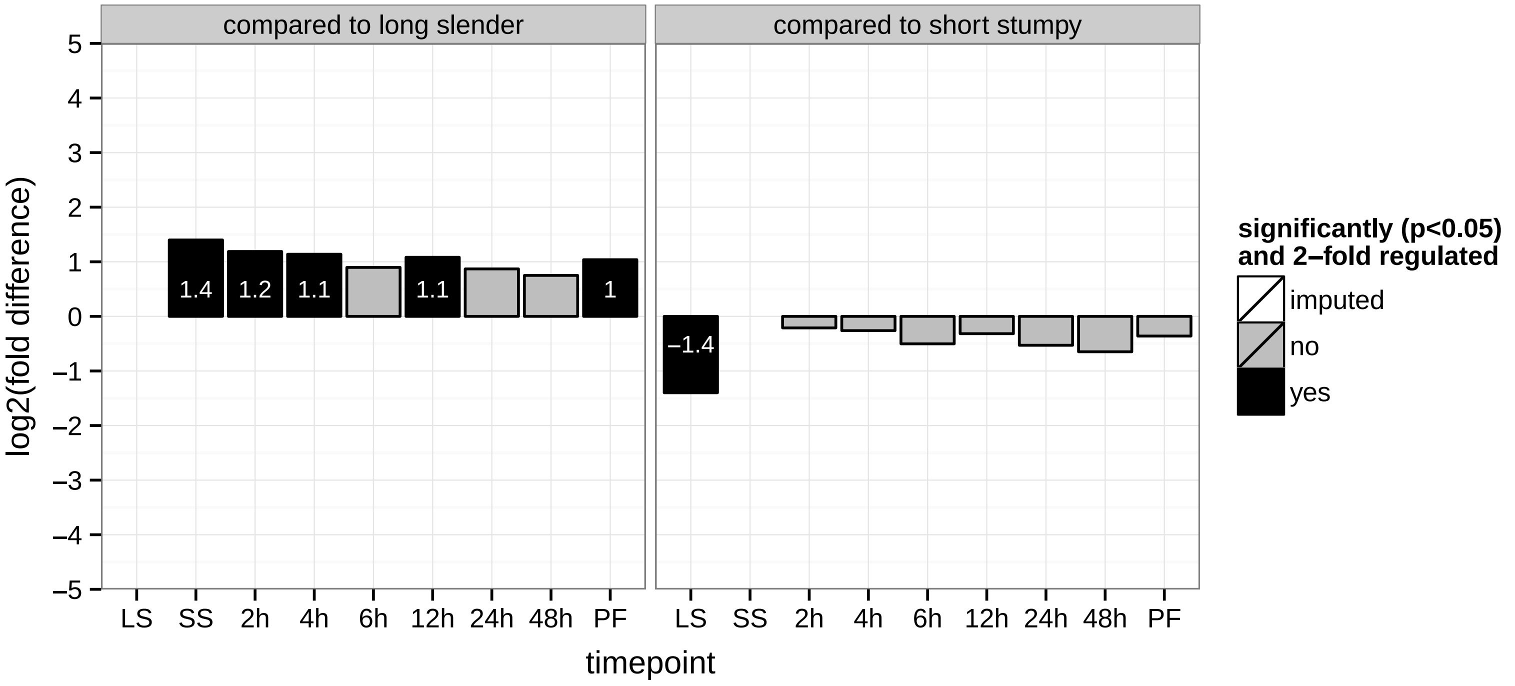

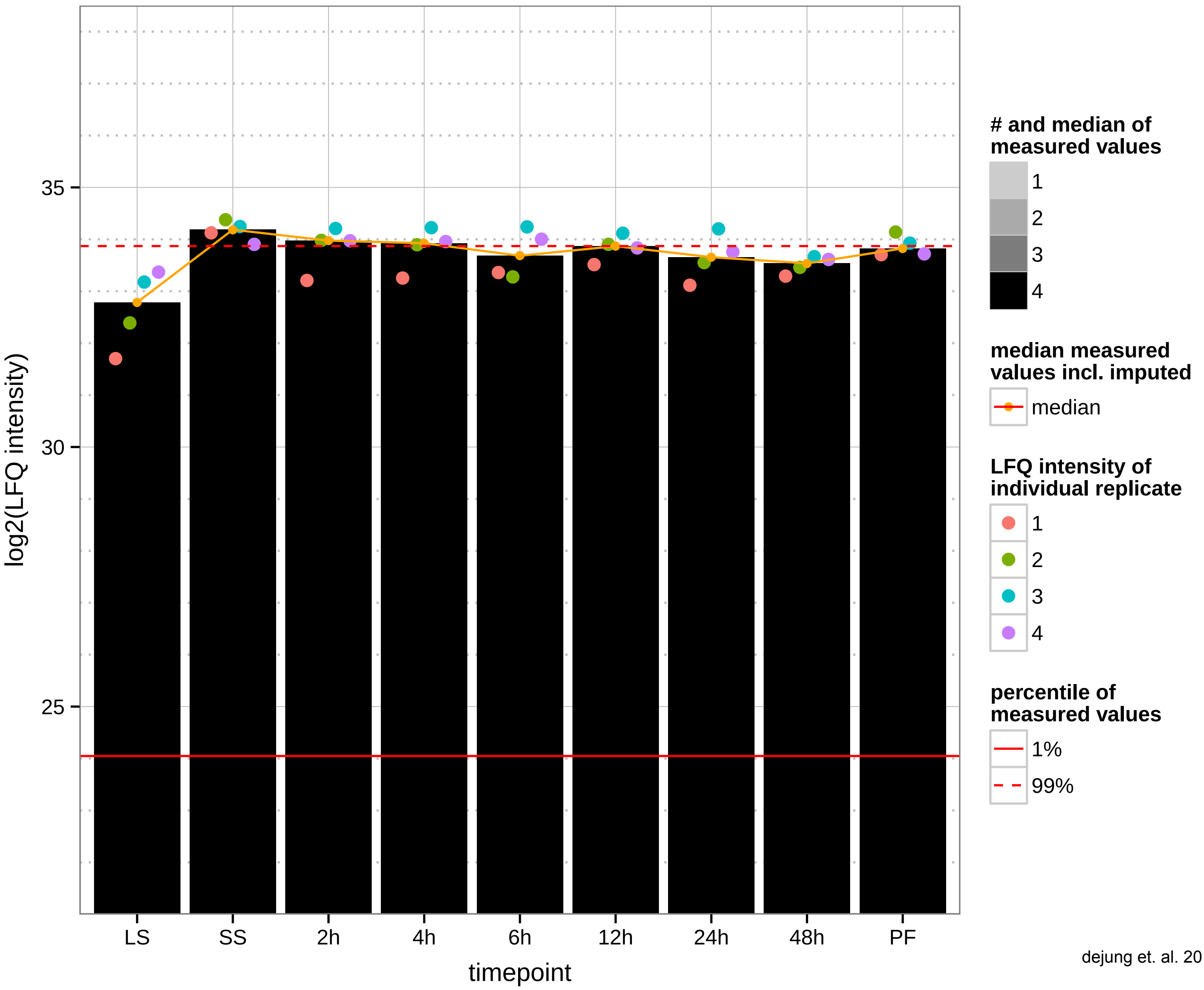

dejung et. al. 2015

hypothetical protein, conserved
Tb927.9.11770
AGOF: null
AGOC: null
AGOP: null
PGOF: null
PGOC: null
PGOP: null

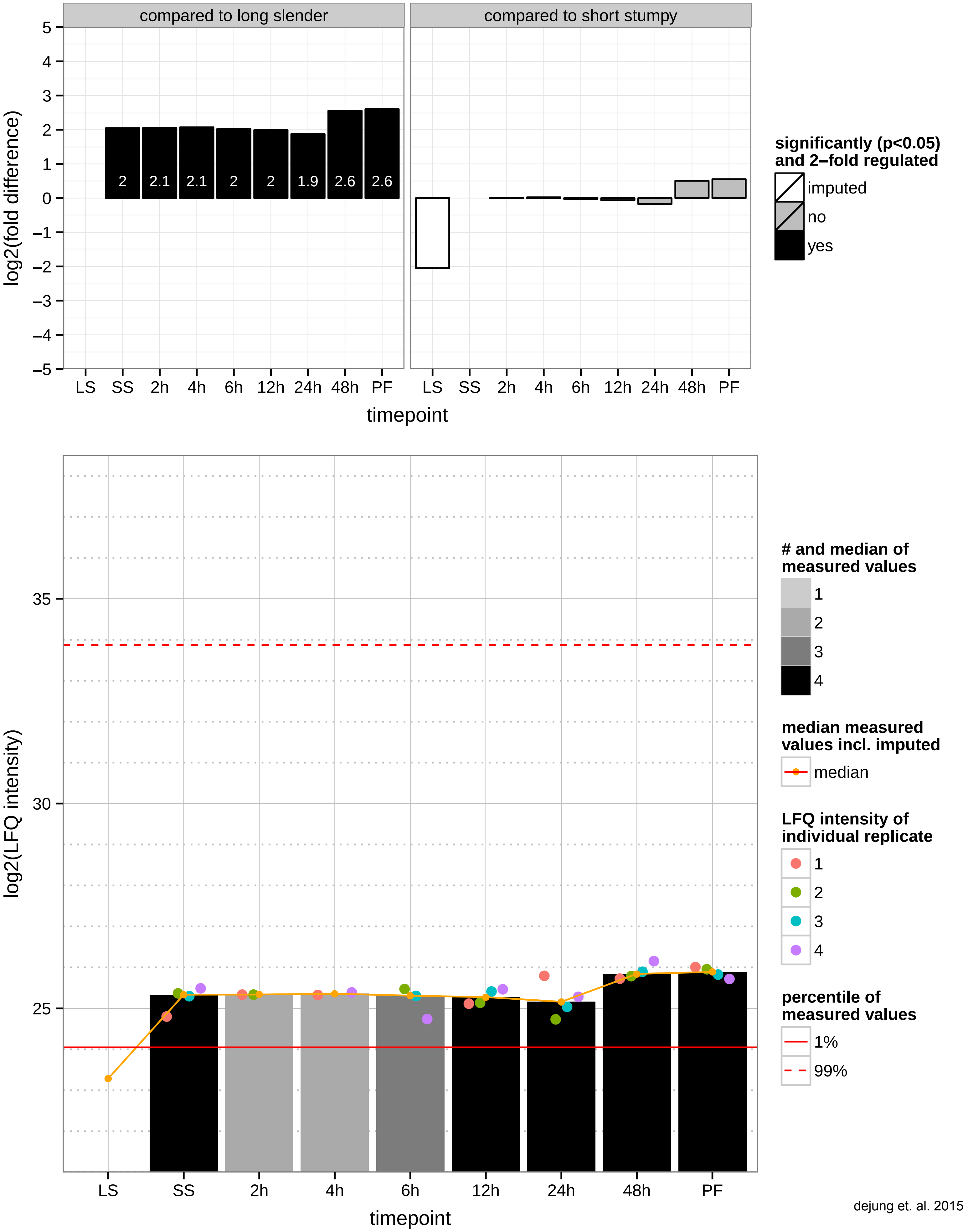

dejung et. al. 2015

structural maintenance of chromosome 1, putative (SMC1)
Tb927.9.11850
AGOF: ATP binding
AGOC: chromosome, condensin complex, nucleus
AGOP: chromosome organization, chromosome segregation, mitotic sister chromatid segregation
PGOF: ATP binding, protein binding
PGOC: chromosome
PGOP: DNA recombination, DNA repair, chromosome condensation, chromosome organization, sister chromatid cohesion

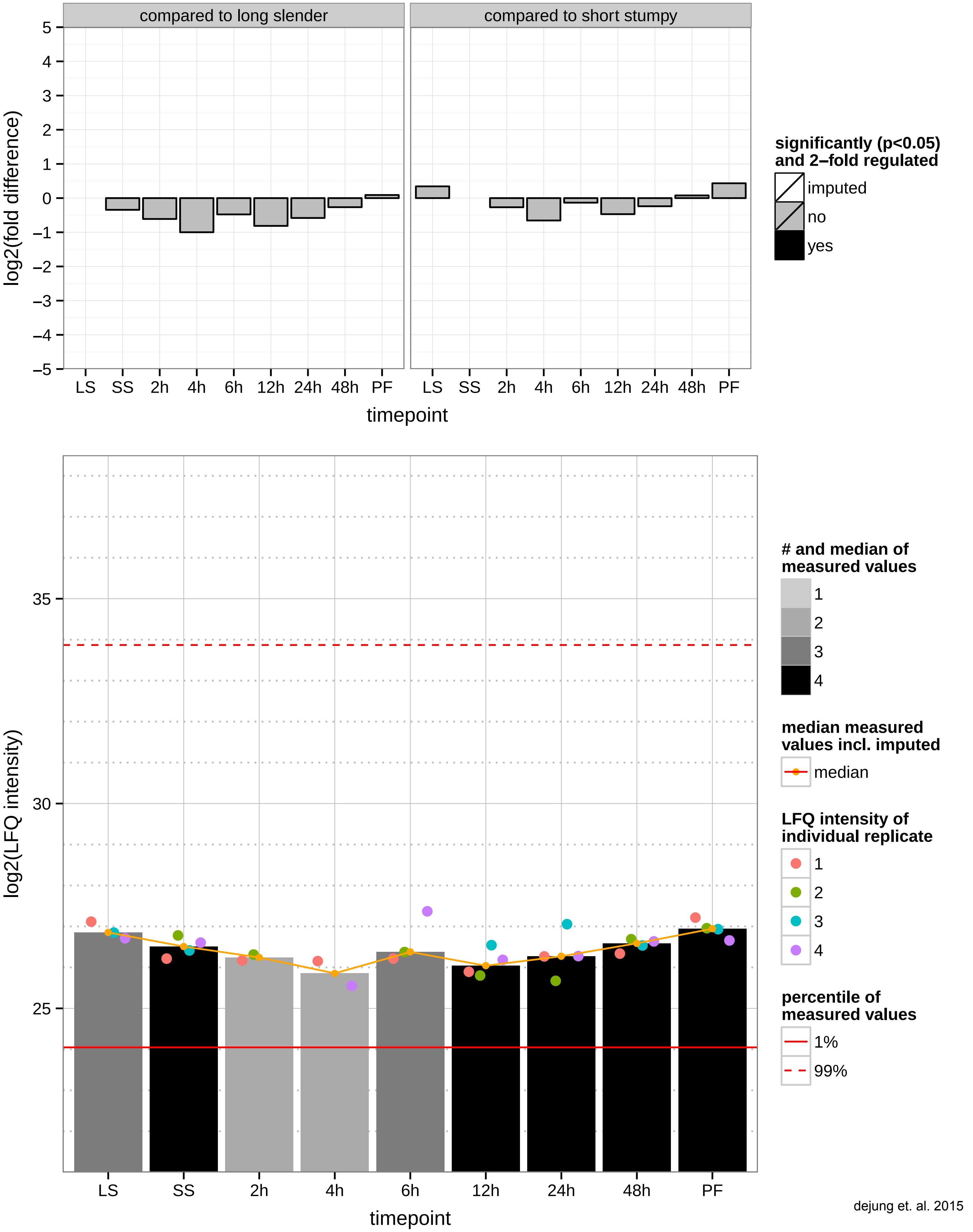

dejung et. al. 2015

acyl transferase–like protein
Tb927.9.11900
AGOF: transferase activity
AGOC: null
AGOP: metabolic process
PGOF: null
PGOC: null
PGOP: metabolic process

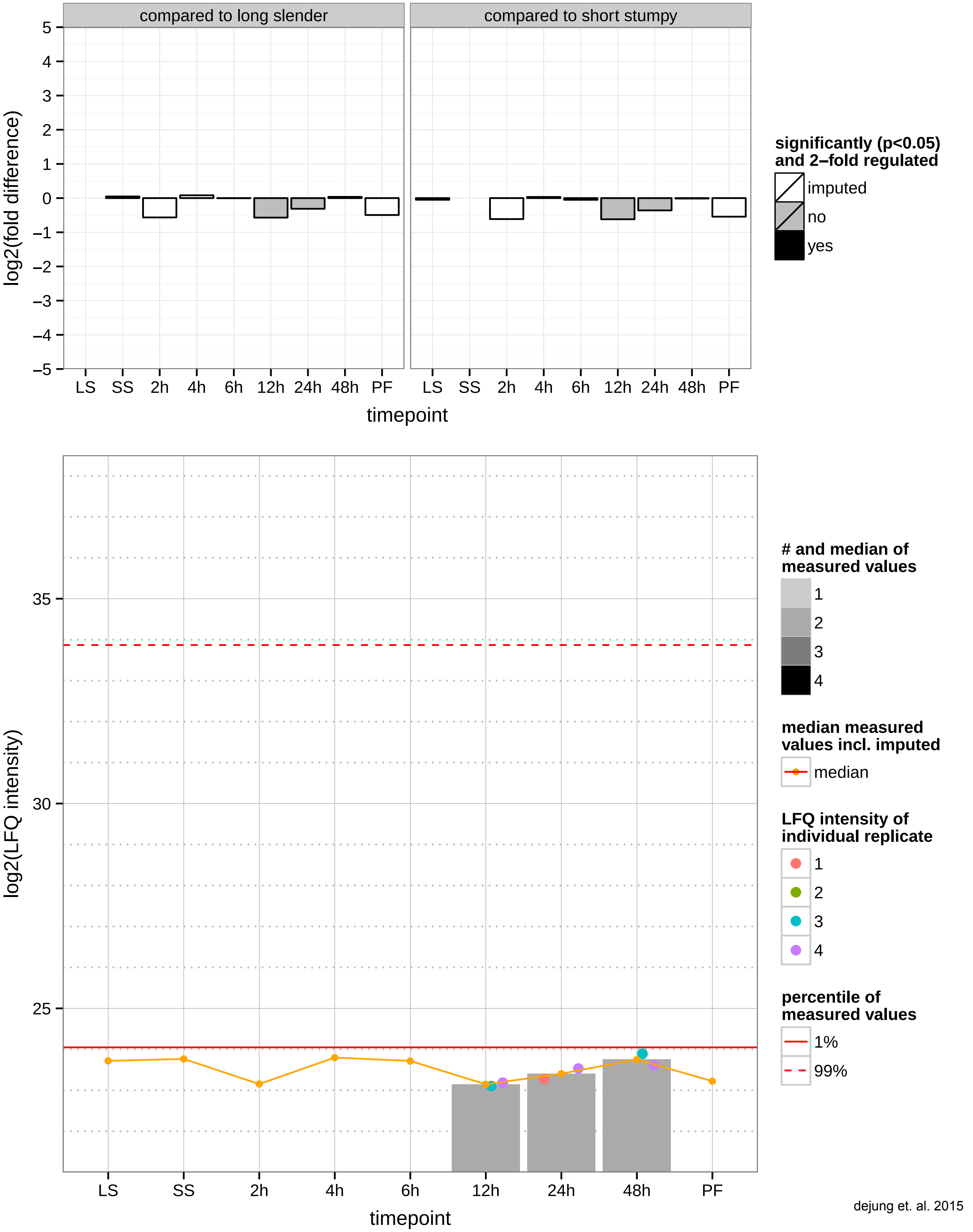

dejung et. al. 2015

hypothetical protein, conserved
Tb927.9.12100
AGOF: methyltransferase activity
AGOC: mitochondrion
AGOP: metabolic process
PGOF: methyltransferase activity
PGOC: null
PGOP: metabolic process

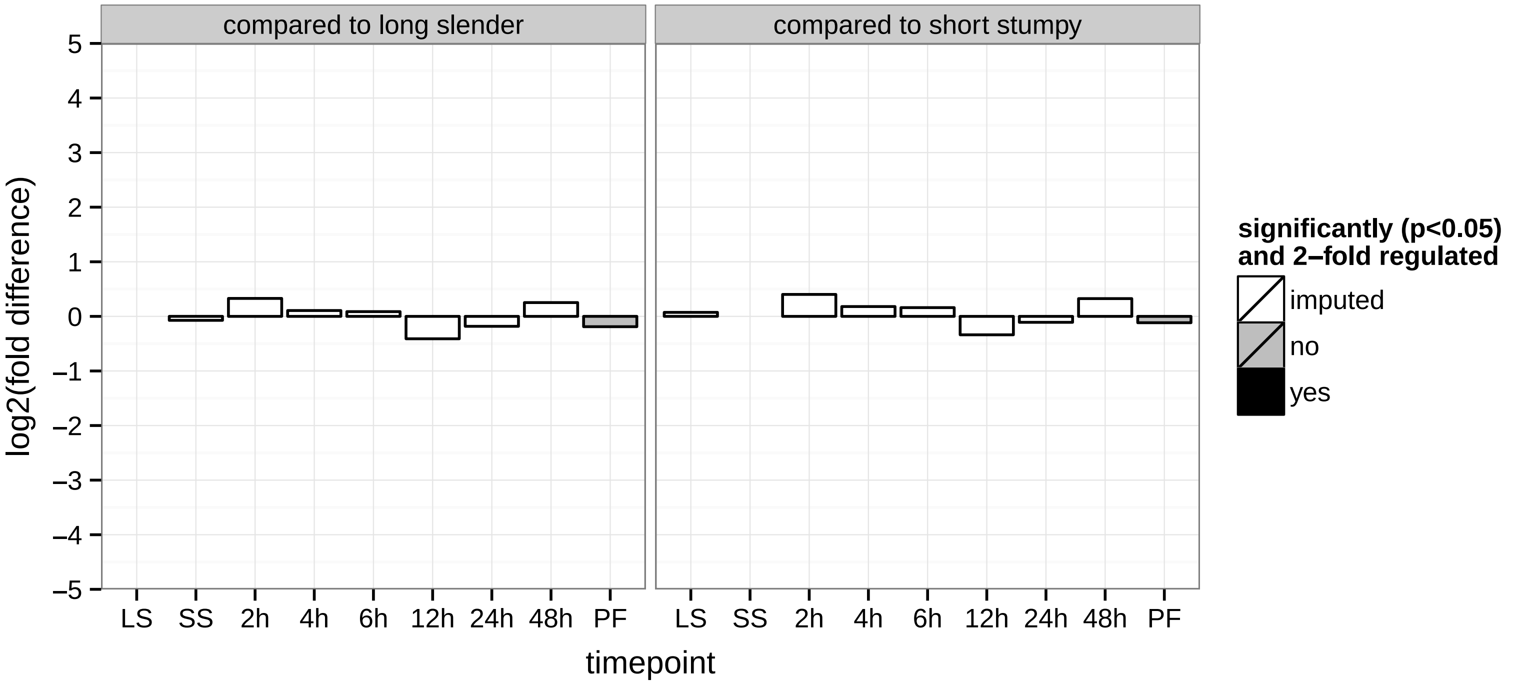

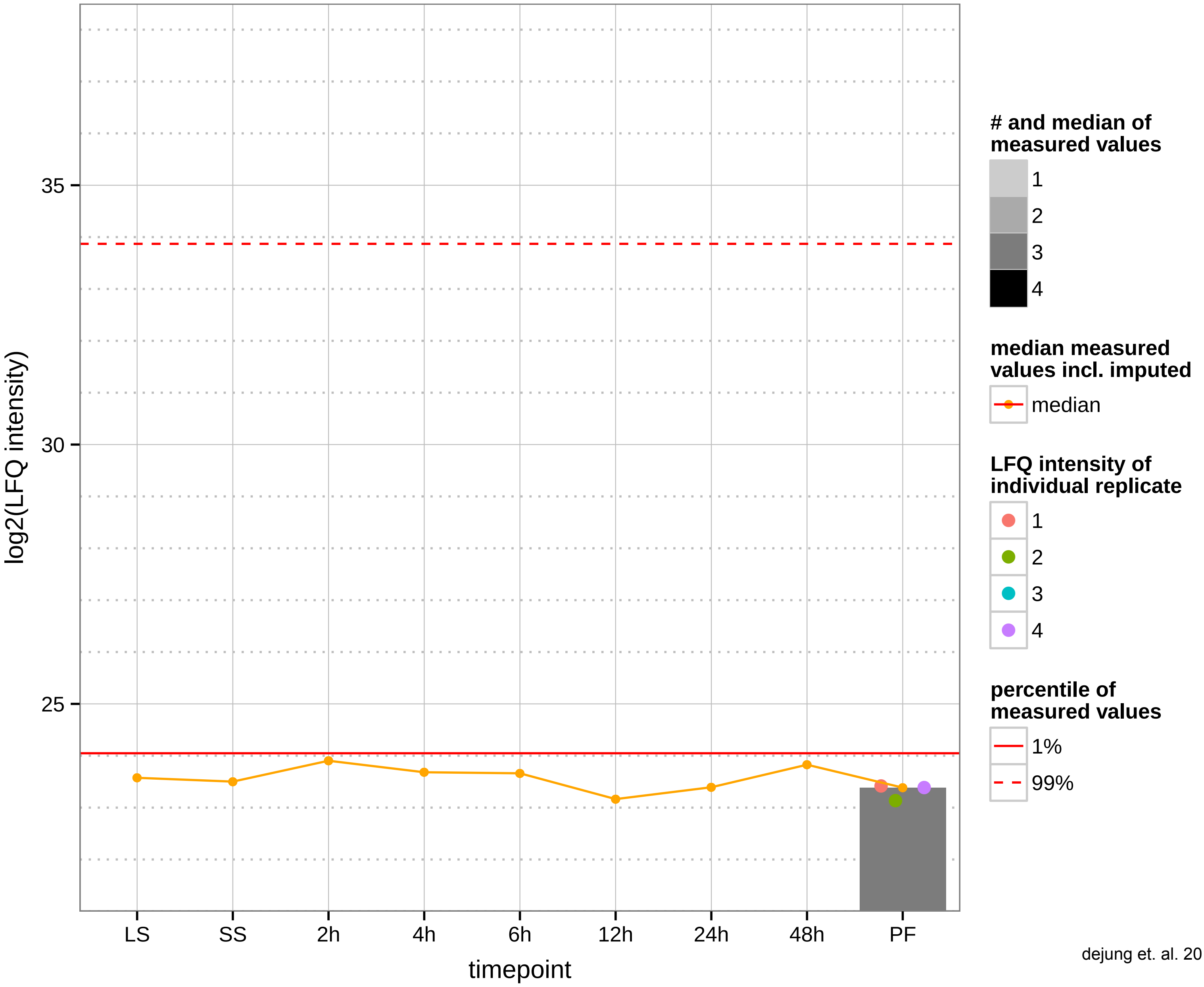

dejung et. al. 2015

transport protein particle (TRAPP) subunit, putative
Tb927.9.12150
AGOF: null
AGOC: TRAPP complex, membrane
AGOP: ER to Golgi vesicle–mediated transport
PGOF: null
PGOC: null
PGOP: null

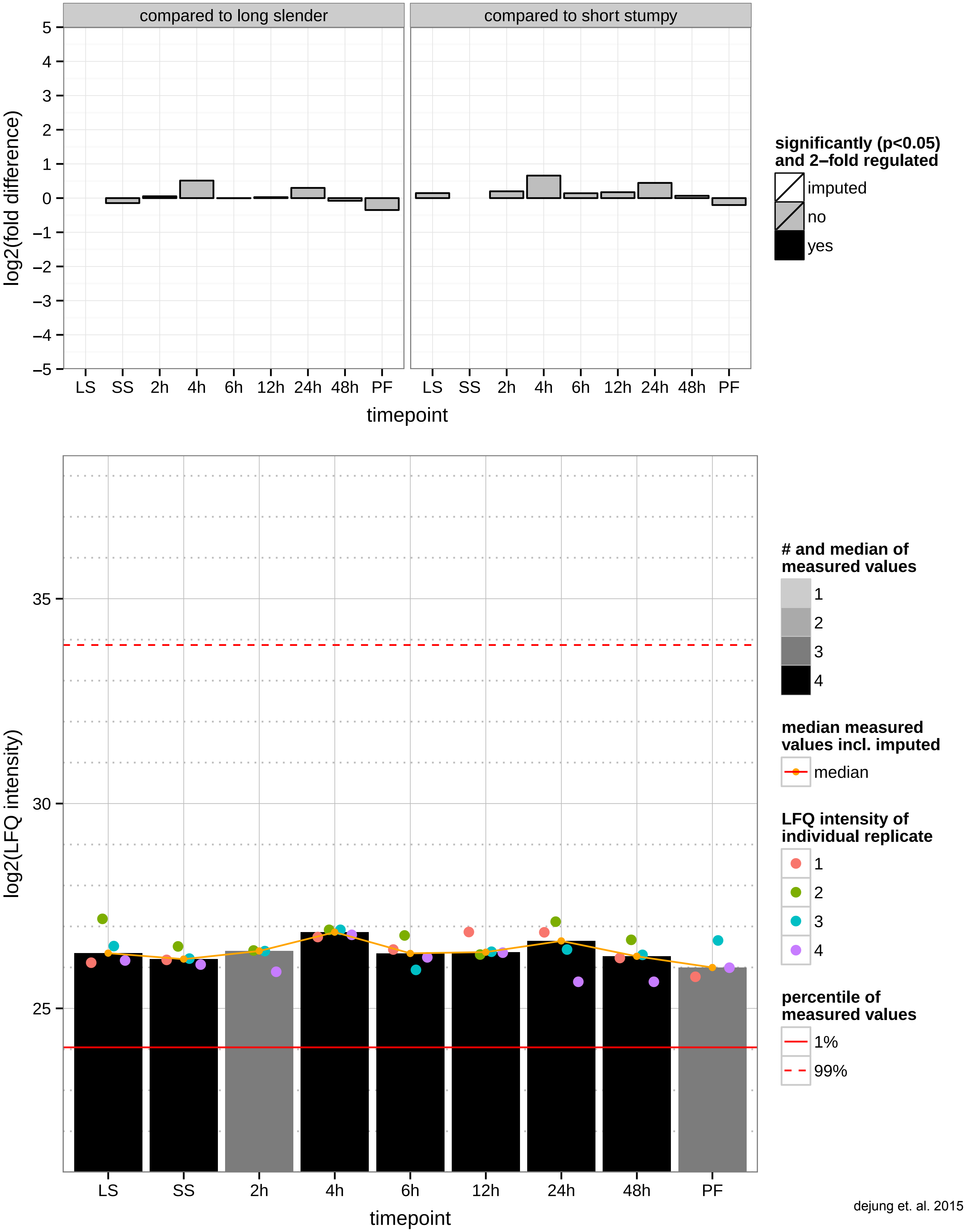

dejung et. al. 2015

unspecified product
Tb927.9.12240;Tb927.9.12200
AGOF: structural constituent of ribosome
AGOC: intracellular, ribosome
AGOP: translation
PGOF: structural constituent of ribosome
PGOC: intracellular, ribosome
PGOP: translation

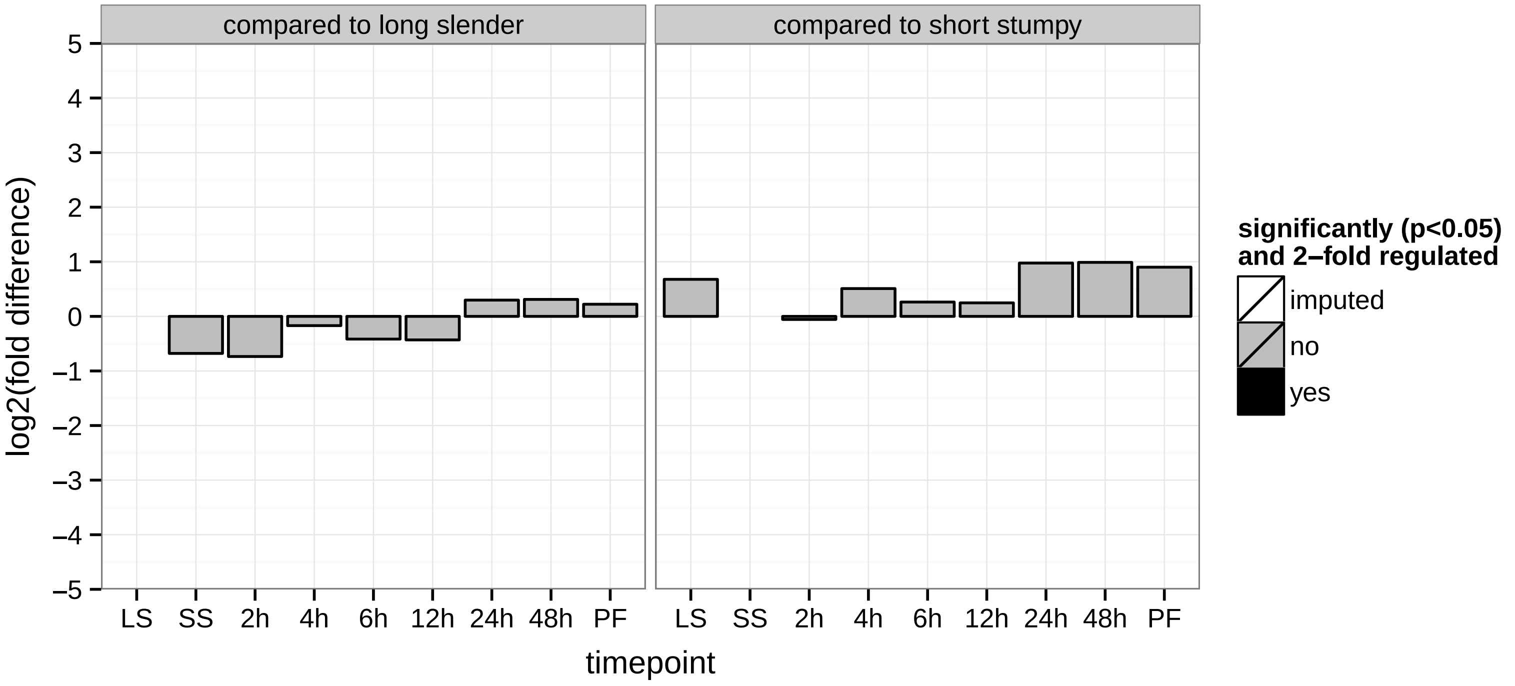

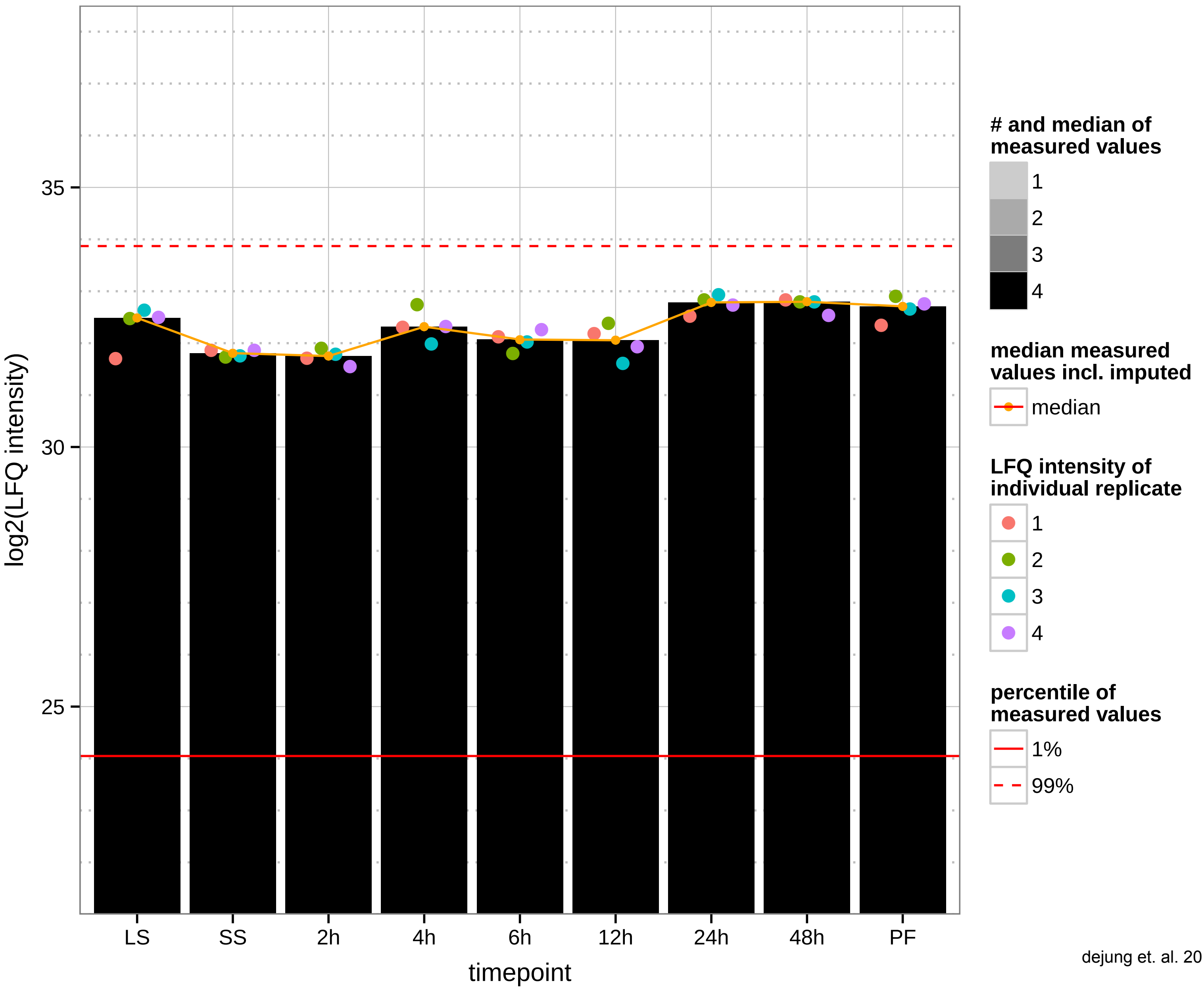

dejung et. al. 2015

Peroxin 19
Tb927.9.12290
AGOF: null
AGOC: peroxisome
AGOP: null
PGOF: null
PGOC: peroxisome
PGOP: null

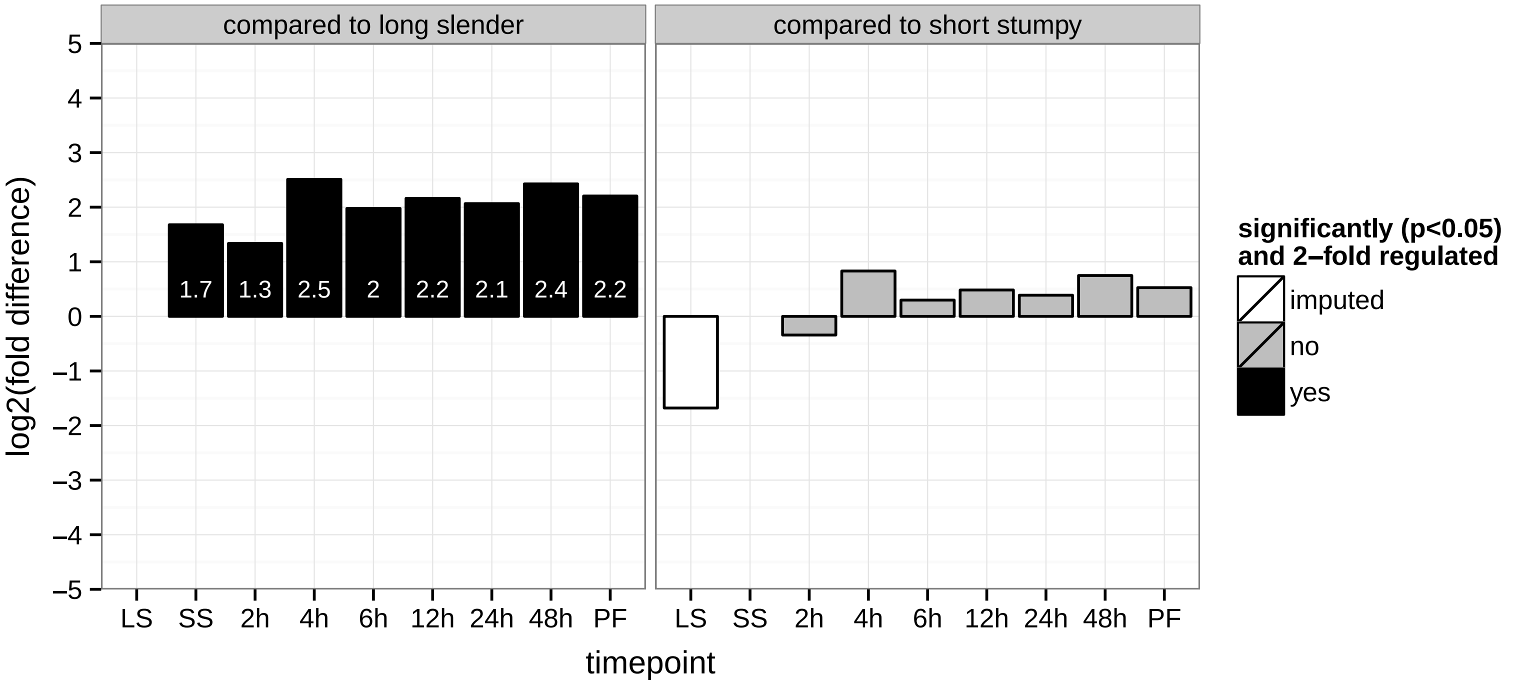

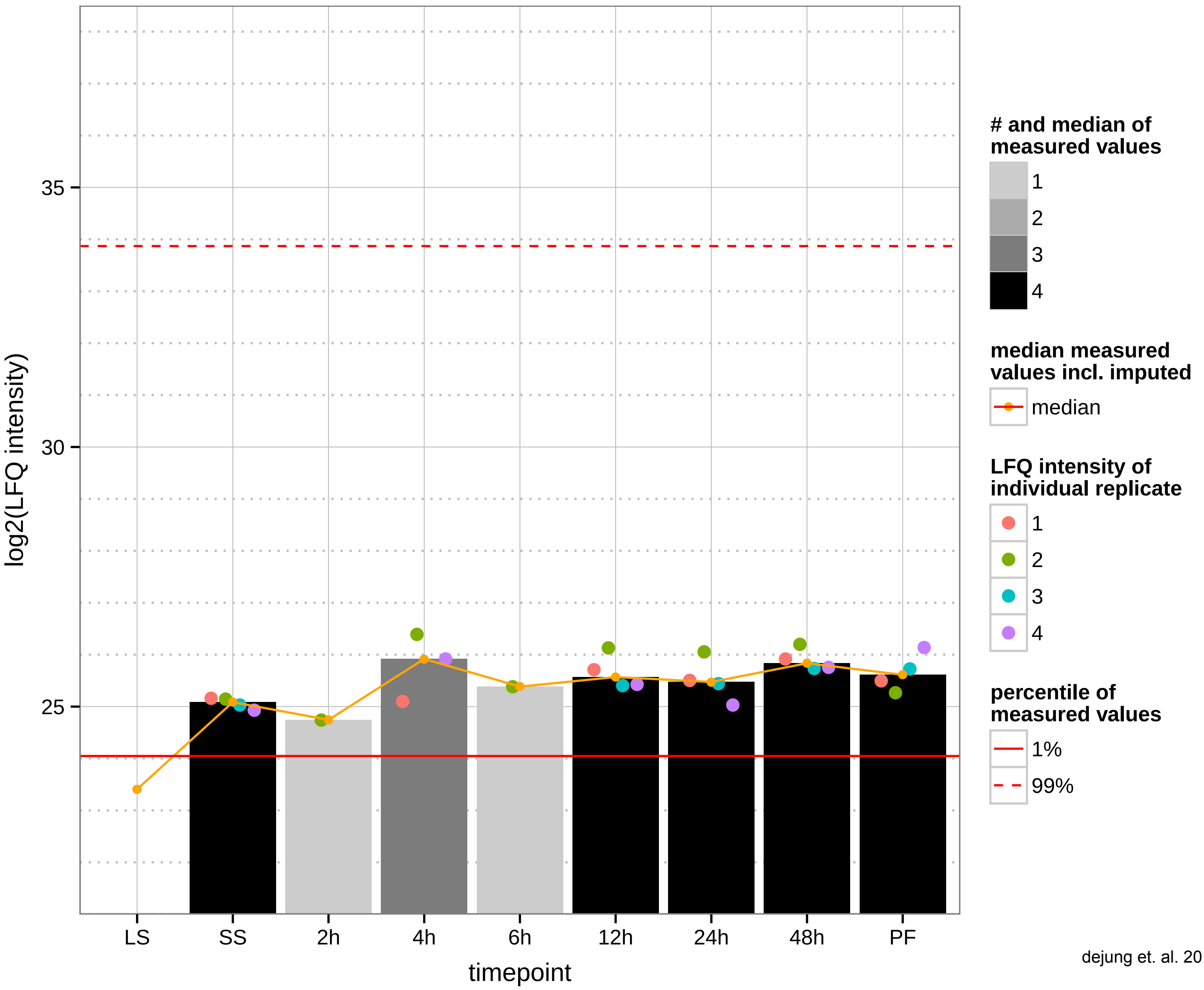

dejung et. al. 2015

replication factor C, subunit 3, putative
Tb927.9.12300
AGOF: ATP binding, DNA binding, DNA clamp loader activity
AGOC: DNA replication factor C complex
AGOP: DNA replication
PGOF: ATP binding, DNA binding, nucleoside–triphosphatase activity, nucleotide binding
PGOC: null
PGOP: DNA replication

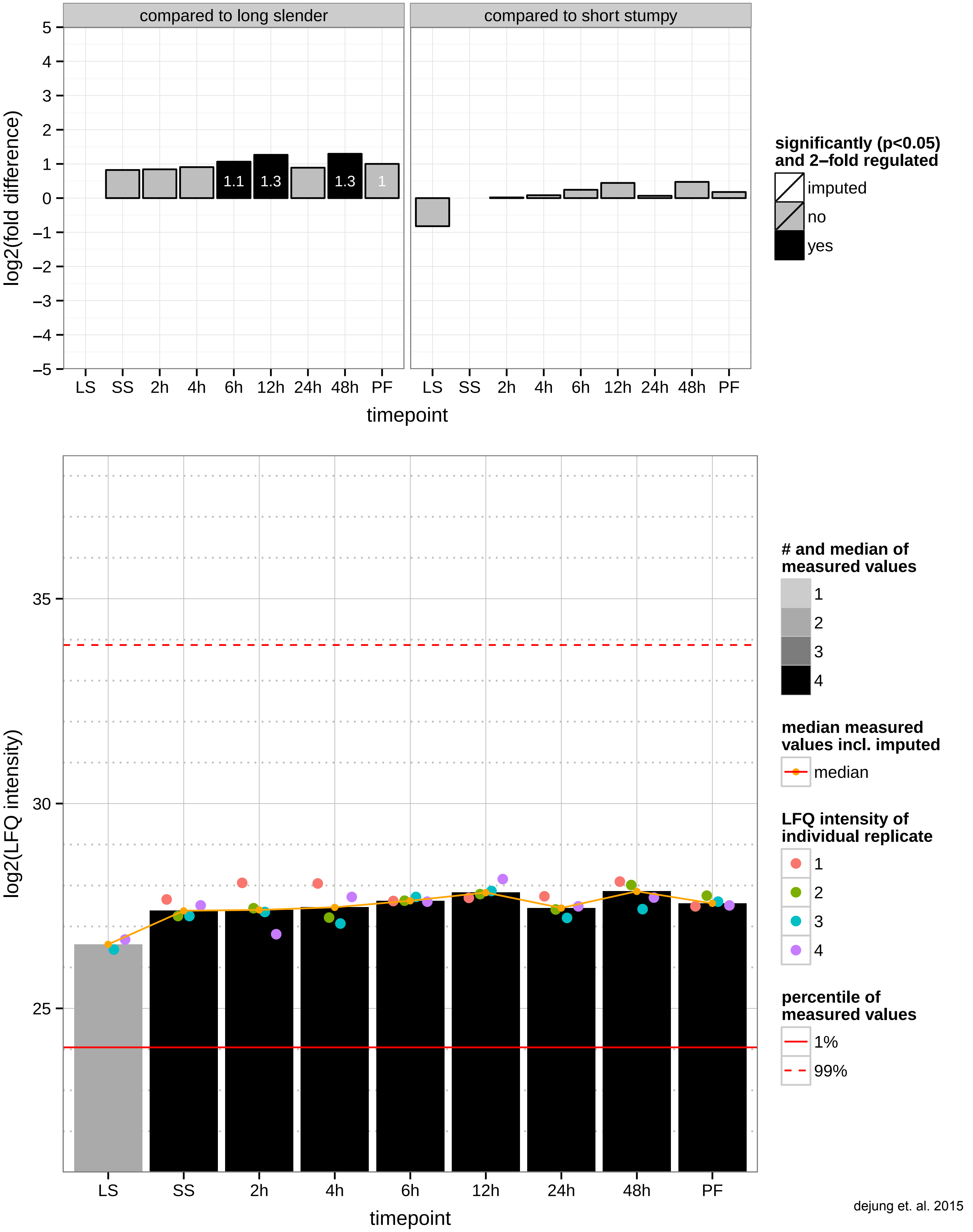

dejung et. al. 2015

hypothetical protein, conserved
Tb927.9.12480
AGOF: microtubule motor activity
AGOC: microtubule associated complex, mitochondrion
AGOP: microtubule–based process
PGOF: null
PGOC: microtubule associated complex
PGOP: microtubule–based process

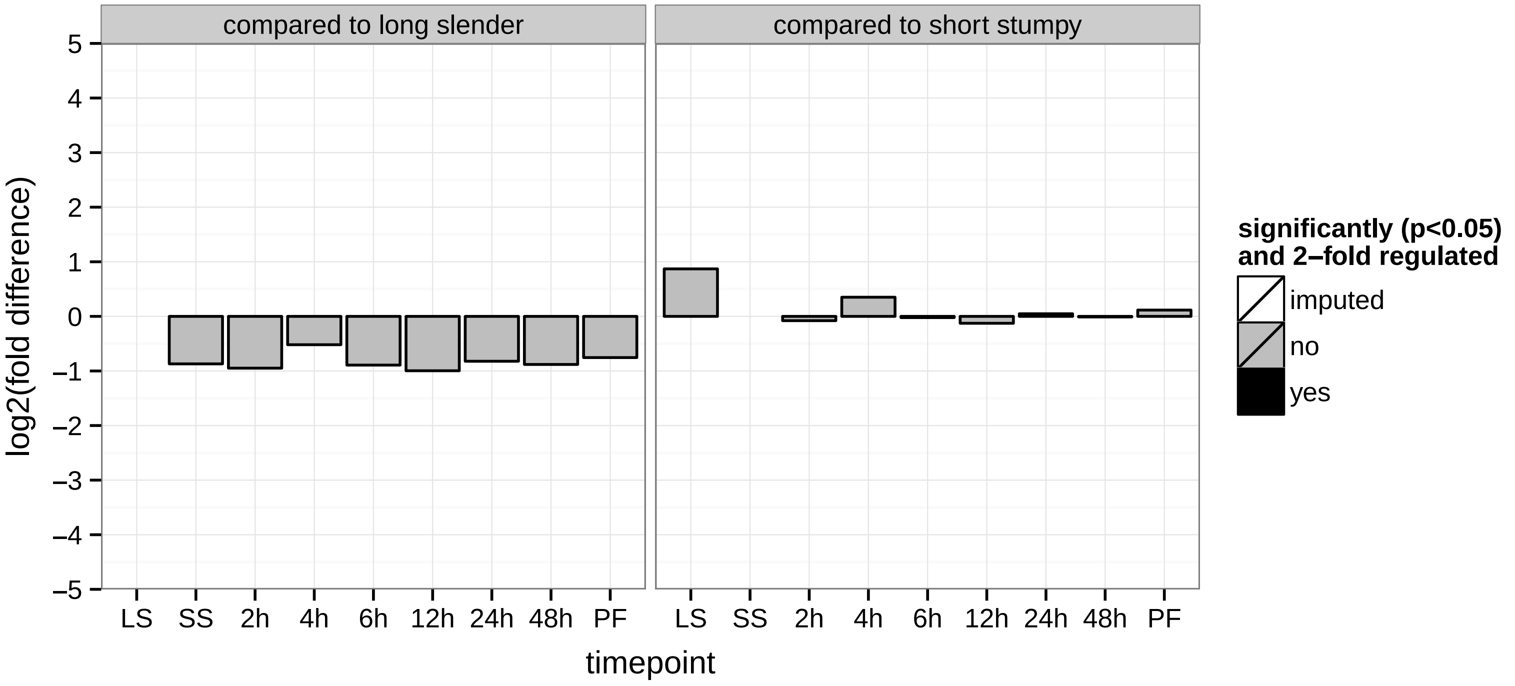

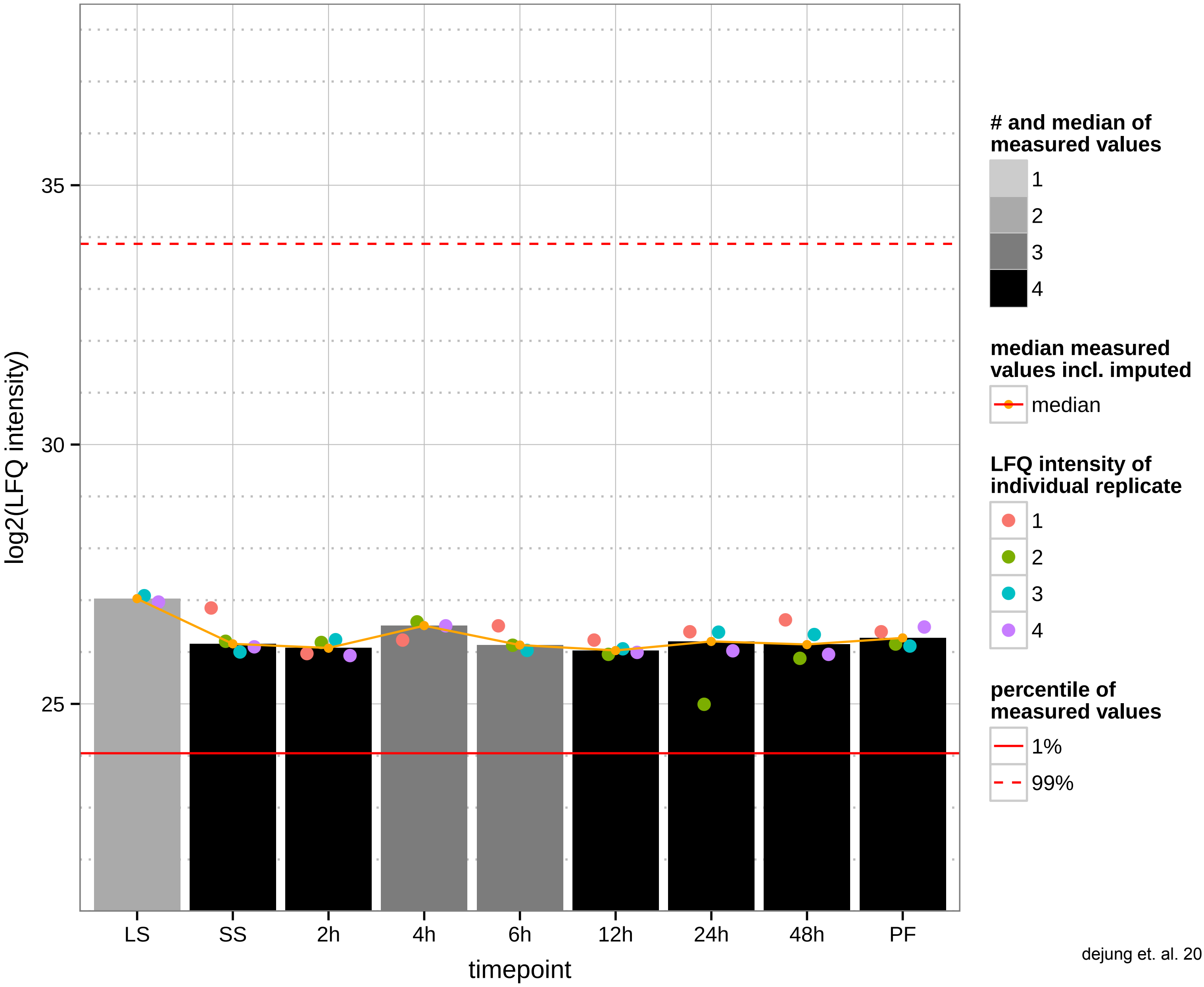

dejung et. al. 2015

hypothetical protein, conserved
Tb927.9.12500
AGOF: null
AGOC: mitochondrion
AGOP: null
PGOF: null
PGOC: null
PGOP: null

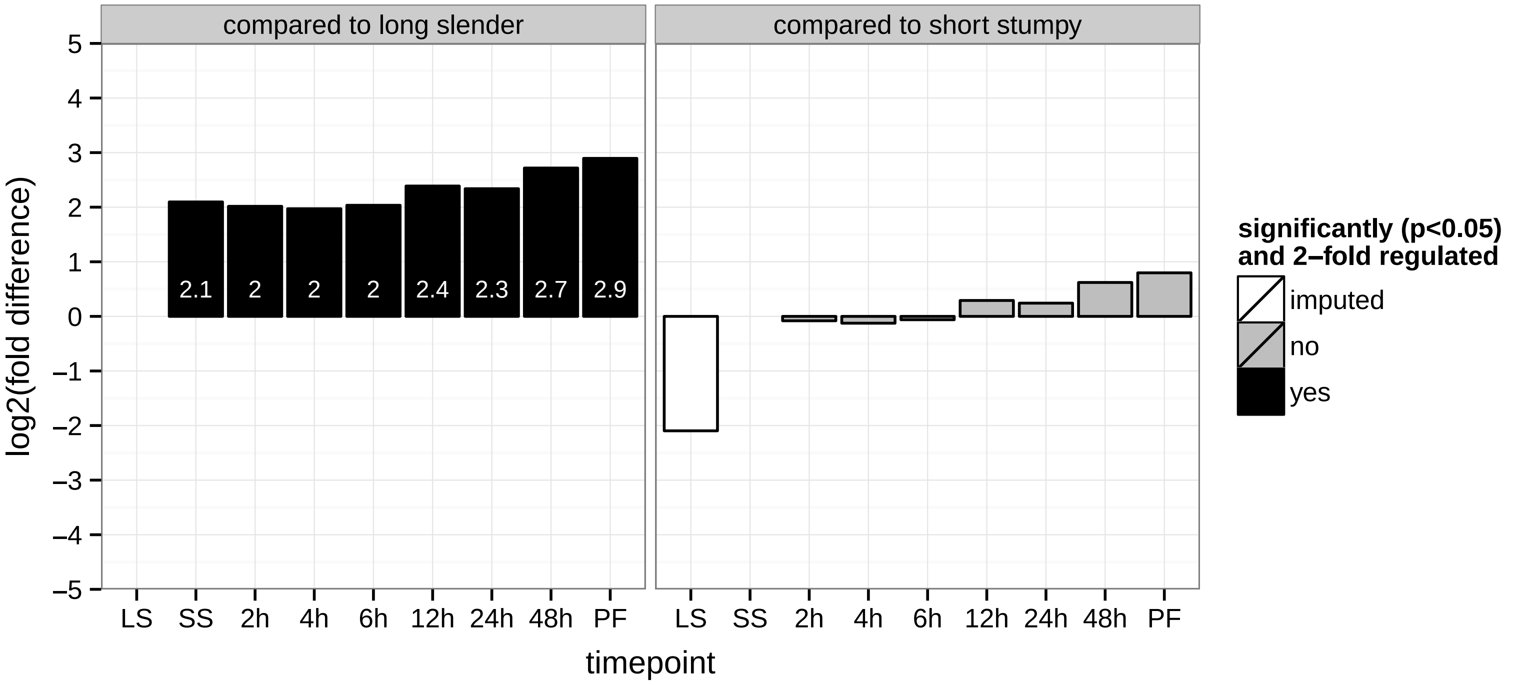

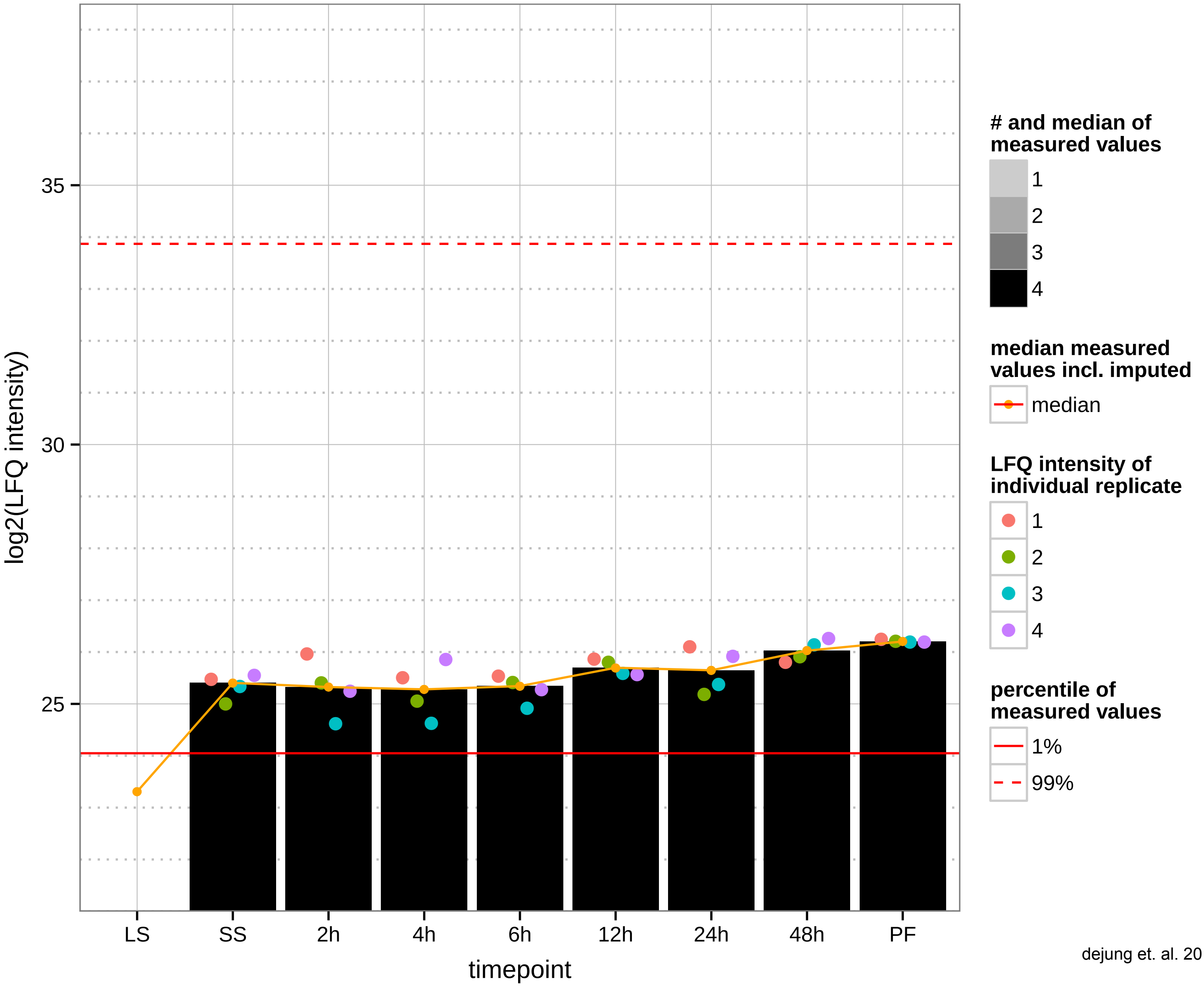

dejung et. al. 2015

phospholipase A1, putative
Tb927.9.12700
AGOF: 1−alkyl−2−acetylglycerophosphocholine esterase activity, phosphatidylcholine 1−acylhydrolase activity
AGOC: extracellular region
AGOP: lipid catabolic process
PGOF: 1−alkyl−2−acetylglycerophosphocholine esterase activity
PGOC: null
PGOP: lipid catabolic process

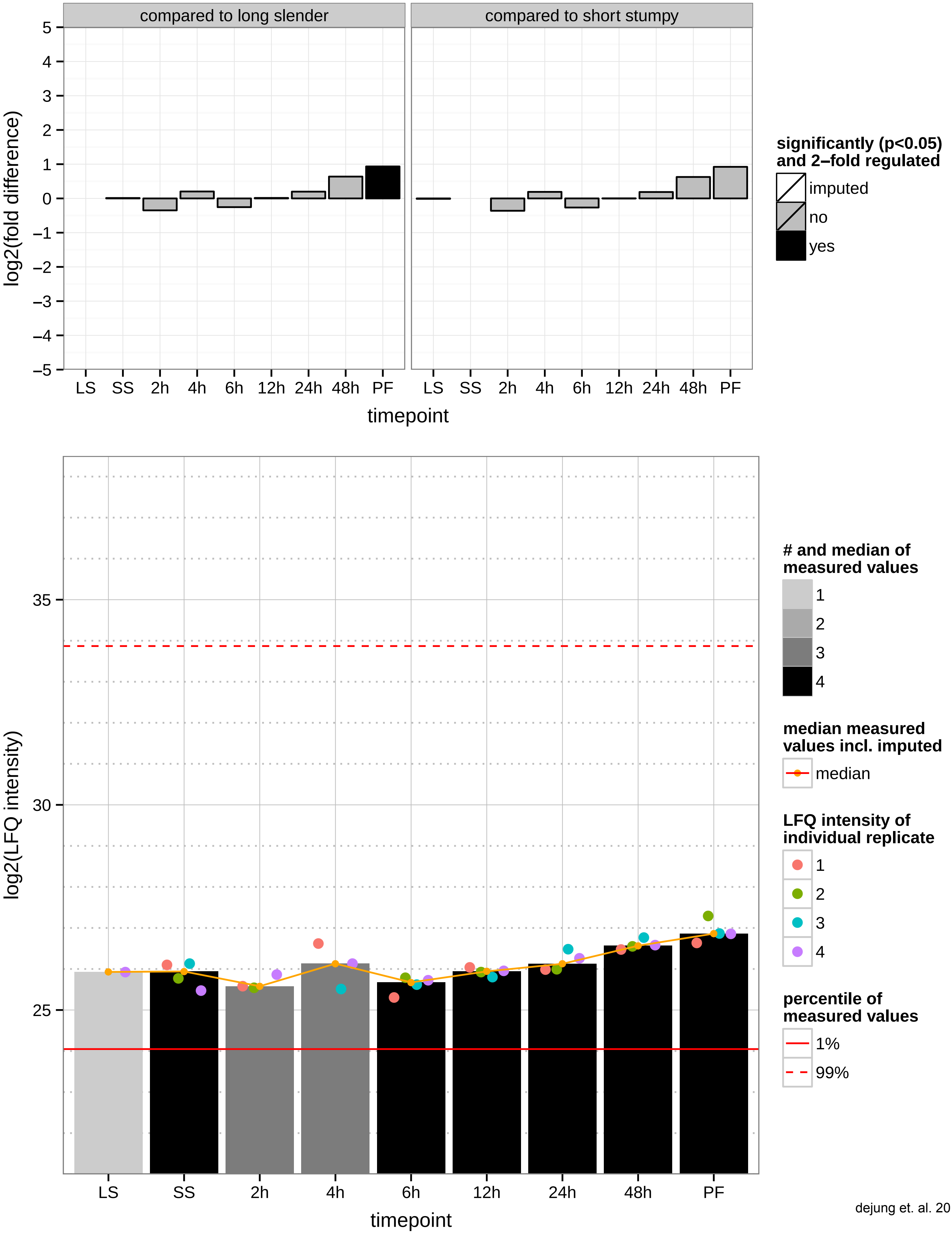

dejung et. al. 2015

hypothetical protein, conserved
Tb927.9.12710
AGOF: null
AGOC: null
AGOP: null
PGOF: null
PGOC: null
PGOP: null

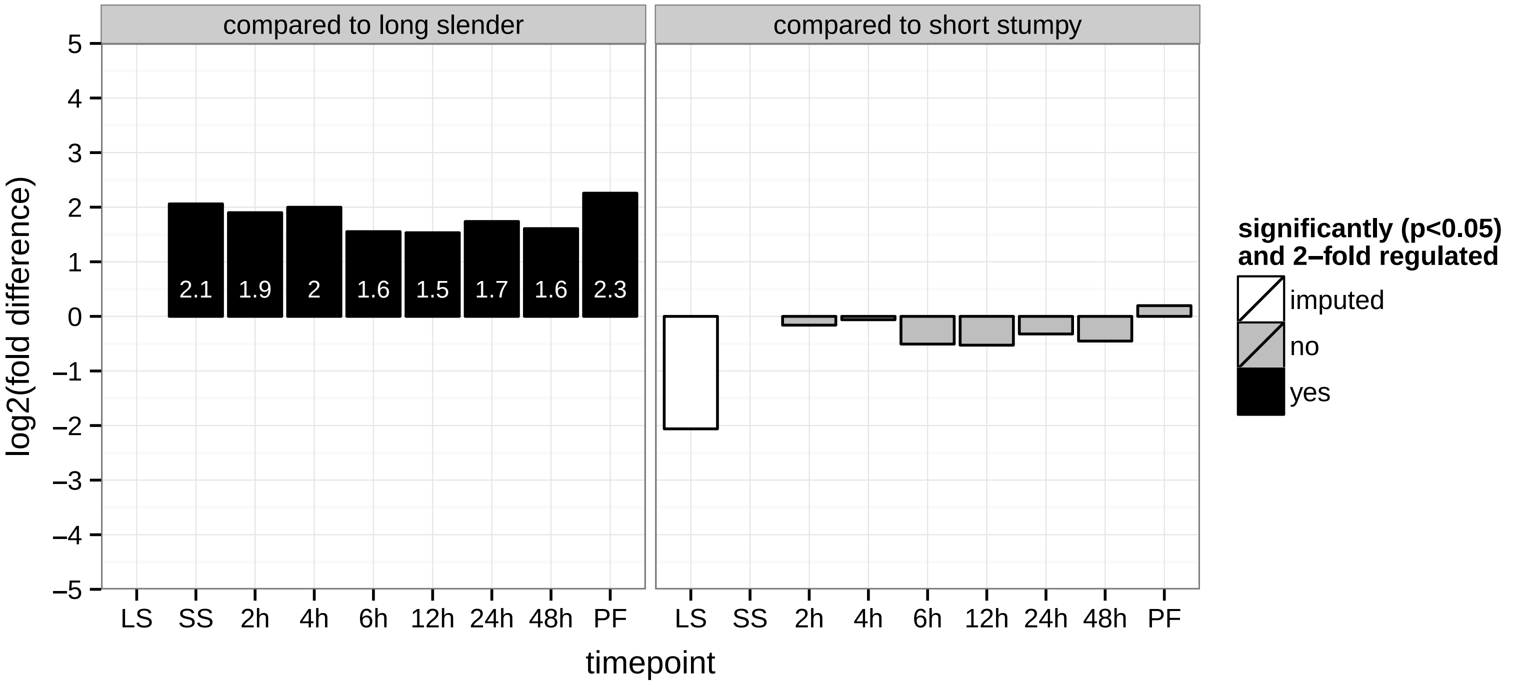

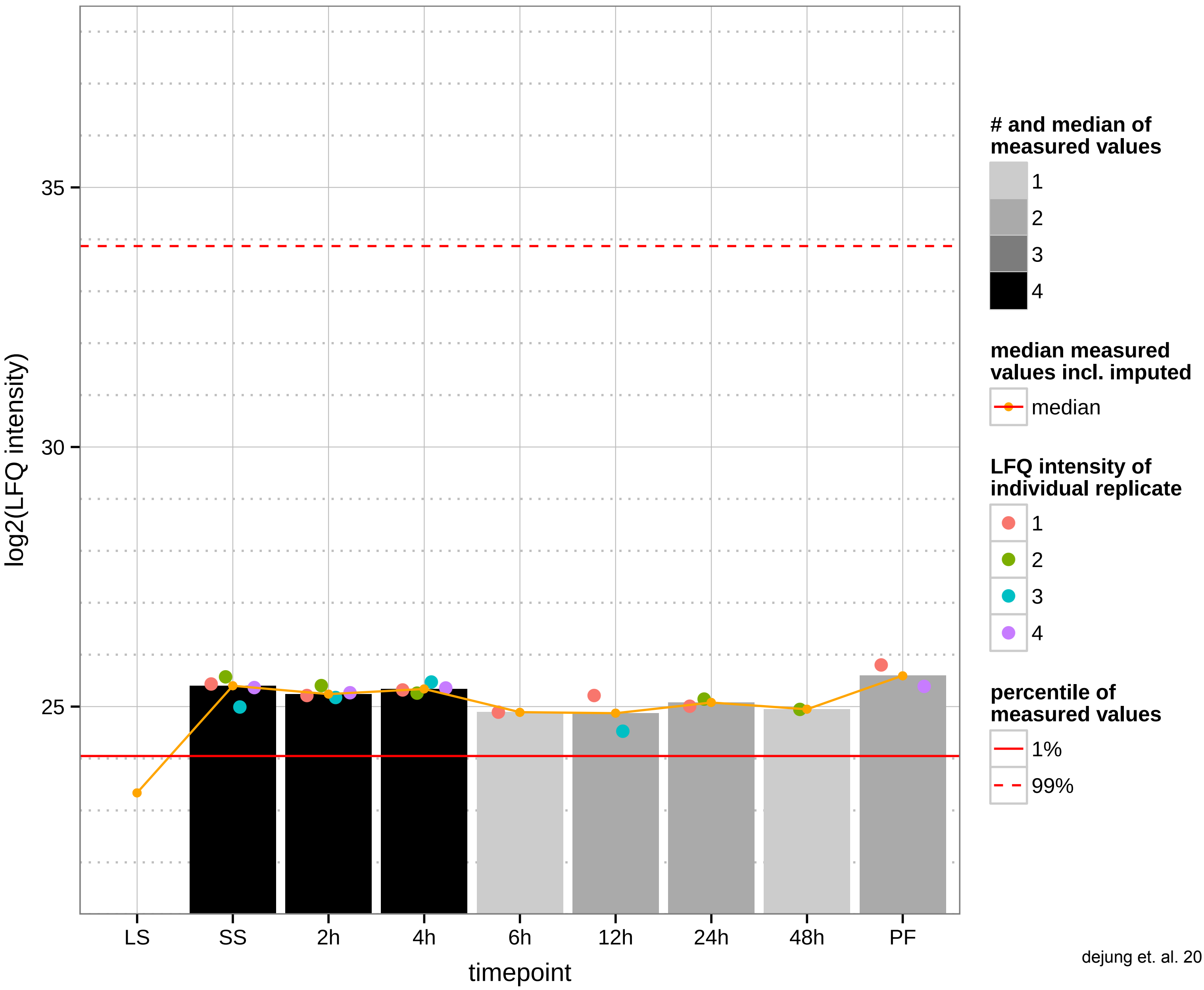

dejung et. al. 2015

chaperone protein DNAj, putative
Tb927.9.12730
AGOF: chaperone binding, heat shock protein binding, unfolded protein binding
AGOC: mitochondrion
AGOP: protein folding
PGOF: heat shock protein binding, unfolded protein binding
PGOC: null
PGOP: protein folding

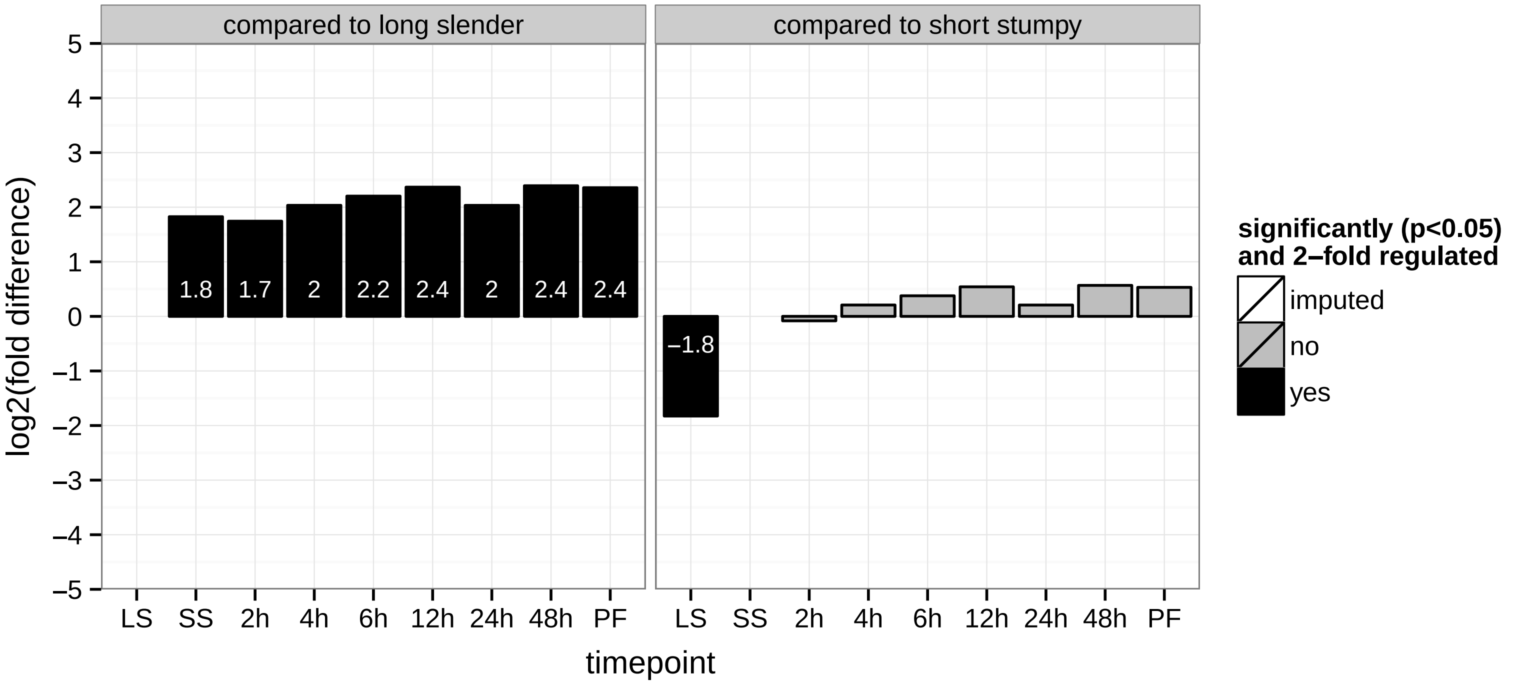

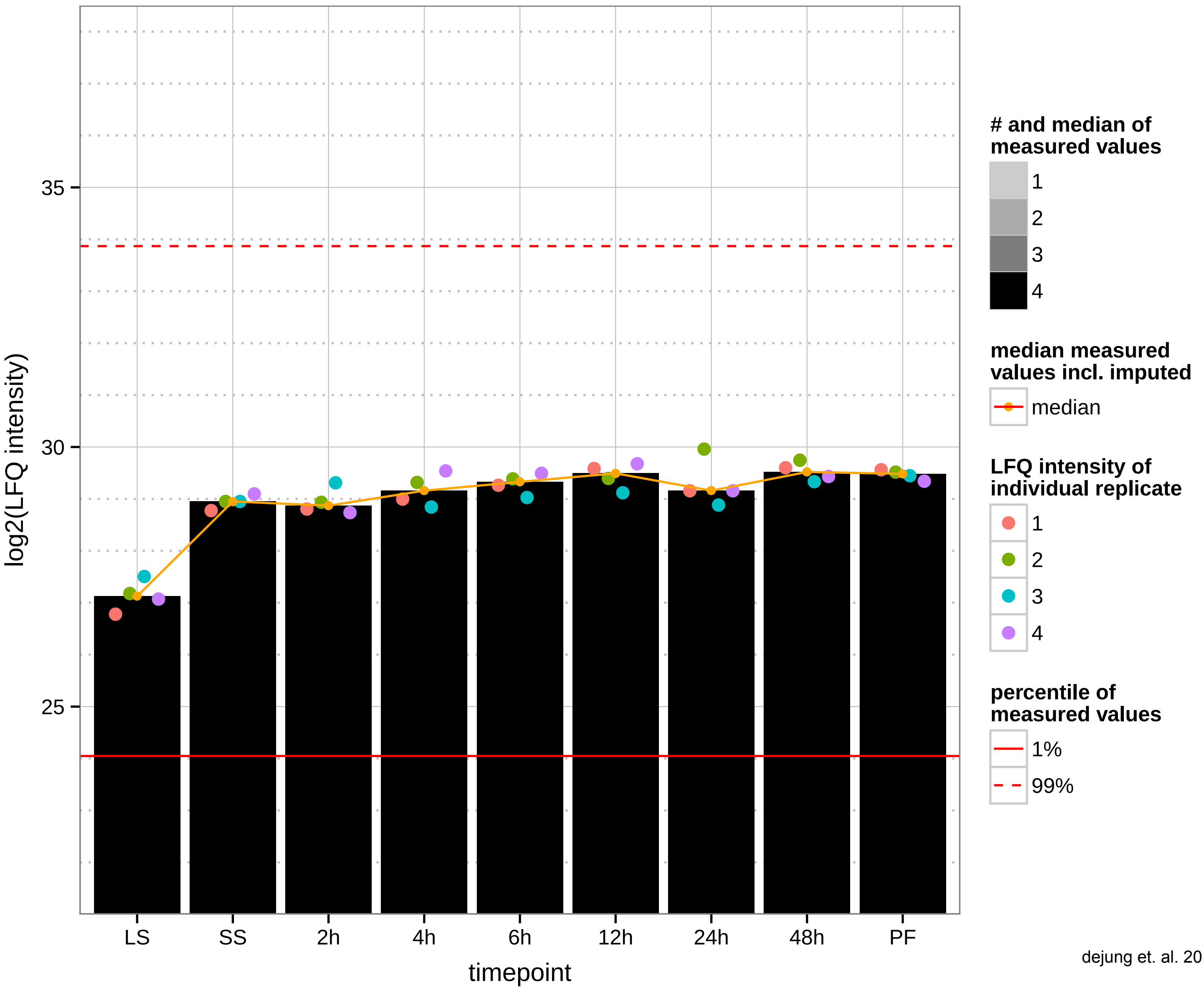

dejung et. al. 2015

dynein light chain, putative, dynein Tctex2 family
Tb927.9.12820
AGOF: ATPase activity, coupled
AGOC: dynein complex
AGOP: microtubule−based movement
PGOF: null
PGOC: null
PGOP: null

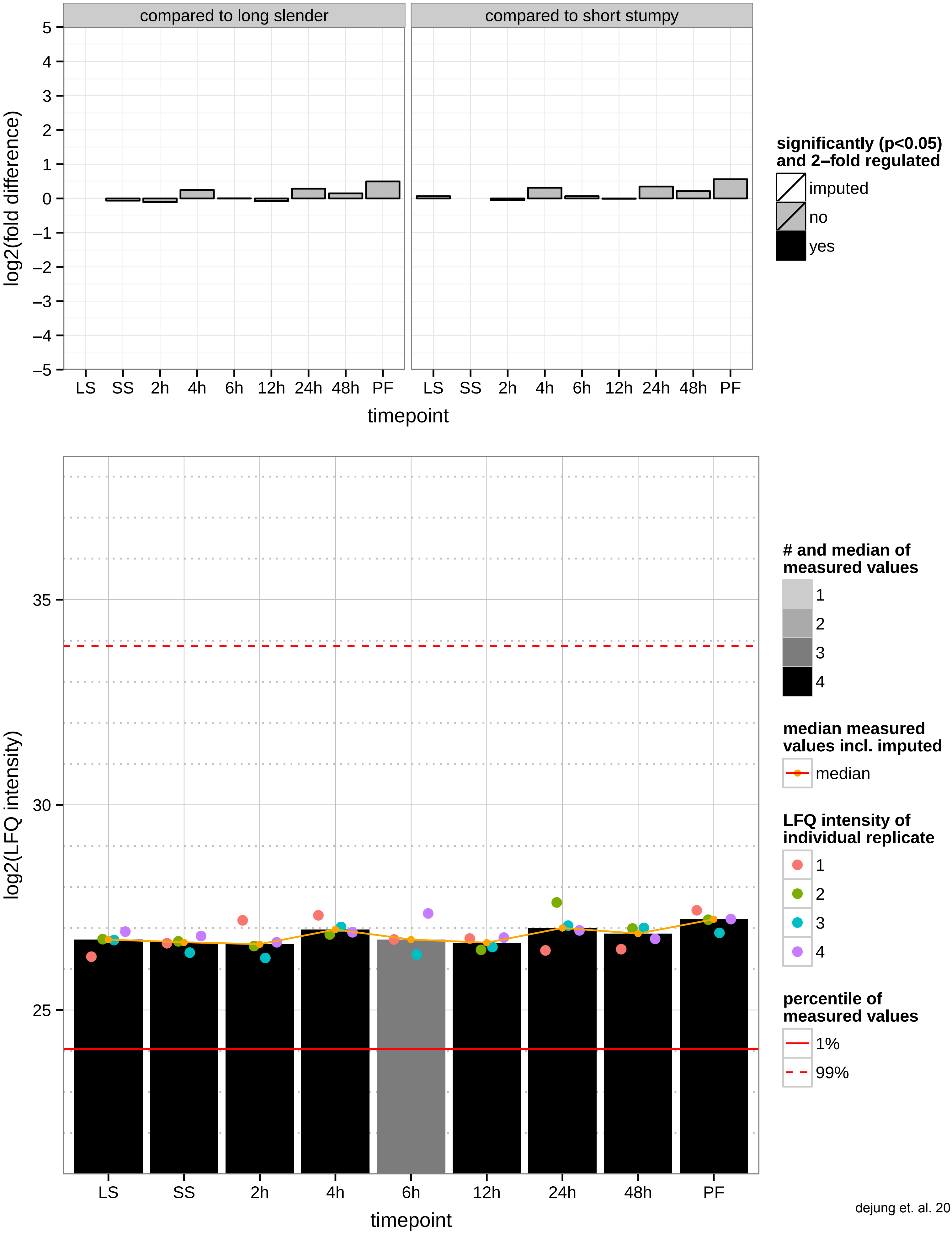

dejung et. al. 2015

RNA polymerase–associated protein LEO1, putative
Tb927.9.12900
AGOF: RNA binding
AGOC: Cdc73/Paf1 complex
AGOP: null
PGOF: null
PGOC: null
PGOP: null

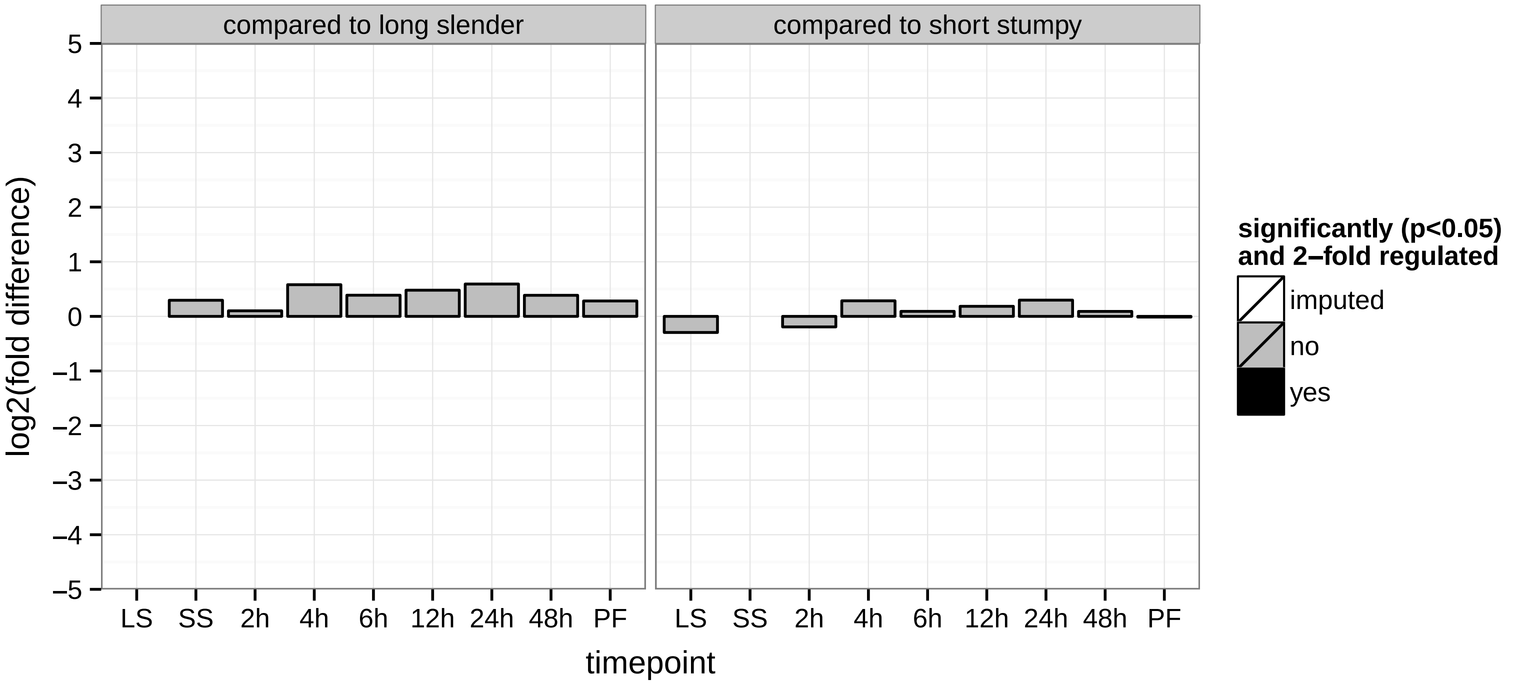

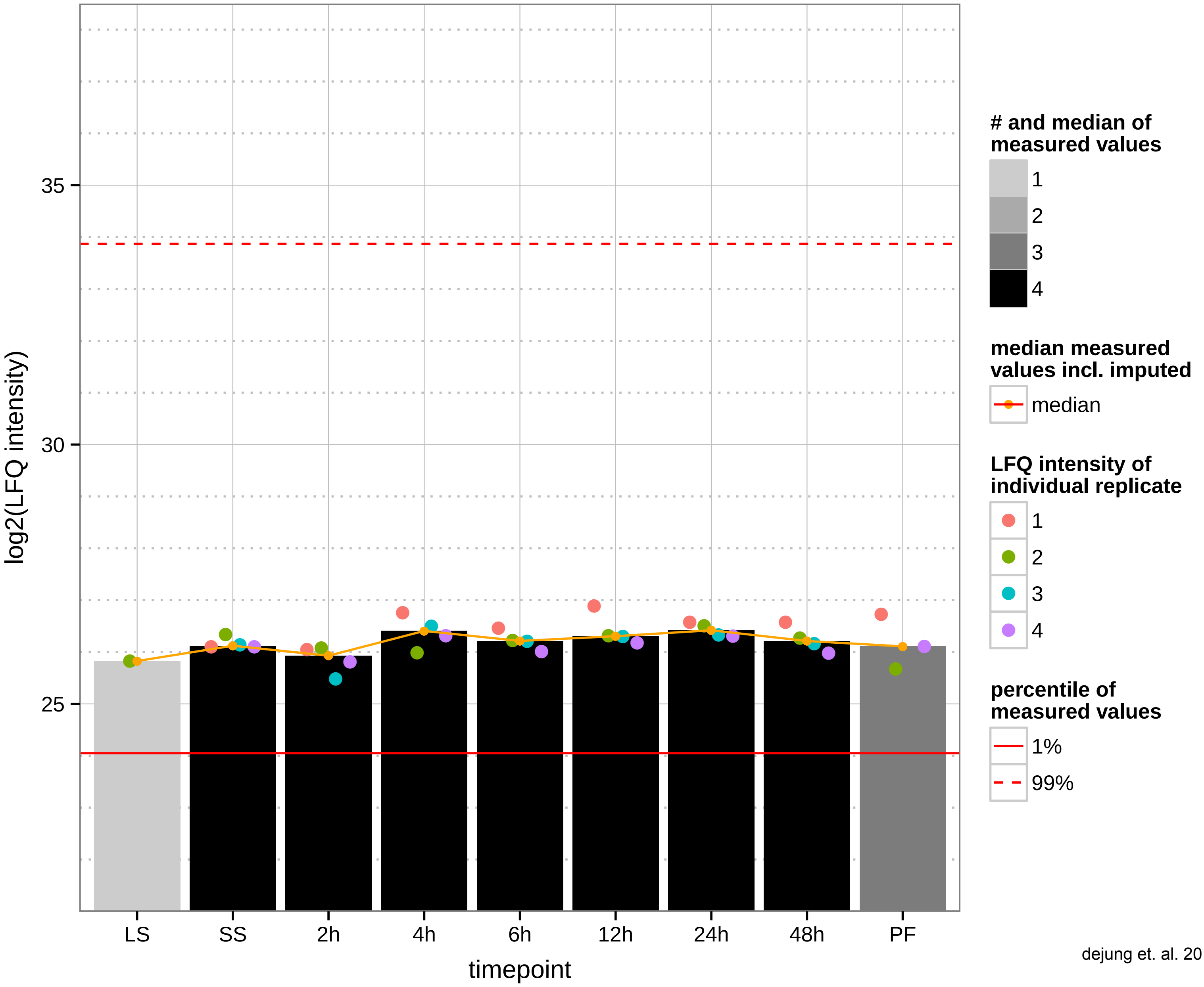

dejung et. al. 2015

hypothetical protein, conserved
Tb927.9.12990
AGOF: null
AGOC: null
AGOP: null
PGOF: null, protein binding
PGOC: null
PGOP: null

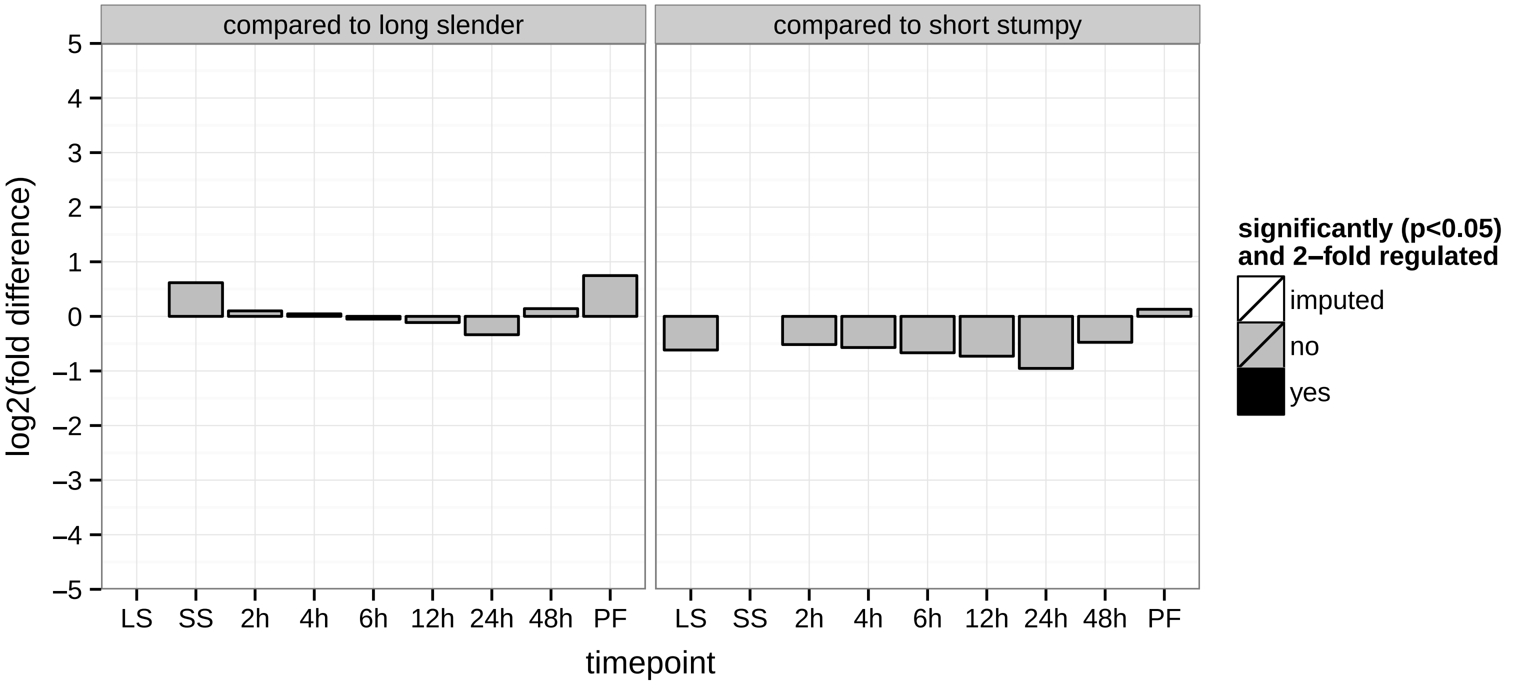

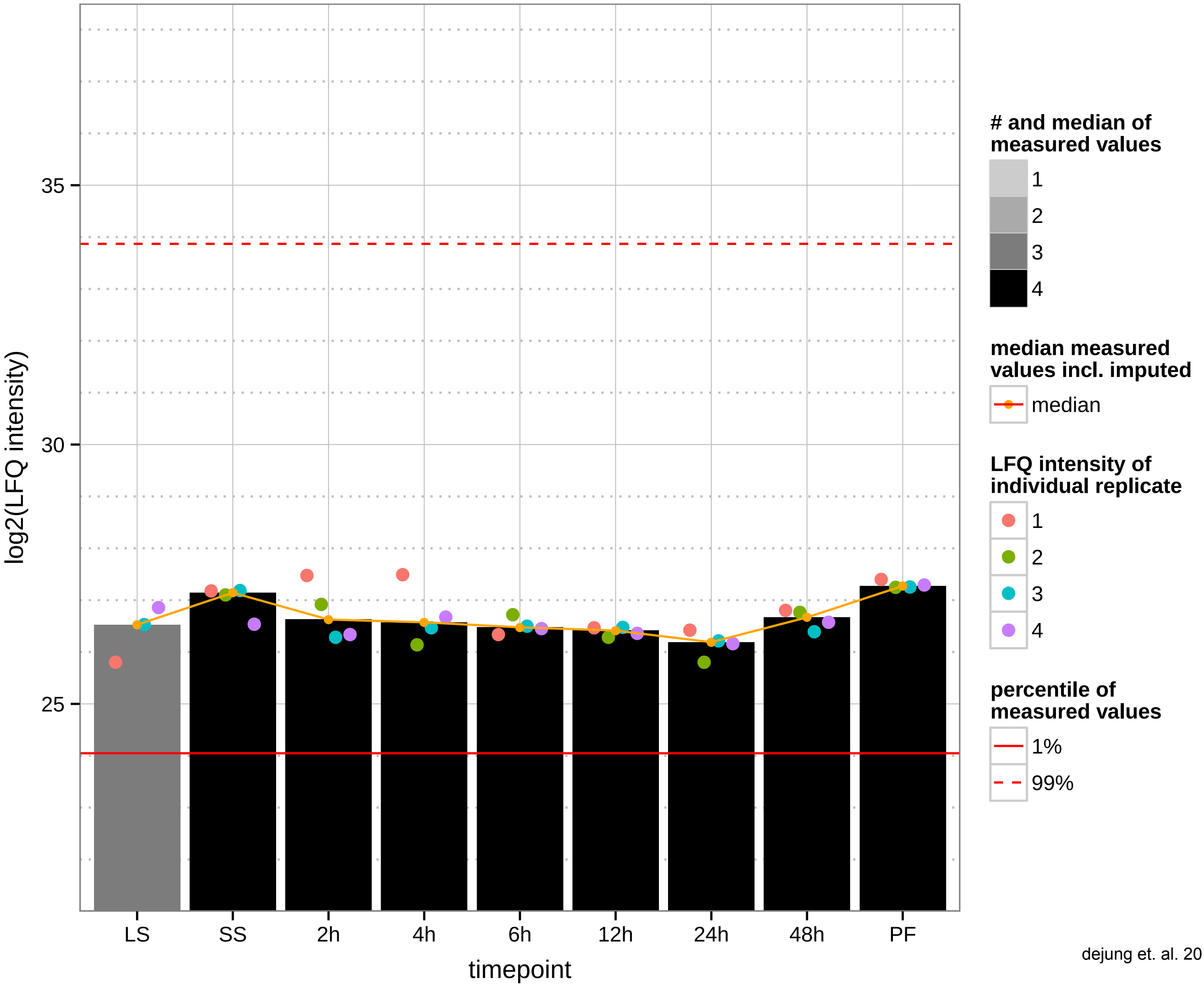

dejung et. al. 2015

hypothetical protein, conserved
Tb927.9.13320
AGOF: null
AGOC: null
AGOP: null
PGOF: null
PGOC: null
PGOP: null

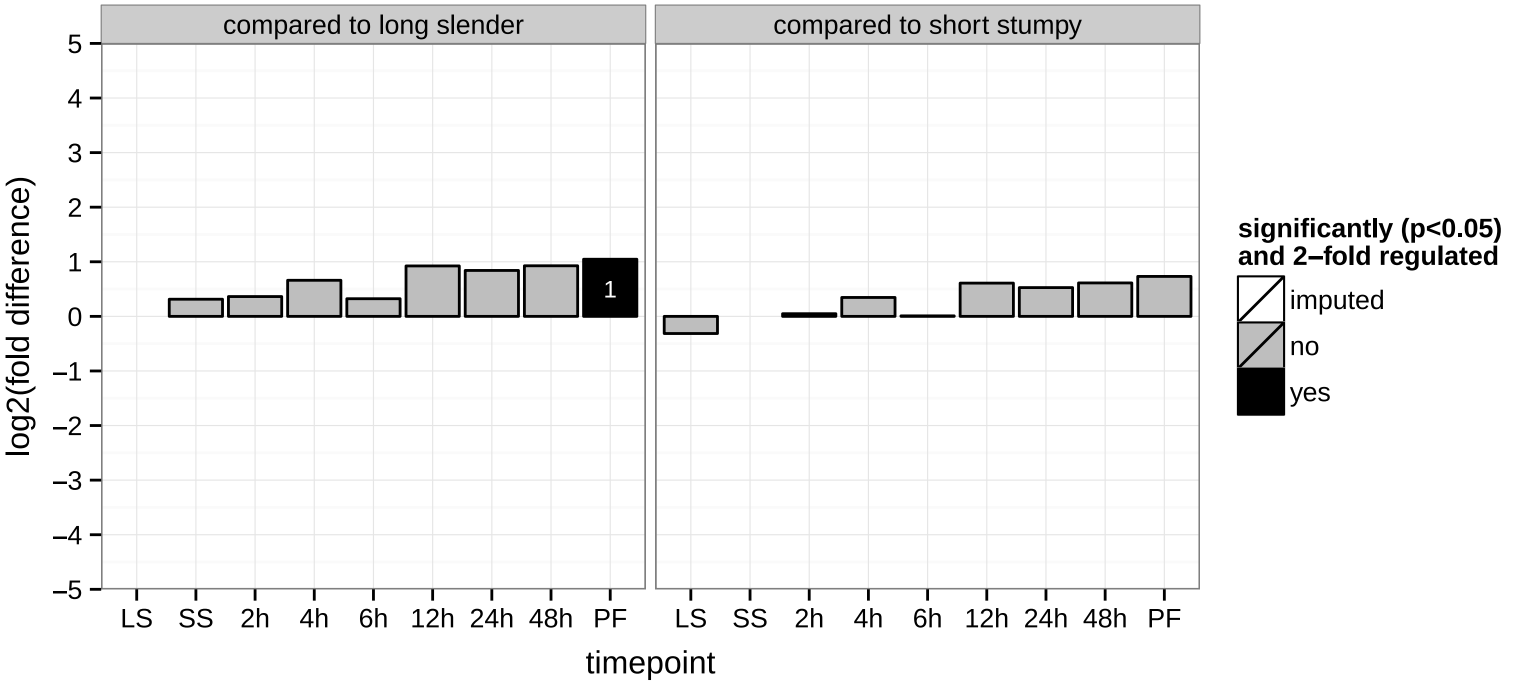

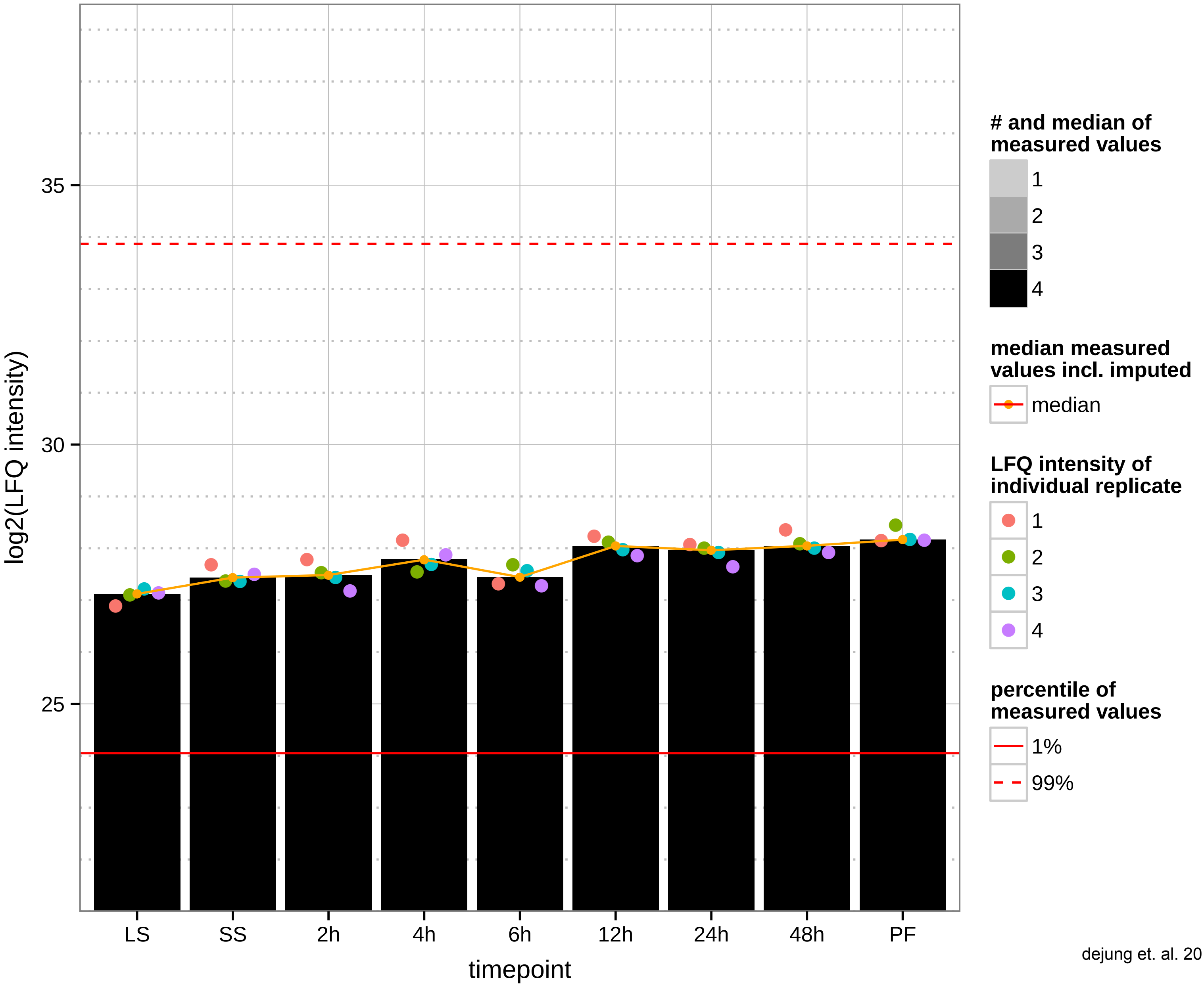

dejung et. al. 2015

hypothetical protein, conserved
Tb927.9.13330
AGOF: null
AGOC: null
AGOP: null
PGOF: binding
PGOC: null
PGOP: null

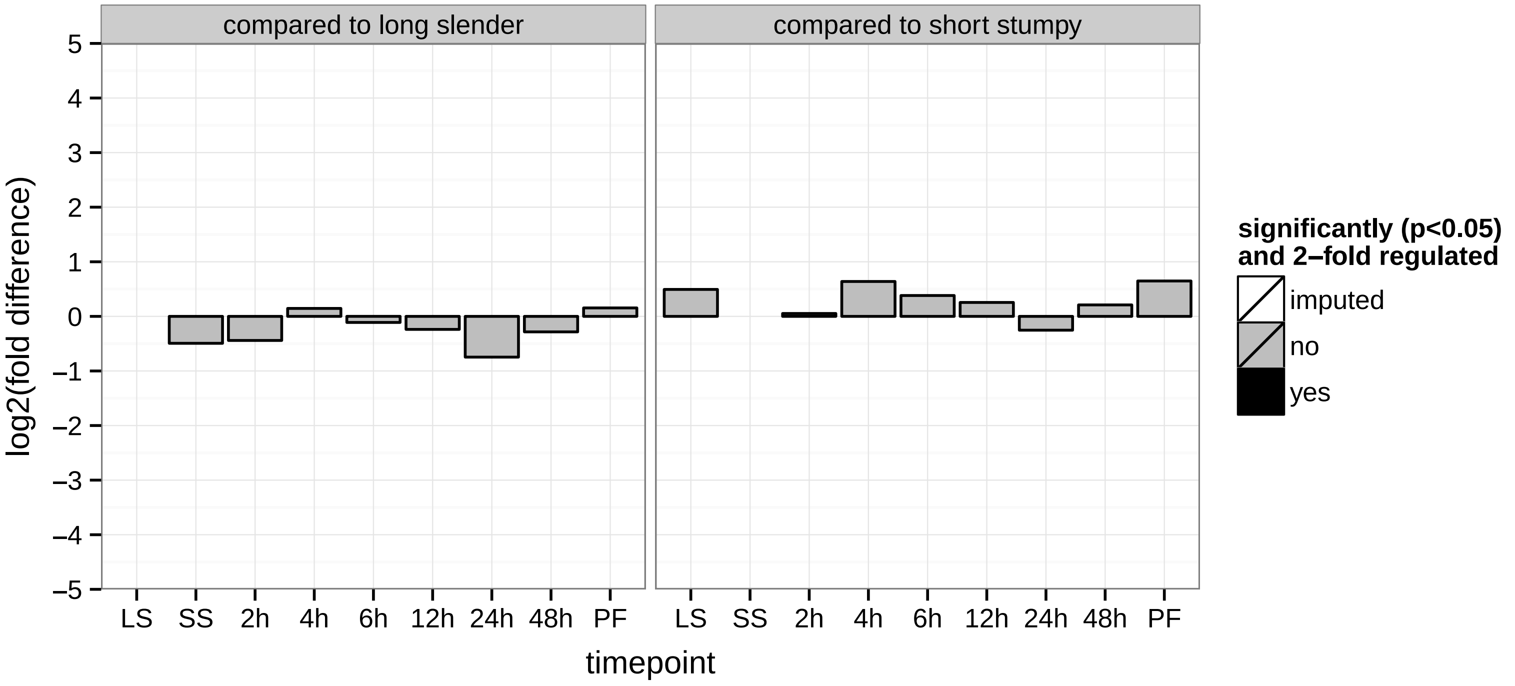

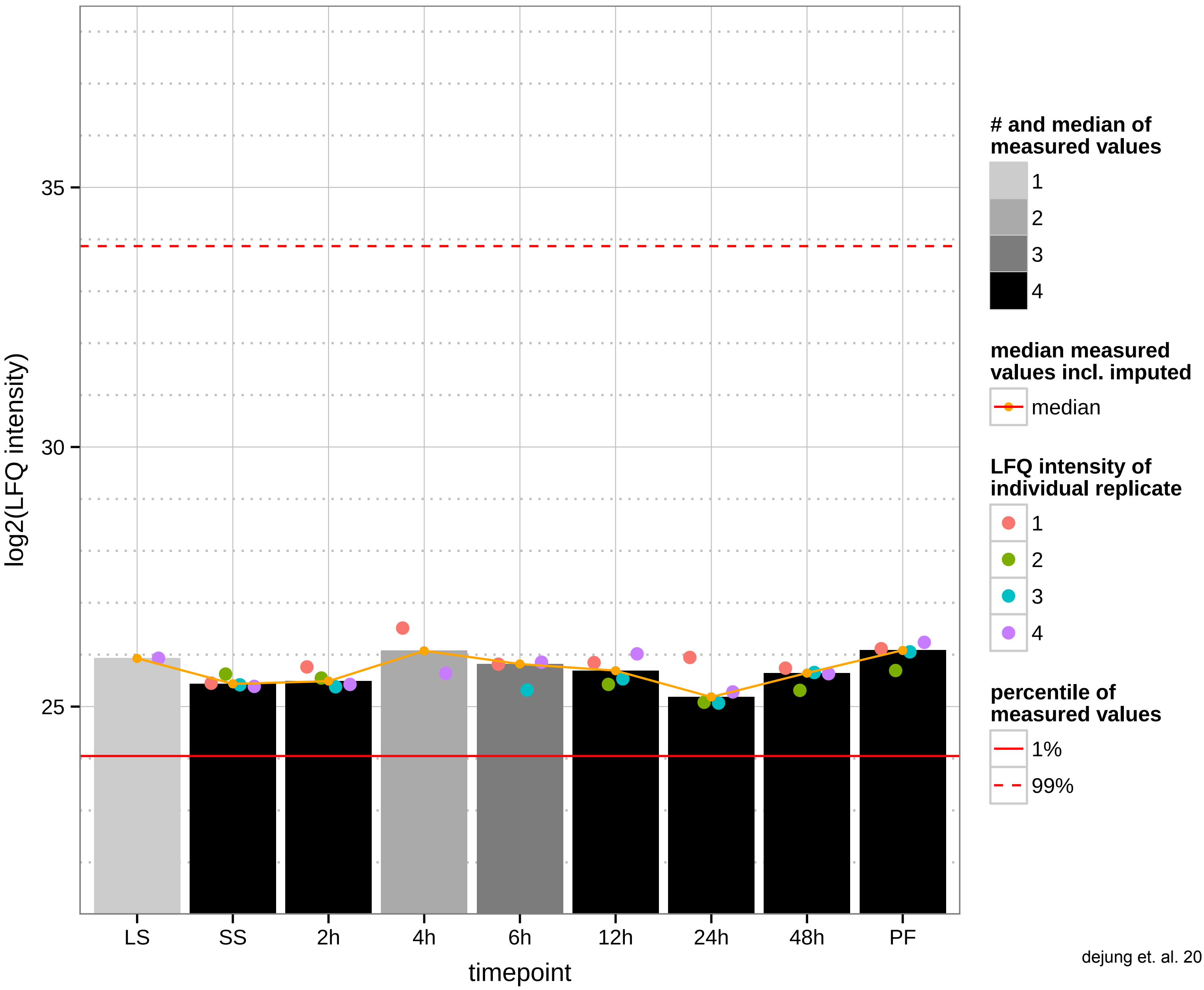

dejung et. al. 2015

hypothetical protein, conserved
Tb927.9.13440
AGOF: null
AGOC: null
AGOP: null
PGOF: protein binding
PGOC: null
PGOP: null

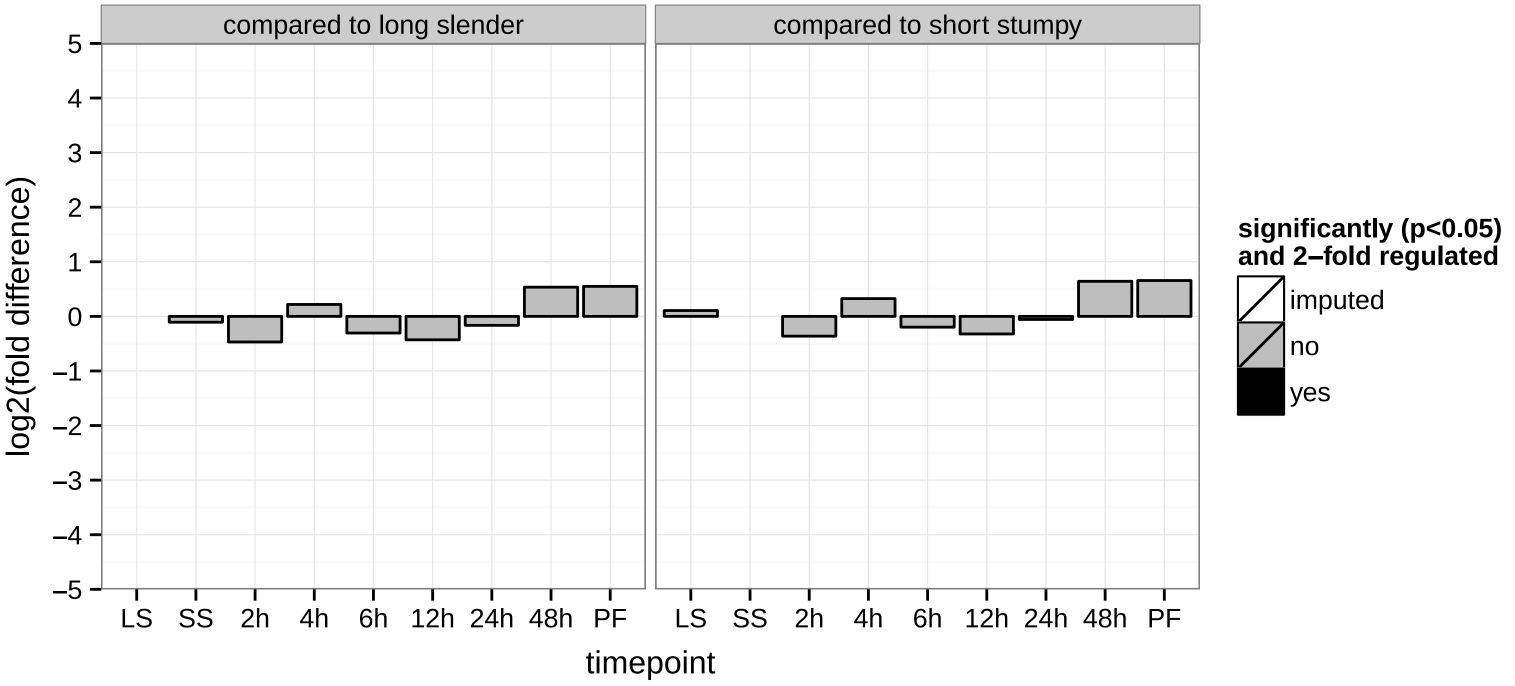

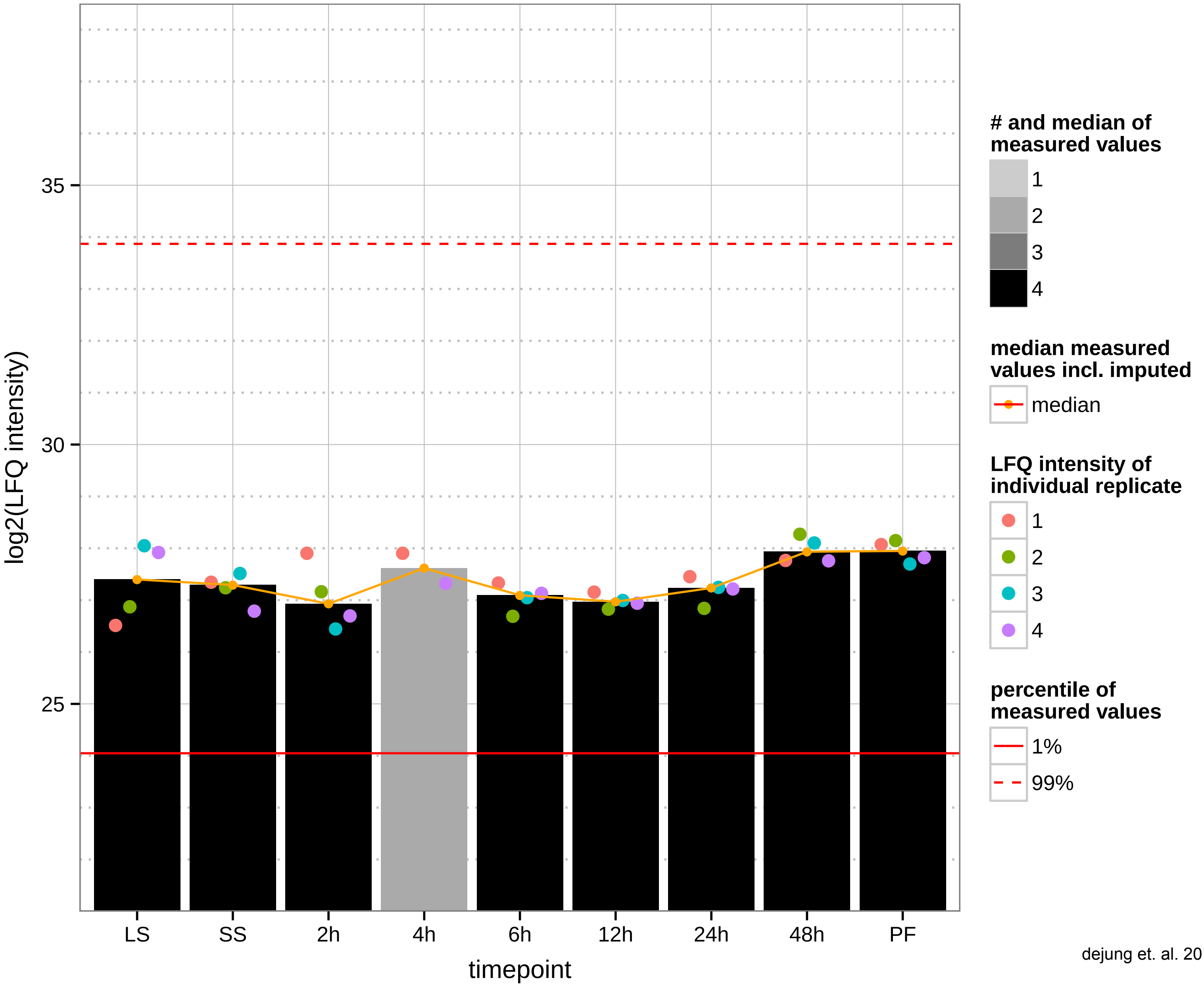

dejung et. al. 2015

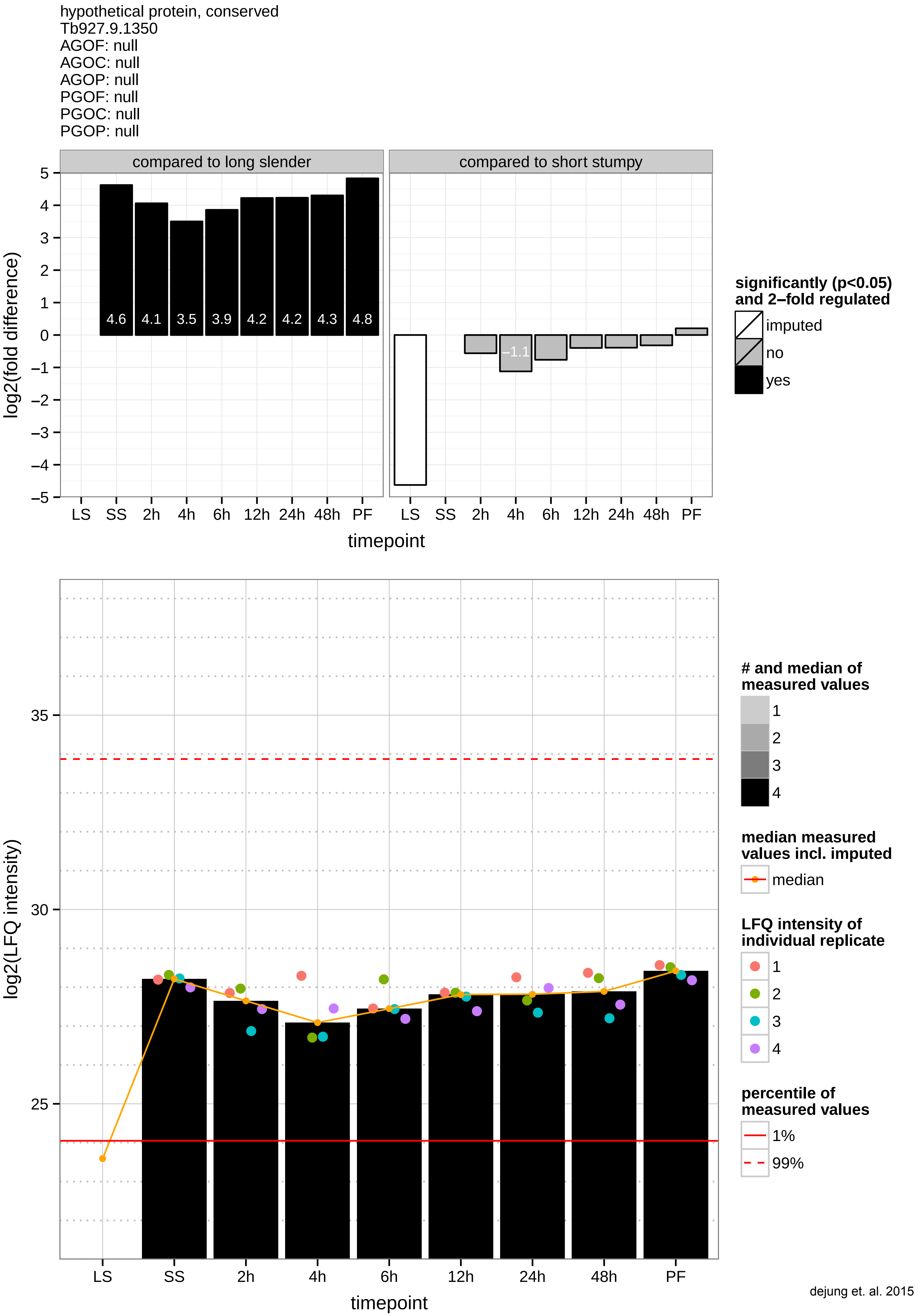
hypothetical protein, conserved
Tb927.9.1350
AGOF: null
AGOC: null
AGOP: null
PGOF: null
PGOC: null
PGOP: null
compared to long slender
compared to short stumpy
log2(fold difference)
4.6
4.1
3.5
3.9
4.2
4.2
4.3
4.8
−1.1
LS
SS
2h
4h
6h
12h
24h
48h
PF
timepoint
significantly (p<0.05) and 2−fold regulated
imputed
no
yes
log2(LFQ intensity)
# and median of measured values
1
2
3
4
median measured values incl. imputed
median
LFQ intensity of individual replicate
percentile of measured values
1%
99%
dejung et. al. 2015

hypothetical protein, conserved
Tb927.9.13540
AGOF: null
AGOC: mitochondrion
AGOP: null
PGOF: null
PGOC: null
PGOP: null

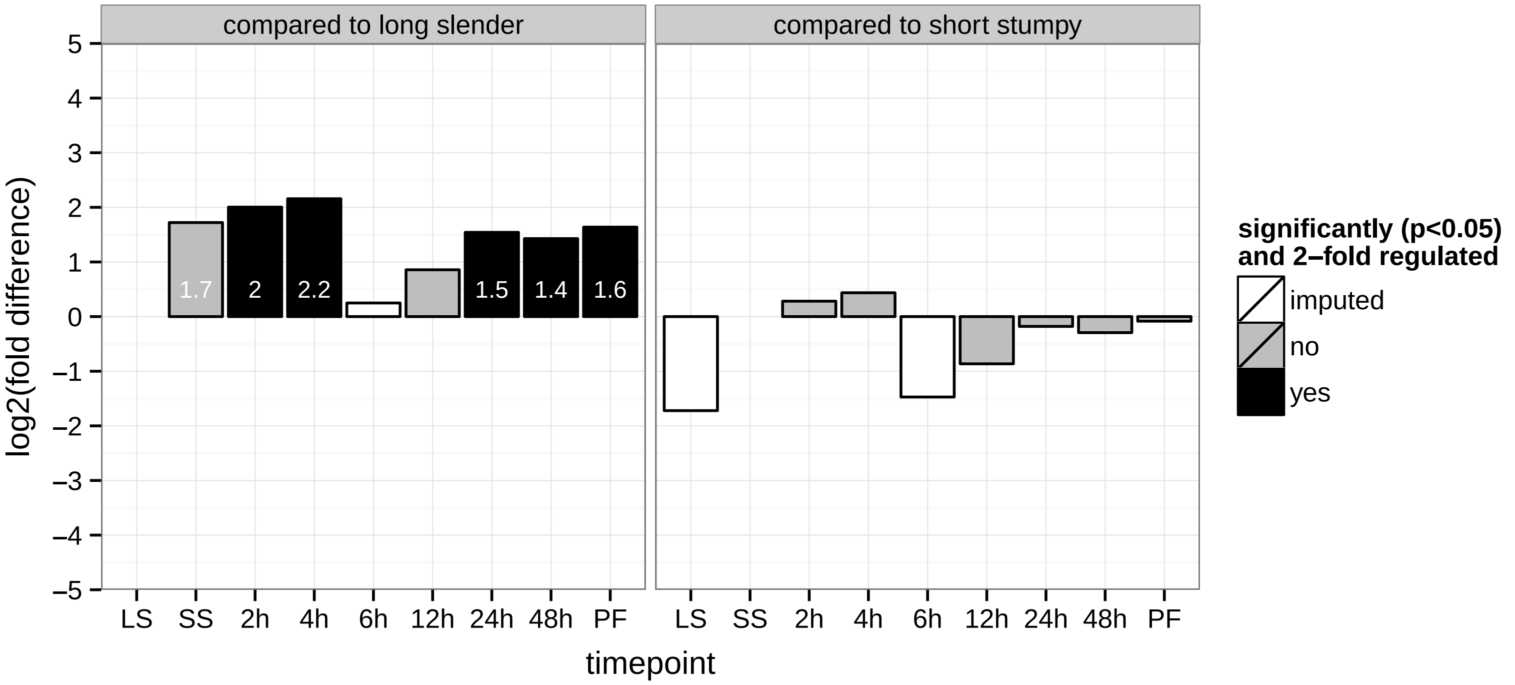

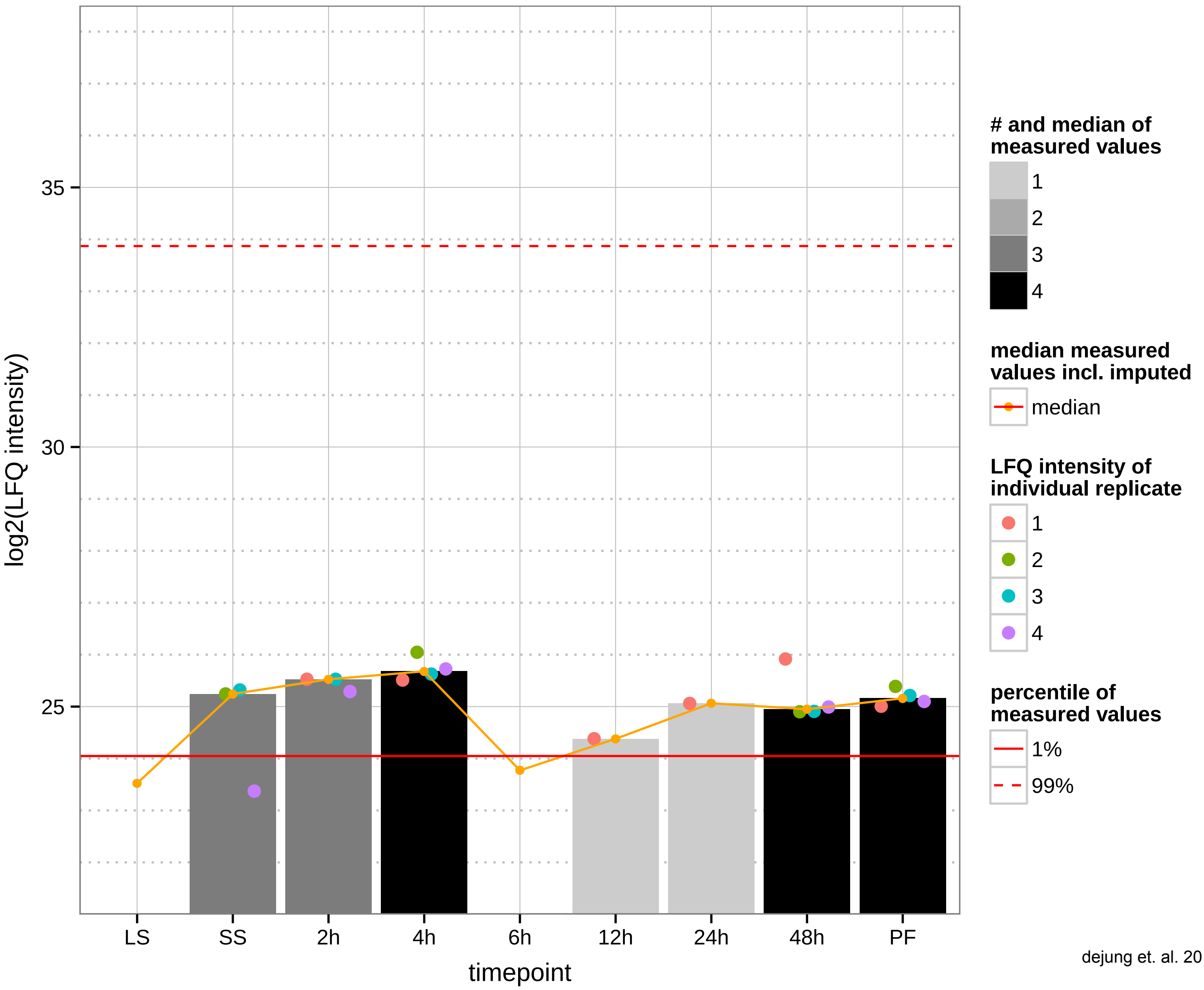

dejung et. al. 2015

helicase, putative
Tb927.9.13610
AGOF: ATP−dependent helicase activity, helicase activity, nucleic acid binding
AGOC: null
AGOP: nucleobase−containing compound metabolic process
PGOF: ATP binding, ATP−dependent helicase activity, helicase activity, nucleic acid binding
PGOC: null
PGOP: null

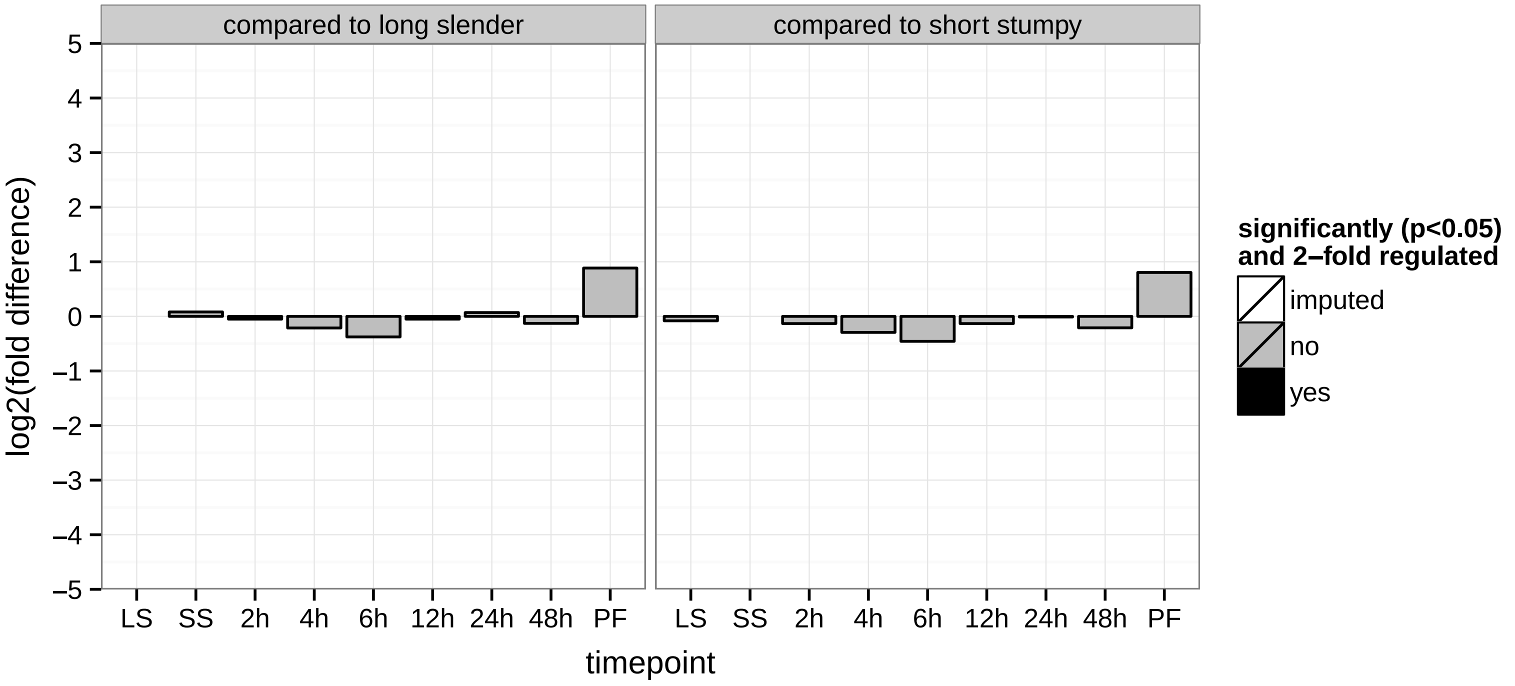

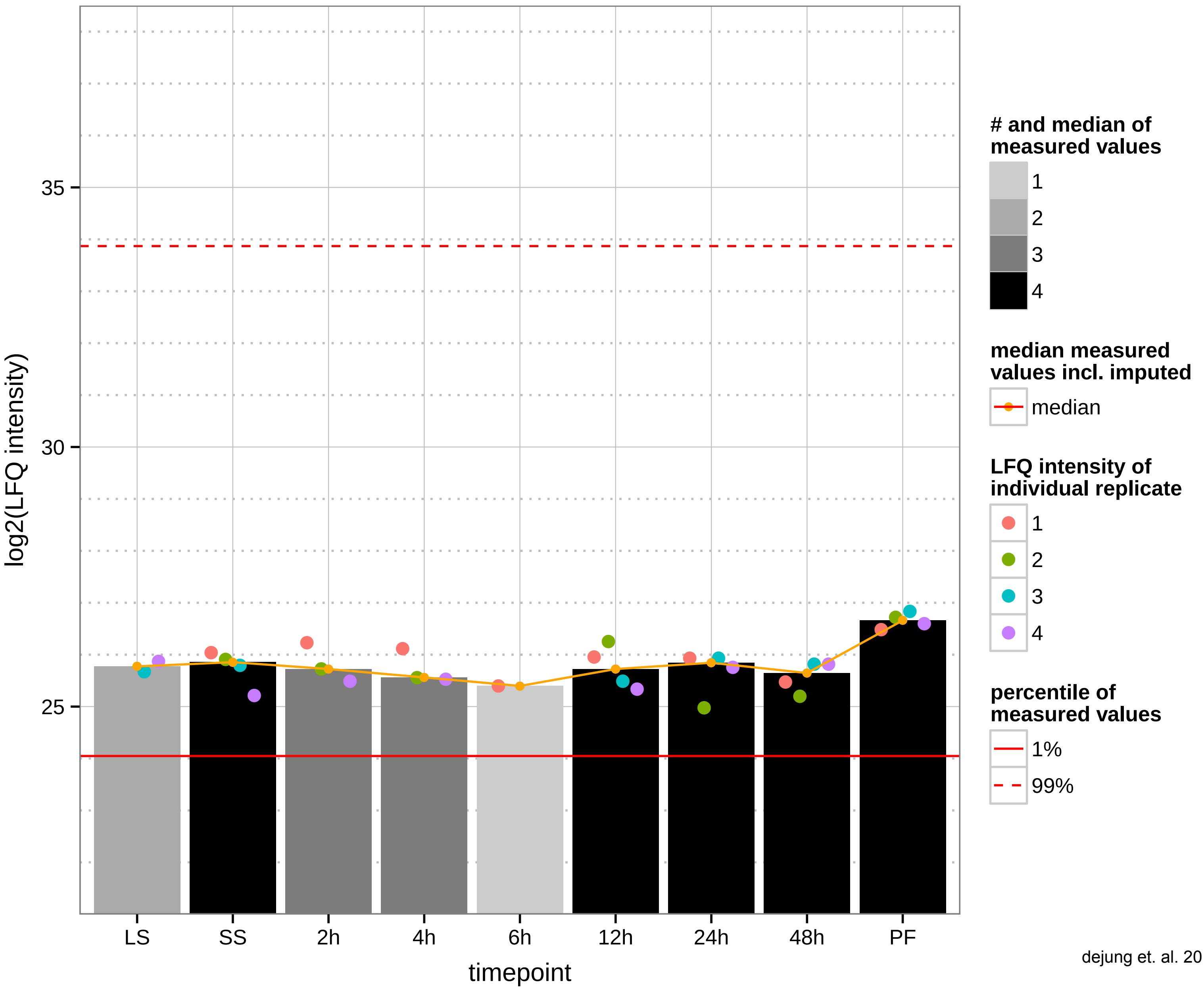

dejung et. al. 2015

ADP–ribosylation factor, putative
Tb927.9.13740;Tb927.9.13710;Tb927.9.13680;Tb927.9.13650
AGOF: GTP binding
AGOC: intracellular
AGOP: intracellular protein transport, small GTPase mediated signal transduction
PGOF: GTP binding, null
PGOC: intracellular, null
PGOP: intracellular protein transport, protein transport, small GTPase mediated signal transduction, null

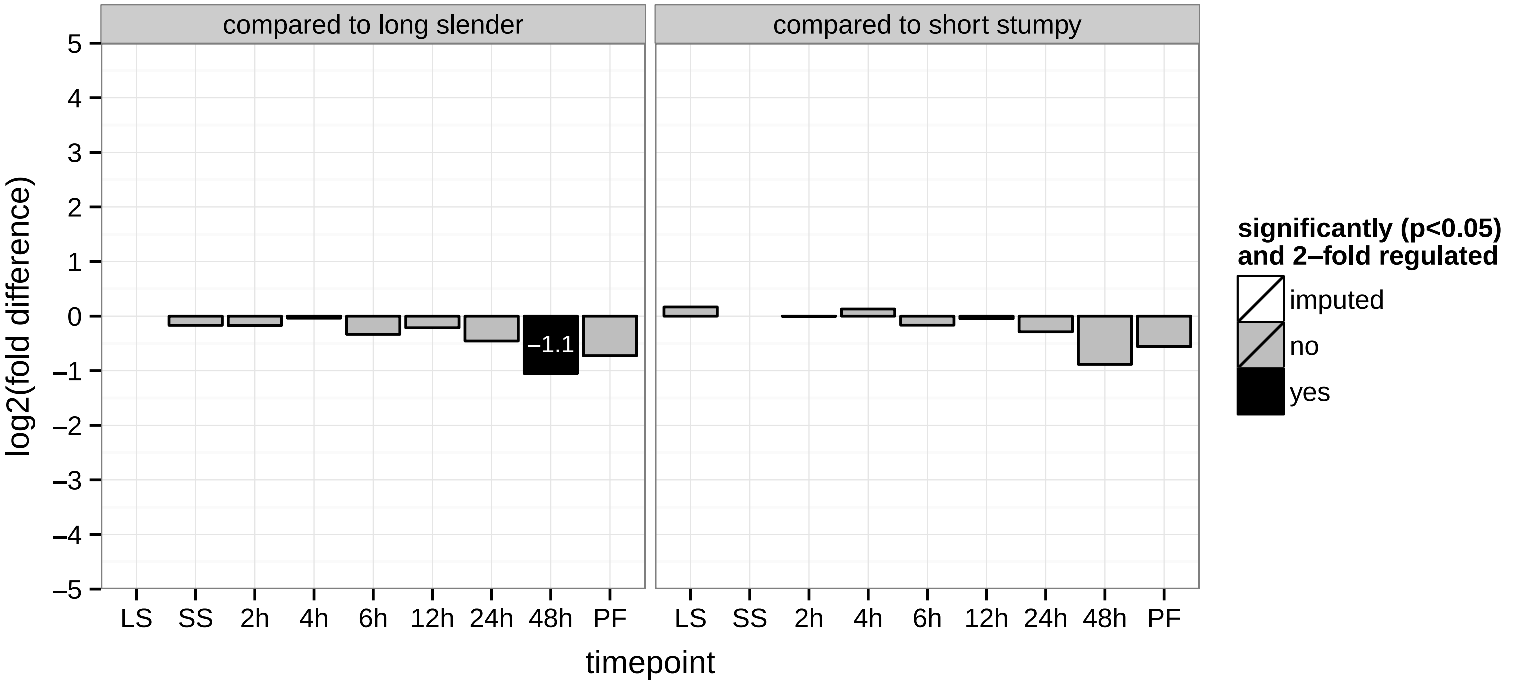

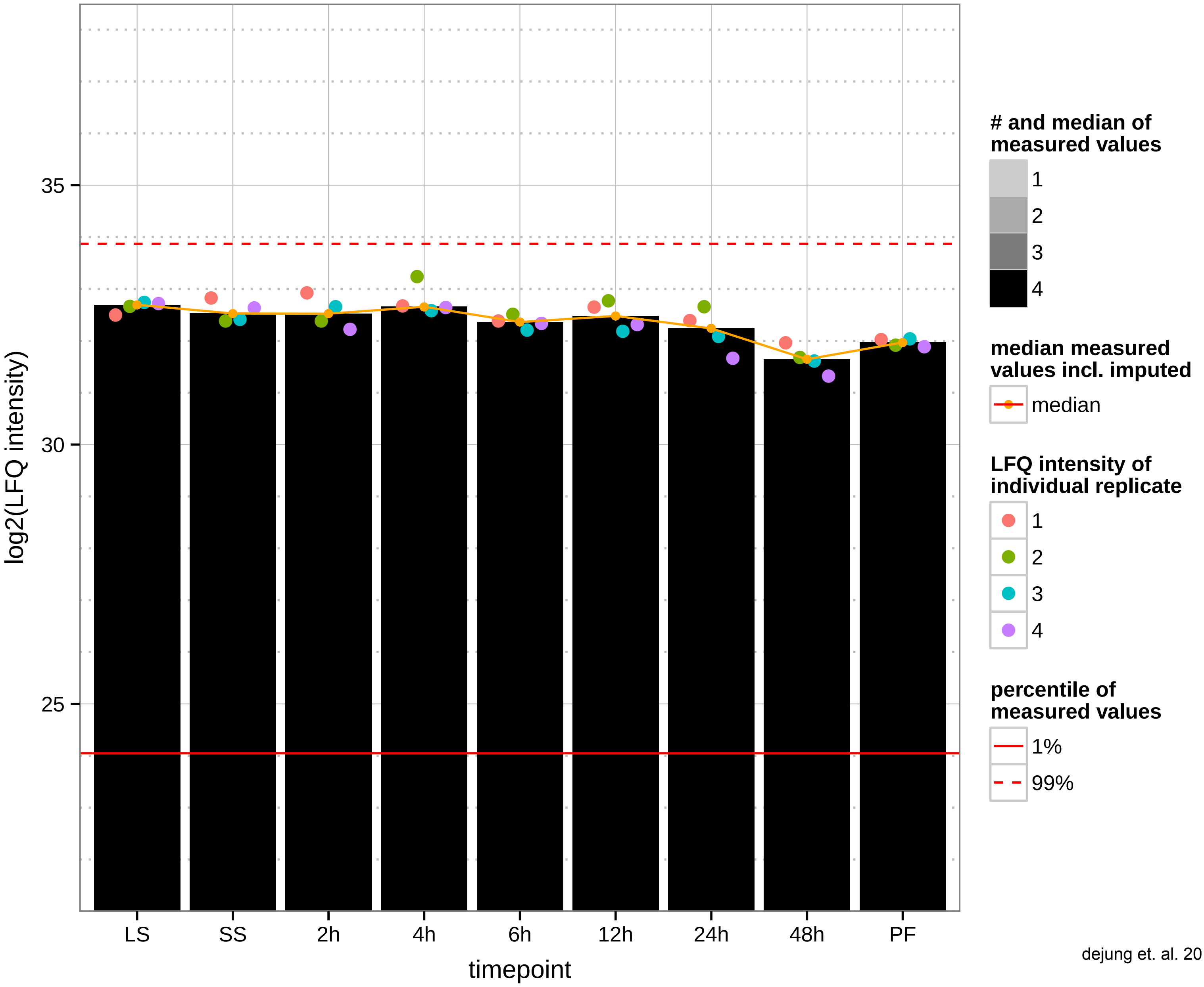

dejung et. al. 2015

hypothetical protein, conserved
Tb927.9.1380
AGOF: null
AGOC: mitochondrion
AGOP: null
PGOF: null
PGOC: null
PGOP: null

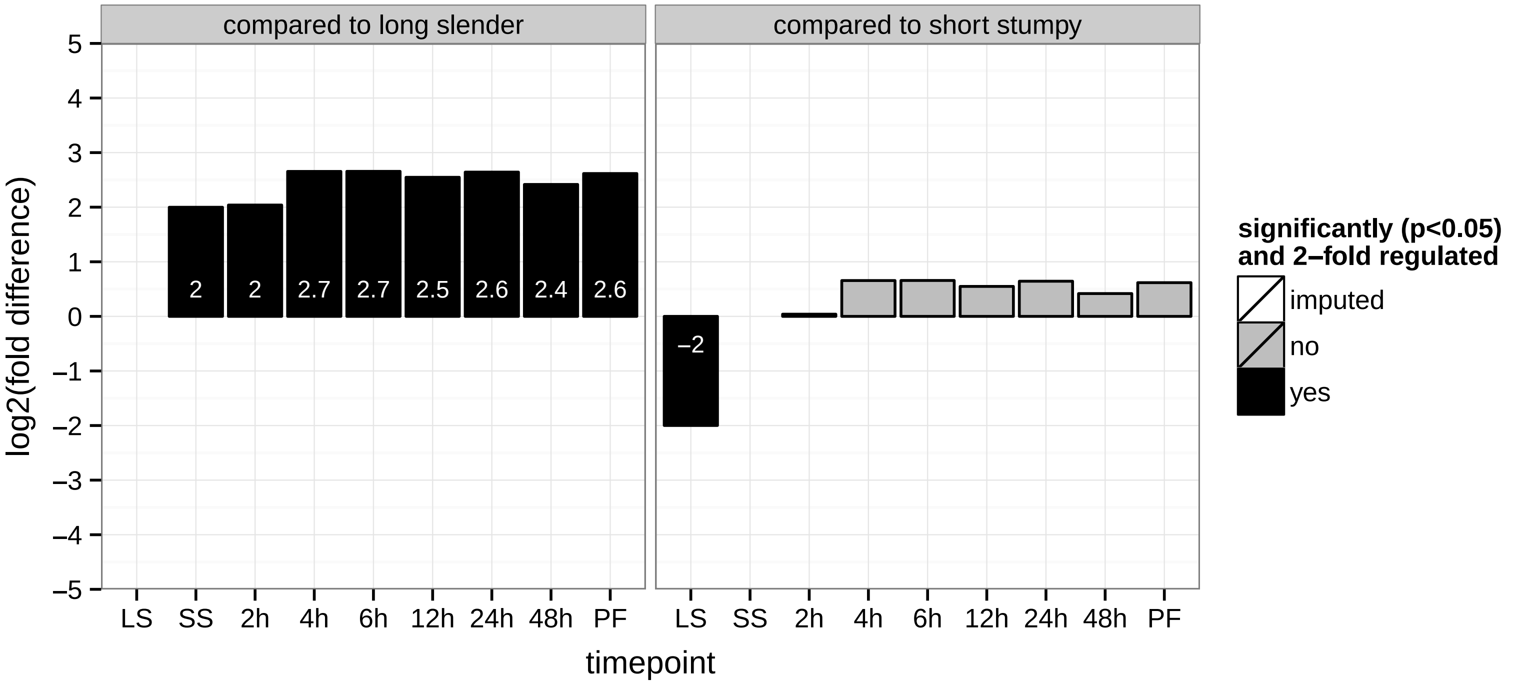

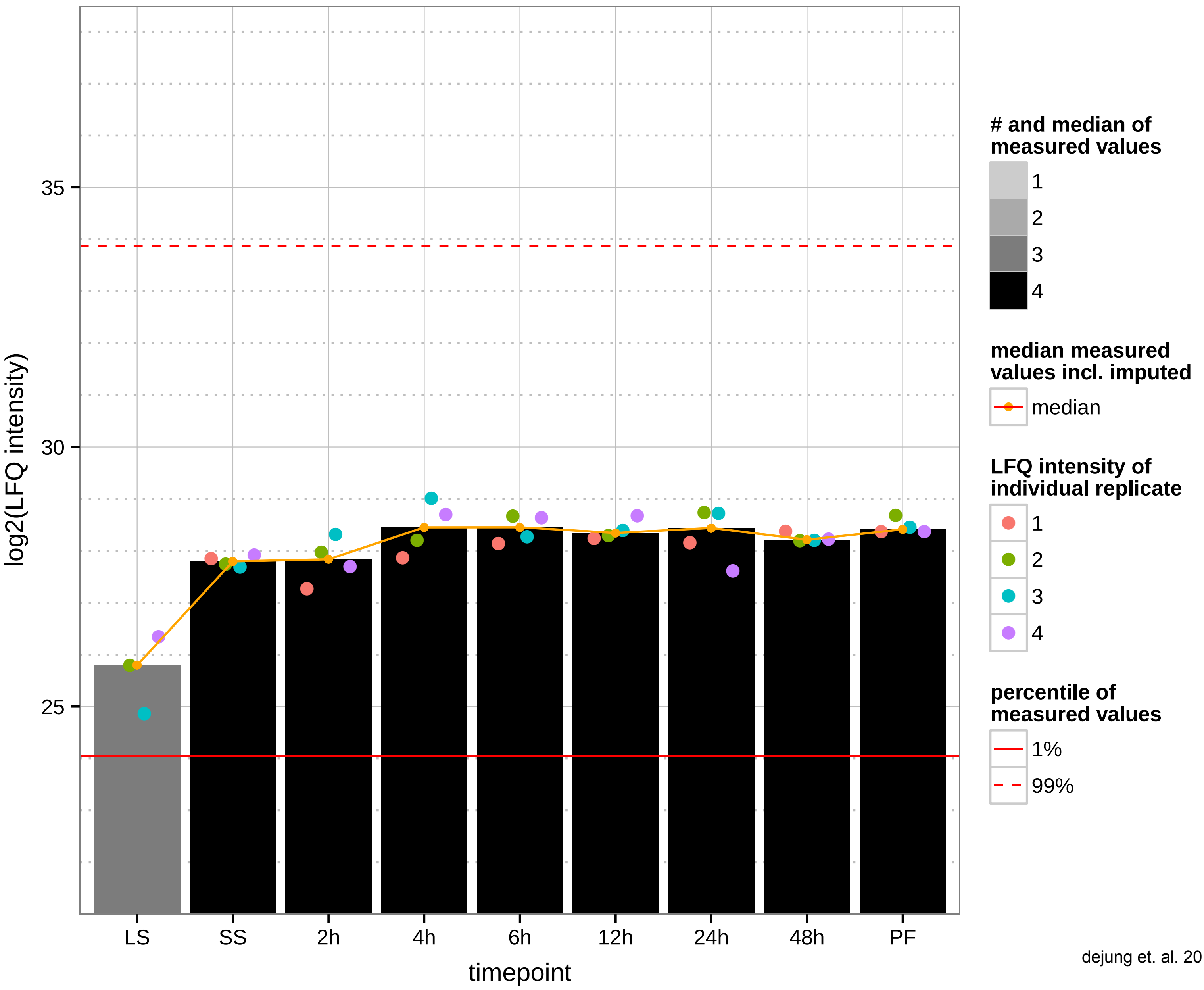

dejung et. al. 2015

RNA–binding protein, putative (DRBD2)
Tb927.9.13990
AGOF: RNA binding
AGOC: null
AGOP: RNA processing
PGOF: nucleic acid binding
PGOC: null
PGOP: null

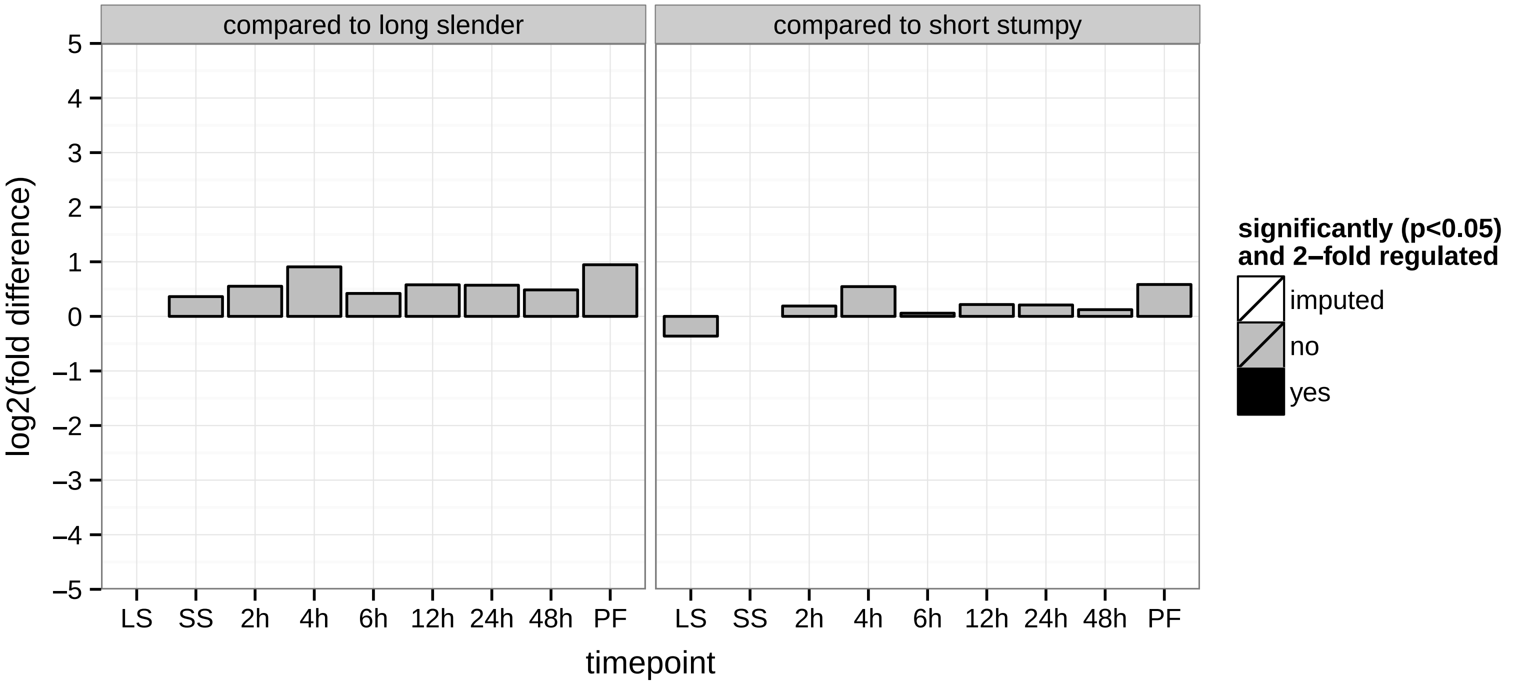

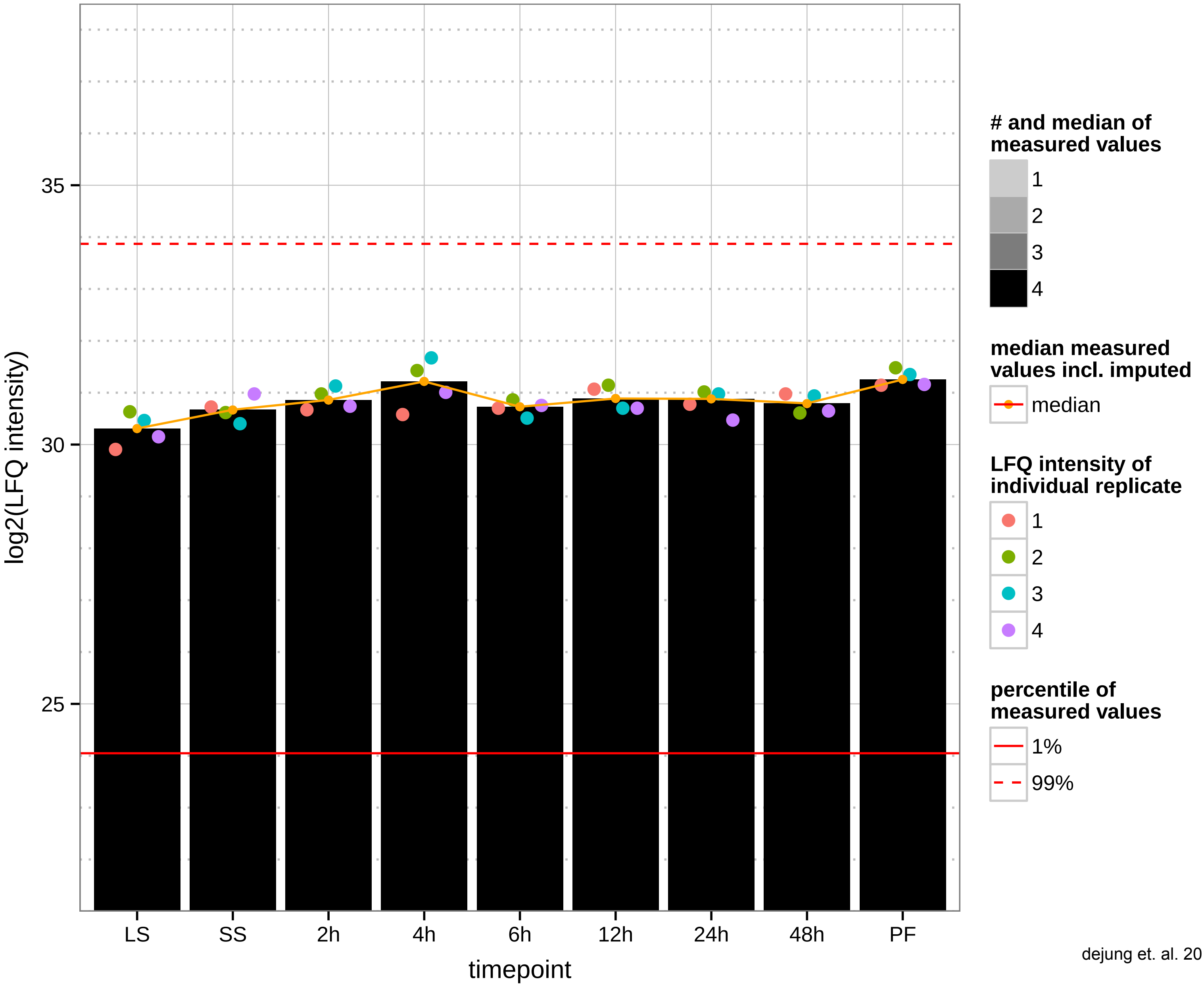

dejung et. al. 2015

Monooxygenase, putative
Tb927.9.1400
AGOF: monooxygenase activity
AGOC: null
AGOP: cellular aromatic compound metabolic process
PGOF: null
PGOC: null
PGOP: null

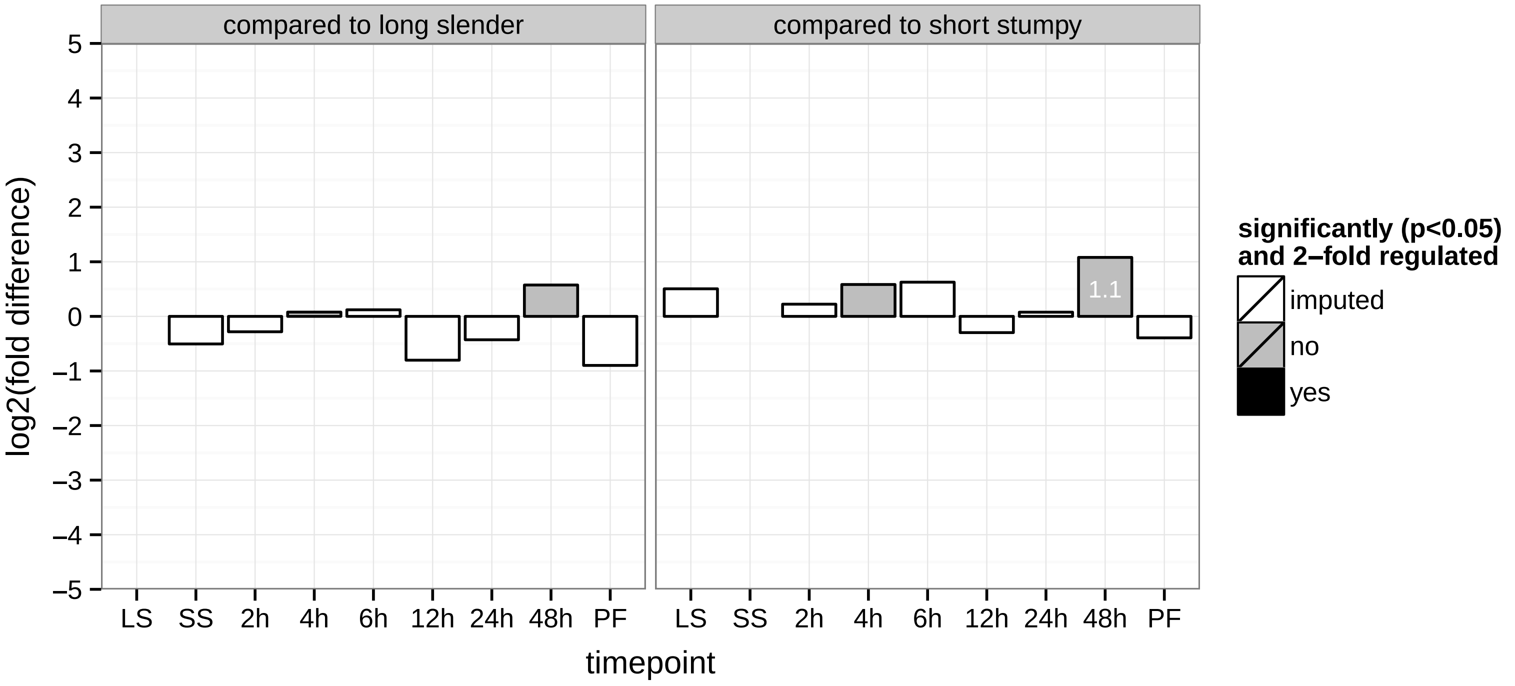

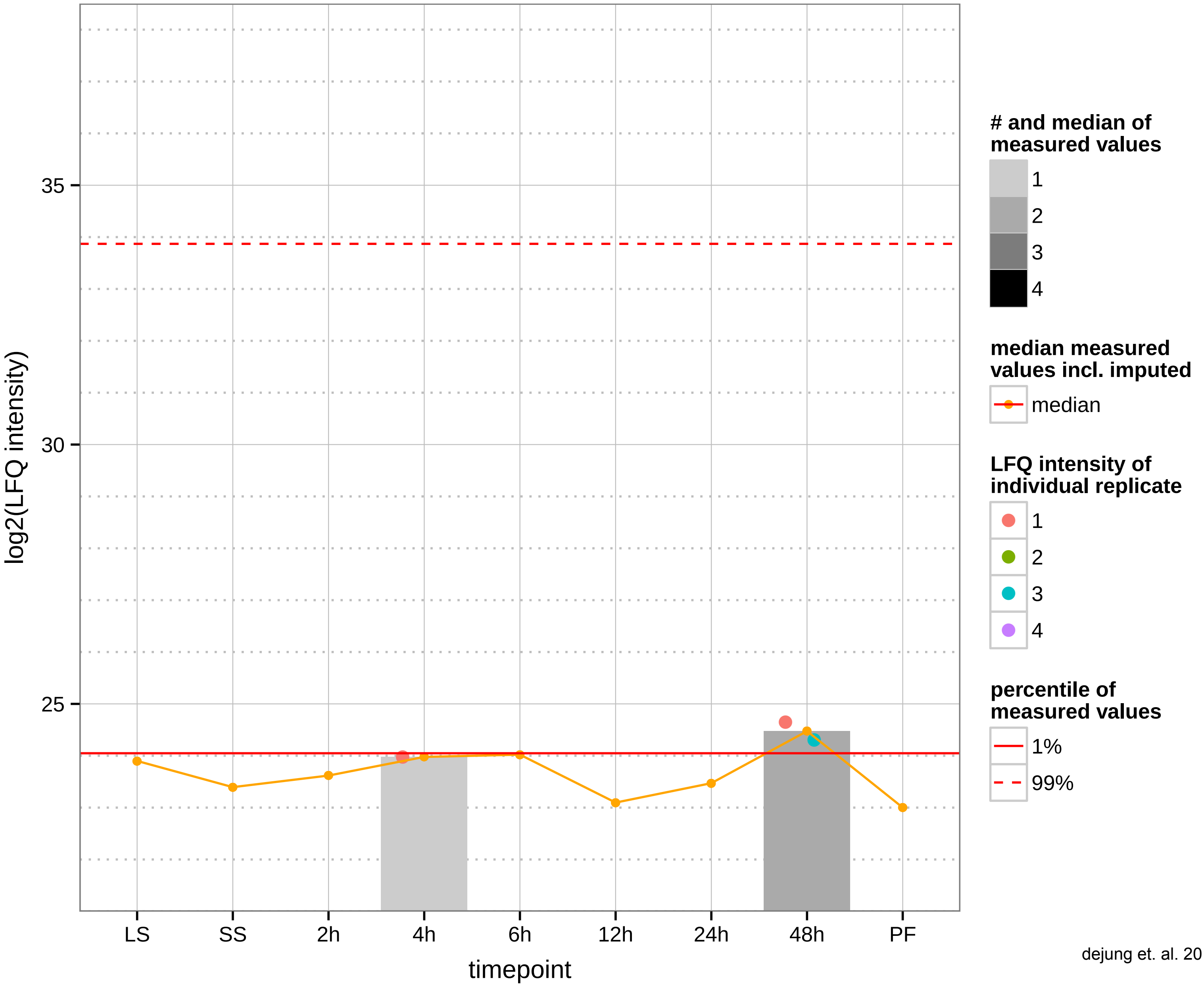

dejung et. al. 2015

short–chain dehydrogenase, putative
Tb927.9.14070
AGOF: oxidoreductase activity
AGOC: null
AGOP: metabolic process
PGOF: oxidoreductase activity
PGOC: null
PGOP: metabolic process

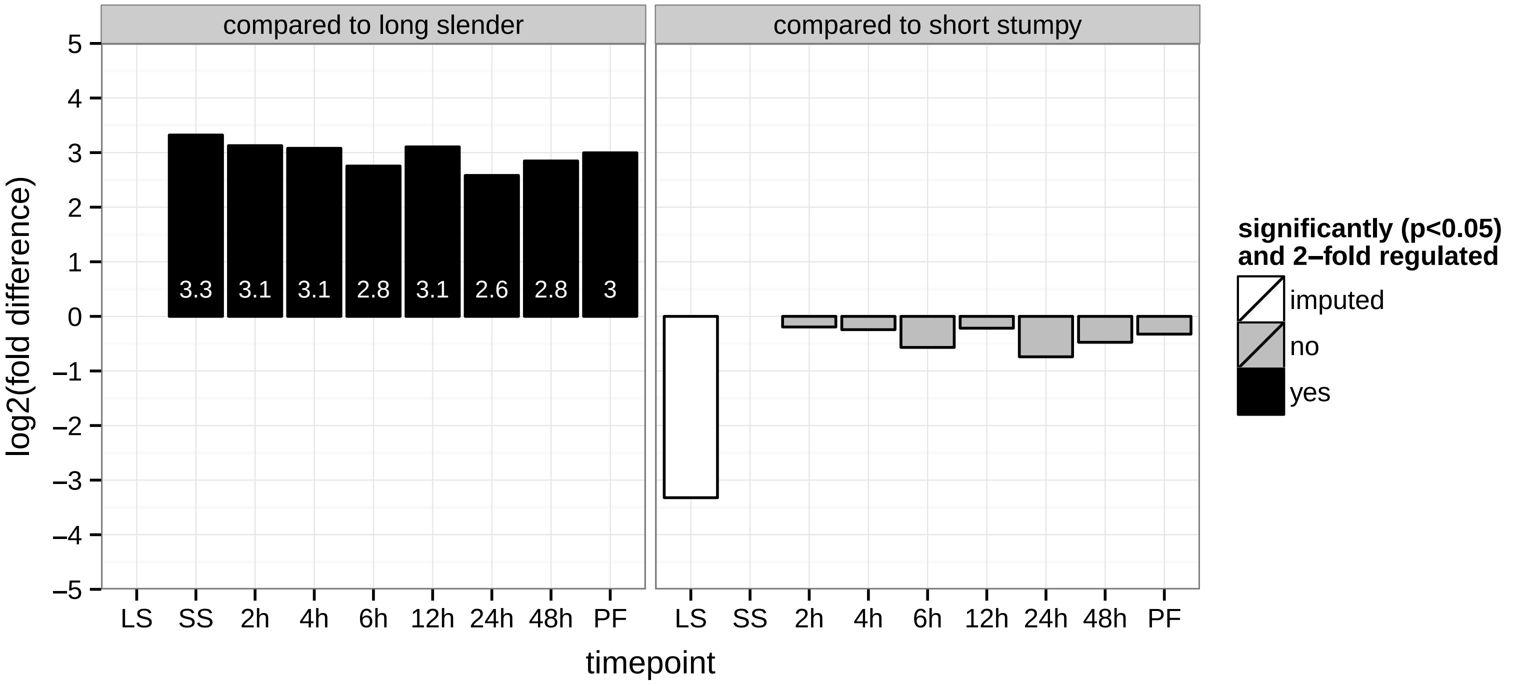

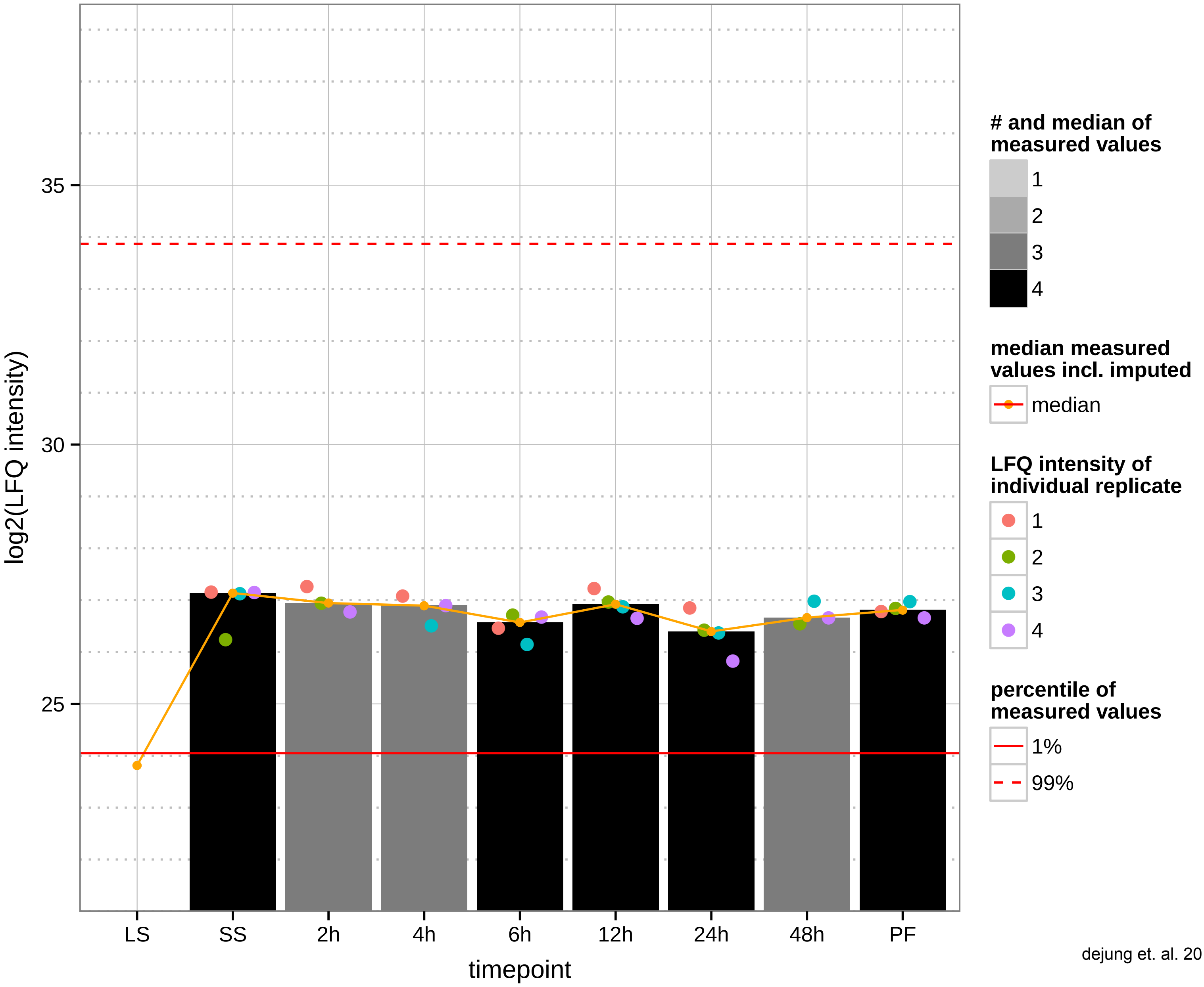

dejung et. al. 2015

vesicle–associated membrane protein, putative
Tb927.9.14080
AGOF: null
AGOC: integral to membrane
AGOP: vesicle–mediated transport
PGOF: null
PGOC: integral to membrane
PGOP: transport, vesicle–mediated transport

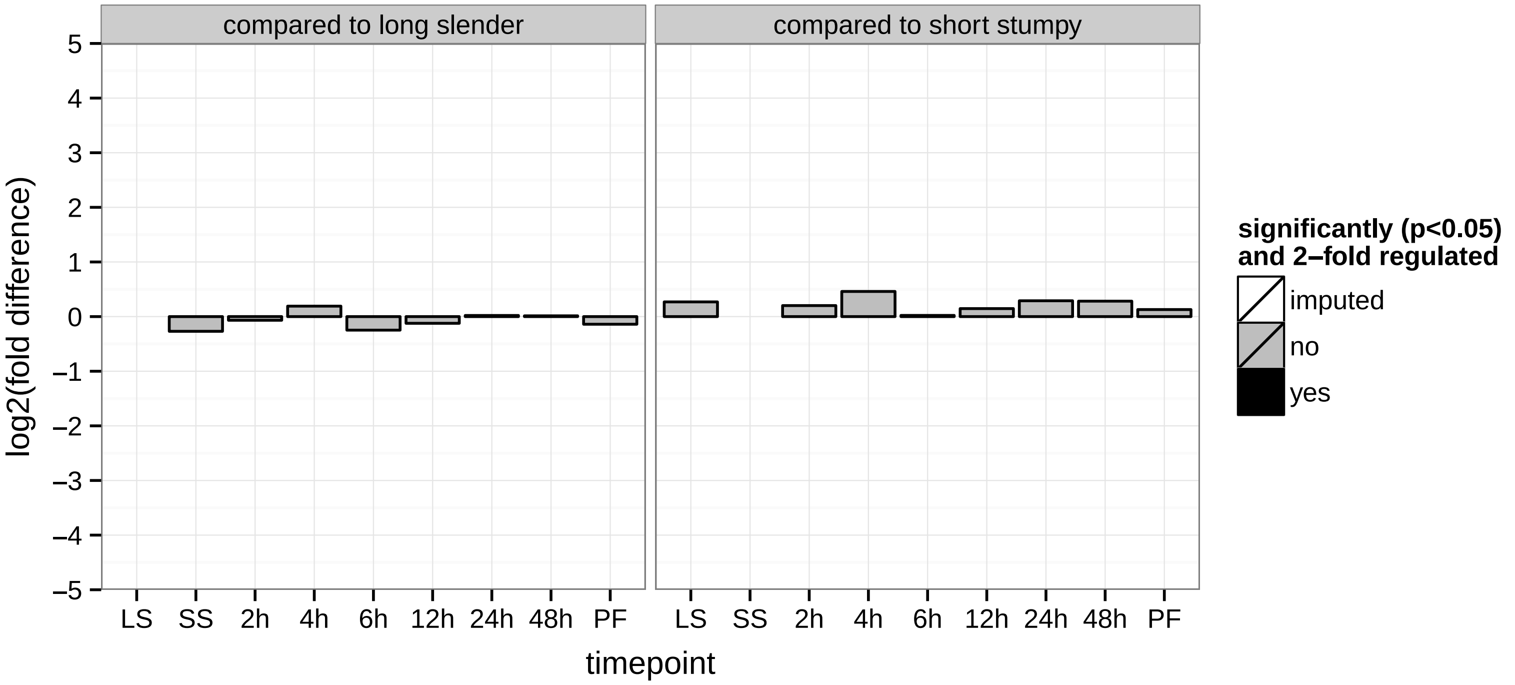

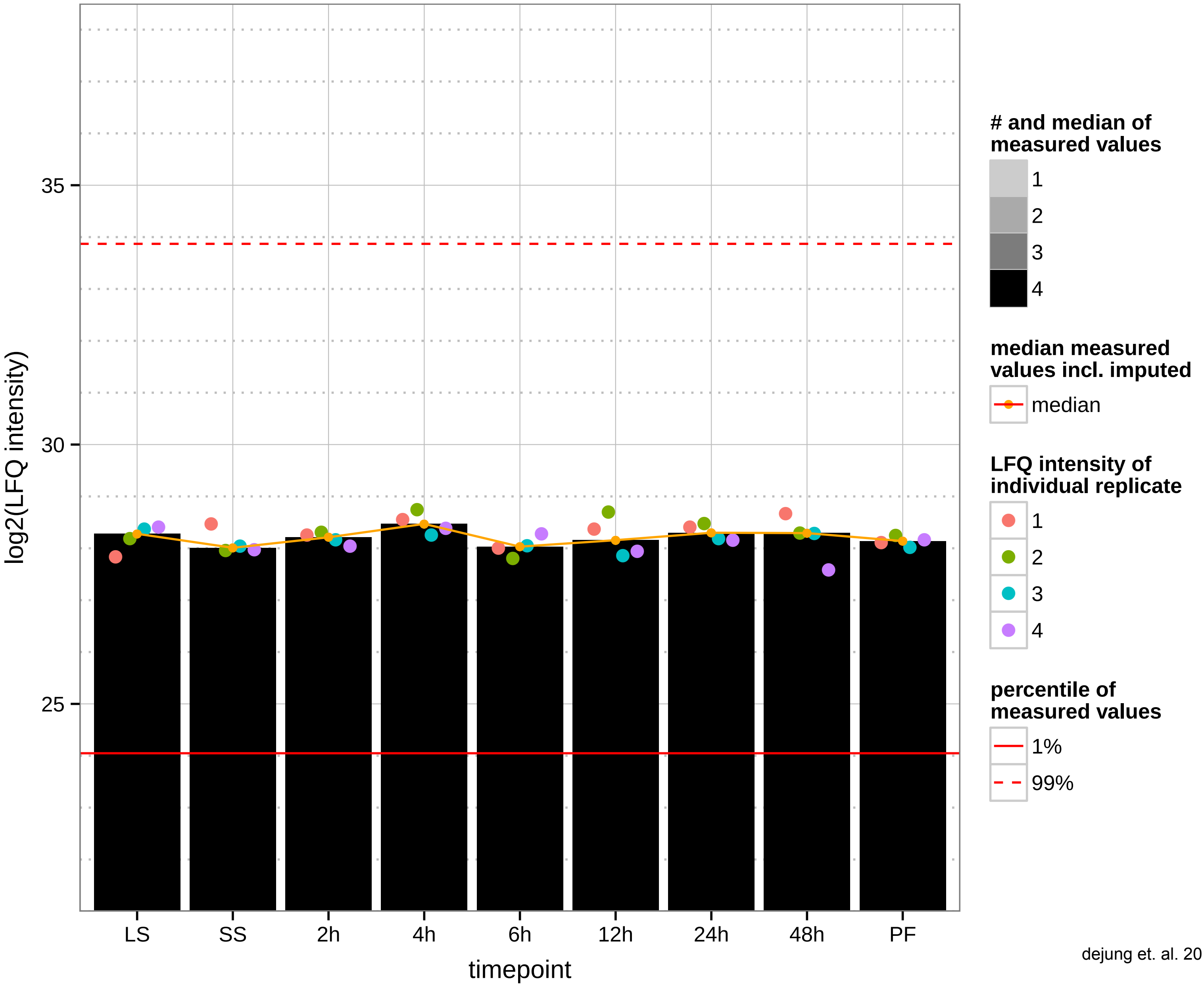

dejung et. al. 2015

hypothetical protein, conserved
Tb927.9.14230
AGOF: null
AGOC: null
AGOP: null
PGOF: null
PGOC: null
PGOP: null

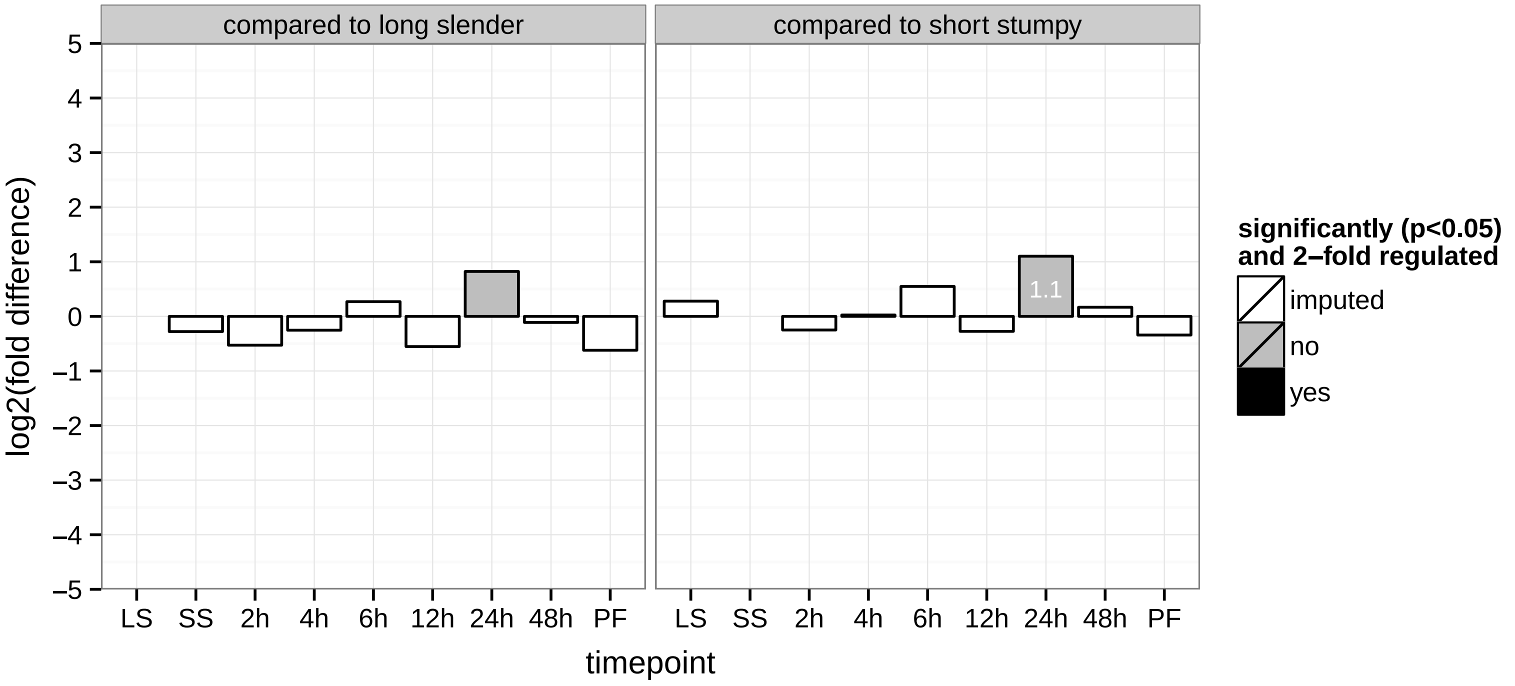

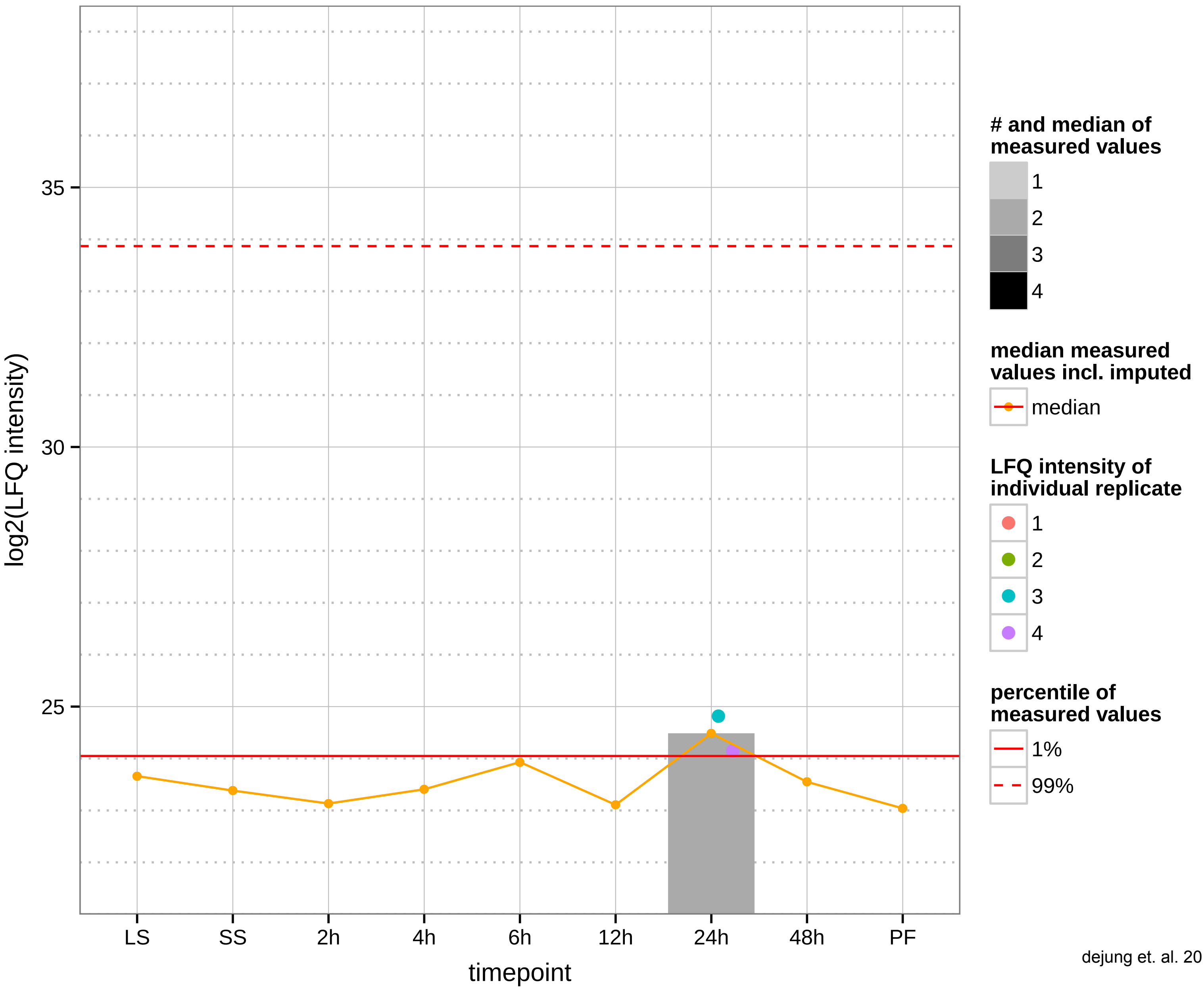

dejung et. al. 2015

hypothetical protein, conserved, Nucleoporin (Nup82)
Tb927.9.14240;Tb11.v5.0621
AGOF: null
AGOC: null, nuclear pore
AGOP: null
PGOF: null
PGOC: null
PGOP: null

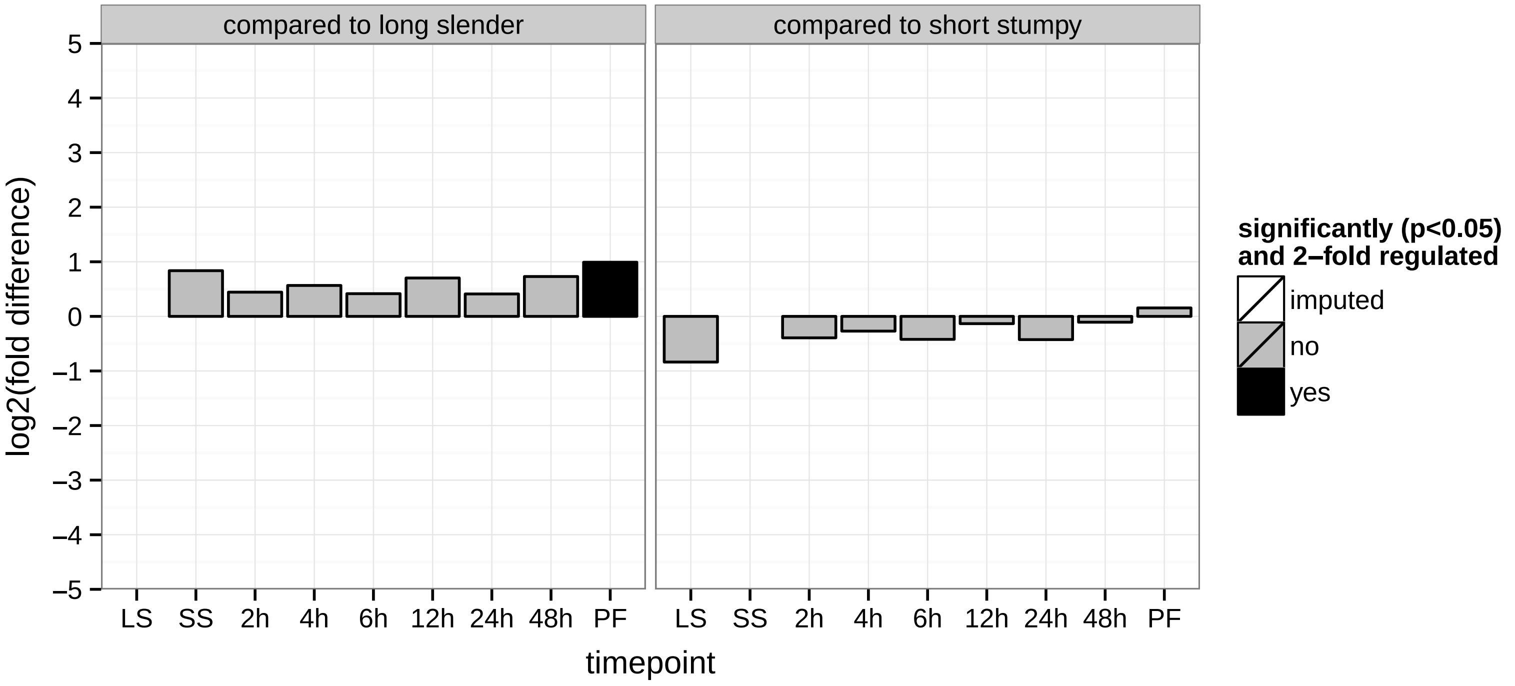

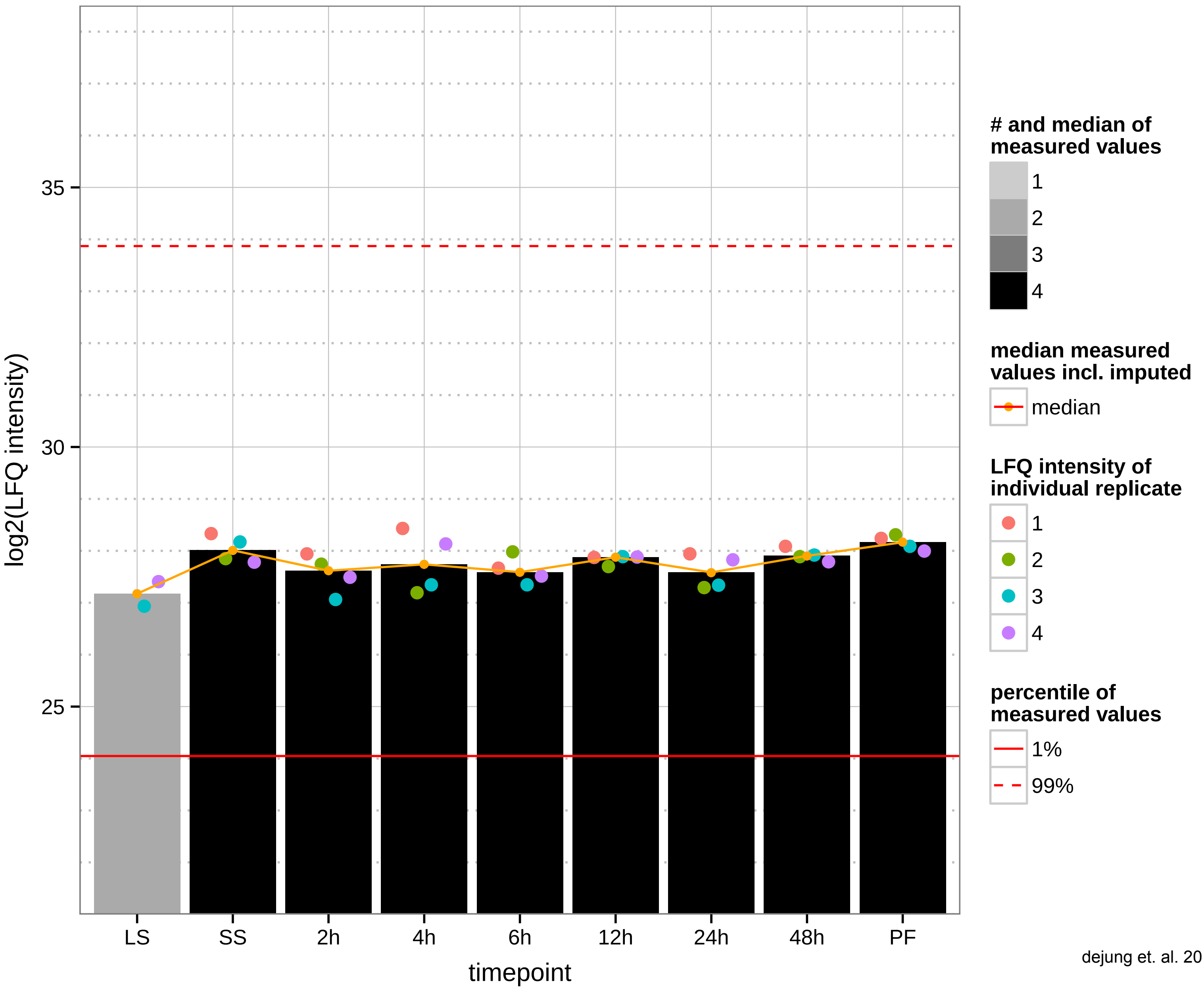

dejung et. al. 2015

casein kinase II, putative
Tb927.9.14430
AGOF: ATP binding, protein serine/threonine kinase activity, protein tyrosine kinase activity
AGOC: nucleolus
AGOP: growth, protein phosphorylation
PGOF: ATP binding, protein kinase activity, transferase activity, transferring phosphorus–containing groups
PGOC: null
PGOP: protein phosphorylation

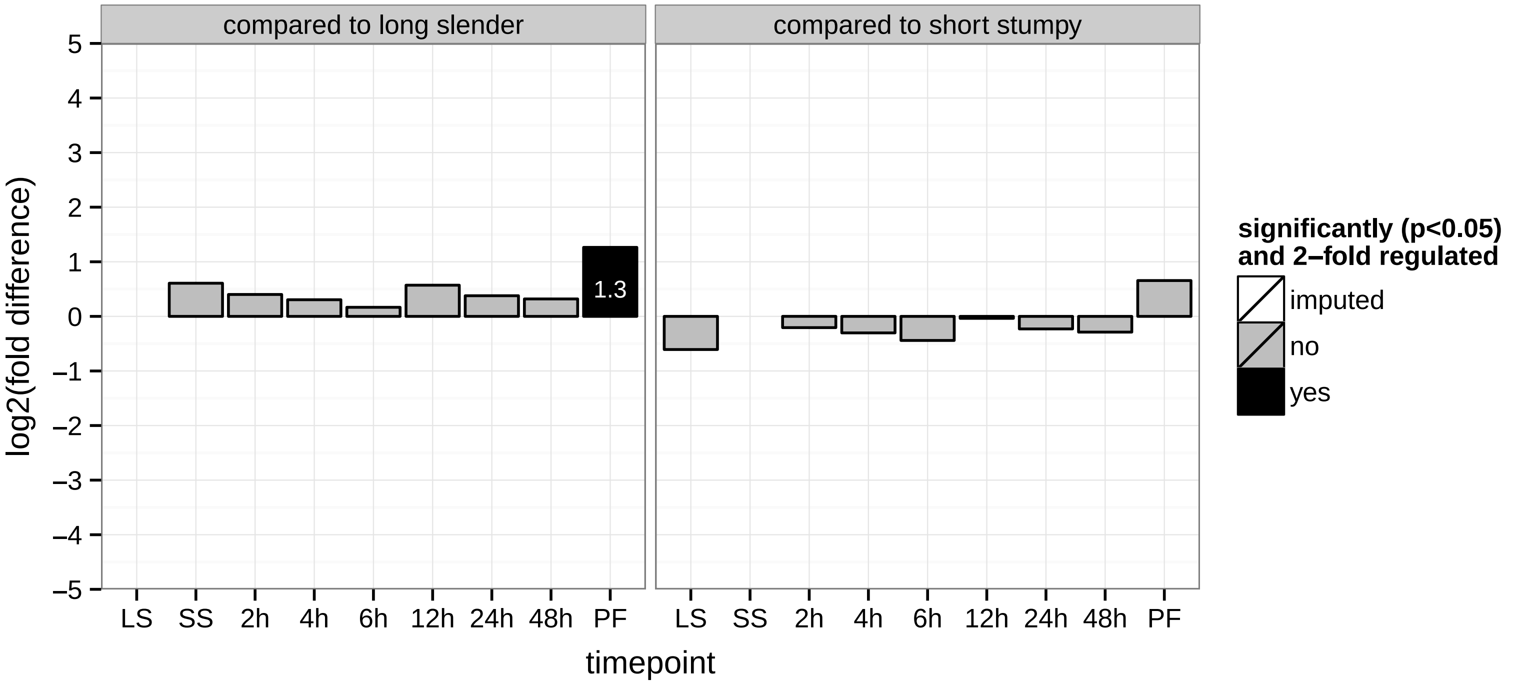

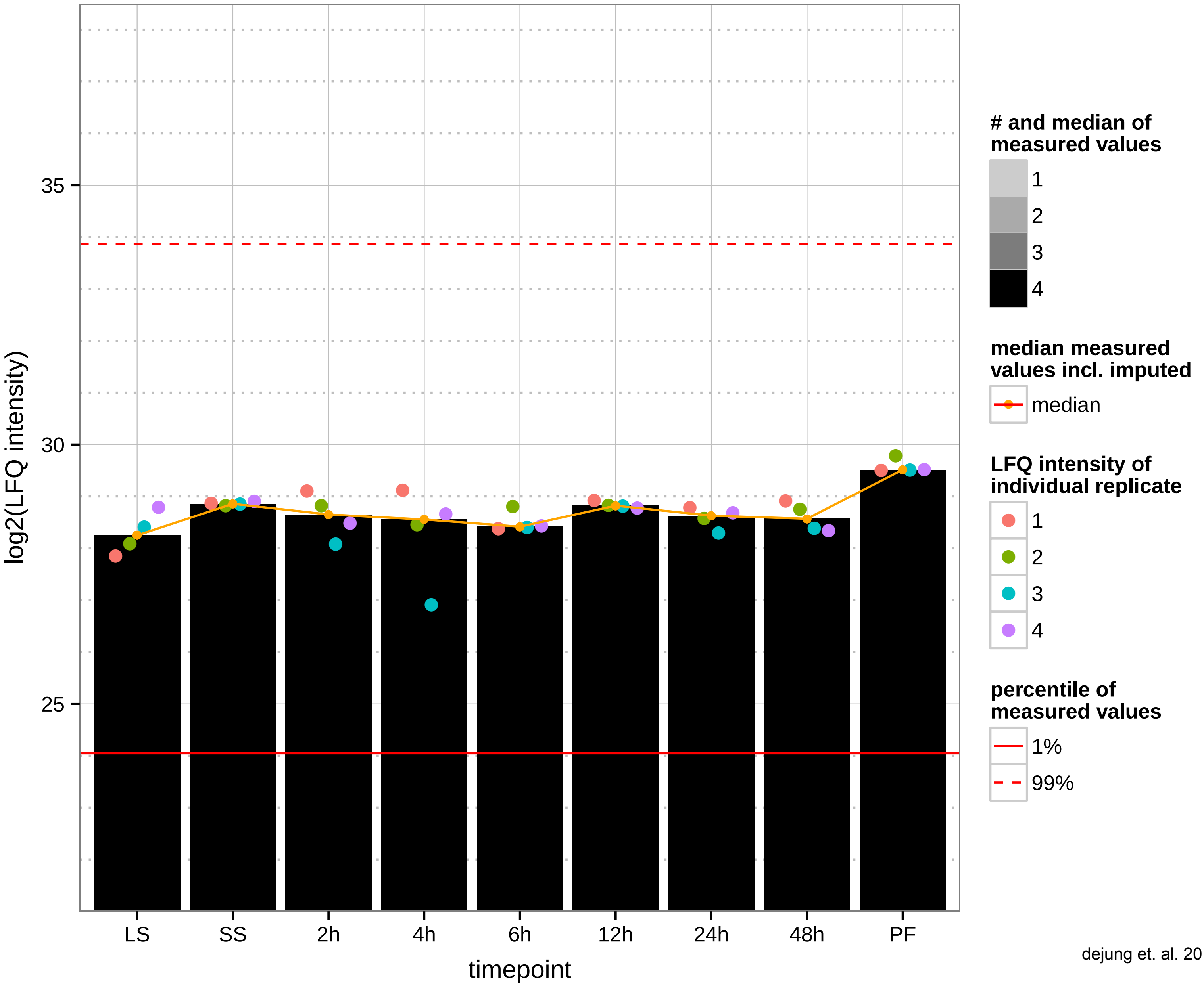

dejung et. al. 2015

dynein–associated protein, putative
Tb927.9.14480
AGOF: null
AGOC: null
AGOP: microtubule–based movement
PGOF: null
PGOC: null
PGOP: null

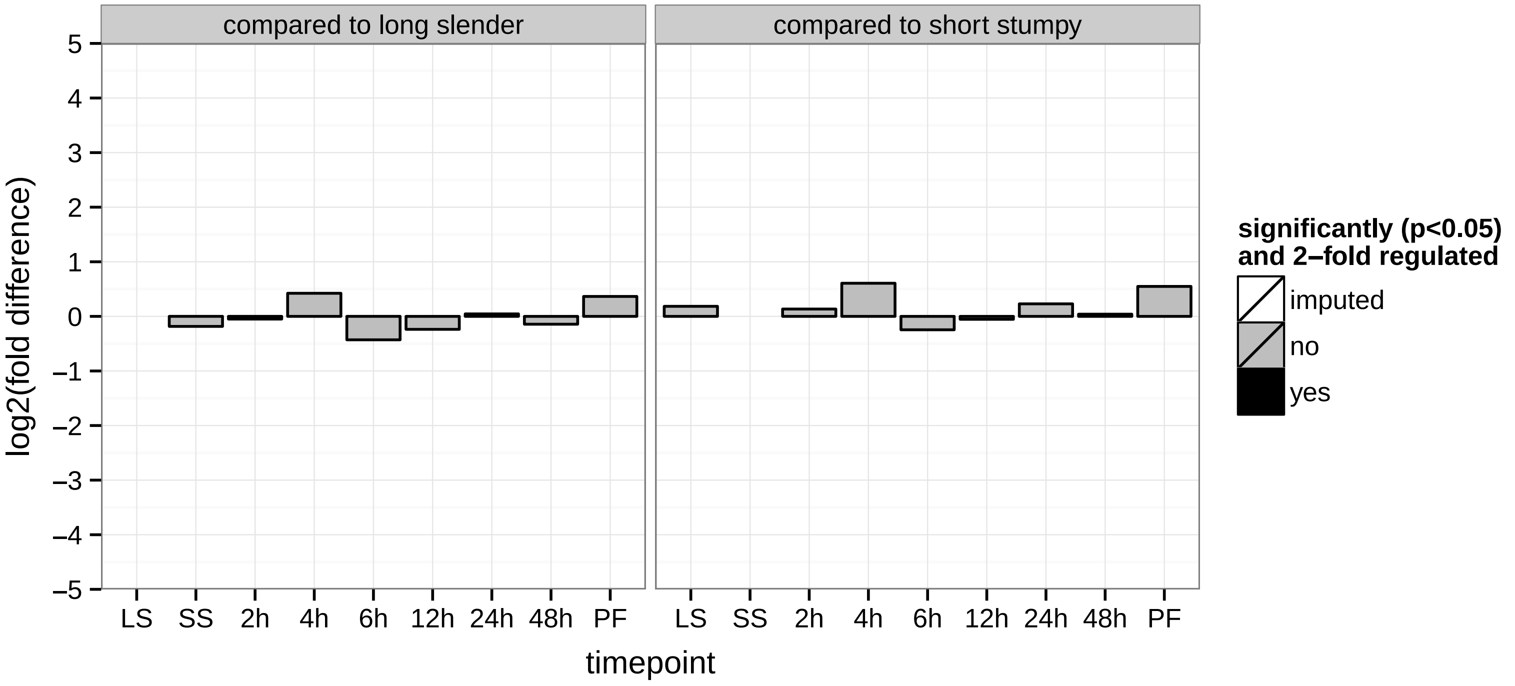

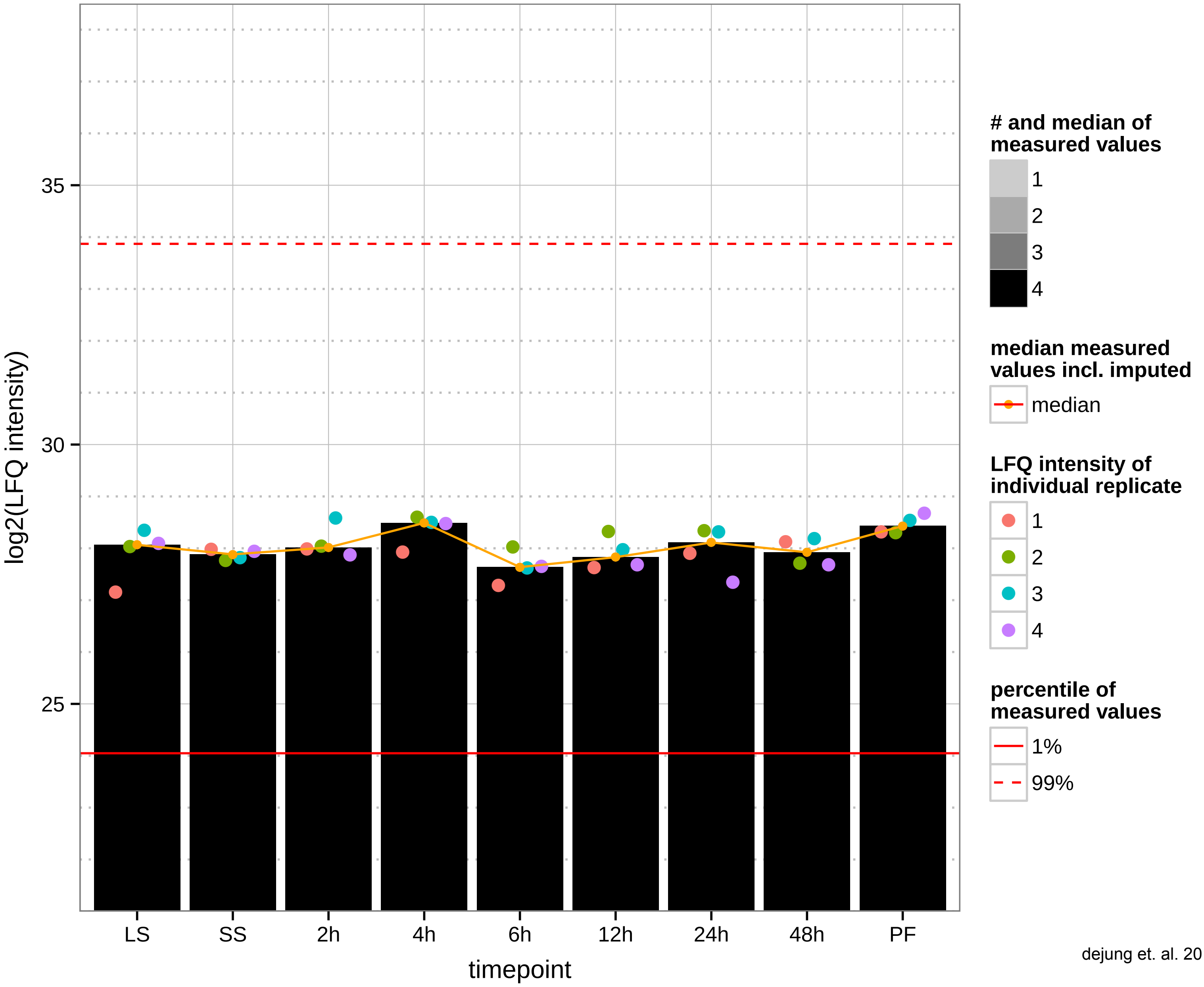

dejung et. al. 2015

hypothetical protein, conserved
Tb927.9.1470
AGOF: null
AGOC: null
AGOP: null
PGOF: null
PGOC: null
PGOP: null

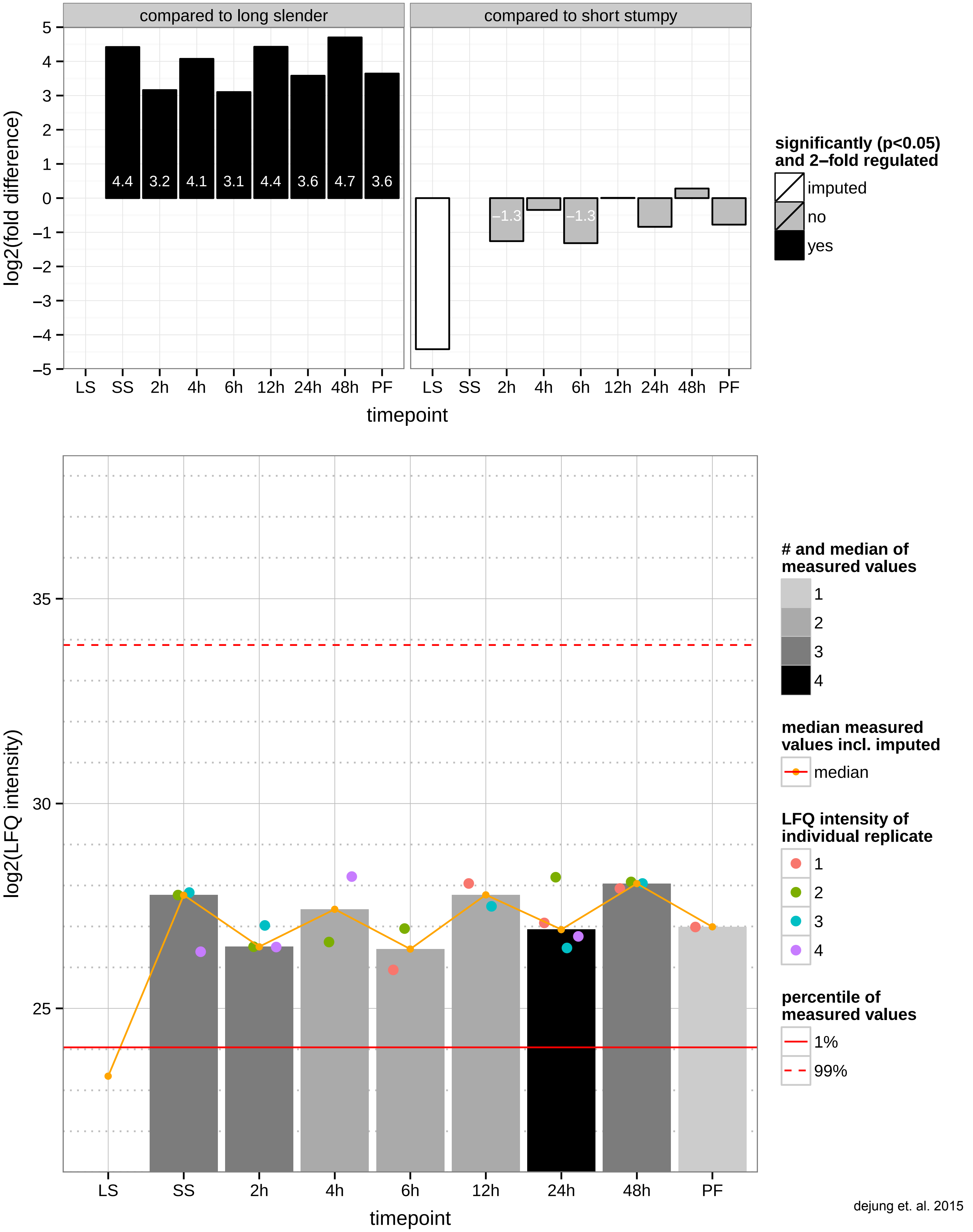

dejung et. al. 2015

DNA polymerase epsilon subunit b, putative
Tb927.9.15030
AGOF: DNA binding, DNA–directed DNA polymerase activity
AGOC: nucleus
AGOP: DNA–dependent DNA replication
PGOF: DNA binding, DNA–directed DNA polymerase activity
PGOC: nucleus
PGOP: DNA replication, DNA–dependent DNA replication

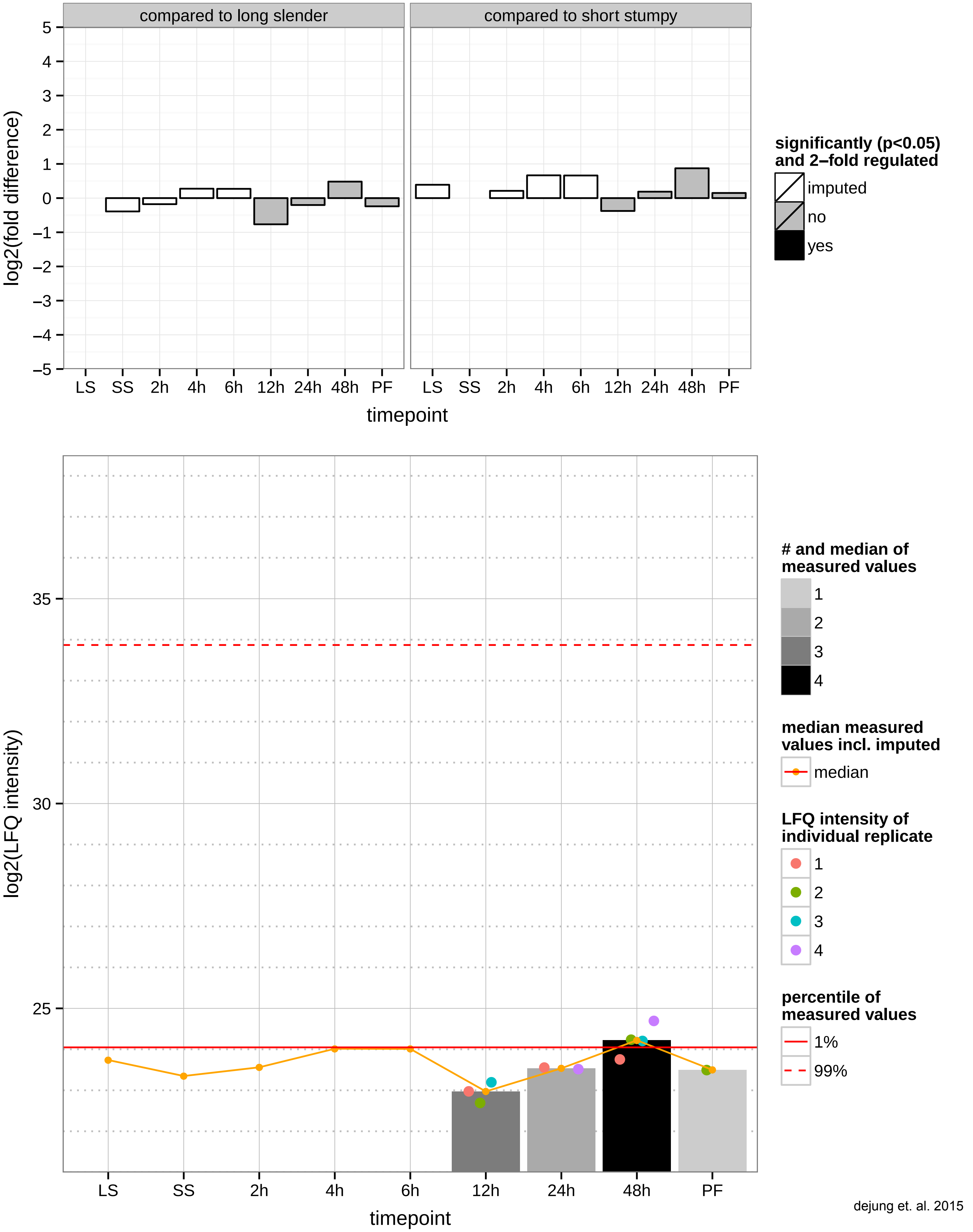

dejung et. al. 2015

trypanin–related protein, putative
Tb927.9.15050
AGOF: null
AGOC: null
AGOP: null
PGOF: null
PGOC: microtubule–based flagellum
PGOP: cell motility

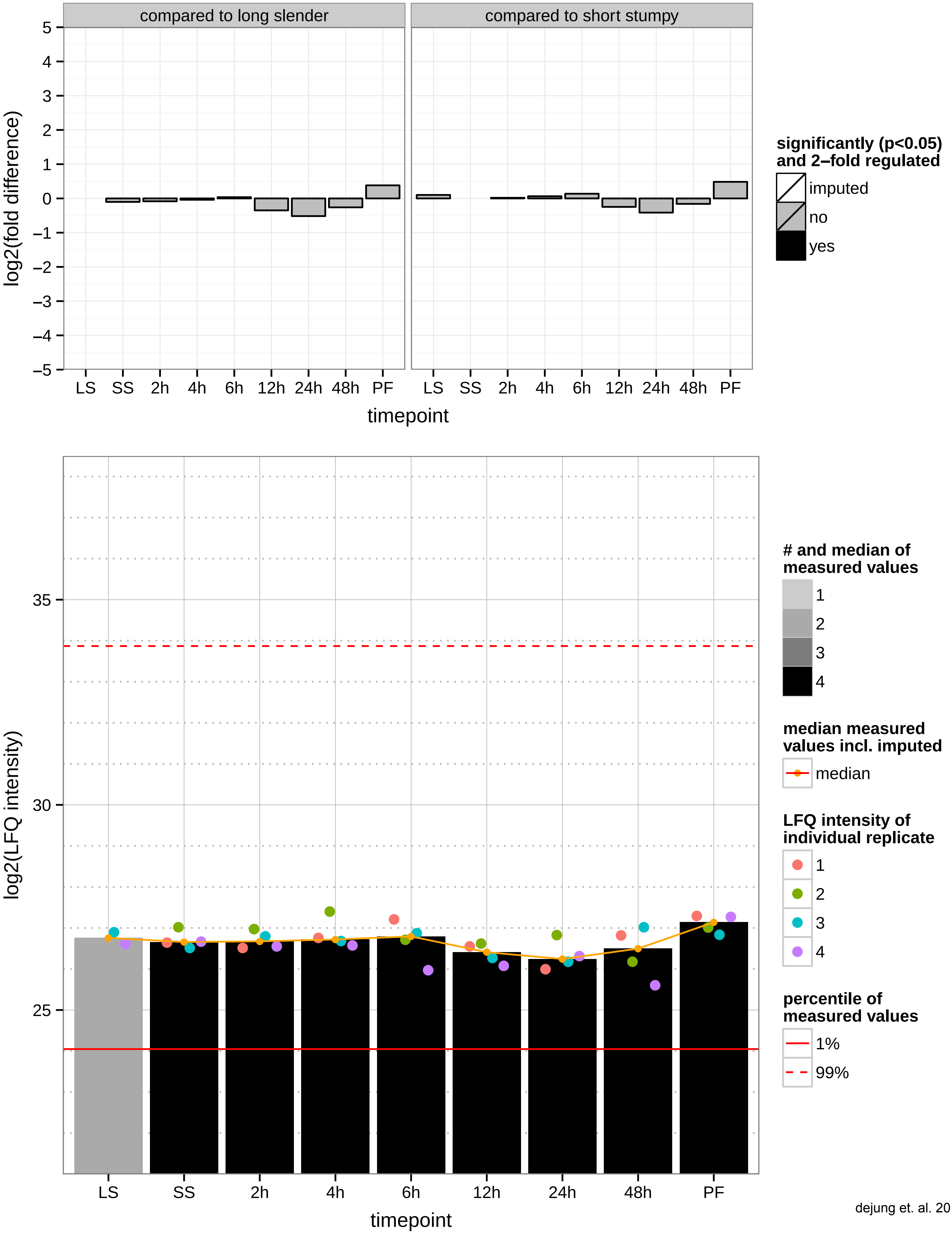

dejung et. al. 2015

rRNA processing protein, putative
Tb927.9.15060
AGOF: null
AGOC: nucleus
AGOP: ribosome biogenesis
PGOF: null
PGOC: null
PGOP: null

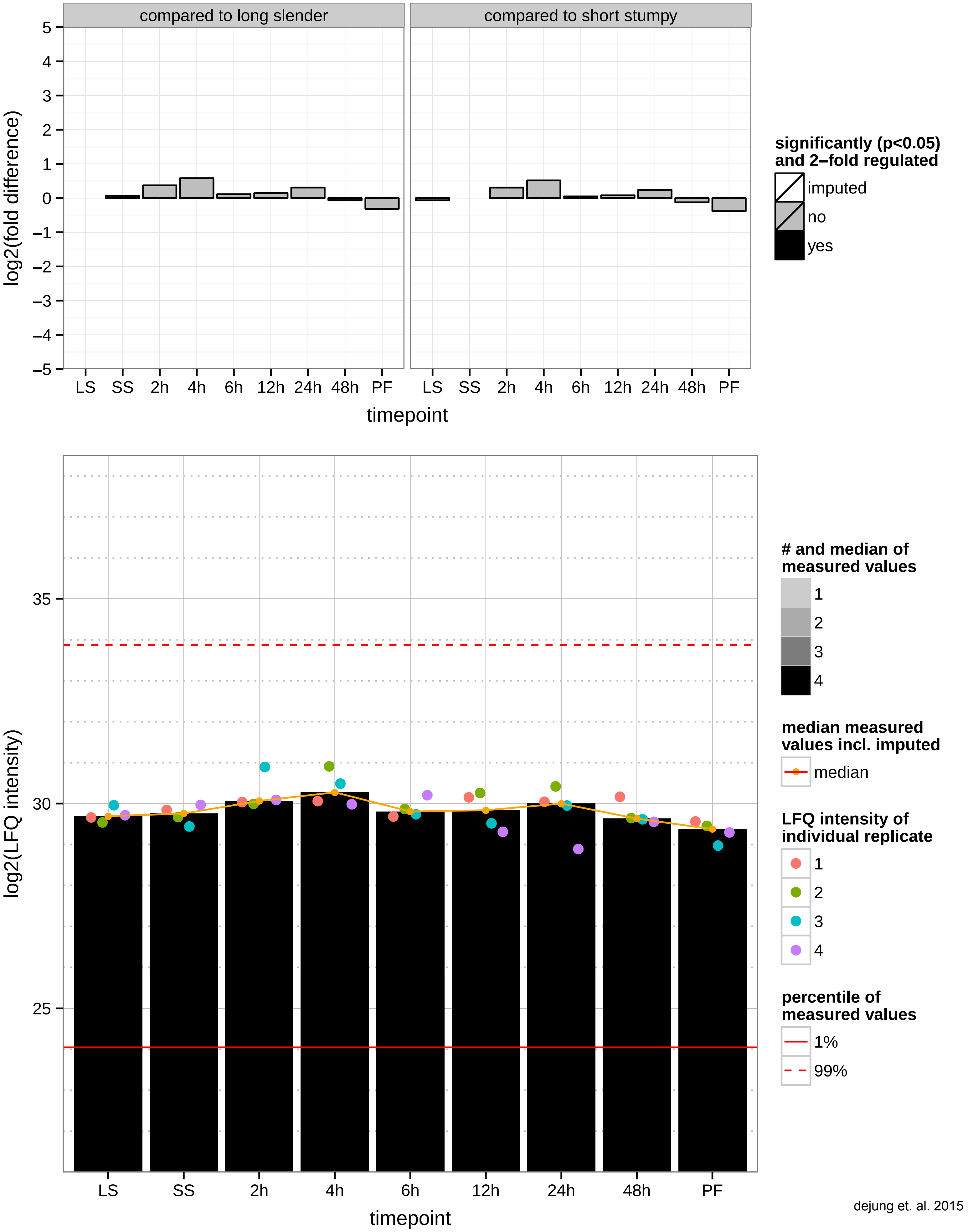

dejung et. al. 2015

hypothetical protein, conserved
Tb927.9.15070
AGOF: null
AGOC: null
AGOP: null
PGOF: null
PGOC: null
PGOP: null

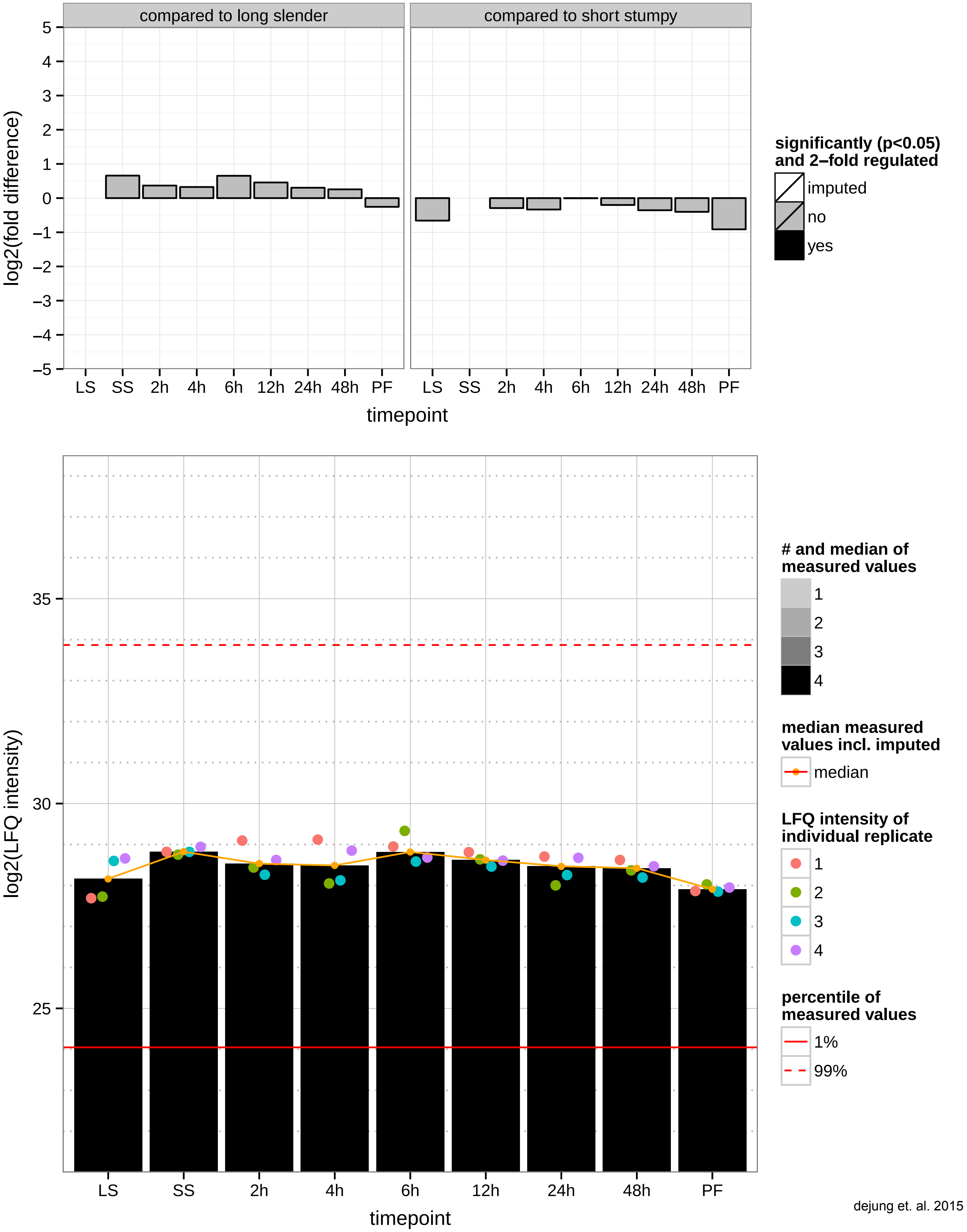

dejung et. al. 2015

cytosolic coat protein, putative
Tb927.9.15090
AGOF: protein transmembrane transporter activity
AGOC: ER to Golgi transport vesicle membrane, integral to membrane
AGOP: transport
PGOF: null
PGOC: integral to membrane
PGOP: transport

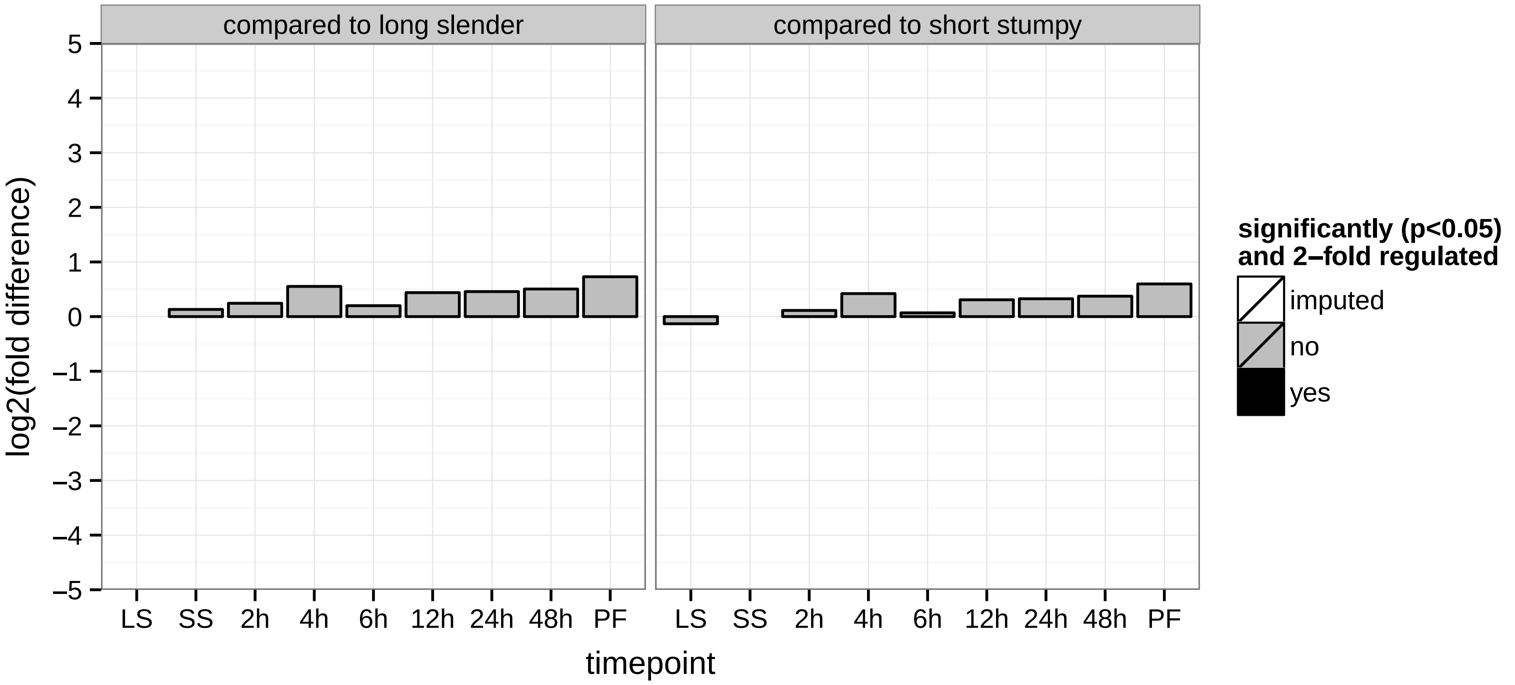

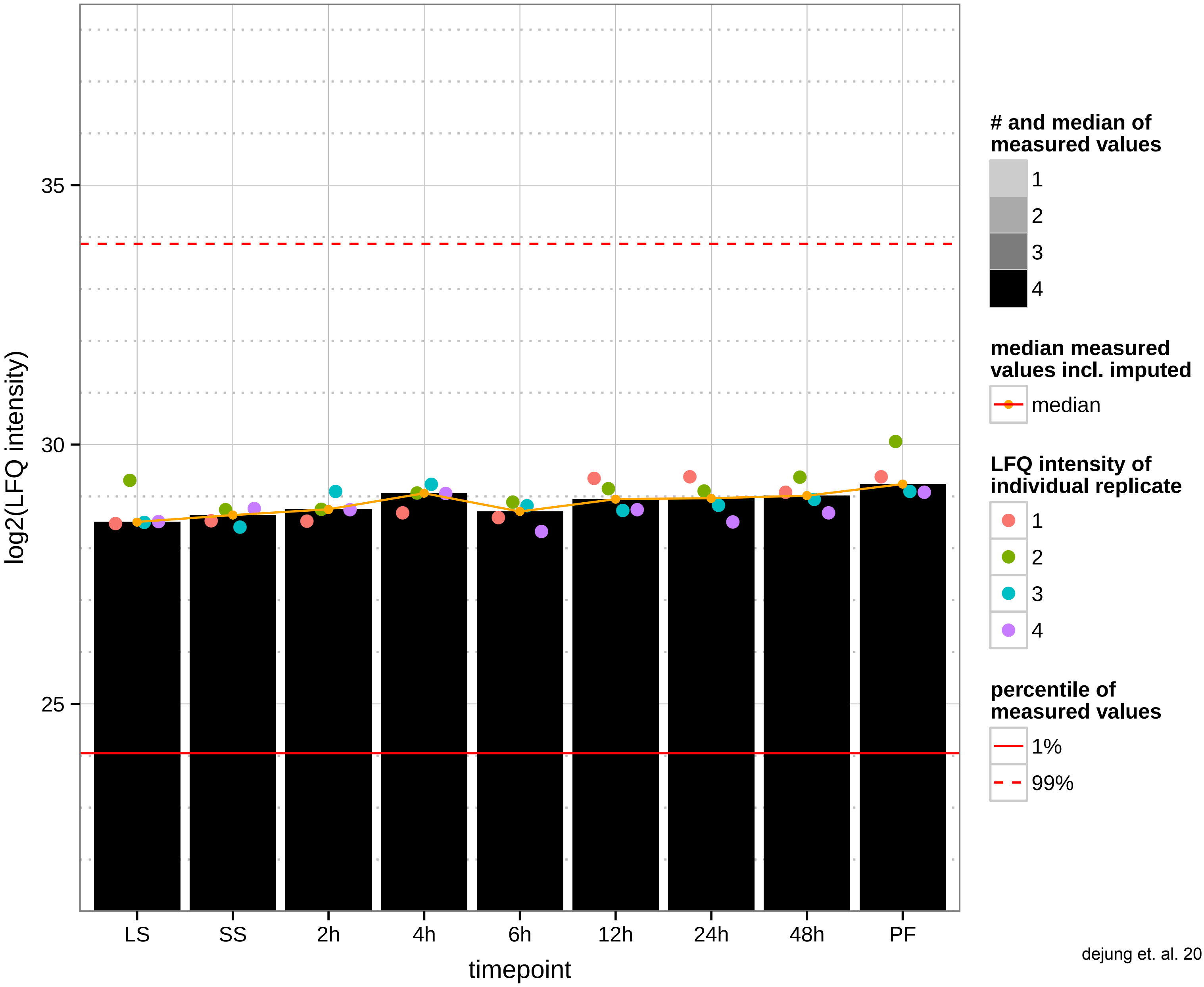

dejung et. al. 2015

hypothetical protein, conserved
Tb927.9.1520
AGOF: null
AGOC: null
AGOP: null
PGOF: null
PGOC: null
PGOP: null

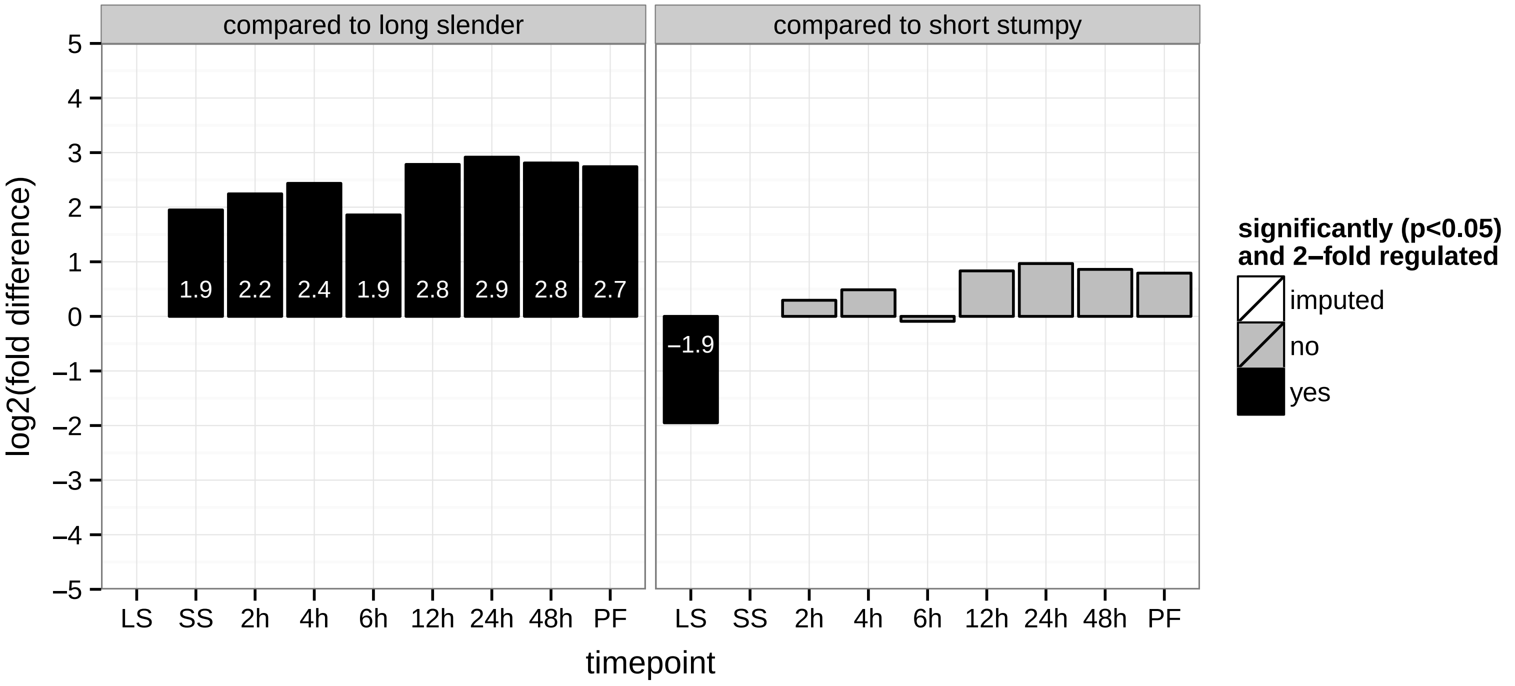

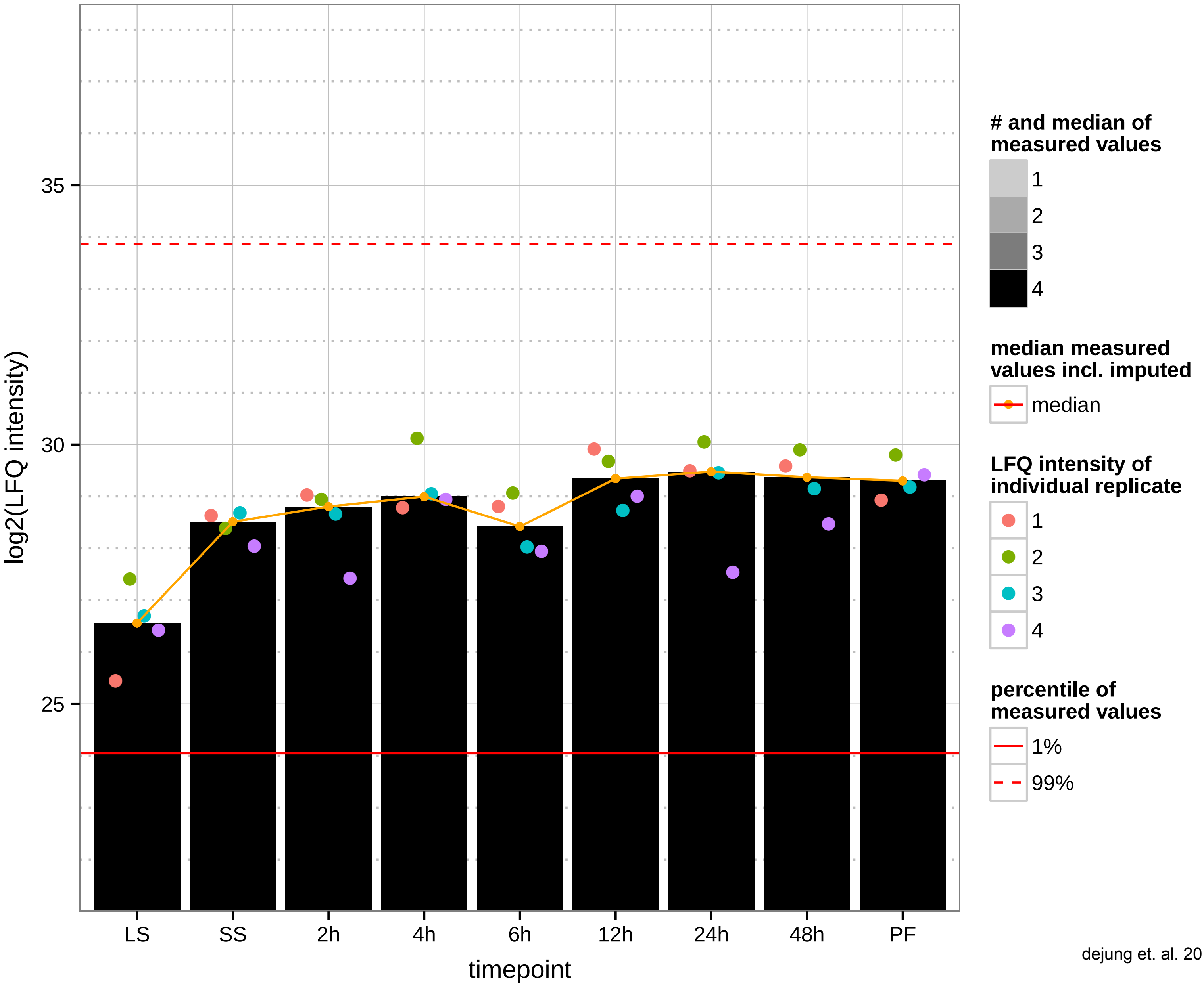

dejung et. al. 2015

hypothetical protein, conserved
Tb927.9.1530
AGOF: null
AGOC: mitochondrion
AGOP: null
PGOF: null
PGOC: null
PGOP: null

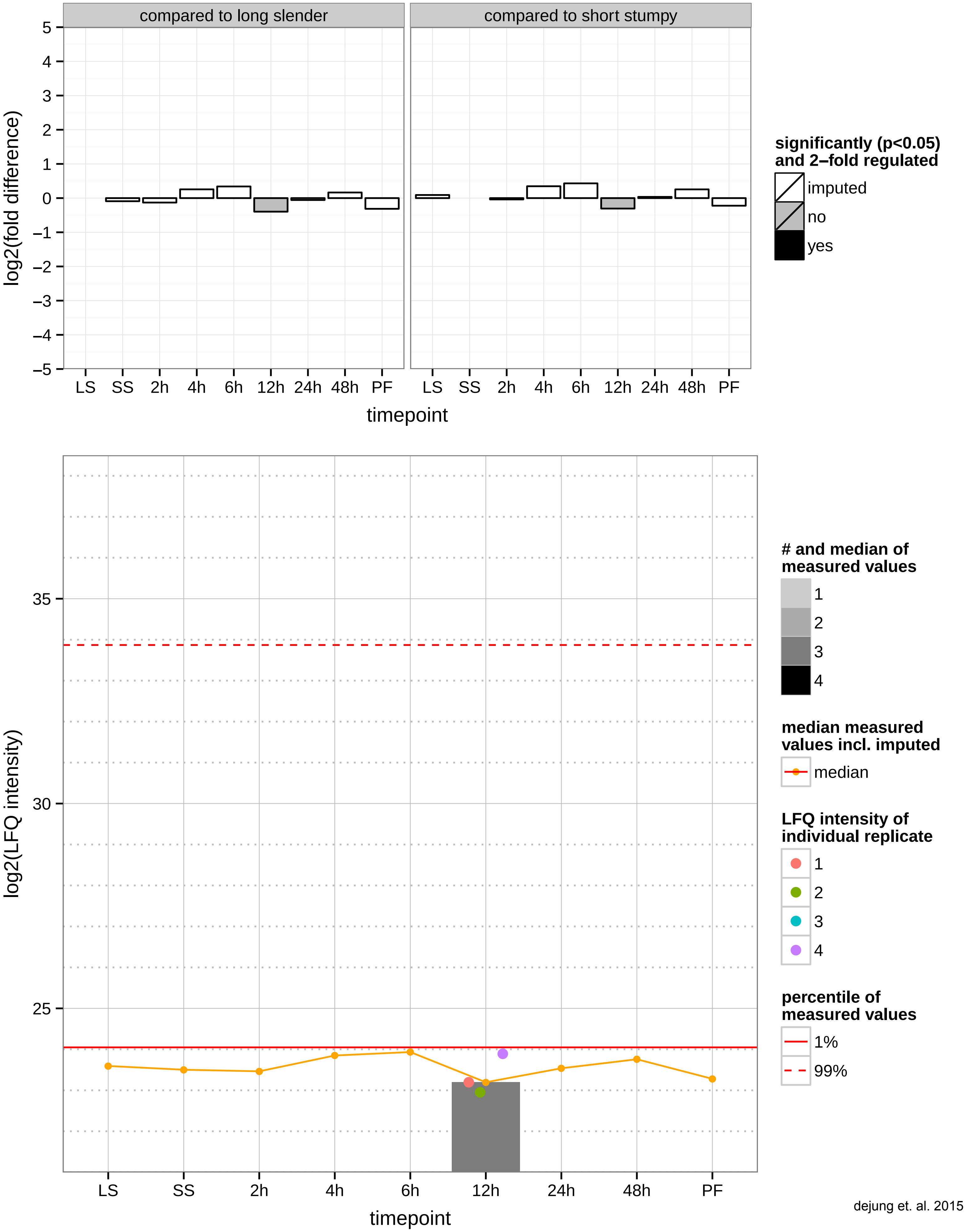

dejung et. al. 2015

40S ribosomal protein S6, putative
Tb927.9.15360
AGOF: null
AGOC: intracellular, ribosome
AGOP: regulation of cell growth, regulation of cell proliferation, translation
PGOF: structural constituent of ribosome
PGOC: intracellular, ribosome
PGOP: translation

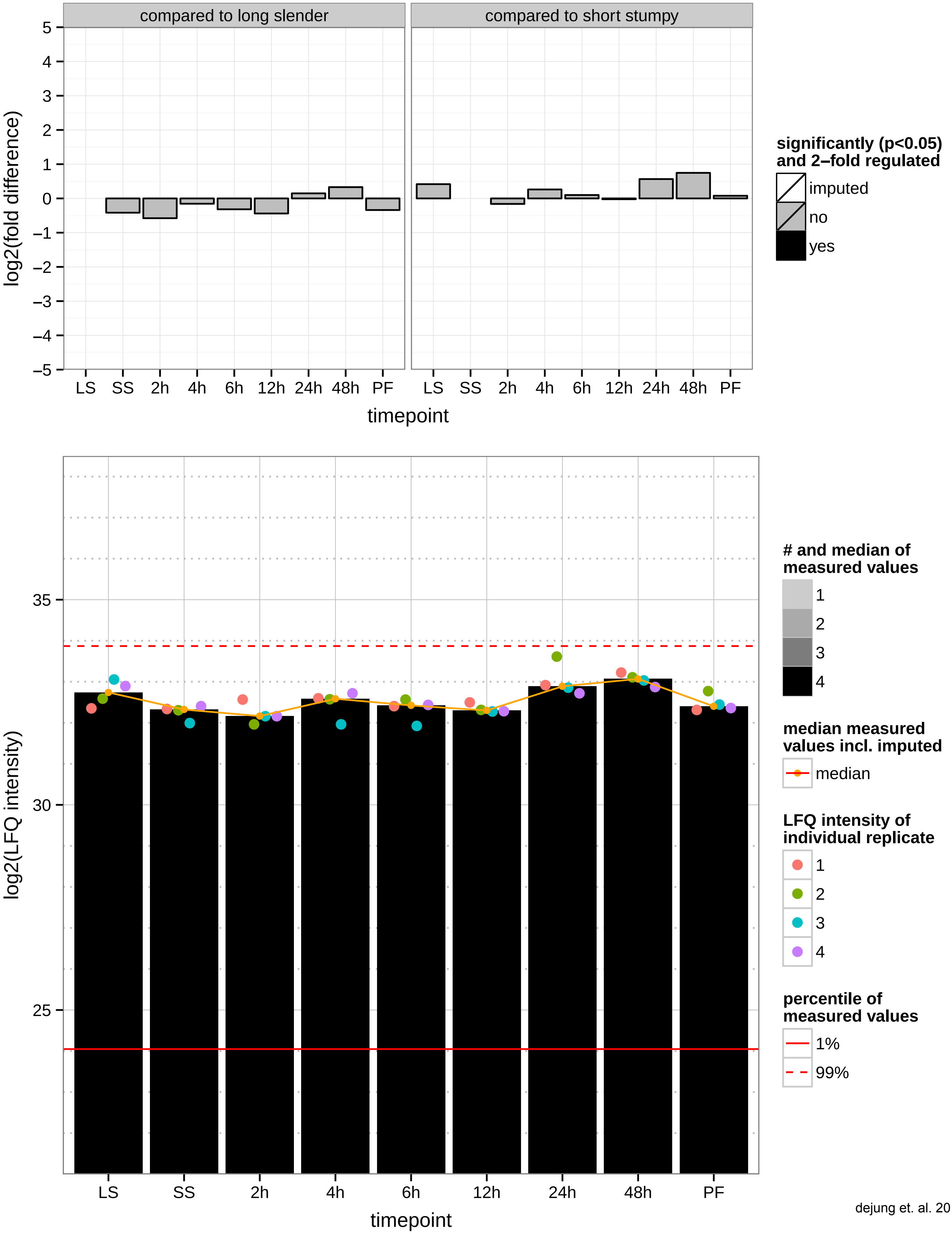

dejung et. al. 2015

kinesin, putative
Tb927.9.15470
AGOF: null, ATP binding, microtubule motor activity, motor activity
AGOC: null, microtubule associated complex
AGOP: null, microtubule–based movement
PGOF: ATP binding, microtubule motor activity
PGOC: null
PGOP: microtubule–based movement

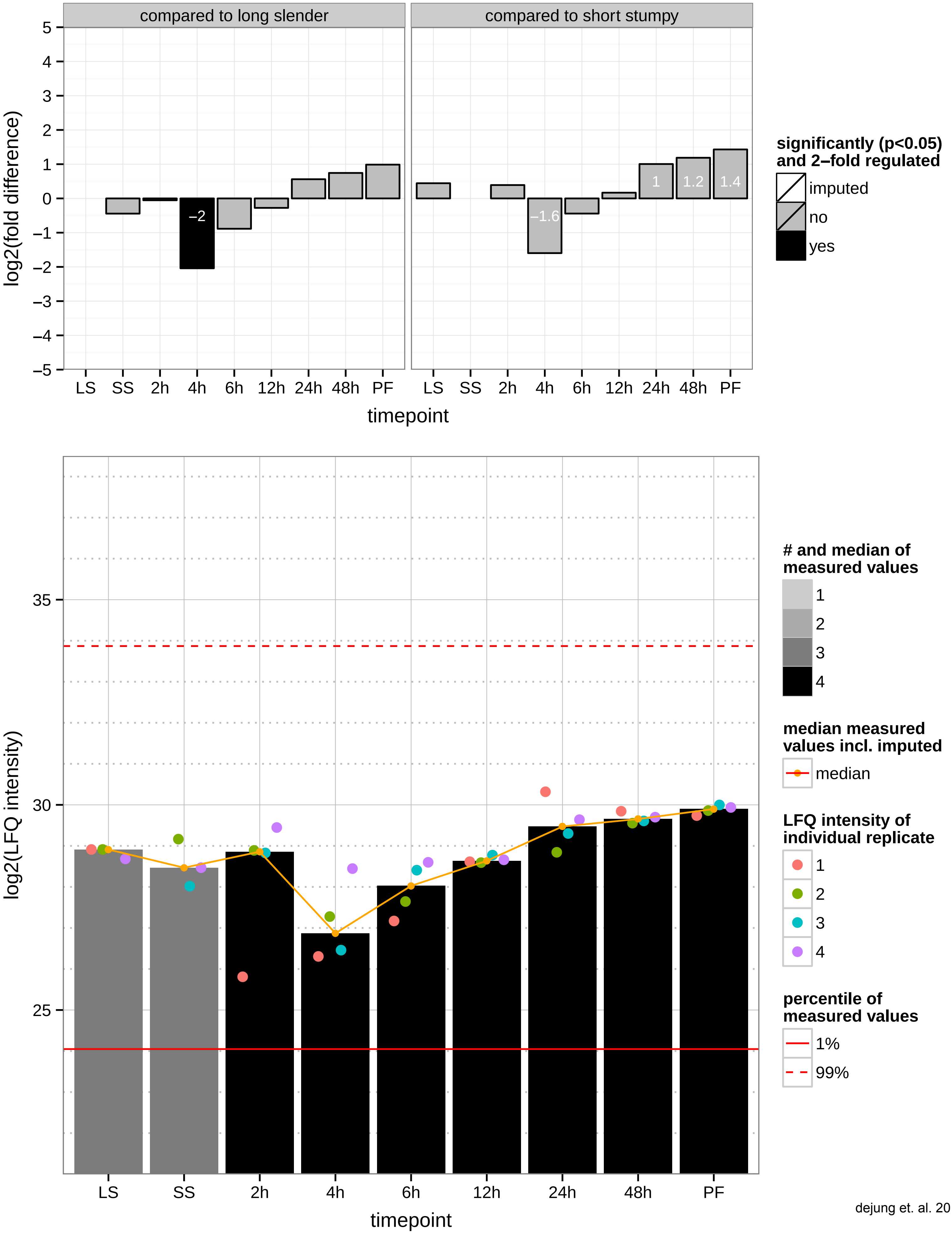

dejung et. al. 2015

hypothetical protein, conserved
Tb927.9.1560
AGOF: heat shock protein binding
AGOC: null
AGOP: null
PGOF: heat shock protein binding
PGOC: null
PGOP: null

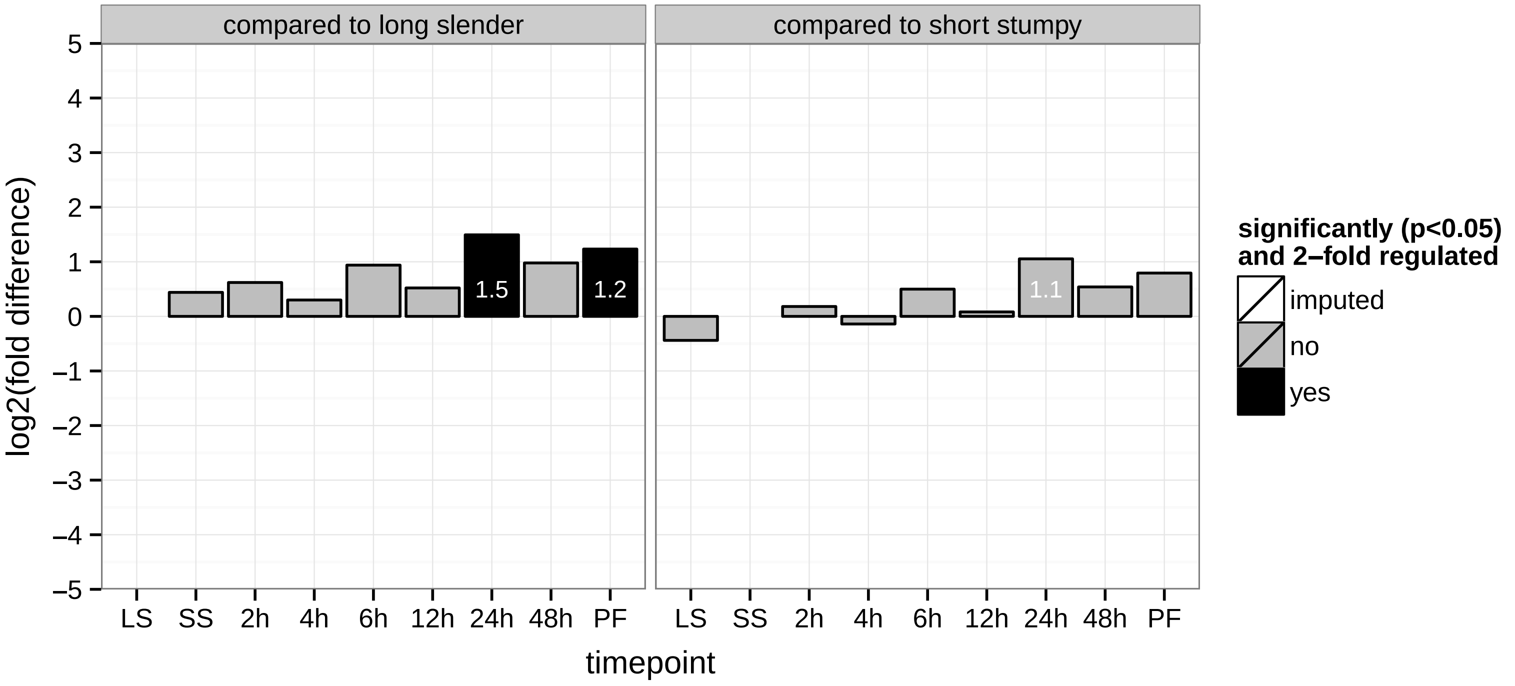

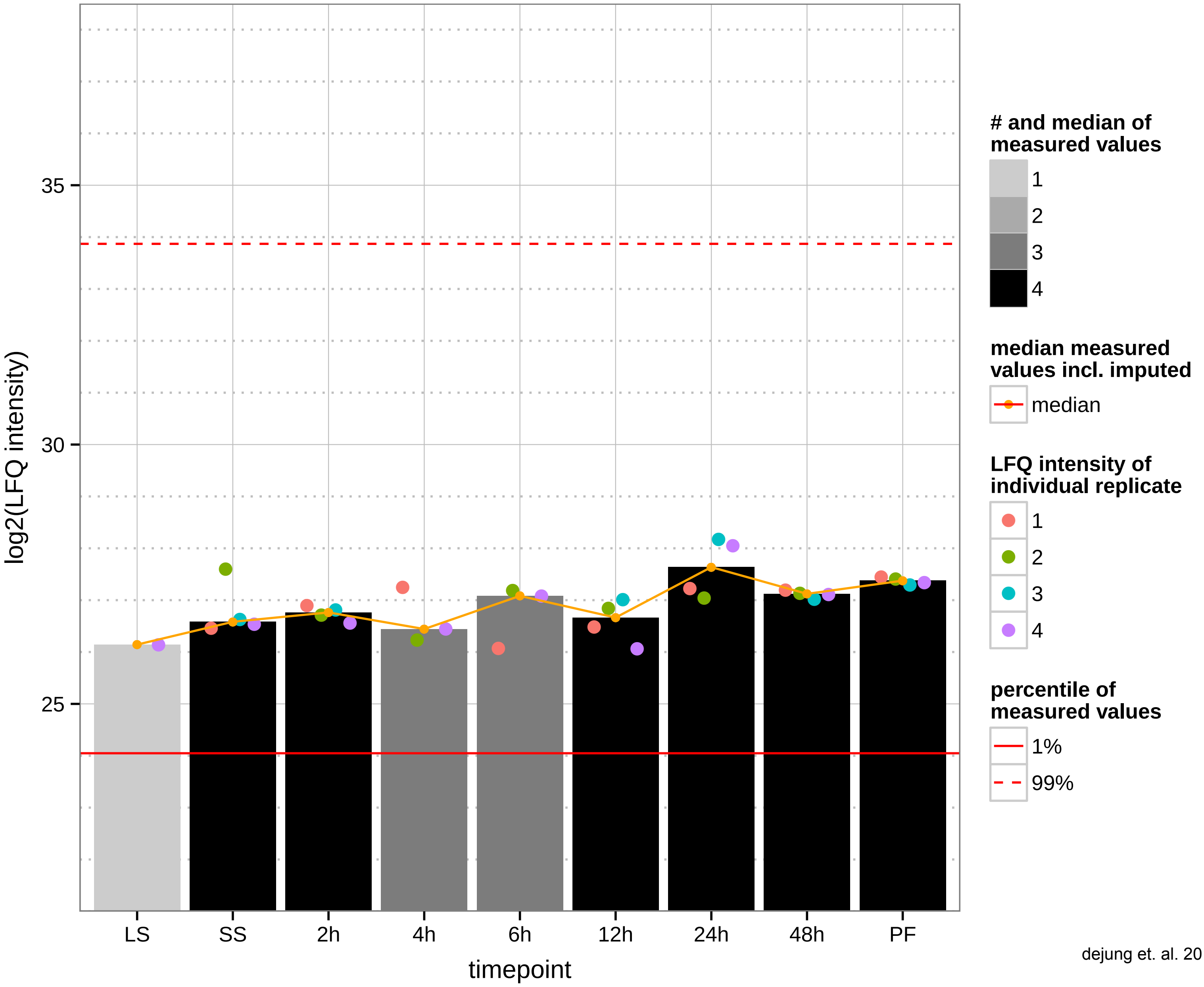

dejung et. al. 2015

hypothetical protein, conserved
Tb927.9.1660
AGOF: null
AGOC: null
AGOP: null
PGOF: null
PGOC: null
PGOP: null

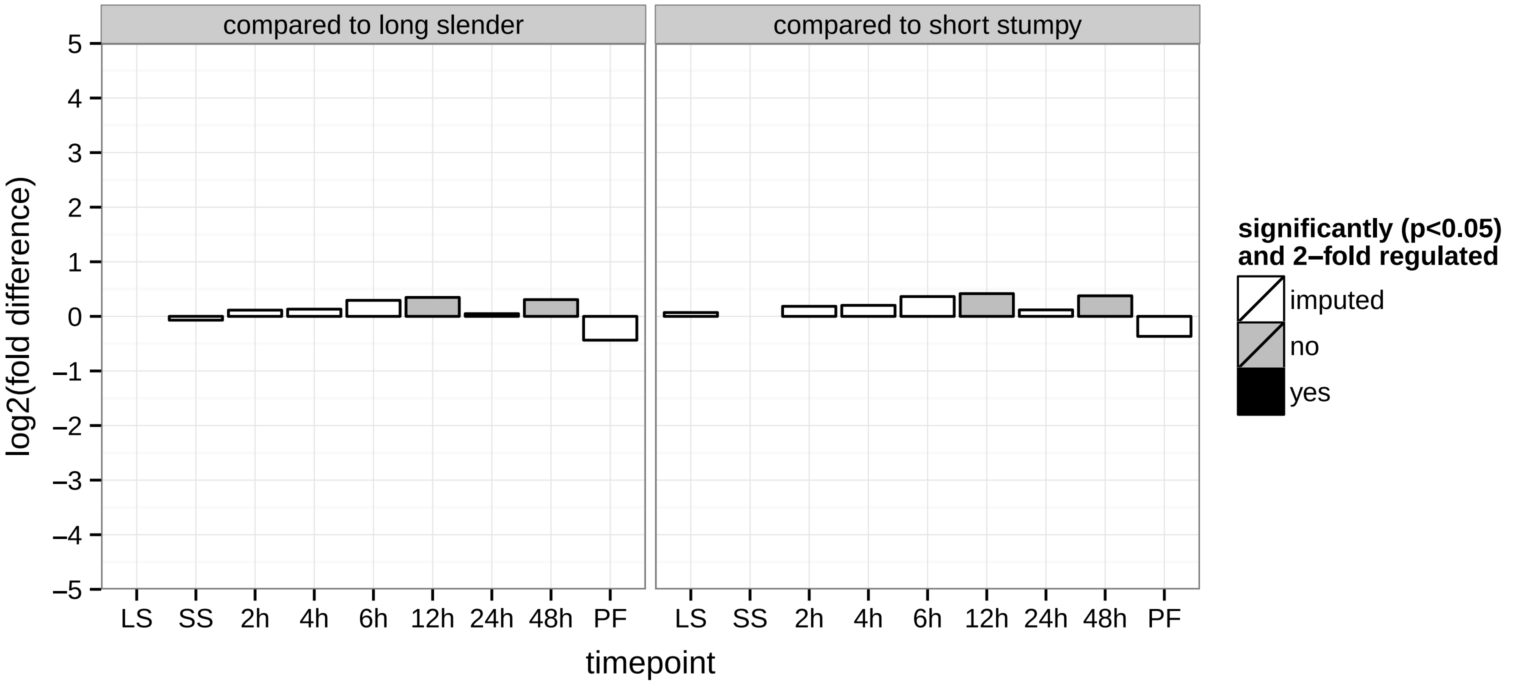

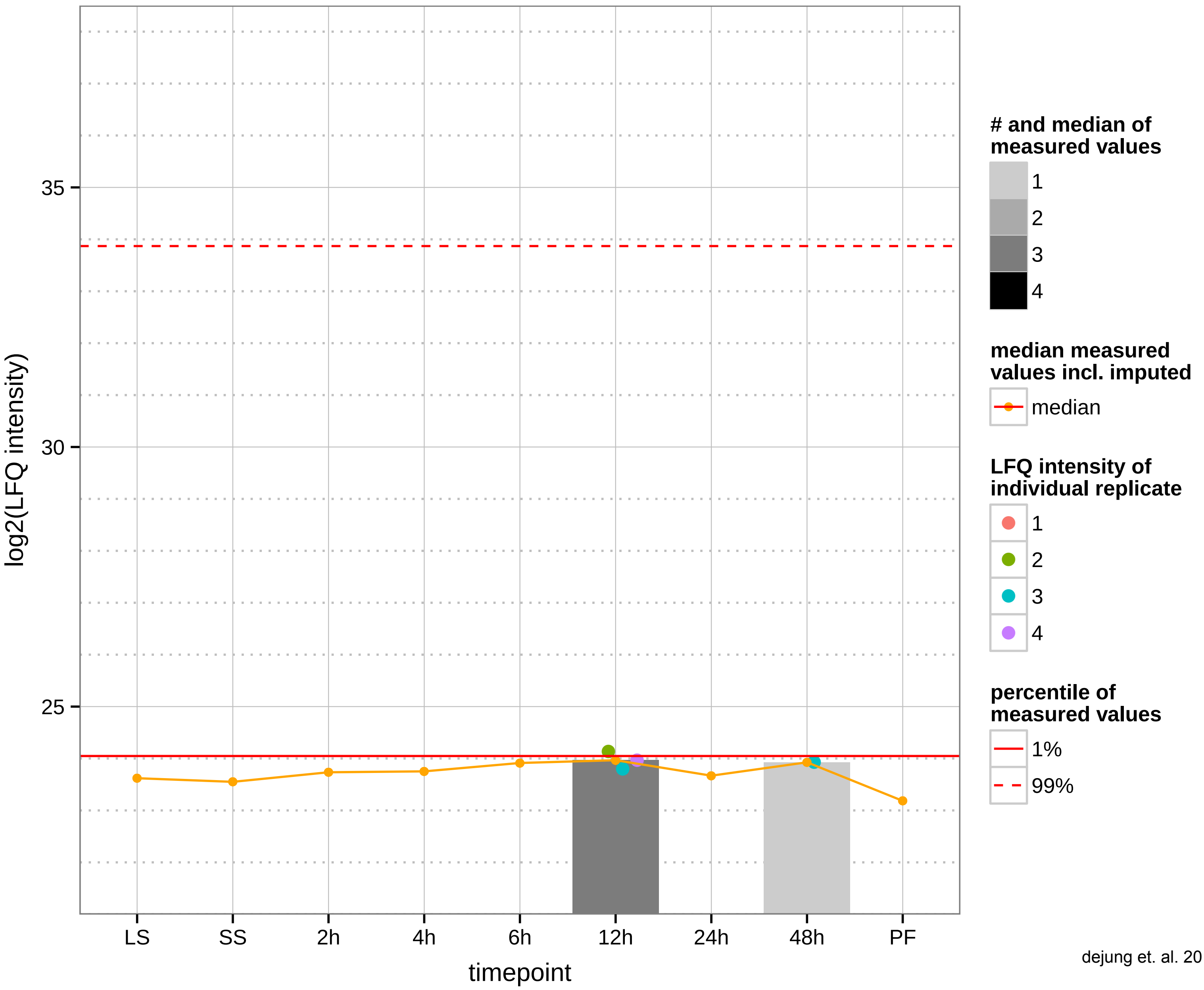

dejung et. al. 2015

hypothetical protein, conserved
Tb927.9.1700
AGOF: null
AGOC: null
AGOP: null
PGOF: protein binding
PGOC: null
PGOP: null

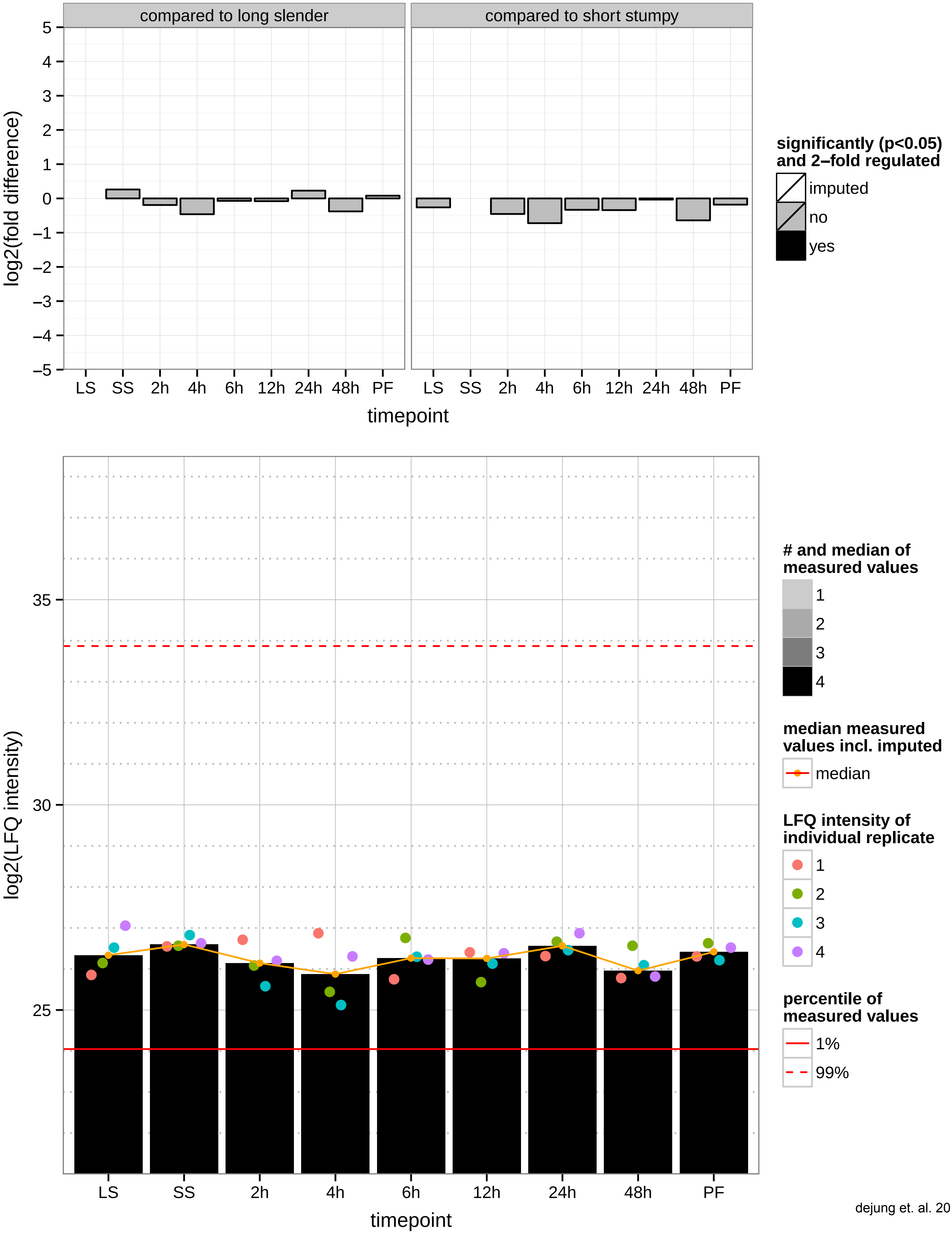

dejung et. al. 2015

peroxisomal membrane protein 4, putative
Tb927.9.1720
AGOF: null
AGOC: null
AGOP: null
PGOF: null
PGOC: null
PGOP: null

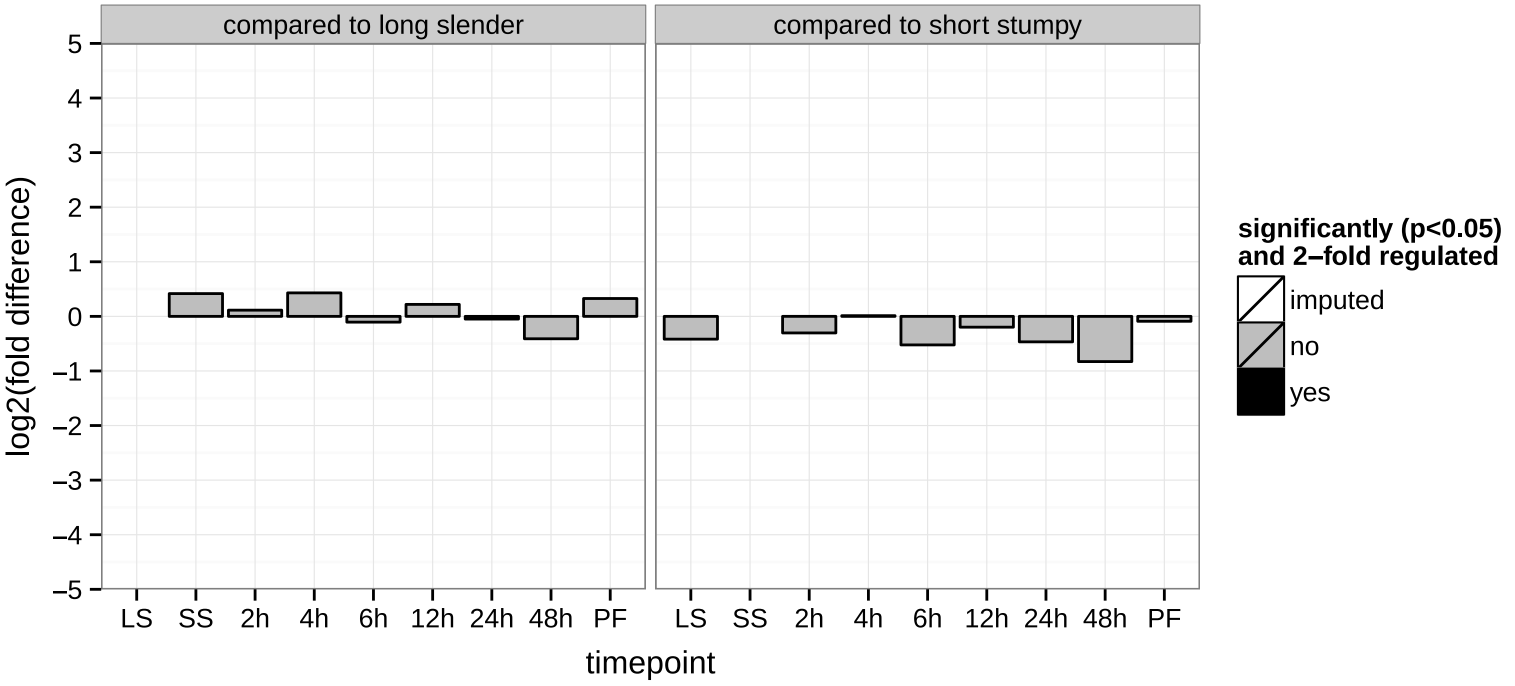

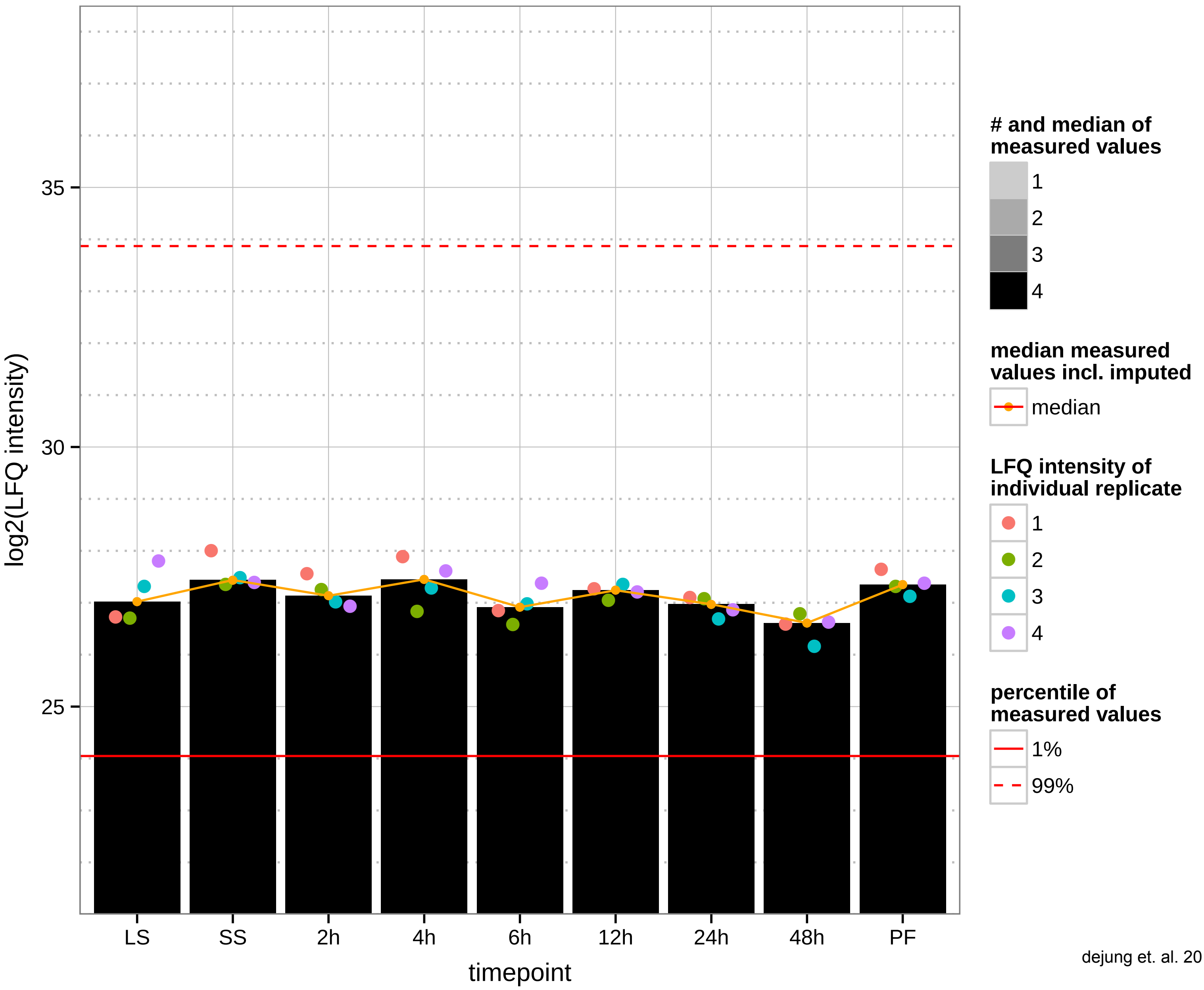

dejung et. al. 2015

hypothetical protein, conserved
Tb927.9.1750
AGOF: null
AGOC: null
AGOP: null
PGOF: protein binding
PGOC: null
PGOP: null

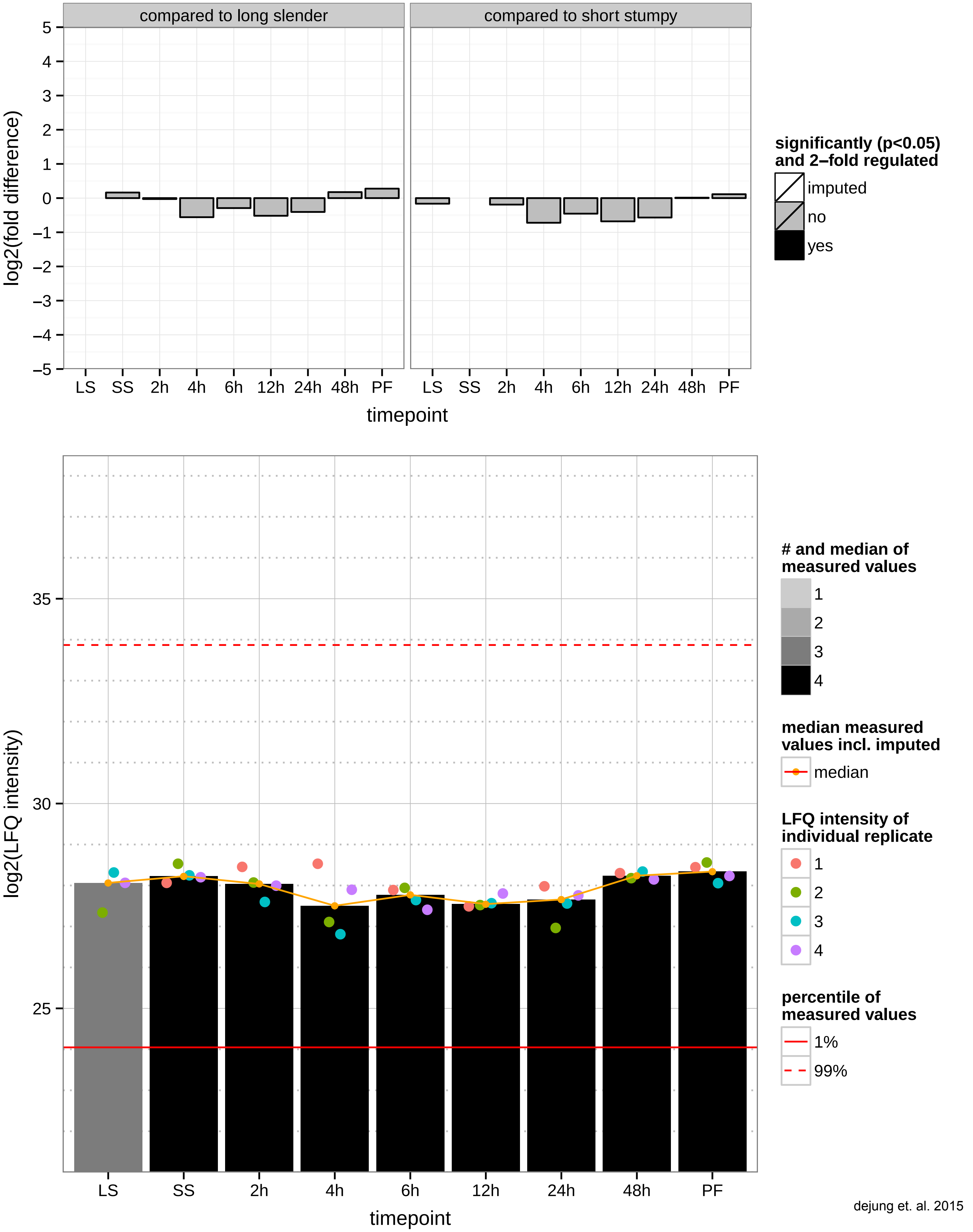

dejung et. al. 2015

hypothetical protein, conserved
Tb927.9.1770
AGOF: acid–amino acid ligase activity, zinc ion binding
AGOC: intracellular
AGOP: cellular protein modification process
PGOF: acid–amino acid ligase activity, protein binding, zinc ion binding
PGOC: intracellular
PGOP: cellular protein modification process

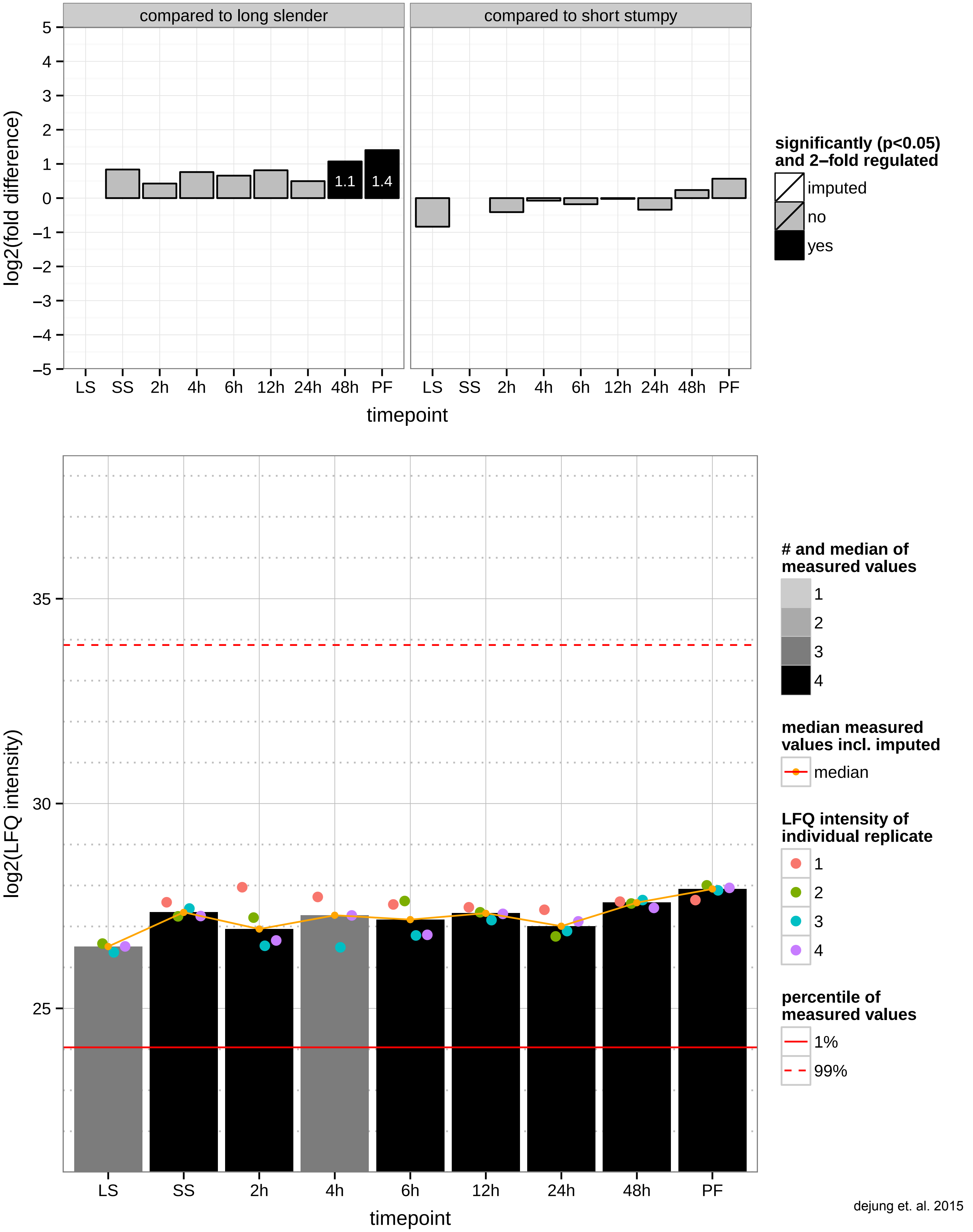

dejung et. al. 2015

sec1 family transport protein, putative (SLY1)
Tb927.9.1780
AGOF: protein transporter activity
AGOC: mitochondrion
AGOP: protein secretion, vesicle docking involved in exocytosis, vesicle–mediated transport
PGOF: null
PGOC: null
PGOP: vesicle docking involved in exocytosis, vesicle–mediated transport

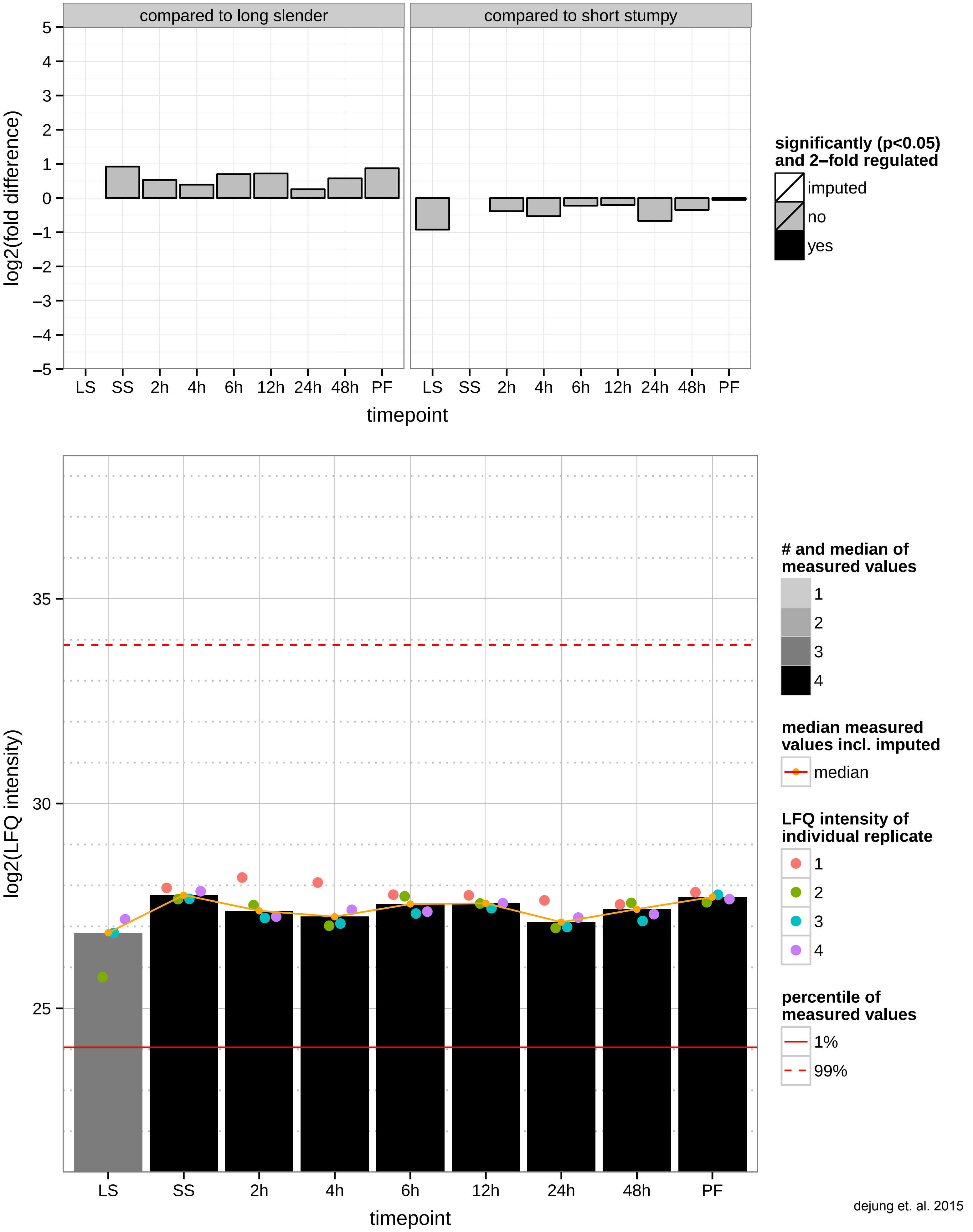

dejung et. al. 2015

hypothetical protein, conserved
Tb927.9.1880
AGOF: null
AGOC: null
AGOP: null
PGOF: binding, protein binding
PGOC: null
PGOP: null

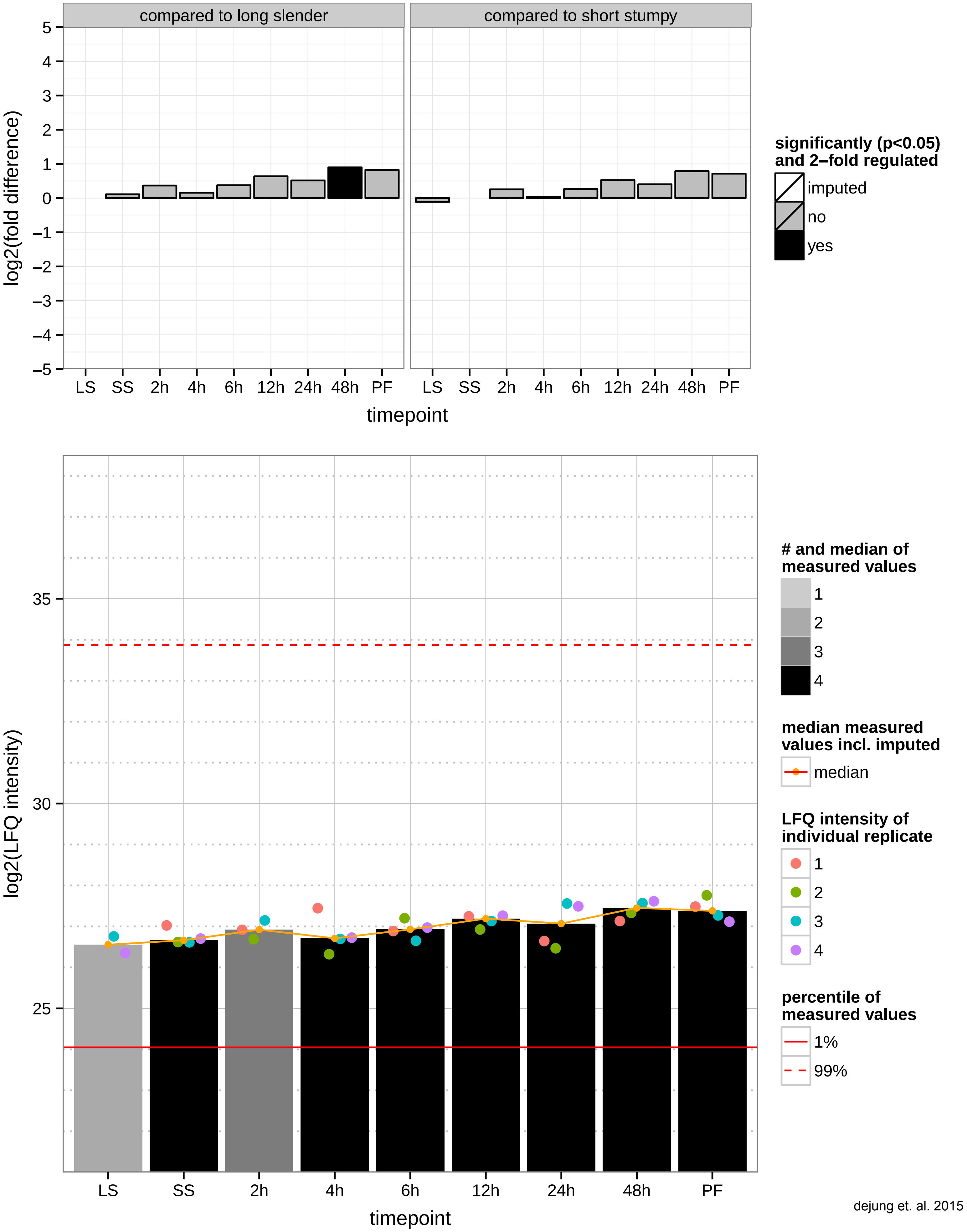

dejung et. al. 2015

hypothetical protein, conserved
Tb927.9.1950
AGOF: null
AGOC: mitochondrion
AGOP: null
PGOF: null
PGOC: null
PGOP: null

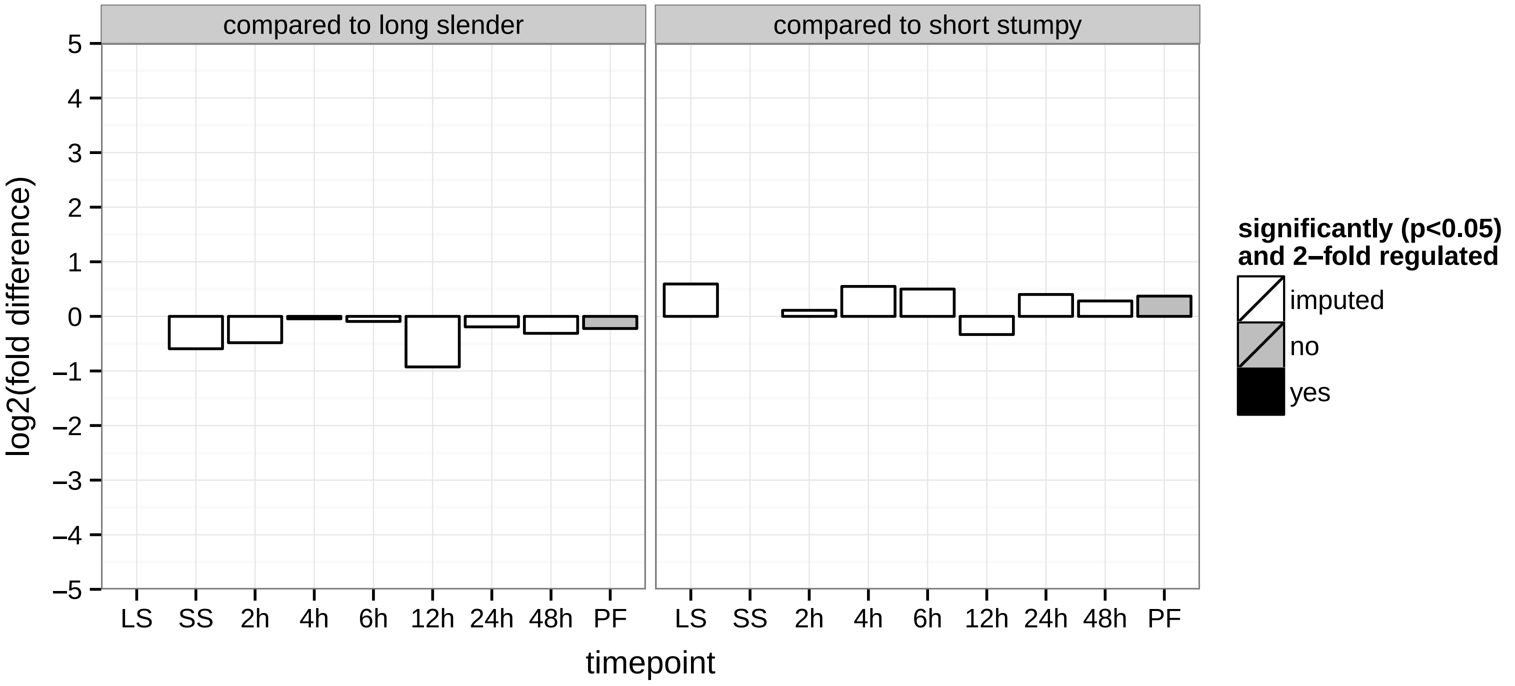

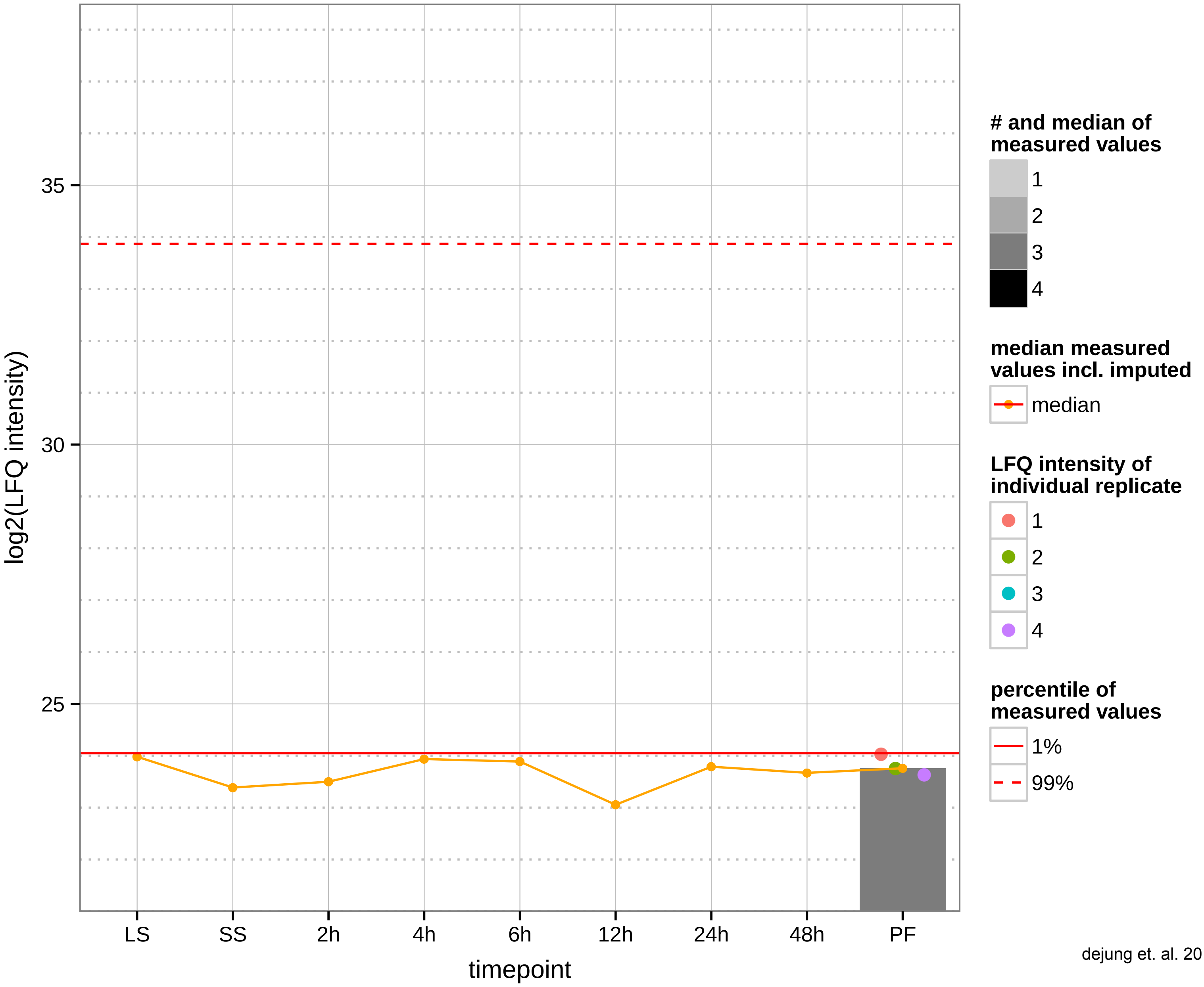

dejung et. al. 2015

hypothetical protein, conserved
Tb927.9.1980
AGOF: null
AGOC: null
AGOP: null
PGOF: null
PGOC: null
PGOP: null

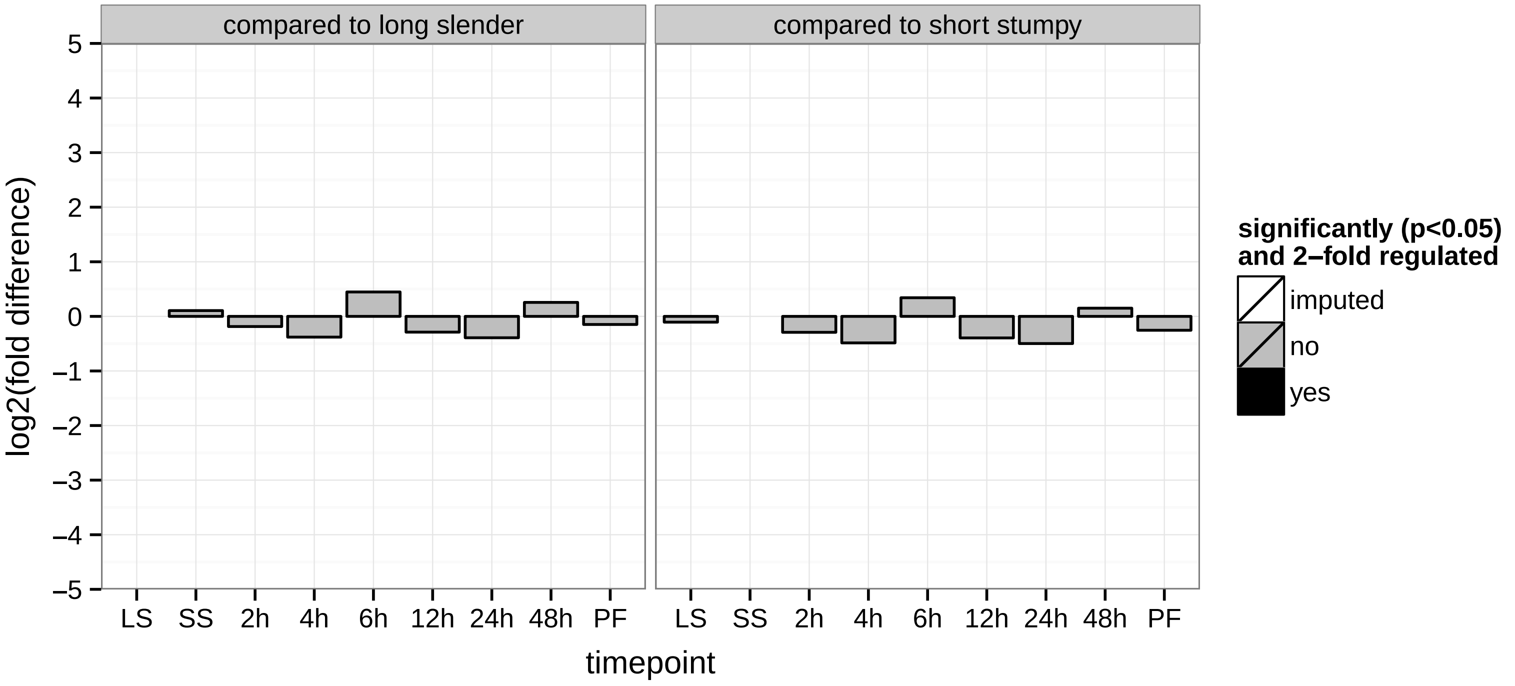

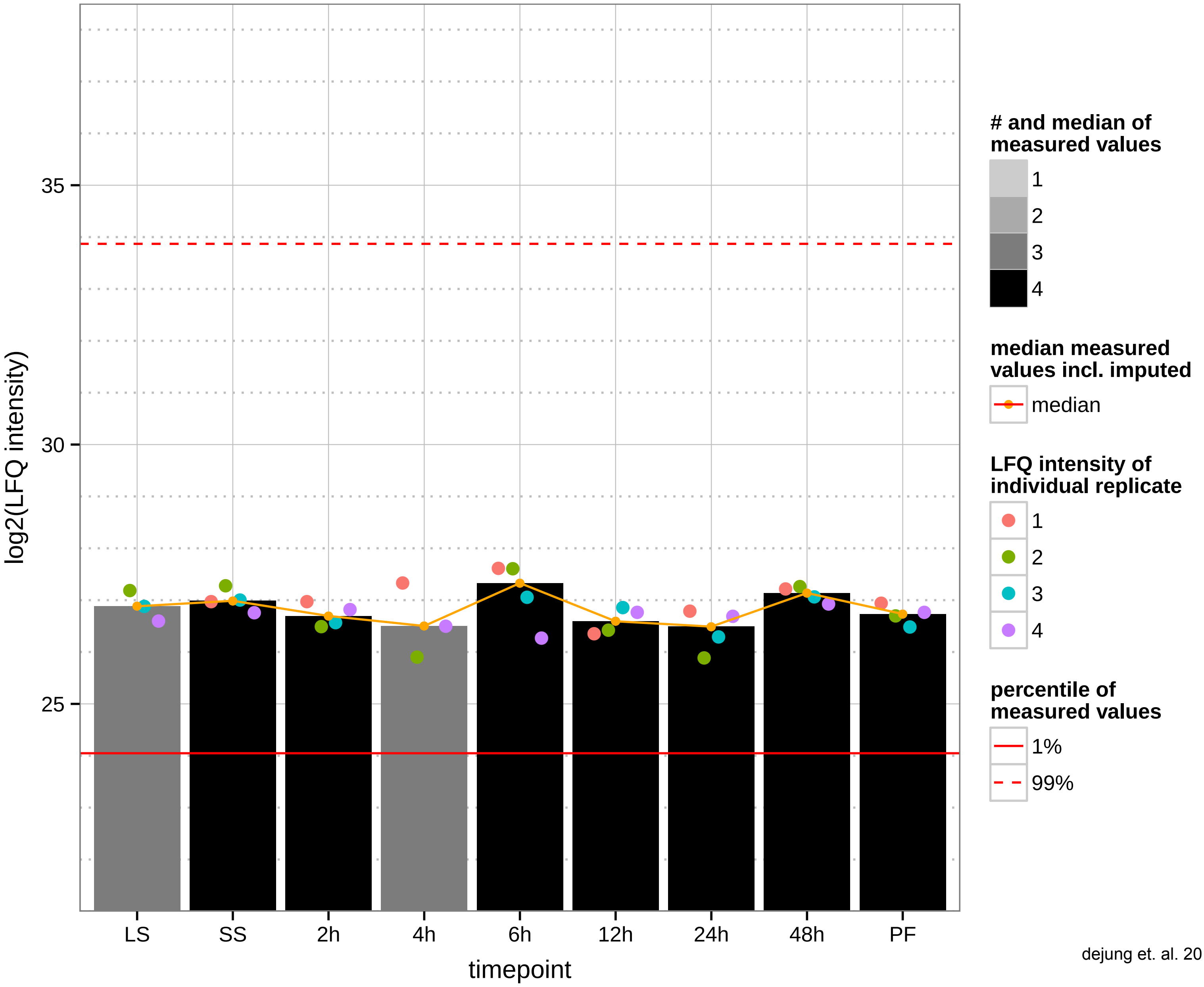

dejung et. al. 2015

kynureninase, putative
Tb927.9.2010
AGOF: kynureninase activity, pyridoxal phosphate binding
AGOC: cytoplasm
AGOP: NAD biosynthetic process, tryptophan catabolic process
PGOF: kynureninase activity, pyridoxal phosphate binding
PGOC: cytoplasm
PGOP: NAD biosynthetic process, metabolic process, tryptophan catabolic process

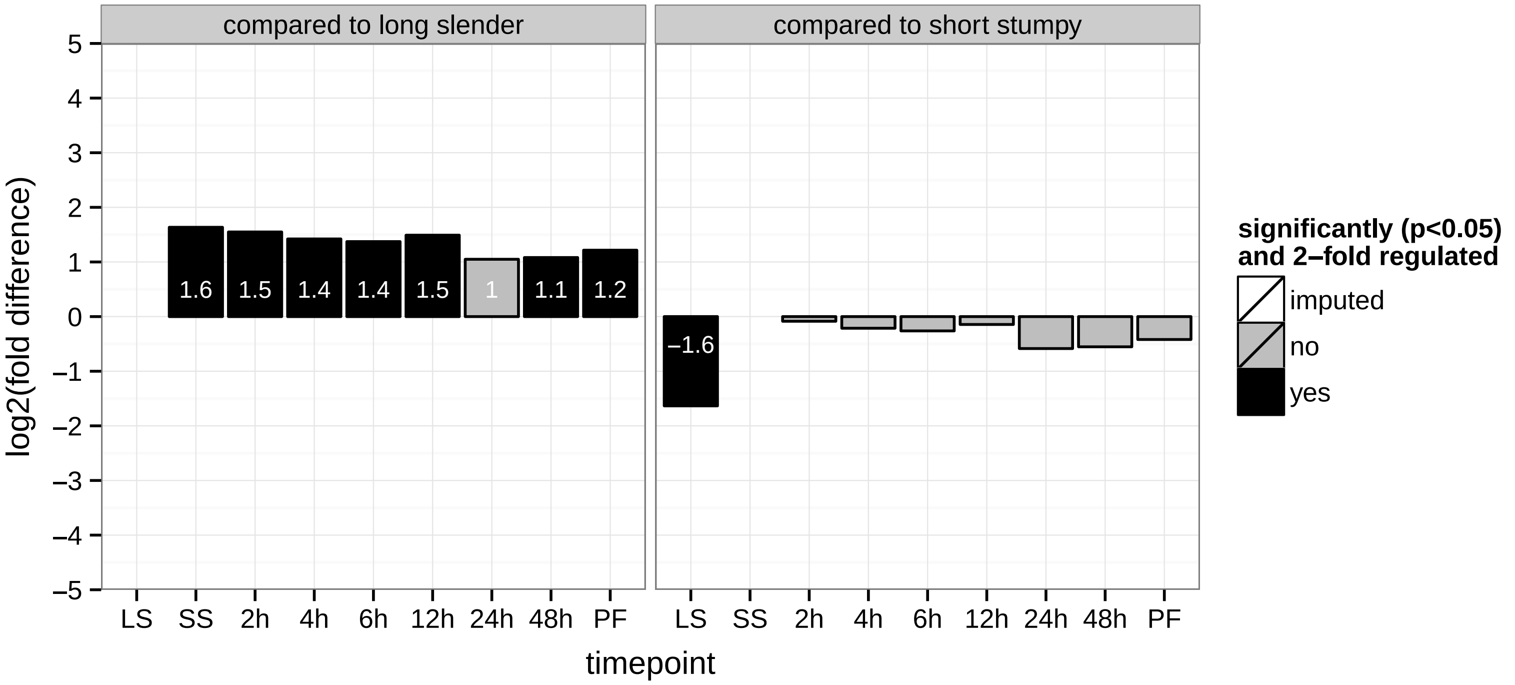

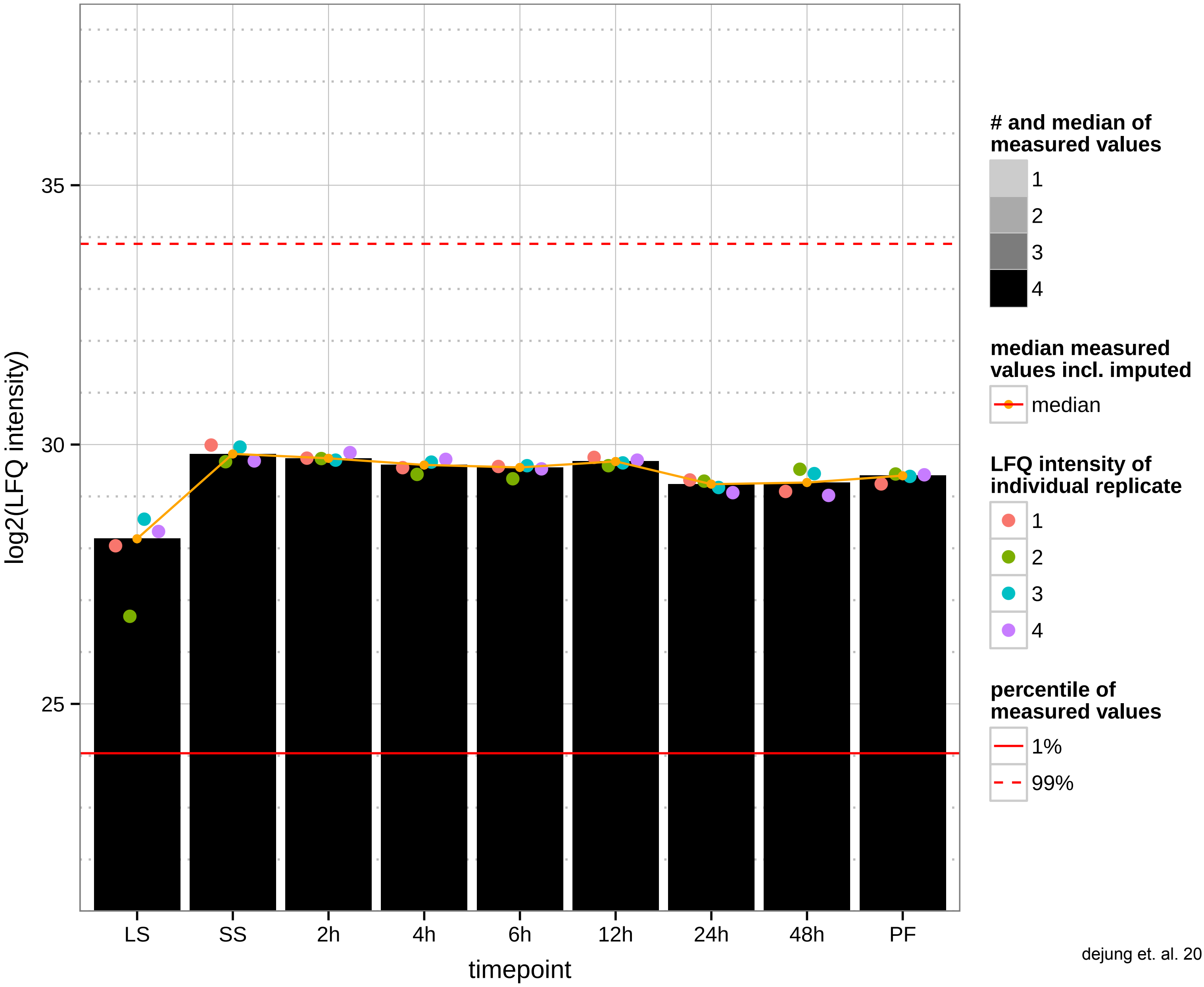

dejung et. al. 2015

hypothetical protein, conserved
Tb927.9.2130
AGOF: null
AGOC: null
AGOP: null
PGOF: transferase activity, transferring phosphorus–containing groups
PGOC: null
PGOP: null

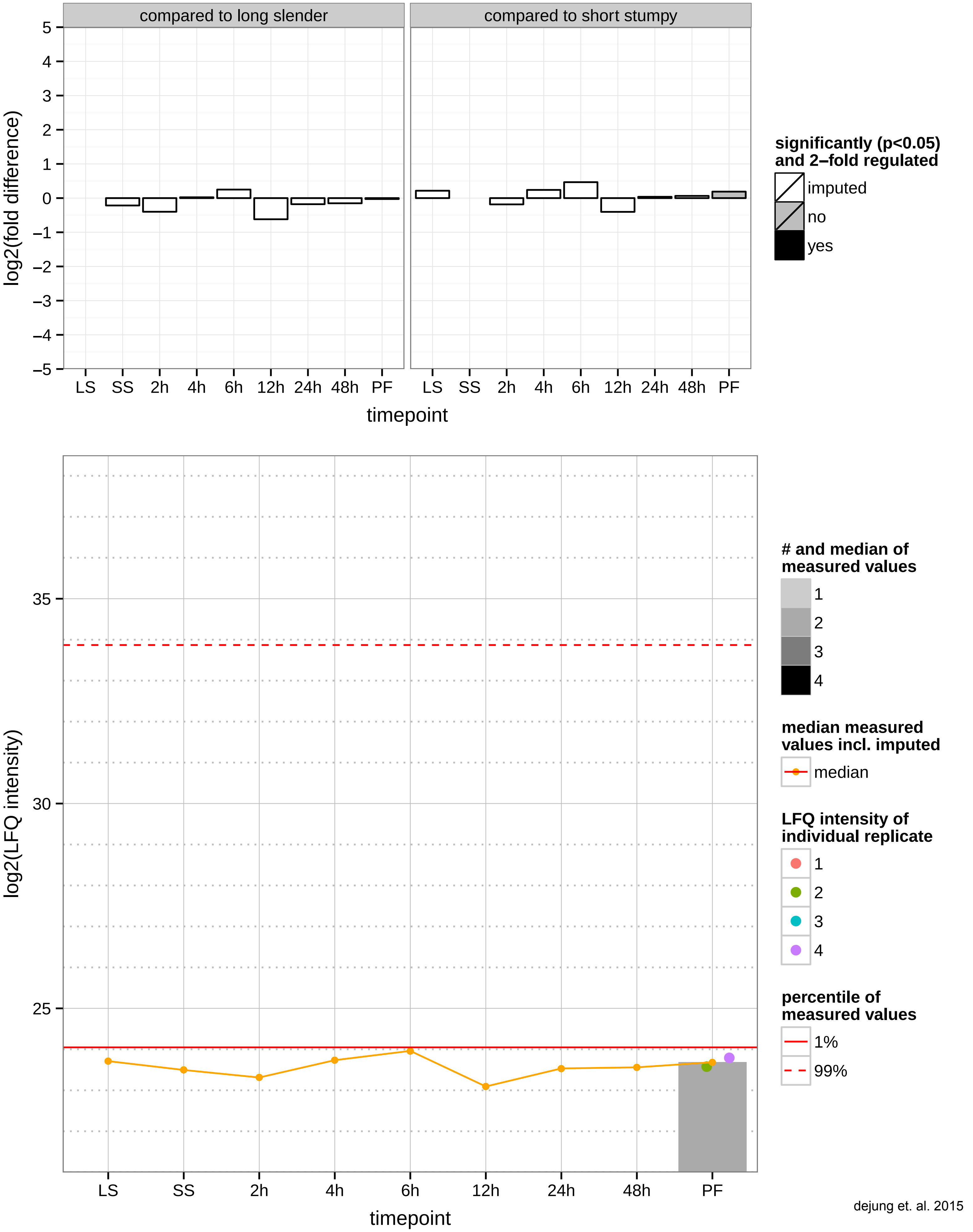

dejung et. al. 2015

hypothetical protein, conserved
Tb927.9.2420
AGOF: null
AGOC: null
AGOP: null
PGOF: null
PGOC: null
PGOP: null

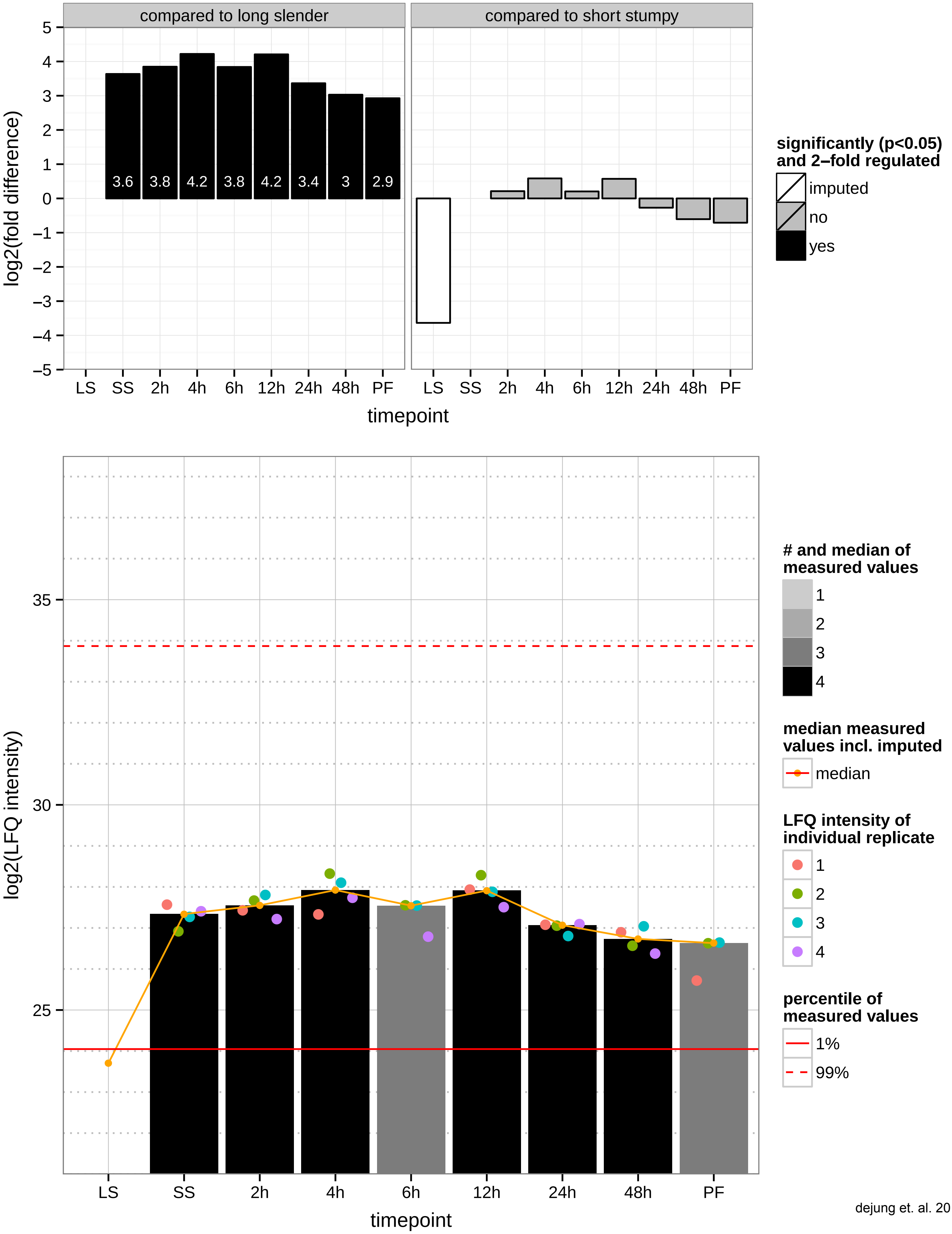

dejung et. al. 2015

nucleolar protein (NOP86)
Tb927.9.2470
AGOF: null
AGOC: nucleolus
AGOP: G2/M transition of mitotic cell cycle
PGOF: null
PGOC: null
PGOP: null

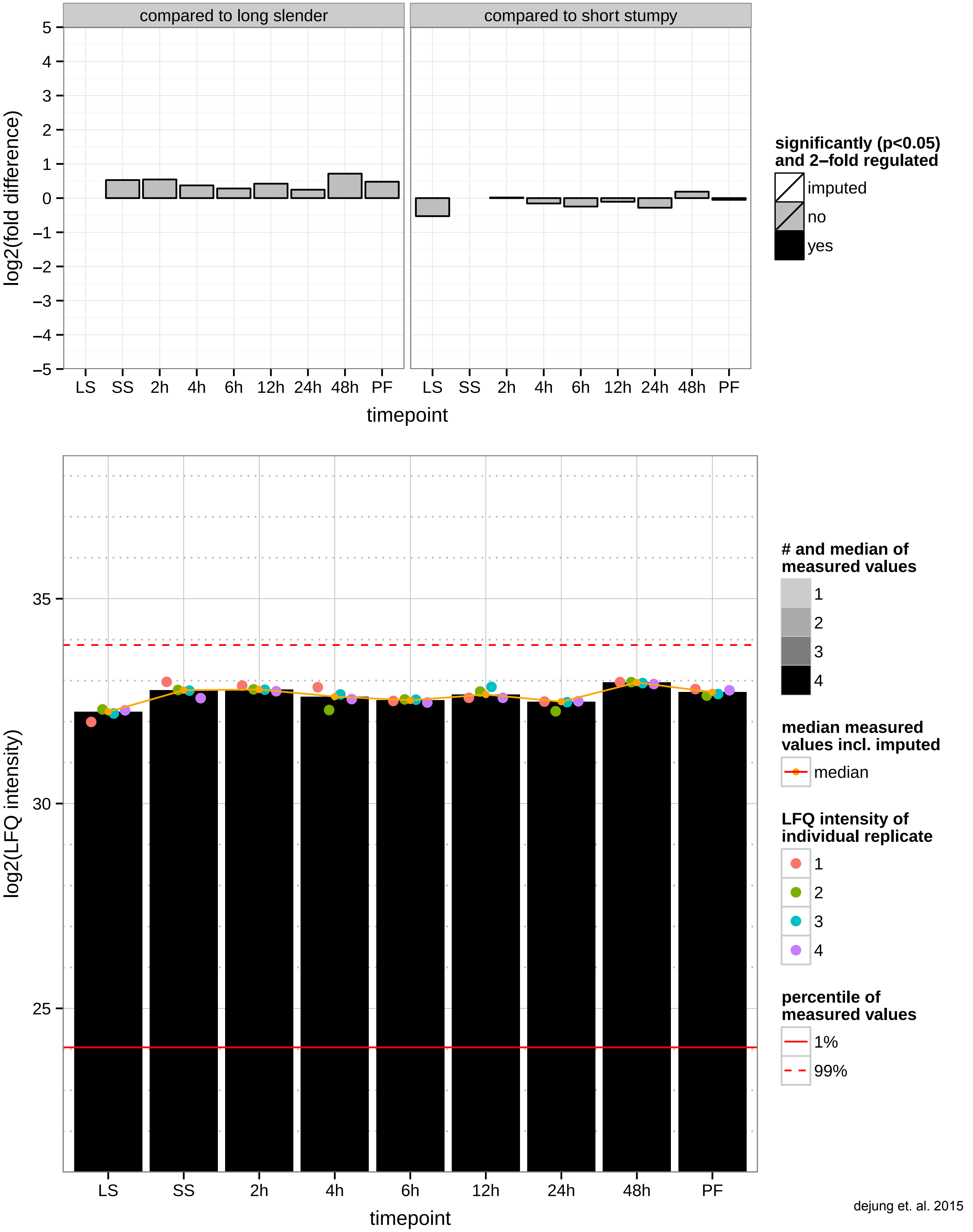

dejung et. al. 2015

mitotubule−associated protein Gb4, putative, microtubule−associated protein (GB4)
Tb927.9.2520;Tb11.v5.0868
AGOF: null
AGOC: null
AGOP: null
PGOF: null
PGOC: null
PGOP: null

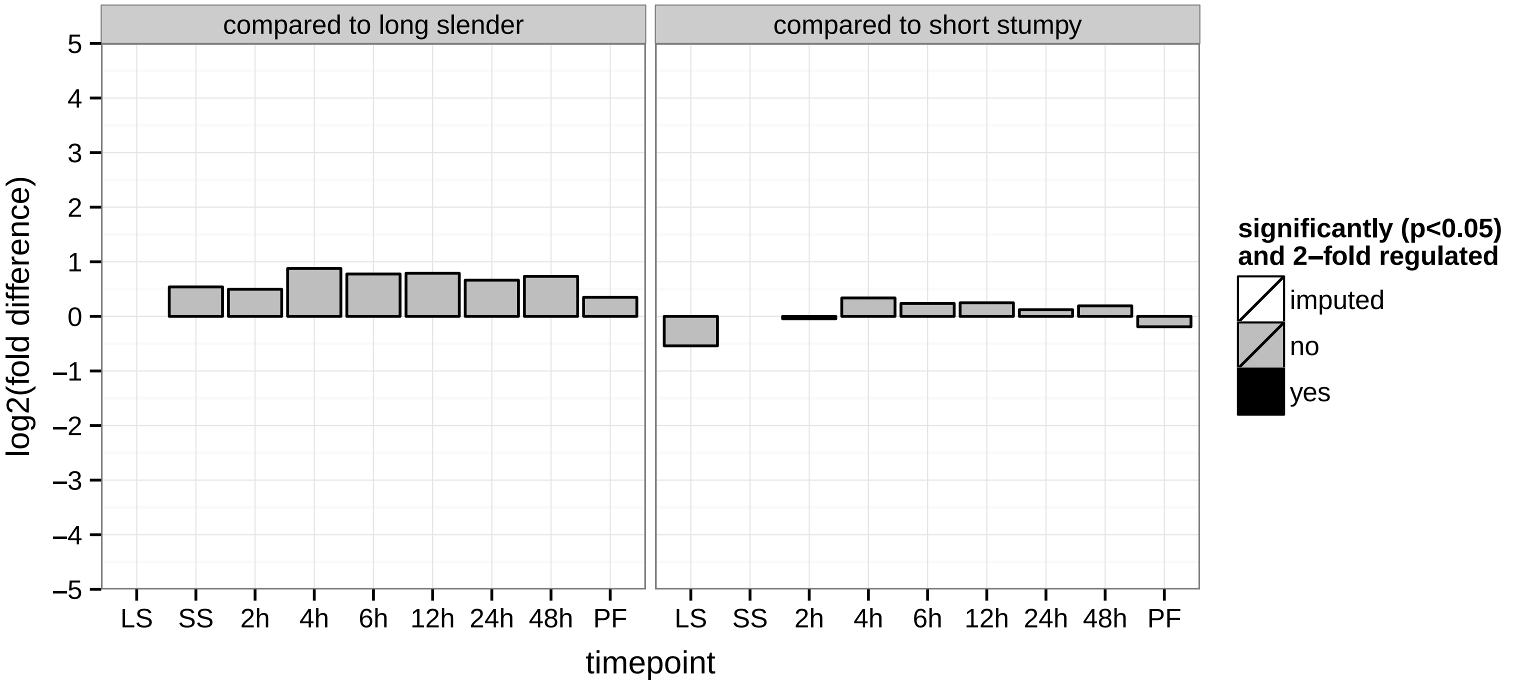

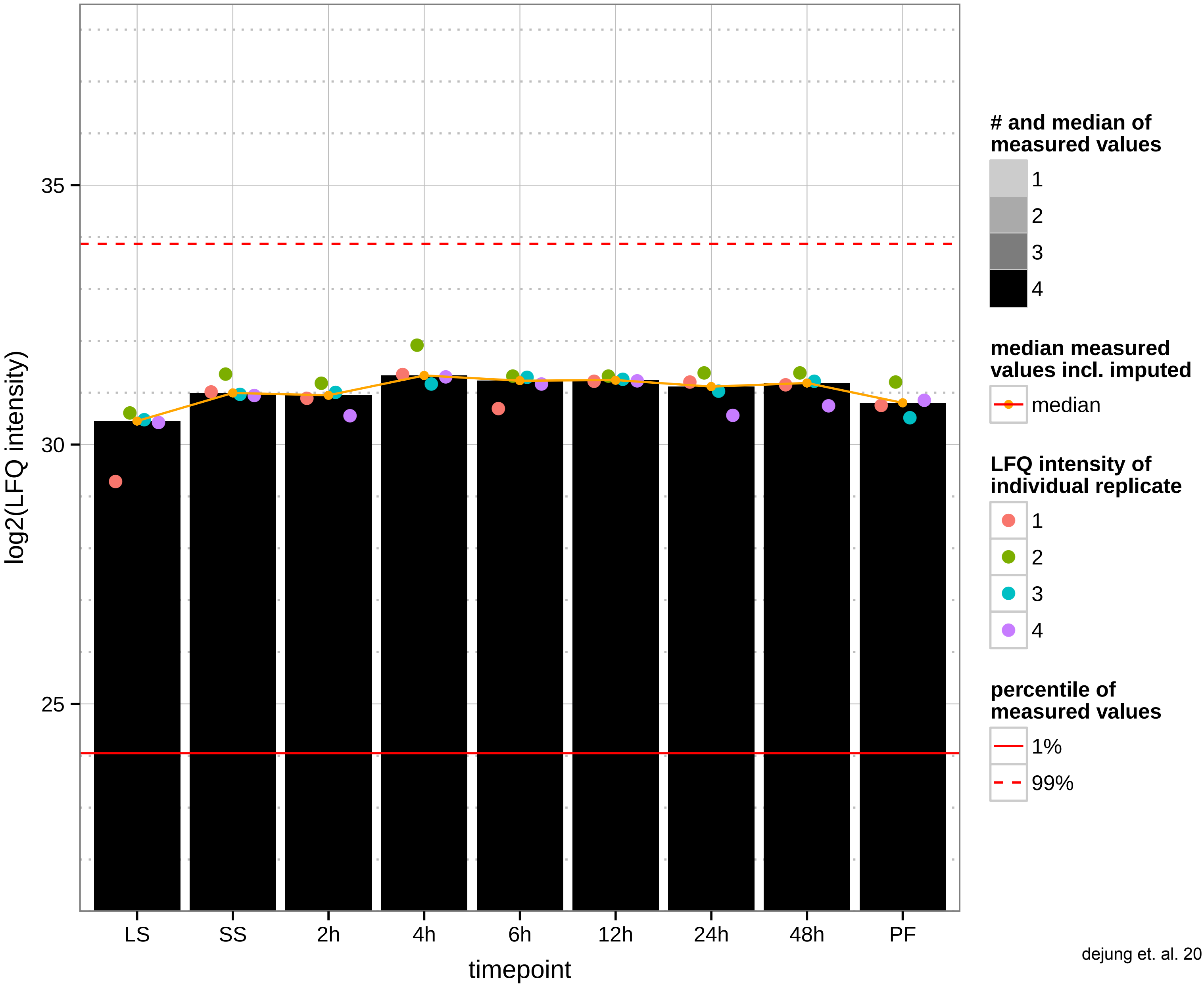

dejung et. al. 2015

deoxyhypusine hydroxylase, putative
Tb927.9.2580
AGOF: deoxyhypusine monooxygenase activity
AGOC: null
AGOP: peptidyl–lysine modification to hypusine
PGOF: binding
PGOC: null
PGOP: null

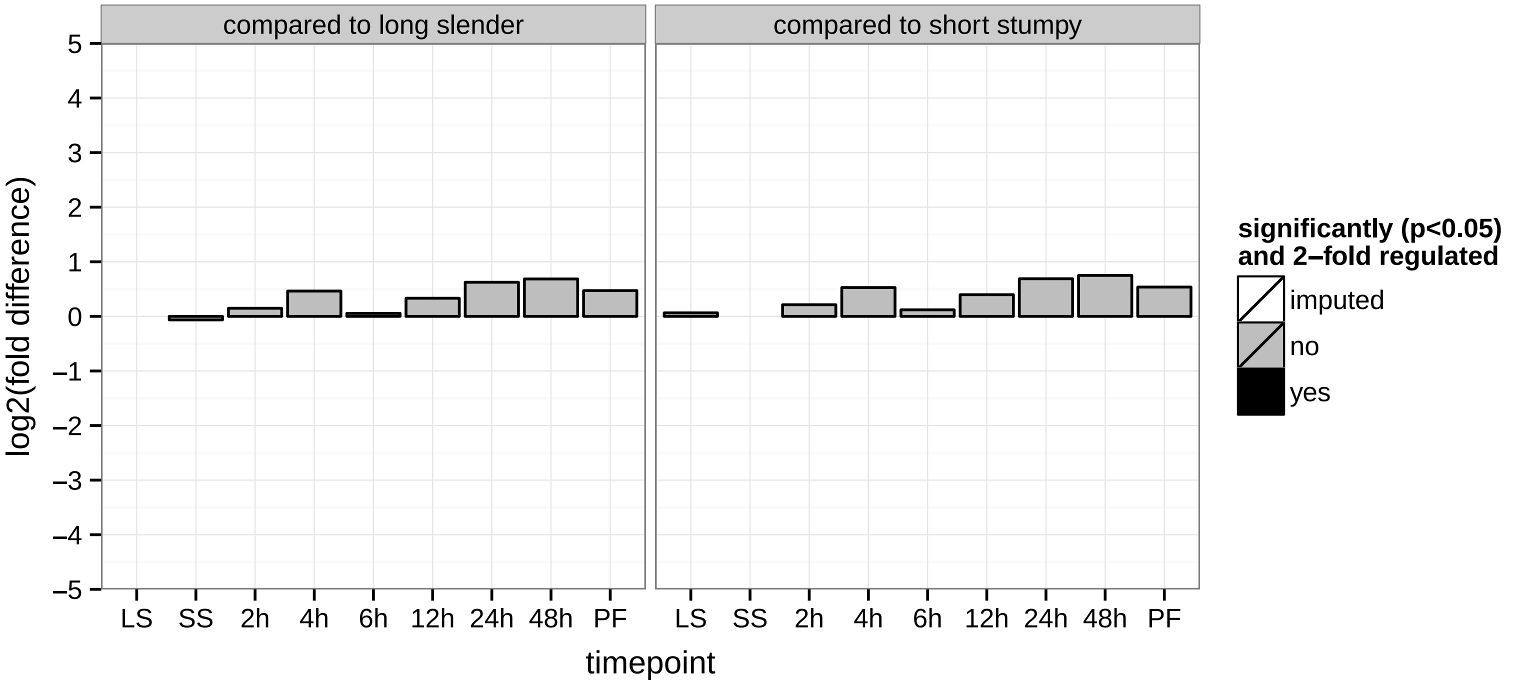

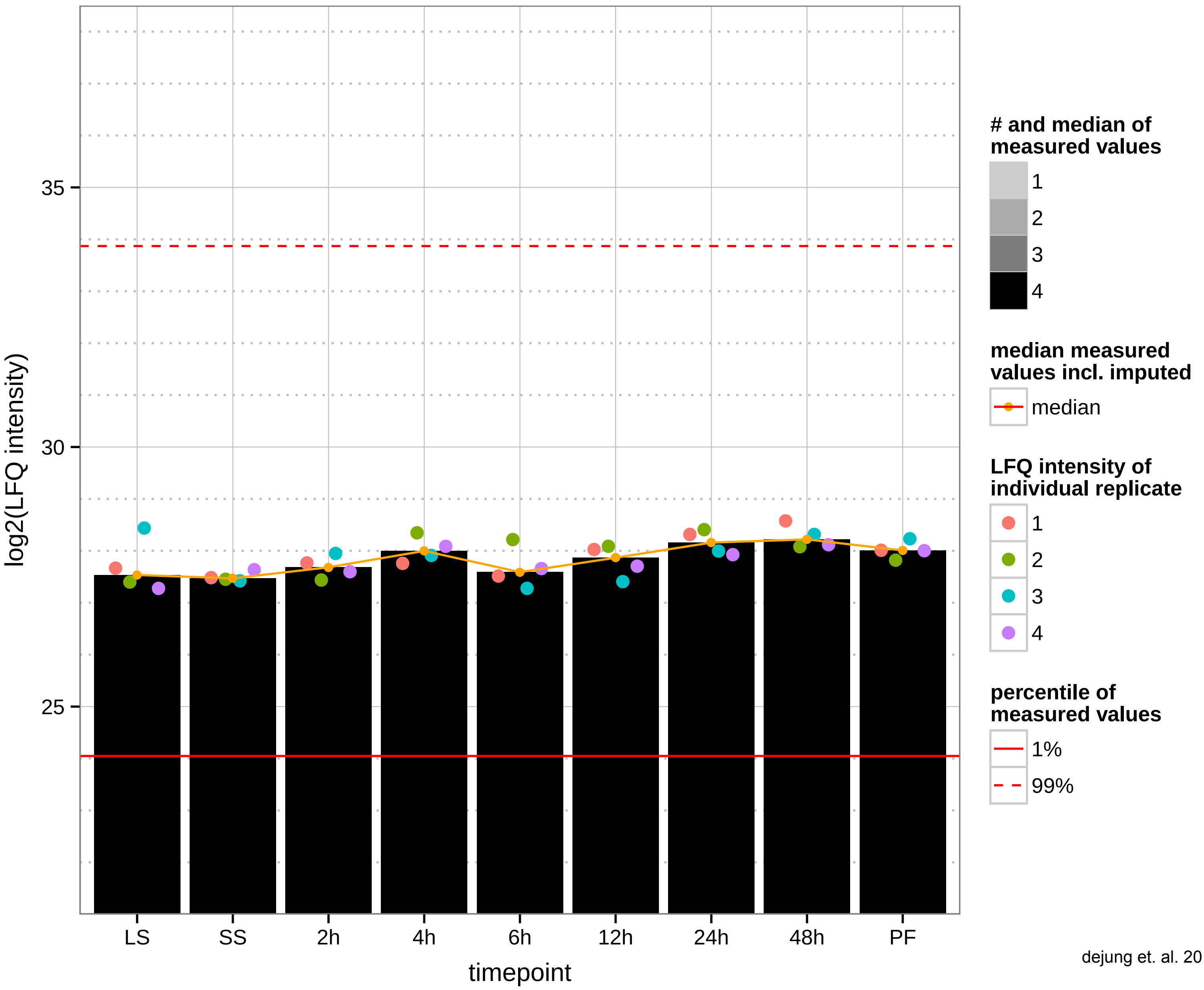

dejung et. al. 2015

hypothetical protein, conserved
Tb927.9.2590
AGOF: null
AGOC: null
AGOP: null
PGOF: binding
PGOC: null
PGOP: null

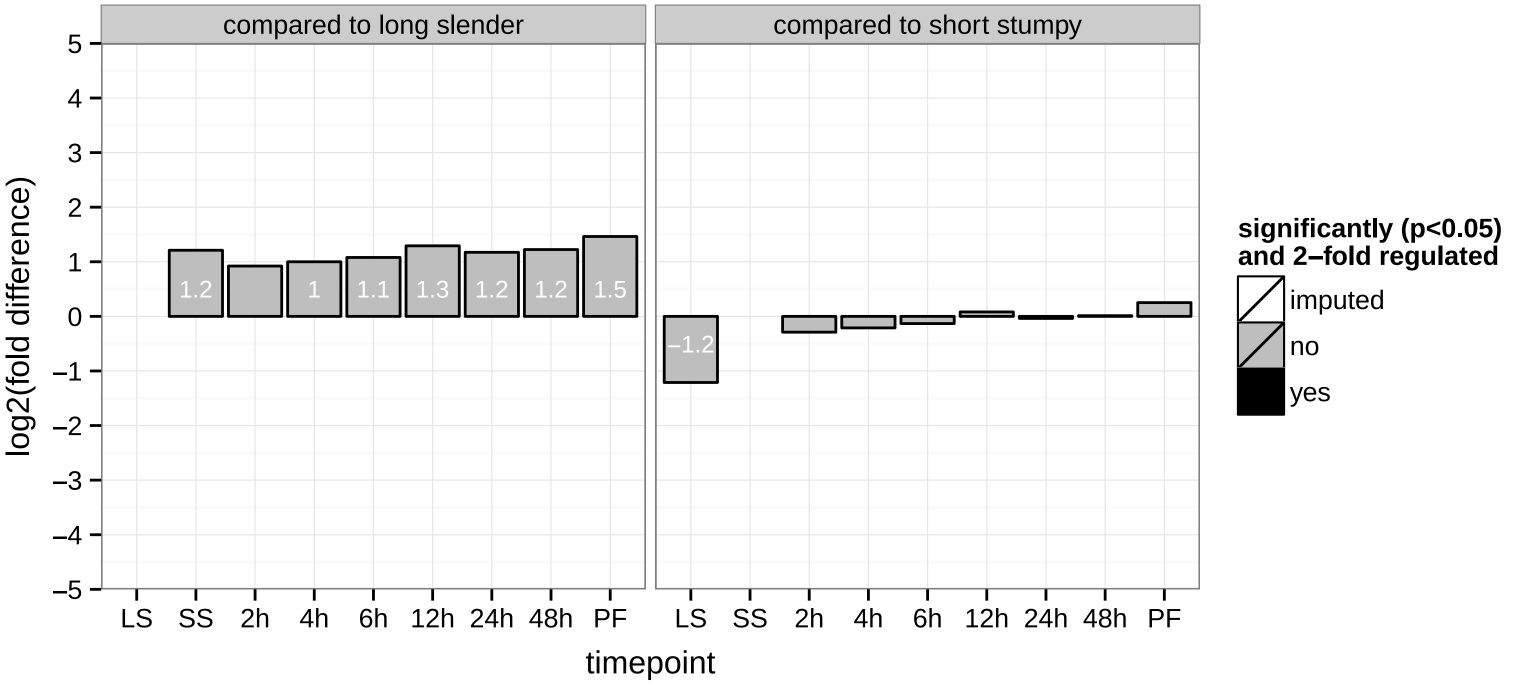

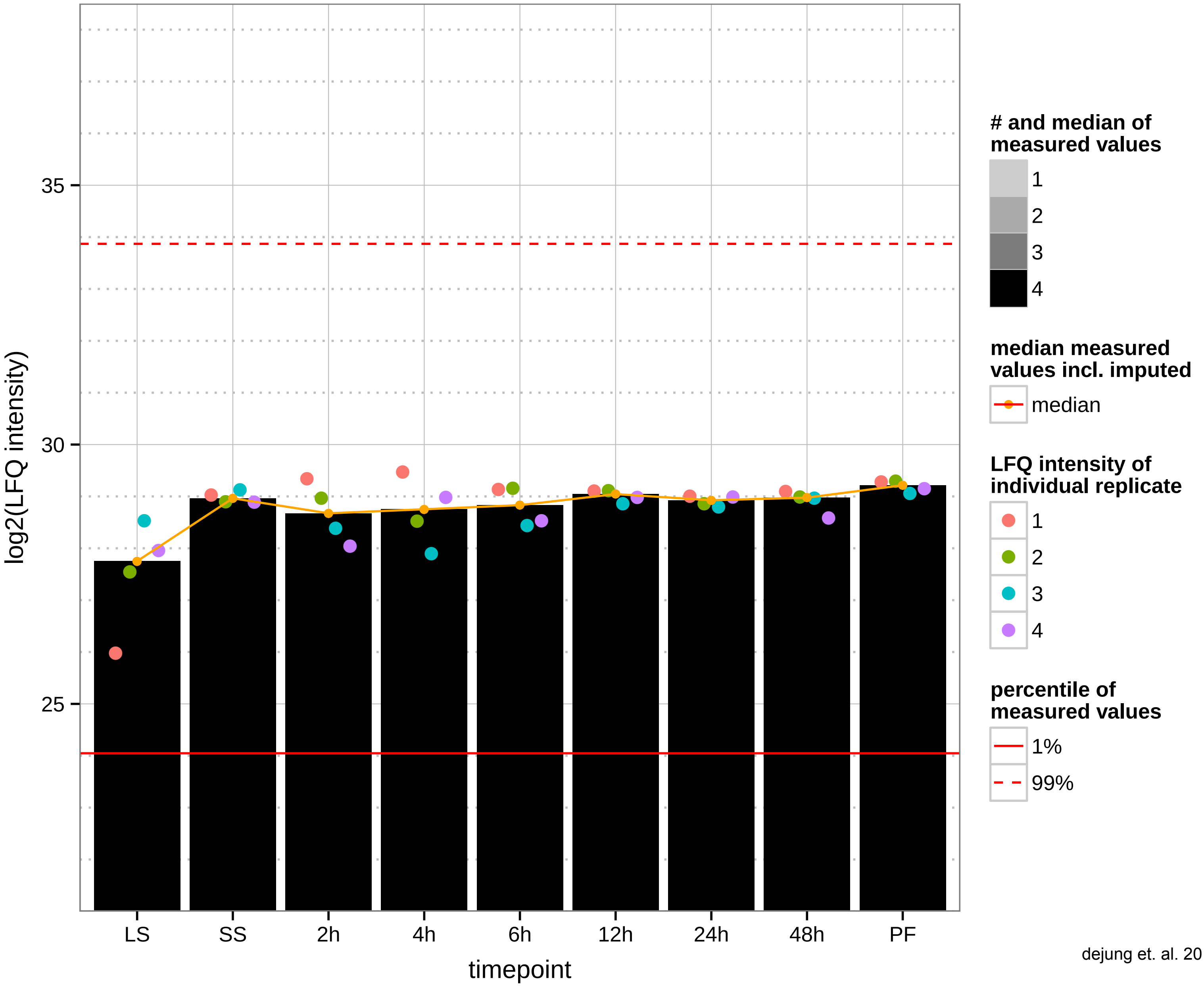

dejung et. al. 2015

hypothetical protein, conserved
Tb927.9.2600
AGOF: null
AGOC: null
AGOP: null
PGOF: null
PGOC: intracellular
PGOP: ER to Golgi vesicle–mediated transport

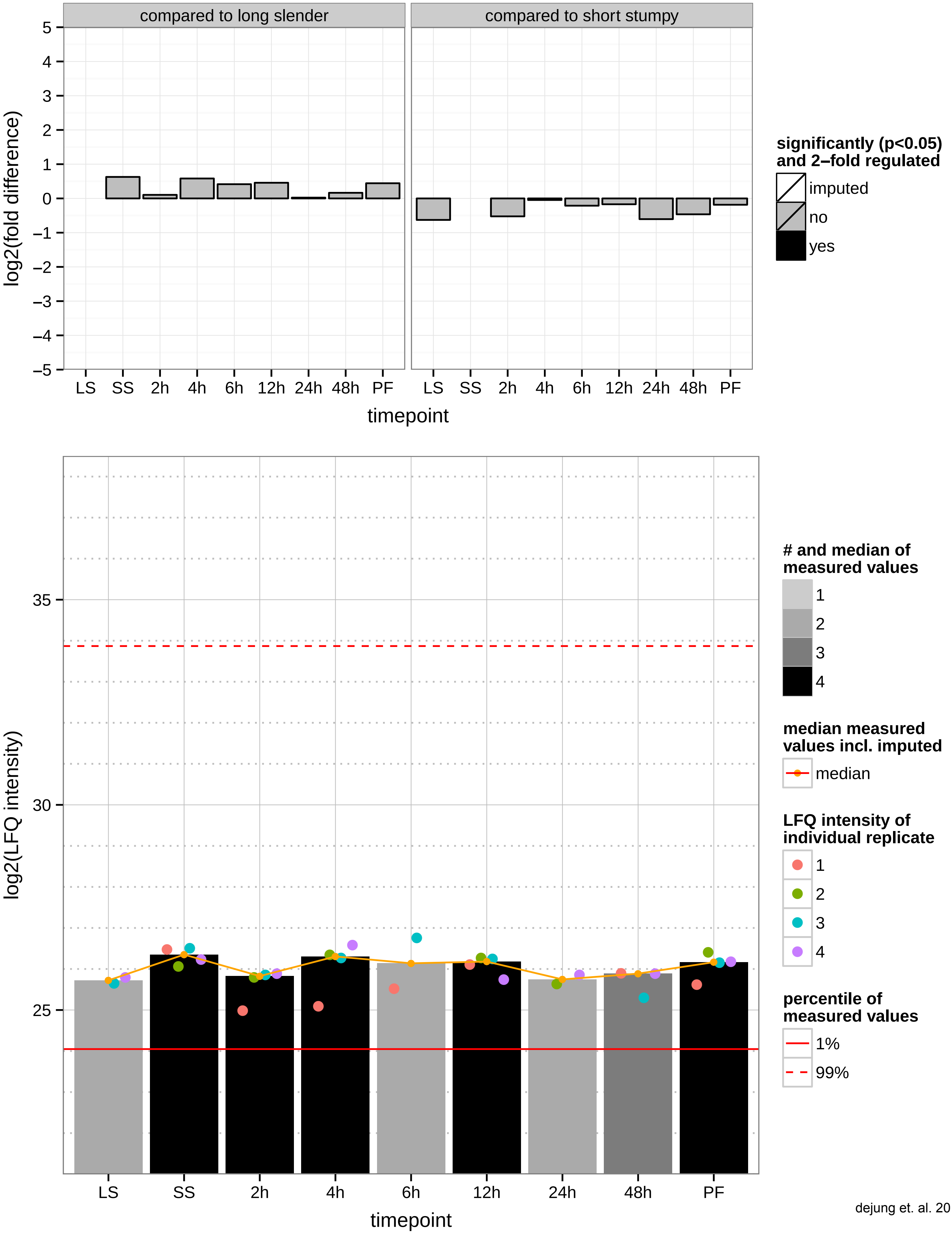

dejung et. al. 2015

hypothetical protein, conserved
Tb927.9.2650
AGOF: null
AGOC: null
AGOP: null
PGOF: null
PGOC: null
PGOP: null

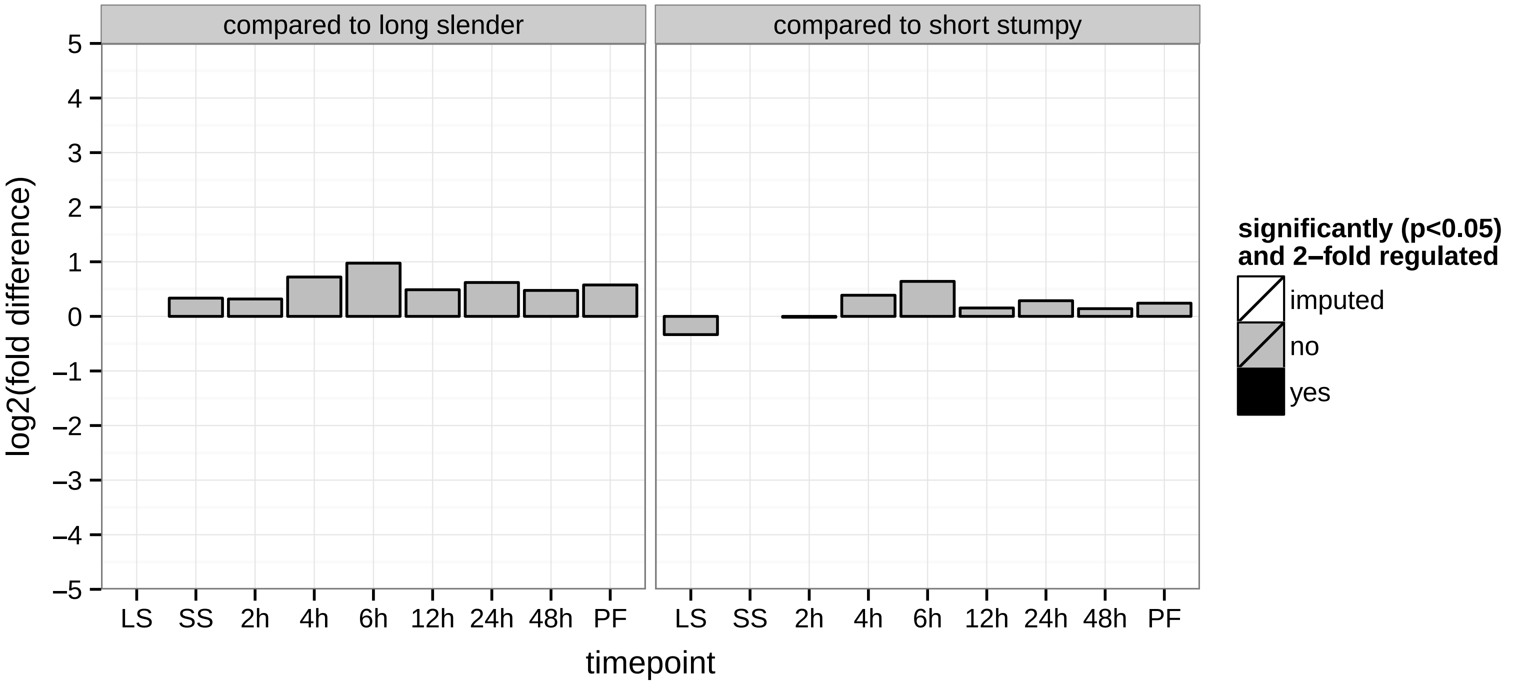

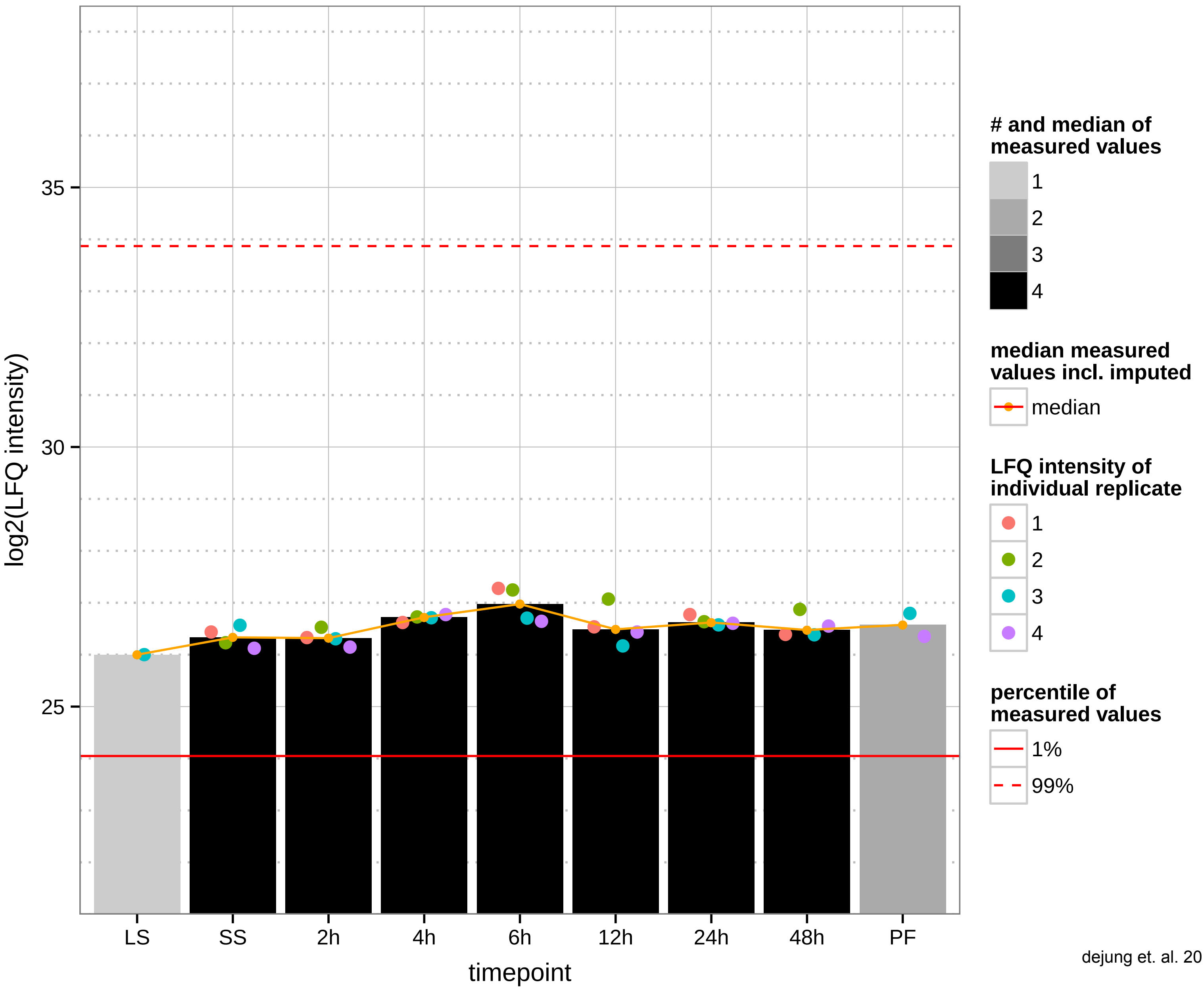

dejung et. al. 2015

hypothetical protein, conserved
Tb927.9.2980
AGOF: null
AGOC: null
AGOP: null
PGOF: cAMP–dependent protein kinase regulator activity
PGOC: null
PGOP: signal transduction

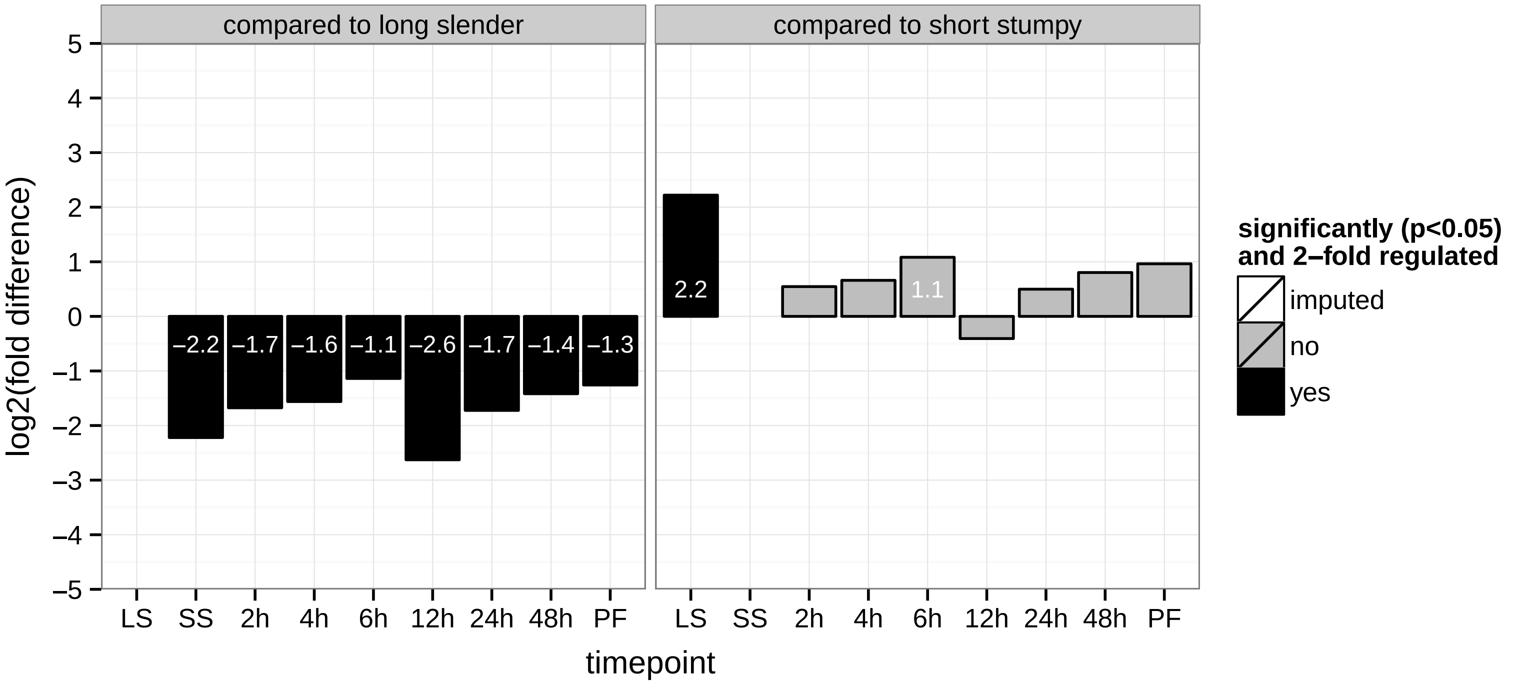

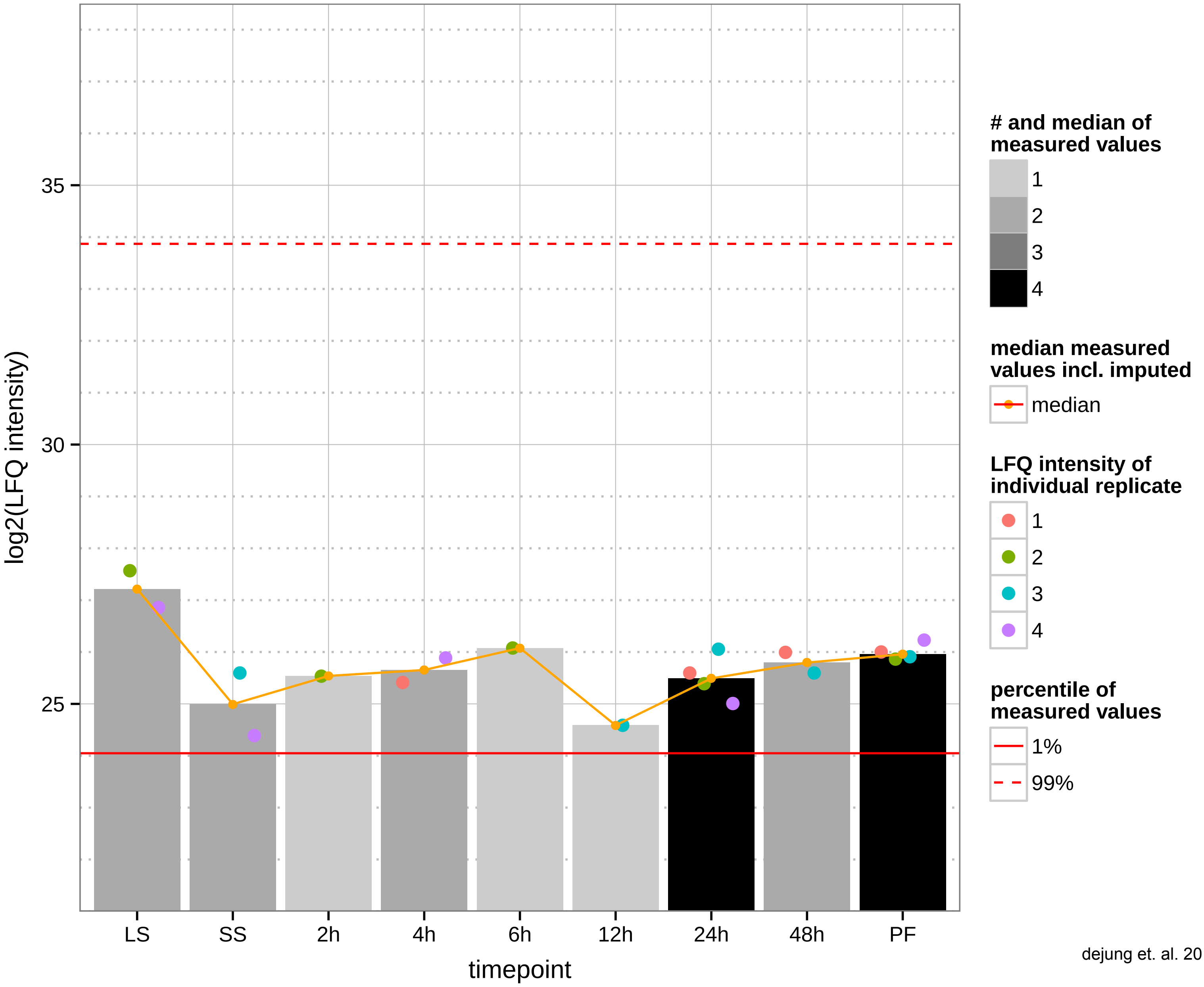

dejung et. al. 2015

cytidine deaminase, putative
Tb927.9.3000
AGOF: cytidine deaminase activity, hydrolase activity, zinc ion binding
AGOC: null
AGOP: cytidine metabolic process
PGOF: catalytic activity, hydrolase activity, zinc ion binding
PGOC: null
PGOP: null

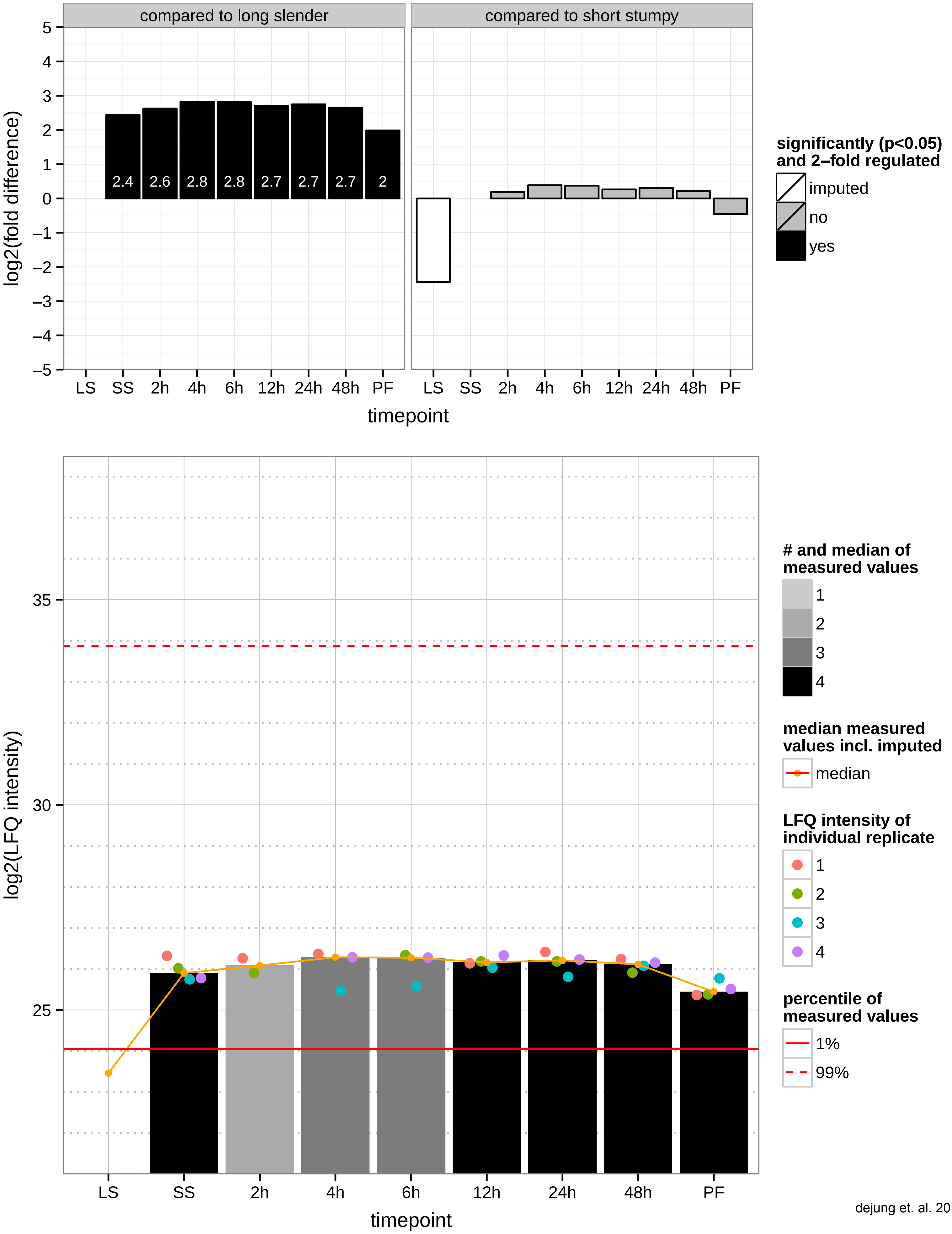

dejung et. al. 2015

acidocalcisomal exopolyphosphatase
Tb927.9.3280
AGOF: exopolyphosphatase activity, manganese ion binding
AGOC: cytoplasm
AGOP: polyphosphate metabolic process
PGOF: hydrolase activity, manganese ion binding, pyrophosphatase activity
PGOC: cytoplasm
PGOP: null

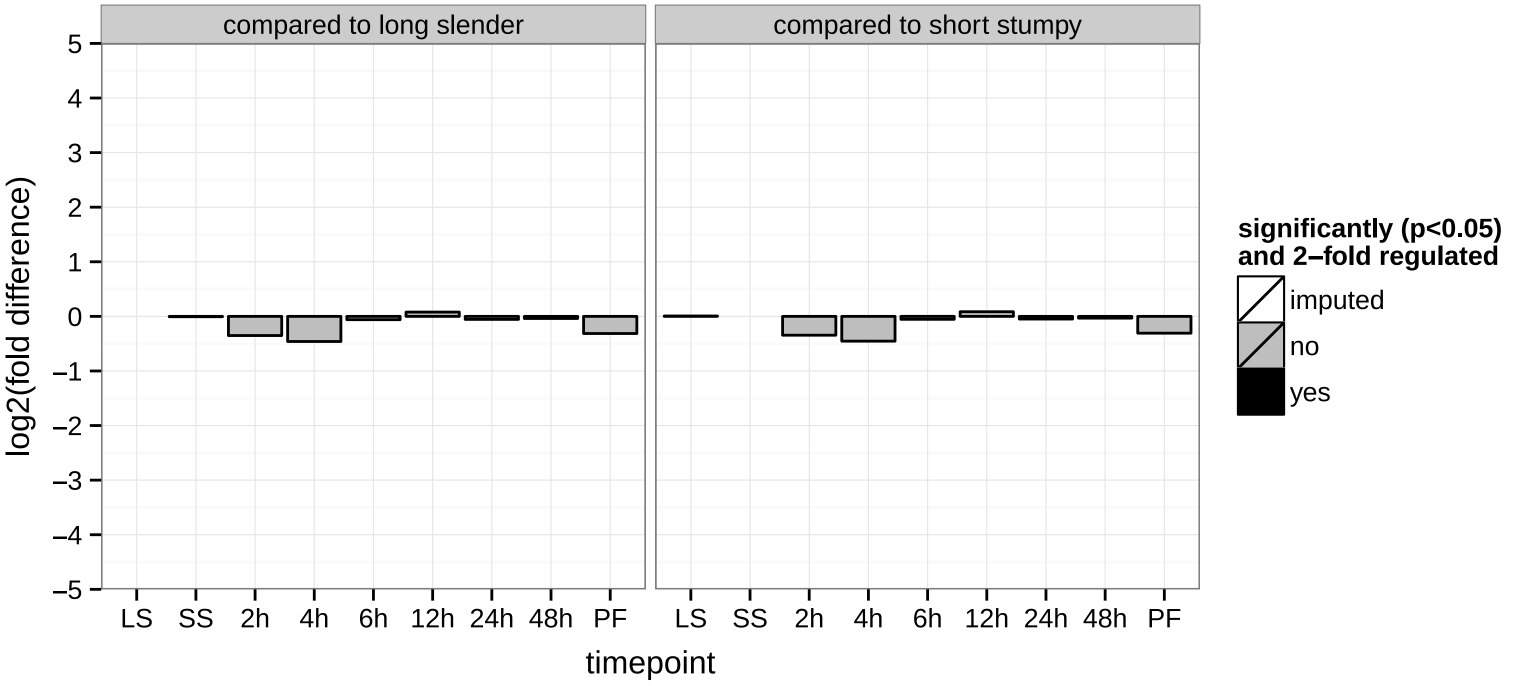

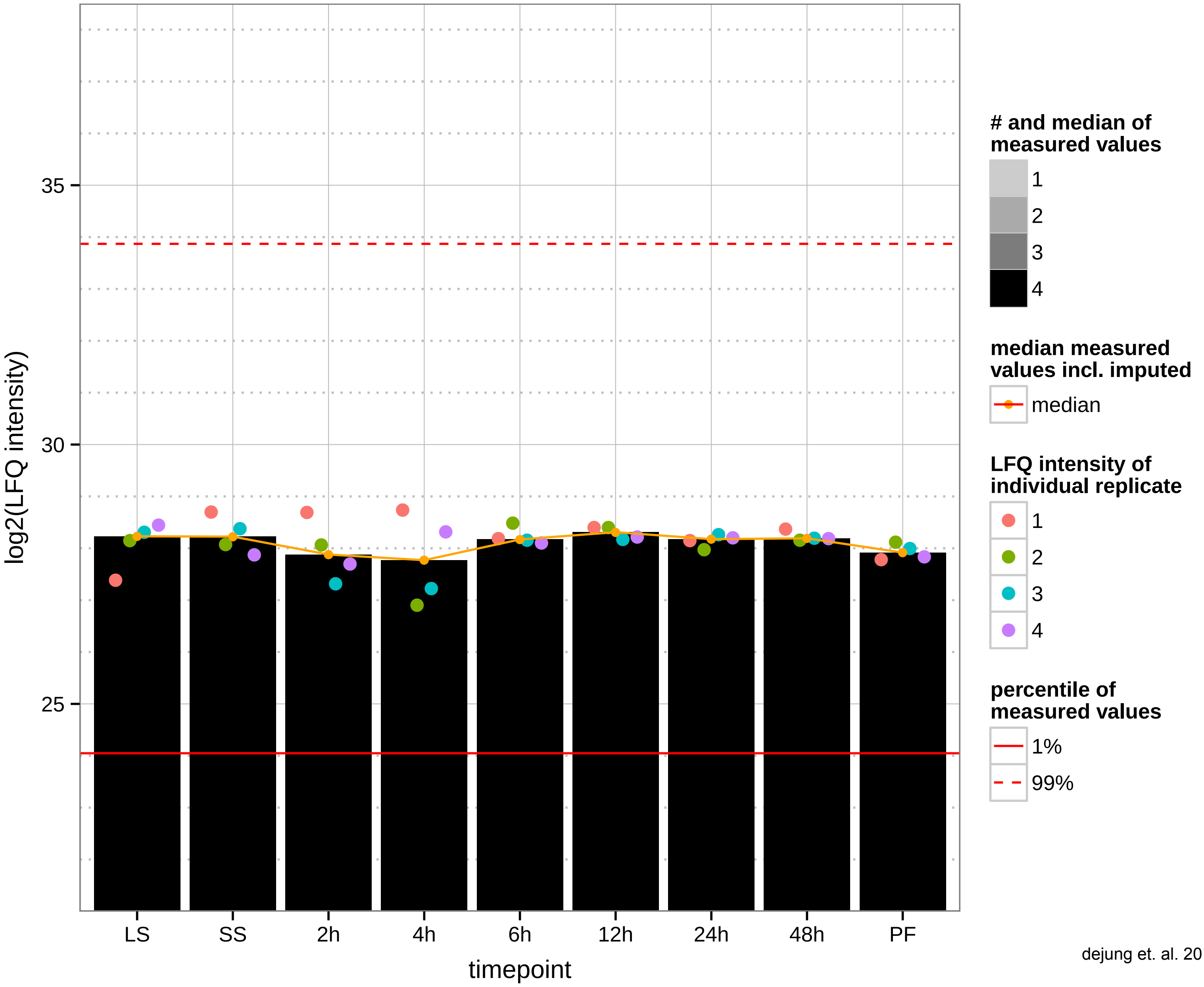

dejung et. al. 2015

endo–beta–N–acetylglucosaminidase, putative
Tb927.9.3400
AGOF: hydrolase activity, acting on glycosyl bonds, mannosyl–glycoprotein endo–beta–N–acetylglucosaminidase activity
AGOC: cytoplasm
AGOP: null
PGOF: mannosyl–glycoprotein endo–beta–N–acetylglucosaminidase activity
PGOC: cytoplasm
PGOP: null

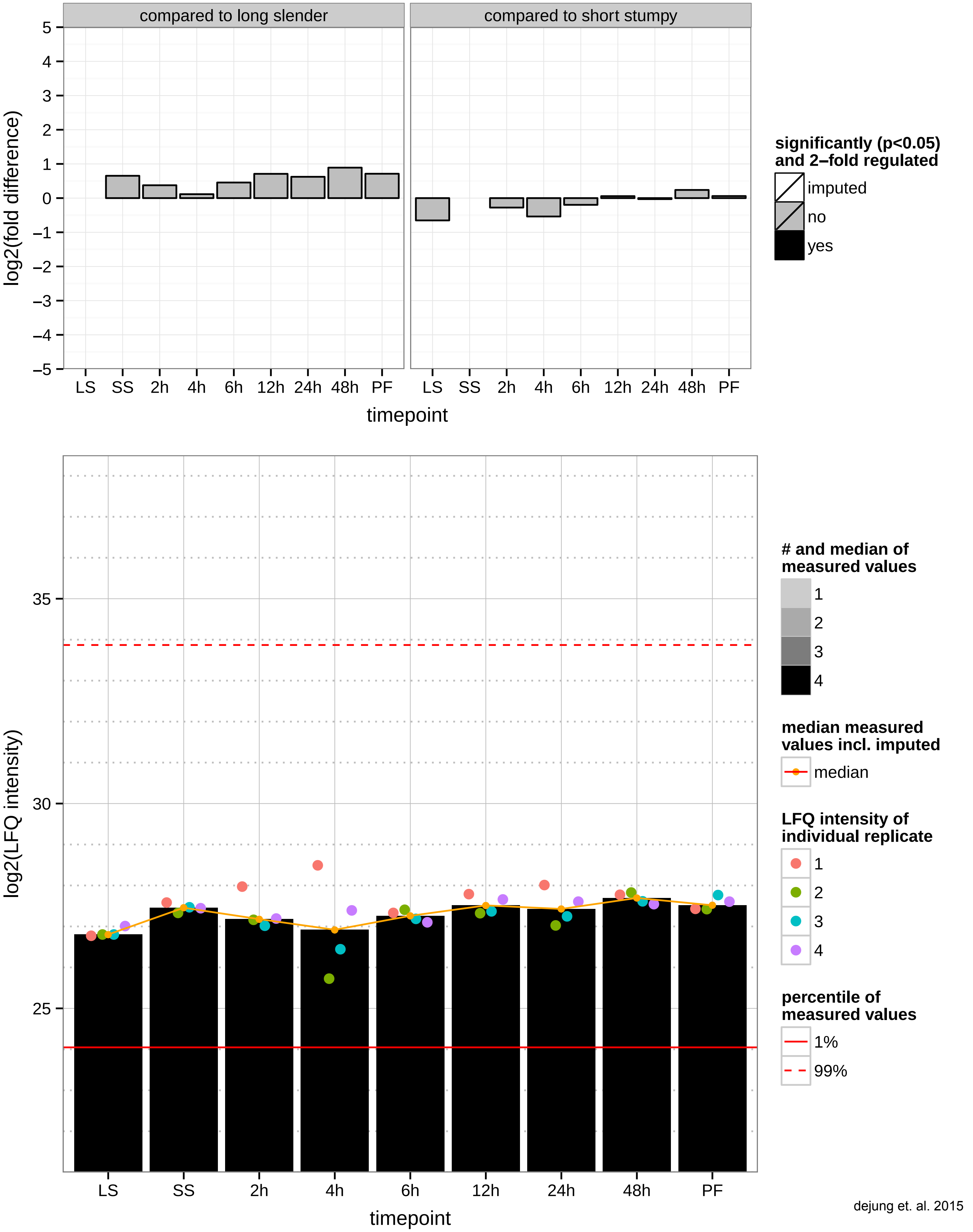

dejung et. al. 2015

cyclophilin type peptidyl–prolyl cis–trans isomerase, putative
Tb927.9.3440
AGOF: peptidyl–prolyl cis–trans isomerase activity
AGOC: null
AGOP: protein folding
PGOF: peptidyl–prolyl cis–trans isomerase activity
PGOC: null
PGOP: protein folding

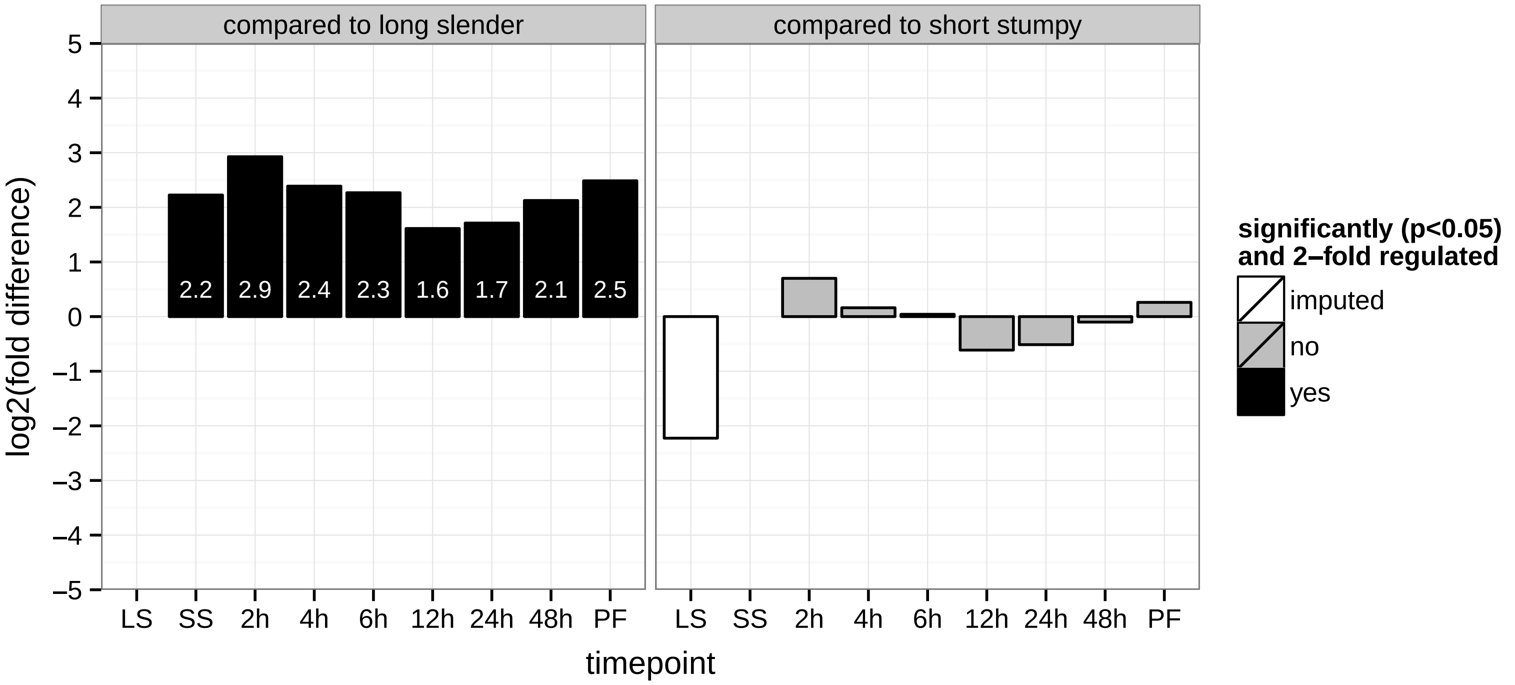

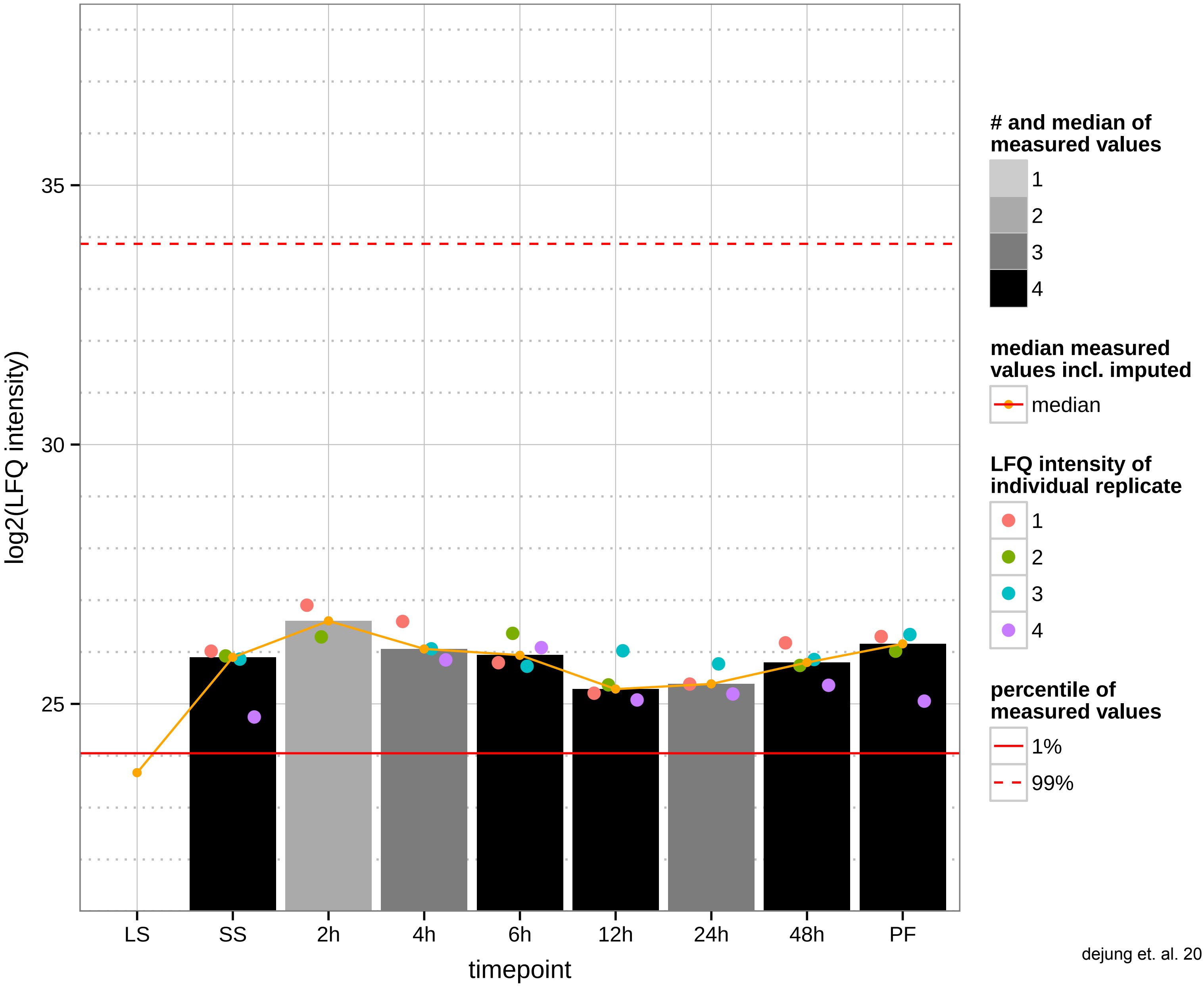

dejung et. al. 2015

low molecular weight protein tyrosine phosphatase, putative
Tb927.9.3470
AGOF: protein tyrosine phosphatase activity
AGOC: mitochondrion
AGOP: protein dephosphorylation
PGOF: null
PGOC: null
PGOP: null

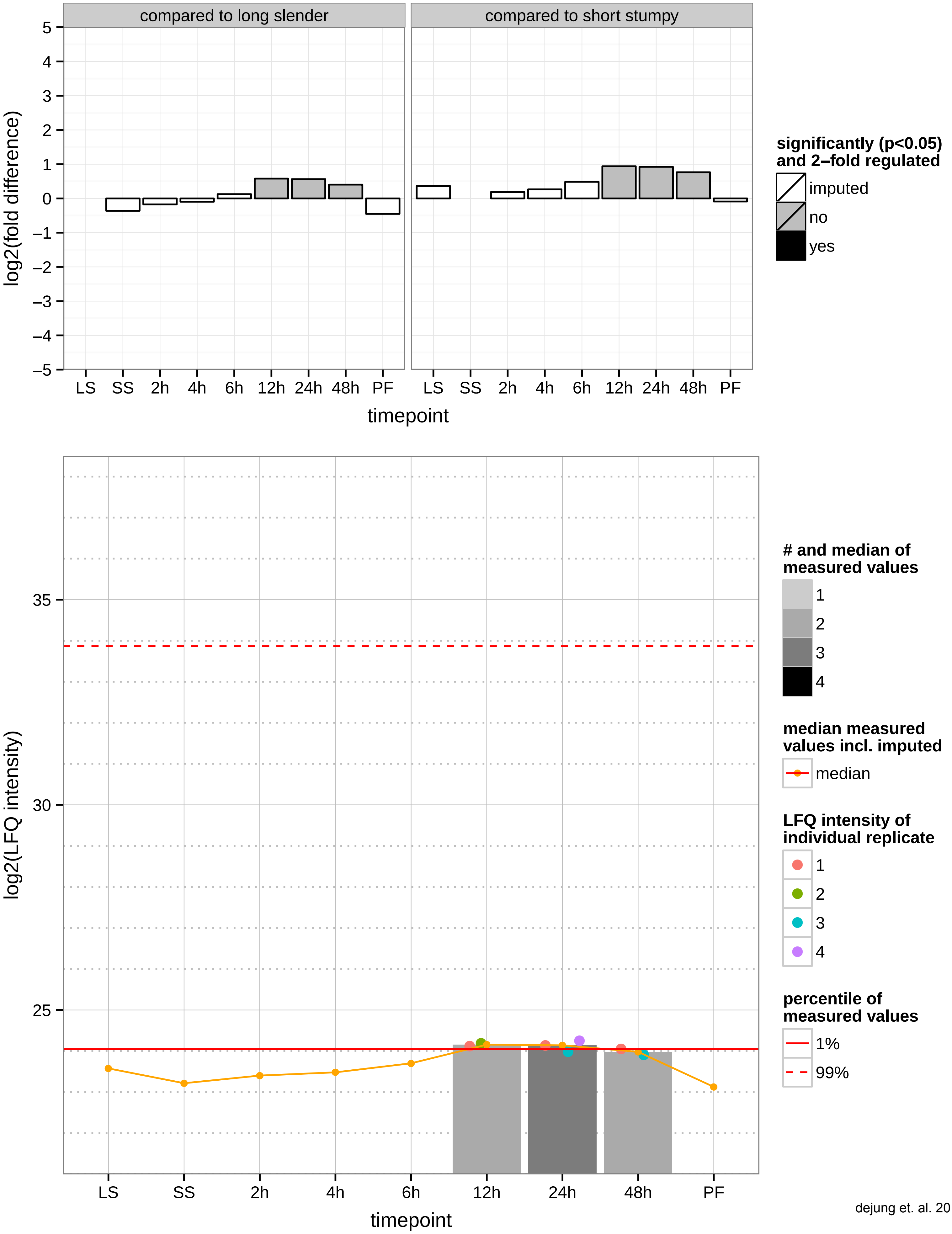

dejung et. al. 2015

hypothetical protein, conserved
Tb927.9.3620
AGOF: null
AGOC: null
AGOP: null
PGOF: null
PGOC: null
PGOP: null

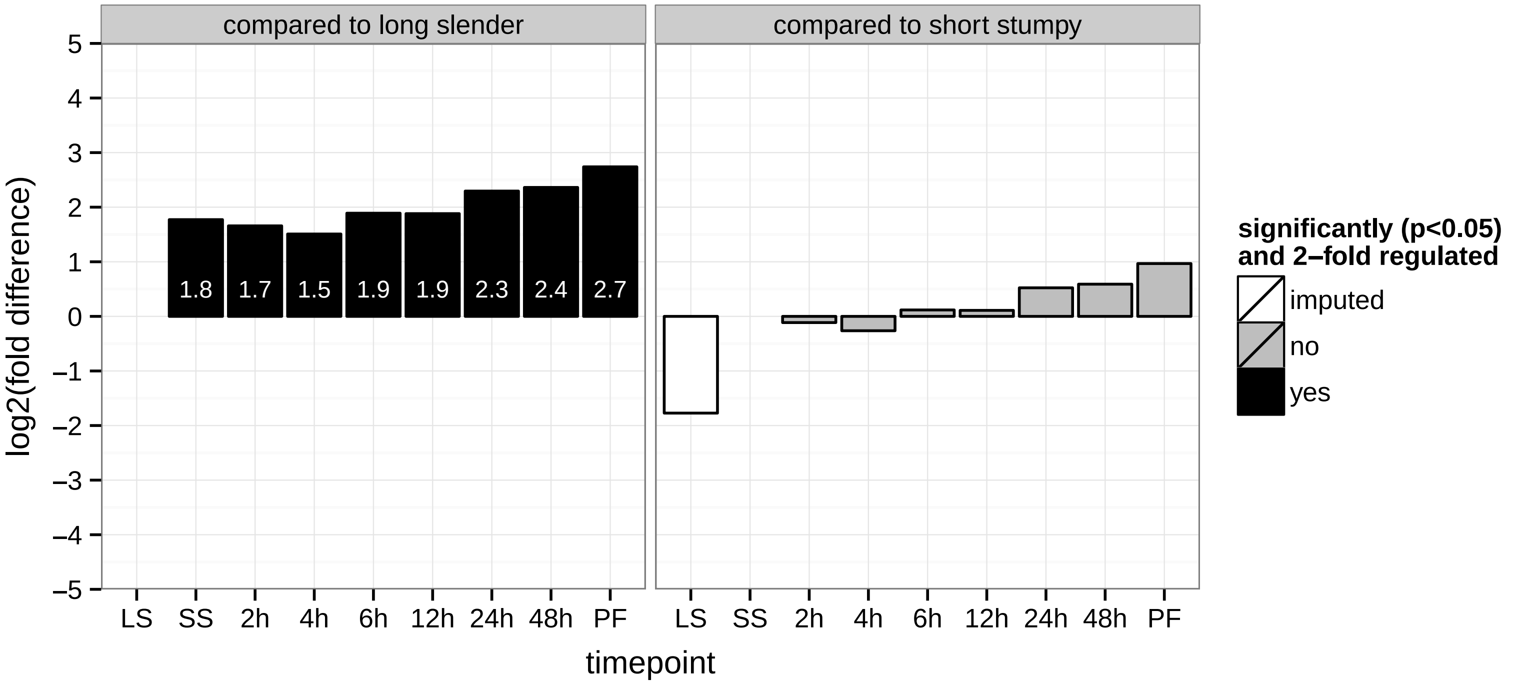

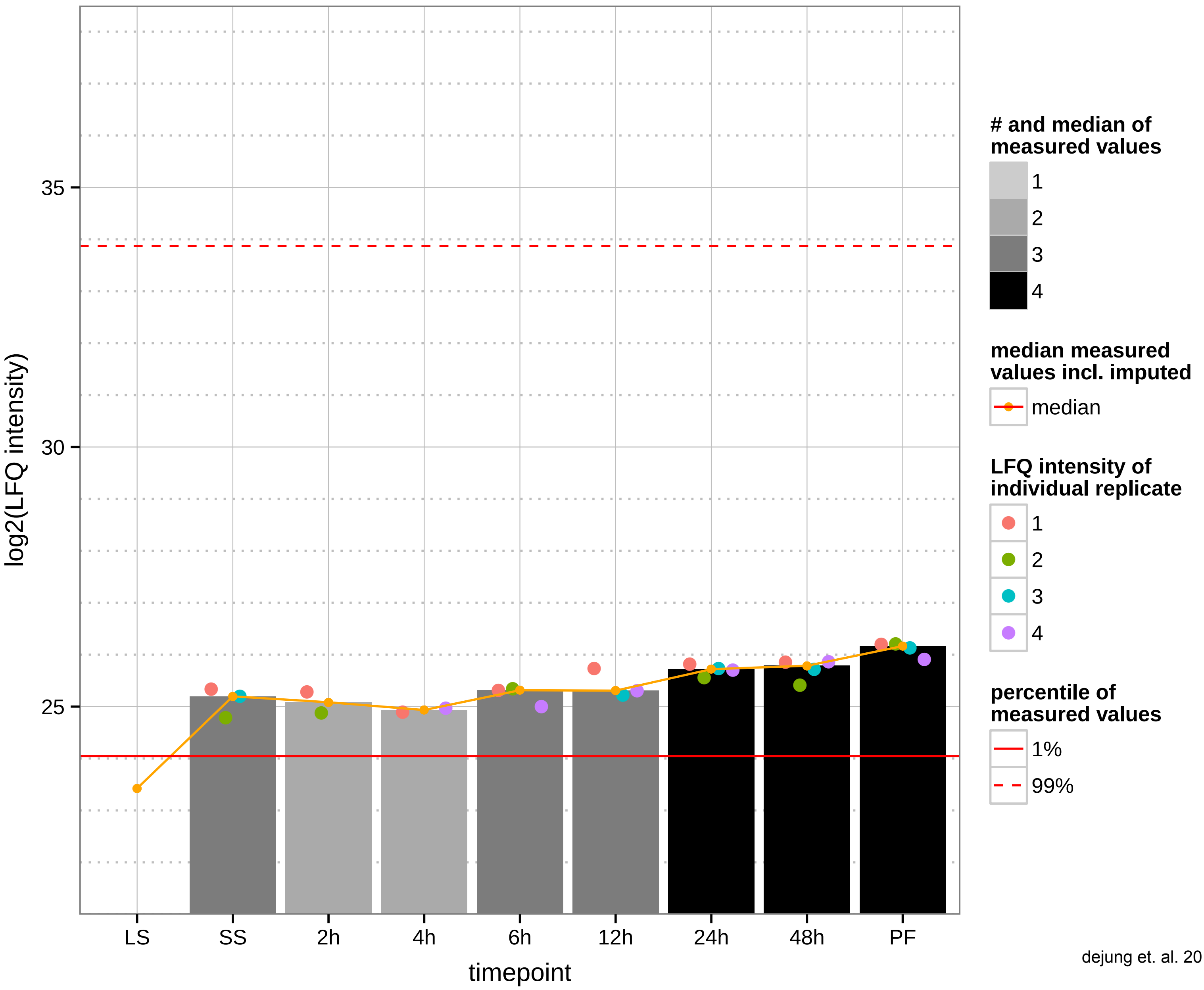

dejung et. al. 2015

hypothetical protein, conserved
Tb927.9.3630;Tb11.v5.0530
AGOF: null
AGOC: null
AGOP: null
PGOF: null
PGOC: null
PGOP: null

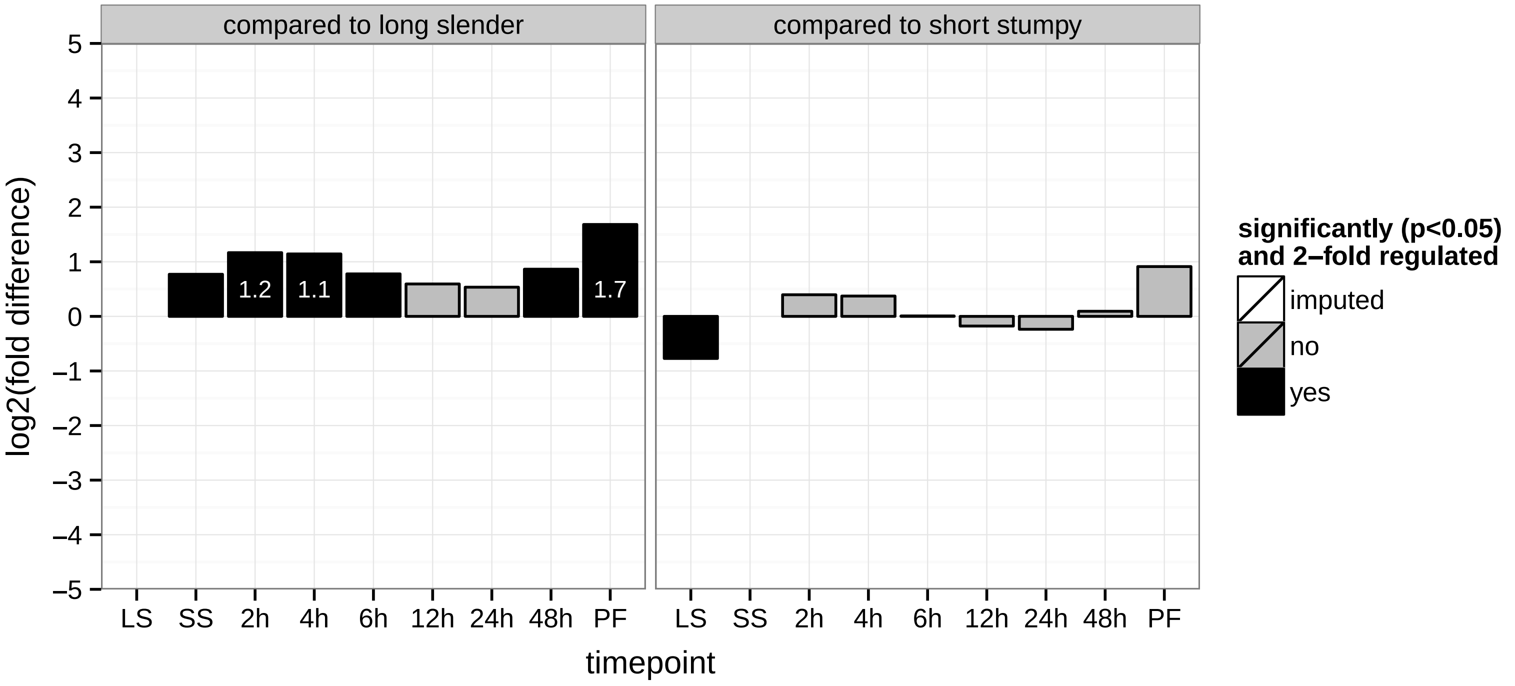

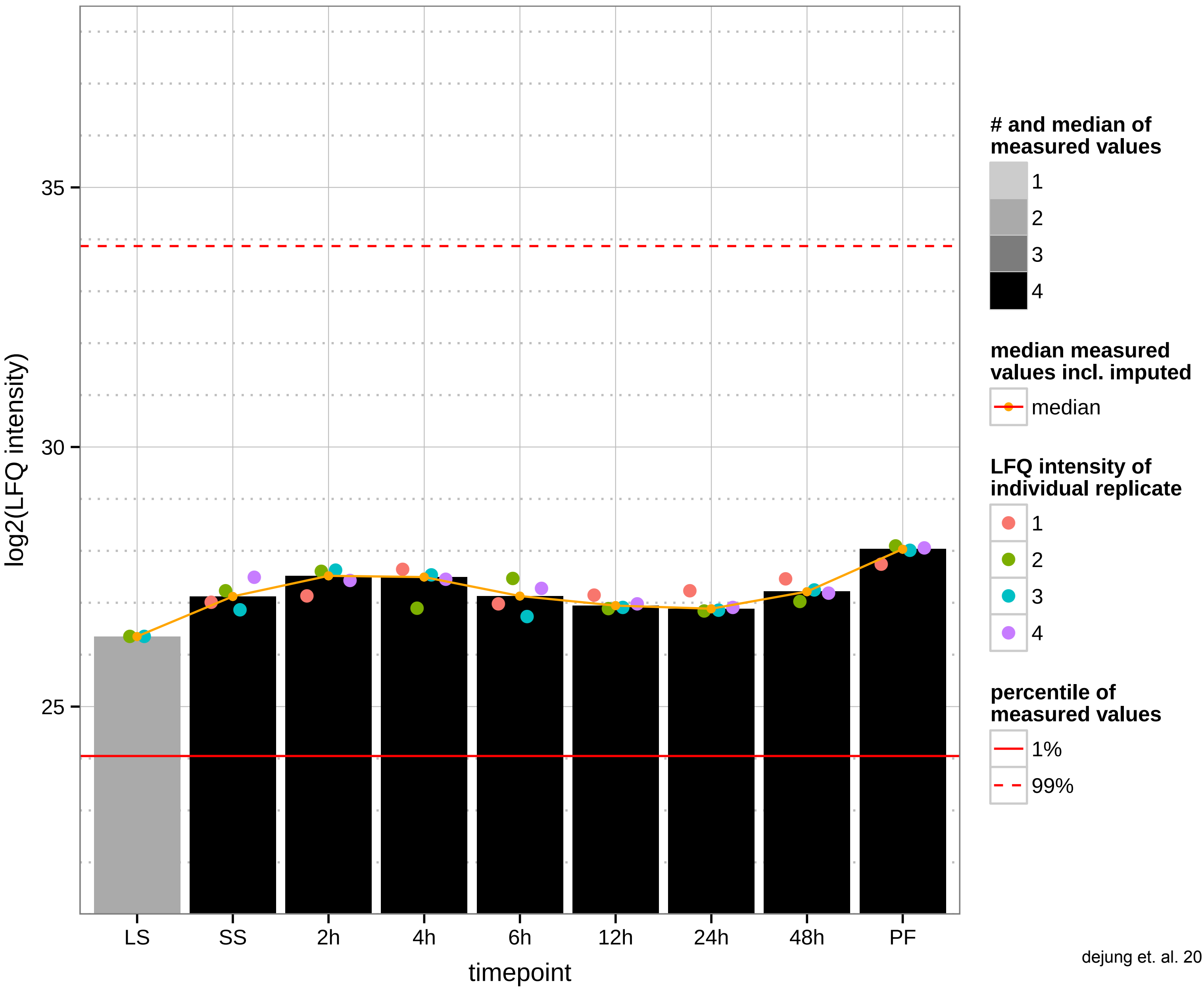

dejung et. al. 2015

hypothetical protein, conserved
Tb927.9.3670
AGOF: null
AGOC: mitochondrion
AGOP: null
PGOF: null
PGOC: null
PGOP: null

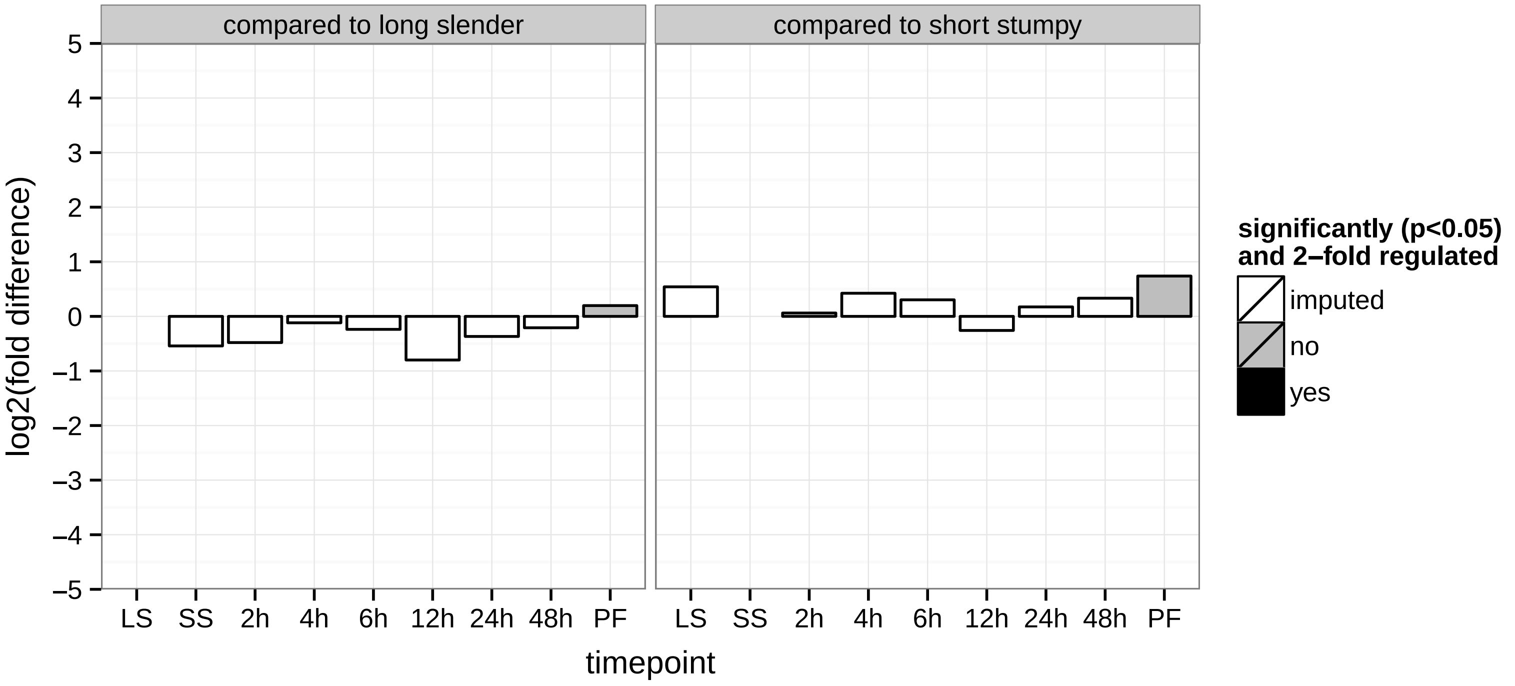

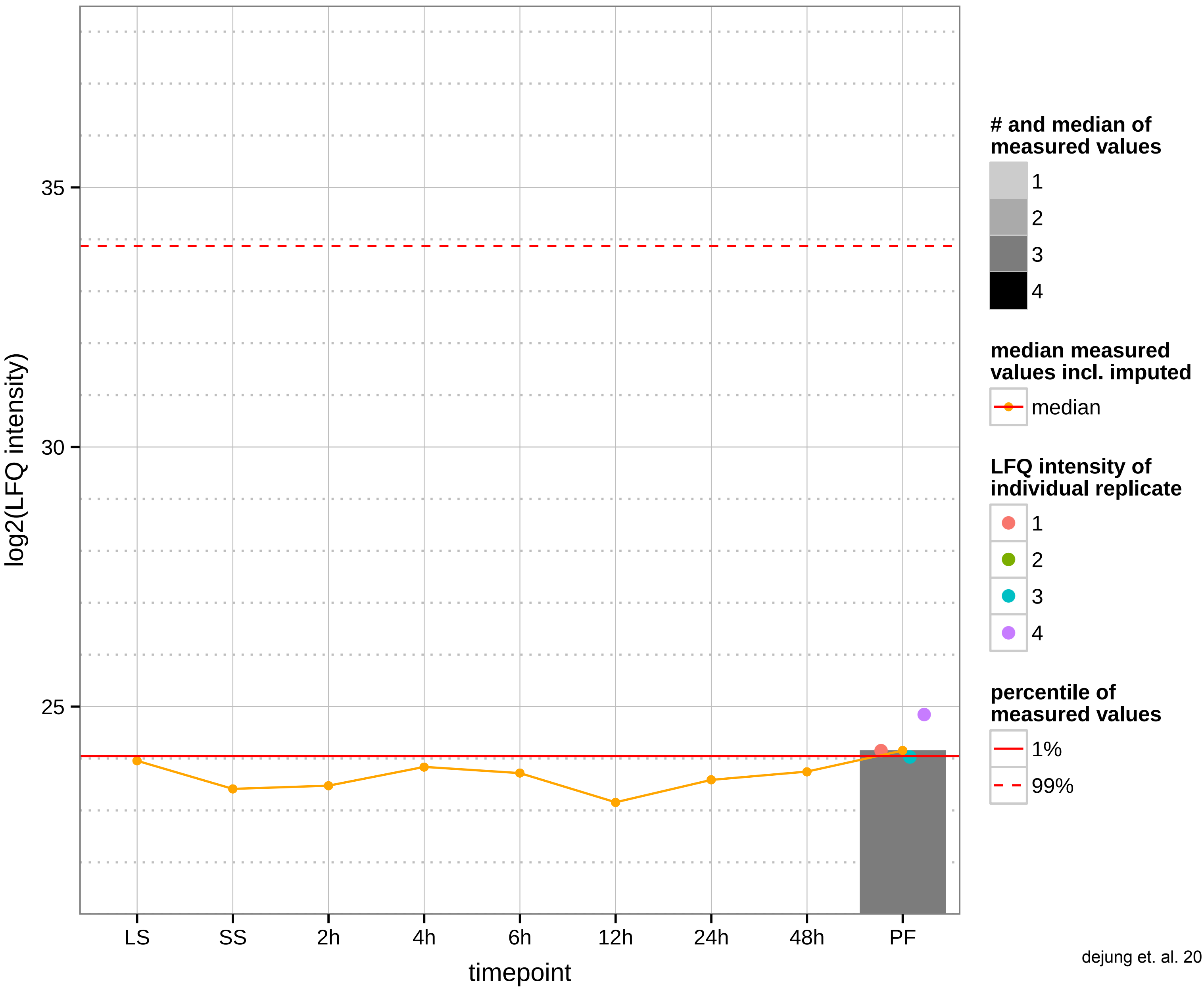

dejung et. al. 2015

poly(A) export protein, putative (TbGLE2)
Tb927.9.3760
AGOF: null
AGOC: null
AGOP: null
PGOF: protein binding
PGOC: null
PGOP: null

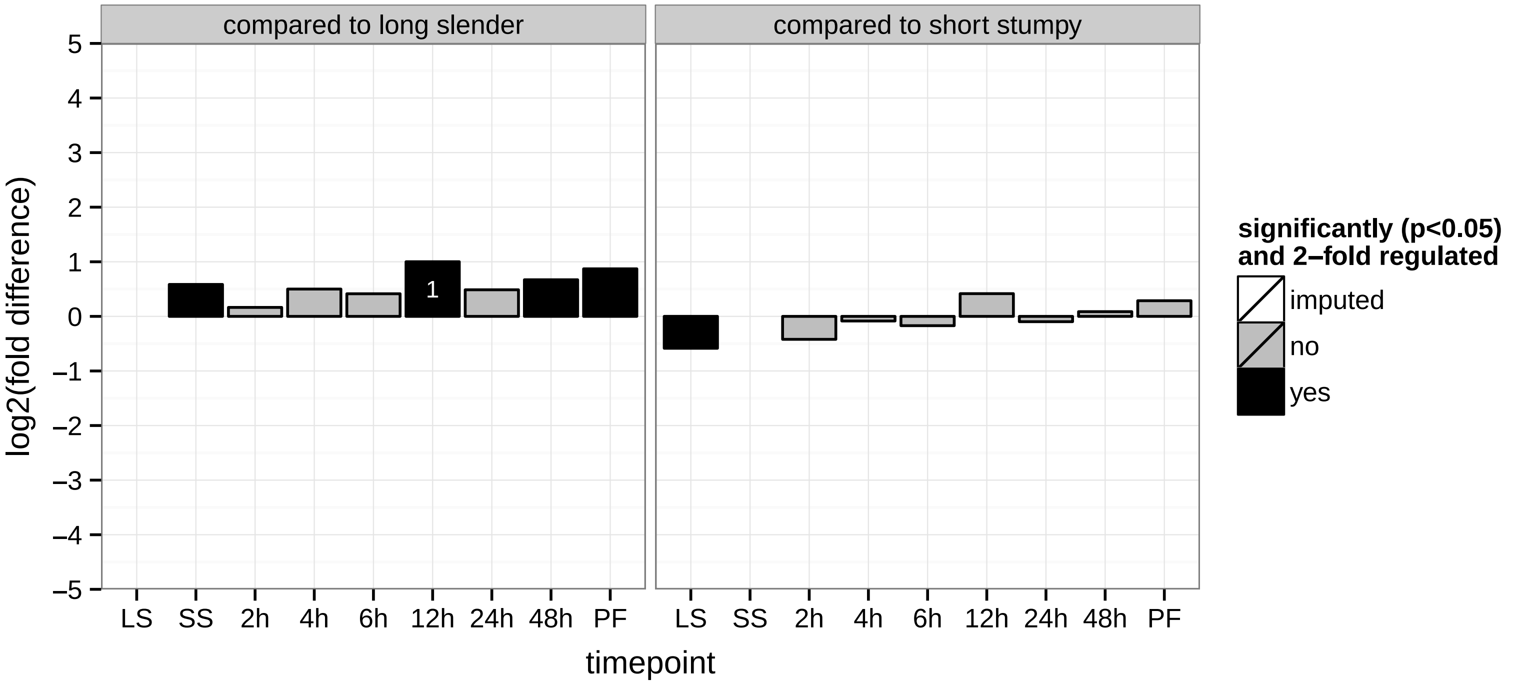

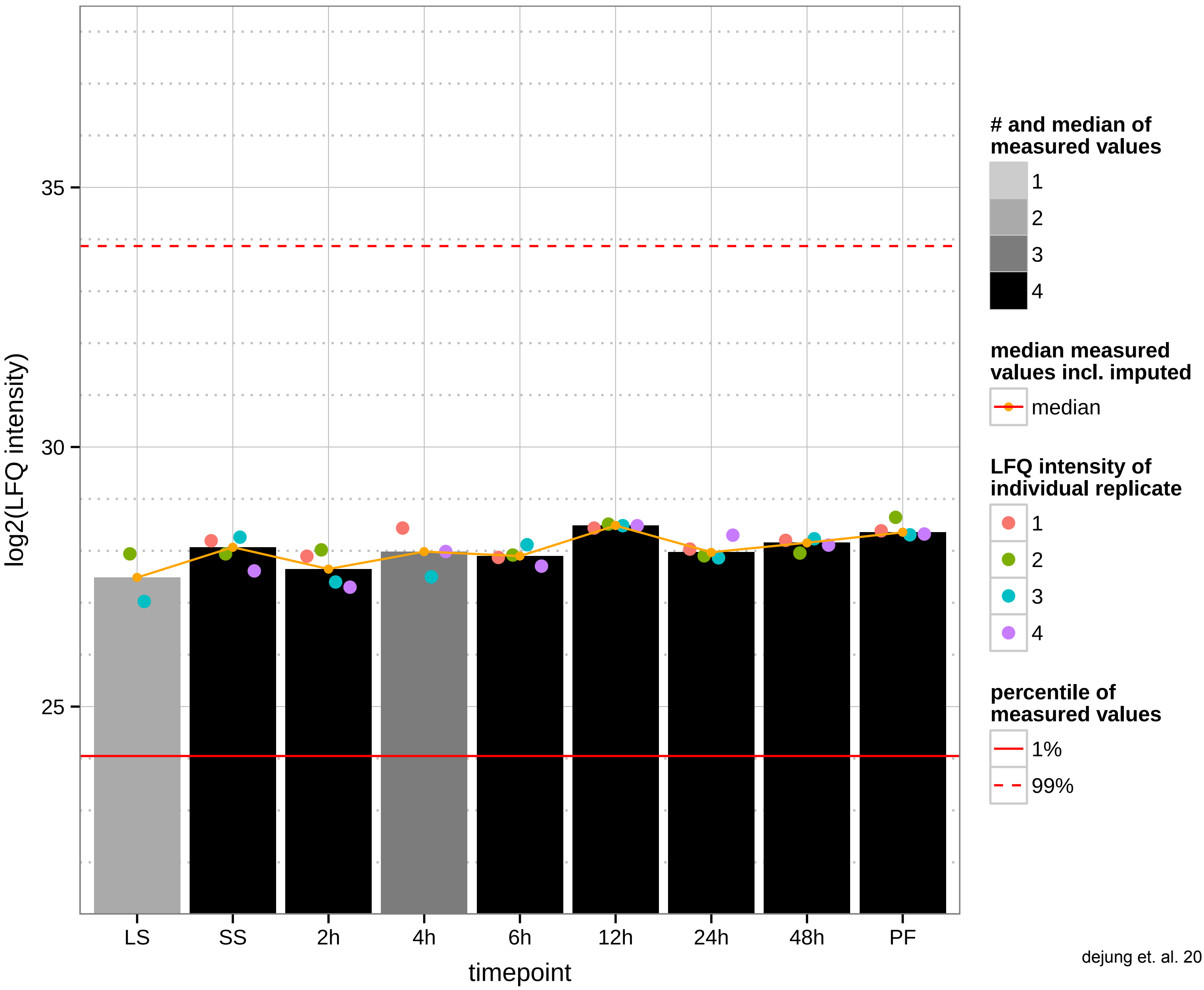

dejung et. al. 2015

hypothetical protein, conserved
Tb927.9.3870
AGOF: null
AGOC: null
AGOP: null
PGOF: null
PGOC: null
PGOP: null

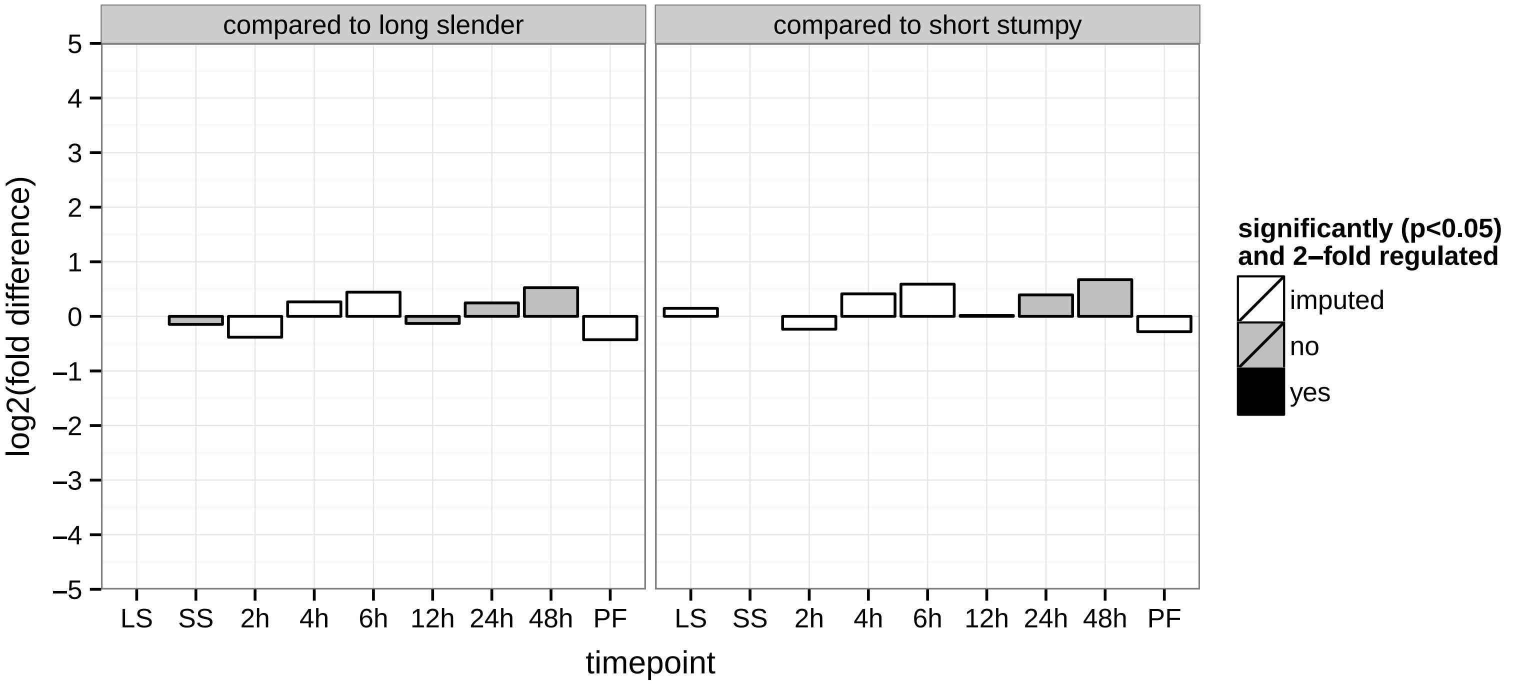

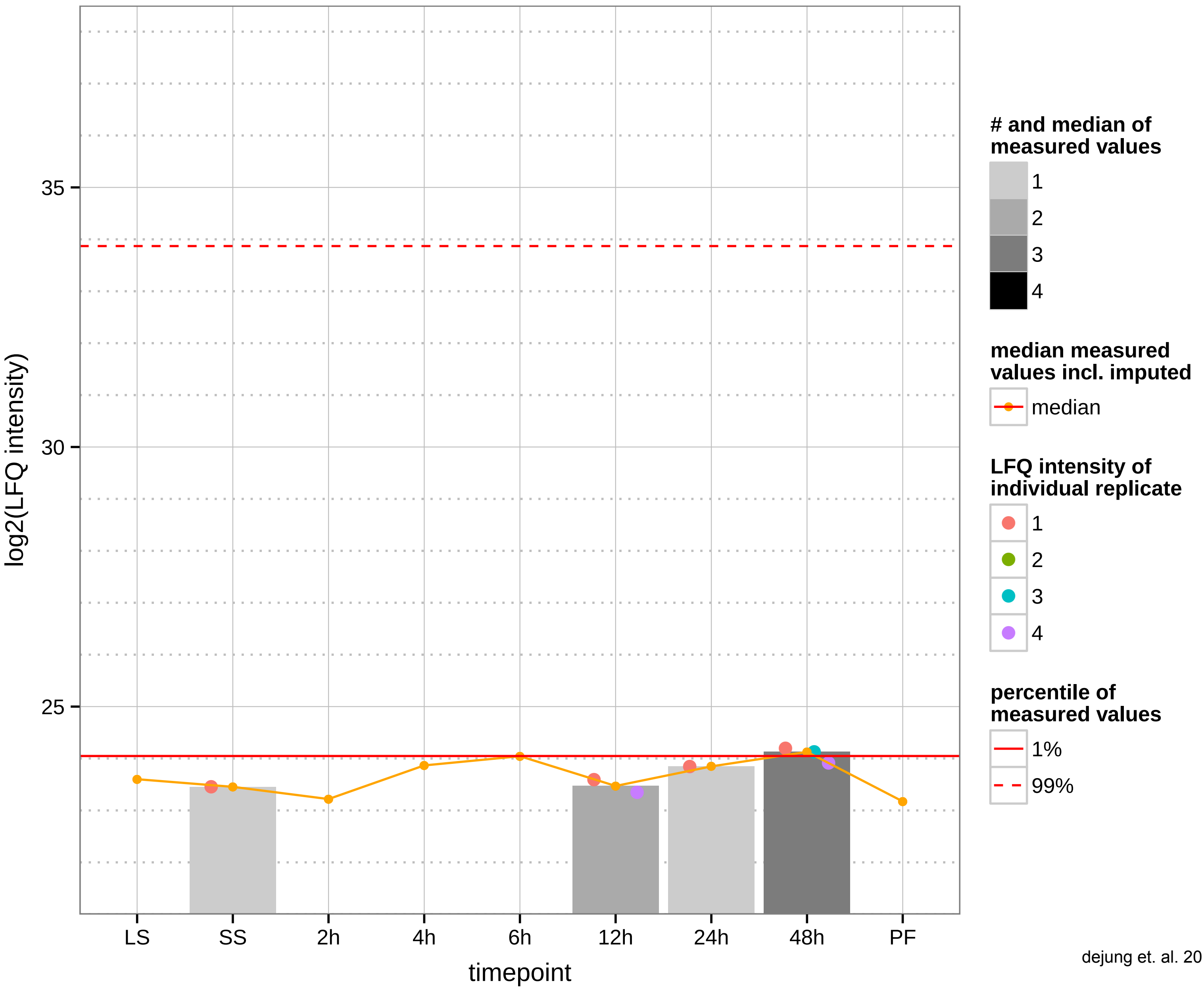

dejung et. al. 2015

nicotinamidase, putative (PNC1)
Tb927.9.4040;Tb927.9.3970
AGOF: hydrolase activity, nicotinamidase activity
AGOC: cytosol
AGOP: NAD biosynthetic process, metabolic process
PGOF: null, catalytic activity
PGOC: null
PGOP: null, metabolic process

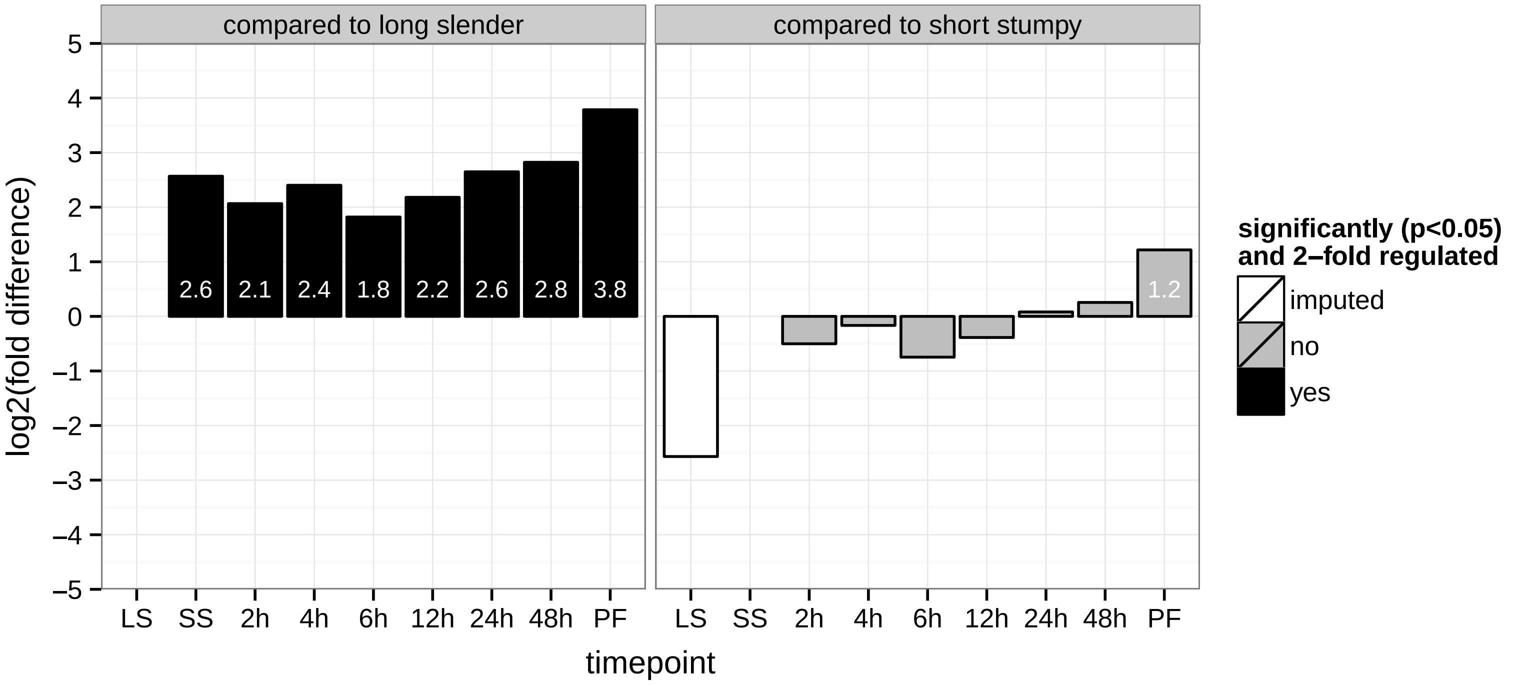

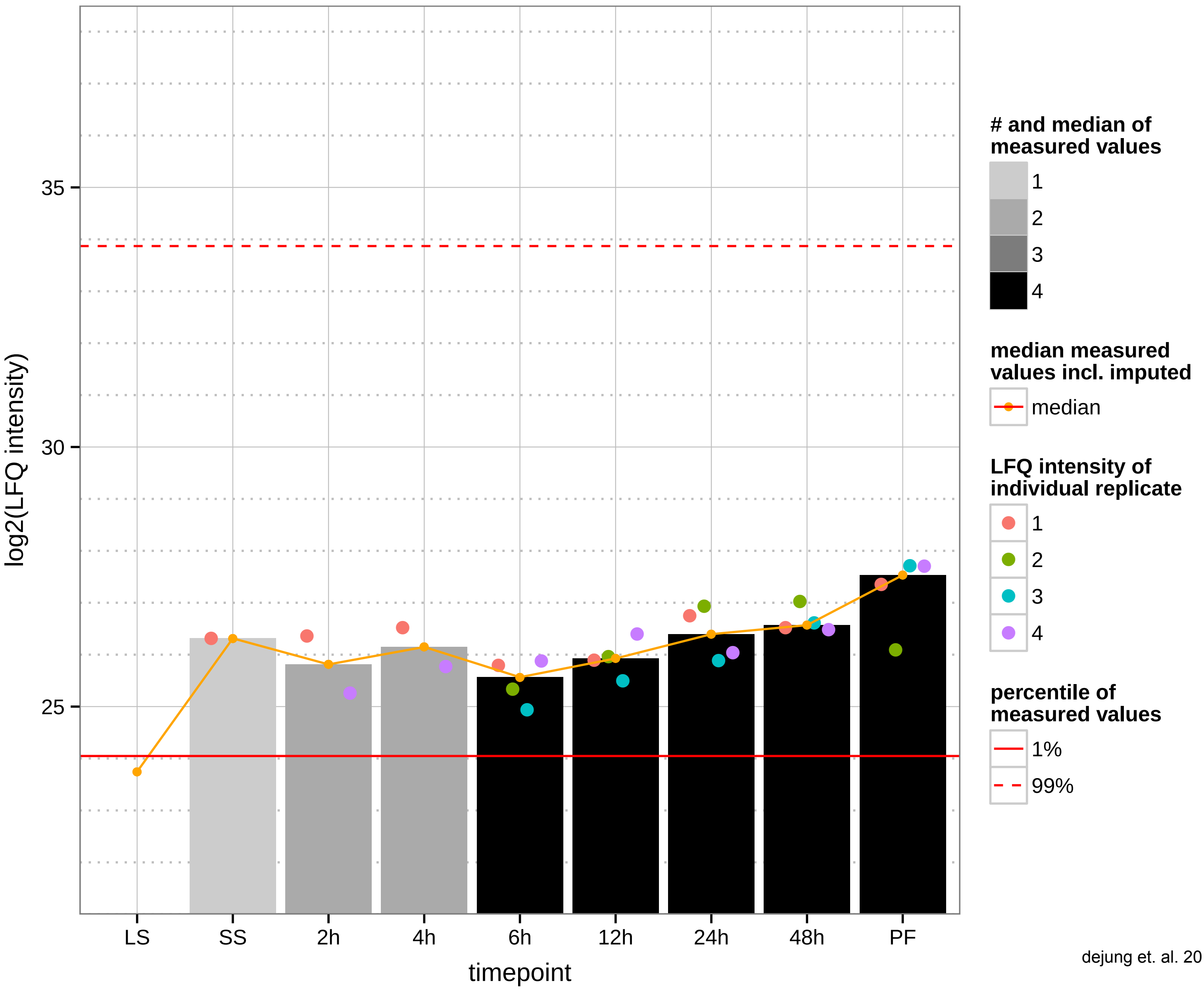

dejung et. al. 2015

ribosomal protein S7, putative
Tb927.9.3990;Tb927.9.3920
AGOF: structural constituent of ribosome
AGOC: intracellular, ribosome
AGOP: translation
PGOF: structural constituent of ribosome
PGOC: intracellular, ribosome
PGOP: translation

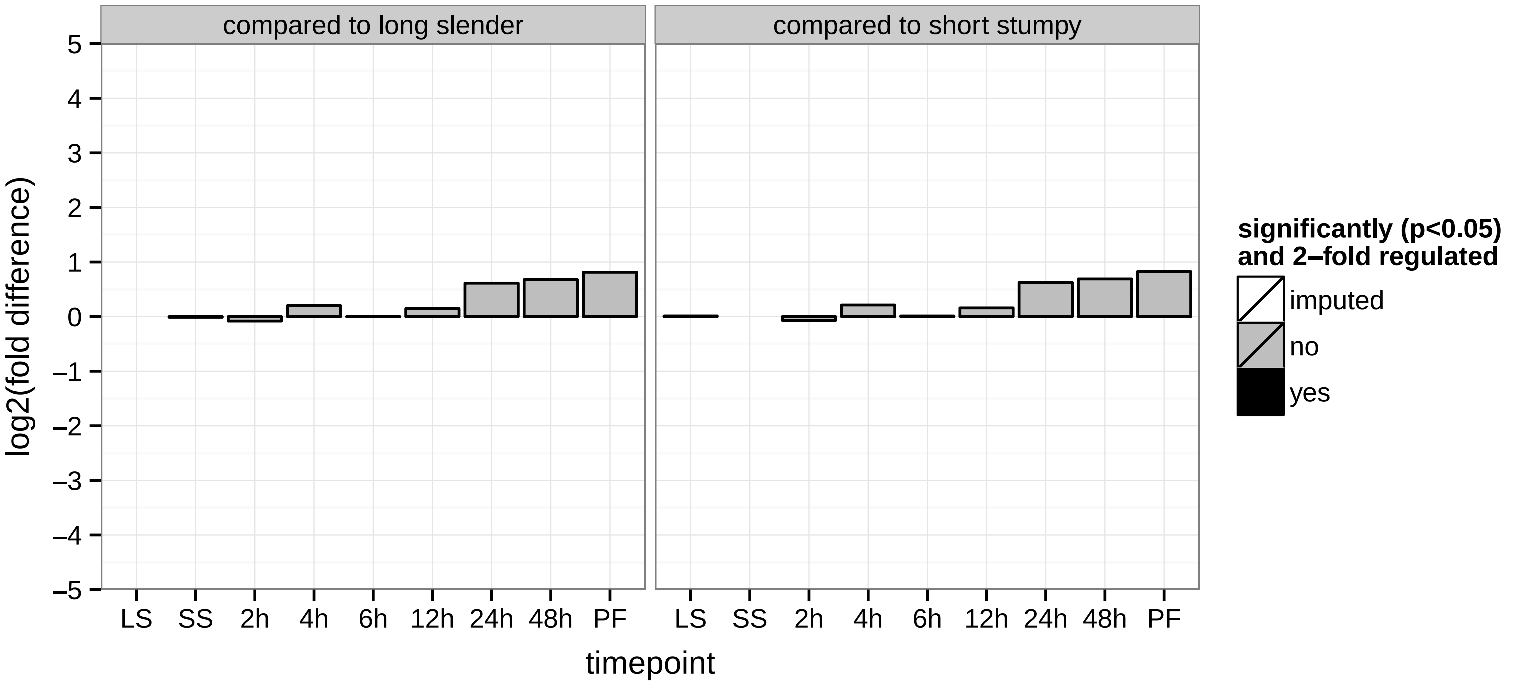

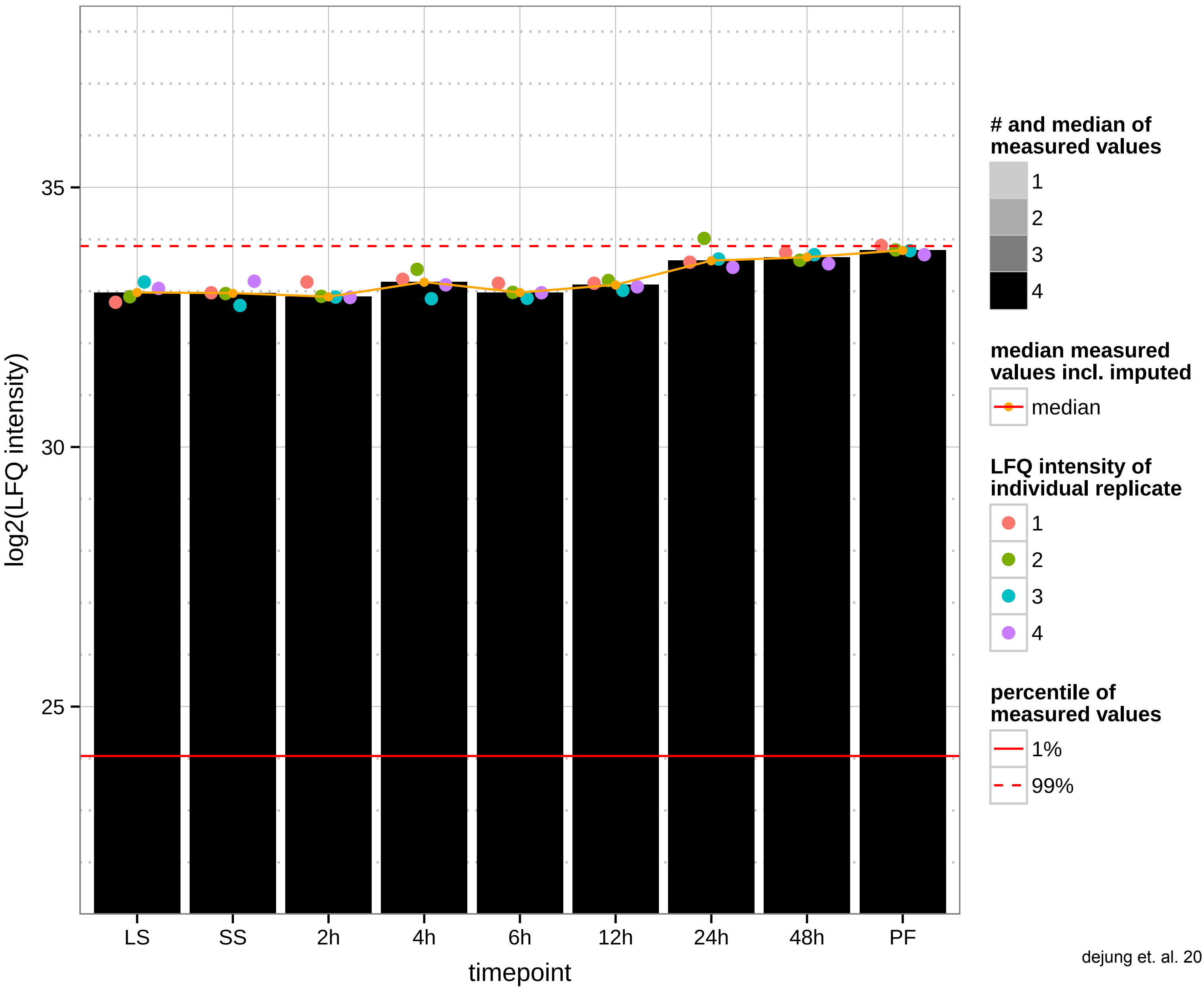

dejung et. al. 2015

hypothetical protein, conserved
Tb927.9.4050
AGOF: zinc ion binding
AGOC: null
AGOP: null
PGOF: protein binding, zinc ion binding
PGOC: null
PGOP: null

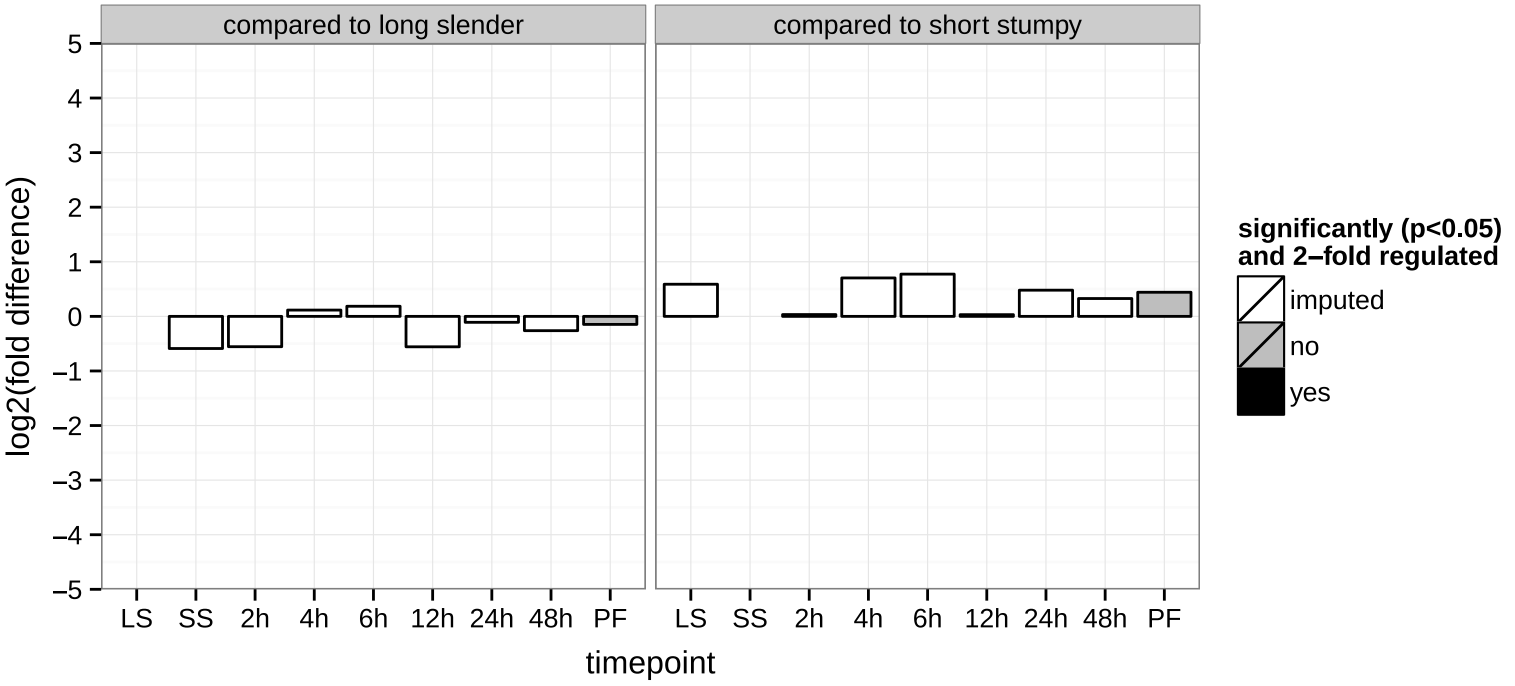

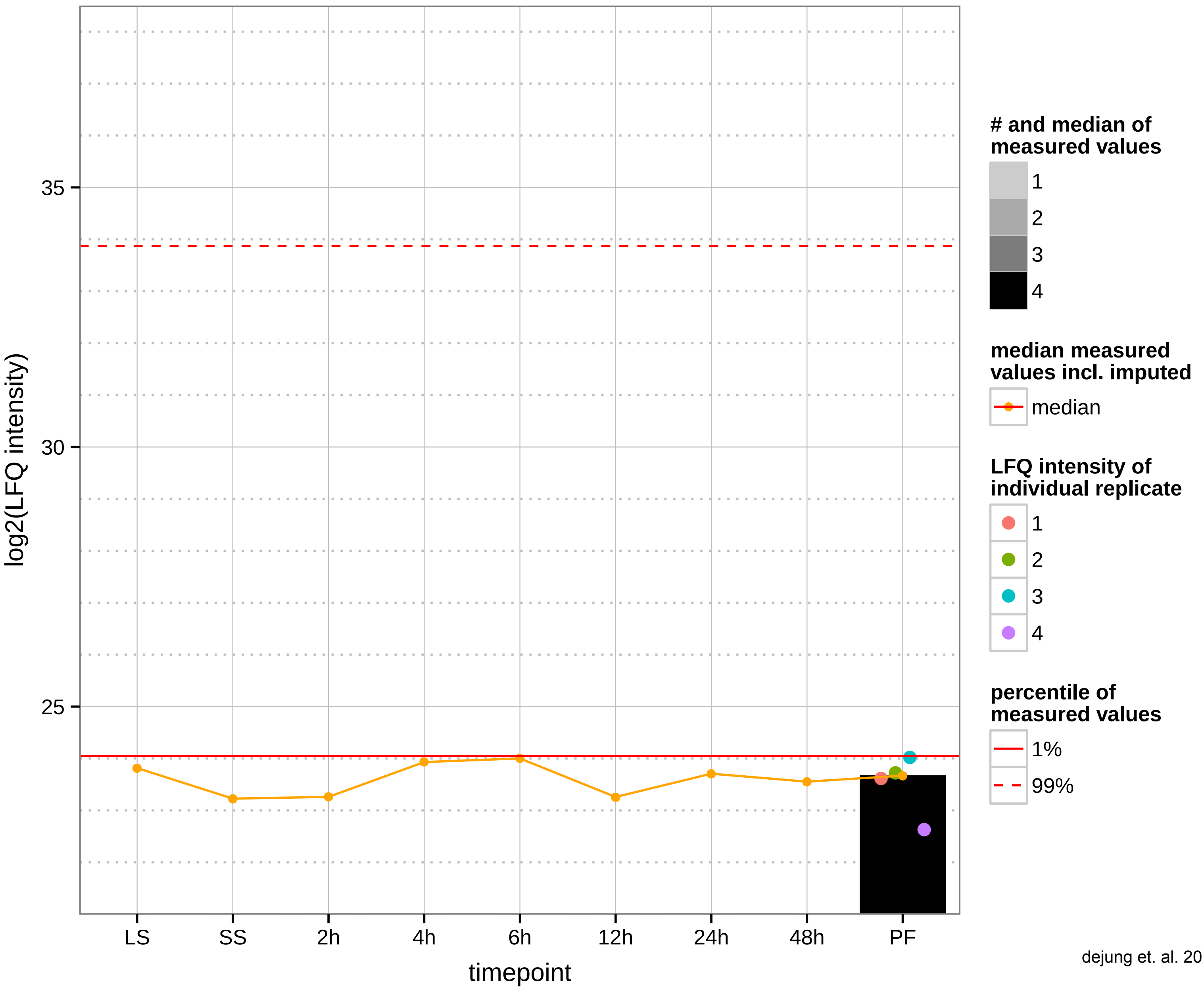

dejung et. al. 2015

conserved protein
Tb927.9.4080
AGOF: zinc ion binding
AGOC: null
AGOP: modulation of development of symbiont involved in interaction with host, quorum sensing involved in interaction with ho
PGOF: zinc ion binding
PGOC: null
PGOP: null

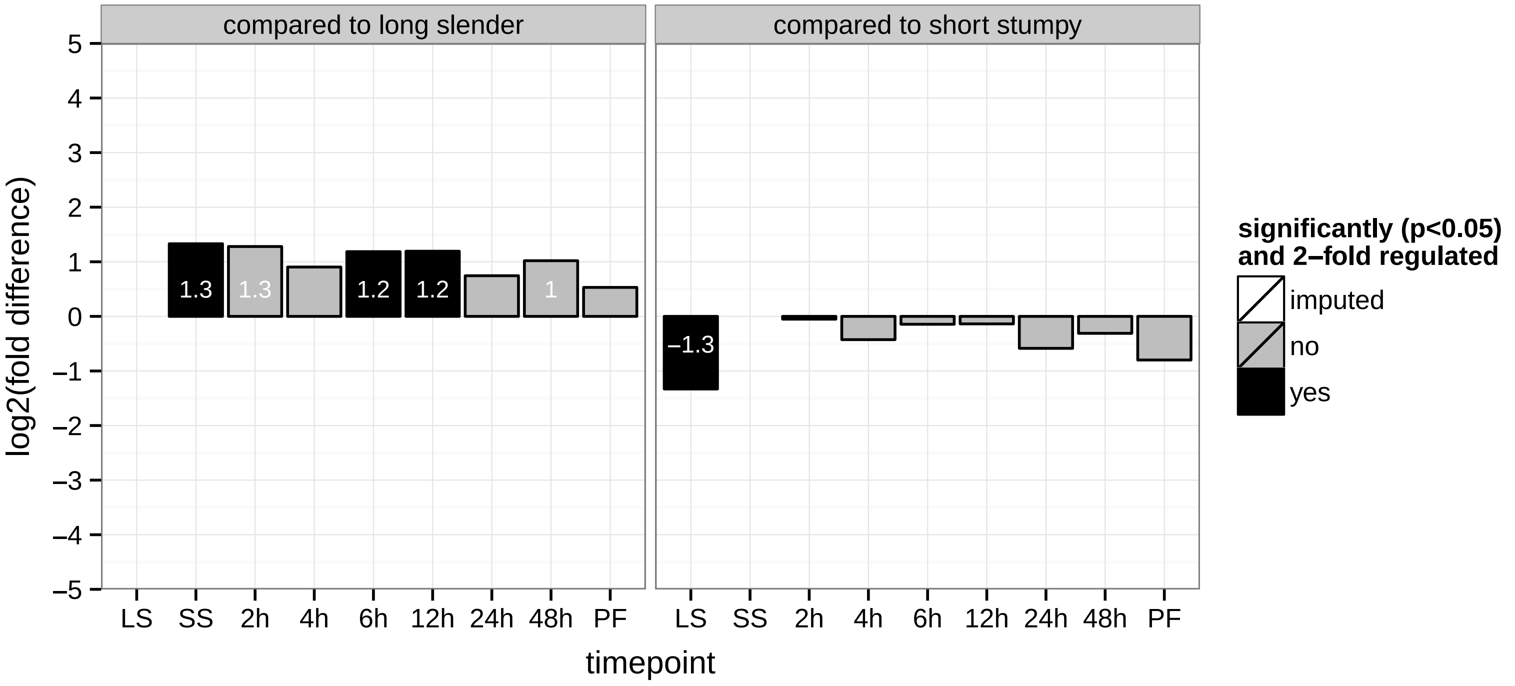

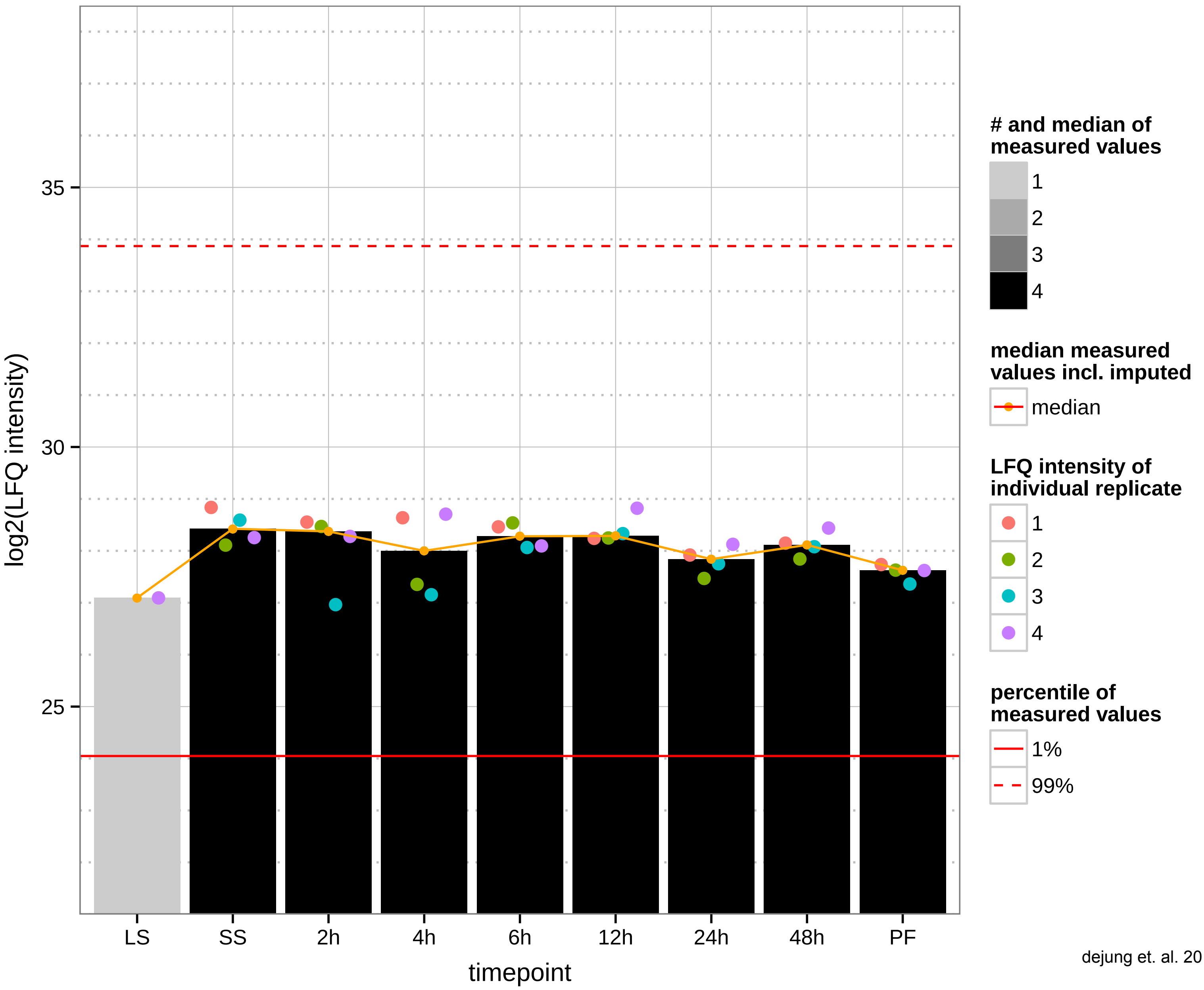

dejung et. al. 2015

fatty acyl CoA syntetase 1 (ACS1)
Tb927.9.4190
AGOF: fatty−acyl−CoA synthase activity
AGOC: membrane
AGOP: fatty acid metabolic process
PGOF: catalytic activity
PGOC: null
PGOP: metabolic process

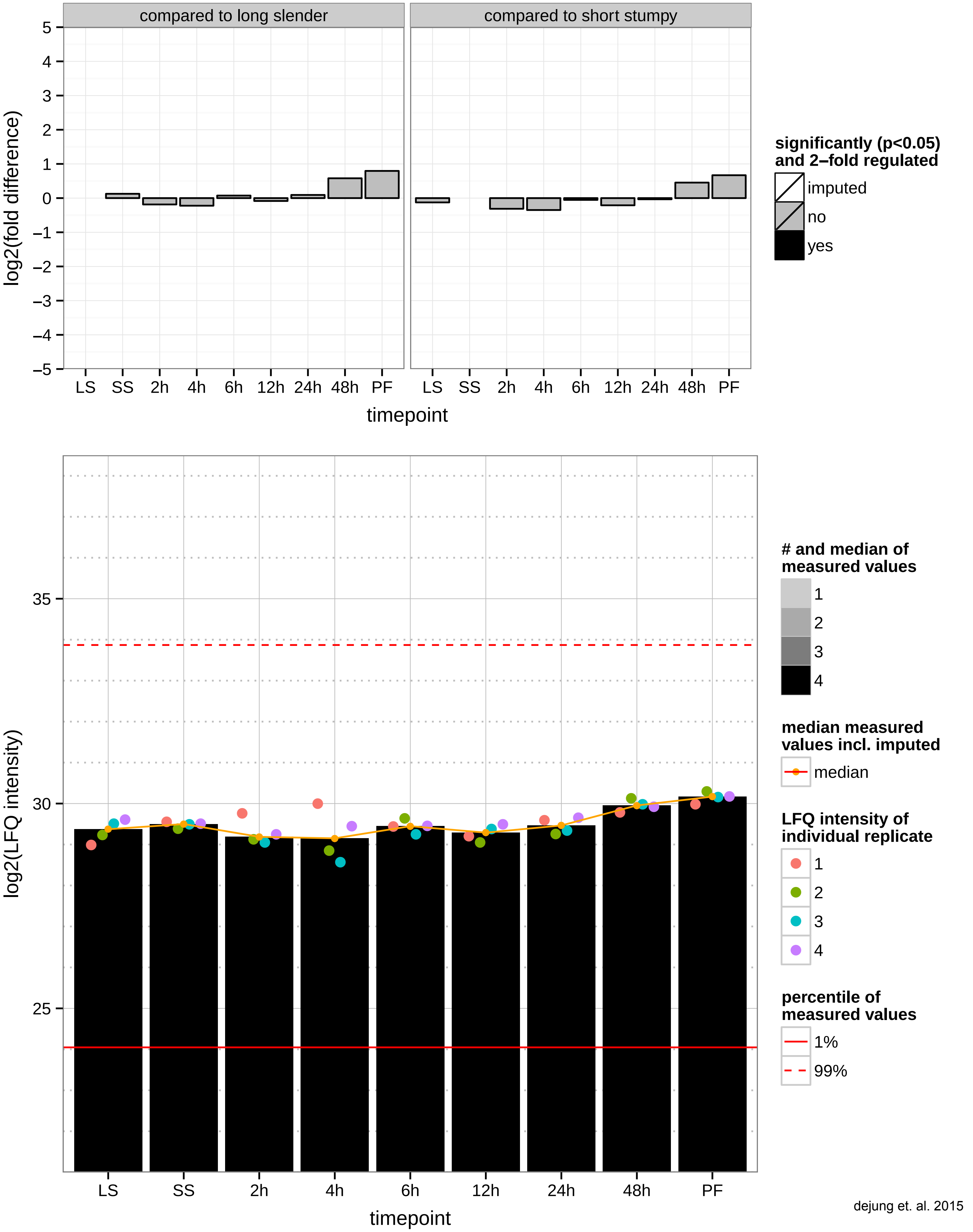

dejung et. al. 2015

fatty acyl CoA synthetase 4 (ACS4)
Tb927.9.4230
AGOF: fatty−acyl−CoA synthase activity
AGOC: membrane
AGOP: fatty acid metabolic process
PGOF: catalytic activity
PGOC: null
PGOP: metabolic process

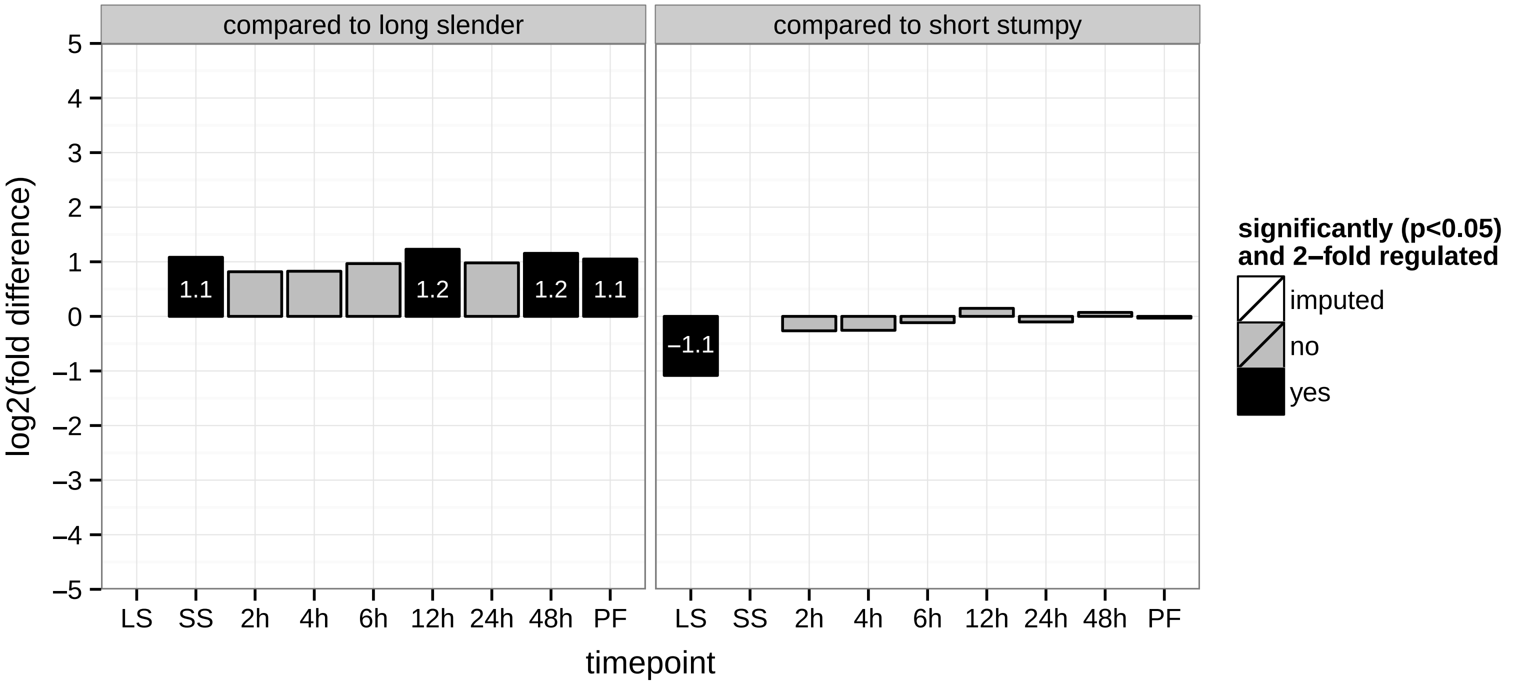

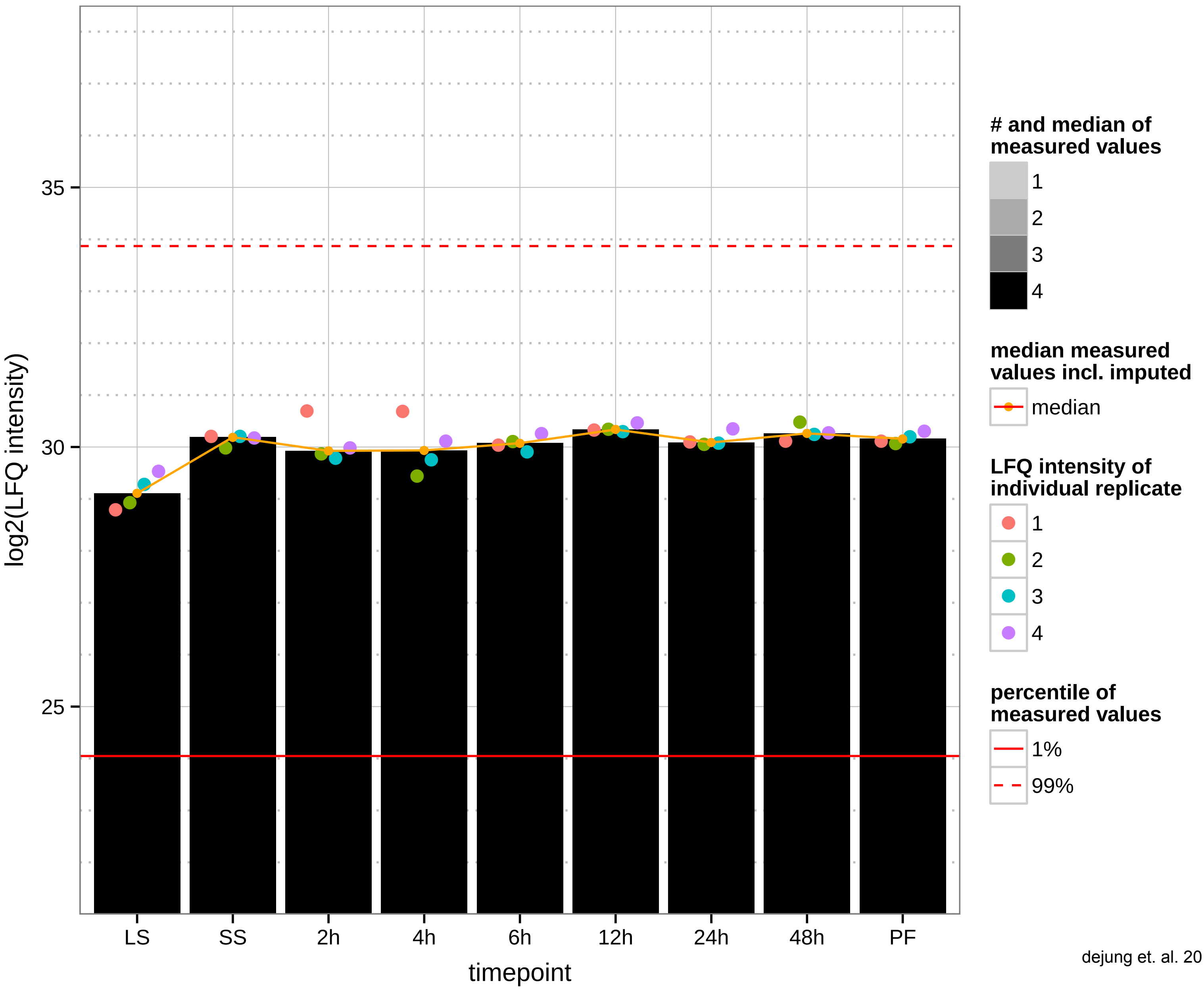

dejung et. al. 2015

hypothetical protein, conserved
Tb927.9.4250
AGOF: null
AGOC: null
AGOP: null
PGOF: null
PGOC: null
PGOP: null

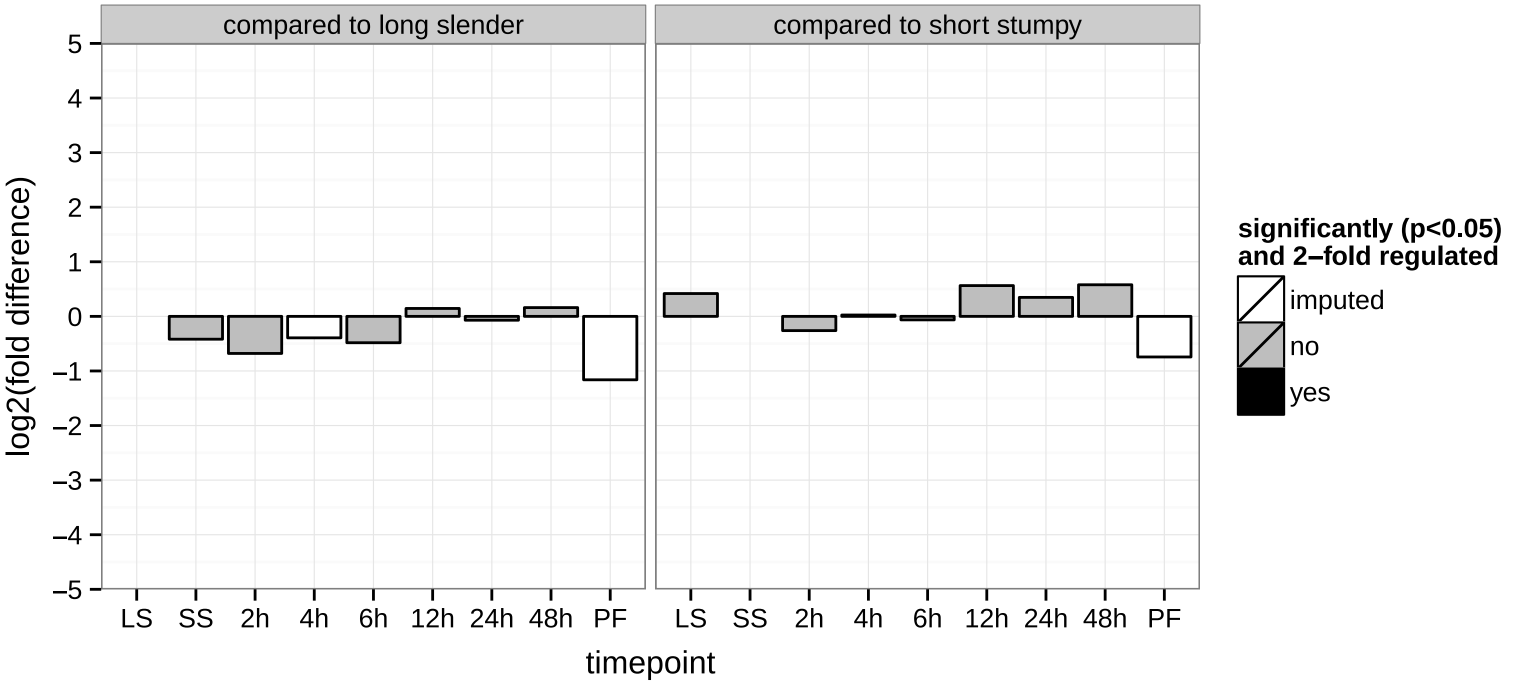

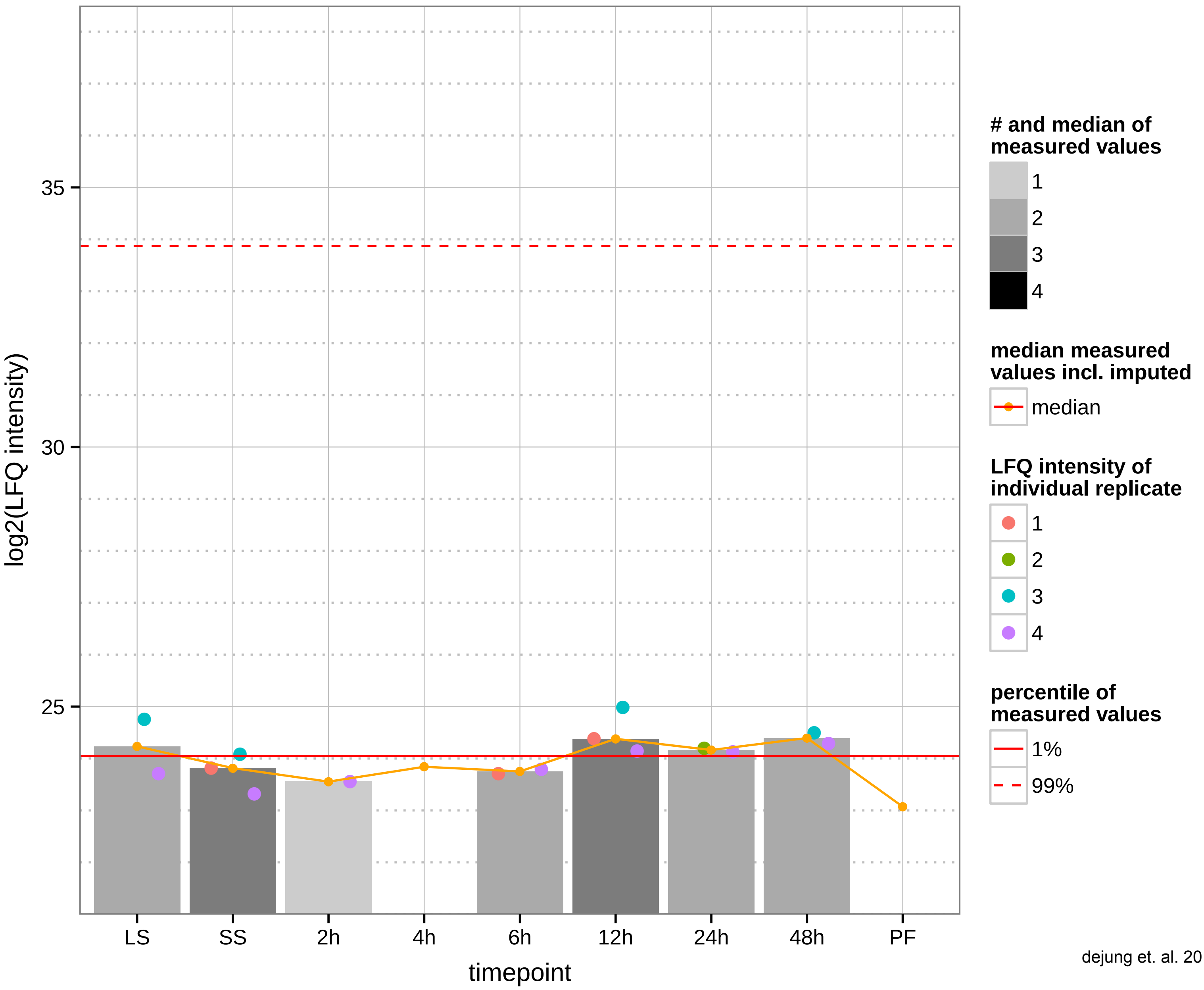

dejung et. al. 2015

DNA–damage inducible protein DDI1–like protein
Tb927.9.4390
AGOF: aspartic–type endopeptidase activity
AGOC: null
AGOP: nucleotide–excision repair, proteolysis
PGOF: aspartic–type endopeptidase activity, protein binding
PGOC: null
PGOP: proteolysis

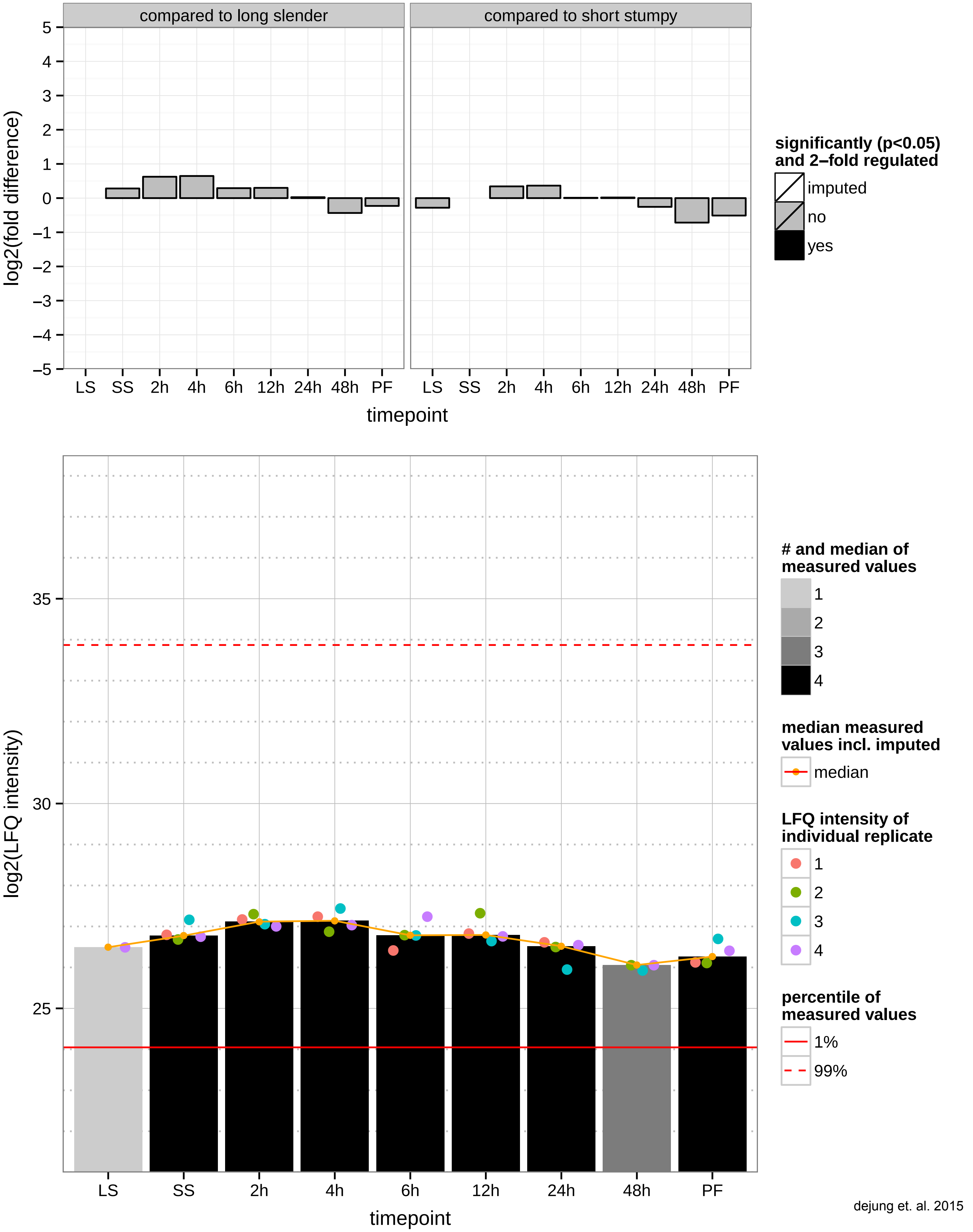

dejung et. al. 2015

hypothetical protein, conserved
Tb927.9.4420
AGOF: null
AGOC: null
AGOP: null
PGOF: null
PGOC: null
PGOP: null

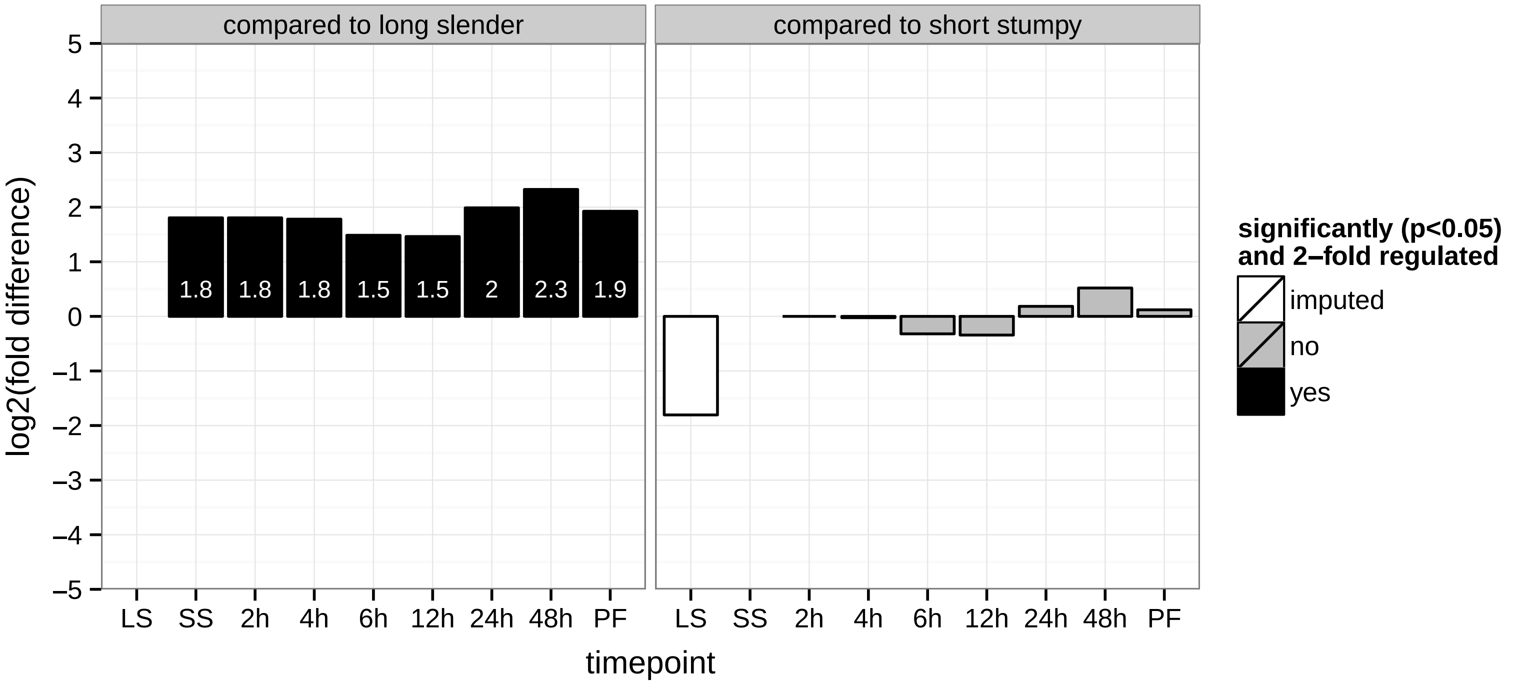

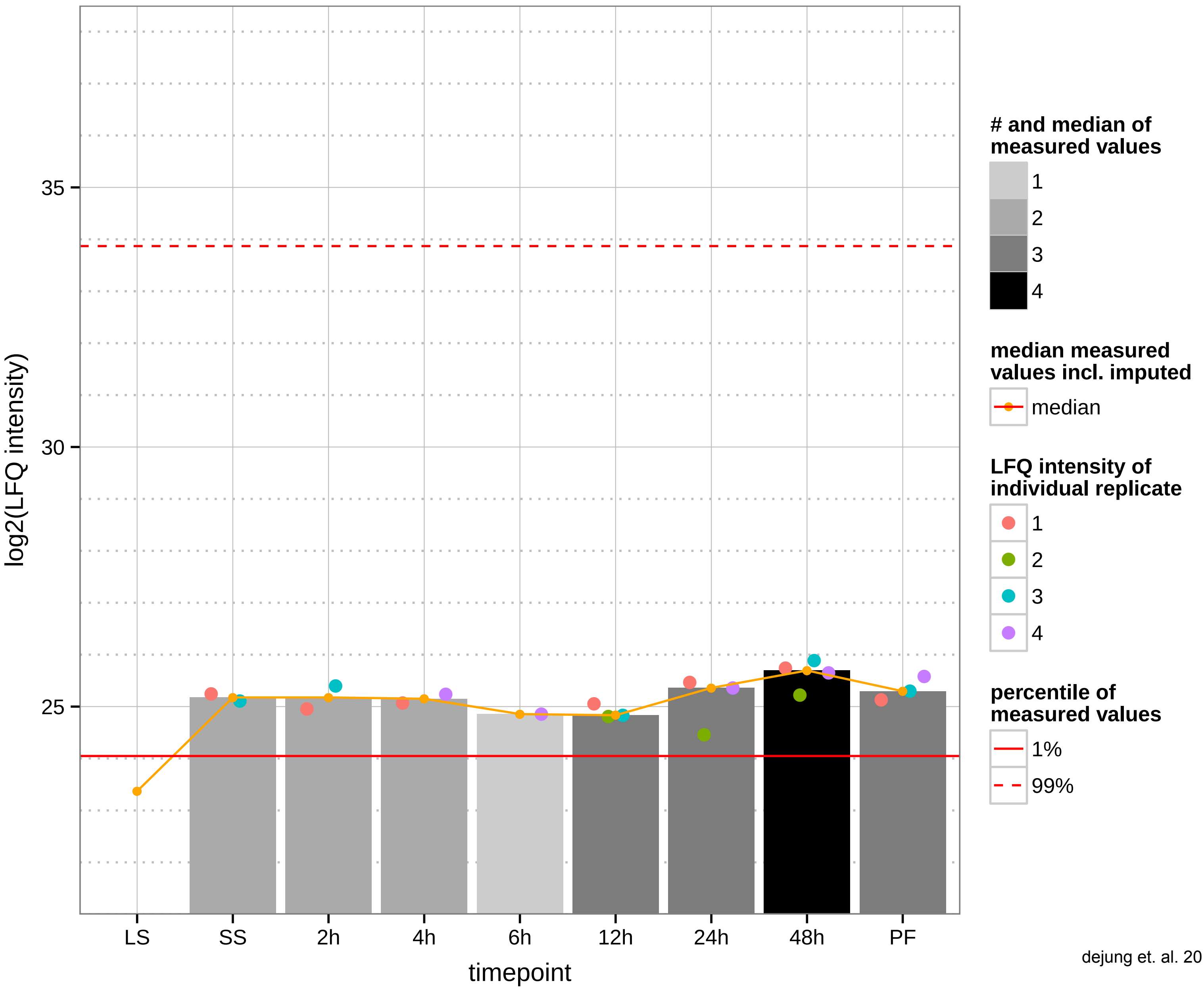

dejung et. al. 2015

mitochondrial processing peptide beta subunit, putative, metallo–peptidase, Clan ME, Family M16
Tb927.9.4520
AGOF: catalytic activity, metalloendopeptidase activity, zinc ion binding
AGOC: mitochondrial processing peptidase complex, mitochondrion
AGOP: protein processing involved in protein targeting to mitochondrion, proteolysis
PGOF: catalytic activity, metal ion binding, metalloendopeptidase activity, zinc ion binding
PGOC: null
PGOP: proteolysis

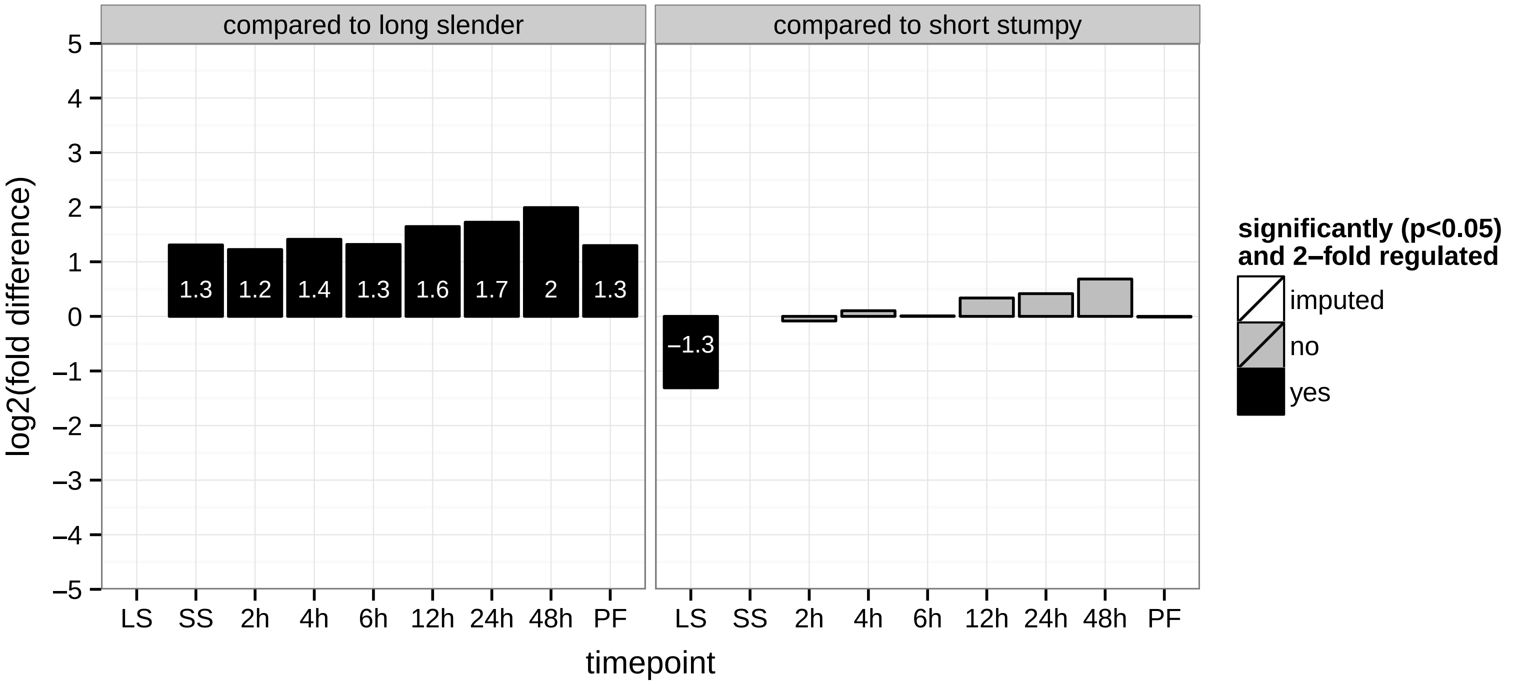

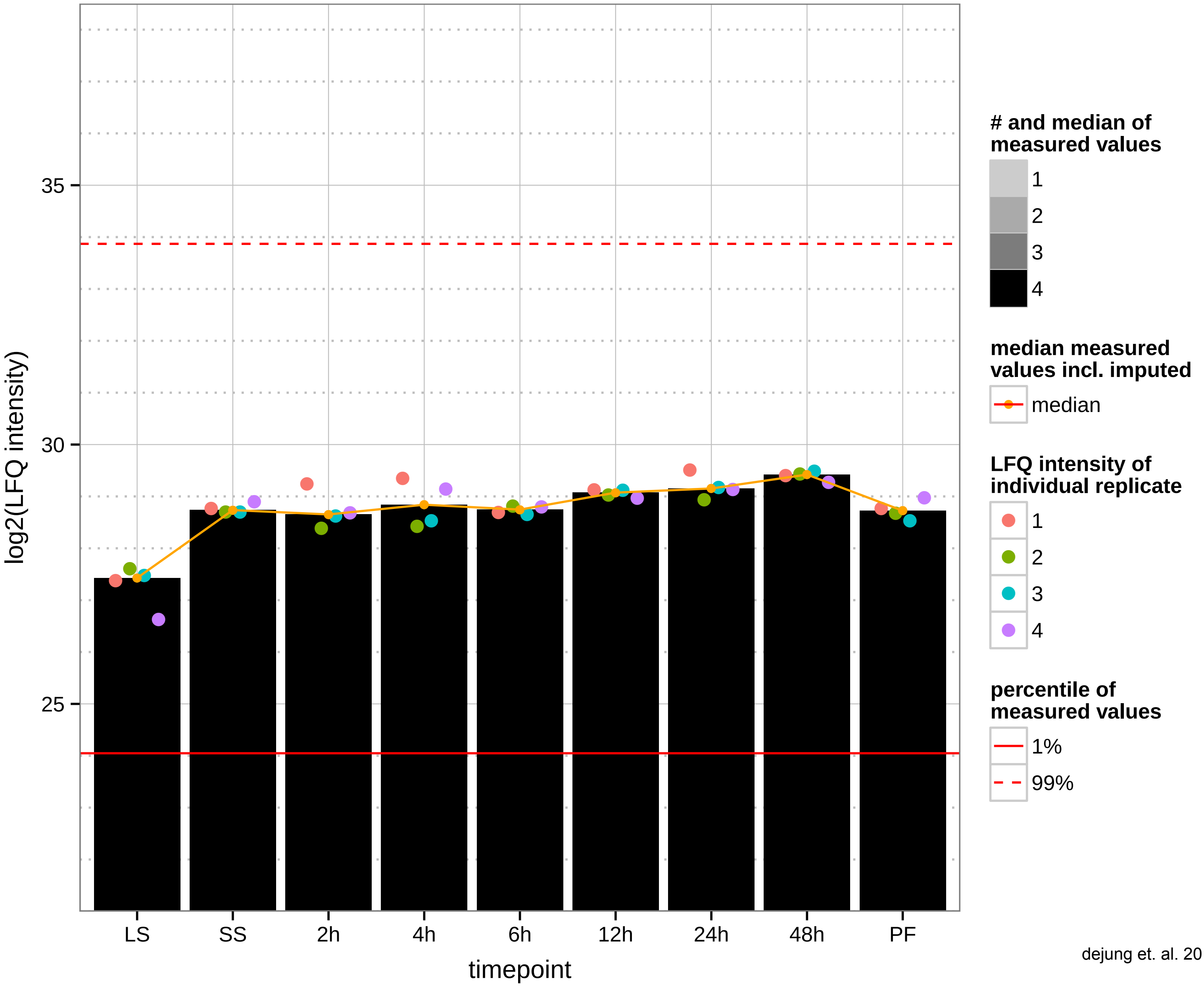

dejung et. al. 2015

hypothetical protein, conserved
Tb927.9.4660
AGOF: null
AGOC: null
AGOP: null
PGOF: null
PGOC: null
PGOP: null

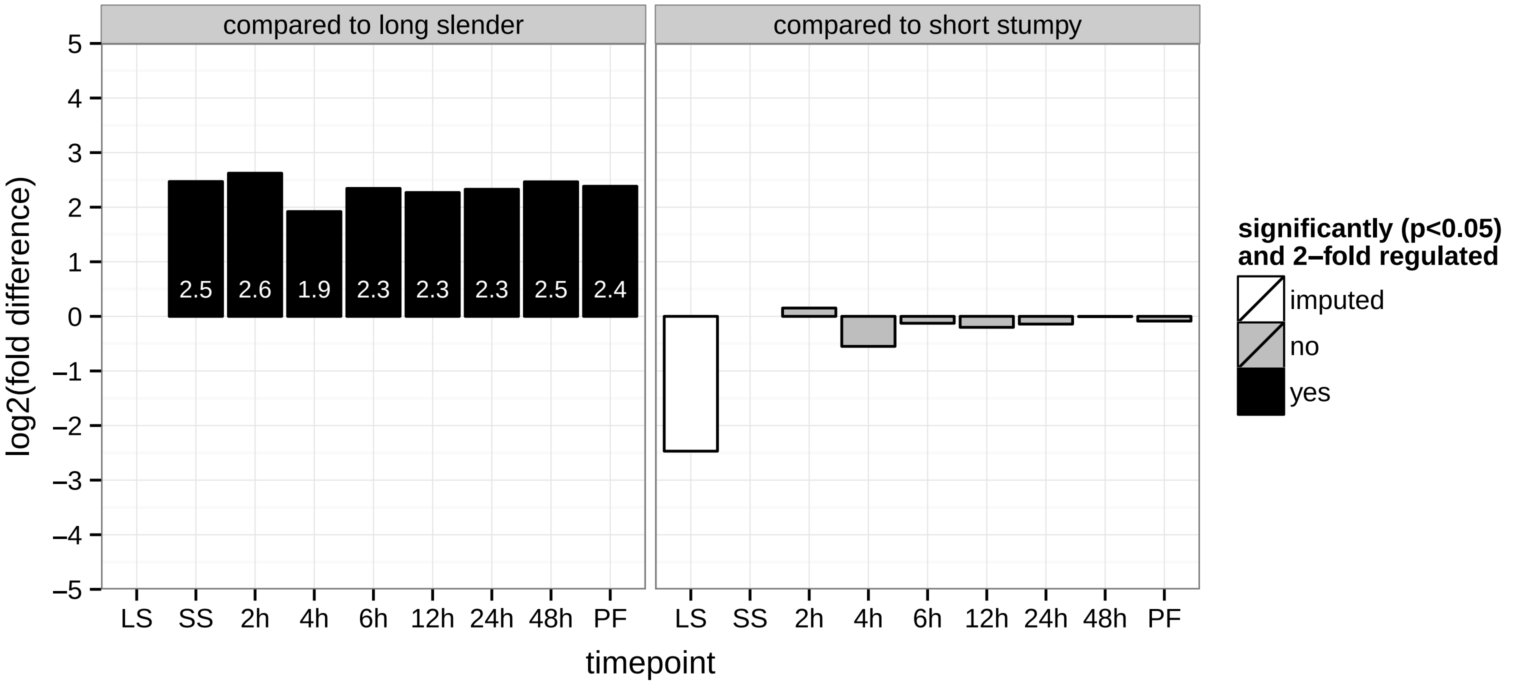

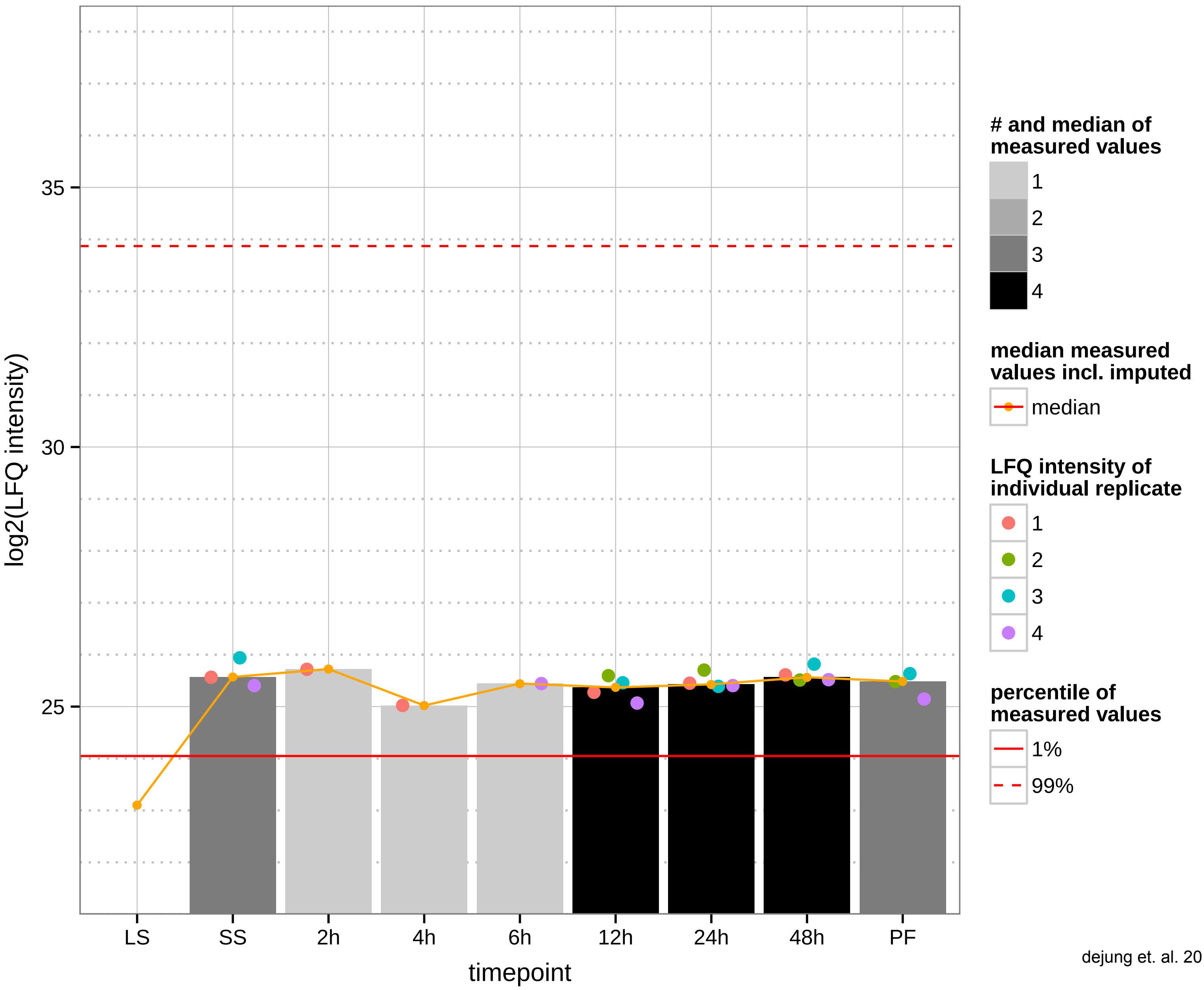

dejung et. al. 2015

ATP−dependent DEAD box helicase, putative, eukaryotic initiation factor 4a
Tb927.9.4680
AGOF: ATP binding, ATP−dependent helicase activity, helicase activity, nucleic acid binding
AGOC: cytosol
AGOP: nucleobase−containing compound metabolic process, translation
PGOF: ATP binding, ATP−dependent helicase activity, helicase activity, nucleic acid binding
PGOC: null
PGOP: null

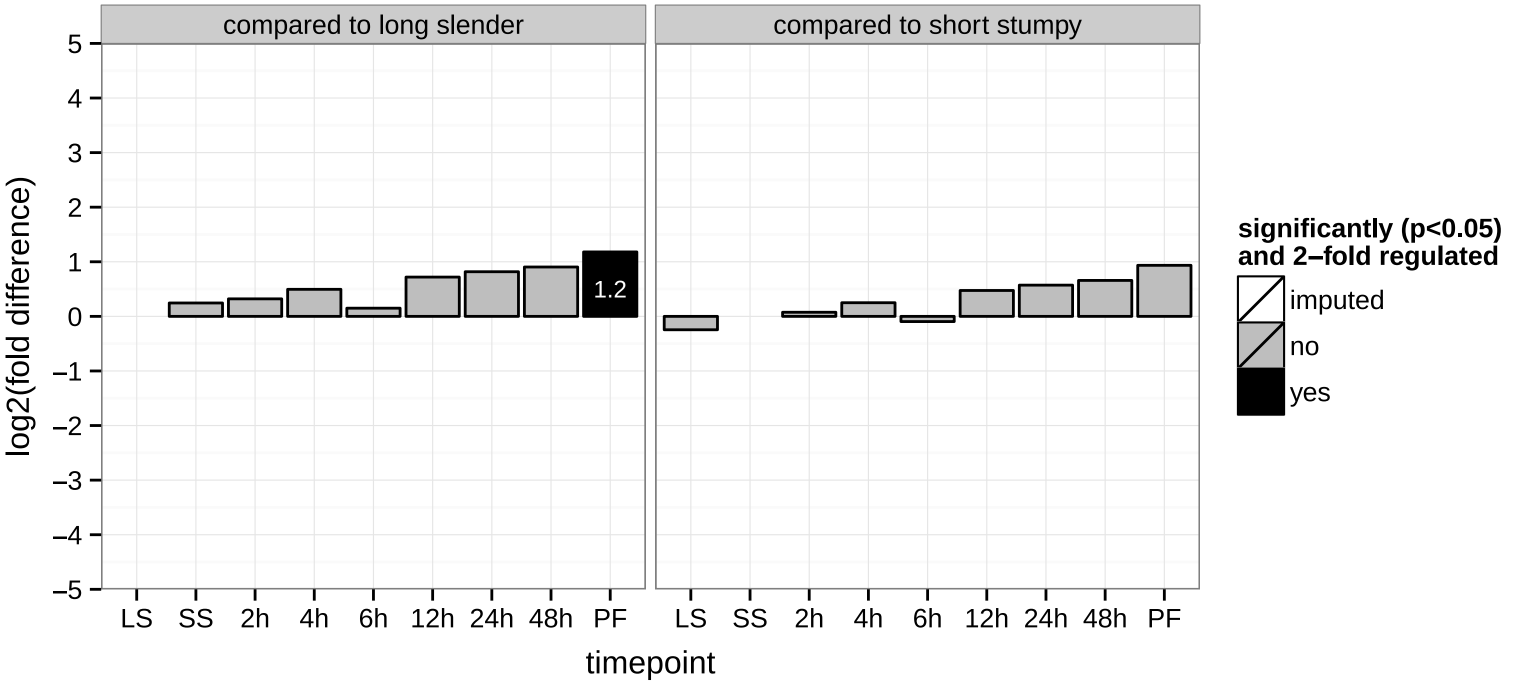

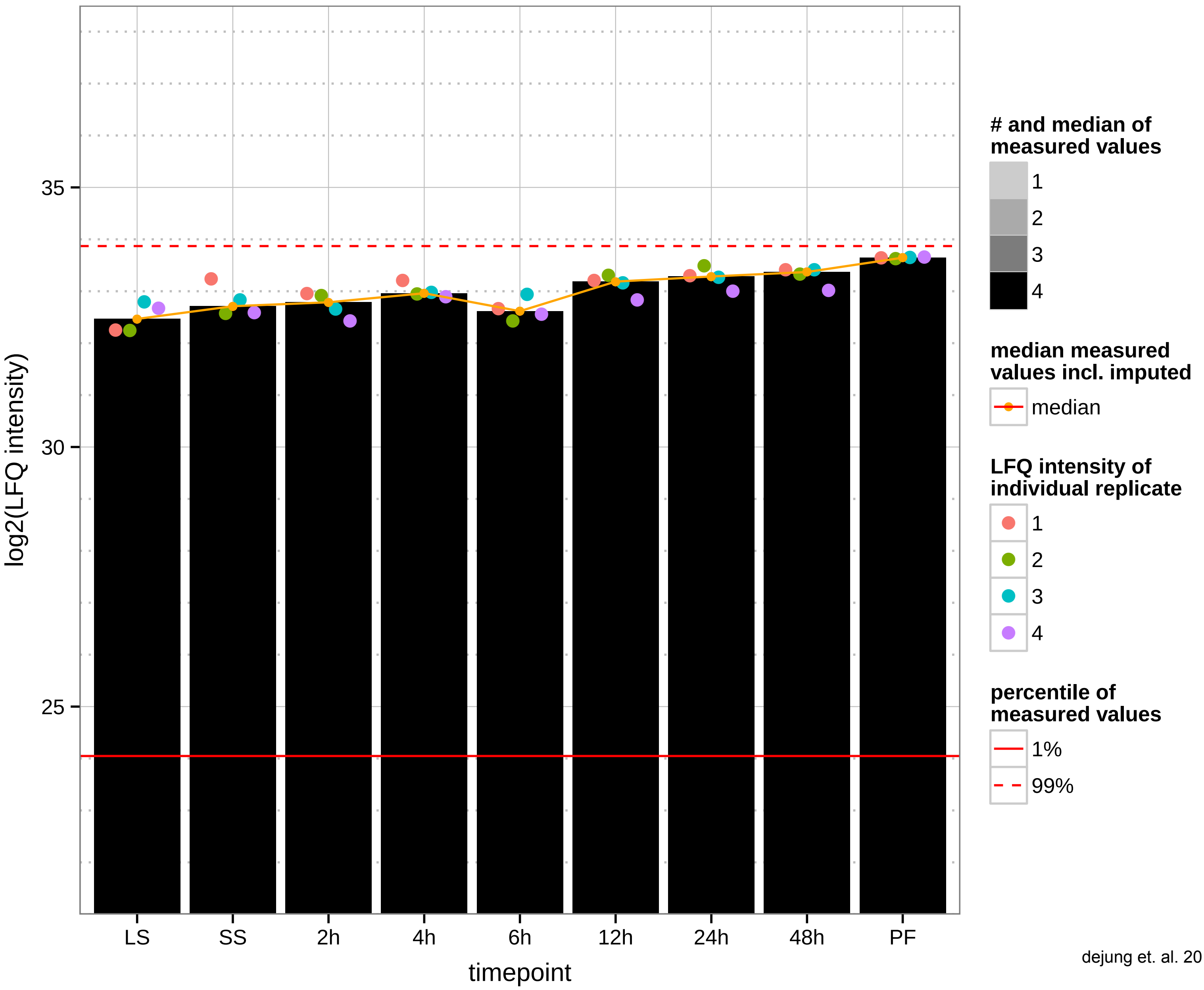

dejung et. al. 2015

hypothetical protein, conserved
Tb927.9.5000
AGOF: null
AGOC: null
AGOP: null
PGOF: null
PGOC: null
PGOP: null

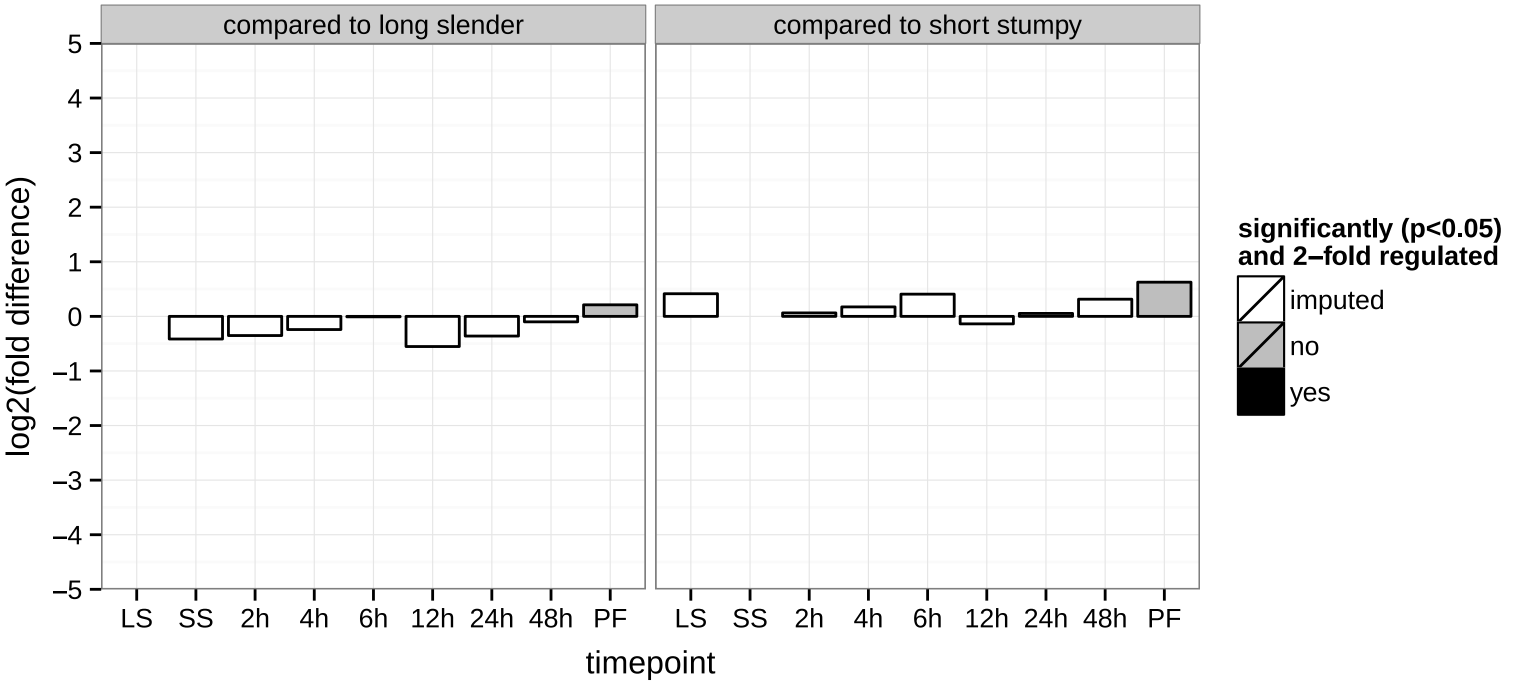

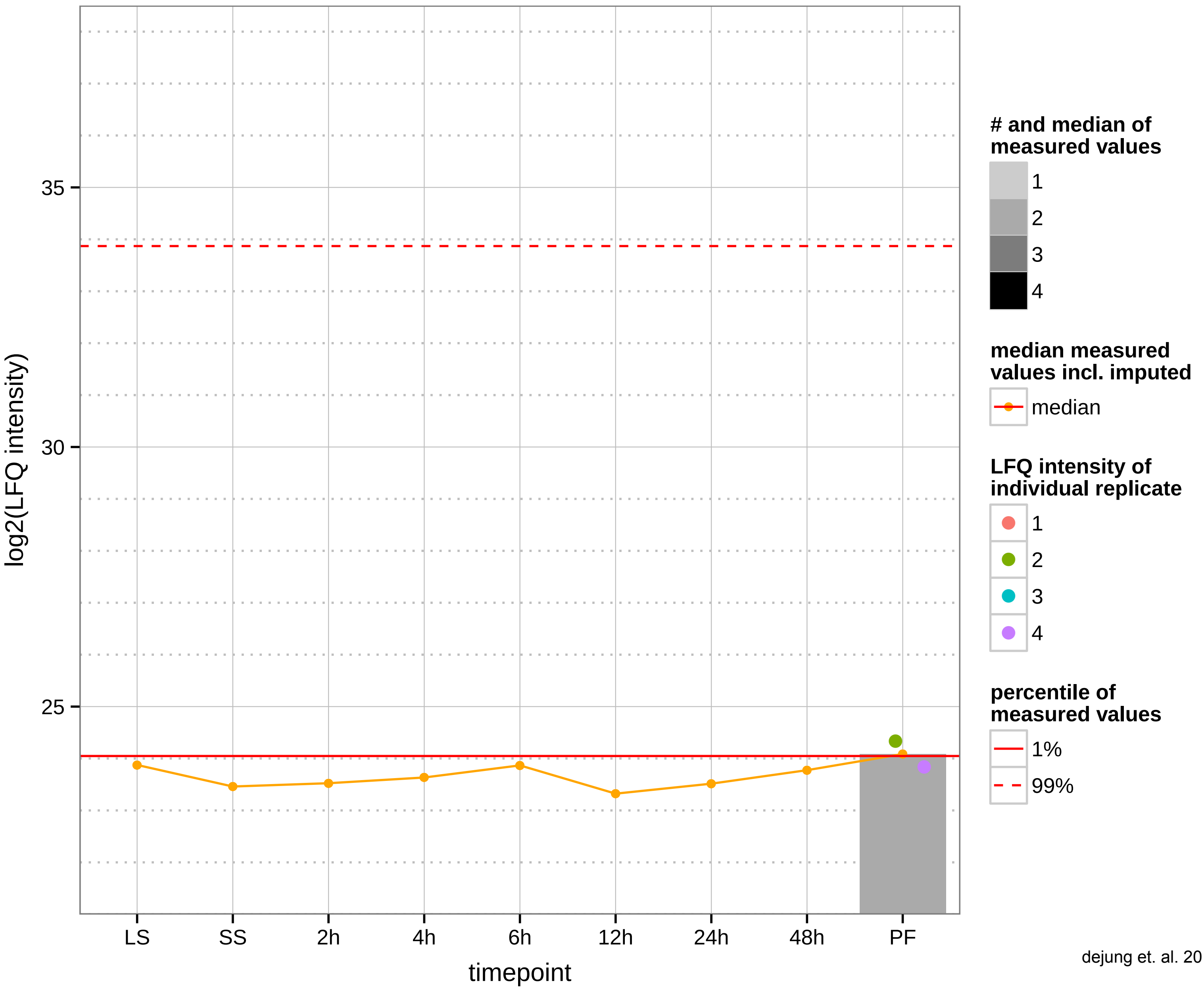

dejung et. al. 2015

hypothetical protein, conserved
Tb927.9.5020
AGOF: null
AGOC: null
AGOP: null
PGOF: protein binding
PGOC: null
PGOP: null

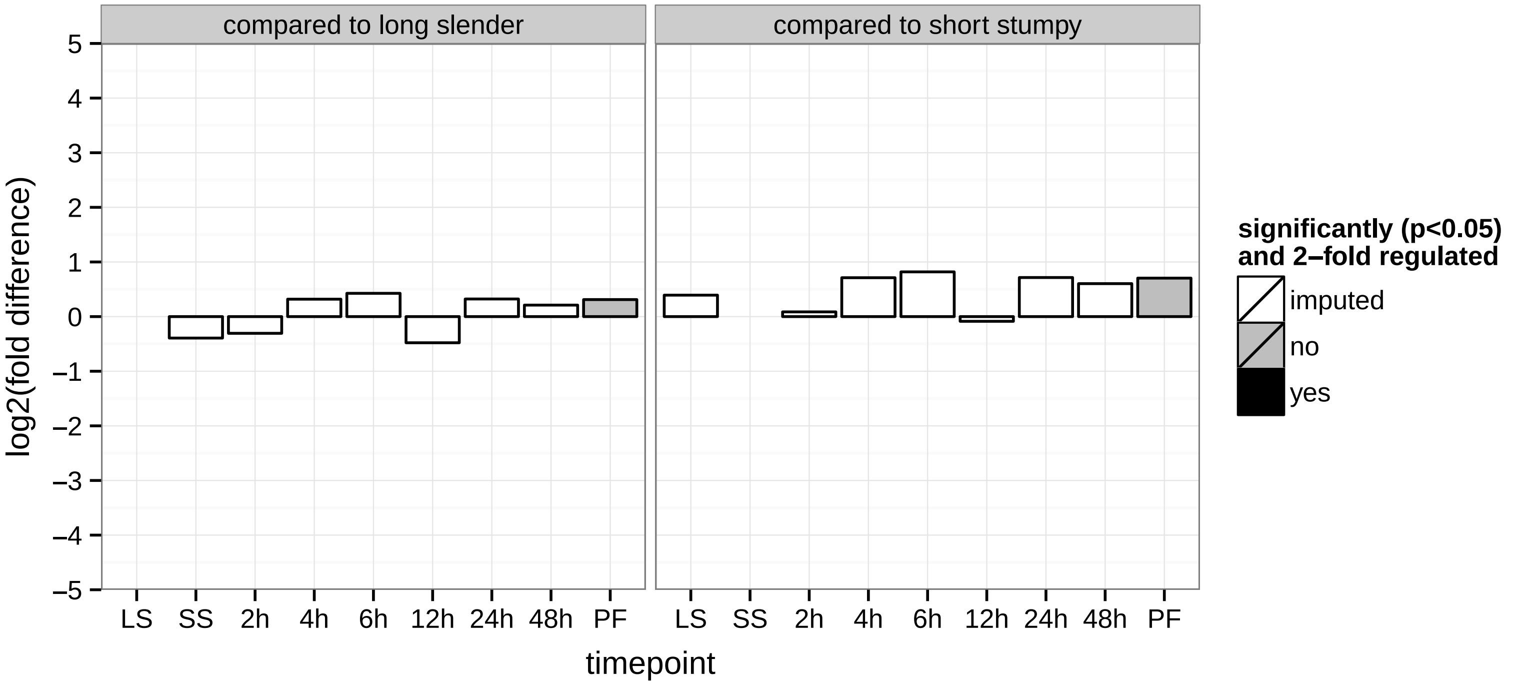

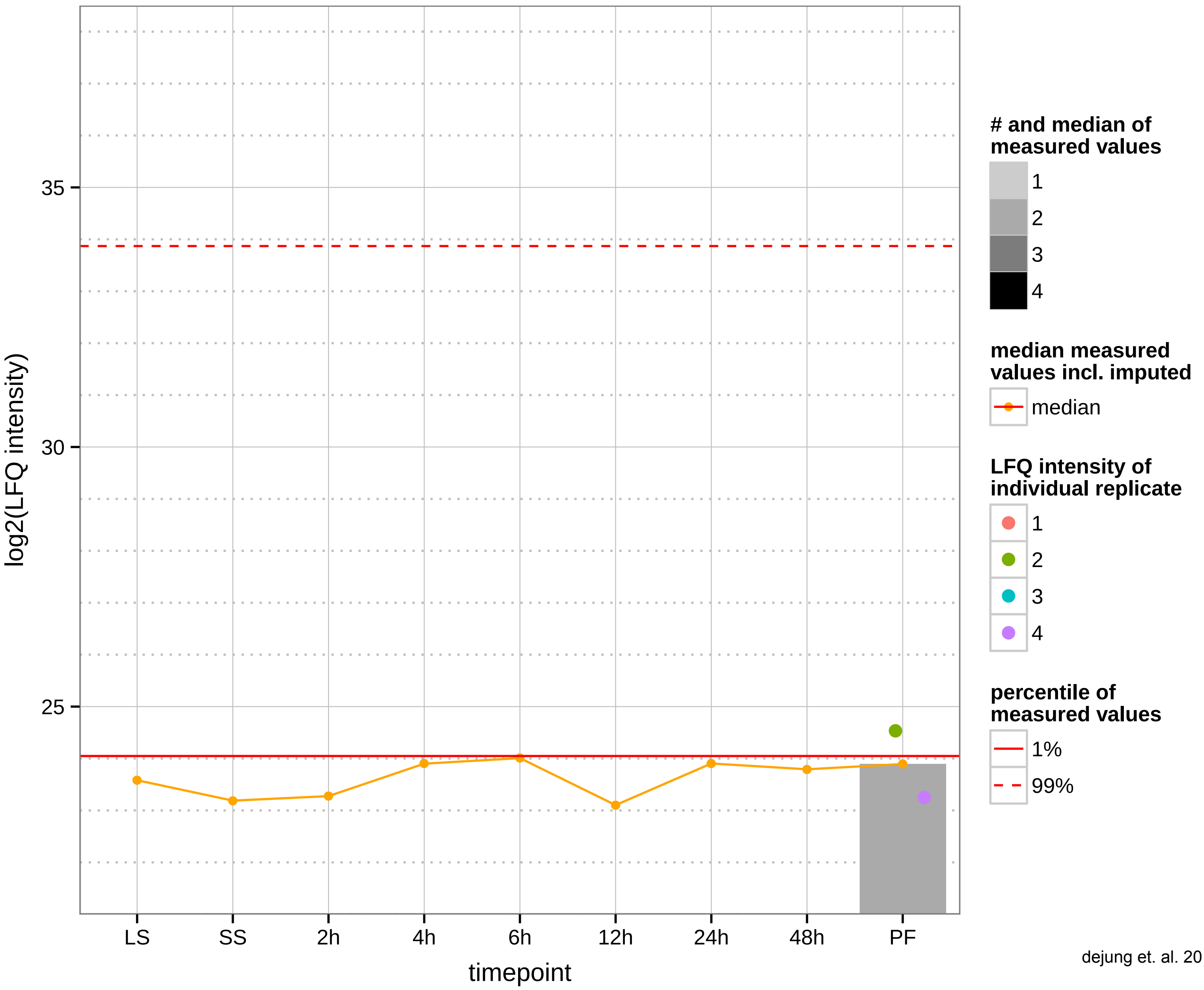

dejung et. al. 2015

ribosomal protein S6, putative, NHP2/RS6–like protein (NHP2)
Tb927.9.5150
AGOF: structural constituent of ribosome
AGOC: ribosome
AGOP: translation
PGOF: null
PGOC: null
PGOP: null

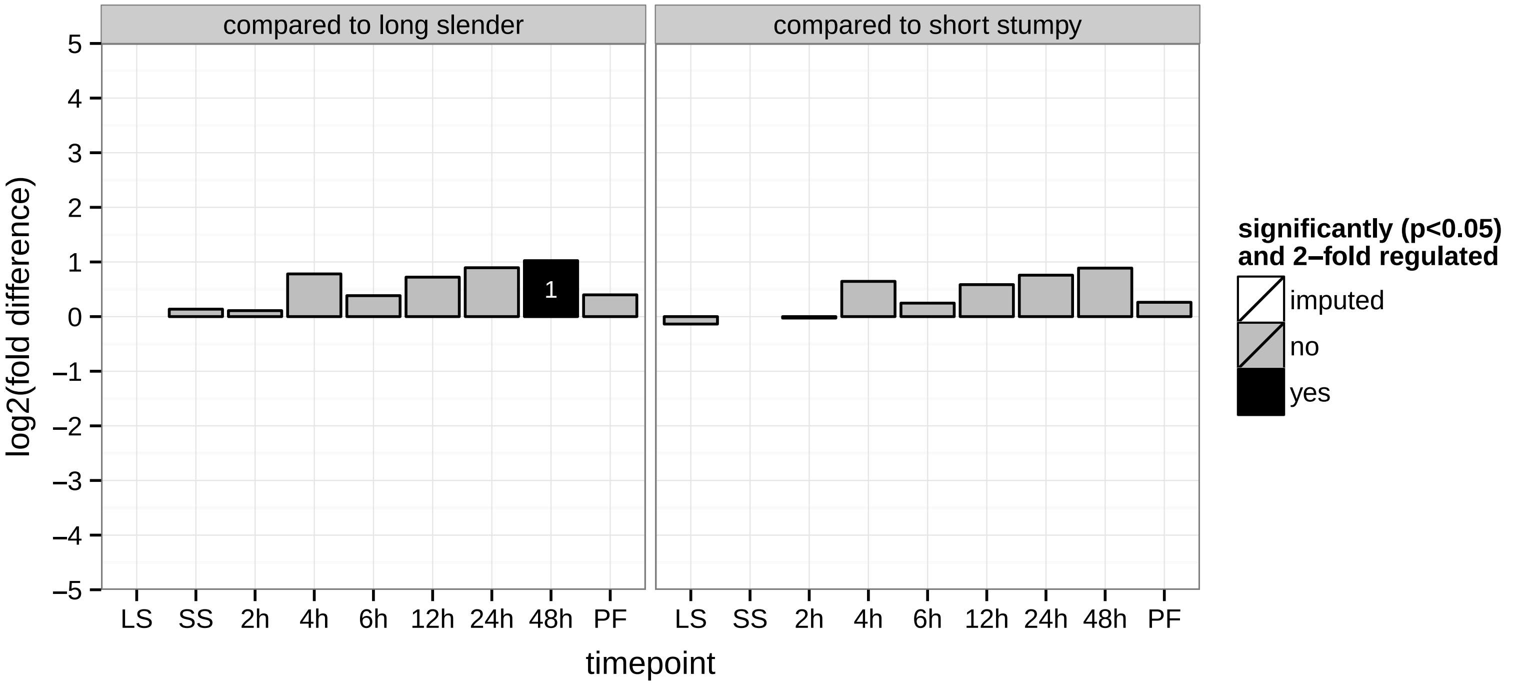

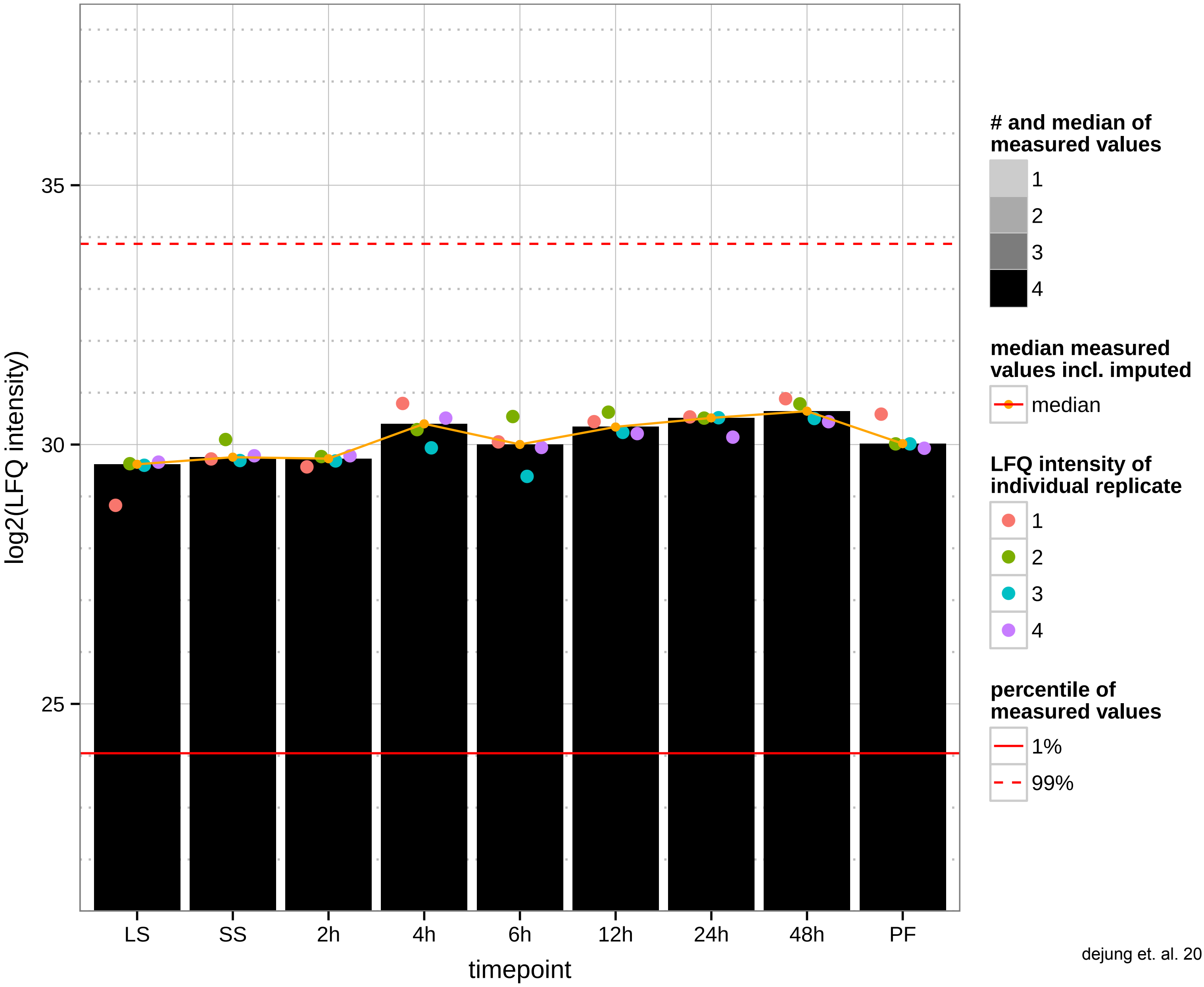

dejung et. al. 2015

phosphomevalonate kinase protein, putative
Tb927.9.5170
AGOF: ATP binding, phosphomevalonate kinase activity
AGOC: cytoplasm, nucleus
AGOP: ergosterol biosynthetic process, farnesyl diphosphate biosynthetic process, mevalonate pathway, isopentenyl diphosp
PGOF: ATP binding
PGOC: null
PGOP: null

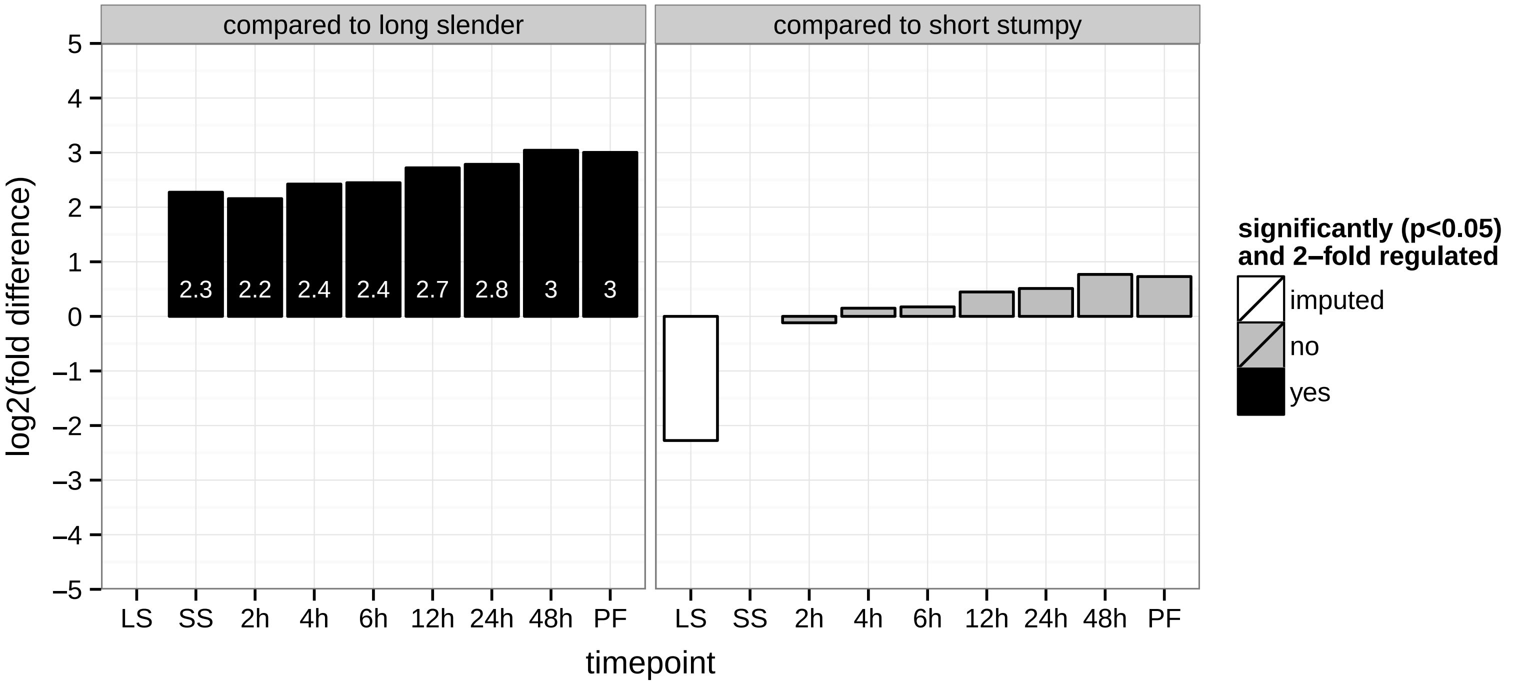

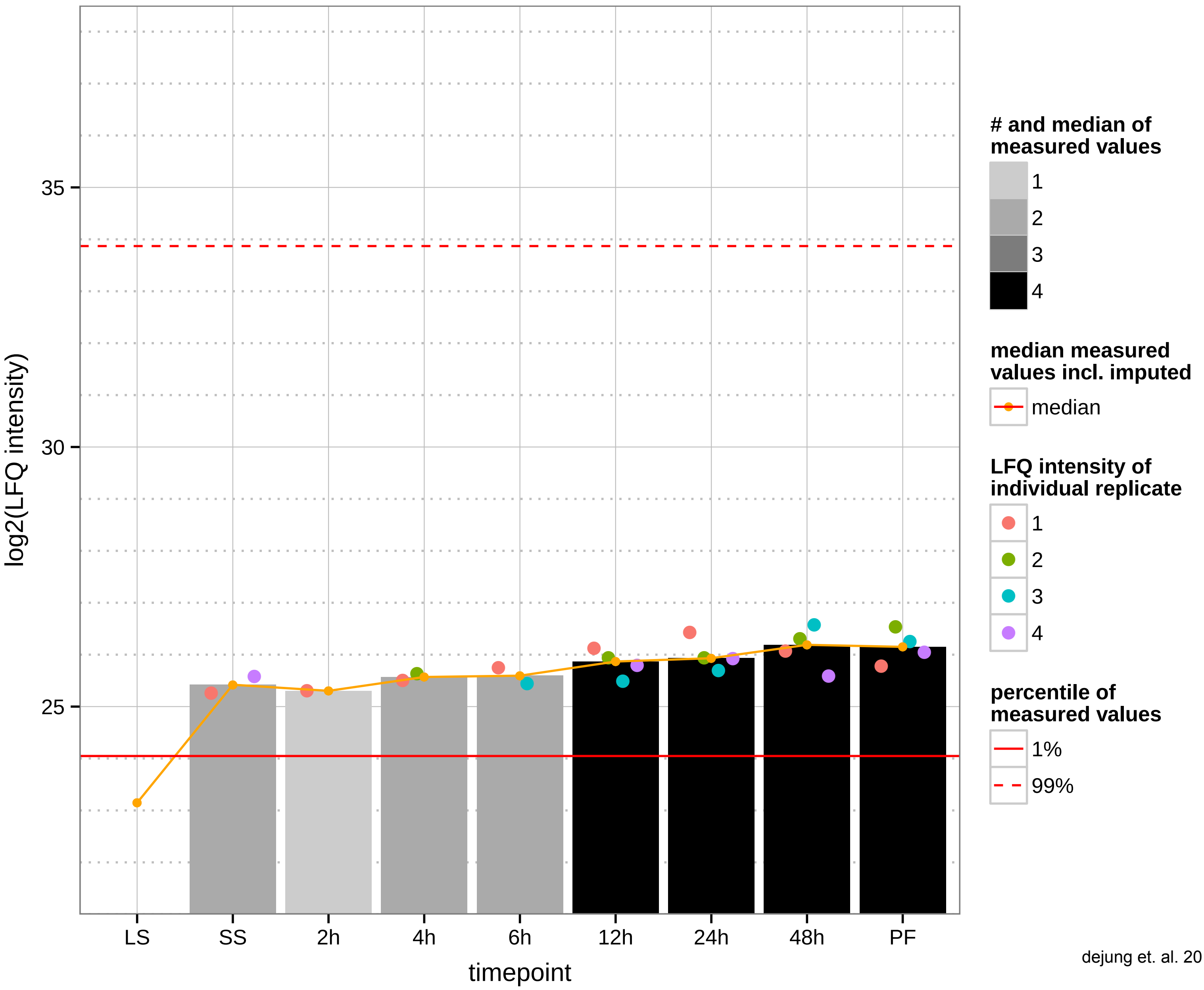

dejung et. al. 2015

proliferative cell nuclear antigen (PCNA), putative
Tb927.9.5190
AGOF: DNA binding, DNA polymerase processivity factor activity
AGOC: PCNA complex
AGOP: regulation of DNA replication
PGOF: DNA binding, DNA polymerase processivity factor activity
PGOC: PCNA complex
PGOP: regulation of DNA replication

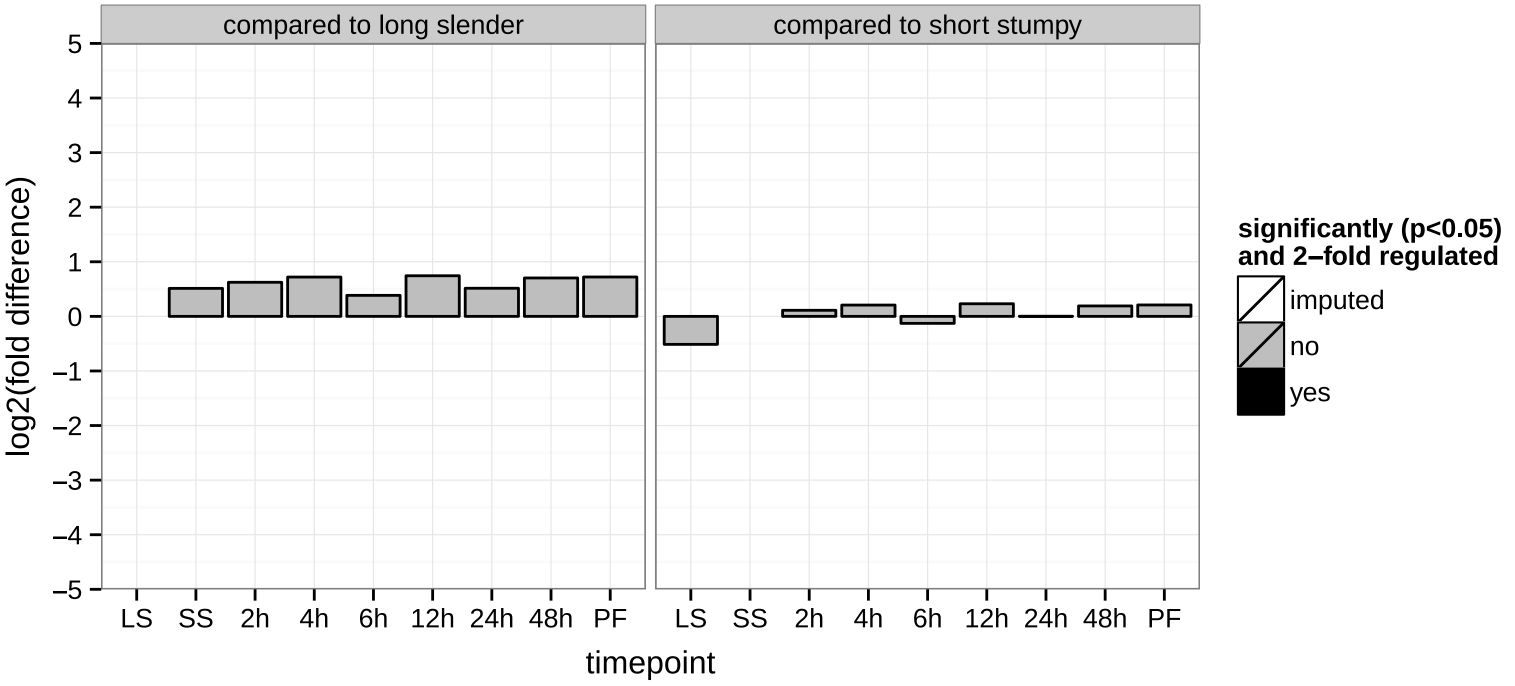

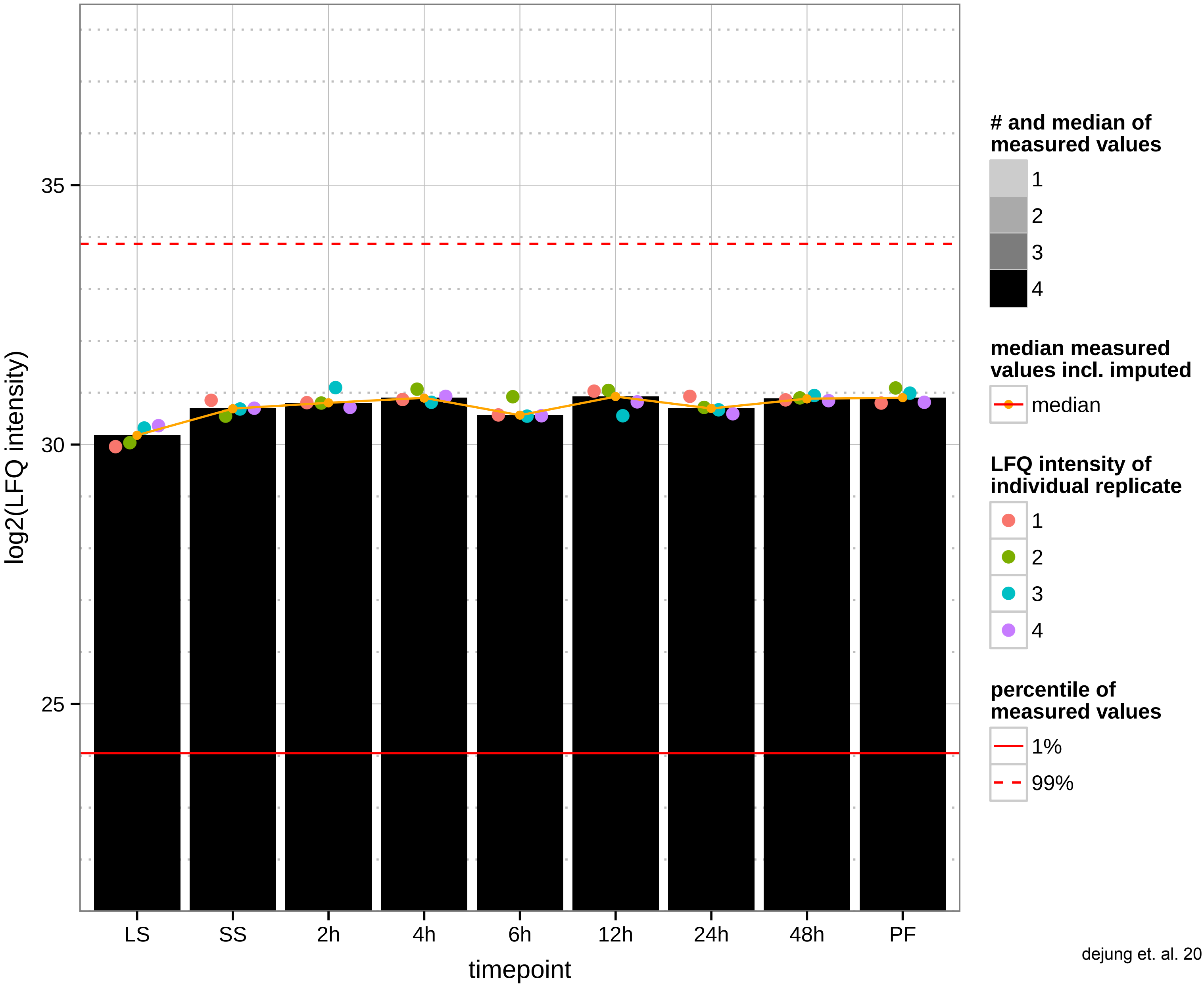

dejung et. al. 2015

glutaminyl–tRNA synthetase, putative
Tb927.9.5210
AGOF: ATP binding, glutamine–tRNA ligase activity
AGOC: cytoplasm
AGOP: glutaminyl–tRNA aminoacylation, translation
PGOF: ATP binding, aminoacyl–tRNA ligase activity, glutamine–tRNA ligase activity, ligase activity, forming aminoacyl–tRNA a
PGOC: cytoplasm
PGOP: glutaminyl–tRNA aminoacylation, tRNA aminoacylation, tRNA aminoacylation for protein translation, translation

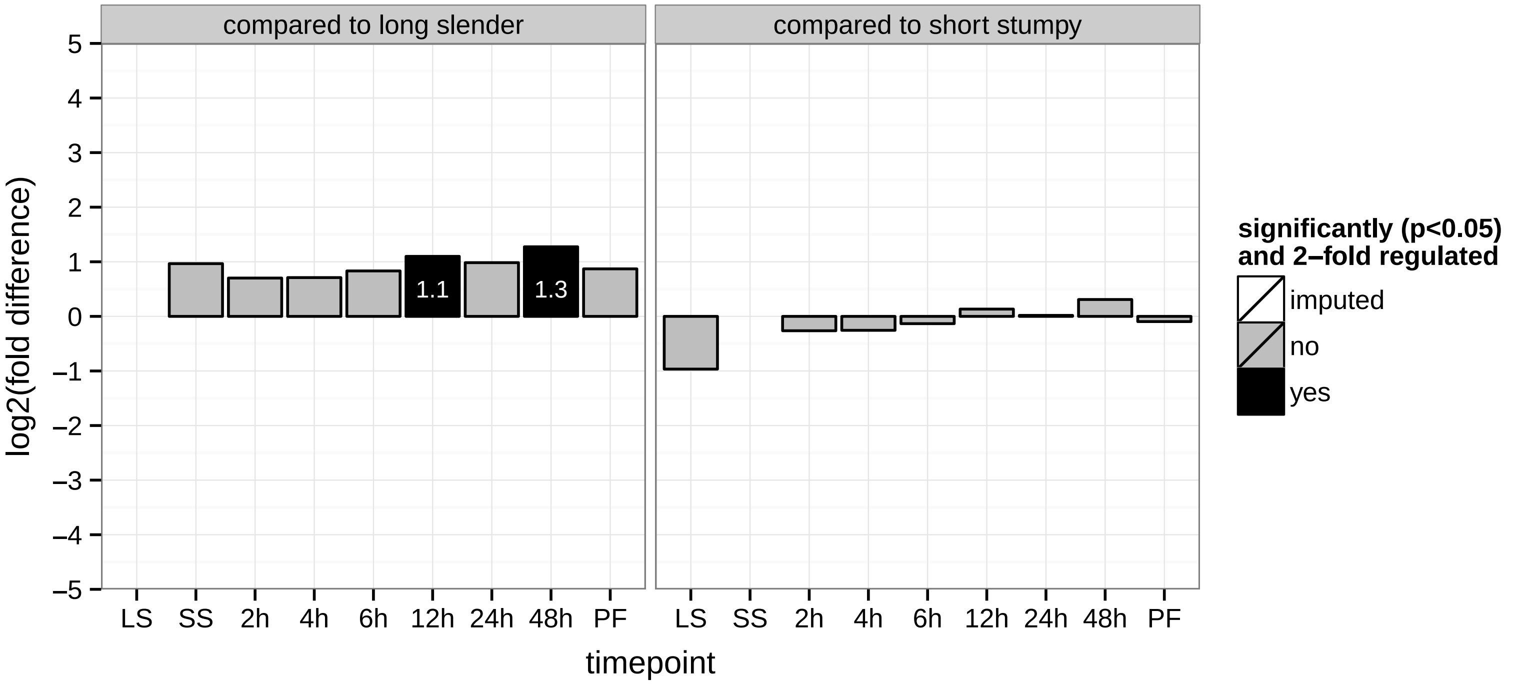

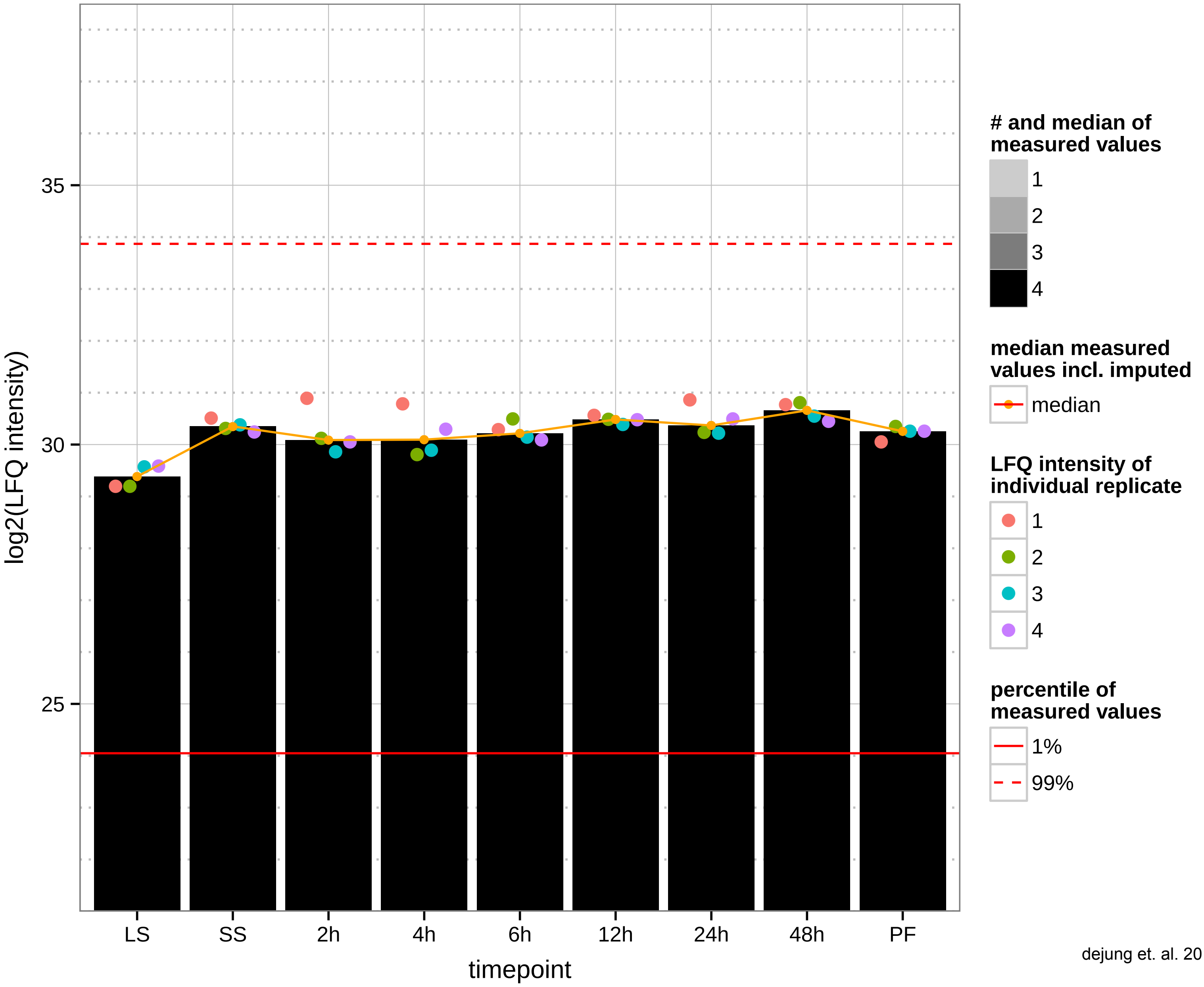

dejung et. al. 2015

nucleolar RNA binding protein, putative
Tb927.9.5320
AGOF: snoRNA binding
AGOC: null
AGOP: nuclear mRNA splicing, via spliceosome
PGOF: null
PGOC: null
PGOP: null

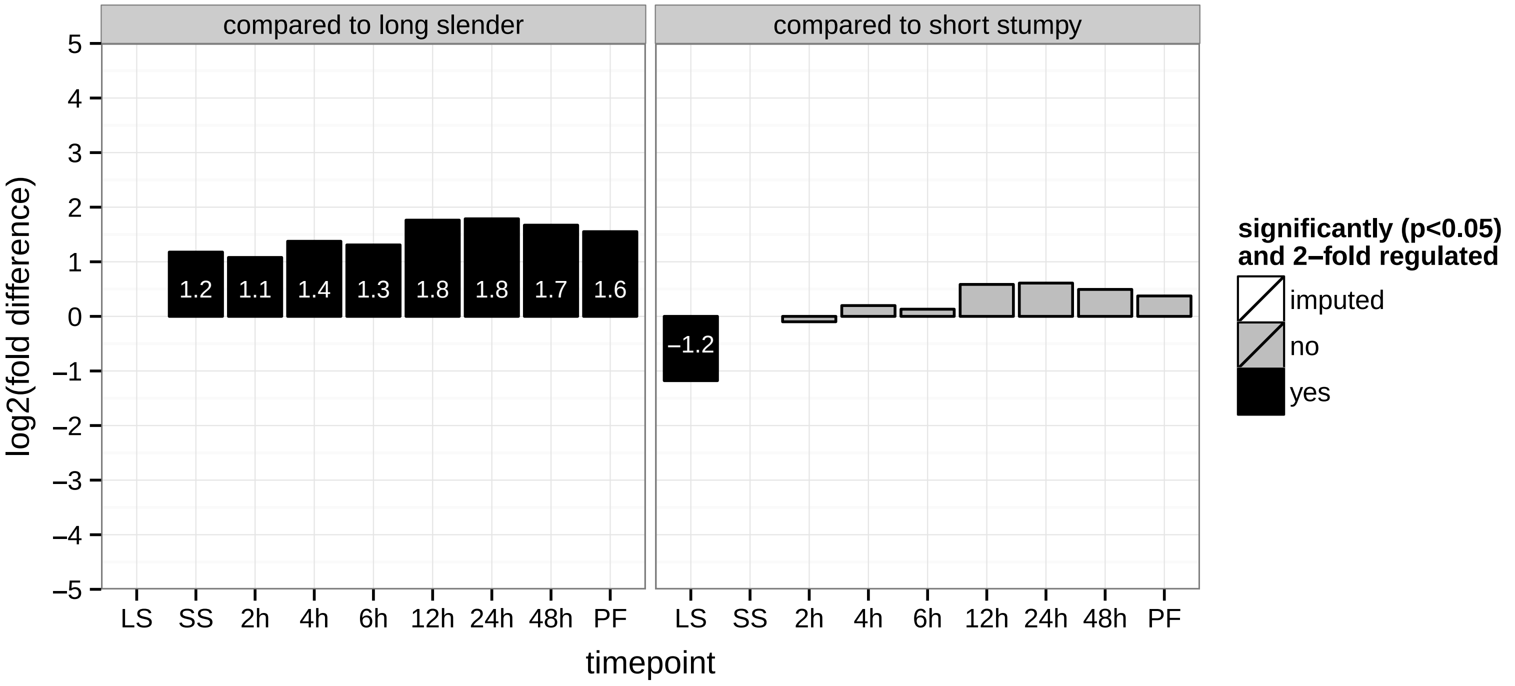

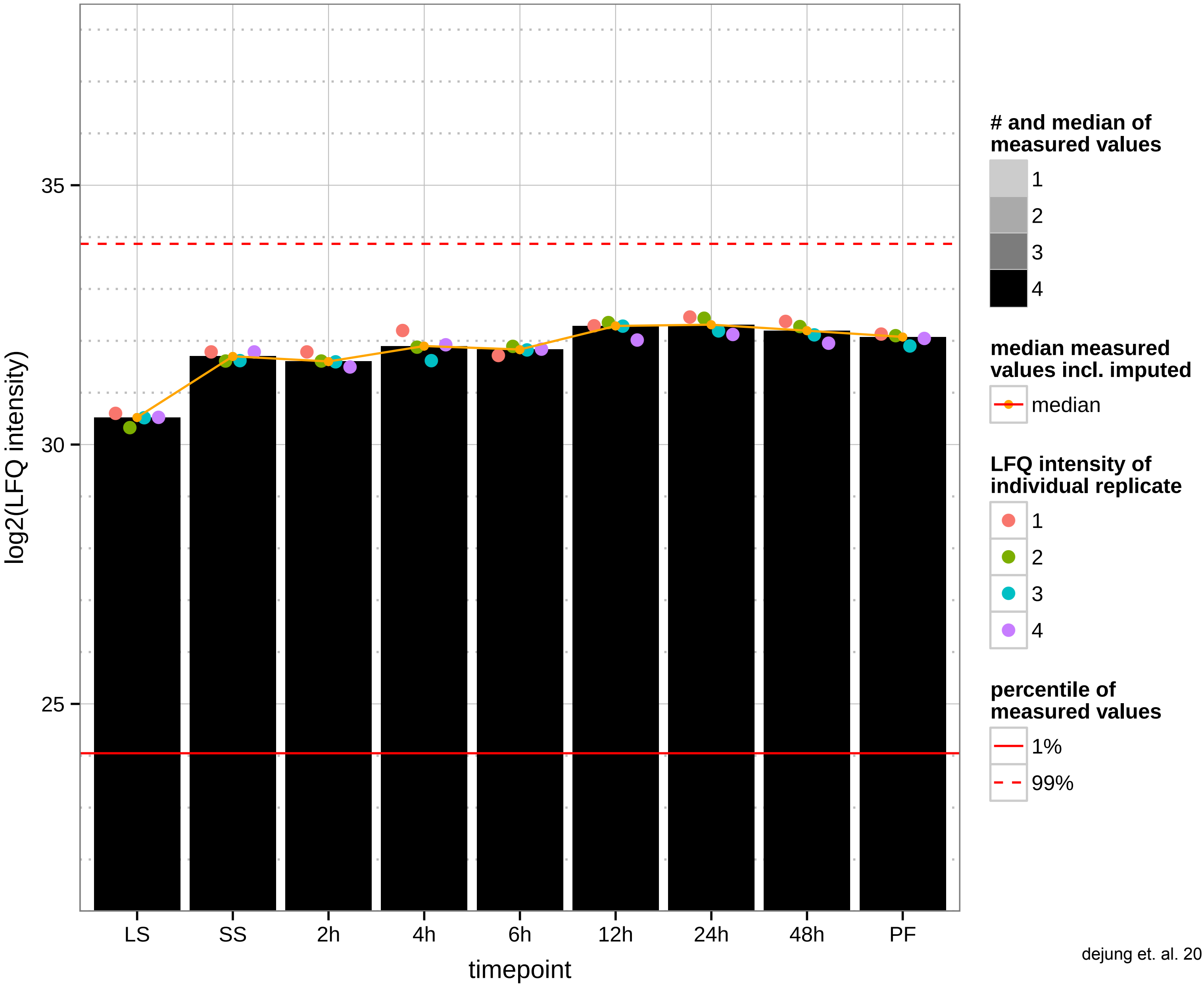

dejung et. al. 2015

hypothetical protein, conserved
Tb927.9.5410
AGOF: null
AGOC: null
AGOP: null
PGOF: null
PGOC: null
PGOP: null

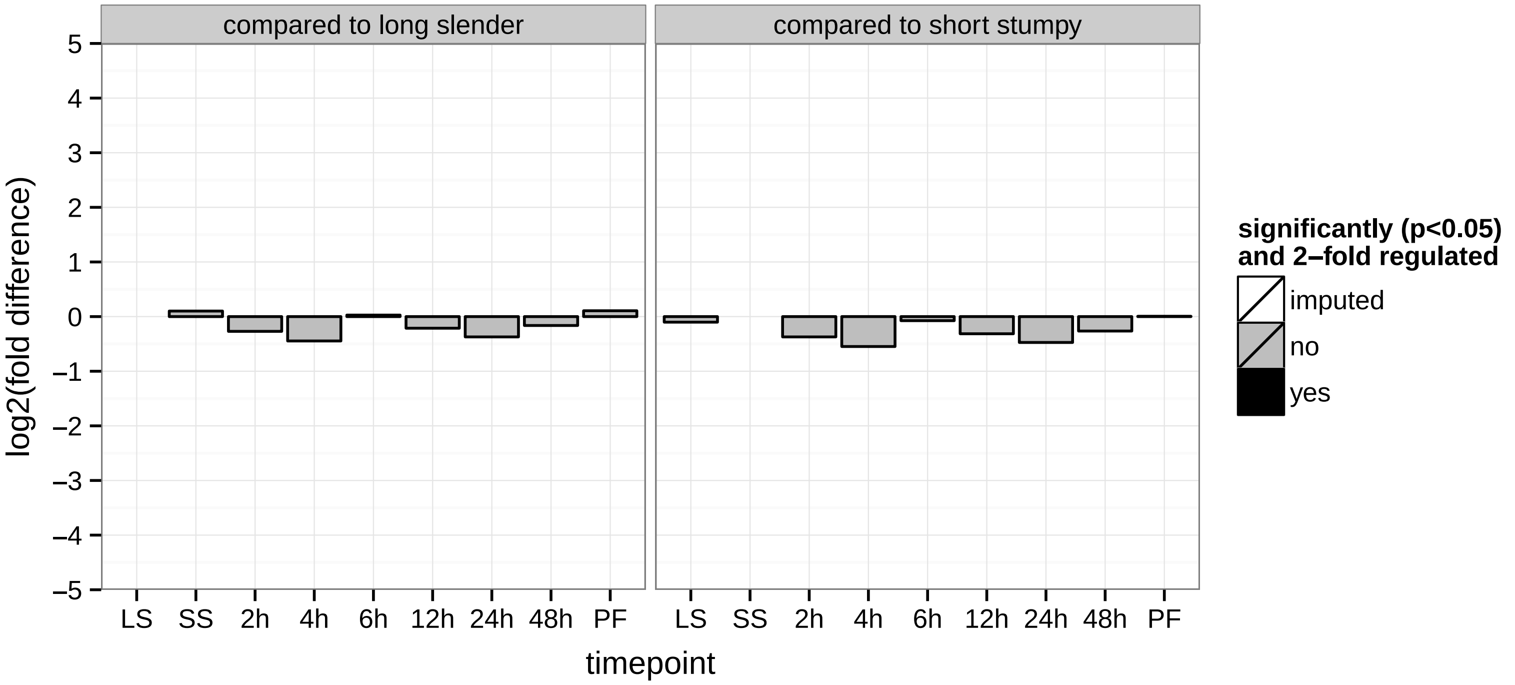

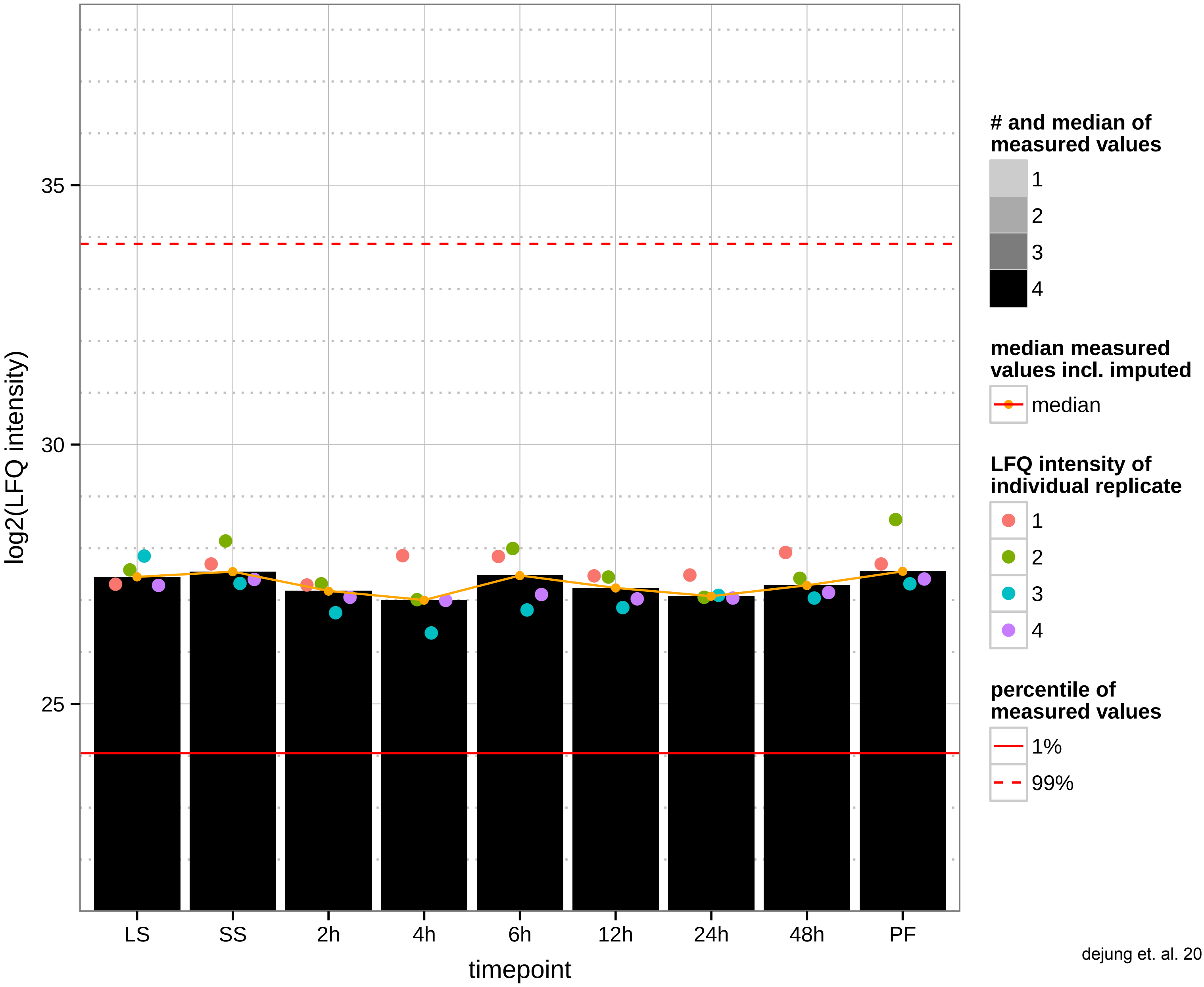

dejung et. al. 2015

Eukaryotic translation initiation factor 4 gamma type 2 (eif4g2)
Tb927.9.5460
AGOF: RNA binding, nucleic acid binding, protein binding
AGOC: eukaryotic translation initiation factor 4F complex
AGOP: RNA metabolic process, nucleobase–containing compound metabolic process, translation
PGOF: DNA binding, RNA binding, binding, protein binding
PGOC: null
PGOP: null

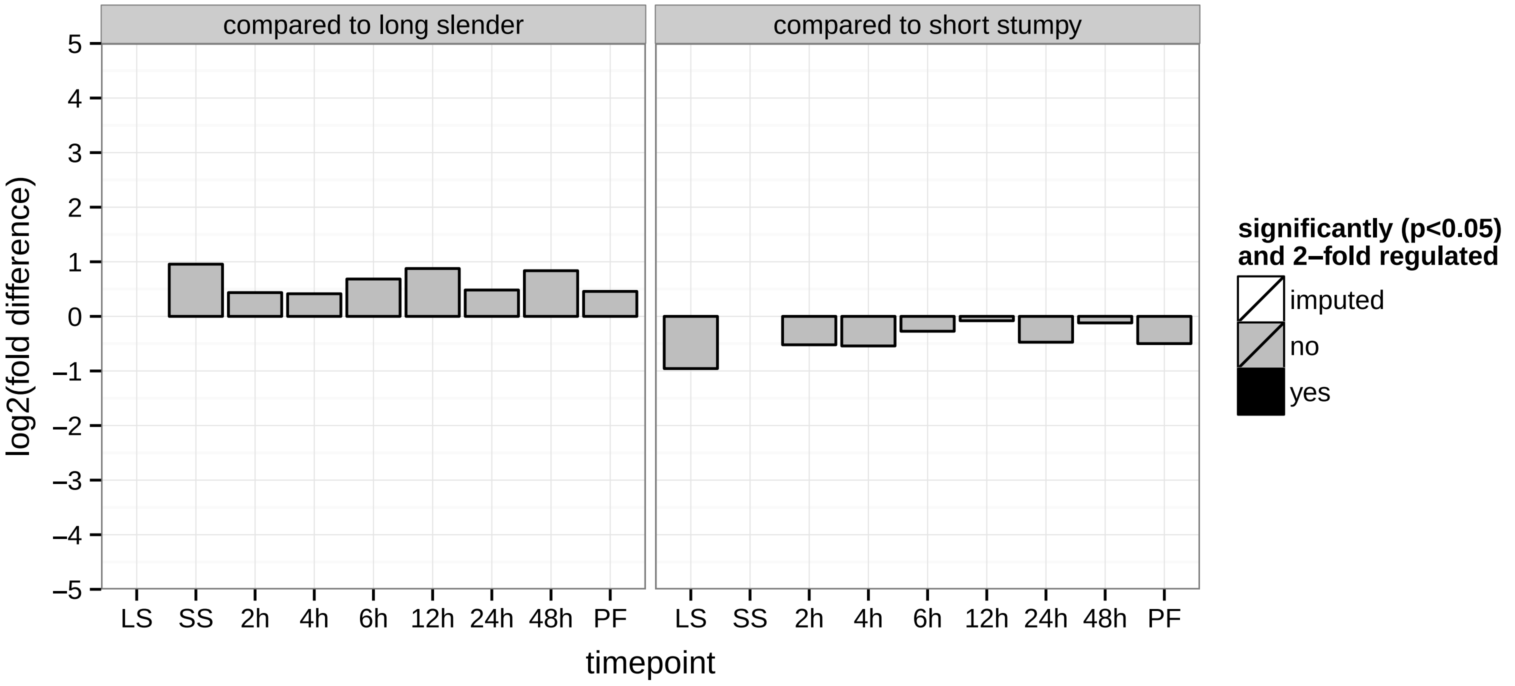

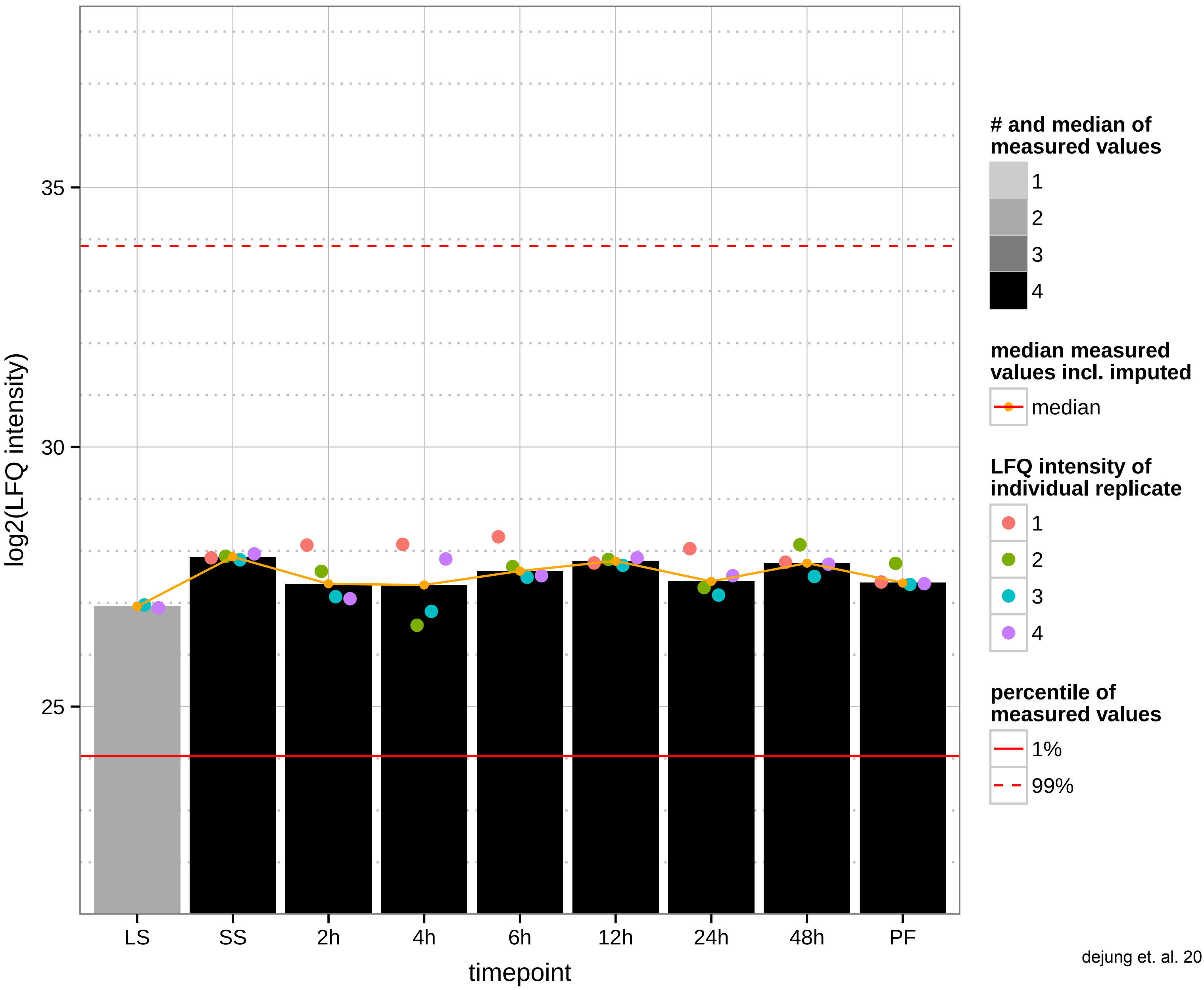

dejung et. al. 2015

DNA topoisomerase ii (TOP2)
Tb927.9.5590
AGOF: ATP binding, DNA topoisomerase (ATP−hydrolyzing) activity
AGOC: chromosome, mitochondrion, nucleus
AGOP: DNA topological change
PGOF: ATP binding, DNA binding, DNA topoisomerase (ATP−hydrolyzing) activity
PGOC: chromosome
PGOP: DNA topological change

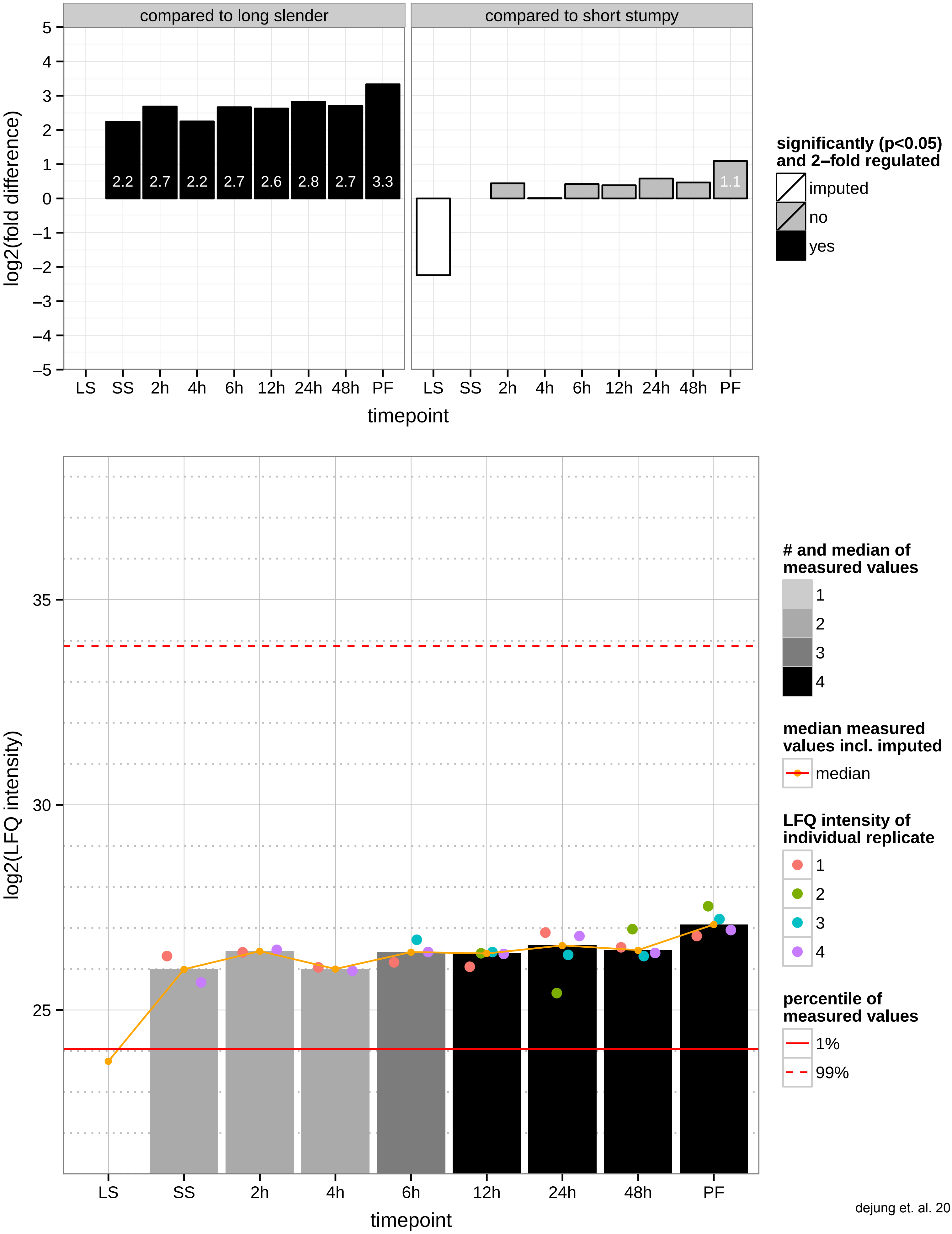

dejung et. al. 2015

unspecified product
Tb927.9.5840;Tb927.9.5750
AGOF: null
AGOC: null
AGOP: null
PGOF: antioxidant activity, oxidoreductase activity, peroxiredoxin activity
PGOC: null
PGOP: oxidation–reduction process

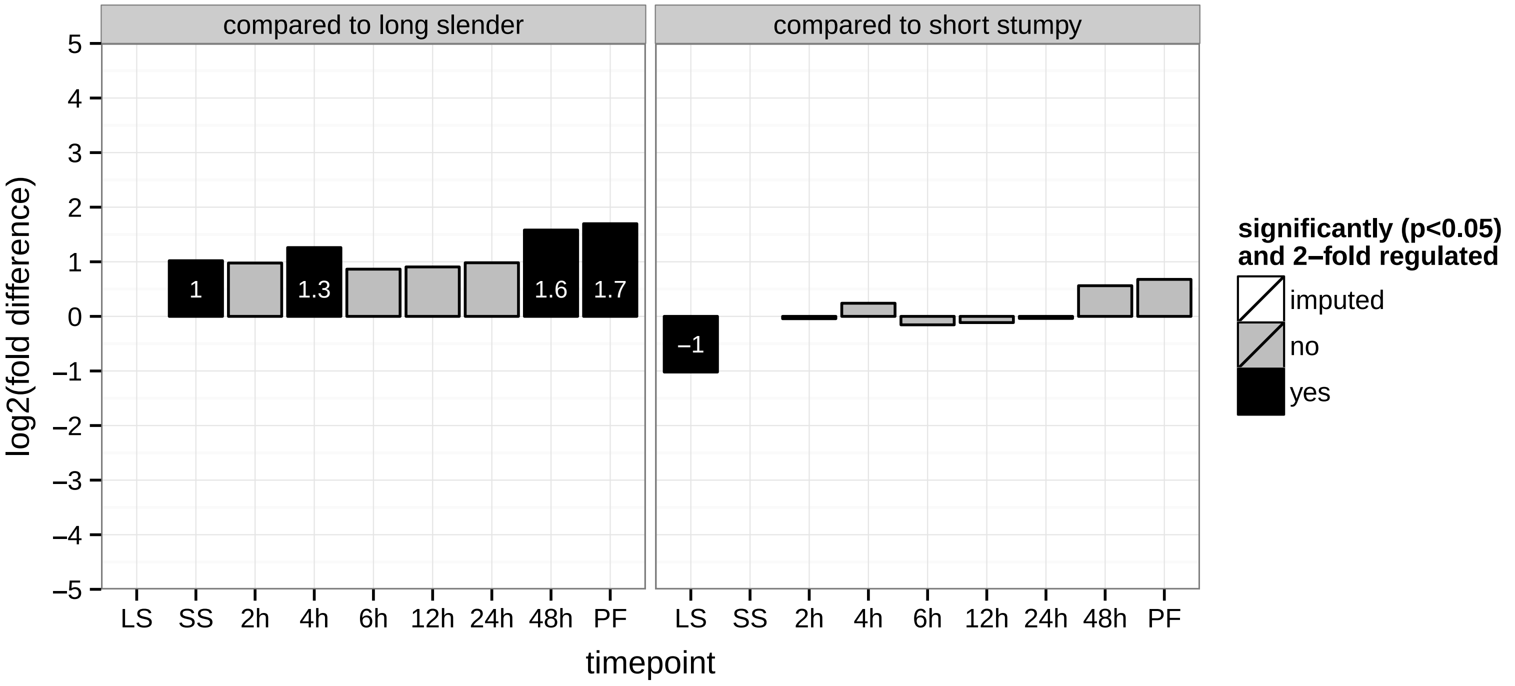

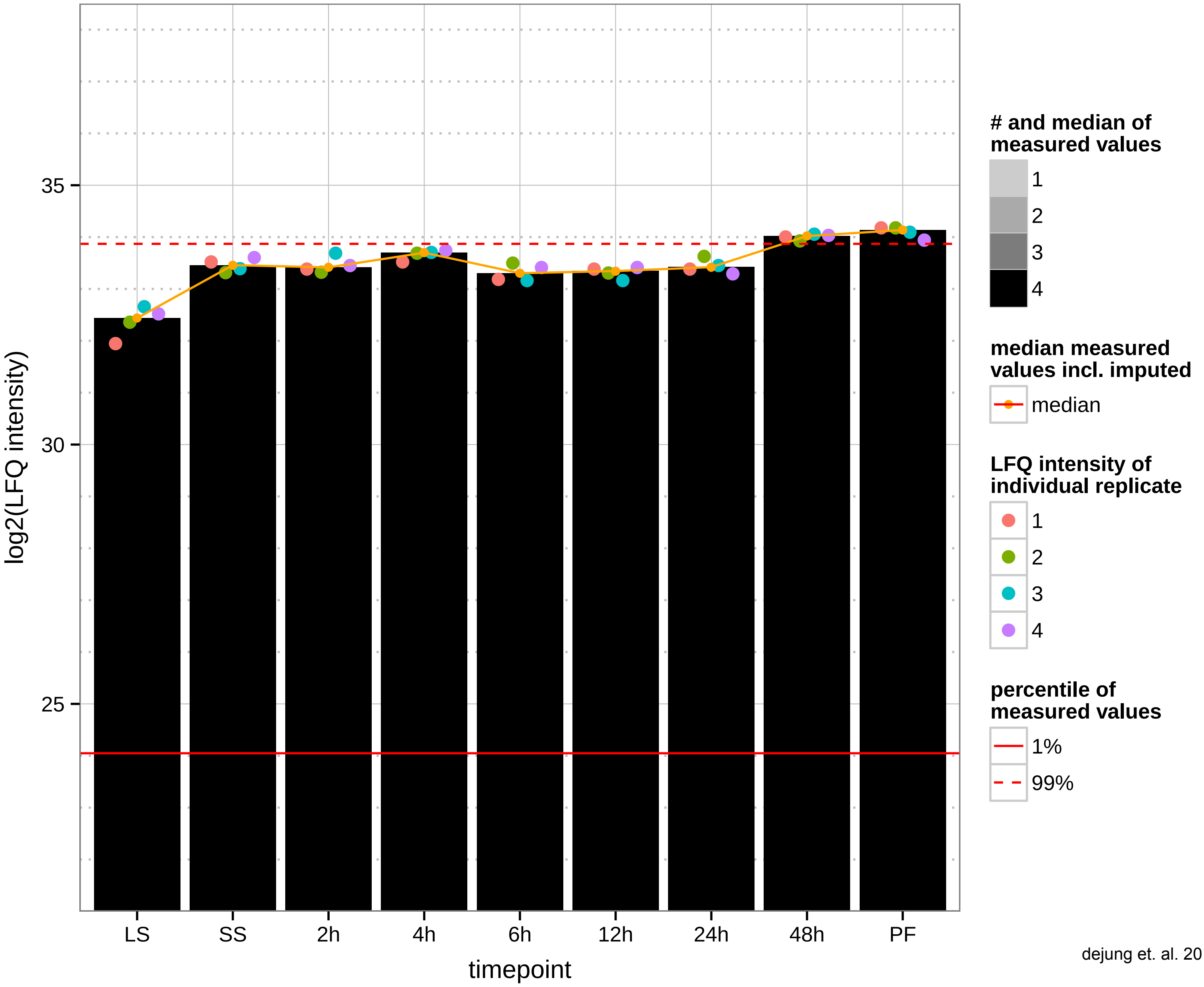

dejung et. al. 2015

E1–like ubiquitin–activating enzyme, putative (UBA5)
Tb927.9.6040
AGOF: catalytic activity, small conjugating protein binding, small protein activating enzyme activity
AGOC: mitochondrion
AGOP: null
PGOF: catalytic activity
PGOC: null
PGOP: null

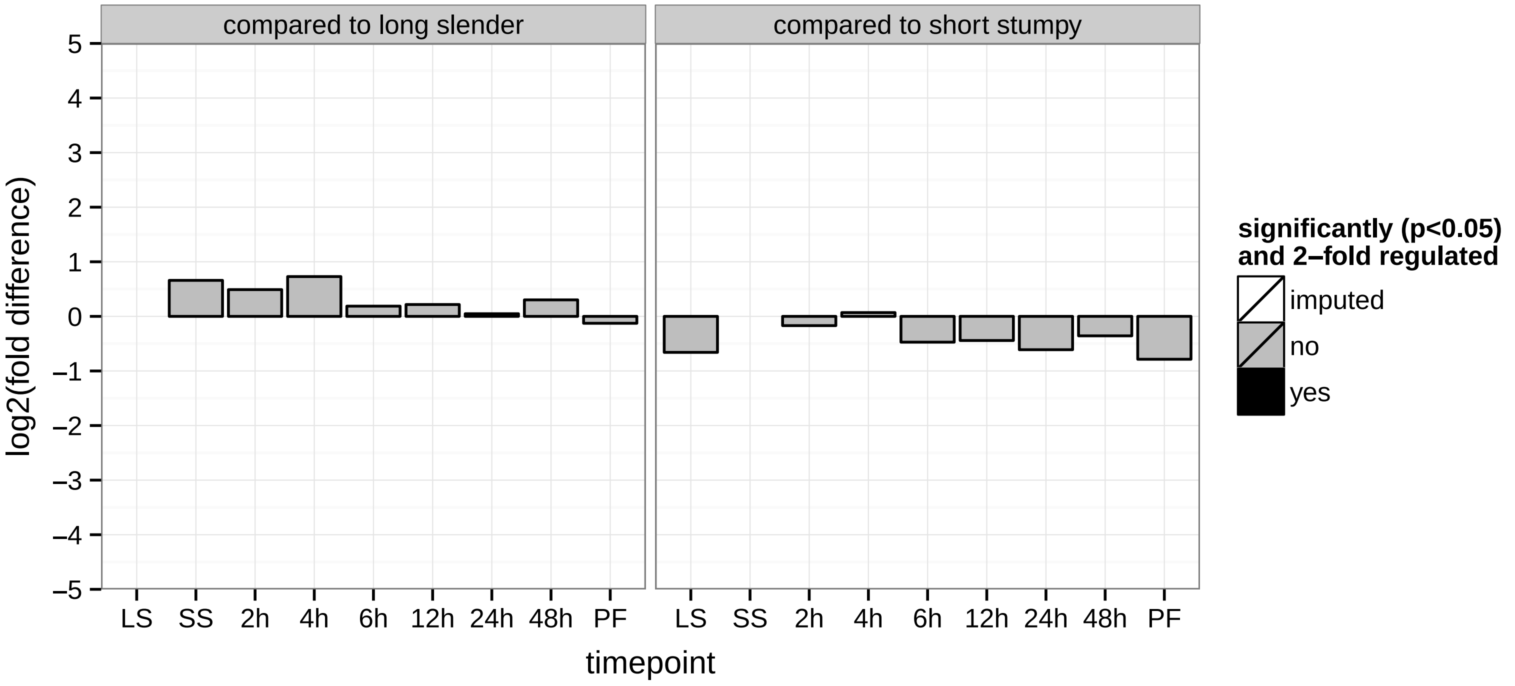

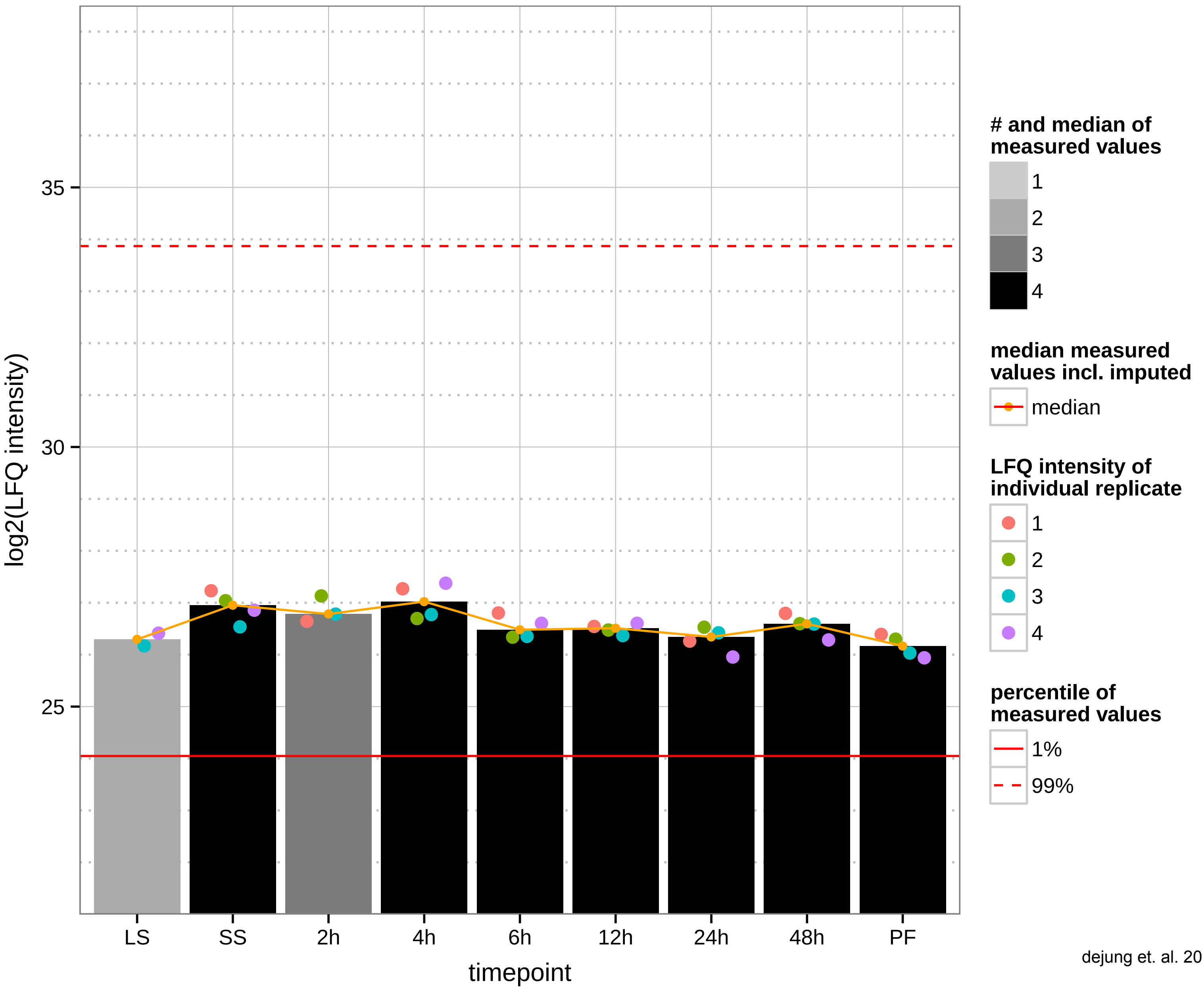

dejung et. al. 2015

calmodulin, putative
Tb927.9.6130
AGOF: calcium ion binding
AGOC: null
AGOP: null
PGOF: calcium ion binding
PGOC: null
PGOP: null

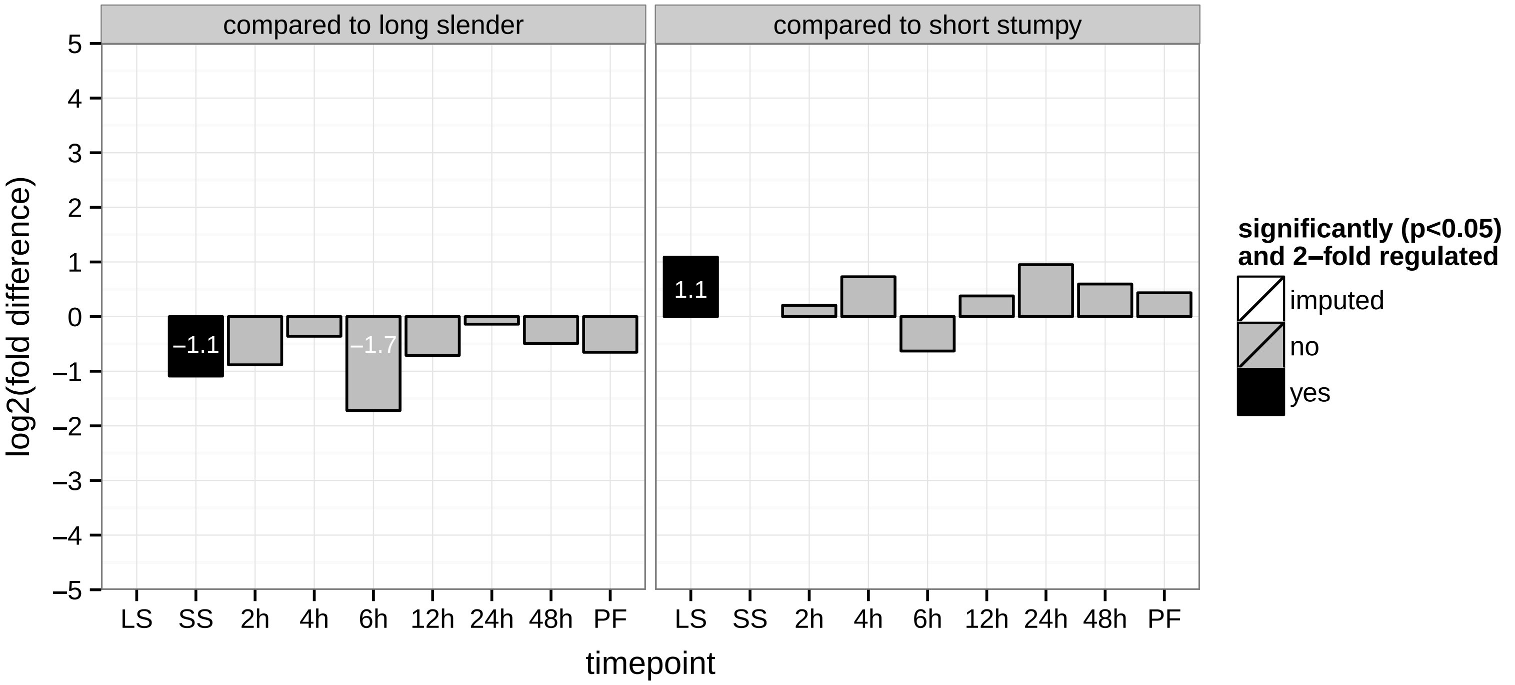

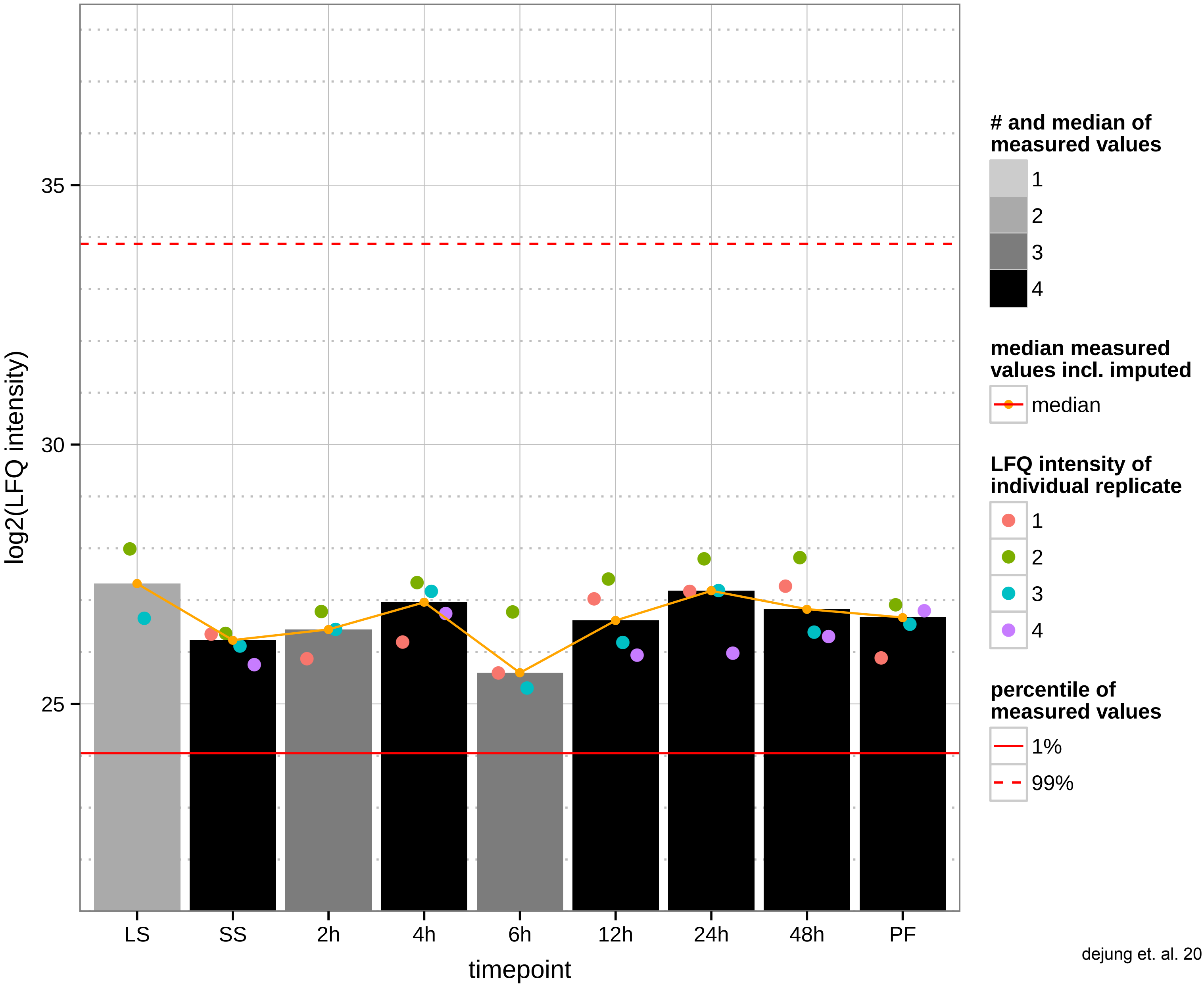

dejung et. al. 2015

unspecified product
Tb927.9.6230;Tb927.9.6170
AGOF: null
AGOC: null
AGOP: null
PGOF: kinase activity, transferase activity, transferring phosphorus–containing groups
PGOC: null
PGOP: null

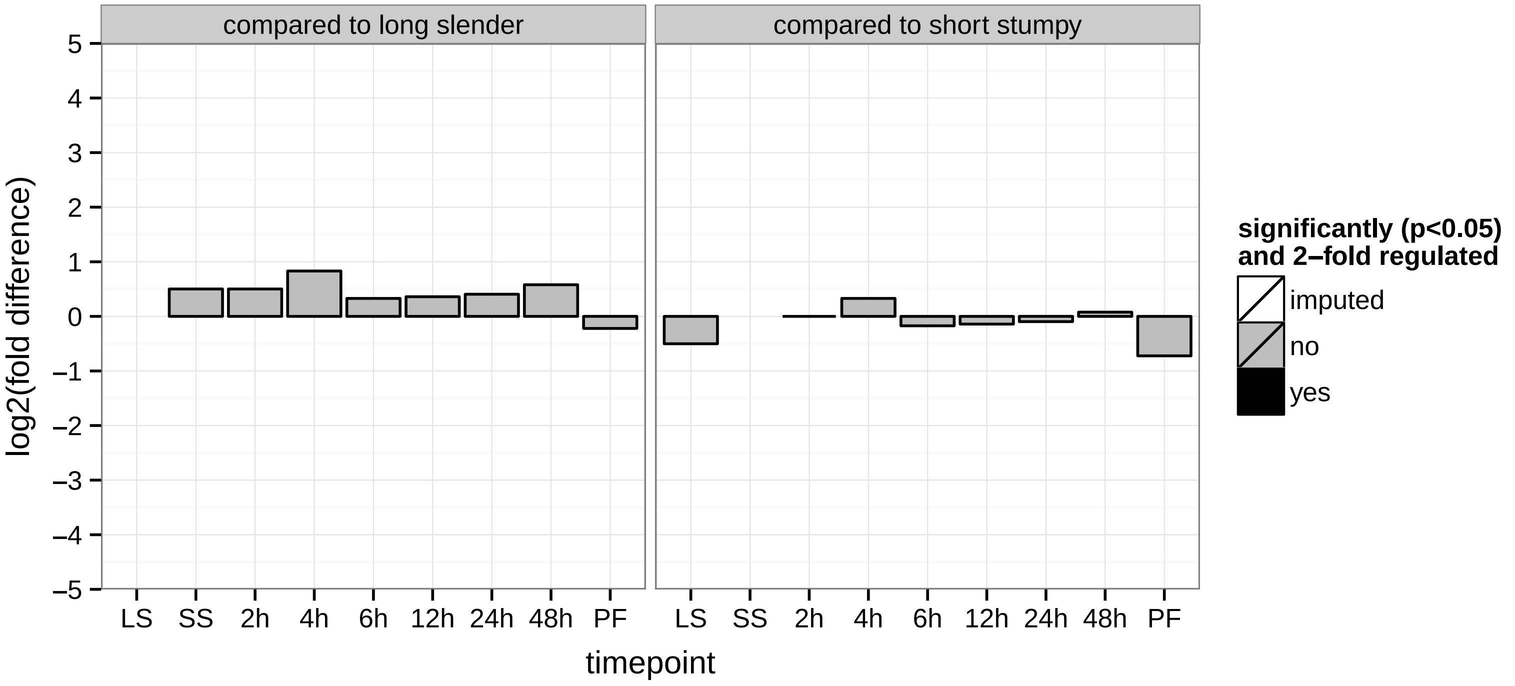

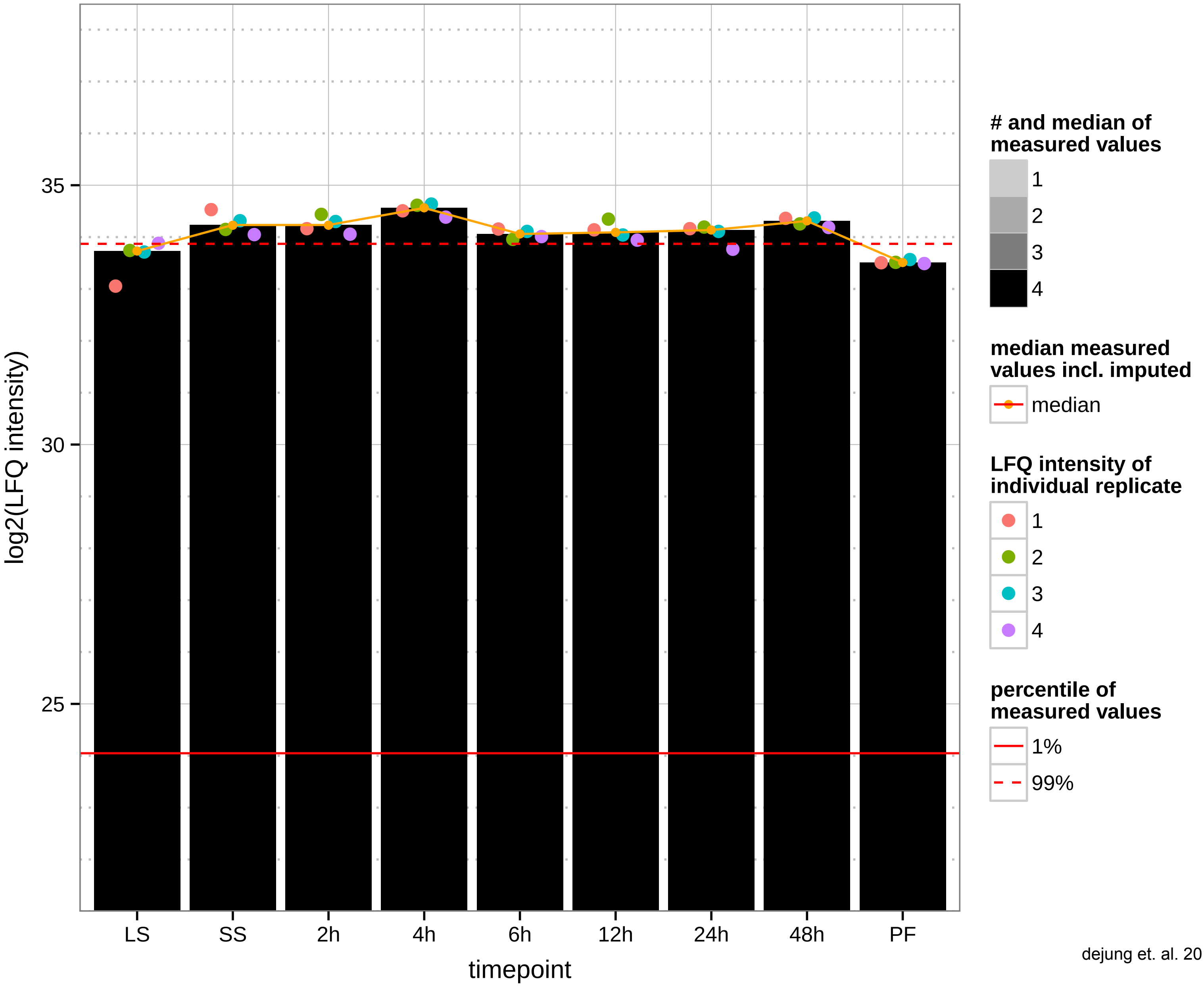

dejung et. al. 2015

Rab geranylgeranyl transferase component A, putative, Rab escort protein 1
Tb927.9.6430
AGOF: Rab GTPase binding
AGOC: Rab–protein geranylgeranyltransferase complex
AGOP: regulation of GTPase activity, vesicle–mediated transport
PGOF: null
PGOC: null
PGOP: null

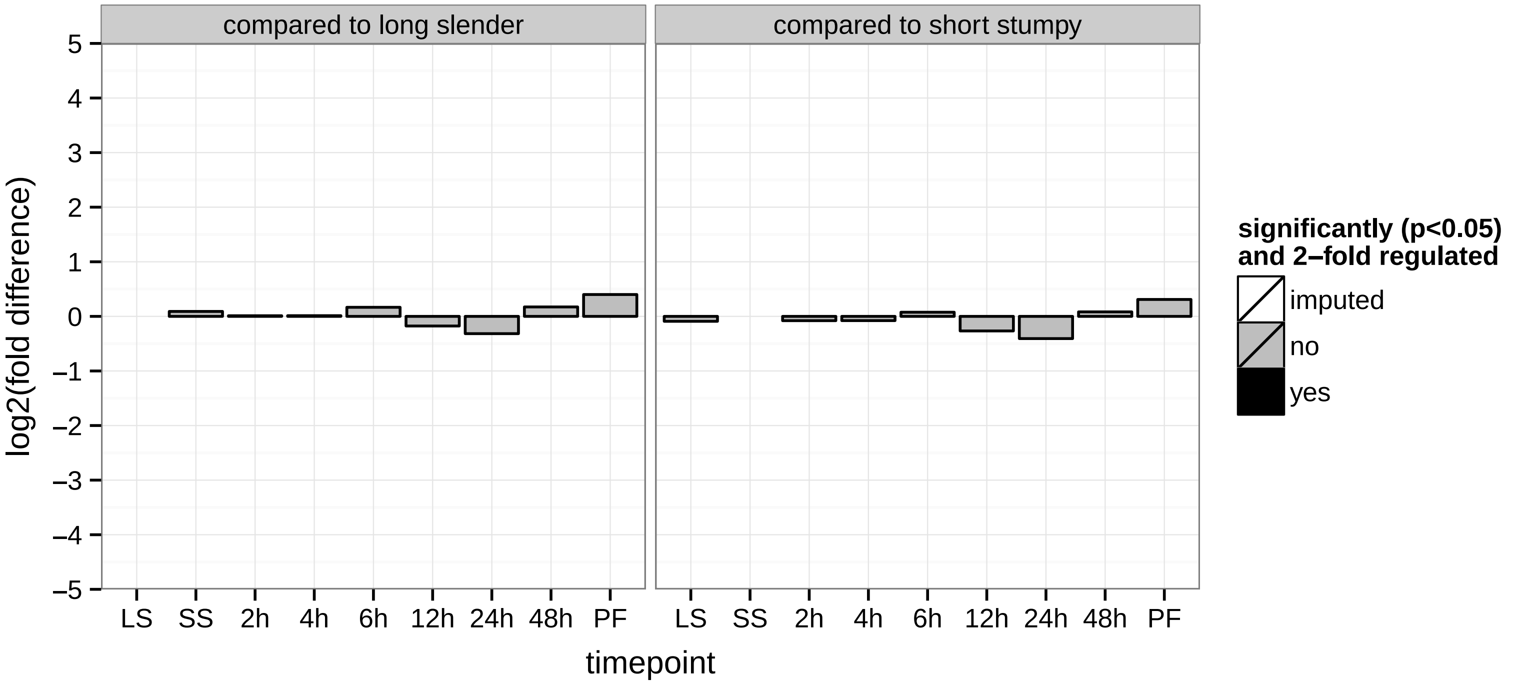

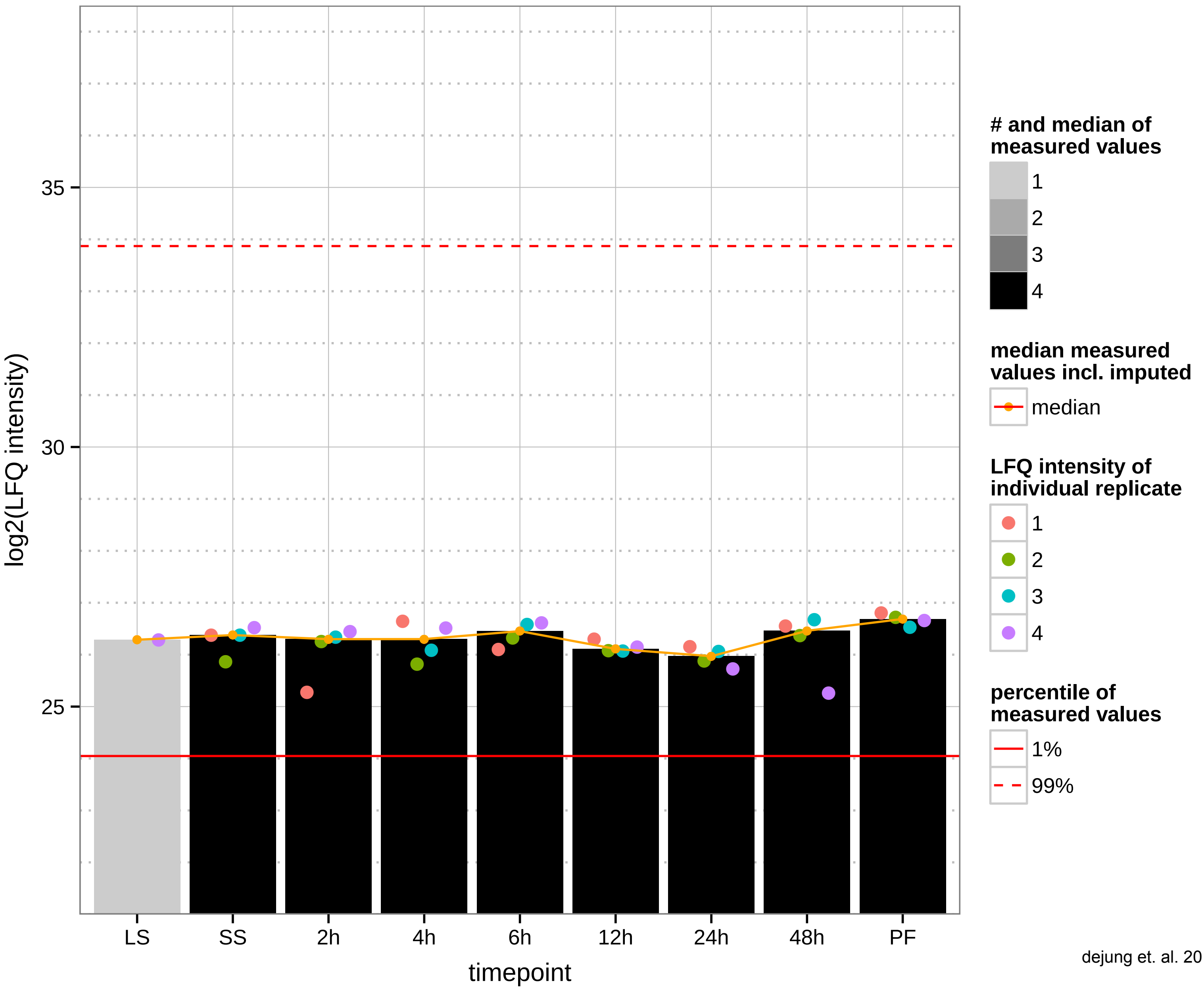

dejung et. al. 2015

hypothetical protein, conserved
Tb927.9.6450
AGOF: null
AGOC: null
AGOP: null
PGOF: null
PGOC: null
PGOP: null

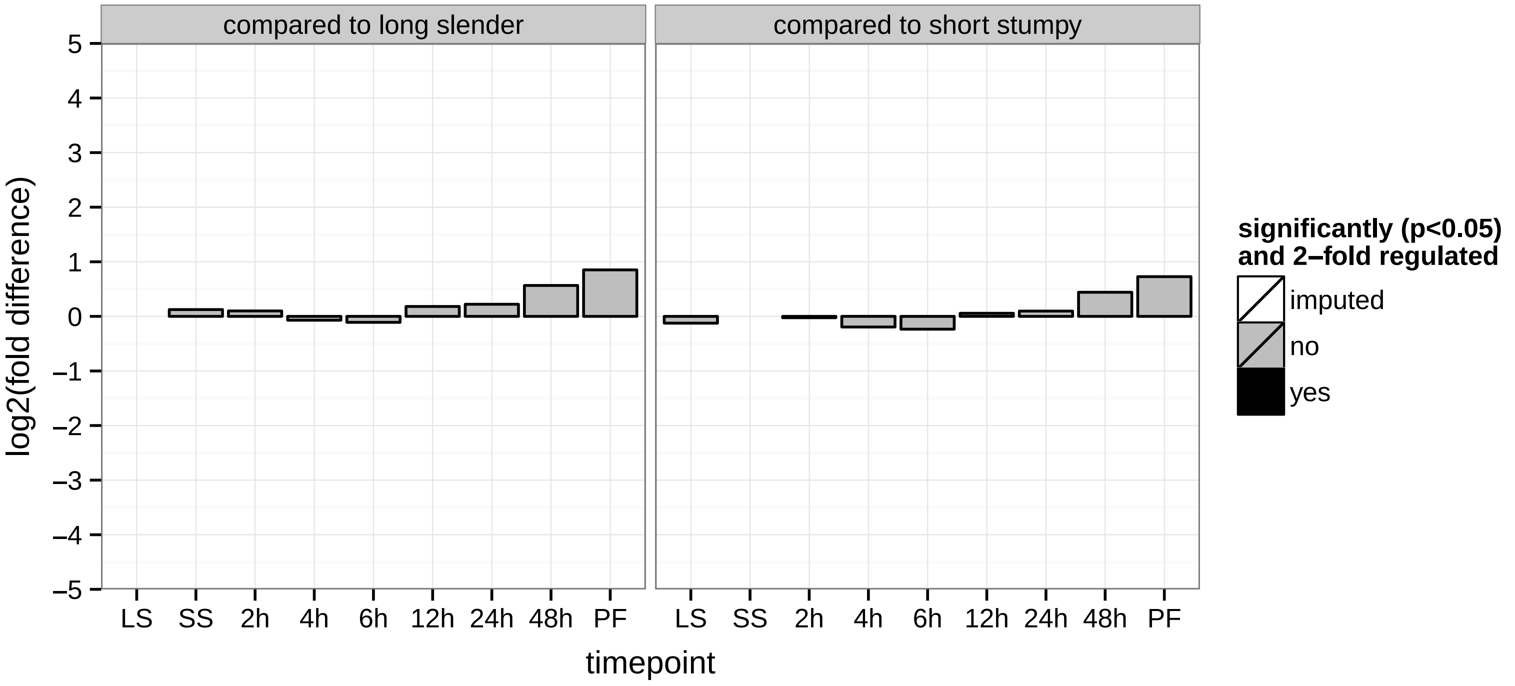

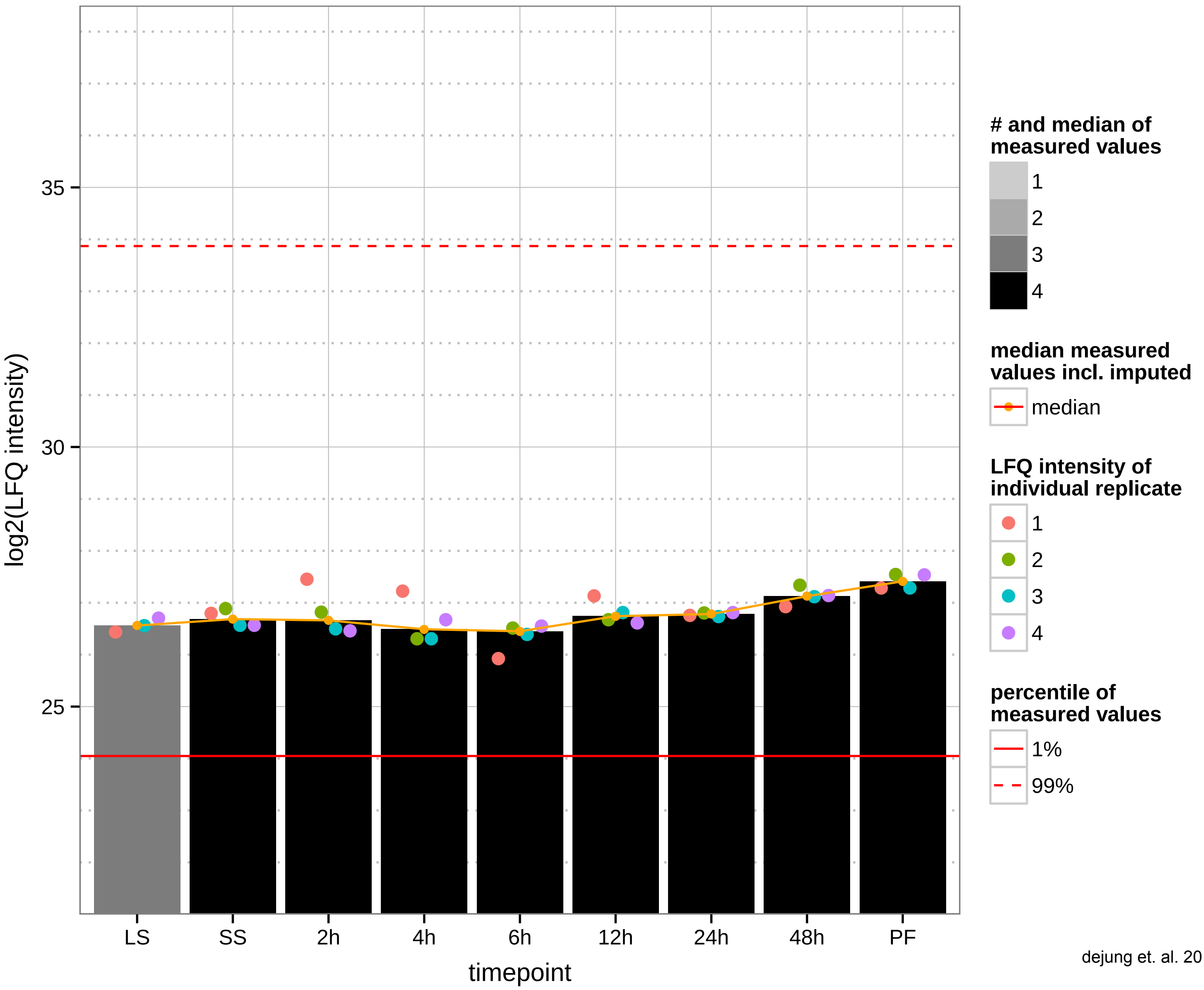

dejung et. al. 2015

hypothetical protein, conserved
Tb927.9.6460
AGOF: null
AGOC: null
AGOP: null
PGOF: null
PGOC: null
PGOP: null

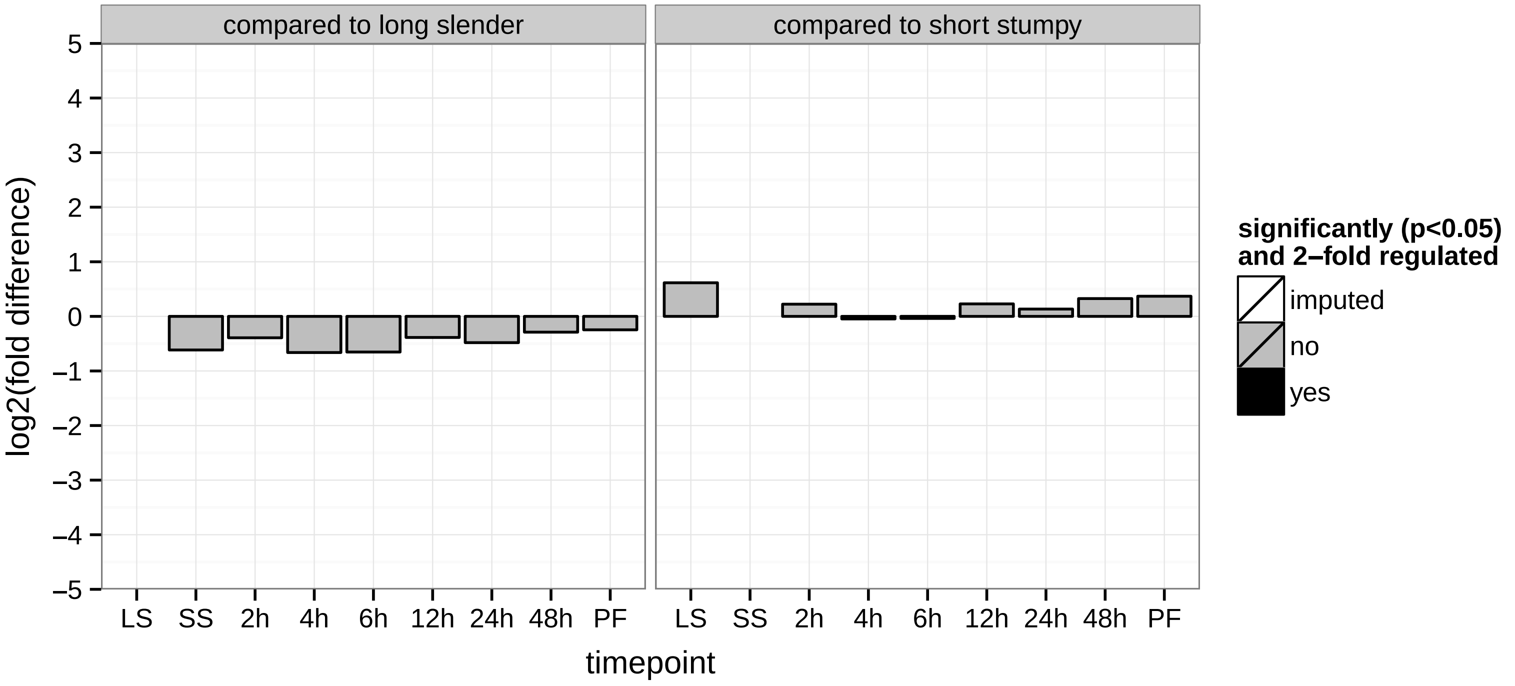

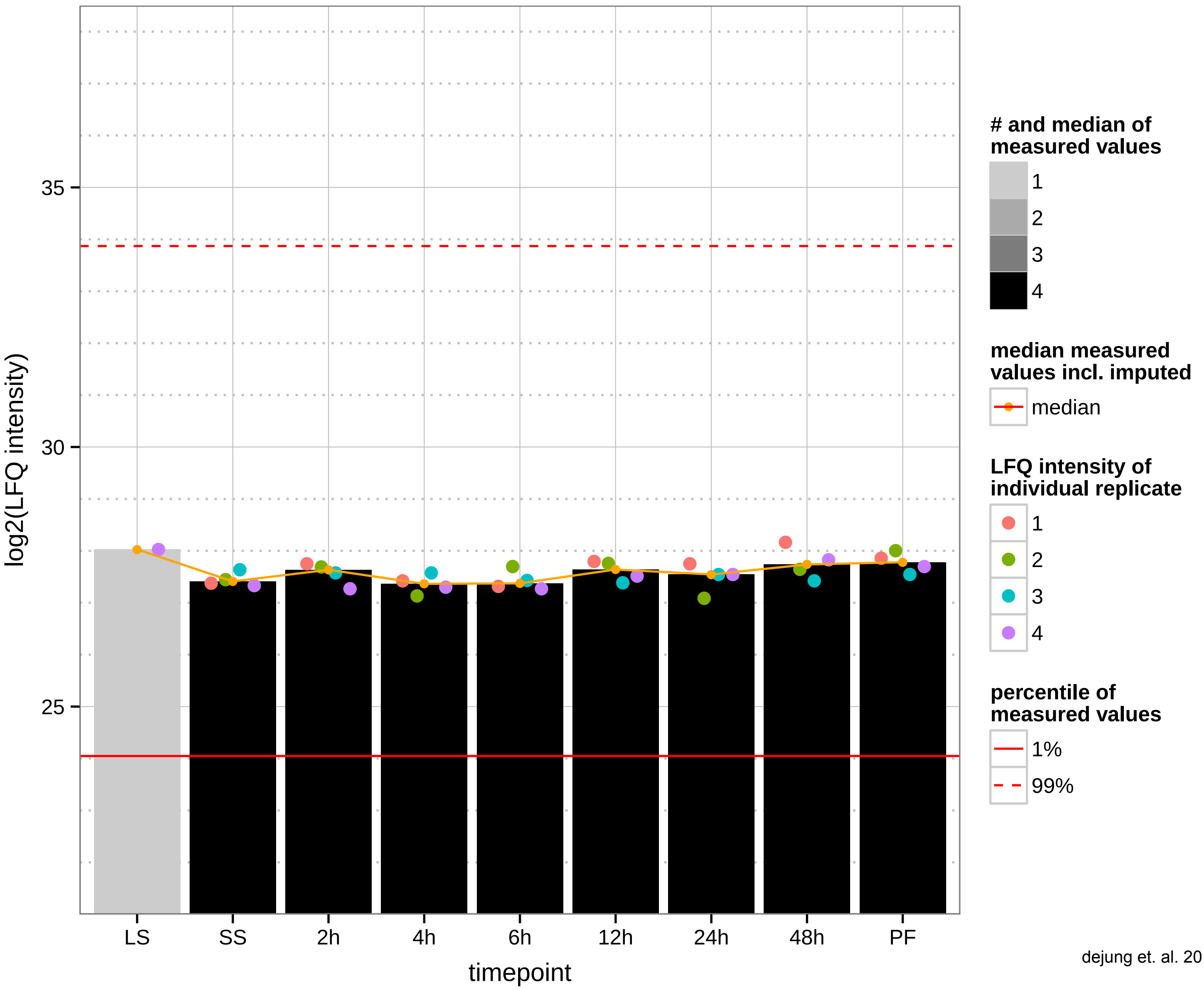

dejung et. al. 2015

hypothetical protein, conserved
Tb927.9.6510
AGOF: null
AGOC: mitochondrion
AGOP: null
PGOF: null
PGOC: null
PGOP: null

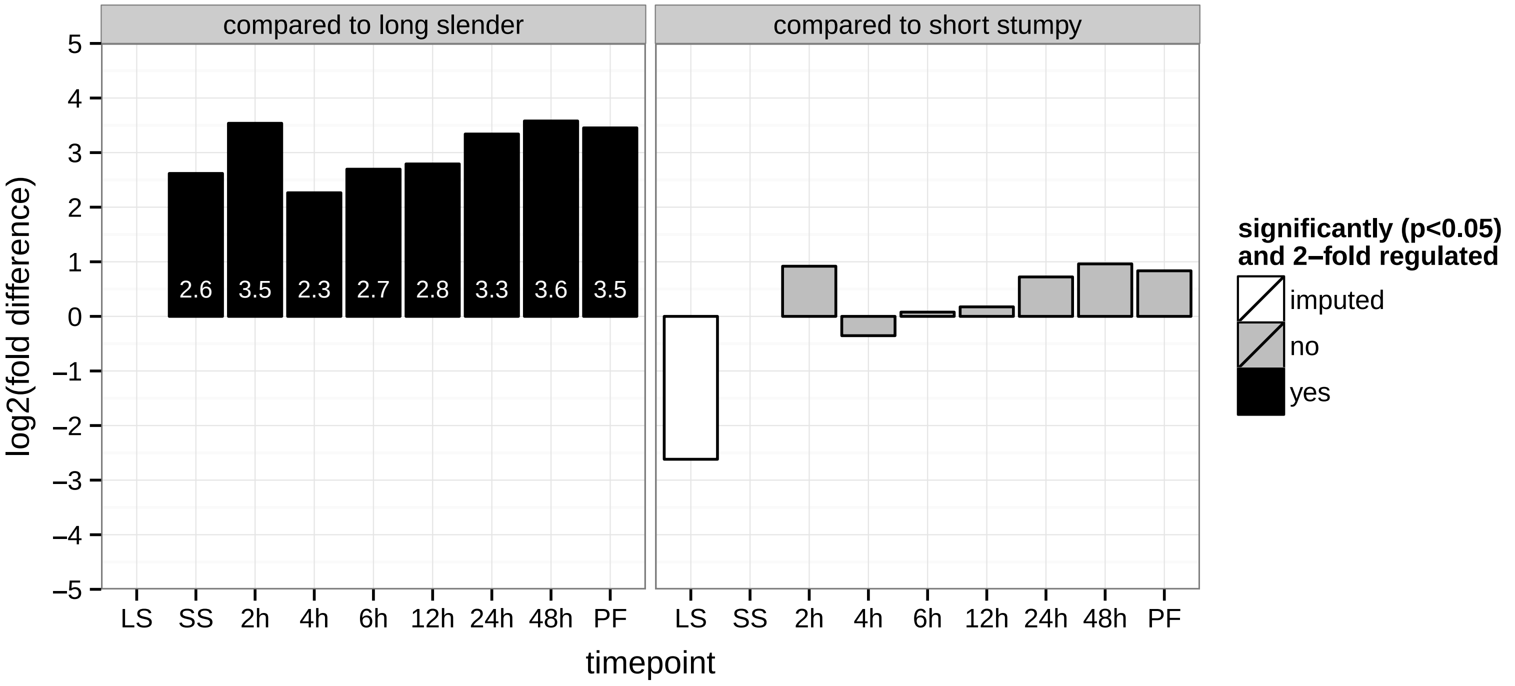

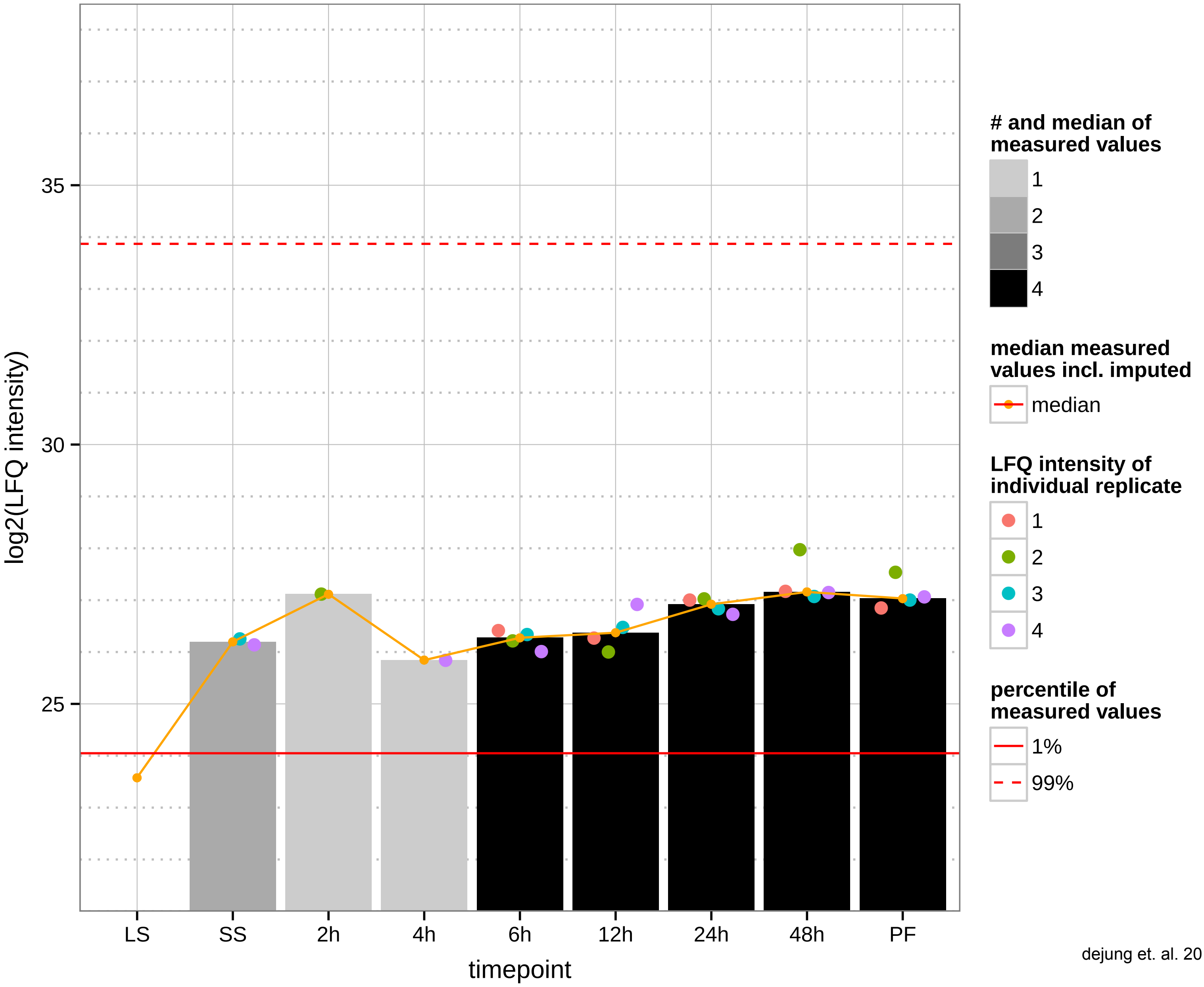

dejung et. al. 2015

hypothetical protein, conserved
Tb927.9.6580
AGOF: null
AGOC: null
AGOP: null
PGOF: null
PGOC: null
PGOP: null

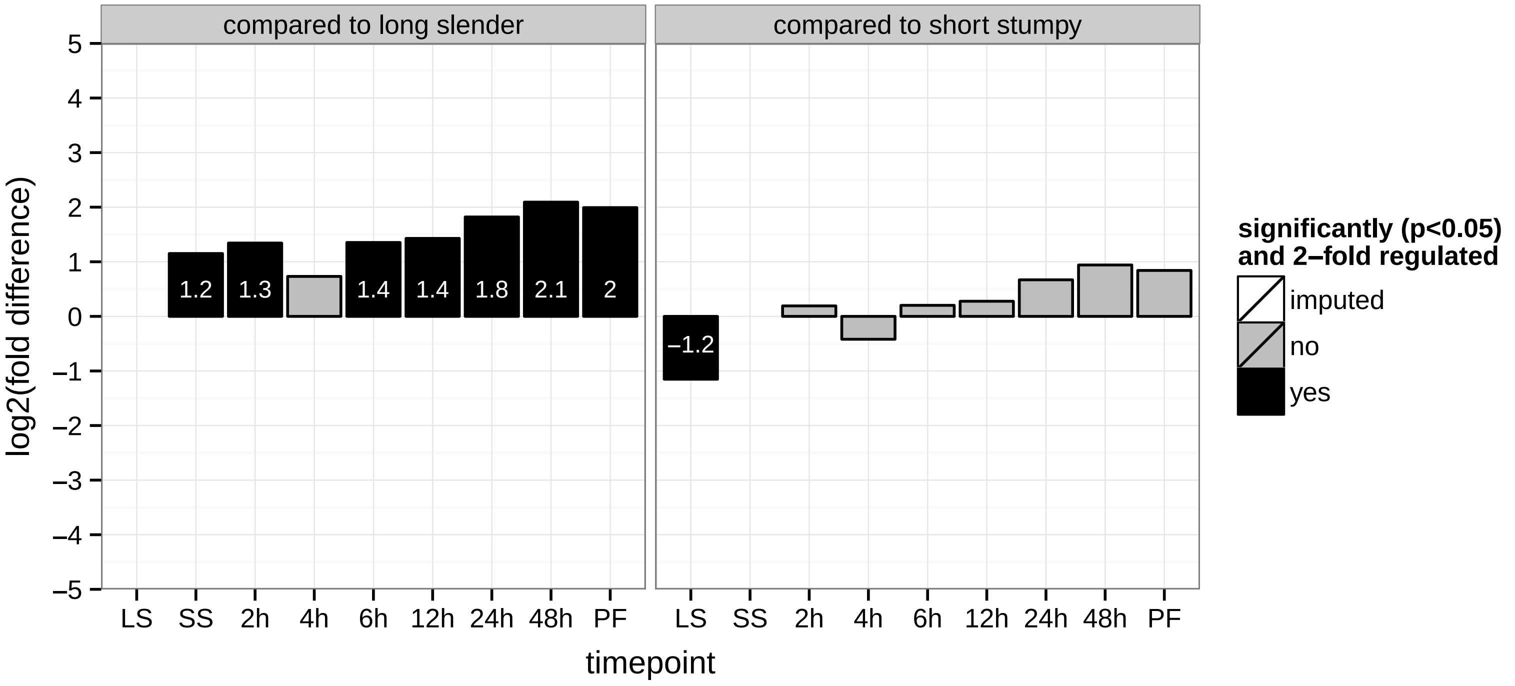

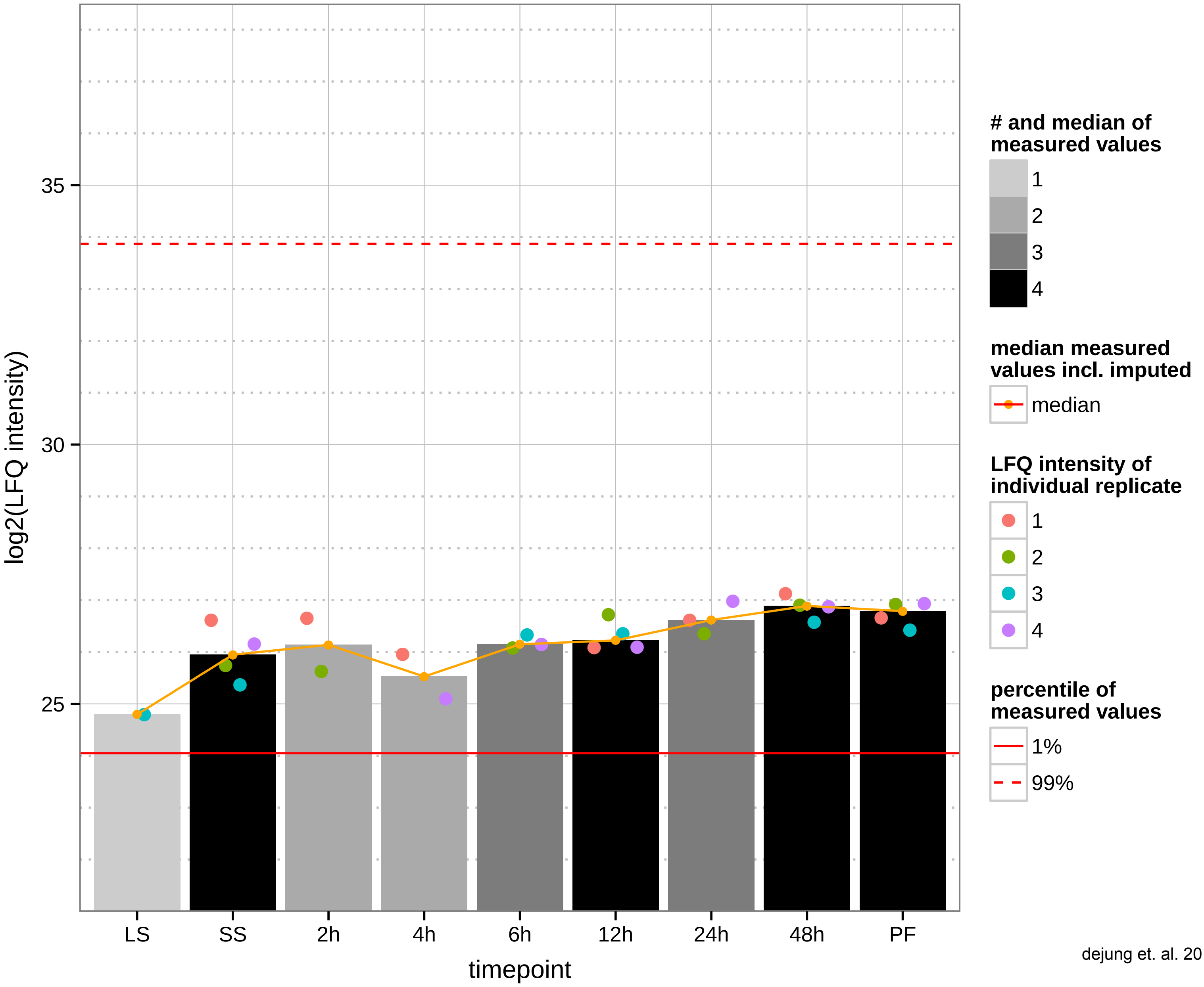

dejung et. al. 2015

hypothetical protein, conserved
Tb927.9.6760
AGOF: null
AGOC: null
AGOP: null
PGOF: null
PGOC: null
PGOP: null

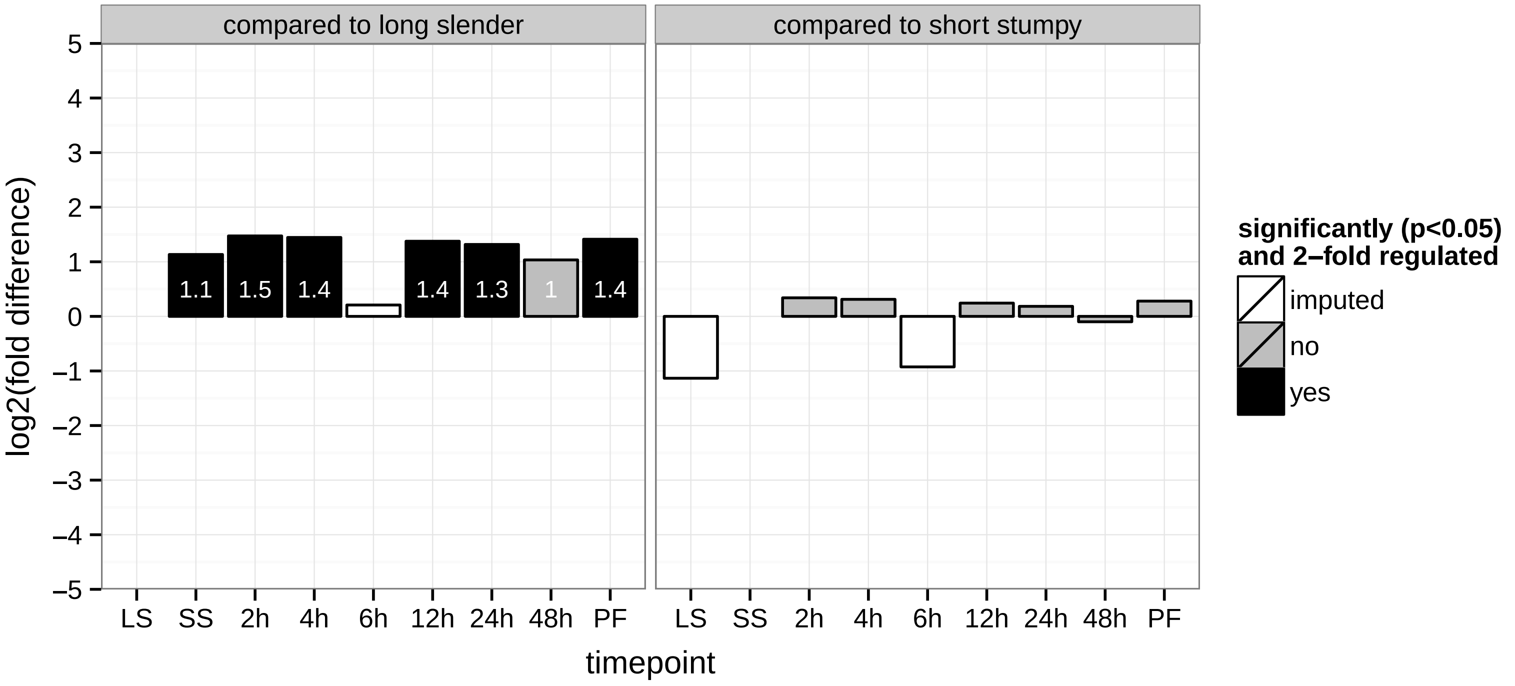

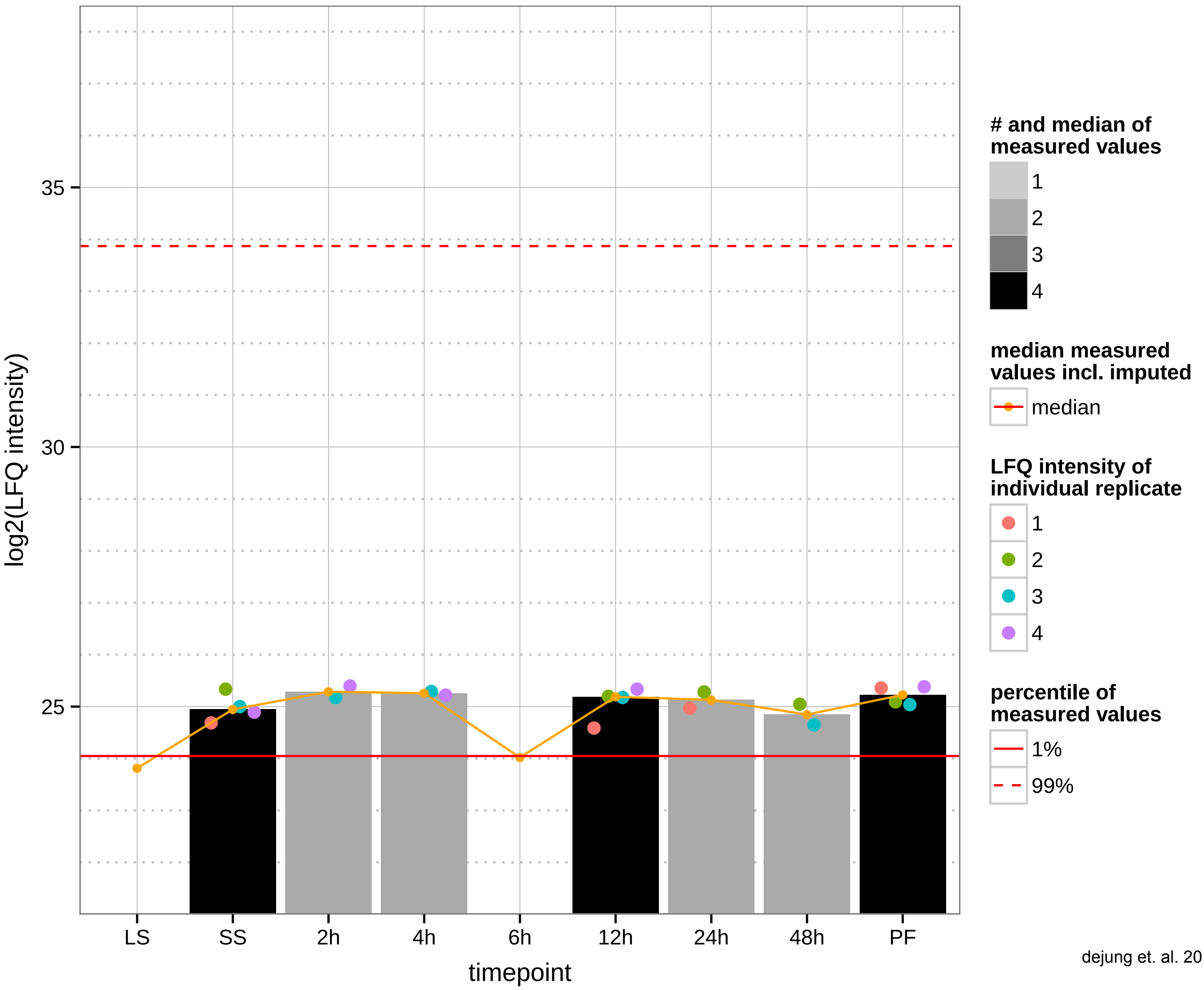

dejung et. al. 2015

cAMP–specific phosphodiesterase, putative
Tb927.9.6880
AGOF: 3', 5'–cyclic–AMP phosphodiesterase activity
AGOC: null
AGOP: signal transduction
PGOF: 3', 5'–cyclic–nucleotide phosphodiesterase activity
PGOC: null
PGOP: signal transduction

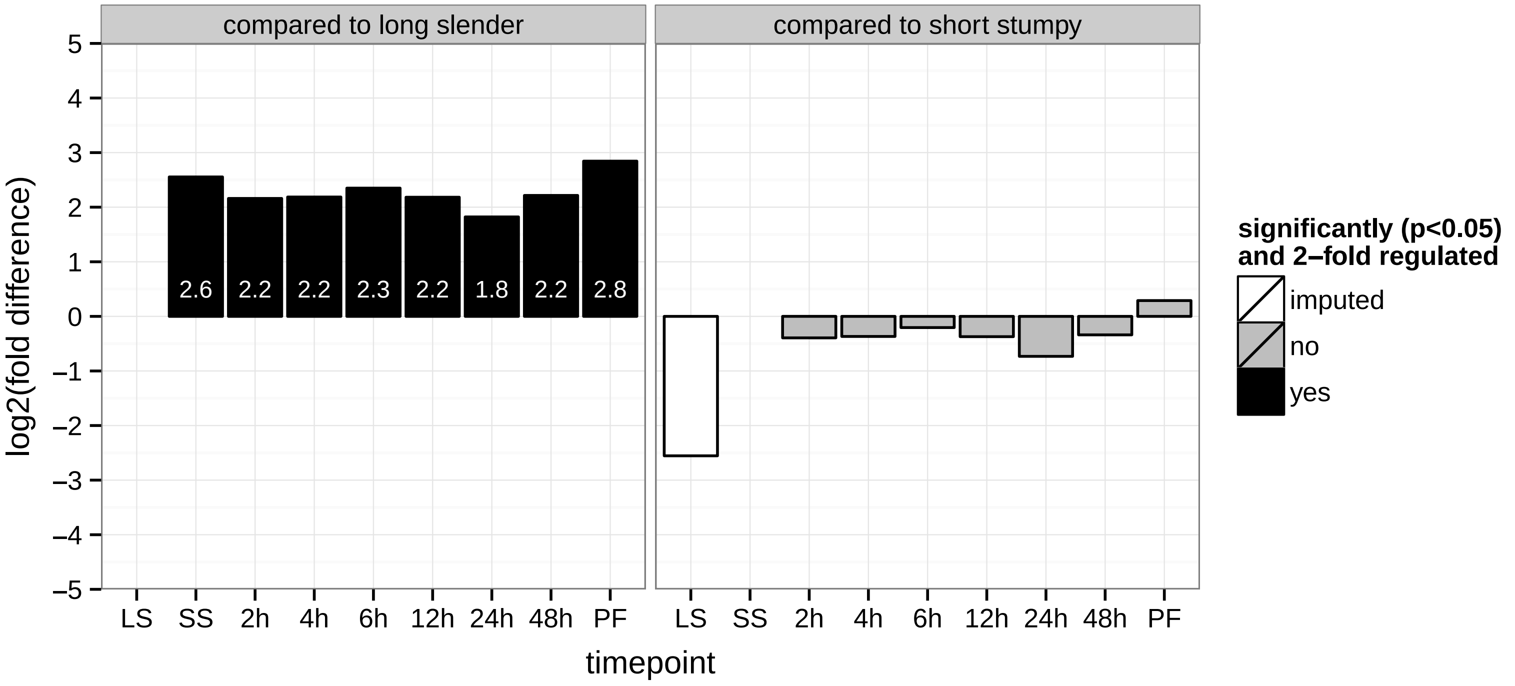

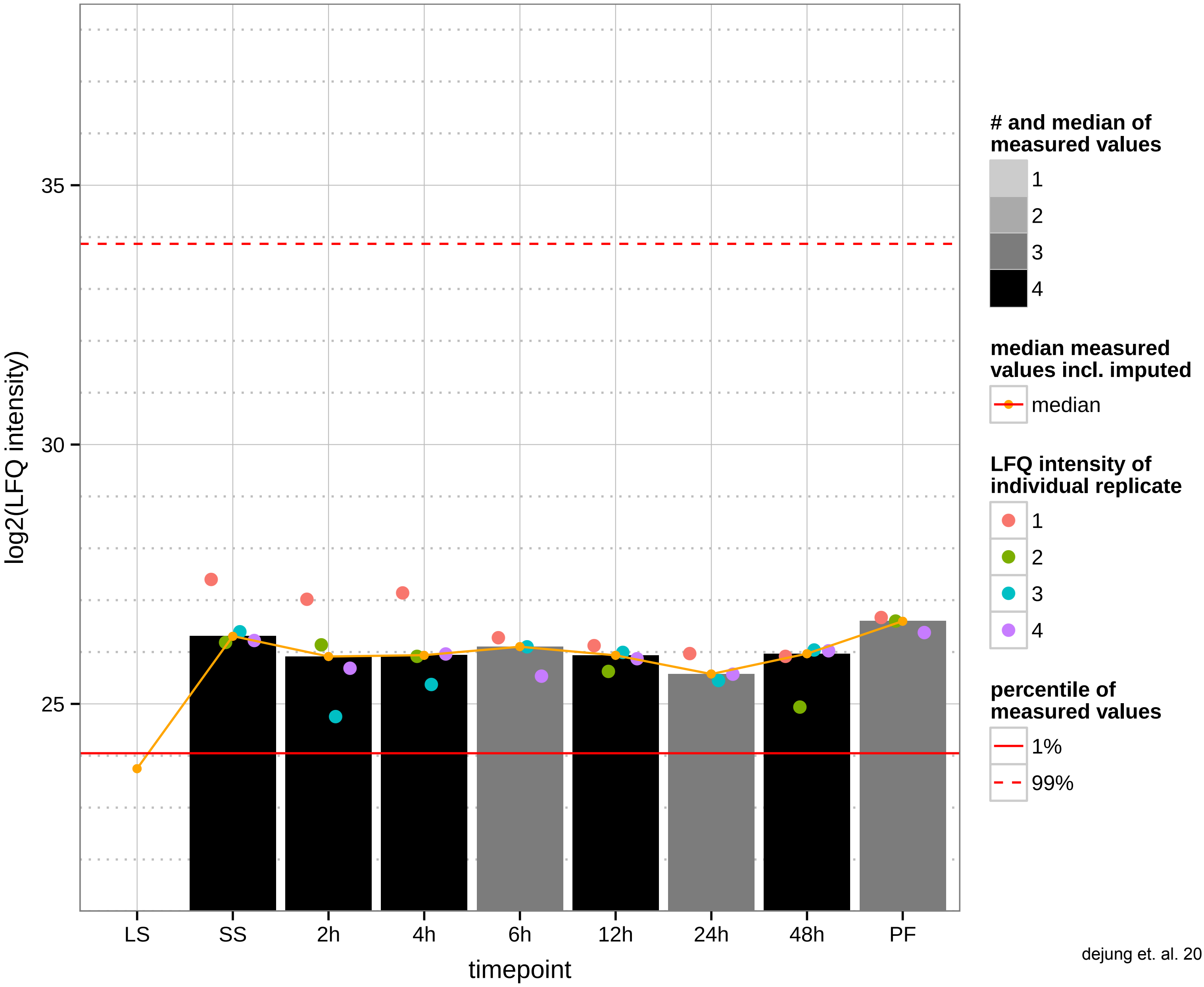

dejung et. al. 2015

DNA topoisomerase type IB small subunit
Tb927.9.6940
AGOF: ATP binding, DNA topoisomerase type I activity, magnesium ion binding
AGOC: nucleus
AGOP: DNA topological change, chromatin assembly or disassembly
PGOF: DNA binding
PGOC: null
PGOP: null

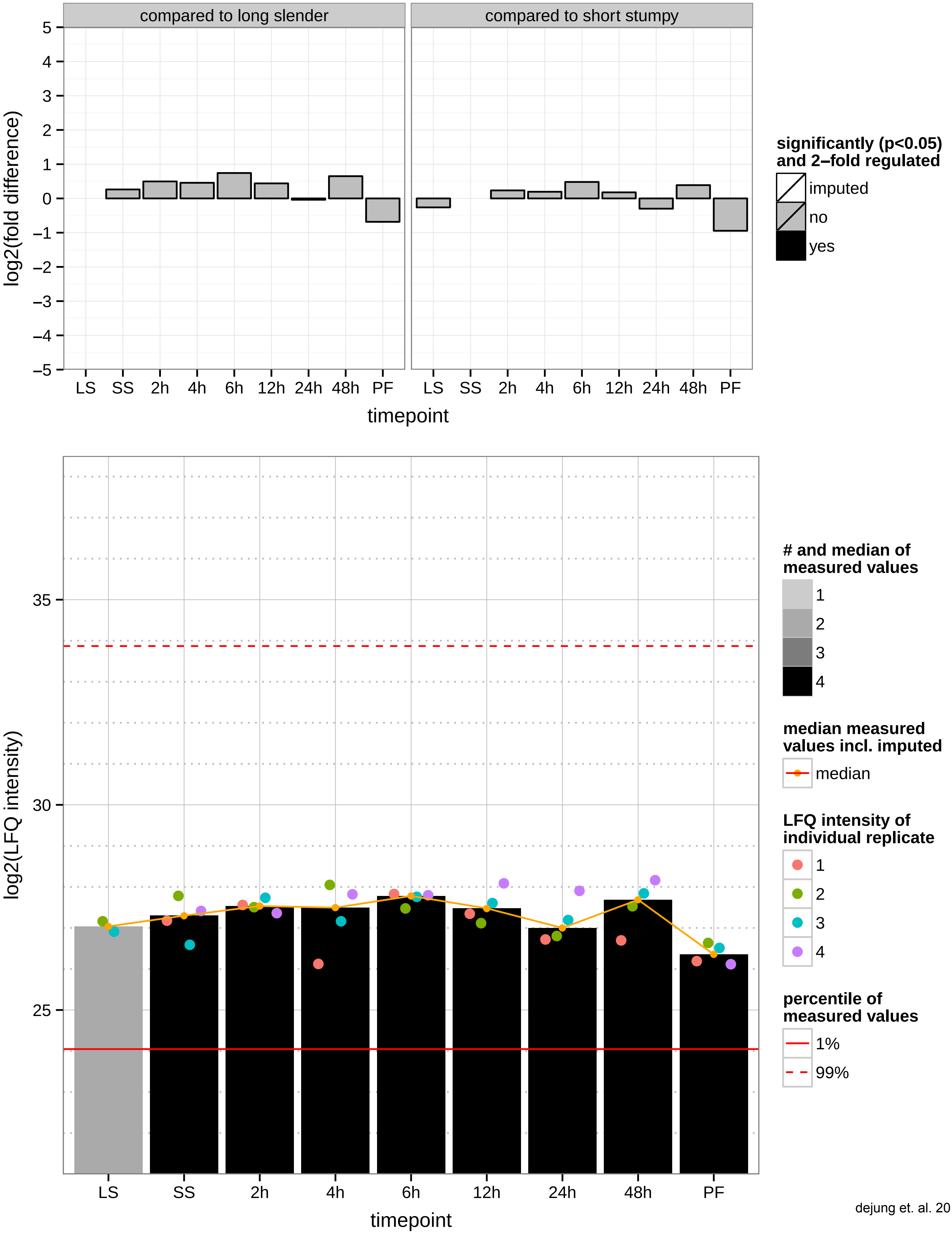

dejung et. al. 2015

RNA pseudouridylate synthase protein, putative
Tb927.9.7150
AGOF: RNA binding, pseudouridine synthase activity
AGOC: null
AGOP: RNA processing, pseudouridine synthesis
PGOF: RNA binding, pseudouridine synthase activity
PGOC: null
PGOP: RNA modification, pseudouridine synthesis

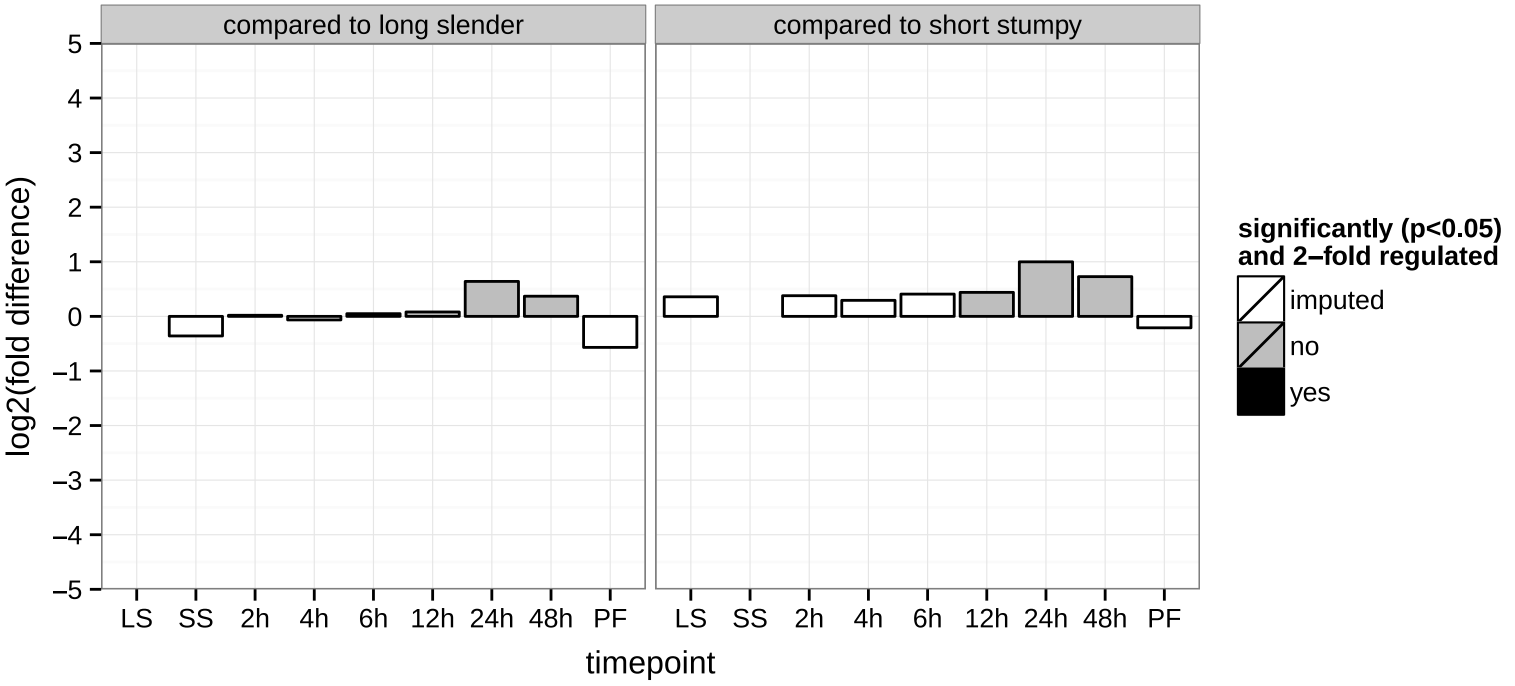

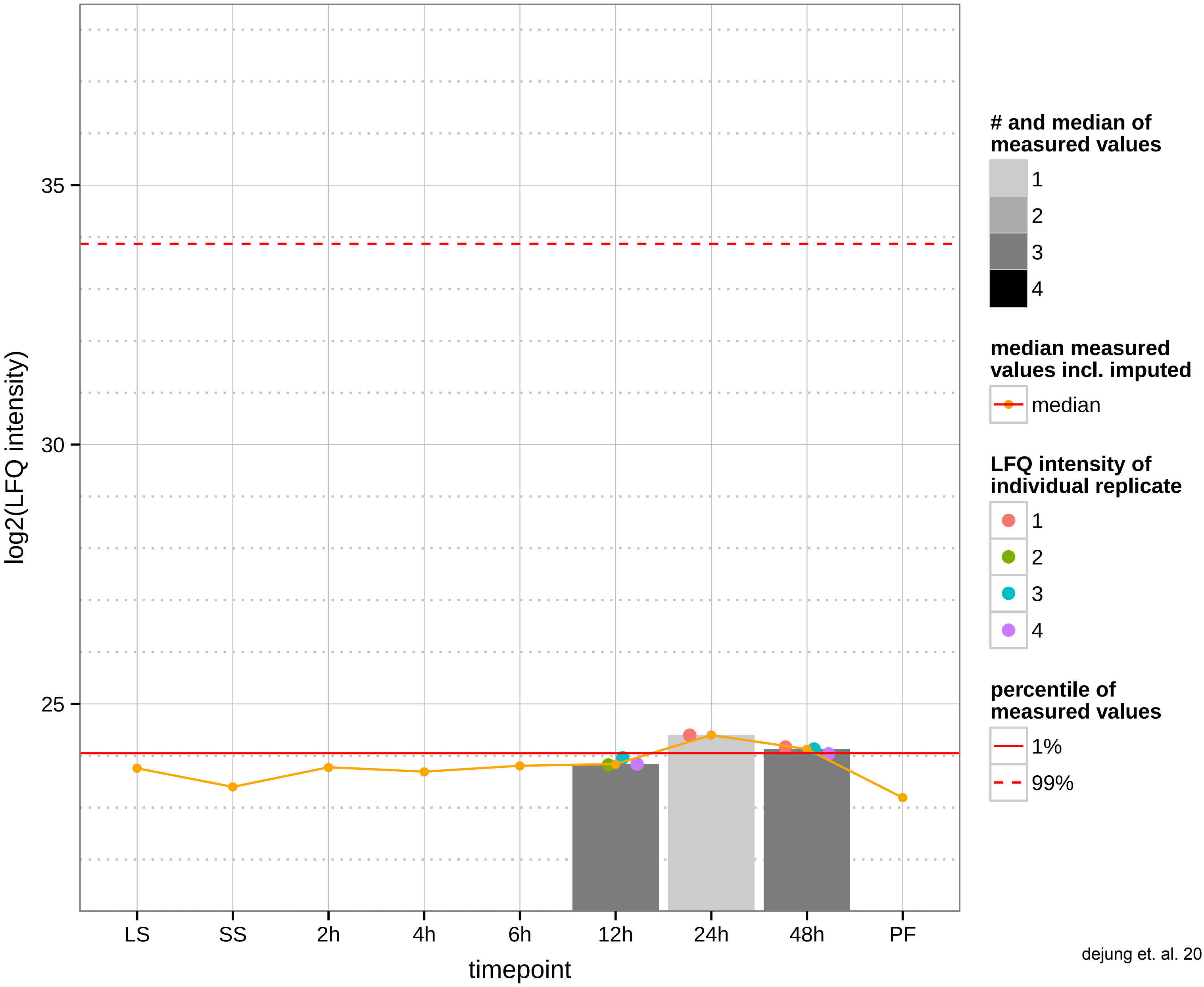

dejung et. al. 2015

adenosine monophosphate deaminase, putative, AMP deaminase
Tb927.9.7180
AGOF: AMP deaminase activity
AGOC: null
AGOP: purine ribonucleoside monophosphate biosynthetic process
PGOF: deaminase activity
PGOC: null
PGOP: purine ribonucleoside monophosphate biosynthetic process

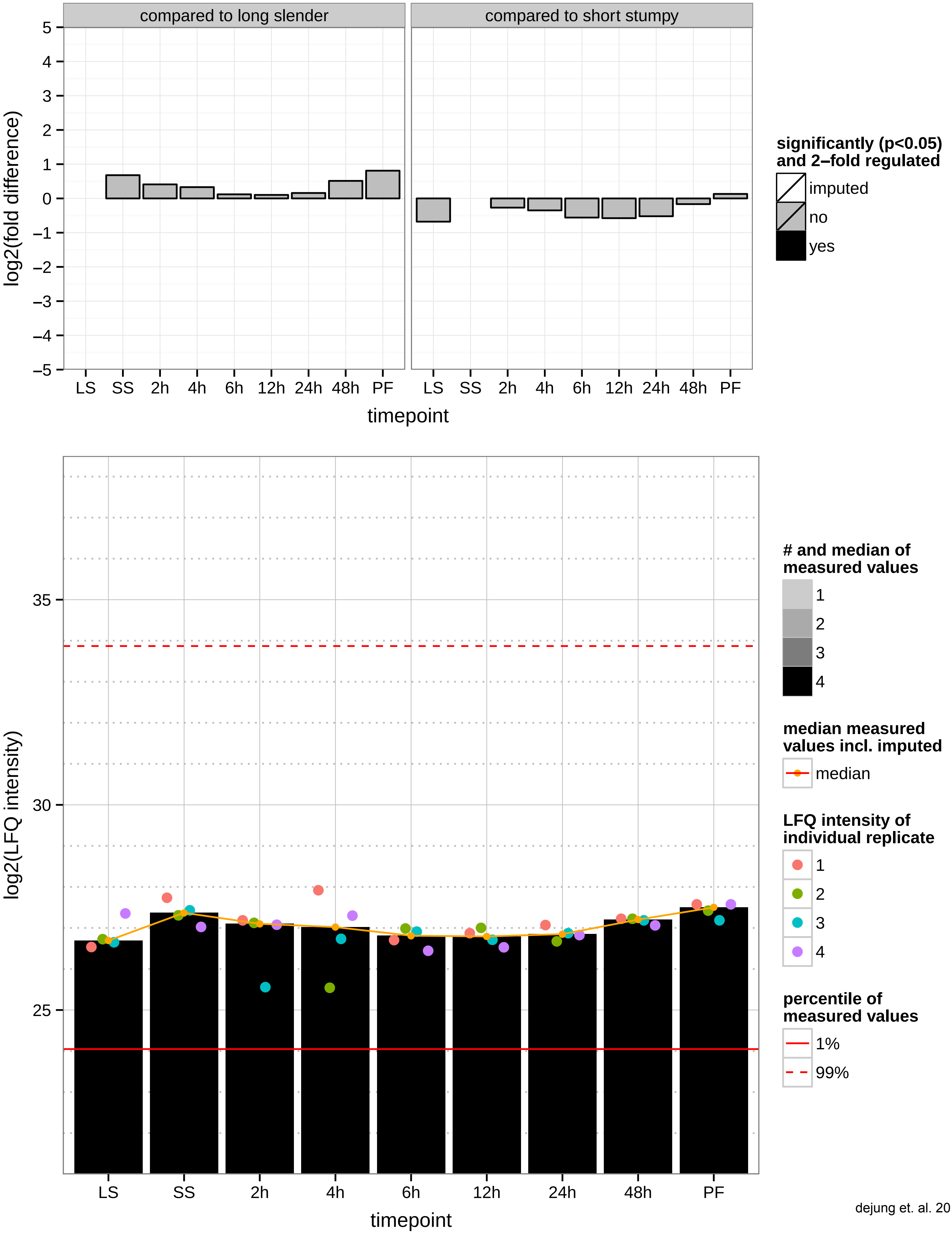

dejung et. al. 2015

mitochondrial exoribonuclease DSS−1, exoribonuclease DSS−1 (EMBL:AY233297)
Tb927.9.7210
AGOF: RNA binding, exoribonuclease activity
AGOC: mitochondrion
AGOP: mitochondrial RNA 5'−end processing
PGOF: RNA binding, ribonuclease activity
PGOC: null
PGOP: null

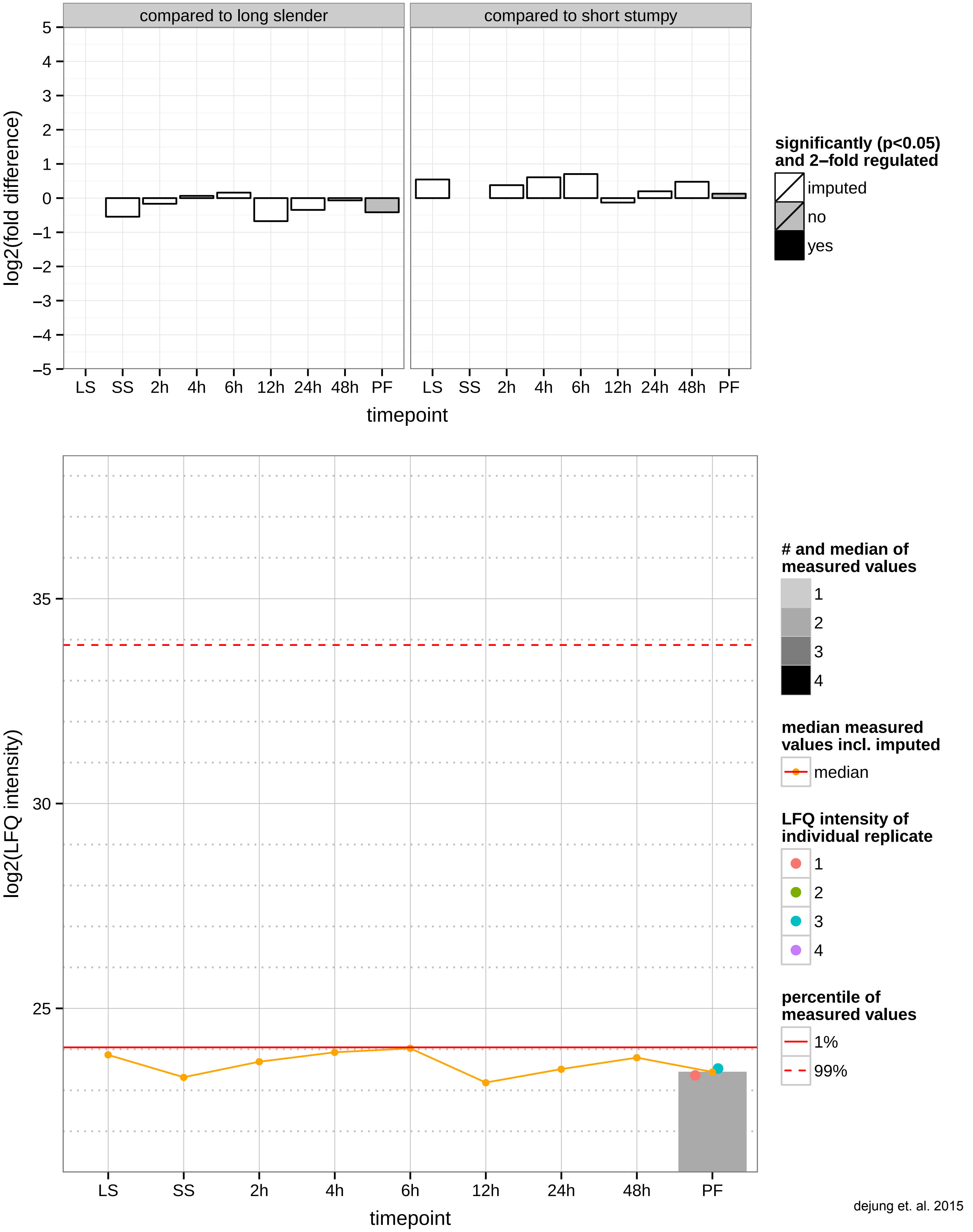

dejung et. al. 2015

calpain–like cysteine peptidase, putative, cysteine peptidase, Clan CA, family C2
Tb927.9.7540
AGOF: calcium–dependent cysteine–type endopeptidase activity
AGOC: intracellular
AGOP: proteolysis
PGOF: calcium–dependent cysteine–type endopeptidase activity
PGOC: intracellular
PGOP: proteolysis

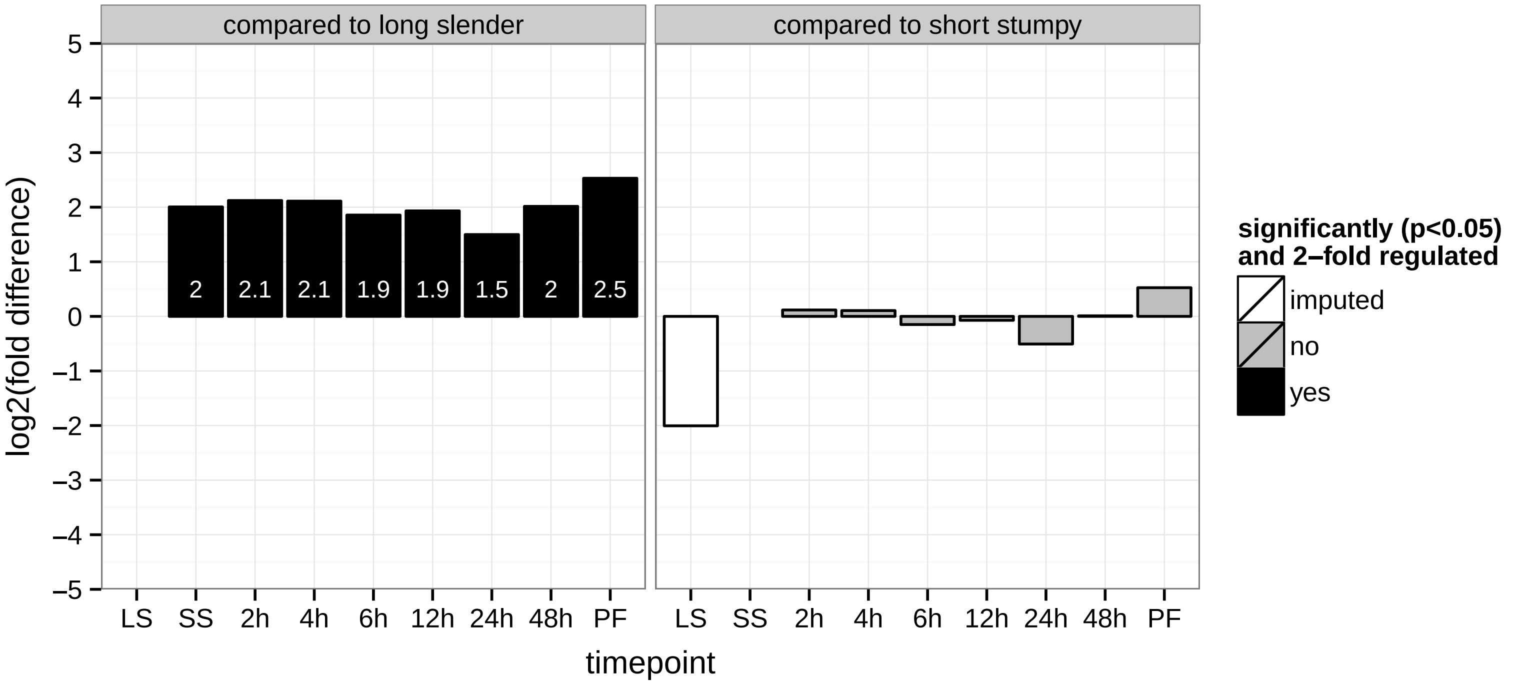

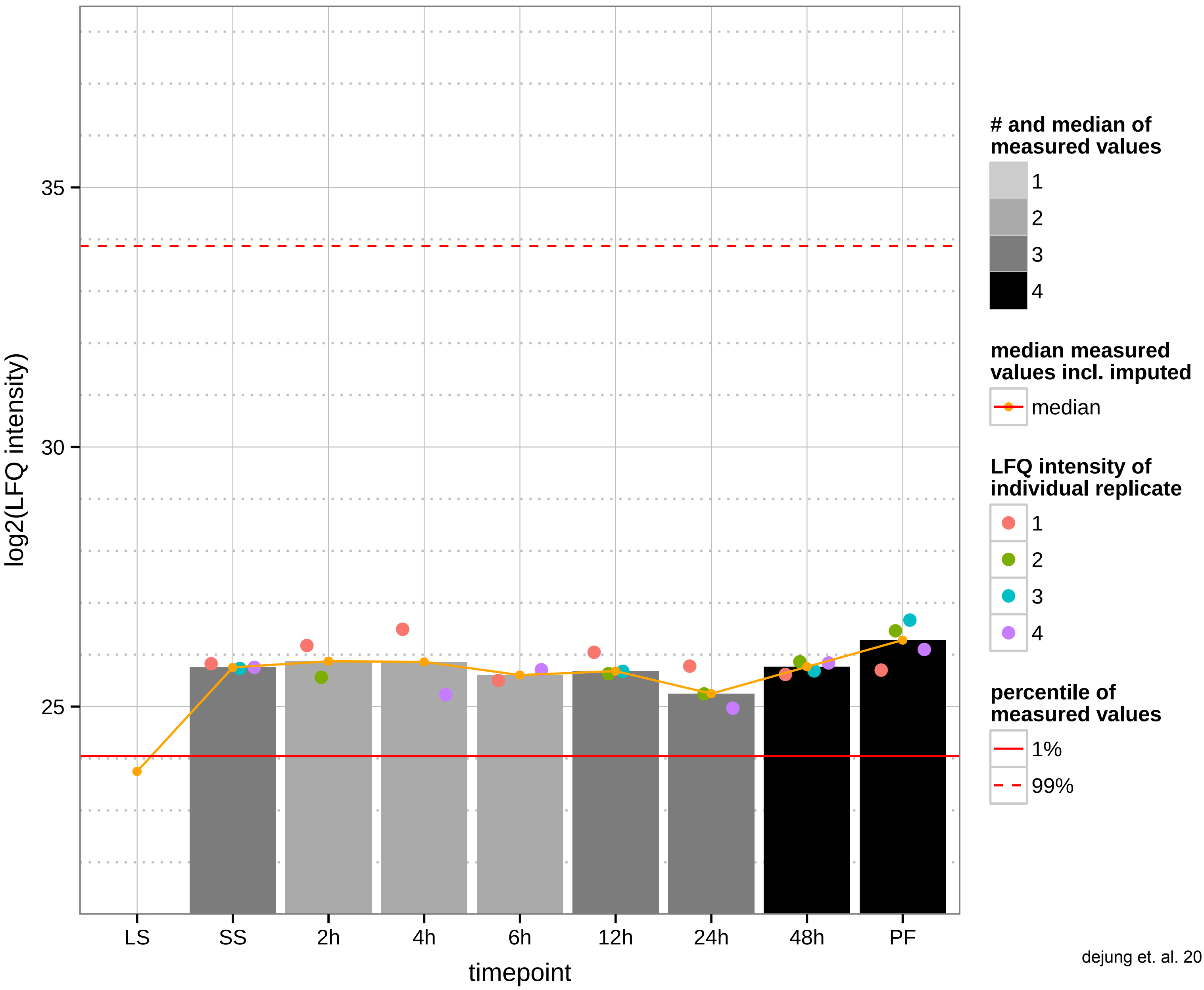

dejung et. al. 2015

ADP–ribosylation factor, putative
Tb927.9.7650
AGOF: GTP binding
AGOC: intracellular
AGOP: intracellular protein transport, small GTPase mediated signal transduction
PGOF: GTP binding
PGOC: intracellular
PGOP: intracellular protein transport, small GTPase mediated signal transduction

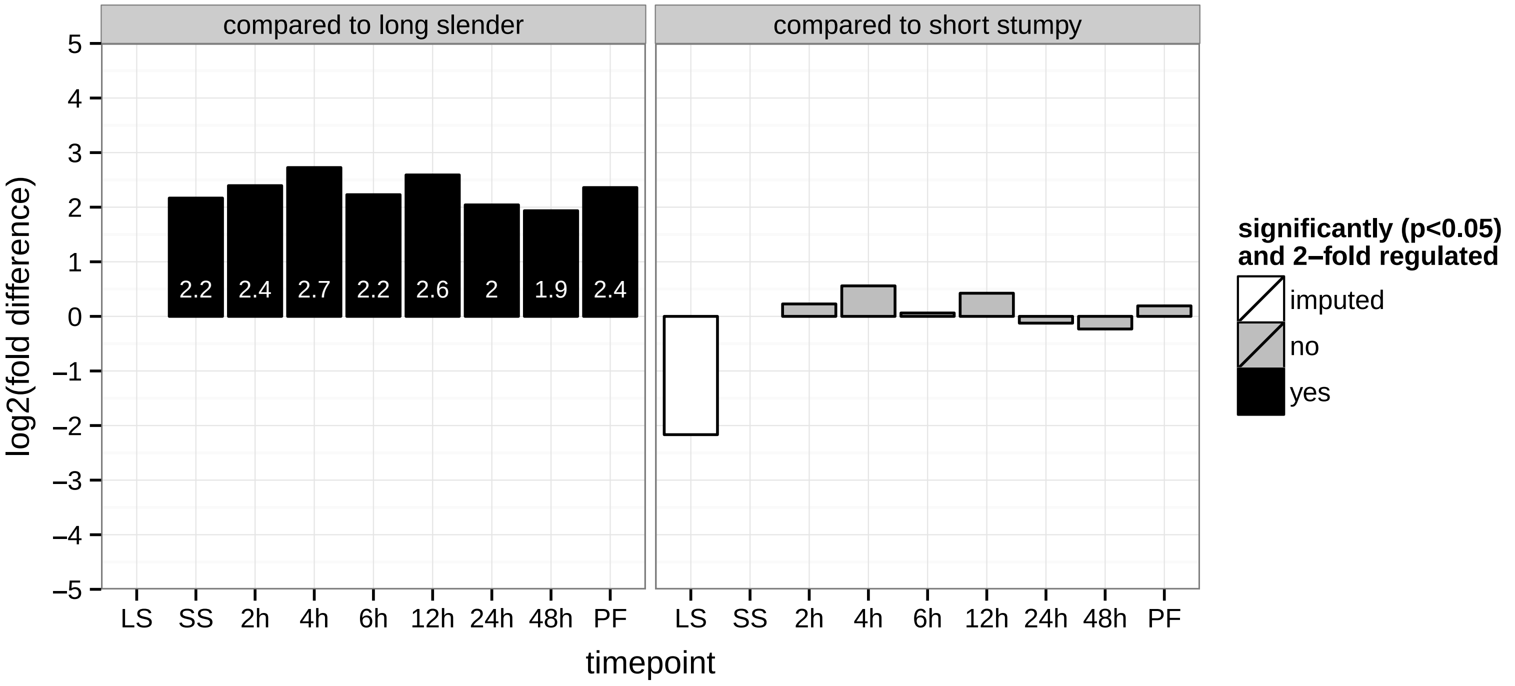

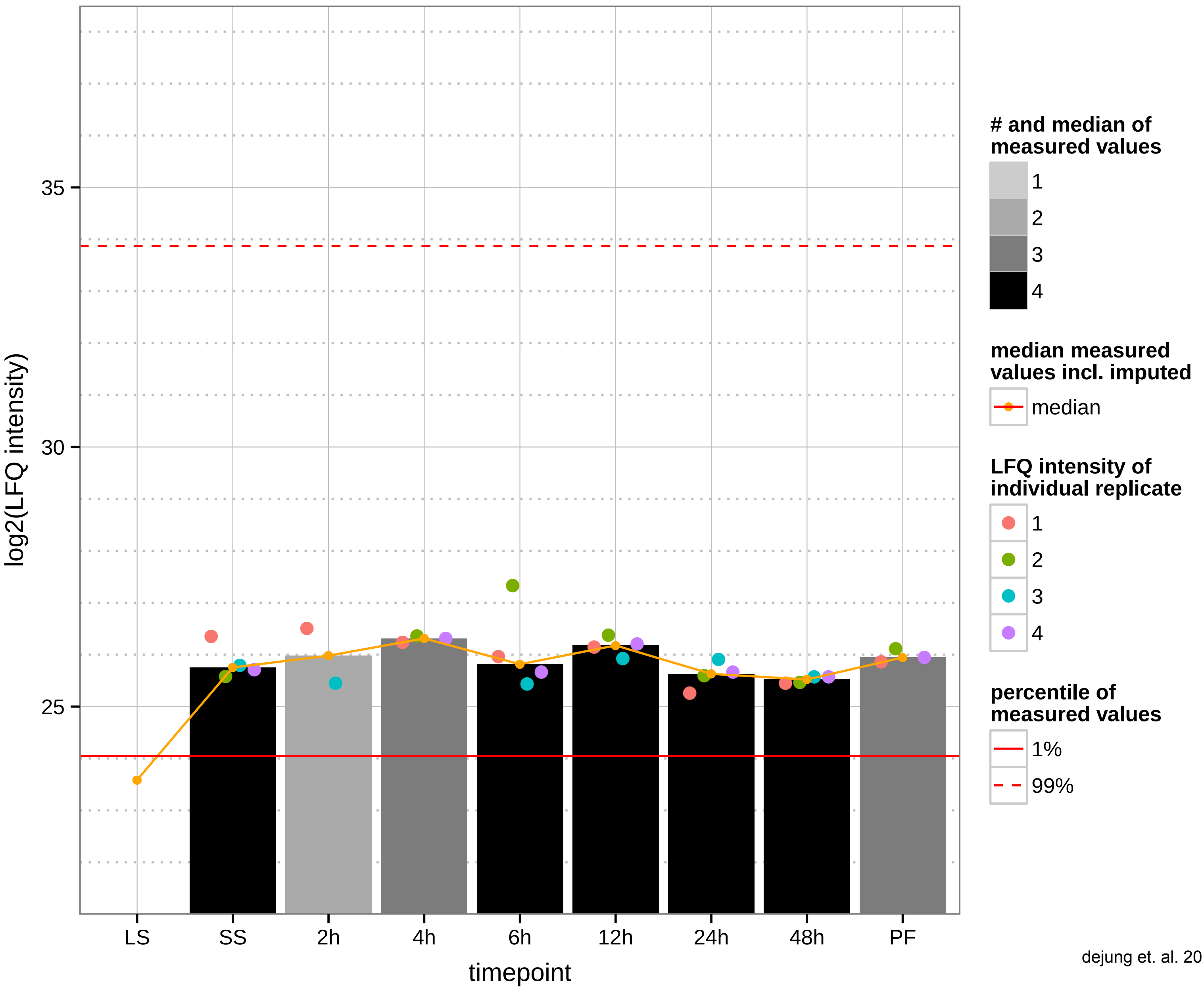

dejung et. al. 2015

predicted tetratricopeptide repeat protein
Tb927.9.7720
AGOF: null
AGOC: null
AGOP: null
PGOF: protein binding
PGOC: null
PGOP: null

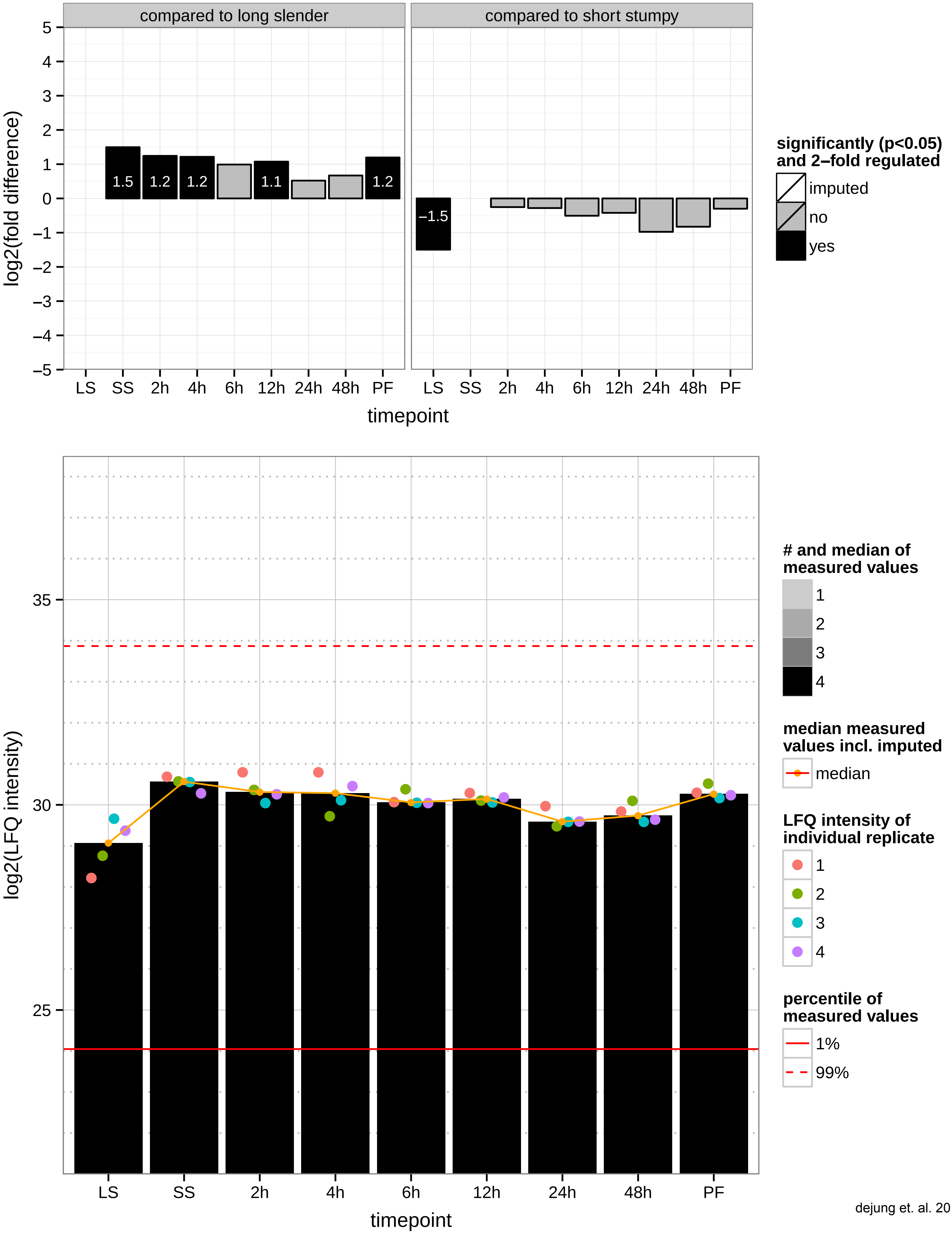

dejung et. al. 2015

spermidine synthase, SPDSYN (SpSyn)
Tb927.9.7770
AGOF: spermidine synthase activity
AGOC: null
AGOP: null
PGOF: catalytic activity
PGOC: null
PGOP: null

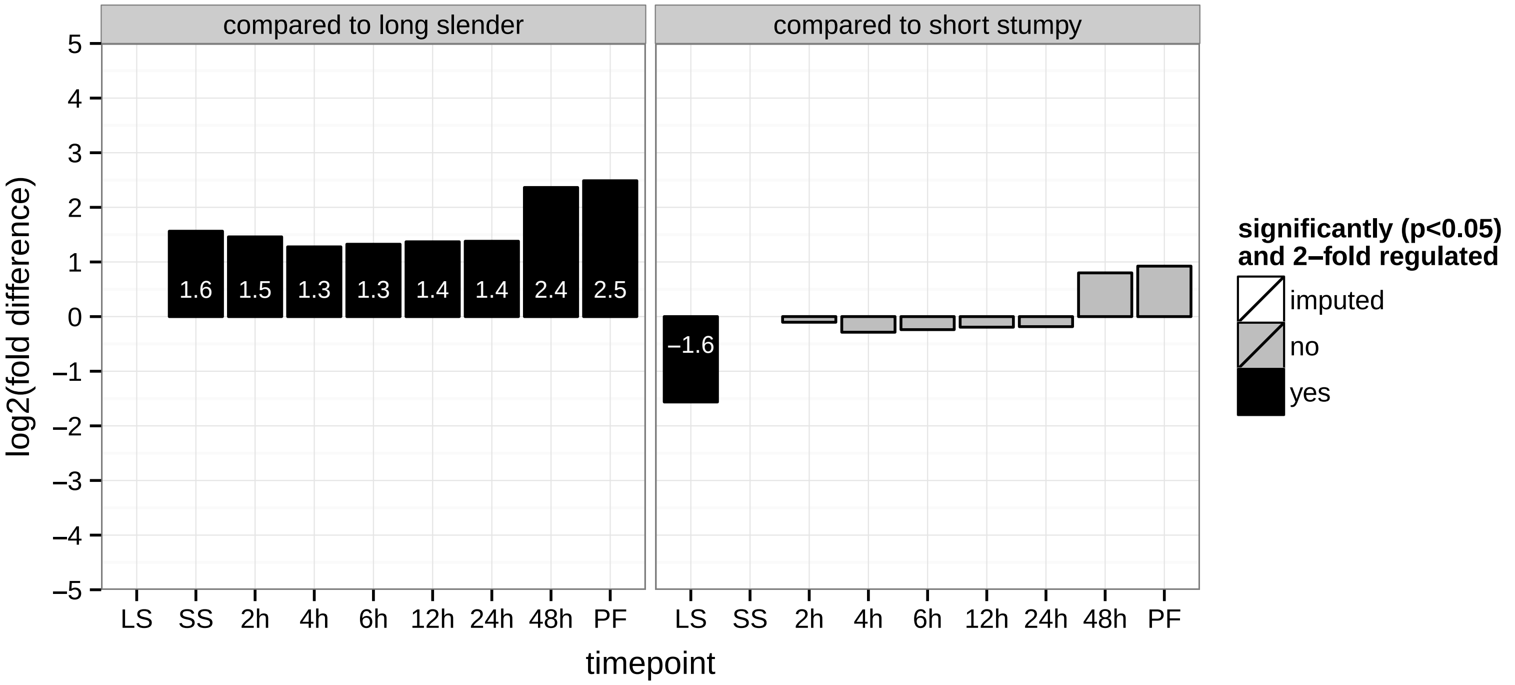

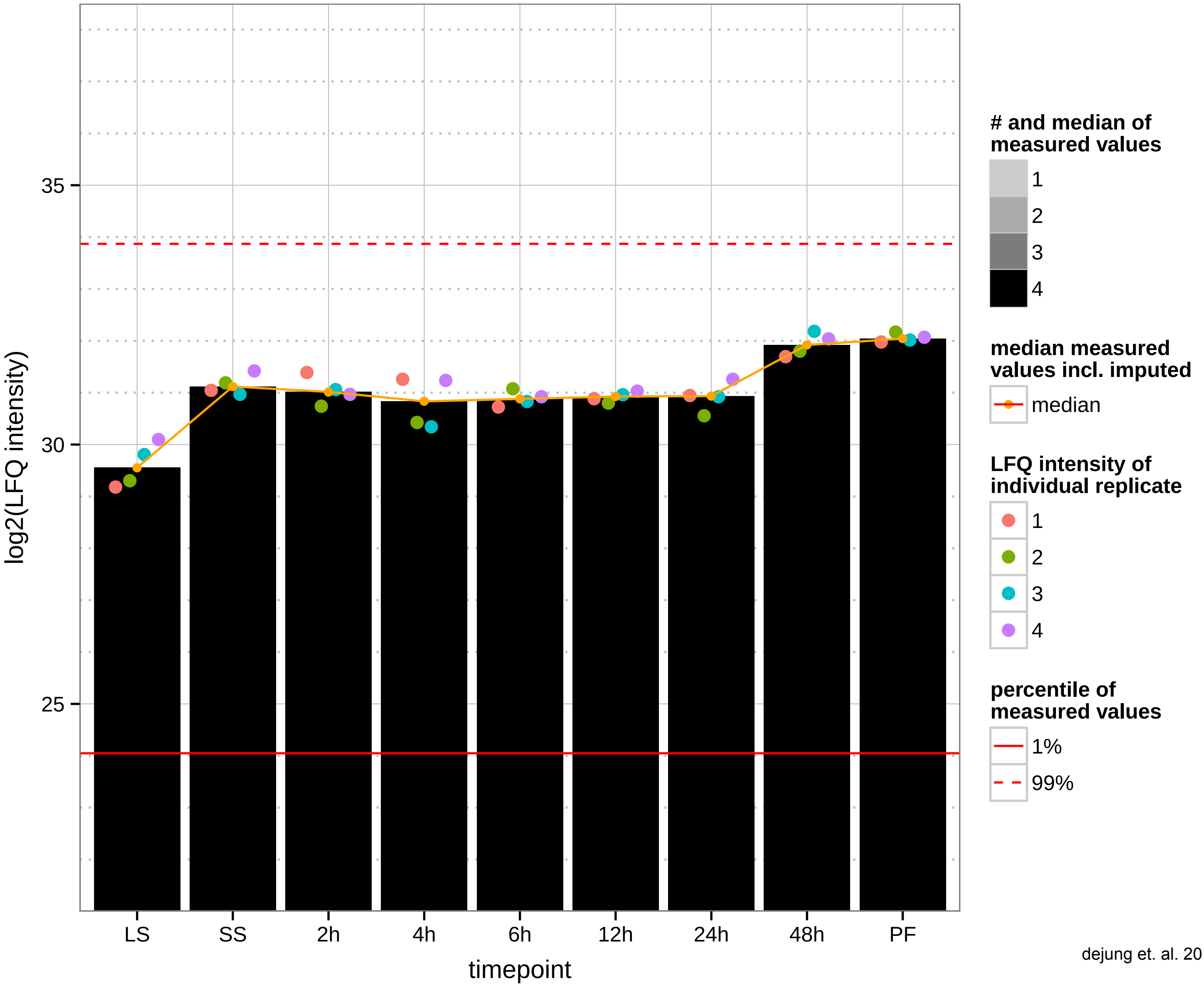

dejung et. al. 2015

hypothetical protein, conserved
Tb927.9.7800
AGOF: null
AGOC: null
AGOP: null
PGOF: null
PGOC: null
PGOP: null

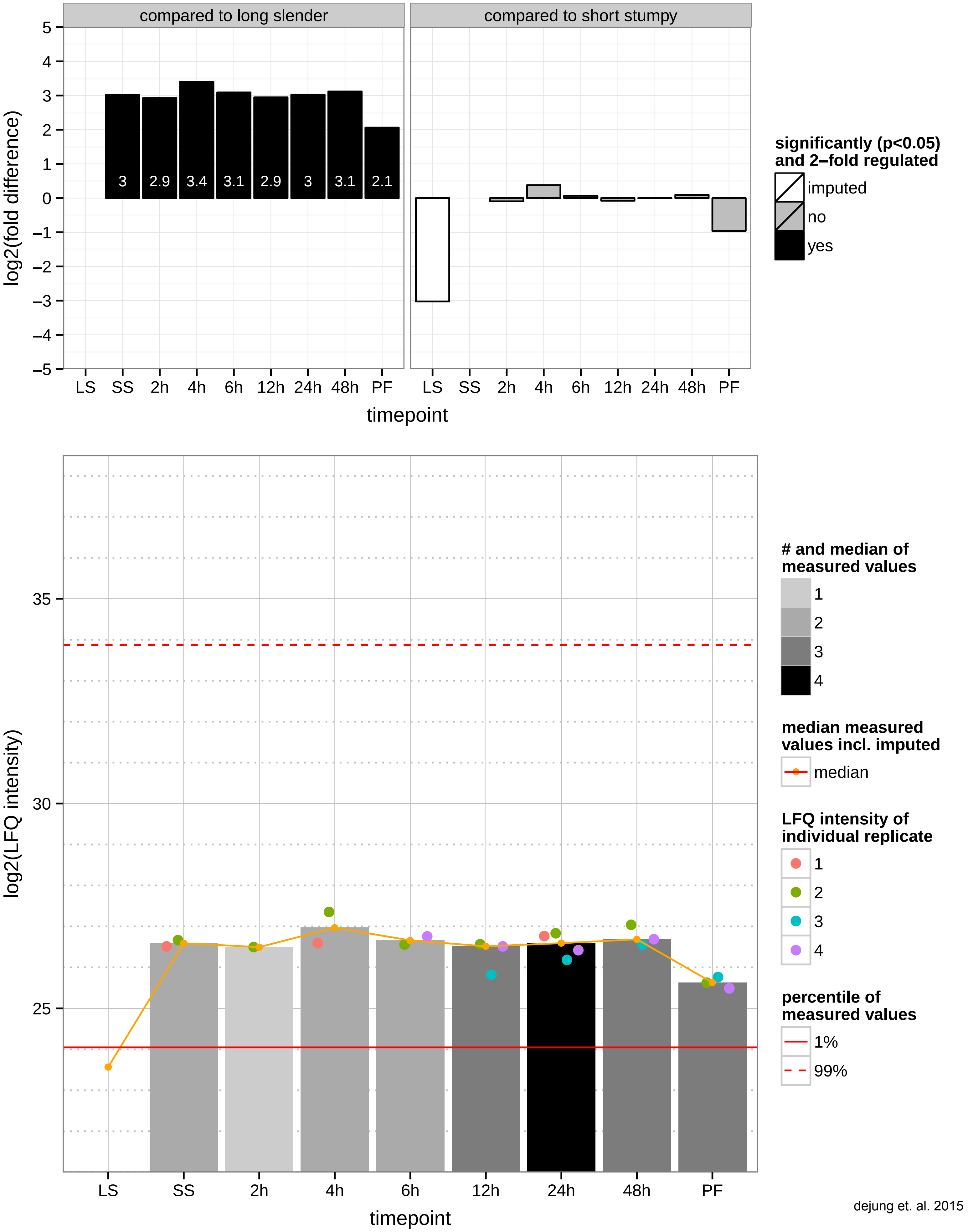

dejung et. al. 2015

hypothetical protein, conserved
Tb927.9.8190
AGOF: null
AGOC: null
AGOP: null
PGOF: null
PGOC: null
PGOP: null

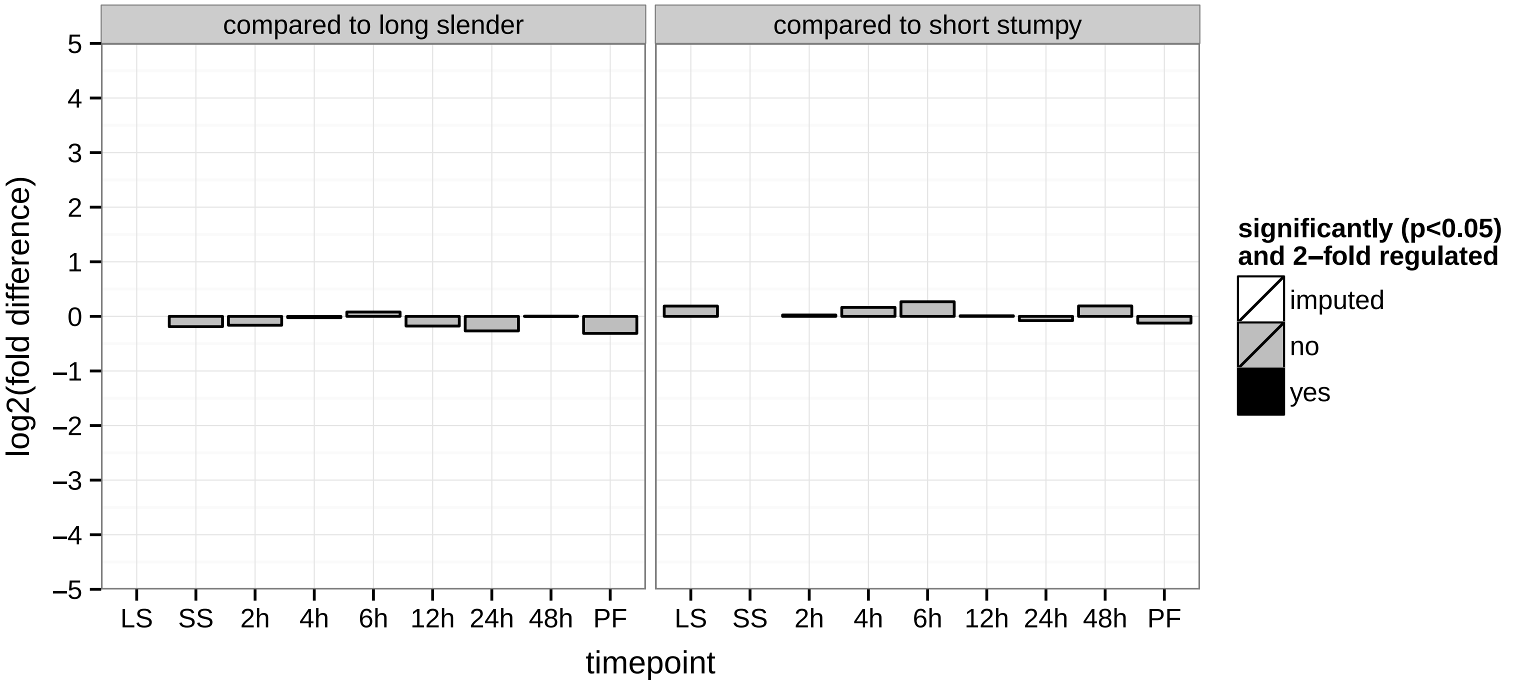

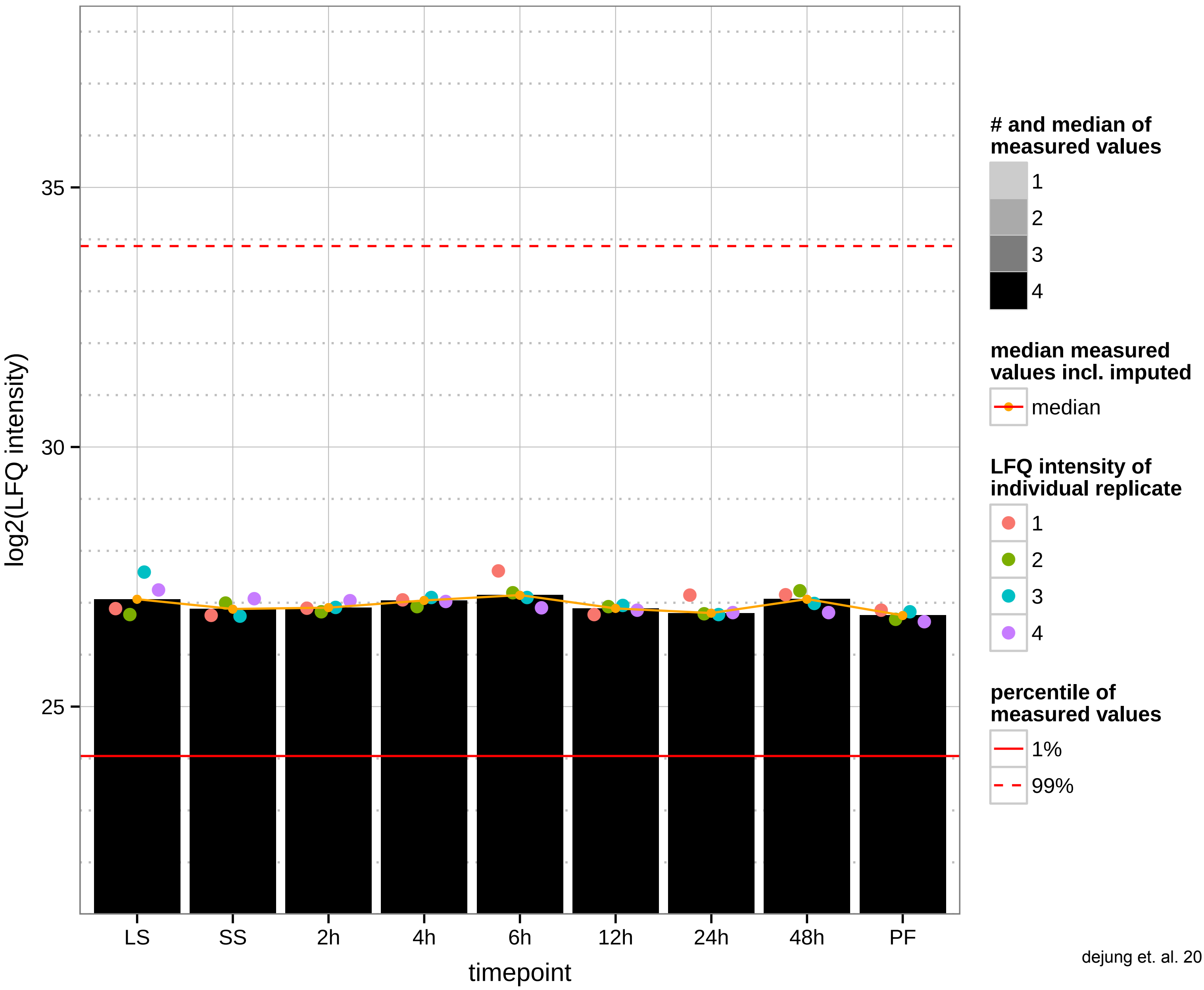

dejung et. al. 2015

hypothetical protein, conserved
Tb927.9.8200;Tb11.v5.0687
AGOF: null
AGOC: null, intracellular, nucleolus
AGOP: null, cell proliferation
PGOF: null
PGOC: nucleolus
PGOP: ribosome biogenesis

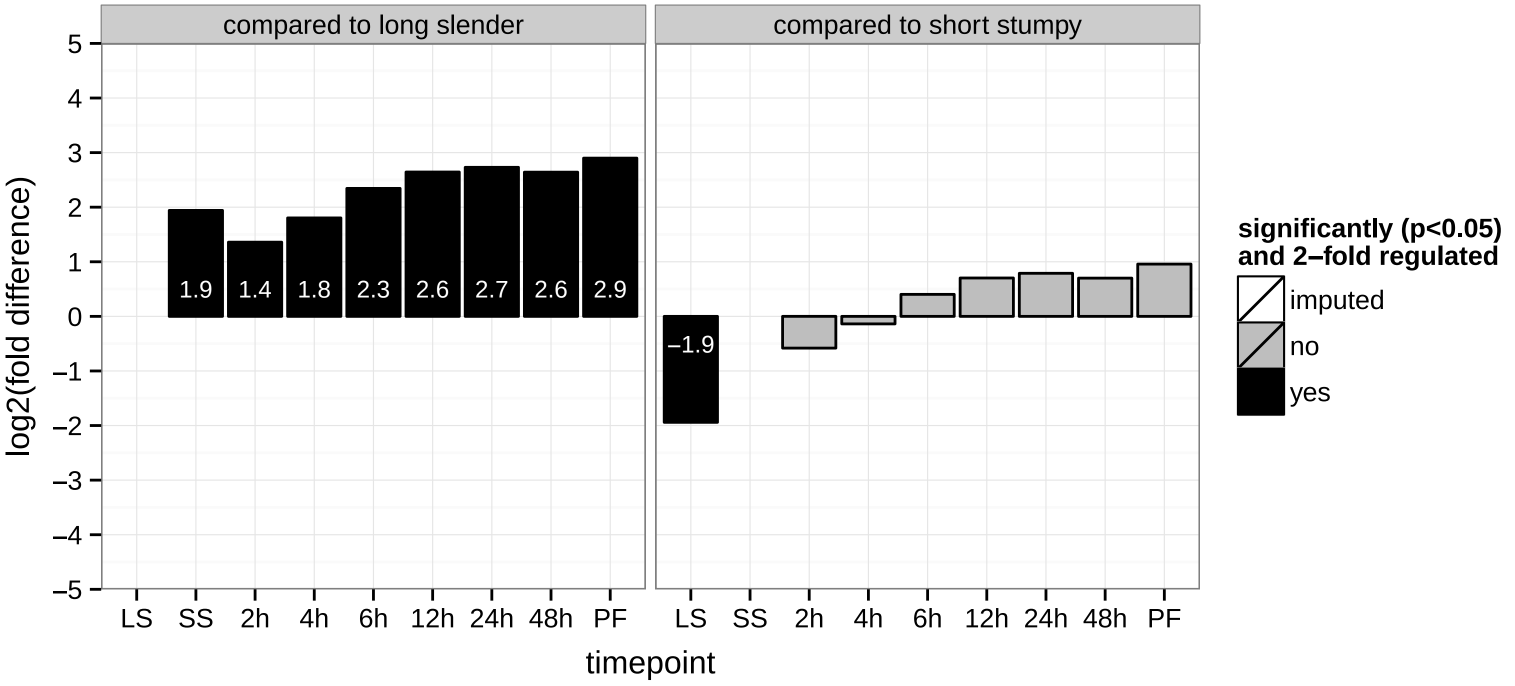

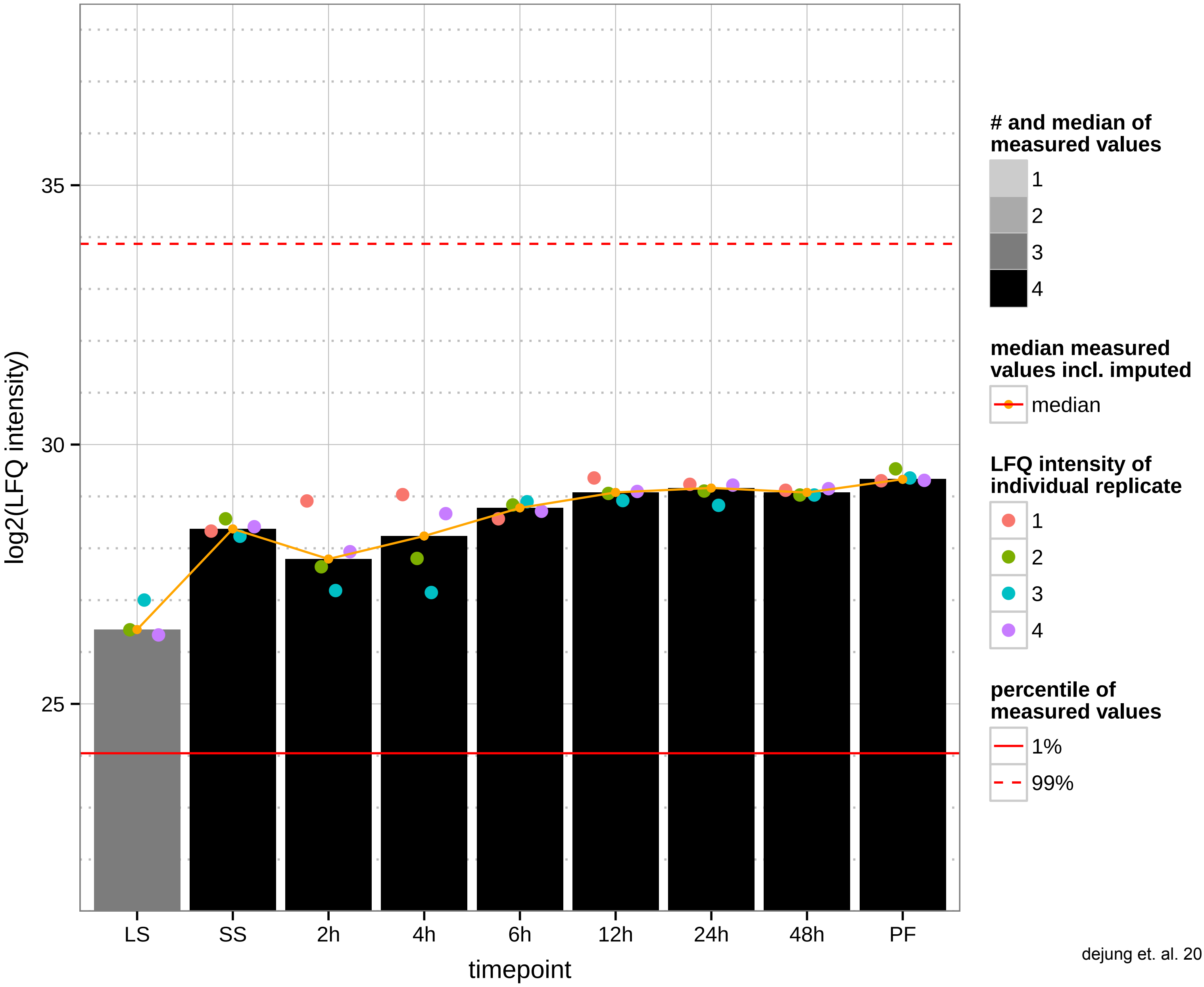

dejung et. al. 2015

chaperone protein DNAj, putative
Tb927.9.8410
AGOF: heat shock protein binding, unfolded protein binding
AGOC: mitochondrion
AGOP: protein folding
PGOF: heat shock protein binding, unfolded protein binding
PGOC: null
PGOP: protein folding

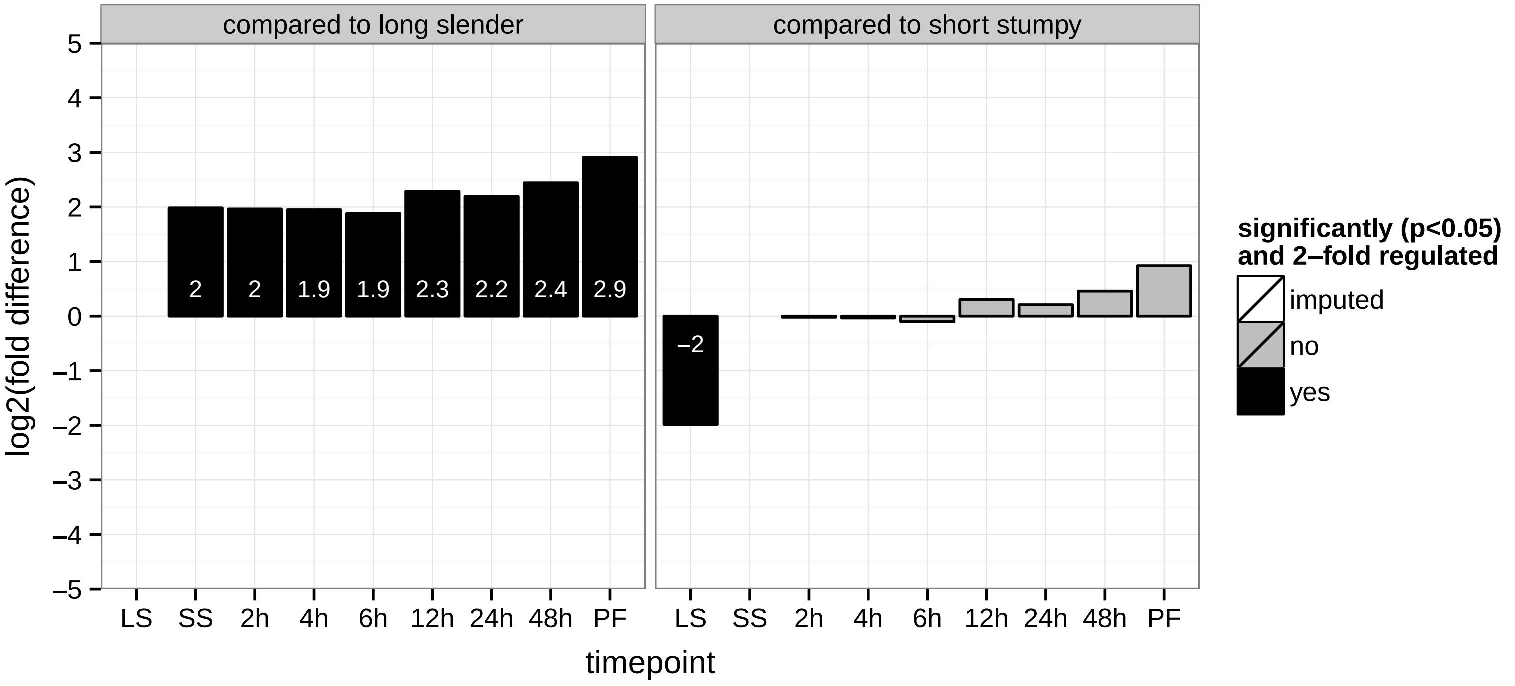

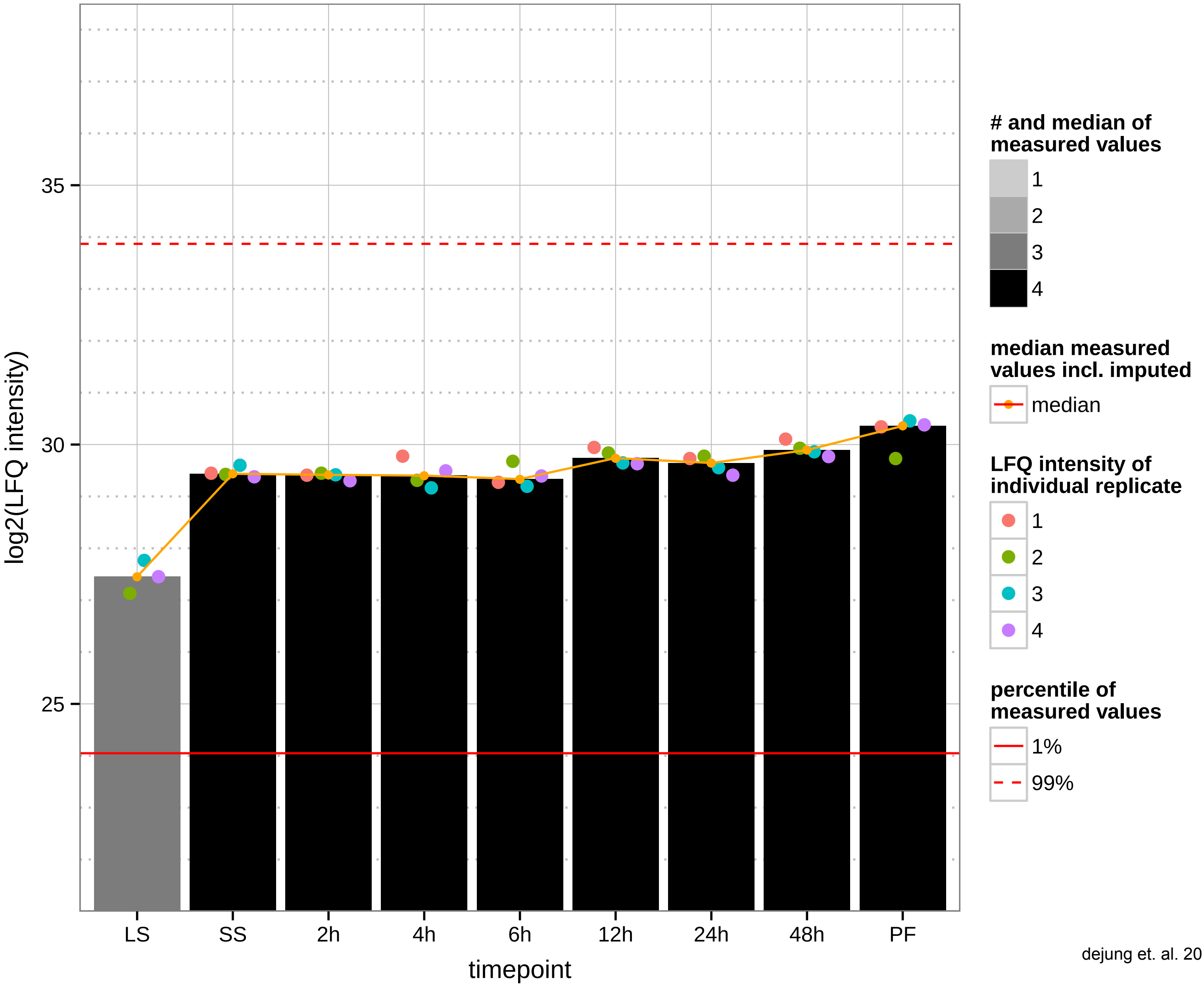

dejung et. al. 2015

tRNA nucleotidyltransferase, putative
Tb927.9.8780
AGOF: RNA binding, nucleotidyltransferase activity
AGOC: null
AGOP: RNA processing
PGOF: RNA binding, nucleotidyltransferase activity
PGOC: null
PGOP: RNA processing

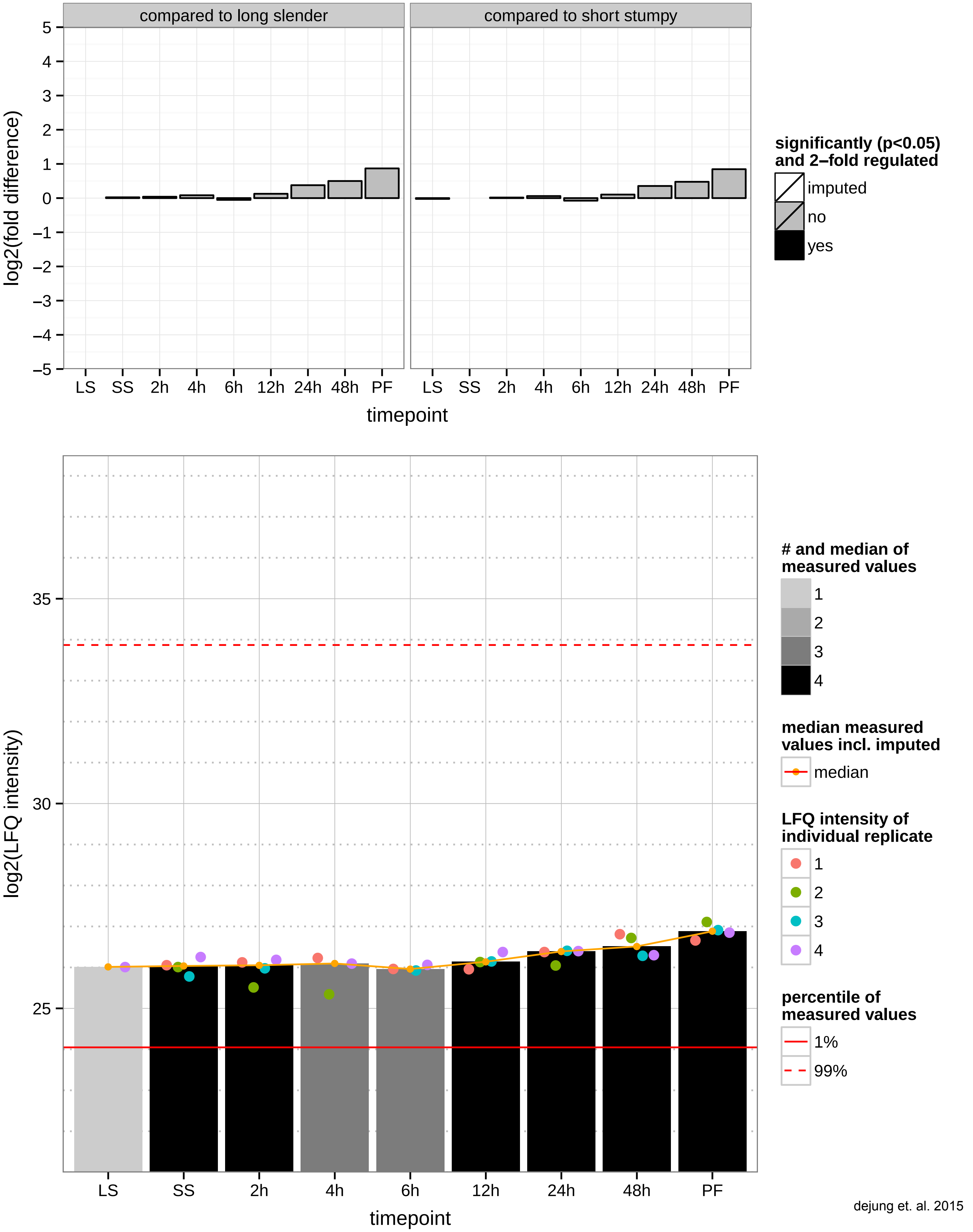

dejung et. al. 2015

hypothetical protein, conserved
Tb927.9.8810
AGOF: null
AGOC: null
AGOP: null
PGOF: null
PGOC: null
PGOP: null

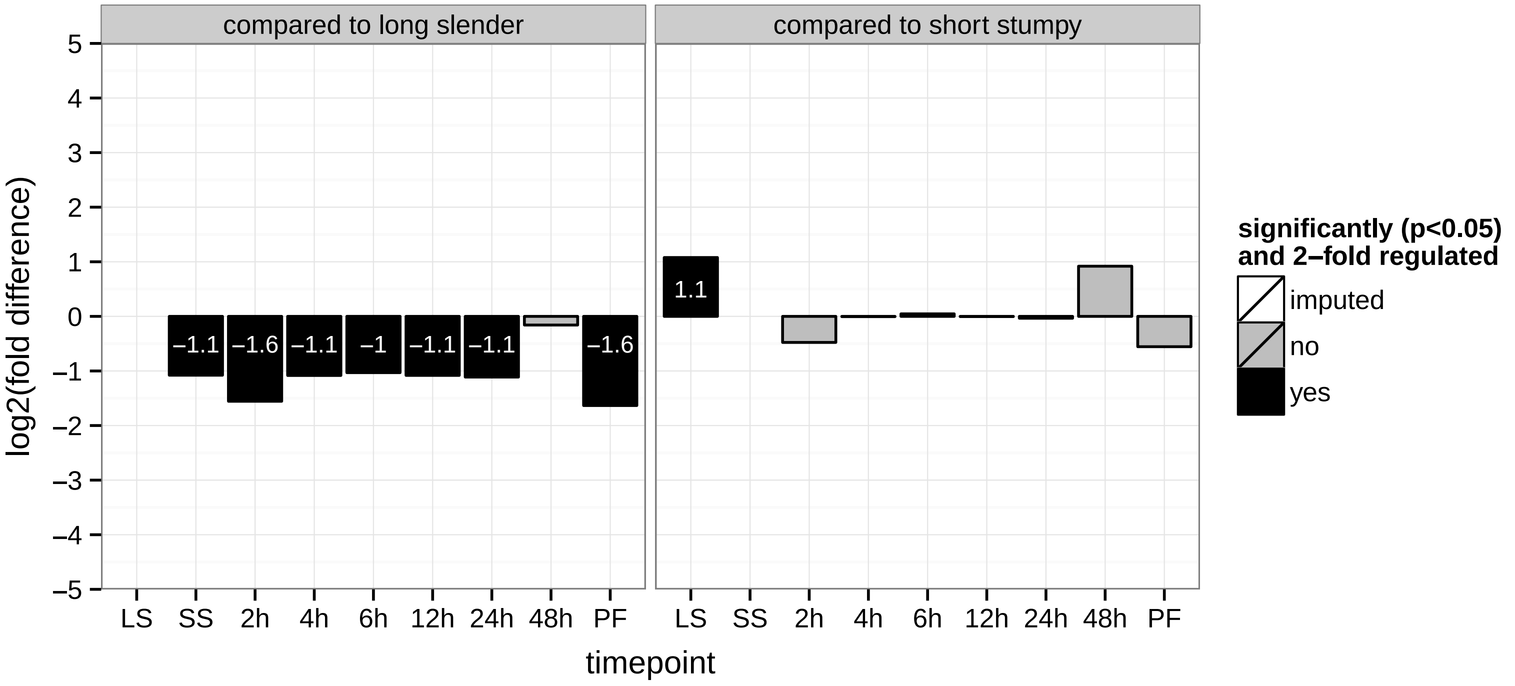

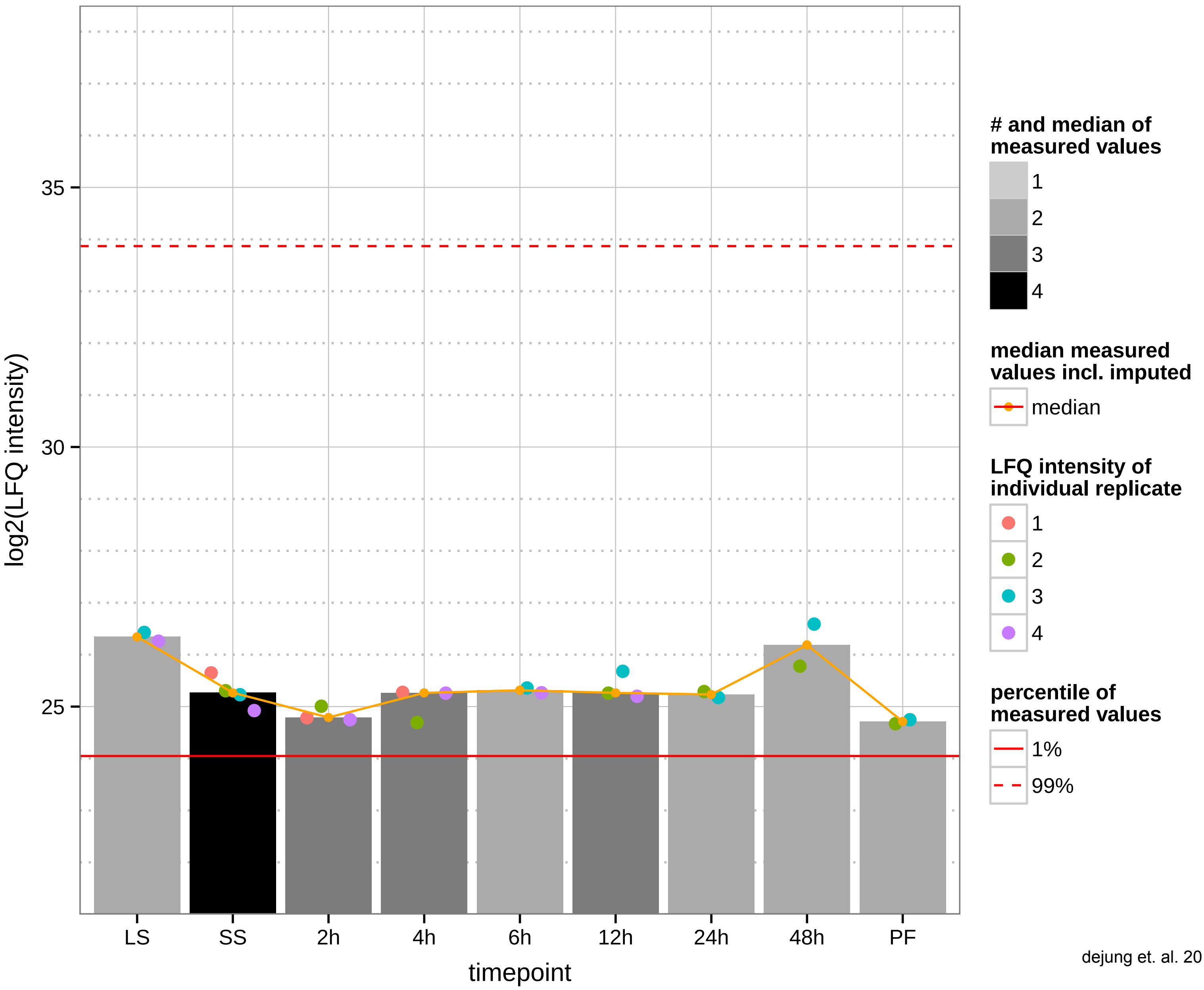

dejung et. al. 2015

actin A
Tb927.9.8880;Tb927.9.8850
AGOF: structural constituent of cytoskeleton
AGOC: actin cytoskeleton, cytoplasm
AGOP: cytoskeleton organization
PGOF: null
PGOC: null
PGOP: null

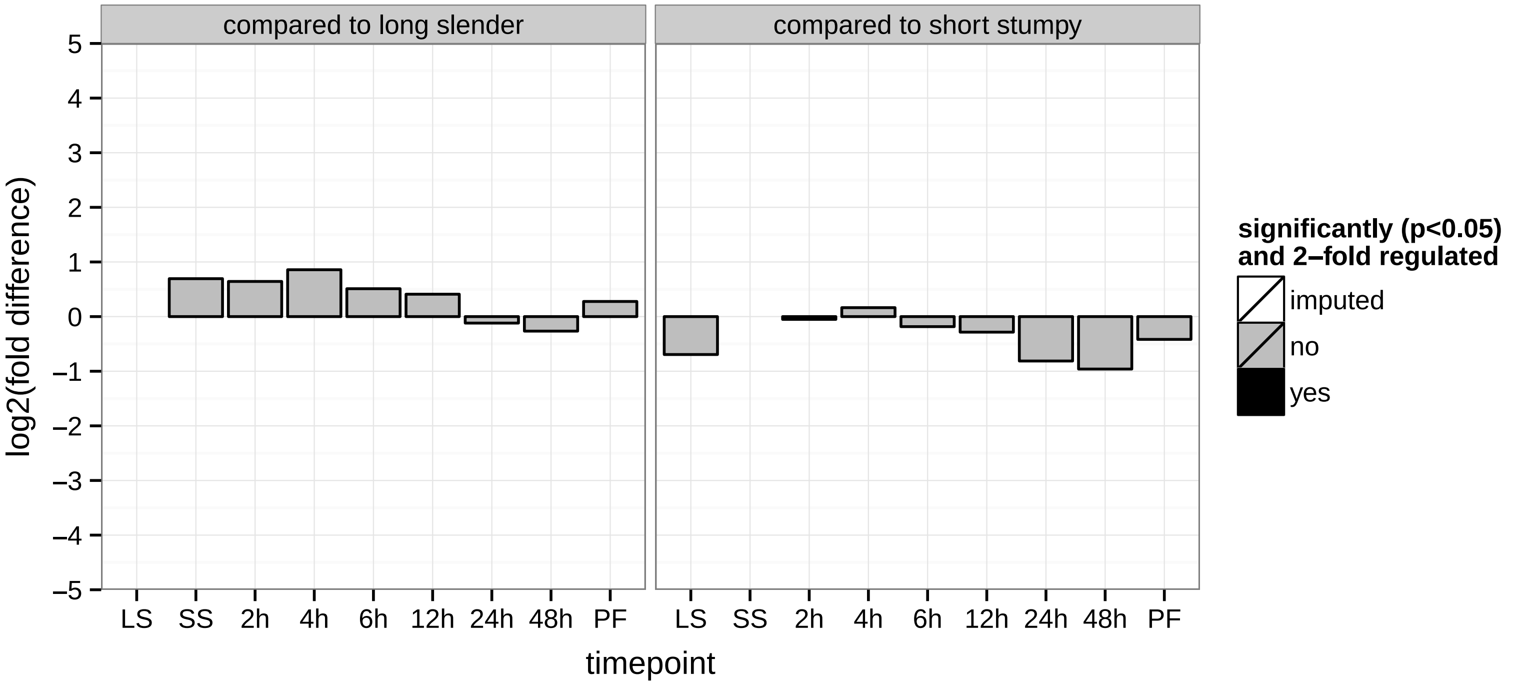

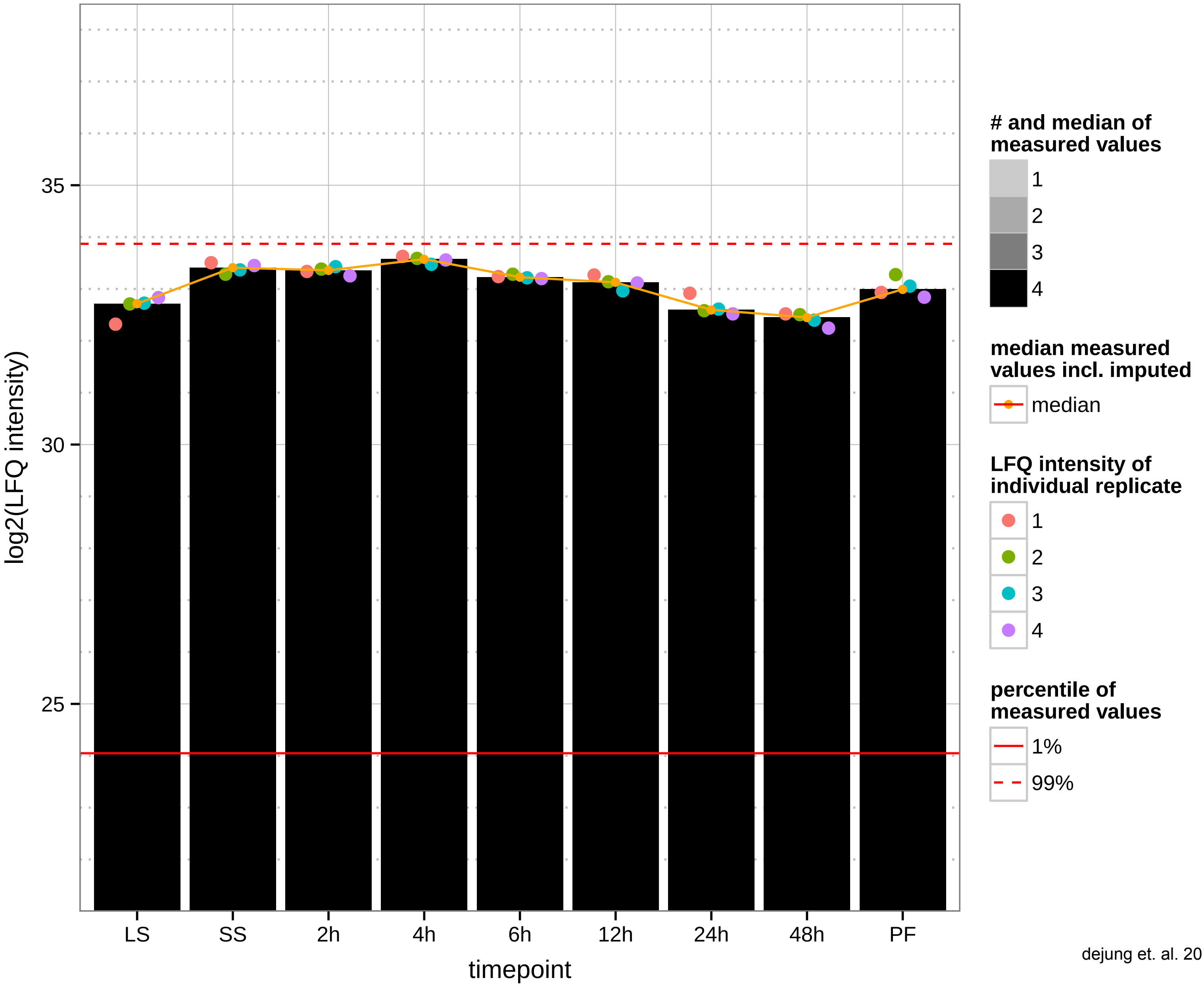

dejung et. al. 2015

CAAX prenyl protease 1, putative, metallo– peptidase, Clan M– Family M48
Tb927.9.8950
AGOF: metalloendopeptidase activity
AGOC: membrane, mitochondrial inner membrane
AGOP: proteolysis
PGOF: metalloendopeptidase activity
PGOC: membrane
PGOP: proteolysis

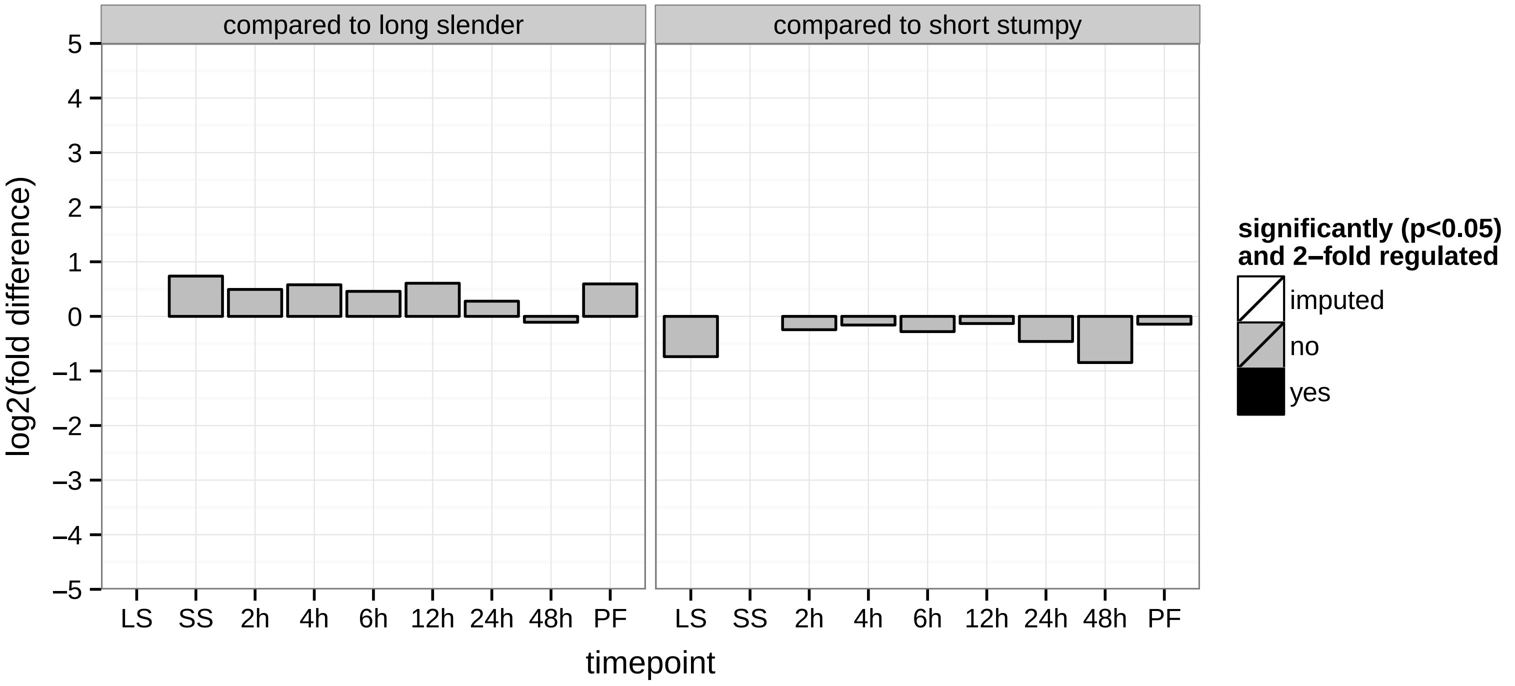

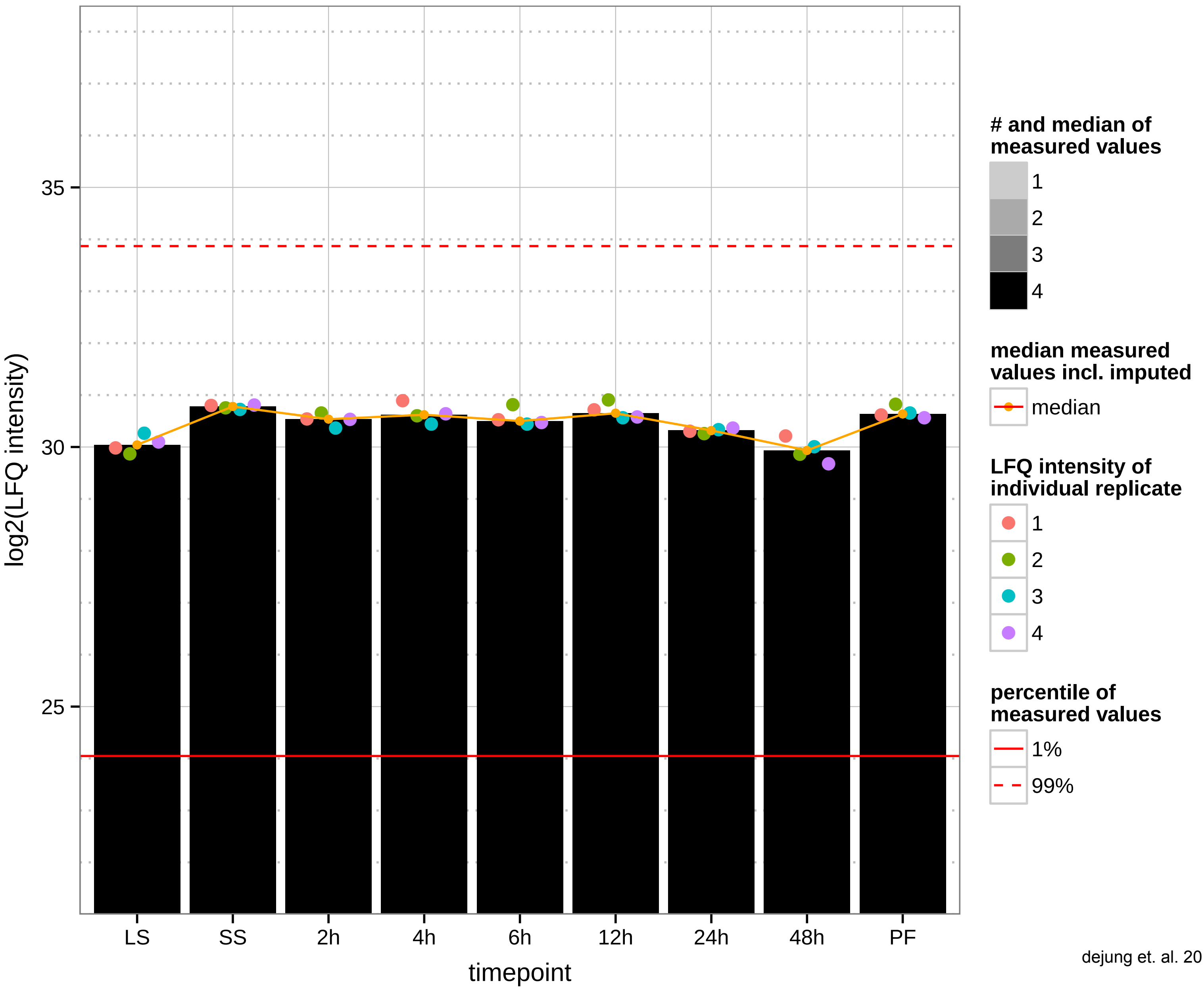

dejung et. al. 2015

hypothetical protein, conserved
Tb927.9.8990
AGOF: null
AGOC: null
AGOP: null
PGOF: protein binding
PGOC: null
PGOP: null

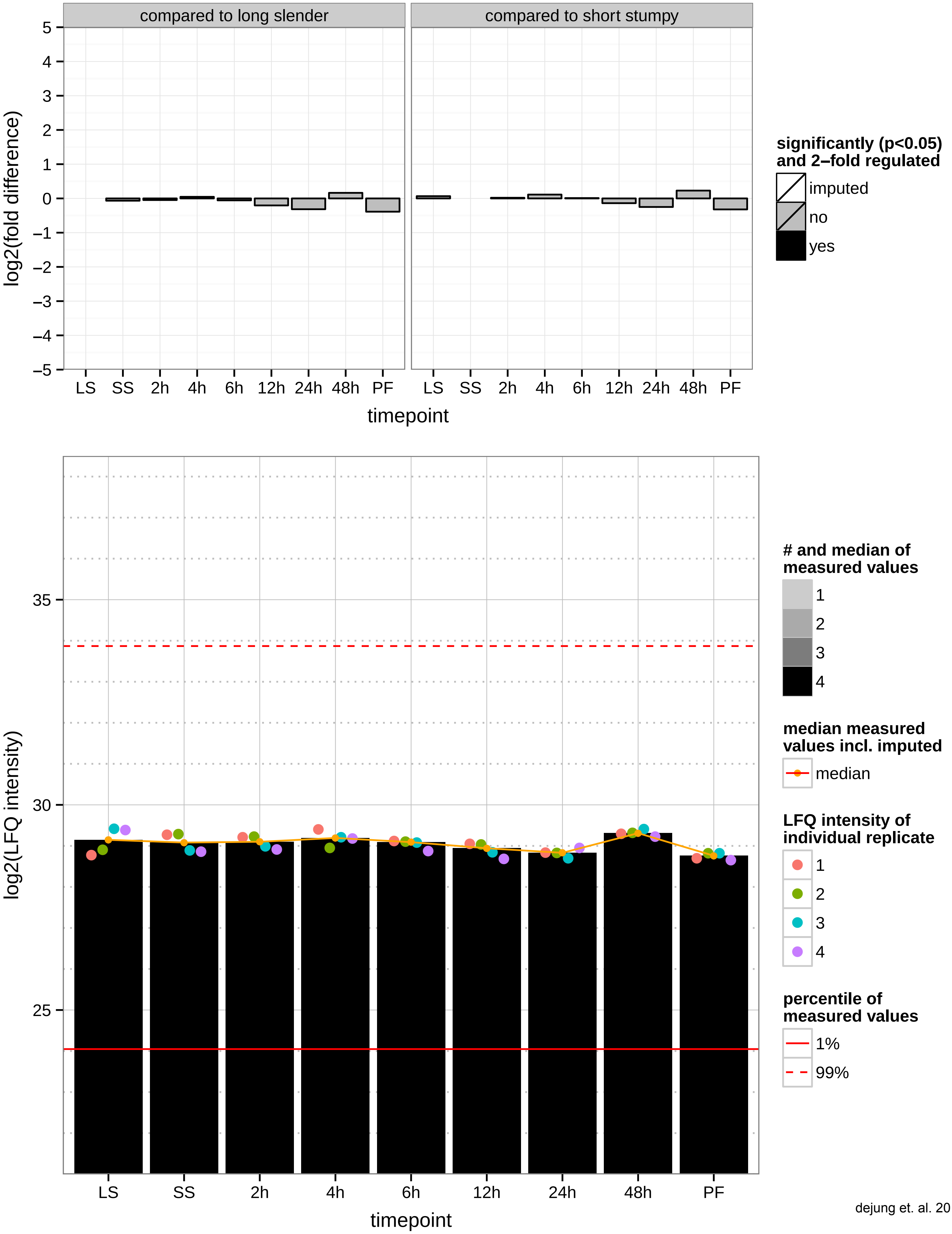

dejung et. al. 2015

hypothetical protein, conserved
Tb927.9.9010
AGOF: null
AGOC: null
AGOP: null
PGOF: null
PGOC: null
PGOP: null

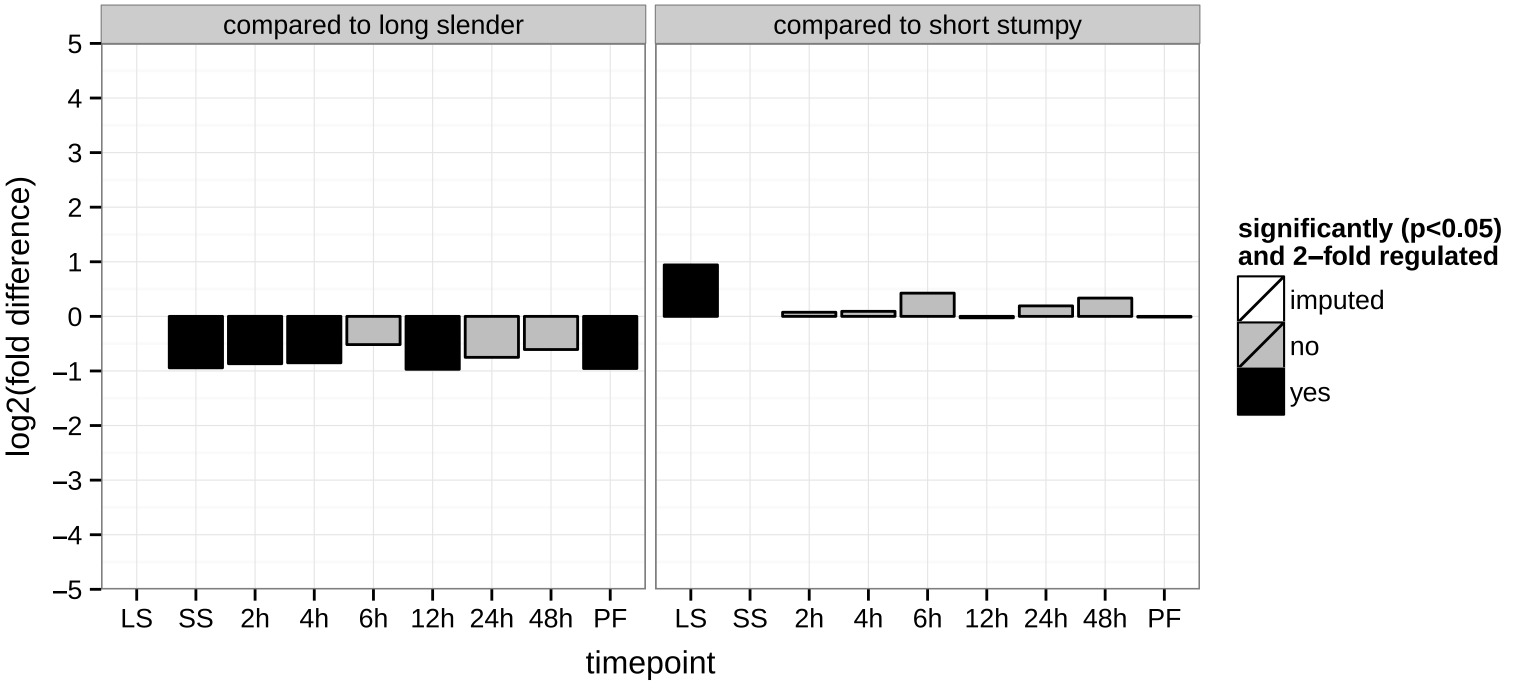

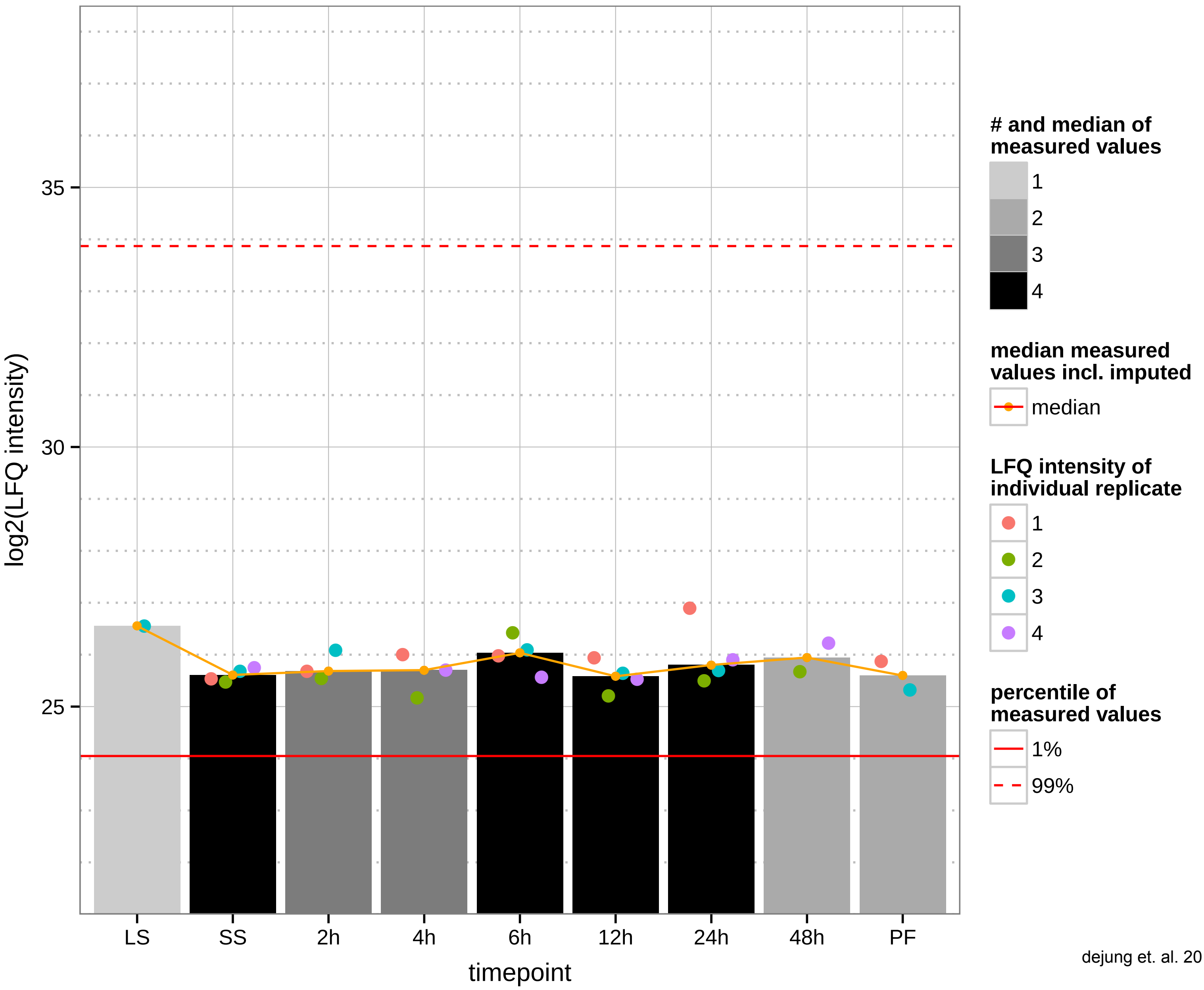

dejung et. al. 2015

Lsm12 protein, putative
Tb927.9.9060
AGOF: null
AGOC: cytoplasmic stress granule
AGOP: null
PGOF: null
PGOC: null
PGOP: null

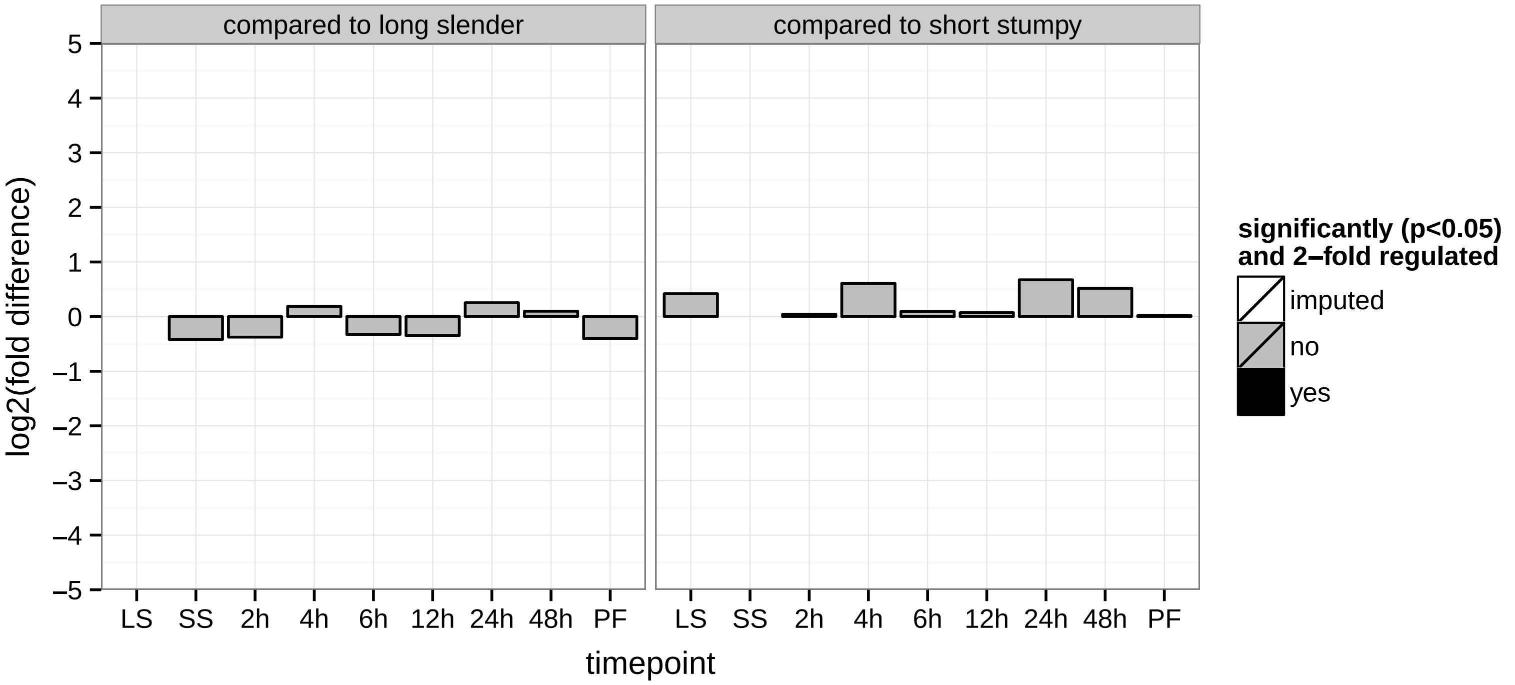

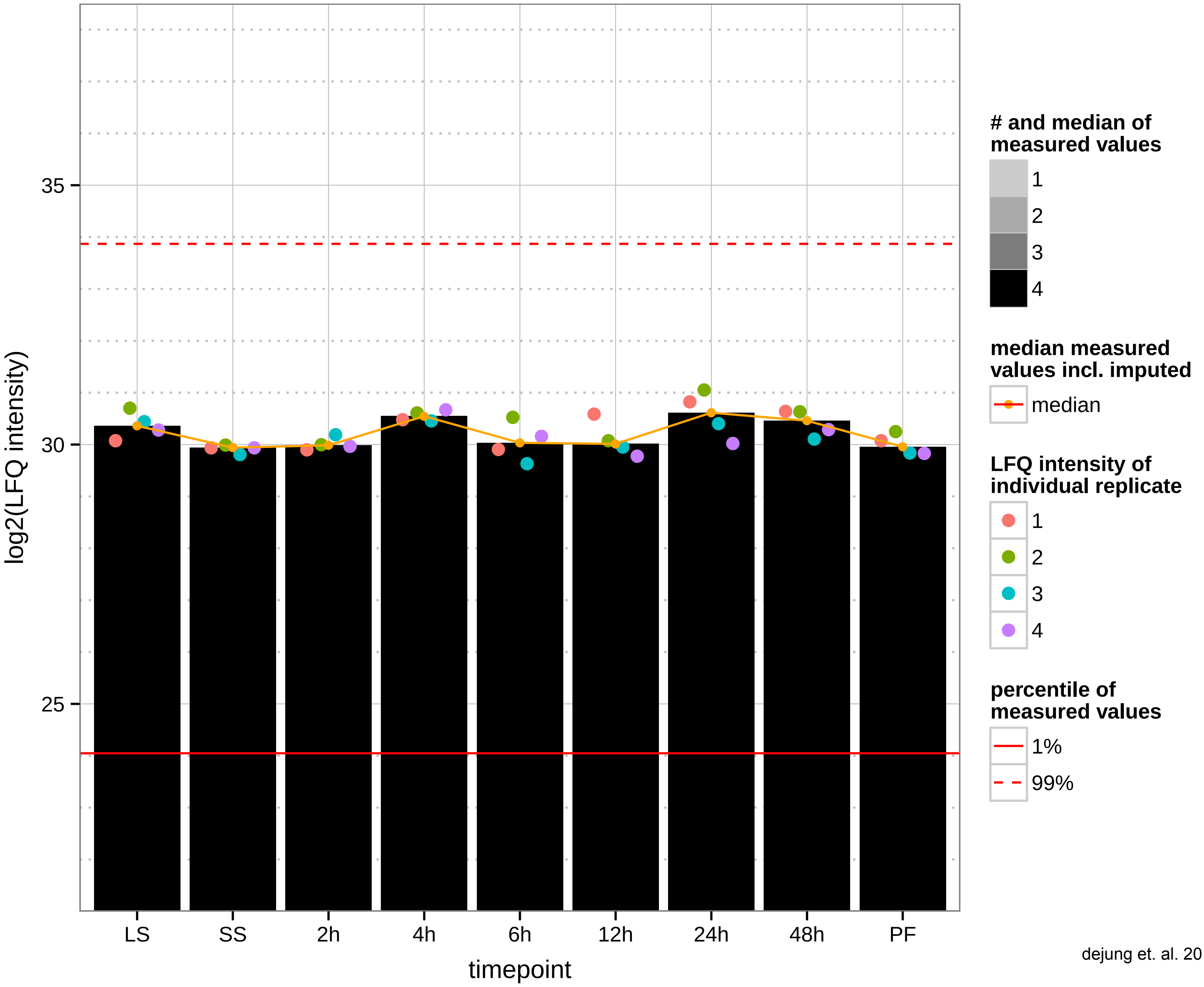

dejung et. al. 2015

tRNA (guanine−N(7)−)−methyltransferase, putative, methyltransferase
Tb927.9.9120
AGOF: tRNA (guanine−N7−)−methyltransferase activity
AGOC: null
AGOP: tRNA modification
PGOF: tRNA (guanine−N7−)−methyltransferase activity
PGOC: null
PGOP: tRNA modification

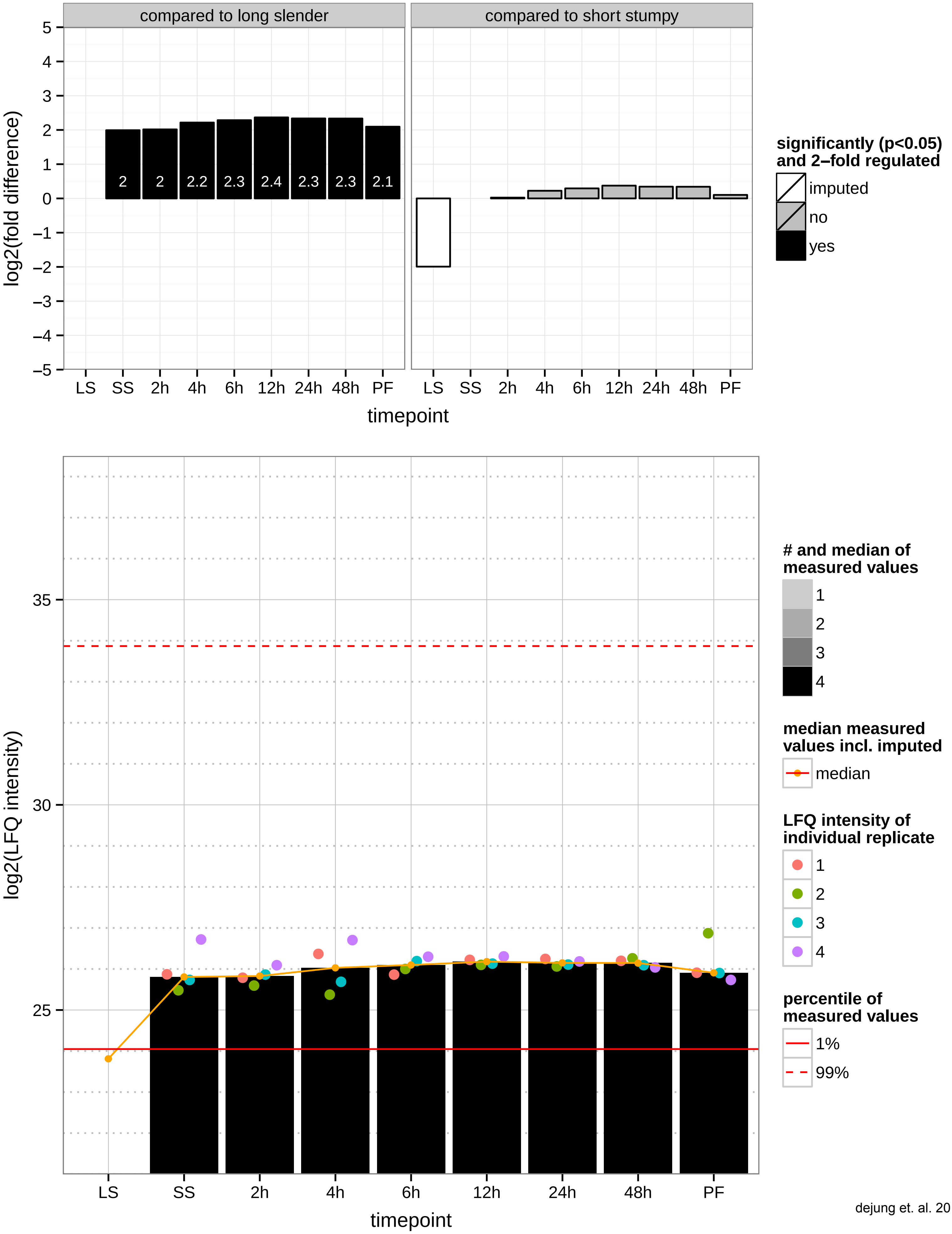

dejung et. al. 2015

GTP–binding protein, putative
Tb927.9.9150
AGOF: GTP binding
AGOC: null
AGOP: null
PGOF: GTP binding
PGOC: null
PGOP: null

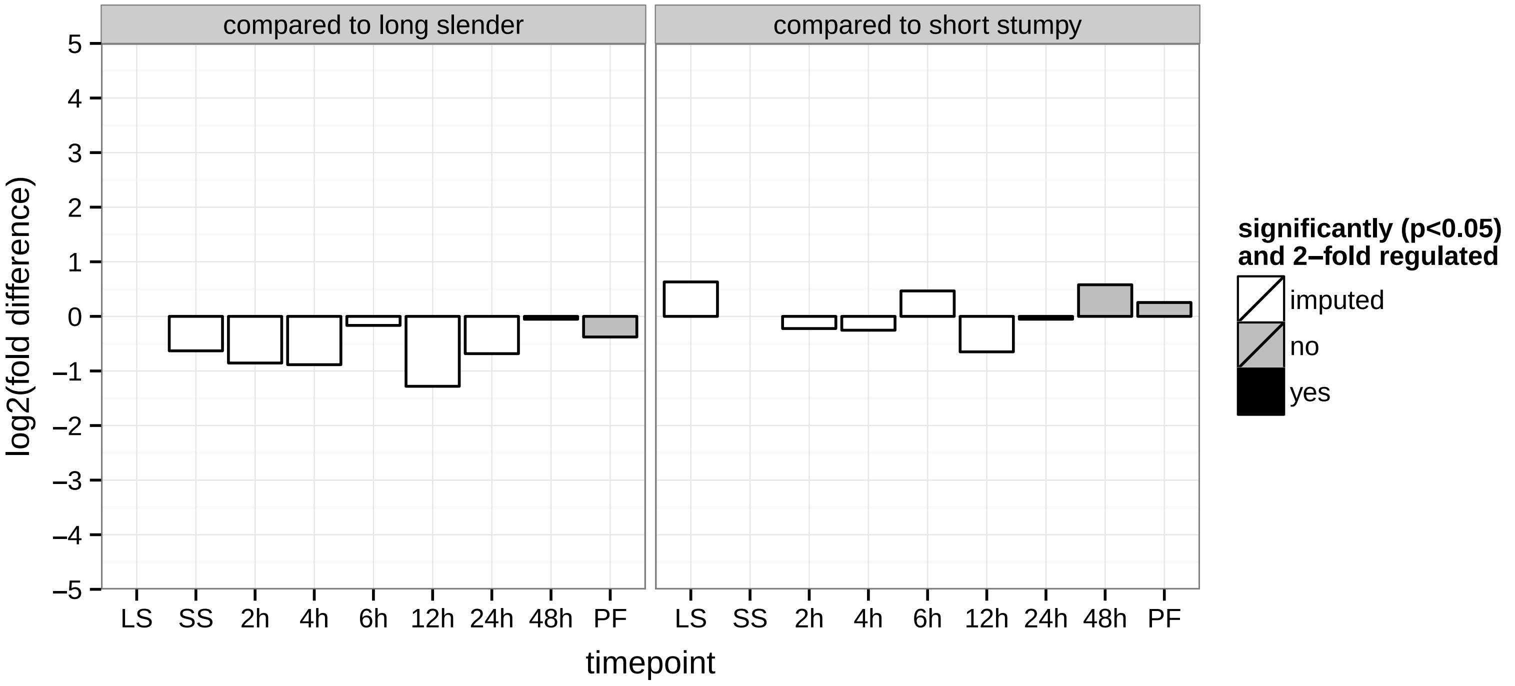

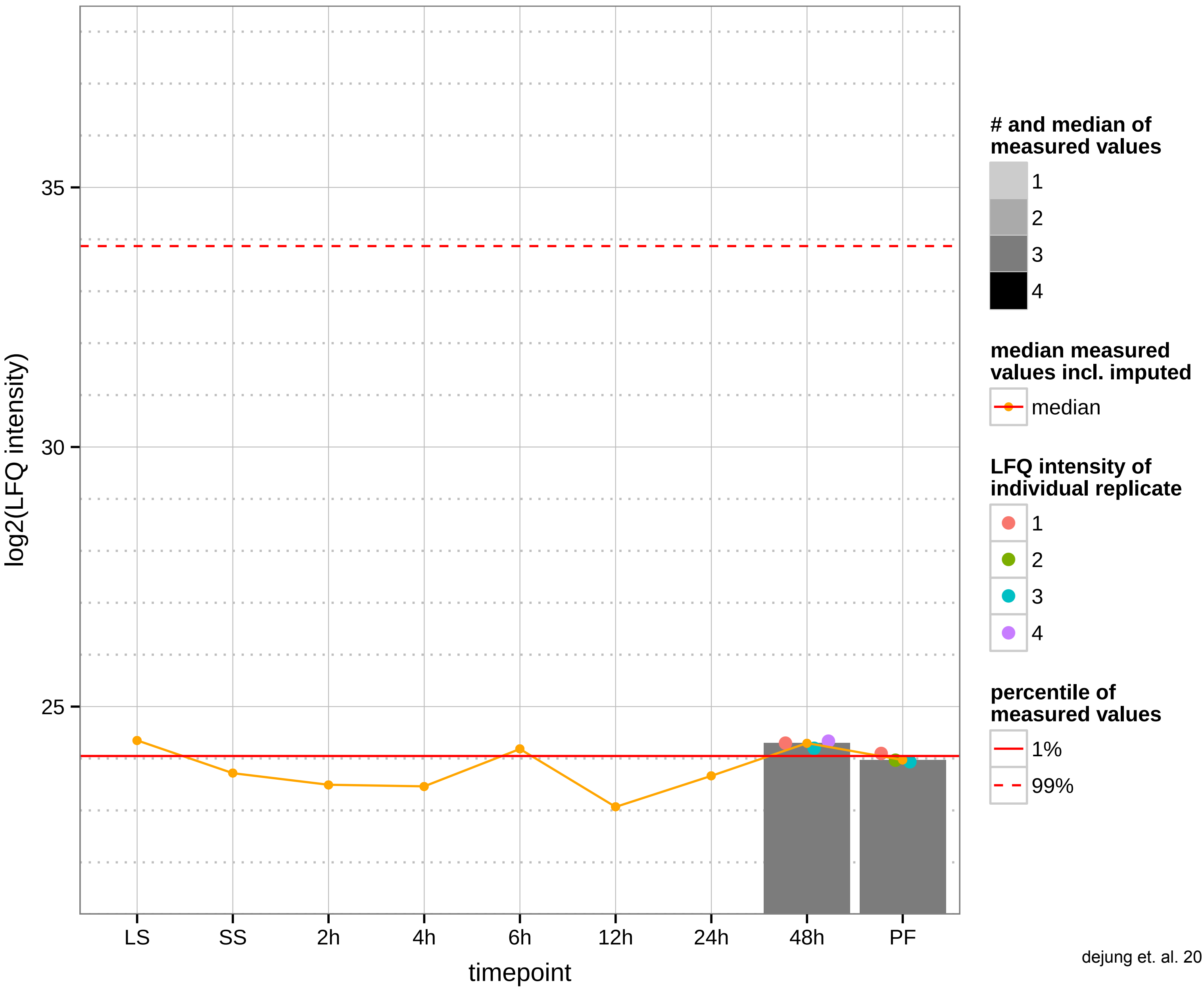

dejung et. al. 2015

hypothetical protein, conserved
Tb927.9.9260
AGOF: null
AGOC: null
AGOP: null
PGOF: null
PGOC: null
PGOP: null

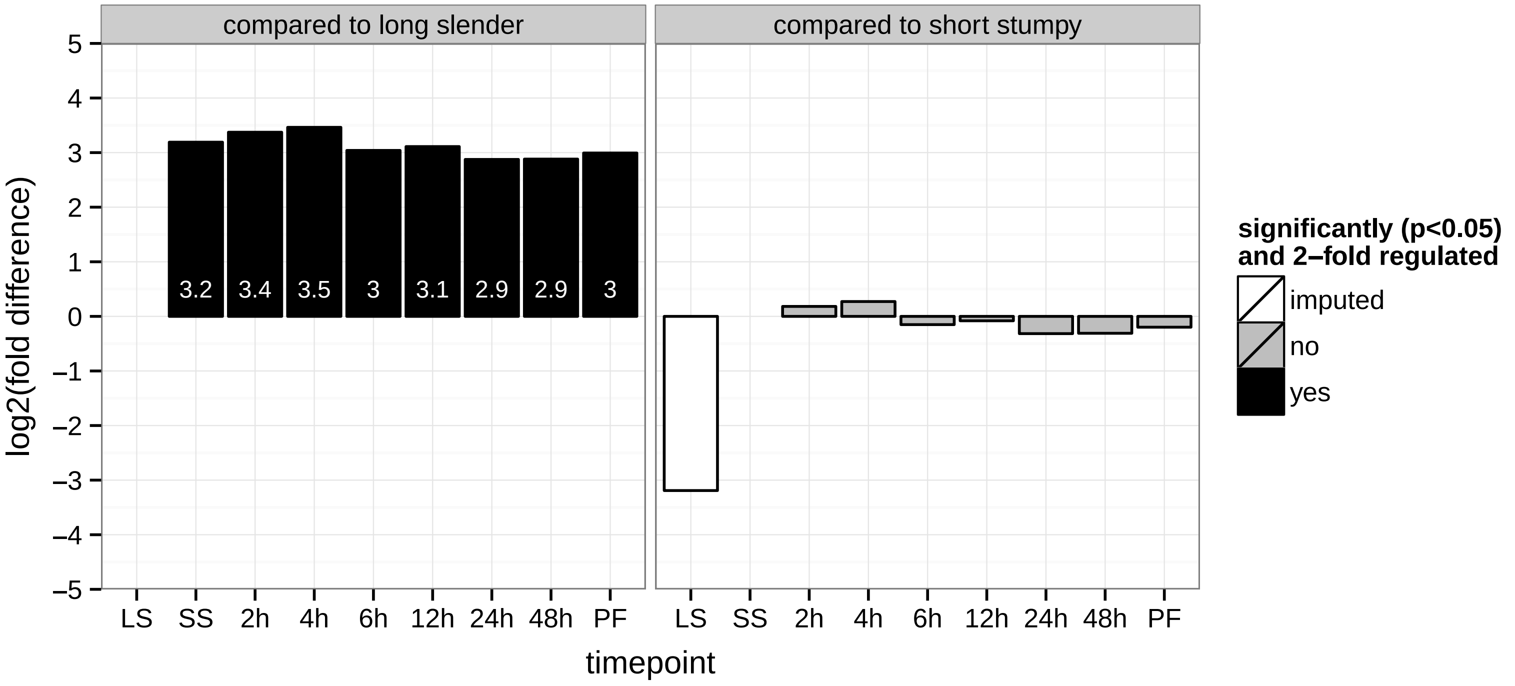

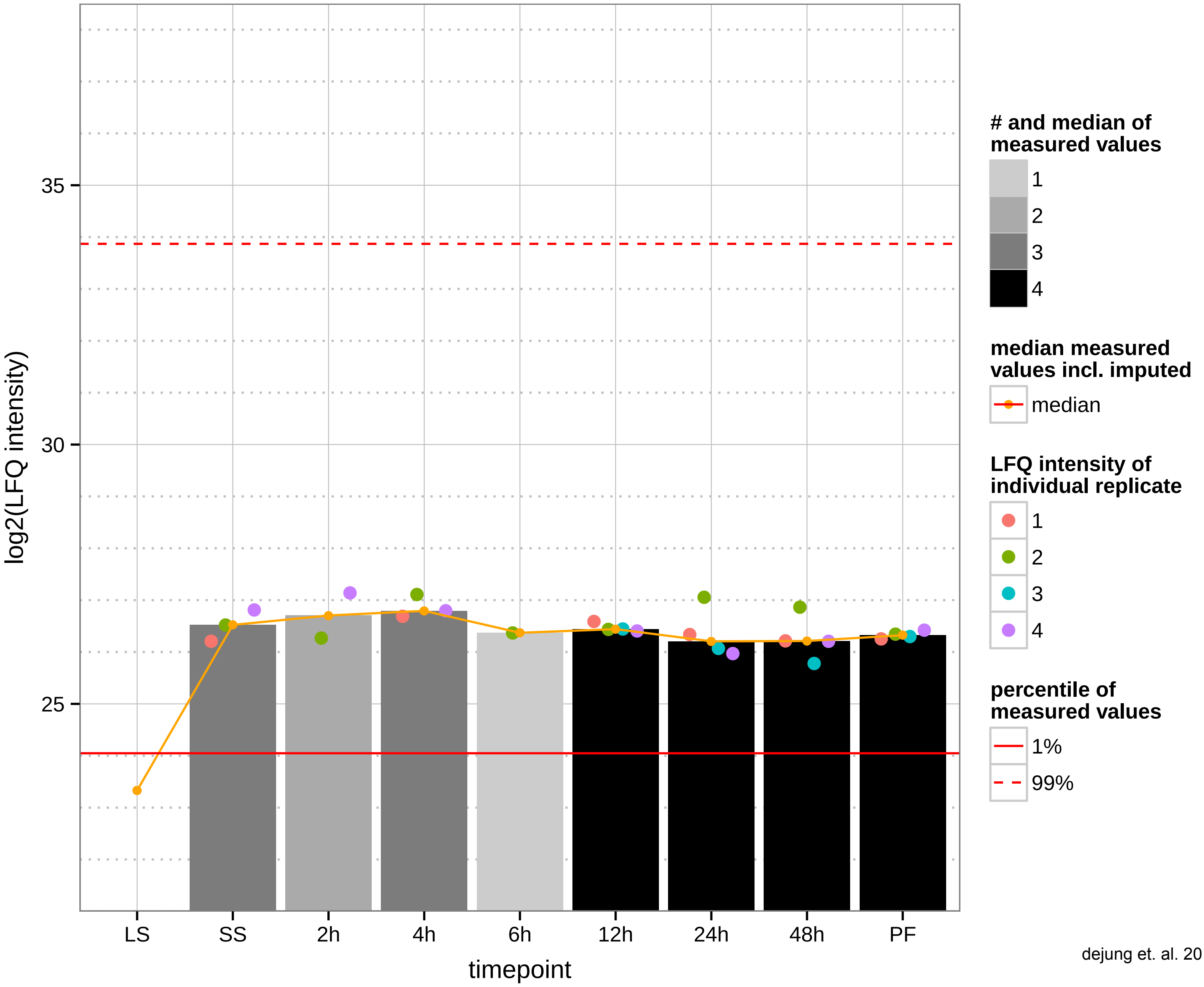

dejung et. al. 2015

Polyadenylate–binding protein 1 (Poly(A)–binding protein 1) (PABP 1) (PABP1)
Tb927.9.9290
AGOF: RNA binding, poly(A) RNA binding
AGOC: cytoplasm, nucleus
AGOP: RNA processing, regulation of translational initiation
PGOF: RNA binding, nucleic acid binding
PGOC: null
PGOP: null

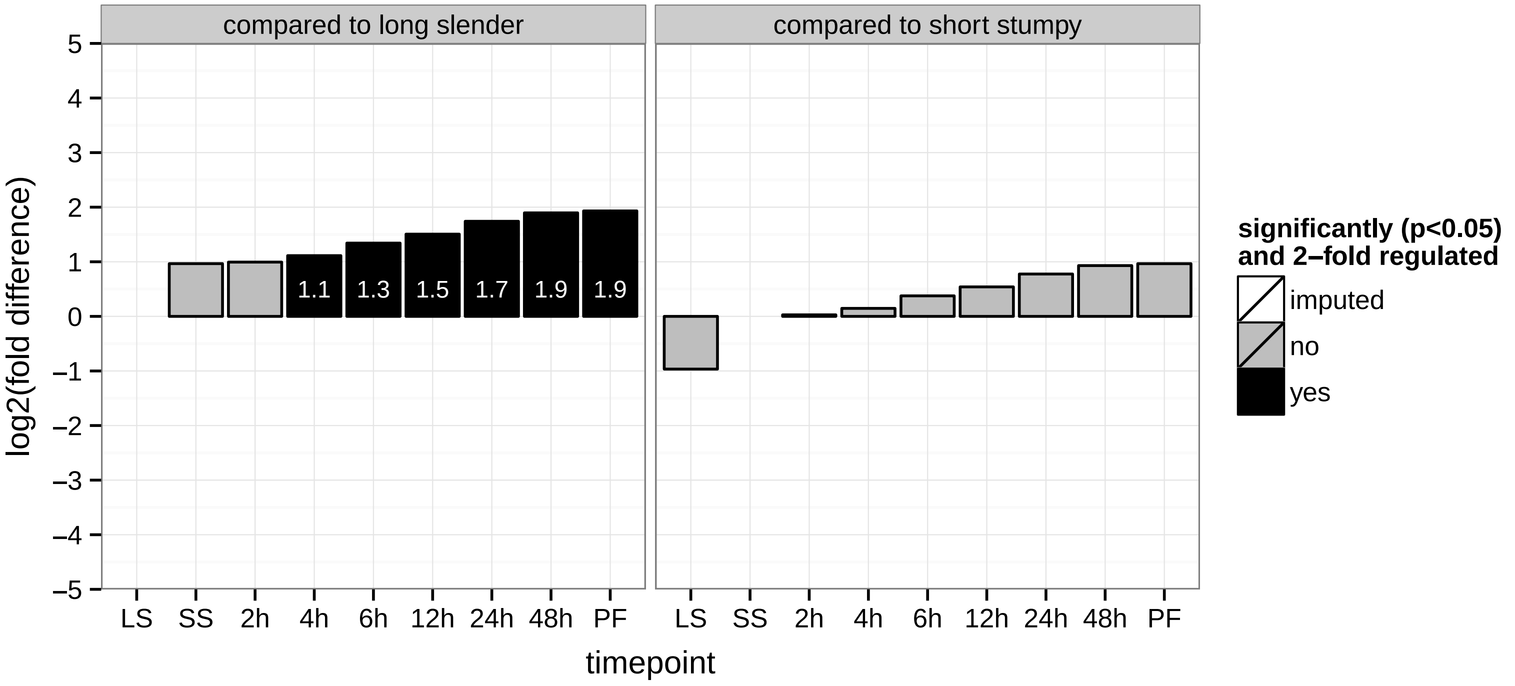

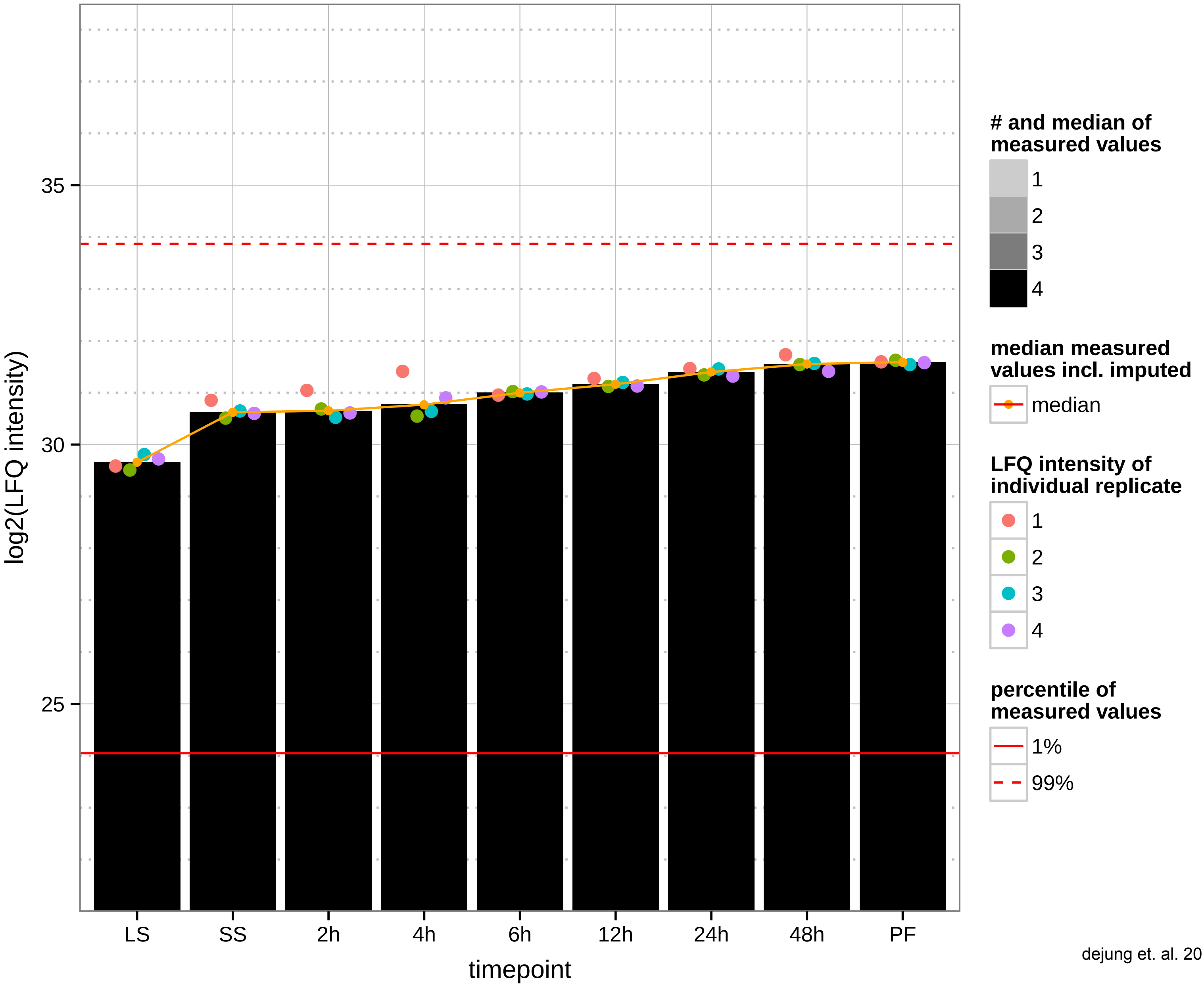

dejung et. al. 2015

tubulin tyrosine ligase protein, putative
Tb927.9.9580
AGOF: null
AGOC: null
AGOP: cellular protein modification process
PGOF: tubulin–tyrosine ligase activity
PGOC: null
PGOP: cellular protein modification process

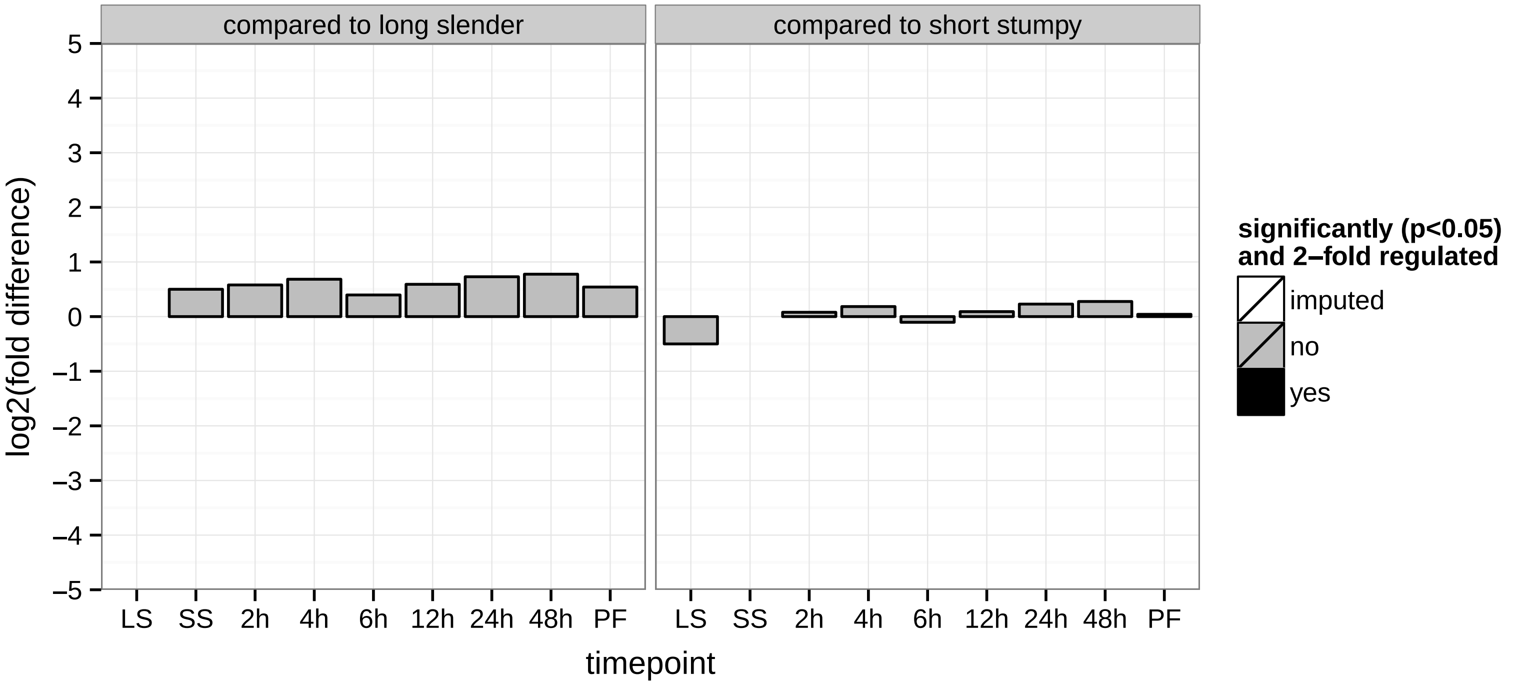

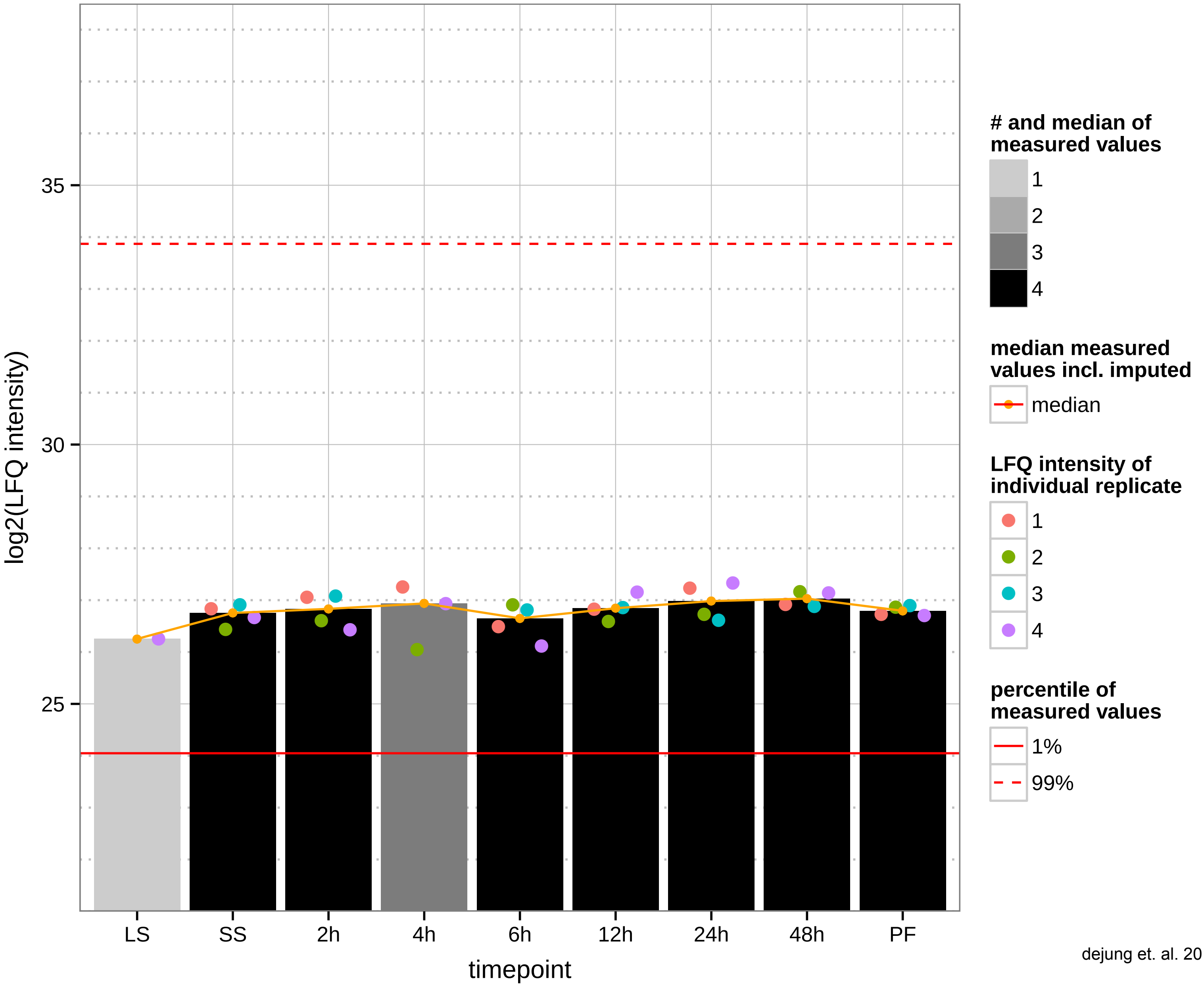

dejung et. al. 2015

proteasome alpha 1 subunit, putative, 20S proteasome subunit alpha−6, (putative) (TbPSA6)
Tb927.9.9670
AGOF: endopeptidase activity, threonine−type endopeptidase activity
AGOC: proteasome core complex
AGOP: ubiquitin−dependent protein catabolic process
PGOF: endopeptidase activity, threonine−type endopeptidase activity
PGOC: proteasome core complex, proteasome core complex, alpha−subunit complex
PGOP: proteolysis involved in cellular protein catabolic process, ubiquitin−dependent protein catabolic process

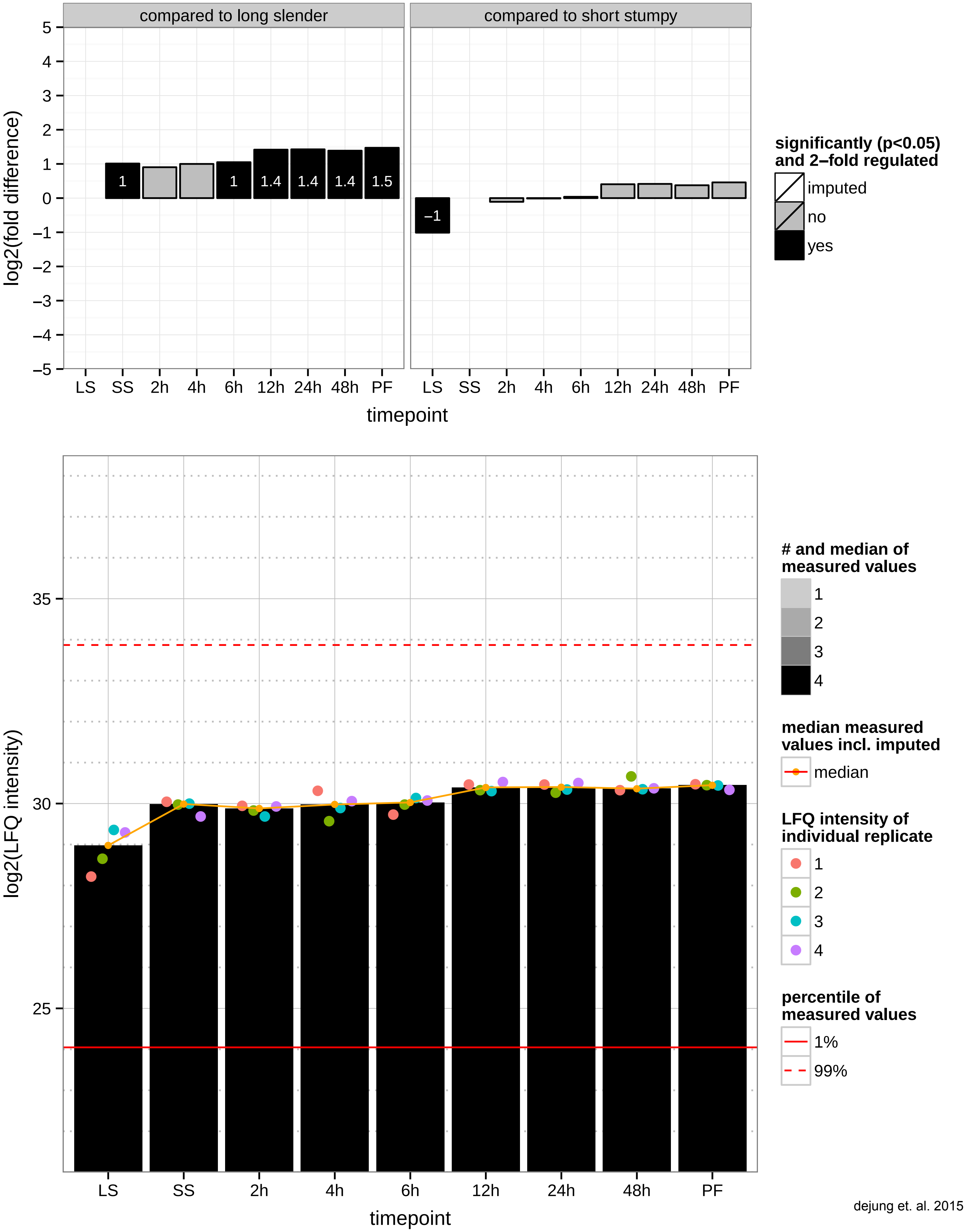

dejung et. al. 2015

hypothetical protein, conserved
Tb927.9.9710
AGOF: null
AGOC: null
AGOP: null
PGOF: null
PGOC: null
PGOP: null

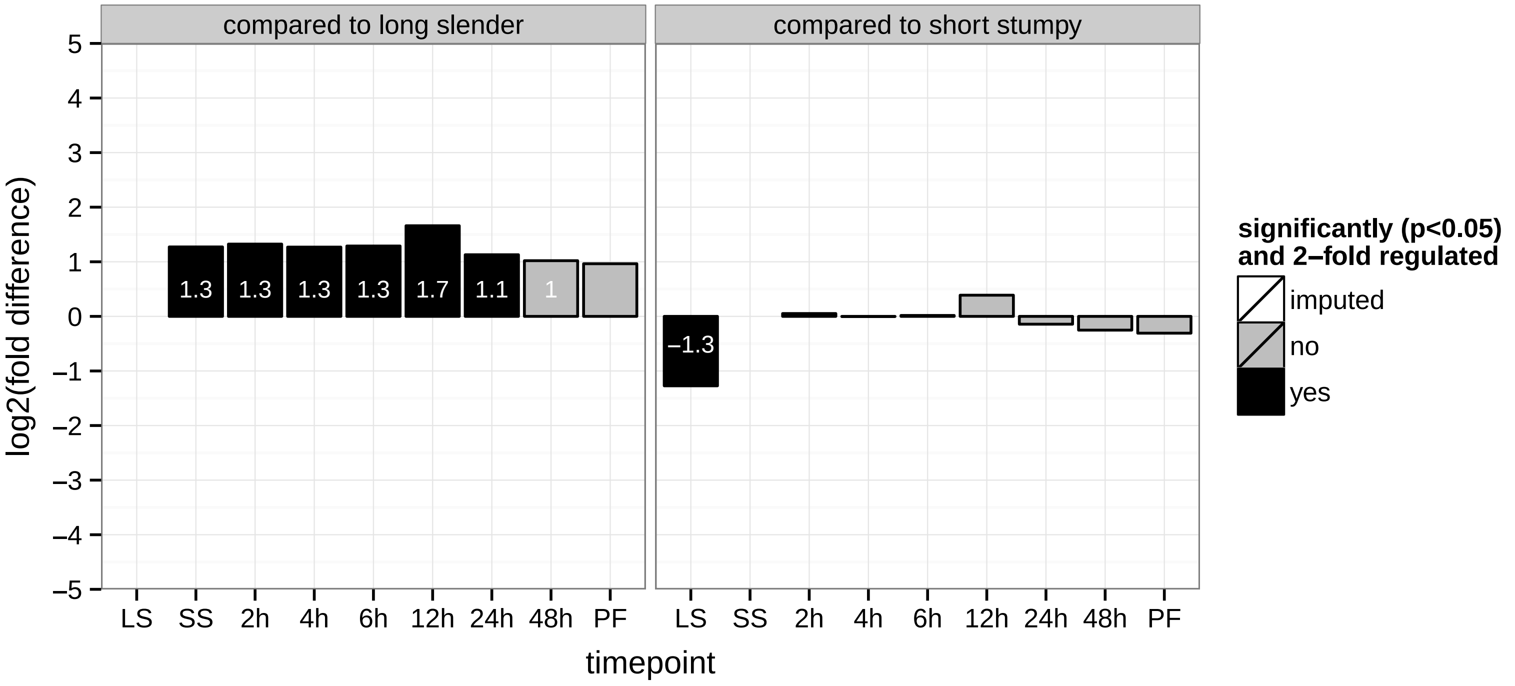

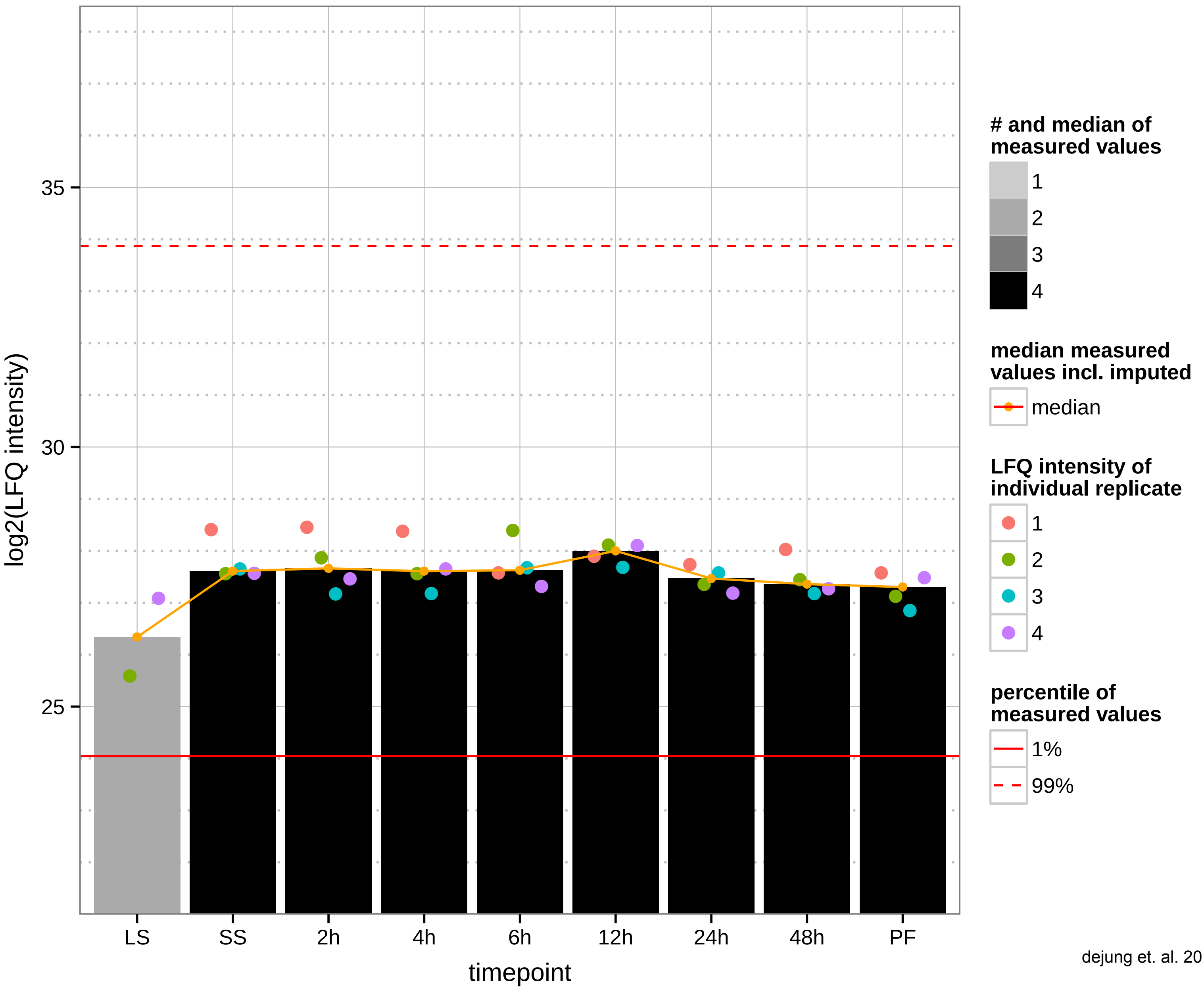

dejung et. al. 2015

AMP deaminase, putative
Tb927.9.9740
AGOF: AMP deaminase activity
AGOC: mitochondrion
AGOP: purine ribonucleoside monophosphate biosynthetic process
PGOF: deaminase activity
PGOC: null
PGOP: purine ribonucleoside monophosphate biosynthetic process

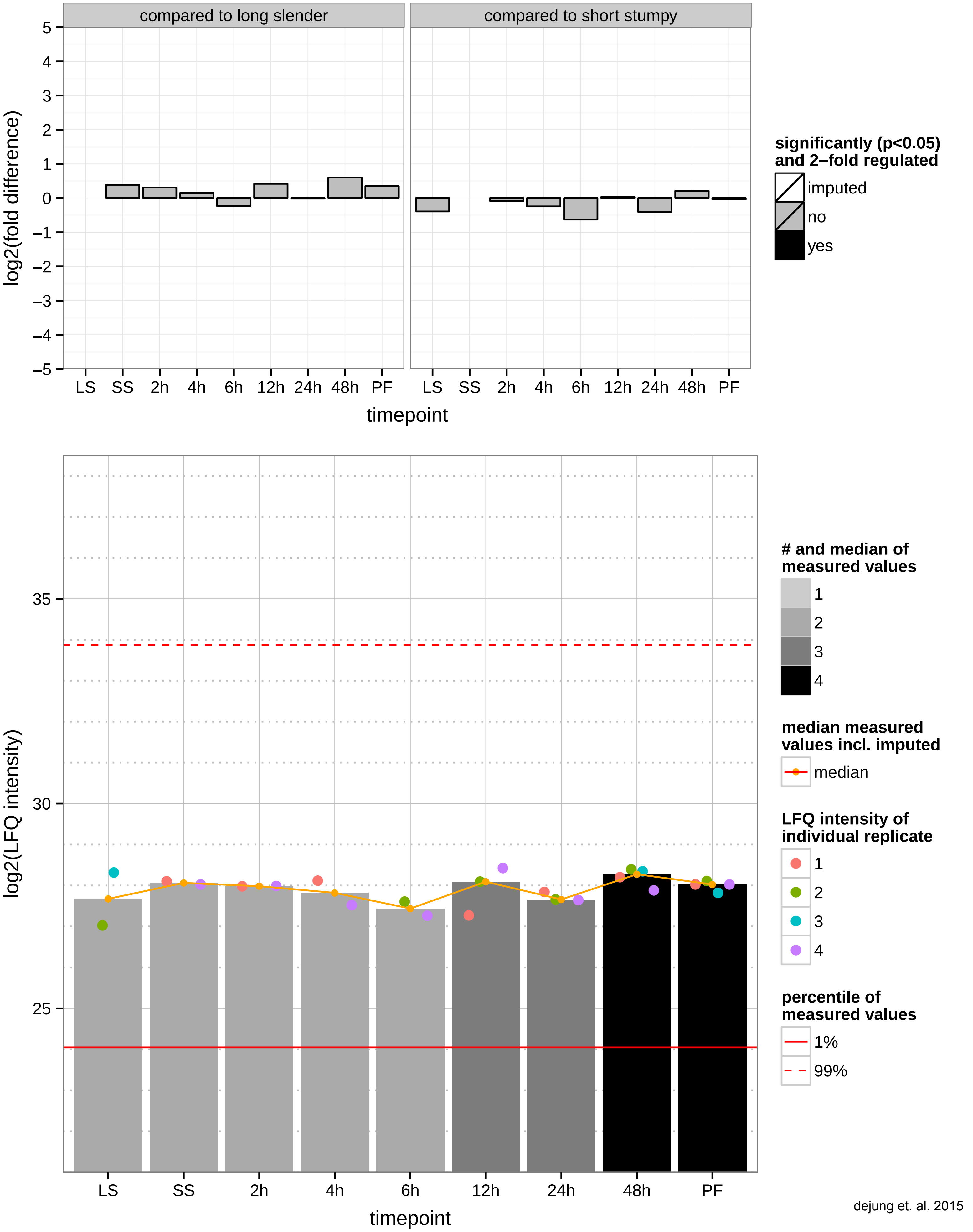

dejung et. al. 2015

membrane- trafficking protein, putative
Tb927.9.9750
AGOF: null
AGOC: integral to membrane
AGOP: protein transport
PGOF: null
PGOC: integral to membrane
PGOP: protein transport

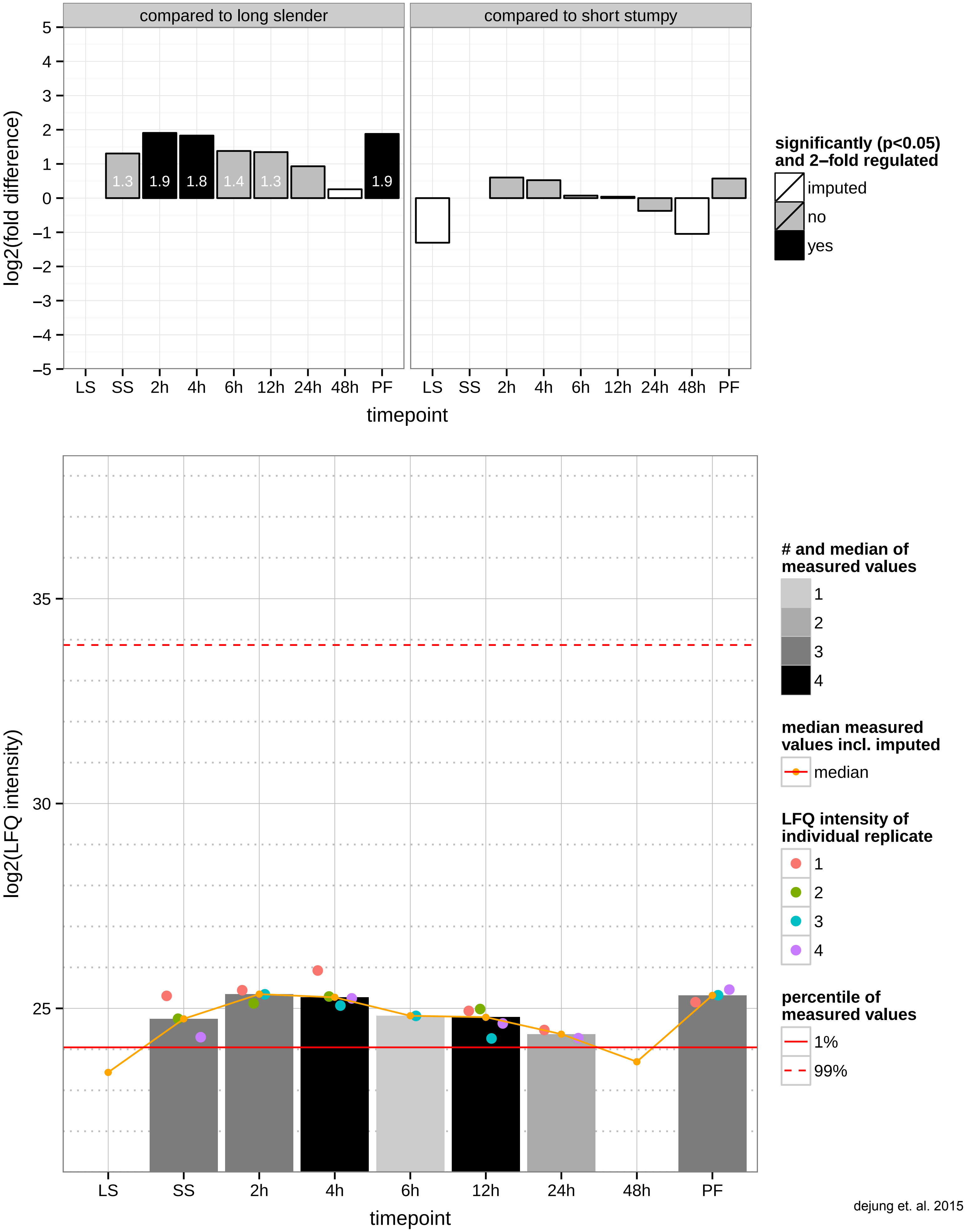

dejung et. al. 2015

hypothetical protein, conserved
Tb927.9.9810
AGOF: null
AGOC: null
AGOP: null
PGOF: null
PGOC: null
PGOP: null

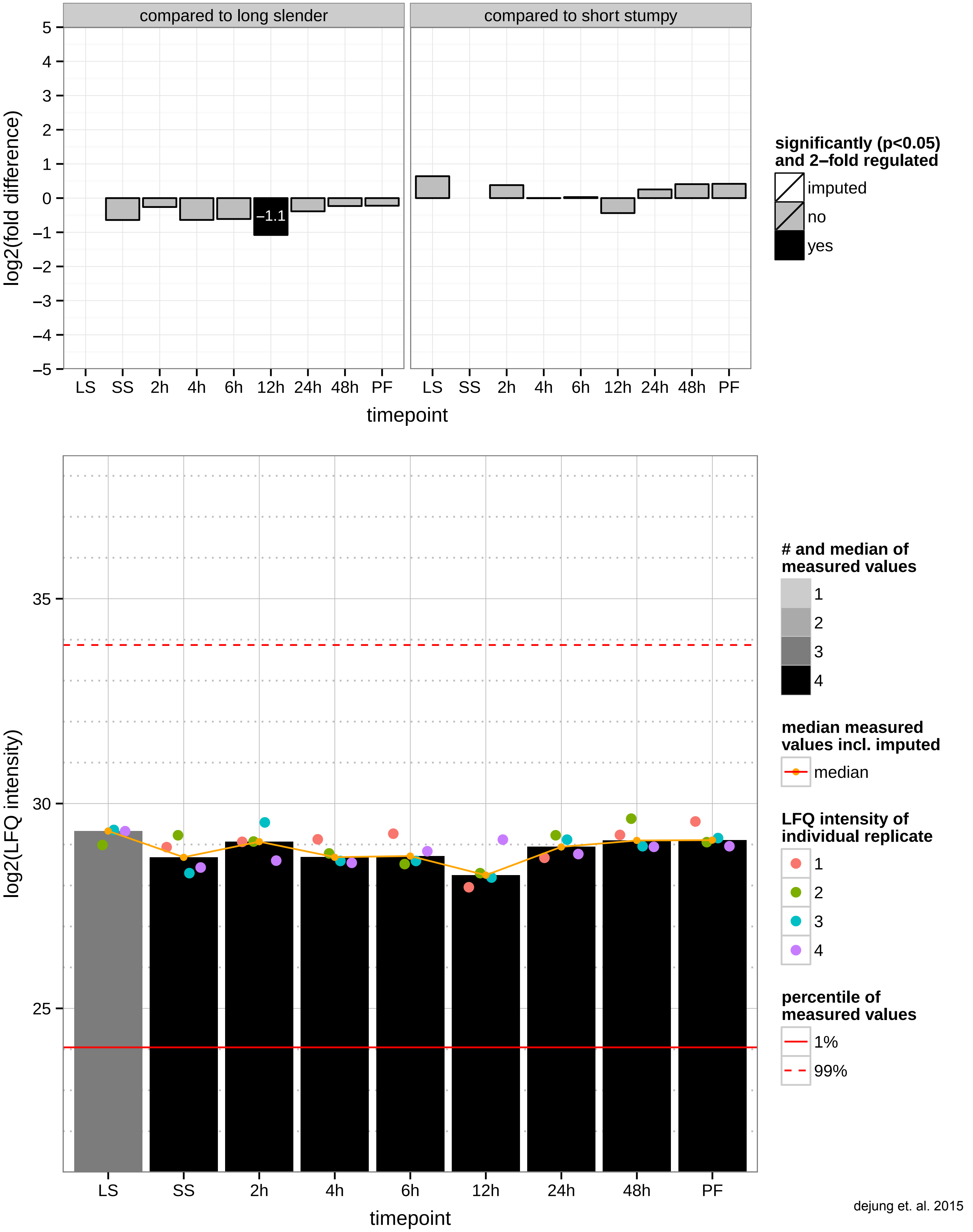

dejung et. al. 2015

glyceraldehyde−3−phosphate dehydrogenase, putative
Tb927.9.9820
AGOF: NAD binding, glyceraldehyde−3−phosphate dehydrogenase (NAD+) (phosphorylating) activity
AGOC: null
AGOP: glycolysis
PGOF: oxidoreductase activity, acting on the aldehyde or oxo group of donors, NAD or NADP as acceptor
PGOC: null
PGOP: oxidation−reduction process

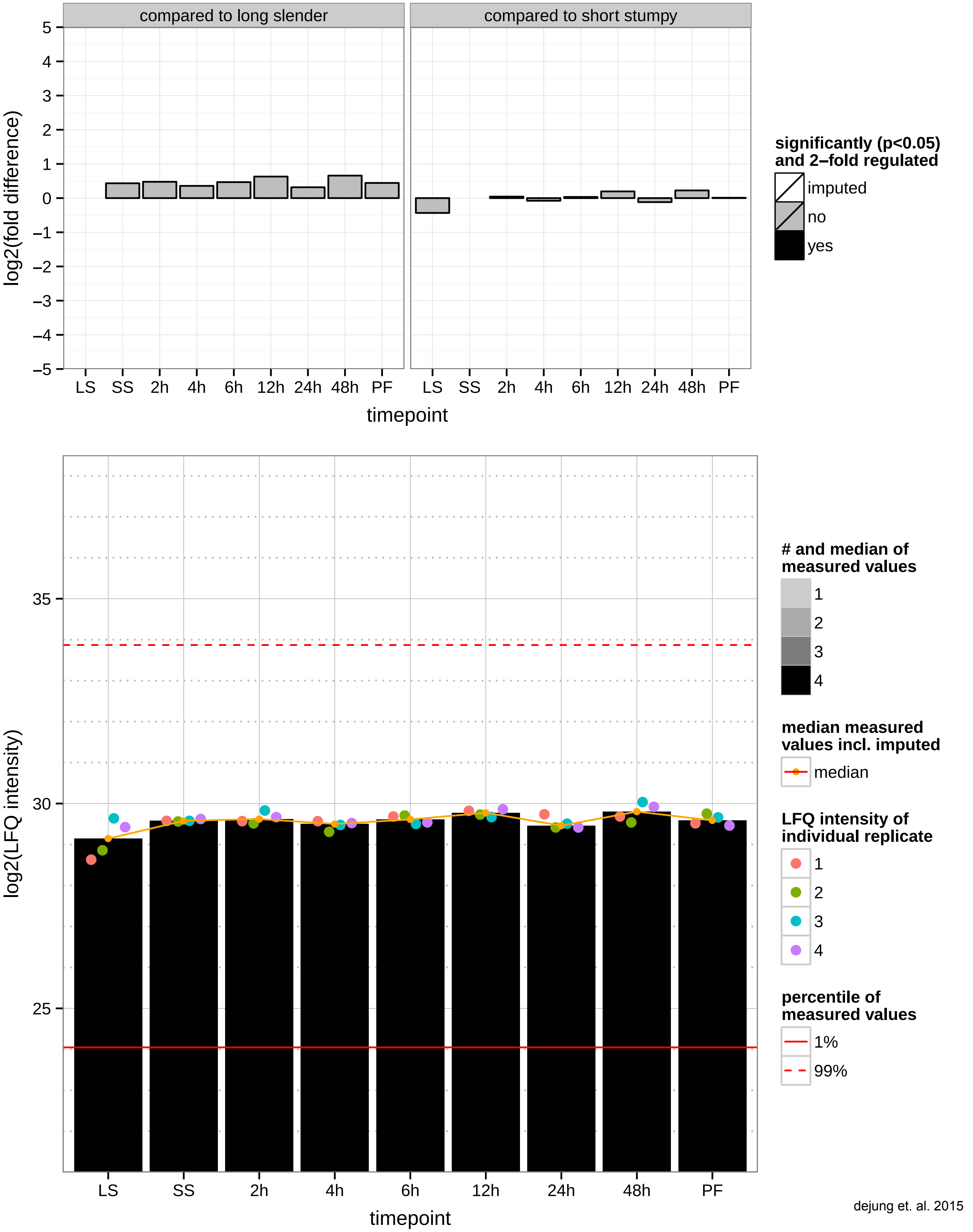

dejung et. al. 2015

lipoic acid containing carrier protein, putative (GCVH)
Tb927.9.9840
AGOF: null
AGOC: glycine cleavage complex, mitochondrion
AGOP: glycine catabolic process
PGOF: null
PGOC: glycine cleavage complex
PGOP: glycine catabolic process, glycine decarboxylation via glycine cleavage system

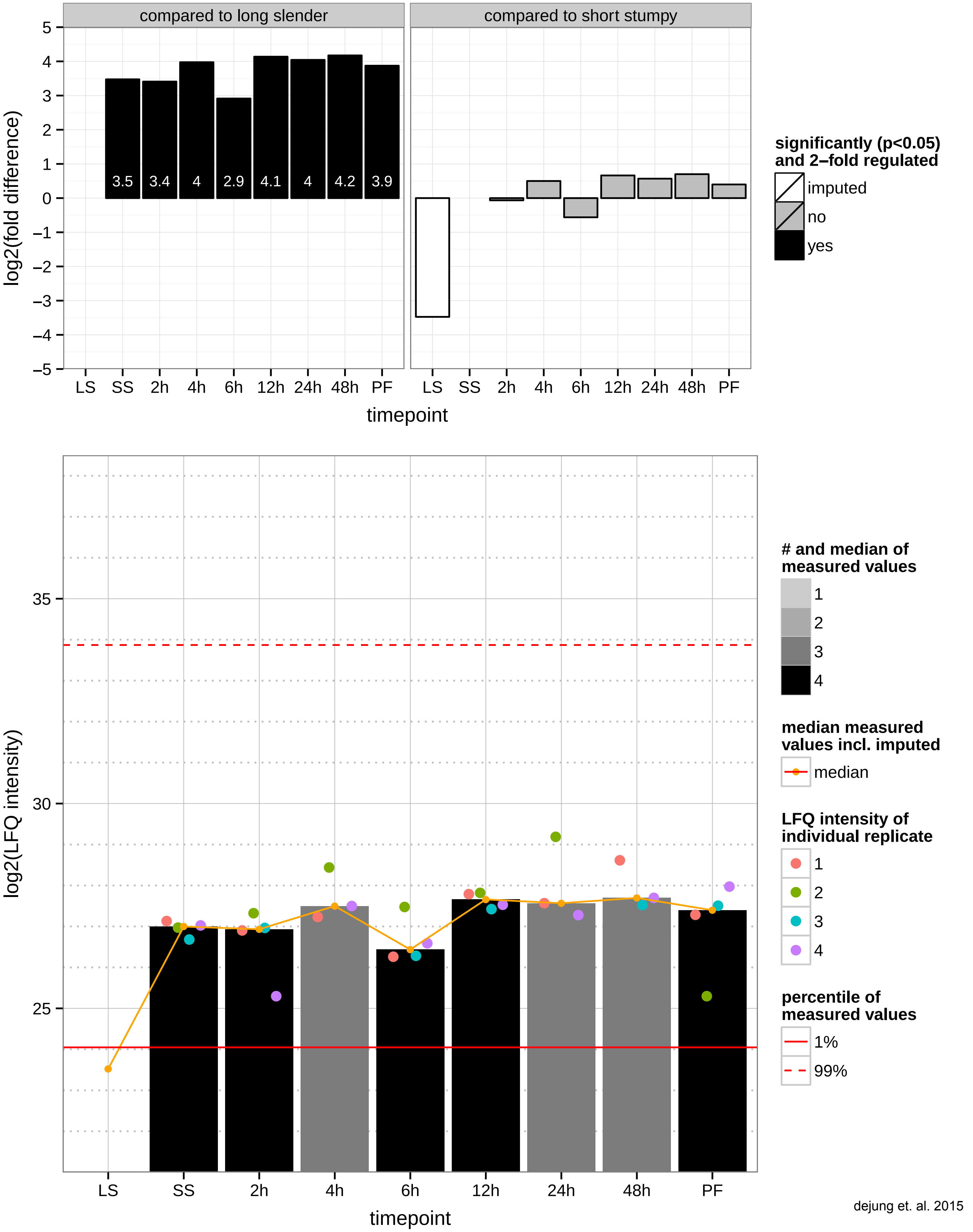

dejung et. al. 2015

hypothetical protein, conserved
Tb927.9.9860
AGOF: ATP binding
AGOC: null
AGOP: protein folding
PGOF: null
PGOC: null
PGOP: null

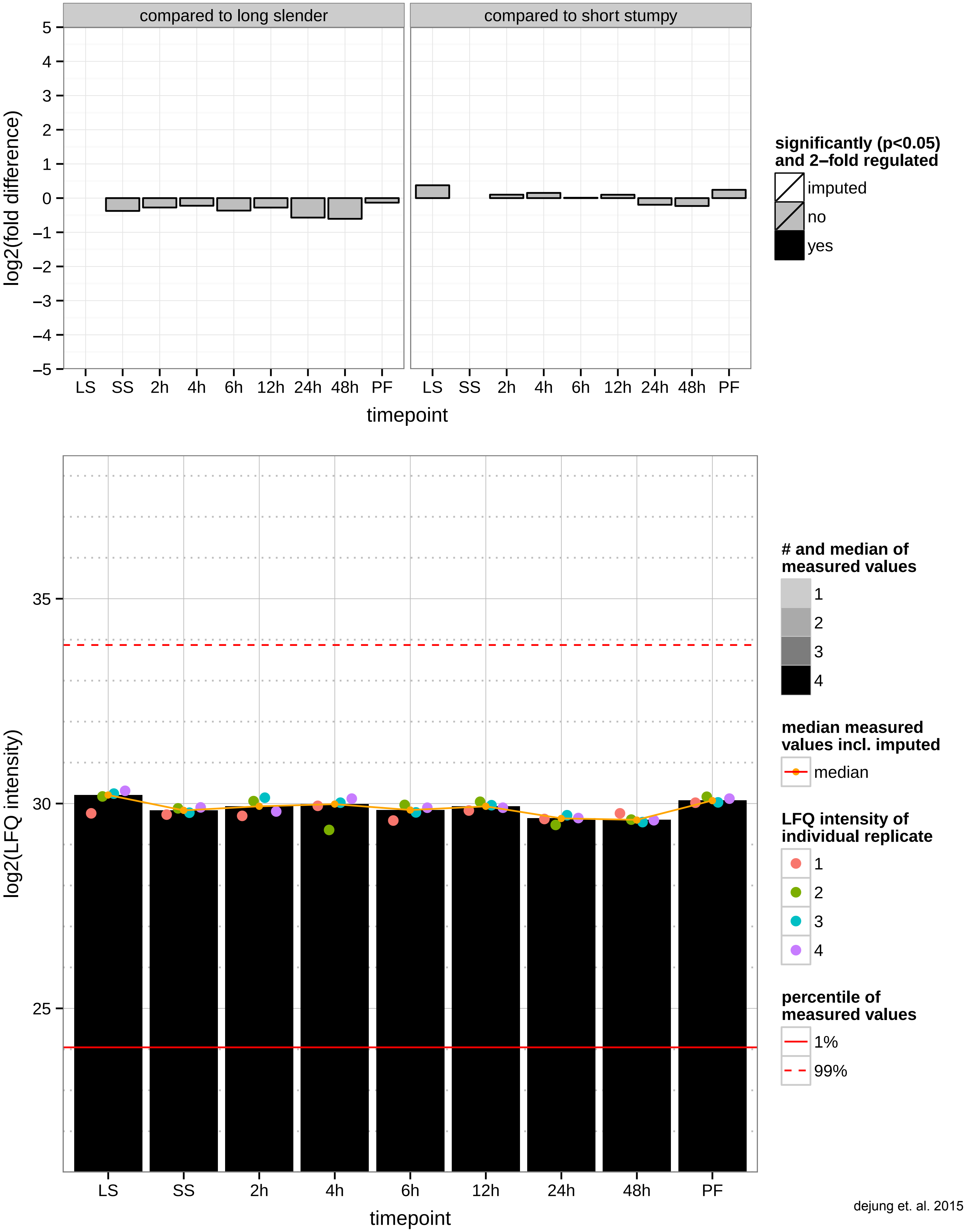

dejung et. al. 2015

MCAK–like kinesin, putative
Tb927.9.9870
AGOF: ATP binding, microtubule motor activity, motor activity
AGOC: microtubule associated complex
AGOP: microtubule–based movement
PGOF: ATP binding, microtubule motor activity
PGOC: null
PGOP: microtubule–based movement

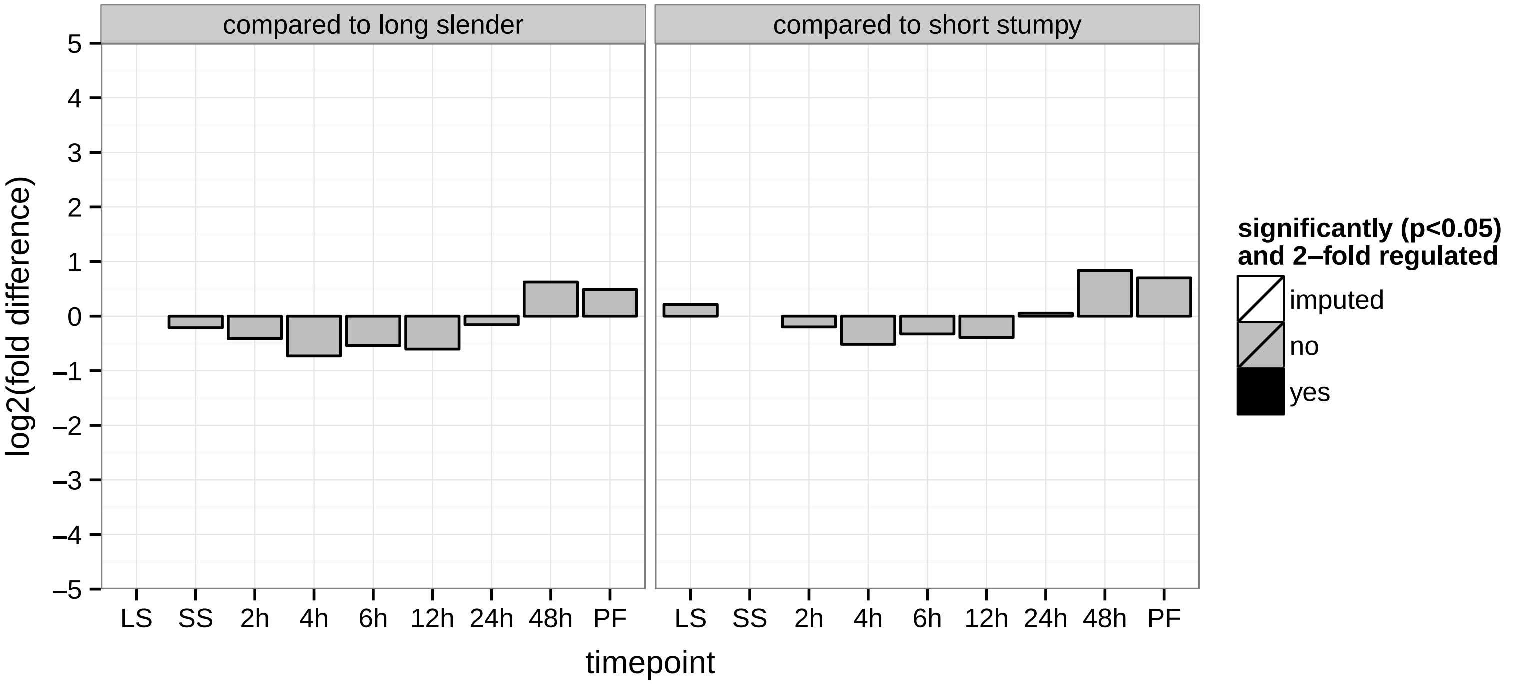

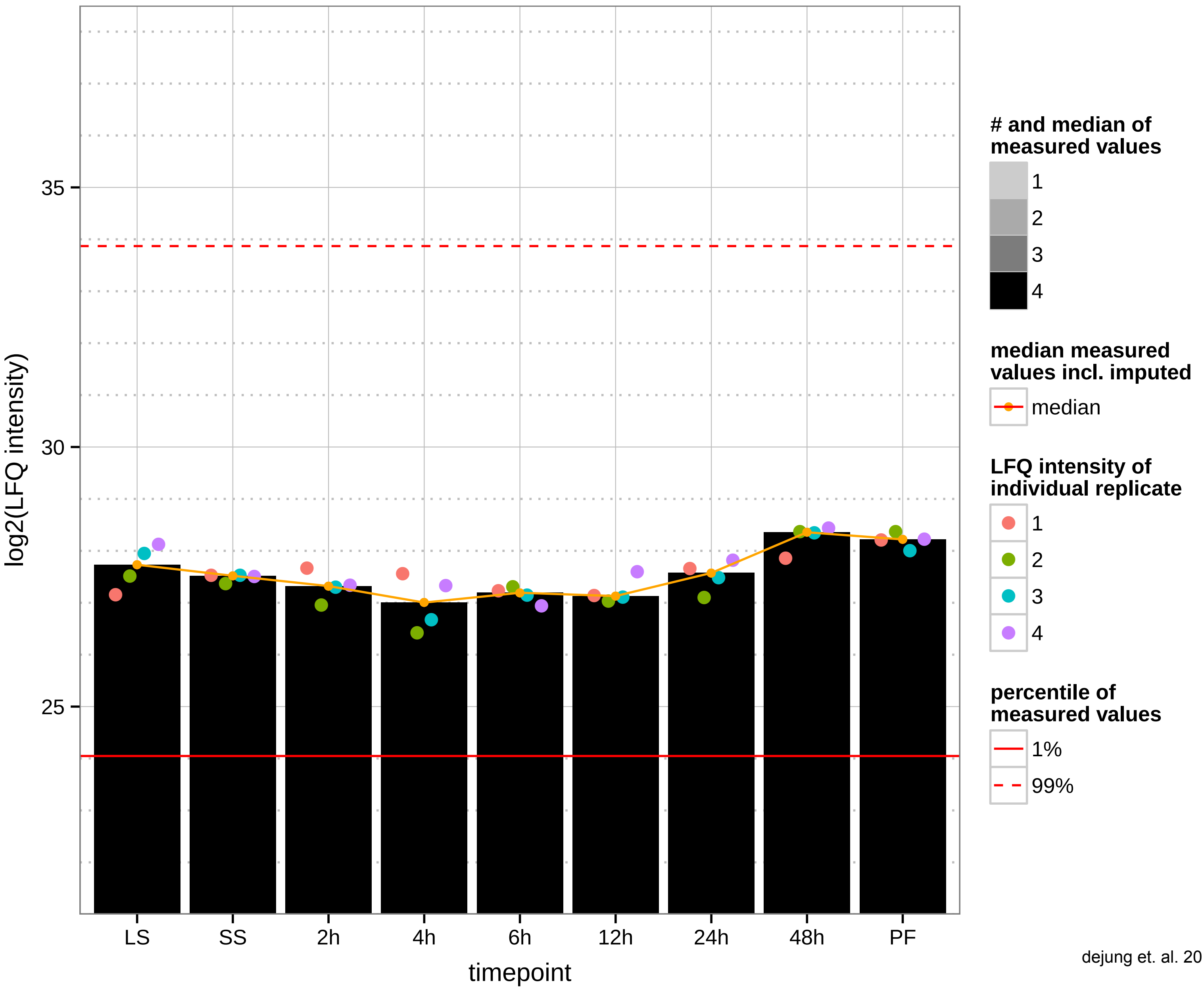

dejung et. al. 2015

PACRGB
Tb927.9.9940
AGOF: null
AGOC: cilium part
AGOP: null
PGOF: binding
PGOC: null
PGOP: null

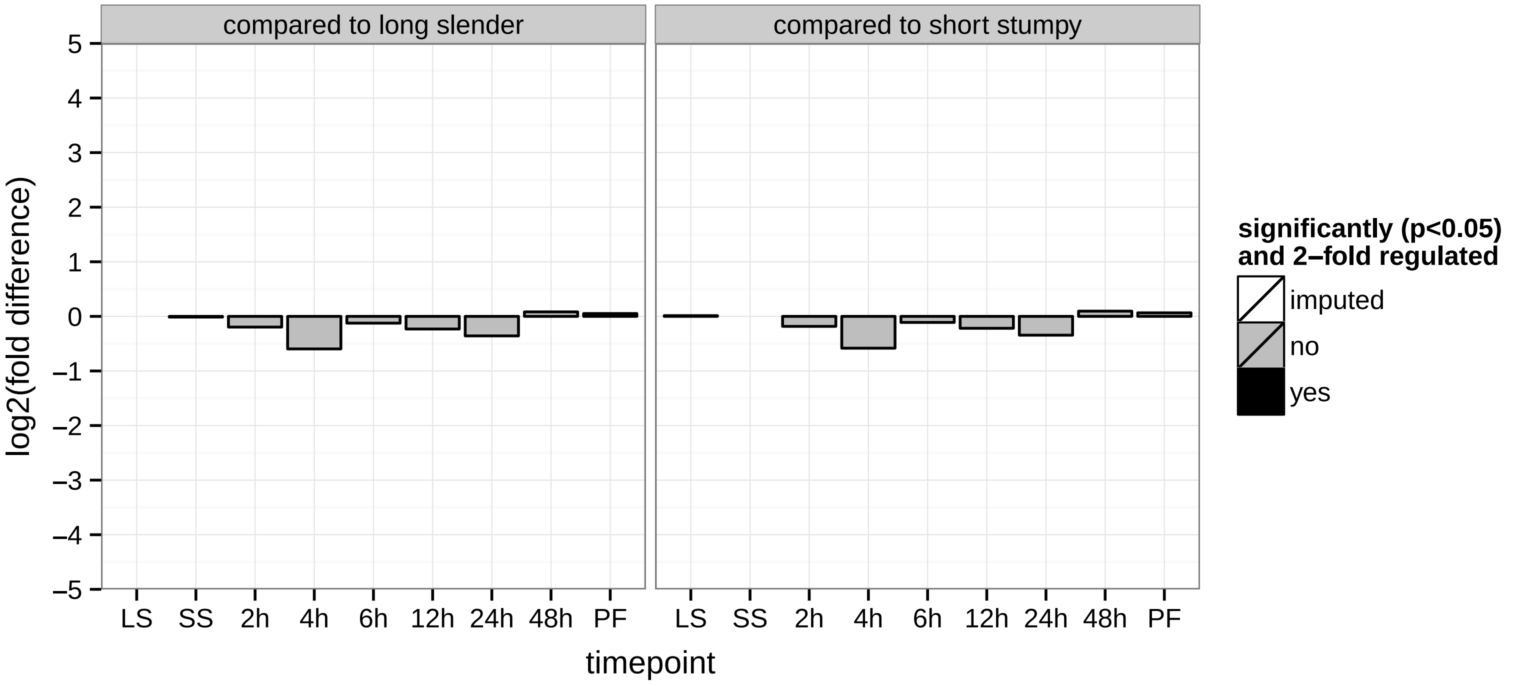

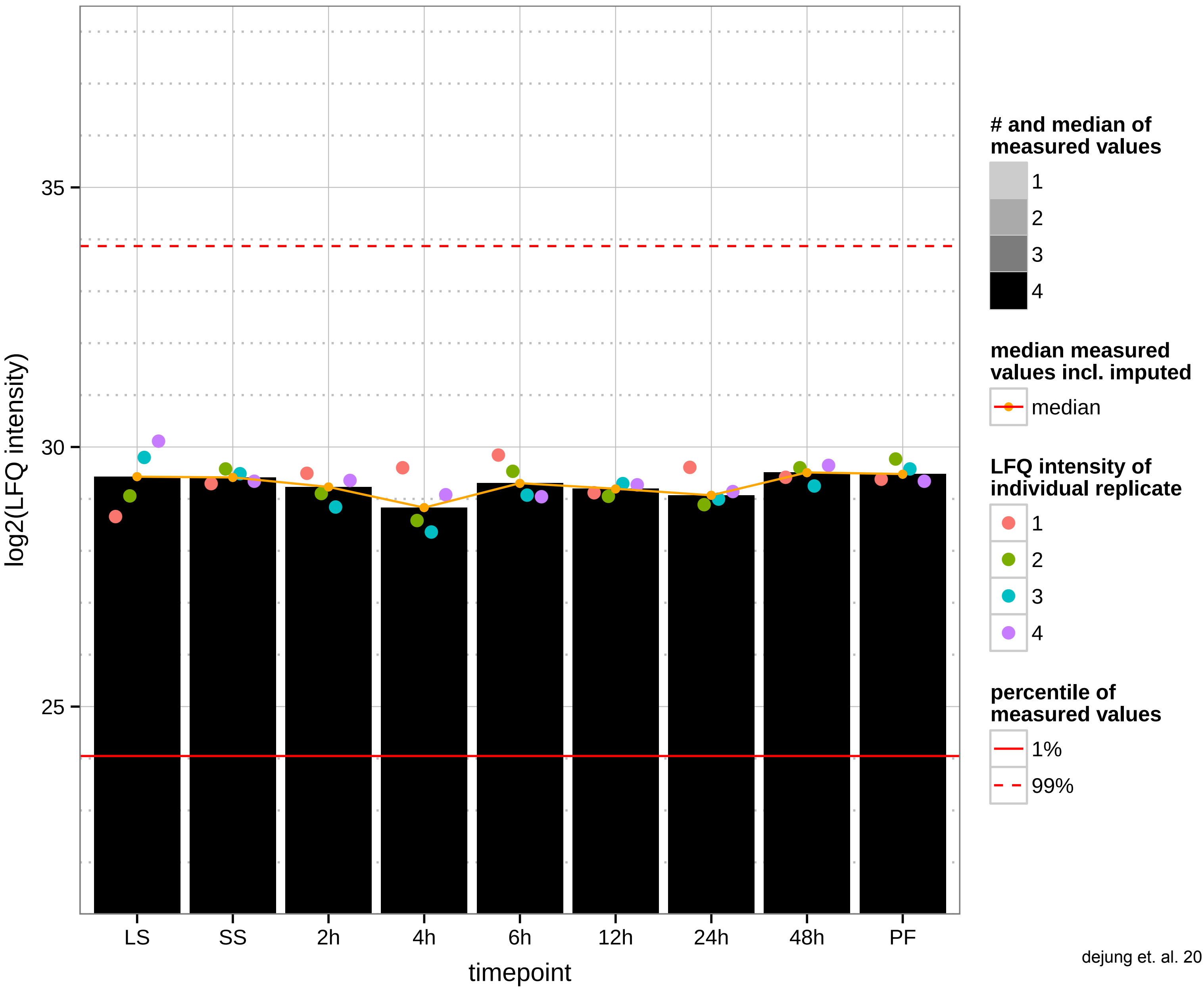

dejung et. al. 2015

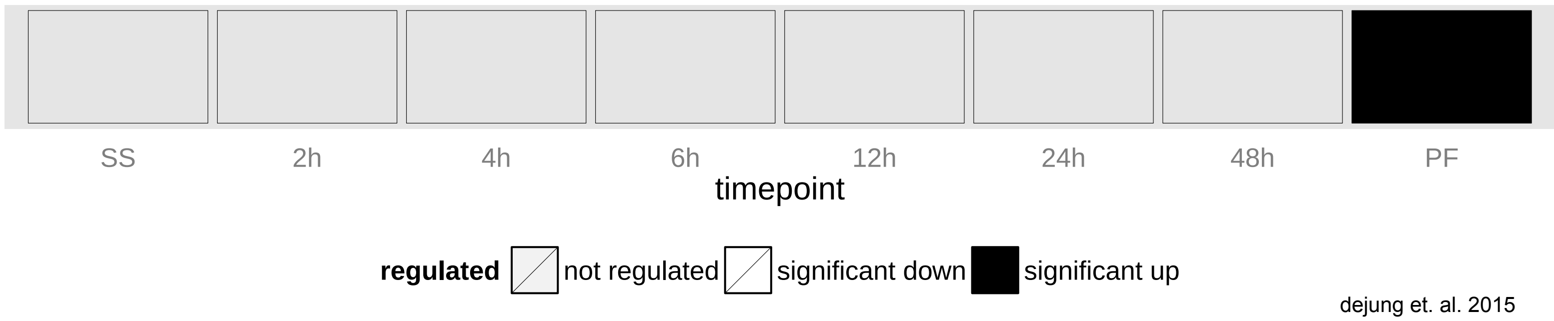
SS
2h
4h
6h
12h
24h
48h
PF
timepoint
regulated
not regulated
significant down
significant up
dejung et. al. 2015

hypothetical protein, conserved, BAC from homologous region on chr5
Tb927.5.4470;Tb05.5K5.120
AGOF: phosphorus–oxygen lyase activity
AGOC: null
AGOP: cyclic nucleotide biosynthetic process, intracellular signal transduction
PGOF: phosphorus–oxygen lyase activity
PGOC: null
PGOP: cyclic nucleotide biosynthetic process, intracellular signal transduction

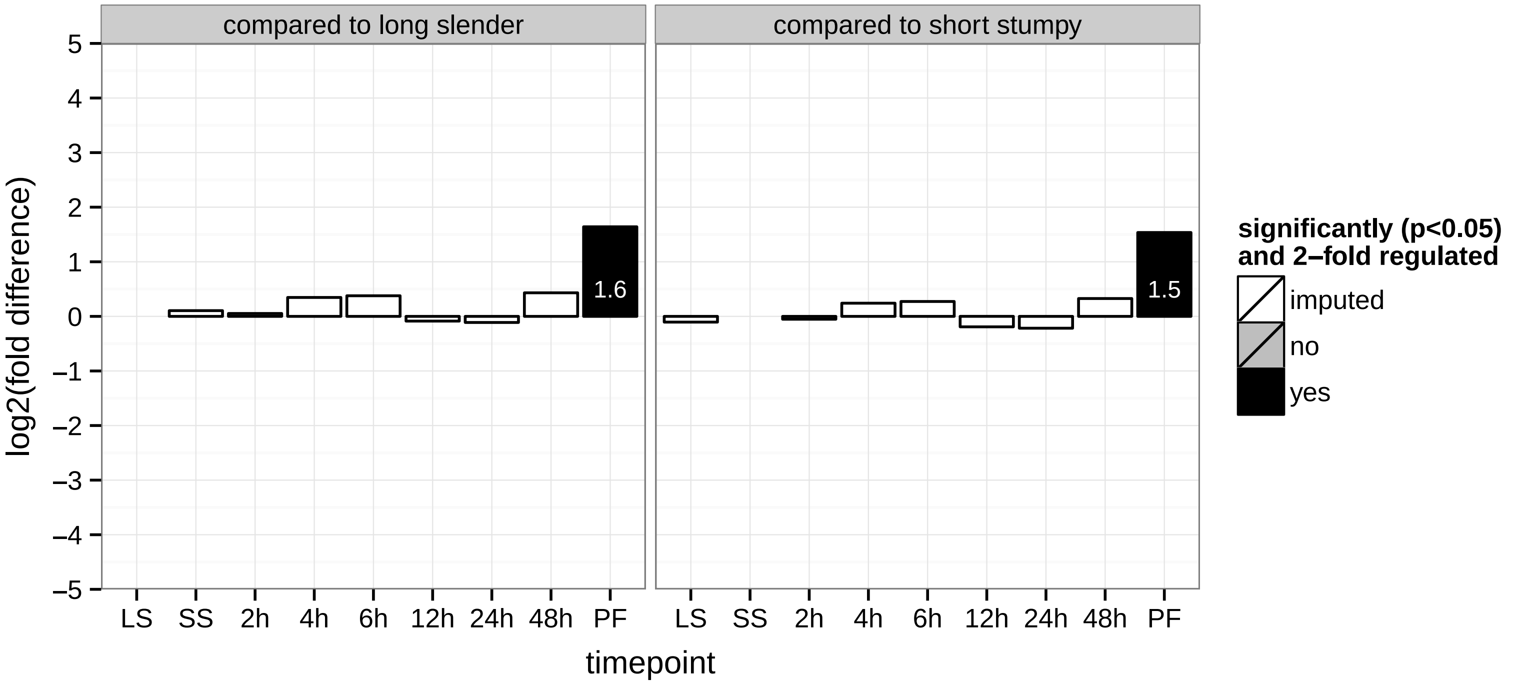

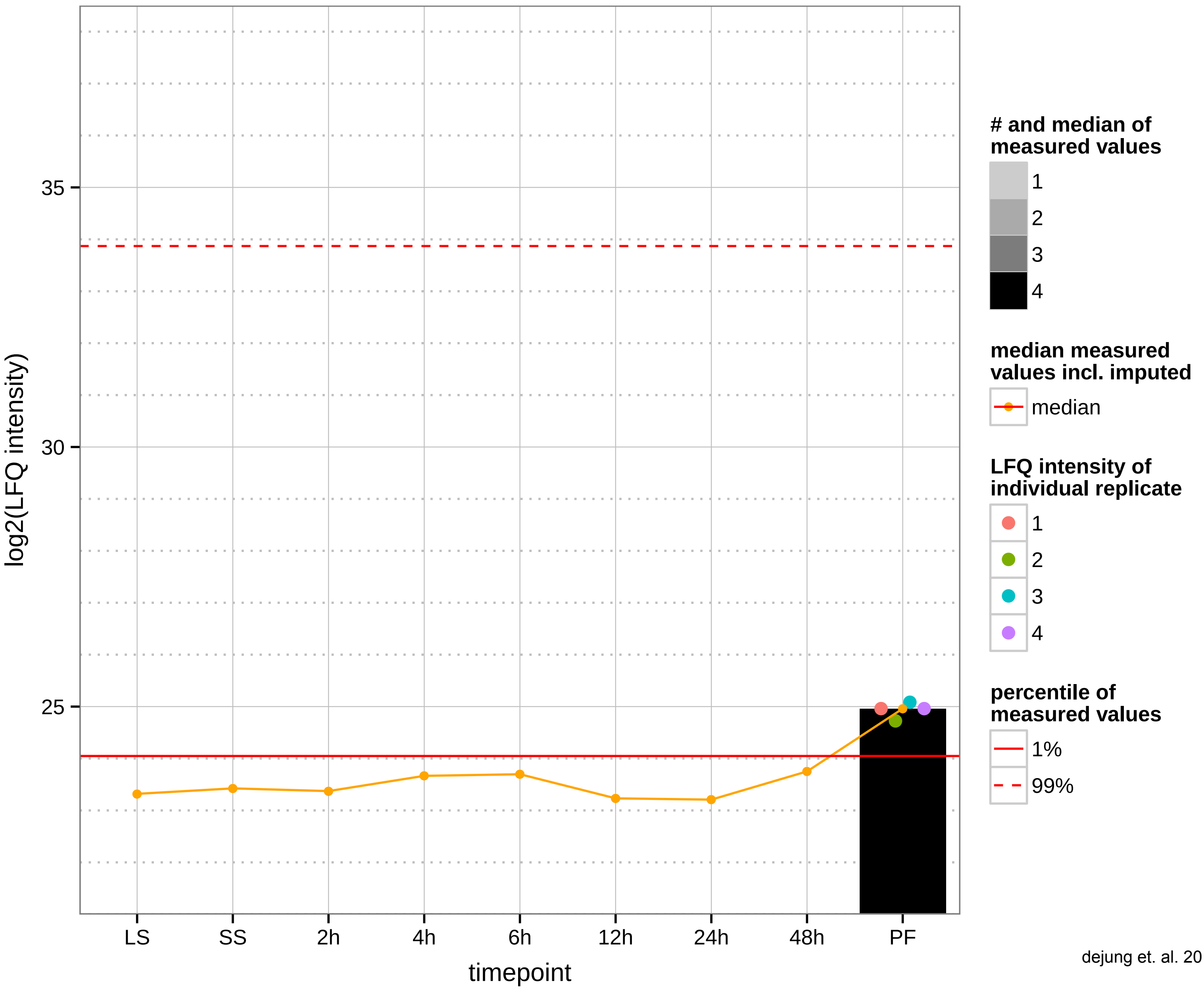

dejung et. al. 2015

hypothetical protein, conserved
Tb11.02.5360b;Tb11.02.5360
AGOF: null
AGOC: null
AGOP: null
PGOF: null
PGOC: null
PGOP: null

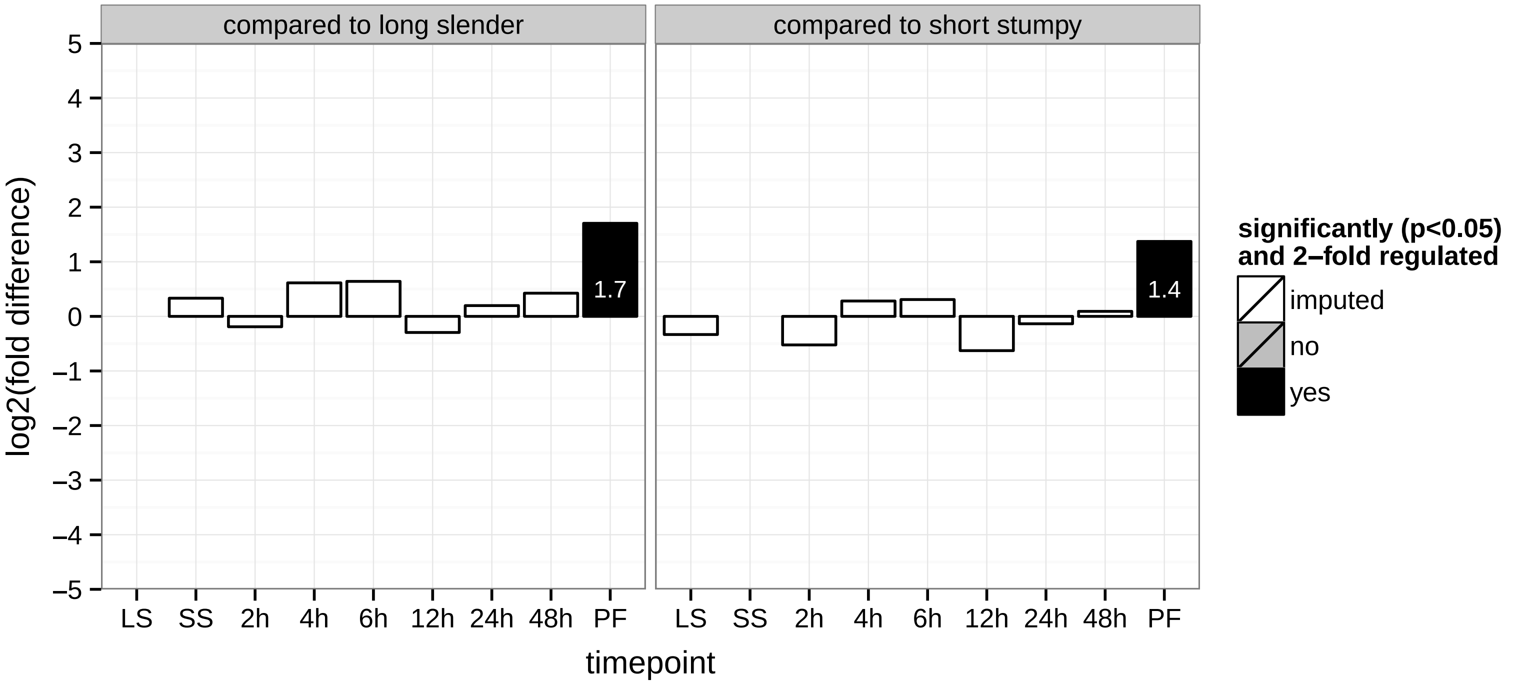

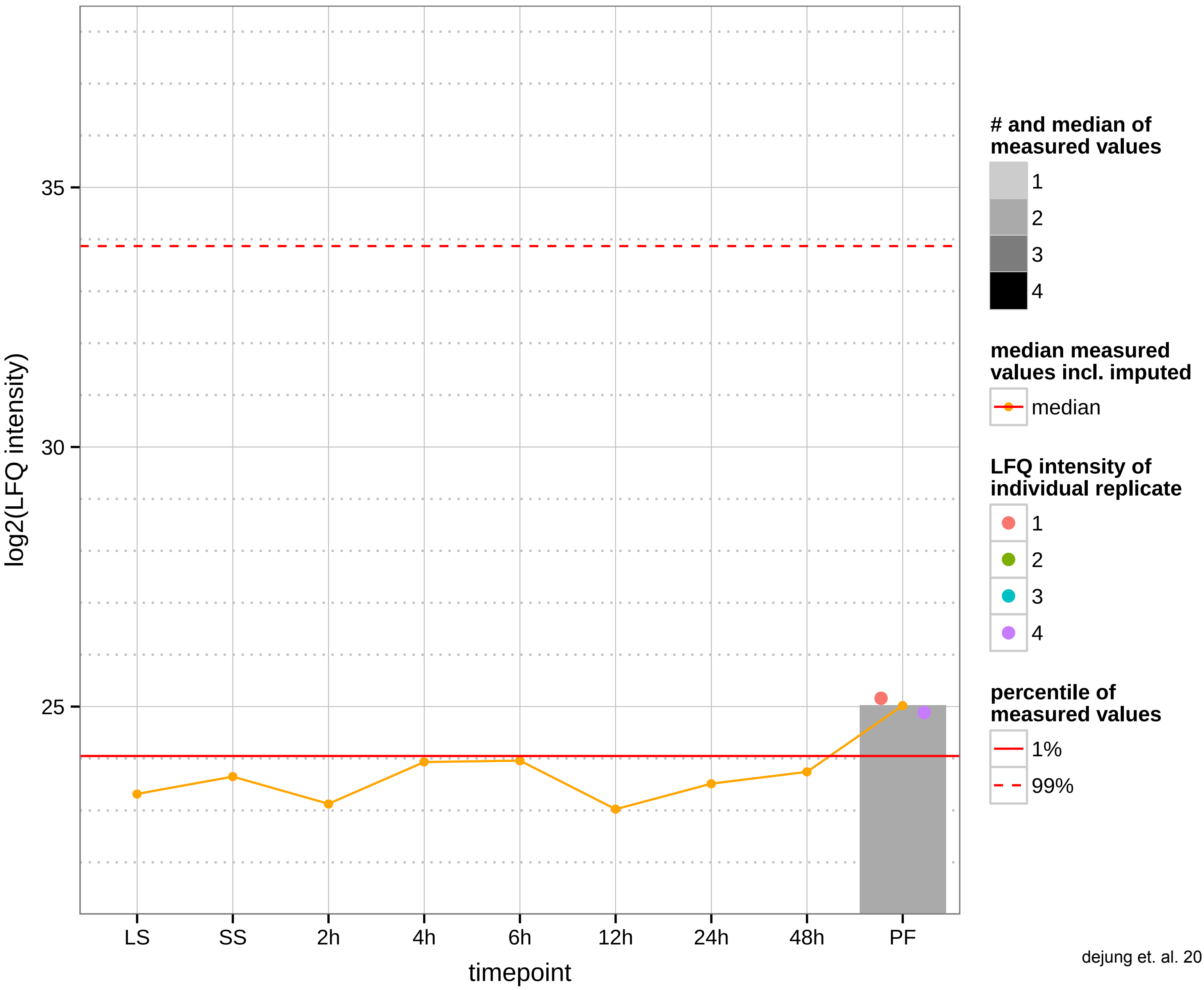

dejung et. al. 2015

exosome complex exonuclease RRP44p homologue (RRP44), exosome complex exonuclease RRP44p homologue, putative
Tb11.02.5380b;Tb11.02.5380
AGOF: RNA binding, guanyl–nucleotide exchange factor activity, ribonuclease activity
AGOC: exosome (RNase complex), mitochondrion
AGOP: rRNA processing
PGOF: RNA binding, ribonuclease activity
PGOC: null
PGOP: null

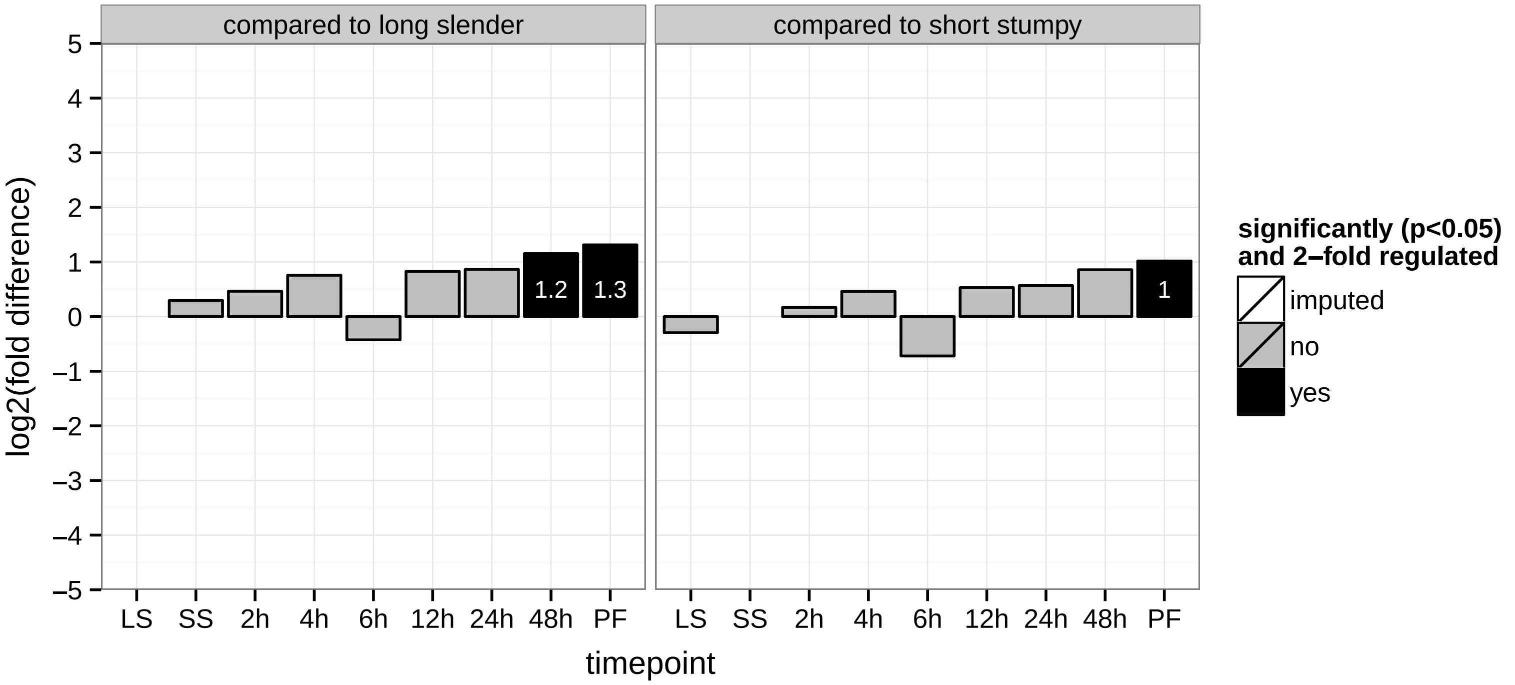

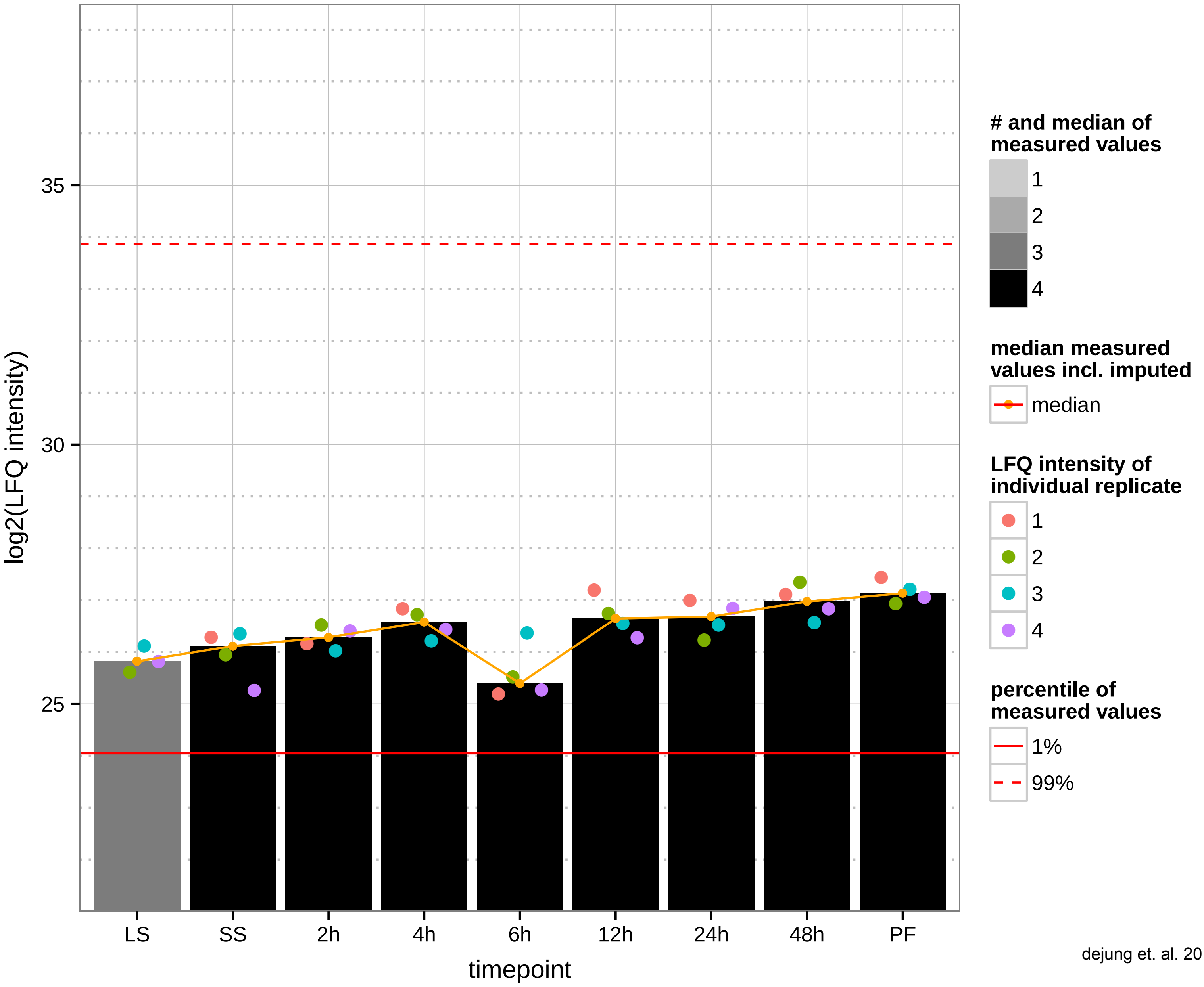

dejung et. al. 2015

mRNA processing protein, putative
Tb11.02.5390b;Tb11.02.5390
AGOF: mRNA binding
AGOC: mitochondrion
AGOP: mitochondrial RNA processing
PGOF: null
PGOC: null
PGOP: null

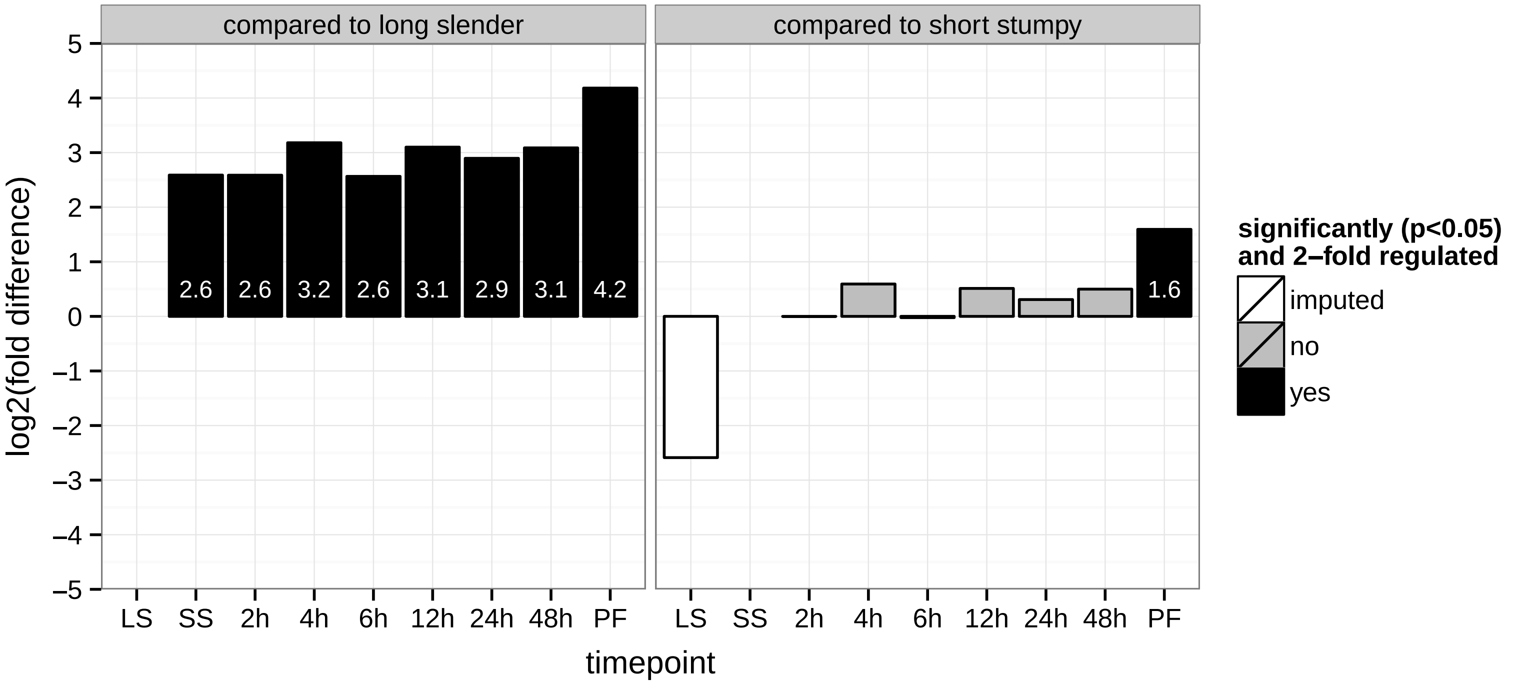

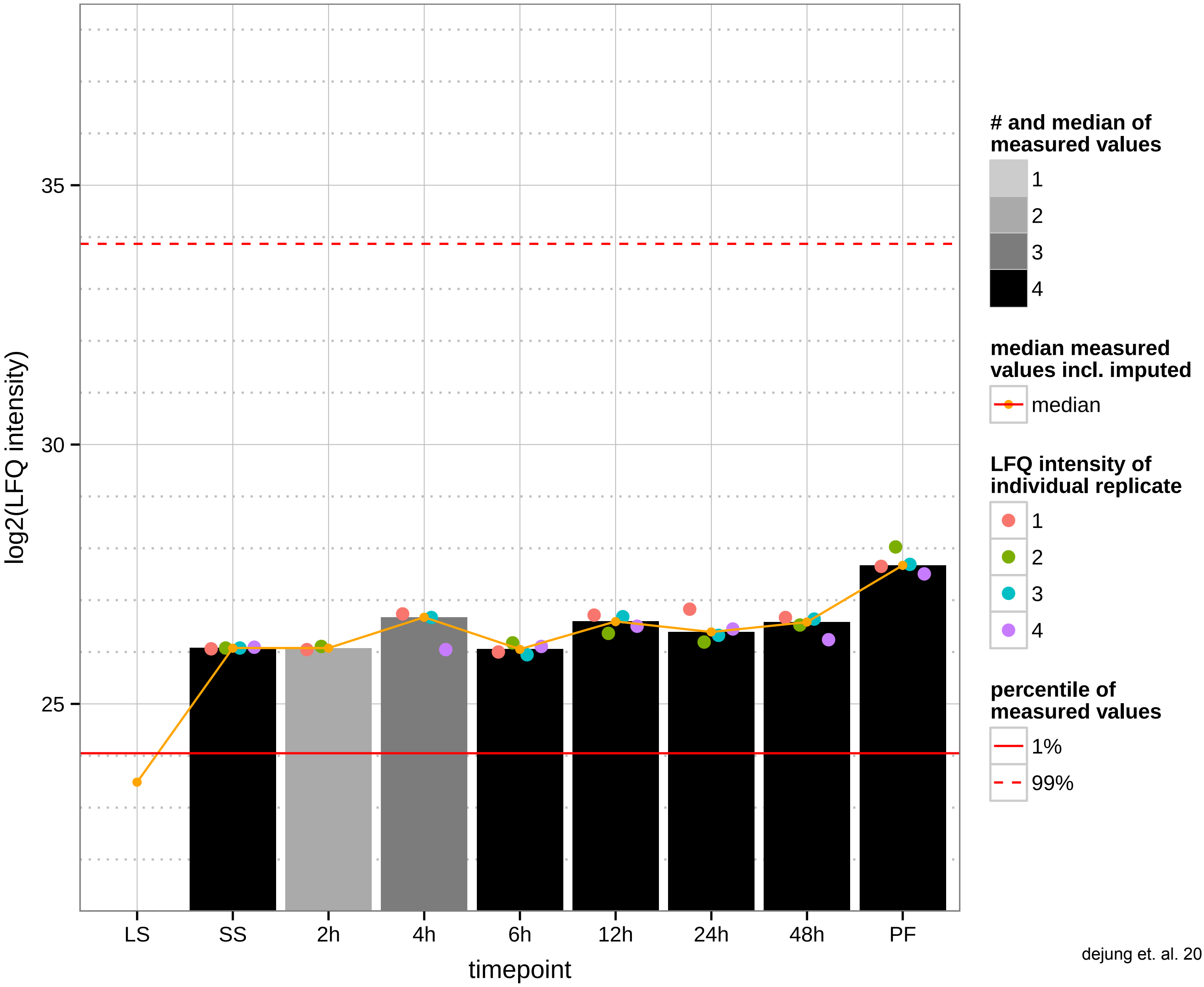

dejung et. al. 2015

ribonuclease H1, putative
Tb927.7.4930;Tb11.v5.0174
AGOF: null, magnesium ion binding, nucleic acid binding, ribonuclease H activity
AGOC: null
AGOP: null
PGOF: magnesium ion binding, nucleic acid binding, ribonuclease H activity
PGOC: null
PGOP: null

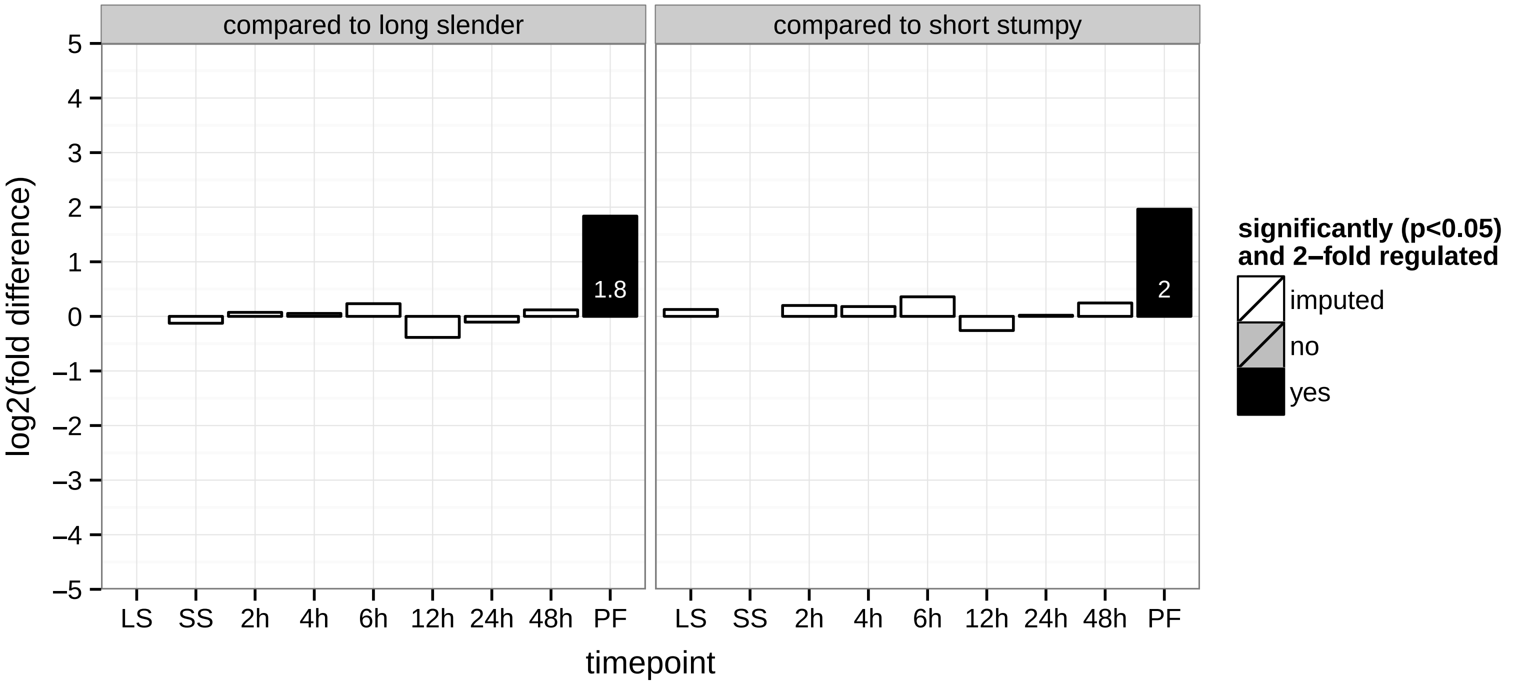

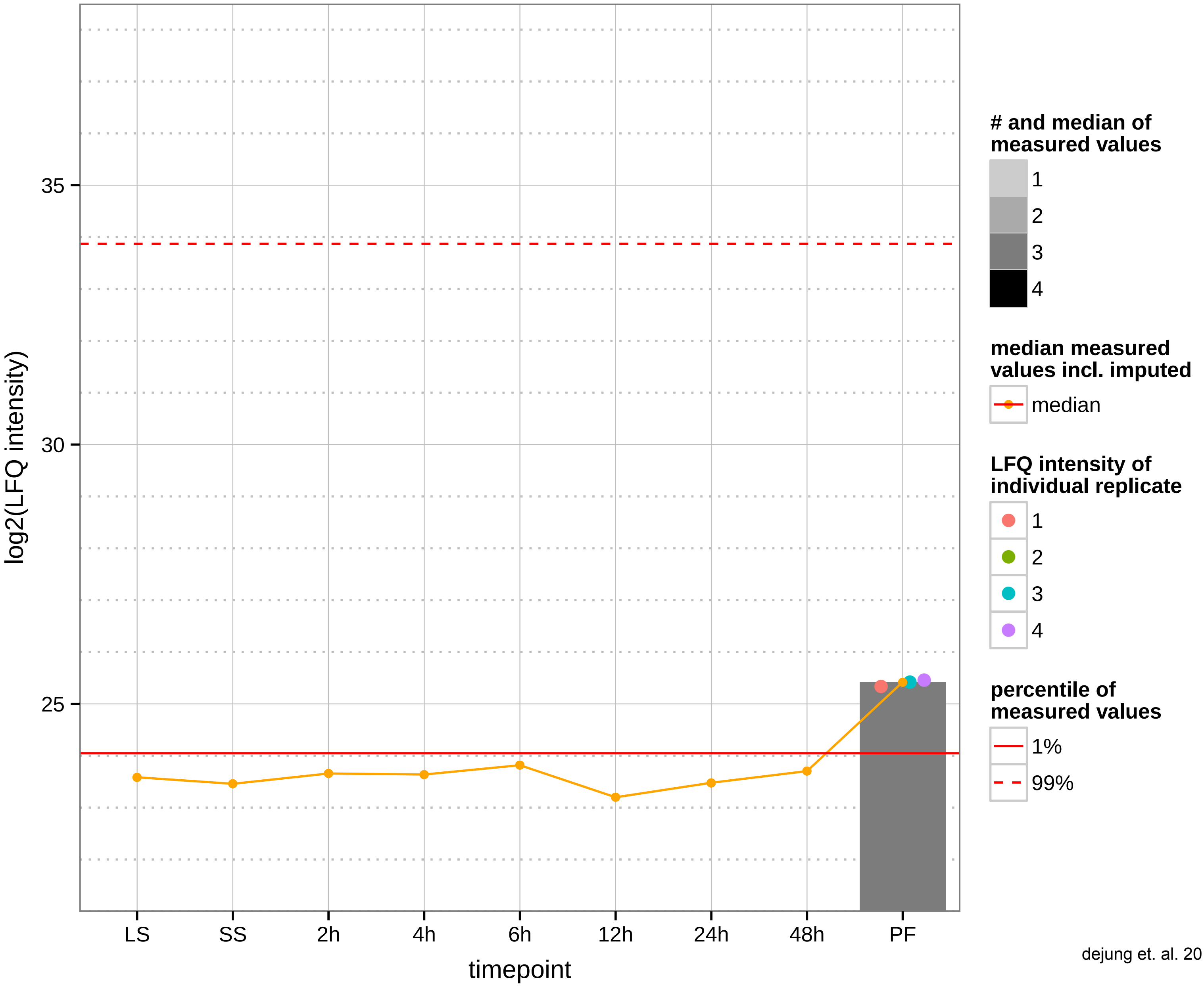

dejung et. al. 2015

hypothetical protein, conserved
Tb927.10.5200;Tb11.v5.0237
AGOF: null
AGOC: null
AGOP: null
PGOF: null
PGOC: null
PGOP: null

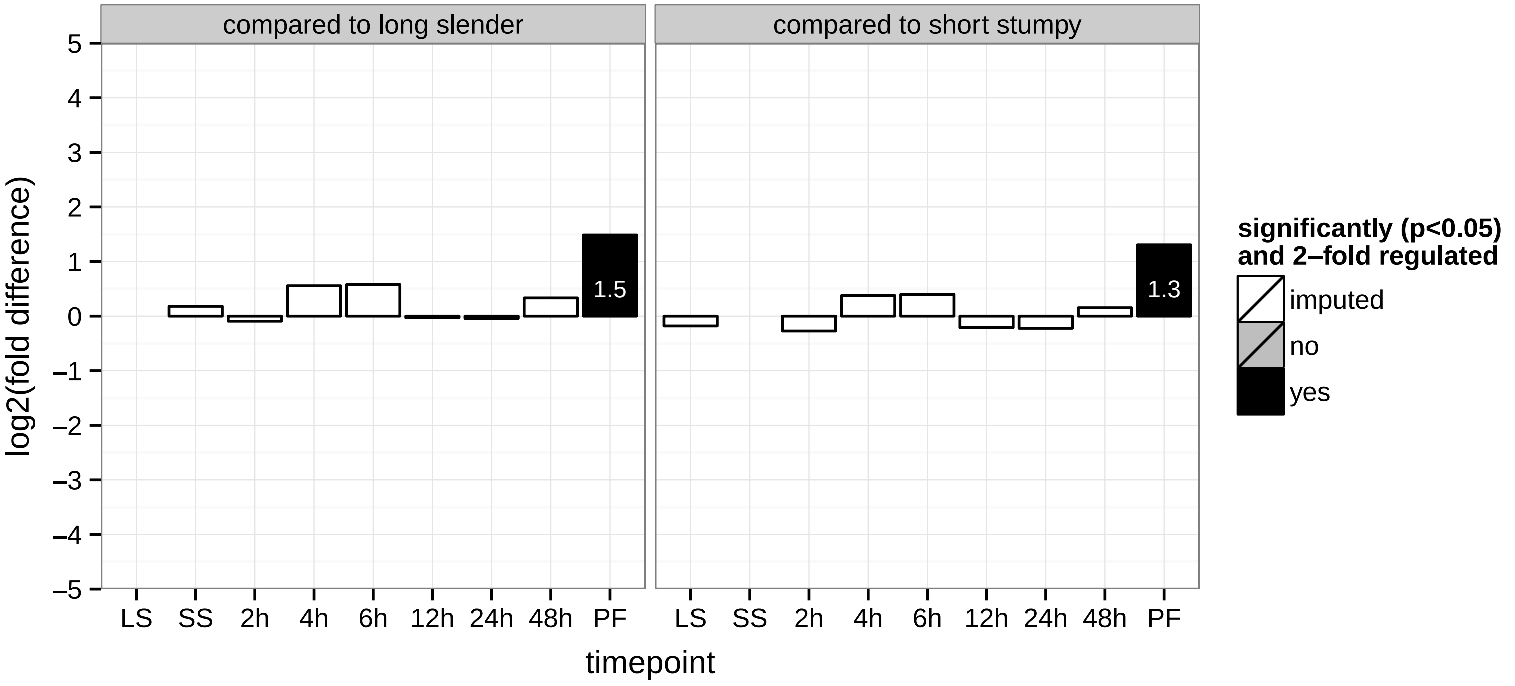

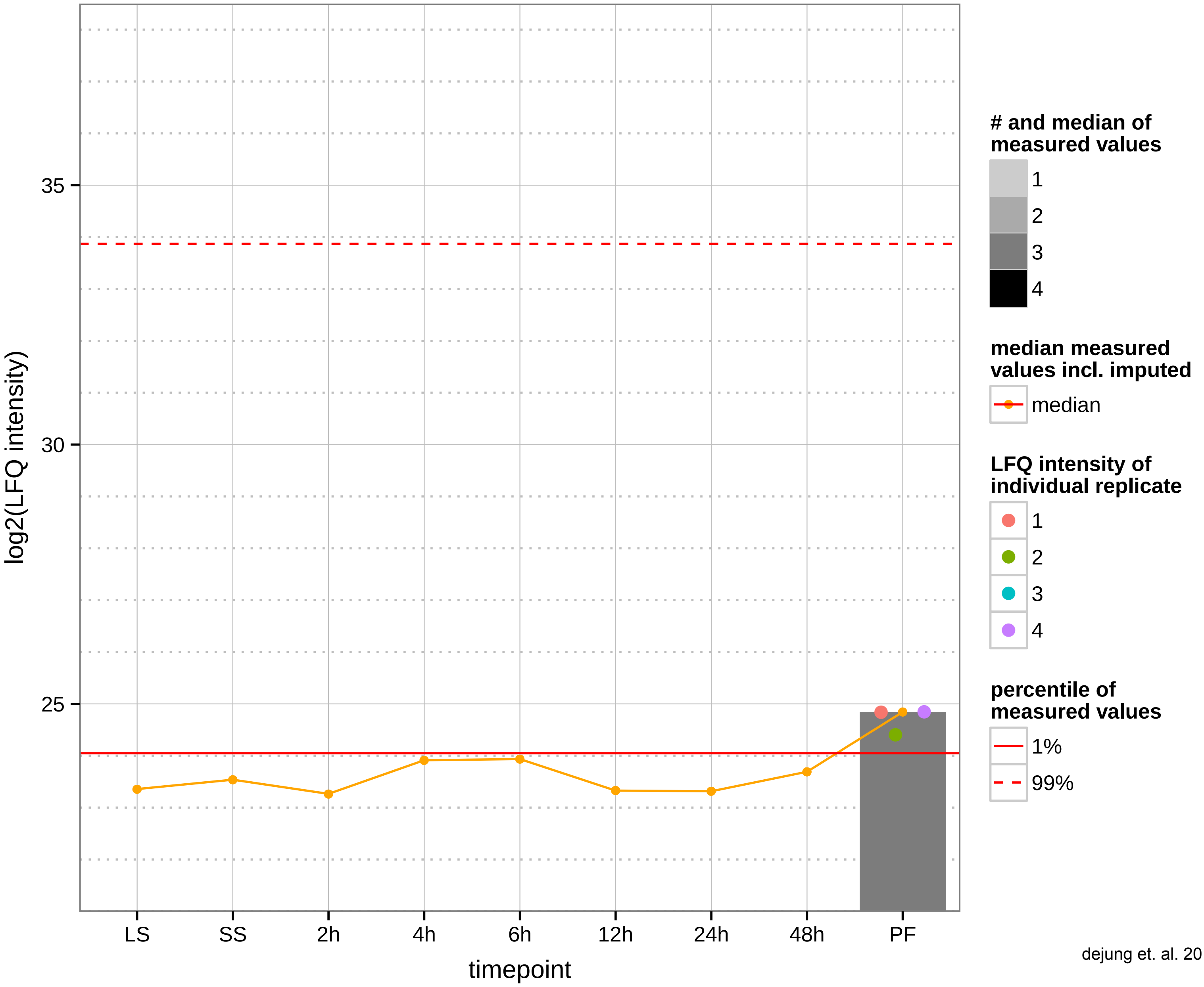

dejung et. al. 2015

hypothetical protein, conserved
Tb927.10.6320;Tb11.v5.0274
AGOF: null
AGOC: null
AGOP: null
PGOF: binding
PGOC: null
PGOP: null

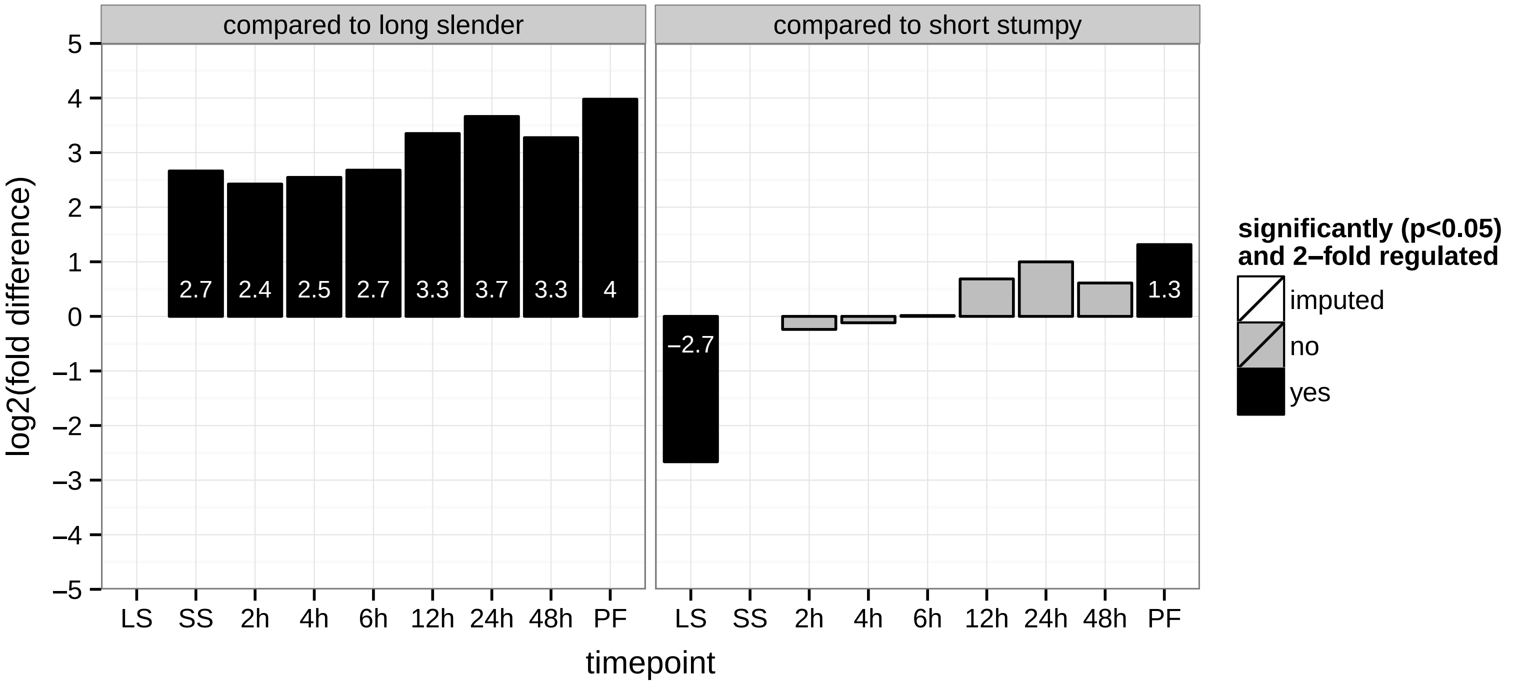

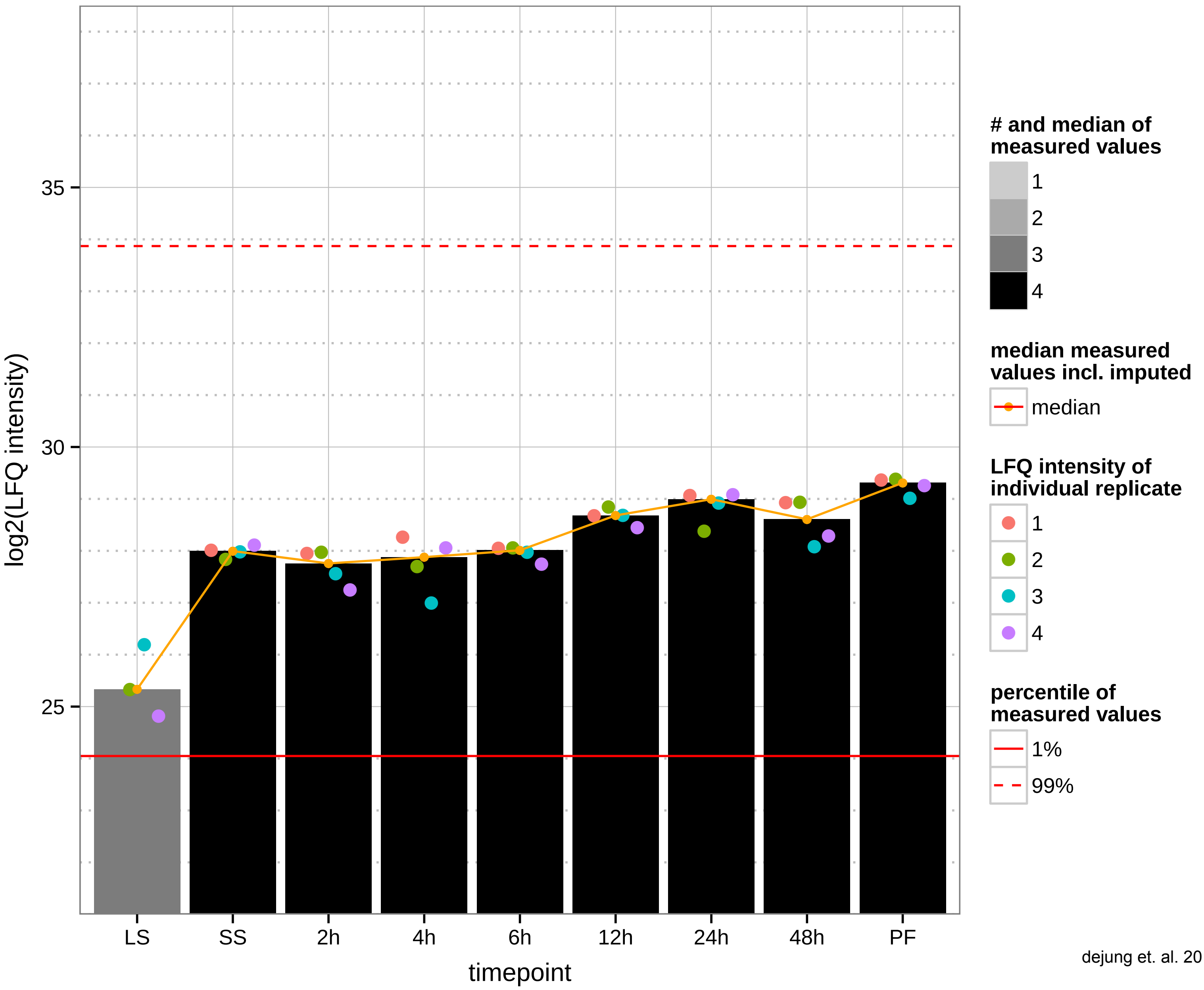

dejung et. al. 2015

cation transporter, putative
Tb927.10.15940;Tb11.v5.0304
AGOF: null, cation transmembrane transporter activity
AGOC: null, membrane, mitochondrion
AGOP: null, cation transport
PGOF: cation transmembrane transporter activity
PGOC: integral to membrane
PGOP: cation transport, transmembrane transport

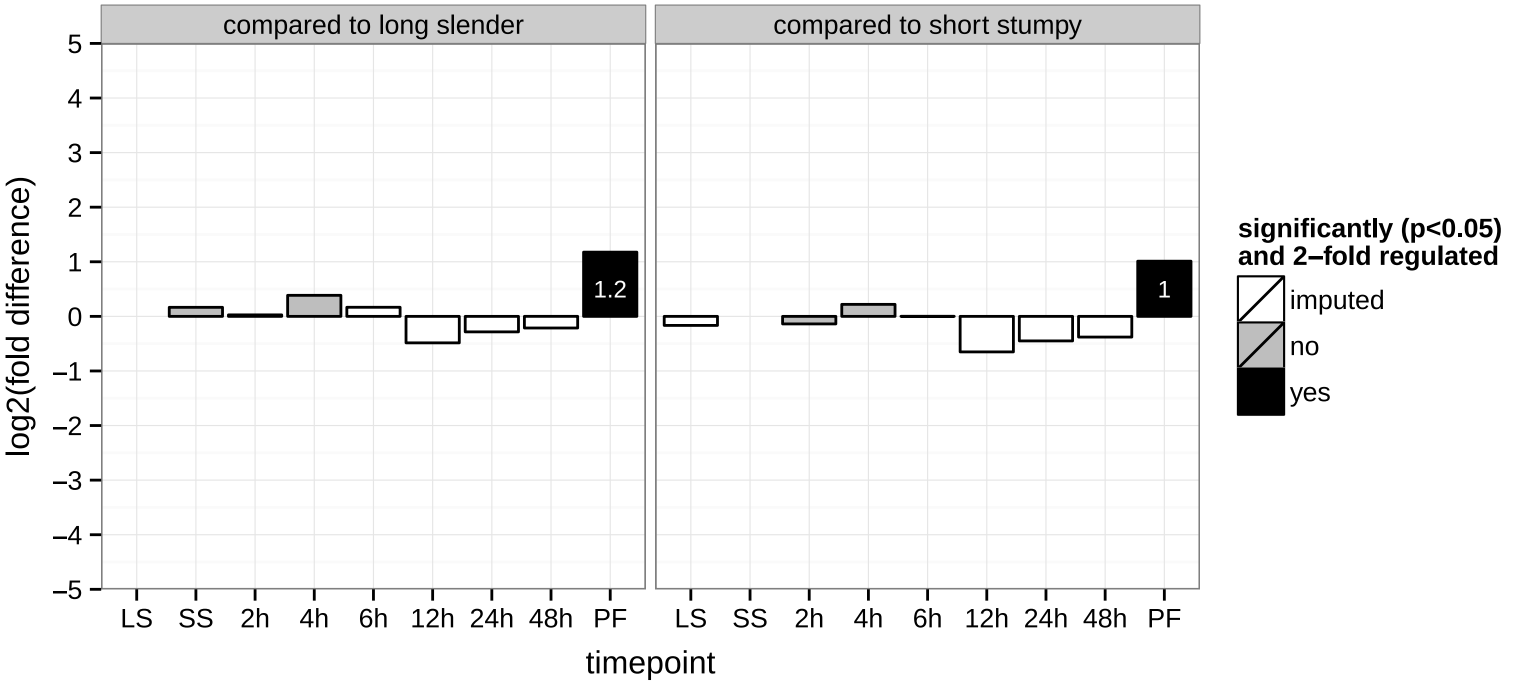

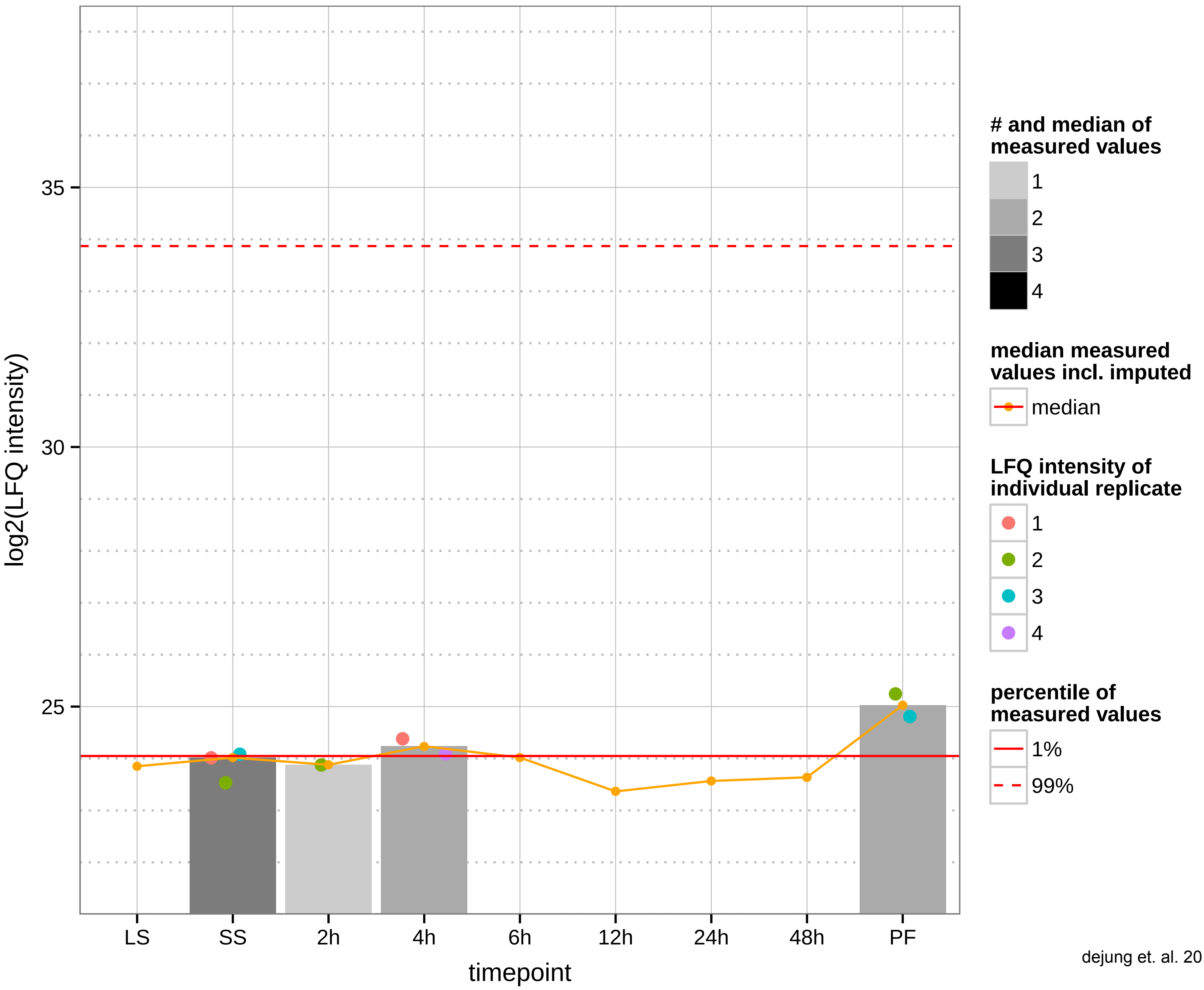

dejung et. al. 2015

hypothetical protein, conserved, Mitochondrial Editosome−like Complex TUTase (MEAT1)
Tb927.1.1330;Tb11.v5.0371
AGOF: null, RNA uridylyltransferase activity
AGOC: null, mitochondrion
AGOP: null, transcription, DNA−dependent
PGOF: null
PGOC: null
PGOP: null

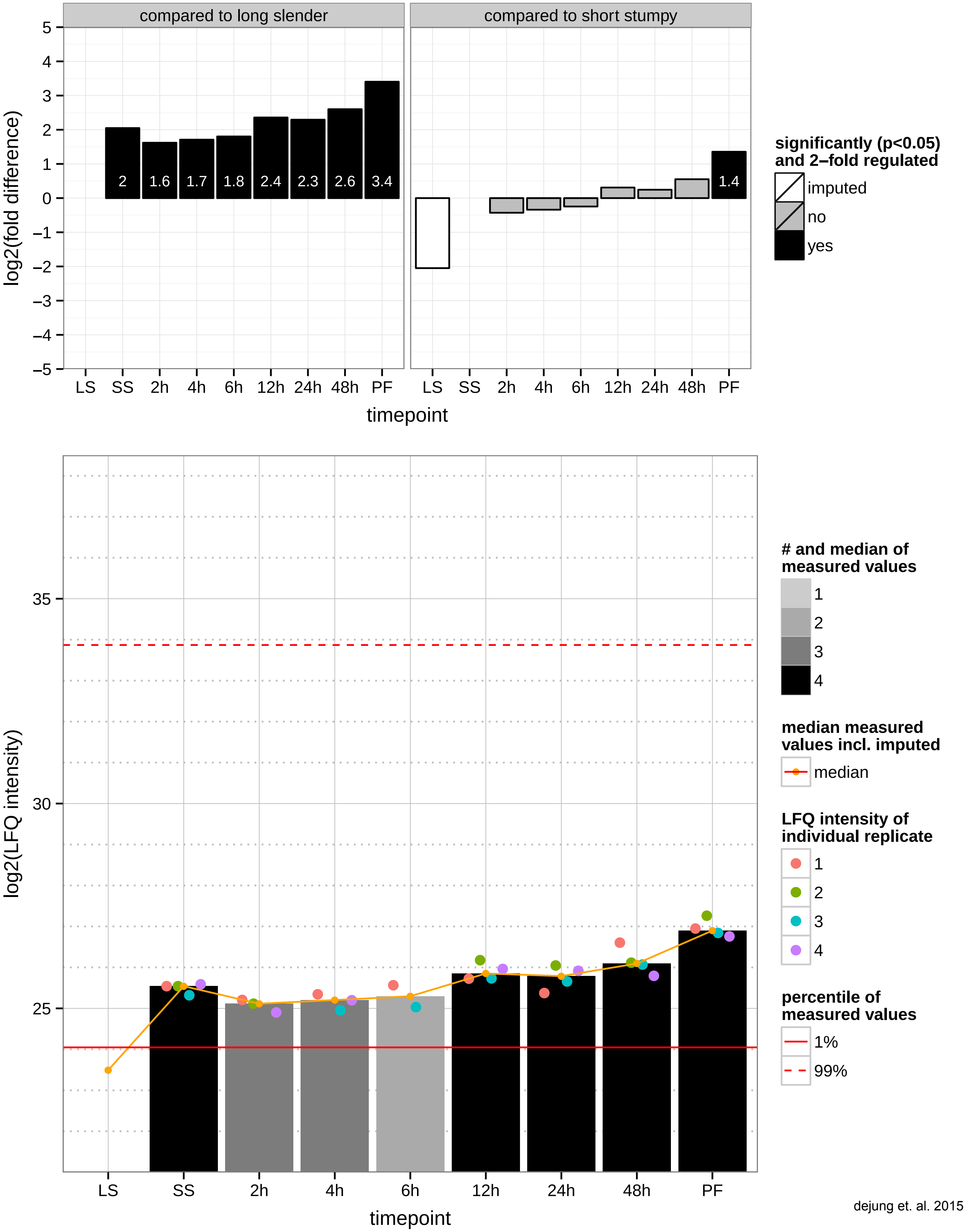

dejung et. al. 2015

ATP−dependent zinc metallopeptidase, putative, metallo−peptidase, Clan MA(E) Family M41
Tb927.10.16150;Tb11.v5.0397
AGOF: null, ATP binding, metalloendopeptidase activity, nucleoside−triphosphatase activity
AGOC: null, mitochondrion
AGOP: null, proteolysis
PGOF: ATP binding, metalloendopeptidase activity, nucleoside−triphosphatase activity, nucleotide binding
PGOC: null
PGOP: proteolysis

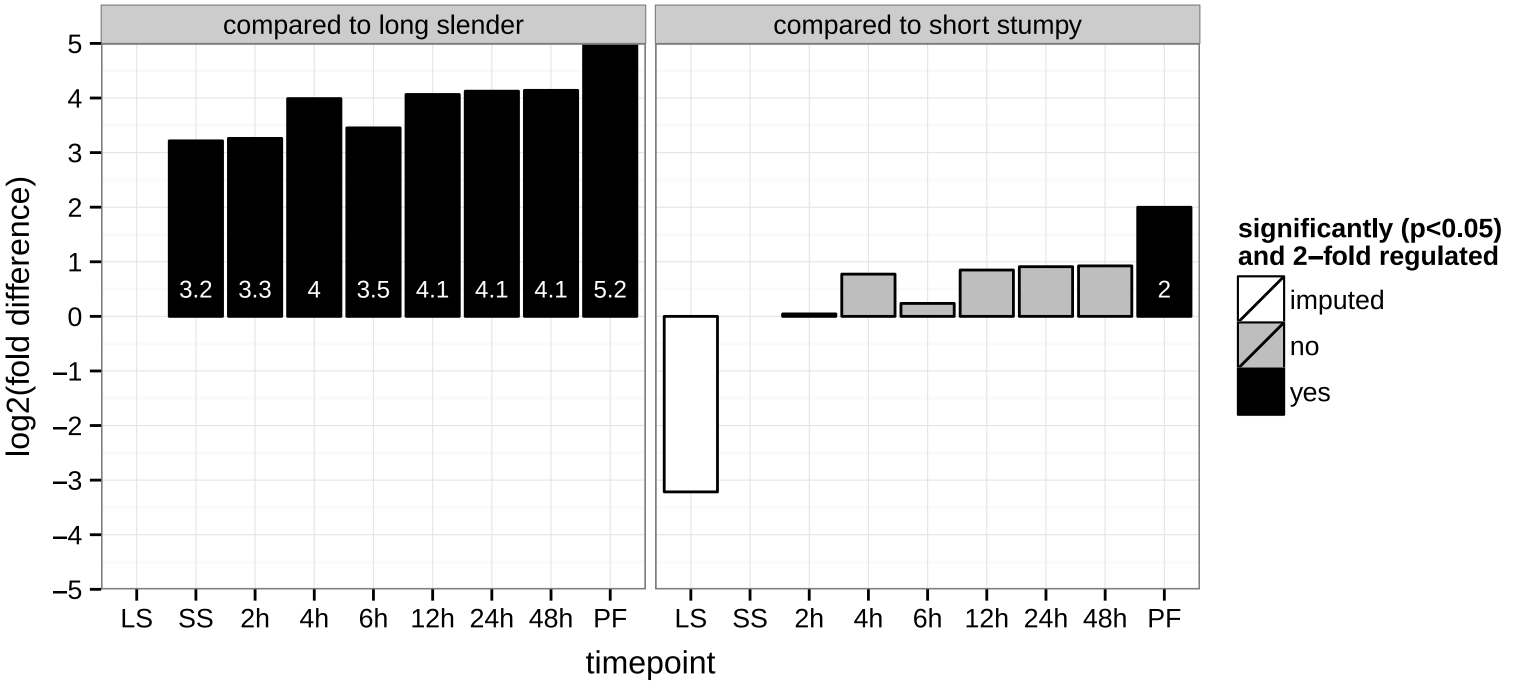

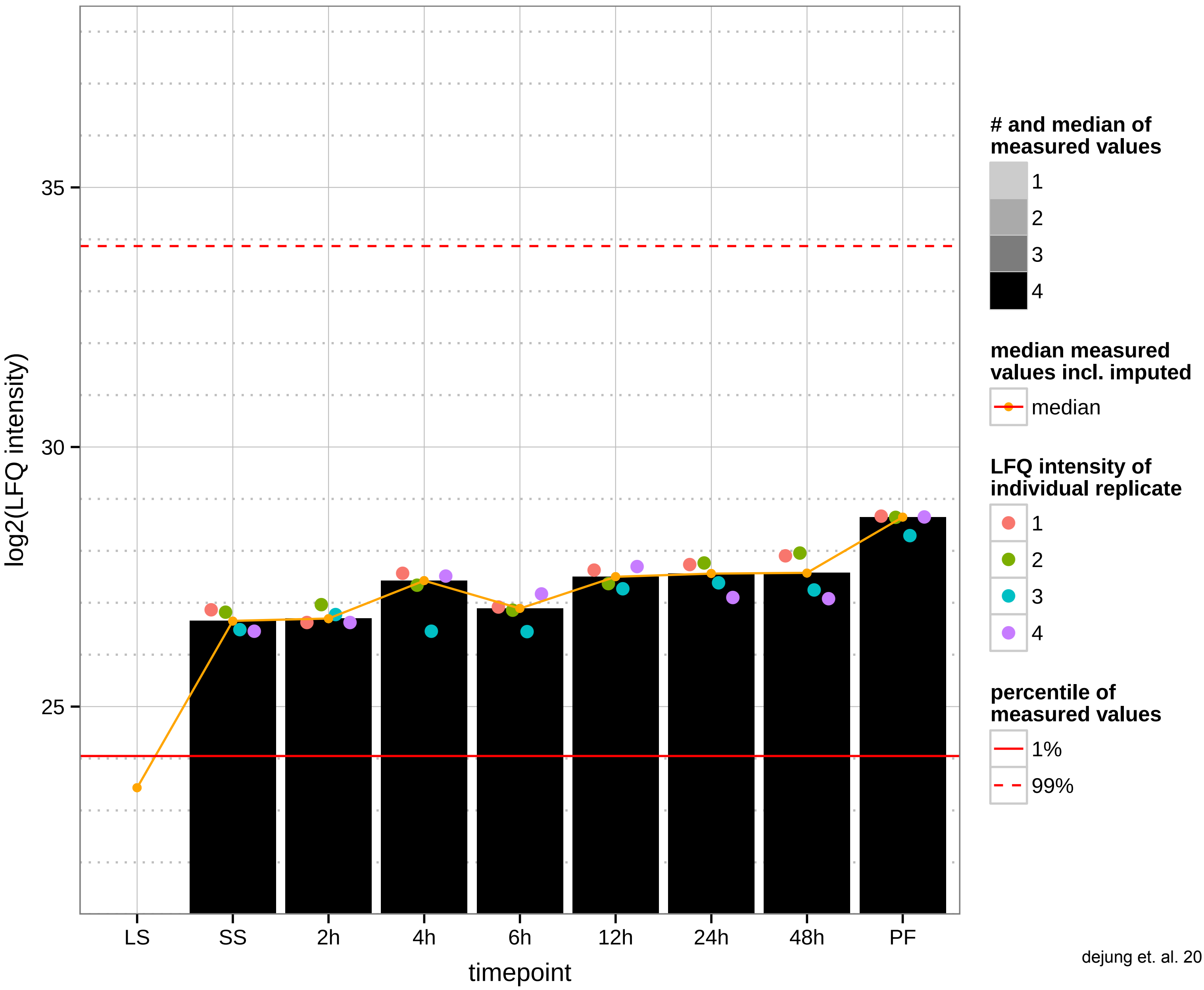

dejung et. al. 2015

hypothetical protein, conserved
Tb11.v5.0517;Tb927.11.4050
AGOF: null
AGOC: null
AGOP: null
PGOF: null
PGOC: null
PGOP: null

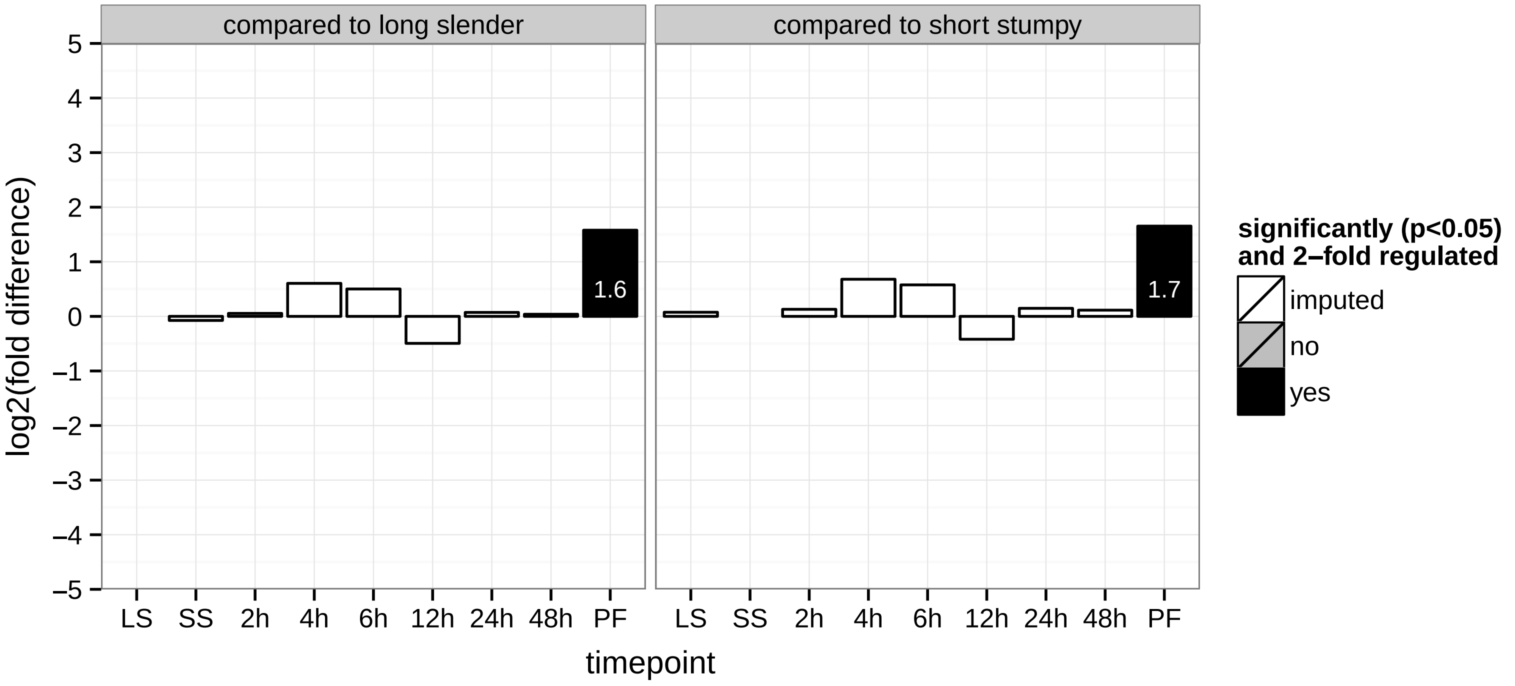

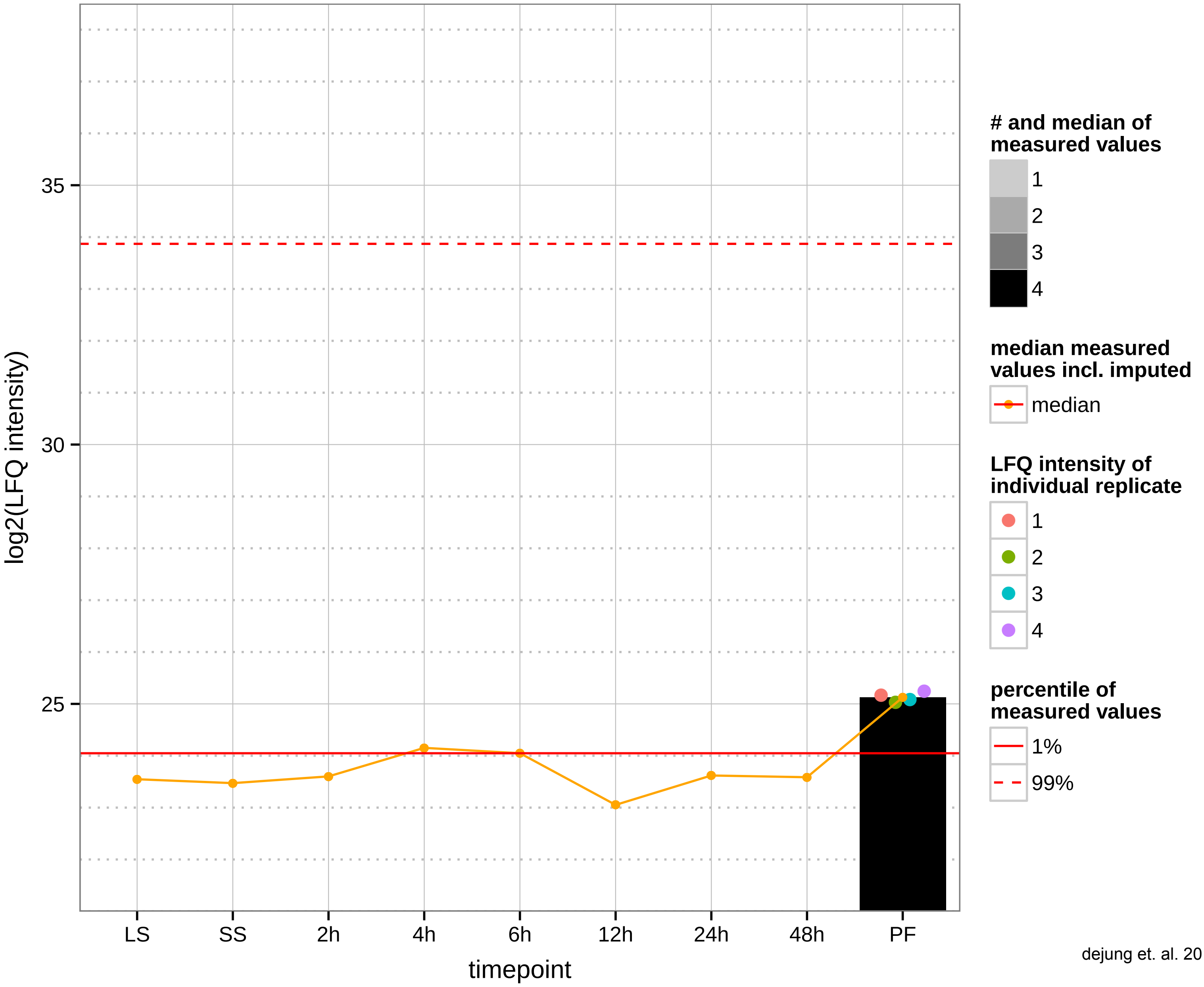

dejung et. al. 2015

hypothetical protein, conserved
Tb927.8.3320;Tb11.v5.0519
AGOF: null
AGOC: null, mitochondrion
AGOP: null
PGOF: protein binding
PGOC: null
PGOP: null

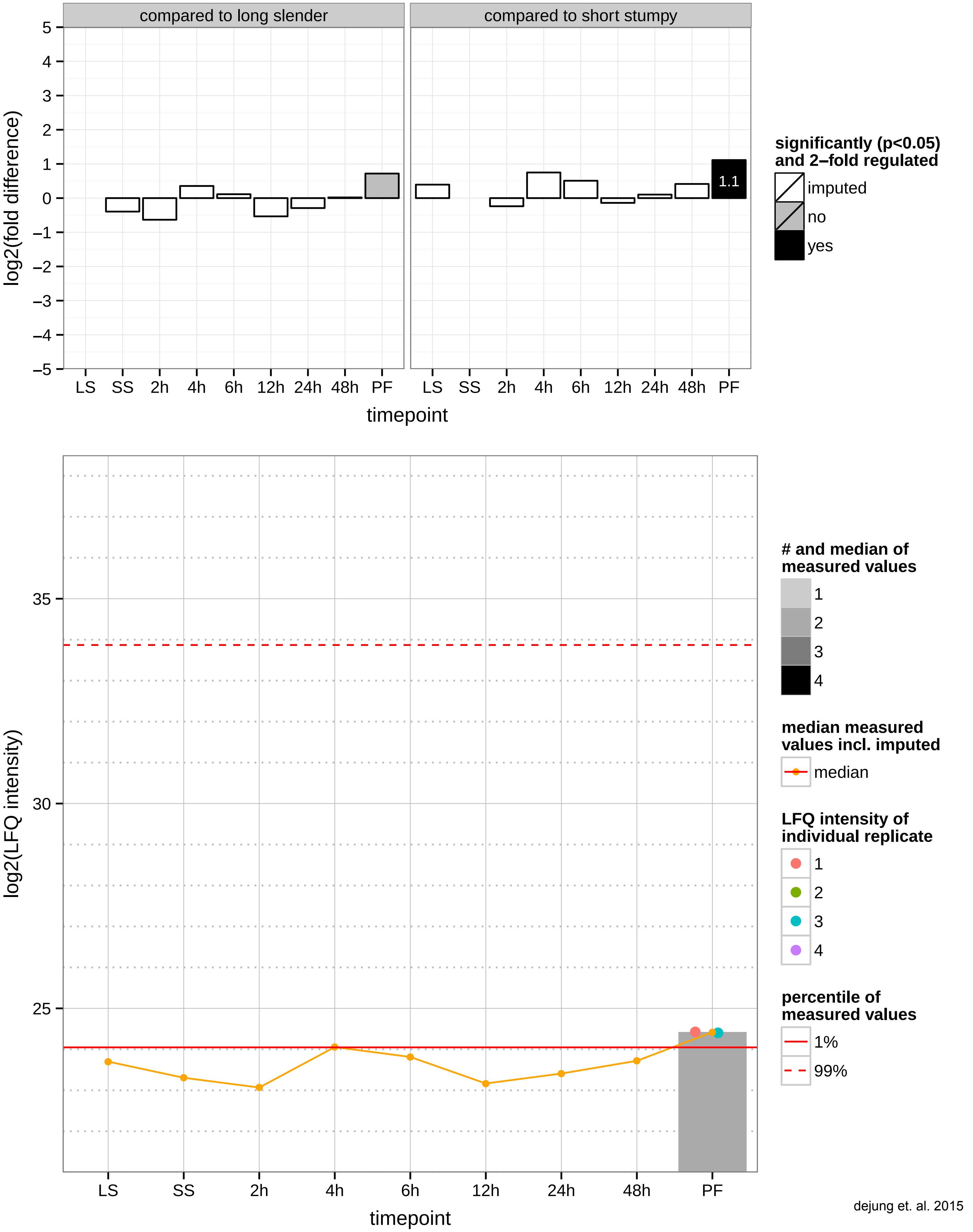

dejung et. al. 2015

hypothetical protein, conserved, leucine−rich repeat−containing protein
Tb927.1.780;Tb11.v5.0523
AGOF: null
AGOC: null
AGOP: null
PGOF: null
PGOC: null
PGOP: null

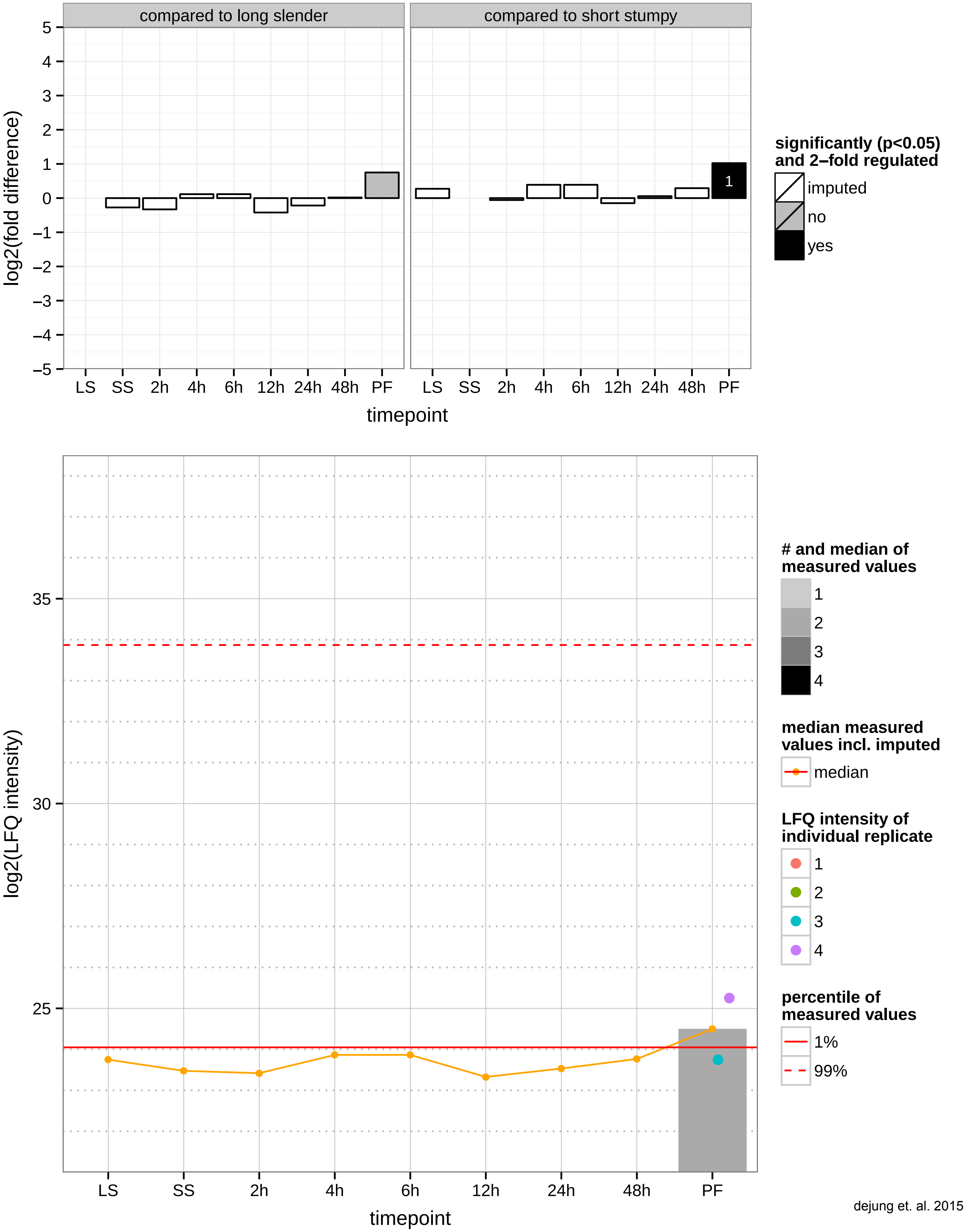

dejung et. al. 2015

protein kinase, putative
Tb927.7.3210;Tb11.v5.0652
AGOF: null, ATP binding, protein kinase activity, protein serine/threonine kinase activity, protein tyrosine kinase activity
AGOC: null
AGOP: null, growth, protein phosphorylation
PGOF: ATP binding, protein kinase activity, transferase activity, transferring phosphorus–containing groups
PGOC: null
PGOP: protein phosphorylation

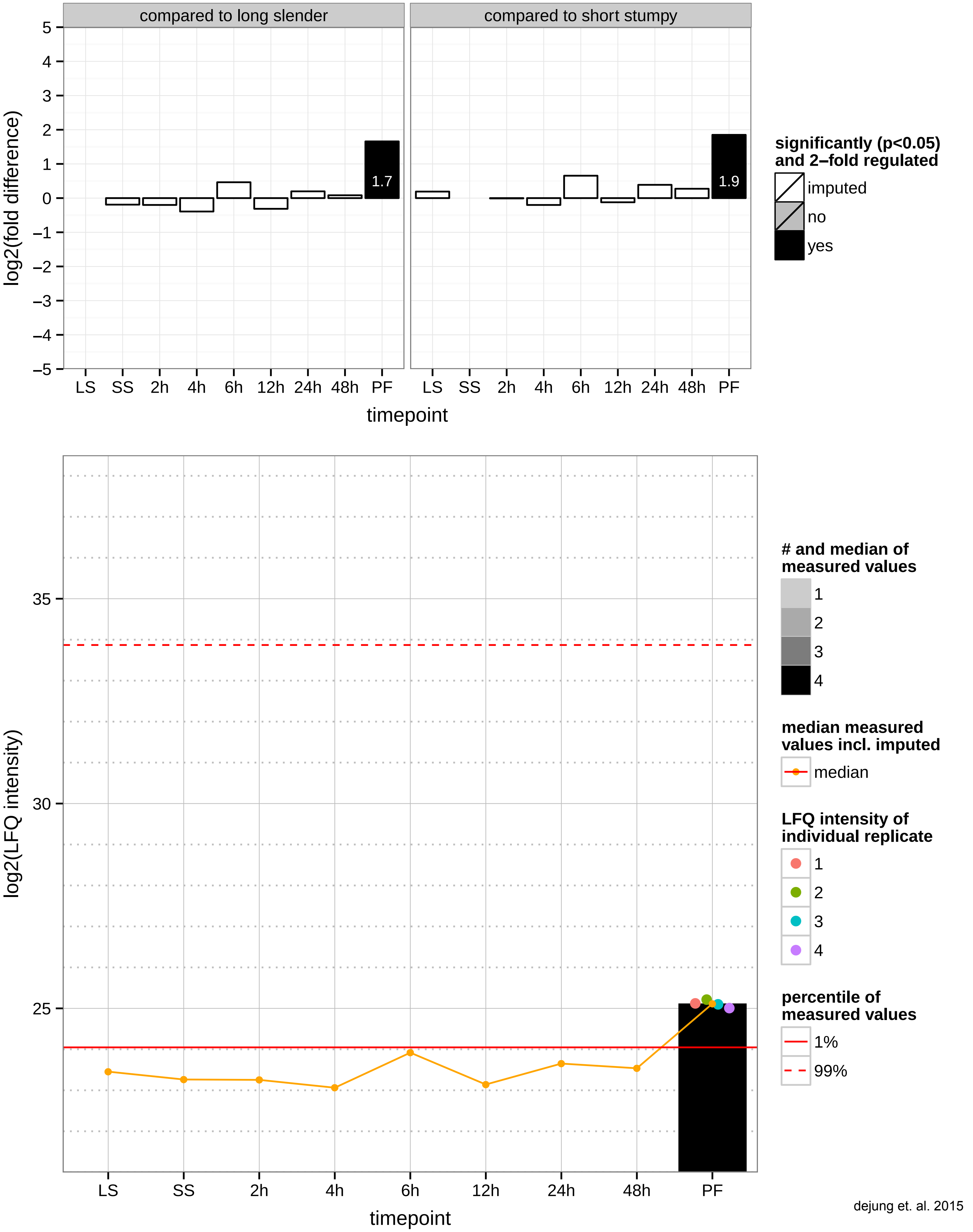

dejung et. al. 2015

tatD related deoxyribonuclease, putative
Tb927.11.6090;Tb11.v5.0746
AGOF: null, deoxyribonuclease activity, endodeoxyribonuclease activity, producing 5'−phosphomonoesters
AGOC: null
AGOP: null, metabolic process
PGOF: endodeoxyribonuclease activity, producing 5'−phosphomonoesters
PGOC: null
PGOP: null

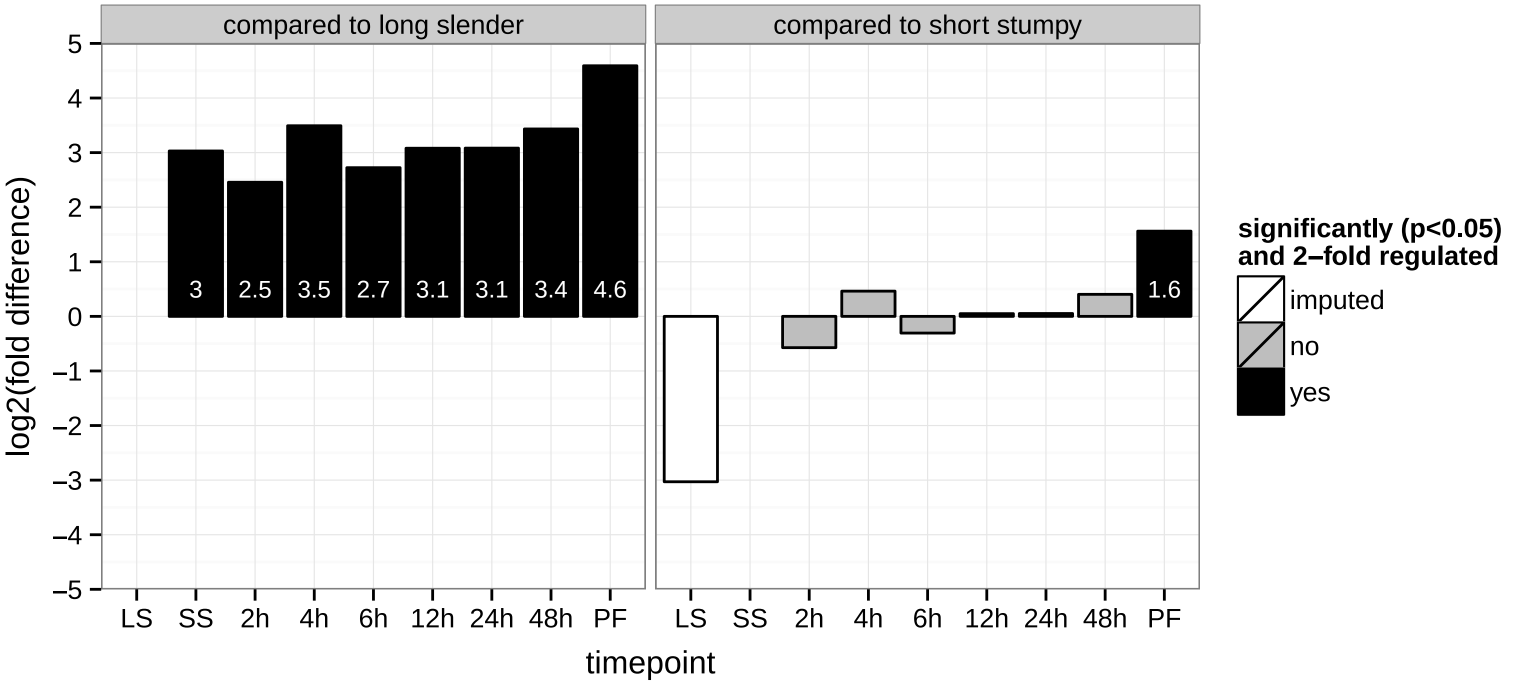

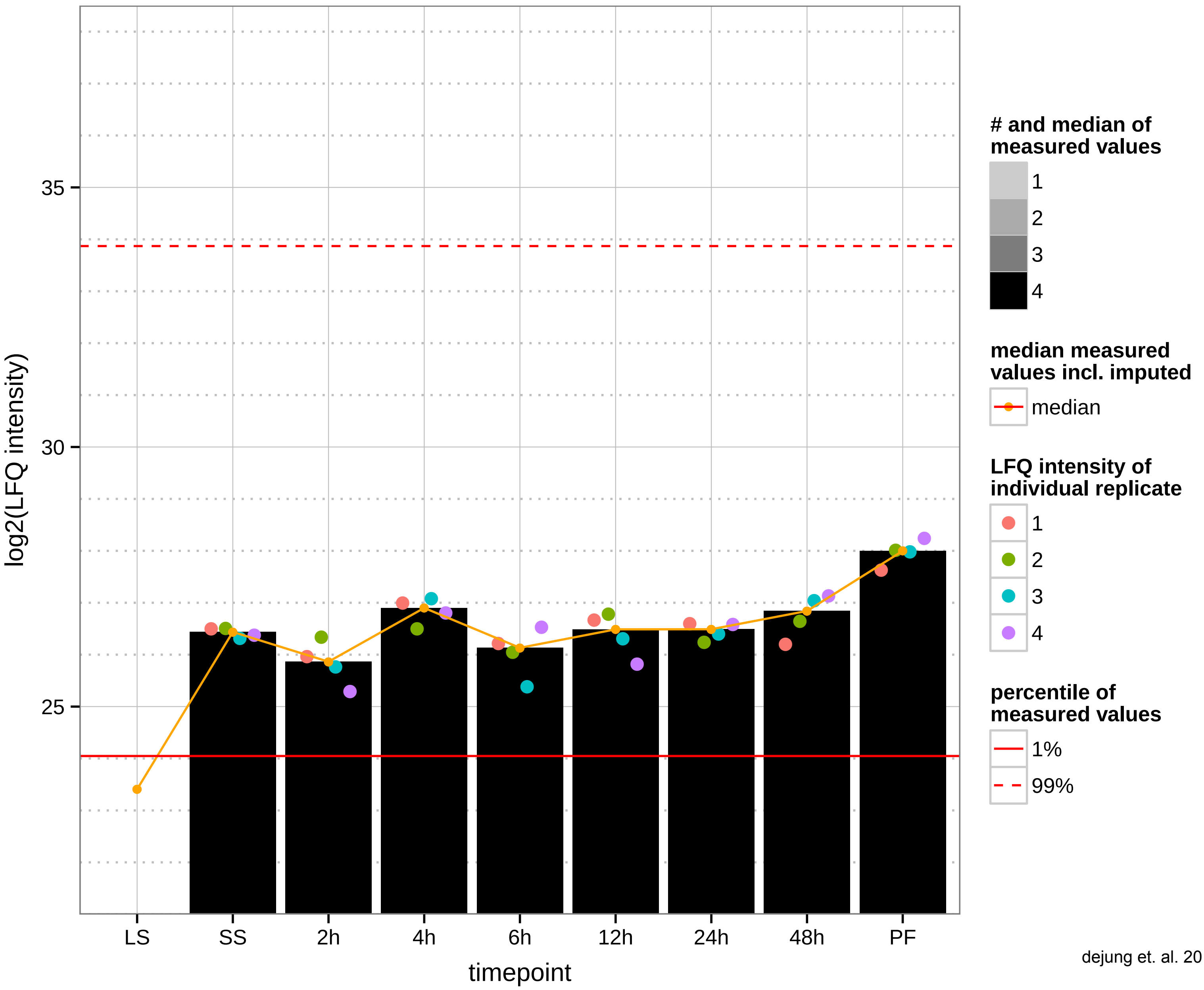

dejung et. al. 2015

UDP–Gal or UDP–GlcNAc–dependent glycosyltransferase, putative
Tb927.4.5300;Tb11.v5.0880;Tb927.4.5290;Tb927.4.5270;Tb927.4.5260;Tb927.4.5250;Tb927.4.5280
AGOF: null, UDP–glycosyltransferase activity, galactosyltransferase activity
AGOC: null, integral to membrane, membrane, membrane
AGOP: null, GPI anchor biosynthetic process, protein glycosylation
PGOF: galactosyltransferase activity, null
PGOC: membrane, null
PGOP: protein glycosylation, null

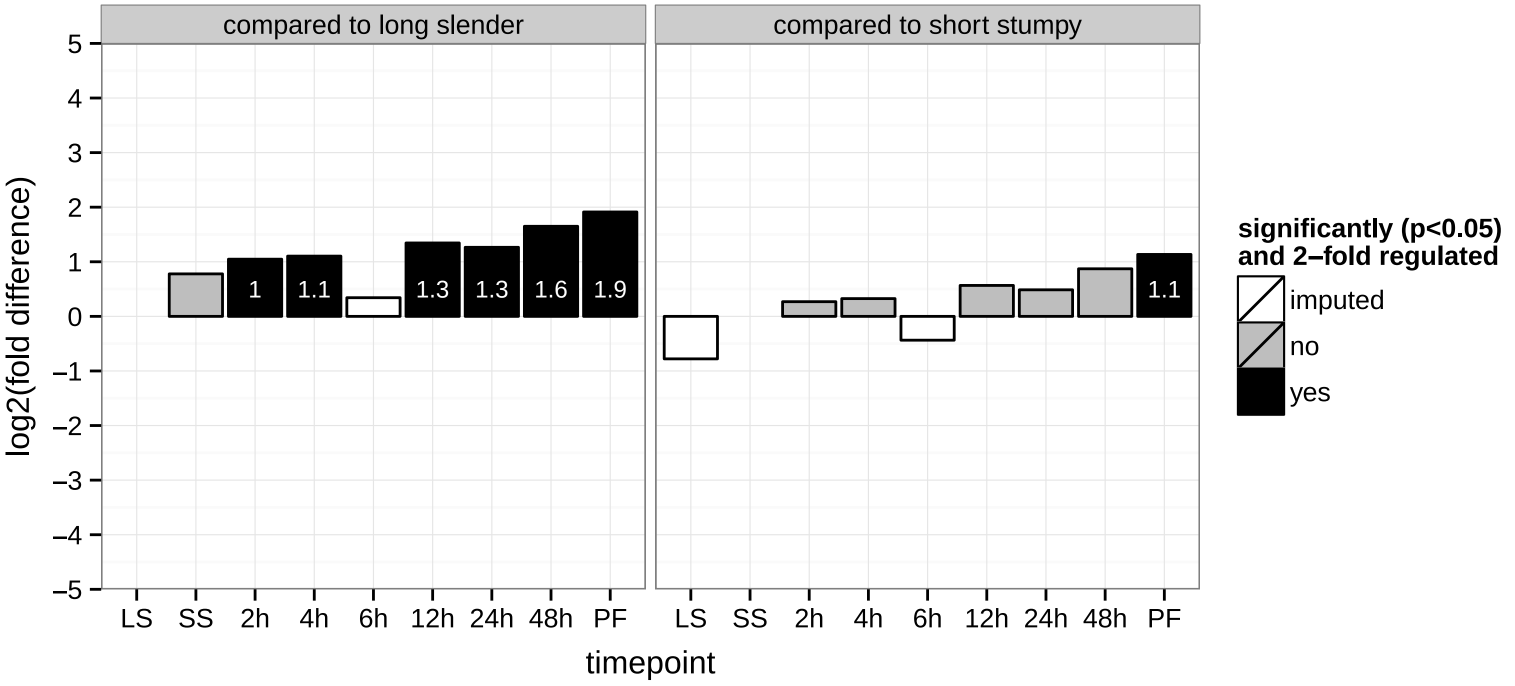

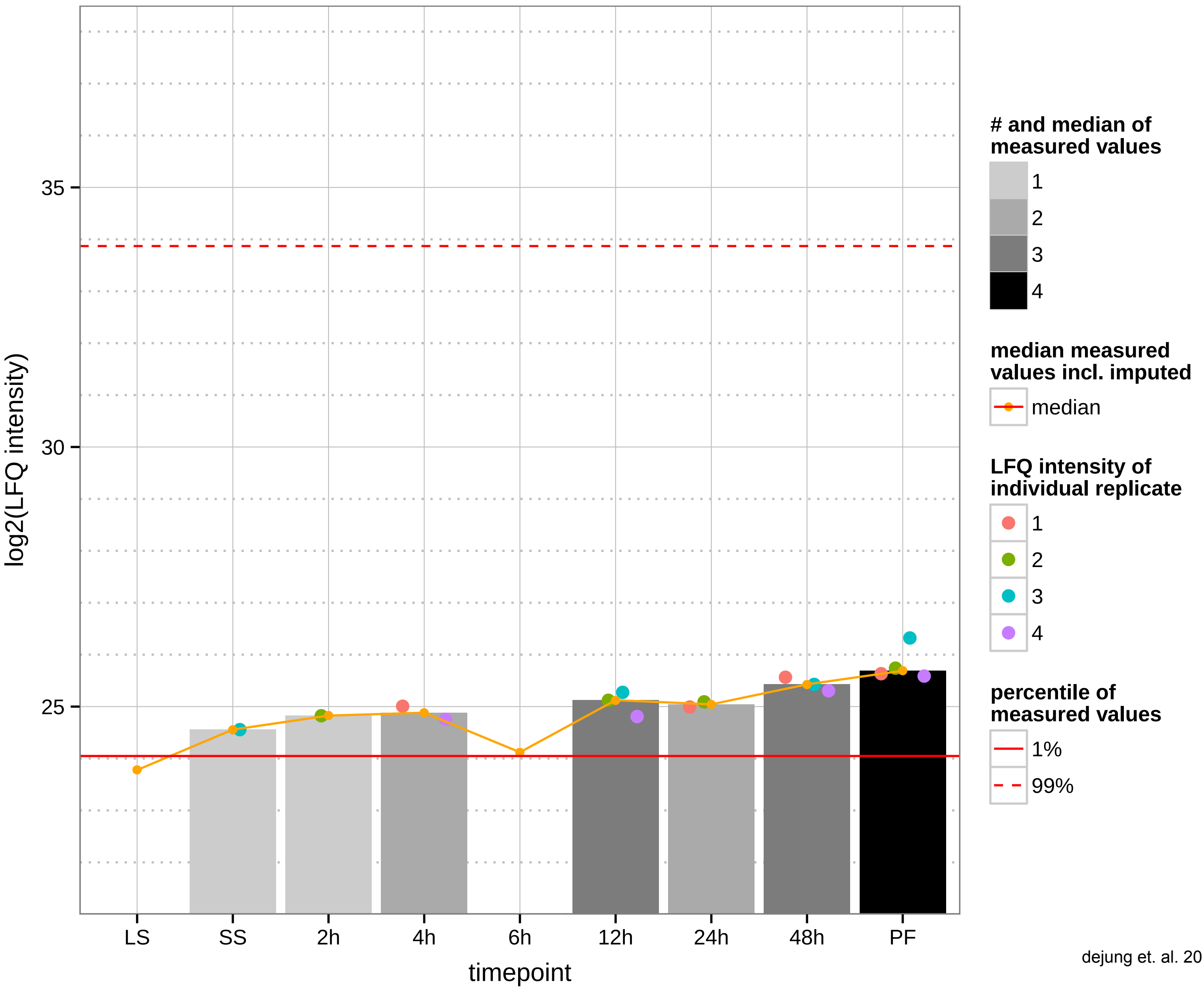

dejung et. al. 2015

hypothetical protein, conserved
Tb927.4.3080;Tb11.v5.0963
AGOF: null
AGOC: null, mitochondrion
AGOP: null
PGOF: null
PGOC: null
PGOP: null

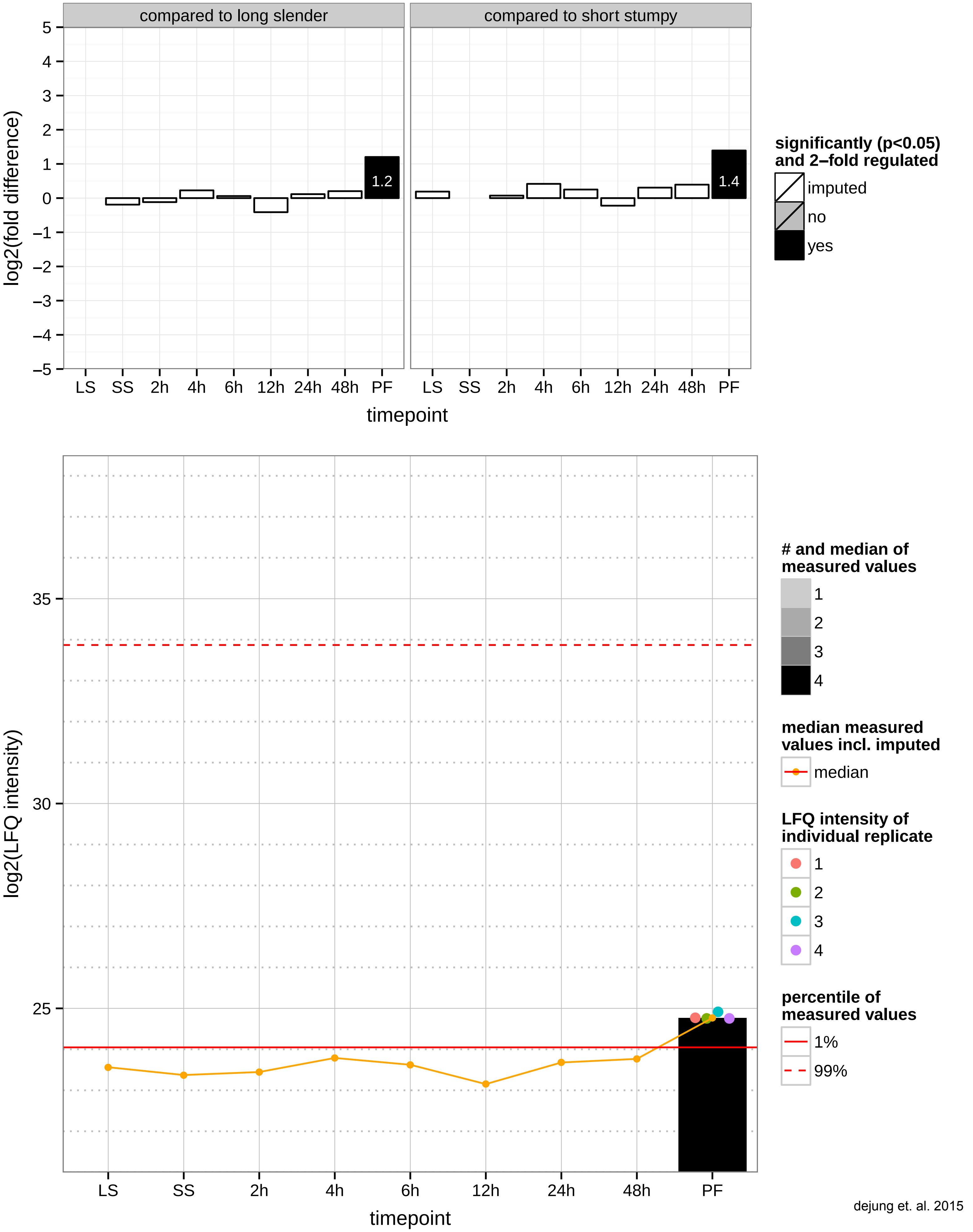

dejung et. al. 2015

hypothetical protein, conserved
Tb927.4.2990;Tb11.v5.0968
AGOF: null, phosphorus–oxygen lyase activity
AGOC: null
AGOP: null, cyclic nucleotide biosynthetic process, intracellular signal transduction
PGOF: phosphorus–oxygen lyase activity
PGOC: null
PGOP: cyclic nucleotide biosynthetic process, intracellular signal transduction

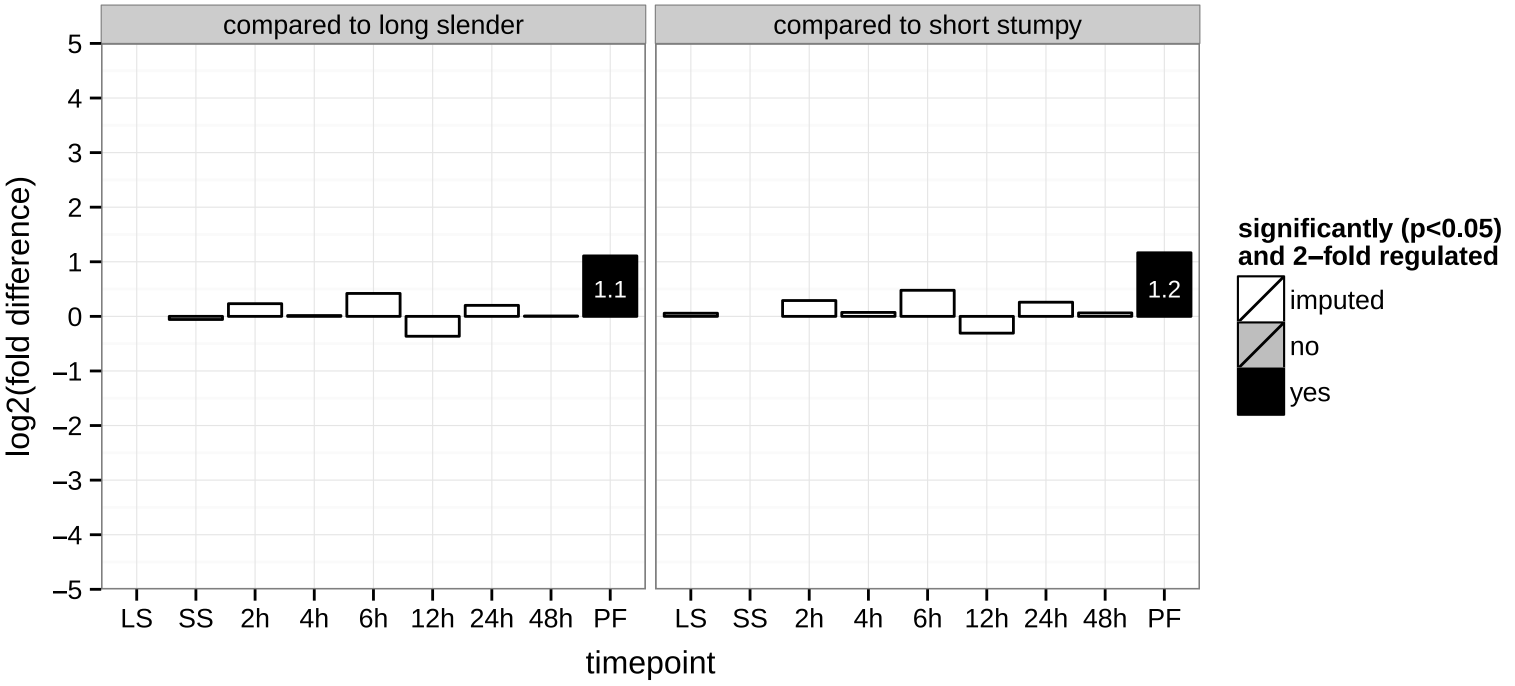

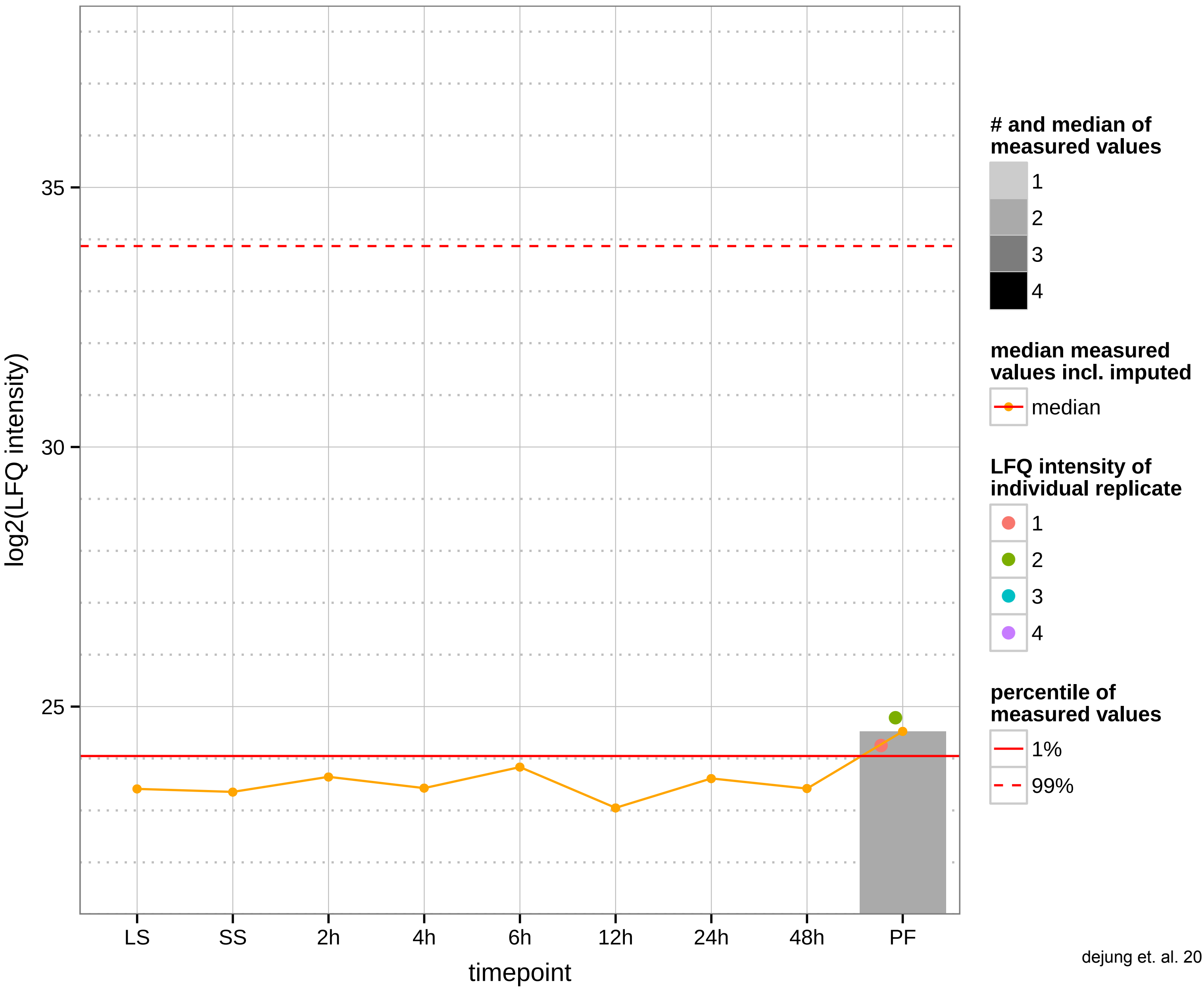

dejung et. al. 2015

protein kinase, putative
Tb927.10.3340;Tb11.v5.1022
AGOF: null, ATP binding, protein serine/threonine kinase activity, protein tyrosine kinase activity
AGOC: null
AGOP: null, protein phosphorylation
PGOF: ATP binding, protein kinase activity, transferase activity, transferring phosphorus–containing groups
PGOC: null
PGOP: protein phosphorylation

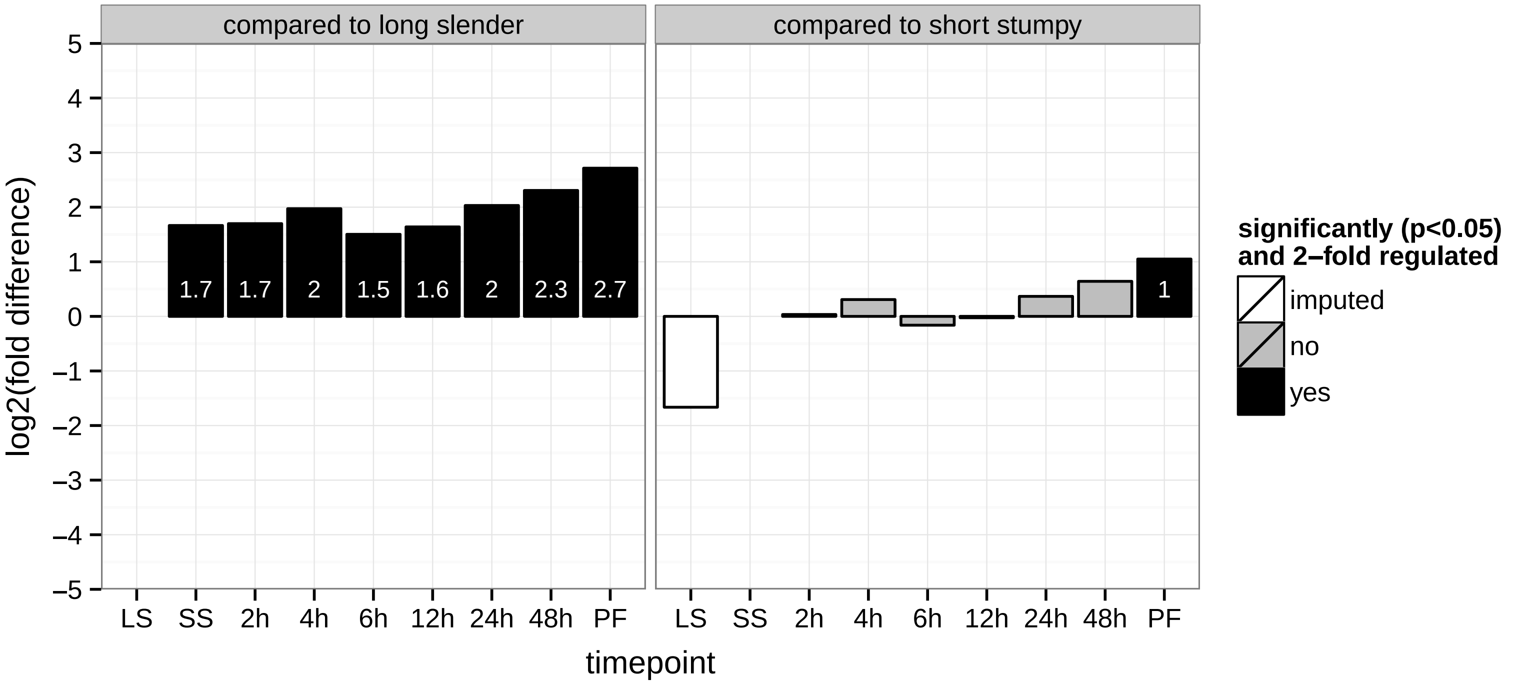

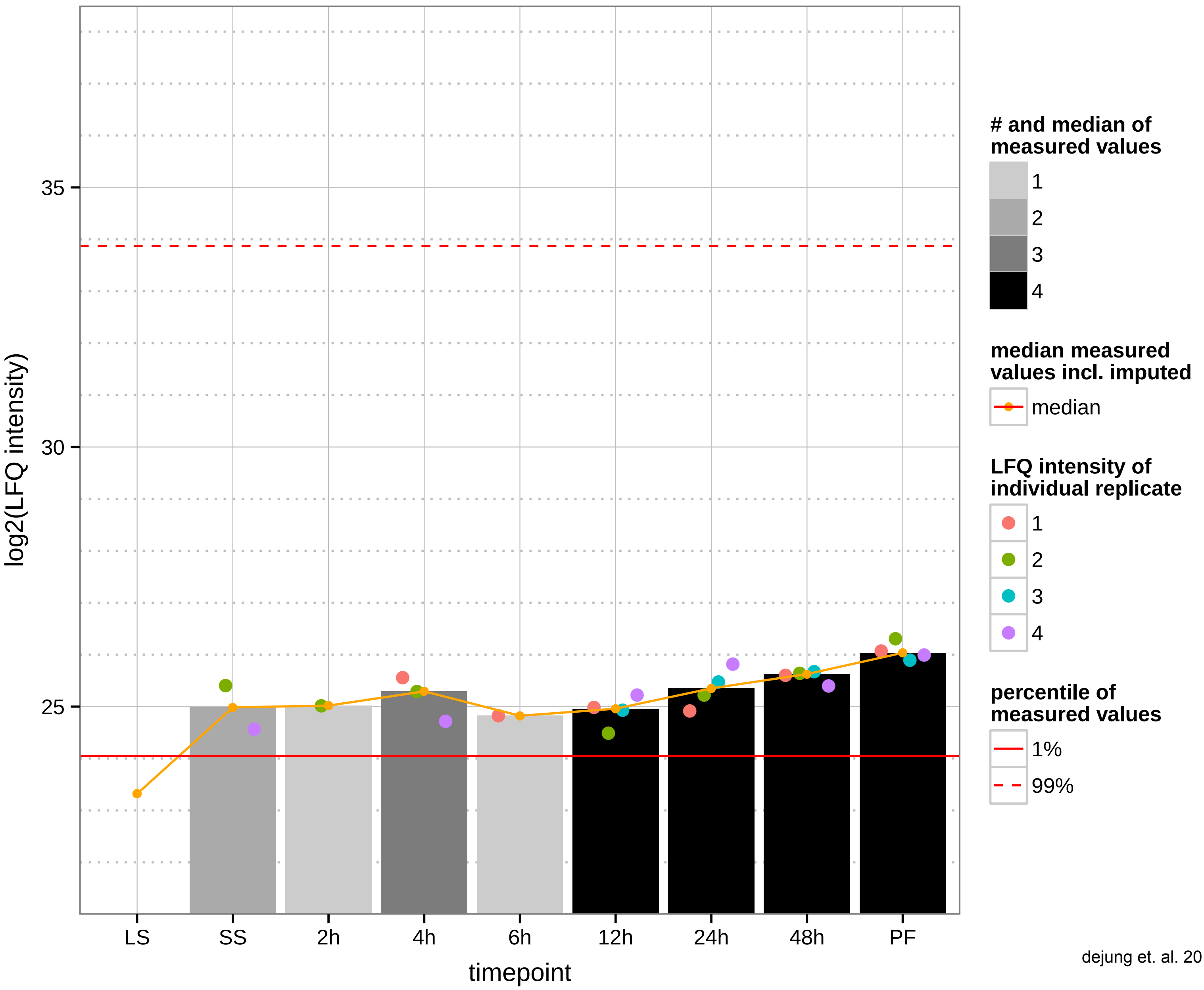

dejung et. al. 2015

40S ribosomal protein S4, putative
Tb927.11.3600;Tb927.11.3590;Tb11.v5.1059
AGOF: null, RNA binding, structural constituent of ribosome
AGOC: null, intracellular, ribosome
AGOP: null, translation
PGOF: RNA binding, structural constituent of ribosome, null
PGOC: intracellular, ribosome, null
PGOP: translation, null

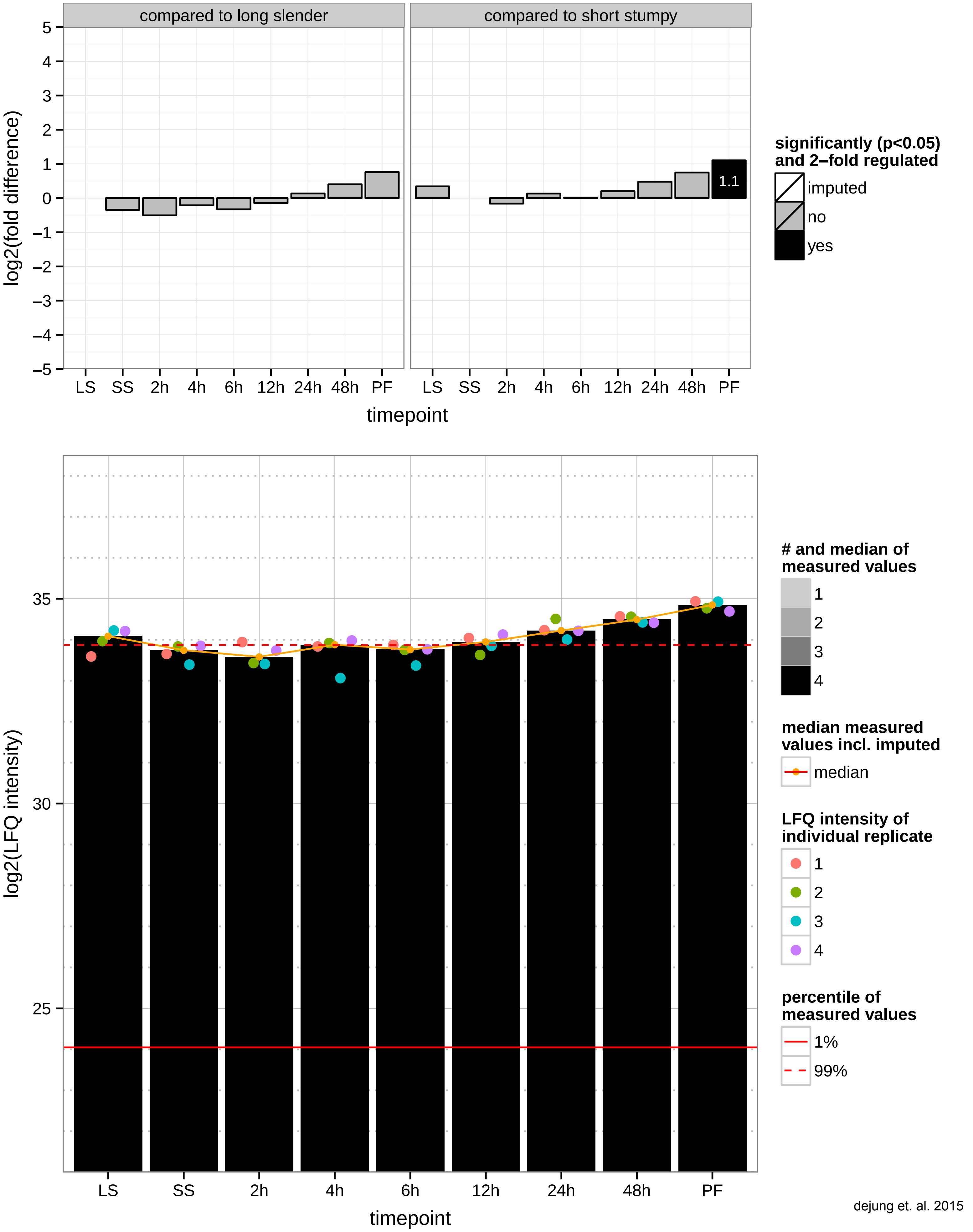

dejung et. al. 2015

Tubulin/FtsZ family, putative
Tb927.1.1540
AGOF: null
AGOC: null
AGOP: null
PGOF: null
PGOC: protein complex
PGOP: protein polymerization

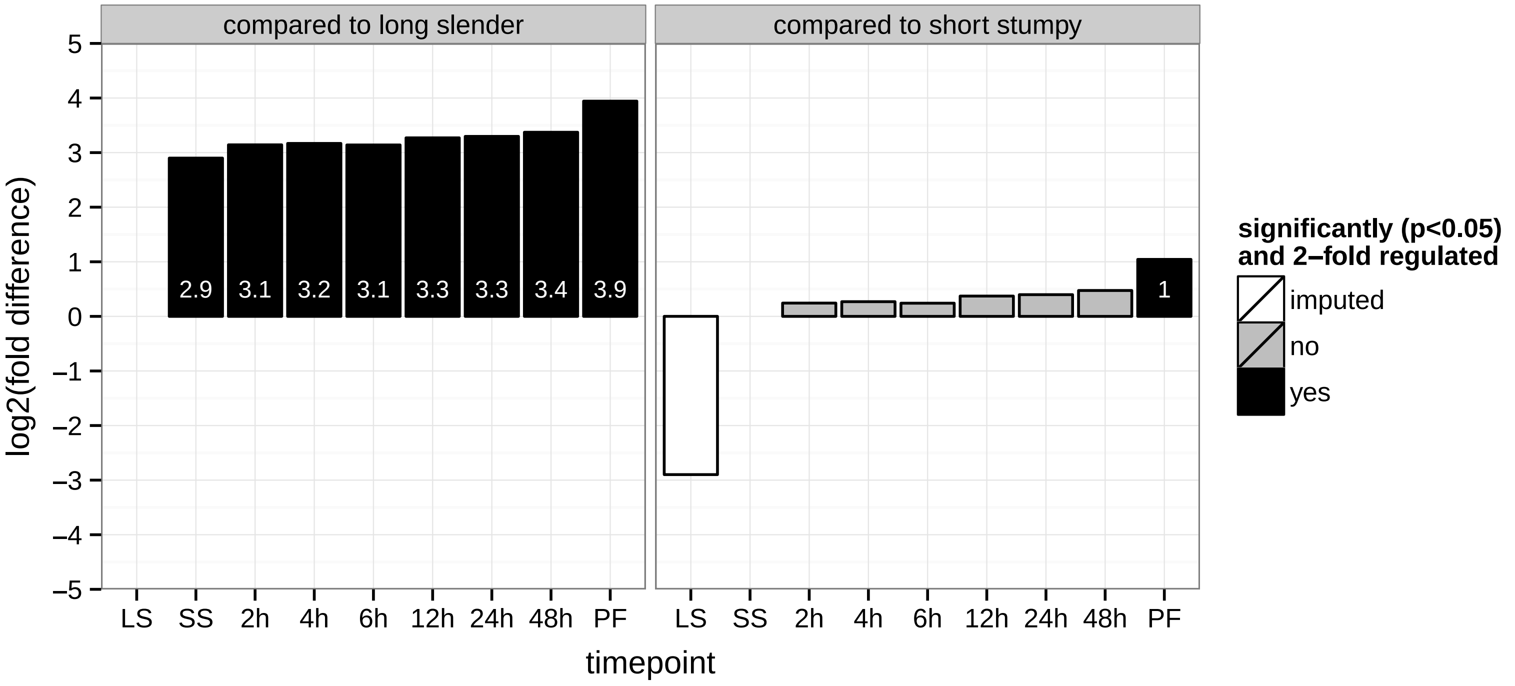

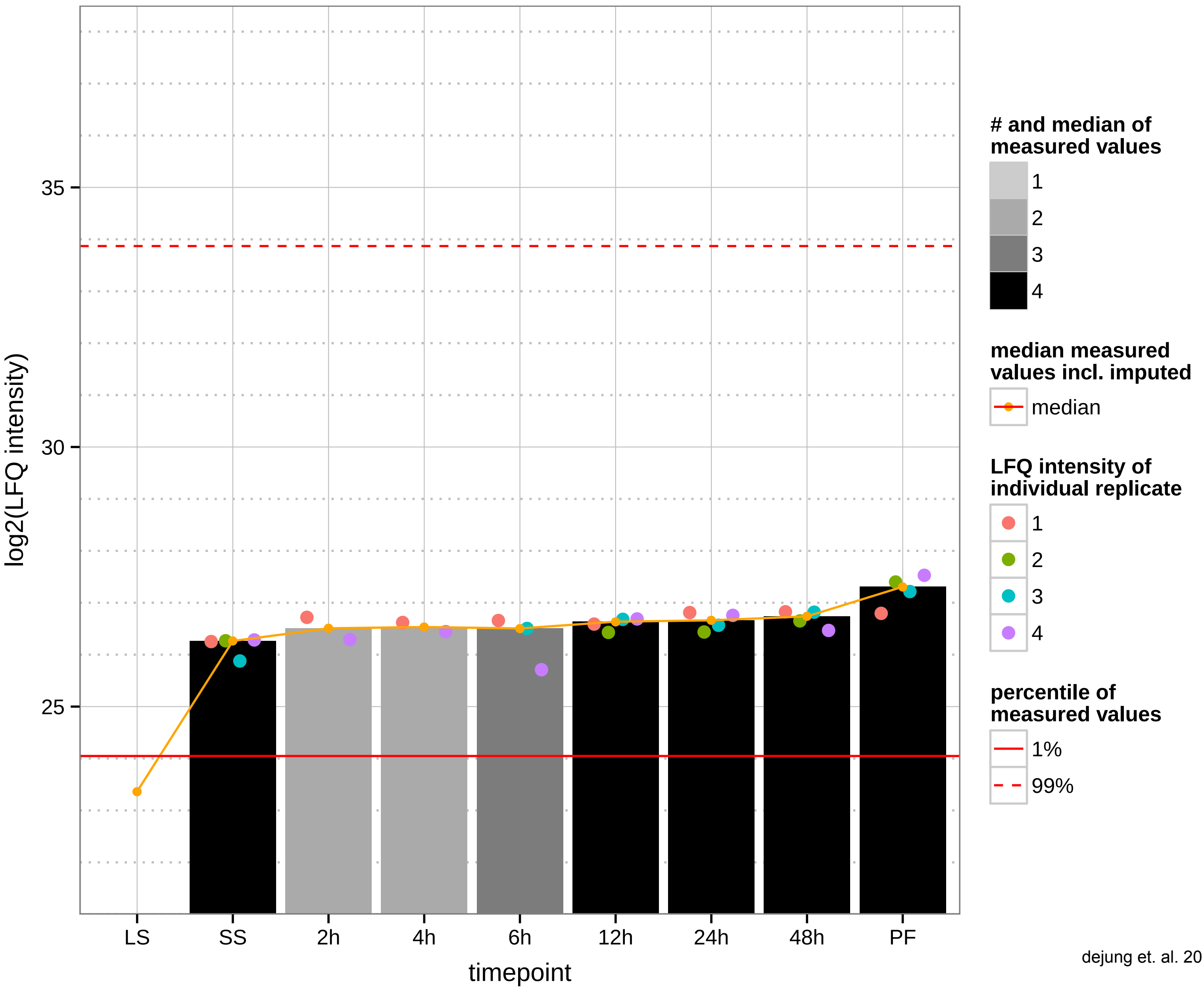

dejung et. al. 2015

tubulin–tyrosine ligase, putative
Tb927.1.1550
AGOF: tubulin–tyrosine ligase activity
AGOC: null
AGOP: cellular protein modification process
PGOF: tubulin–tyrosine ligase activity
PGOC: null
PGOP: cellular protein modification process

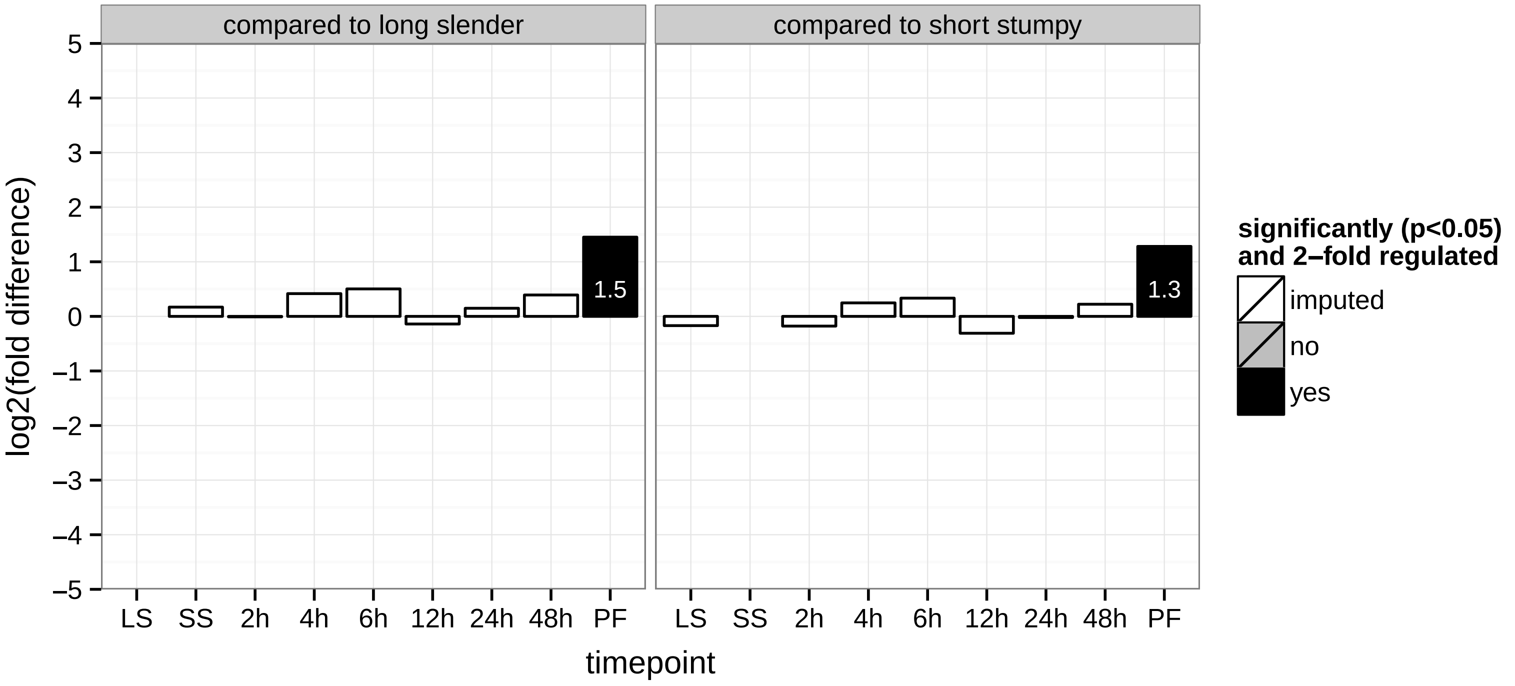

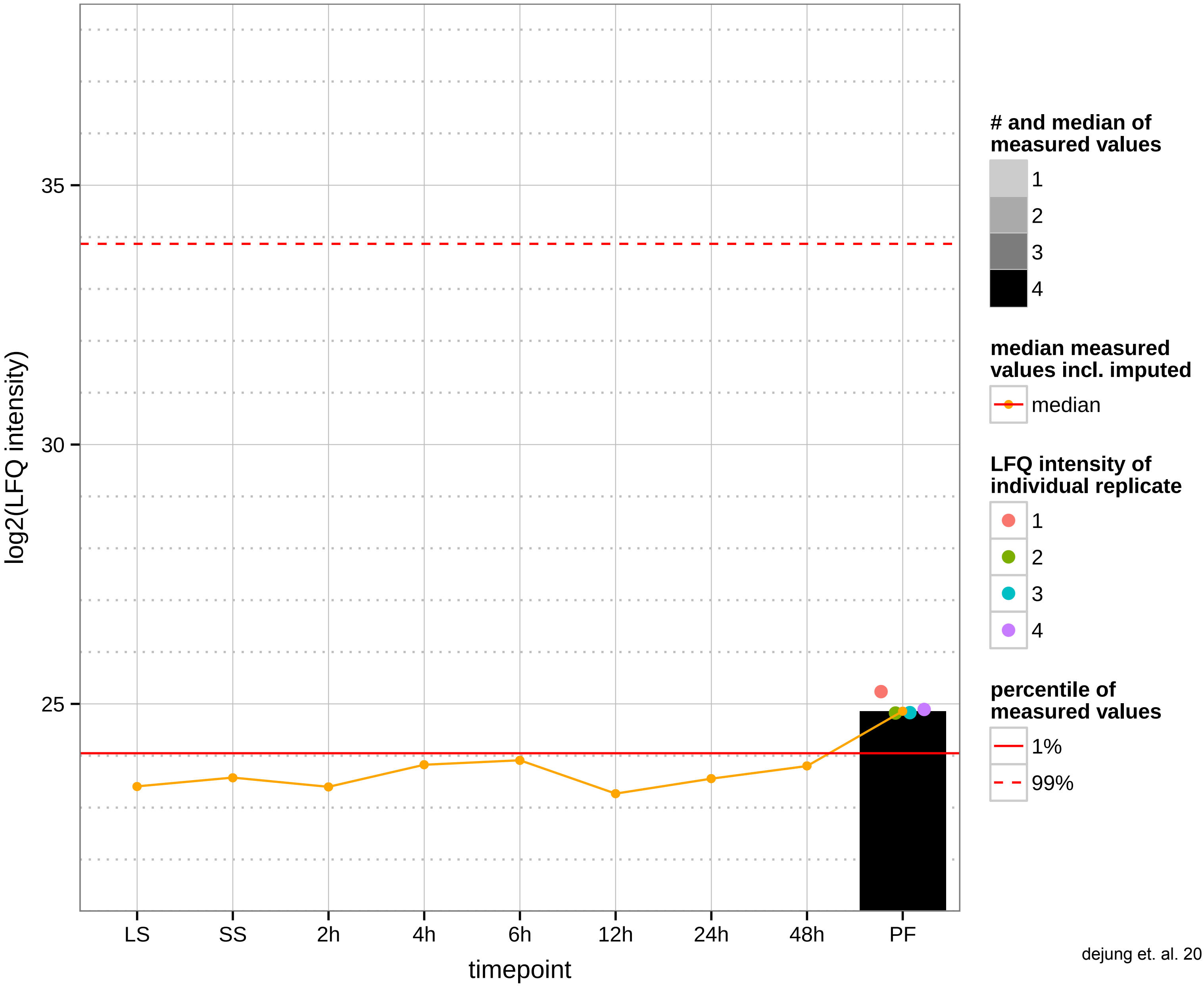

dejung et. al. 2015

RNasePH–like protein, exosome–associated protein 1, Rrp42 homologue (TbEAP1)
Tb927.1.2580
AGOF: 3'–5'–exoribonuclease activity, RNA binding
AGOC: null
AGOP: RNA processing
PGOF: null
PGOC: null
PGOP: null

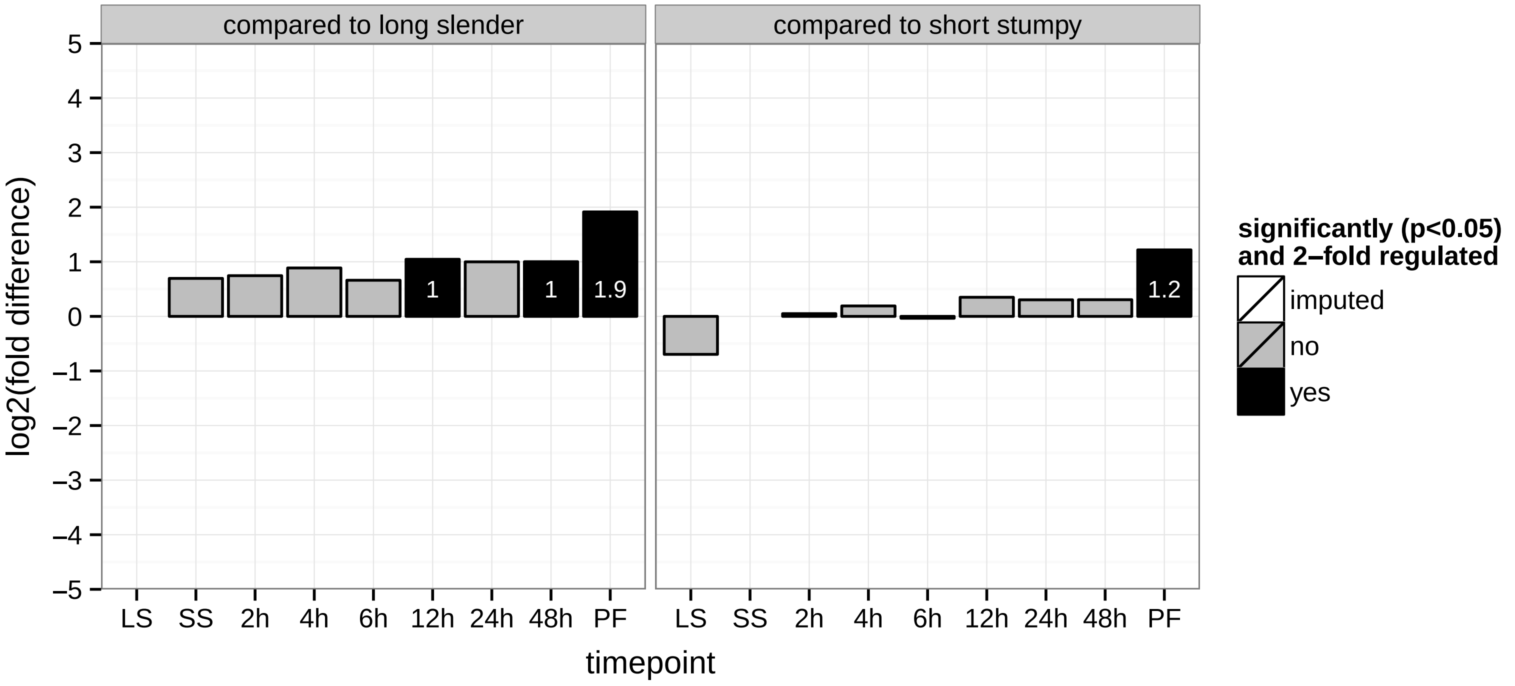

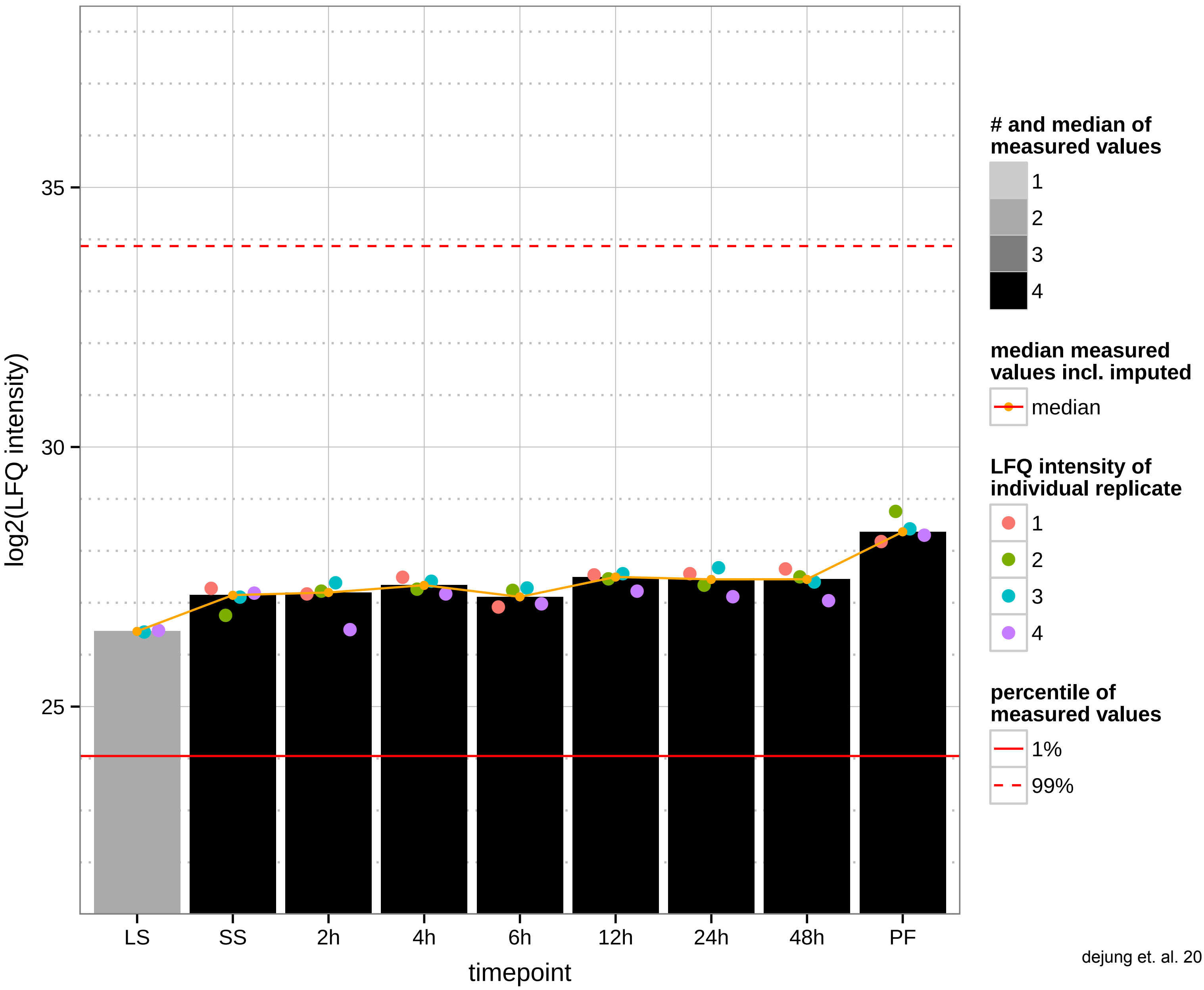

dejung et. al. 2015

hypothetical protein, conserved
Tb927.1.2750
AGOF: null
AGOC: null
AGOP: null
PGOF: null
PGOC: null
PGOP: null

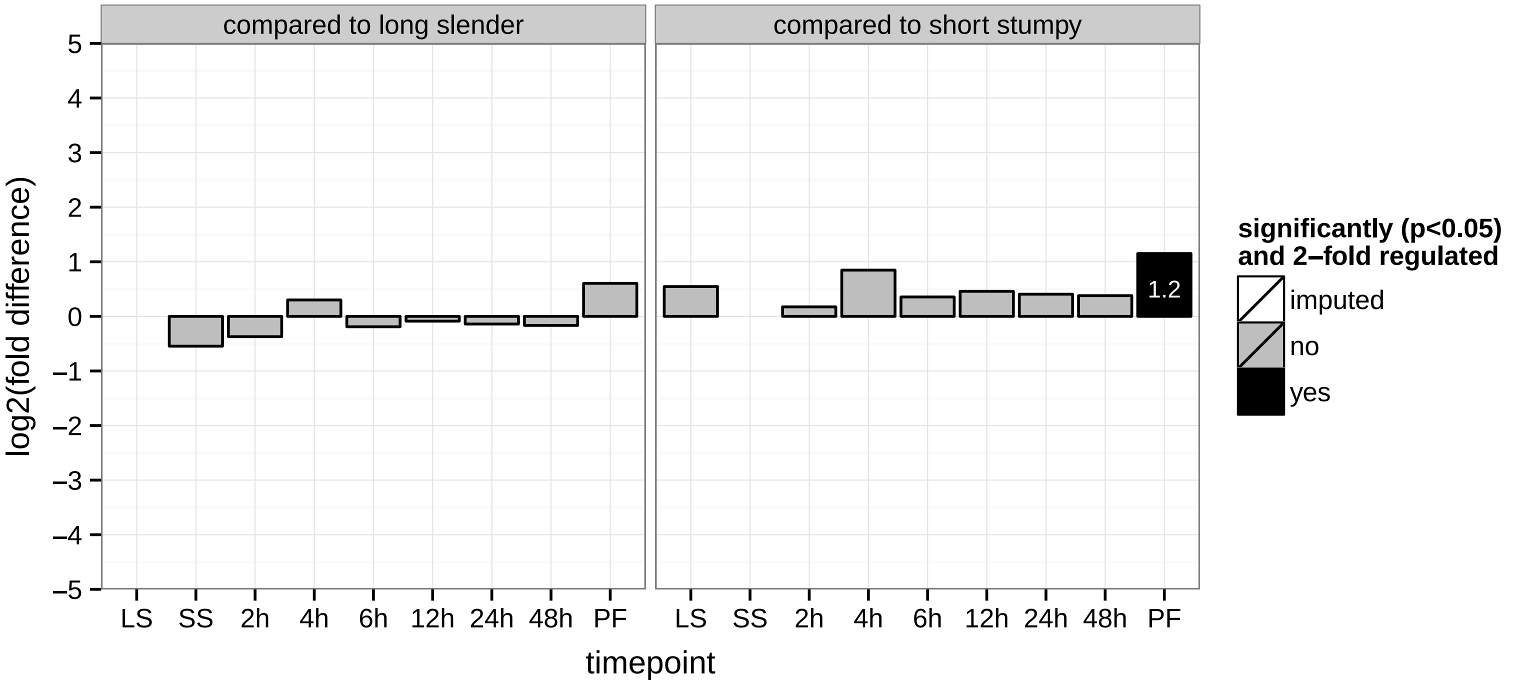

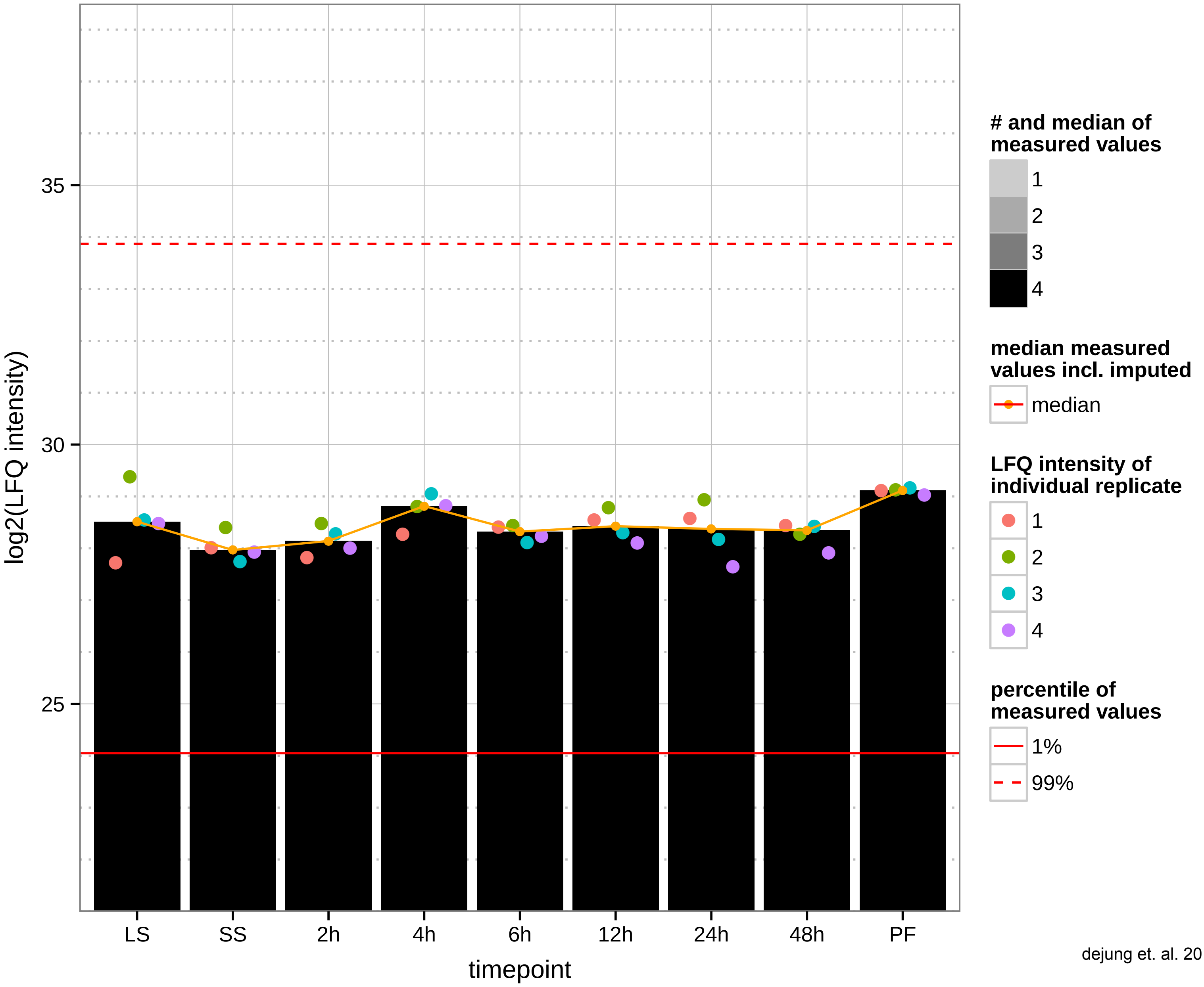

dejung et. al. 2015

mRNA processing protein, putative
Tb927.1.3010
AGOF: mRNA binding
AGOC: mitochondrion
AGOP: mRNA processing
PGOF: null
PGOC: null
PGOP: null

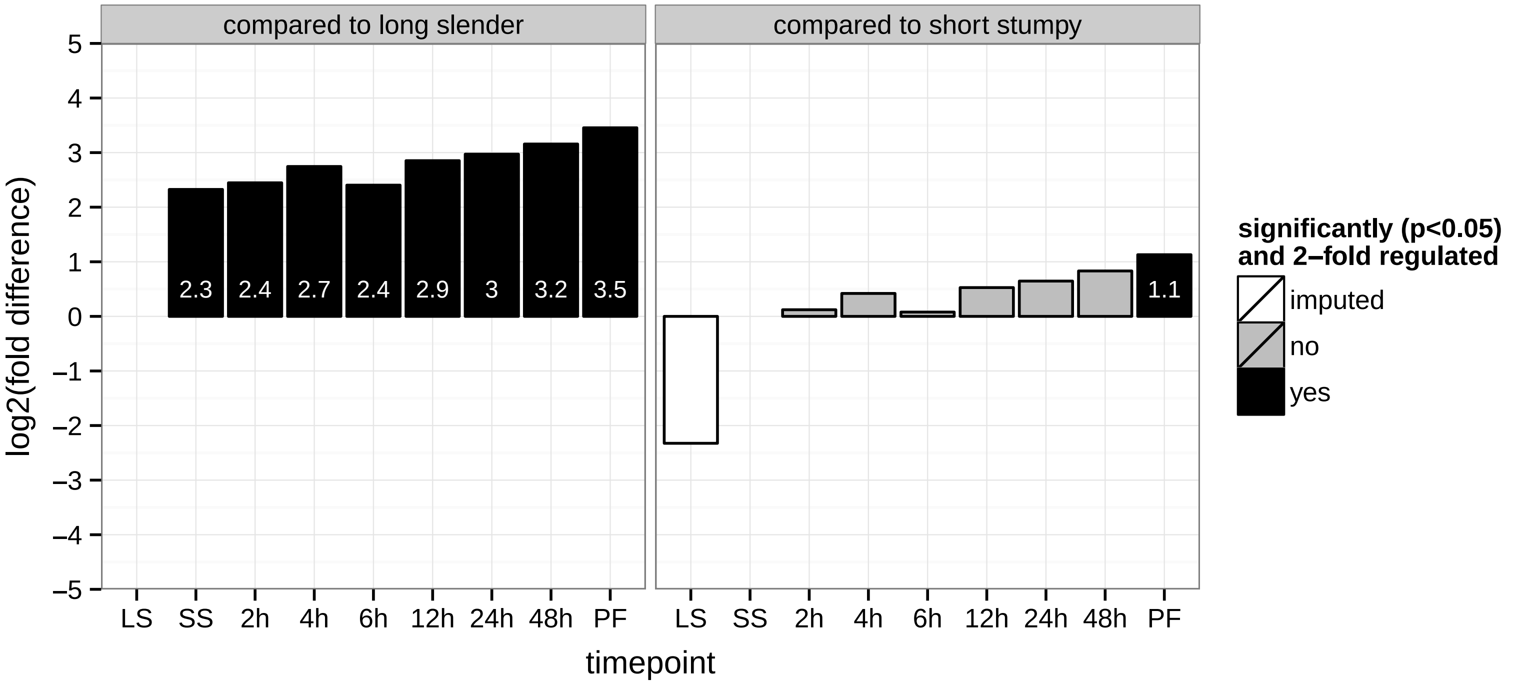

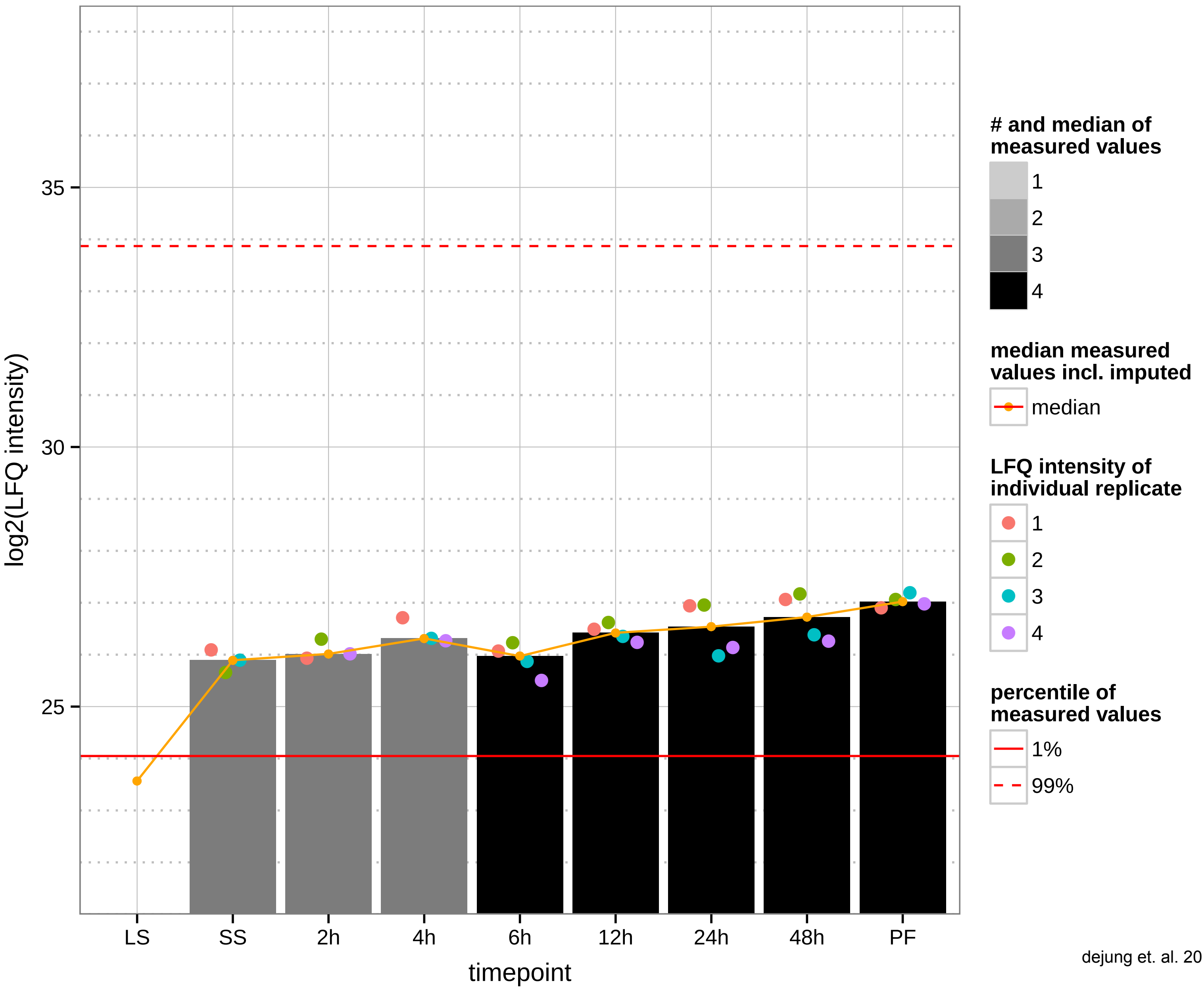

dejung et. al. 2015

hypothetical protein, conserved
Tb927.1.3050
AGOF: methyltransferase activity
AGOC: null
AGOP: metabolic process
PGOF: RNA methyltransferase activity
PGOC: null
PGOP: RNA processing

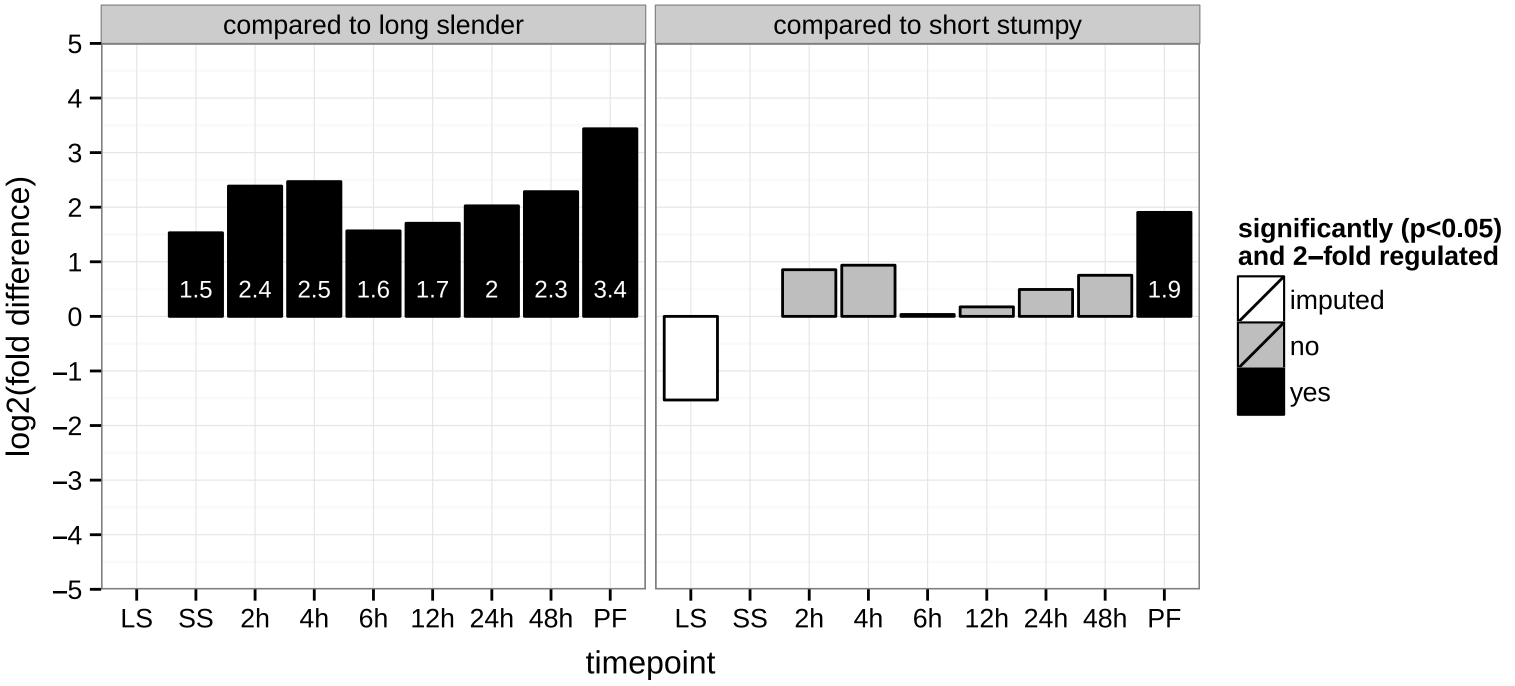

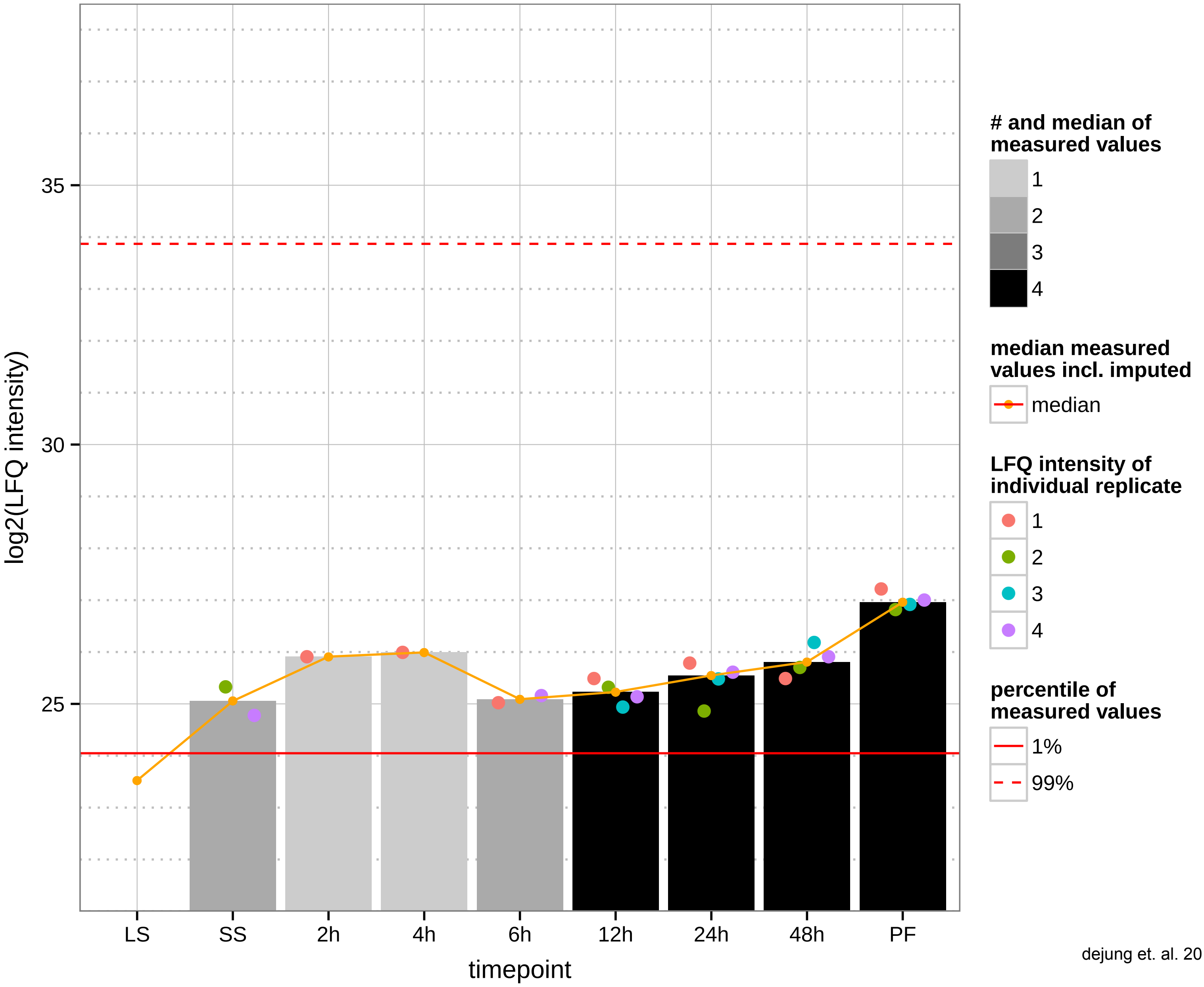

dejung et. al. 2015

conserved protein, unknown function
Tb927.1.650
AGOF: null
AGOC: null
AGOP: chromatin organization
PGOF: null
PGOC: null
PGOP: null

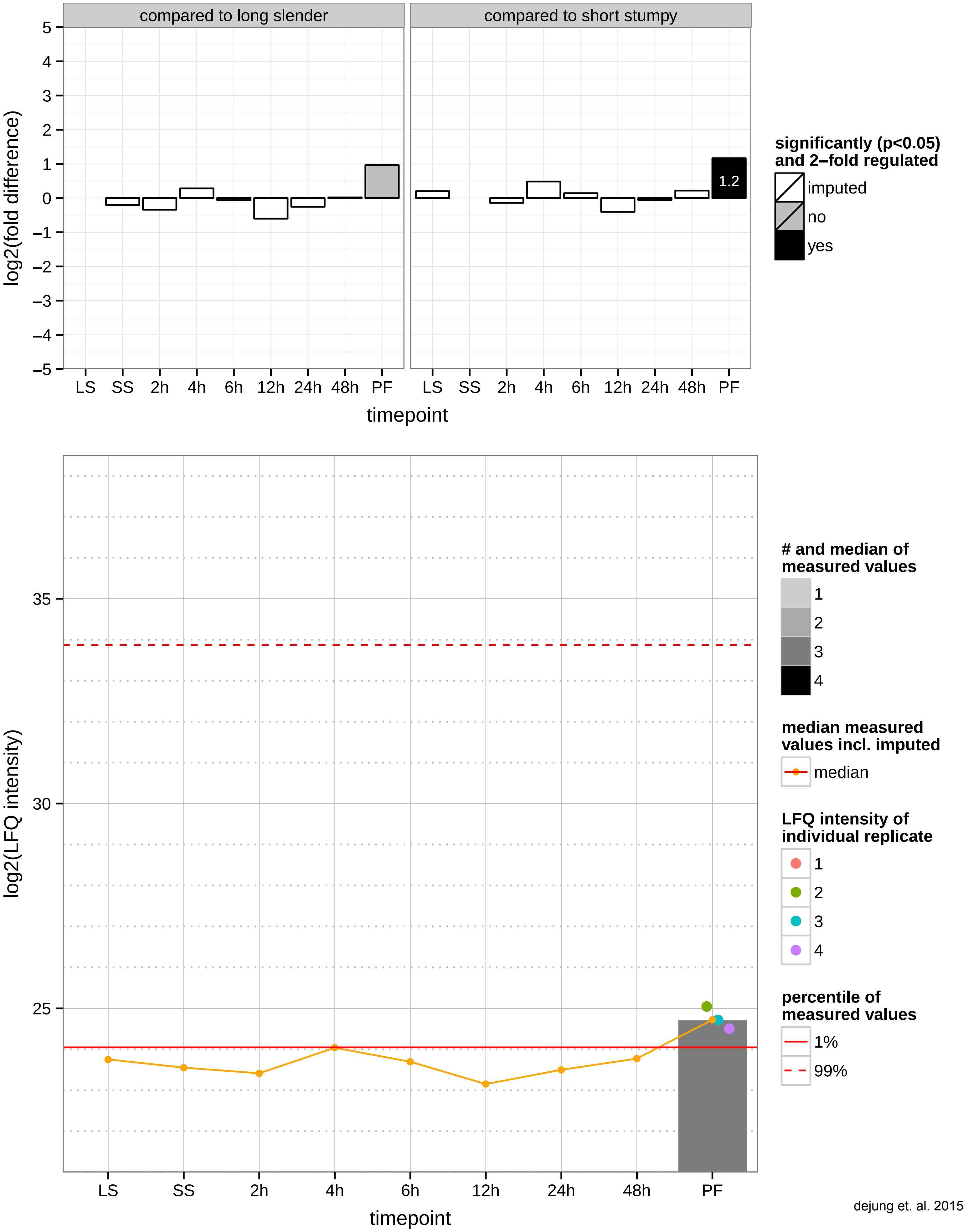

dejung et. al. 2015

phosphoglycerate kinase (PGKA)
Tb927.1.720
AGOF: phosphoglycerate kinase activity
AGOC: glycosome
AGOP: glycolysis
PGOF: phosphoglycerate kinase activity
PGOC: null
PGOP: glycolysis

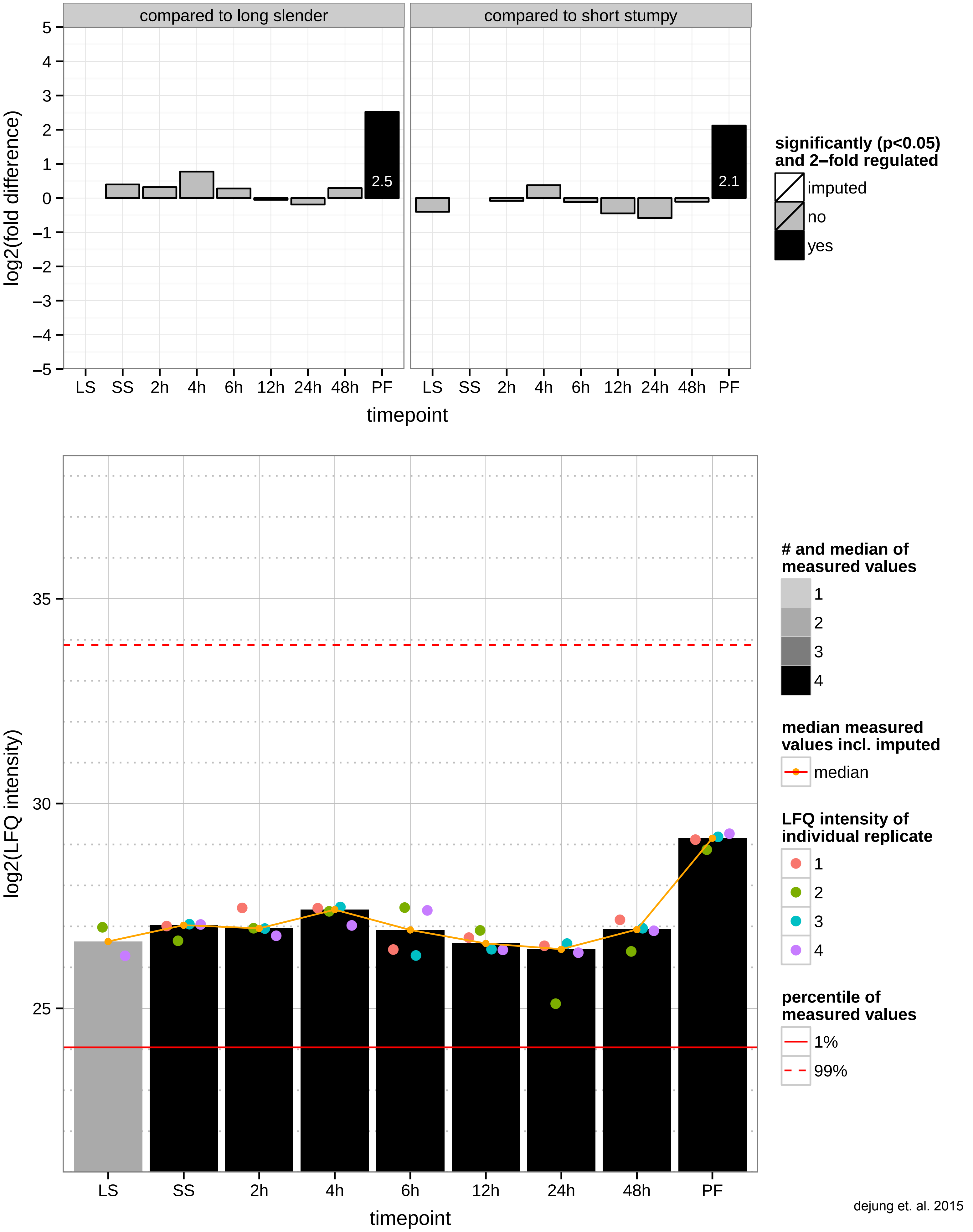

dejung et. al. 2015

predicted WD40 repeat protein
Tb927.10.10170
AGOF: null
AGOC: null
AGOP: null
PGOF: protein binding
PGOC: null
PGOP: null

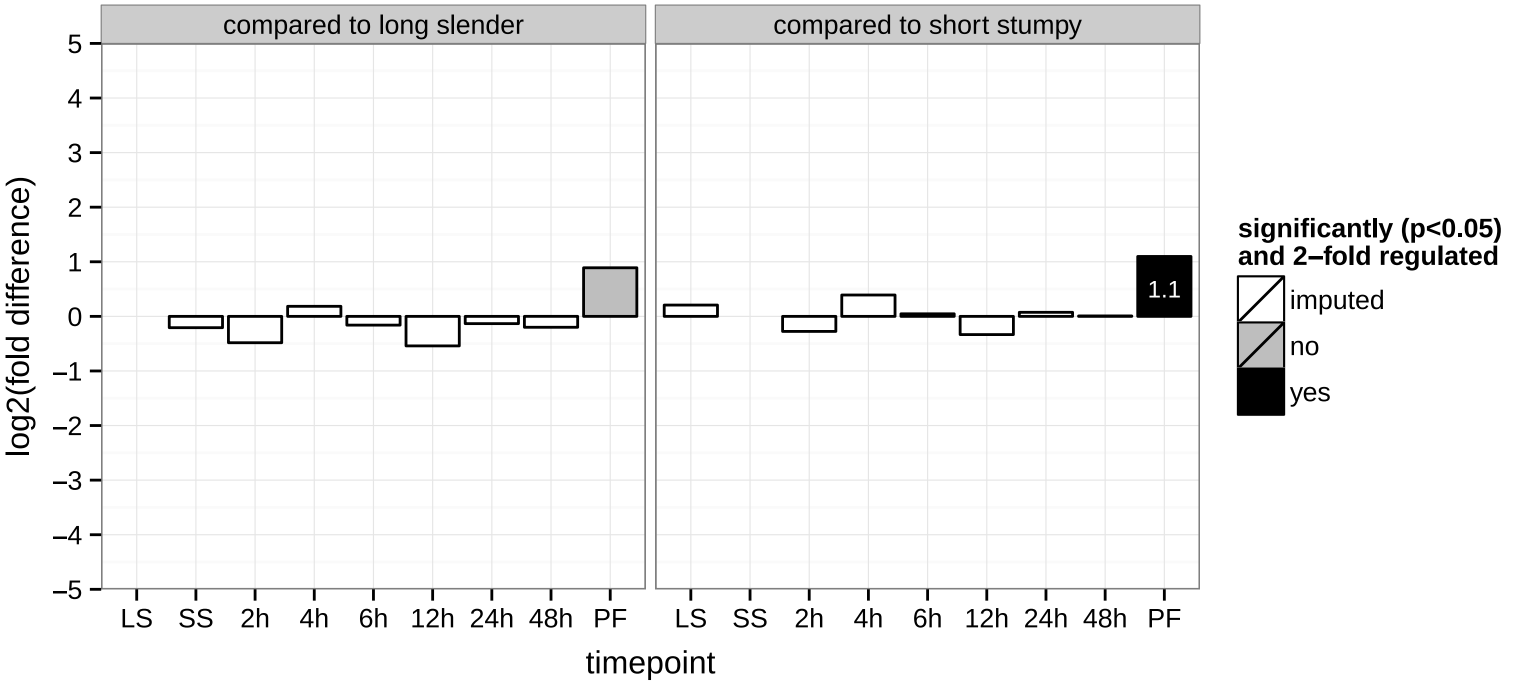

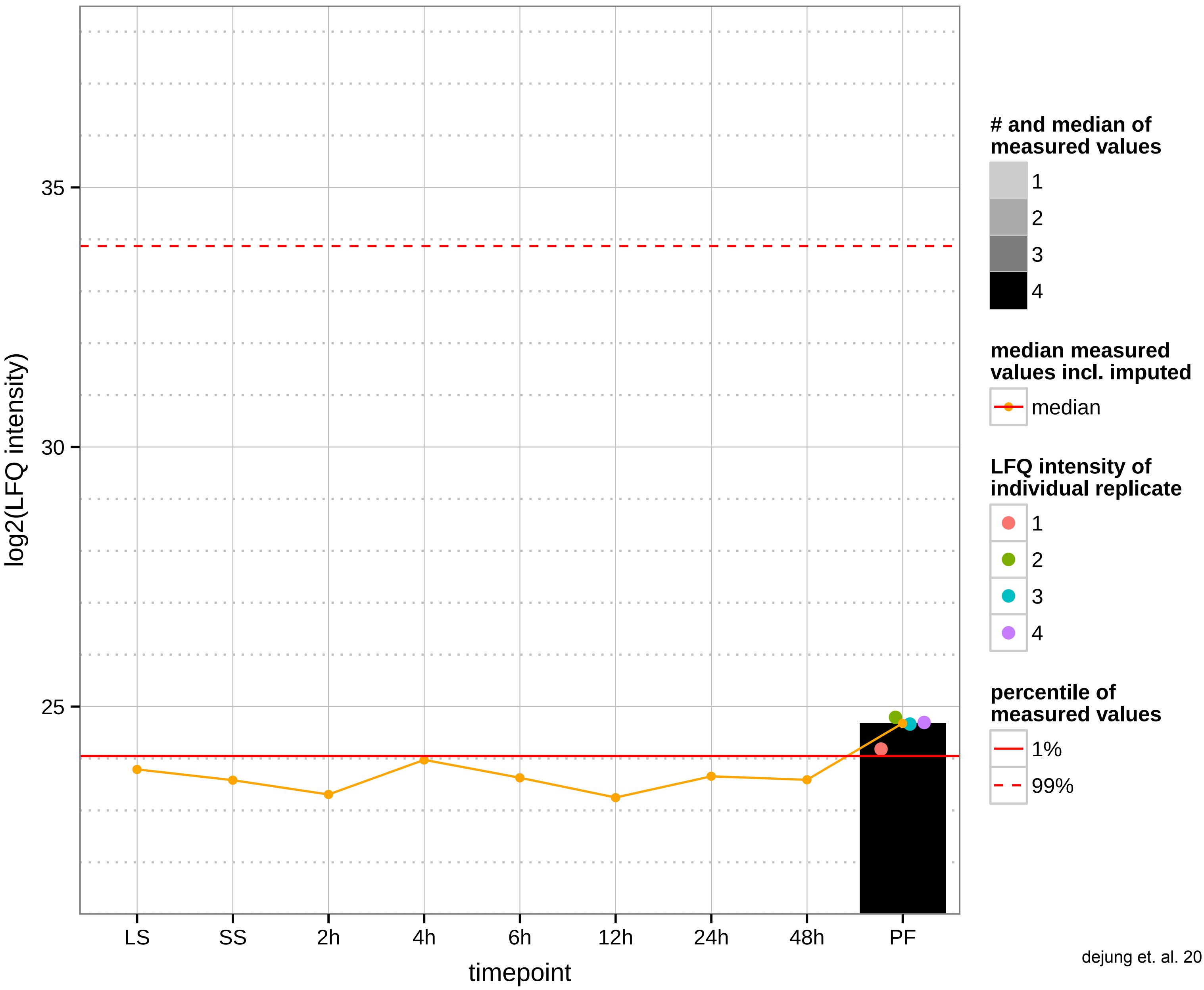

dejung et. al. 2015

hypothetical protein, conserved
Tb927.10.10690
AGOF: null
AGOC: null
AGOP: null
PGOF: null
PGOC: null
PGOP: null

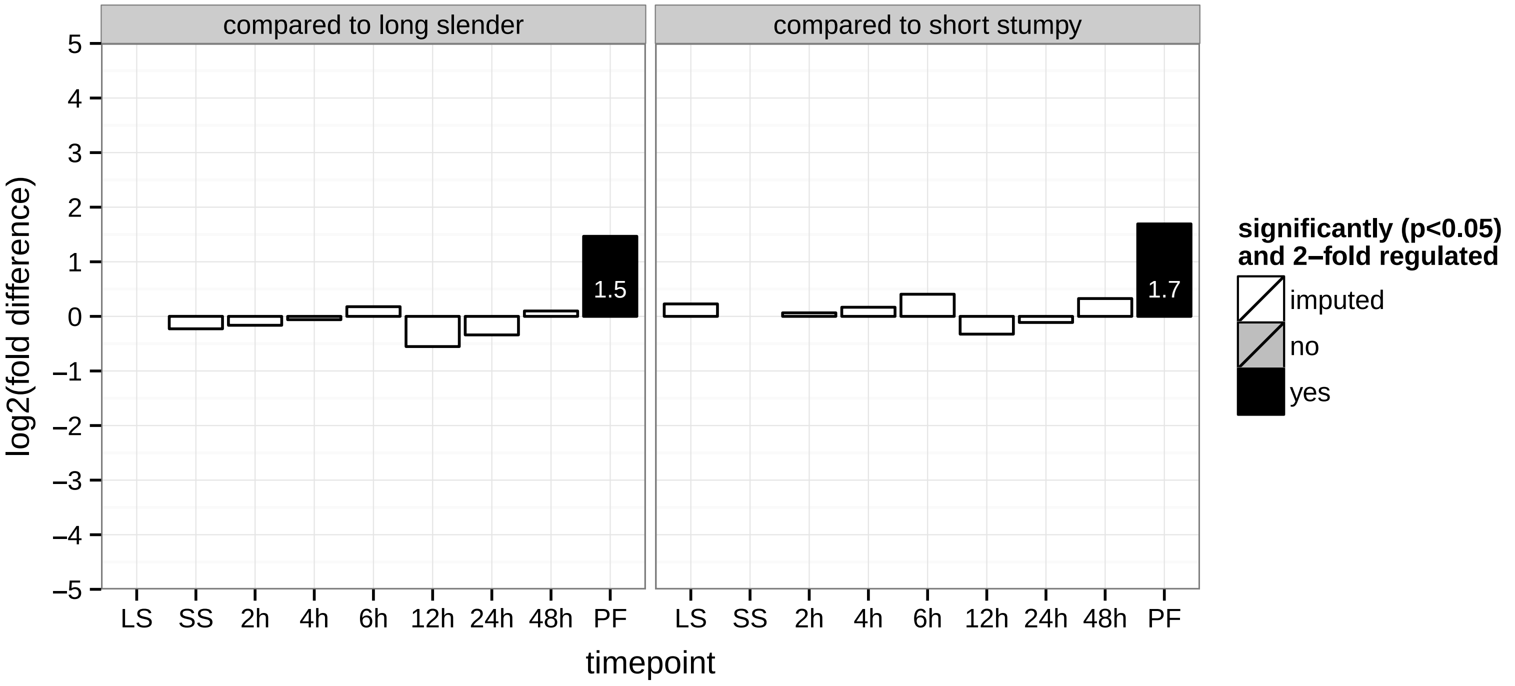

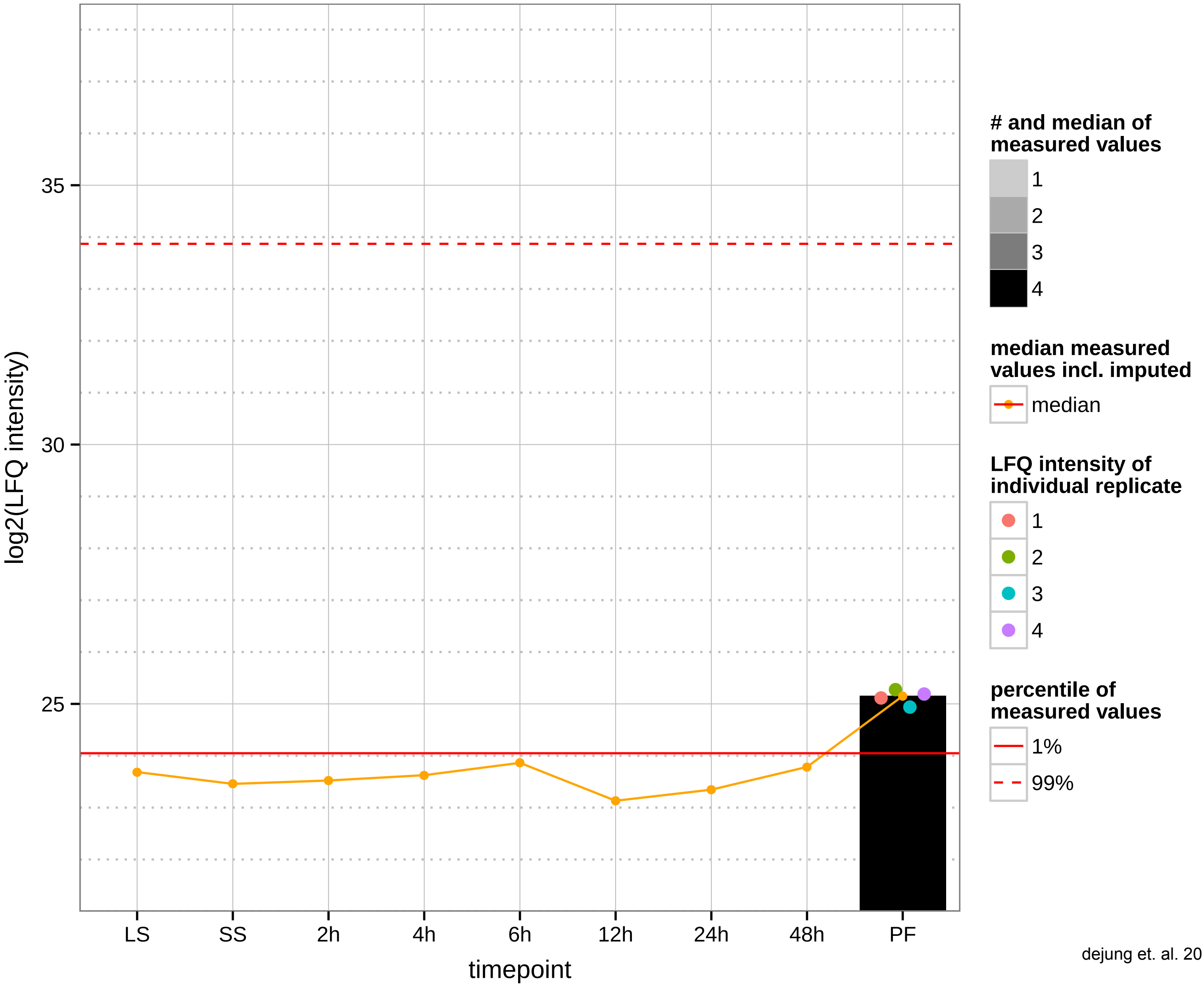

dejung et. al. 2015

hypothetical protein, conserved
Tb927.10.11230
AGOF: null
AGOC: null
AGOP: null
PGOF: null
PGOC: null
PGOP: null

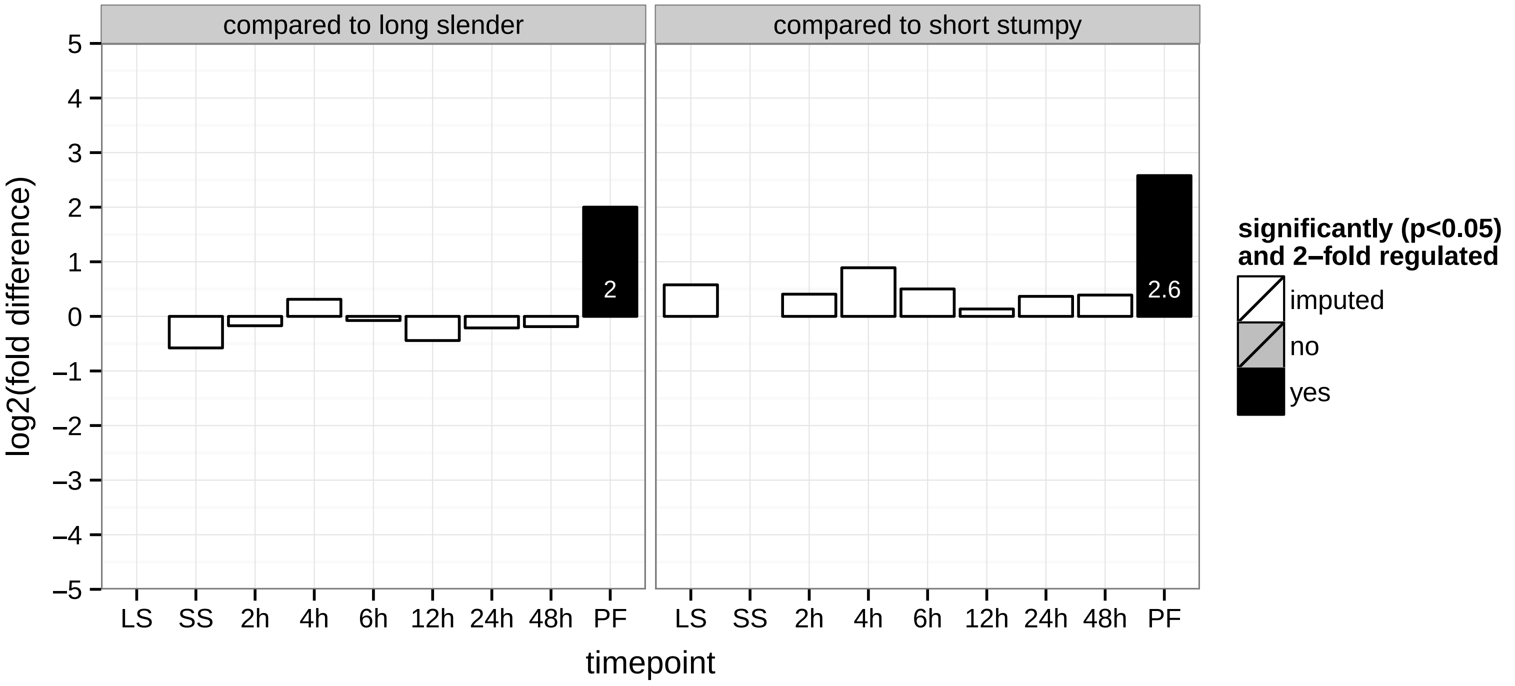

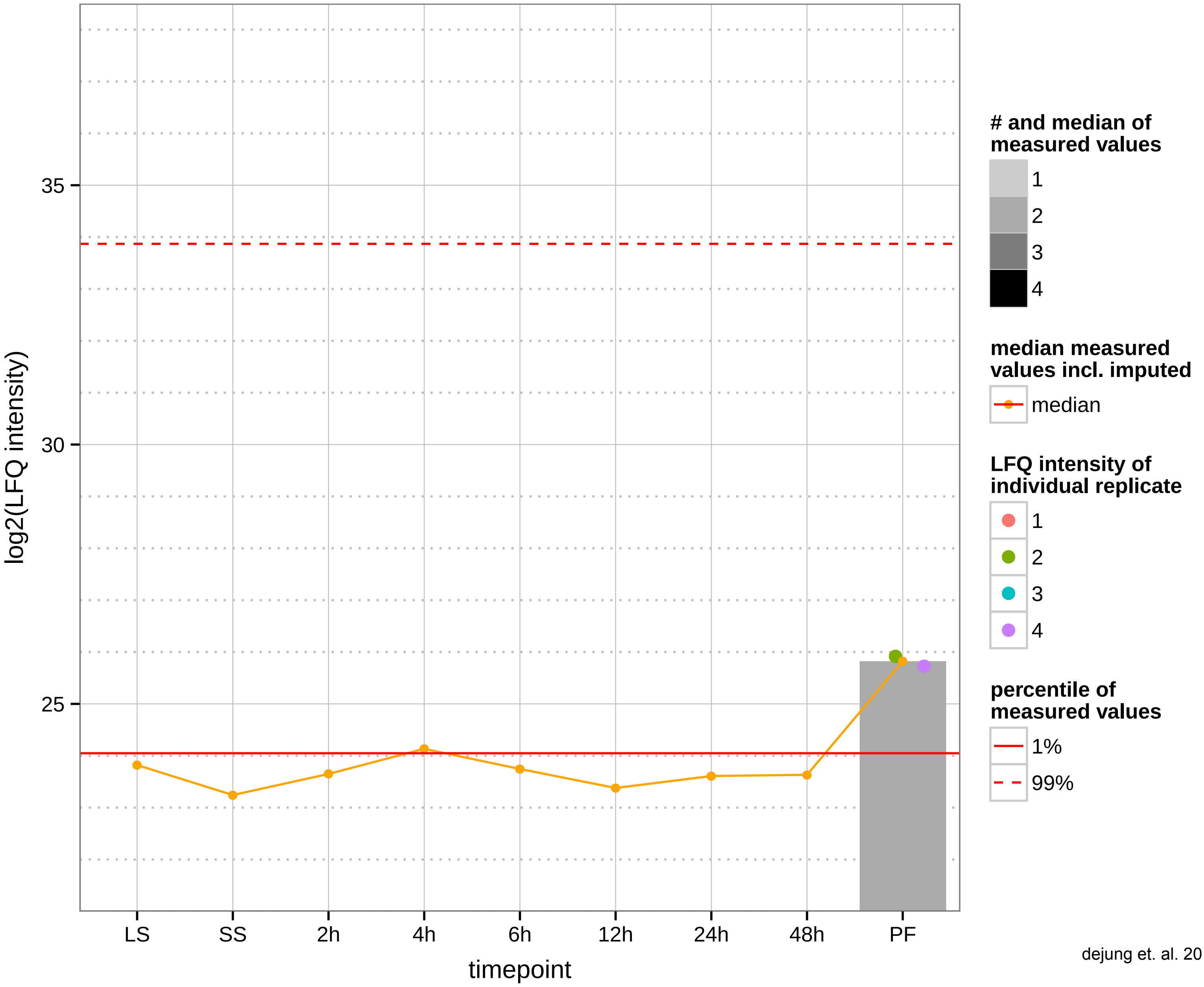

dejung et. al. 2015

hypothetical protein, conserved
Tb927.10.11340;Tb11.v5.0604
AGOF: null
AGOC: null
AGOP: null
PGOF: null
PGOC: null
PGOP: null

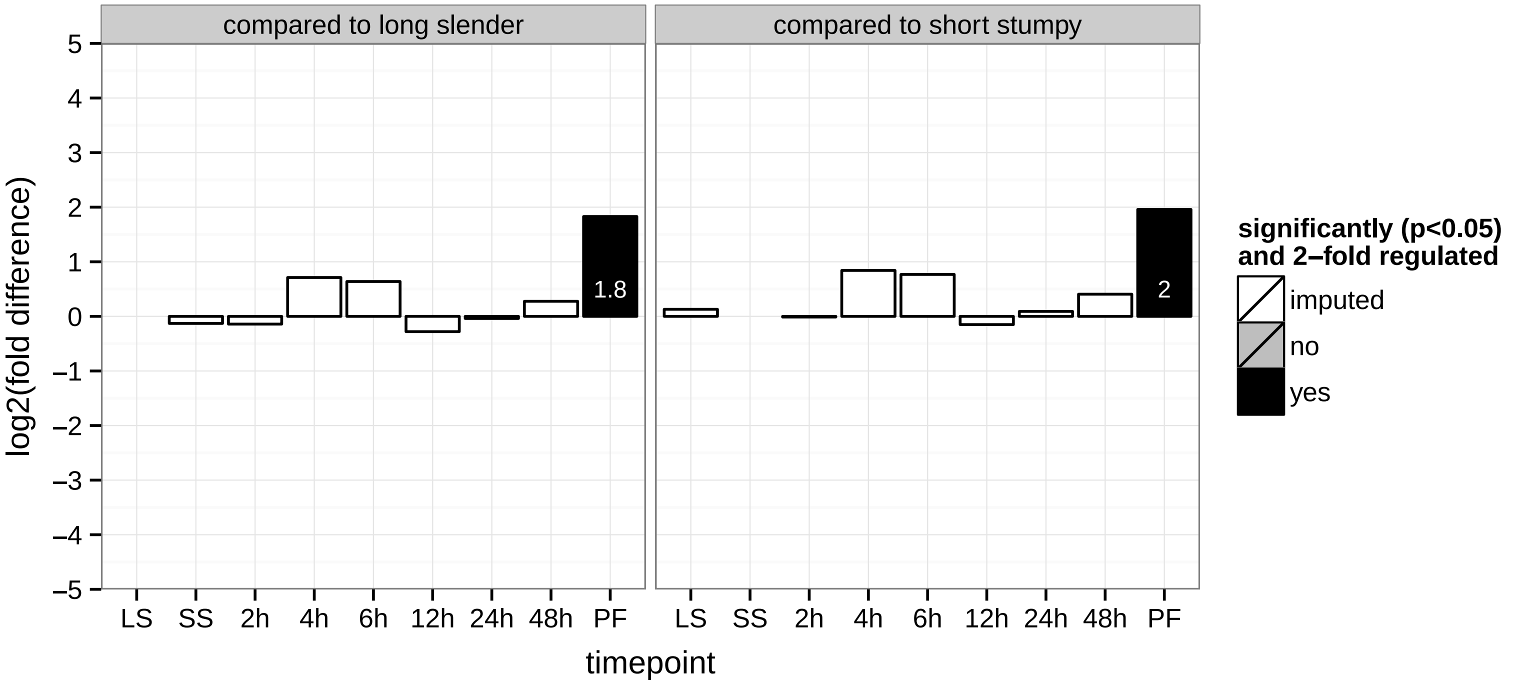

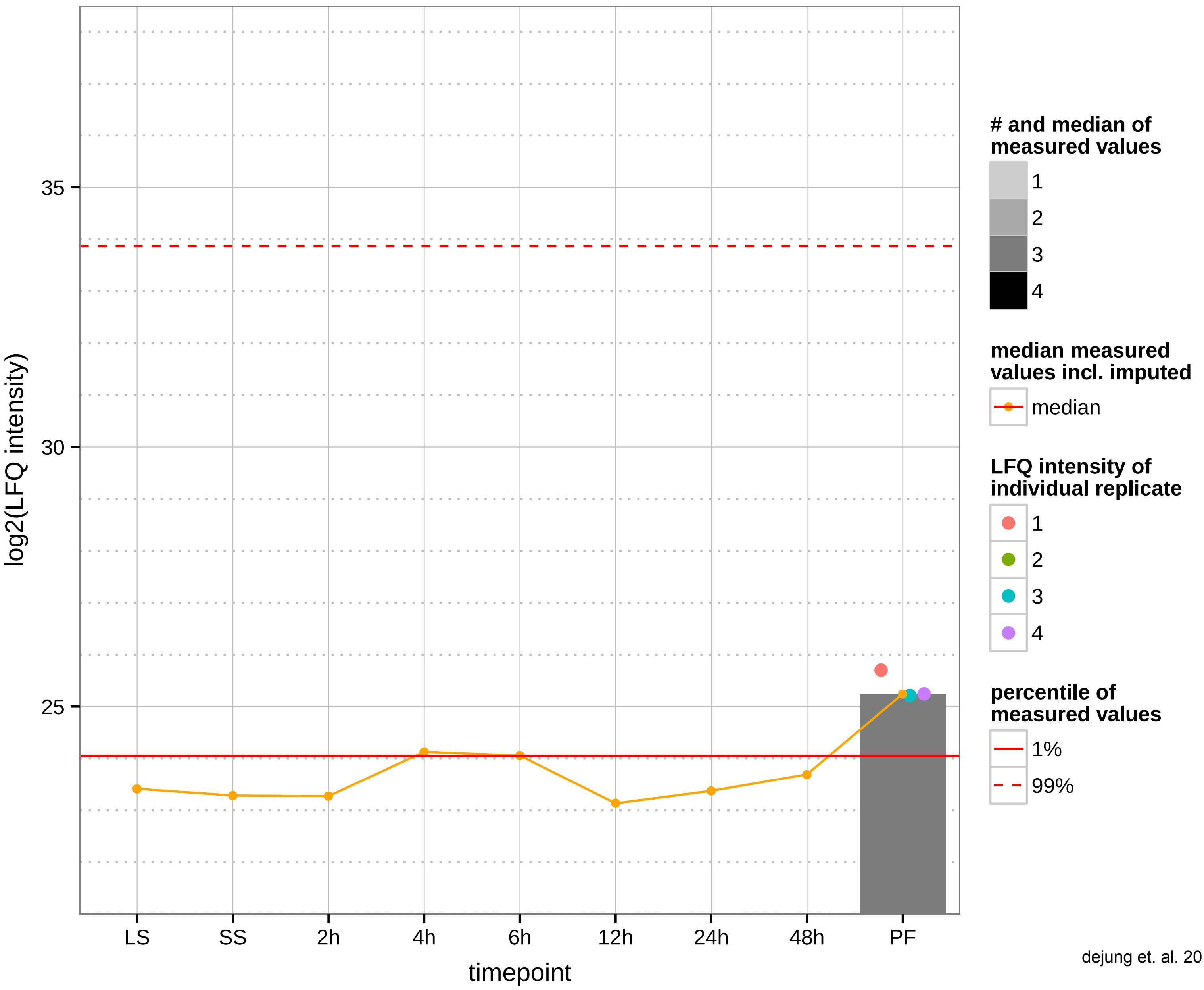

dejung et. al. 2015

hypothetical protein, conserved
Tb927.10.11410
AGOF: null
AGOC: null
AGOP: null
PGOF: null
PGOC: null
PGOP: null

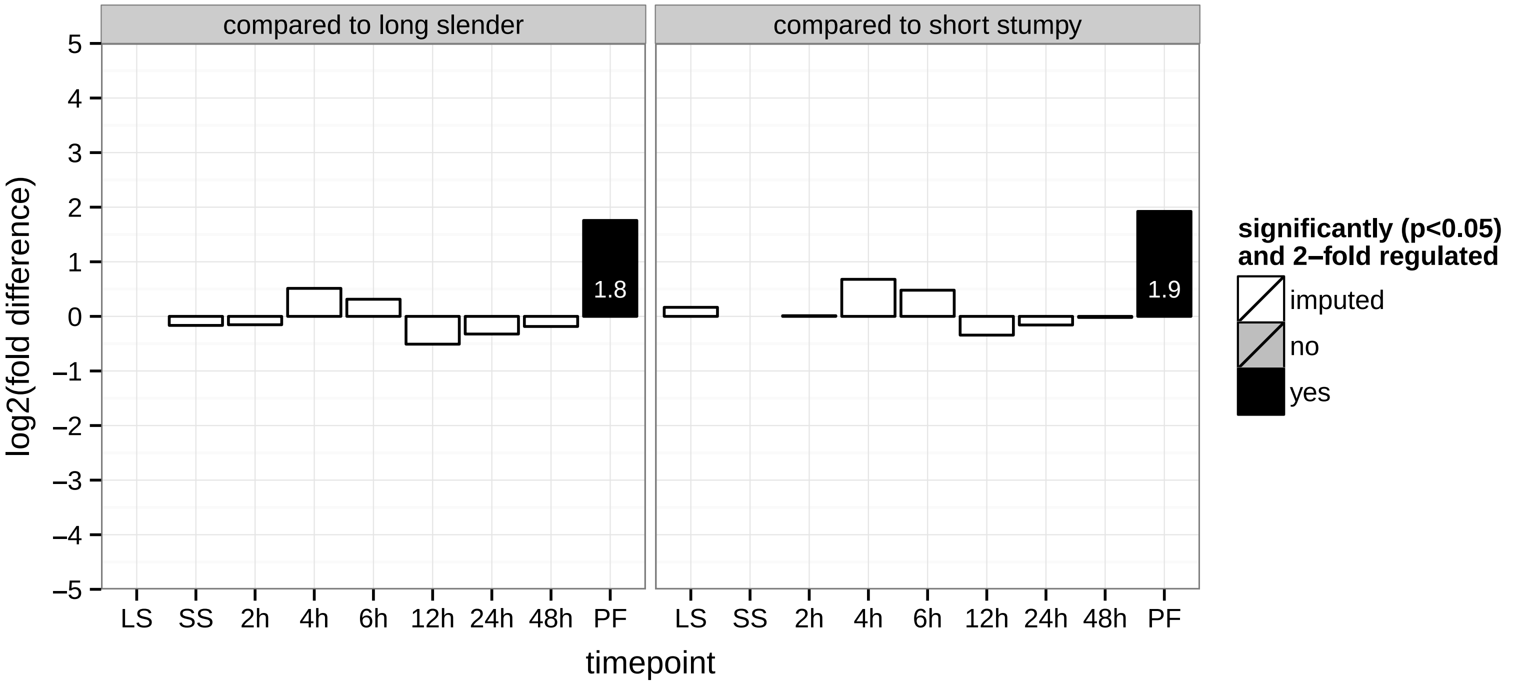

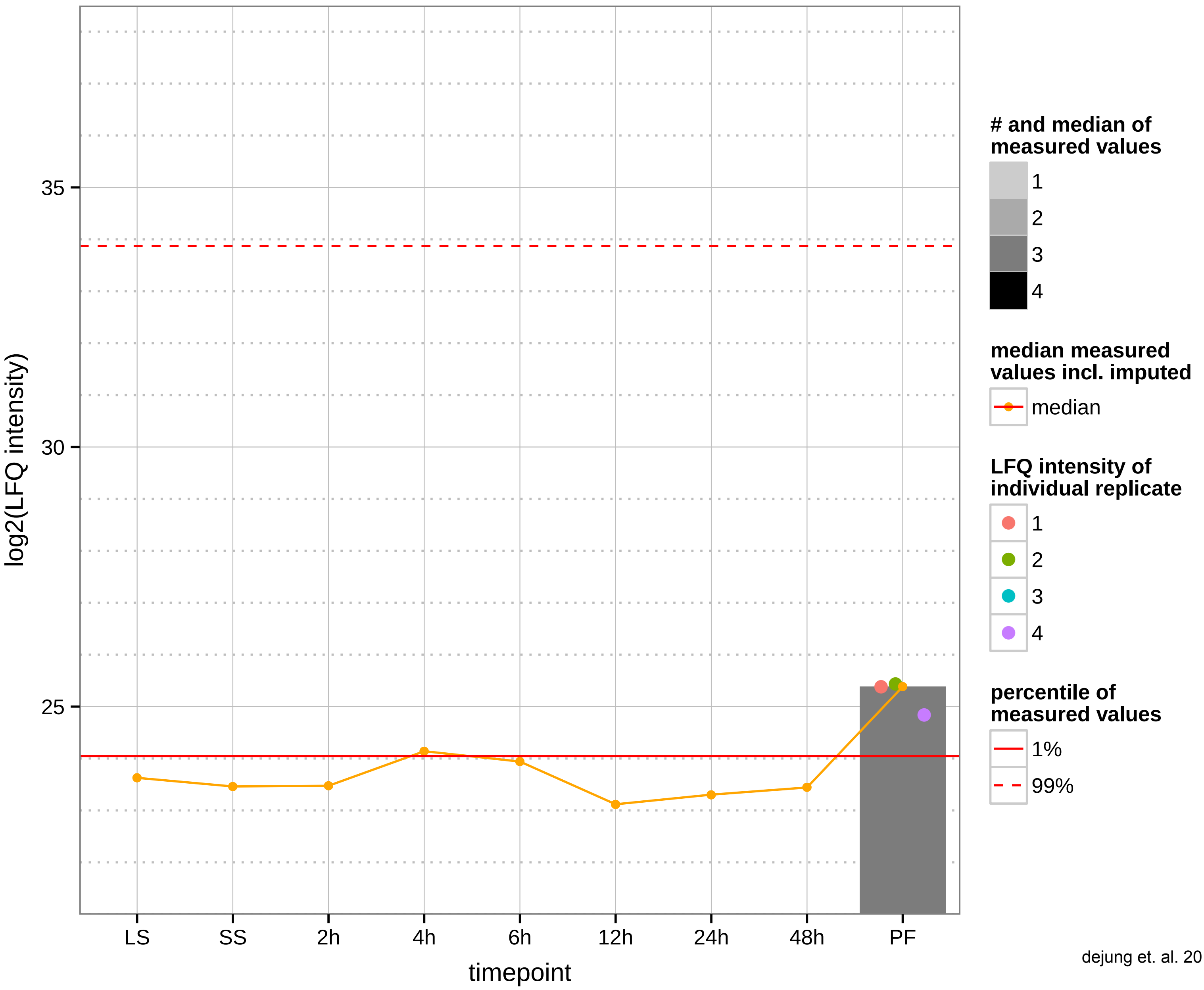

dejung et. al. 2015

hypothetical protein, conserved
Tb927.10.11810
AGOF: null
AGOC: null
AGOP: null
PGOF: null
PGOC: null
PGOP: null

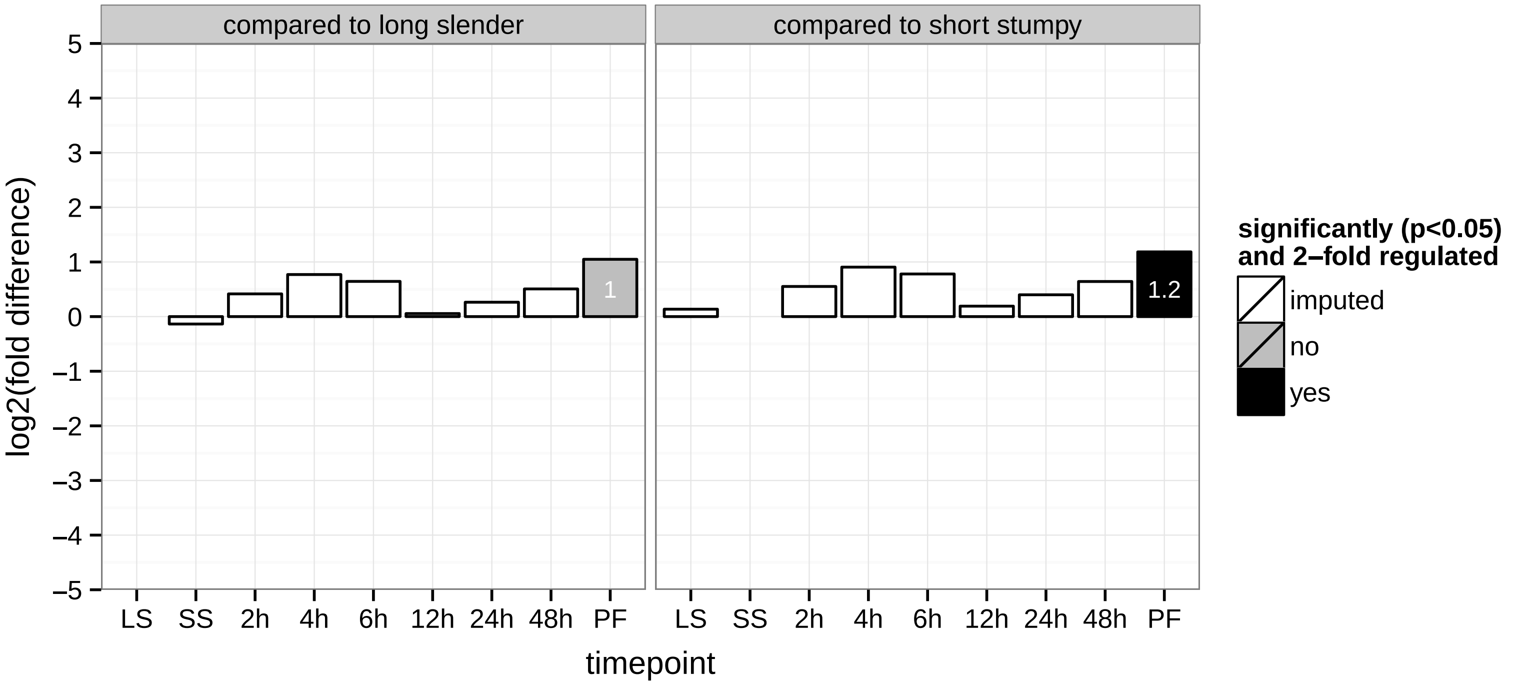

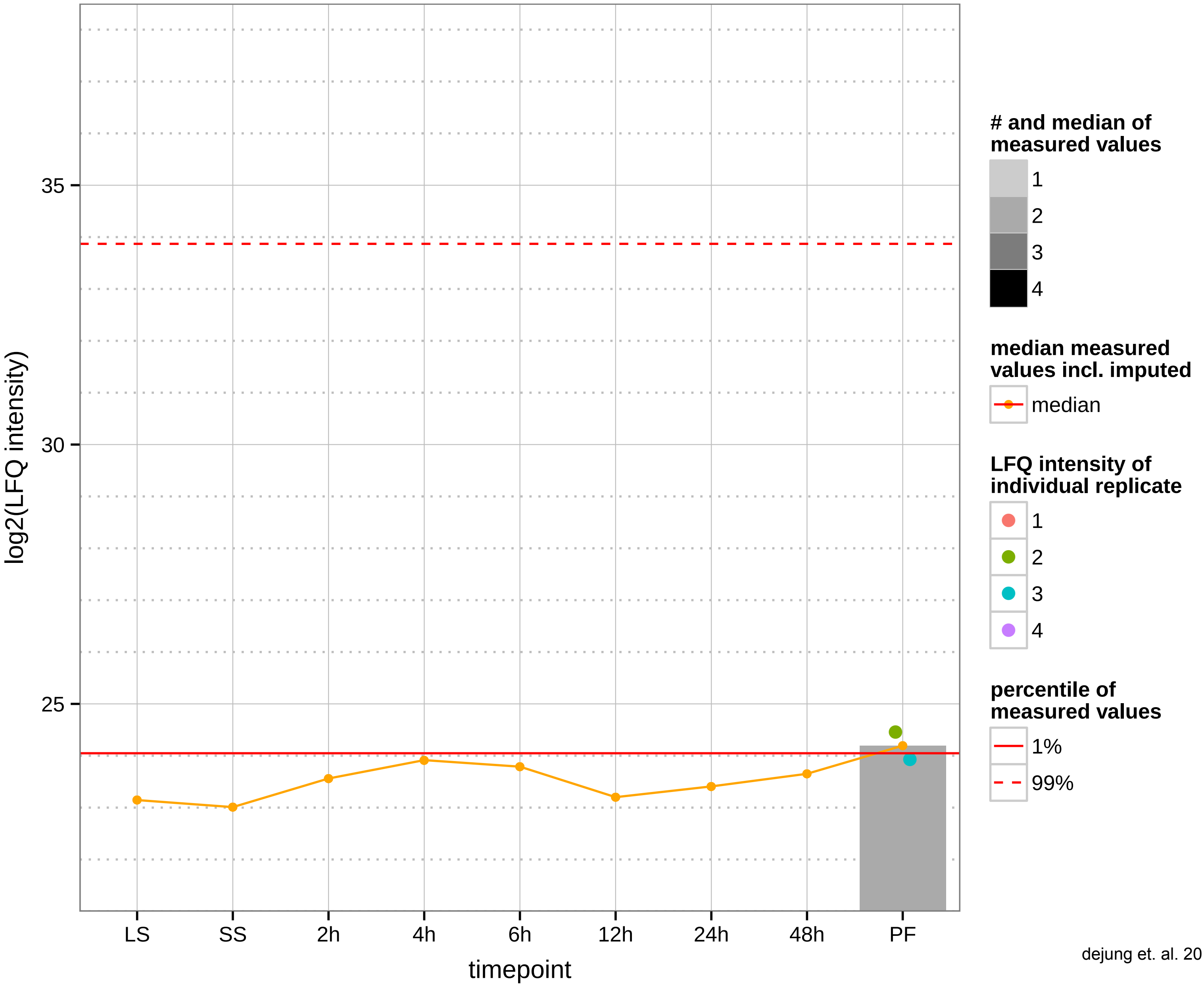

dejung et. al. 2015

hypothetical protein
Tb927.10.11840
AGOF: null
AGOC: null
AGOP: null
PGOF: protein binding
PGOC: null
PGOP: null

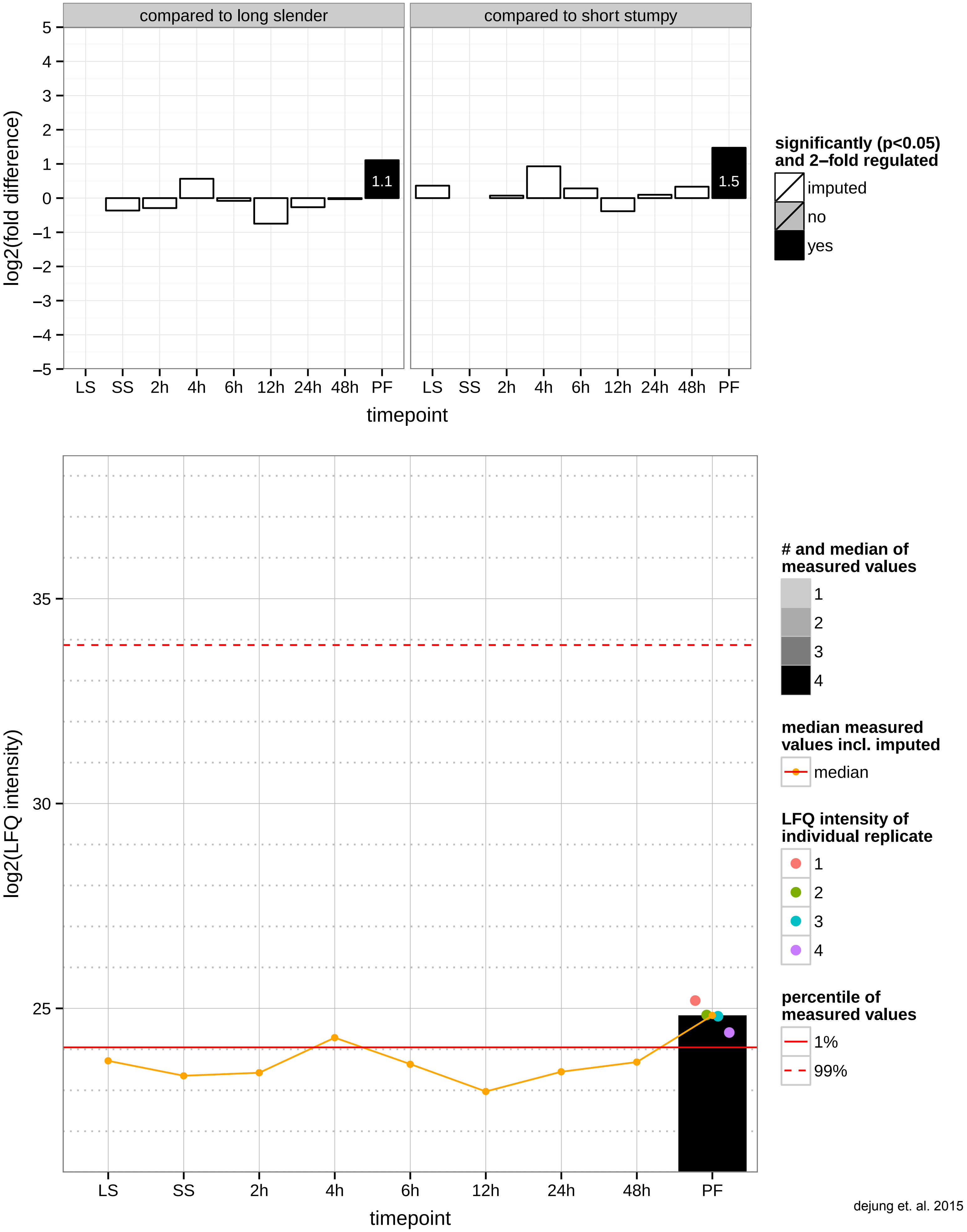

dejung et. al. 2015

hypothetical protein, conserved
Tb927.10.12180
AGOF: null
AGOC: null
AGOP: null
PGOF: null
PGOC: null
PGOP: null

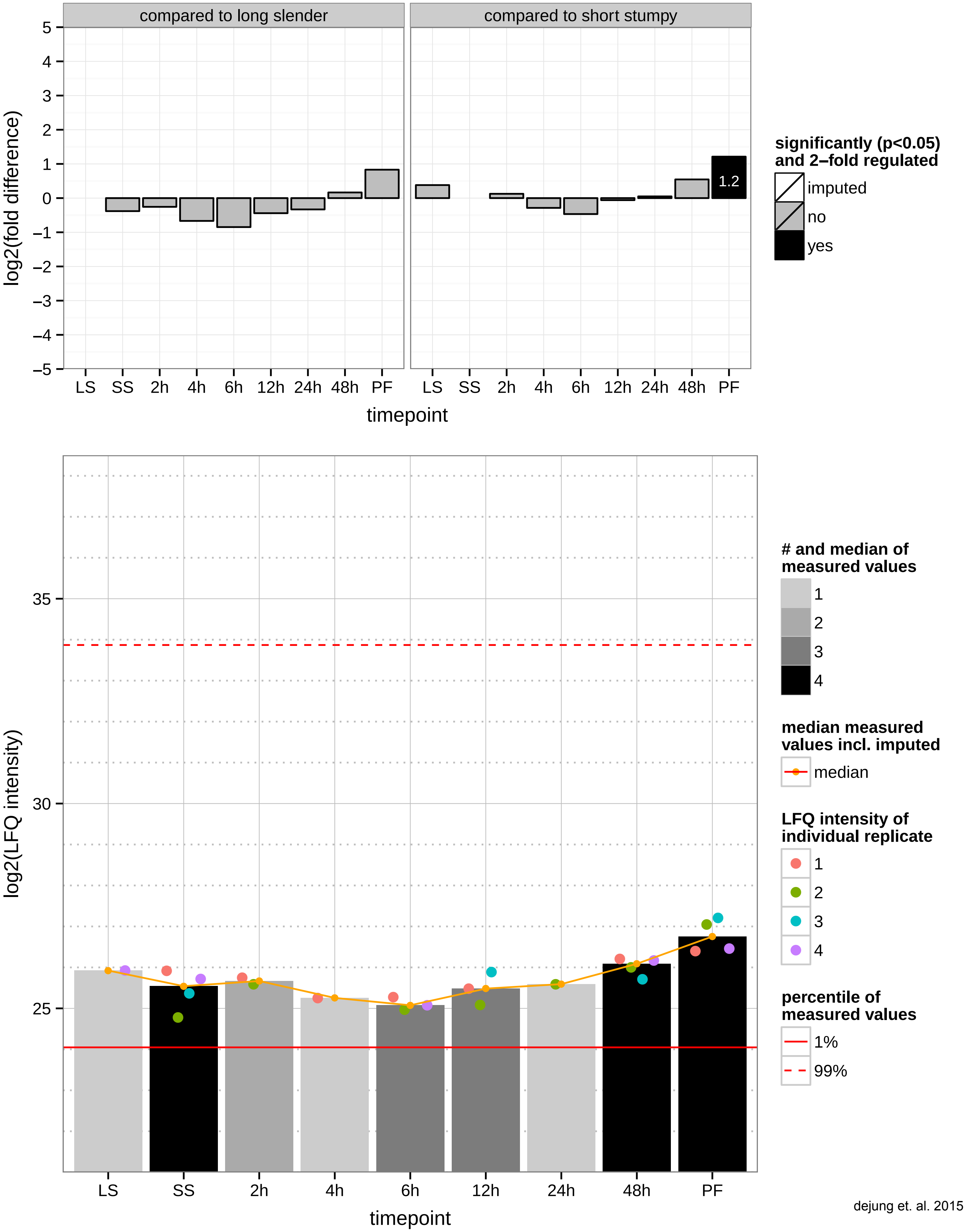

dejung et. al. 2015

hypothetical protein, conserved
Tb927.10.12930
AGOF: null
AGOC: mitochondrion
AGOP: null
PGOF: null
PGOC: null
PGOP: null

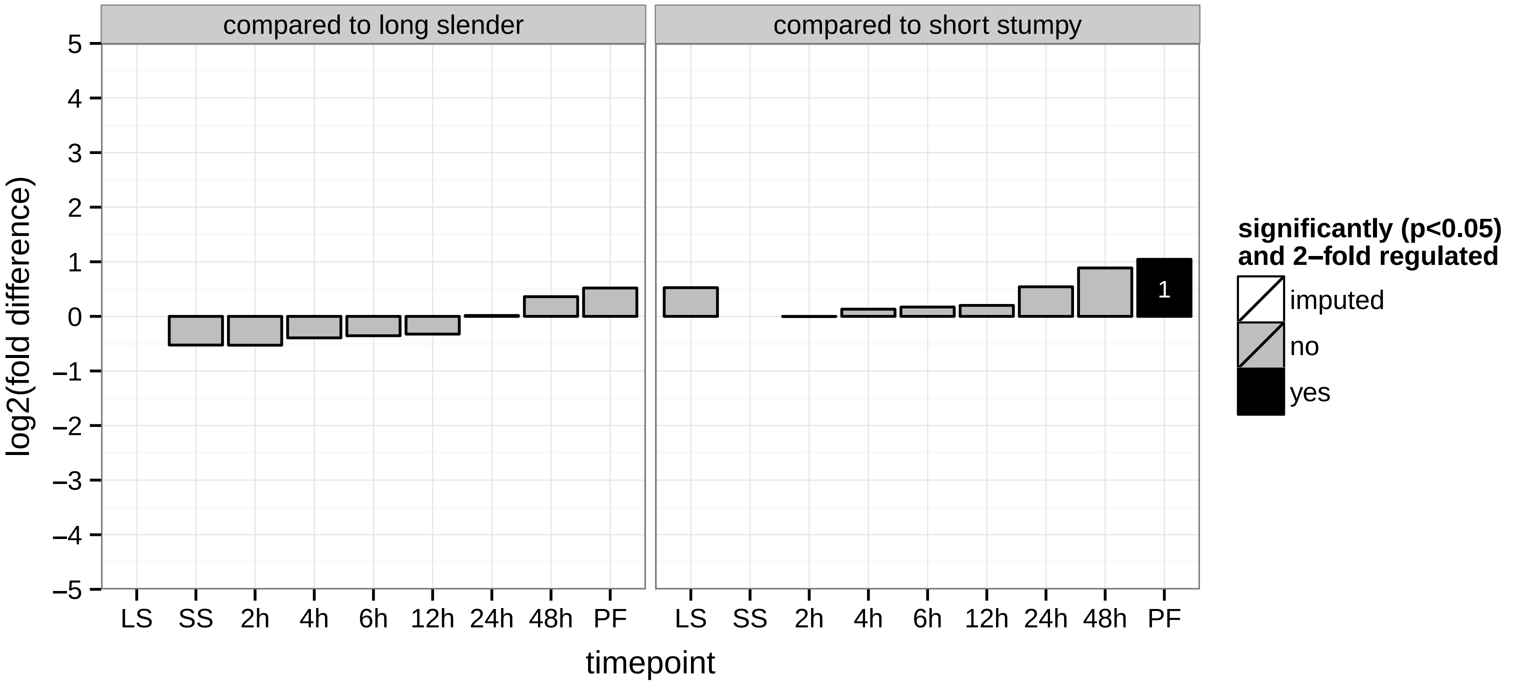

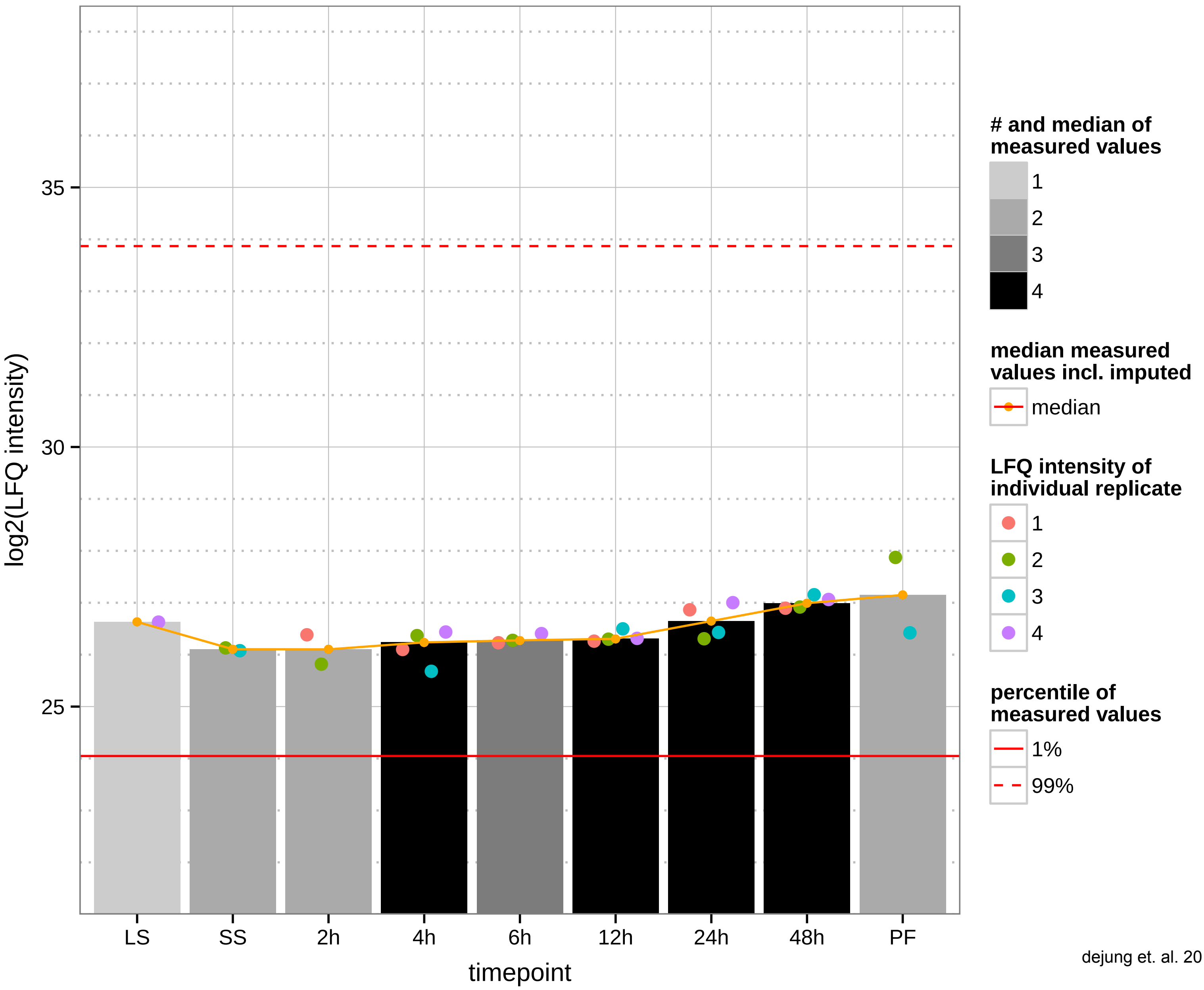

dejung et. al. 2015

hypothetical protein, conserved
Tb927.10.13180
AGOF: null
AGOC: null
AGOP: null
PGOF: null
PGOC: null
PGOP: null

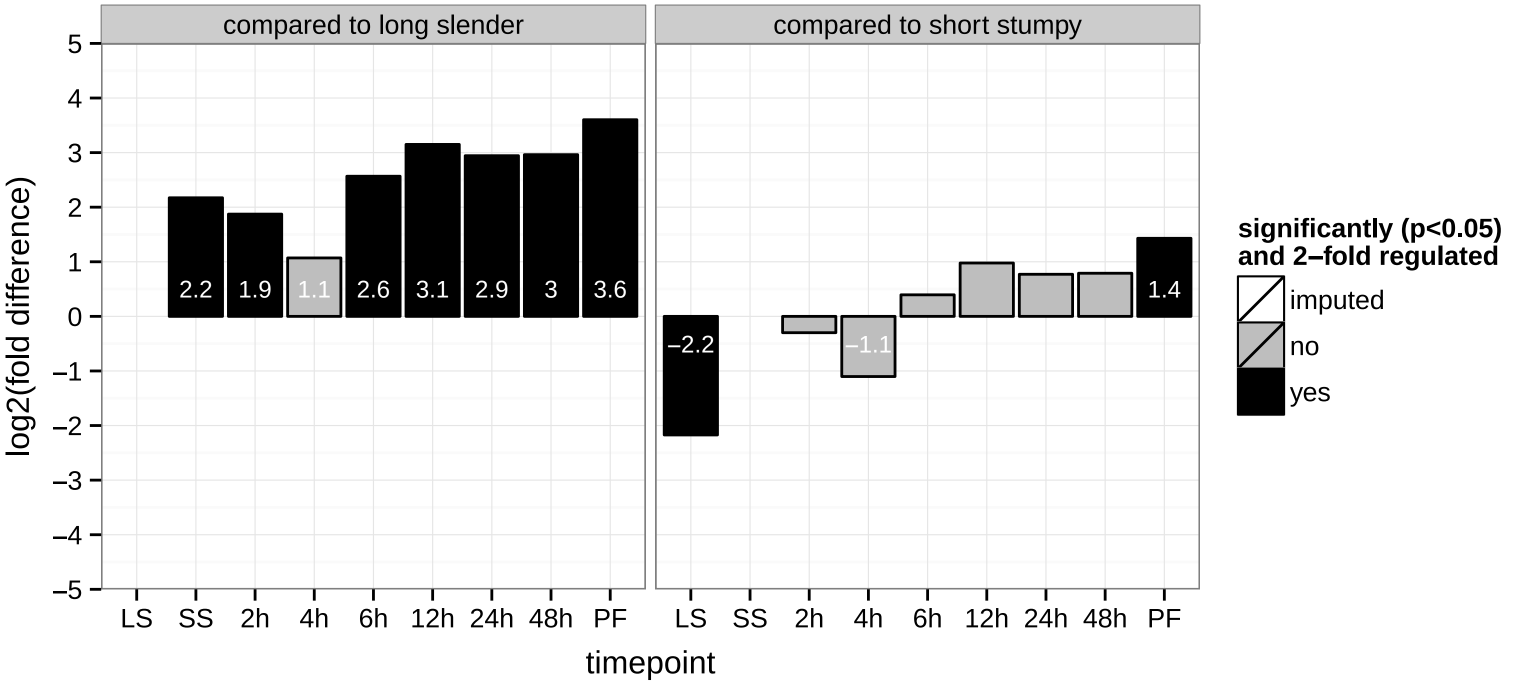

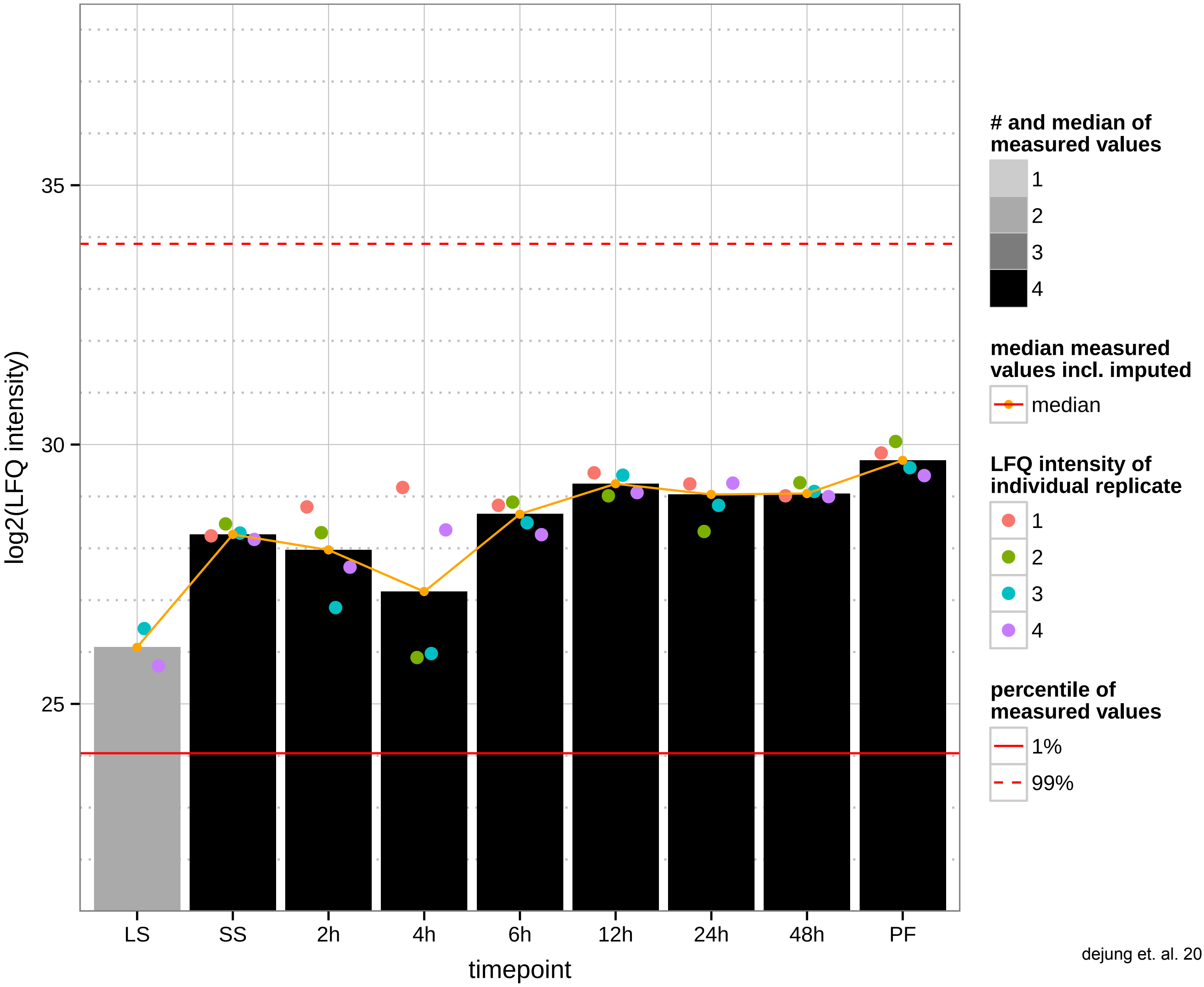

dejung et. al. 2015

hypothetical protein, conserved
Tb927.10.13230
AGOF: null
AGOC: null
AGOP: null
PGOF: null
PGOC: null
PGOP: null

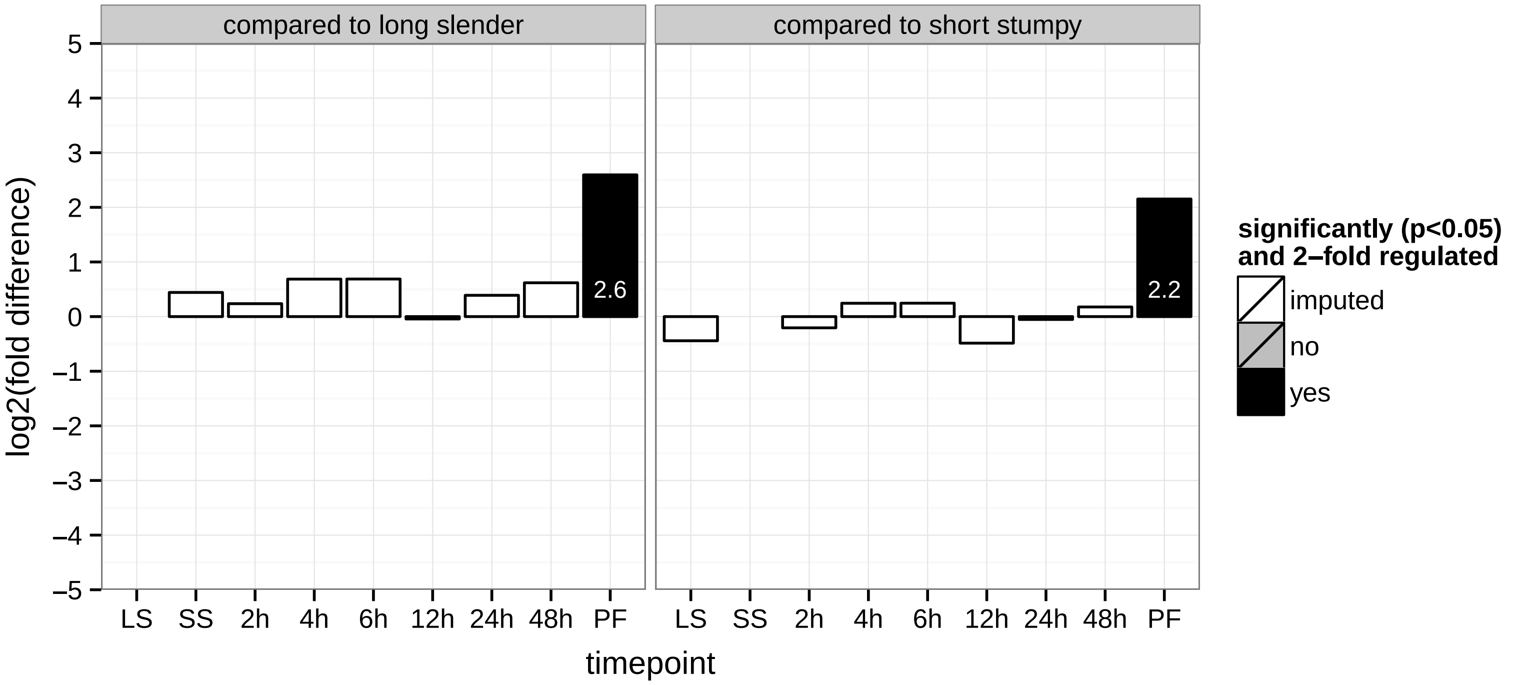

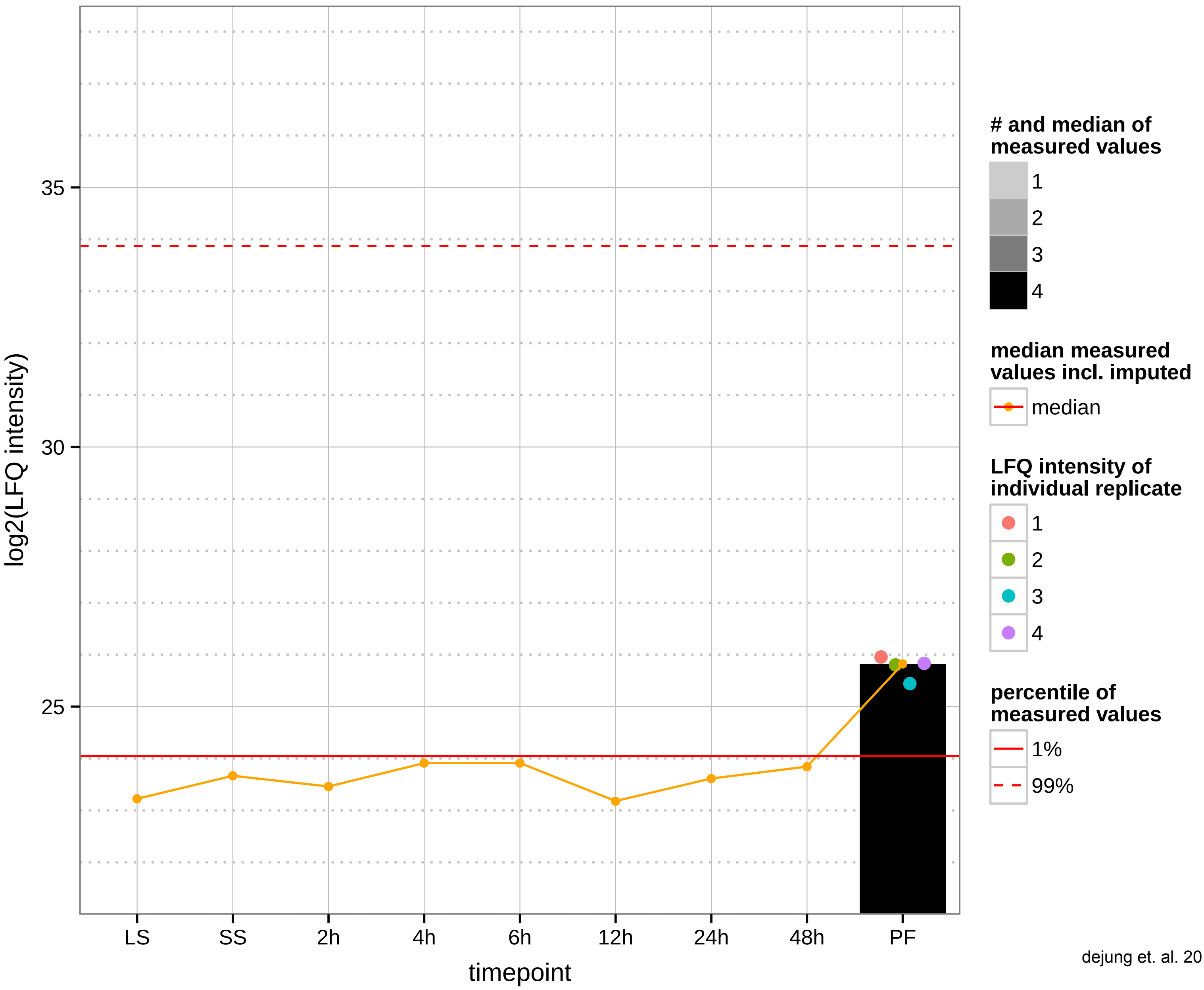

dejung et. al. 2015

hypothetical protein, conserved
Tb927.10.13280
AGOF: null
AGOC: null
AGOP: null
PGOF: binding
PGOC: null
PGOP: null

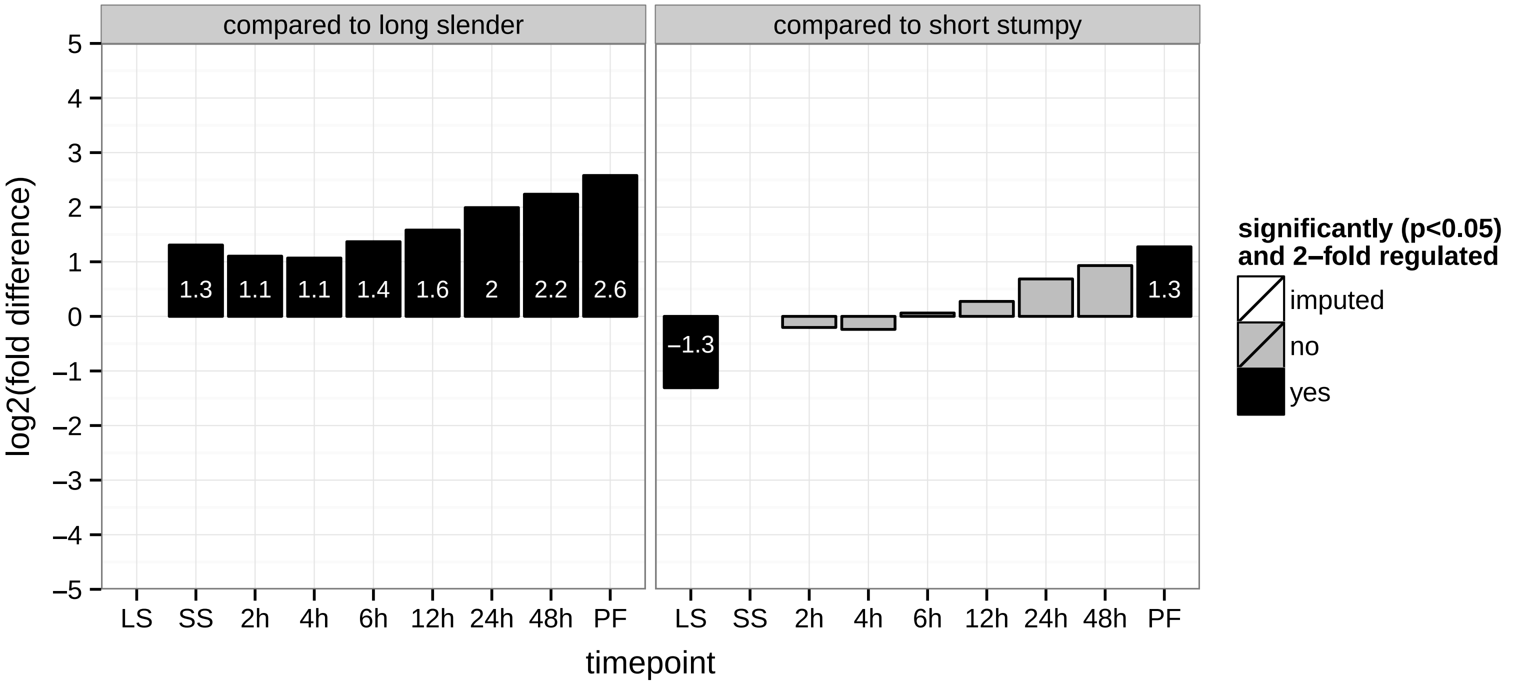

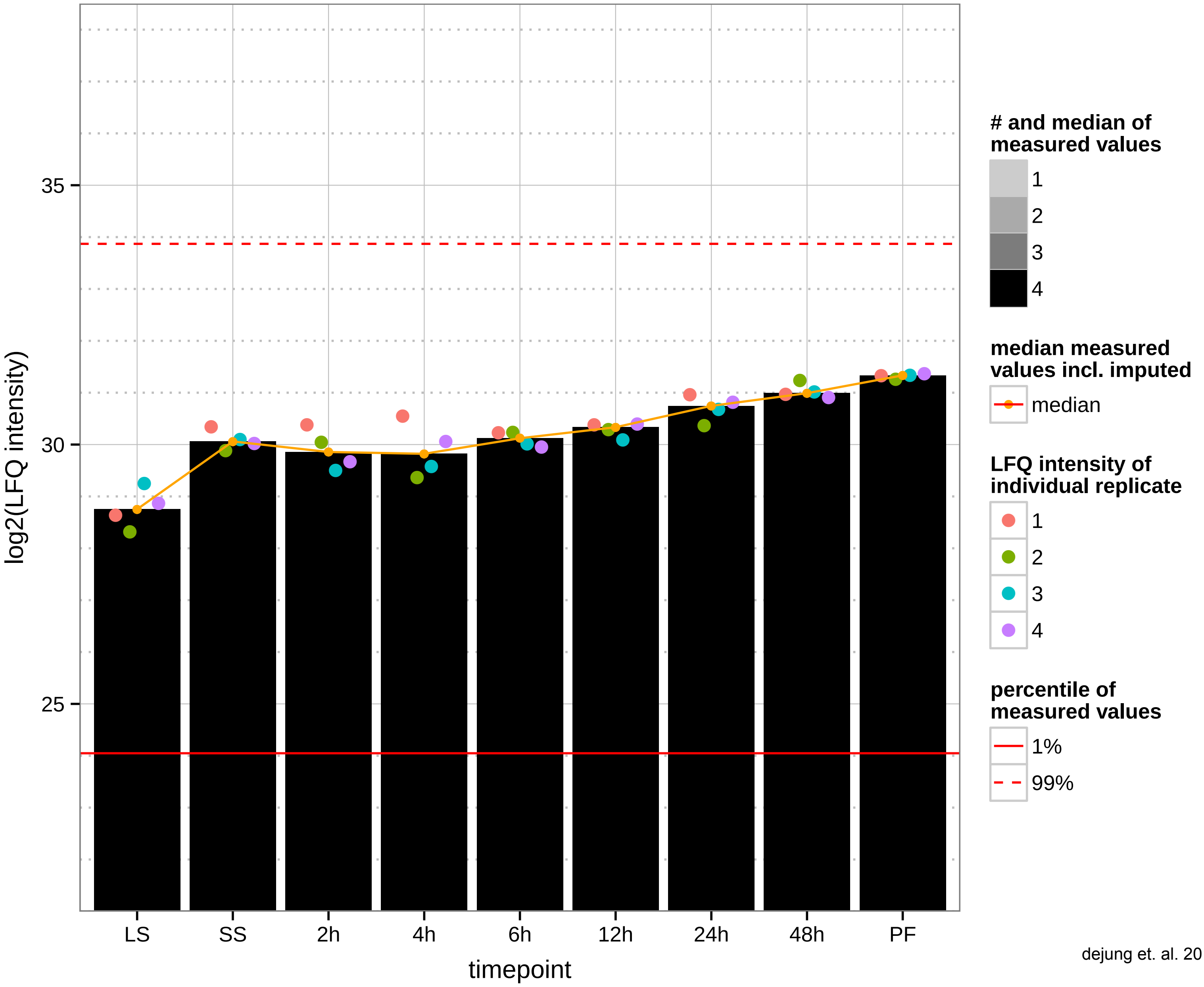

dejung et. al. 2015

Dynein light chain Tctex–type, putative
Tb927.10.13370
AGOF: microtubule motor activity
AGOC: cytoplasmic dynein complex
AGOP: microtubule–based process
PGOF: null
PGOC: null
PGOP: null

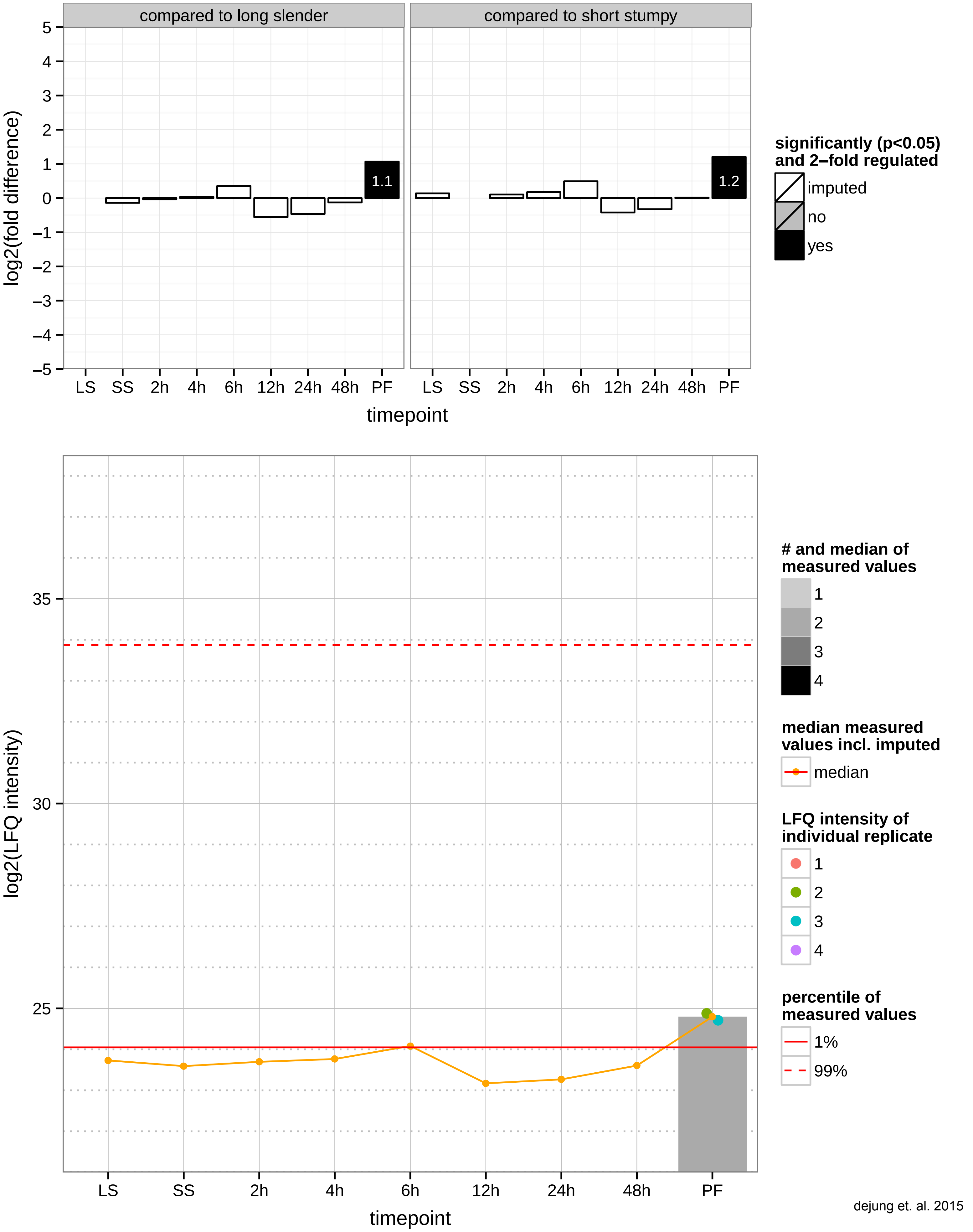

dejung et. al. 2015

60S ribosomal protein L10a, putative (RPL10A)
Tb927.11.9710;Tb927.10.13500
AGOF: RNA binding, structural constituent of ribosome
AGOC: ribosome, large ribosomal subunit
AGOP: RNA processing, translation
PGOF: RNA binding, structural constituent of ribosome
PGOC: large ribosomal subunit
PGOP: translation

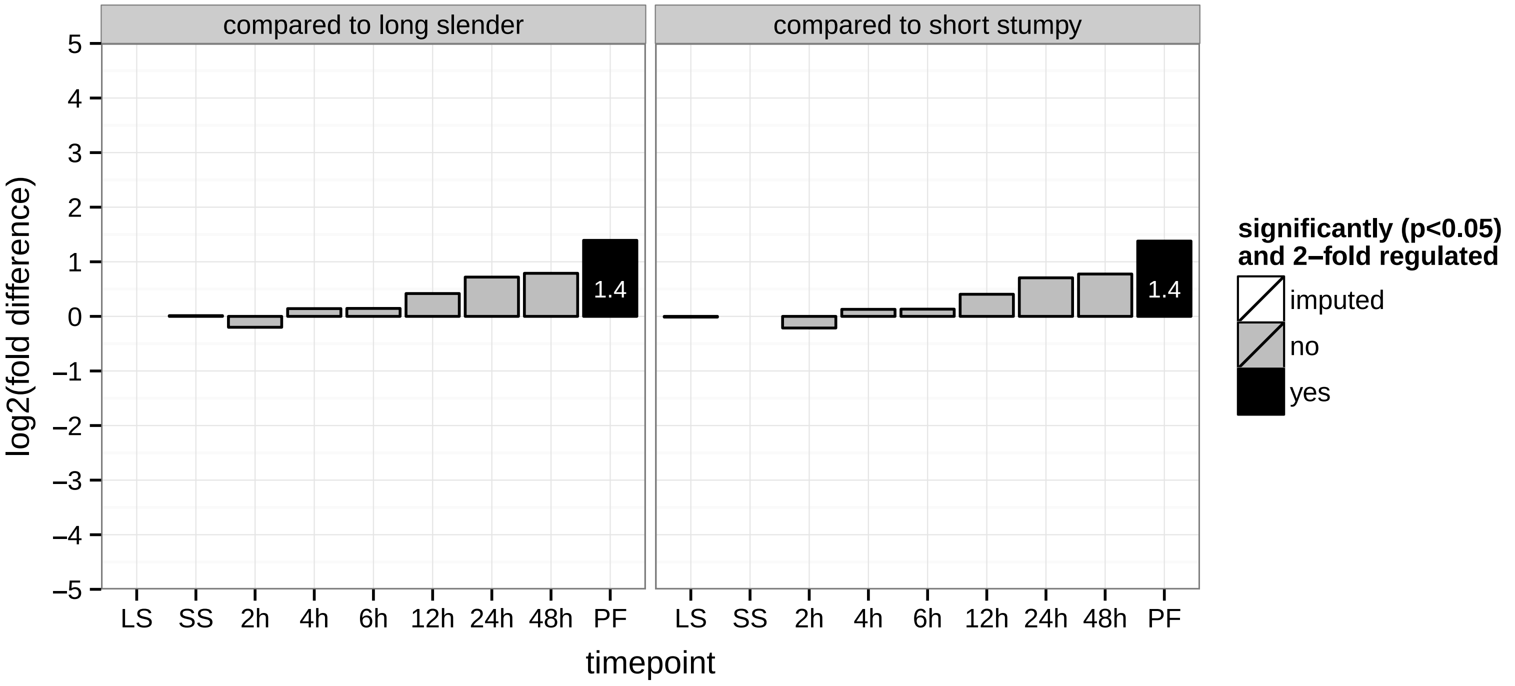

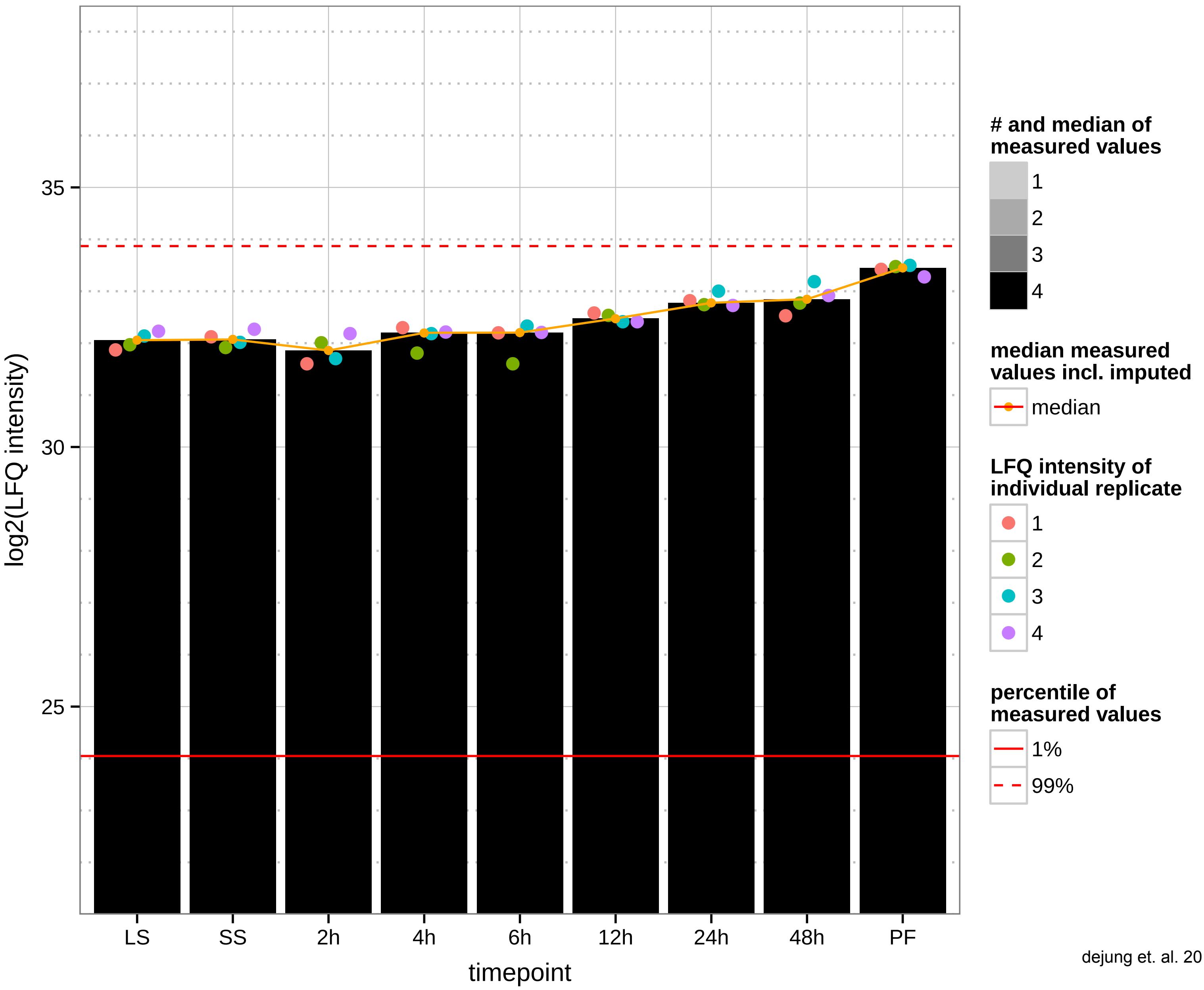

dejung et. al. 2015

hypothetical protein, conserved
Tb927.10.13660
AGOF: null
AGOC: null
AGOP: null
PGOF: null
PGOC: null
PGOP: null

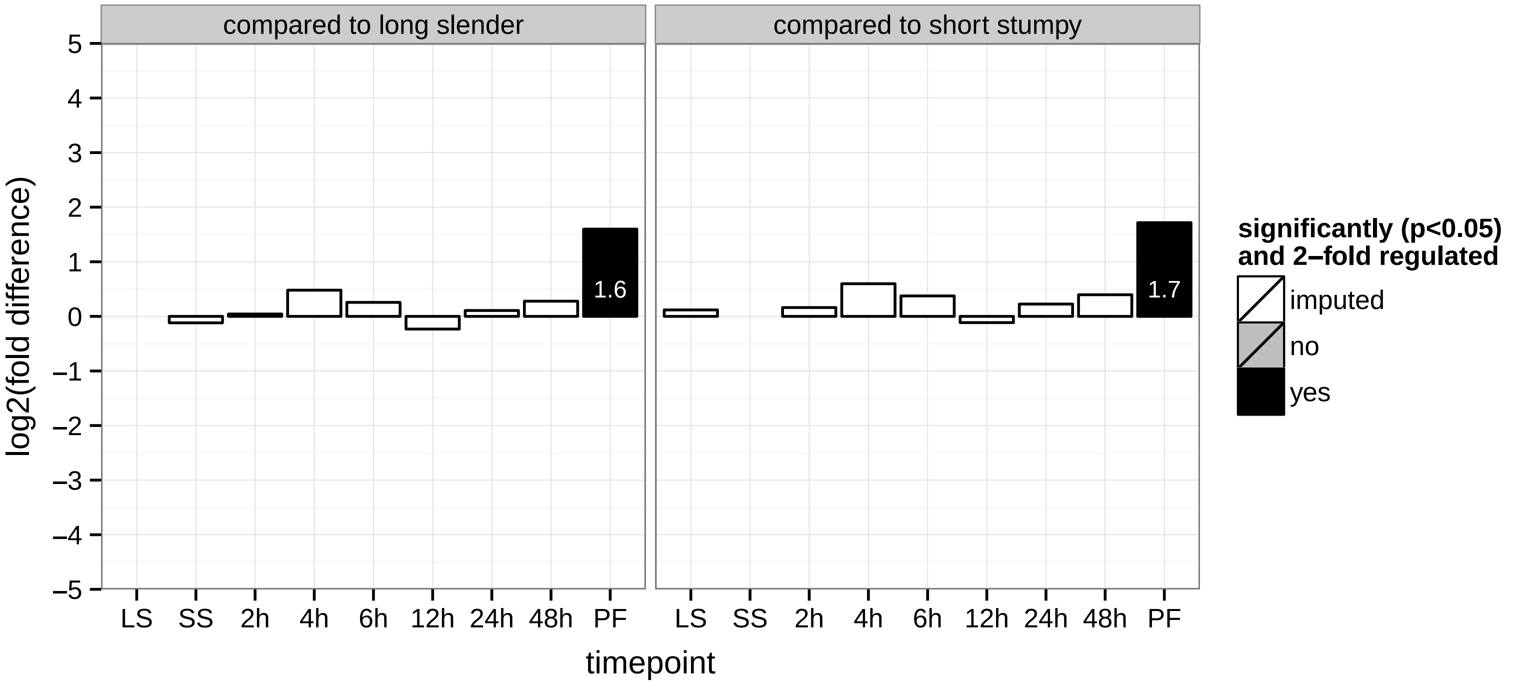

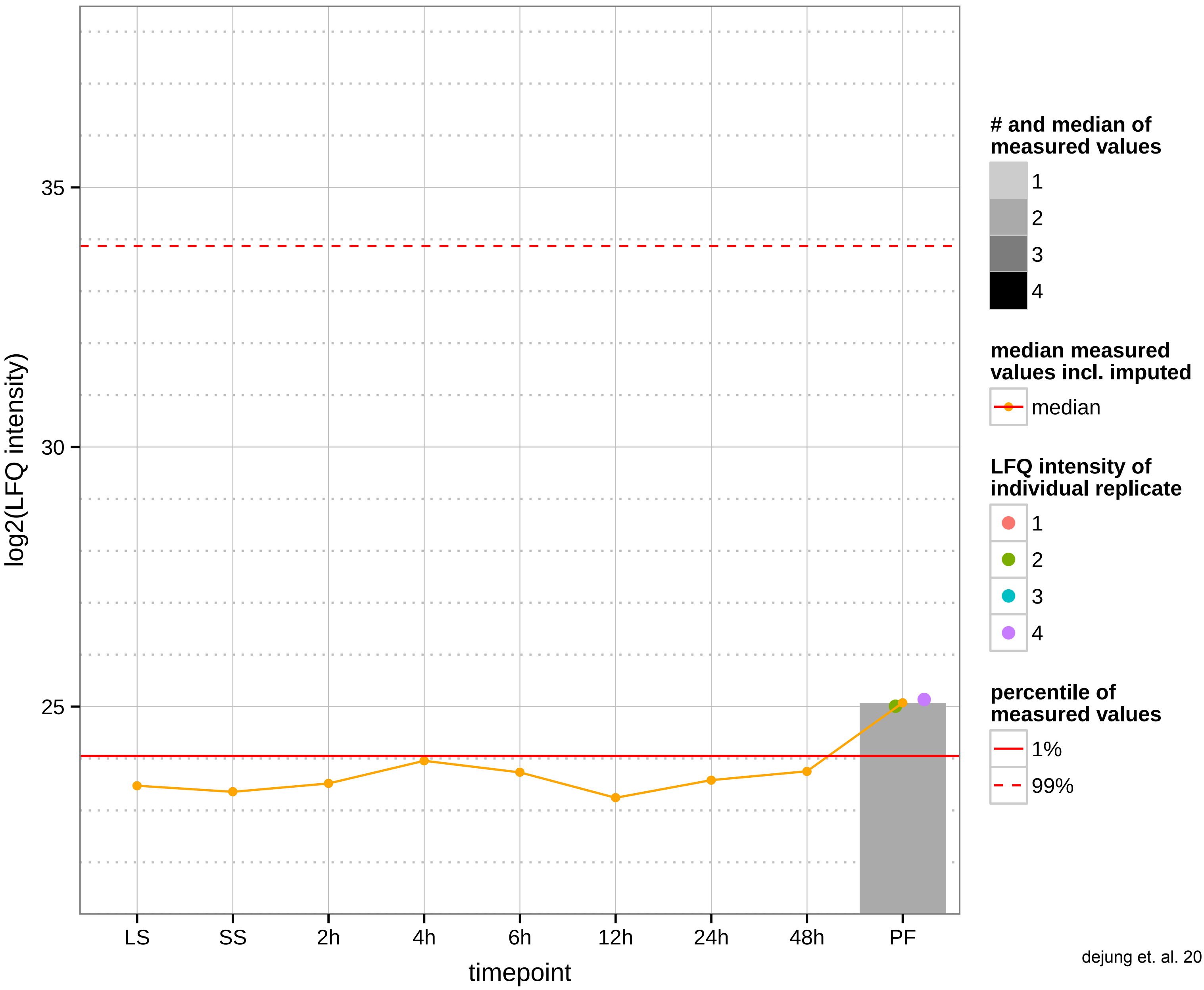

dejung et. al. 2015

NA, aconitase (ACO)
Tb927.10.14000
AGOF: NA, aconitate hydratase activity
AGOC: NA, cytosol, mitochondrion
AGOP: NA, tricarboxylic acid cycle
PGOF: NA, 4 iron, 4 sulfur cluster binding
PGOC: NA, null
PGOP: NA, metabolic process

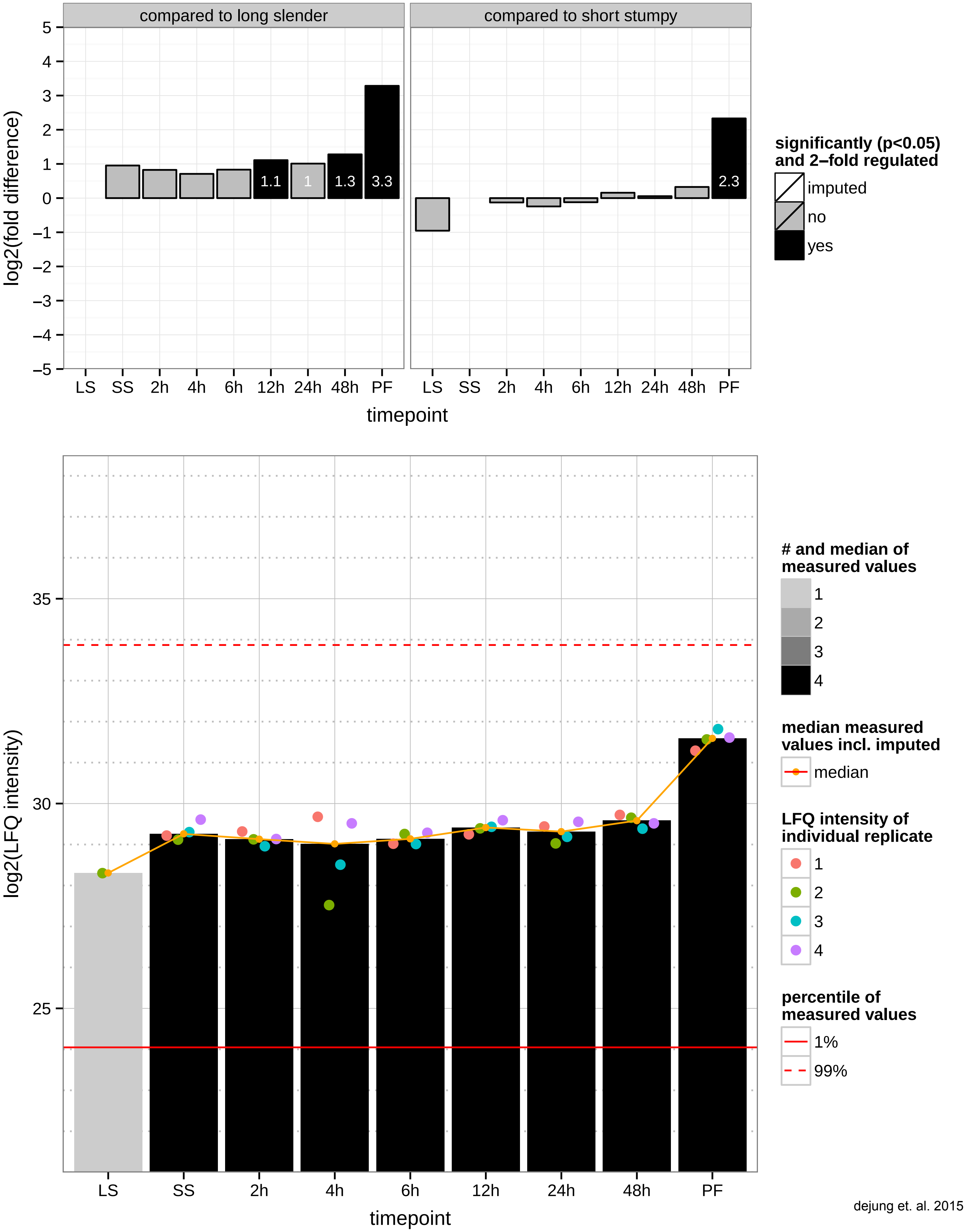

dejung et. al. 2015

hypothetical protein, conserved
Tb927.10.14030
AGOF: null
AGOC: null
AGOP: null
PGOF: null
PGOC: null
PGOP: null

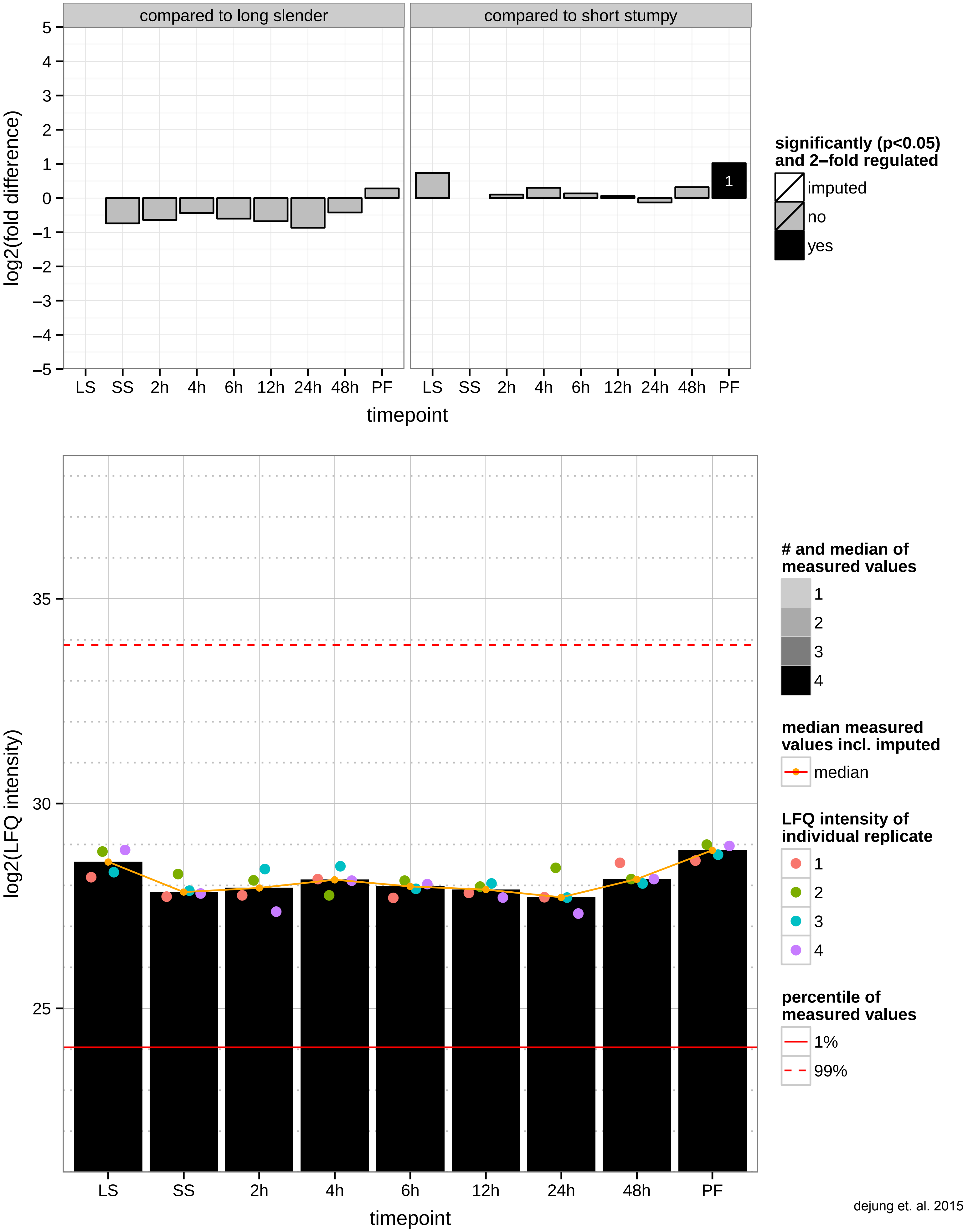

dejung et. al. 2015

nuclear segregation protein, putative
Tb927.10.14150
AGOF: RNA binding
AGOC: nuclear outer membrane–endoplasmic reticulum membrane network, polysome, ribonucleoprotein complex
AGOP: chromosome segregation, meiosis, regulation of mitosis
PGOF: null
PGOC: null
PGOP: null

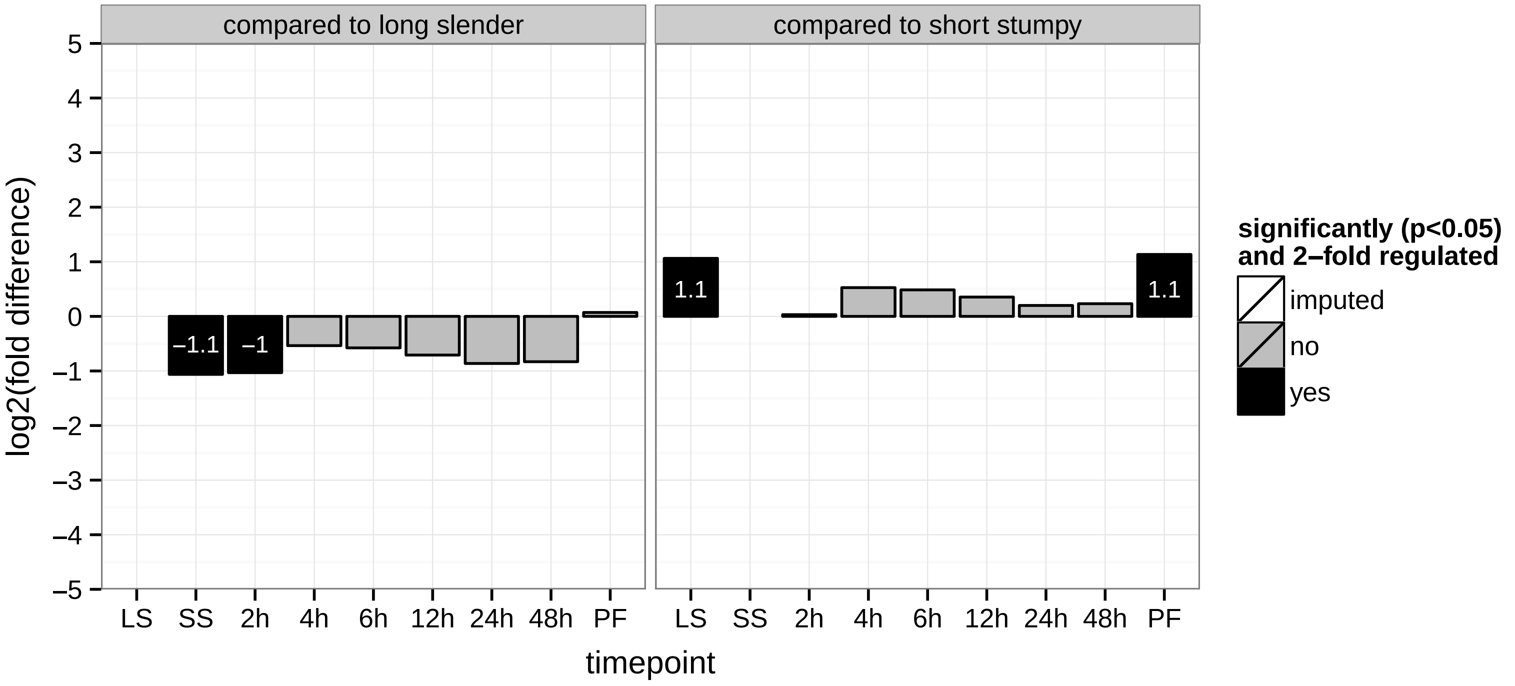

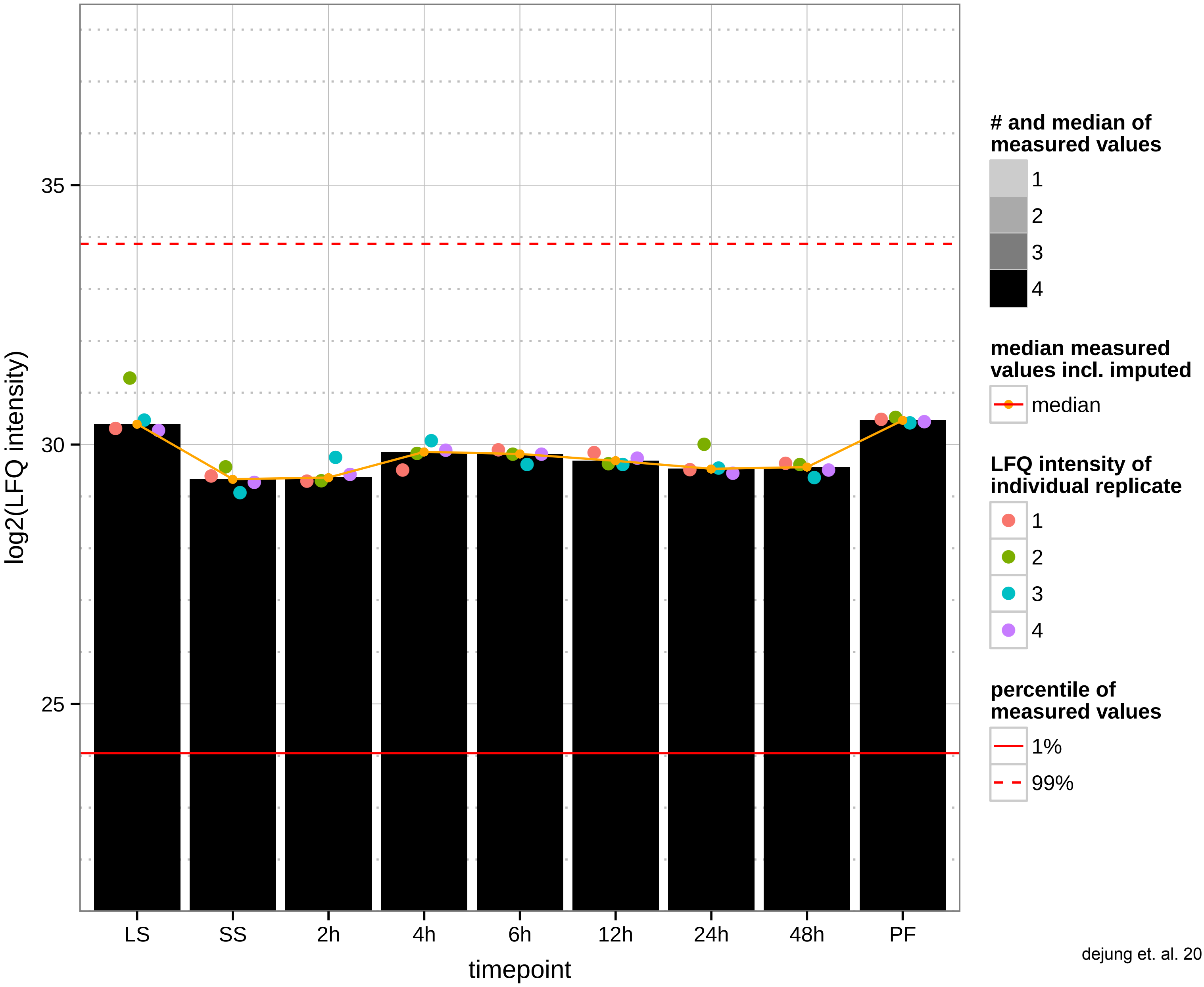

dejung et. al. 2015

hypothetical protein, conserved
Tb927.10.14320
AGOF: null
AGOC: null
AGOP: null
PGOF: binding, protein binding
PGOC: null
PGOP: null

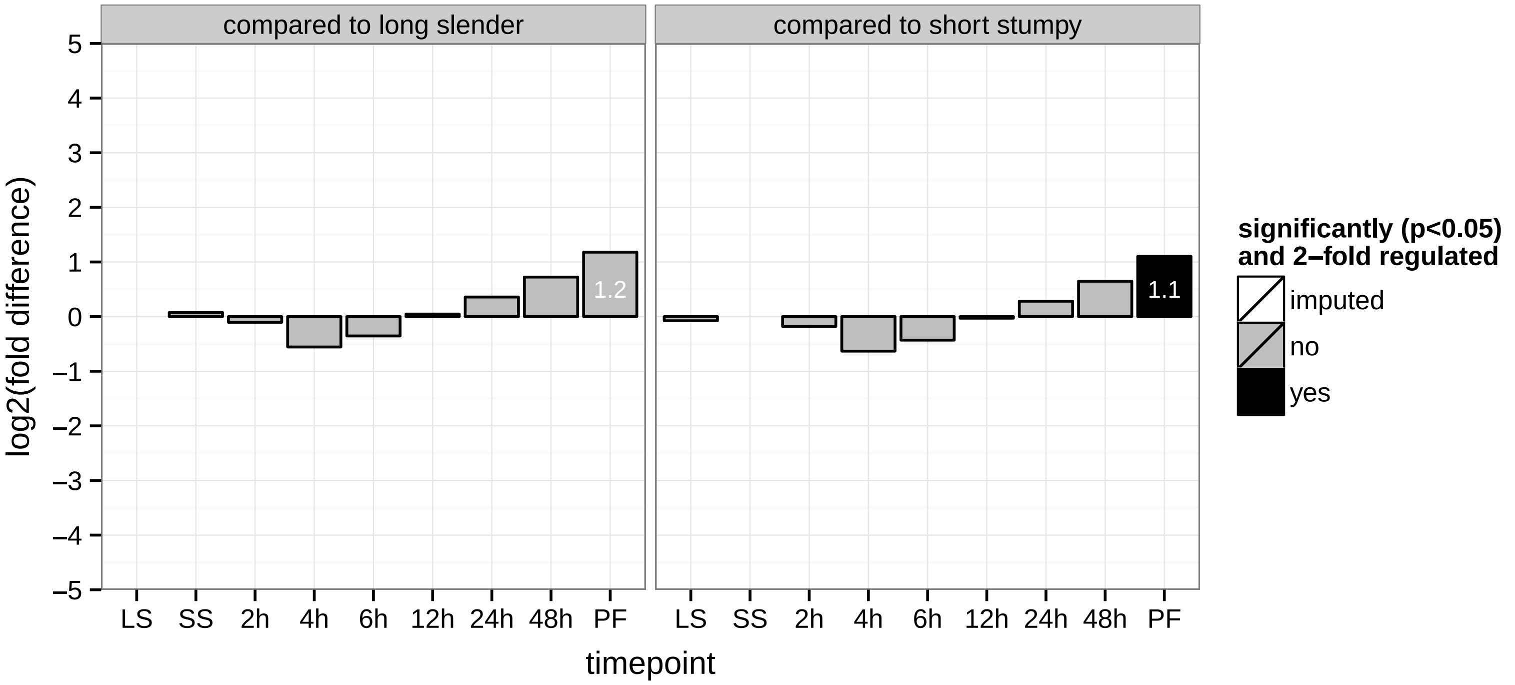

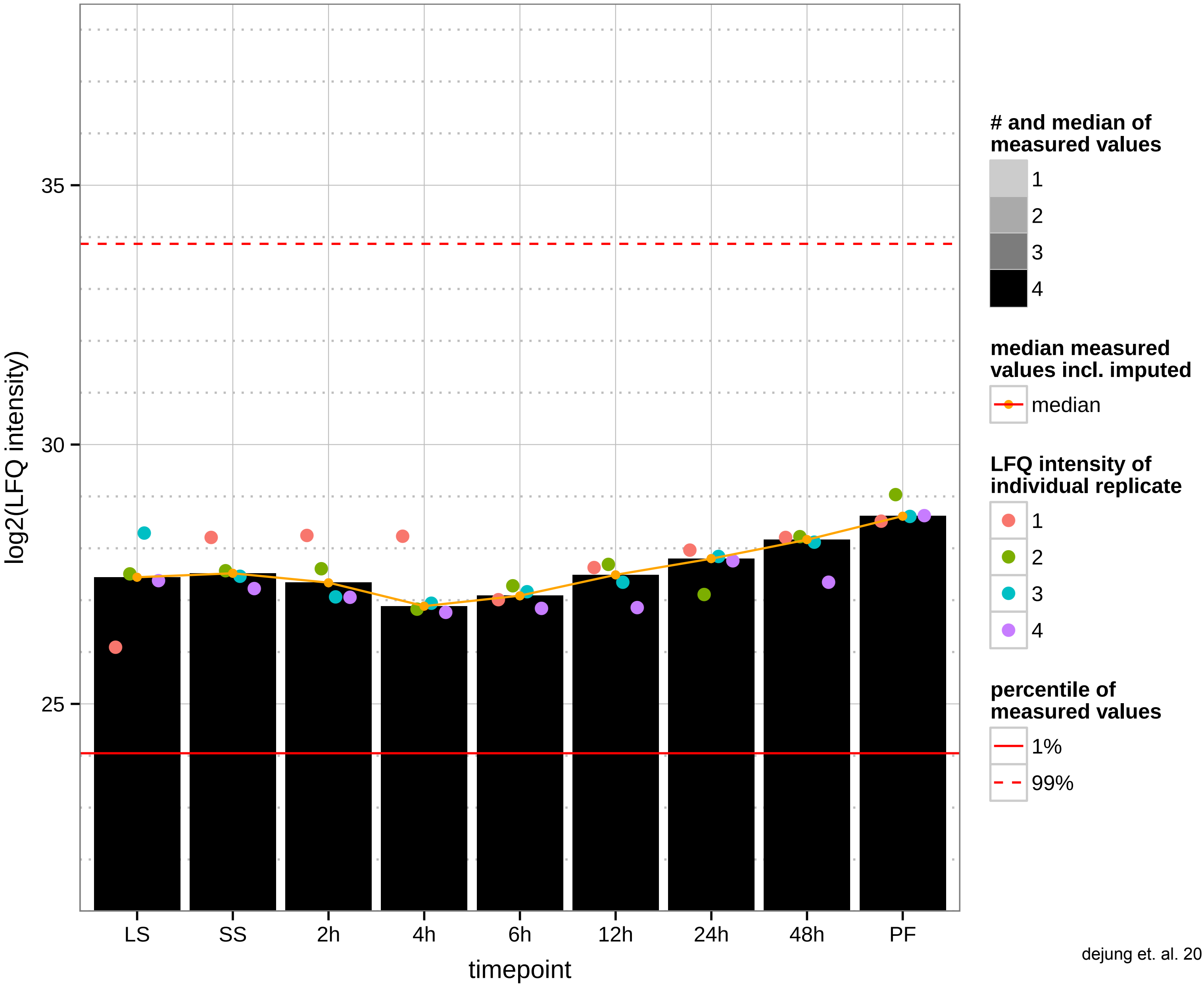

dejung et. al. 2015

hypothetical protein, conserved
Tb927.10.14380
AGOF: ATP binding
AGOC: null
AGOP: null
PGOF: null
PGOC: null
PGOP: null

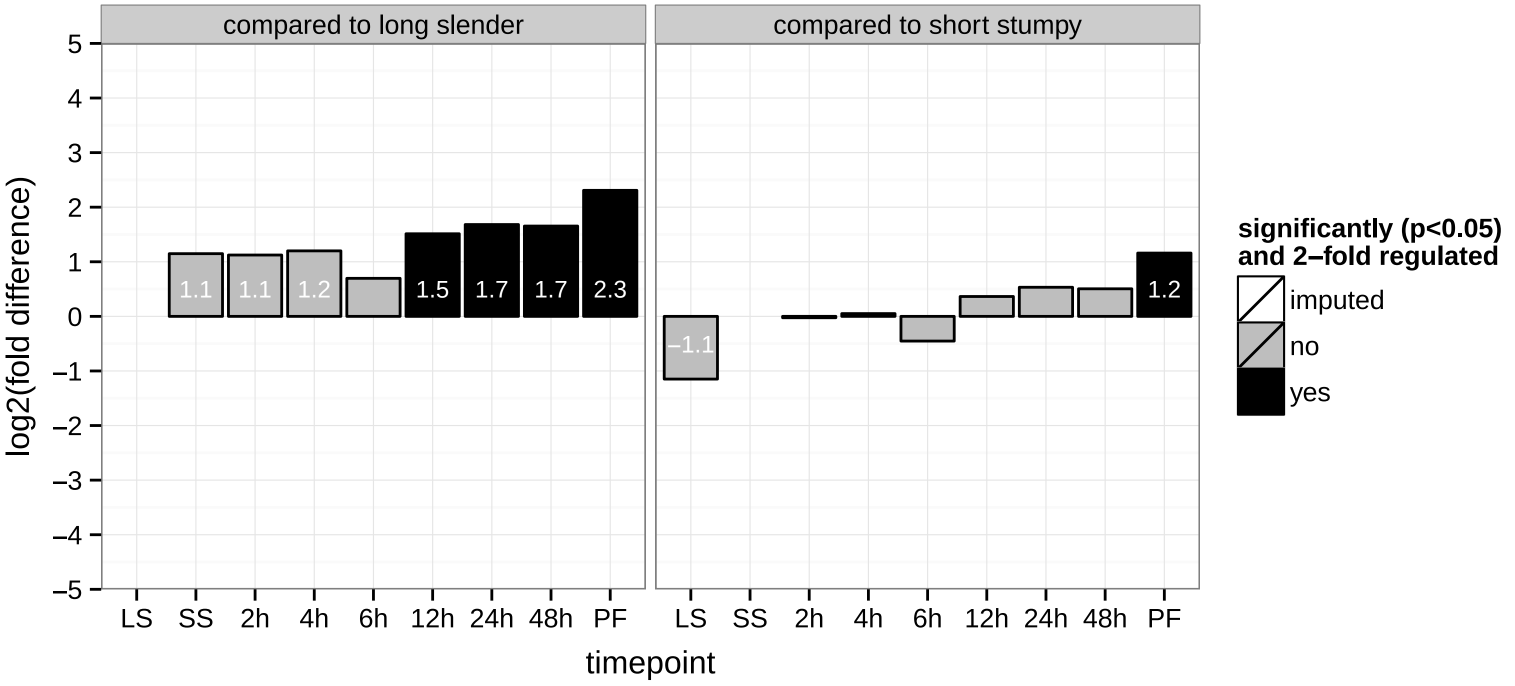

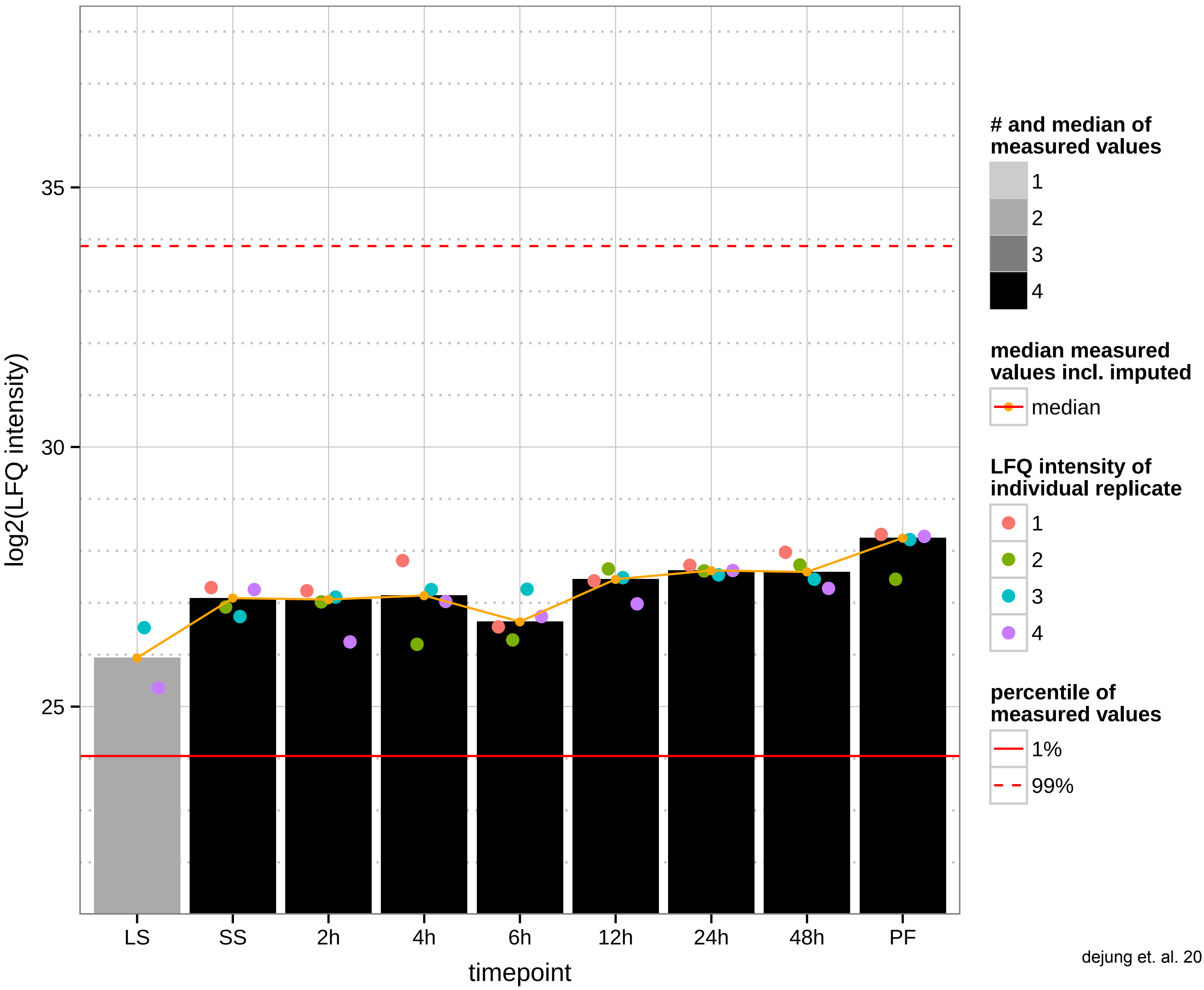

dejung et. al. 2015

hypothetical protein, conserved
Tb927.10.14430
AGOF: null
AGOC: null
AGOP: null
PGOF: null
PGOC: null
PGOP: null

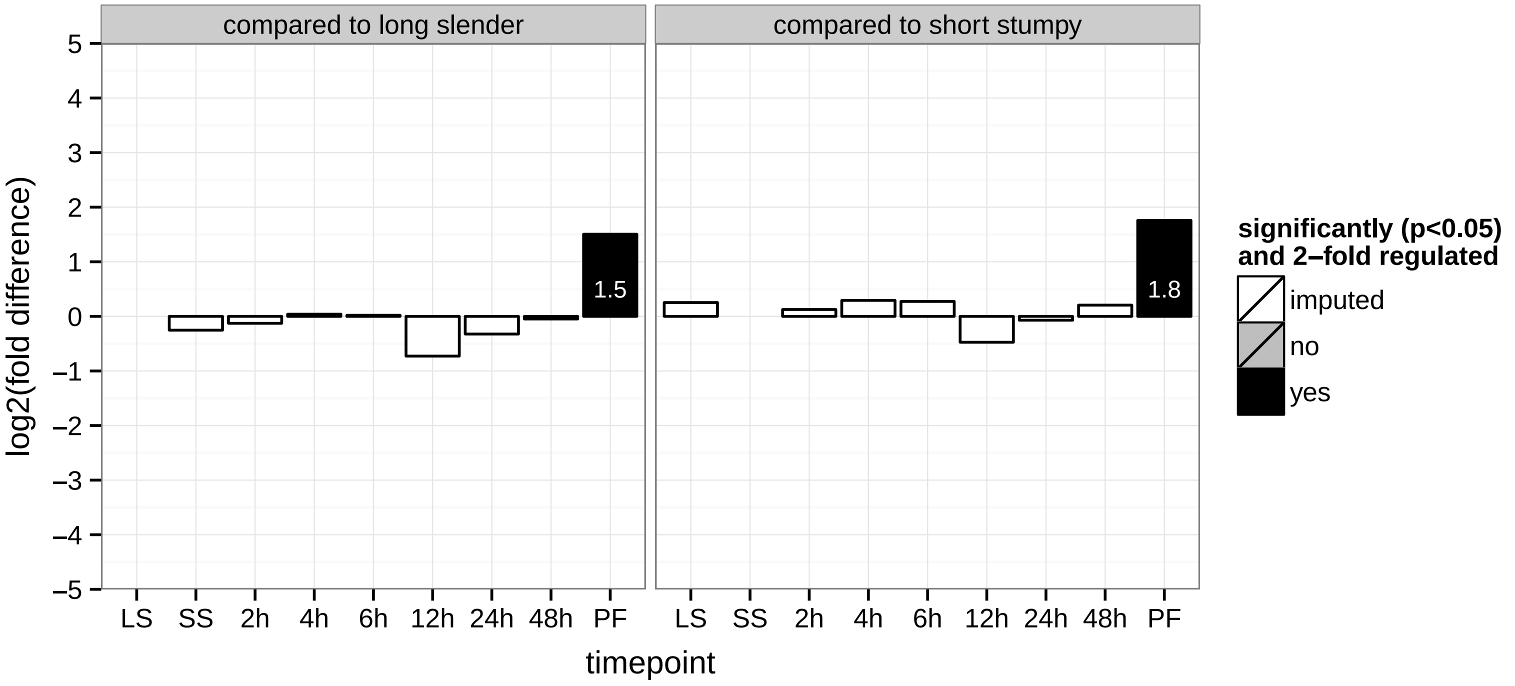

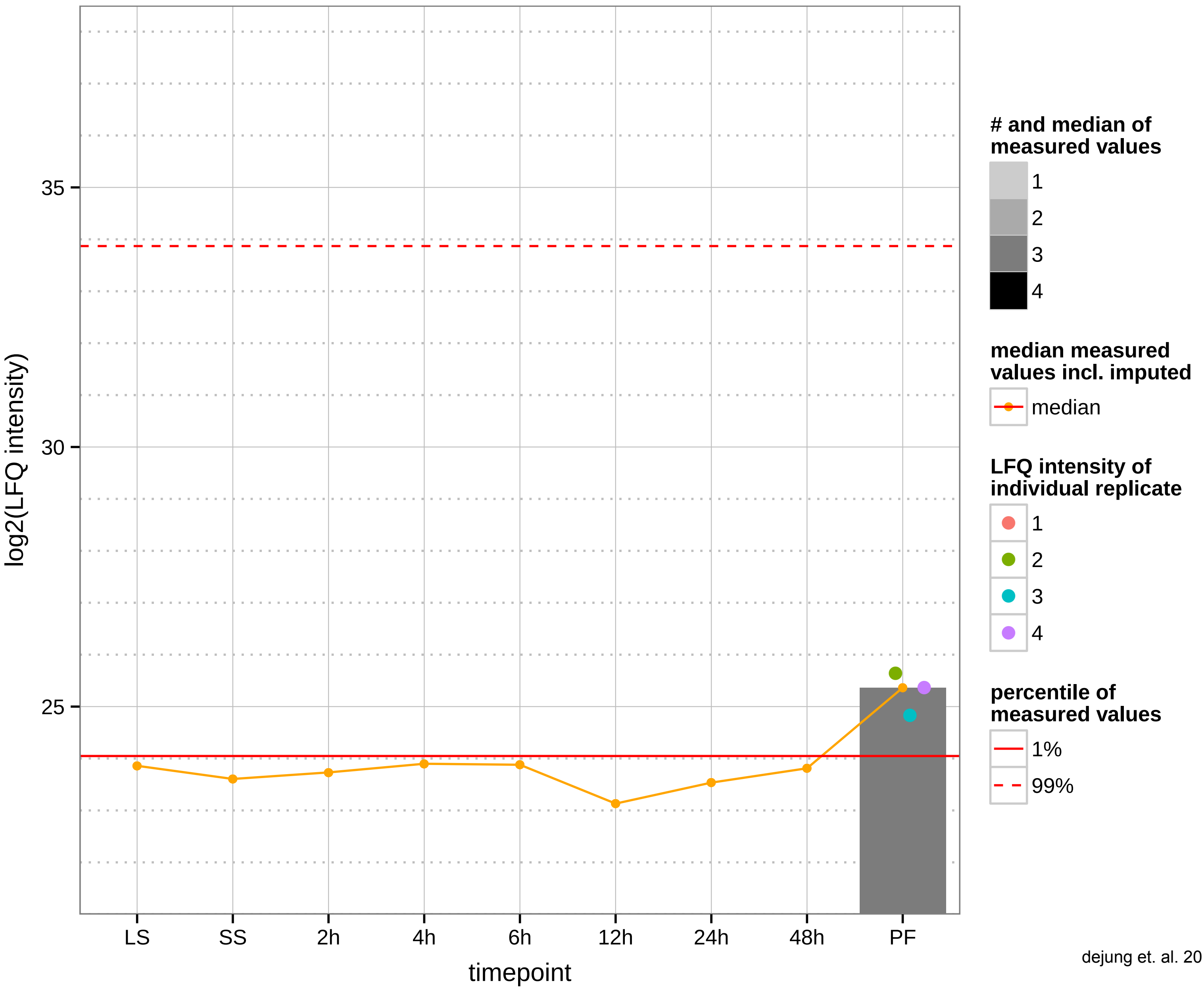

dejung et. al. 2015

hypothetical protein, conserved
Tb927.10.14870
AGOF: null
AGOC: null
AGOP: null
PGOF: null
PGOC: null
PGOP: null

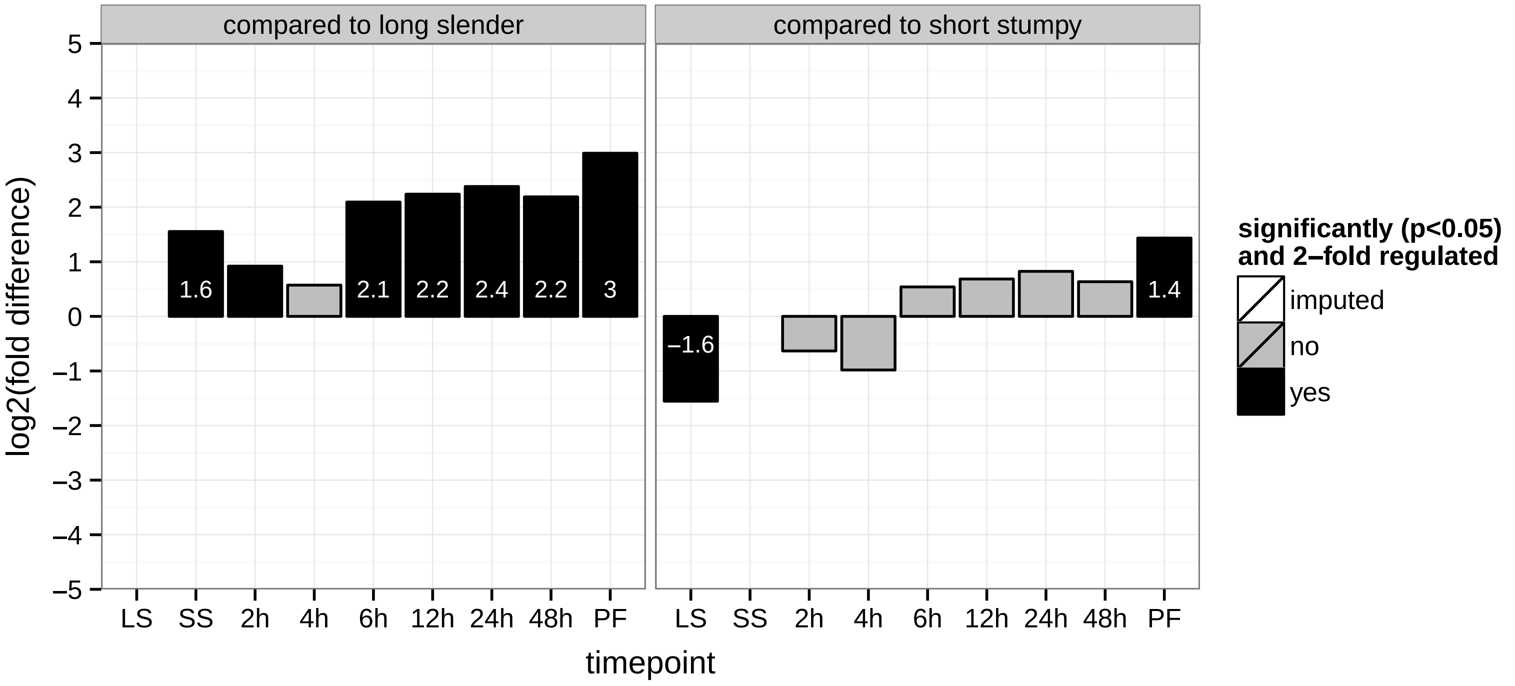

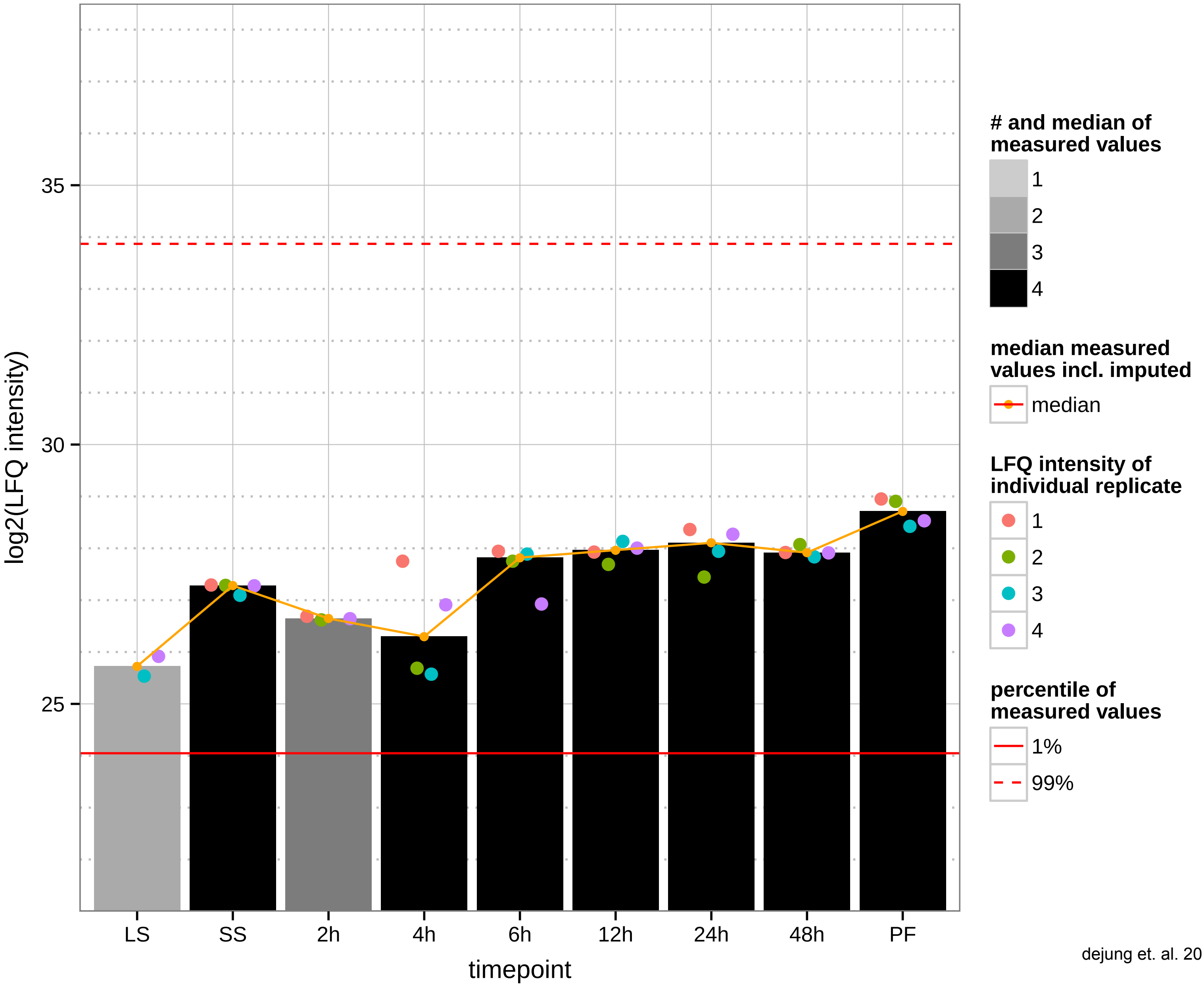

dejung et. al. 2015

ATP−dependent DEAD/H RNA helicase, putative
Tb927.10.1720
AGOF: ATP binding, ATP−dependent helicase activity, helicase activity, nucleic acid binding
AGOC: null
AGOP: nucleobase−containing compound metabolic process
PGOF: ATP binding, ATP−dependent helicase activity, helicase activity, nucleic acid binding
PGOC: null
PGOP: null

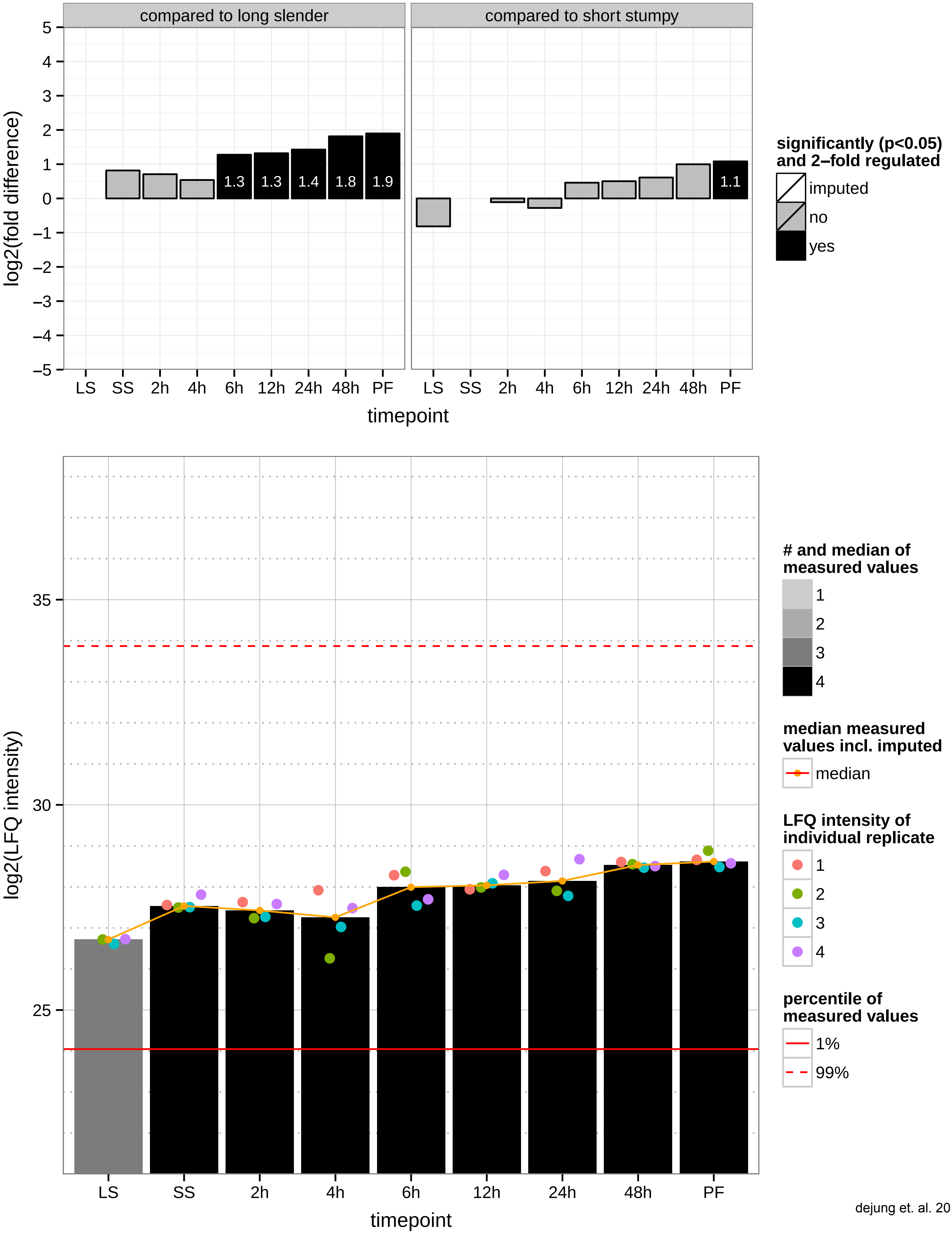

dejung et. al. 2015

chaperone protein DNAj, putative
Tb927.10.2290
AGOF: chaperone binding, heat shock protein binding, unfolded protein binding
AGOC: null
AGOP: protein folding
PGOF: heat shock protein binding, unfolded protein binding
PGOC: null
PGOP: protein folding

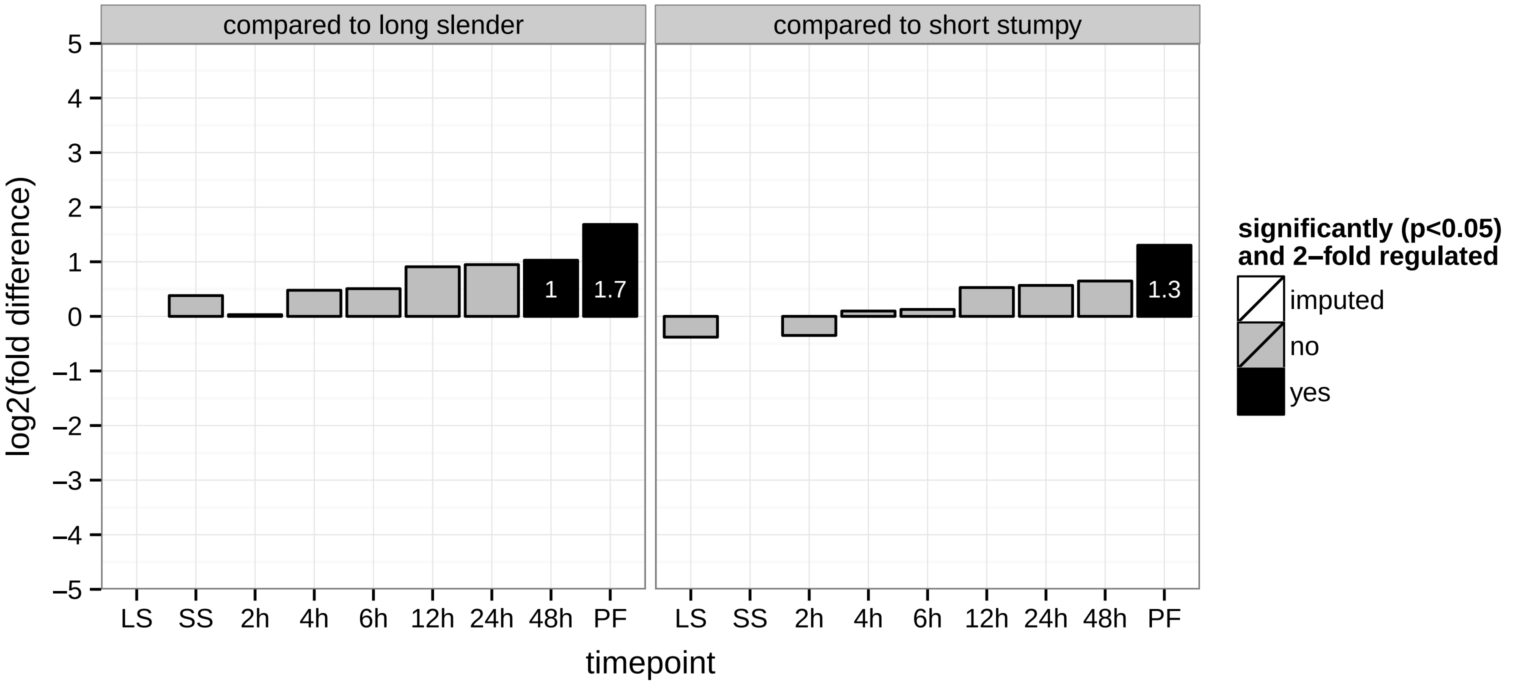

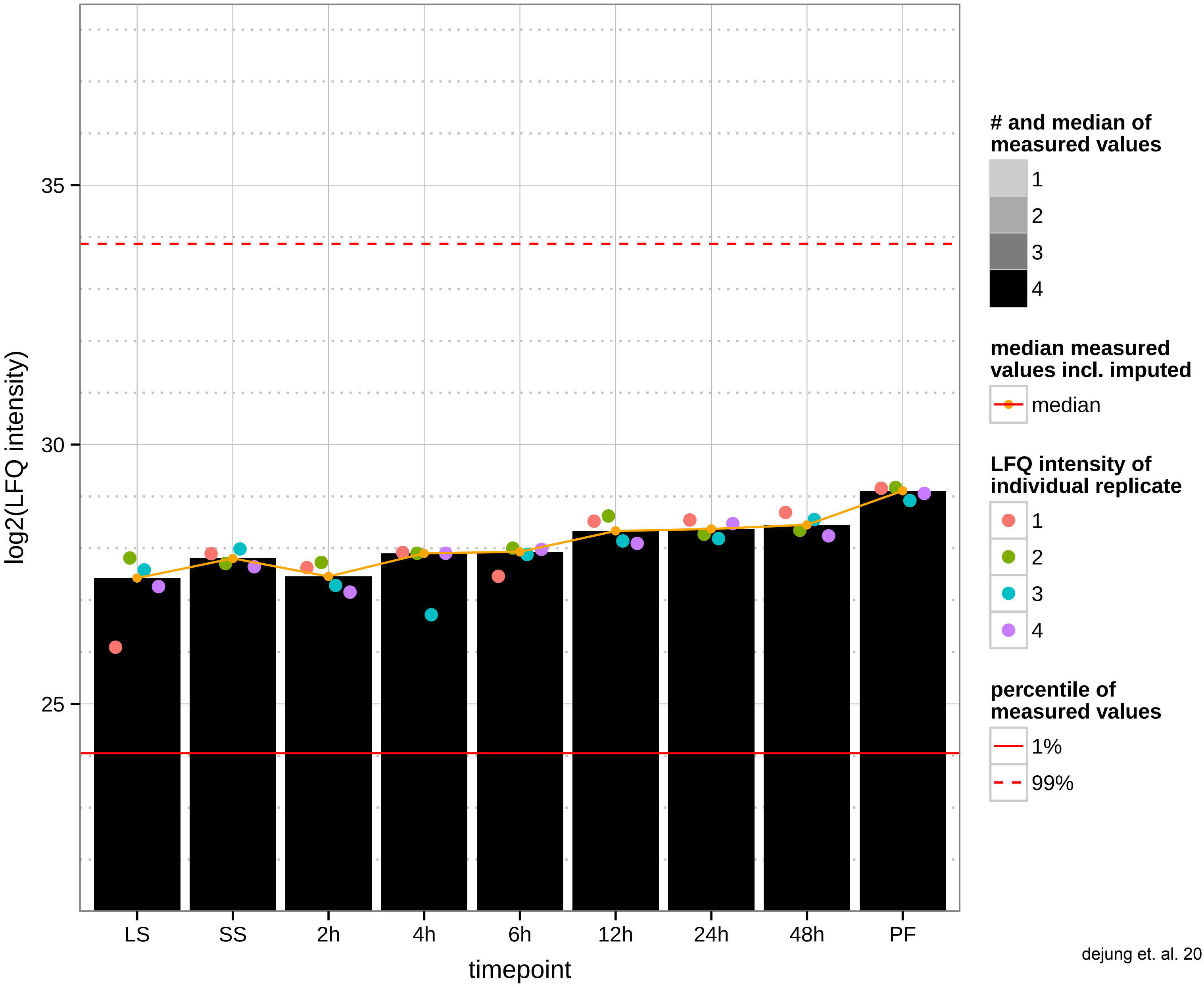

dejung et. al. 2015

hypothetical protein, conserved
Tb927.10.2320
AGOF: null
AGOC: null
AGOP: null
PGOF: protein binding
PGOC: null
PGOP: null

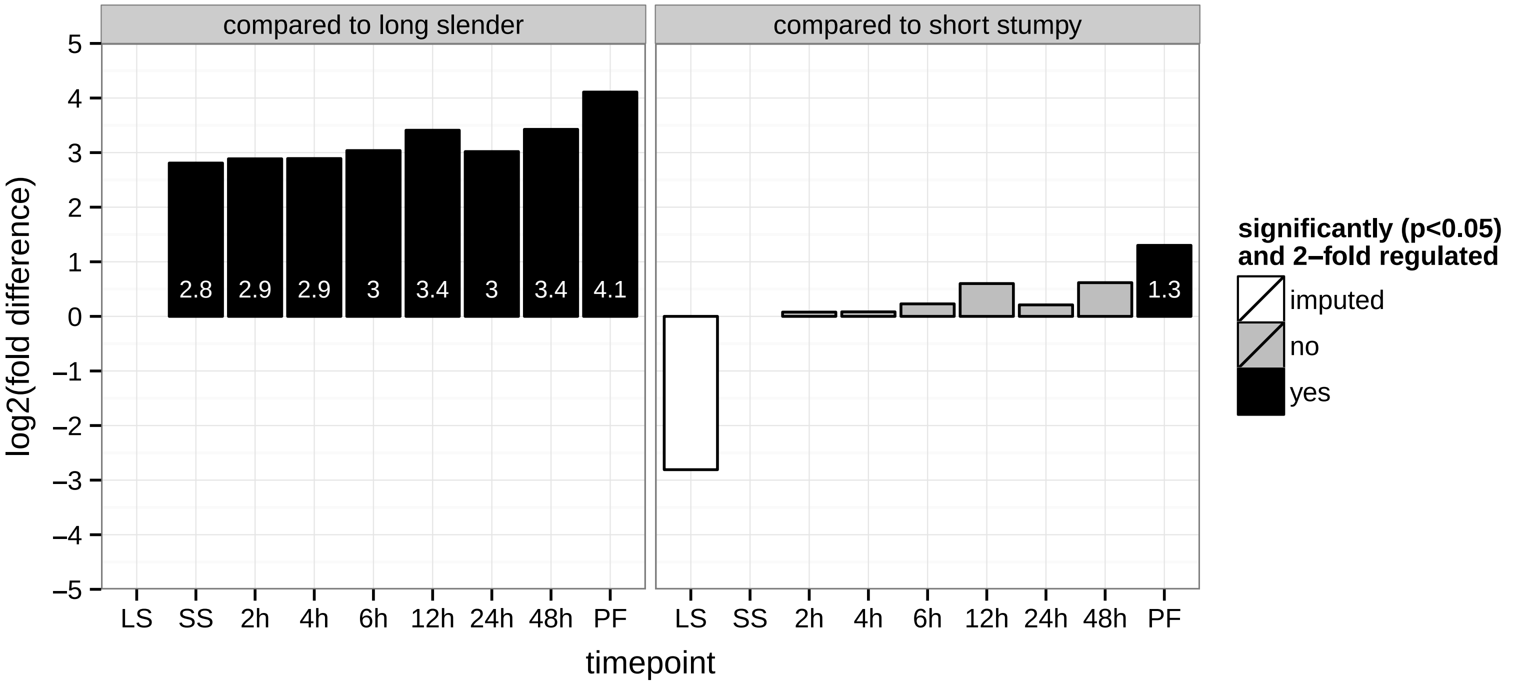

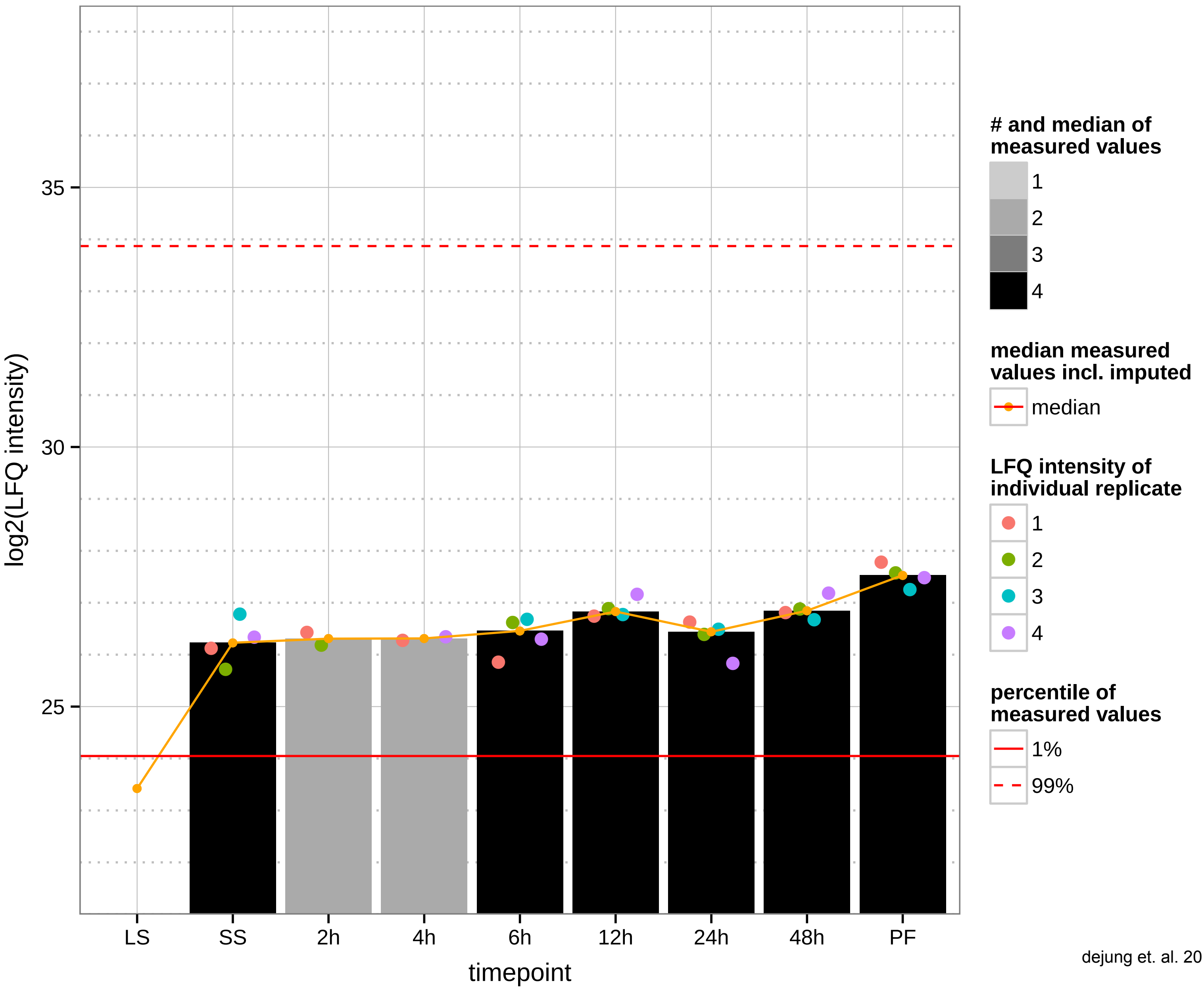

dejung et. al. 2015

peroxin 14, putative (PEX14)
Tb927.10.240
AGOF: null
AGOC: glycosome membrane, peroxisome
AGOP: protein targeting to vacuole
PGOF: null
PGOC: null
PGOP: null

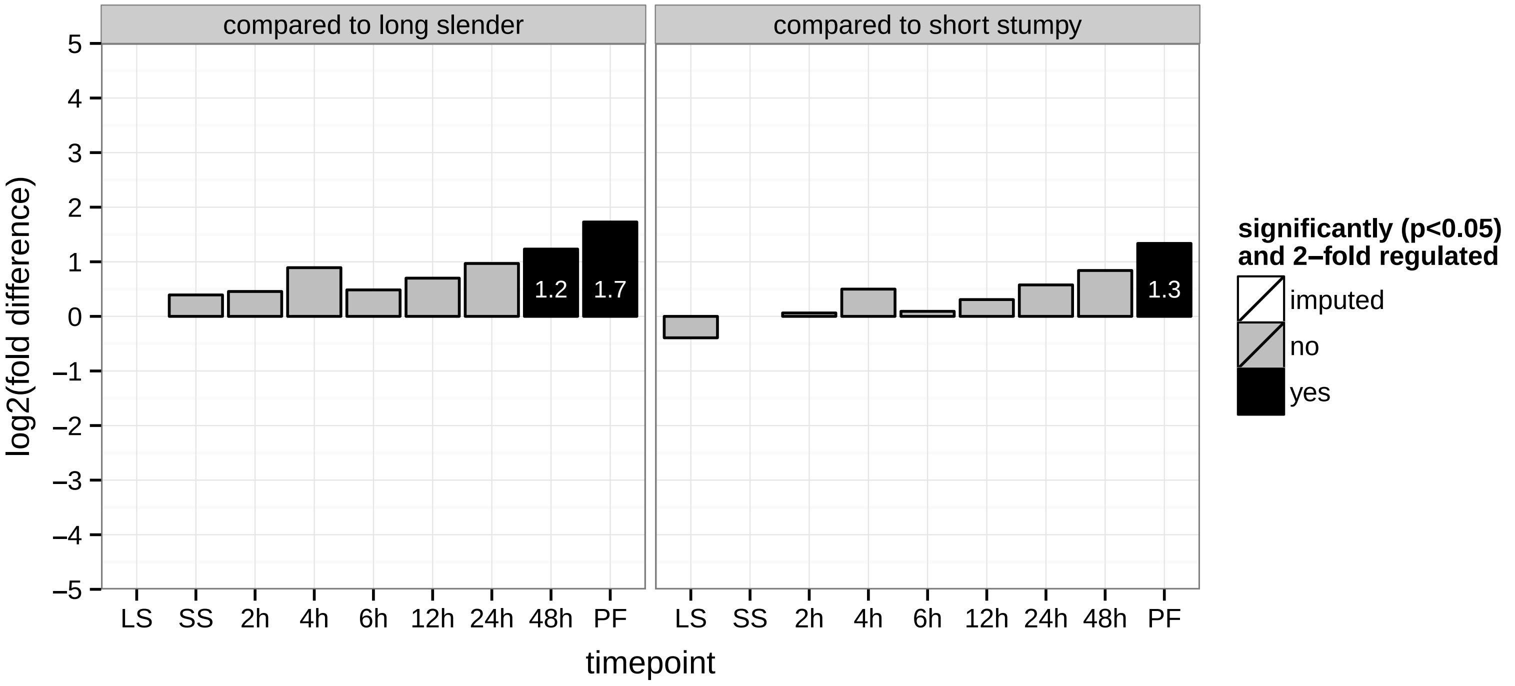

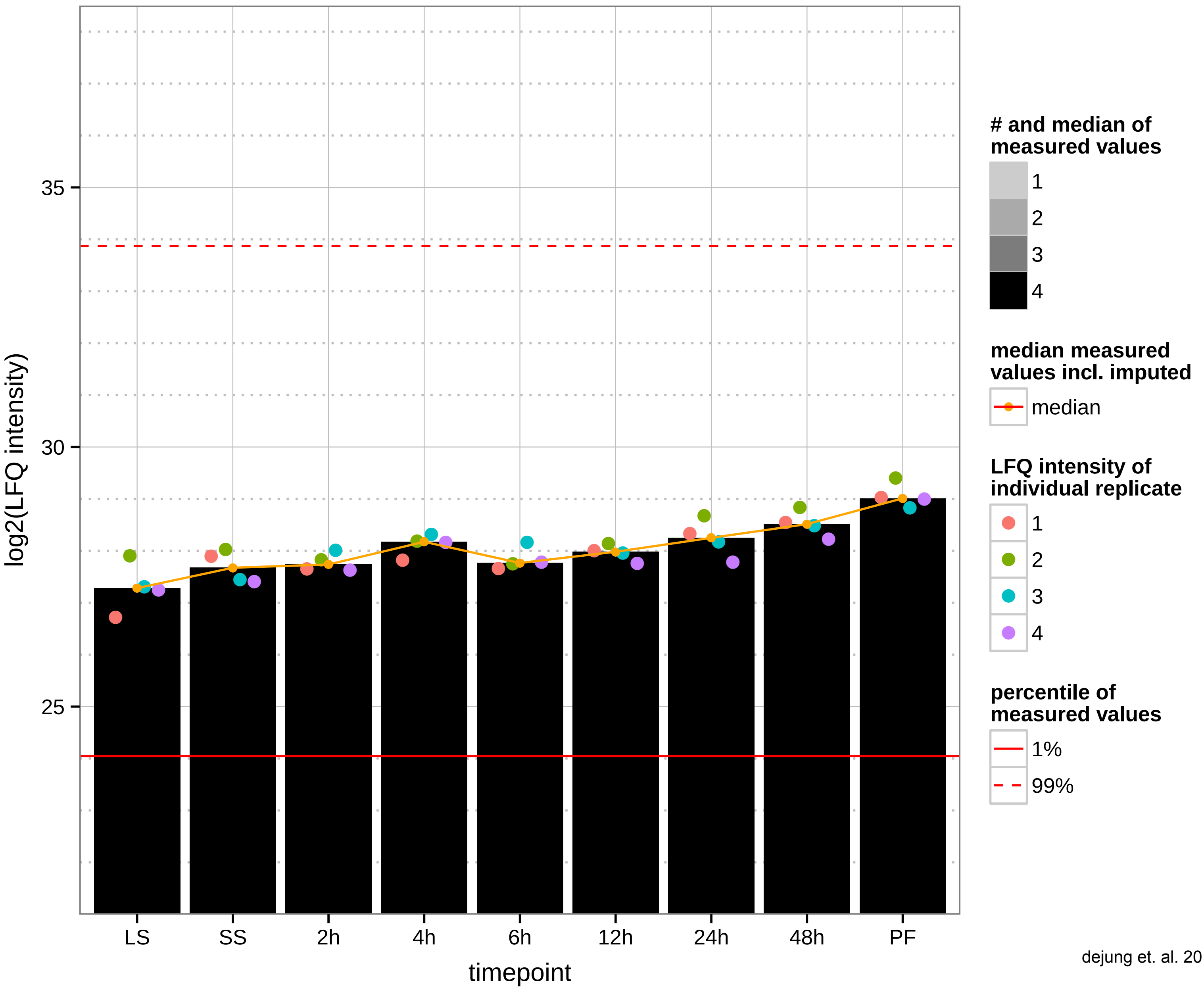

dejung et. al. 2015

nuclear cap binding complex subunit CBP110, putative, hypothetical protein, conserved (CBP110), nuclear cap binding compl
Tb927.10.2990;Tb11.v5.1039
AGOF: null
AGOC: nuclear cap binding complex
AGOP: null
PGOF: null
PGOC: null
PGOP: null

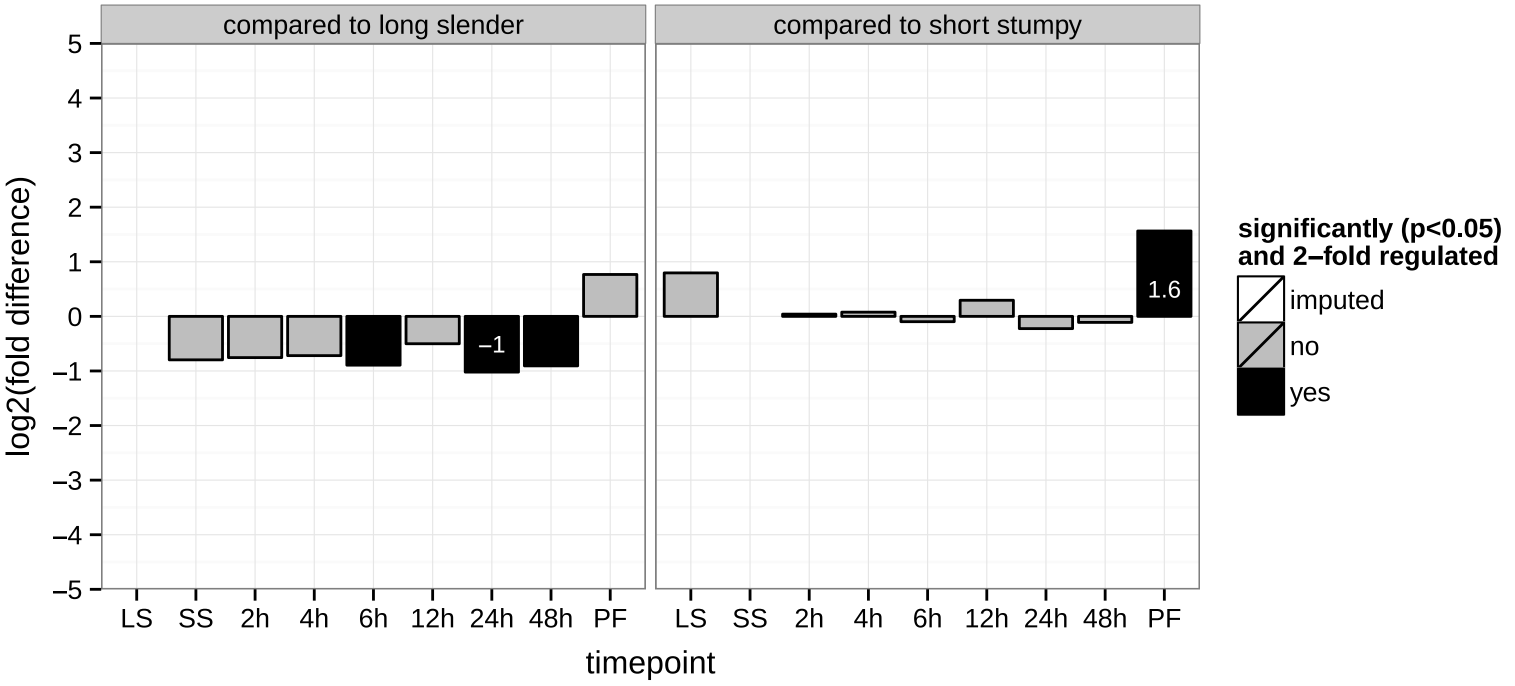

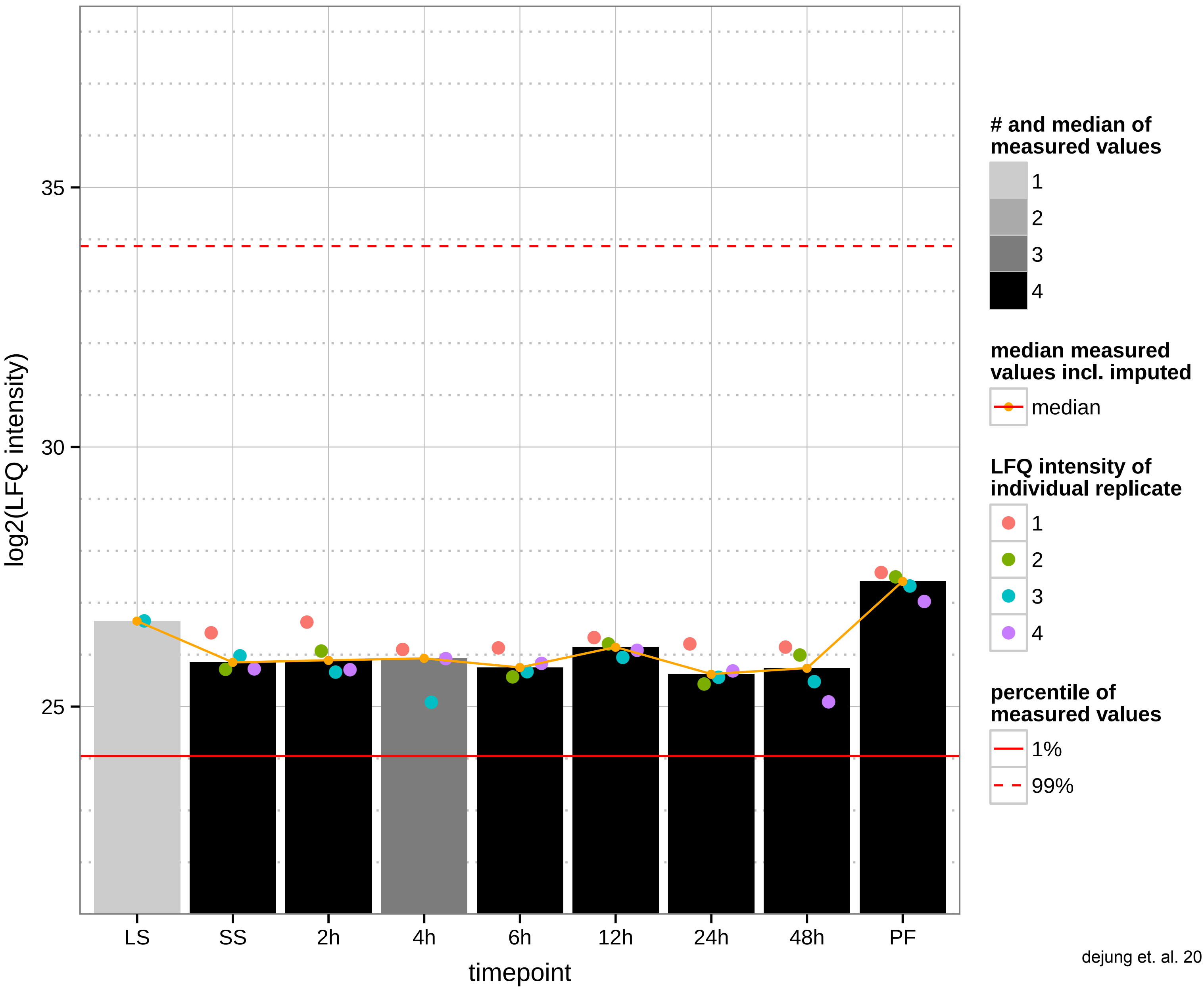

dejung et. al. 2015

cytochrome c oxidase assembly protein, putative
Tb927.10.3120
AGOF: copper ion binding
AGOC: mitochondrion
AGOP: aerobic respiration
PGOF: copper ion binding
PGOC: null
PGOP: null

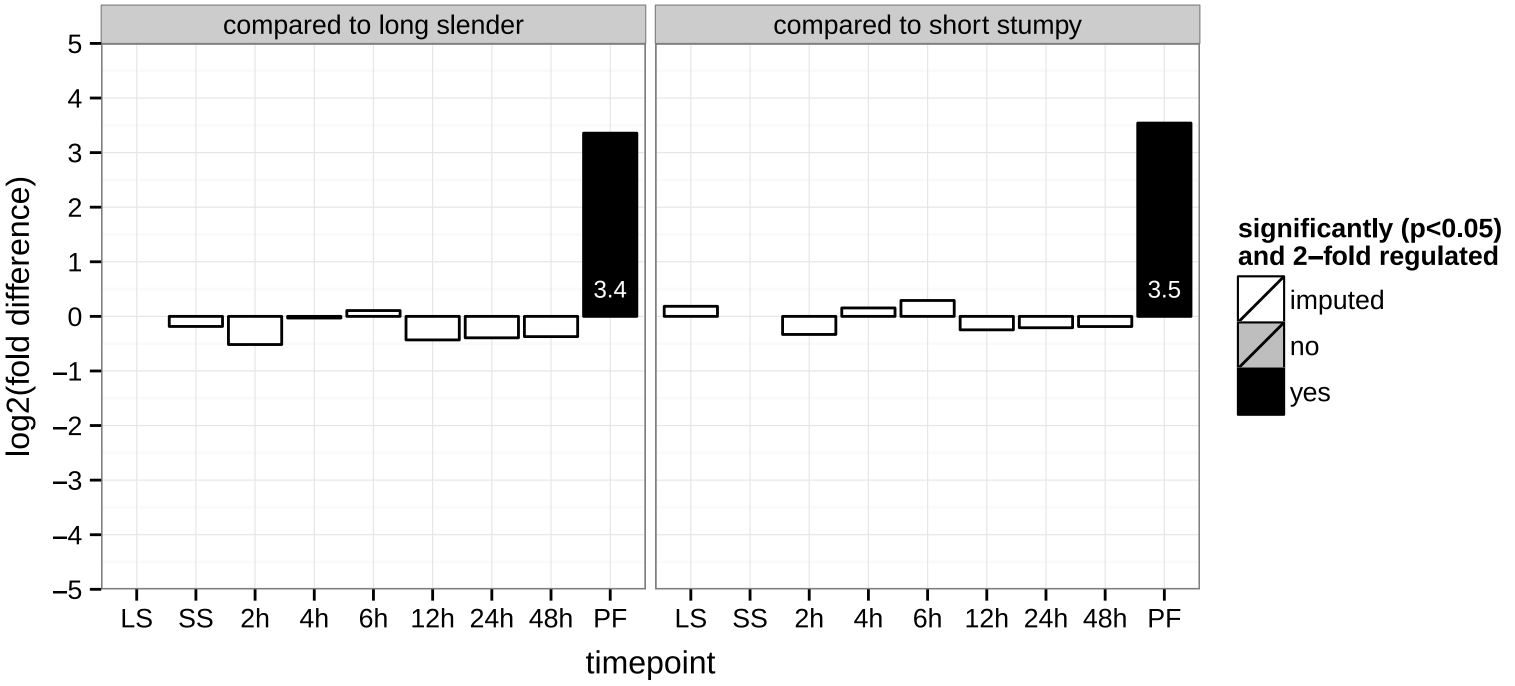

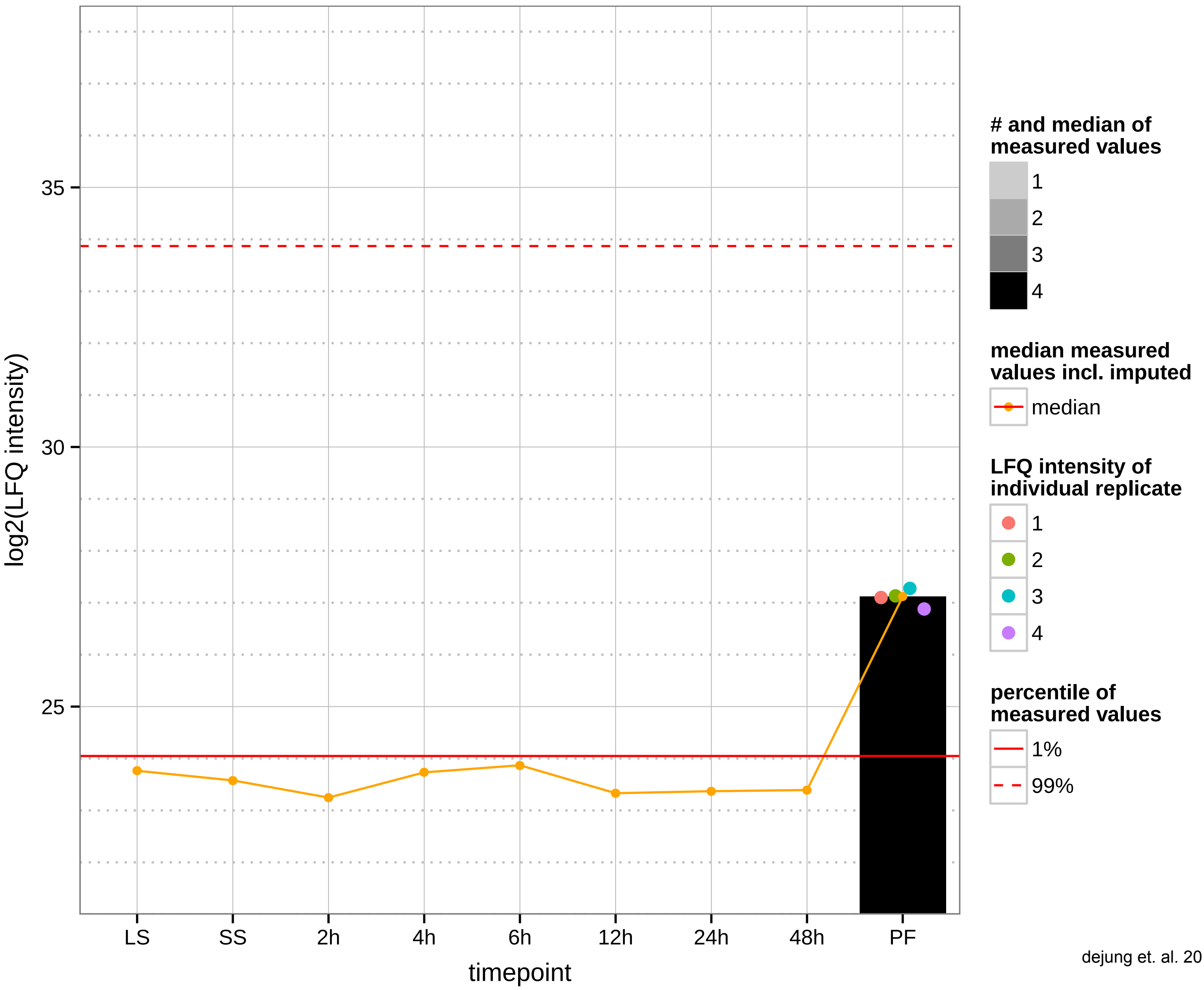

dejung et. al. 2015

60S acidic ribosomal protein P2, putative
Tb927.10.3380;Tb927.10.3370
AGOF: structural constituent of ribosome
AGOC: intracellular, ribosome
AGOP: translation, translational elongation
PGOF: null, structural constituent of ribosome
PGOC: null, intracellular, ribosome
PGOP: null, translational elongation

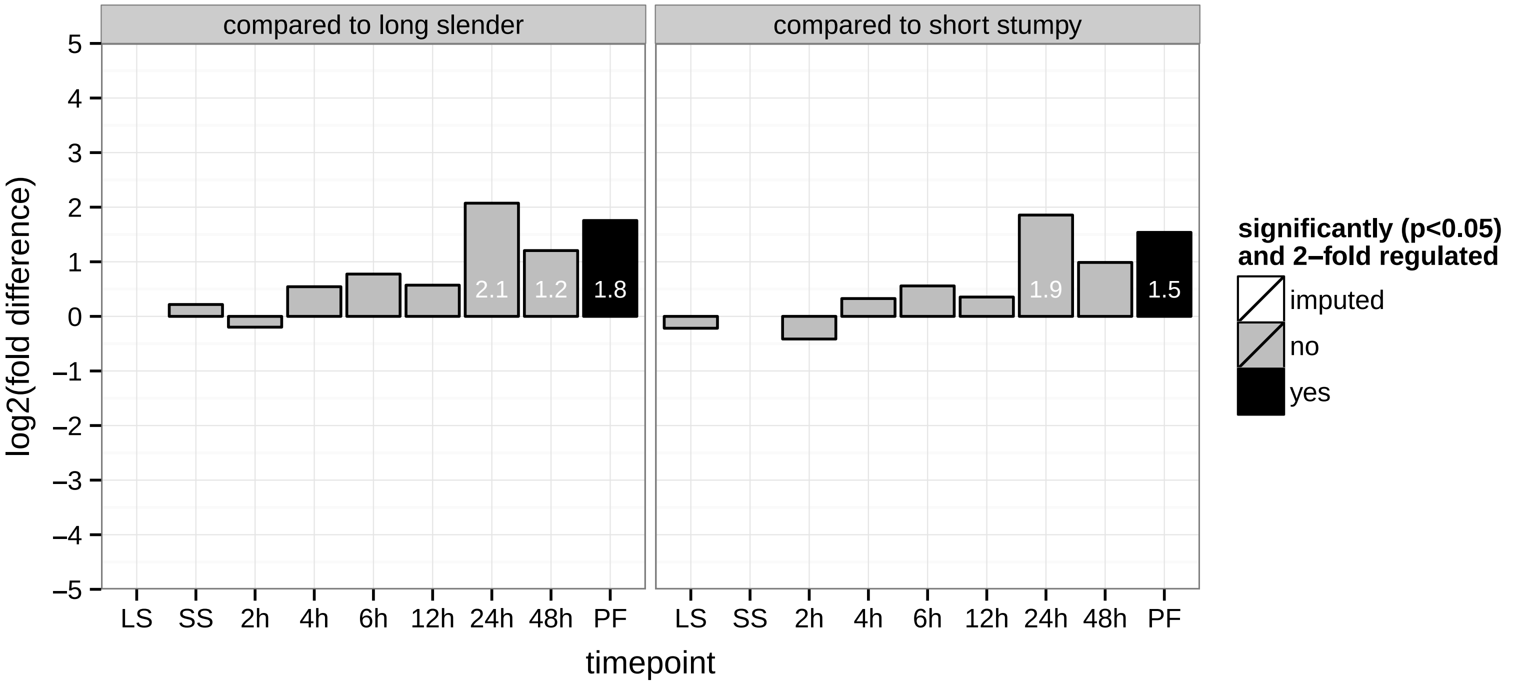

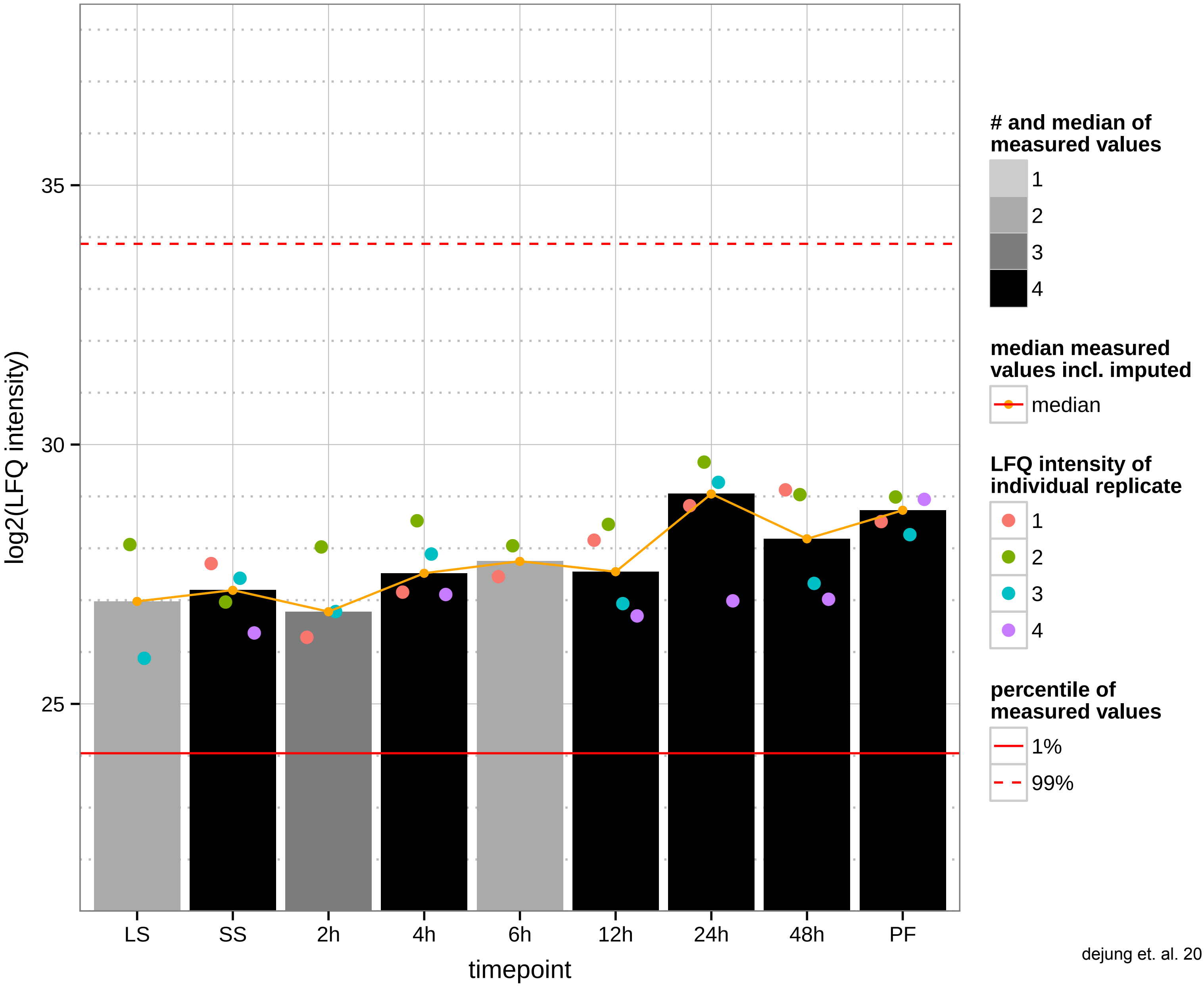

dejung et. al. 2015

hypothetical protein, conserved
Tb927.10.3430
AGOF: null
AGOC: null
AGOP: null
PGOF: null
PGOC: null
PGOP: null

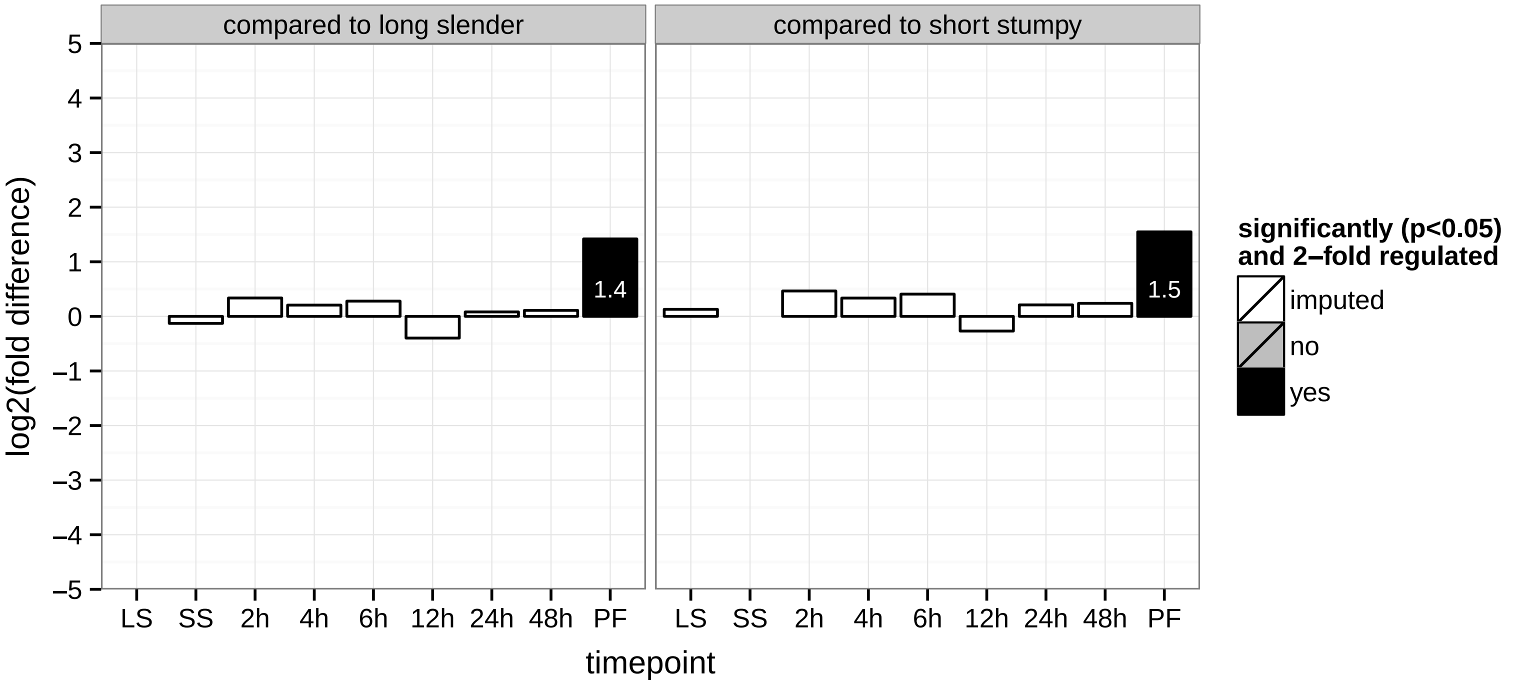

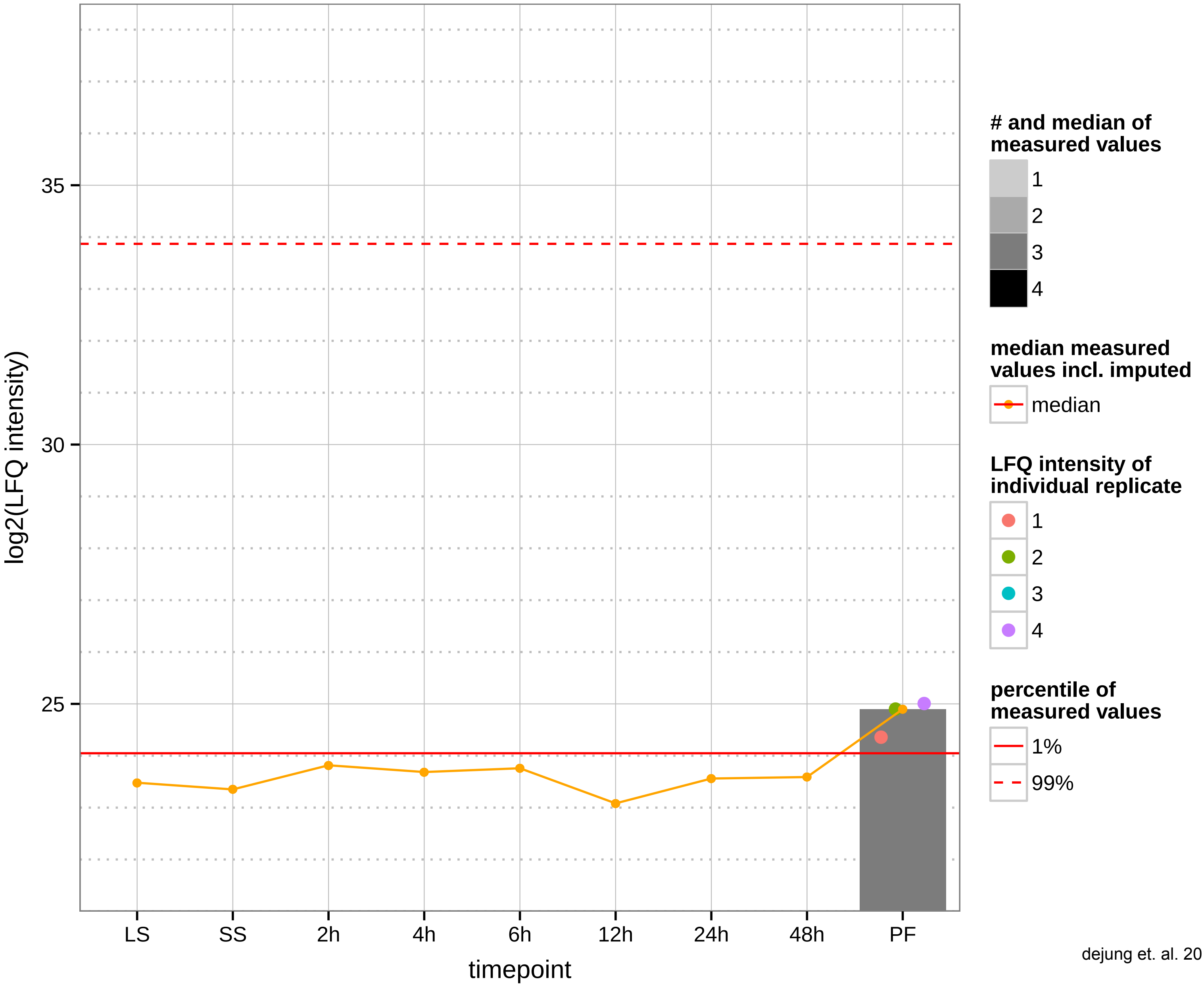

dejung et. al. 2015

serine palmitoyltransferase, putative
Tb927.10.4050;Tb11.v5.0649
AGOF: null, pyridoxal phosphate binding, serine C–palmitoyltransferase activity, transferase activity, transferring nitrogenous g
AGOC: null
AGOP: null, biosynthetic process
PGOF: pyridoxal phosphate binding, transferase activity
PGOC: null
PGOP: biosynthetic process

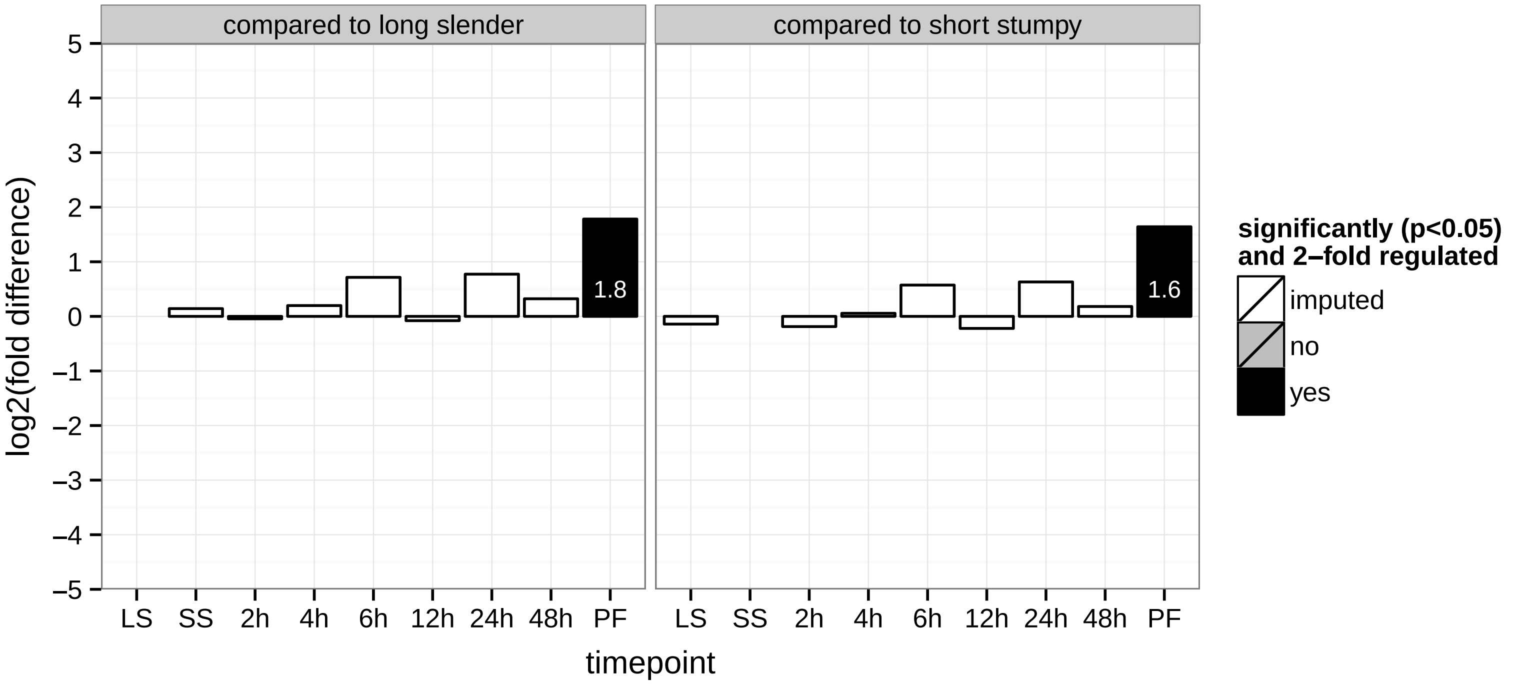

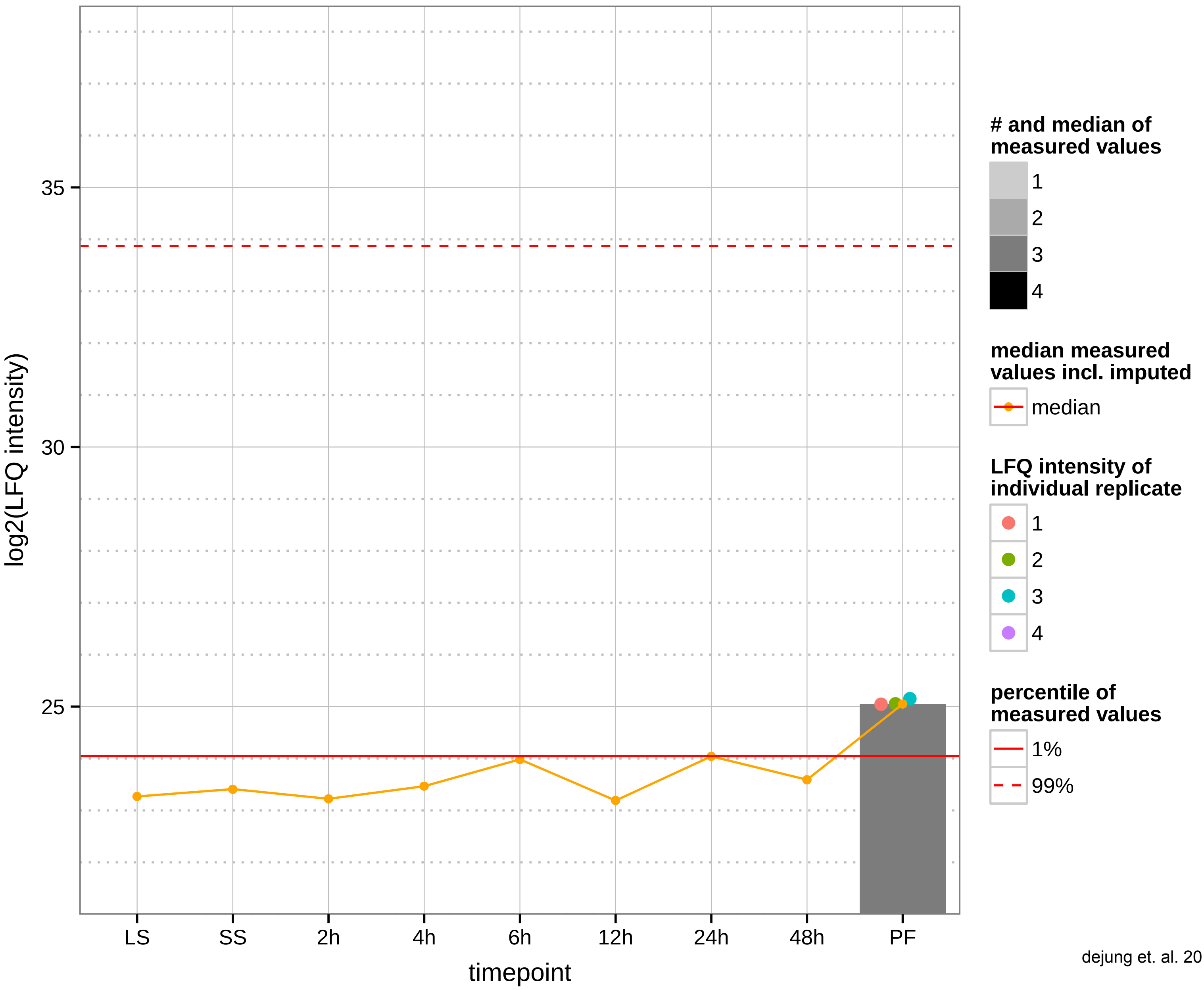

dejung et. al. 2015

elongation factor 2
Tb927.10.4570;Tb927.10.4560
AGOF: GTP binding, GTPase activity
AGOC: null
AGOP: translation
PGOF: null, GTP binding, GTPase activity
PGOC: null
PGOP: null

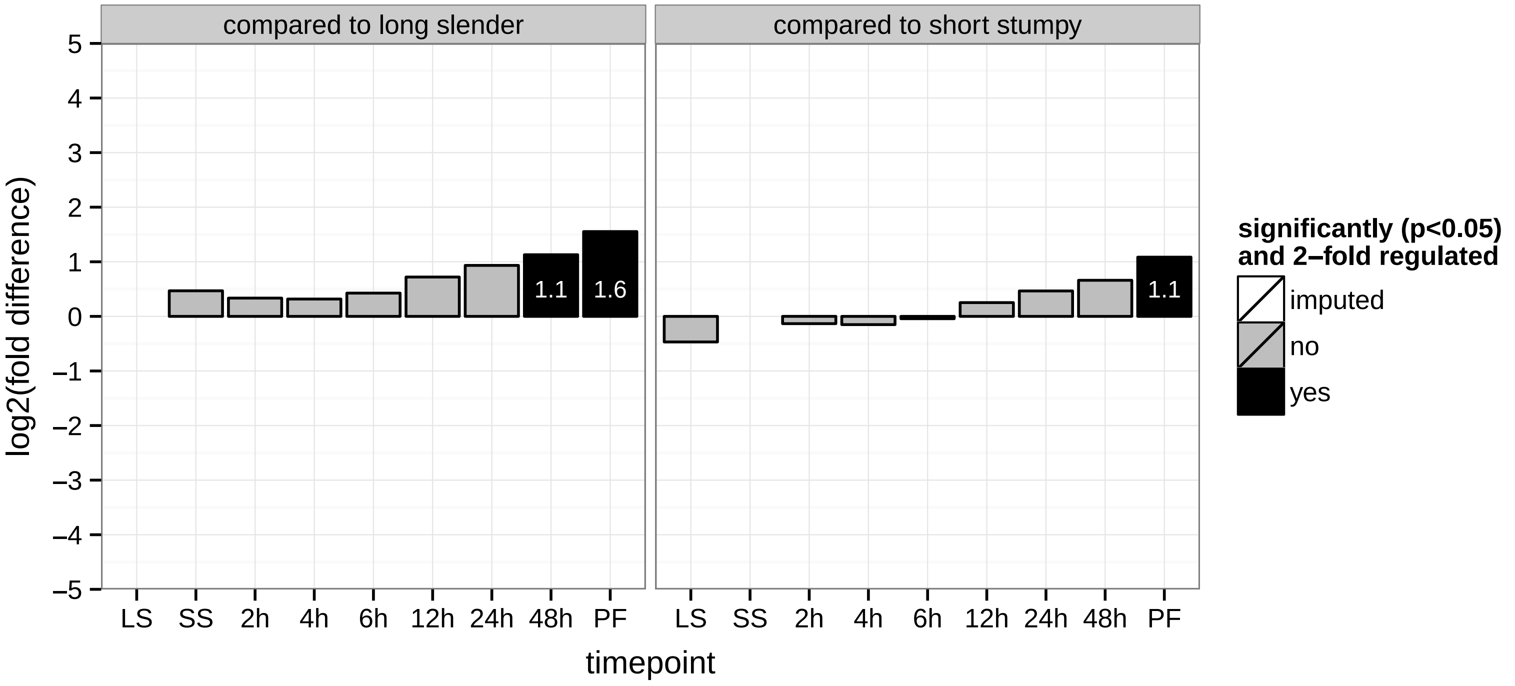

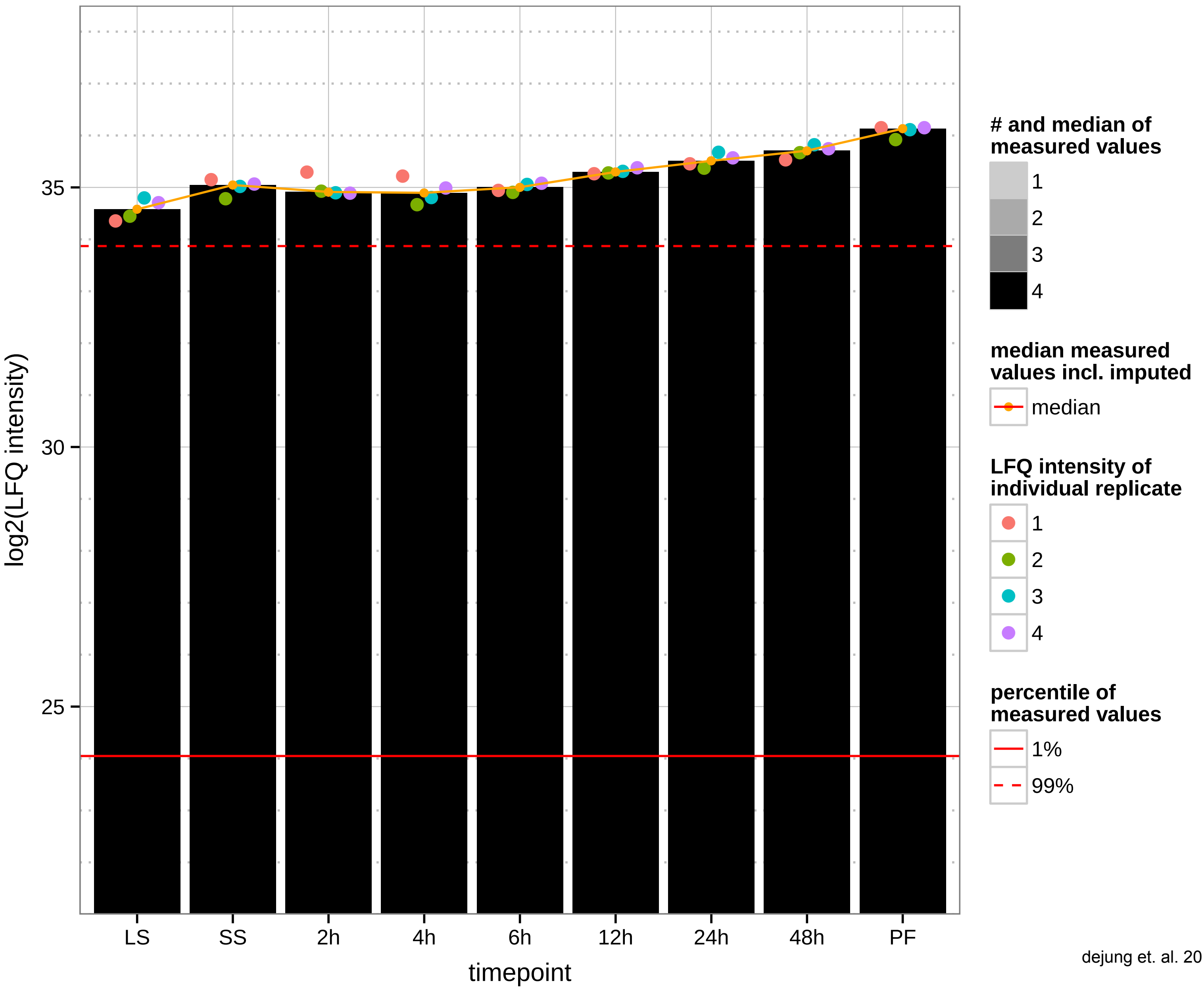

dejung et. al. 2015

nucleolar RNA–binding protein, putative
Tb927.10.4740
AGOF: RNA binding
AGOC: nucleolus
AGOP: rRNA metabolic process
PGOF: null
PGOC: null
PGOP: null

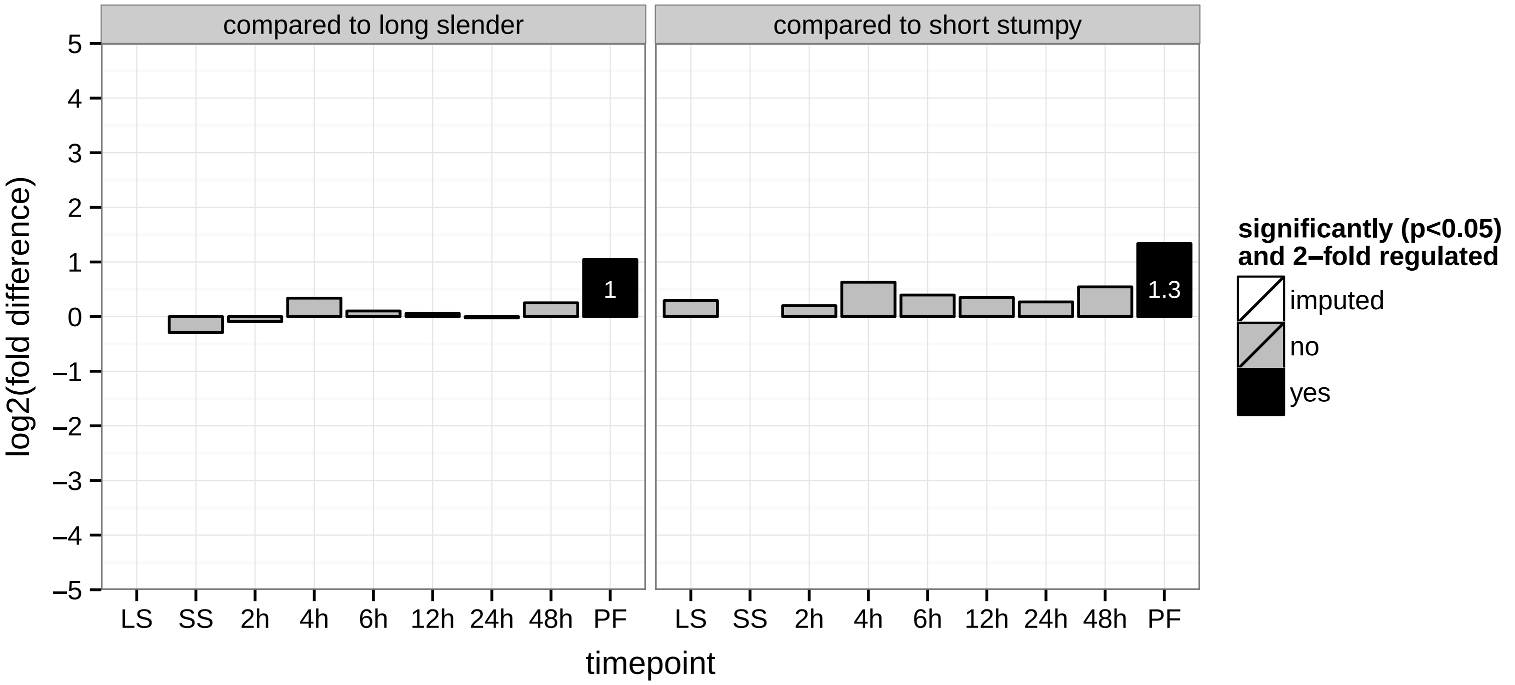

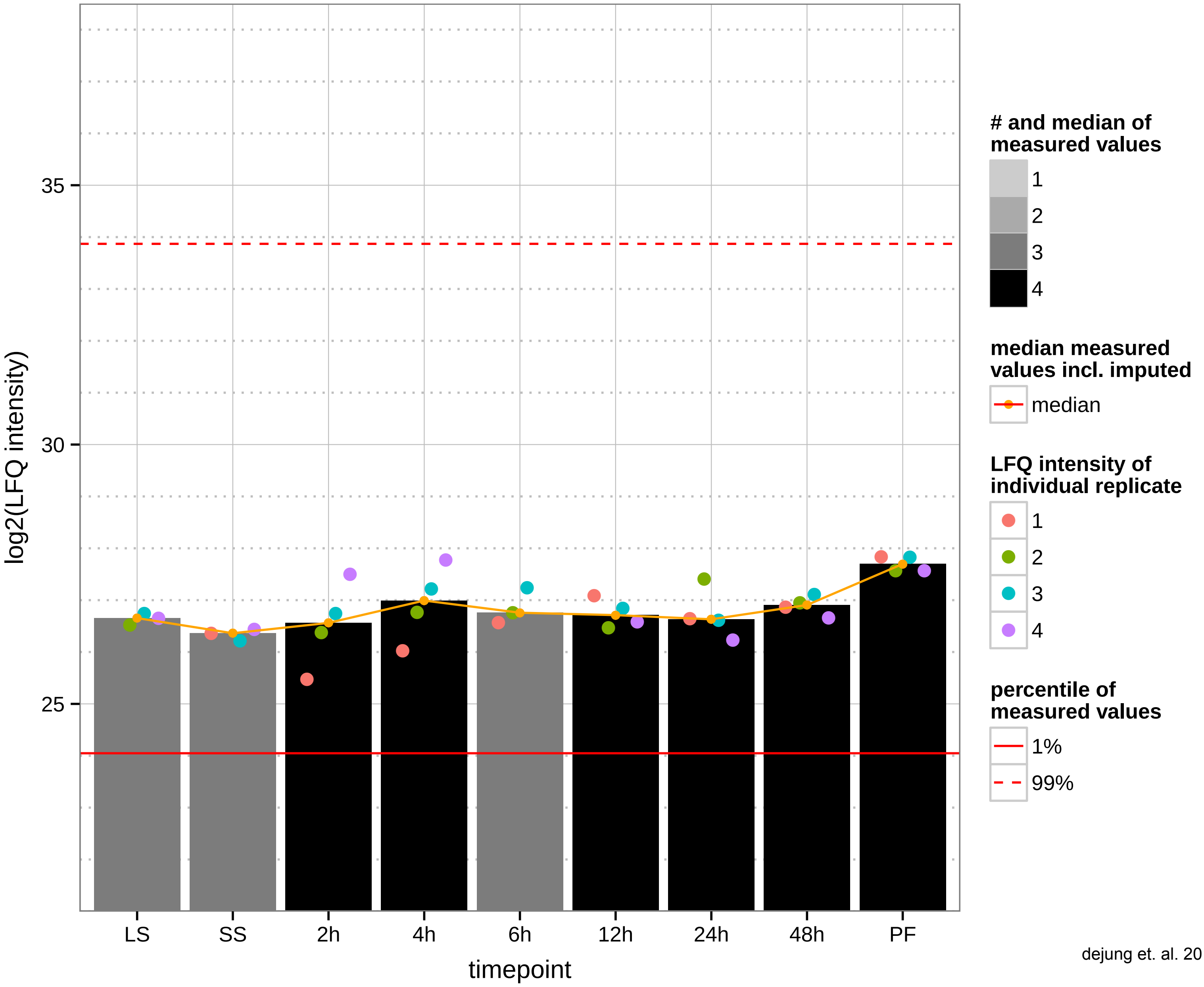

dejung et. al. 2015

hypothetical protein, conserved
Tb927.10.4760
AGOF: null
AGOC: null
AGOP: null
PGOF: null
PGOC: membrane
PGOP: null

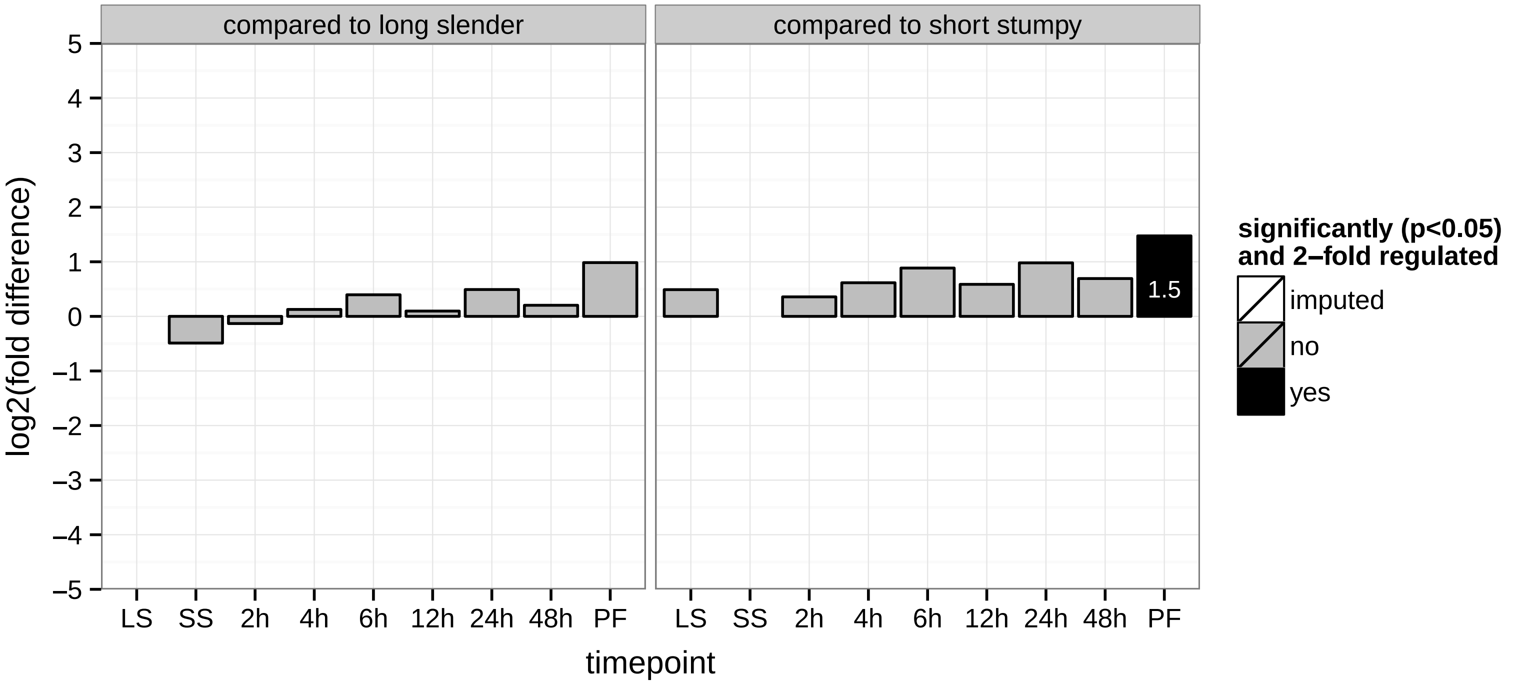

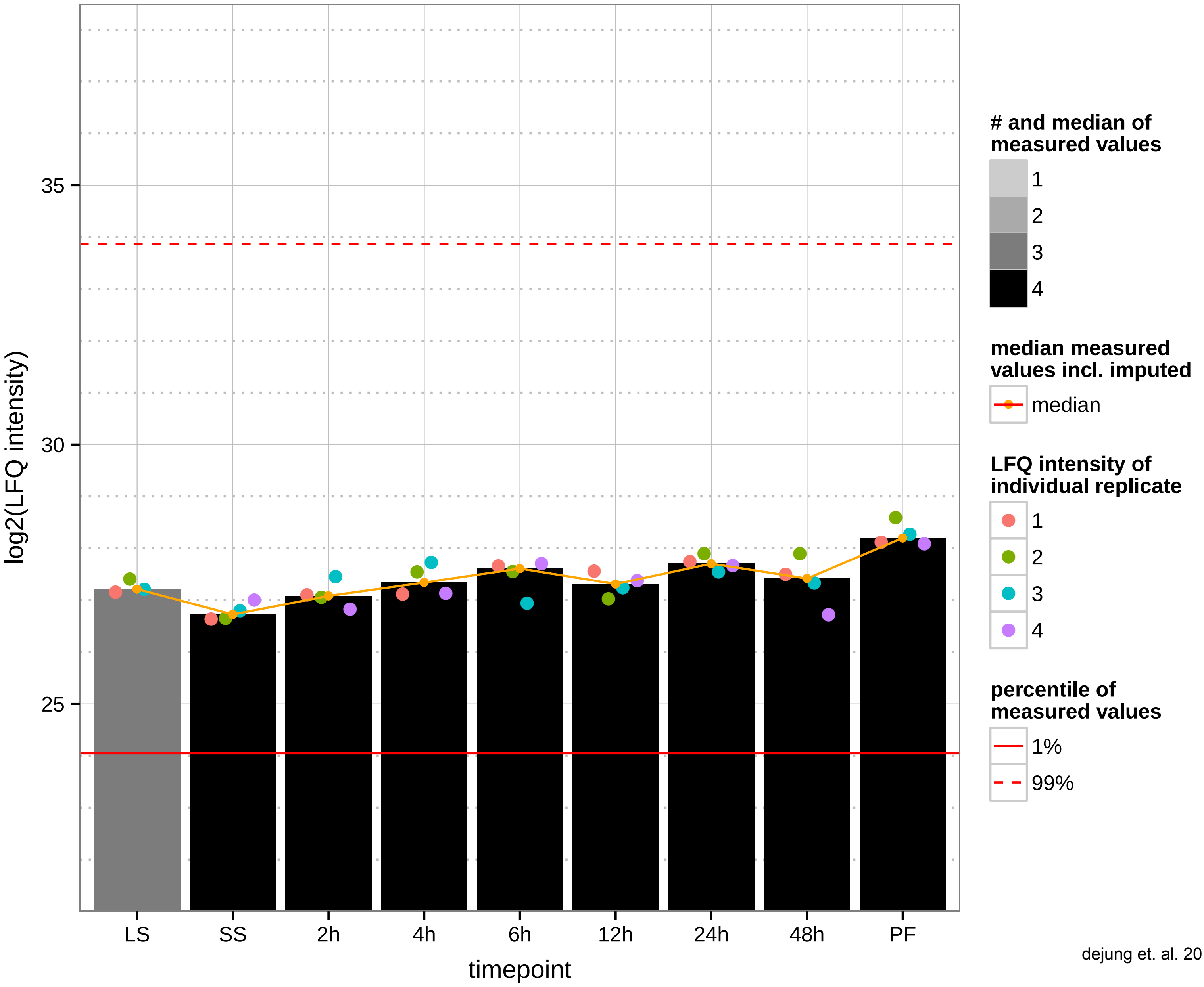

dejung et. al. 2015

Eukaryotic translation initiation factor 4E type 5 (eif4e5)
Tb927.10.5020
AGOF: RNA 7–methylguanosine cap binding, RNA cap 4 binding, protein binding, translation initiation factor activity
AGOC: cytoplasm, eukaryotic translation initiation factor 4F complex
AGOP: cell motility, translation, translational initiation
PGOF: RNA binding, translation initiation factor activity
PGOC: cytoplasm
PGOP: translational initiation

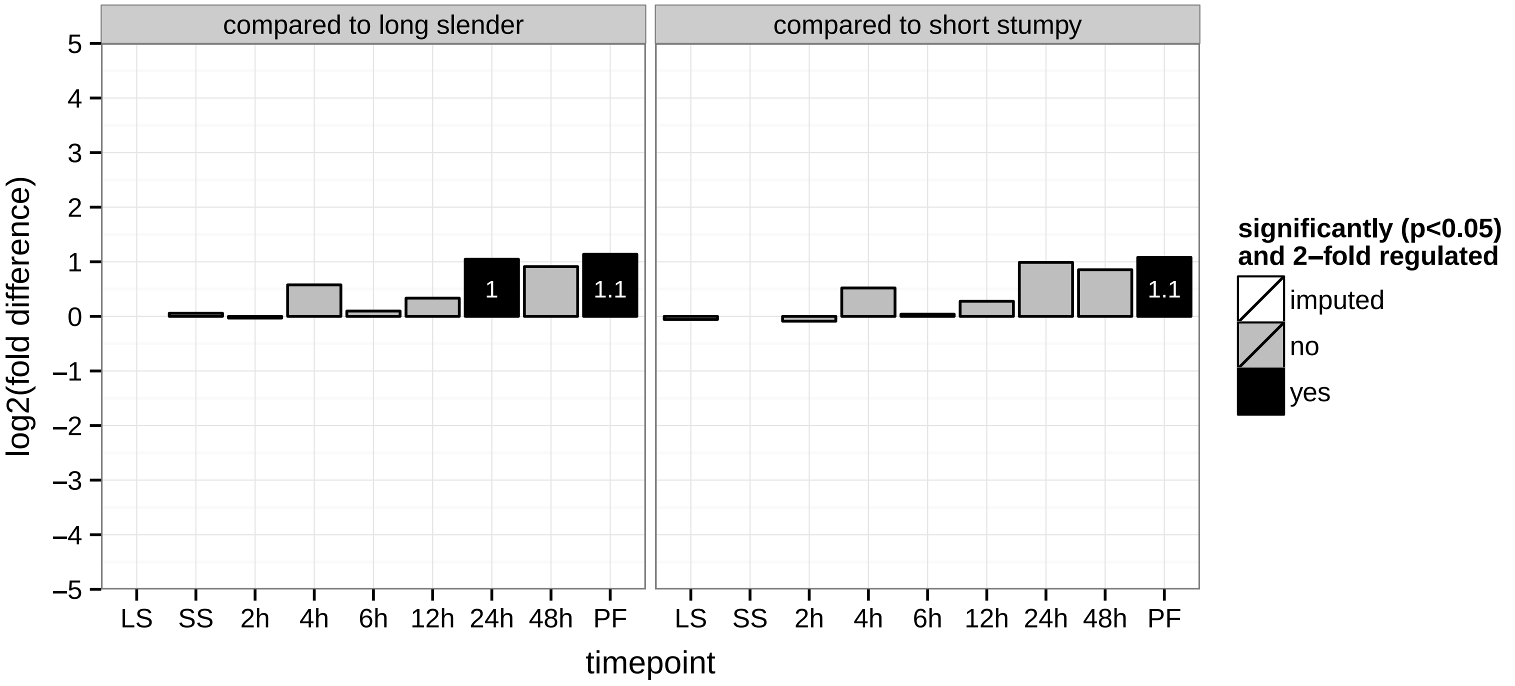

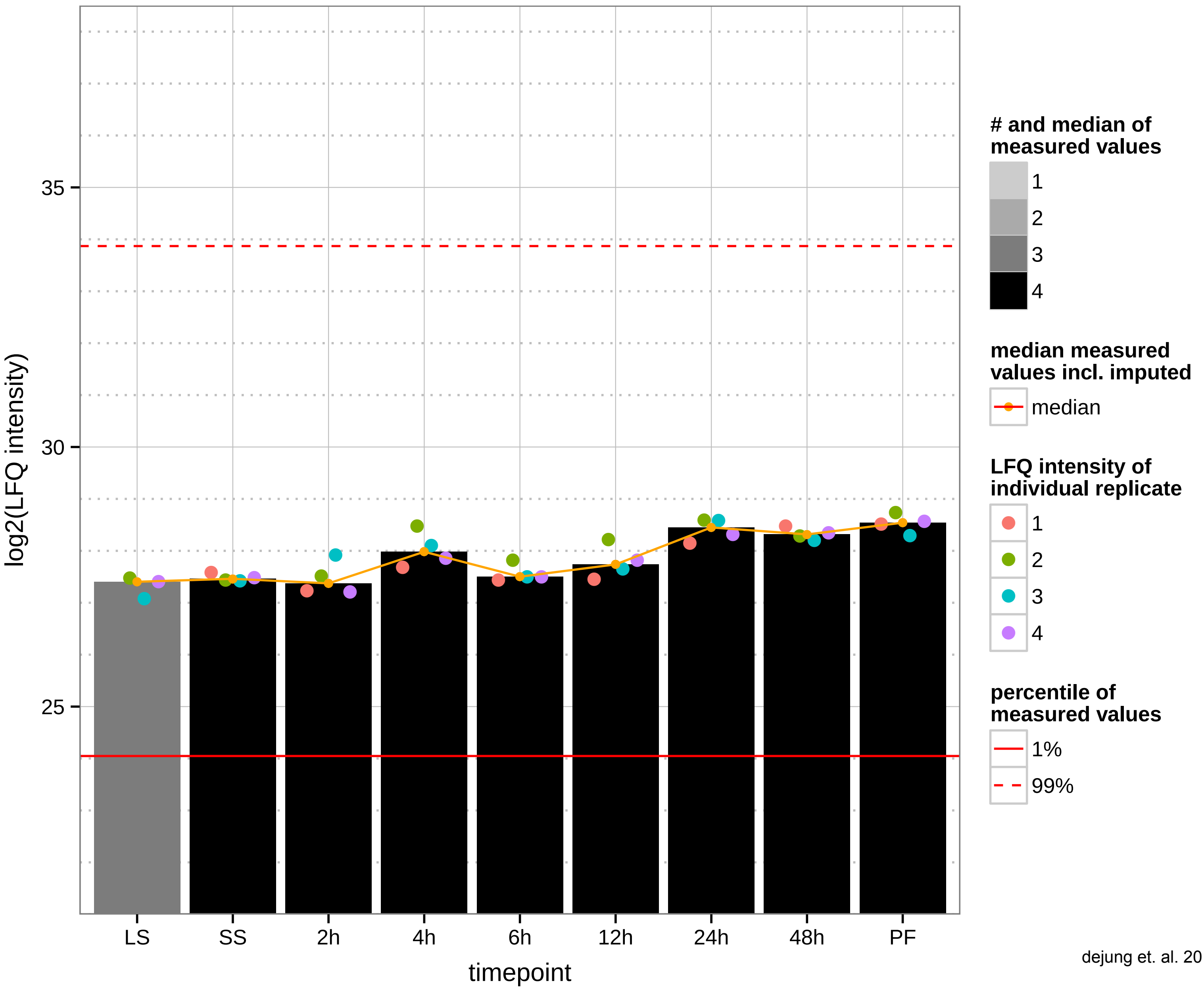

dejung et. al. 2015

ubiquitin/ribosomal protein S27a, putative
Tb927.10.5030;Tb927.7.3680
AGOF: heme binding, structural constituent of ribosome, structural constituent of ribosome
AGOC: intracellular, ribosome
AGOP: ribosome biogenesis, translation, translation
PGOF: structural constituent of ribosome
PGOC: intracellular, ribosome
PGOP: translation

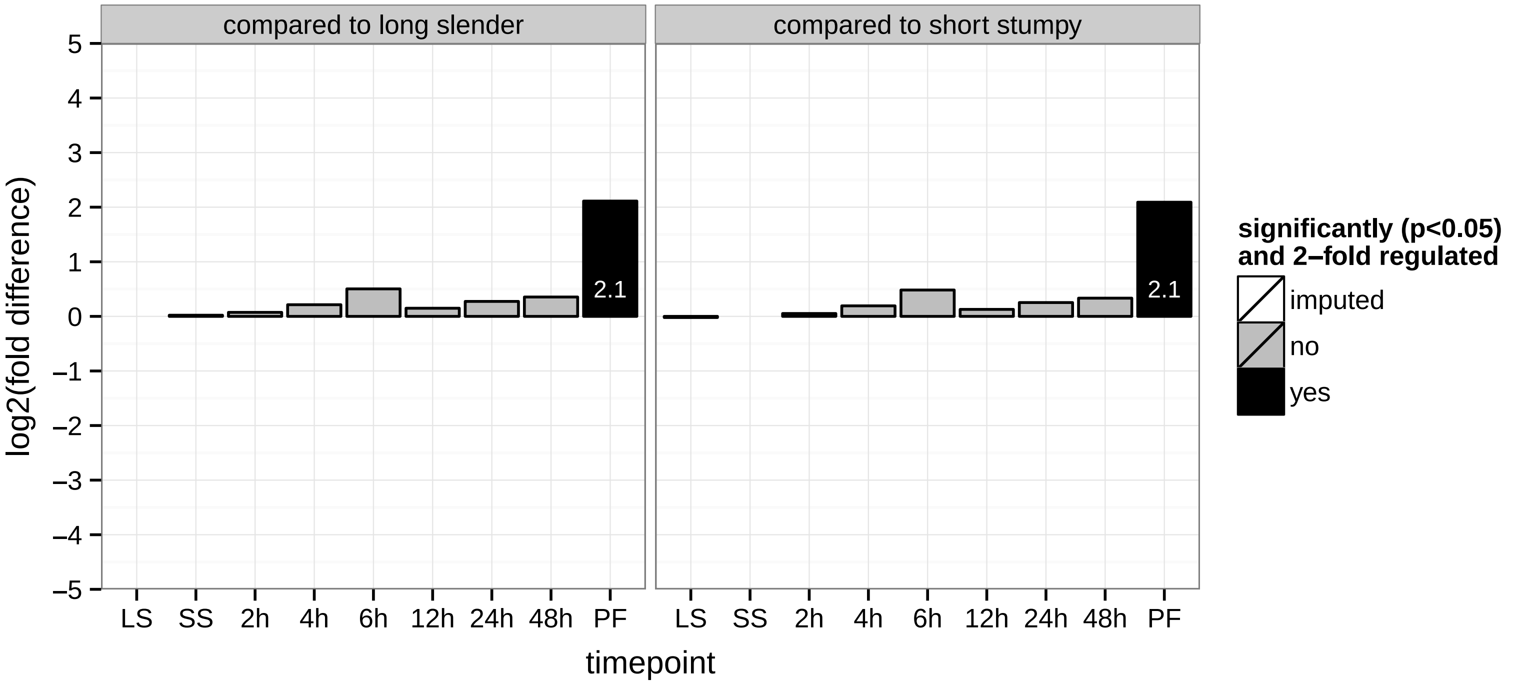

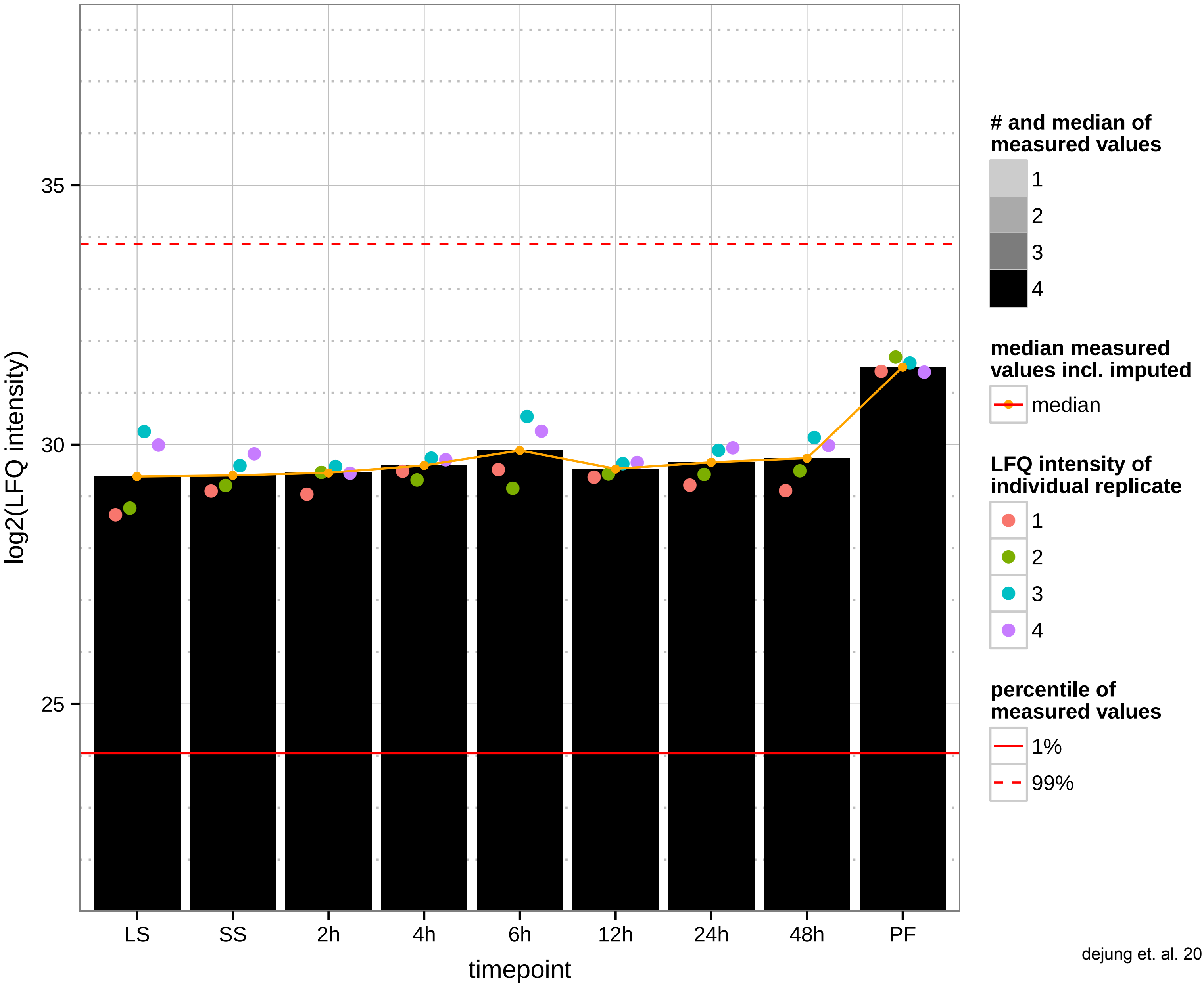

dejung et. al. 2015

hypothetical protein, conserved
Tb927.10.510;Tb11.v5.0374
AGOF: null
AGOC: null
AGOP: null
PGOF: null
PGOC: null
PGOP: null

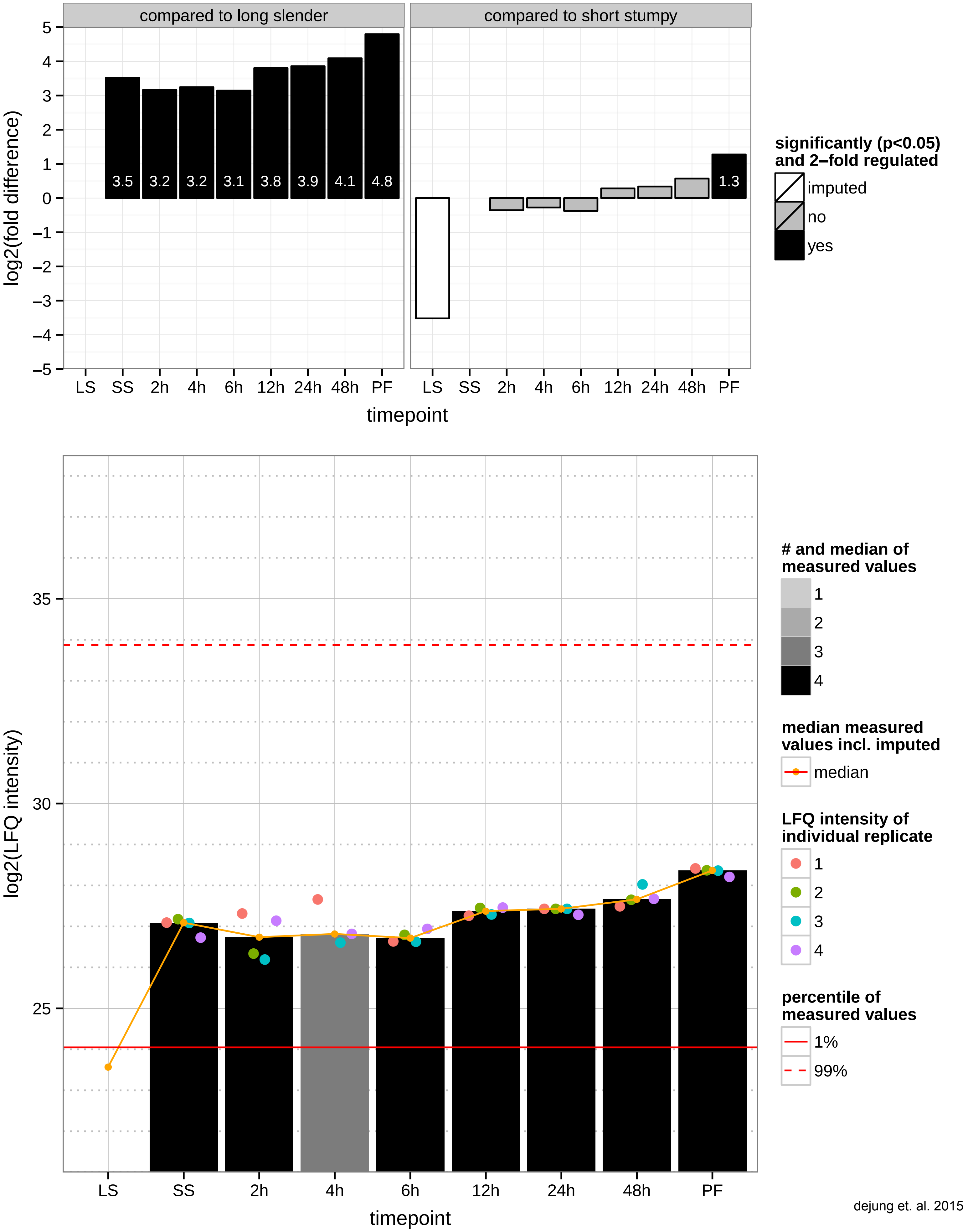

dejung et. al. 2015

hypothetical protein, conserved
Tb927.10.5260;Tb11.v5.0497
AGOF: null
AGOC: null
AGOP: null
PGOF: protein binding
PGOC: null
PGOP: null

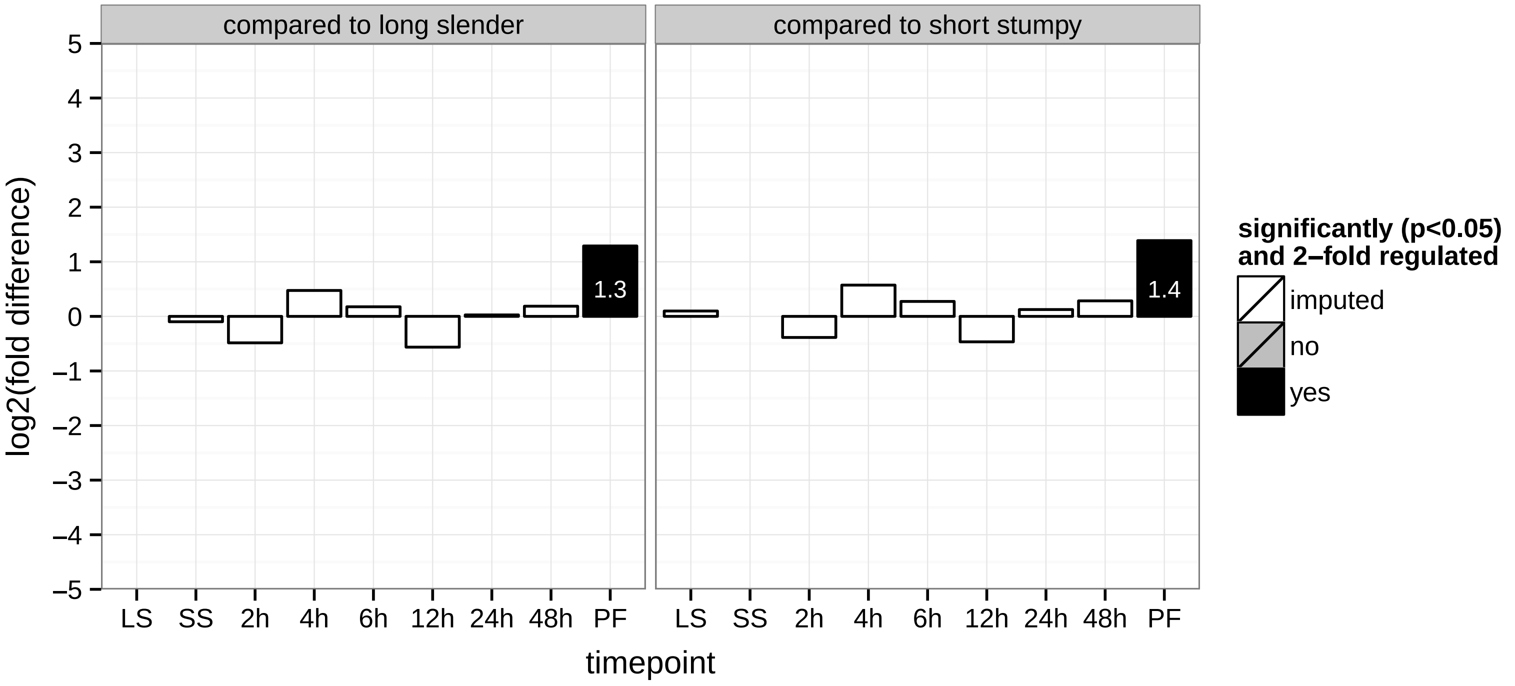

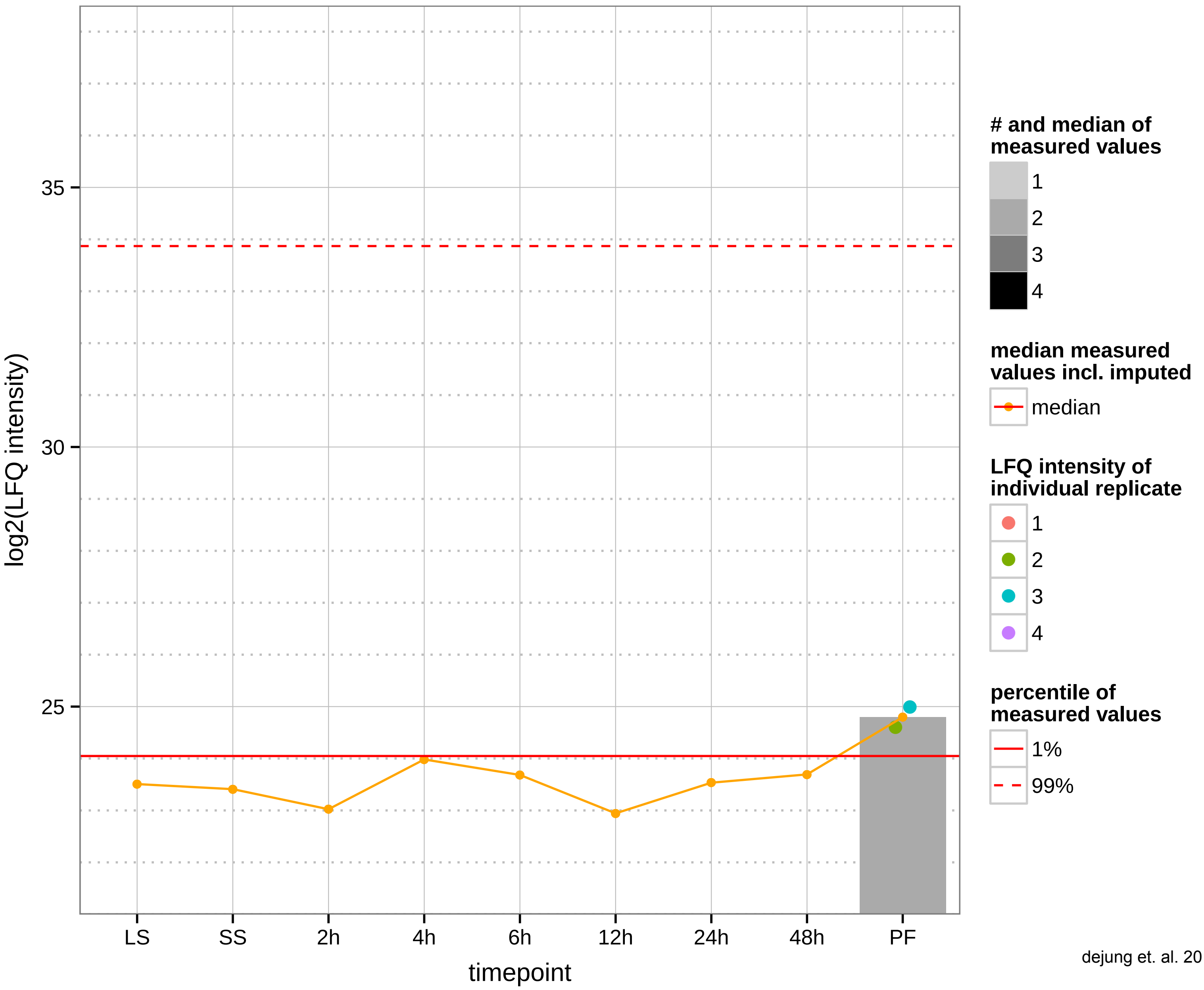

dejung et. al. 2015

heat shock protein, putative
Tb927.10.5660
AGOF: heat shock protein binding
AGOC: mitochondrion
AGOP: protein folding
PGOF: heat shock protein binding
PGOC: null
PGOP: null

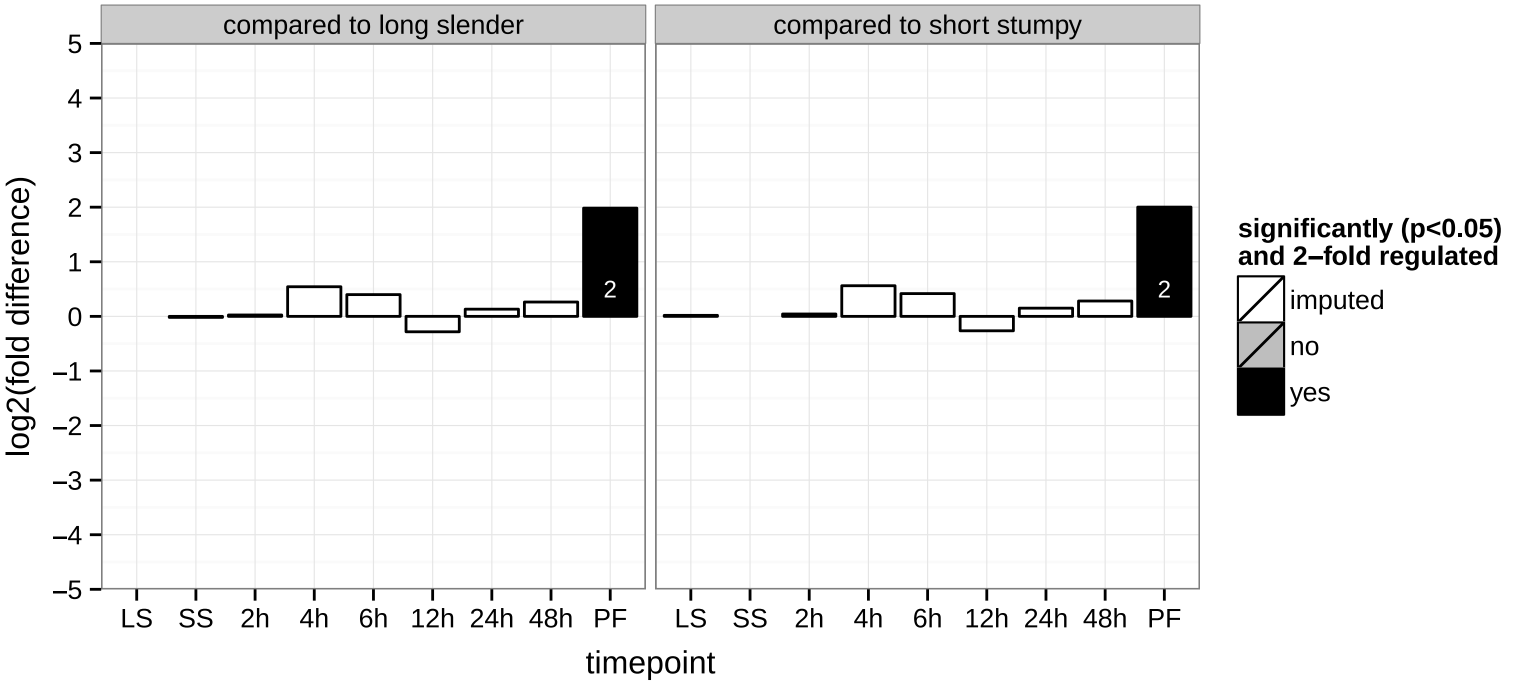

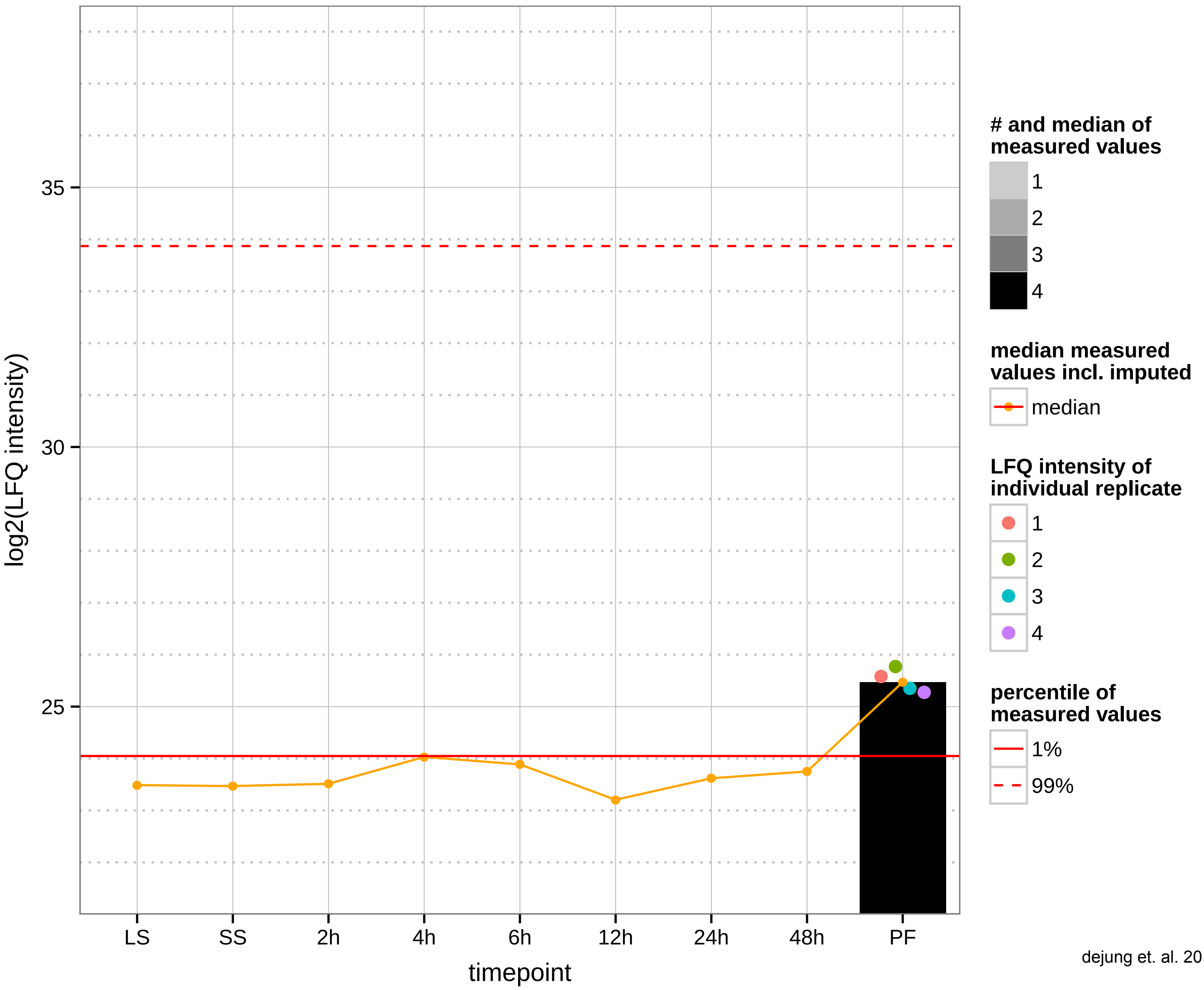

dejung et. al. 2015

N–acetyltransferase subunit Nat1, putative (NAT1)
Tb927.10.5670
AGOF: N–acetyltransferase activity
AGOC: null
AGOP: cellular protein modification process
PGOF: protein binding
PGOC: null
PGOP: null

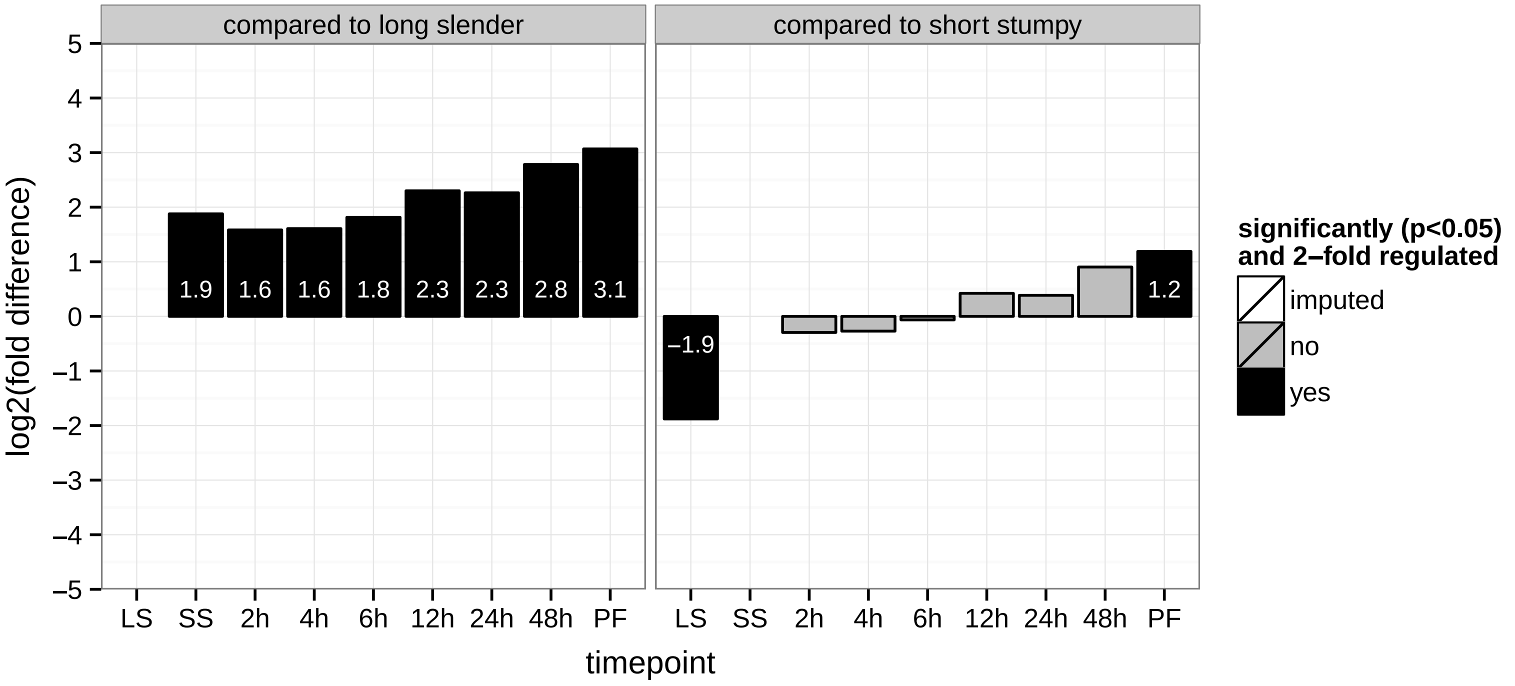

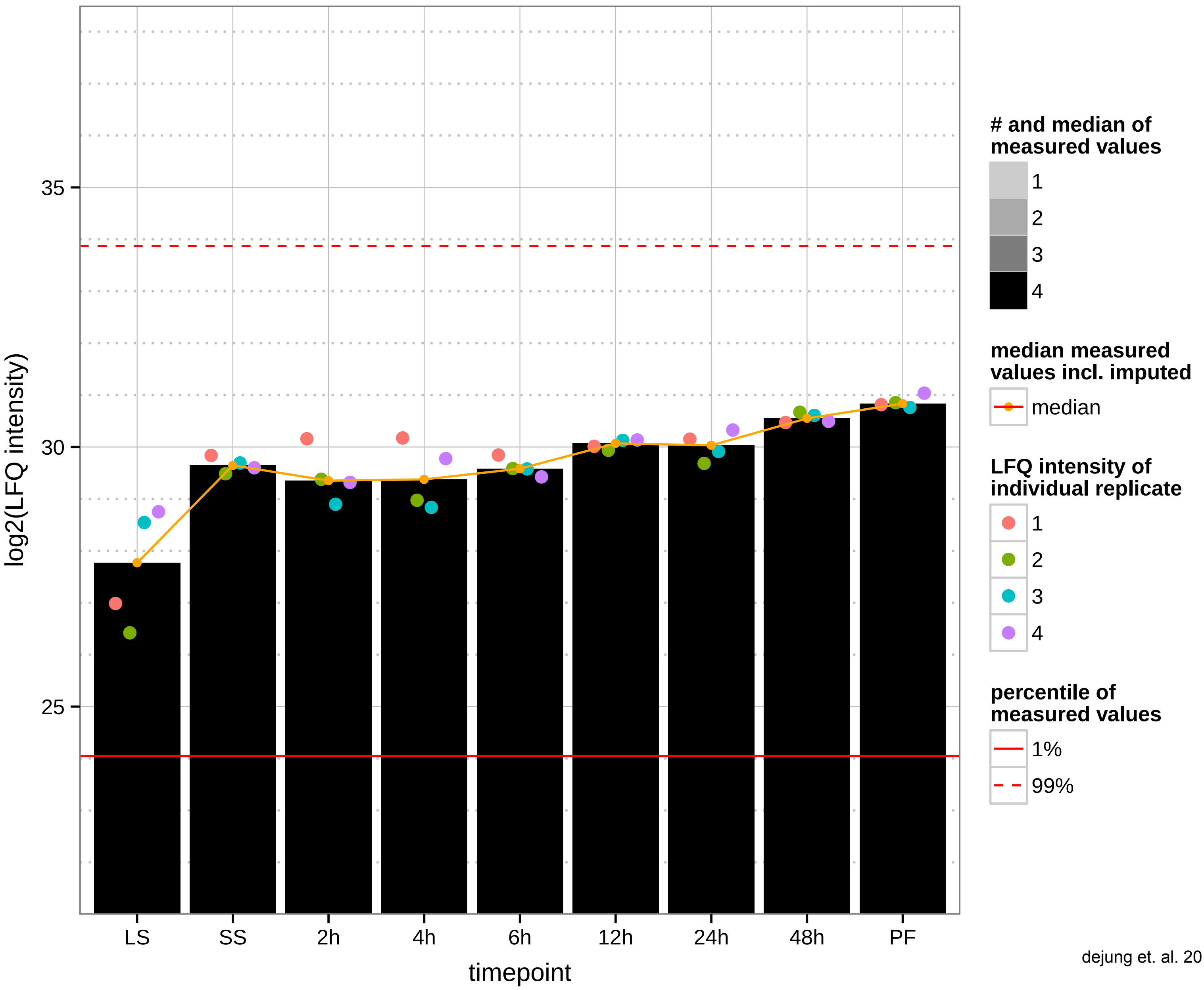

dejung et. al. 2015

hypothetical protein, conserved
Tb927.10.5920
AGOF: null
AGOC: null
AGOP: null
PGOF: null
PGOC: null
PGOP: null

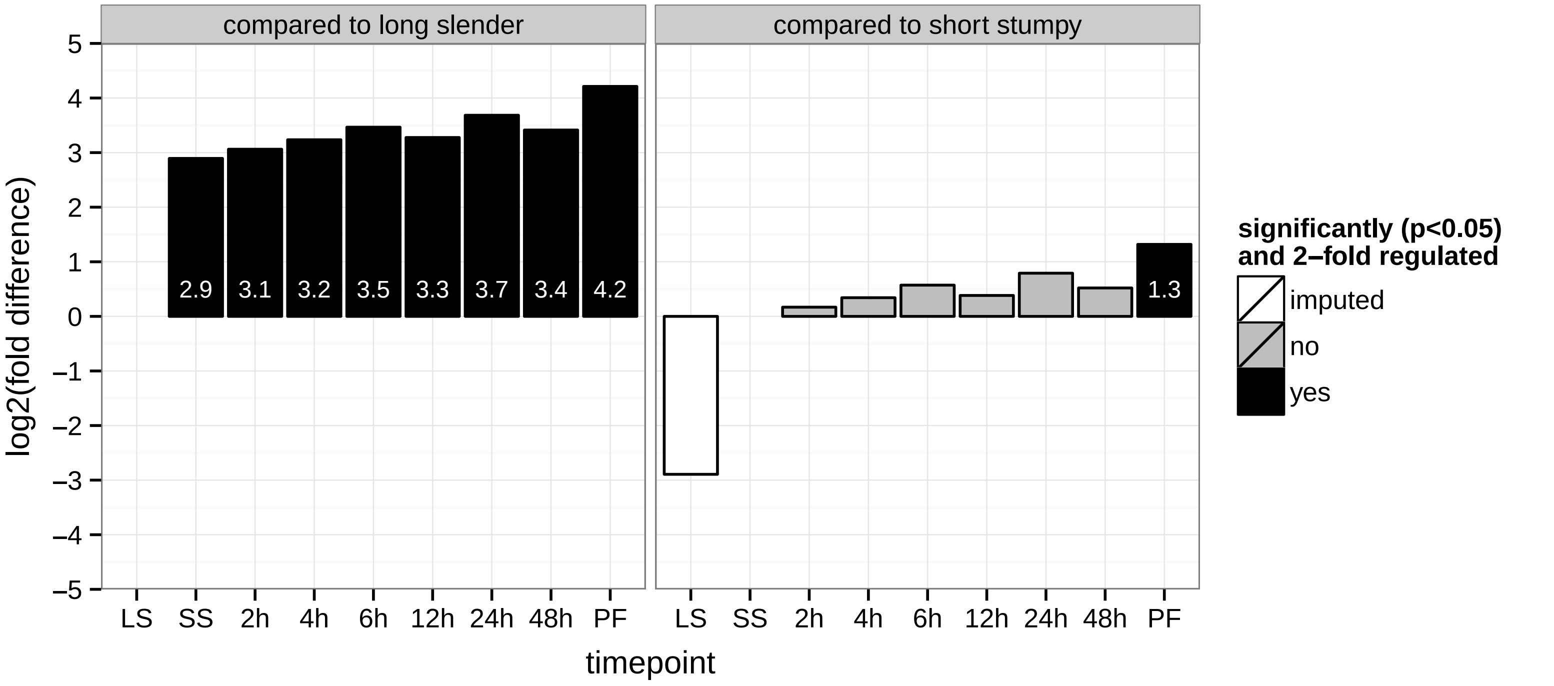

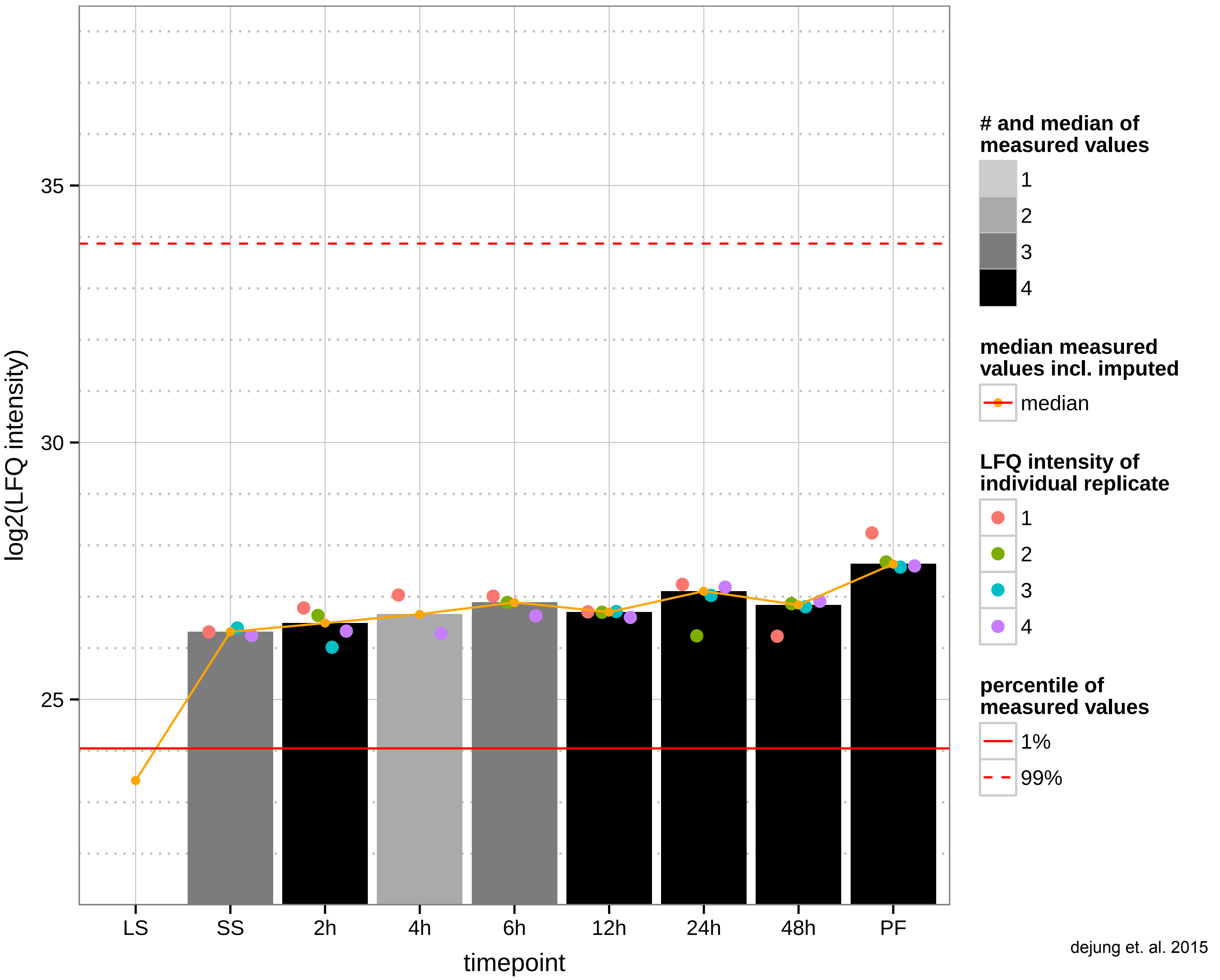

dejung et. al. 2015

hypothetical protein, conserved
Tb927.10.6180
AGOF: null
AGOC: null
AGOP: null
PGOF: null
PGOC: null
PGOP: null

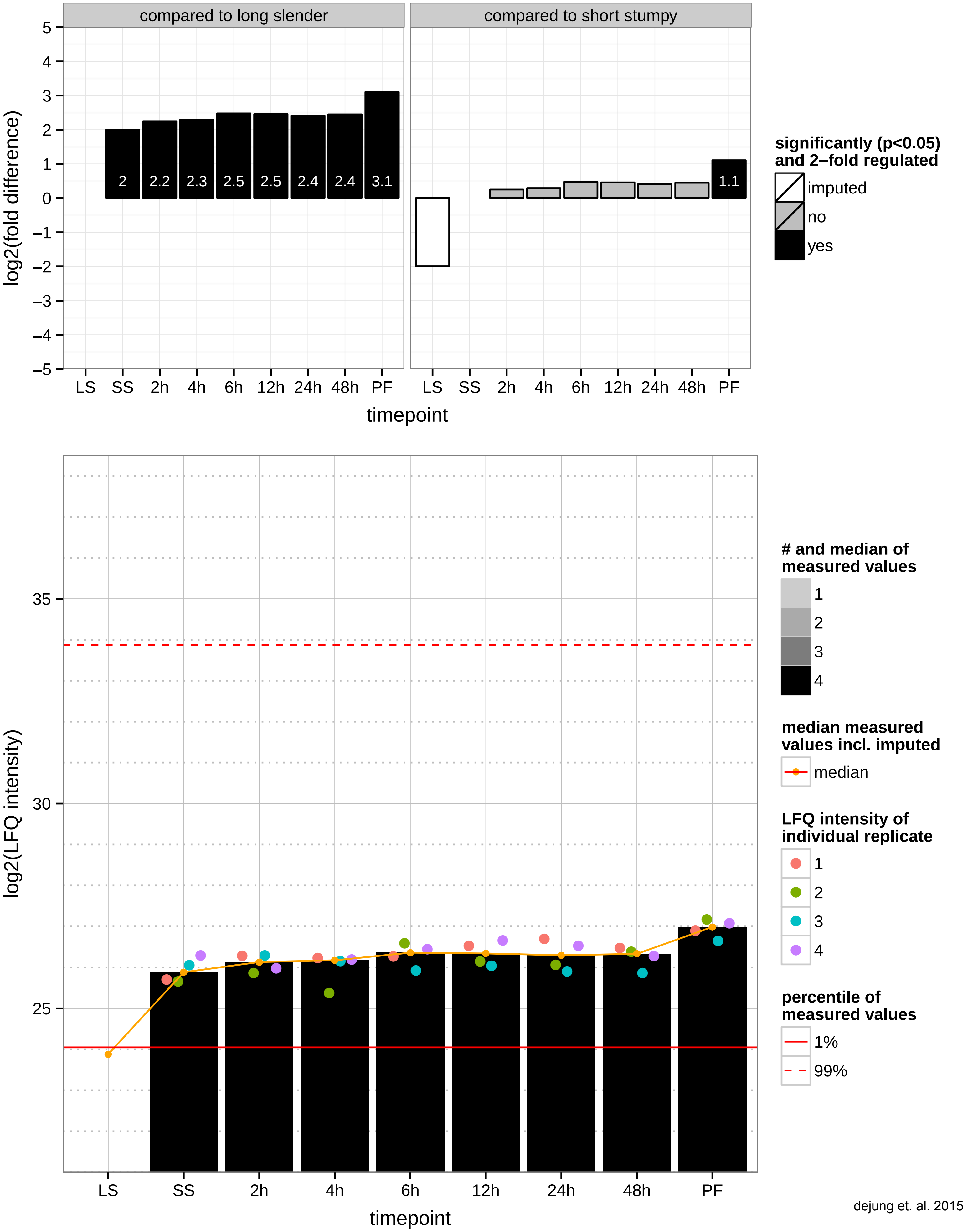

dejung et. al. 2015

member of the NOL1/NOP2/sun family of proteins
Tb927.10.6680
AGOF: null
AGOC: null
AGOP: null
PGOF: null
PGOC: null
PGOP: null

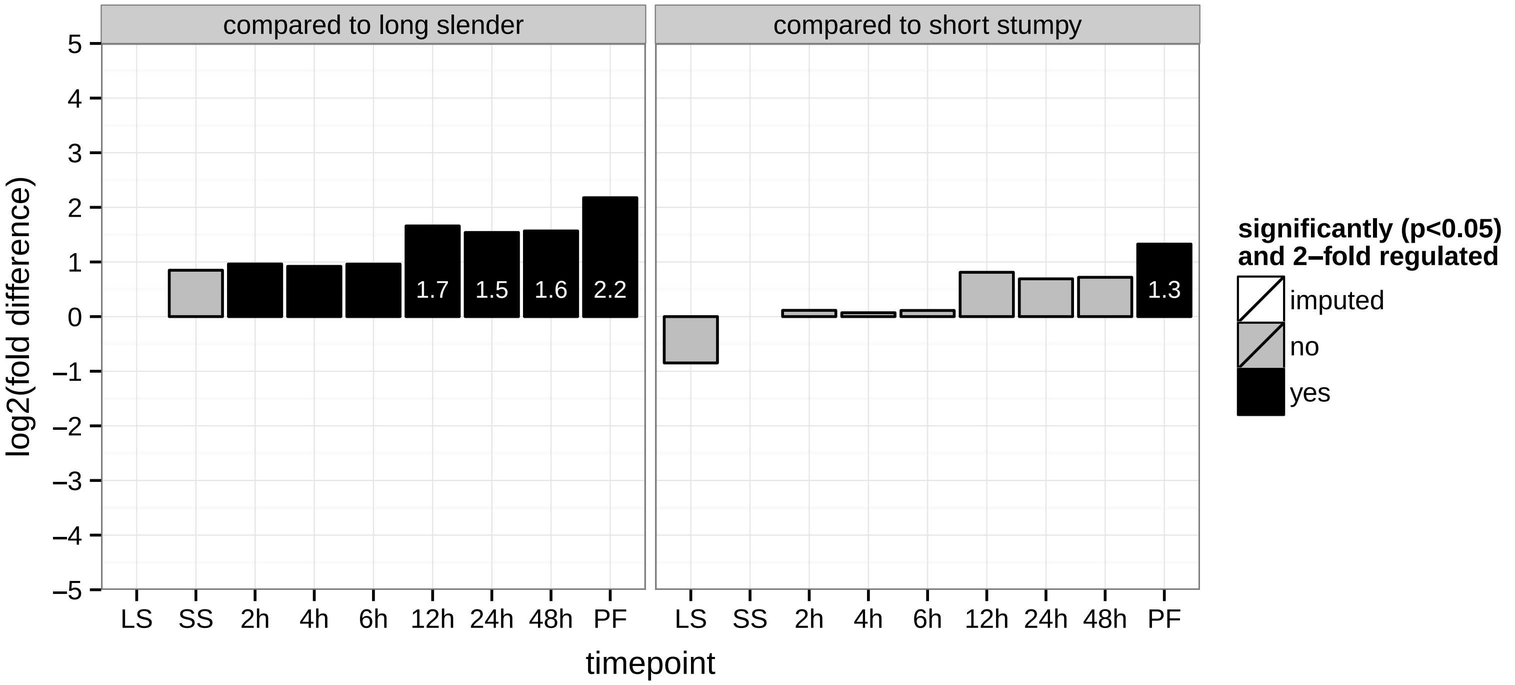

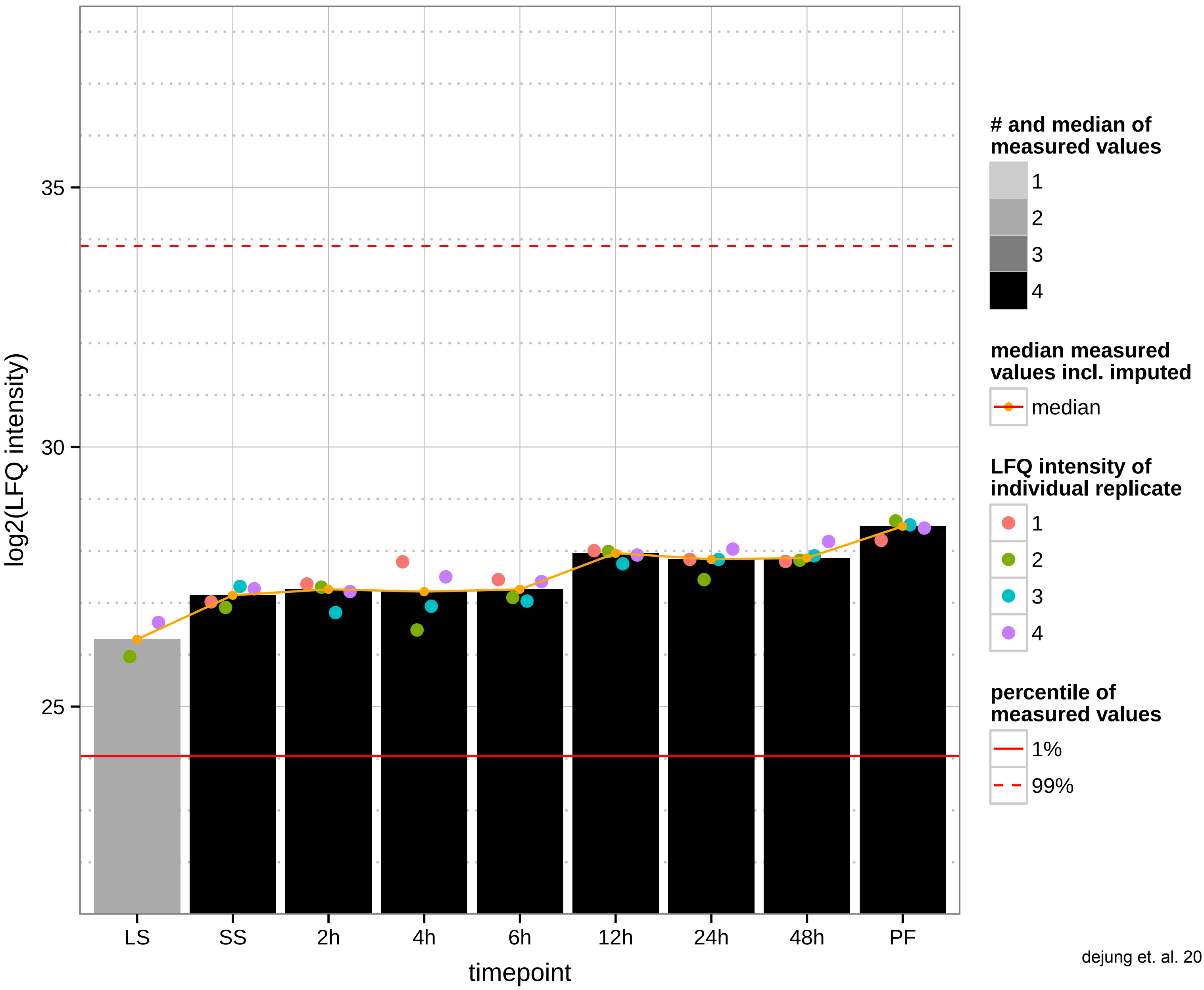

dejung et. al. 2015

hypothetical protein, conserved
Tb927.10.6710
AGOF: null
AGOC: null
AGOP: null
PGOF: null
PGOC: null
PGOP: null

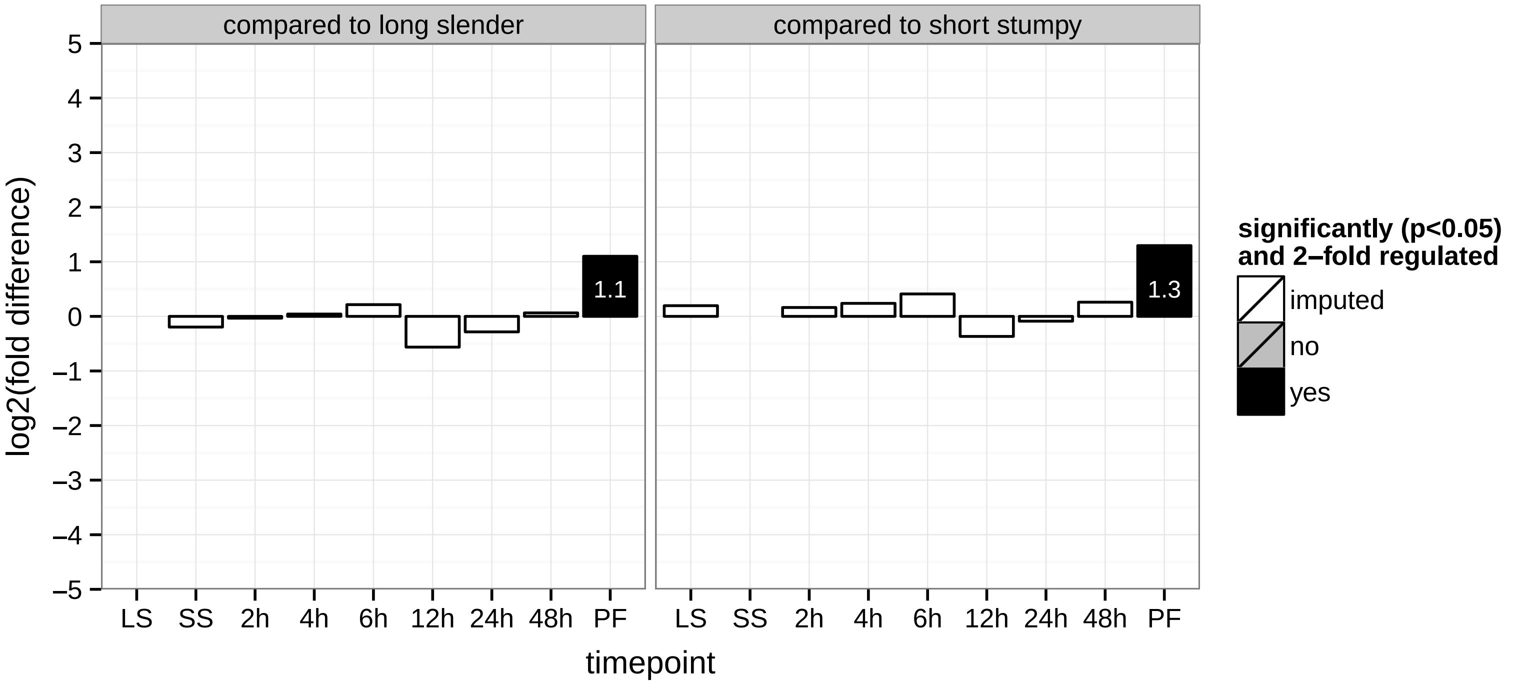

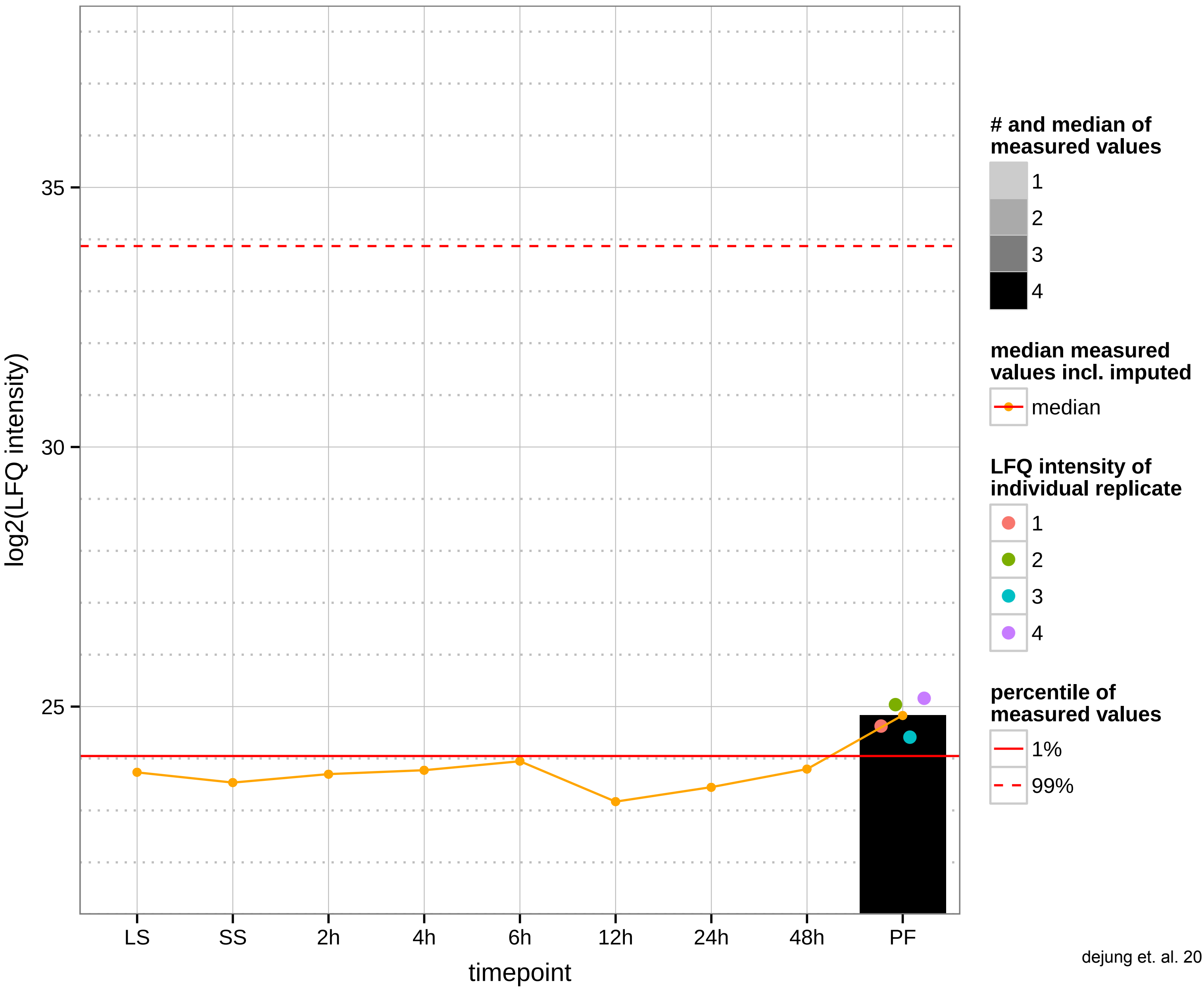

dejung et. al. 2015

chromatin assembly factor 1 subunit B, putative
Tb927.10.7050
AGOF: histone binding, unfolded protein binding
AGOC: null
AGOP: chromatin assembly, chromatin assembly or disassembly, nucleosome assembly
PGOF: protein binding
PGOC: null
PGOP: null

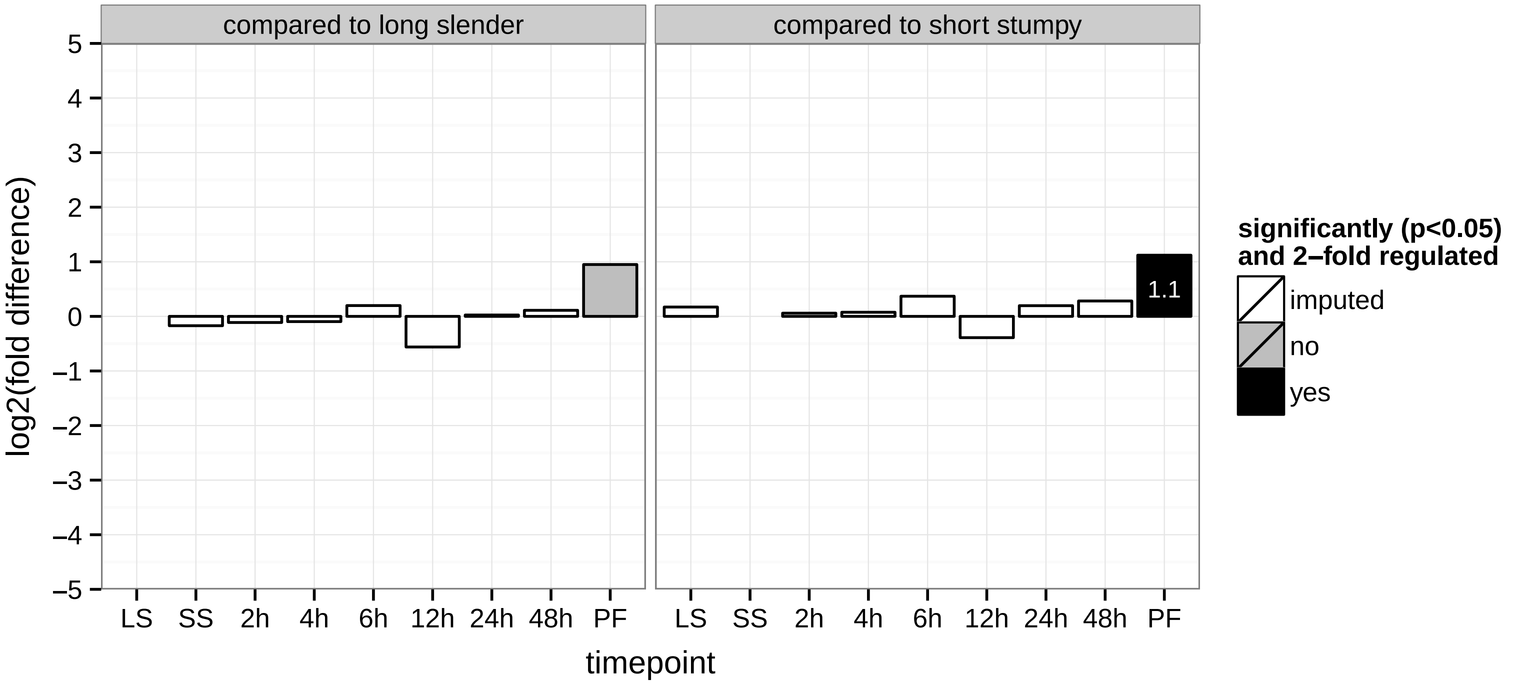

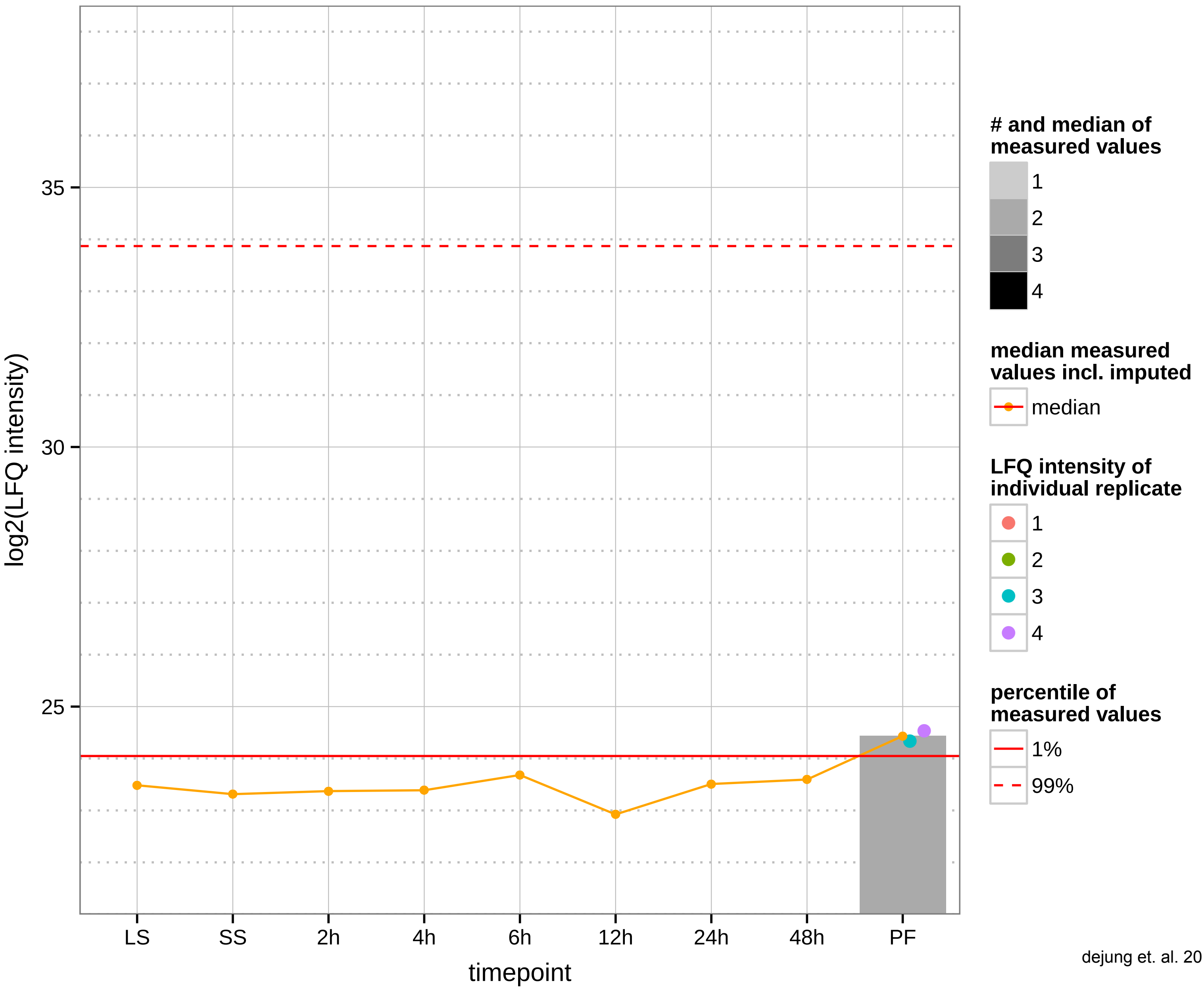

dejung et. al. 2015

hypothetical protein, conserved
Tb927.10.7270
AGOF: null
AGOC: null
AGOP: null
PGOF: null
PGOC: null
PGOP: null

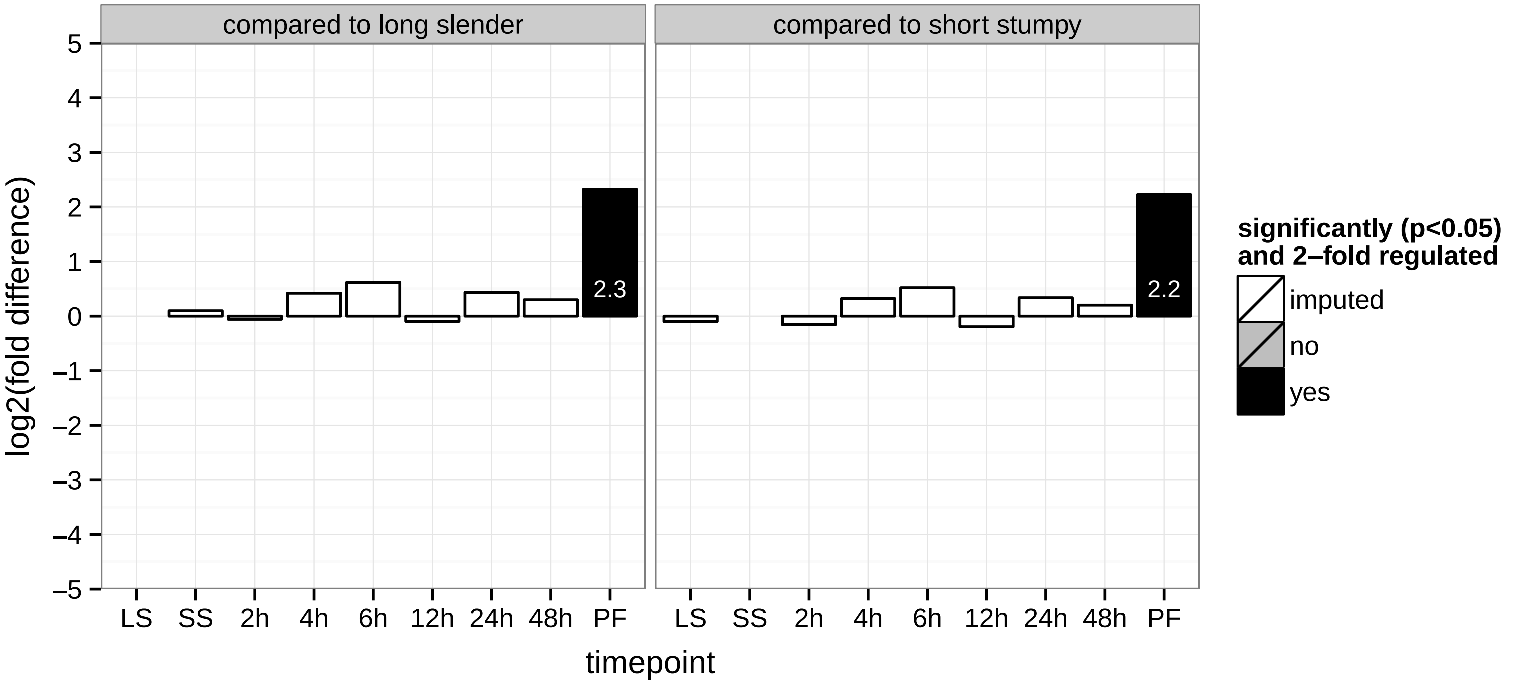

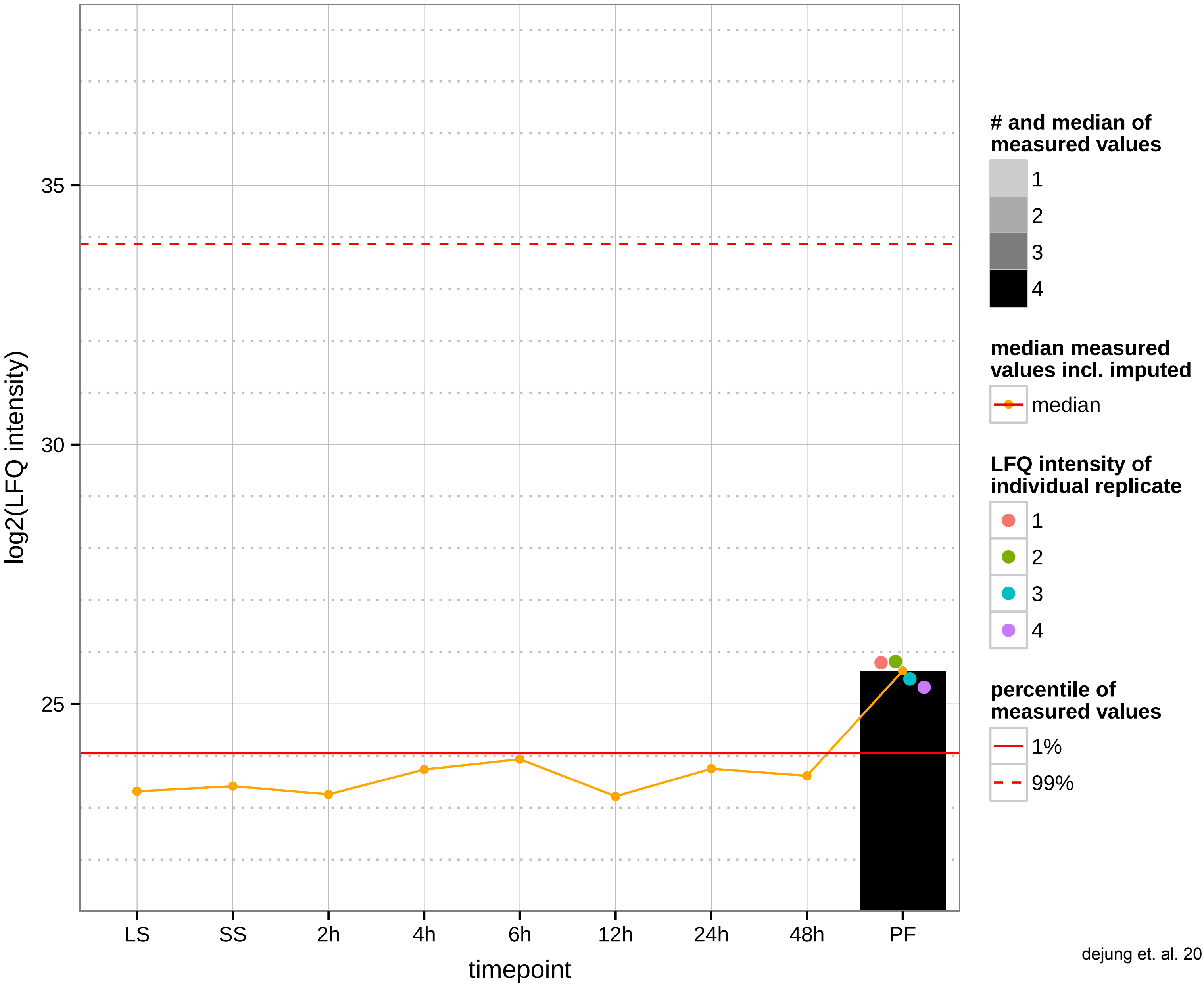

dejung et. al. 2015

terminal uridylyltransferase 3, putative (TUT3)
Tb927.10.7310
AGOF: null
AGOC: mitochondrion
AGOP: null
PGOF: null
PGOC: null
PGOP: null

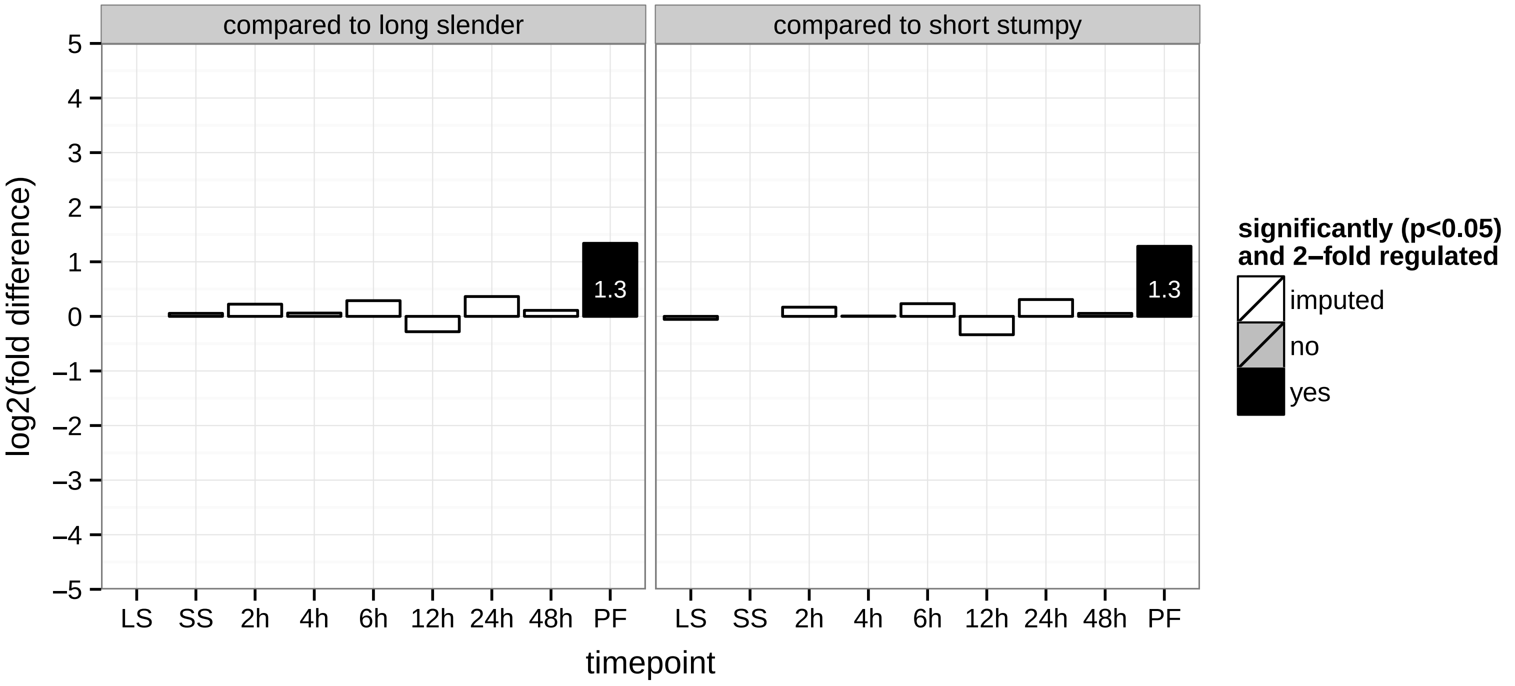

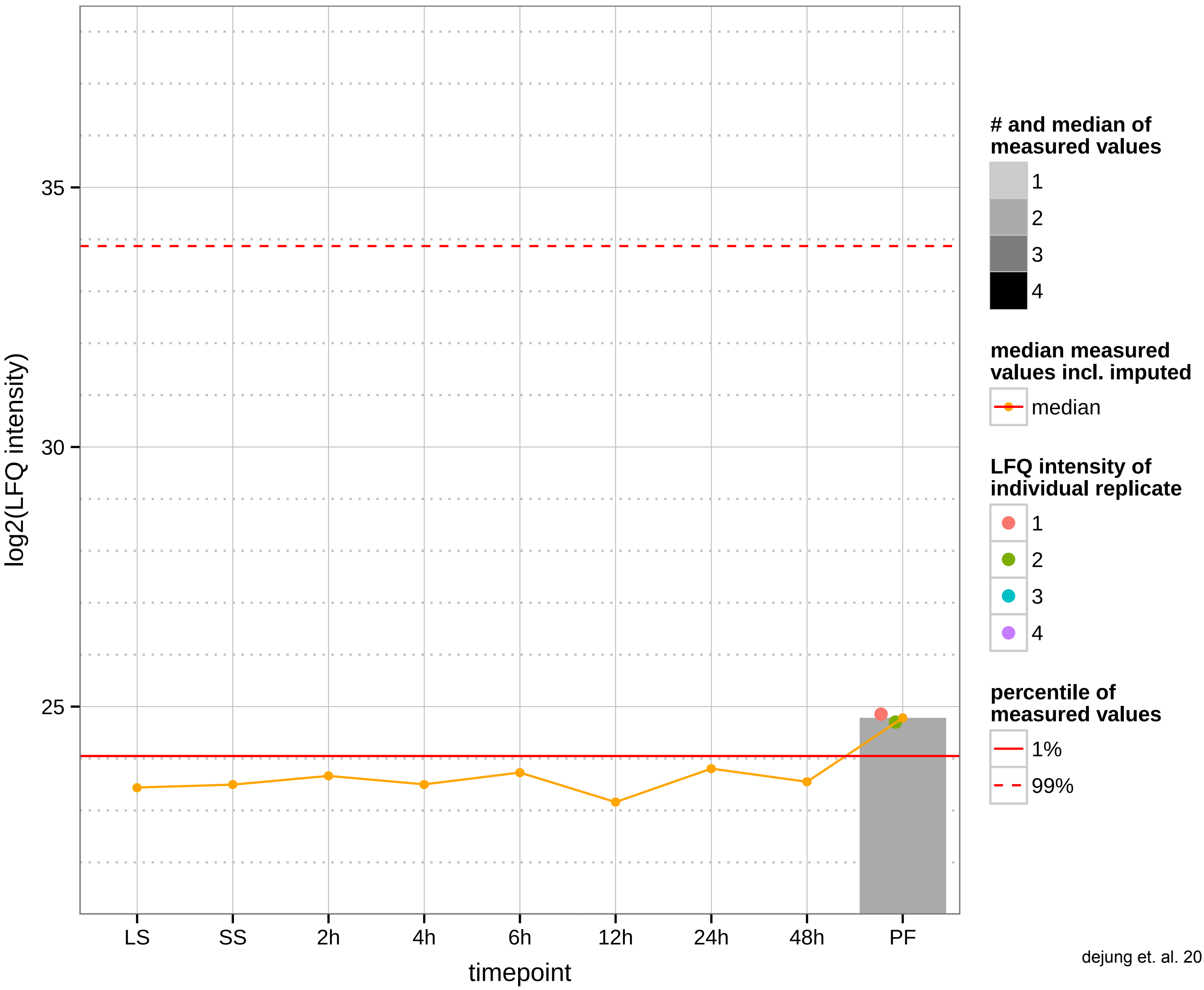

dejung et. al. 2015

40S ribosomal protein S24E, putative
Tb927.10.7340;Tb927.10.7330
AGOF: structural constituent of ribosome
AGOC: intracellular, ribosome
AGOP: translation
PGOF: null, structural constituent of ribosome
PGOC: null, intracellular, ribosome
PGOP: null, translation

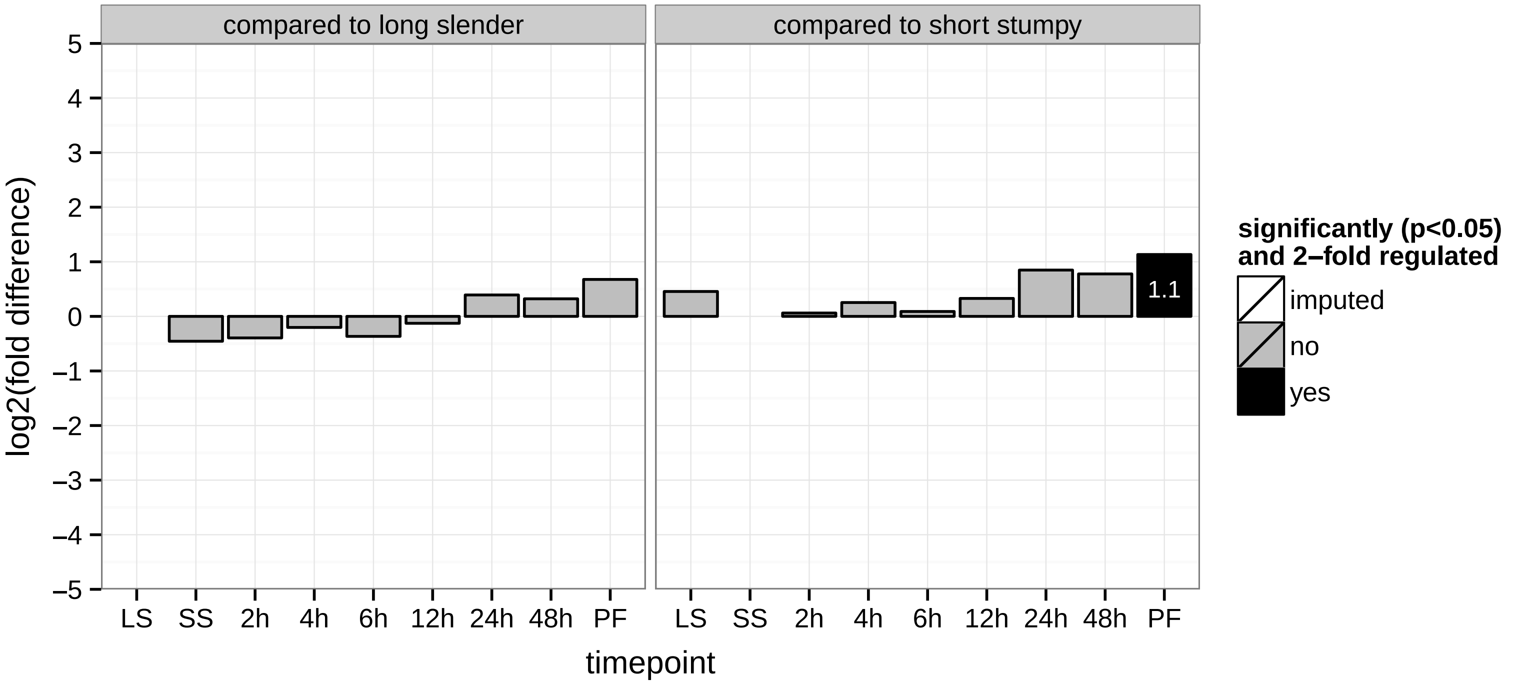

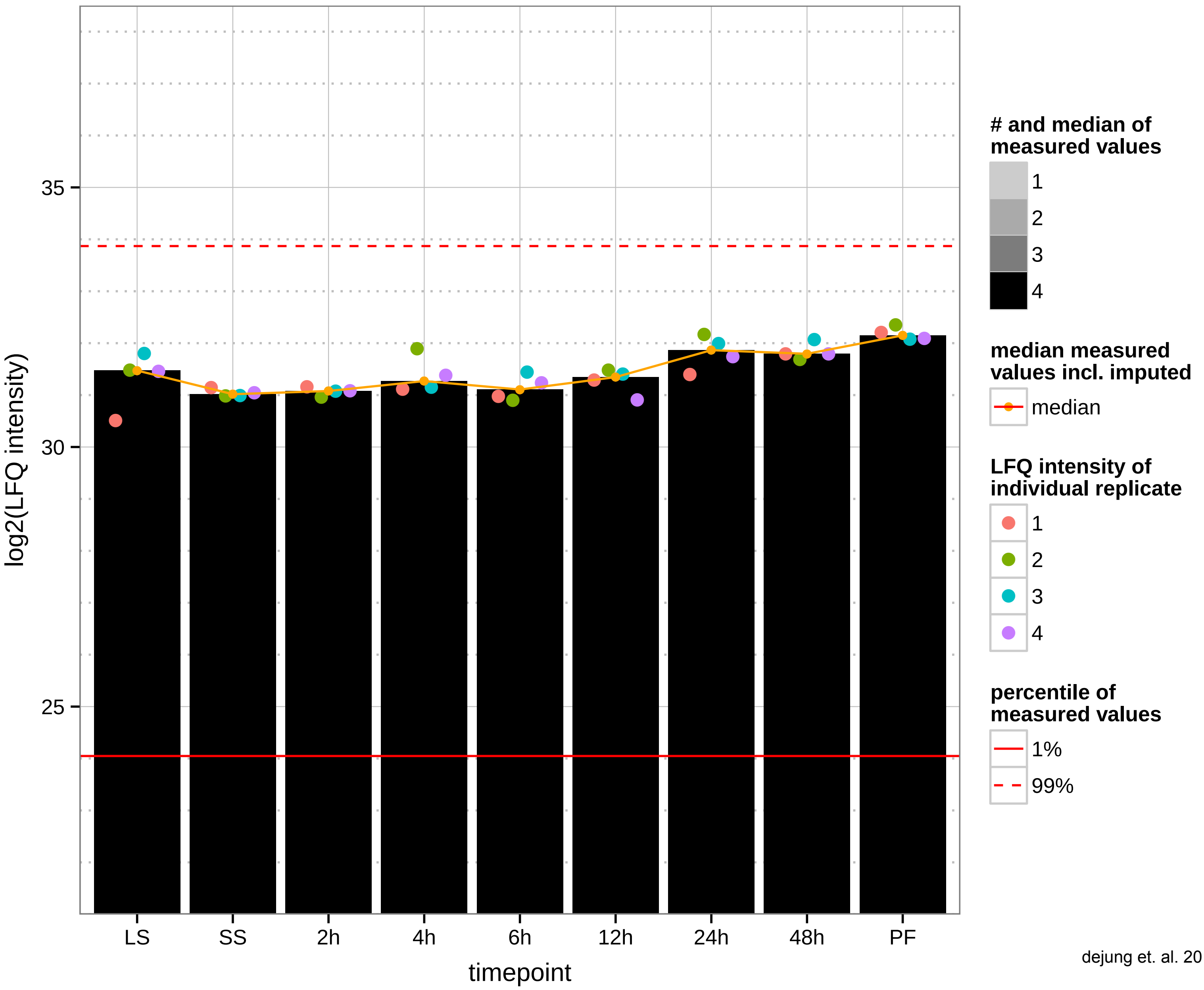

dejung et. al. 2015

hypothetical protein, conserved
Tb927.10.7600
AGOF: null
AGOC: null
AGOP: null
PGOF: null
PGOC: null
PGOP: null

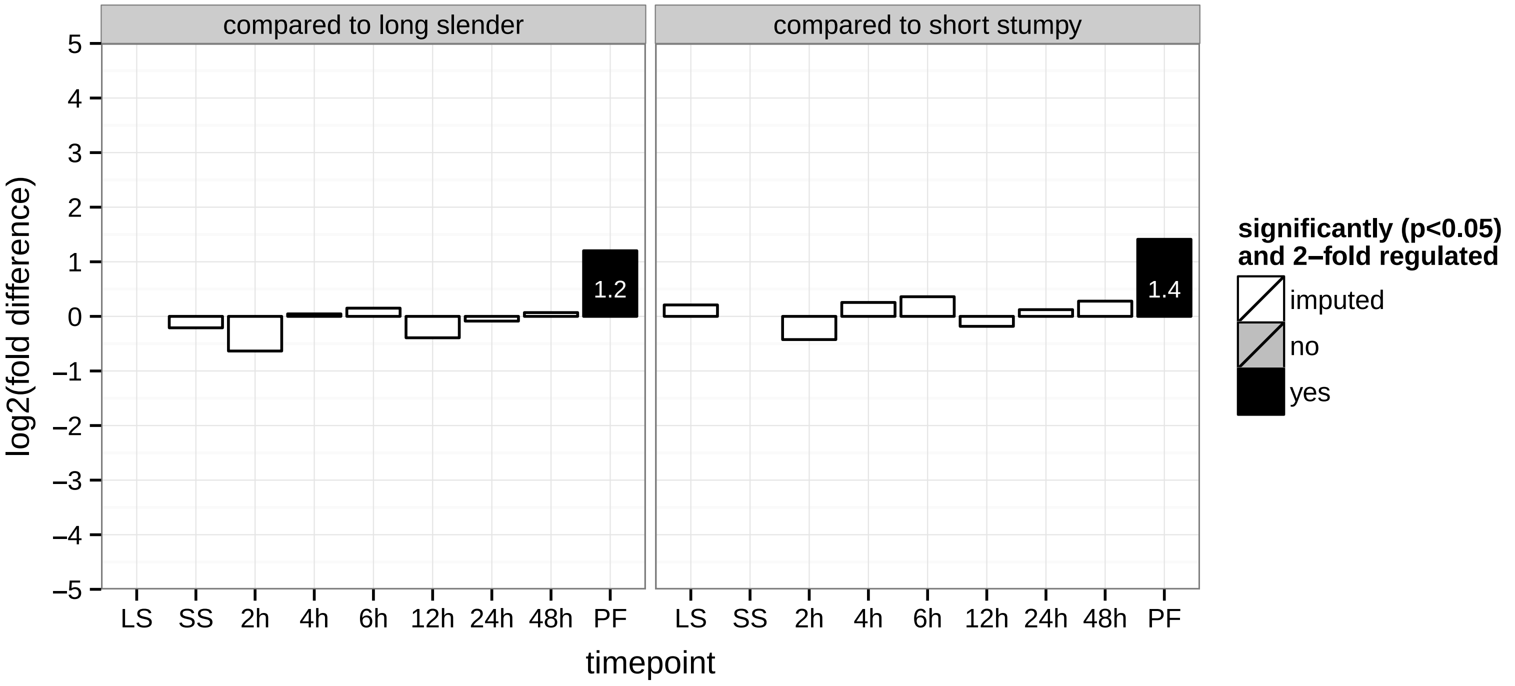

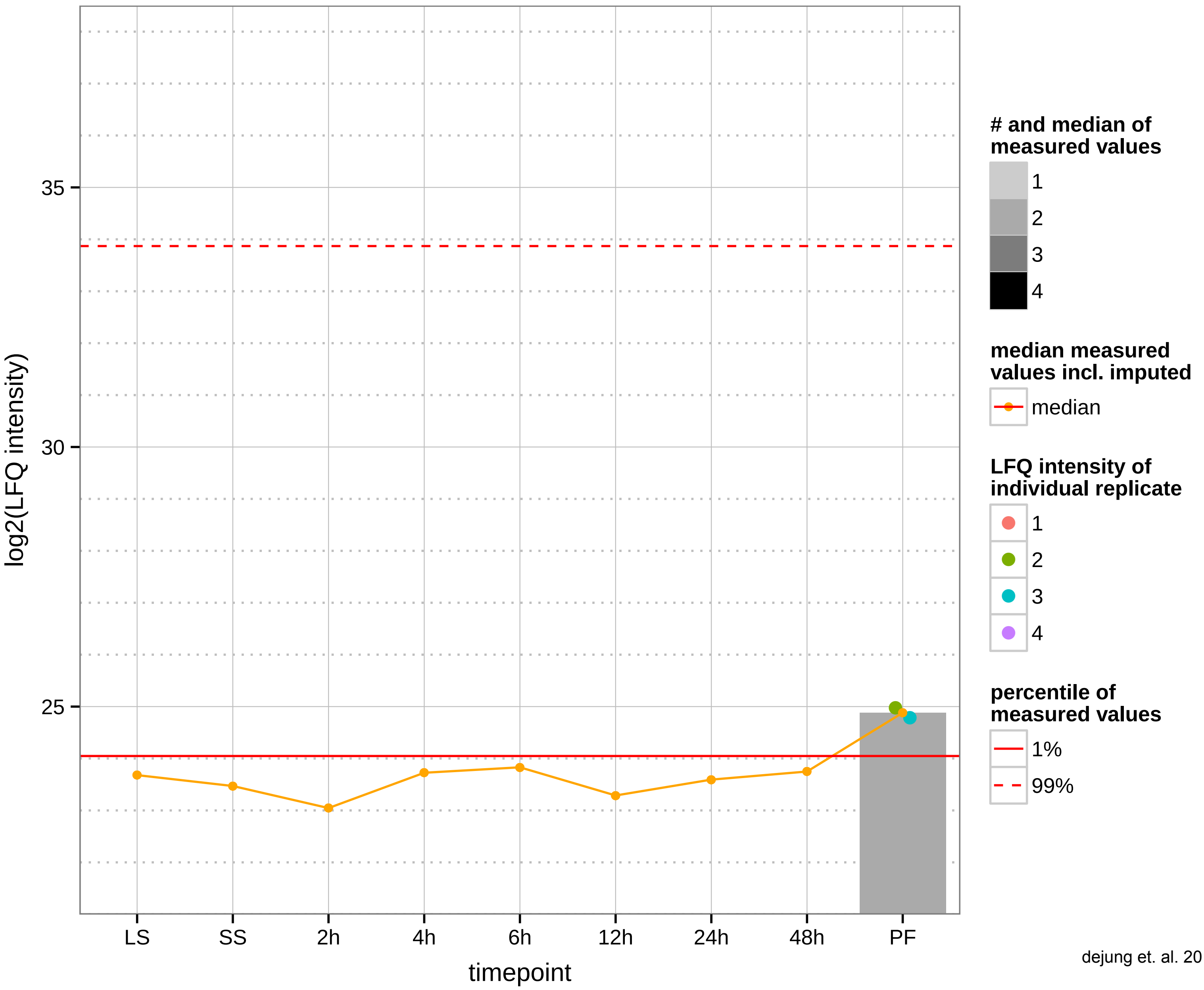

dejung et. al. 2015

GTPase activating protein, putative
Tb927.10.7680
AGOF: Rab GTPase activator activity
AGOC: intracellular
AGOP: regulation of Rab GTPase activity
PGOF: Rab GTPase activator activity
PGOC: intracellular
PGOP: regulation of Rab GTPase activity

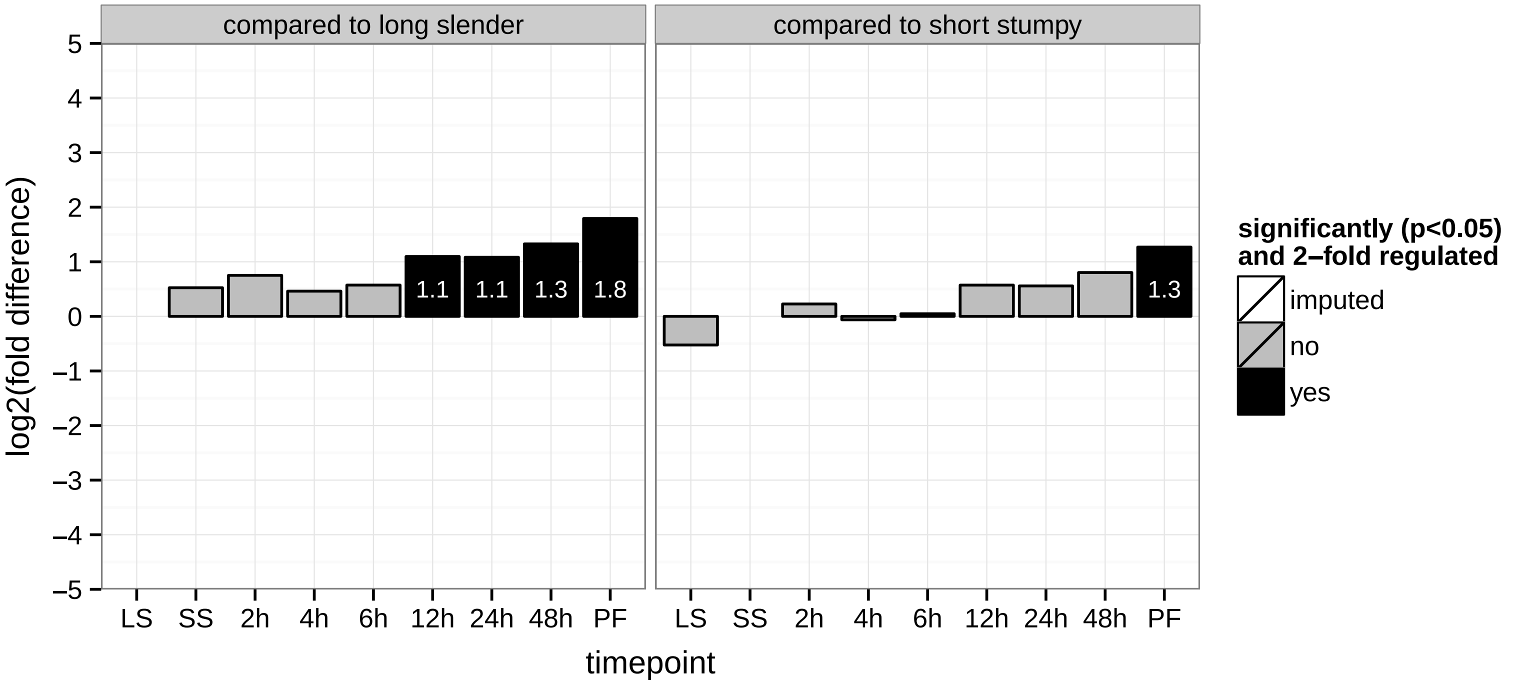

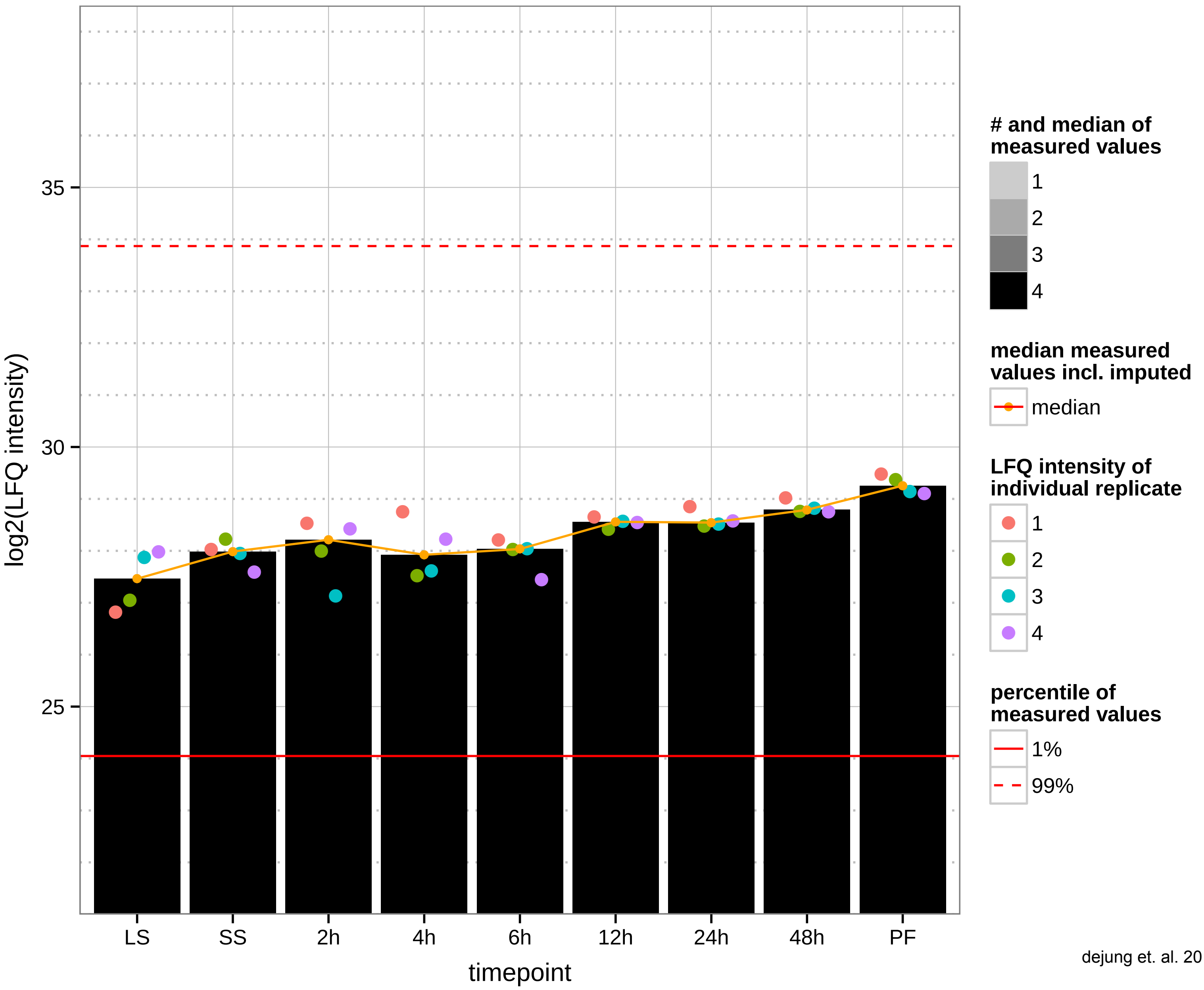

dejung et. al. 2015

prolyl oligopeptidase, putative, serine peptidase clan SC, family S9A, putative (POP)
Tb927.10.8020
AGOF: APG8−specific protease activity, oligopeptidase activity, serine−type endopeptidase activity
AGOC: cytoplasm, nucleus
AGOP: proteolysis
PGOF: serine−type endopeptidase activity, serine−type peptidase activity
PGOC: null
PGOP: proteolysis

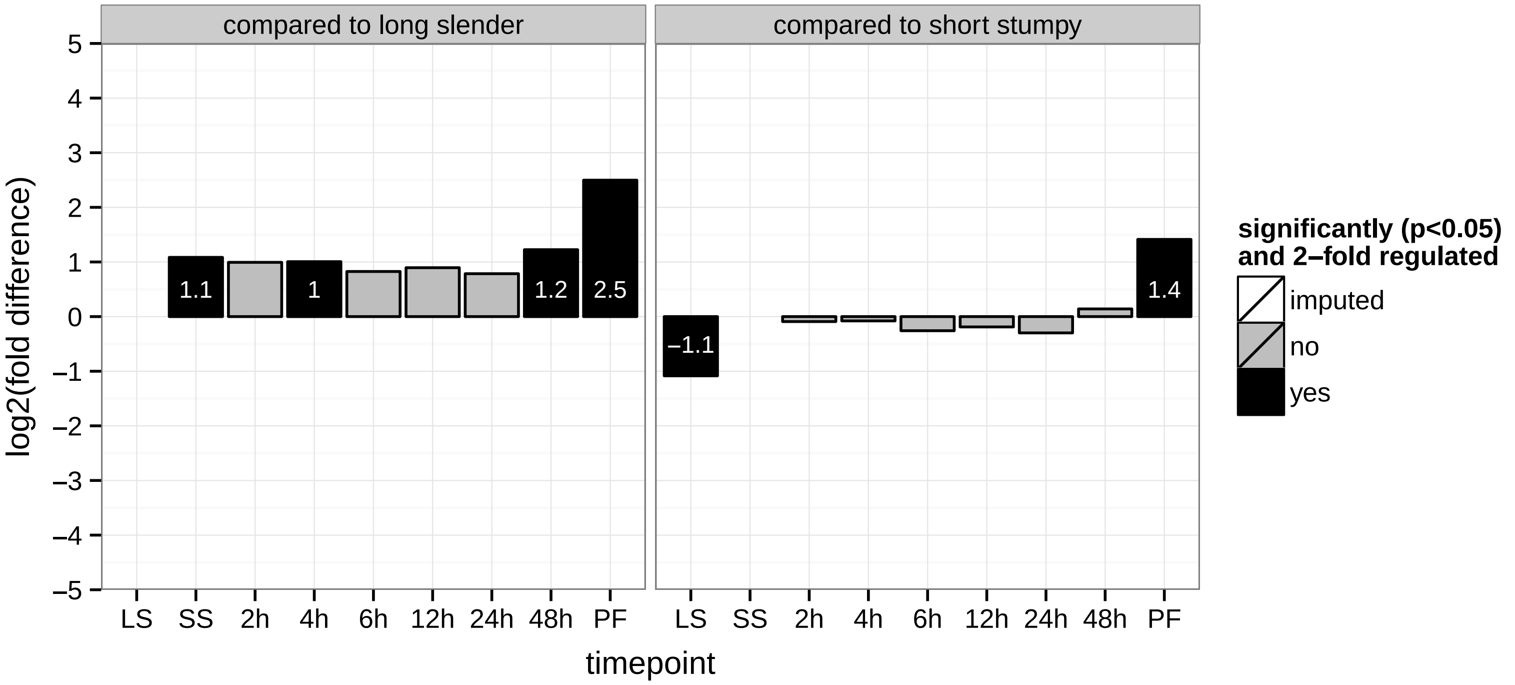

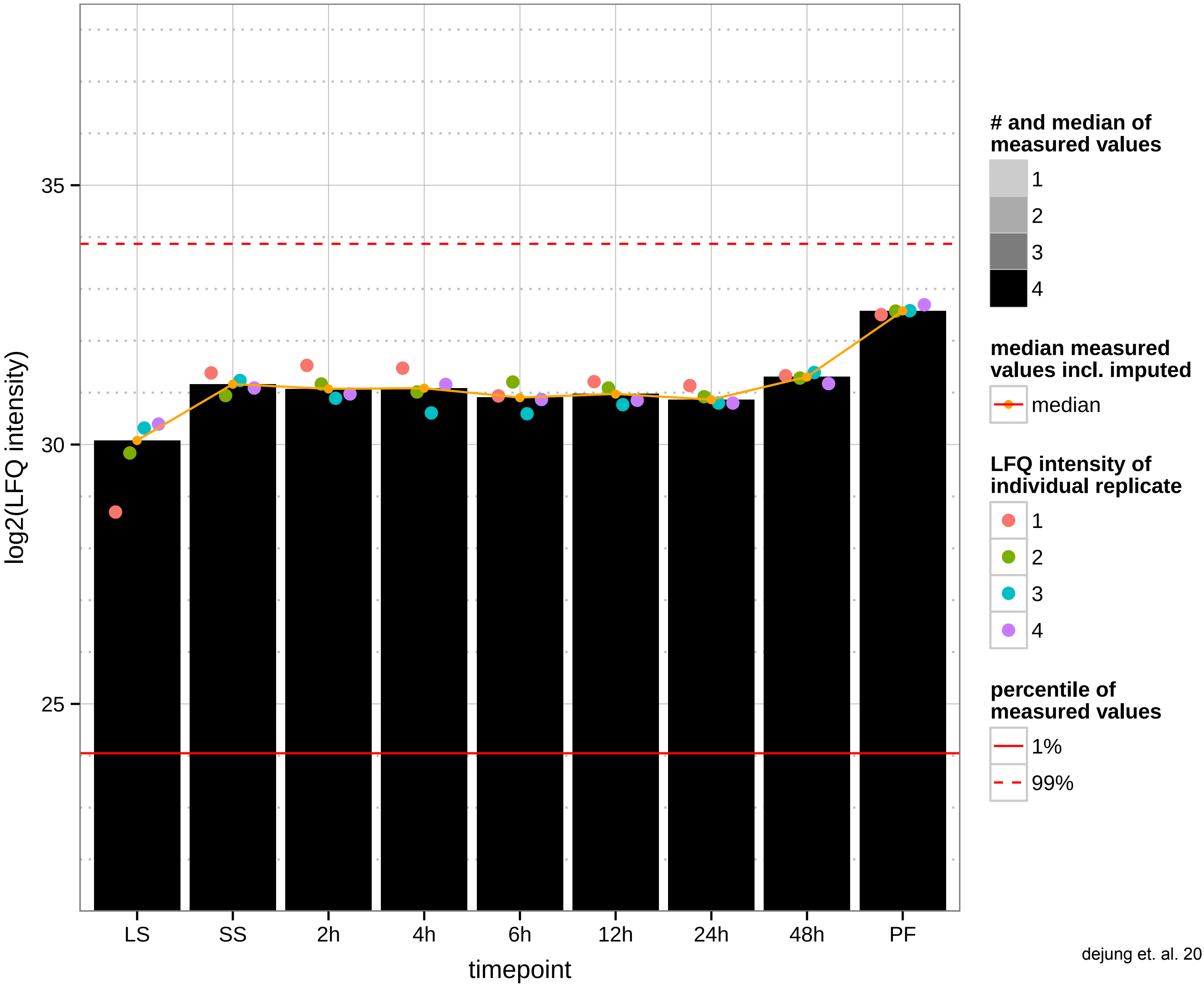

dejung et. al. 2015

hypothetical protein, conserved
Tb927.10.8200
AGOF: RNA binding
AGOC: null
AGOP: RNA processing
PGOF: RNA binding, structural constituent of ribosome
PGOC: large ribosomal subunit
PGOP: translation

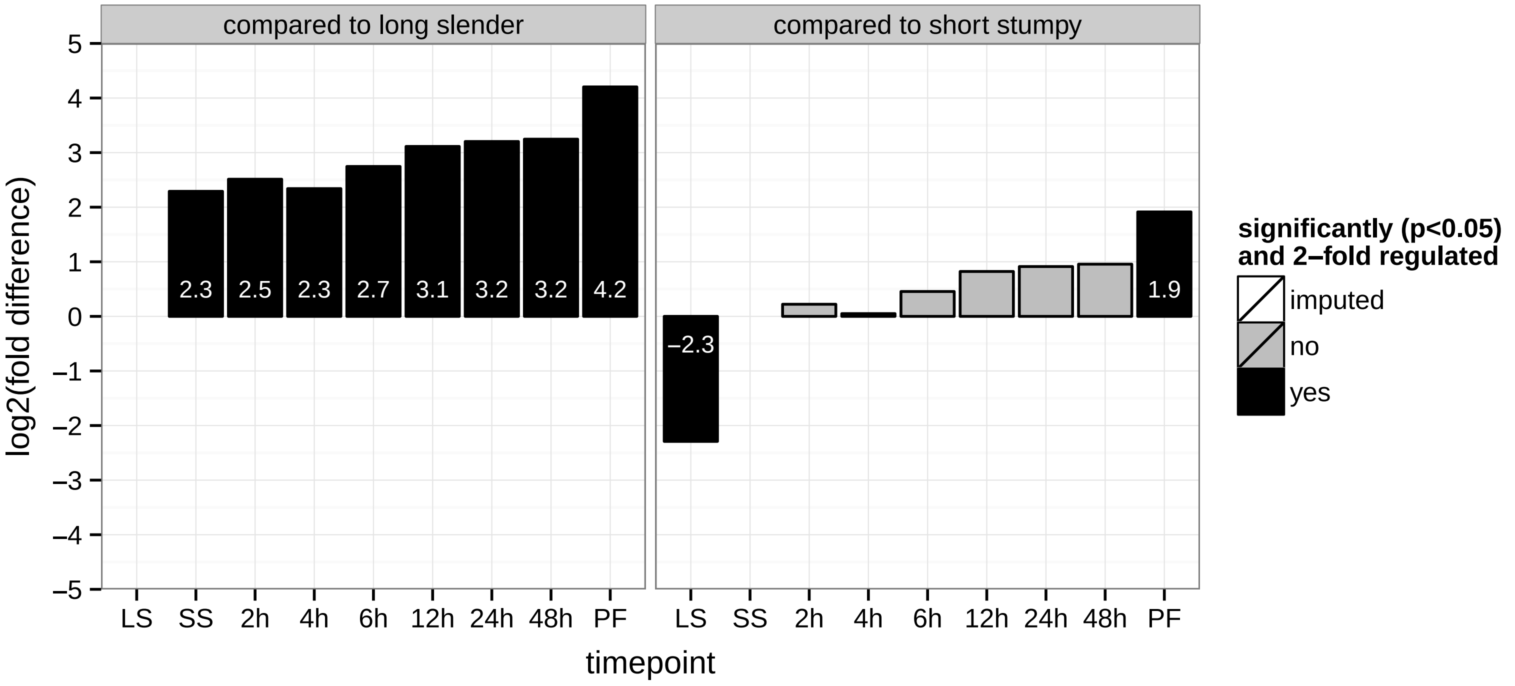

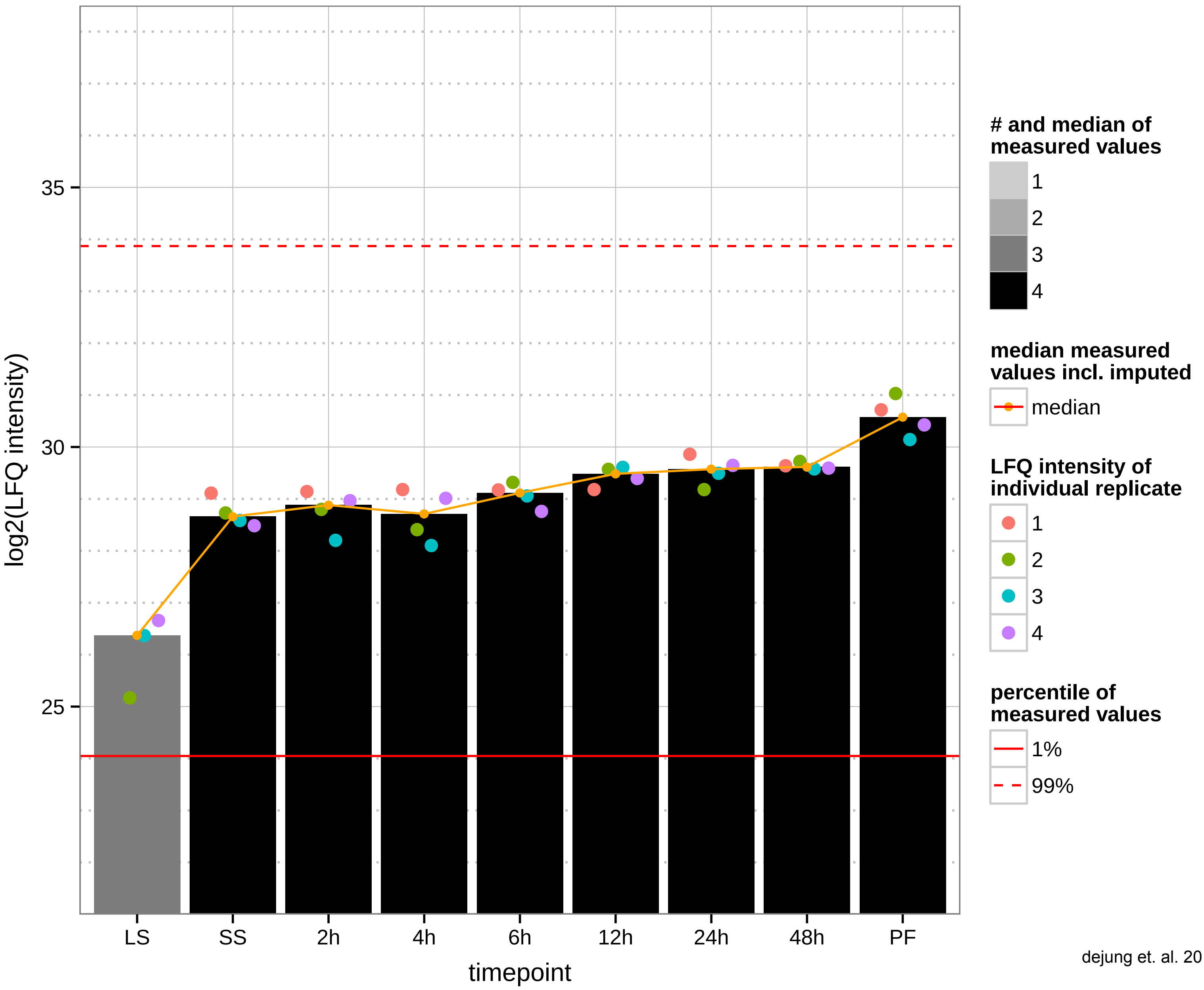

dejung et. al. 2015

S. cerevisiae PSP1 homologue, putative
Tb927.10.8330
AGOF: null
AGOC: null
AGOP: null
PGOF: null
PGOC: null
PGOP: null

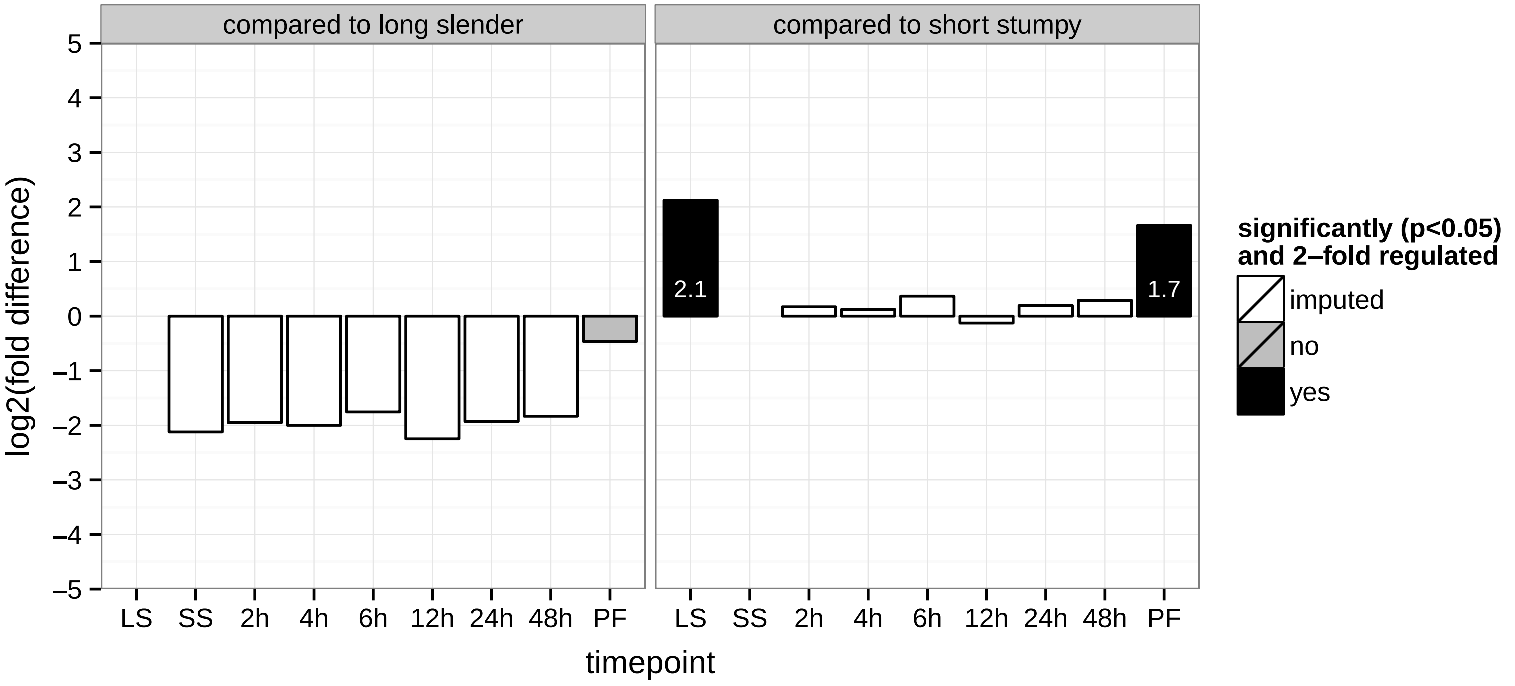

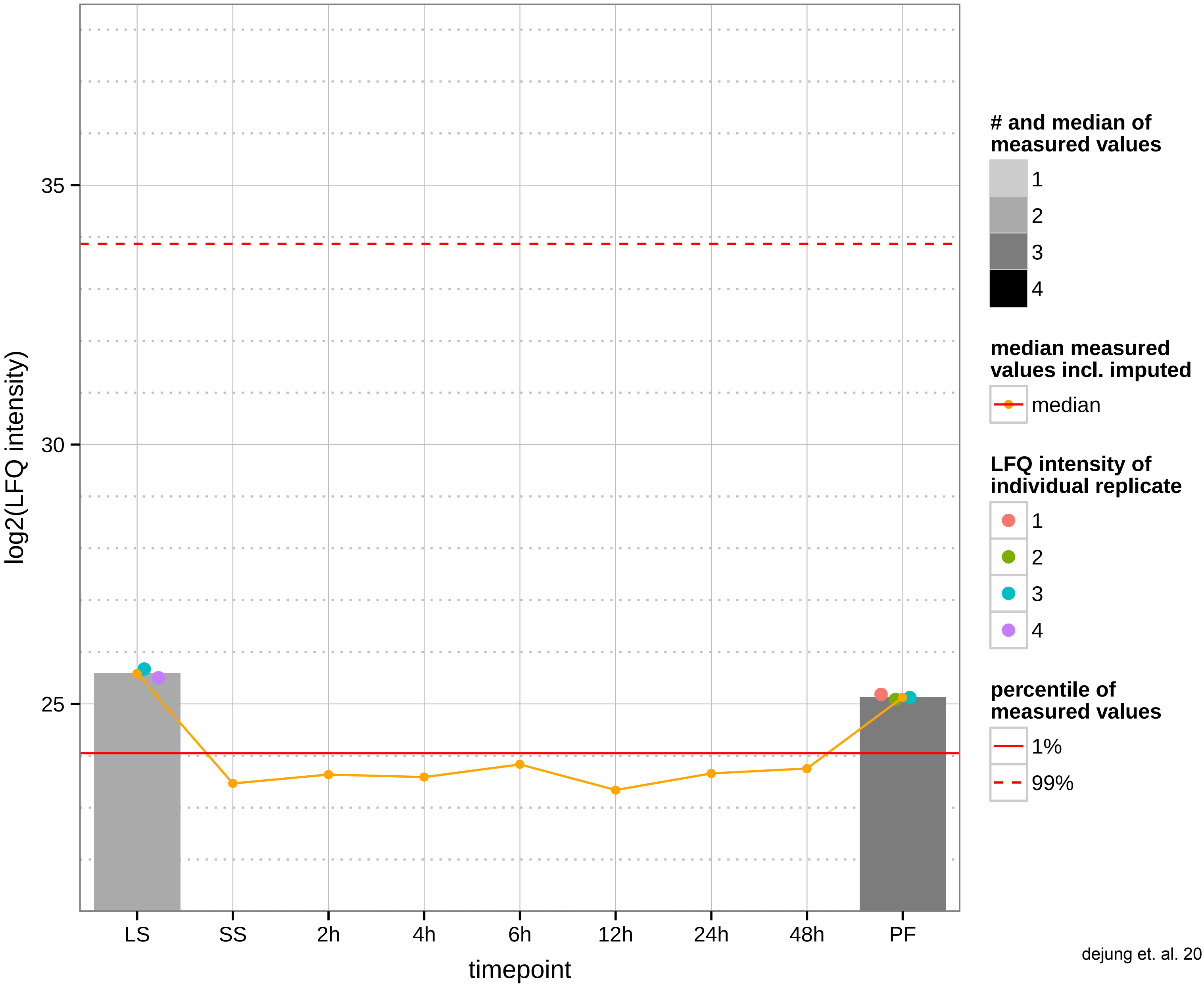

dejung et. al. 2015

ADP−ribosylation factor 3, putative, ADP−ribosylation factor−like protein, putative (ARL3)
Tb927.10.8580
AGOF: GTP binding
AGOC: intracellular
AGOP: intracellular protein transport, small GTPase mediated signal transduction
PGOF: GTP binding
PGOC: intracellular
PGOP: intracellular protein transport, protein transport, small GTPase mediated signal transduction

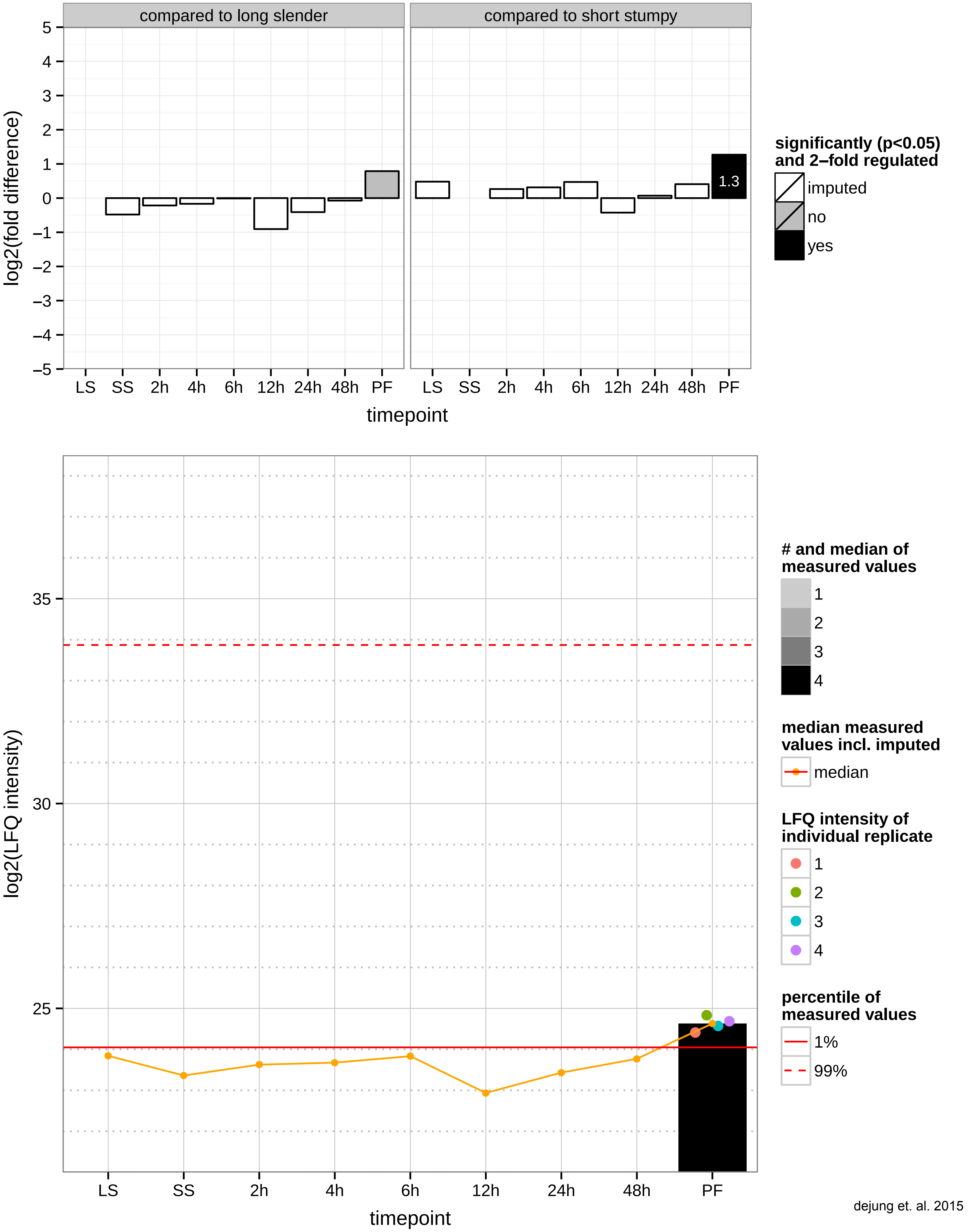

dejung et. al. 2015

nucleoporin, putative (Nup181)
Tb927.10.8910
AGOF: null
AGOC: nuclear pore
AGOP: intracellular transport
PGOF: null
PGOC: null
PGOP: null

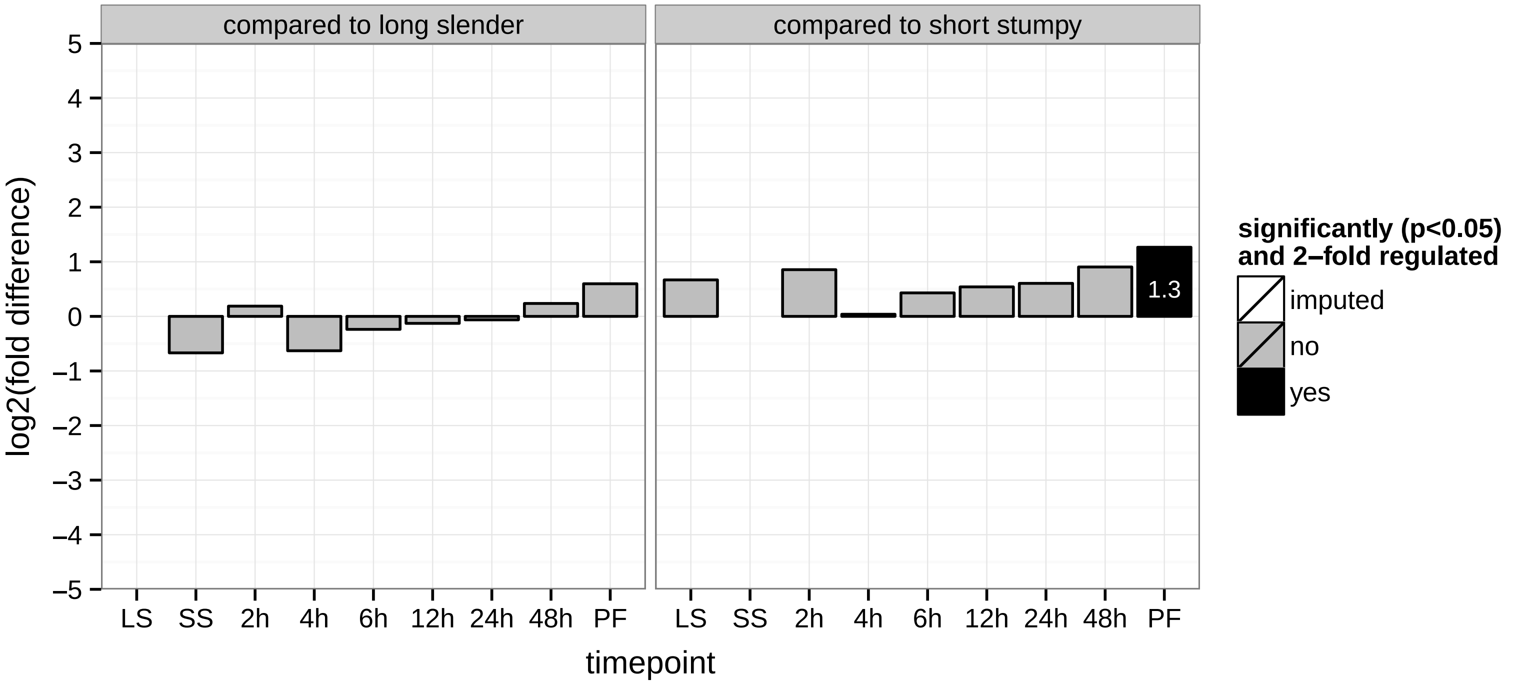

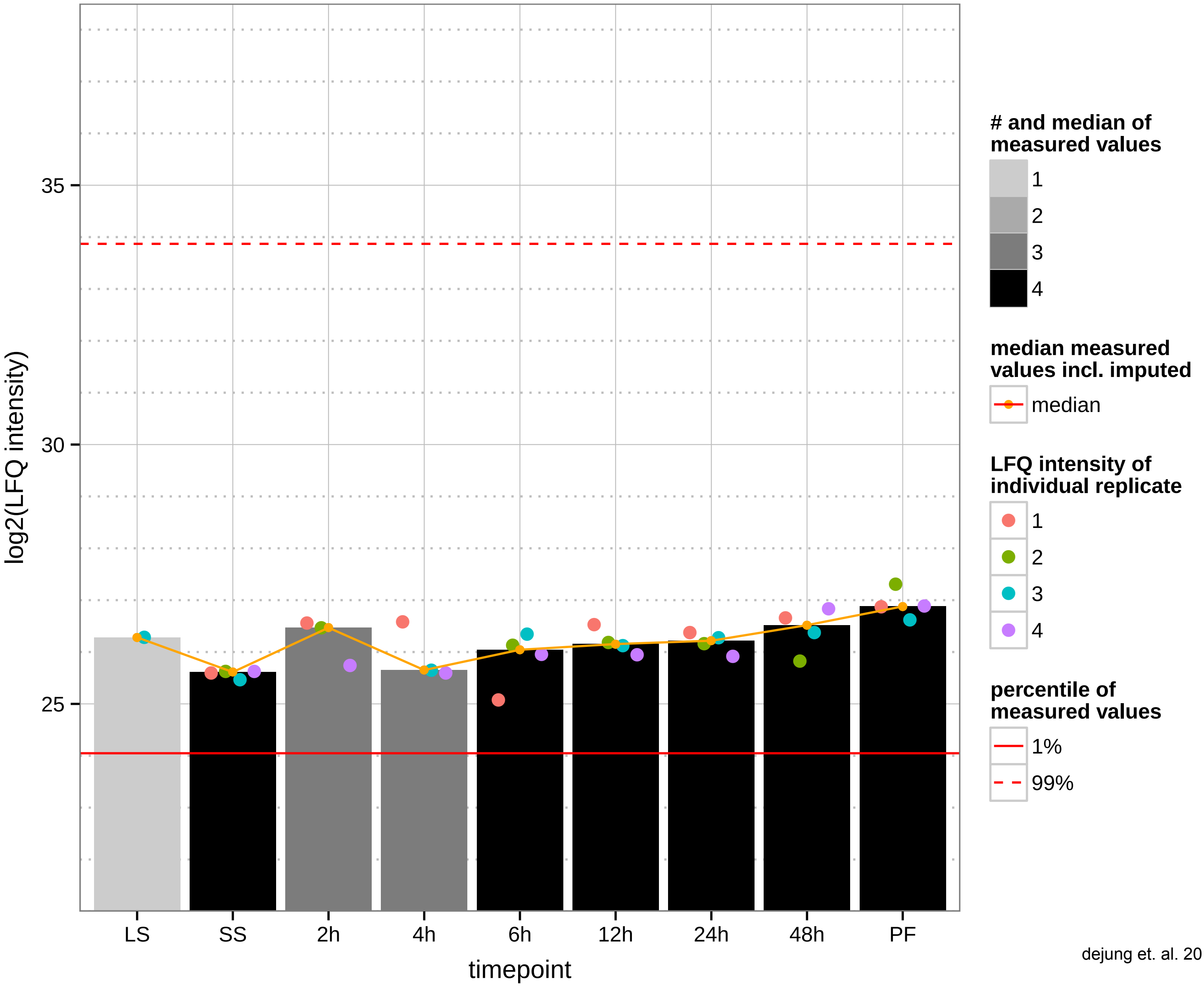

dejung et. al. 2015

hypothetical protein, conserved
Tb927.10.9070
AGOF: null
AGOC: null
AGOP: null
PGOF: null
PGOC: null
PGOP: null

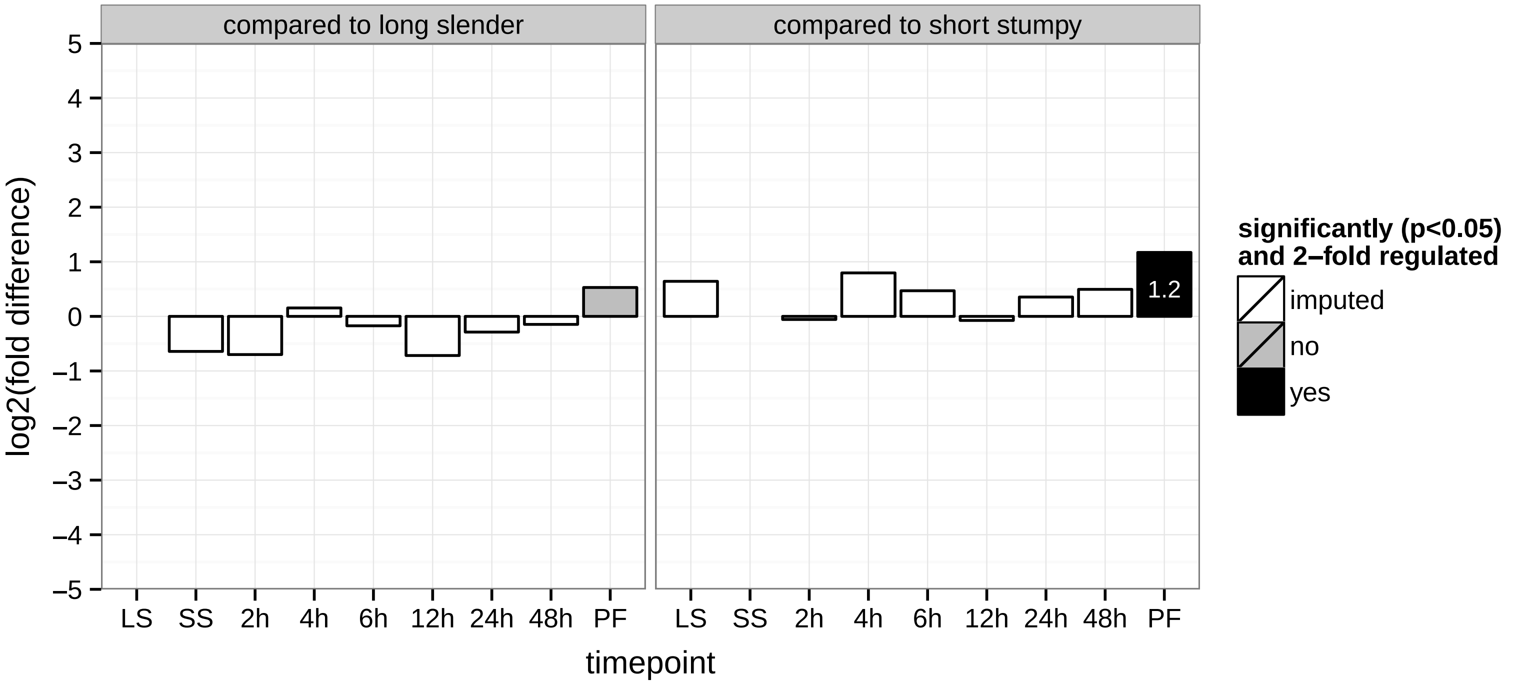

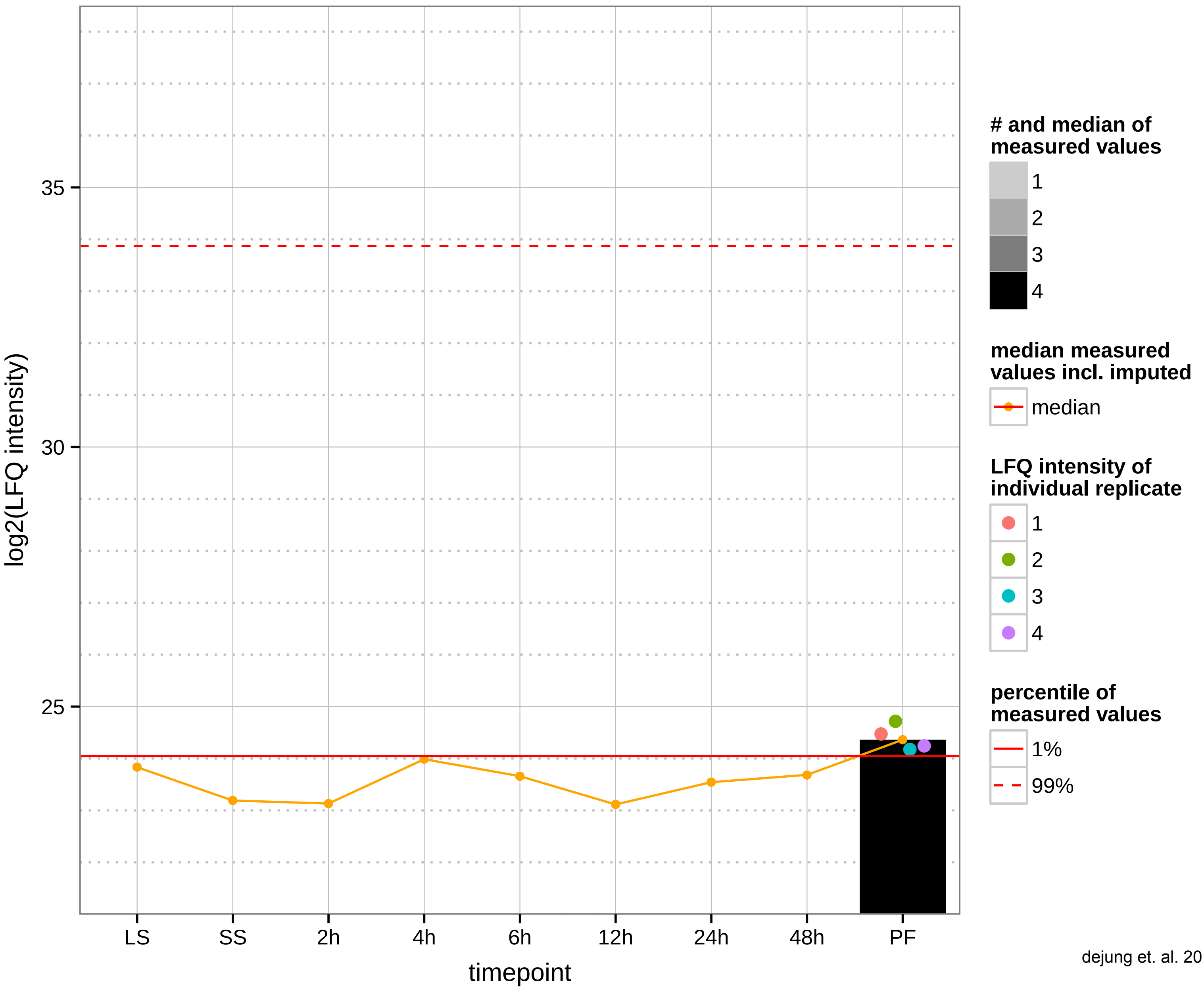

dejung et. al. 2015

hypothetical protein, conserved
Tb927.10.9120
AGOF: null
AGOC: mitochondrion
AGOP: null
PGOF: null
PGOC: null
PGOP: null

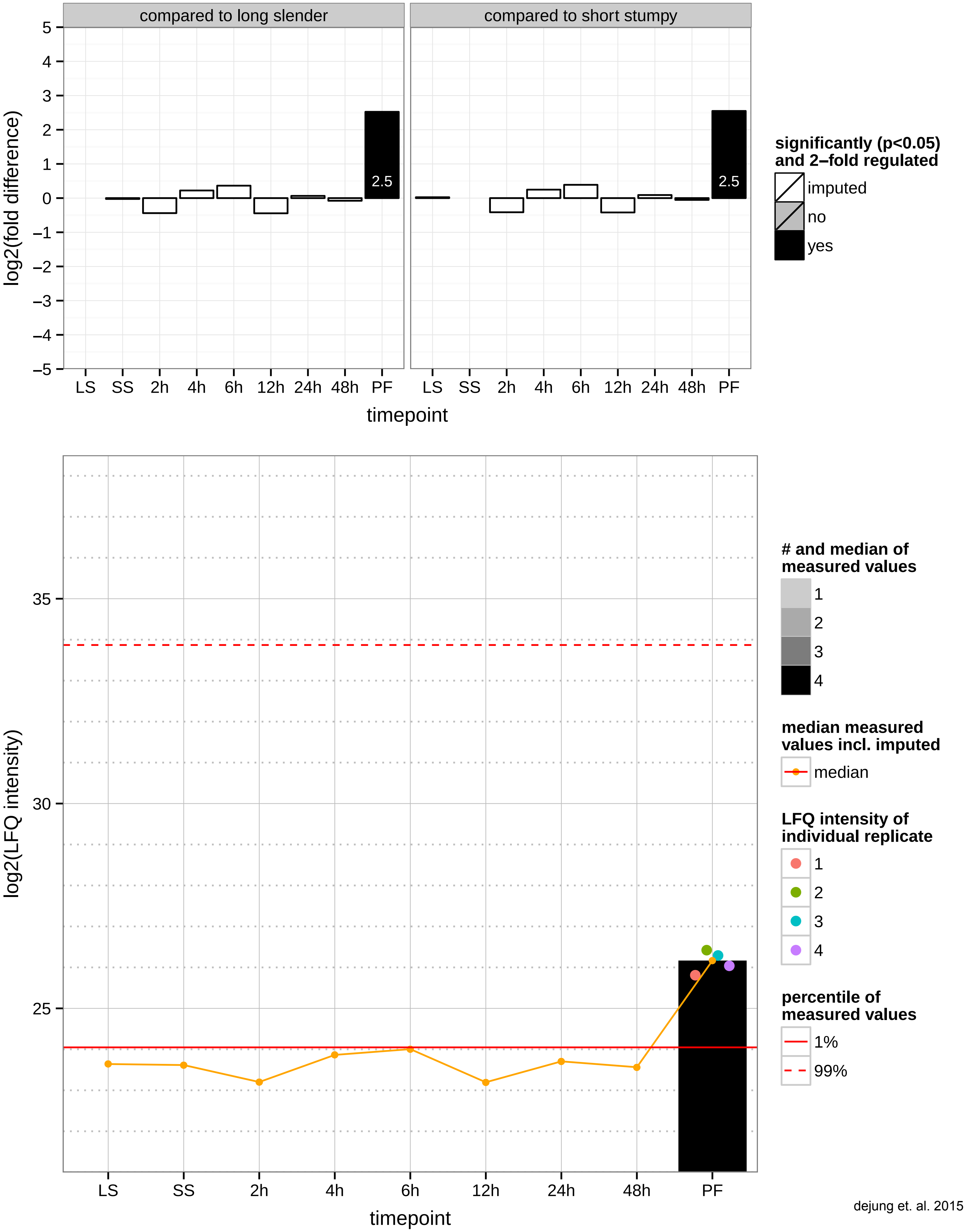

dejung et. al. 2015

hypothetical protein, conserved
Tb927.10.9150
AGOF: null
AGOC: null
AGOP: null
PGOF: null
PGOC: null
PGOP: null

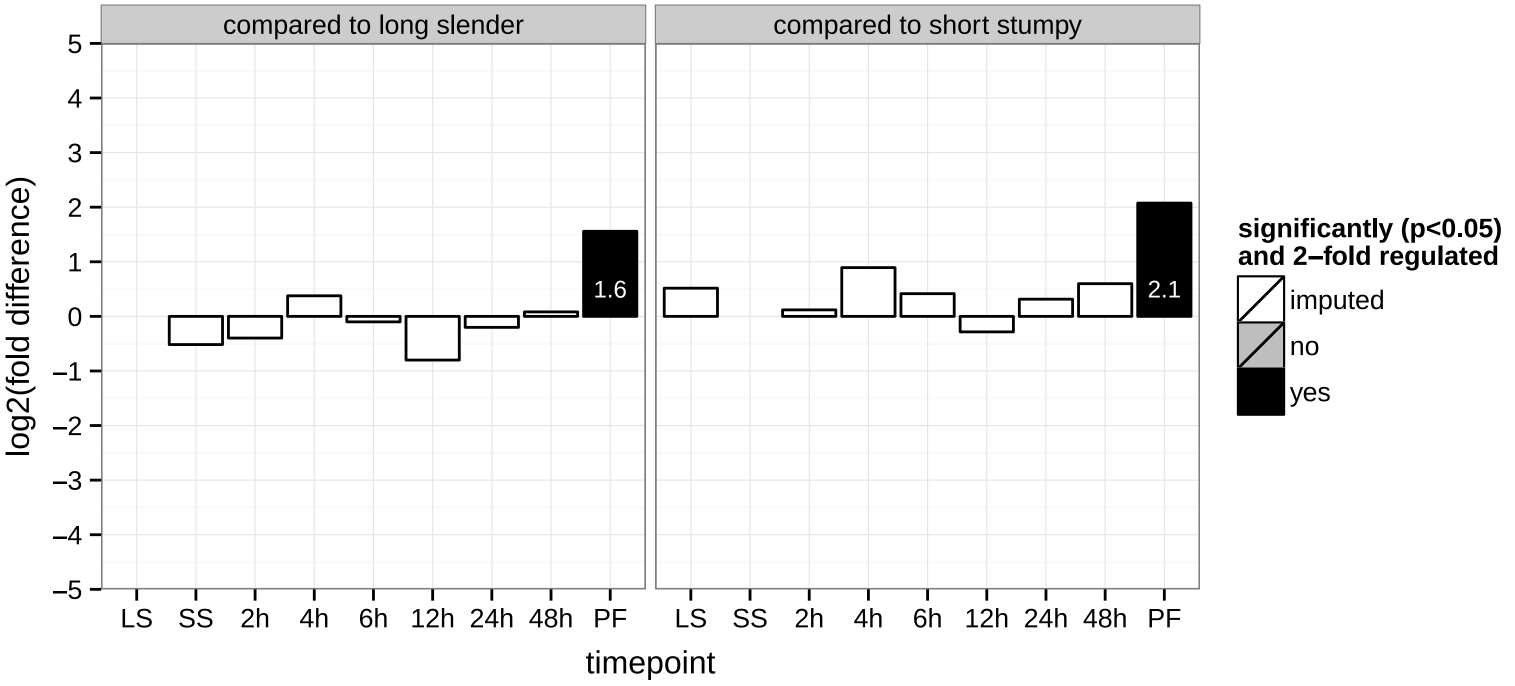

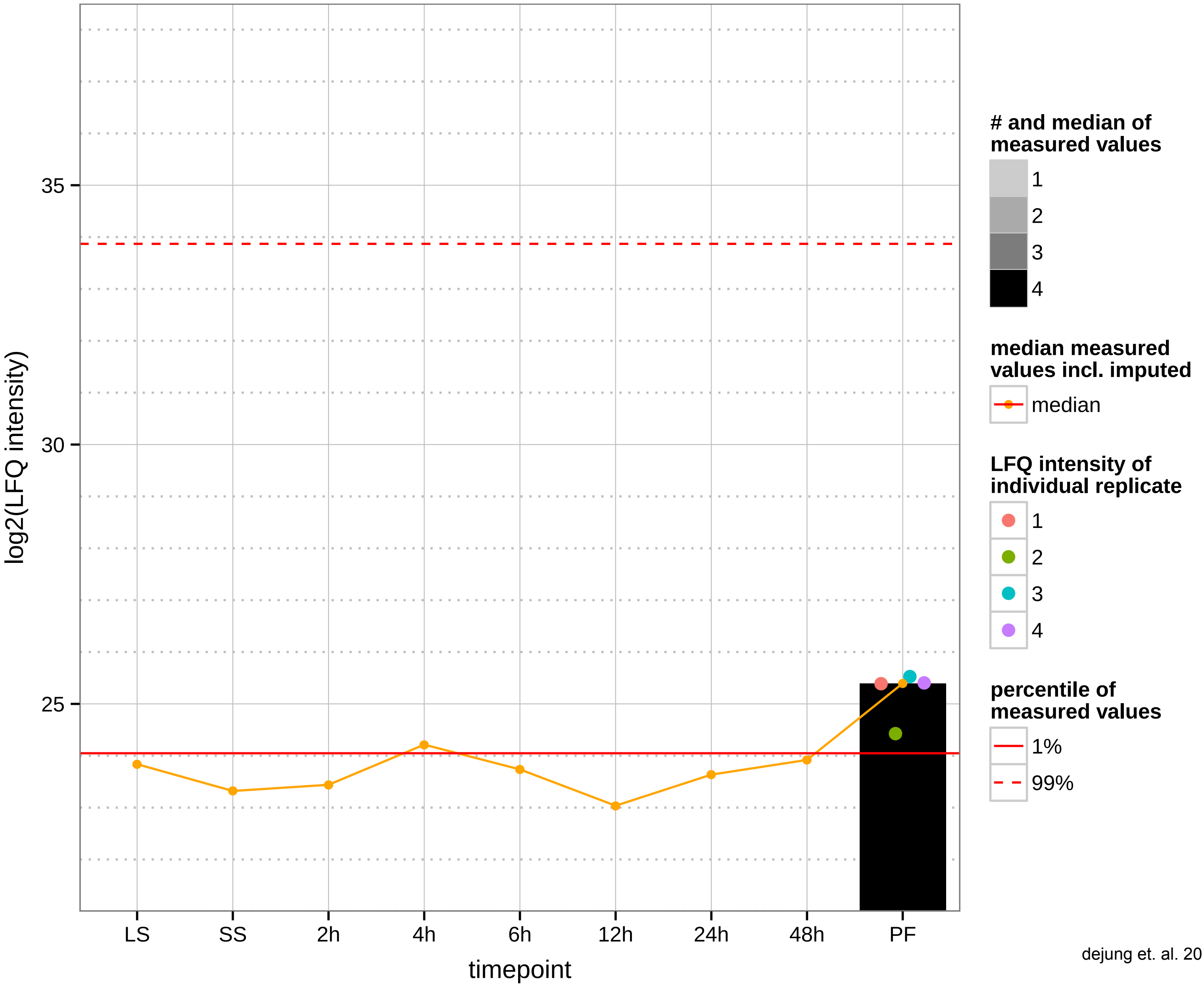

dejung et. al. 2015

60S ribosomal protein L18, putative (TbL18LP), putative (RPL18)
Tb927.11.4300;Tb927.10.9880
AGOF: structural constituent of ribosome
AGOC: intracellular, ribosome
AGOP: translation, null
PGOF: null
PGOC: null
PGOP: null

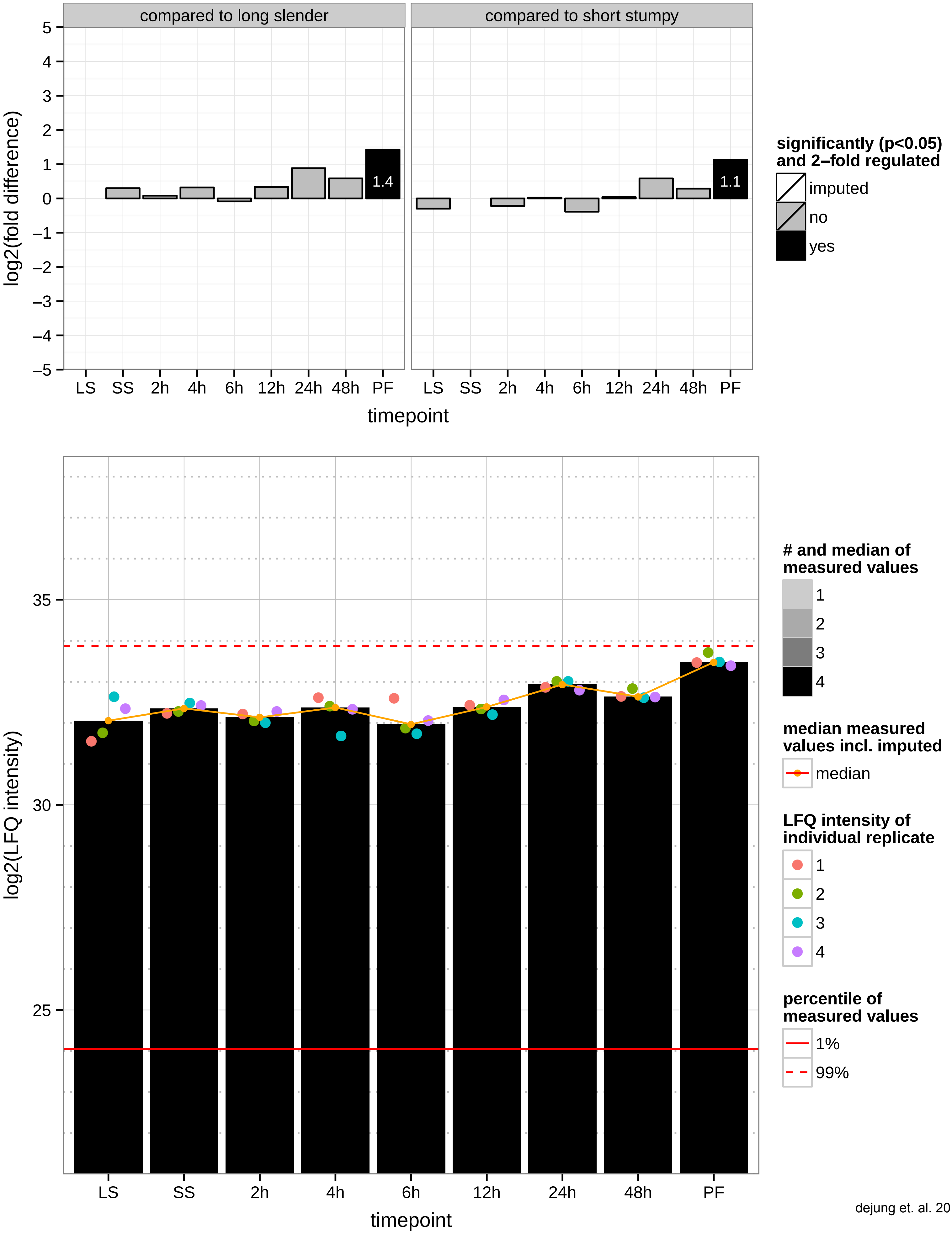

dejung et. al. 2015

hypothetical protein, conserved
Tb927.10.9960
AGOF: null
AGOC: null
AGOP: null
PGOF: null
PGOC: null
PGOP: null

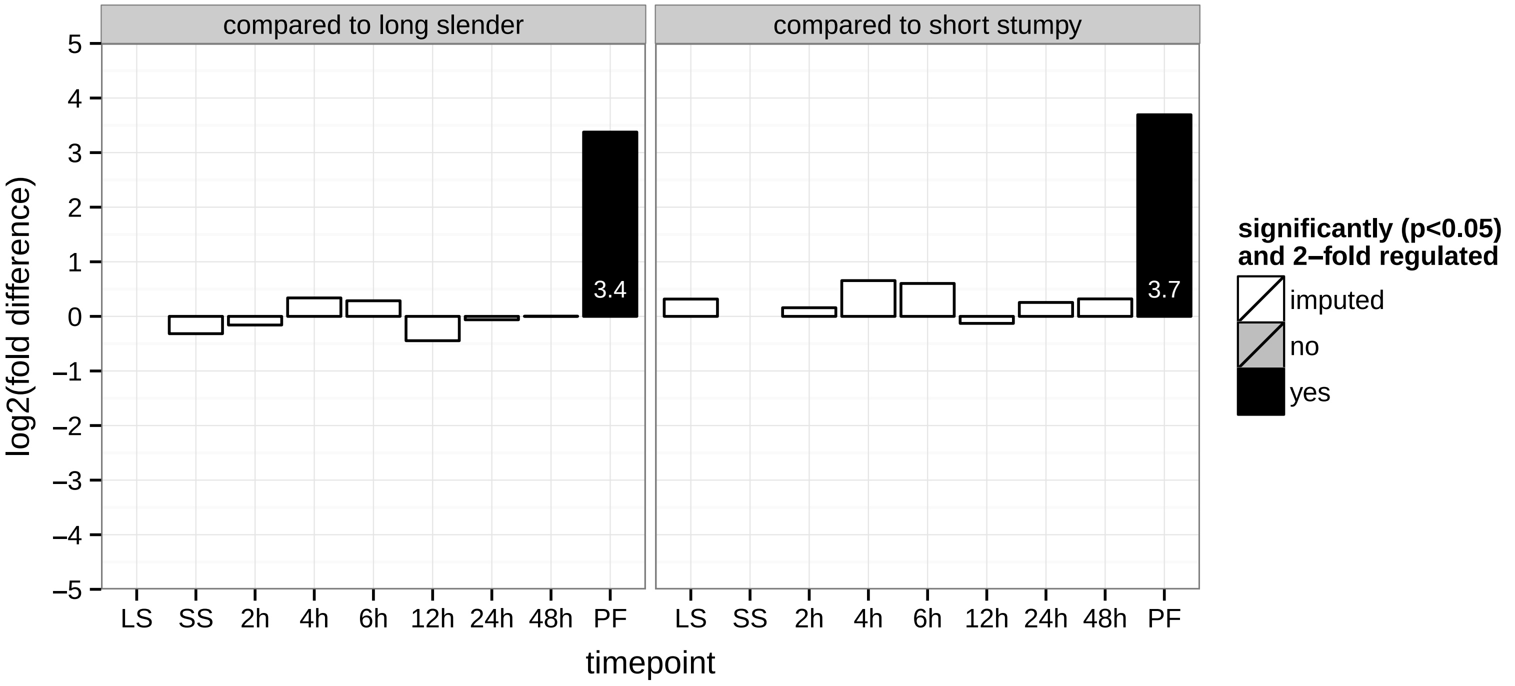

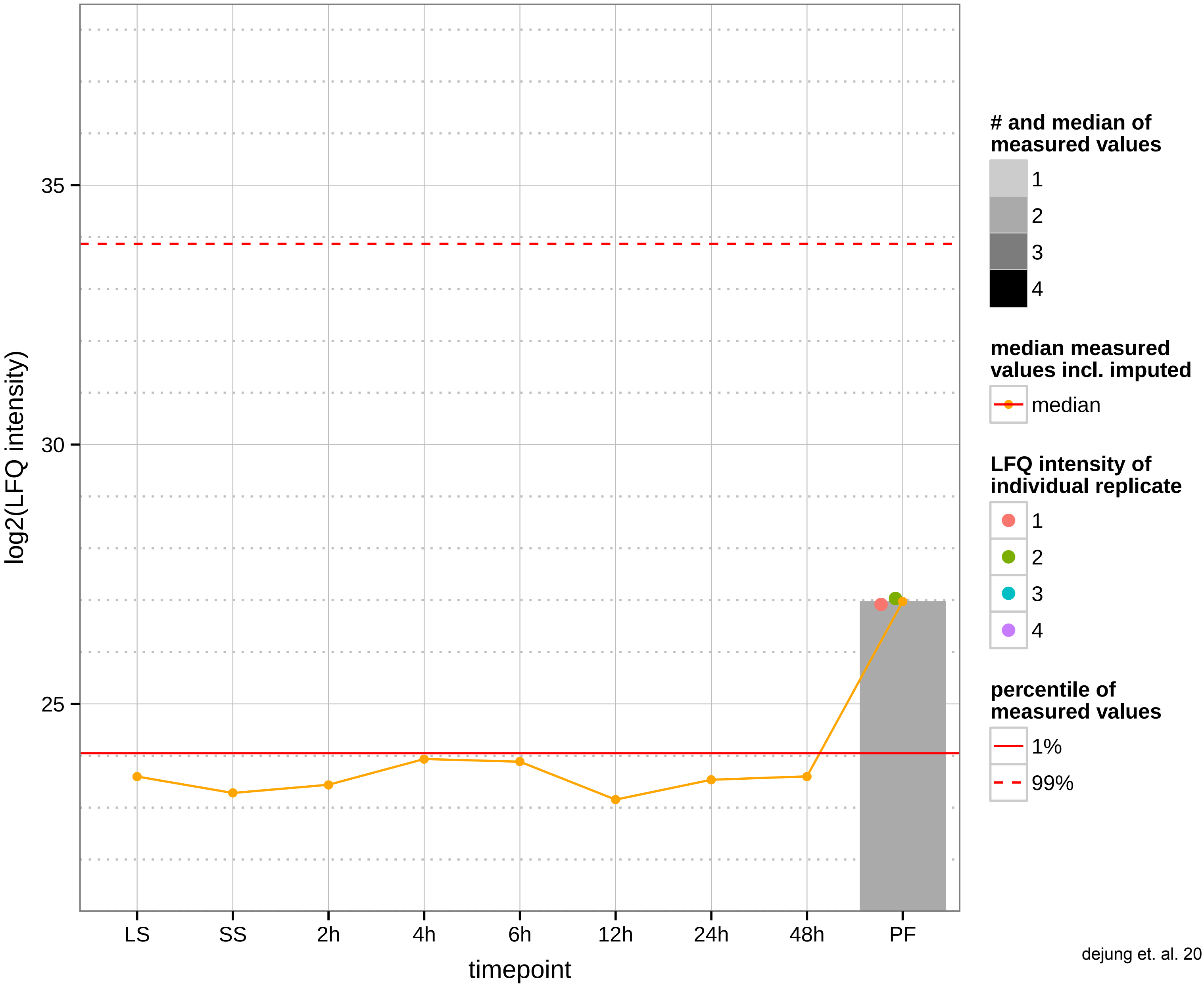

dejung et. al. 2015

60S ribosomal protein L29, putative
Tb927.11.10030
AGOF: structural constituent of ribosome
AGOC: intracellular, ribosome
AGOP: translation
PGOF: structural constituent of ribosome
PGOC: intracellular, ribosome
PGOP: translation

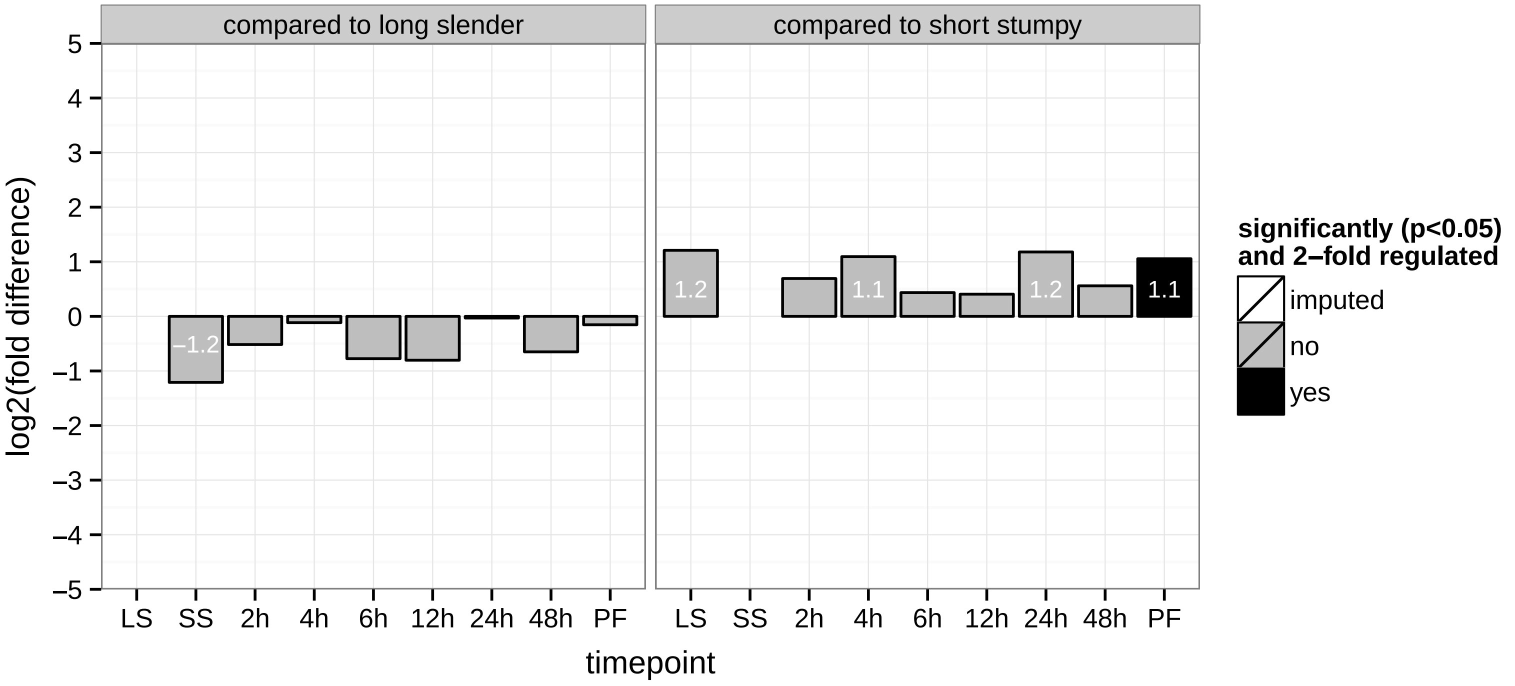

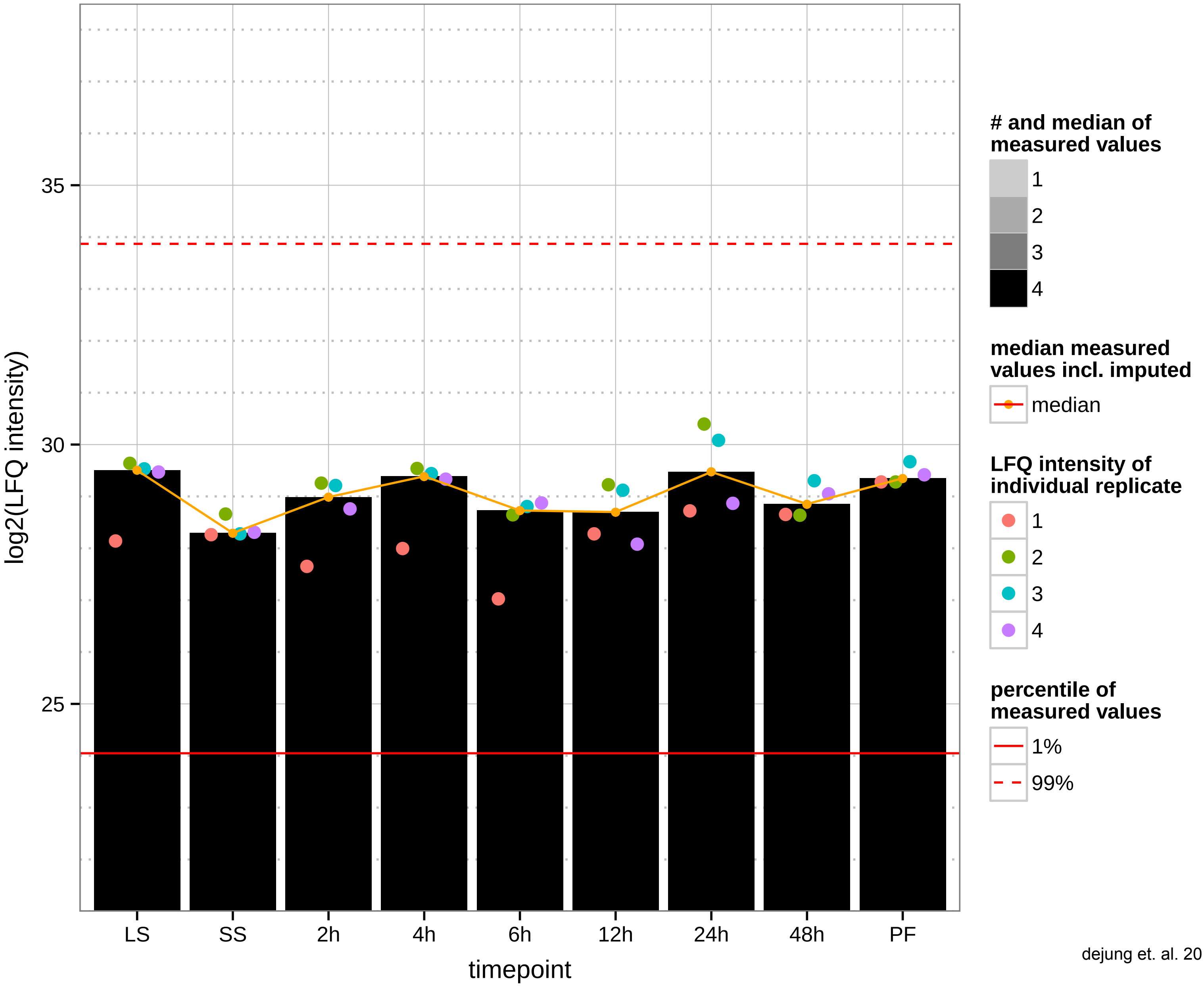

dejung et. al. 2015

hypothetical protein, conserved
Tb927.11.10080
AGOF: null
AGOC: mitochondrion
AGOP: null
PGOF: null
PGOC: null
PGOP: null

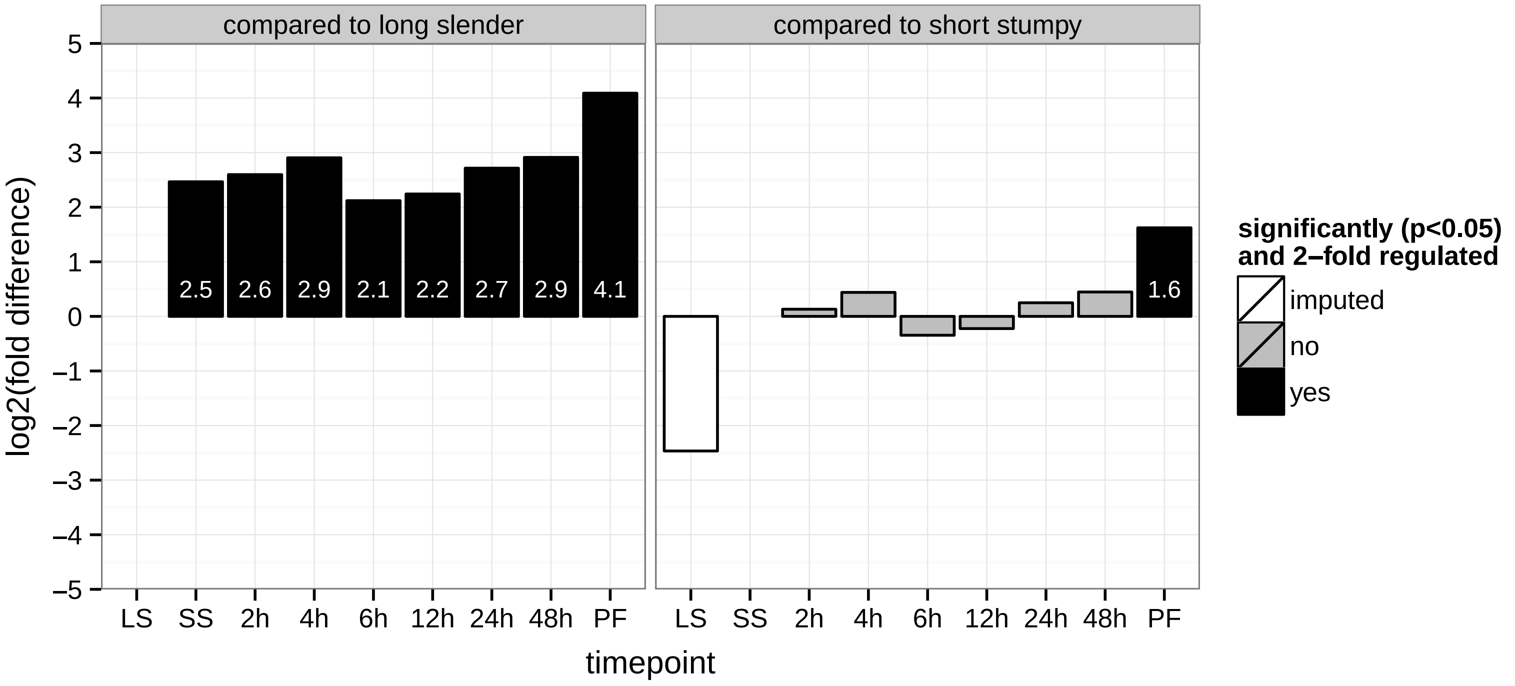

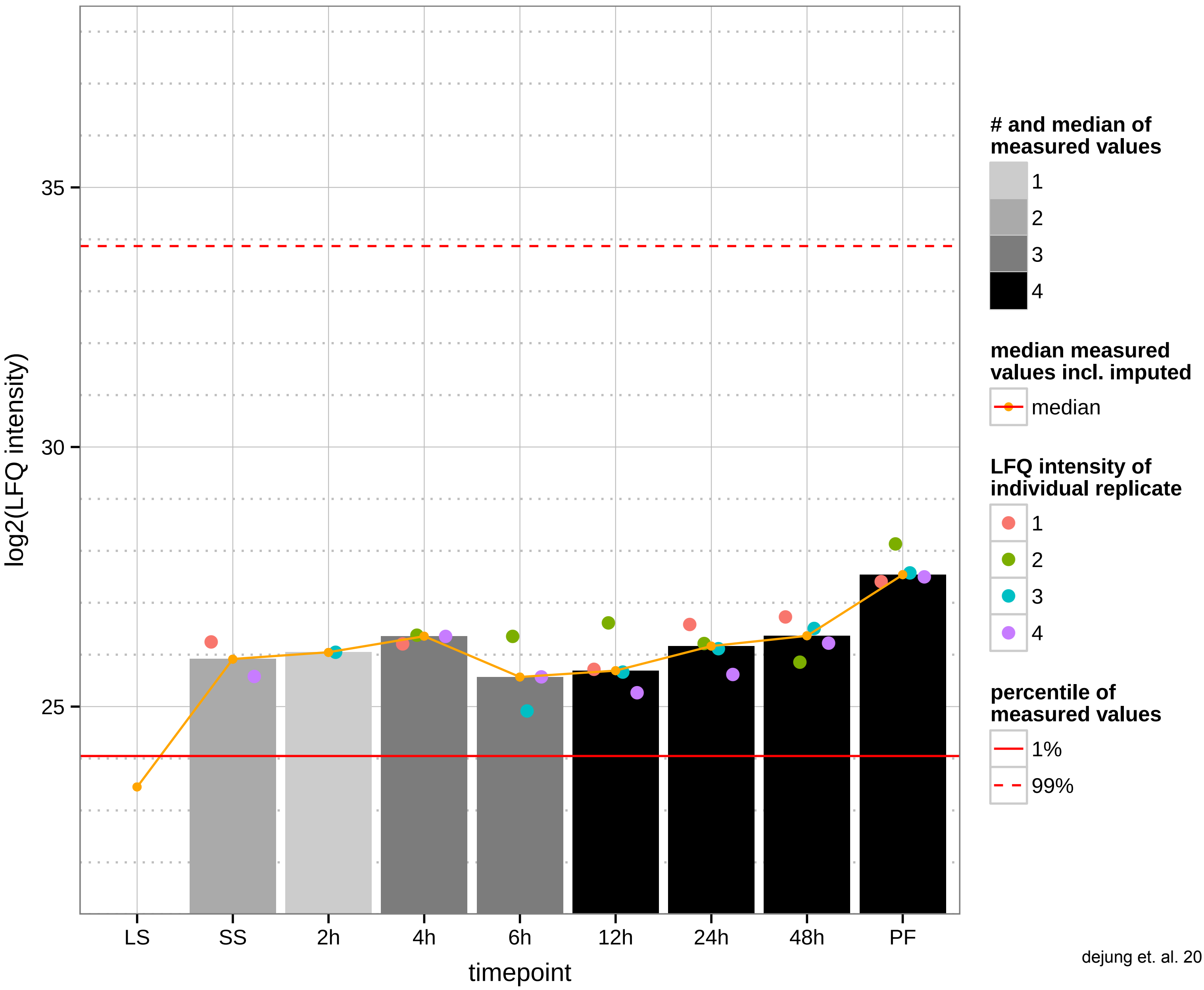

dejung et. al. 2015

hypothetical protein, conserved
Tb927.11.1040
AGOF: null
AGOC: null
AGOP: null
PGOF: null
PGOC: null
PGOP: null

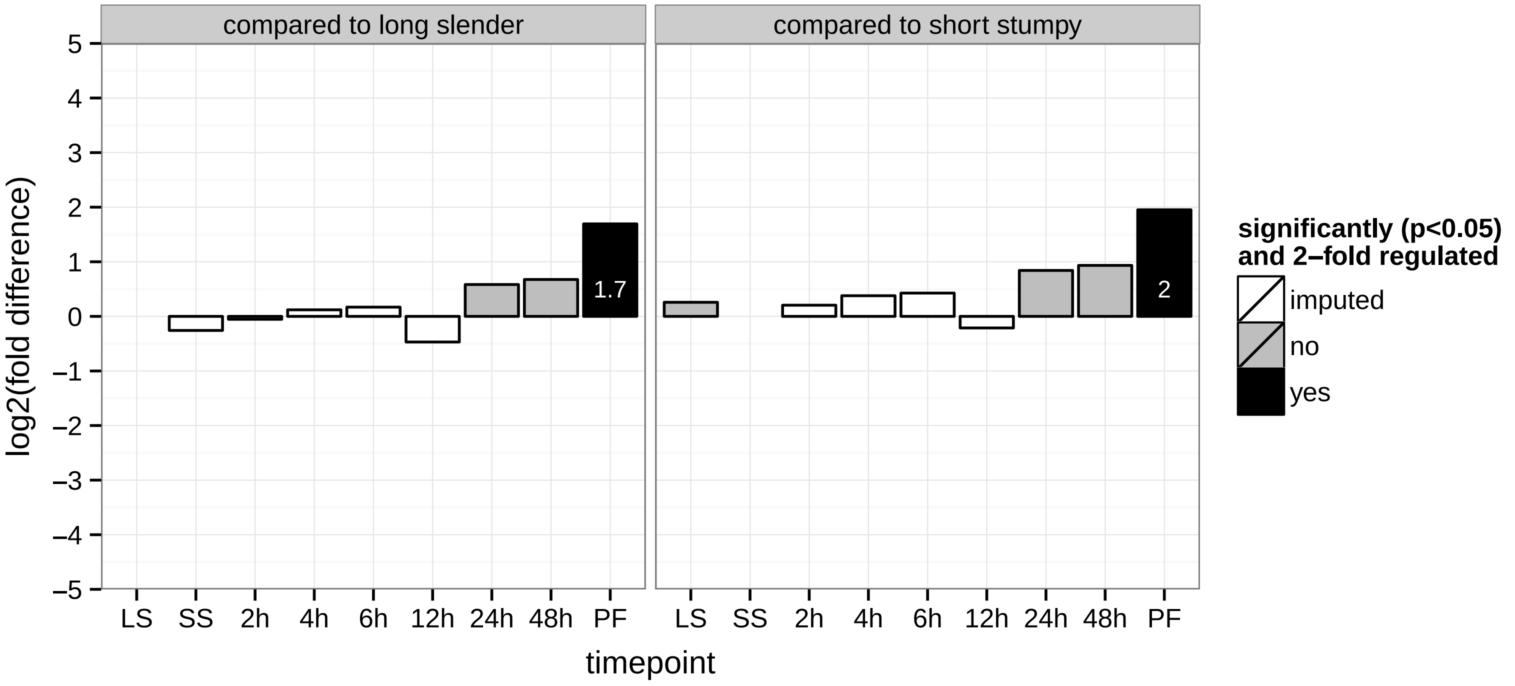

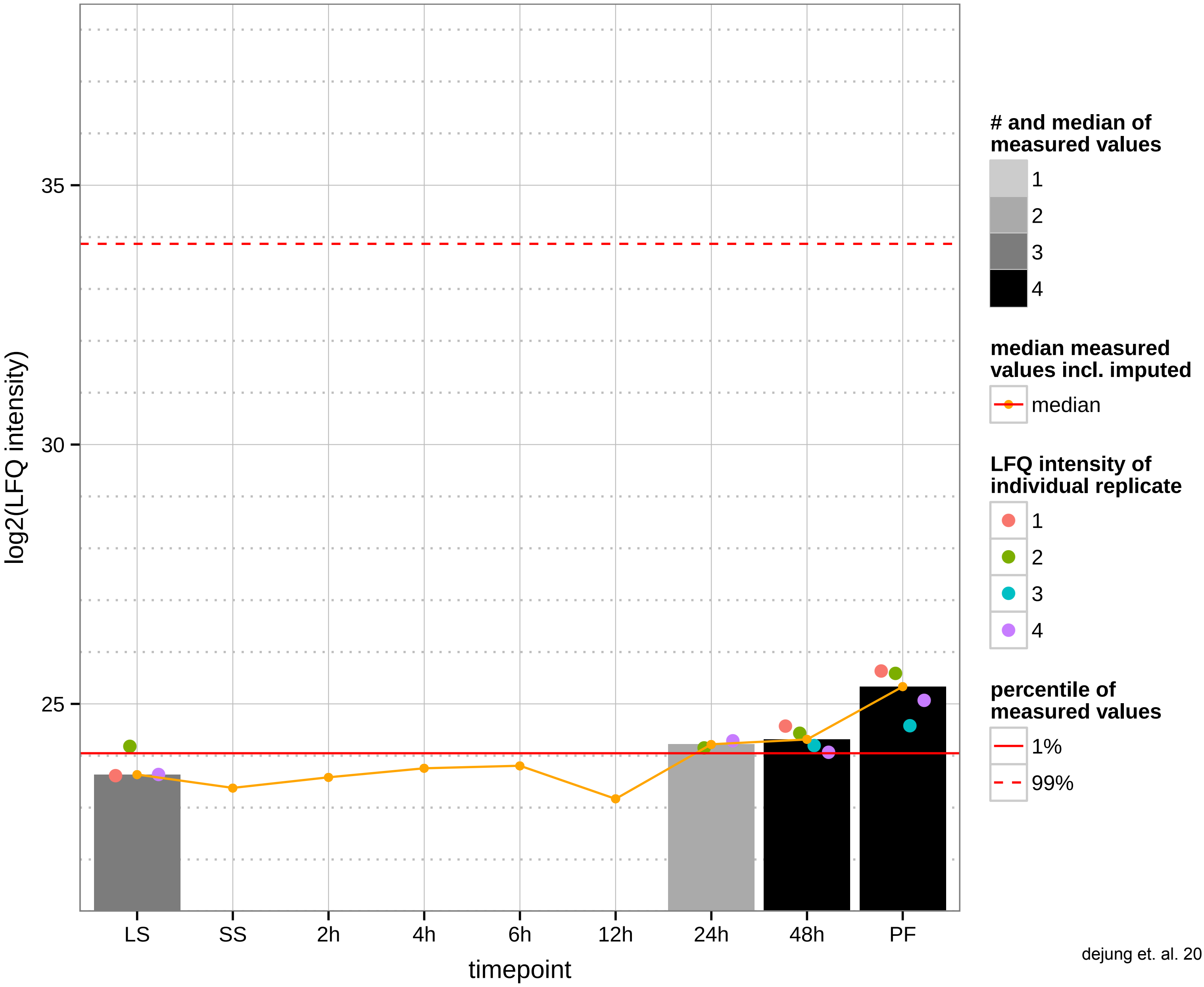

dejung et. al. 2015

hypothetical protein, conserved
Tb927.11.10460
AGOF: null
AGOC: nucleus
AGOP: DNA replication
PGOF: null
PGOC: null
PGOP: null

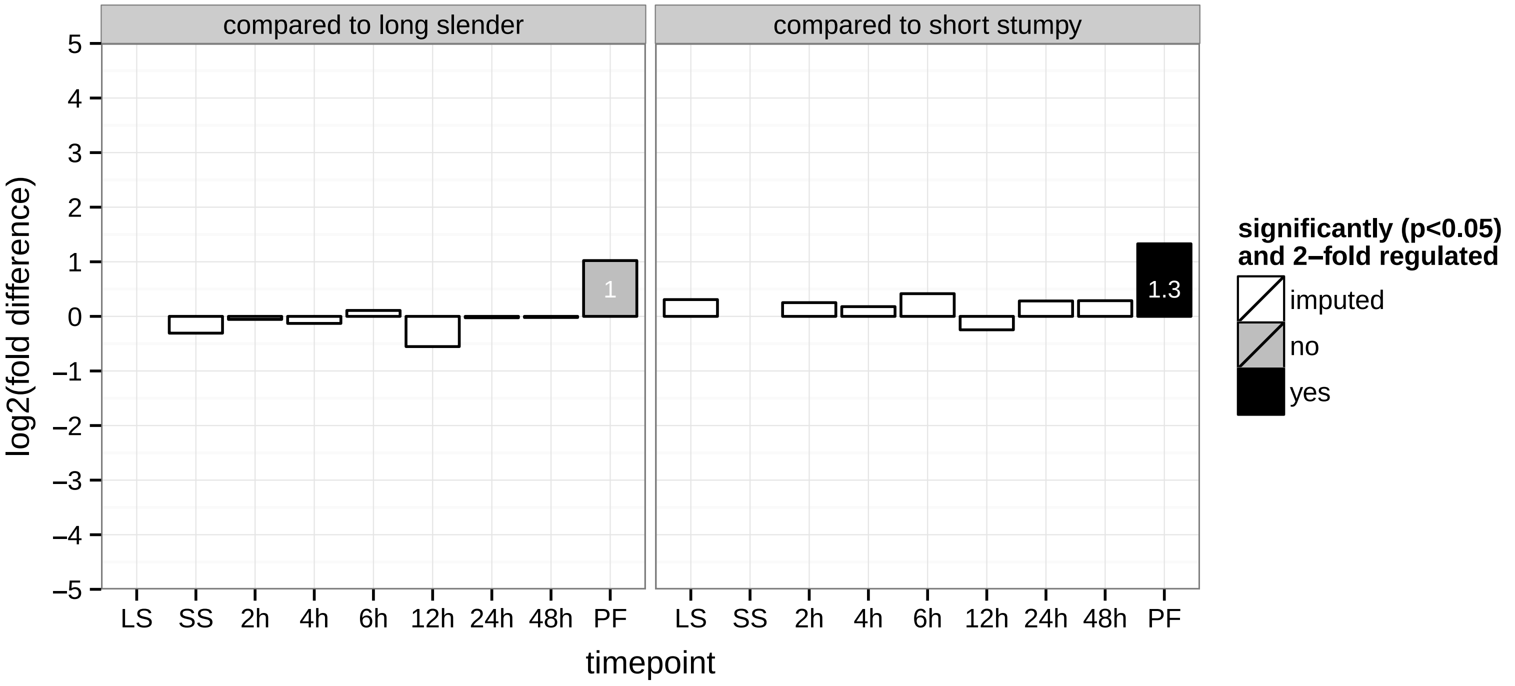

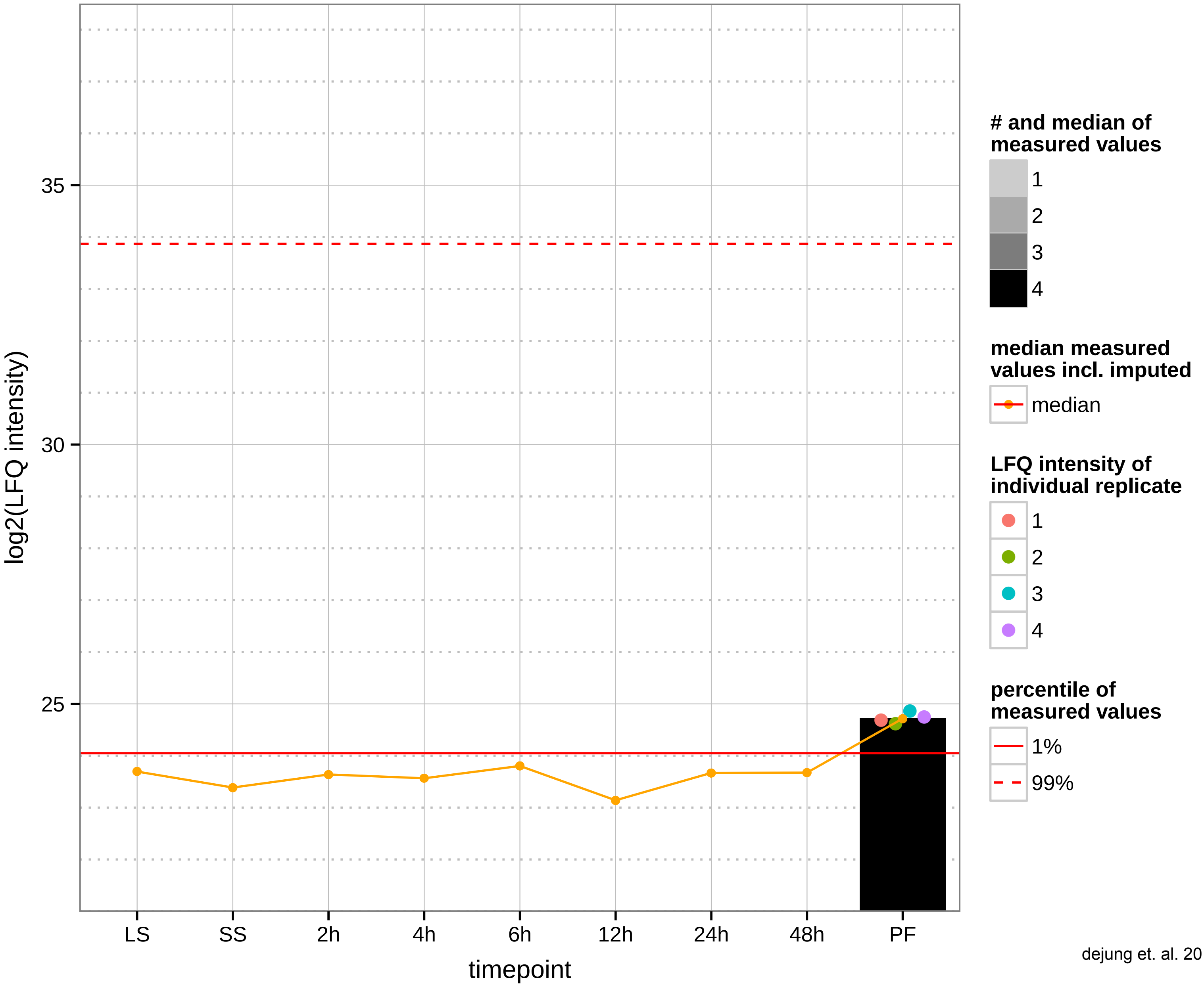

dejung et. al. 2015

hypothetical protein, conserved
Tb927.11.10470
AGOF: null
AGOC: null
AGOP: null
PGOF: null
PGOC: null
PGOP: null

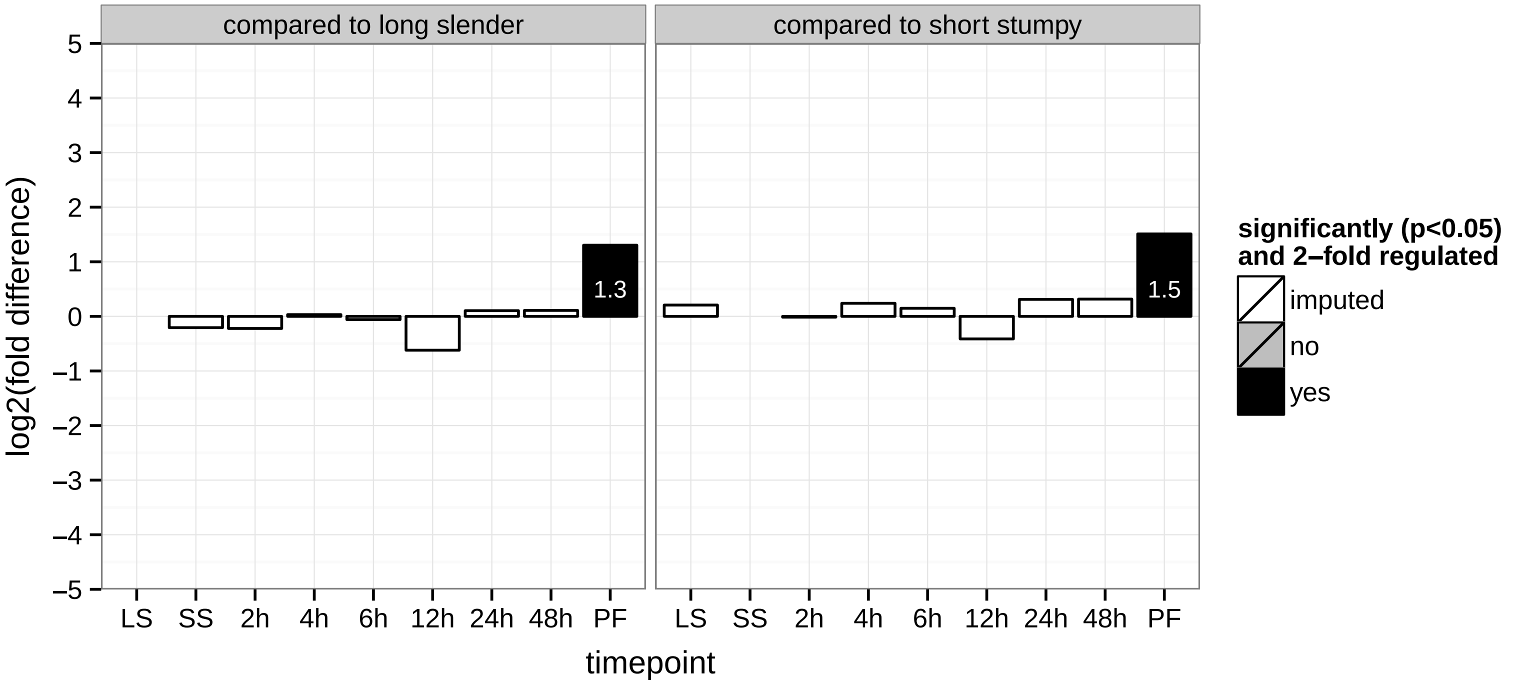

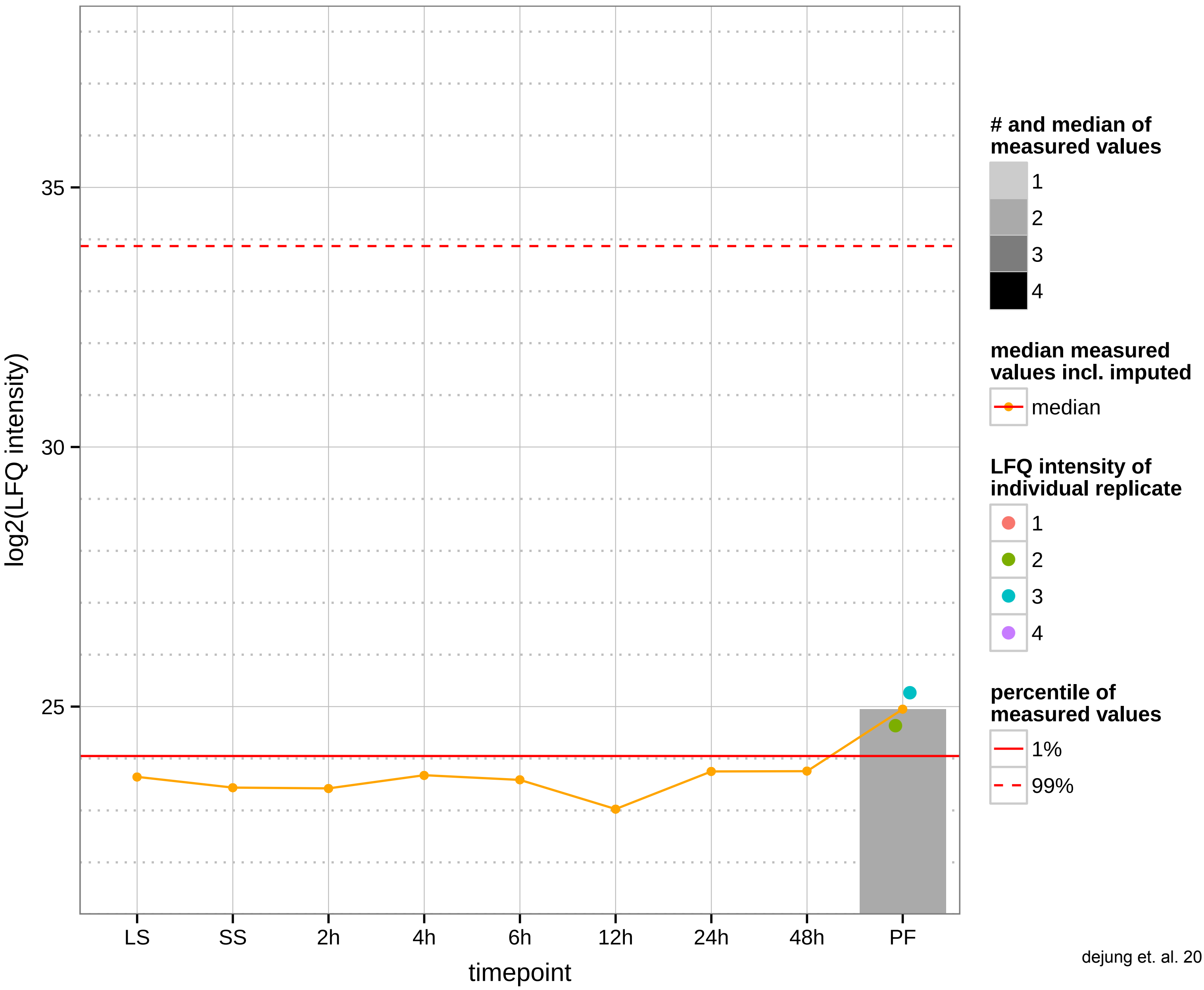

dejung et. al. 2015

kinetoplastid kinetochore protein 2, serine/threonine−protein kinase, putative, putative (kkt2)
Tb927.11.10520
AGOF: ATP binding, protein serine/threonine kinase activity
AGOC: kinetochore
AGOP: chromosome segregation, protein phosphorylation
PGOF: ATP binding, protein kinase activity, transferase activity, transferring phosphorus−containing groups
PGOC: null
PGOP: protein phosphorylation

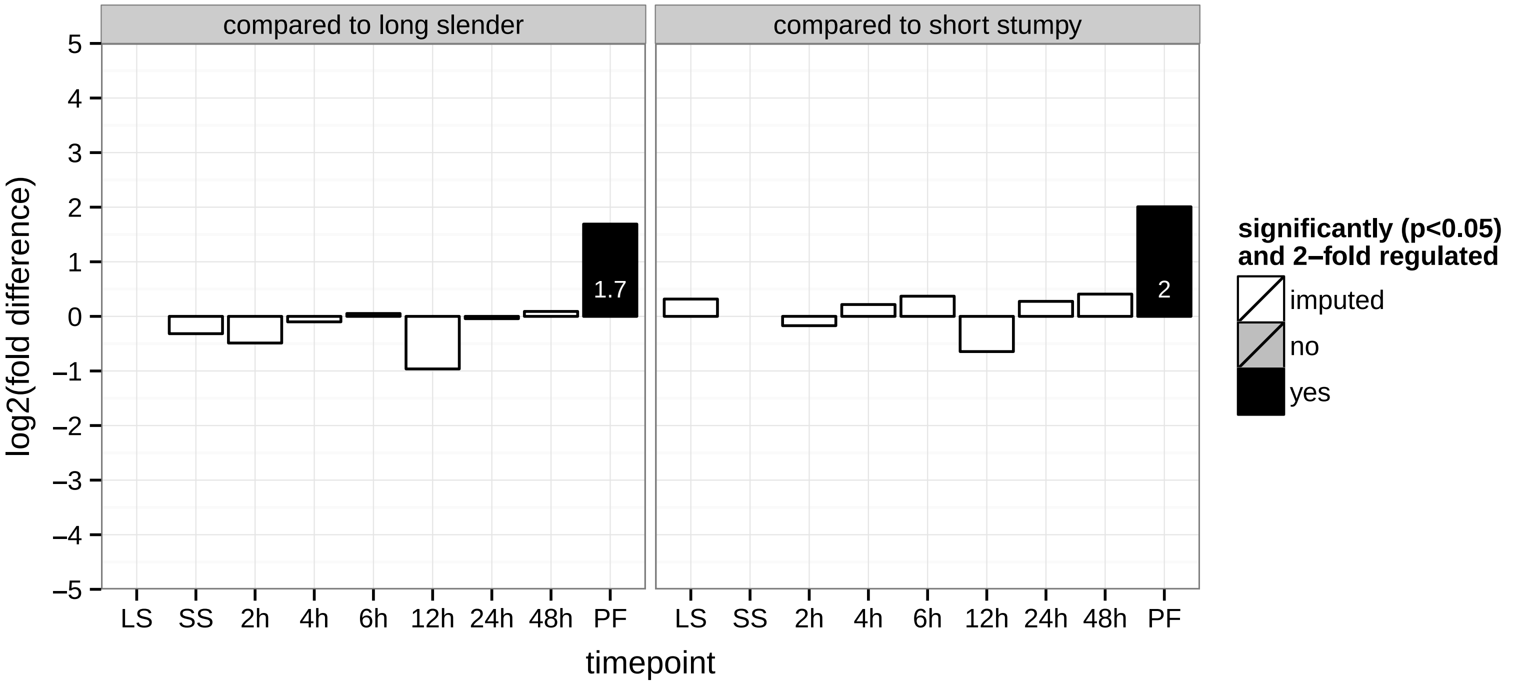

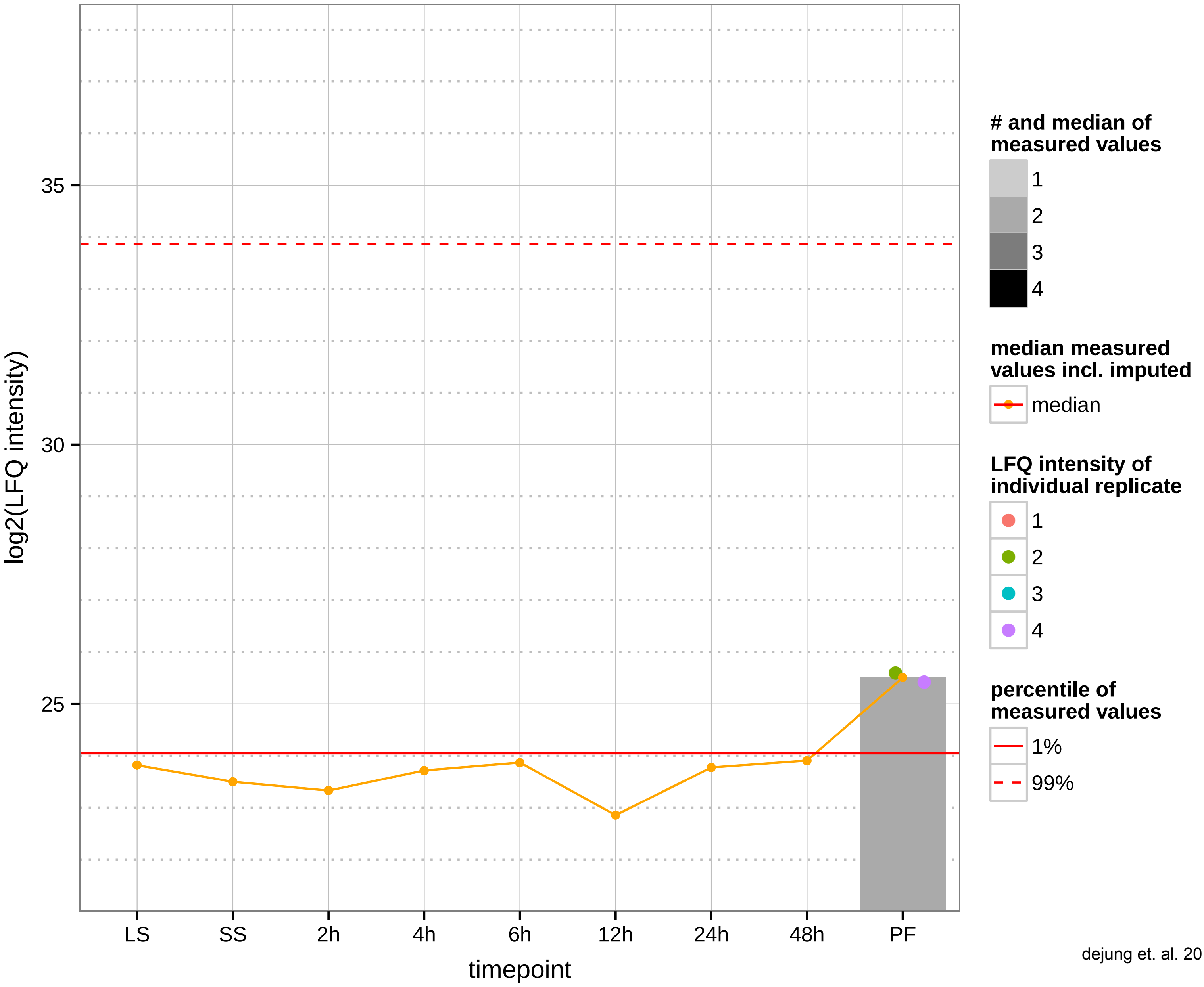

dejung et. al. 2015

hypothetical protein, conserved
Tb927.11.10590
AGOF: null
AGOC: mitochondrion
AGOP: null
PGOF: null
PGOC: null
PGOP: null

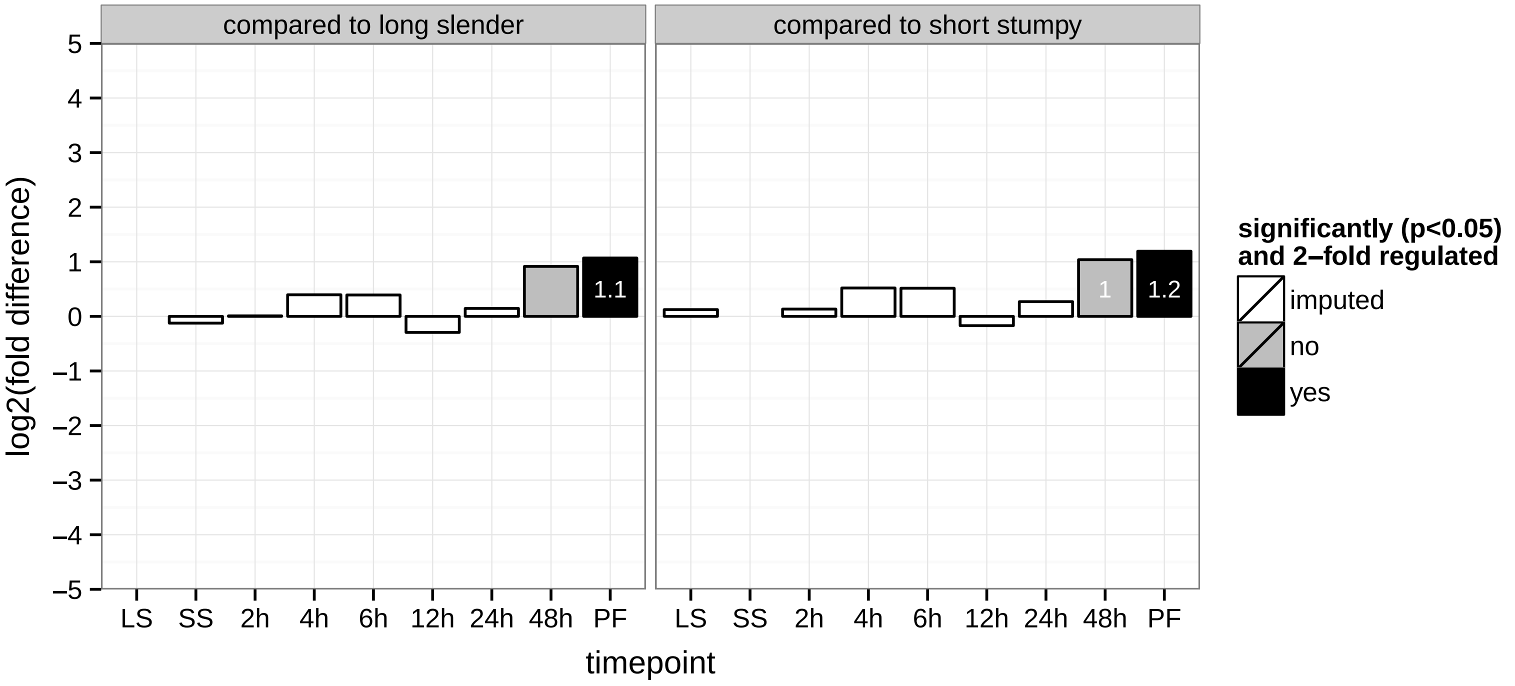

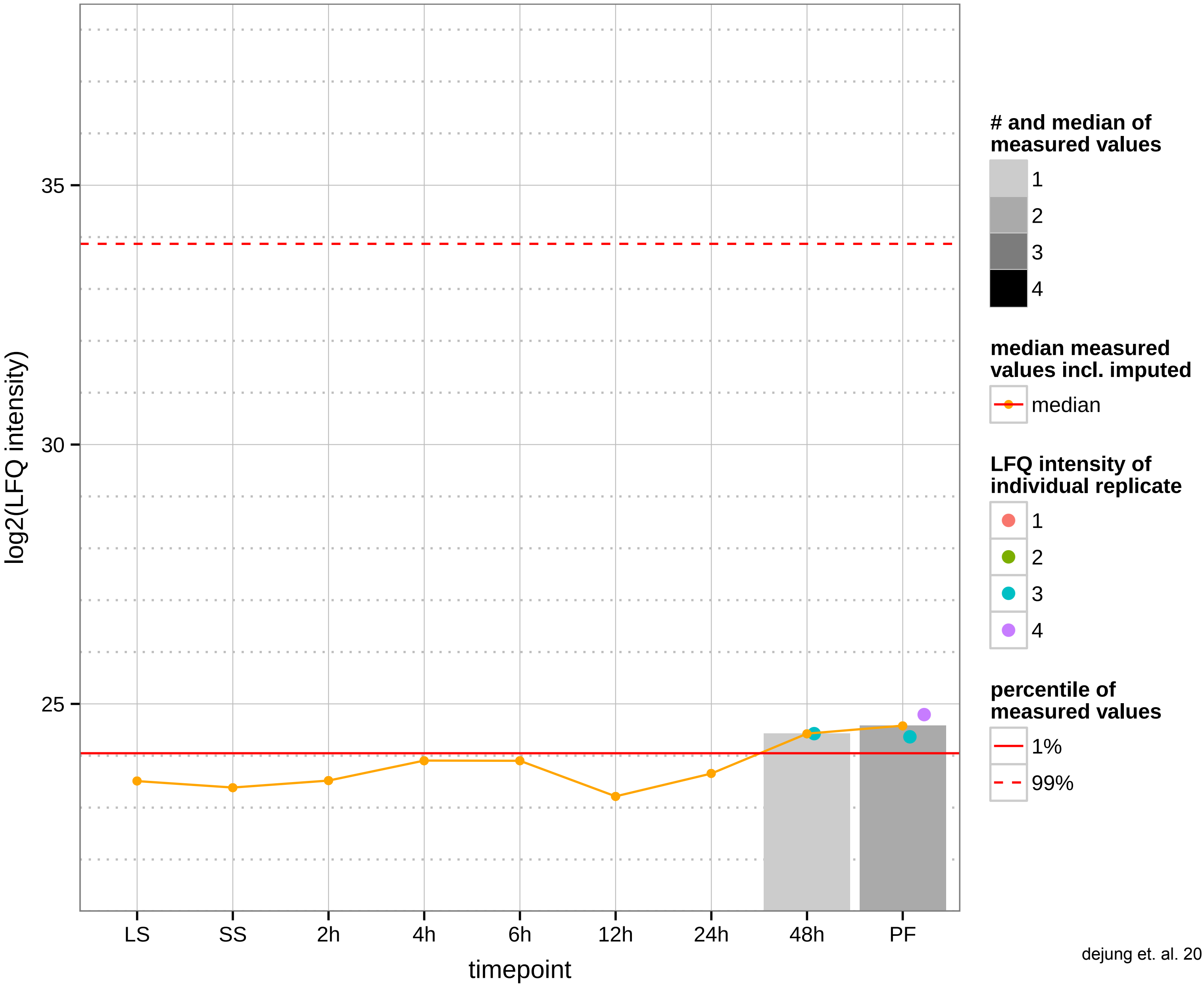

dejung et. al. 2015

hypothetical protein, conserved
Tb927.11.10660;Tb11.v5.0377;Tb11.v5.0762;Tb11.v5.0378
AGOF: null
AGOC: null
AGOP: null, protein metabolic process
PGOF: null
PGOC: null
PGOP: null

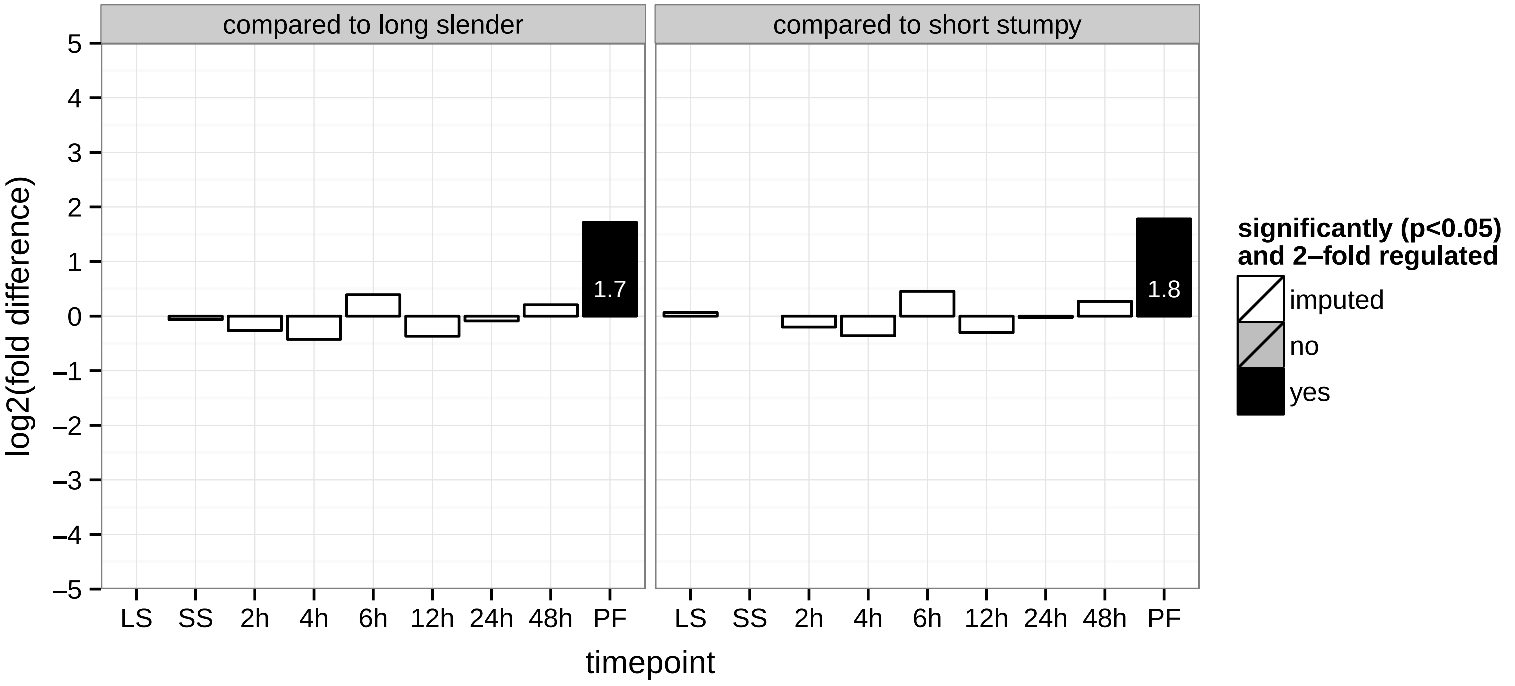

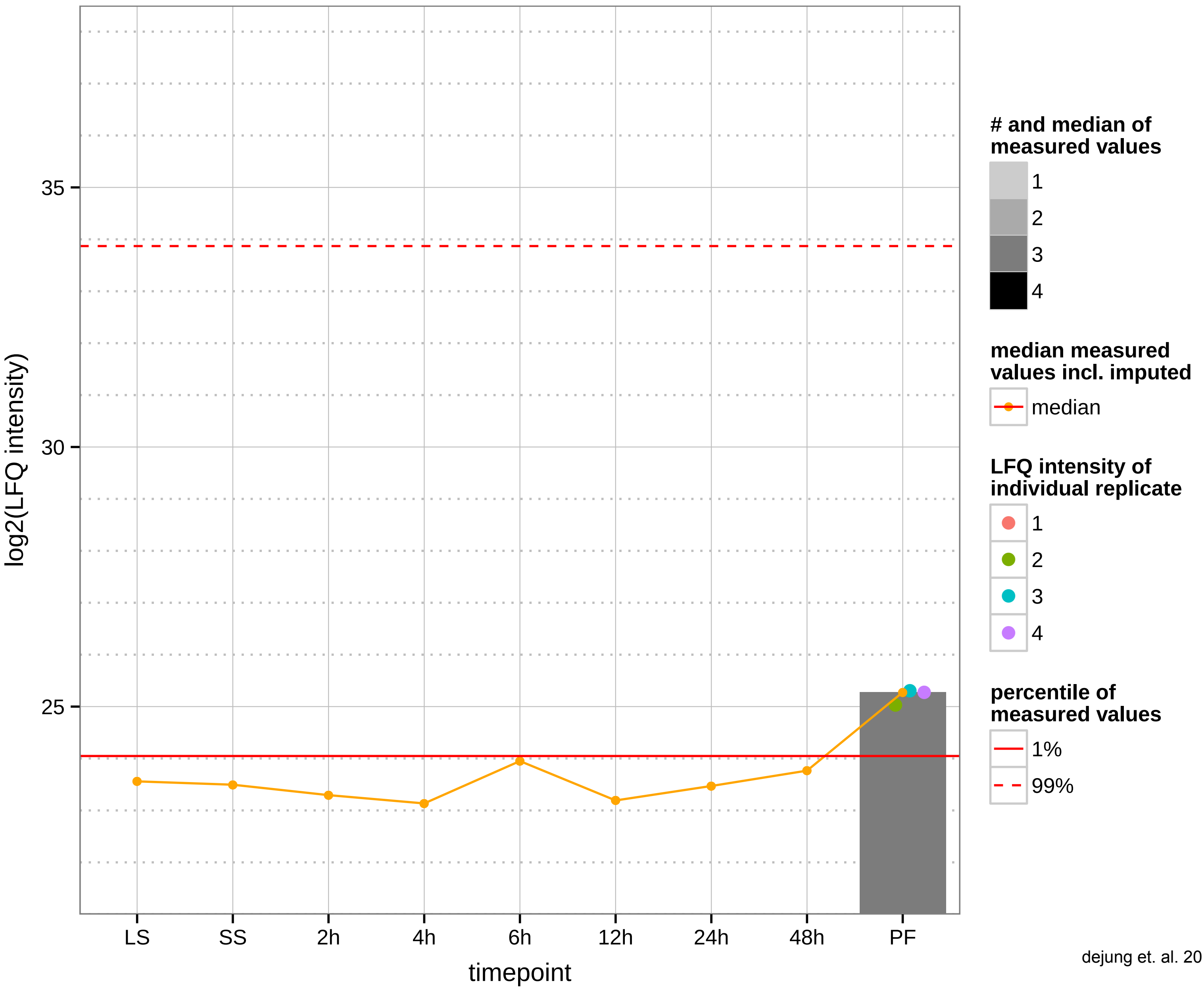

dejung et. al. 2015

receptor for activated C kinase 1 (RACK1)
Tb927.11.11370;Tb927.11.11360
AGOF: protein kinase C binding
AGOC: ribosome
AGOP: apoptotic process, regulation of cytokinesis
PGOF: null, protein binding
PGOC: null
PGOP: null

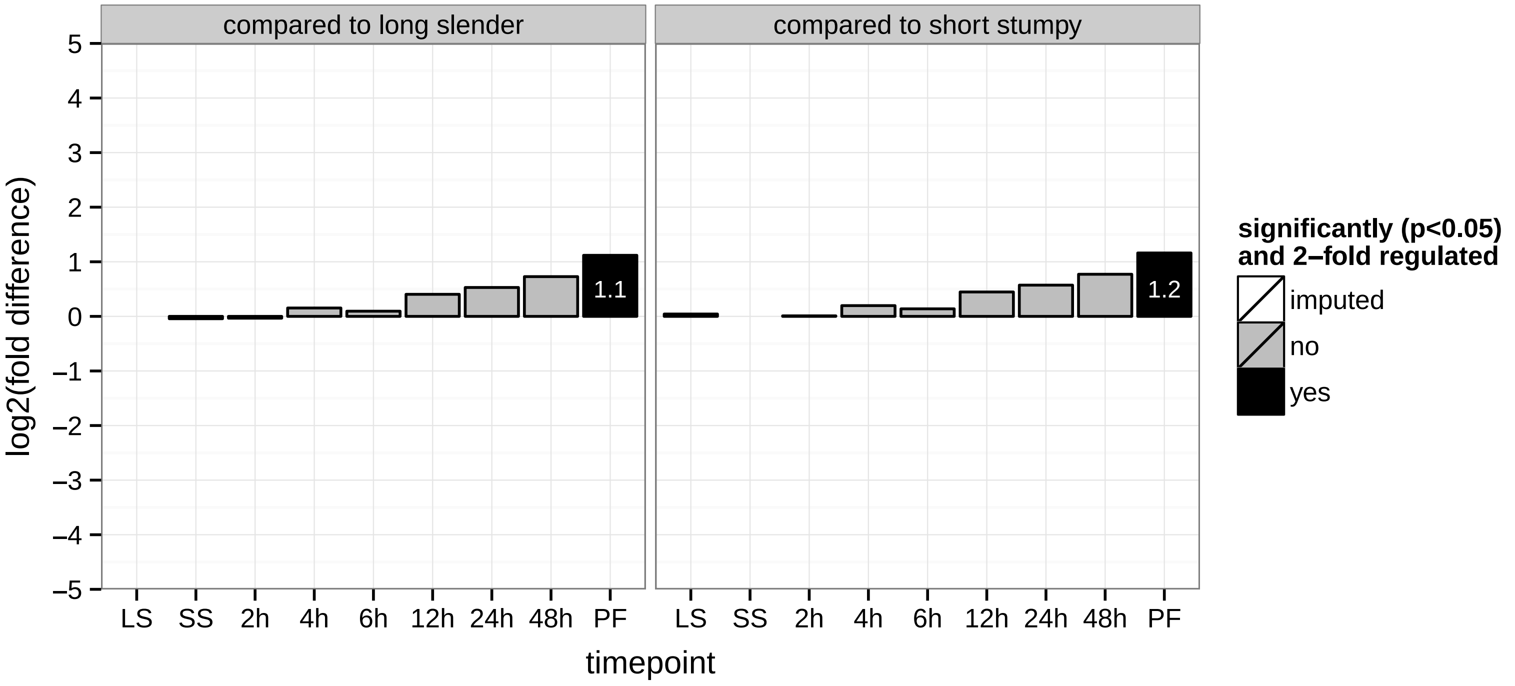

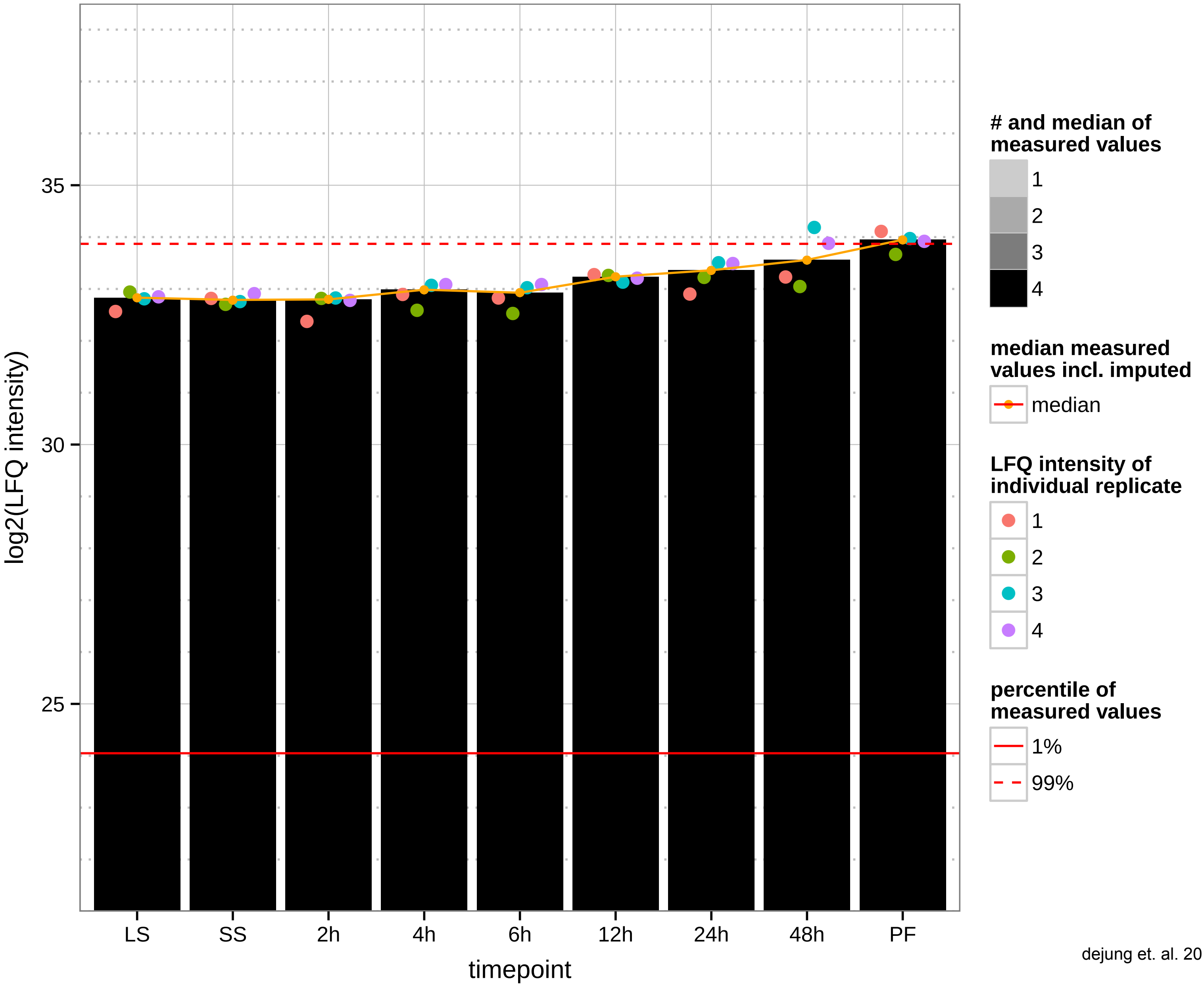

dejung et. al. 2015

DNA replication licensing factor MCM6, putative
Tb927.11.11640
AGOF: ATP binding, DNA binding
AGOC: nucleus
AGOP: DNA replication, DNA–dependent DNA replication initiation
PGOF: ATP binding, DNA binding
PGOC: null
PGOP: DNA replication

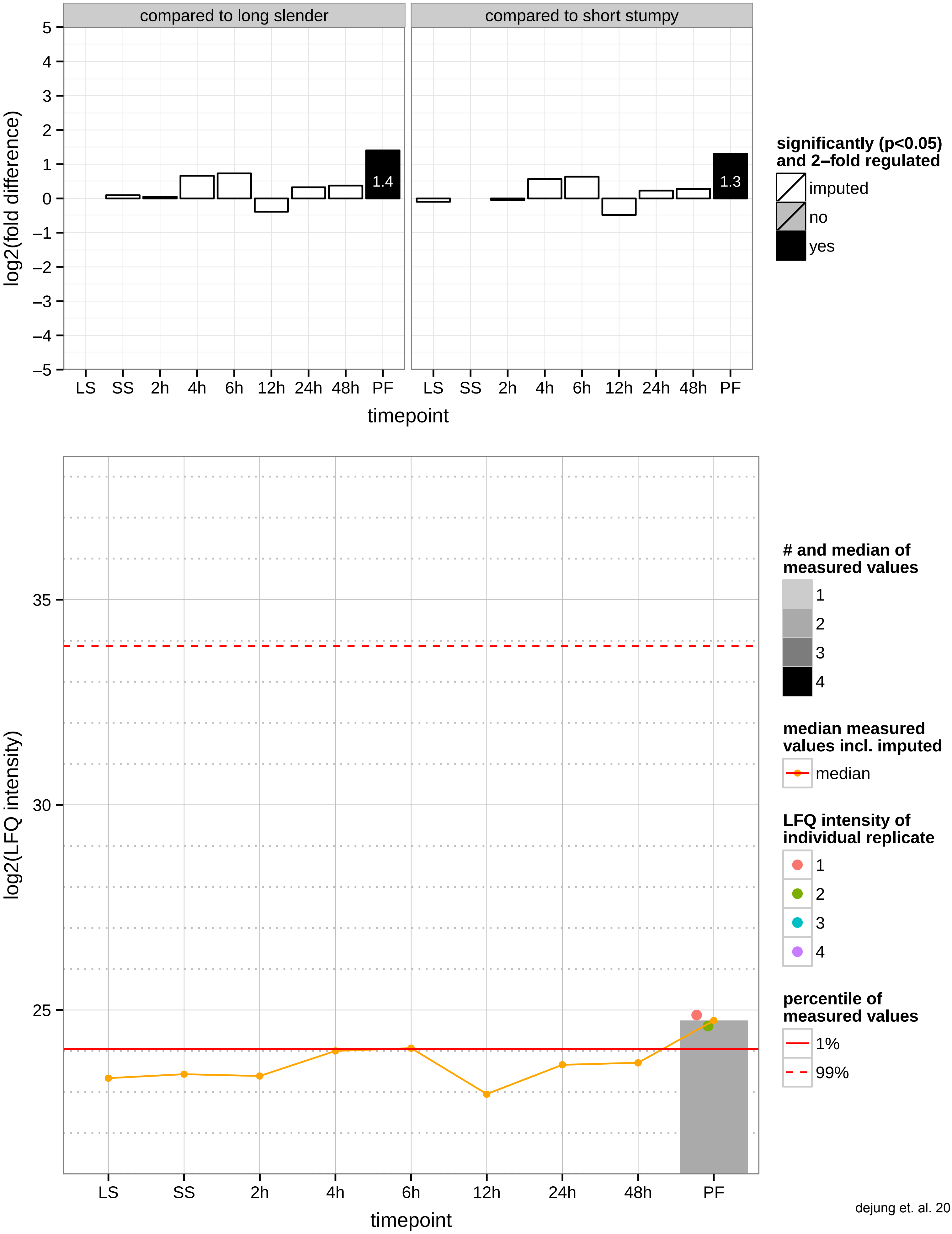

dejung et. al. 2015

acyl–CoA dehydrogenase, putative
Tb927.11.11780
AGOF: oxidoreductase activity, oxidoreductase activity, acting on the CH–CH group of donors
AGOC: null
AGOP: metabolic process, oxidation–reduction process
PGOF: oxidoreductase activity, acting on the CH–CH group of donors
PGOC: null
PGOP: metabolic process, oxidation–reduction process

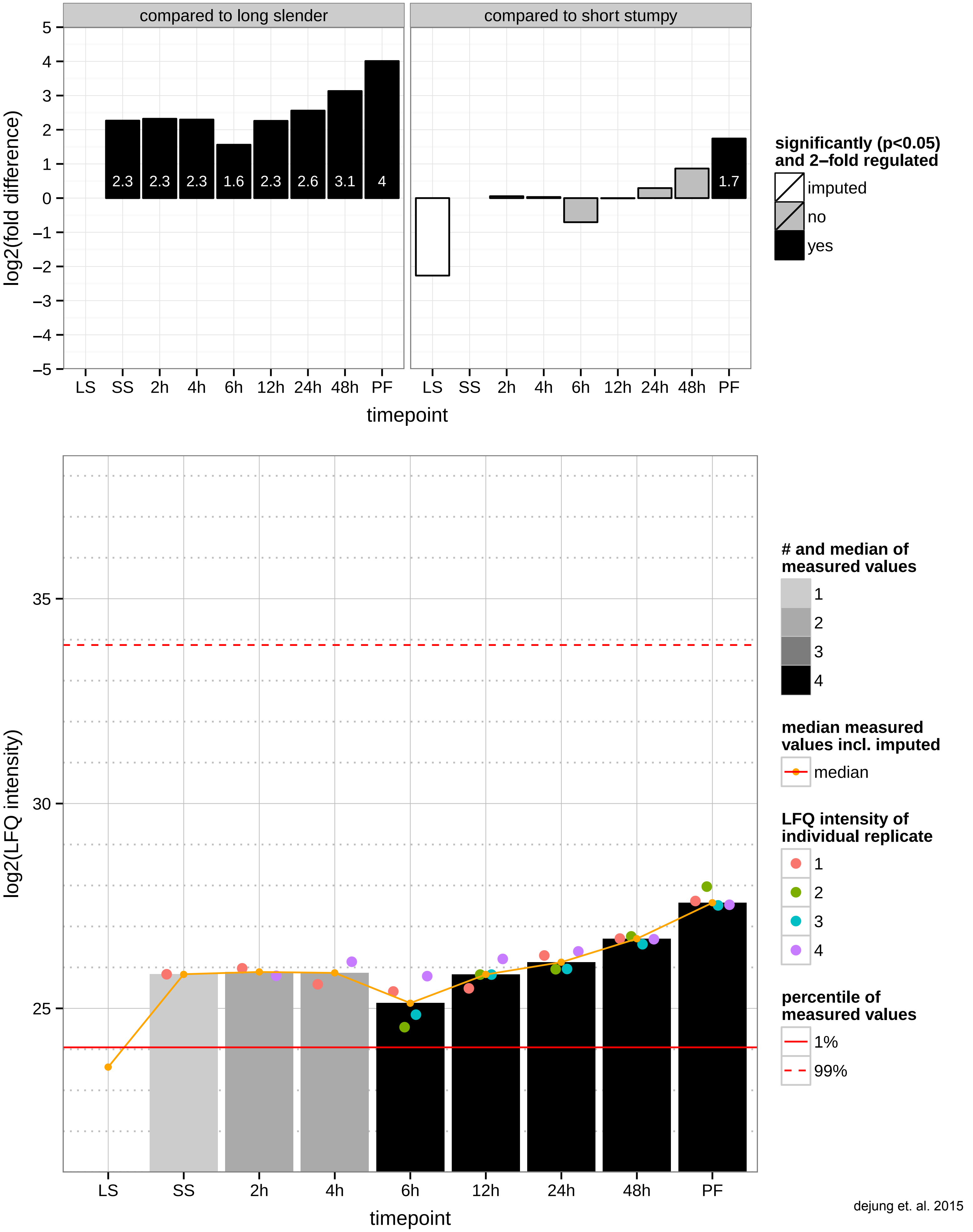

dejung et. al. 2015

40S ribosomal protein S17, putative
Tb927.11.11830;Tb927.11.11820
AGOF: structural constituent of ribosome
AGOC: intracellular, ribosome
AGOP: translation
PGOF: structural constituent of ribosome, null
PGOC: intracellular, ribosome, null
PGOP: translation, null

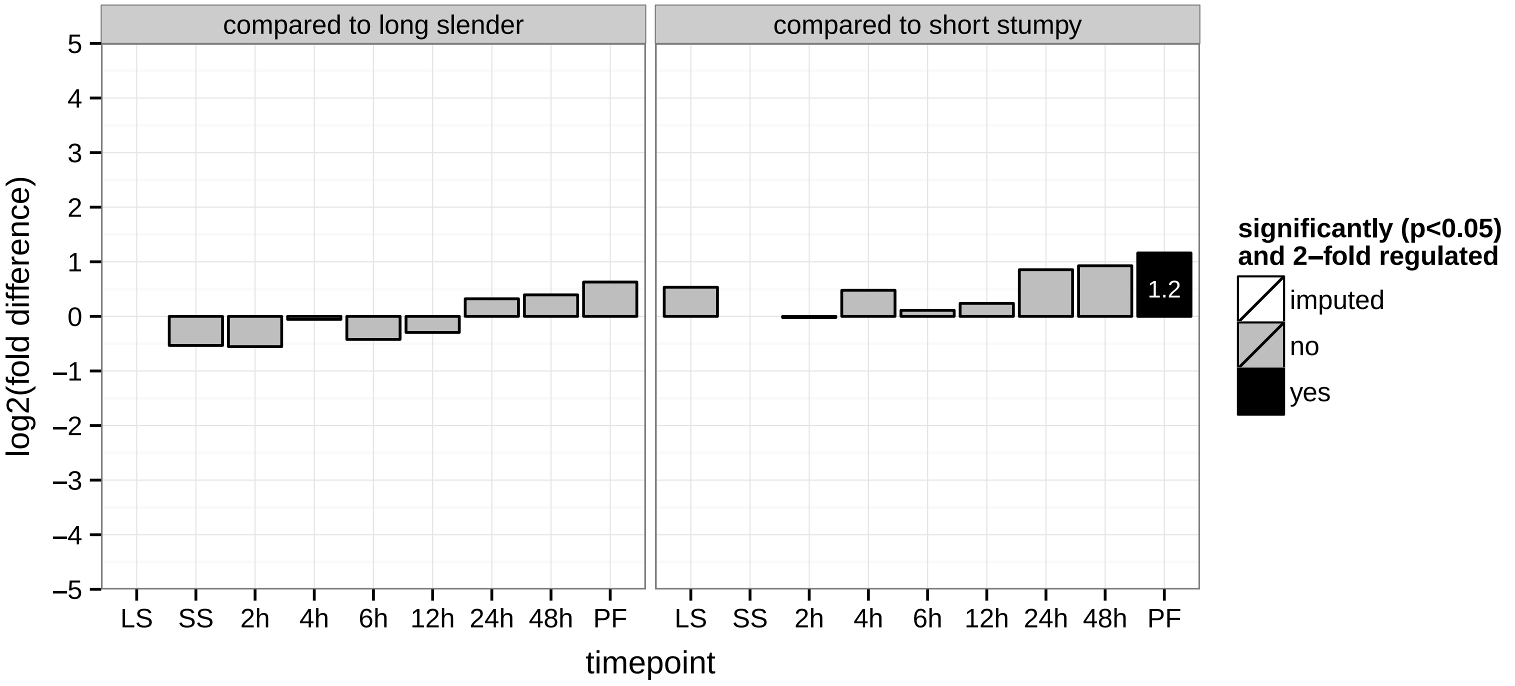

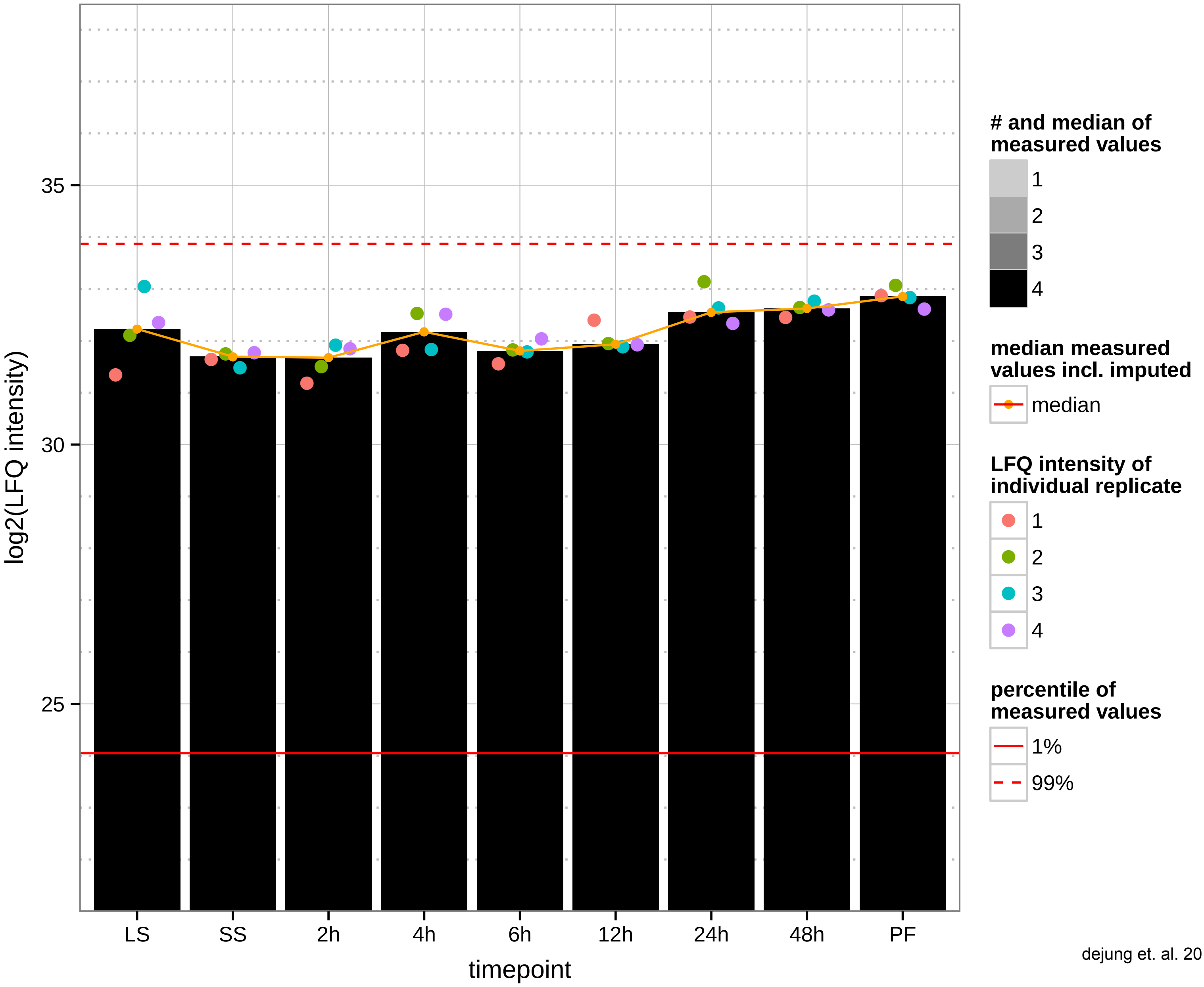

dejung et. al. 2015

hypothetical protein, conserved
Tb927.11.12030
AGOF: null
AGOC: null
AGOP: null
PGOF: protein binding
PGOC: null
PGOP: null

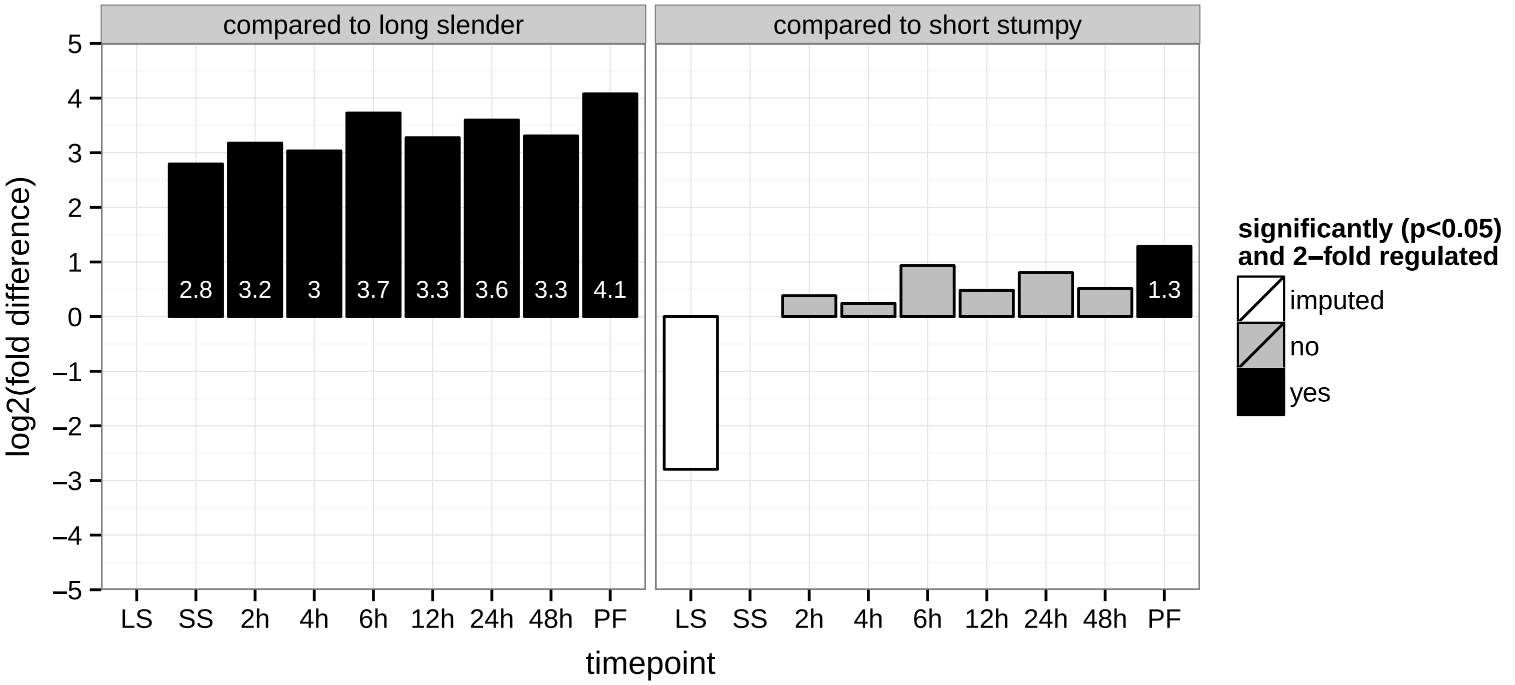

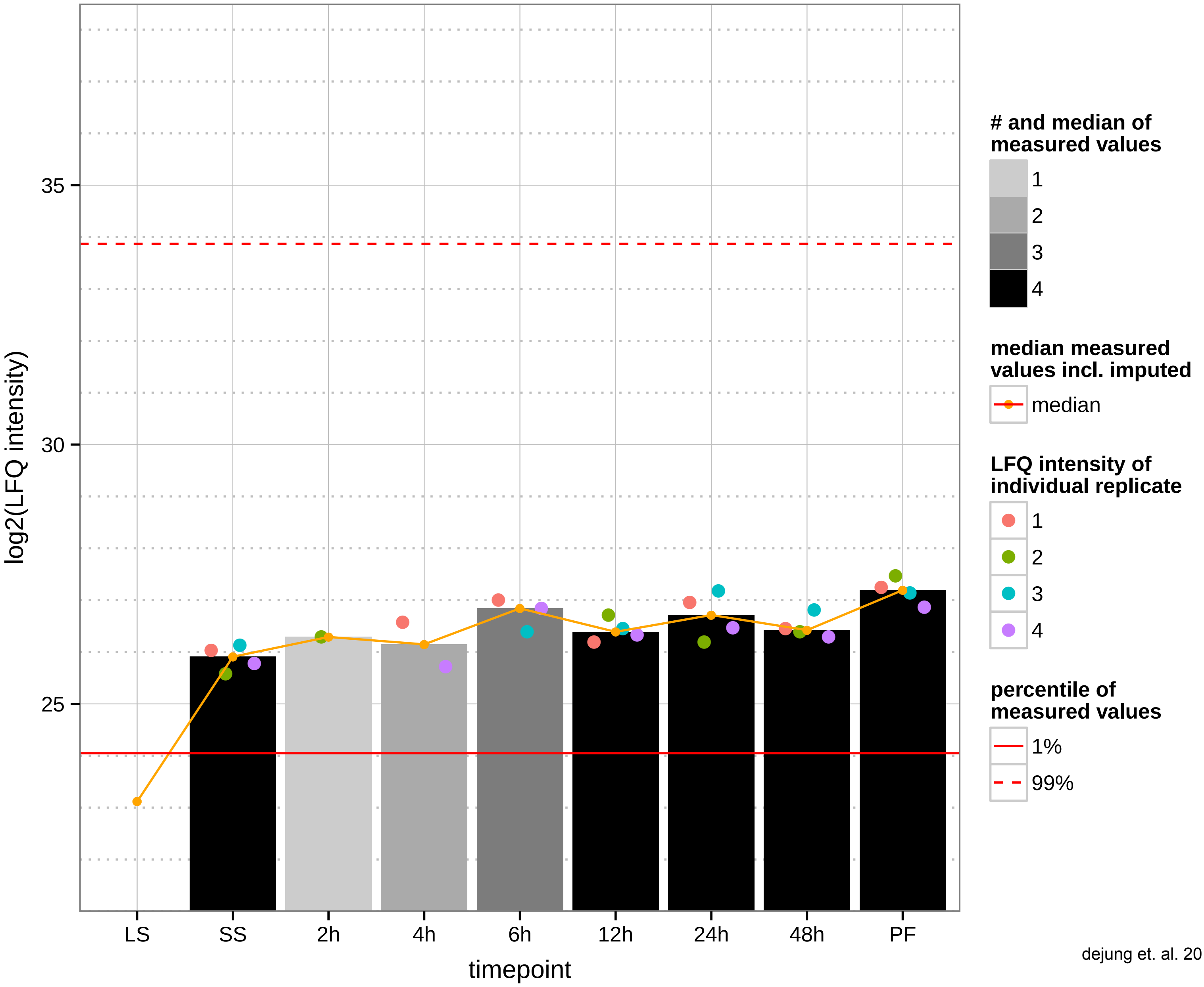

dejung et. al. 2015

flagellar protein essential for flagellar pocket biogenesis (BILBO1)
Tb927.11.12150
AGOF: calcium ion binding
AGOC: null
AGOP: null
PGOF: calcium ion binding
PGOC: null
PGOP: null

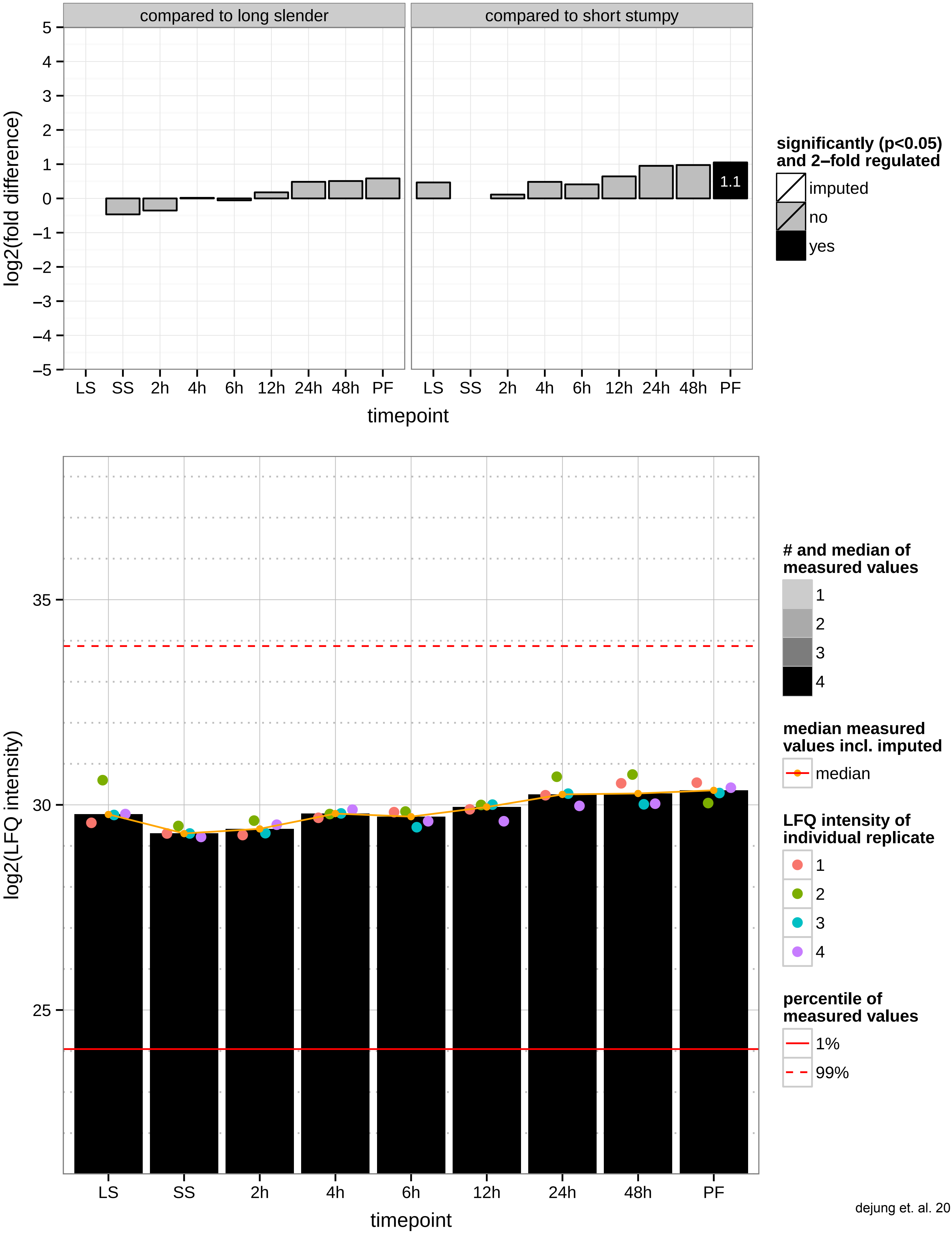

dejung et. al. 2015

hypothetical protein, conserved
Tb927.11.12160
AGOF: null
AGOC: mitochondrion
AGOP: null
PGOF: null
PGOC: null
PGOP: null

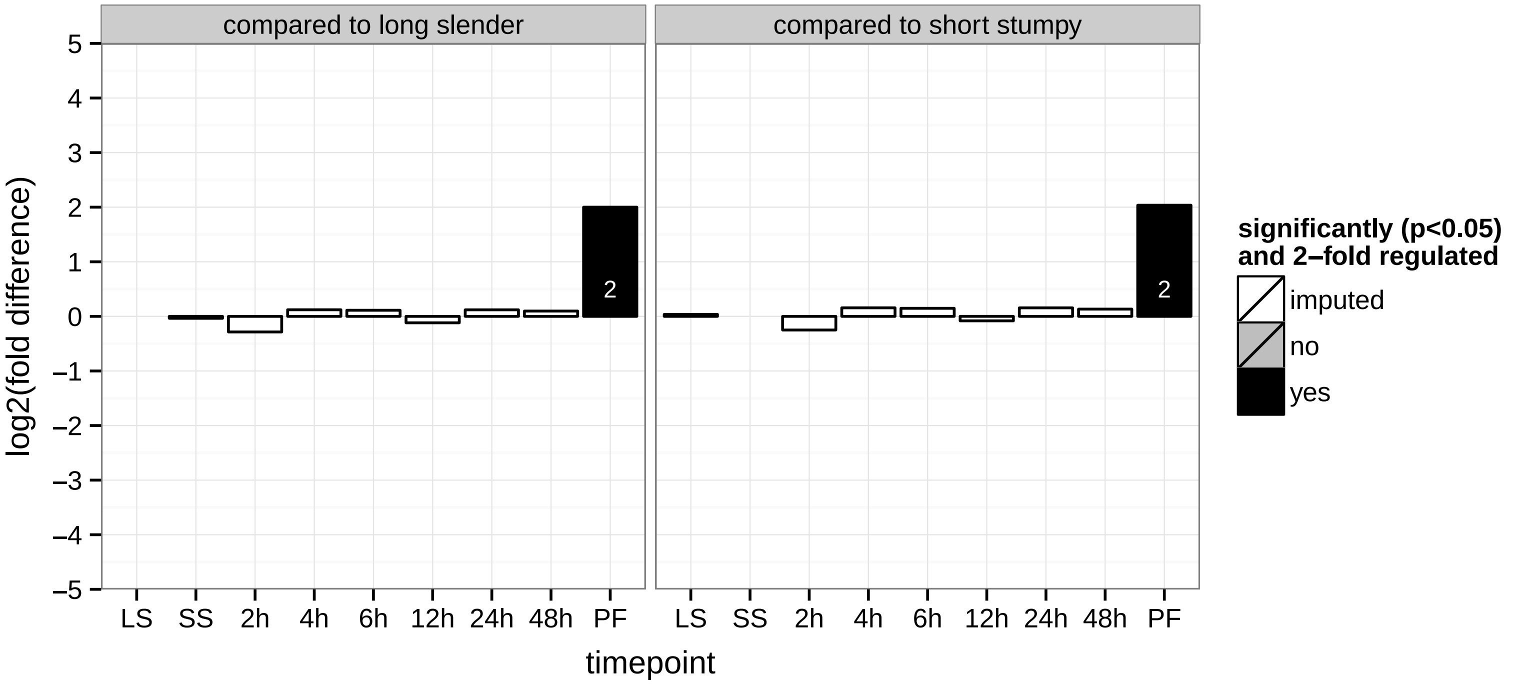

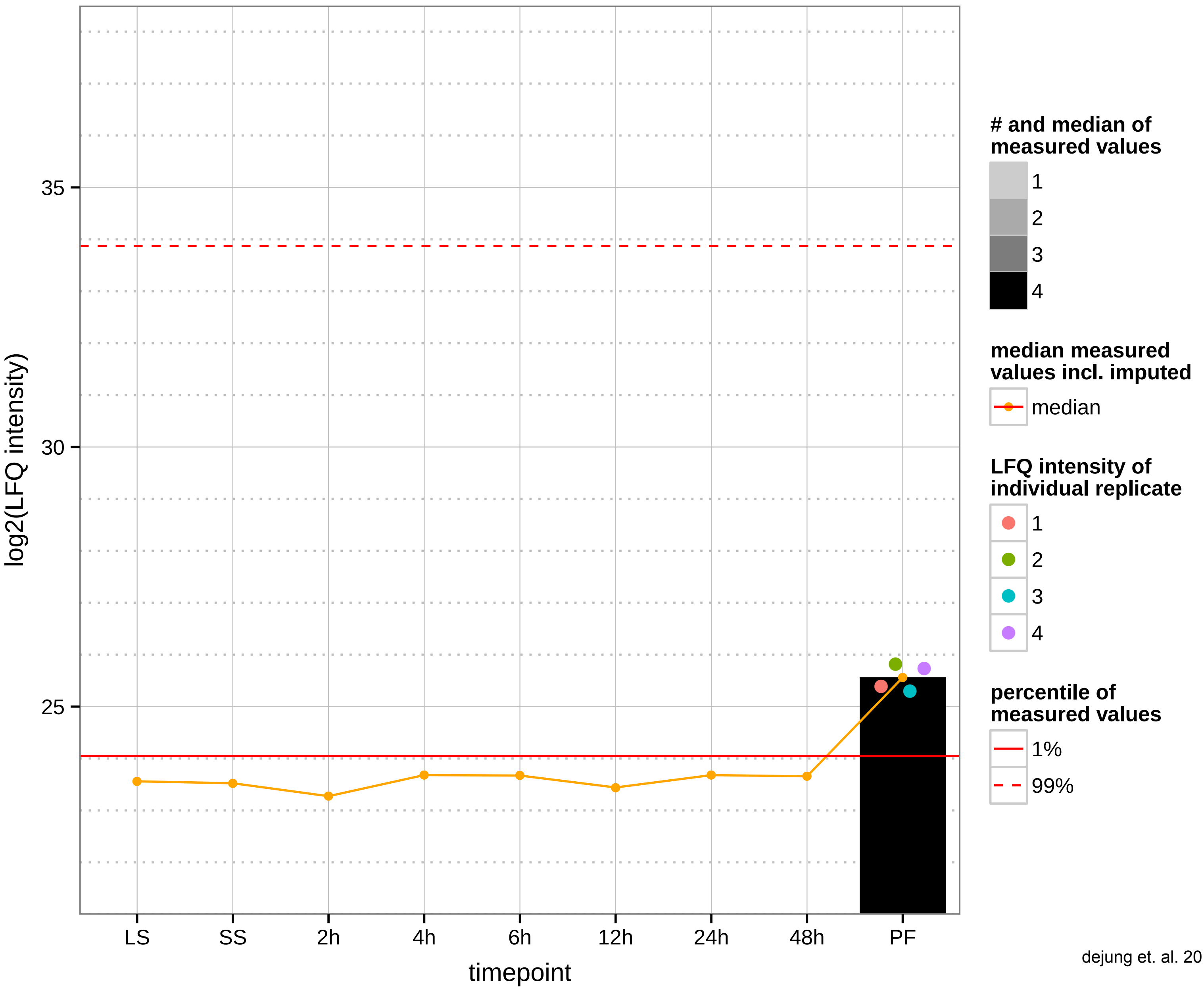

dejung et. al. 2015

kinetoplastid kinetochore protein 10 (kkt10)
Tb927.11.12410
AGOF: ATP binding, protein serine/threonine kinase activity, protein tyrosine kinase activity
AGOC: null
AGOP: cell proliferation, mitotic cell cycle, protein phosphorylation
PGOF: ATP binding, protein kinase activity, transferase activity, transferring phosphorus−containing groups
PGOC: null
PGOP: protein phosphorylation

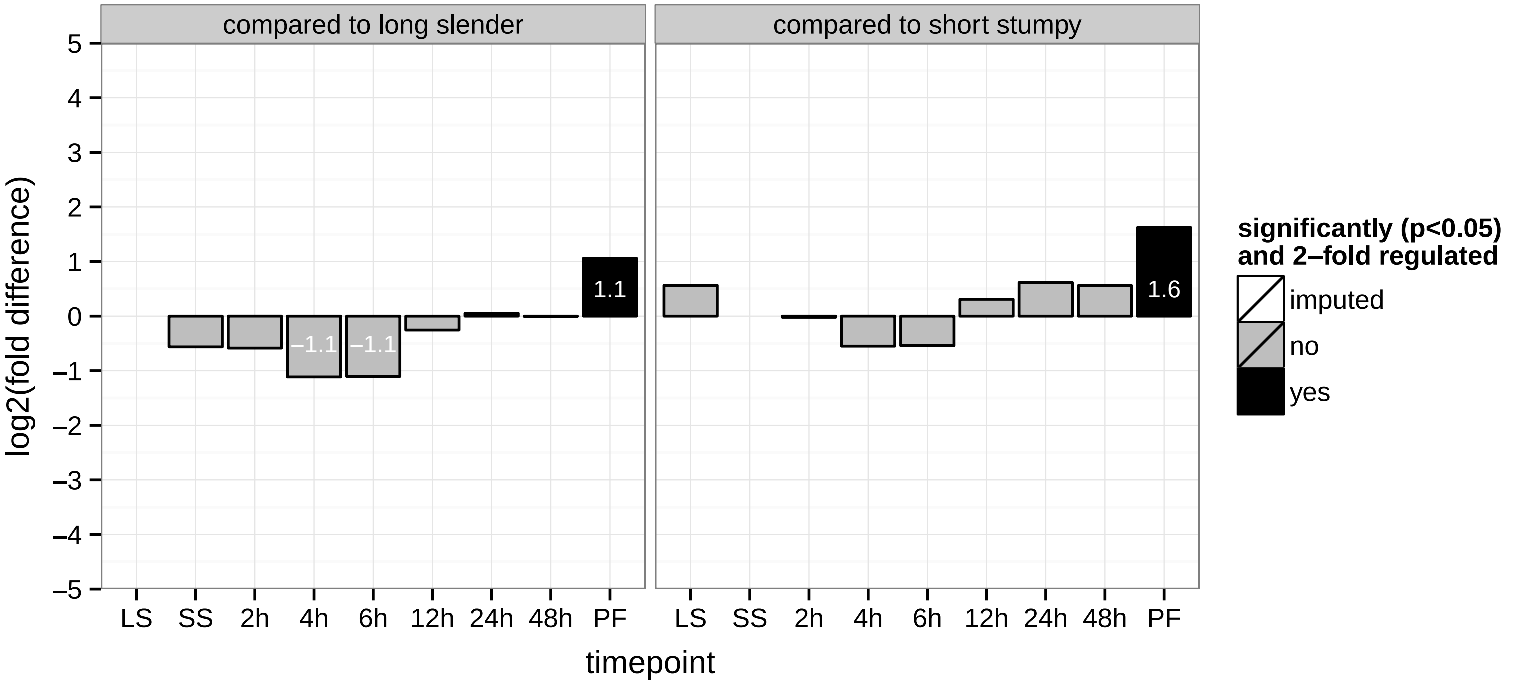

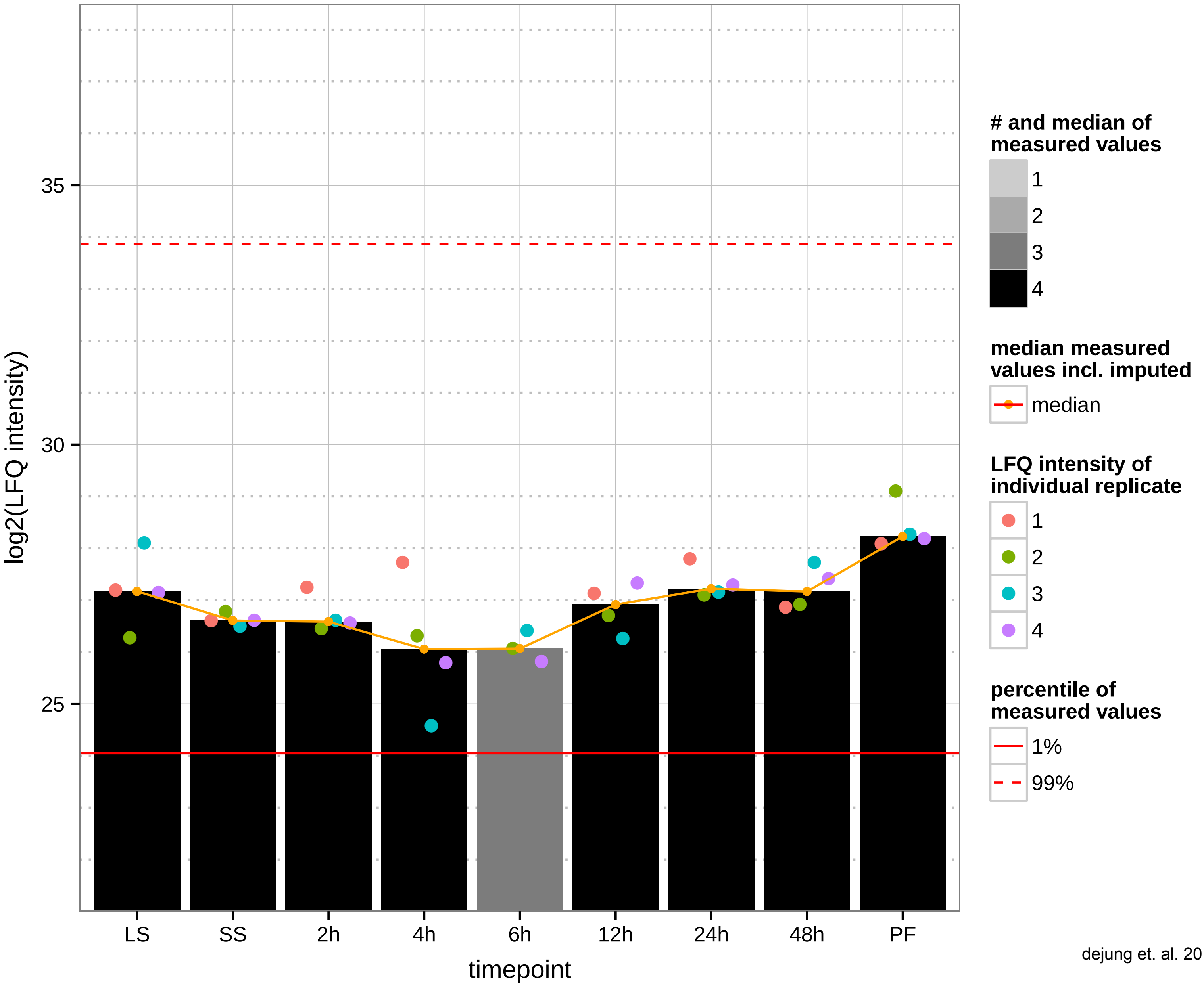

dejung et. al. 2015

hypothetical protein
Tb927.11.12560
AGOF: null
AGOC: null
AGOP: null
PGOF: null
PGOC: null
PGOP: null

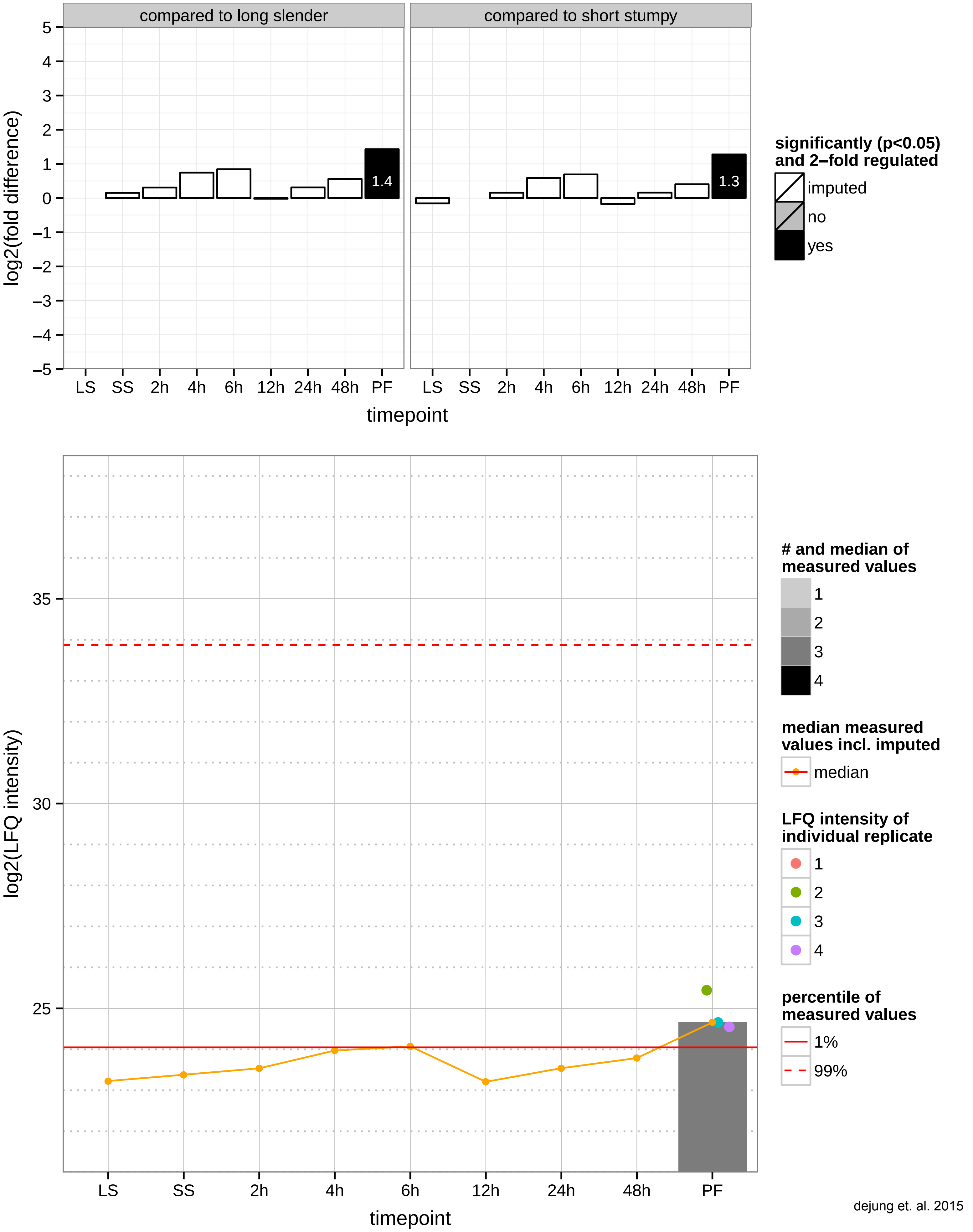

dejung et. al. 2015

oligopeptidase b, serine peptidase, clan SC, family S9A–like protein (OPB)
Tb927.11.12850
AGOF: serine–type endopeptidase activity, serine–type peptidase activity
AGOC: null
AGOP: proteolysis
PGOF: serine–type endopeptidase activity, serine–type peptidase activity
PGOC: null
PGOP: proteolysis

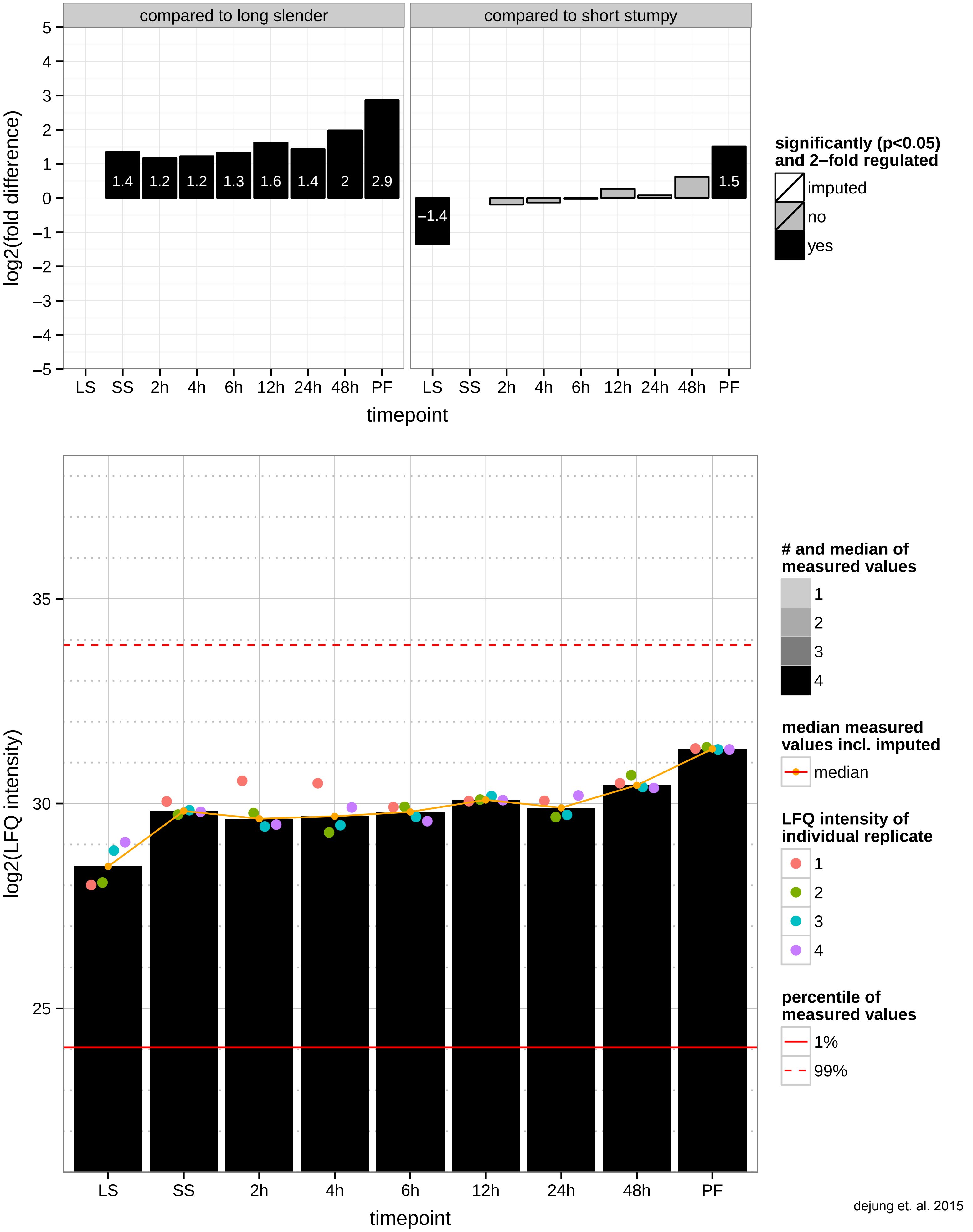

dejung et. al. 2015

translation–associated element 2, putative
Tb927.11.13060
AGOF: null
AGOC: cytoplasm
AGOP: translation
PGOF: null
PGOC: null
PGOP: null

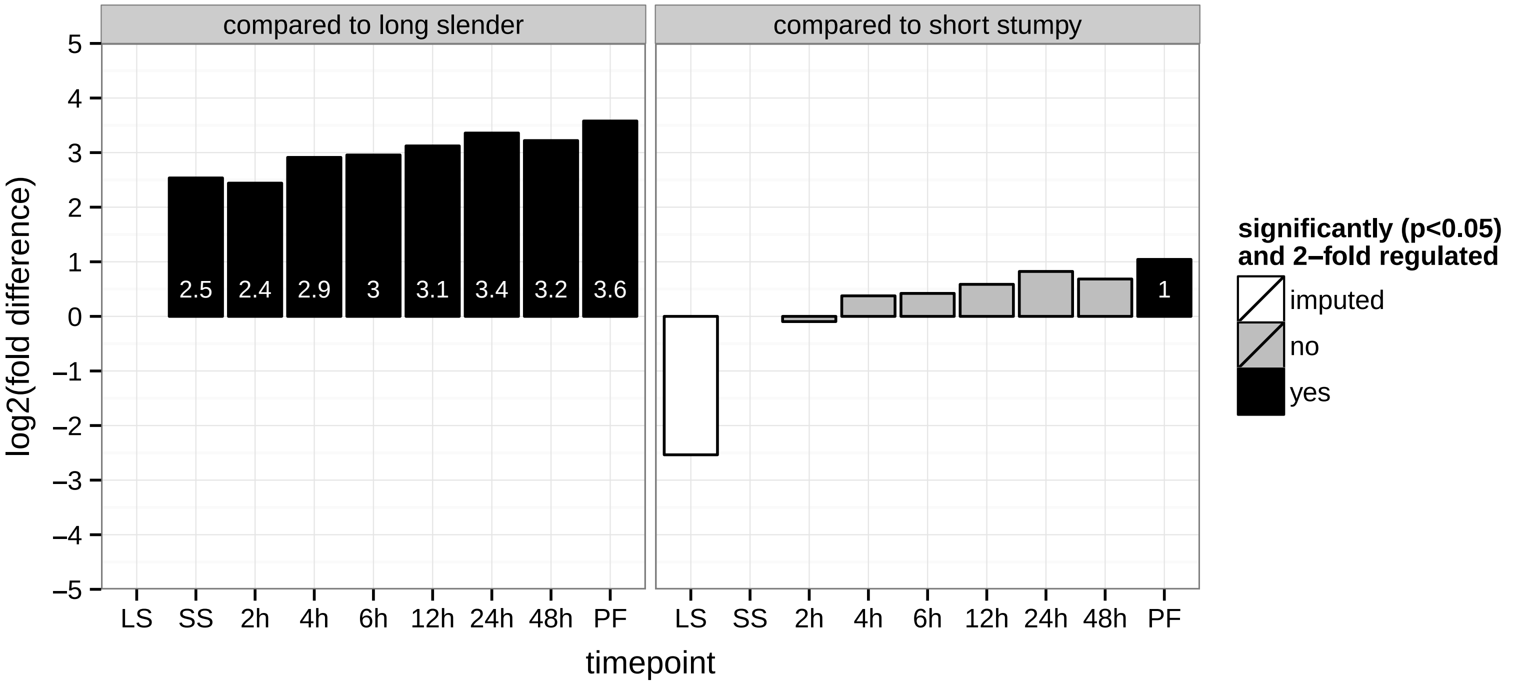

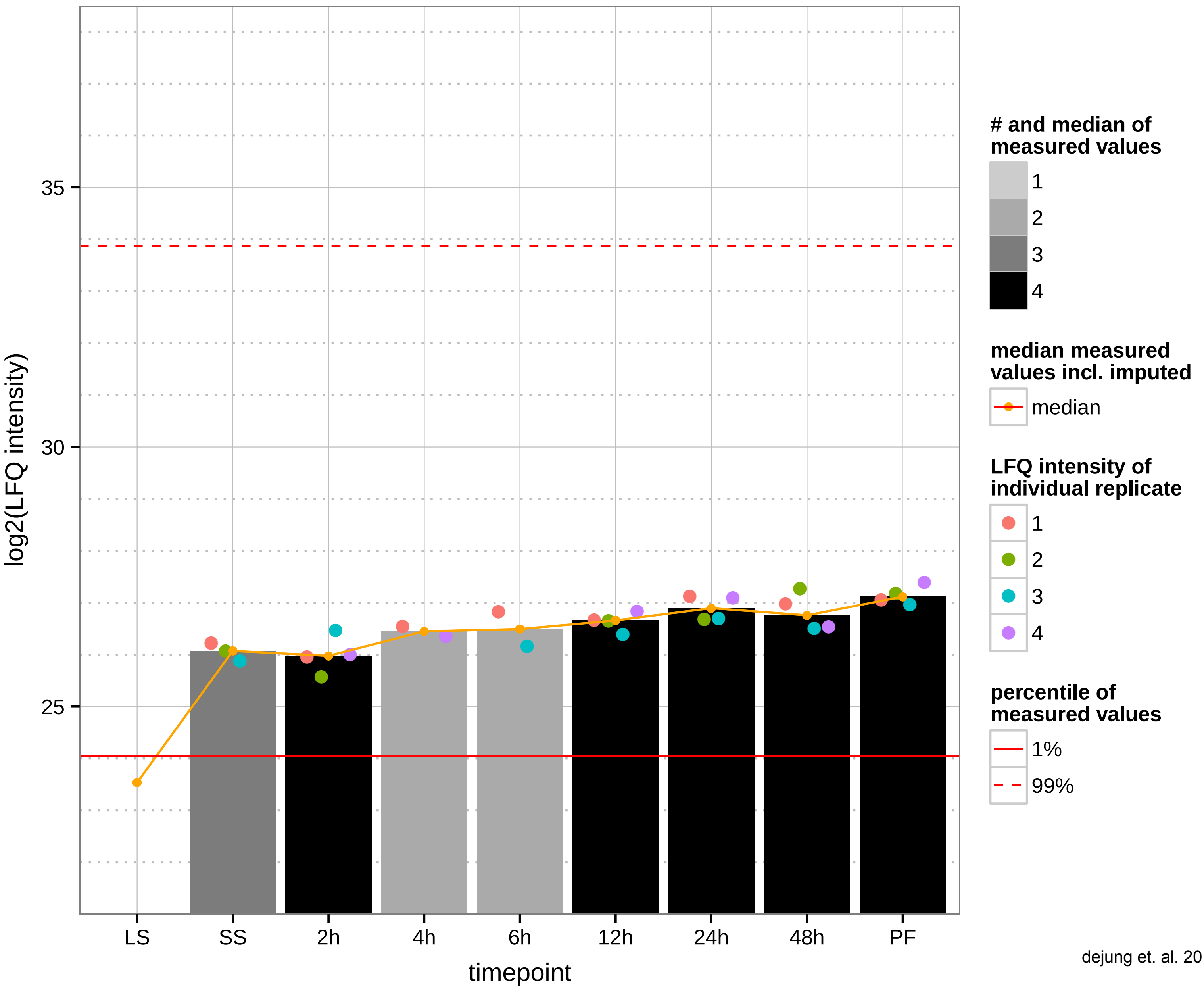

dejung et. al. 2015

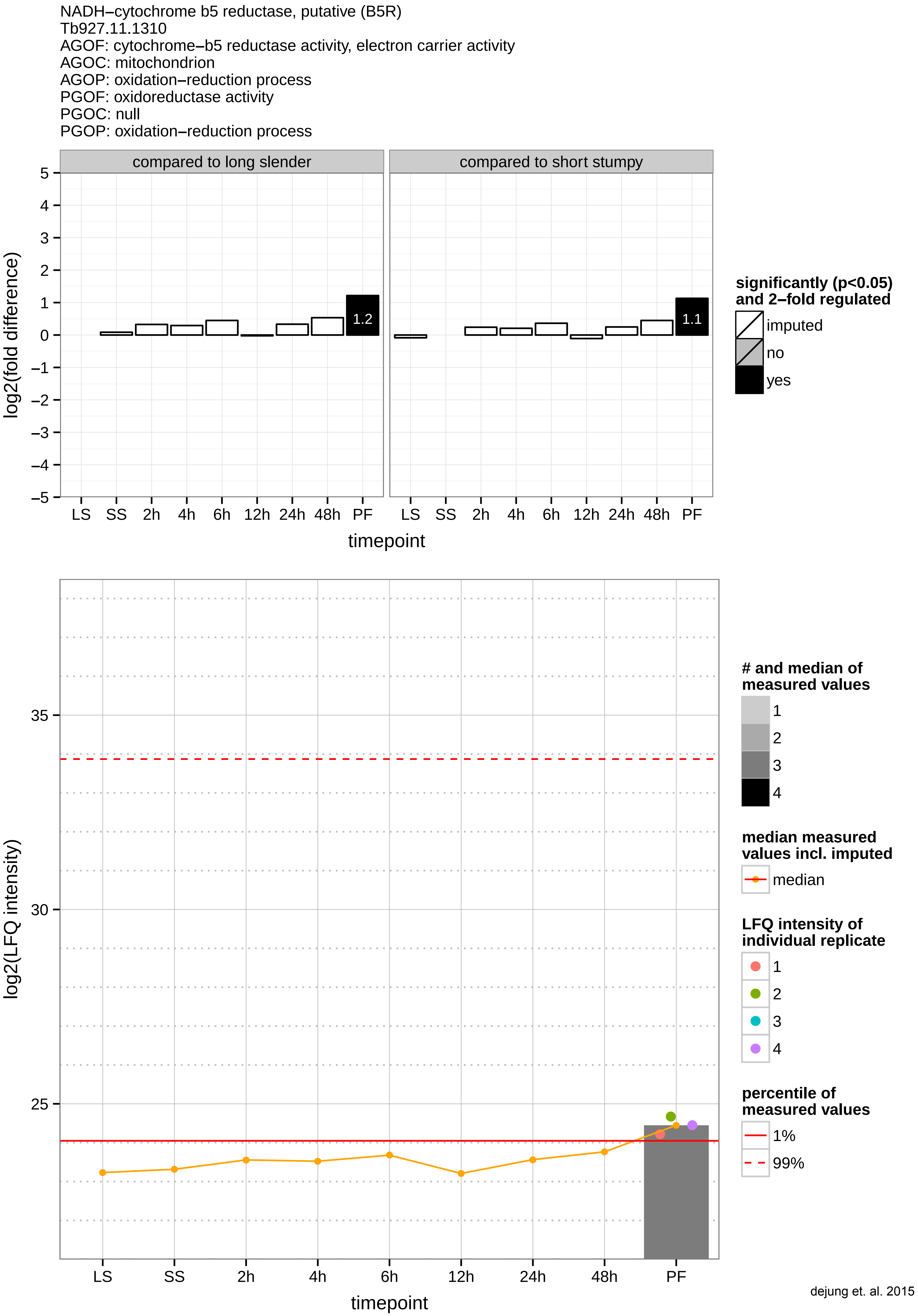
NADH–cytochrome b5 reductase, putative (B5R)
Tb927.11.1310
AGOF: cytochrome–b5 reductase activity, electron carrier activity
AGOC: mitochondrion
AGOP: oxidation–reduction process
PGOF: oxidoreductase activity
PGOC: null
PGOP: oxidation–reduction process
compared to long slender
compared to short stumpy
log2(fold difference)
1.2
1.1
LS SS 2h 4h 6h 12h 24h 48h PF
timepoint
significantly (p<0.05) and 2–fold regulated
imputed
no
yes
log2(LFQ intensity)
# and median of measured values
1
2
3
4
median measured values incl. imputed
median
LFQ intensity of individual replicate
percentile of measured values
1%
99%
dejung et. al. 2015

hypothetical protein, conserved
Tb927.11.13200
AGOF: null
AGOC: membrane
AGOP: null
PGOF: null
PGOC: membrane
PGOP: null

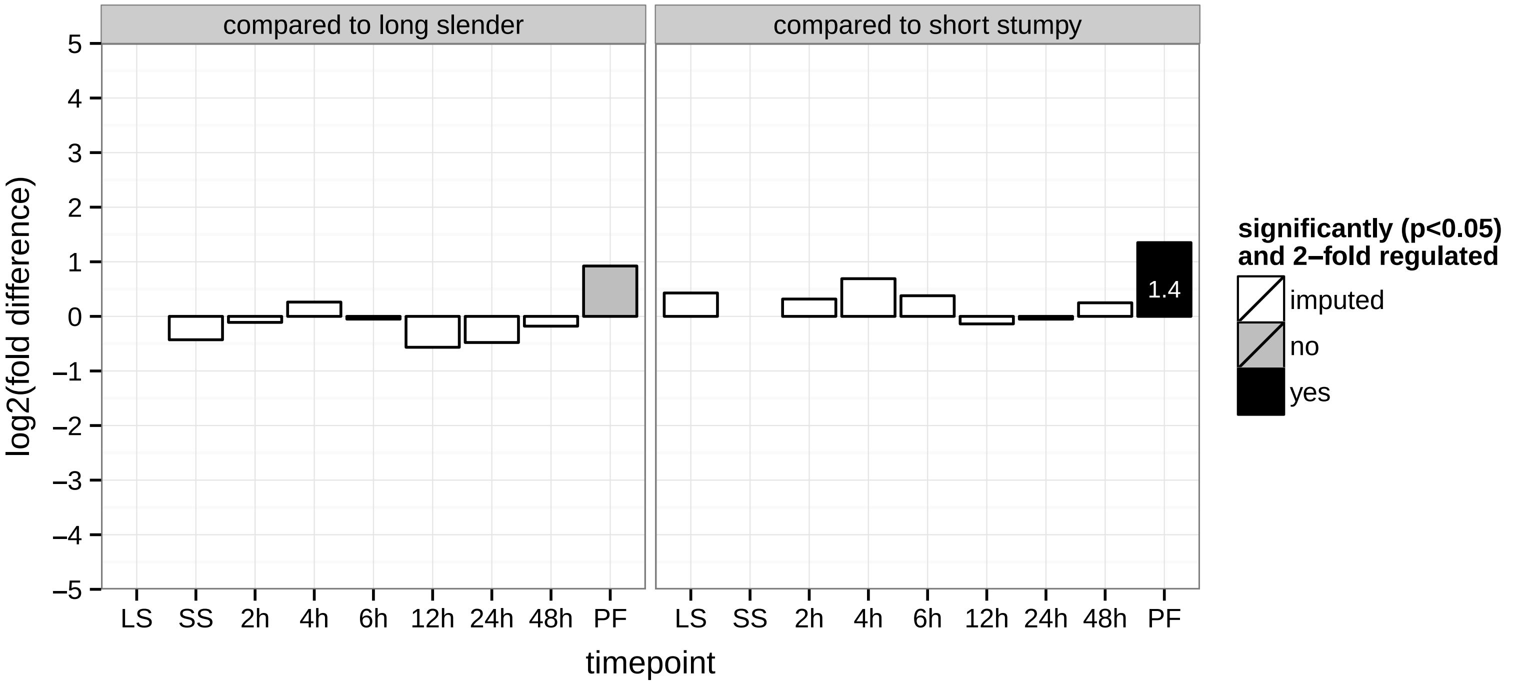

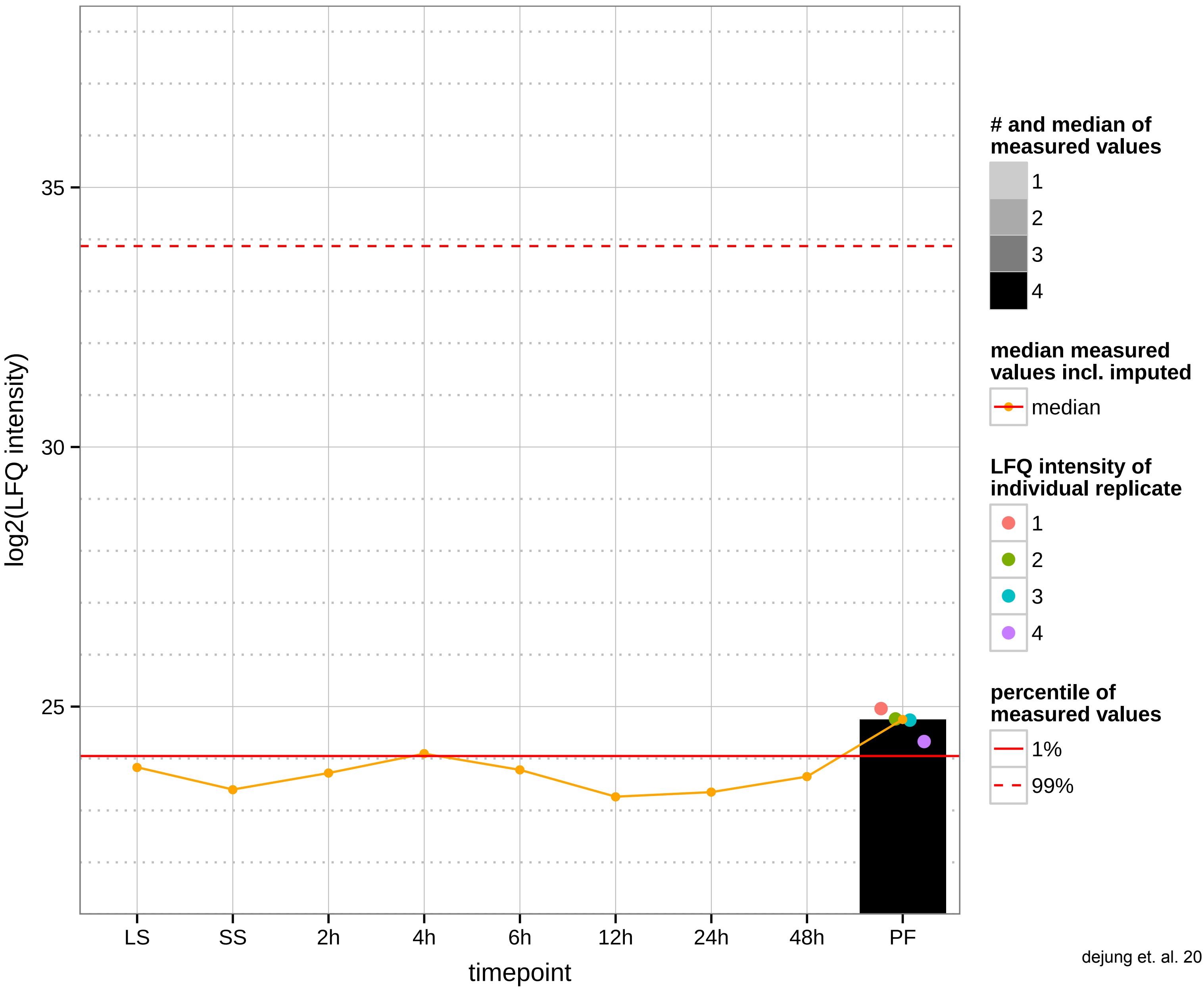

dejung et. al. 2015

hypothetical protein, conserved
Tb927.11.13310
AGOF: null
AGOC: null
AGOP: null
PGOF: null
PGOC: null
PGOP: null

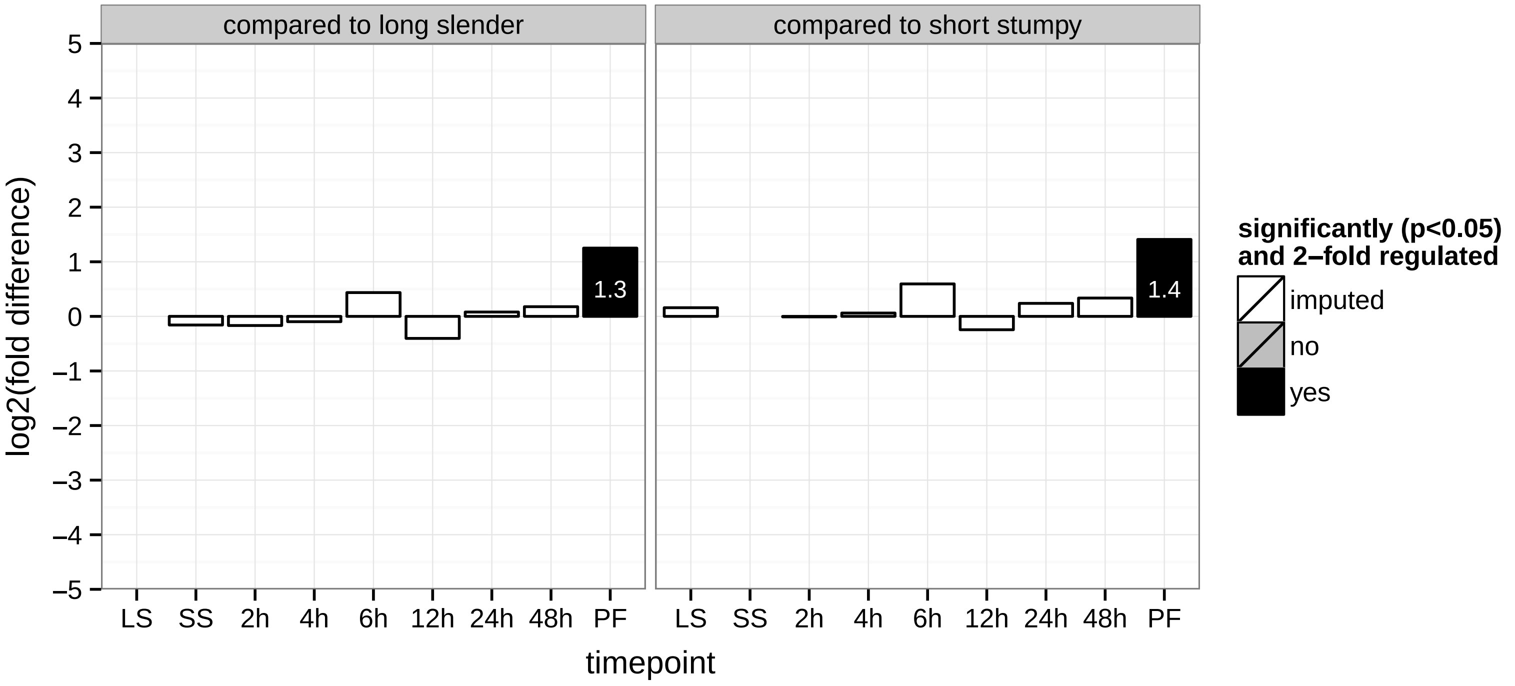

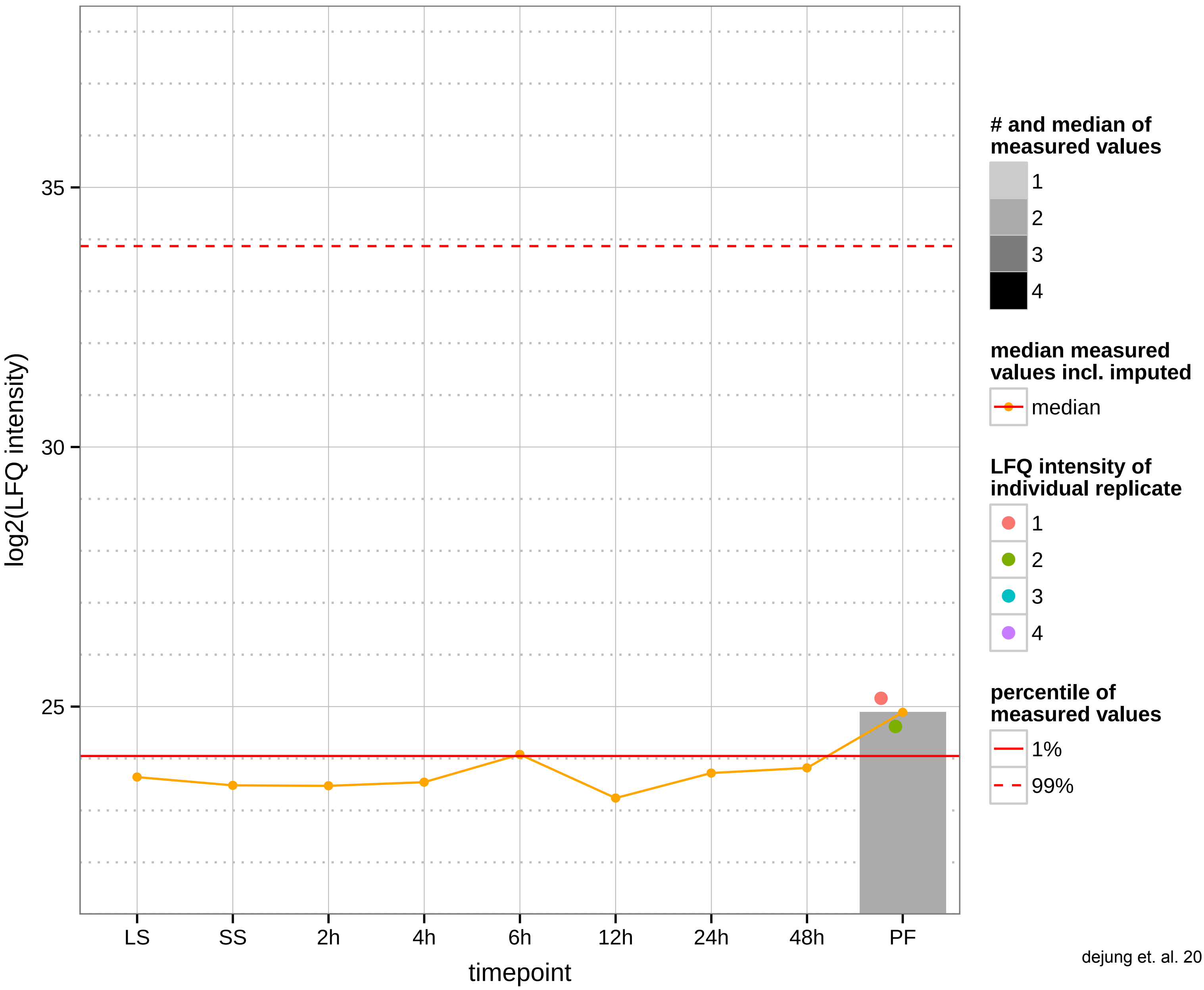

dejung et. al. 2015

ATP−dependent helicase, putative
Tb927.11.13380
AGOF: nucleoside−triphosphatase activity, nucleotide binding
AGOC: null
AGOP: null
PGOF: nucleoside−triphosphatase activity, nucleotide binding
PGOC: null
PGOP: null

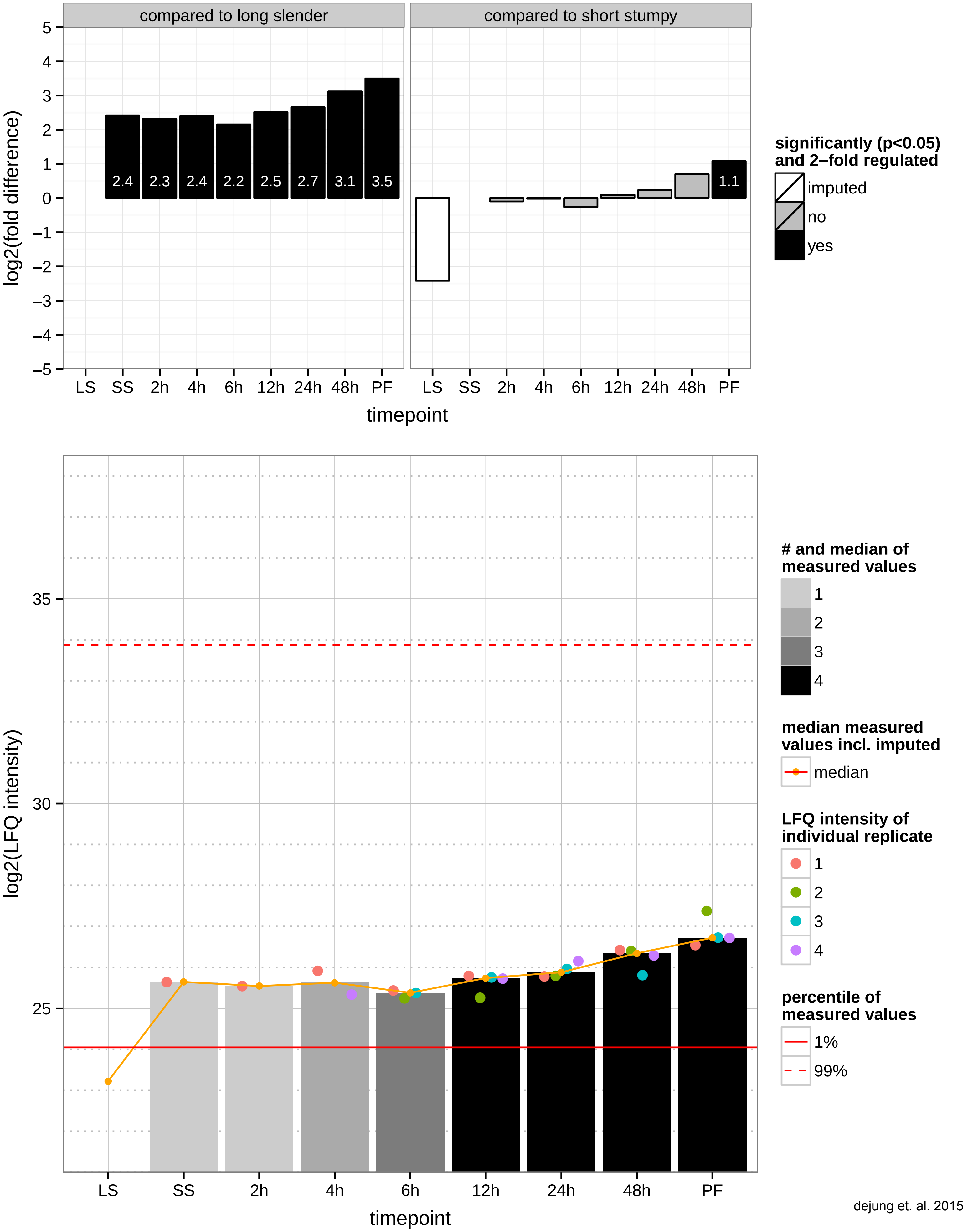

dejung et. al. 2015

hypothetical protein, conserved
Tb927.11.13520
AGOF: null
AGOC: null
AGOP: null
PGOF: null
PGOC: null
PGOP: null

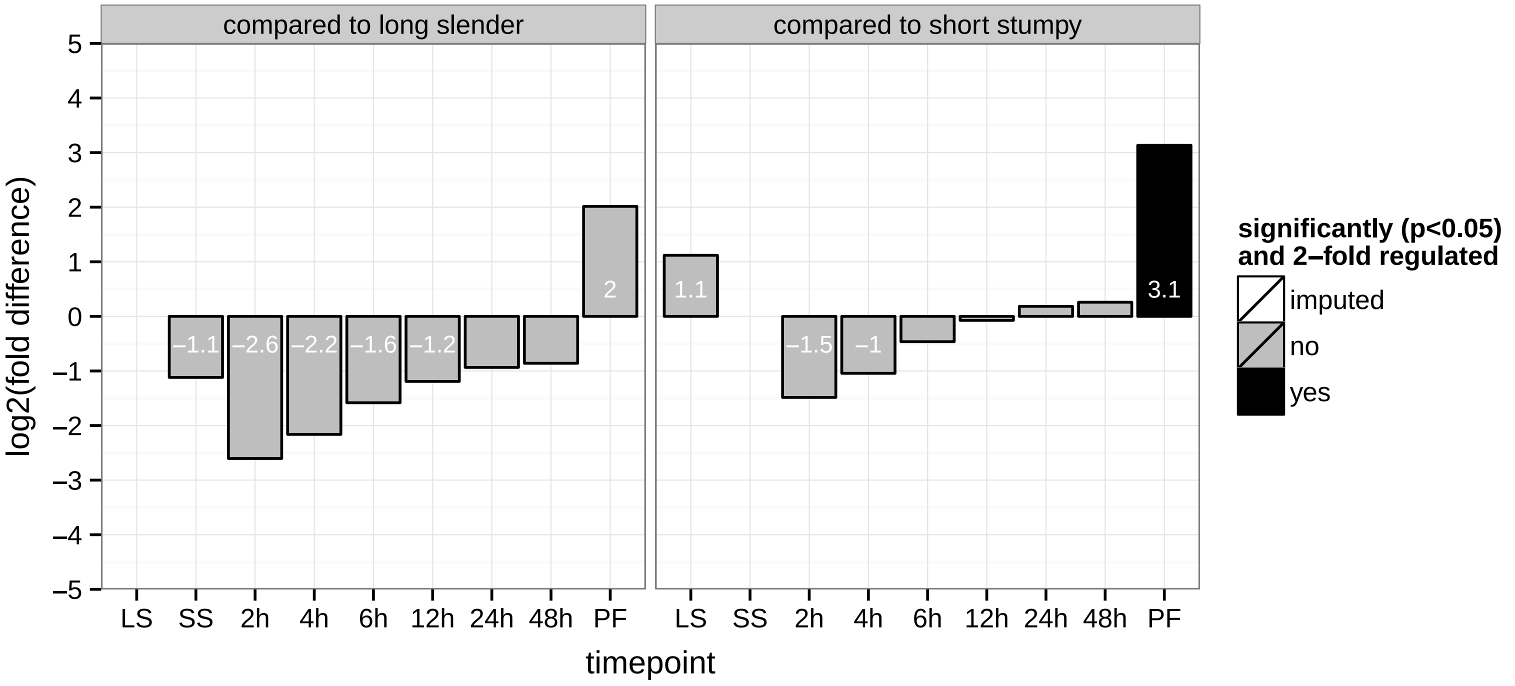

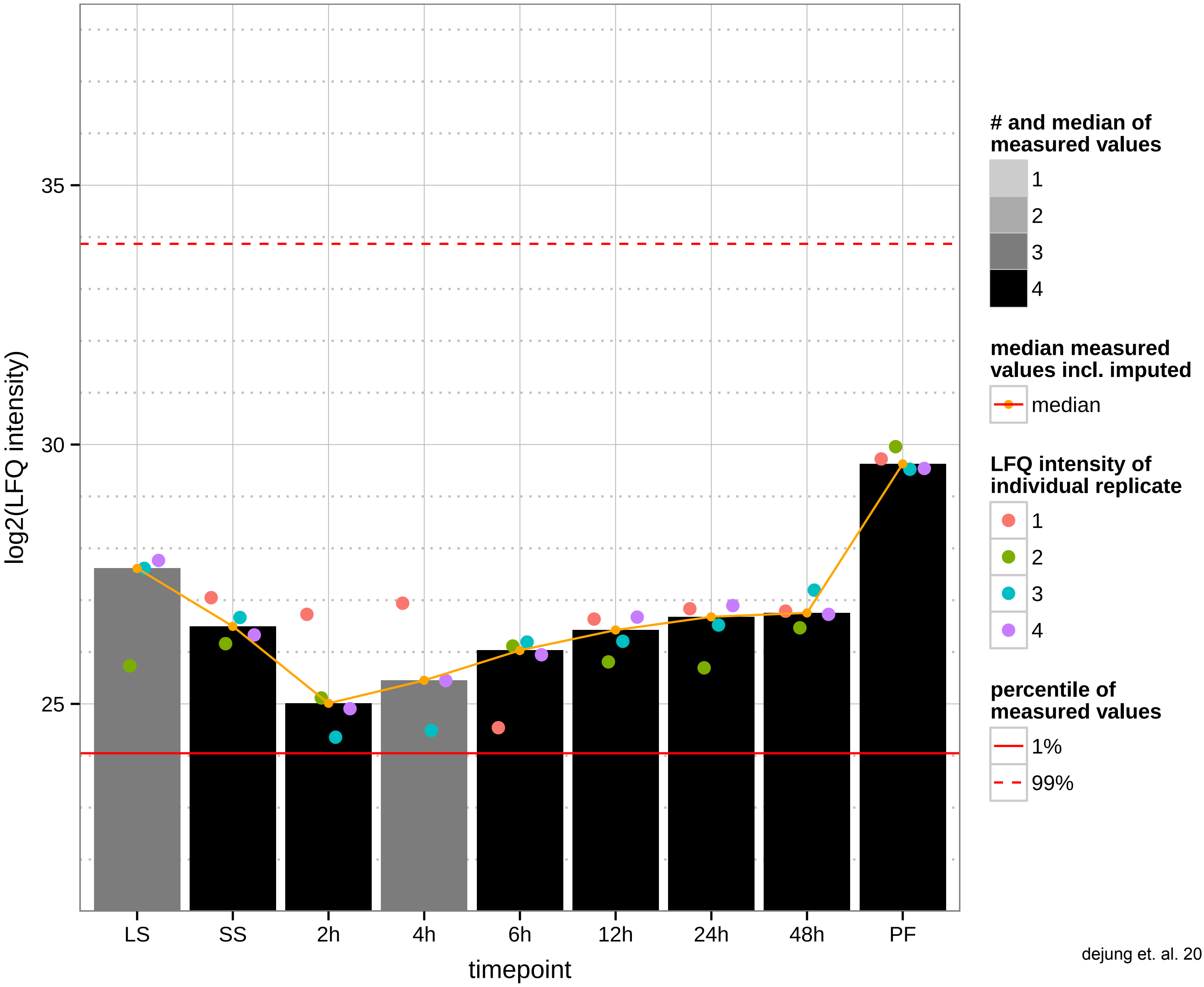

dejung et. al. 2015

hypothetical protein, conserved
Tb927.11.13680
AGOF: null
AGOC: null
AGOP: null
PGOF: null
PGOC: null
PGOP: null

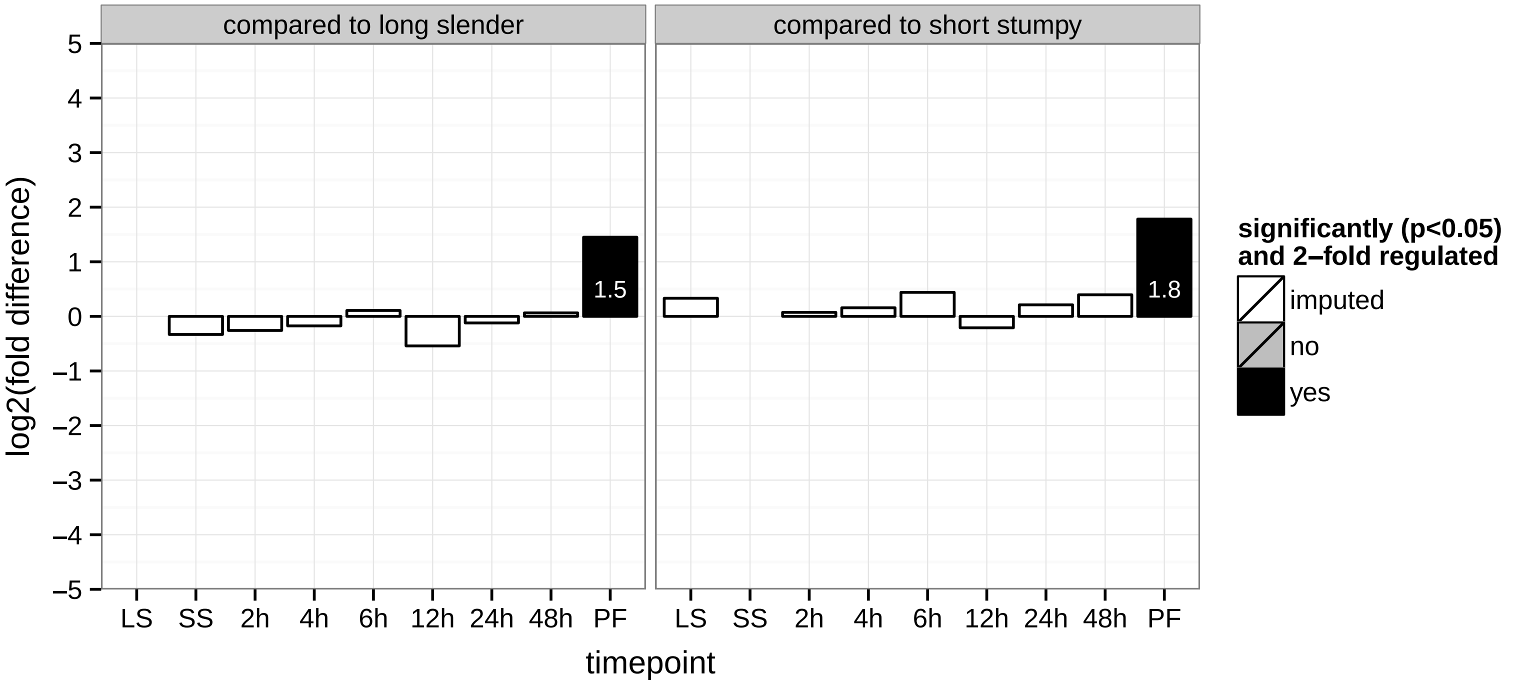

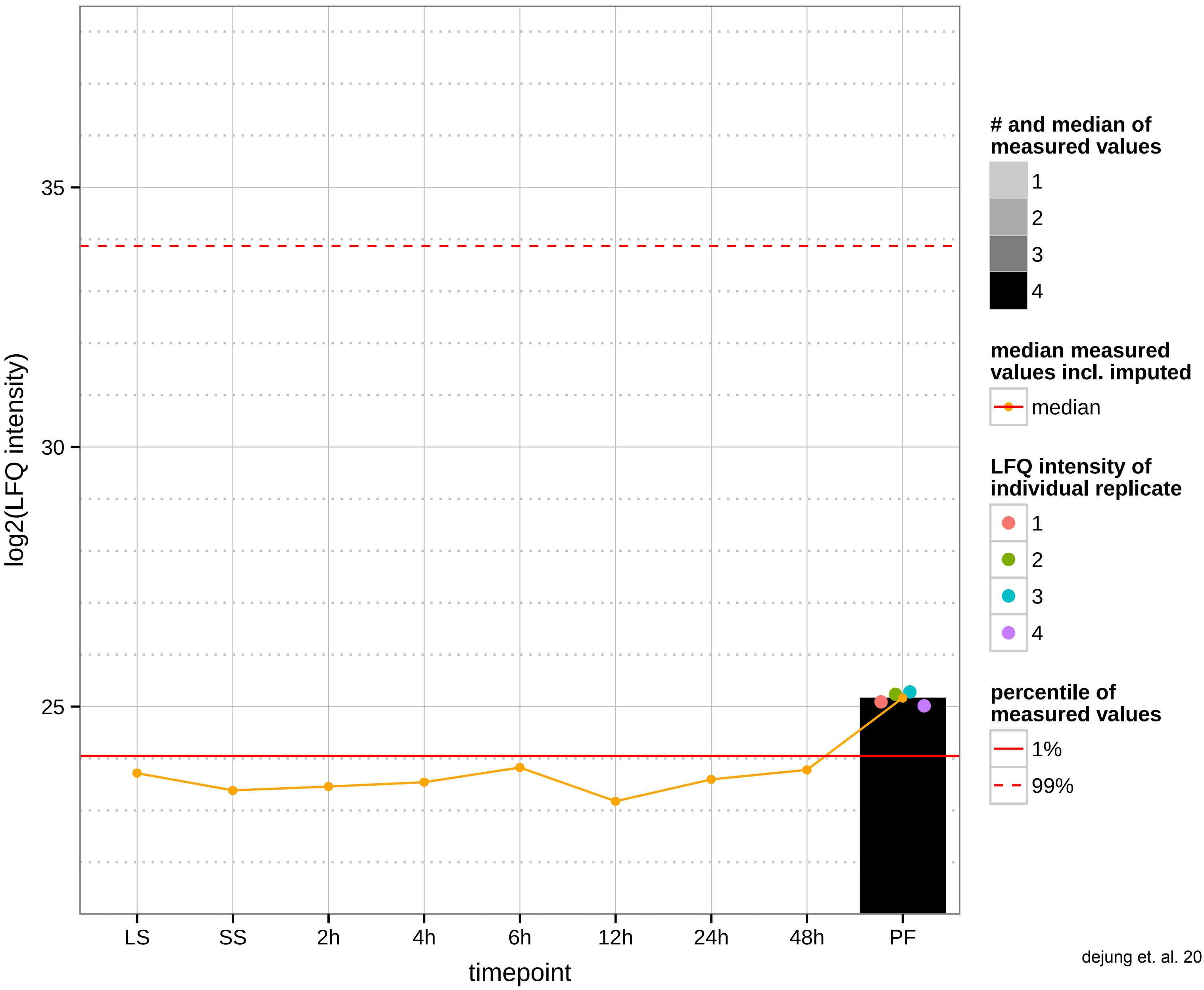

dejung et. al. 2015

hypothetical protein, conserved
Tb927.11.13870
AGOF: null
AGOC: null
AGOP: null
PGOF: null
PGOC: null
PGOP: null

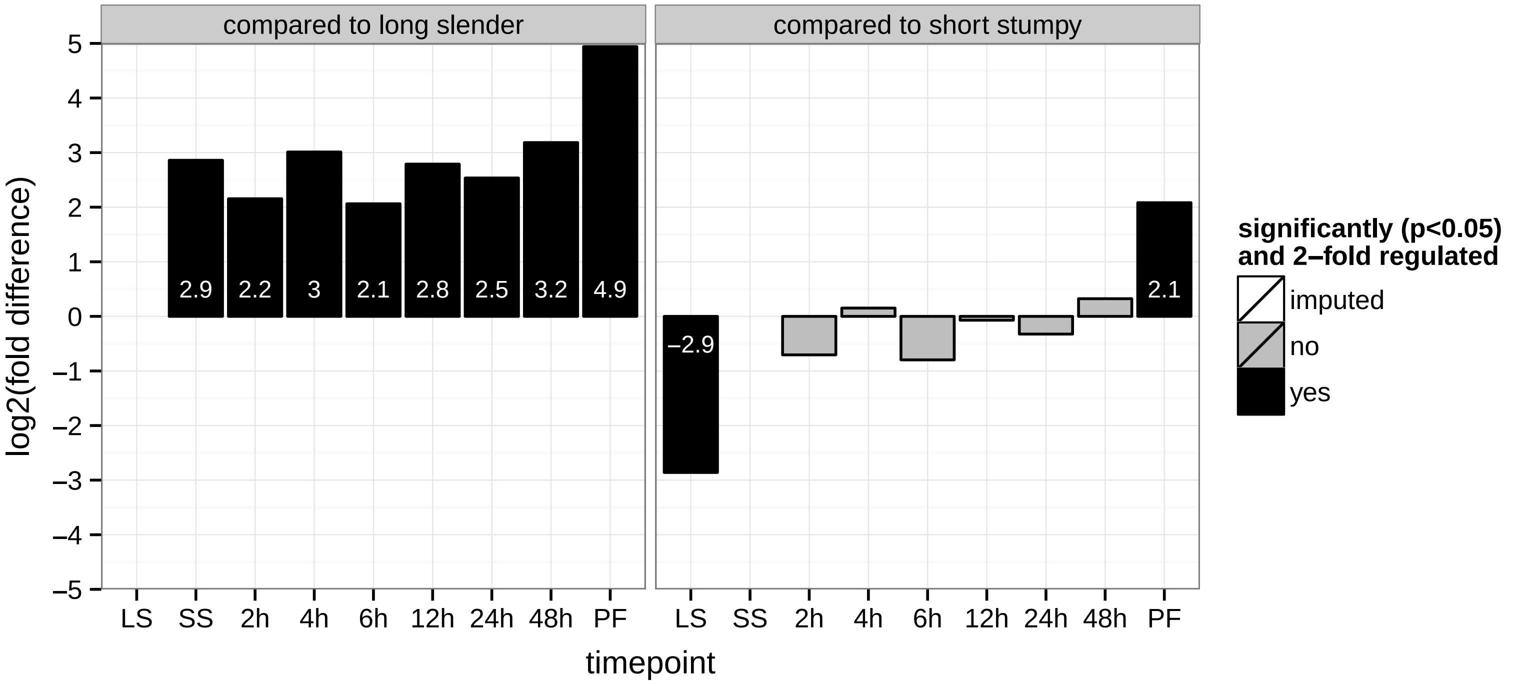

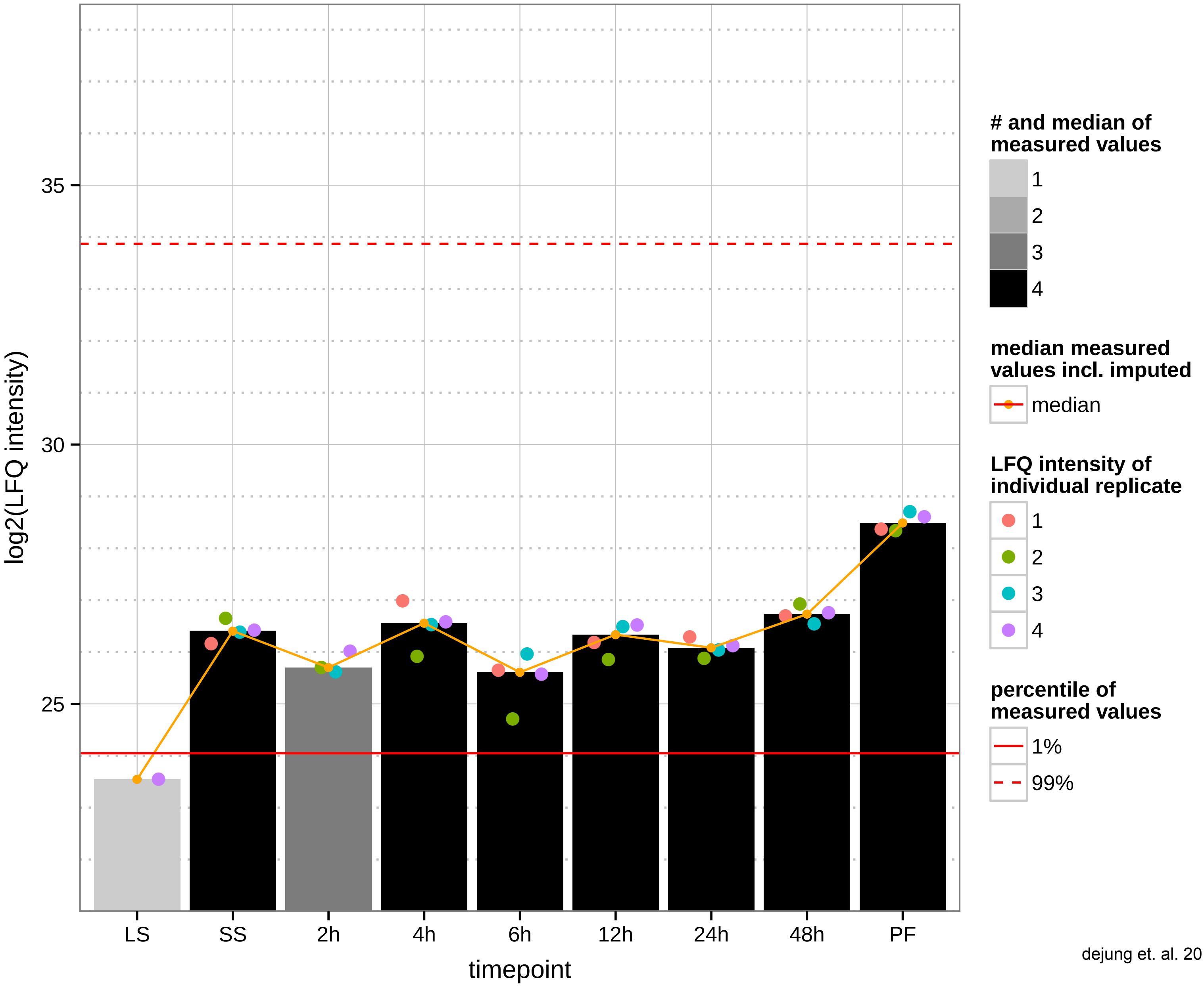

dejung et. al. 2015

Pab1p–dependent poly(A) ribonuclease subunit, putative, PAN3
Tb927.11.13970
AGOF: null
AGOC: null
AGOP: null
PGOF: transferase activity, transferring phosphorus–containing groups
PGOC: null
PGOP: null

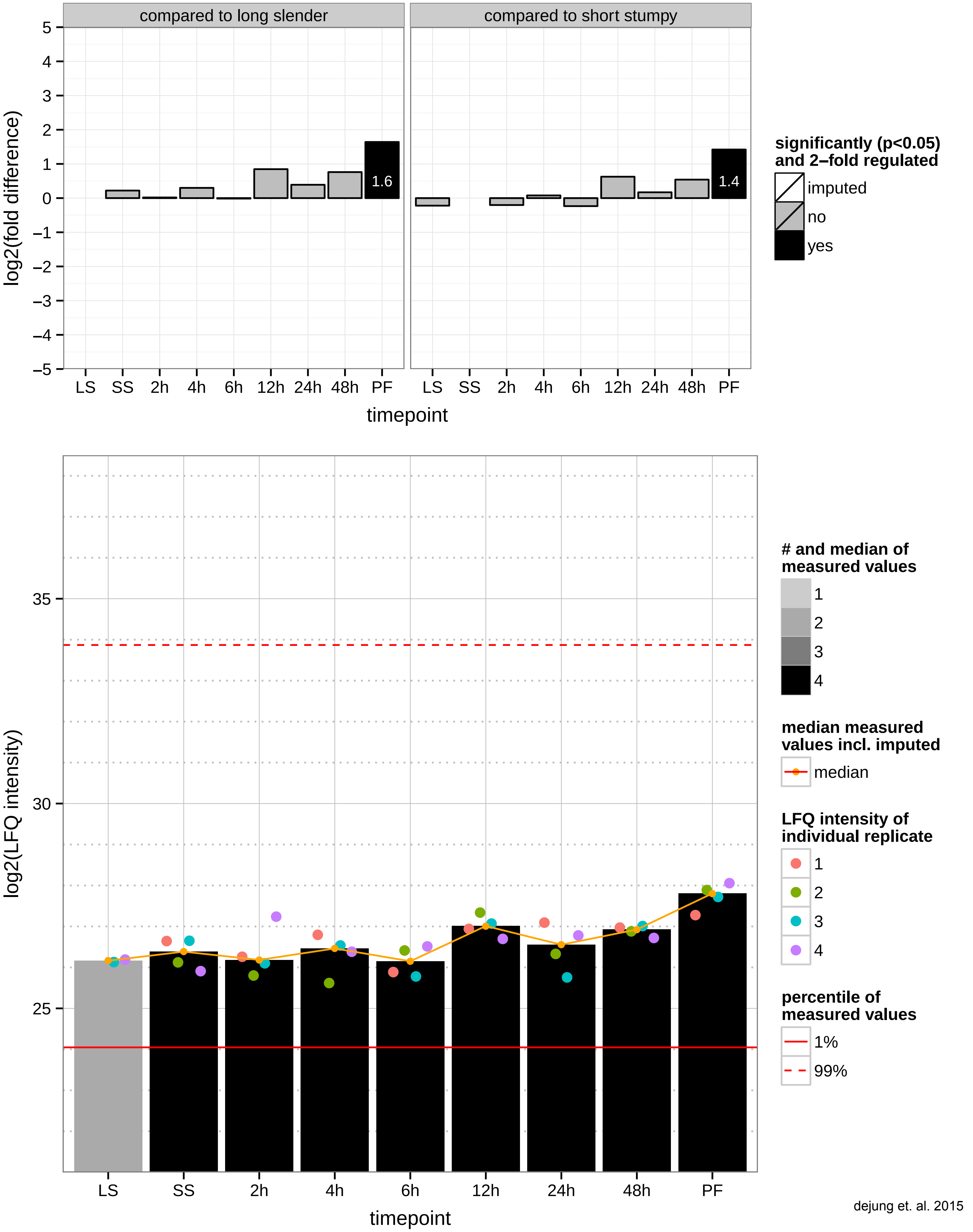

dejung et. al. 2015

Repressor of differentiation kinase 1 (RDK1)
Tb927.11.14070
AGOF: ATP binding, protein serine/threonine kinase activity, protein tyrosine kinase activity
AGOC: cilium, integral to membrane, plasma membrane
AGOP: growth, modulation of development of symbiont involved in interaction with host, protein phosphorylation
PGOF: ATP binding, protein kinase activity, transferase activity, transferring phosphorus–containing groups
PGOC: null
PGOP: protein phosphorylation

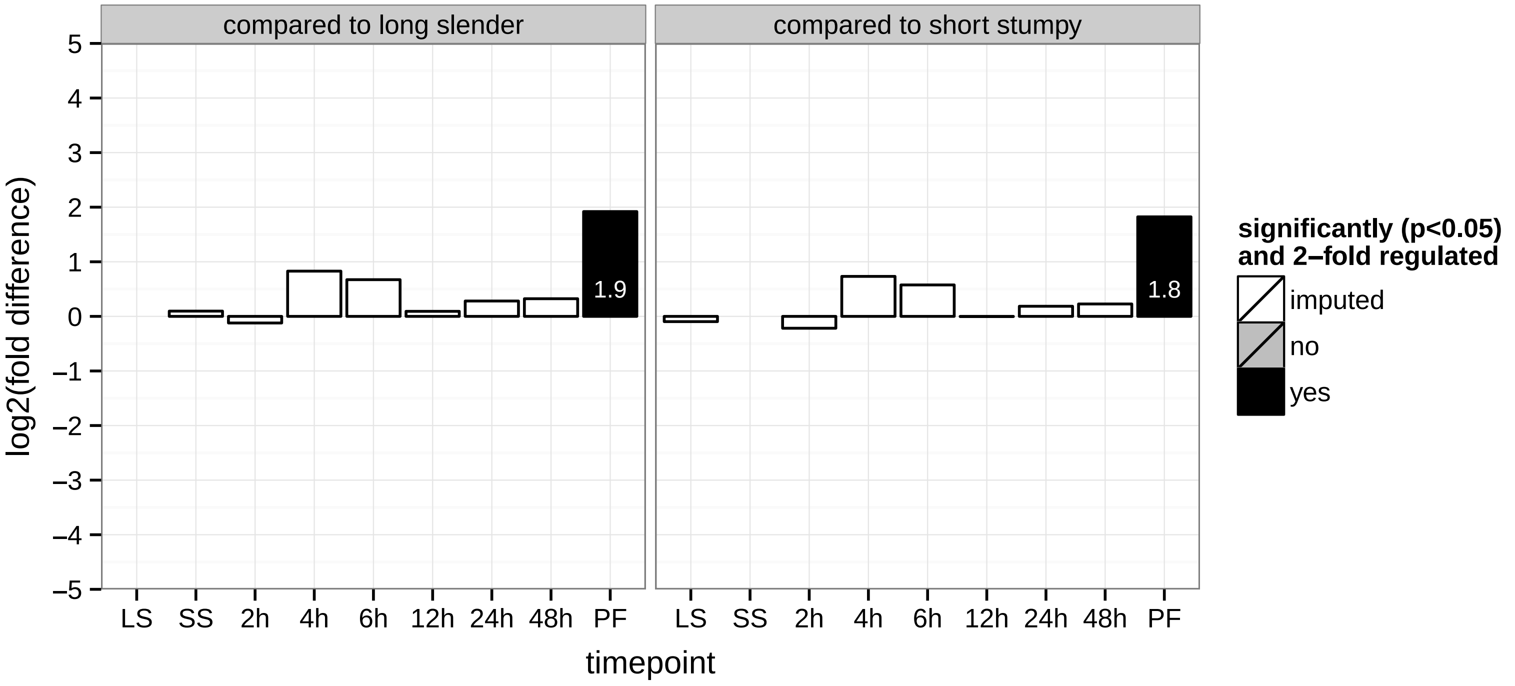

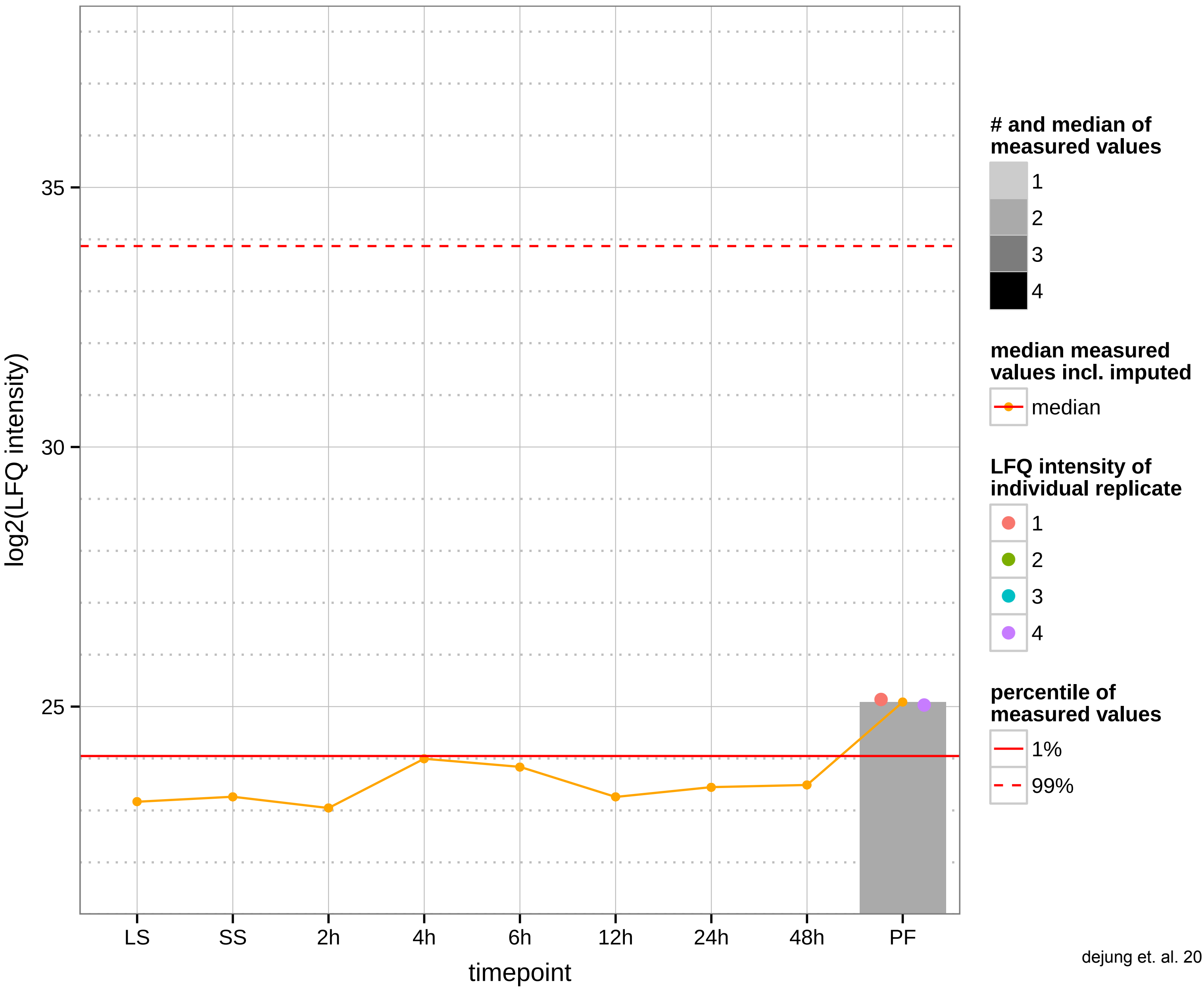

dejung et. al. 2015

conserved protein
Tb927.11.14210
AGOF: calcium−dependent cysteine−type endopeptidase activity
AGOC: intracellular
AGOP: proteolysis
PGOF: calcium−dependent cysteine−type endopeptidase activity
PGOC: intracellular
PGOP: proteolysis

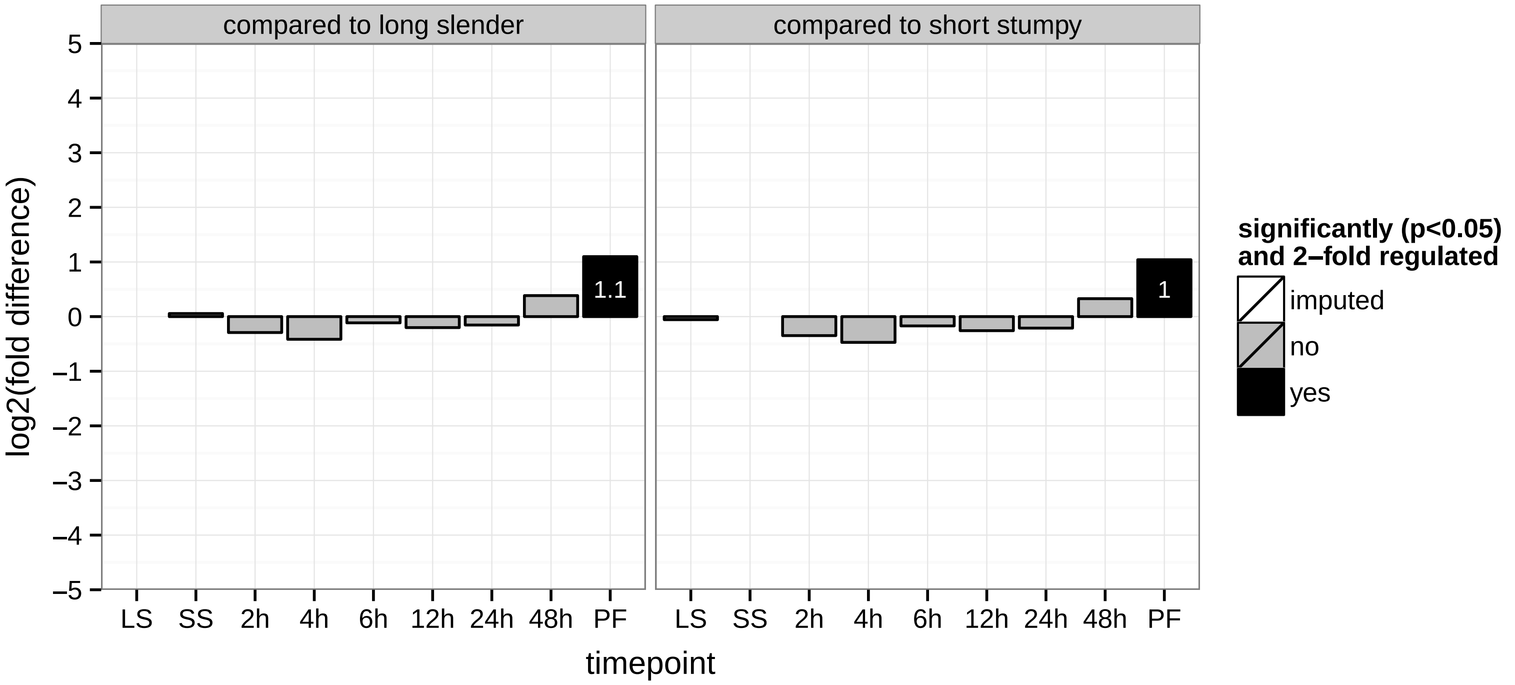

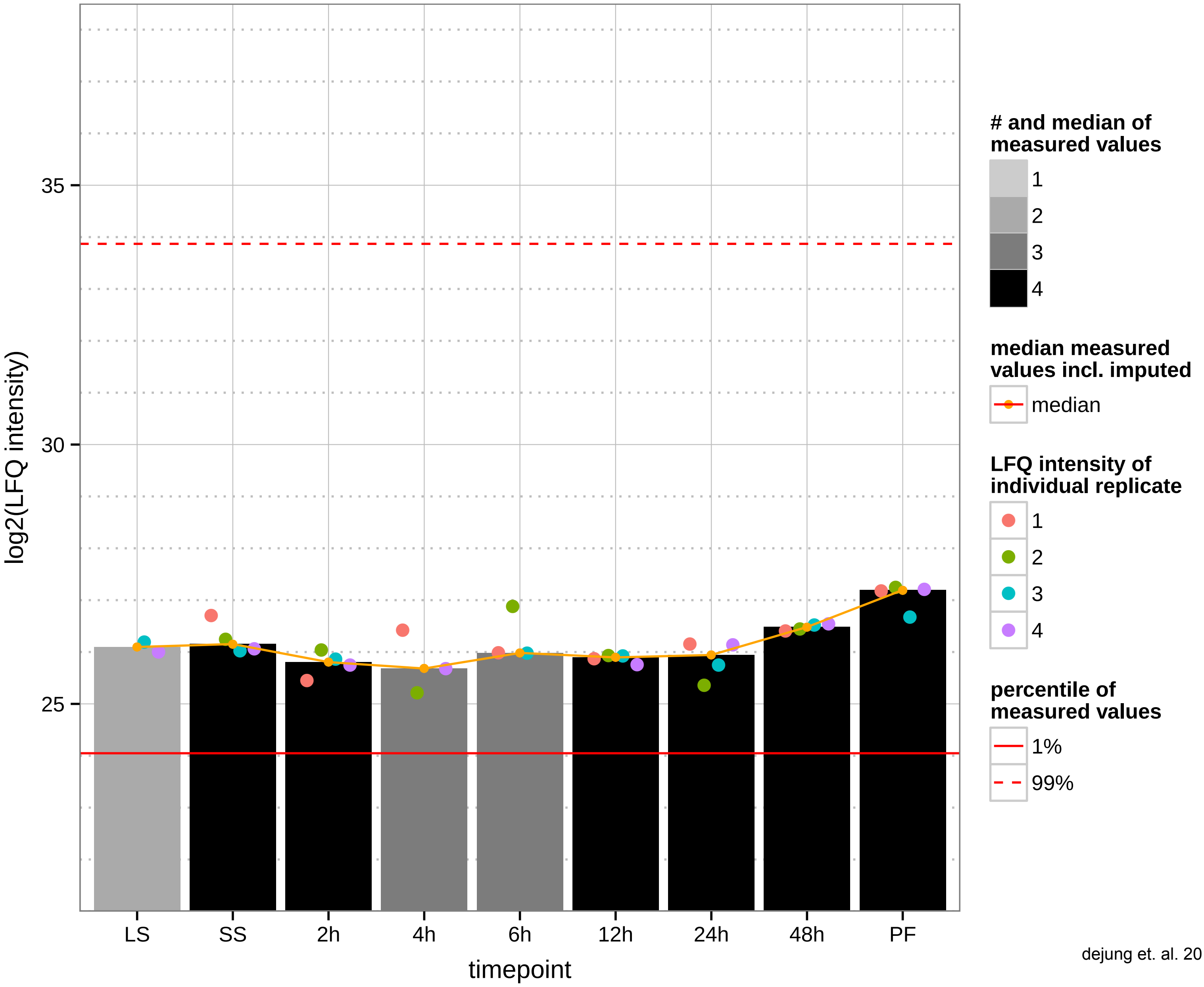

dejung et. al. 2015

RNA guanylyltransferase
Tb927.11.14580
AGOF: mRNA guanylyltransferase activity
AGOC: nucleus
AGOP: 7−methylguanosine mRNA capping
PGOF: mRNA guanylyltransferase activity
PGOC: null
PGOP: 7−methylguanosine mRNA capping, mRNA processing

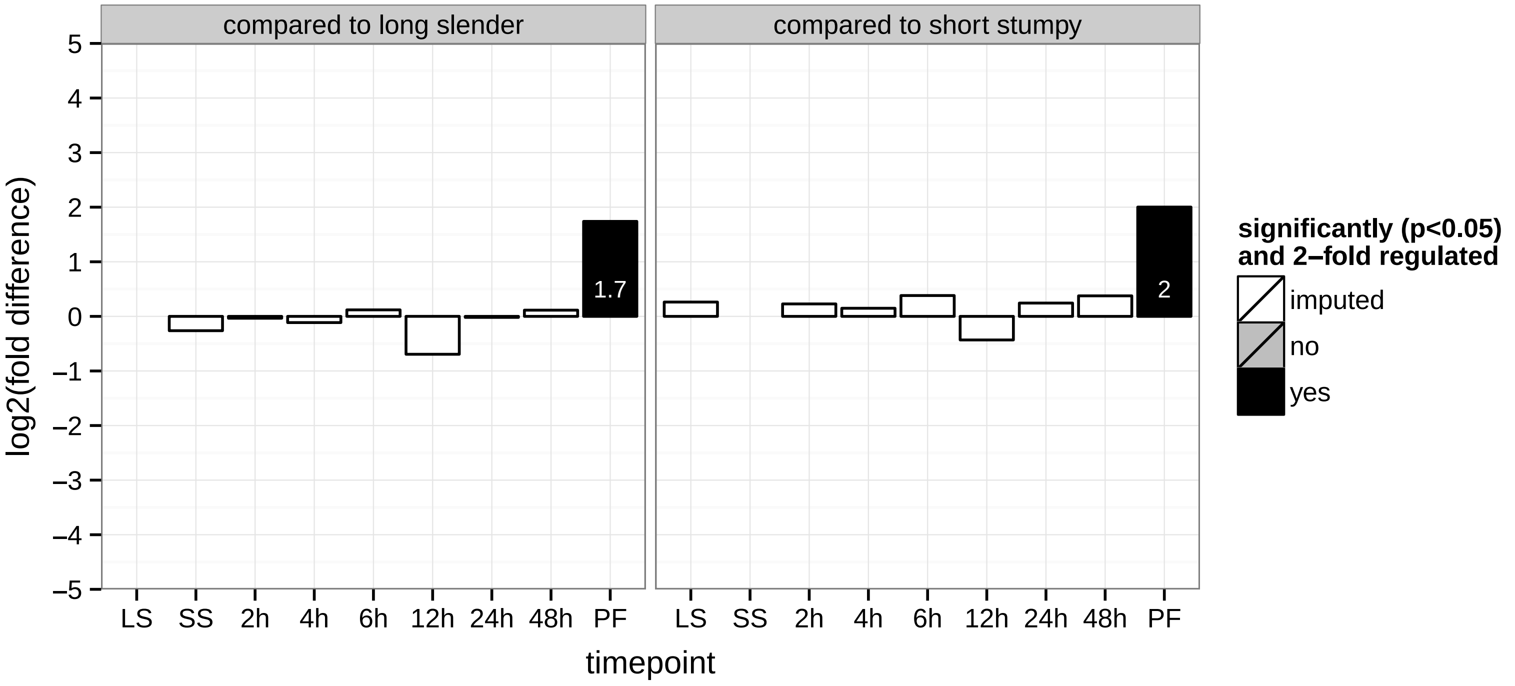

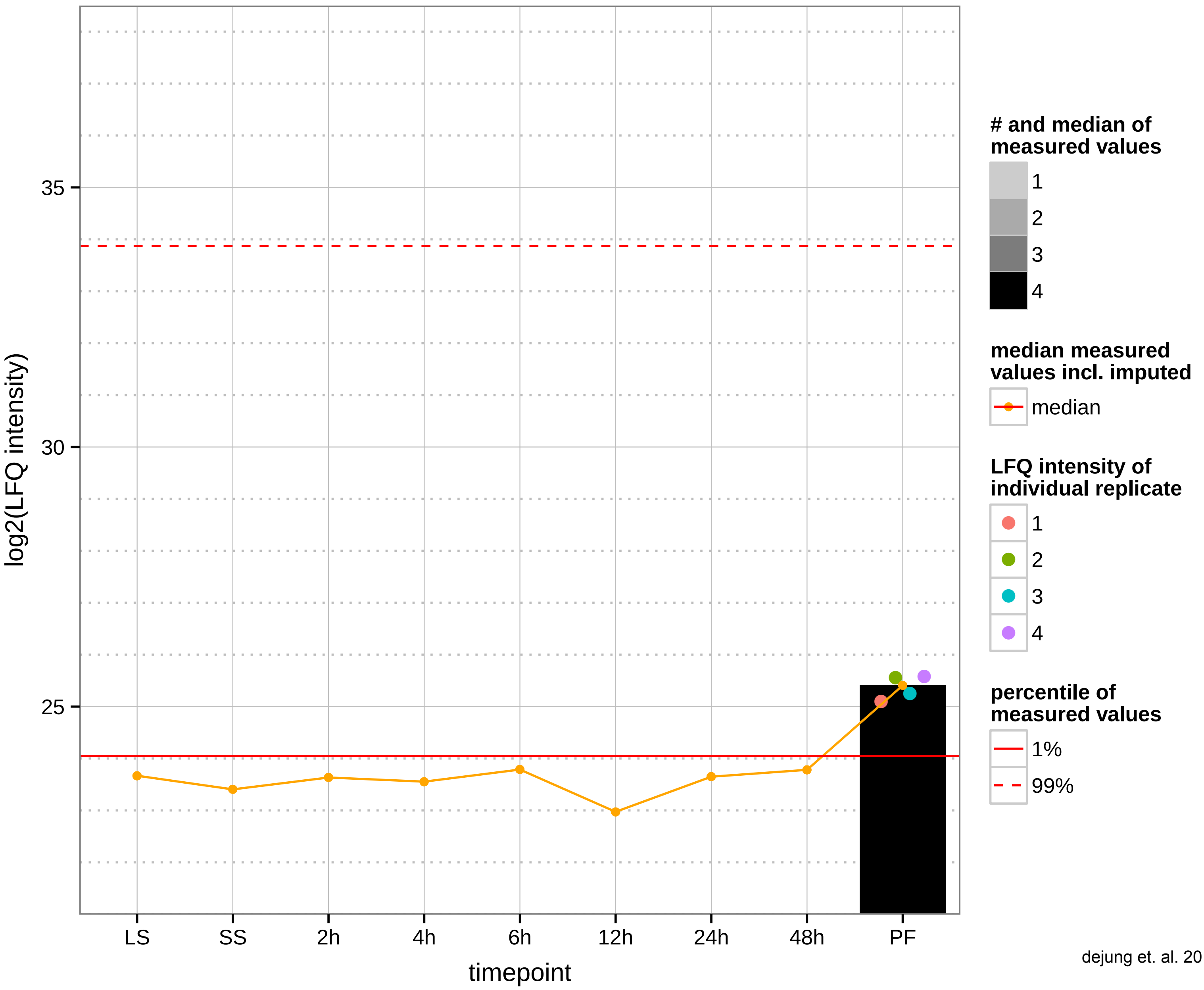

dejung et. al. 2015

pumilio/PUF RNA binding protein 7, putative (PUF7)
Tb927.11.14960
AGOF: RNA binding, protein binding
AGOC: nucleolus
AGOP: mRNA metabolic process, rRNA processing
PGOF: RNA binding, binding
PGOC: null
PGOP: null

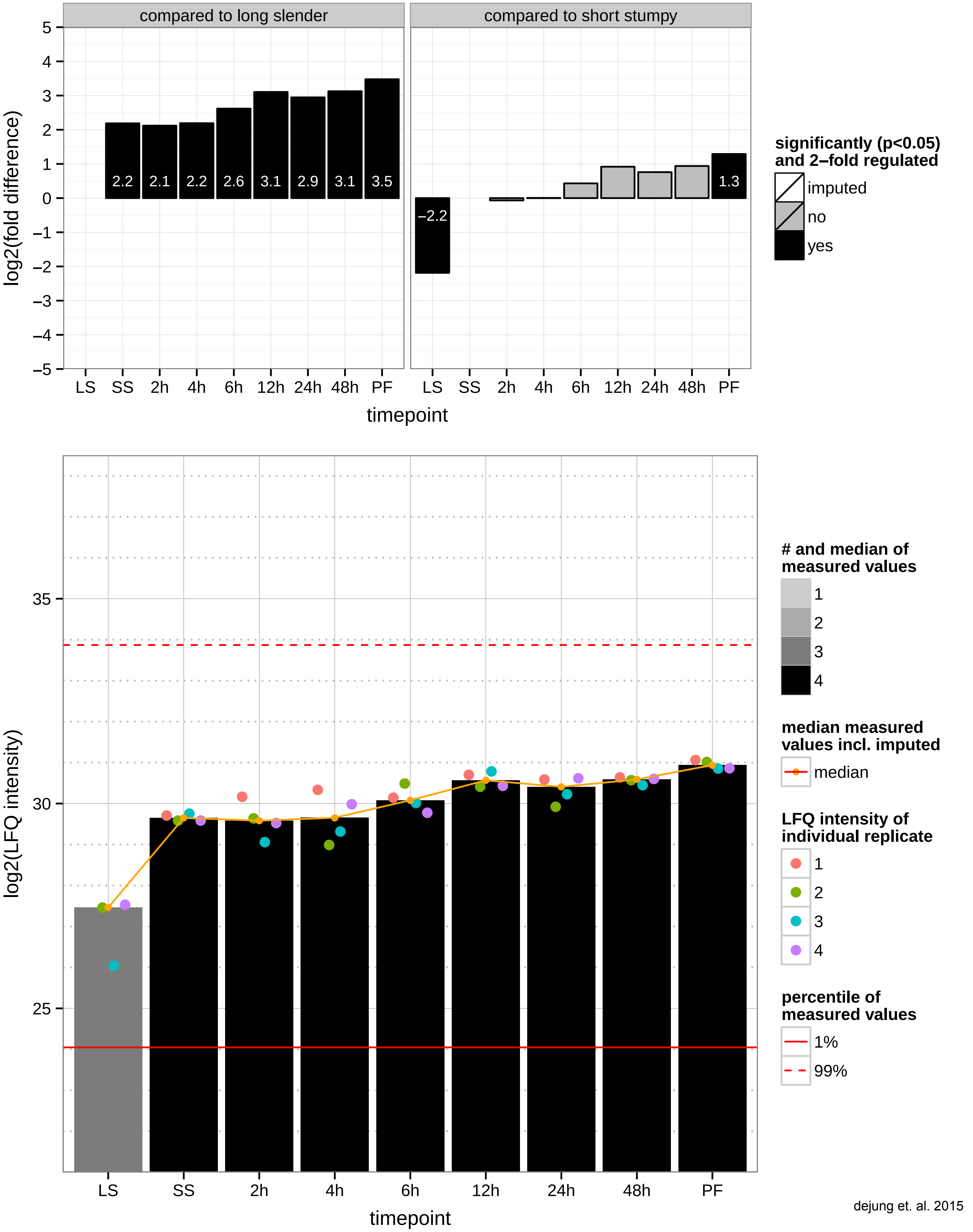

dejung et. al. 2015

hypothetical protein, conserved
Tb927.11.15050
AGOF: null
AGOC: null
AGOP: null
PGOF: cation transmembrane transporter activity
PGOC: integral to membrane
PGOP: cation transport, transmembrane transport

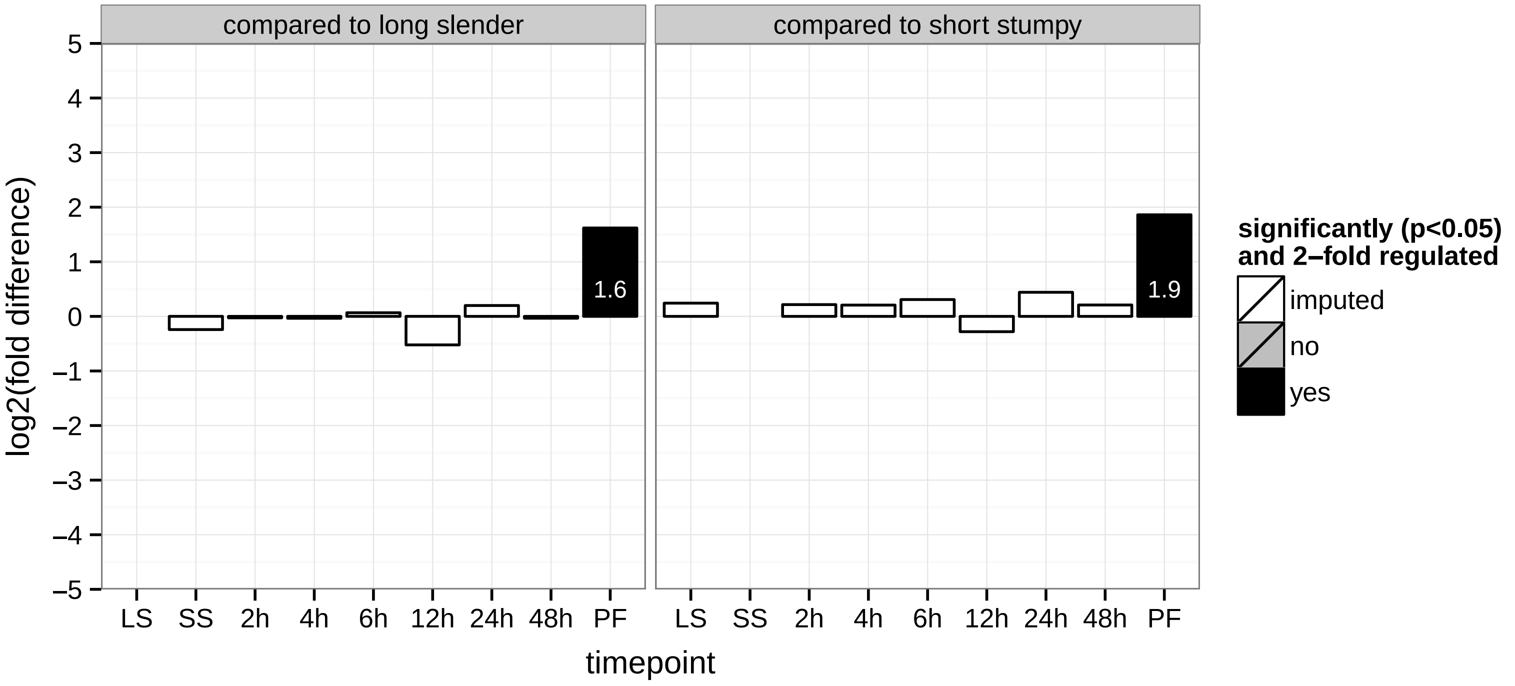

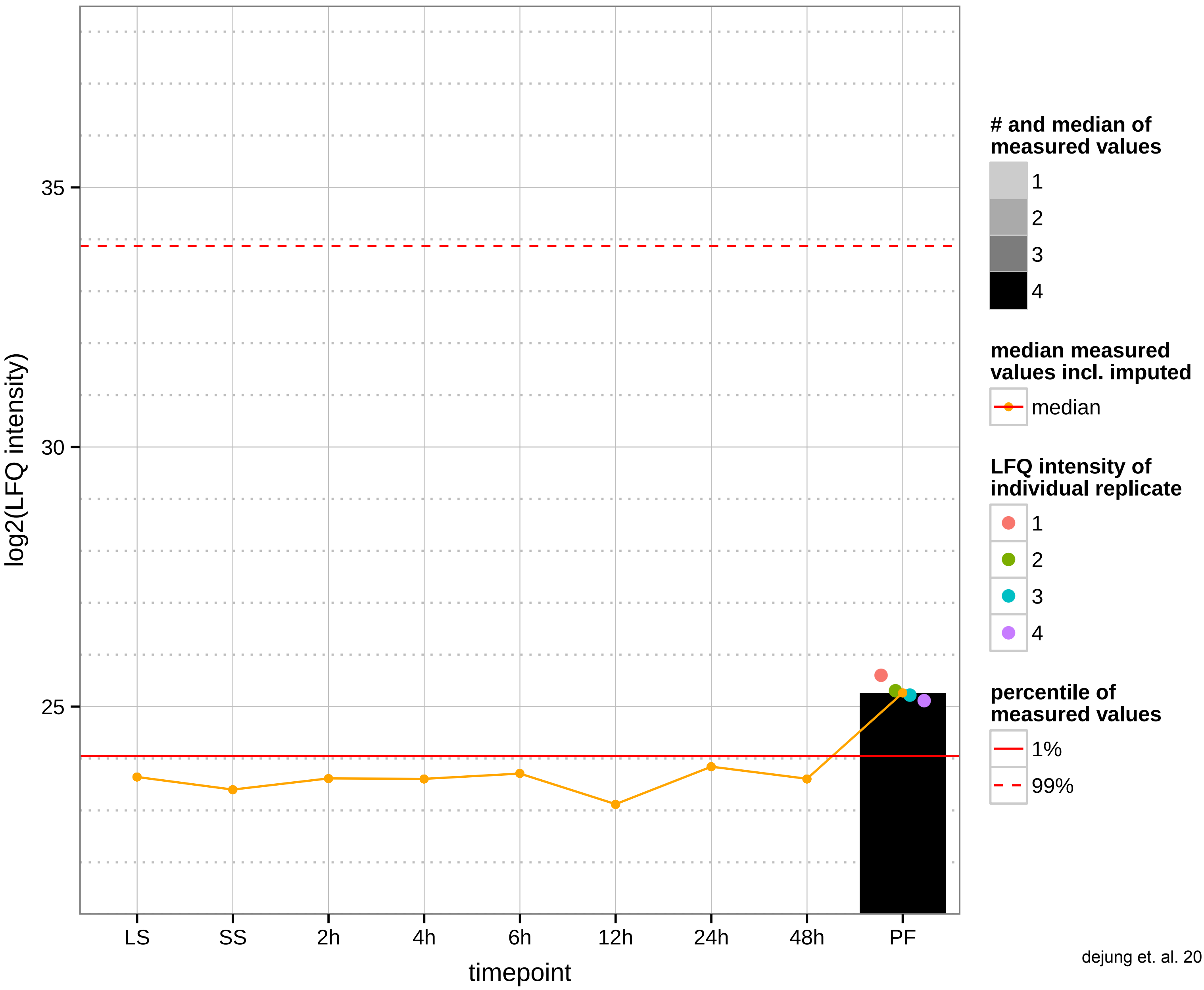

dejung et. al. 2015

hypothetical protein, conserved
Tb927.11.15220
AGOF: null
AGOC: null
AGOP: null
PGOF: null
PGOC: null
PGOP: null

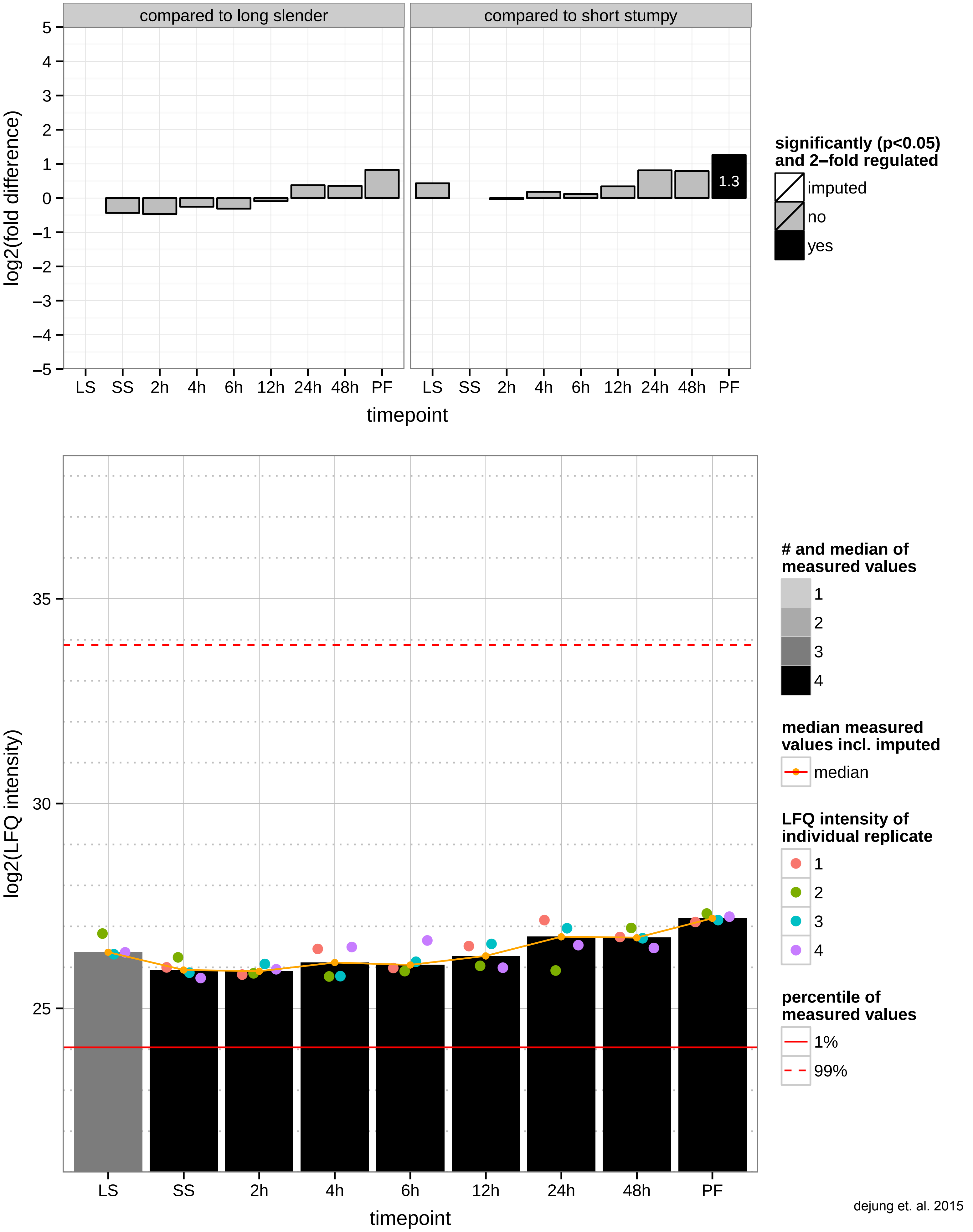

dejung et. al. 2015

eRF1 methyltransferase catalytic subunit, putative
Tb927.11.15290
AGOF: protein methyltransferase activity
AGOC: cytoplasm
AGOP: regulation of translation
PGOF: null
PGOC: null
PGOP: null

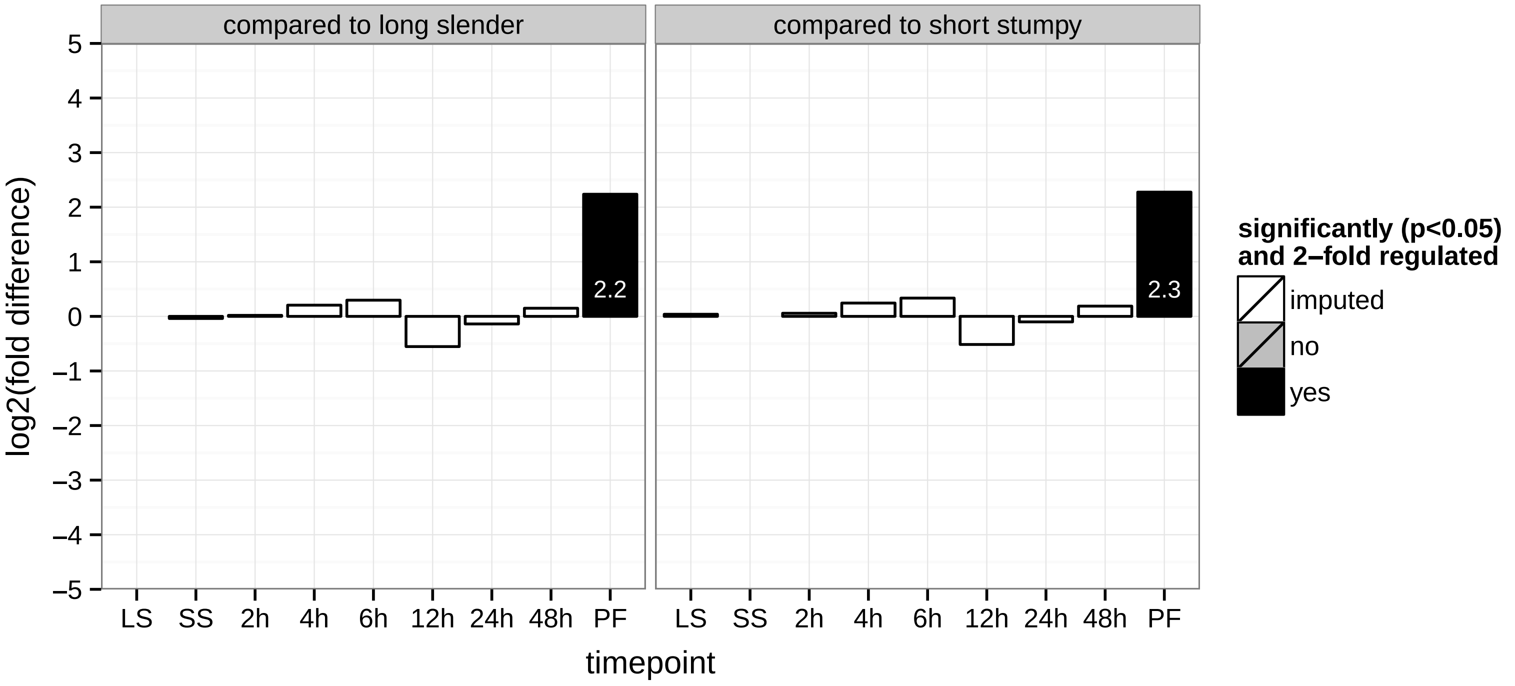

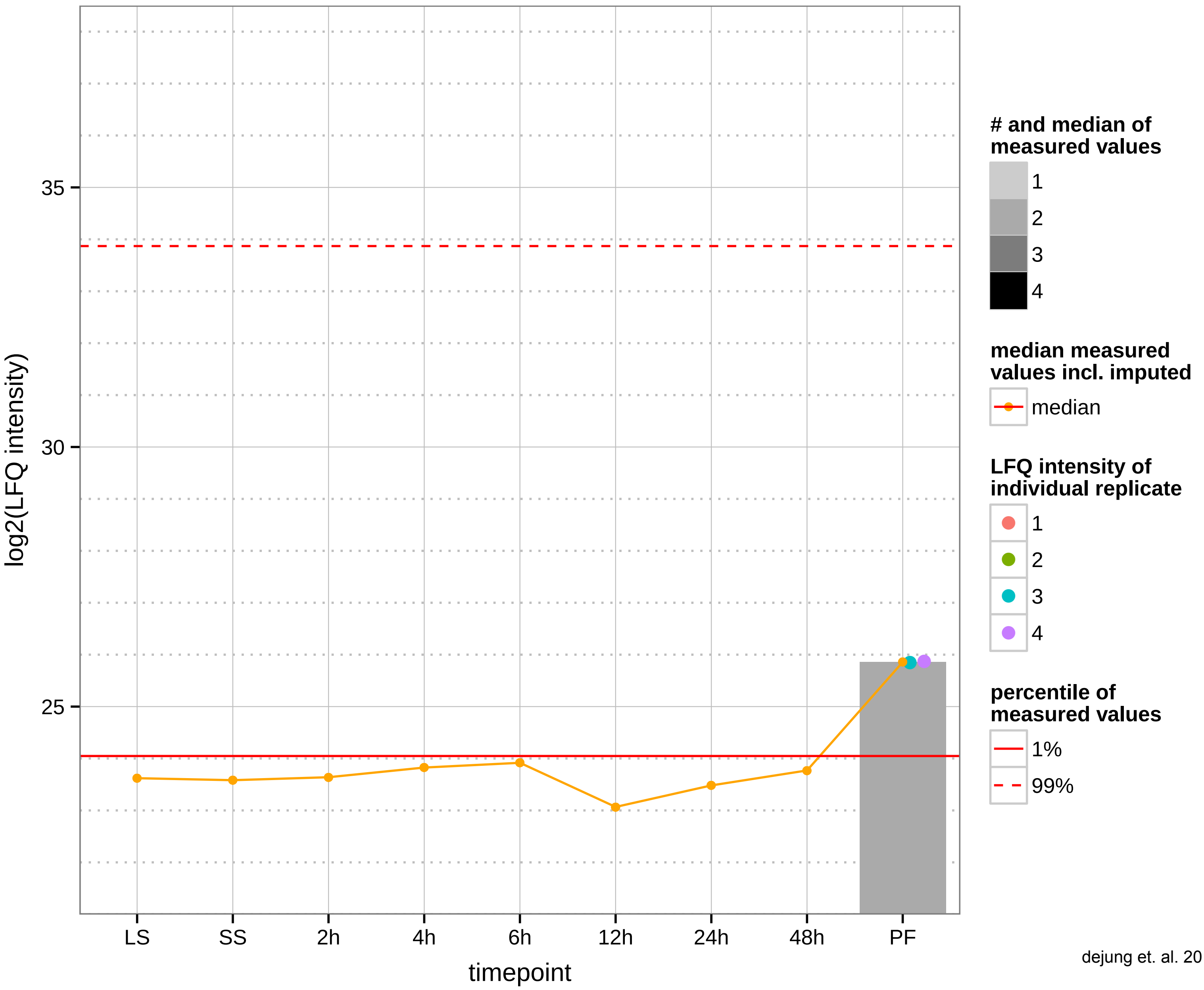

dejung et. al. 2015

hypothetical protein, conserved (TbKap123)
Tb927.11.15370
AGOF: null
AGOC: null
AGOP: null
PGOF: binding
PGOC: membrane coat
PGOP: intracellular protein transport, vesicle–mediated transport

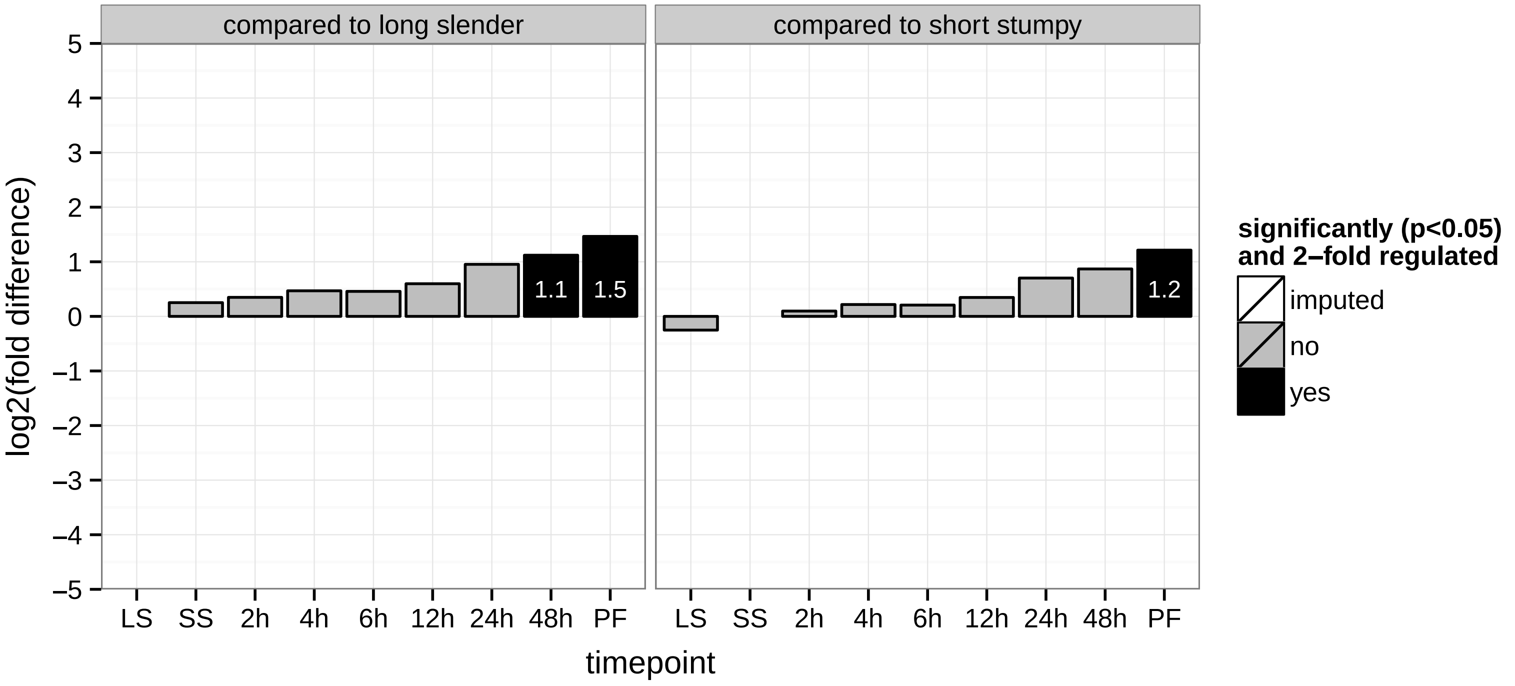

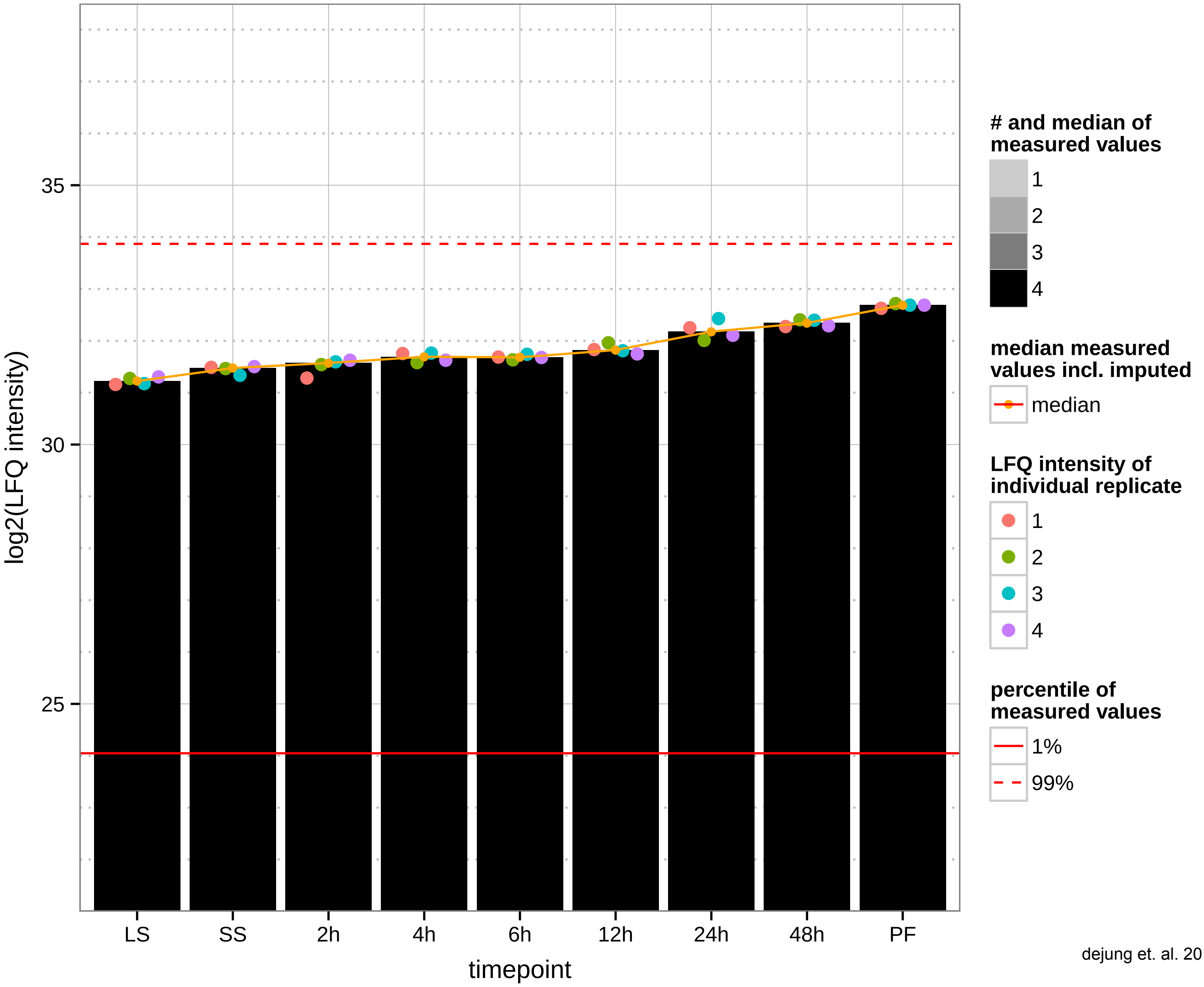

dejung et. al. 2015

poly(A) polymerase, putative
Tb927.11.15650;Tb11.v5.0667
AGOF: null
AGOC: null, mitochondrion
AGOP: null
PGOF: null
PGOC: null
PGOP: null

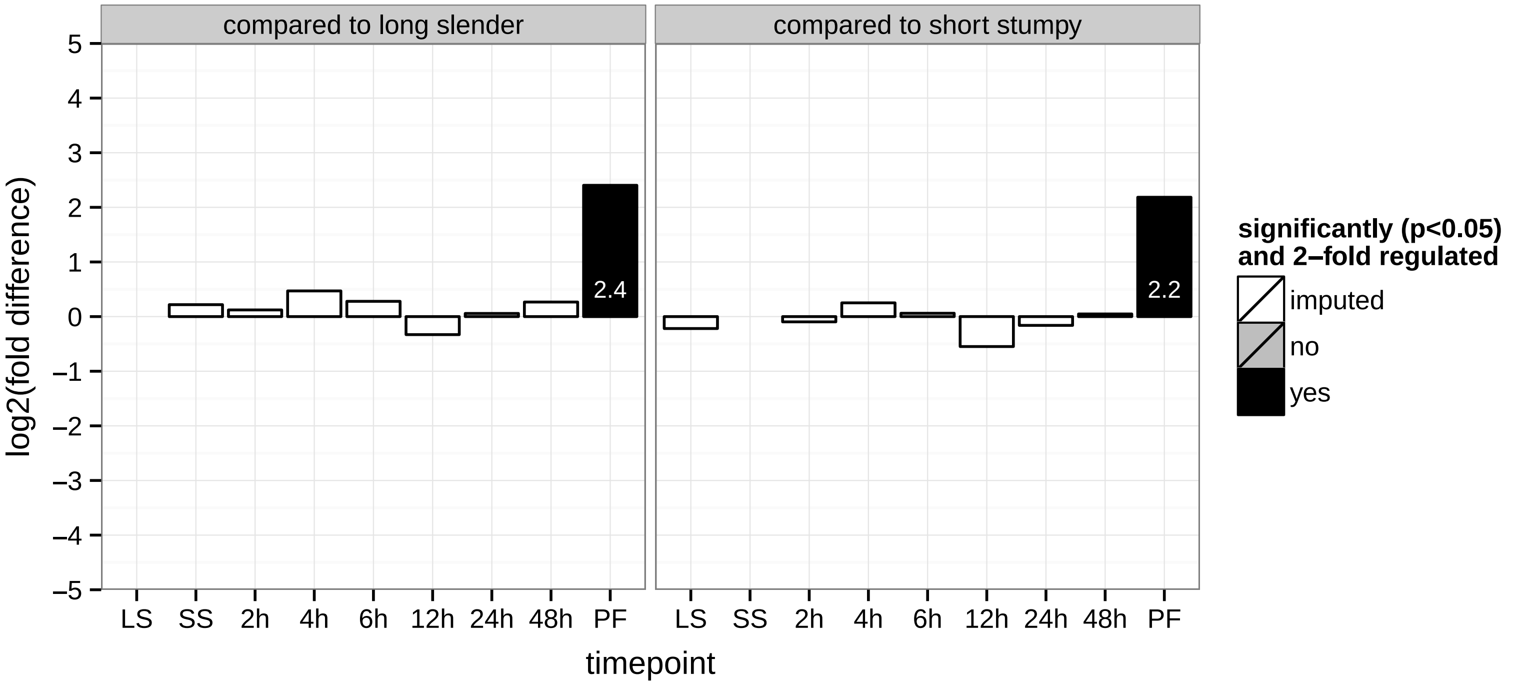

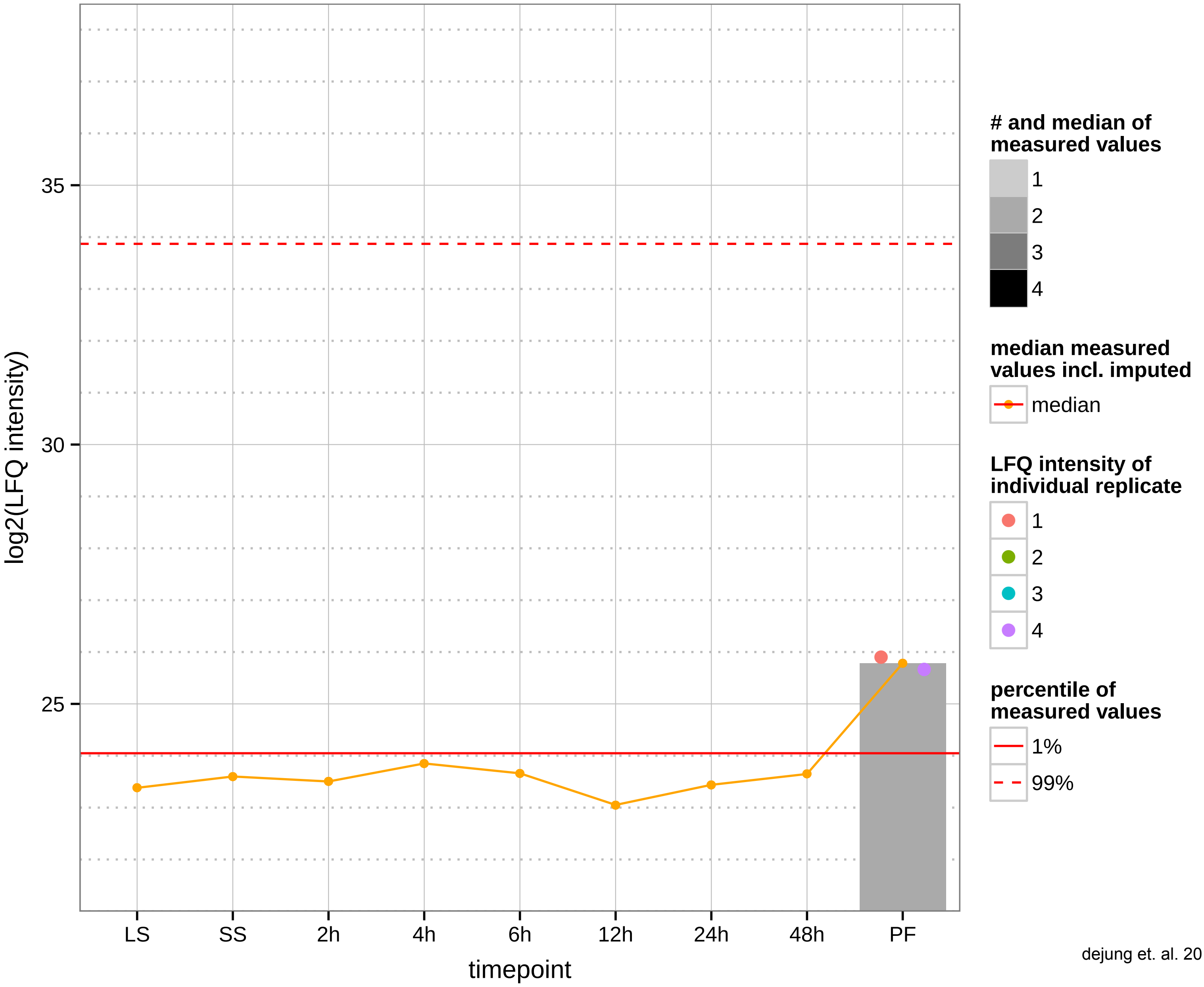

dejung et. al. 2015

microtubule–associated protein, corset–associated protein 17 (CAP17)
Tb927.11.16200
AGOF: null
AGOC: microtubule cytoskeleton
AGOP: cytoskeleton organization
PGOF: null
PGOC: null
PGOP: null

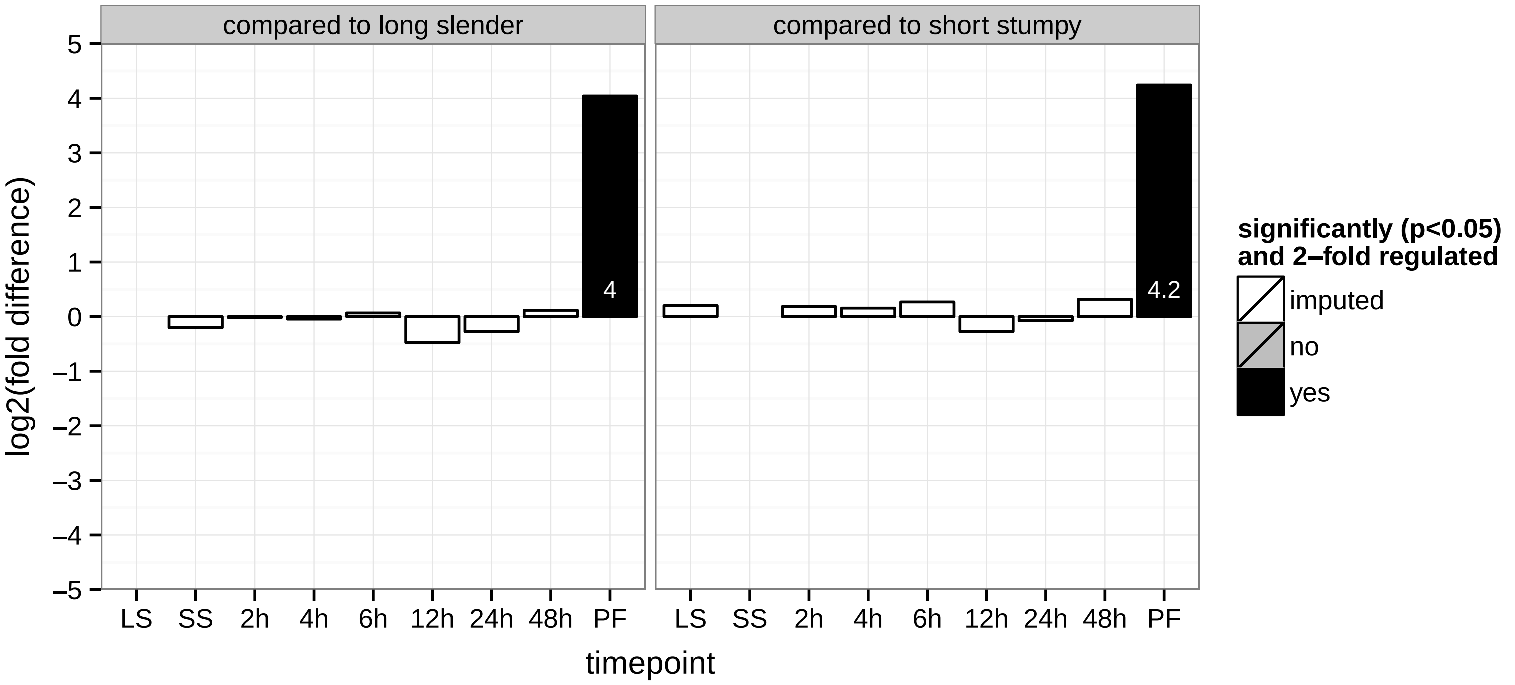

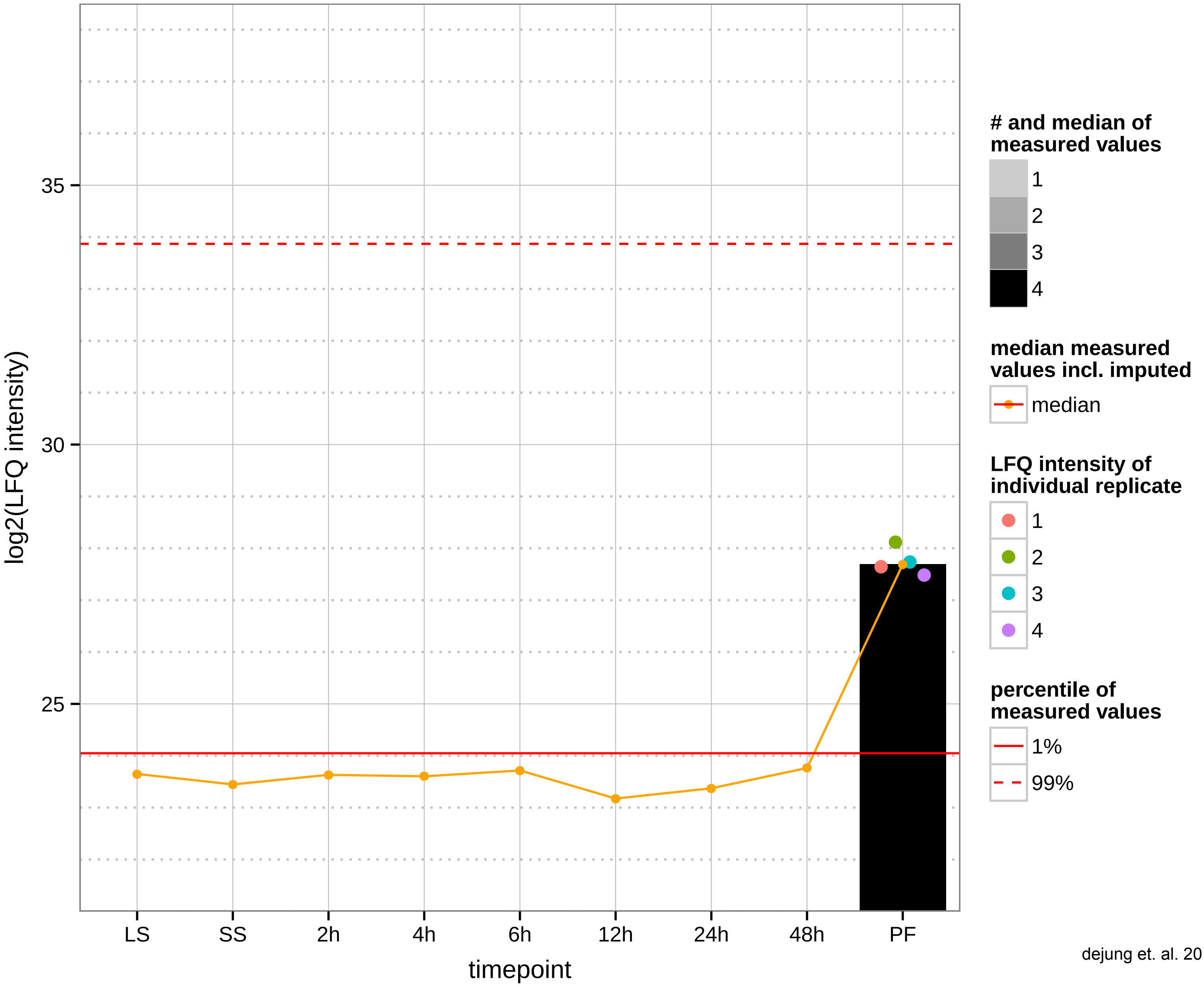

dejung et. al. 2015

60S ribosomal protein L2, putative, 60S ribosomal protein L8
Tb927.5.1110;Tb927.11.16280
AGOF: structural constituent of ribosome
AGOC: intracellular, ribosome
AGOP: translation
PGOF: structural constituent of ribosome
PGOC: intracellular, ribosome
PGOP: translation

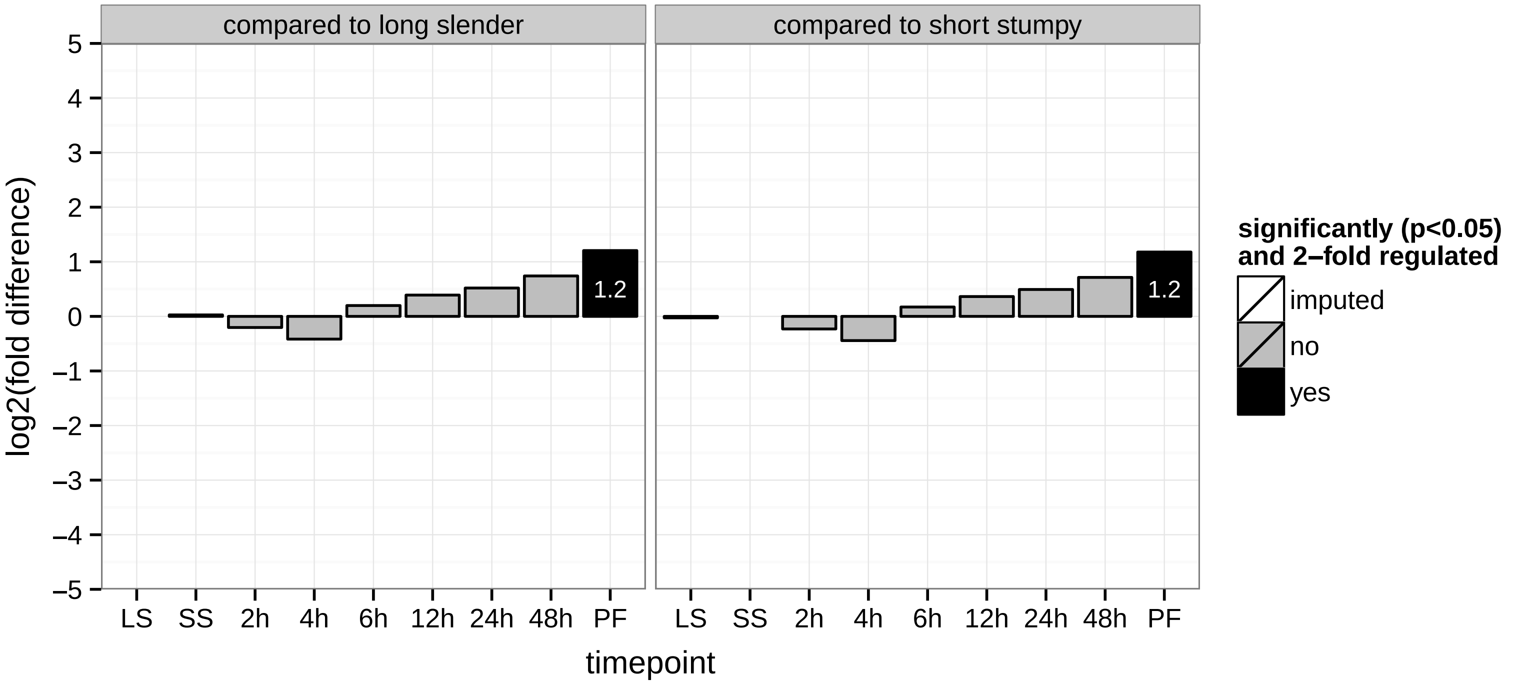

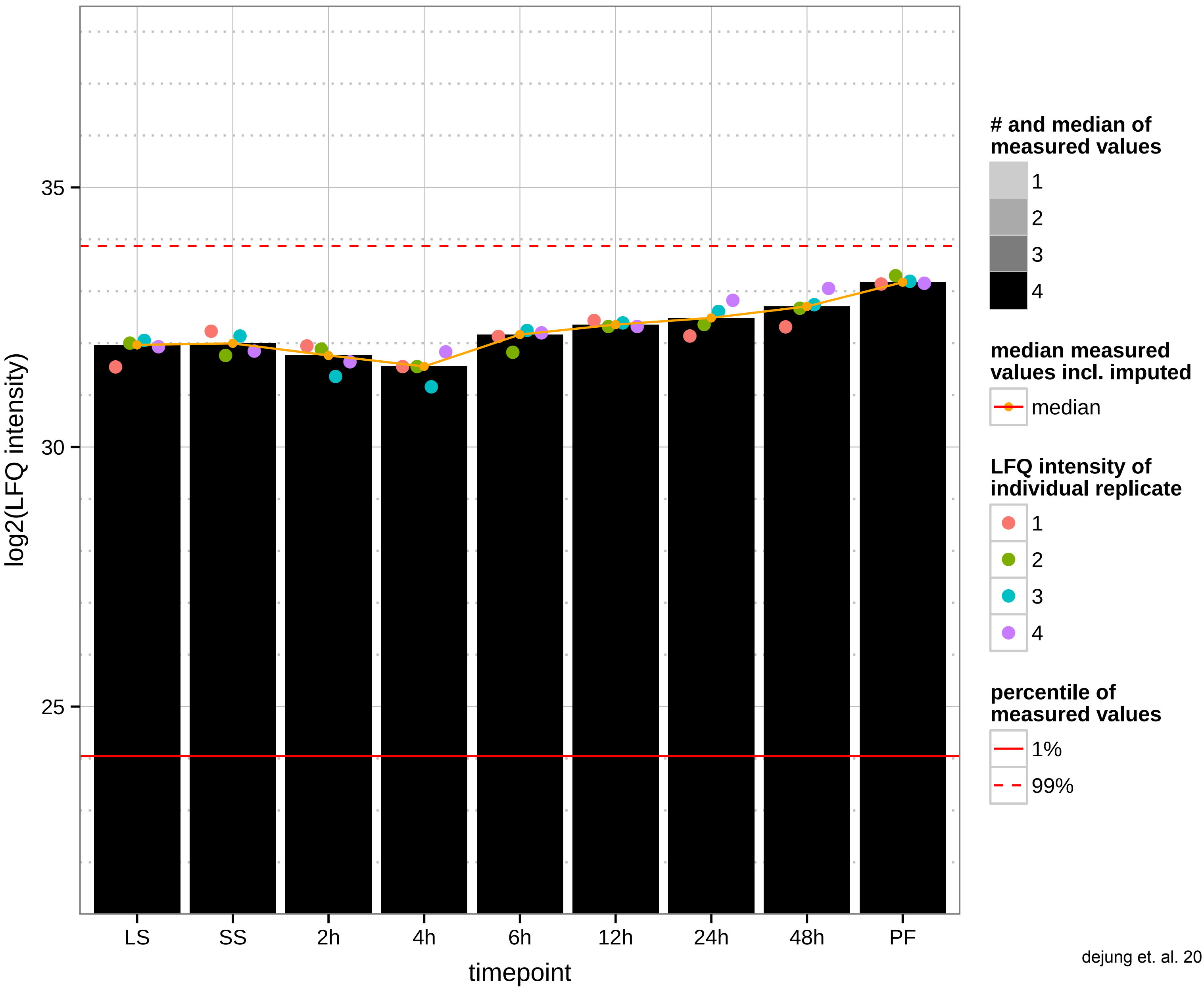

dejung et. al. 2015

hypothetical protein, conserved
Tb927.11.16530
AGOF: null
AGOC: mitochondrion
AGOP: null
PGOF: null
PGOC: null
PGOP: null

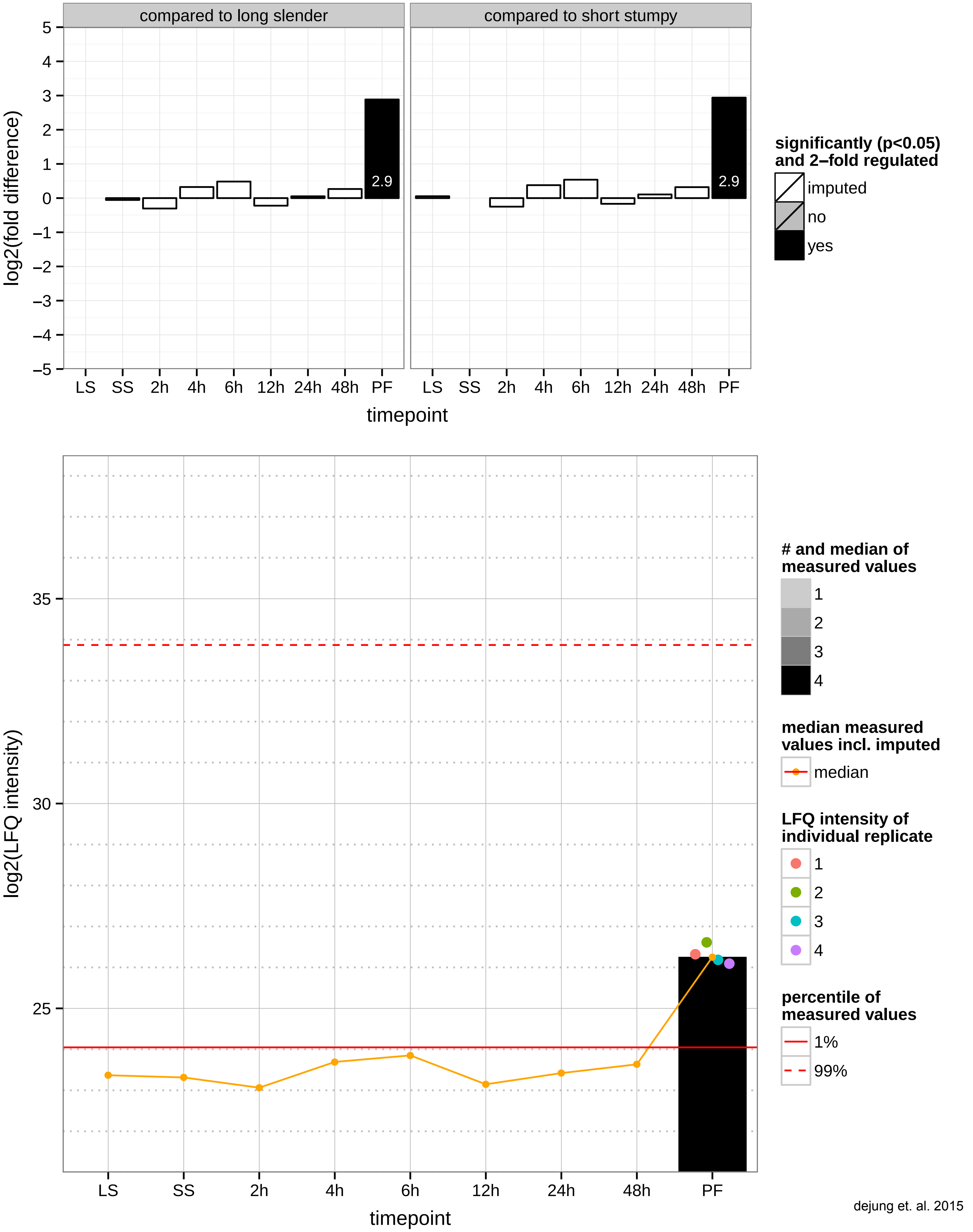

dejung et. al. 2015

hypothetical protein, conserved
Tb927.11.16670
AGOF: zinc ion binding
AGOC: null
AGOP: null
PGOF: zinc ion binding
PGOC: null
PGOP: null

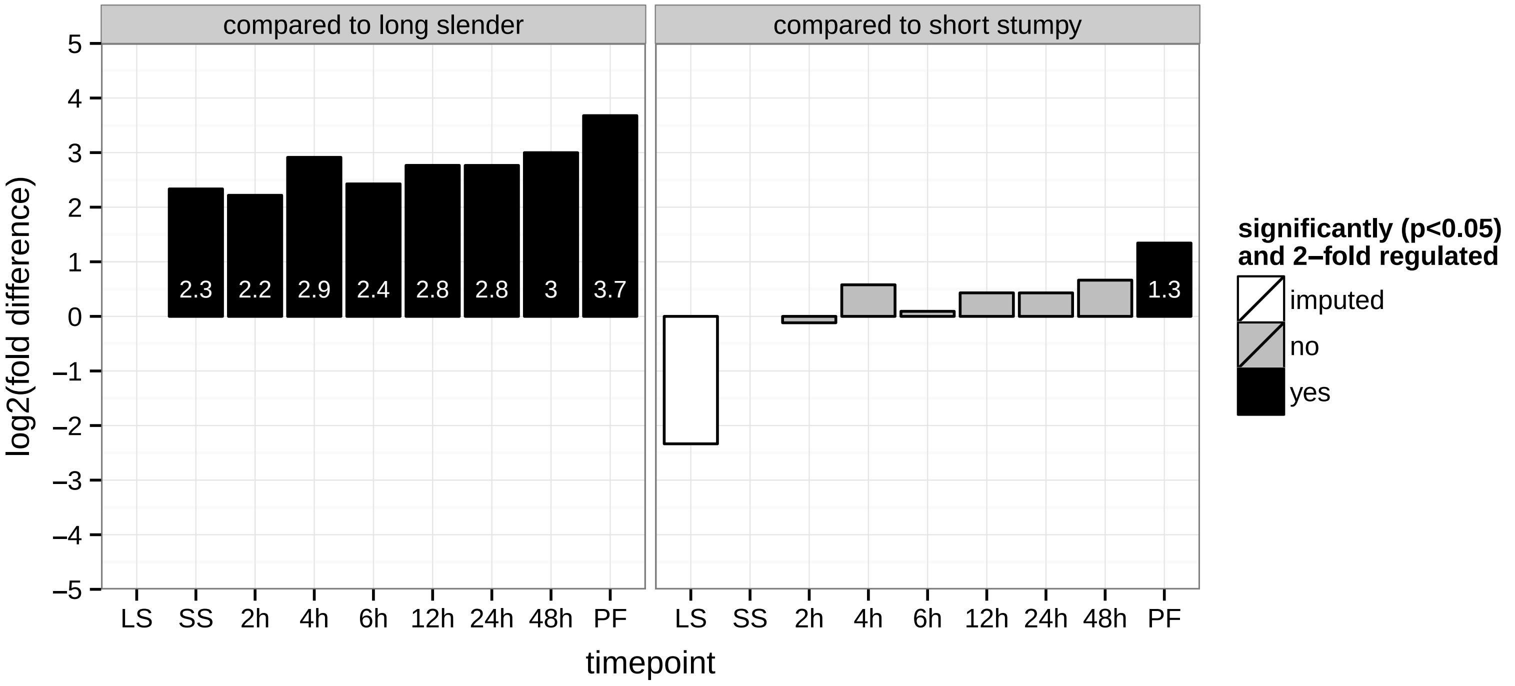

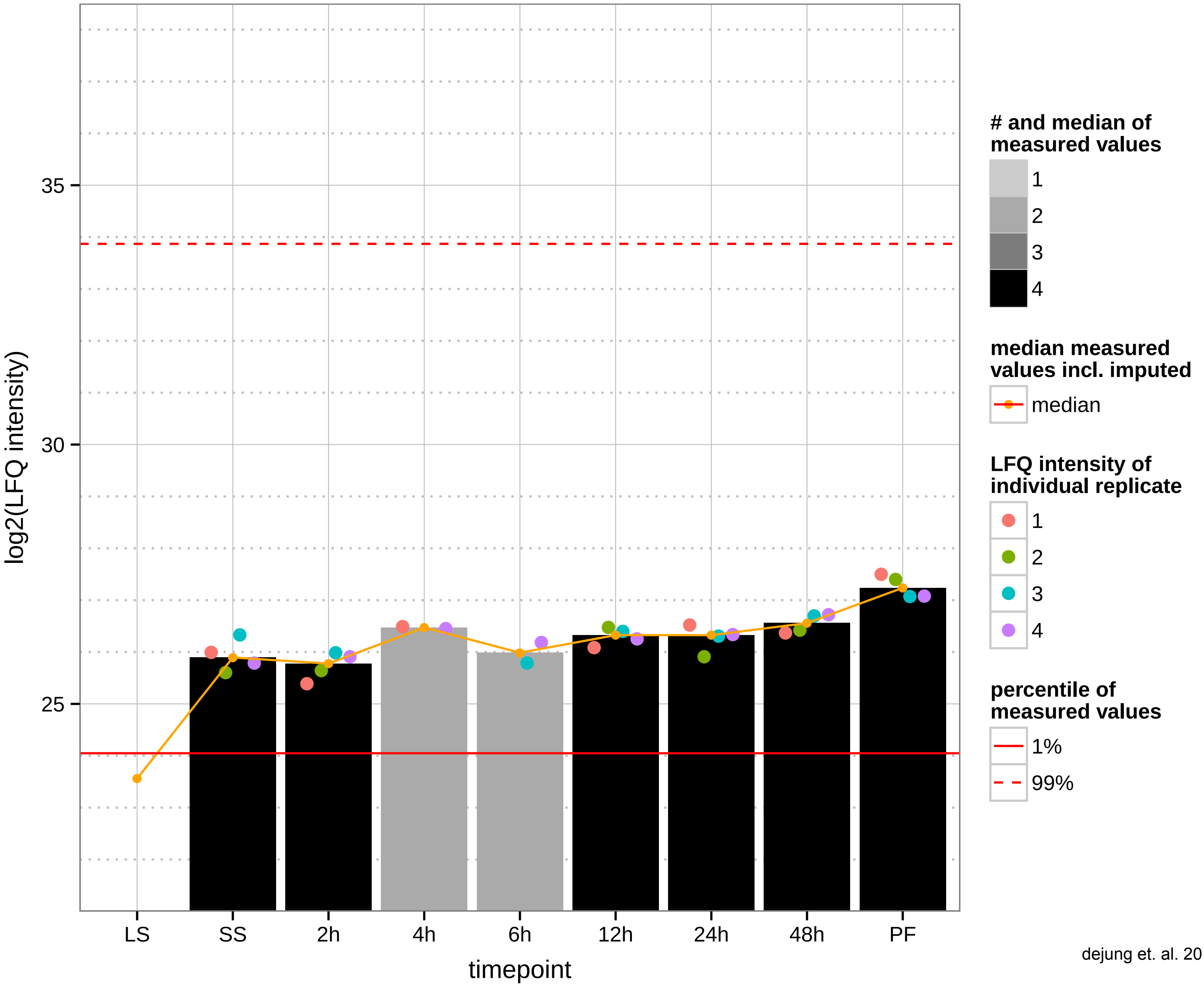

dejung et. al. 2015

mitotic cyclin 6, cyclin 6 (CYC6)
Tb927.11.16720
AGOF: protein binding
AGOC: nuclear cyclin–dependent protein kinase holoenzyme complex, nucleus
AGOP: cytokinesis, regulation of cell cycle
PGOF: protein kinase binding
PGOC: nucleus
PGOP: regulation of cell cycle, regulation of cyclin–dependent protein kinase activity

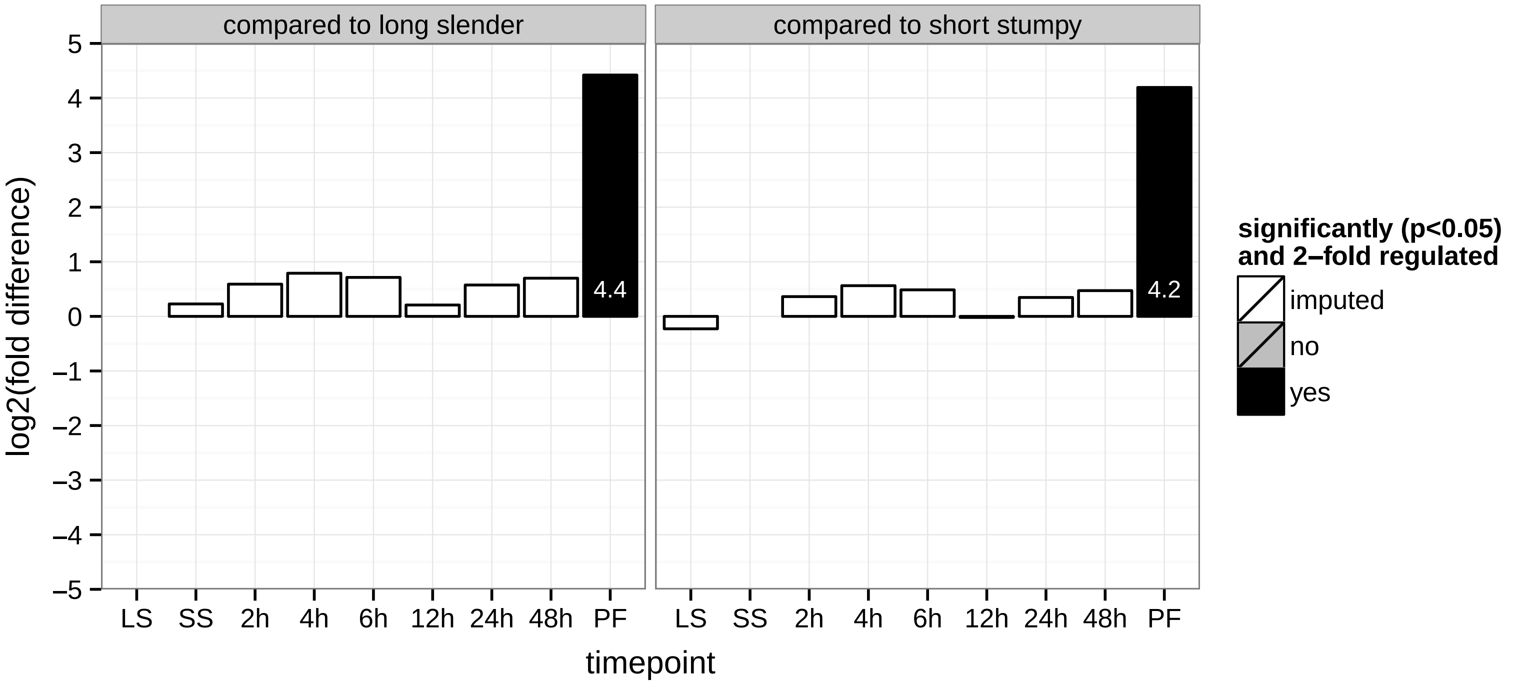

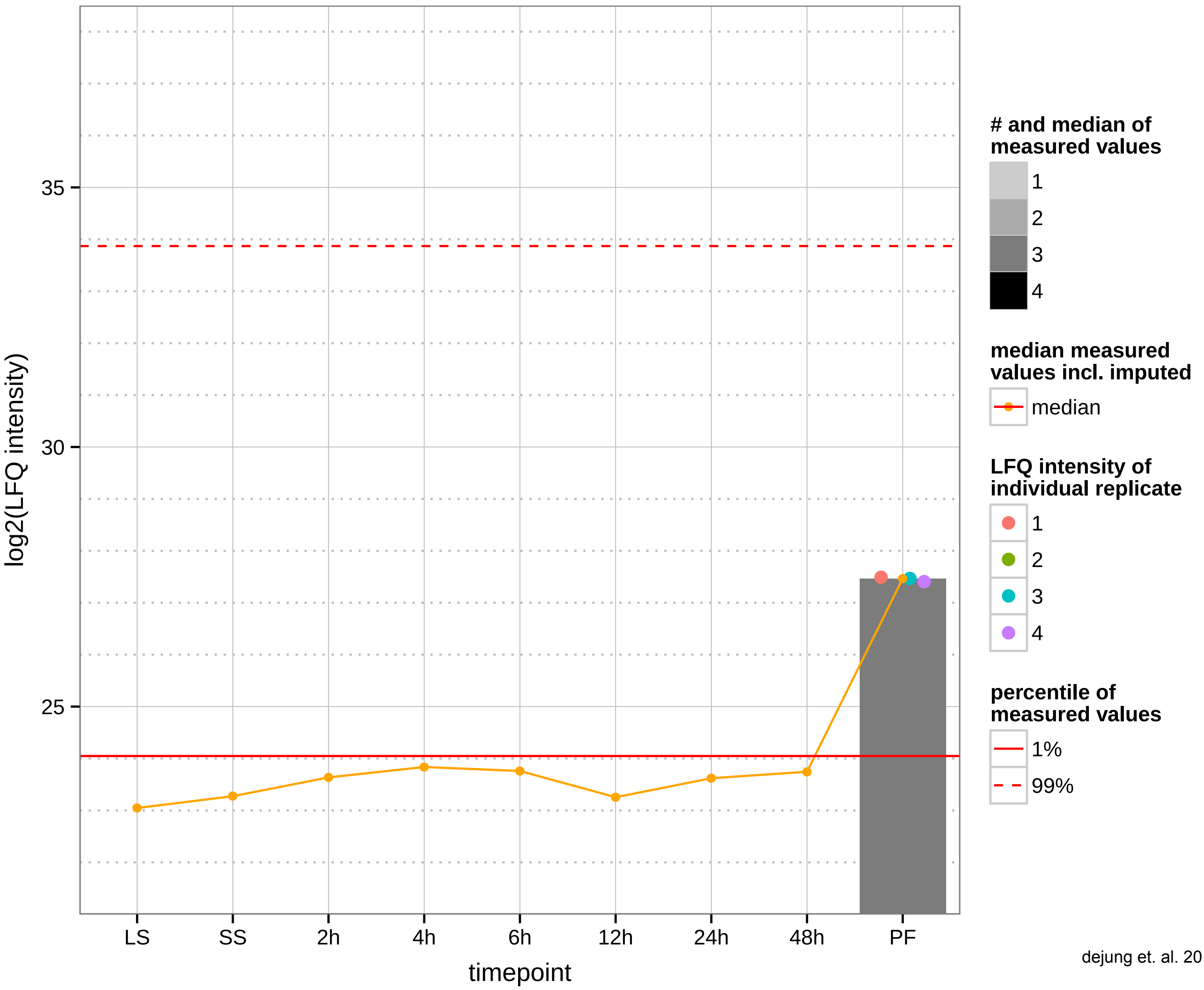

dejung et. al. 2015

hypothetical protein, conserved
Tb927.11.16840.1;Tb927.11.16840.2
AGOF: null
AGOC: null
AGOP: null
PGOF: null
PGOC: null
PGOP: null

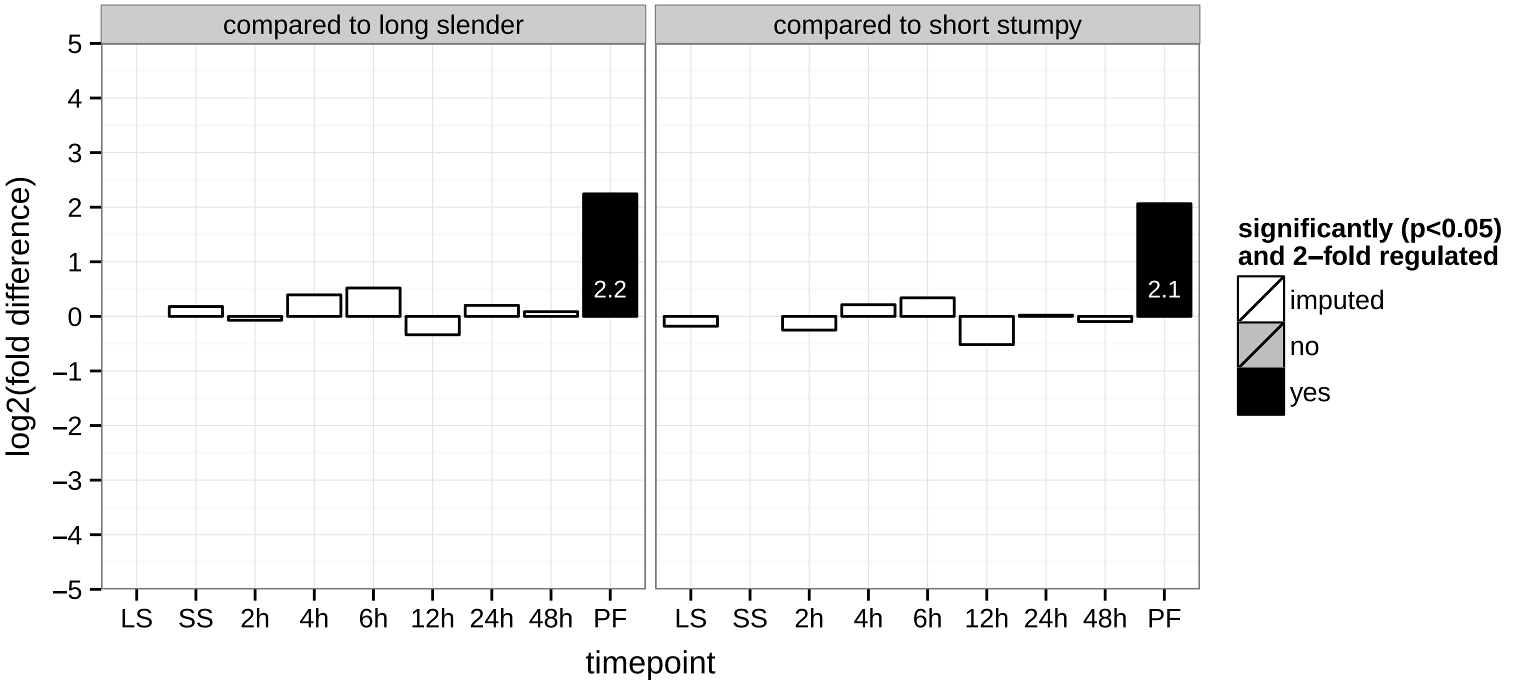

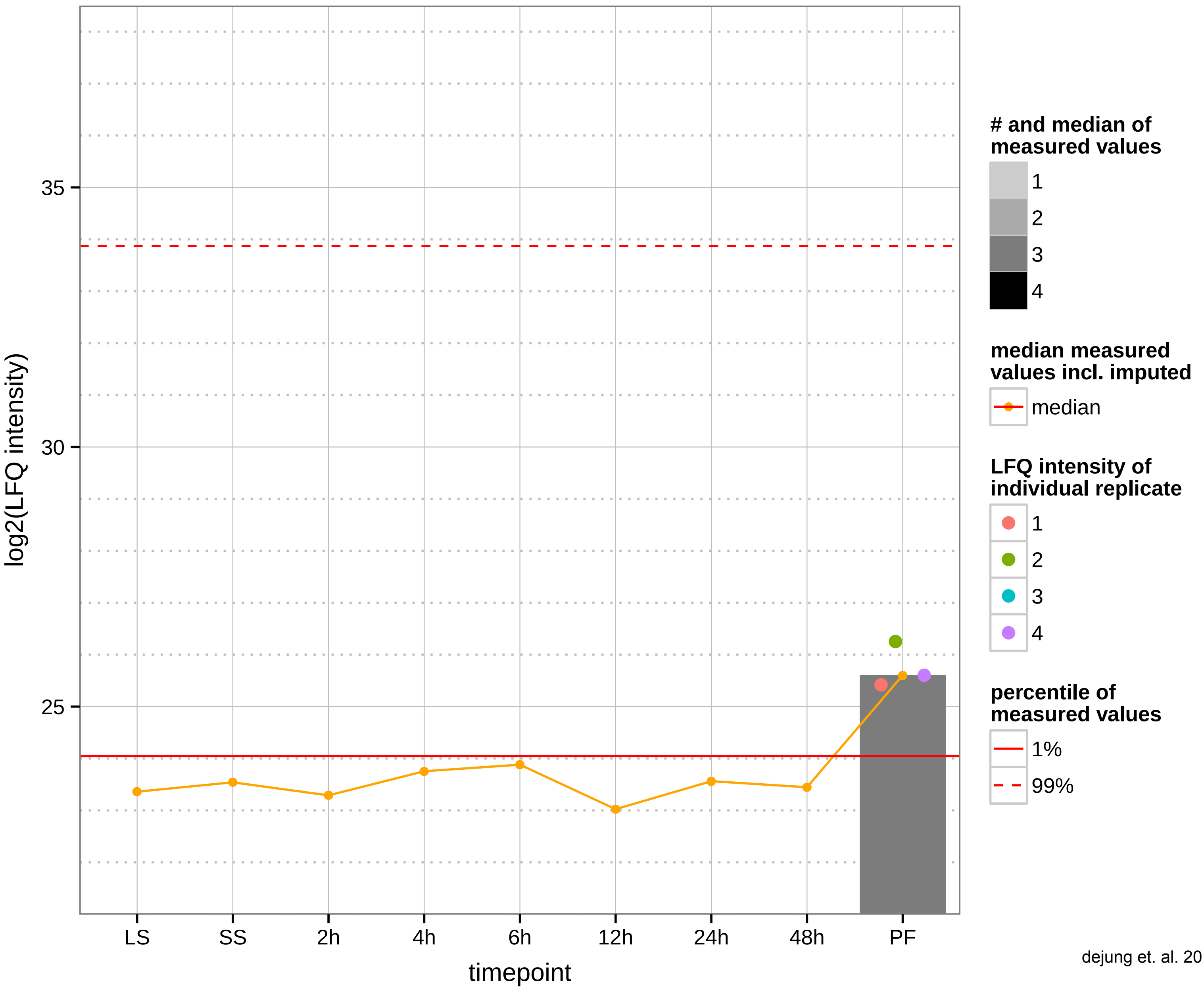

dejung et. al. 2015

hypothetical protein
Tb927.11.1730
AGOF: null
AGOC: null
AGOP: null
PGOF: null
PGOC: null
PGOP: null

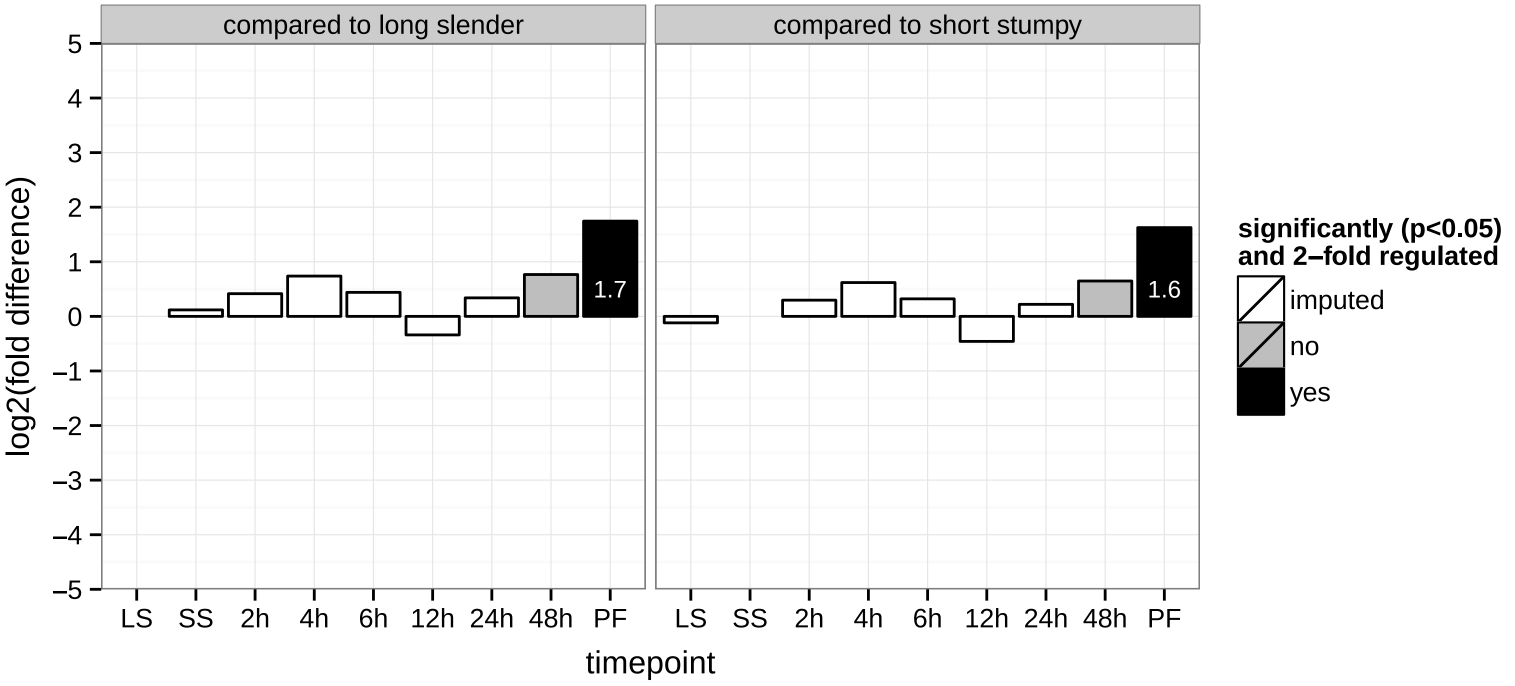

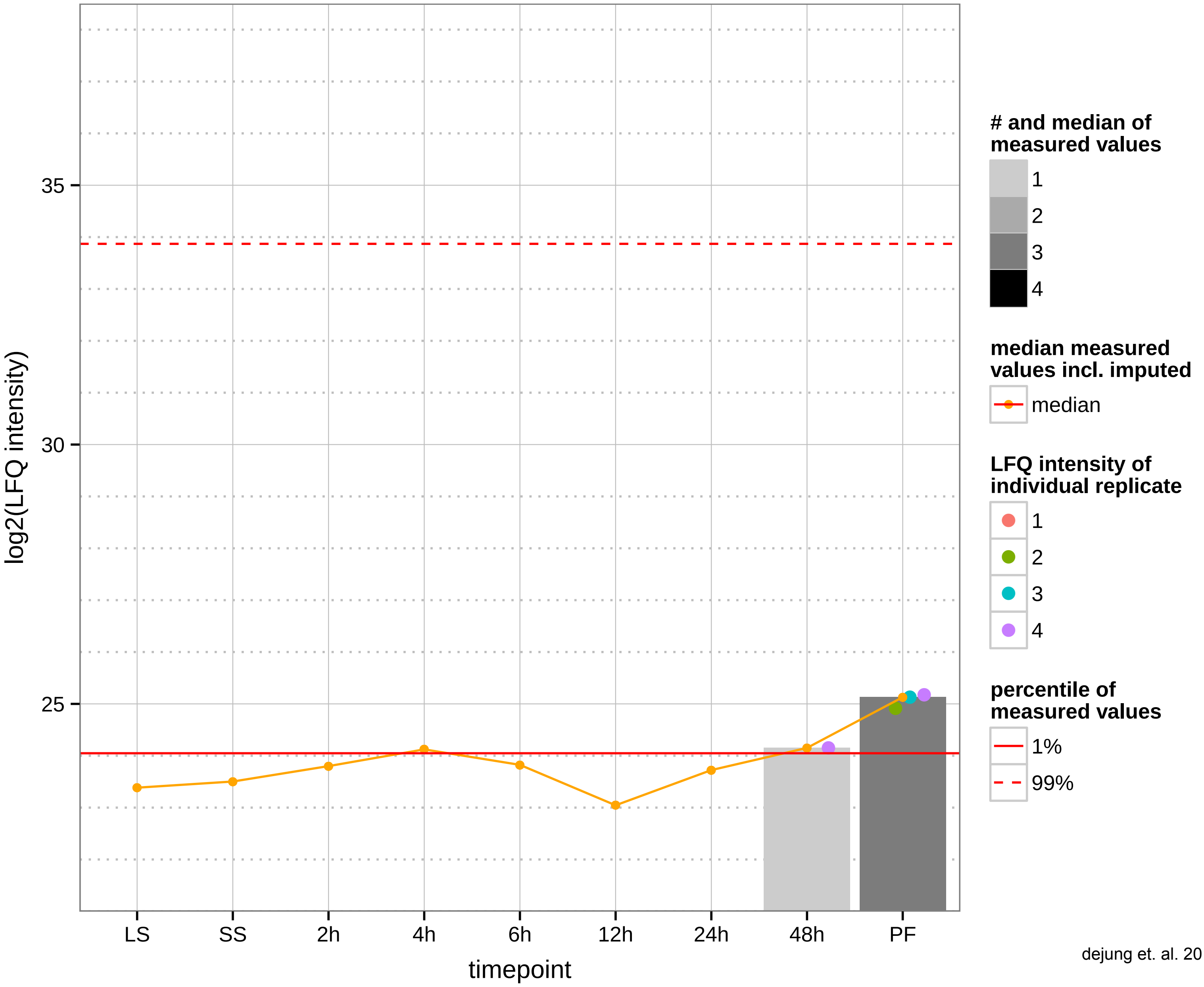

dejung et. al. 2015

hypothetical protein, conserved
Tb927.11.2000
AGOF: null
AGOC: mitochondrion
AGOP: null
PGOF: protein binding
PGOC: null
PGOP: null

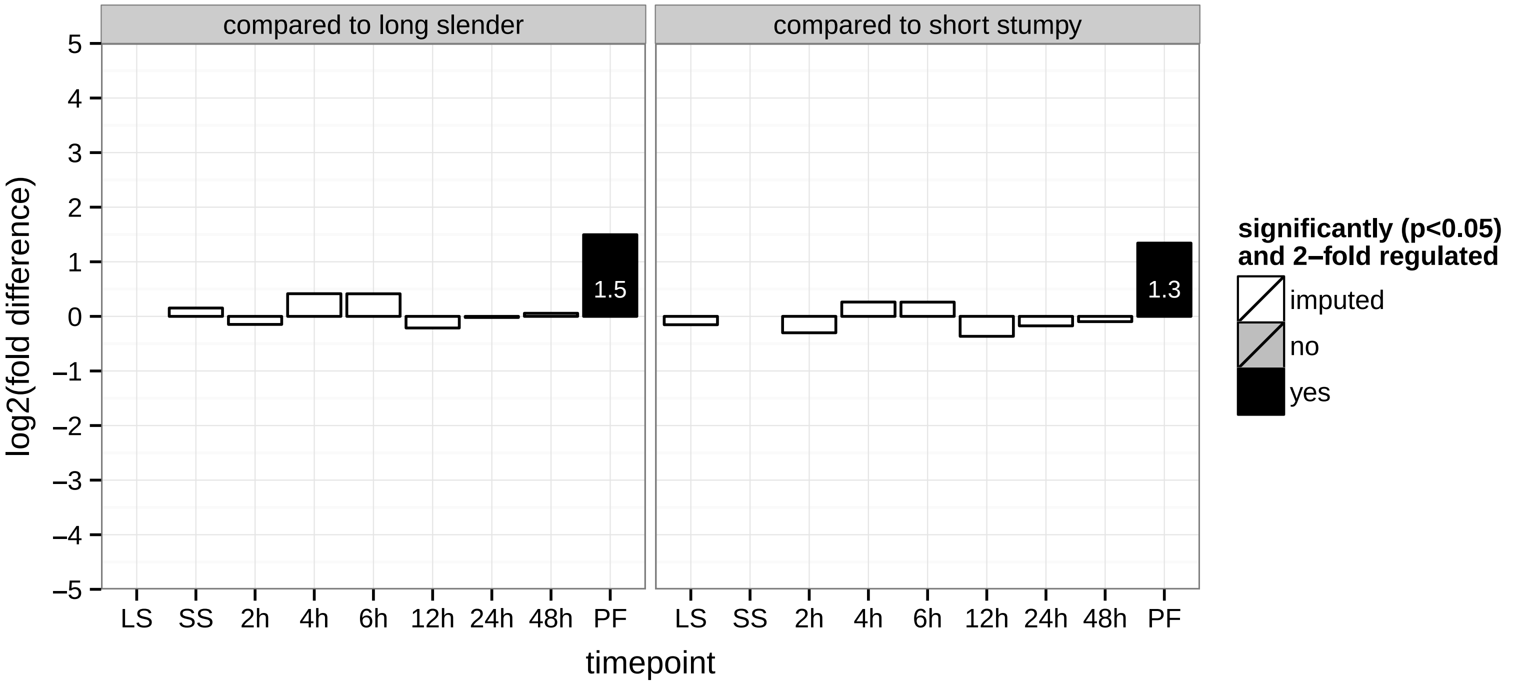

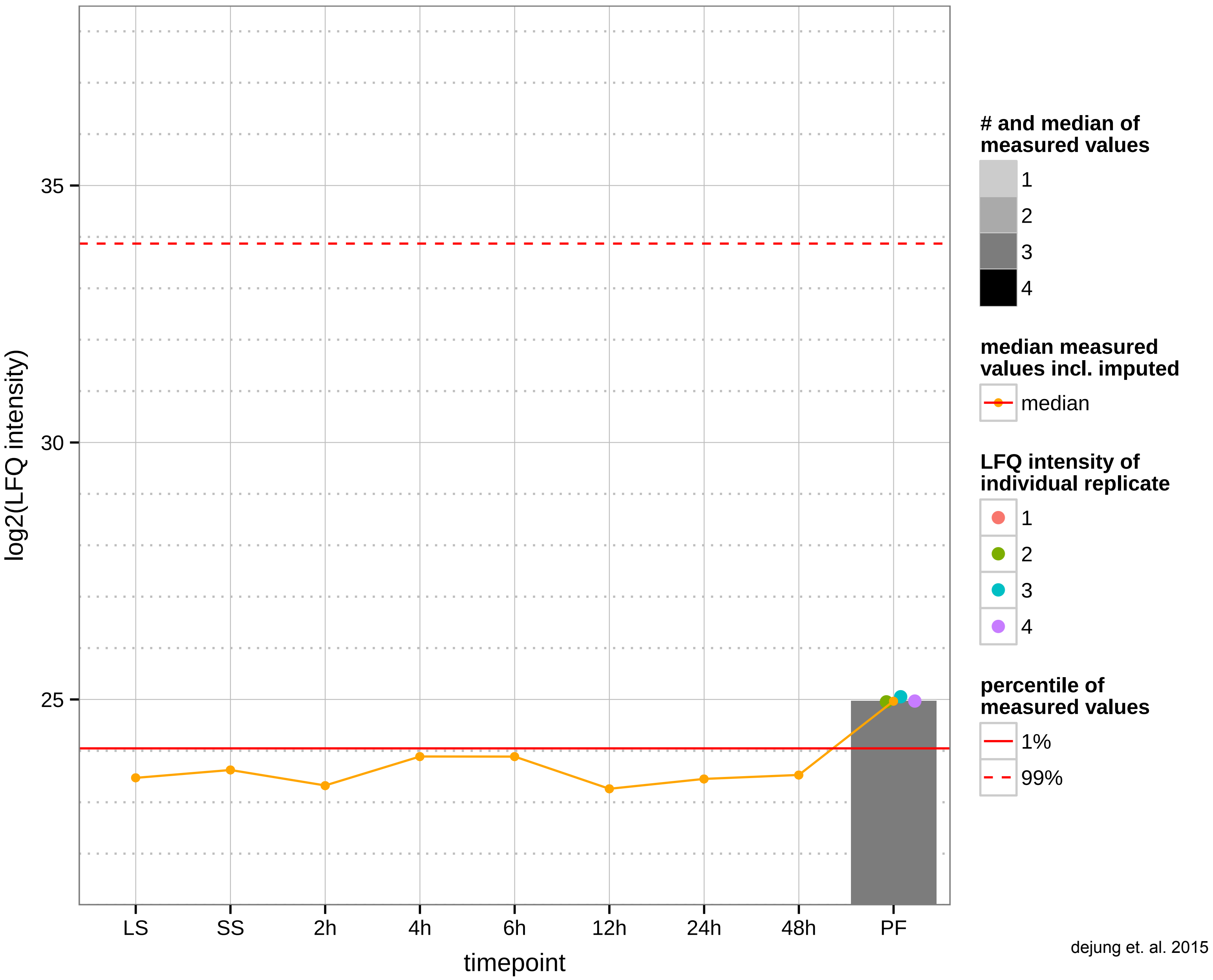

dejung et. al. 2015

carnitine O–acetyltransferase, putative (CAT)
Tb927.11.2230
AGOF: carnitine O–acetyltransferase activity
AGOC: integral to membrane
AGOP: generation of precursor metabolites and energy
PGOF: transferase activity, transferring acyl groups
PGOC: null
PGOP: null

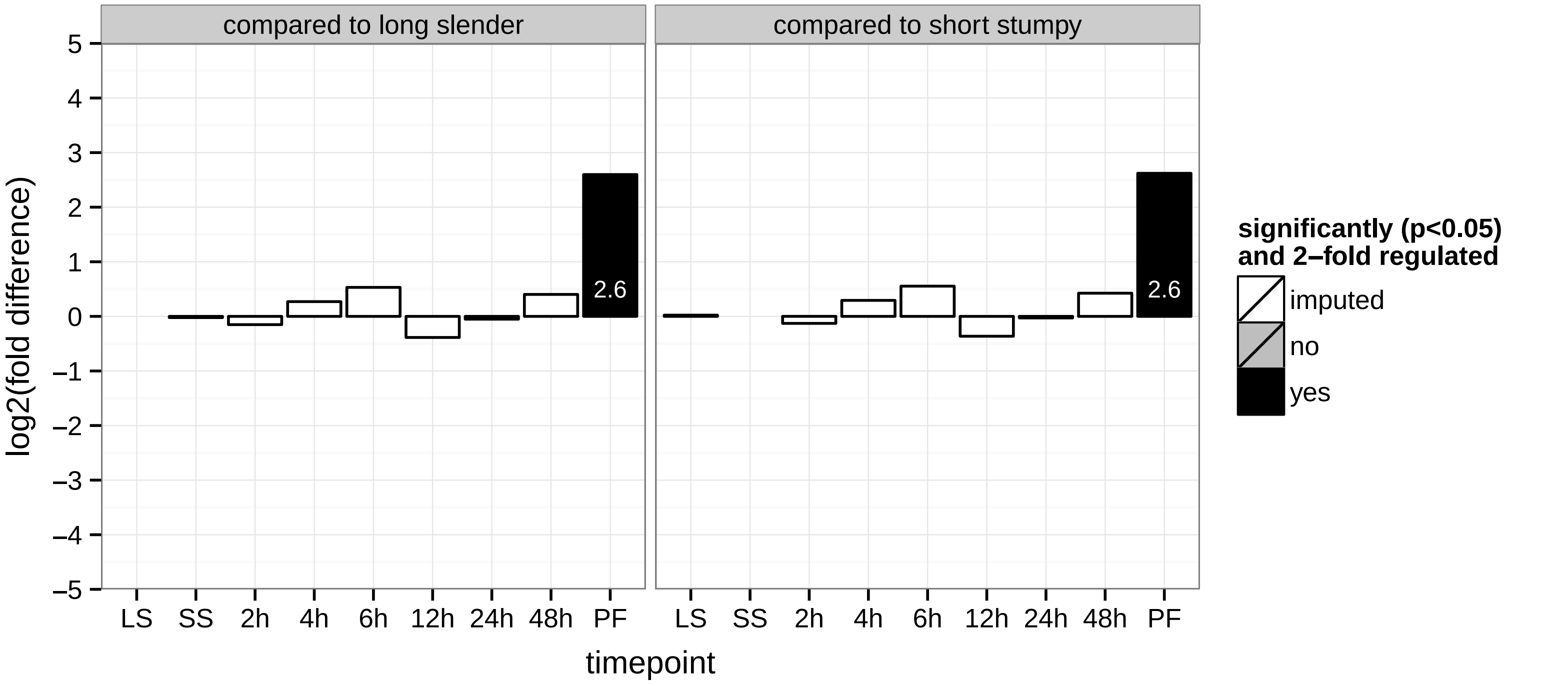

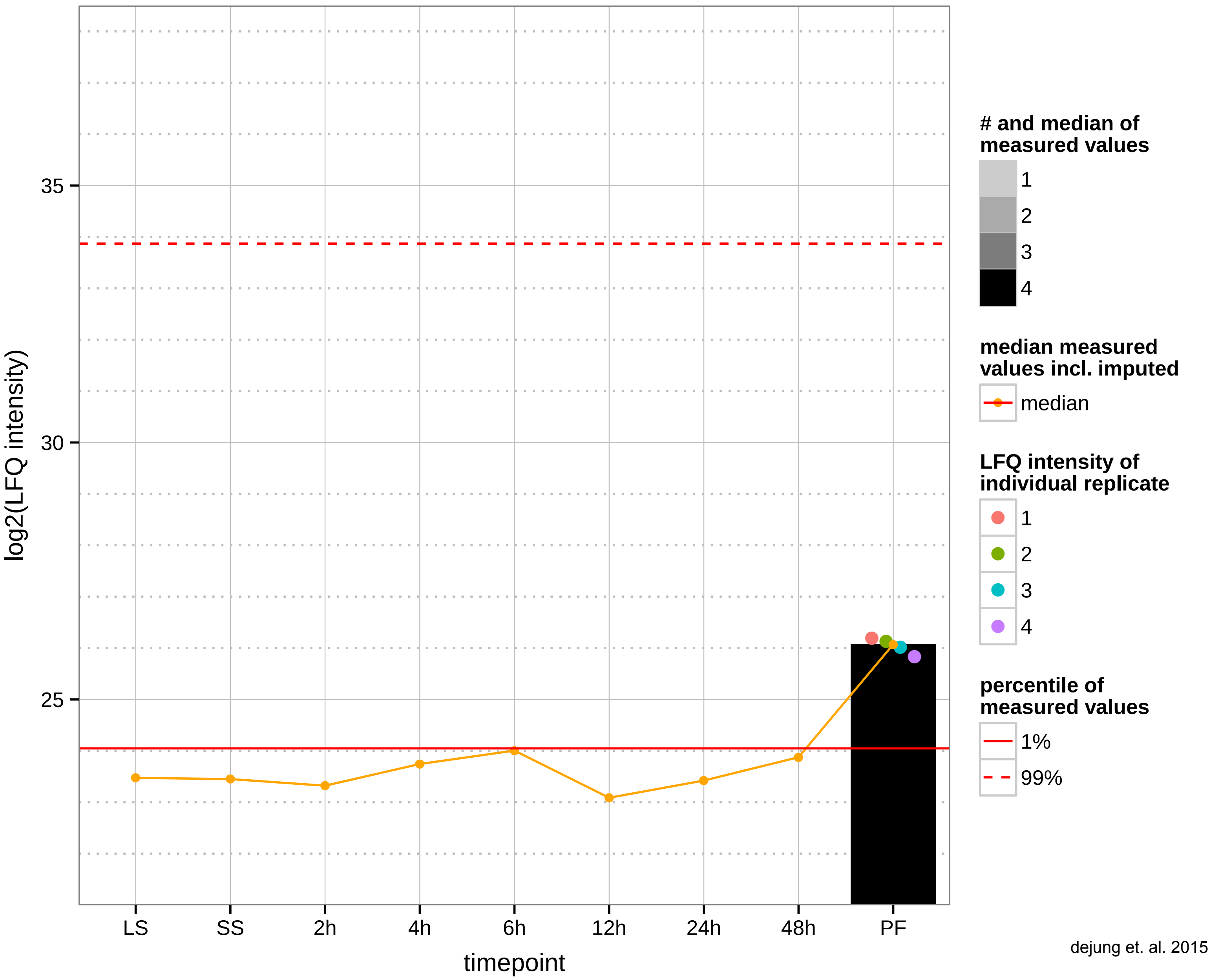

dejung et. al. 2015

hypothetical protein, conserved
Tb927.11.2250
AGOF: null
AGOC: null
AGOP: modulation of development of symbiont involved in interaction with host, quorum sensing involved in interaction with ho
PGOF: null
PGOC: null
PGOP: null

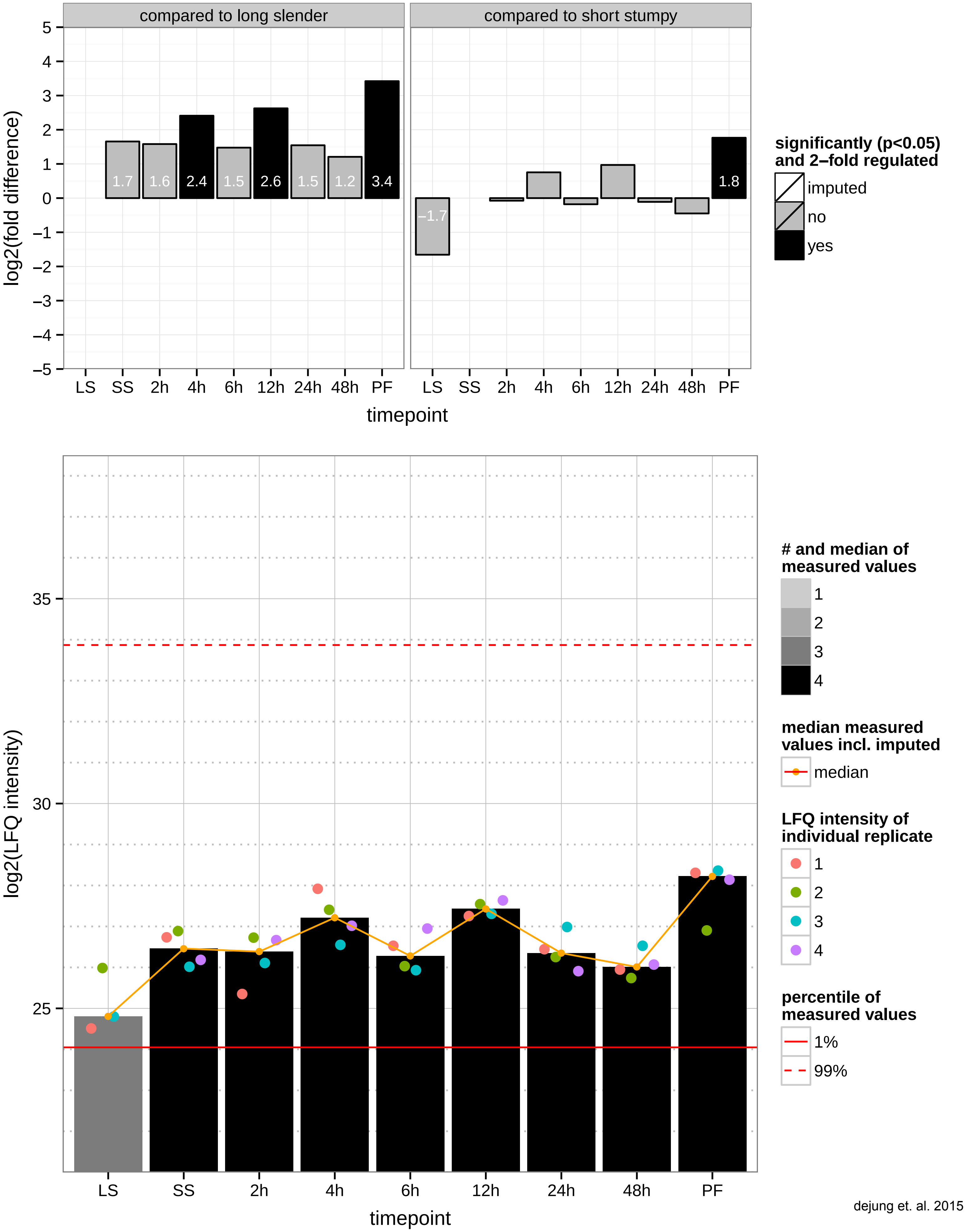

dejung et. al. 2015

aminopeptidase, putative, metallo–peptidase, Clan MF, Family M17
Tb927.11.2470
AGOF: aminopeptidase activity
AGOC: intracellular
AGOP: proteolysis
PGOF: aminopeptidase activity
PGOC: intracellular
PGOP: proteolysis

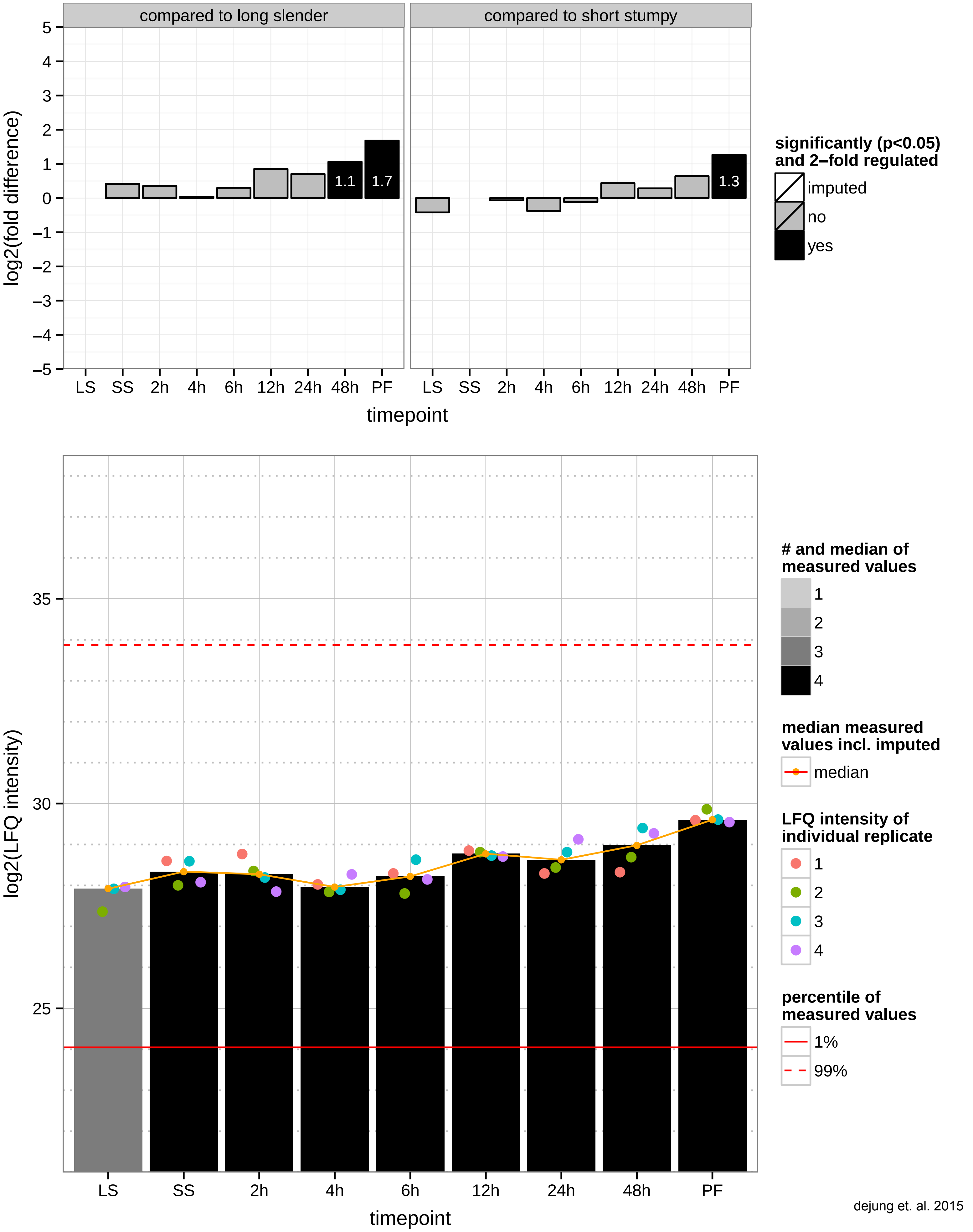

dejung et. al. 2015

carboxypeptidase, putative, metallo–peptidase, Clan MA(E) Family M32
Tb927.11.2500
AGOF: metallocarboxypeptidase activity
AGOC: cytosol
AGOP: peptide metabolic process, proteolysis
PGOF: metallocarboxypeptidase activity
PGOC: null
PGOP: proteolysis

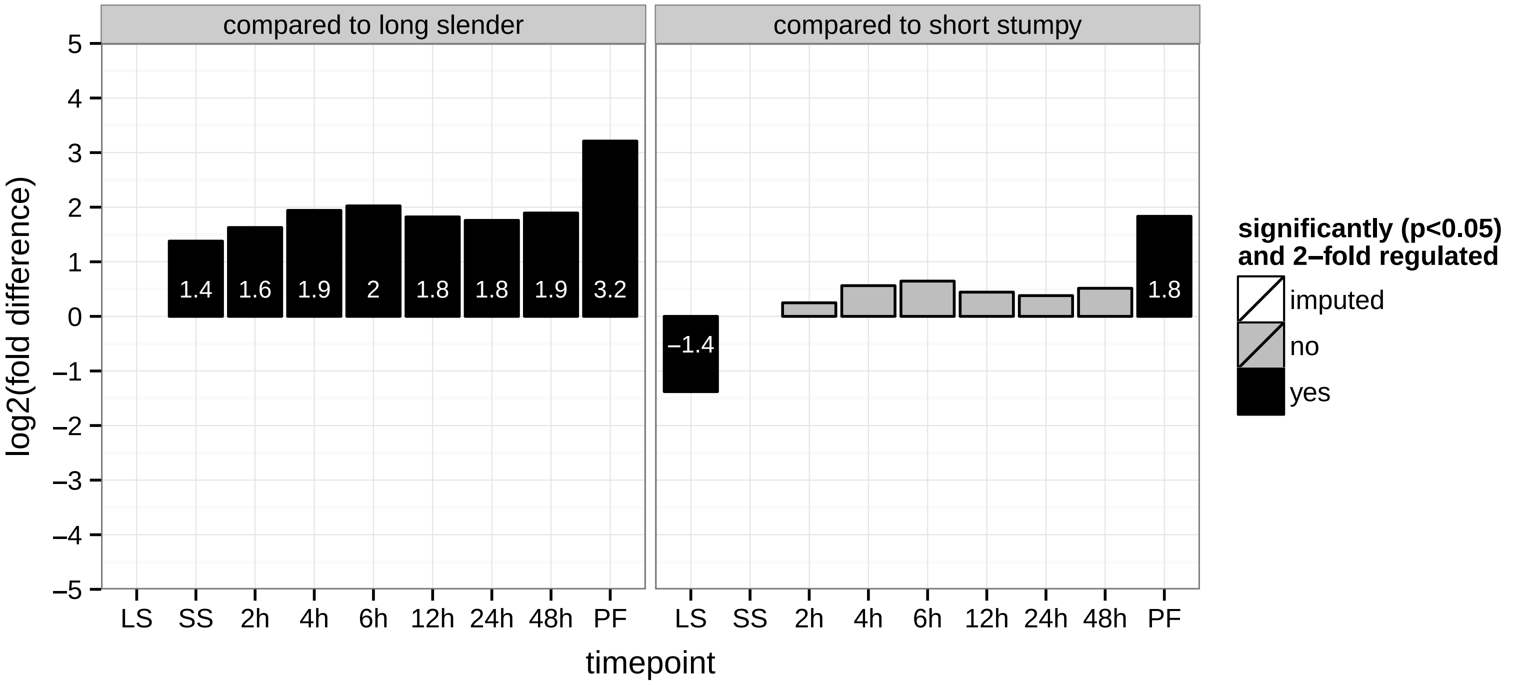

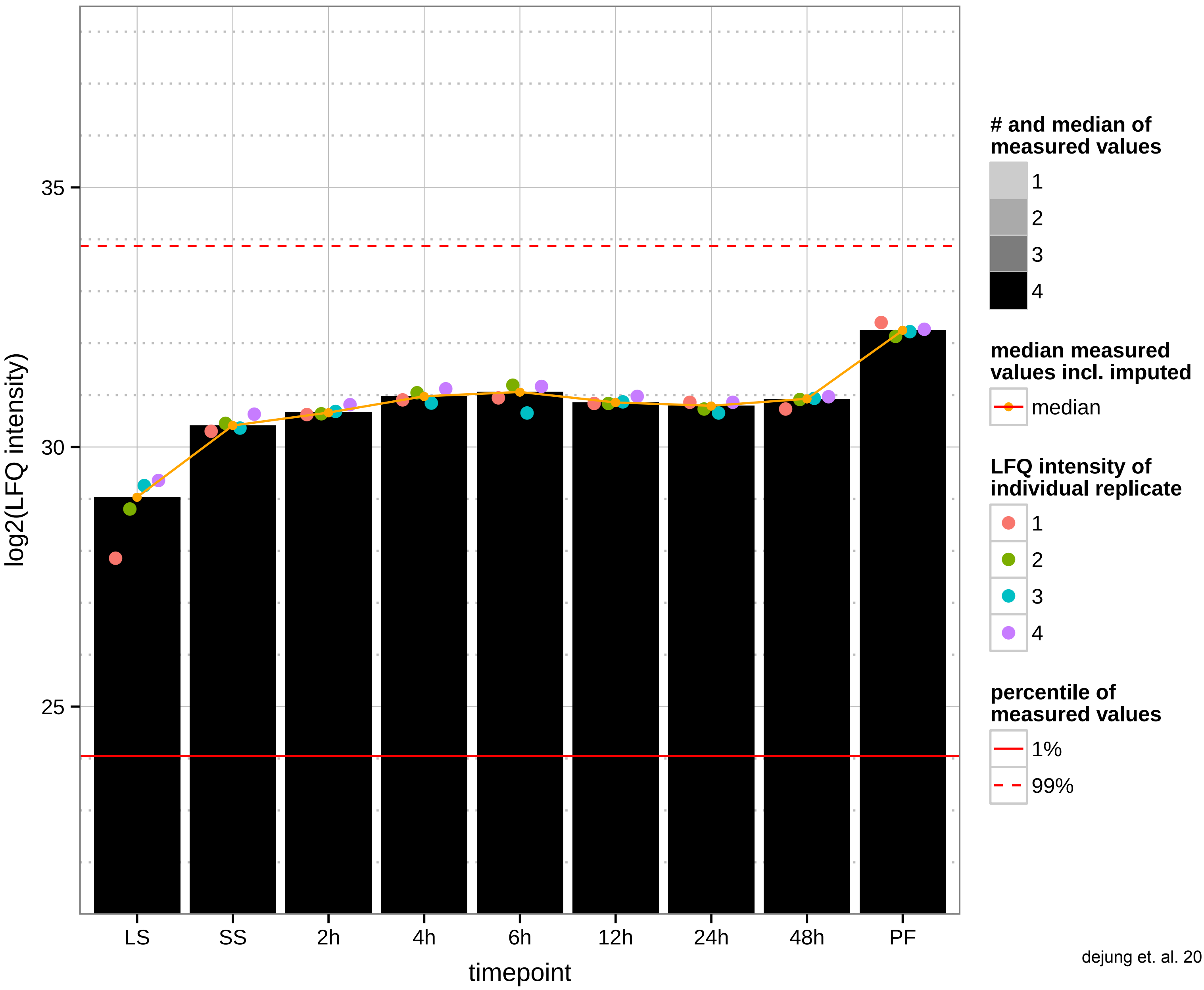

dejung et. al. 2015

mitochondrial carrier protein (MCP10)
Tb927.11.270
AGOF: transporter activity
AGOC: mitochondrial inner membrane, mitochondrial part
AGOP: transport
PGOF: null
PGOC: null
PGOP: null

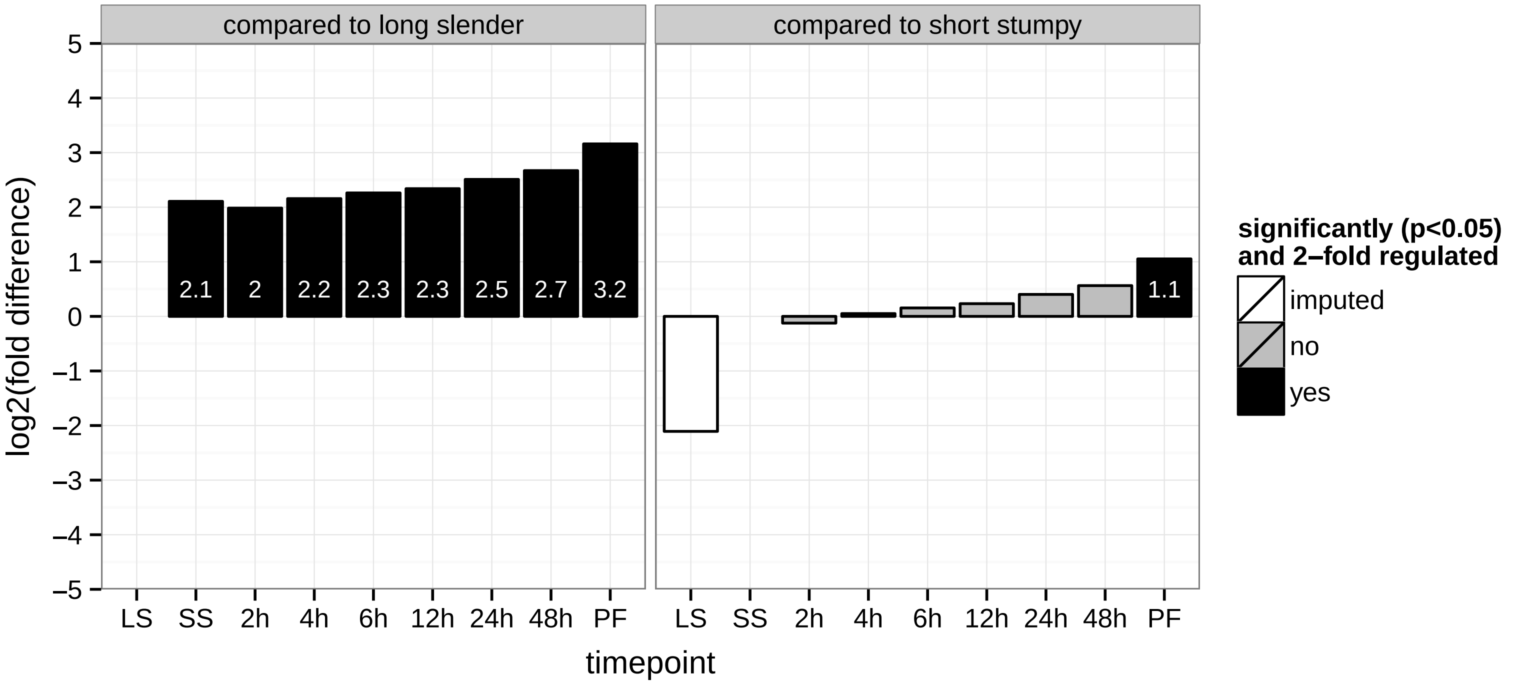

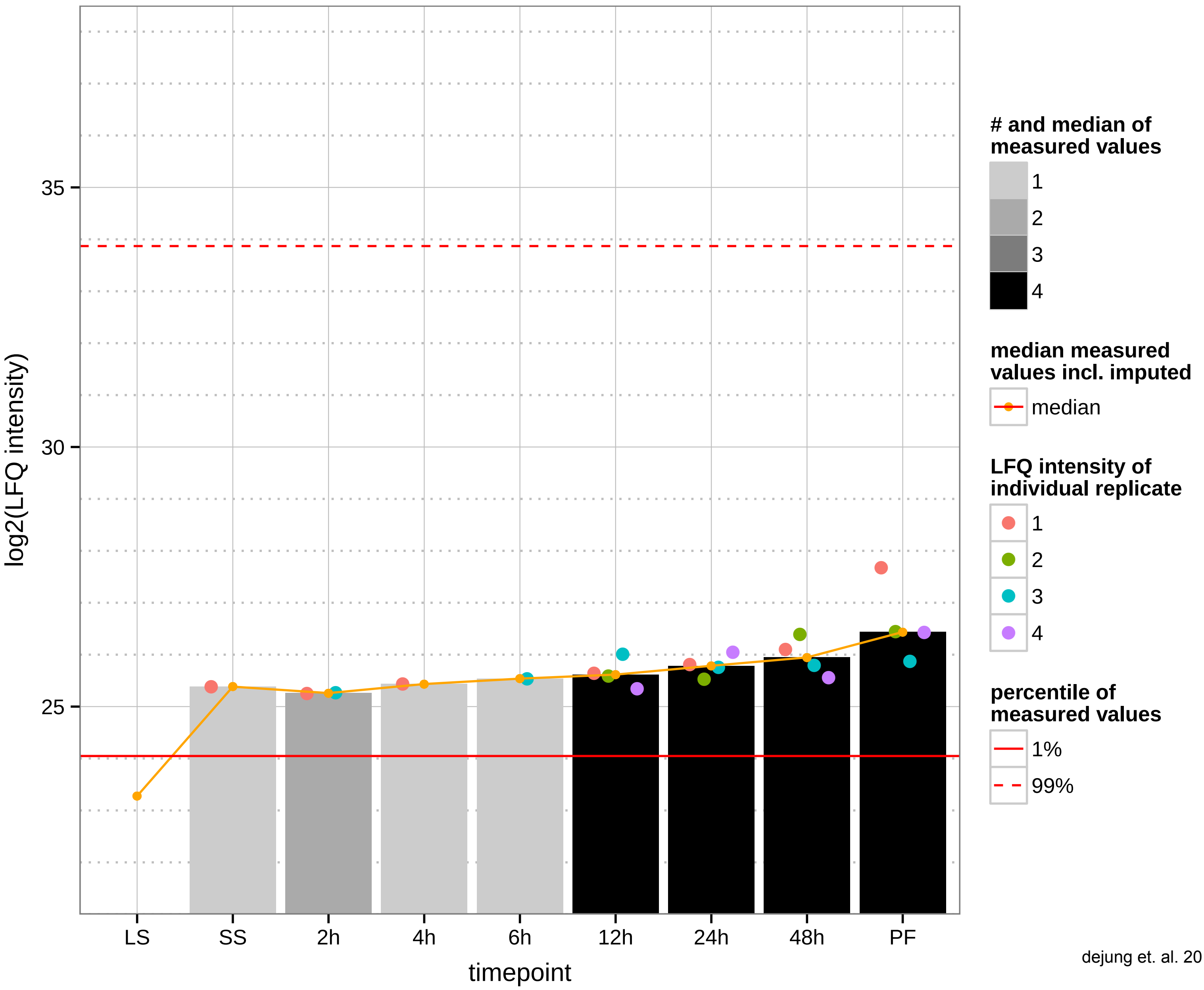

dejung et. al. 2015

hypothetical protein, conserved
Tb927.11.2940
AGOF: null
AGOC: null
AGOP: null
PGOF: binding
PGOC: null
PGOP: null

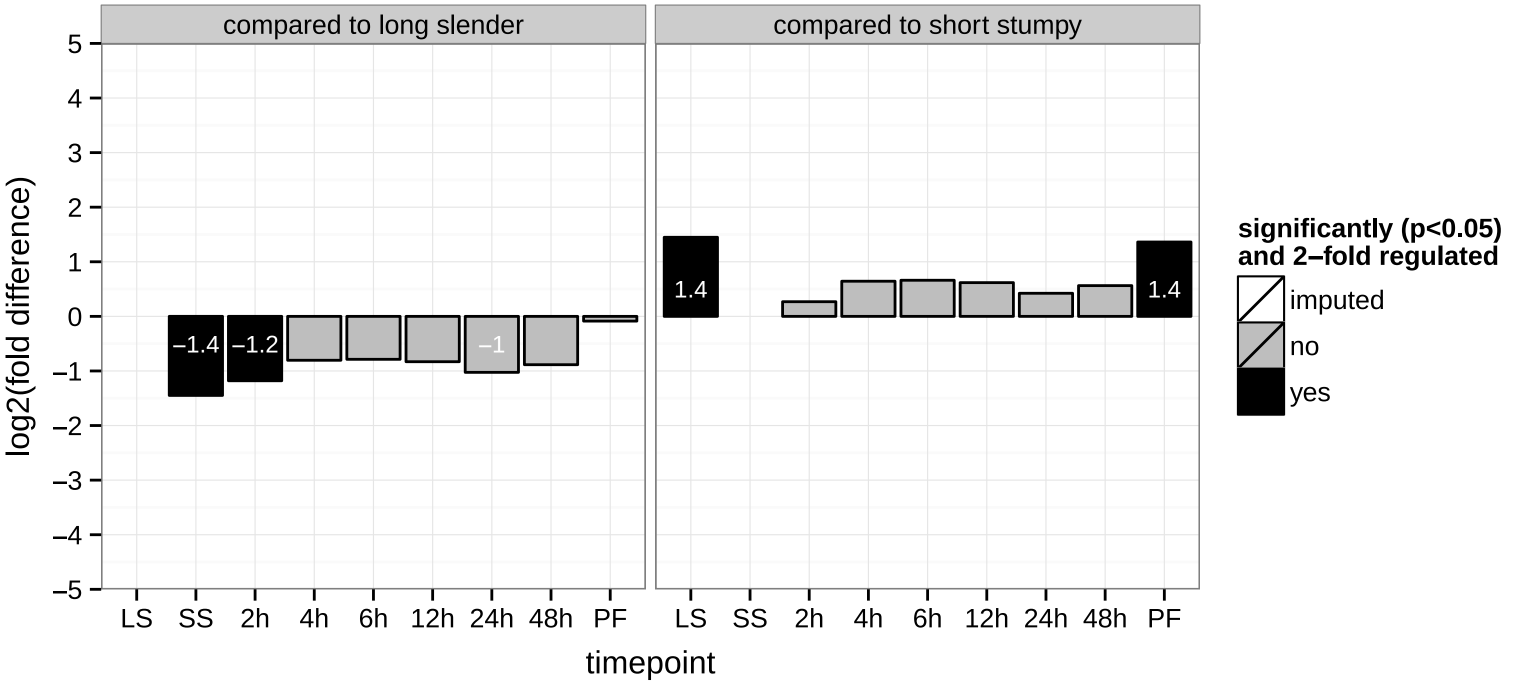

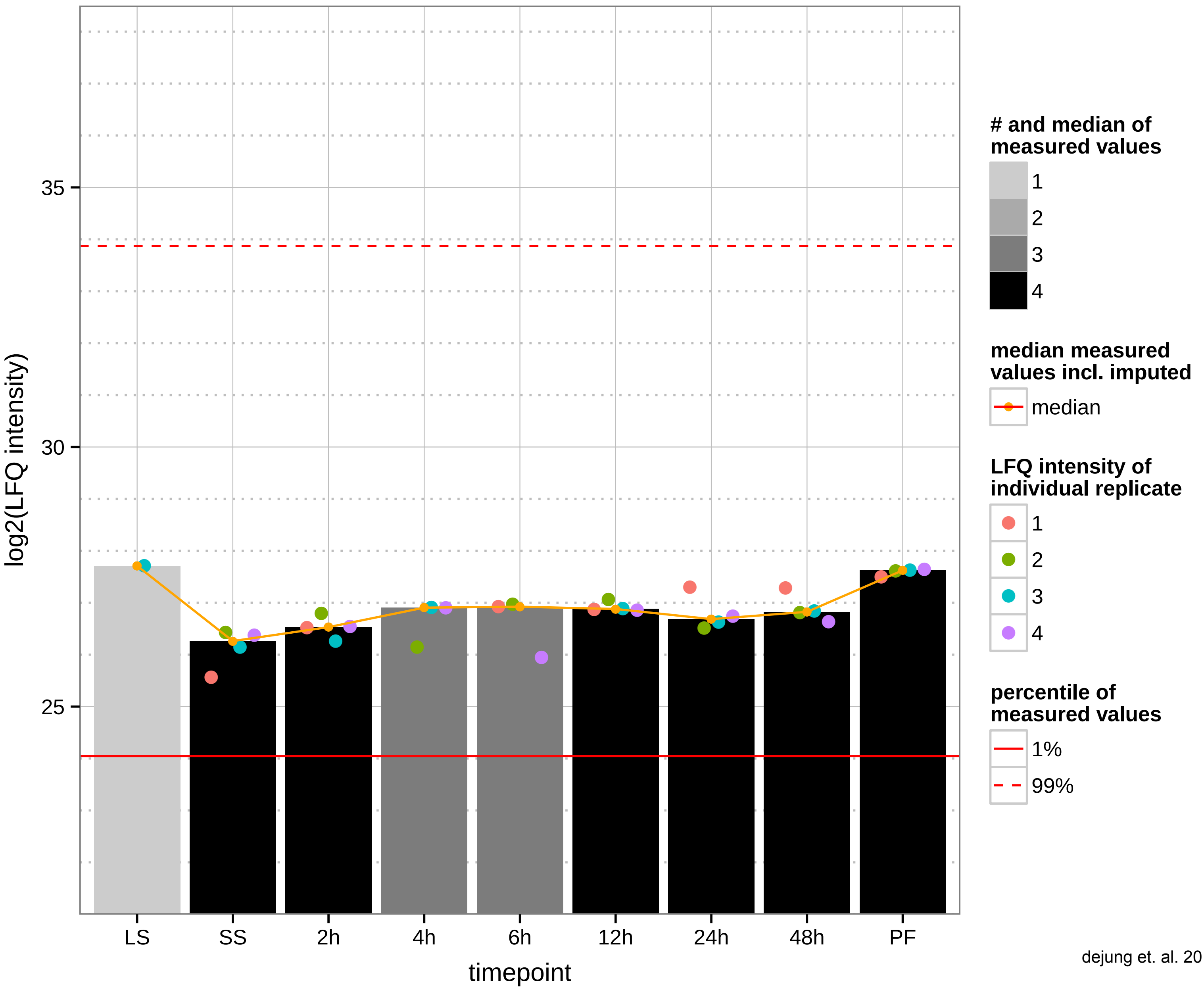

dejung et. al. 2015

hypothetical protein, conserved
Tb927.11.3060
AGOF: null
AGOC: null
AGOP: null
PGOF: null
PGOC: null
PGOP: null

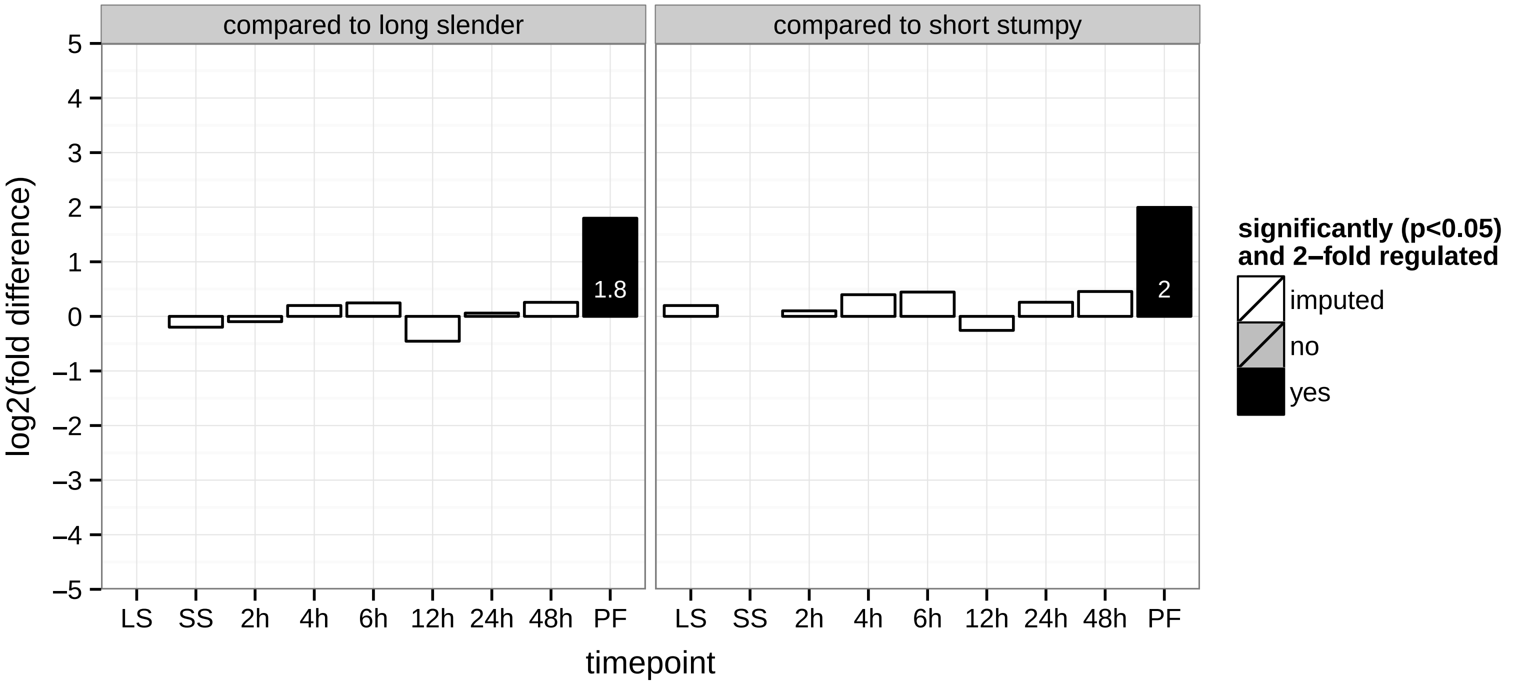

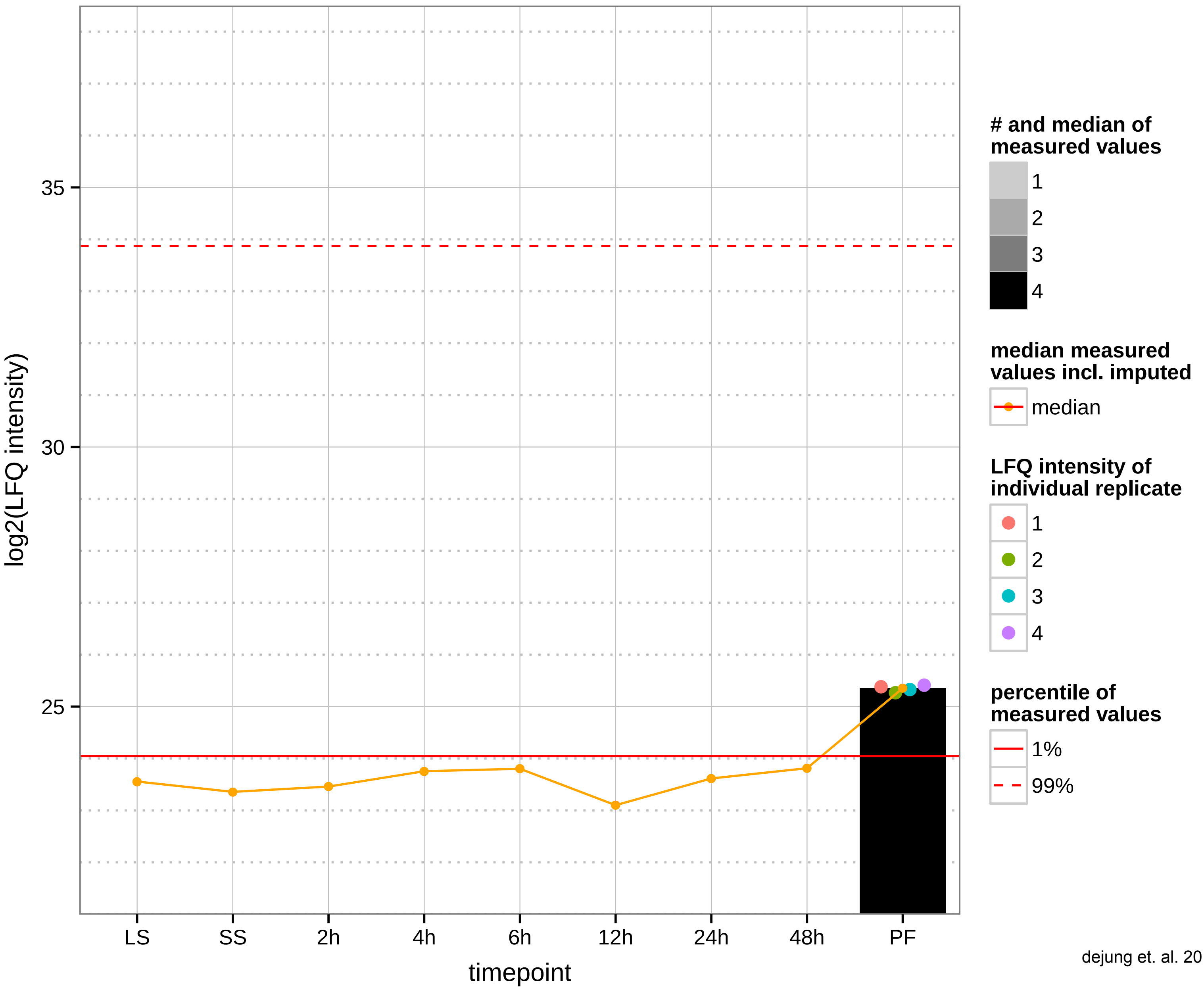

dejung et. al. 2015

hypothetical protein, conserved
Tb927.11.3110
AGOF: null
AGOC: null
AGOP: null
PGOF: protein binding
PGOC: null
PGOP: null

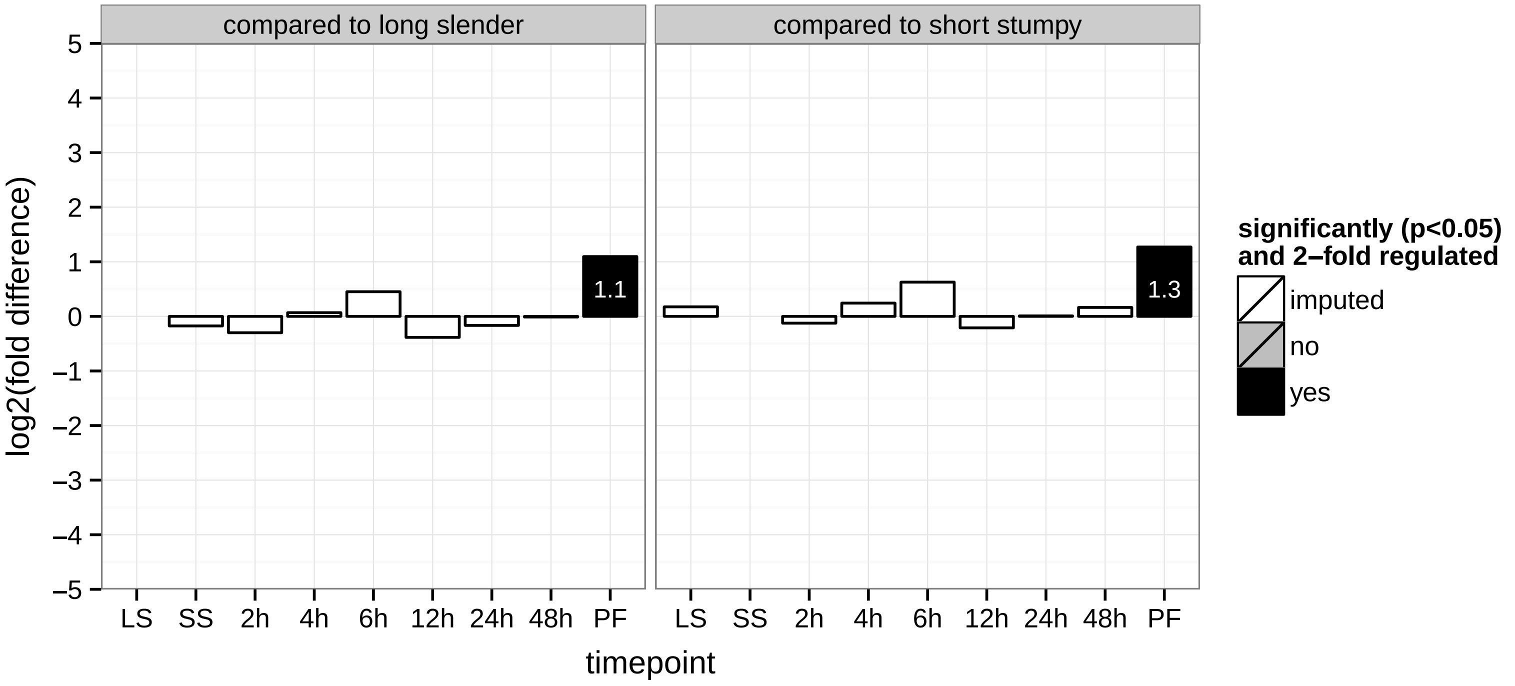

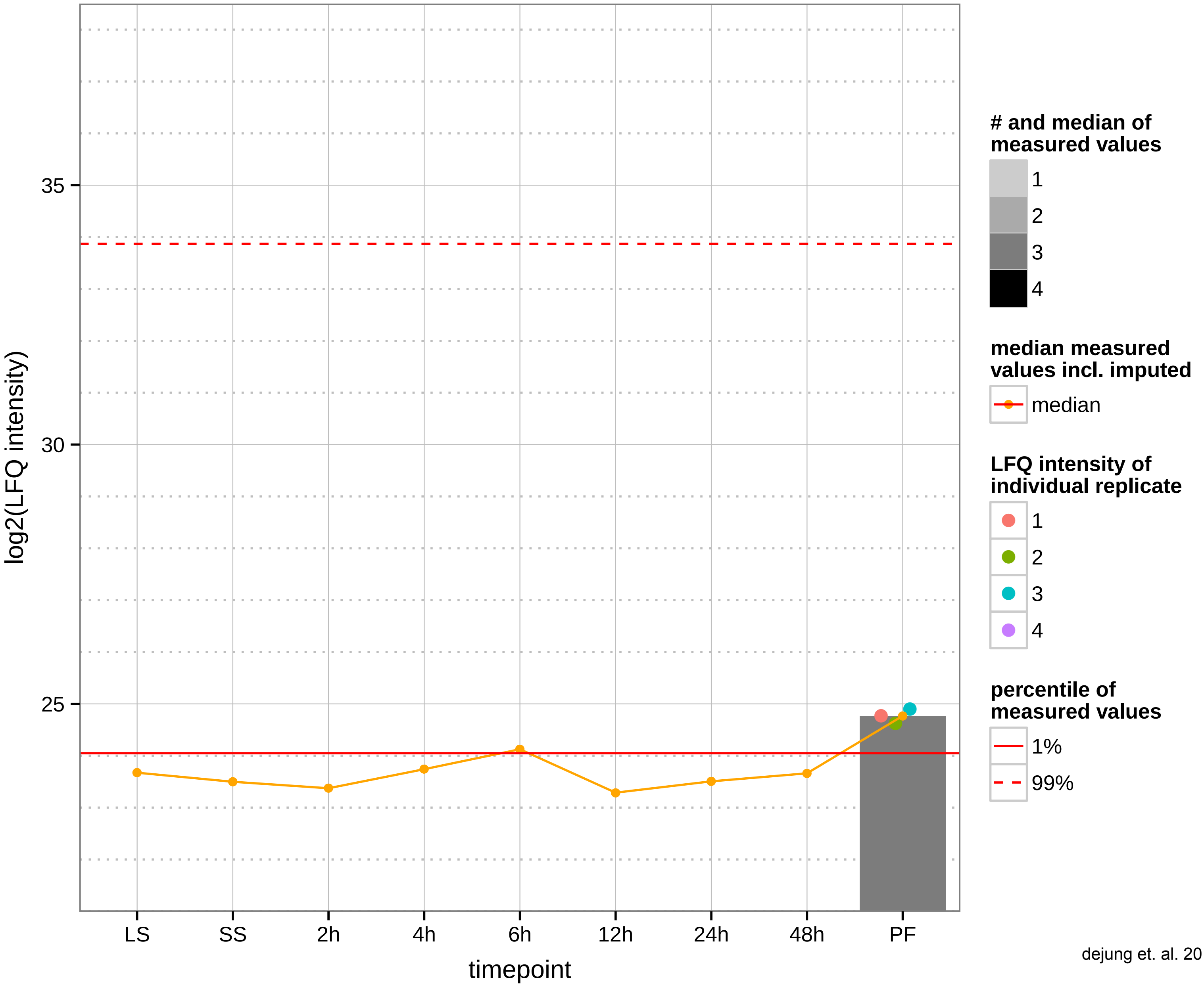

dejung et. al. 2015

Nucleoporin, Myosin–like protein (TbMlp–1)
Tb927.11.330
AGOF: null
AGOC: nuclear pore
AGOP: null
PGOF: null
PGOC: null
PGOP: null

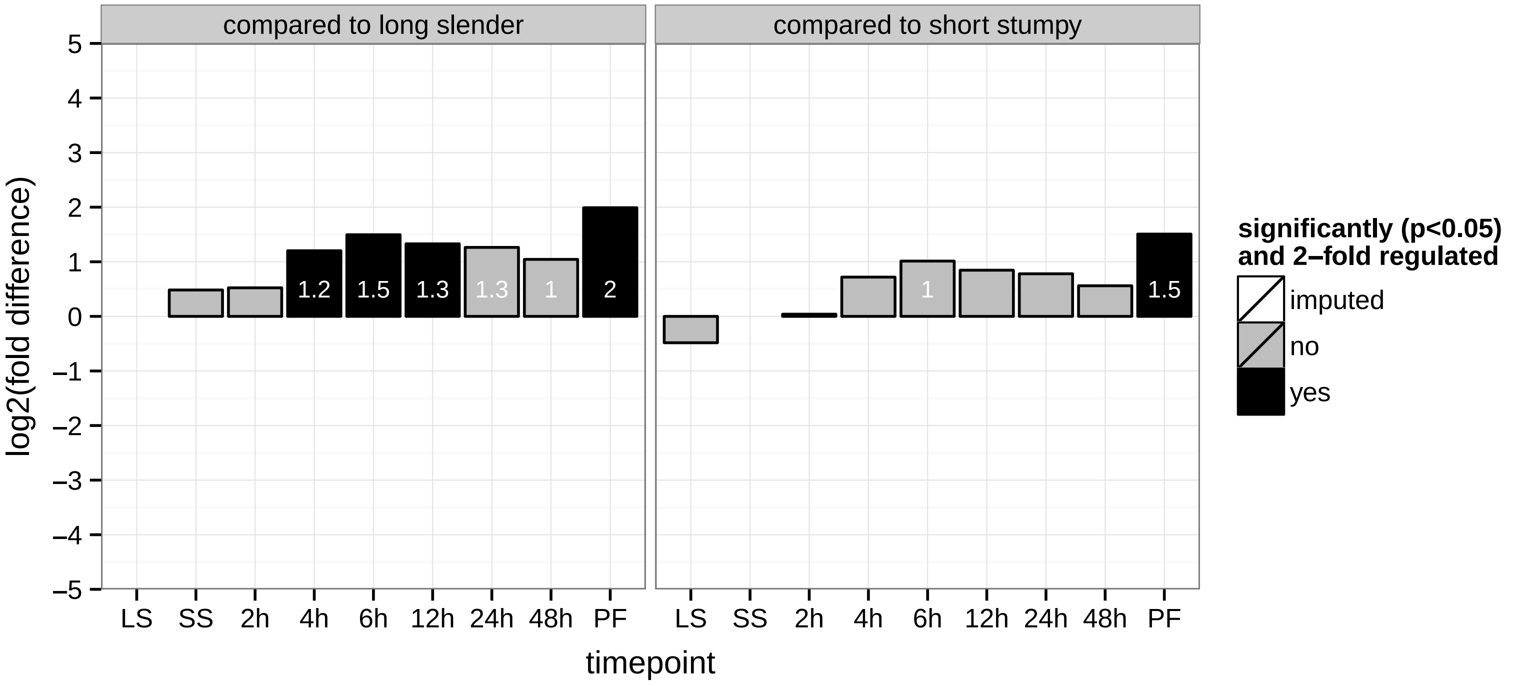

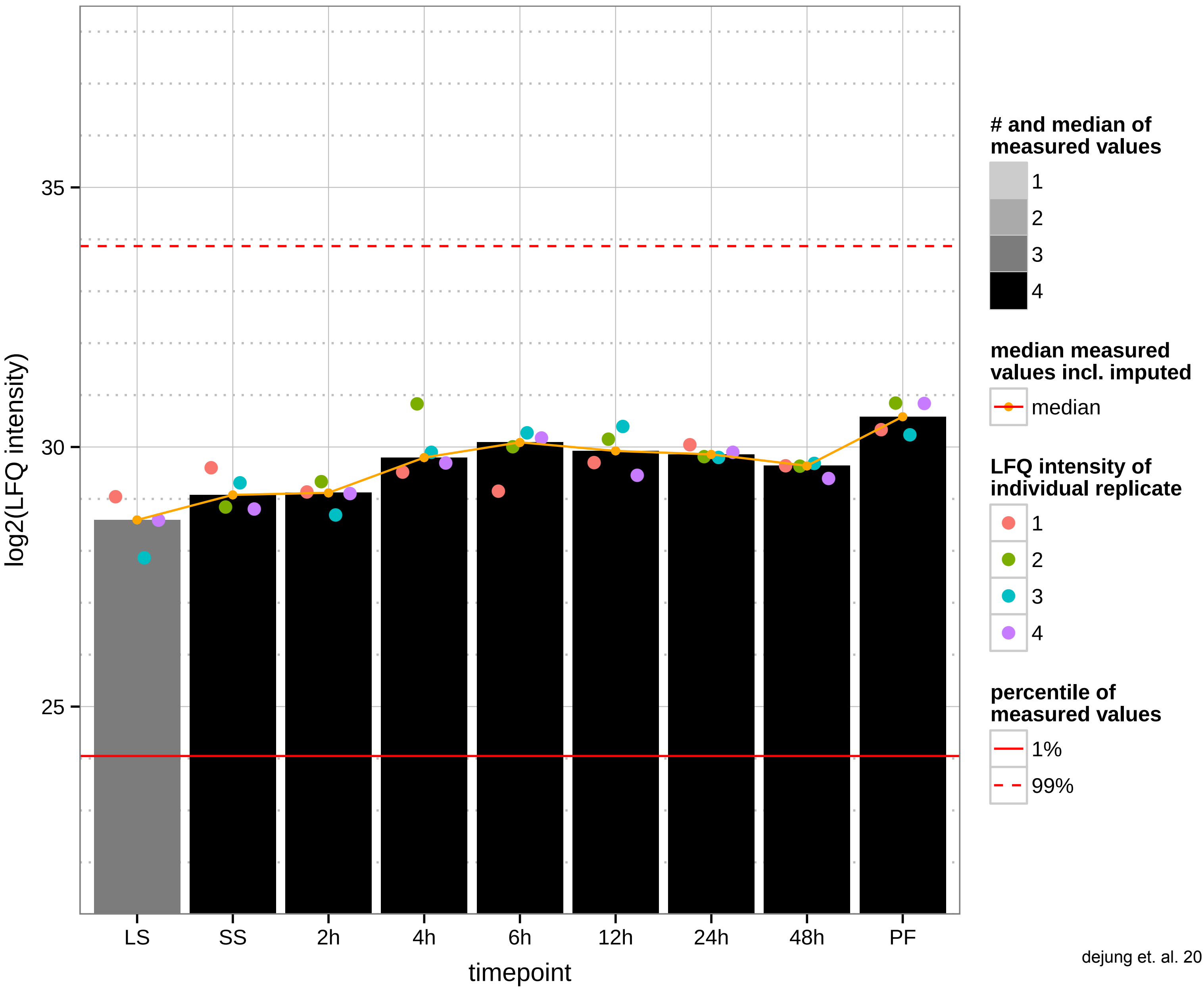

dejung et. al. 2015

hypothetical protein, conserved
Tb927.11.3460
AGOF: null
AGOC: null
AGOP: null
PGOF: null
PGOC: null
PGOP: null

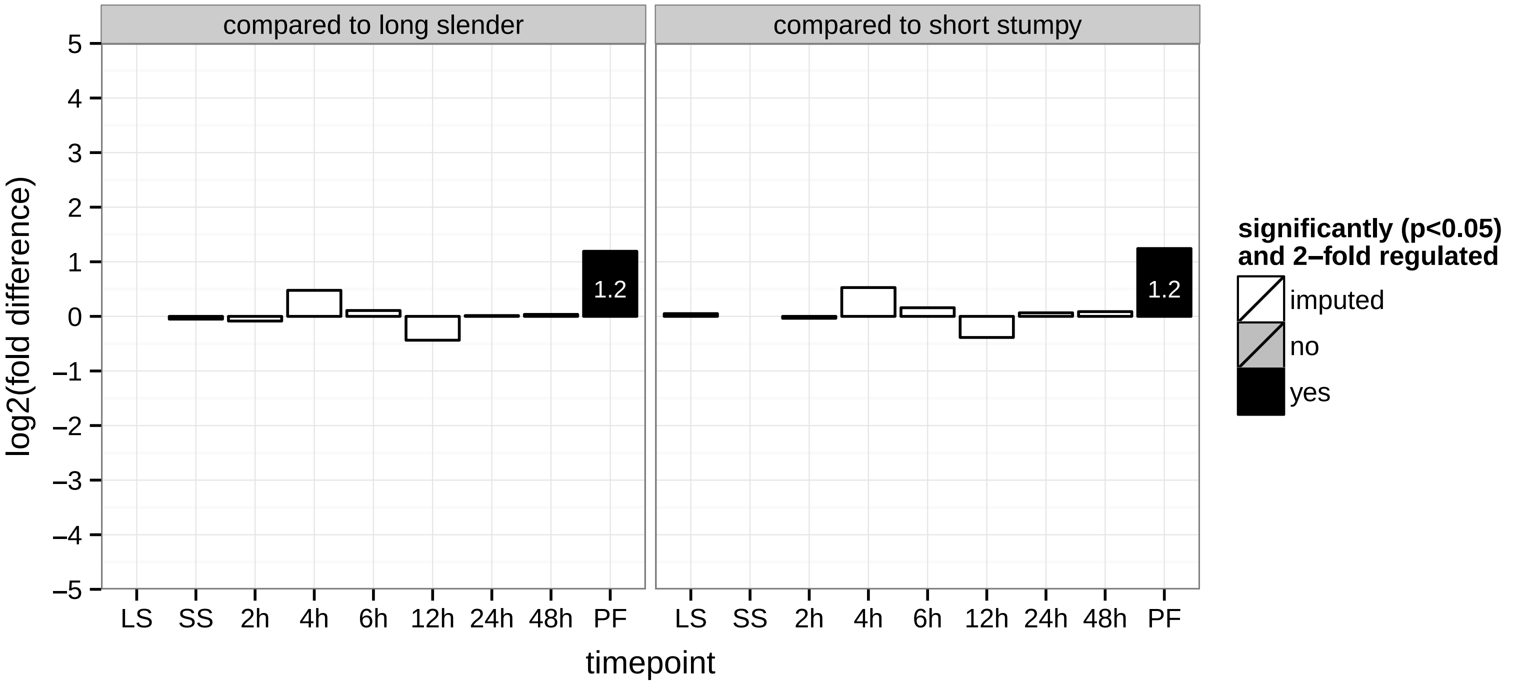

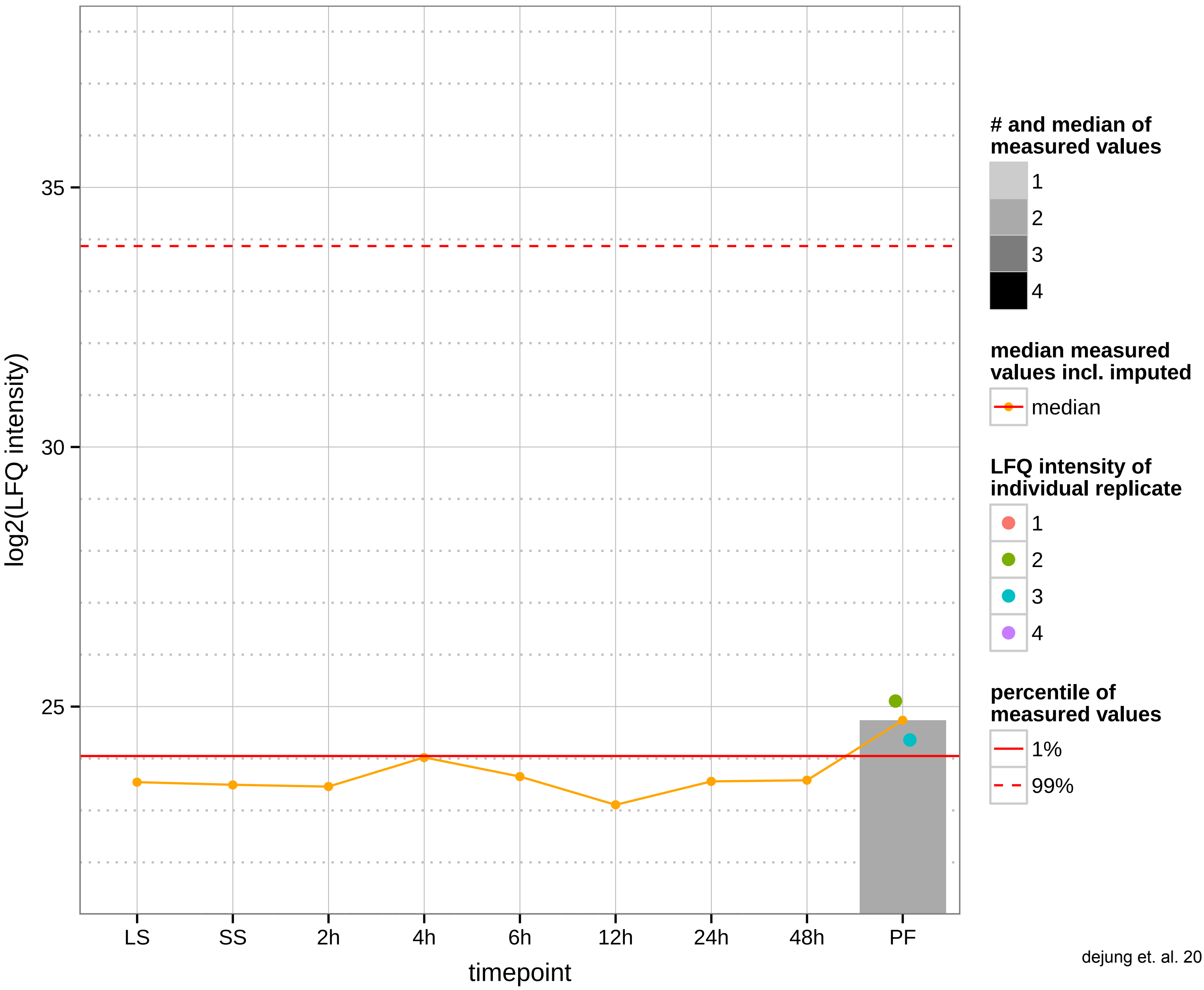

dejung et. al. 2015

predicted C2 domain protein
Tb927.11.4160
AGOF: null
AGOC: null
AGOP: null
PGOF: protein binding
PGOC: null
PGOP: null

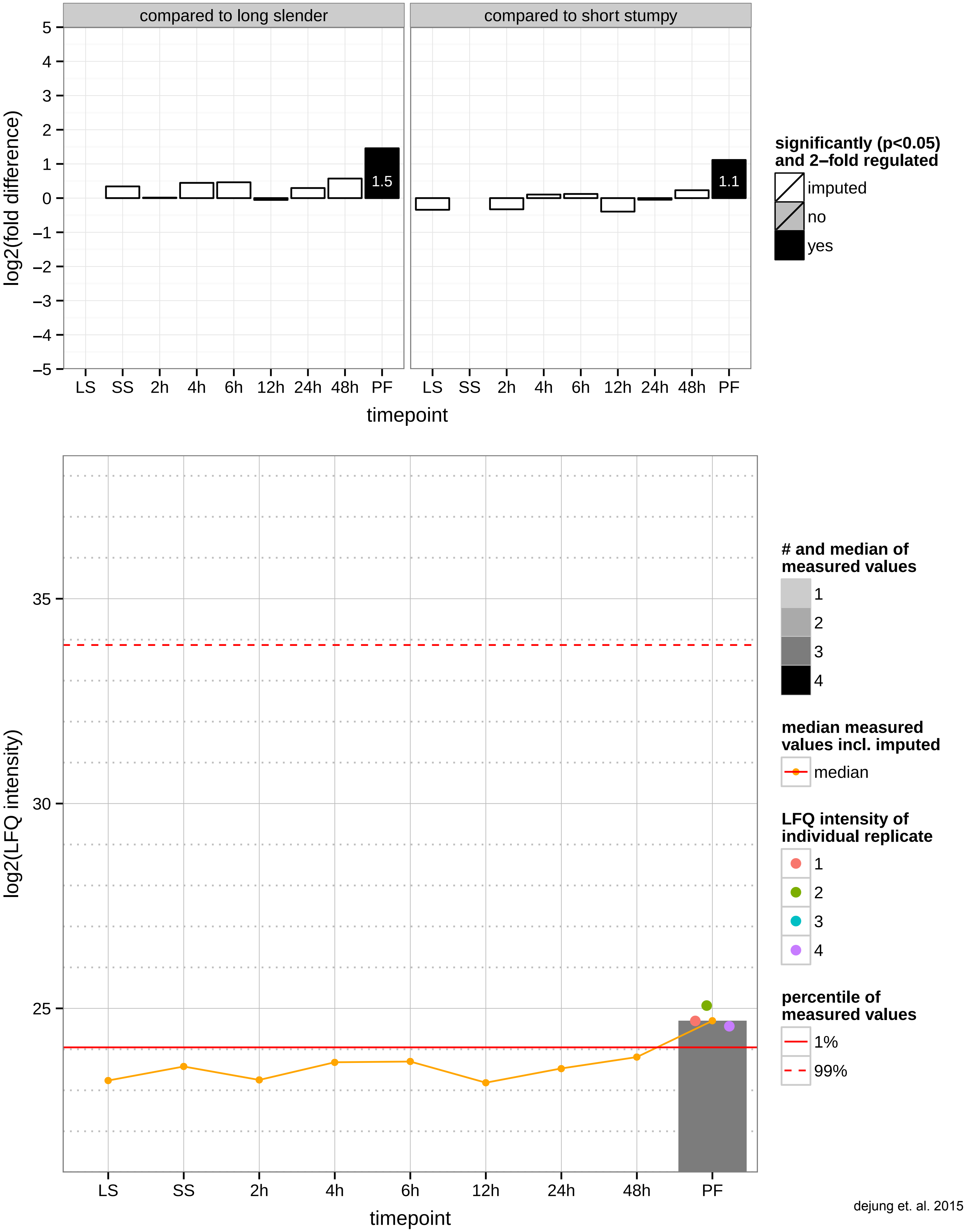

dejung et. al. 2015

conserved protein
Tb927.11.440
AGOF: translation initiation factor activity
AGOC: integral to membrane
AGOP: translational initiation
PGOF: translation initiation factor activity
PGOC: null
PGOP: translational initiation

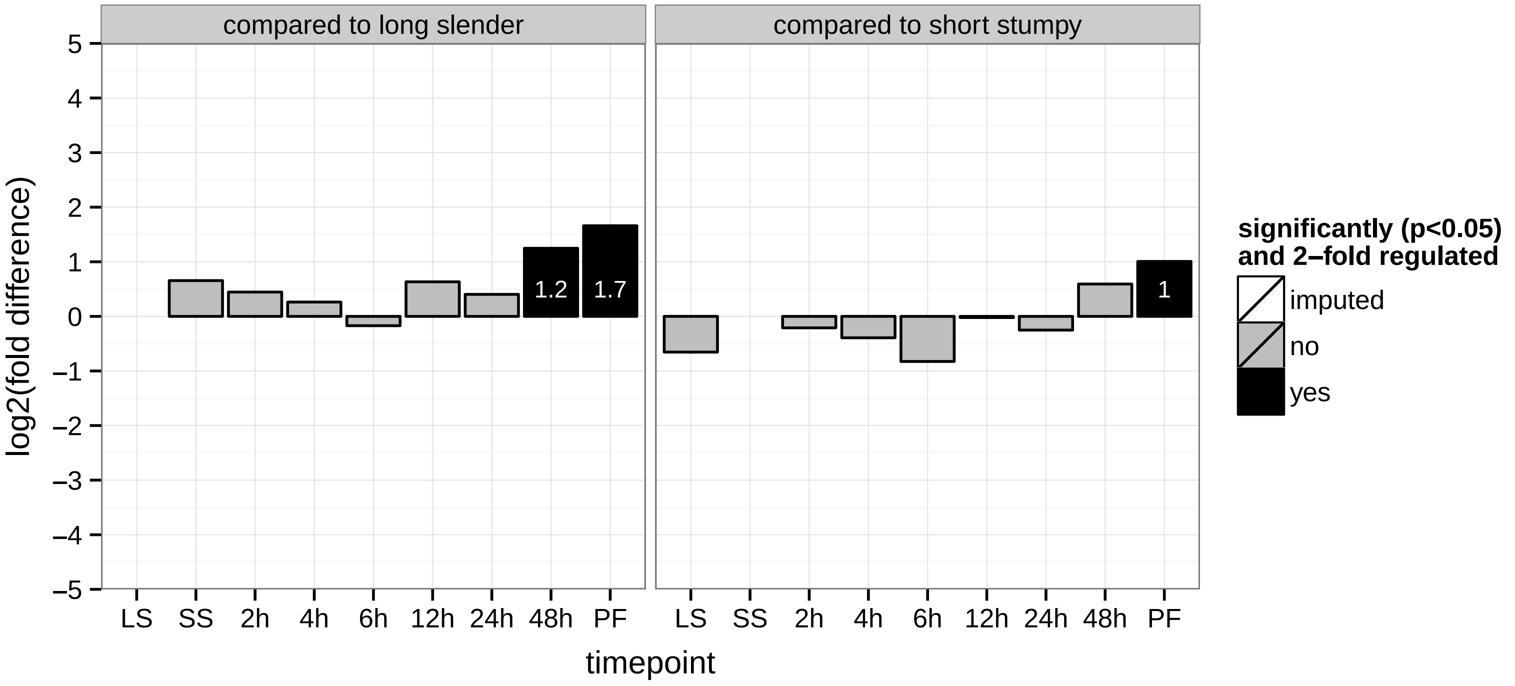

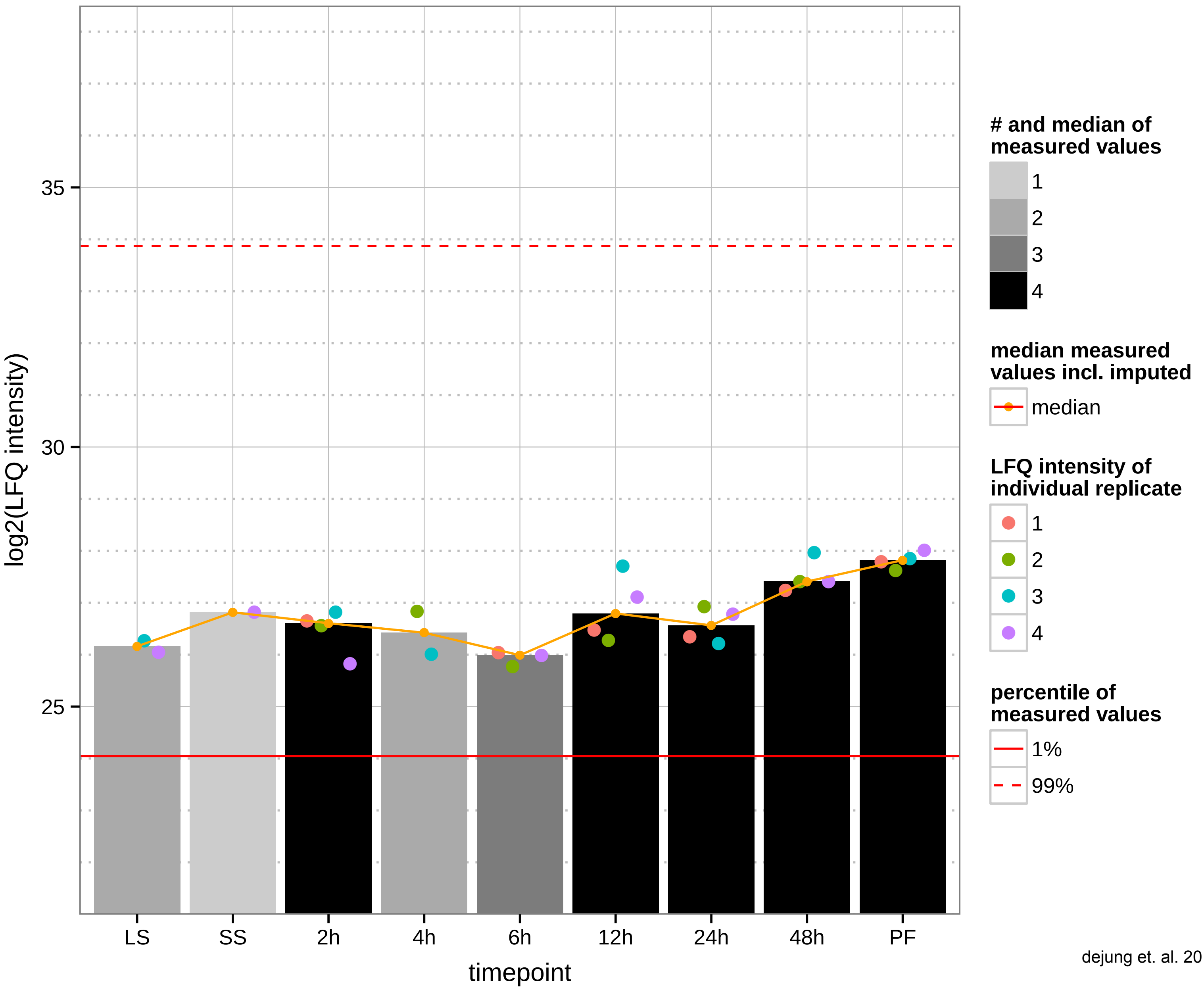

dejung et. al. 2015

hypothetical protein, conserved
Tb927.11.4500
AGOF: null
AGOC: null
AGOP: null
PGOF: null
PGOC: null
PGOP: null

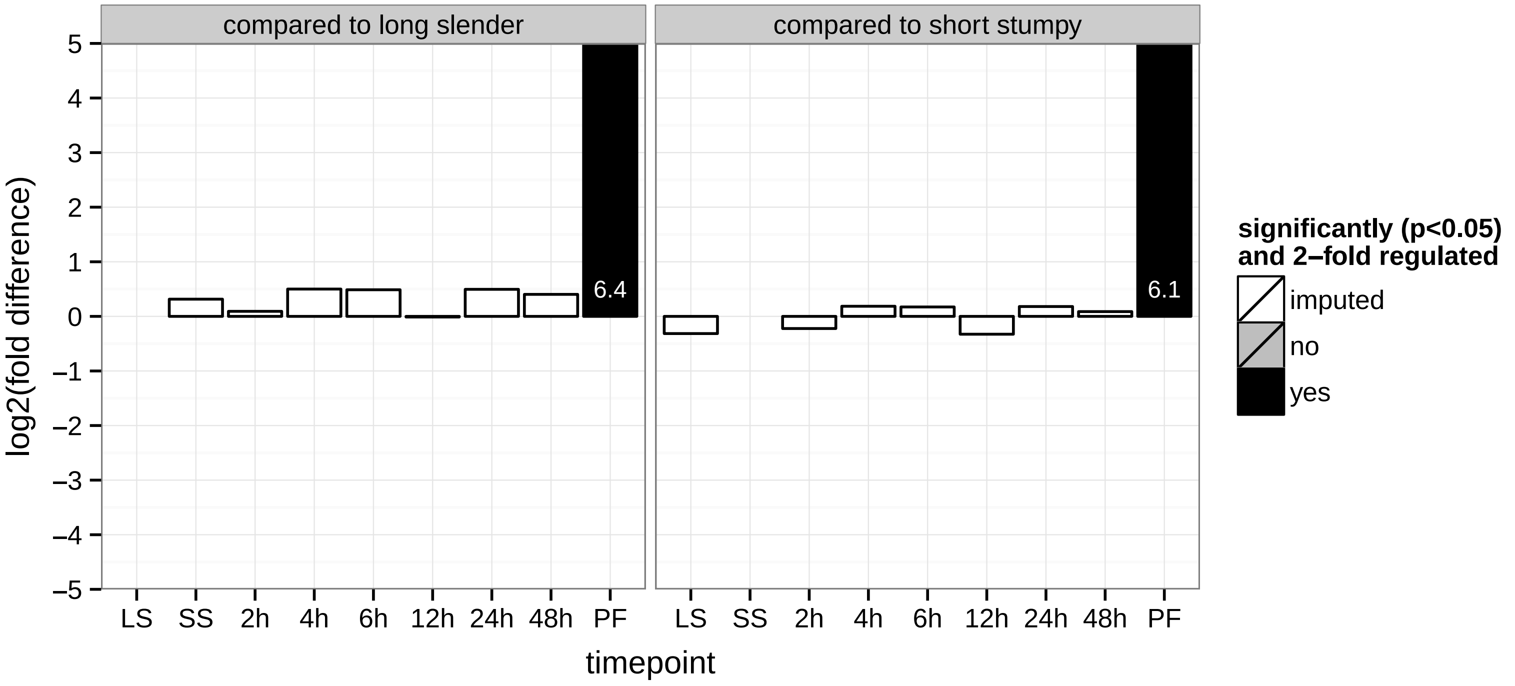

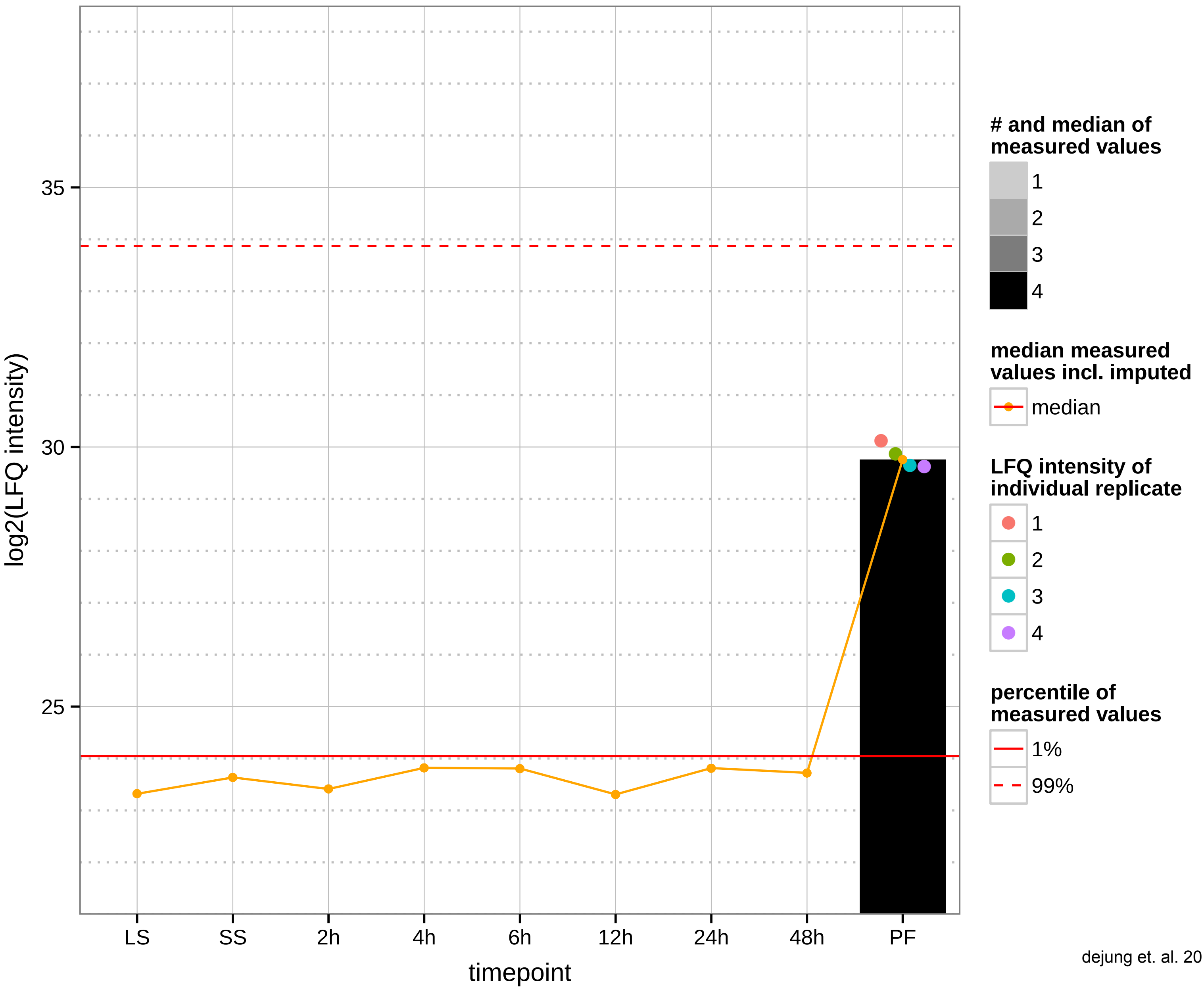

dejung et. al. 2015

N–acetyltransferase subunit ARD1 (ARD1)
Tb927.11.4530
AGOF: N–acetyltransferase activity, catalytic activity
AGOC: null
AGOP: N–terminal protein amino acid acetylation, cell growth
PGOF: N–acetyltransferase activity
PGOC: null
PGOP: null

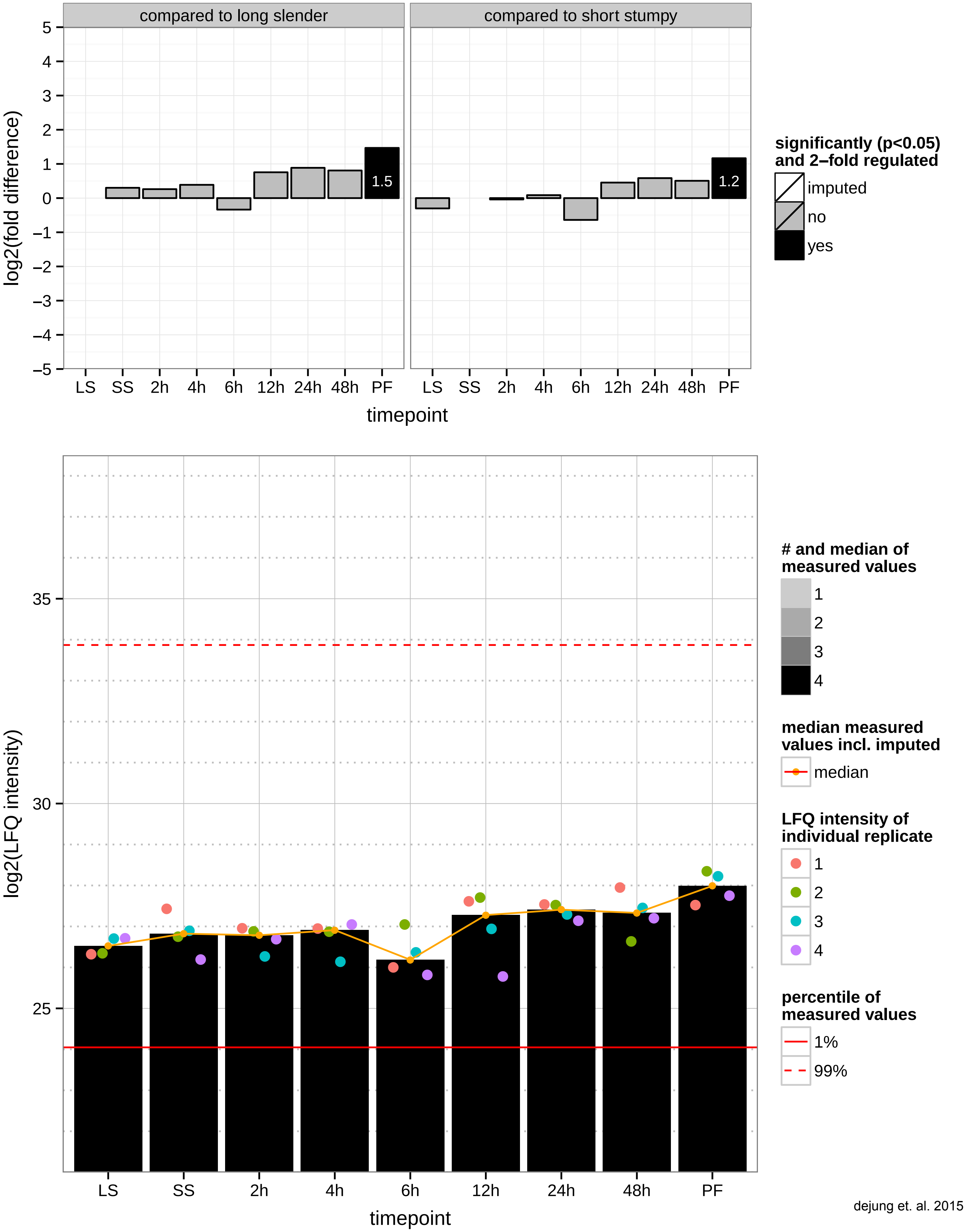

dejung et. al. 2015

hypothetical protein, conserved, predicted WD40 repeat protein
Tb927.11.460;Tb11.v5.0726
AGOF: null
AGOC: null, small−subunit processome
AGOP: null, rRNA processing
PGOF: protein binding
PGOC: small−subunit processome
PGOP: rRNA processing

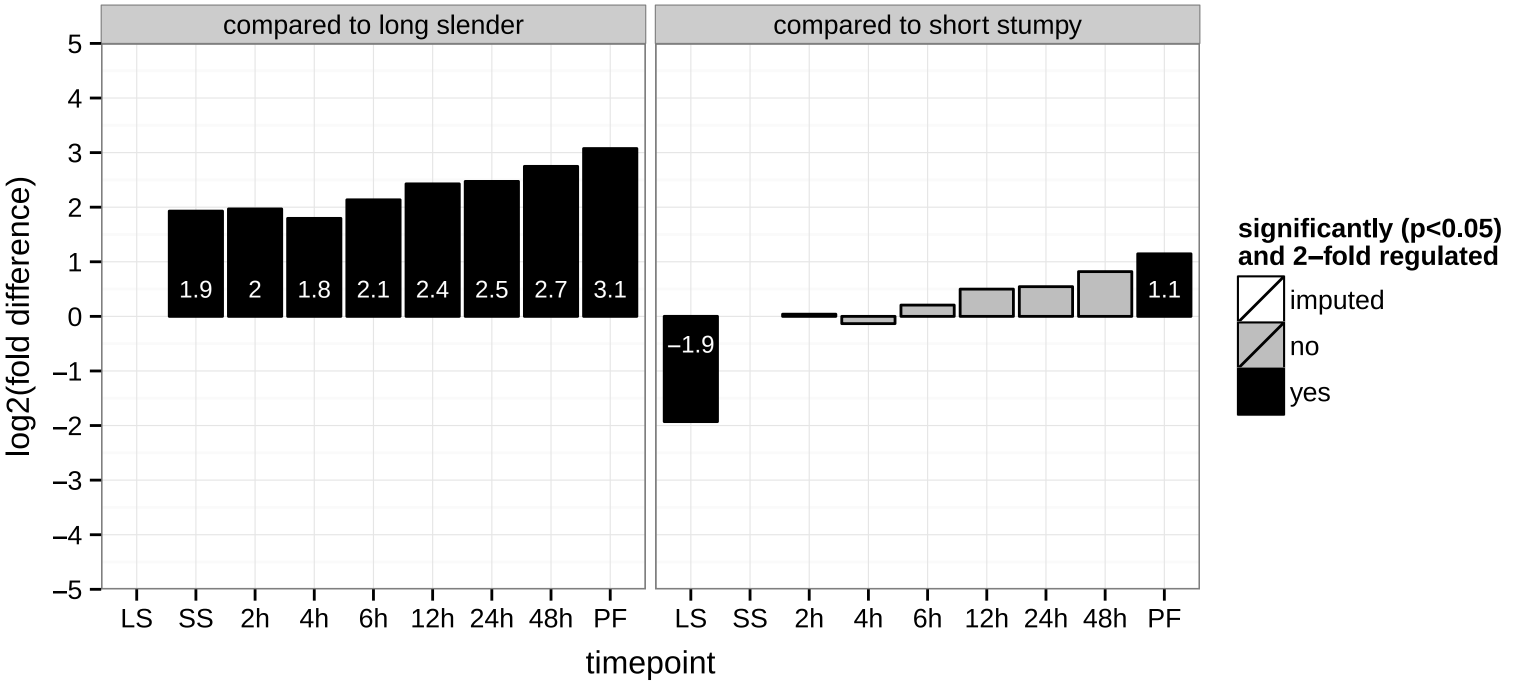

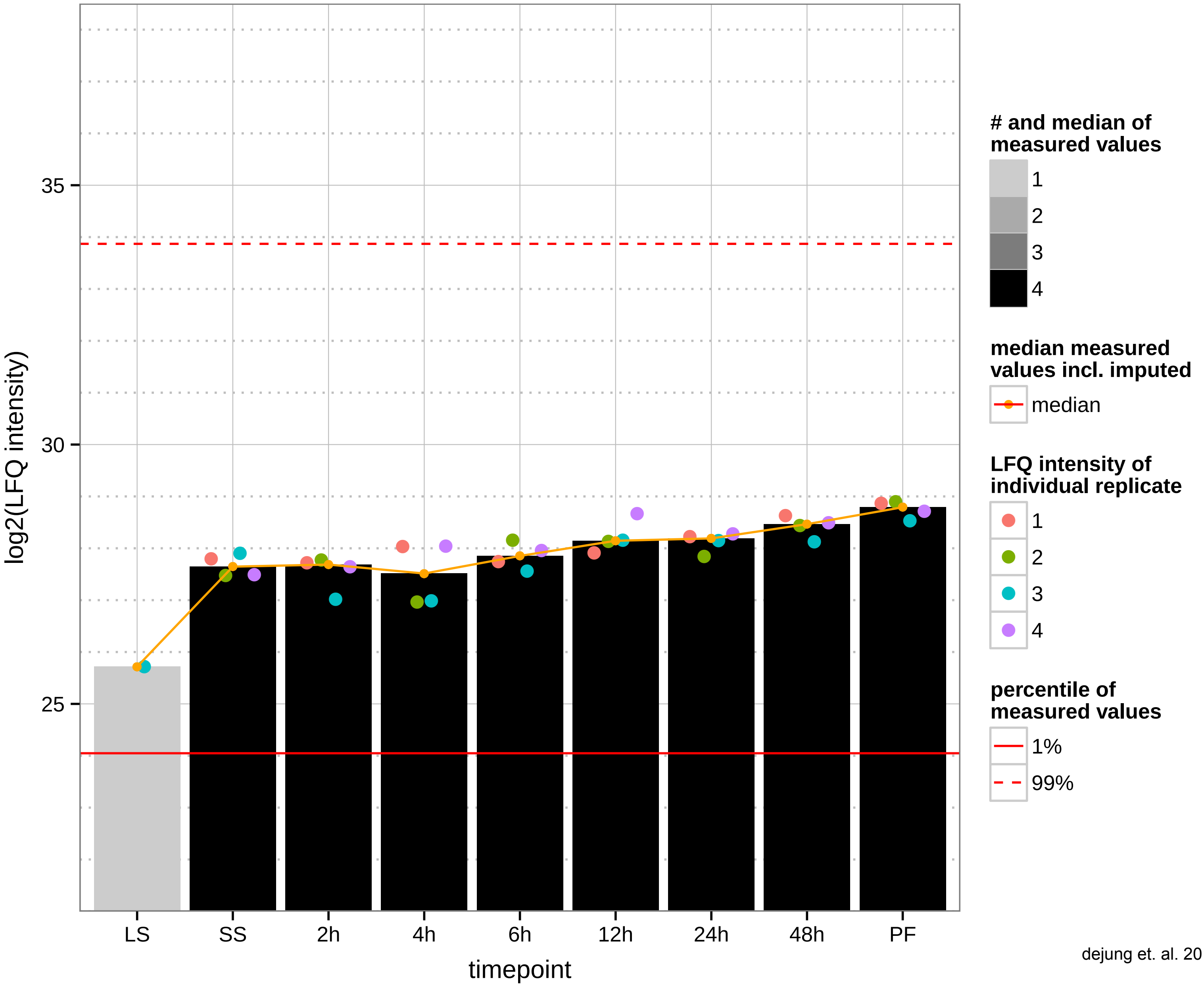

dejung et. al. 2015

hypothetical protein, conserved
Tb927.11.4870
AGOF: null
AGOC: mitochondrion
AGOP: null
PGOF: null
PGOC: null
PGOP: null

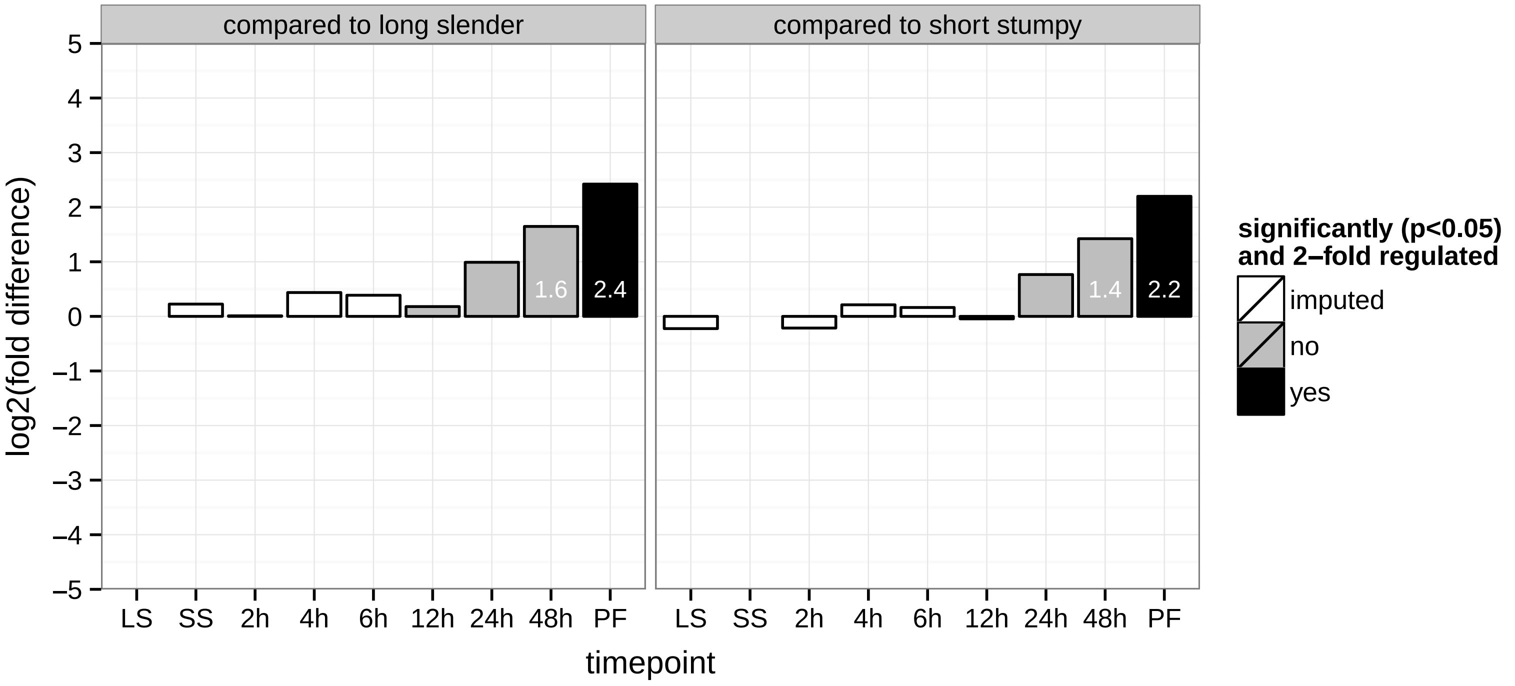

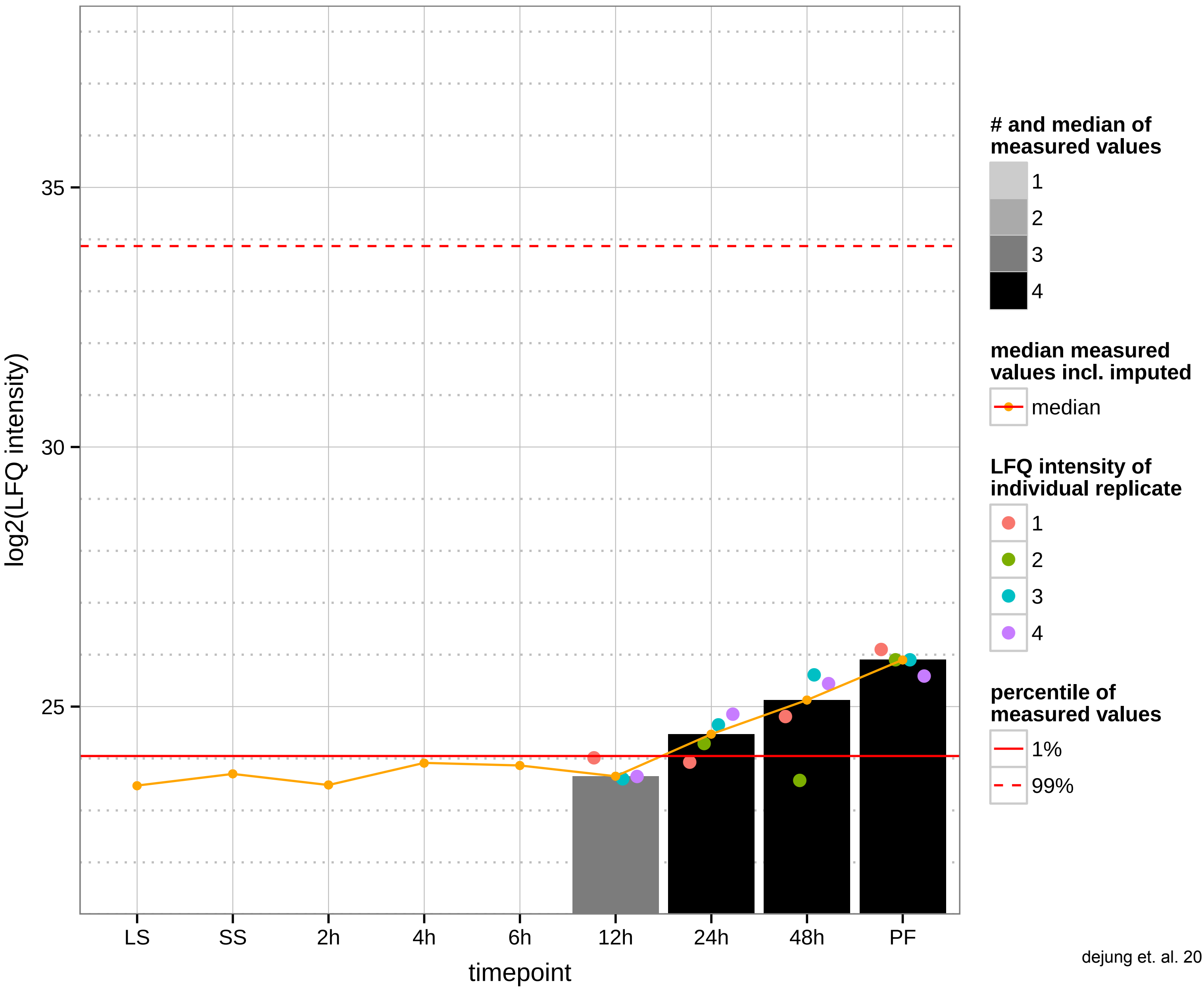

dejung et. al. 2015

hypothetical protein, conserved
Tb927.11.5020
AGOF: zinc ion binding
AGOC: null
AGOP: null
PGOF: binding, protein binding, zinc ion binding
PGOC: null
PGOP: null

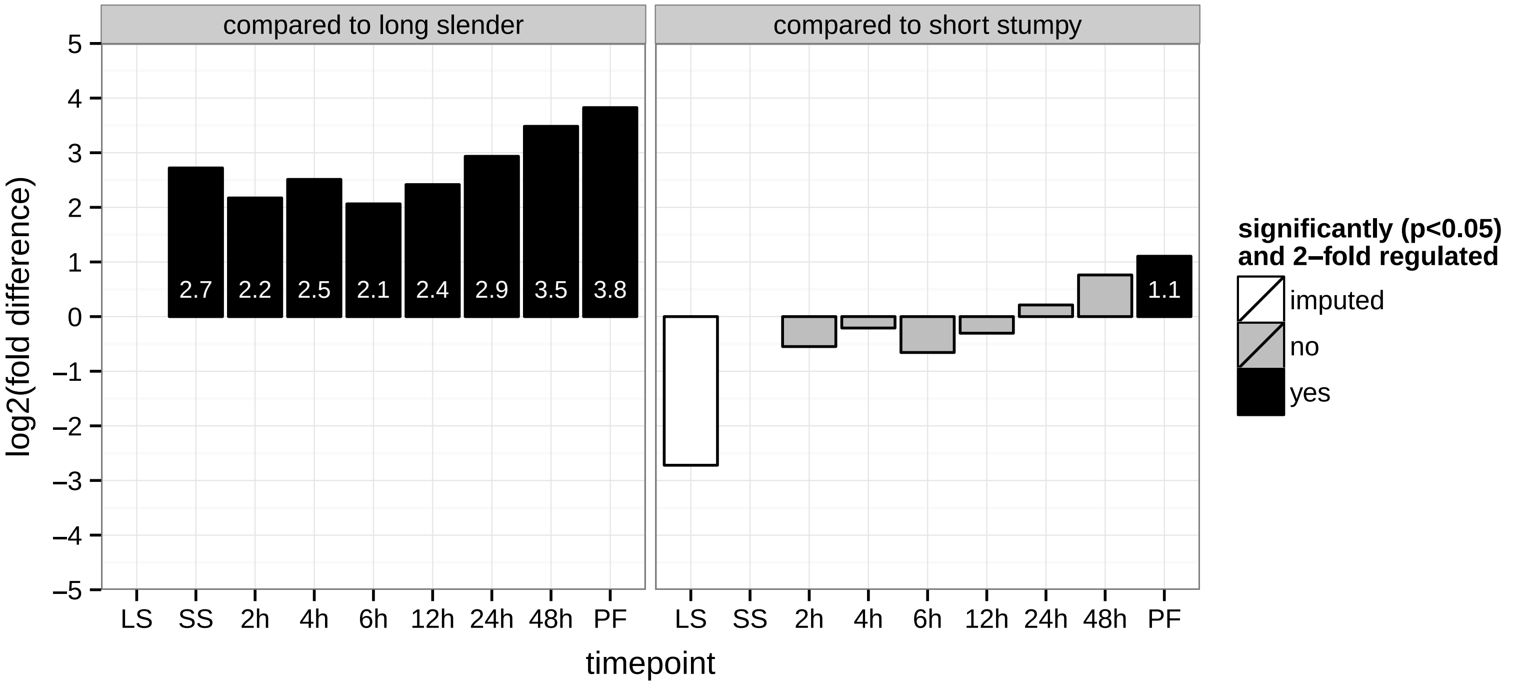

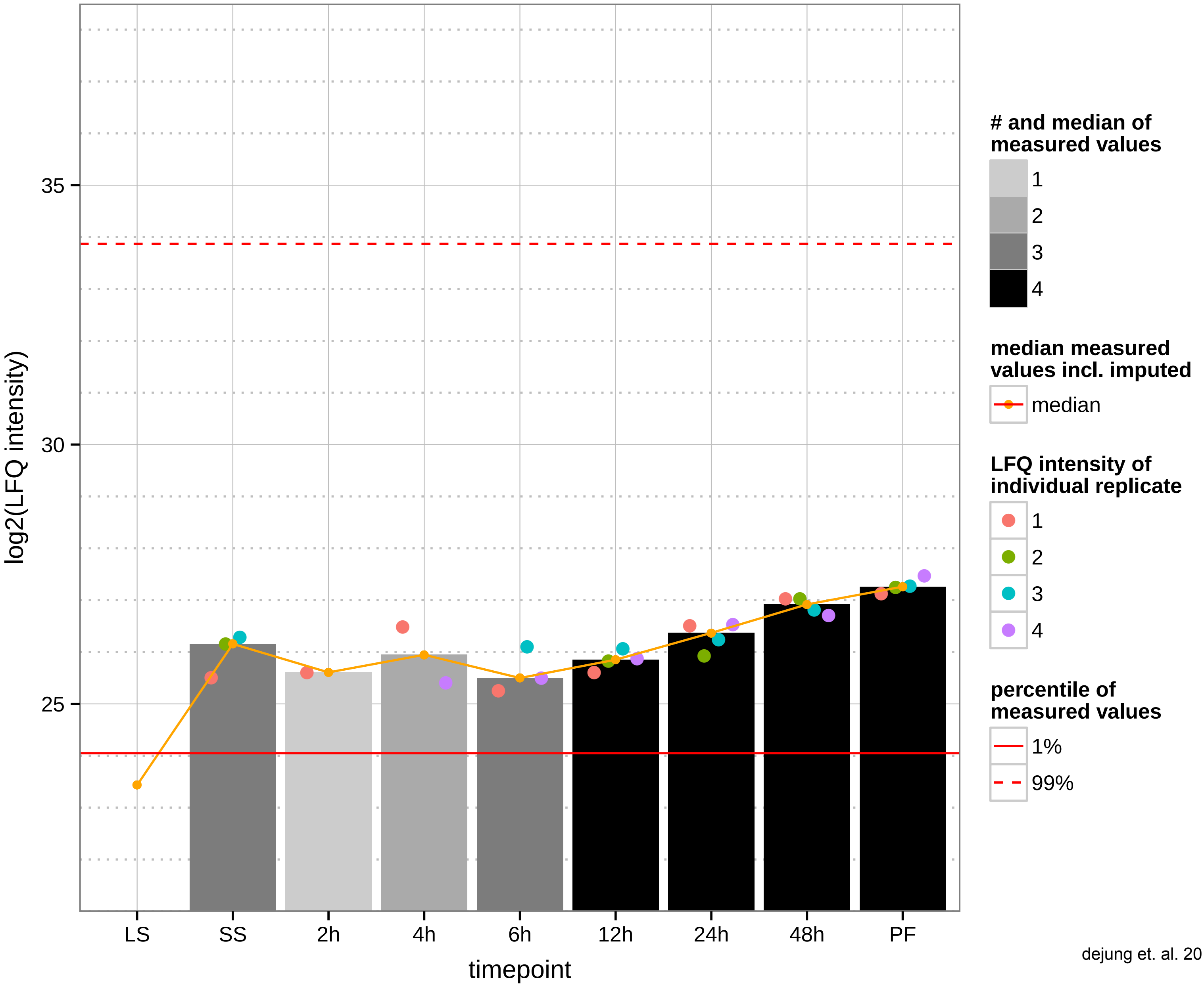

dejung et. al. 2015

ubiquitin carboxyl–terminal hydrolase, putative, cysteine peptidase, Clan CA, family C12
Tb927.11.5140
AGOF: ubiquitin thiolesterase activity
AGOC: cytoplasm, intracellular, proteasome regulatory particle
AGOP: ubiquitin–dependent protein catabolic process
PGOF: omega peptidase activity, ubiquitin thiolesterase activity
PGOC: intracellular
PGOP: ubiquitin–dependent protein catabolic process

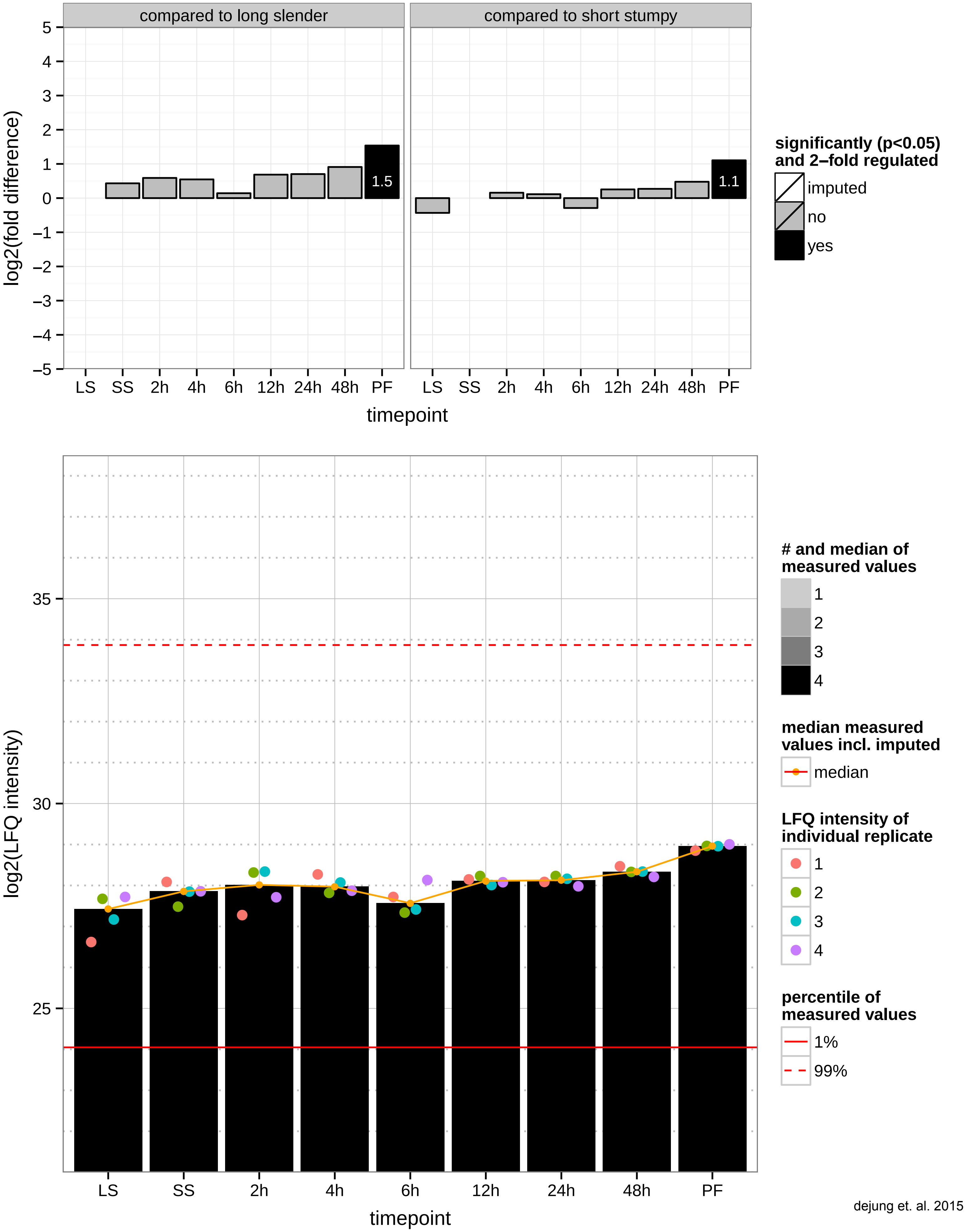

dejung et. al. 2015

hypothetical protein, conserved
Tb927.11.5250
AGOF: null
AGOC: null
AGOP: null
PGOF: null
PGOC: null
PGOP: null

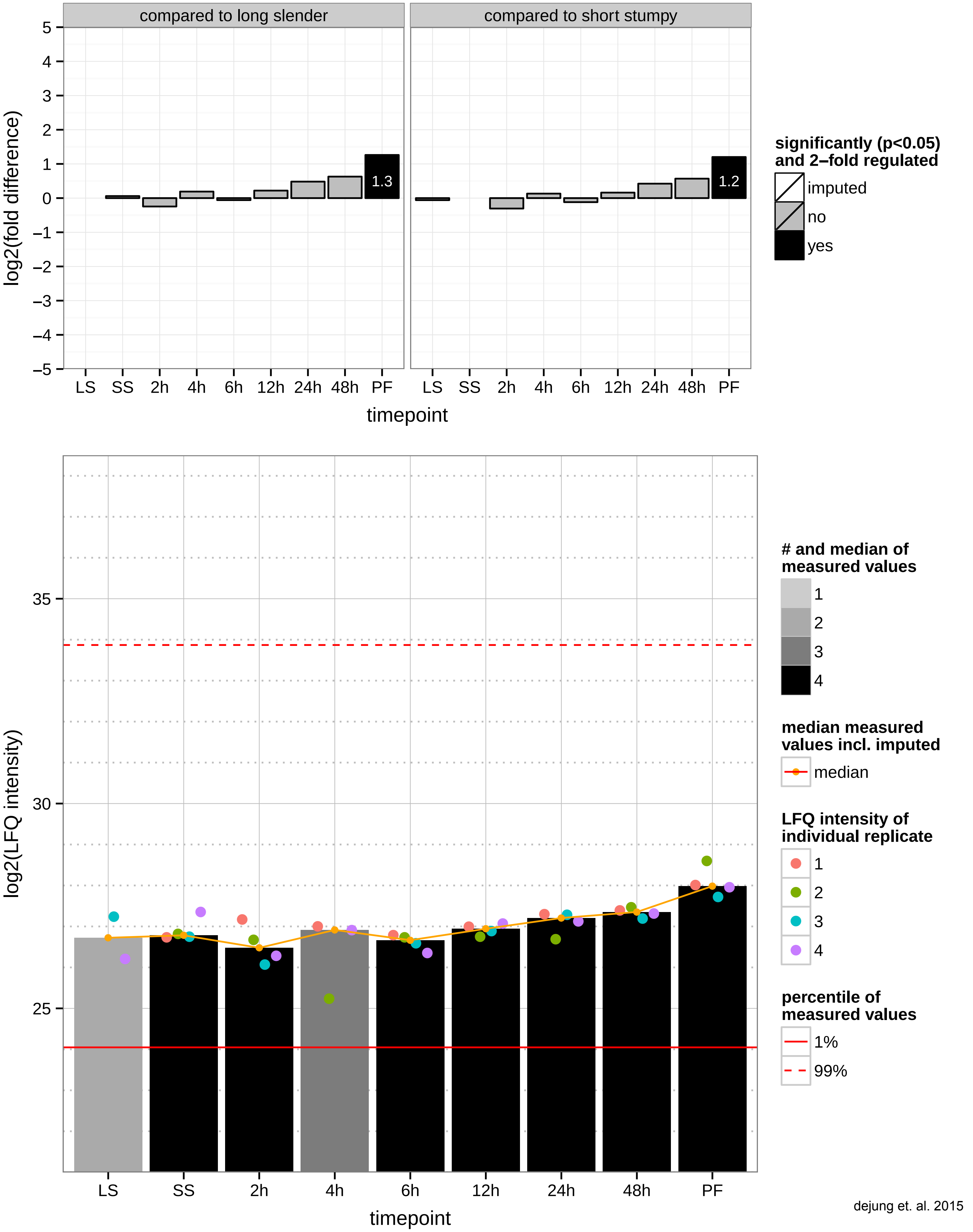

dejung et. al. 2015

hypothetical protein, conserved
Tb927.11.560
AGOF: null
AGOC: null
AGOP: null
PGOF: binding
PGOC: null
PGOP: null

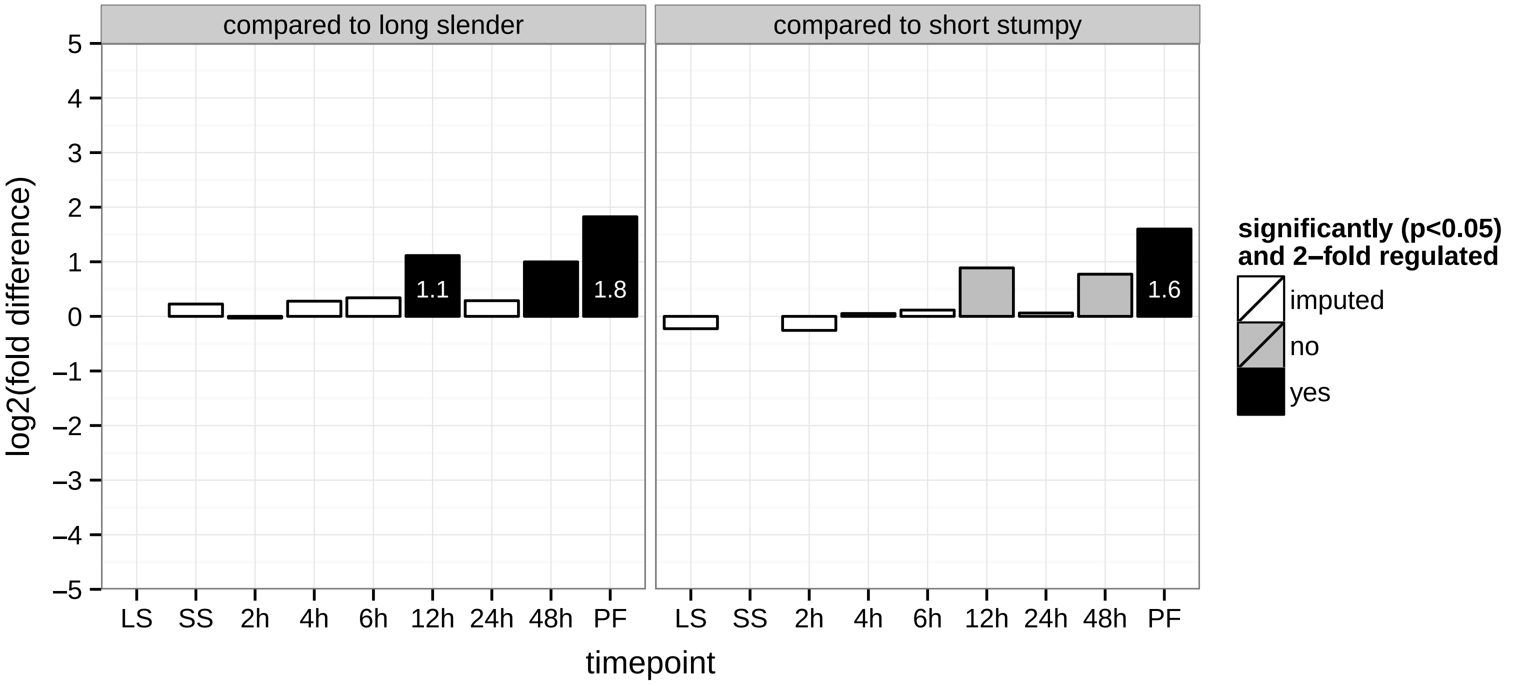

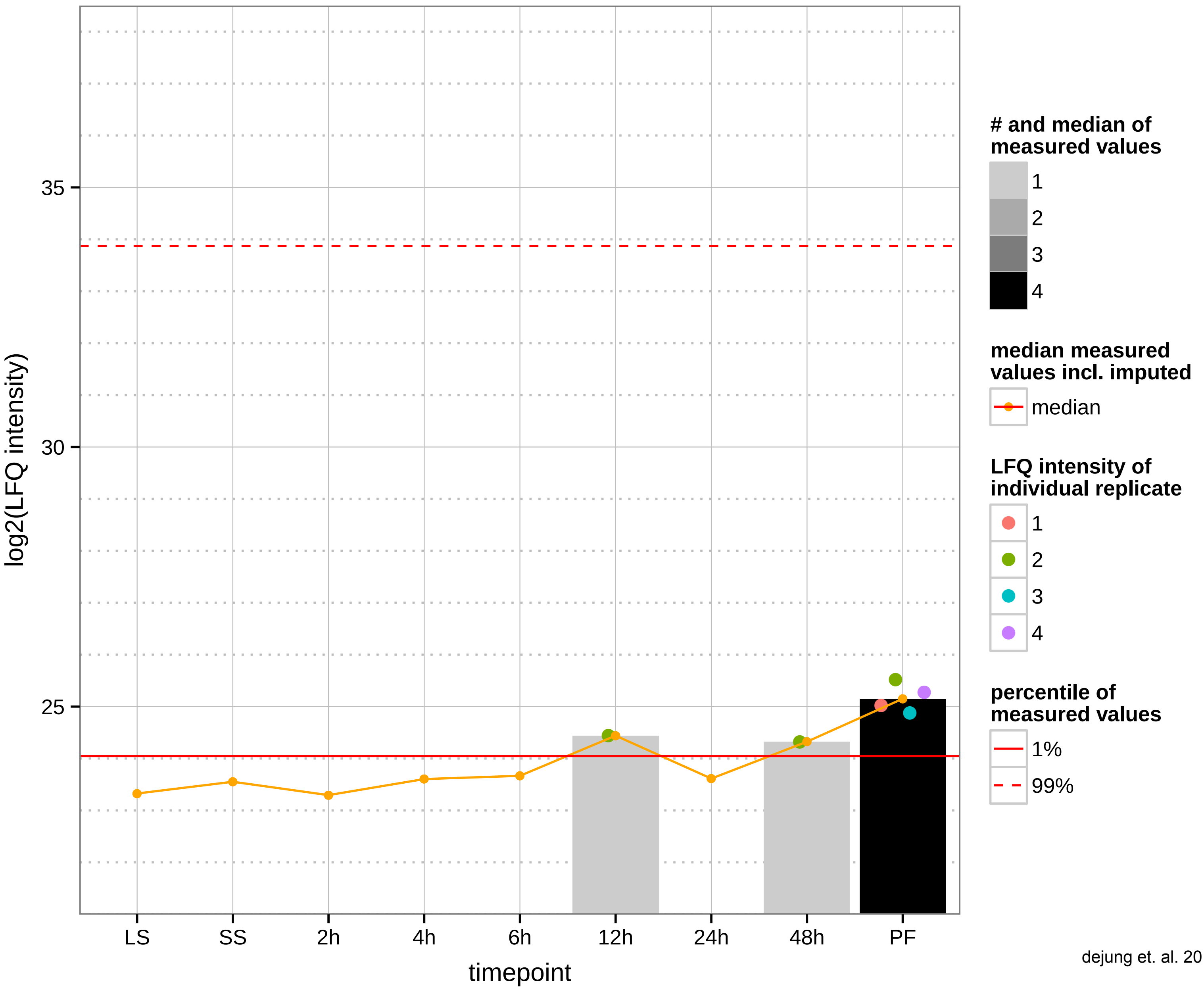

dejung et. al. 2015

dynein light chain, putative
Tb927.11.5680
AGOF: motor activity
AGOC: dynein complex
AGOP: microtubule−based movement
PGOF: protein binding
PGOC: null
PGOP: null

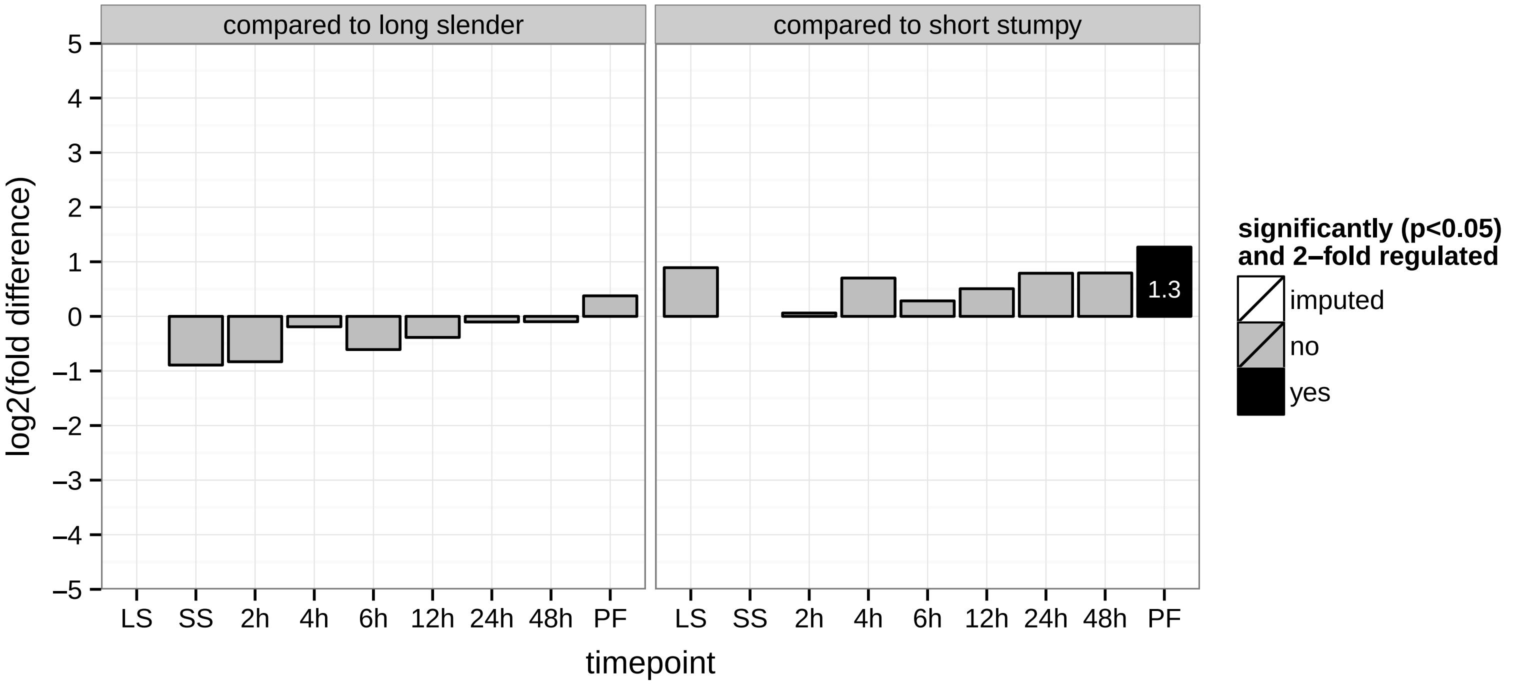

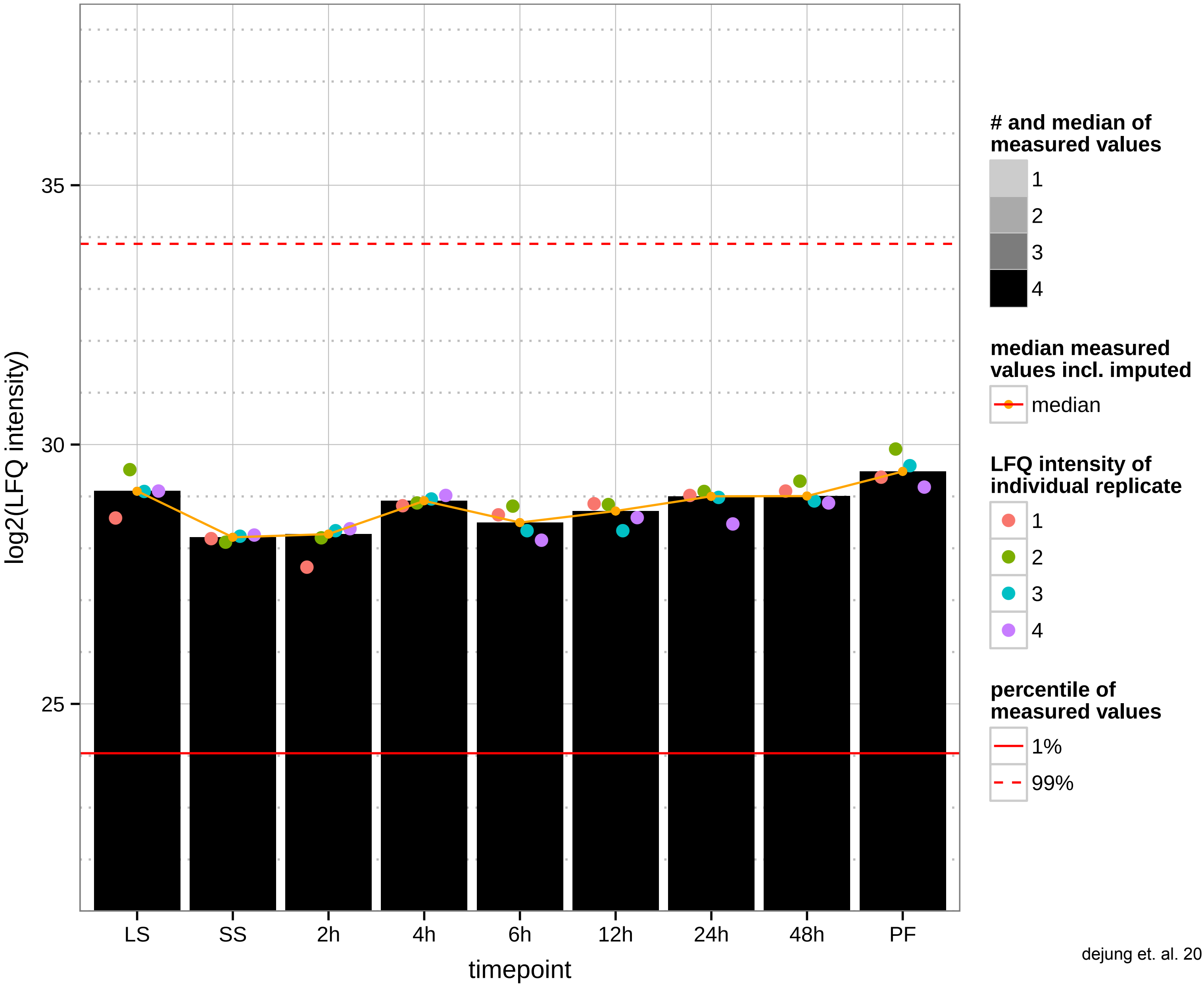

dejung et. al. 2015

hypothetical protein, conserved
Tb927.11.5900
AGOF: null
AGOC: null
AGOP: null
PGOF: null
PGOC: null
PGOP: null

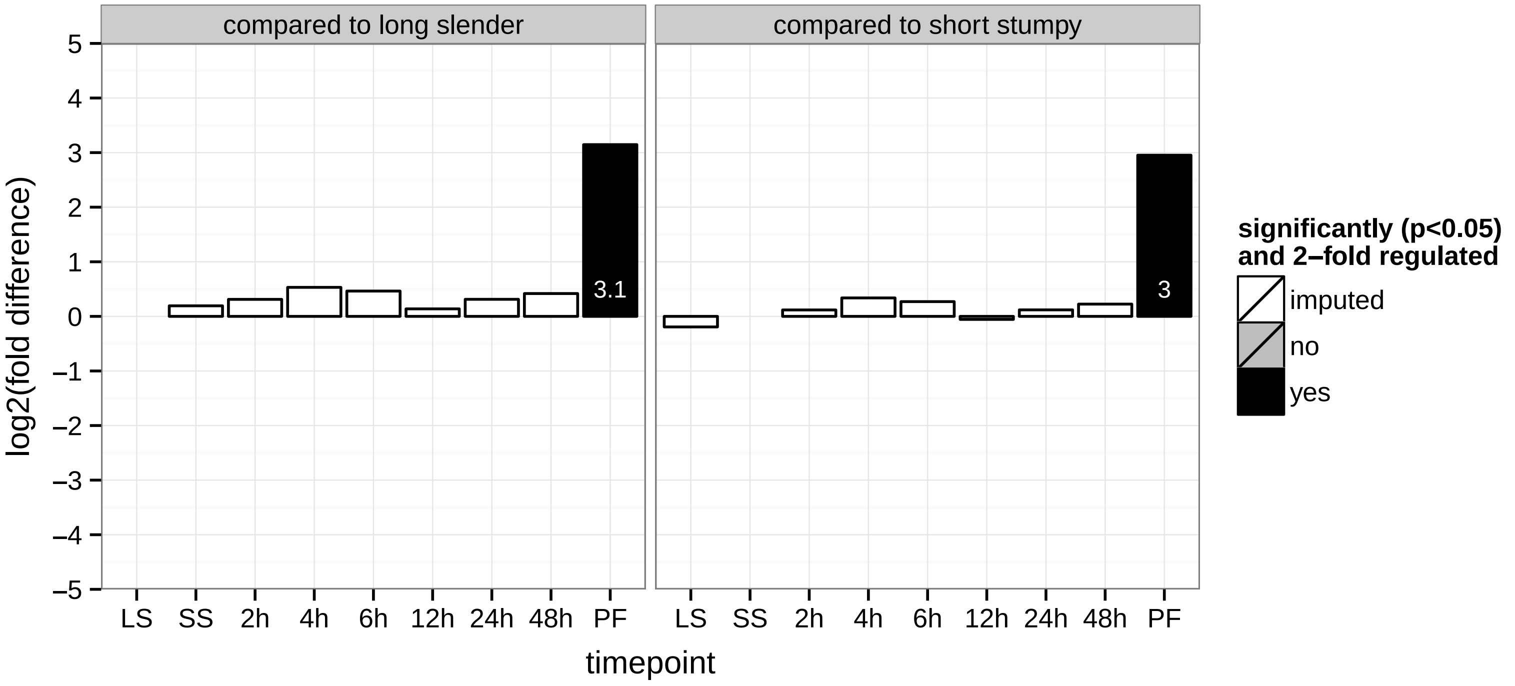

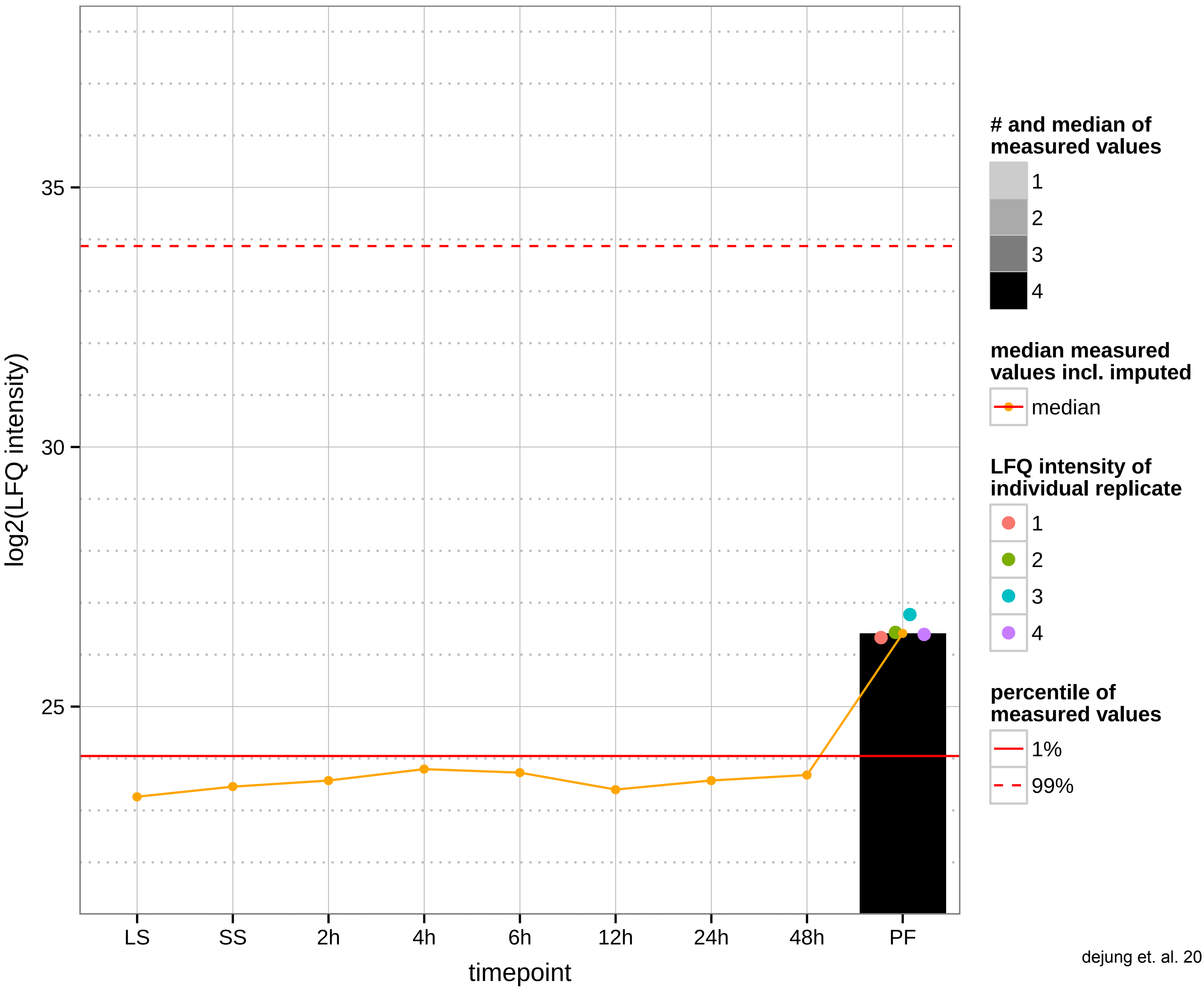

dejung et. al. 2015

hypothetical protein, conserved
Tb927.11.5990
AGOF: RNA binding, pseudouridine synthase activity
AGOC: mitochondrion
AGOP: pseudouridine synthesis
PGOF: RNA binding, pseudouridine synthase activity
PGOC: null
PGOP: RNA modification, pseudouridine synthesis

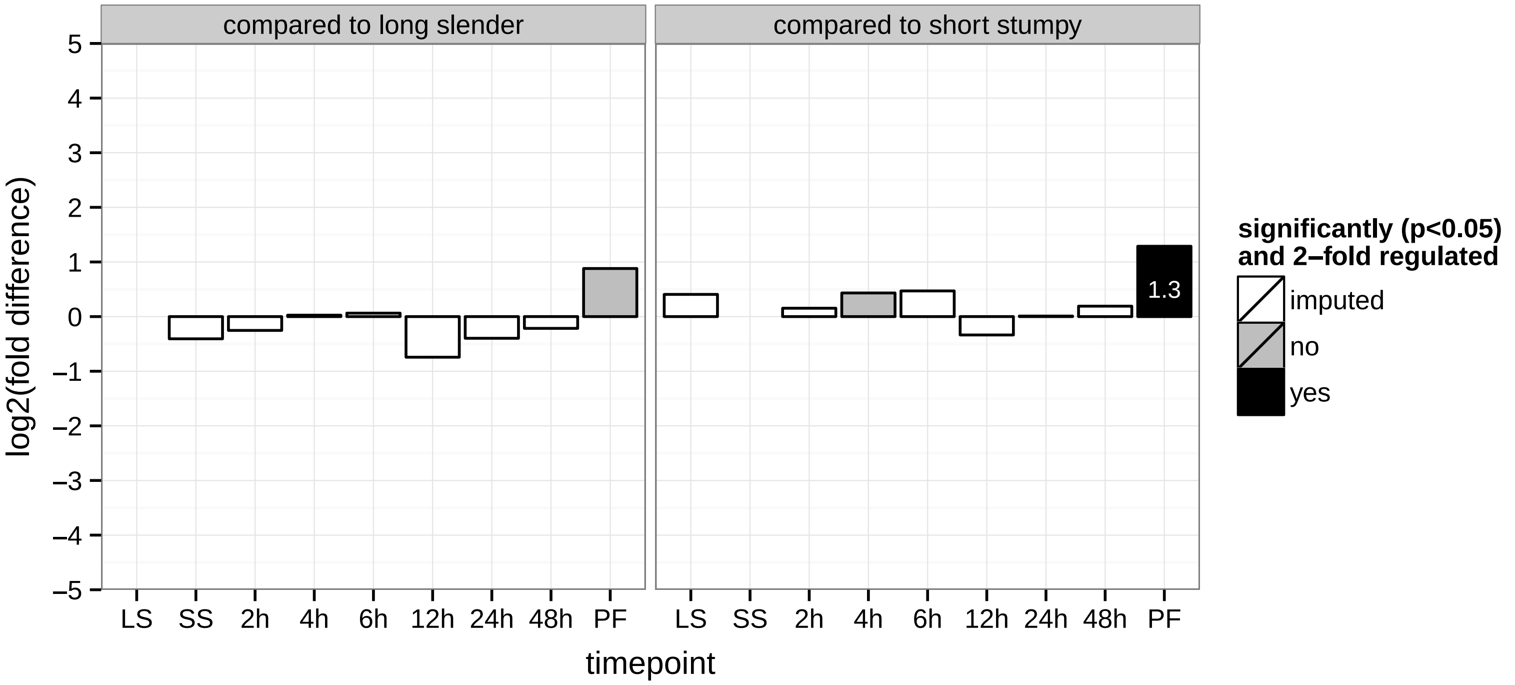

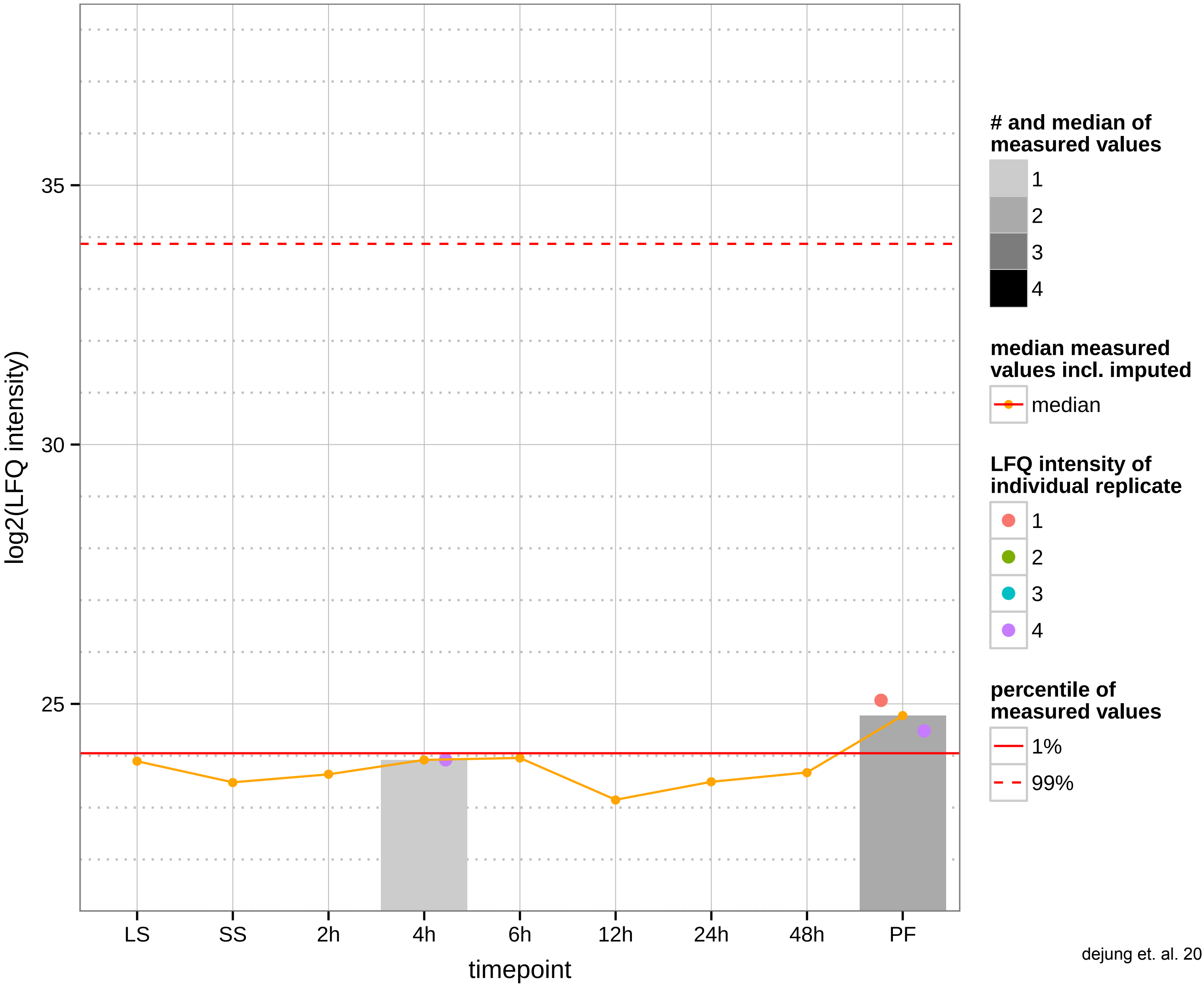

dejung et. al. 2015

hypothetical protein, conserved
Tb927.11.6150
AGOF: null
AGOC: integral to membrane, mitochondrion
AGOP: protein insertion into membrane
PGOF: null
PGOC: integral to membrane
PGOP: protein insertion into membrane

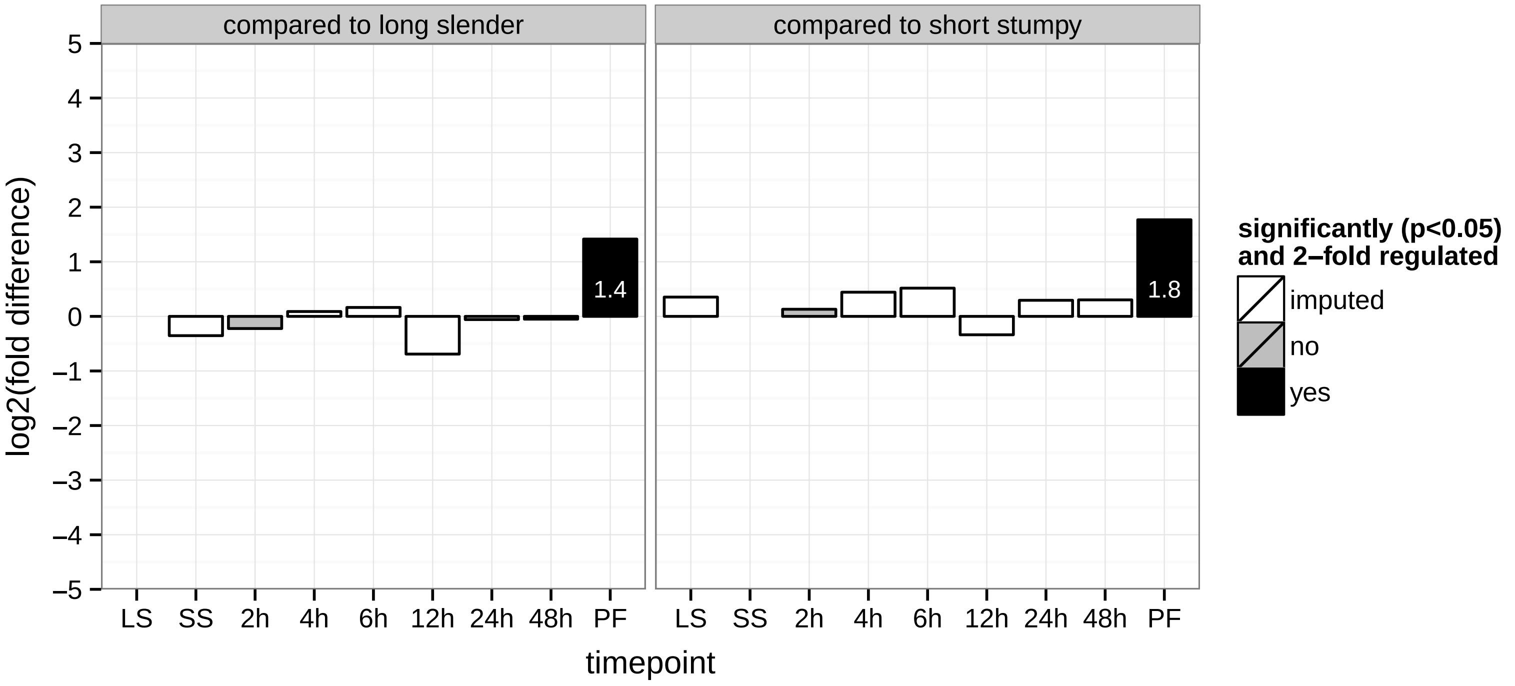

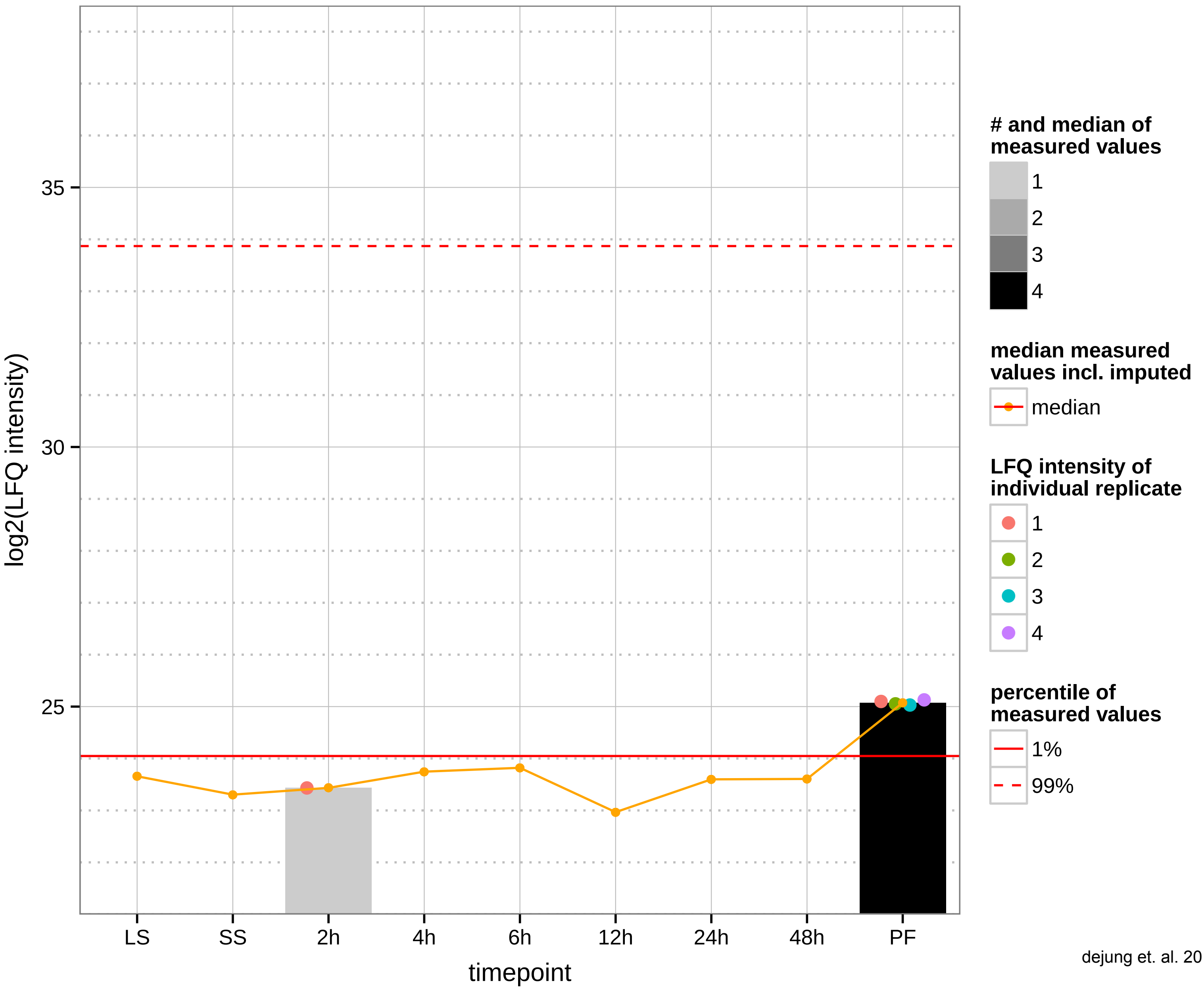

dejung et. al. 2015

eukaryotic release factor 3, putative (ERF3)
Tb927.11.6160
AGOF: GTP binding, GTPase activity, translation elongation factor activity
AGOC: null
AGOP: translation, translational elongation
PGOF: GTP binding, GTPase activity
PGOC: null
PGOP: null

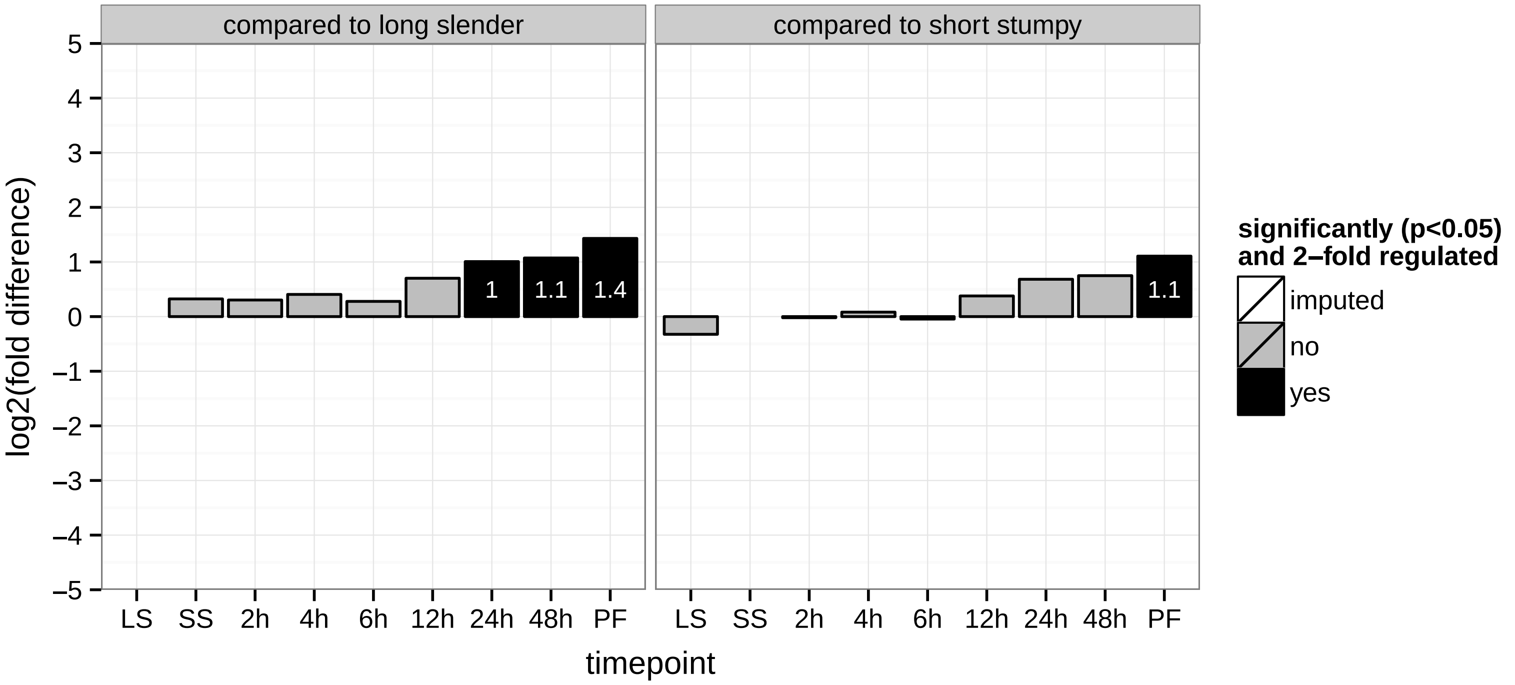

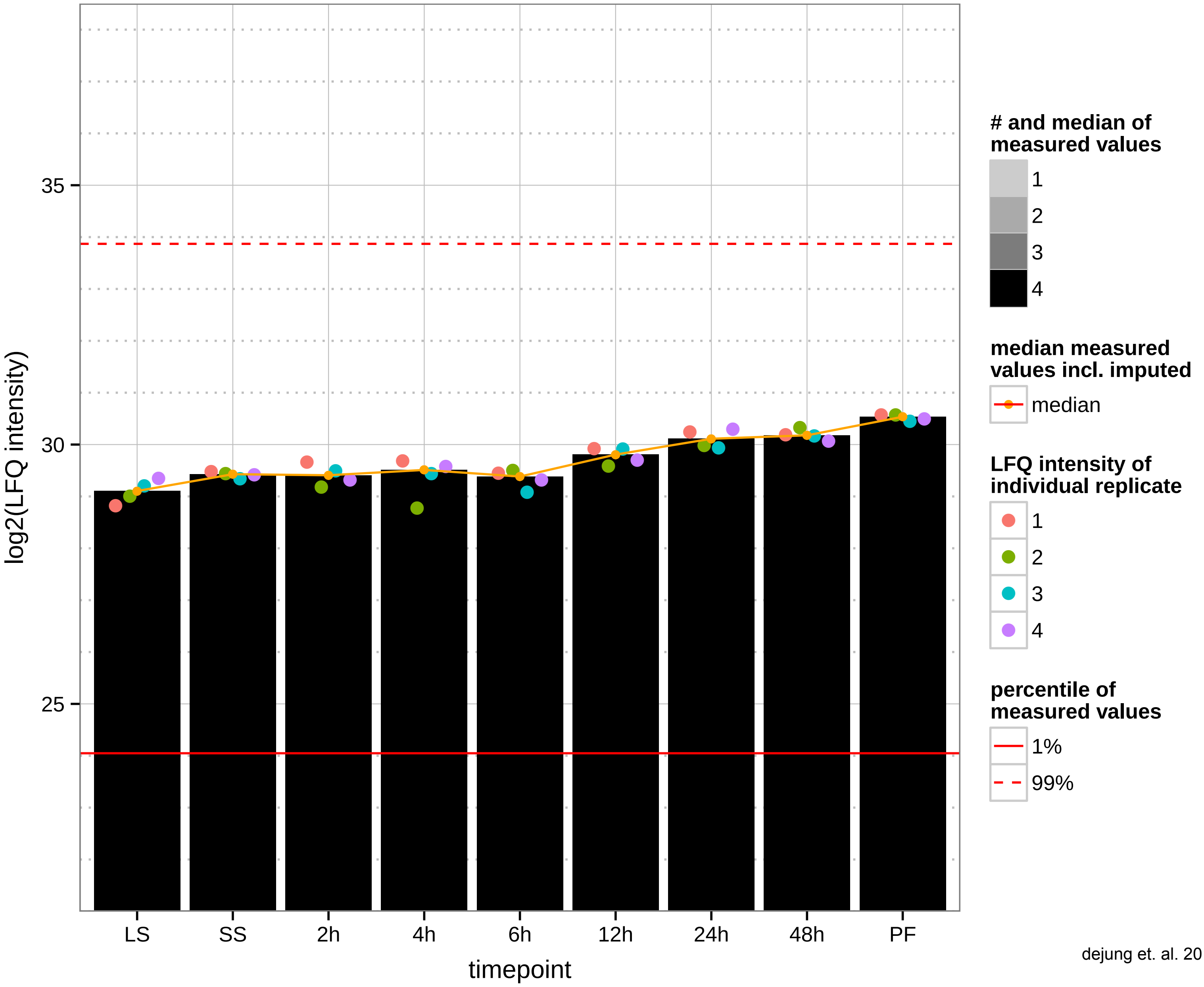

dejung et. al. 2015

inositol–1, 4, 5–trisphosphate (IP3) 5–phosphatase, putative
Tb927.11.6270
AGOF: inositol–polyphosphate 5–phosphatase activity
AGOC: null
AGOP: intracellular signal transduction
PGOF: null
PGOC: null
PGOP: phosphatidylinositol phosphorylation

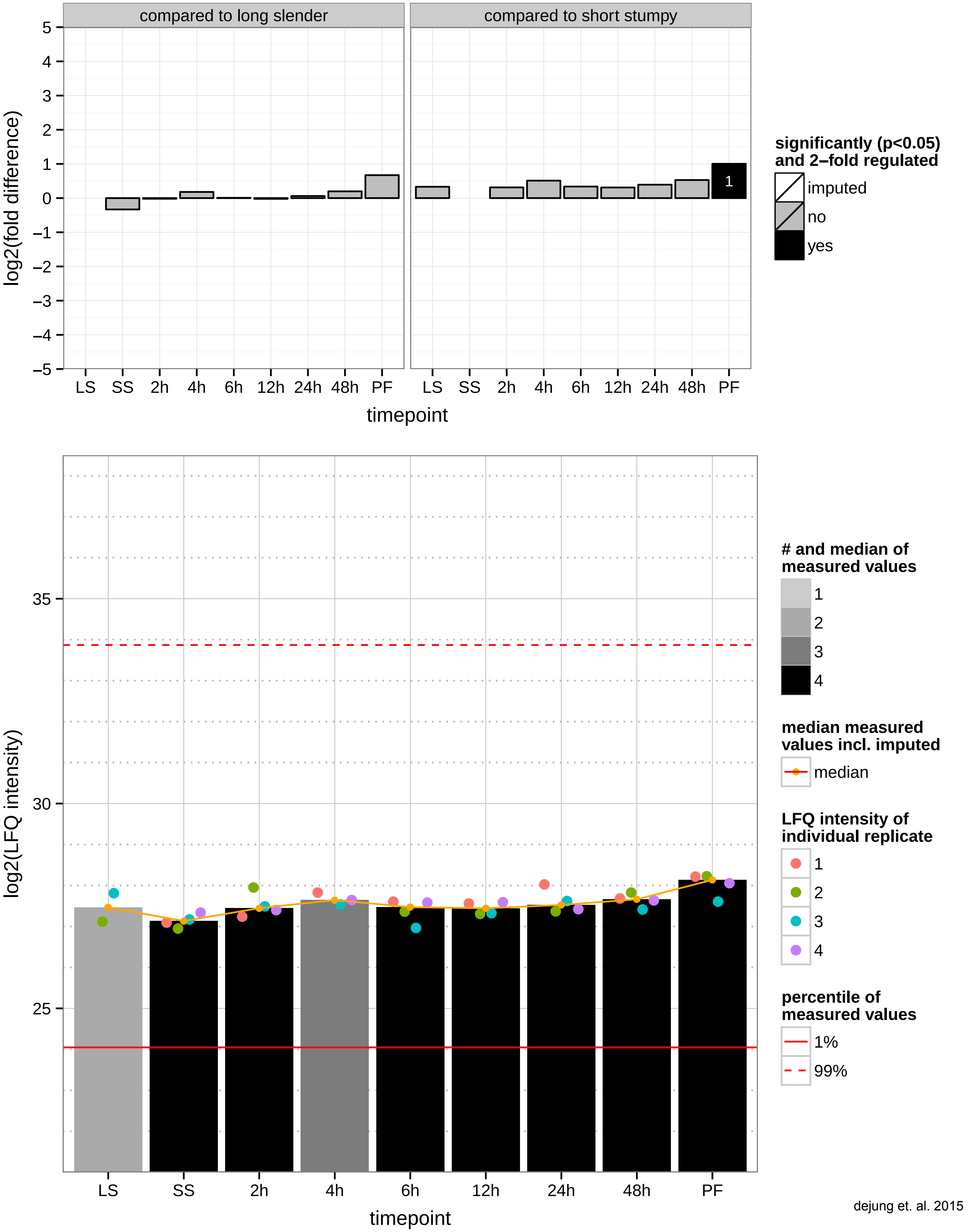

dejung et. al. 2015

hypothetical protein, conserved
Tb927.11.6490
AGOF: null
AGOC: null
AGOP: null
PGOF: null
PGOC: null
PGOP: null

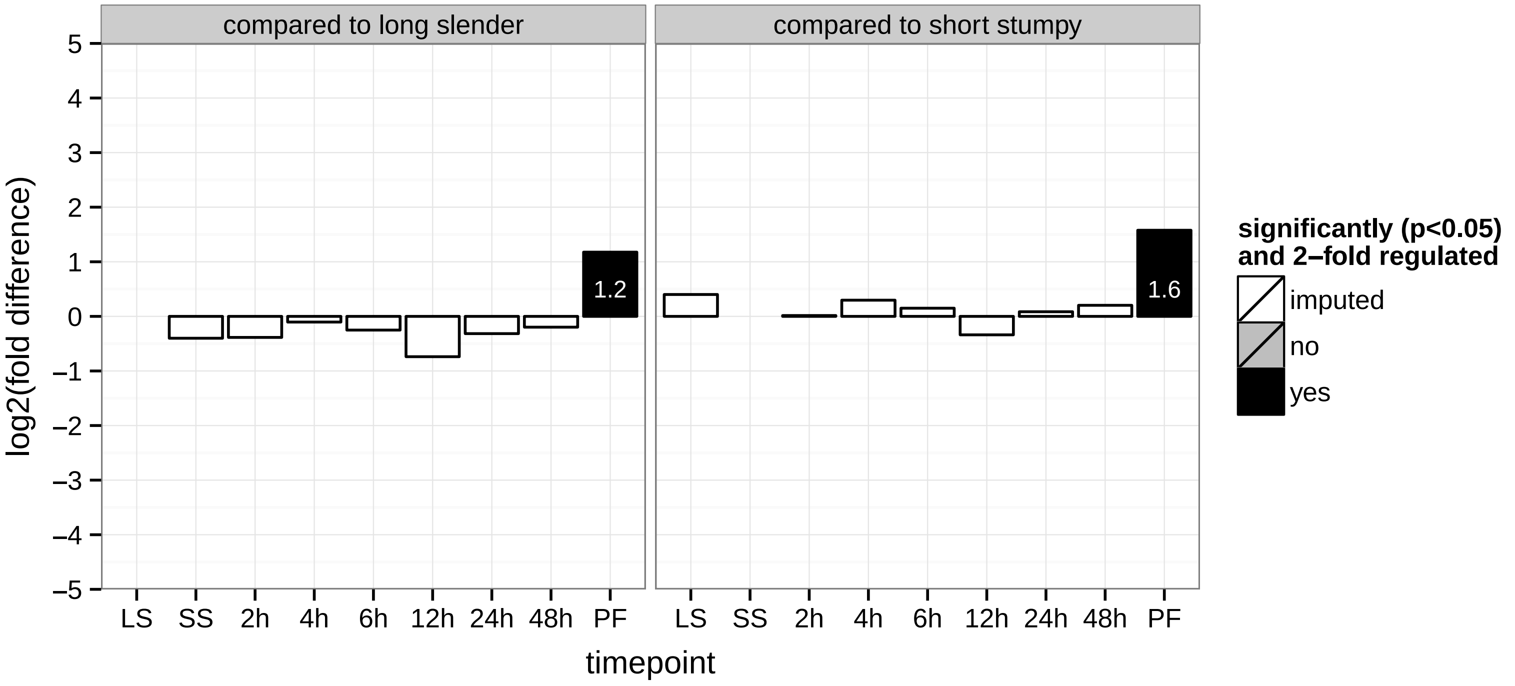

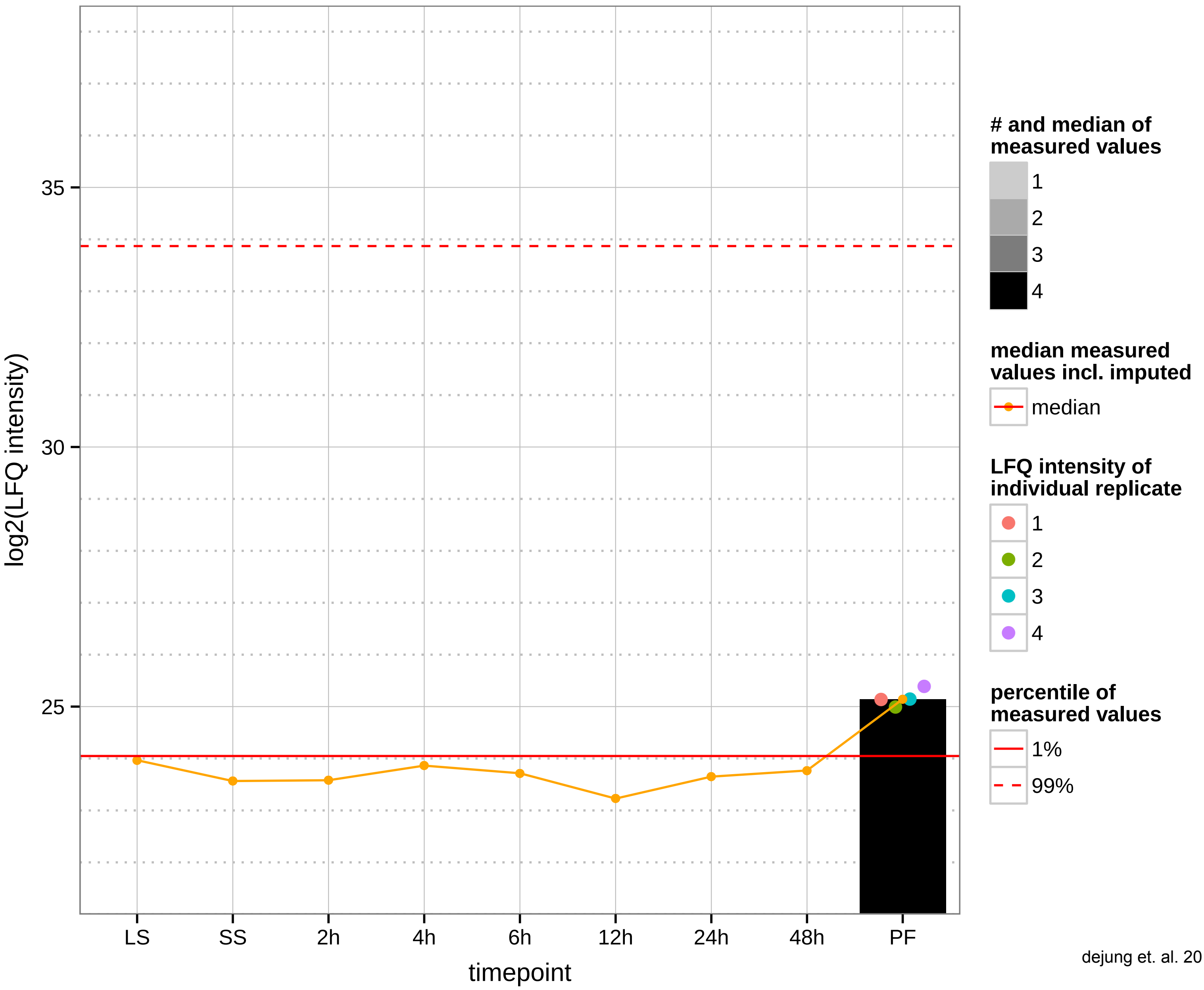

dejung et. al. 2015

aminopeptidase, putative, metallo–peptidase, Clan MF, Family M17
Tb927.11.6590
AGOF: null, aminopeptidase activity
AGOC: null, intracellular
AGOP: null, proteolysis
PGOF: aminopeptidase activity
PGOC: intracellular
PGOP: proteolysis

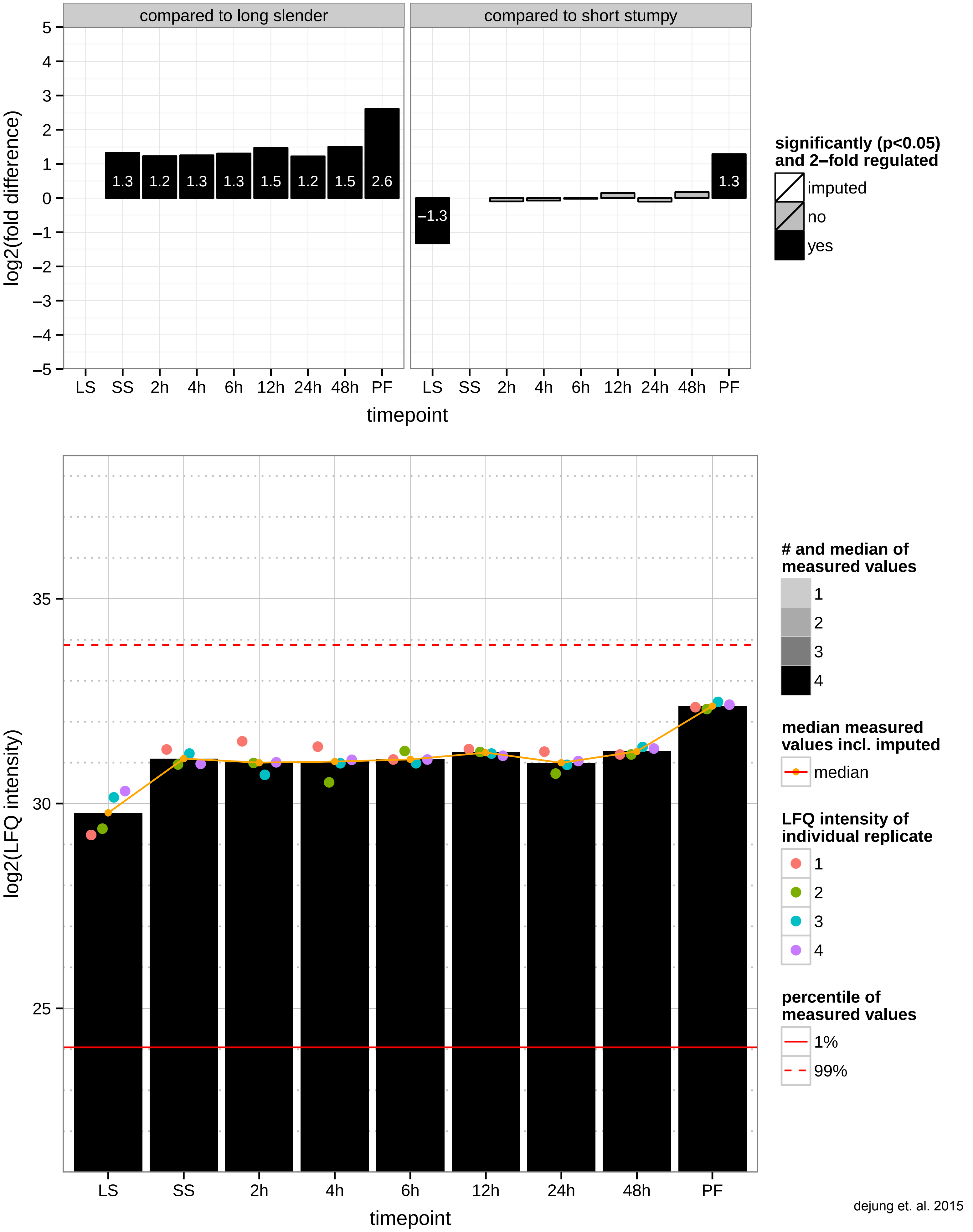

dejung et. al. 2015

hypothetical protein, conserved
Tb927.11.6980
AGOF: phosphoenolpyruvate carboxykinase activity, purine nucleotide binding
AGOC: mitochondrion
AGOP: gluconeogenesis
PGOF: phosphoenolpyruvate carboxykinase activity, purine nucleotide binding
PGOC: null
PGOP: gluconeogenesis

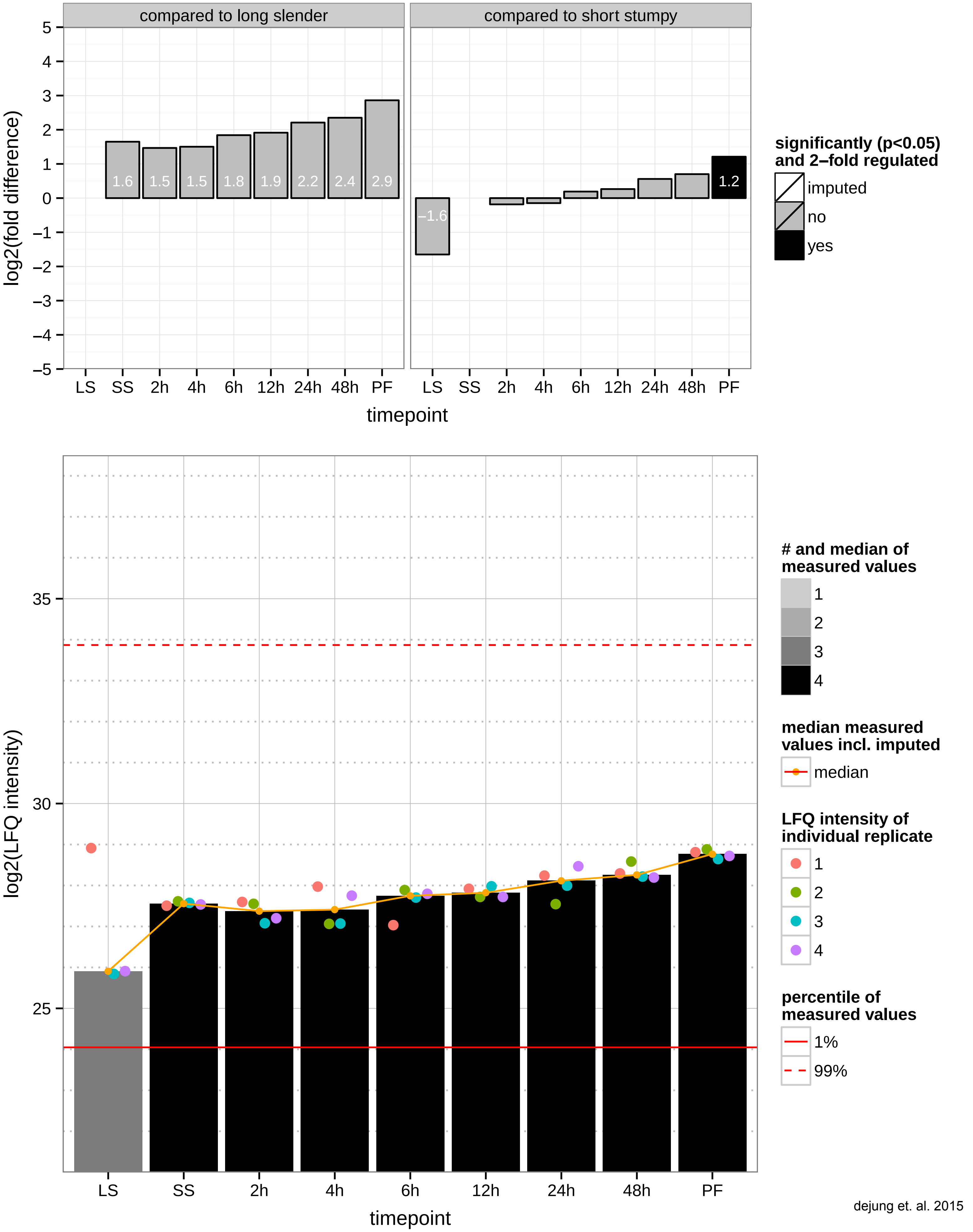

dejung et. al. 2015

hypothetical protein, conserved
Tb927.11.7050
AGOF: null
AGOC: null
AGOP: null
PGOF: null
PGOC: null
PGOP: null

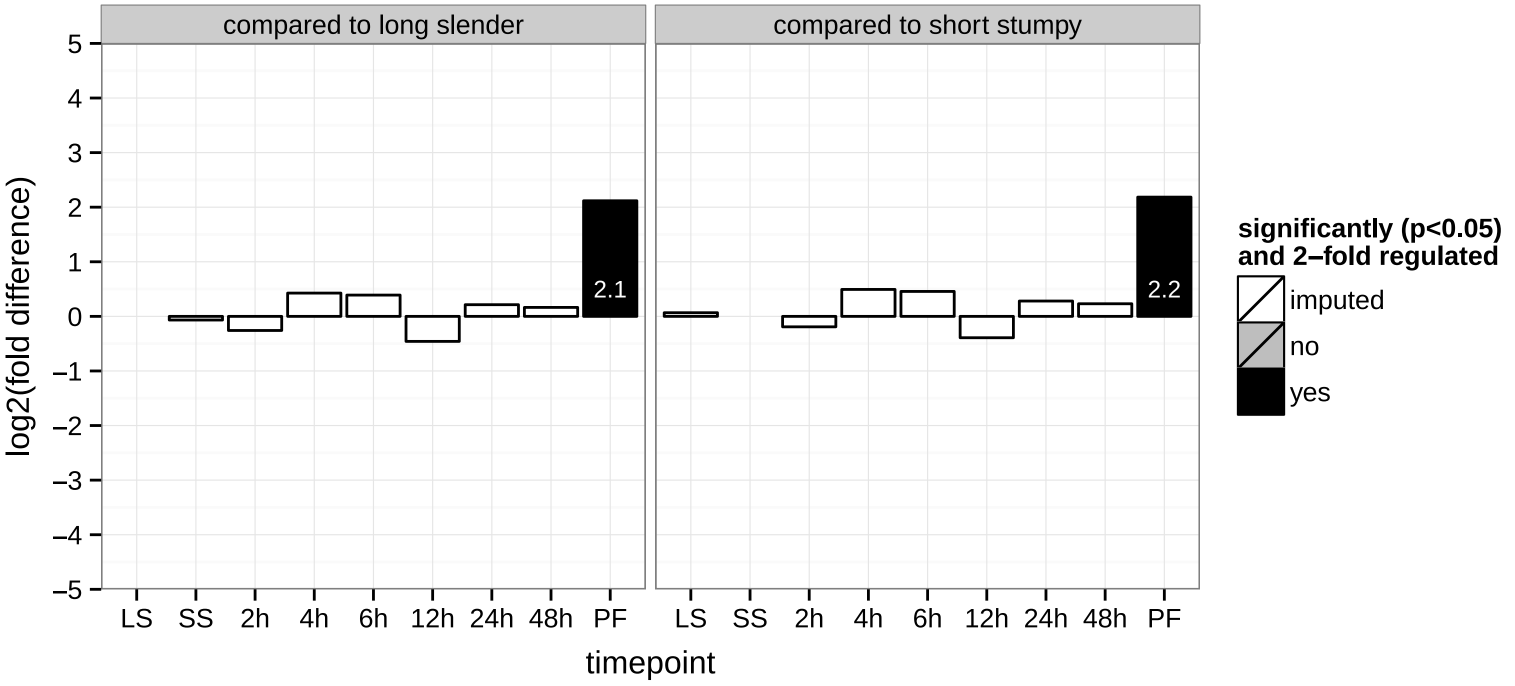

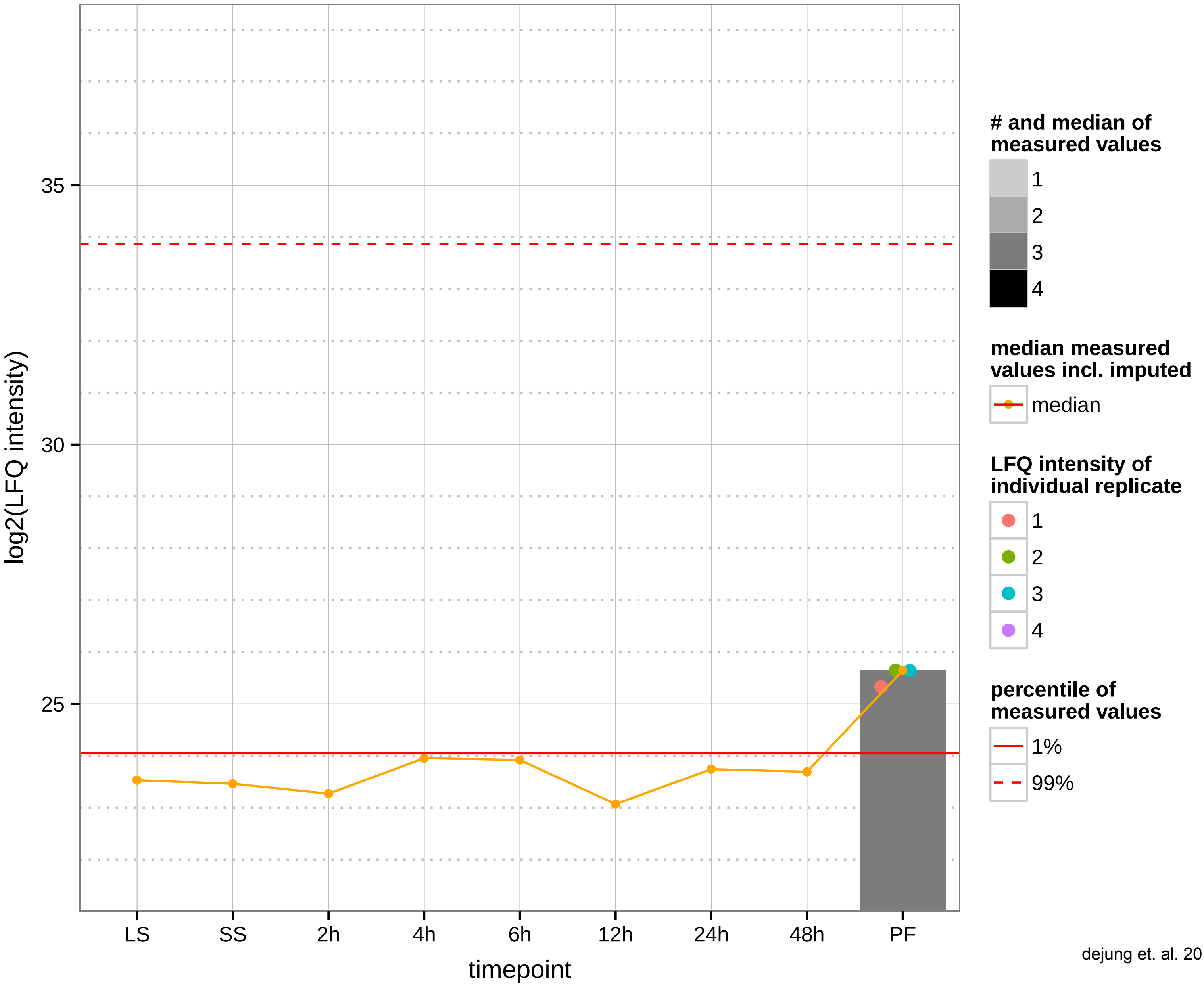

dejung et. al. 2015

protein phosphatase 1, putative
Tb927.11.8090
AGOF: hydrolase activity, protein serine/threonine phosphatase activity
AGOC: null
AGOP: cellular process
PGOF: hydrolase activity
PGOC: null
PGOP: null

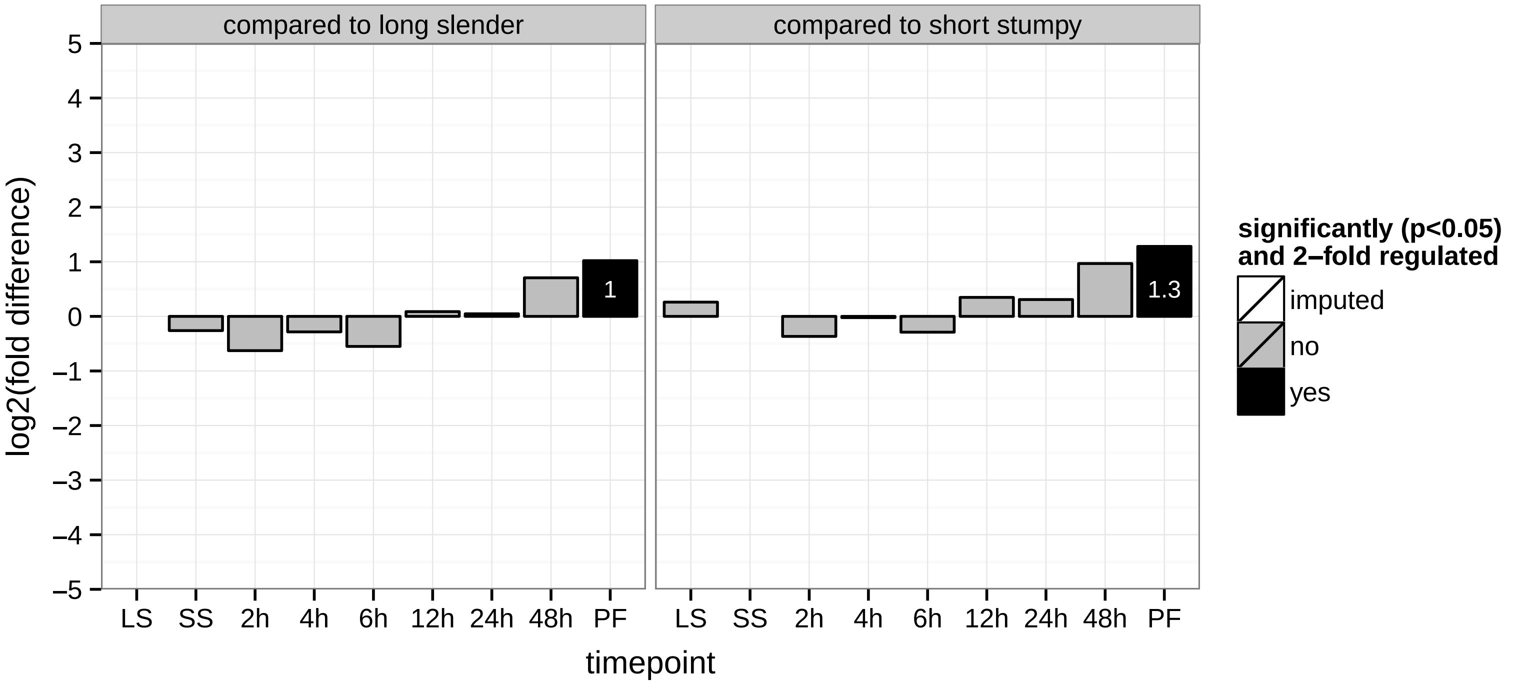

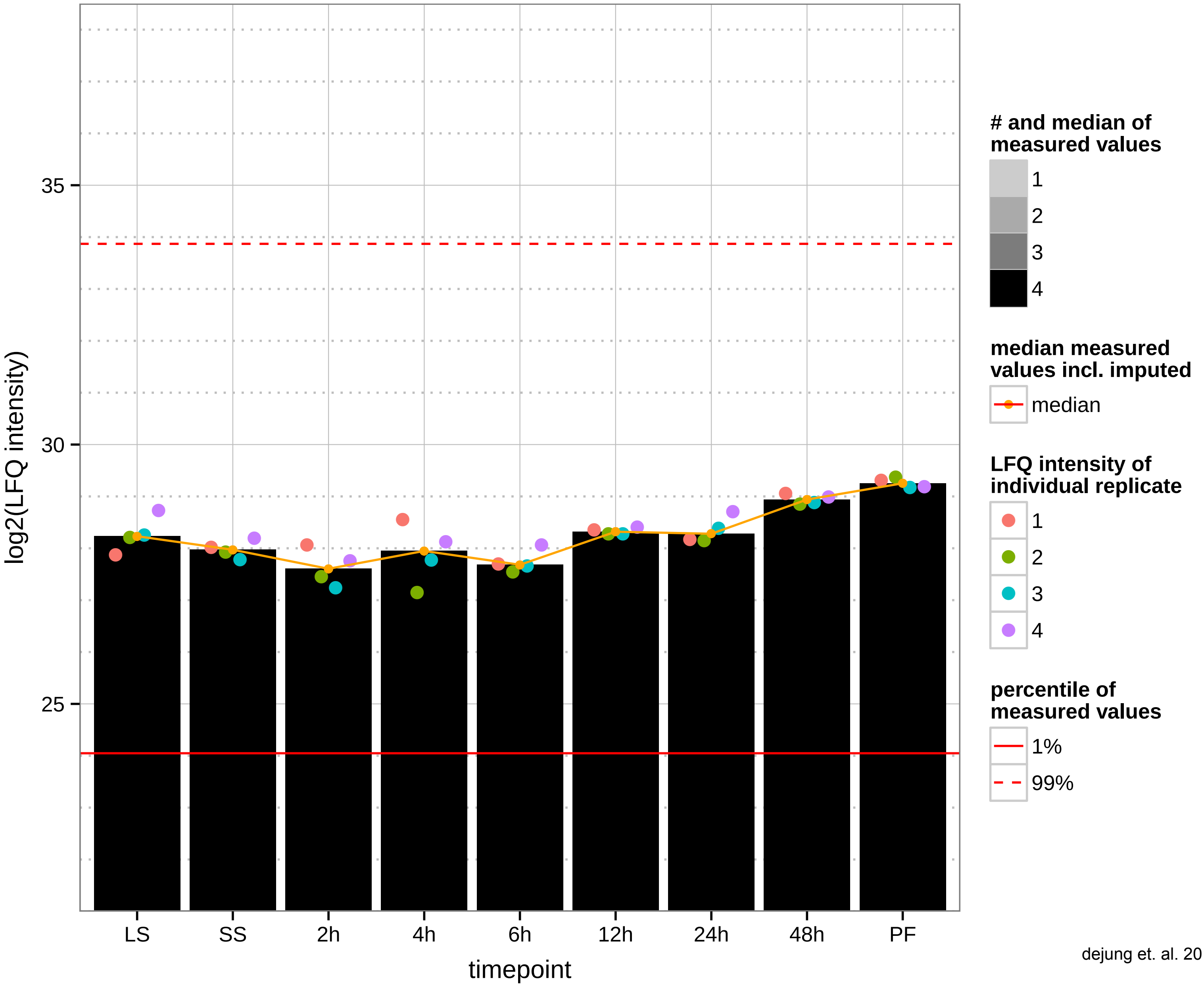

dejung et. al. 2015

DNA repair protein RAD51 (RAD51)
Tb927.11.8190
AGOF: ATP binding, ATPase activity, damaged DNA binding, double−stranded DNA binding, double−stranded DNA−depende
AGOC: nucleus, site of double−strand break
AGOP: DNA recombination, double−strand break repair via homologous recombination, evasion or tolerance of host immune
PGOF: ATP binding, DNA binding, DNA−dependent ATPase activity, damaged DNA binding, nucleoside−triphosphatase activi
PGOC: null
PGOP: DNA metabolic process, DNA repair

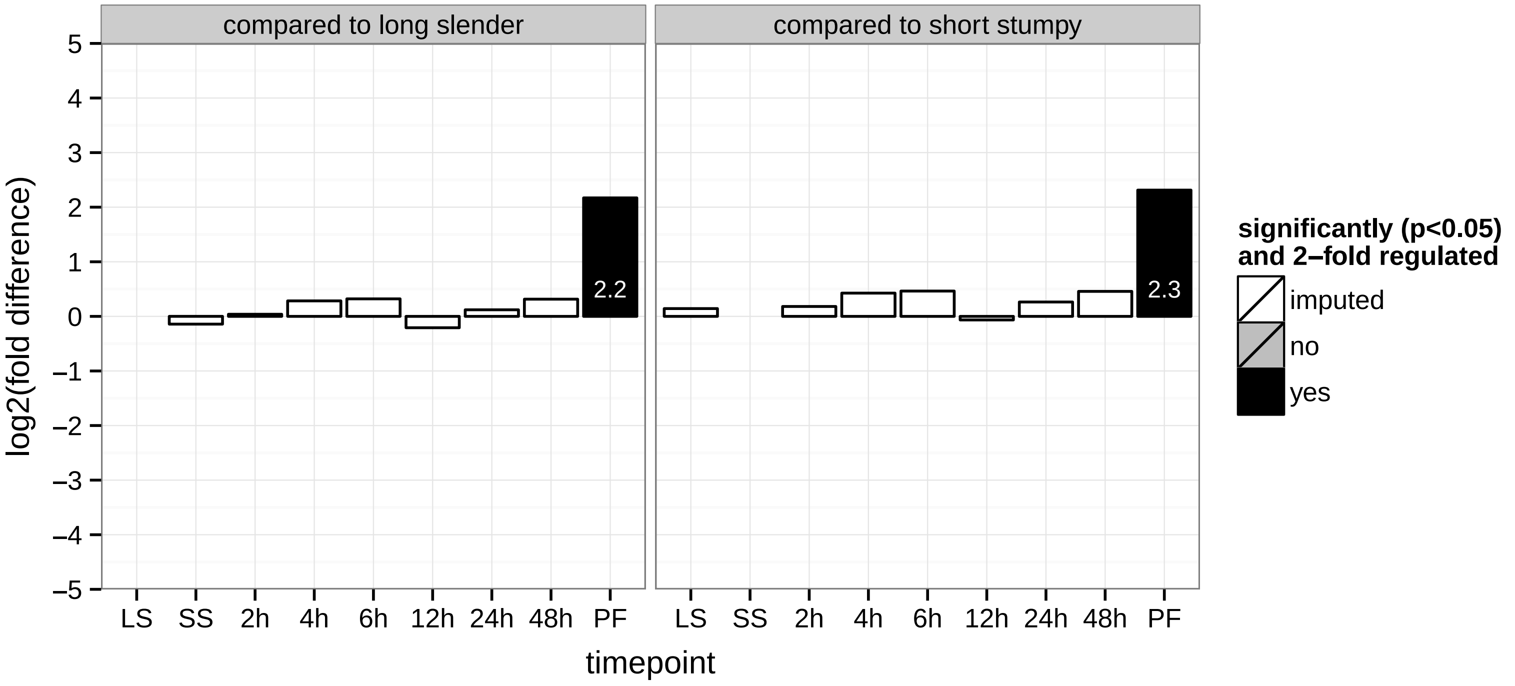

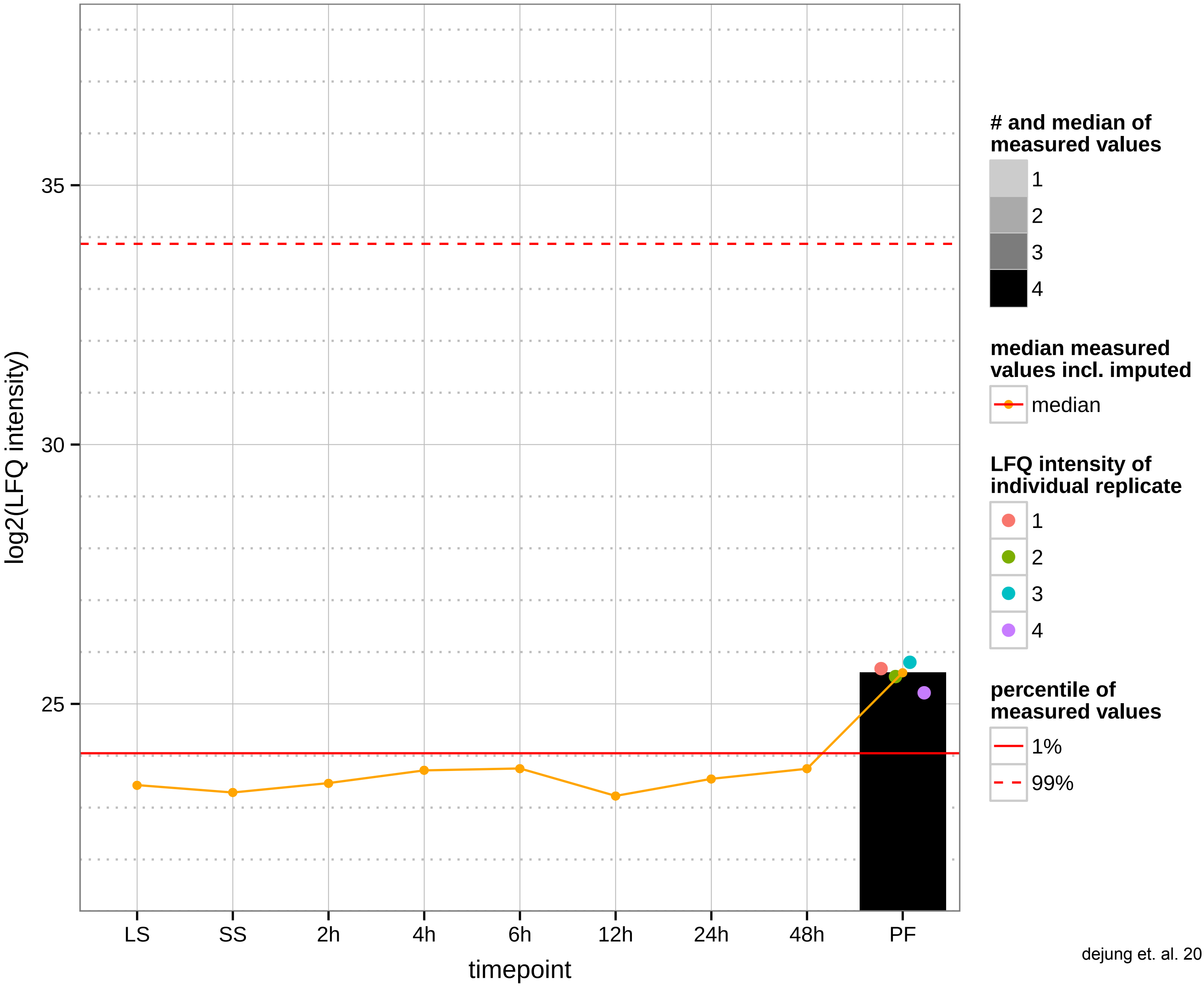

dejung et. al. 2015

hypothetical protein, conserved
Tb927.11.8230
AGOF: null
AGOC: null
AGOP: null
PGOF: null
PGOC: null
PGOP: null

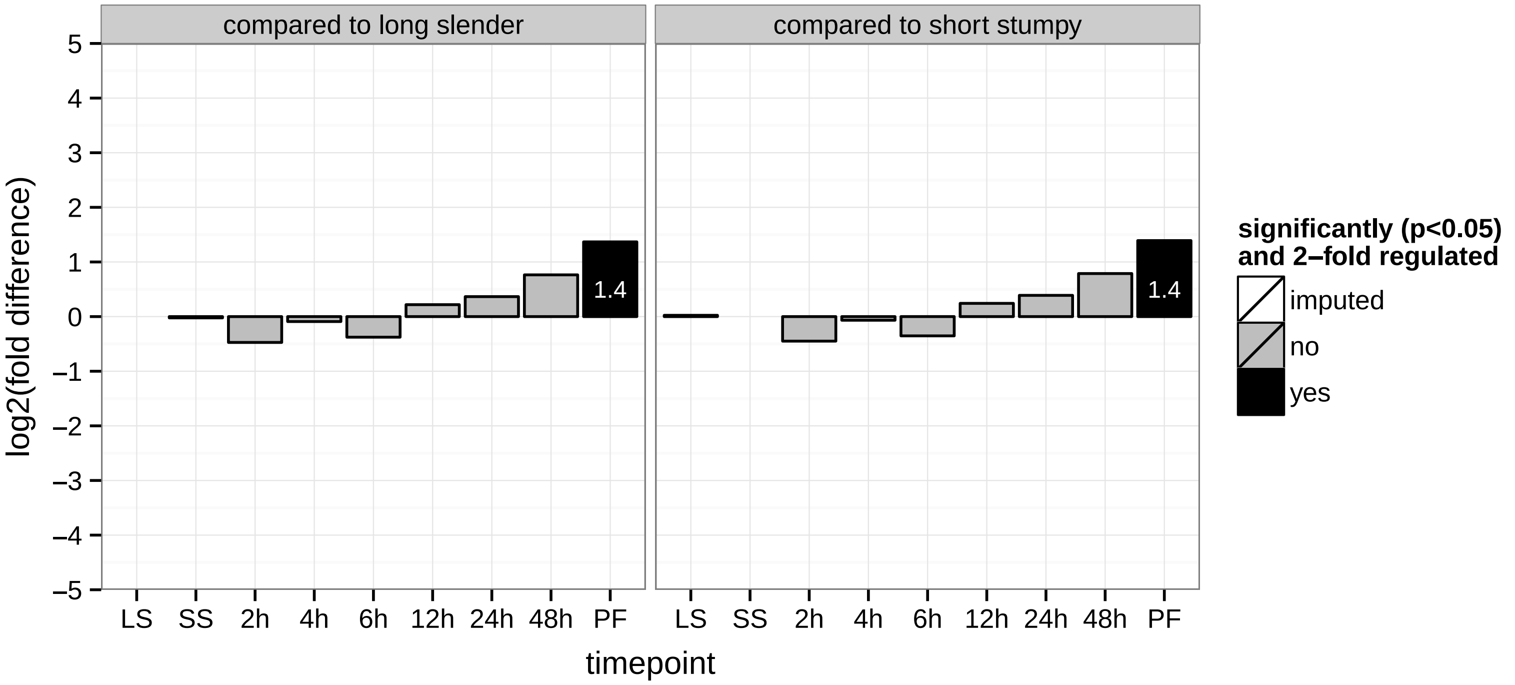

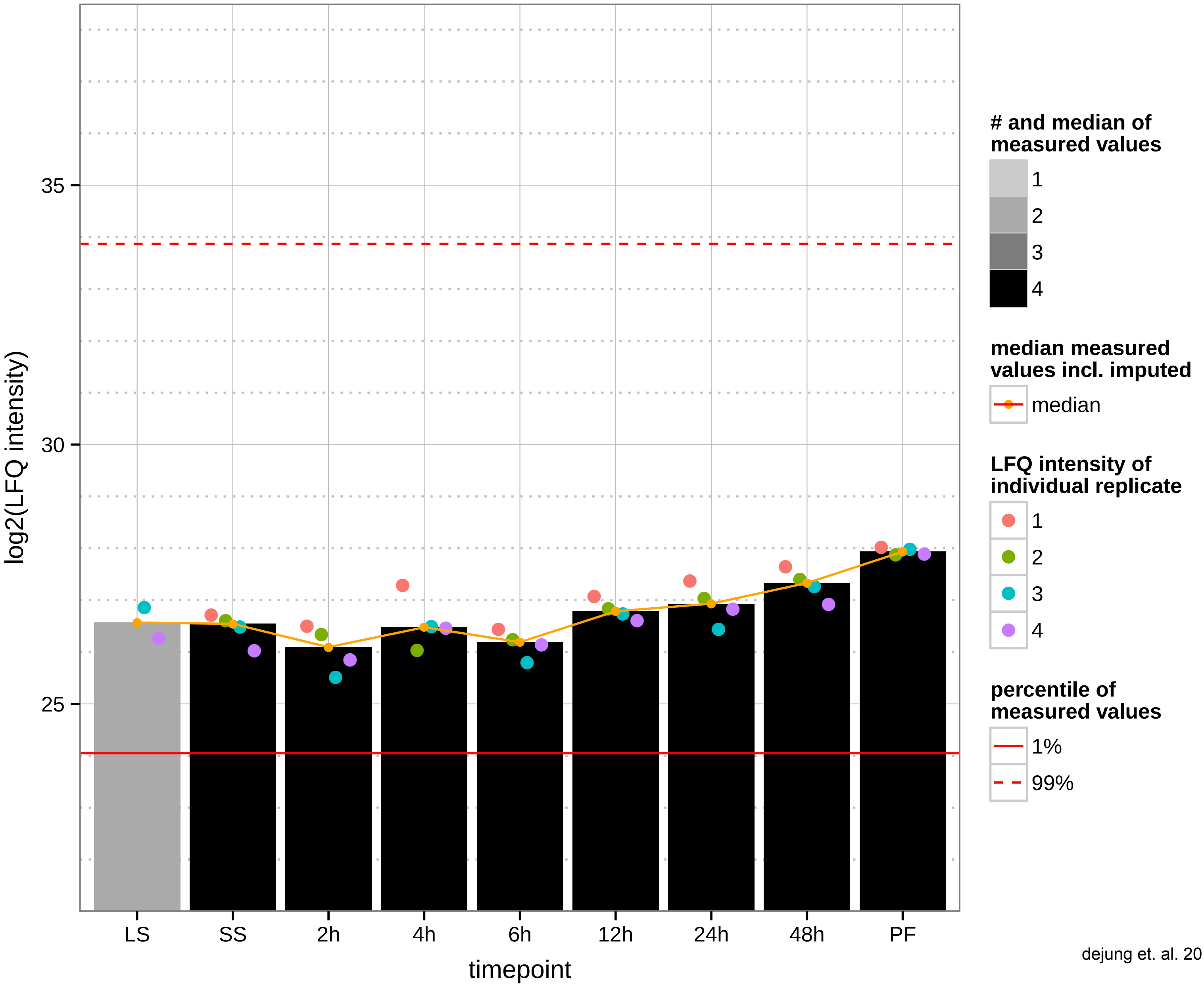

dejung et. al. 2015

hypothetical protein, conserved
Tb927.11.8270
AGOF: null
AGOC: null
AGOP: null
PGOF: protein binding
PGOC: null
PGOP: null

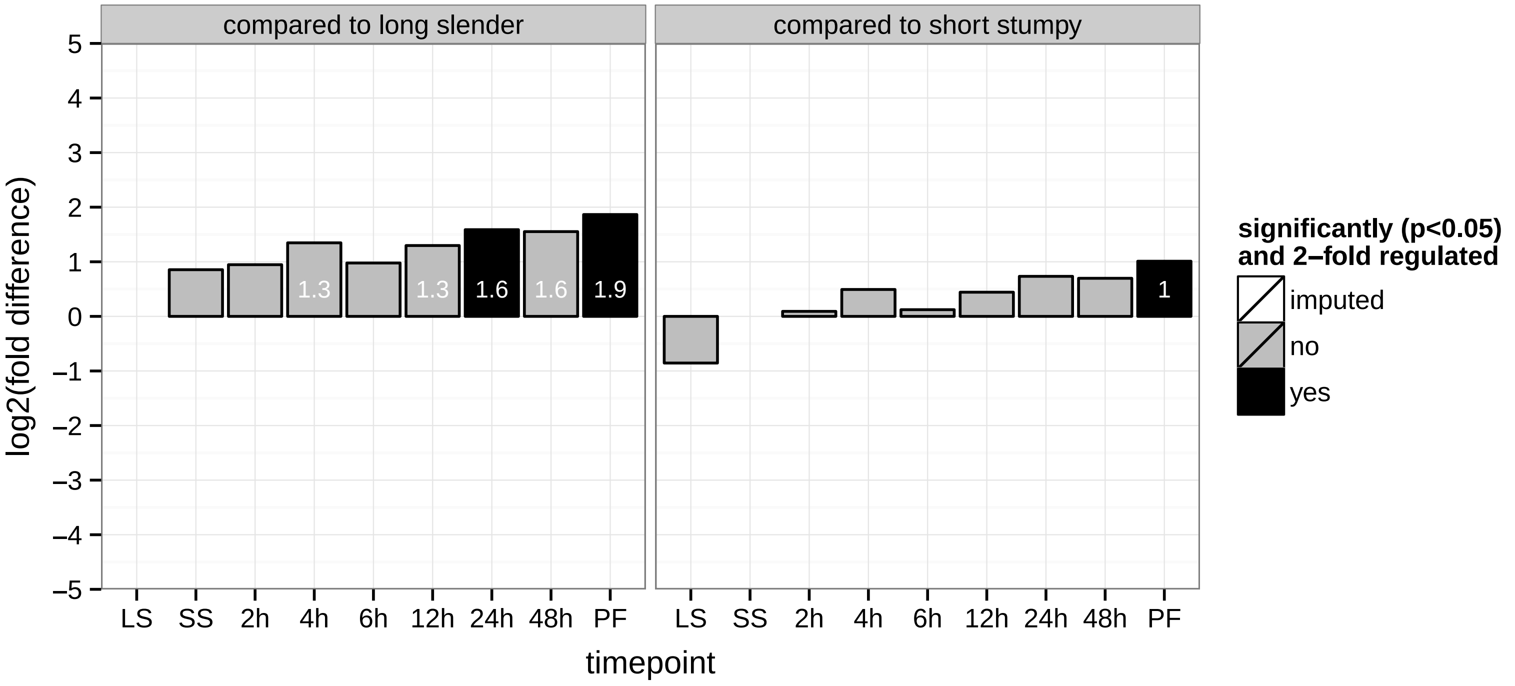

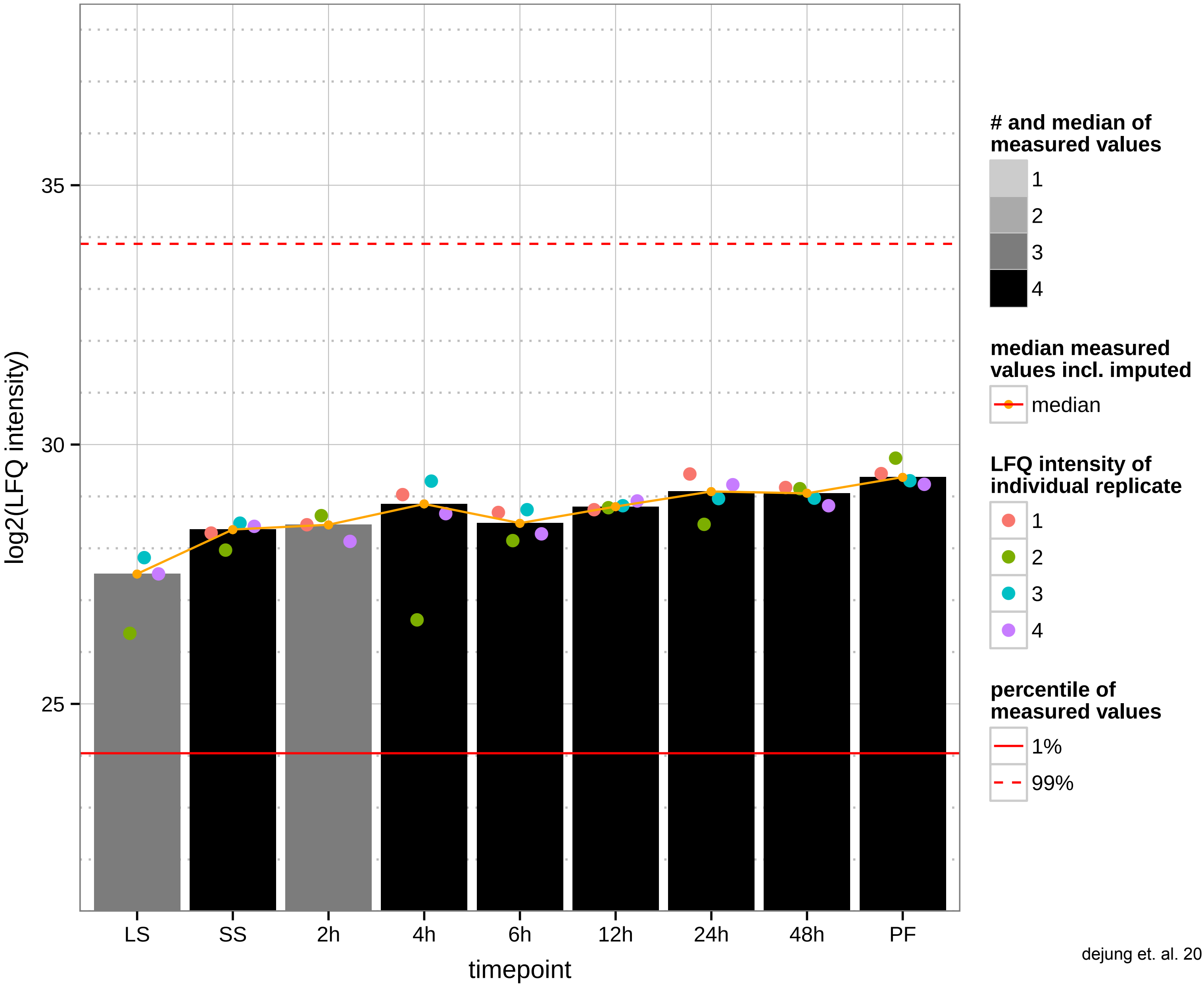

dejung et. al. 2015

hypothetical protein, conserved
Tb927.11.9120
AGOF: zinc ion binding
AGOC: null
AGOP: null
PGOF: ATP binding, DNA binding, protein binding, zinc ion binding
PGOC: null
PGOP: null

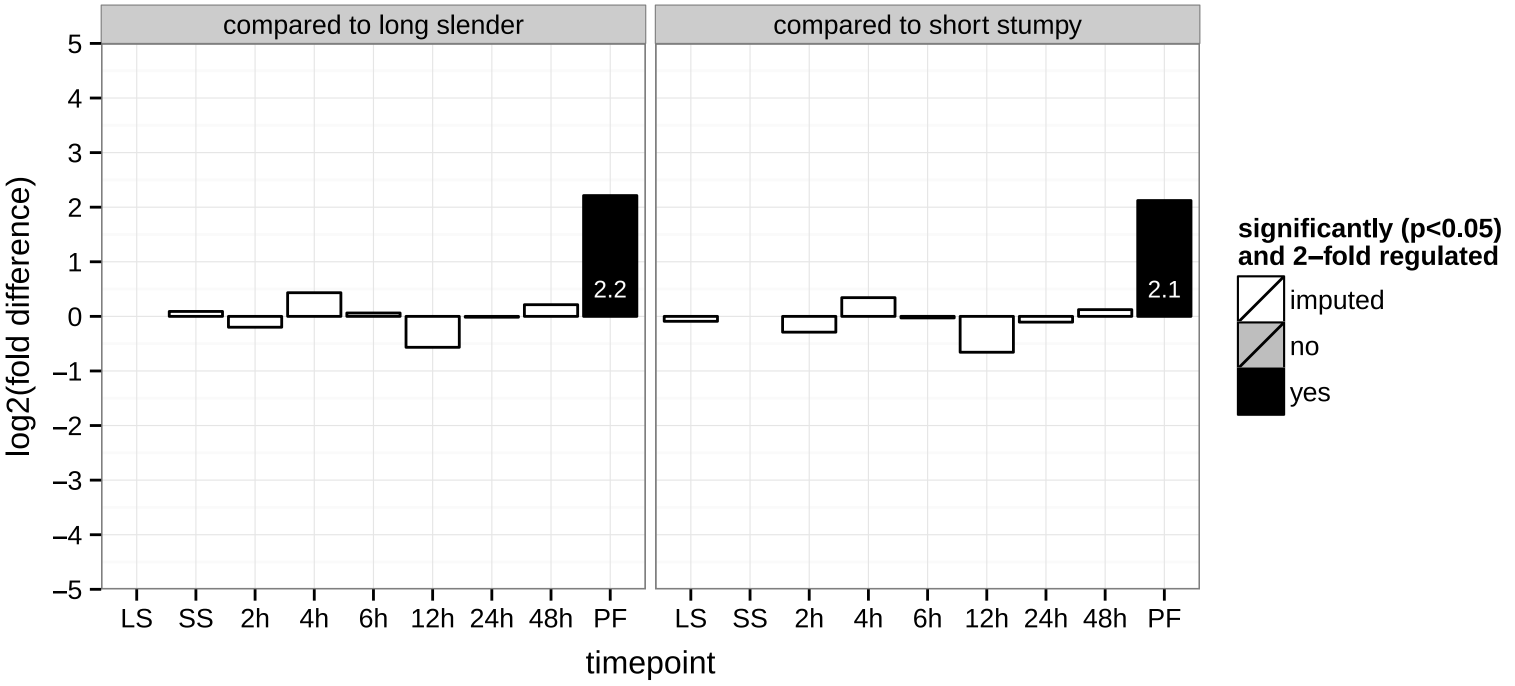

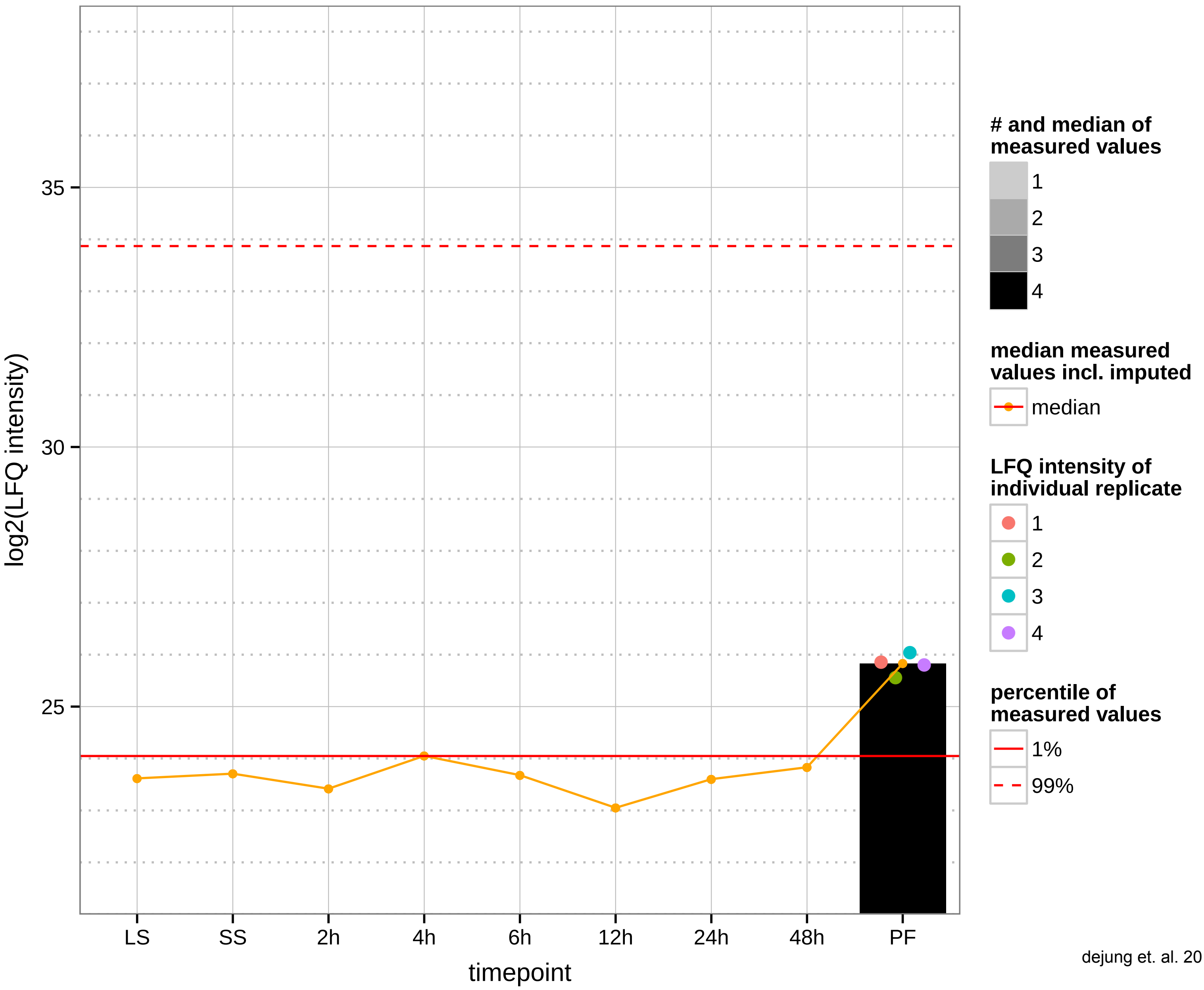

dejung et. al. 2015

cyclophilin type peptidyl–prolyl cis–trans isomerase, putative (PPIase)
Tb927.11.9450
AGOF: peptidyl–prolyl cis–trans isomerase activity
AGOC: null
AGOP: protein folding
PGOF: peptidyl–prolyl cis–trans isomerase activity
PGOC: null
PGOP: protein folding

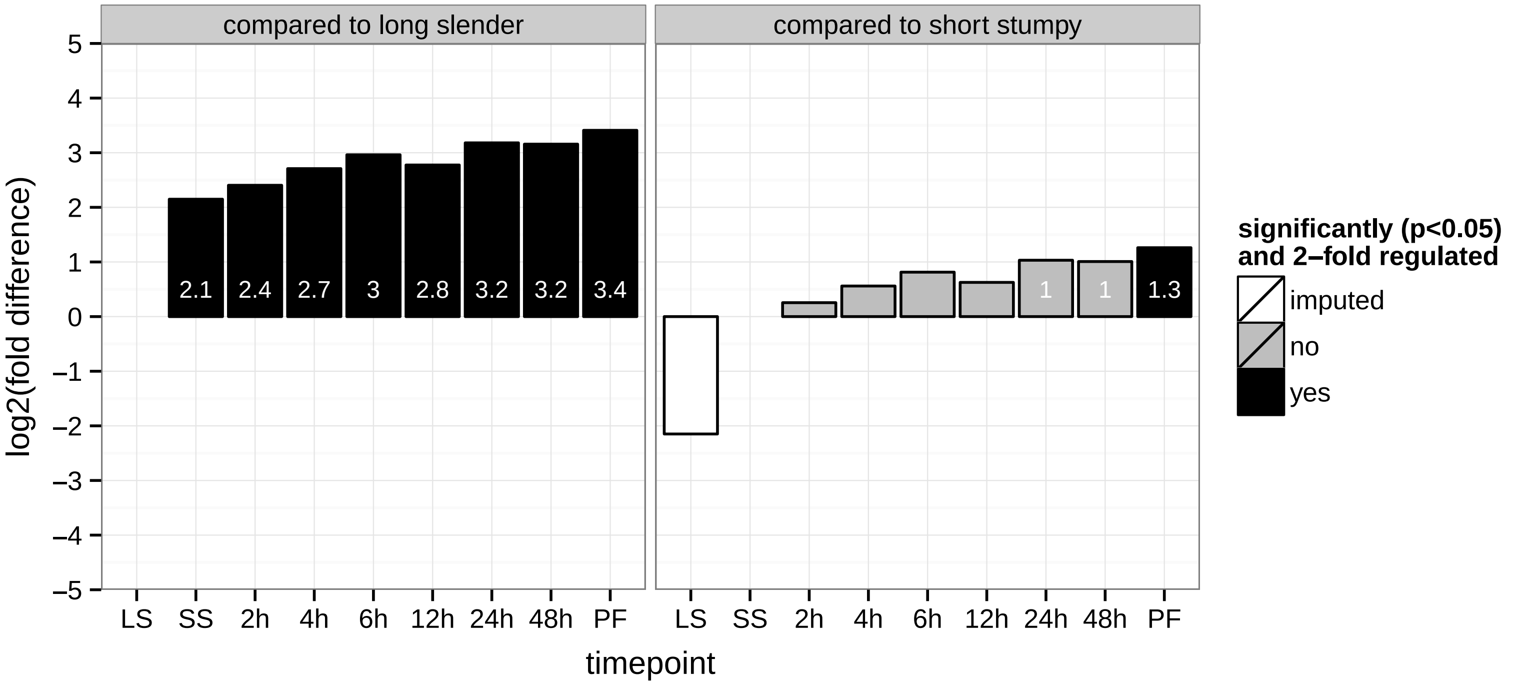

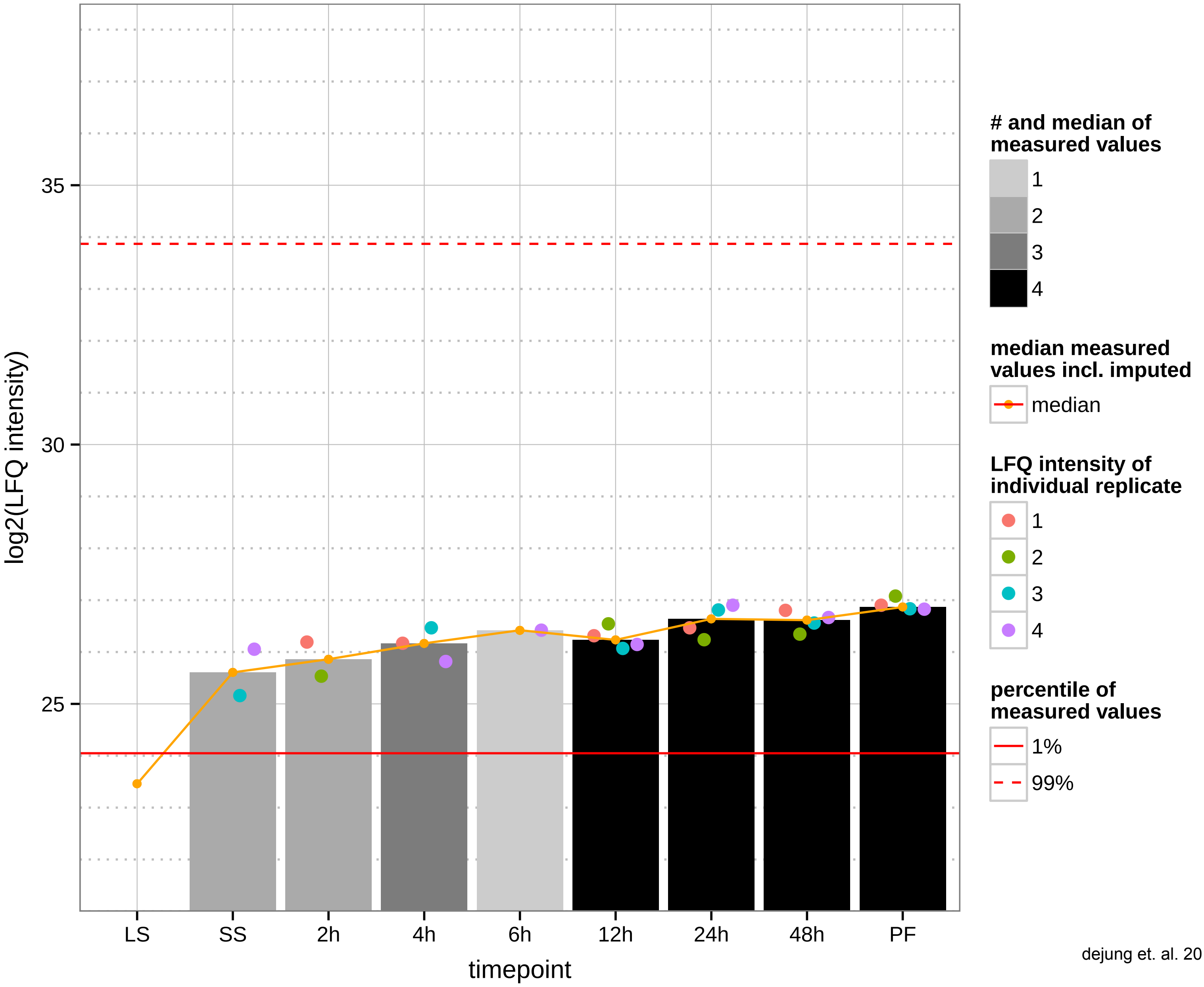

dejung et. al. 2015

S–adenosylhomocysteine hydrolase, putative
Tb927.11.9590
AGOF: adenosylhomocysteinase activity
AGOC: null
AGOP: one–carbon metabolic process
PGOF: adenosylhomocysteinase activity
PGOC: null
PGOP: one–carbon metabolic process

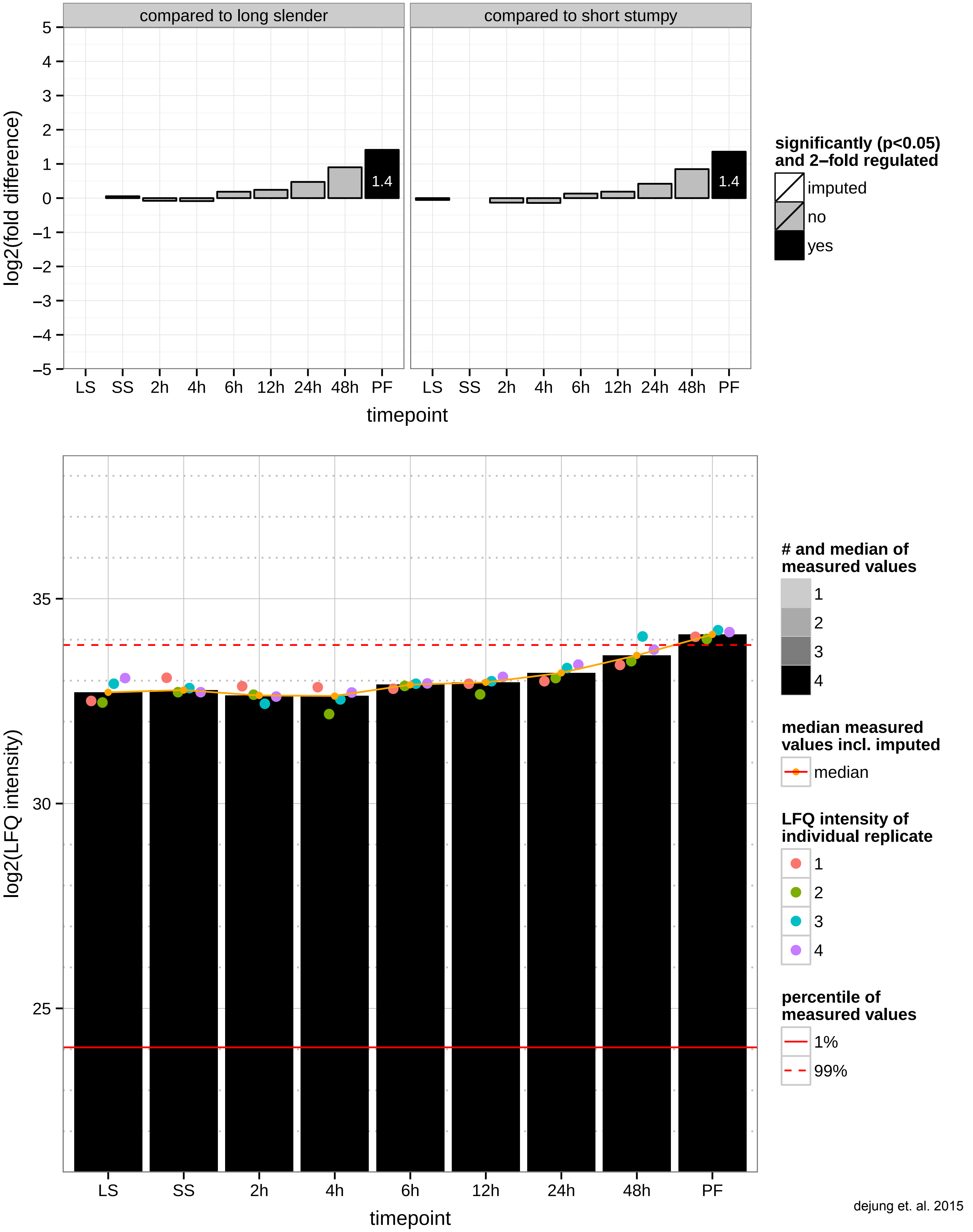

dejung et. al. 2015

conserved protein
Tb927.11.9780
AGOF: null
AGOC: null
AGOP: null
PGOF: null
PGOC: null
PGOP: null

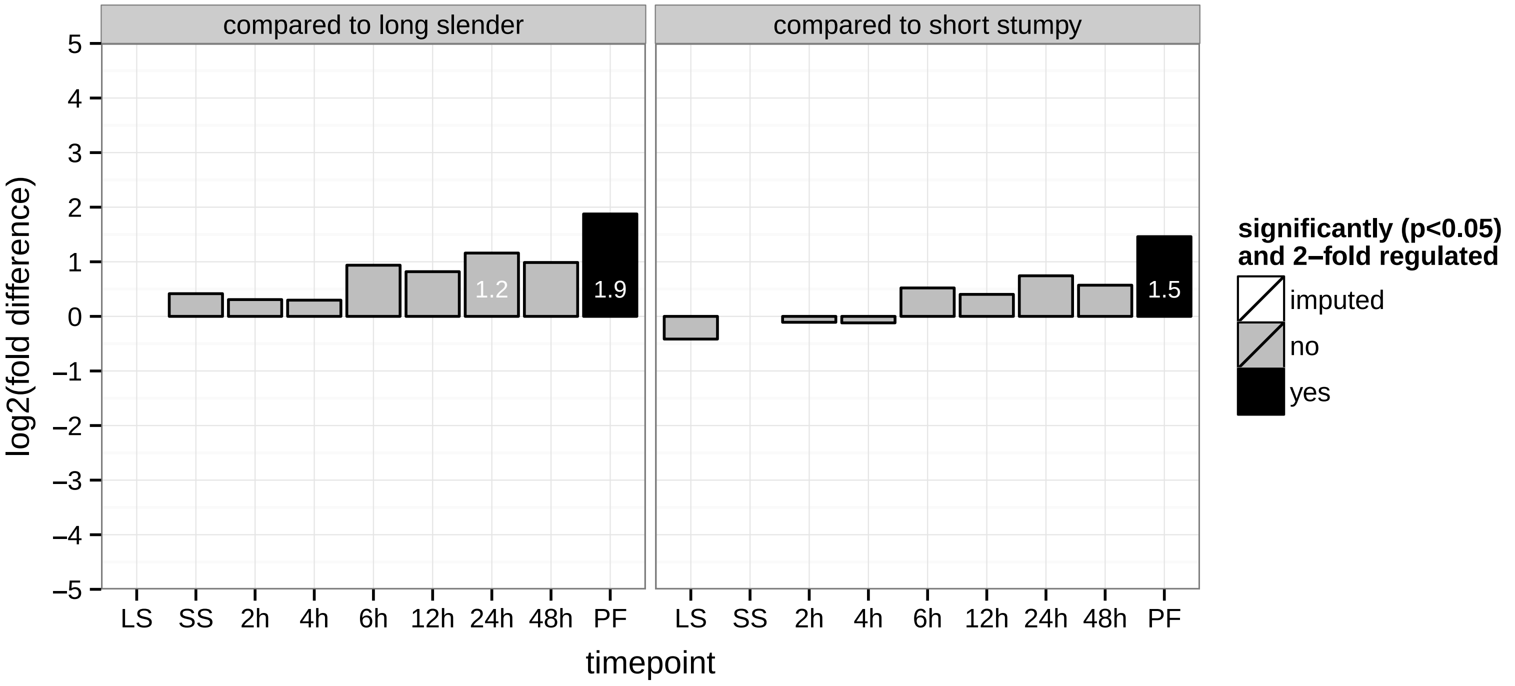

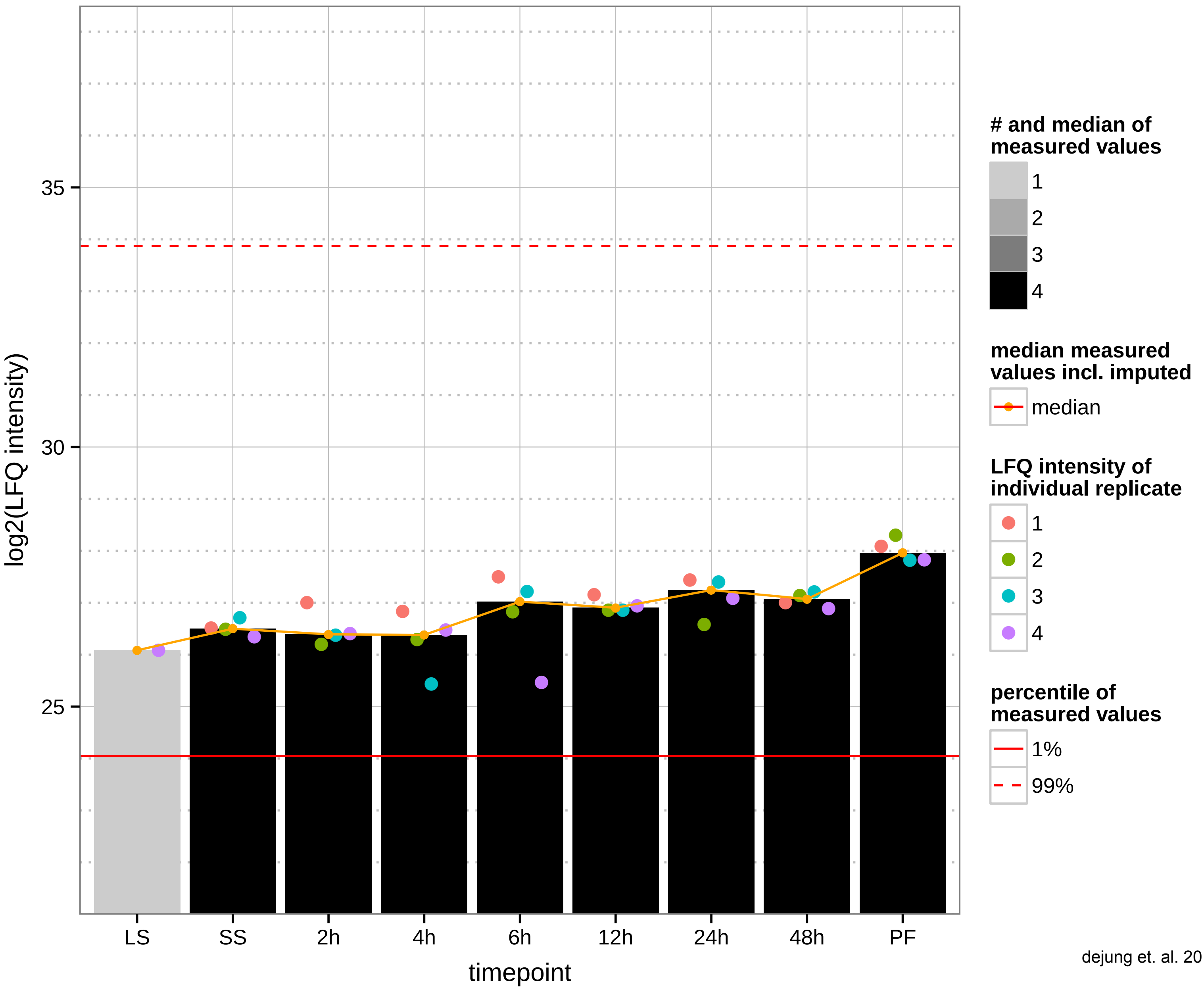

dejung et. al. 2015

hypothetical protein, conserved
Tb927.11.9870
AGOF: null
AGOC: null
AGOP: null
PGOF: null
PGOC: null
PGOP: null

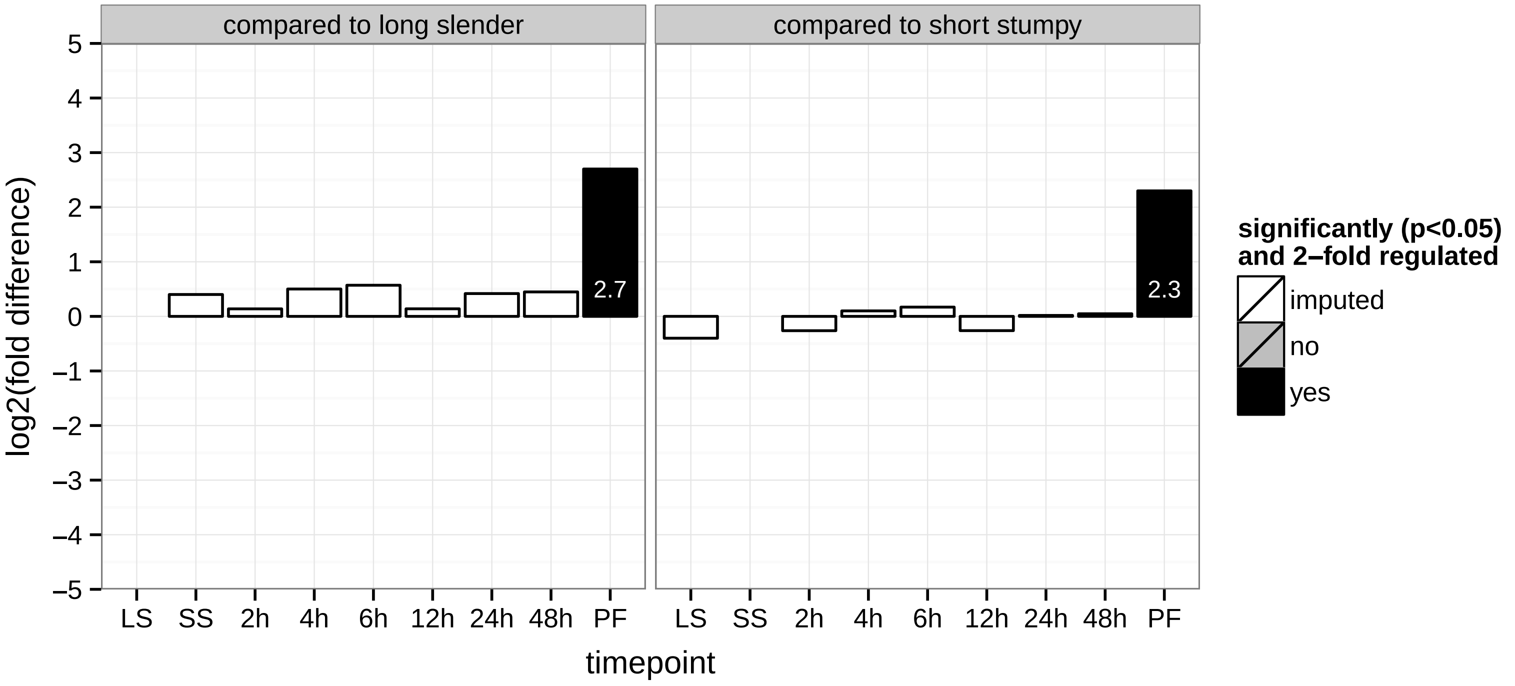

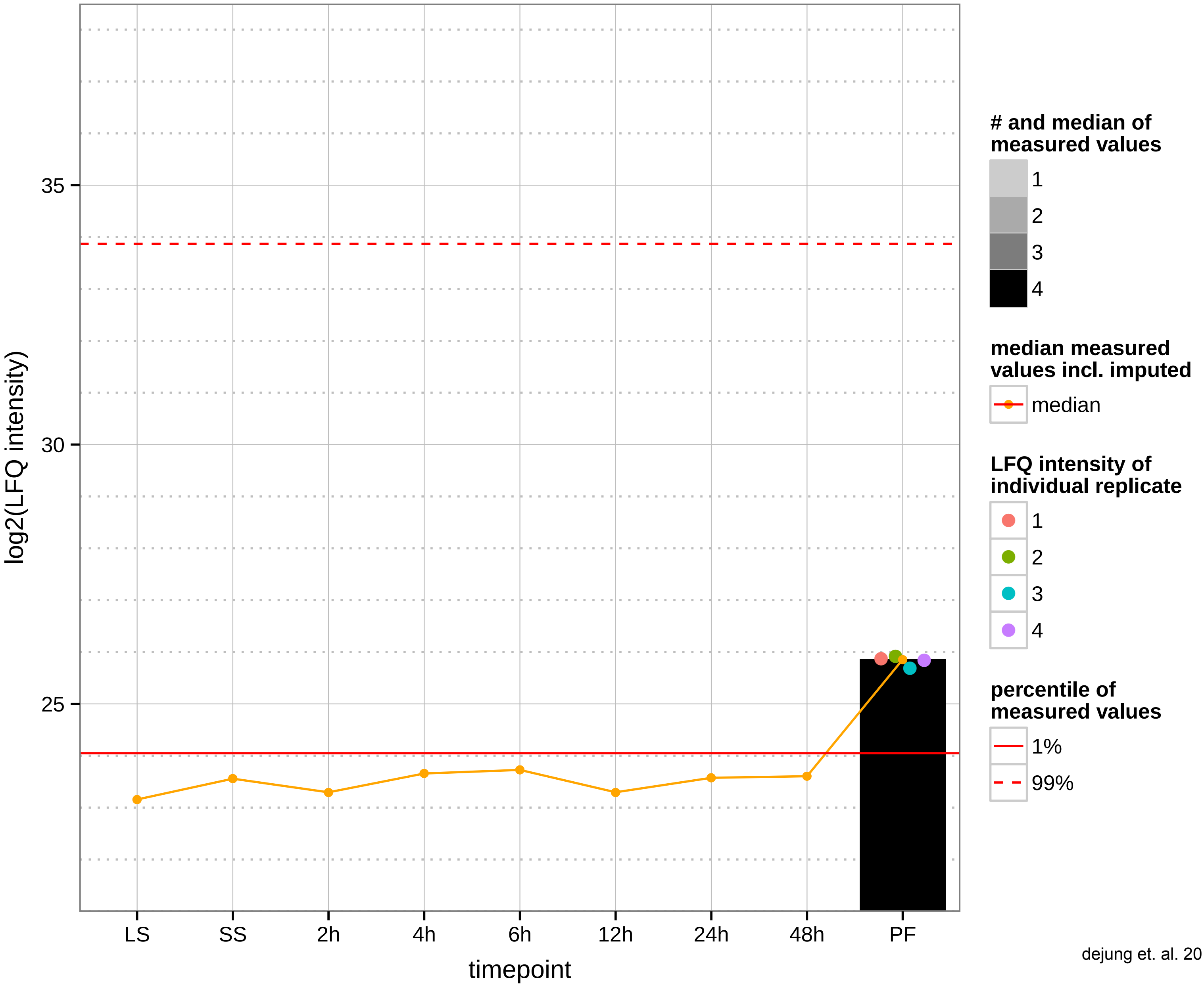

dejung et. al. 2015

signal recognition particle receptor alpha subunit, putative
Tb927.11.9890
AGOF: GTP binding, nucleoside–triphosphatase activity, signal recognition particle binding
AGOC: signal recognition particle receptor complex
AGOP: SRP–dependent cotranslational protein targeting to membrane, cotranslational protein targeting to membrane
PGOF: GTP binding, nucleoside–triphosphatase activity, nucleotide binding
PGOC: null
PGOP: SRP–dependent cotranslational protein targeting to membrane, transport

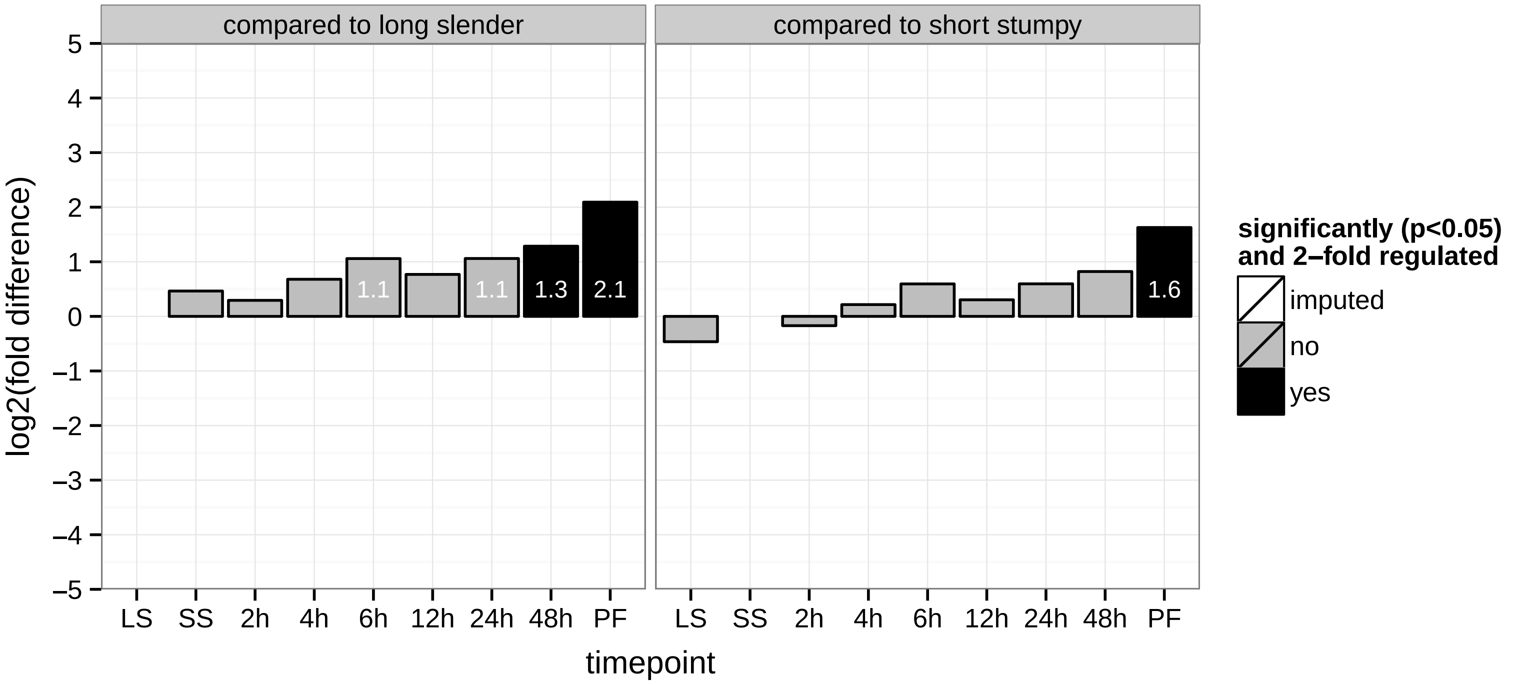

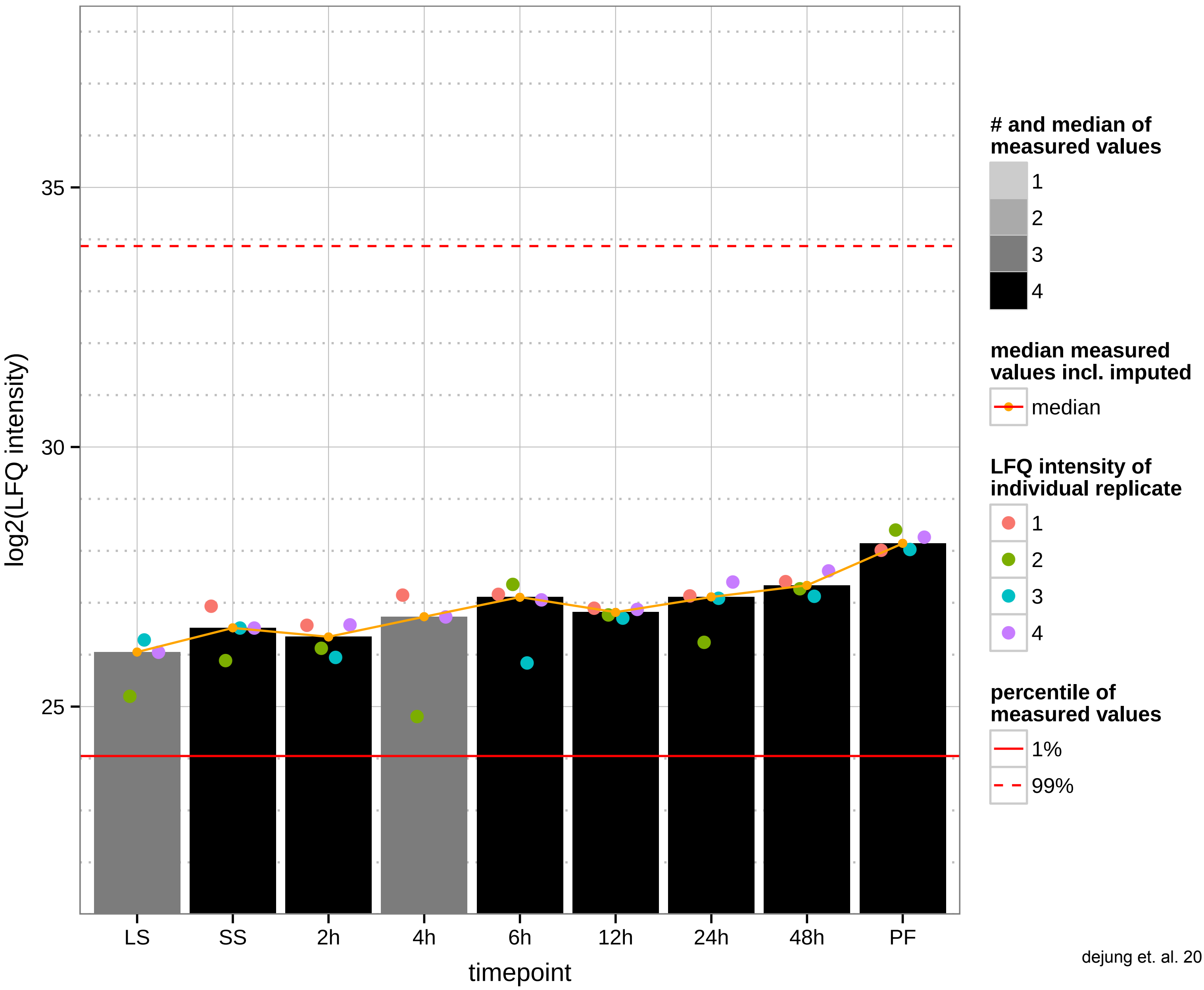

dejung et. al. 2015

hypothetical protein, conserved
Tb927.2.1760
AGOF: null
AGOC: null
AGOP: null
PGOF: protein binding
PGOC: null
PGOP: null

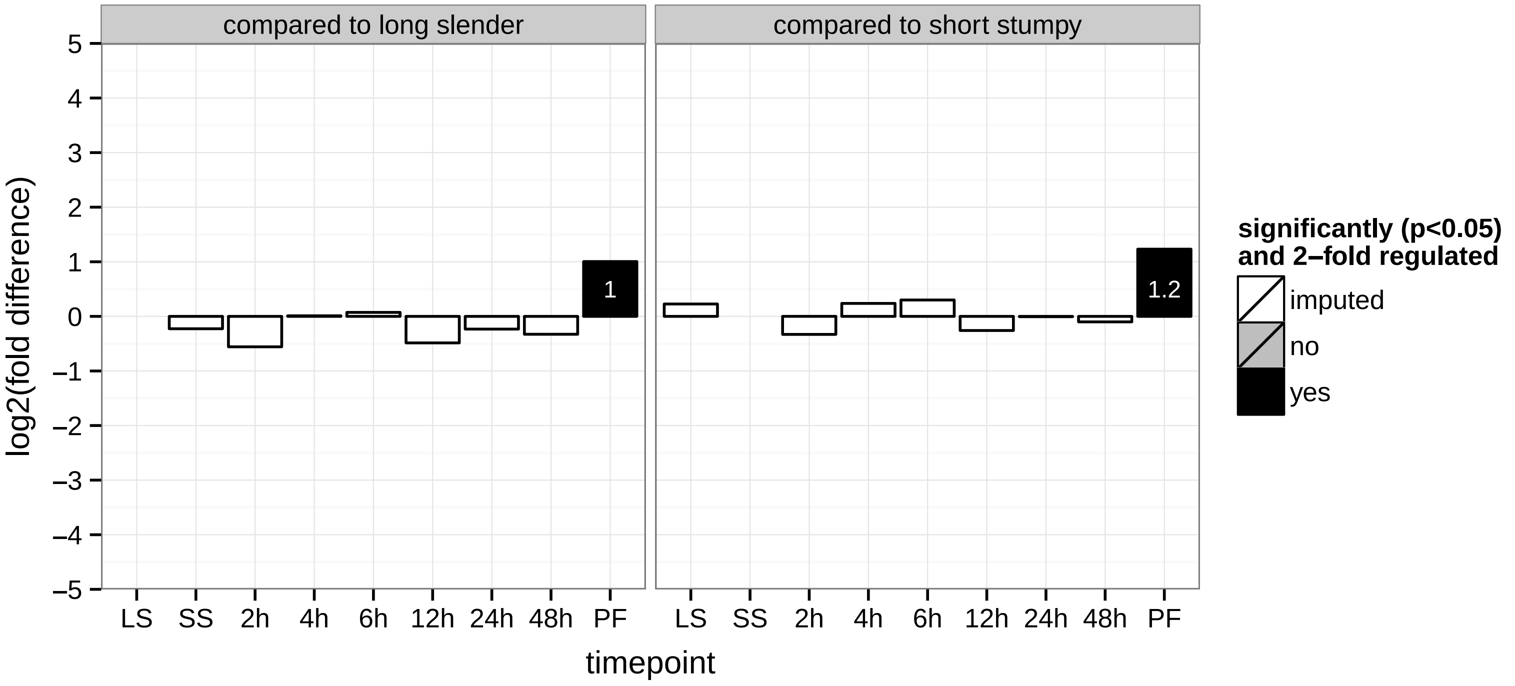

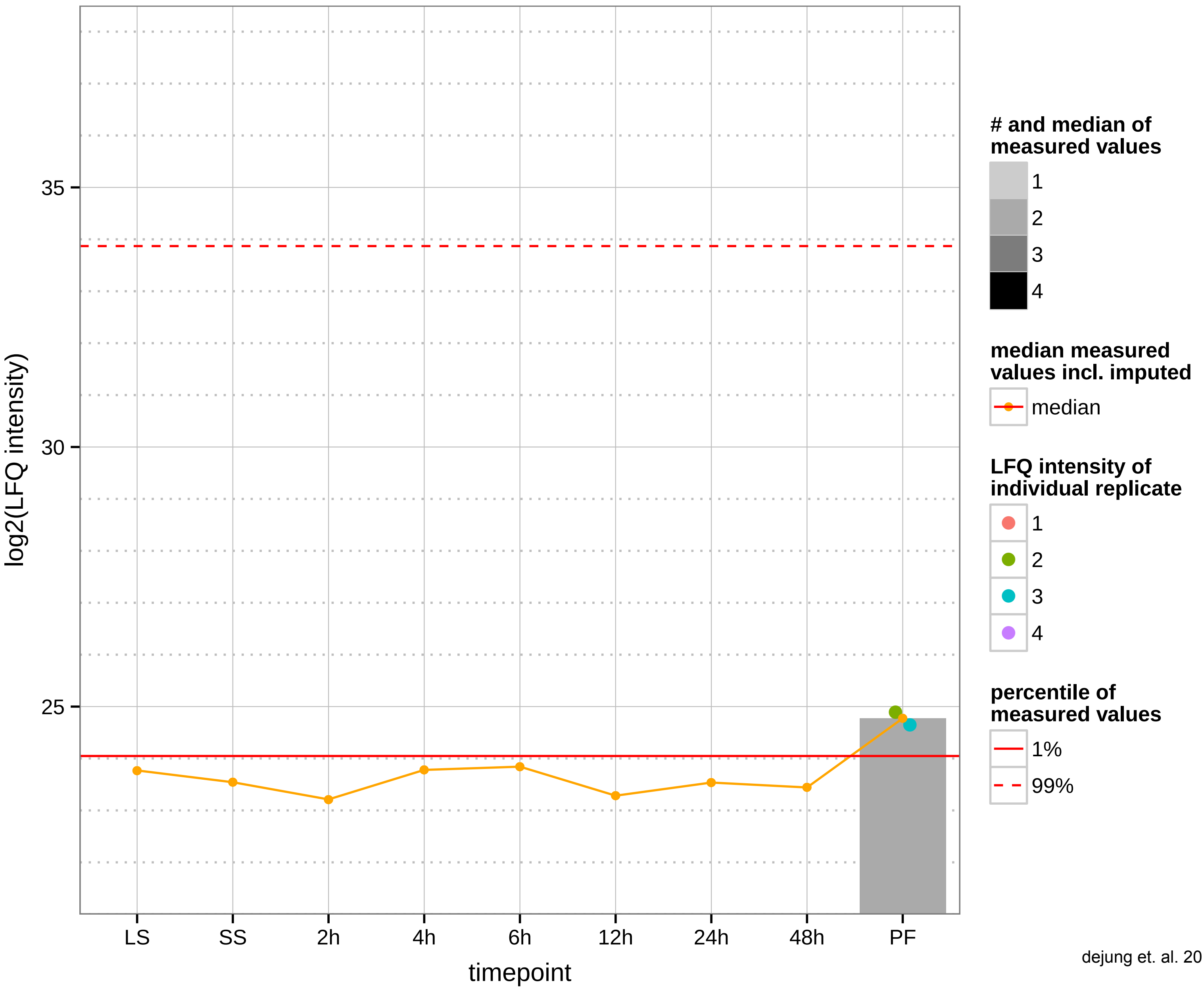

dejung et. al. 2015

hypothetical protein, conserved
Tb927.2.2950
AGOF: null
AGOC: null
AGOP: null
PGOF: null
PGOC: null
PGOP: null

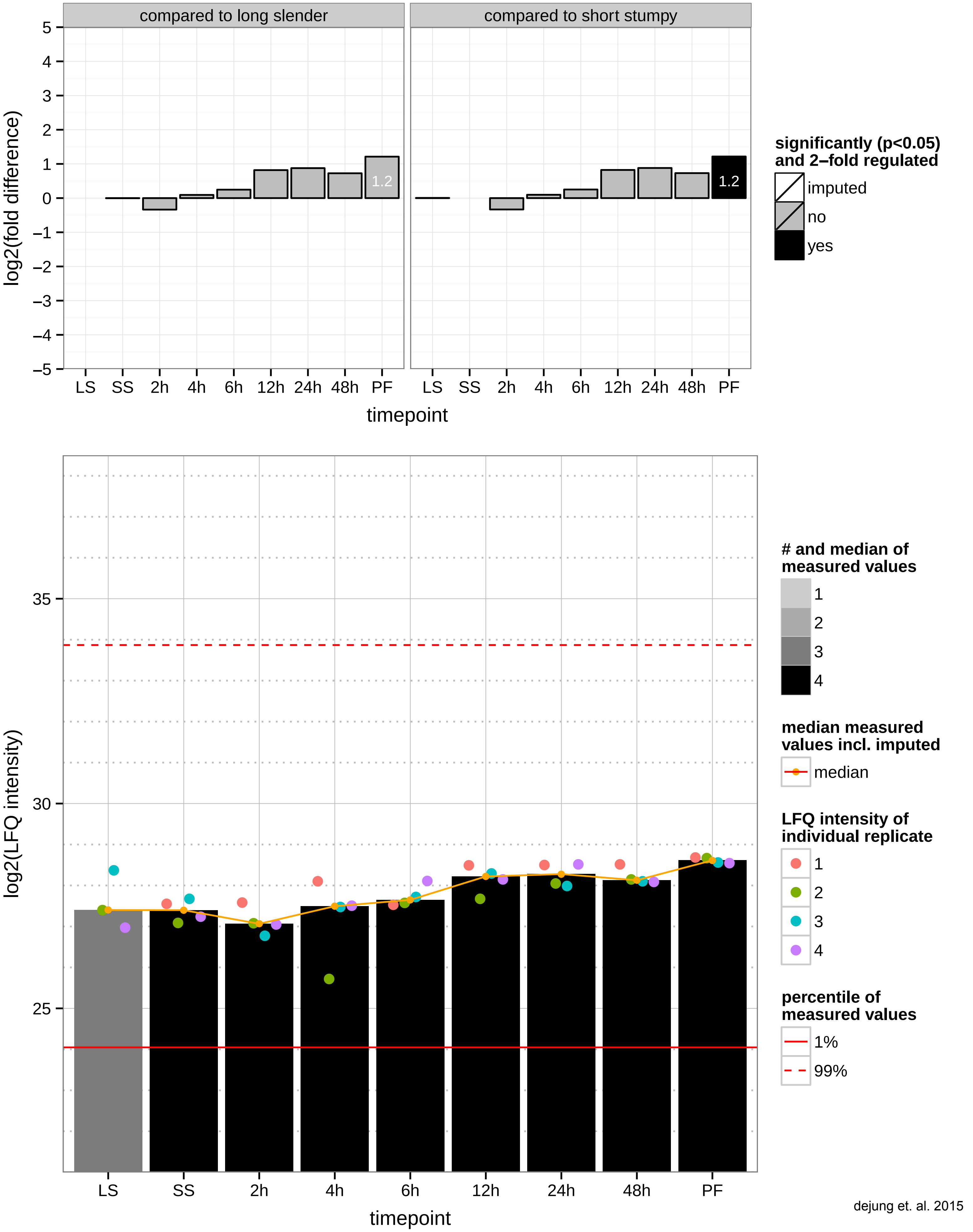

dejung et. al. 2015

translation initiation factor IF–2, putative
Tb927.2.3780
AGOF: GTP binding, GTPase activity, translation initiation factor activity
AGOC: null
AGOP: translation
PGOF: GTP binding, GTPase activity
PGOC: null
PGOP: null

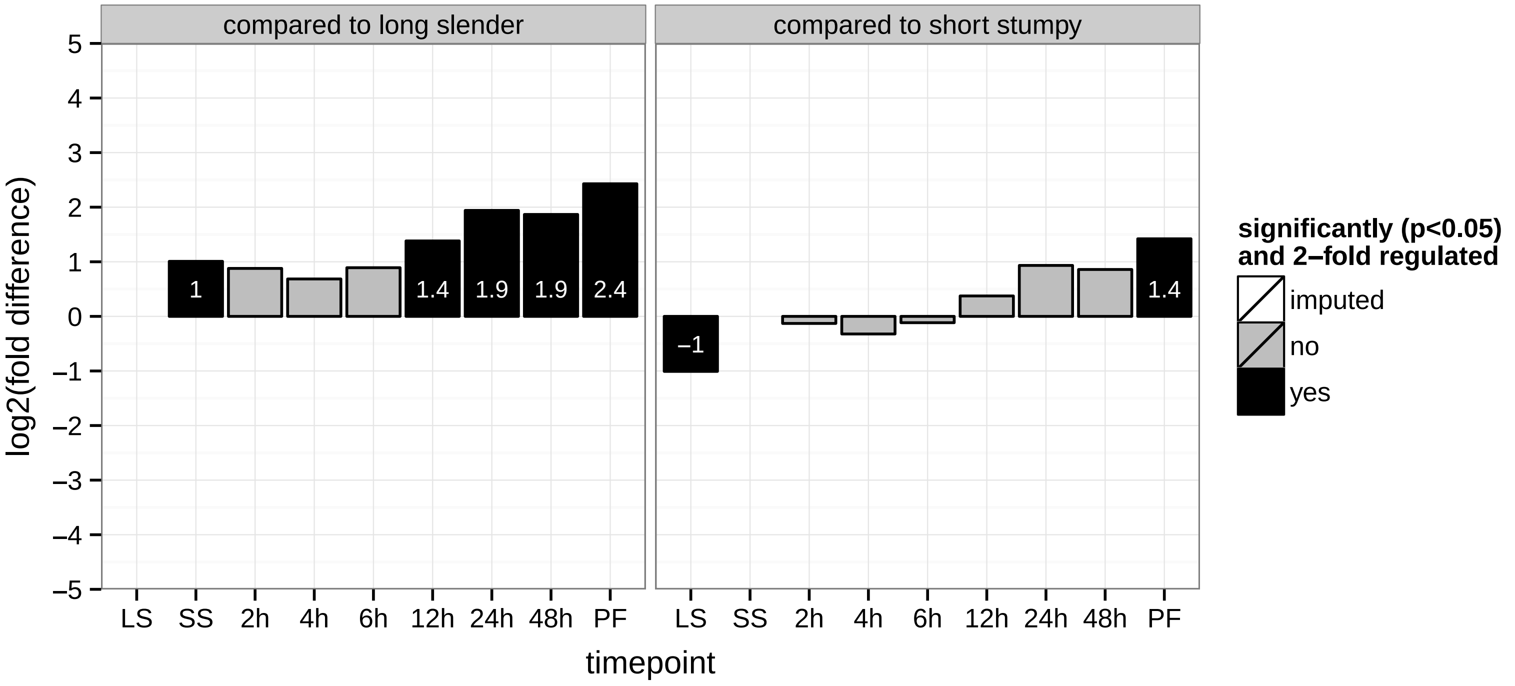

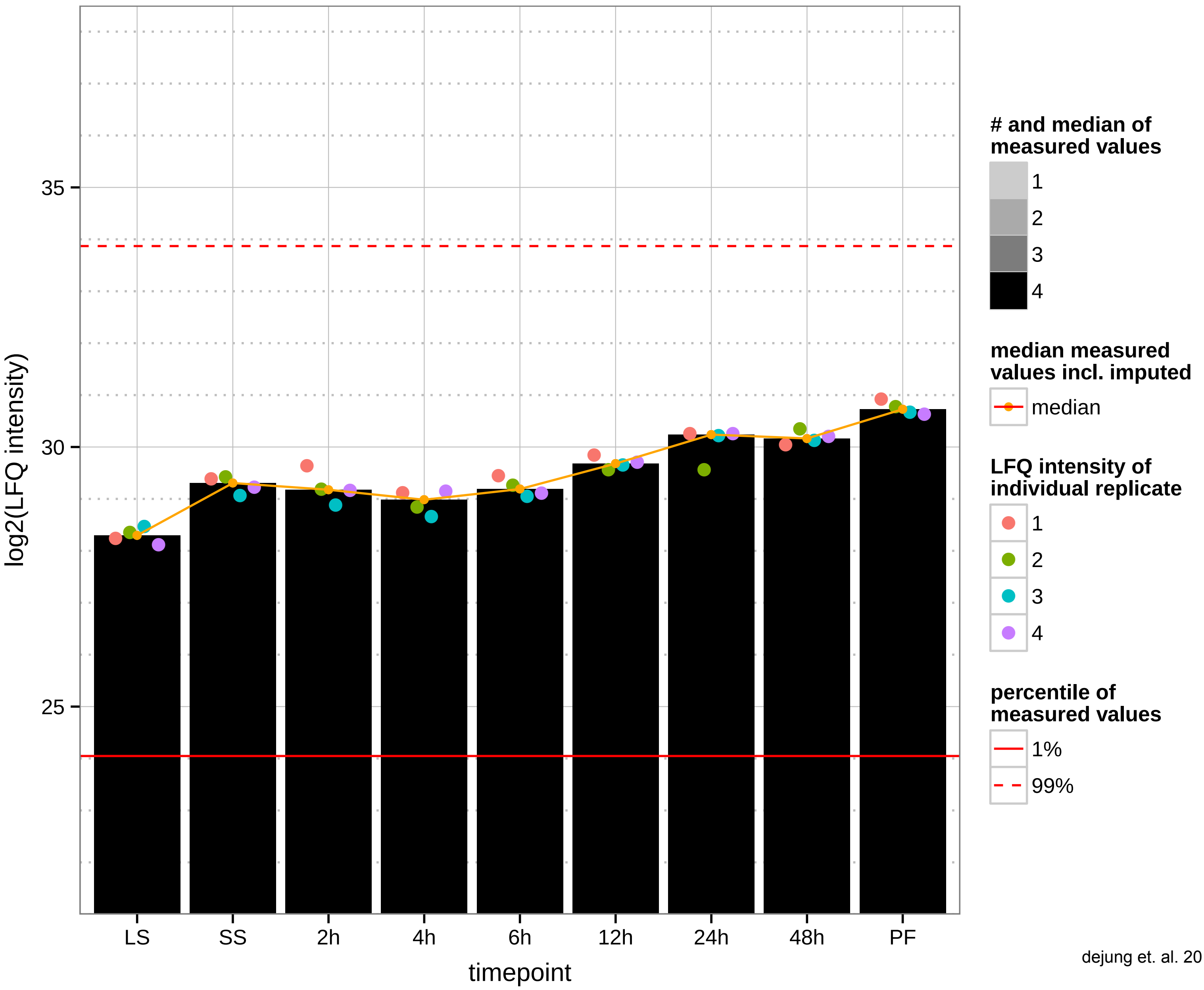

dejung et. al. 2015

TFIIF–stimulated CTD phosphatase, putative
Tb927.2.4830
AGOF: null
AGOC: integral to membrane, mitochondrion
AGOP: null
PGOF: protein binding
PGOC: null
PGOP: null

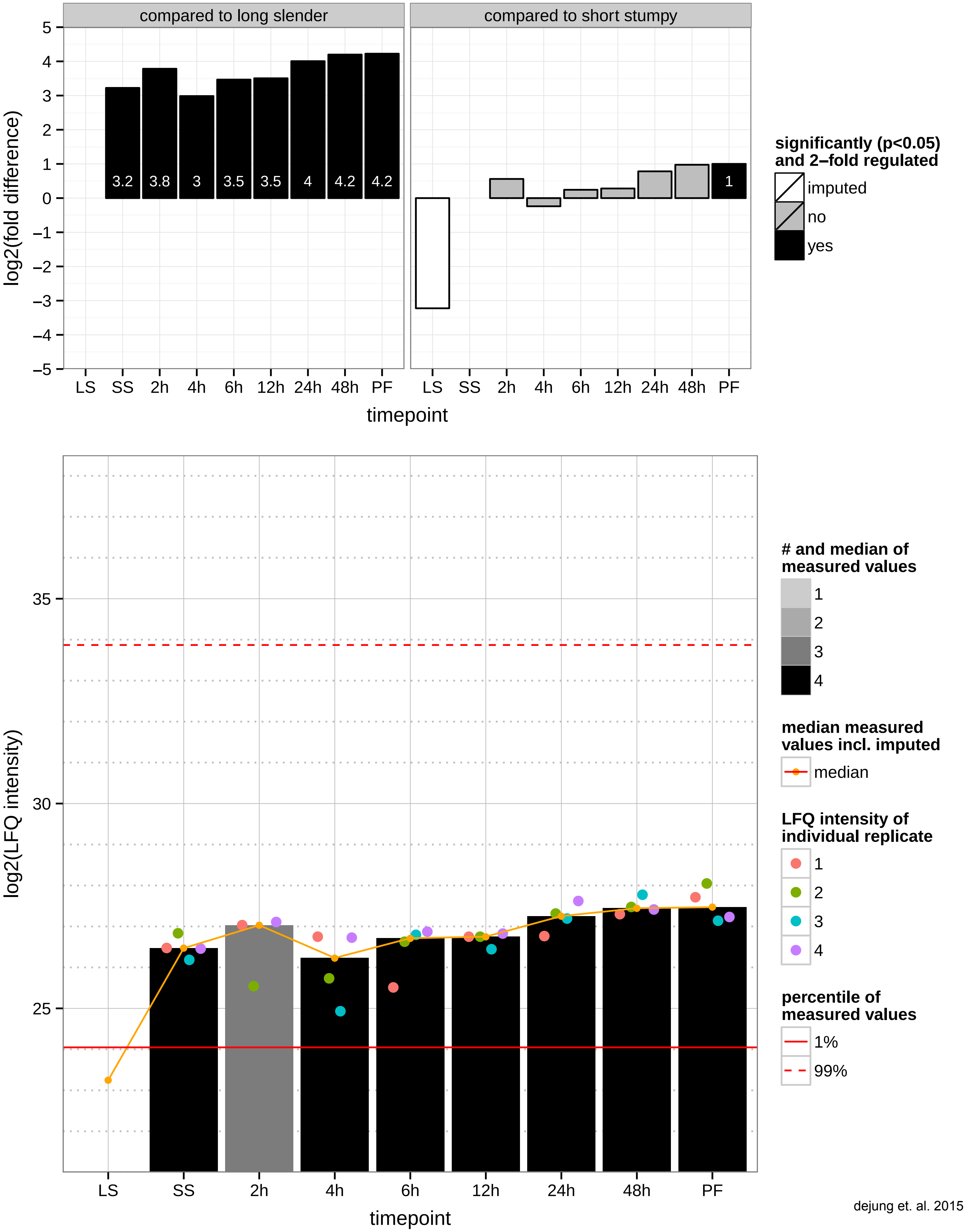

dejung et. al. 2015

hypothetical protein, conserved
Tb927.2.5130
AGOF: null
AGOC: null
AGOP: null
PGOF: null
PGOC: null
PGOP: null

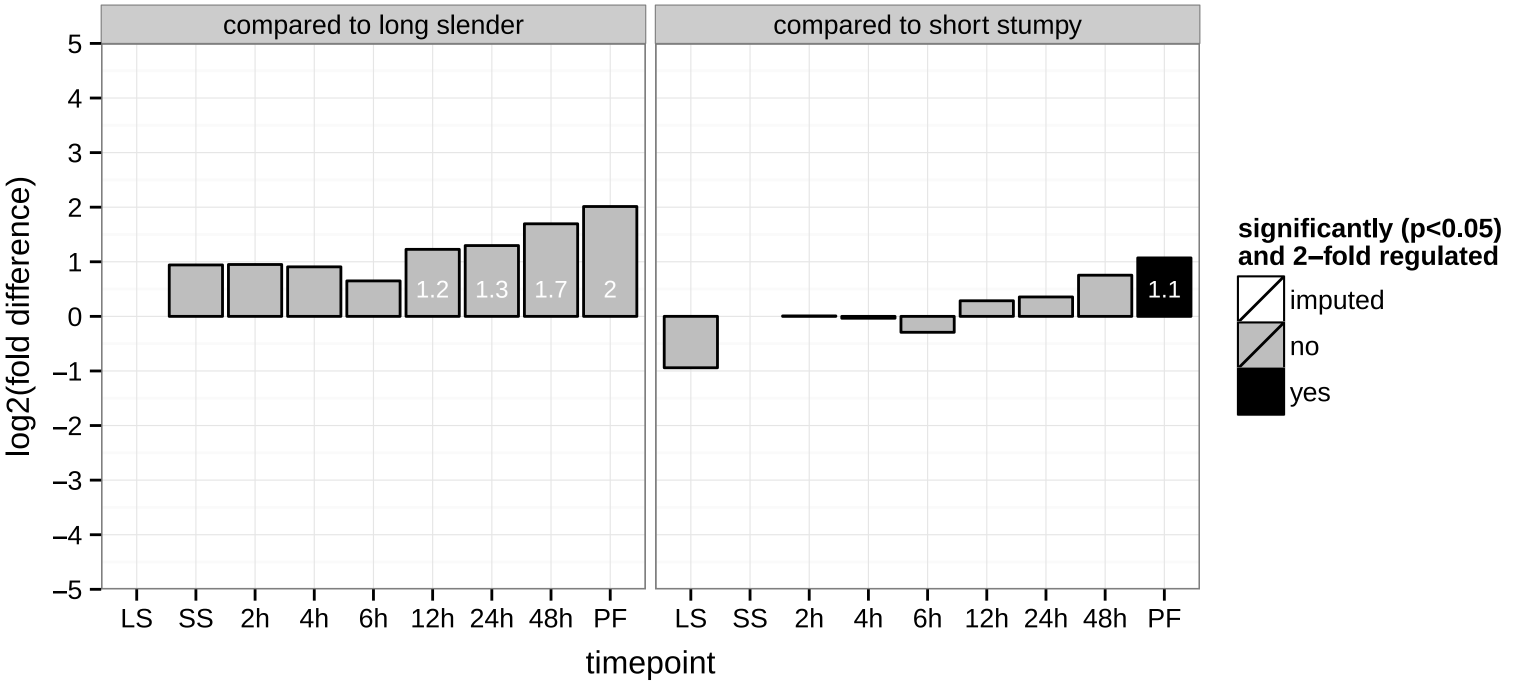

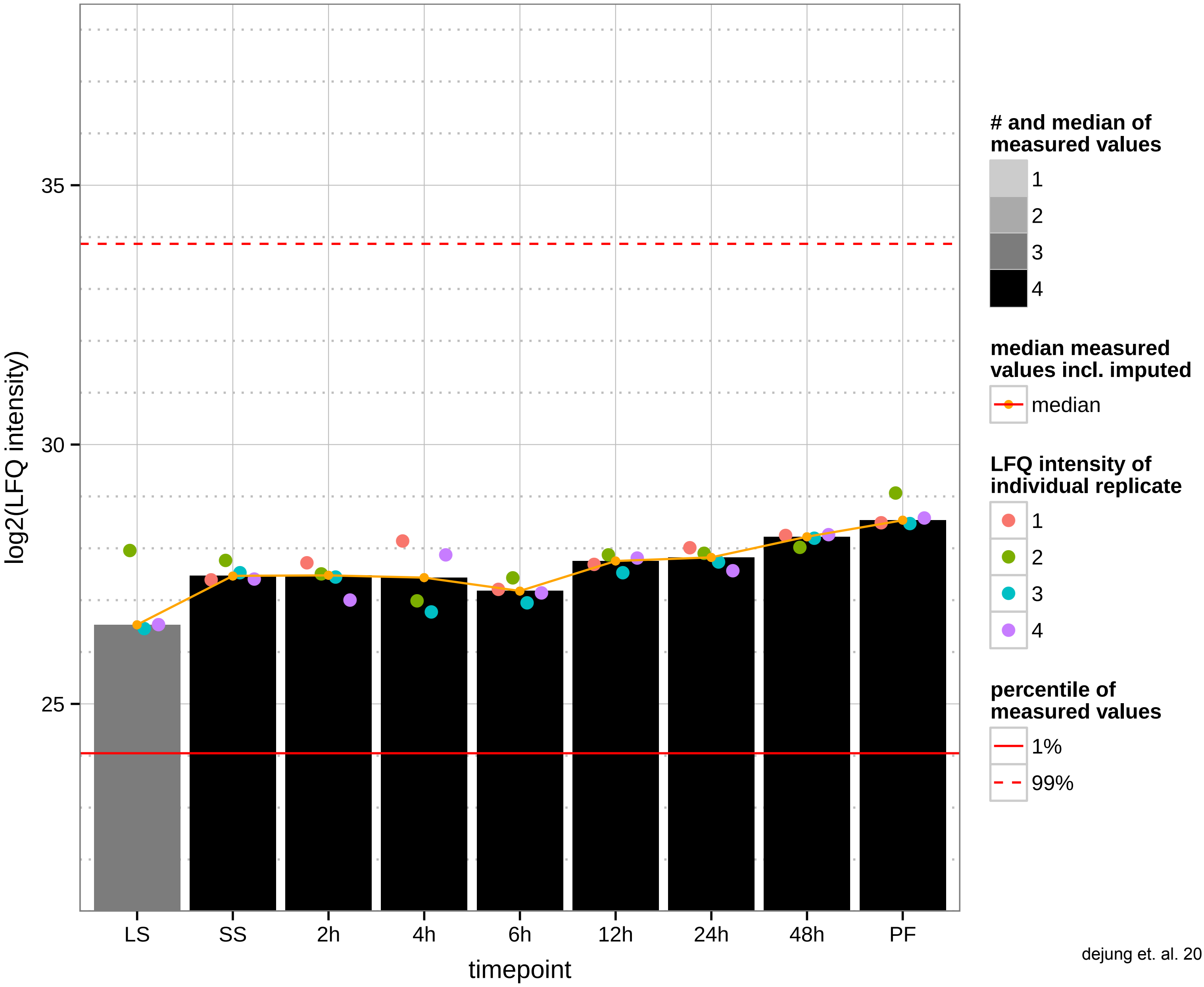

dejung et. al. 2015

trans–sialidase, putative
Tb927.2.5280
AGOF: exo–alpha–sialidase activity
AGOC: null
AGOP: pathogenesis
PGOF: null
PGOC: null
PGOP: null

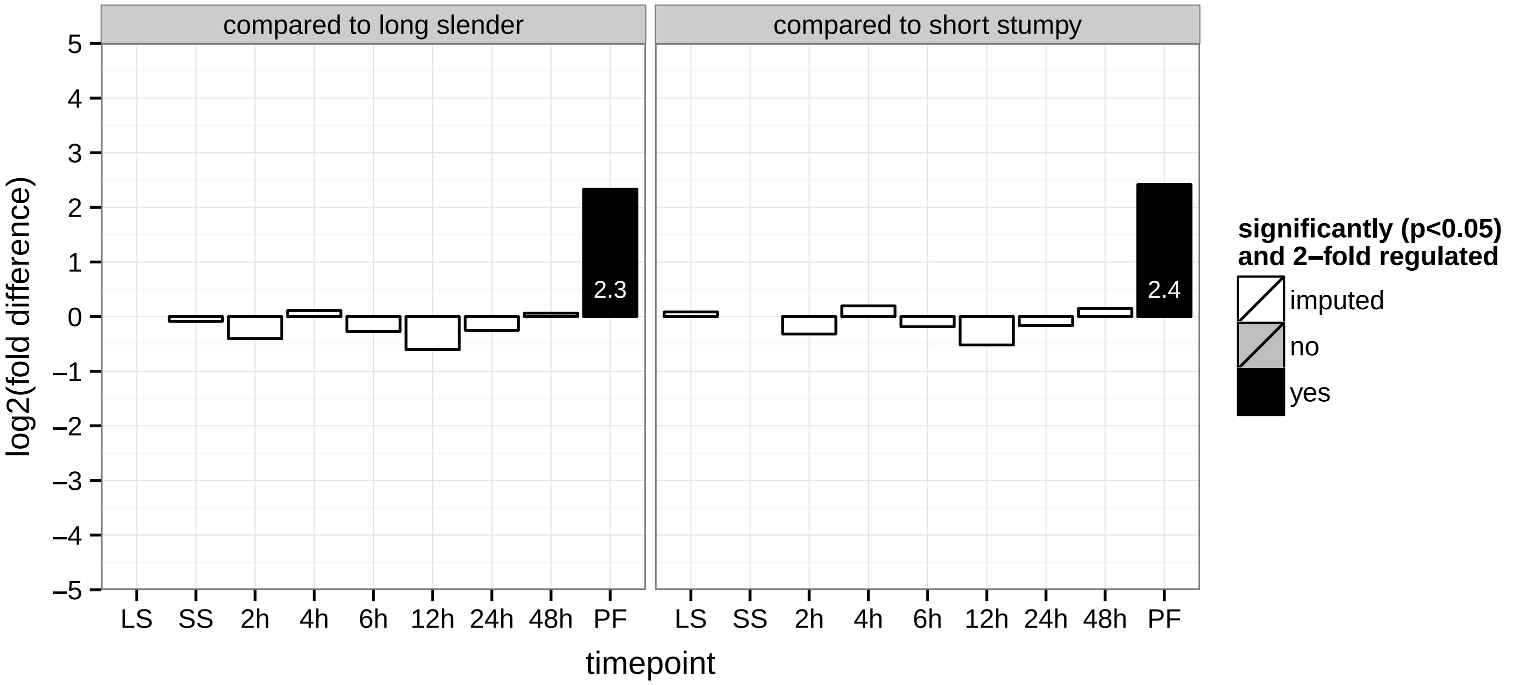

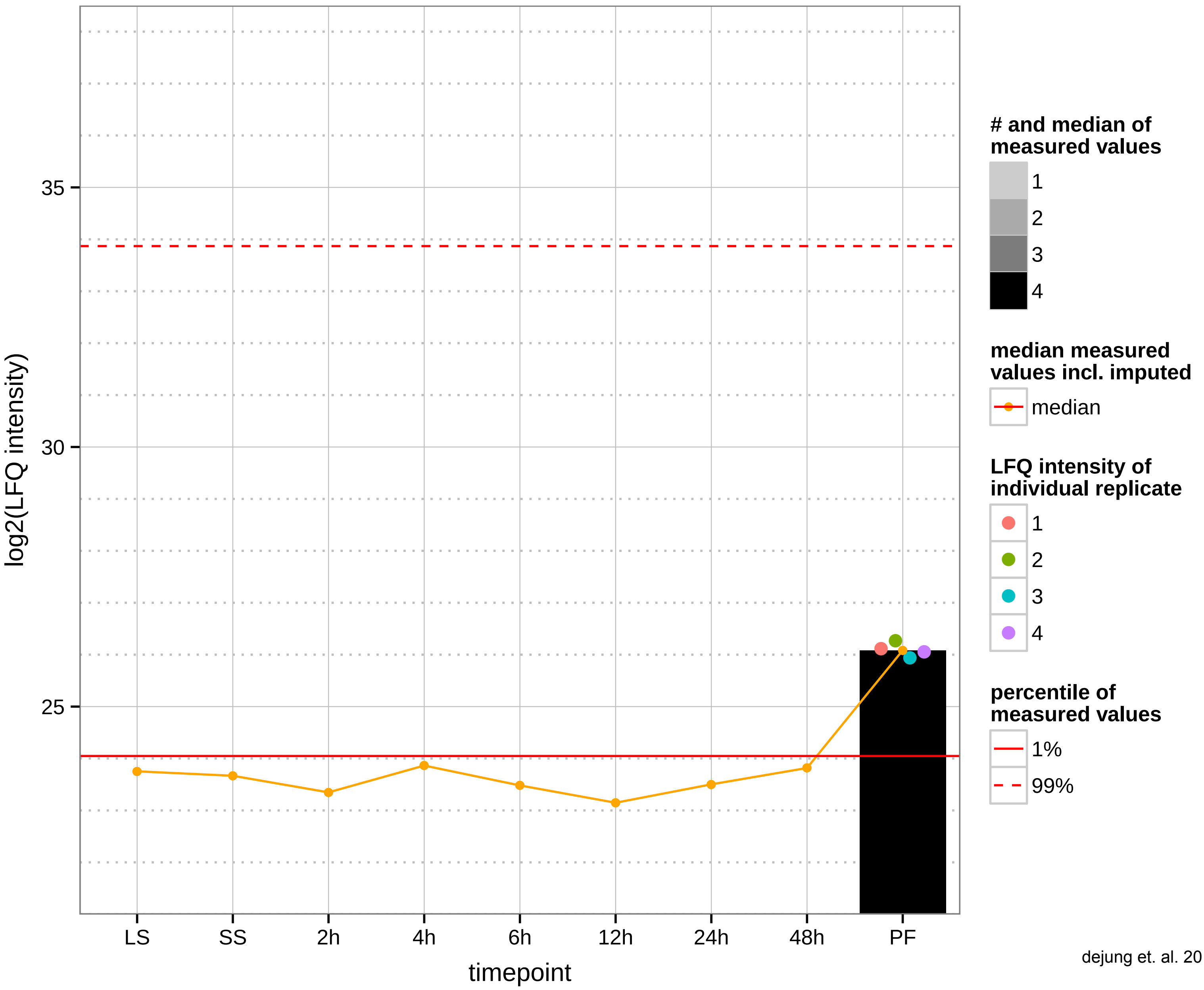

dejung et. al. 2015

hypothetical protein, conserved
Tb927.3.1020
AGOF: null
AGOC: null
AGOP: null
PGOF: null
PGOC: null
PGOP: null

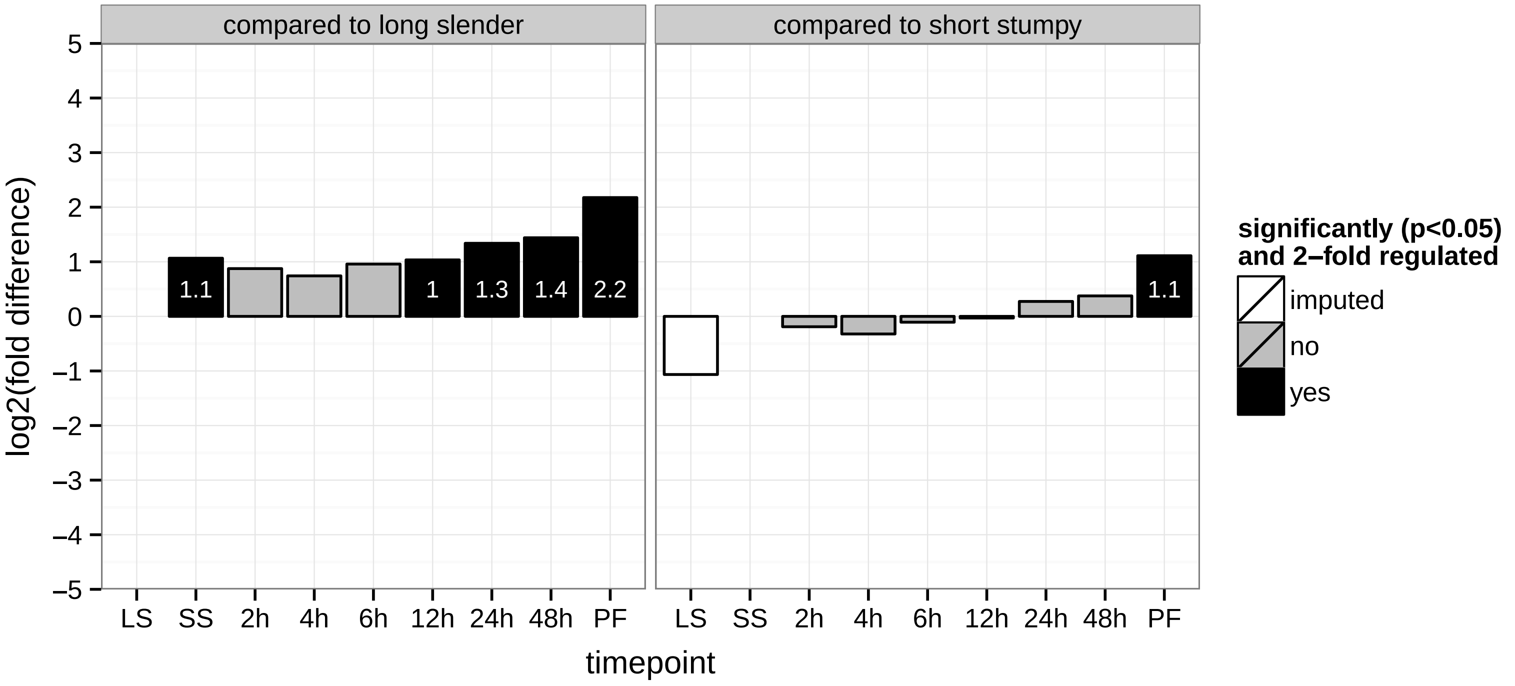

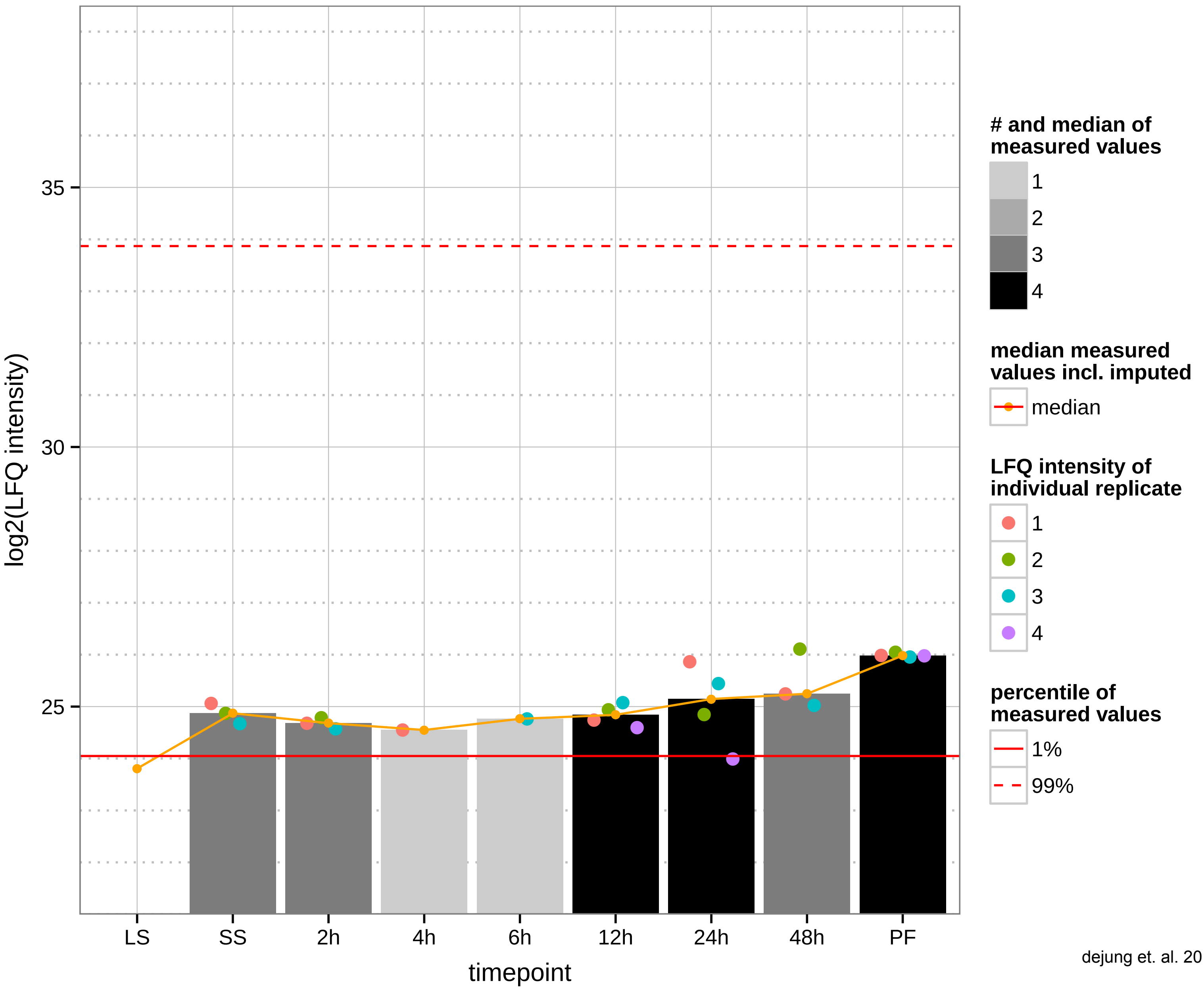

dejung et. al. 2015

protein phosphatase 2A, putative
Tb927.3.1240
AGOF: hydrolase activity, protein serine/threonine phosphatase activity
AGOC: null
AGOP: protein dephosphorylation
PGOF: hydrolase activity
PGOC: null
PGOP: null

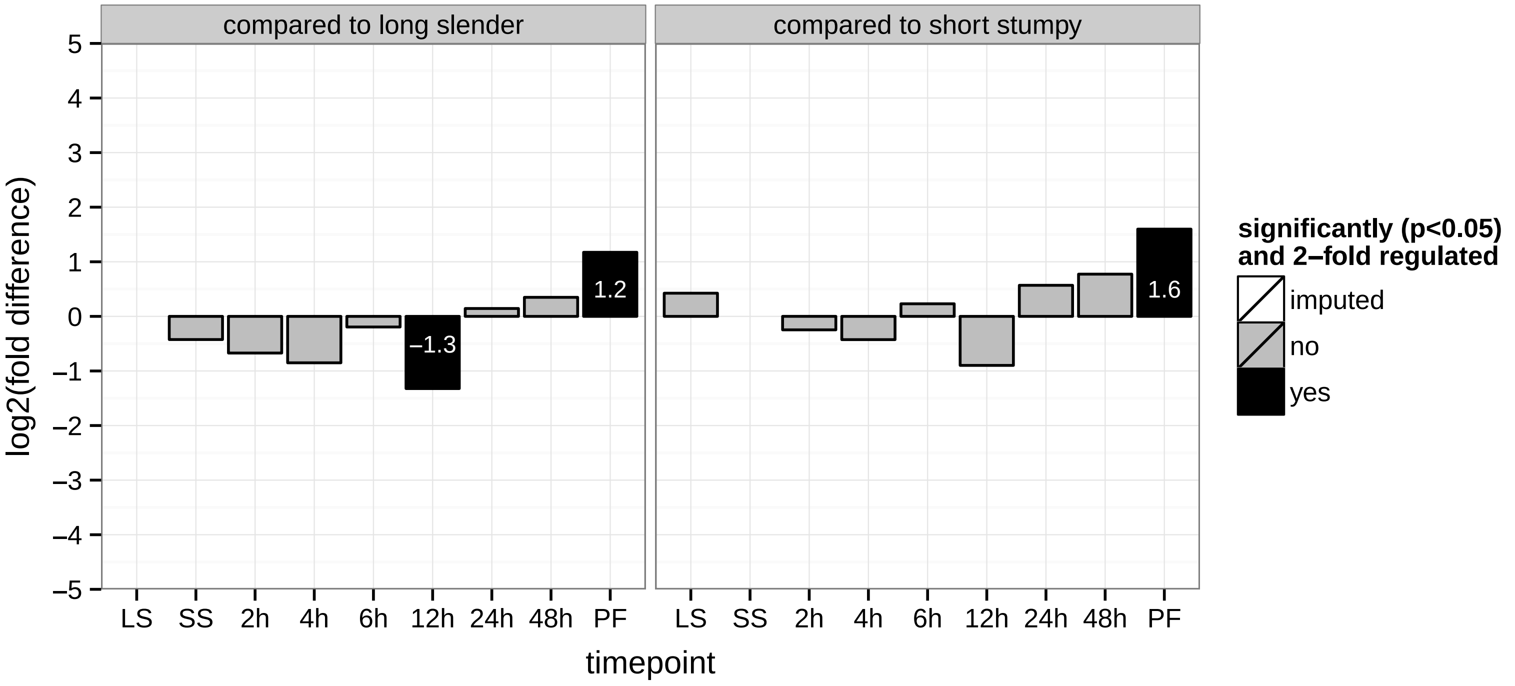

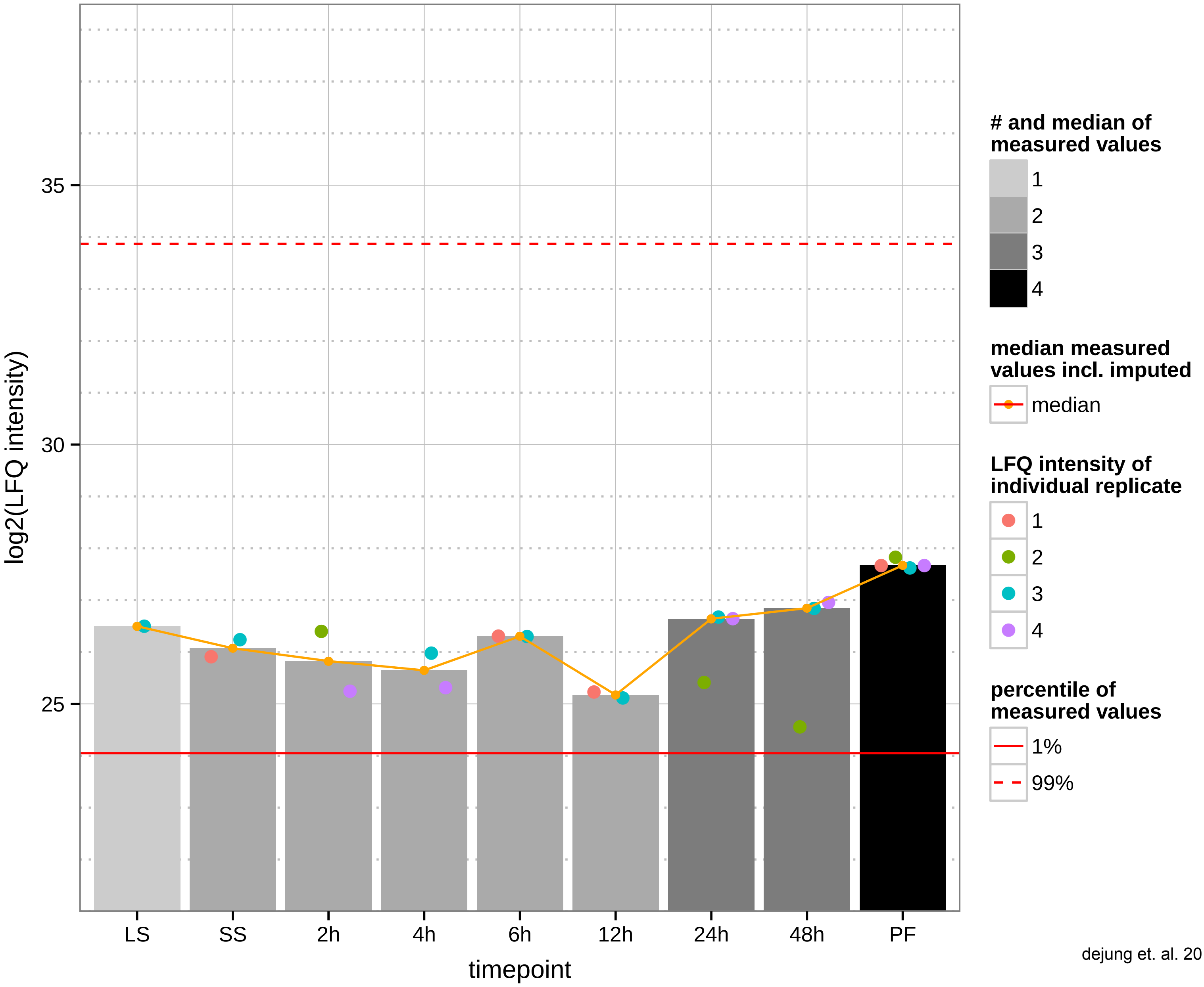

dejung et. al. 2015

hypothetical protein, conserved
Tb927.3.1350
AGOF: null
AGOC: null
AGOP: null
PGOF: protein binding
PGOC: null
PGOP: null

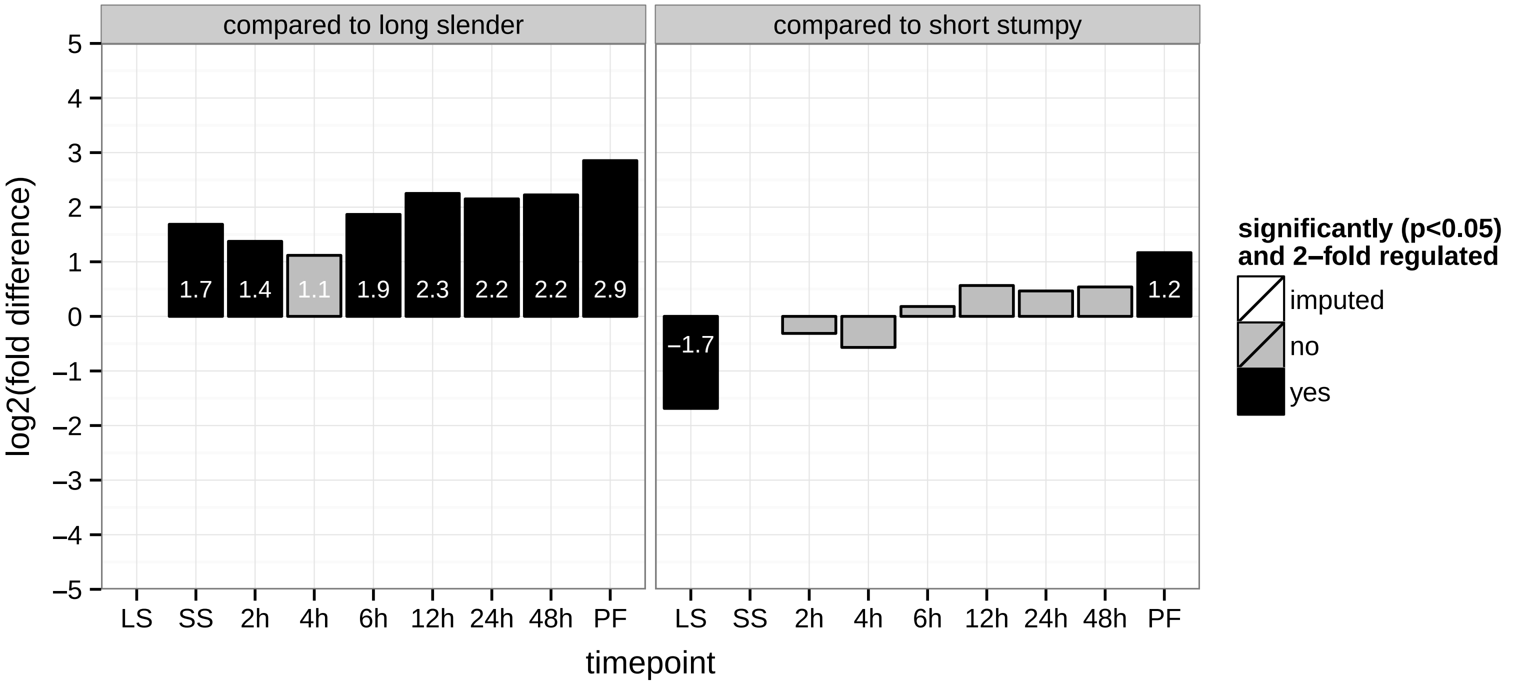

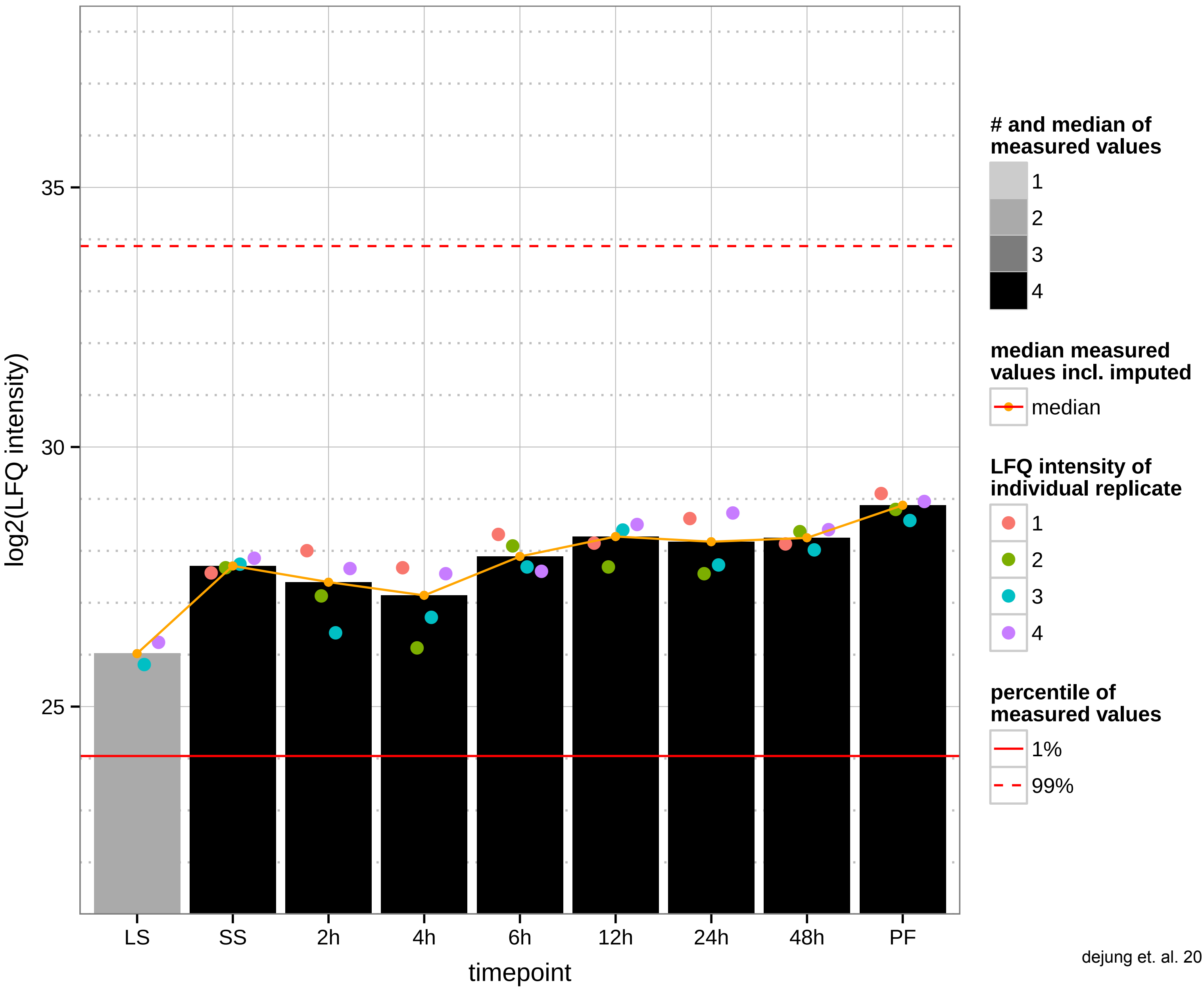

dejung et. al. 2015

hypothetical protein, conserved
Tb927.3.1550
AGOF: null
AGOC: null
AGOP: null
PGOF: null
PGOC: null
PGOP: null

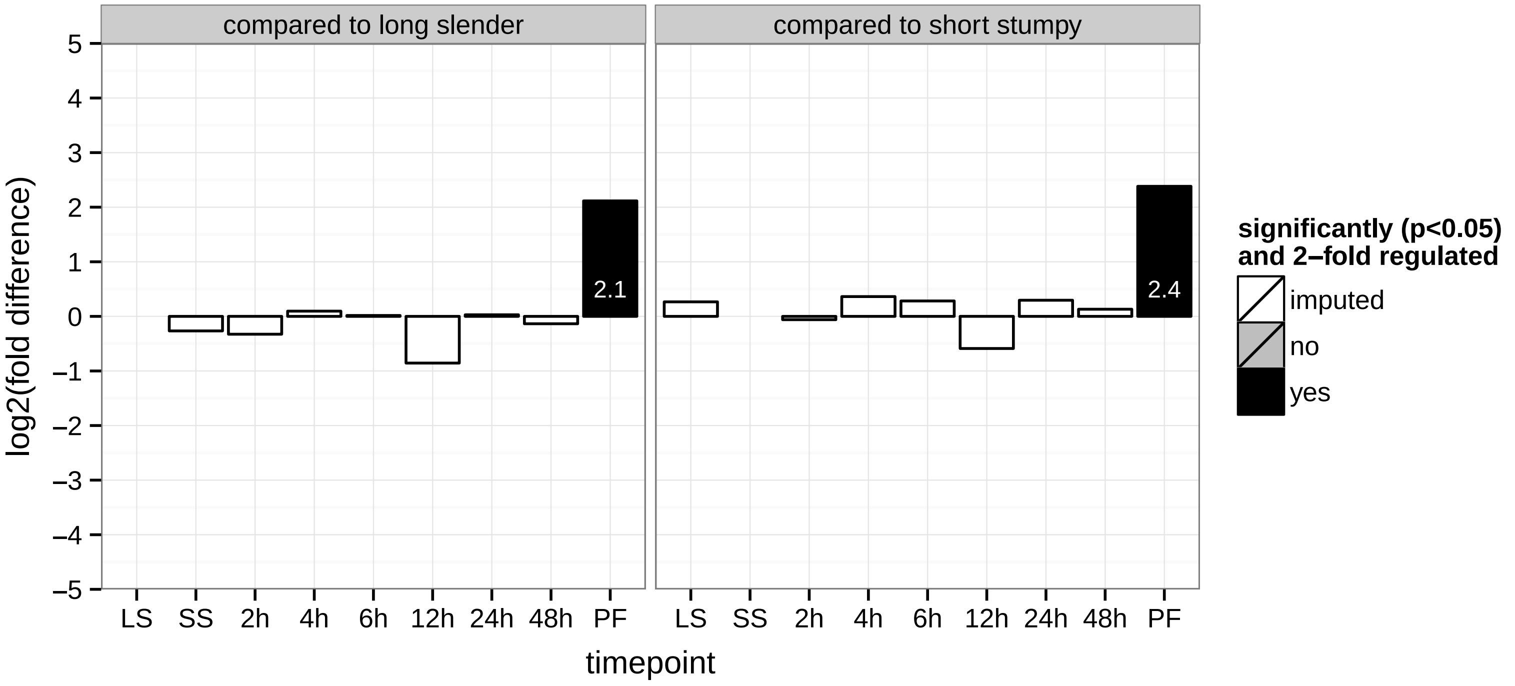

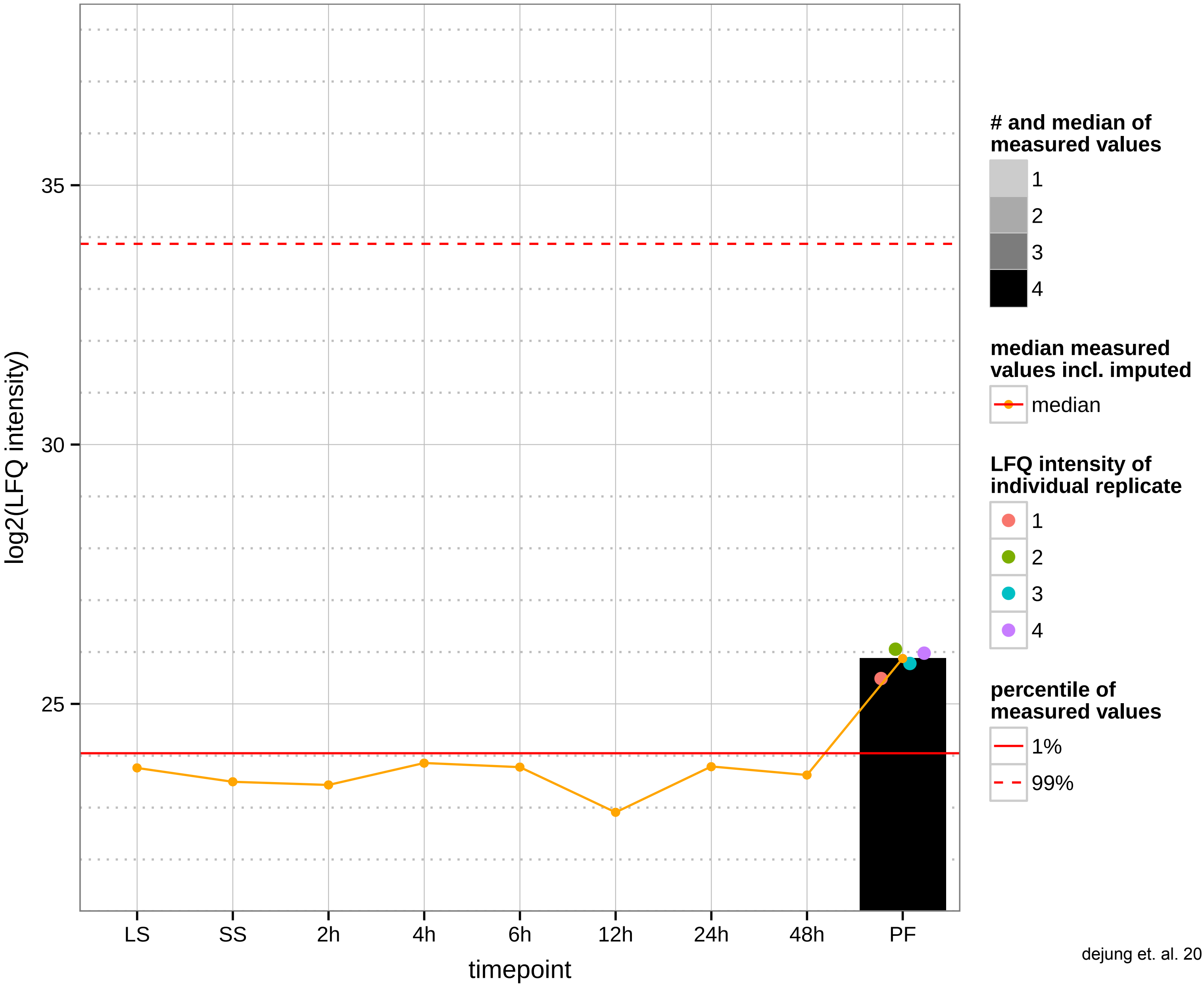

dejung et. al. 2015

pumilio RNA binding protein, putative (PUF8)
Tb927.3.2470
AGOF: RNA binding
AGOC: null
AGOP: null
PGOF: RNA binding, binding
PGOC: null
PGOP: null

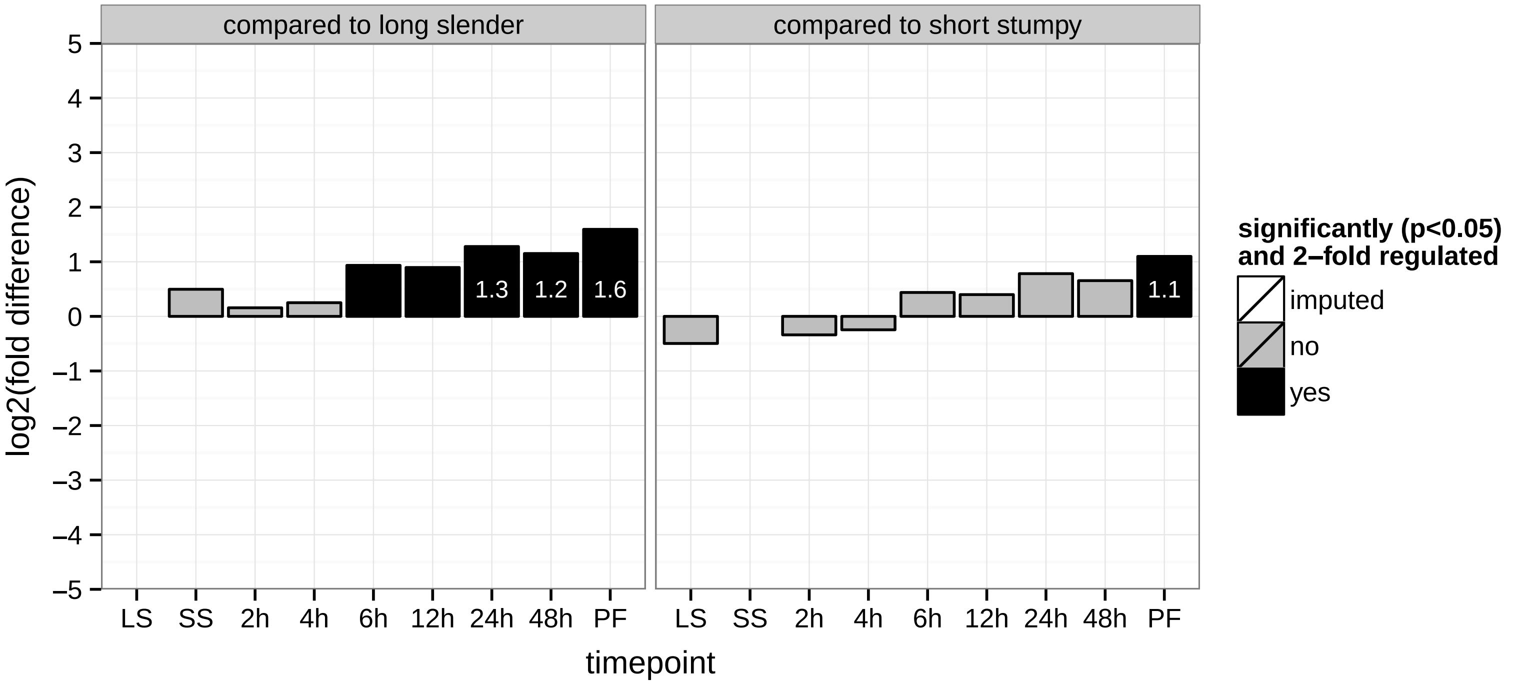

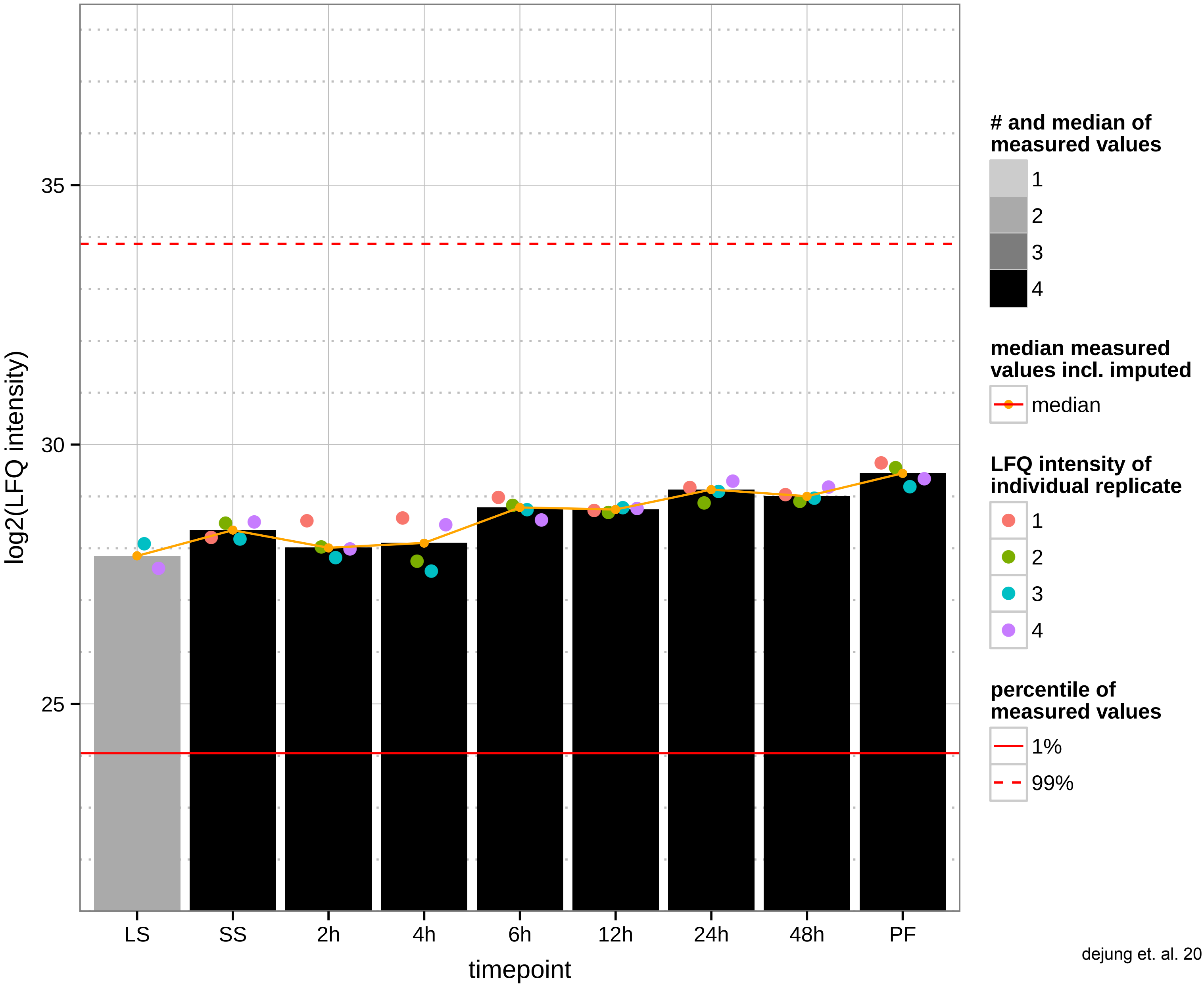

dejung et. al. 2015

hypothetical protein, conserved
Tb927.3.2670
AGOF: null
AGOC: mitochondrion
AGOP: null
PGOF: null
PGOC: null
PGOP: null

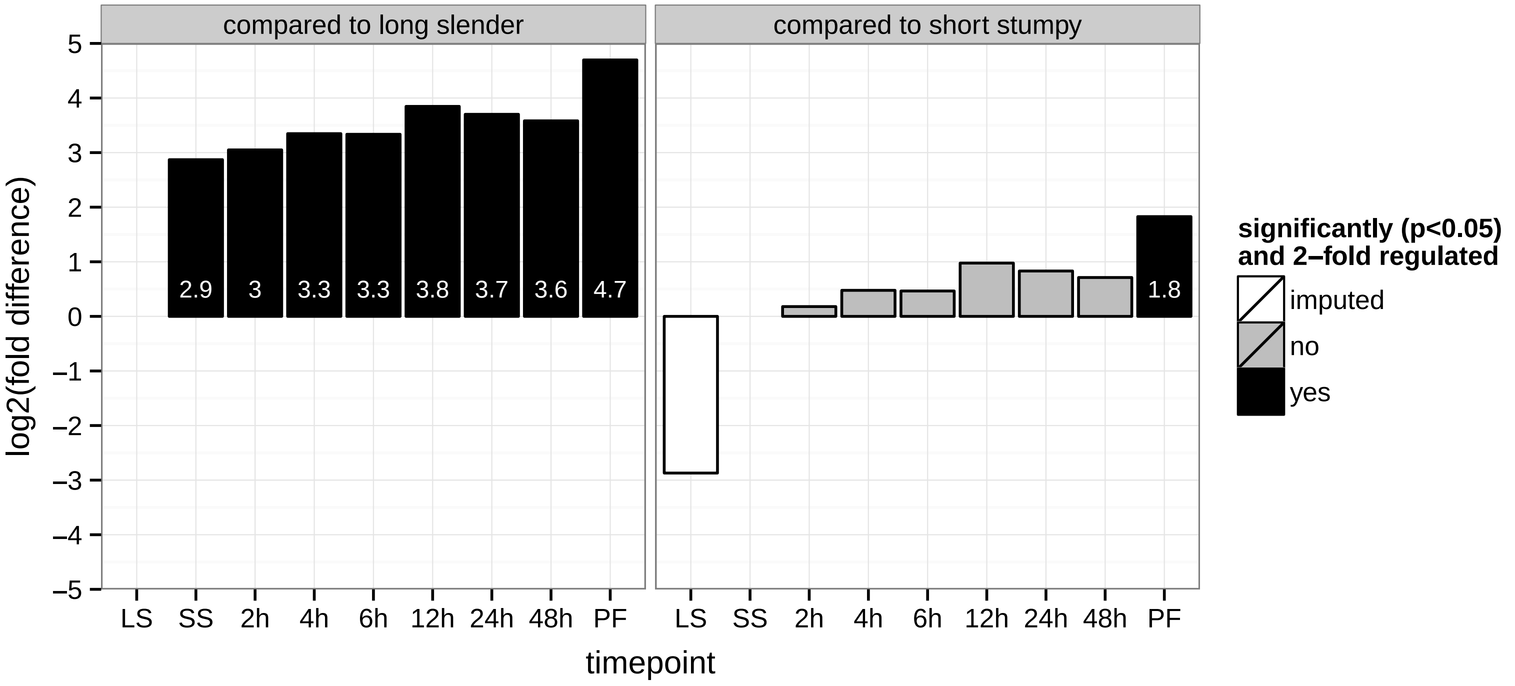

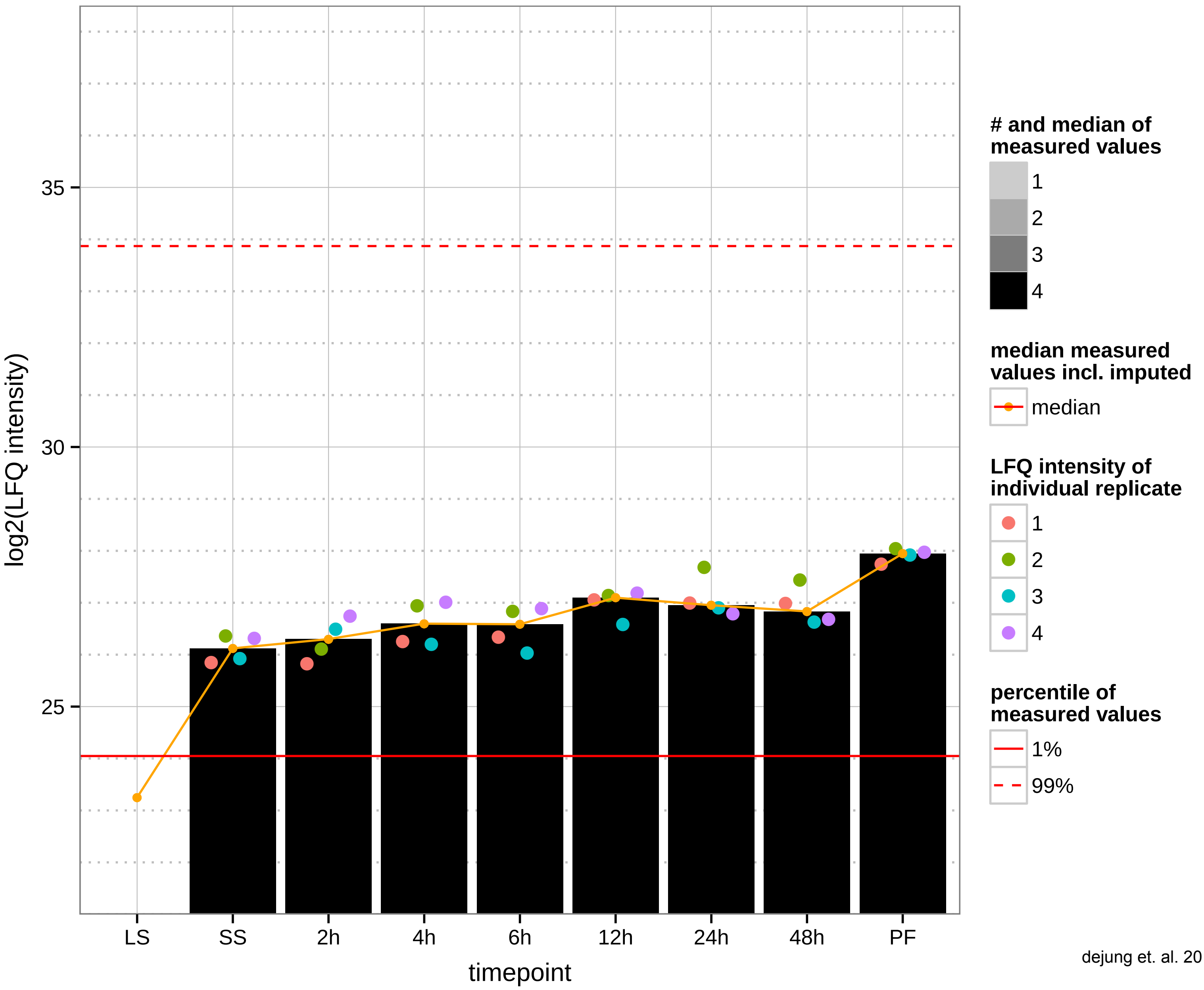

dejung et. al. 2015

hypothetical protein, conserved
Tb927.3.3370
AGOF: null
AGOC: null
AGOP: null
PGOF: protein binding
PGOC: null
PGOP: null

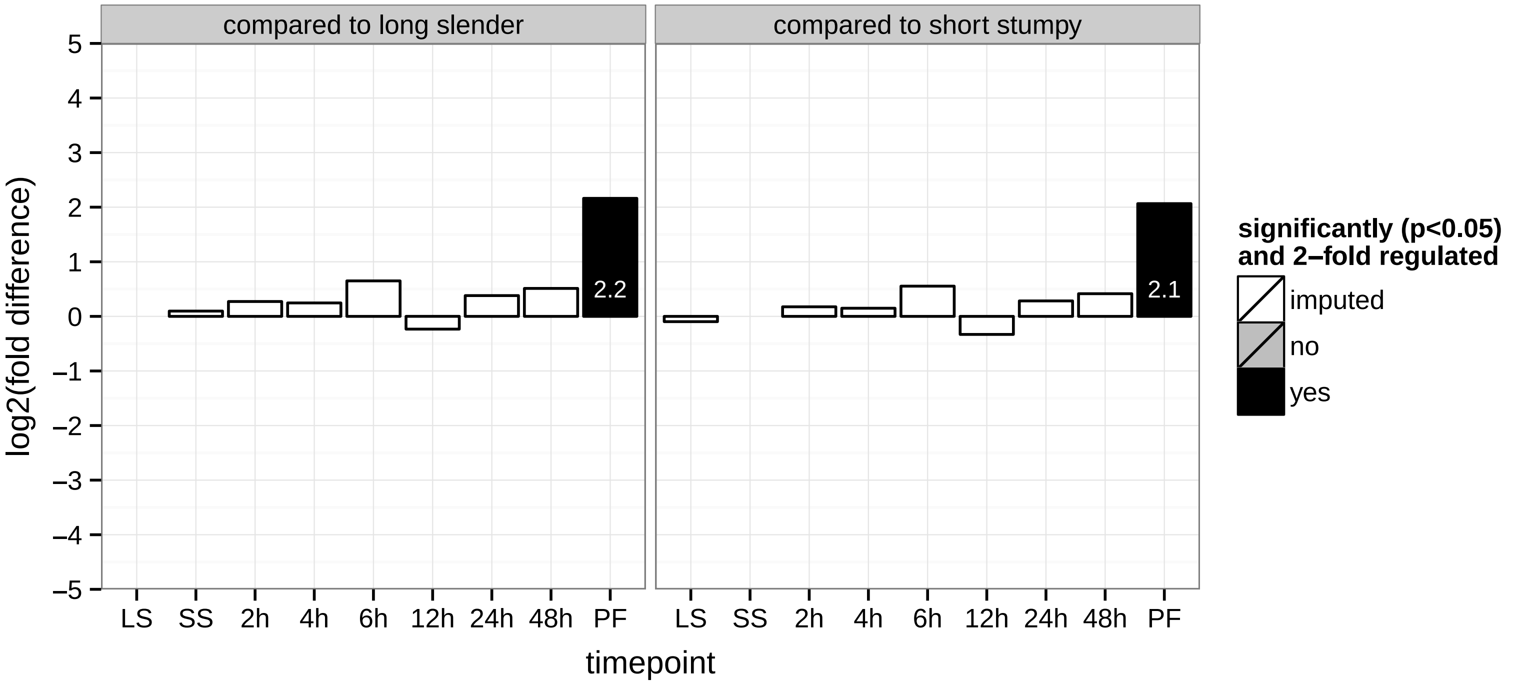

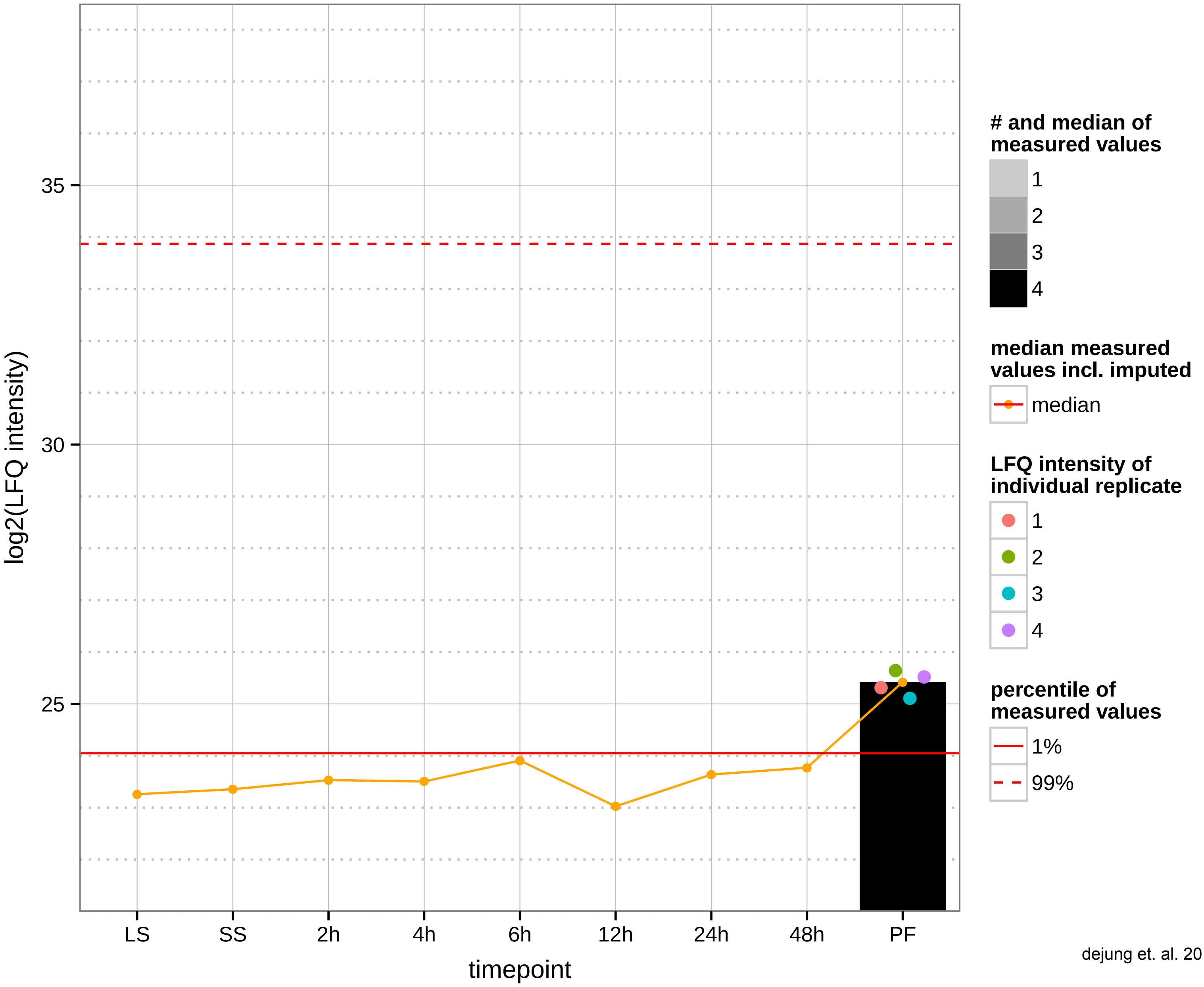

dejung et. al. 2015

hypothetical protein, conserved
Tb927.3.3510
AGOF: null
AGOC: null
AGOP: null
PGOF: null
PGOC: null
PGOP: null

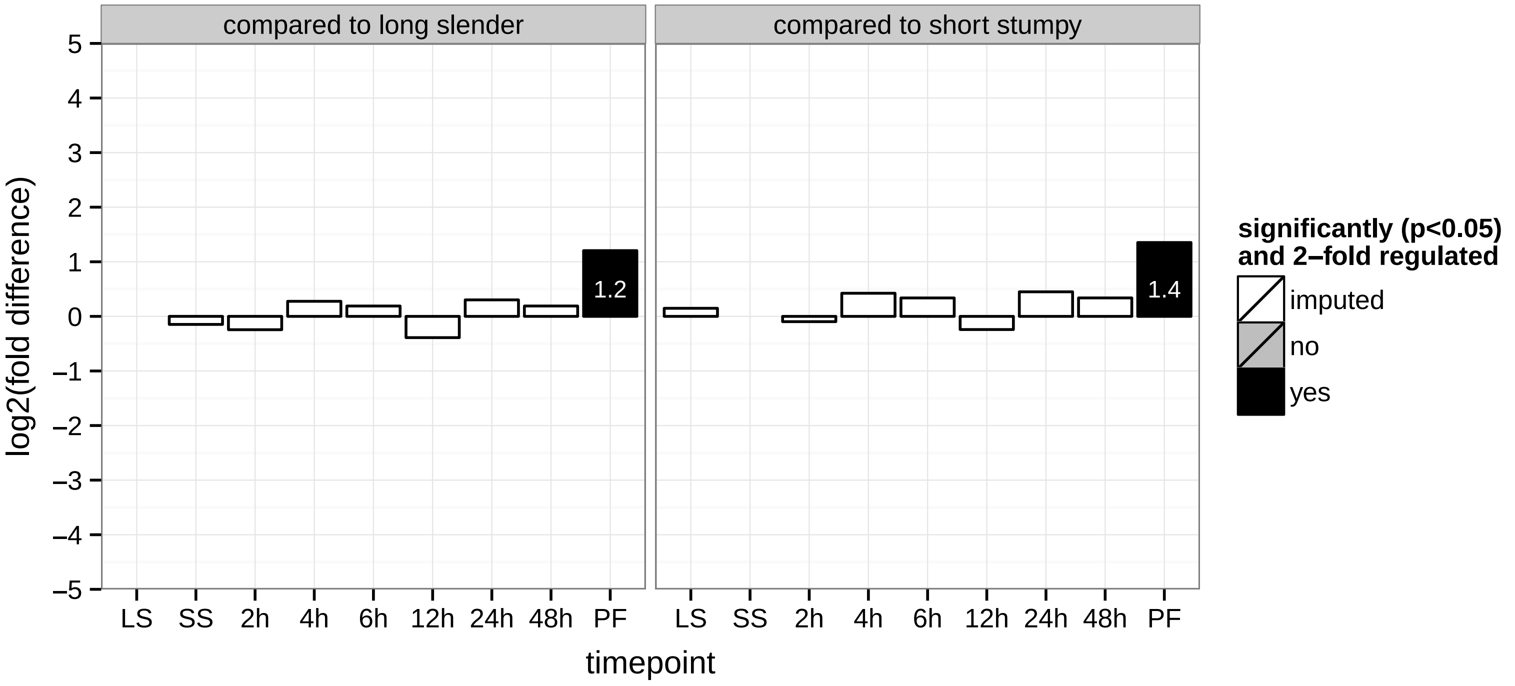

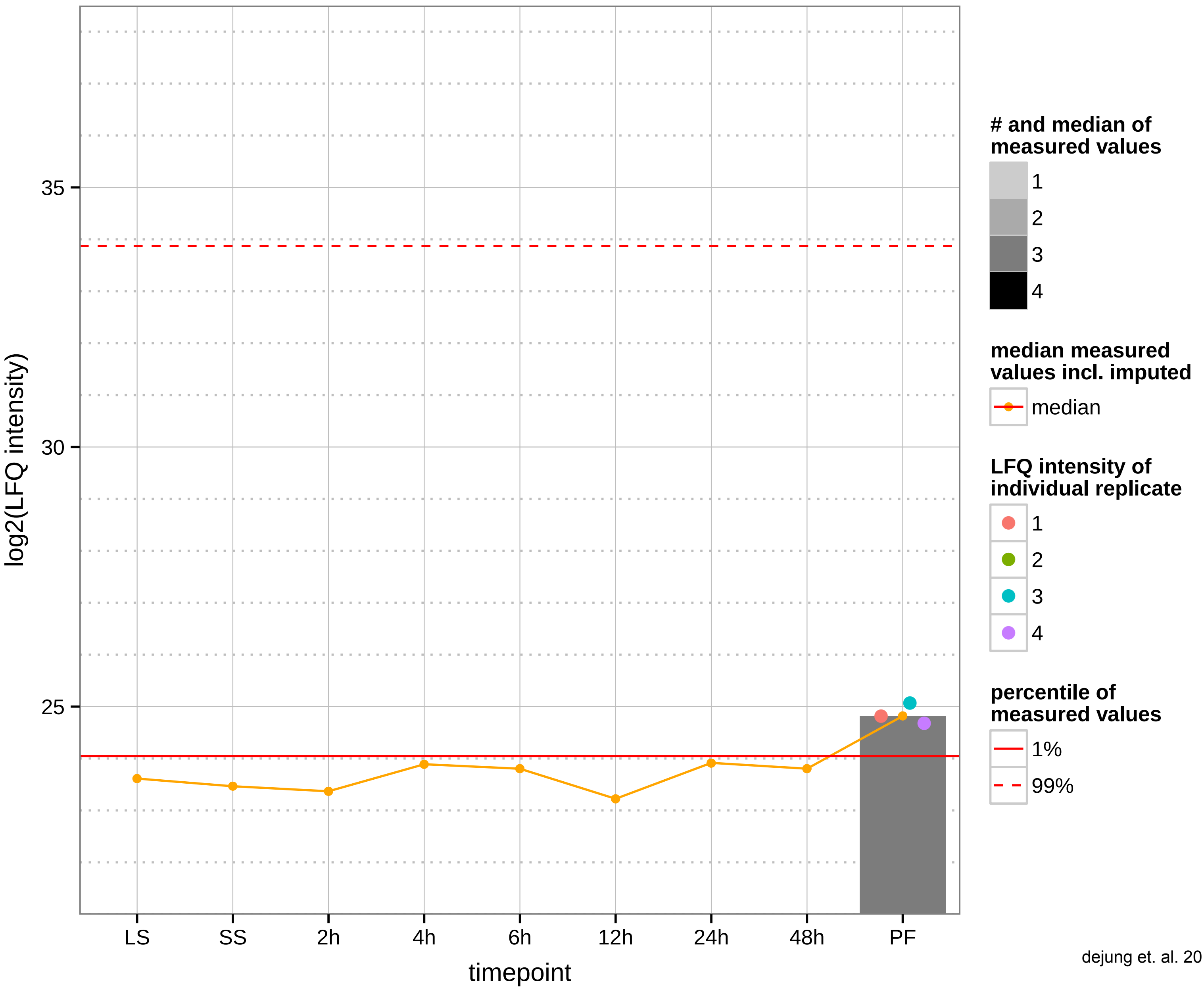

dejung et. al. 2015

hypothetical protein, conserved
Tb927.3.3710
AGOF: catalytic activity
AGOC: null
AGOP: null
PGOF: catalytic activity
PGOC: null
PGOP: null

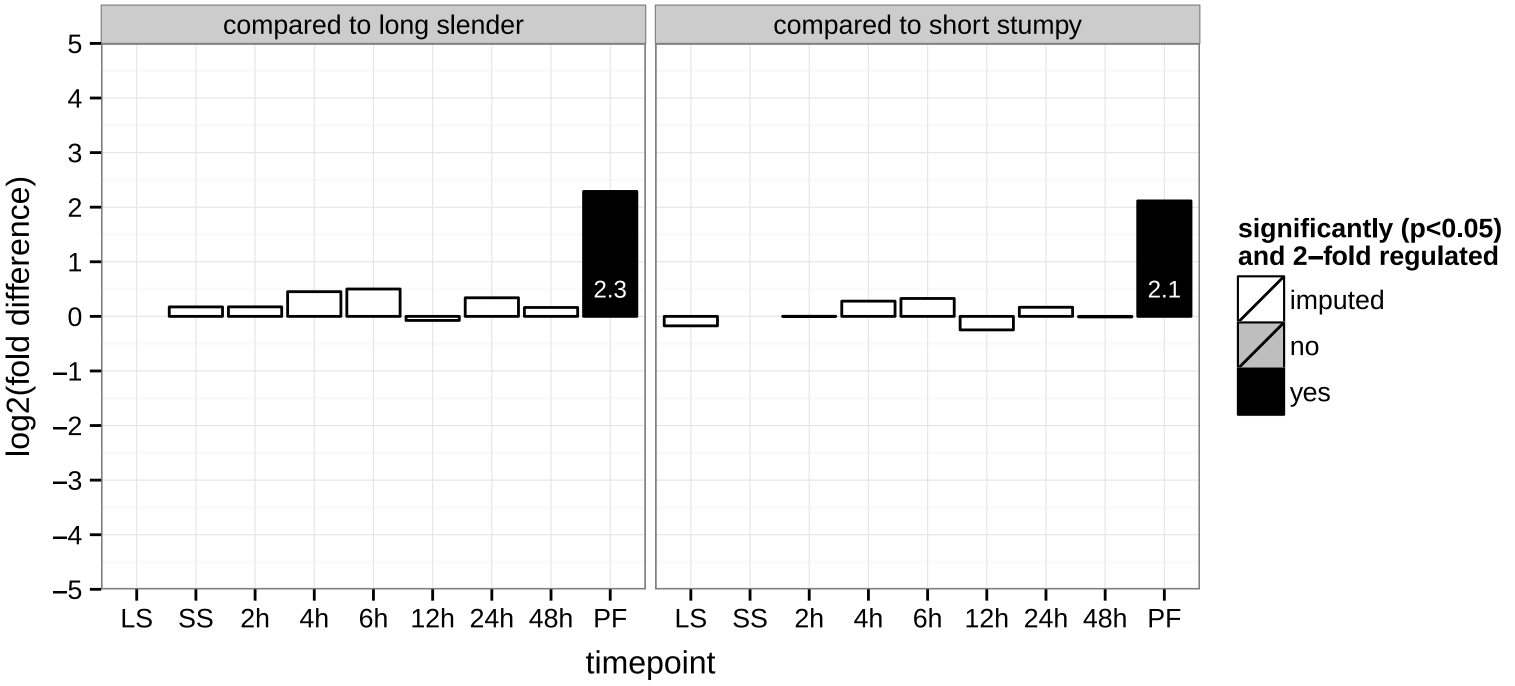

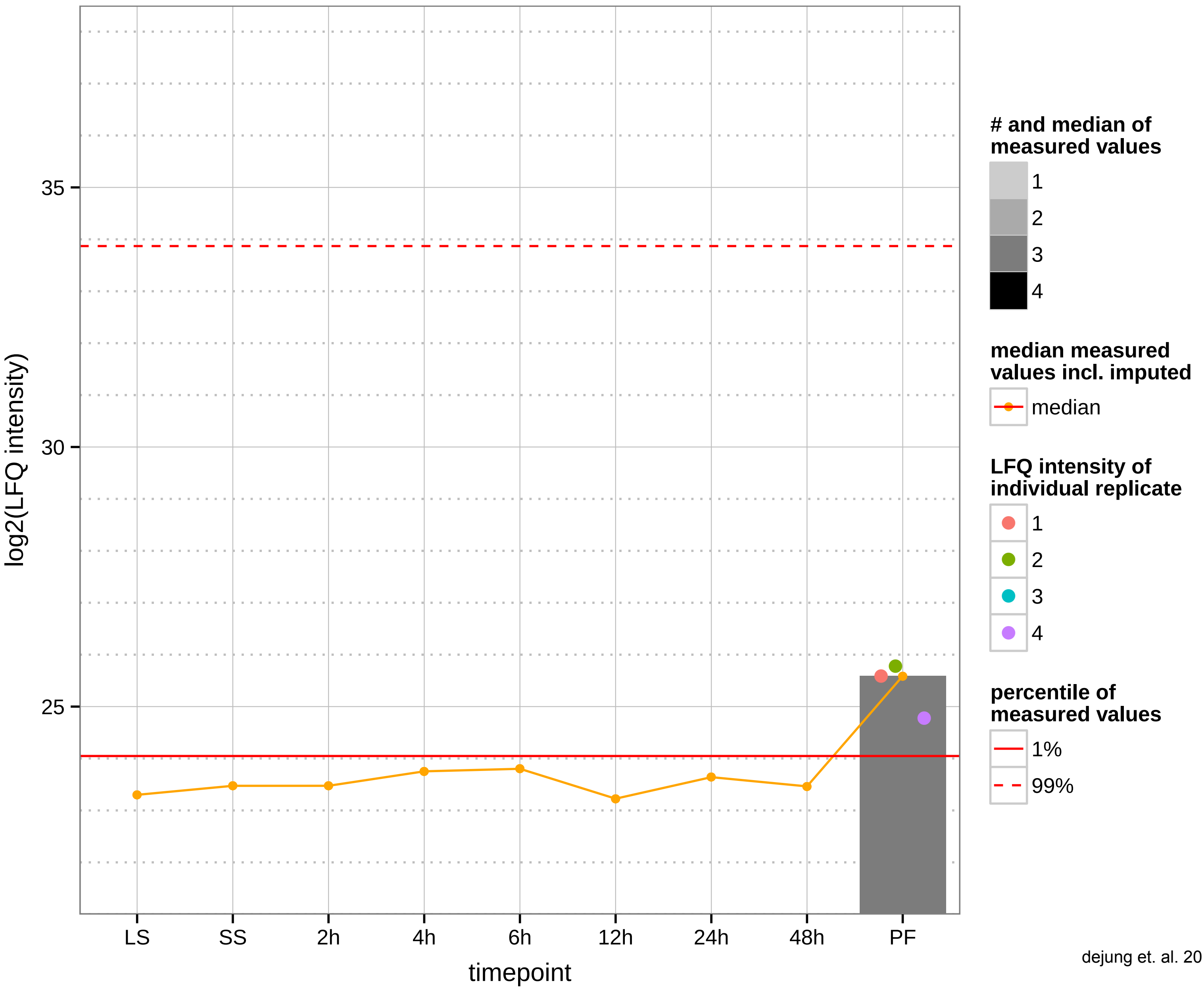

dejung et. al. 2015

hypothetical protein, conserved
Tb927.3.3880
AGOF: null
AGOC: null
AGOP: null
PGOF: null
PGOC: null
PGOP: null

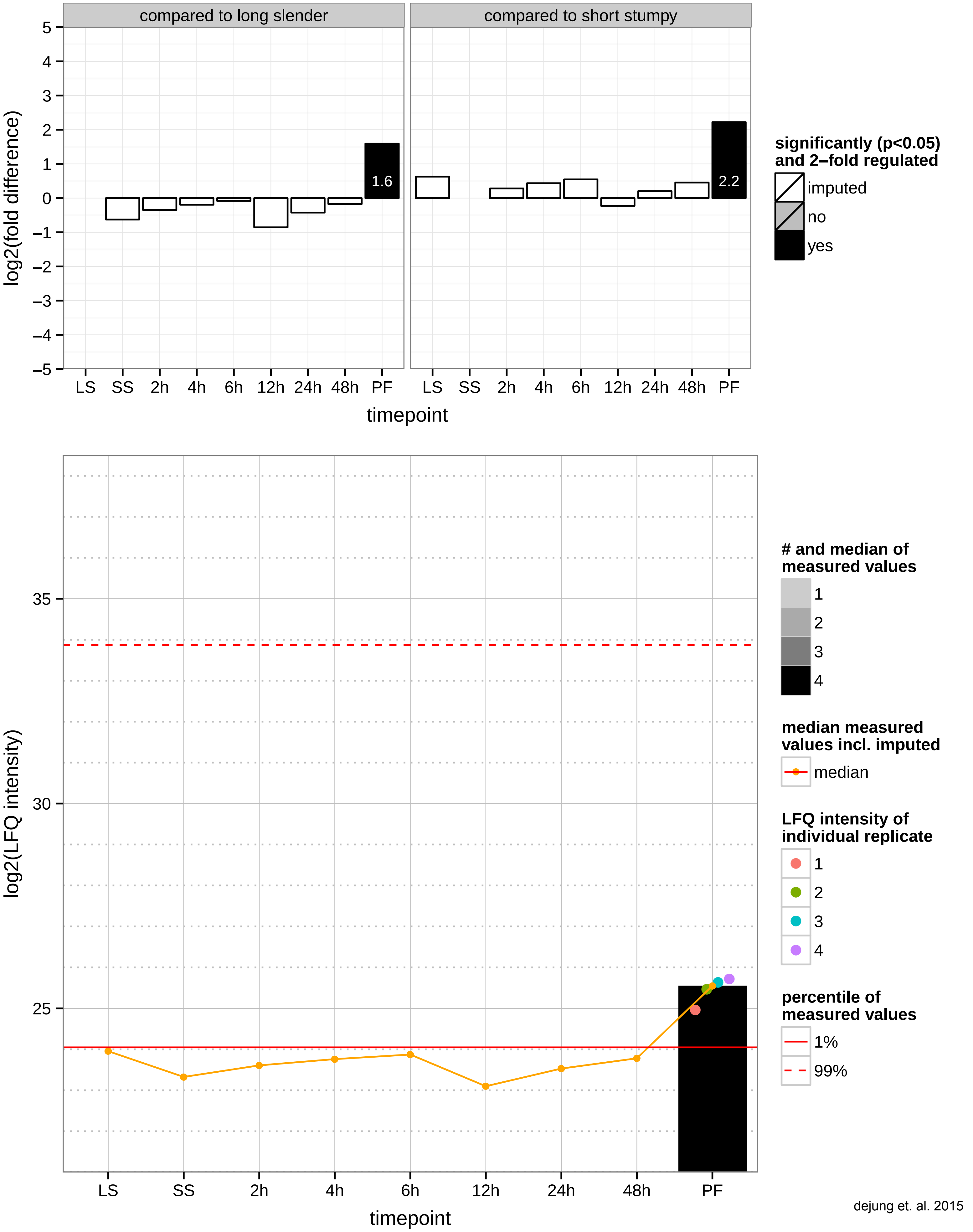

dejung et. al. 2015

protein farnesyltransferase alpha subunit, putative
Tb927.3.4490
AGOF: protein farnesyltransferase activity
AGOC: null
AGOP: protein prenylation
PGOF: protein prenyltransferase activity
PGOC: null
PGOP: protein prenylation

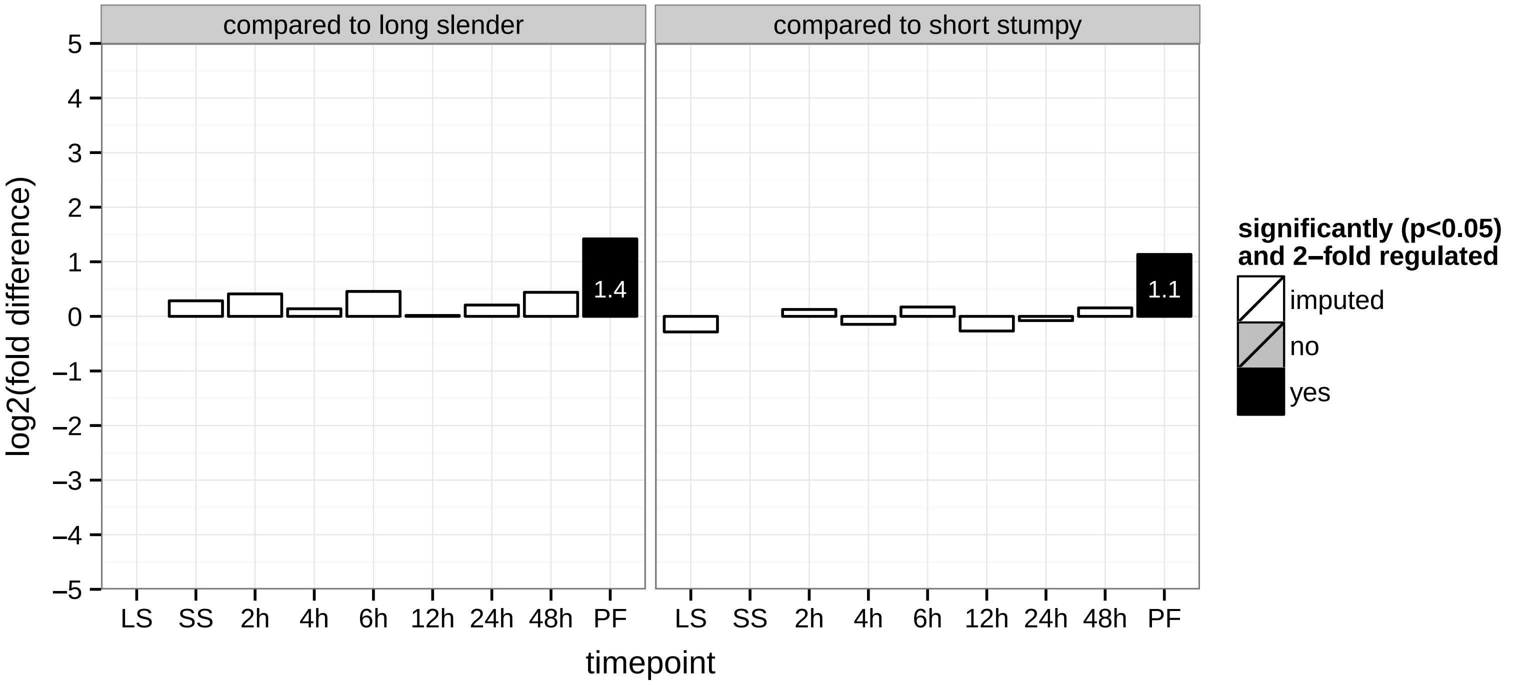

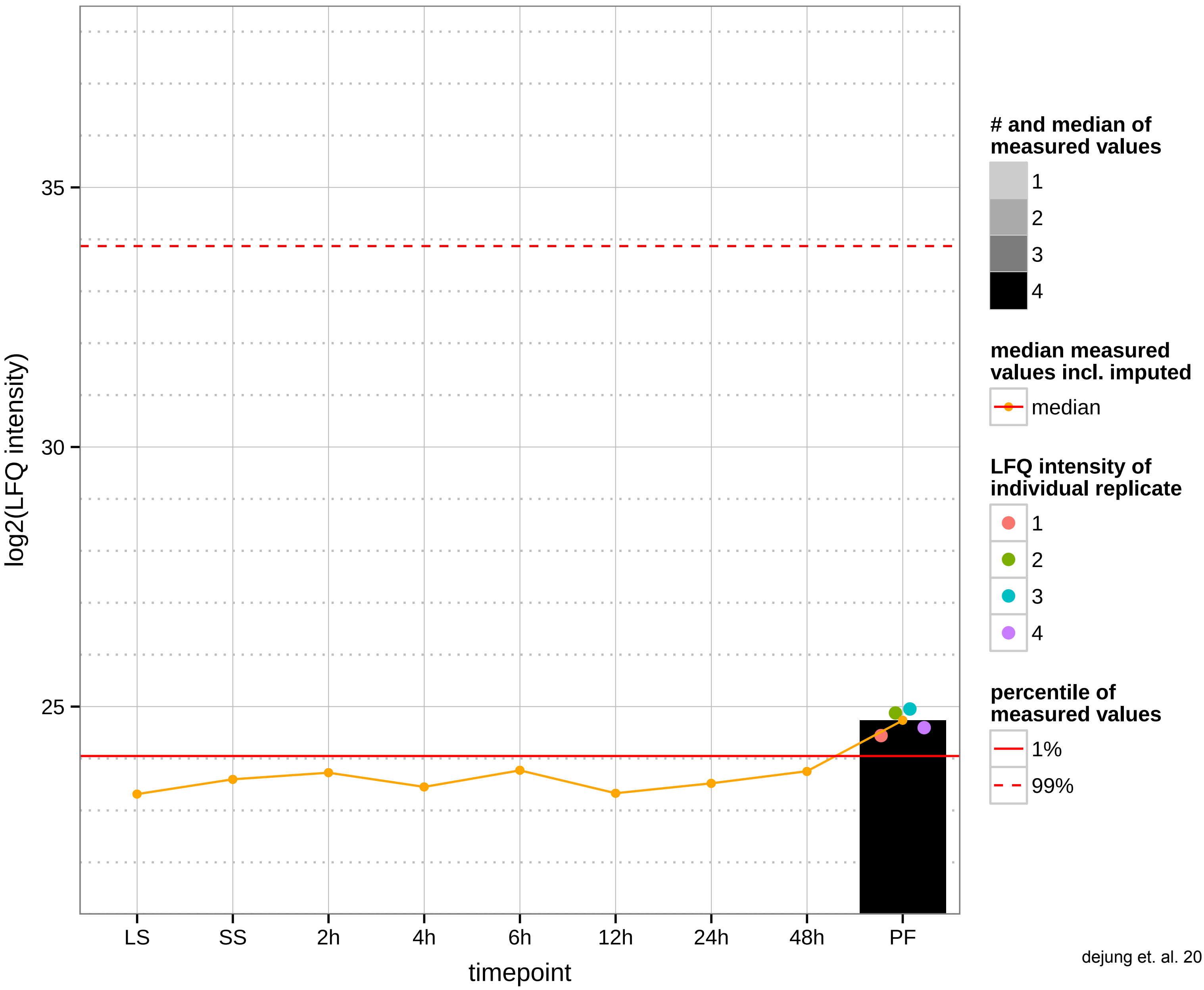

dejung et. al. 2015

hypothetical protein, conserved
Tb927.3.4950
AGOF: null
AGOC: mitochondrion
AGOP: null
PGOF: null
PGOC: null
PGOP: null

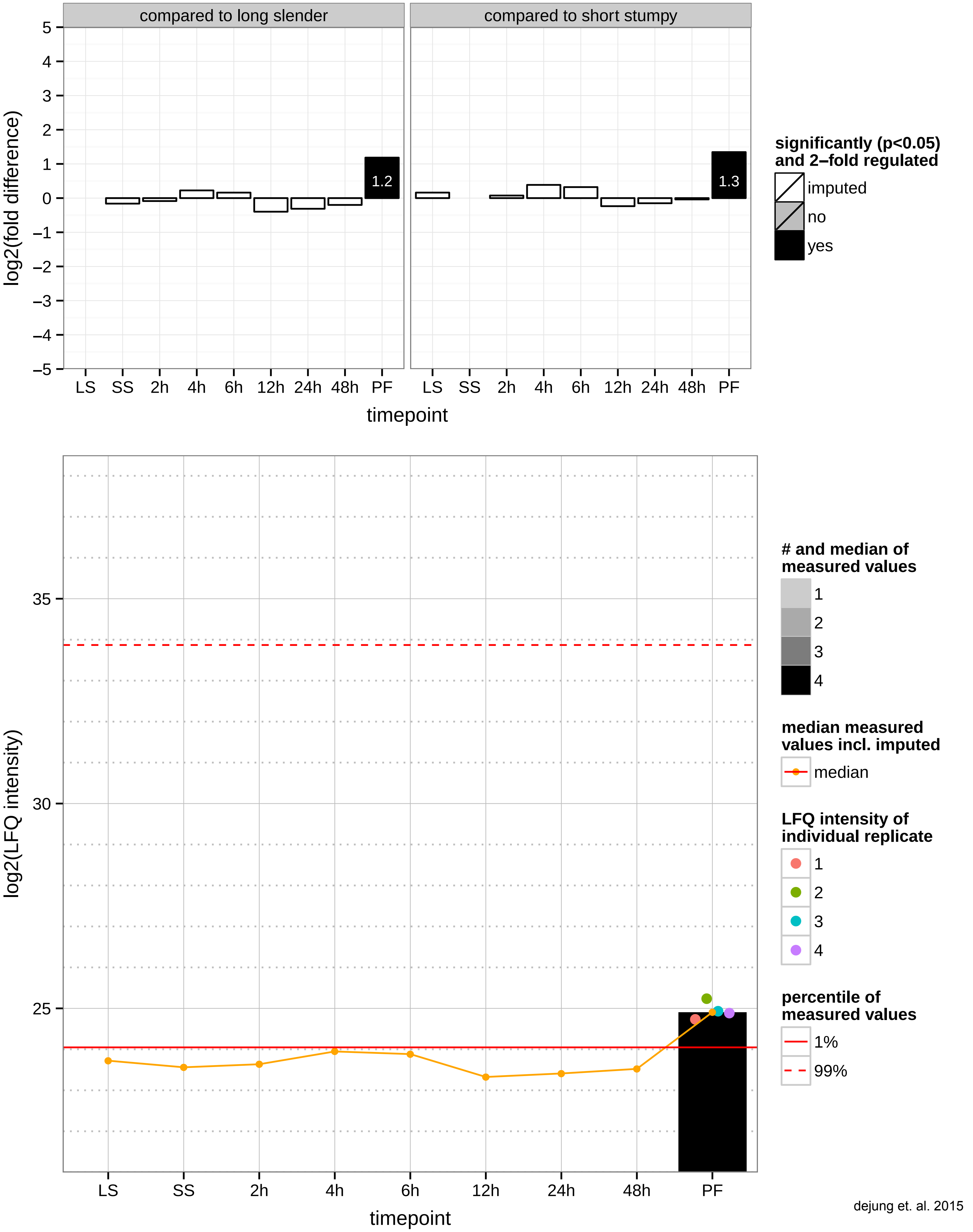

dejung et. al. 2015

60S ribosomal protein L4
Tb927.3.5050
AGOF: structural constituent of ribosome
AGOC: intracellular, ribosome
AGOP: translation
PGOF: structural constituent of ribosome
PGOC: ribosome
PGOP: translation

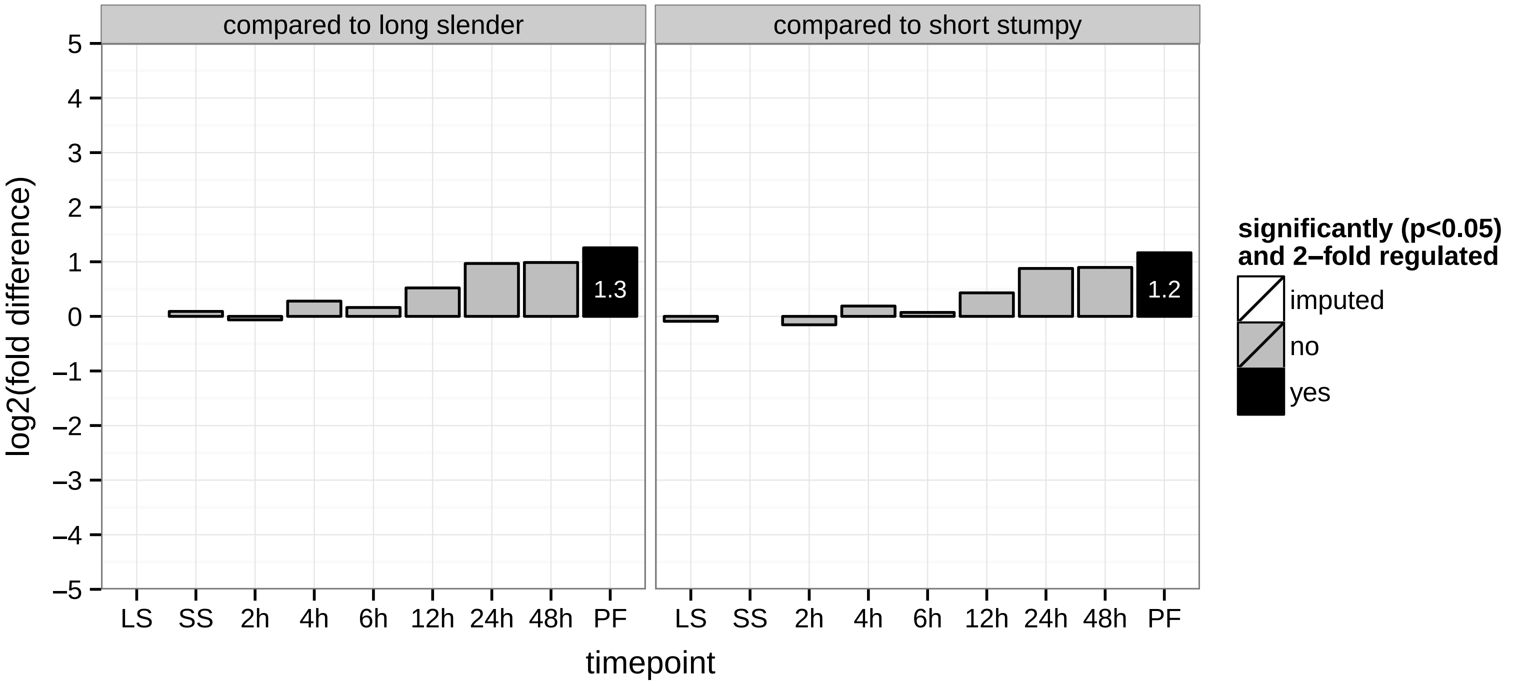

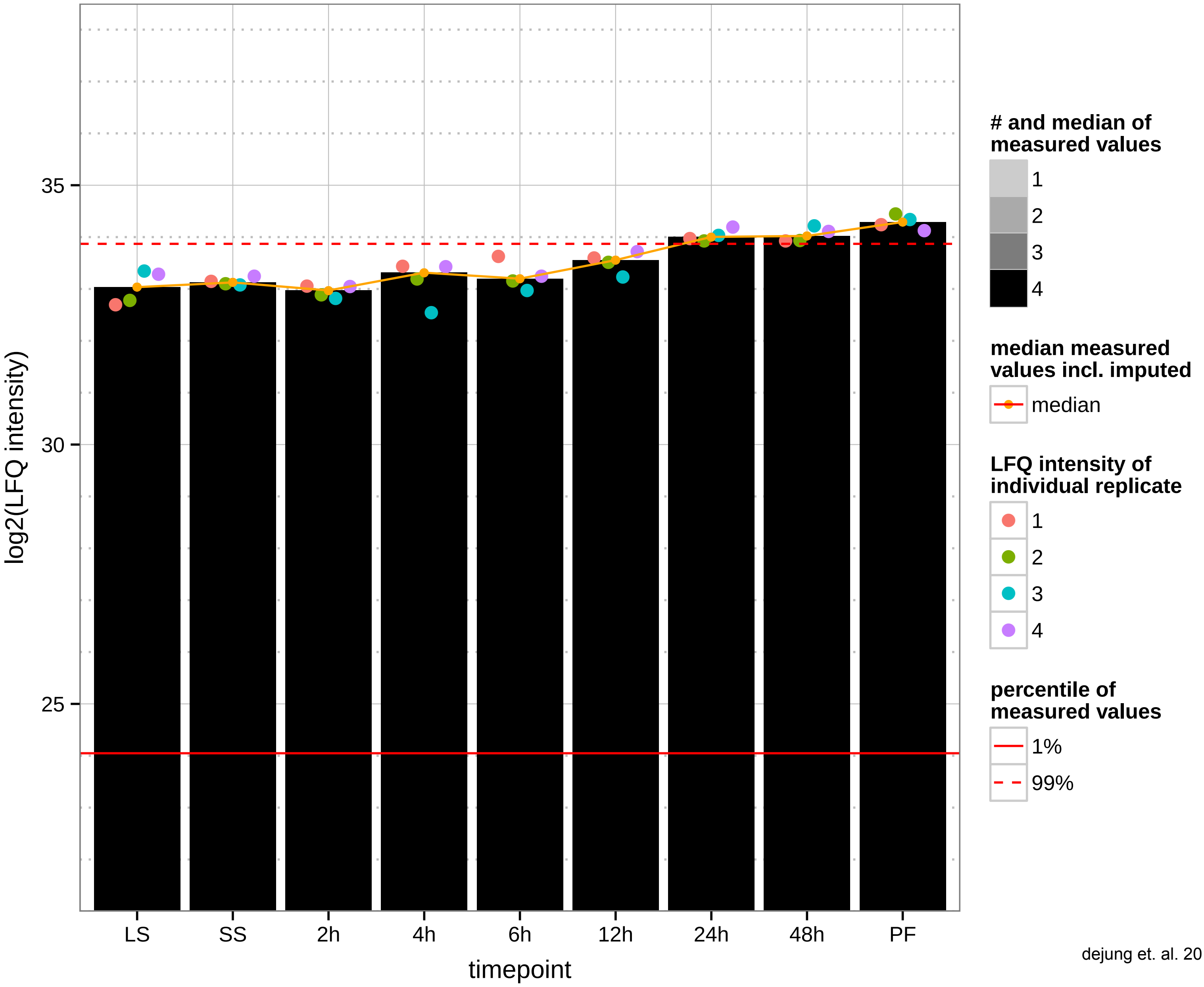

dejung et. al. 2015

UBX domain–containing protein
Tb927.3.5320
AGOF: ubiquitin binding, ubiquitin protein ligase binding
AGOC: cytoplasm, nucleus
AGOP: null
PGOF: null
PGOC: null
PGOP: null

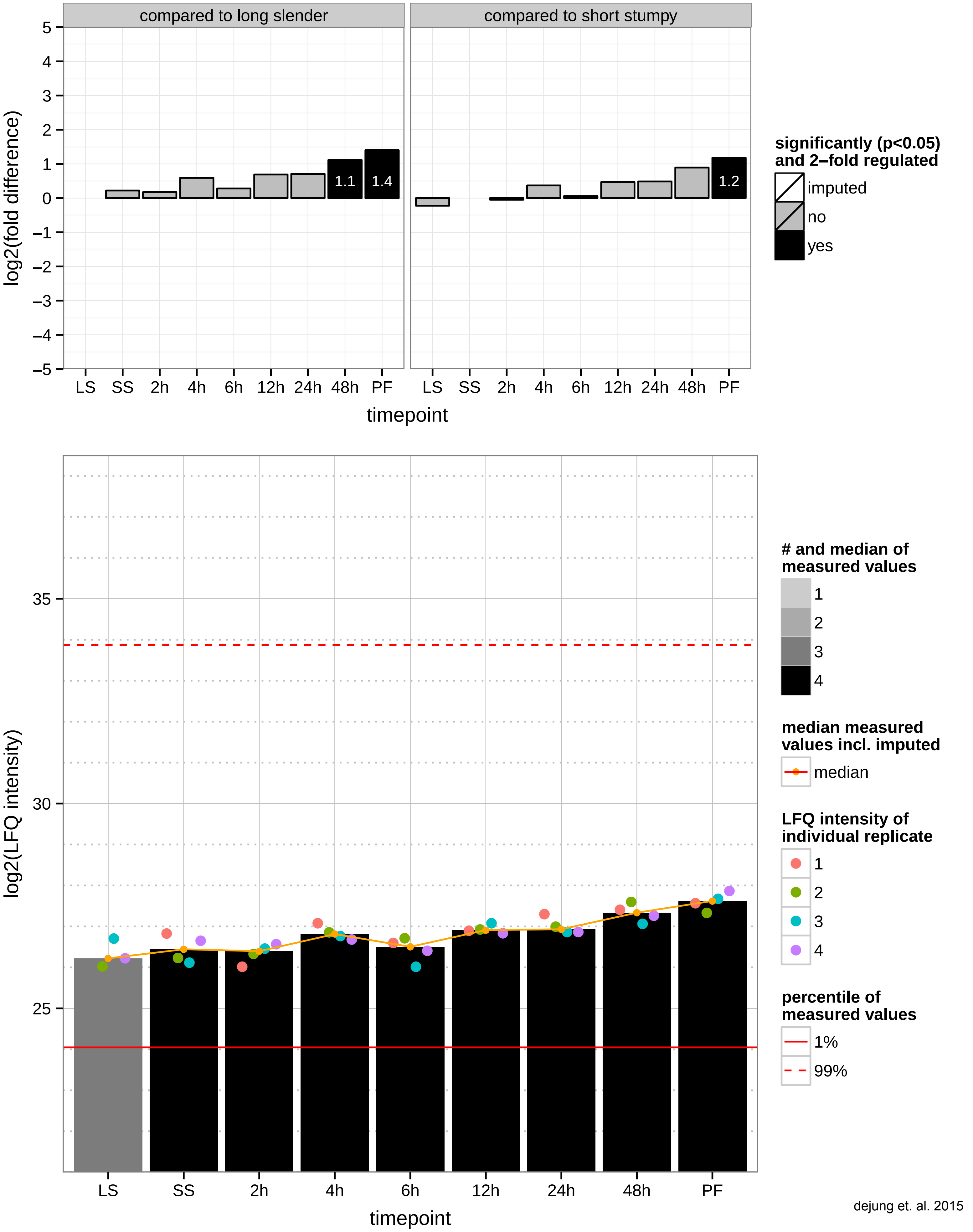

dejung et. al. 2015

hypothetical protein, conserved
Tb927.3.5430
AGOF: null
AGOC: null
AGOP: null
PGOF: null
PGOC: null
PGOP: null

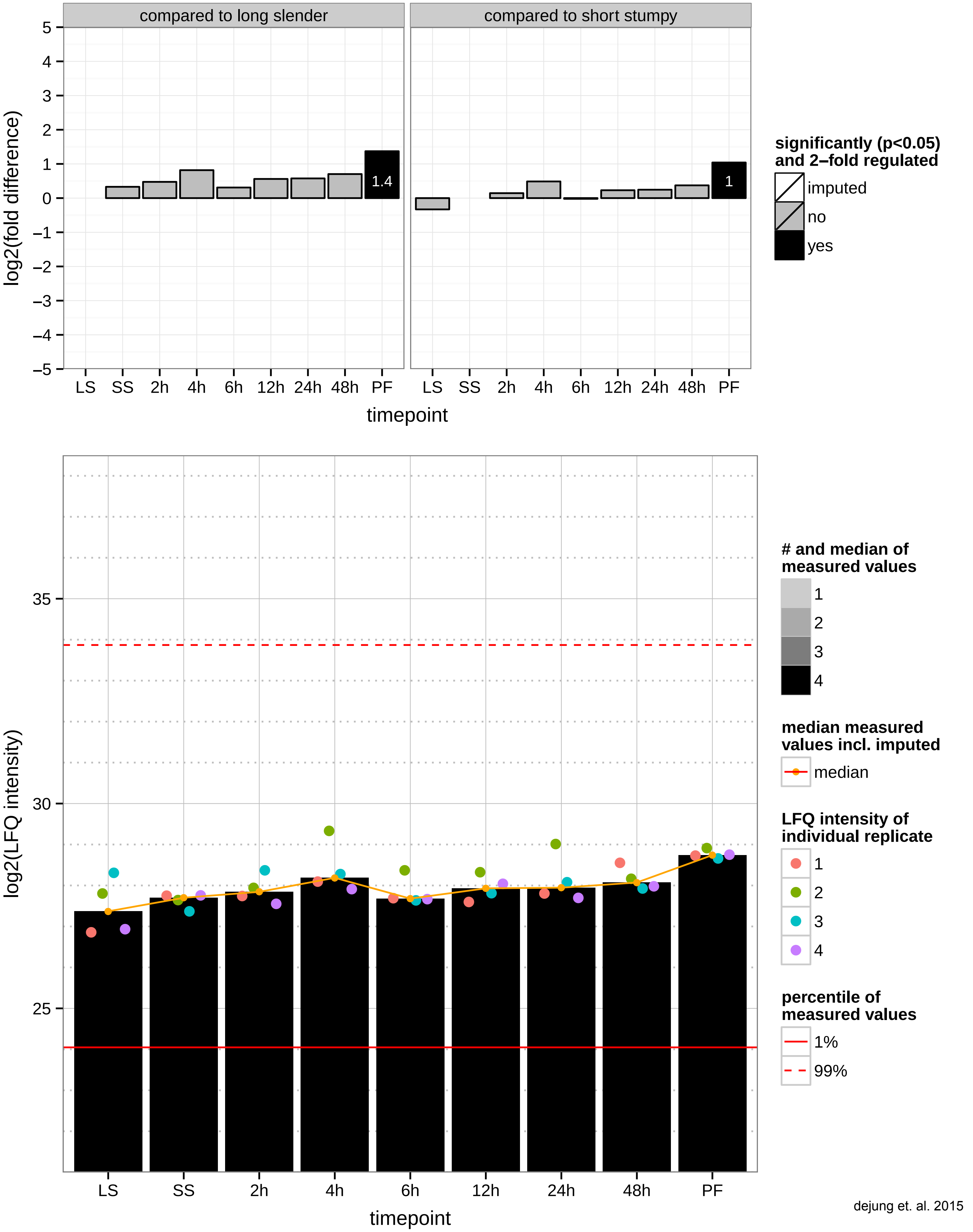

dejung et. al. 2015

hypothetical protein, conserved
Tb927.3.5600
AGOF: null
AGOC: mitochondrion
AGOP: null
PGOF: null
PGOC: null
PGOP: null

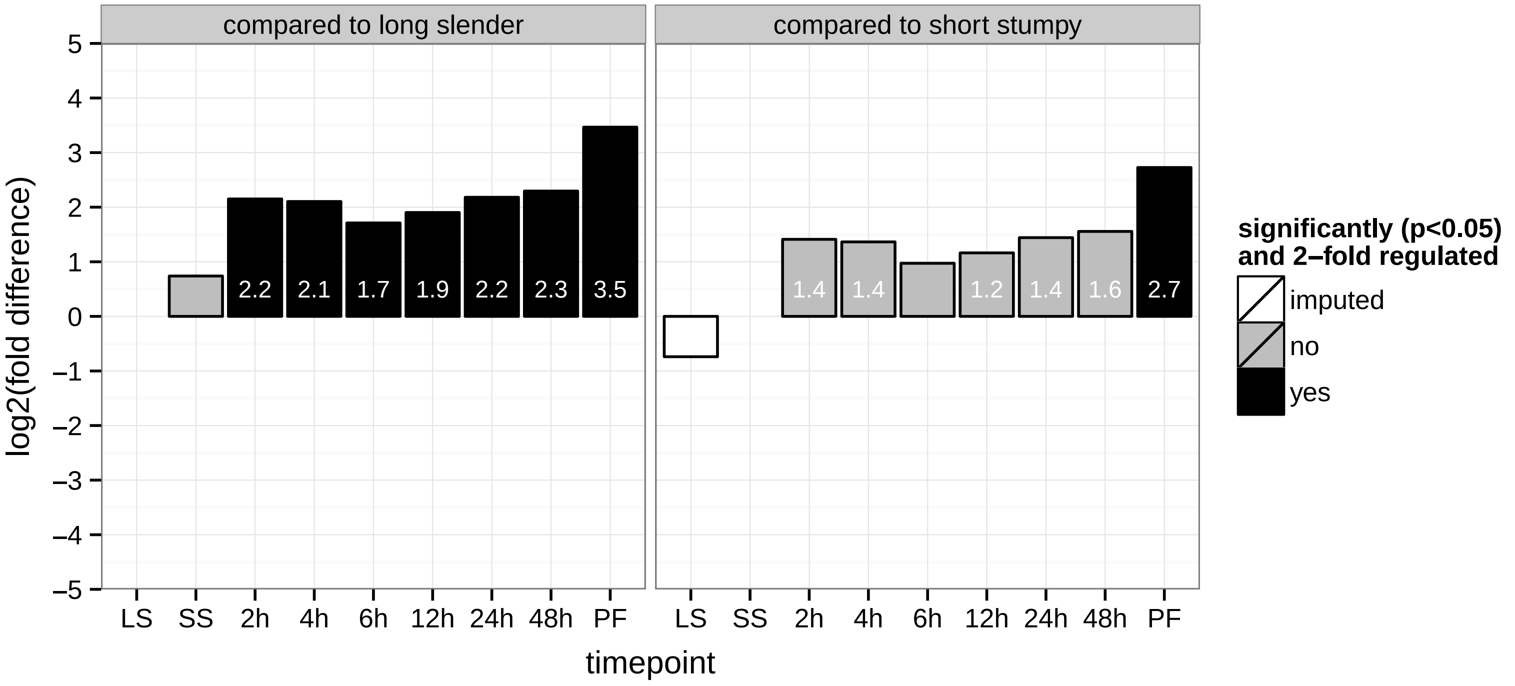

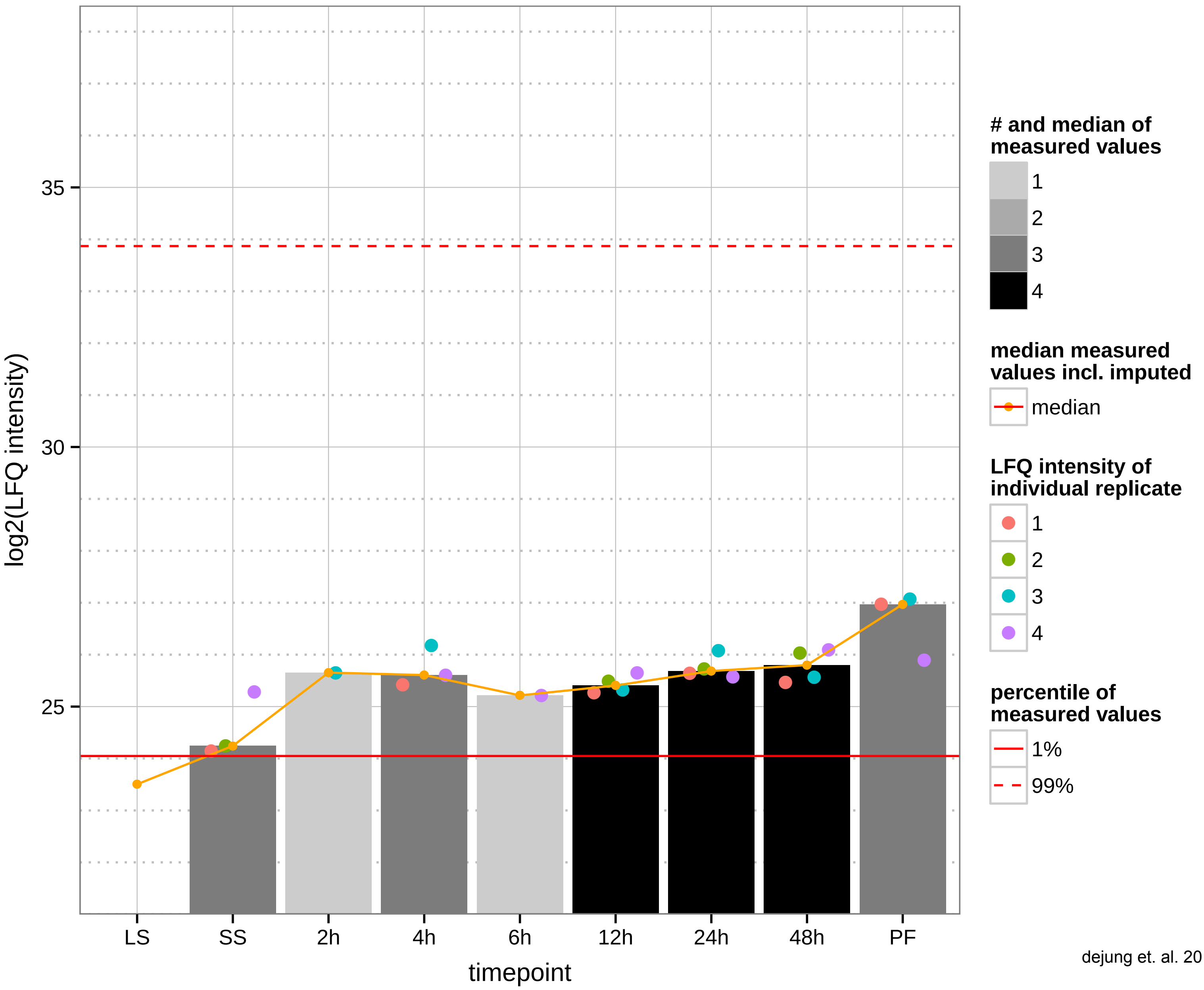

dejung et. al. 2015

ATP−dependent DEAD/H RNA helicase, putative
Tb927.3.620
AGOF: ATP binding, ATP−dependent helicase activity, helicase activity, nucleic acid binding
AGOC: null
AGOP: nucleobase−containing compound metabolic process
PGOF: ATP binding, ATP−dependent helicase activity, helicase activity, nucleic acid binding
PGOC: null
PGOP: null

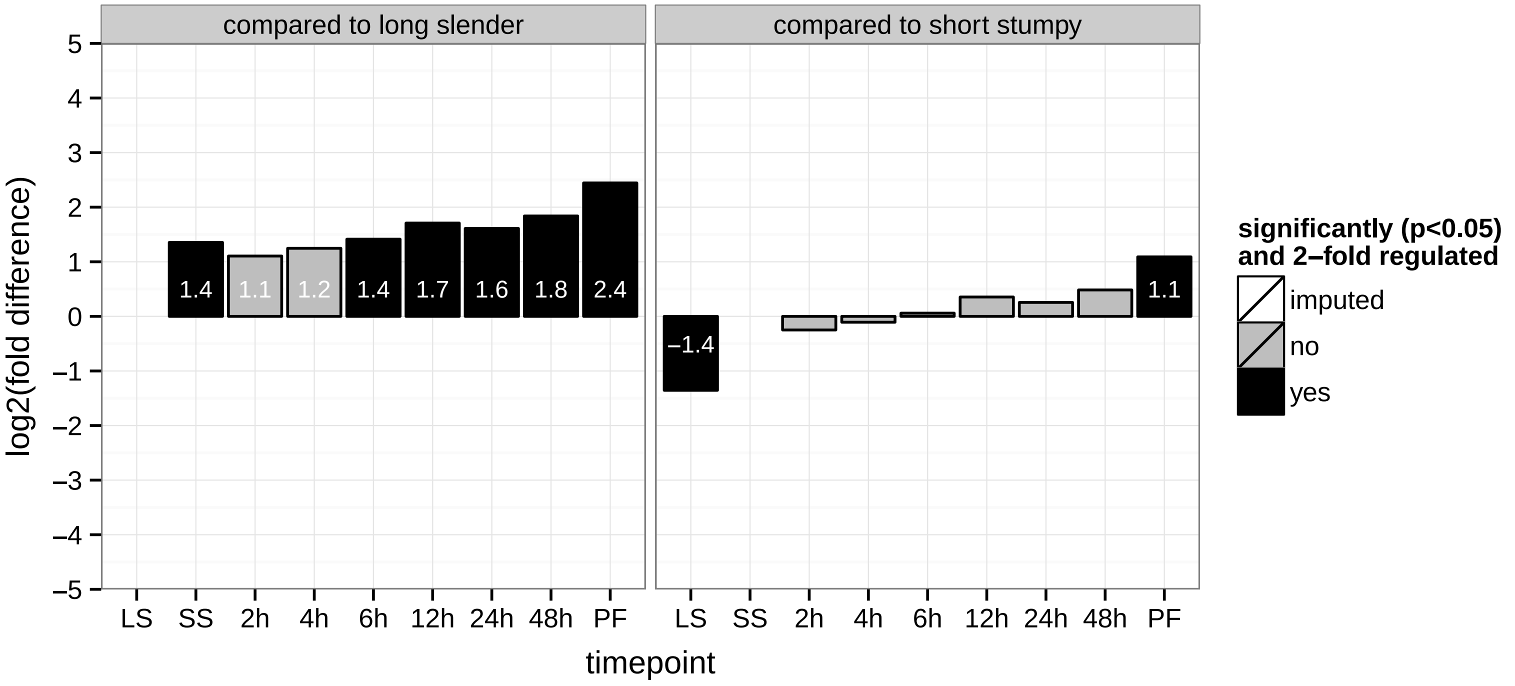

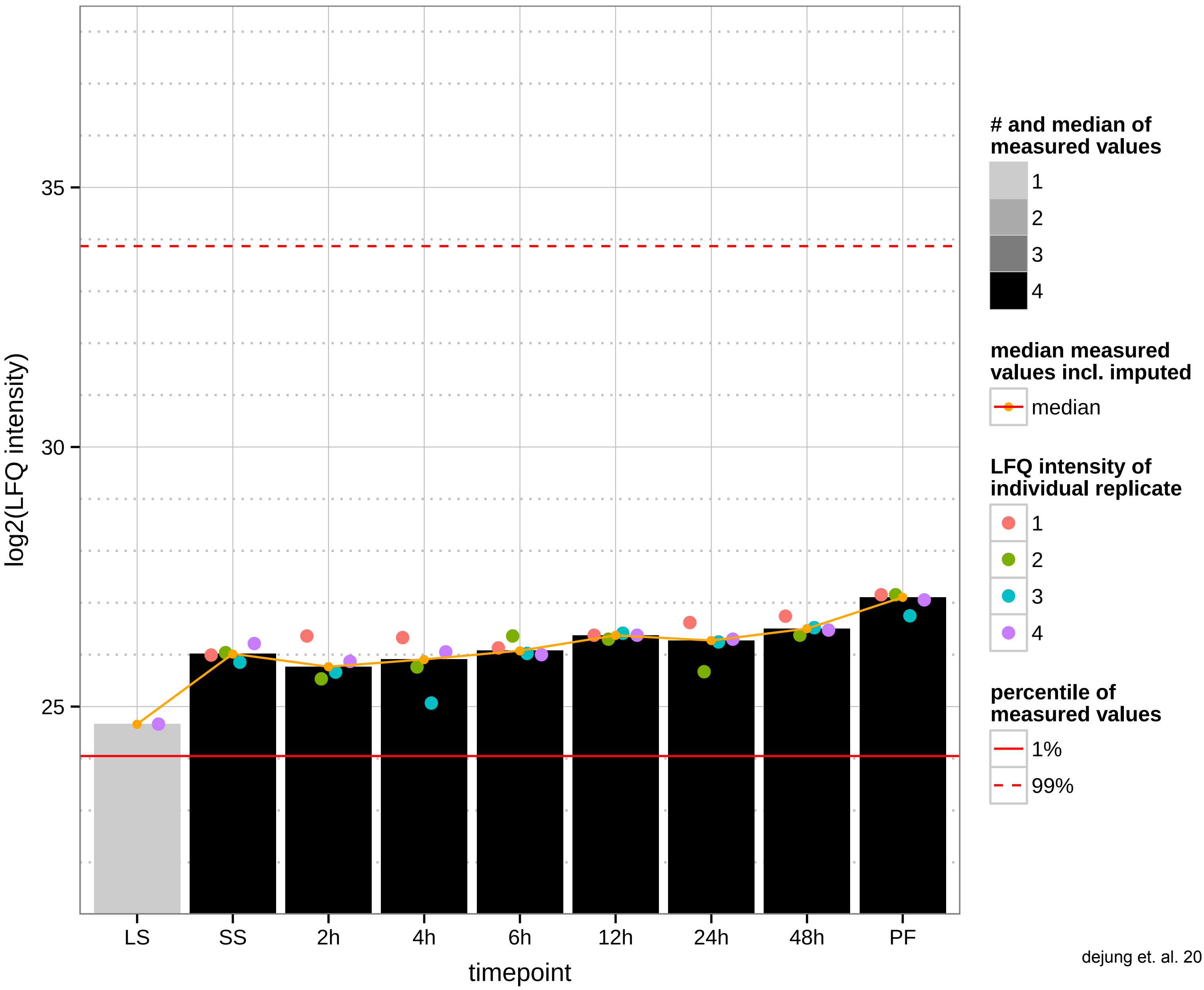

dejung et. al. 2015

ZFP family member, putative (ZC3H5)
Tb927.3.740
AGOF: RNA binding, zinc ion binding
AGOC: null
AGOP: null
PGOF: nucleic acid binding, zinc ion binding
PGOC: null
PGOP: null

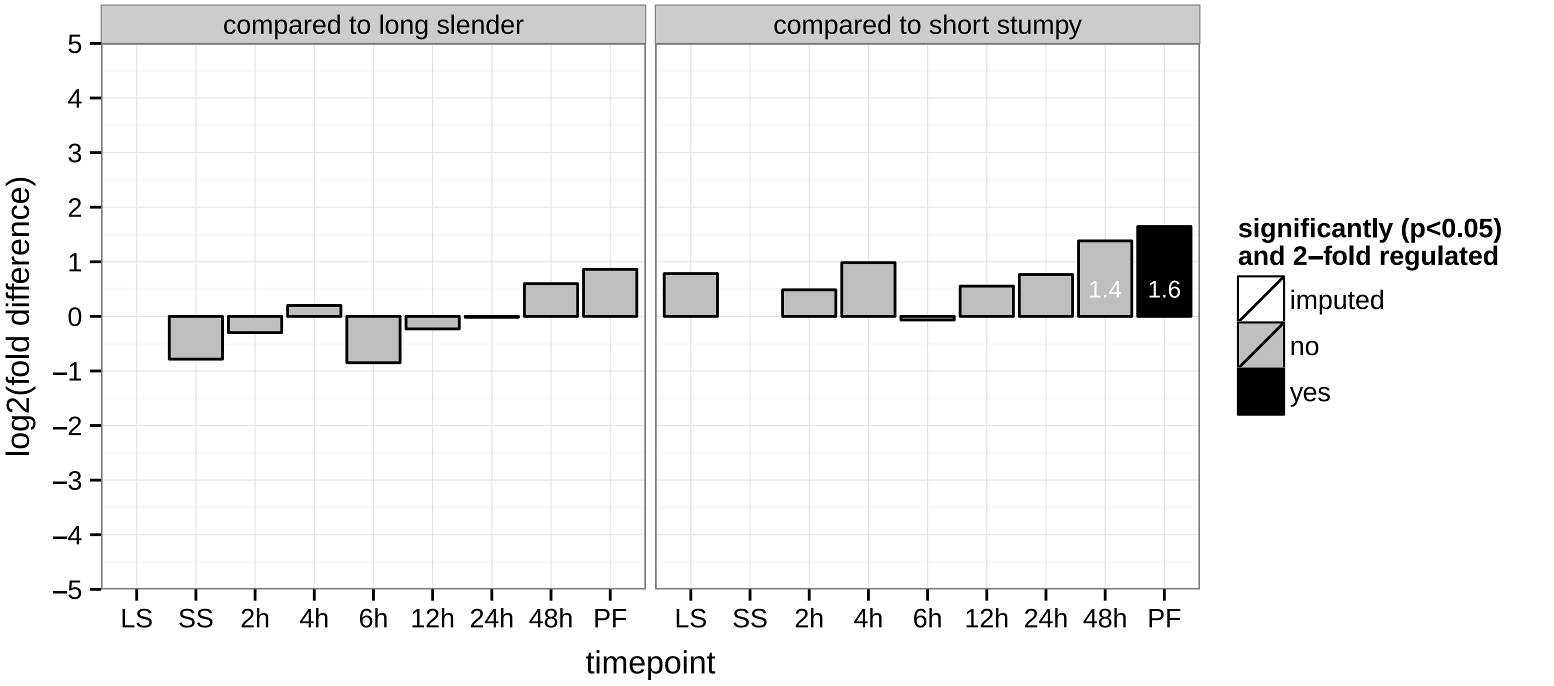

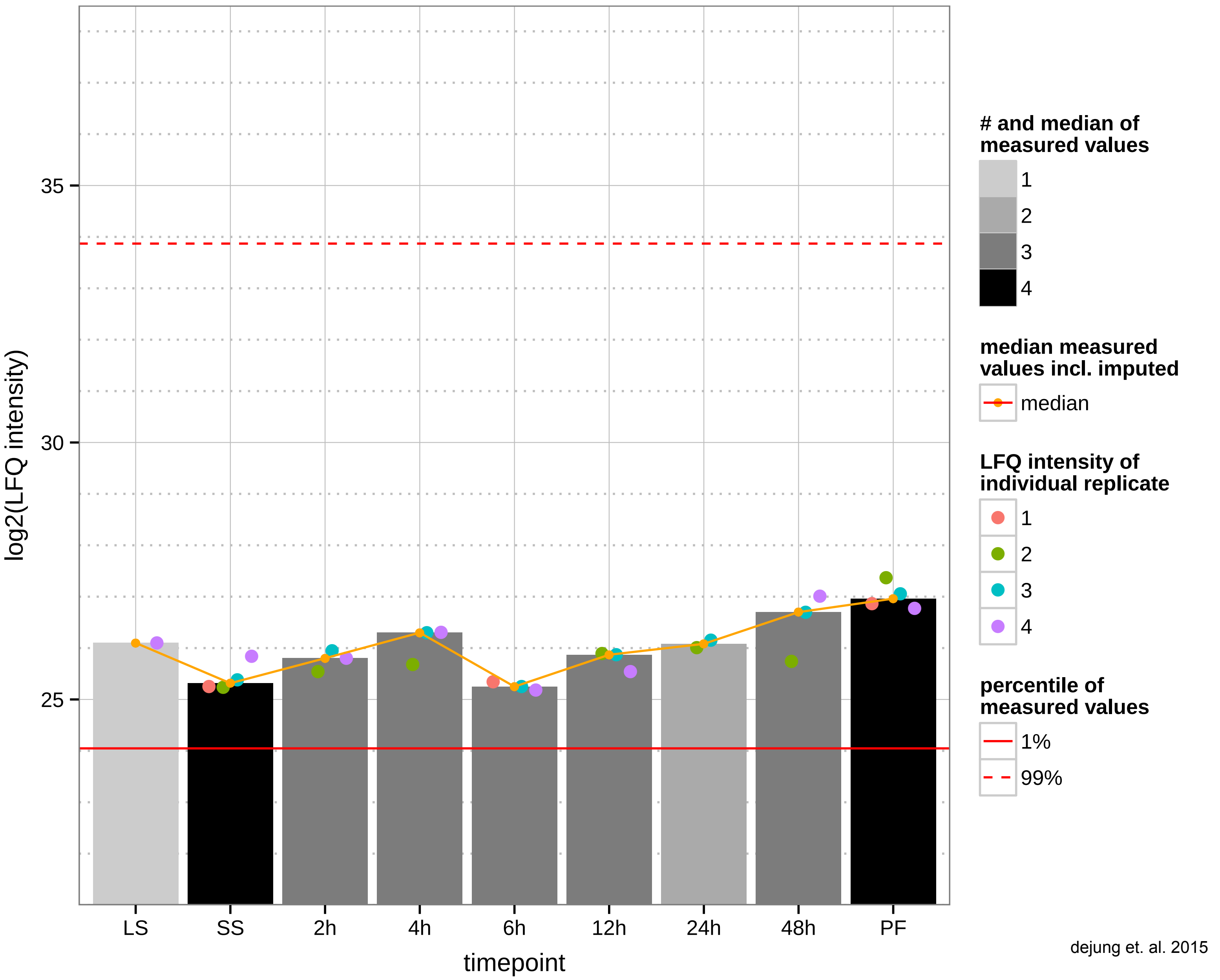

dejung et. al. 2015

flap endonuclease−1 (FEN−1), putative
Tb927.3.830
AGOF: DNA binding, nuclease activity
AGOC: null
AGOP: DNA repair
PGOF: DNA binding, catalytic activity, nuclease activity
PGOC: null
PGOP: DNA repair

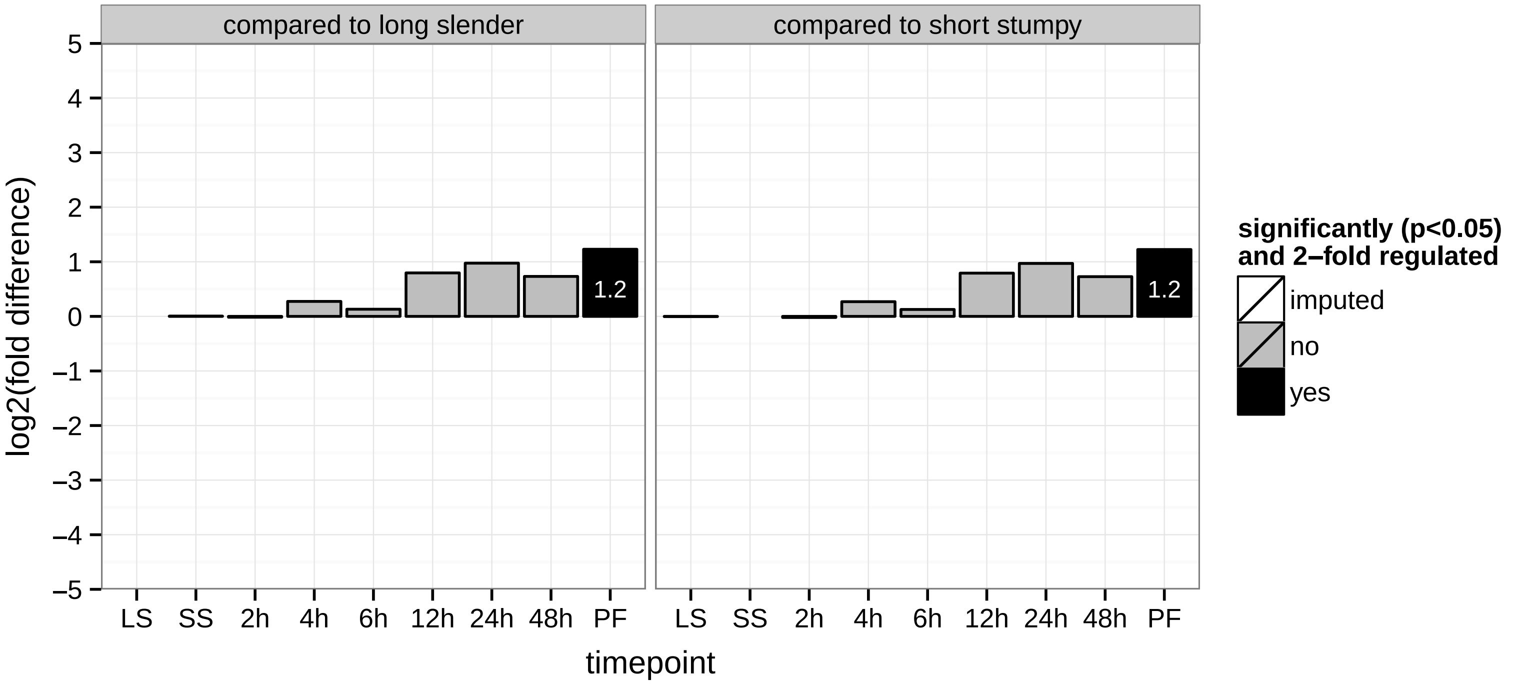

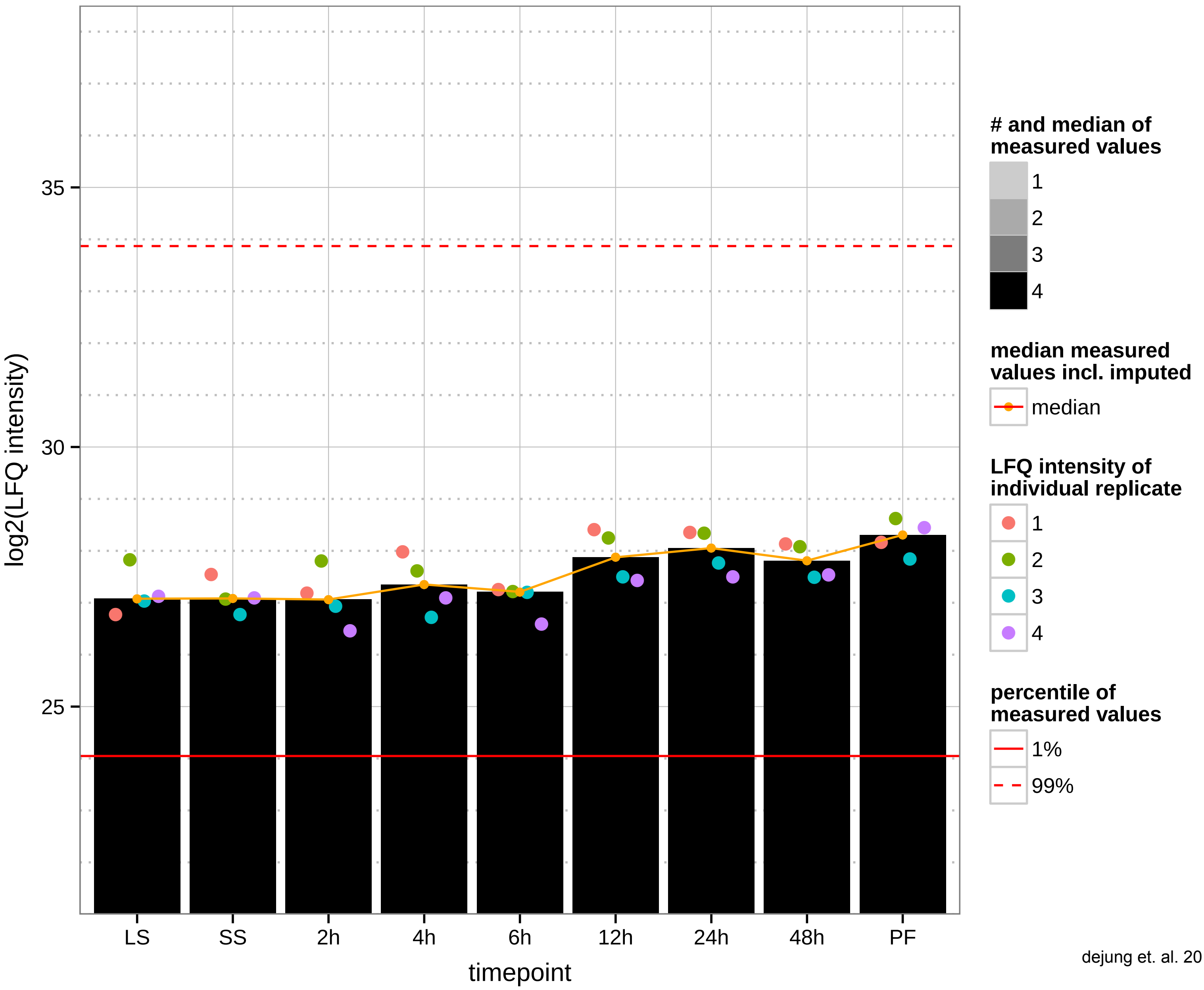

dejung et. al. 2015

protein transport protein Sec61 gamma subunit, putative
Tb927.3.960
AGOF: P–P–bond–hydrolysis–driven protein transmembrane transporter activity
AGOC: membrane
AGOP: protein targeting
PGOF: P–P–bond–hydrolysis–driven protein transmembrane transporter activity
PGOC: membrane
PGOP: intracellular protein transport, protein targeting, protein transport

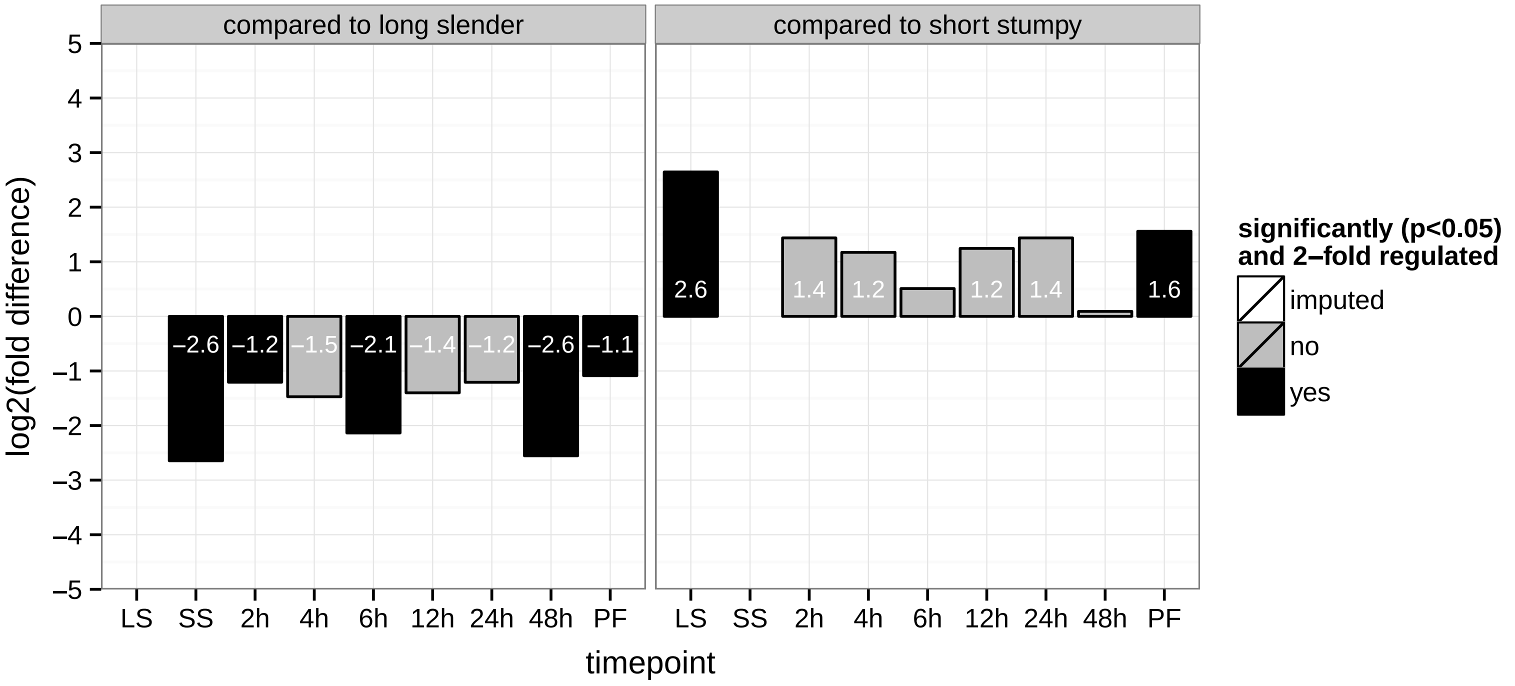

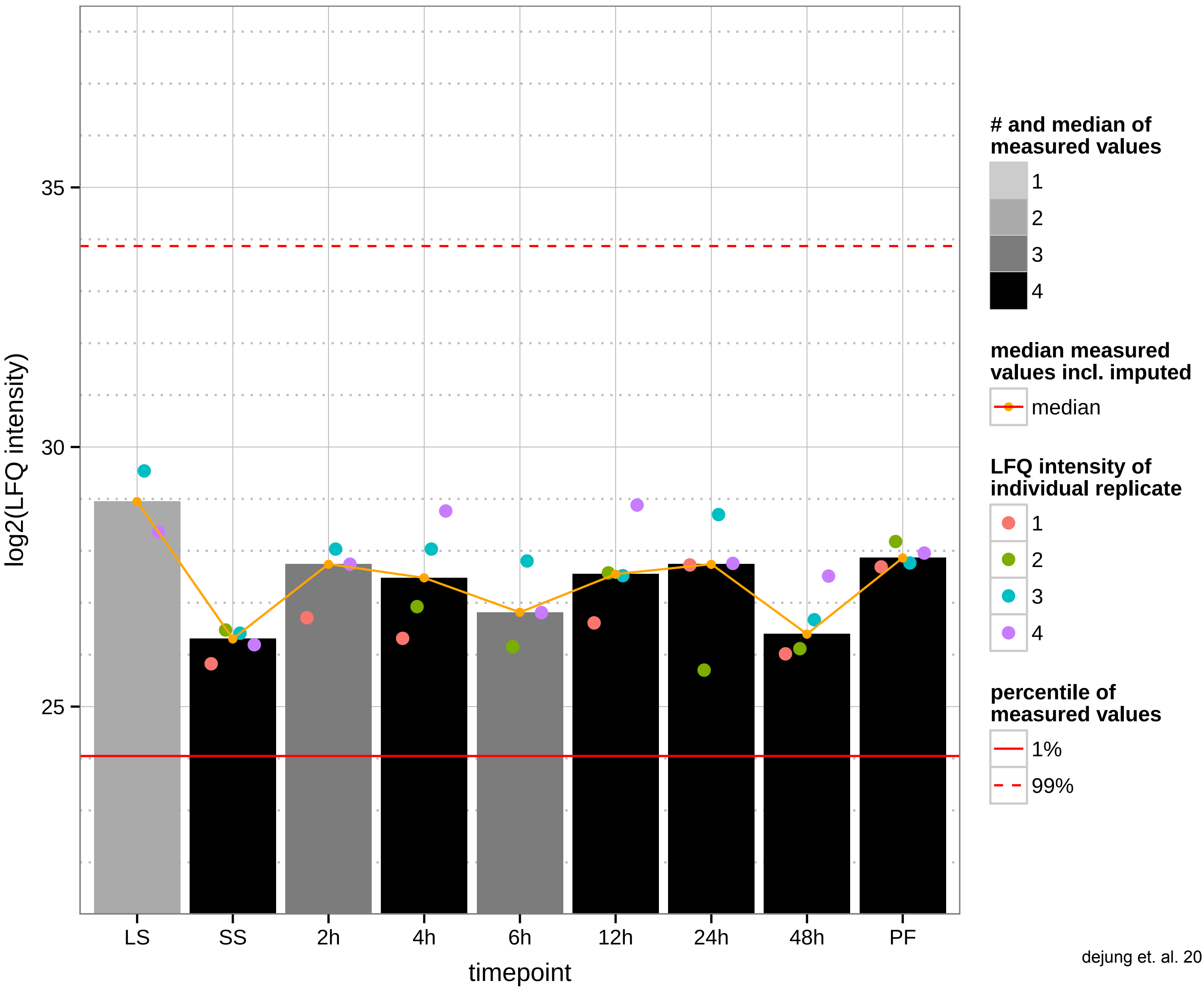

dejung et. al. 2015

serine–palmitoyl–CoA transferase, putative
Tb927.4.1020
AGOF: pyridoxal phosphate binding, transferase activity, transferring nitrogenous groups
AGOC: endoplasmic reticulum
AGOP: biosynthetic process, membrane lipid metabolic process
PGOF: pyridoxal phosphate binding, transferase activity
PGOC: null
PGOP: biosynthetic process

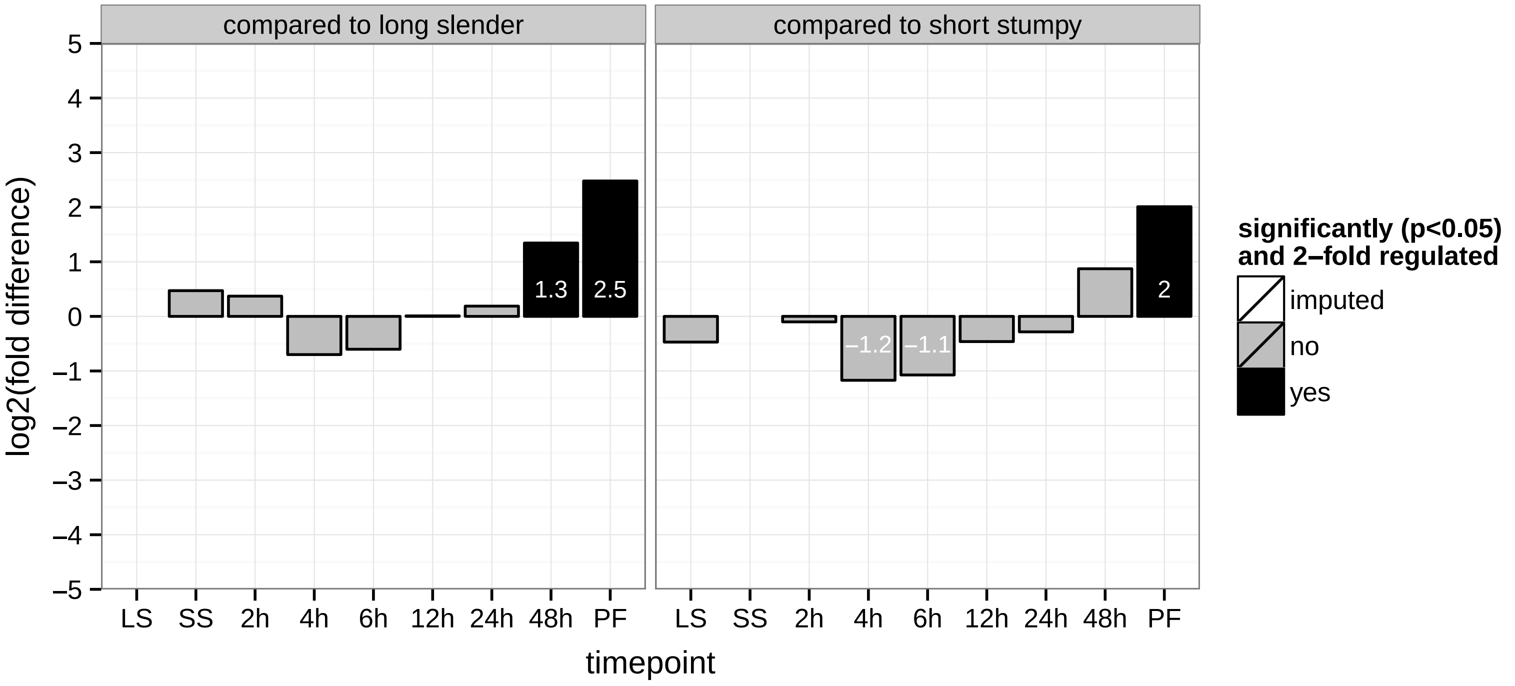

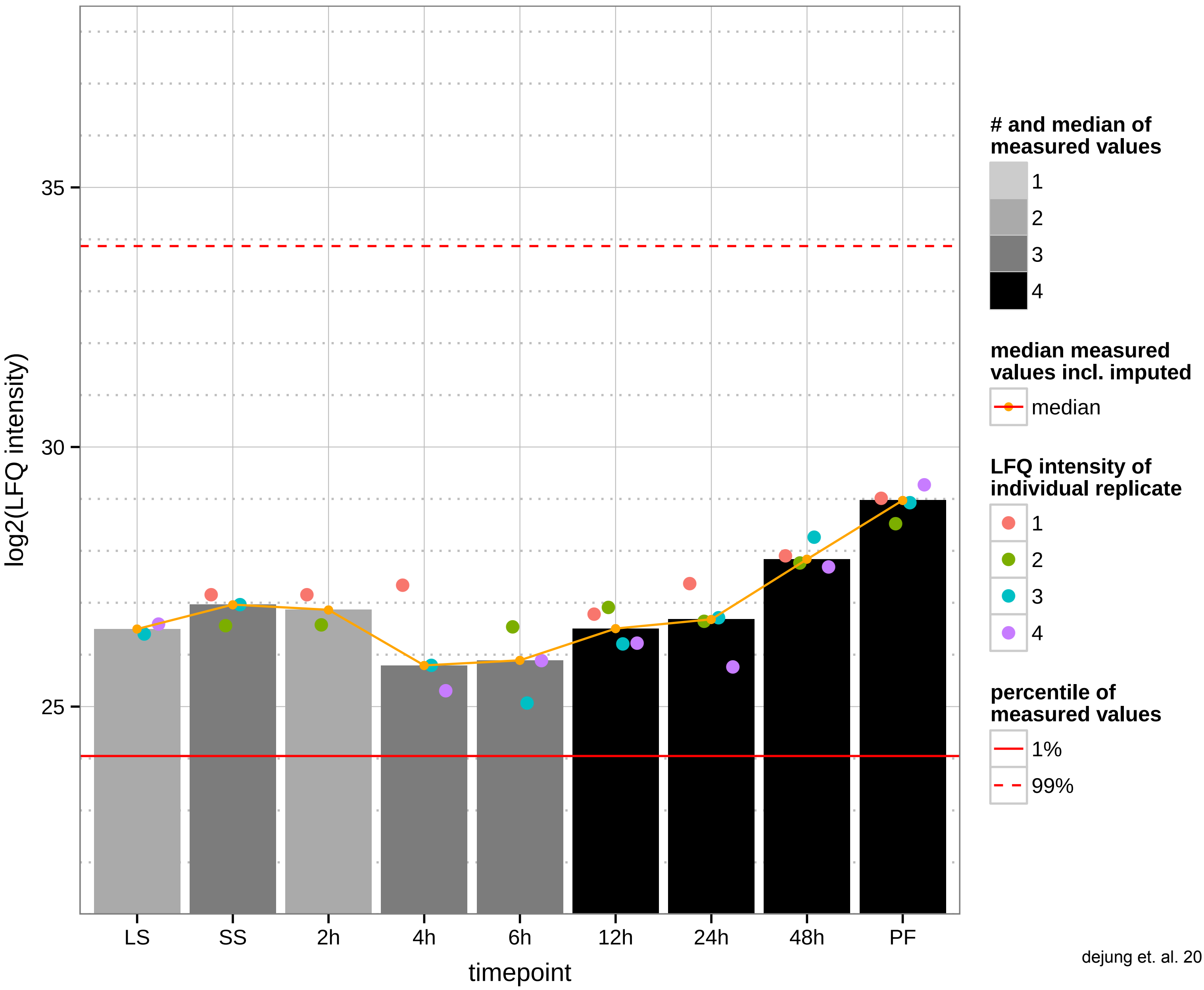

dejung et. al. 2015

endosomal integral membrane protein, putative
Tb927.4.1090
AGOF: null
AGOC: integral to membrane
AGOP: null
PGOF: null
PGOC: integral to membrane
PGOP: null

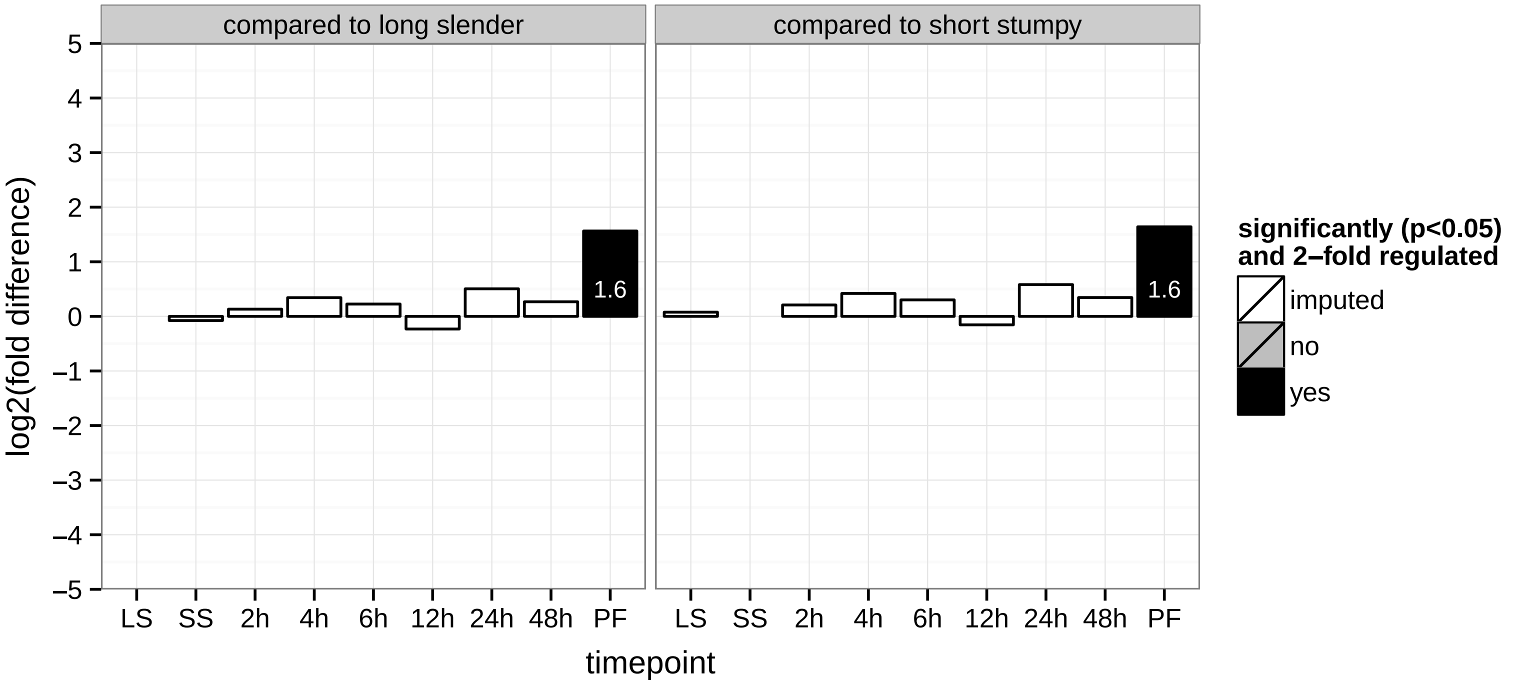

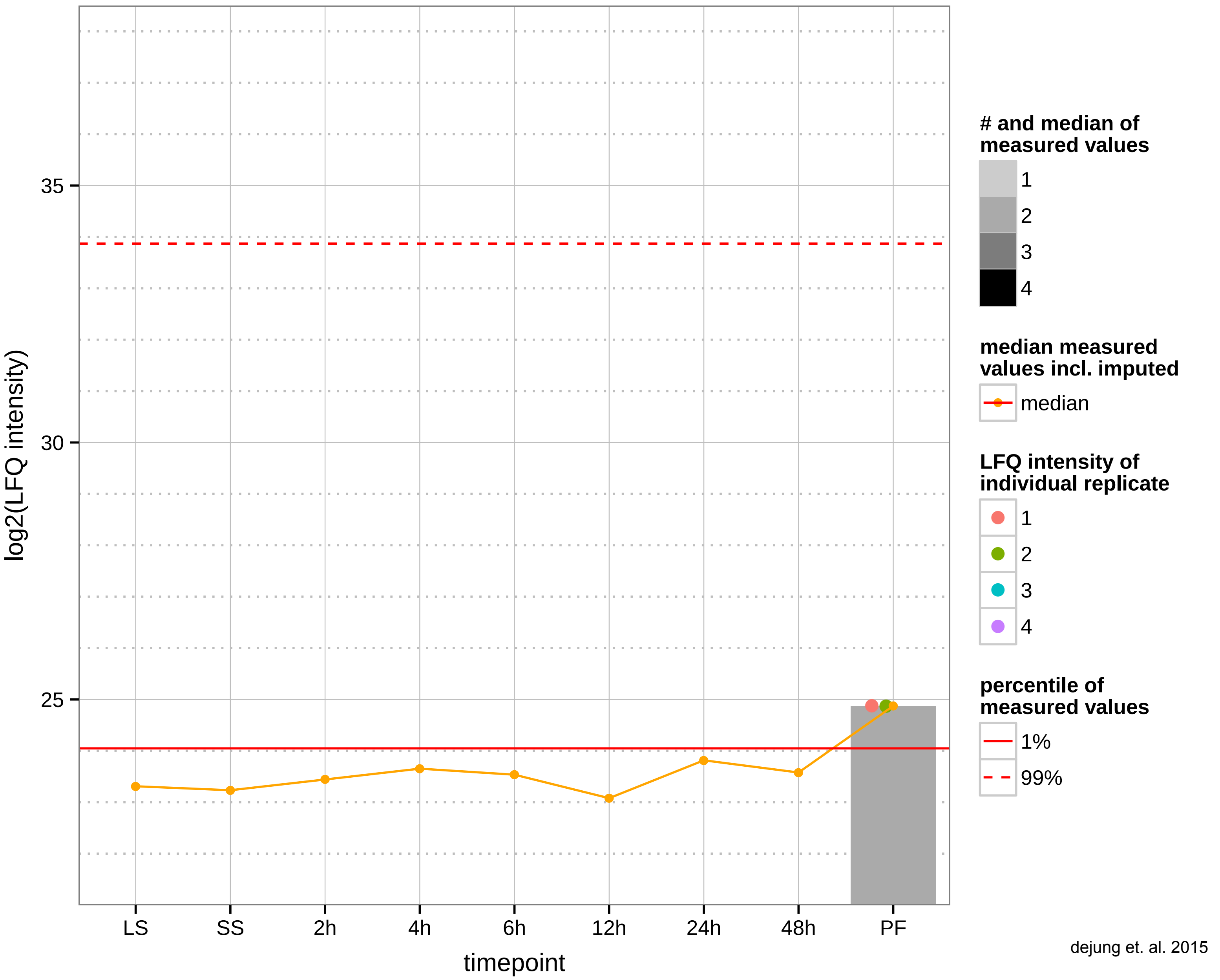

dejung et. al. 2015

ZFP family member, putative (ZC3H9)
Tb927.4.1310
AGOF: RNA binding, zinc ion binding
AGOC: intracellular
AGOP: null
PGOF: nucleic acid binding, zinc ion binding
PGOC: intracellular
PGOP: null

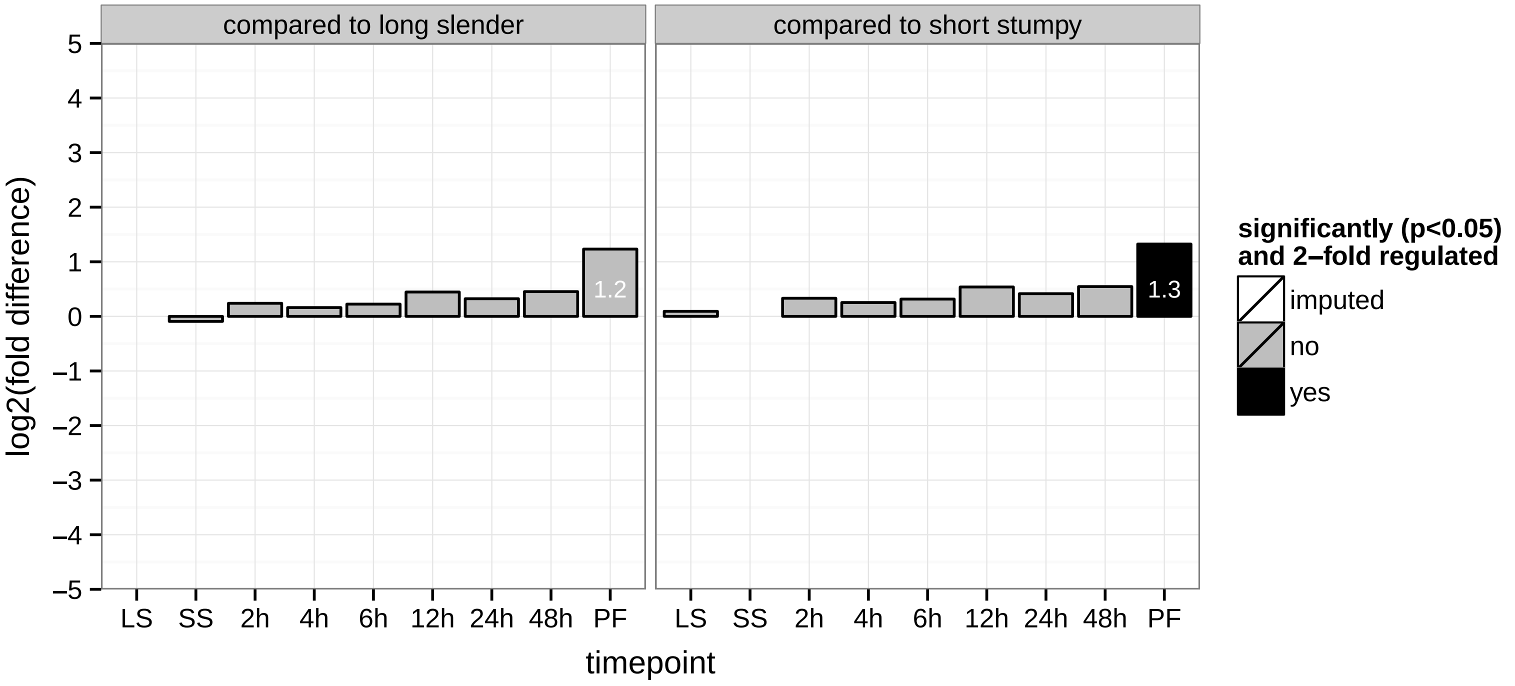

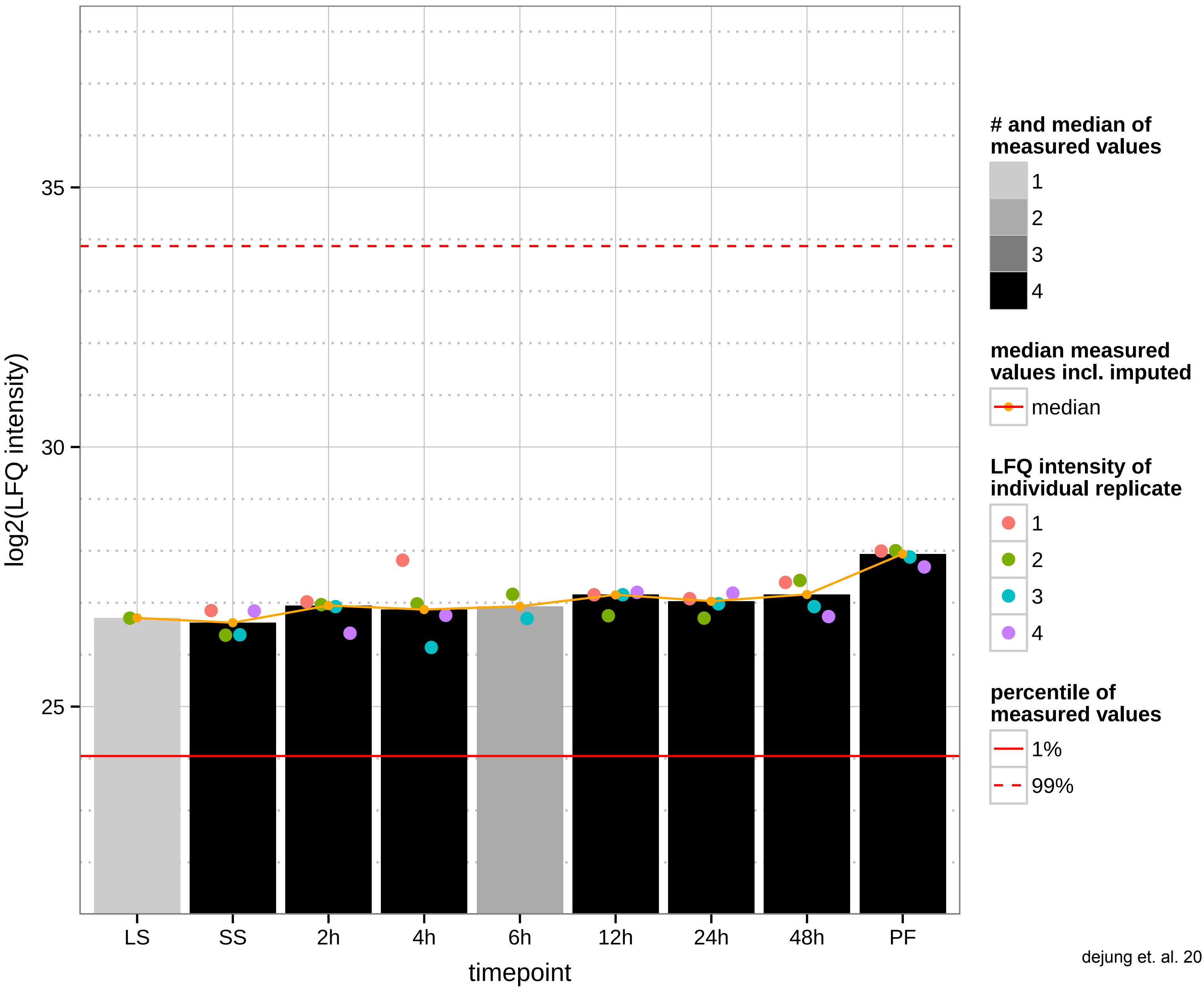

dejung et. al. 2015

hypothetical protein, conserved
Tb927.4.1380
AGOF: hydrolase activity, acting on ester bonds
AGOC: cytoplasm
AGOP: D–amino acid catabolic process
PGOF: hydrolase activity, acting on ester bonds
PGOC: cytoplasm
PGOP: D–amino acid catabolic process

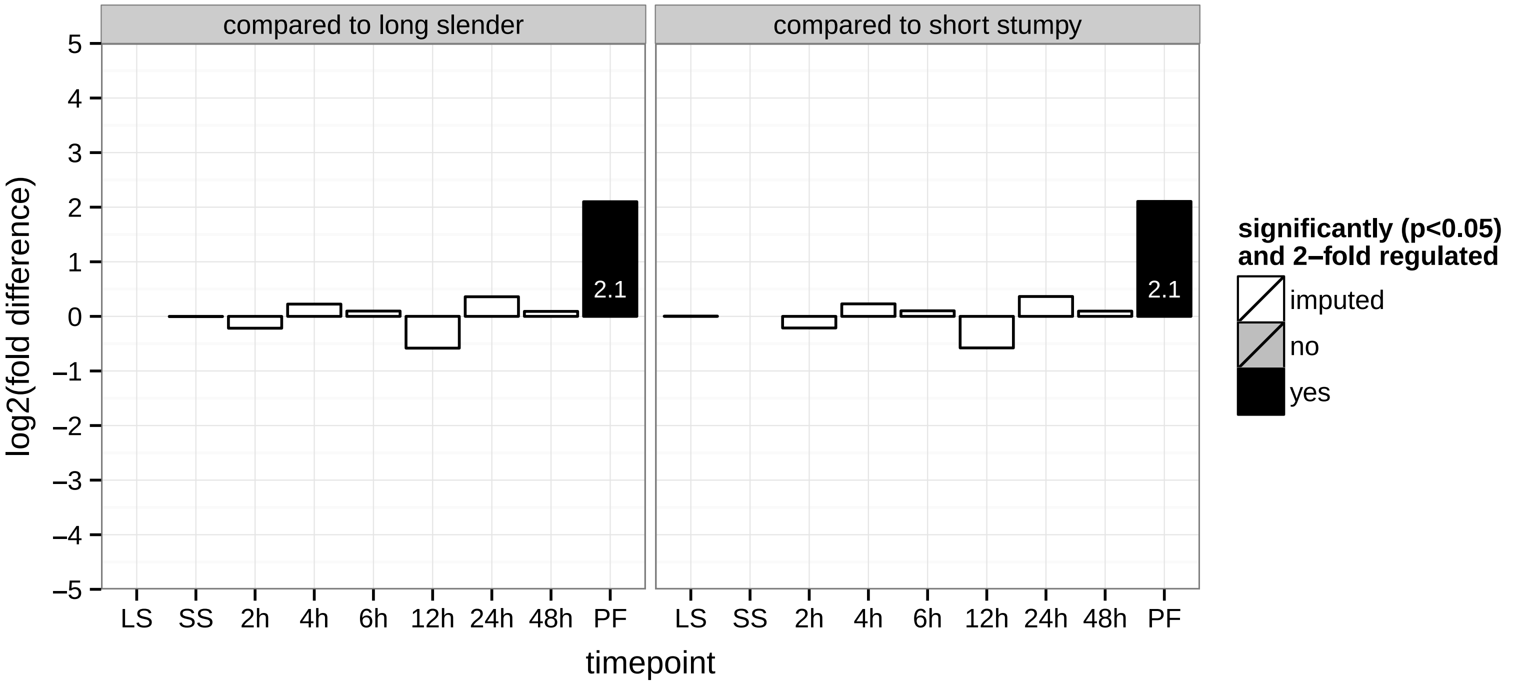

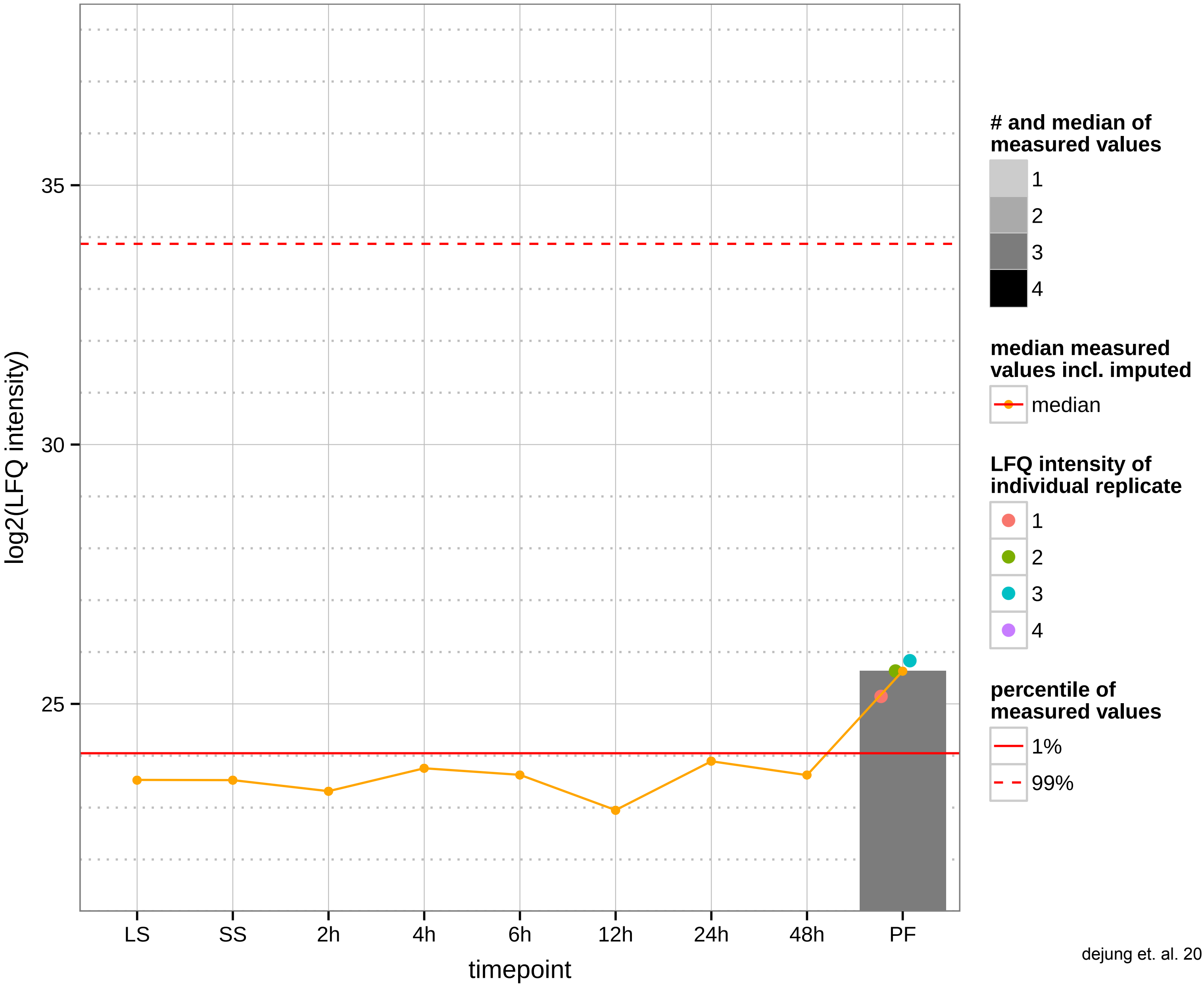

dejung et. al. 2015

hypothetical protein, conserved
Tb927.4.1440
AGOF: null
AGOC: mitochondrion
AGOP: null
PGOF: metal ion binding
PGOC: null
PGOP: null

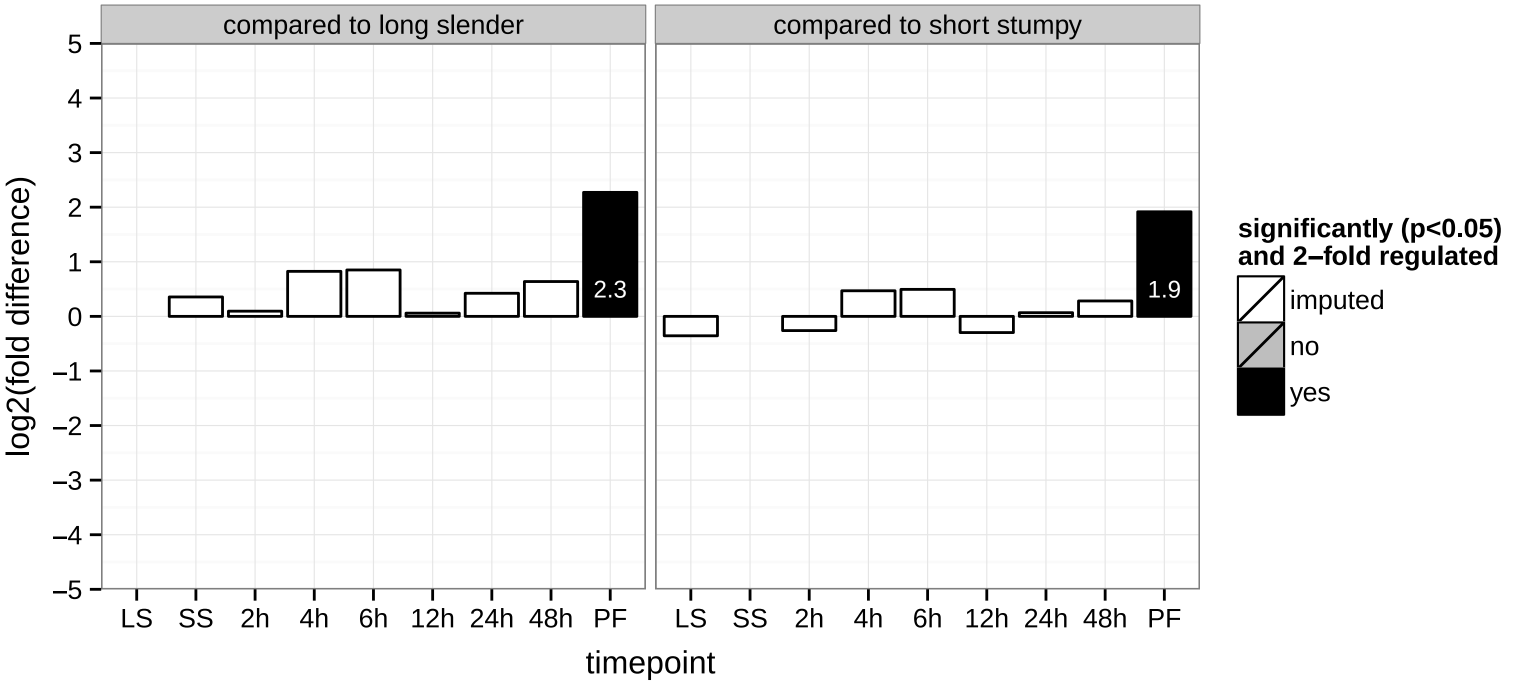

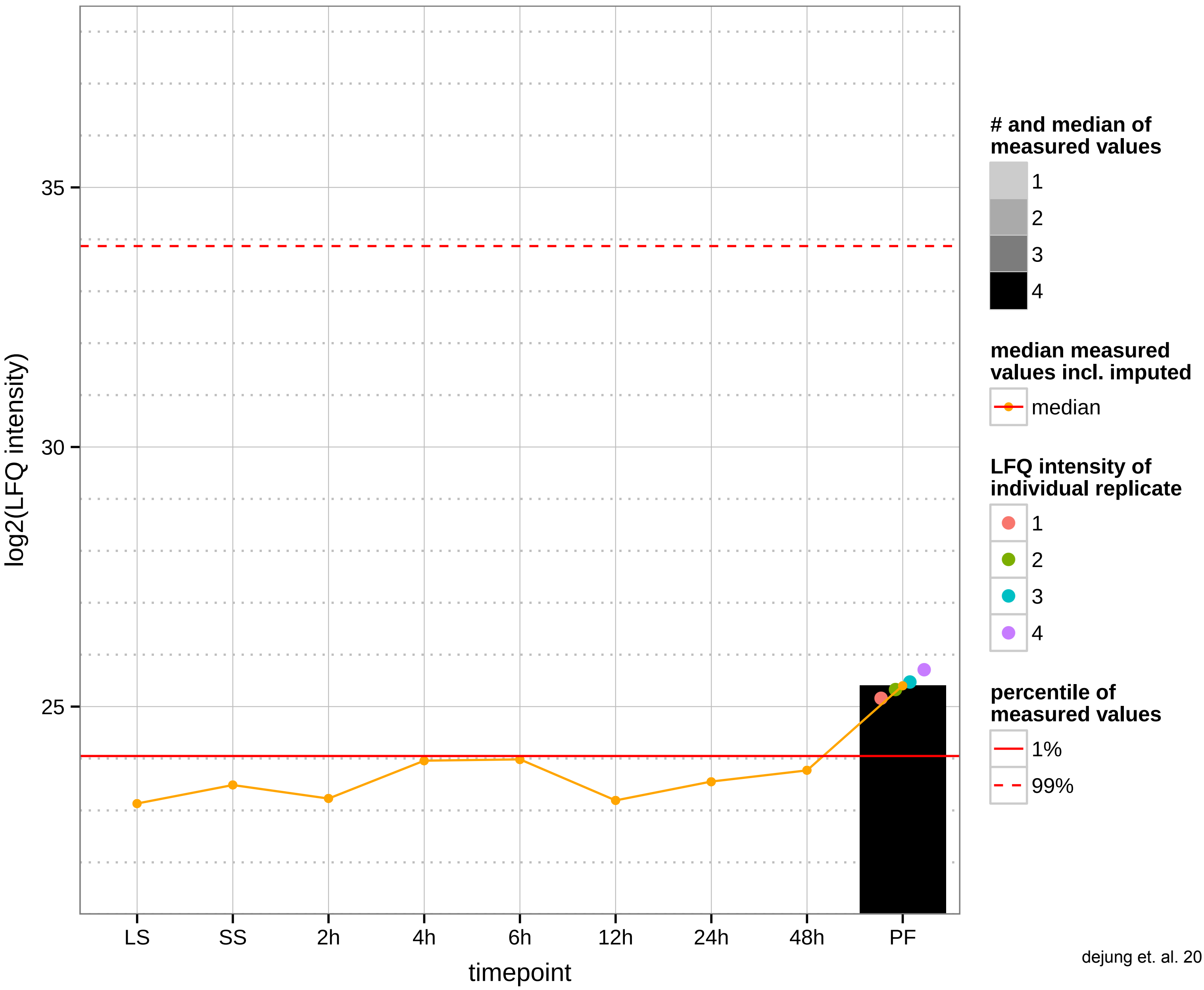

dejung et. al. 2015

hypothetical protein, conserved
Tb927.4.1590.1;Tb927.4.1590.2
AGOF: null
AGOC: null
AGOP: null
PGOF: null
PGOC: null
PGOP: null

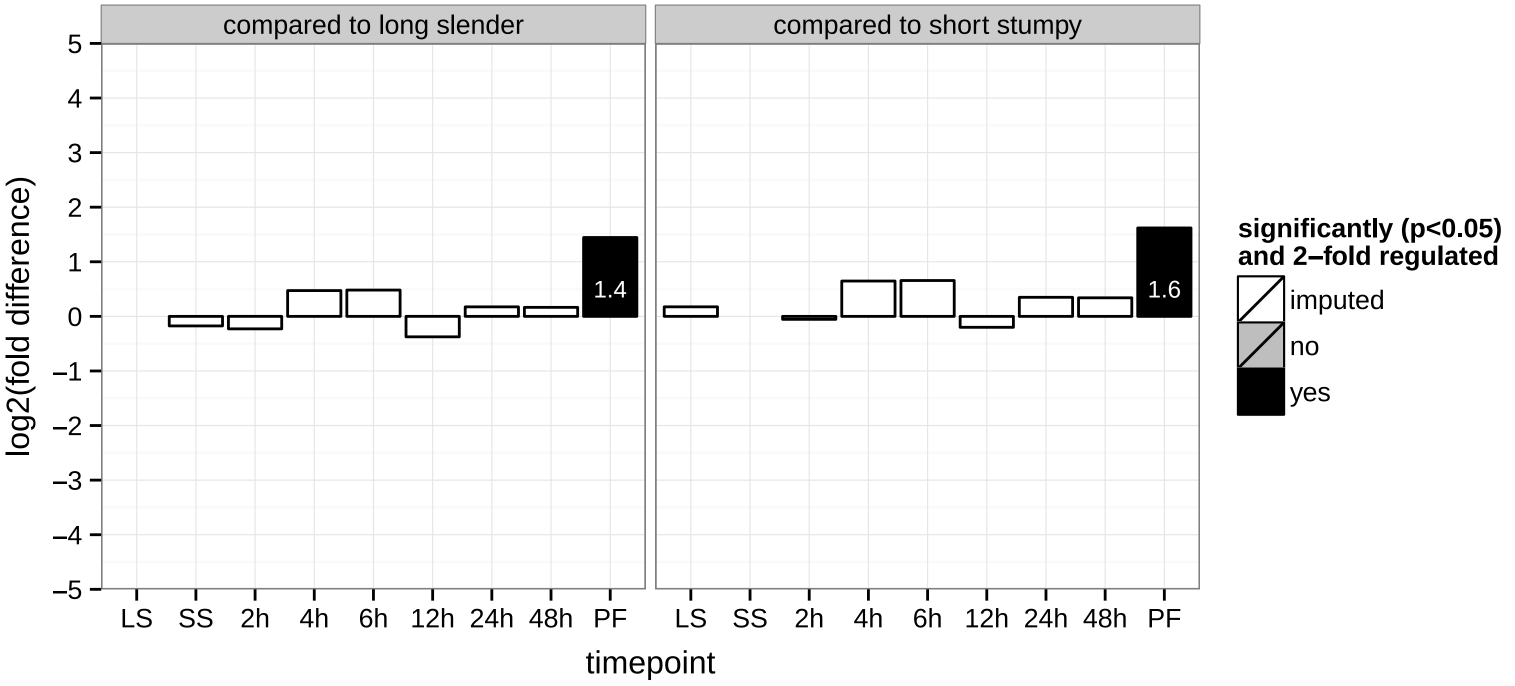

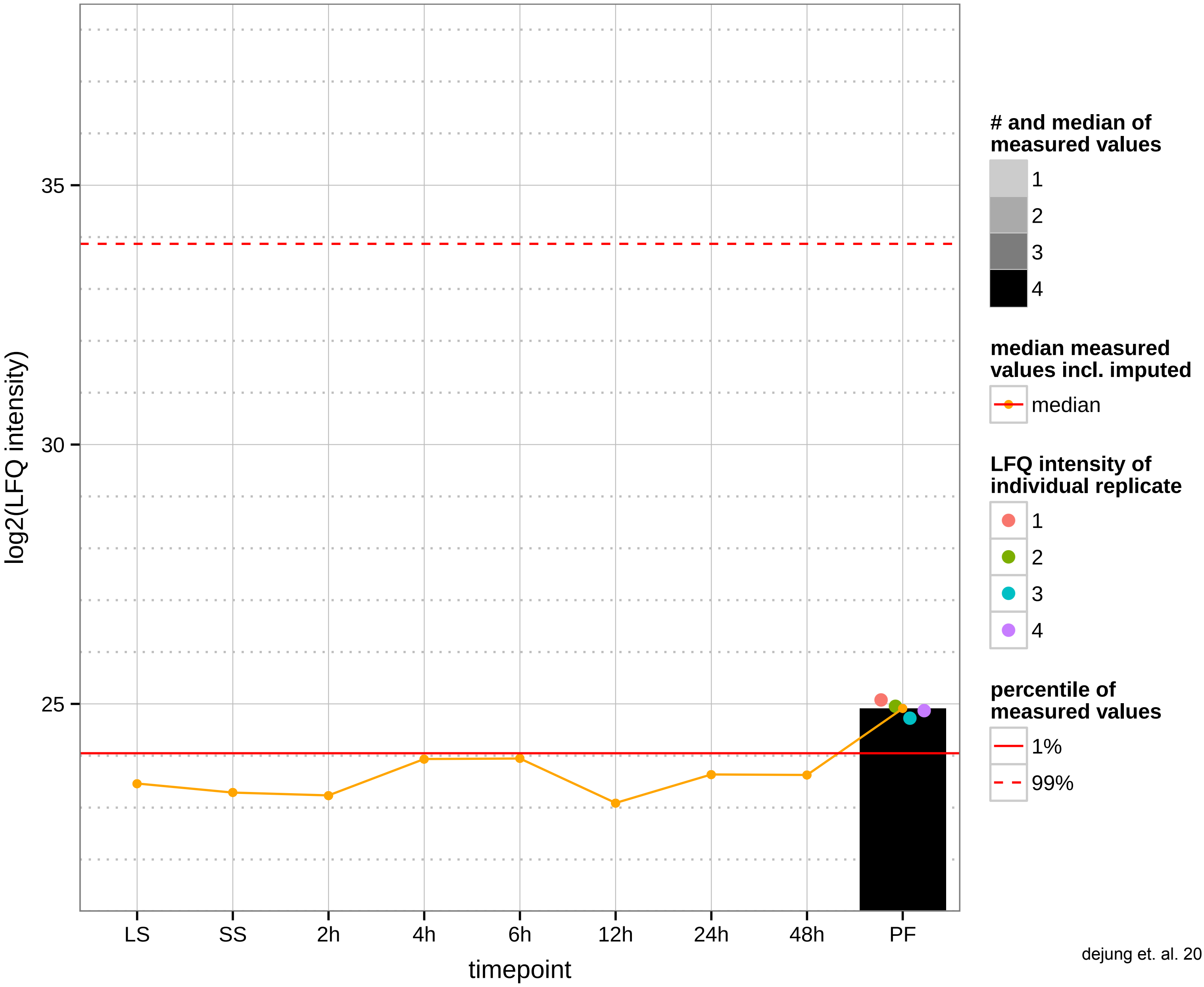

dejung et. al. 2015

hypothetical protein, conserved
Tb927.4.1690
AGOF: null
AGOC: null
AGOP: null
PGOF: null
PGOC: null
PGOP: null

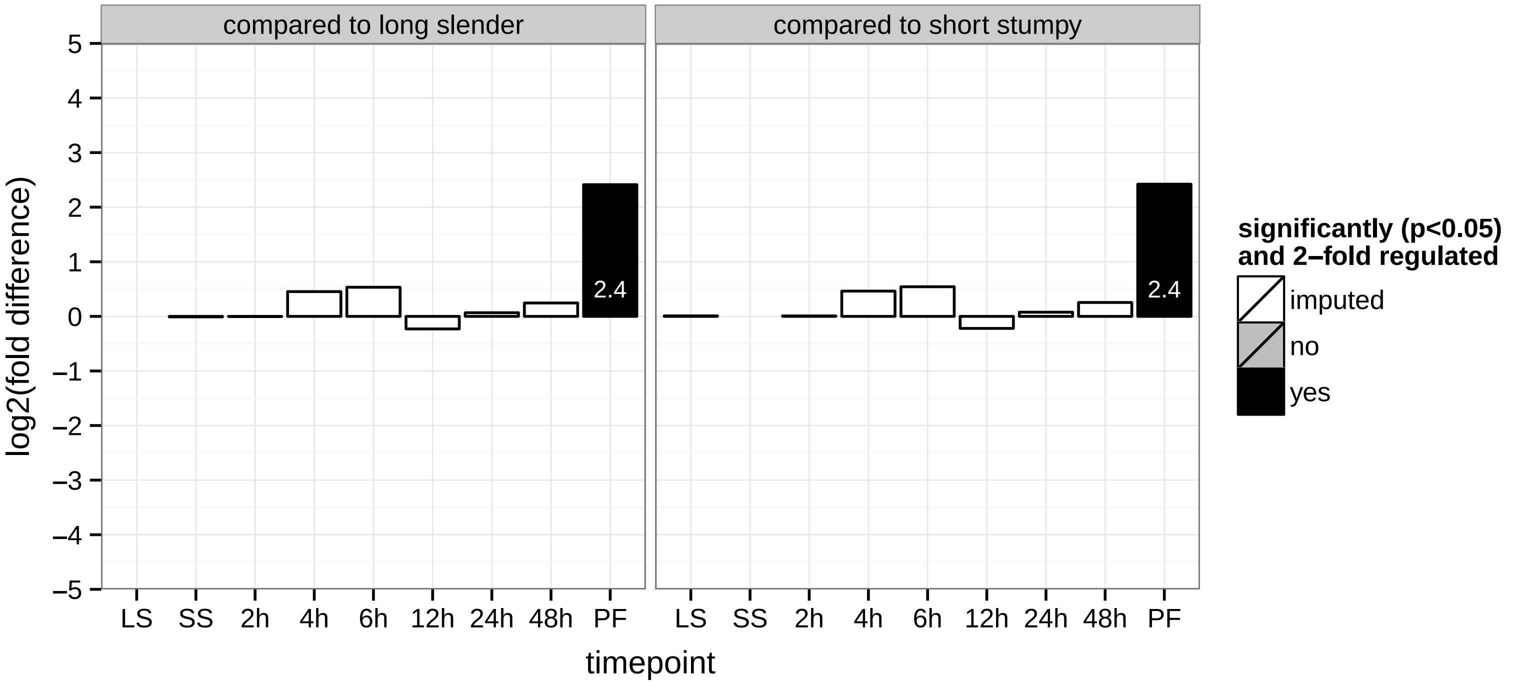

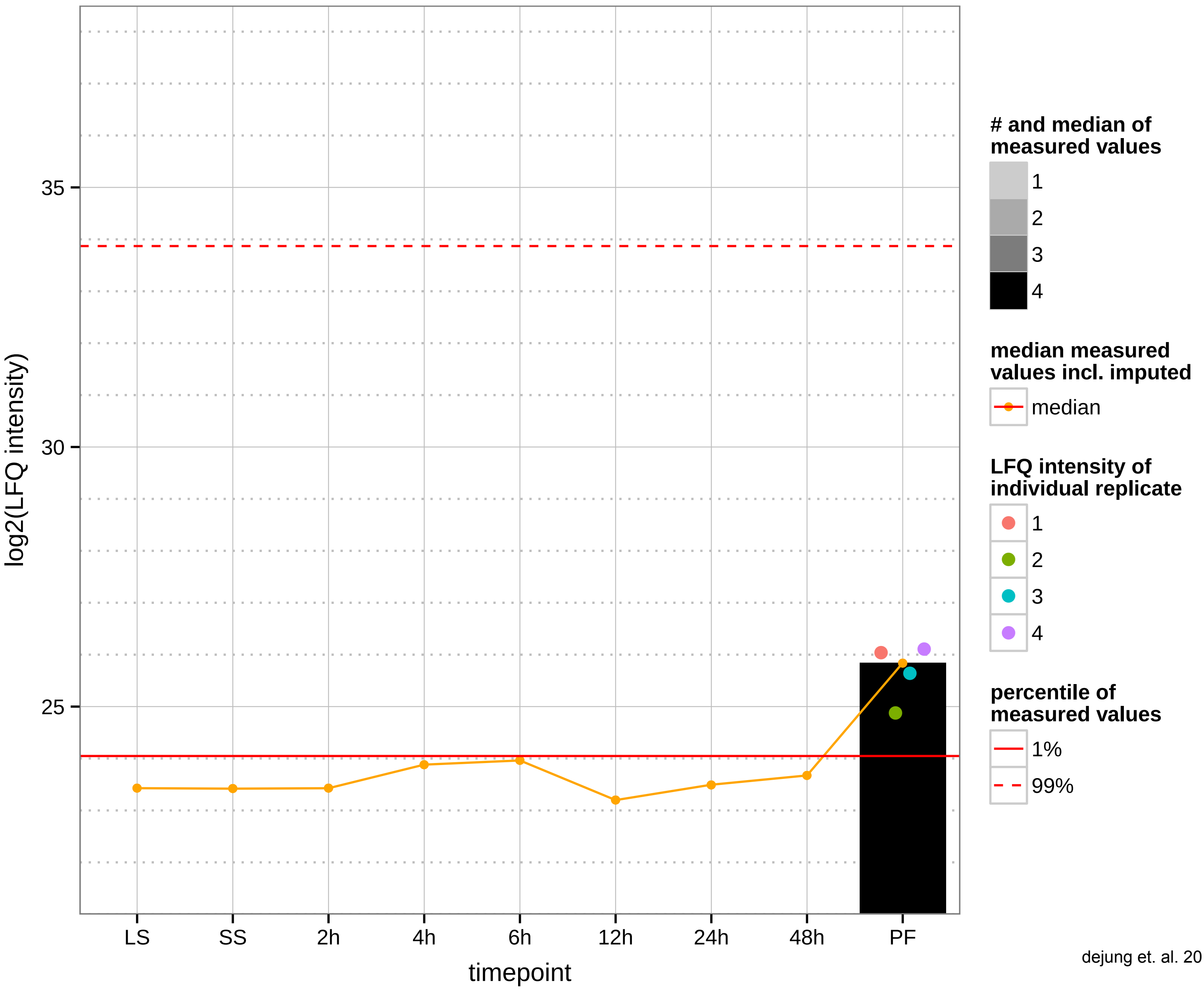

dejung et. al. 2015

hypothetical protein, conserved
Tb927.4.1710
AGOF: null
AGOC: null
AGOP: null
PGOF: null
PGOC: null
PGOP: null

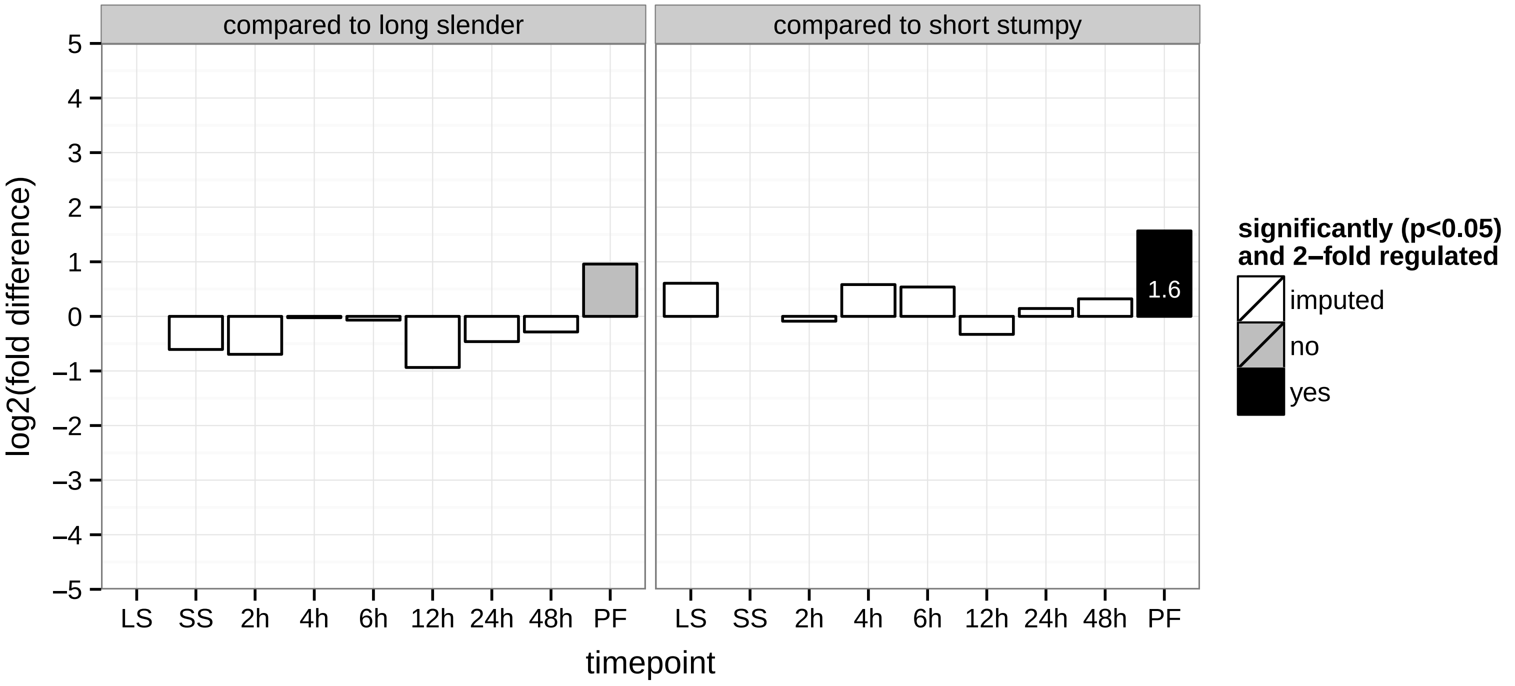

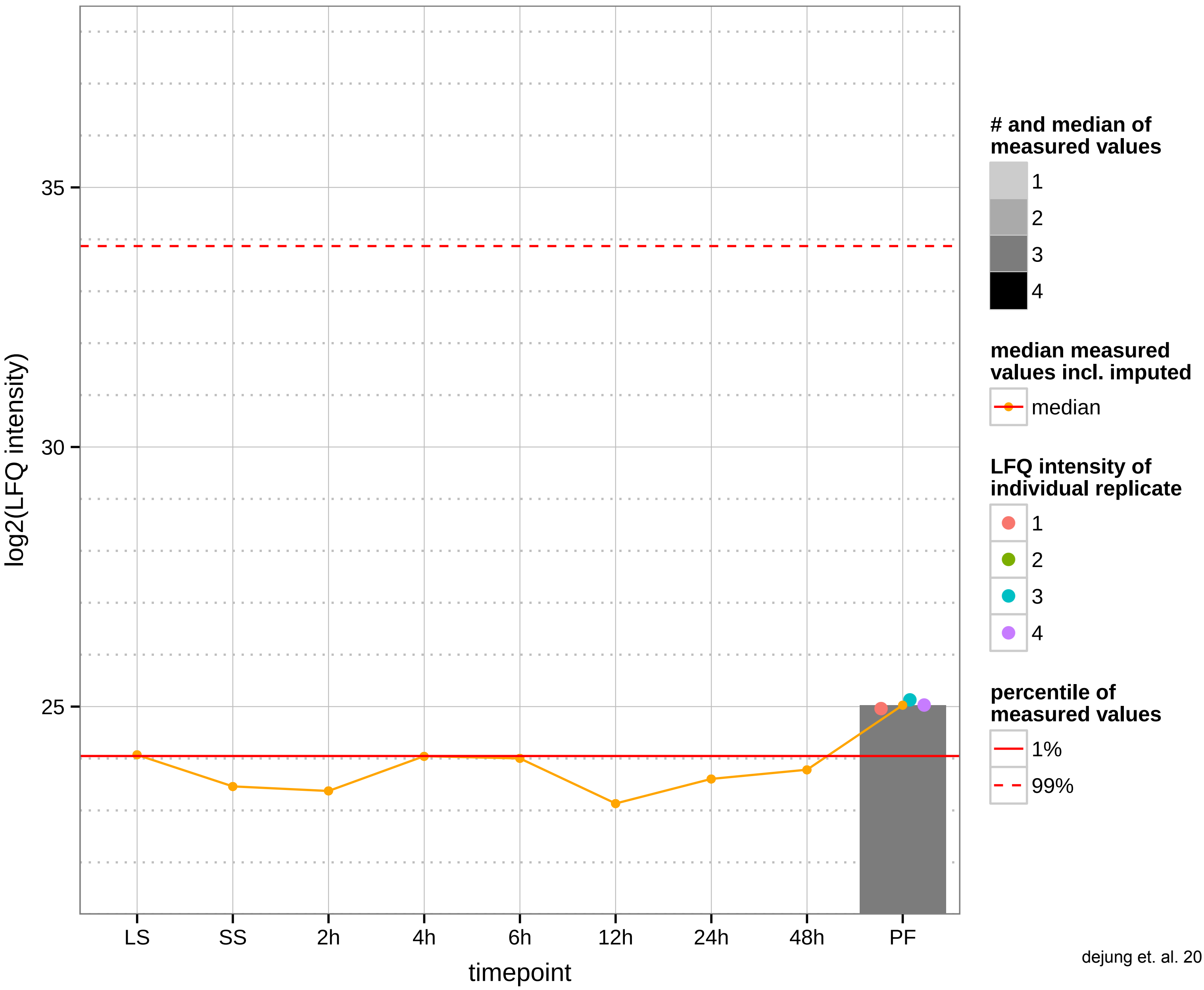

dejung et. al. 2015

retrotransposon hot spot protein 1 (RHS1), putative
Tb927.4.200
AGOF: null
AGOC: null
AGOP: null
PGOF: null
PGOC: null
PGOP: null

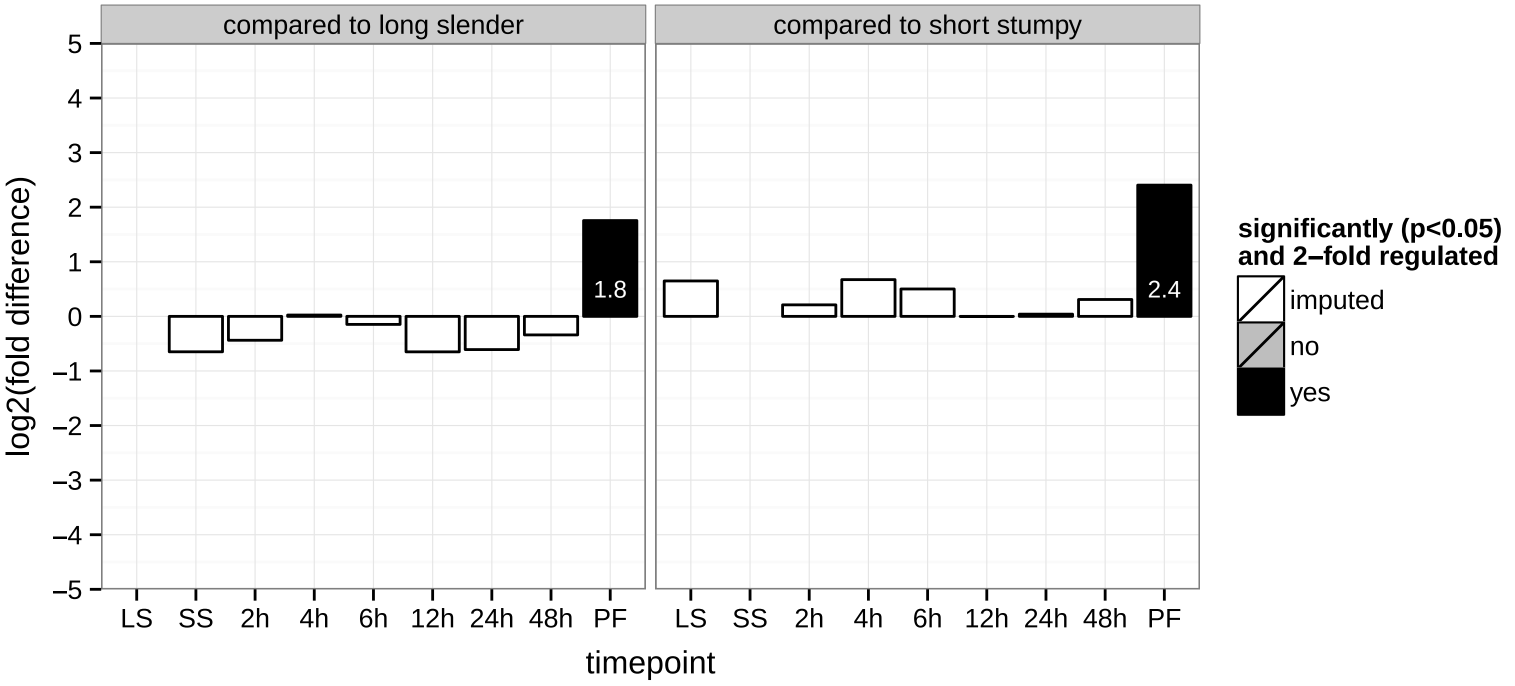

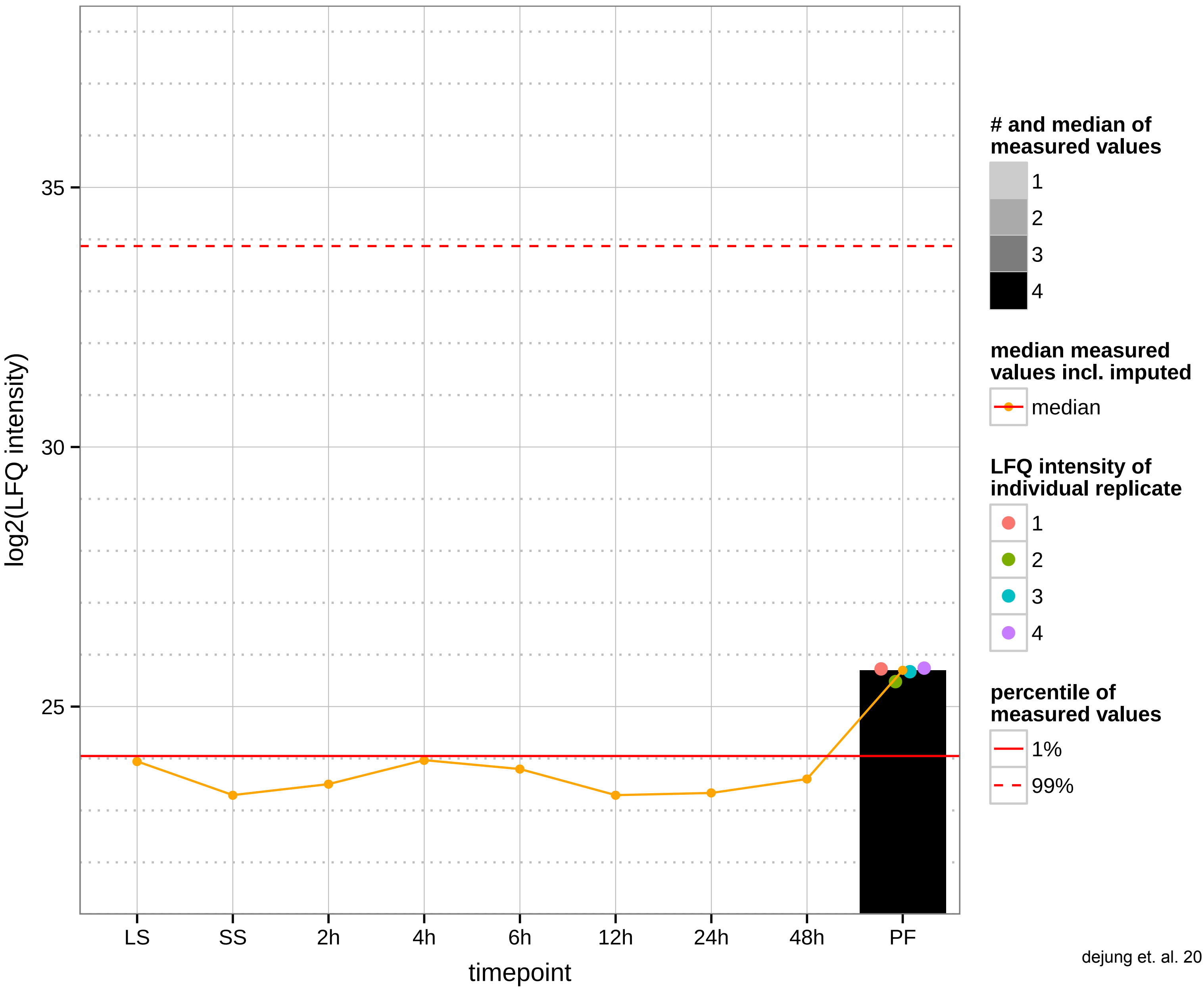

dejung et. al. 2015

hypothetical protein, conserved
Tb927.4.2410
AGOF: catalytic activity
AGOC: null
AGOP: carbohydrate metabolic process
PGOF: catalytic activity
PGOC: null
PGOP: carbohydrate metabolic process

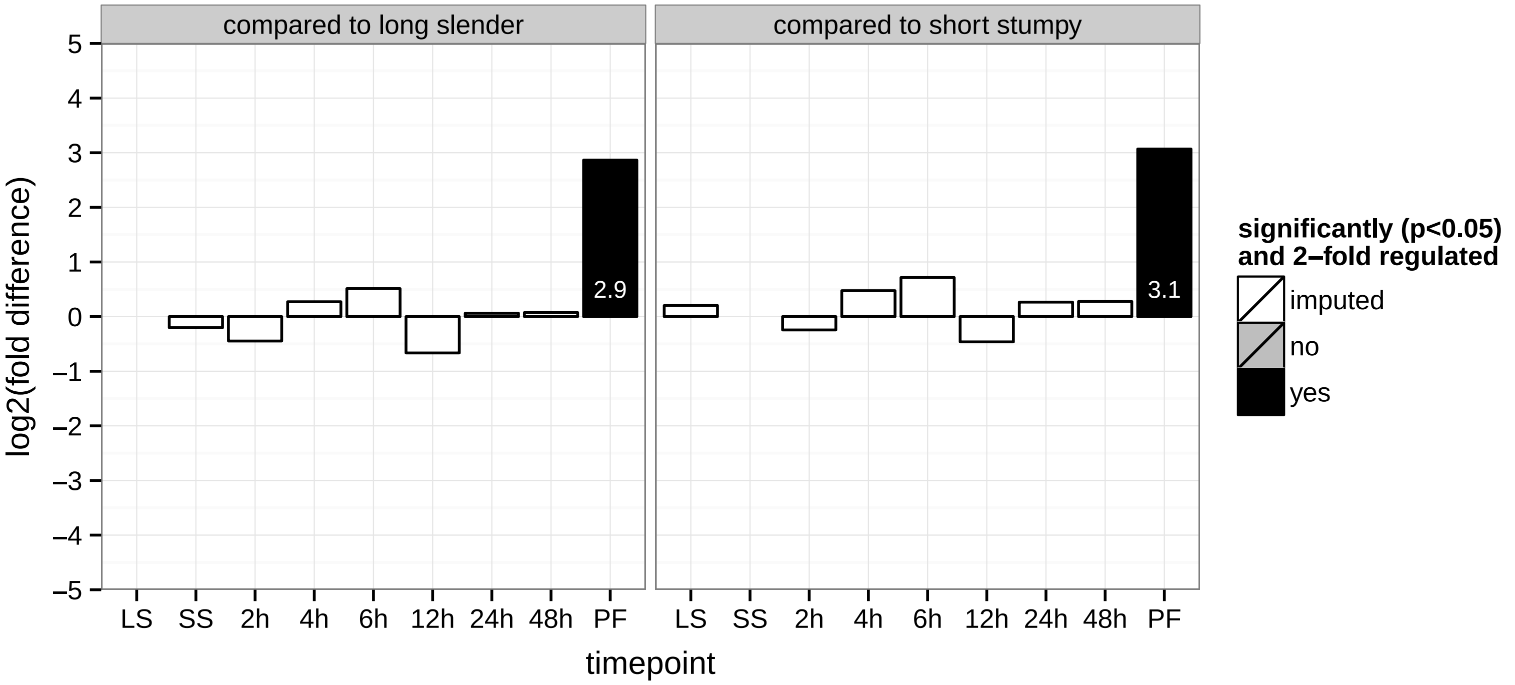

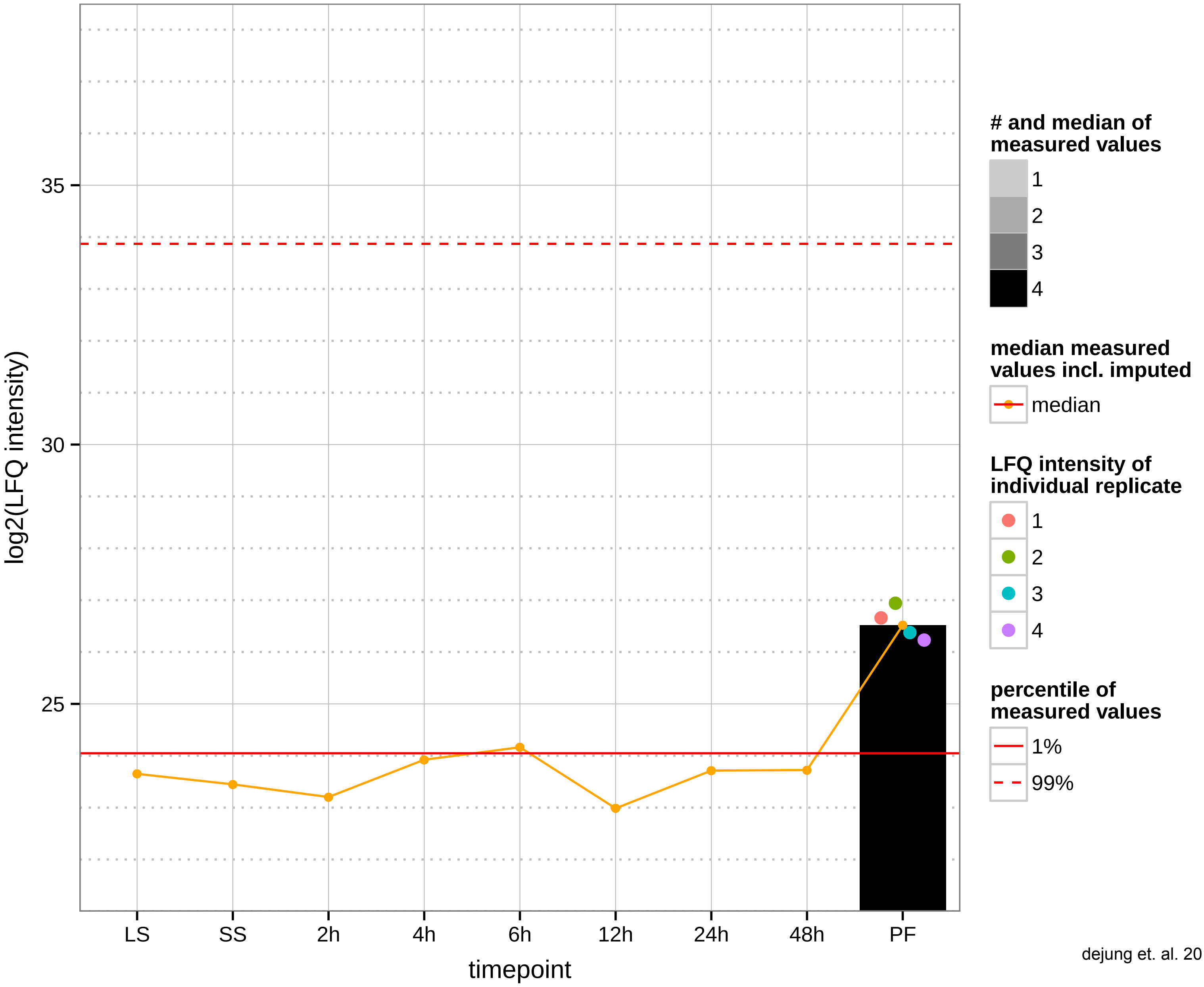

dejung et. al. 2015

pyroglutamyl–peptidase I (PGP), putative, cysteine peptidase, Clan CF, family C15
Tb927.4.2670
AGOF: null
AGOC: null
AGOP: null
PGOF: null
PGOC: null
PGOP: proteolysis

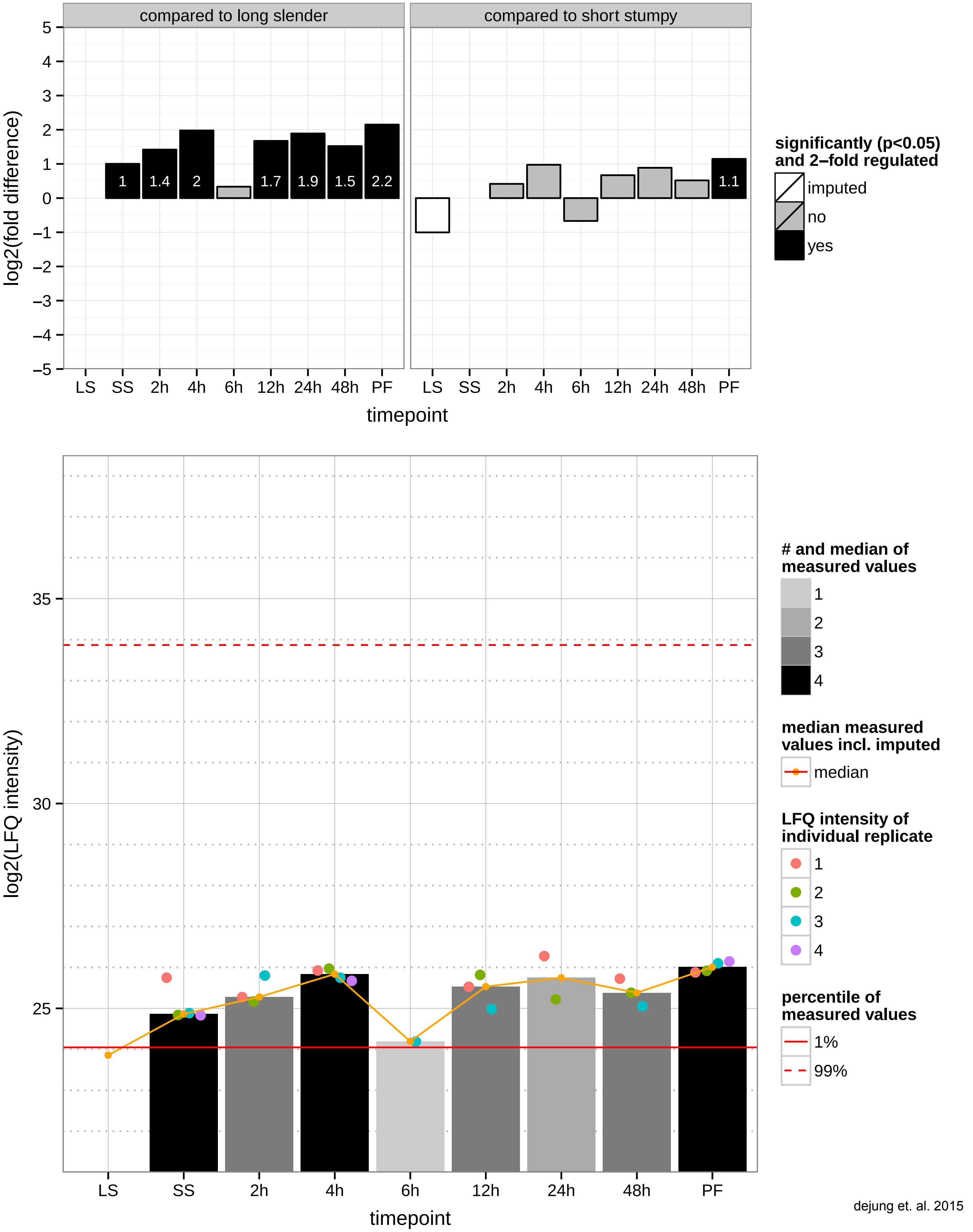

dejung et. al. 2015

hypothetical protein, conserved
Tb927.4.2790
AGOF: null
AGOC: mitochondrion
AGOP: null
PGOF: null
PGOC: null
PGOP: null

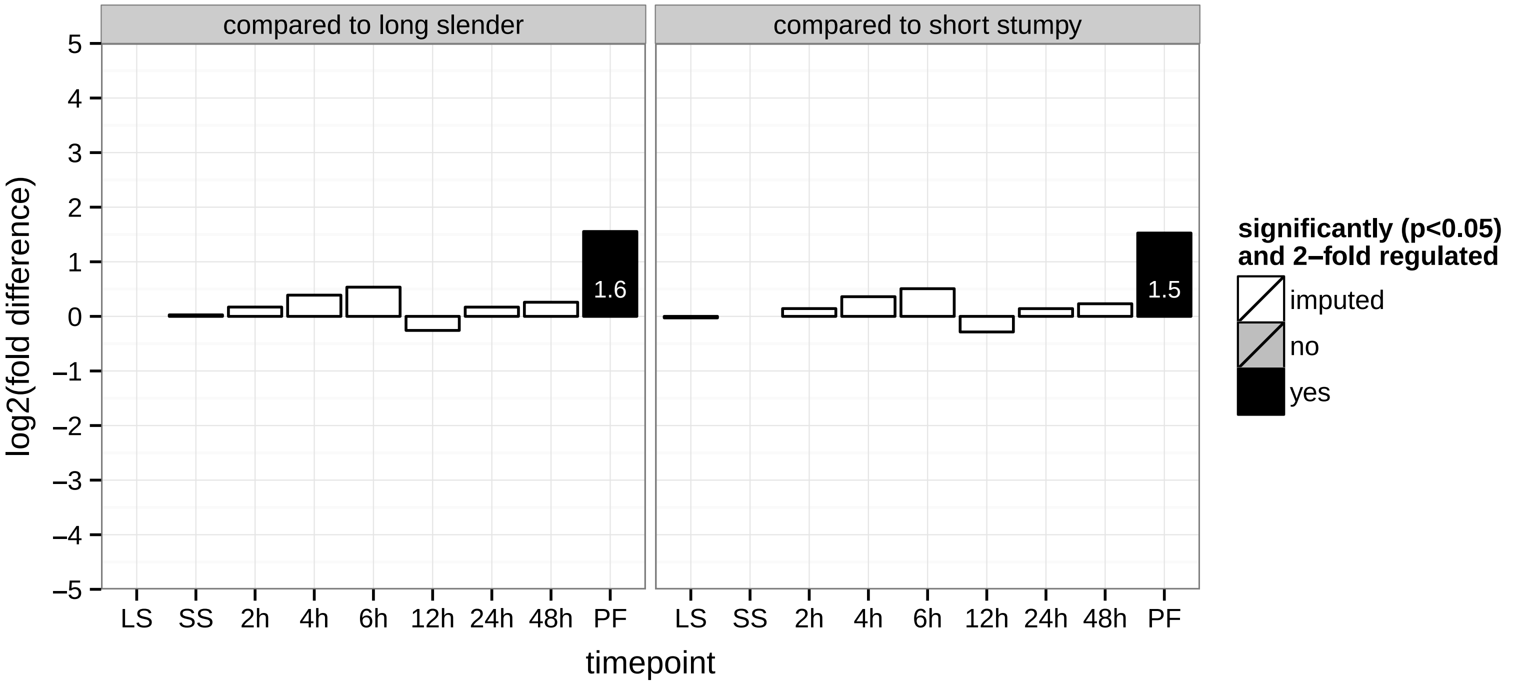

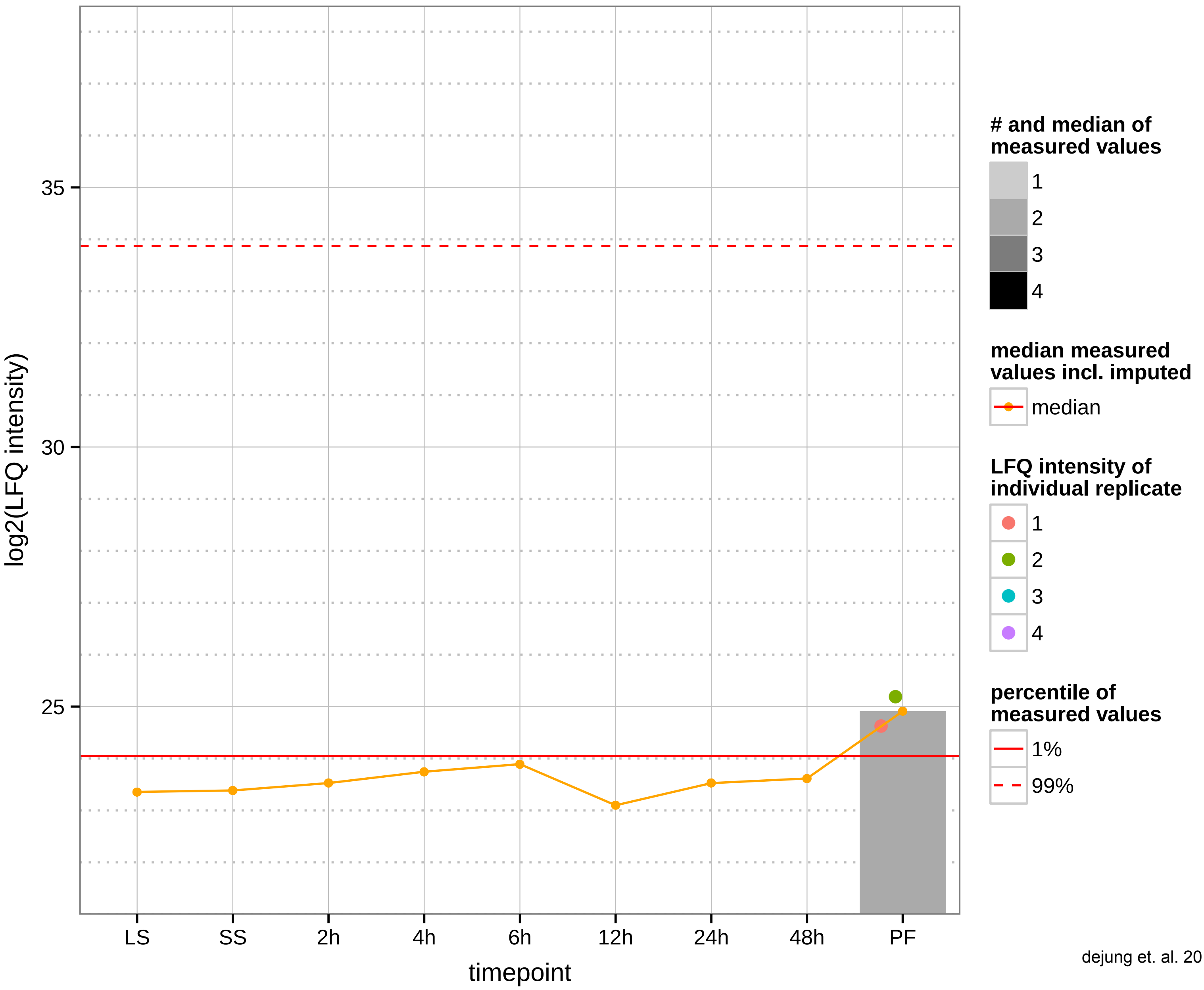

dejung et. al. 2015

Nucleoporin (TbNup225)
Tb927.4.2880
AGOF: null
AGOC: nuclear pore
AGOP: null
PGOF: null
PGOC: null
PGOP: null

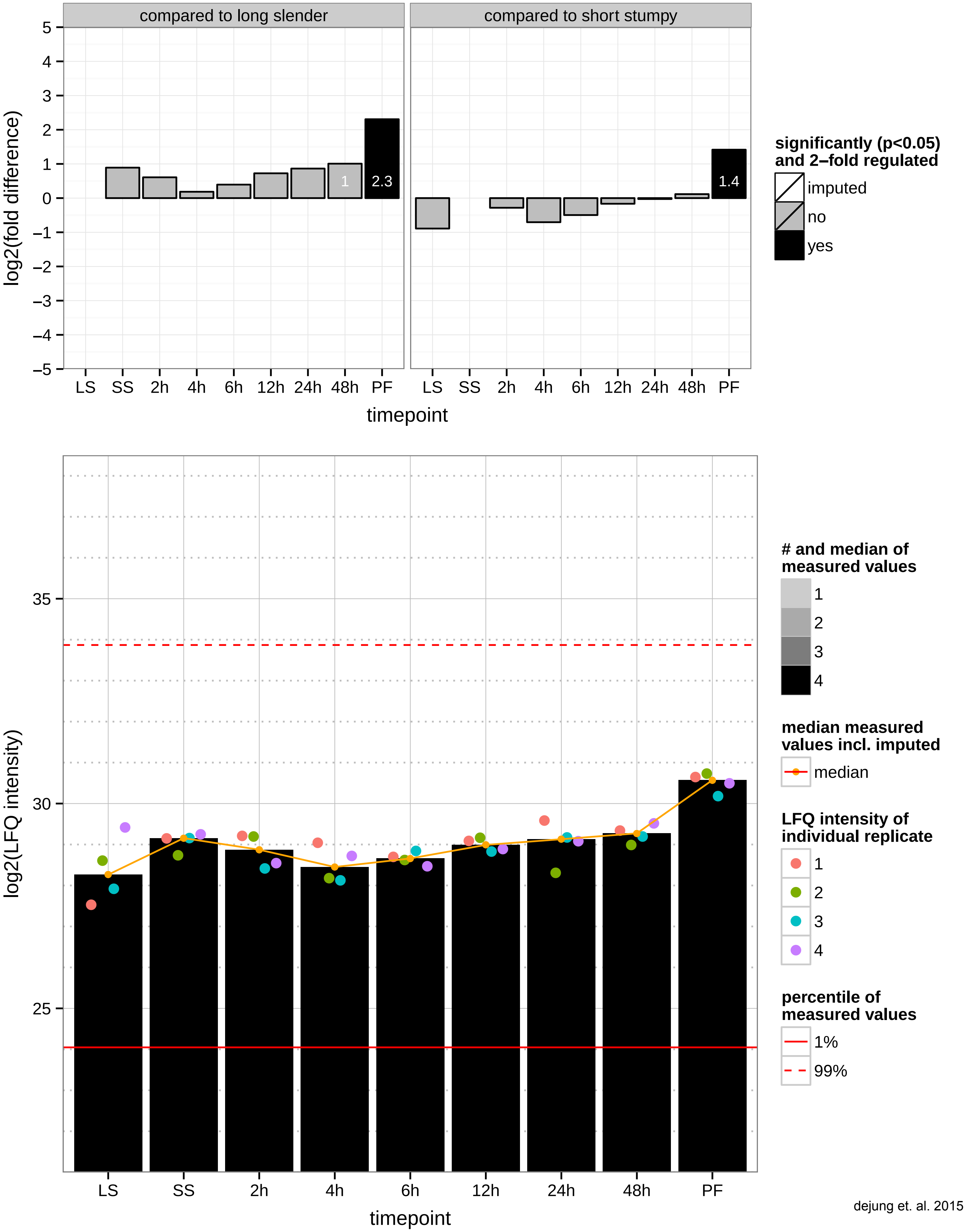

dejung et. al. 2015

hypothetical protein, conserved
Tb927.4.3330
AGOF: calcium ion binding
AGOC: null
AGOP: null
PGOF: calcium ion binding
PGOC: null
PGOP: null

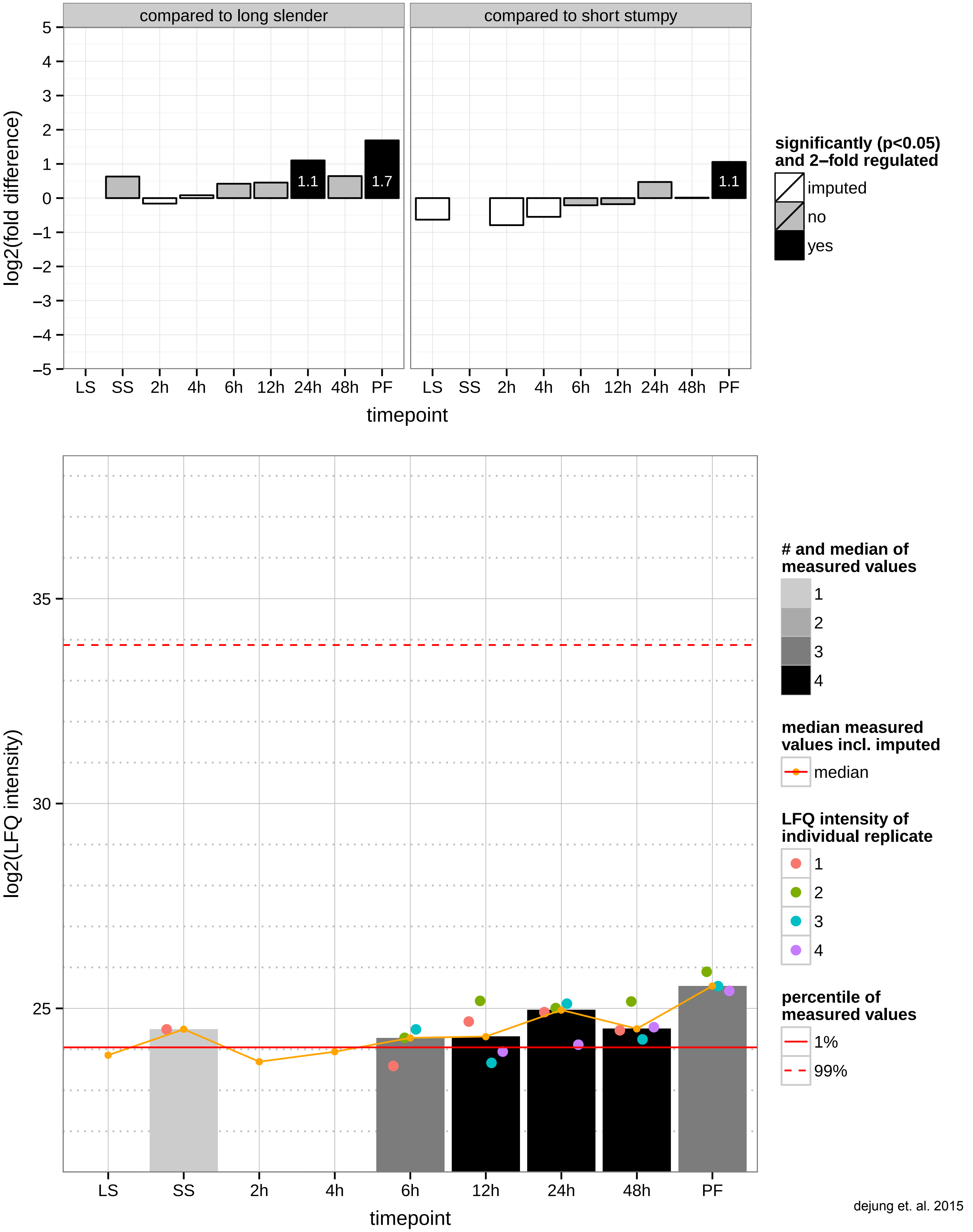

dejung et. al. 2015

translation elongation factor 1–beta, putative
Tb927.4.3590;Tb927.4.3570
AGOF: translation elongation factor activity
AGOC: eukaryotic translation elongation factor 1 complex
AGOP: translational elongation
PGOF: translation elongation factor activity
PGOC: eukaryotic translation elongation factor 1 complex
PGOP: translational elongation

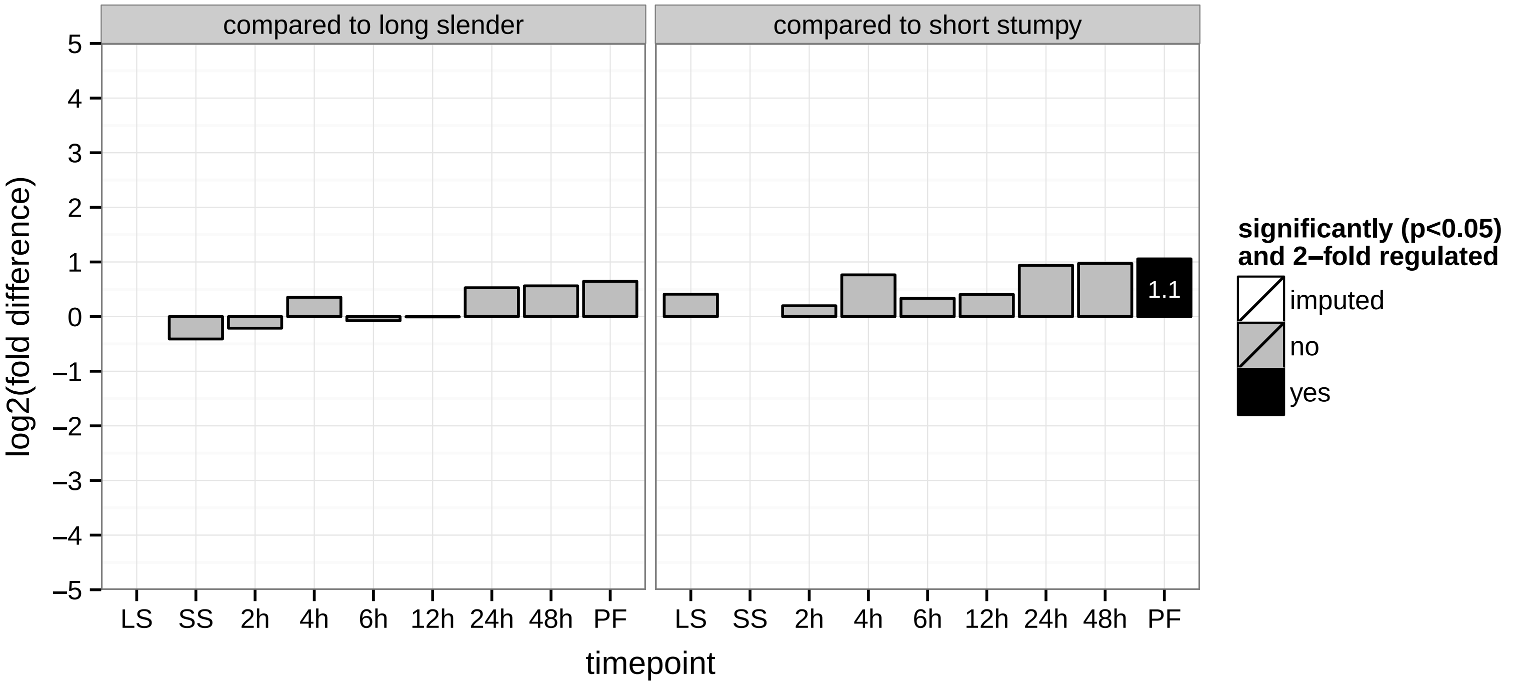

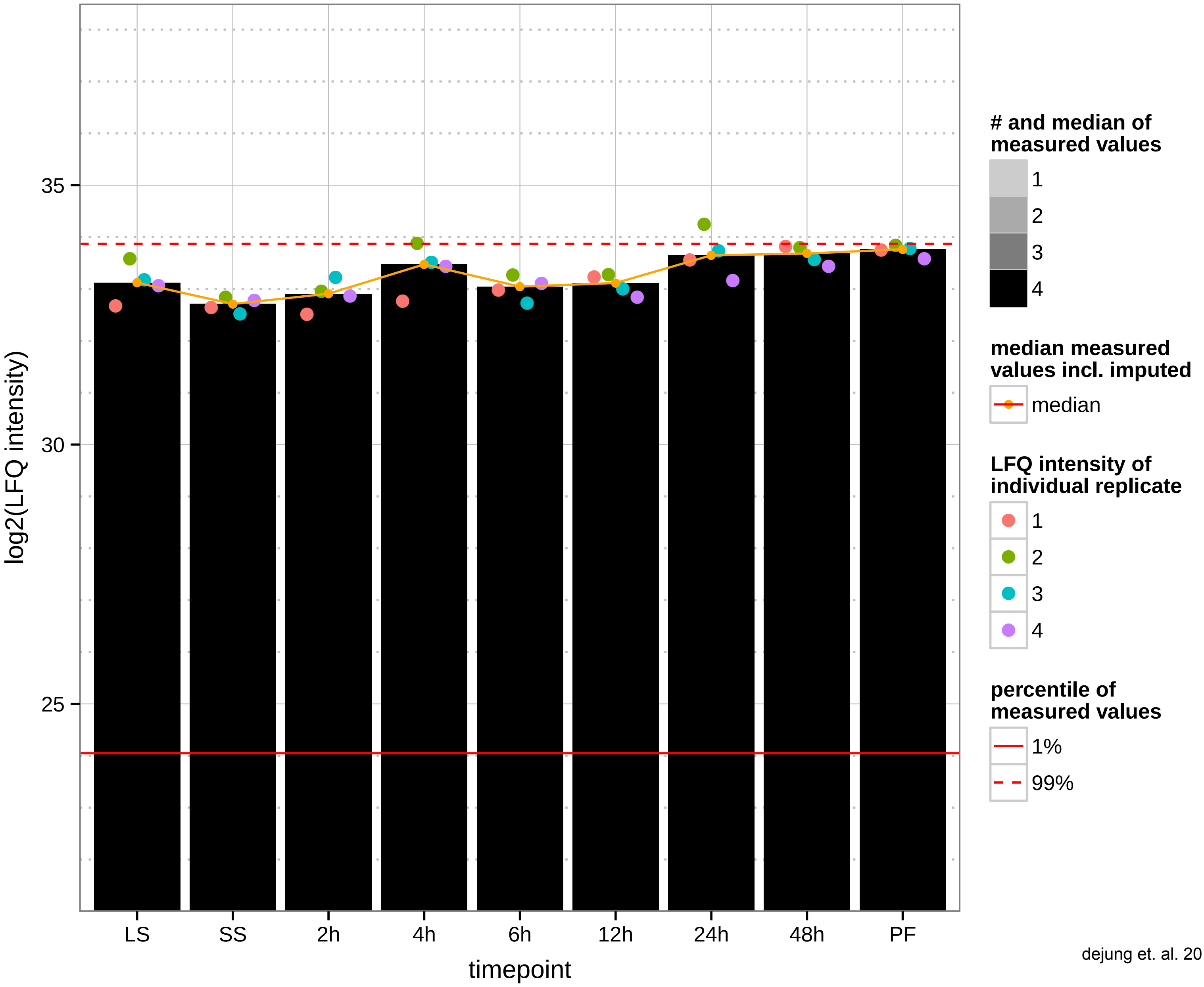

dejung et. al. 2015

hypothetical protein, conserved
Tb927.4.3690
AGOF: metal ion binding, superoxide dismutase activity
AGOC: mitochondrion
AGOP: oxidation–reduction process, superoxide metabolic process
PGOF: metal ion binding, superoxide dismutase activity
PGOC: null
PGOP: oxidation–reduction process, superoxide metabolic process

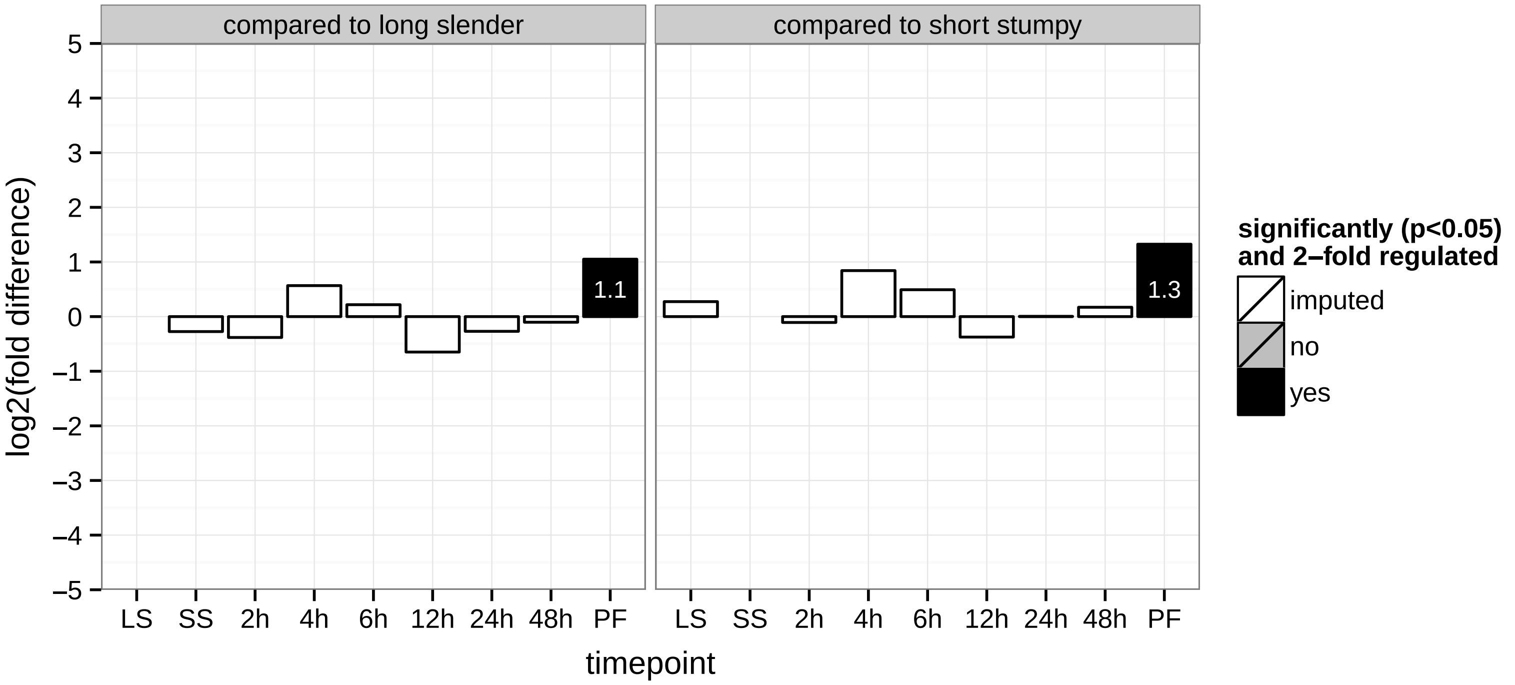

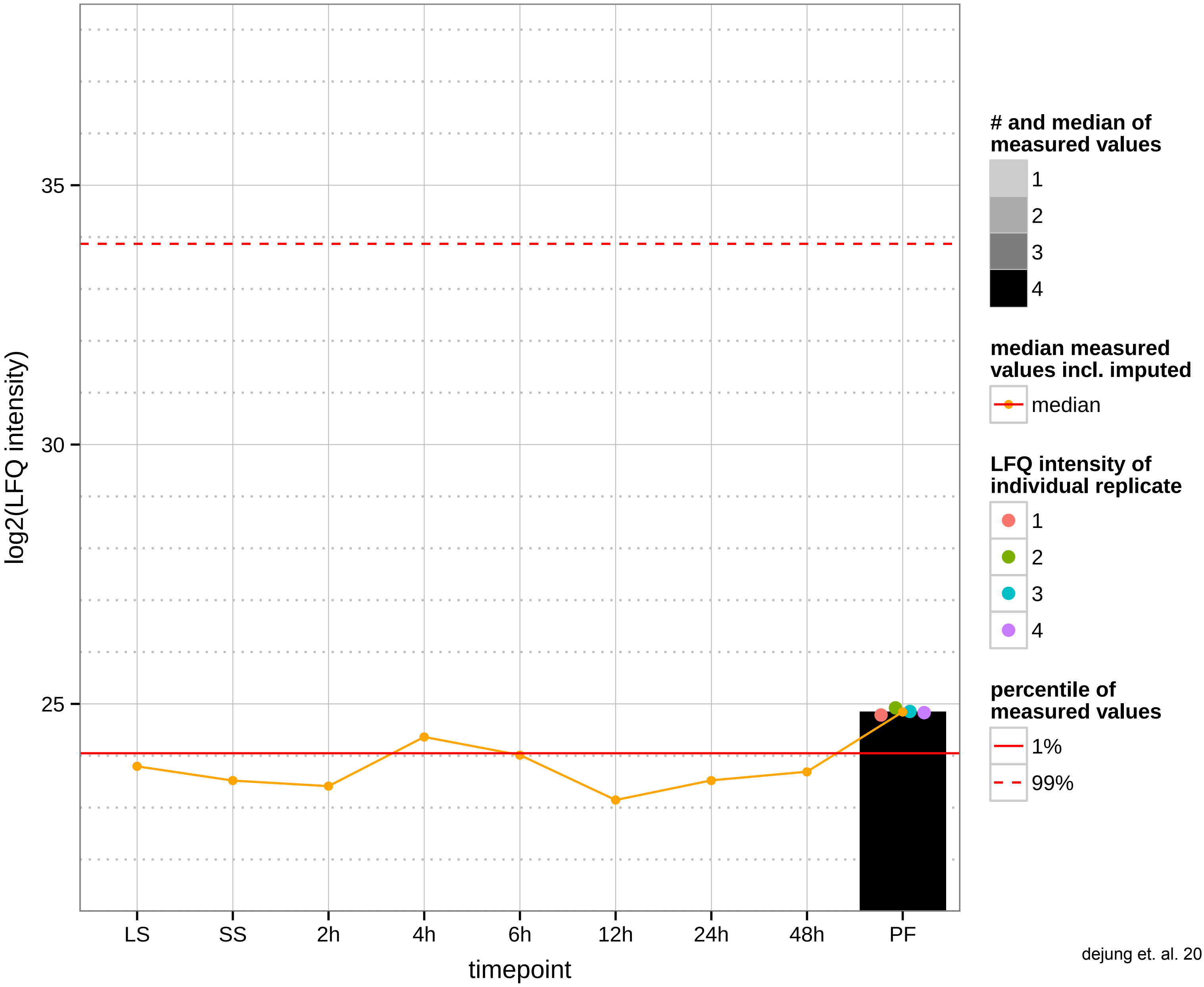

dejung et. al. 2015

protein kinase, putative
Tb927.4.3770
AGOF: ATP binding, protein kinase activity, protein serine/threonine kinase activity, protein tyrosine kinase activity, zinc ion bin
AGOC: null
AGOP: protein phosphorylation
PGOF: ATP binding, protein kinase activity, transferase activity, transferring phosphorus–containing groups, zinc ion binding
PGOC: null
PGOP: protein phosphorylation

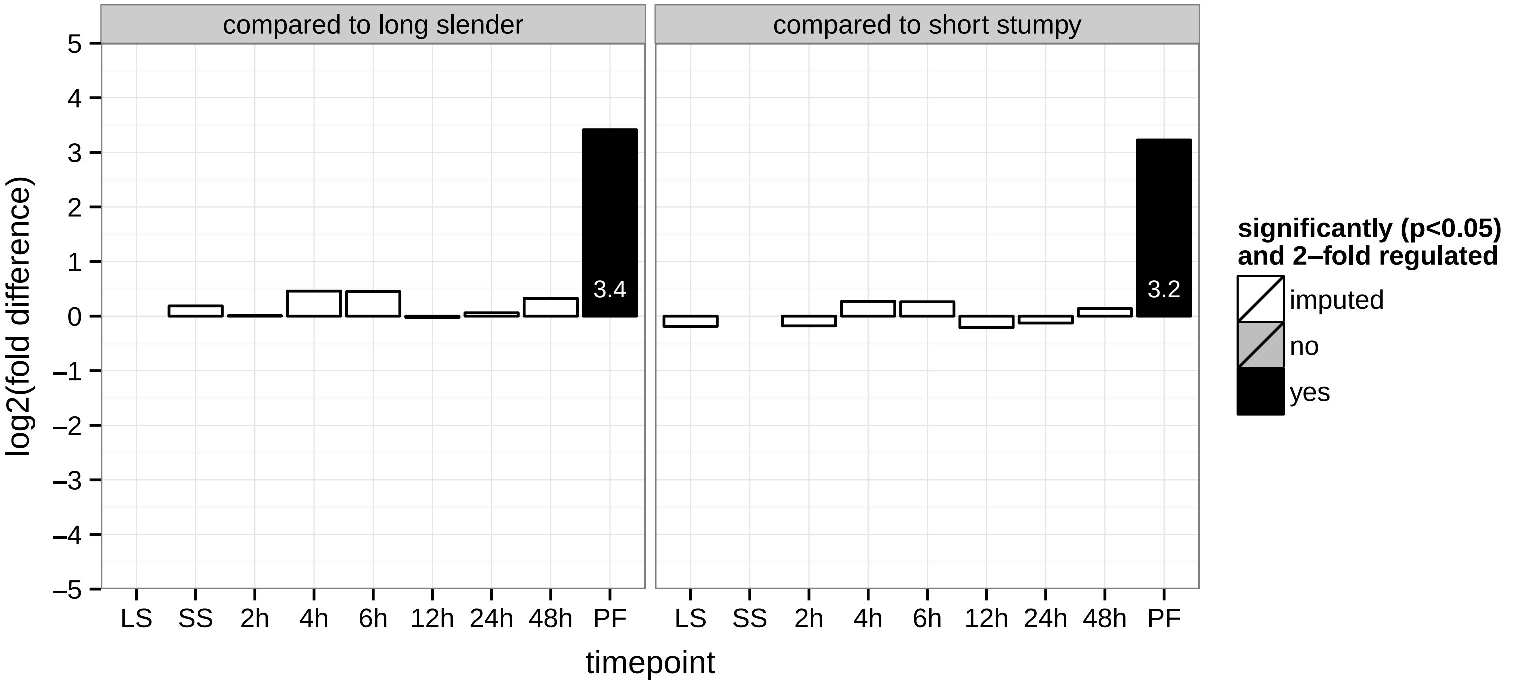

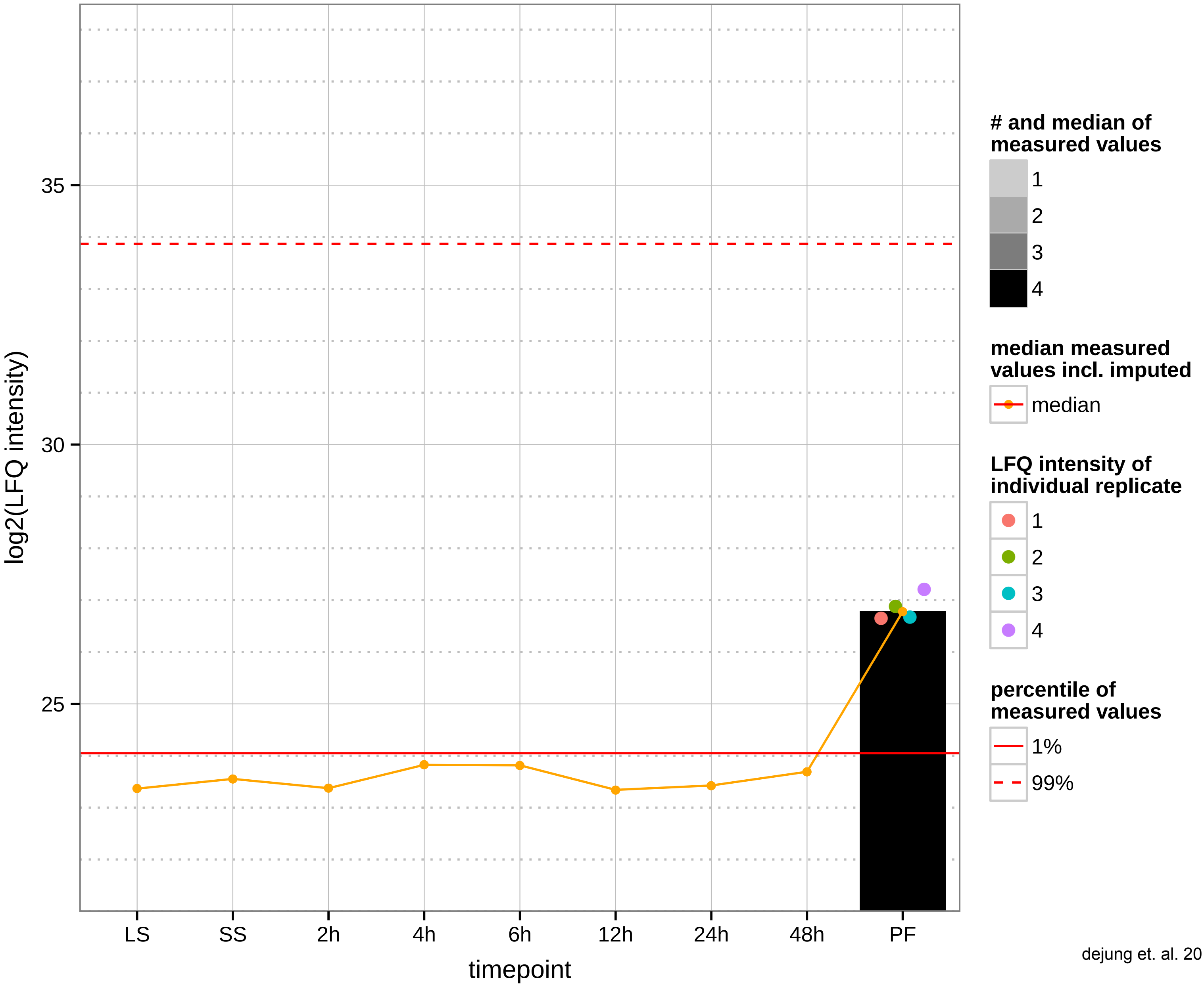

dejung et. al. 2015

calpain–like cysteine peptidase, putative, cysteine peptidase, Clan CA, family C2, putative (TbCALP2)
Tb927.4.3940
AGOF: calcium–dependent cysteine–type endopeptidase activity
AGOC: intracellular
AGOP: proteolysis
PGOF: calcium–dependent cysteine–type endopeptidase activity
PGOC: intracellular
PGOP: proteolysis

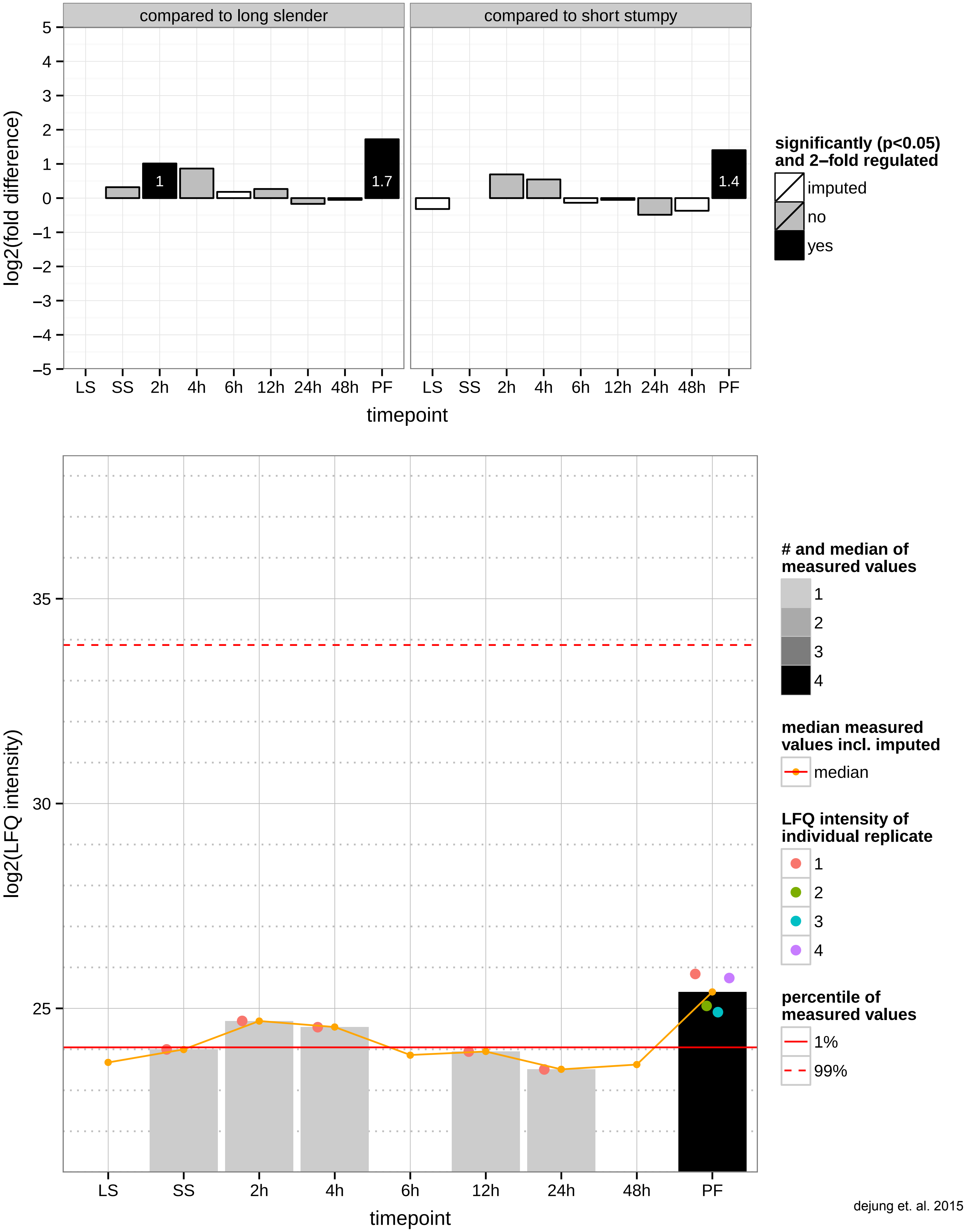

dejung et. al. 2015

proteasome beta 7 subunit
Tb927.4.430
AGOF: endopeptidase activity, threonine−type endopeptidase activity
AGOC: proteasome core complex, proteasome core complex, beta−subunit complex
AGOP: protein metabolic process, proteolysis involved in cellular protein catabolic process
PGOF: threonine−type endopeptidase activity
PGOC: proteasome core complex
PGOP: proteolysis involved in cellular protein catabolic process

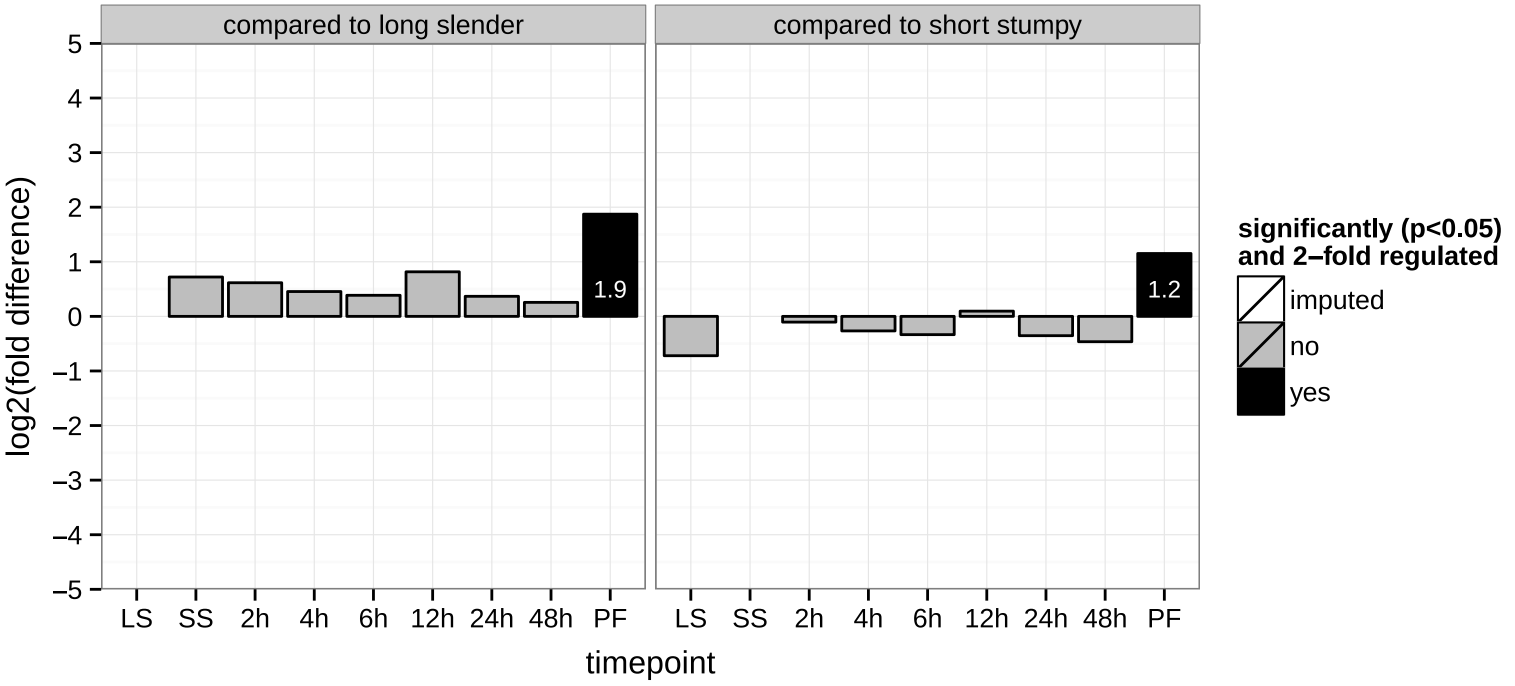

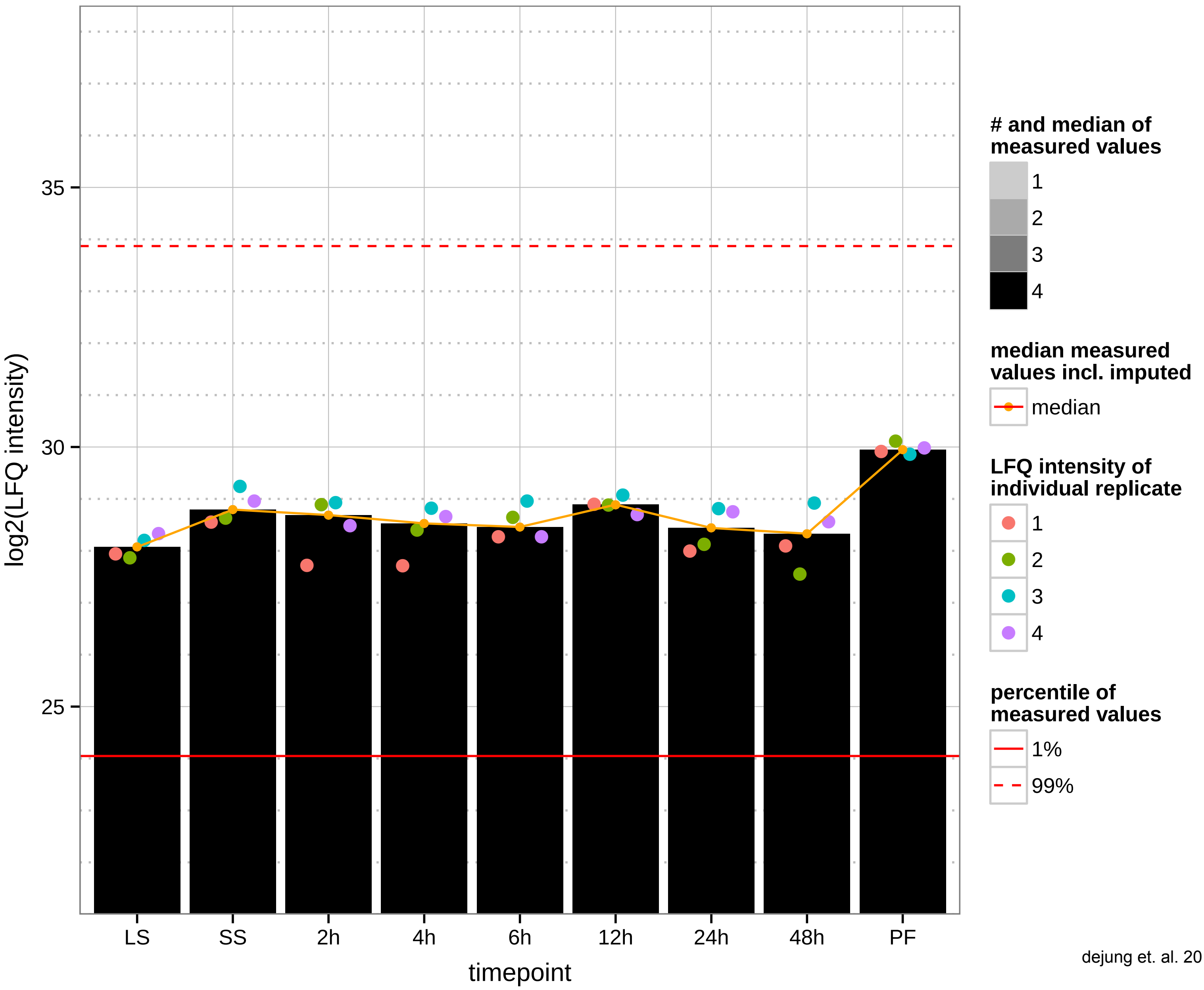

dejung et. al. 2015

hypothetical protein, conserved
Tb927.4.4350
AGOF: null
AGOC: integral to membrane
AGOP: transport
PGOF: null
PGOC: integral to membrane
PGOP: transport

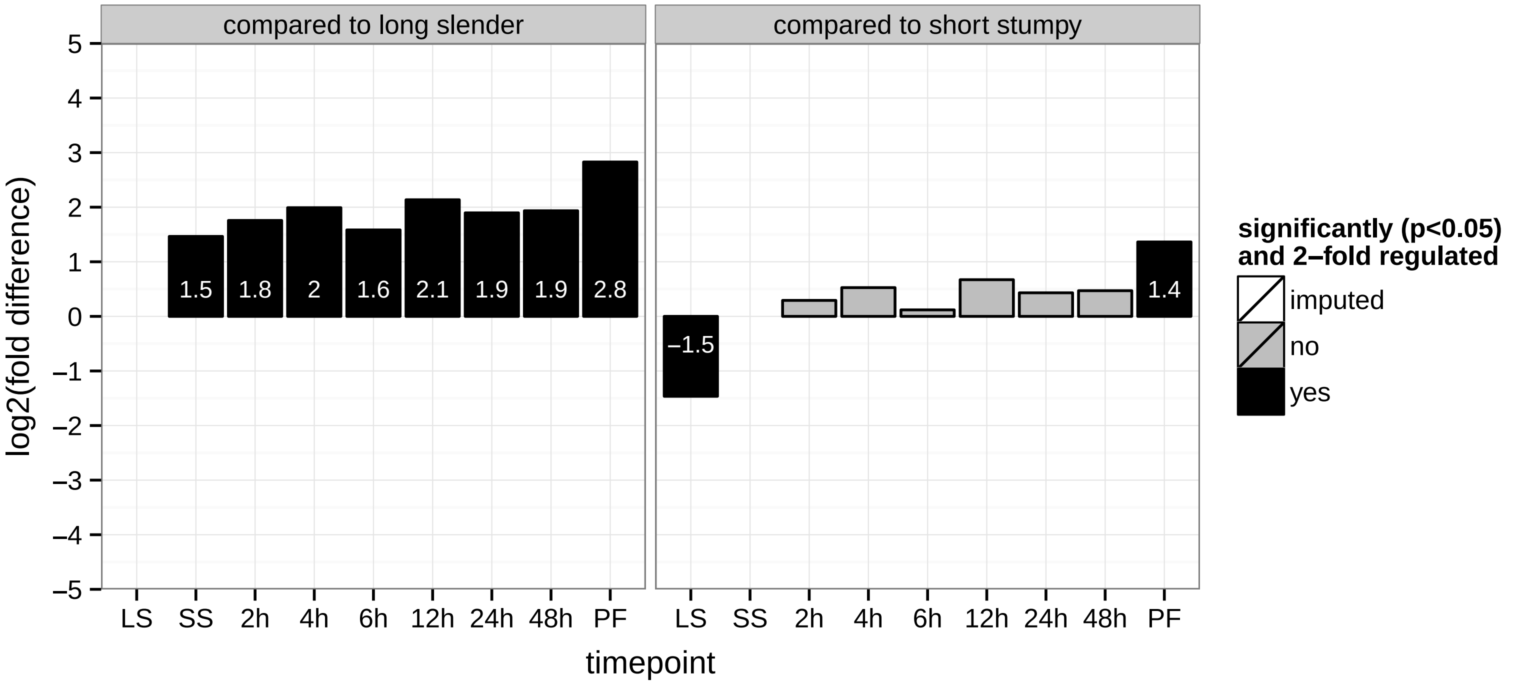

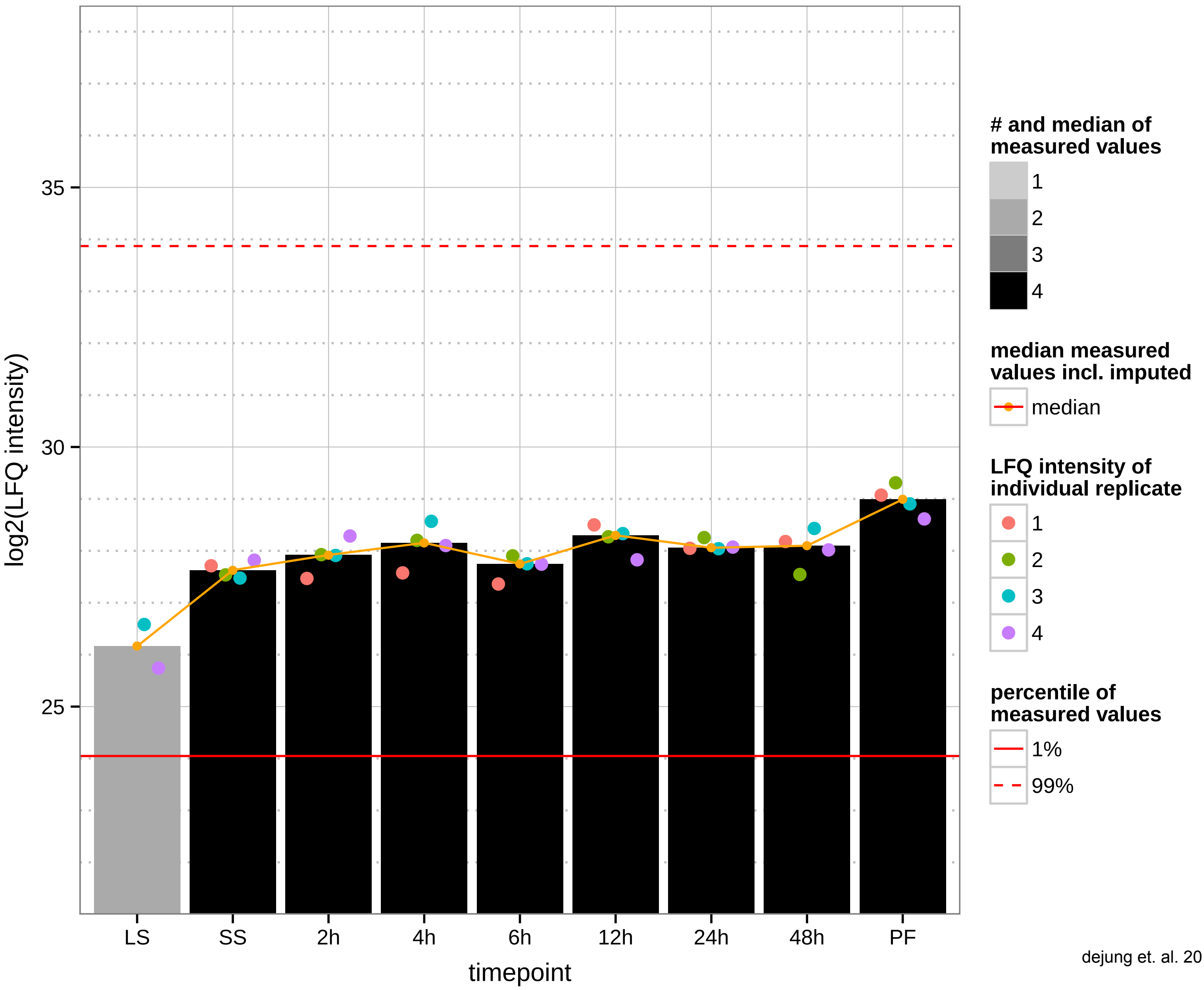

dejung et. al. 2015

hypothetical protein, conserved
Tb927.4.4600
AGOF: null
AGOC: mitochondrion
AGOP: null
PGOF: null
PGOC: null
PGOP: null

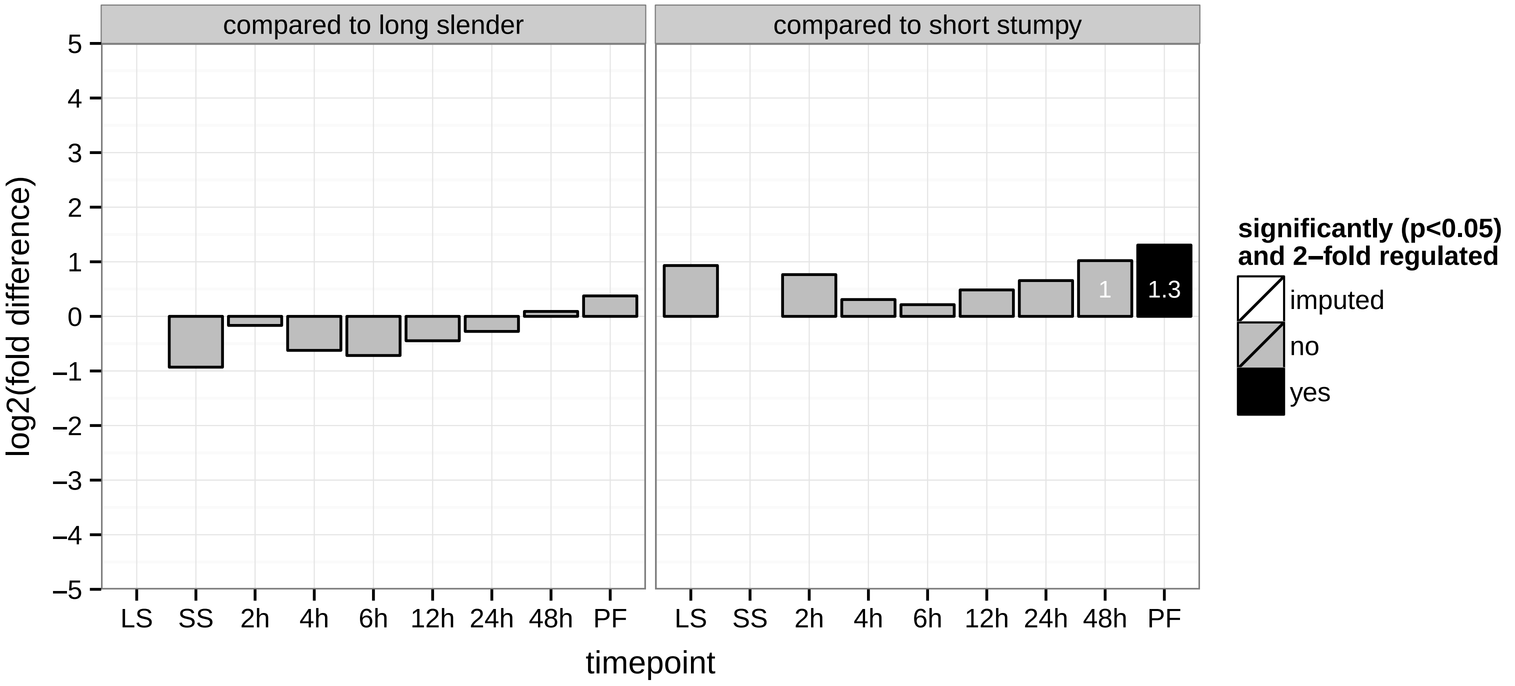

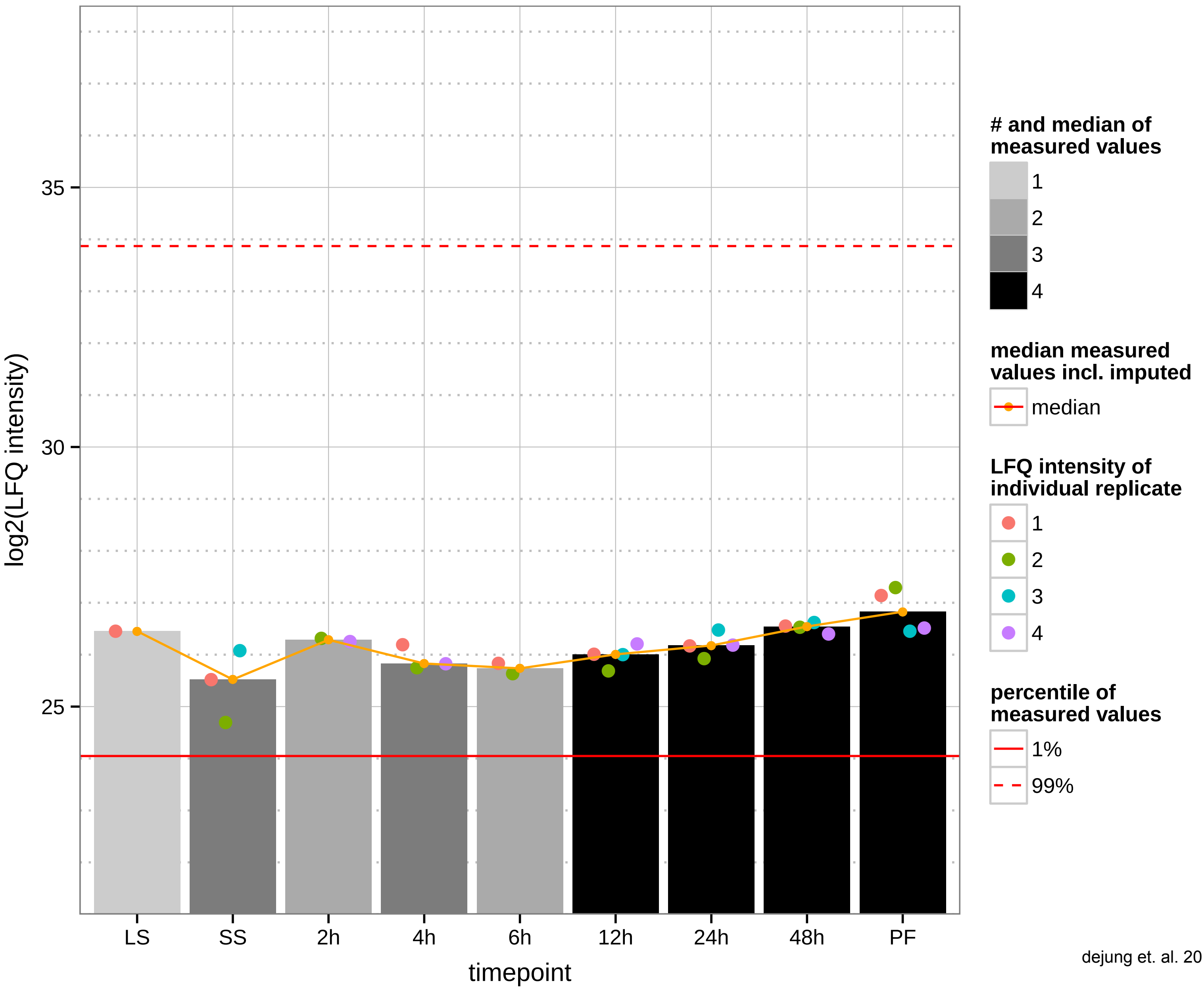

dejung et. al. 2015

hypothetical protein, conserved
Tb927.4.590
AGOF: null
AGOC: null
AGOP: null
PGOF: null
PGOC: null
PGOP: null

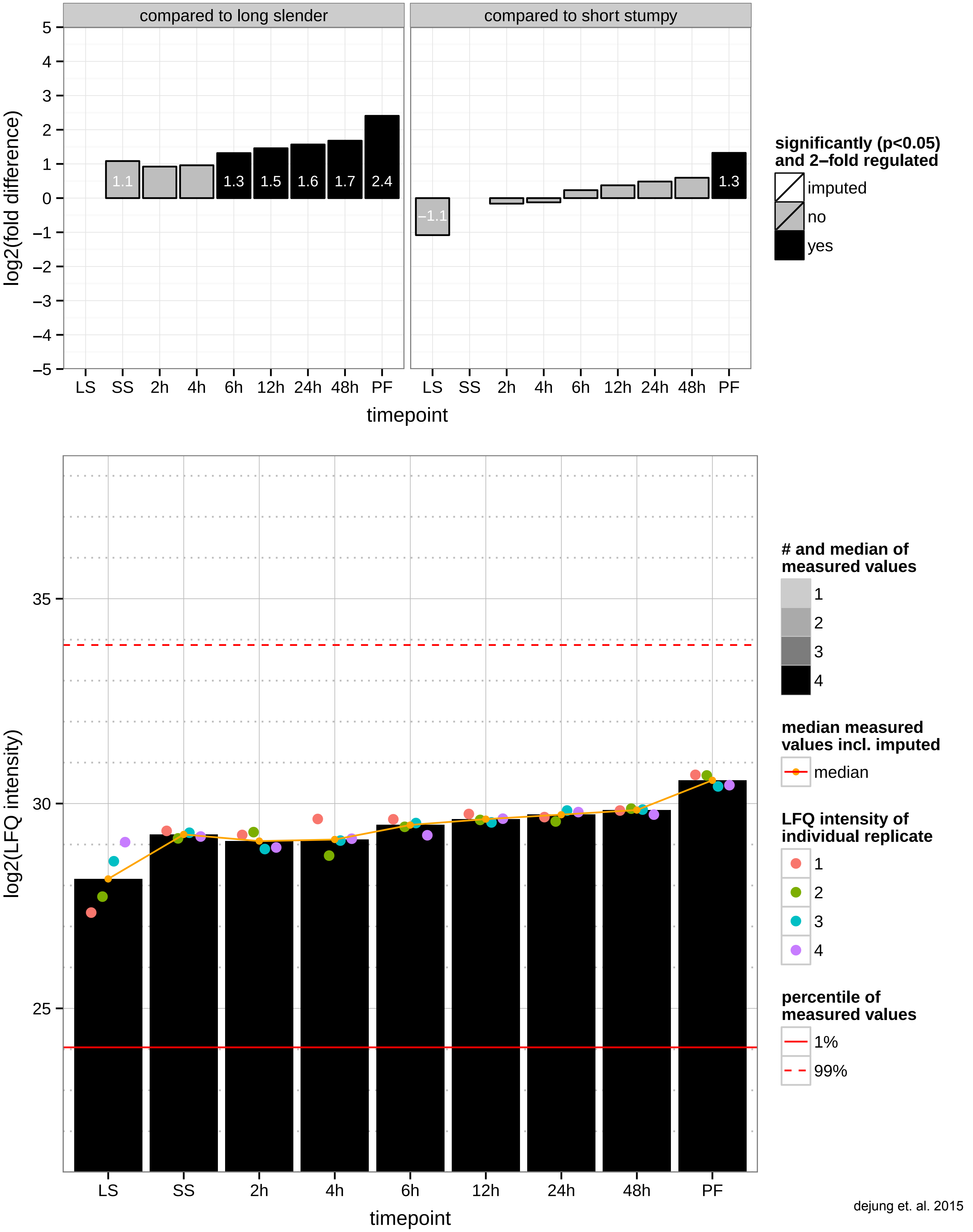

dejung et. al. 2015

hypothetical protein, conserved
Tb927.4.790
AGOF: null
AGOC: null
AGOP: null
PGOF: null
PGOC: null
PGOP: null

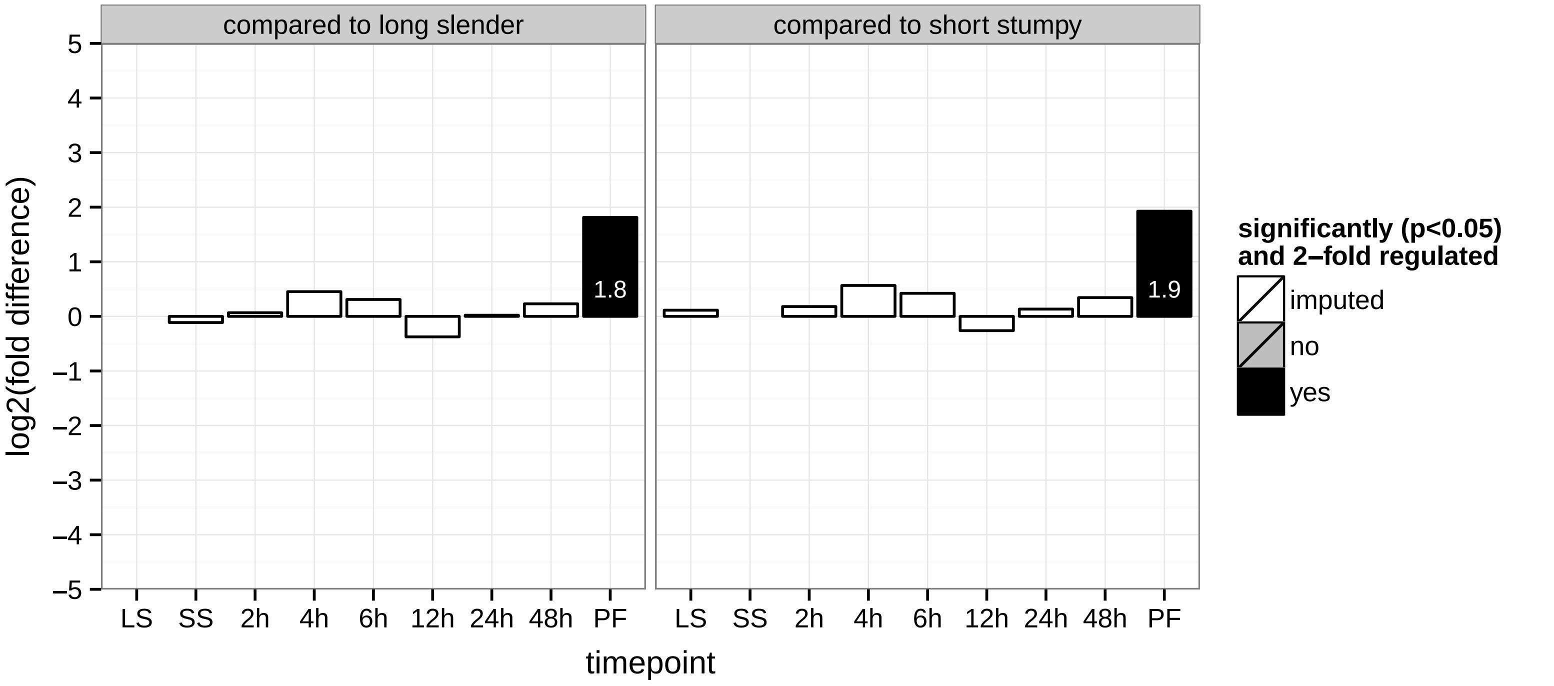

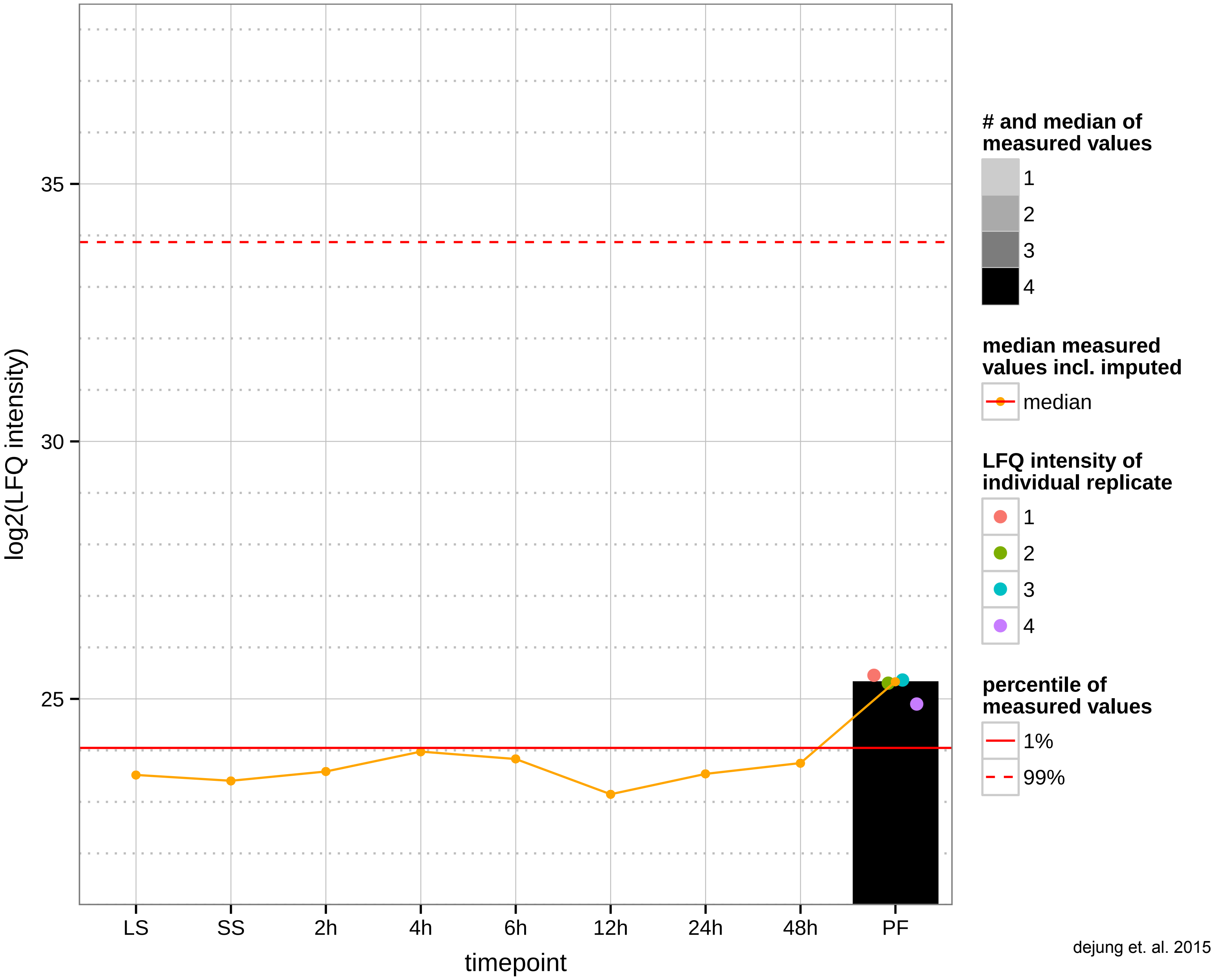

dejung et. al. 2015

actin, putative
Tb927.4.980
AGOF: null
AGOC: null
AGOP: null
PGOF: null
PGOC: null
PGOP: null

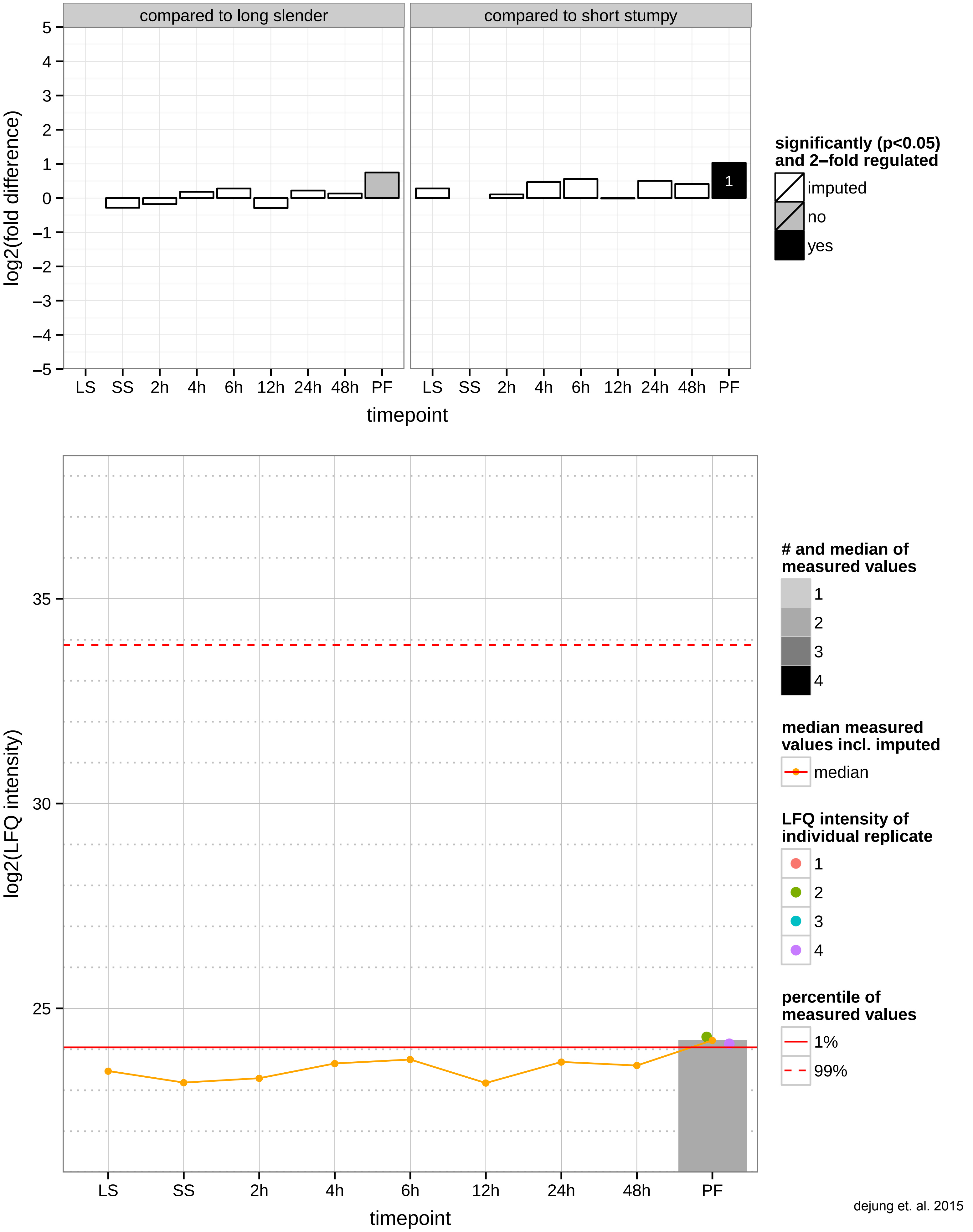

dejung et. al. 2015

threonyl–tRNA synthetase, putative
Tb927.5.1090
AGOF: ATP binding, aminoacyl–tRNA ligase activity, threonine–tRNA ligase activity
AGOC: null
AGOP: threonyl–tRNA aminoacylation, translation
PGOF: ATP binding, aminoacyl–tRNA ligase activity, ligase activity, forming aminoacyl–tRNA and related compounds, nucleot
PGOC: cytoplasm
PGOP: tRNA aminoacylation, tRNA aminoacylation for protein translation, threonyl–tRNA aminoacylation

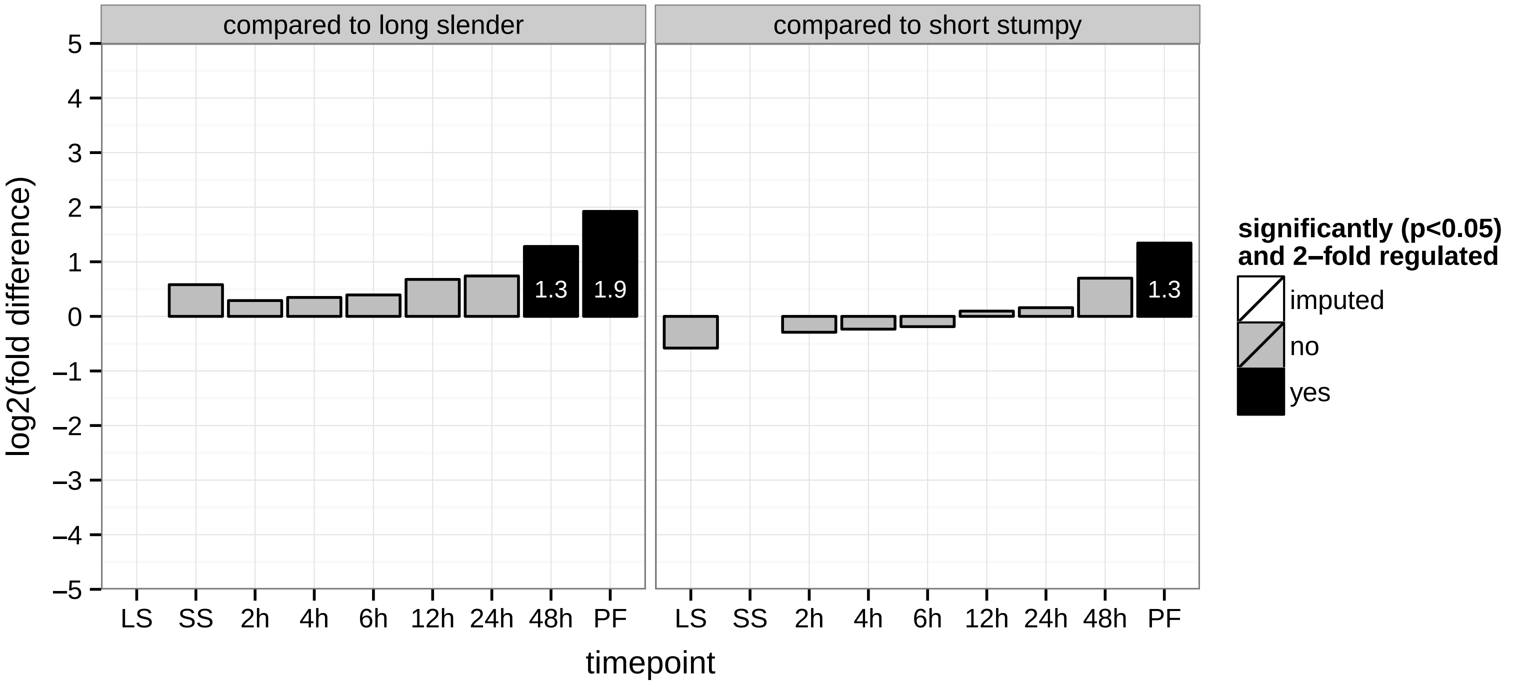

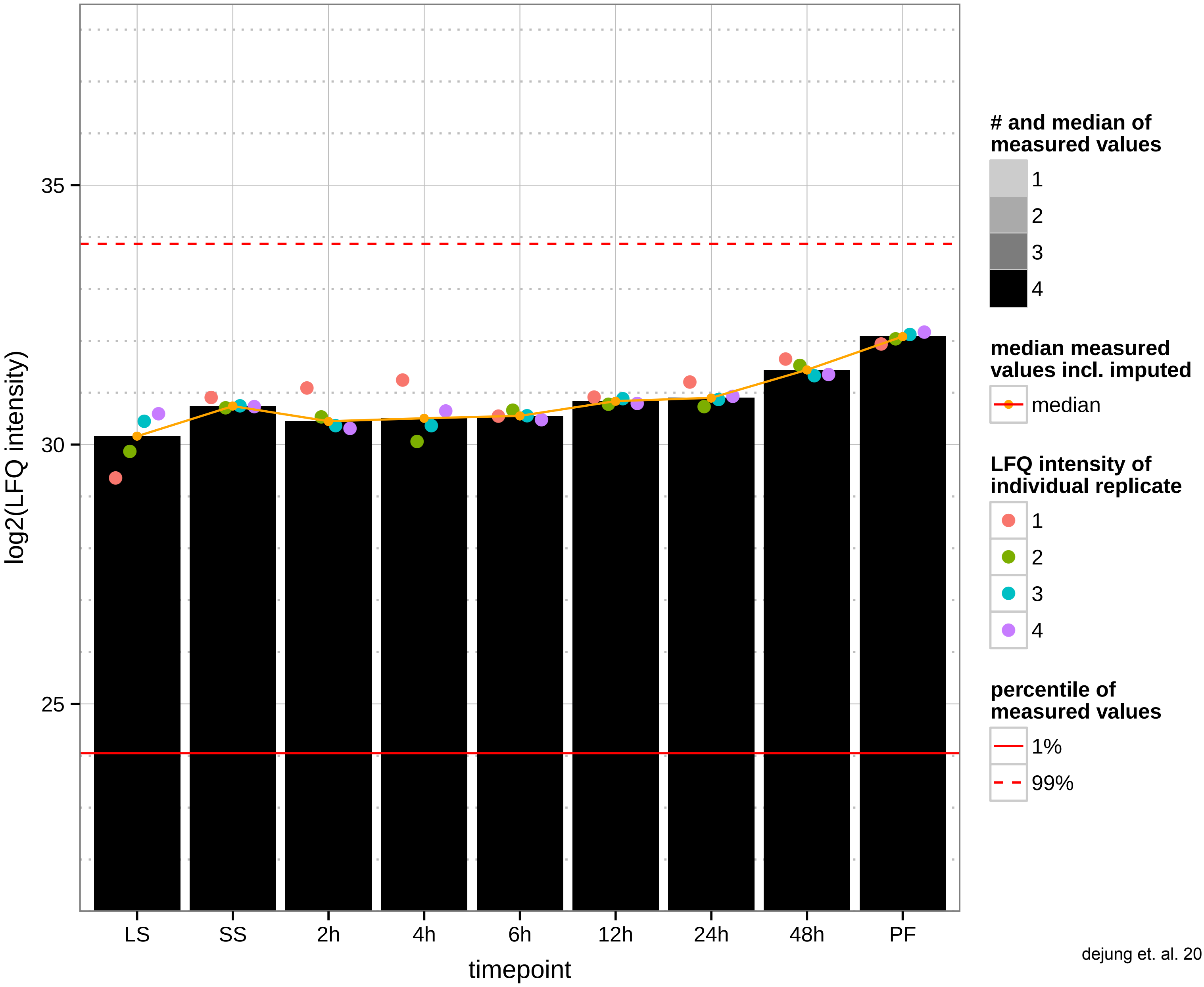

dejung et. al. 2015

hypothetical protein, conserved
Tb927.5.1240
AGOF: null
AGOC: mitochondrion
AGOP: null
PGOF: null
PGOC: null
PGOP: null

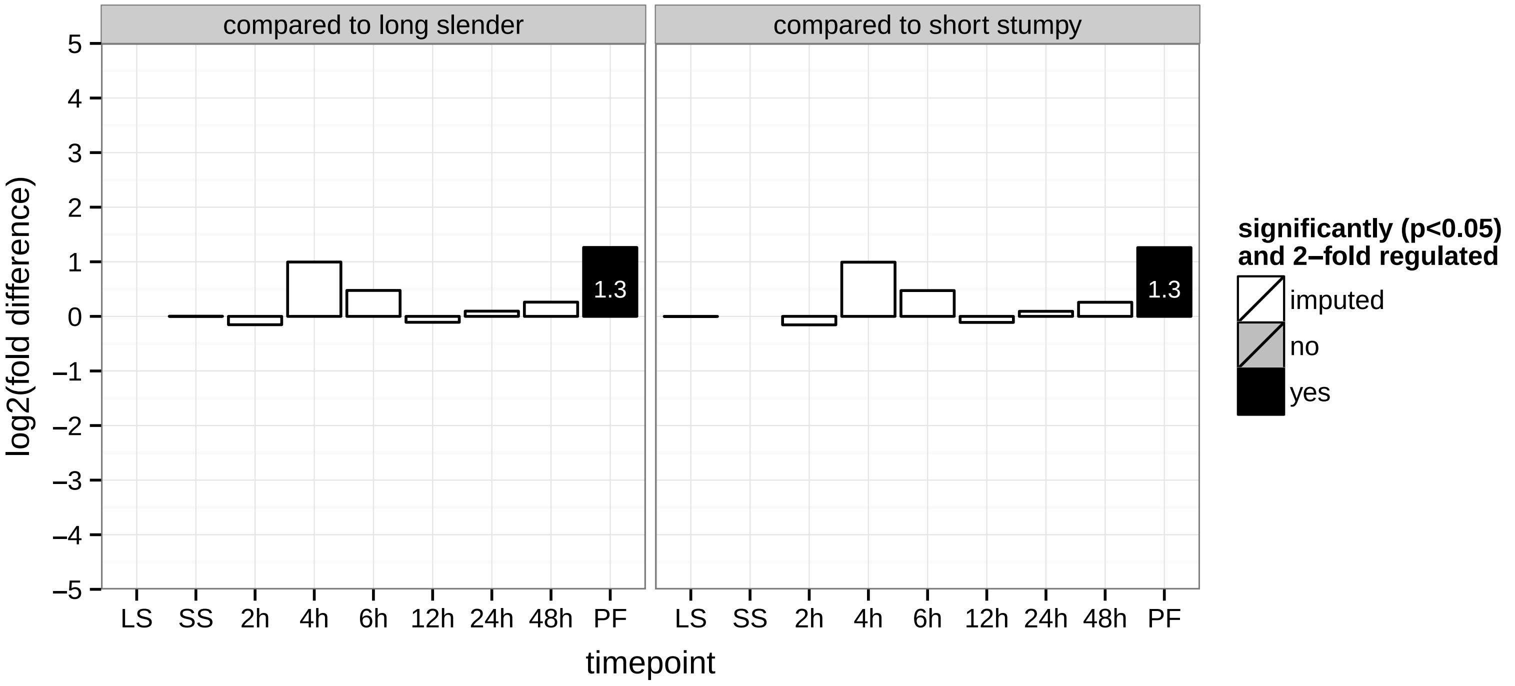

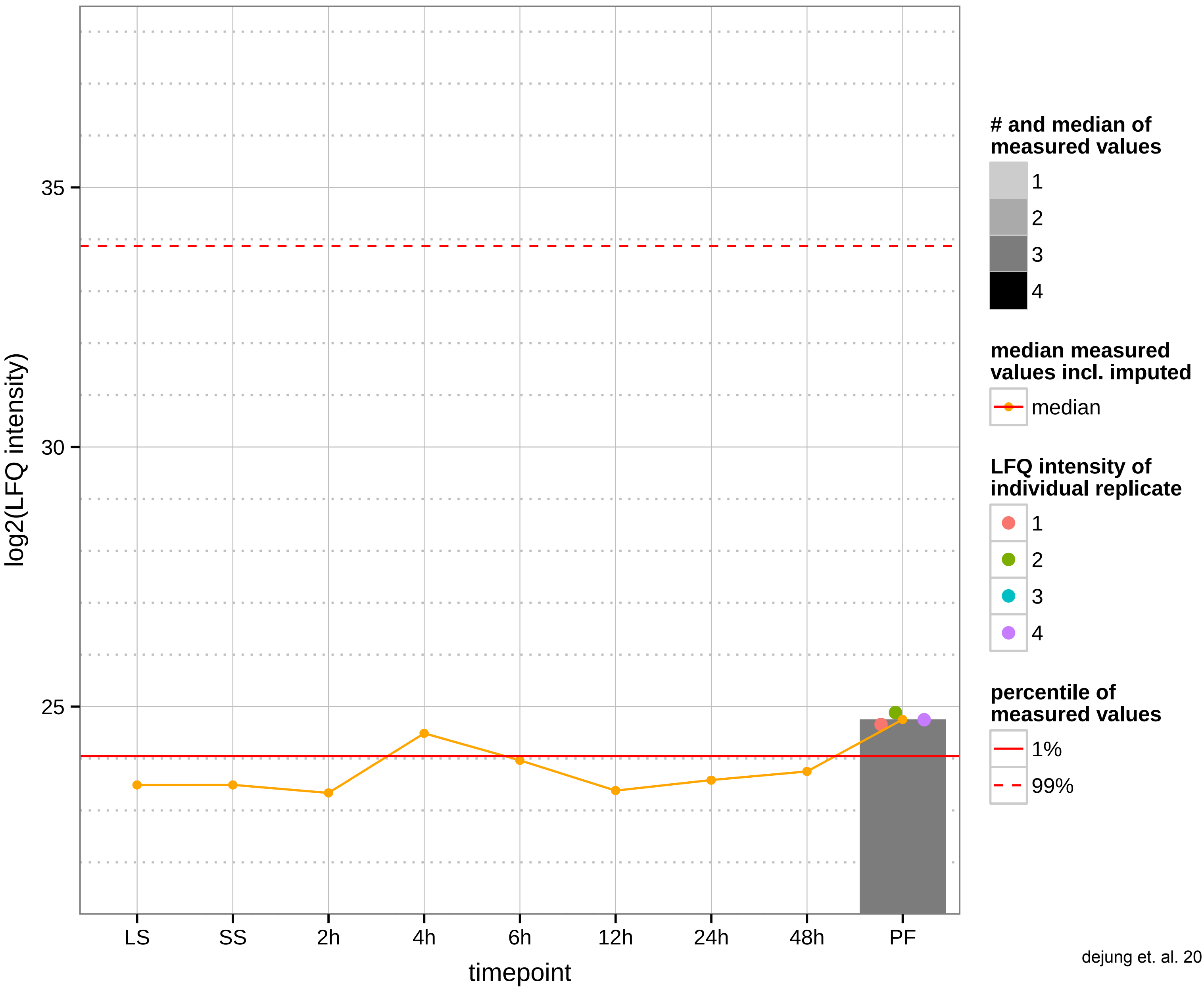

dejung et. al. 2015

heat shock protein HslVU, ATPase subunit HslU, putative
Tb927.5.1520
AGOF: ATP binding, ATPase activity, peptidase activity, acting on L−amino acid peptides
AGOC: HslUV protease complex, cytoplasm, mitochondrion
AGOP: rolling circle DNA replication
PGOF: ATP binding, ATPase activity, nucleoside−triphosphatase activity, nucleotide binding, peptidase activity, acting on L−an
PGOC: HslUV protease complex, cytoplasm
PGOP: null

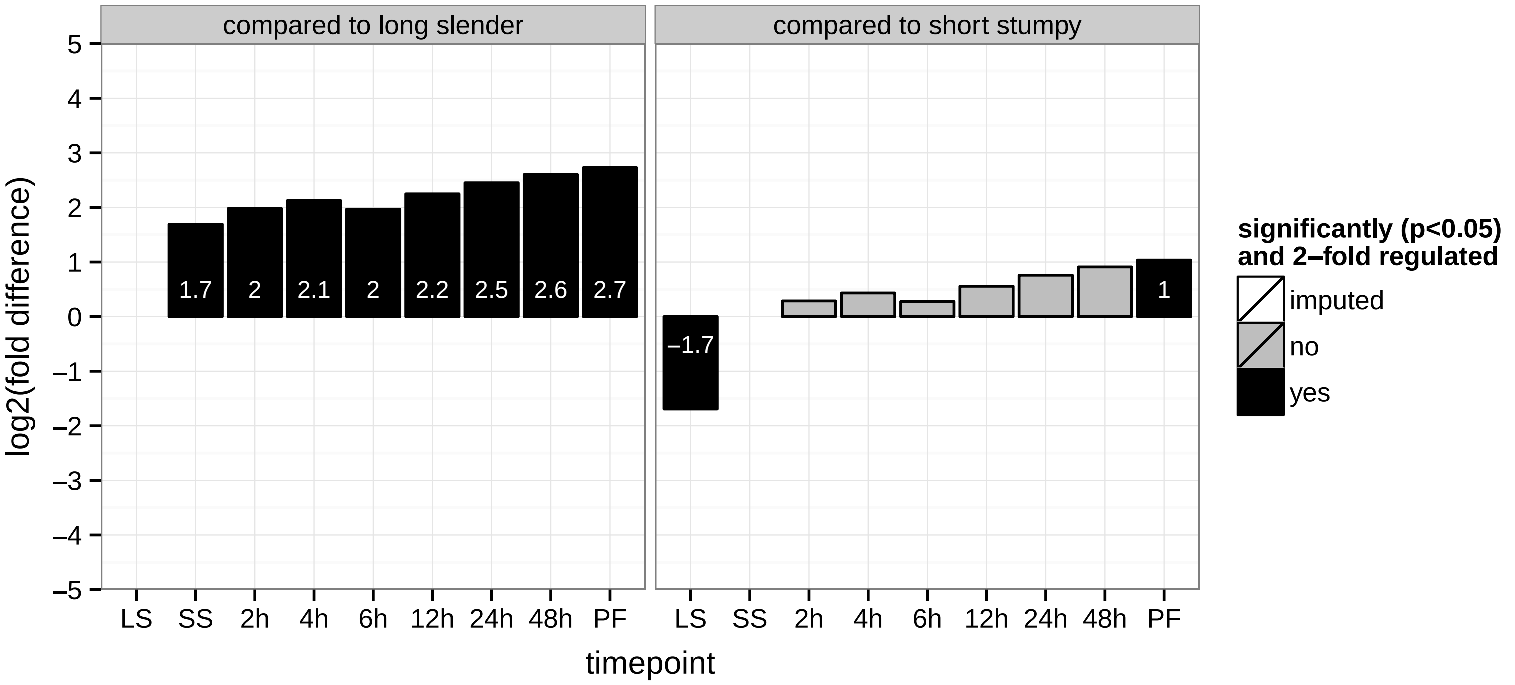

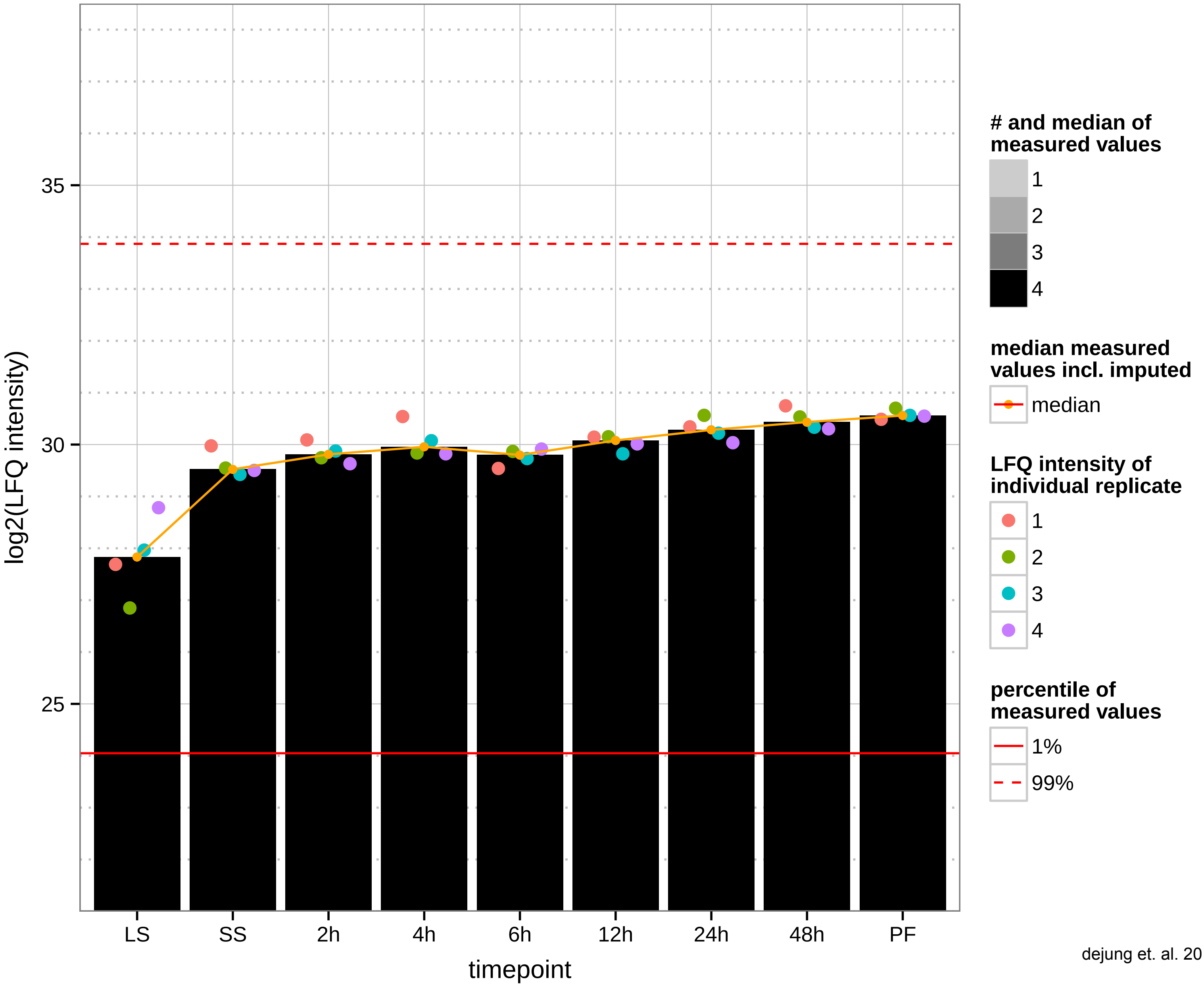

dejung et. al. 2015

hypothetical protein, conserved
Tb927.5.1670
AGOF: null
AGOC: null
AGOP: null
PGOF: protein binding
PGOC: null
PGOP: null

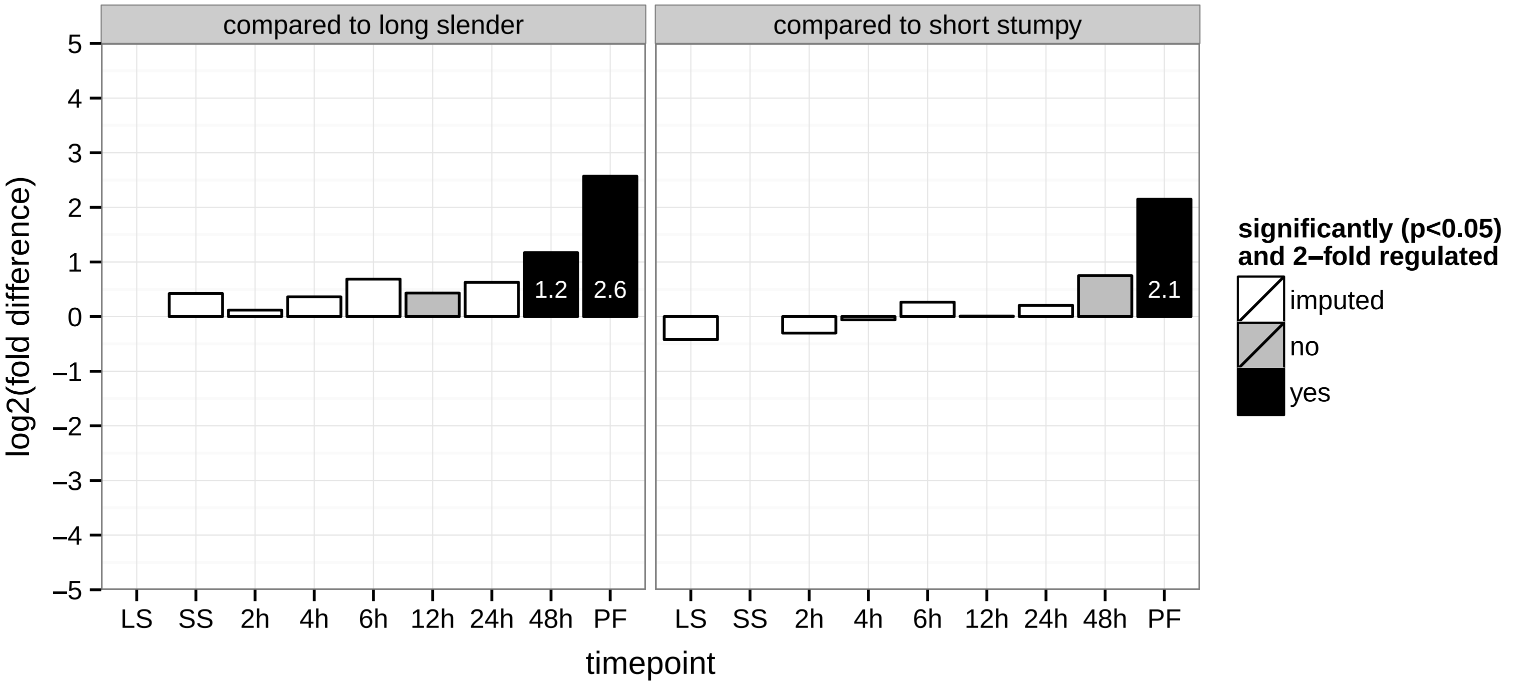

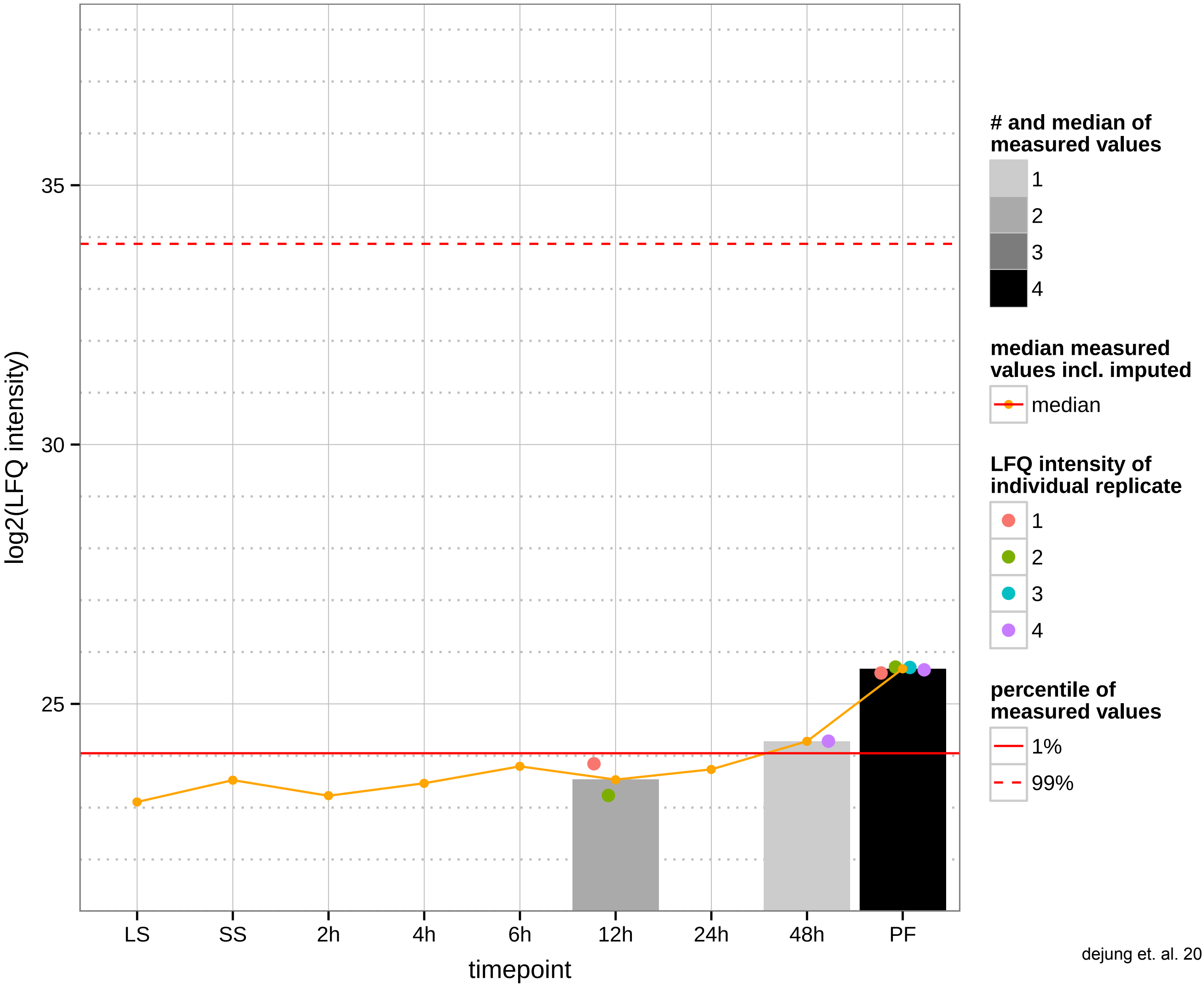

dejung et. al. 2015

hypothetical protein, conserved
Tb927.5.1720
AGOF: null
AGOC: mitochondrion
AGOP: null
PGOF: null
PGOC: null
PGOP: null

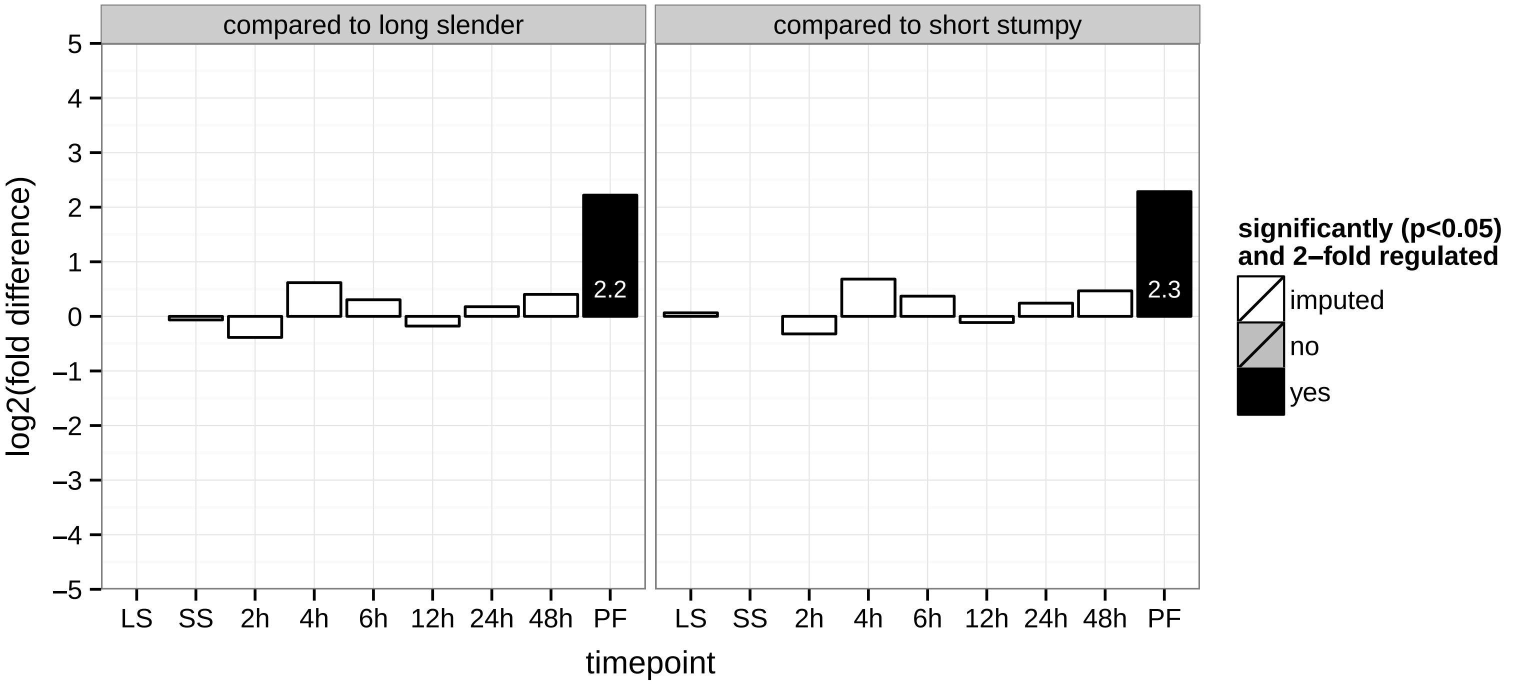

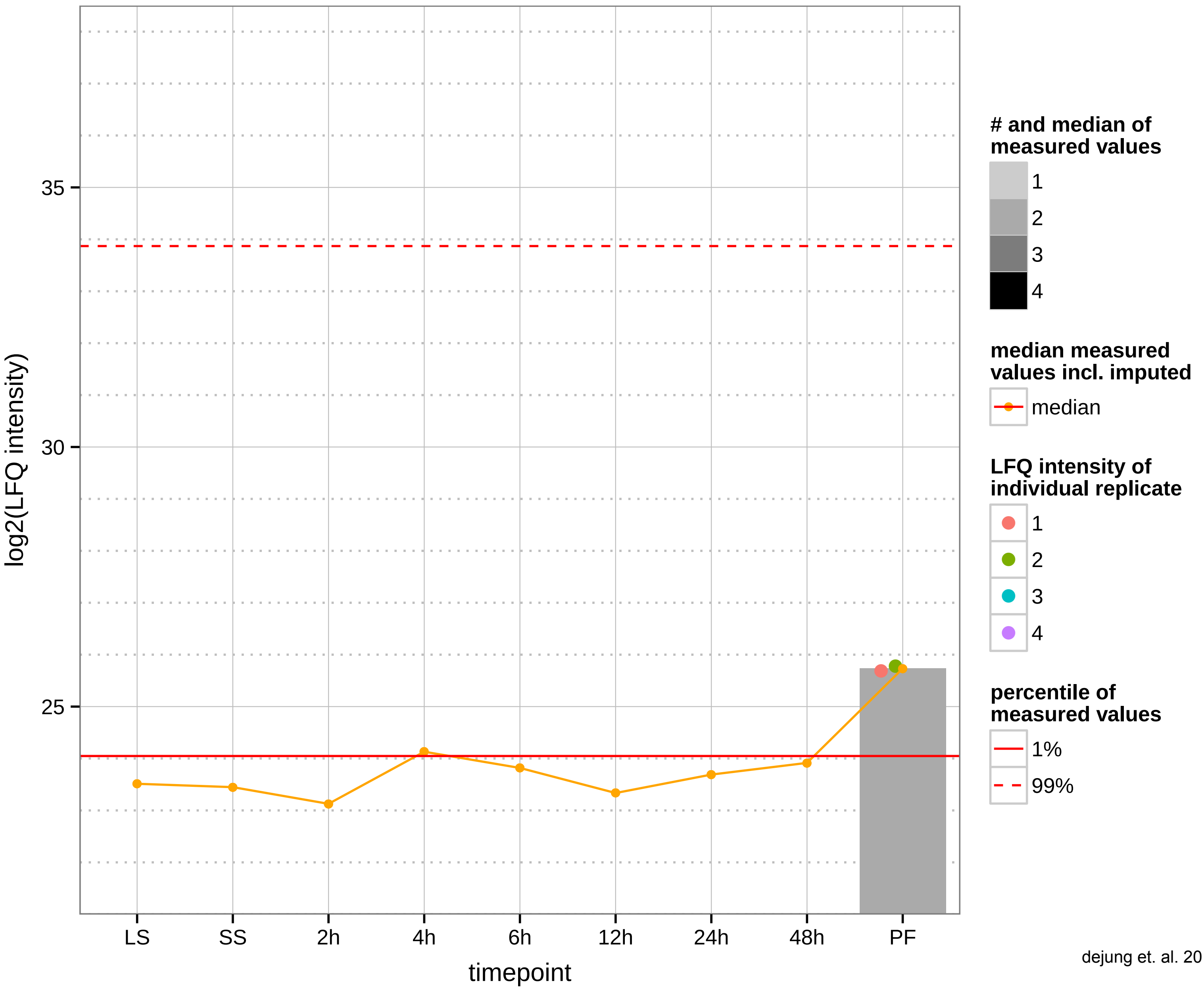

dejung et. al. 2015

hypothetical protein, conserved
Tb927.5.2130
AGOF: hydrolase activity
AGOC: null
AGOP: null
PGOF: hydrolase activity, acting on ester bonds
PGOC: null
PGOP: mRNA processing

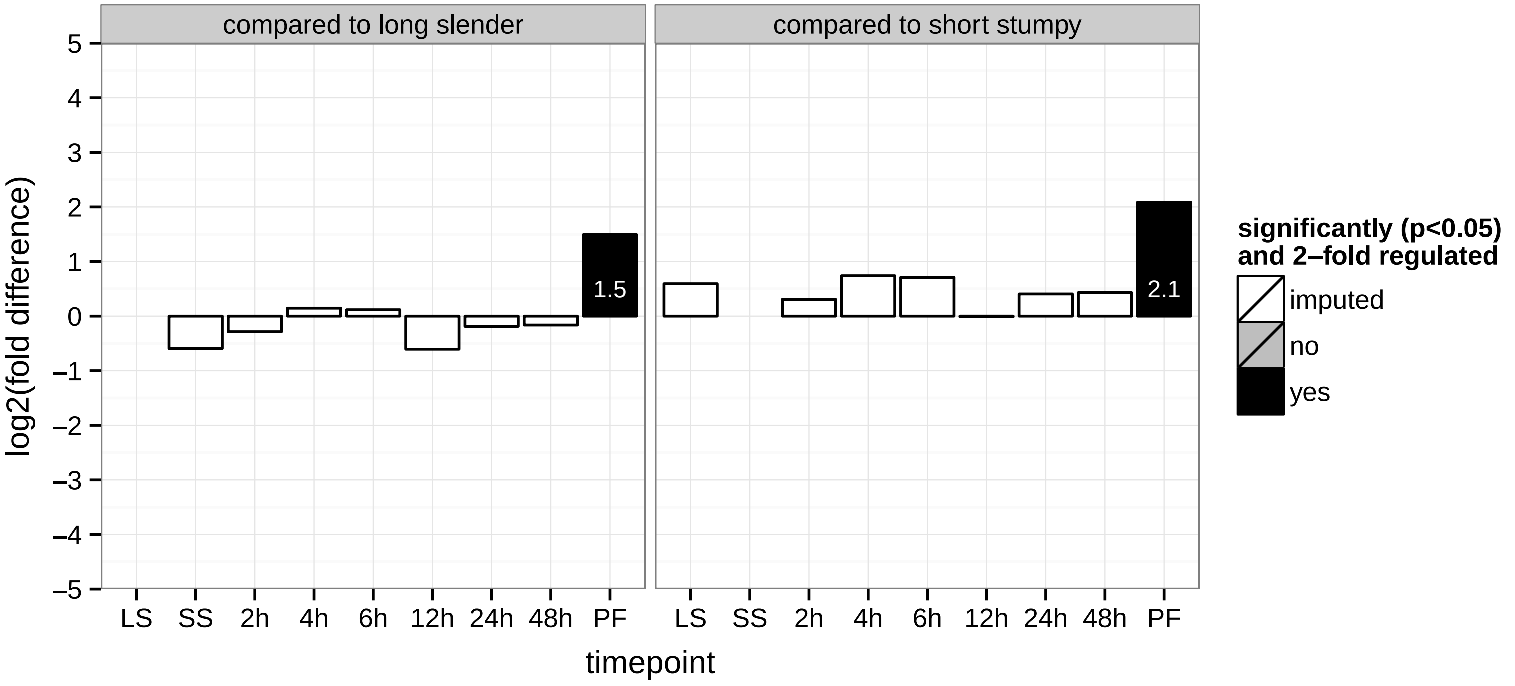

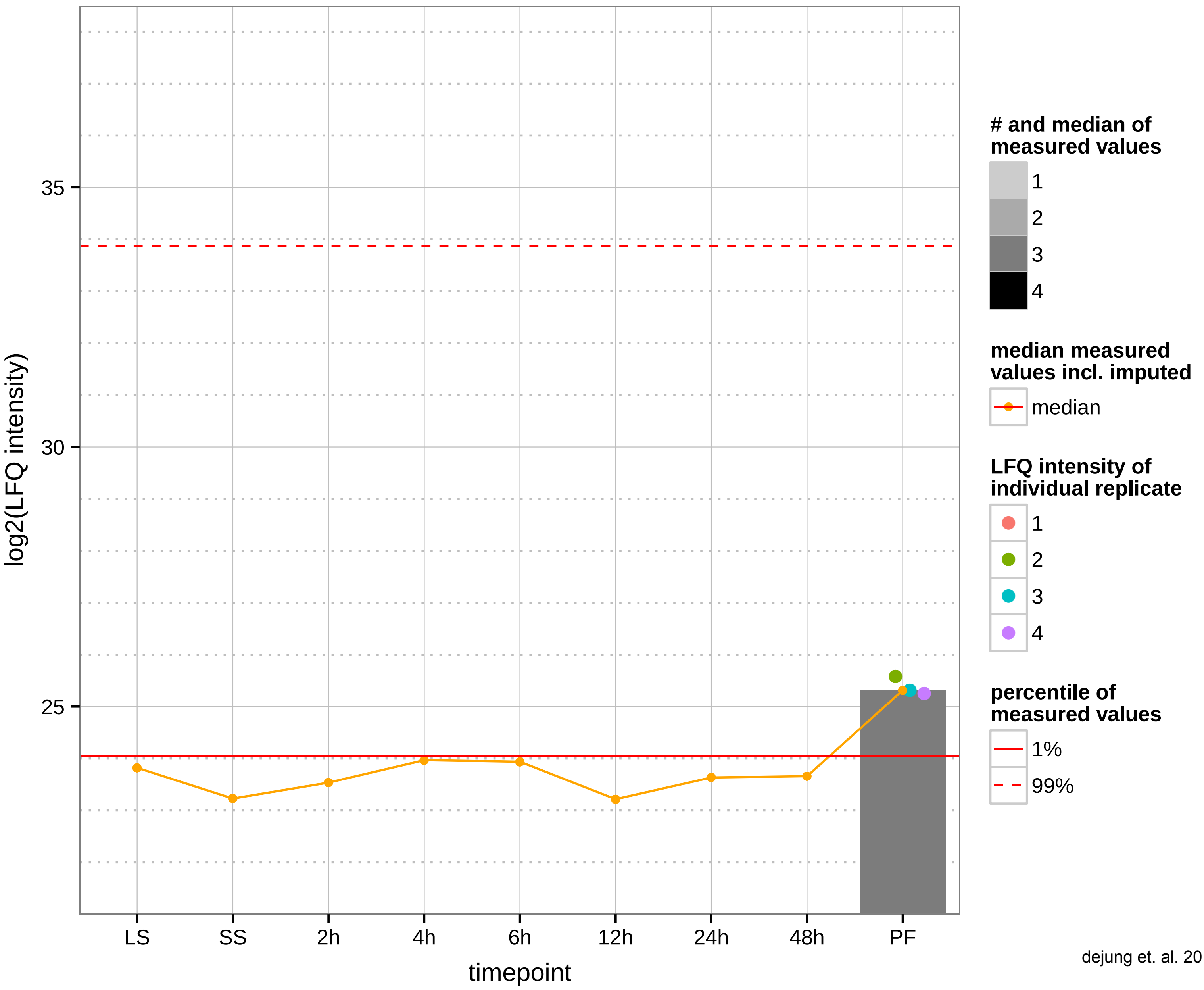

dejung et. al. 2015

ATP−dependent RNA helicase, putative
Tb927.5.2290
AGOF: ATP binding, ATP−dependent helicase activity, helicase activity, nucleic acid binding
AGOC: null
AGOP: null
PGOF: ATP binding, ATP−dependent helicase activity, helicase activity, nucleic acid binding
PGOC: null
PGOP: null

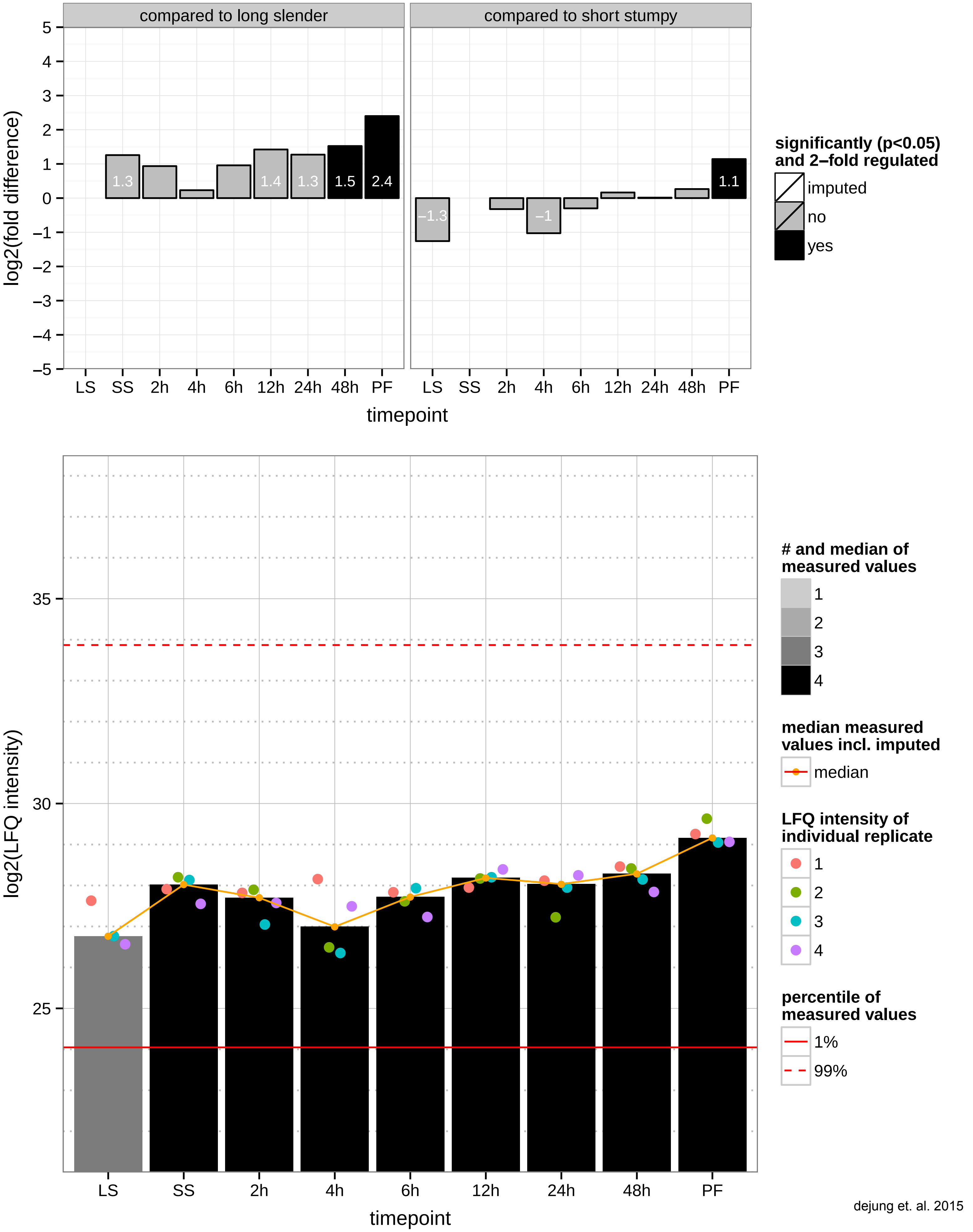

dejung et. al. 2015

hypothetical protein, conserved
Tb927.5.2530
AGOF: null
AGOC: null
AGOP: null
PGOF: null
PGOC: null
PGOP: null

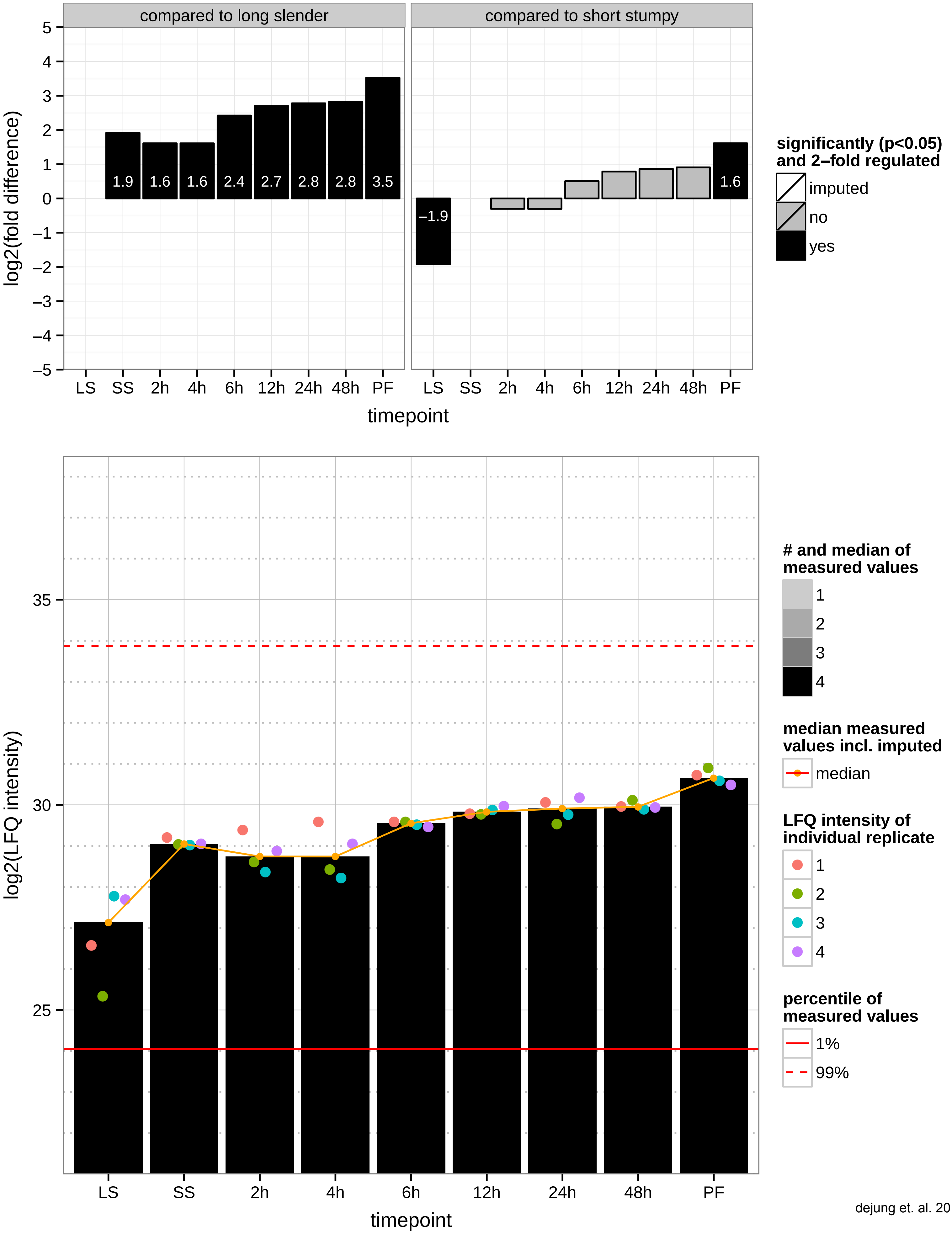

dejung et. al. 2015

hypothetical protein, conserved
Tb927.5.2550
AGOF: null
AGOC: mitochondrion
AGOP: null
PGOF: null
PGOC: null
PGOP: null

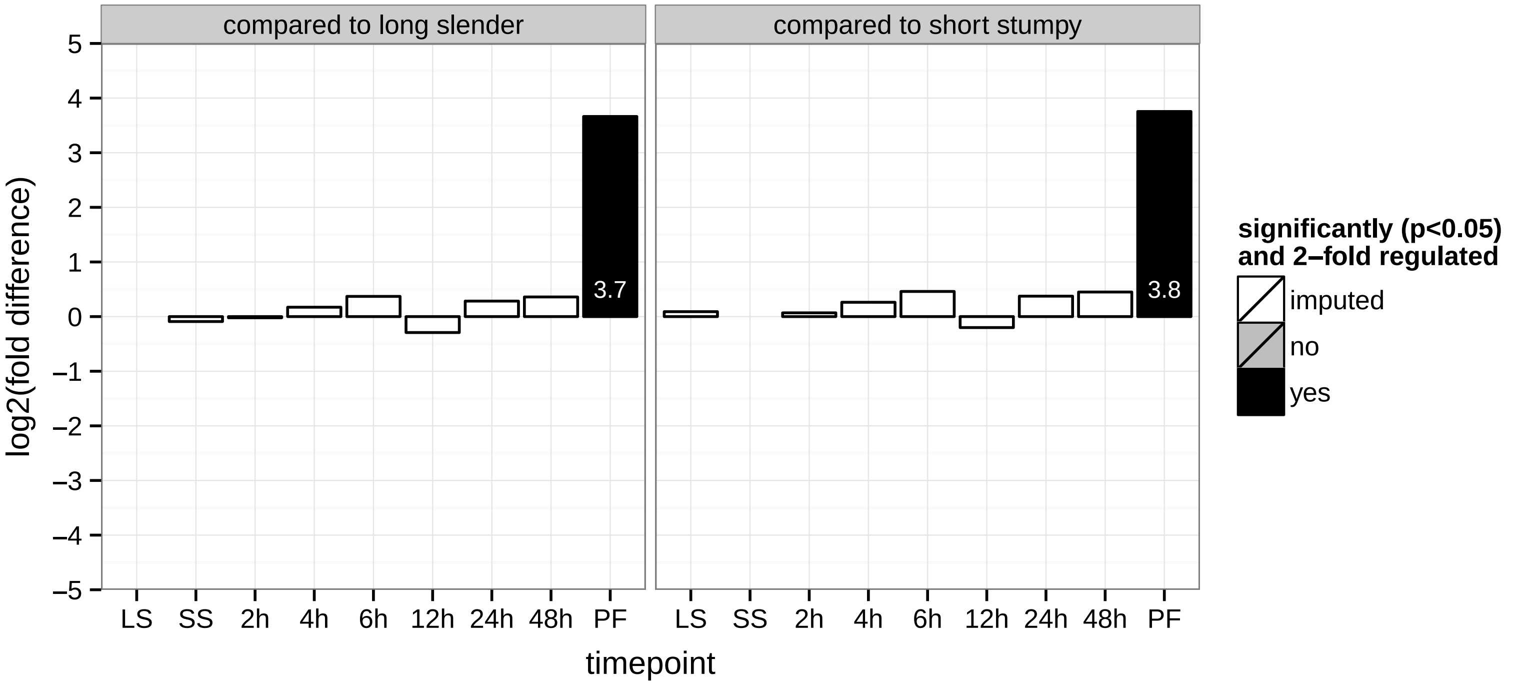

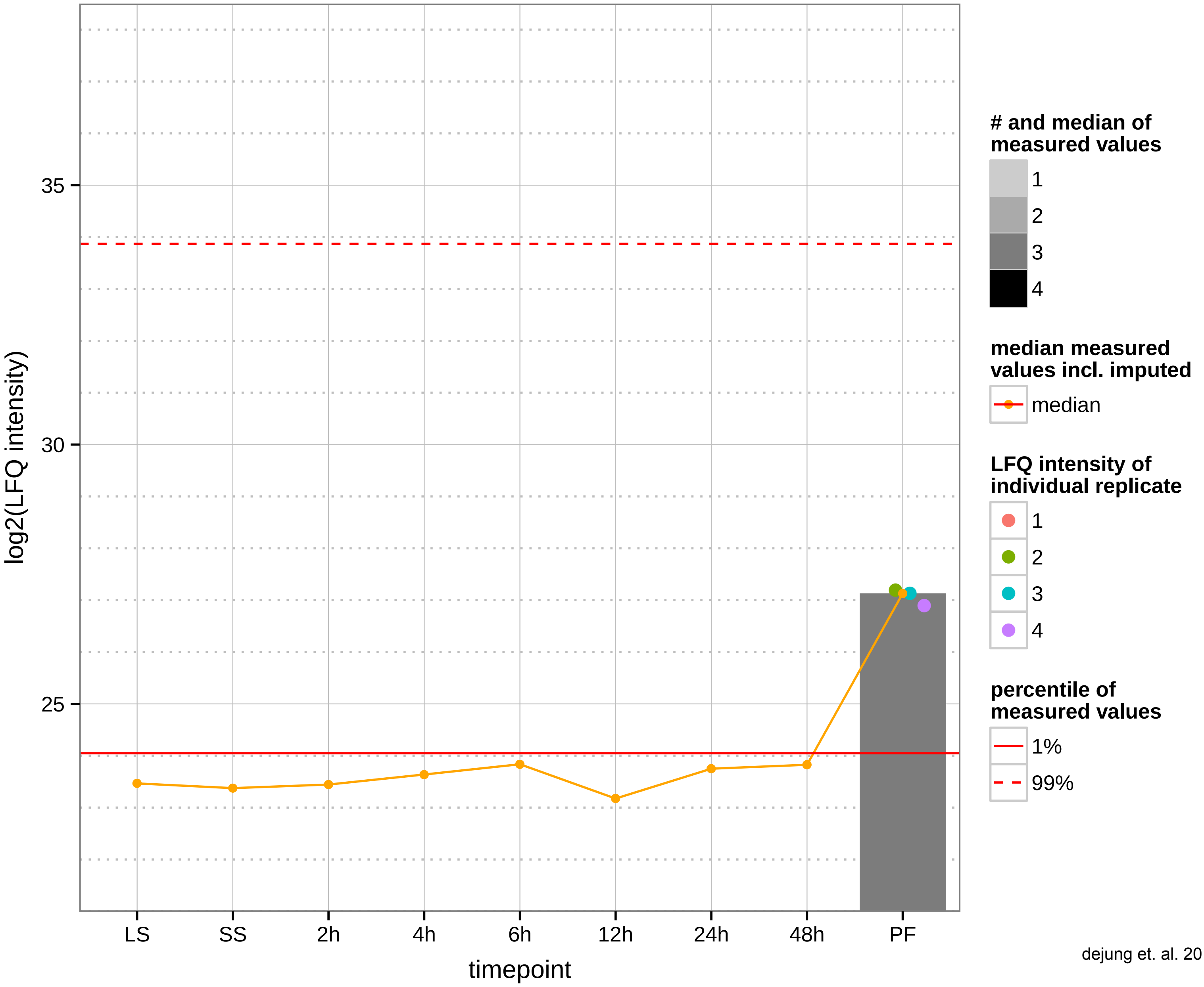

dejung et. al. 2015

zinc−finger protein ZPR1, putative
Tb927.5.2720
AGOF: zinc ion binding
AGOC: null
AGOP: null
PGOF: zinc ion binding
PGOC: null
PGOP: null

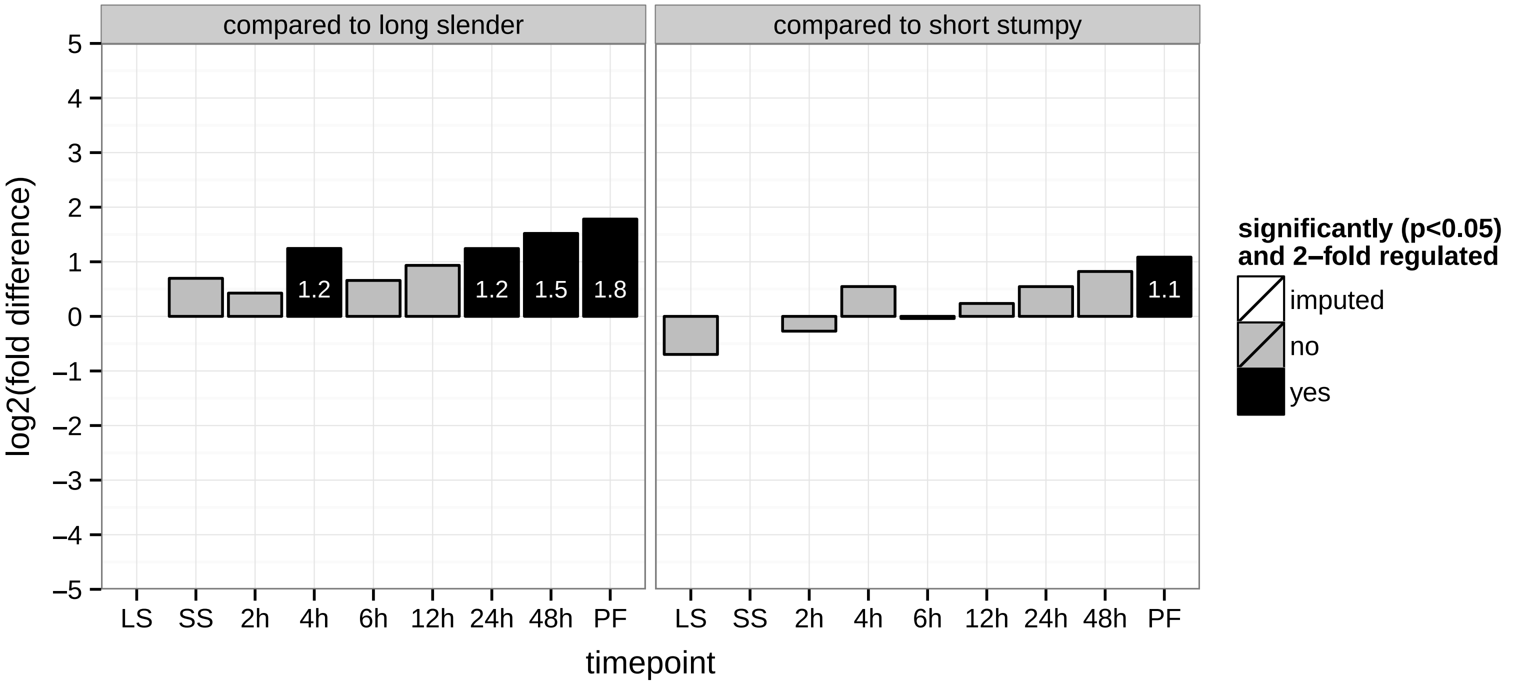

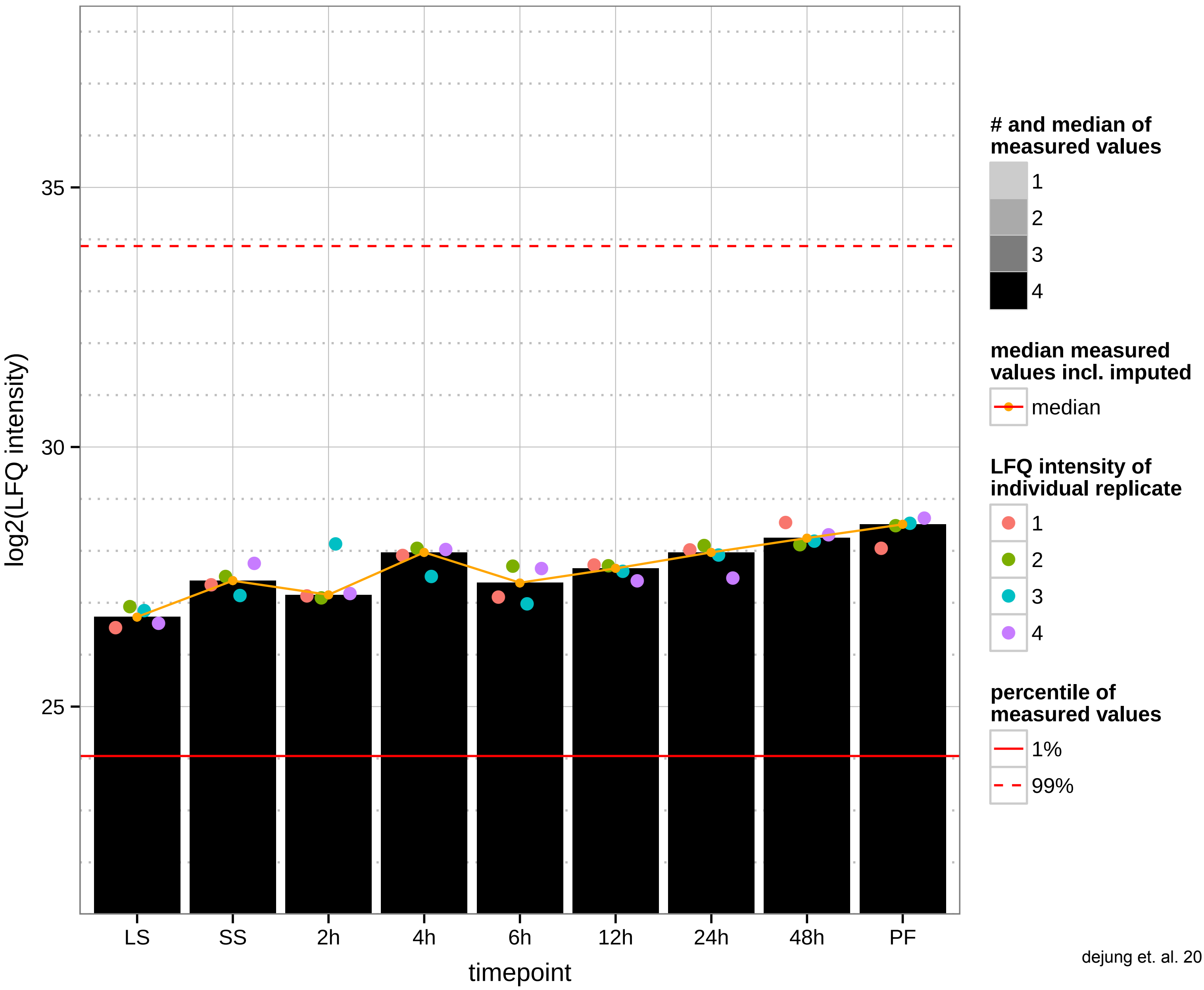

dejung et. al. 2015

hypothetical protein, conserved
Tb927.5.2890
AGOF: ATP binding, ATPase activity
AGOC: null
AGOP: null
PGOF: ATP binding, ATPase activity, nucleoside−triphosphatase activity, nucleotide binding, protein binding
PGOC: null
PGOP: null

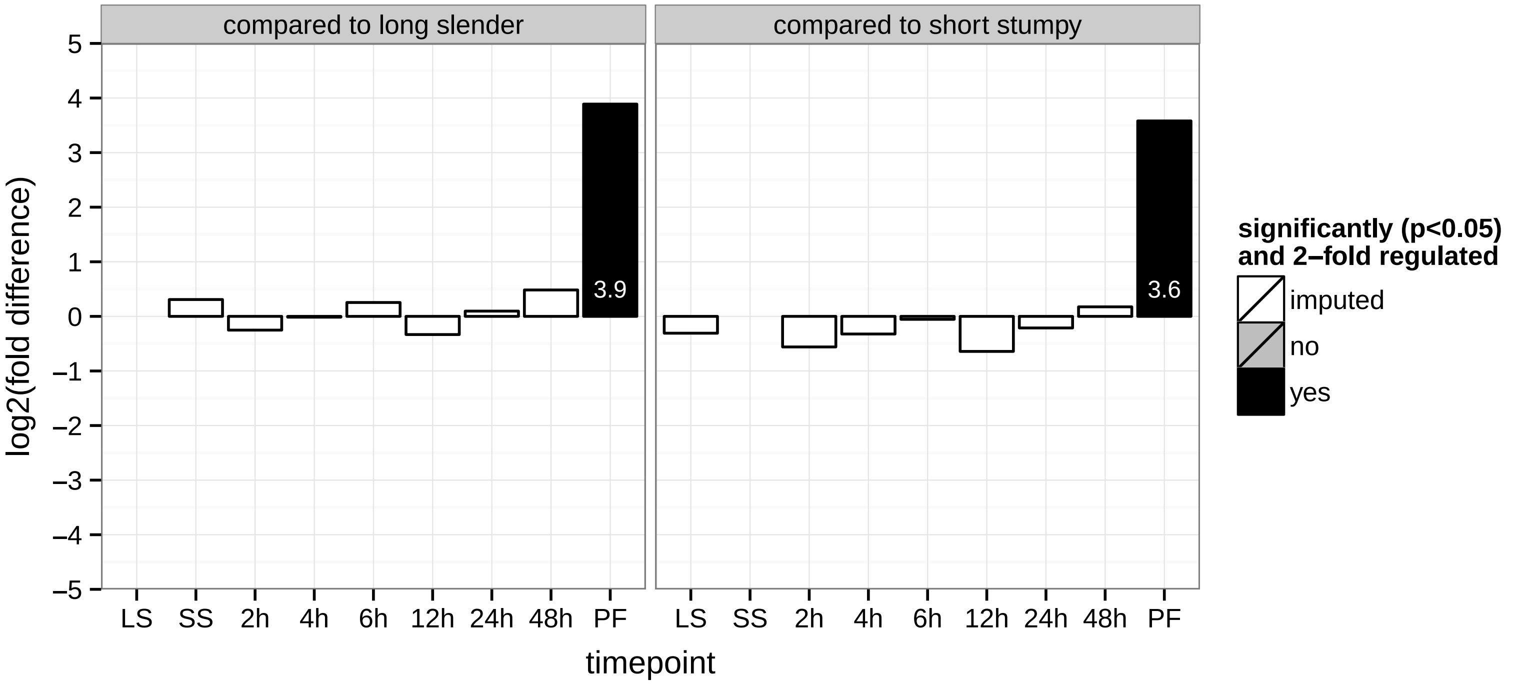

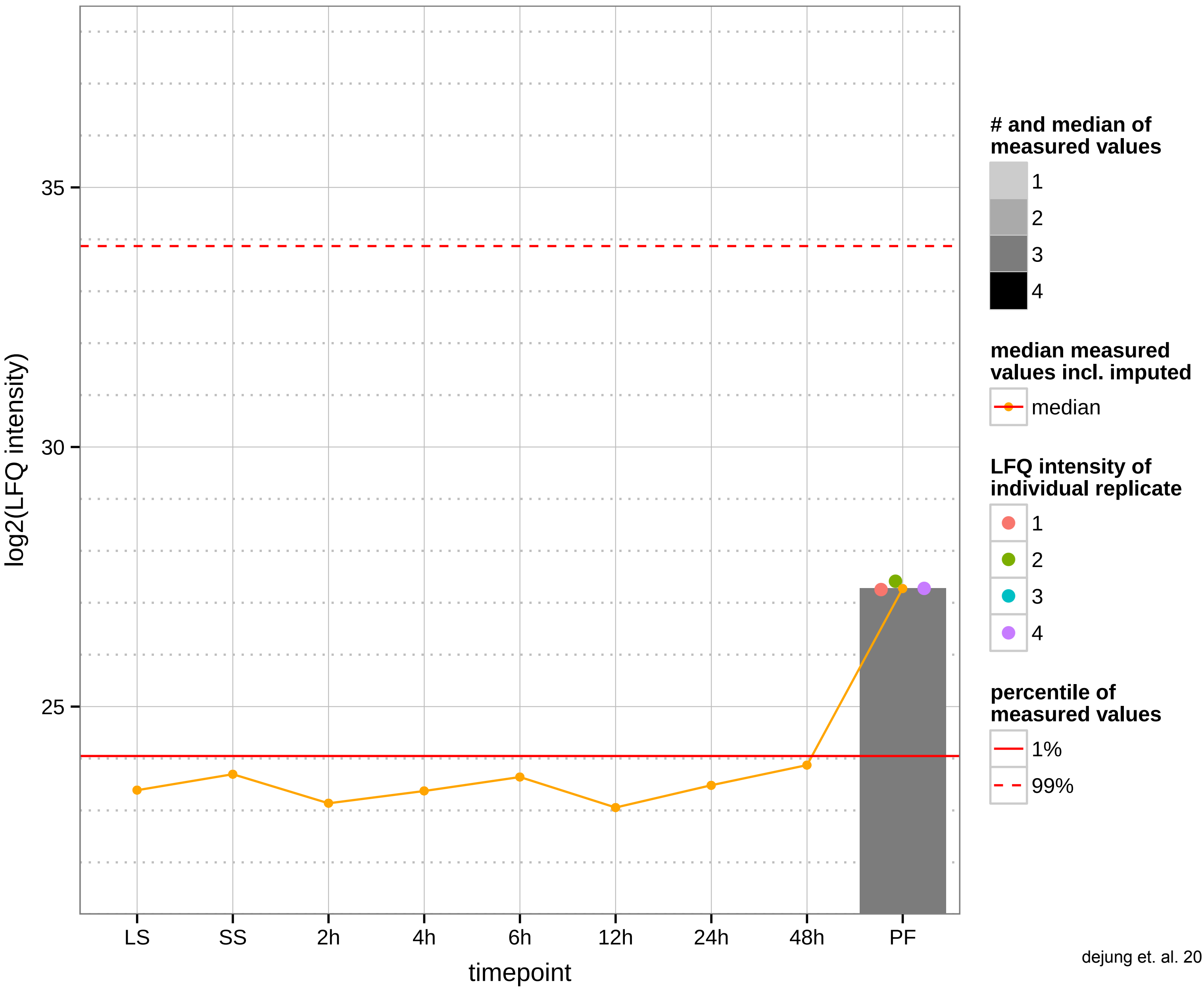

dejung et. al. 2015

hypothetical protein, conserved
Tb927.5.3230
AGOF: null
AGOC: null
AGOP: null
PGOF: null
PGOC: null
PGOP: null

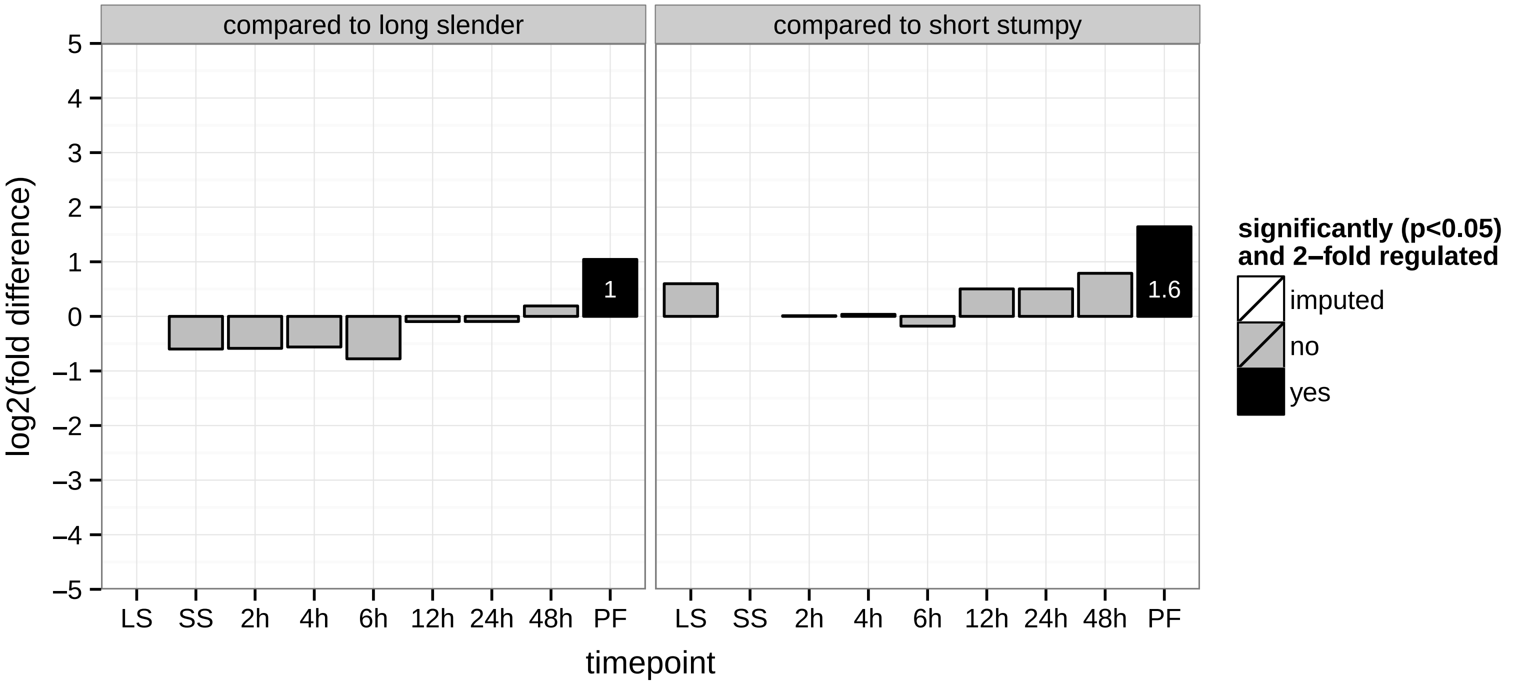

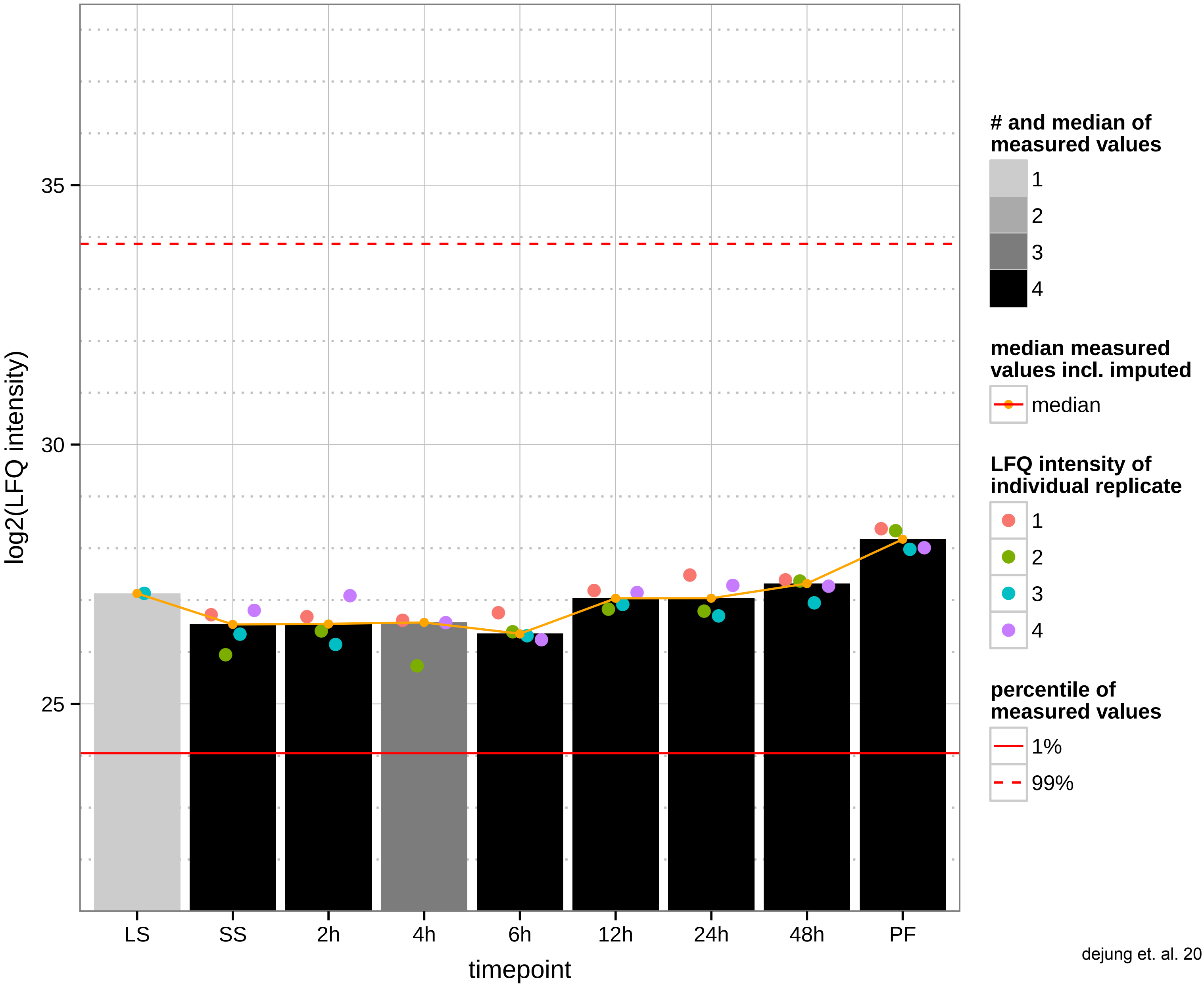

dejung et. al. 2015

queuine tRNA–ribosyltransferase, putative
Tb927.5.3520
AGOF: queuine tRNA–ribosyltransferase activity
AGOC: null
AGOP: queuosine biosynthetic process
PGOF: queuine tRNA–ribosyltransferase activity
PGOC: null
PGOP: queuosine biosynthetic process, tRNA modification

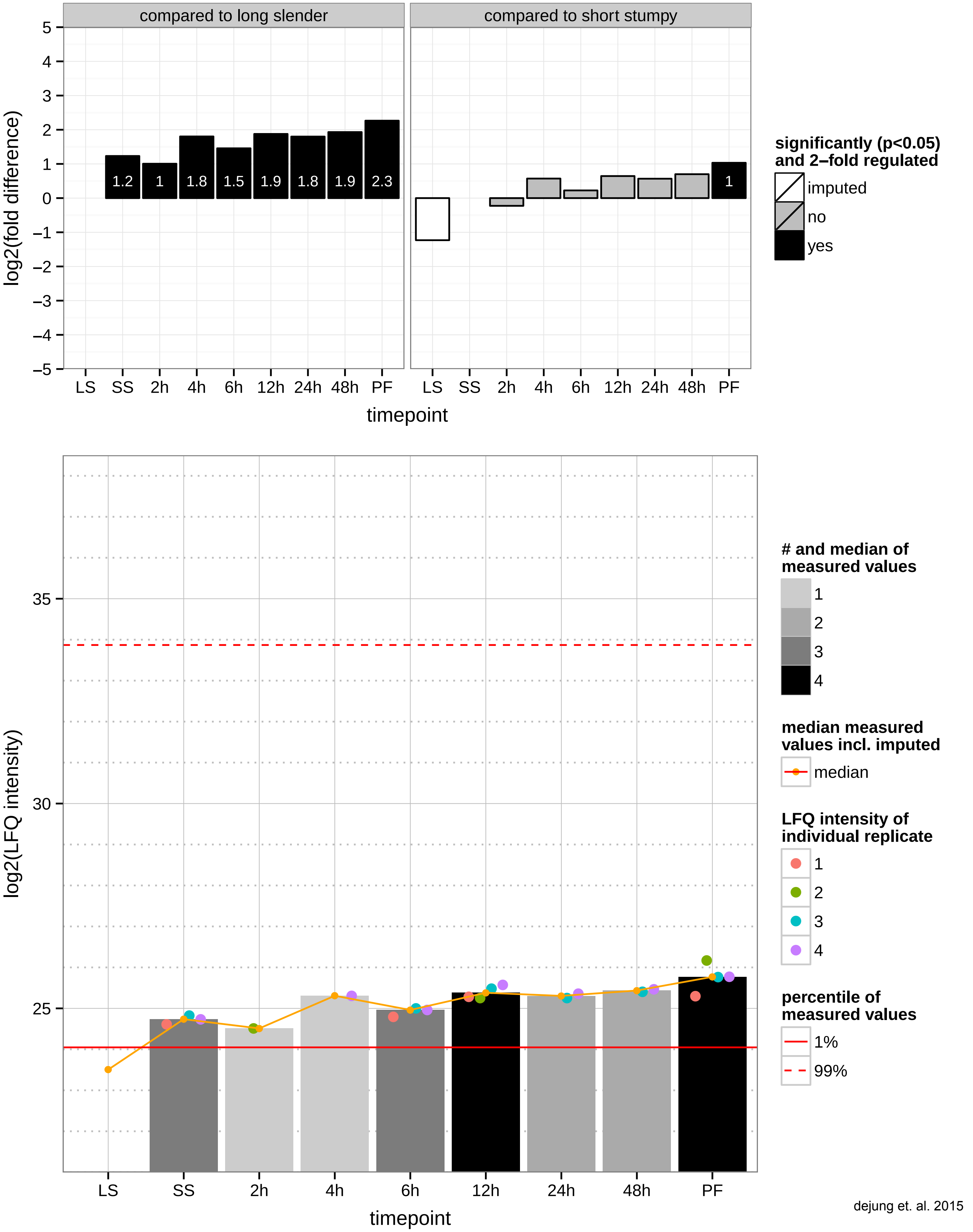

dejung et. al. 2015

glutamine hydrolysing (not ammonia–dependent) carbomoyl phosphate synthase, putative
Tb927.5.3800
AGOF: ATP binding, argininosuccinate synthase activity
AGOC: null
AGOP: arginine biosynthetic process, glutamine metabolic process, nitrogen compound metabolic process
PGOF: ATP binding, argininosuccinate synthase activity, catalytic activity, metal ion binding
PGOC: null
PGOP: arginine biosynthetic process, carbamoyl phosphate biosynthetic process, glutamine catabolic process, metabolic proc

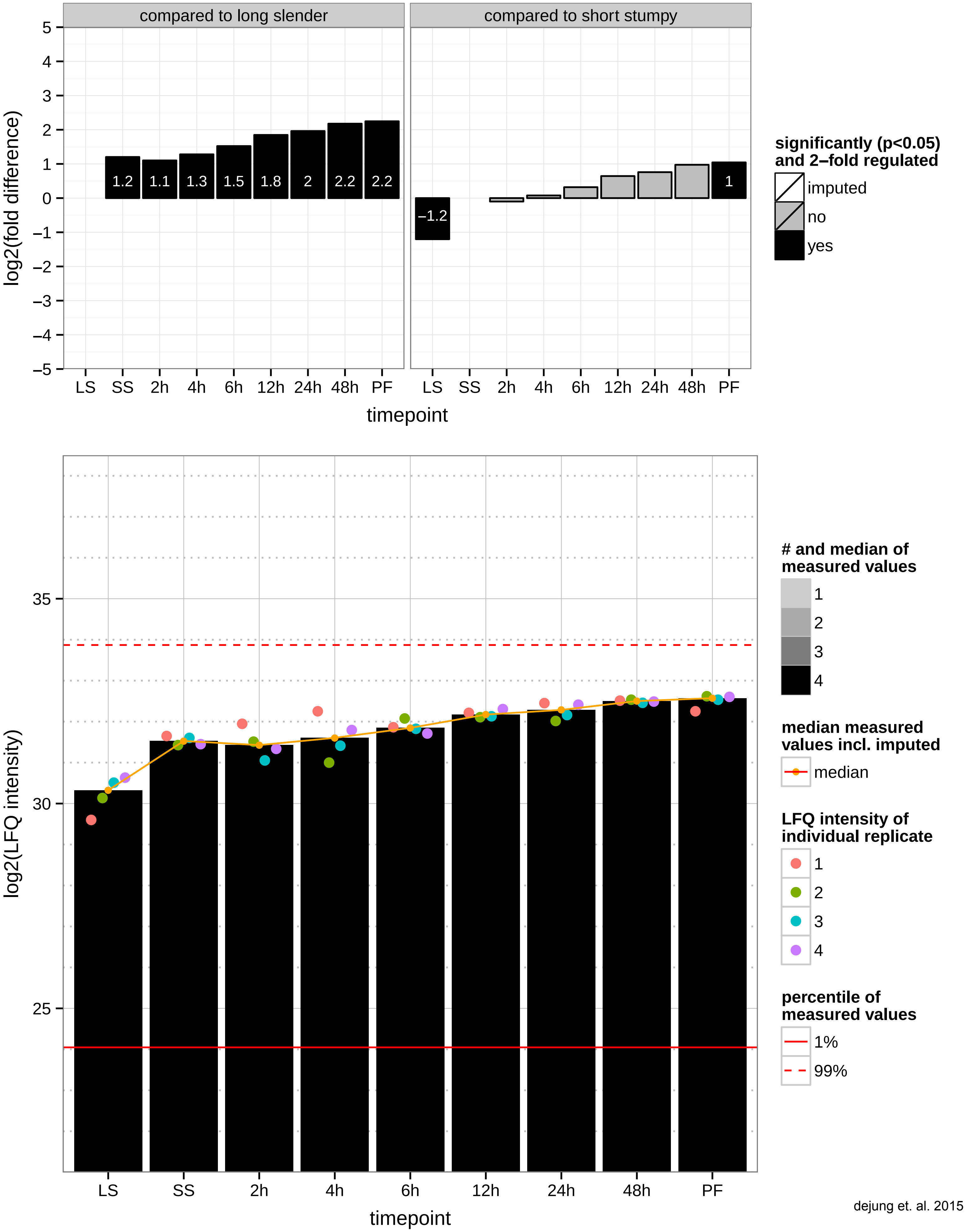

dejung et. al. 2015

tubulin tyrosine ligase, putative
Tb927.5.3860
AGOF: tubulin–tyrosine ligase activity
AGOC: null
AGOP: cellular protein modification process
PGOF: tubulin–tyrosine ligase activity
PGOC: null
PGOP: cellular protein modification process

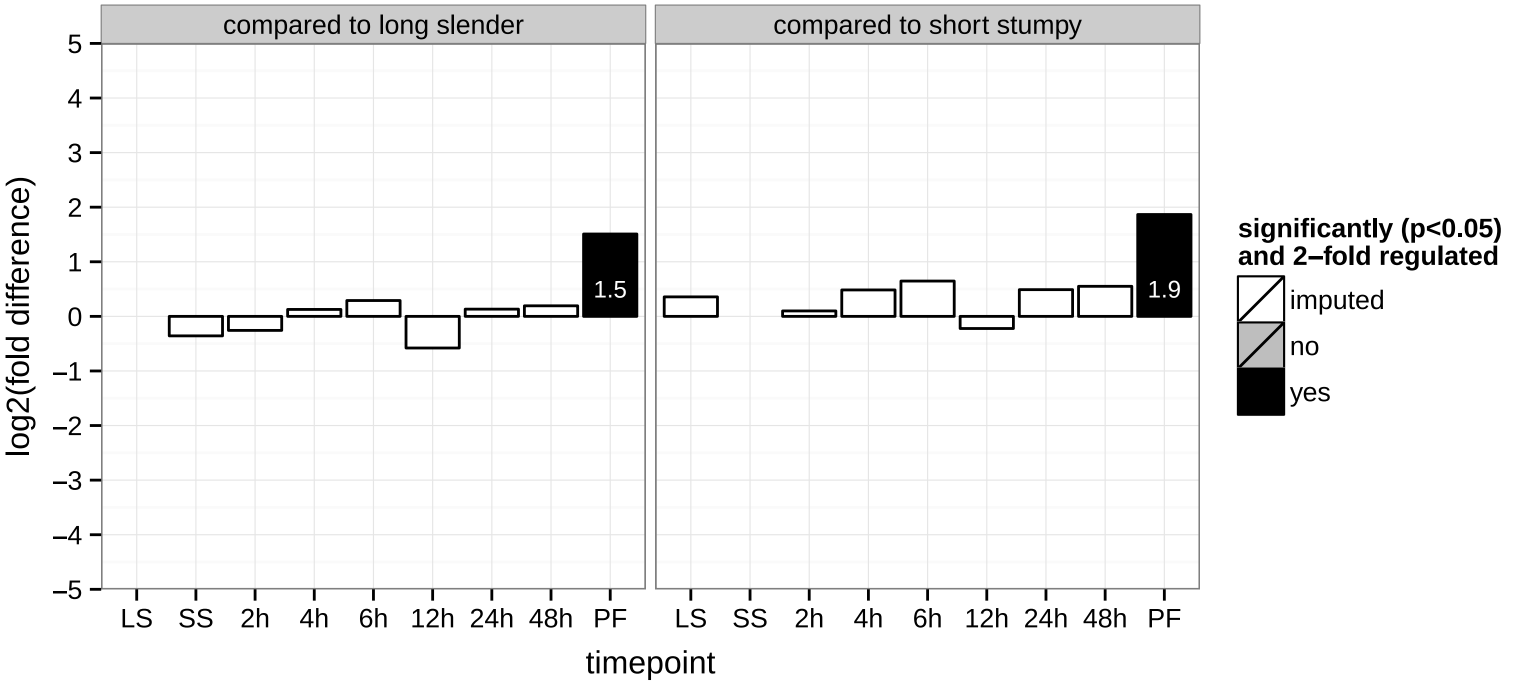

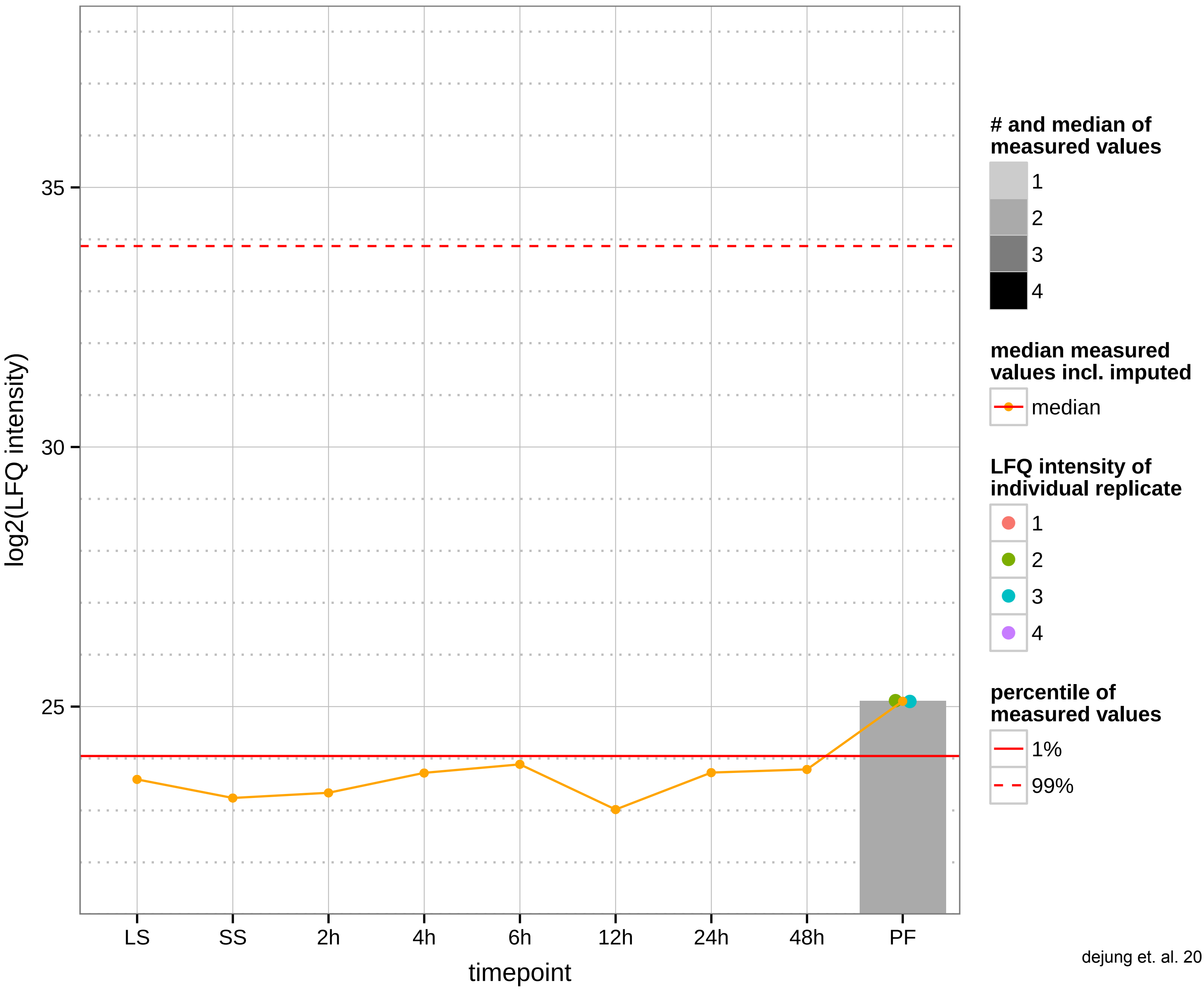

dejung et. al. 2015

hypothetical protein, conserved
Tb927.5.4060
AGOF: null
AGOC: integral to membrane
AGOP: protein processing
PGOF: null
PGOC: integral to membrane
PGOP: protein processing

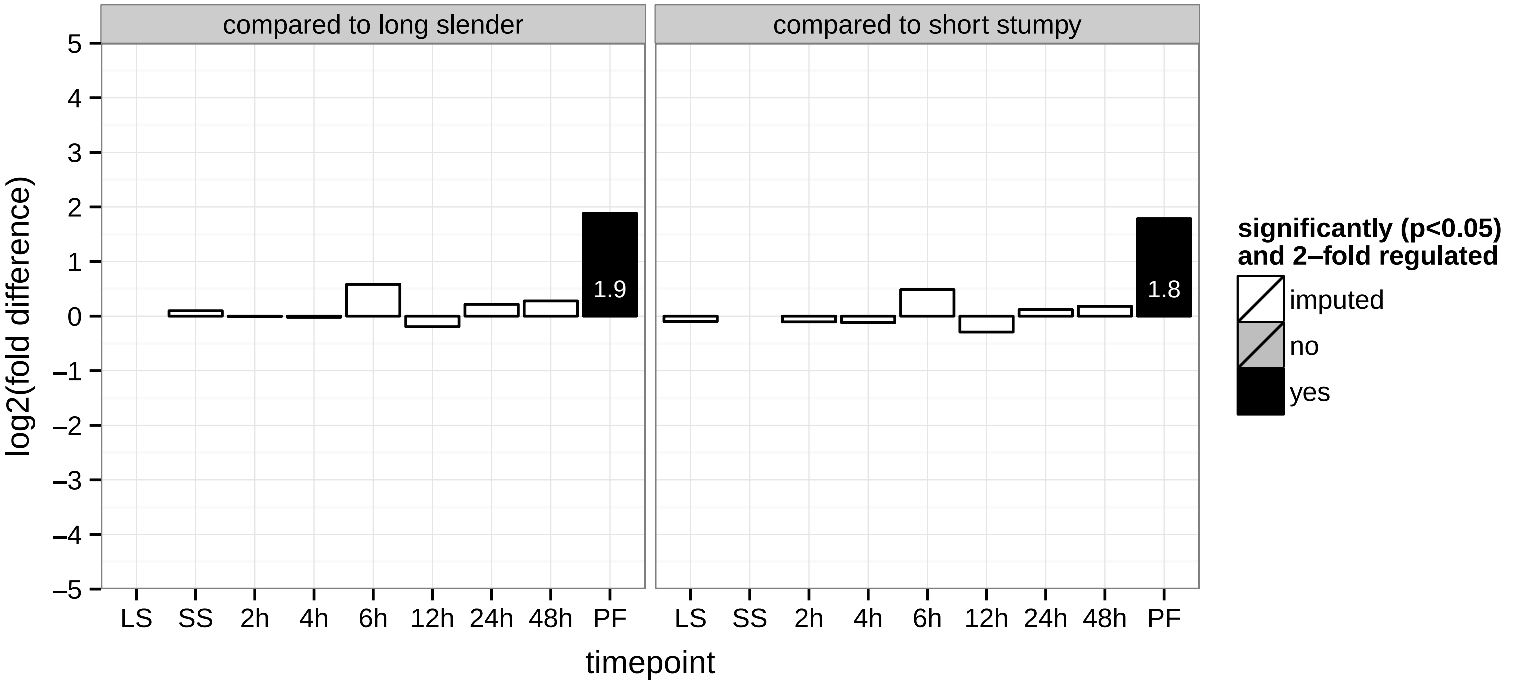

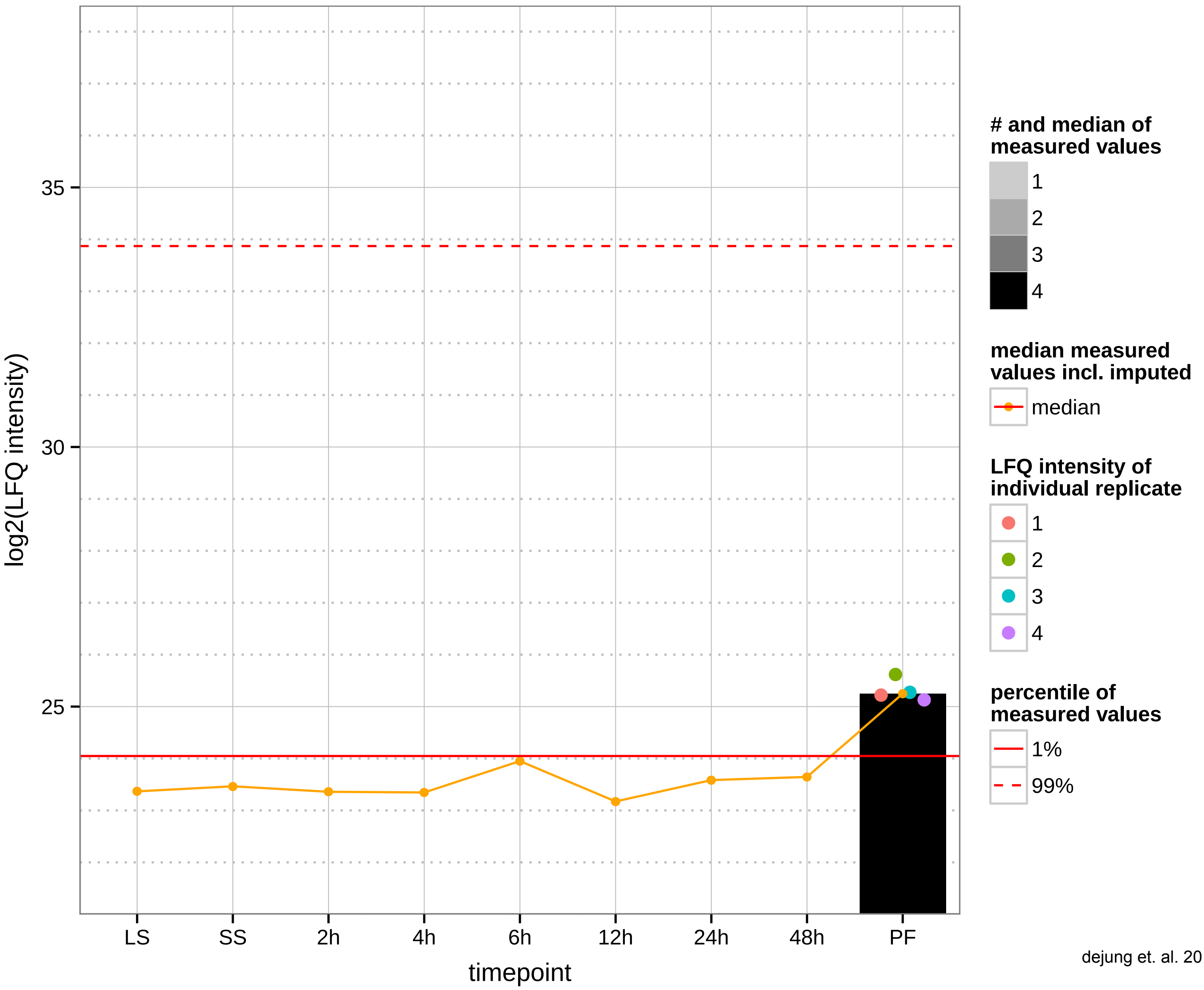

dejung et. al. 2015

stomatin–like protein, putative
Tb927.5.520
AGOF: null
AGOC: integral to membrane, membrane
AGOP: null
PGOF: null
PGOC: null
PGOP: null

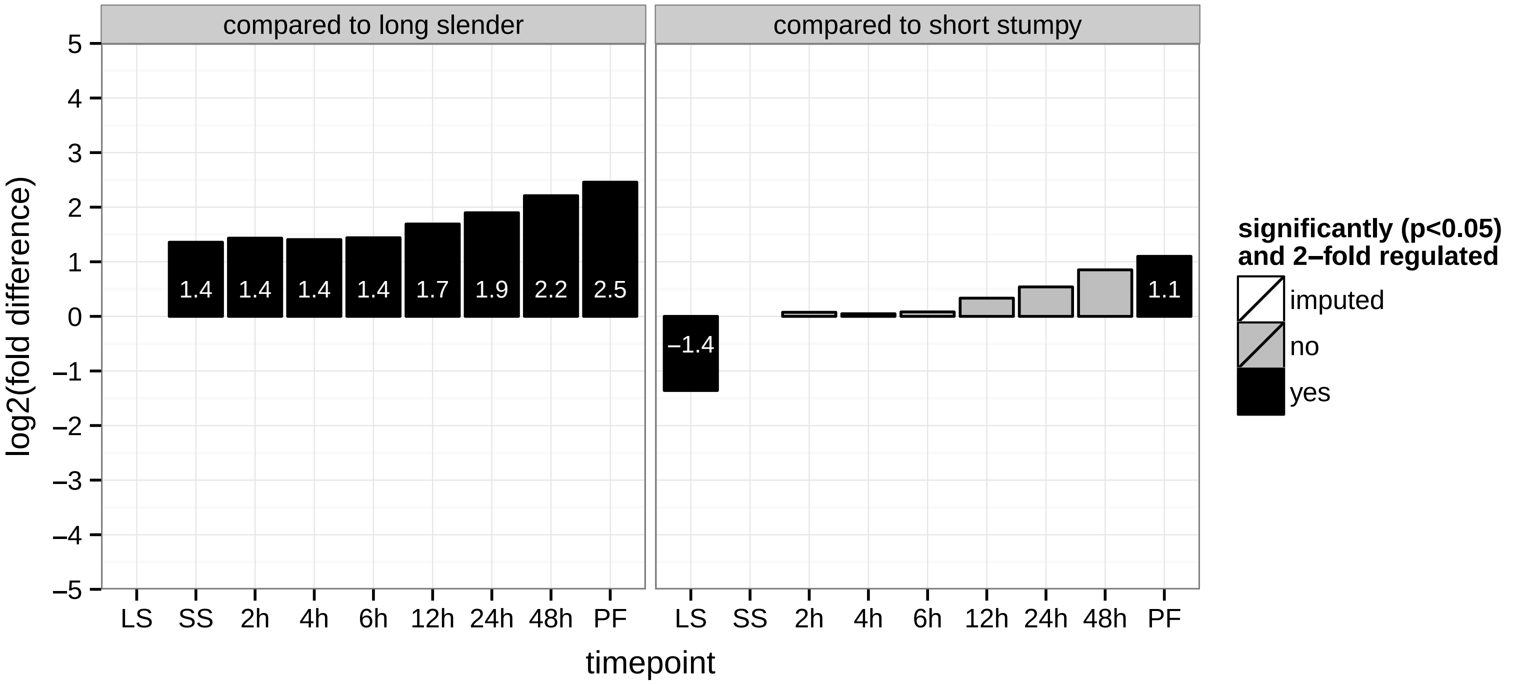

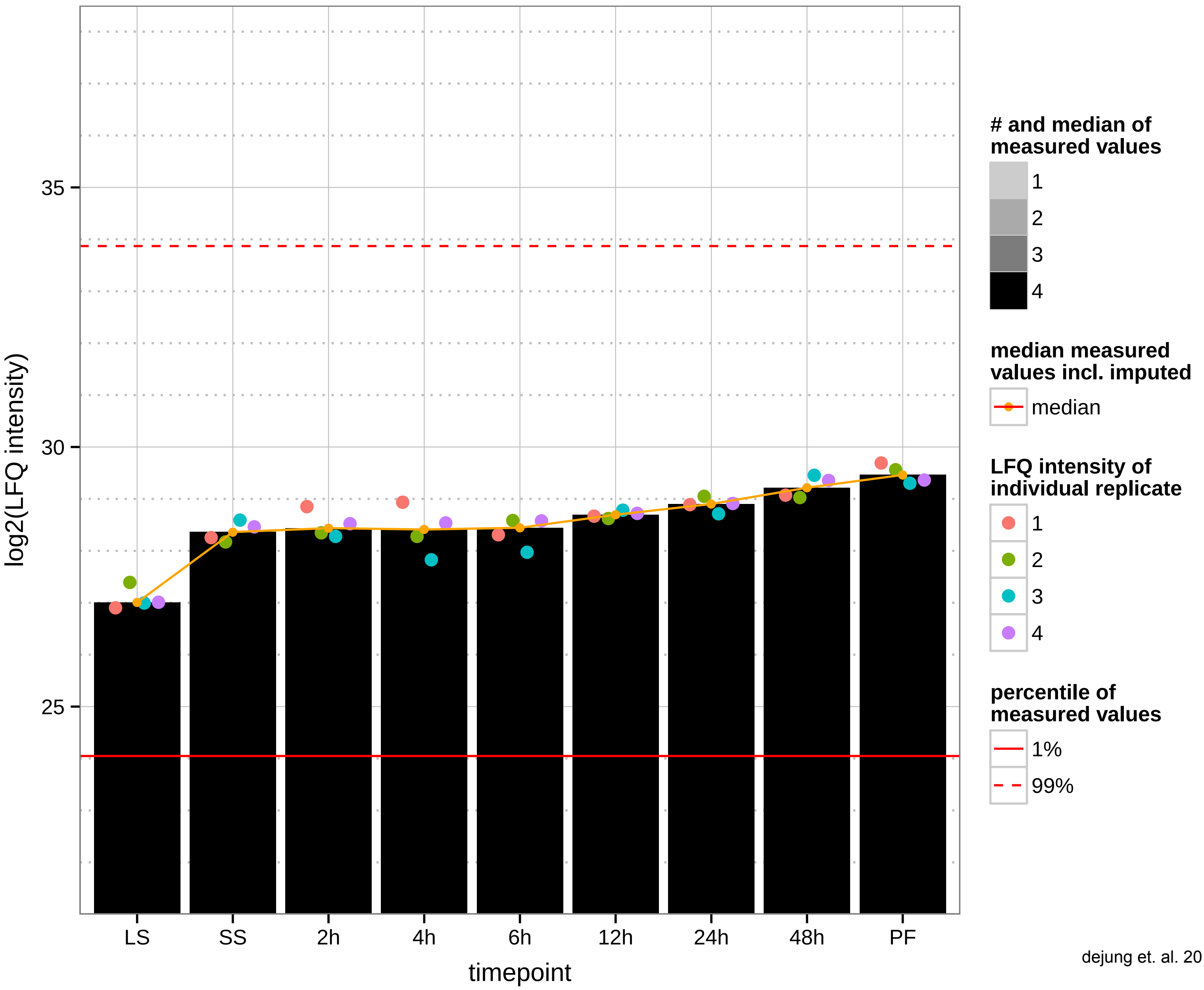

dejung et. al. 2015

hypothetical protein, conserved
Tb927.5.600
AGOF: null
AGOC: null
AGOP: null
PGOF: null
PGOC: null
PGOP: null

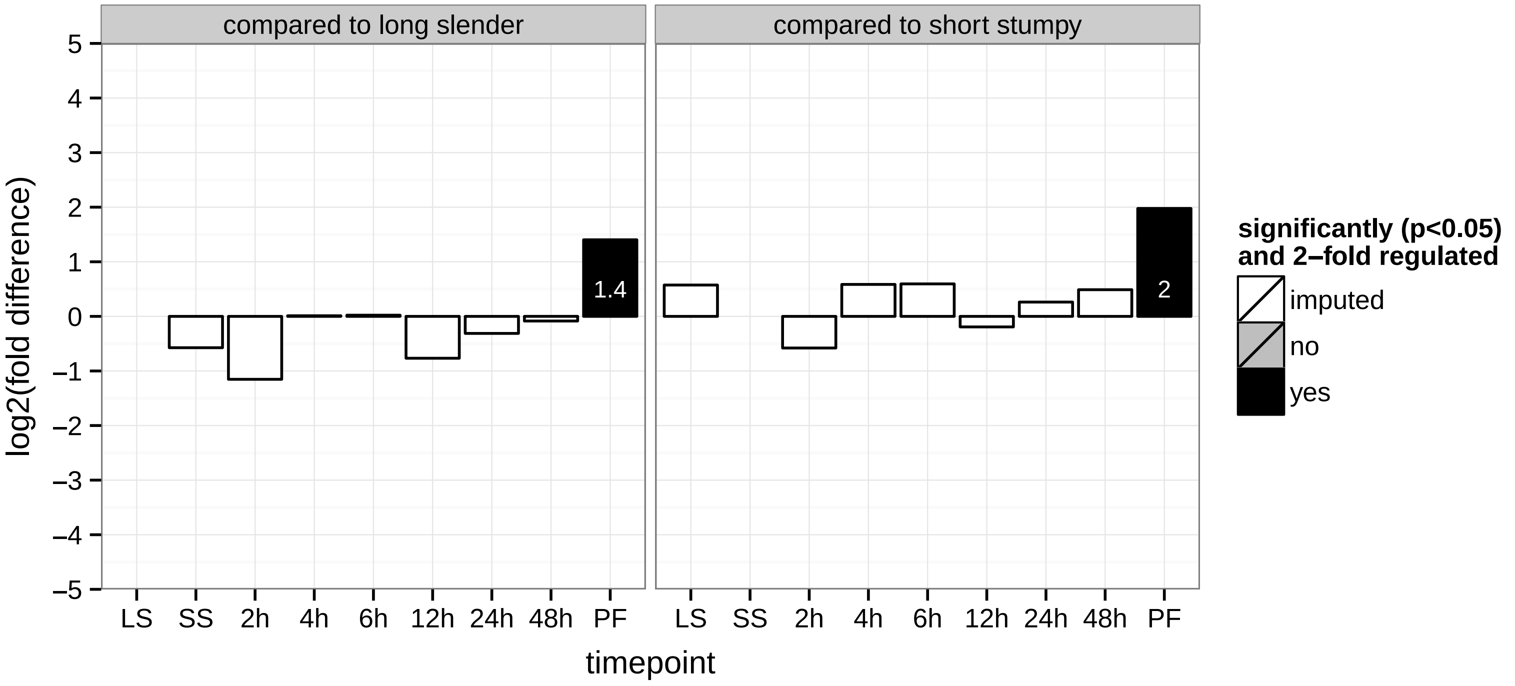

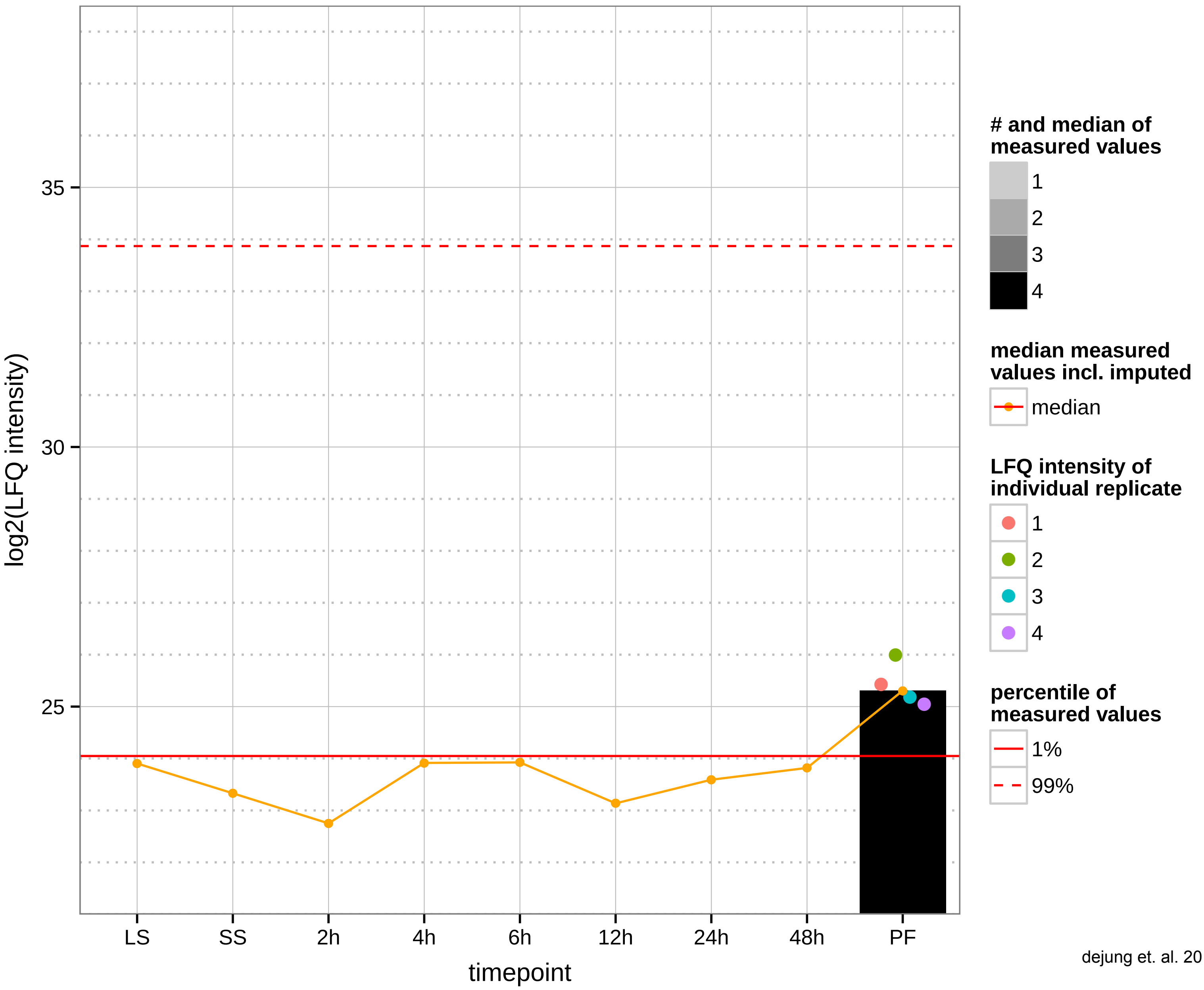

dejung et. al. 2015

hypothetical protein, conserved
Tb927.6.1200
AGOF: null
AGOC: mitochondrion
AGOP: null
PGOF: null
PGOC: null
PGOP: null

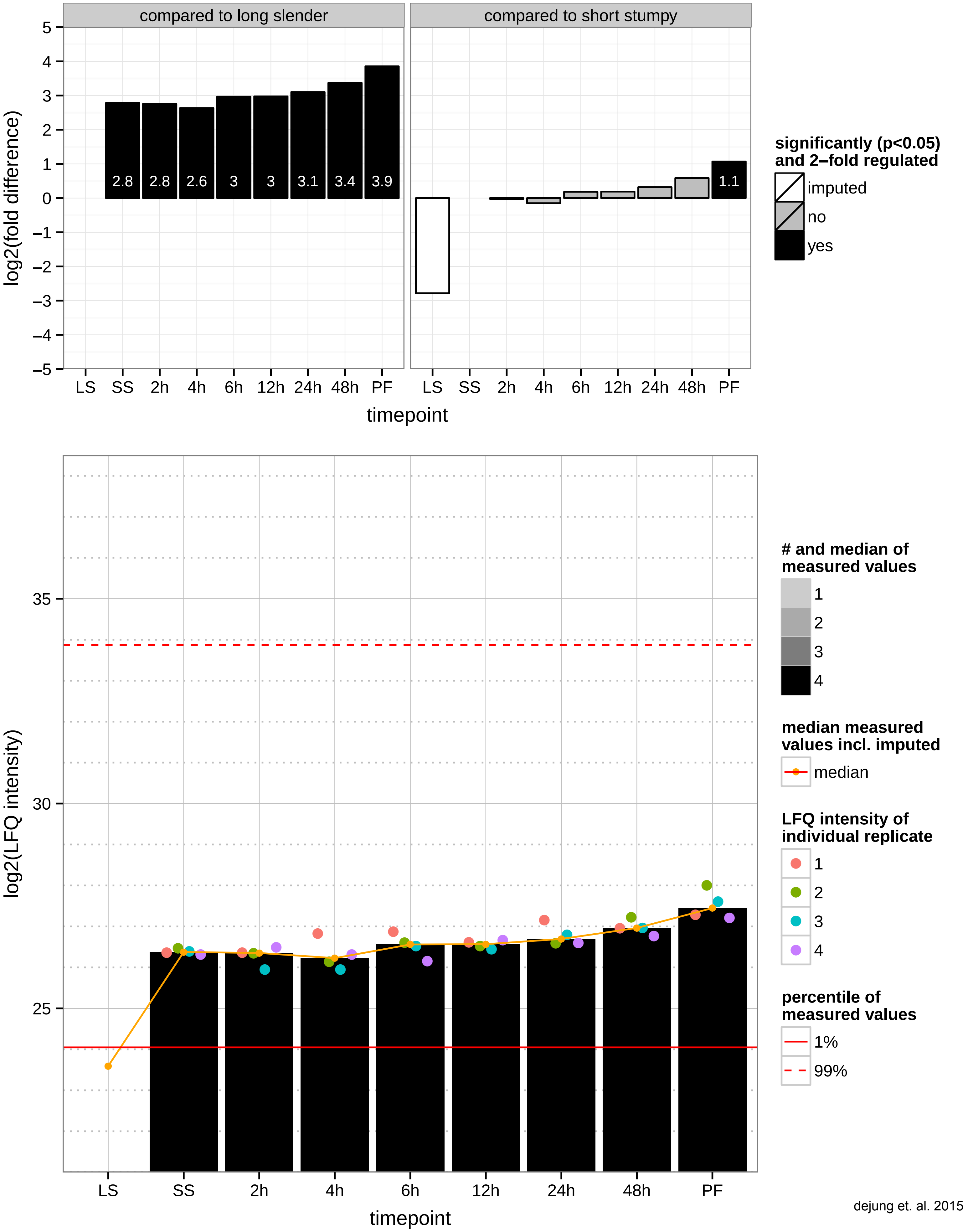

dejung et. al. 2015

hypothetical protein, conserved
Tb927.6.1290
AGOF: null
AGOC: null
AGOP: null
PGOF: binding
PGOC: null
PGOP: null

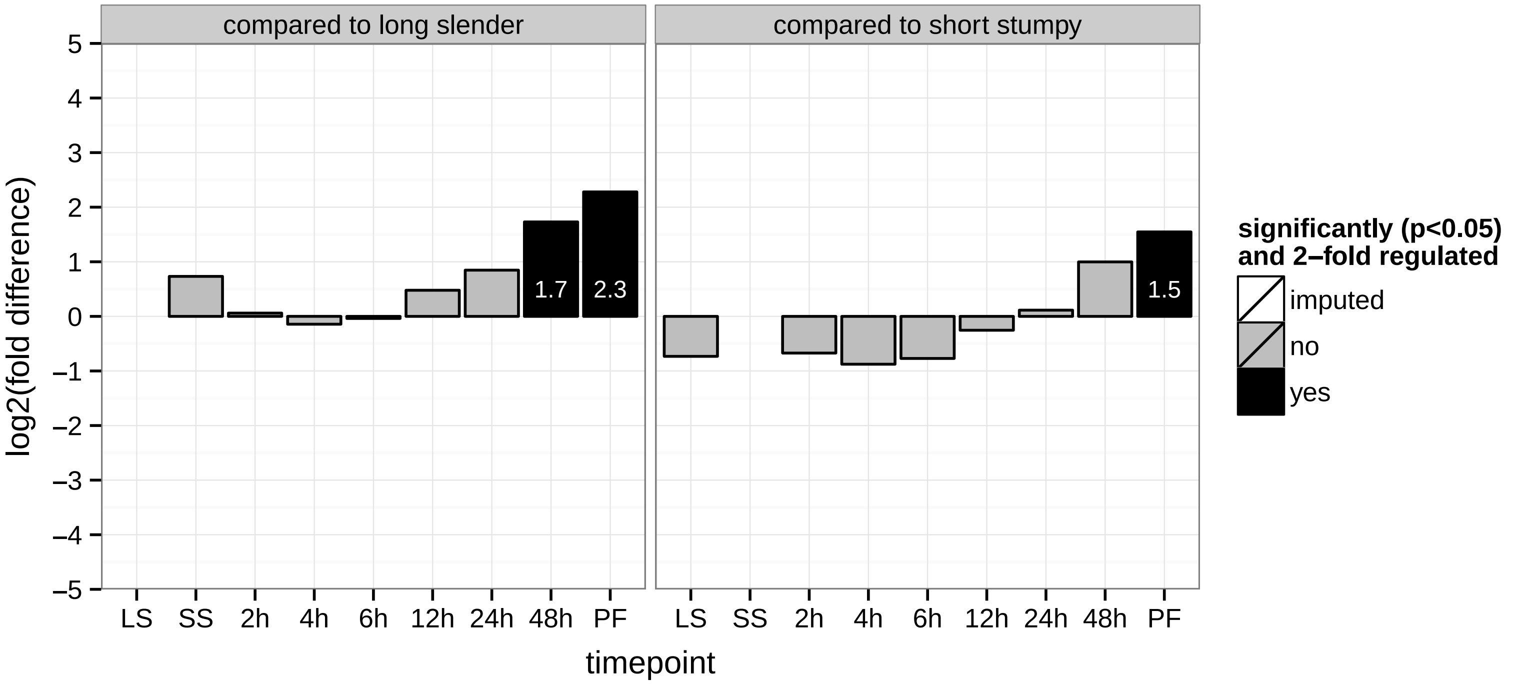

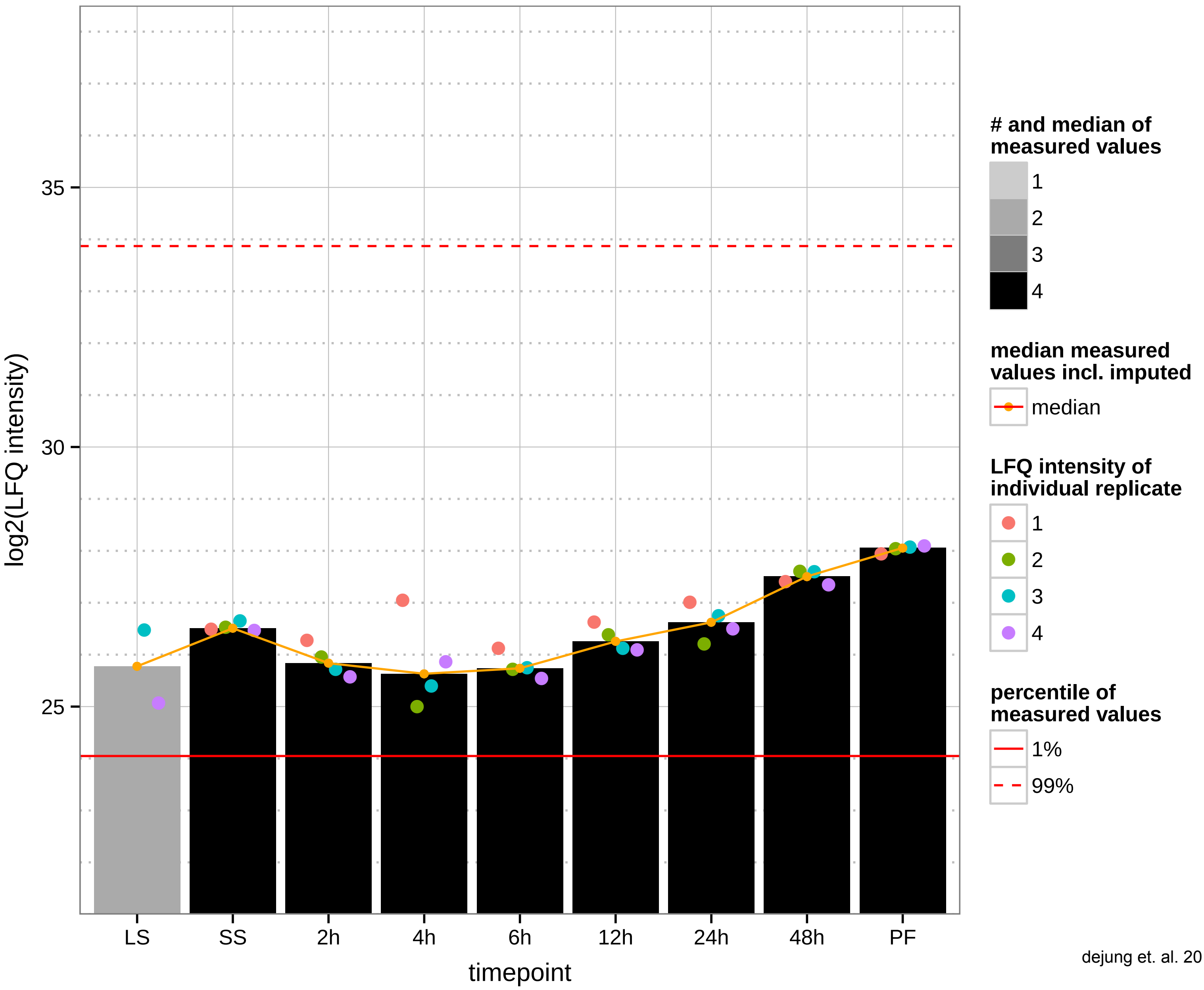

dejung et. al. 2015

cyclophilin–type peptidyl–prolyl cis–trans isomerase, putative (PPIase)
Tb927.6.1400;Tb927.6.1380;Tb927.6.1360;Tb927.6.1320;Tb927.6.1340
AGOF: peptidyl–prolyl cis–trans isomerase activity
AGOC: null
AGOP: protein folding
PGOF: peptidyl–prolyl cis–trans isomerase activity
PGOC: null
PGOP: protein folding

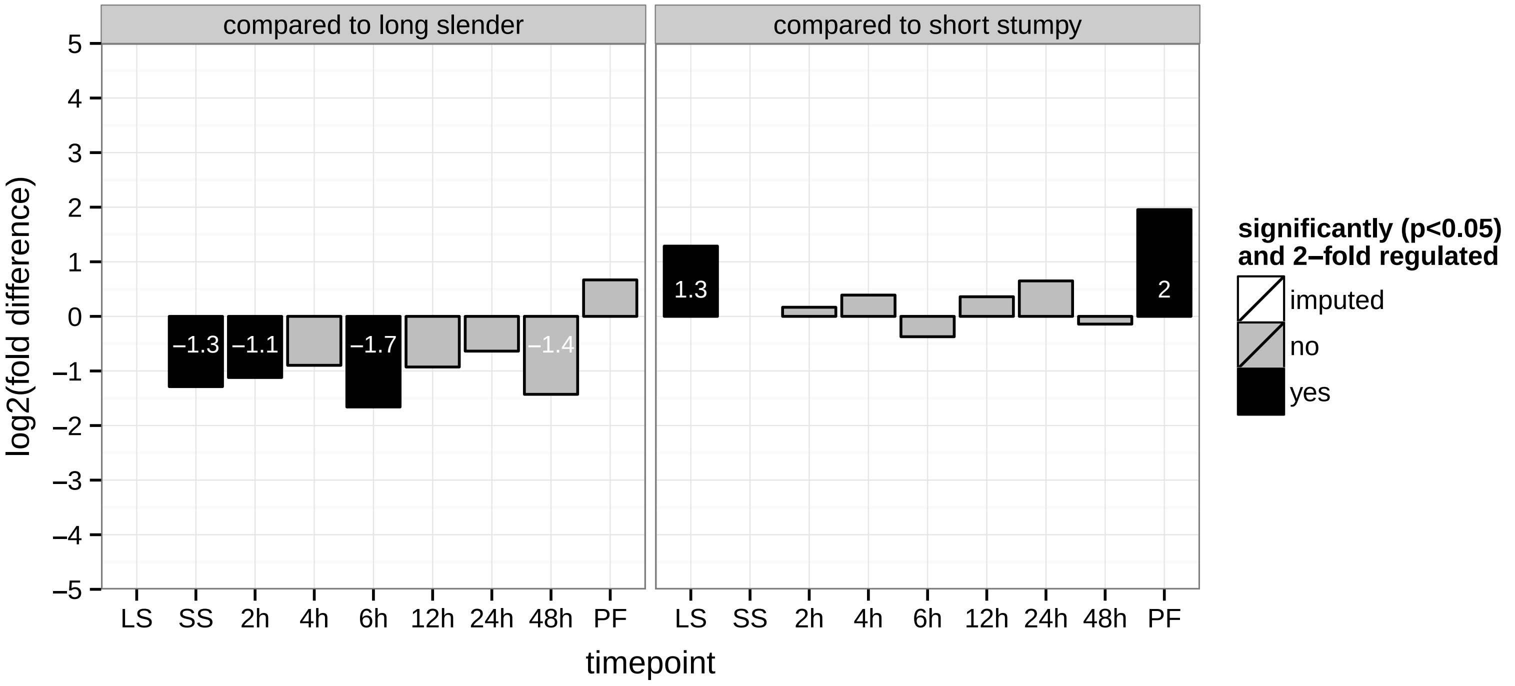

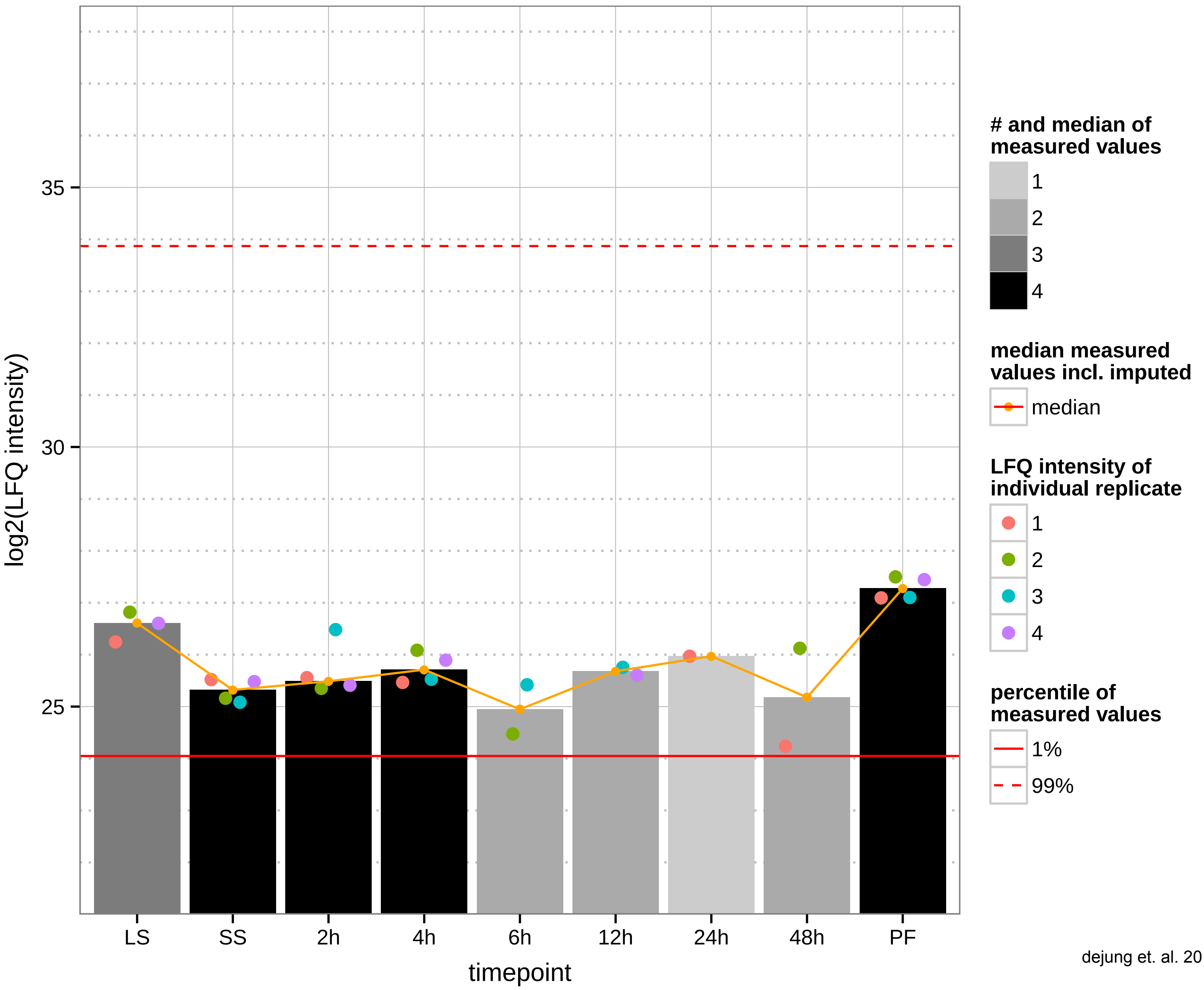

dejung et. al. 2015

alkyl–dihydroxyacetone phosphate synthase, alkylglycerone–phosphate synthase, alkyl–DHAP synthase (DHAP)
Tb927.6.1500
AGOF: alkylglycerone–phosphate synthase activity, flavin adenine dinucleotide binding, oxidoreductase activity
AGOC: glycosome
AGOP: ether lipid biosynthetic process
PGOF: UDP–N–acetylmuramate dehydrogenase activity, catalytic activity, flavin adenine dinucleotide binding, oxidoreductase
PGOC: null
PGOP: oxidation–reduction process

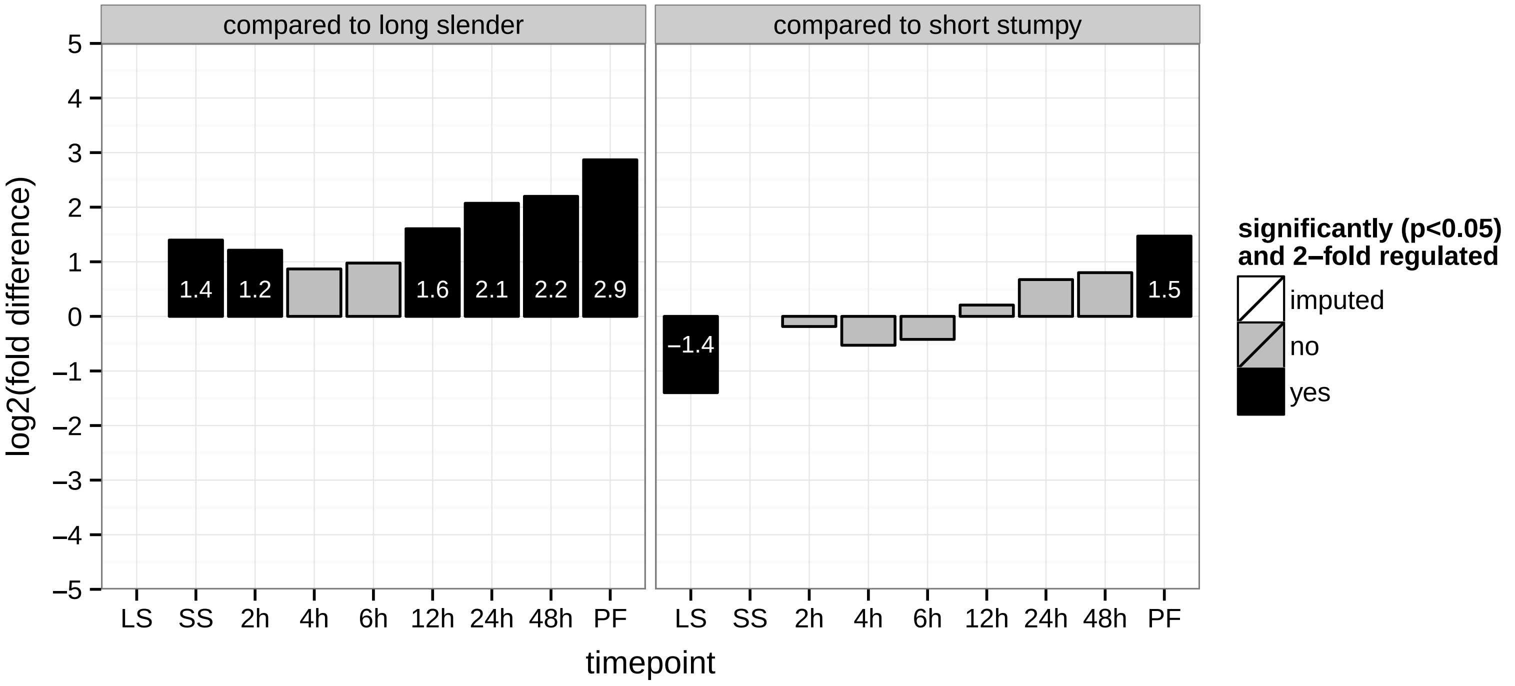

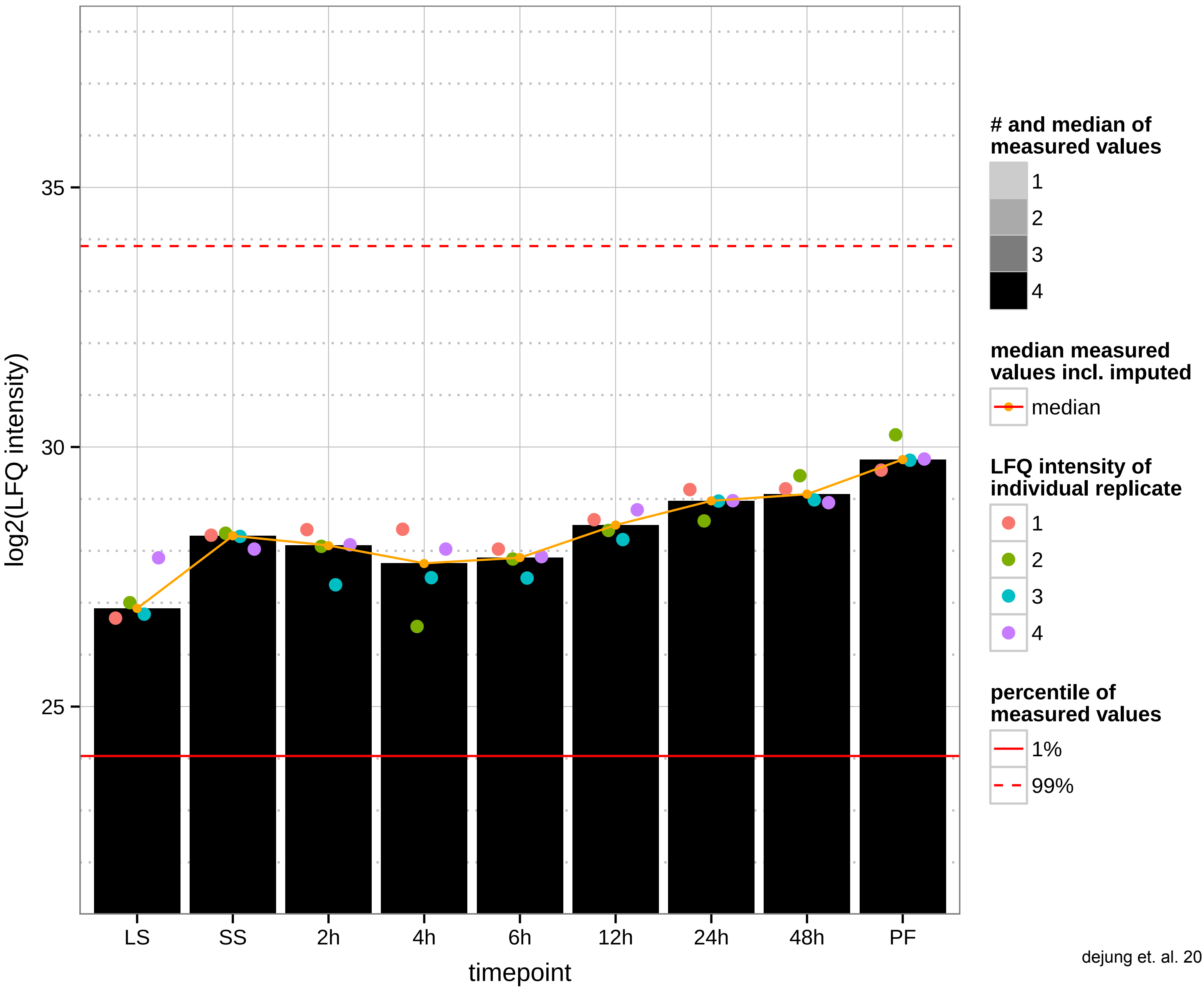

dejung et. al. 2015

hypothetical protein, conserved
Tb927.6.1700
AGOF: zinc ion binding
AGOC: null
AGOP: null
PGOF: zinc ion binding
PGOC: null
PGOP: null

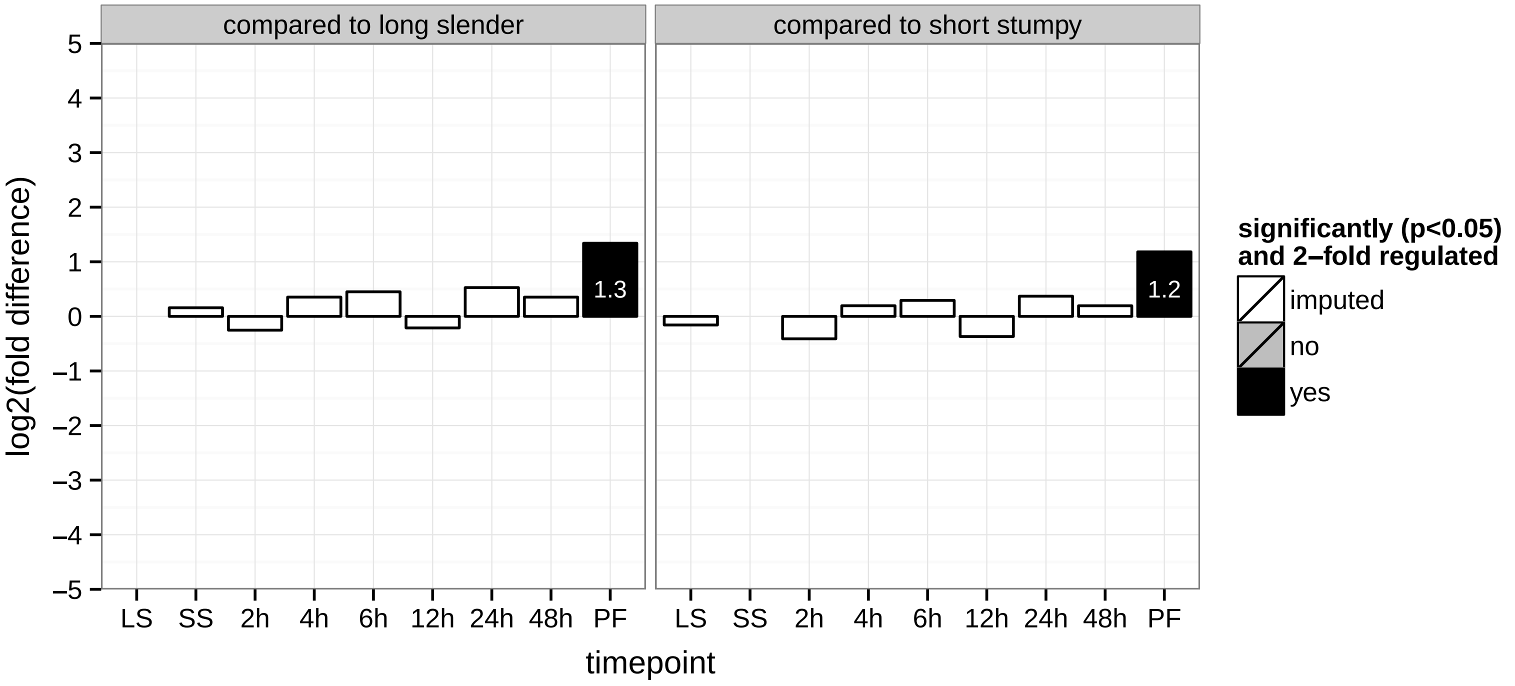

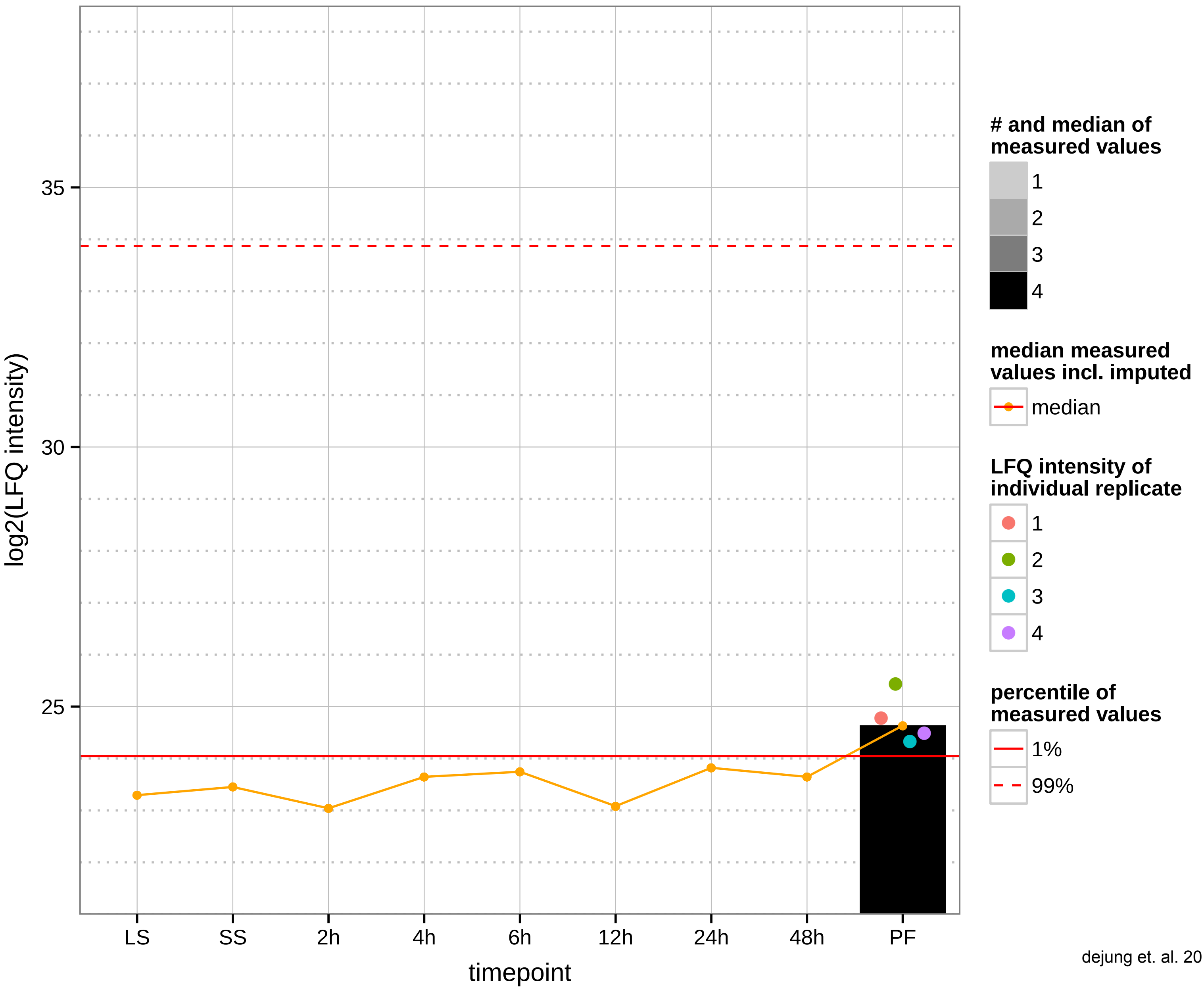

dejung et. al. 2015

hypothetical protein, conserved
Tb927.6.1810
AGOF: null
AGOC: null
AGOP: null
PGOF: null
PGOC: null
PGOP: null

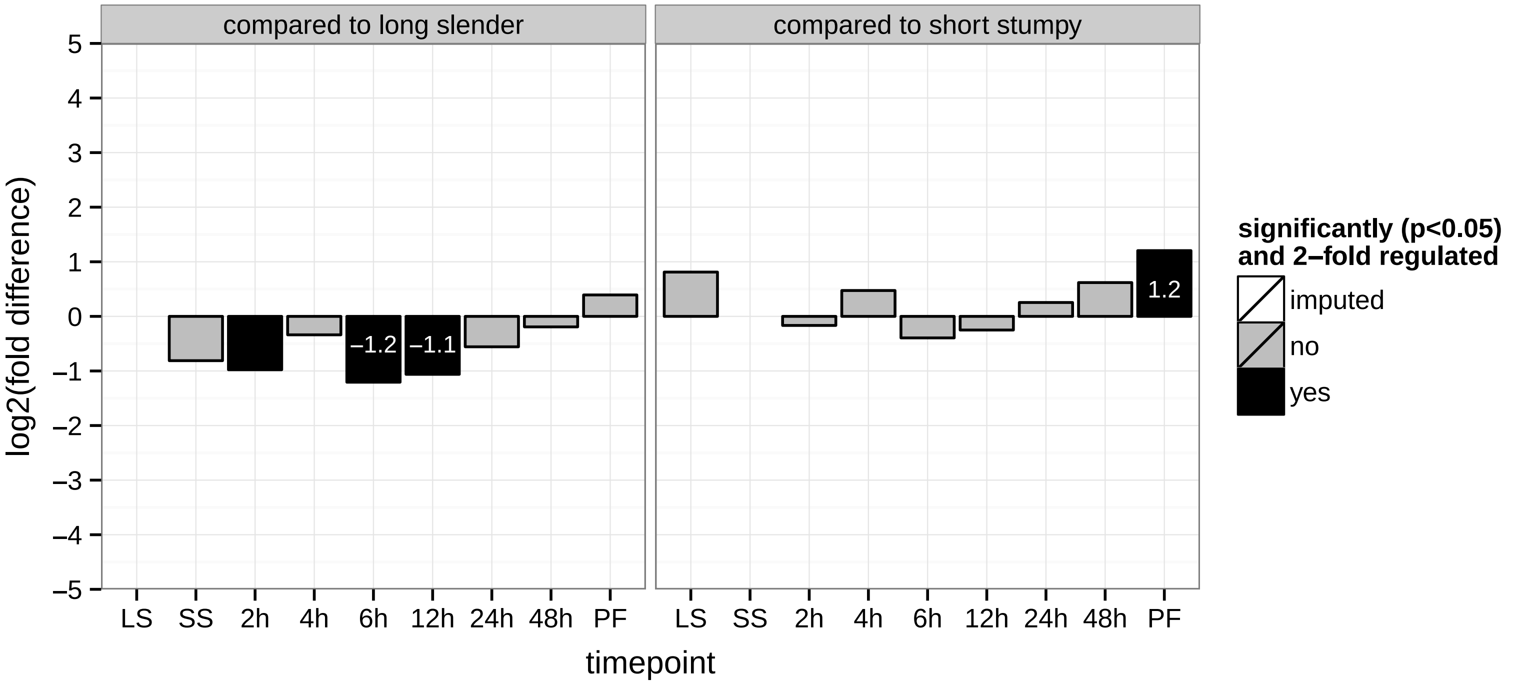

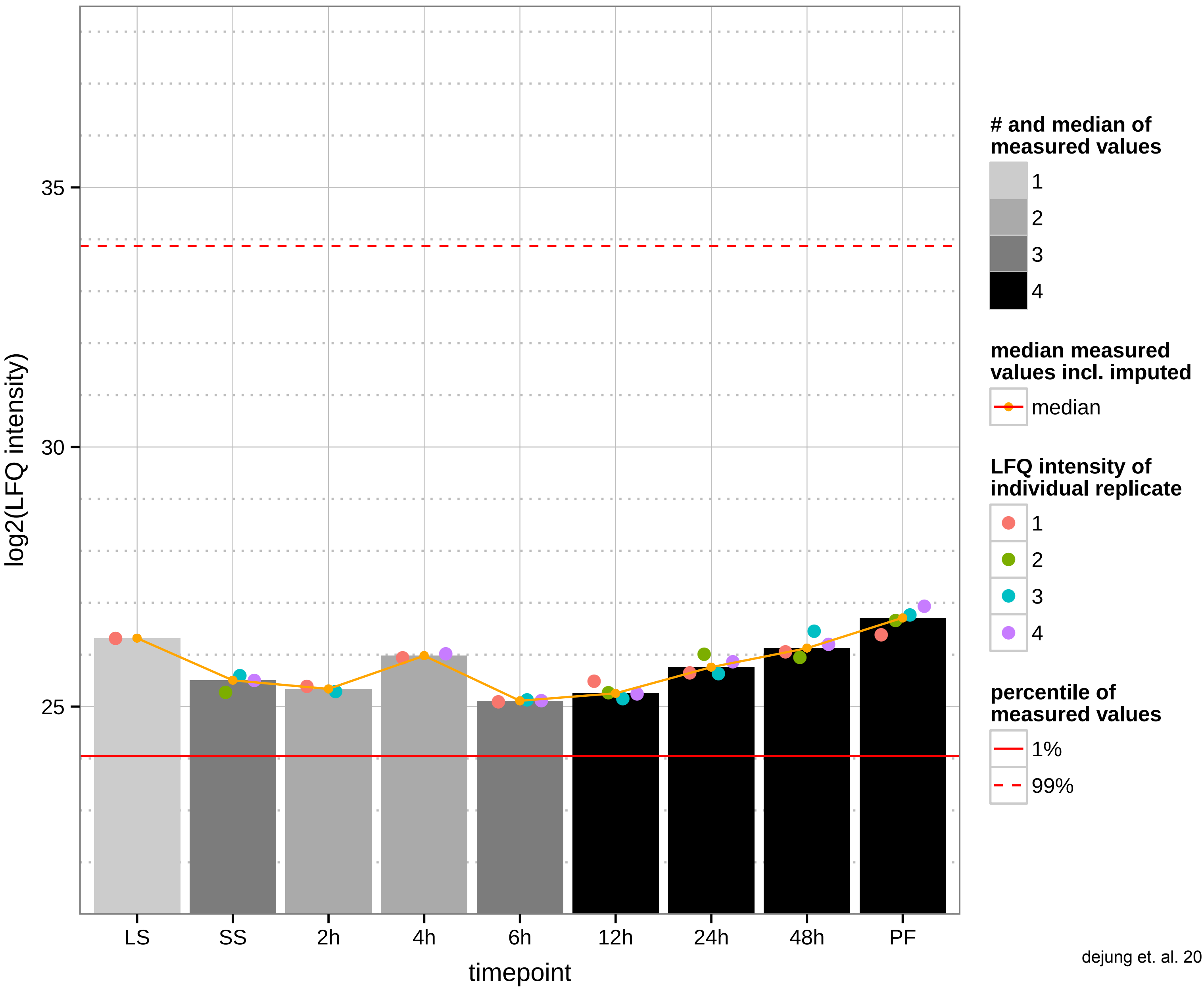

dejung et. al. 2015

quiescin sulfhydryl oxidase (QSOX)
Tb927.6.1850
AGOF: flavin–linked sulfhydryl oxidase activity, thiol oxidase activity
AGOC: null
AGOP: cell redox homeostasis, oxidation–reduction process, protein folding
PGOF: thiol oxidase activity
PGOC: null
PGOP: cell redox homeostasis, oxidation–reduction process

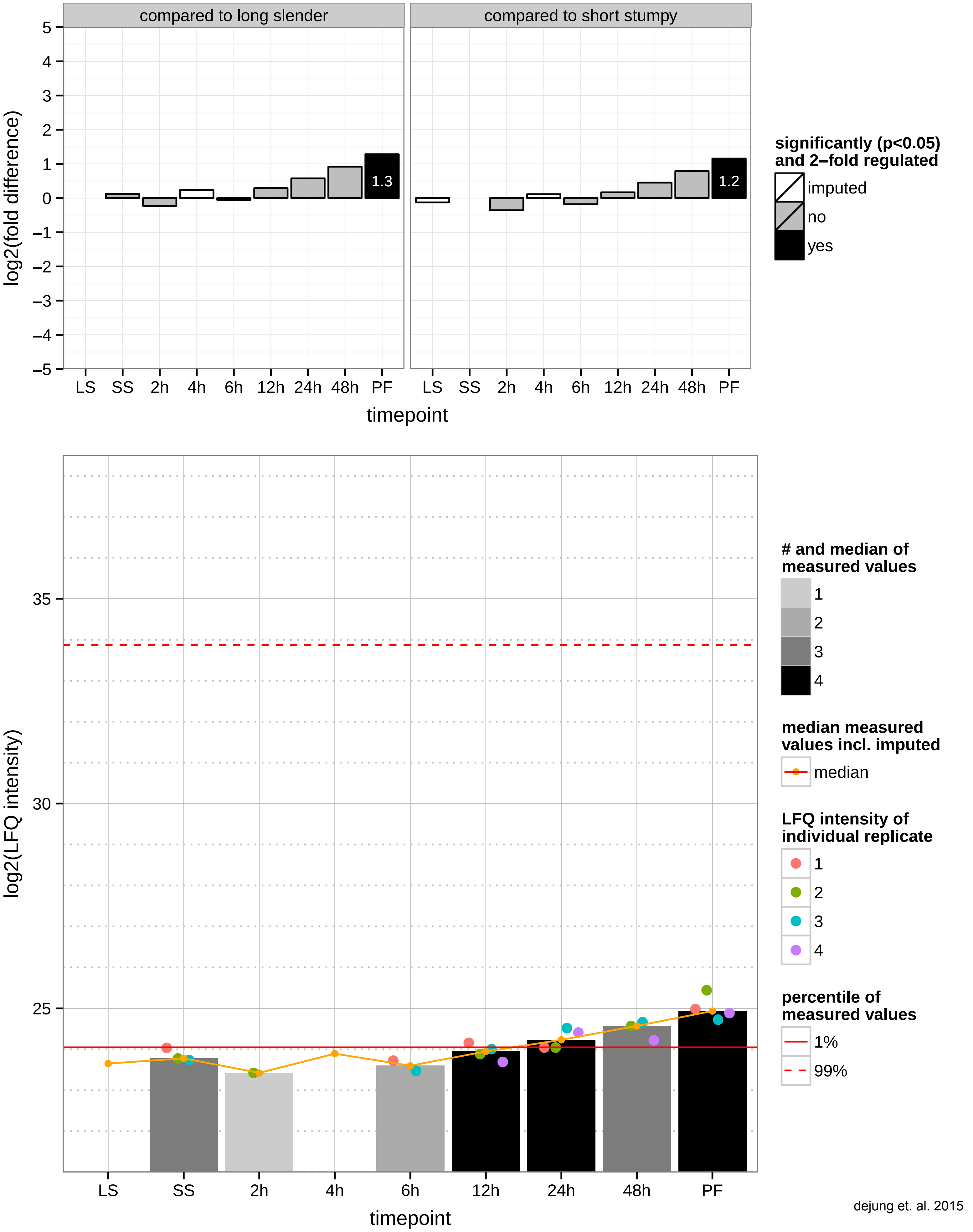

dejung et. al. 2015

eukaryotic translation initiation factor 4e, putative
Tb927.6.1870
AGOF: RNA cap binding, translation initiation factor activity
AGOC: cytoplasm
AGOP: translational initiation
PGOF: RNA binding, translation initiation factor activity
PGOC: cytoplasm
PGOP: translational initiation

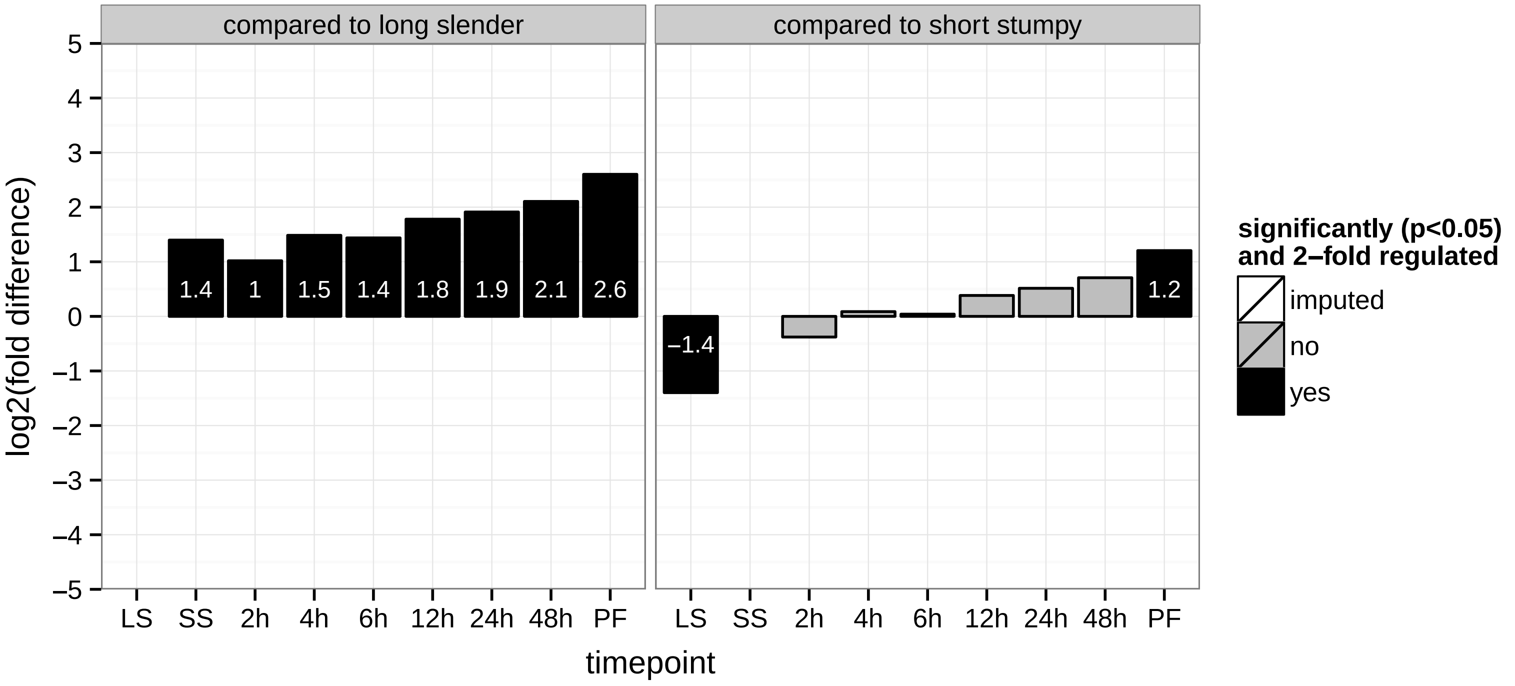

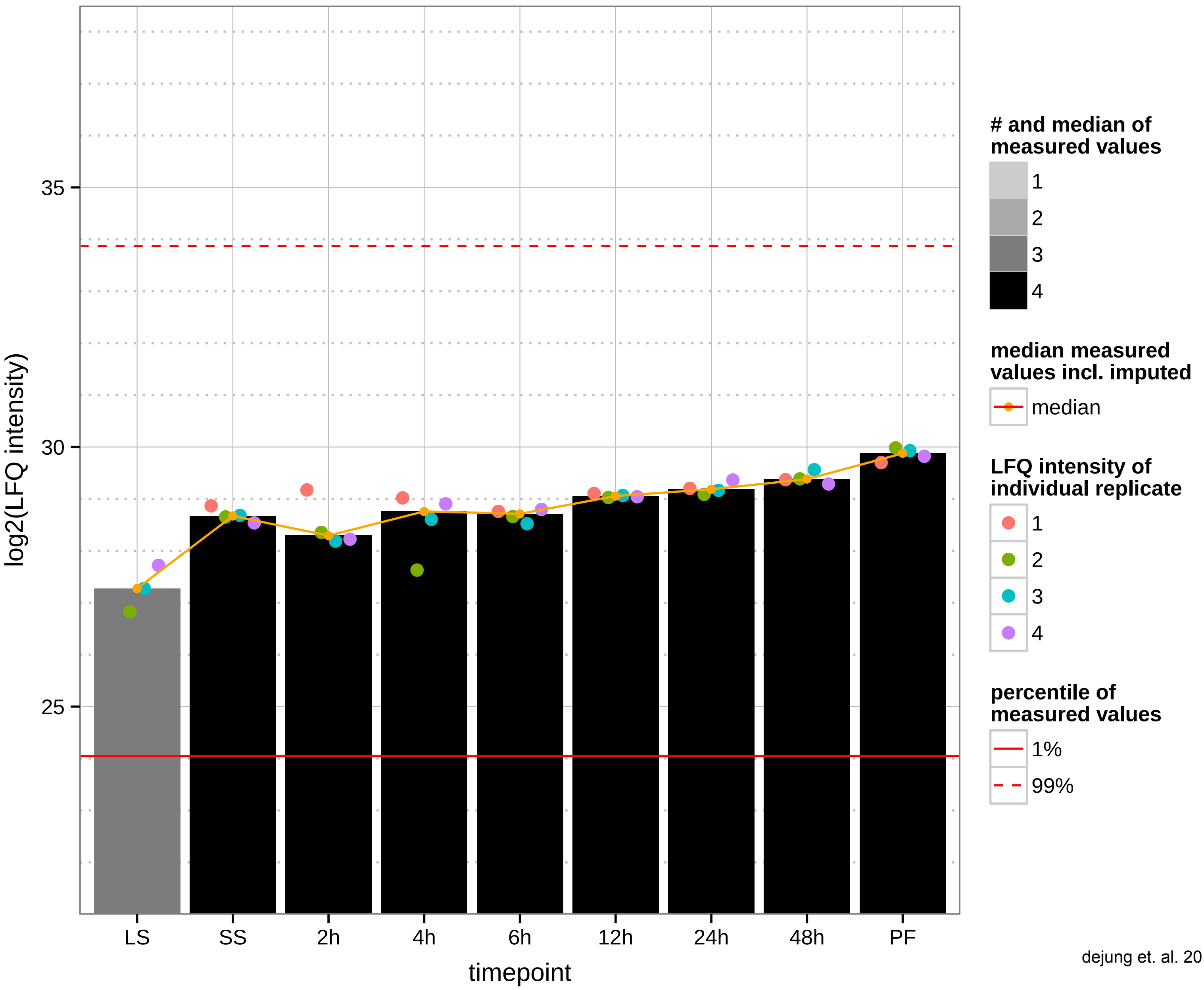

dejung et. al. 2015

hypothetical protein, conserved
Tb927.6.2140
AGOF: catalytic activity
AGOC: mitochondrion
AGOP: metabolic process
PGOF: catalytic activity
PGOC: null
PGOP: metabolic process

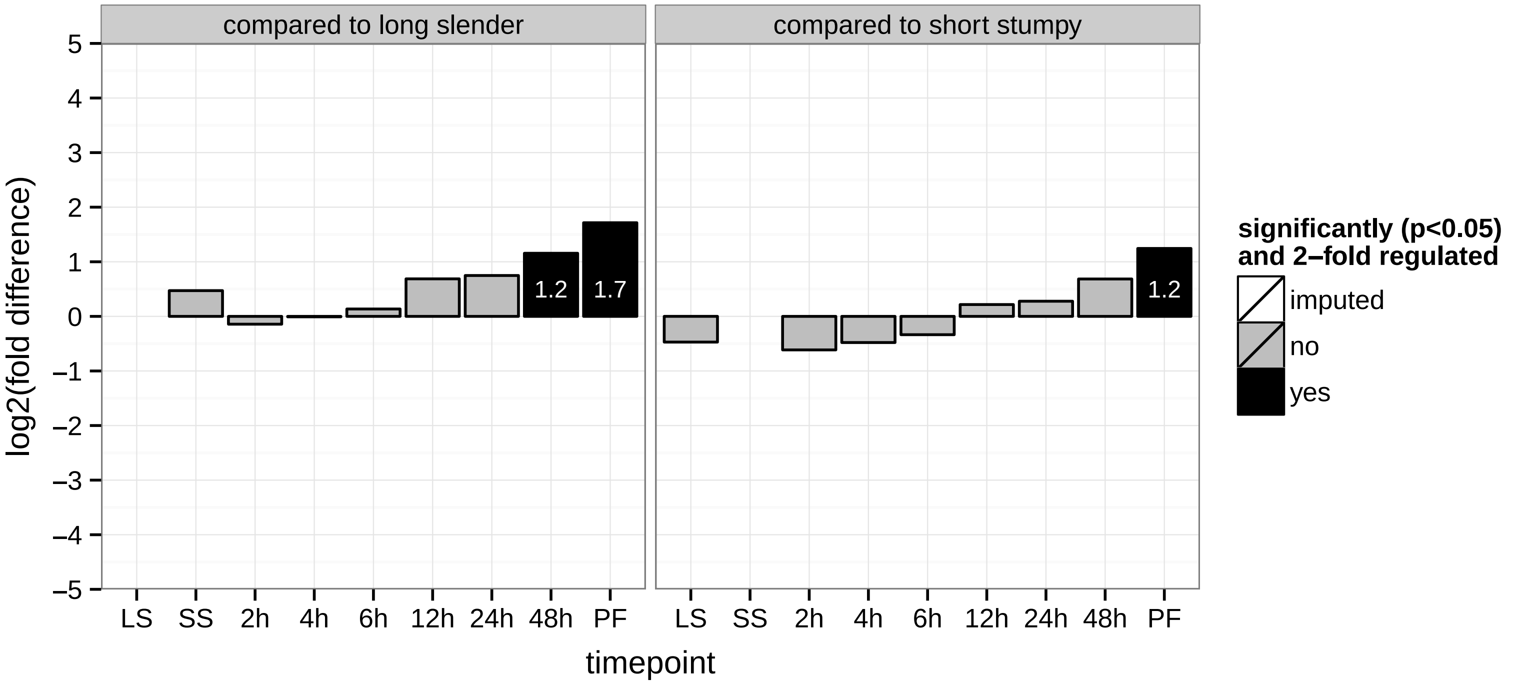

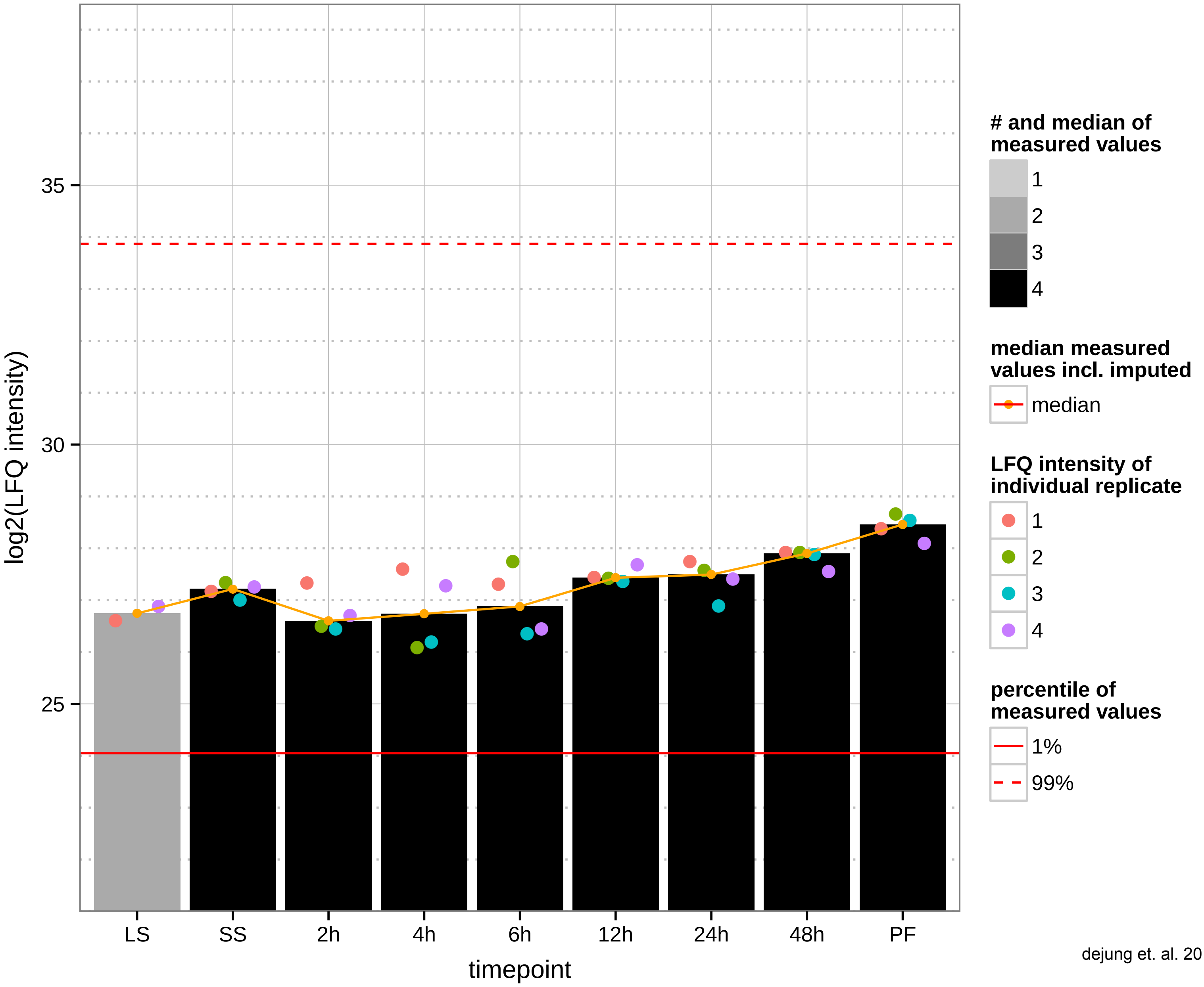

dejung et. al. 2015

hypothetical protein, conserved, RGG protein (RGG1)
Tb927.6.2330;Tb927.6.2270
AGOF: methyltransferase activity, protein binding
AGOC: null
AGOP: null
PGOF: methyltransferase activity
PGOC: null
PGOP: null

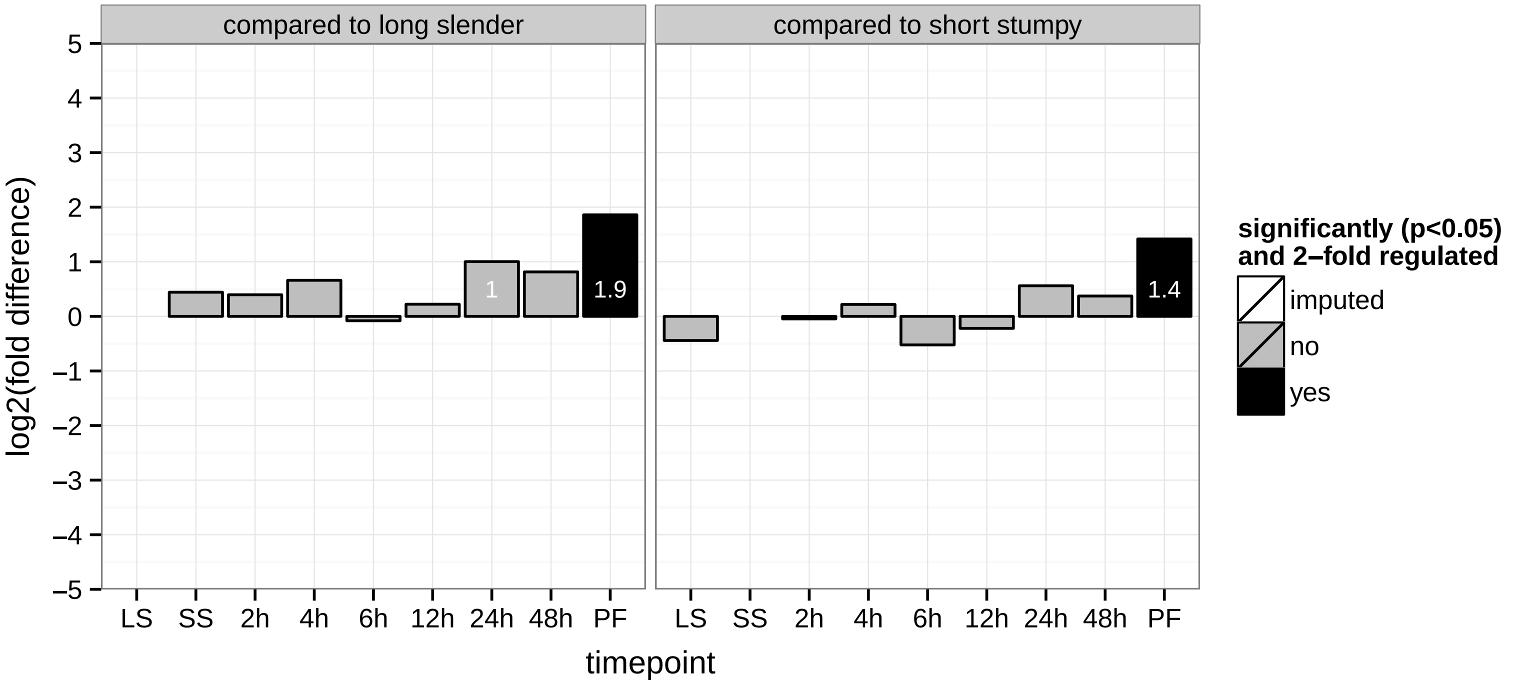

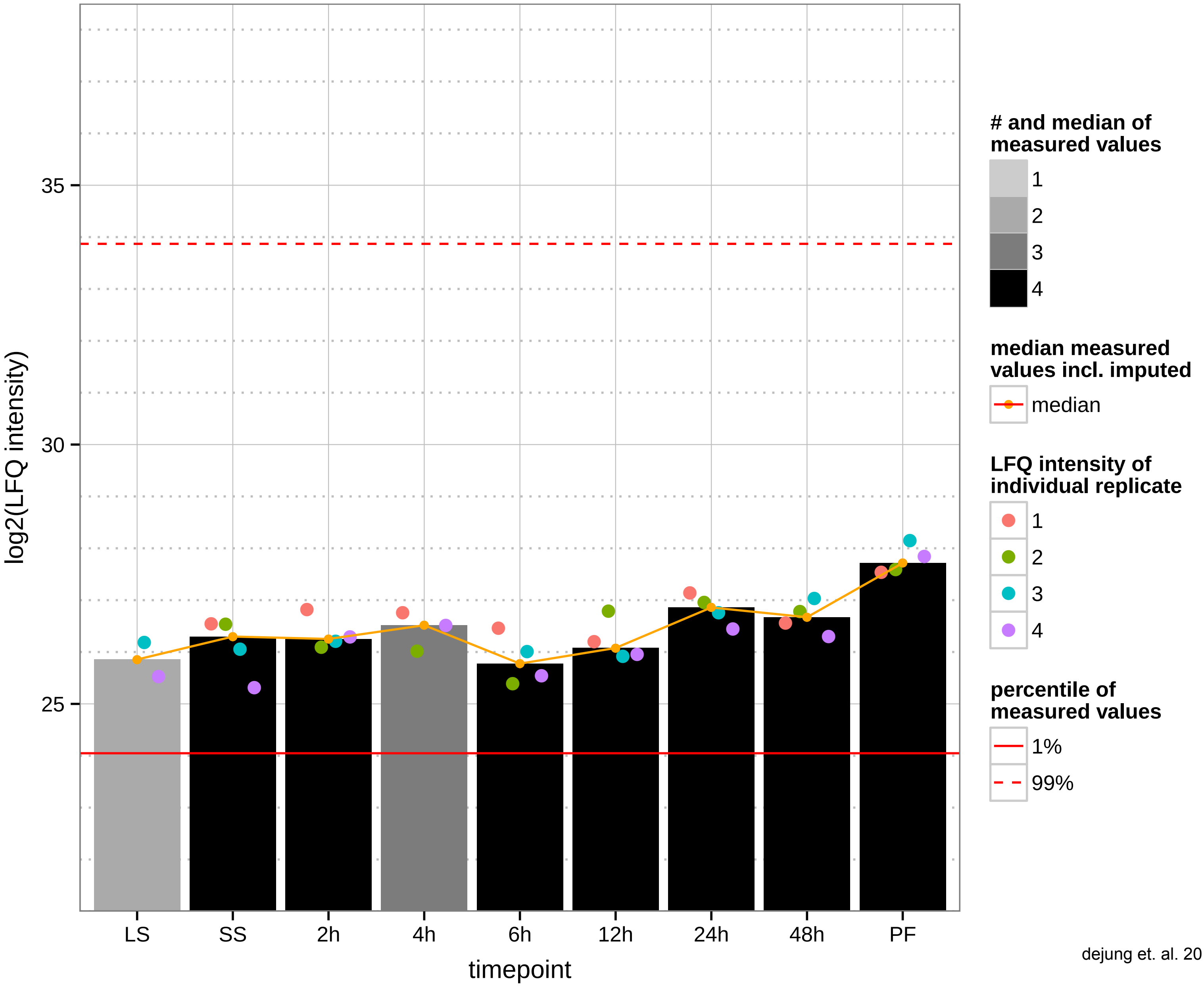

dejung et. al. 2015

DREV methyltransferase, putative
Tb927.6.2540
AGOF: null
AGOC: mitochondrion
AGOP: null
PGOF: null
PGOC: null
PGOP: null

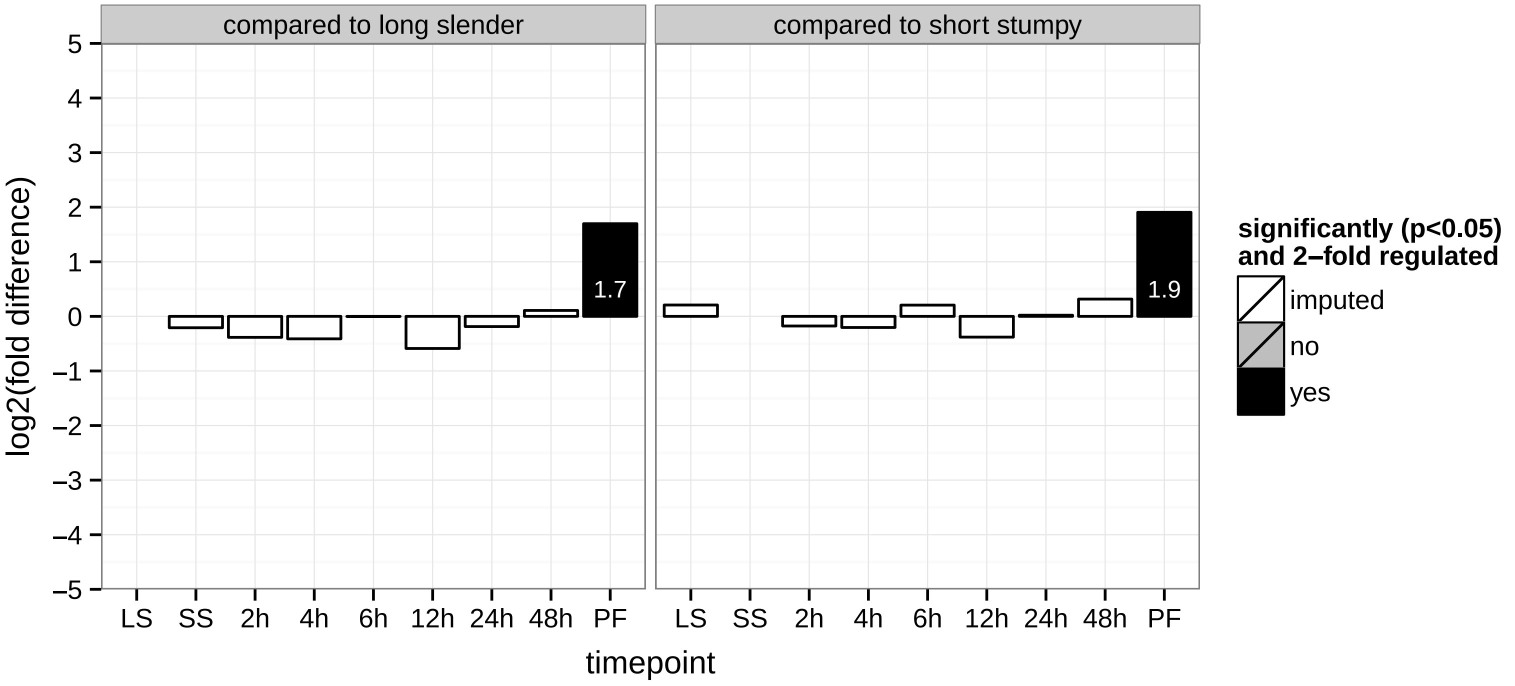

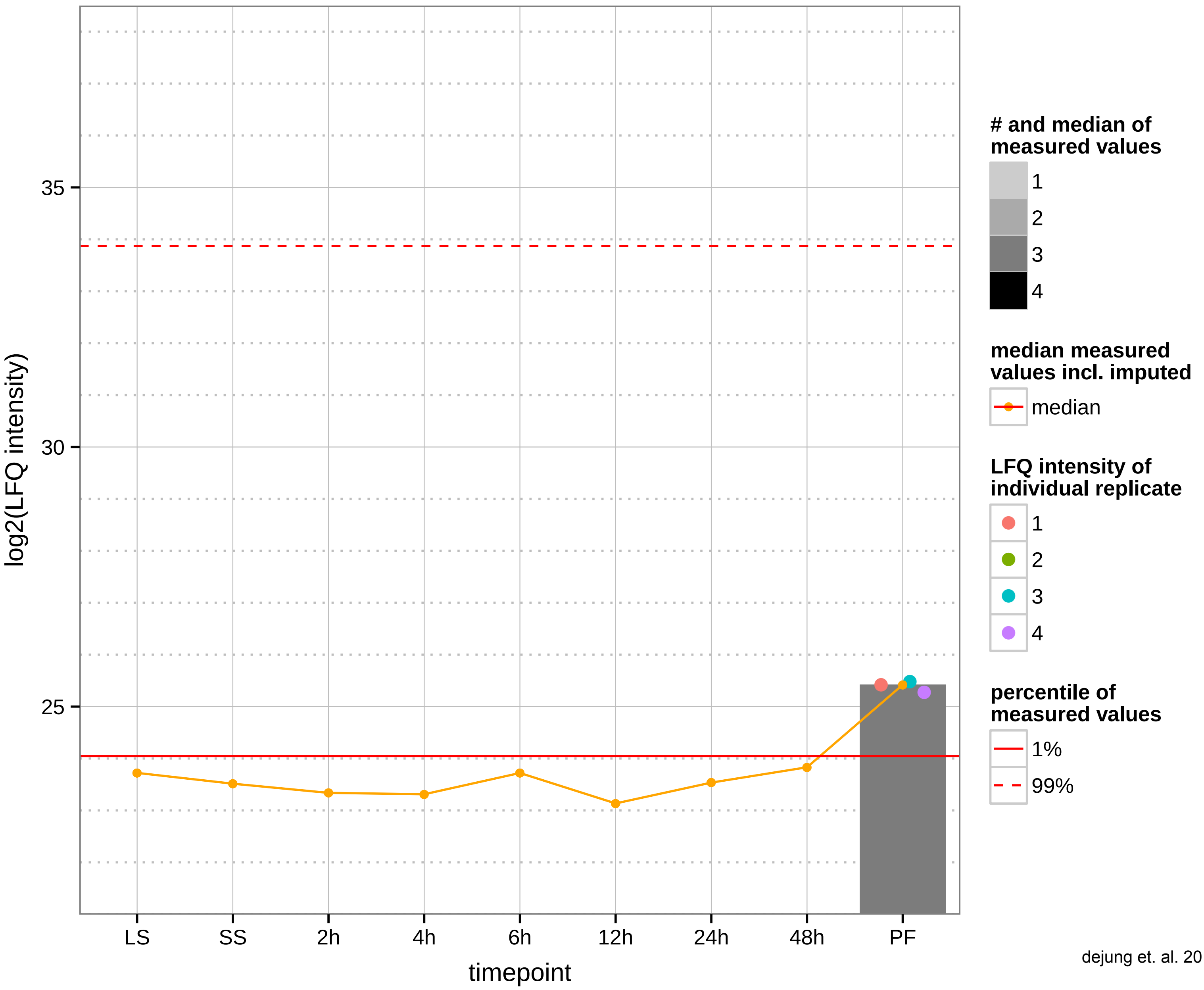

dejung et. al. 2015

dendritic cell−derived IFNG−induced protein, SAM domain and HD domain−containing protein 1
Tb927.6.2900
AGOF: catalytic activity
AGOC: null
AGOP: null
PGOF: catalytic activity, metal ion binding, phosphoric diester hydrolase activity
PGOC: null
PGOP: null

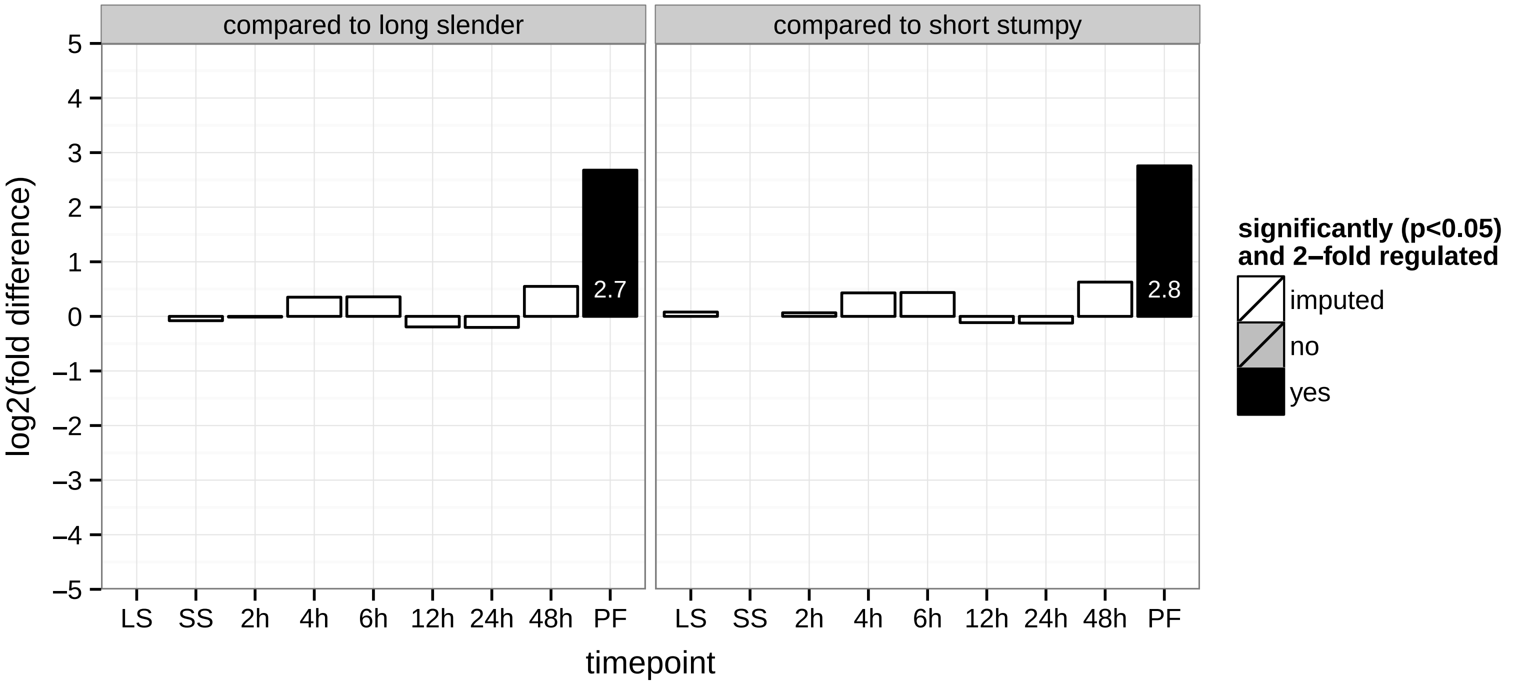

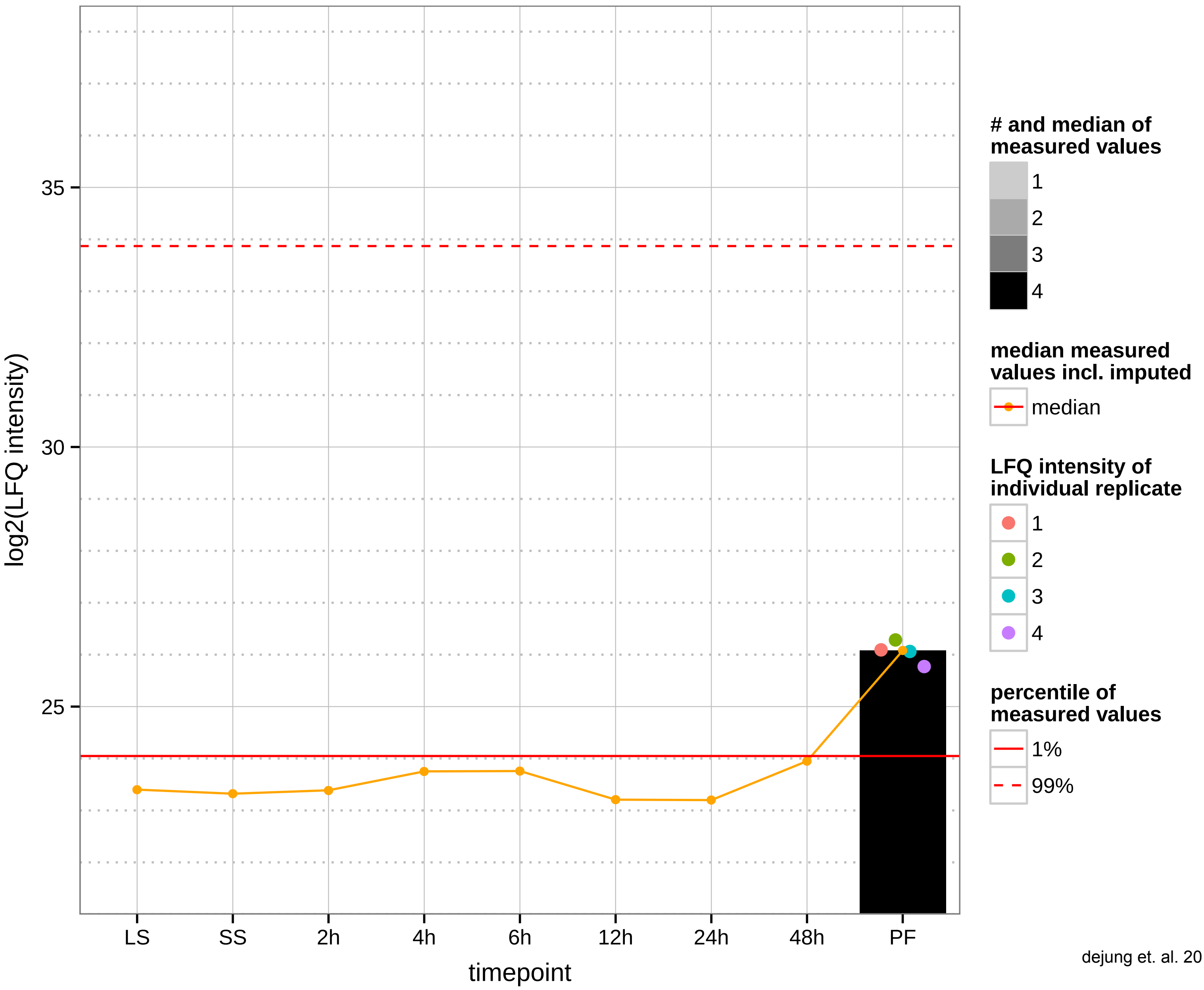

dejung et. al. 2015

sphingosine 1−phosphate lyase, putative, sphingosine phosphate lyase−like protein, putative (SPL)
Tb927.6.3630
AGOF: carboxy−lyase activity, pyridoxal phosphate binding, sphinganine−1−phosphate aldolase activity
AGOC: null
AGOP: carboxylic acid metabolic process, ethanolamine biosynthetic process
PGOF: carboxy−lyase activity, pyridoxal phosphate binding
PGOC: null
PGOP: carboxylic acid metabolic process

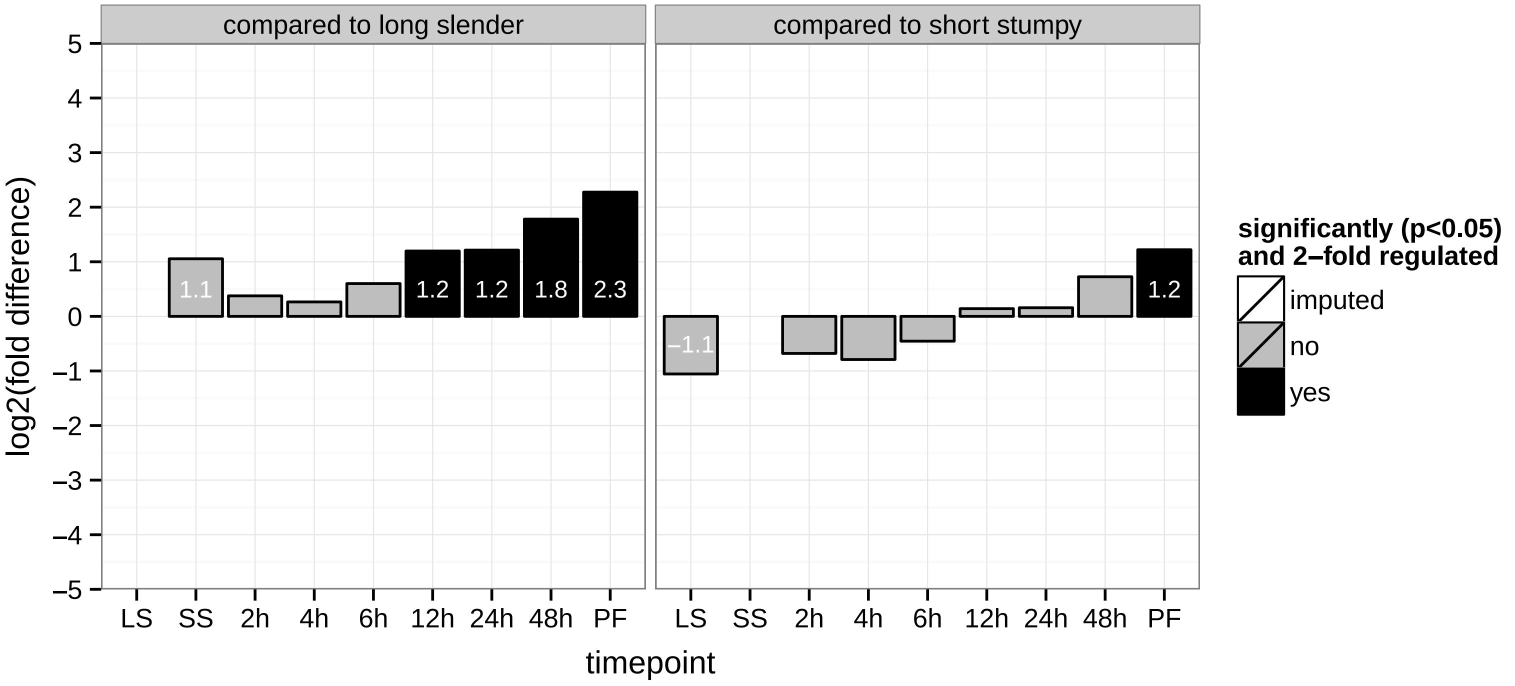

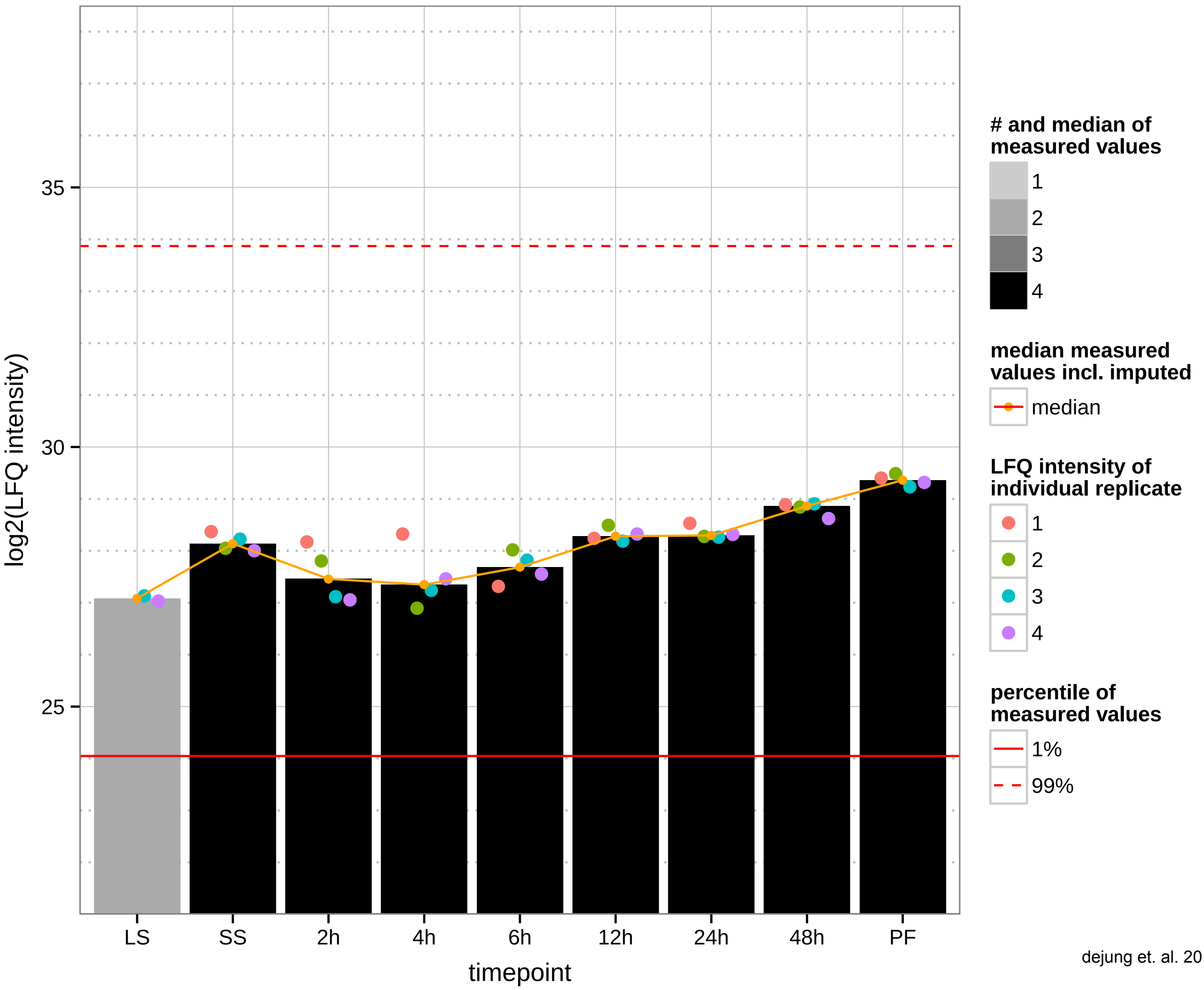

dejung et. al. 2015

hypothetical protein, conserved
Tb927.6.4360
AGOF: null
AGOC: null
AGOP: null
PGOF: null
PGOC: null
PGOP: null

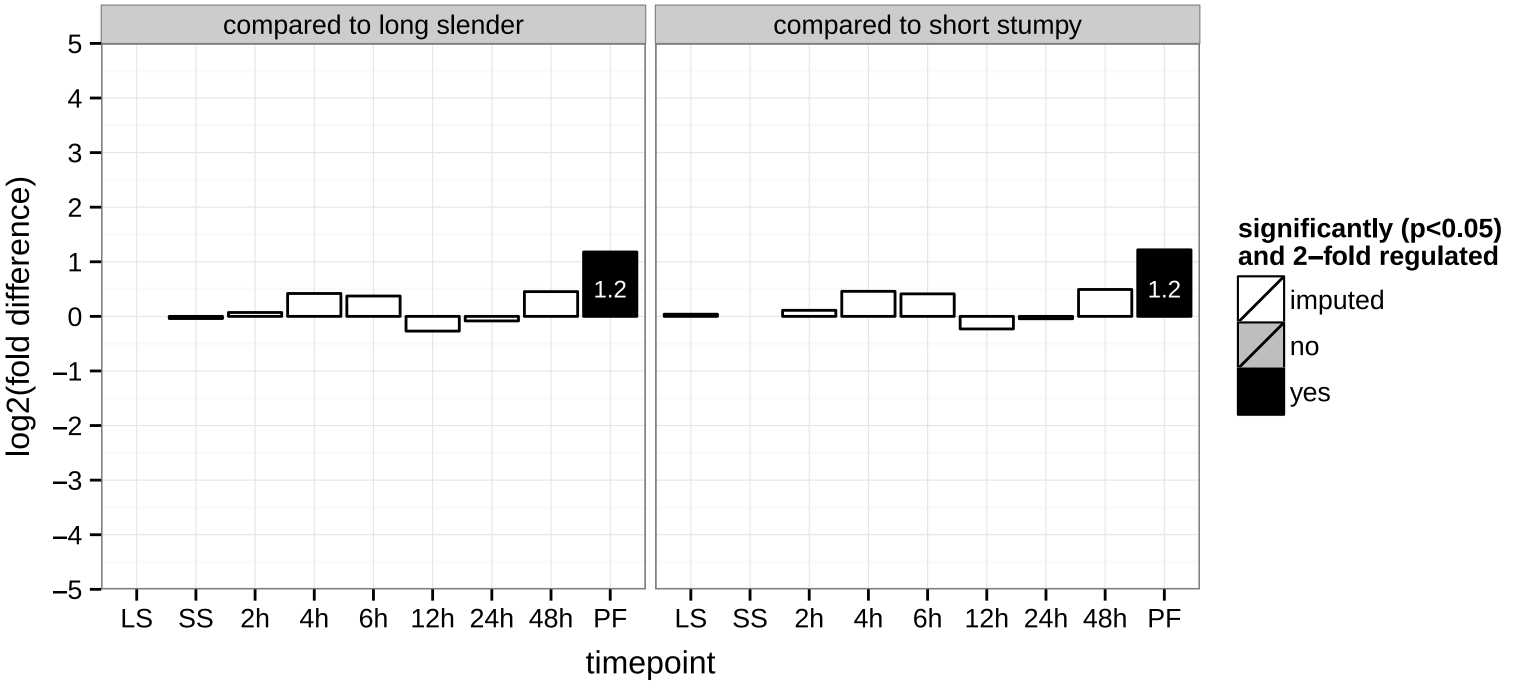

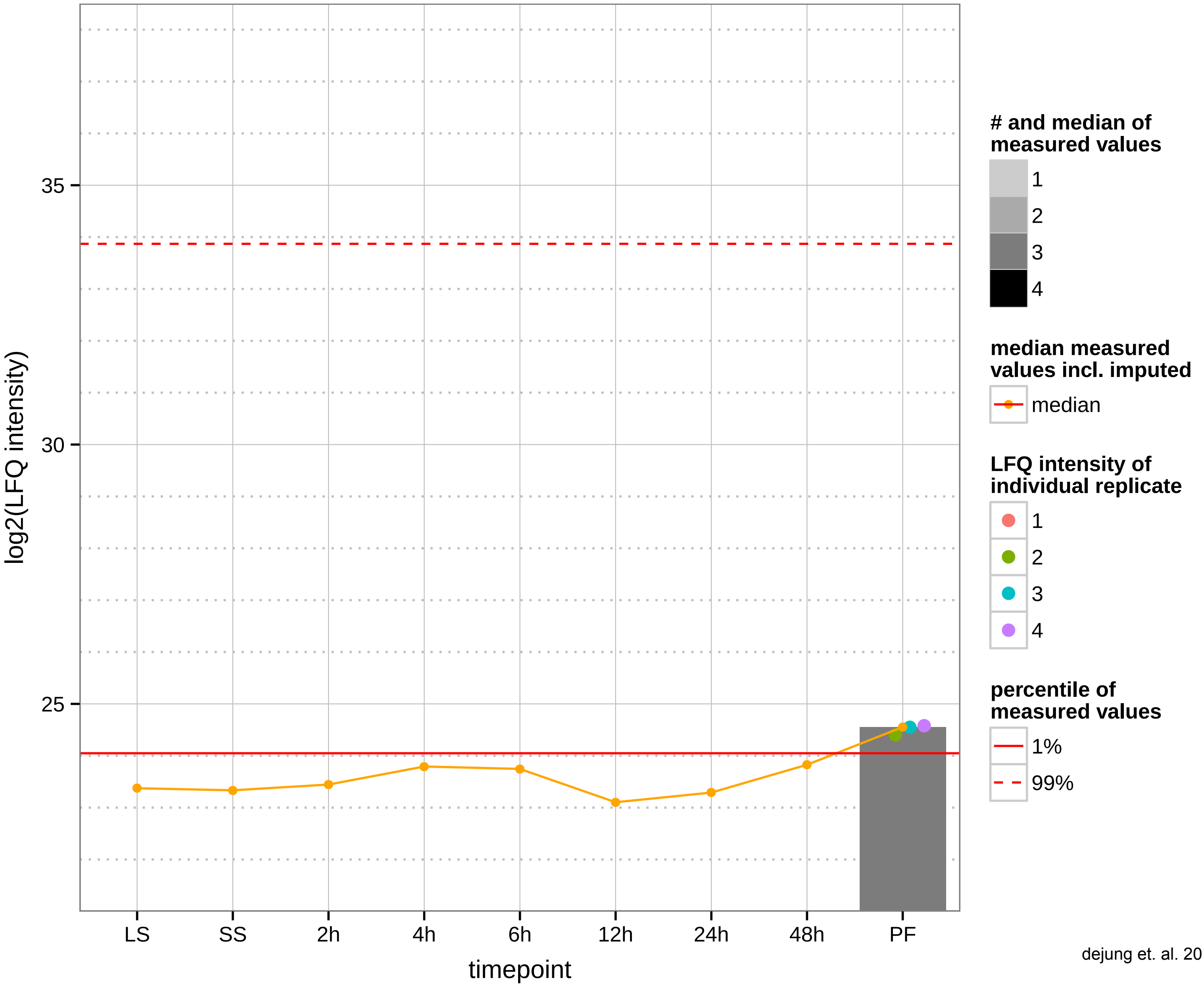

dejung et. al. 2015

hypothetical protein, conserved
Tb927.6.4380
AGOF: null
AGOC: null
AGOP: null
PGOF: null
PGOC: null
PGOP: null

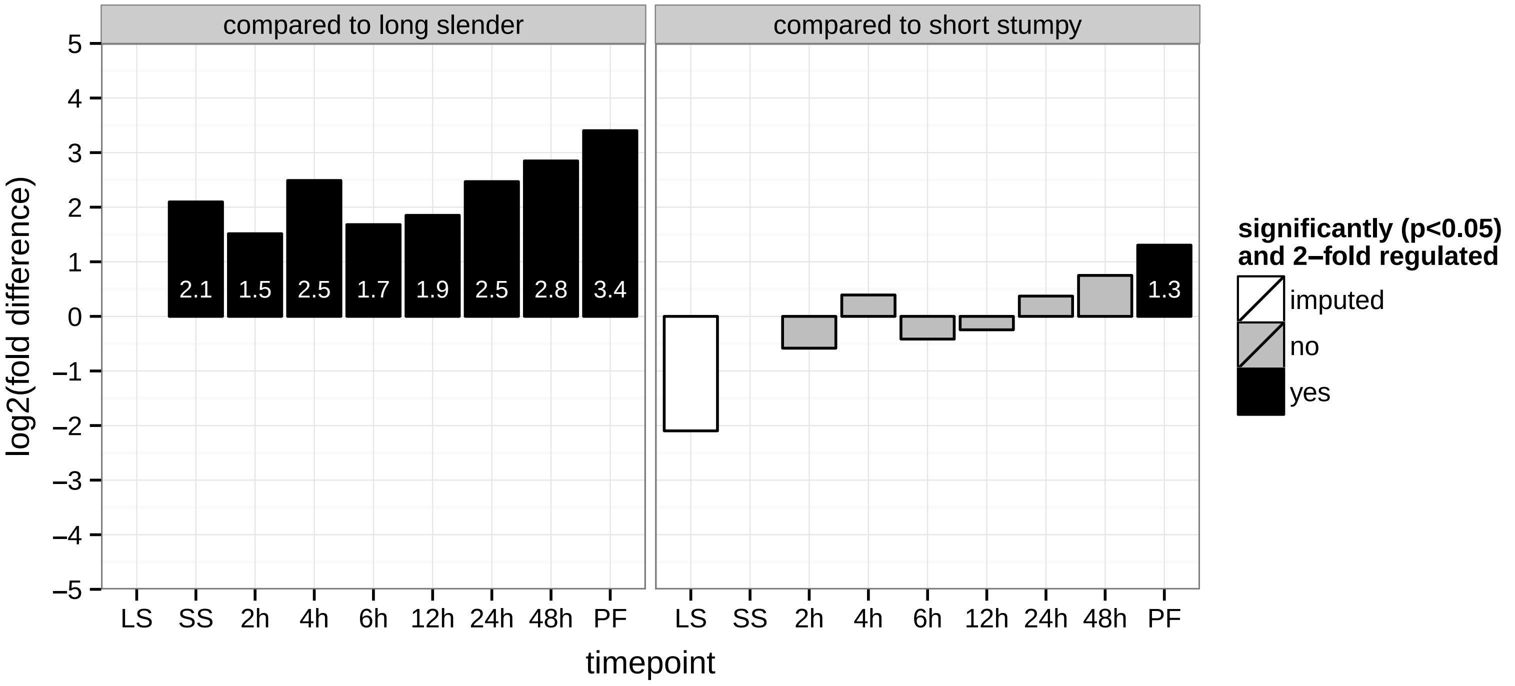

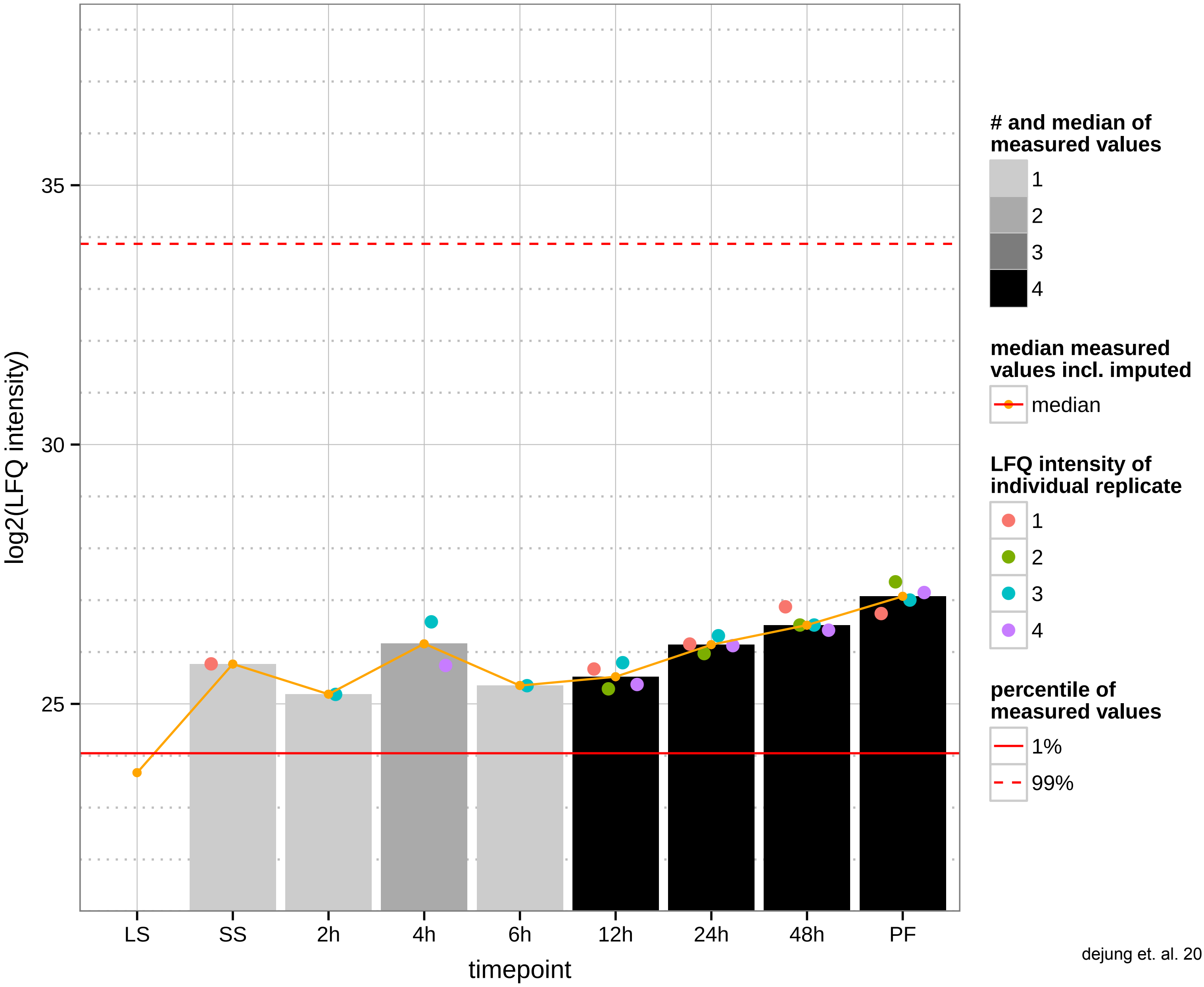

dejung et. al. 2015

hypothetical protein, conserved
Tb927.6.4510
AGOF: null
AGOC: null
AGOP: null
PGOF: null
PGOC: null
PGOP: null

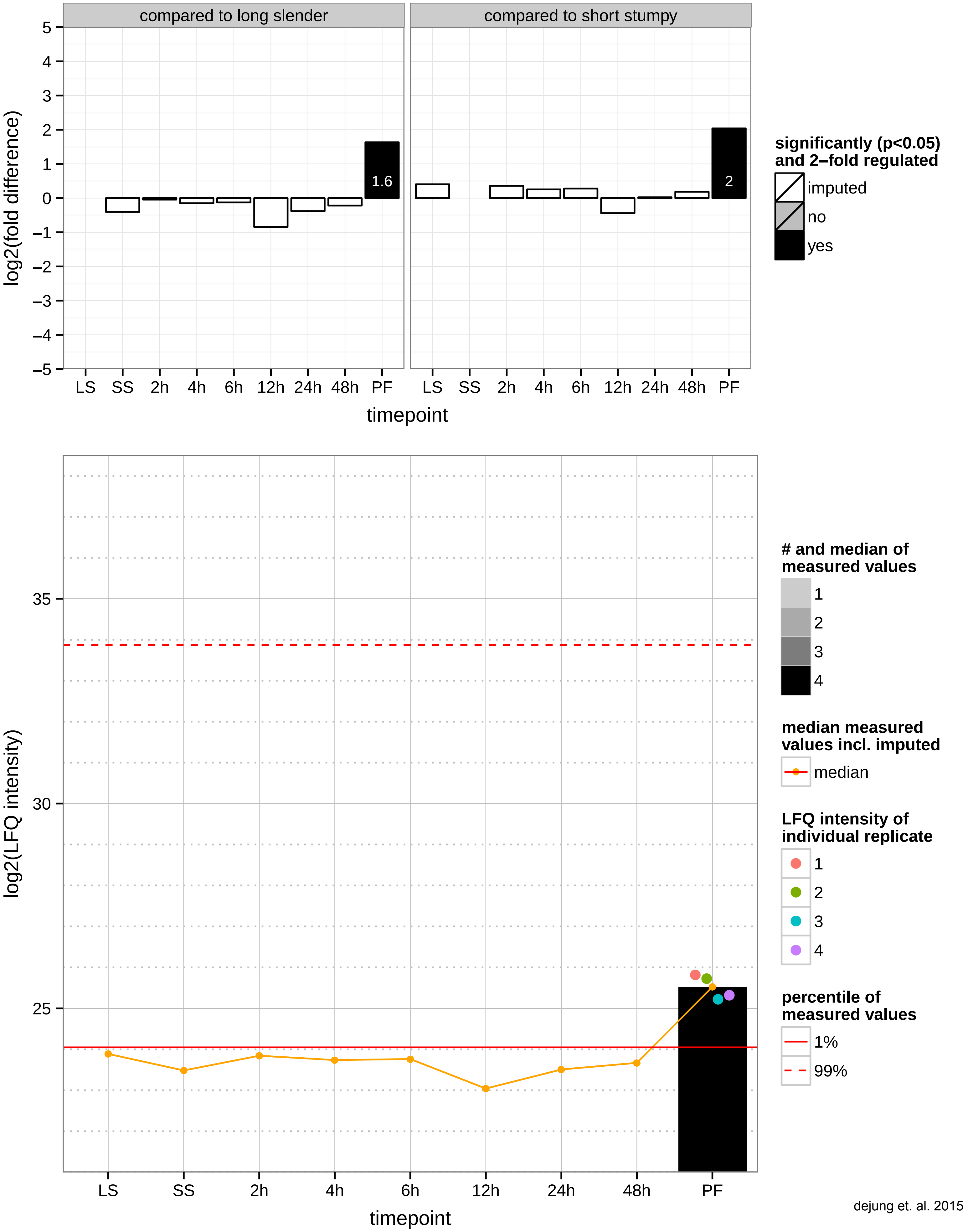

dejung et. al. 2015

kinetoplastid−specific phospho−protein phosphatase, putative
Tb927.6.4630
AGOF: hydrolase activity, protein serine/threonine phosphatase activity
AGOC: null
AGOP: protein dephosphorylation
PGOF: hydrolase activity
PGOC: null
PGOP: null

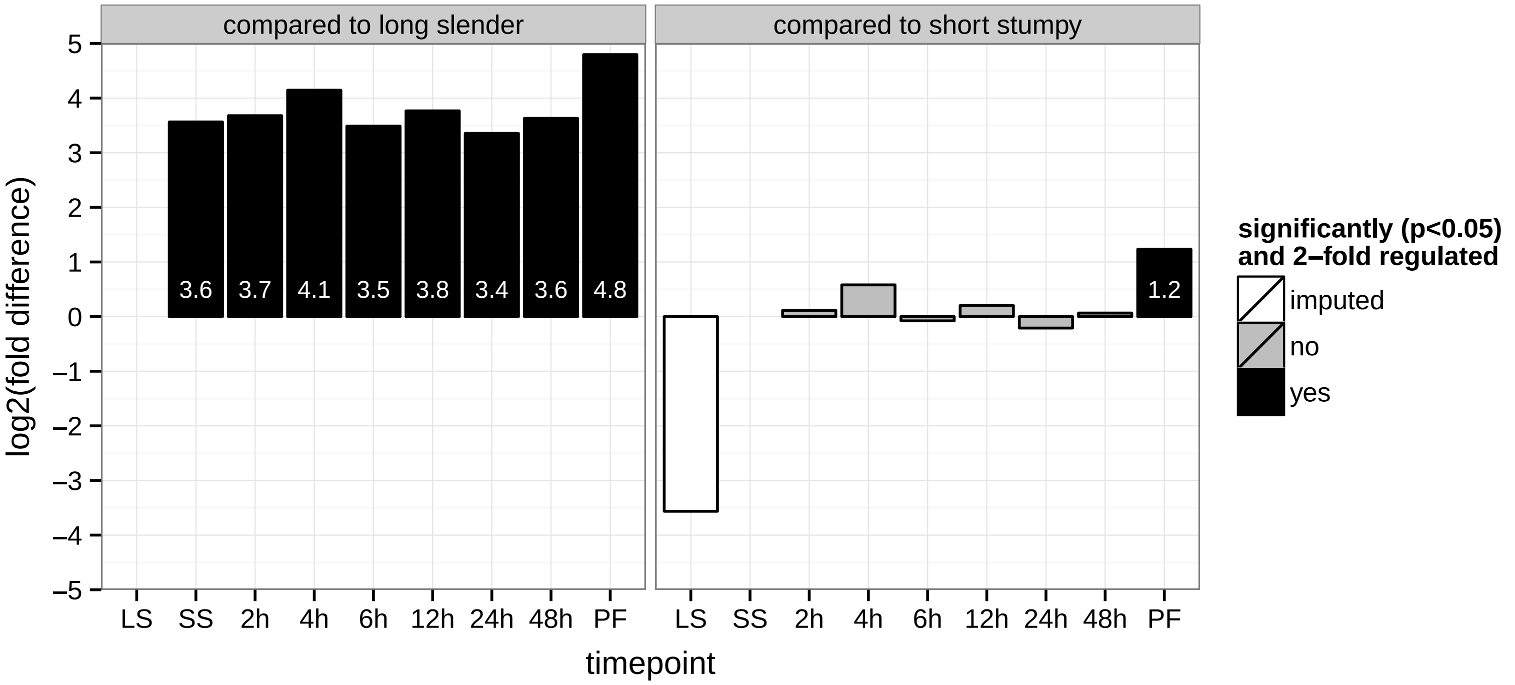

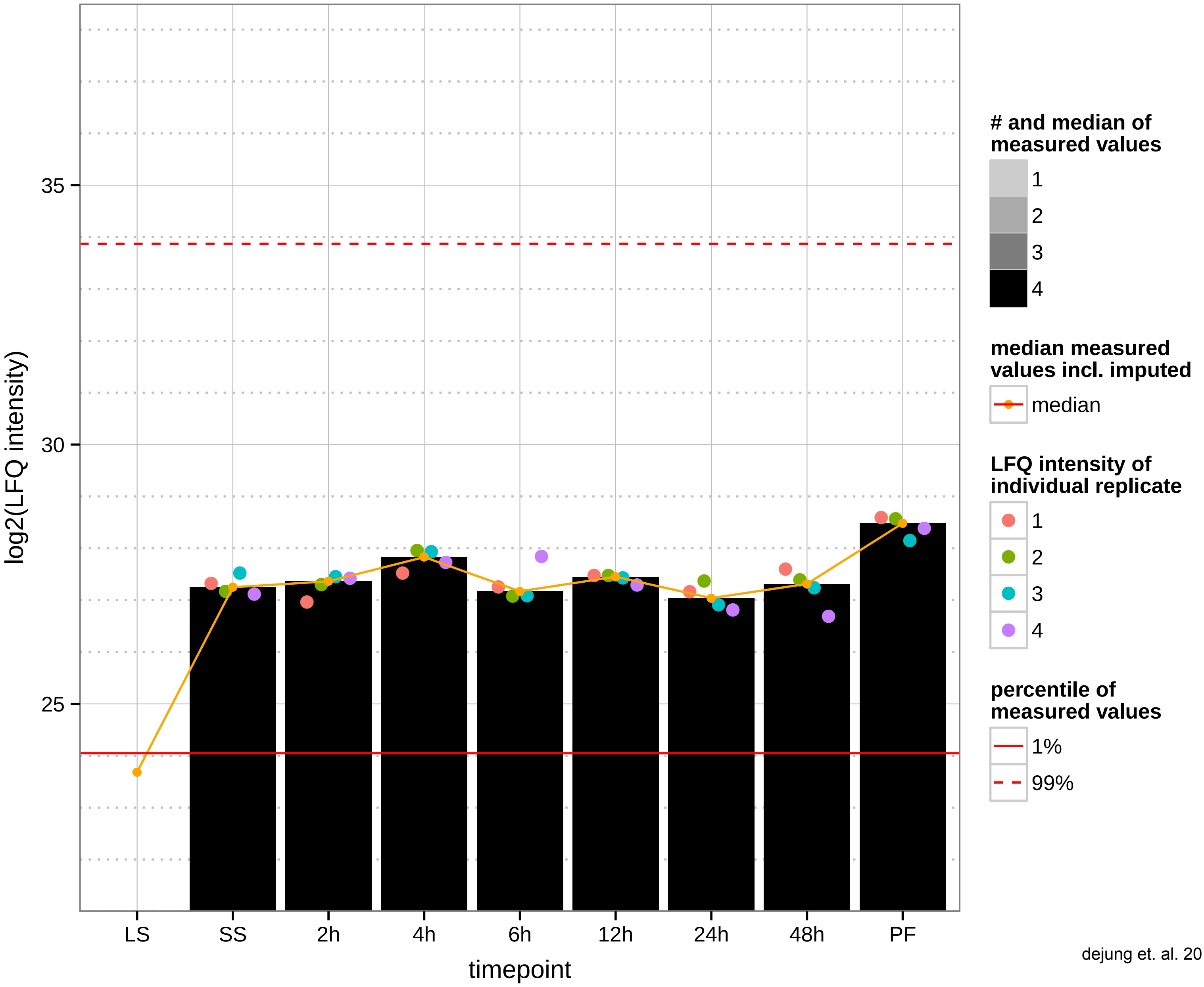

dejung et. al. 2015

hypothetical protein, conserved
Tb927.6.4700
AGOF: null
AGOC: mitochondrion
AGOP: null
PGOF: null
PGOC: null
PGOP: null

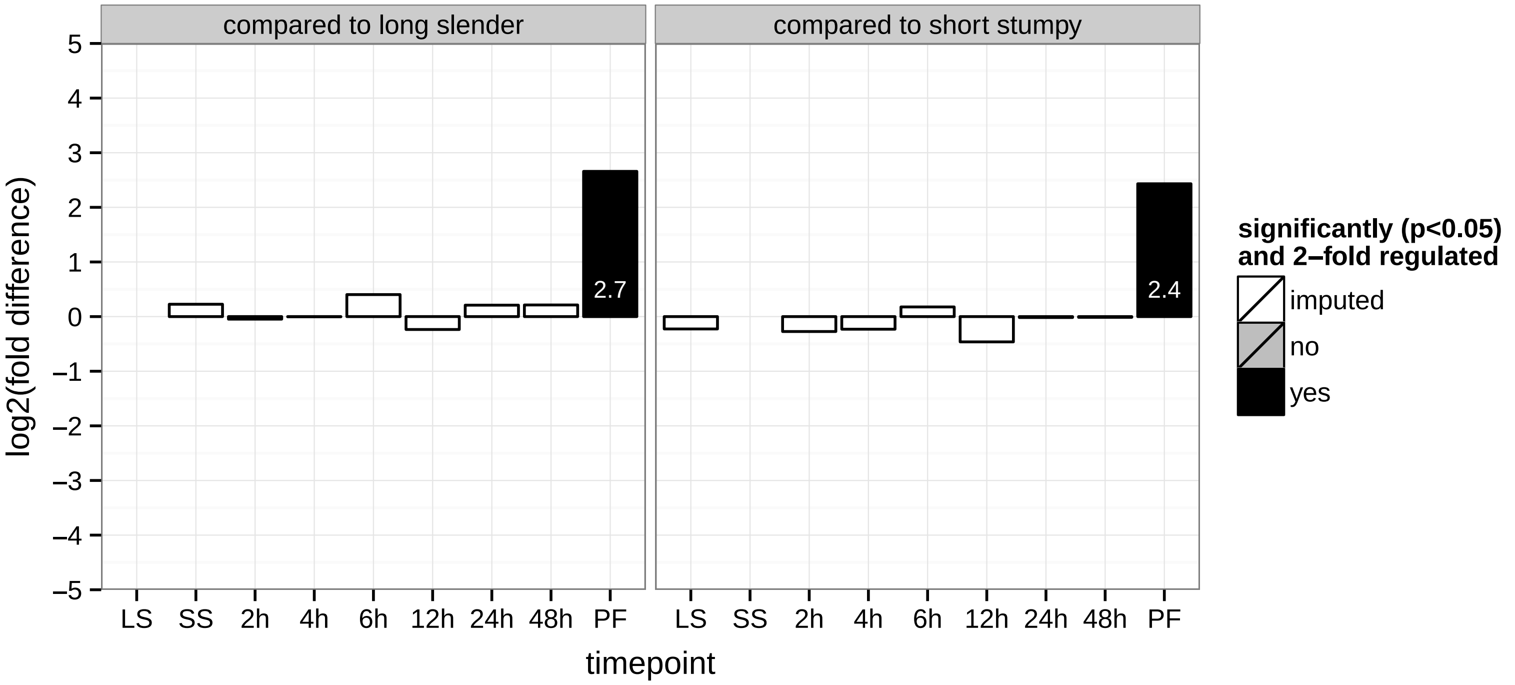

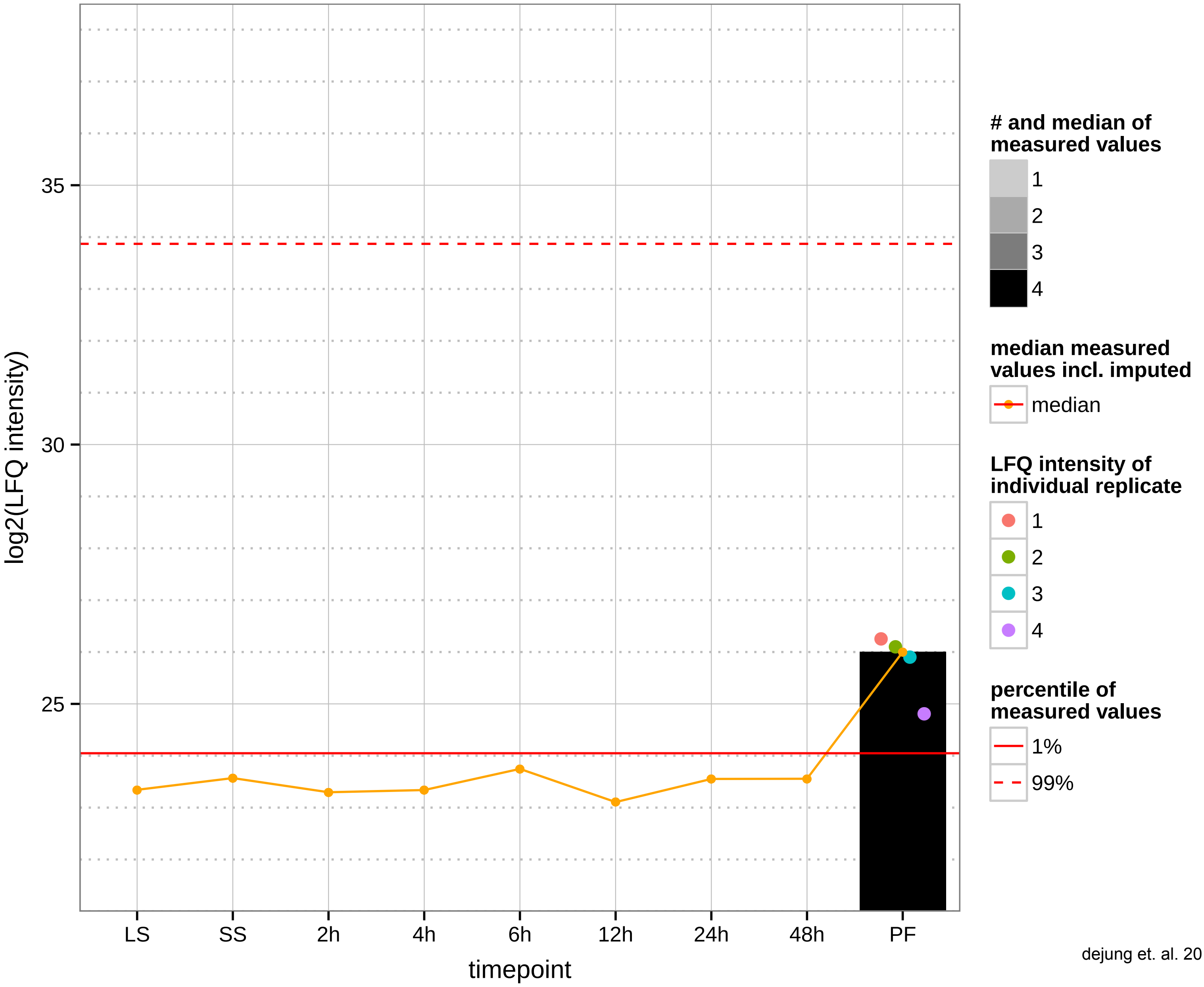

dejung et. al. 2015

CCR4 associated factor, putative, CAF1 family ribonuclease, putative (CAF1)
Tb927.6.600
AGOF: poly(A) RNA binding
AGOC: cytoplasm, nucleus
AGOP: mRNA catabolic process
PGOF: nucleic acid binding
PGOC: nucleus
PGOP: null

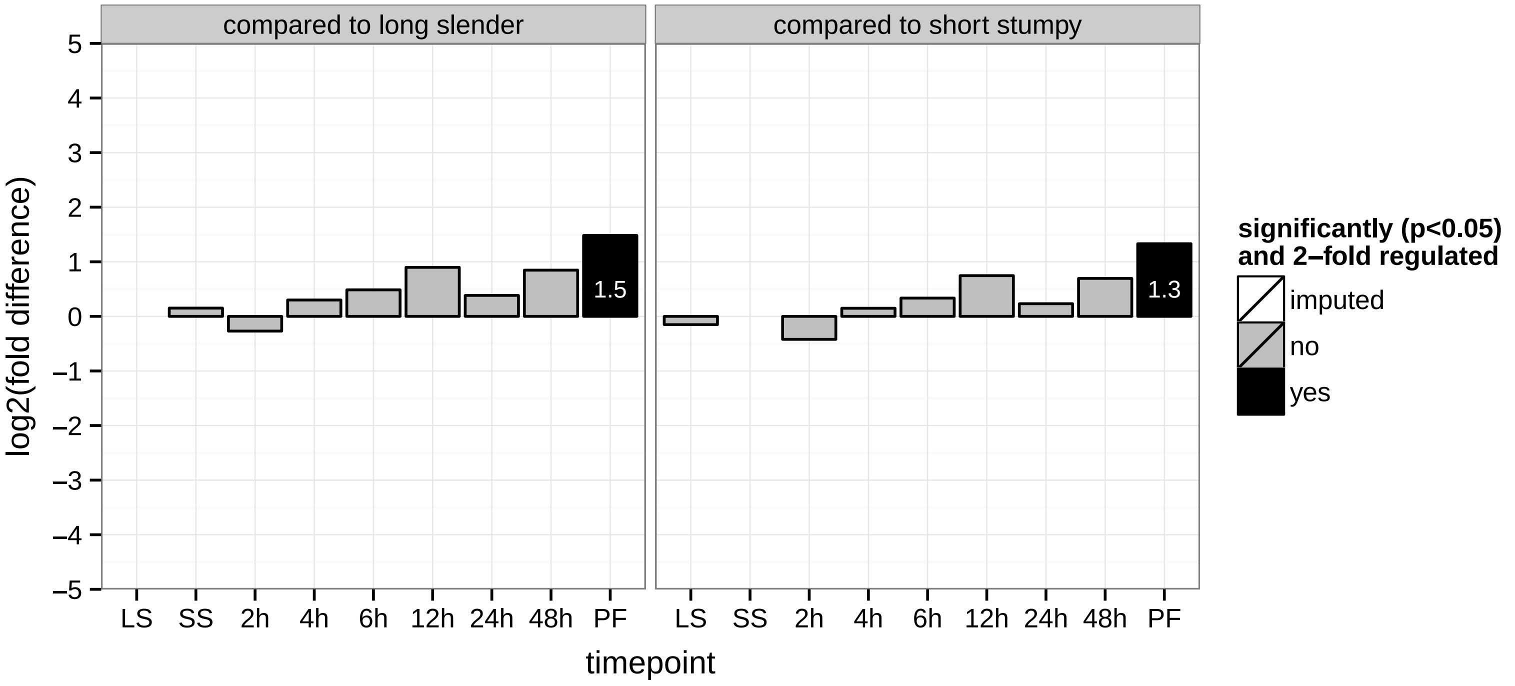

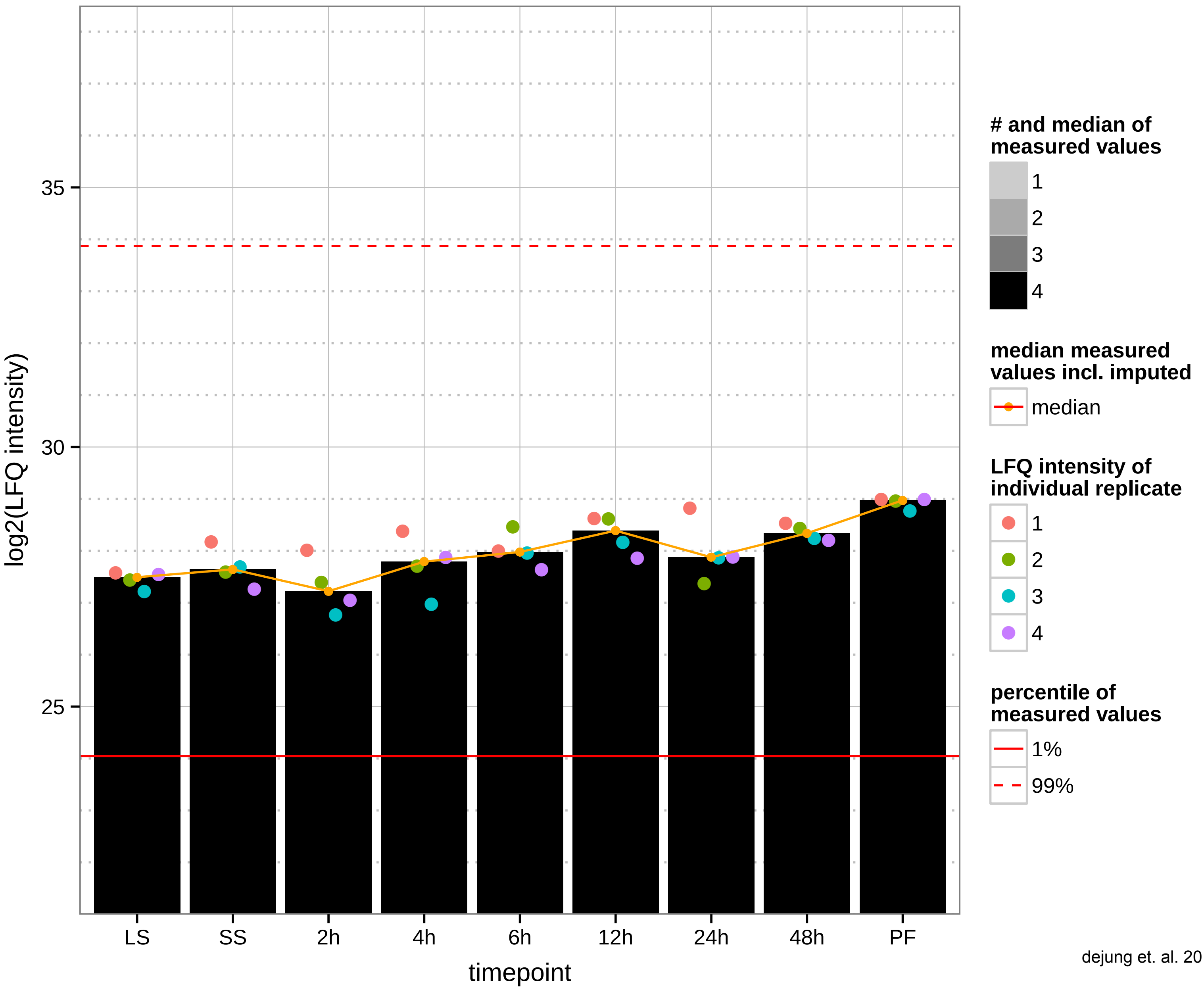

dejung et. al. 2015

hypothetical protein, conserved
Tb927.6.890
AGOF: null
AGOC: null
AGOP: null
PGOF: null
PGOC: null
PGOP: null

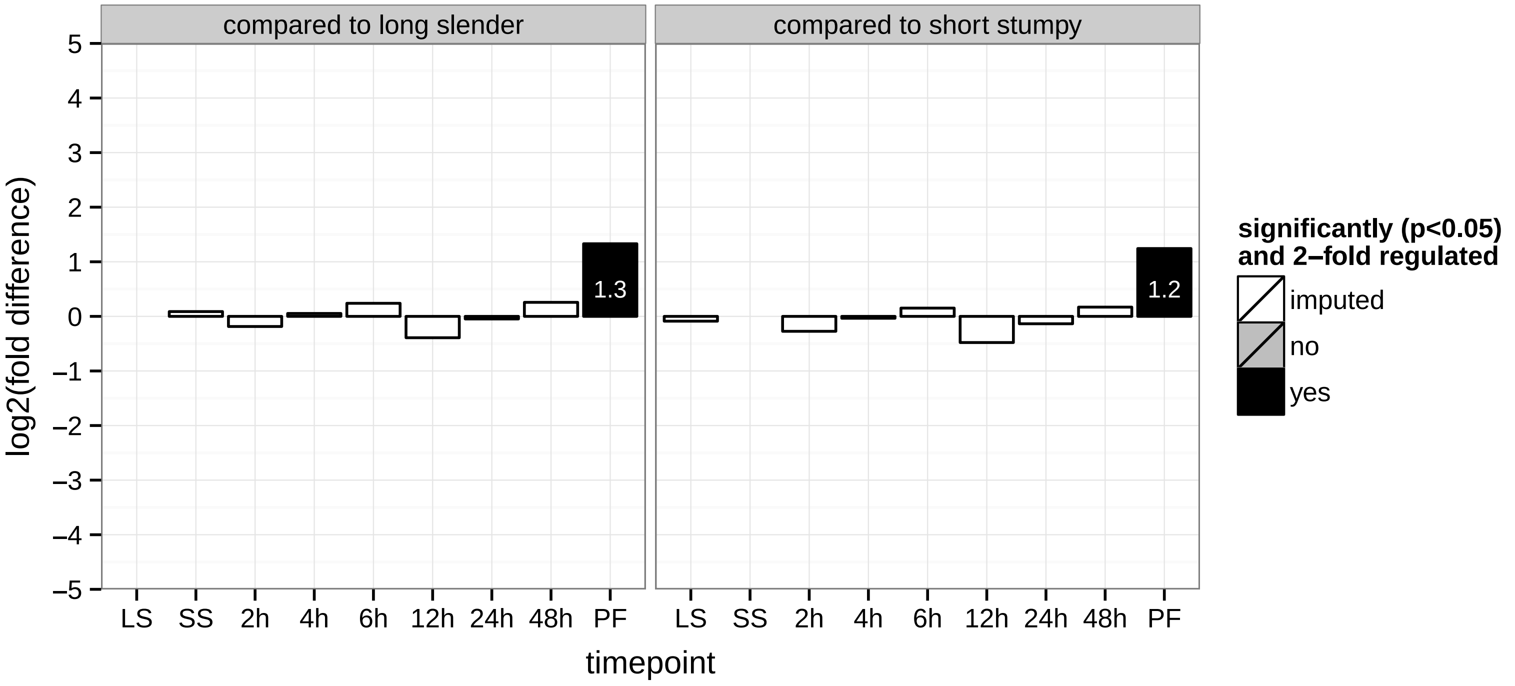

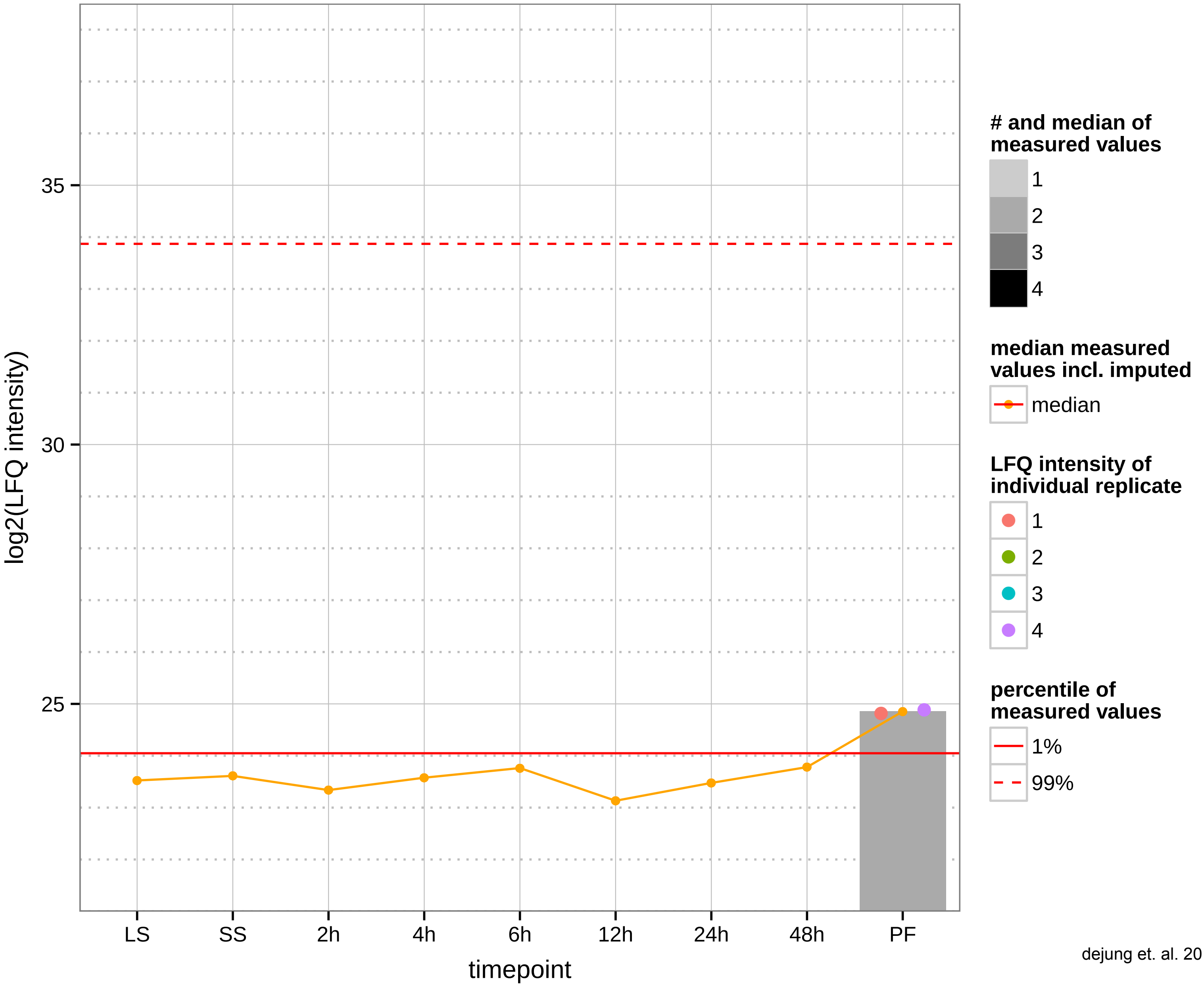

dejung et. al. 2015

cysteinyl–tRNA synthetase, putative
Tb927.6.950
AGOF: ATP binding, cysteine–tRNA ligase activity
AGOC: cytoplasm
AGOP: cysteinyl–tRNA aminoacylation, translation
PGOF: ATP binding, aminoacyl–tRNA ligase activity, cysteine–tRNA ligase activity, nucleotide binding
PGOC: cytoplasm
PGOP: cysteinyl–tRNA aminoacylation, tRNA aminoacylation for protein translation

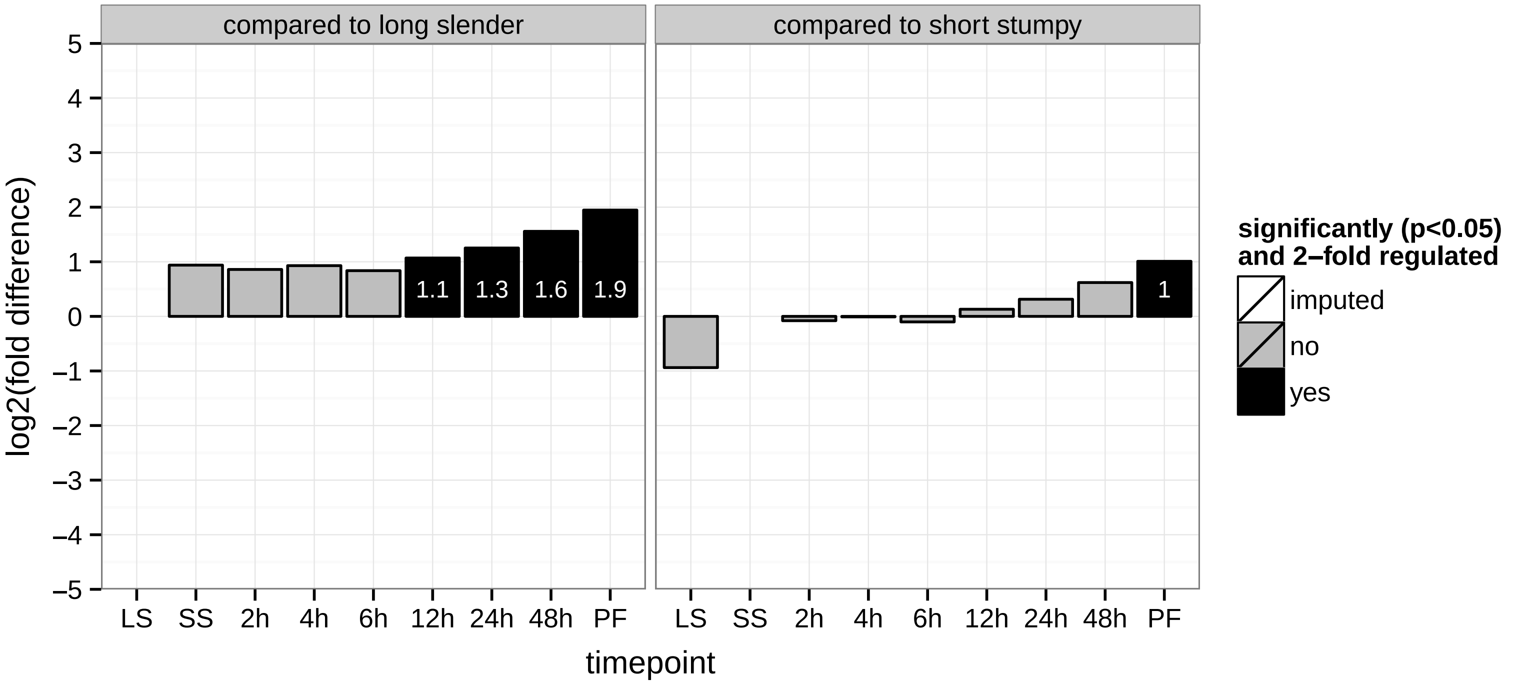

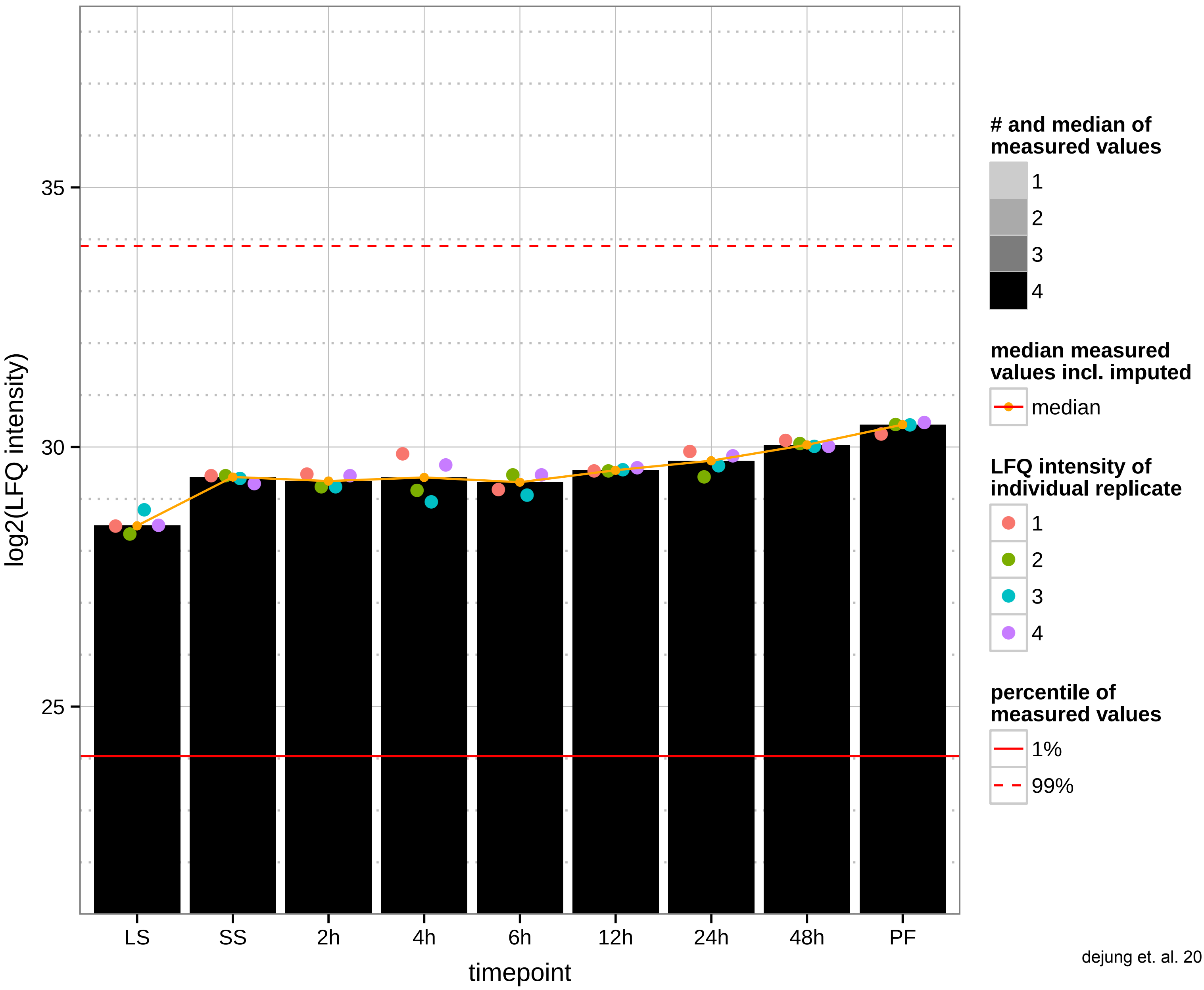

dejung et. al. 2015

hypothetical protein, conserved
Tb927.7.1220
AGOF: null
AGOC: null
AGOP: null
PGOF: null
PGOC: null
PGOP: null

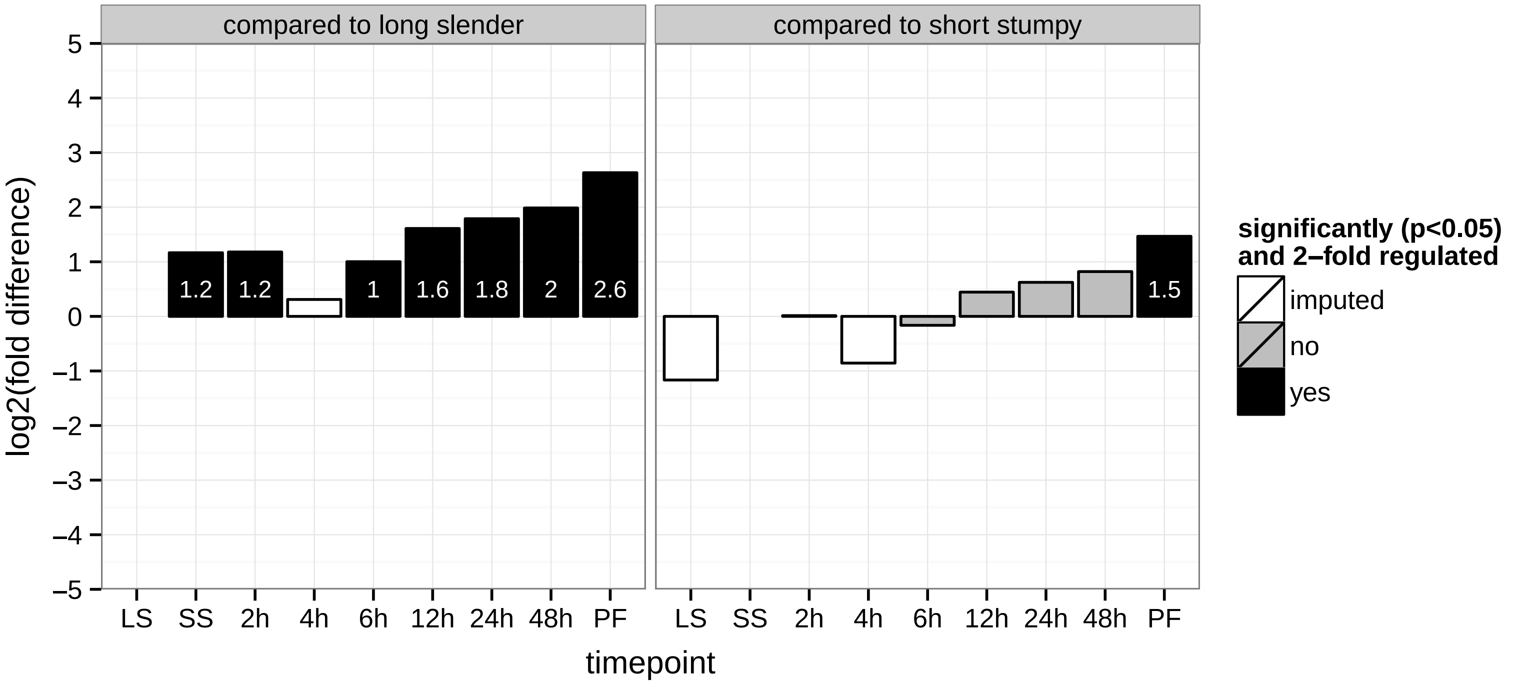

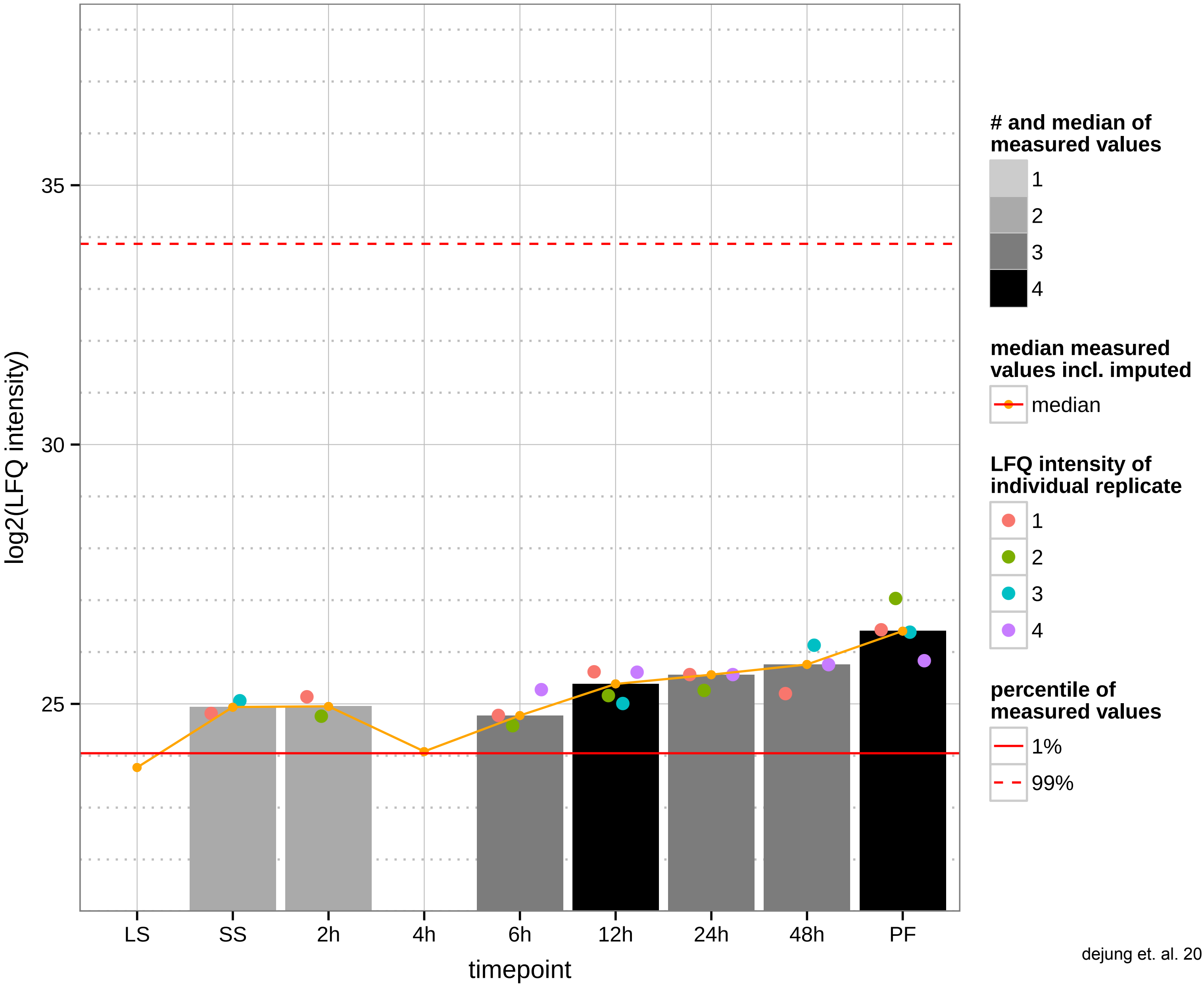

dejung et. al. 2015

hypothetical protein, conserved
Tb927.7.1460
AGOF: protein transporter activity
AGOC: cytoplasm, nuclear pore, nucleus
AGOP: intracellular protein transport, protein import into nucleus, docking
PGOF: binding, protein transporter activity
PGOC: null
PGOP: intracellular protein transport

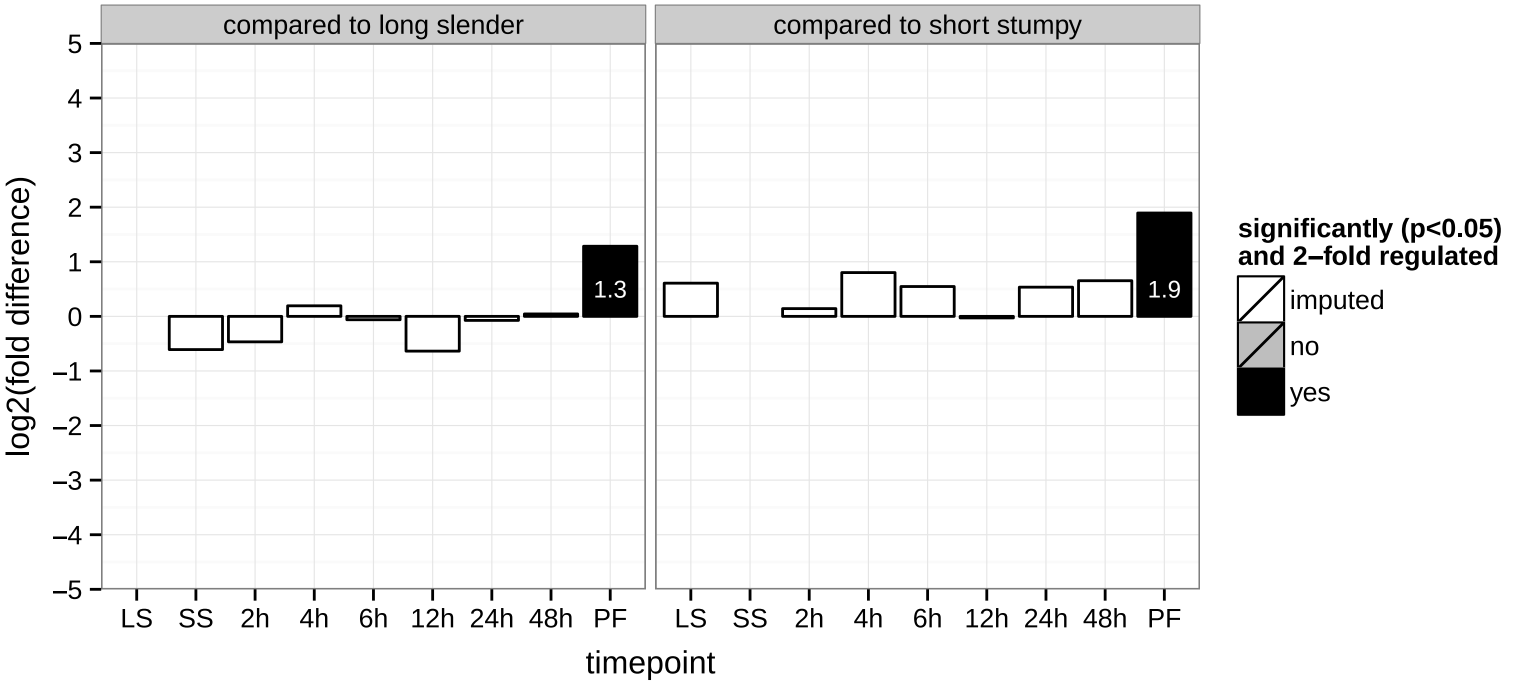

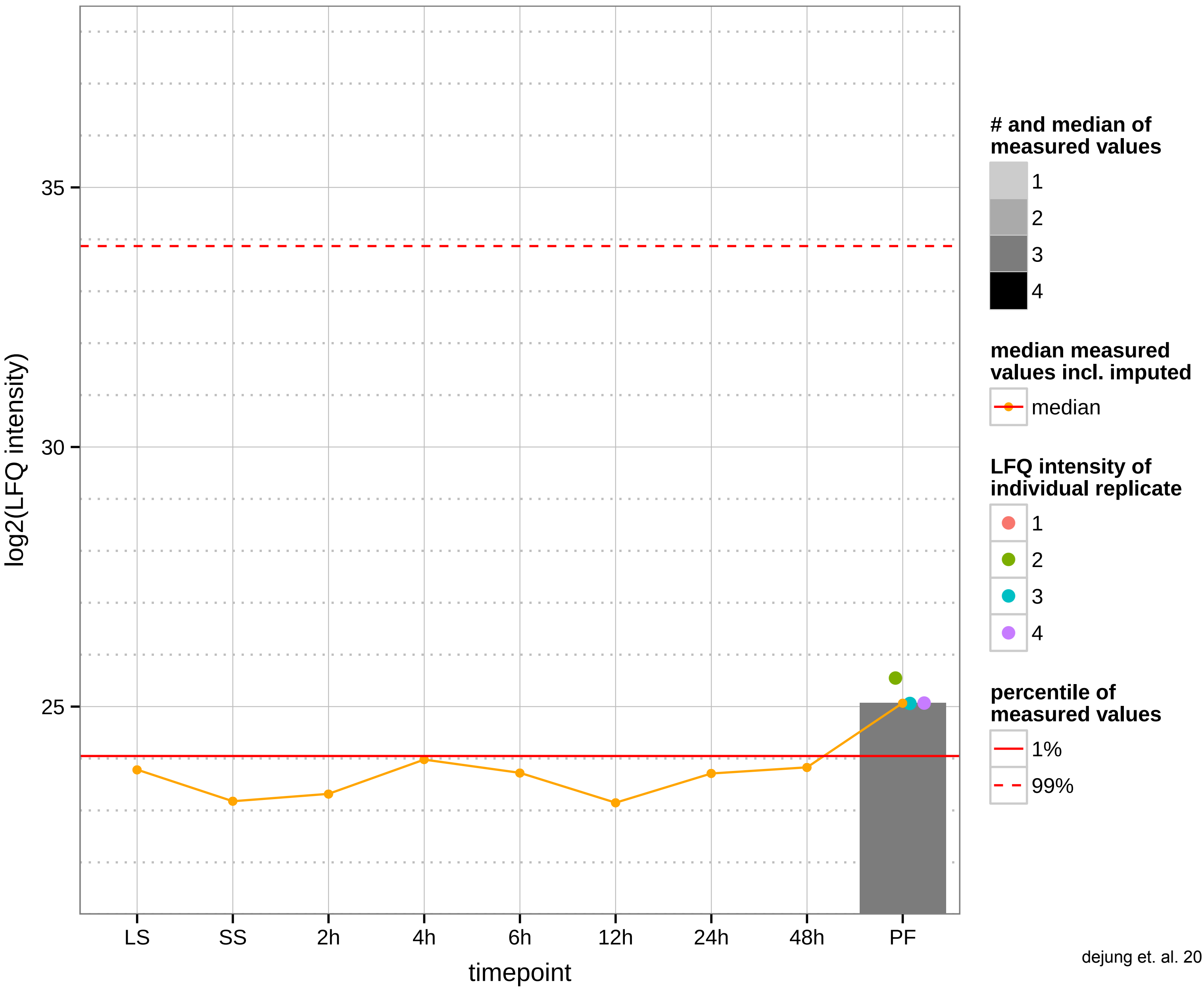

dejung et. al. 2015

KRET2, RNA–editing 3' terminal uridylyl transferase 2, RNA–editing complex protein (KRET2)
Tb927.7.1550
AGOF: null
AGOC: mitochondrial mRNA editing complex, mitochondrion
AGOP: null
PGOF: nucleotidyltransferase activity
PGOC: null
PGOP: null

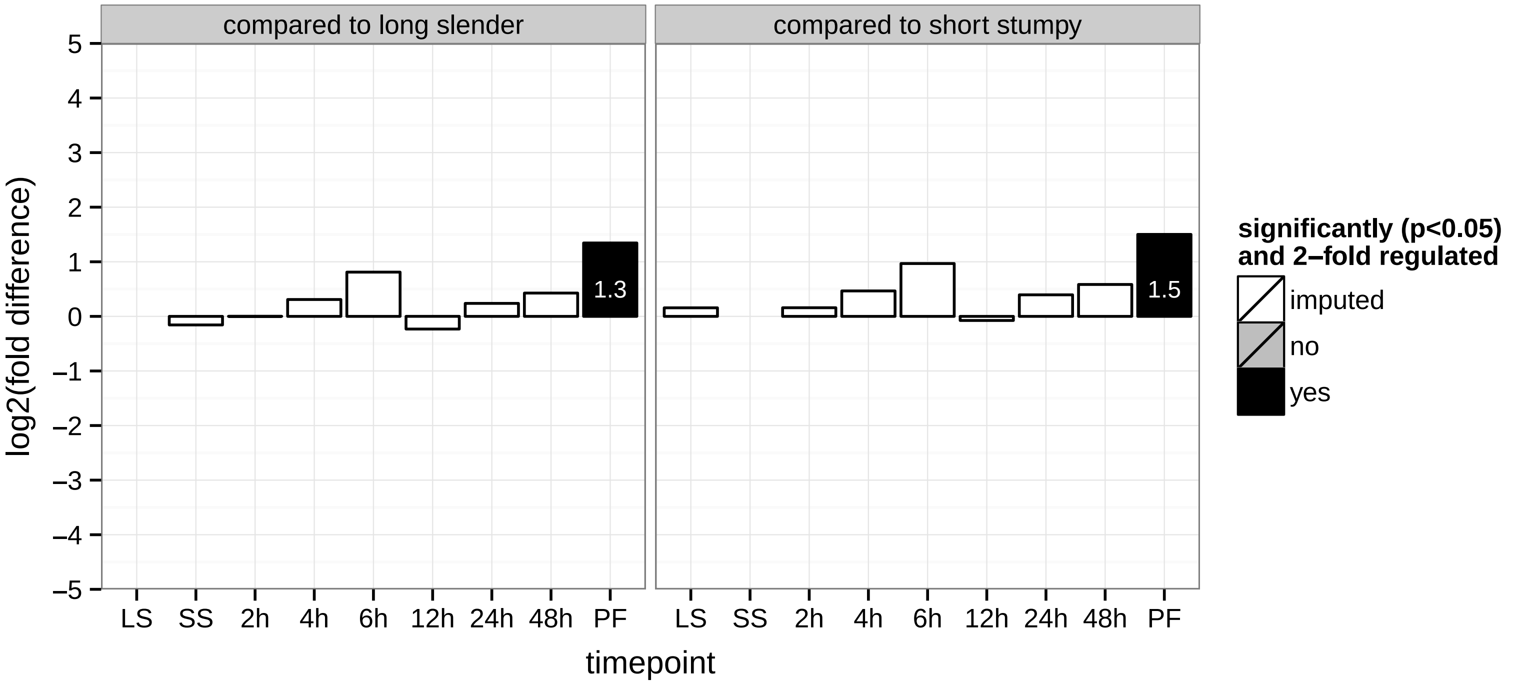

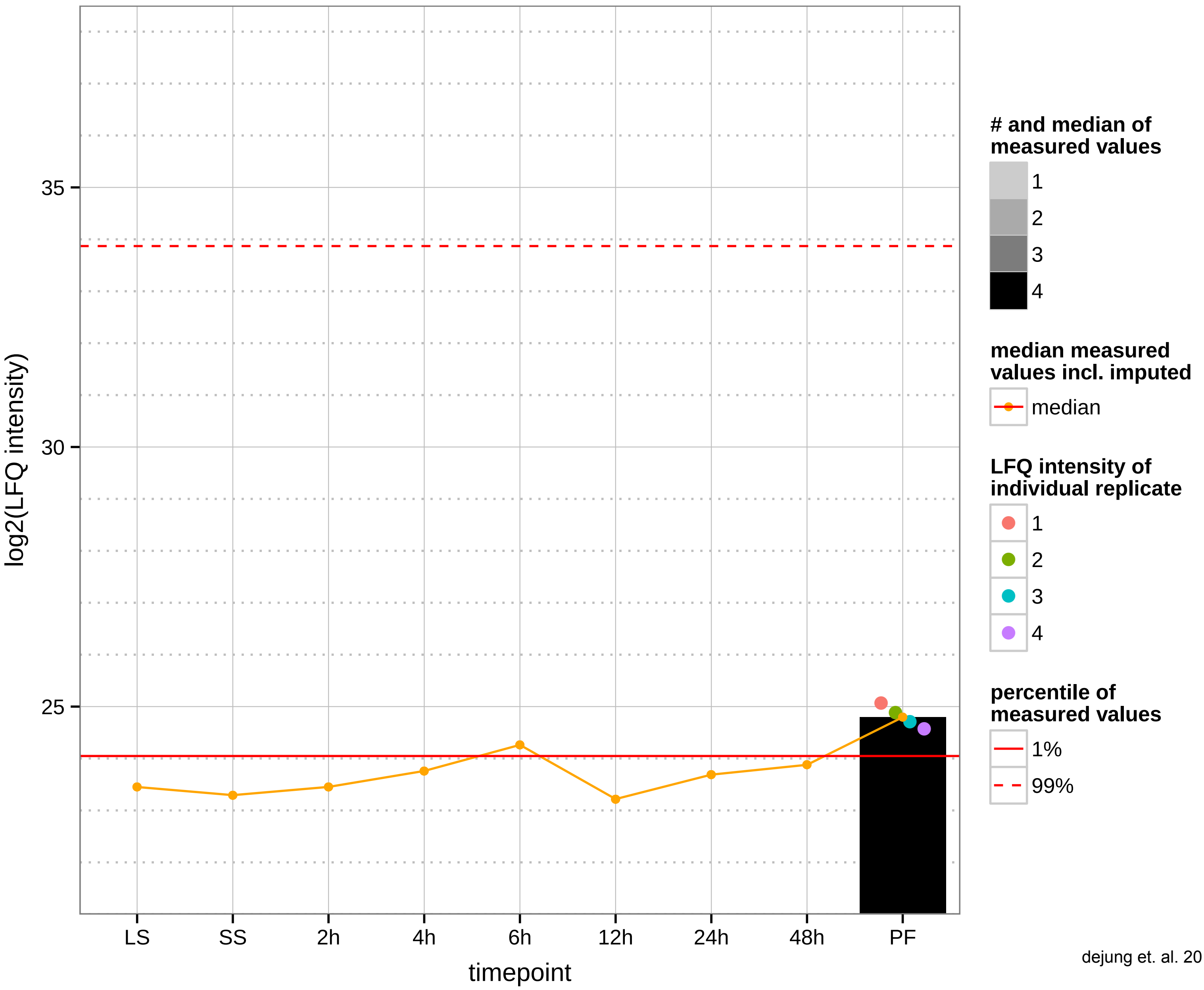

dejung et. al. 2015

hypothetical protein, conserved
Tb927.7.1700
AGOF: null
AGOC: null
AGOP: null
PGOF: null
PGOC: null
PGOP: null

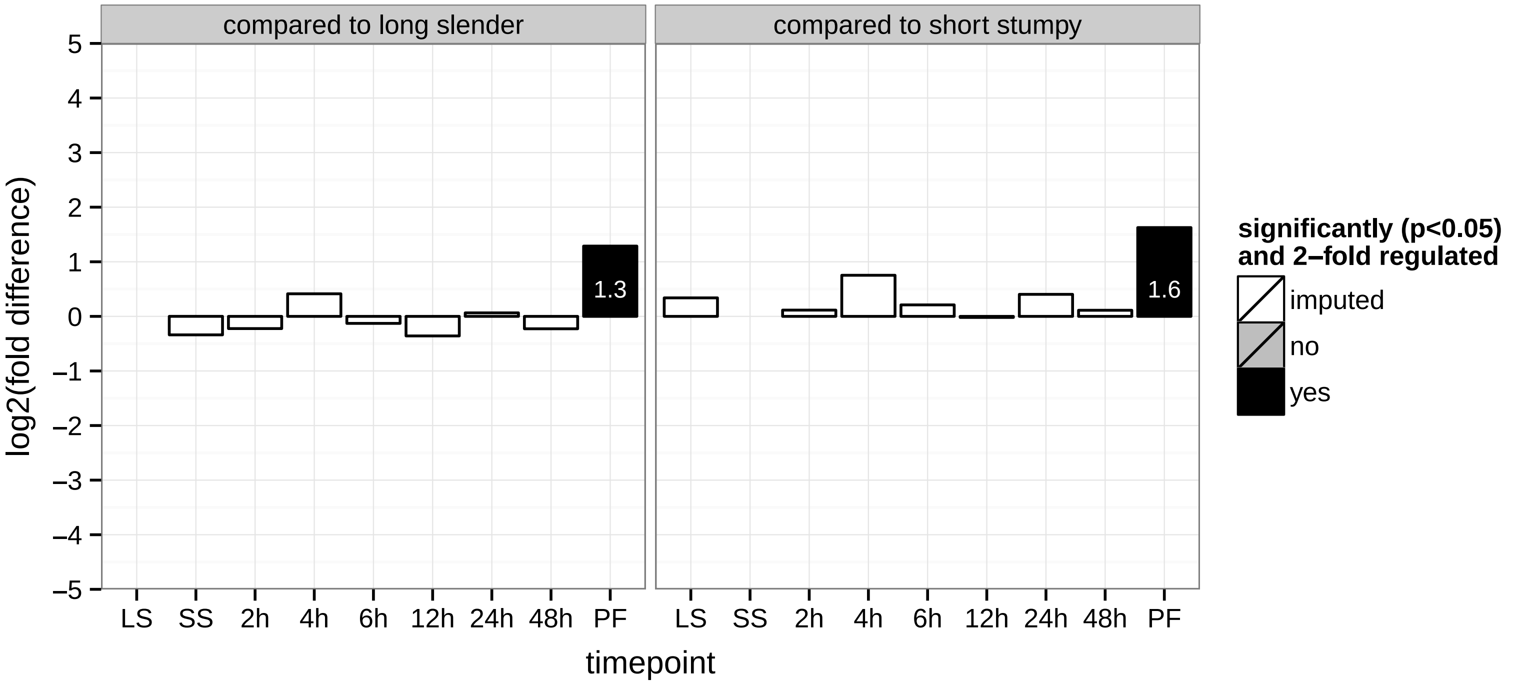

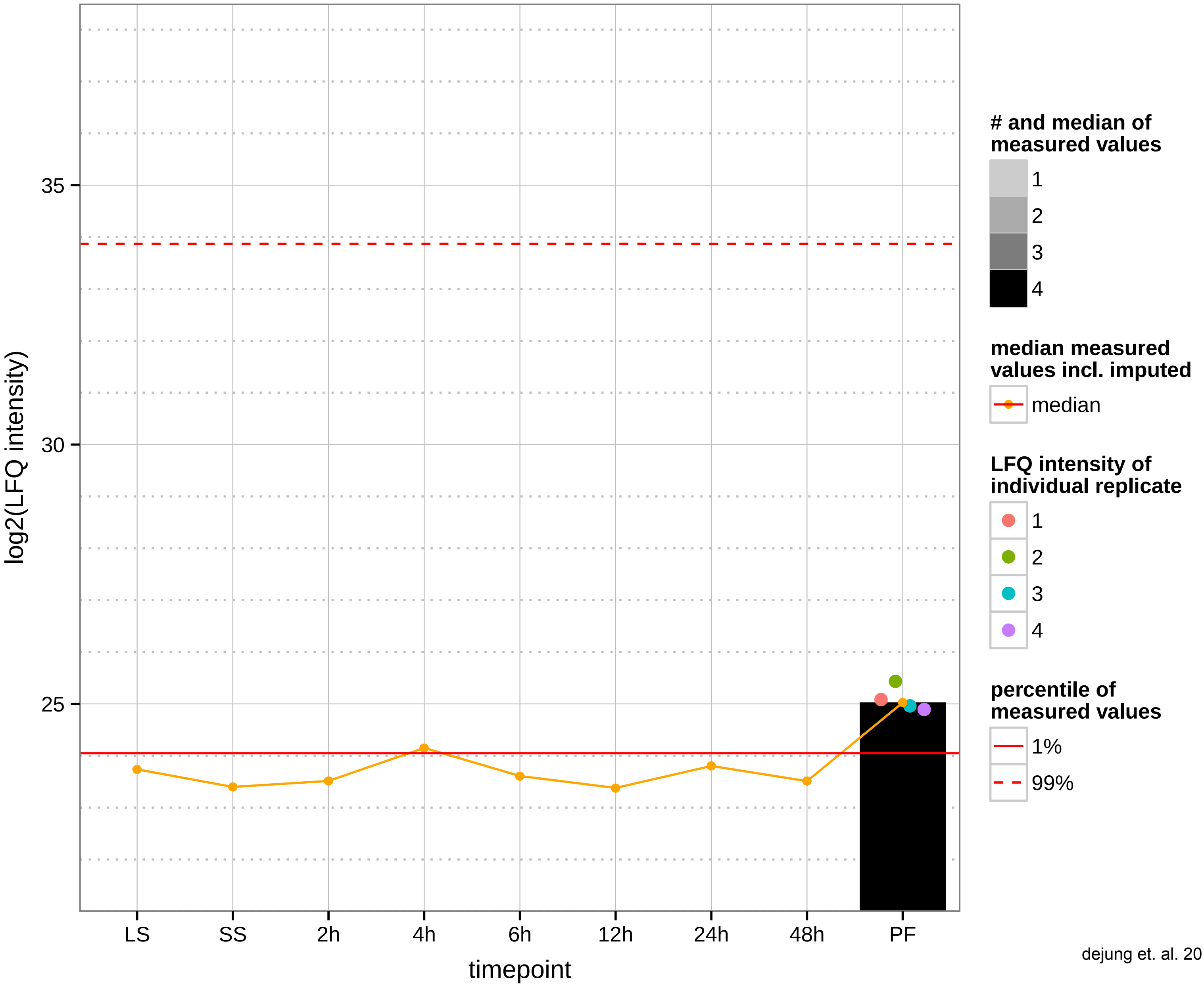

dejung et. al. 2015

hypothetical protein, conserved
Tb927.7.1860;Tb927.7.1820
AGOF: null
AGOC: null
AGOP: null
PGOF: null
PGOC: null
PGOP: null

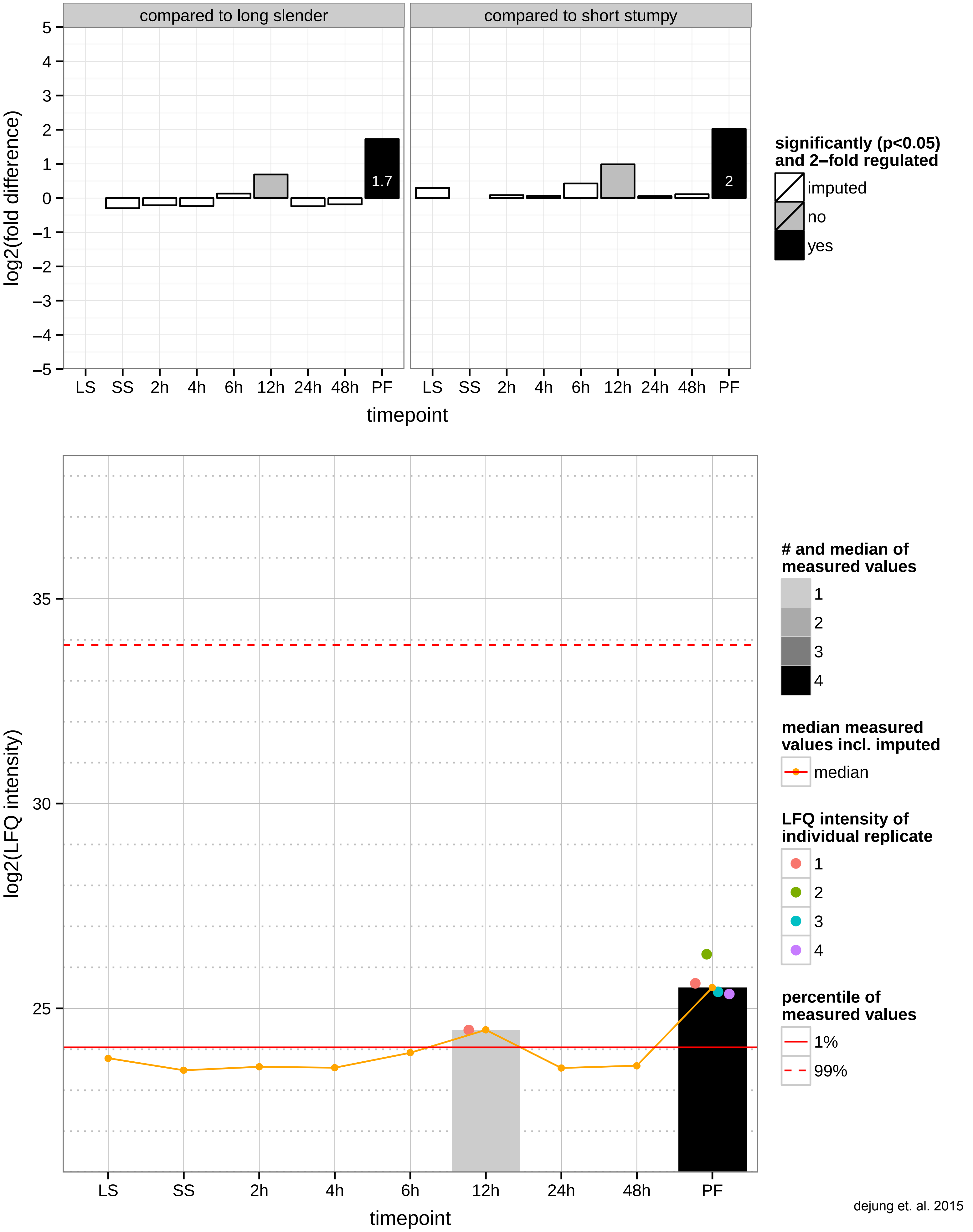

dejung et. al. 2015

hypothetical protein, conserved
Tb927.7.1870;Tb927.7.1830
AGOF: null
AGOC: null
AGOP: null
PGOF: null
PGOC: null
PGOP: null

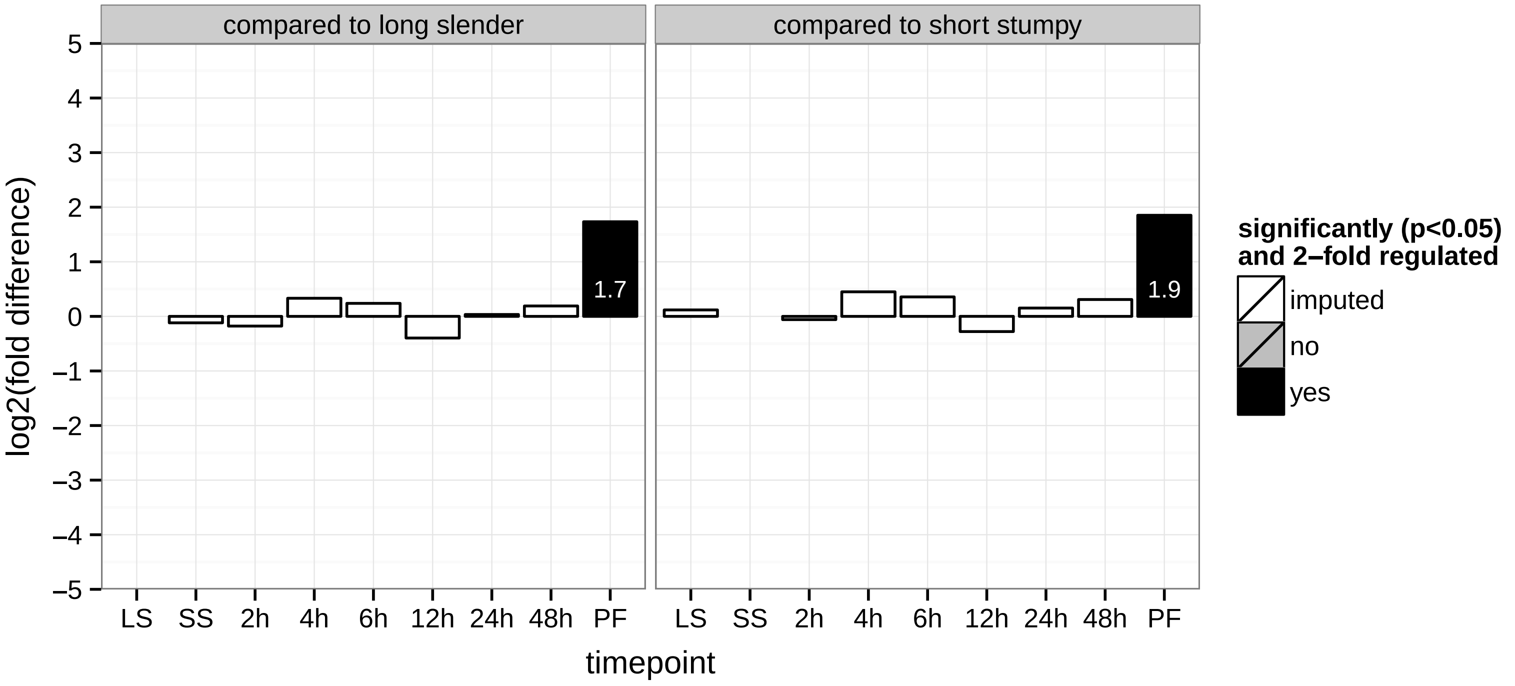

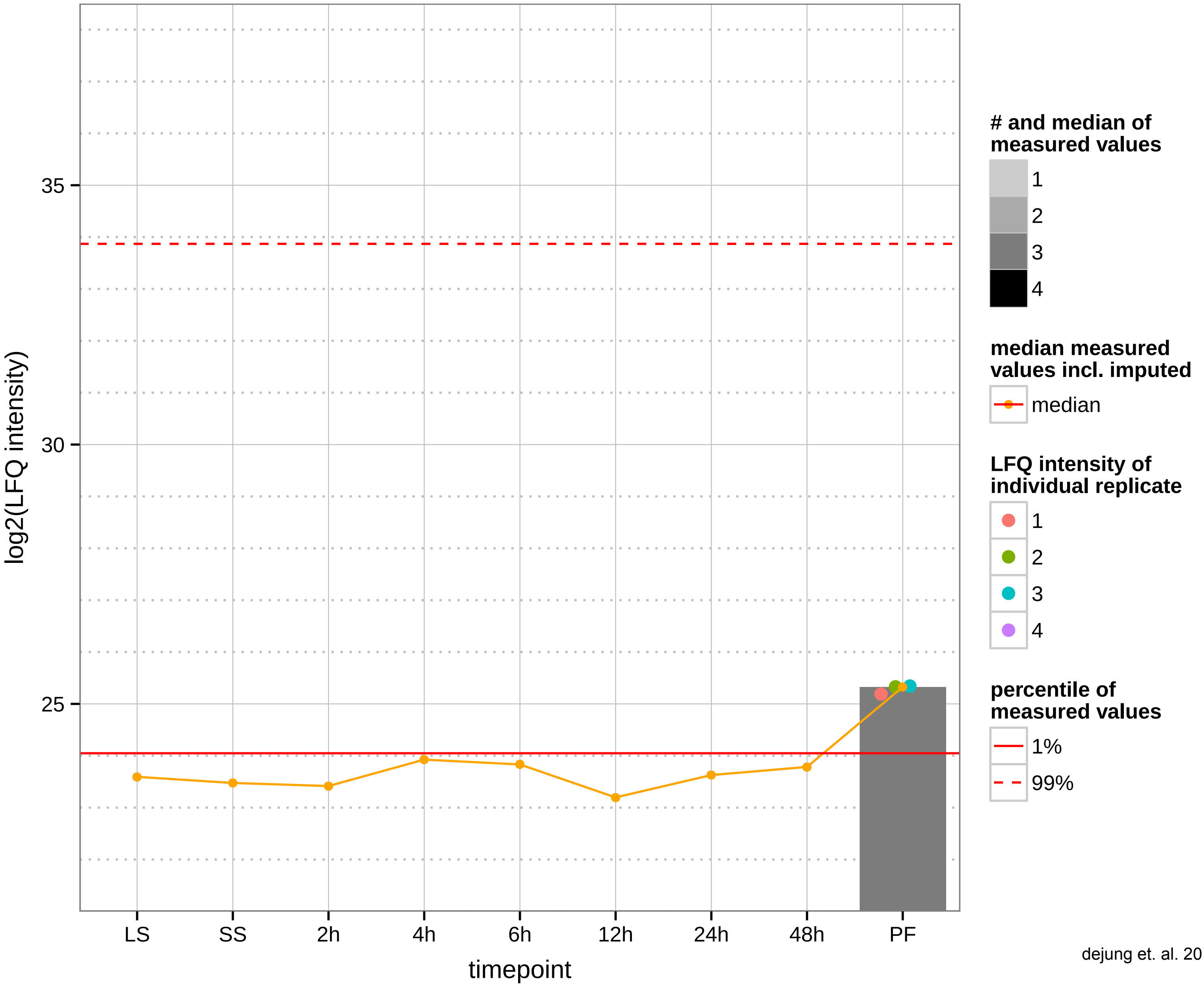

dejung et. al. 2015

pyridoxal phosphate containing glycine decarboxylase, putative, glycine dehydrogenase, putative (GCVP)
Tb927.7.1910
AGOF: glycine dehydrogenase (decarboxylating) activity
AGOC: mitochondrion
AGOP: glycine decarboxylation via glycine cleavage system, oxidation–reduction process
PGOF: glycine dehydrogenase (decarboxylating) activity, lyase activity
PGOC: null
PGOP: cellular amino acid metabolic process, glycine metabolic process, oxidation–reduction process

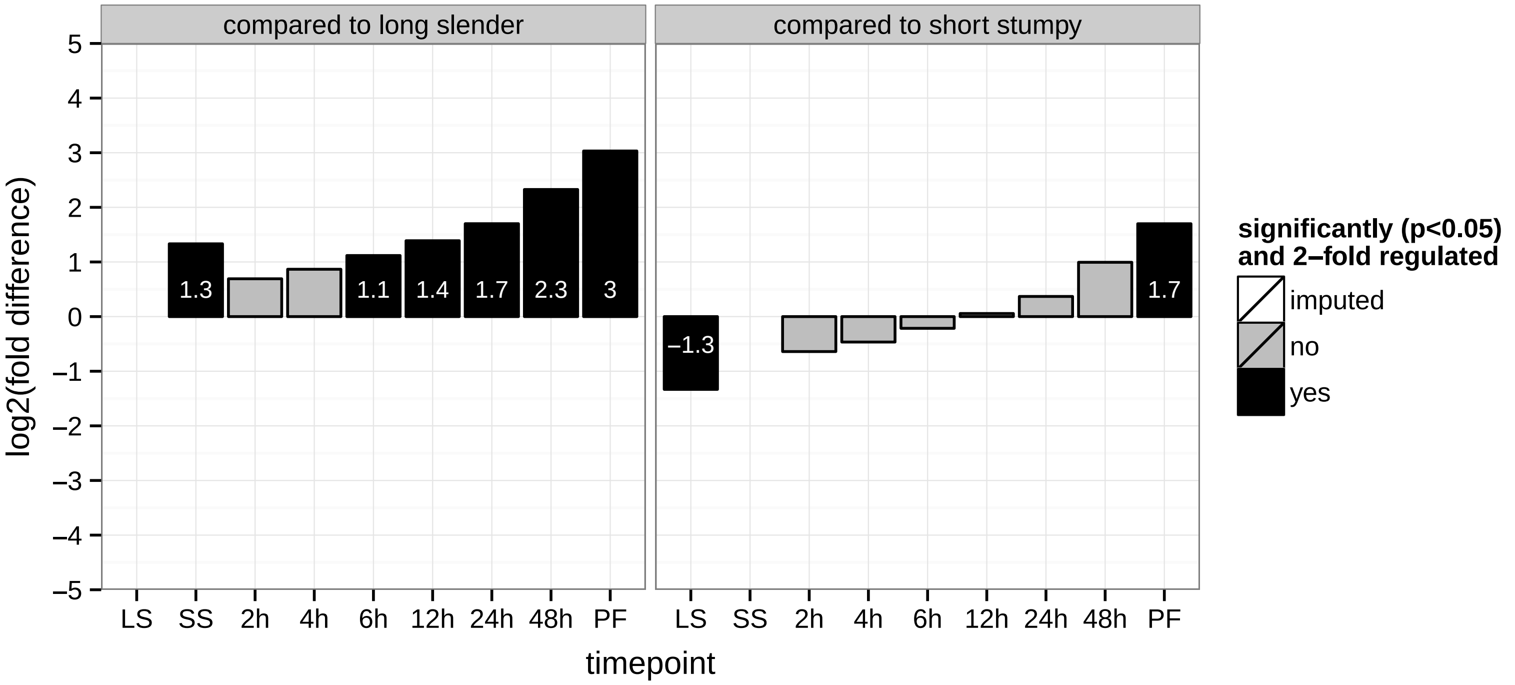

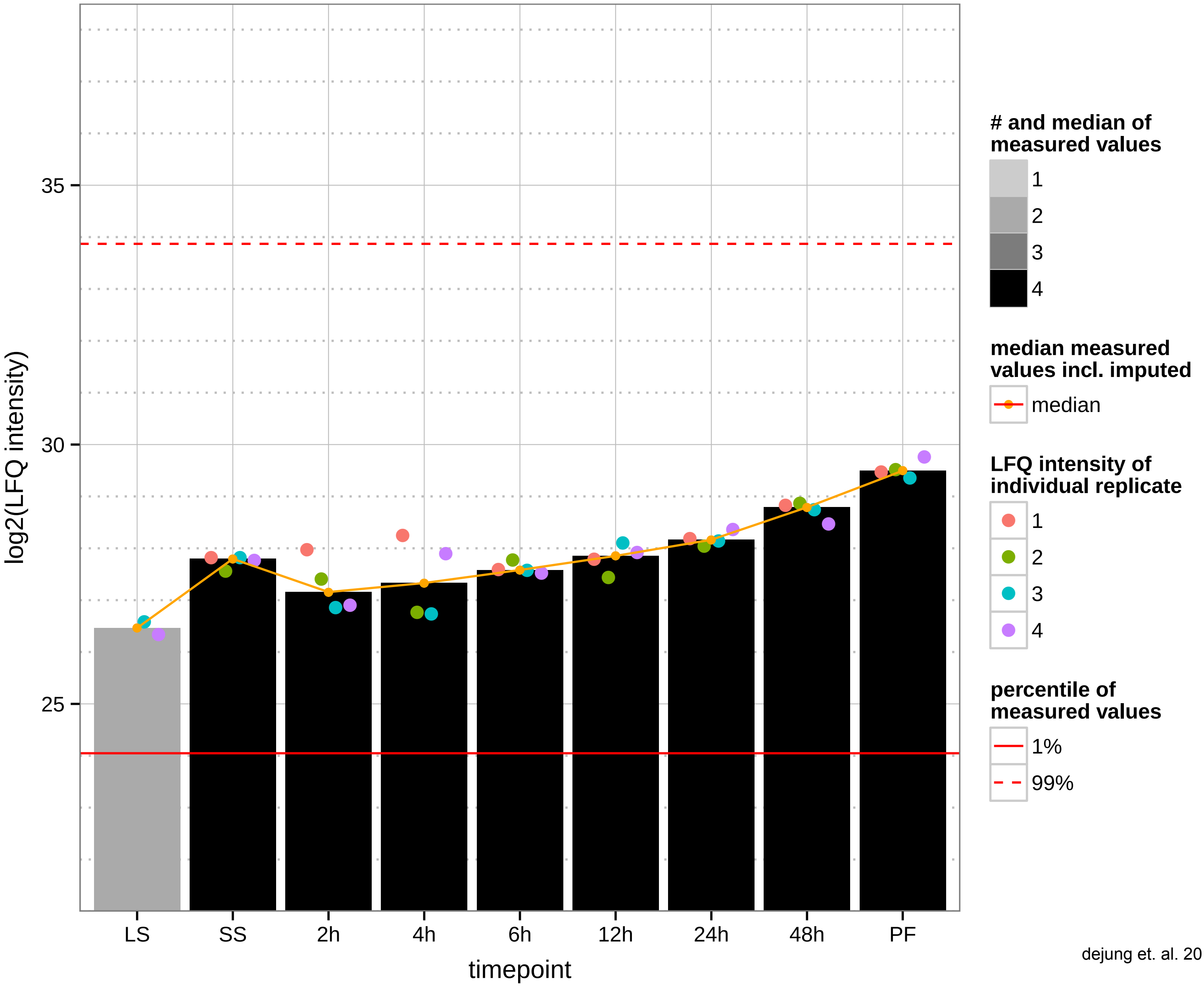

dejung et. al. 2015

retrotransposon hot spot protein 7 (RHS7), putative, retrotransposon hot spot protein (RHS, pseudogene), point mutation
Tb927.7.1970;Tb927.7.1950;Tb927.7.1990;Tb927.7.1980;Tb927.7.1960
AGOF: null
AGOC: null
AGOP: null
PGOF: null
PGOC: null
PGOP: null

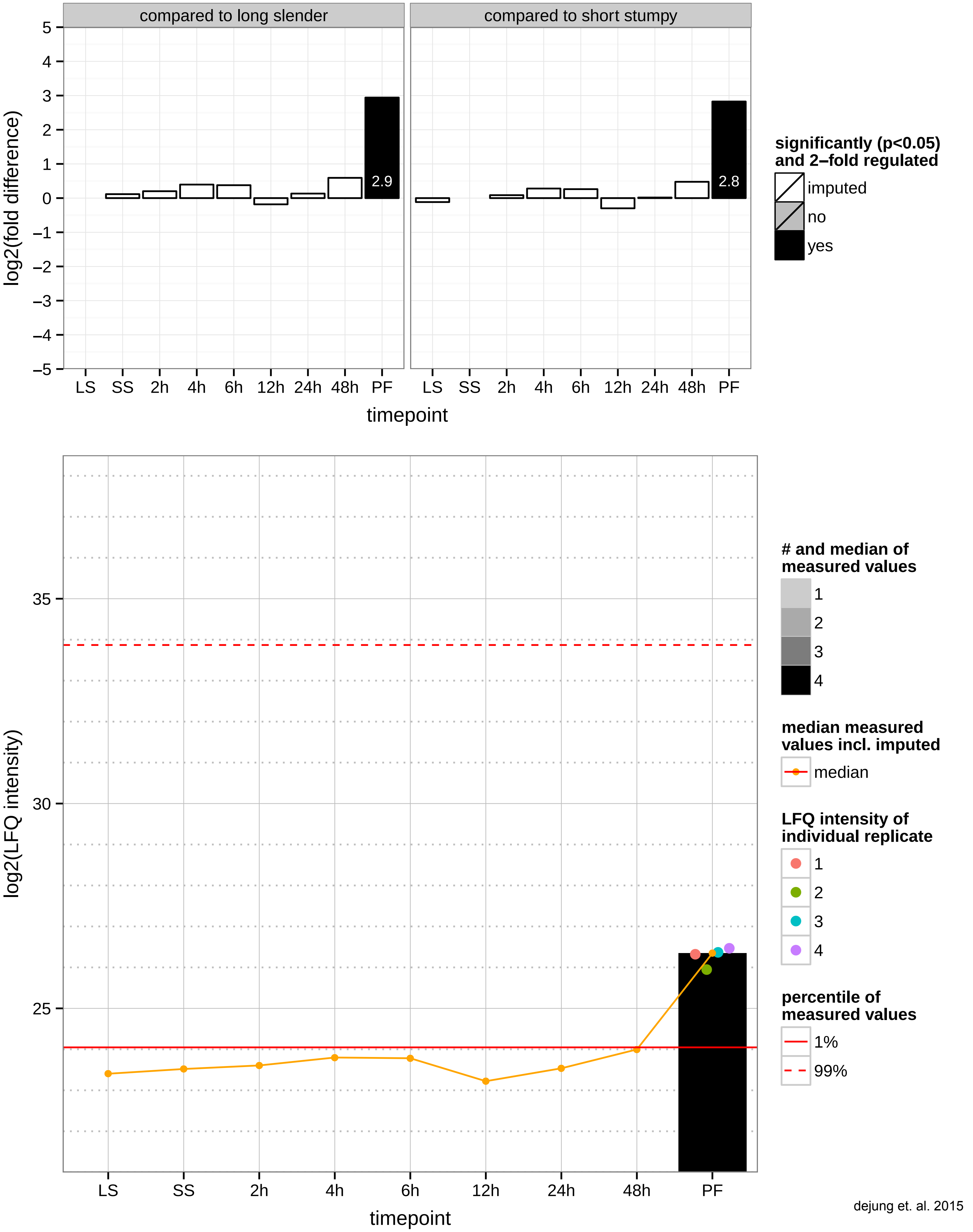

dejung et. al. 2015

hypothetical protein, conserved
Tb927.7.2170
AGOF: null
AGOC: null
AGOP: null
PGOF: null
PGOC: null
PGOP: null

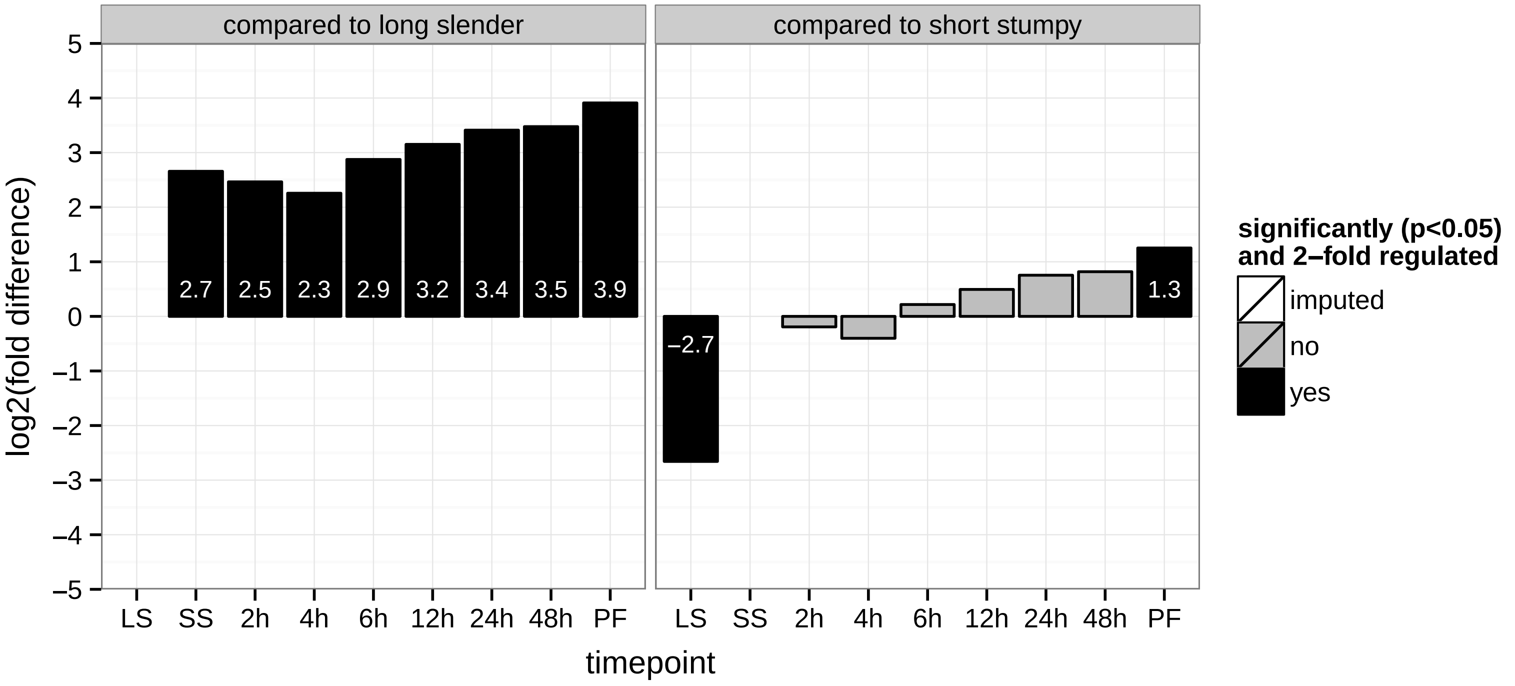

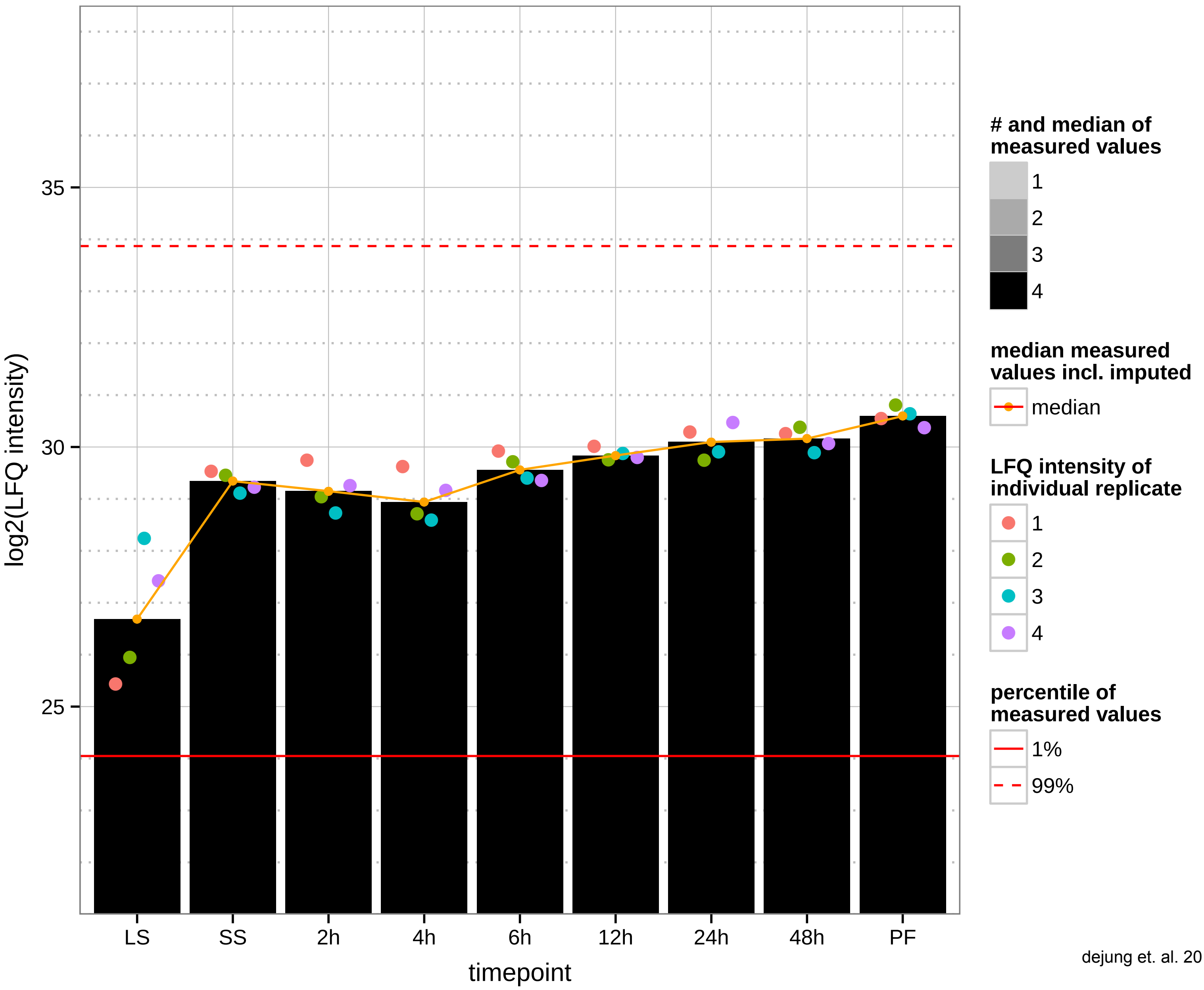

dejung et. al. 2015

40S ribosomal protein S15, putative
Tb927.7.2340;Tb927.7.2370
AGOF: structural constituent of ribosome
AGOC: intracellular, ribosome, small ribosomal subunit
AGOP: translation
PGOF: structural constituent of ribosome
PGOC: ribosome, small ribosomal subunit
PGOP: translation

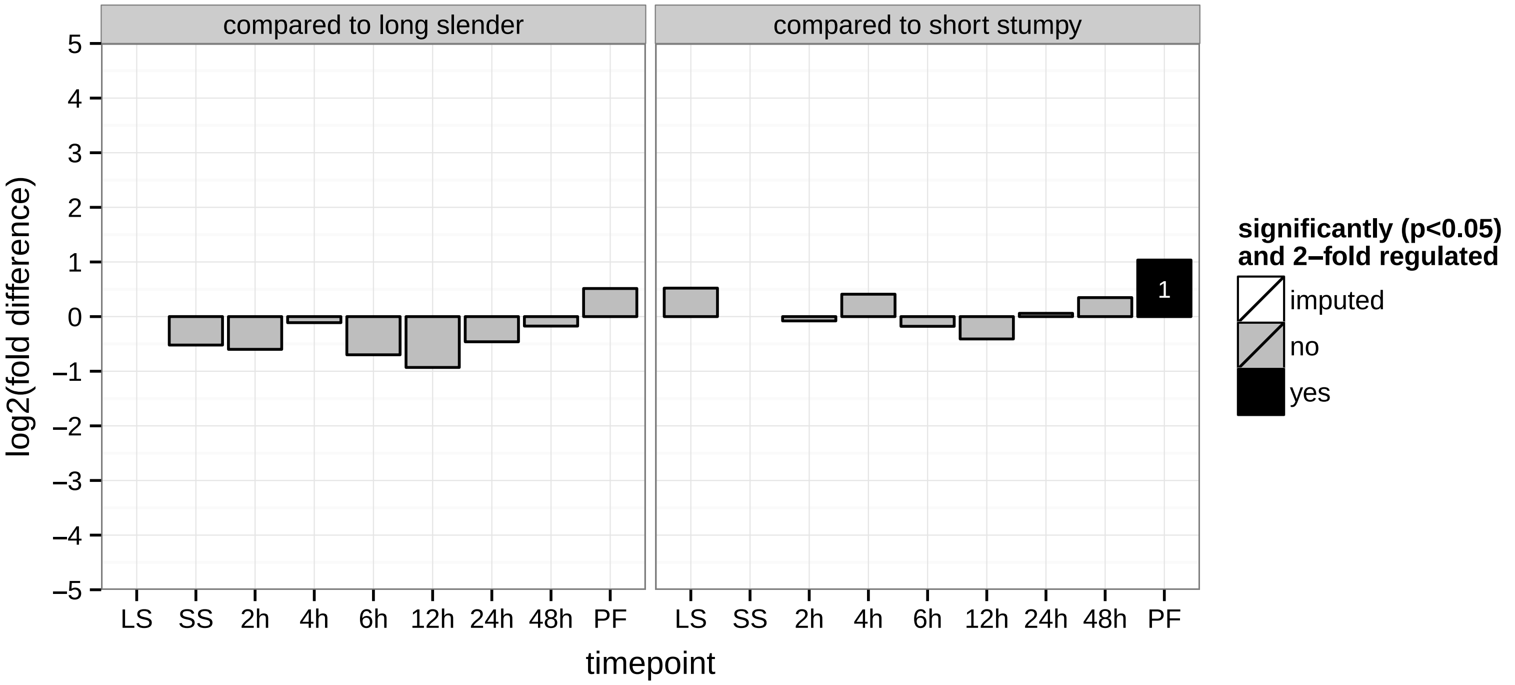

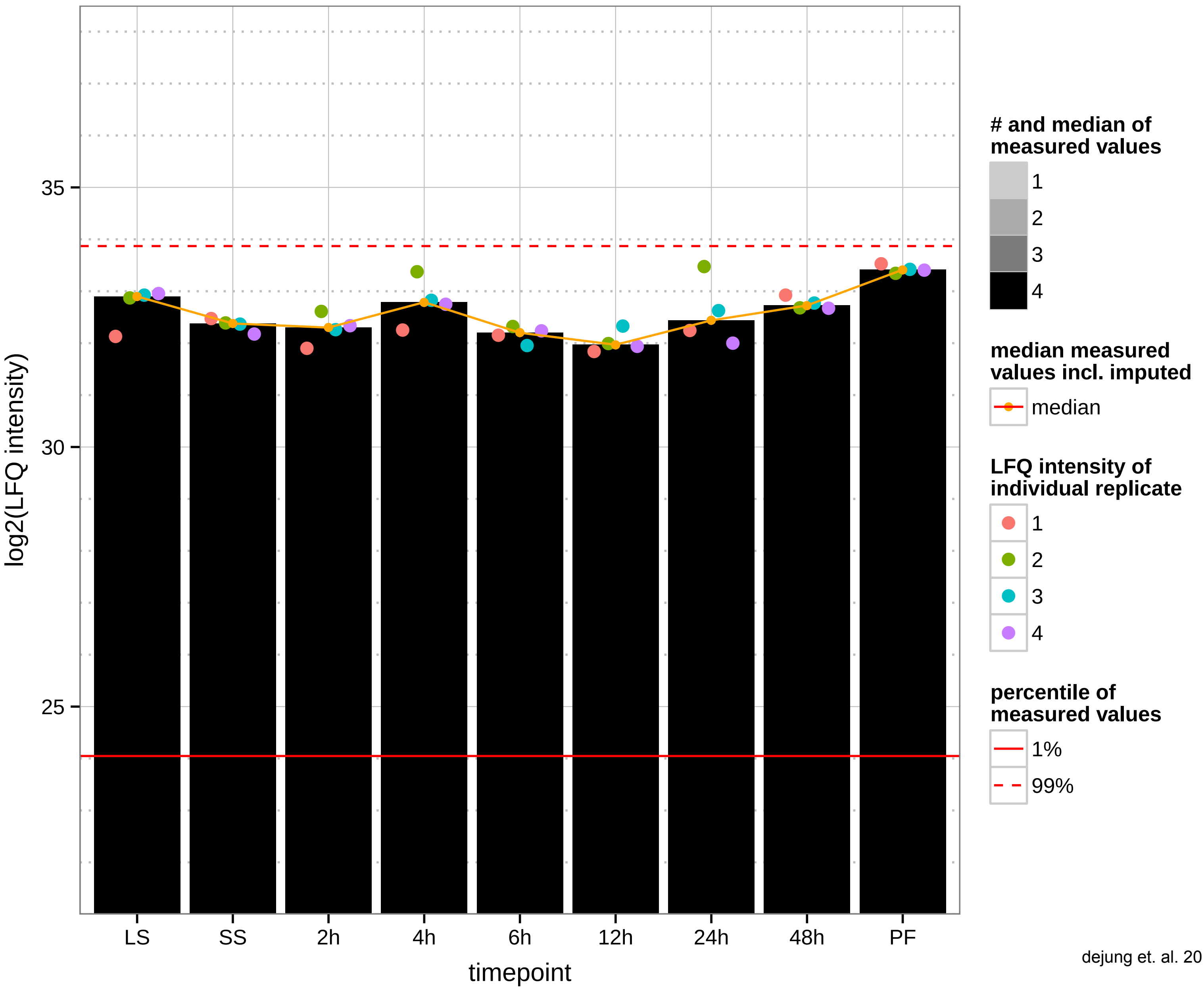

dejung et. al. 2015

hypothetical protein, conserved
Tb927.7.3050
AGOF: null
AGOC: mitochondrion
AGOP: null
PGOF: null
PGOC: null
PGOP: null

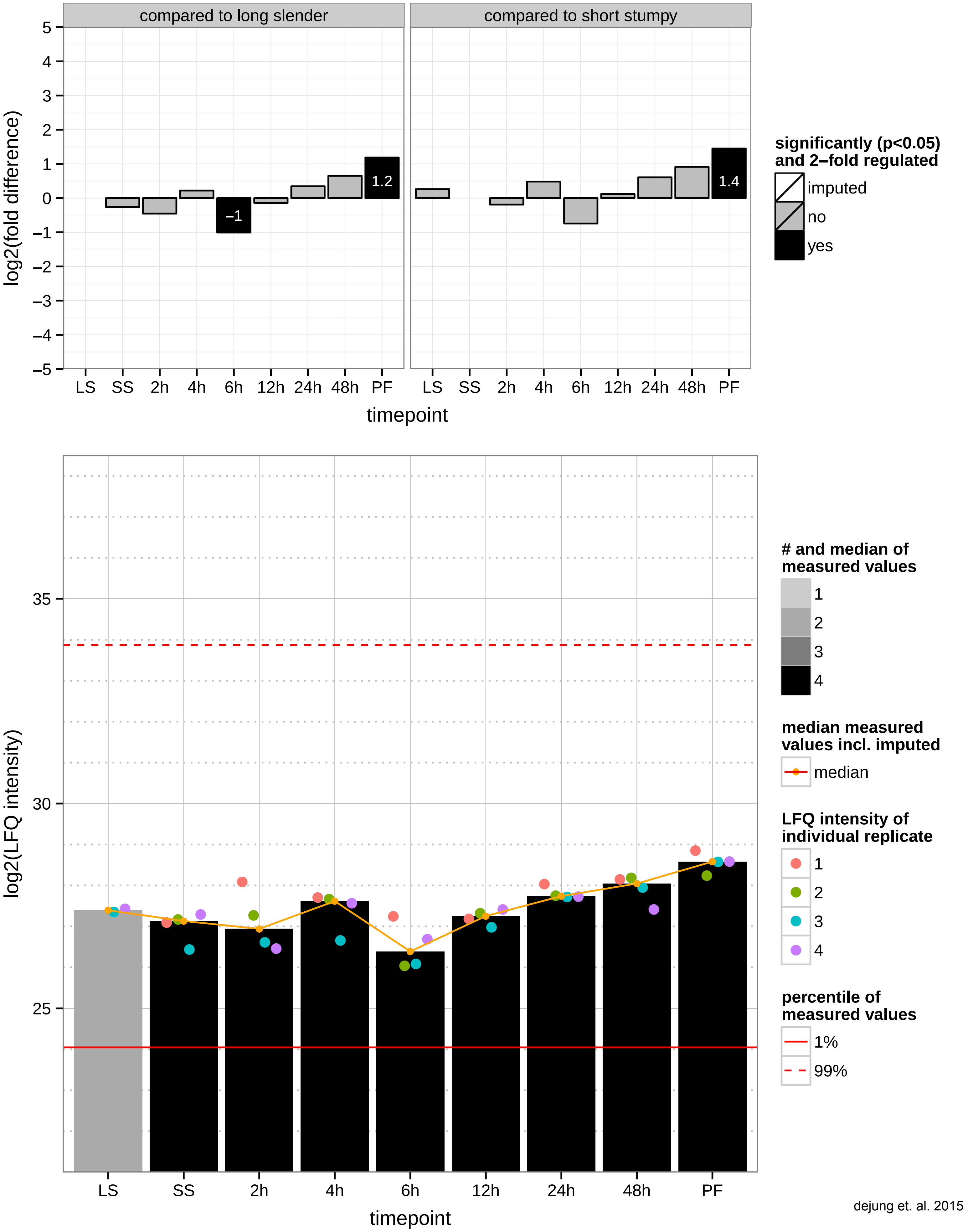

hypothetical protein, conserved
Tb927.7.3220
AGOF: null
AGOC: null
AGOP: null
PGOF: null
PGOC: null
PGOP: null

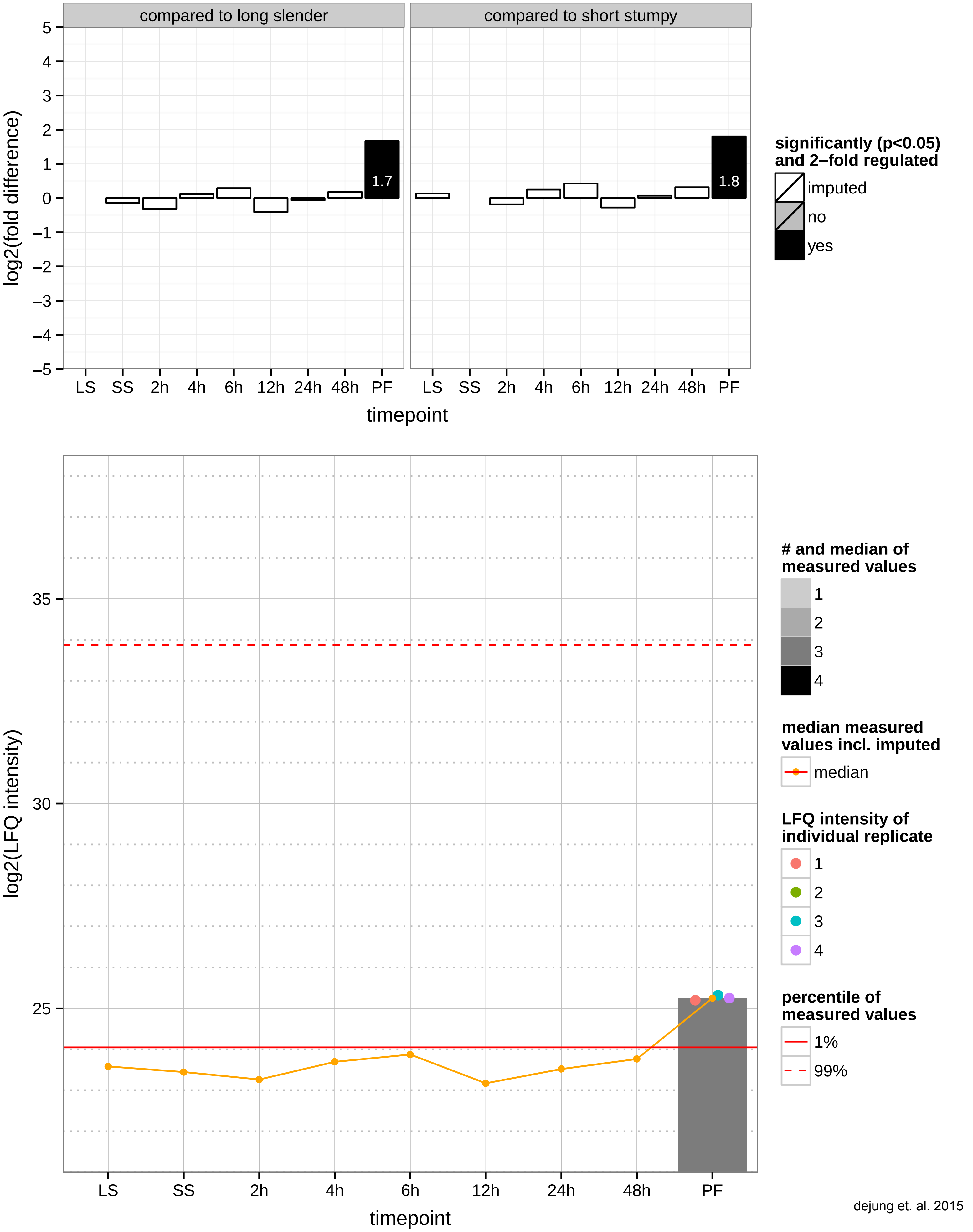

dejung et. al. 2015

hypothetical protein, conserved
Tb927.7.3240
AGOF: null
AGOC: mitochondrion
AGOP: null
PGOF: null
PGOC: null
PGOP: null

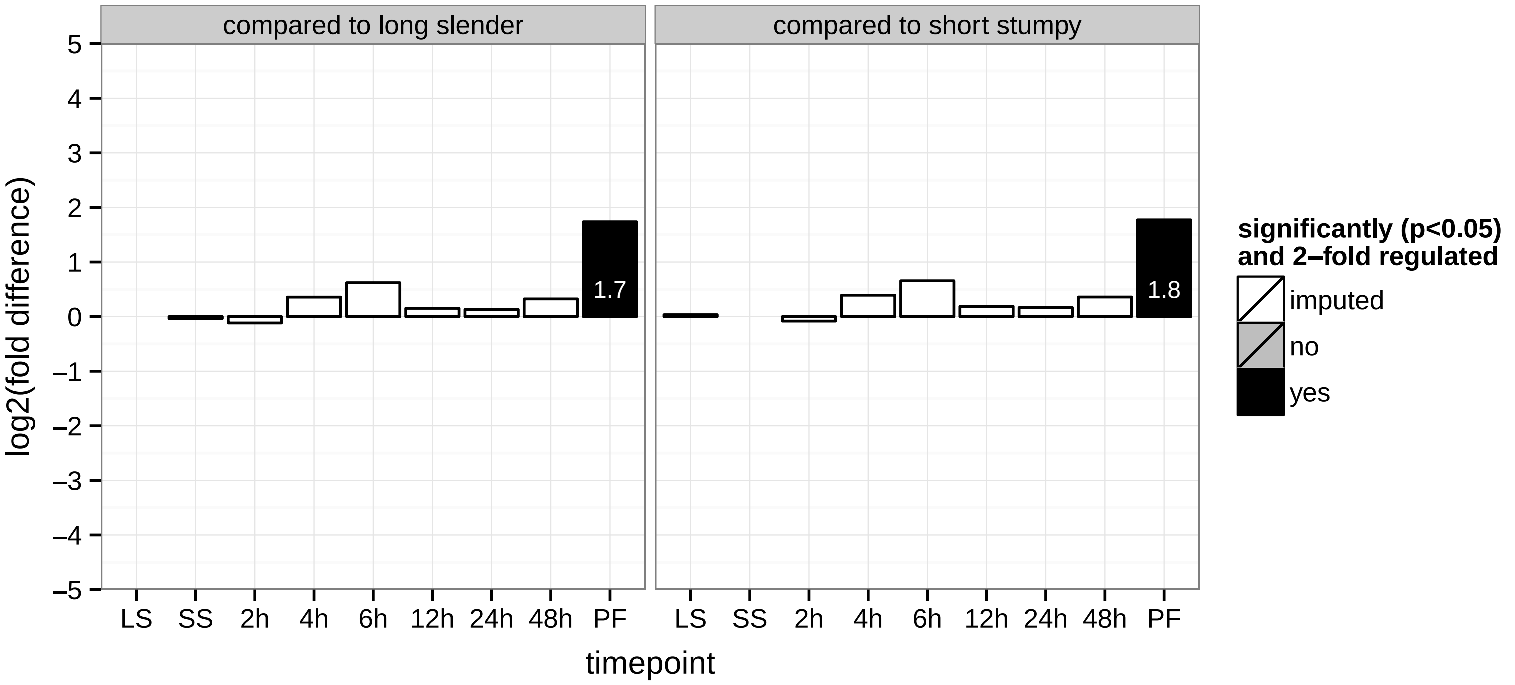

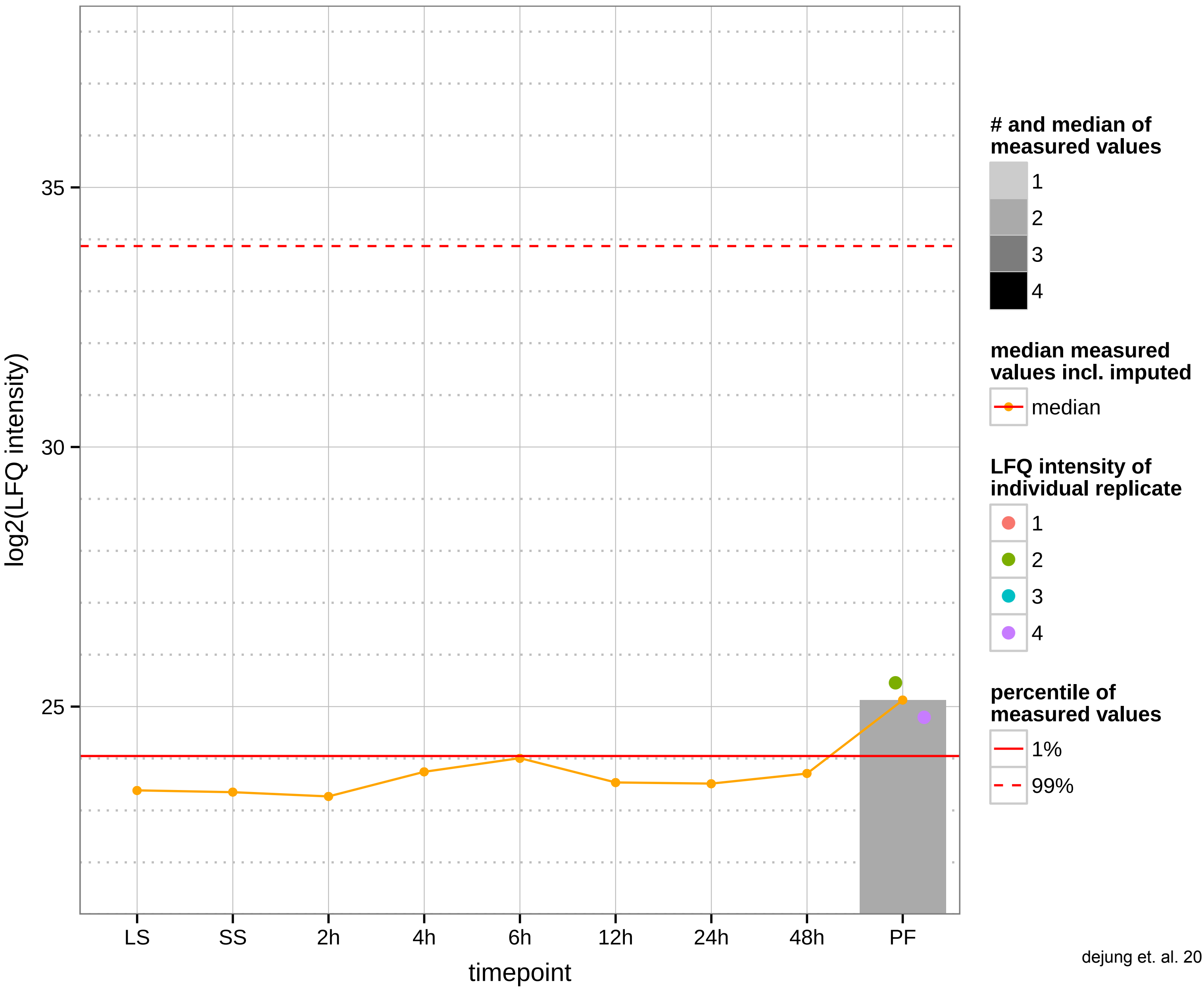

dejung et. al. 2015

cyclophilin–type peptidyl–prolyl cis–trans isomerase, putative (PPIase)
Tb927.7.3430
AGOF: peptidyl–prolyl cis–trans isomerase activity
AGOC: null
AGOP: protein folding
PGOF: peptidyl–prolyl cis–trans isomerase activity
PGOC: null
PGOP: protein folding

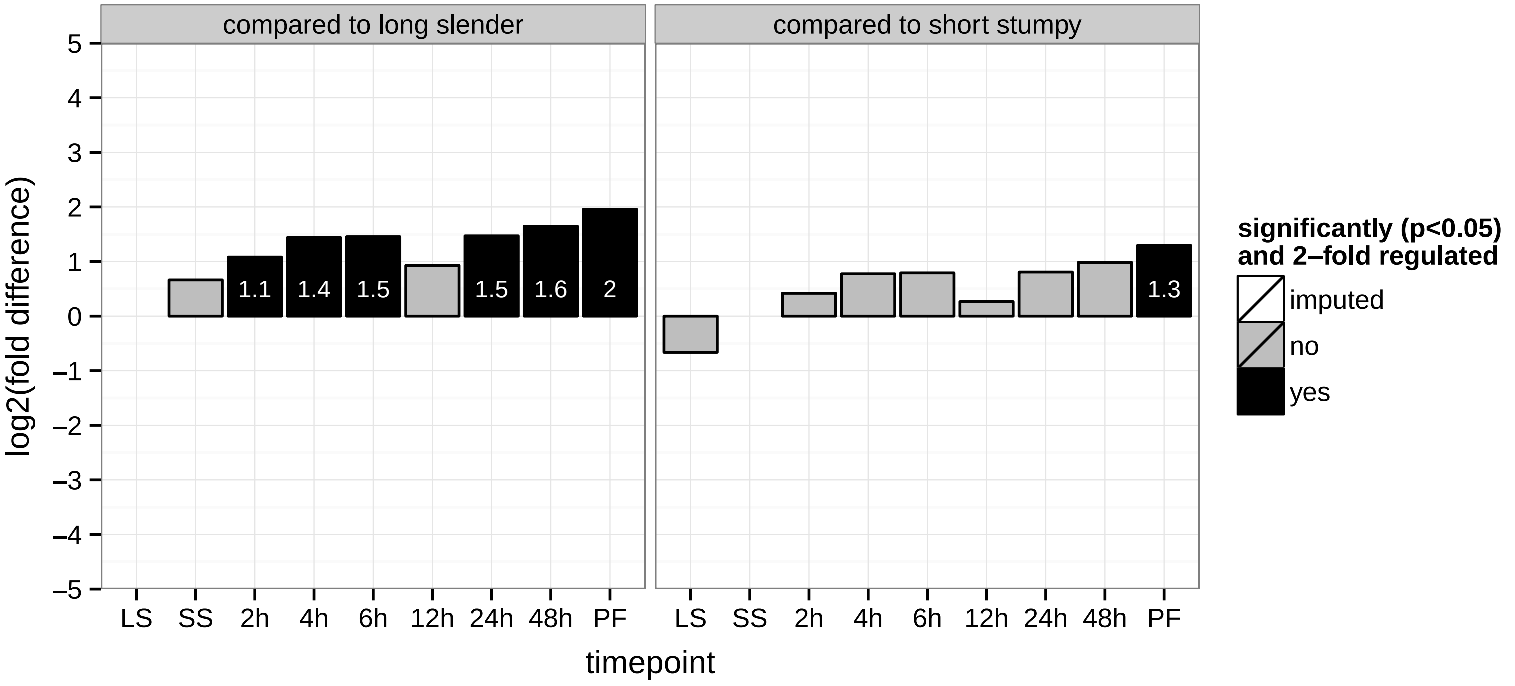

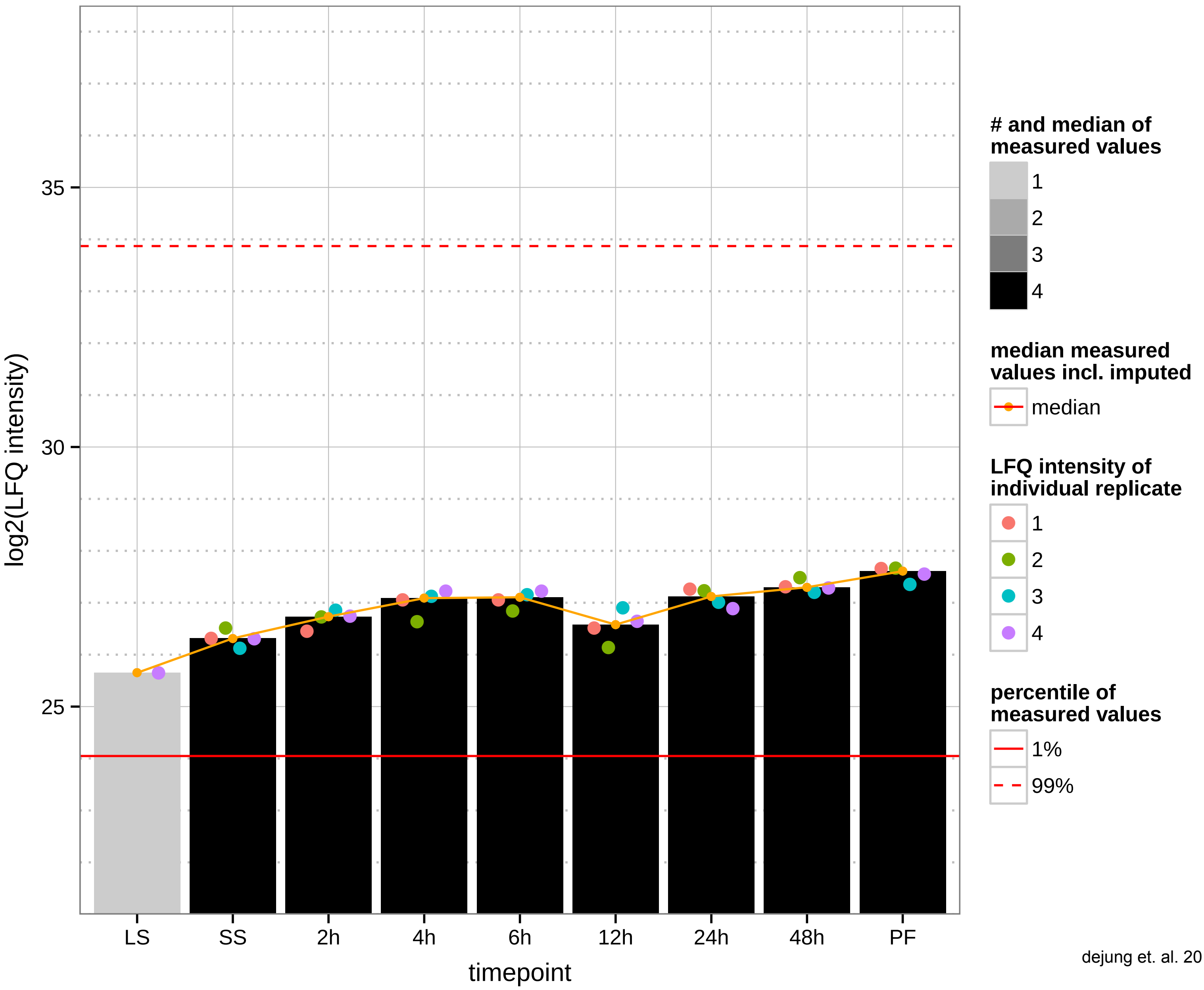

dejung et. al. 2015

hypothetical protein, conserved
Tb927.7.3460
AGOF: null
AGOC: mitochondrion
AGOP: null
PGOF: null
PGOC: null
PGOP: null

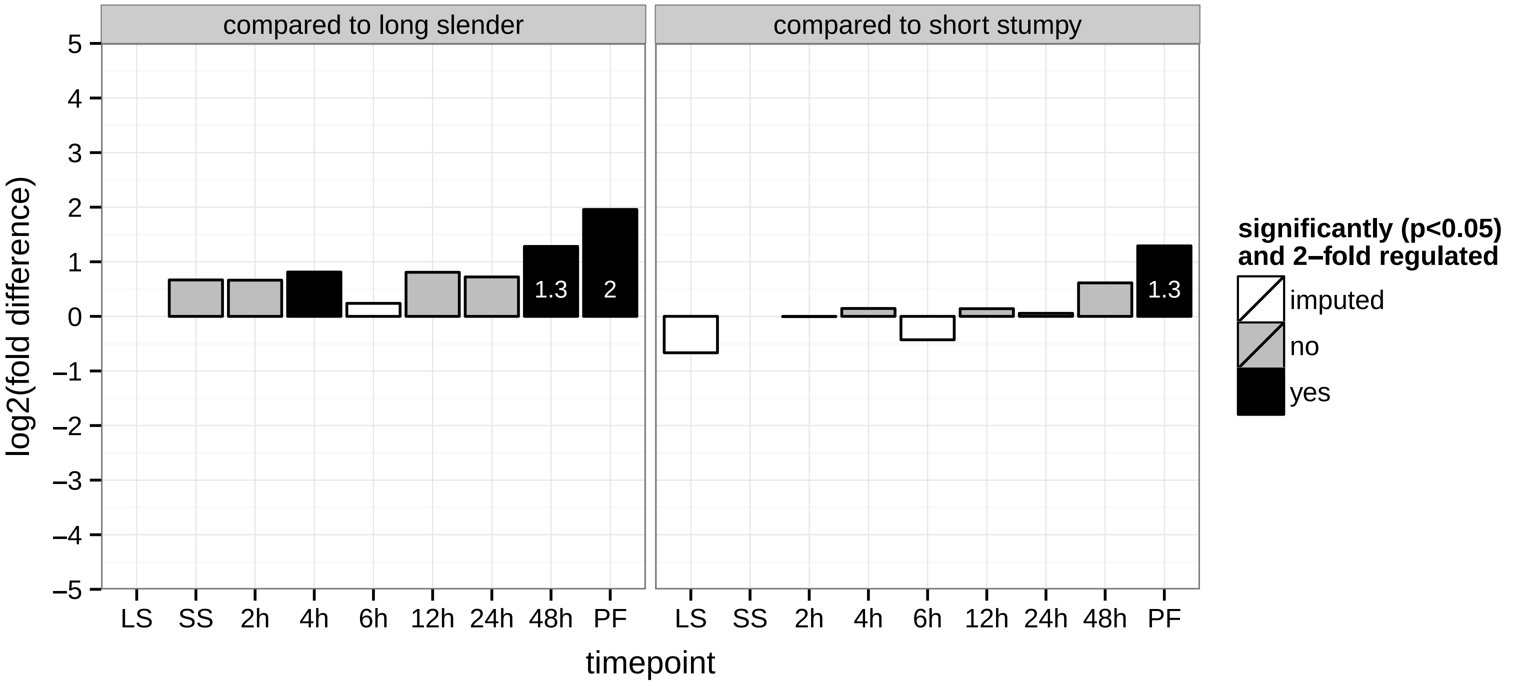

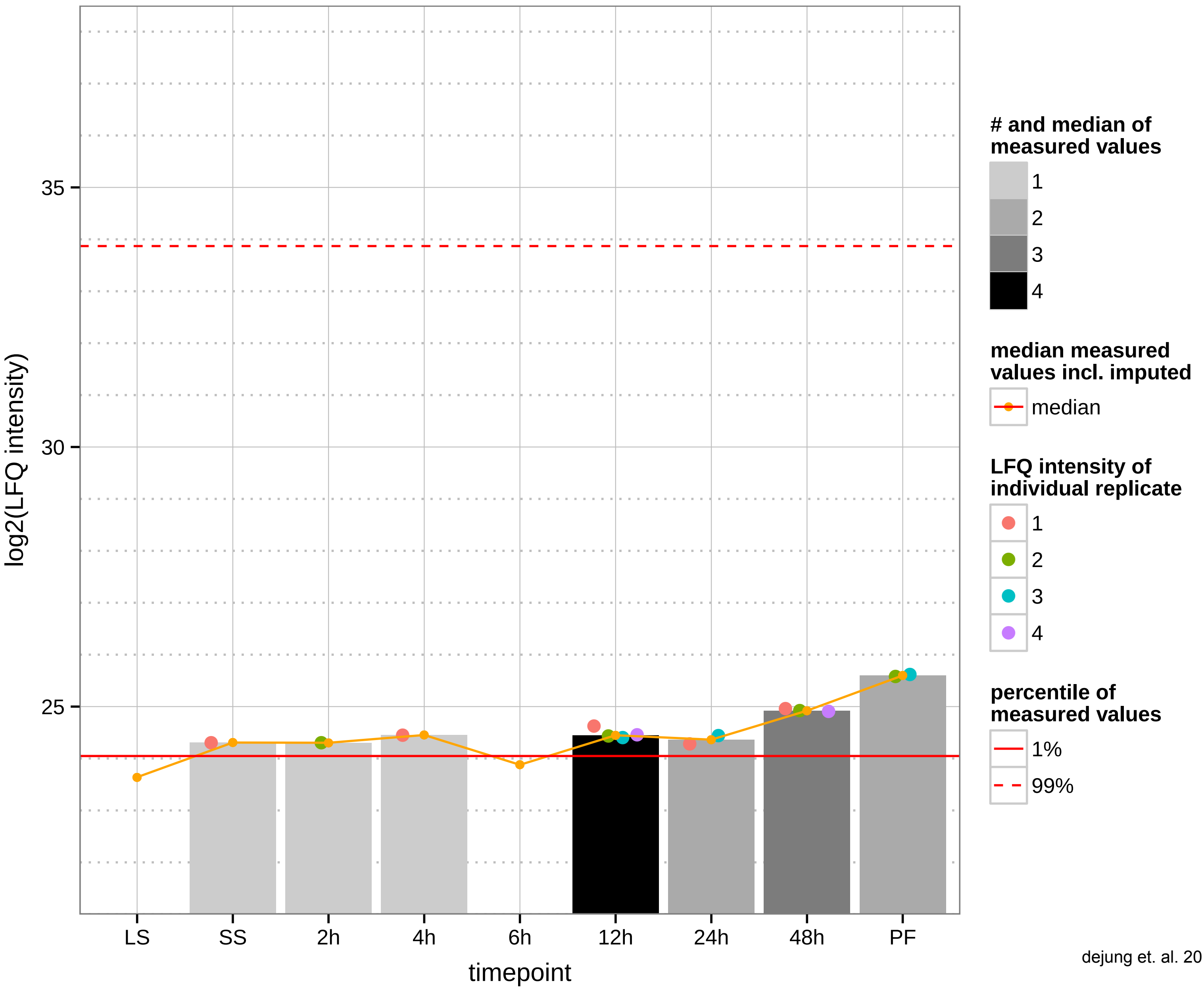

dejung et. al. 2015

poly(A) polymerase, putative
Tb927.7.3780
AGOF: RNA binding, nucleotidyltransferase activity, polynucleotide adenylyltransferase activity
AGOC: nucleus
AGOP: RNA polyadenylation, transcription, DNA–dependent
PGOF: RNA binding, nucleotidyltransferase activity, polynucleotide adenylyltransferase activity
PGOC: nucleus
PGOP: RNA 3'–end processing, RNA polyadenylation, transcription, DNA–dependent

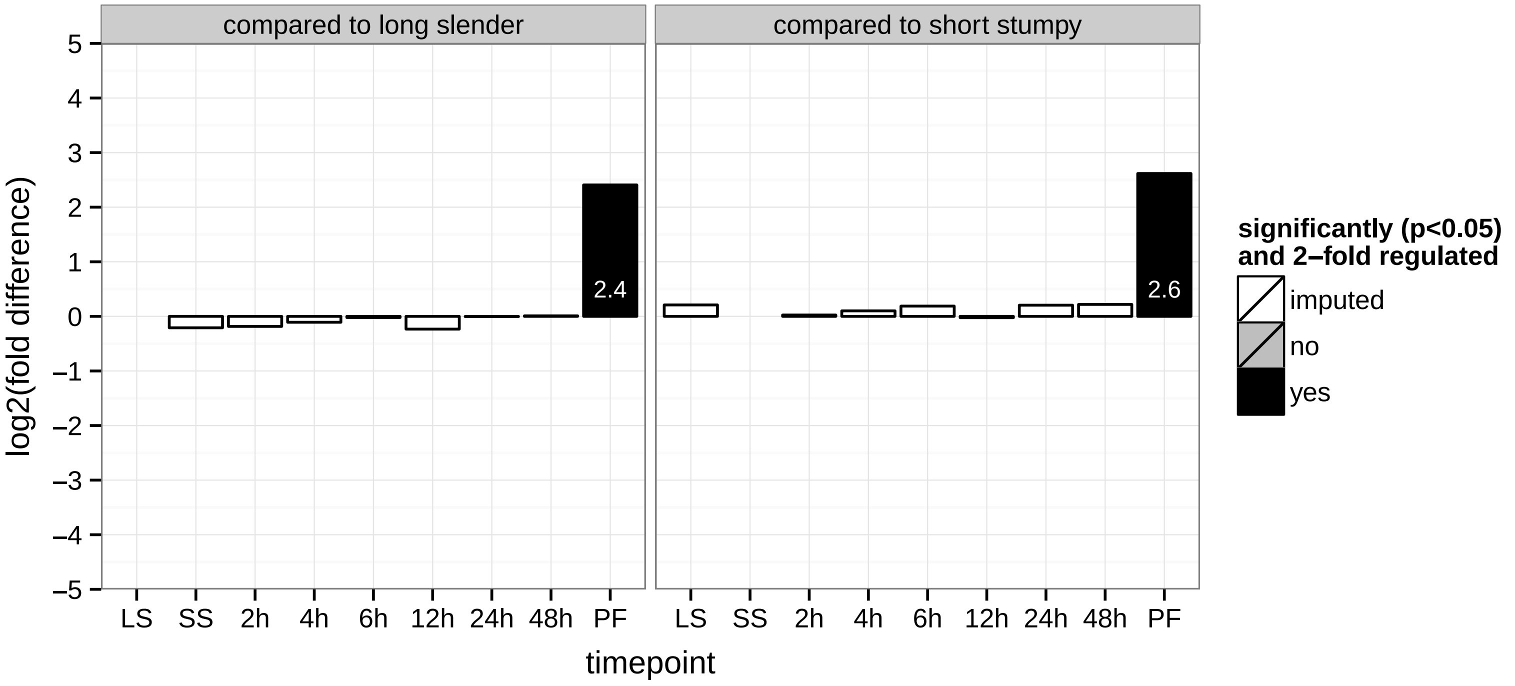

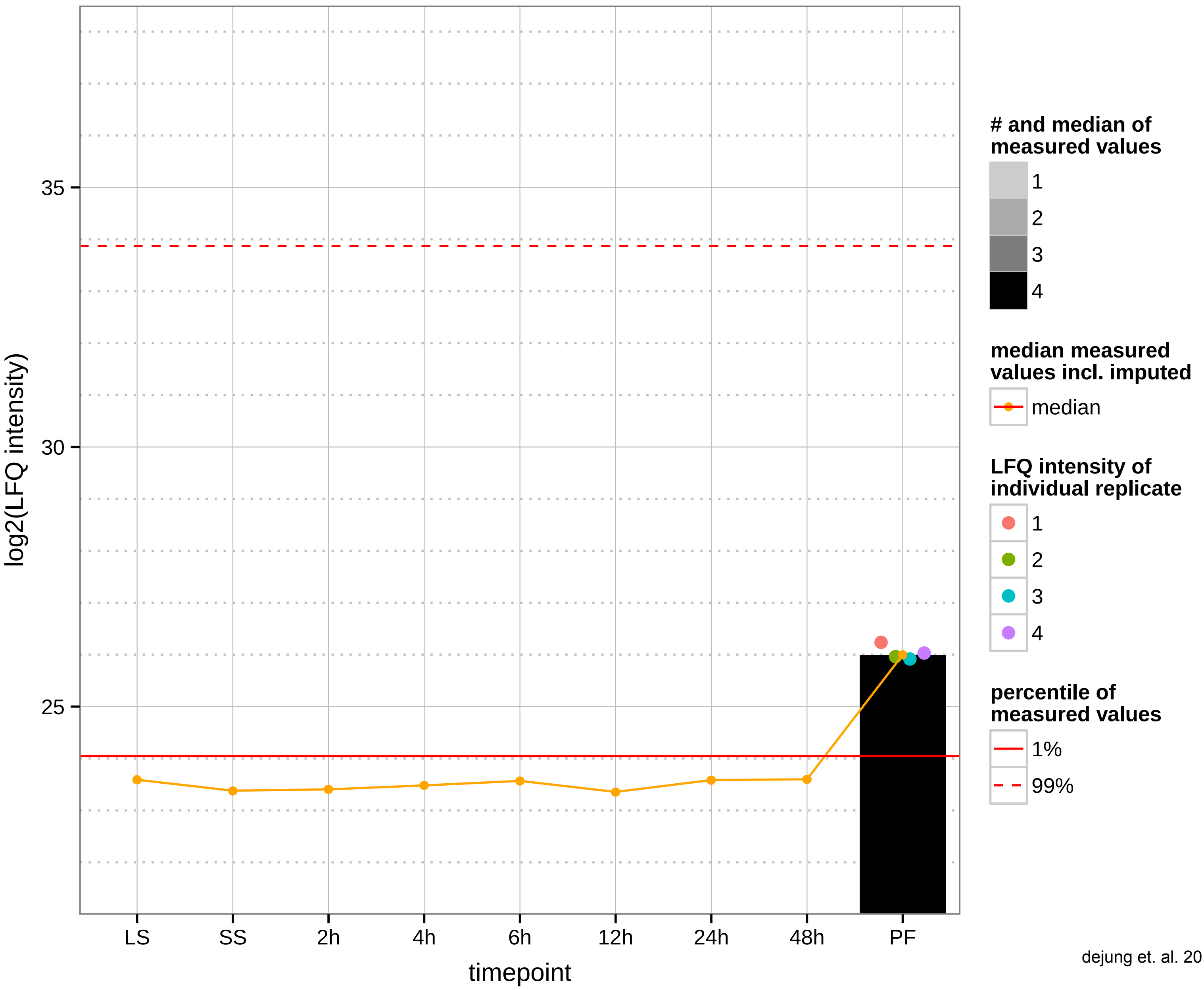

dejung et. al. 2015

hypothetical protein, conserved
Tb927.7.4120
AGOF: null
AGOC: null
AGOP: null
PGOF: null
PGOC: null
PGOP: null

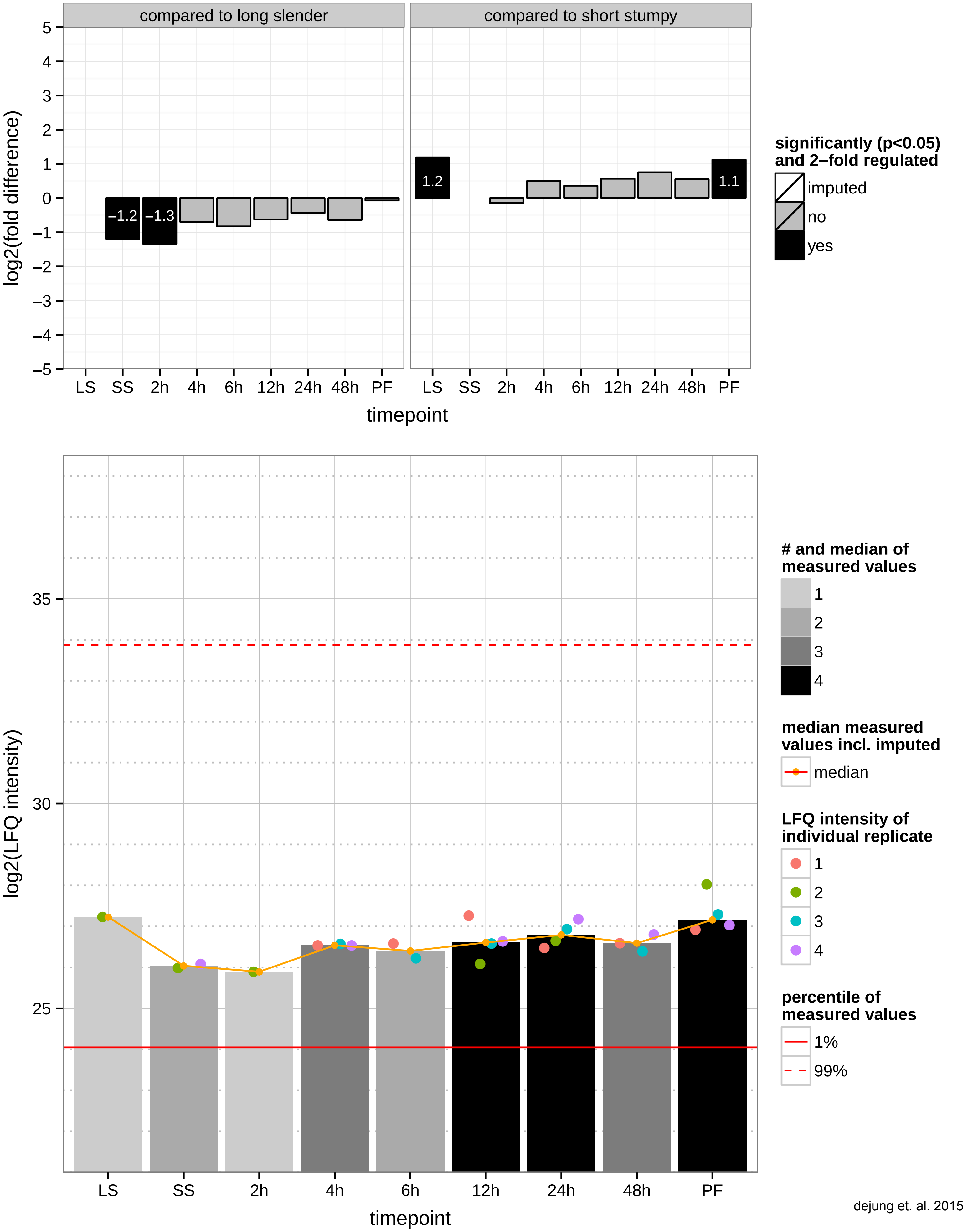

dejung et. al. 2015

hypothetical protein, conserved
Tb927.7.5090;Tb11.v5.0189
AGOF: null
AGOC: null
AGOP: null
PGOF: null
PGOC: null
PGOP: null

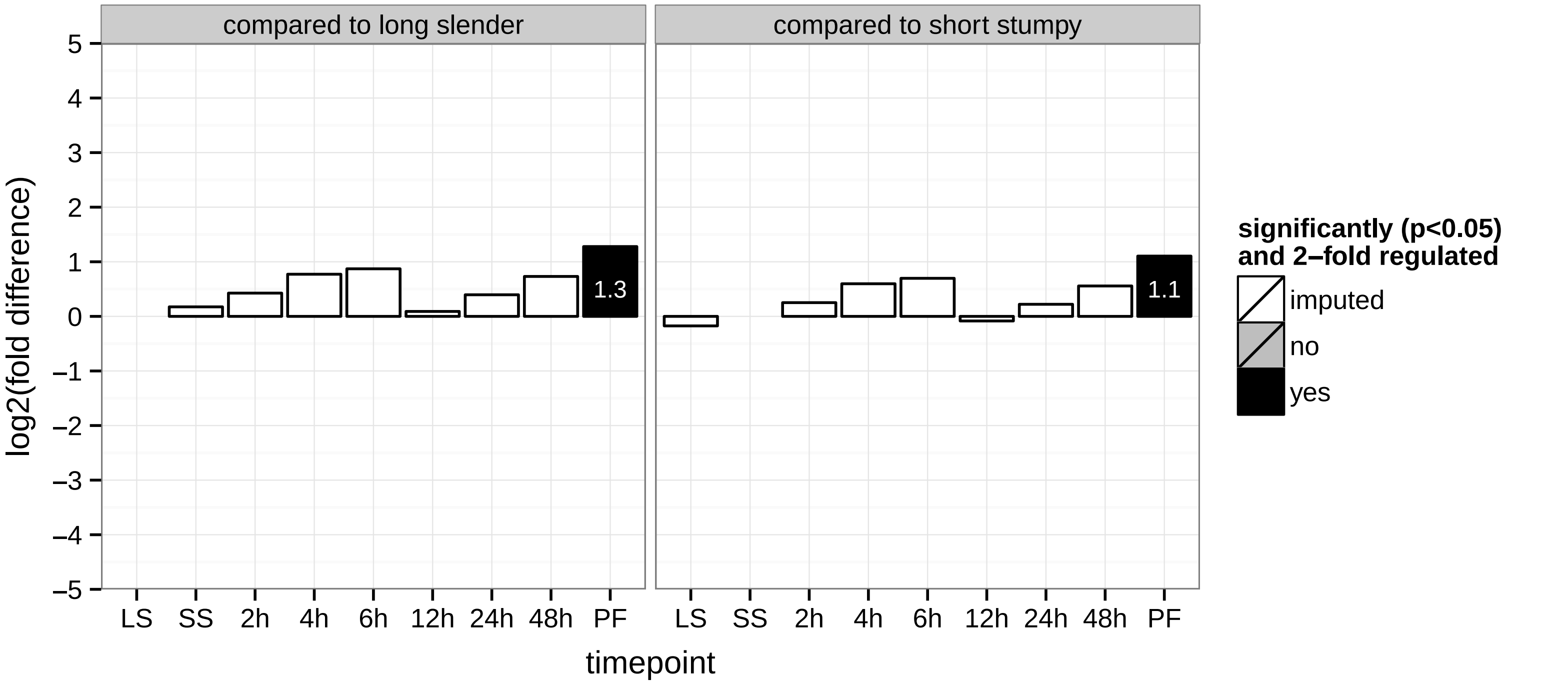

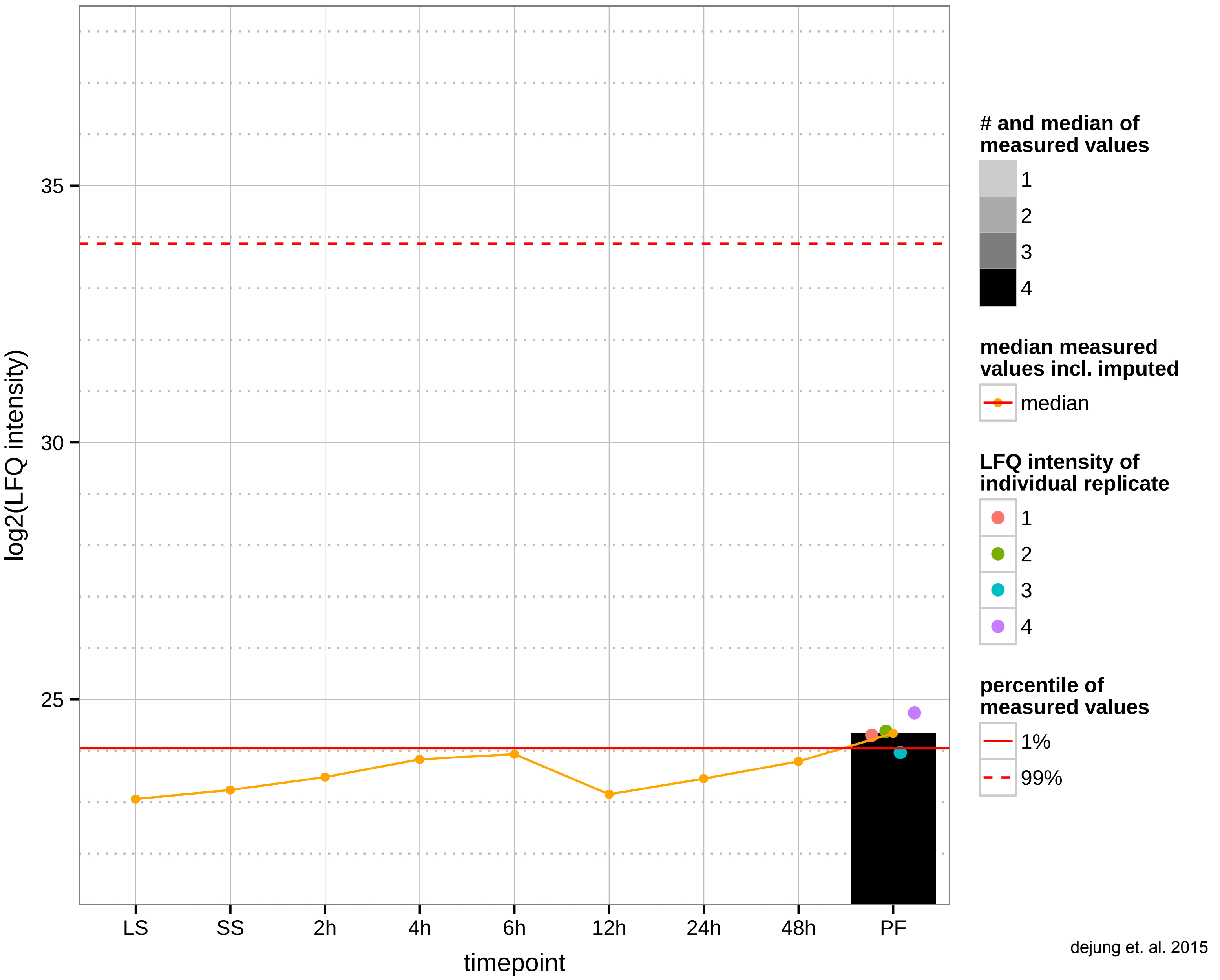

dejung et. al. 2015

hypothetical protein, conserved
Tb927.7.5130
AGOF: null
AGOC: mitochondrion
AGOP: null
PGOF: null
PGOC: null
PGOP: null

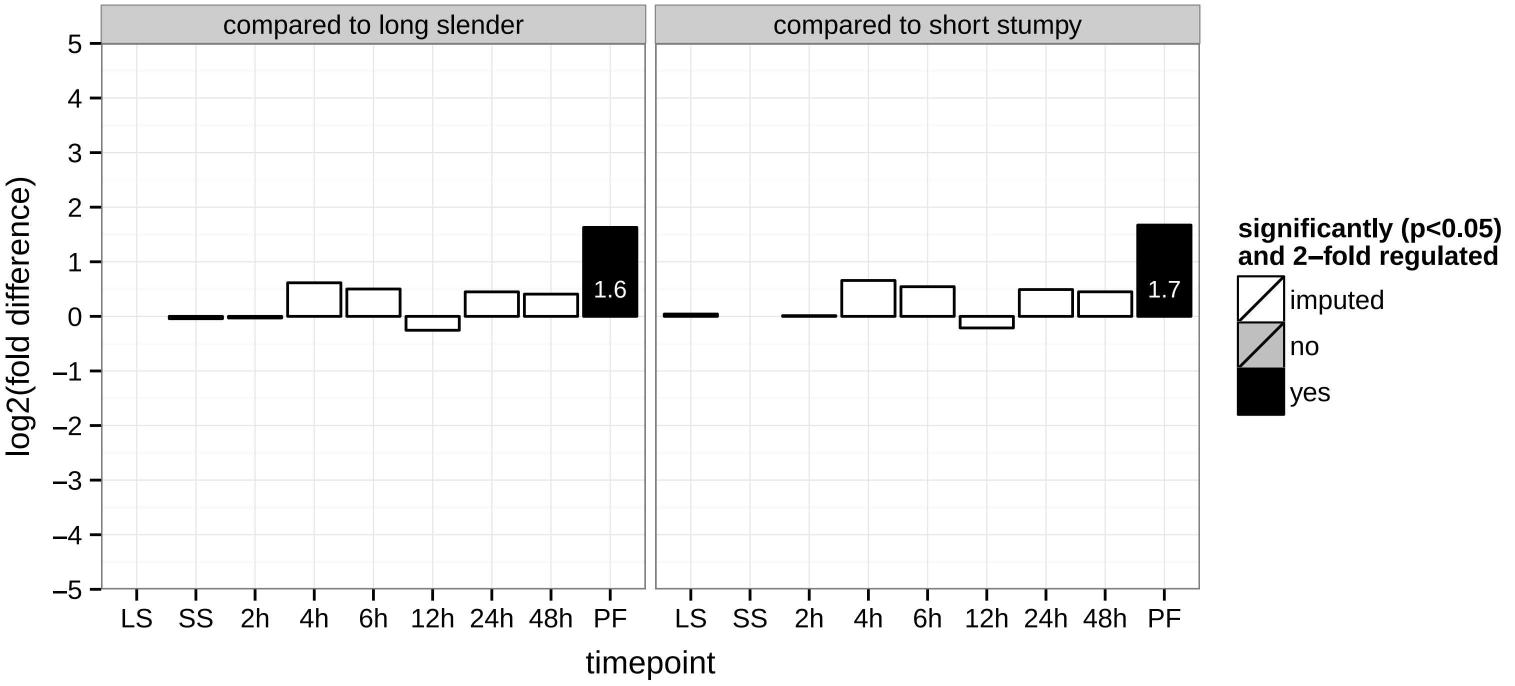

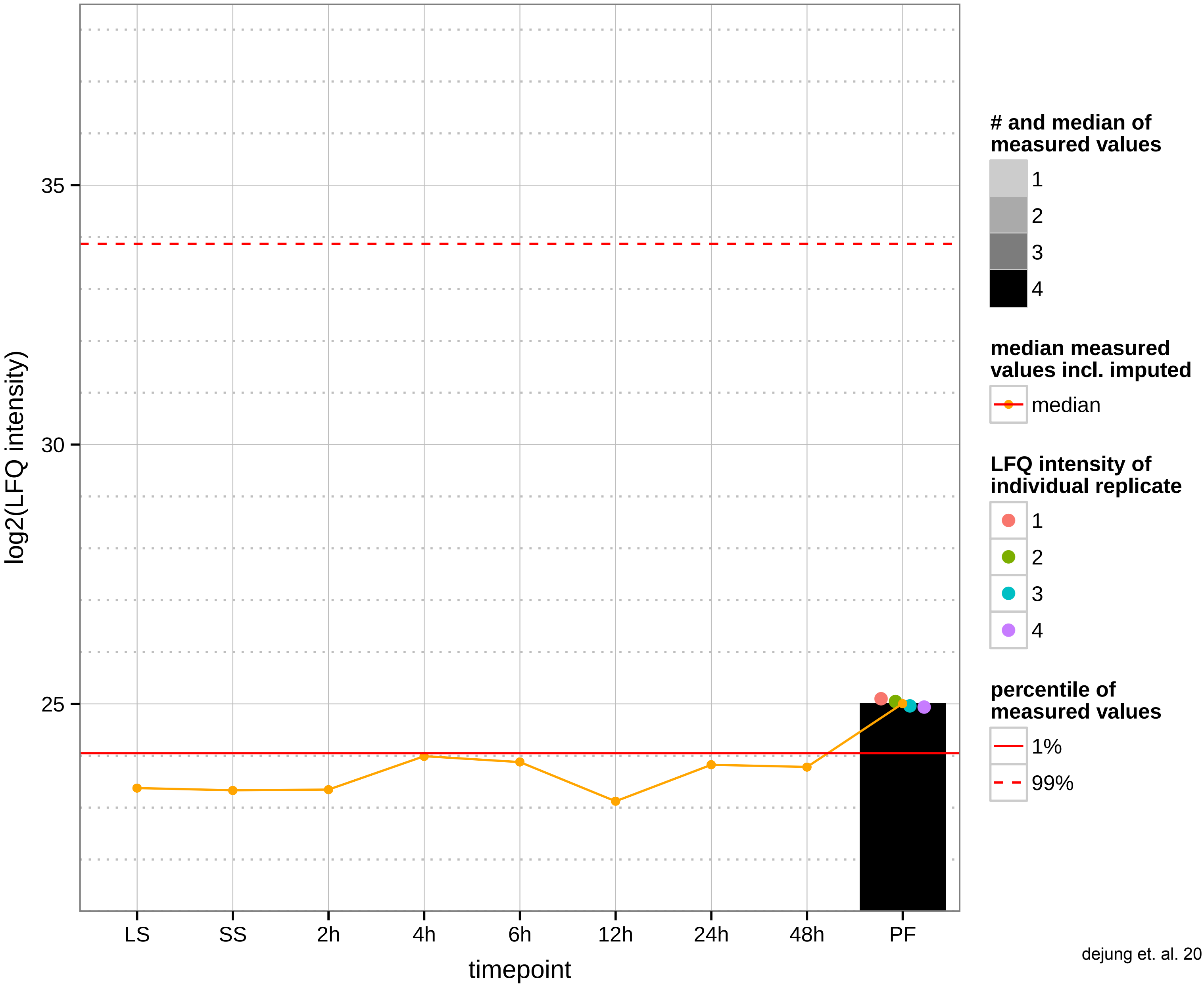

dejung et. al. 2015

hypothetical protein, conserved
Tb927.7.5260
AGOF: null
AGOC: mitochondrion
AGOP: null
PGOF: null
PGOC: null
PGOP: null

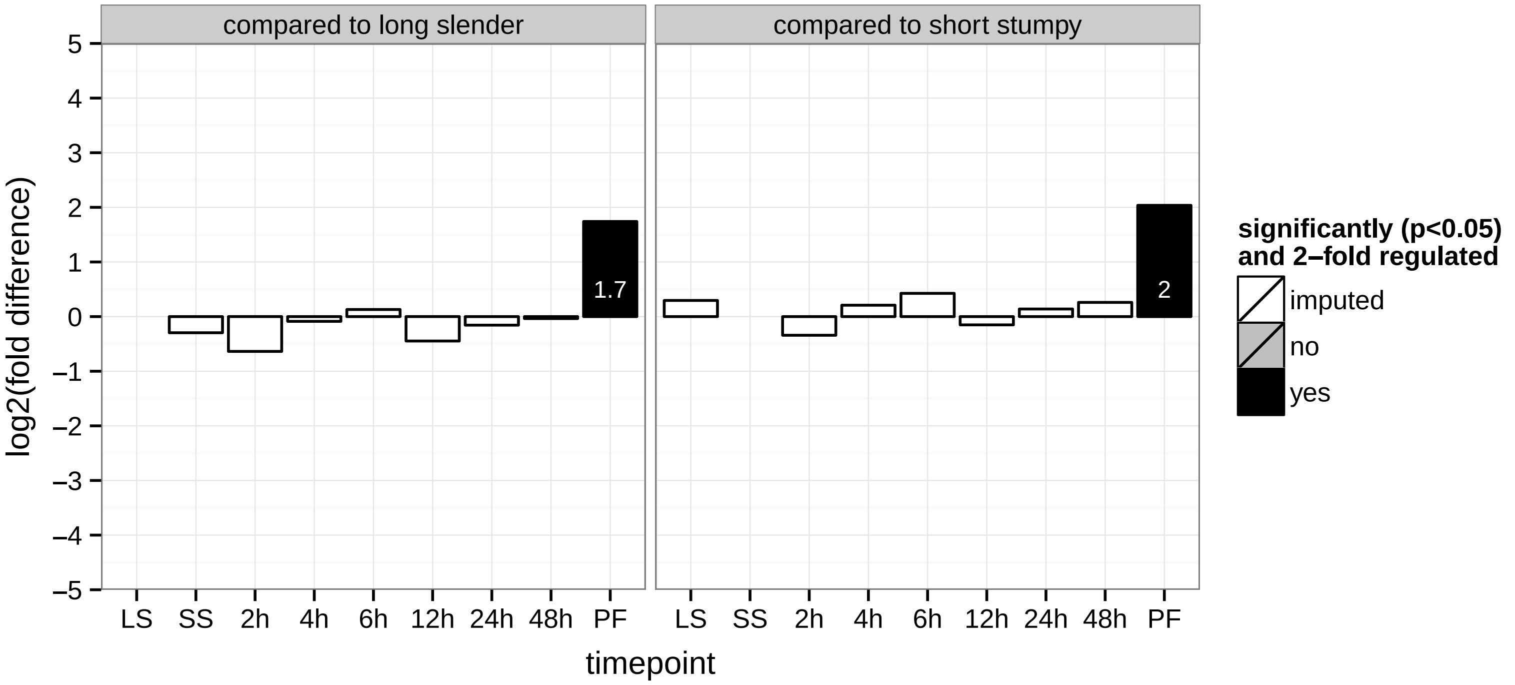

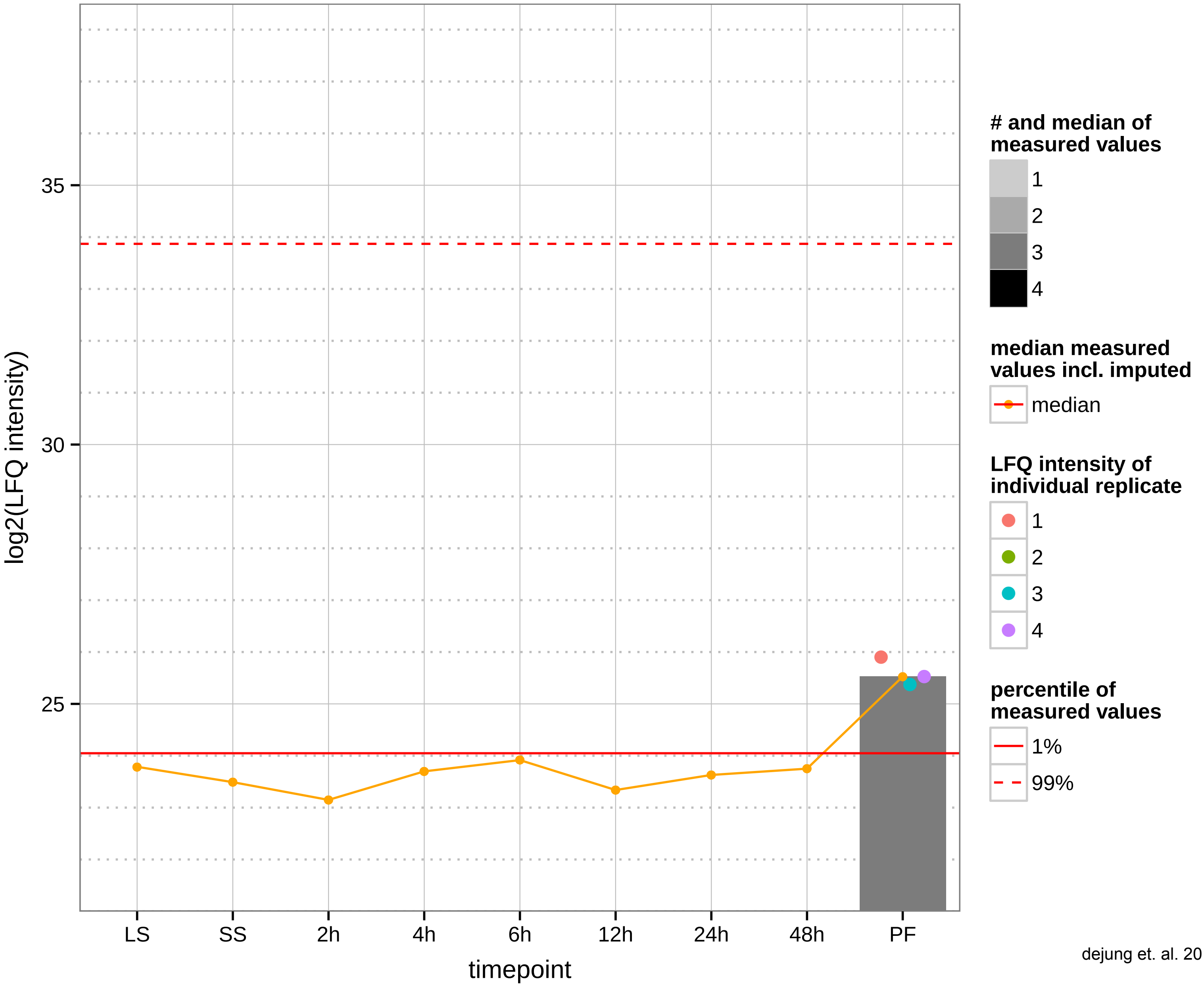

dejung et. al. 2015

hypothetical protein, conserved
Tb927.7.5300
AGOF: null
AGOC: null
AGOP: null
PGOF: protein binding
PGOC: null
PGOP: null

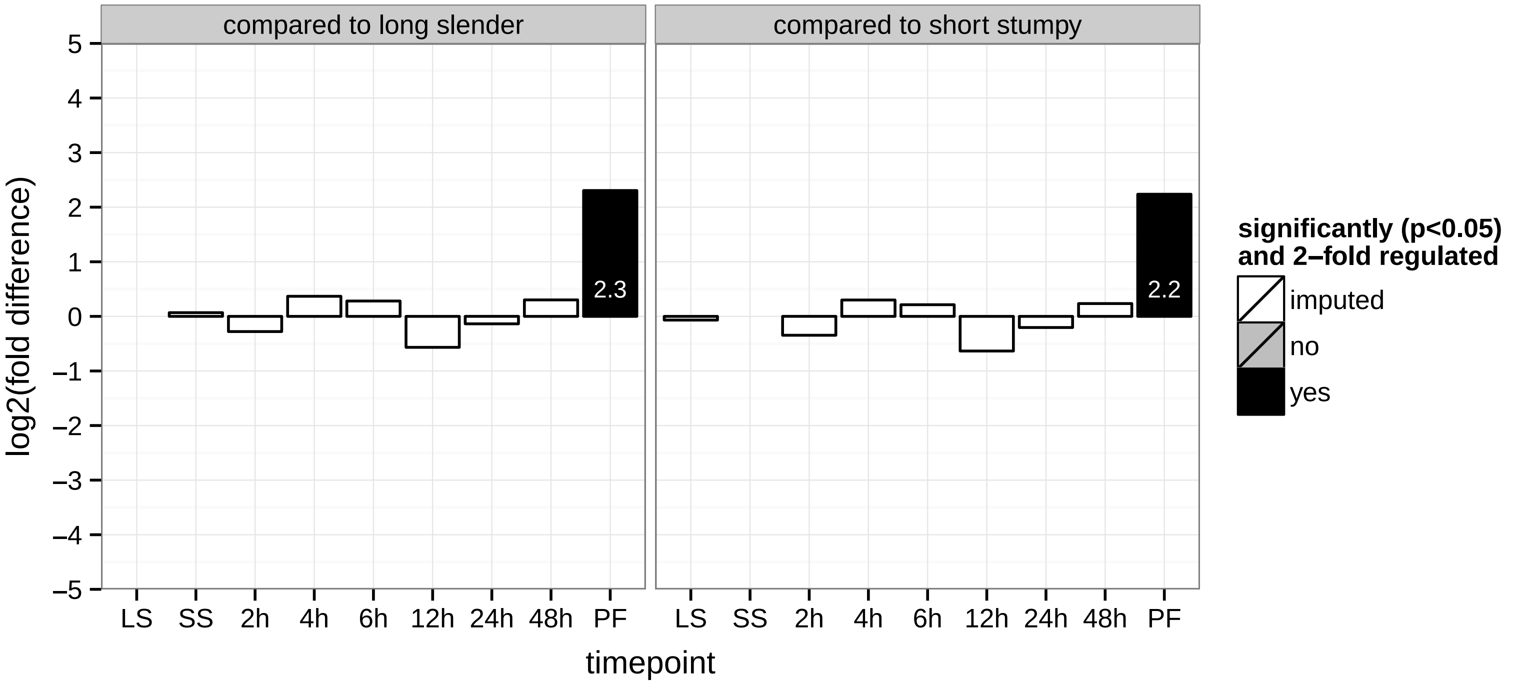

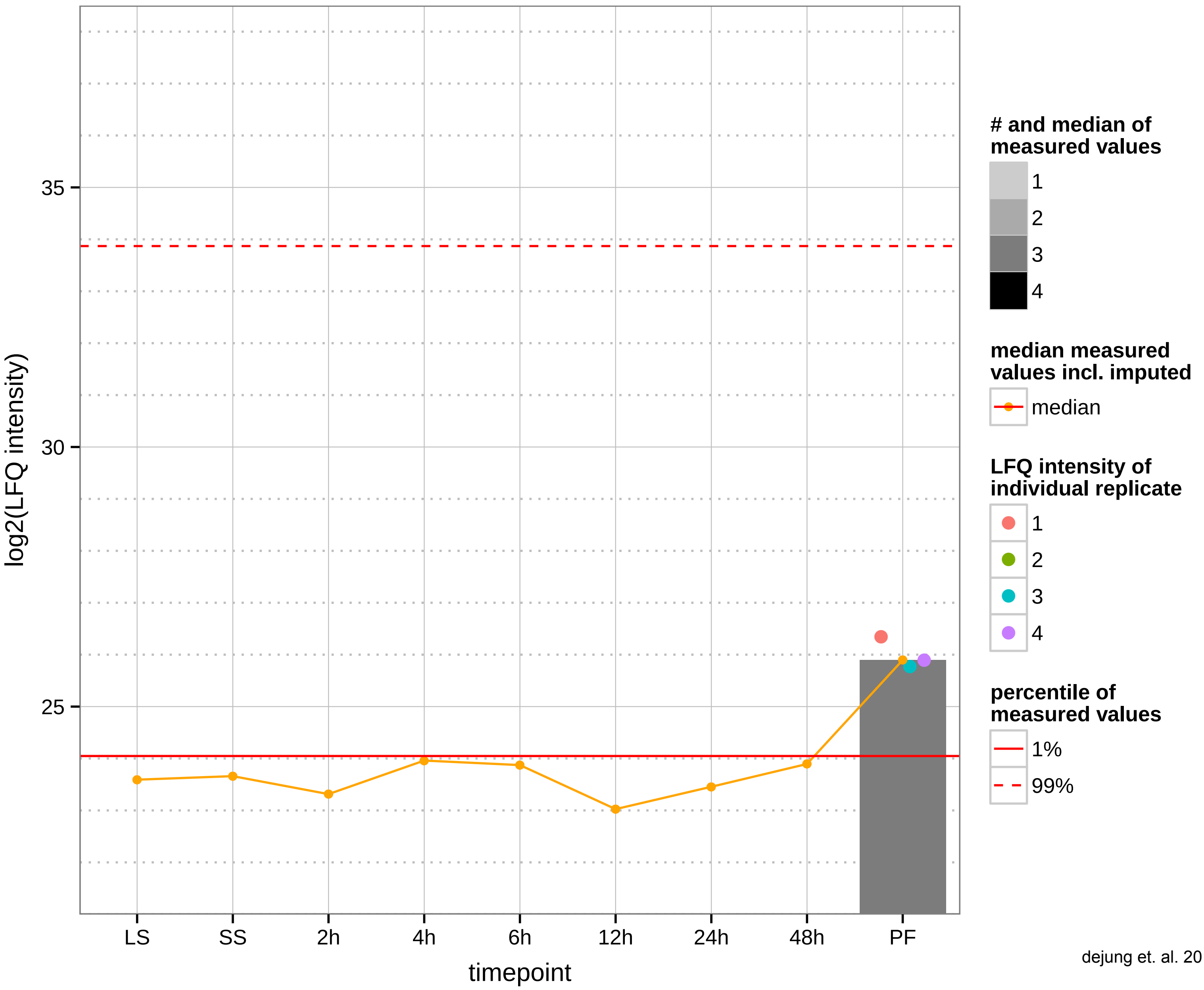

dejung et. al. 2015

prefoldin, putative
Tb927.7.570
AGOF: unfolded protein binding
AGOC: prefoldin complex
AGOP: protein folding
PGOF: unfolded protein binding
PGOC: prefoldin complex
PGOP: protein folding

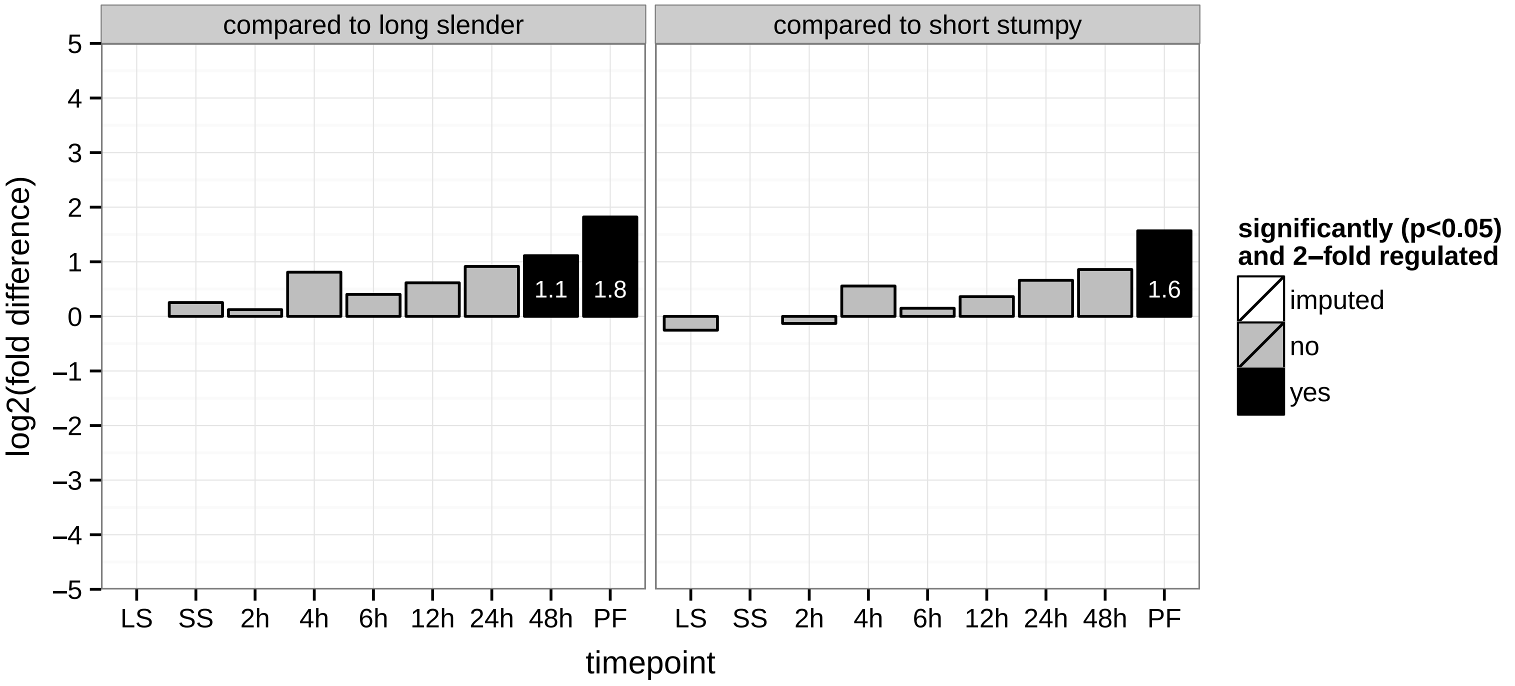

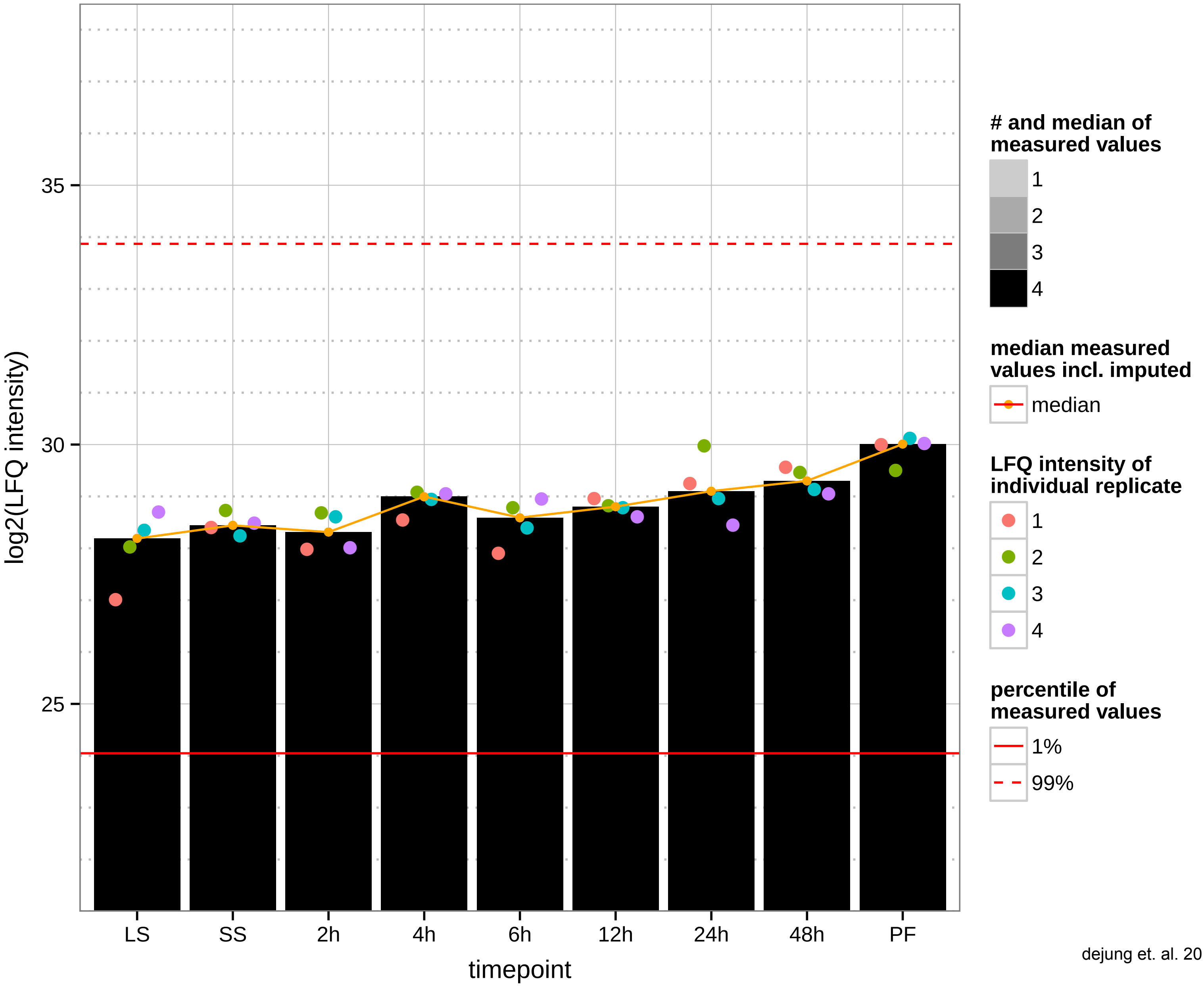

dejung et. al. 2015

hypothetical protein, conserved
Tb927.7.6260
AGOF: null
AGOC: null
AGOP: null
PGOF: protein binding
PGOC: null
PGOP: null

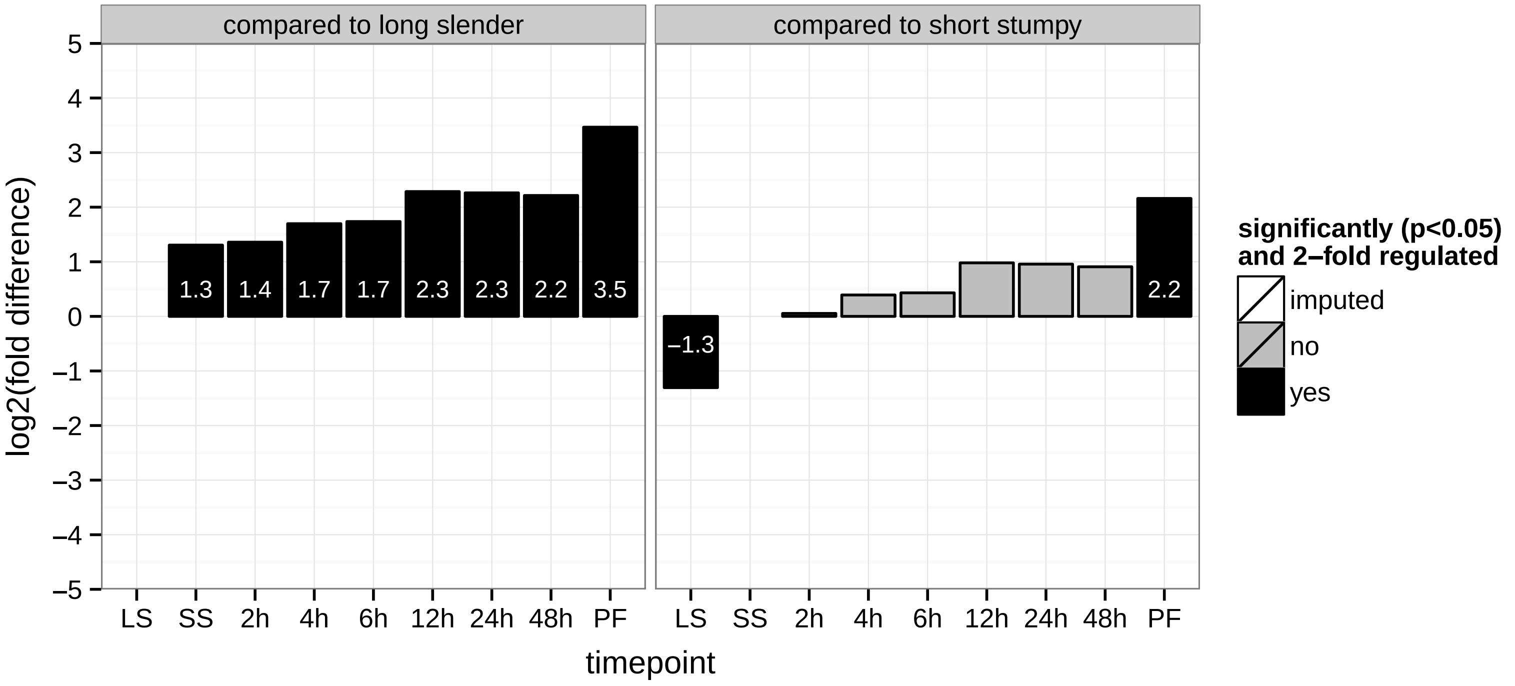

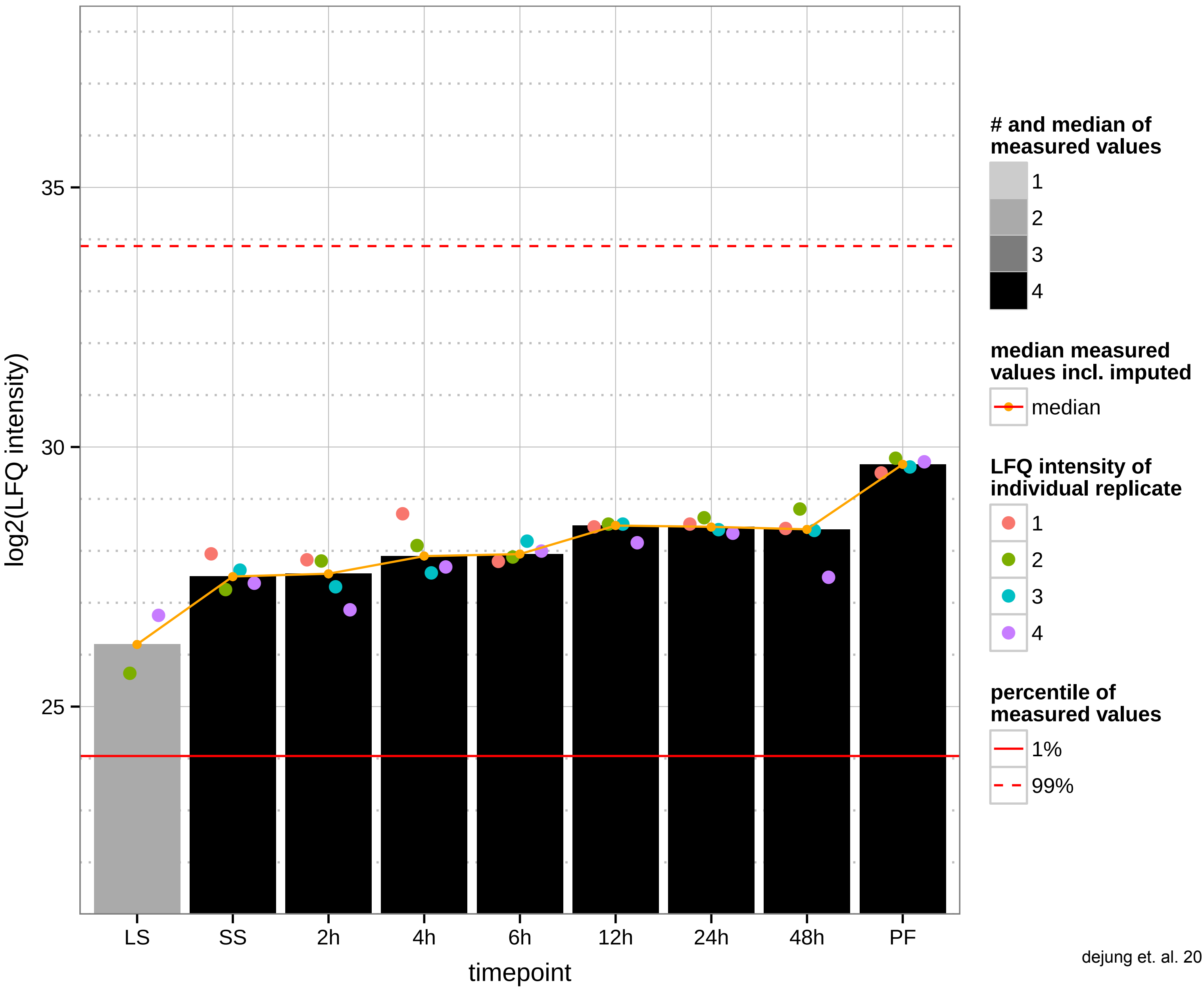

dejung et. al. 2015

FG–GAP repeat protein, putative, intergrin alpha chain protein
Tb927.7.6460
AGOF: null
AGOC: null
AGOP: null
PGOF: null
PGOC: null
PGOP: null

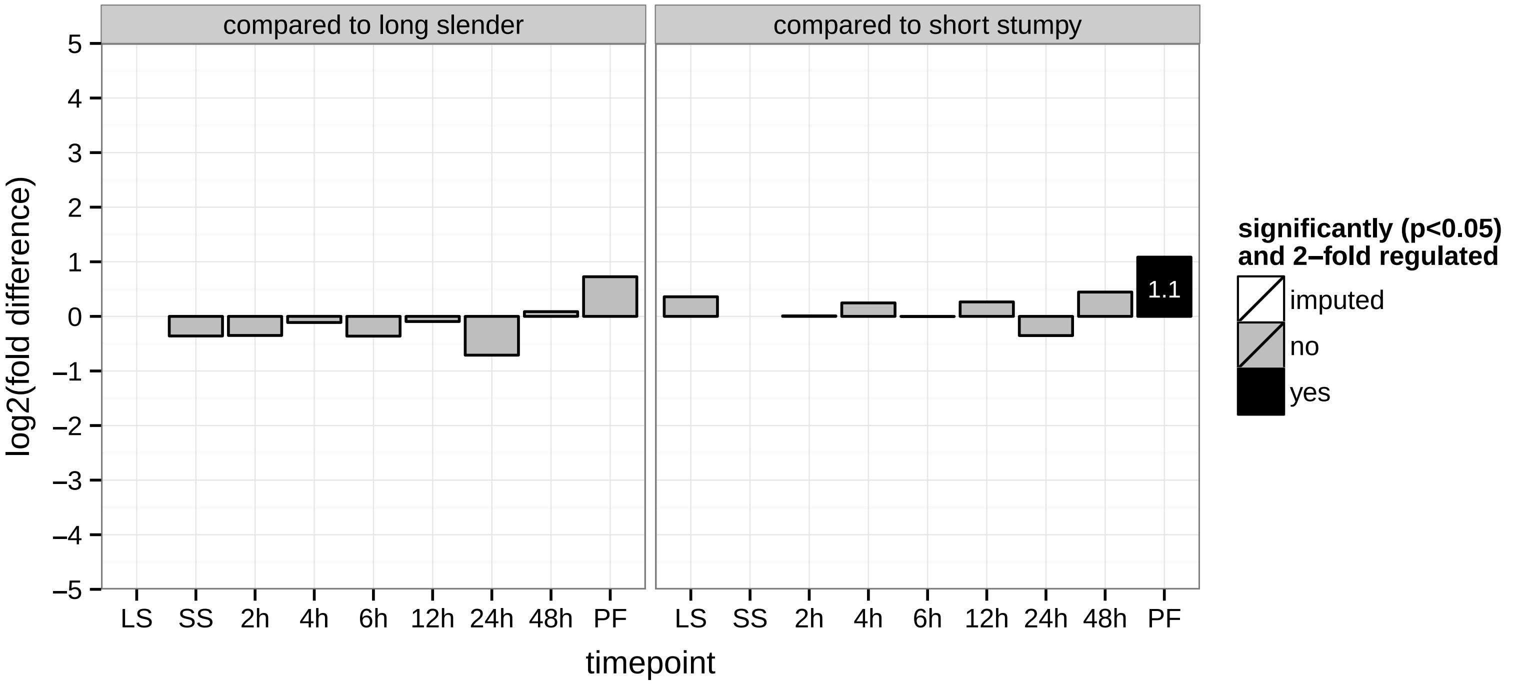

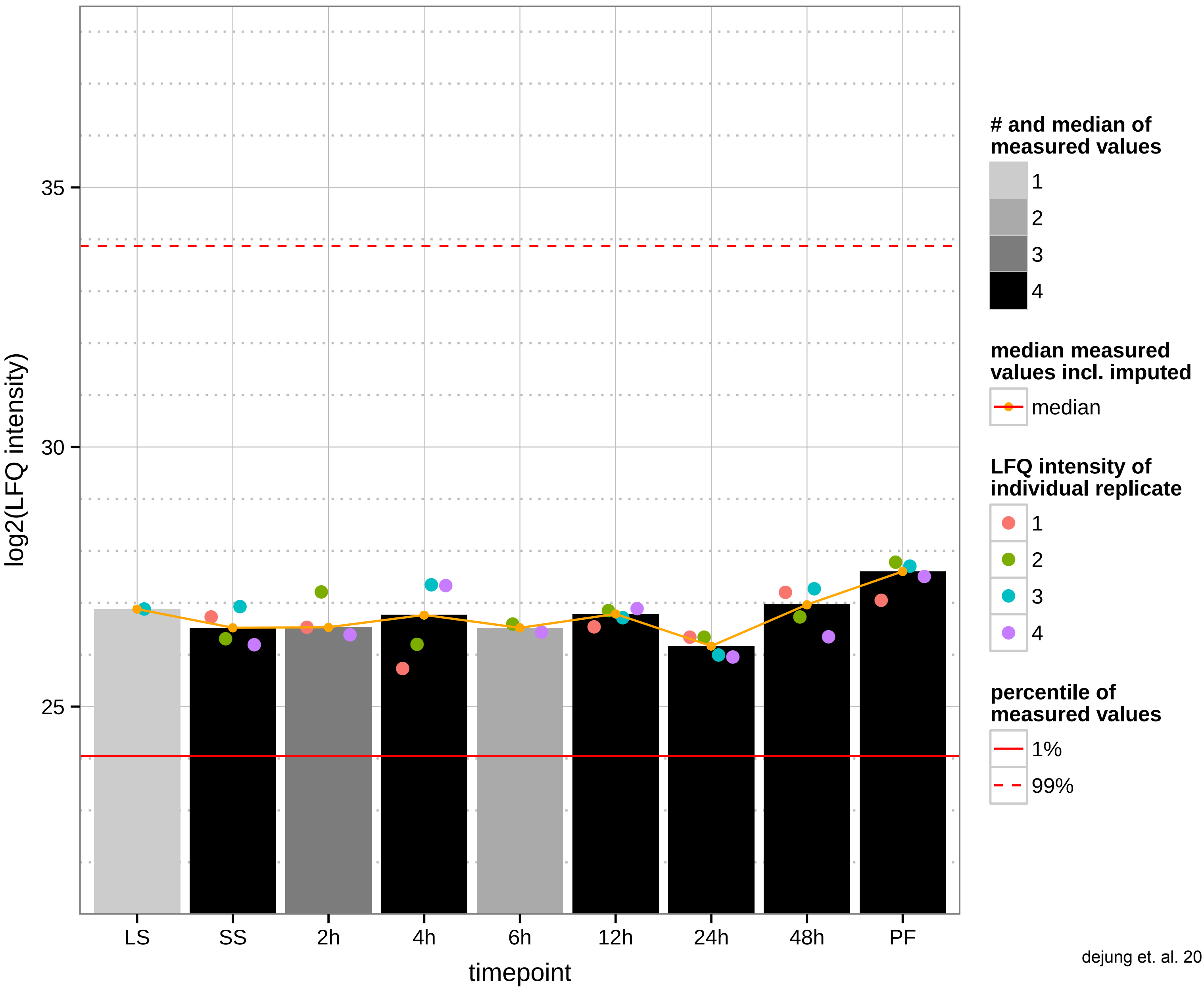

dejung et. al. 2015

hypothetical protein, conserved
Tb927.7.650
AGOF: null
AGOC: null
AGOP: null
PGOF: null
PGOC: null
PGOP: null

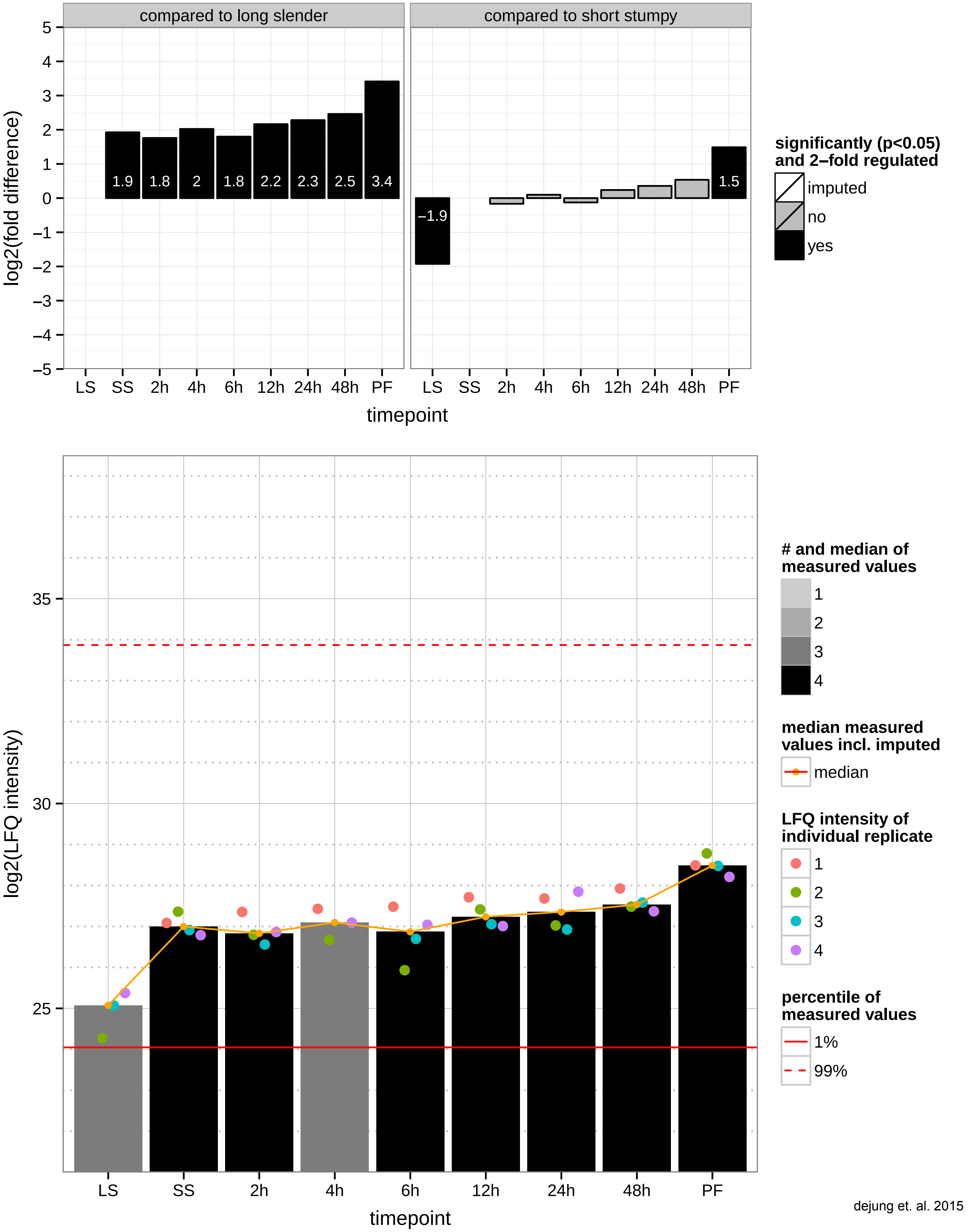

dejung et. al. 2015

trans–sialidase, putative
Tb927.7.6830
AGOF: exo–alpha–sialidase activity
AGOC: null
AGOP: pathogenesis
PGOF: null
PGOC: null
PGOP: null

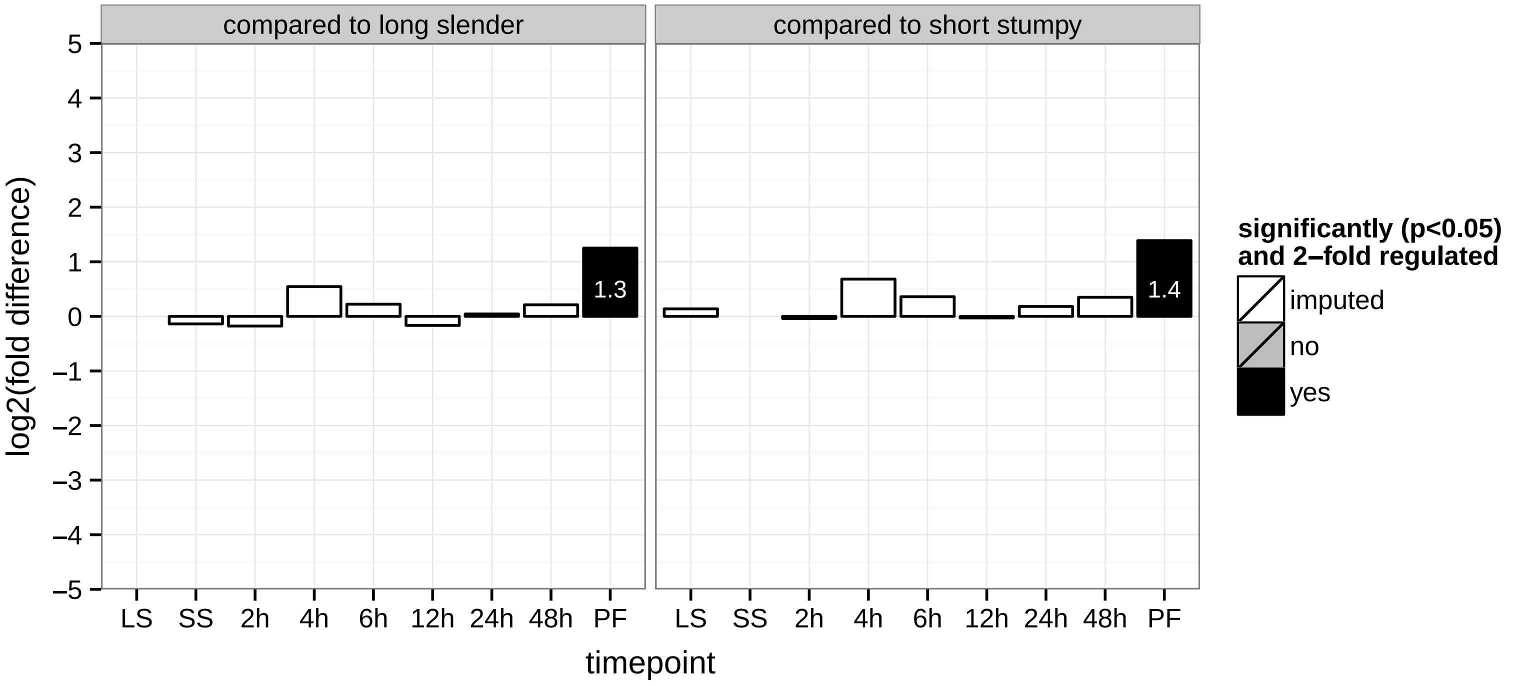

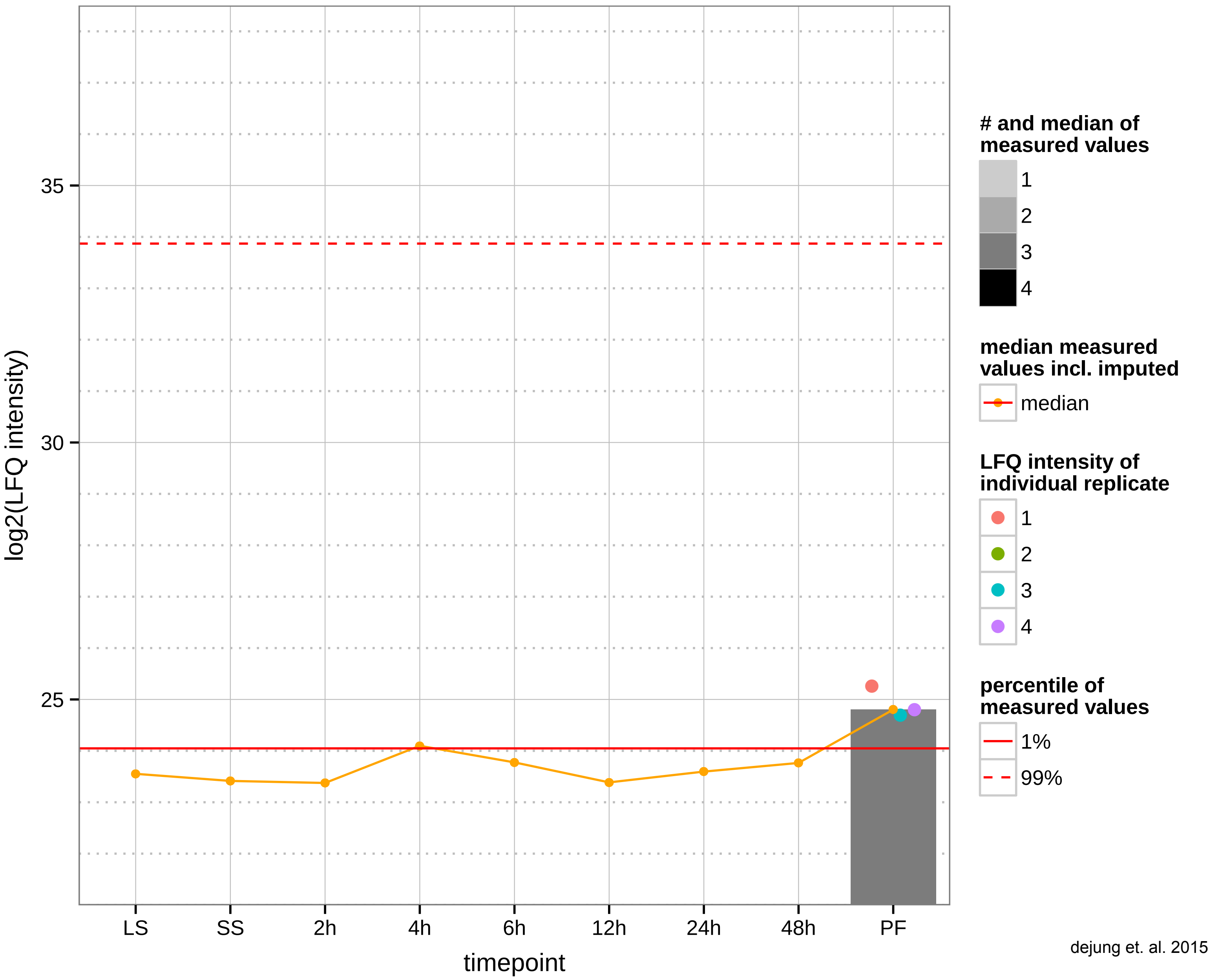

dejung et. al. 2015

hypothetical protein, conserved
Tb927.7.6890
AGOF: null
AGOC: null
AGOP: null
PGOF: null
PGOC: null
PGOP: null

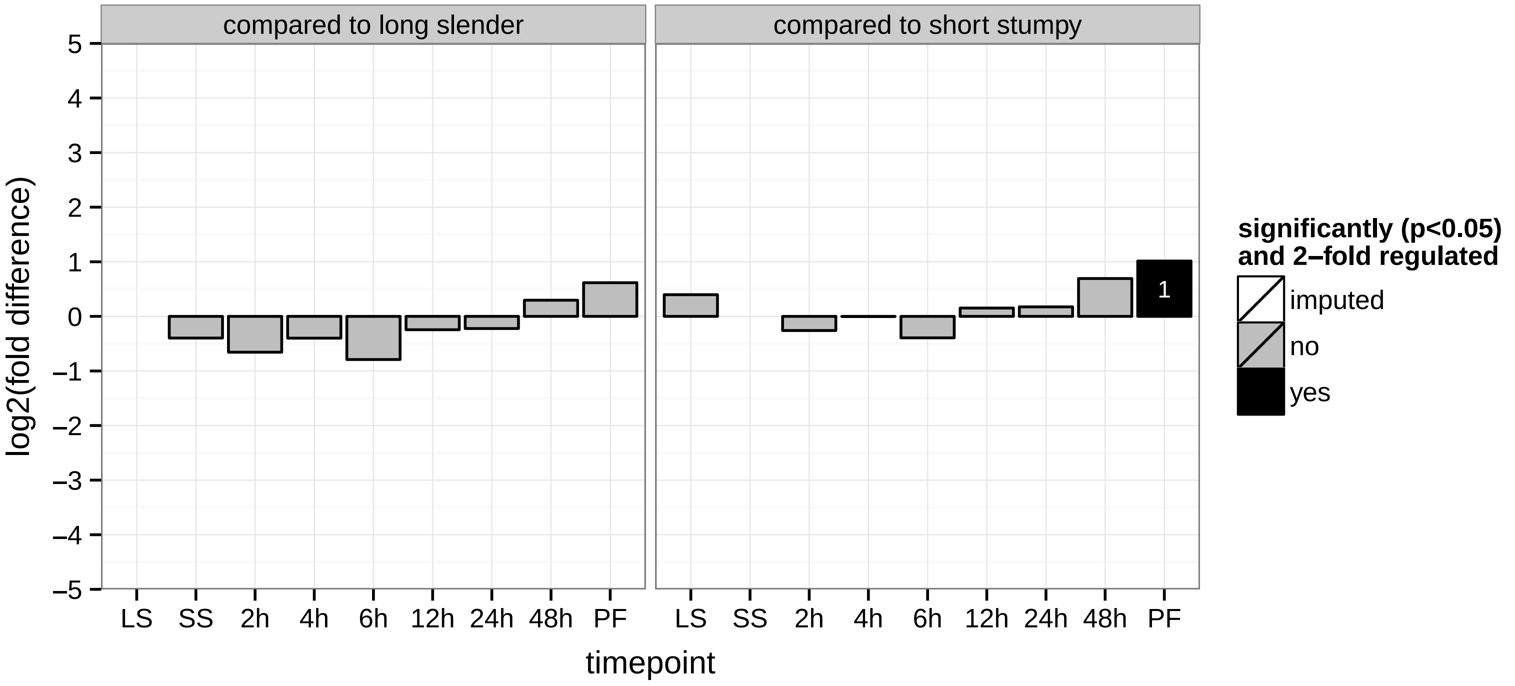

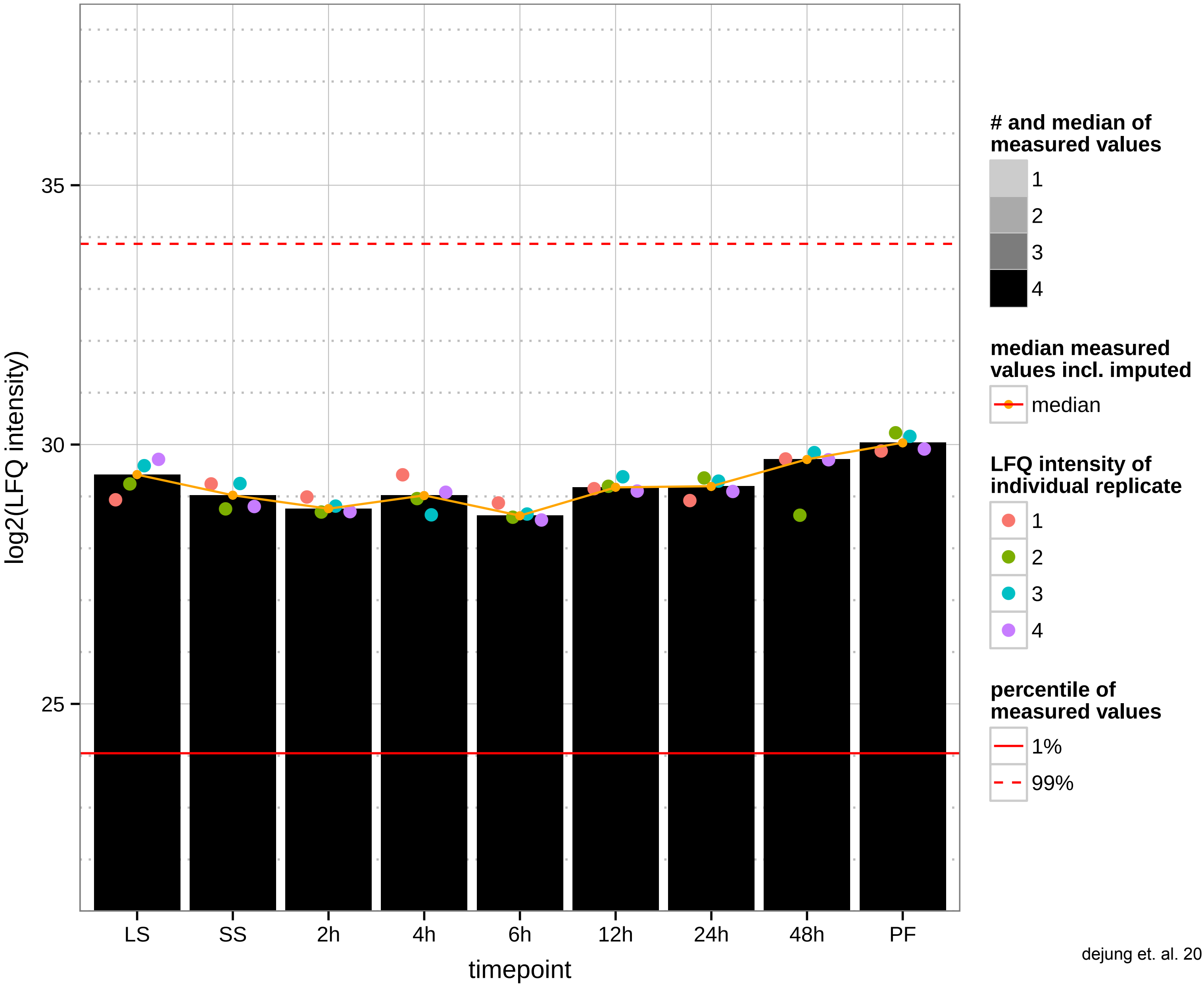

dejung et. al. 2015

RNA–binding protein, putative
Tb927.7.7280
AGOF: RNA binding
AGOC: null
AGOP: RNA processing
PGOF: RNA binding
PGOC: null
PGOP: RNA processing

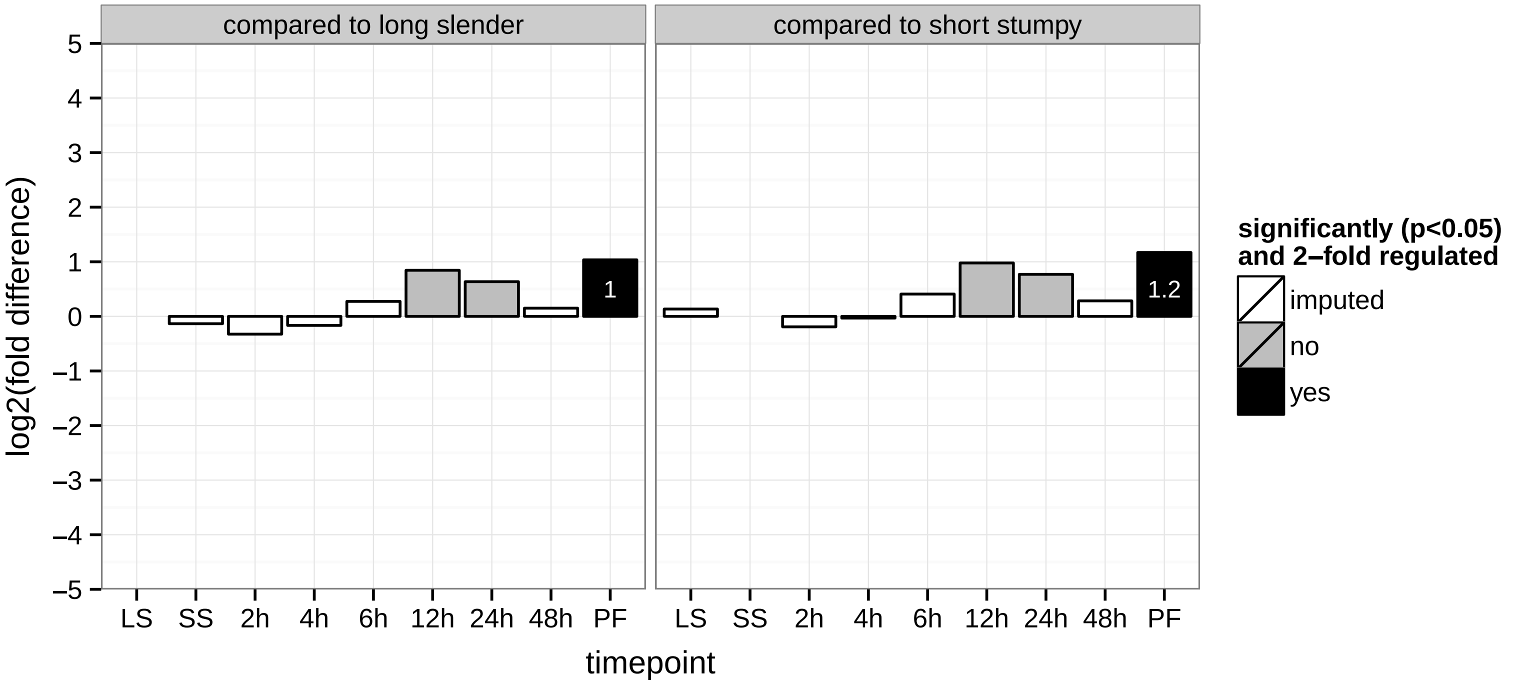

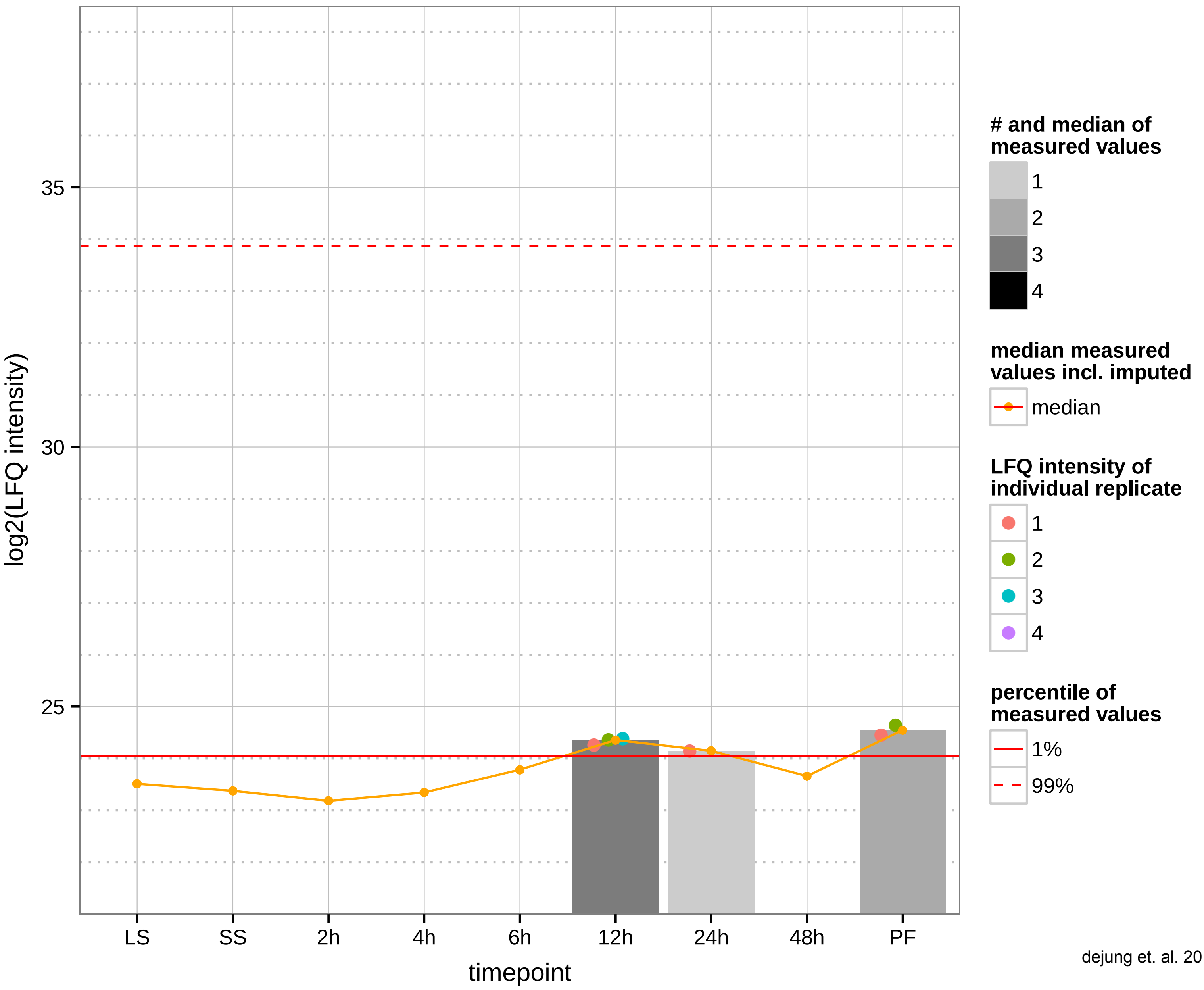

dejung et. al. 2015

cdc2–related kinase 2, cell division control protein 2 homolog 2 (CRK2)
Tb927.7.7360
AGOF: ATP binding, protein serine/threonine kinase activity, protein tyrosine kinase activity
AGOC: mitochondrion
AGOP: cell cycle, growth, protein phosphorylation
PGOF: ATP binding, protein kinase activity, transferase activity, transferring phosphorus–containing groups
PGOC: null
PGOP: protein phosphorylation

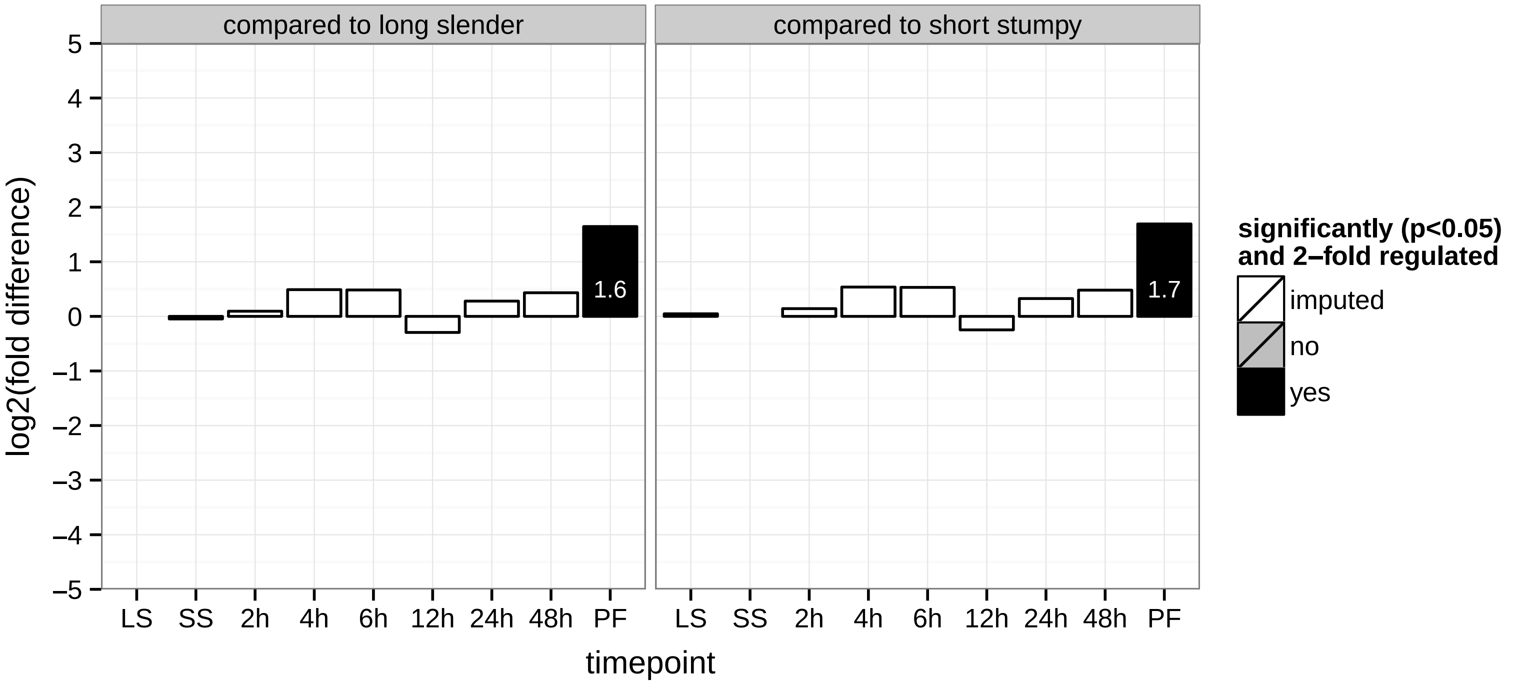

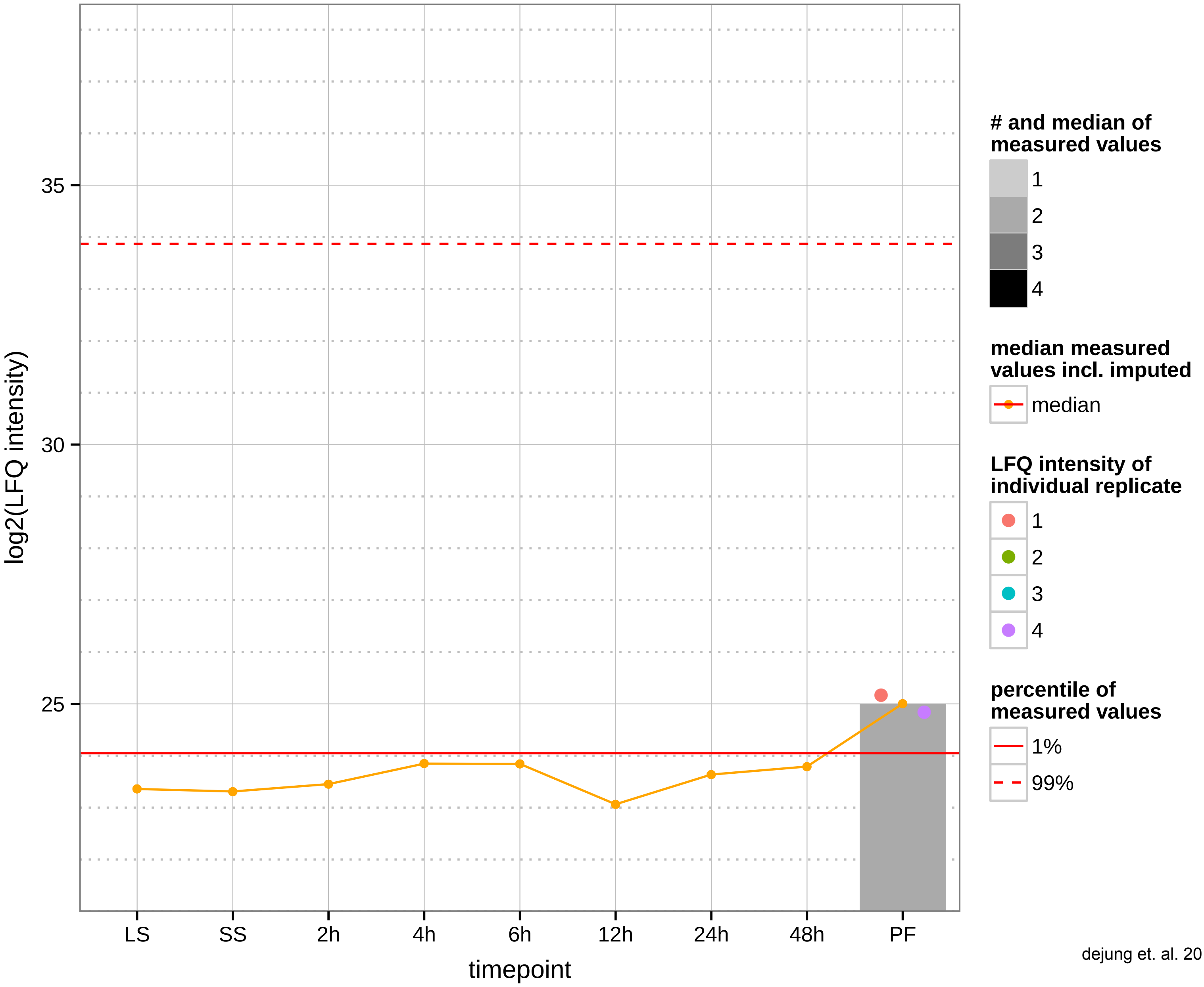

dejung et. al. 2015

mitochondrial RNA binding complex 1 subunit (MRB800)
Tb927.7.800
AGOF: RNA binding
AGOC: mitochondrion
AGOP: cytidine to uridine editing, mRNA stabilization
PGOF: null
PGOC: null
PGOP: null

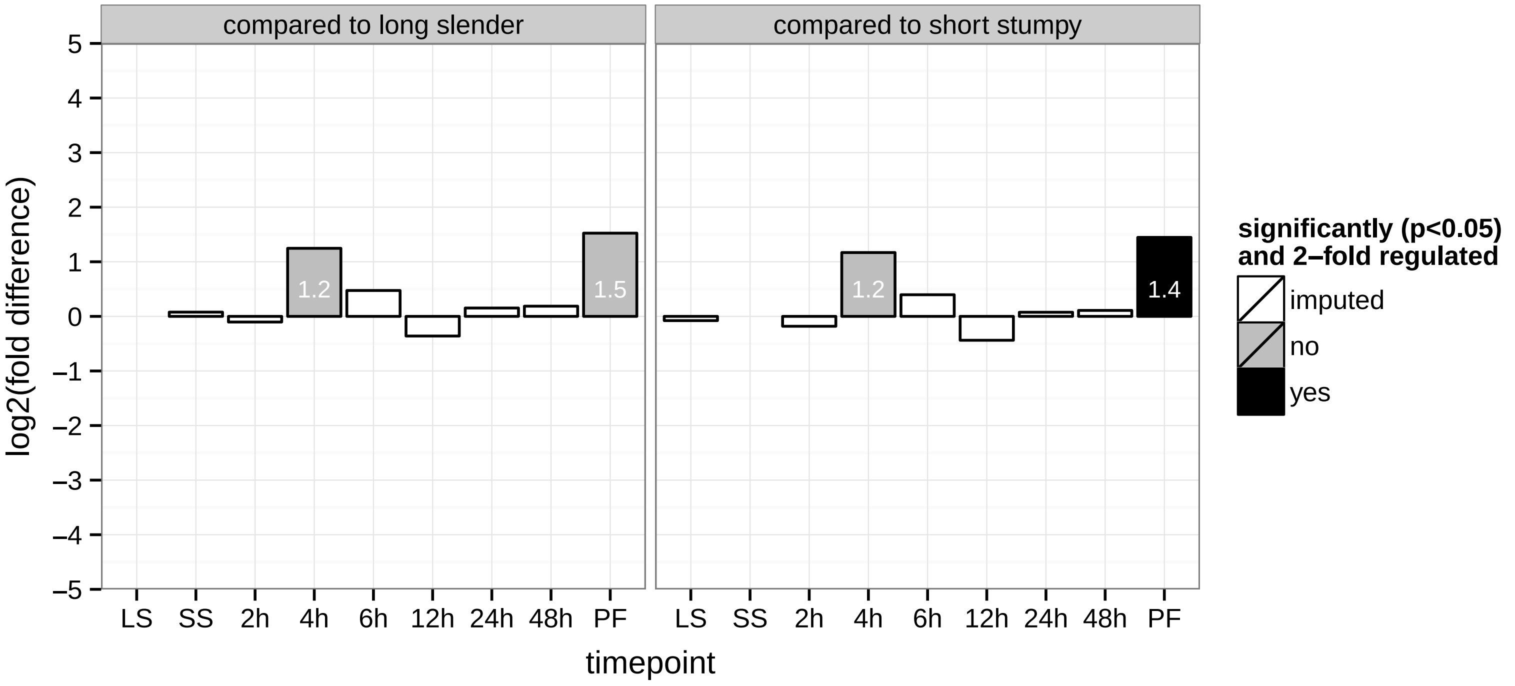

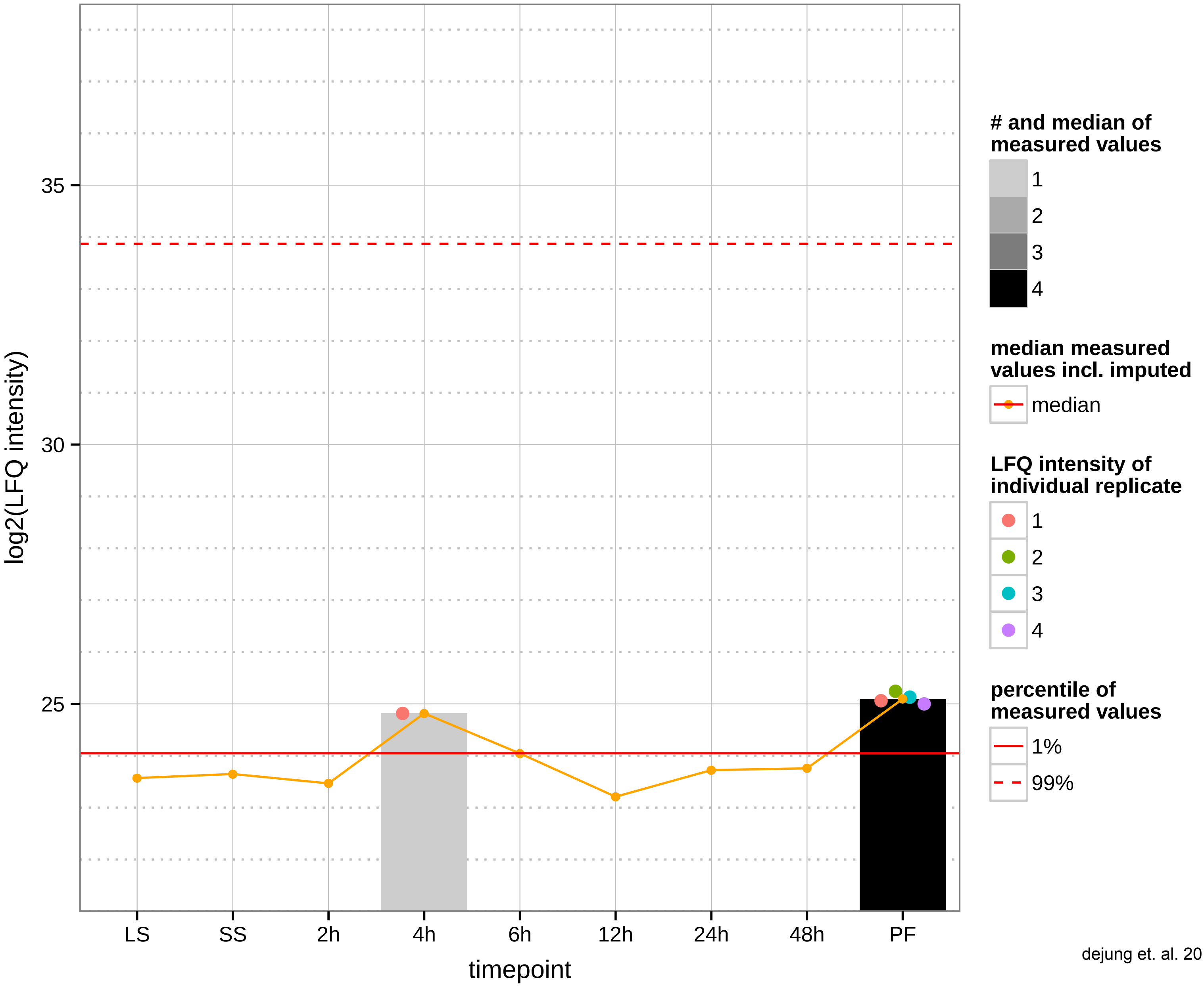

dejung et. al. 2015

hypothetical protein, conserved
Tb927.7.910;Tb11.v5.0841
AGOF: null
AGOC: null, mitochondrion
AGOP: null
PGOF: null
PGOC: null
PGOP: null

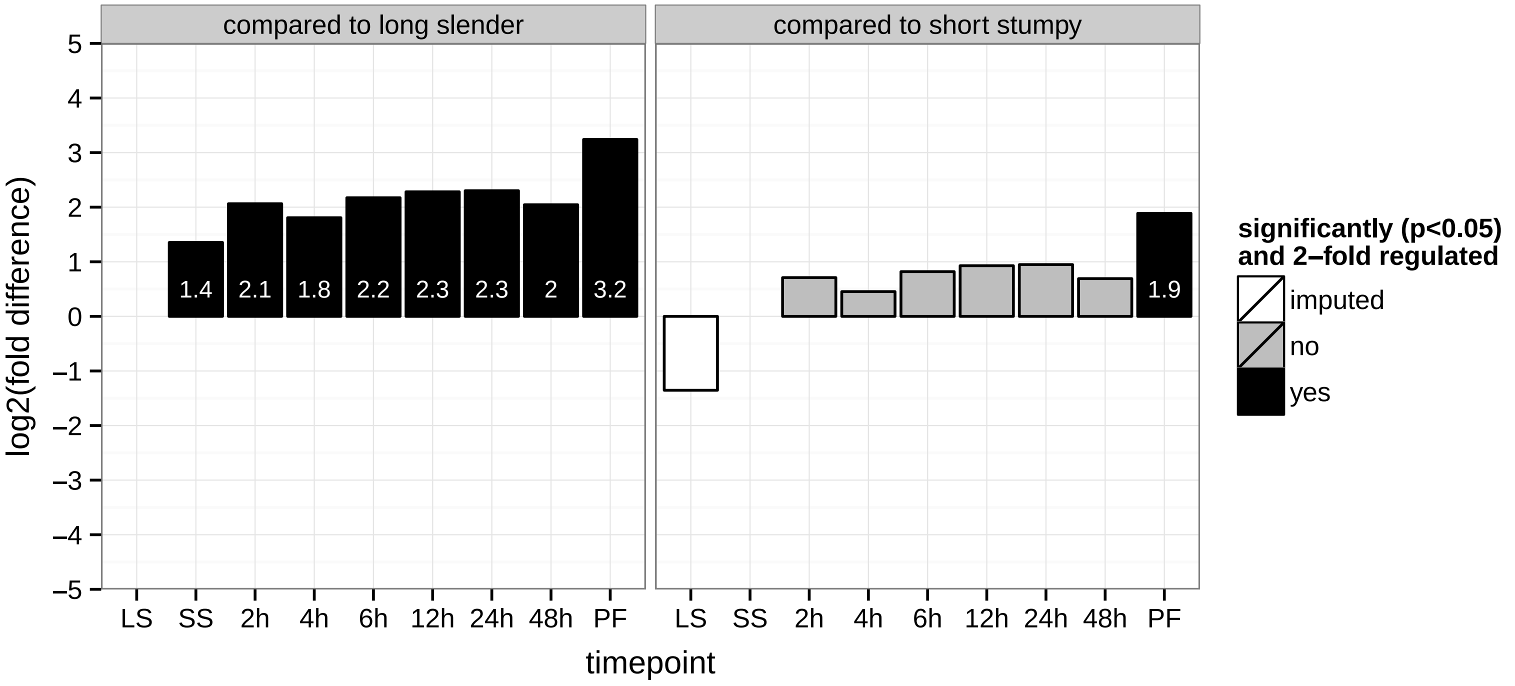

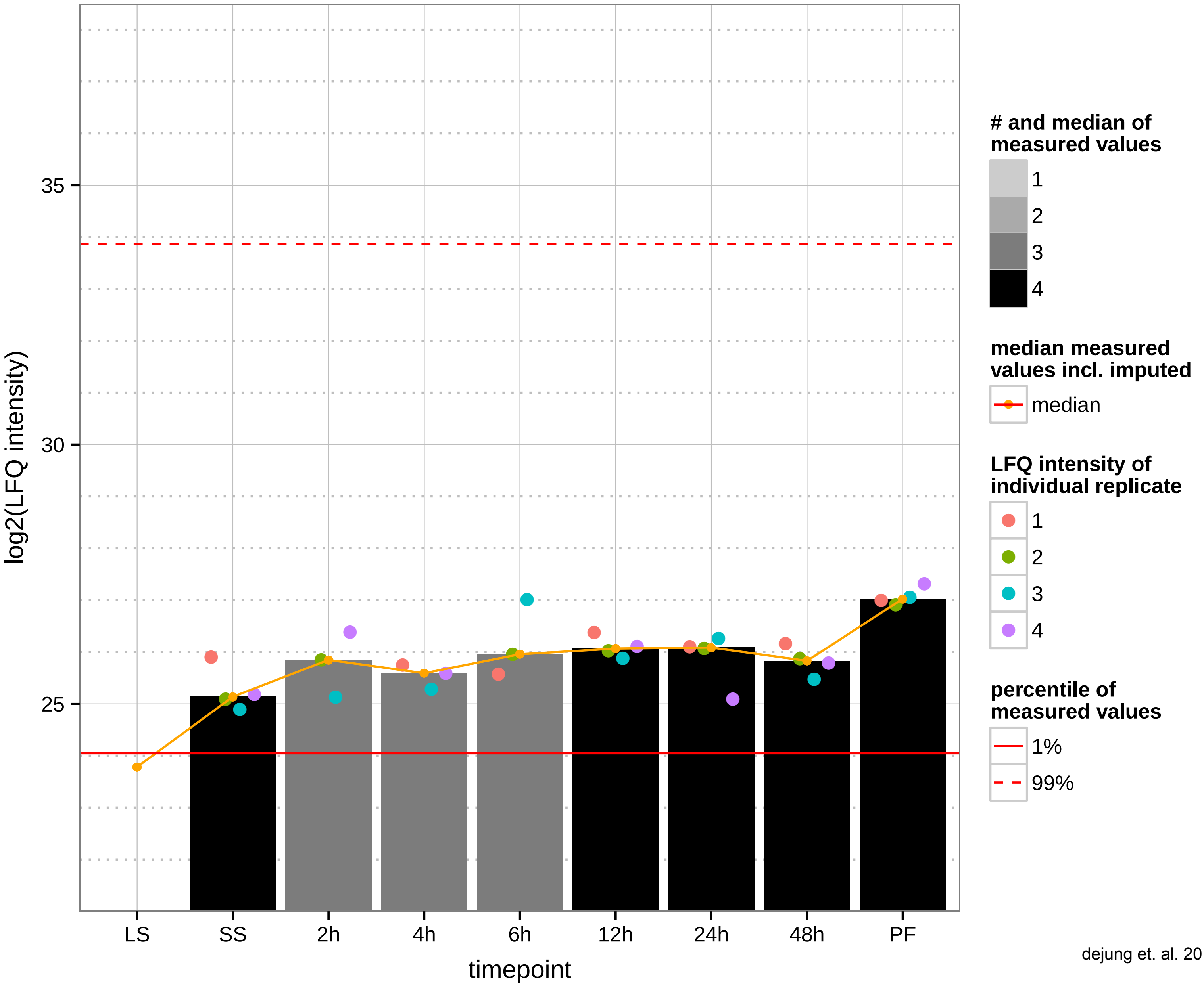

dejung et. al. 2015

hypothetical protein, conserved
Tb927.7.970
AGOF: null
AGOC: null
AGOP: null
PGOF: null
PGOC: null
PGOP: null

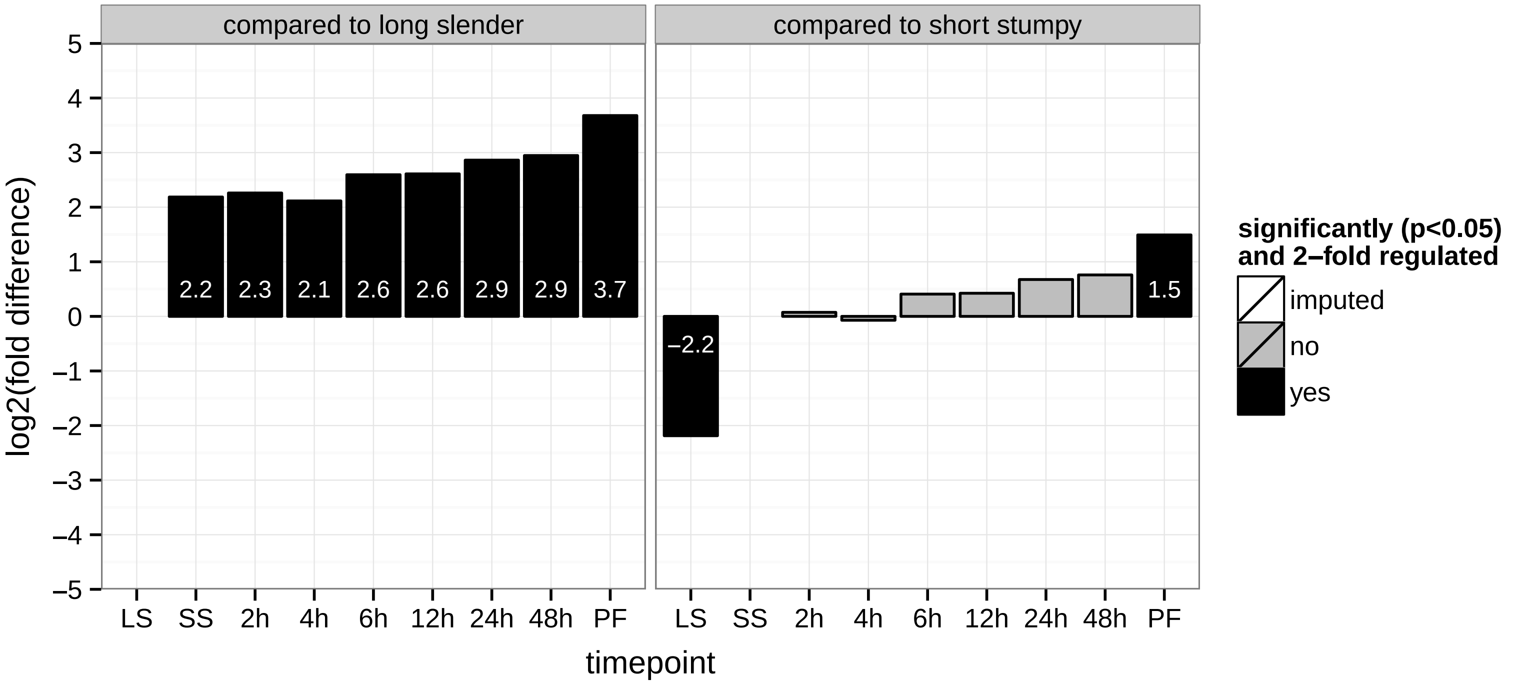

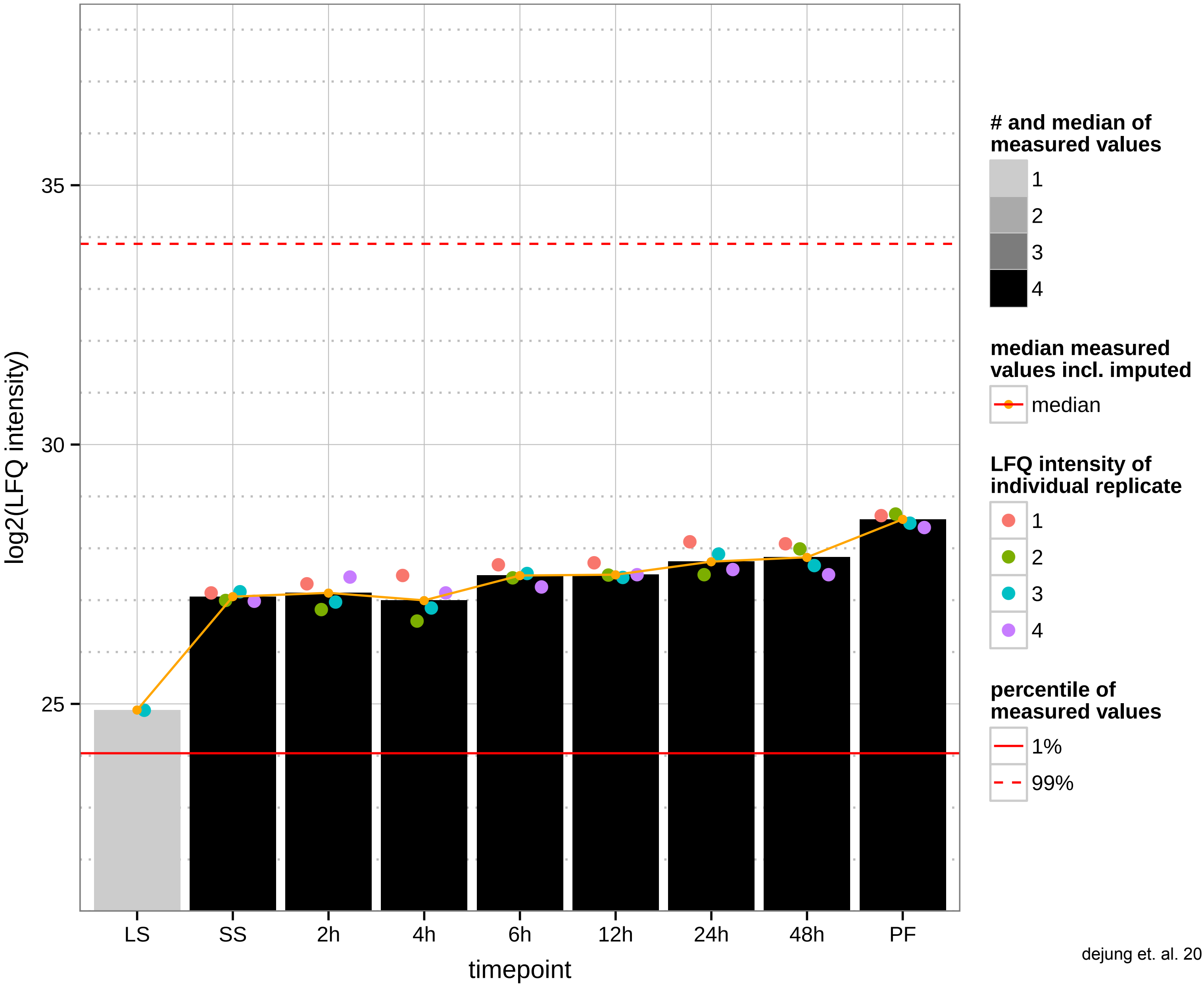

dejung et. al. 2015

chaperone protein DNAj, putative
Tb927.7.990
AGOF: heat shock protein binding, unfolded protein binding
AGOC: null
AGOP: null
PGOF: heat shock protein binding
PGOC: null
PGOP: null

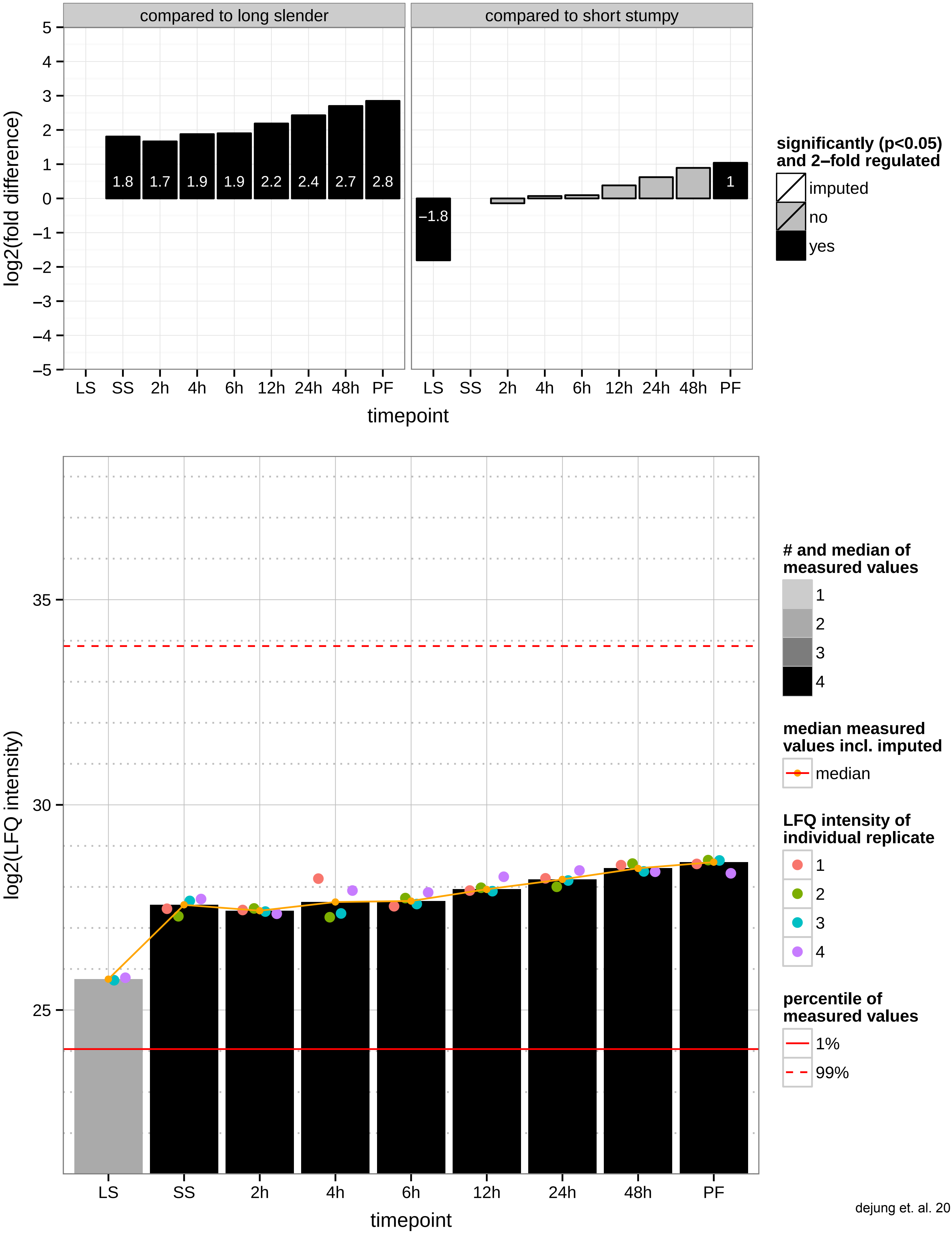

dejung et. al. 2015

hypothetical protein, conserved
Tb927.8.1230
AGOF: null
AGOC: null
AGOP: null
PGOF: null
PGOC: null
PGOP: null

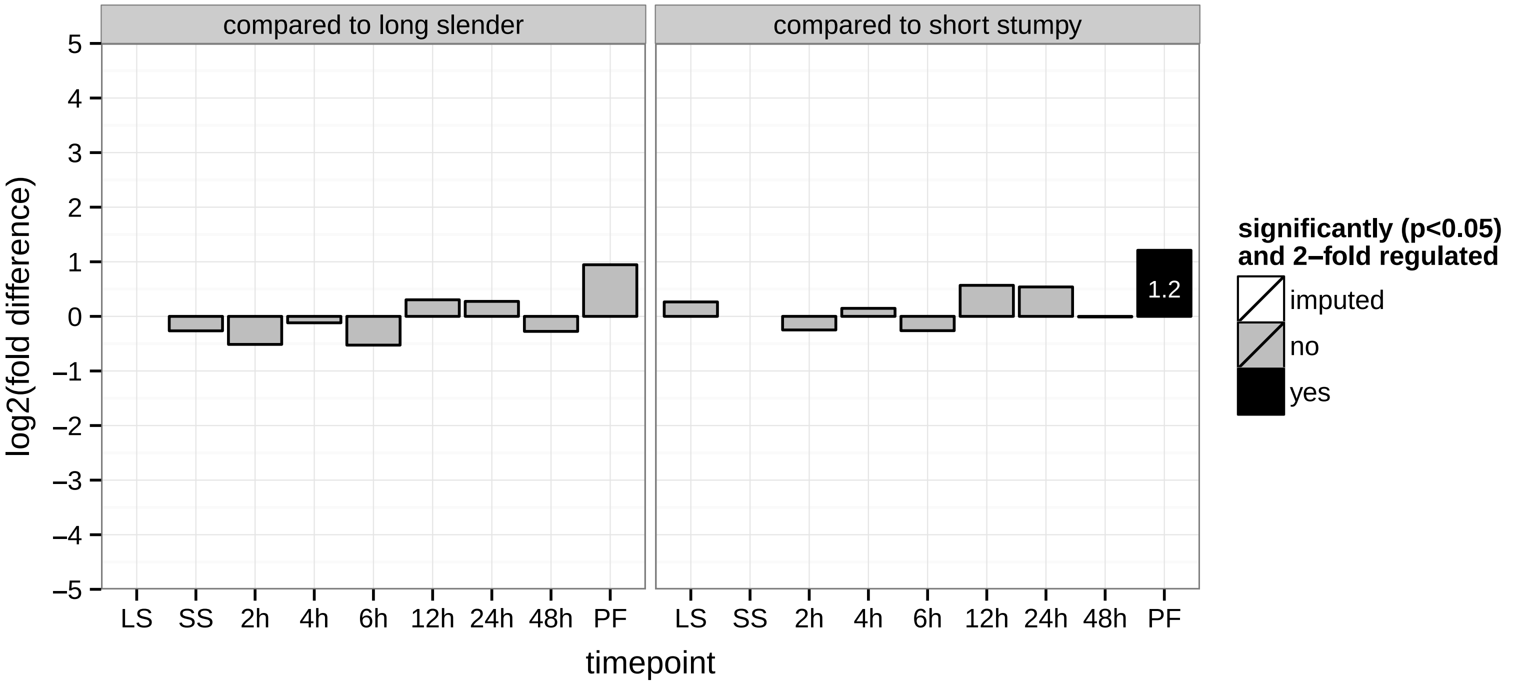

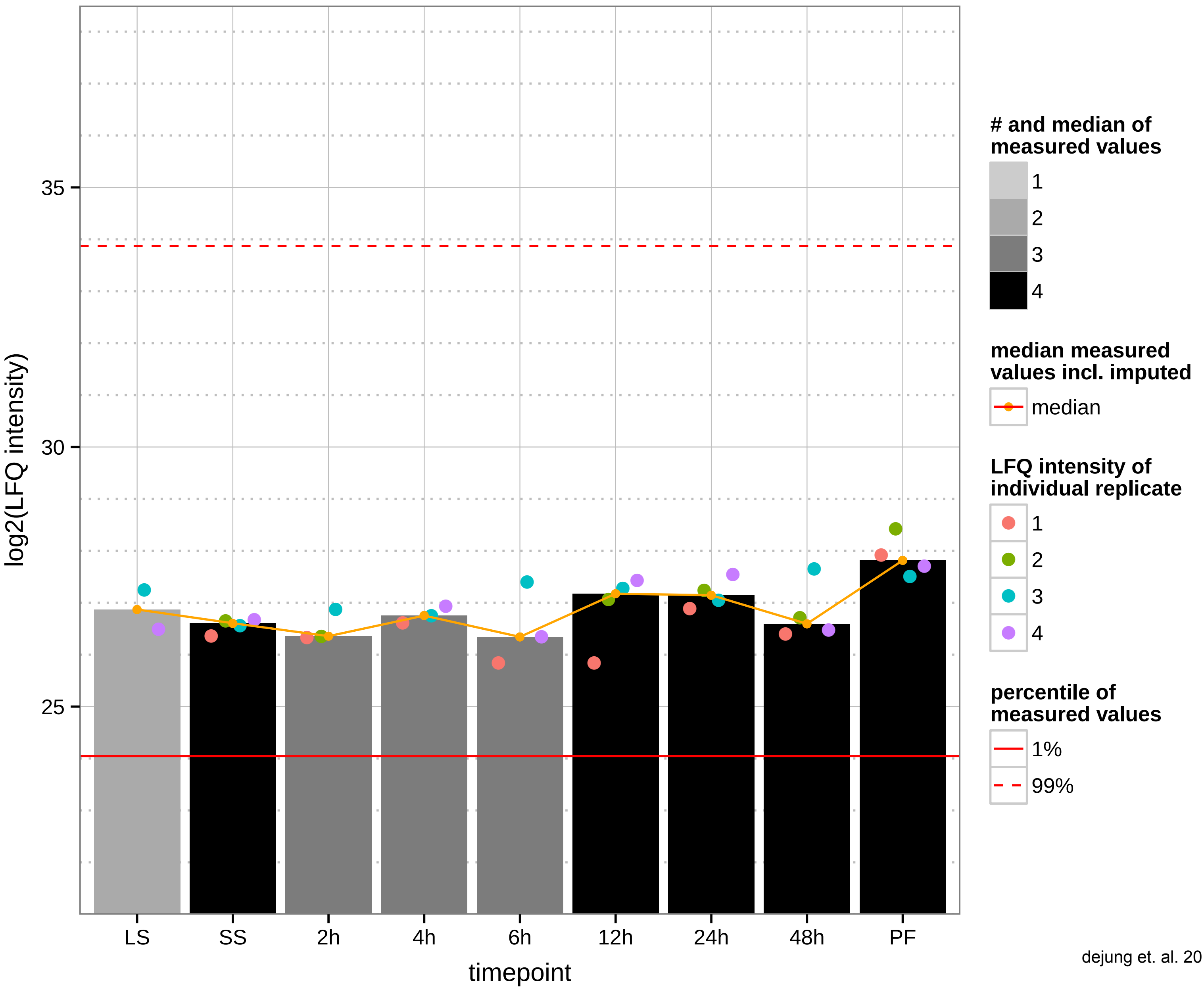

dejung et. al. 2015

hypothetical protein, conserved
Tb927.8.1430
AGOF: null
AGOC: mitochondrion
AGOP: null
PGOF: null
PGOC: null
PGOP: null

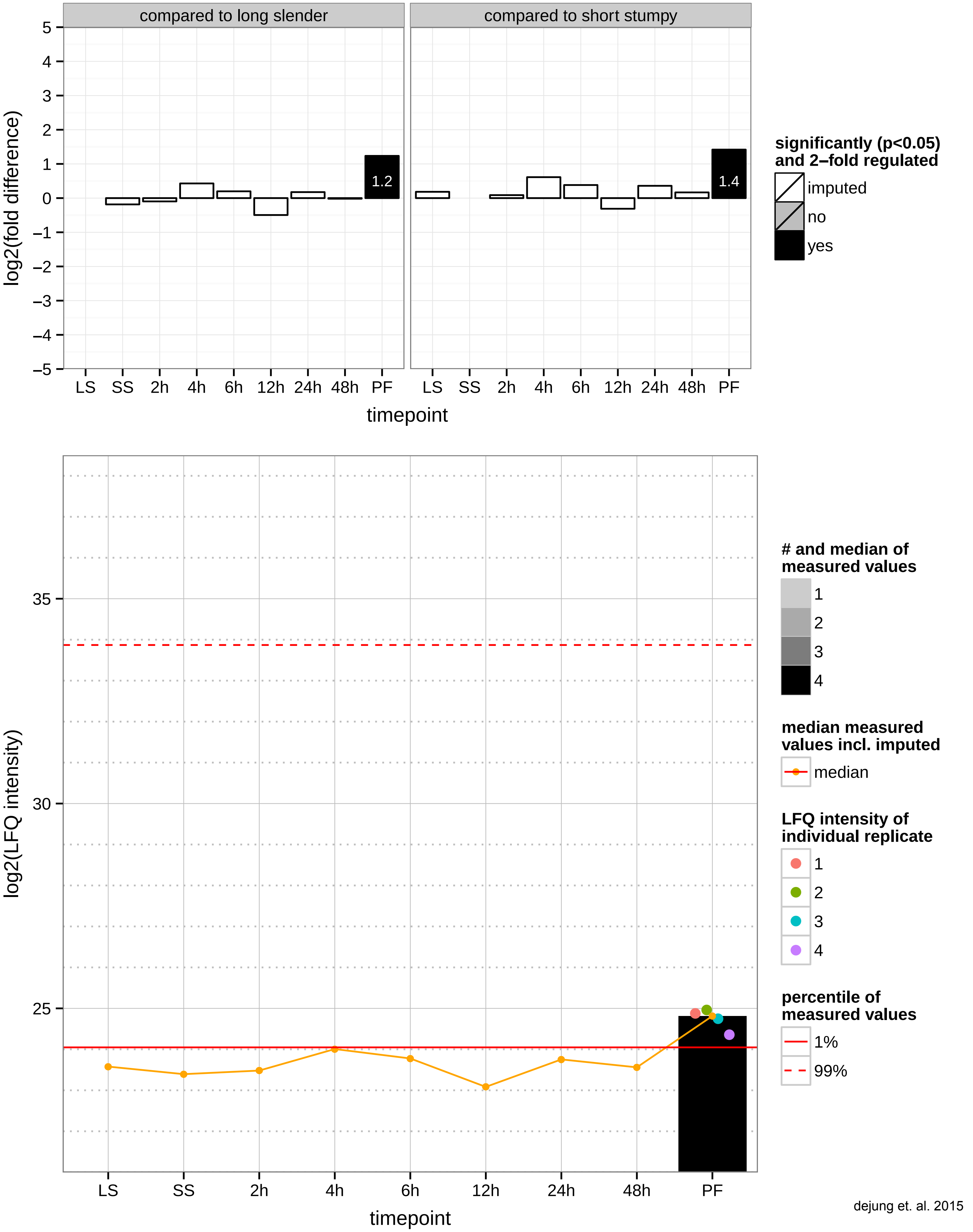

dejung et. al. 2015

ubiquitin–protein ligase, putative (upl3)
Tb927.8.1590
AGOF: RNA binding, ubiquitin–protein ligase activity, zinc ion binding
AGOC: intracellular, mitochondrion
AGOP: cellular protein modification process
PGOF: acid–amino acid ligase activity
PGOC: intracellular
PGOP: cellular protein modification process

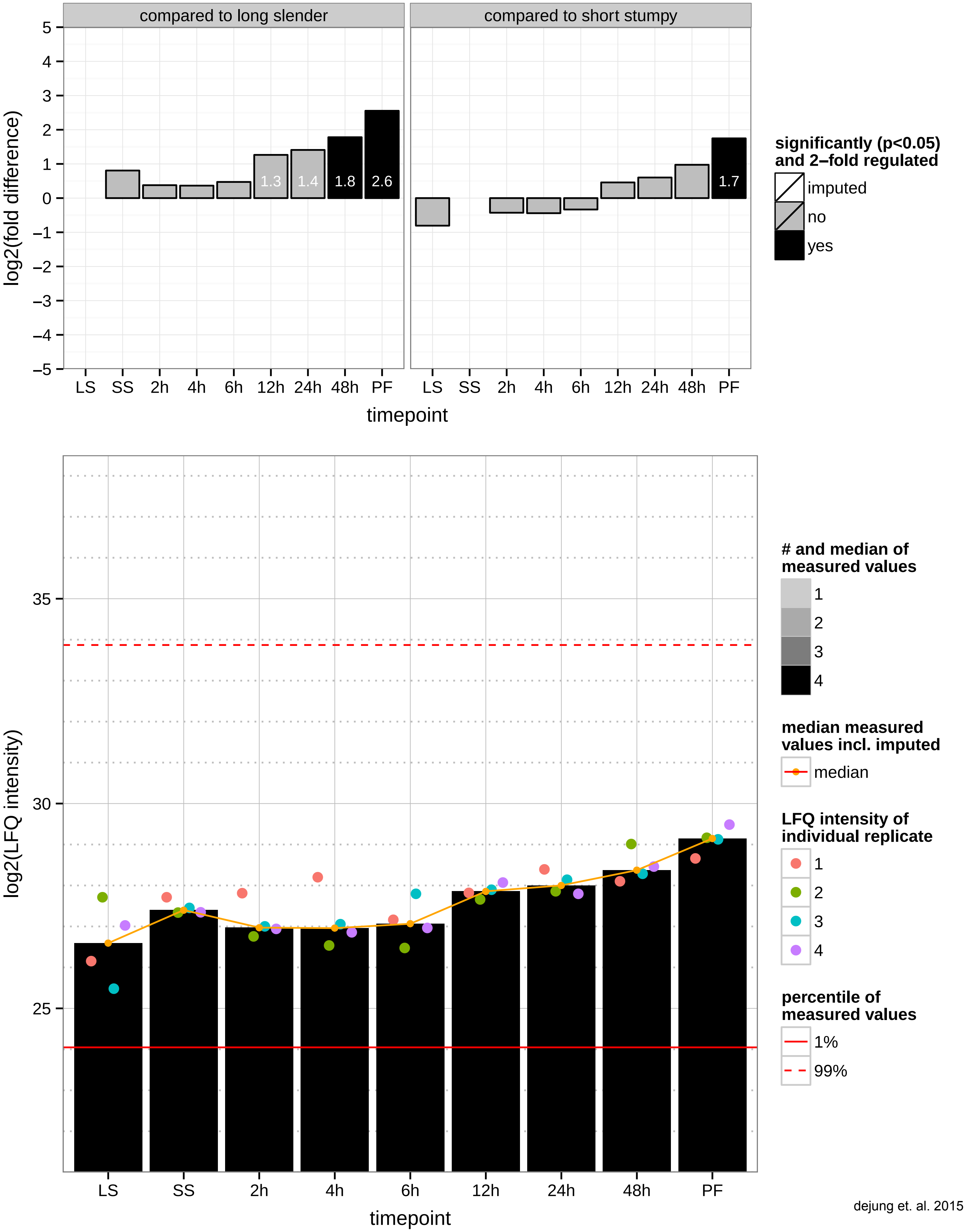

dejung et. al. 2015

pitrilysin–like metalloprotease, metallo–peptidase, Clan ME, Family M16C
Tb927.8.1860
AGOF: catalytic activity, metalloendopeptidase activity, zinc ion binding
AGOC: mitochondrion
AGOP: proteolysis
PGOF: catalytic activity, metal ion binding, metalloendopeptidase activity, metallopeptidase activity, zinc ion binding
PGOC: null
PGOP: proteolysis

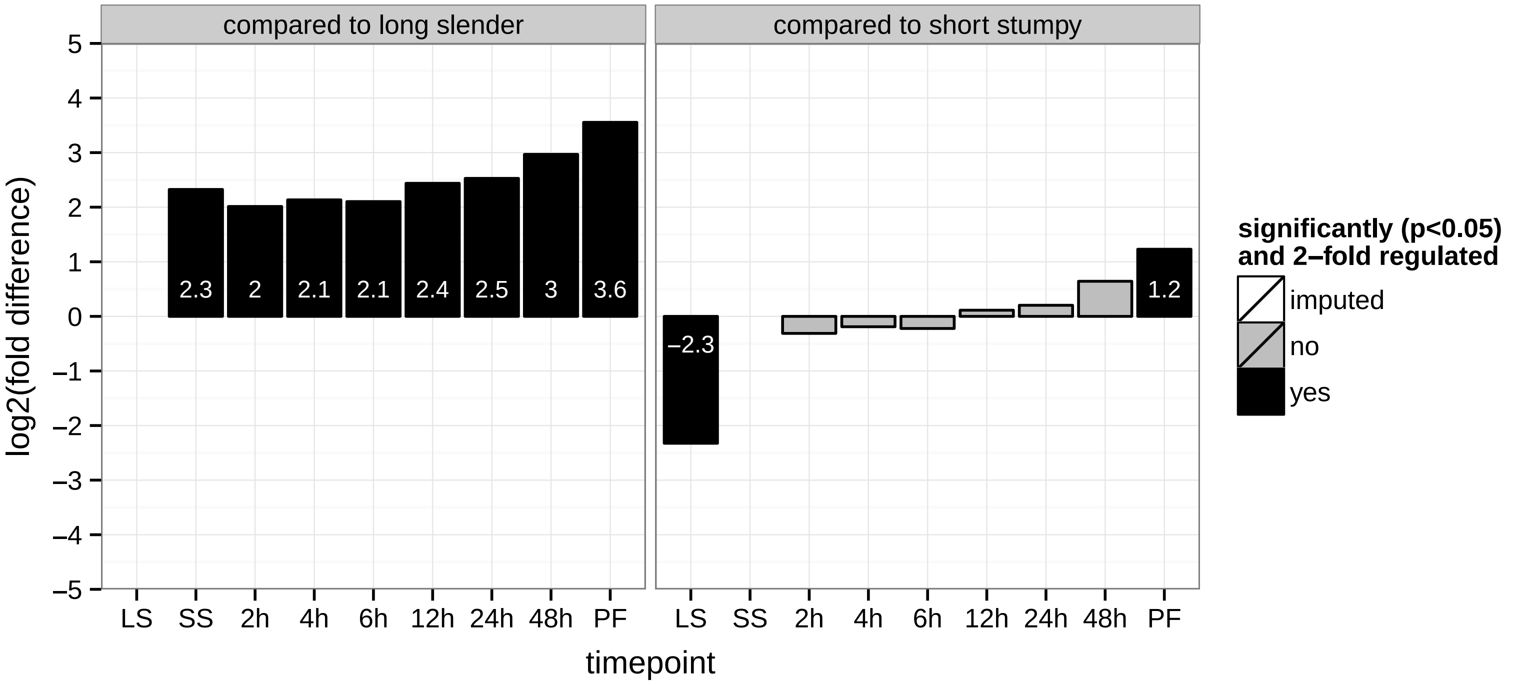

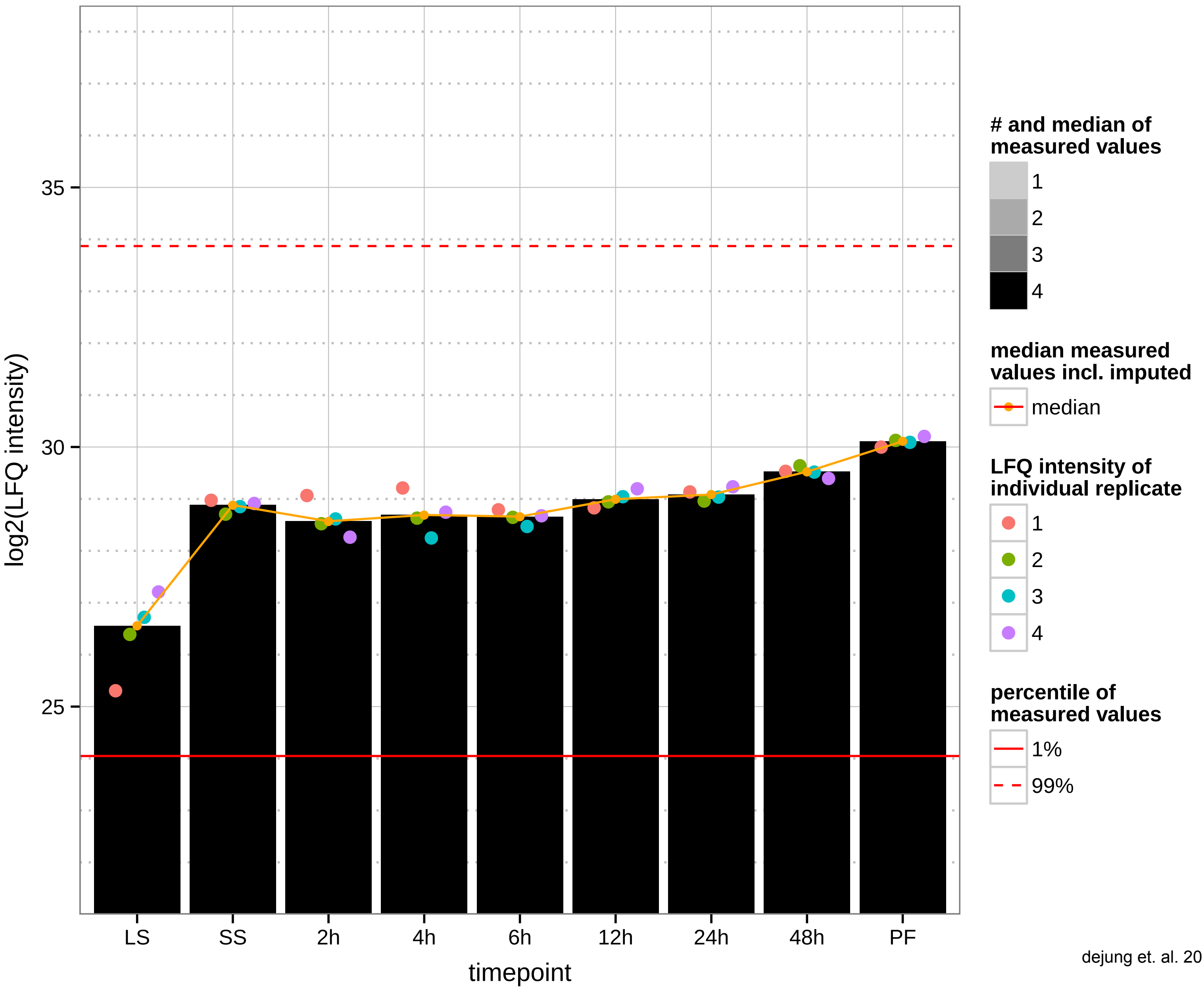

dejung et. al. 2015

hypothetical protein, conserved
Tb927.8.2130
AGOF: null
AGOC: null
AGOP: null
PGOF: null
PGOC: null
PGOP: null

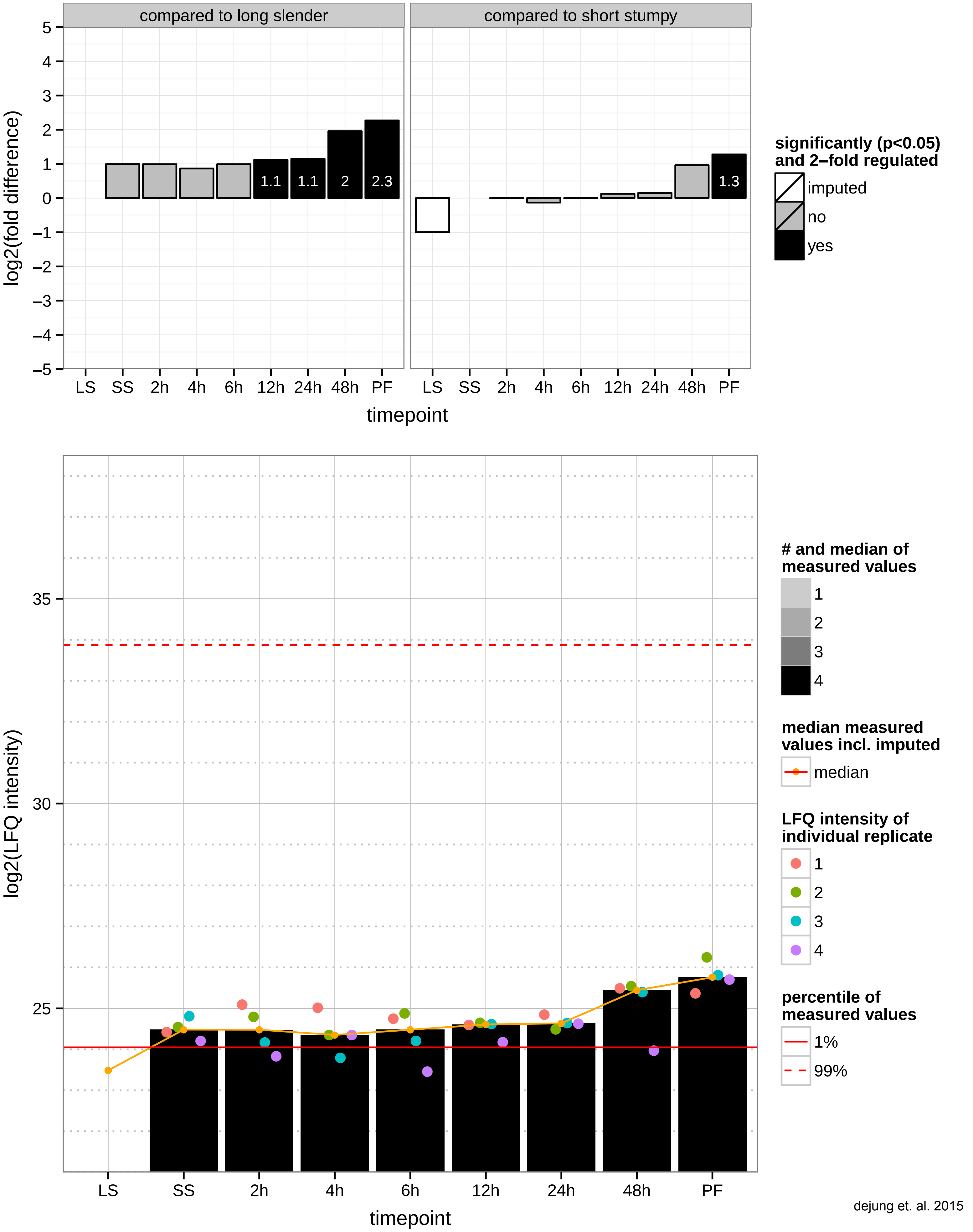

dejung et. al. 2015

hypothetical protein, conserved
Tb927.8.2300
AGOF: null
AGOC: mitochondrion
AGOP: null
PGOF: null
PGOC: null
PGOP: null

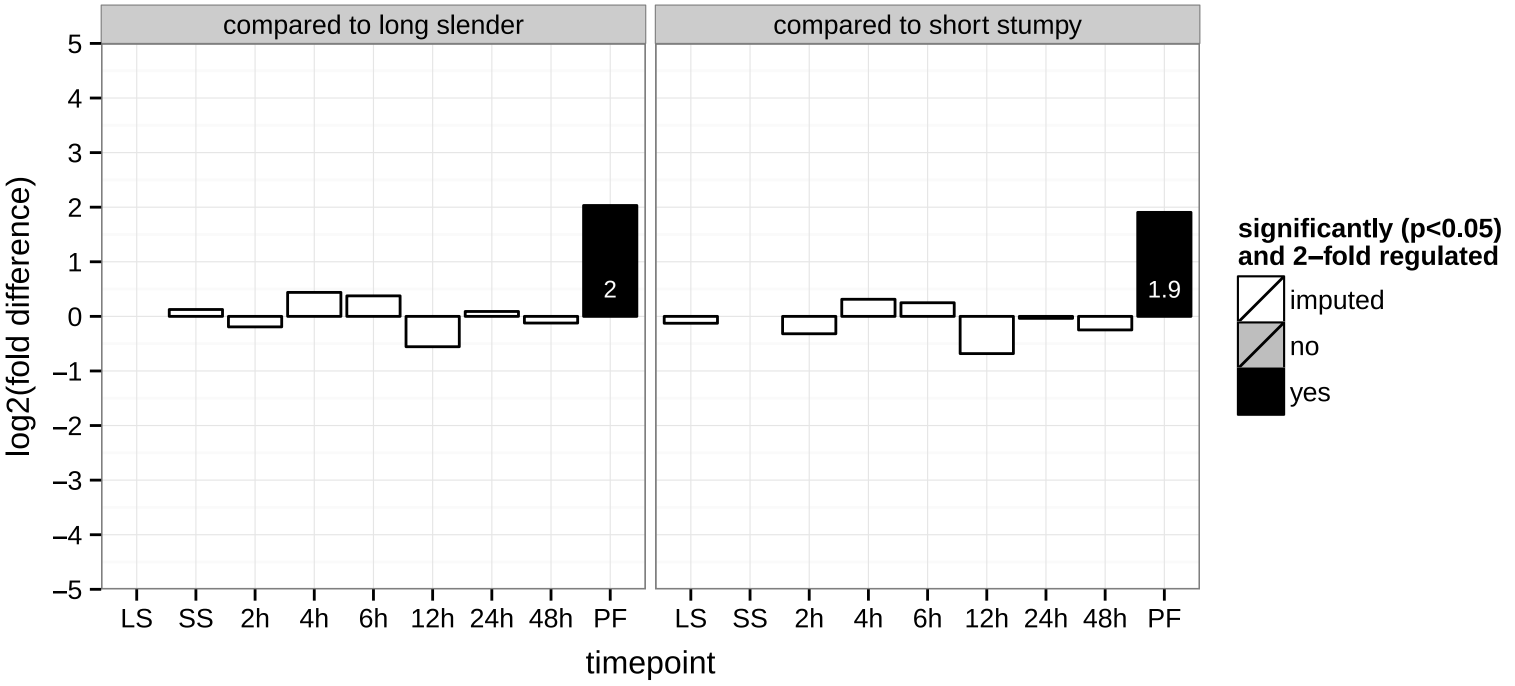

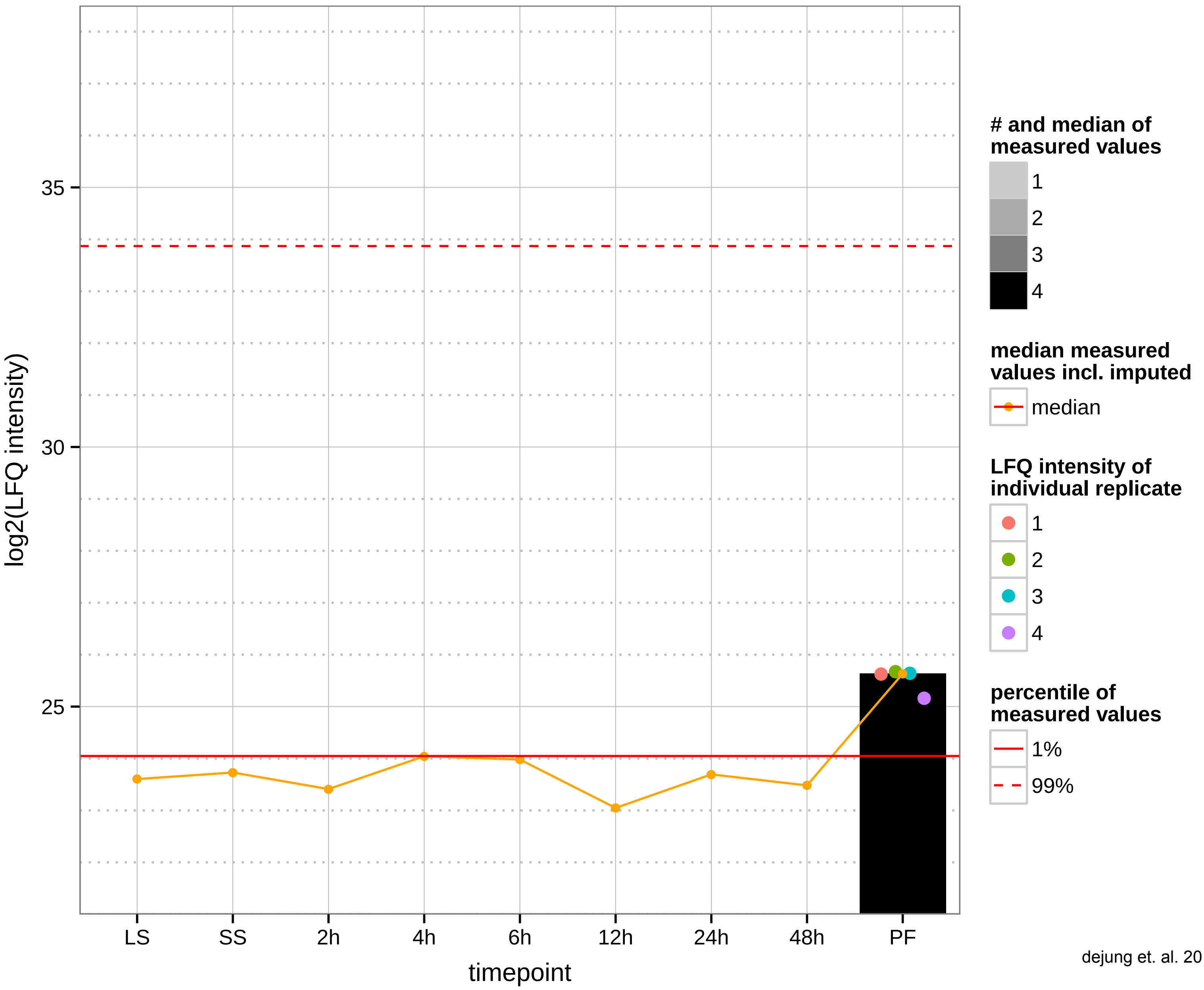

dejung et. al. 2015

acetyl–CoA synthetase, putative, acetyl–CoA synthetase (pseudogene), truncated
Tb927.8.2520
AGOF: AMP binding, acetate–CoA ligase activity, catalytic activity, catalytic activity
AGOC: integral to membrane, null
AGOP: metabolic process
PGOF: AMP binding, acetate–CoA ligase activity, catalytic activity, catalytic activity
PGOC: null
PGOP: metabolic process

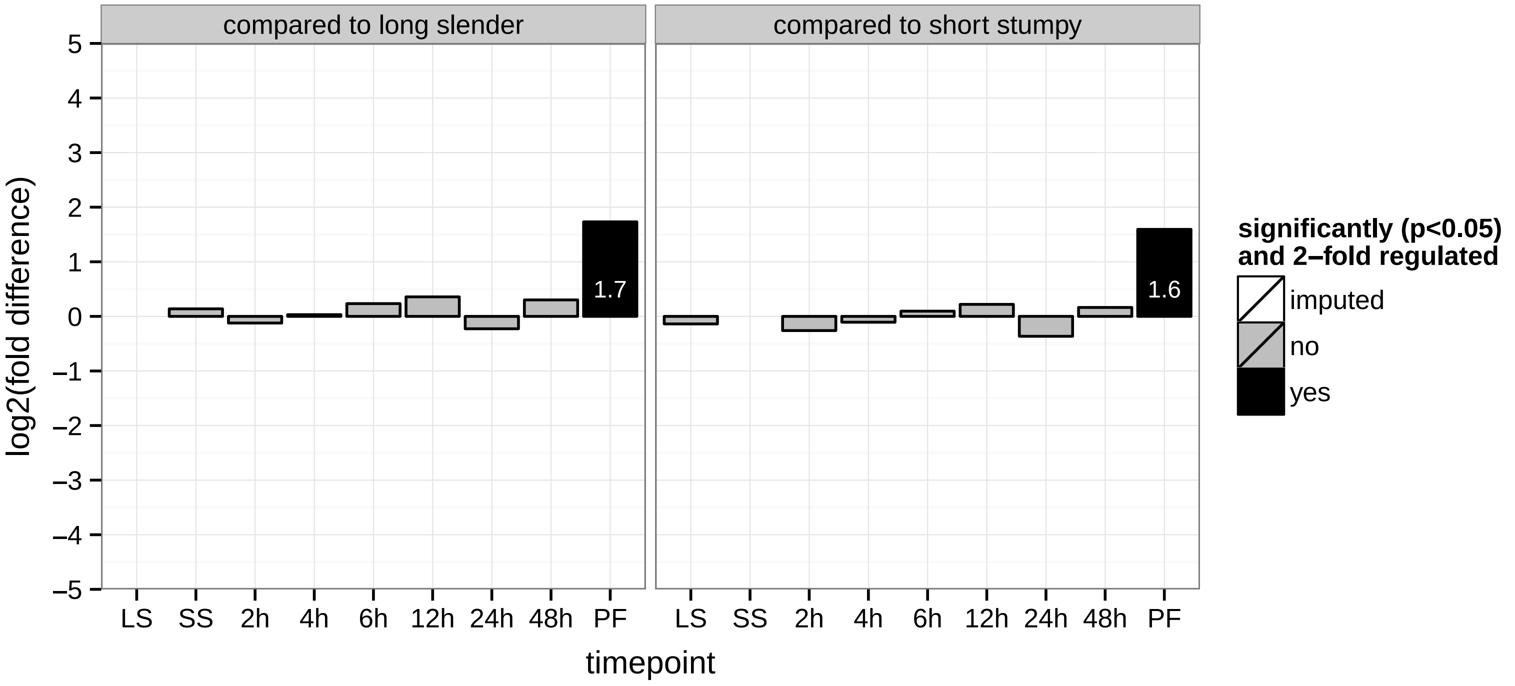

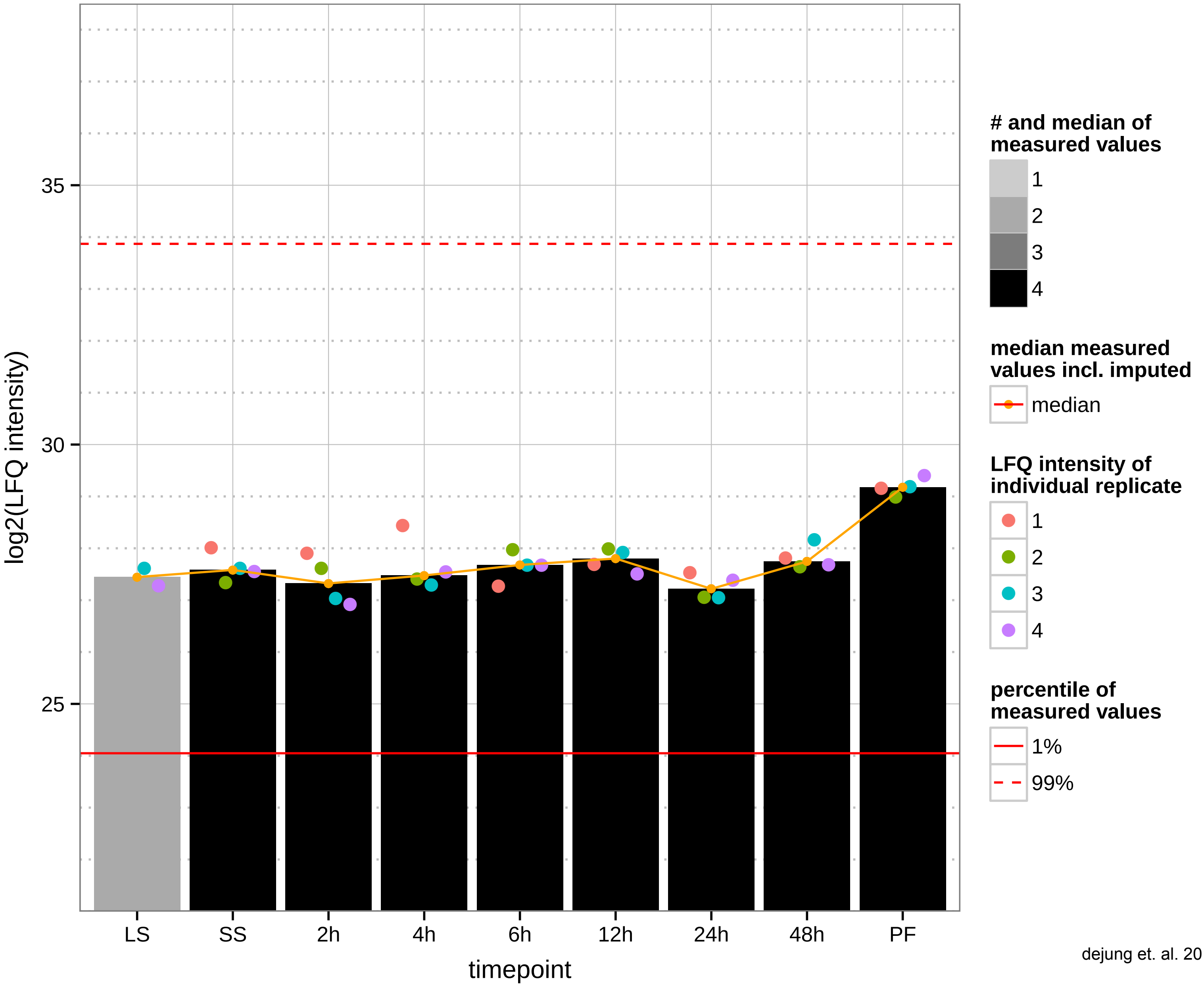

dejung et. al. 2015

hypothetical protein, conserved
Tb927.8.2750
AGOF: null
AGOC: mitochondrion
AGOP: null
PGOF: null
PGOC: null
PGOP: null

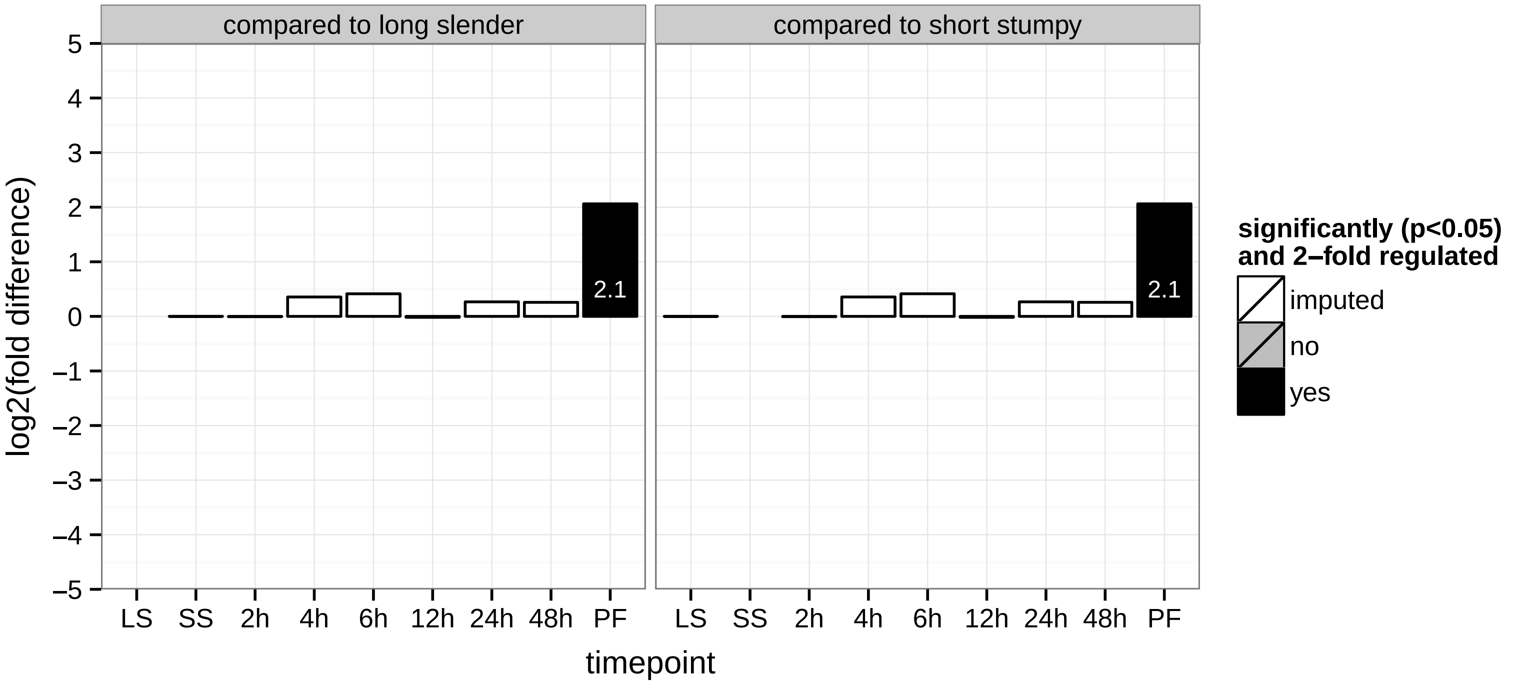

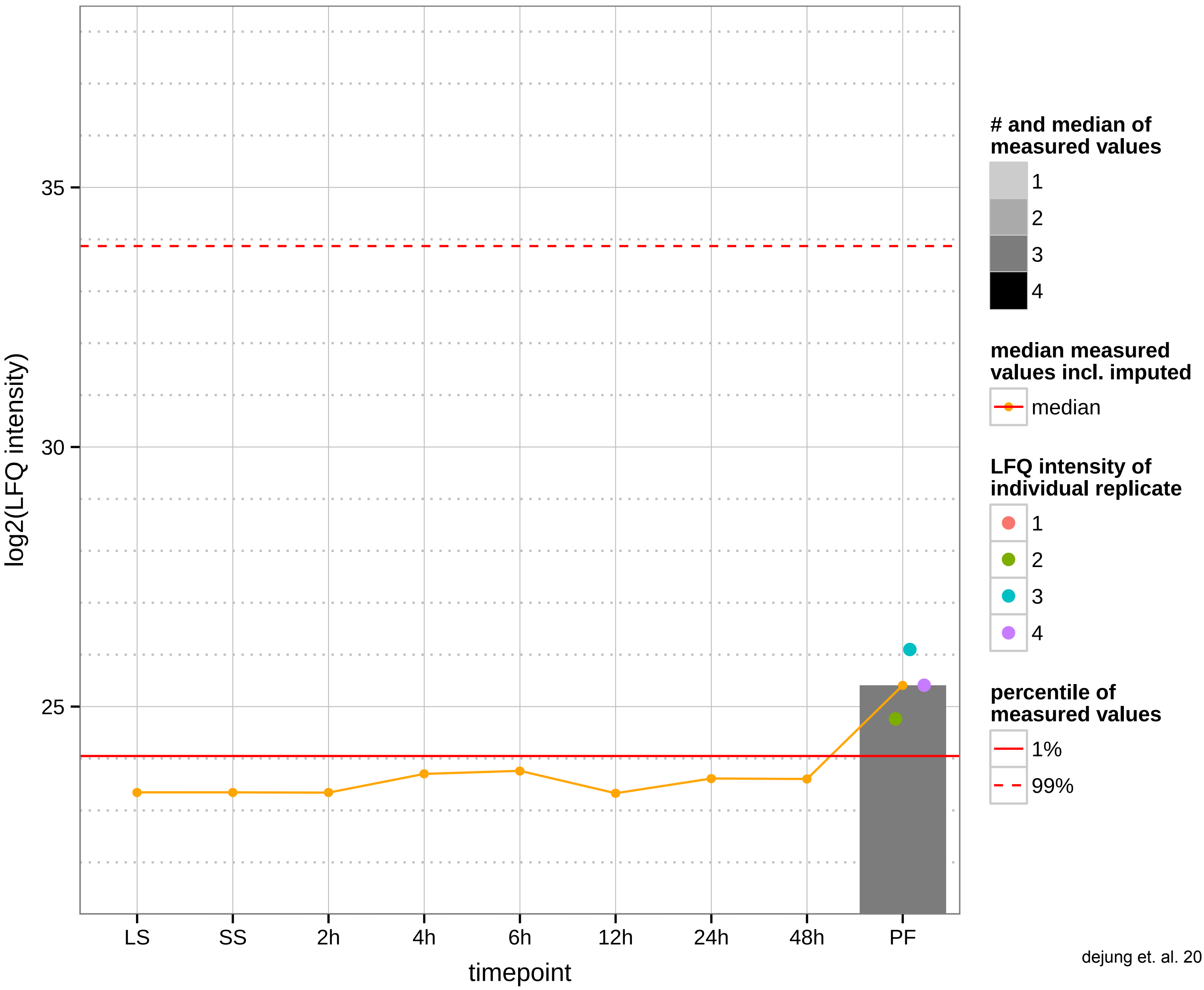

dejung et. al. 2015

mannosyl–oligosaccharide 1, 2–alpha–mannosidase IB, putative
Tb927.8.2920;Tb927.8.2930;Tb927.8.2940
AGOF: calcium ion binding, mannosyl–oligosaccharide 1, 2–alpha–mannosidase activity
AGOC: endoplasmic reticulum, membrane
AGOP: protein N–linked glycosylation
PGOF: calcium ion binding, mannosyl–oligosaccharide 1, 2–alpha–mannosidase activity
PGOC: membrane
PGOP: null

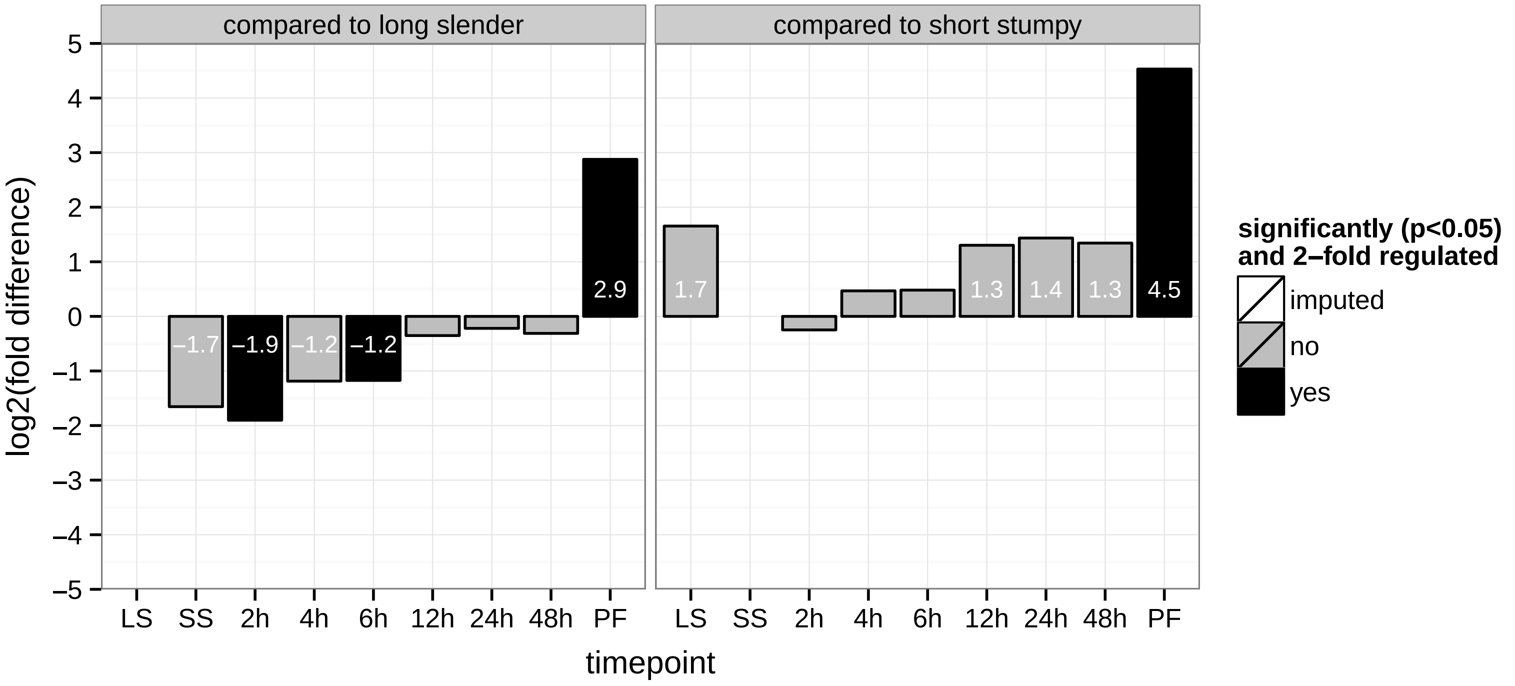

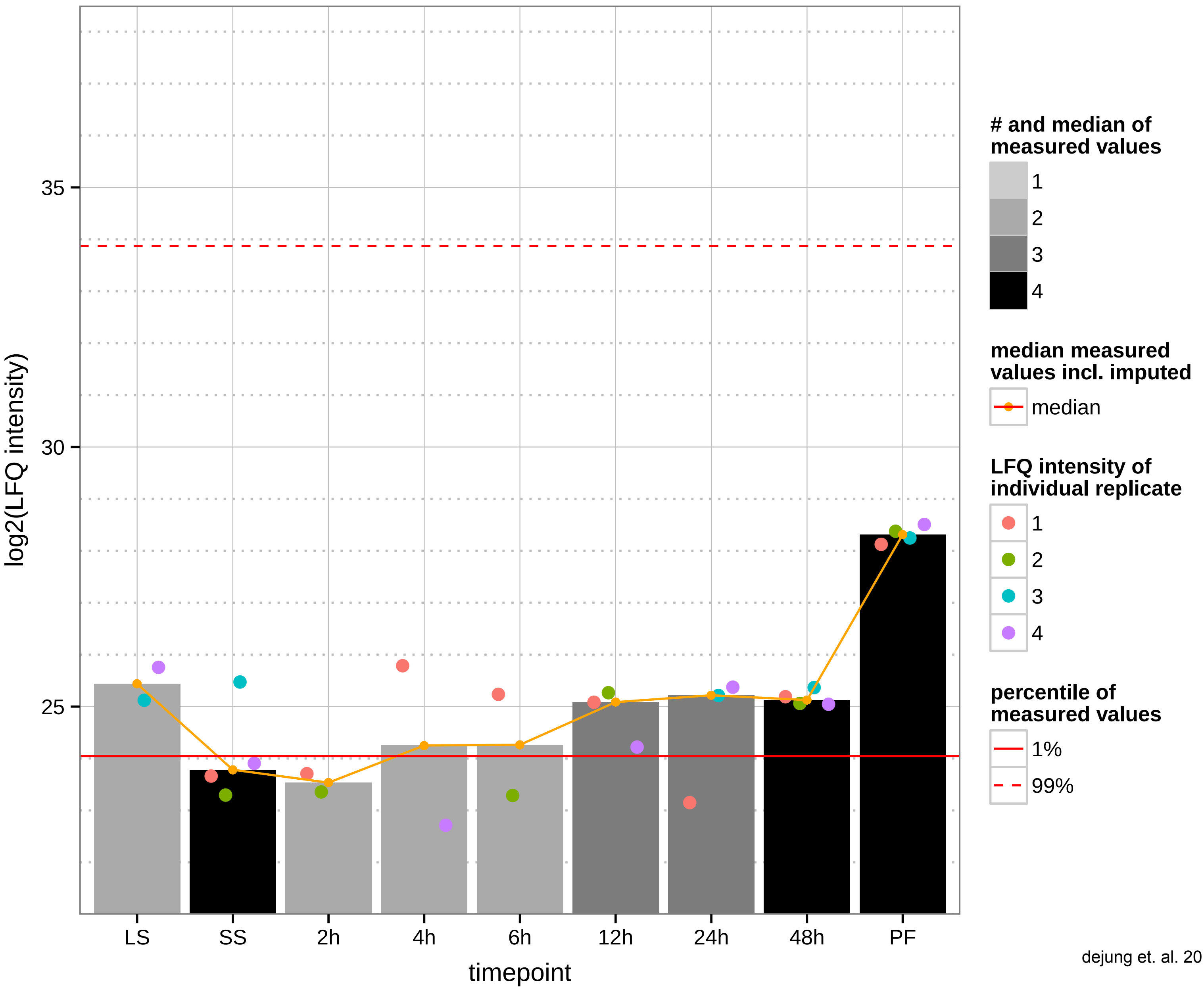

dejung et. al. 2015

hypothetical protein, conserved
Tb927.8.2950
AGOF: null
AGOC: null
AGOP: null
PGOF: null
PGOC: null
PGOP: null

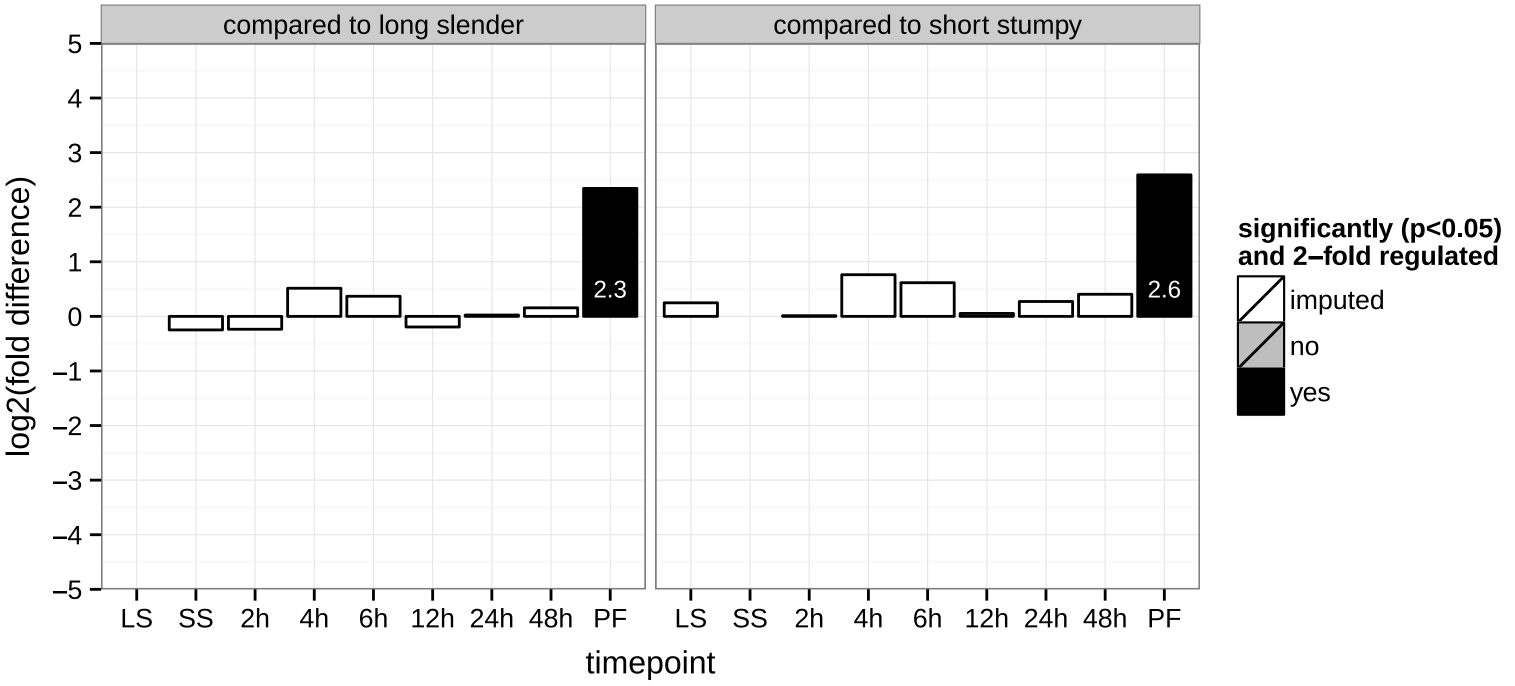

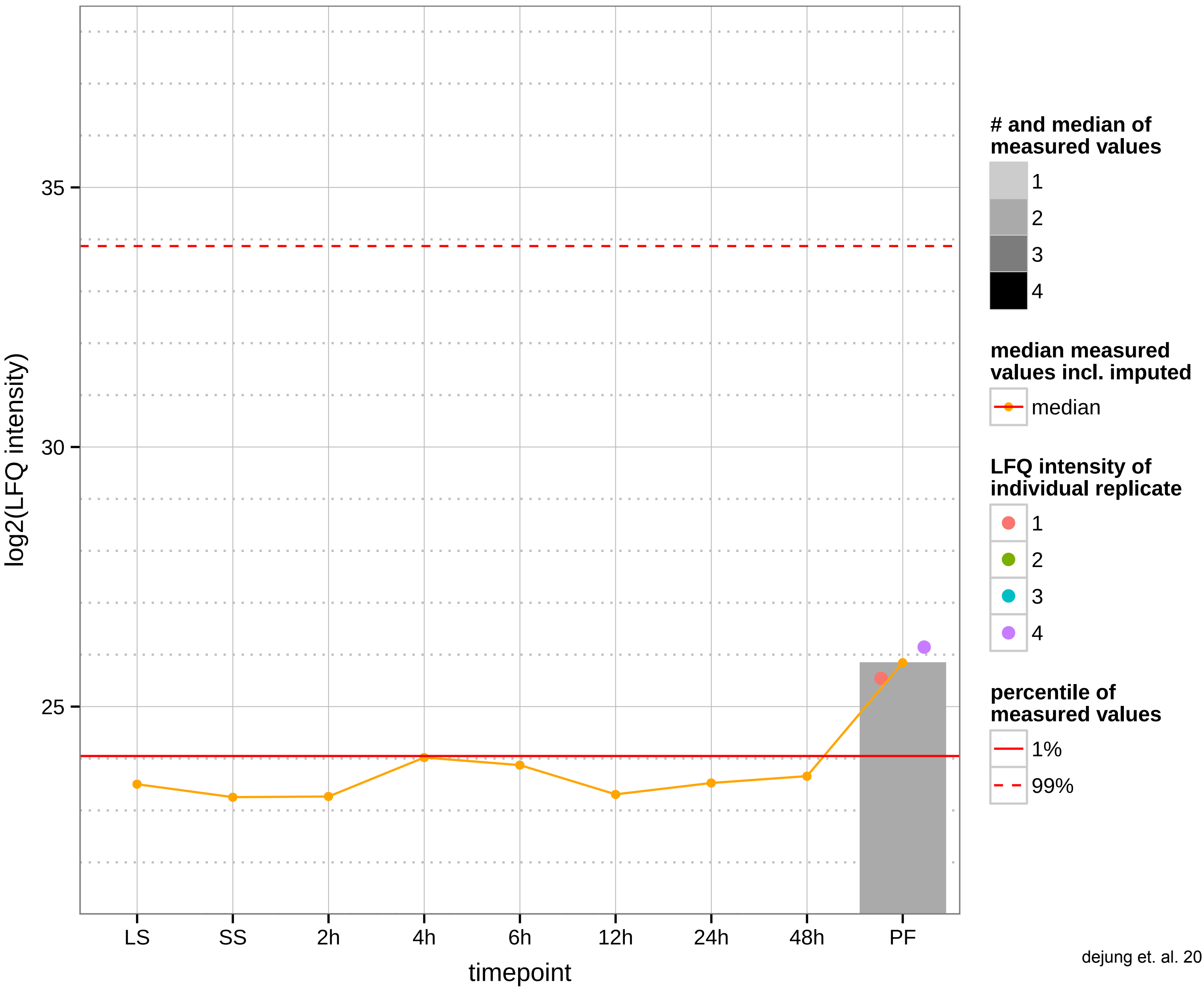

dejung et. al. 2015

hypothetical protein, conserved
Tb927.8.3090
AGOF: null
AGOC: mitochondrion
AGOP: null
PGOF: null
PGOC: null
PGOP: null

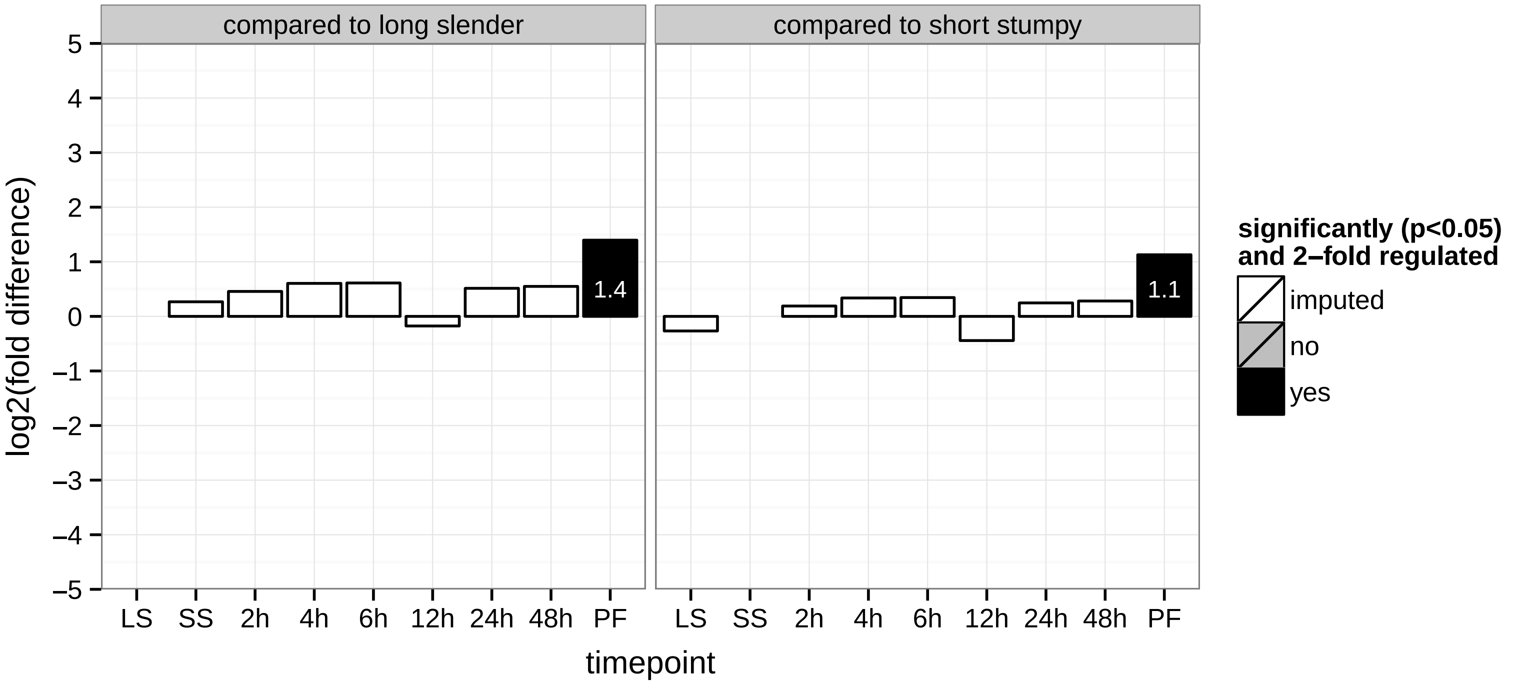

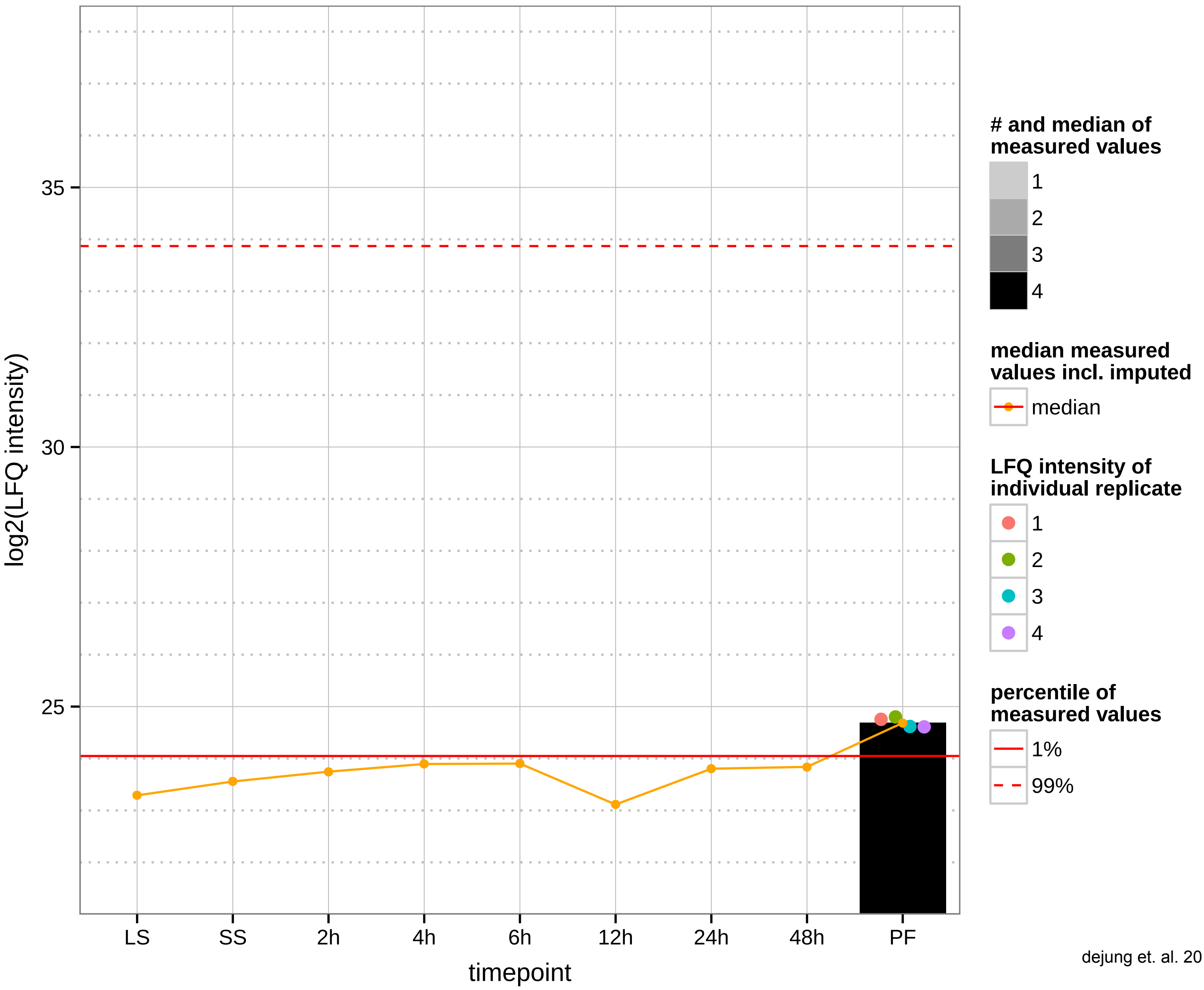

dejung et. al. 2015

lathosterol oxidase, putative
Tb927.8.3240
AGOF: C–5 sterol desaturase activity, iron ion binding, oxidoreductase activity
AGOC: endoplasmic reticulum, integral to membrane
AGOP: fatty acid biosynthetic process, lipid metabolic process, oxidation–reduction process
PGOF: iron ion binding, oxidoreductase activity
PGOC: null
PGOP: fatty acid biosynthetic process, oxidation–reduction process

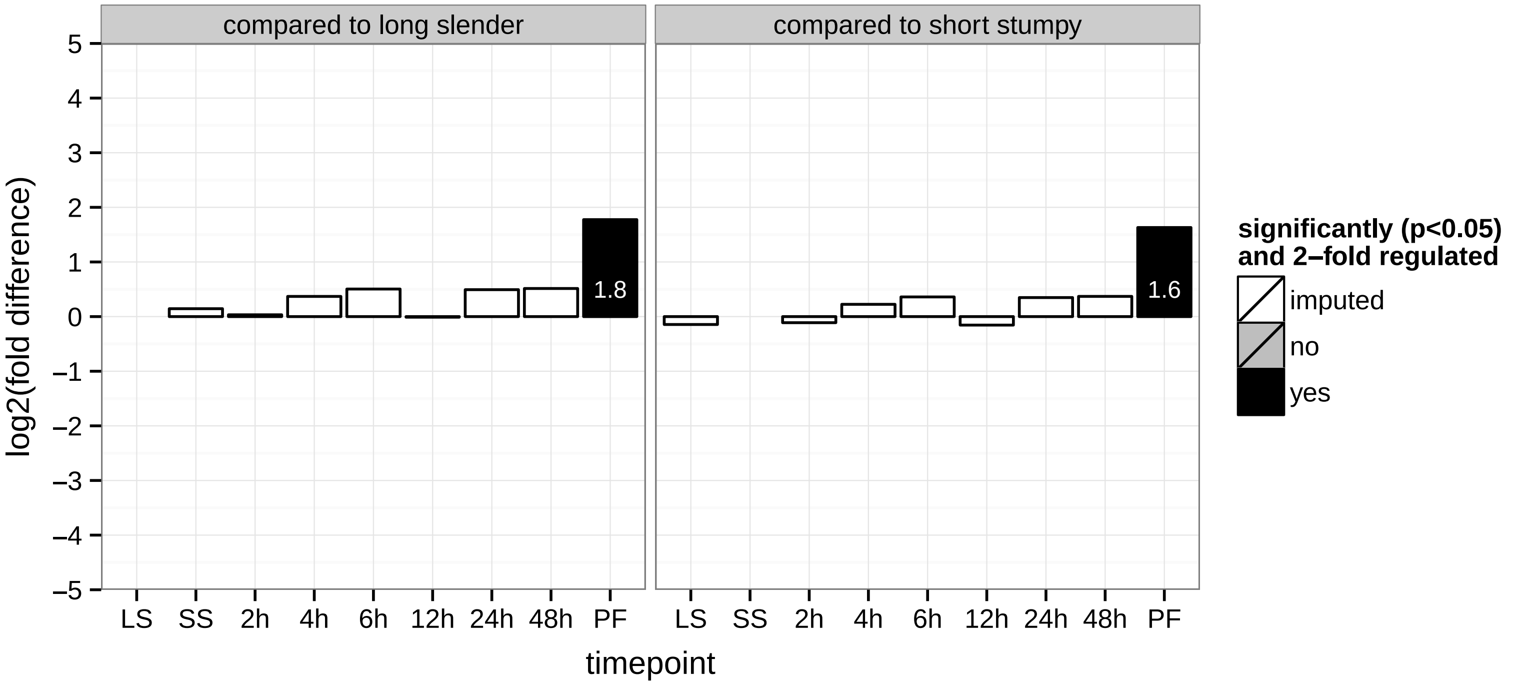

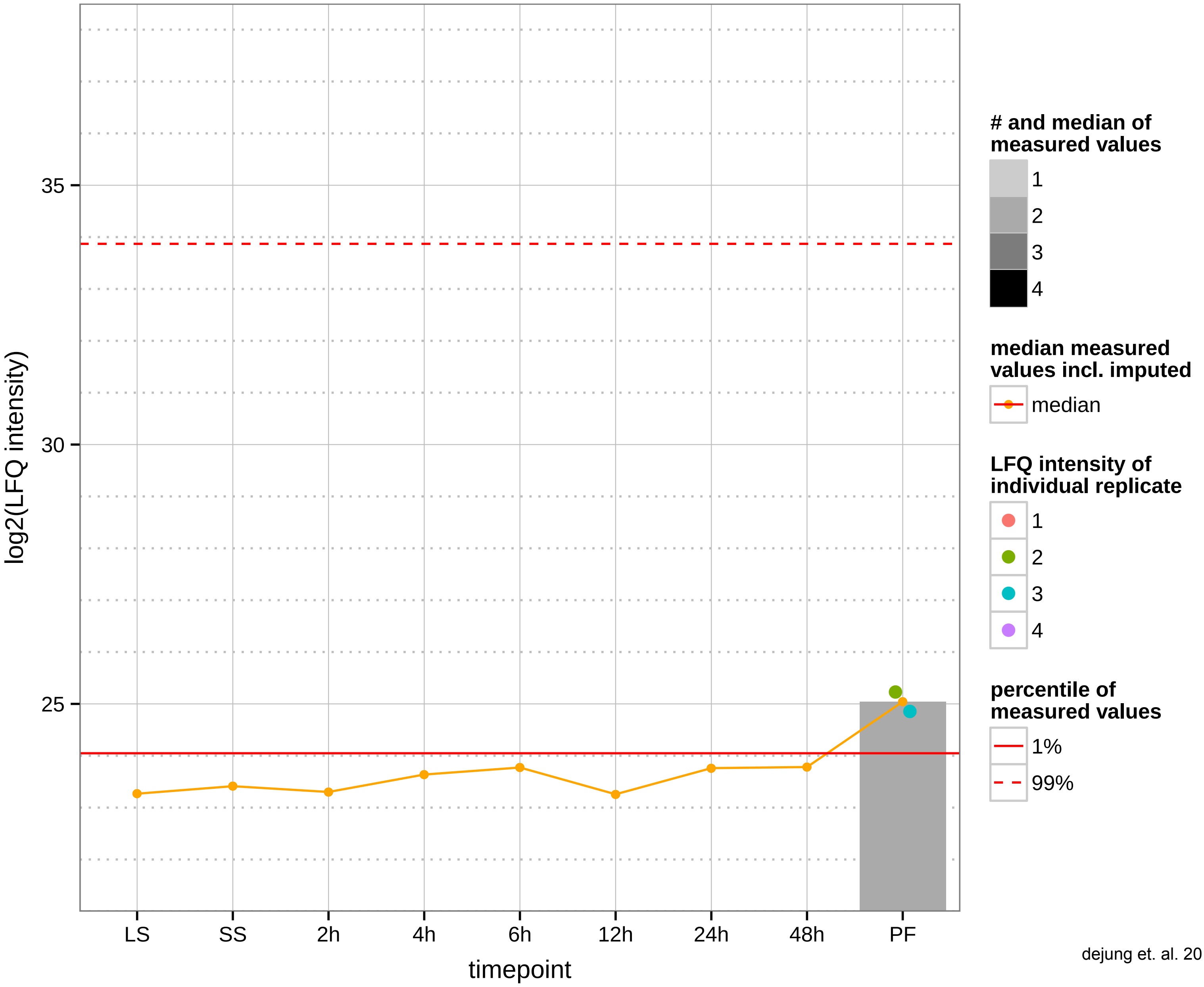

dejung et. al. 2015

hypothetical protein, conserved
Tb927.8.3490
AGOF: null
AGOC: null
AGOP: null
PGOF: null
PGOC: null
PGOP: null

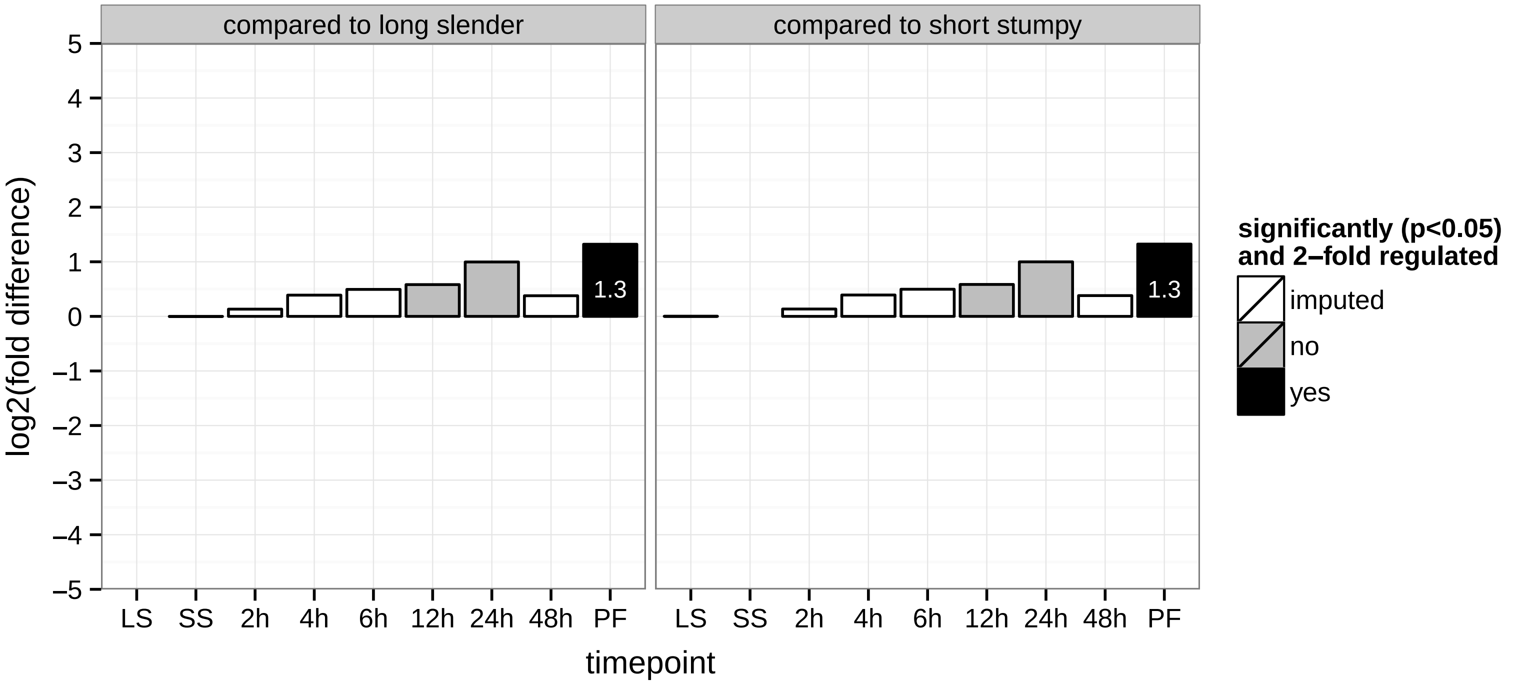

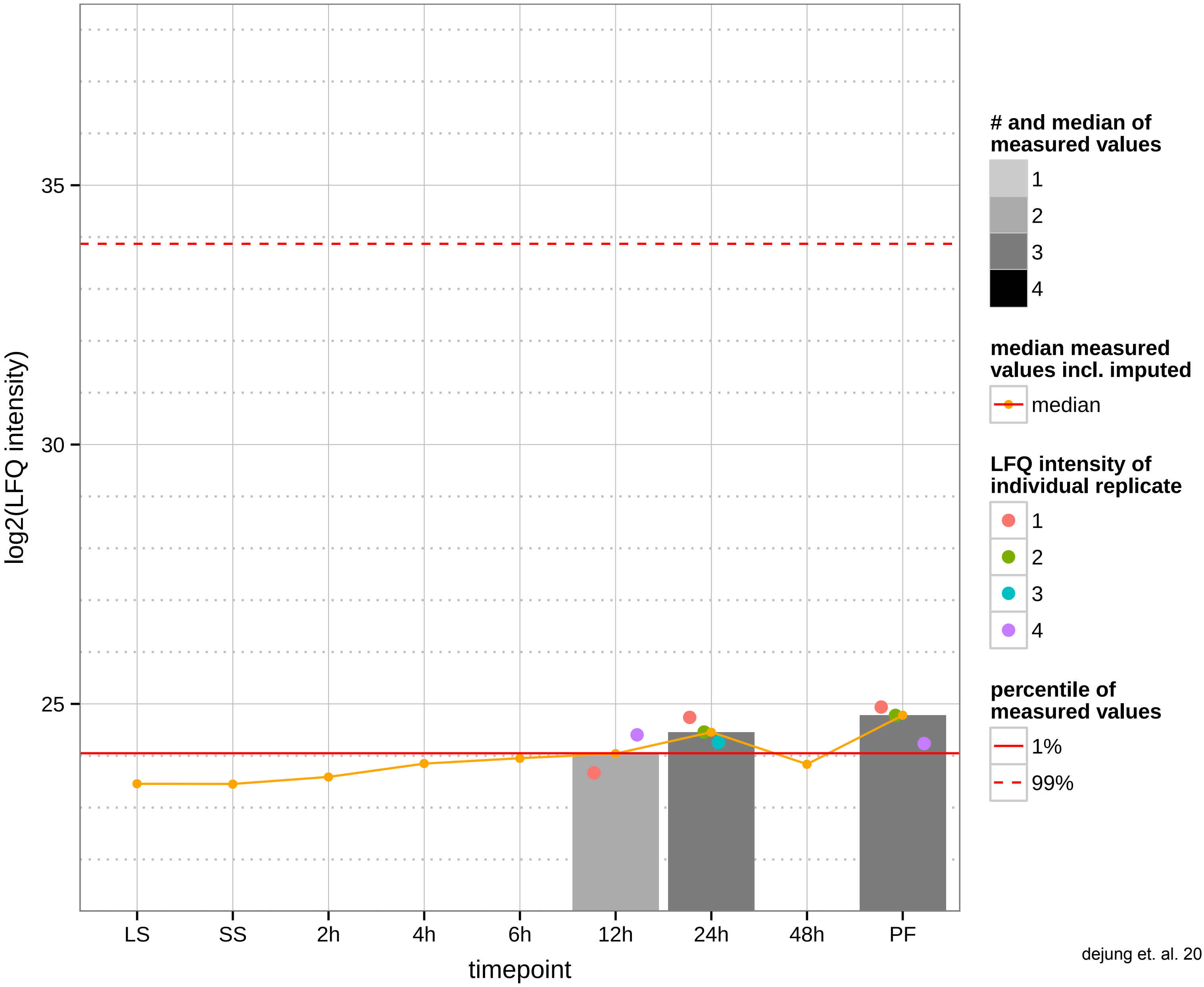

dejung et. al. 2015

hypothetical protein, conserved
Tb927.8.3590
AGOF: null
AGOC: null
AGOP: null
PGOF: null
PGOC: null
PGOP: null

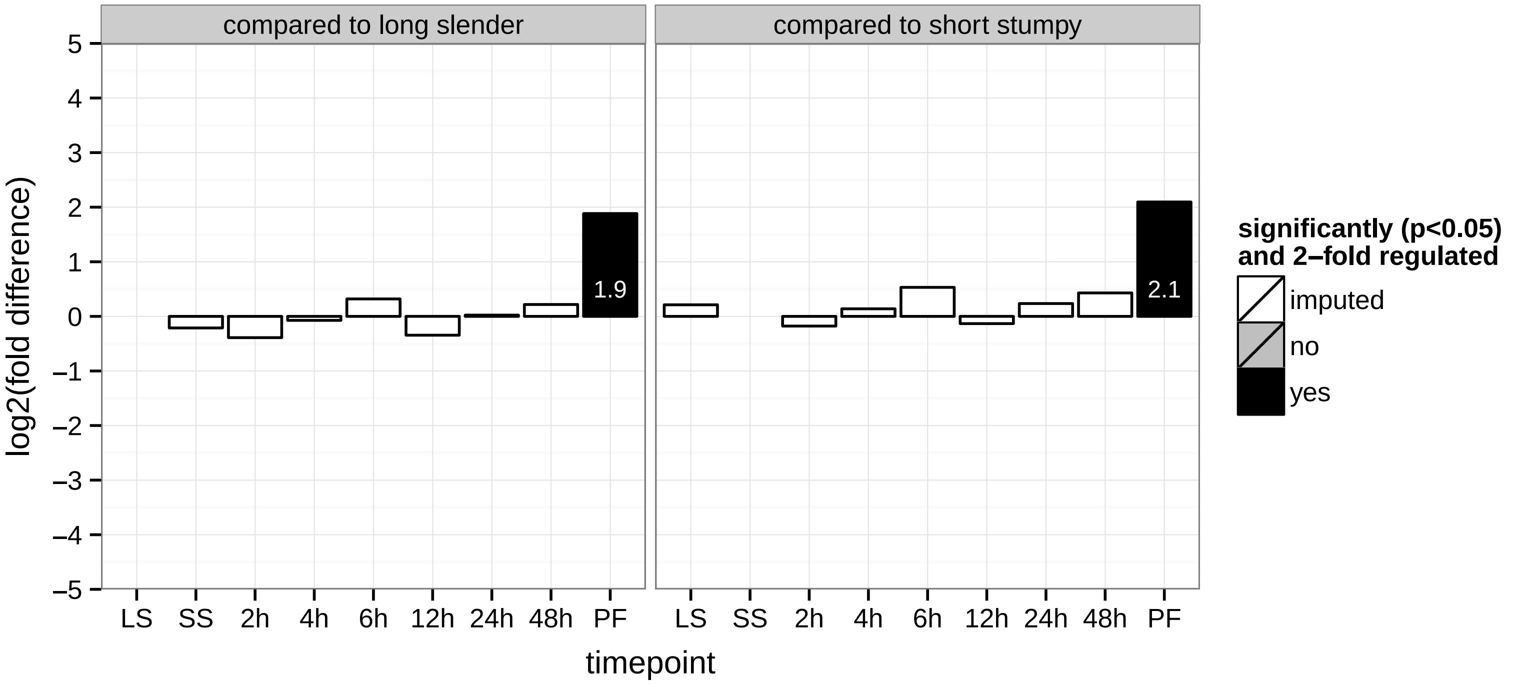

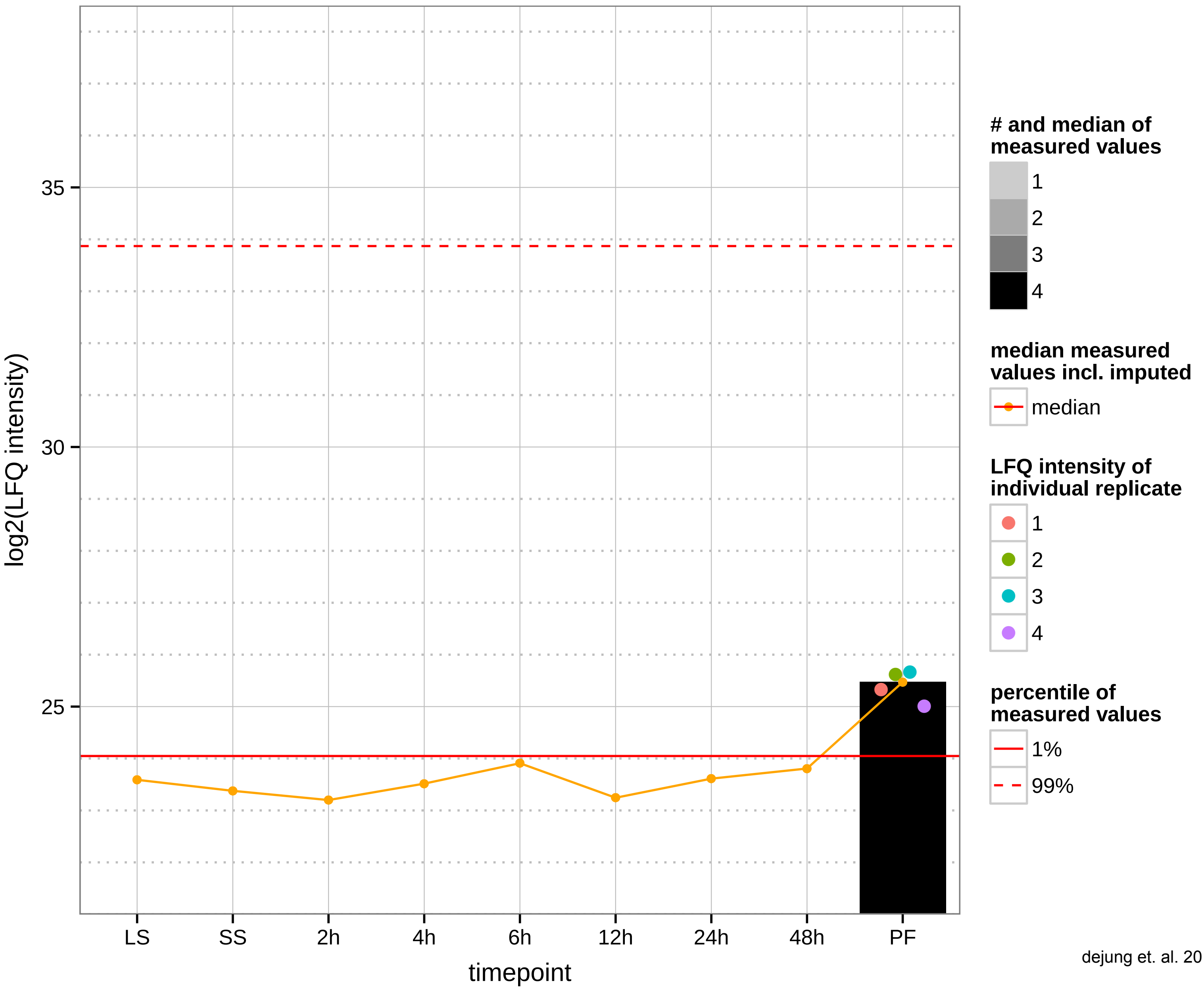

dejung et. al. 2015

hypothetical protein, conserved
Tb927.8.3820
AGOF: null
AGOC: null
AGOP: null
PGOF: null
PGOC: null
PGOP: null

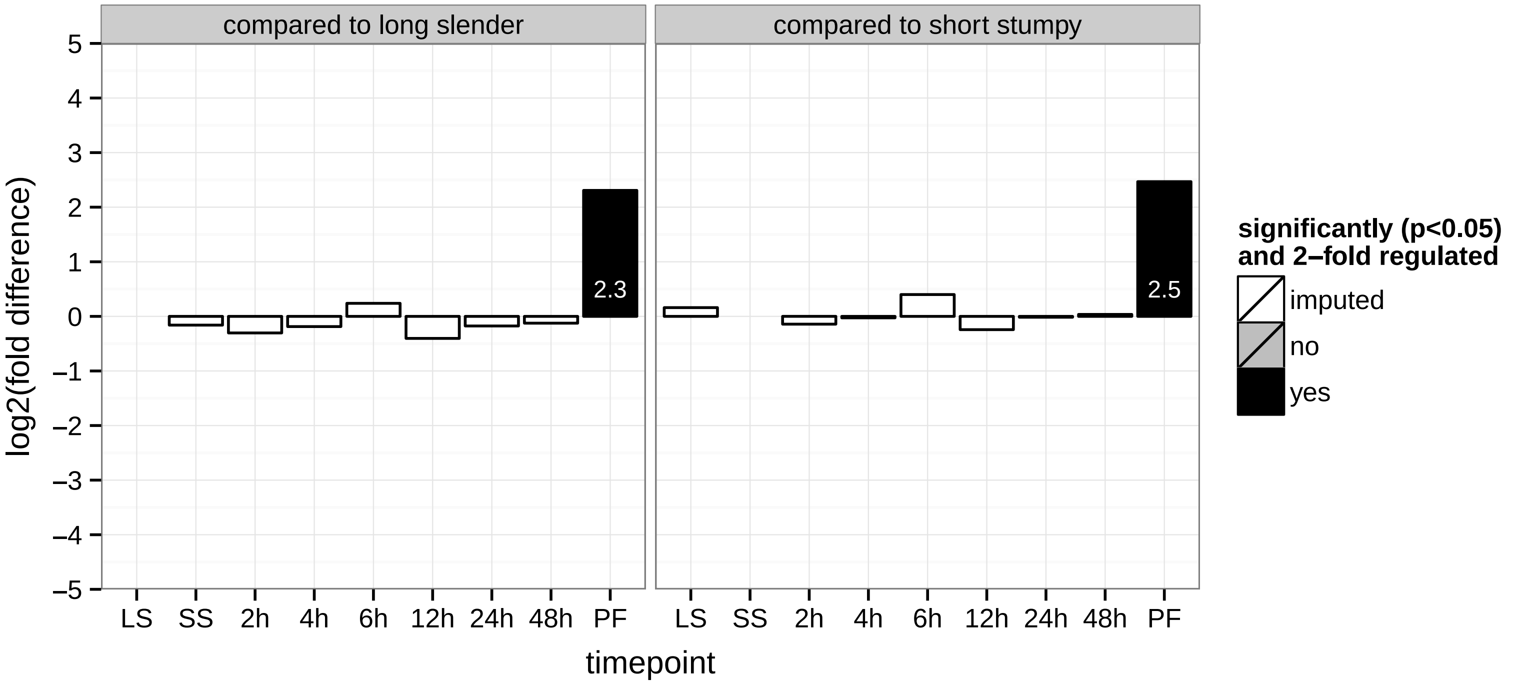

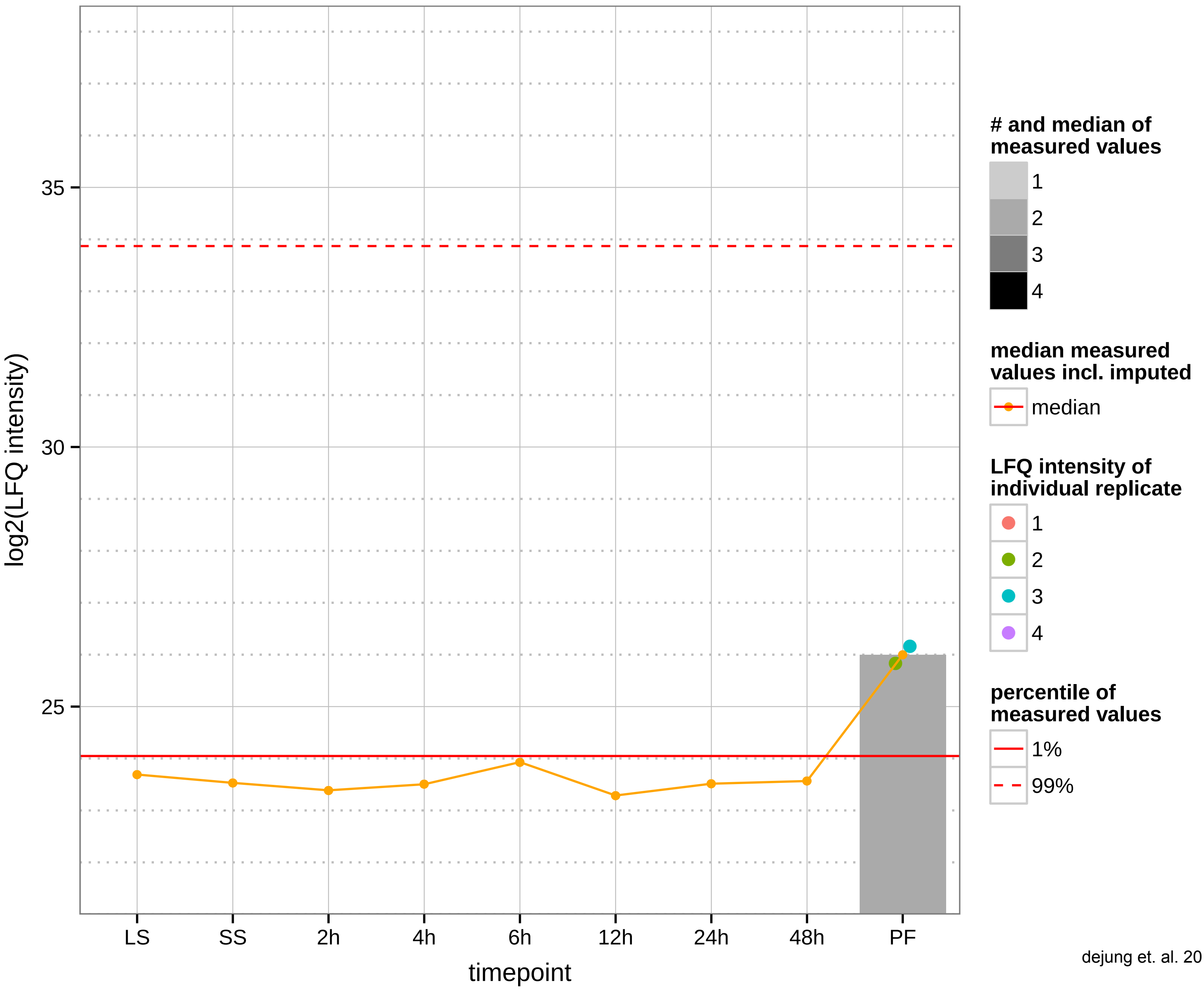

dejung et. al. 2015

hypothetical protein, conserved
Tb927.8.4240
AGOF: null
AGOC: mitochondrion
AGOP: null
PGOF: null
PGOC: null
PGOP: null

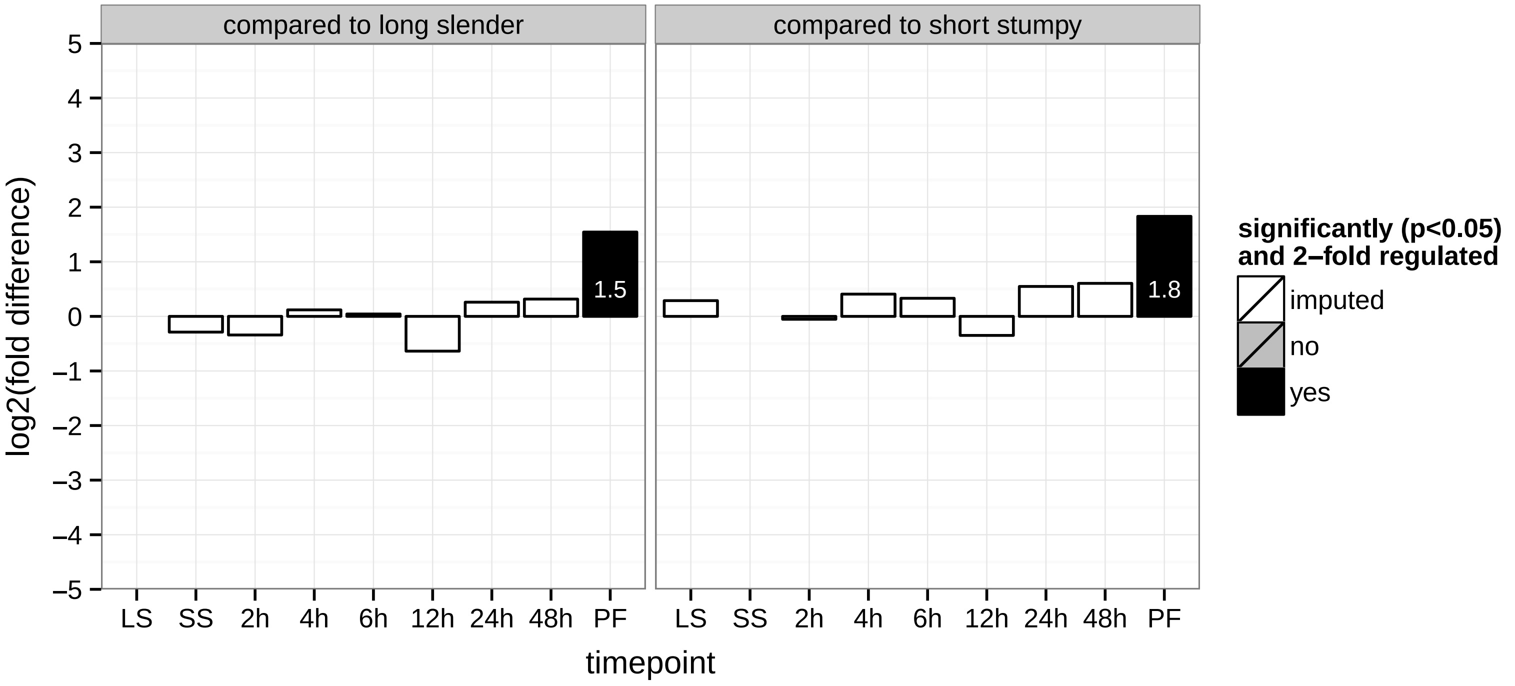

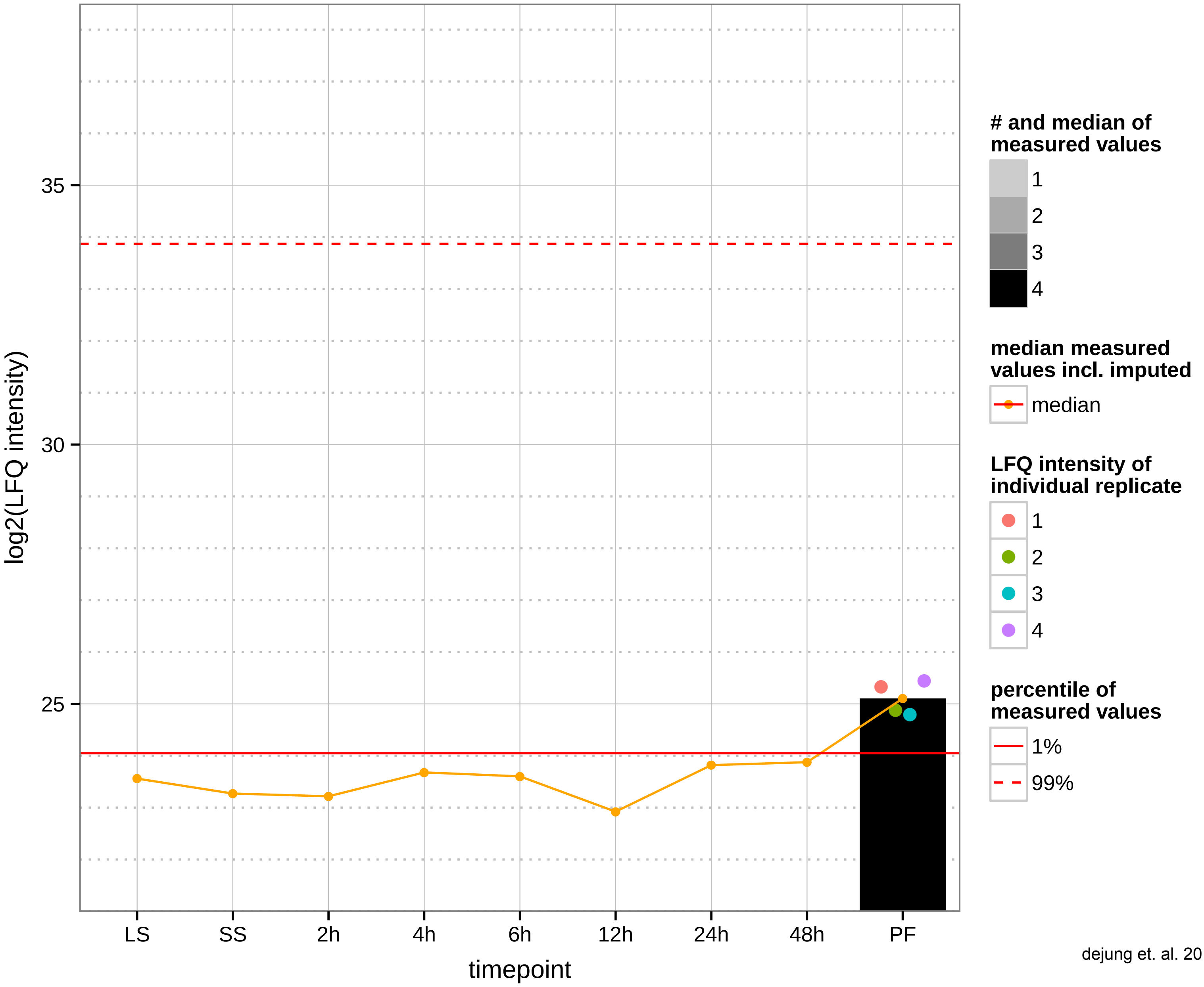

dejung et. al. 2015

Translation initiation factor eIF−2B subunit beta, putative (EIF2BB)
Tb927.8.4390
AGOF: GTP binding, translation initiation factor activity
AGOC: eukaryotic translation initiation factor 2B complex
AGOP: cellular metabolic process, translational initiation
PGOF: null
PGOC: null
PGOP: cellular metabolic process

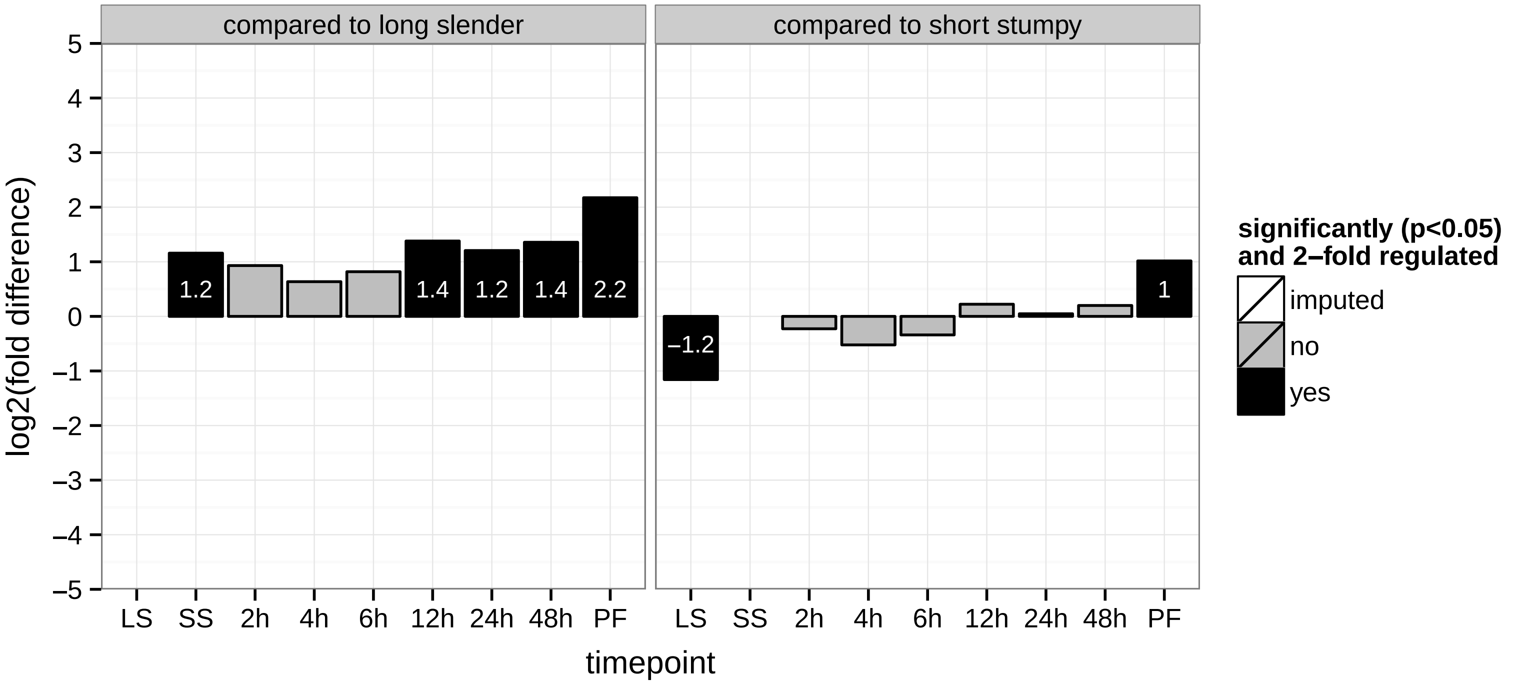

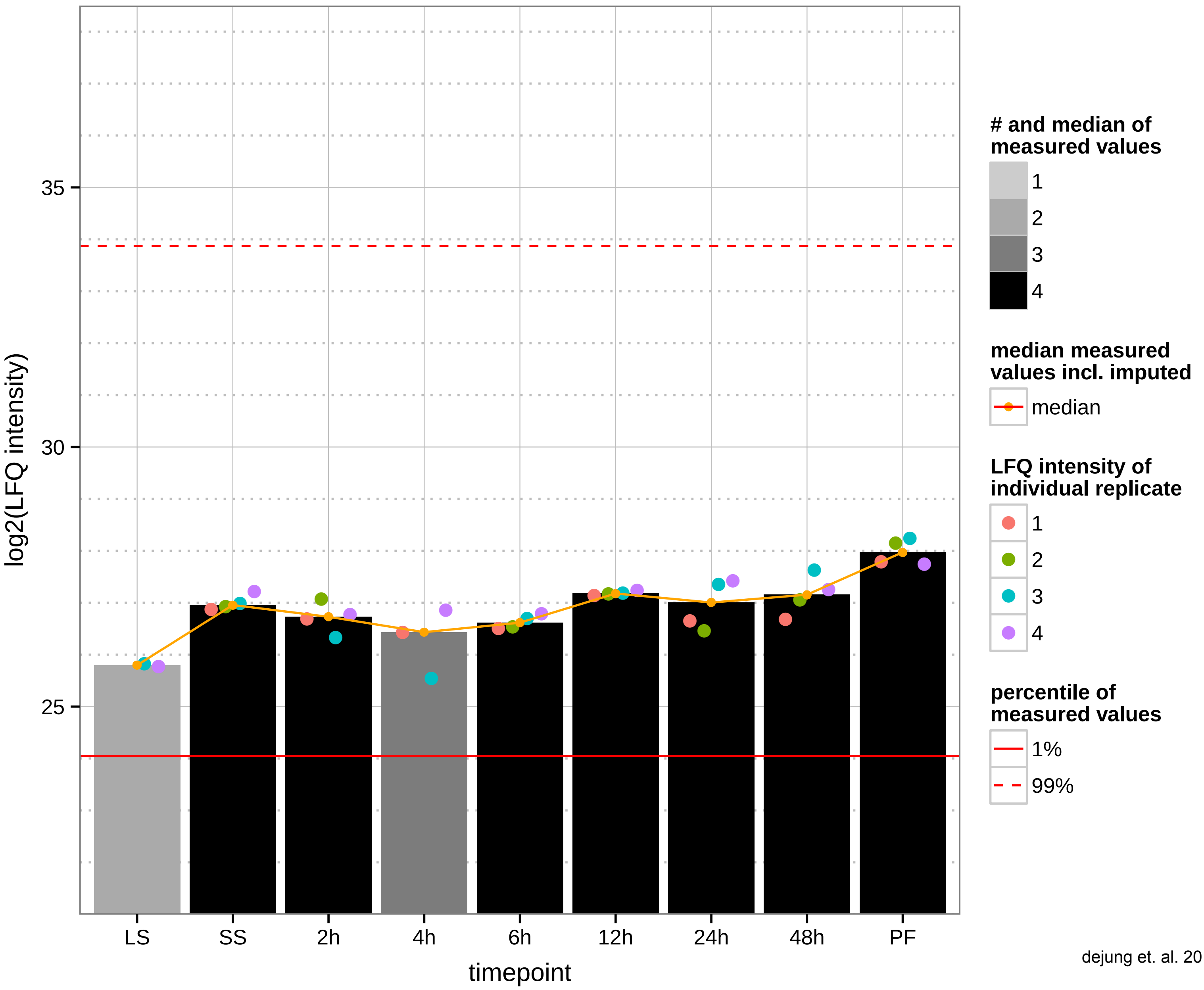

dejung et. al. 2015

hypothetical protein, conserved
Tb927.8.4800
AGOF: null
AGOC: null
AGOP: null
PGOF: protein binding
PGOC: null
PGOP: null

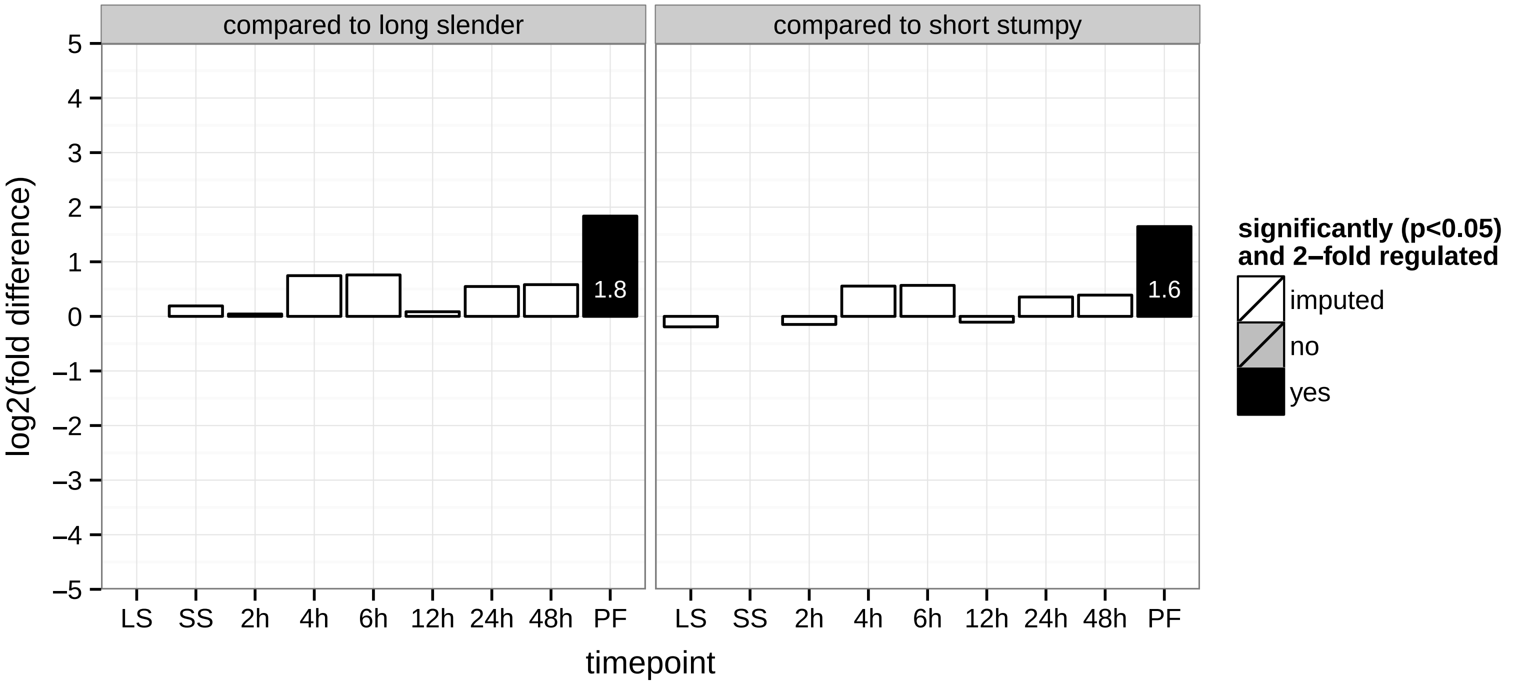

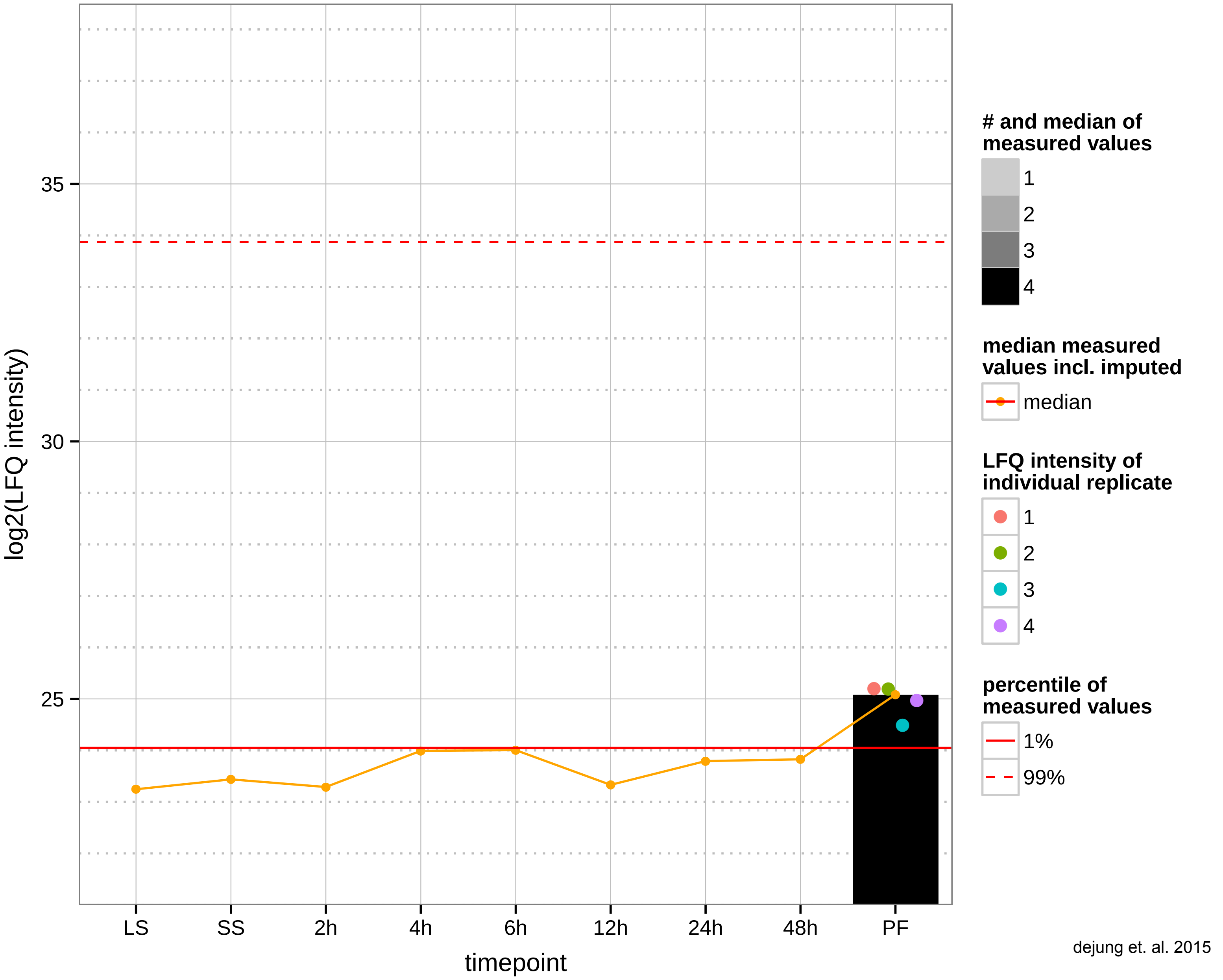

dejung et. al. 2015

hypothetical protein, conserved
Tb927.8.4930
AGOF: null
AGOC: mitochondrion
AGOP: null
PGOF: null
PGOC: null
PGOP: null

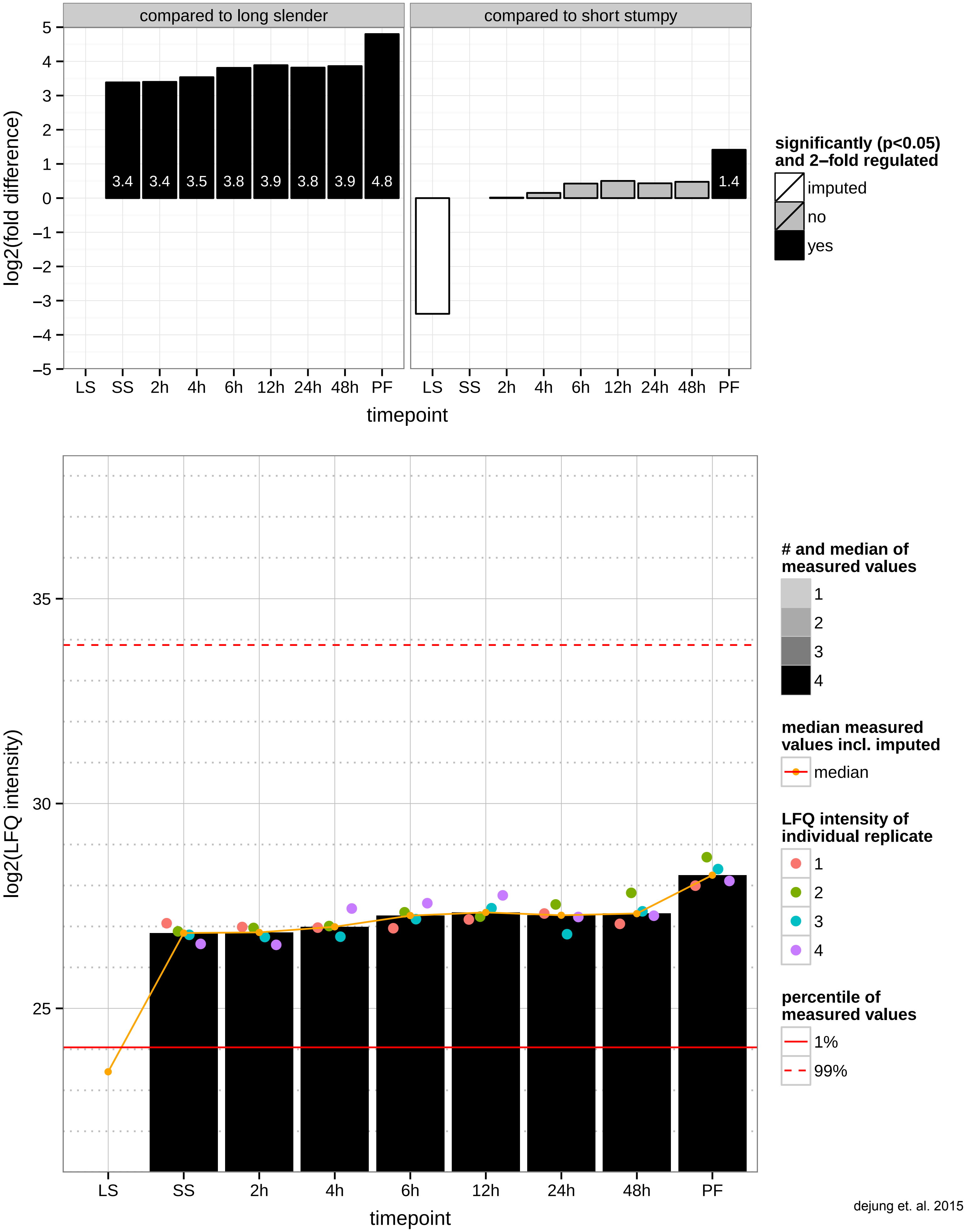

dejung et. al. 2015

hypothetical protein, conserved
Tb927.8.5040
AGOF: null
AGOC: nucleus
AGOP: null
PGOF: methyltransferase activity
PGOC: null
PGOP: null

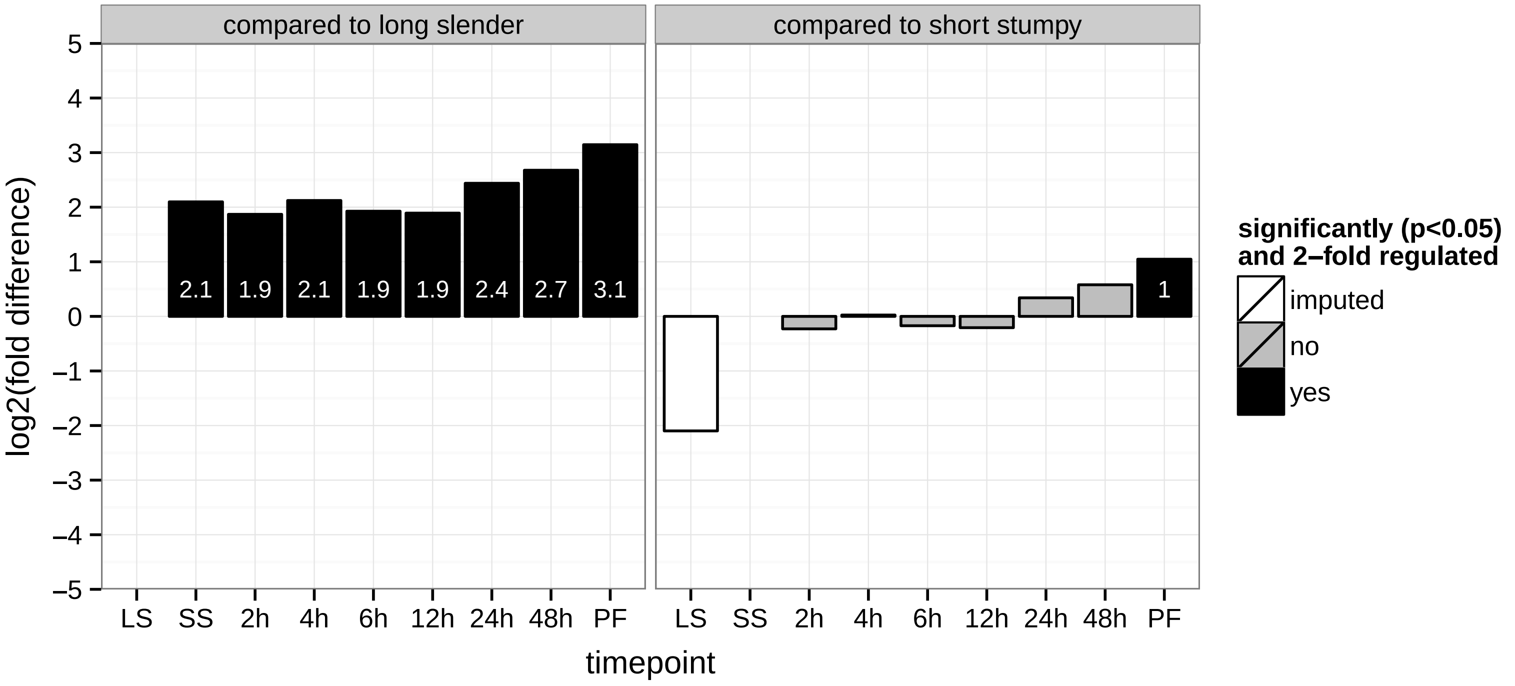

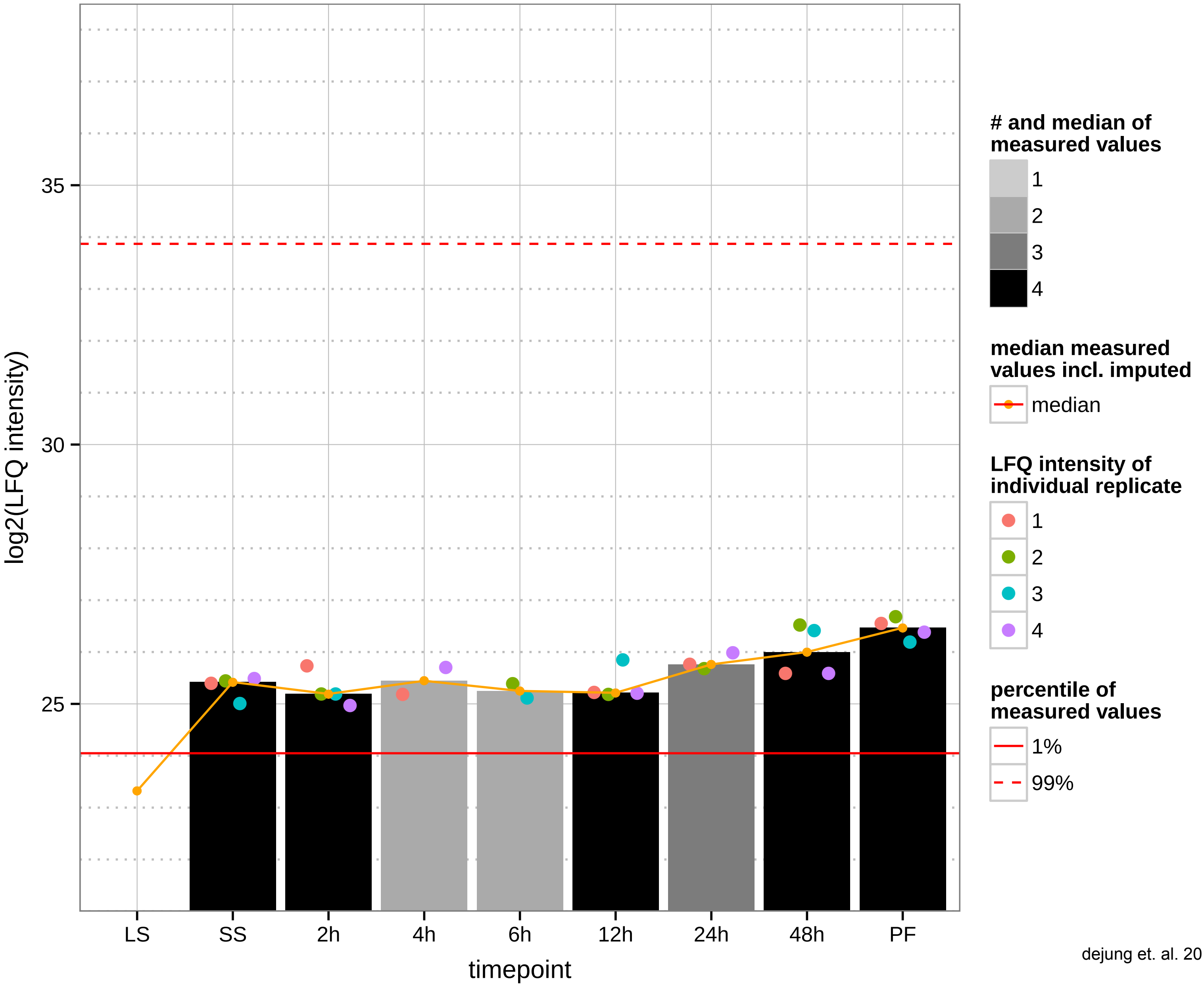

dejung et. al. 2015

hypothetical protein, conserved
Tb927.8.5100
AGOF: null
AGOC: mitochondrion
AGOP: null
PGOF: null
PGOC: null
PGOP: null

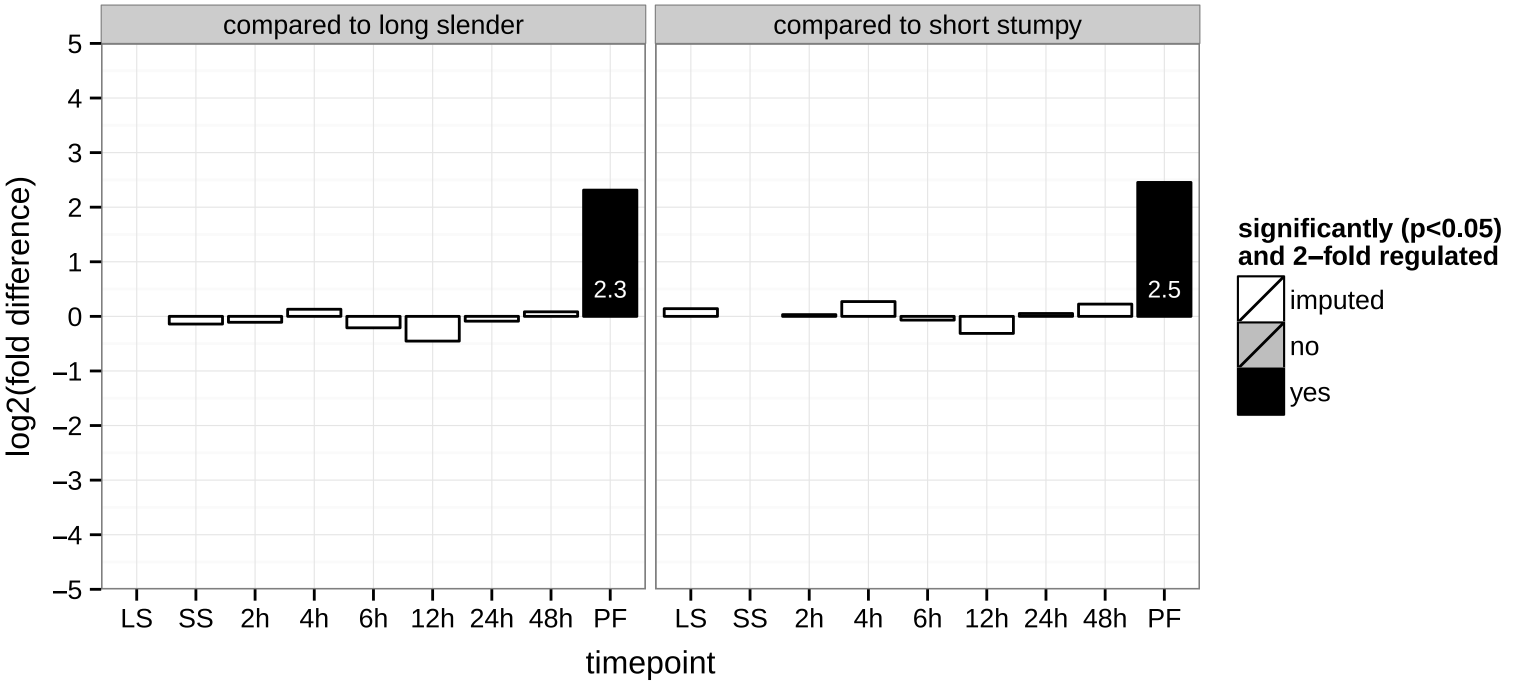

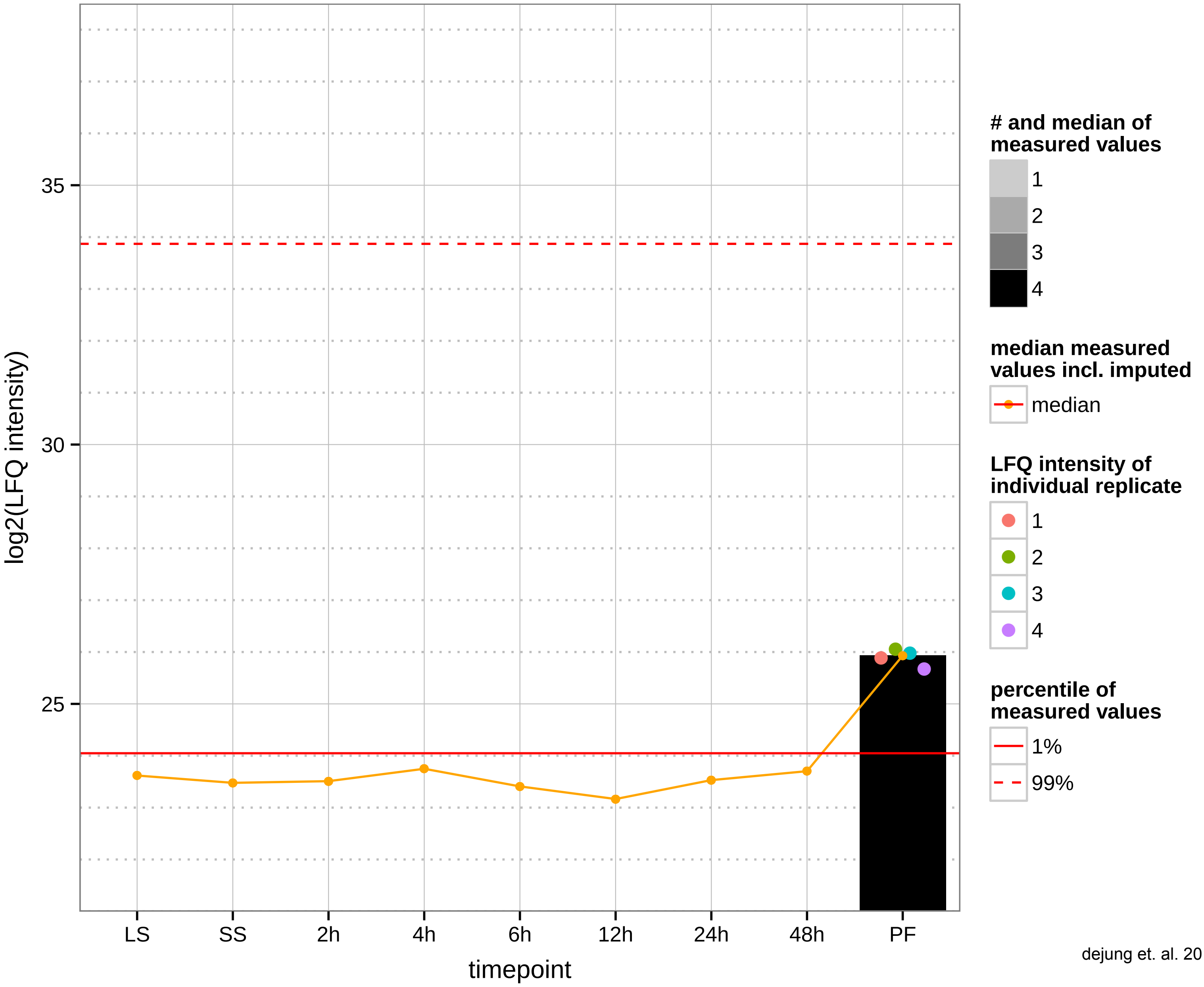

dejung et. al. 2015

hypothetical protein, conserved
Tb927.8.5320
AGOF: null
AGOC: null
AGOP: null
PGOF: null
PGOC: null
PGOP: null

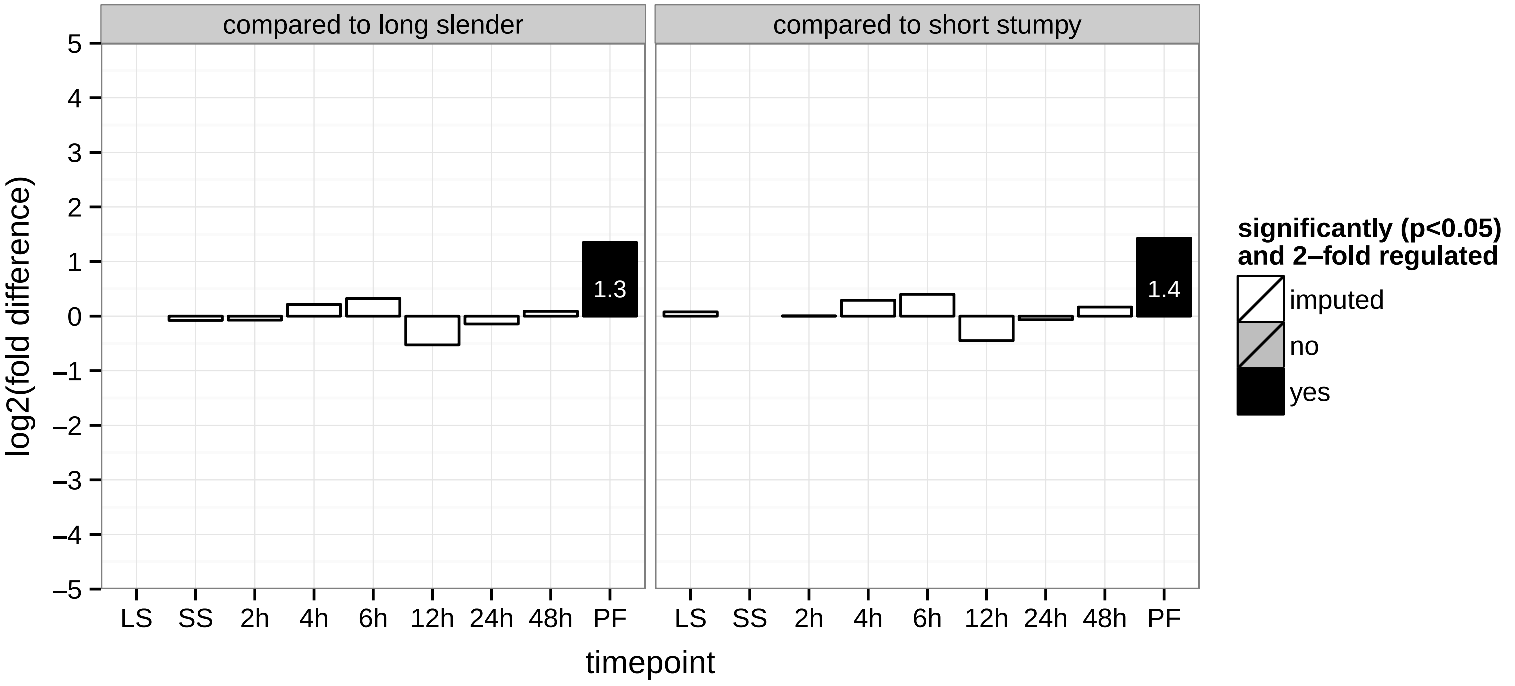

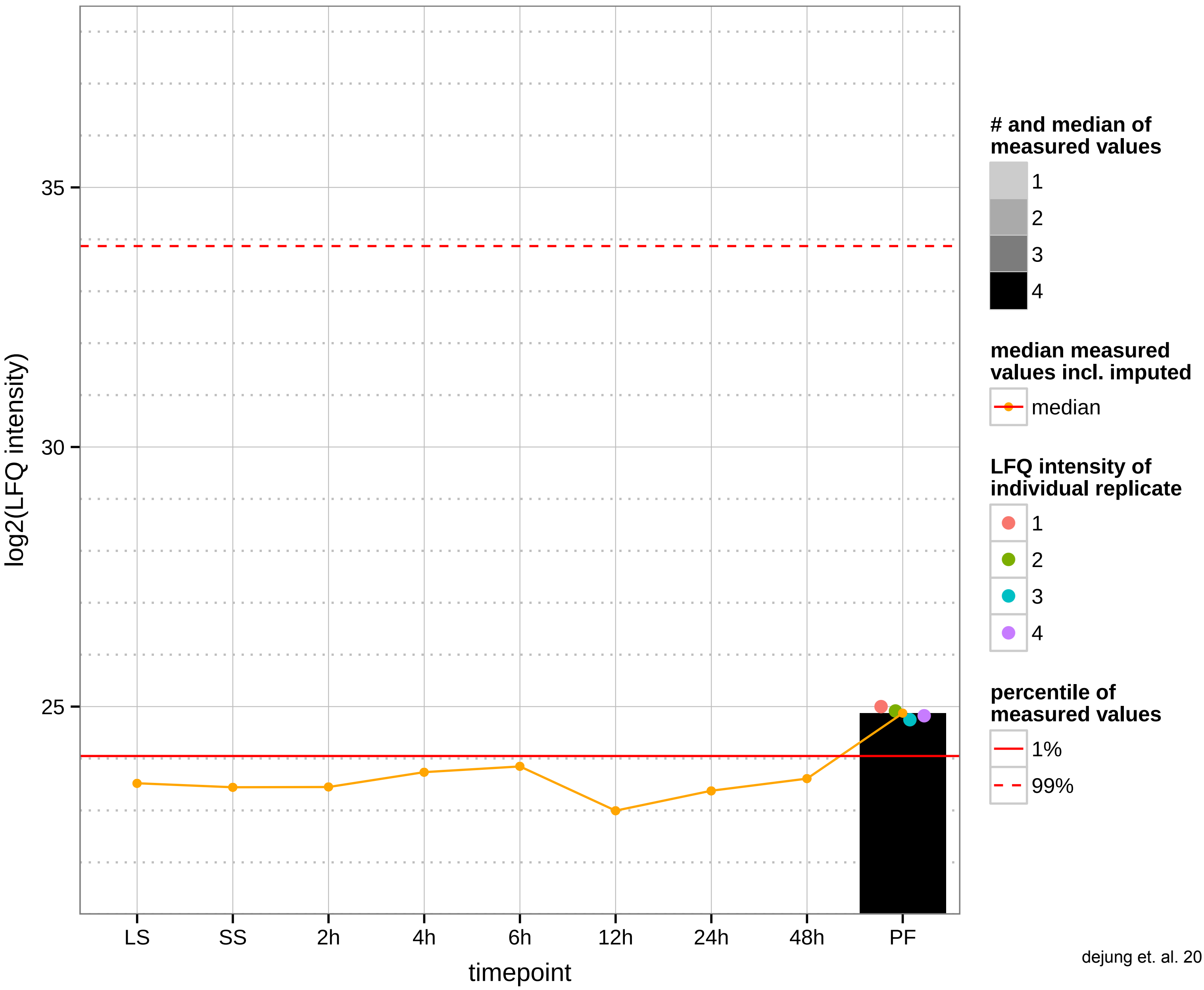

dejung et. al. 2015

amino acid permease 24 (AAT6)
Tb927.8.5450
AGOF: L−proline transmembrane transporter activity, alanine transmembrane transporter activity, drug transmembrane transp
AGOC: integral to membrane, plasma membrane
AGOP: amino acid homeostasis, drug transport, hypotonic response, proline transmembrane transport, regulation of L−glutam
PGOF: null
PGOC: null
PGOP: null

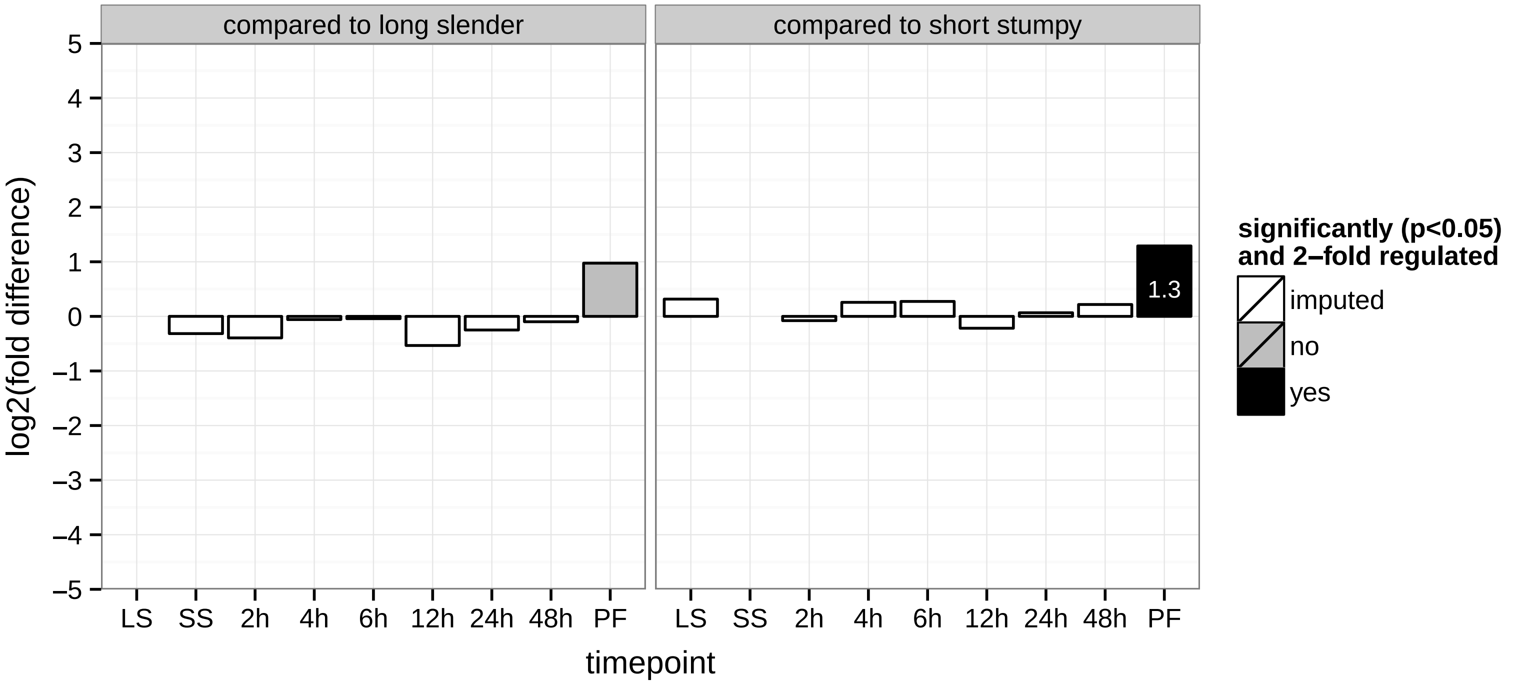

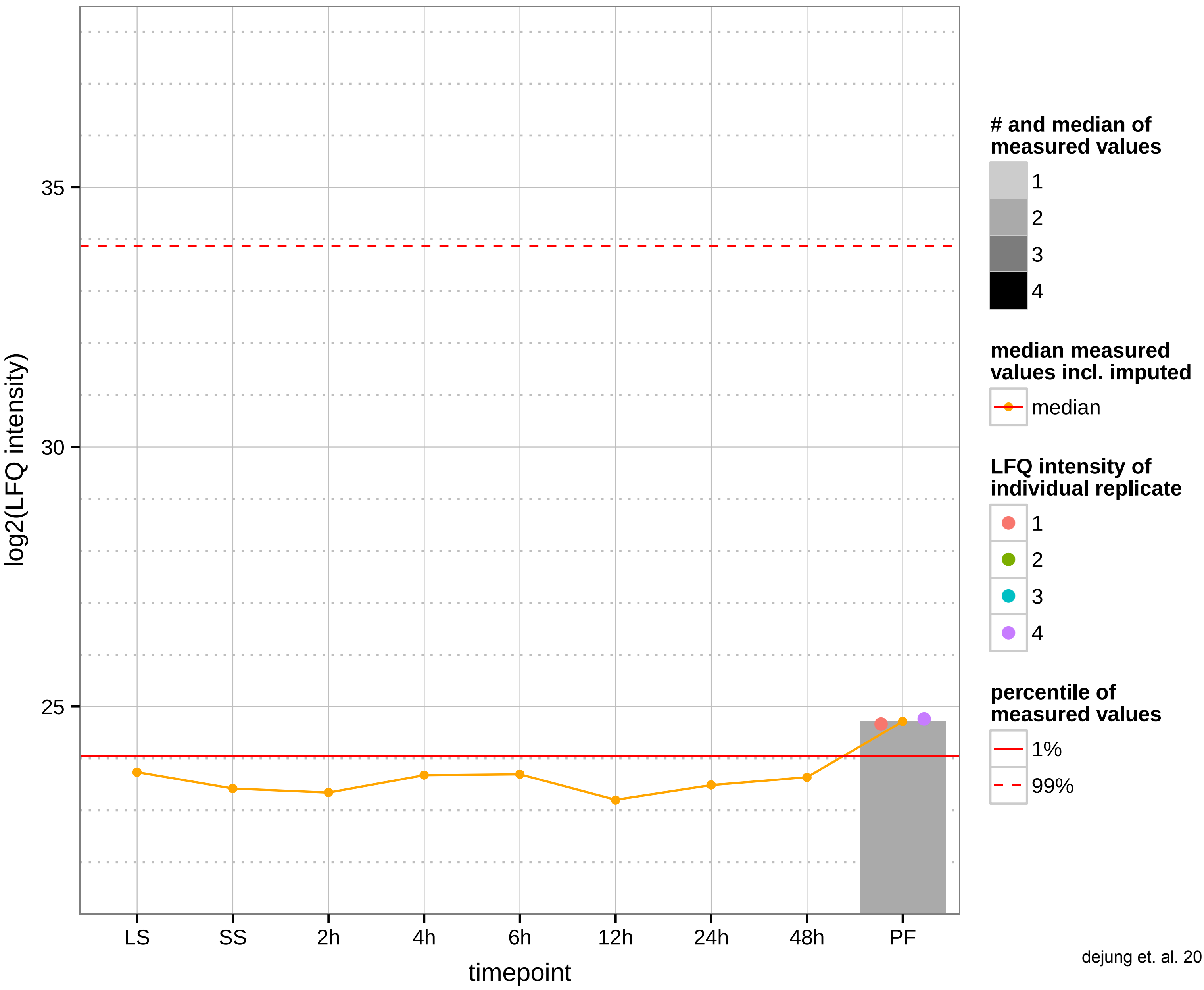

dejung et. al. 2015

hypothetical protein, conserved
Tb927.8.560
AGOF: calcium ion binding
AGOC: integral to membrane
AGOP: null
PGOF: null
PGOC: null
PGOP: null

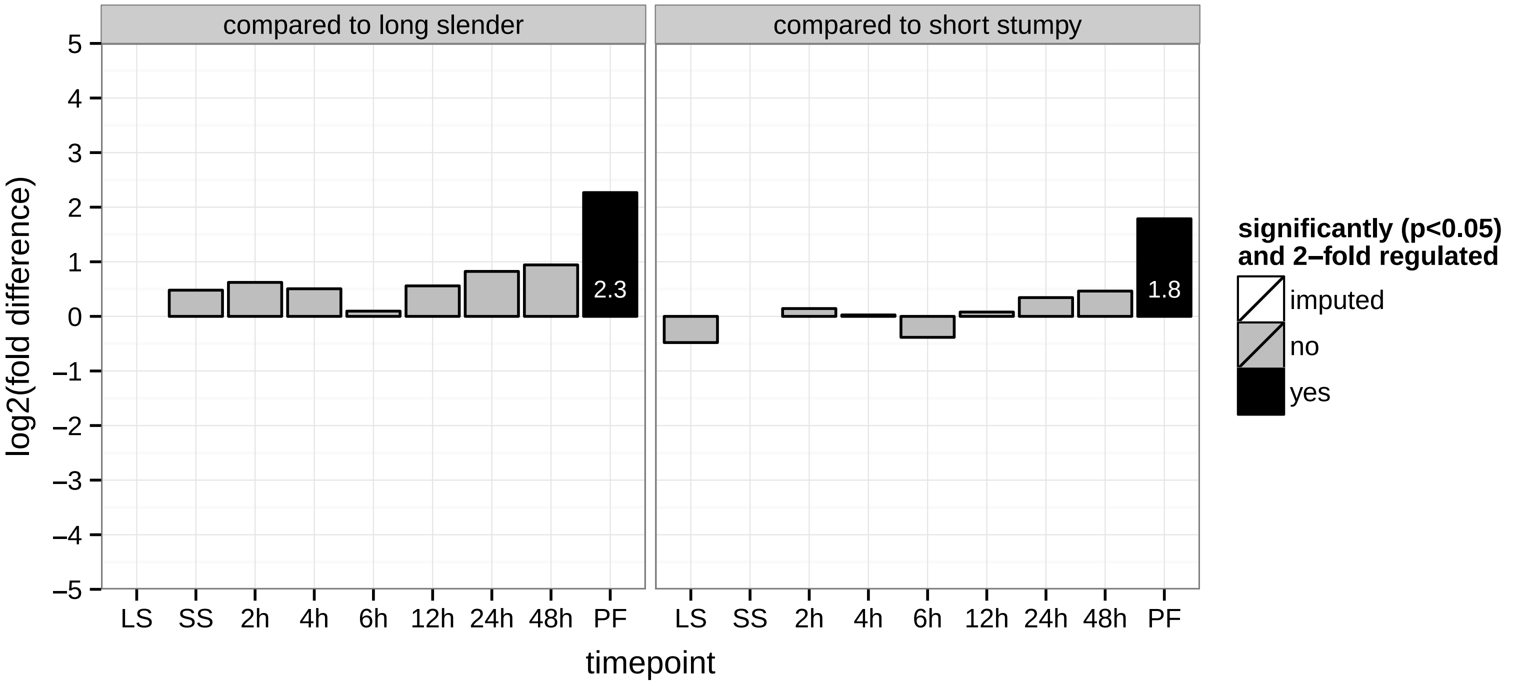

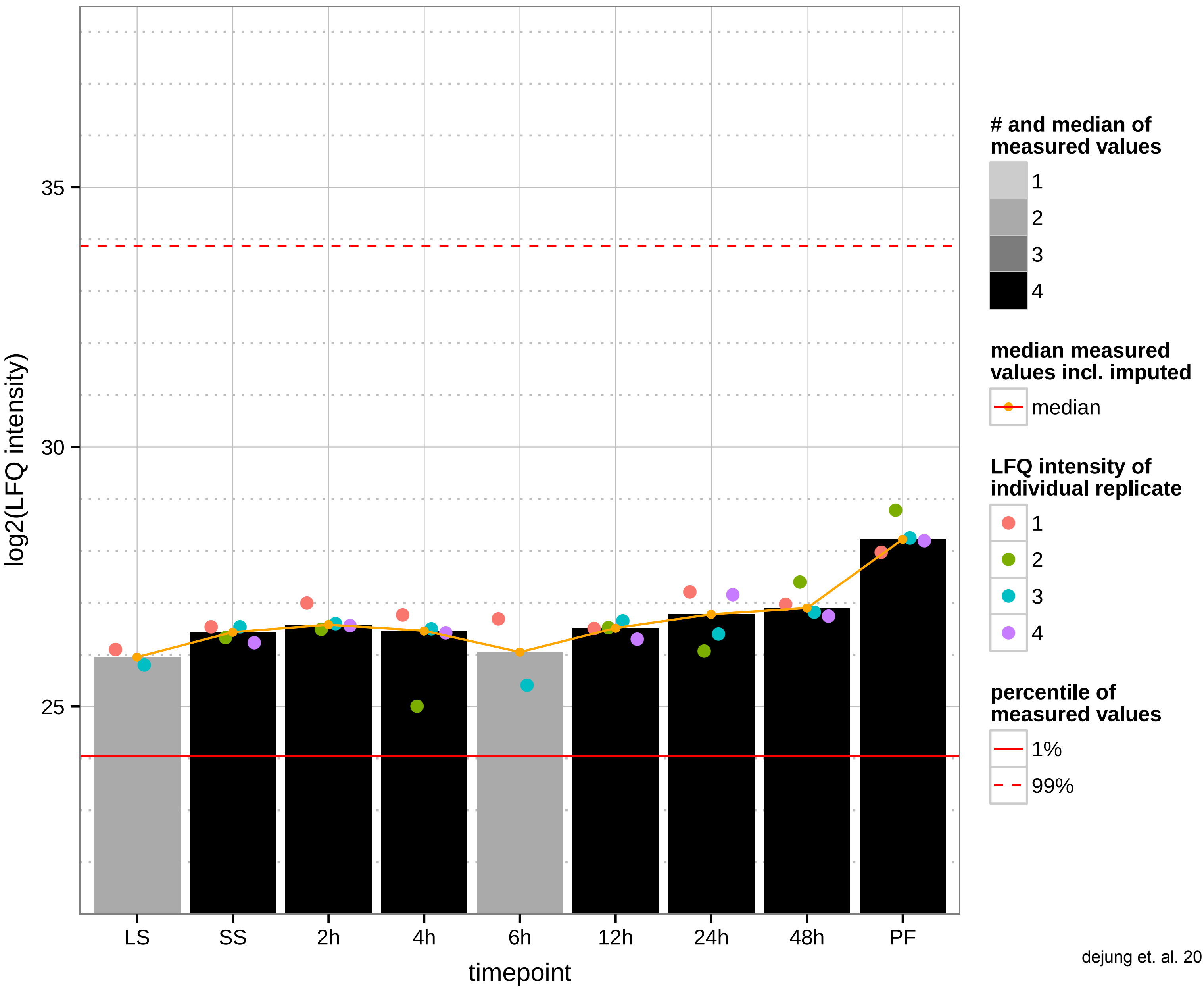

dejung et. al. 2015

ubiquitin carboxyl–terminal hydrolase, putative
Tb927.8.5620
AGOF: cysteine–type endopeptidase activity, ubiquitin thiolesterase activity, zinc ion binding
AGOC: intracellular
AGOP: ubiquitin–dependent protein catabolic process
PGOF: ubiquitin thiolesterase activity, zinc ion binding
PGOC: intracellular
PGOP: ubiquitin–dependent protein catabolic process

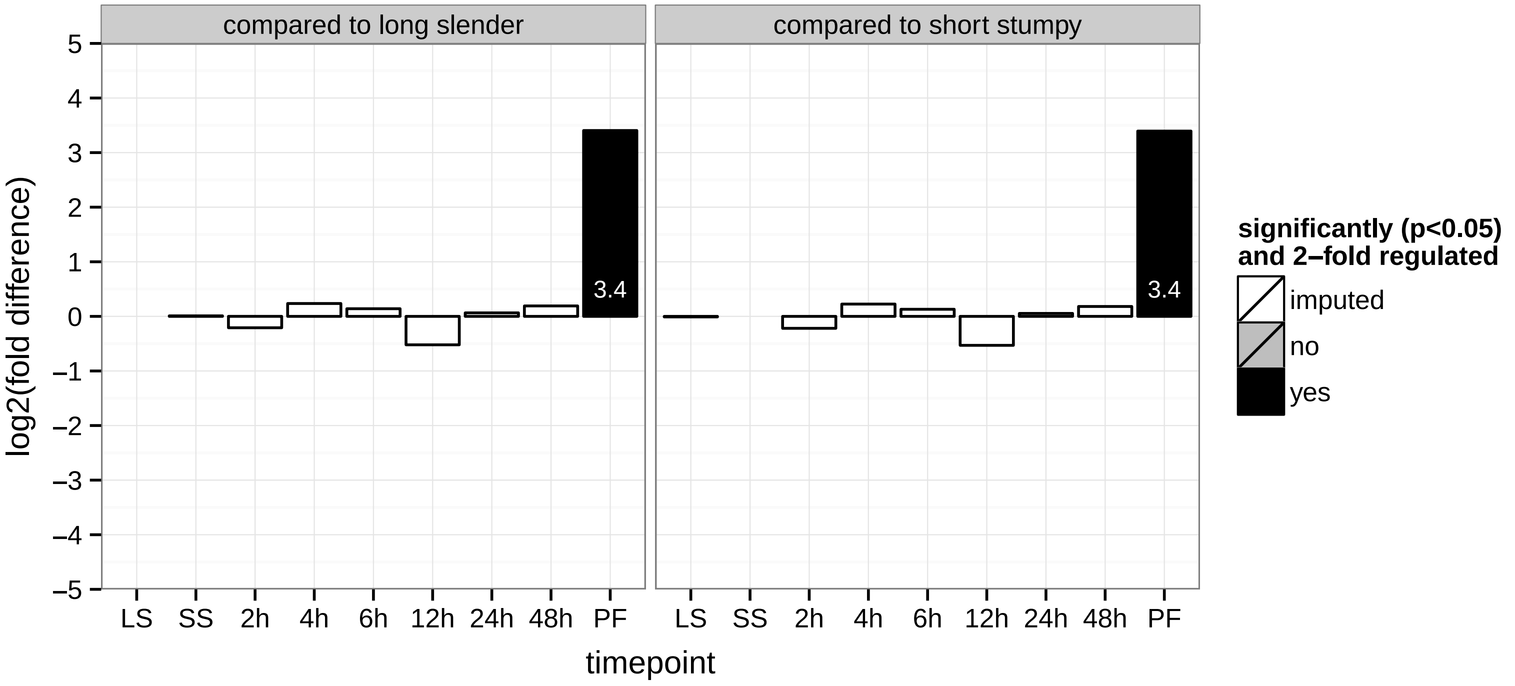

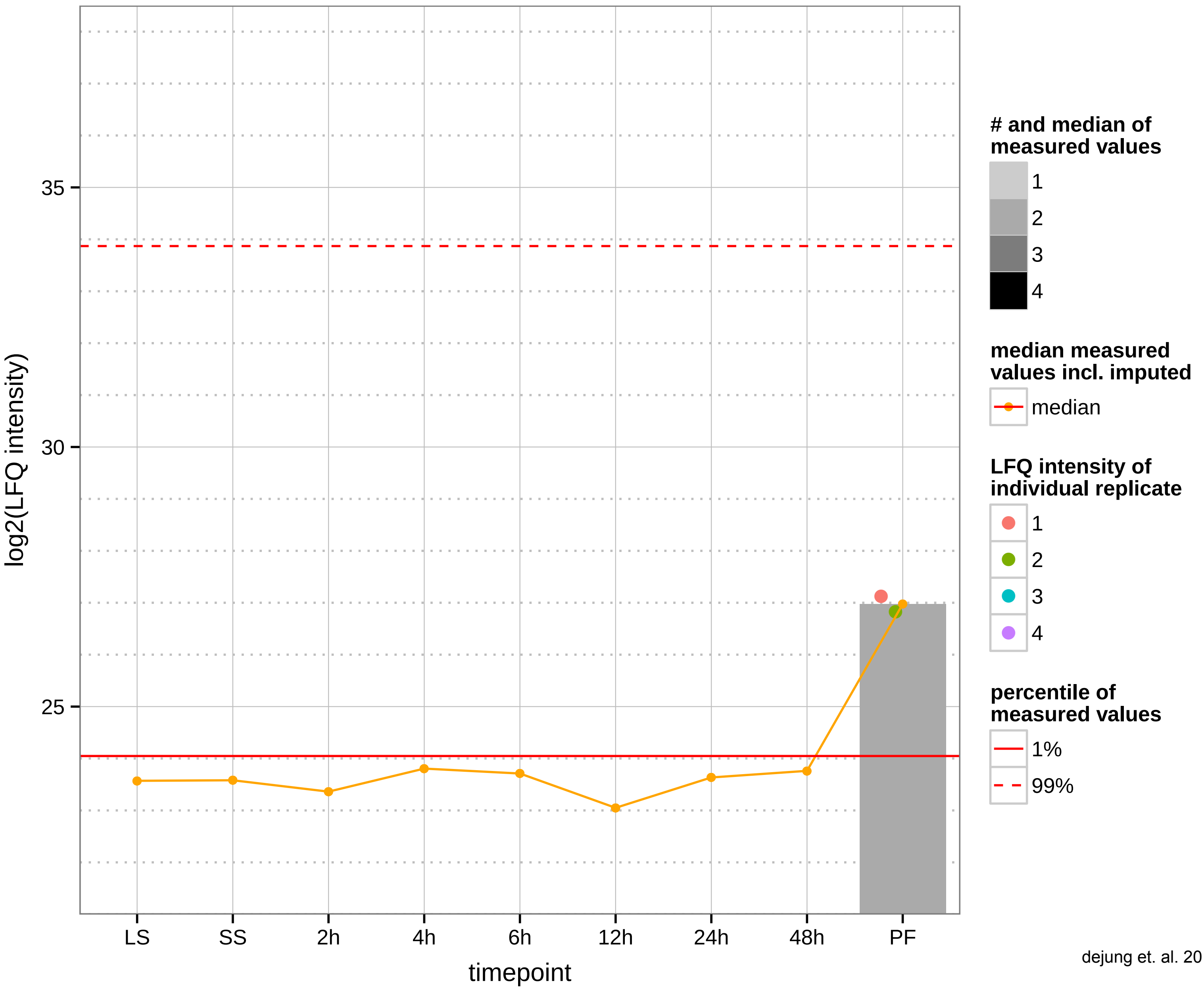

dejung et. al. 2015

hypothetical protein, conserved
Tb927.8.580
AGOF: null
AGOC: mitochondrial inner membrane, mitochondrion
AGOP: null
PGOF: null
PGOC: null
PGOP: null

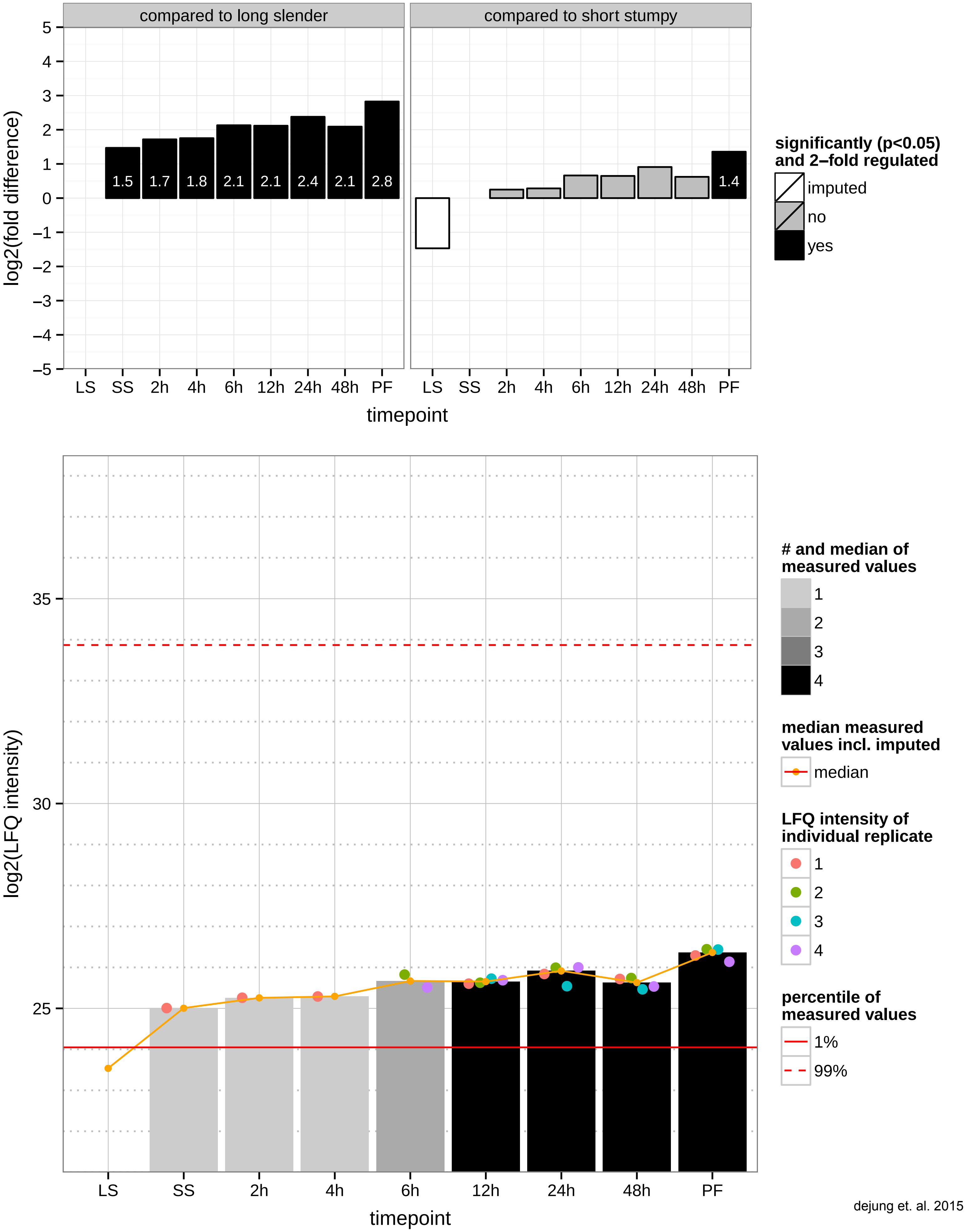

dejung et. al. 2015

hypothetical protein, conserved
Tb927.8.5800
AGOF: null
AGOC: null
AGOP: null
PGOF: null
PGOC: null
PGOP: null

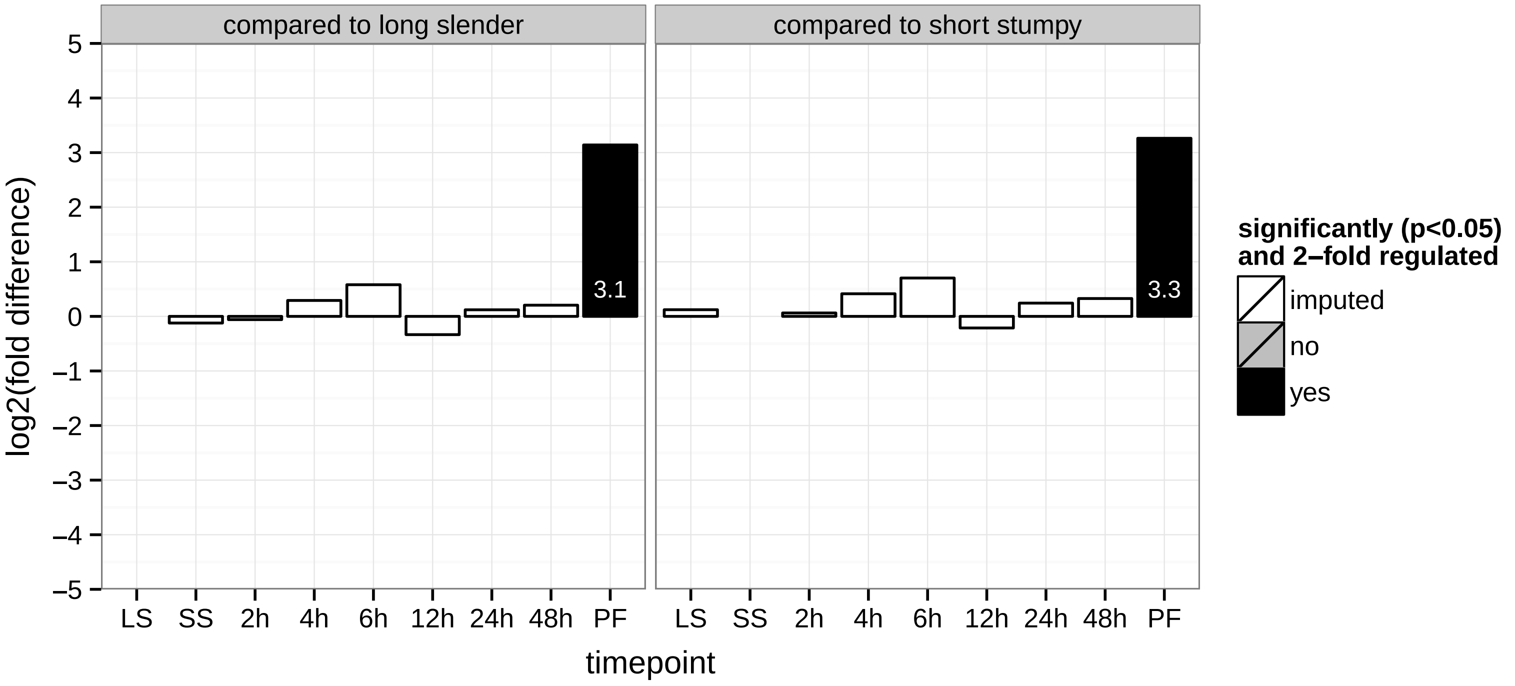

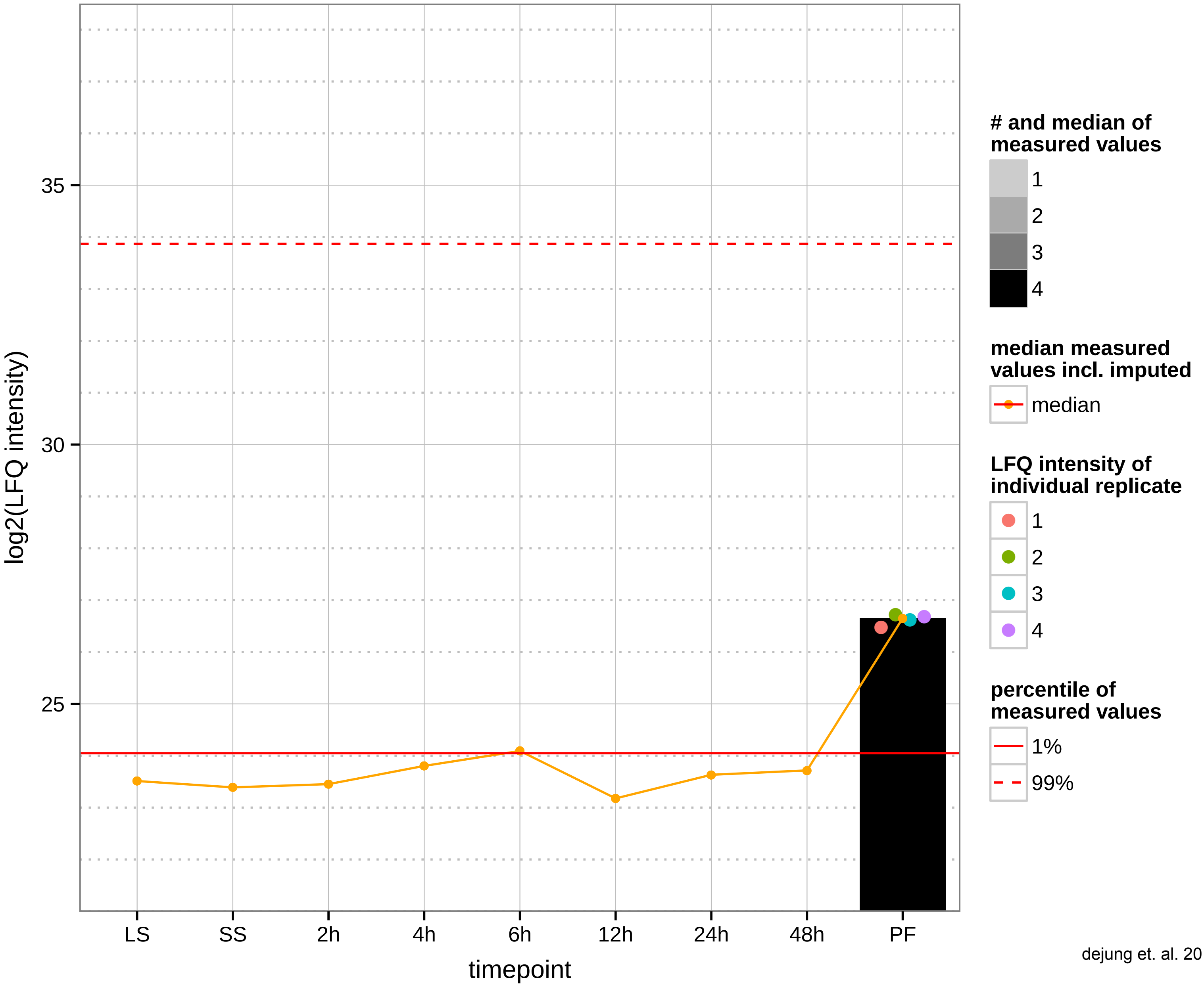

dejung et. al. 2015

protein kinase, putative
Tb927.8.5950
AGOF: ATP binding, protein kinase activity, protein serine/threonine kinase activity, protein tyrosine kinase activity
AGOC: null
AGOP: protein phosphorylation
PGOF: ATP binding, protein kinase activity, transferase activity, transferring phosphorus–containing groups
PGOC: null
PGOP: protein phosphorylation

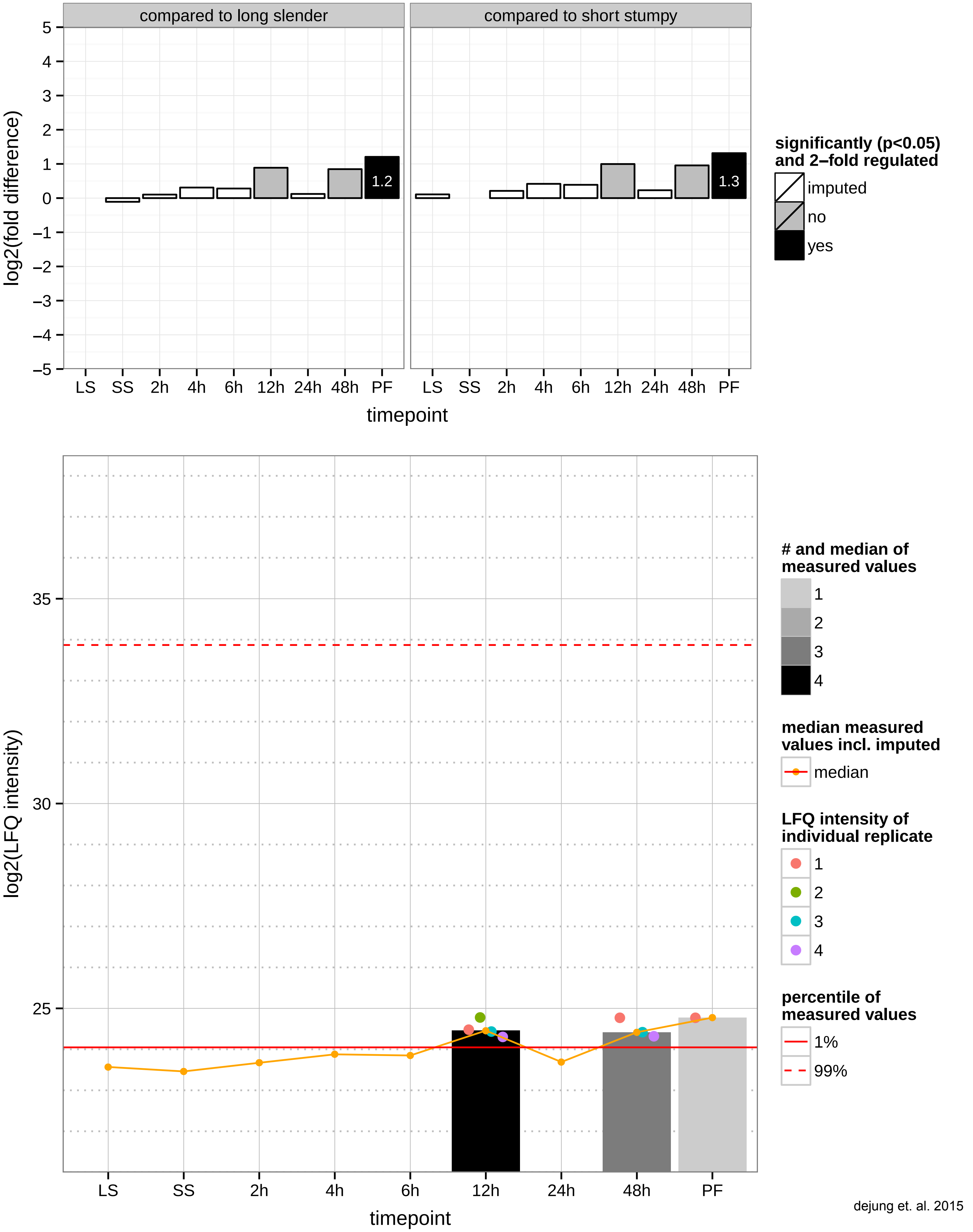

dejung et. al. 2015

60S ribosomal protein L26, putative
Tb927.9.14370;Tb927.8.6180
AGOF: structural constituent of ribosome
AGOC: large ribosomal subunit, ribosome
AGOP: translation
PGOF: structural constituent of ribosome
PGOC: large ribosomal subunit
PGOP: translation

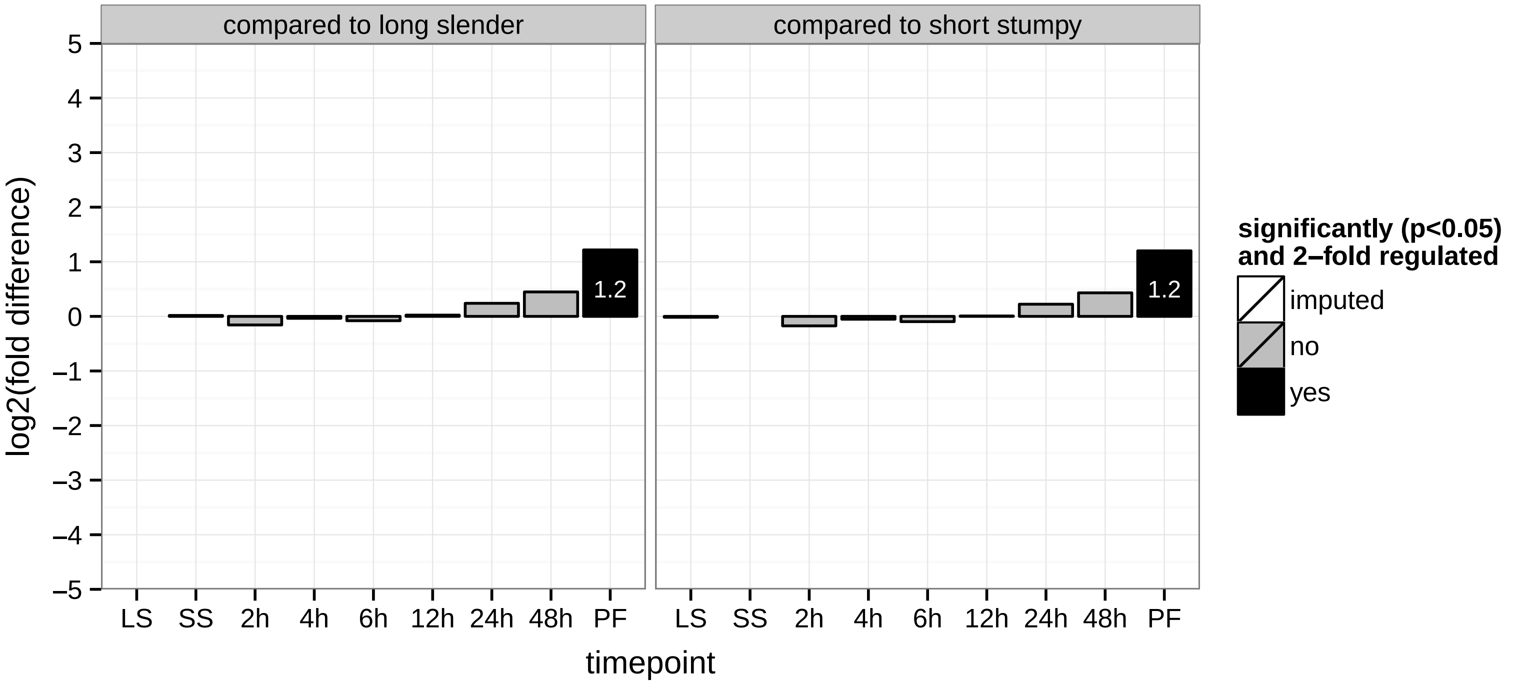

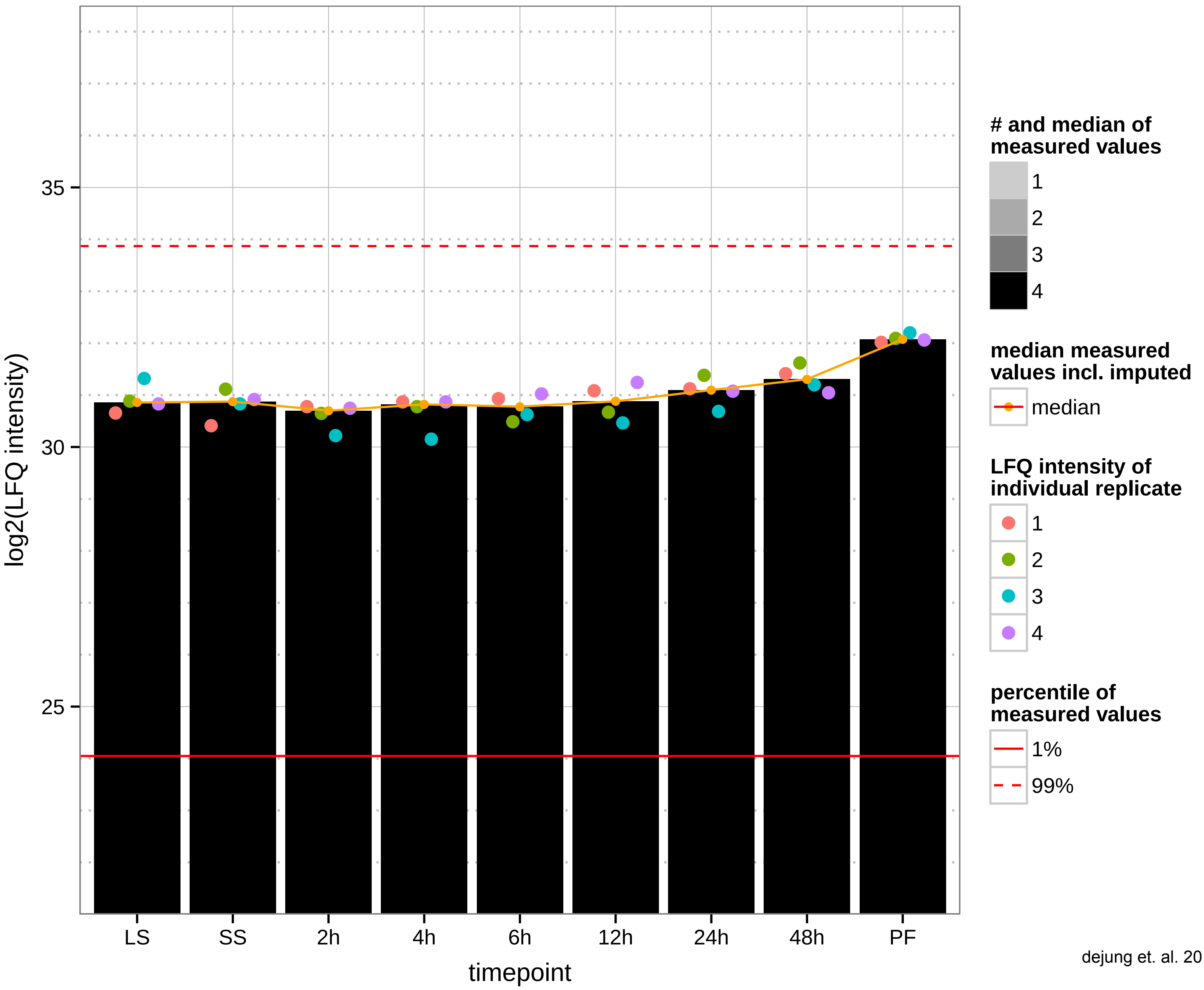

dejung et. al. 2015

tubulin folding cofactor D, putative
Tb927.8.6200
AGOF: microtubule binding
AGOC: null
AGOP: protein folding
PGOF: binding
PGOC: null
PGOP: null

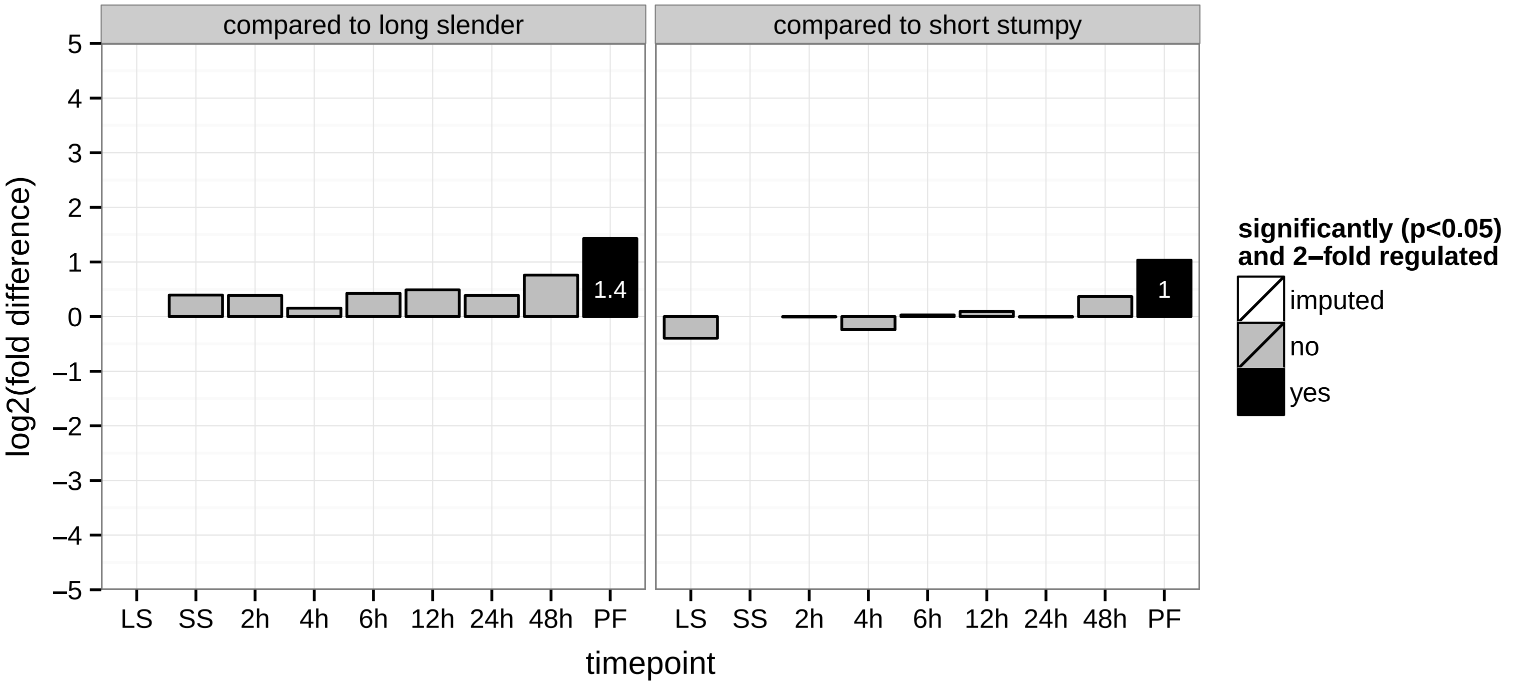

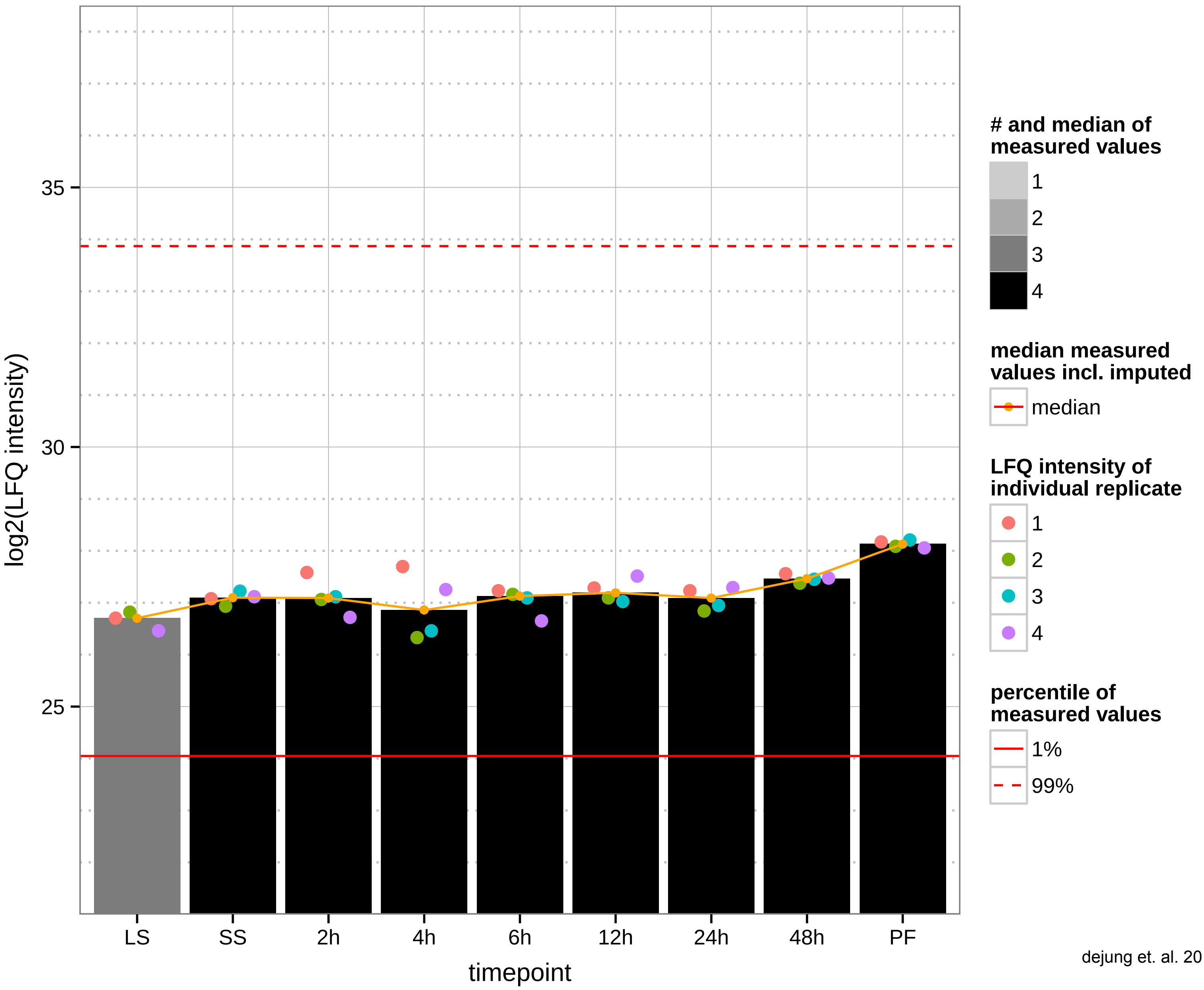

dejung et. al. 2015

hypothetical protein, conserved
Tb927.8.6220
AGOF: null
AGOC: null
AGOP: null
PGOF: null
PGOC: null
PGOP: null

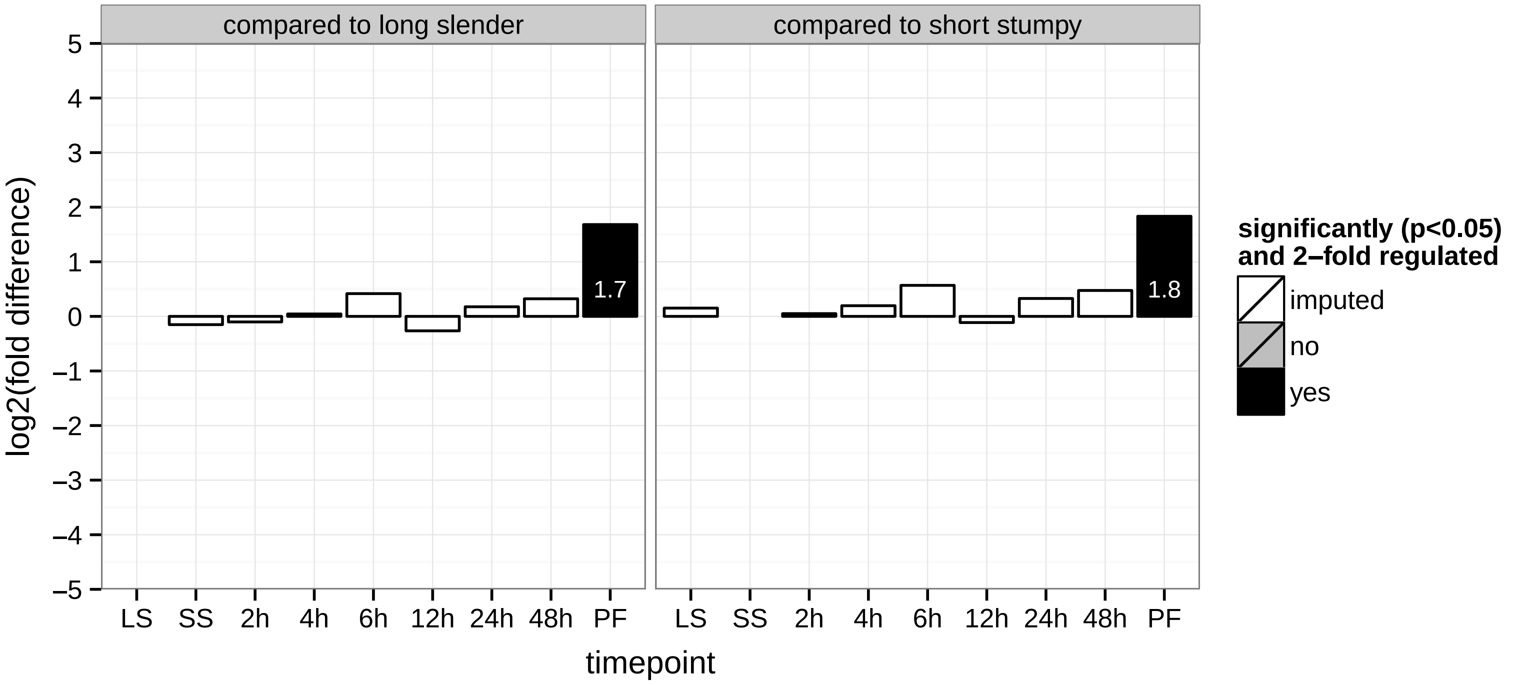

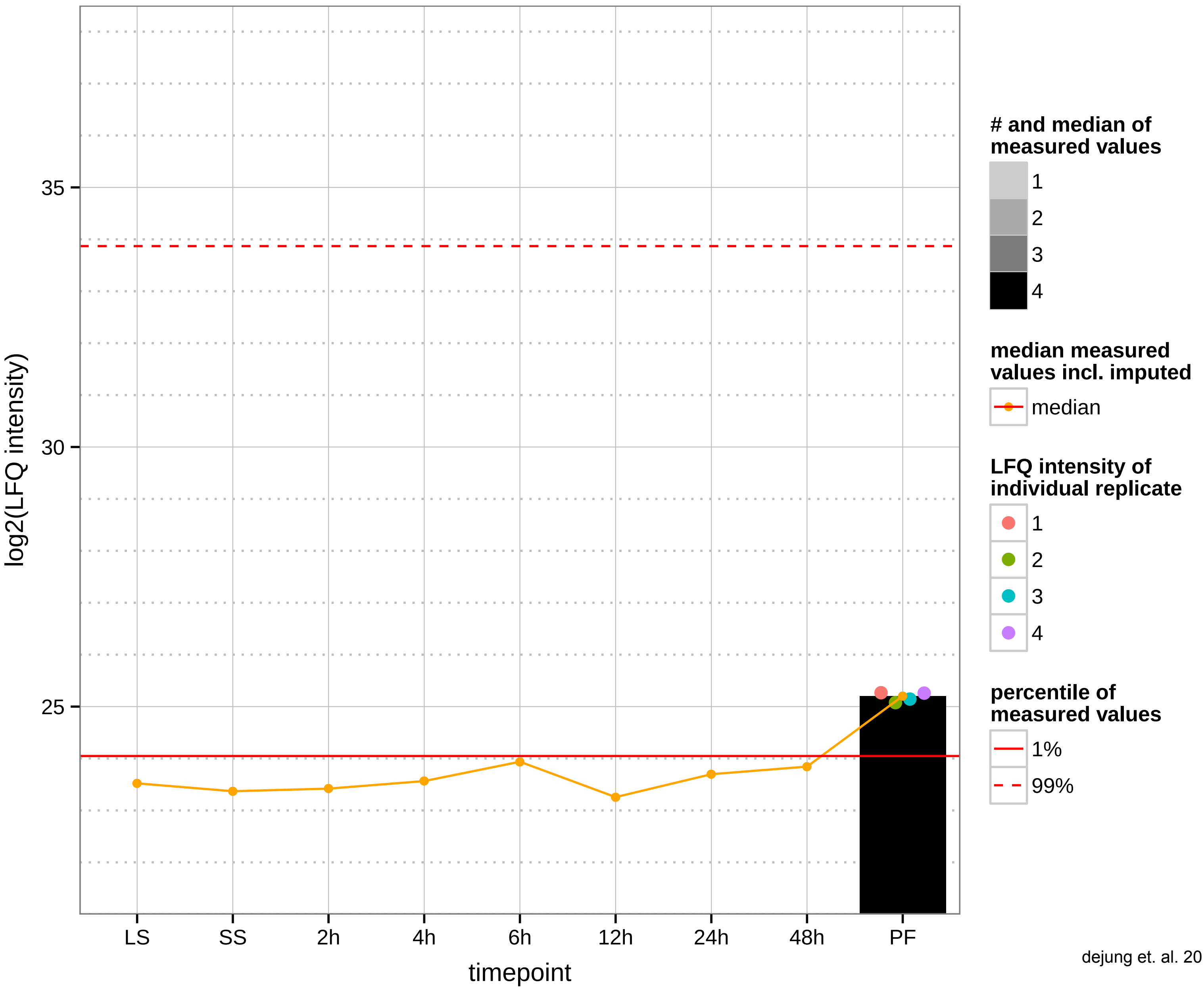

dejung et. al. 2015

cation–transporting ATPase, putative
Tb927.8.650
AGOF: ATP binding, ATPase activity, coupled to transmembrane movement of ions, phosphorylative mechanism, hydrolase ac
AGOC: integral to membrane, membrane
AGOP: ATP biosynthetic process, cation transport, metabolic process
PGOF: ATPase activity, hydrolase activity, acting on acid anhydrides, catalyzing transmembrane movement of substances, me
PGOC: integral to membrane
PGOP: cation transport

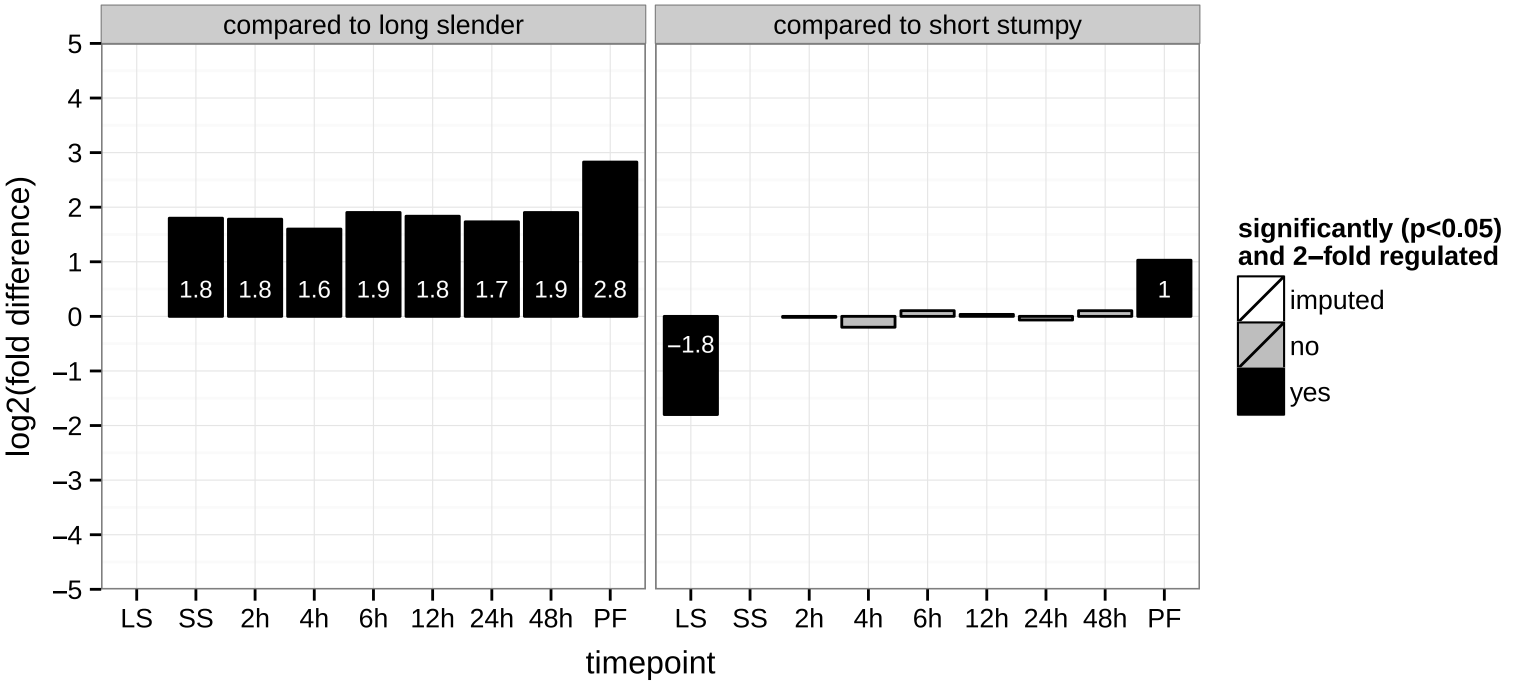

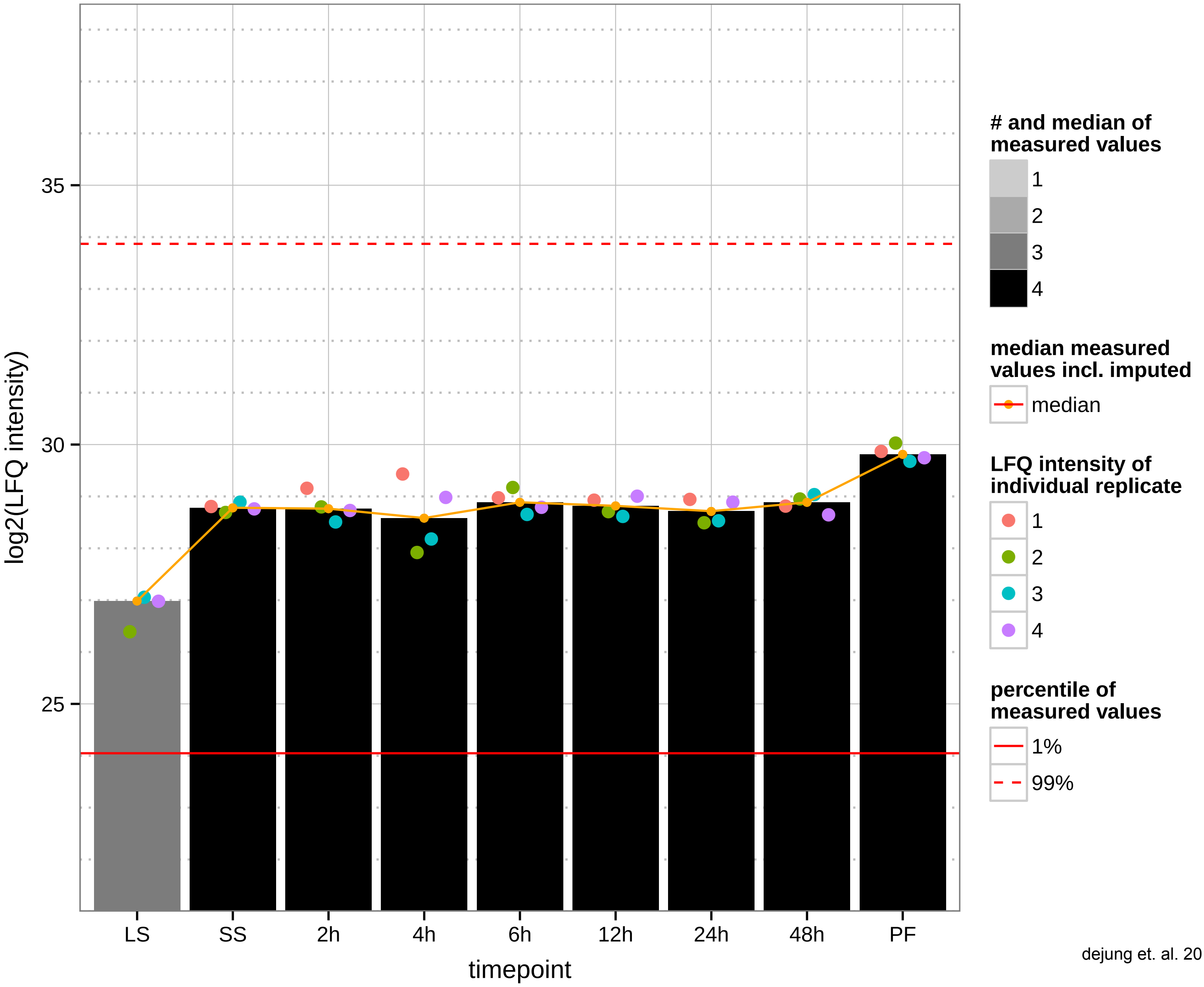

dejung et. al. 2015

hypothetical protein, conserved
Tb927.8.6820
AGOF: carbon–carbon lyase activity
AGOC: mitochondrion
AGOP: cellular aromatic compound metabolic process
PGOF: carbon–carbon lyase activity, catalytic activity
PGOC: null
PGOP: cellular aromatic compound metabolic process

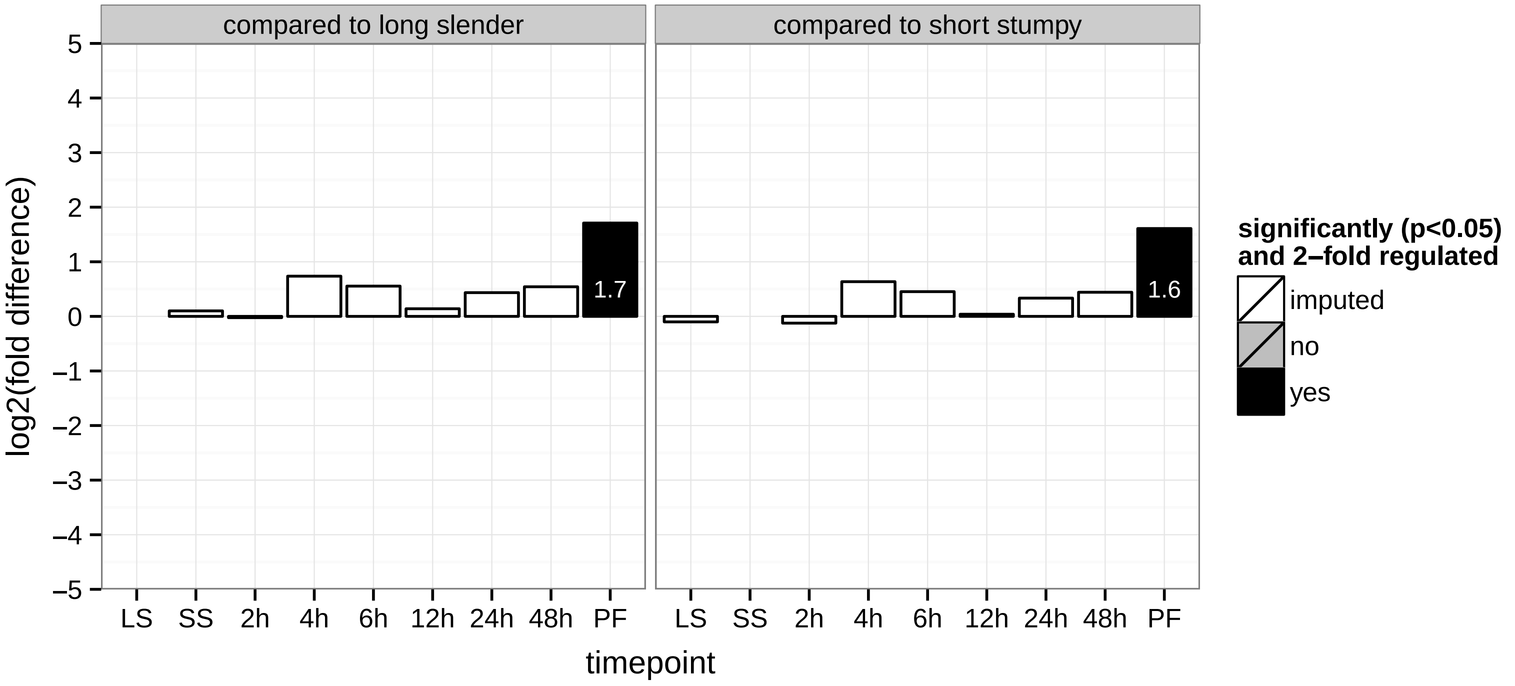

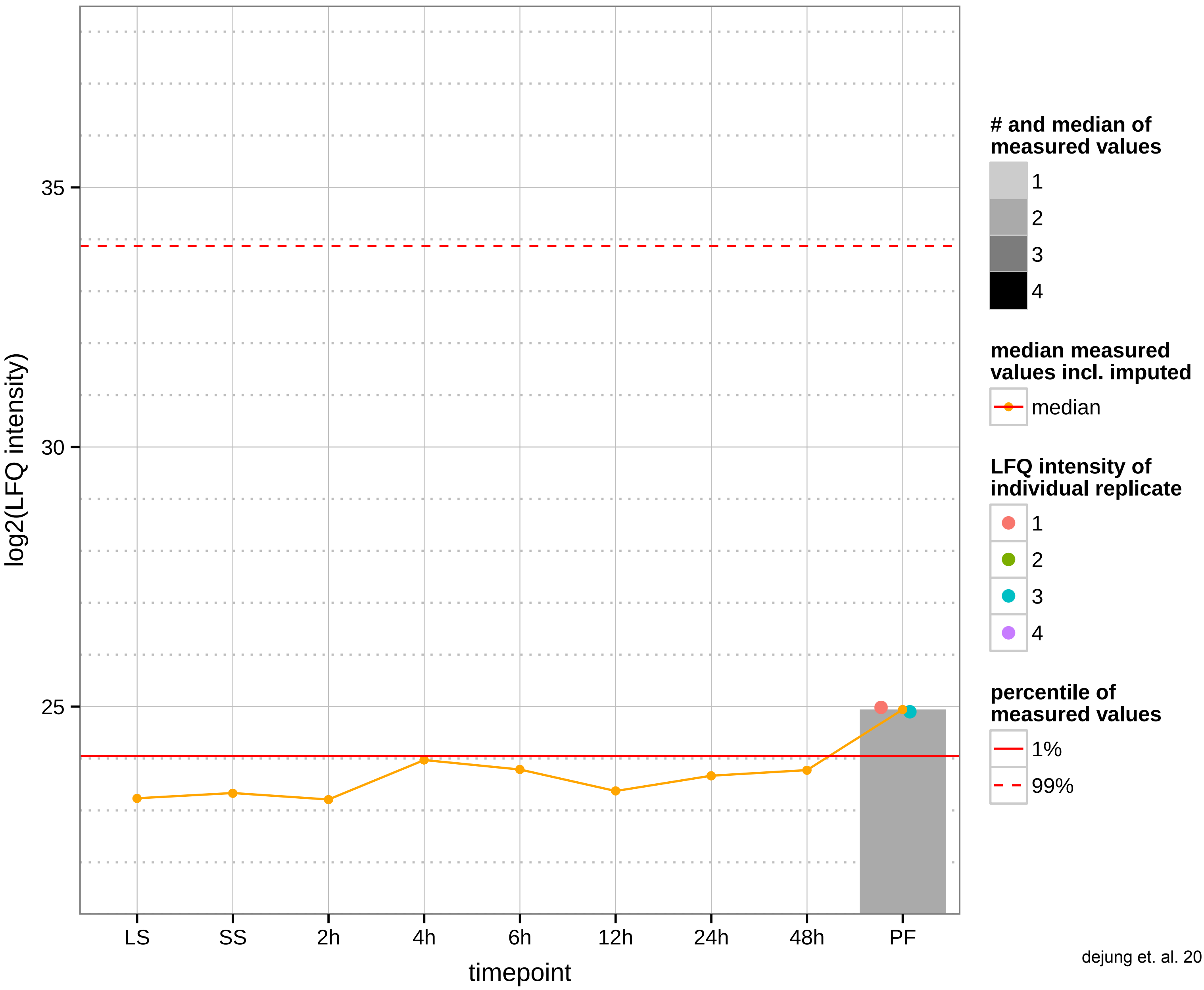

dejung et. al. 2015

chaperone protein DNAj, putative
Tb927.8.7010
AGOF: heat shock protein binding, unfolded protein binding
AGOC: null
AGOP: protein folding
PGOF: heat shock protein binding
PGOC: null
PGOP: null

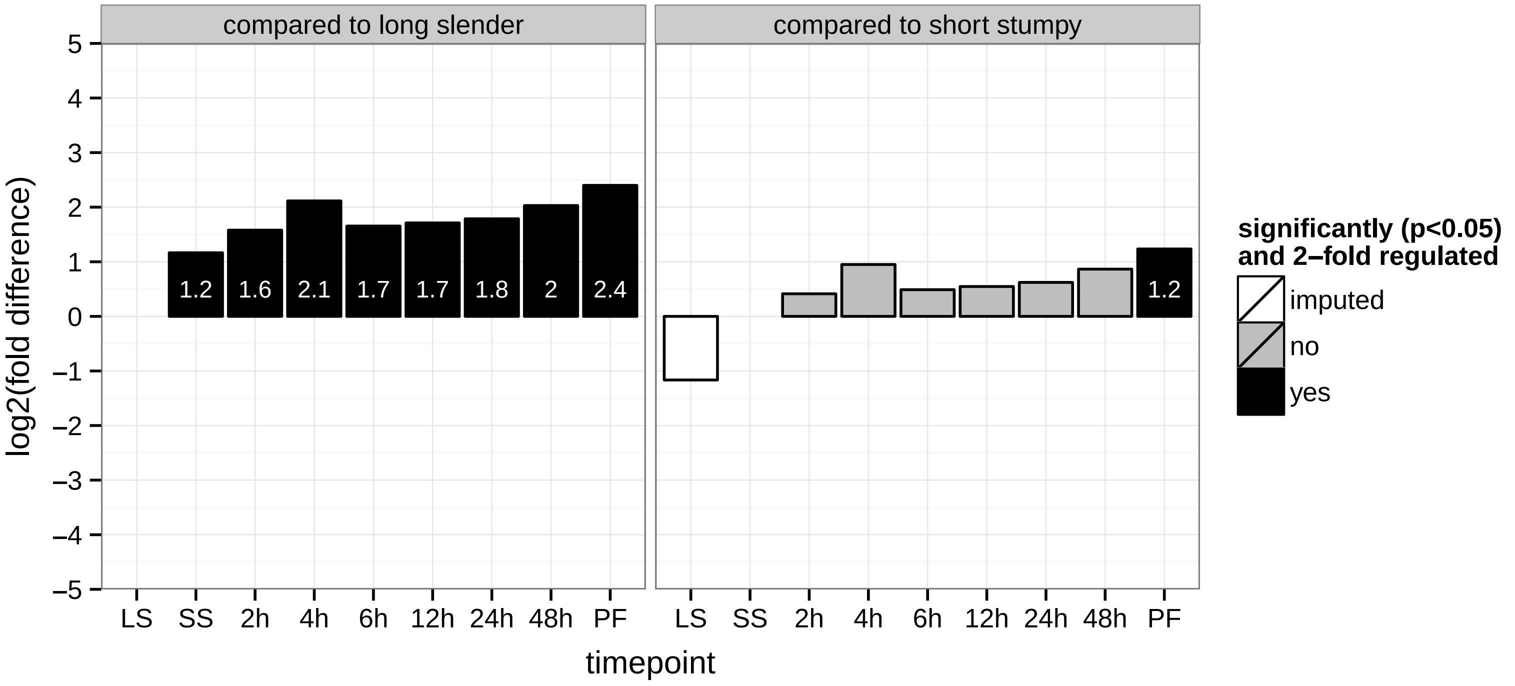

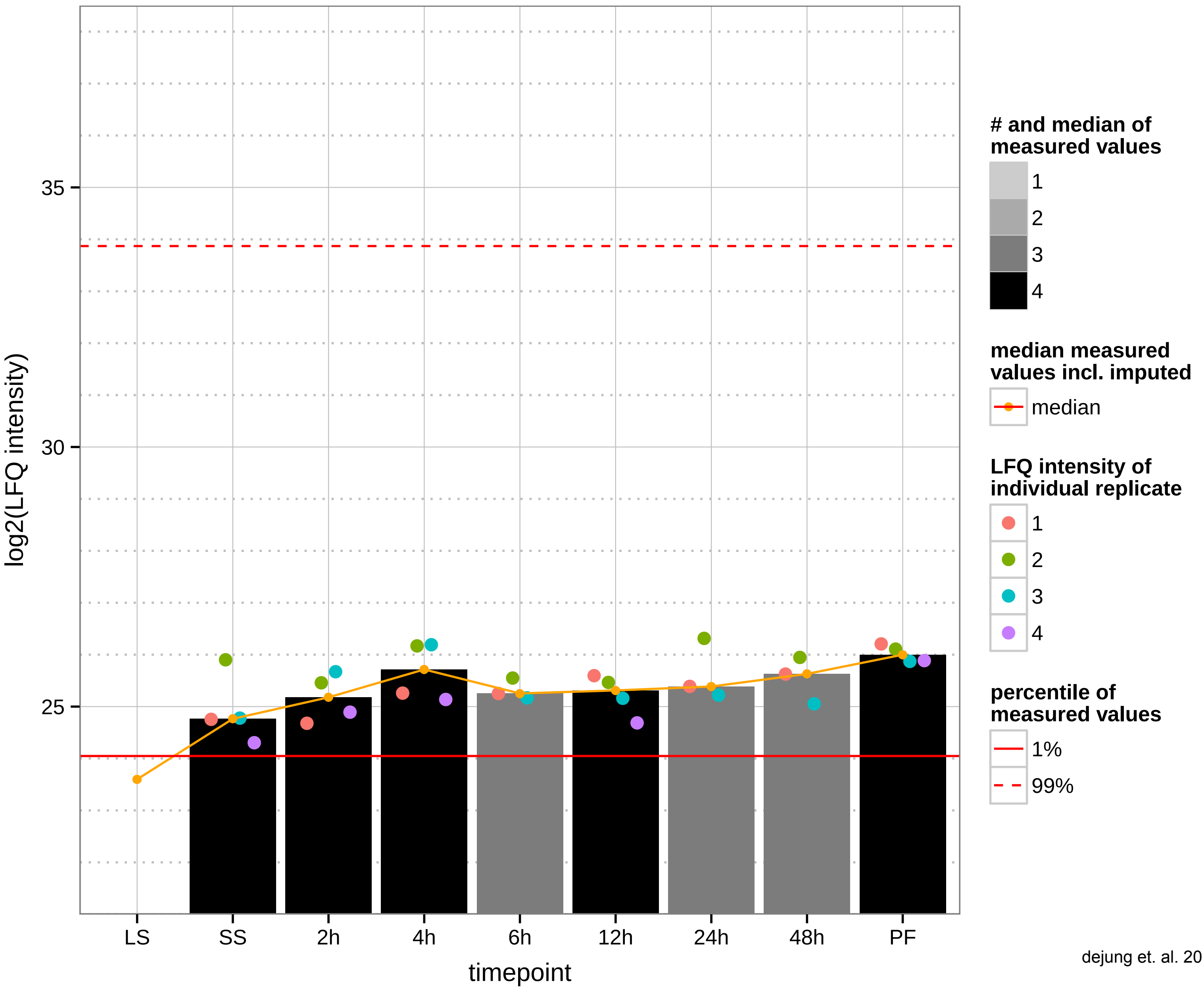

dejung et. al. 2015

hypothetical protein, conserved
Tb927.8.7090
AGOF: null
AGOC: null
AGOP: null
PGOF: null
PGOC: null
PGOP: null

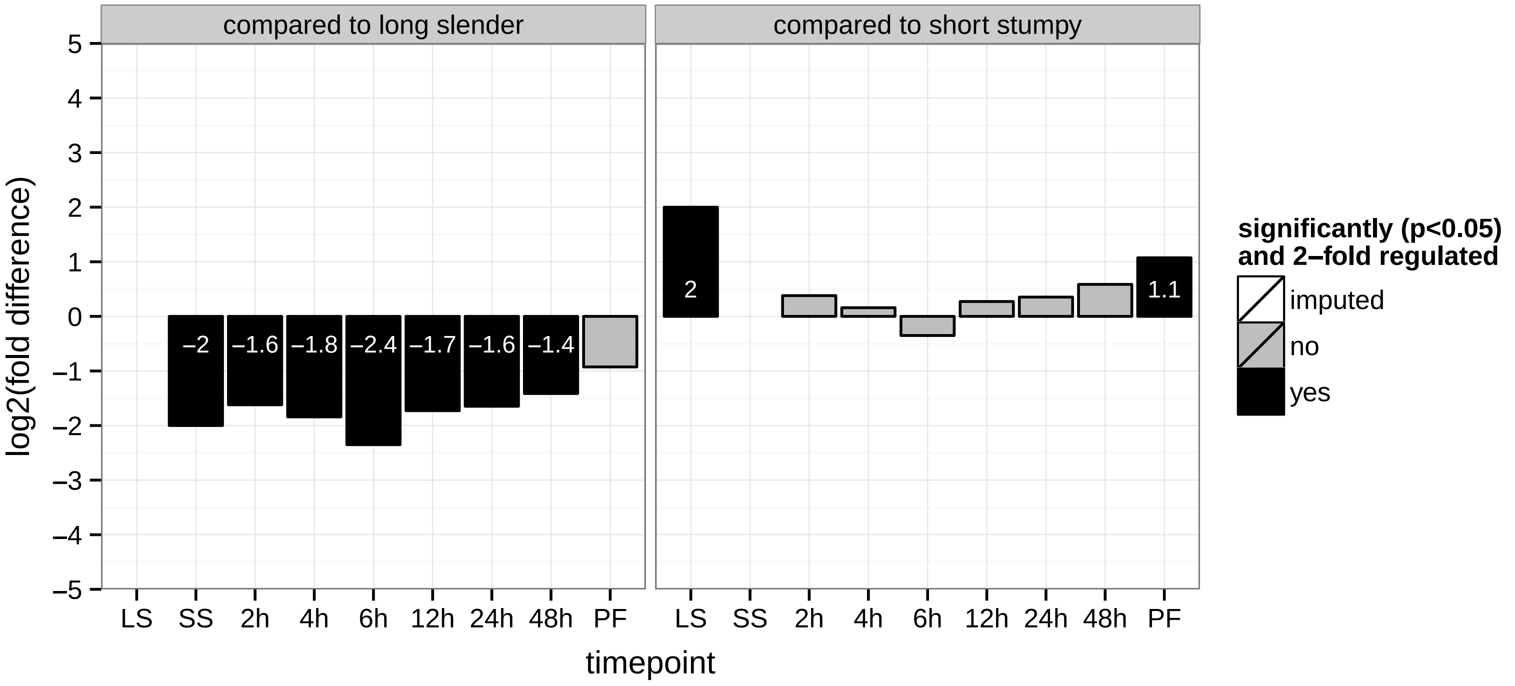

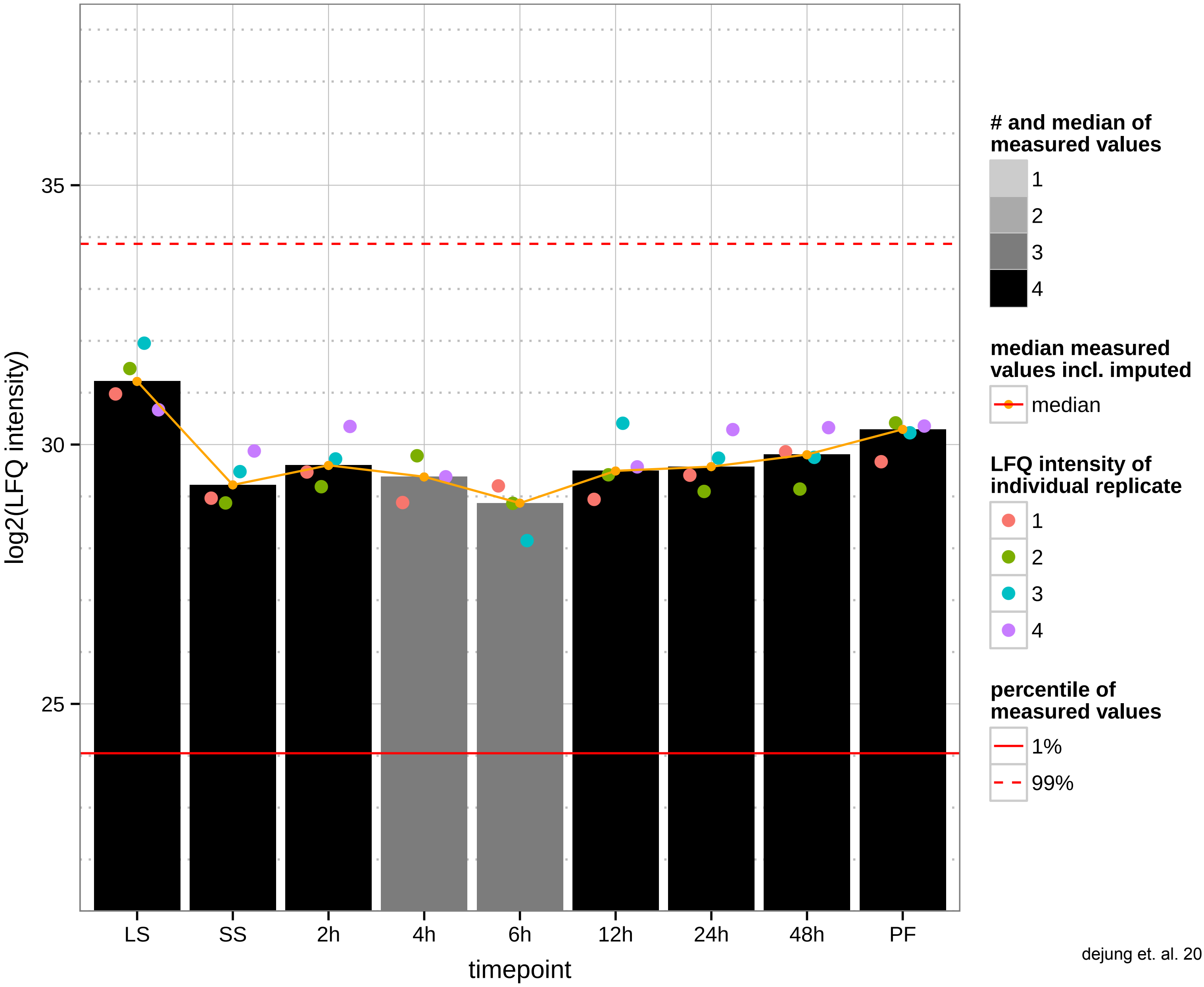

dejung et. al. 2015

RNA–binding protein, putative (DRBD17)
Tb927.8.710
AGOF: RNA binding
AGOC: null
AGOP: RNA processing
PGOF: nucleic acid binding
PGOC: null
PGOP: null

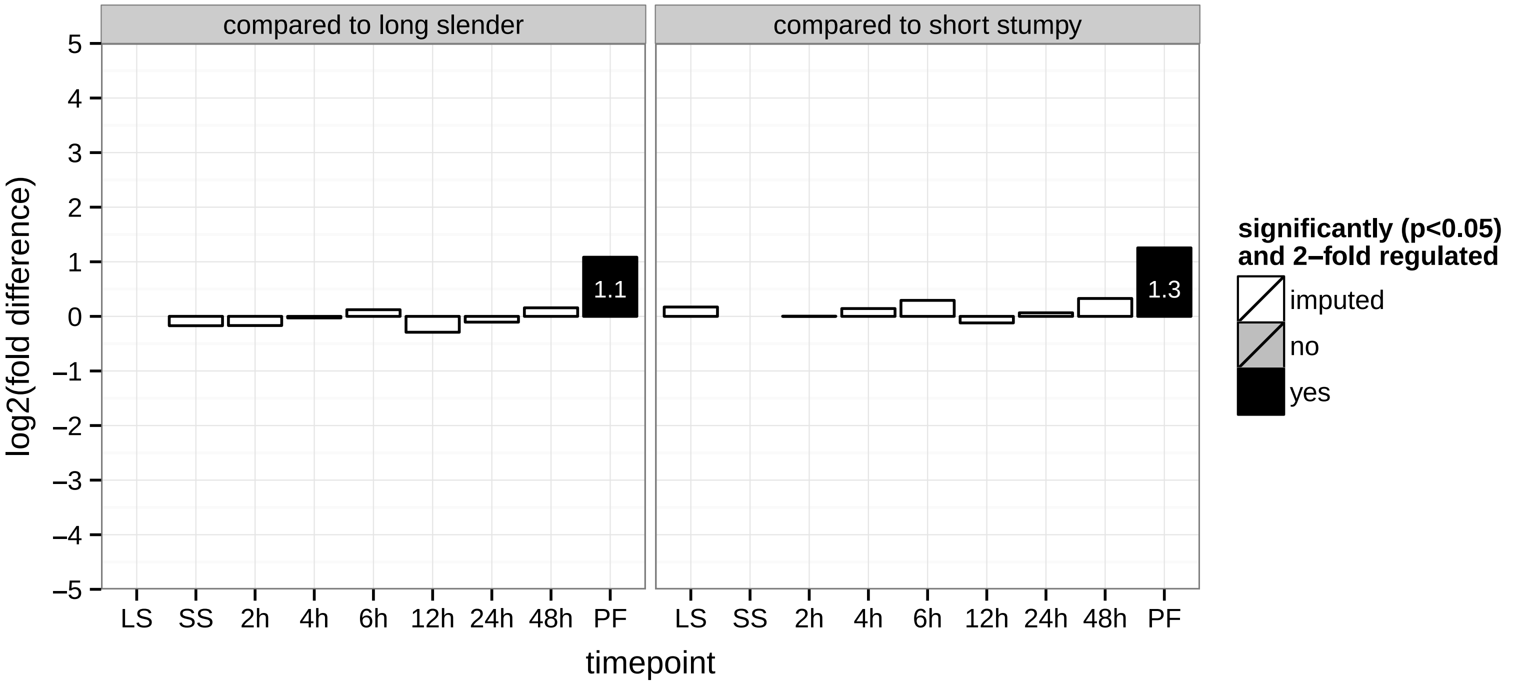

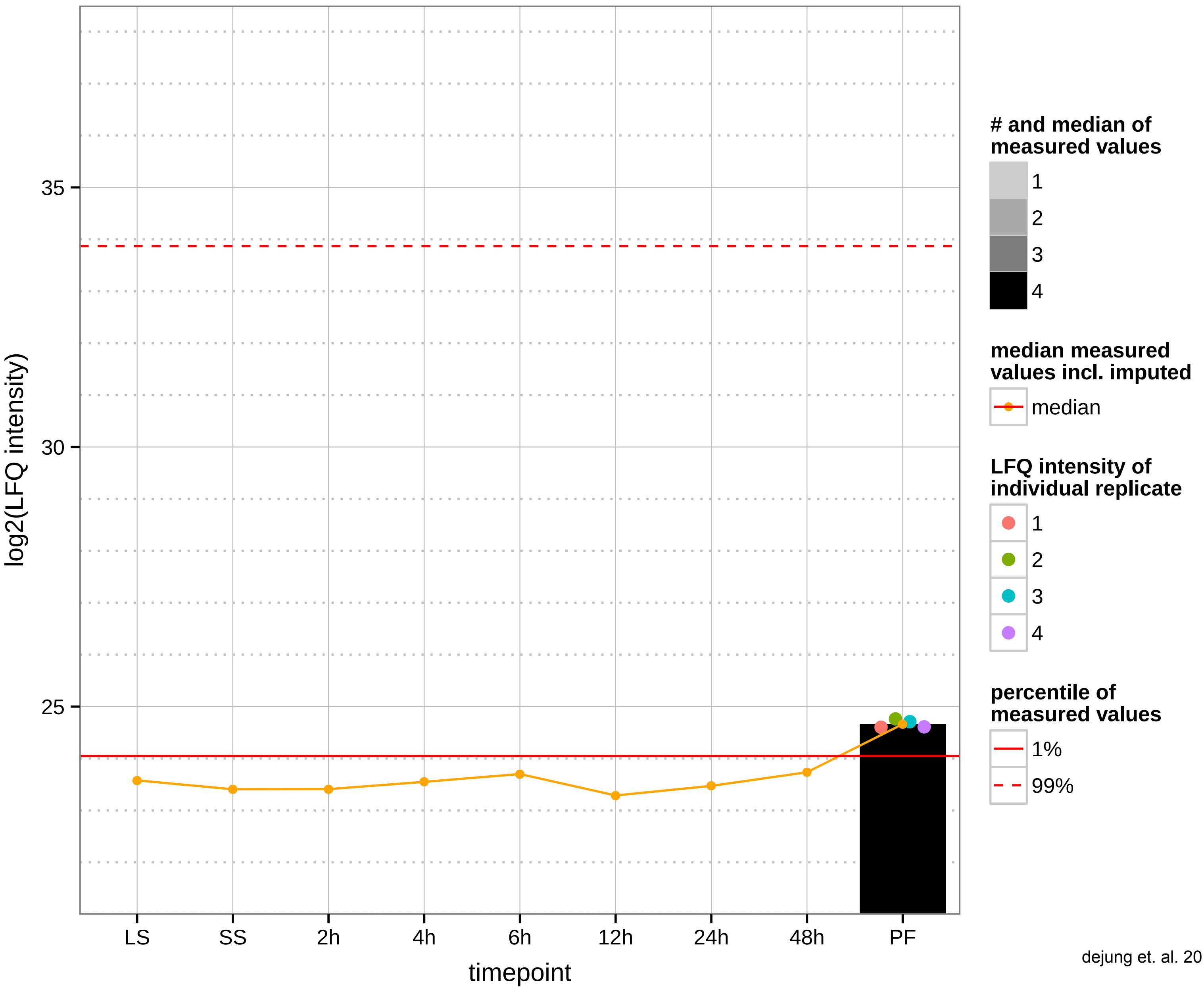

dejung et. al. 2015

hypothetical protein
Tb927.8.7370
AGOF: null
AGOC: null
AGOP: null
PGOF: null
PGOC: null
PGOP: null

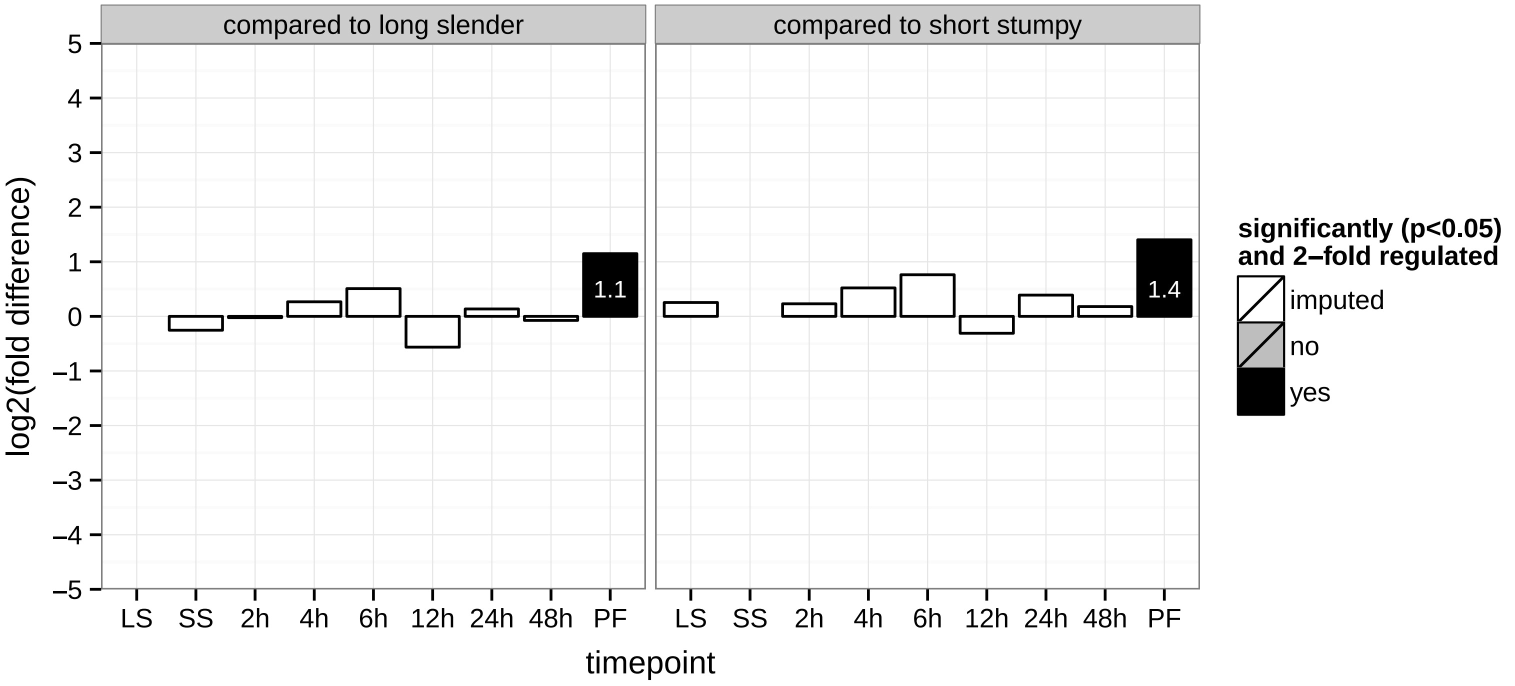

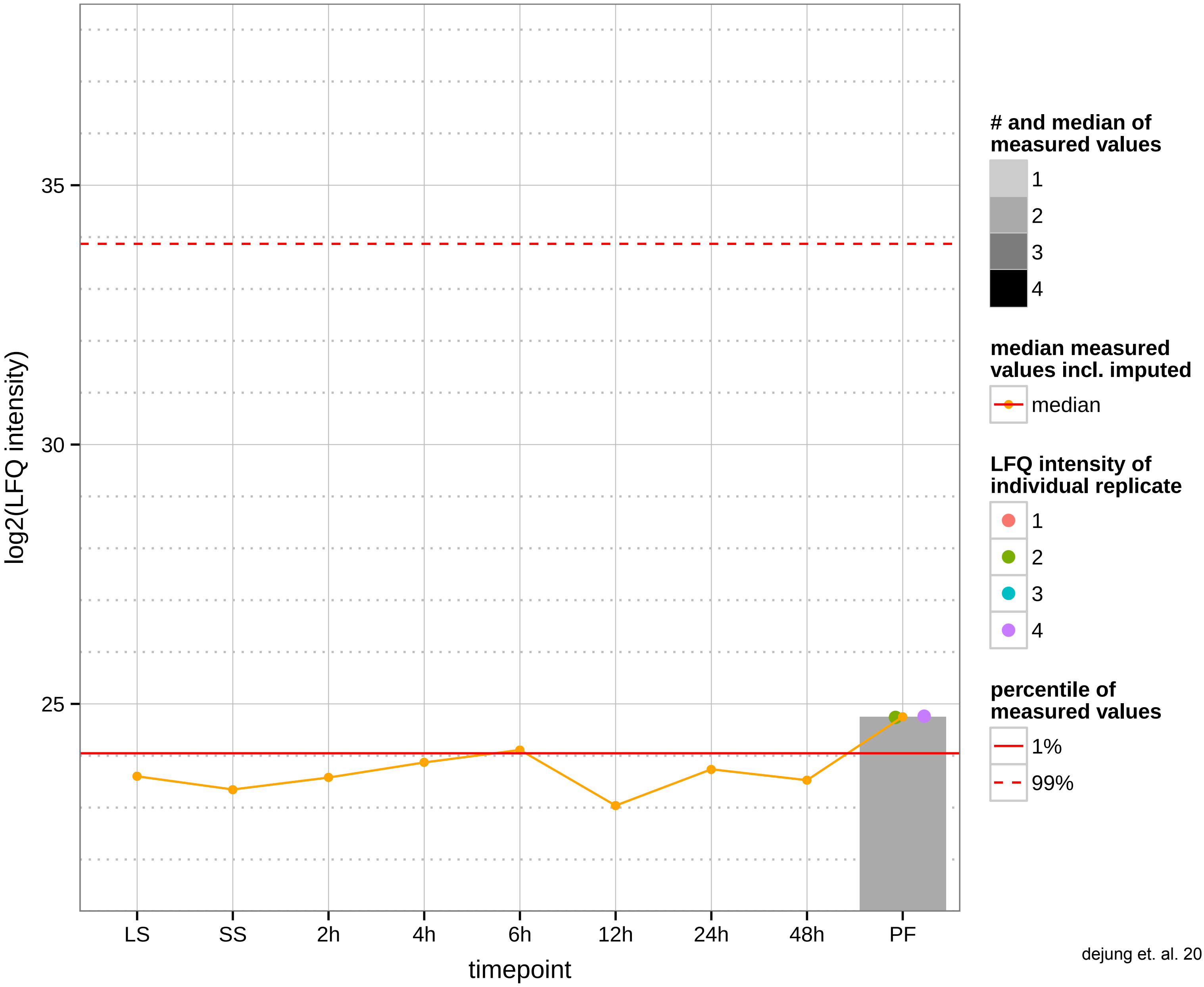

dejung et. al. 2015

amino acid transporter 1, putative (AATP1)
Tb927.8.7640;Tb927.8.7610
AGOF: amine transmembrane transporter activity
AGOC: membrane, null
AGOP: amino acid transport
PGOF: null
PGOC: null
PGOP: null

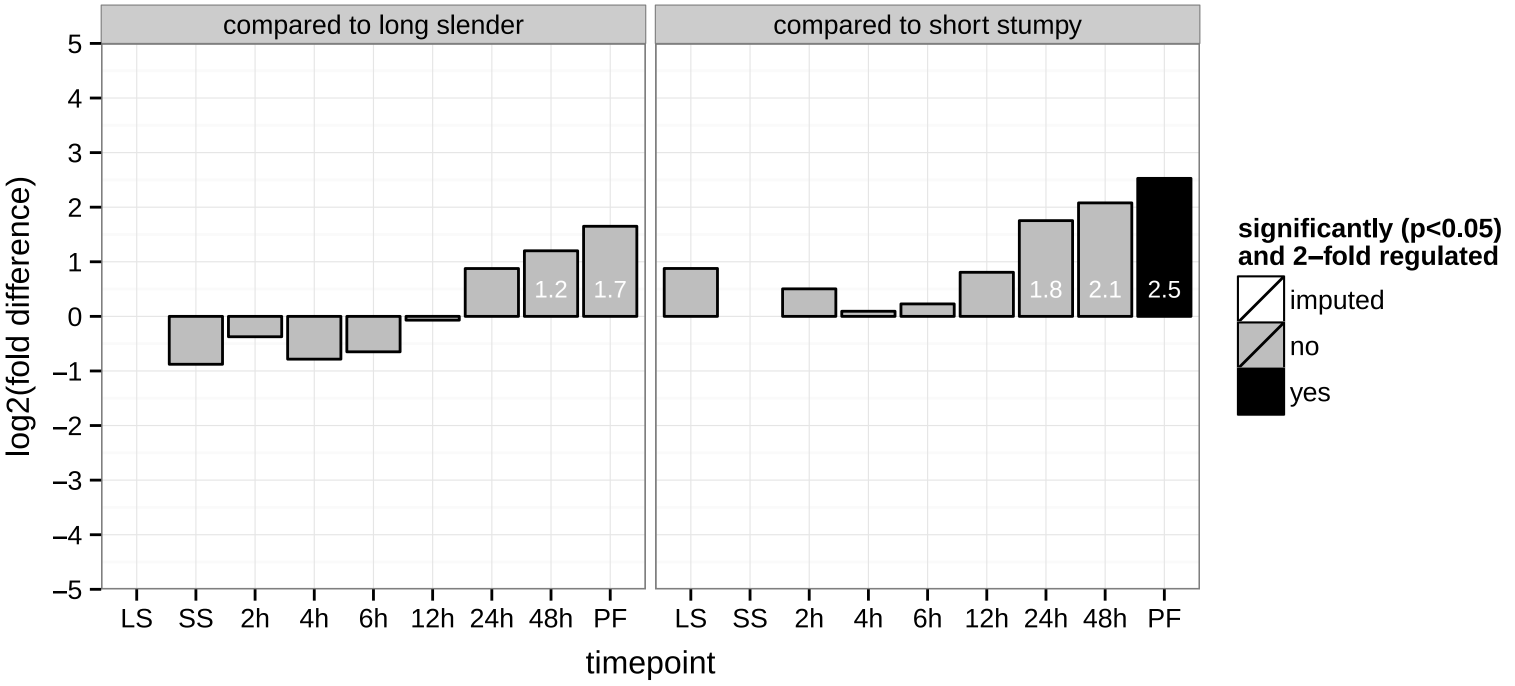

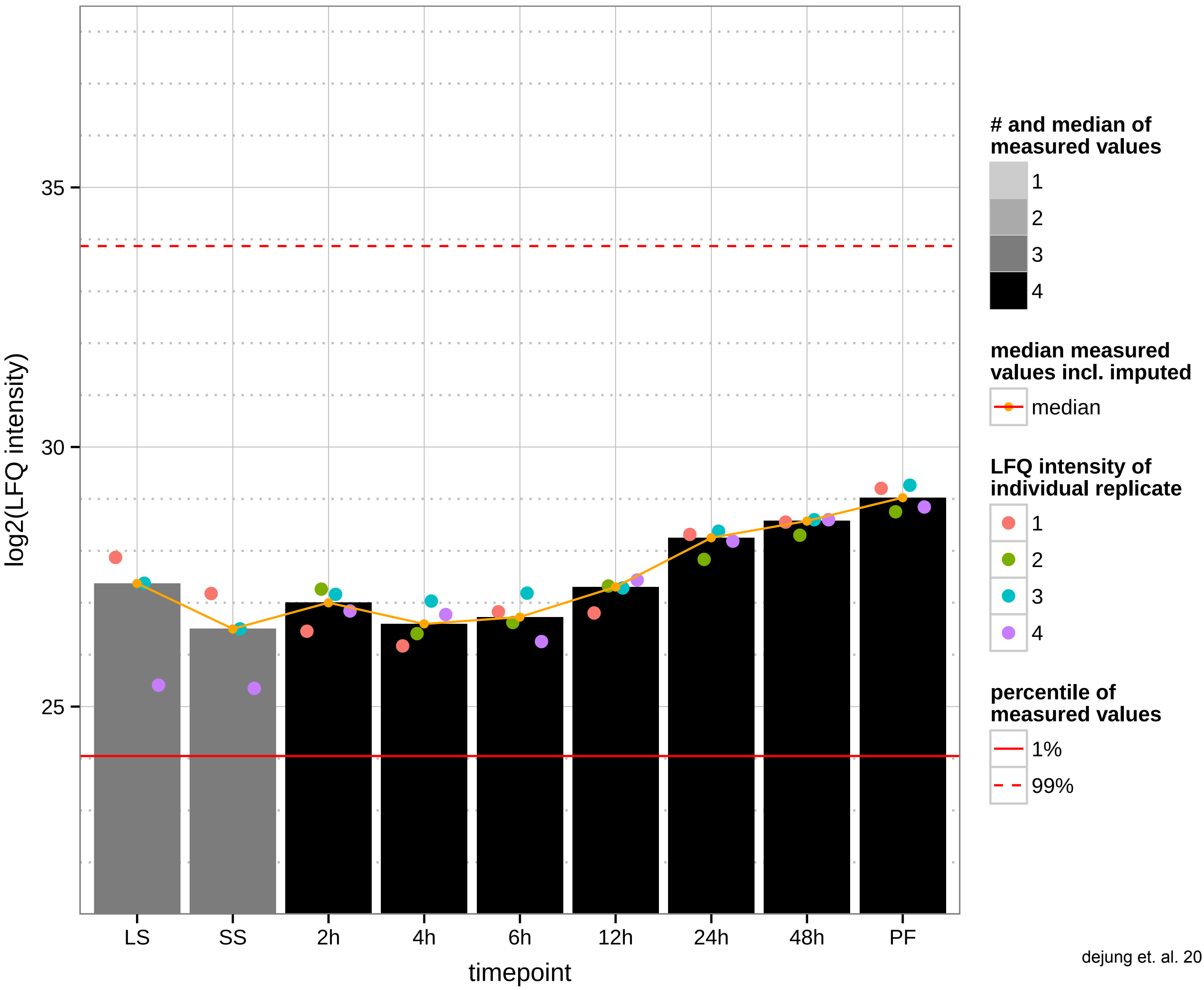

dejung et. al. 2015

amino acid transporter, putative, amino acid transporter (pseudogene)
Tb927.8.7670;Tb927.8.7650
AGOF: amine transmembrane transporter activity, null
AGOC: membrane, null
AGOP: null, amino acid transport
PGOF: null
PGOC: null
PGOP: null

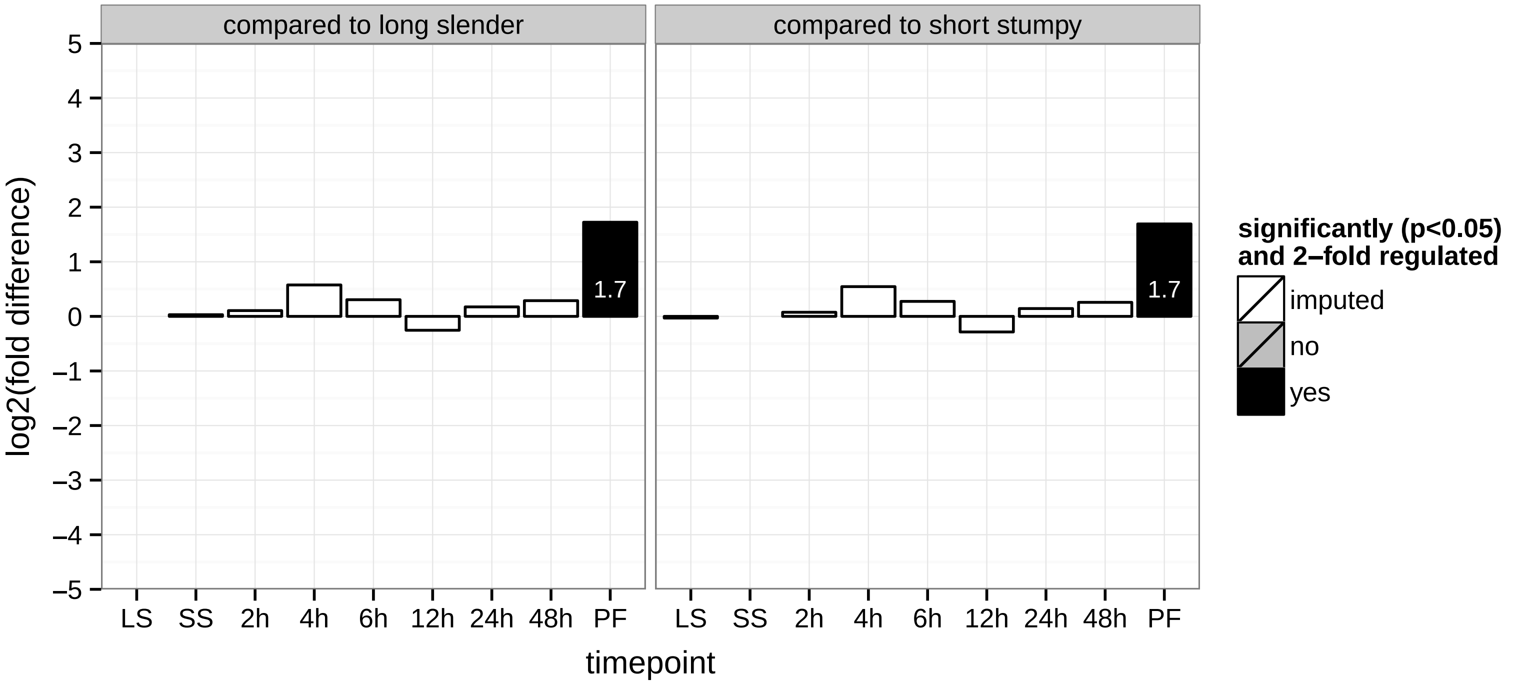

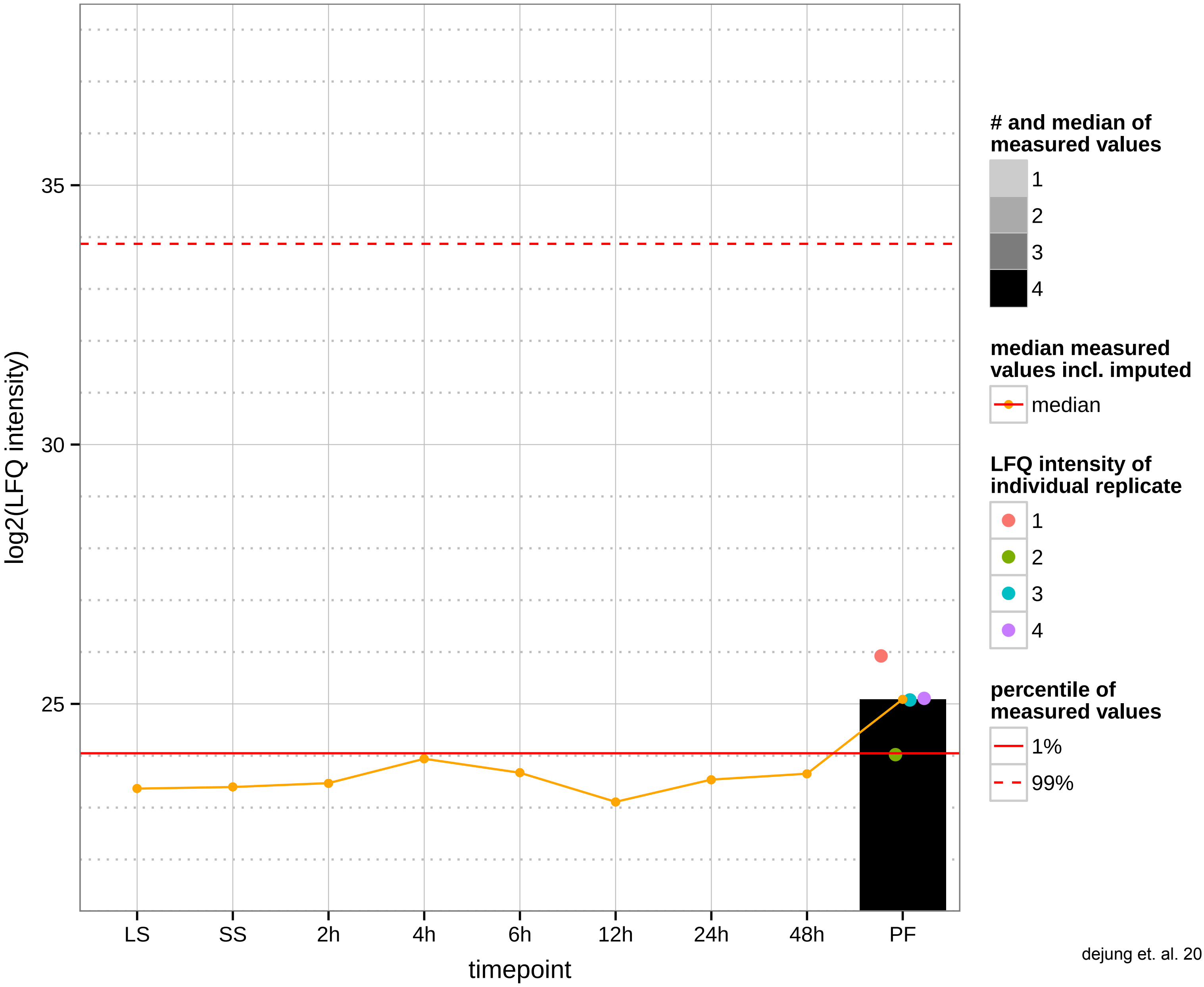

dejung et. al. 2015

hypothetical protein, conserved
Tb927.8.820
AGOF: null
AGOC: null
AGOP: null
PGOF: null
PGOC: null
PGOP: null

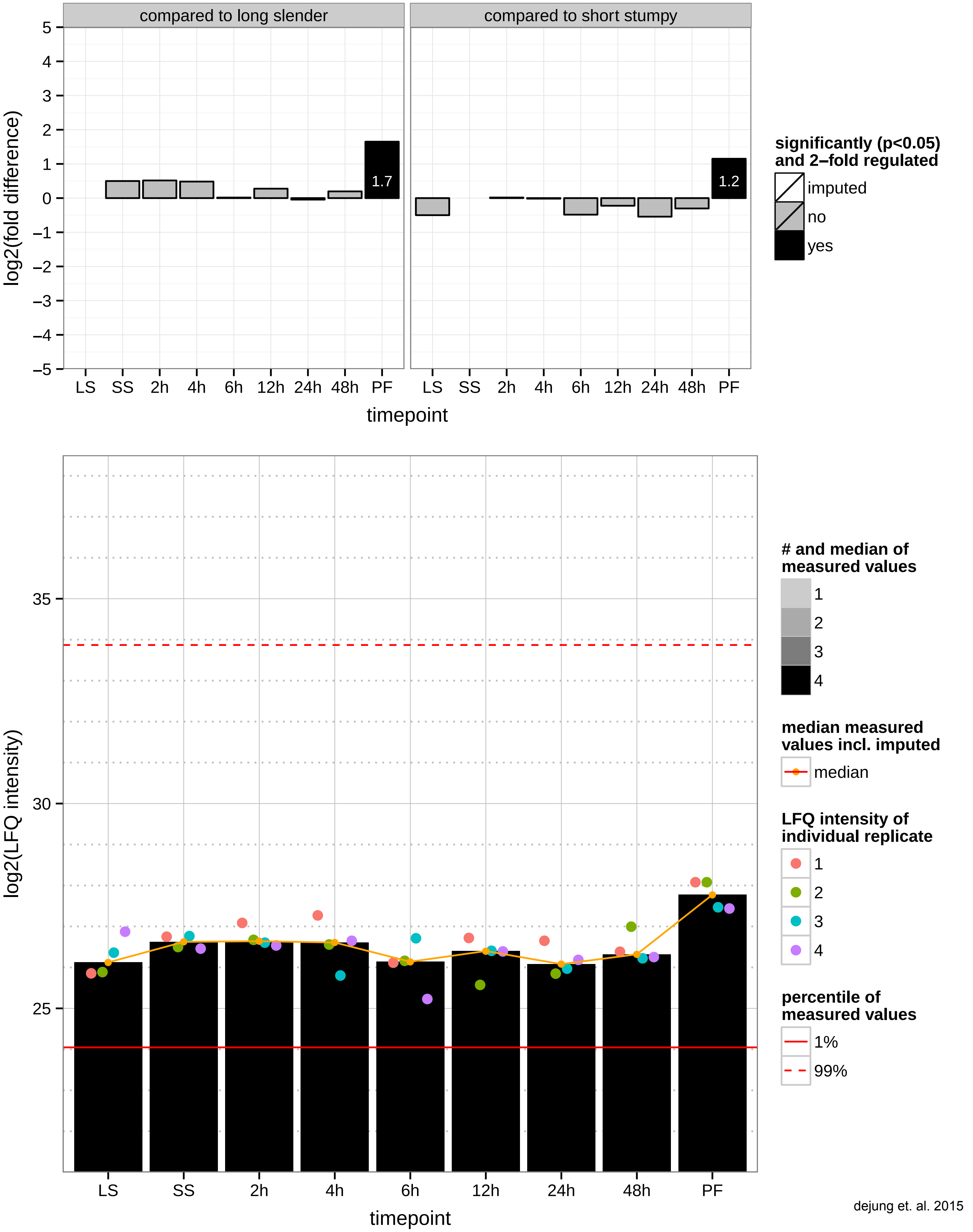

dejung et. al. 2015

hypothetical protein, conserved
Tb927.9.10050
AGOF: null
AGOC: integral to membrane
AGOP: protein insertion into membrane
PGOF: null
PGOC: integral to membrane
PGOP: protein insertion into membrane

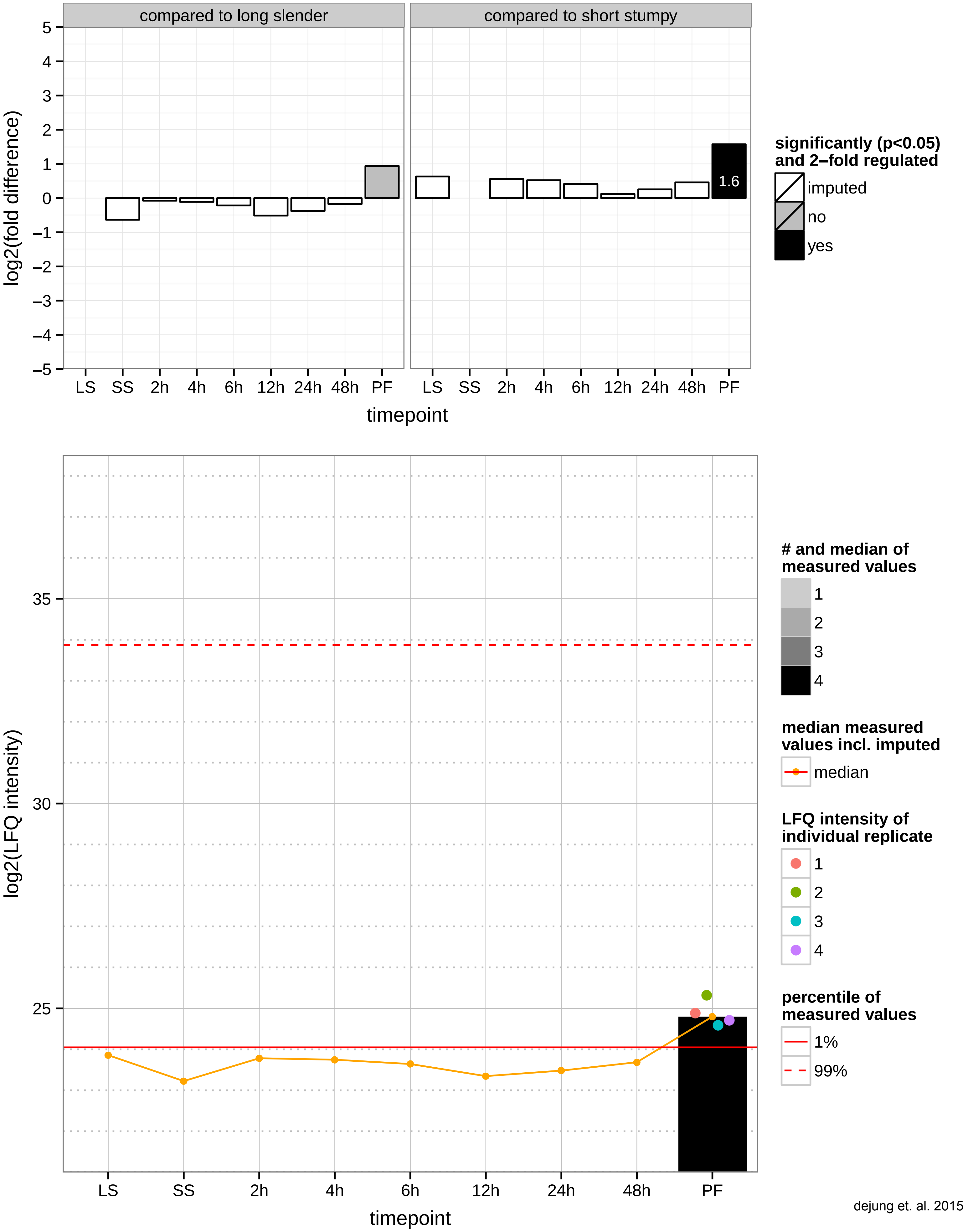

dejung et. al. 2015

hypothetical protein, conserved
Tb927.9.10200
AGOF: null
AGOC: null
AGOP: null
PGOF: null
PGOC: null
PGOP: null

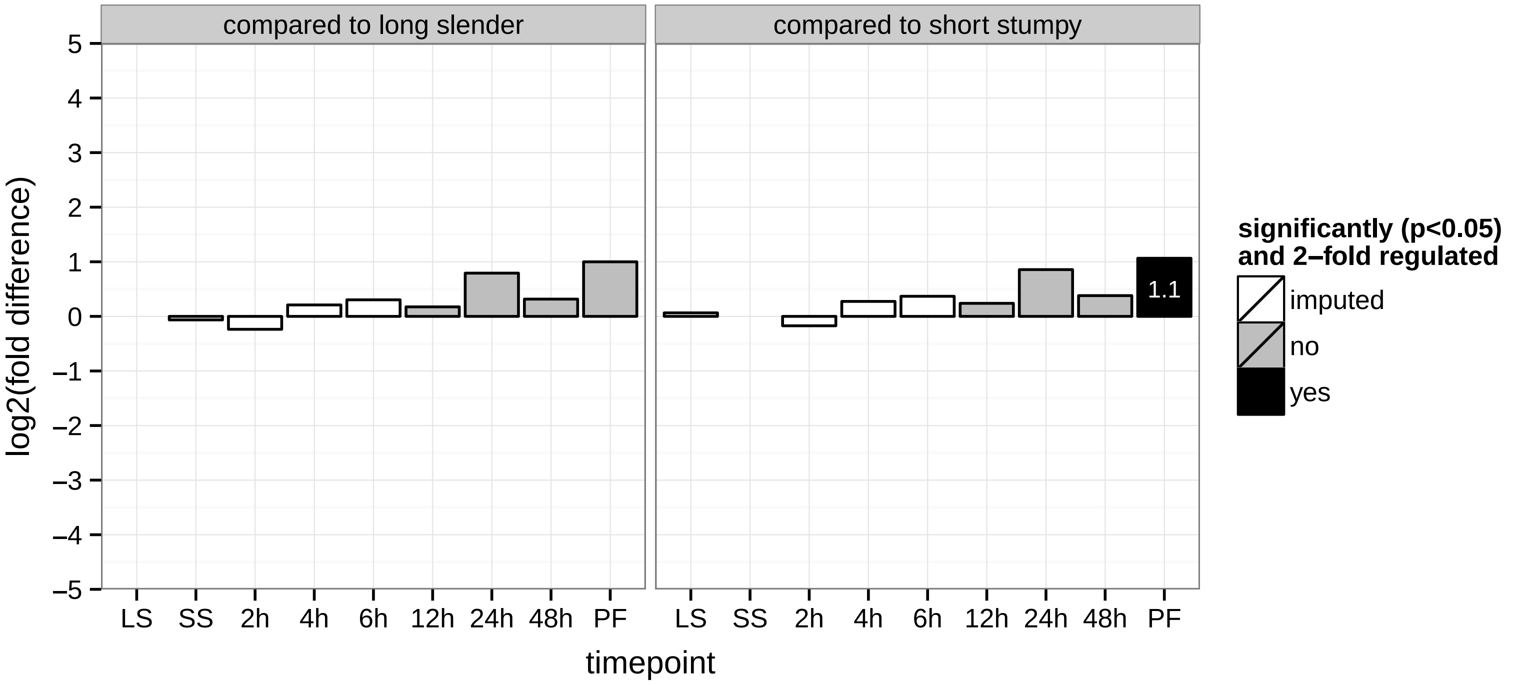

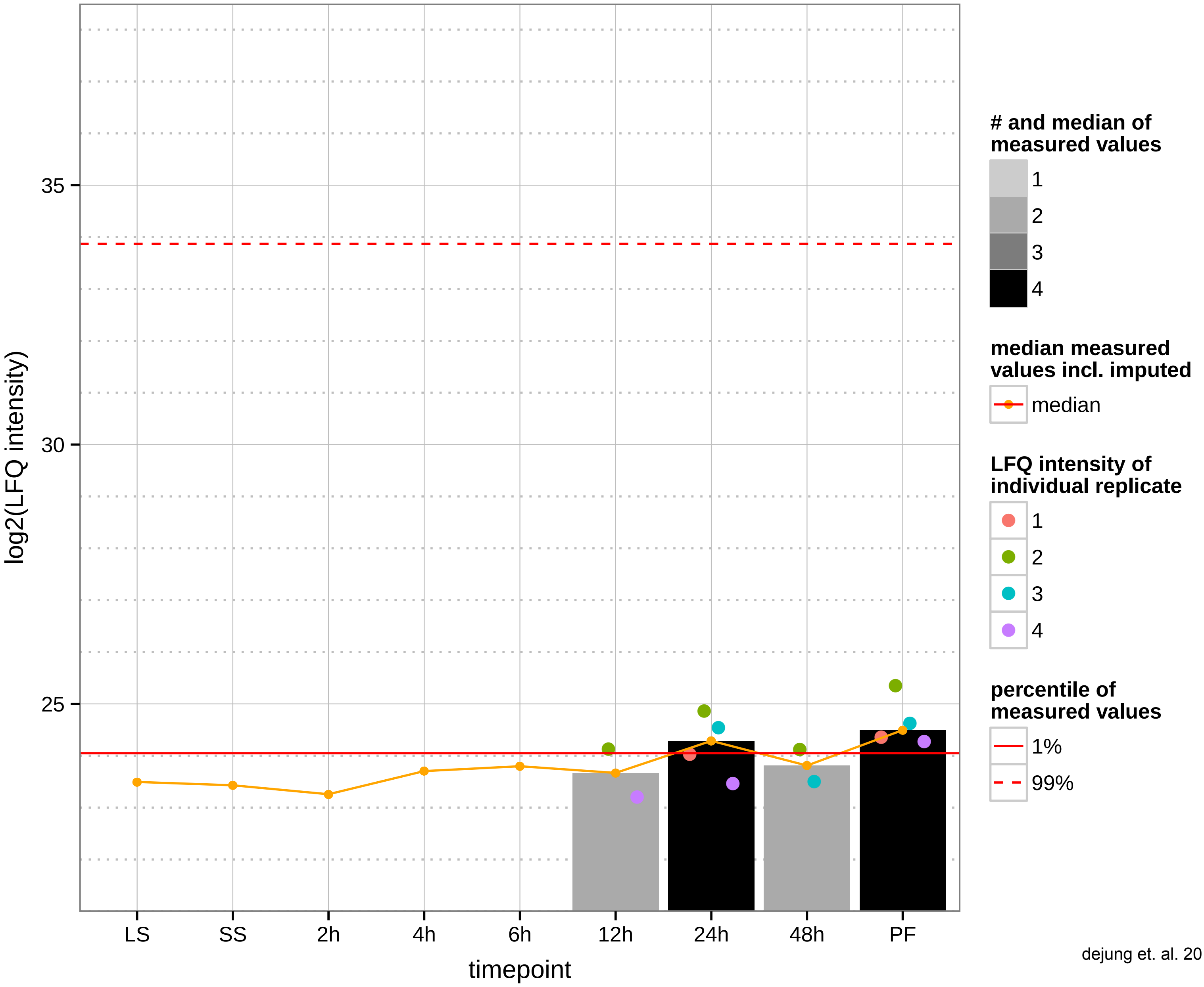

dejung et. al. 2015

TAX−1
Tb927.9.10370
AGOF: null
AGOC: cilium part
AGOP: null
PGOF: null
PGOC: null
PGOP: null

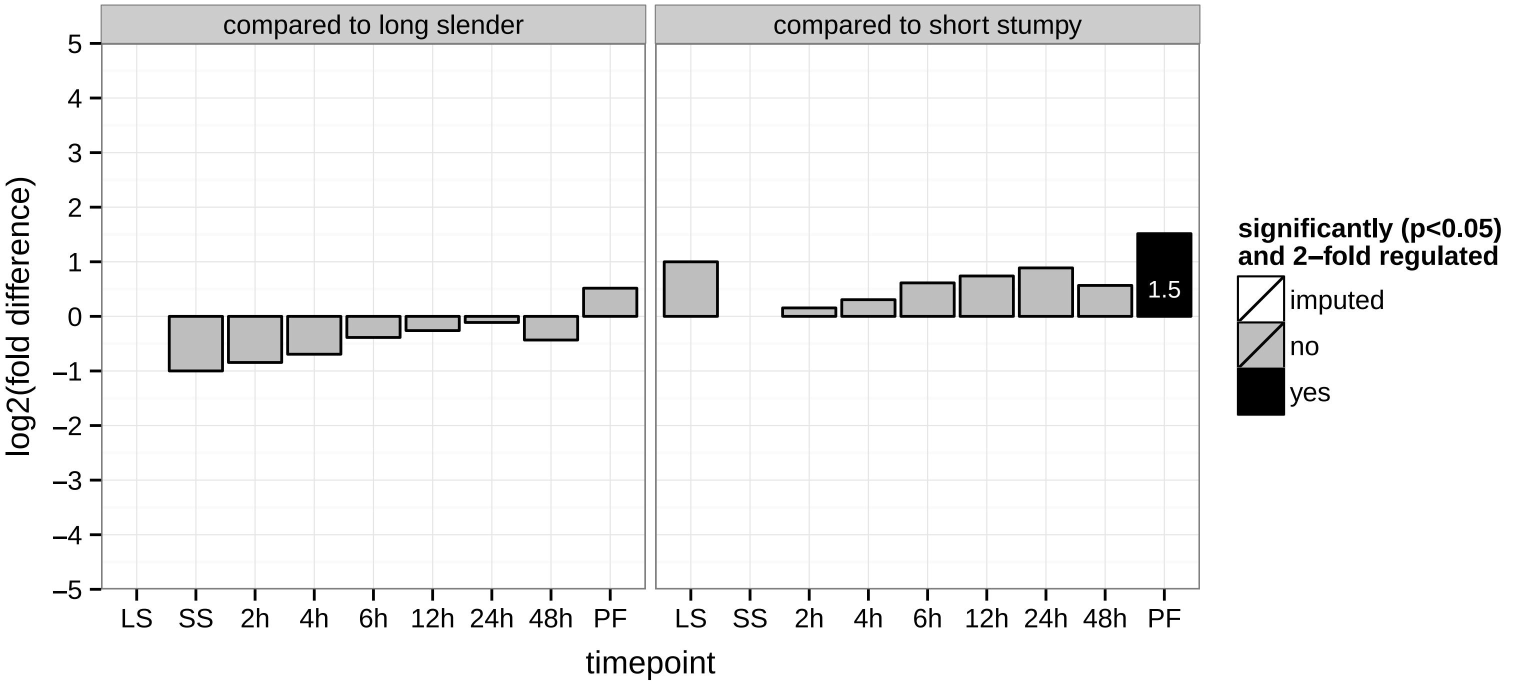

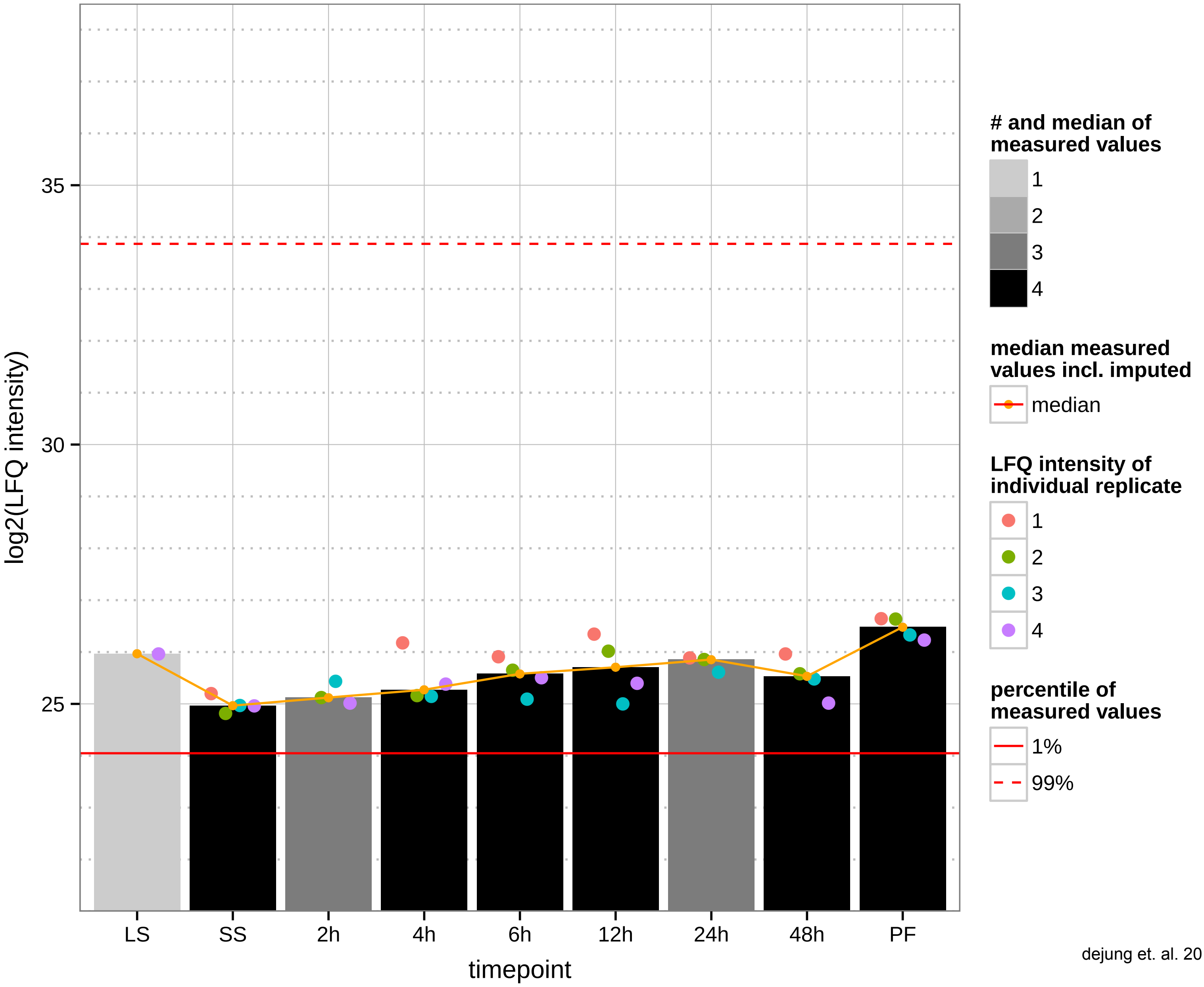

dejung et. al. 2015

hypothetical protein, conserved
Tb927.9.10830
AGOF: null
AGOC: null
AGOP: null
PGOF: null
PGOC: null
PGOP: null

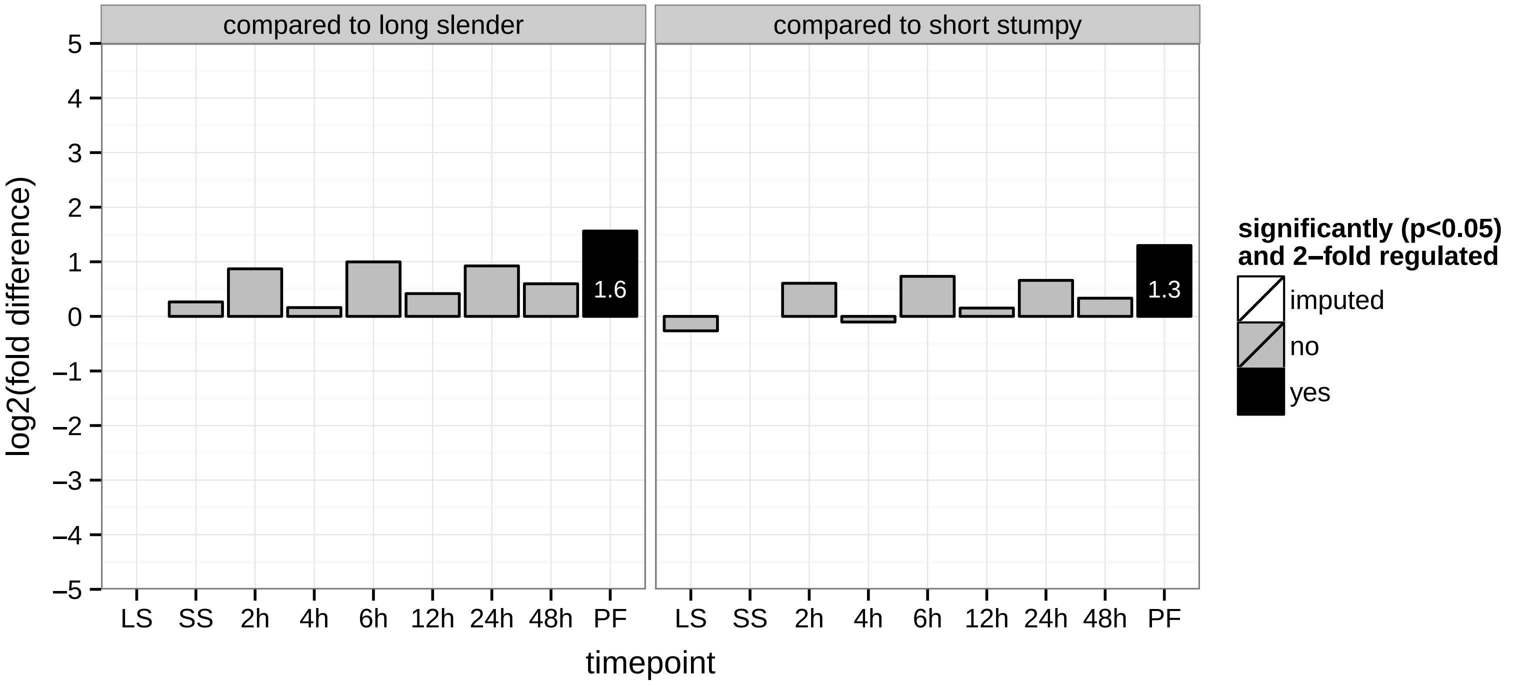

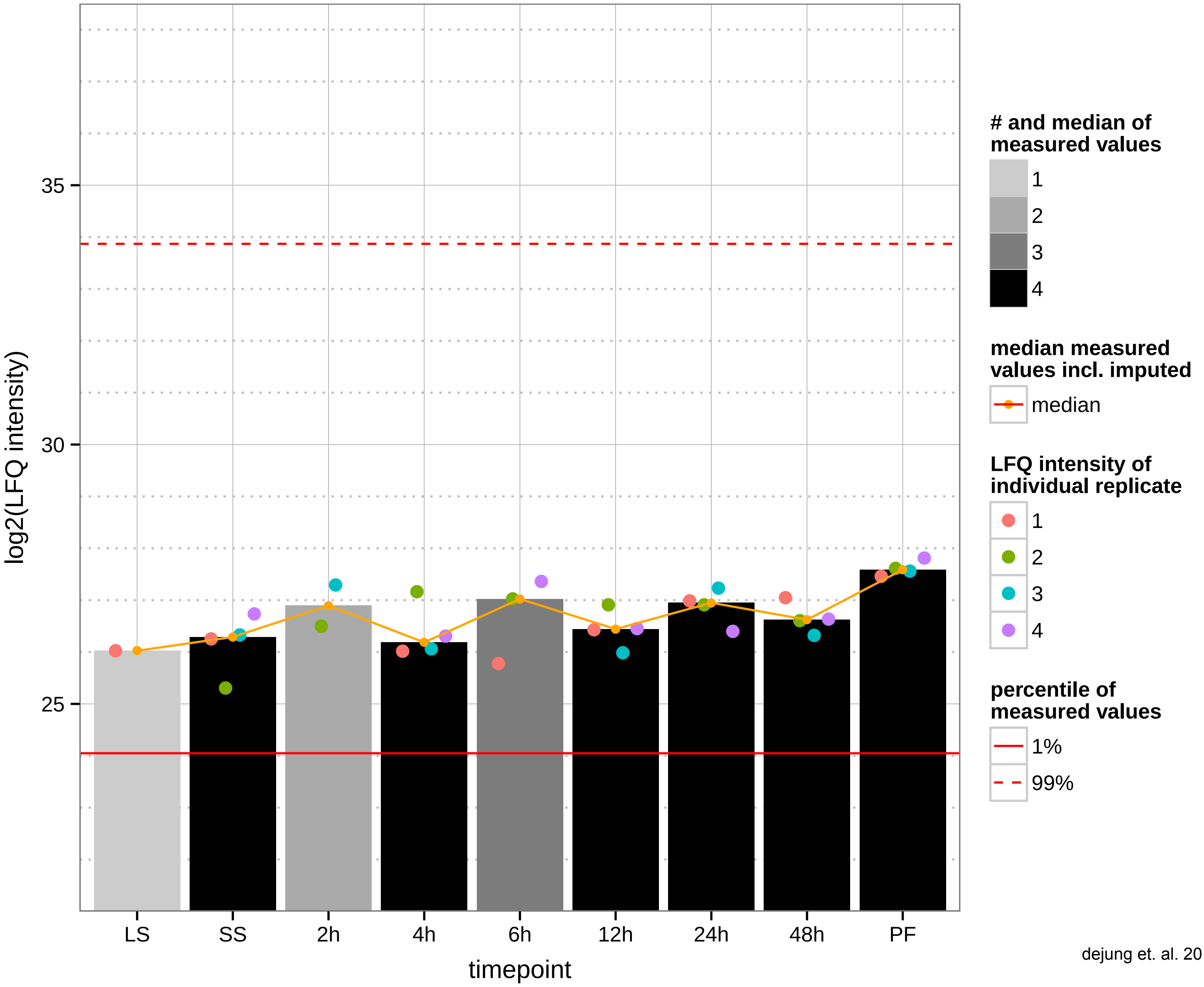

dejung et. al. 2015

T−complex protein 1, eta subunit, putative, t− complex protein 1 (eta subunit), putative (TCP−1−eta)
Tb927.9.11270
AGOF: ATP binding, unfolded protein binding
AGOC: chaperonin−containing T−complex
AGOP: protein folding, regulation of cell cycle
PGOF: ATP binding, unfolded protein binding
PGOC: null
PGOP: cellular protein metabolic process, protein folding

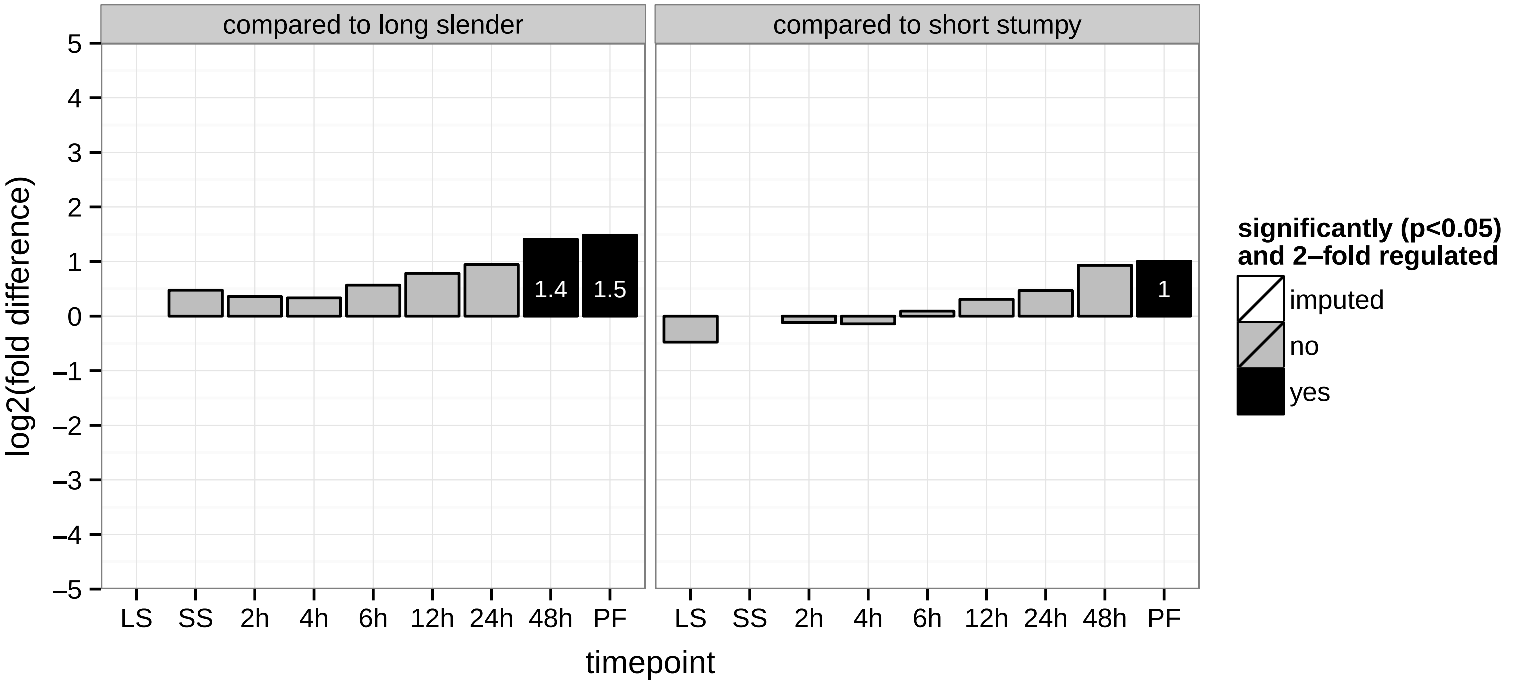

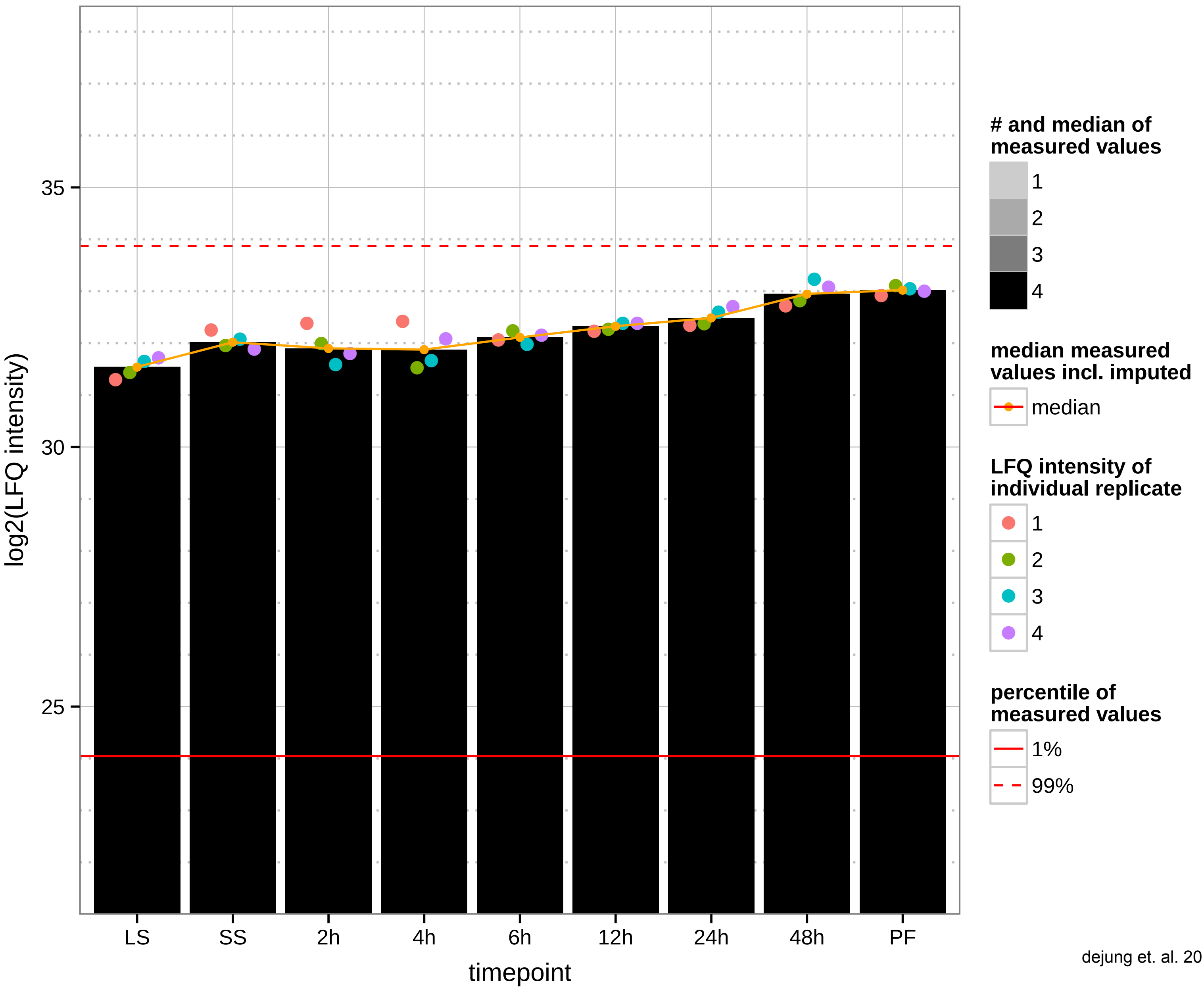

dejung et. al. 2015

hypothetical protein, conserved
Tb927.9.11500
AGOF: ubiquitin protein ligase binding
AGOC: cullin–RING ubiquitin ligase complex
AGOP: ubiquitin–dependent protein catabolic process
PGOF: ubiquitin protein ligase binding
PGOC: cullin–RING ubiquitin ligase complex
PGOP: ubiquitin–dependent protein catabolic process

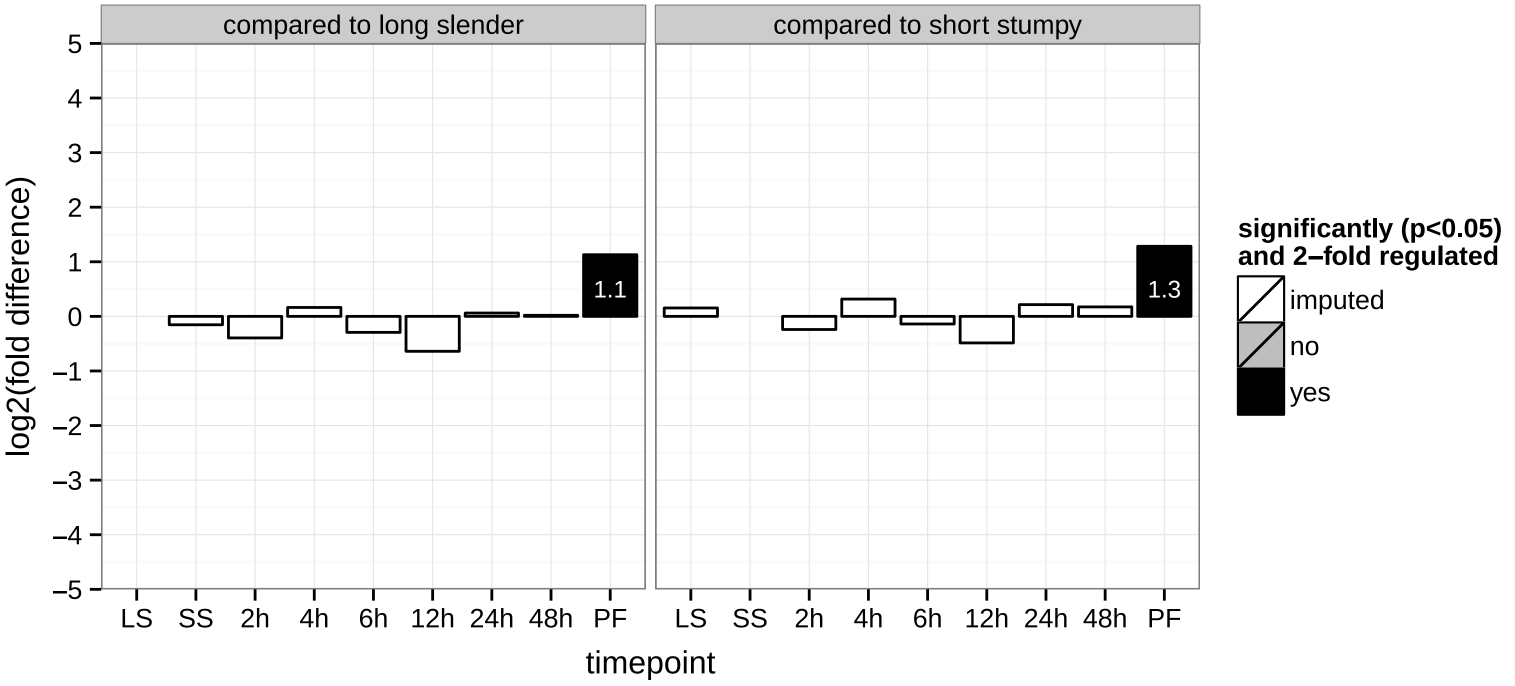

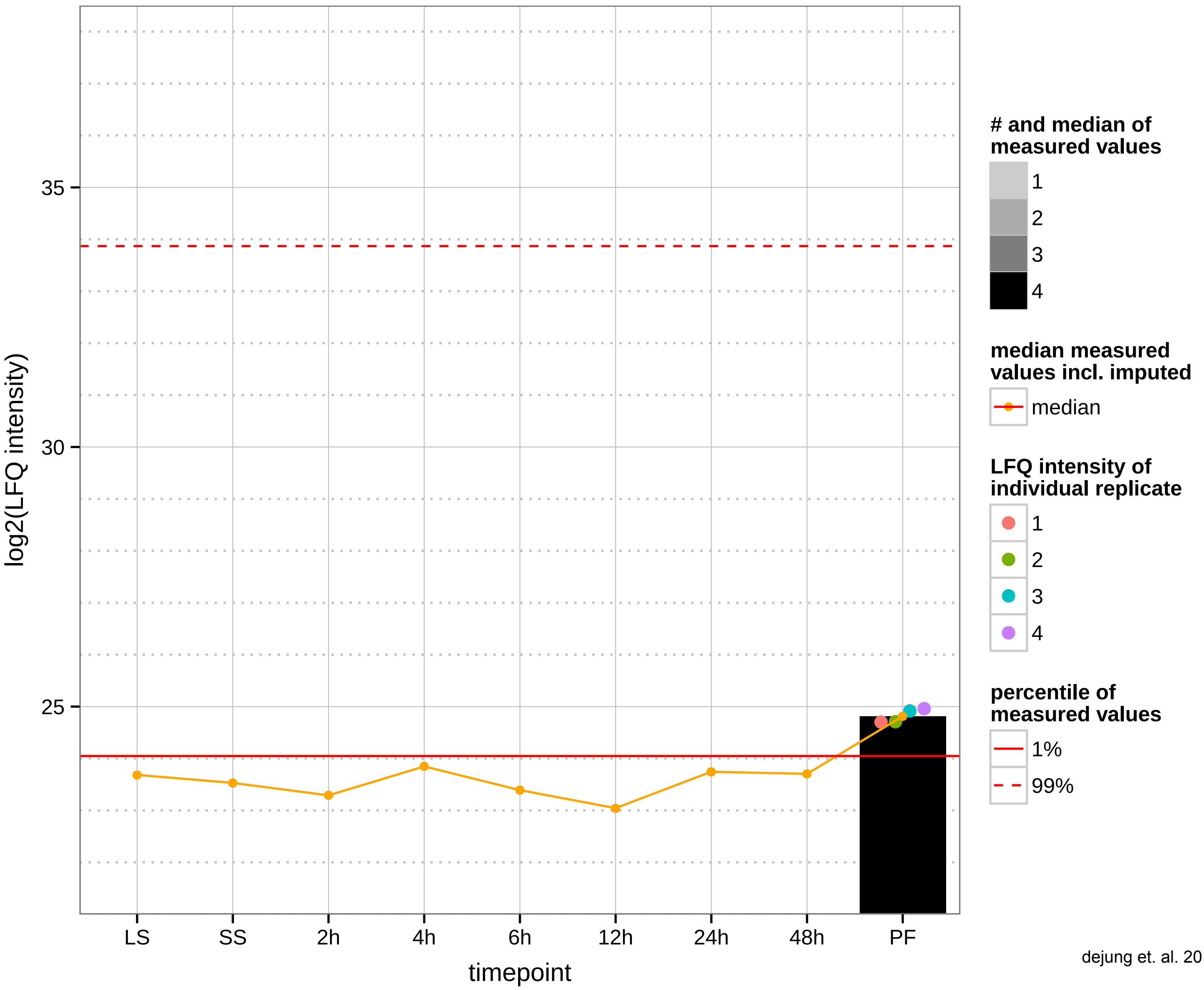

dejung et. al. 2015

Pre–rRNA–processing protein PNO1, putative (PNO1)
Tb927.9.11840
AGOF: RNA binding
AGOC: nucleolus
AGOP: null
PGOF: RNA binding
PGOC: null
PGOP: null

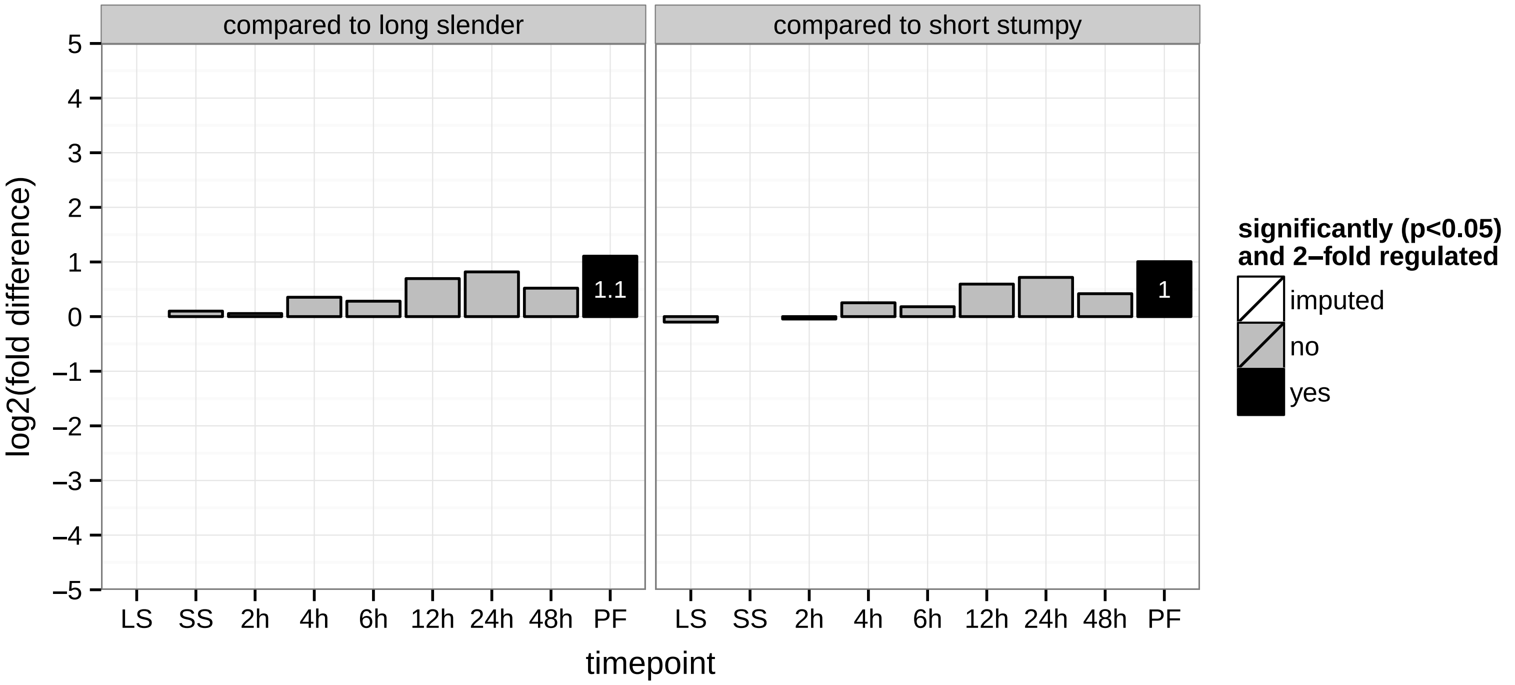

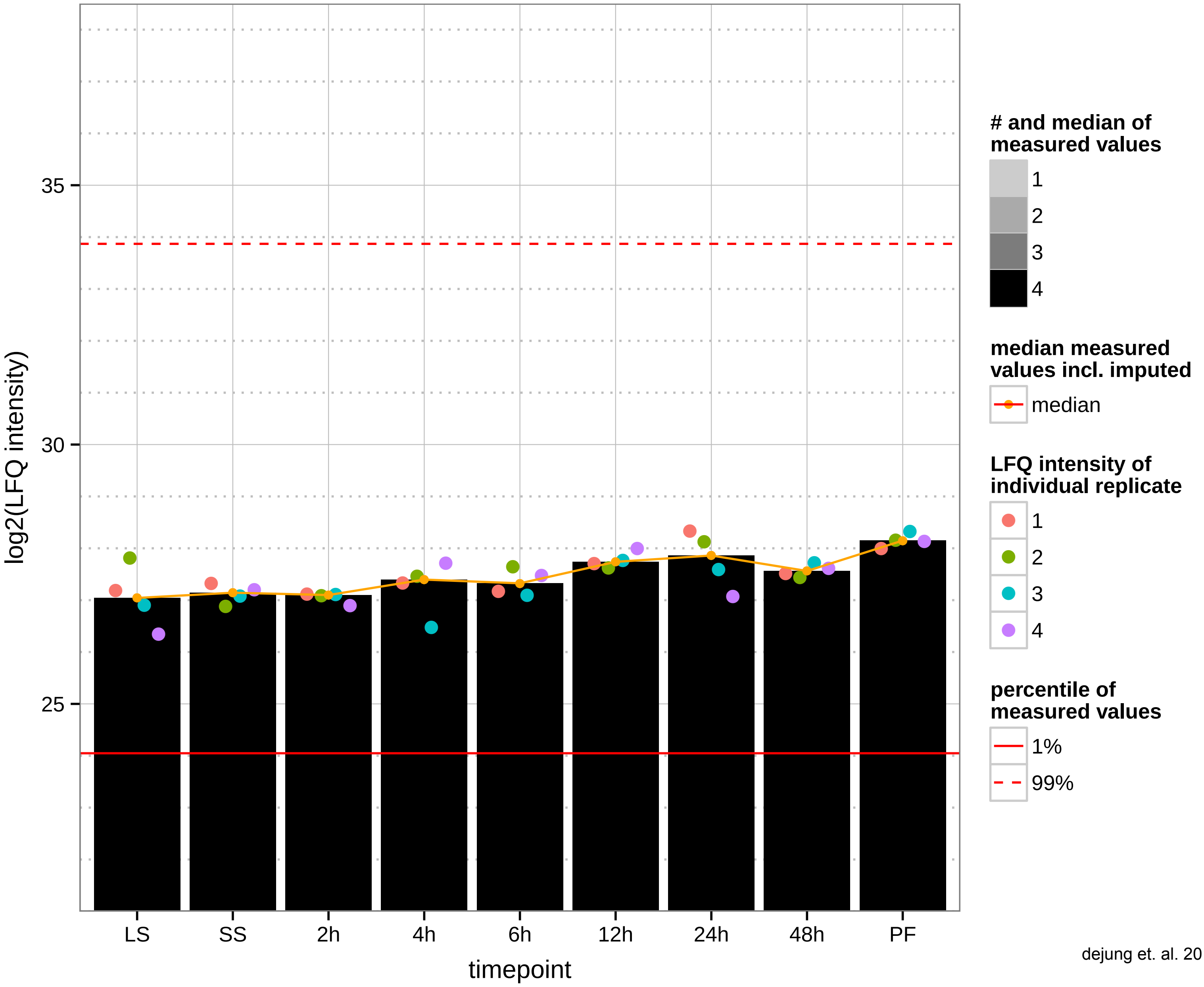

dejung et. al. 2015

hypothetical protein, conserved
Tb927.9.12070
AGOF: null
AGOC: null
AGOP: null
PGOF: null
PGOC: null
PGOP: null

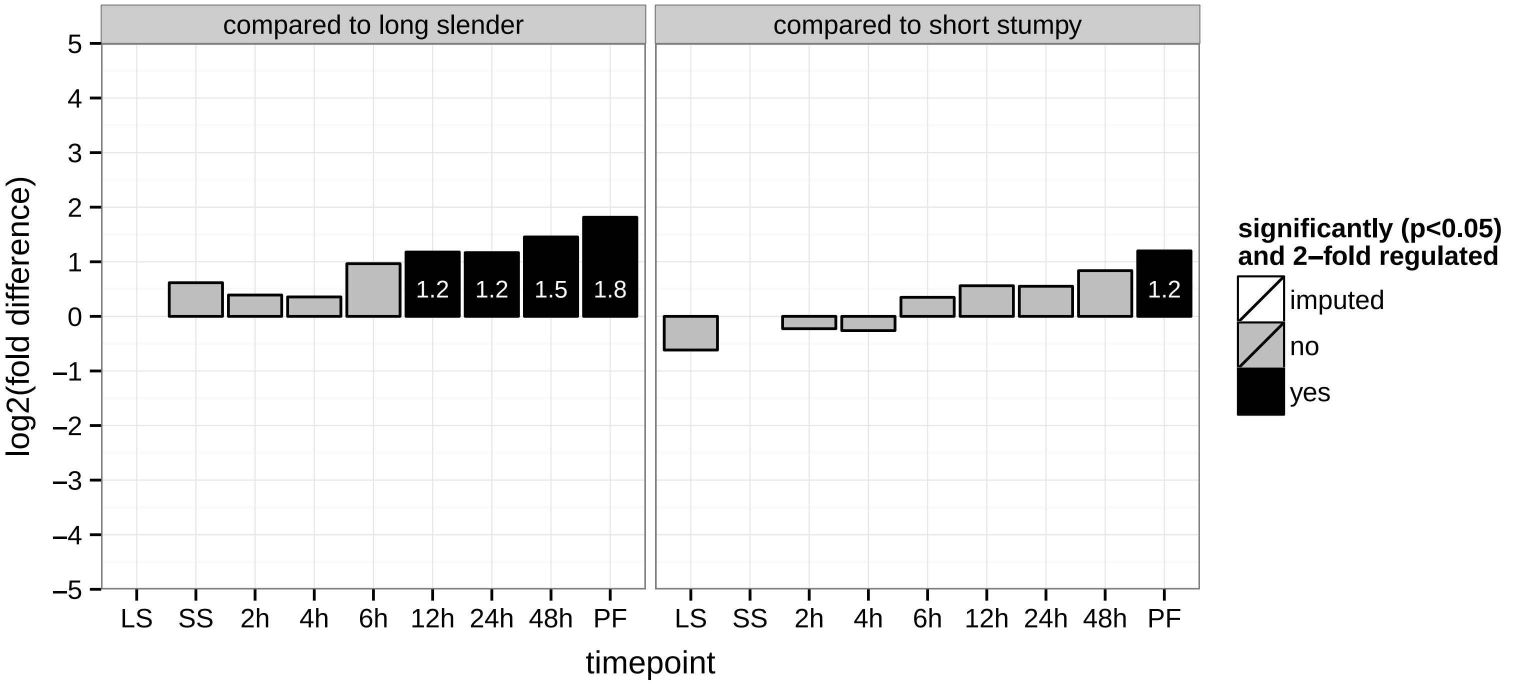

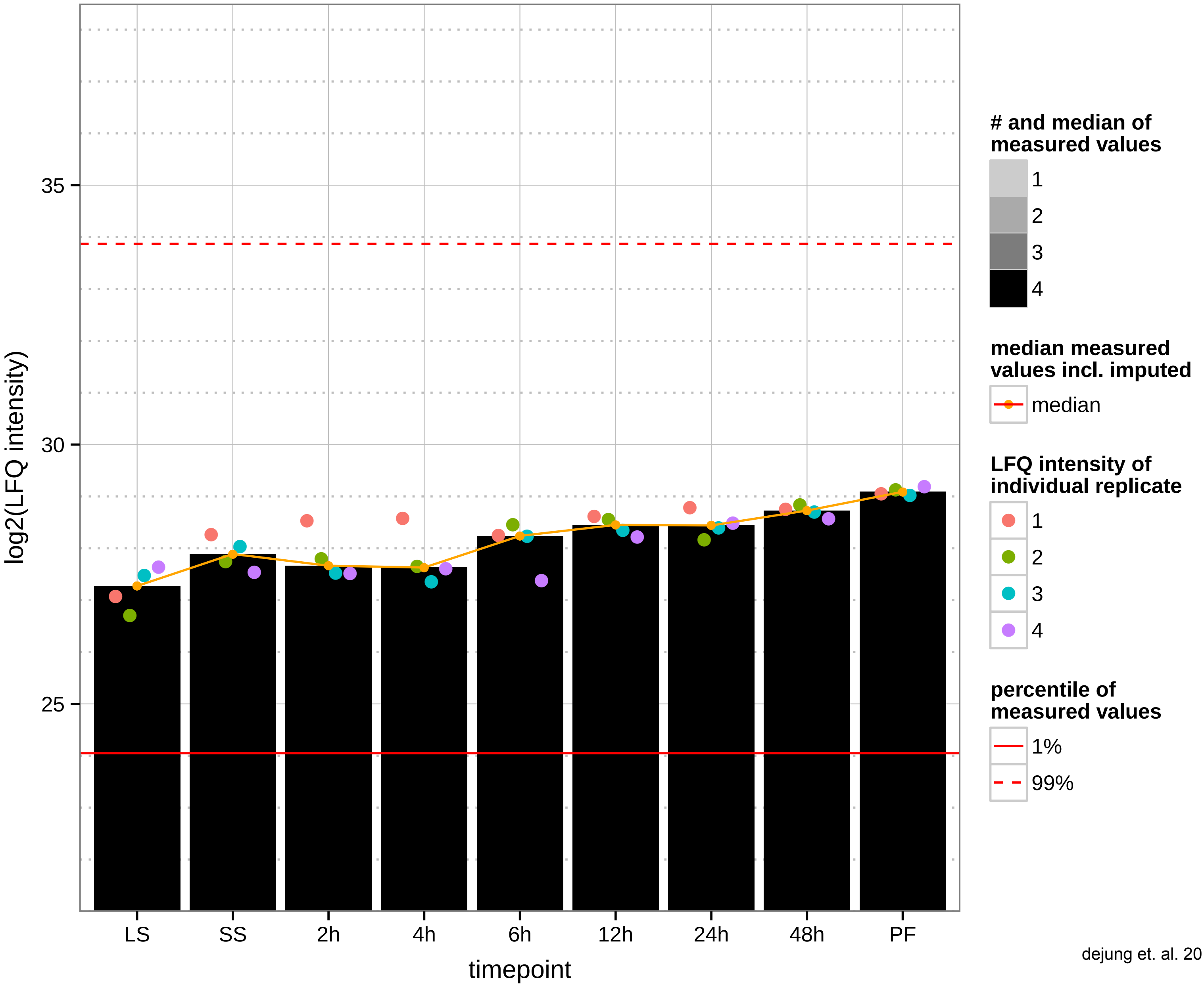

dejung et. al. 2015

class I transcription factor A, subunit 2 (CITFA−2)
Tb927.9.12450
AGOF: null
AGOC: null
AGOP: null
PGOF: null
PGOC: null
PGOP: null

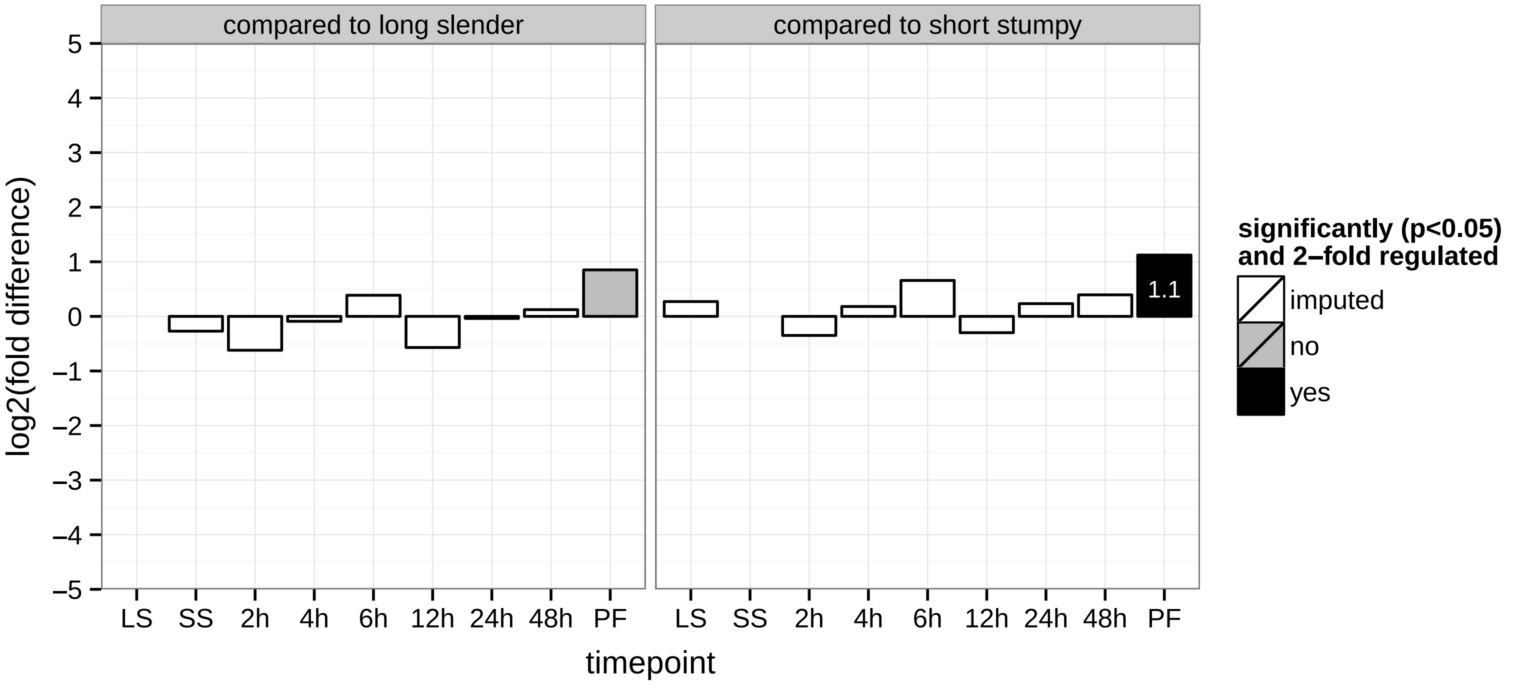

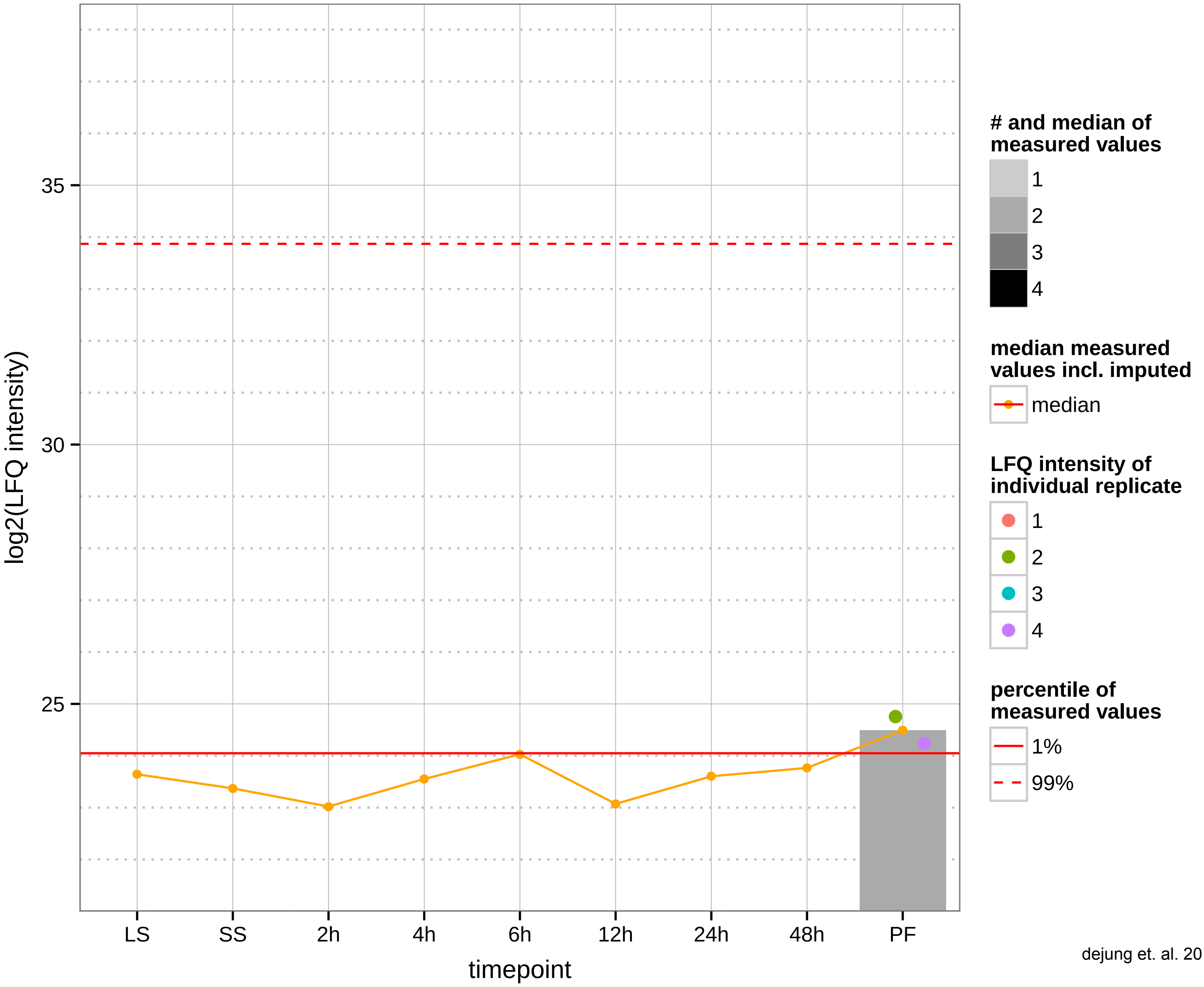

dejung et. al. 2015

hypothetical protein, conserved
Tb927.9.12830
AGOF: null
AGOC: null
AGOP: null
PGOF: null
PGOC: null
PGOP: null

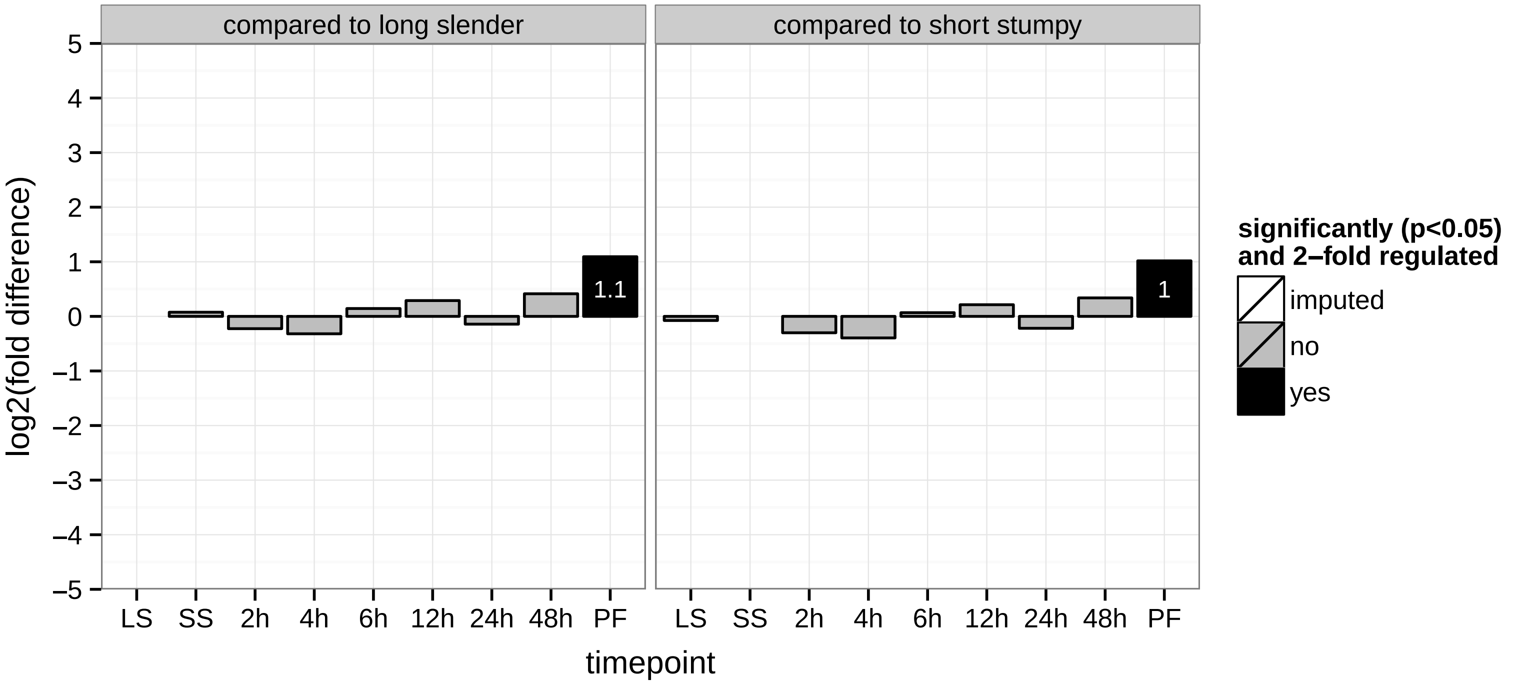

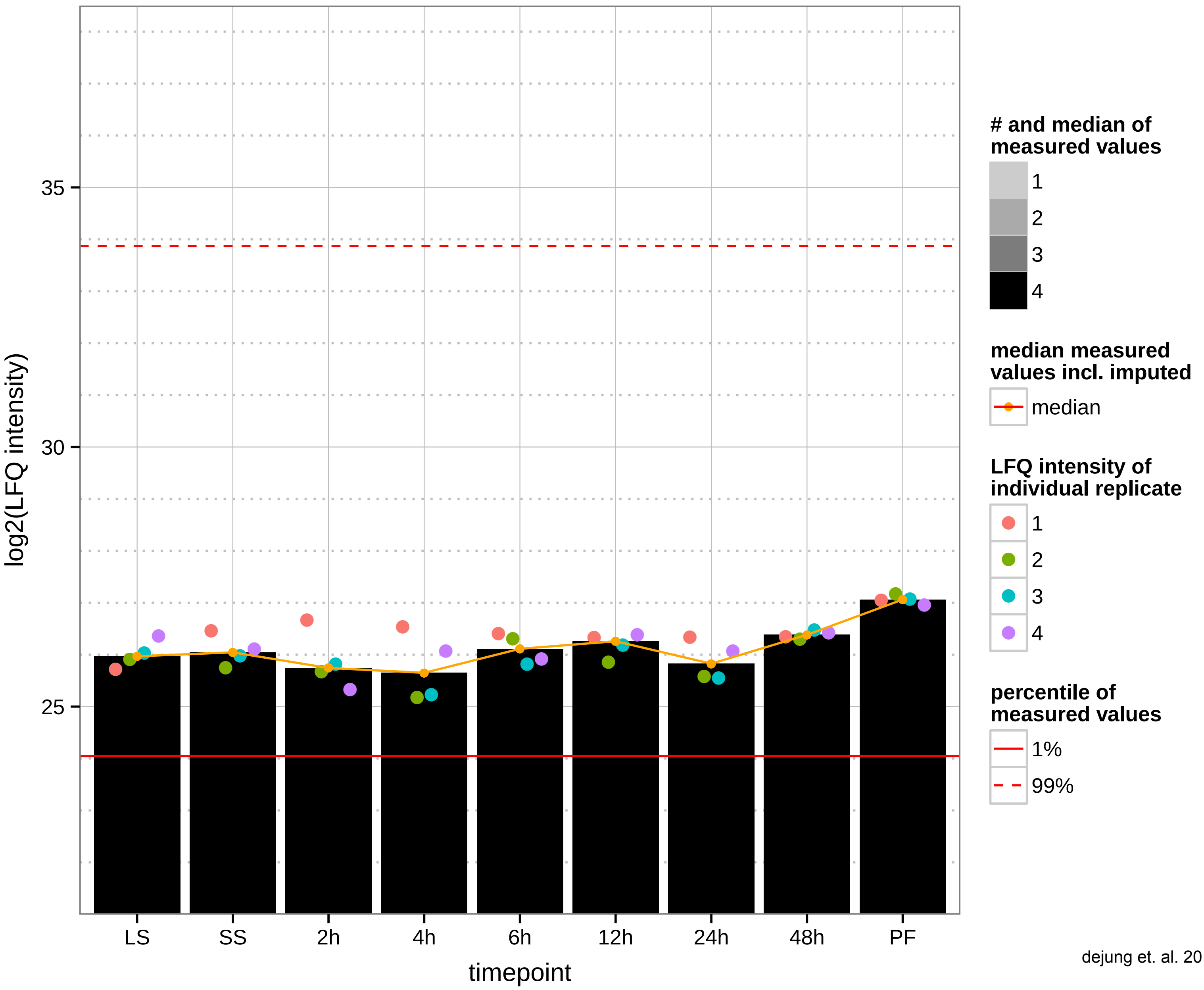

dejung et. al. 2015

hypothetical protein, conserved
Tb927.9.12980
AGOF: null
AGOC: null
AGOP: null
PGOF: null
PGOC: null
PGOP: null

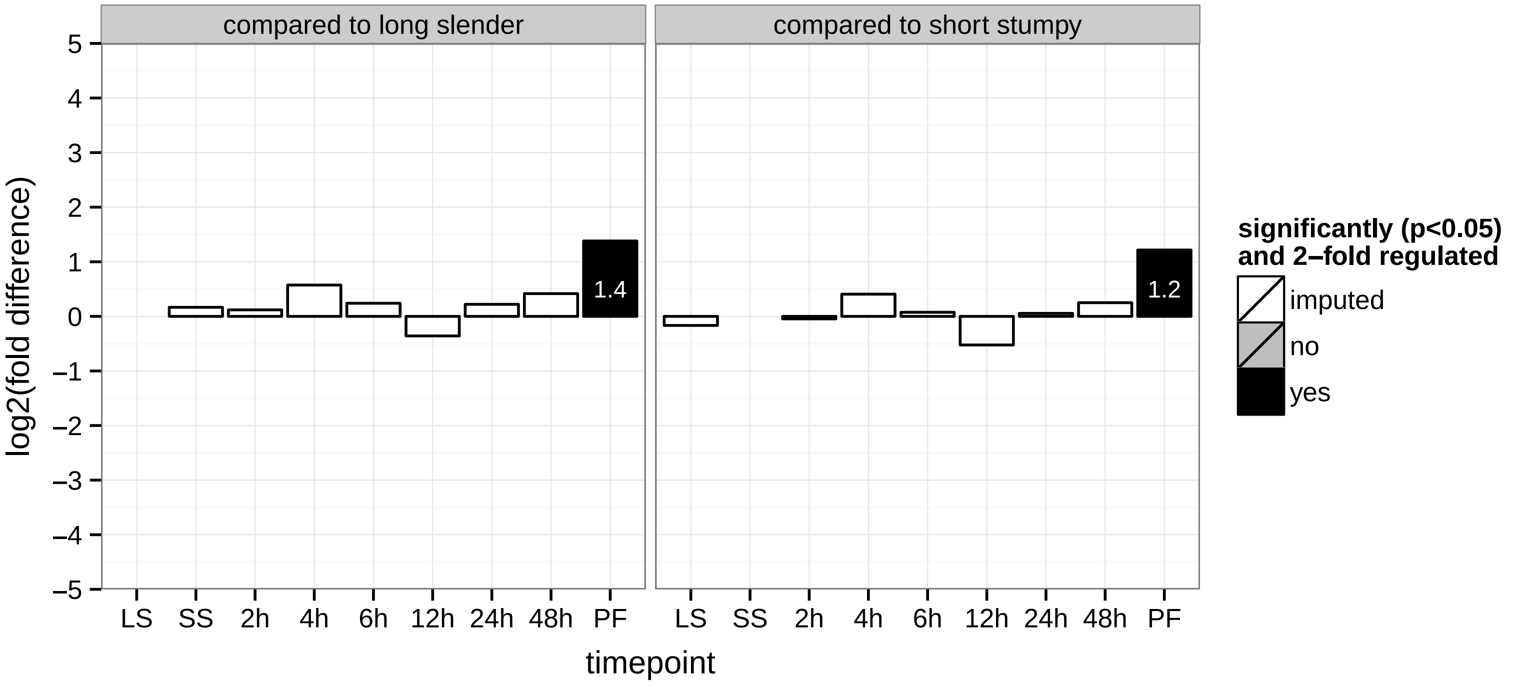

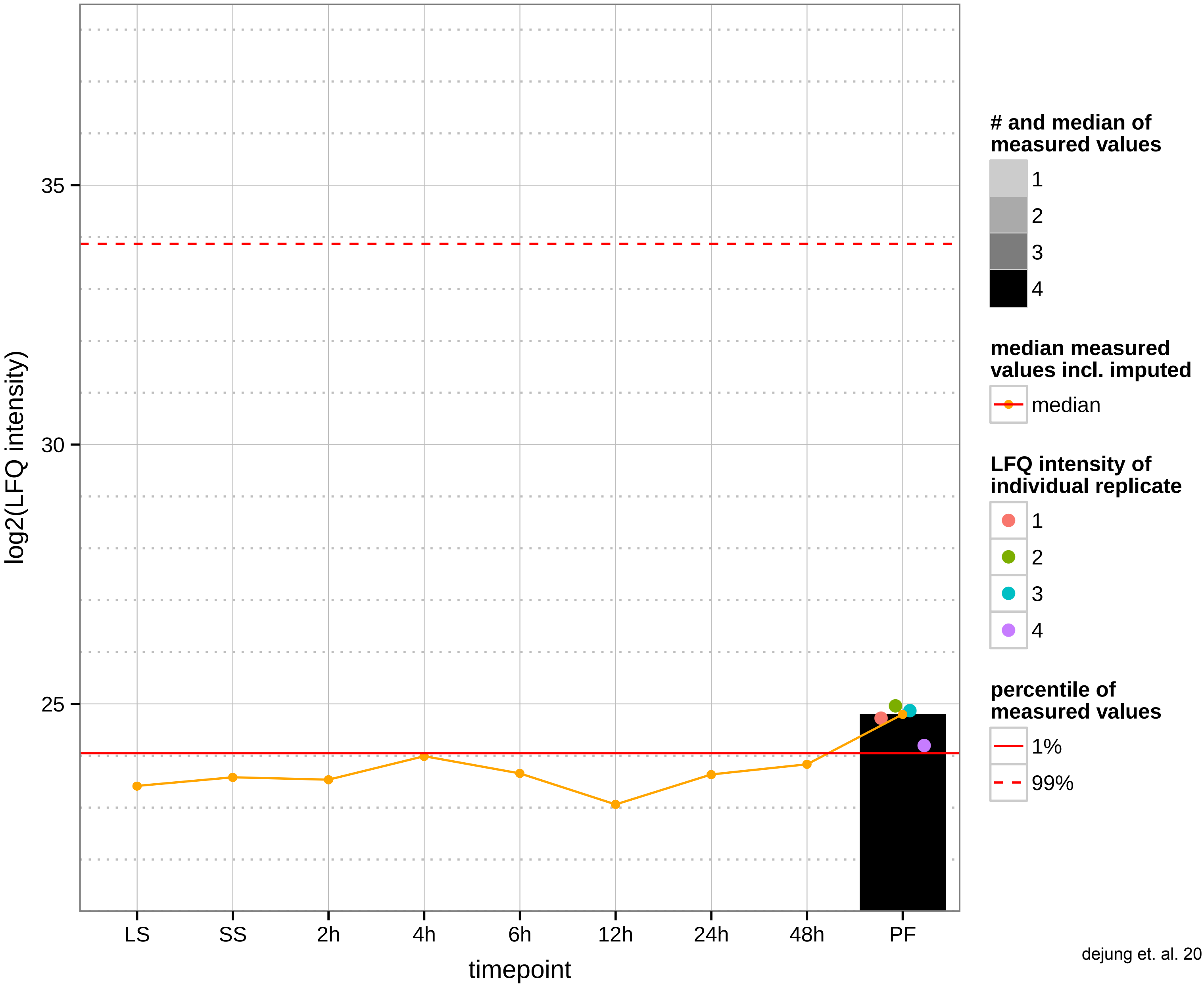

dejung et. al. 2015

hypothetical protein, conserved
Tb927.9.13350
AGOF: null
AGOC: null
AGOP: null
PGOF: null
PGOC: null
PGOP: null

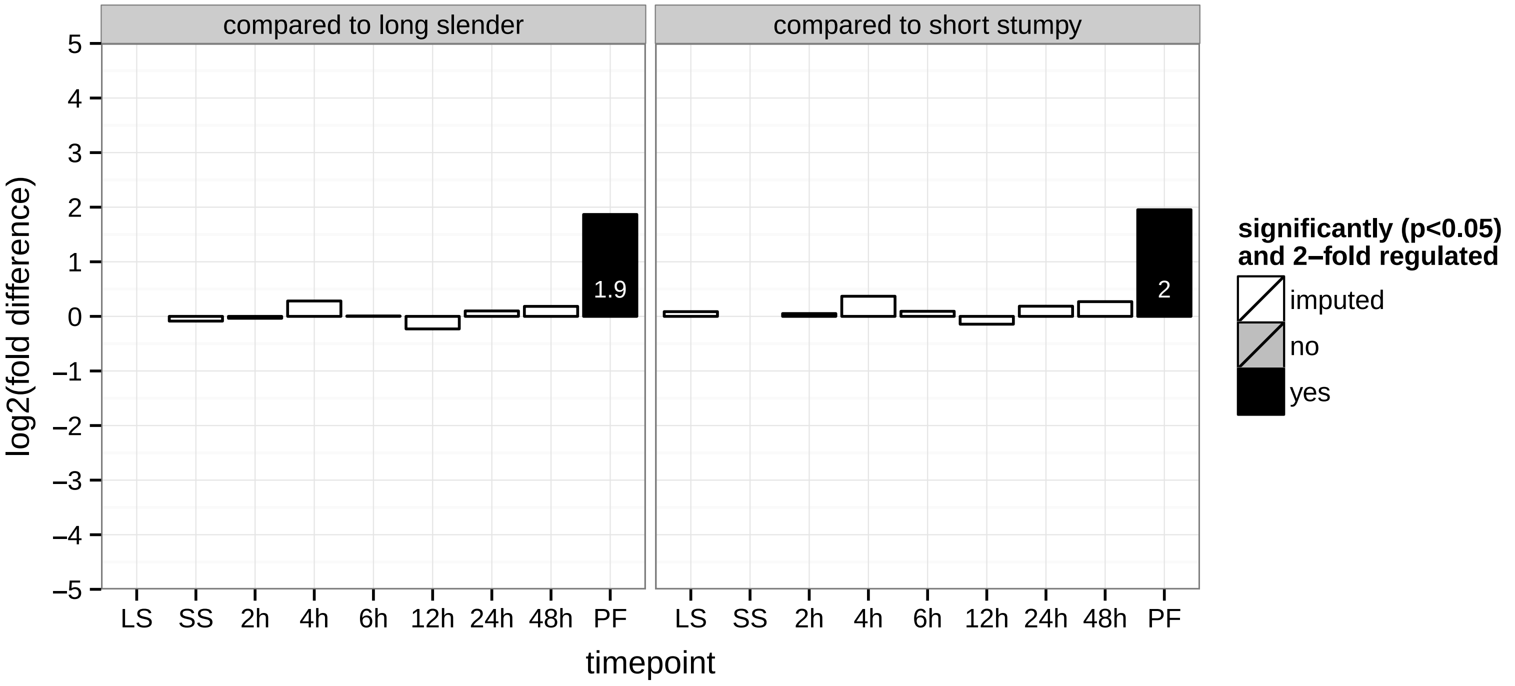

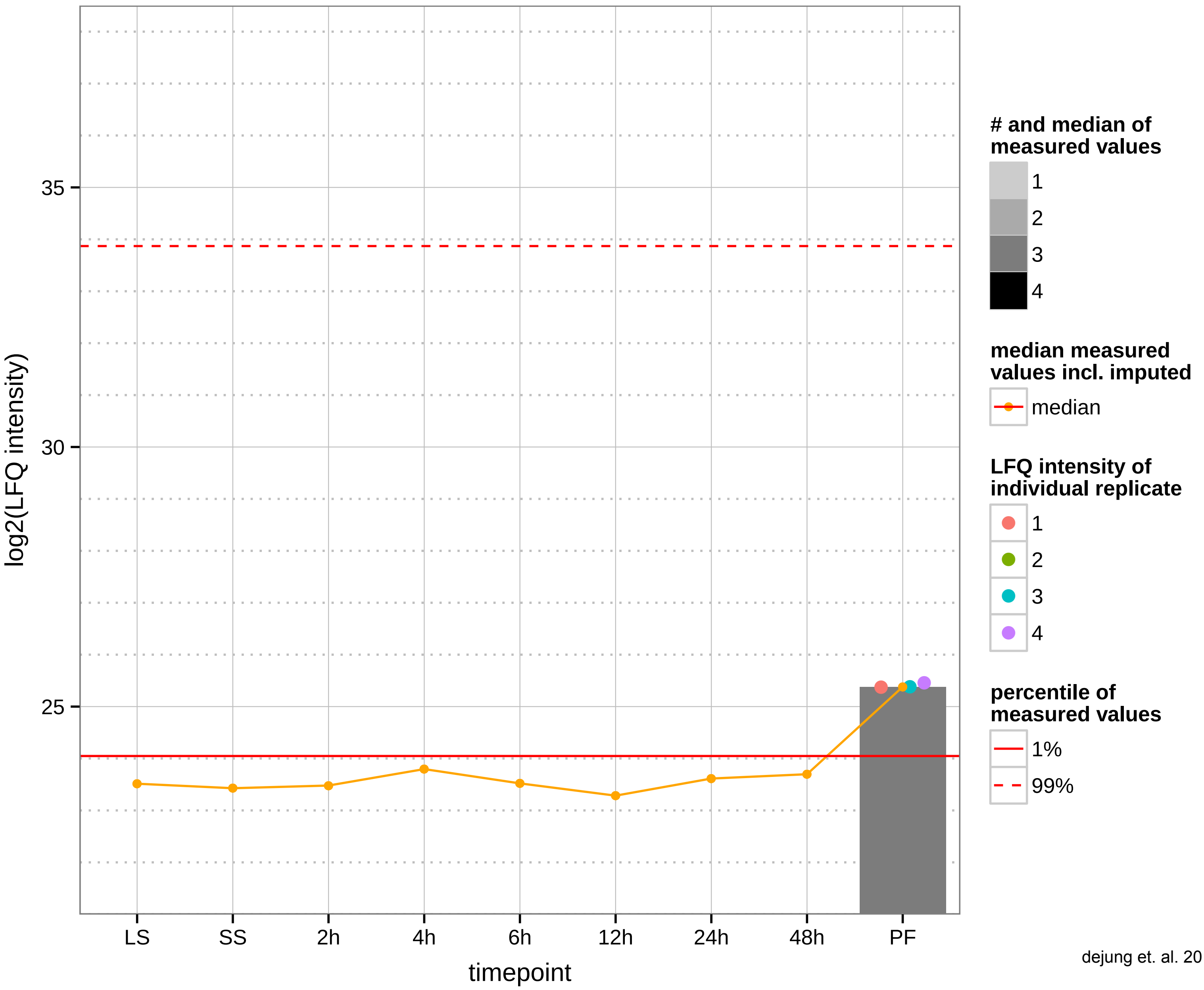

dejung et. al. 2015

Myosin–like protein, Nucleoporin (TbMlp–2)
Tb927.9.1340
AGOF: null
AGOC: intracellular, nuclear pore
AGOP: null
PGOF: null
PGOC: null
PGOP: null

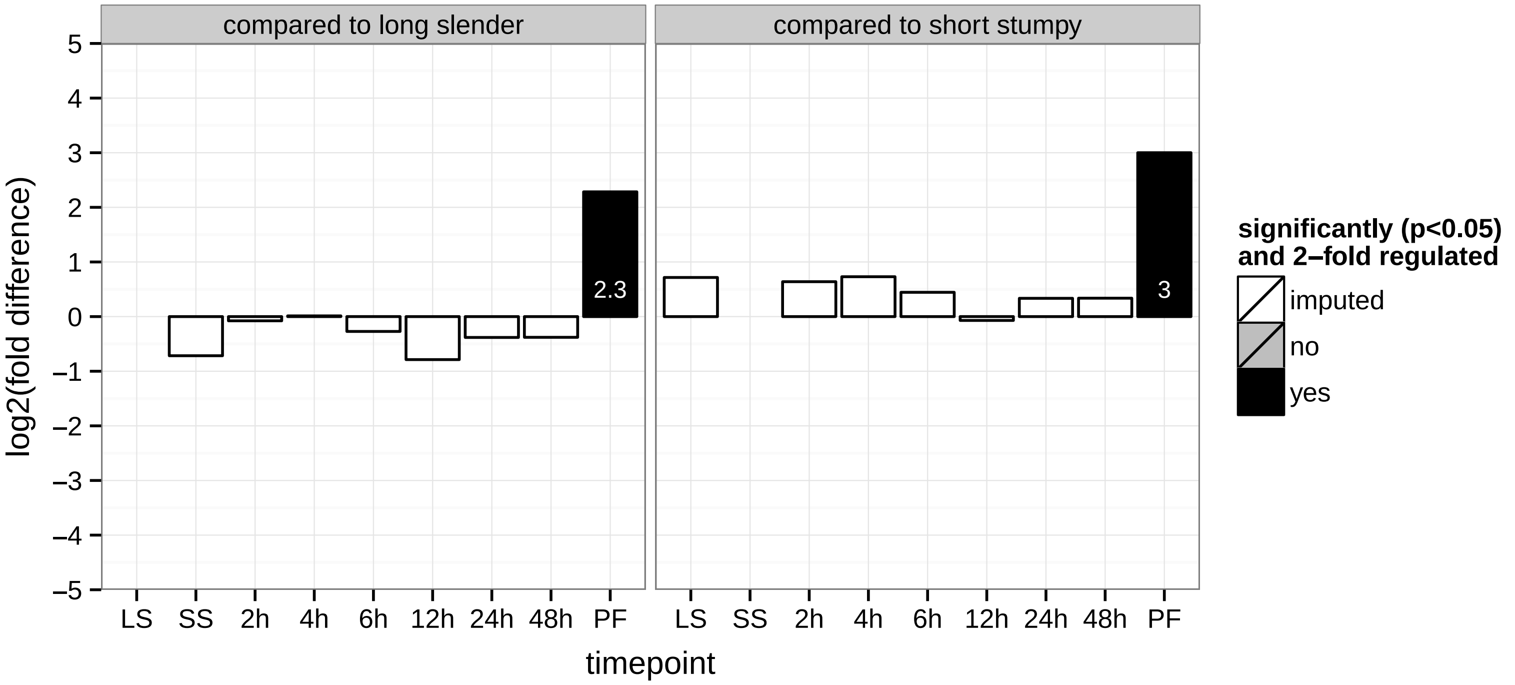

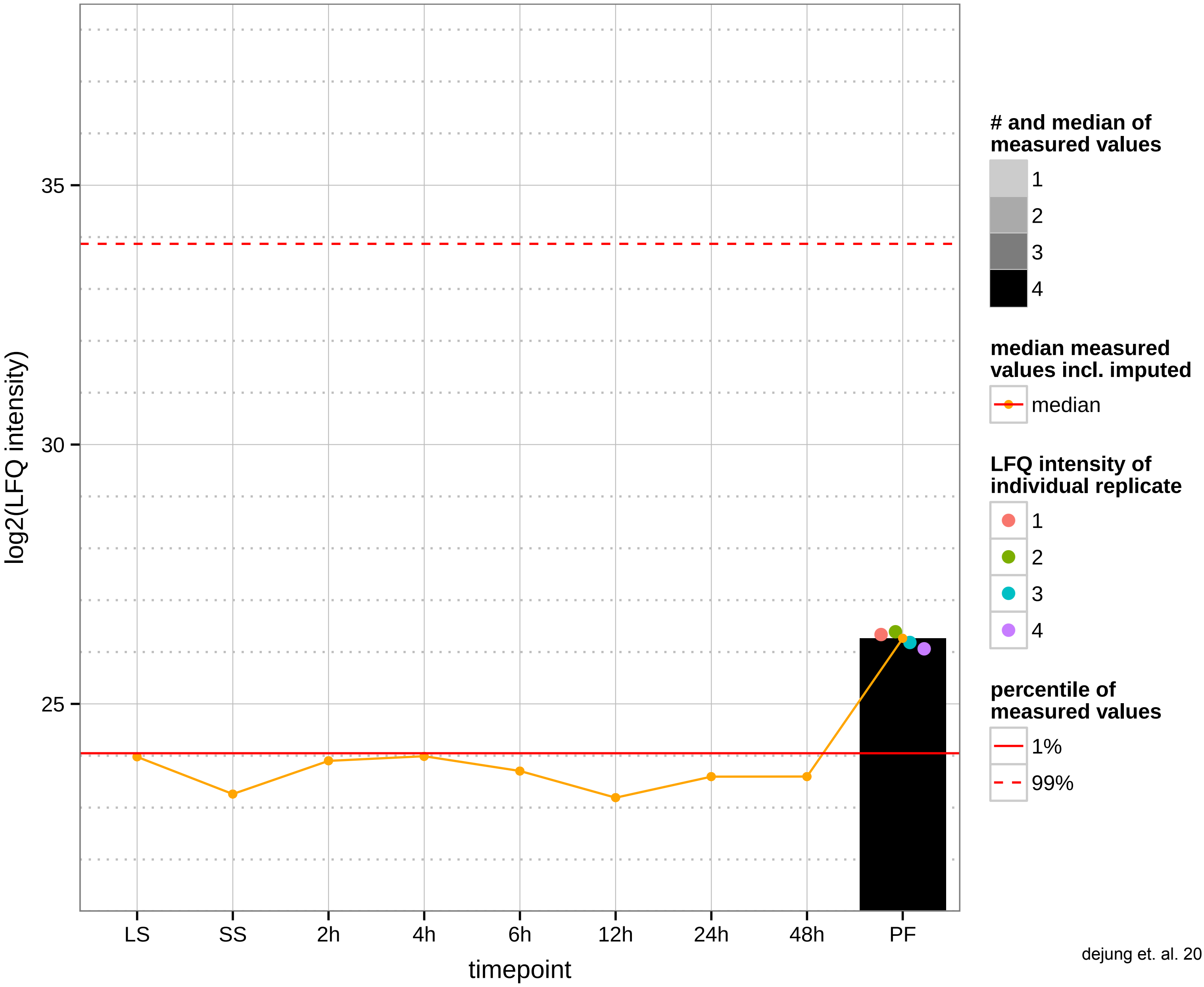

dejung et. al. 2015

hypothetical protein, conserved
Tb927.9.13970
AGOF: null
AGOC: null
AGOP: null
PGOF: null
PGOC: null
PGOP: null

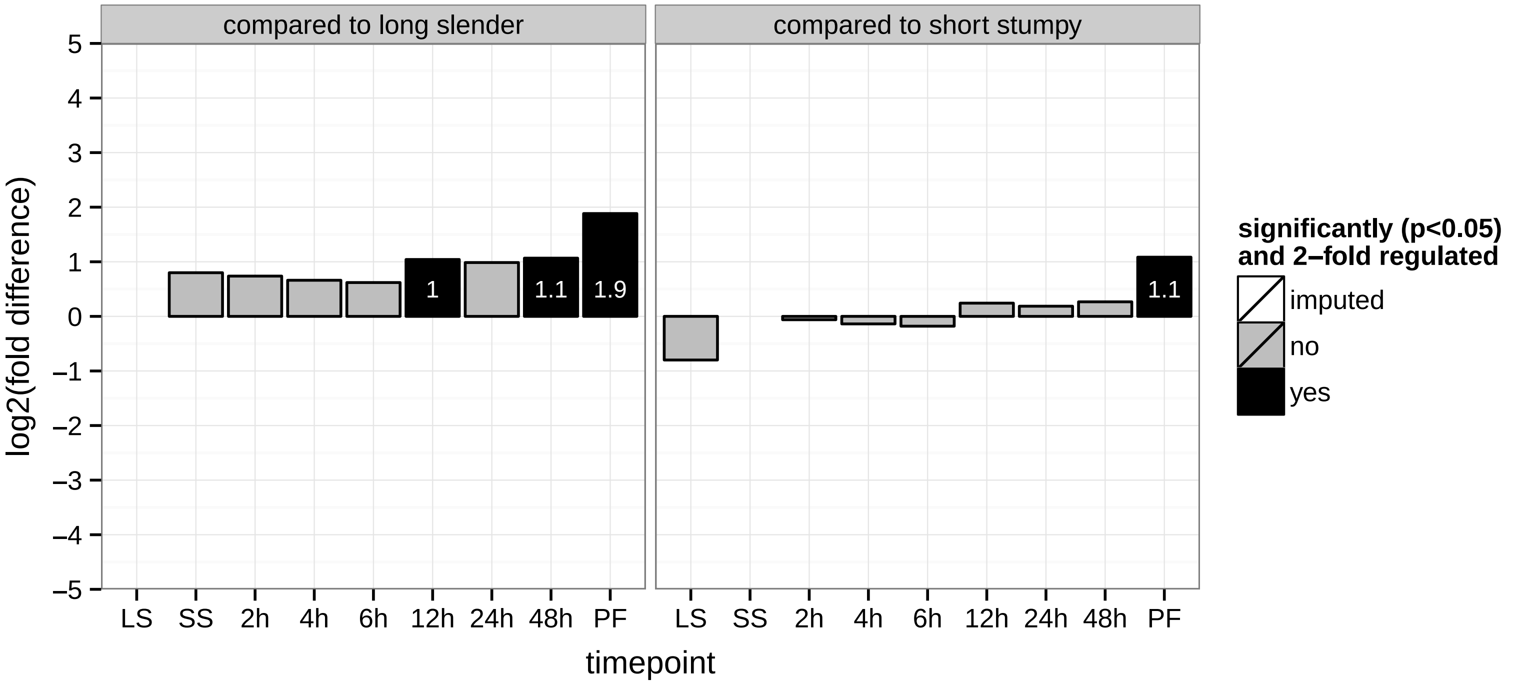

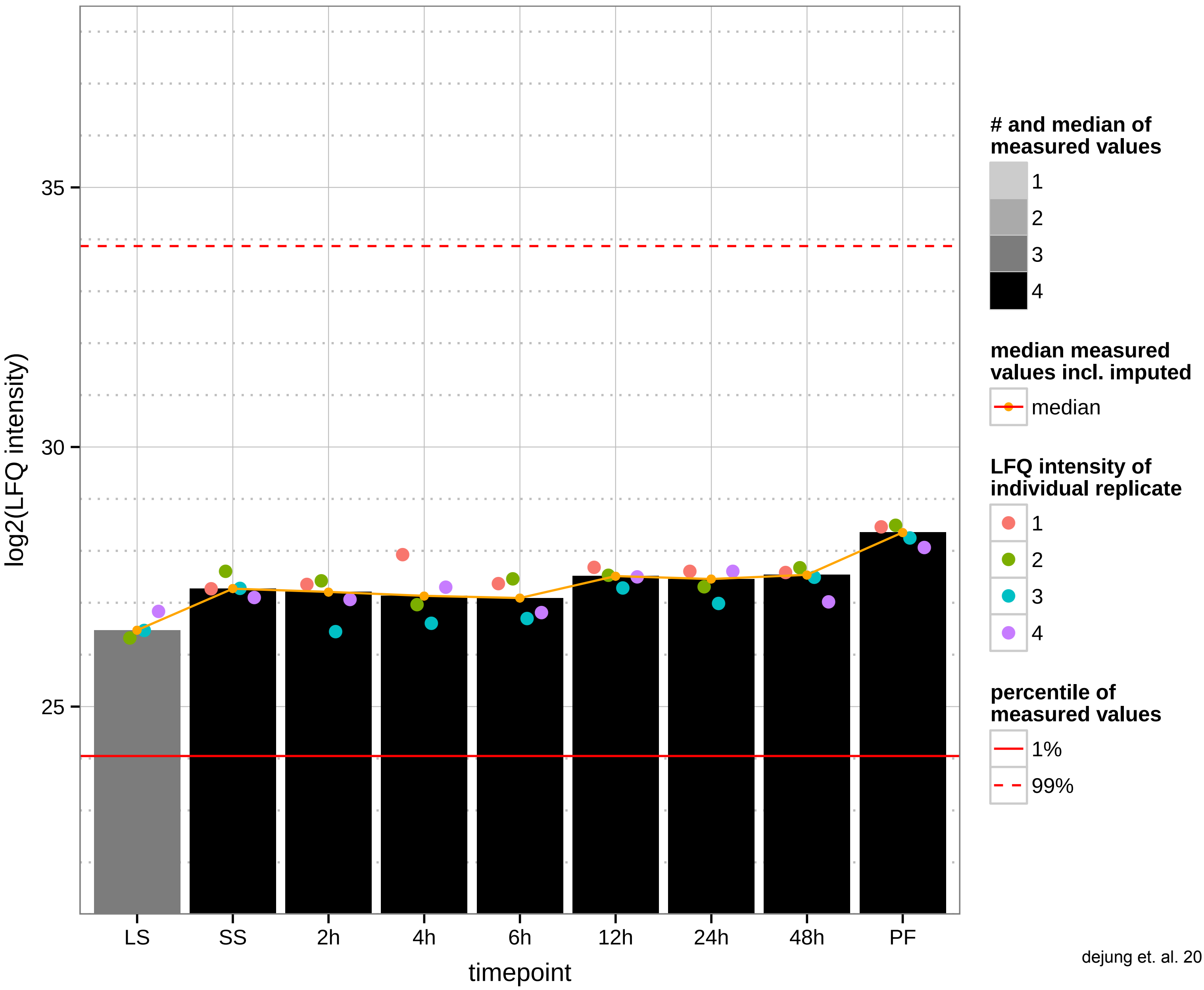

dejung et. al. 2015

hypothetical protein, conserved
Tb927.9.1410
AGOF: null
AGOC: null
AGOP: null
PGOF: null
PGOC: null
PGOP: null

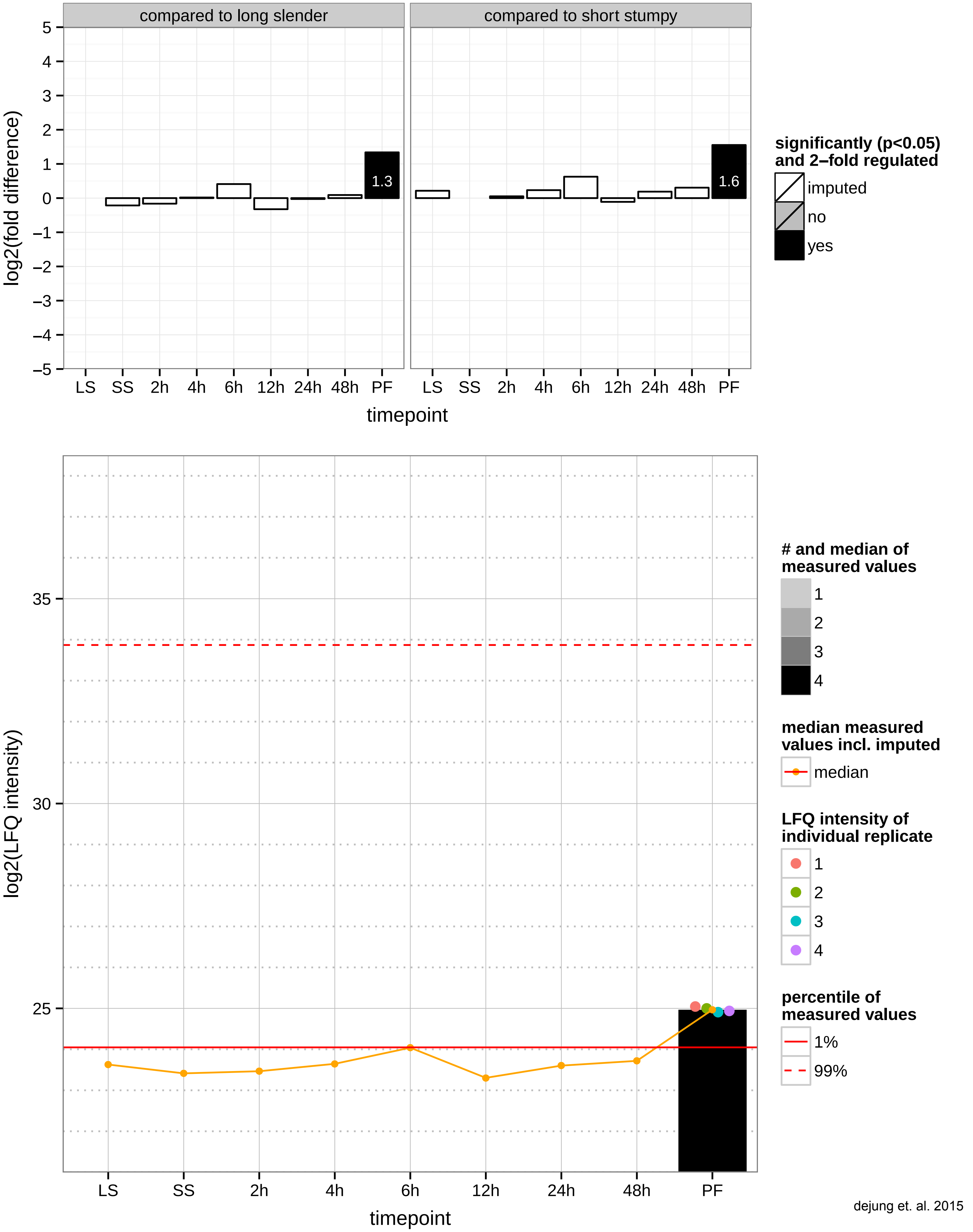

dejung et. al. 2015

hypothetical protein, conserved
Tb927.9.15000
AGOF: null
AGOC: cytoplasm, nucleus
AGOP: null
PGOF: null
PGOC: cytoplasm, nucleus
PGOP: null

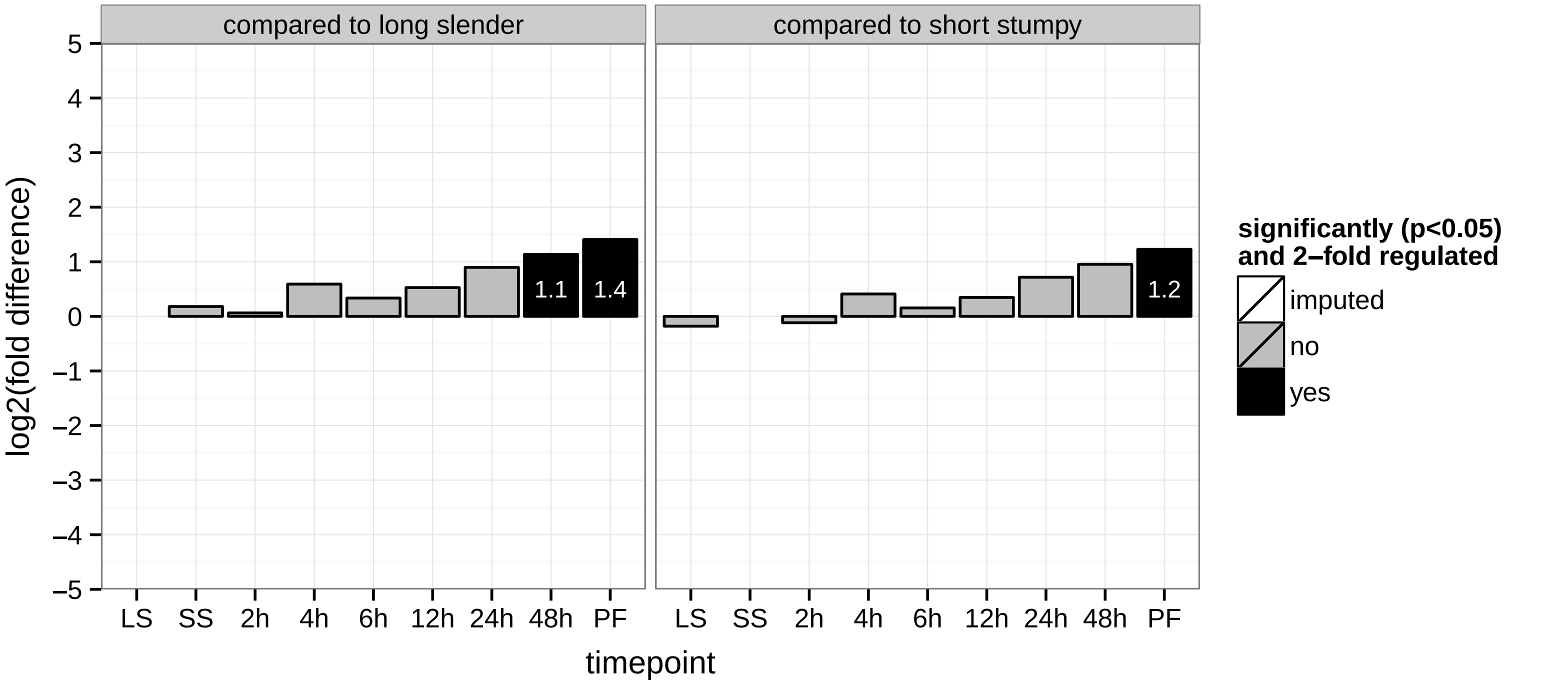

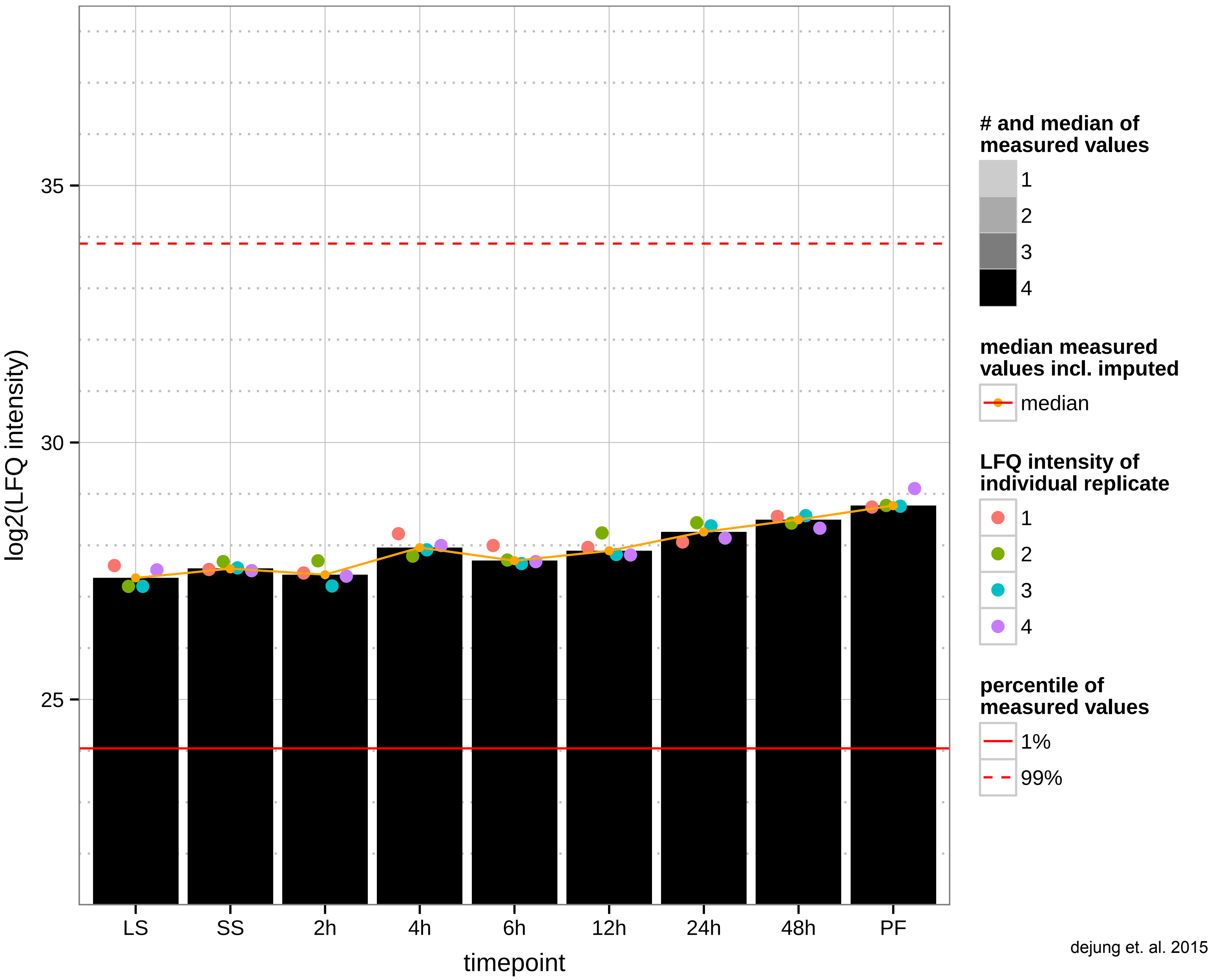

dejung et. al. 2015

unspecified product
Tb927.9.15150;Tb927.9.15110
AGOF: 5S rRNA binding, structural constituent of ribosome, null
AGOC: intracellular, ribosome, null
AGOP: ribosome assembly, translation, null
PGOF: null, structural constituent of ribosome
PGOC: null, intracellular, ribosome
PGOP: null, translation

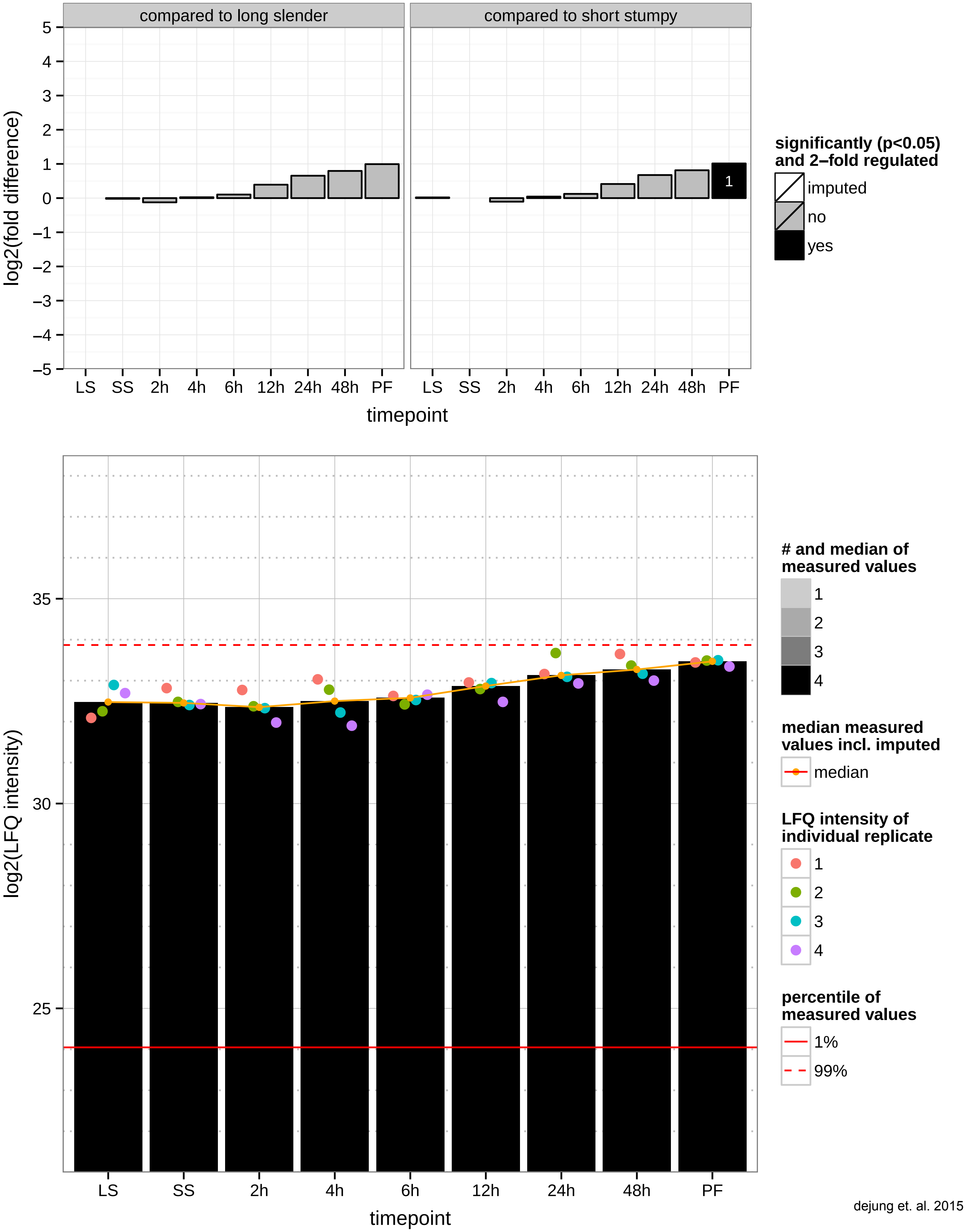

dejung et. al. 2015

nitrilase, putative
Tb927.9.1960
AGOF: hydrolase activity, acting on carbon–nitrogen (but not peptide) bonds
AGOC: null
AGOP: nitrogen compound metabolic process
PGOF: hydrolase activity, acting on carbon–nitrogen (but not peptide) bonds
PGOC: null
PGOP: nitrogen compound metabolic process

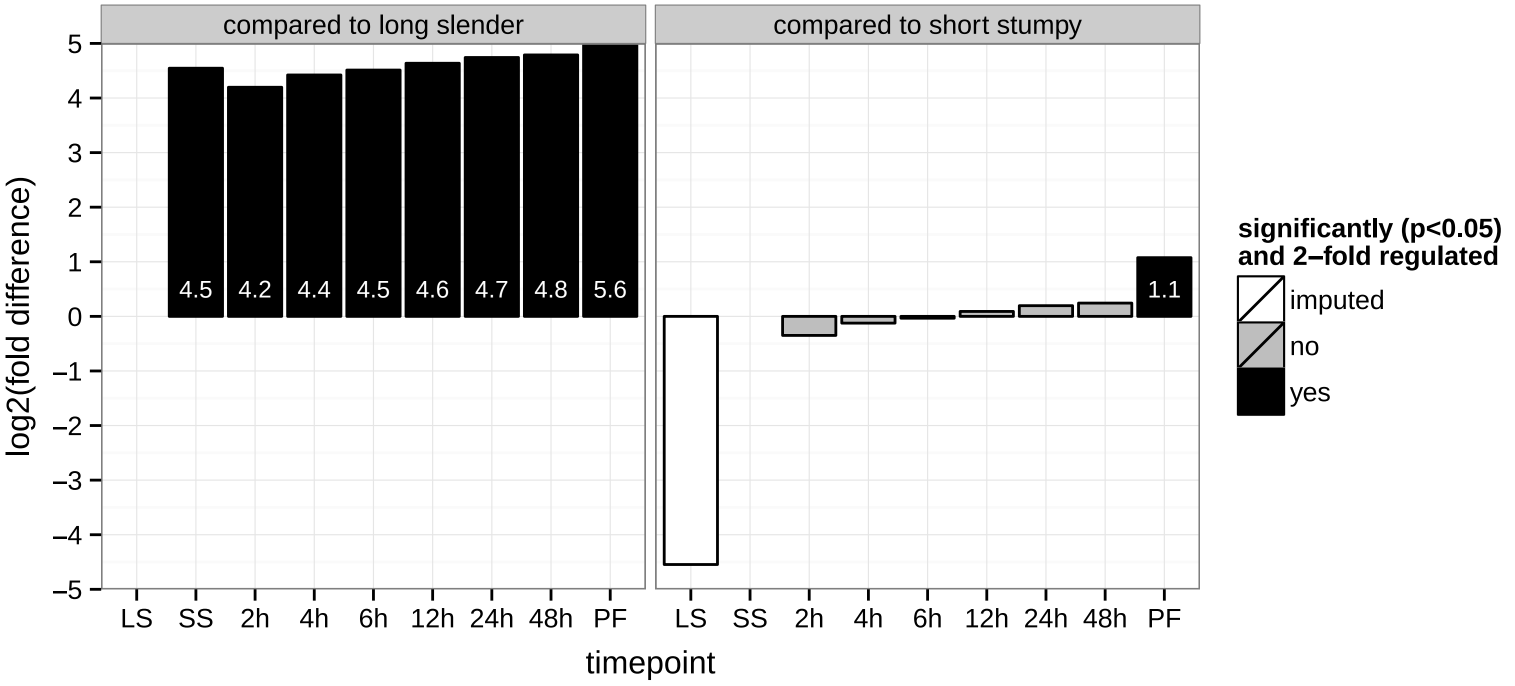

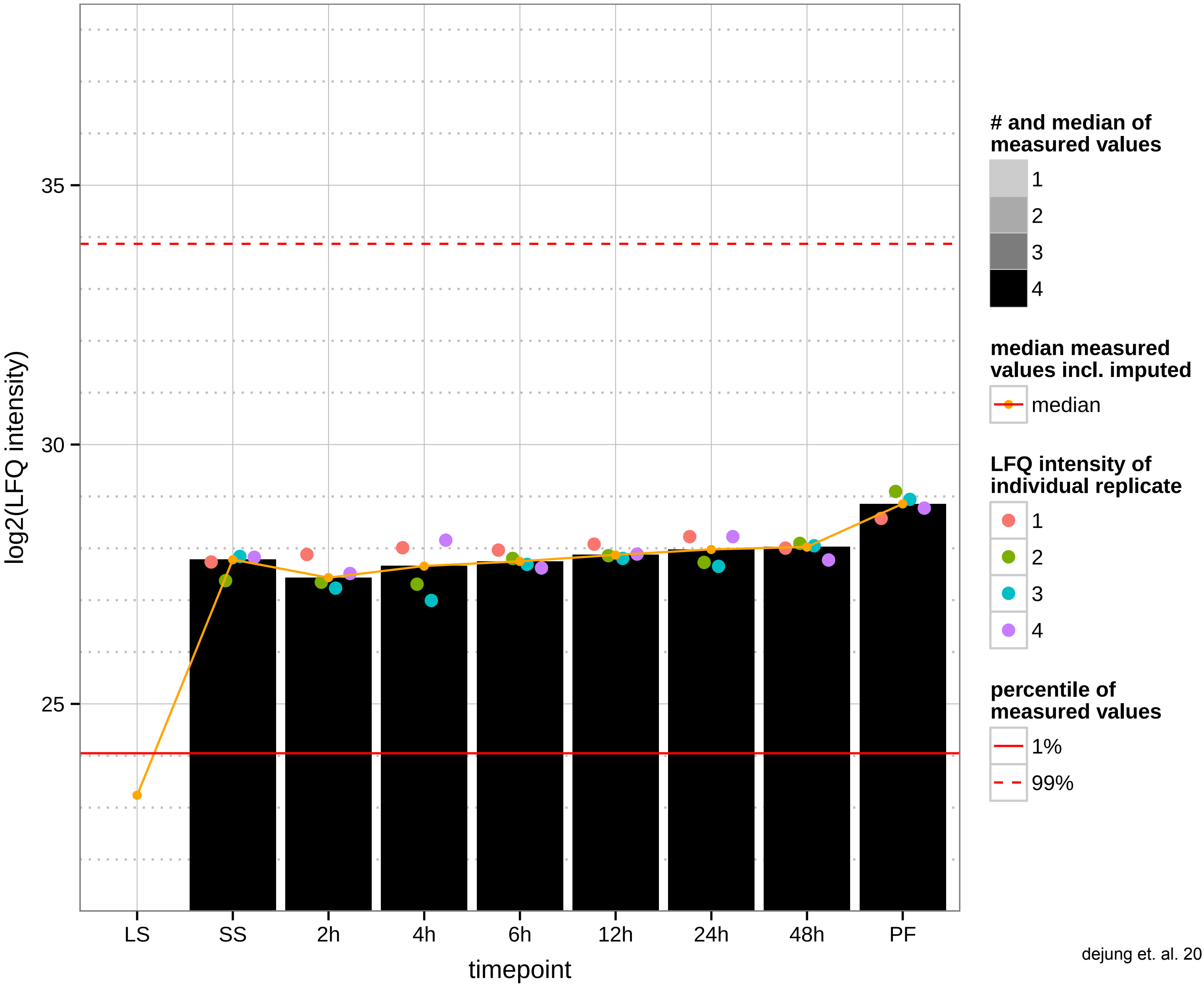

dejung et. al. 2015

membrane protein YIP1, putative
Tb927.9.2050
AGOF: null
AGOC: Golgi apparatus, integral to membrane
AGOP: vesicle–mediated transport
PGOF: null
PGOC: null
PGOP: null

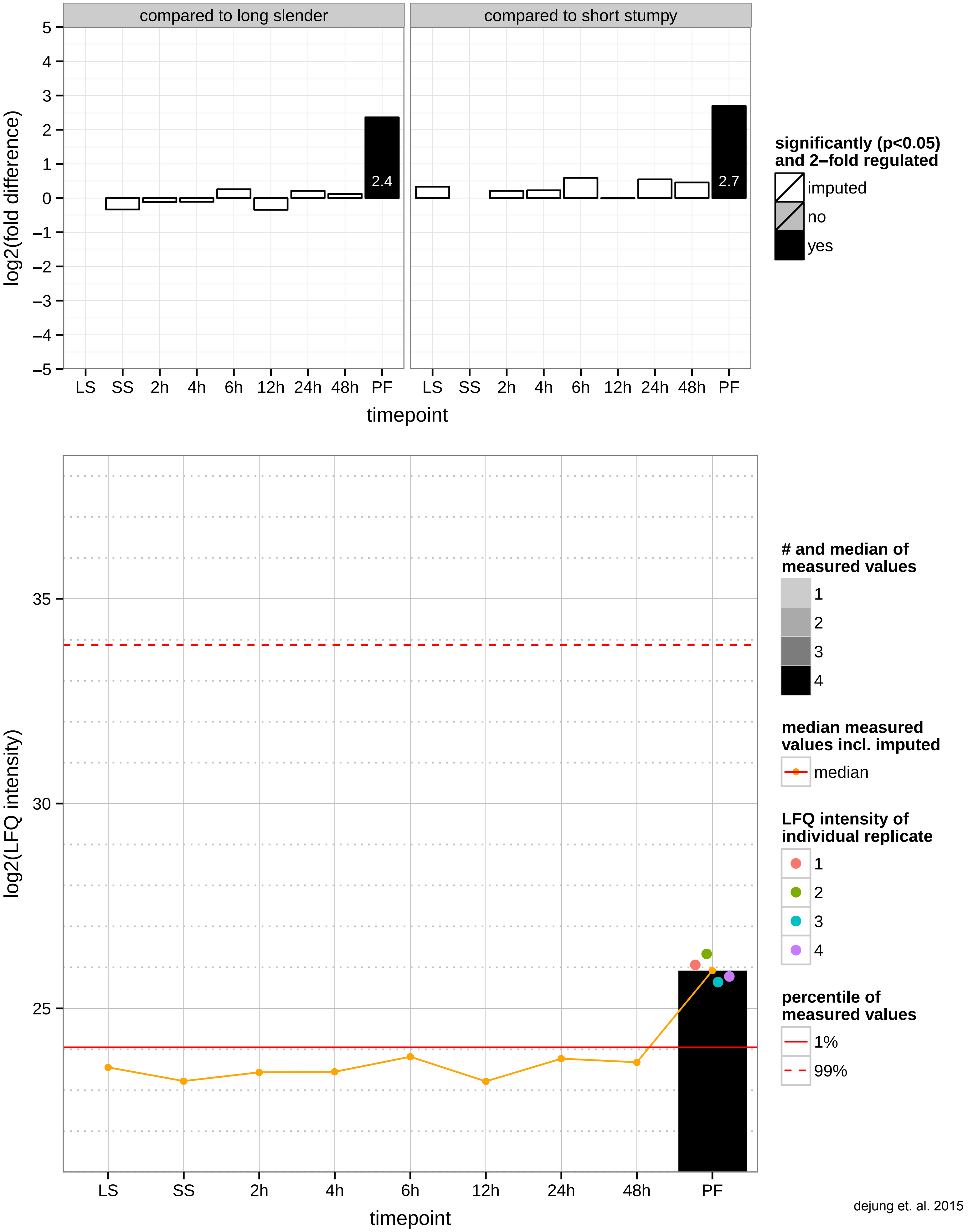

dejung et. al. 2015

hypothetical protein, conserved
Tb927.9.3580
AGOF: null
AGOC: null
AGOP: null
PGOF: null
PGOC: null
PGOP: null

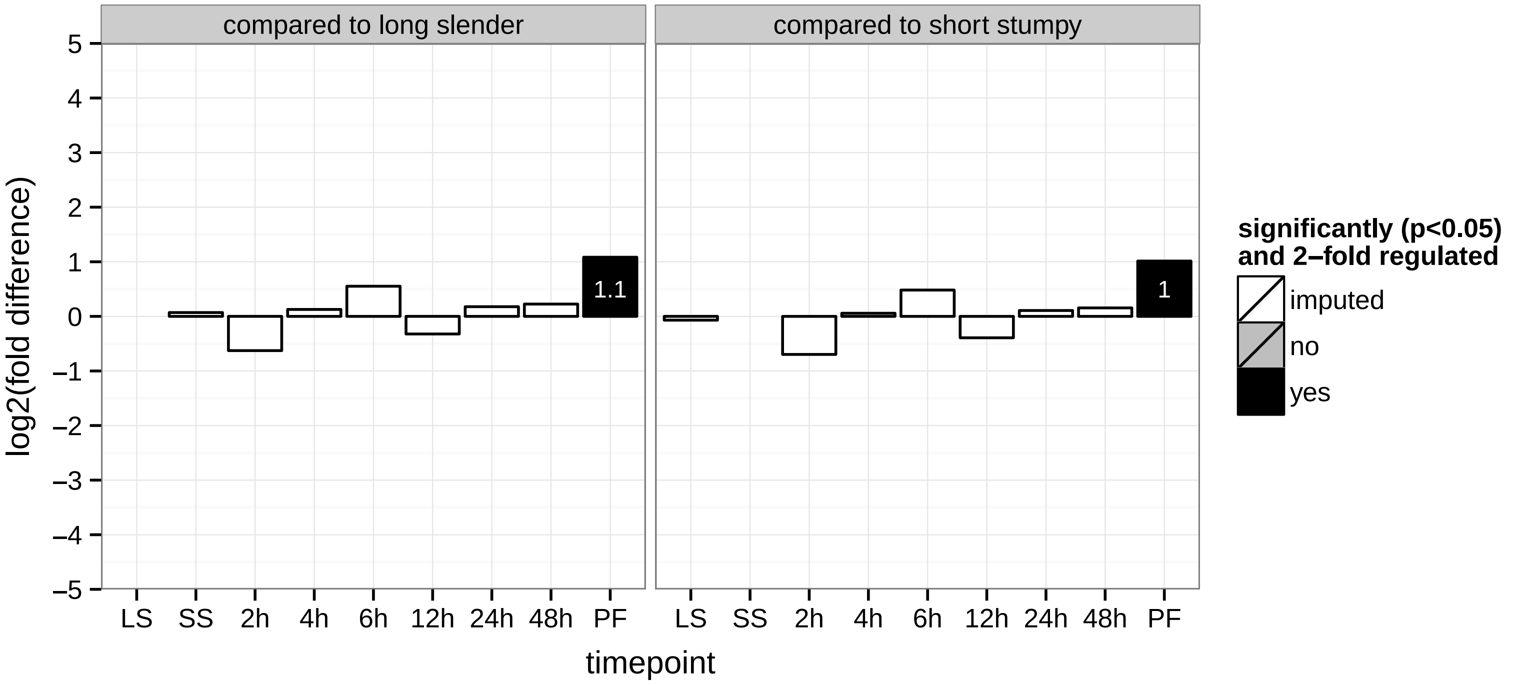

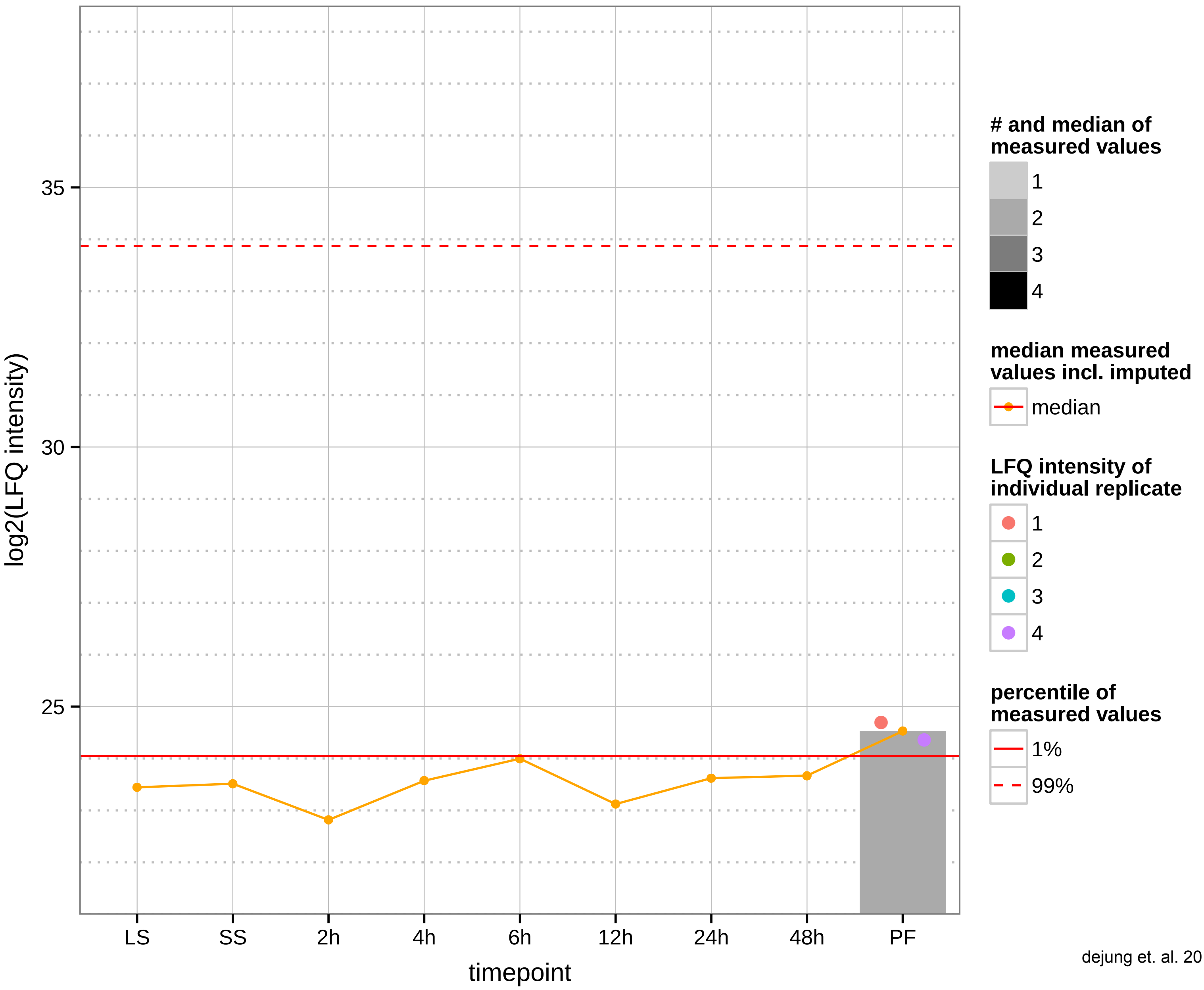

dejung et. al. 2015

hypothetical protein, conserved
Tb927.9.4290
AGOF: null
AGOC: null
AGOP: null
PGOF: null
PGOC: null
PGOP: null

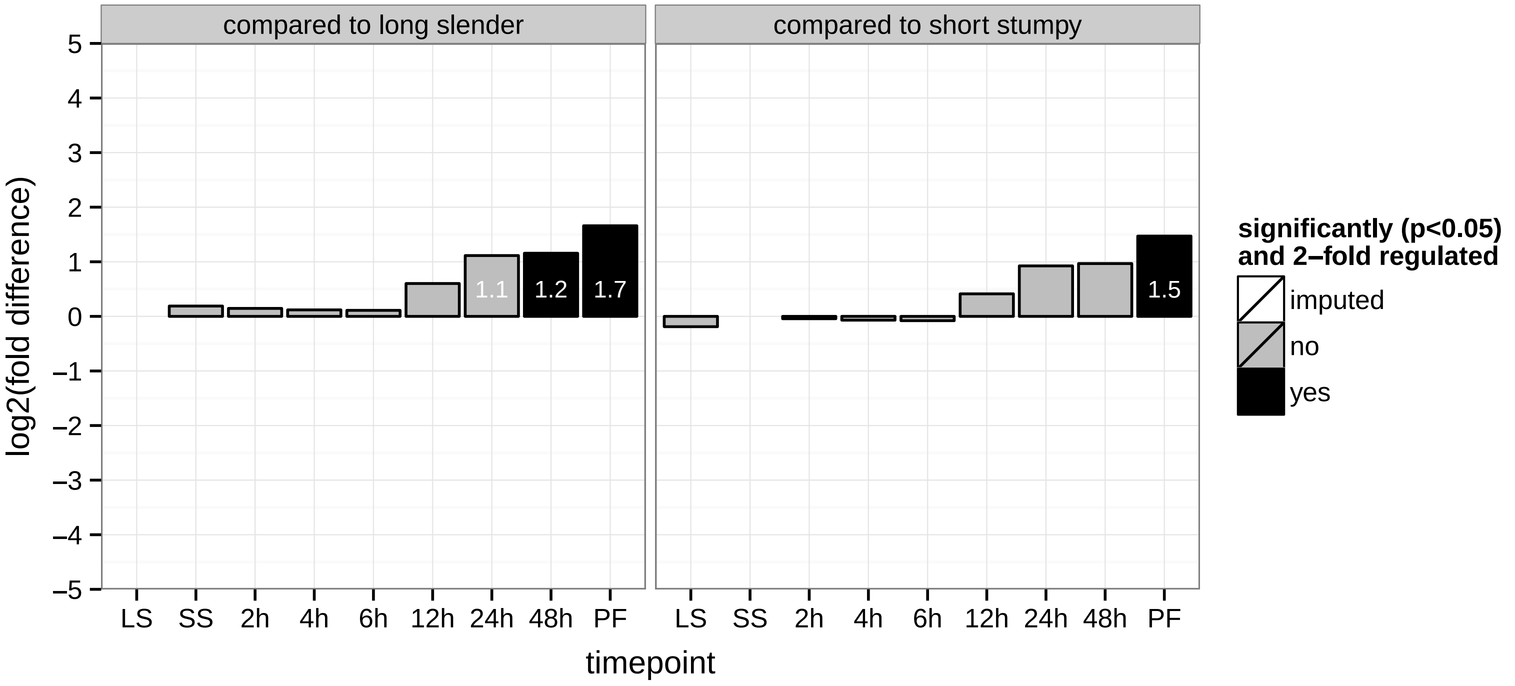

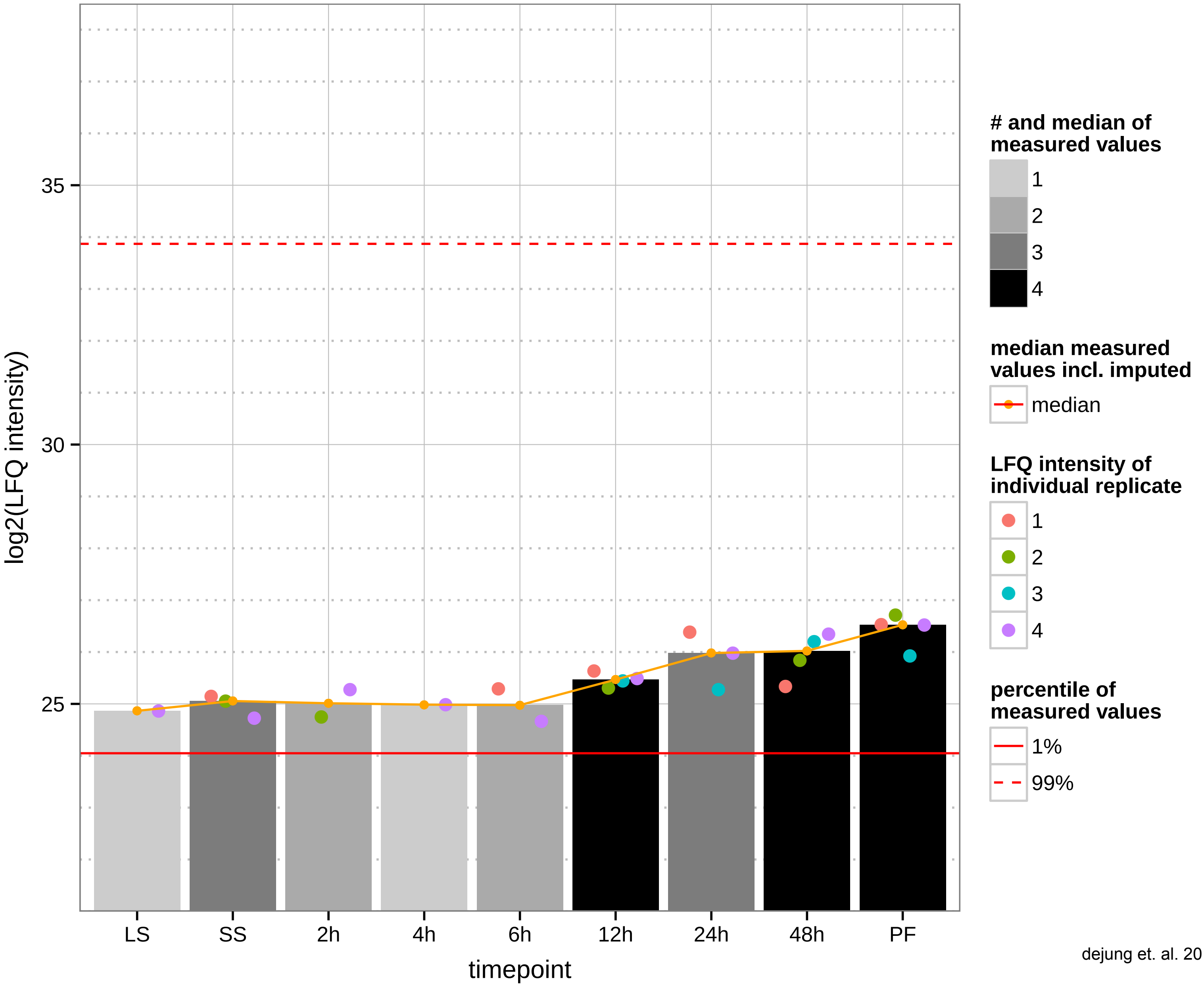

dejung et. al. 2015

KREL1, RNA editing ligase, RNA–editing complex protein, mitochondrial RNA editing ligase 1 (KREL1)
Tb927.9.4360
AGOF: ATP binding, RNA ligase (ATP) activity
AGOC: mitochondrial mRNA editing complex, mitochondrion
AGOP: RNA modification
PGOF: ATP binding, RNA ligase (ATP) activity, ligase activity
PGOC: null
PGOP: null

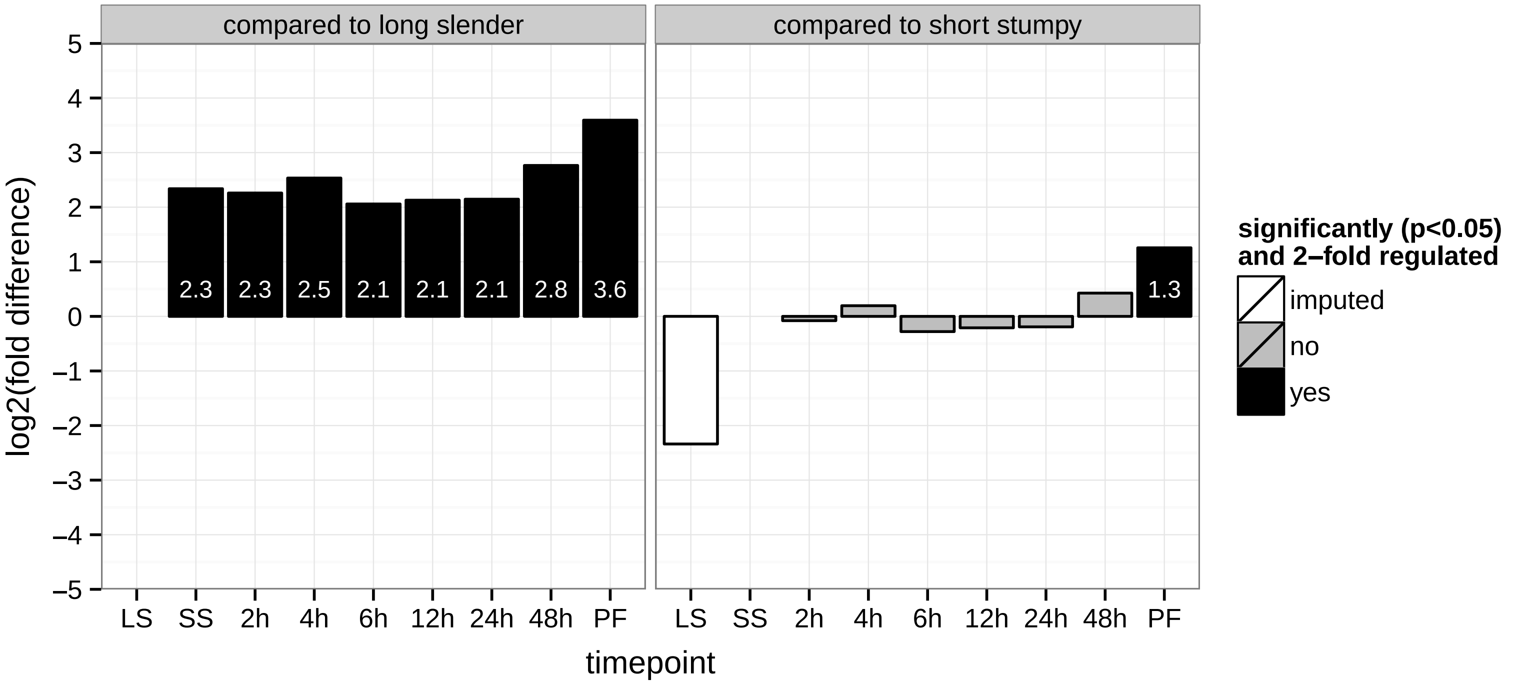

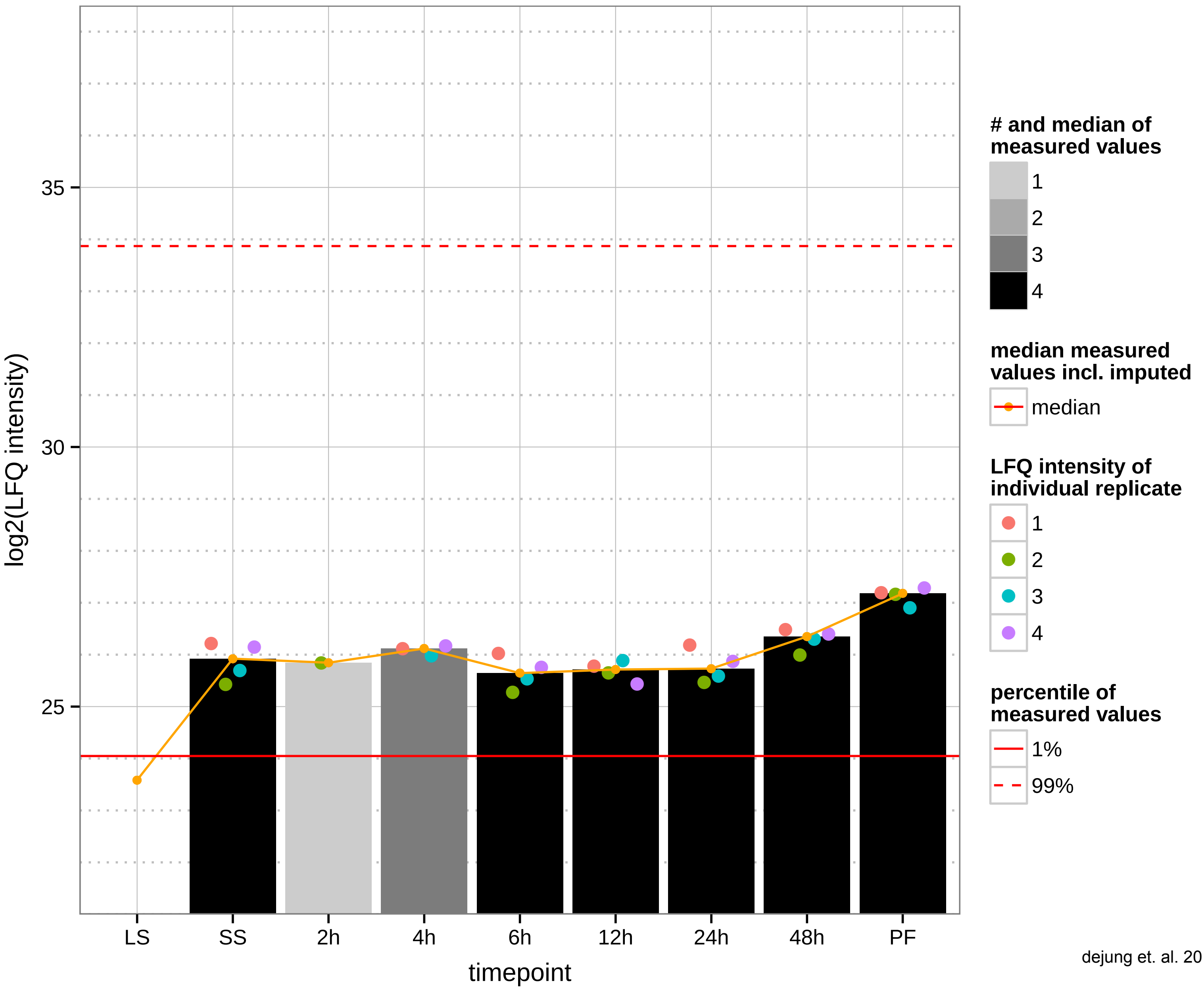

dejung et. al. 2015

leucine−rich repeat protein (LRRP), putative
Tb927.9.4850
AGOF: null
AGOC: null
AGOP: null
PGOF: null
PGOC: null
PGOP: null

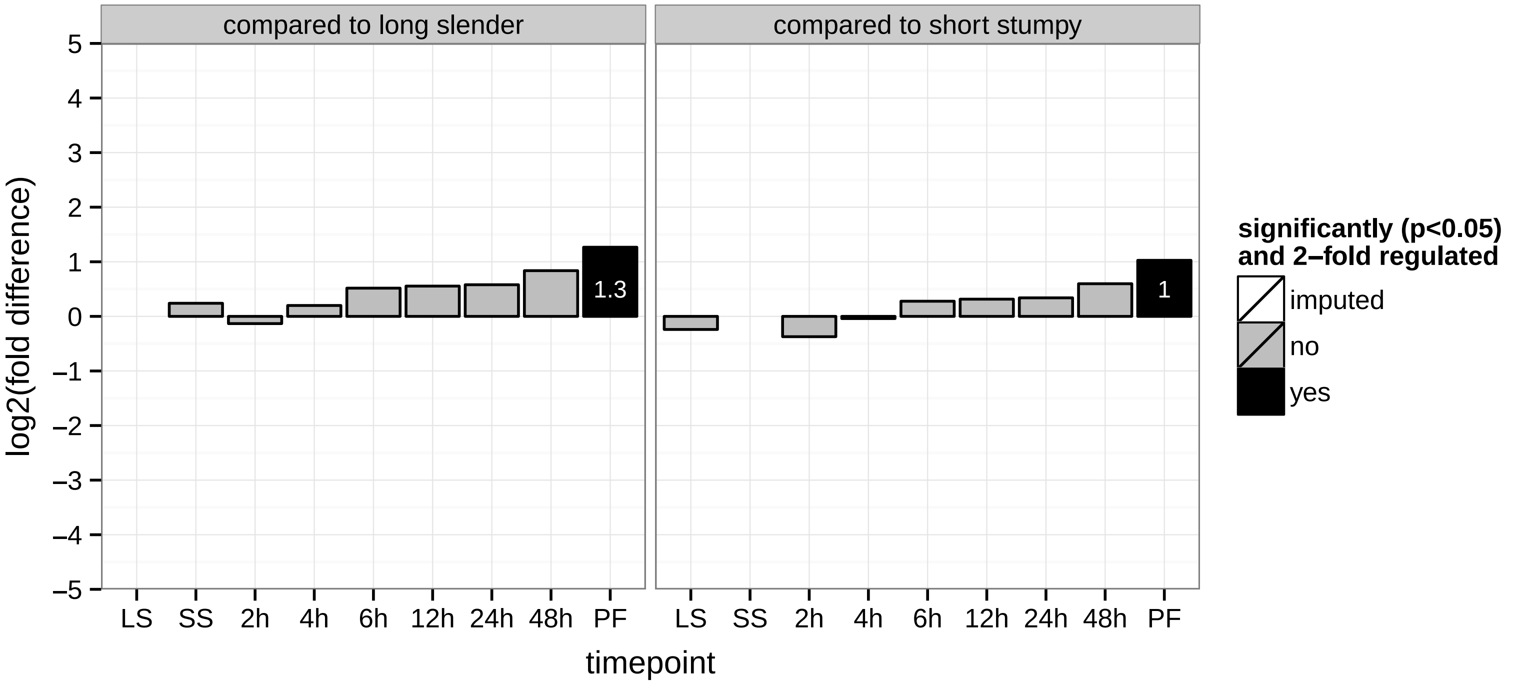

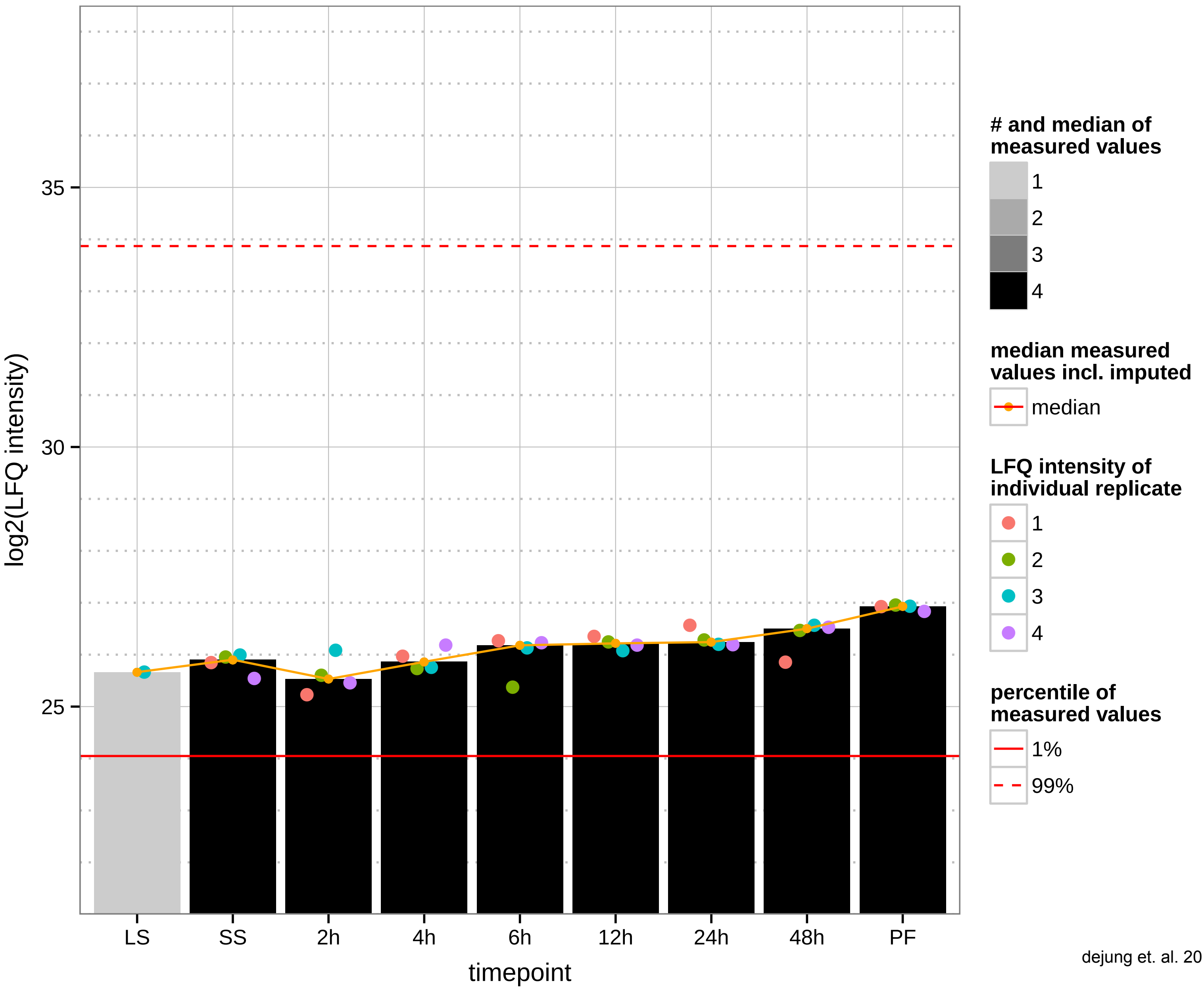

dejung et. al. 2015

protein kinase, putative
Tb927.9.4910
AGOF: ATP binding, protein serine/threonine kinase activity, protein tyrosine kinase activity, zinc ion binding
AGOC: null
AGOP: growth, protein phosphorylation
PGOF: ATP binding, metal ion binding, protein kinase activity, transferase activity, transferring phosphorus–containing groups
PGOC: null
PGOP: protein phosphorylation

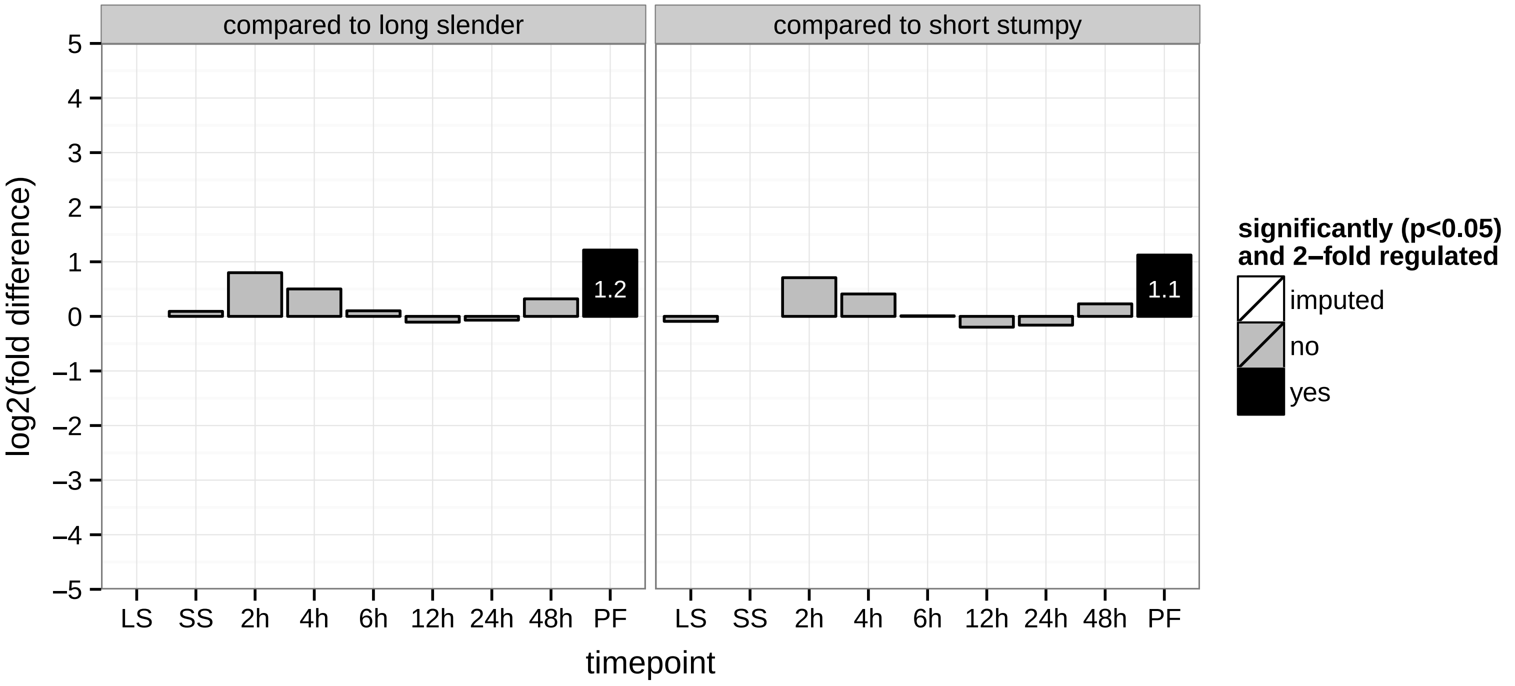

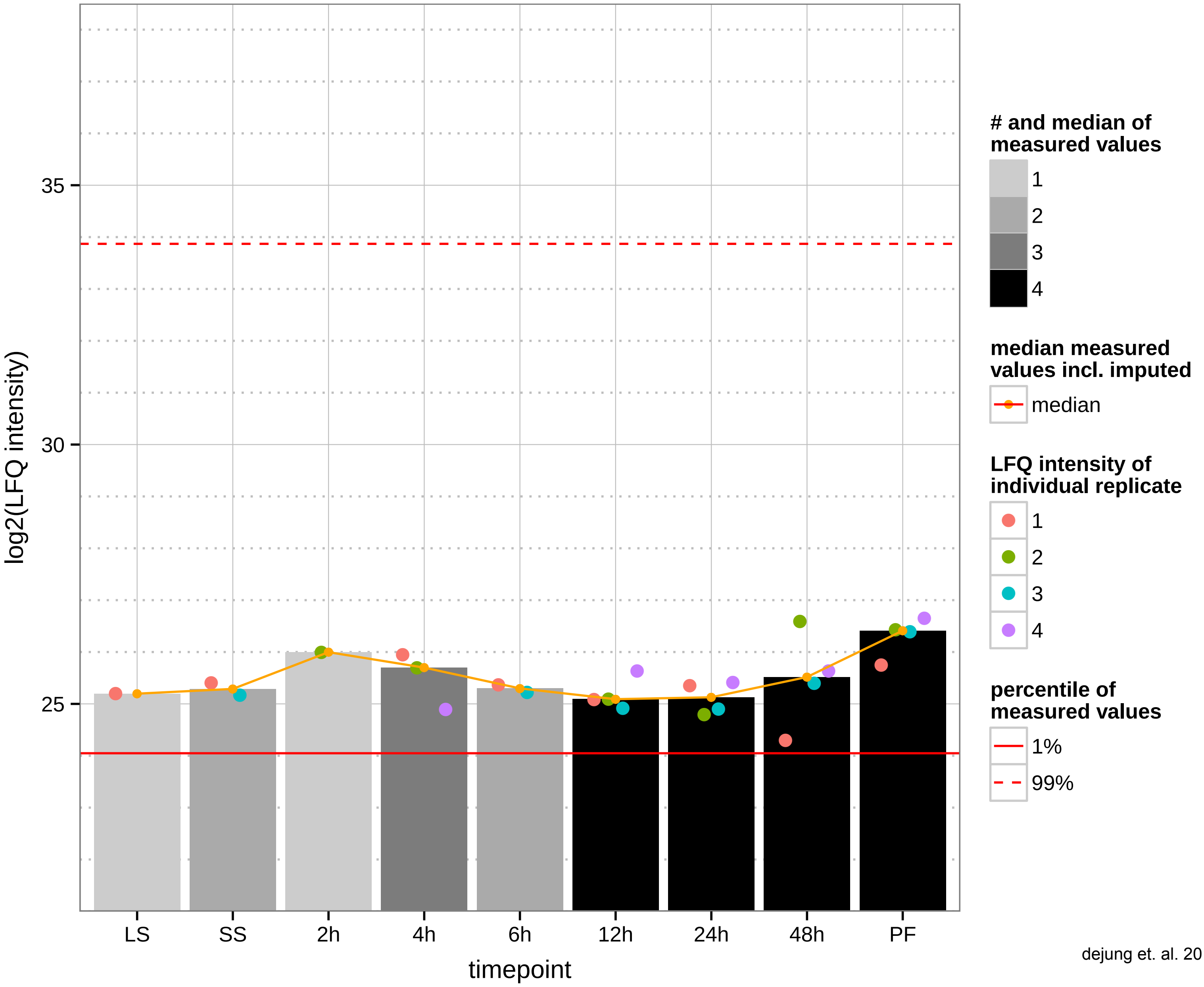

dejung et. al. 2015

unspecified product
Tb927.9.5280
AGOF: null
AGOC: null
AGOP: null
PGOF: null
PGOC: null
PGOP: null

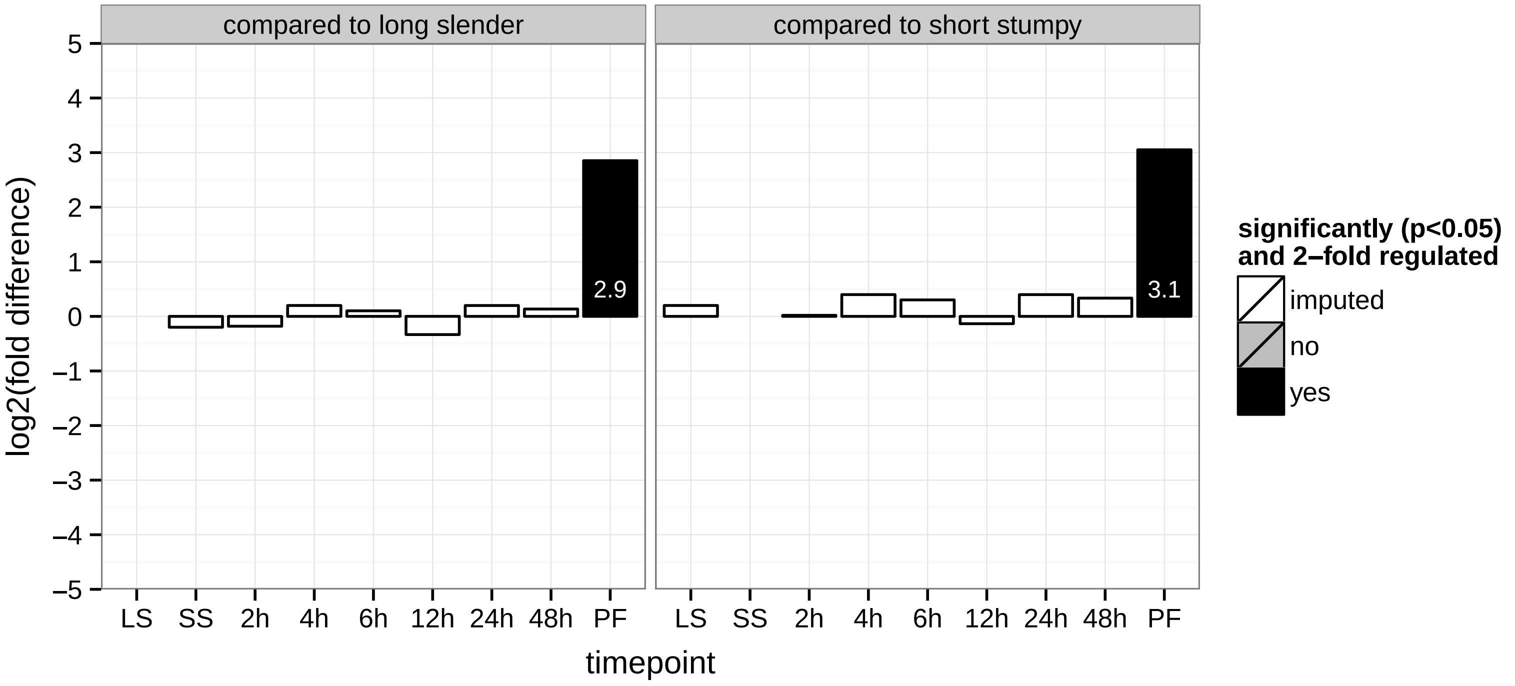

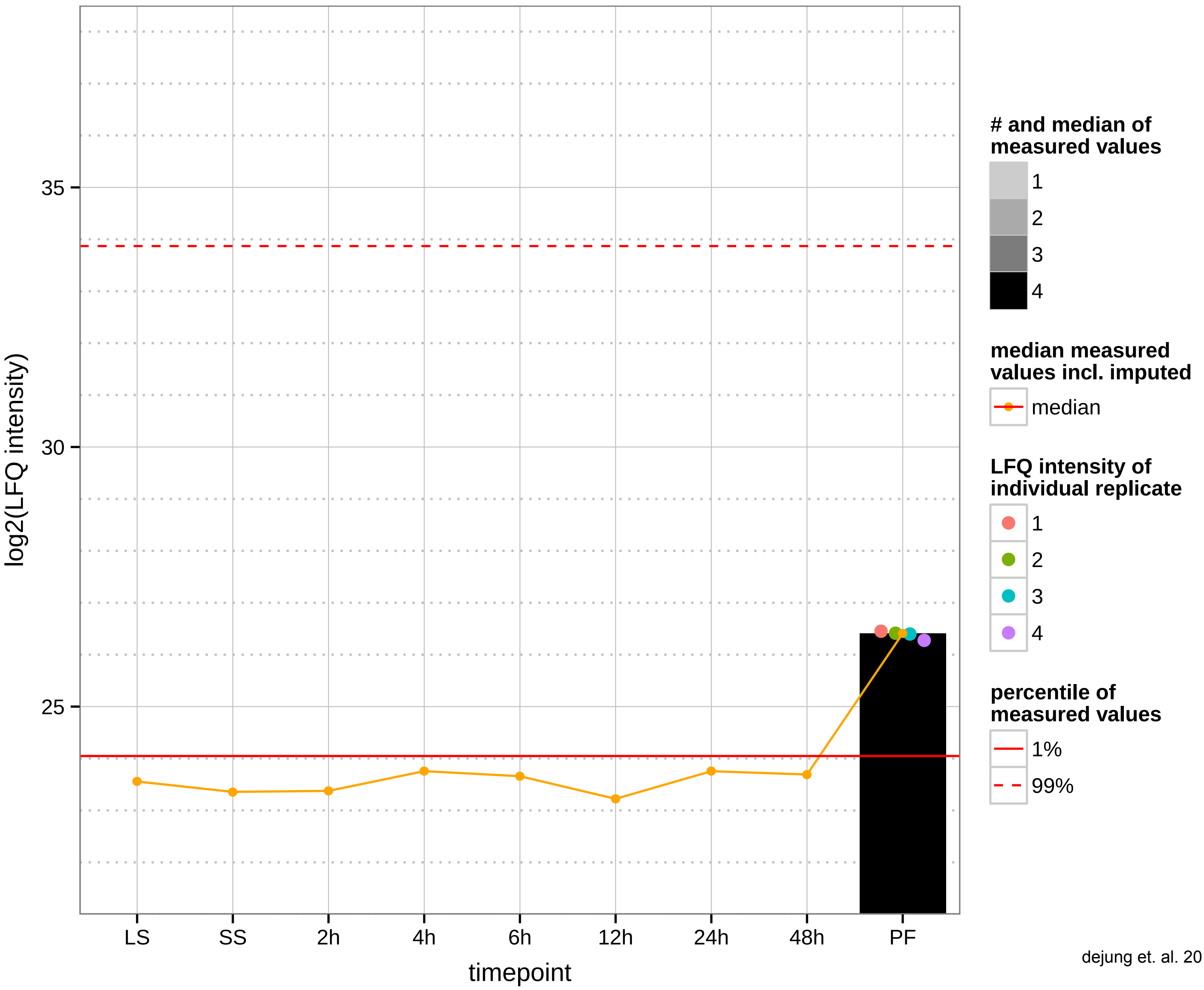

dejung et. al. 2015

hypothetical protein, conserved
Tb927.9.5380
AGOF: Rab GTPase activator activity
AGOC: intracellular
AGOP: regulation of Rab GTPase activity
PGOF: Rab GTPase activator activity
PGOC: intracellular
PGOP: regulation of Rab GTPase activity

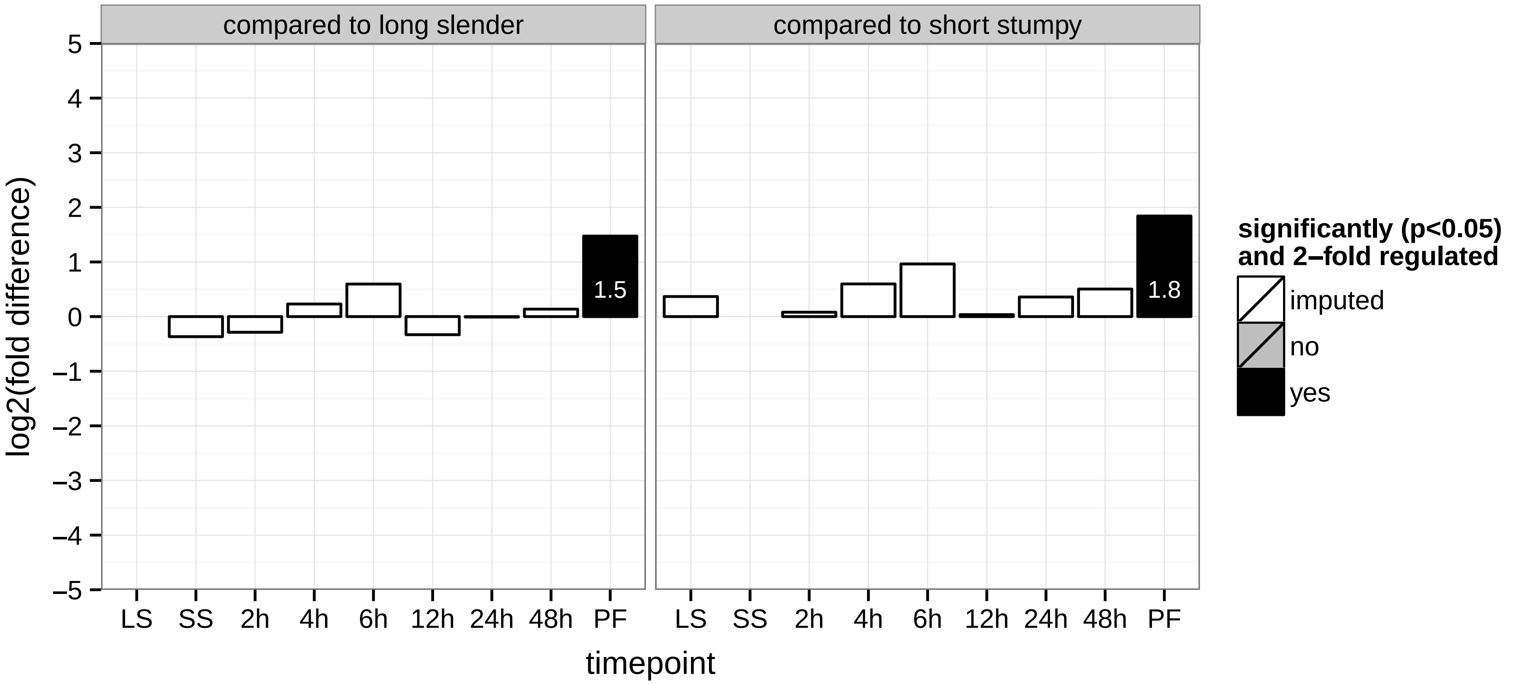

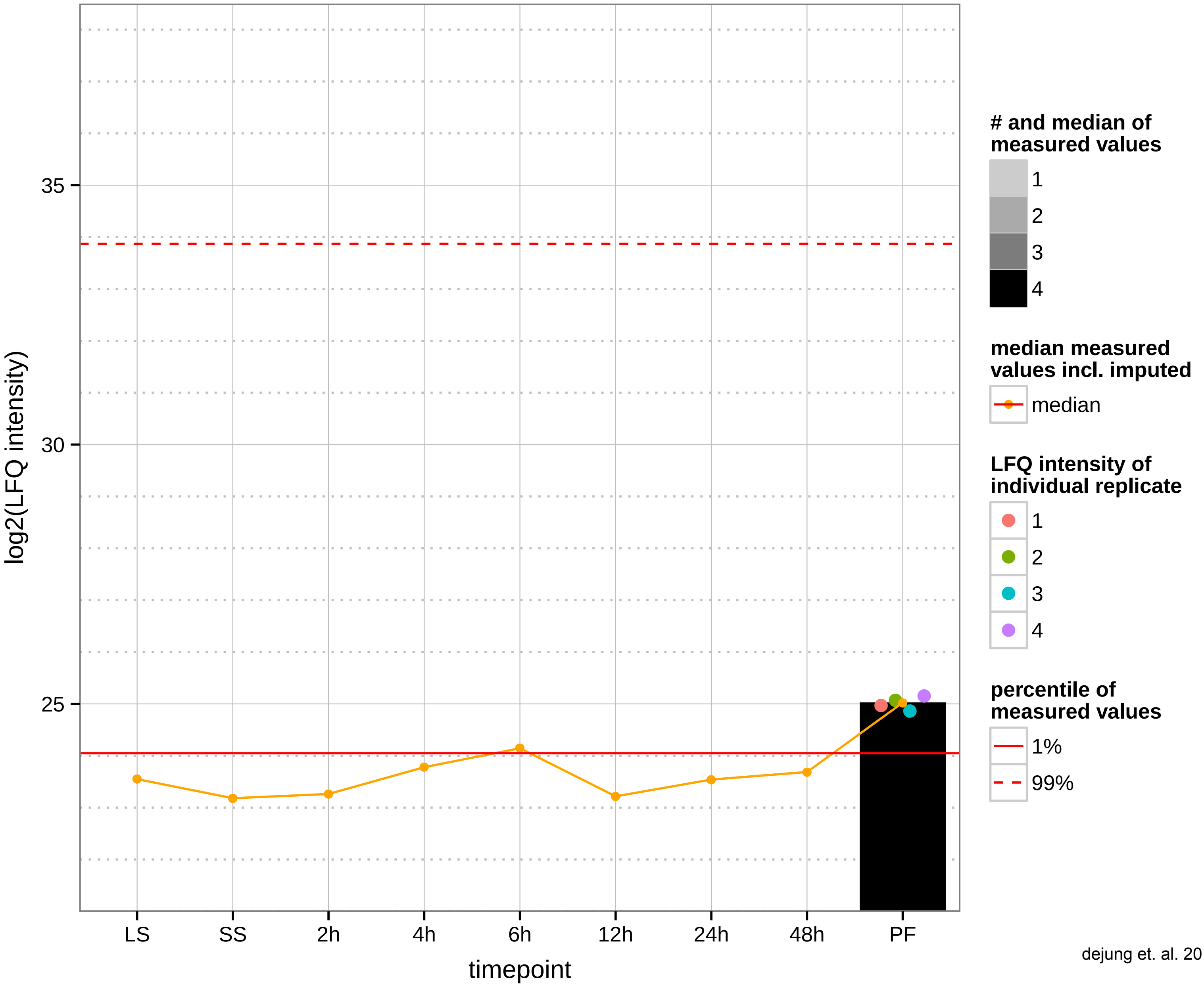

dejung et. al. 2015

E2–like ubiquitin–conjugation enzyme, putative (UFC1)
Tb927.9.5650
AGOF: small conjugating protein binding
AGOC: mitochondrion
AGOP: null
PGOF: null
PGOC: null
PGOP: null

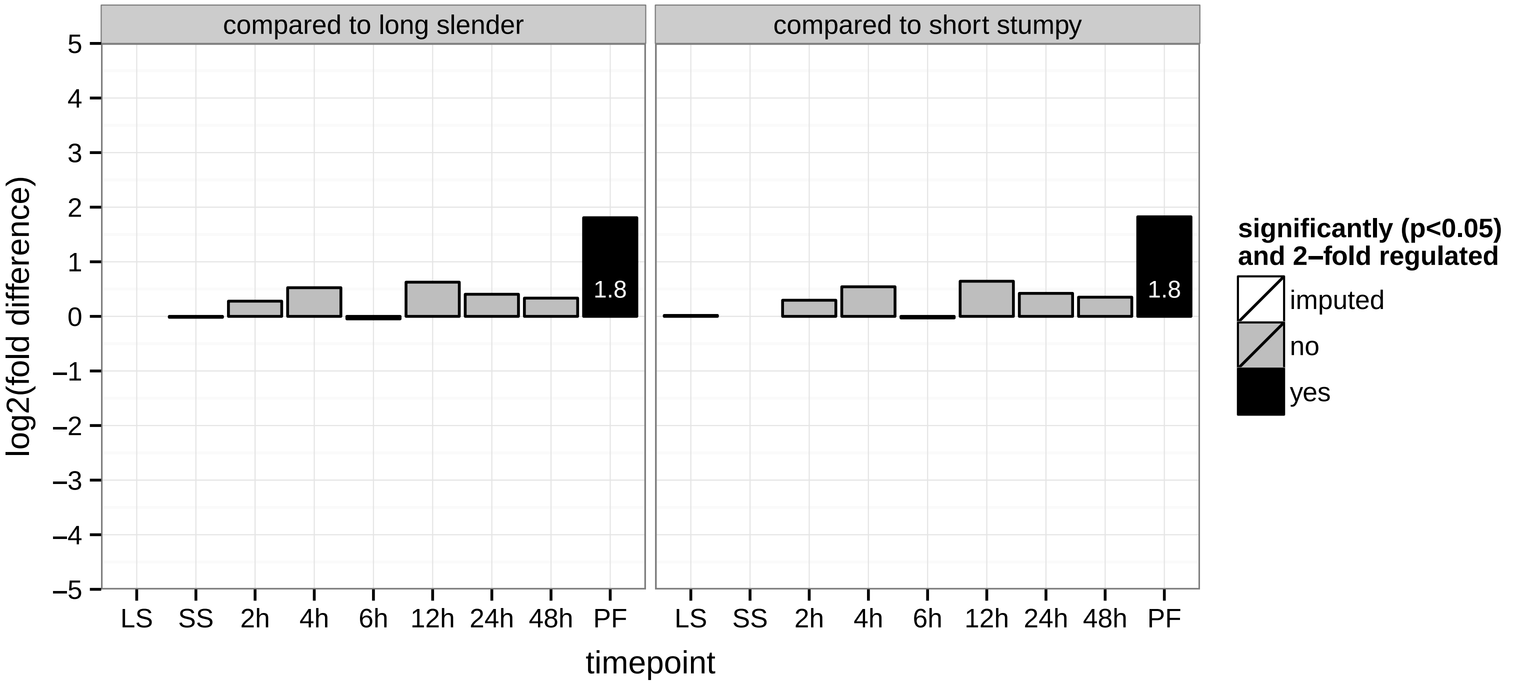

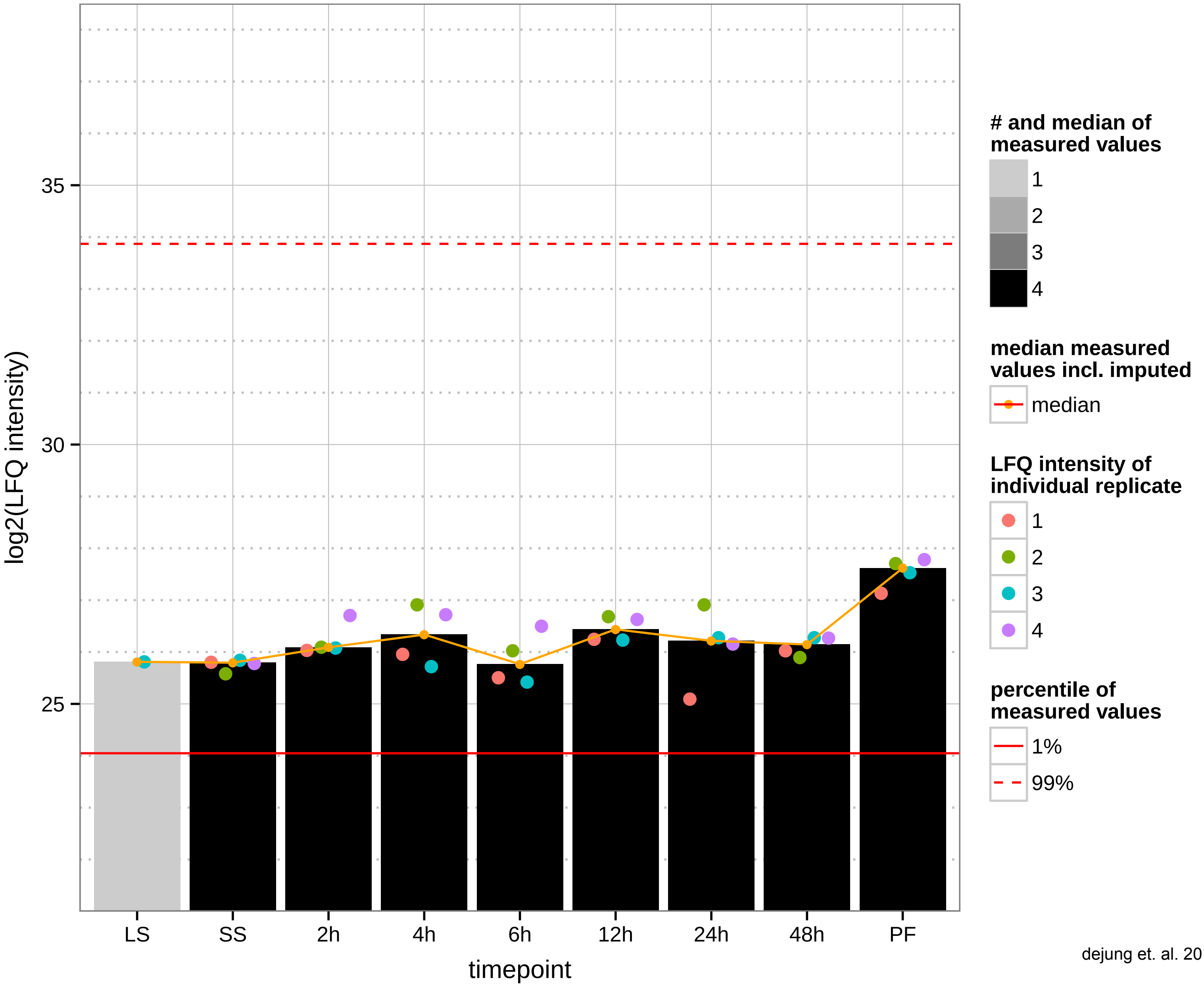

dejung et. al. 2015

hypothetical protein, conserved
Tb927.9.6270
AGOF: null
AGOC: null
AGOP: null
PGOF: null
PGOC: null
PGOP: null

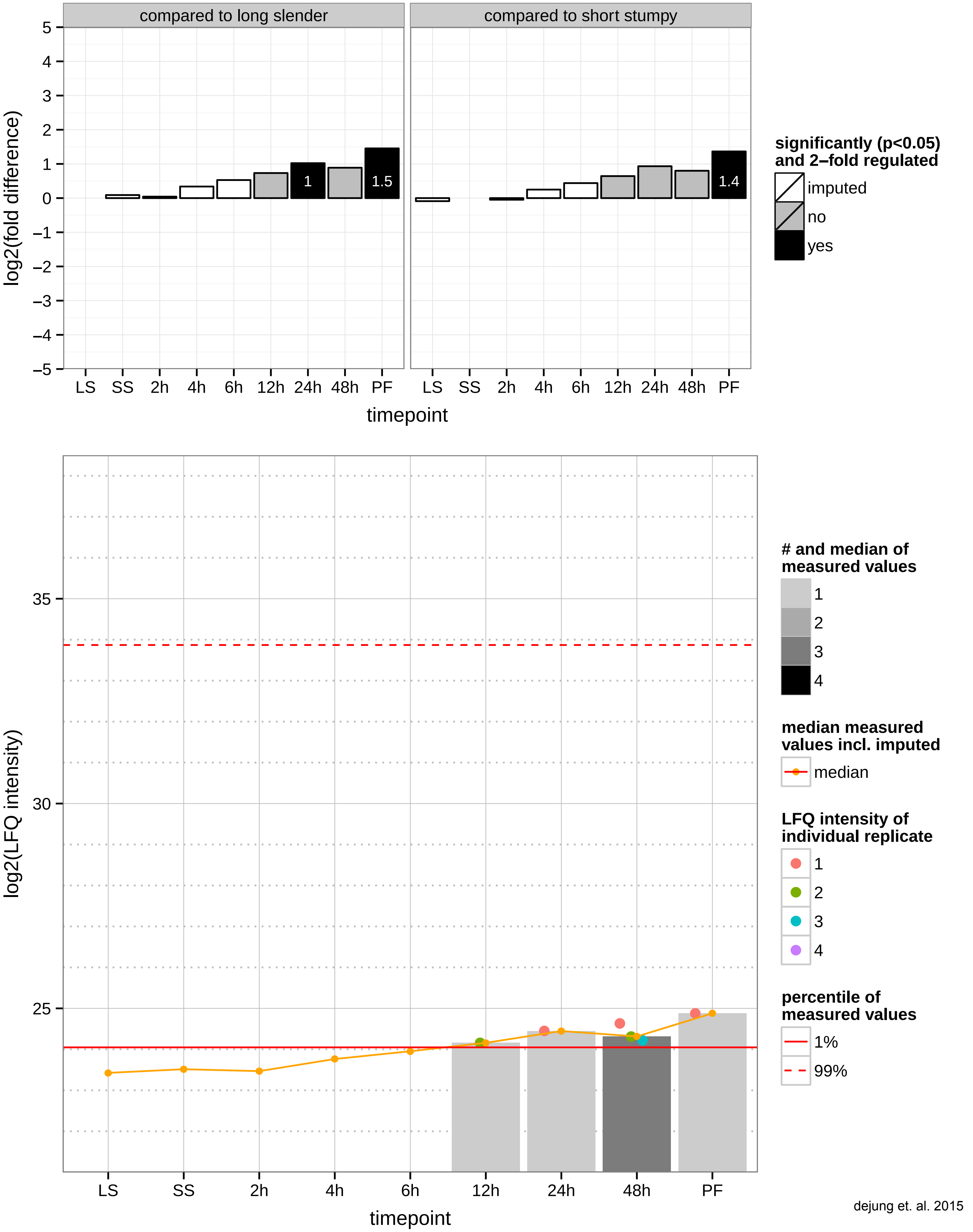

dejung et. al. 2015

RNA–binding protein, putative (RBSR1)
Tb927.9.6870
AGOF: RNA binding
AGOC: null
AGOP: RNA processing
PGOF: nucleic acid binding
PGOC: null
PGOP: null

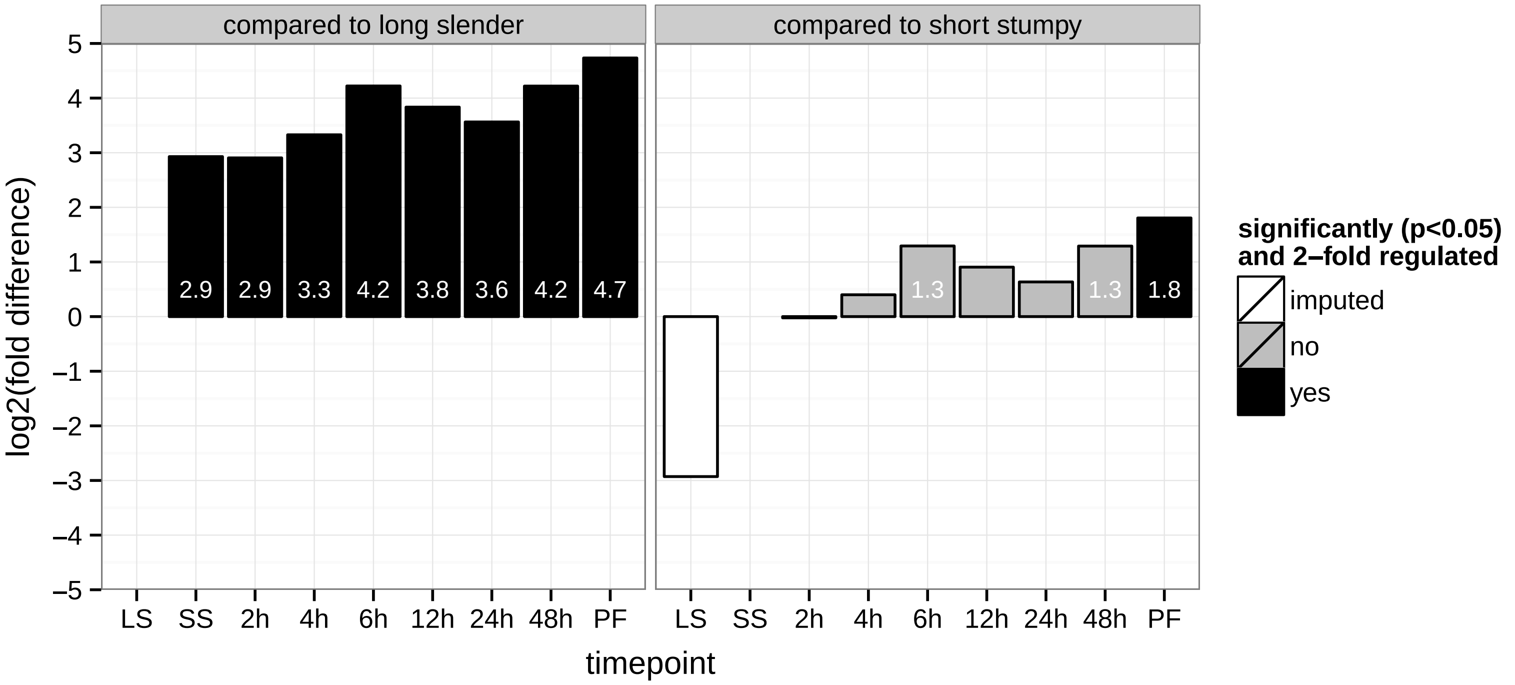

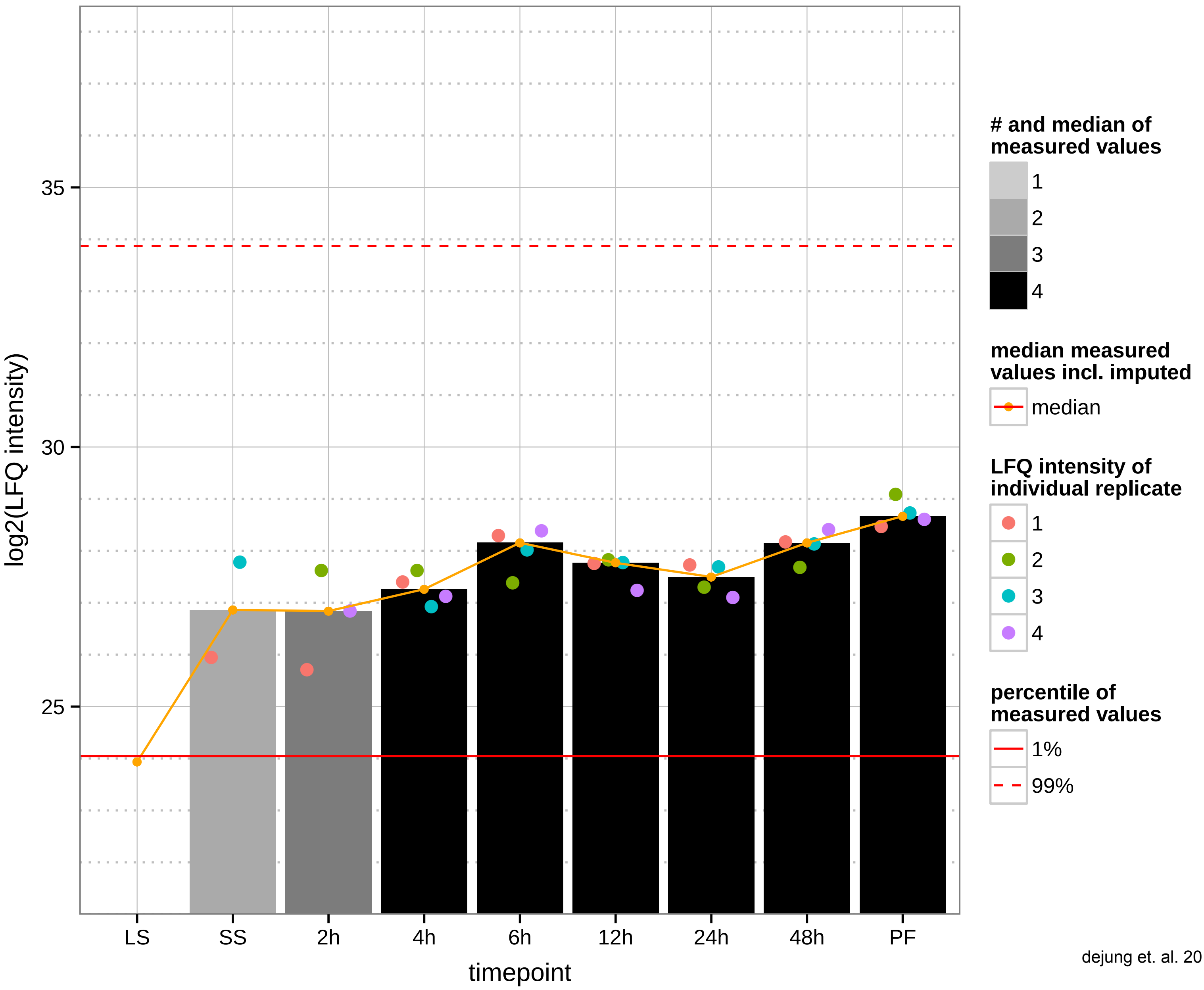

dejung et. al. 2015

hypothetical protein, conserved
Tb927.9.7200
AGOF: null
AGOC: mitochondrion
AGOP: null
PGOF: null
PGOC: null
PGOP: null

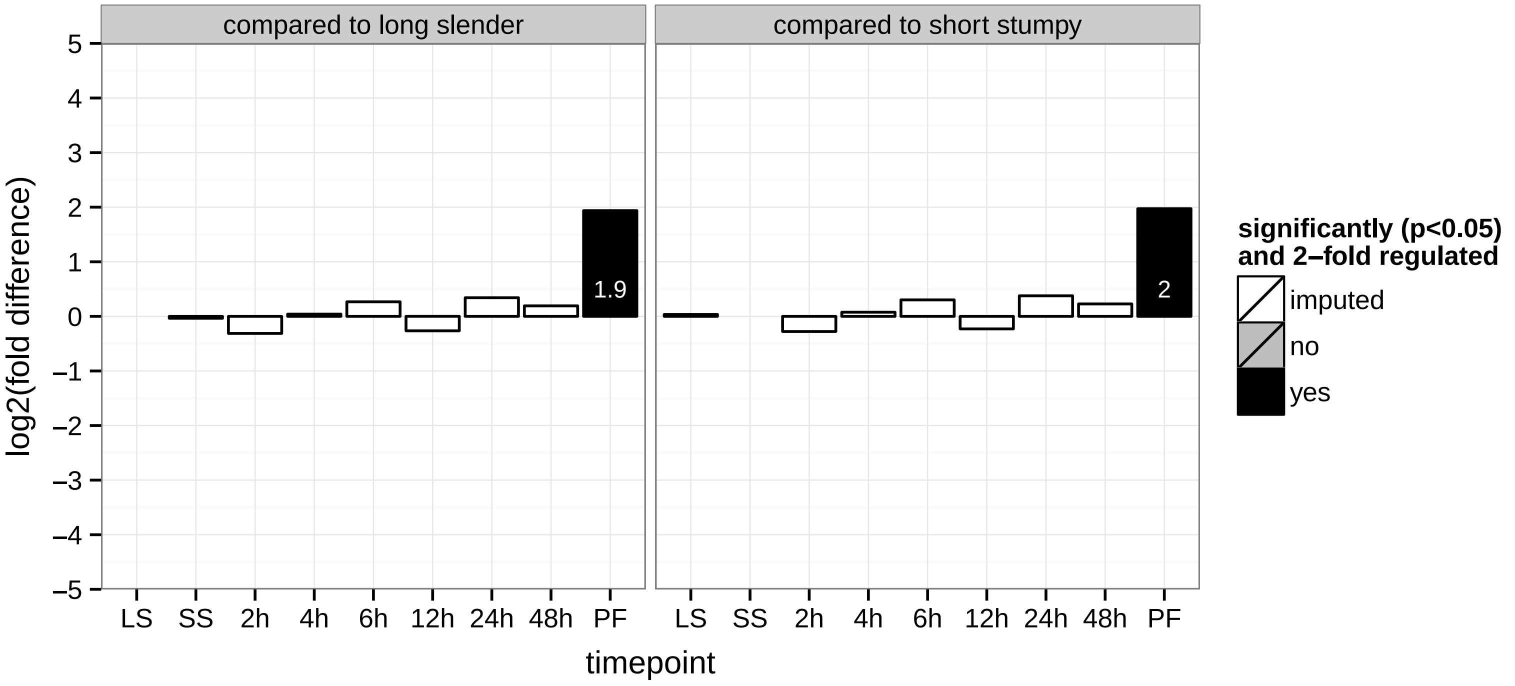

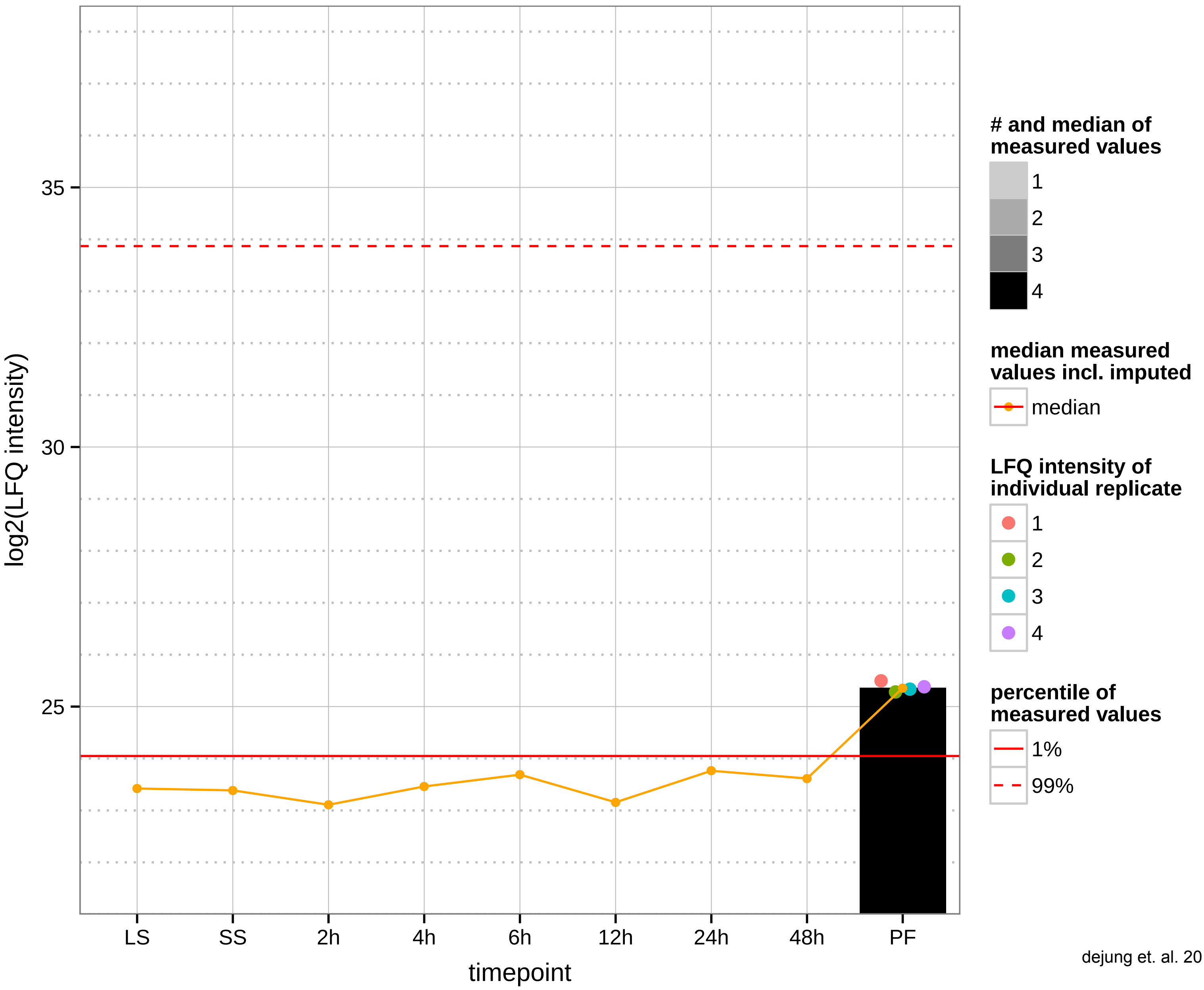

dejung et. al. 2015

hypothetical protein, conserved
Tb927.9.7250
AGOF: null
AGOC: null
AGOP: null
PGOF: null
PGOC: null
PGOP: null

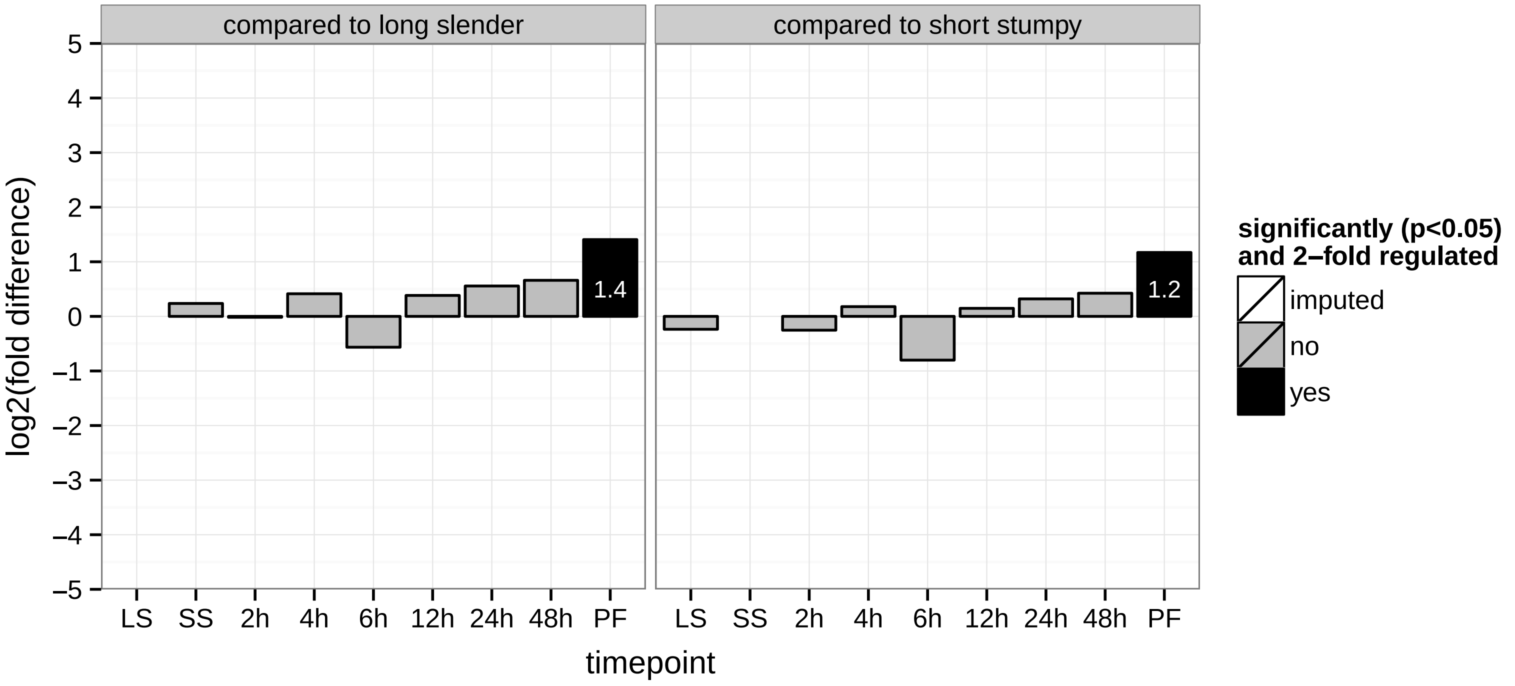

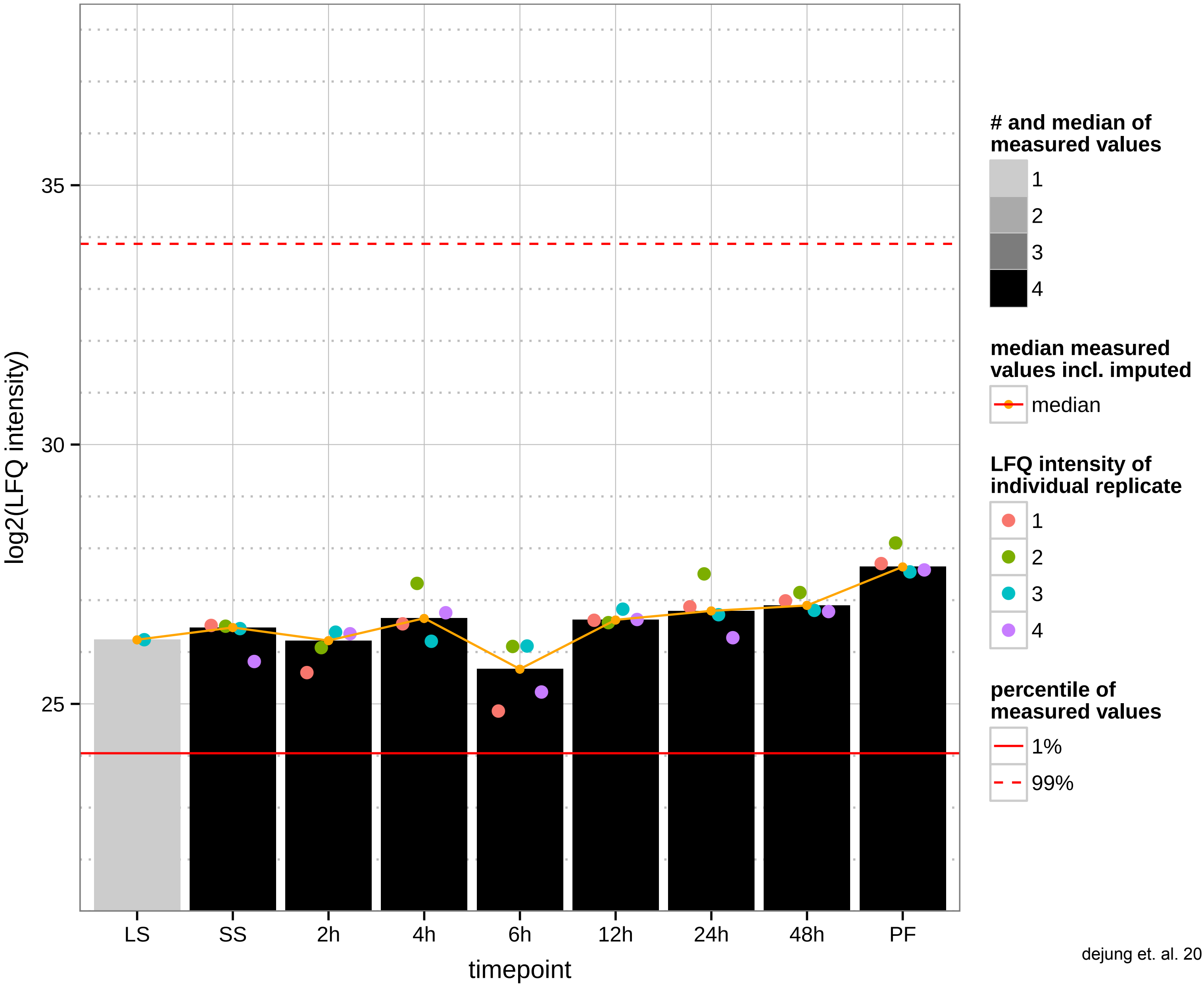

dejung et. al. 2015

hypothetical protein, conserved
Tb927.9.7260
AGOF: null
AGOC: mitochondrion
AGOP: null
PGOF: null
PGOC: null
PGOP: null

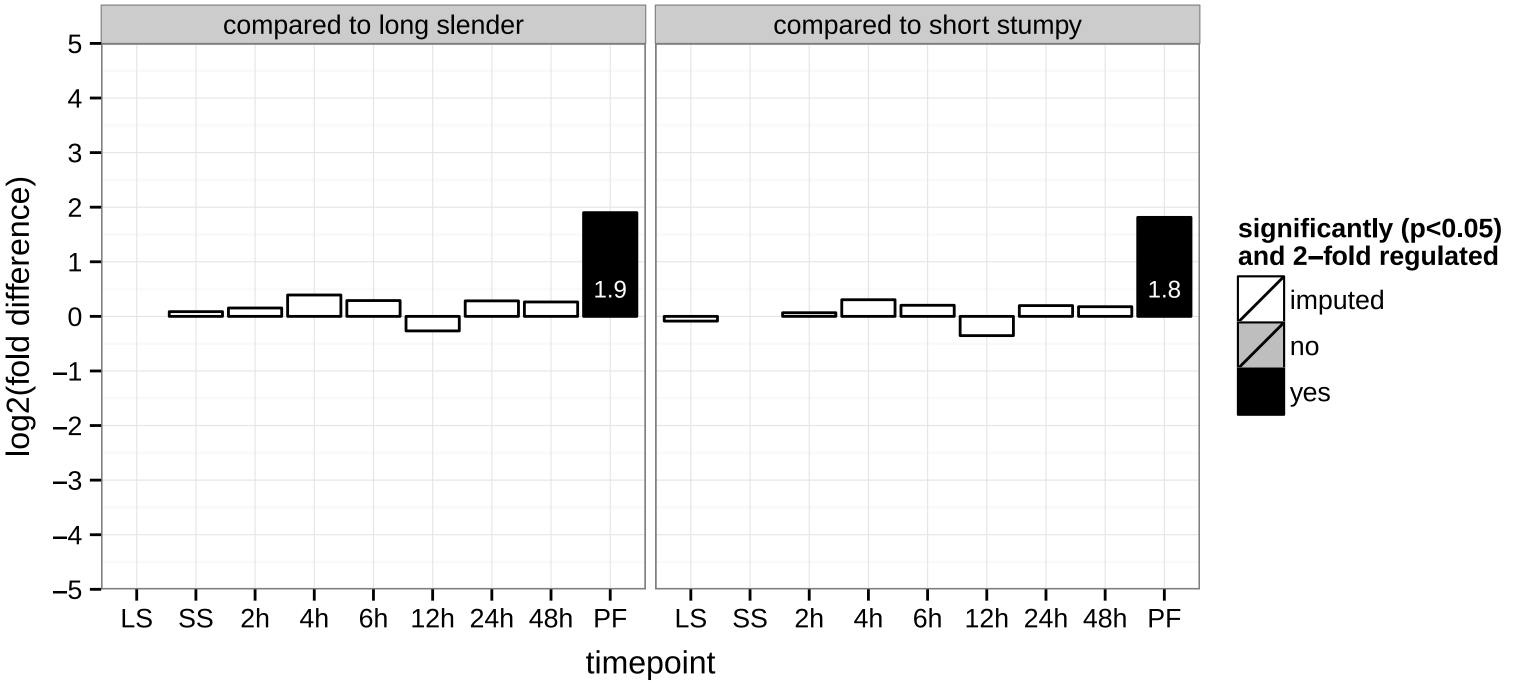

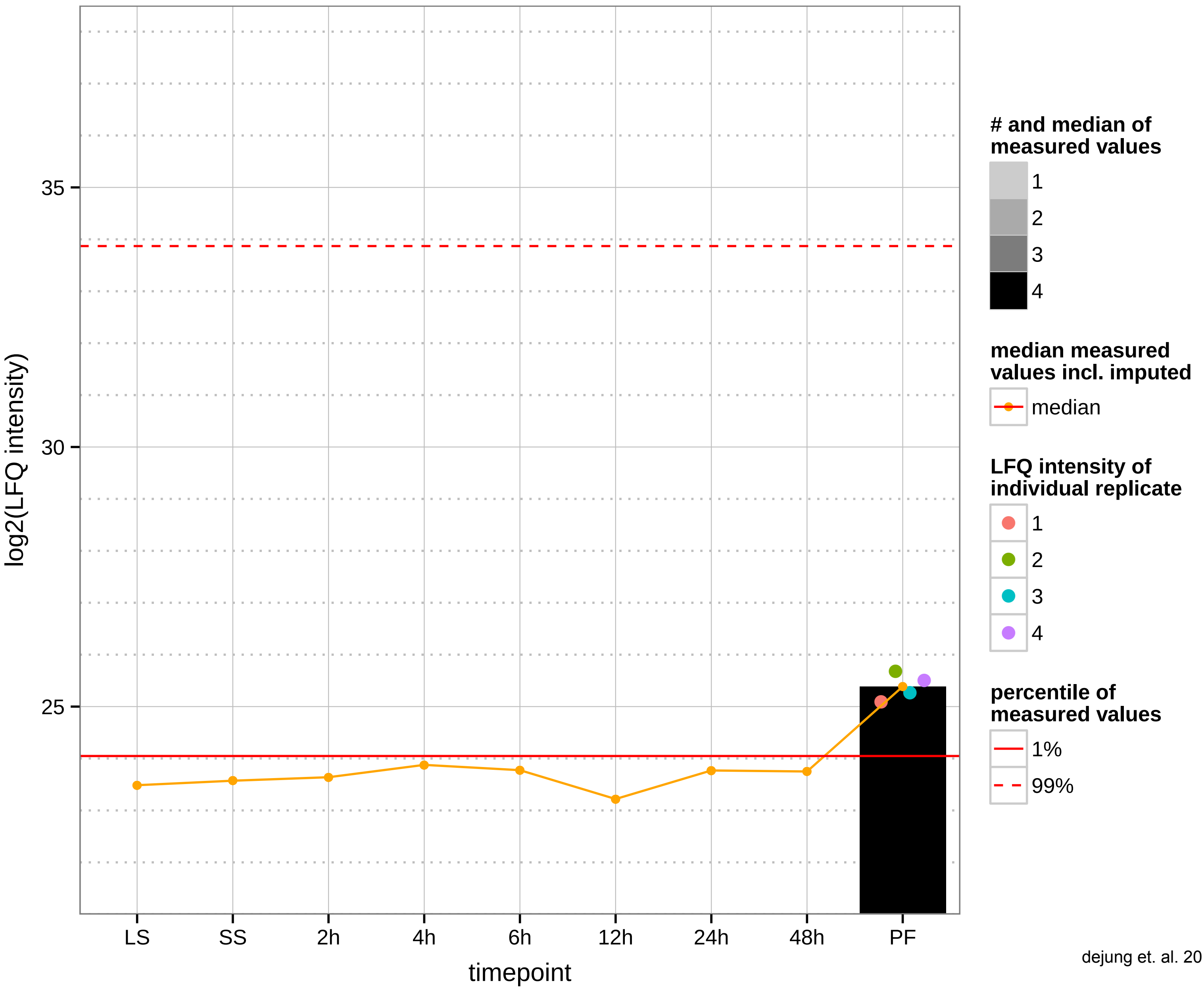

dejung et. al. 2015

60S ribosomal protein L11, putative
Tb927.9.7620;Tb927.9.7590
AGOF: structural constituent of ribosome
AGOC: intracellular, organellar large ribosomal subunit, ribosome
AGOP: translation
PGOF: null, structural constituent of ribosome
PGOC: null, intracellular, ribosome
PGOP: null, translation

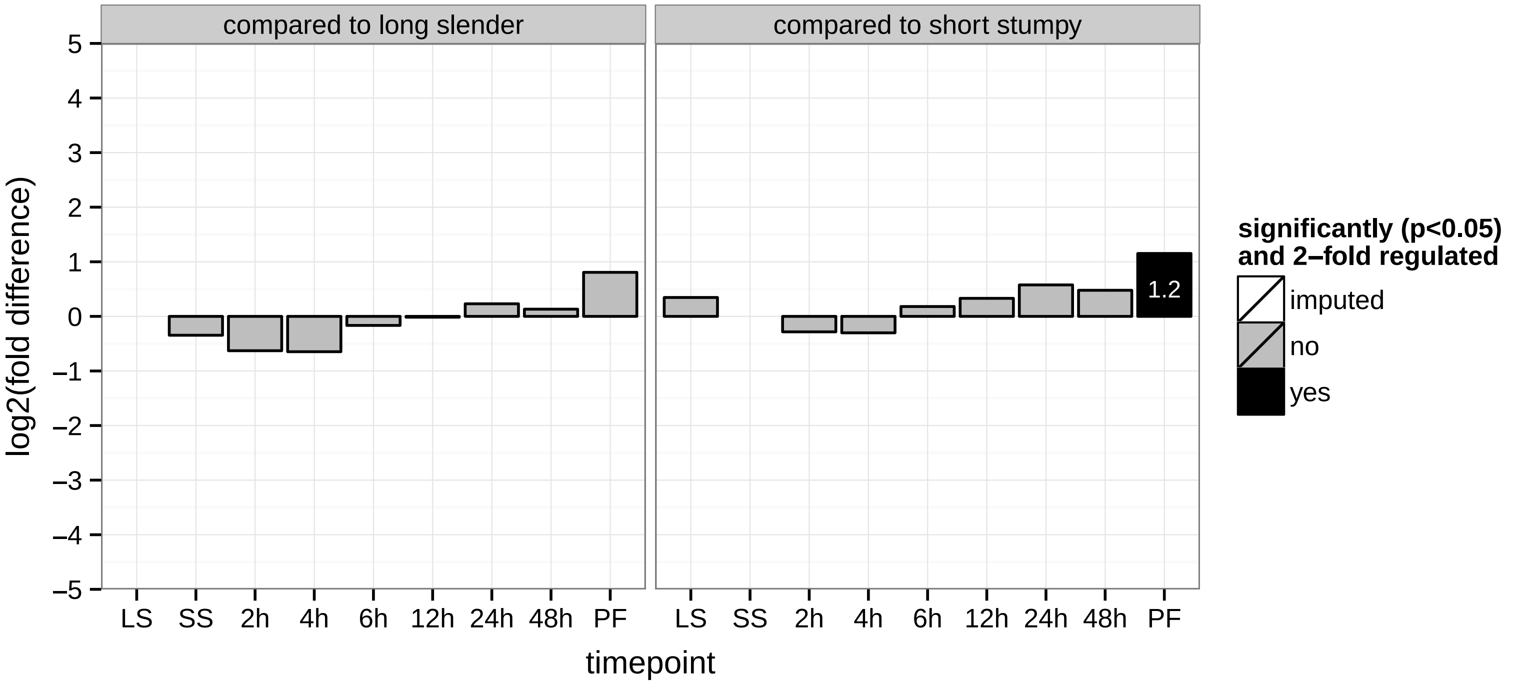

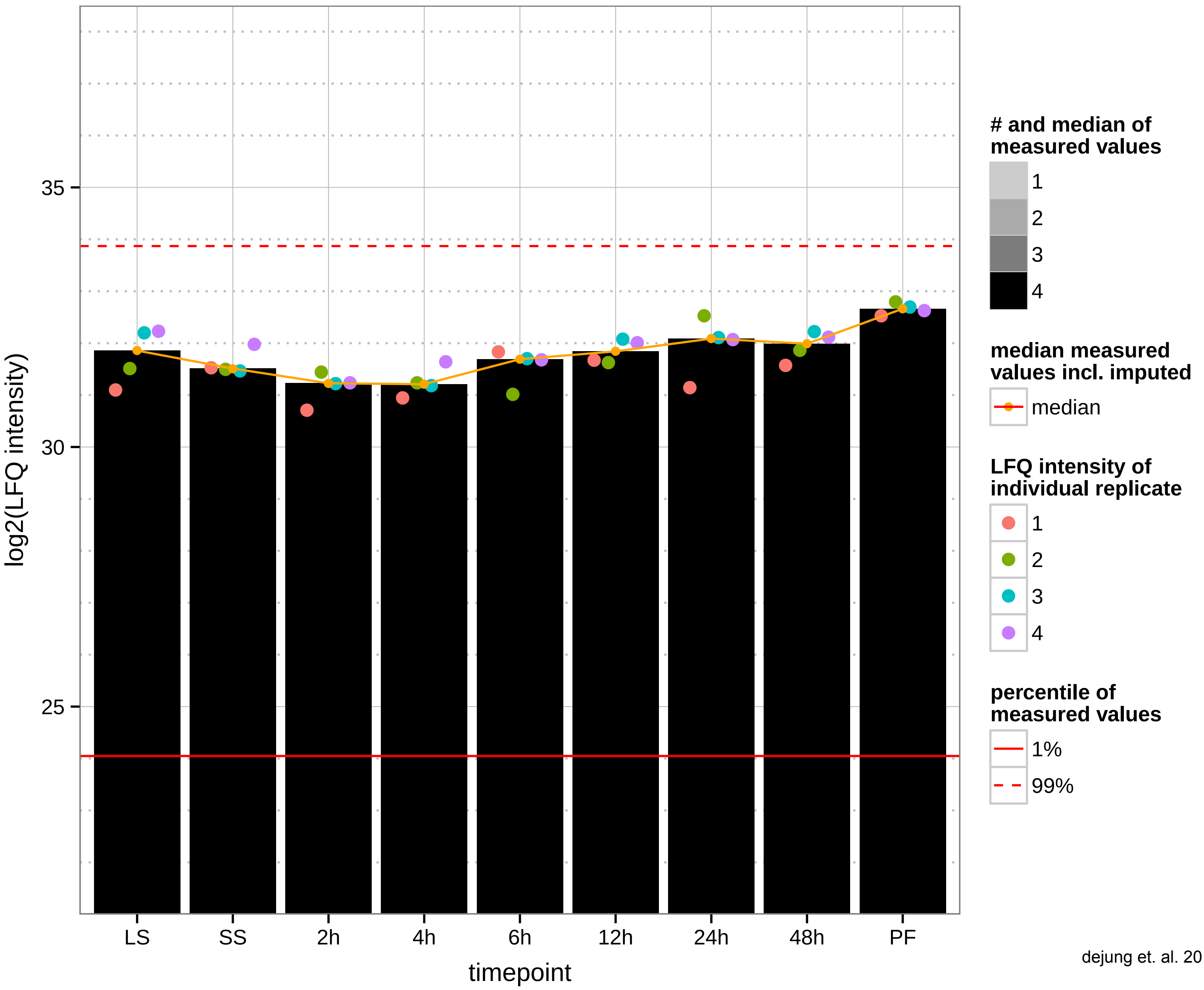

dejung et. al. 2015

rhomboid–like protein, serine peptidase, Clan S–, family S54, putative
Tb927.9.8260
AGOF: null
AGOC: mitochondrial inner membrane, mitochondrion
AGOP: null
PGOF: null
PGOC: null
PGOP: null

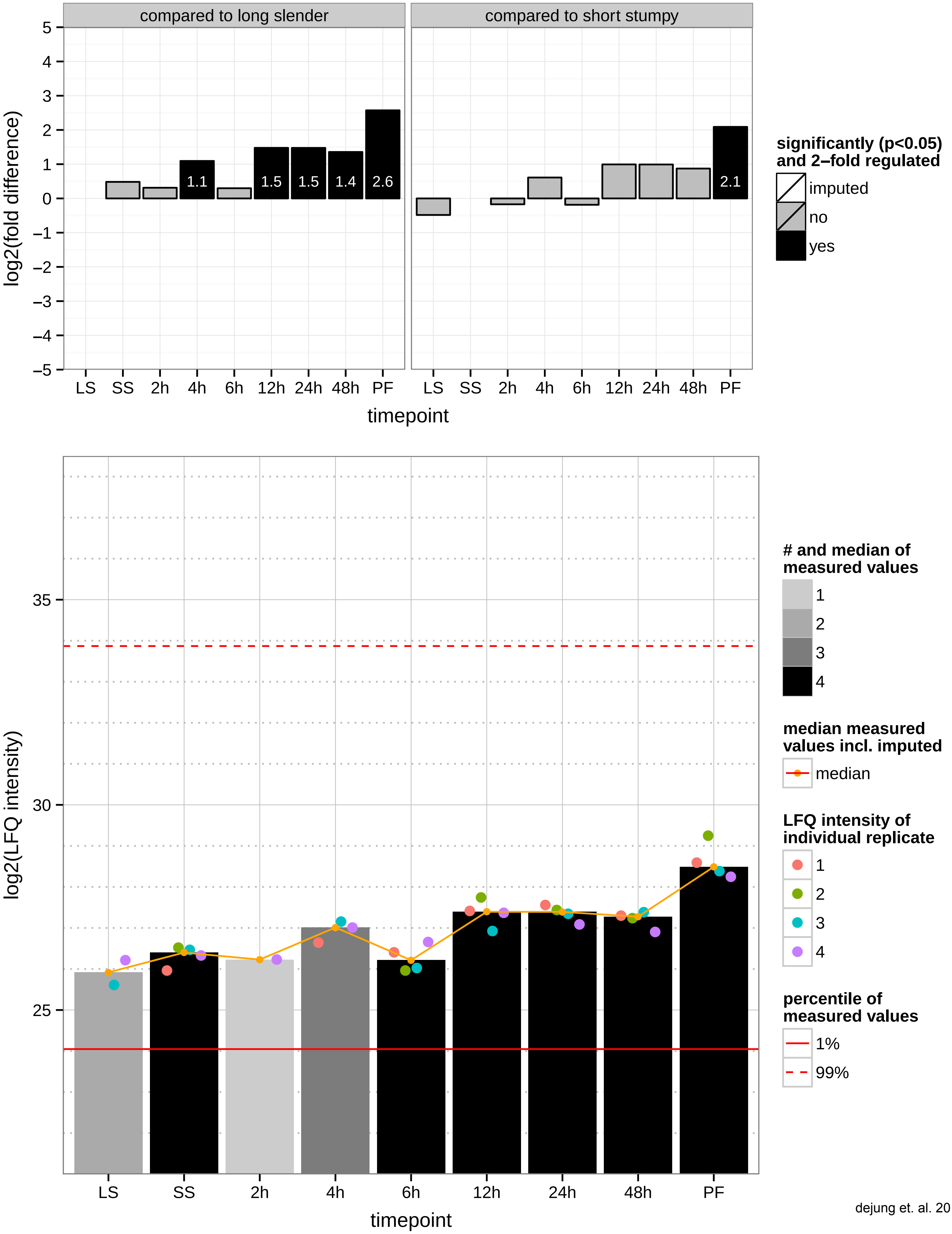

dejung et. al. 2015

RNA–binding protein (DRBD3)
Tb927.9.8740
AGOF: mRNA 3'–UTR binding, mRNA binding
AGOC: cytosol, nucleus, ribonucleoprotein complex
AGOP: RNA processing, mRNA stabilization
PGOF: nucleic acid binding
PGOC: null
PGOP: null

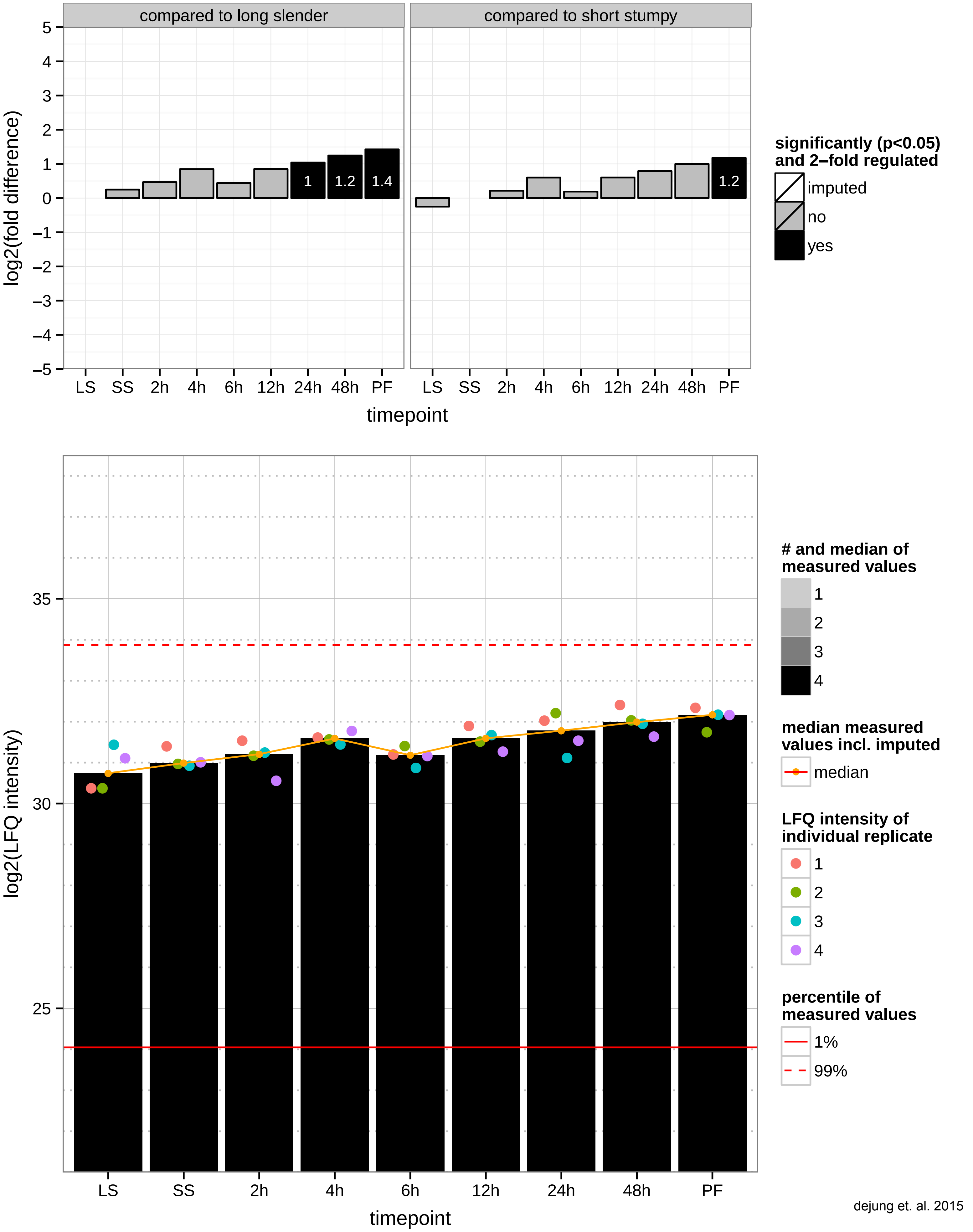

dejung et. al. 2015

hypothetical protein, conserved
Tb927.9.9080
AGOF: null
AGOC: mitochondrion
AGOP: null
PGOF: null
PGOC: null
PGOP: null

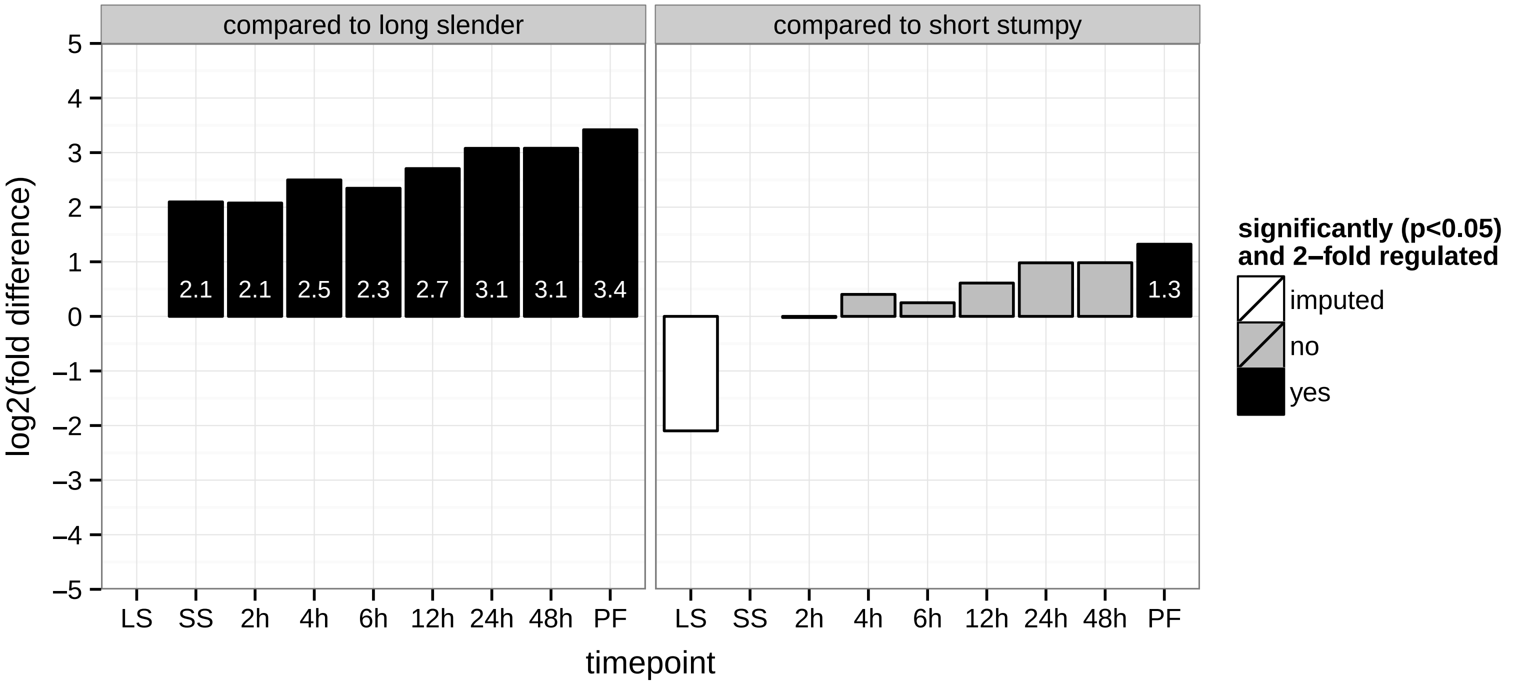

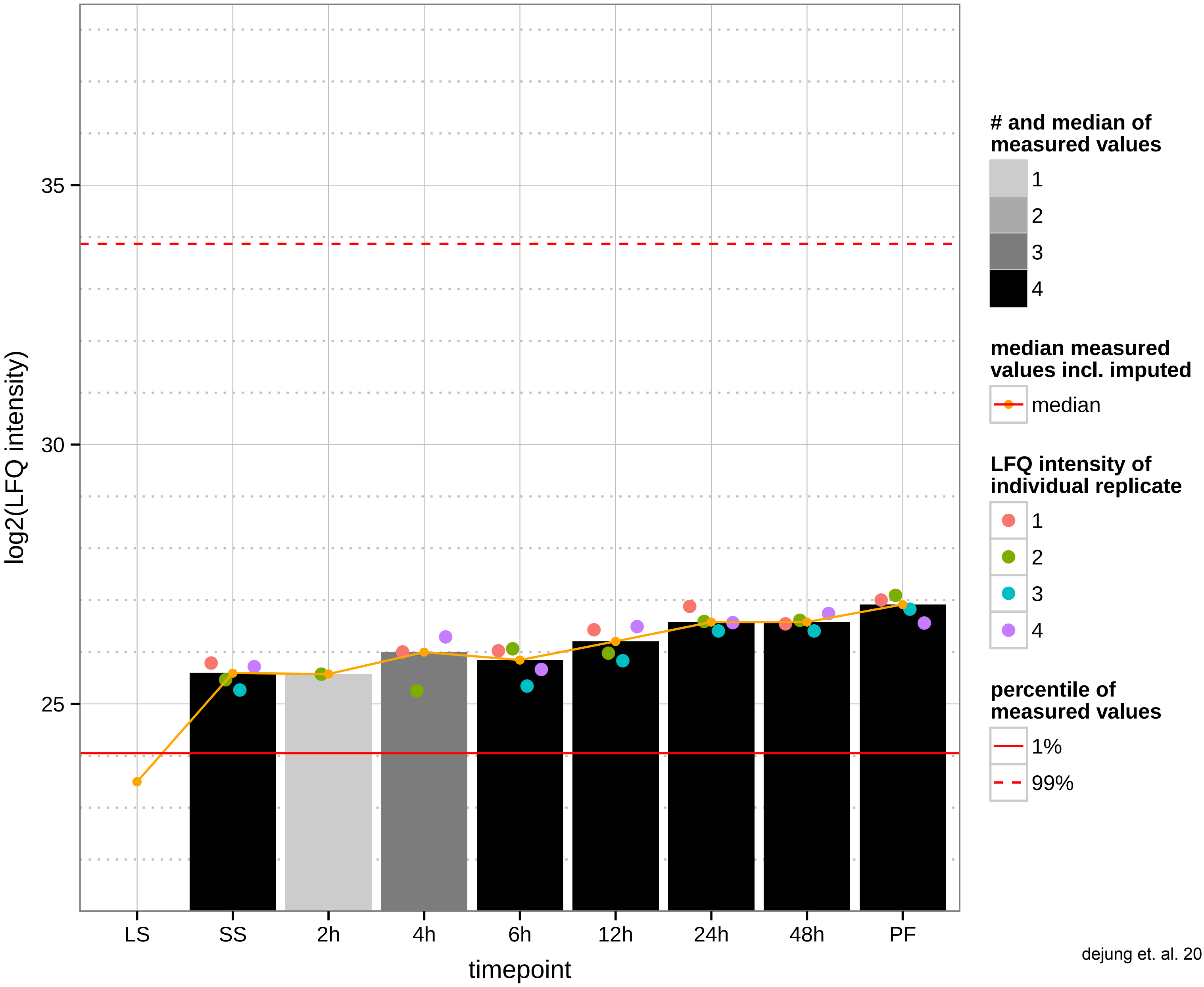

dejung et. al. 2015

hypothetical protein, conserved
Tb927.9.9550
AGOF: null
AGOC: null
AGOP: null
PGOF: null
PGOC: null
PGOP: null

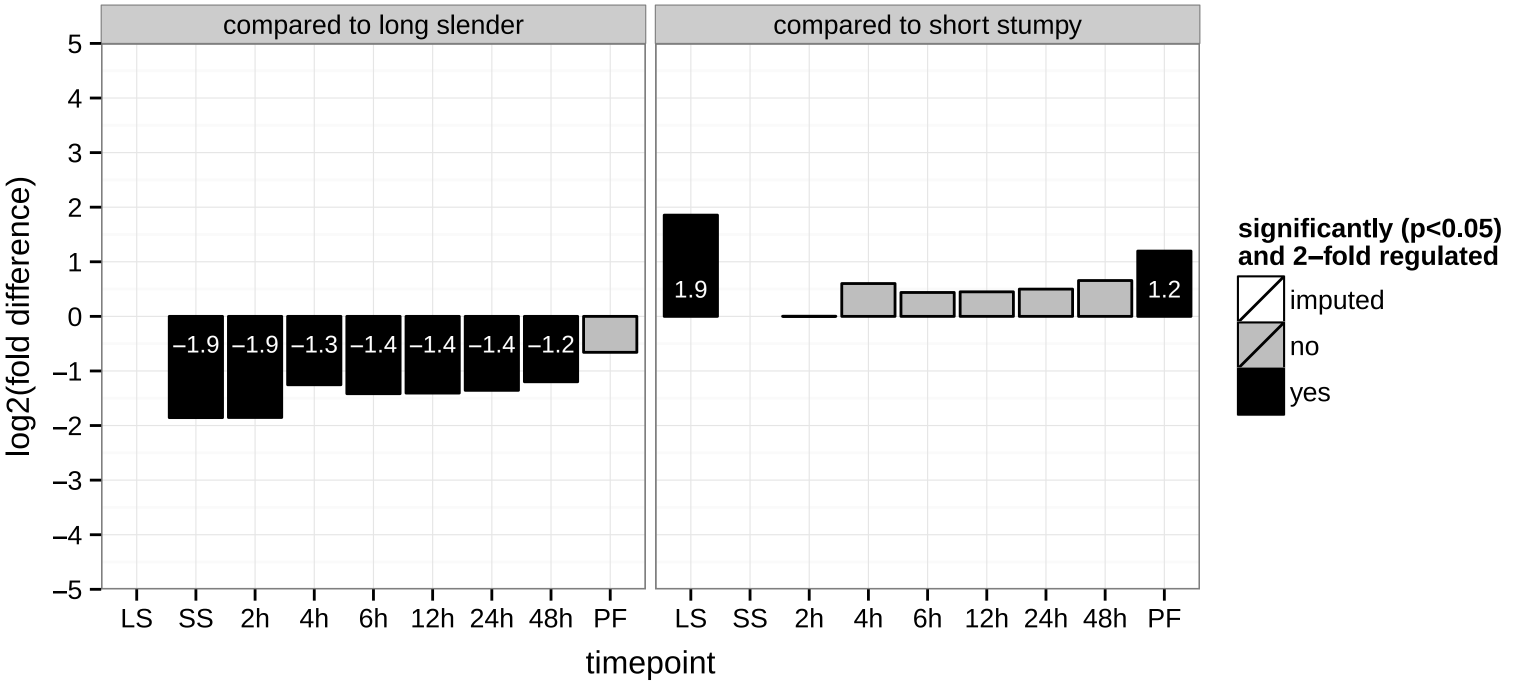

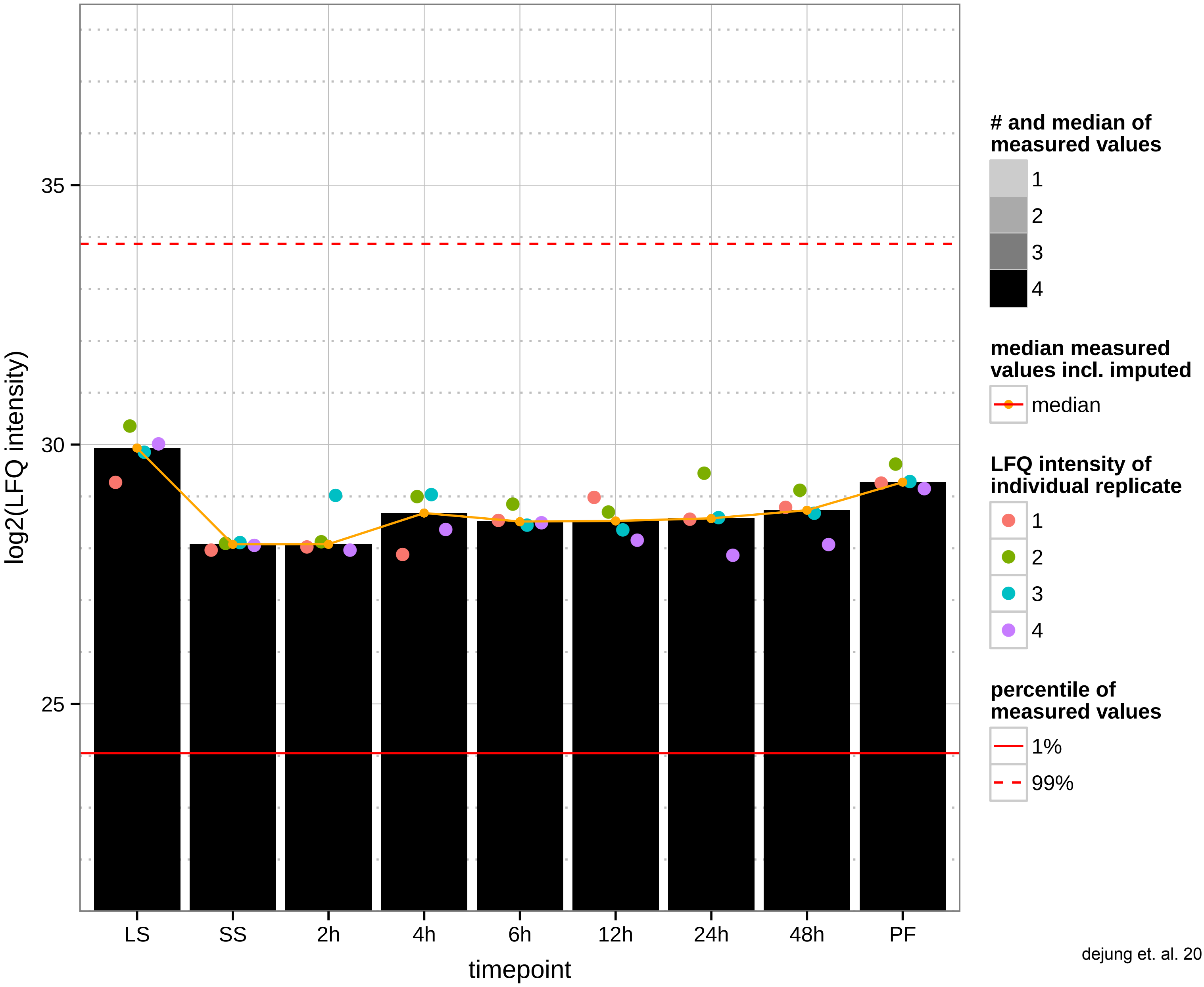

dejung et. al. 2015

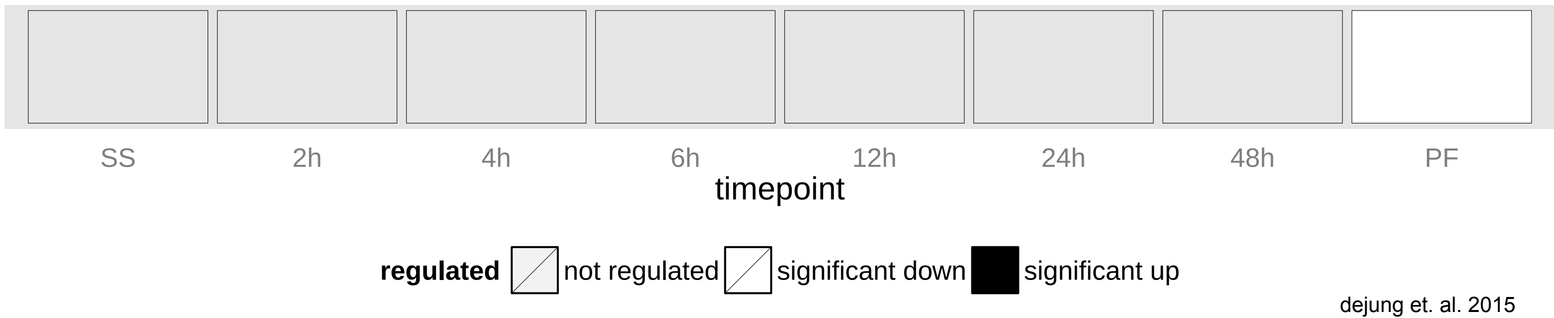
SS
2h
4h
6h
12h
24h
48h
PF
timepoint
regulated
not regulated
significant down
significant up
dejung et. al. 2015

small GTP–binding protein, putative, BAC from homologous region on chr5, ras–like small GTPase, putative (TbSAR1)
Tb927.5.4500;Tb05.5K5.150
AGOF: GTP binding
AGOC: intracellular
AGOP: intracellular protein transport, small GTPase mediated signal transduction
PGOF: GTP binding
PGOC: intracellular
PGOP: intracellular protein transport, small GTPase mediated signal transduction

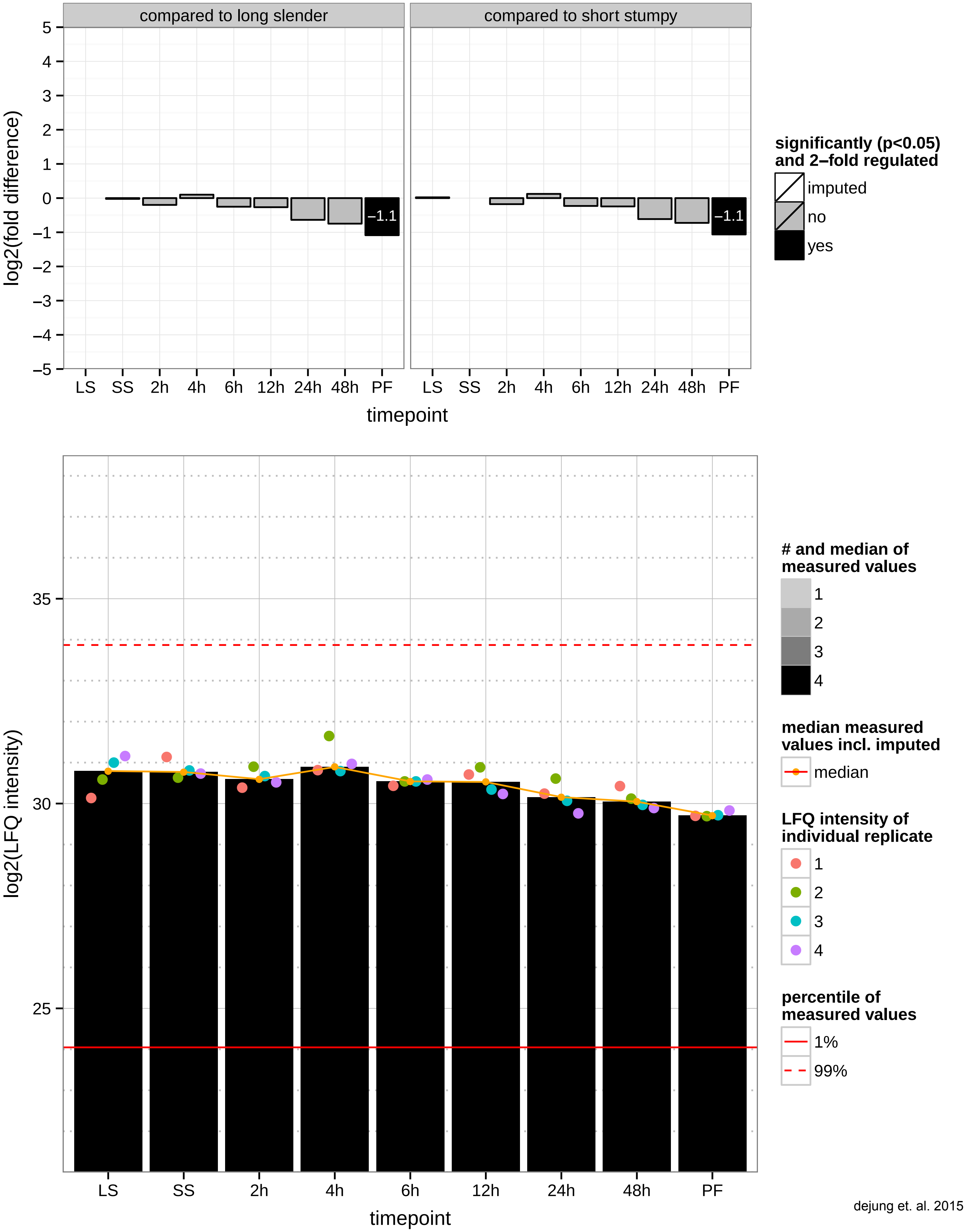

dejung et. al. 2015

pumillo RNA binding protein PUF1, chrX additional, unordered contigs (PUF1), variant surface glycoprotein, fragment, putative
Tb927.10.4430;Tb10.v4.0033;Tb11.v5.0767
AGOF: RNA binding, null
AGOC: null, cytoplasm
AGOP: null, cell growth, mRNA metabolic process
PGOF: RNA binding, binding, null
PGOC: null
PGOP: null

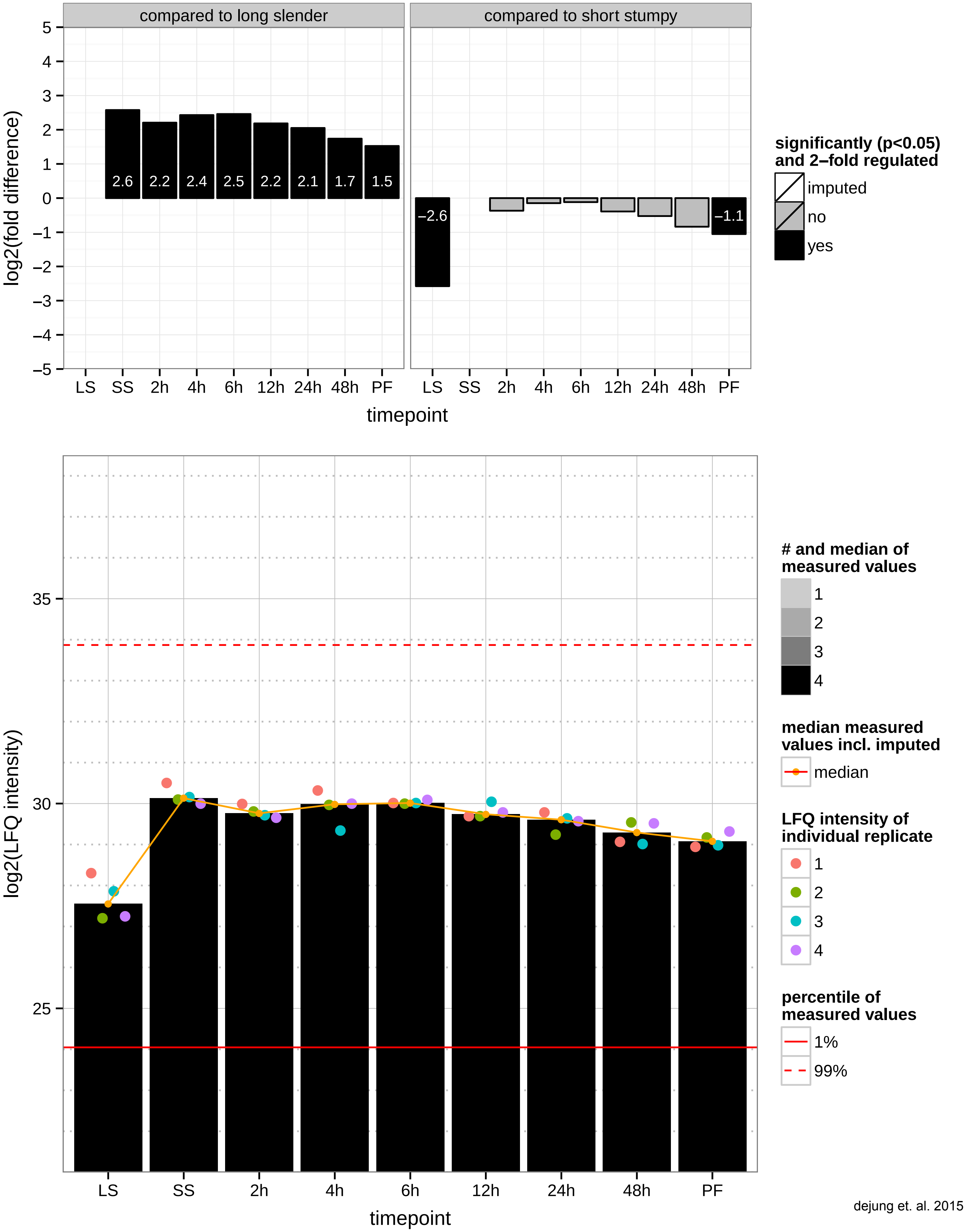

dejung et. al. 2015

hypothetical protein, conserved
Tb927.11.7213;Tb11.02.5080b
AGOF: null
AGOC: null
AGOP: null
PGOF: null
PGOC: null
PGOP: null

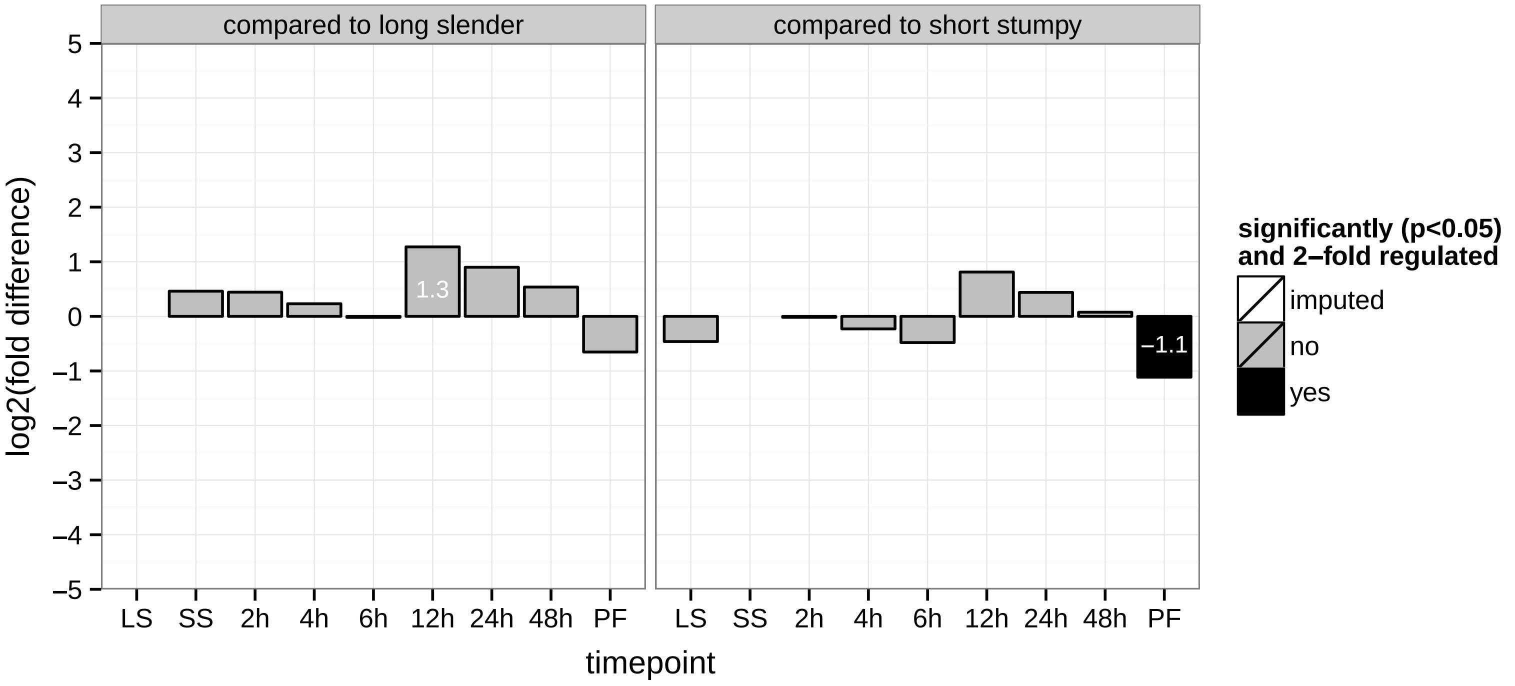

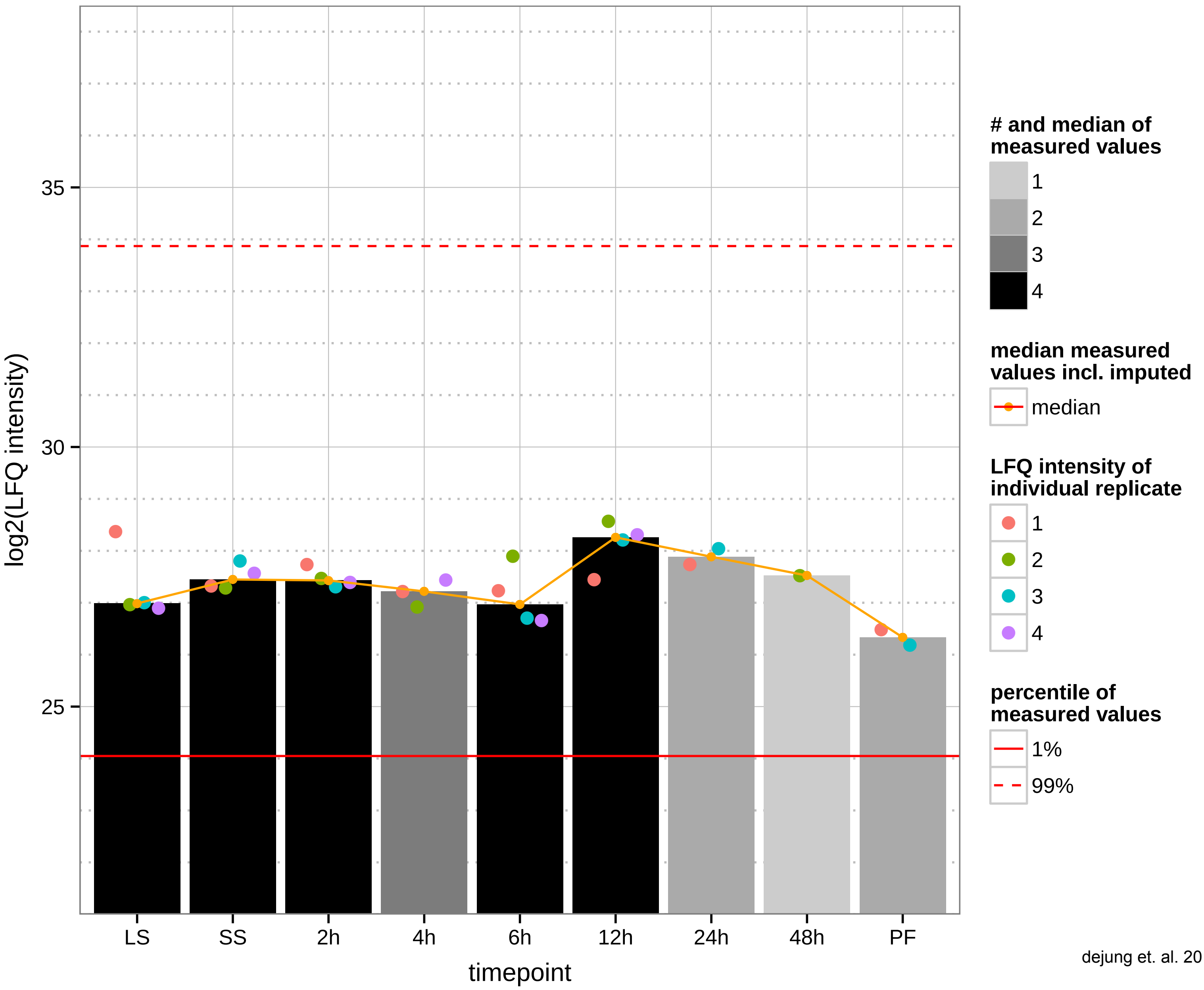

dejung et. al. 2015

ubiquitin activating enzyme, putative
Tb11.02.5410b;Tb11.02.5410
AGOF: catalytic activity
AGOC: null
AGOP: null
PGOF: catalytic activity
PGOC: null
PGOP: null

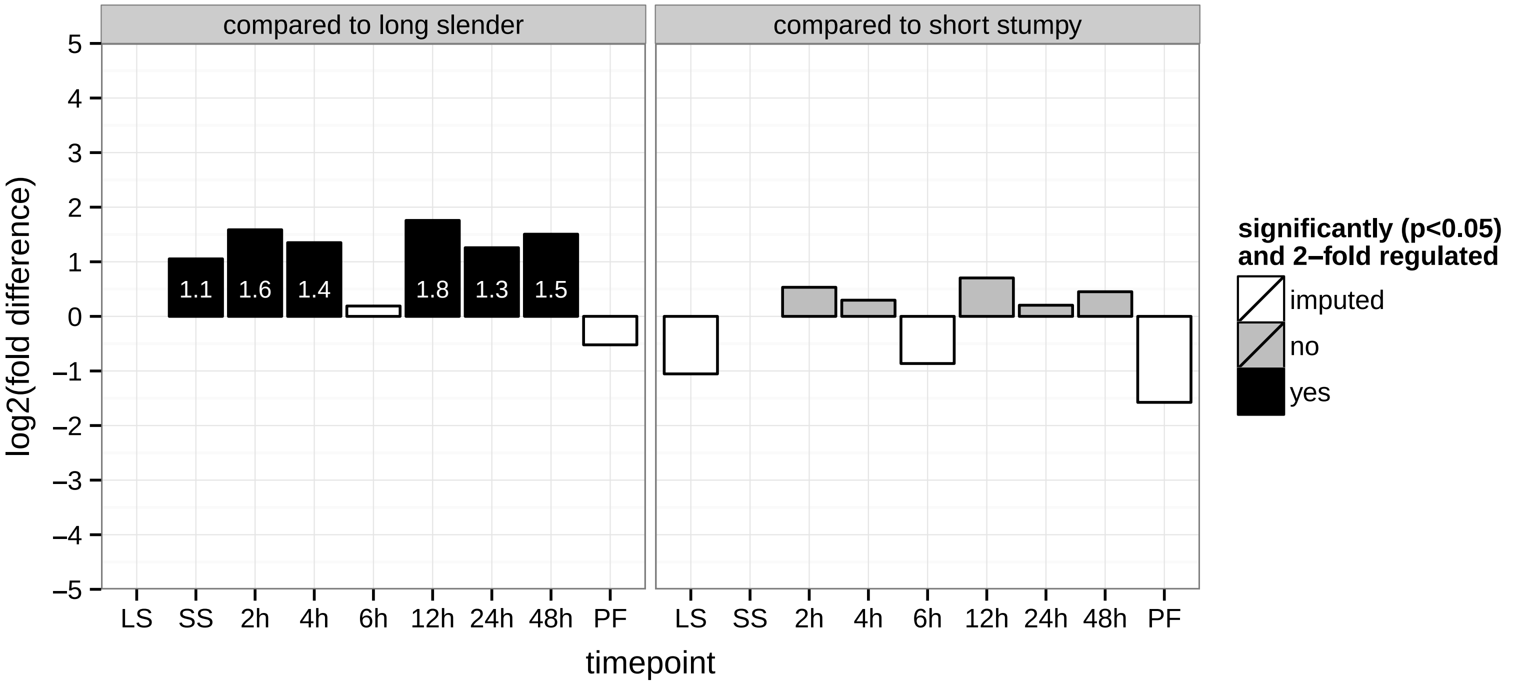

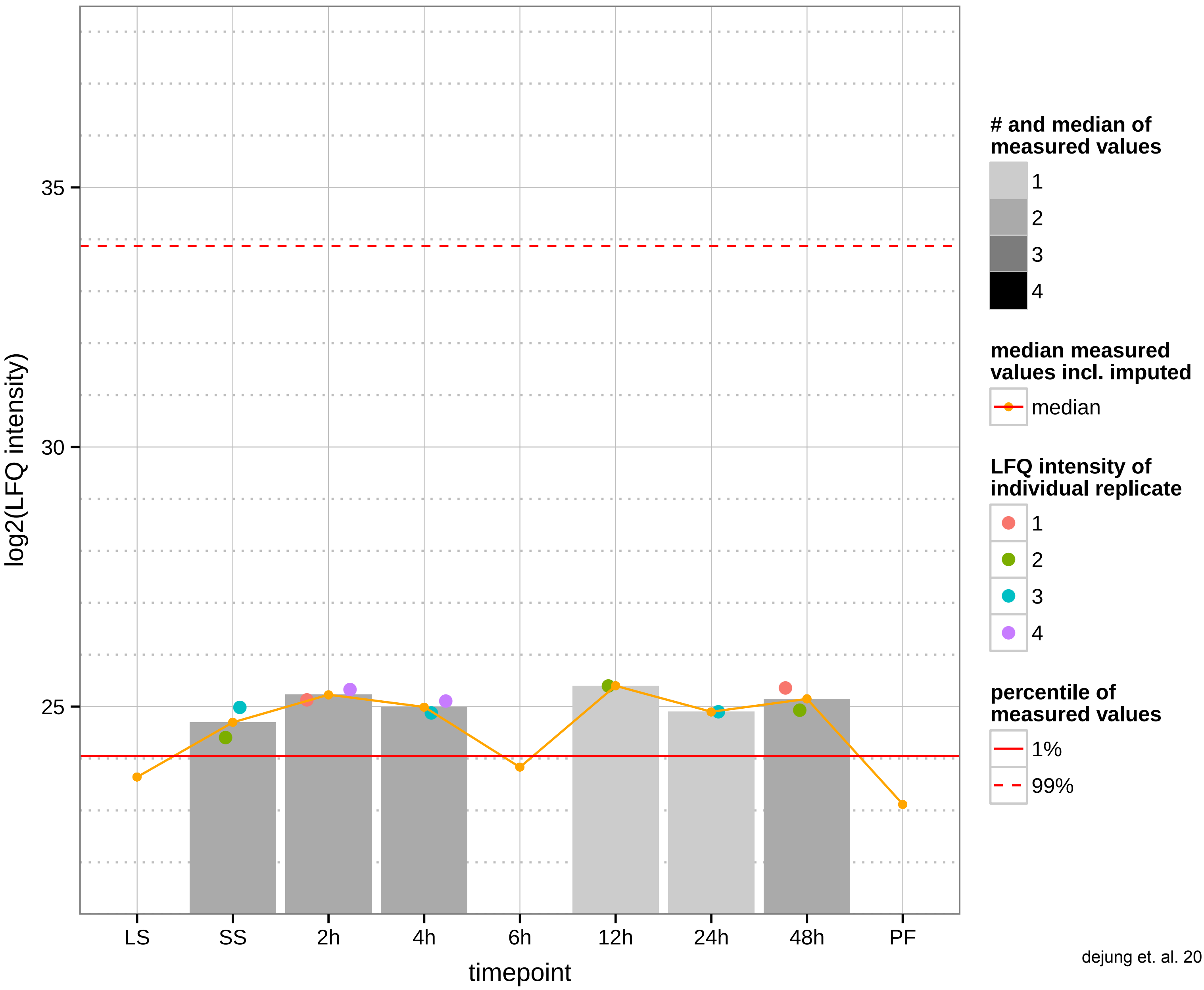

dejung et. al. 2015

hypothetical protein, chrXI additional, unordered contigs, conserved
Tb11.0850;Tb927.11.710
AGOF: null
AGOC: integral to membrane
AGOP: protein transport
PGOF: null
PGOC: integral to membrane
PGOP: protein transport

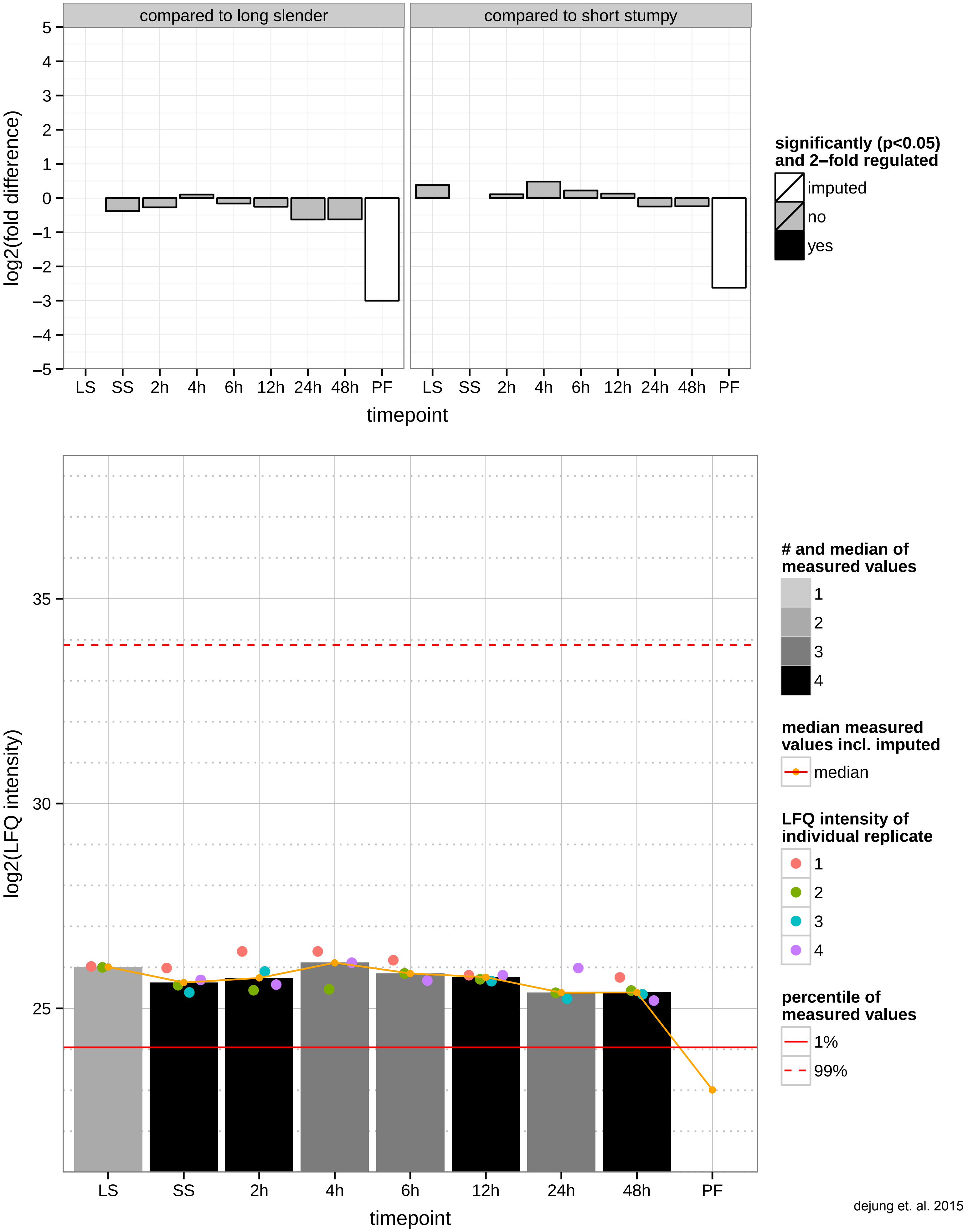

dejung et. al. 2015

hypothetical protein, conserved
Tb927.7.4810;Tb11.v5.0163
AGOF: null, catalytic activity
AGOC: null
AGOP: null
PGOF: catalytic activity, metal ion binding, phosphoric diester hydrolase activity
PGOC: null
PGOP: null

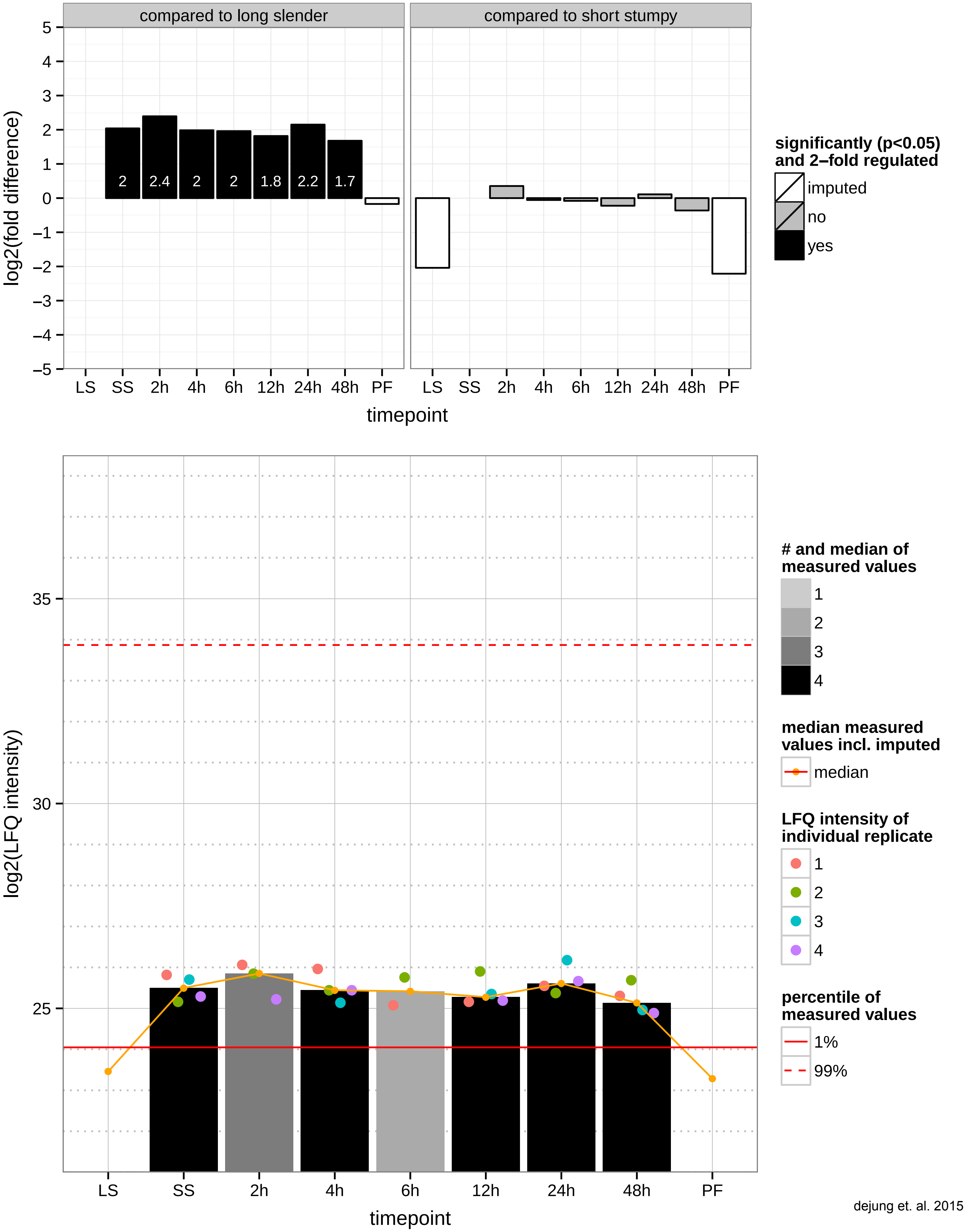

dejung et. al. 2015

hypothetical protein, conserved
Tb927.10.5990;Tb11.v5.0193
AGOF: null
AGOC: null
AGOP: null
PGOF: null
PGOC: null
PGOP: null

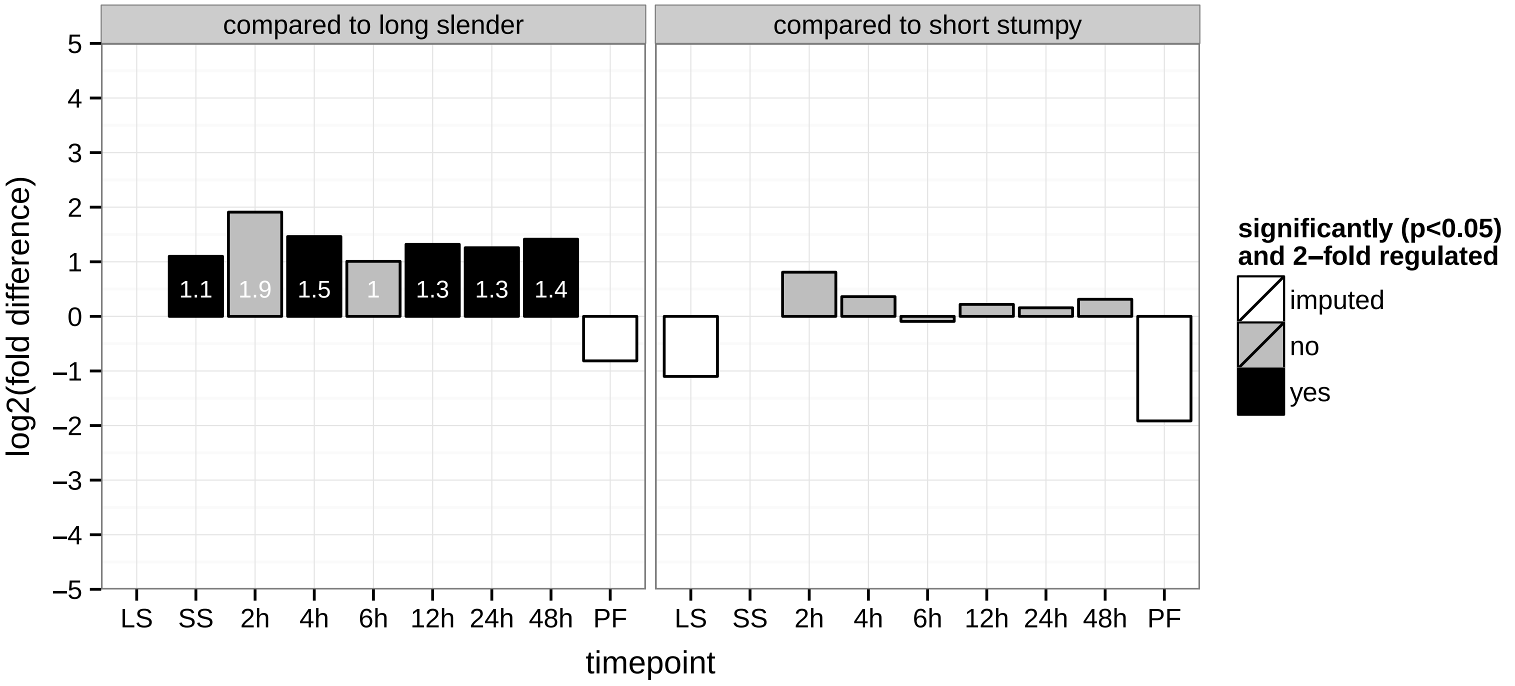

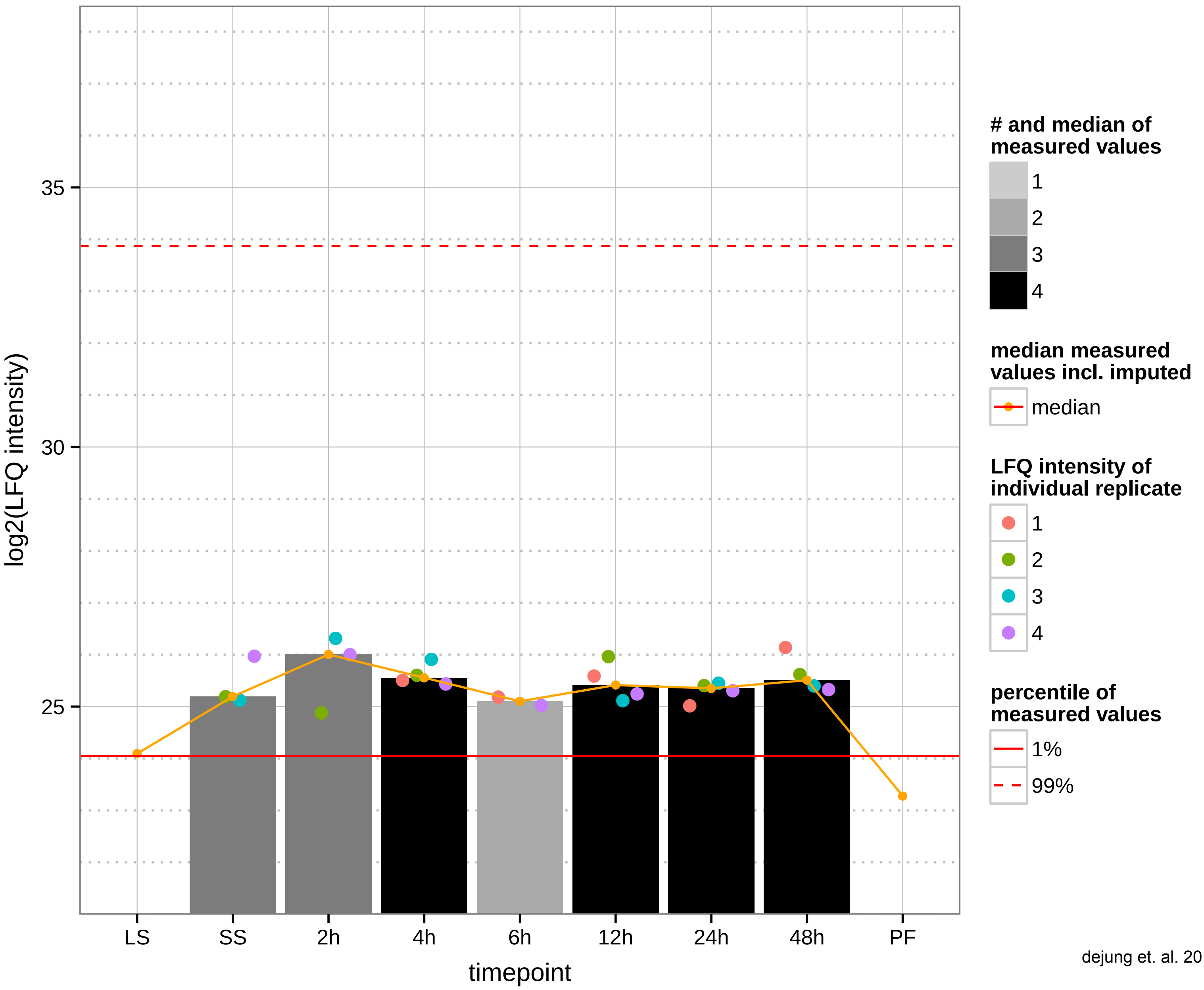

dejung et. al. 2015

pre–mRNA splicing factor ATP–dependent RNA helicase, putative, DEAH–box RNA helicase
Tb927.10.5280;Tb11.v5.0248
AGOF: null, ATP binding, ATP–dependent RNA helicase activity, nucleic acid binding
AGOC: null, spliceosomal complex
AGOP: null, RNA splicing, RNA splicing, via transesterification reactions
PGOF: ATP binding, ATP–dependent helicase activity, helicase activity, nucleic acid binding
PGOC: null
PGOP: null

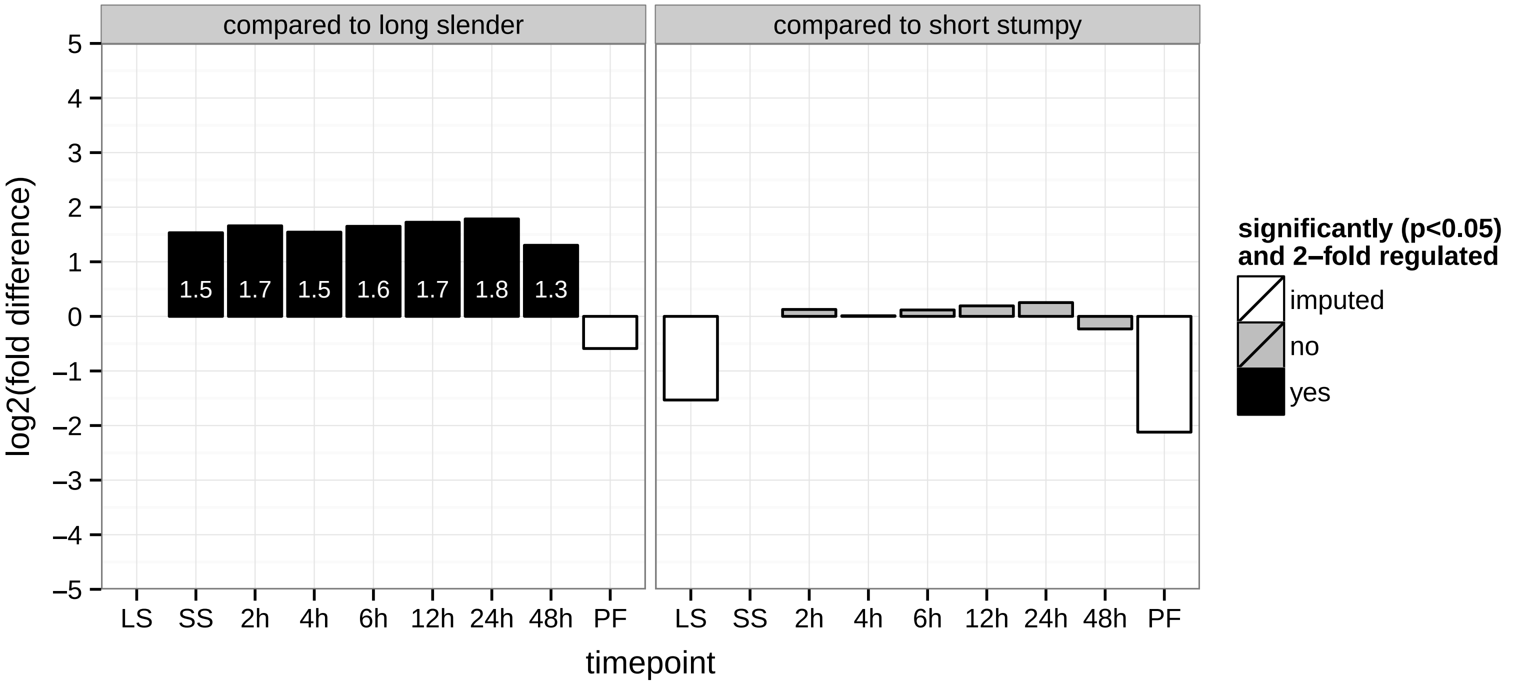

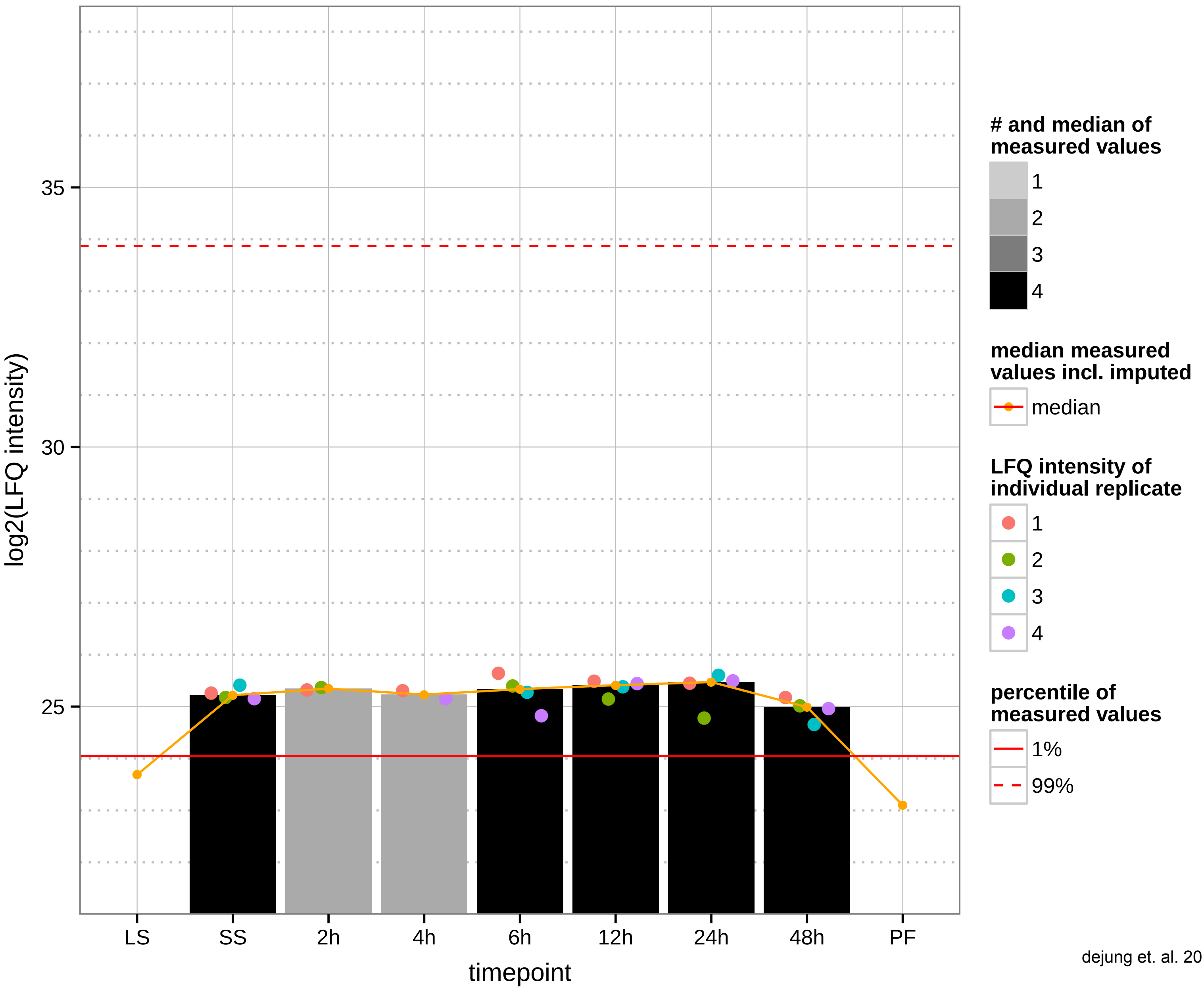

dejung et. al. 2015

hypothetical protein, conserved
Tb927.11.7300;Tb11.v5.0253
AGOF: null, transporter activity
AGOC: null, integral to membrane
AGOP: null, transport
PGOF: null
PGOC: integral to membrane
PGOP: transmembrane transport

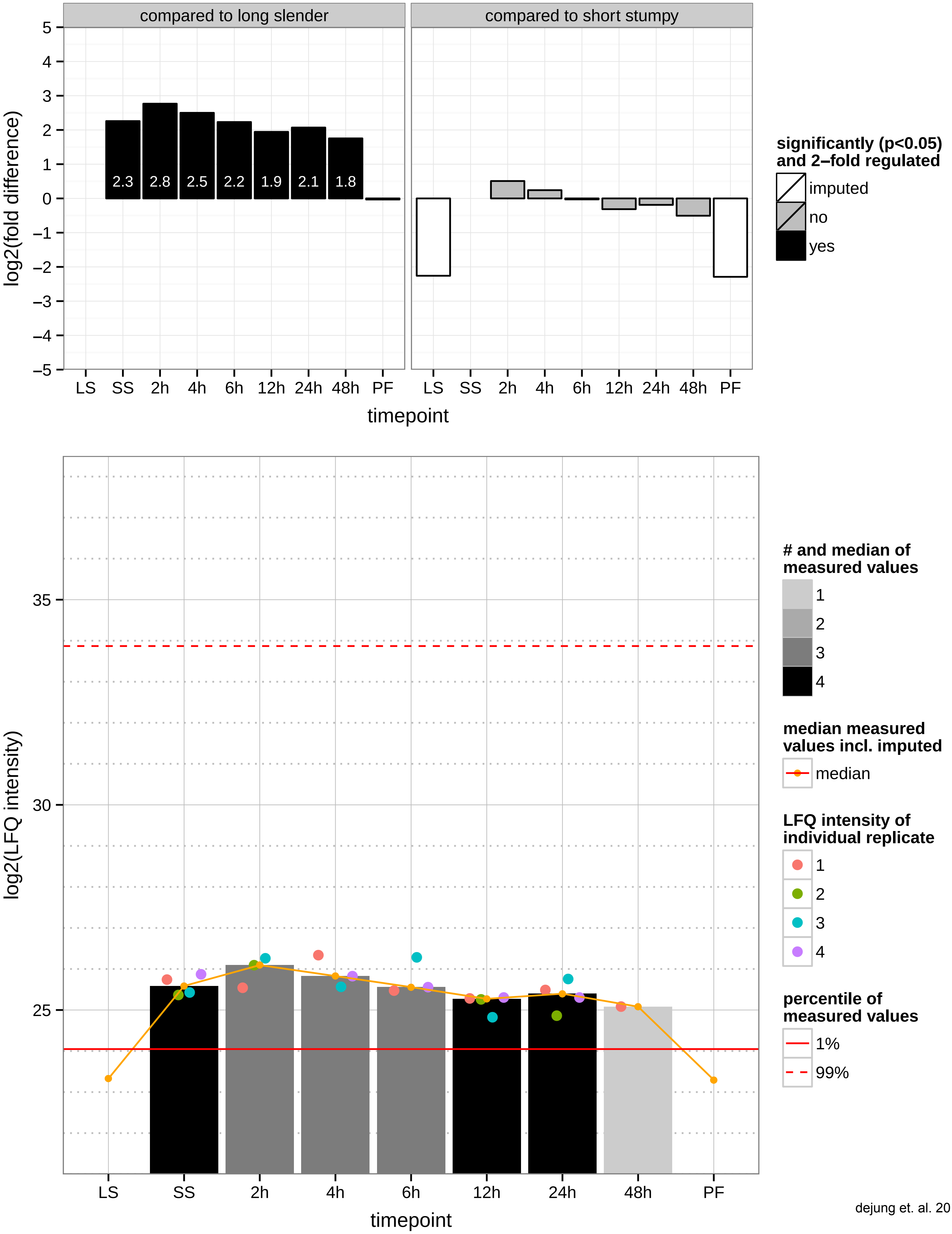

dejung et. al. 2015

hypothetical protein, conserved, GPI transamidase component Tta2 (TTA2)
Tb927.10.5080;Tb11.v5.0339
AGOF: null, GPI–anchor transamidase activity
AGOC: null, GPI–anchor transamidase complex
AGOP: null
PGOF: null
PGOC: null
PGOP: null

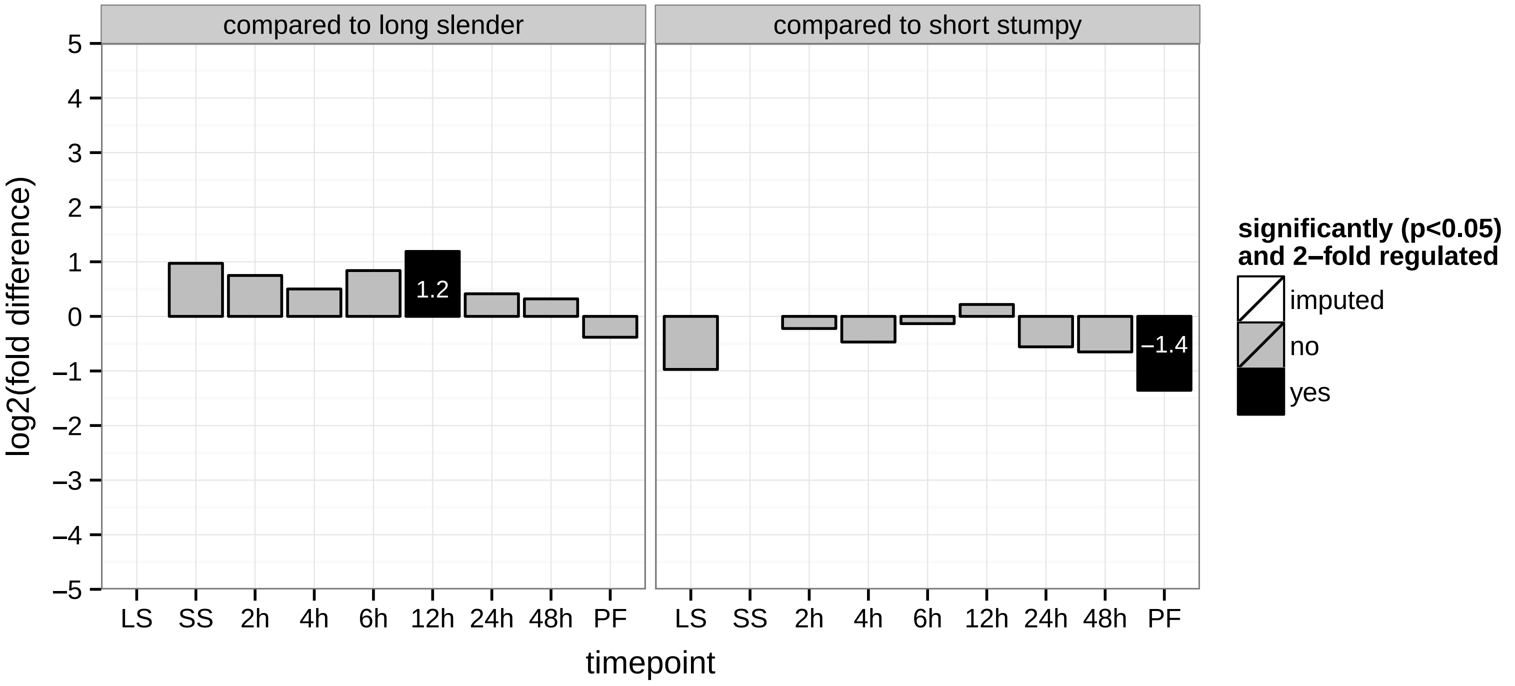

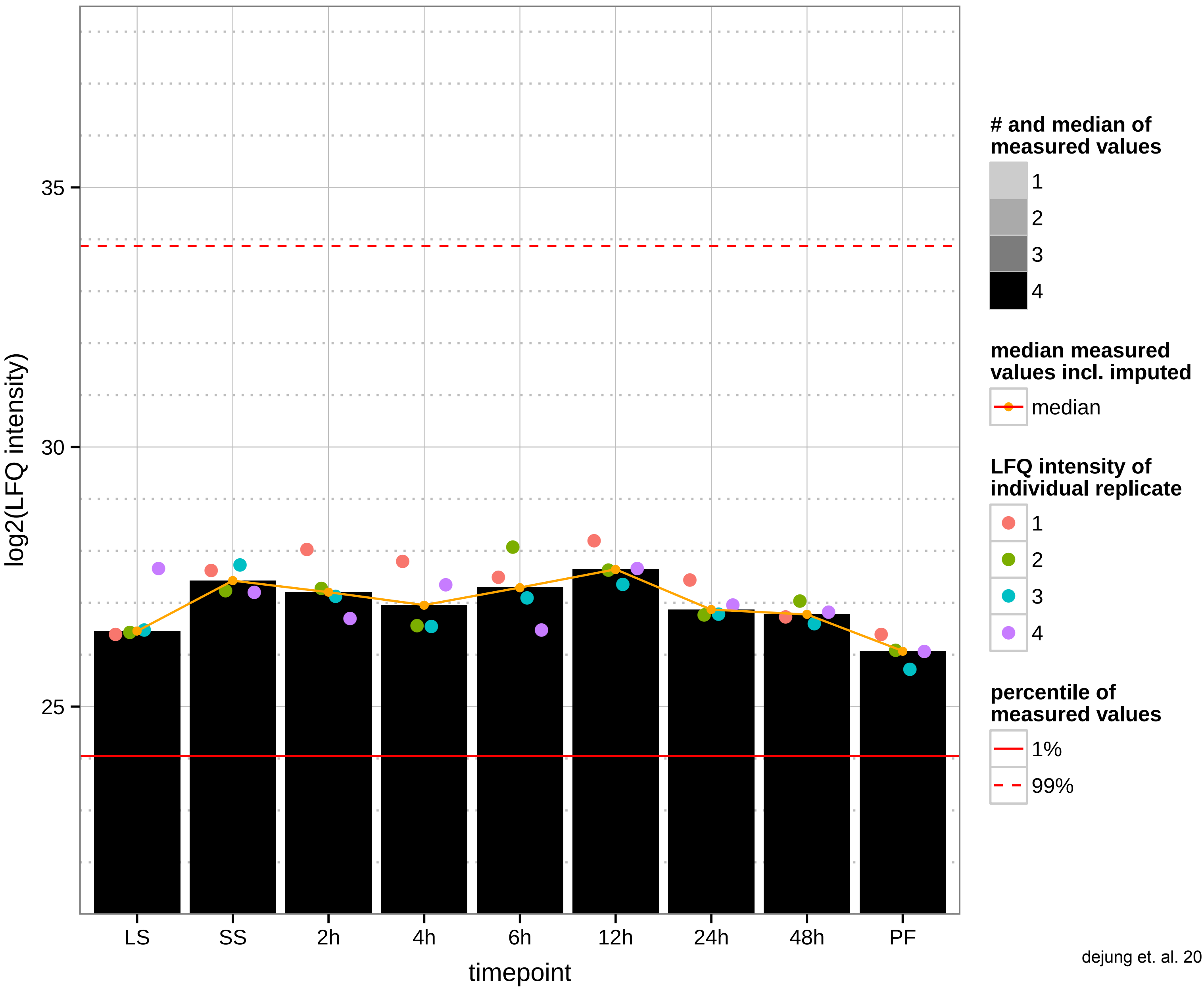

dejung et. al. 2015

glyceraldehyde 3−phosphate dehydrogenase, cytosolic, putative, cytosolic (GAP)
Tb927.10.6880;Tb11.v5.0516
AGOF: null, glyceraldehyde−3−phosphate dehydrogenase (NAD+) (phosphorylating) activity
AGOC: null, cytosol
AGOP: null, glycolysis
PGOF: NAD binding, NADP binding, oxidoreductase activity, acting on the aldehyde or oxo group of donors, NAD or NADP as
PGOC: null
PGOP: glucose metabolic process, oxidation−reduction process

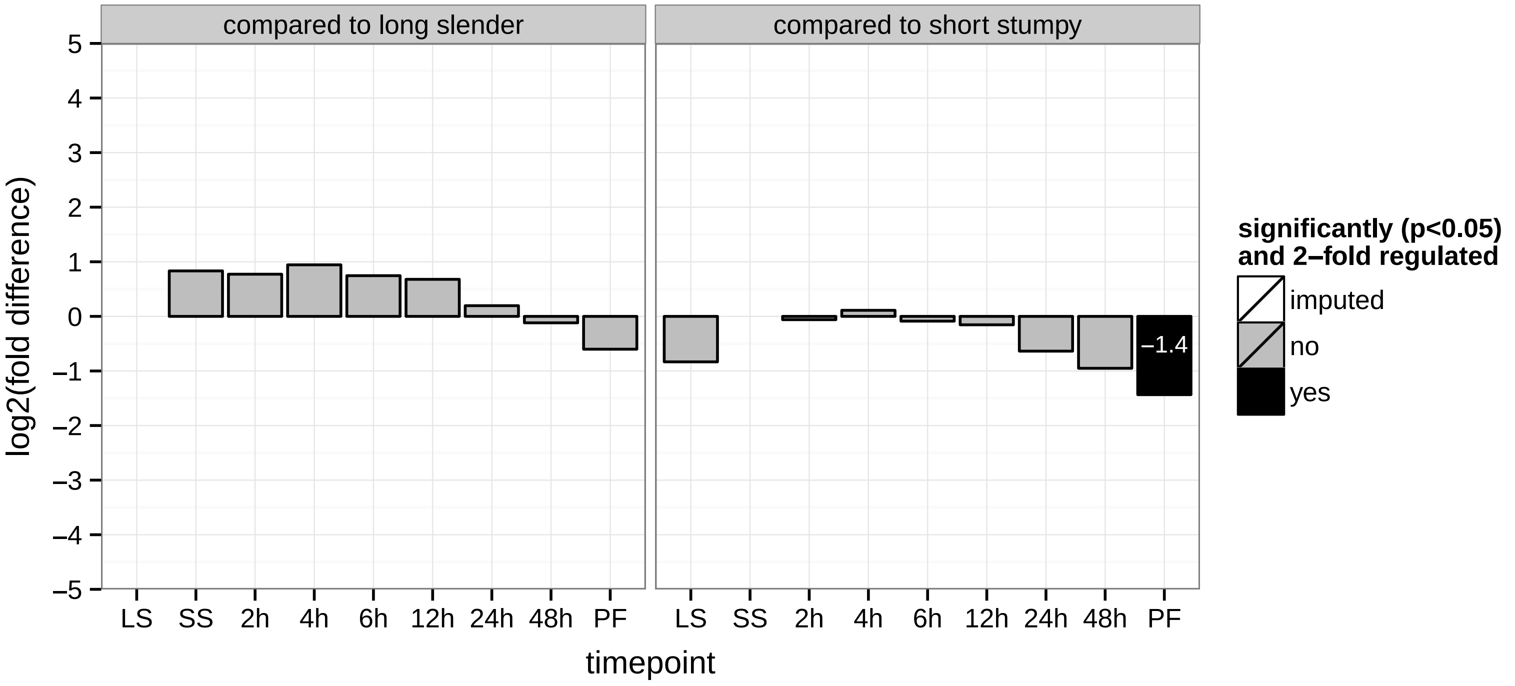

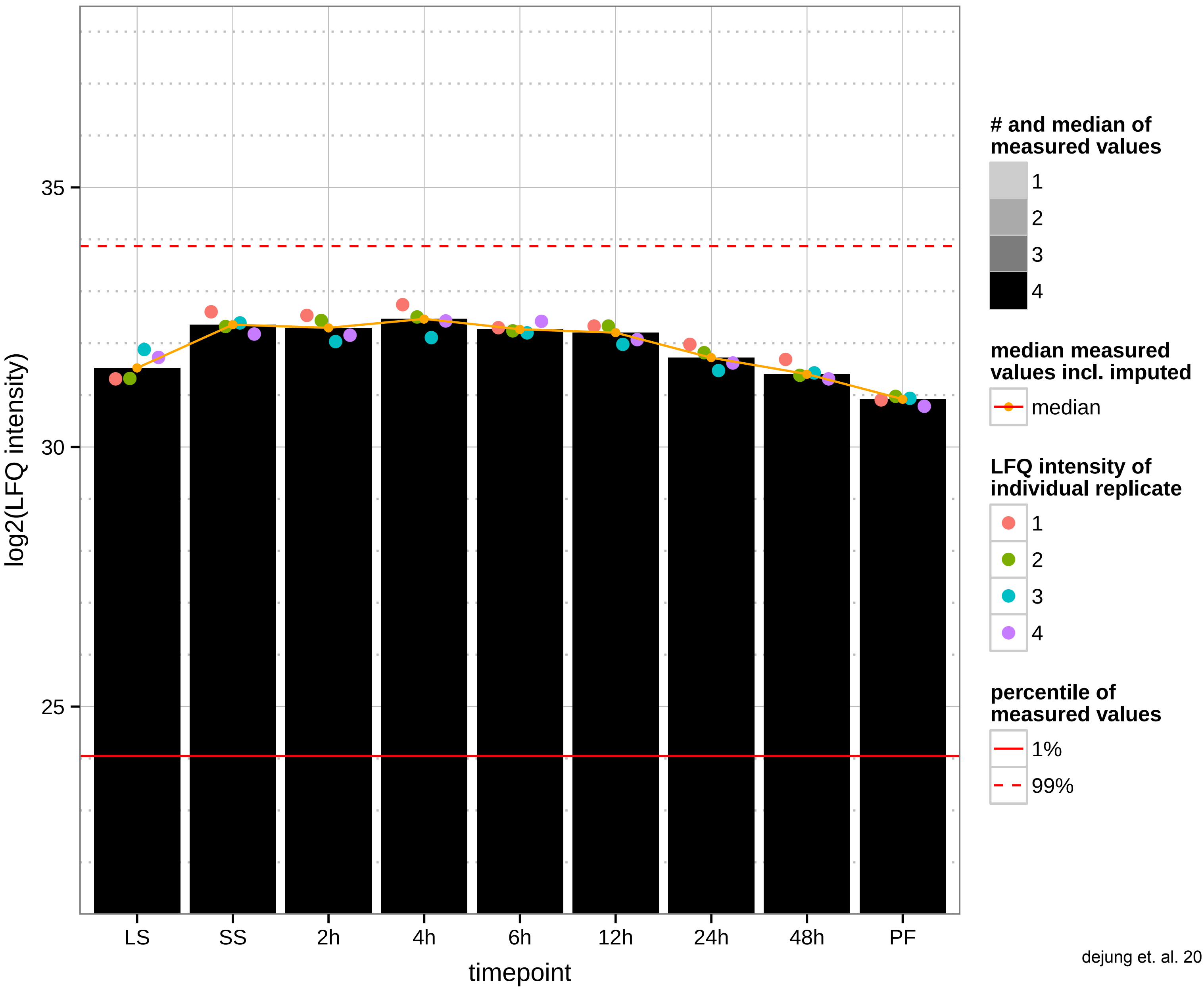

dejung et. al. 2015

polyubiquitin, putative
Tb11.v5.0537;Tb927.11.9920;Tb11.01.1680.v5
AGOF: null
AGOC: null
AGOP: null, cellular protein modification process
PGOF: protein binding
PGOC: null
PGOP: null

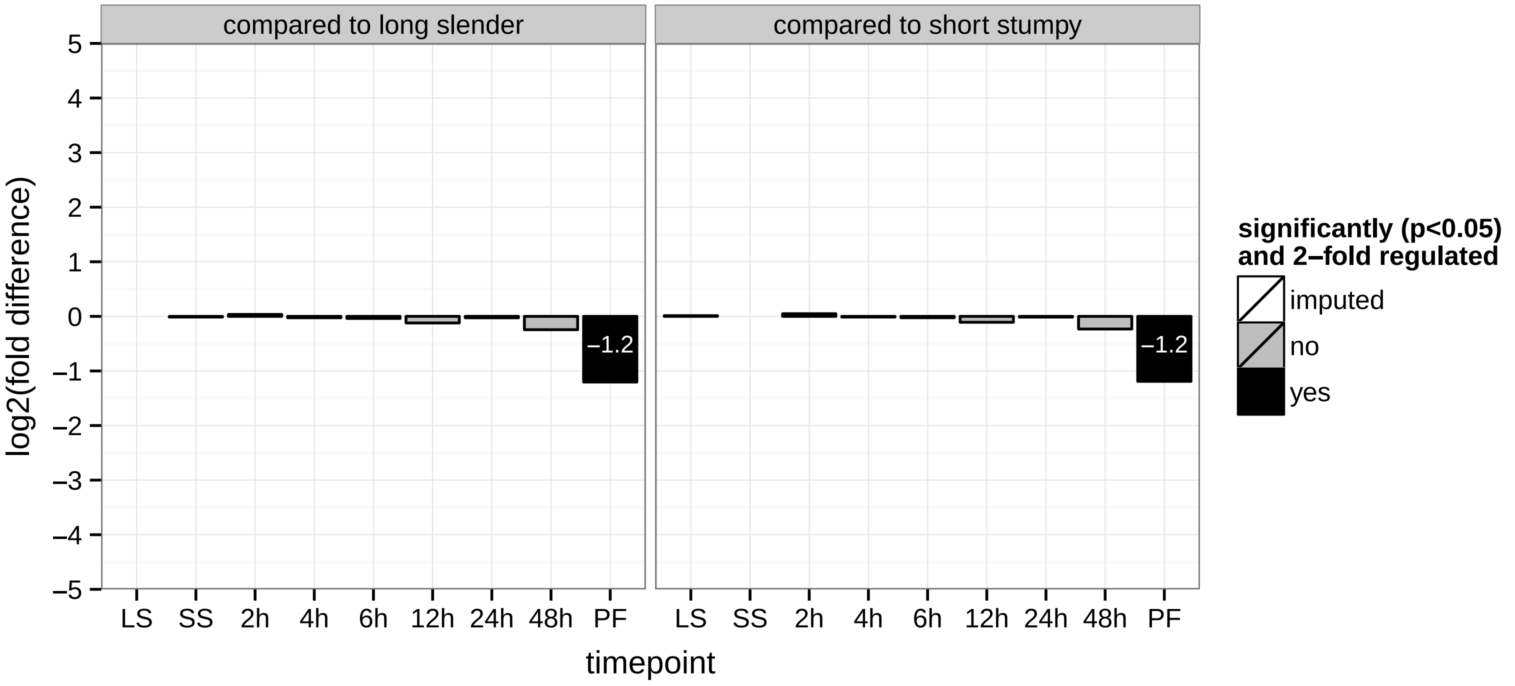

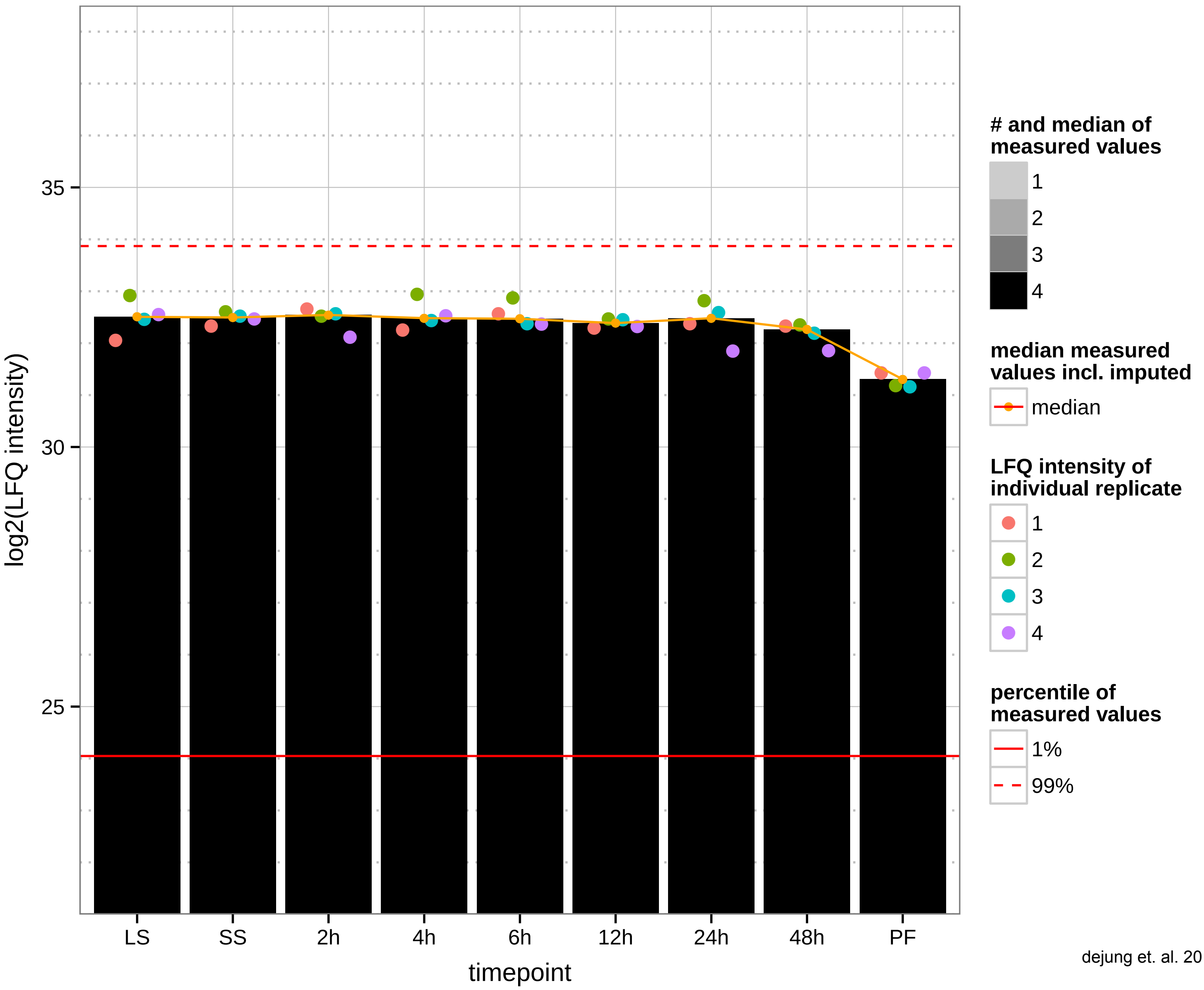

dejung et. al. 2015

hypothetical protein, conserved, U4/U6−specific protein PRP4
Tb927.10.960;Tb11.v5.0631
AGOF: null, pre−mRNA 5'−splice site binding
AGOC: null, cytoplasm, nucleus
AGOP: null, RNA splicing
PGOF: protein binding
PGOC: null
PGOP: RNA splicing

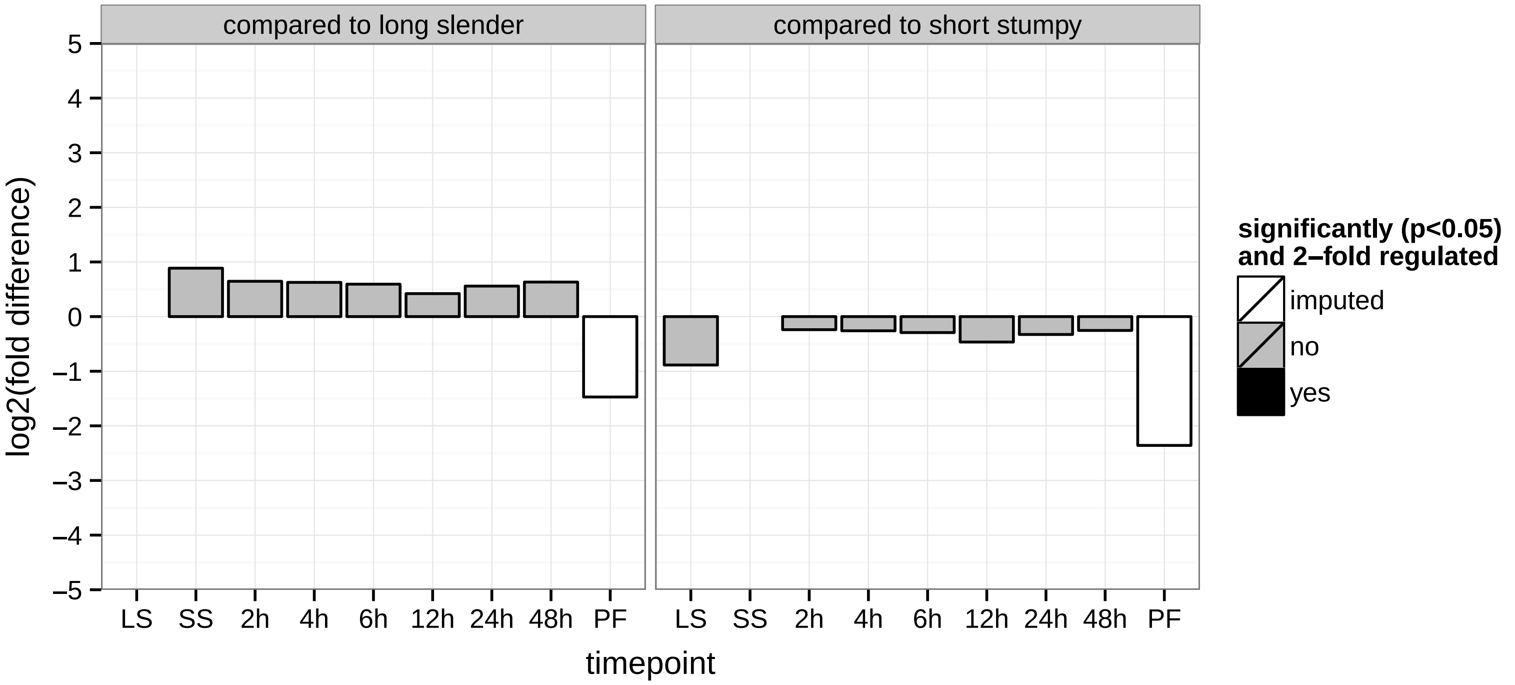

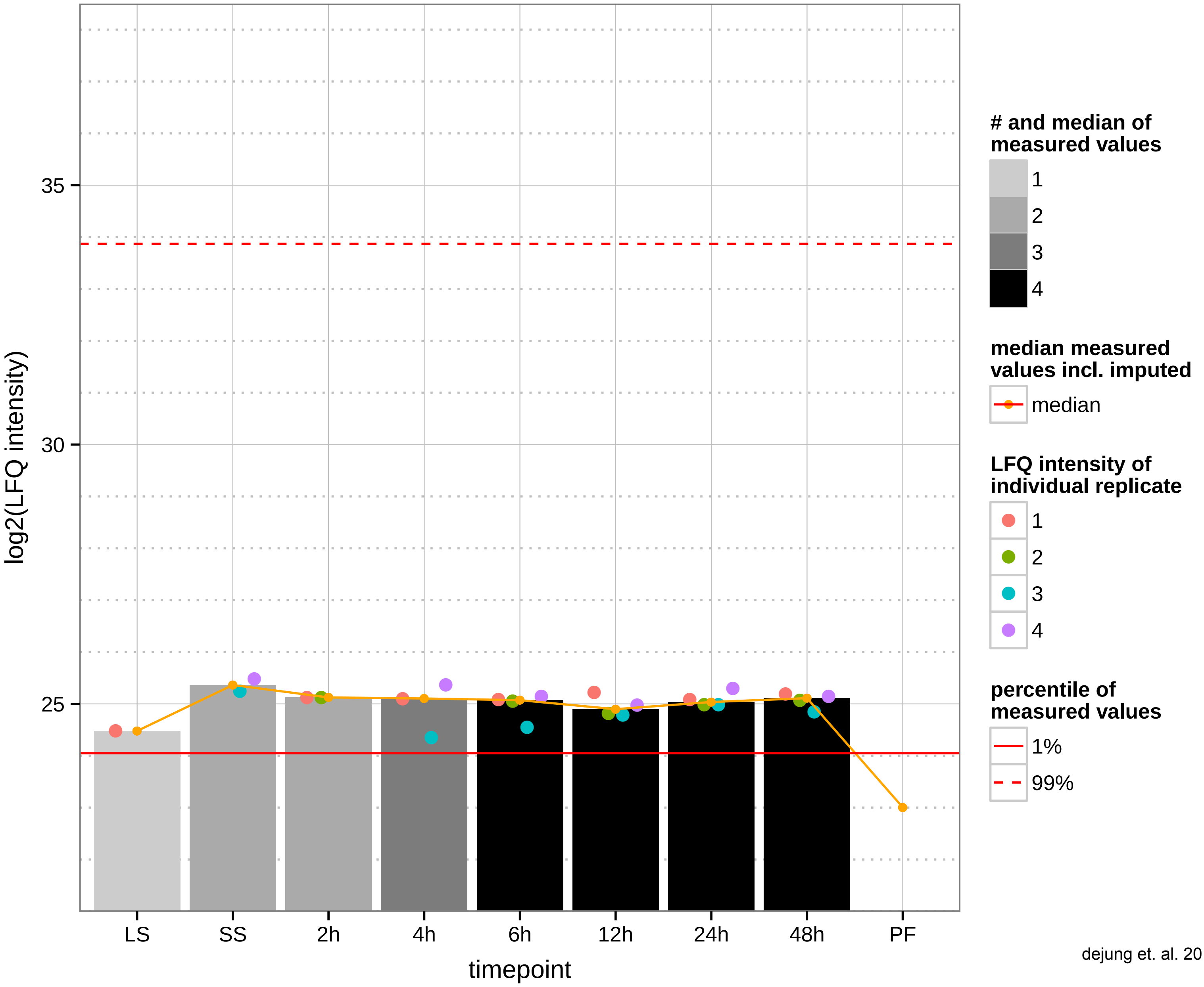

dejung et. al. 2015

glutaredoxin−like protein, putative, monothiol glutaredoxin
Tb927.9.3590;Tb11.v5.0904
AGOF: null, electron carrier activity, glutathione−disulfide reductase activity, protein disulfide oxidoreductase activity
AGOC: null, mitochondrion
AGOP: null, cell redox homeostasis, oxidation−reduction process
PGOF: electron carrier activity, protein disulfide oxidoreductase activity
PGOC: null
PGOP: cell redox homeostasis

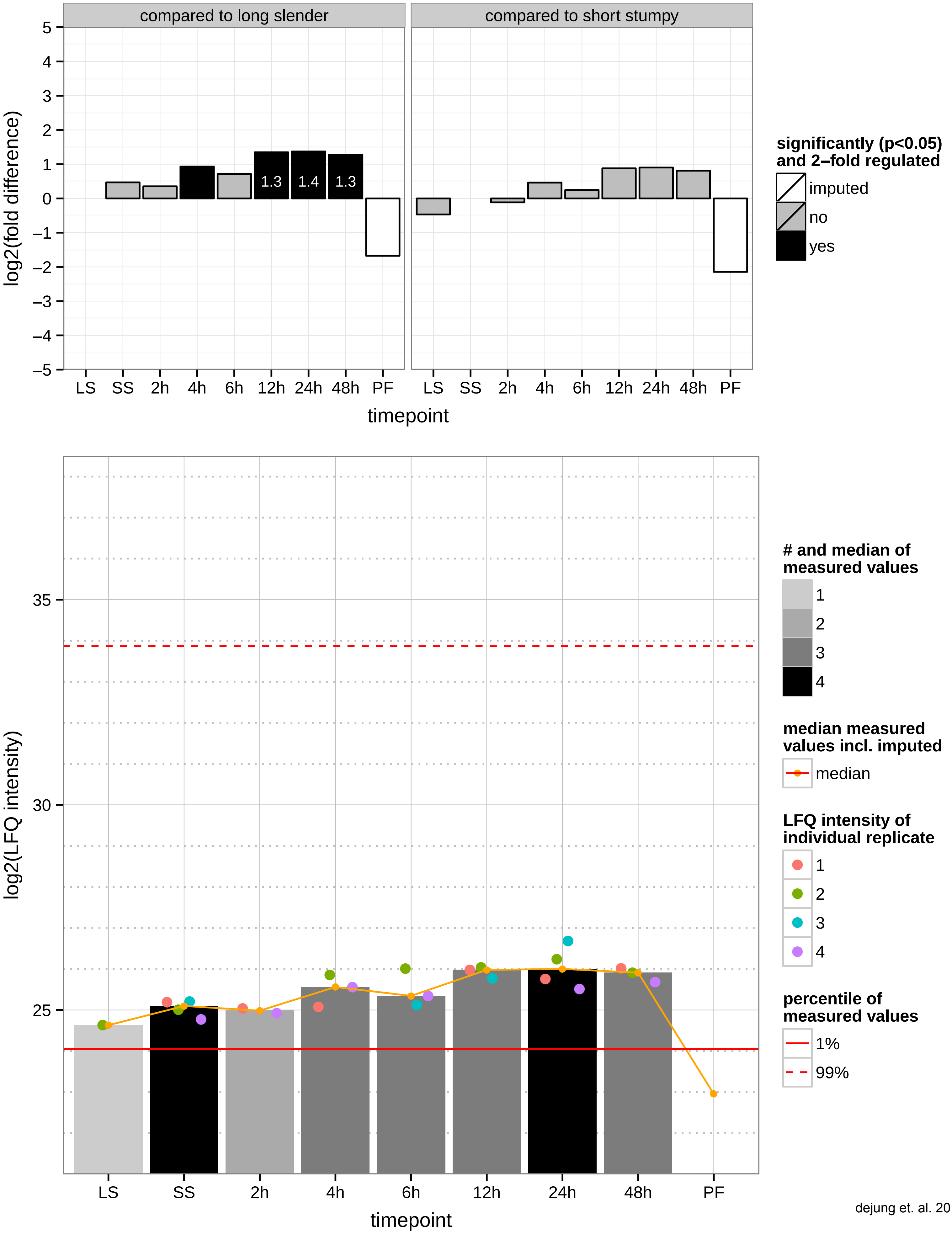

dejung et. al. 2015

FG−GAP repeat protein, putative, intergrin alpha chain protein
Tb927.1.1340
AGOF: null
AGOC: null
AGOP: null
PGOF: null
PGOC: null
PGOP: null

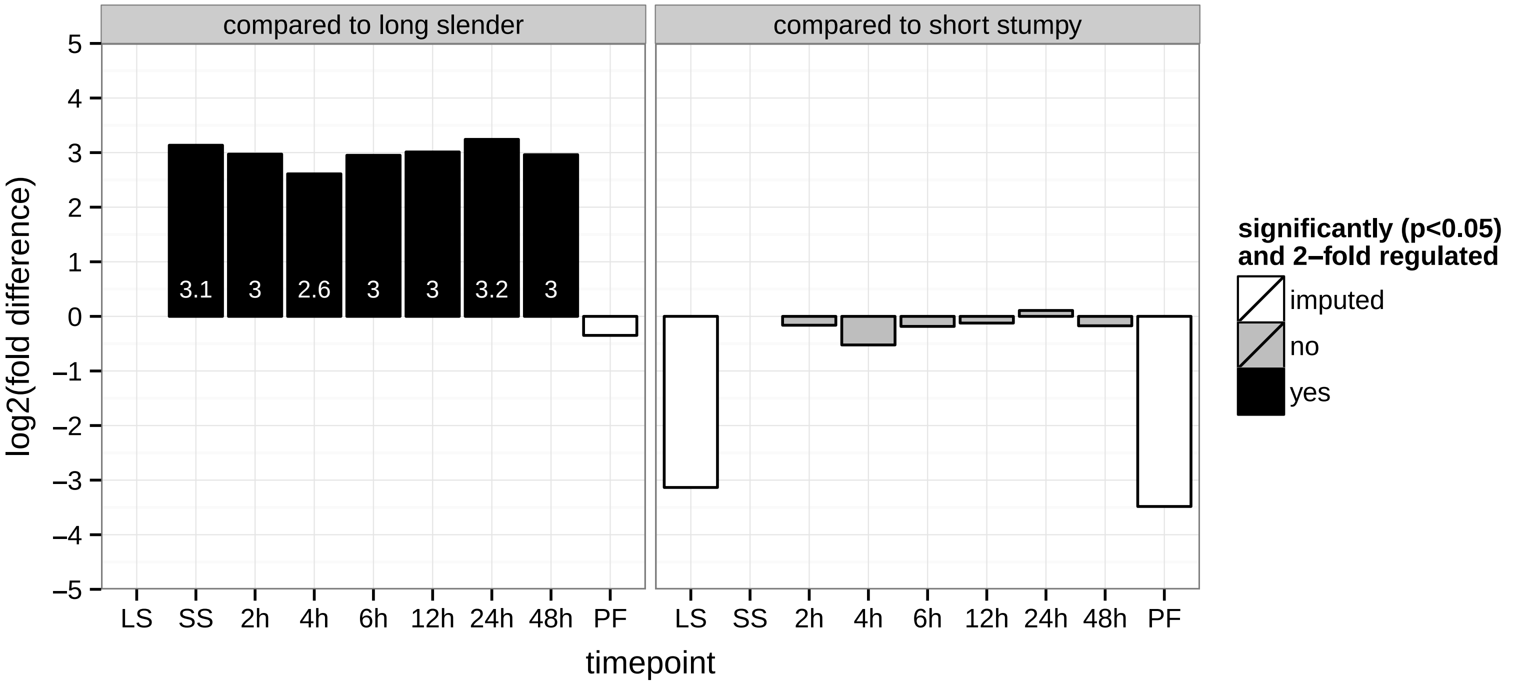

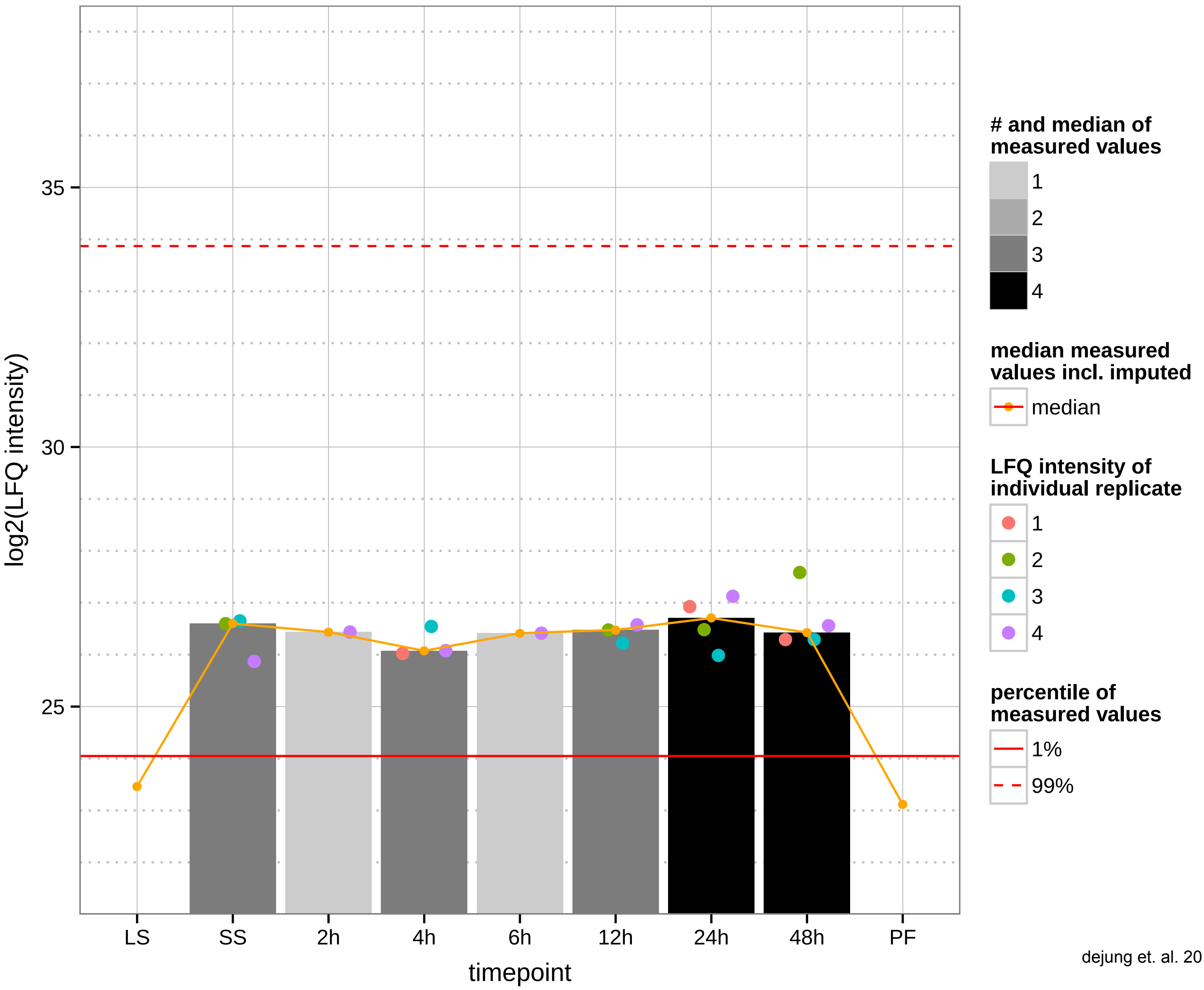

dejung et. al. 2015

Overproducer of inositol protein 10 homolog, putative
Tb927.1.1400
AGOF: null
AGOC: null
AGOP: null
PGOF: null
PGOC: null
PGOP: null

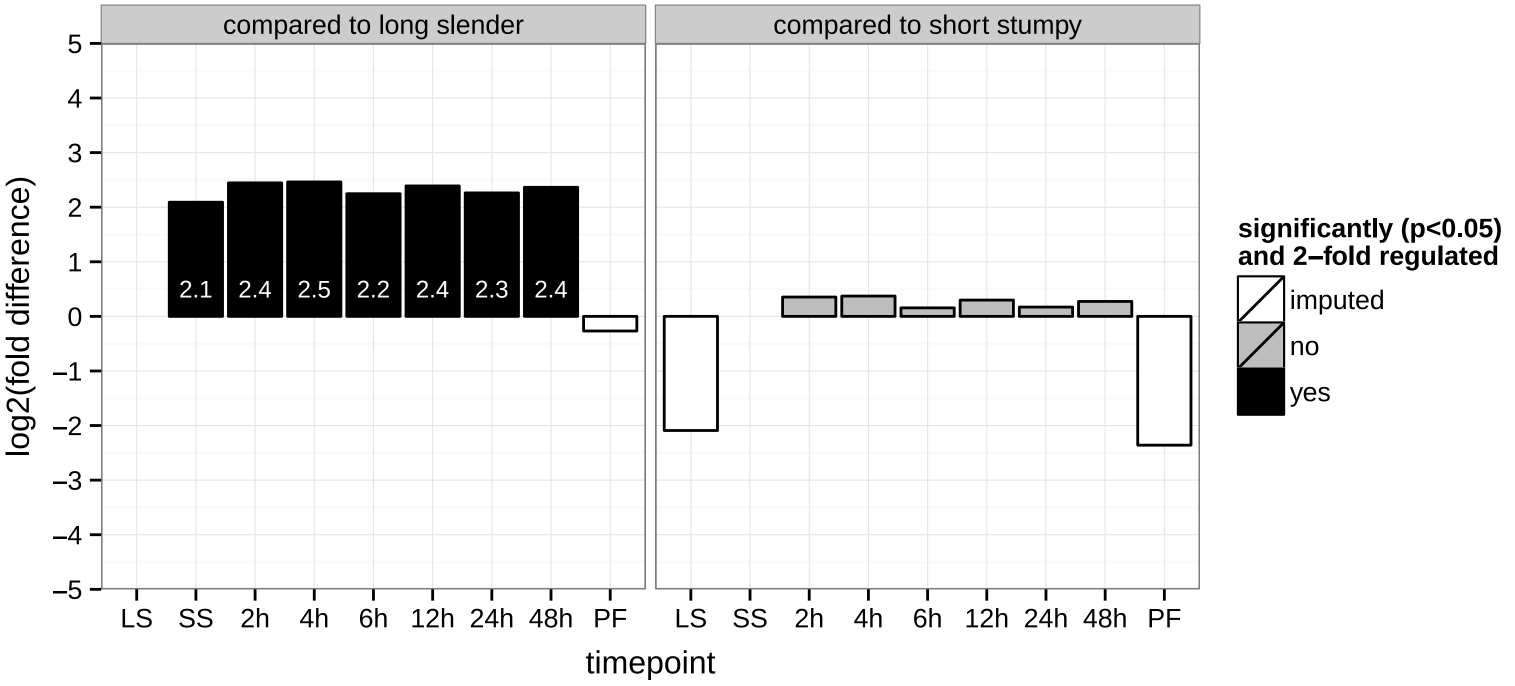

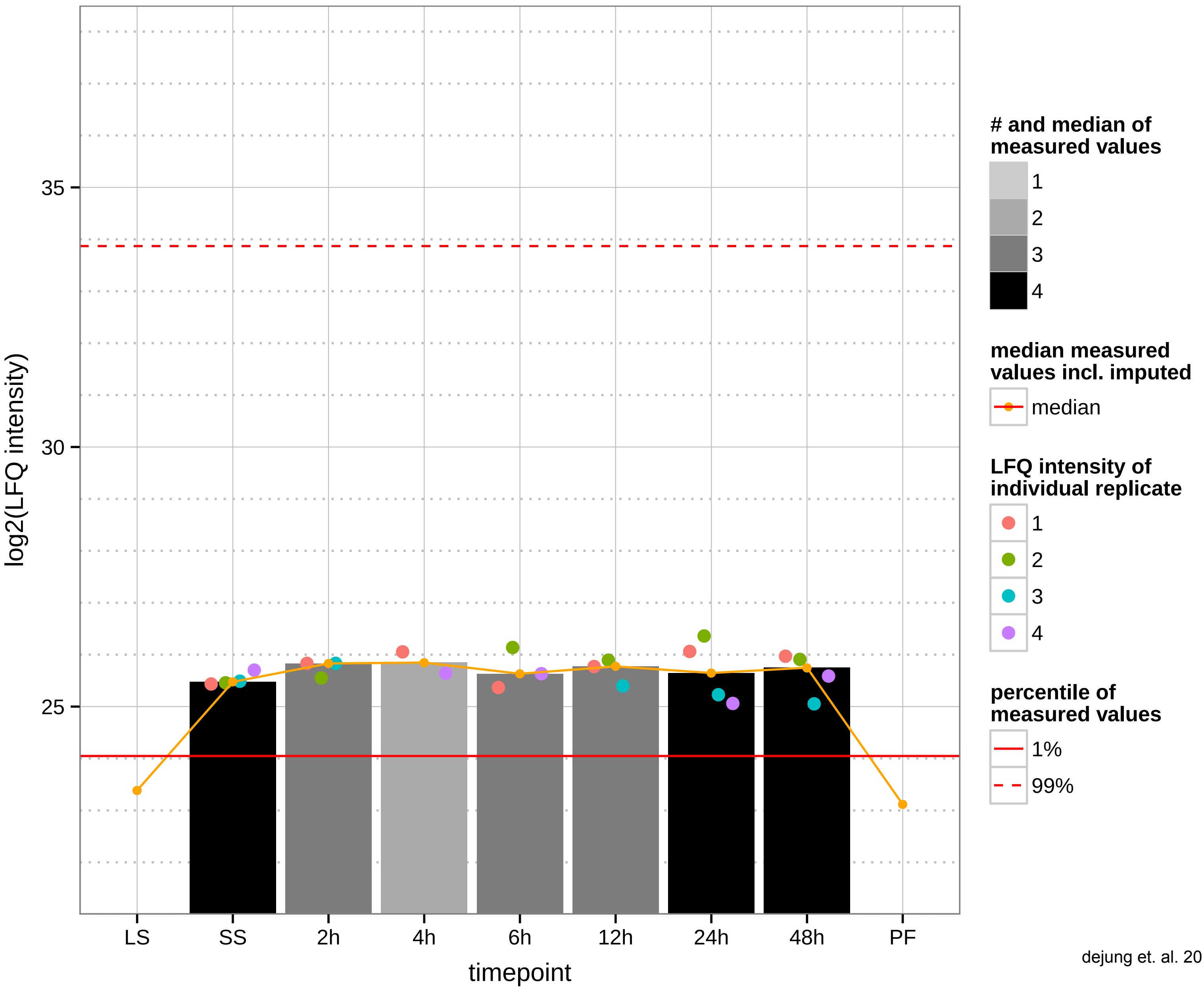

dejung et. al. 2015

hypothetical protein, conserved
Tb927.1.3390
AGOF: null
AGOC: null
AGOP: null
PGOF: null
PGOC: null
PGOP: null

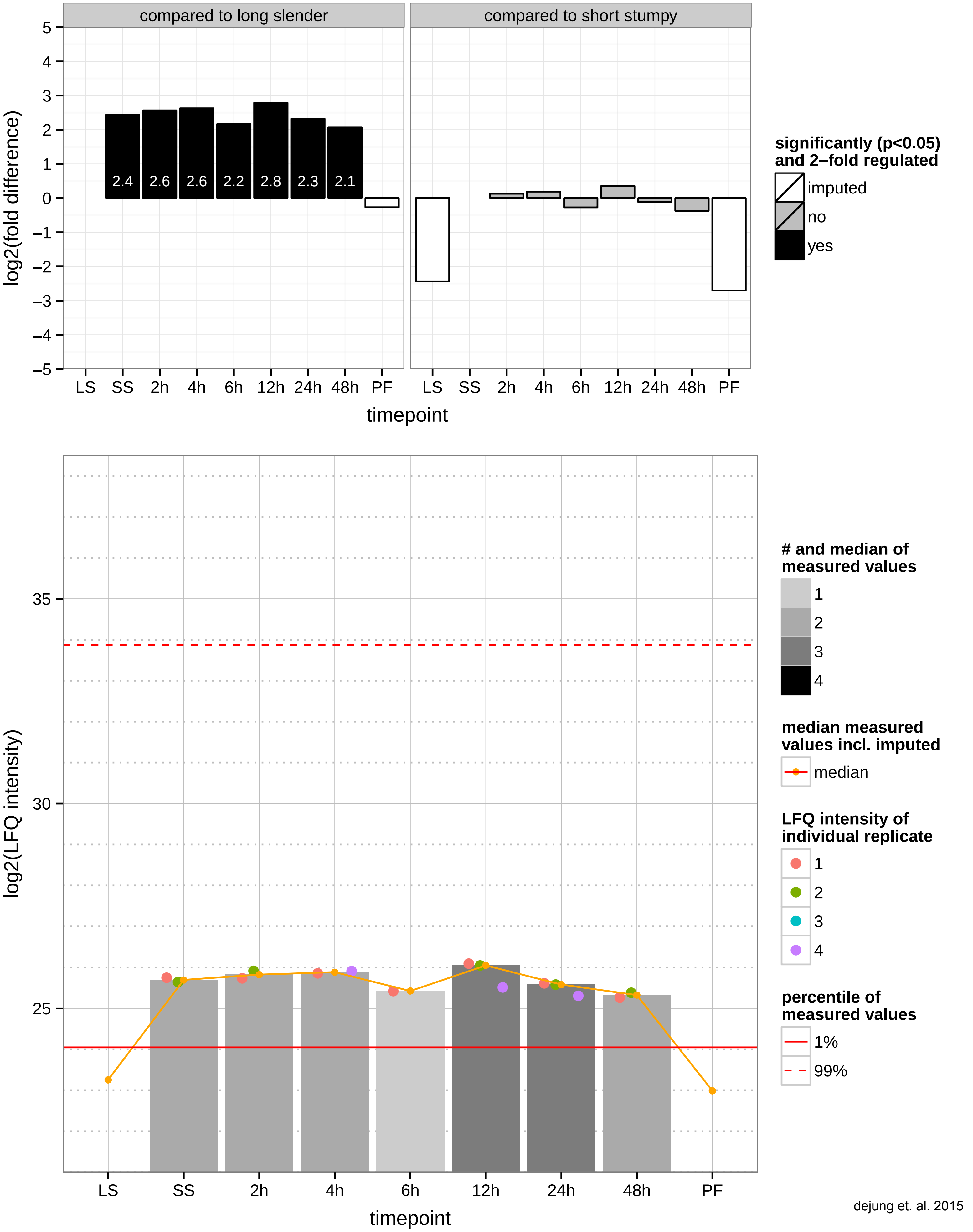

dejung et. al. 2015

hypothetical protein, conserved
Tb927.1.3410
AGOF: null
AGOC: null
AGOP: null
PGOF: null
PGOC: null
PGOP: null

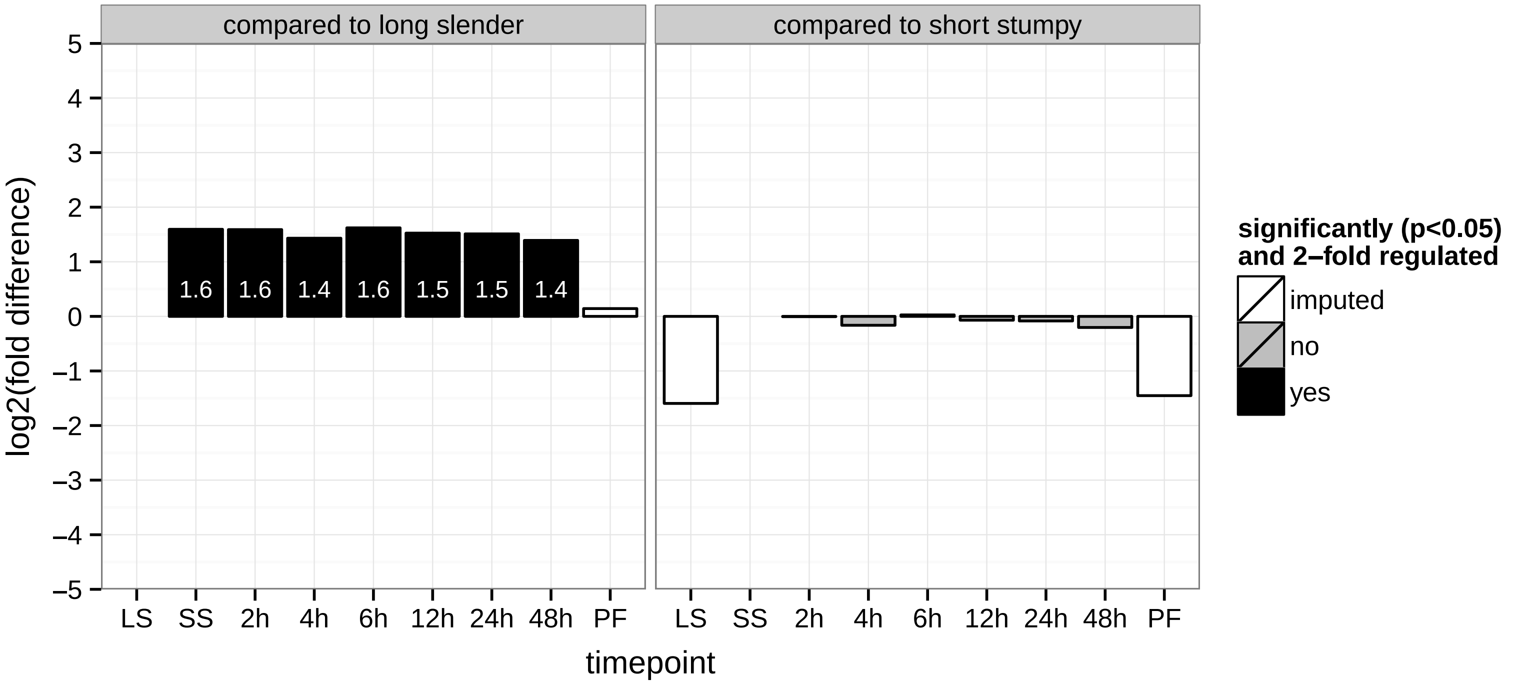

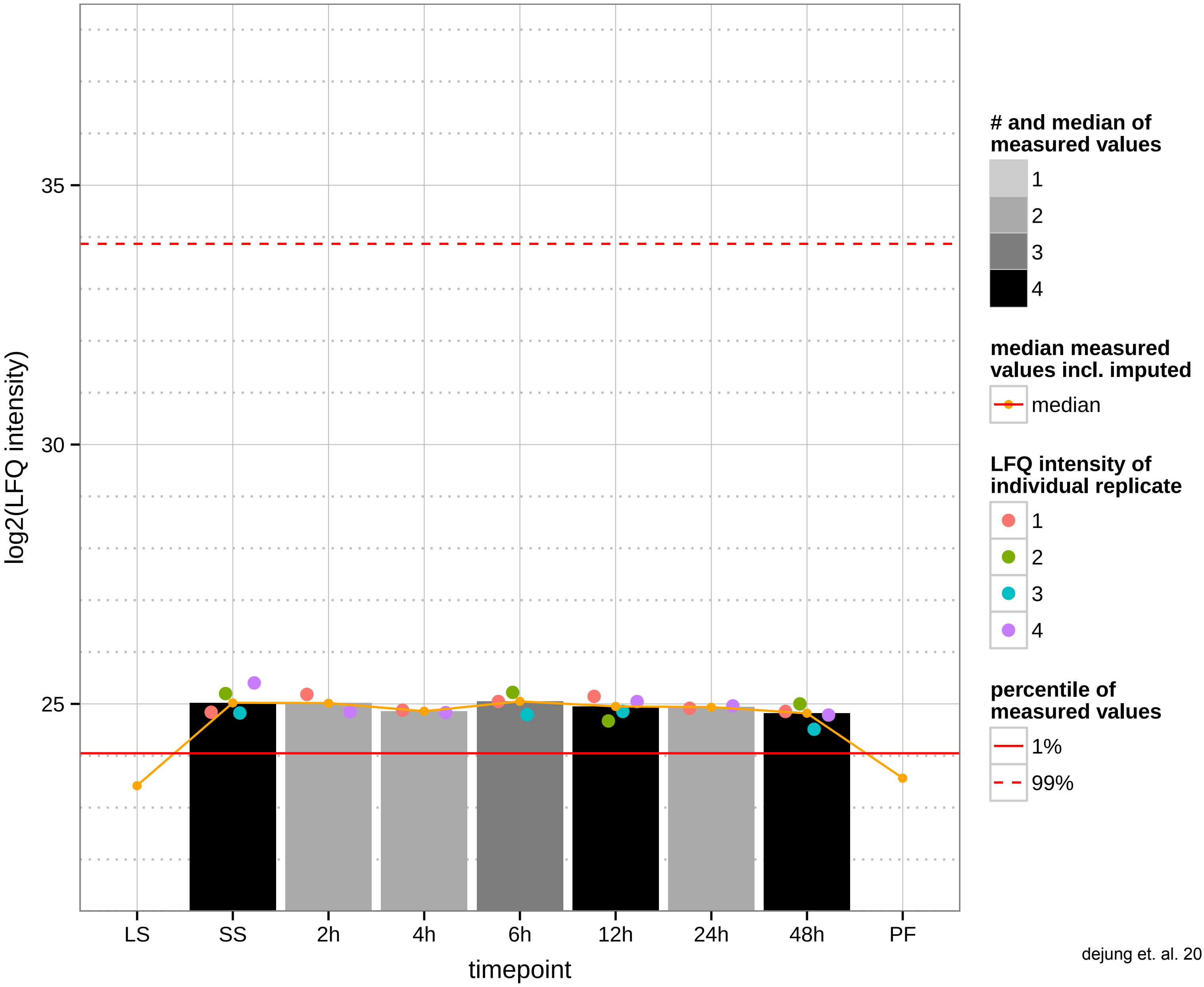

dejung et. al. 2015

hypothetical protein, conserved
Tb927.1.3880
AGOF: null
AGOC: null
AGOP: null
PGOF: null
PGOC: null
PGOP: null

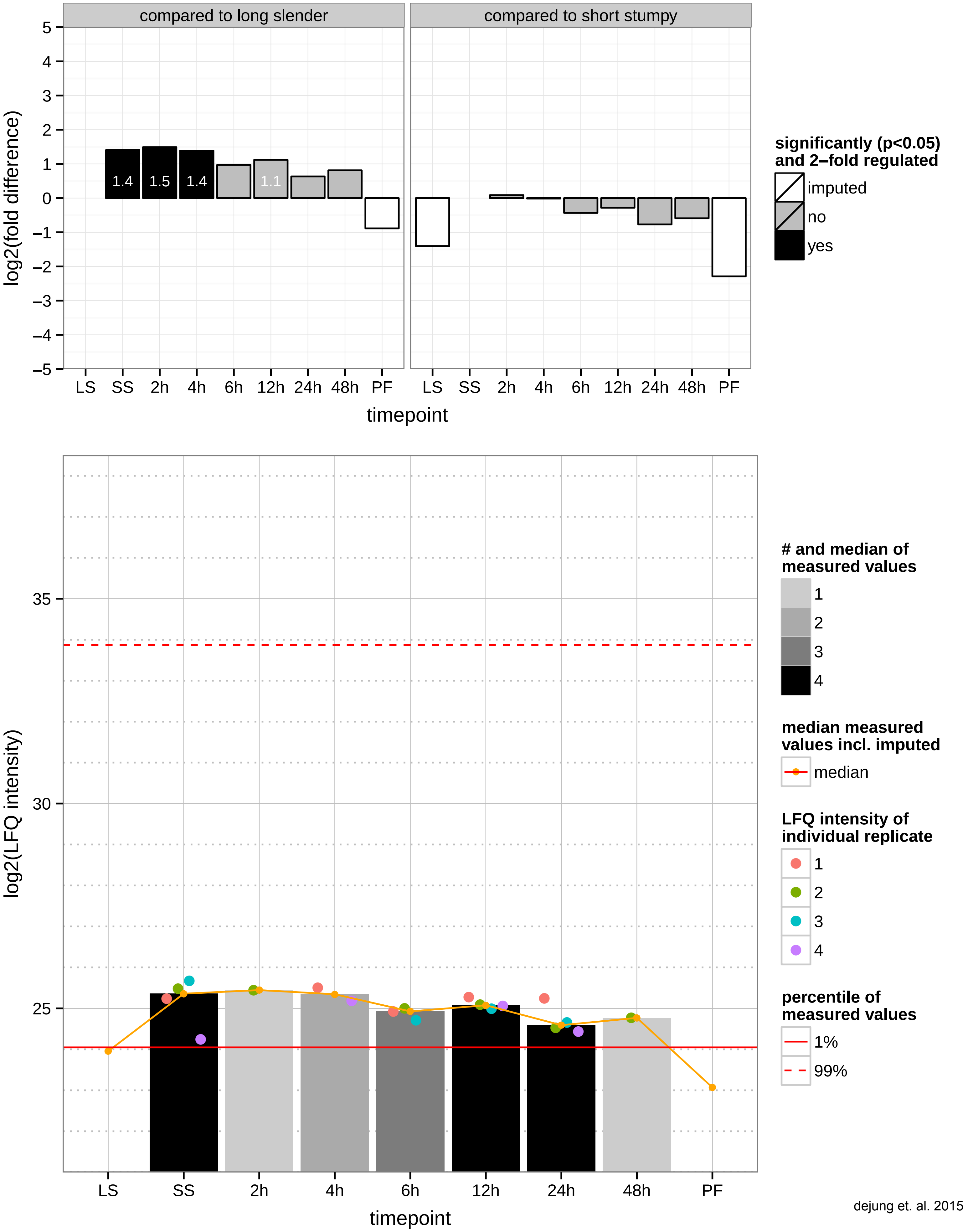

dejung et. al. 2015

alanine aminotransferase (ALAT)
Tb927.1.3950
AGOF: 1−aminocyclopropane−1−carboxylate synthase activity, L−alanine:2−oxoglutarate aminotransferase activity, pyridoxal
AGOC: cytosol
AGOP: alanine catabolic process, biosynthetic process, proline metabolic process
PGOF: pyridoxal phosphate binding, transferase activity
PGOC: null
PGOP: biosynthetic process

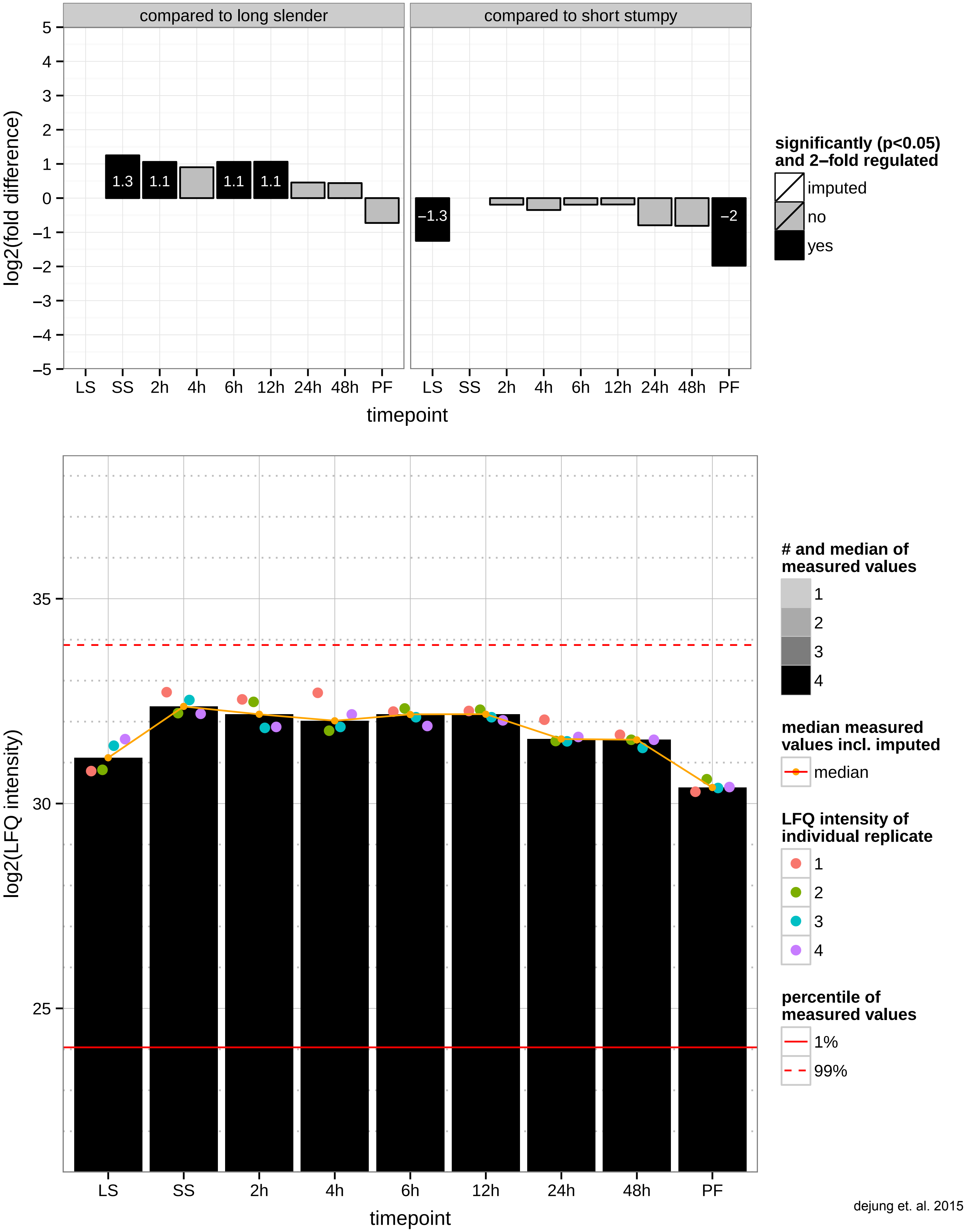

dejung et. al. 2015

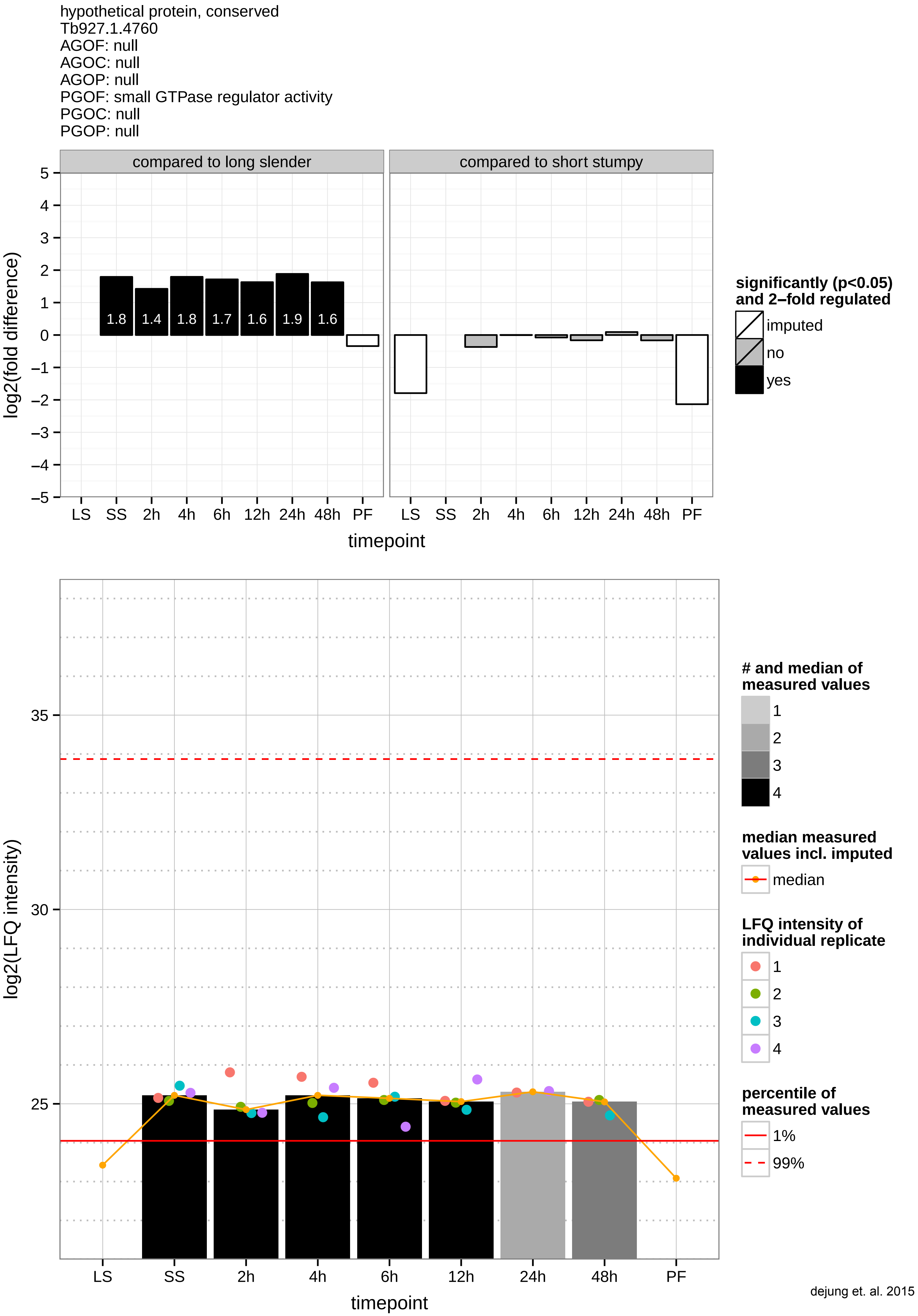
hypothetical protein, conserved
Tb927.1.4760
AGOF: null
AGOC: null
AGOP: null
PGOF: small GTPase regulator activity
PGOC: null
PGOP: null
compared to long slender
compared to short stumpy
log2(fold difference)
1.8
1.4
1.8
1.7
1.6
1.9
1.6
LS SS 2h 4h 6h 12h 24h 48h PF
timepoint
significantly (p<0.05) and 2–fold regulated
imputed
no
yes
log2(LFQ intensity)
# and median of measured values
1
2
3
4
median measured values incl. imputed
median
LFQ intensity of individual replicate
1
2
3
4
percentile of measured values
1%
99%
dejung et. al. 2015

serine peptidase, putative, Clan SC, Family S9D
Tb927.1.4780
AGOF: null
AGOC: null
AGOP: null
PGOF: null
PGOC: null
PGOP: null

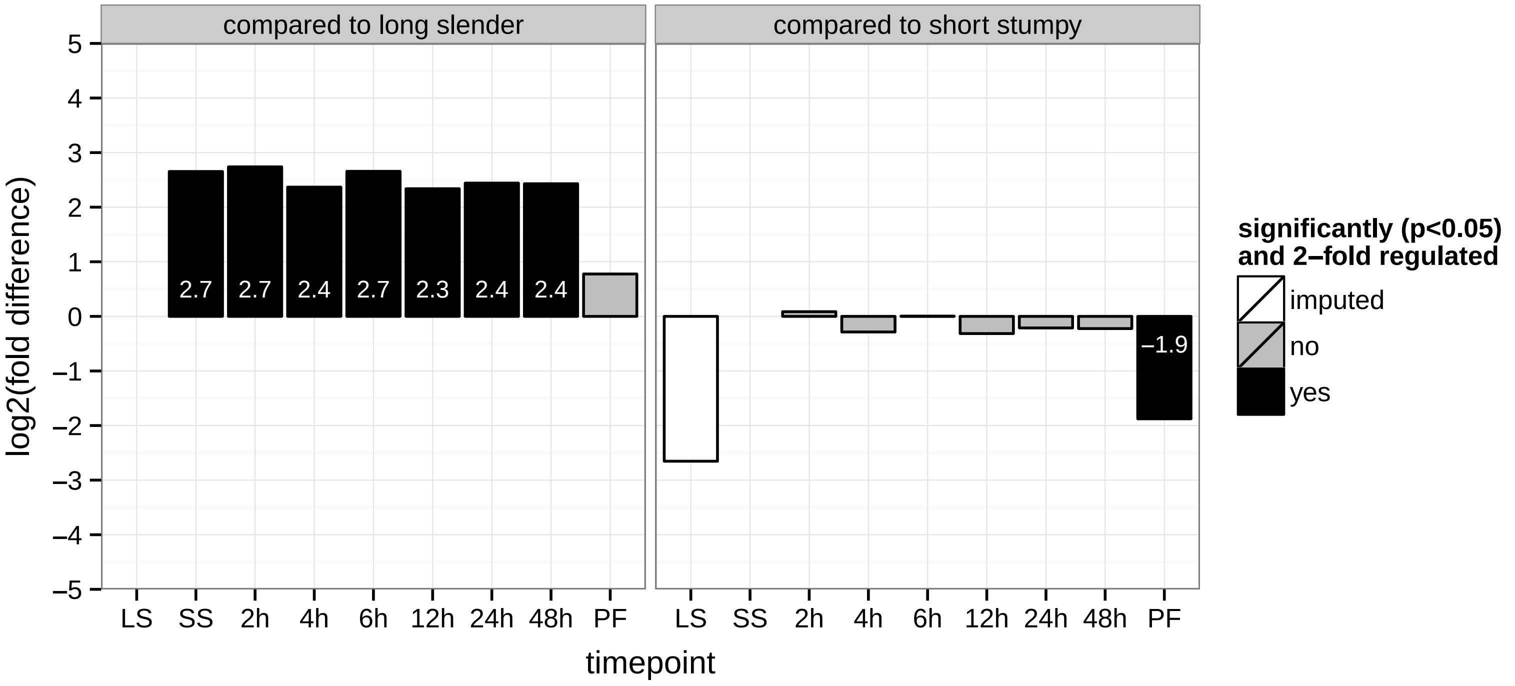

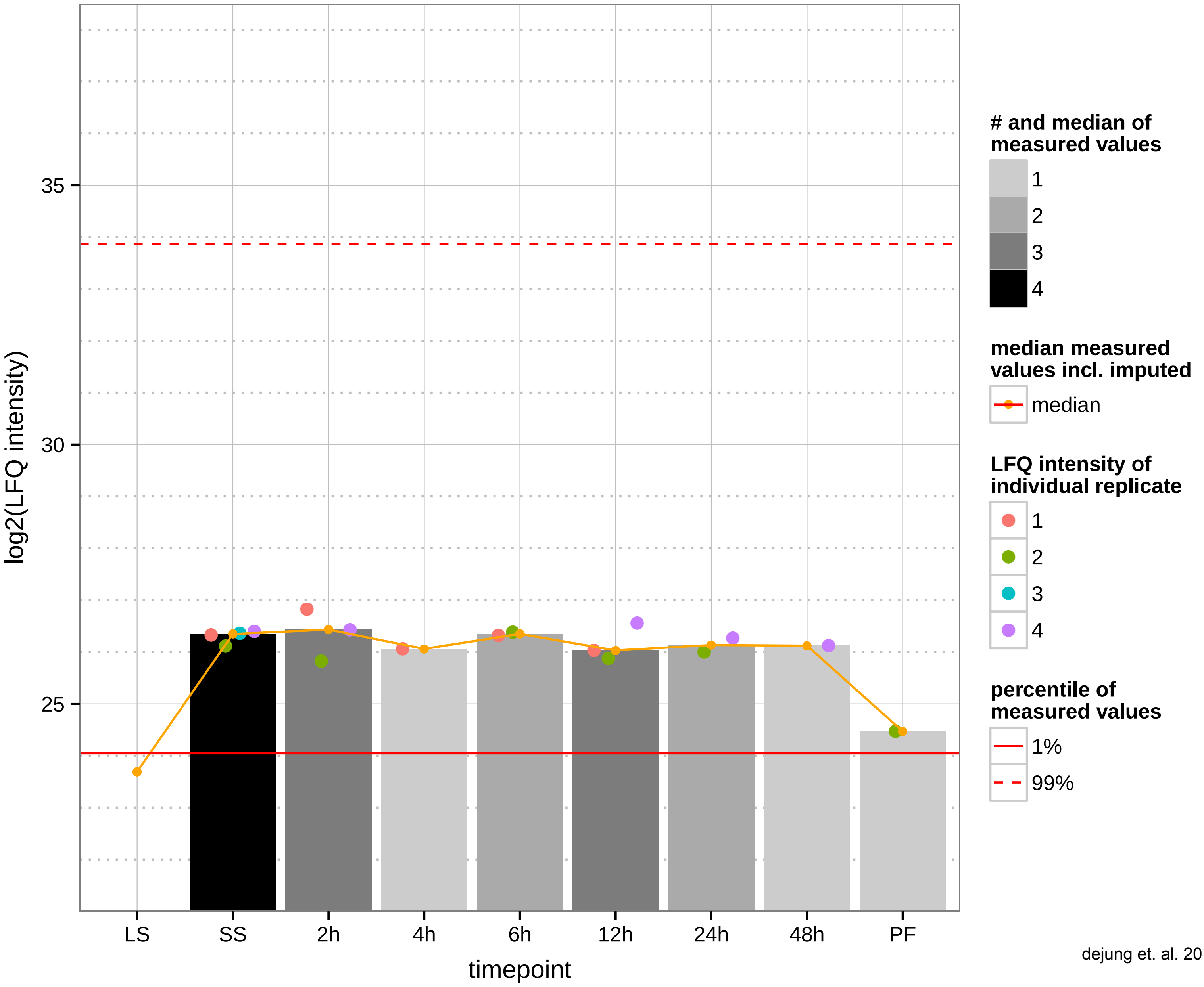

dejung et. al. 2015

phospholipase A1 (PLA1)
Tb927.1.4830
AGOF: phosphatidylcholine 1−acylhydrolase activity
AGOC: null
AGOP: CDP−choline pathway
PGOF: null
PGOC: null
PGOP: null

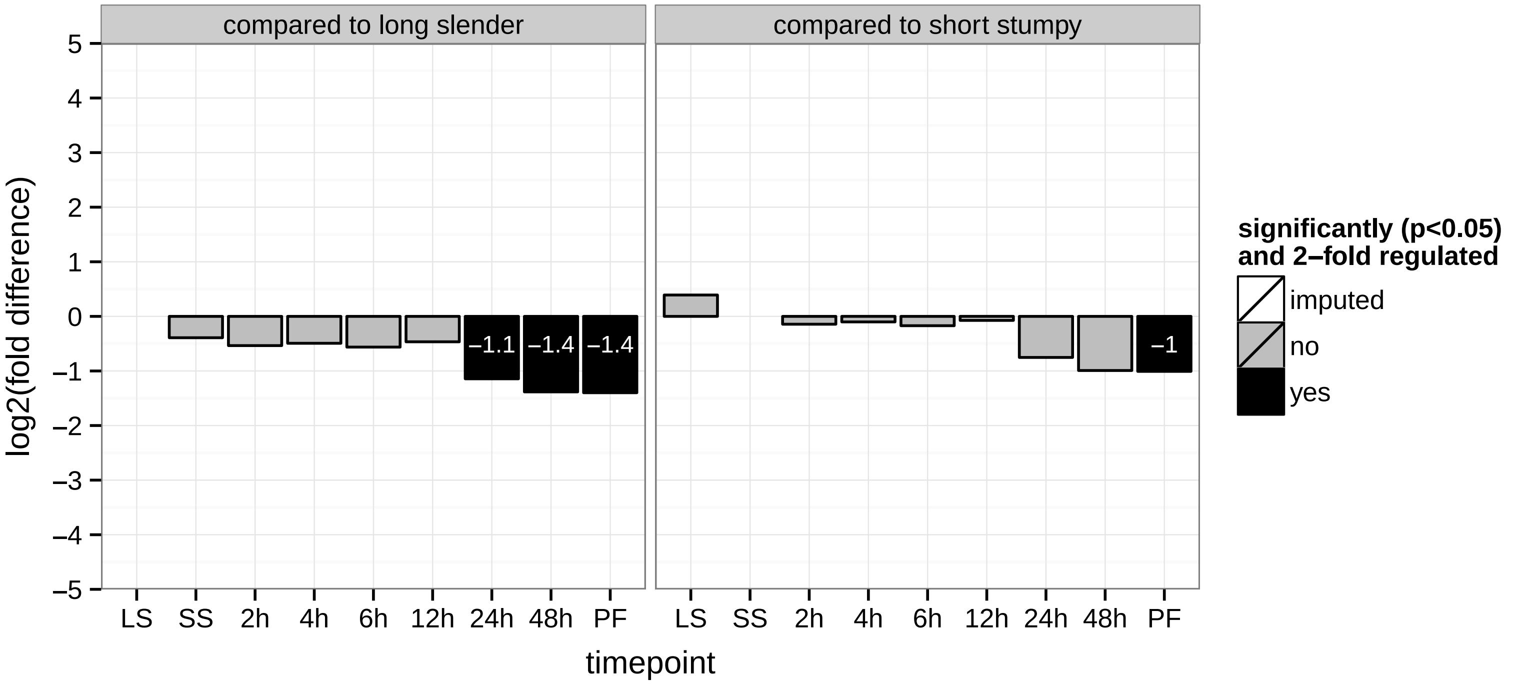

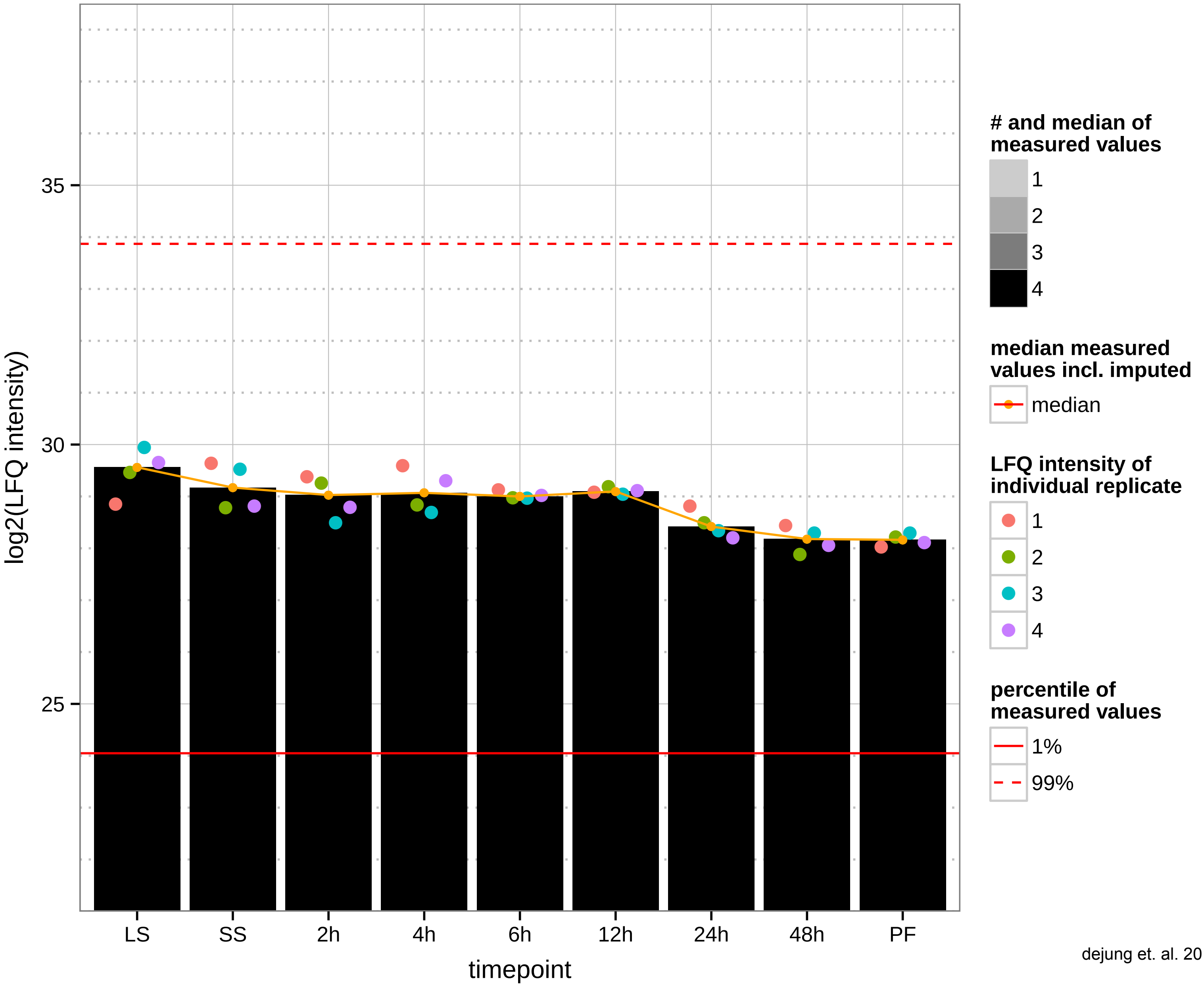

dejung et. al. 2015

hypothetical protein, conserved
Tb927.1.690
AGOF: null
AGOC: null
AGOP: null
PGOF: null
PGOC: null
PGOP: null

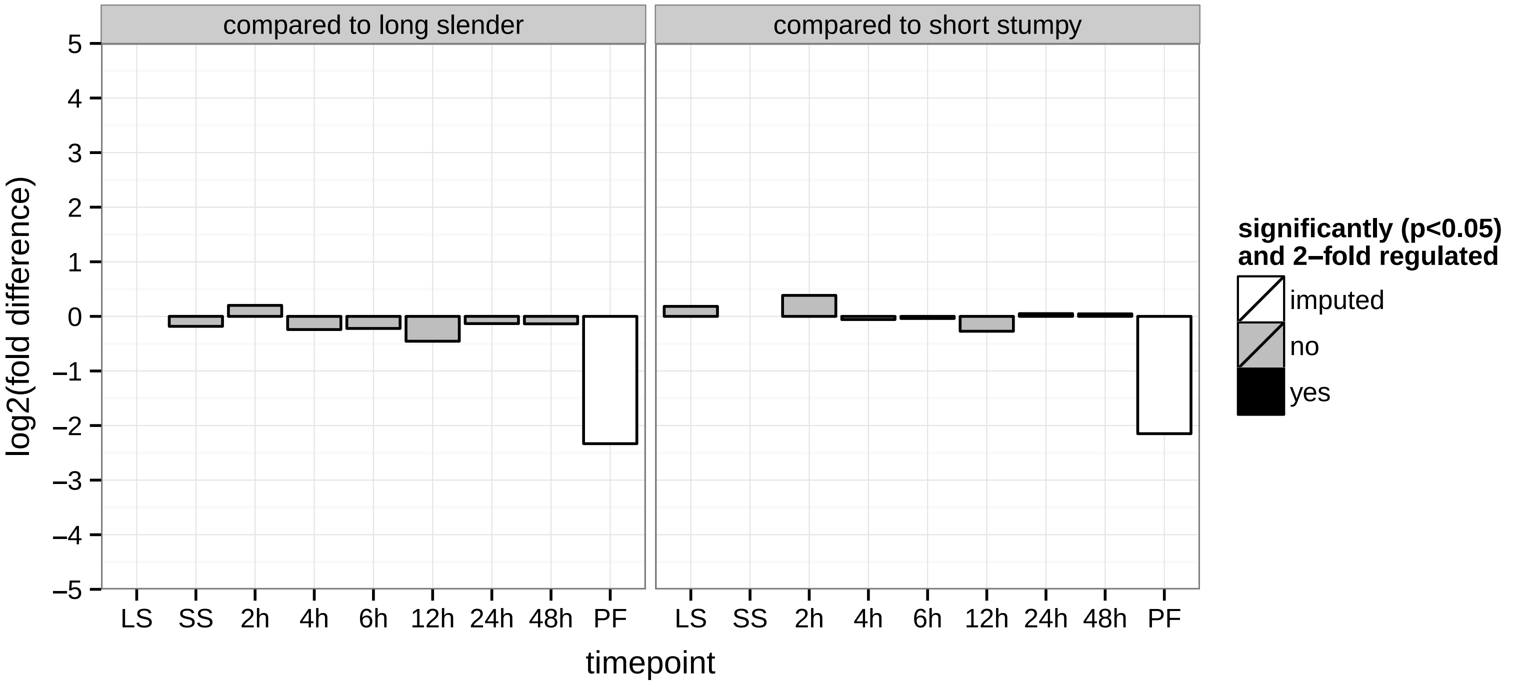

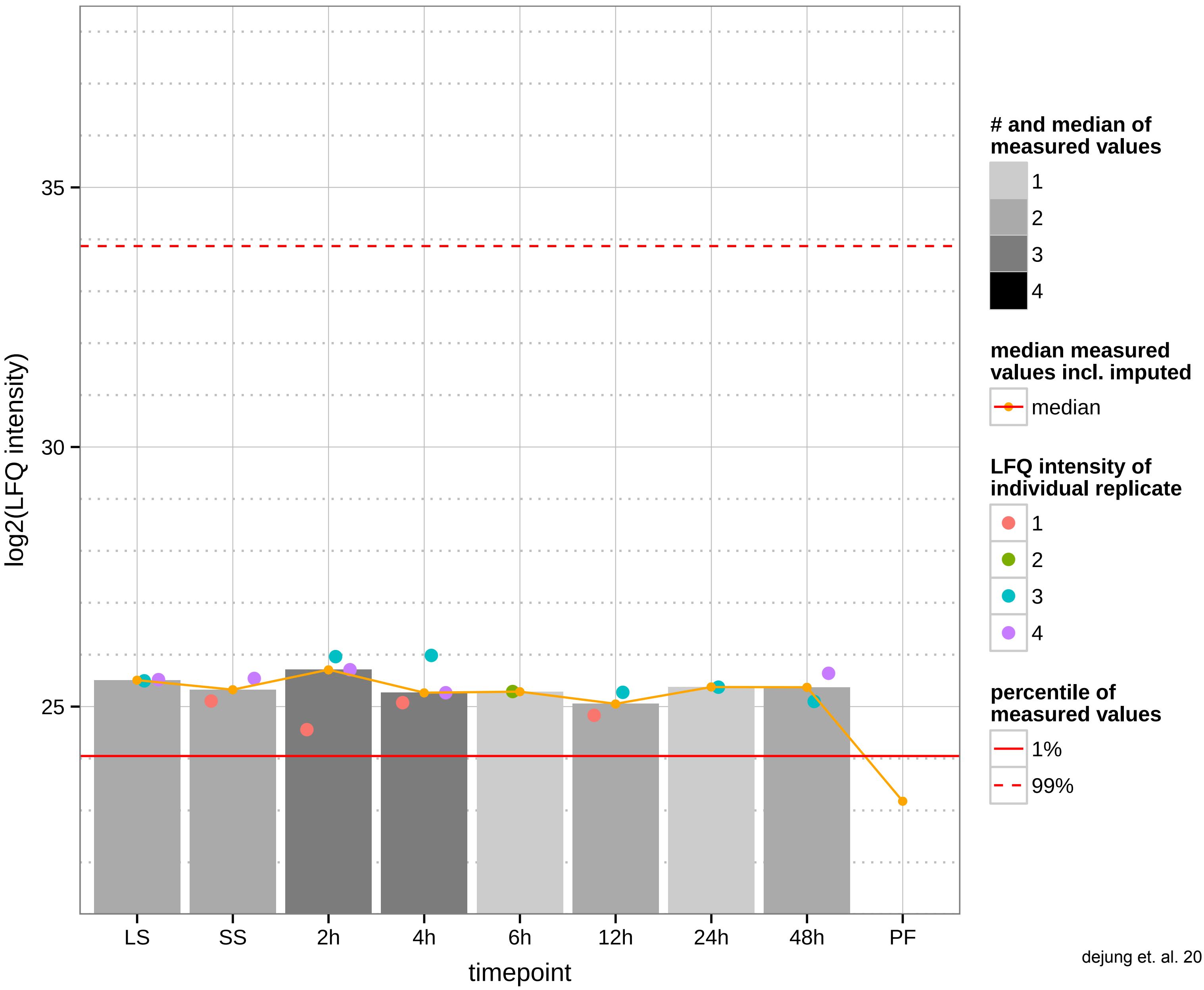

dejung et. al. 2015

retrotransposon hot spot protein (RHS, pseudogene), putative
Tb927.1.90
AGOF: null
AGOC: null
AGOP: null
PGOF: null
PGOC: null
PGOP: null

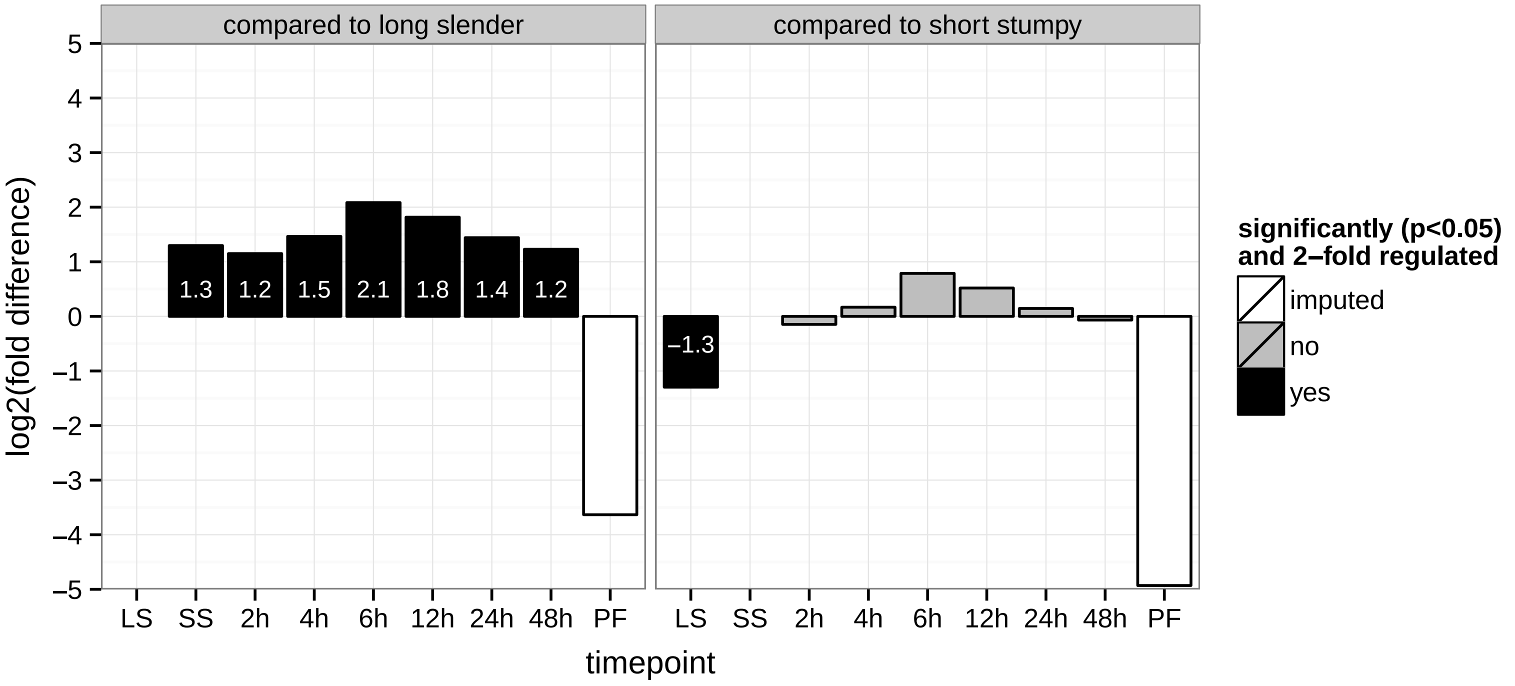

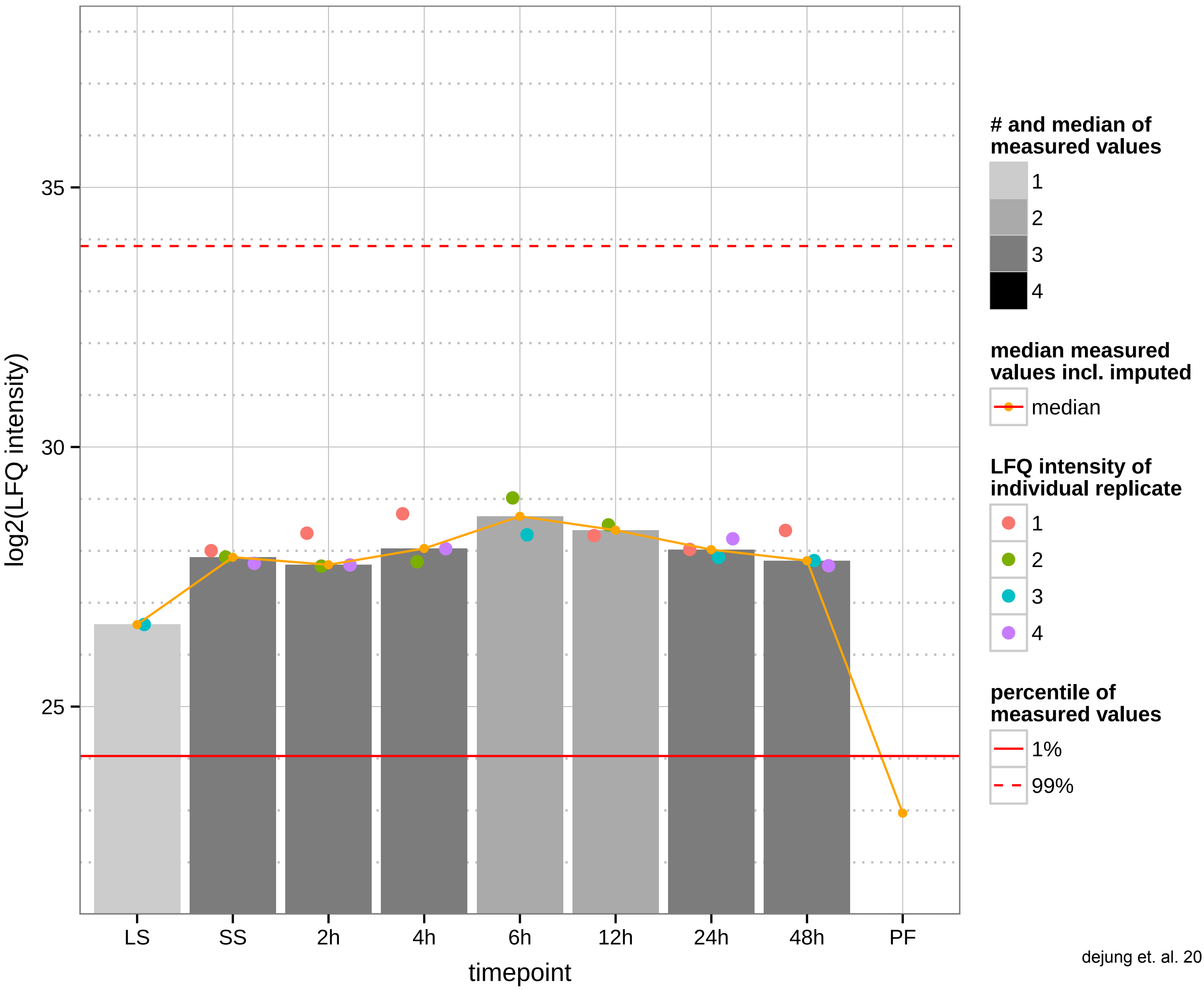

dejung et. al. 2015

hypothetical protein, conserved
Tb927.10.10020;Tb11.v5.0853
AGOF: null
AGOC: null
AGOP: null
PGOF: null
PGOC: null
PGOP: null

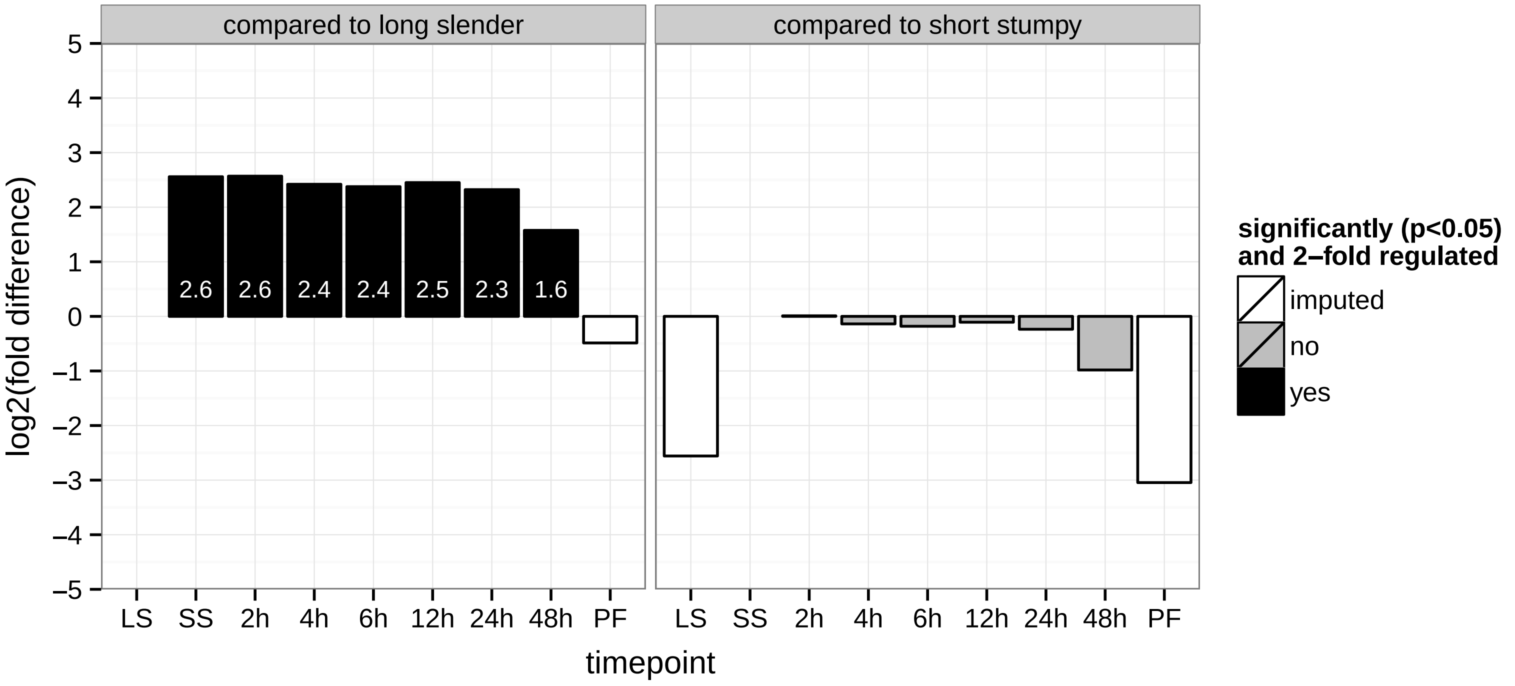

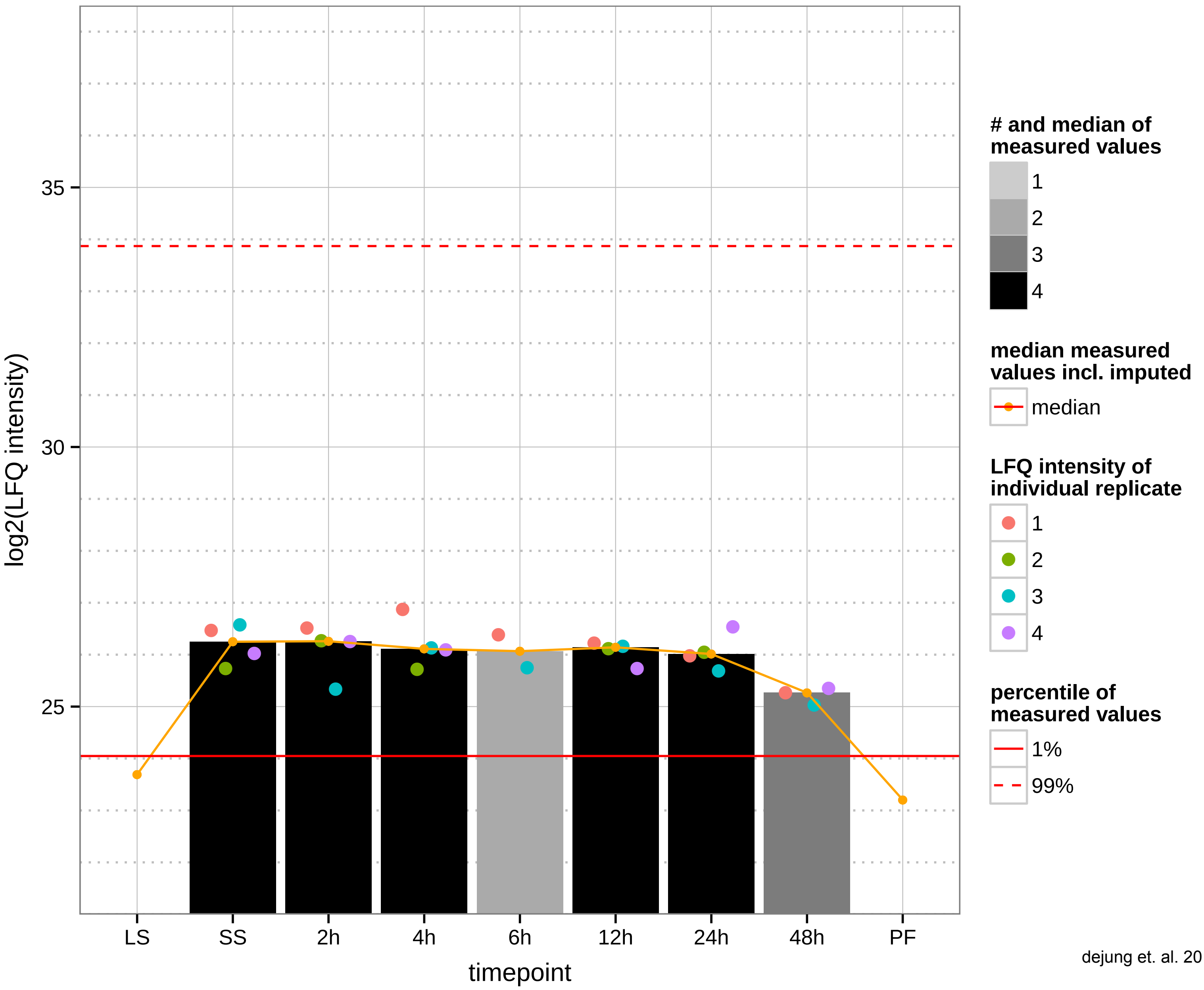

dejung et. al. 2015

microtubule–associated protein, putative
Tb927.10.10280
AGOF: microtubule binding
AGOC: cytoplasm
AGOP: null
PGOF: null
PGOC: null
PGOP: null

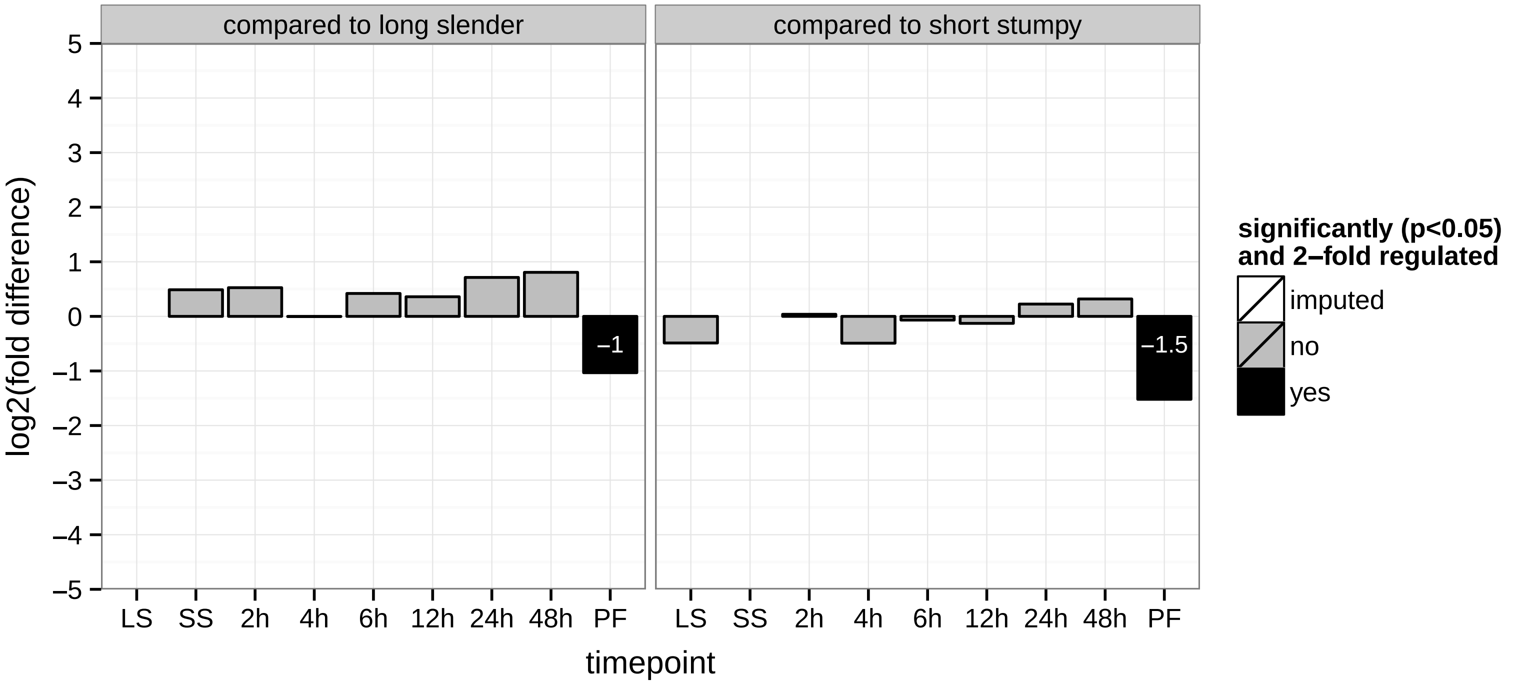

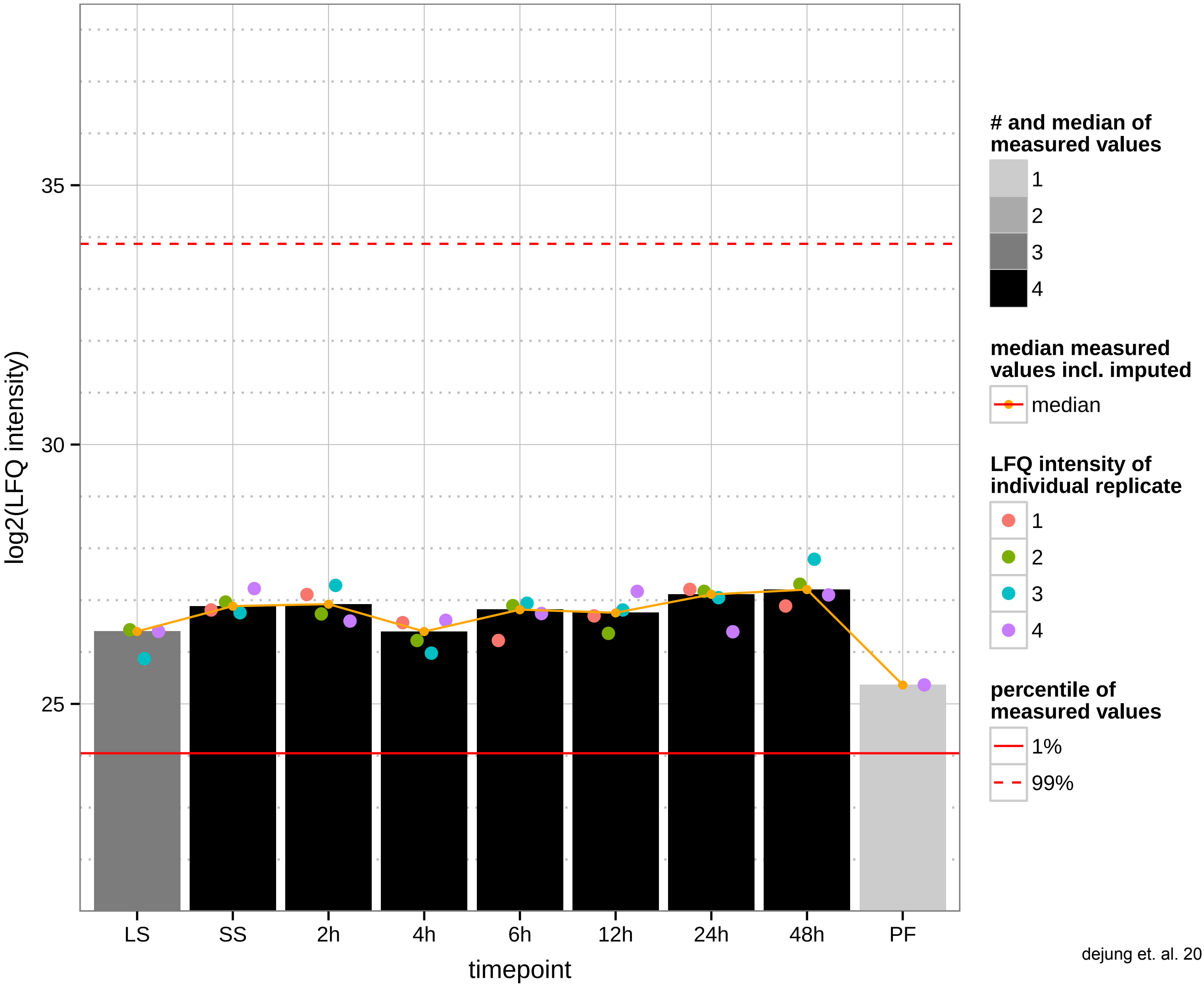

dejung et. al. 2015

hypothetical protein, conserved
Tb927.10.12030
AGOF: null
AGOC: null
AGOP: null
PGOF: null
PGOC: null
PGOP: null

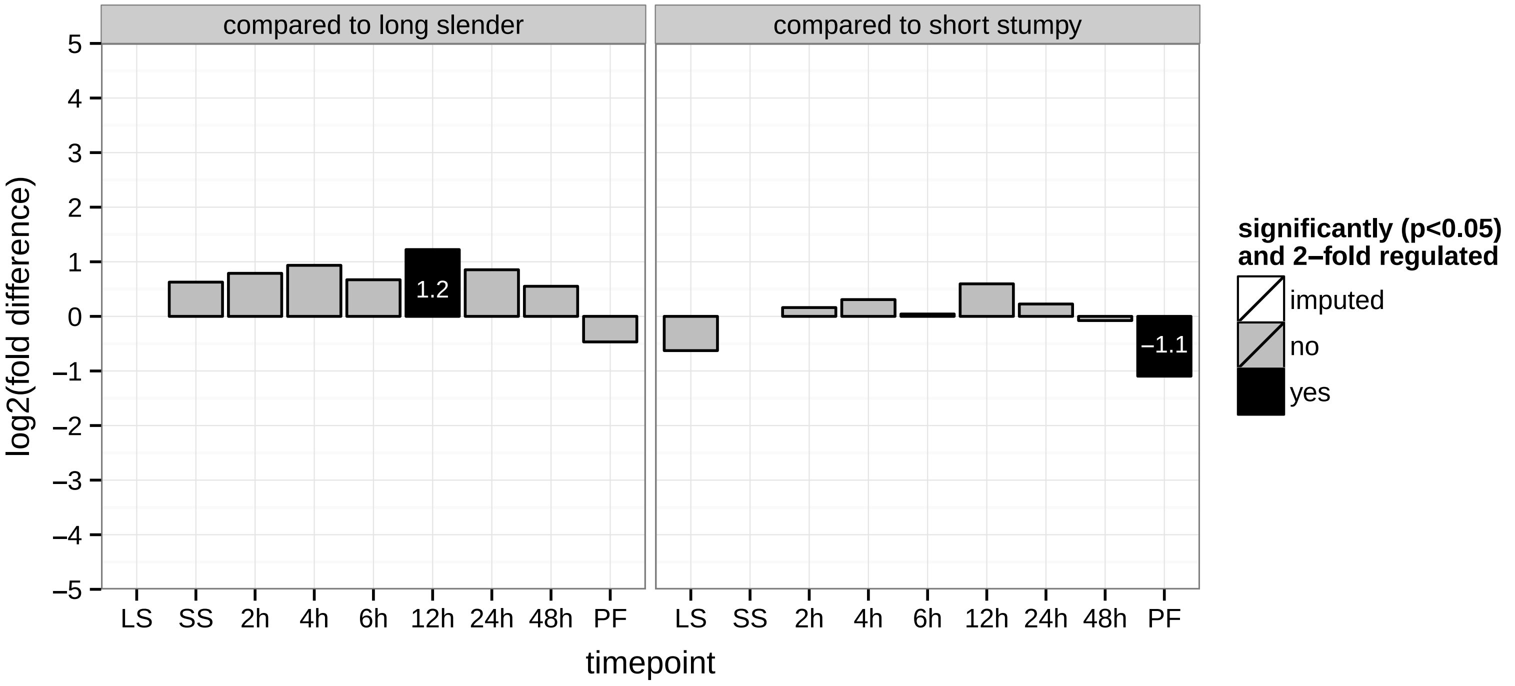

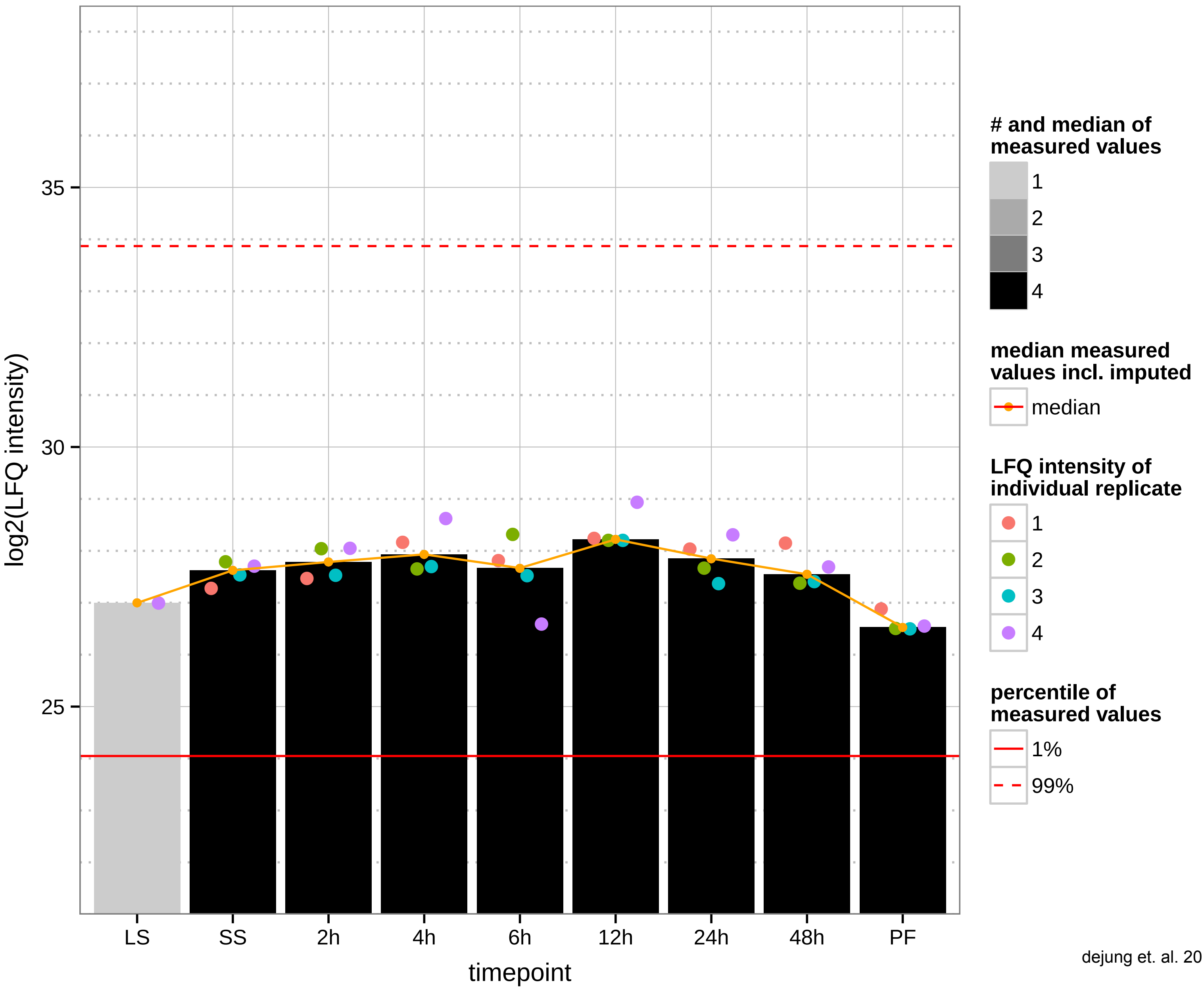

dejung et. al. 2015

SNARE domain−containing protein, putative
Tb927.10.12450
AGOF: null
AGOC: contractile vacuole, integral to membrane
AGOP: vesicle−mediated transport
PGOF: null
PGOC: null
PGOP: transport

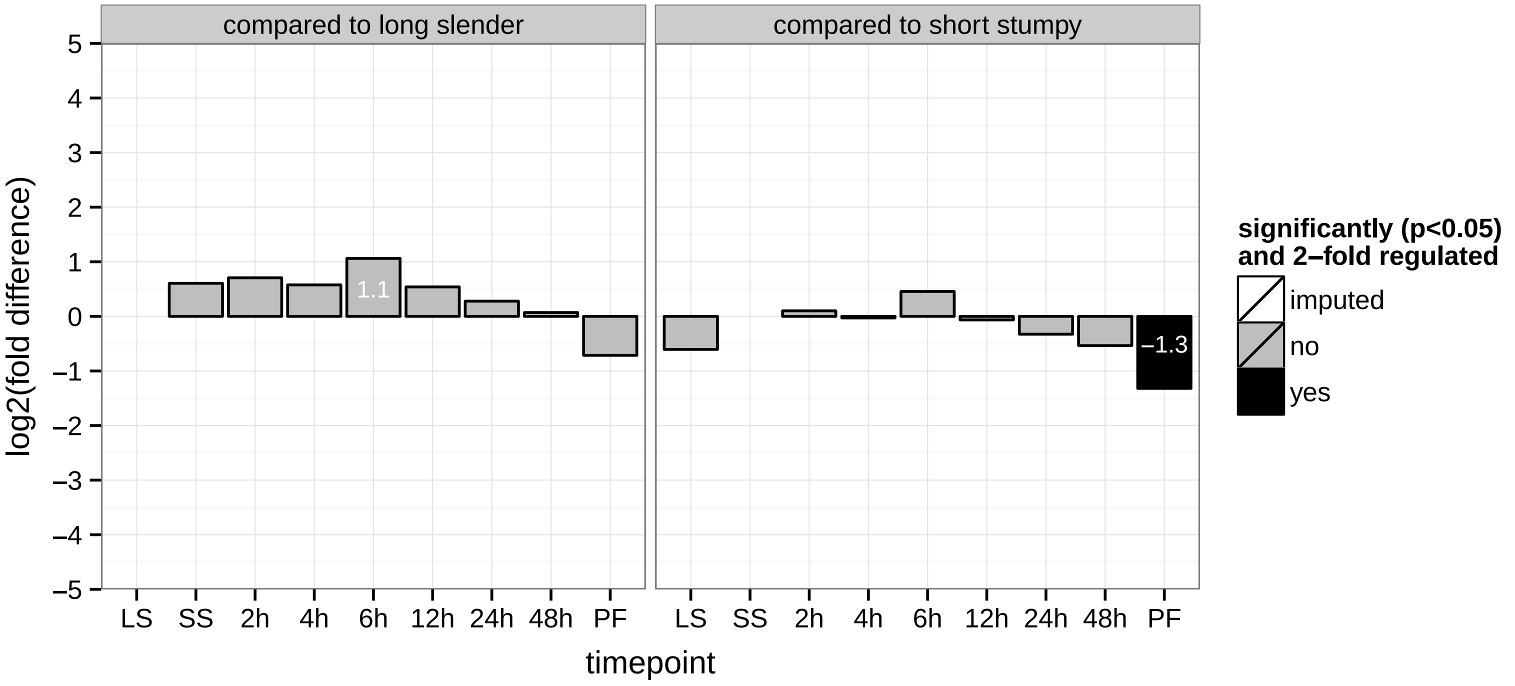

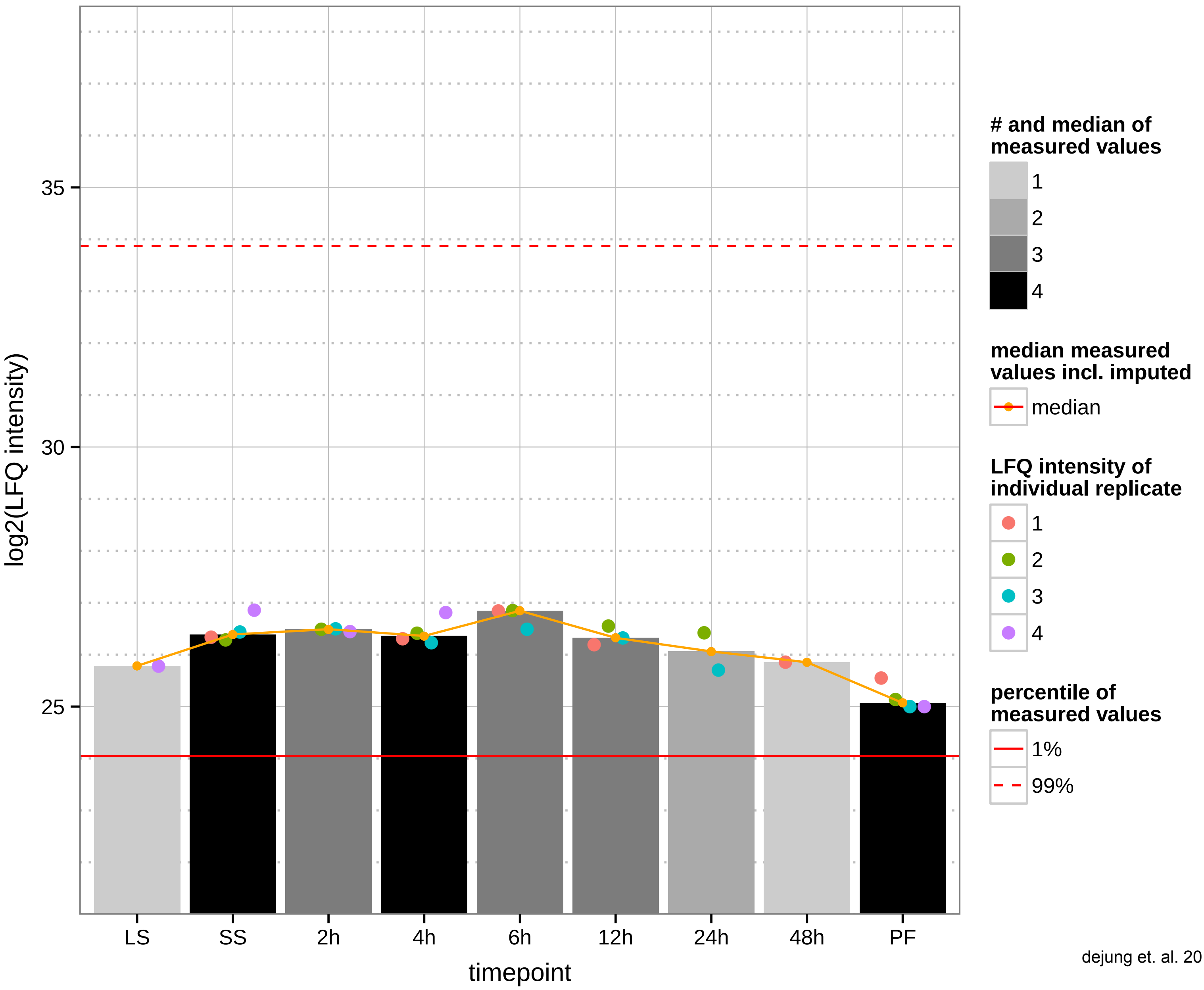

dejung et. al. 2015

hypothetical protein, conserved
Tb927.10.12720
AGOF: null
AGOC: null
AGOP: null
PGOF: null
PGOC: null
PGOP: null

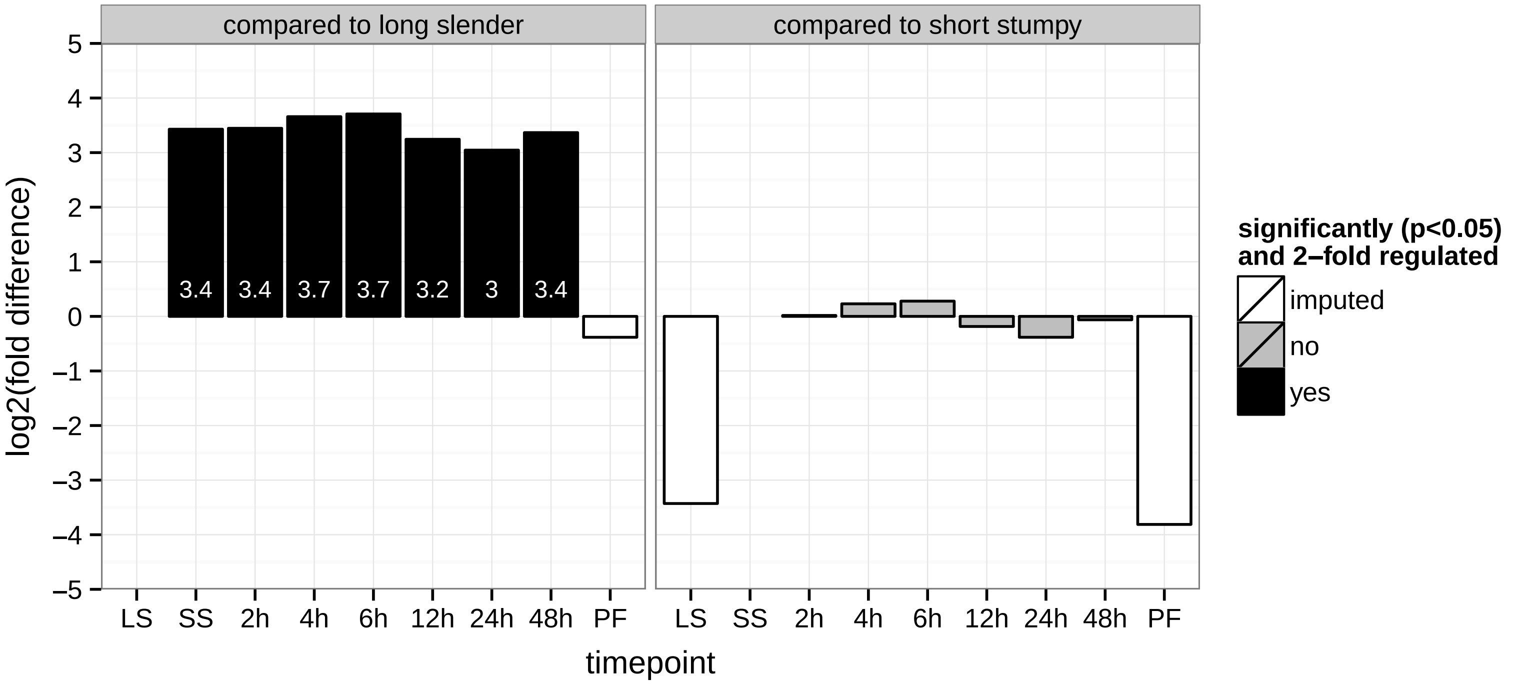

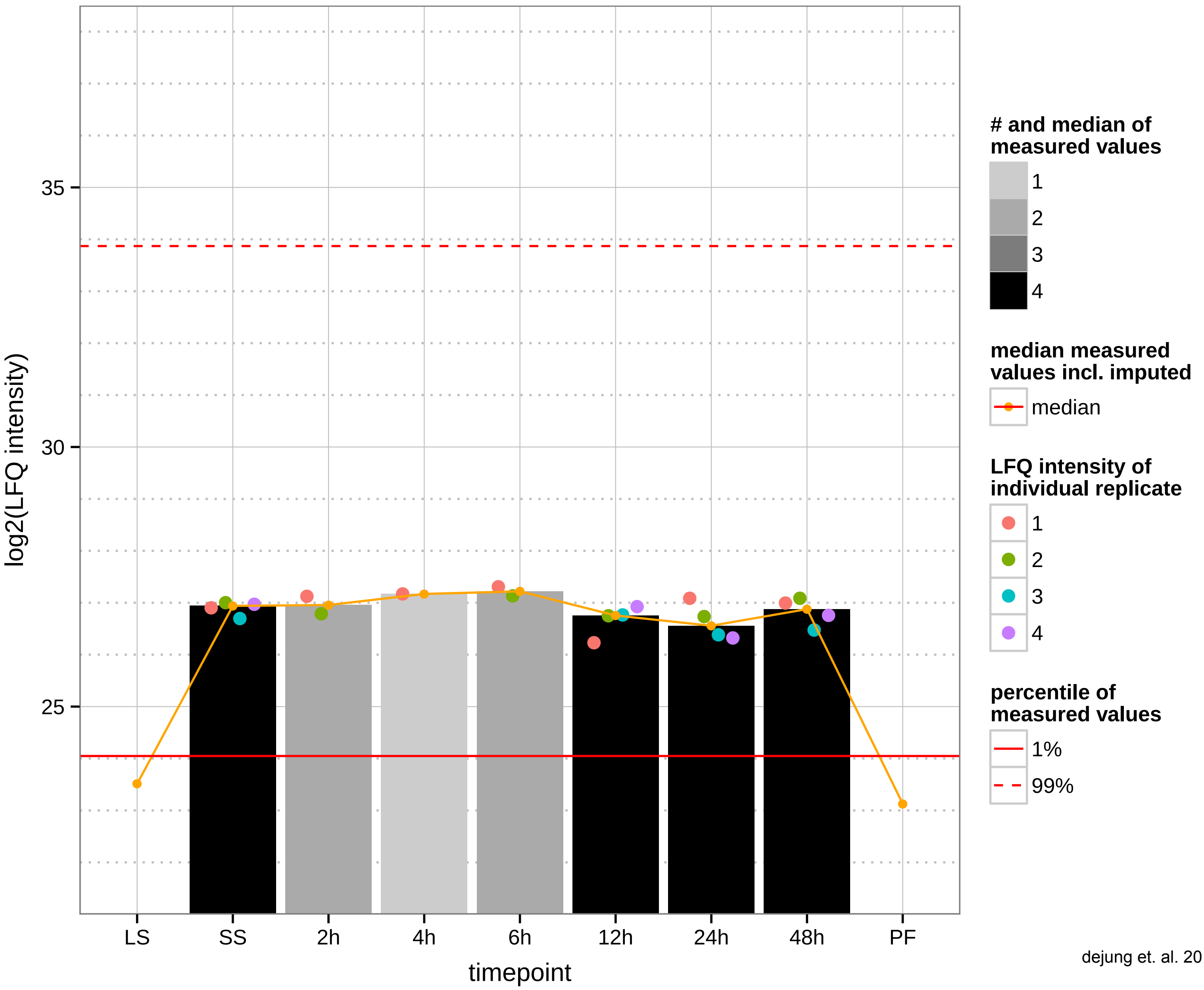

dejung et. al. 2015

hypothetical protein, conserved
Tb927.10.12970
AGOF: zinc ion binding
AGOC: null
AGOP: null
PGOF: zinc ion binding
PGOC: null
PGOP: null

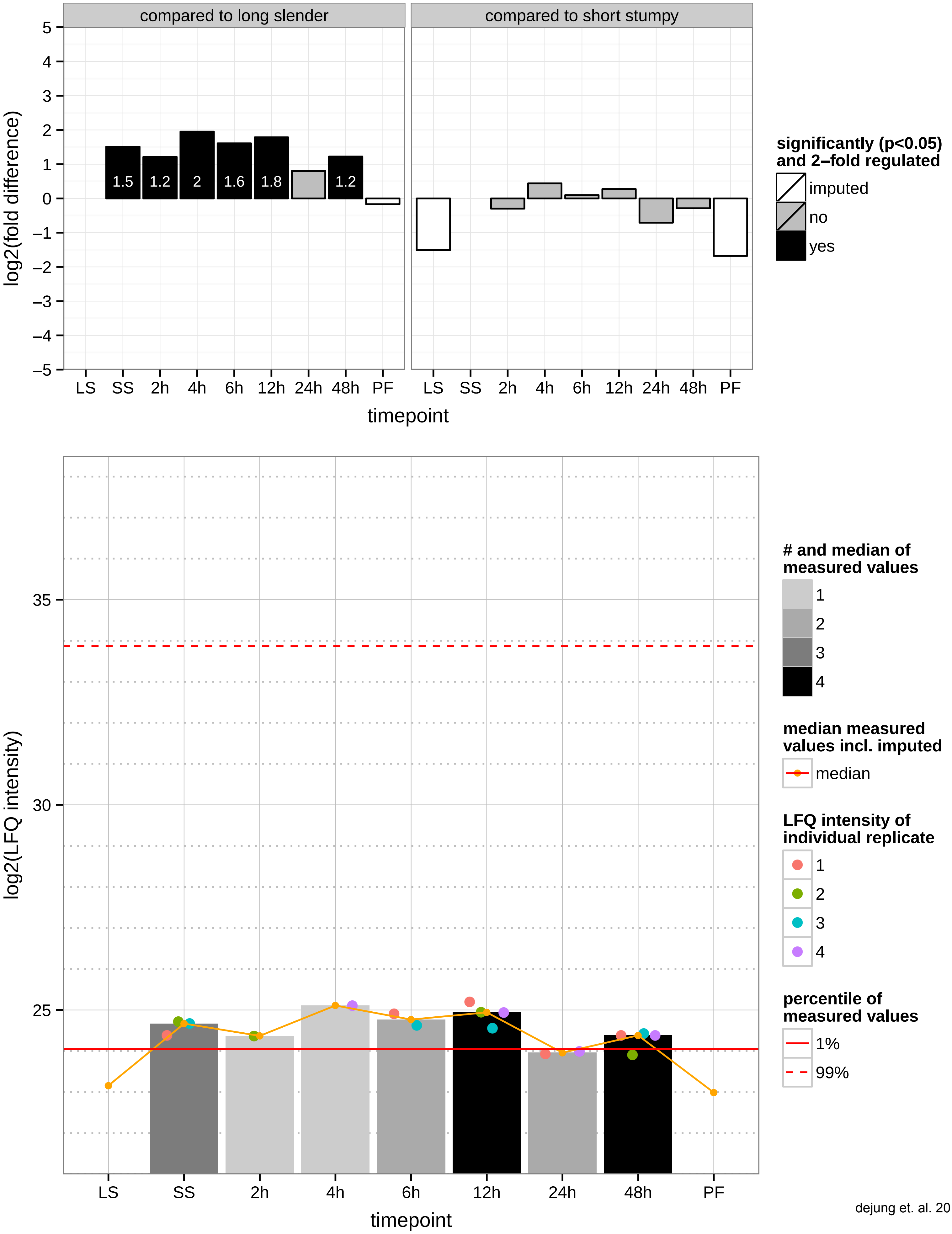

dejung et. al. 2015

Vacuolar protein sorting–associated protein 16 homolog, putative
Tb927.10.13020
AGOF: null
AGOC: cytoplasm
AGOP: intracellular protein transport
PGOF: null
PGOC: cytoplasm
PGOP: intracellular protein transport

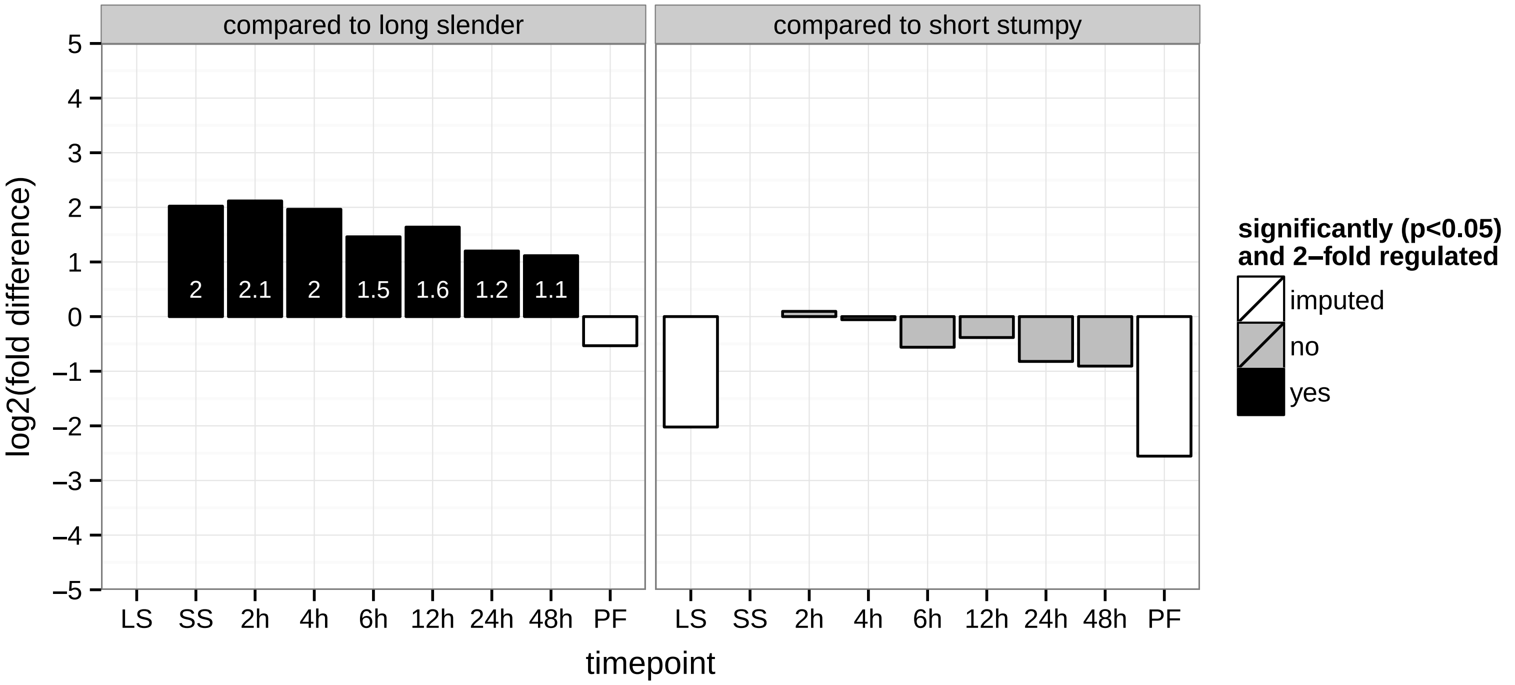

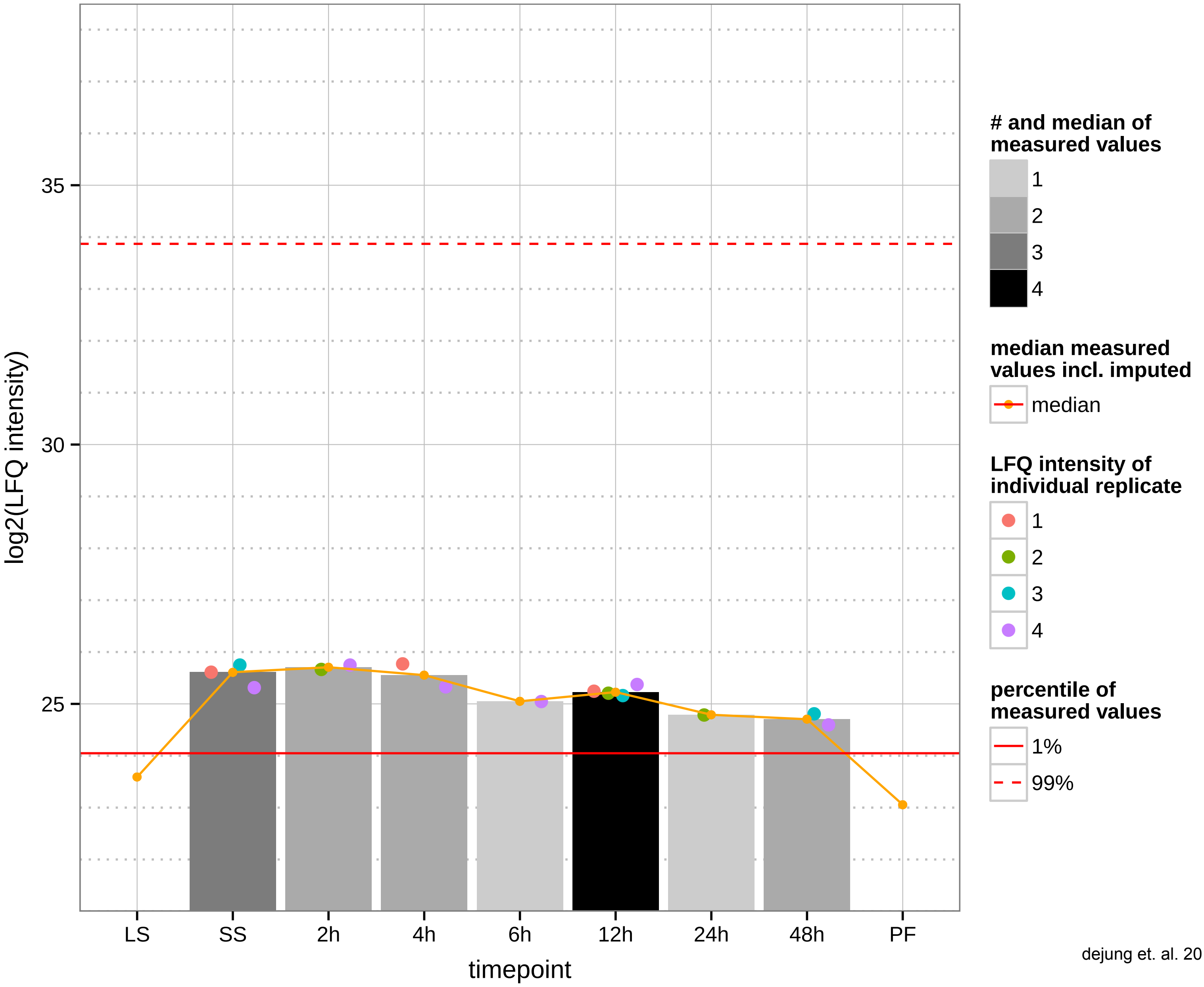

dejung et. al. 2015

cysteine peptidase, Clan CA, family C2, putative, calpain–like cysteine peptidase
Tb927.10.13030
AGOF: calcium–dependent cysteine–type endopeptidase activity
AGOC: intracellular
AGOP: proteolysis
PGOF: calcium–dependent cysteine–type endopeptidase activity
PGOC: intracellular
PGOP: proteolysis

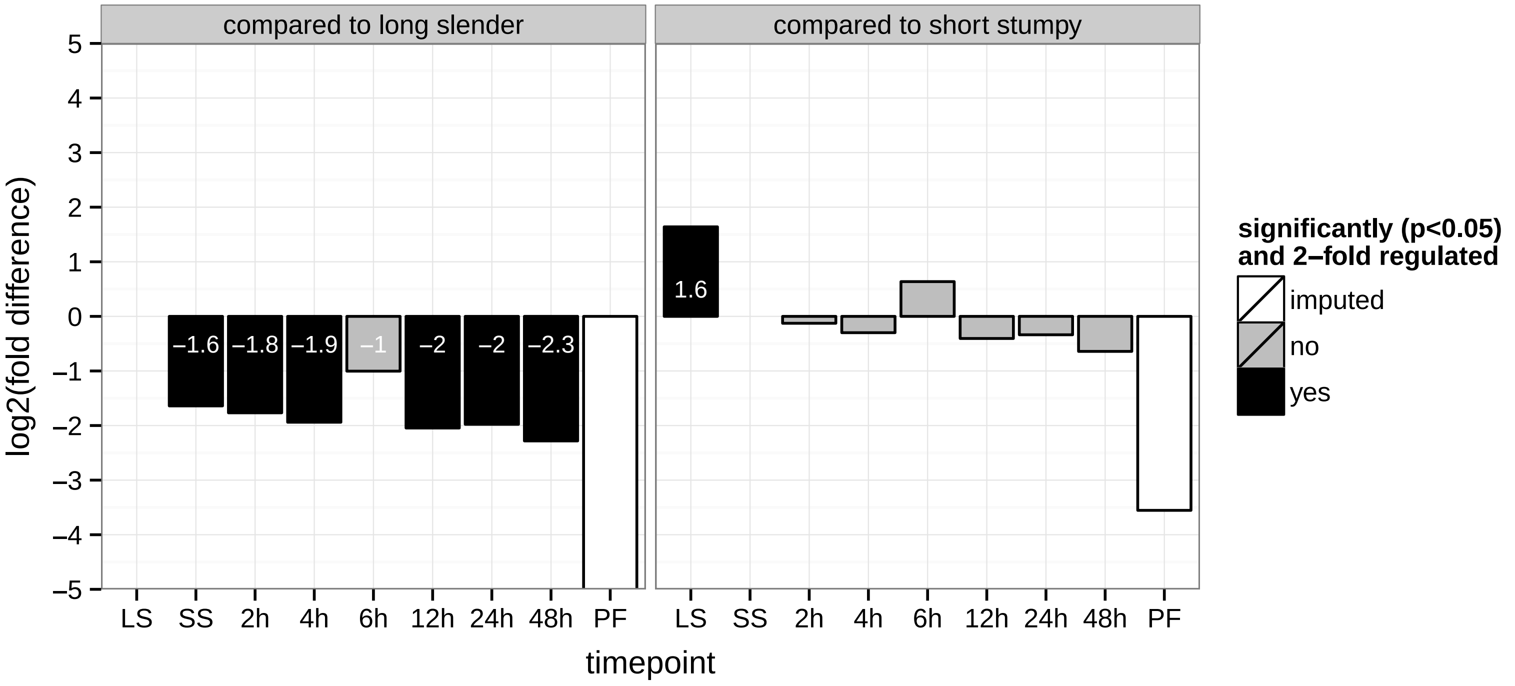

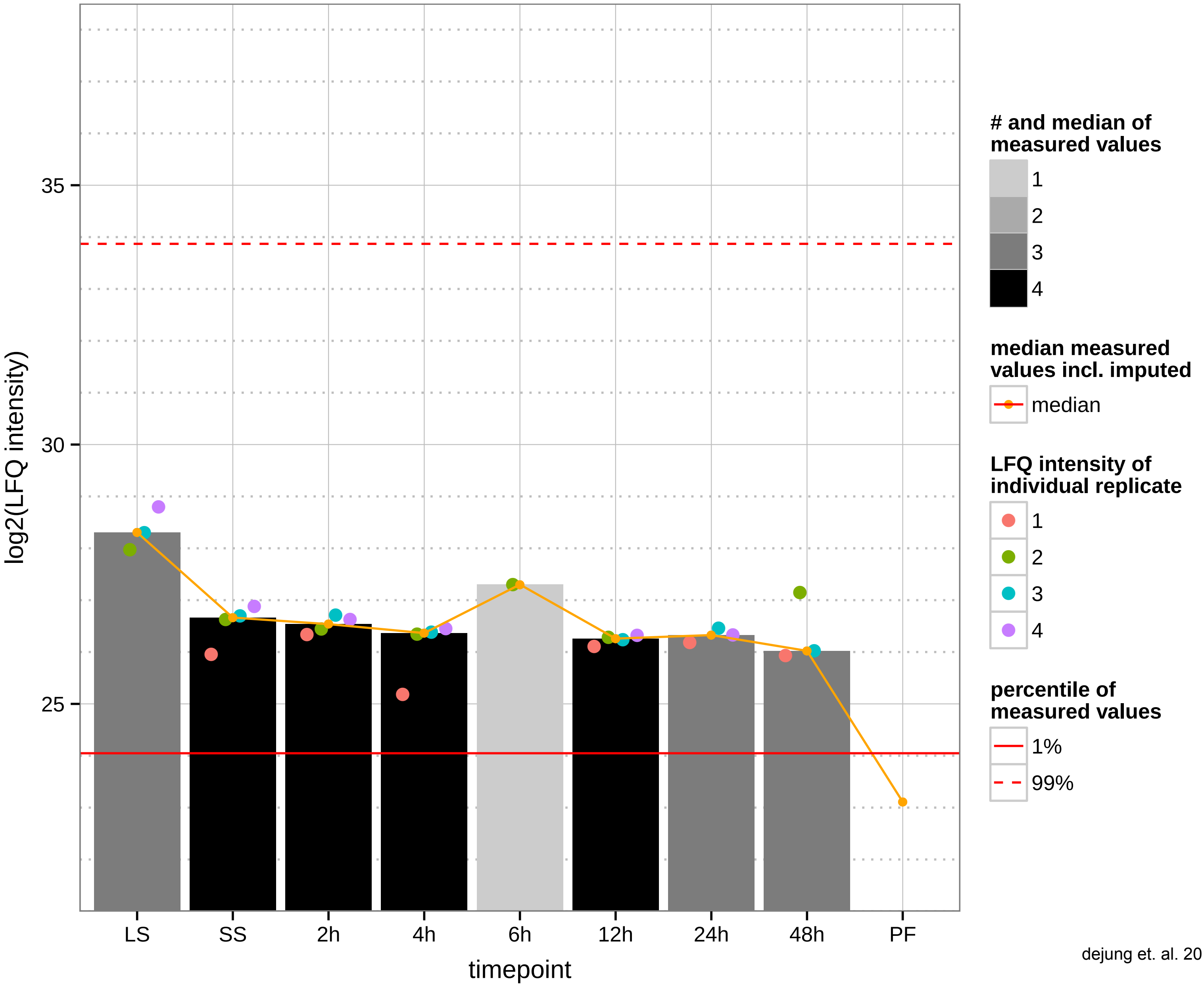

dejung et. al. 2015

flagellar protein PF20
Tb927.10.13960
AGOF: null
AGOC: axoneme
AGOP: ciliary or flagellar motility
PGOF: protein binding
PGOC: null
PGOP: null

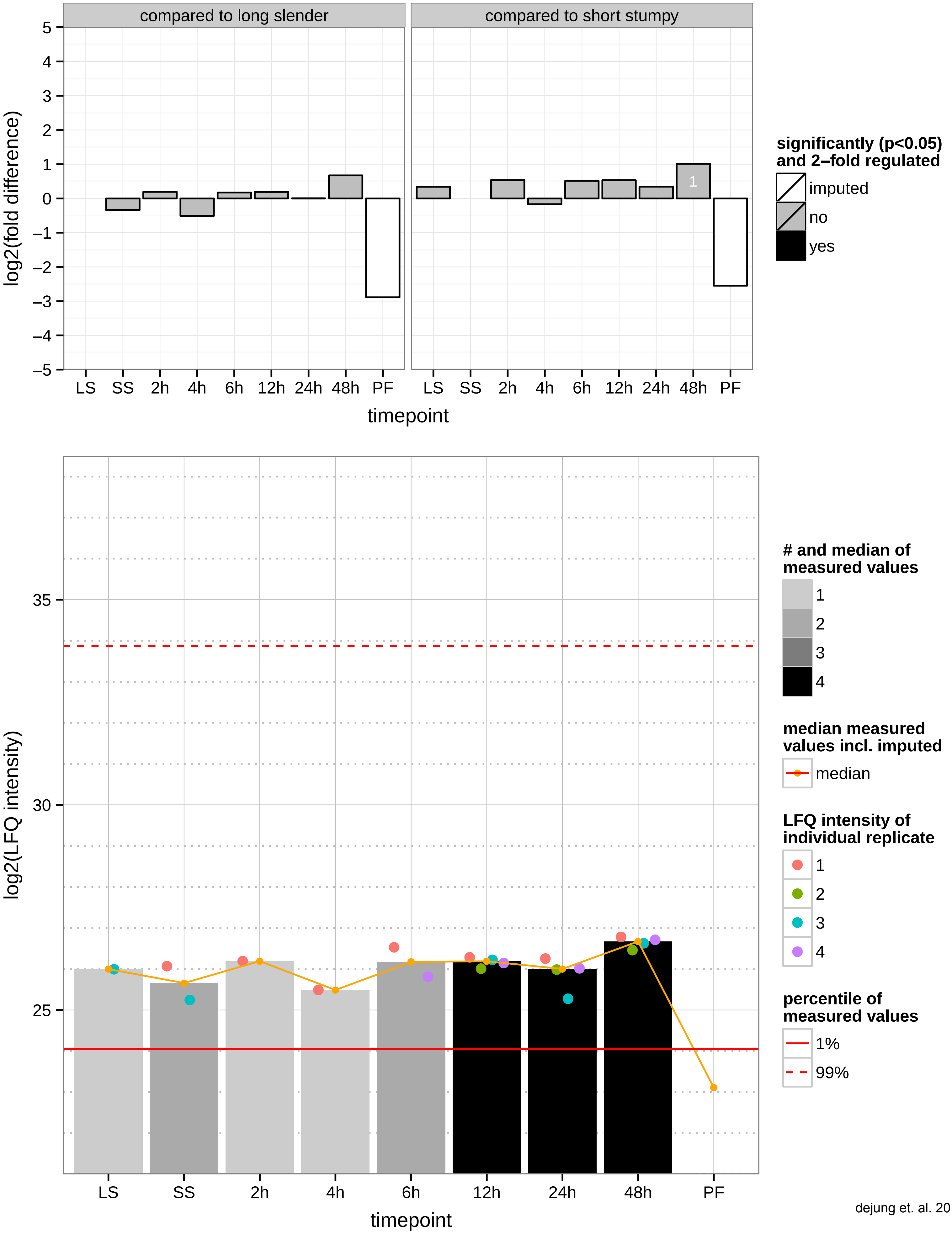

dejung et. al. 2015

hypothetical protein, conserved
Tb927.10.13990
AGOF: null
AGOC: null
AGOP: null
PGOF: null
PGOC: null
PGOP: null

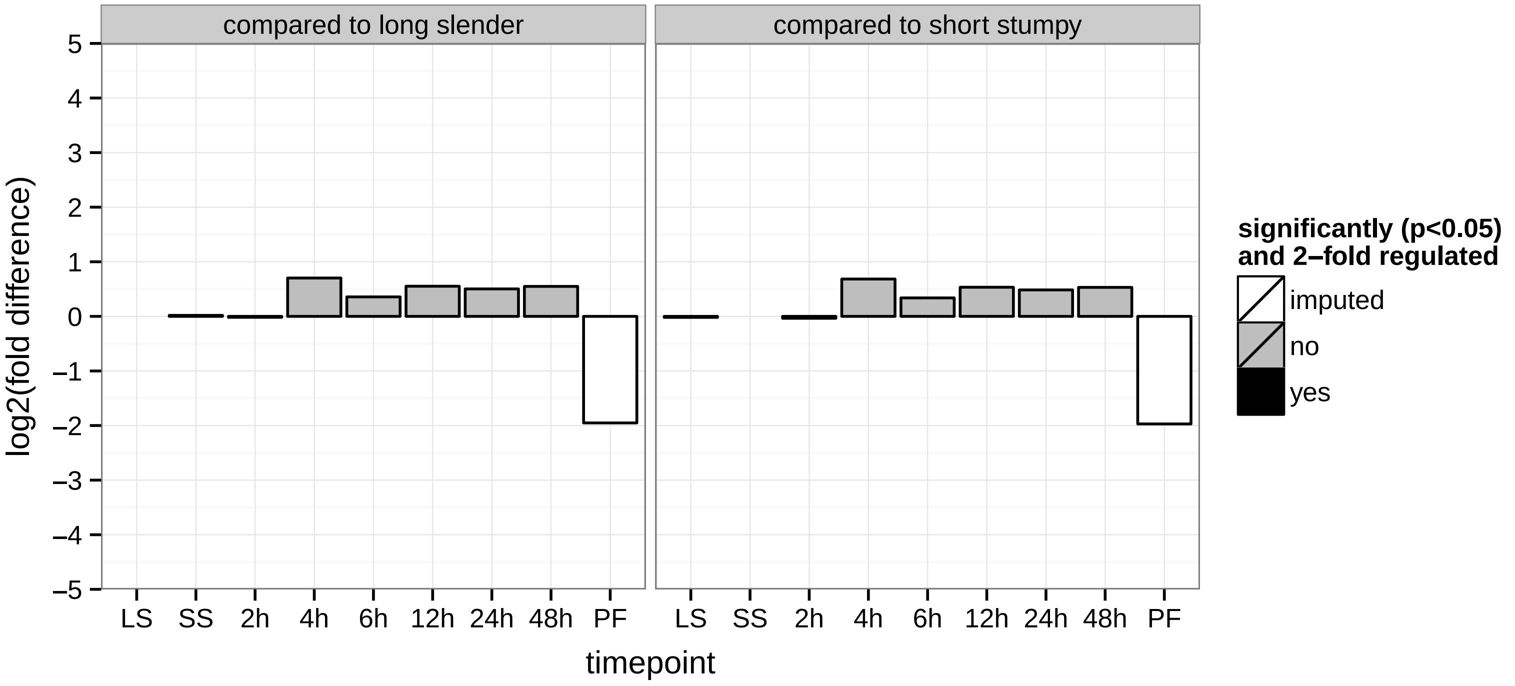

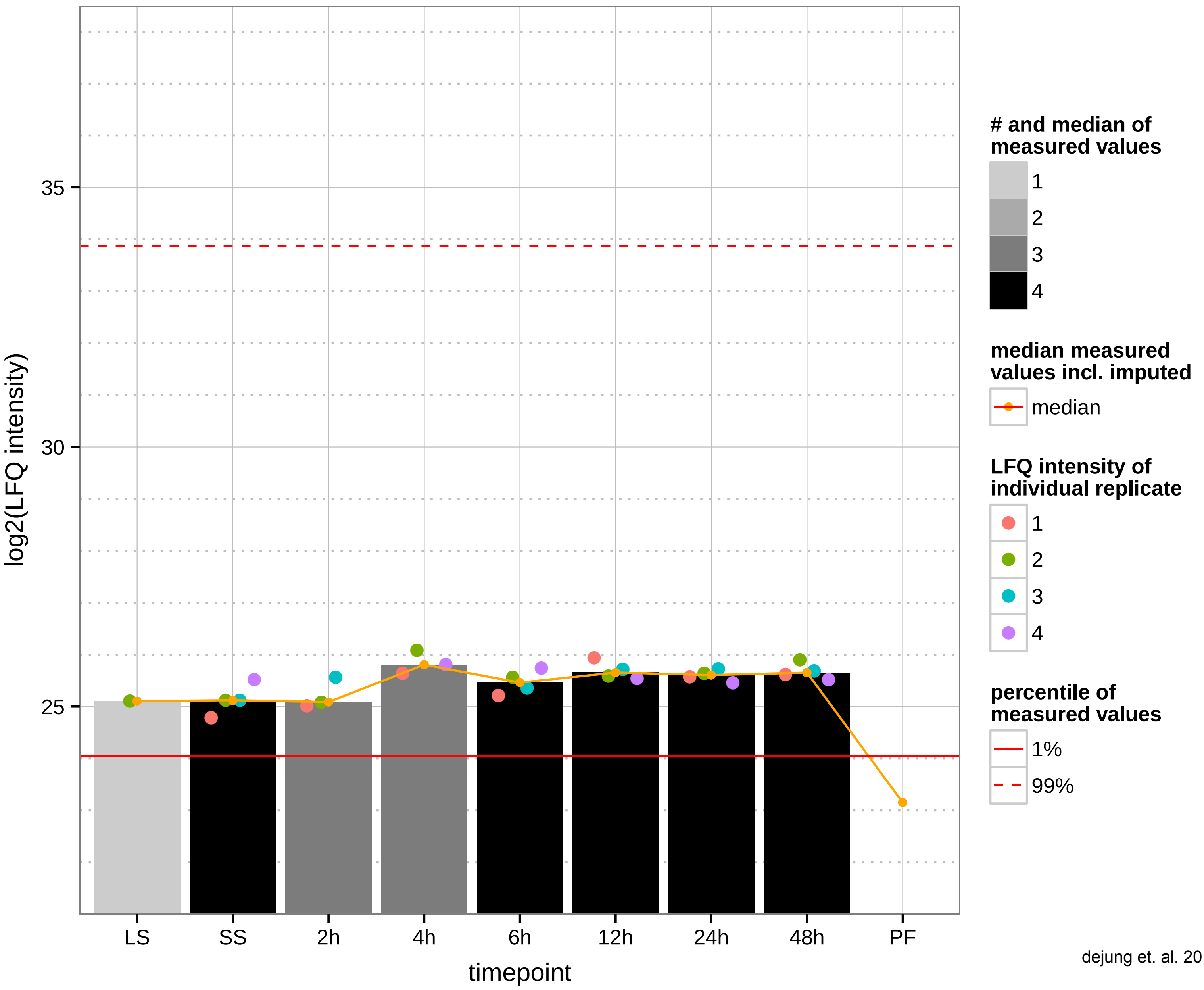

dejung et. al. 2015

hypothetical protein, conserved
Tb927.10.14220
AGOF: null
AGOC: null
AGOP: null
PGOF: null
PGOC: null
PGOP: null

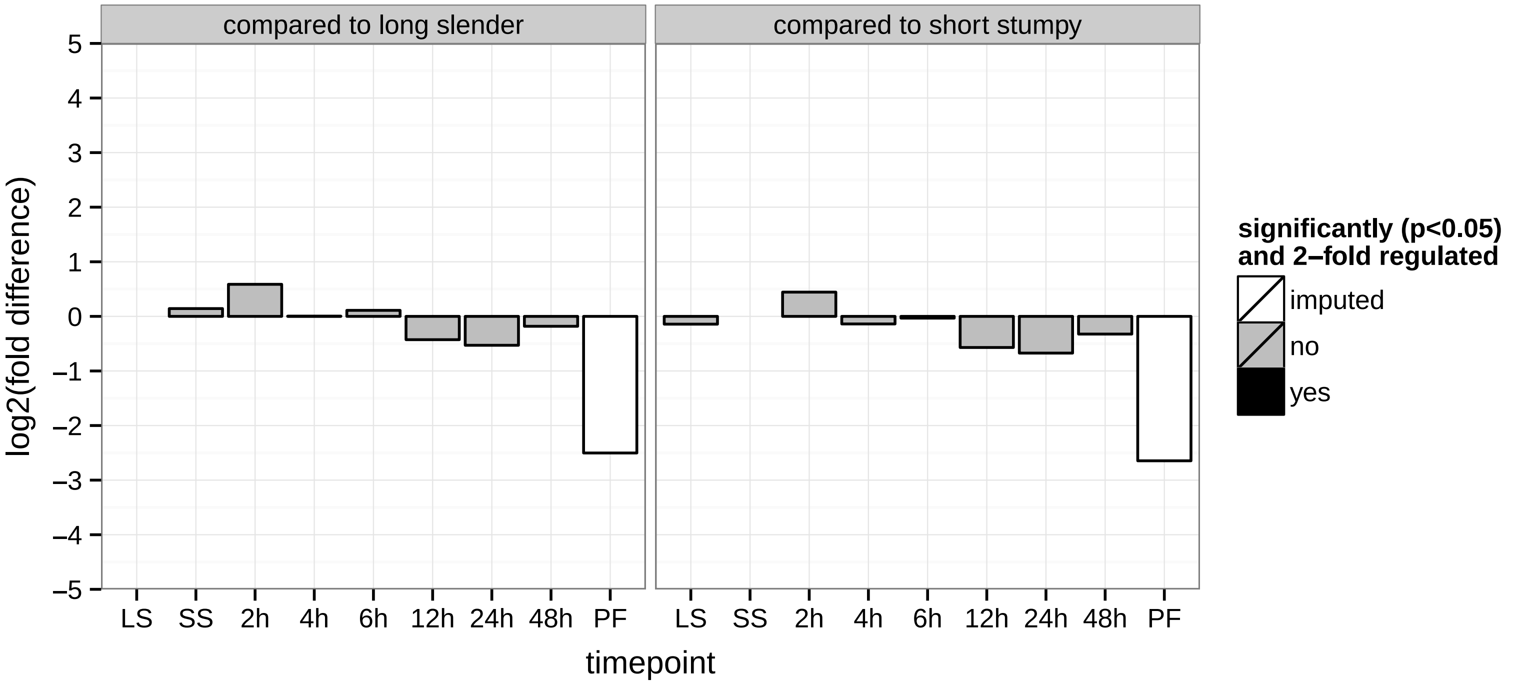

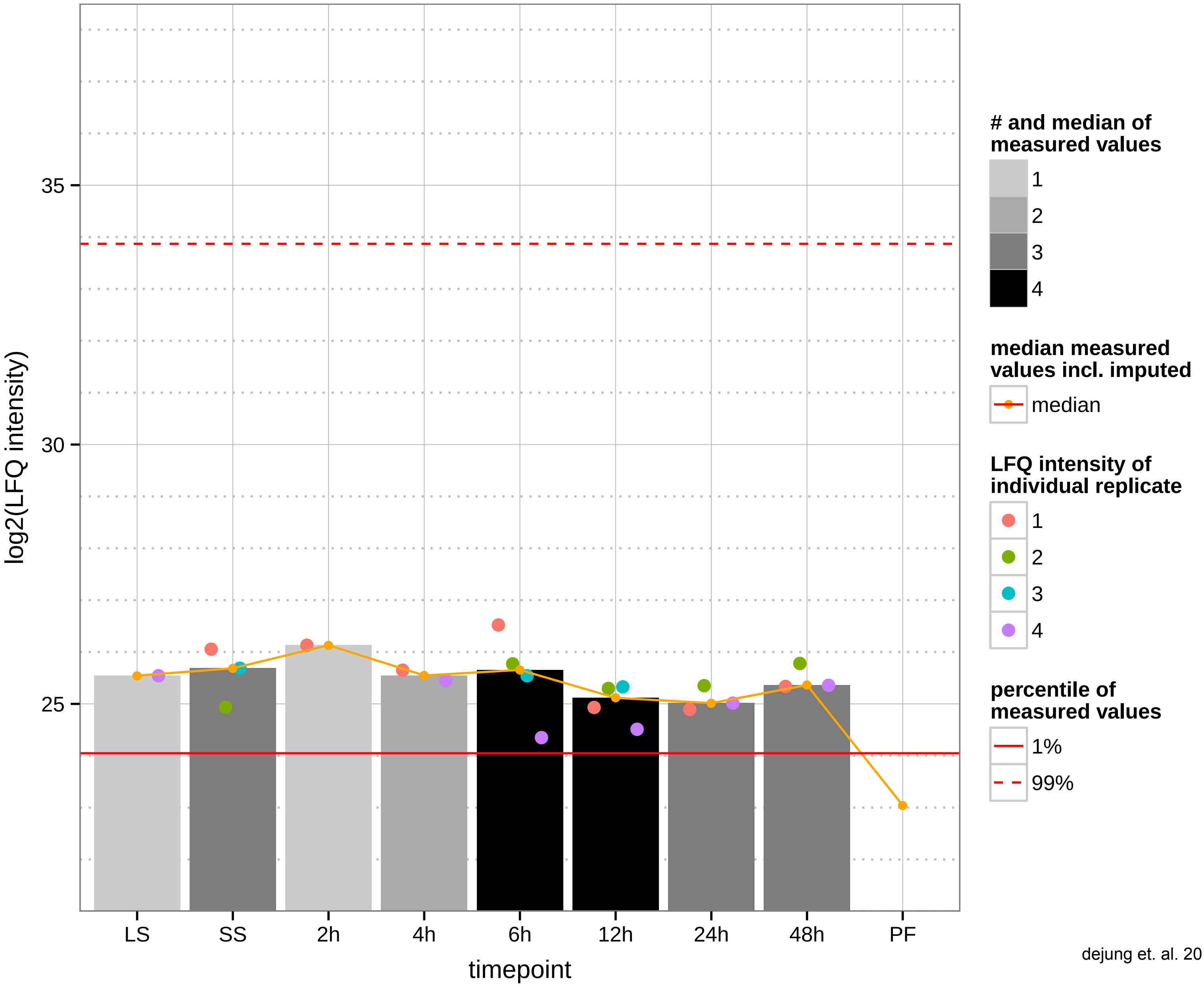

dejung et. al. 2015

hypothetical protein, conserved
Tb927.10.14340
AGOF: null
AGOC: null
AGOP: null
PGOF: null
PGOC: null
PGOP: null

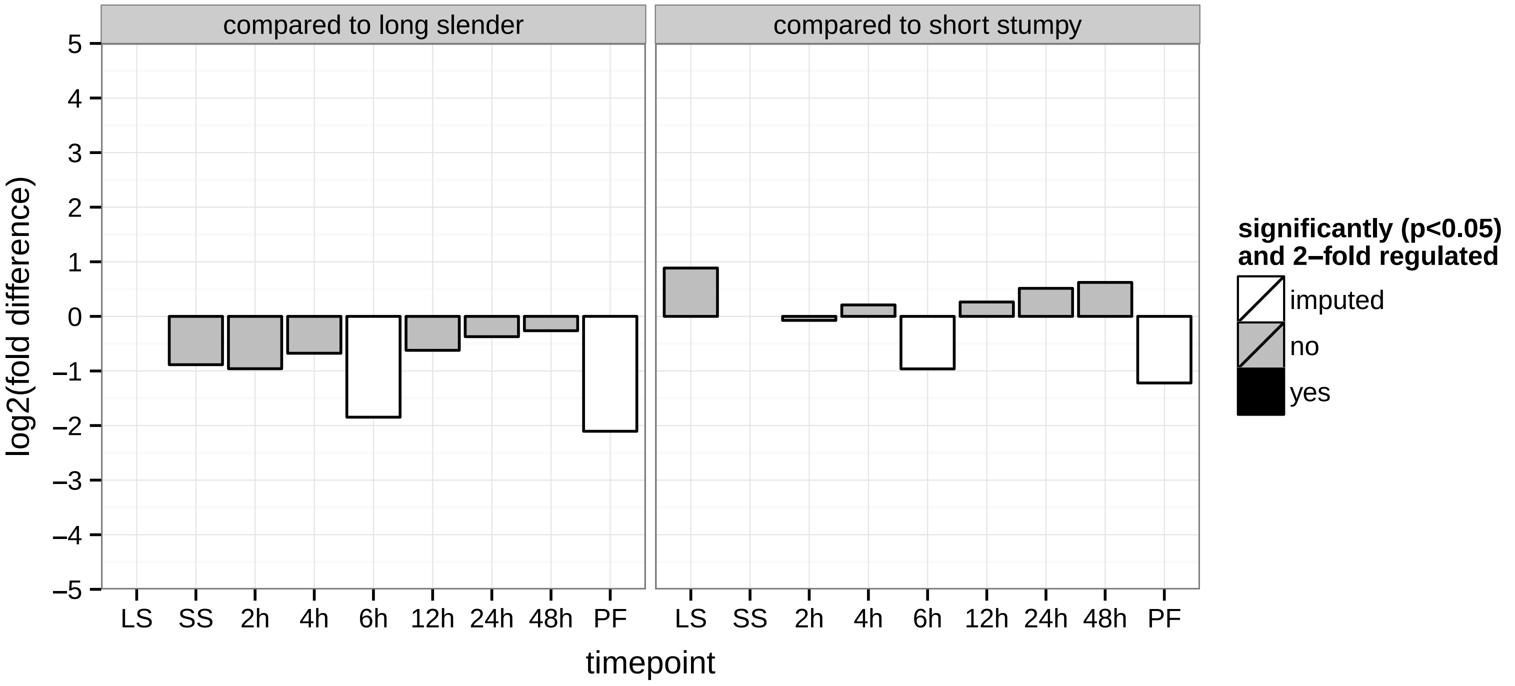

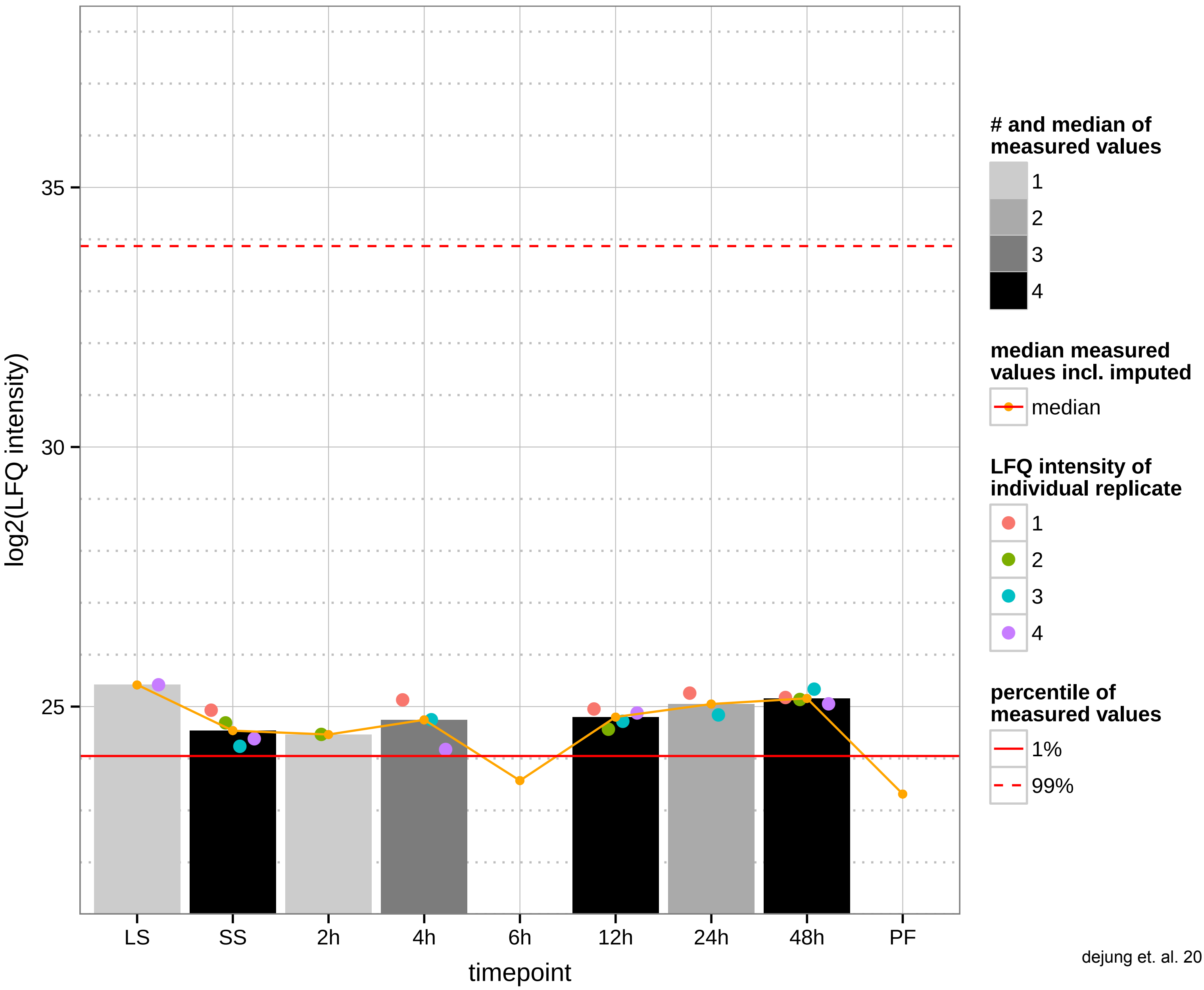

dejung et. al. 2015

hypothetical protein, conserved
Tb927.10.14640
AGOF: null
AGOC: null
AGOP: null
PGOF: null
PGOC: null
PGOP: null

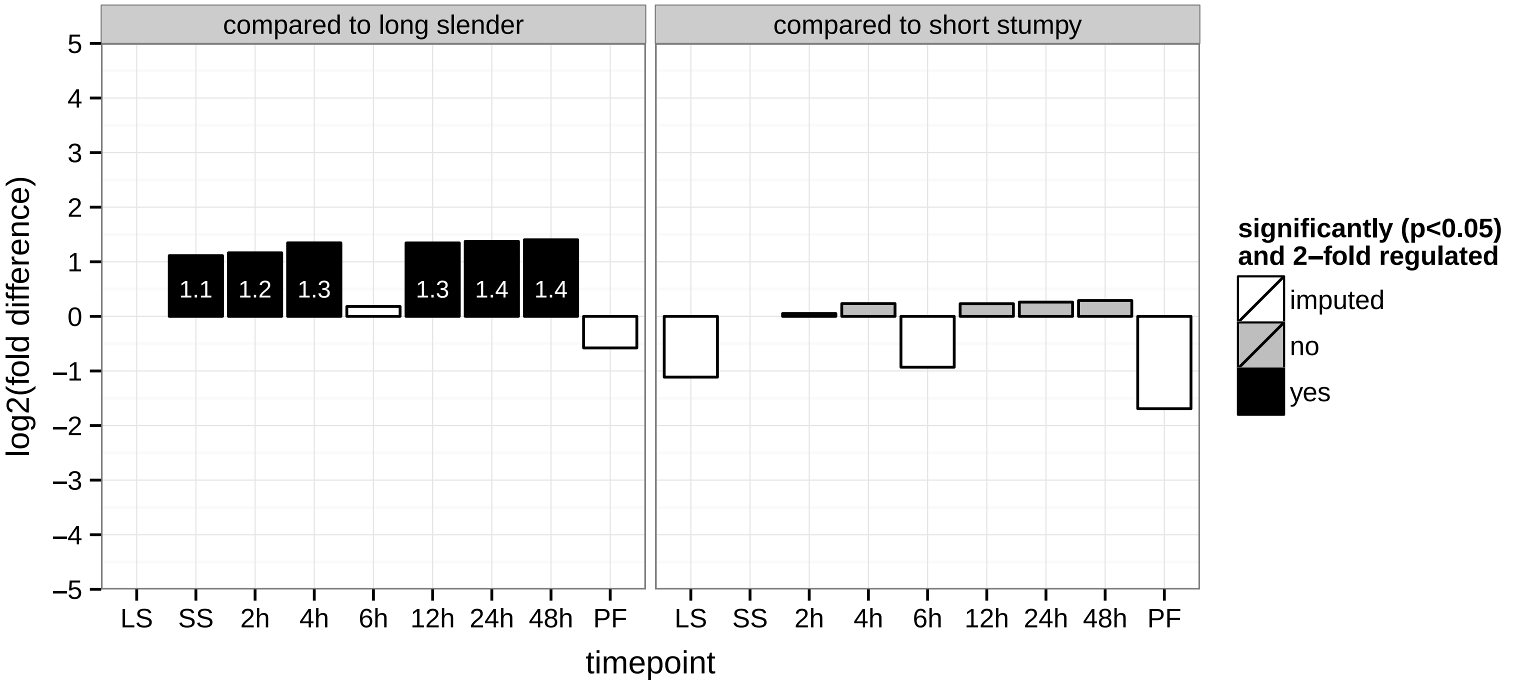

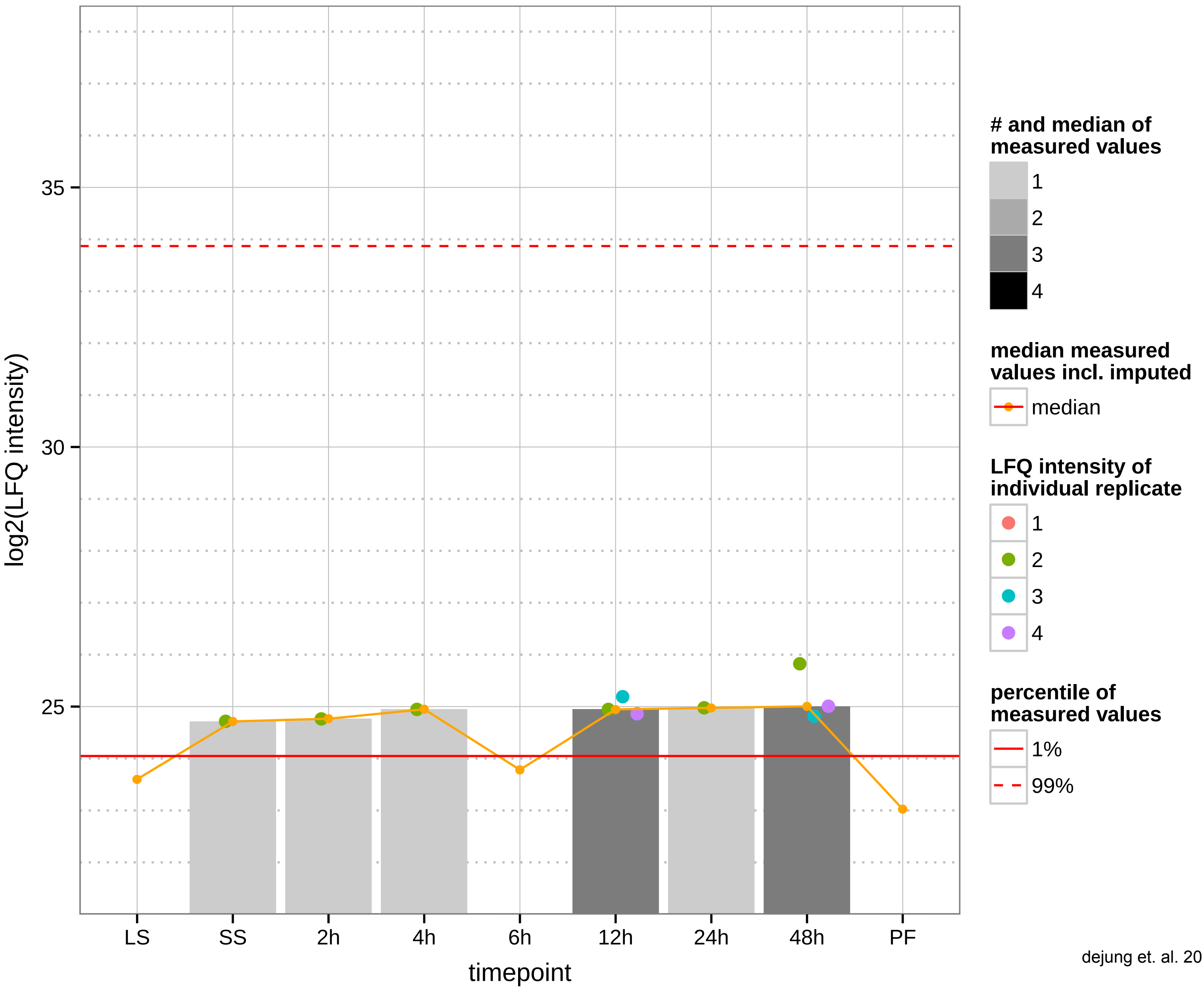

dejung et. al. 2015

intraflagellar transport protein IFT80/CHE2, putative (IFT80)
Tb927.10.14990
AGOF: null
AGOC: null
AGOP: null
PGOF: protein binding
PGOC: null
PGOP: null

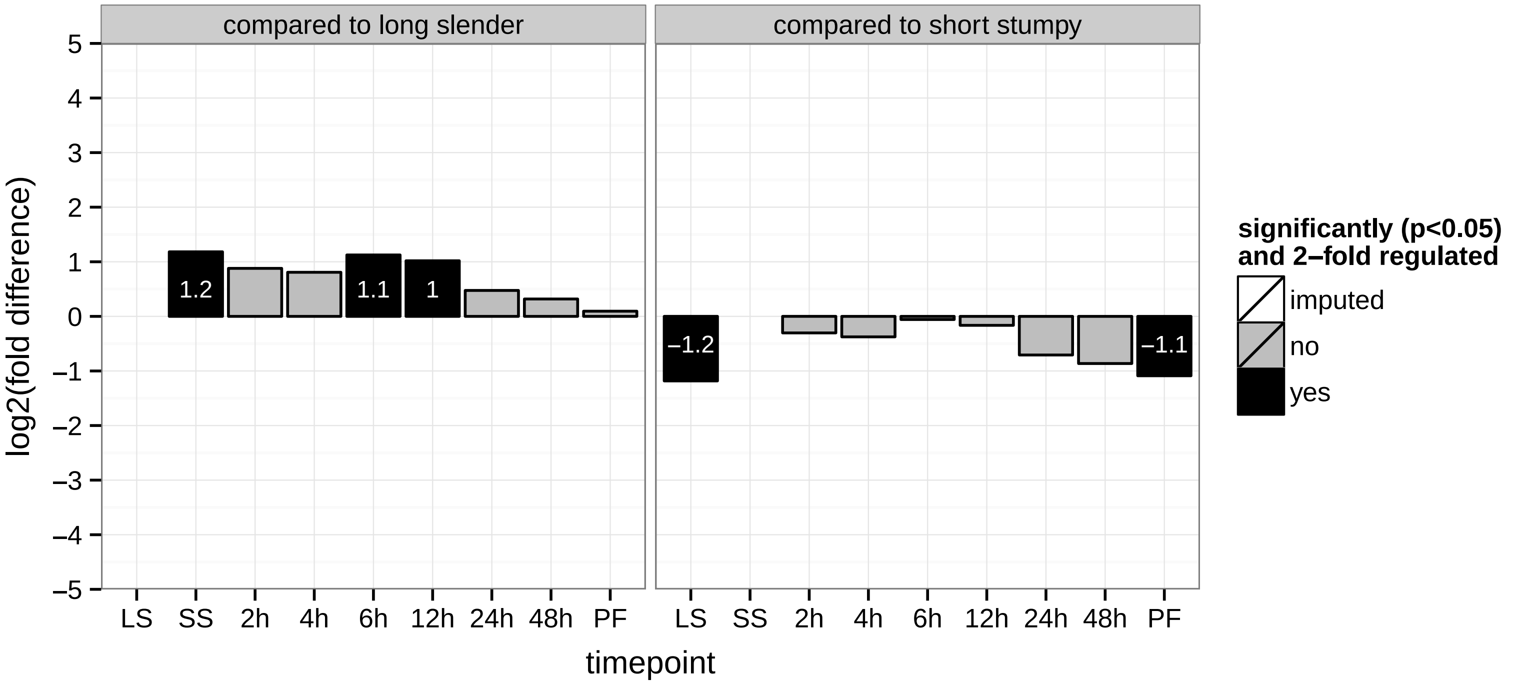

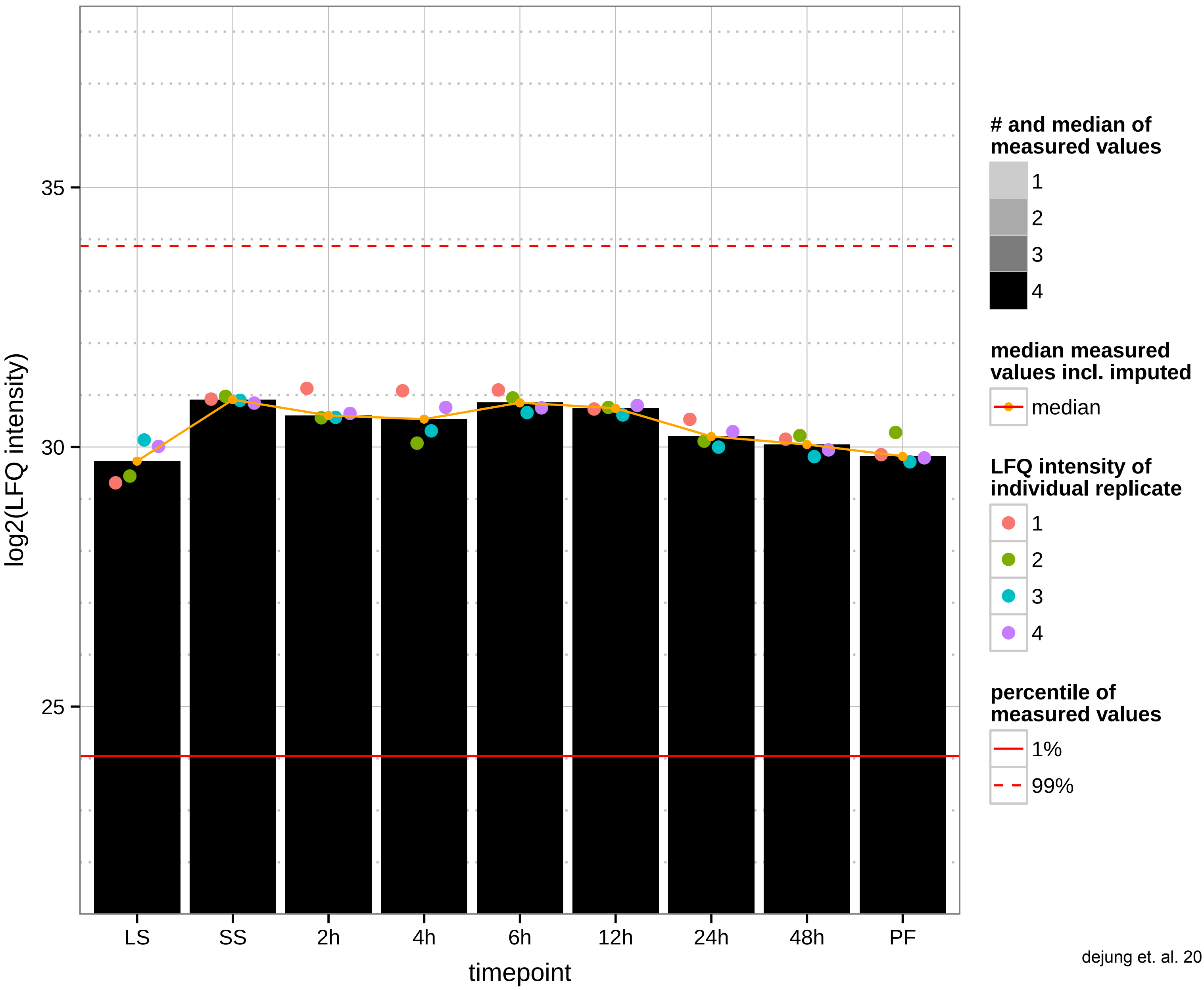

dejung et. al. 2015

hypothetical protein, conserved, predicted VPS53–like domain protein
Tb927.10.15540;Tb11.v5.0651
AGOF: null
AGOC: null
AGOP: null
PGOF: null
PGOC: null
PGOP: null

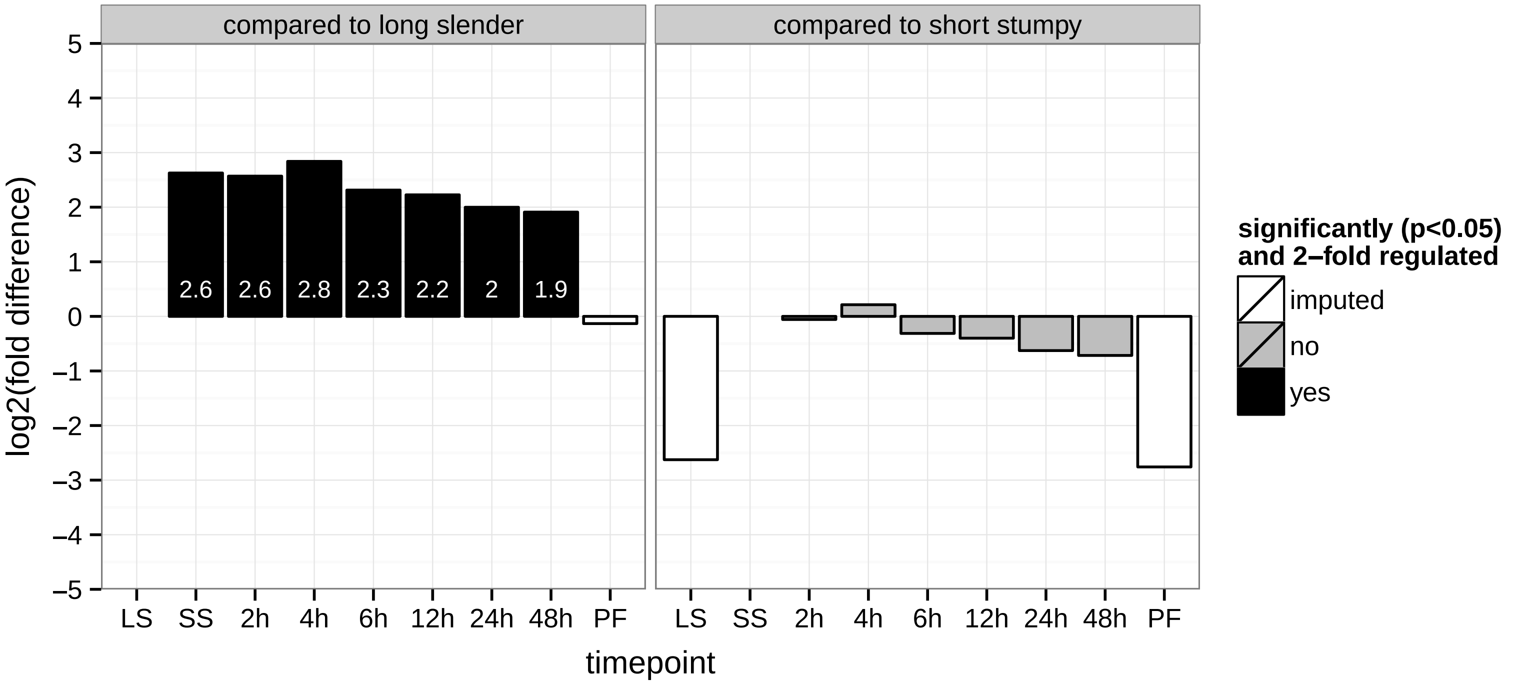

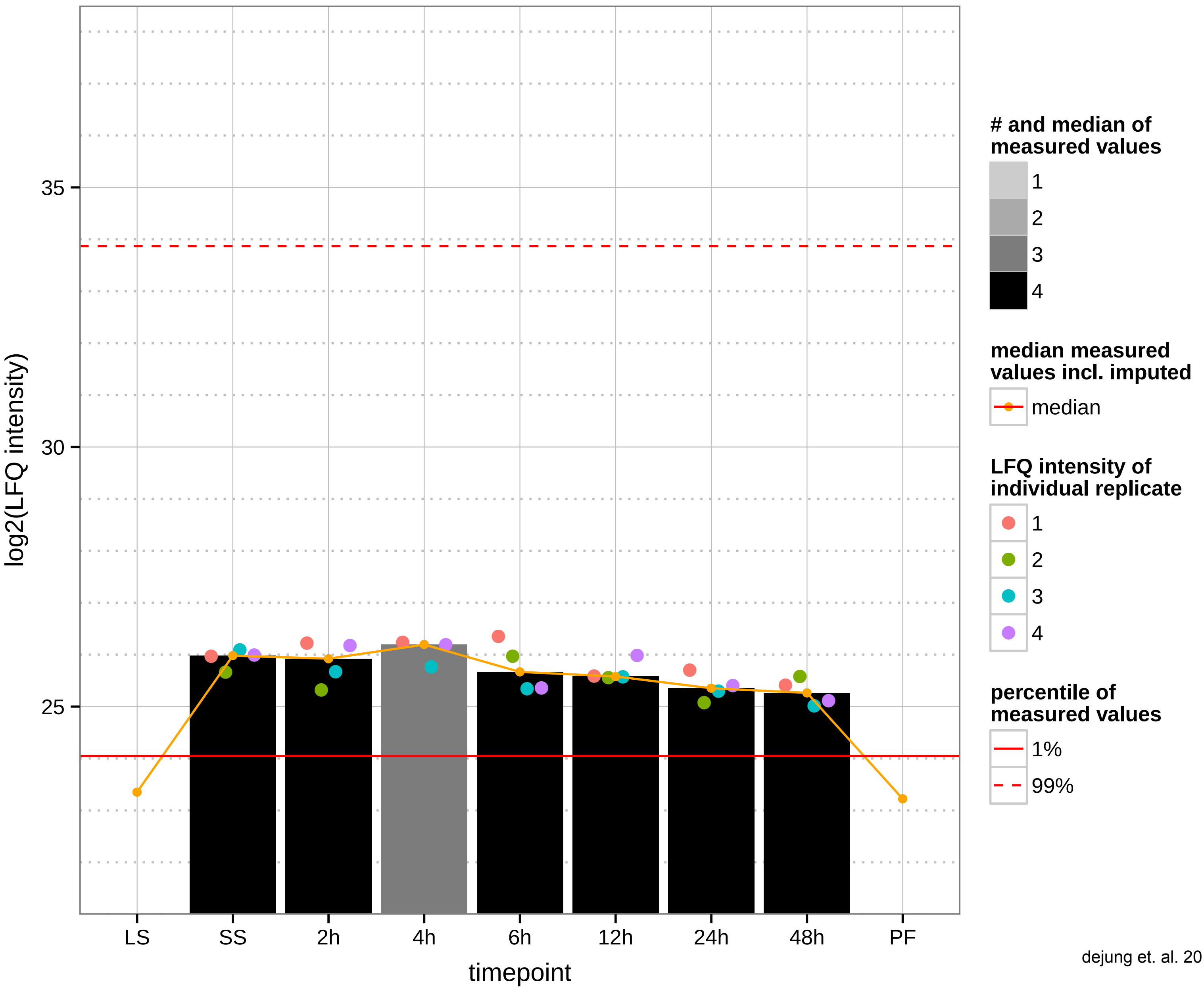

dejung et. al. 2015

macrophage infectivity potentiator, precursor, putative, FKBP–type peptidylprolyl cis–trans isomerase, putative (MIP)
Tb927.10.15590
AGOF: FK506 binding, peptidyl–prolyl cis–trans isomerase activity
AGOC: null
AGOP: protein folding
PGOF: null
PGOC: null
PGOP: protein folding

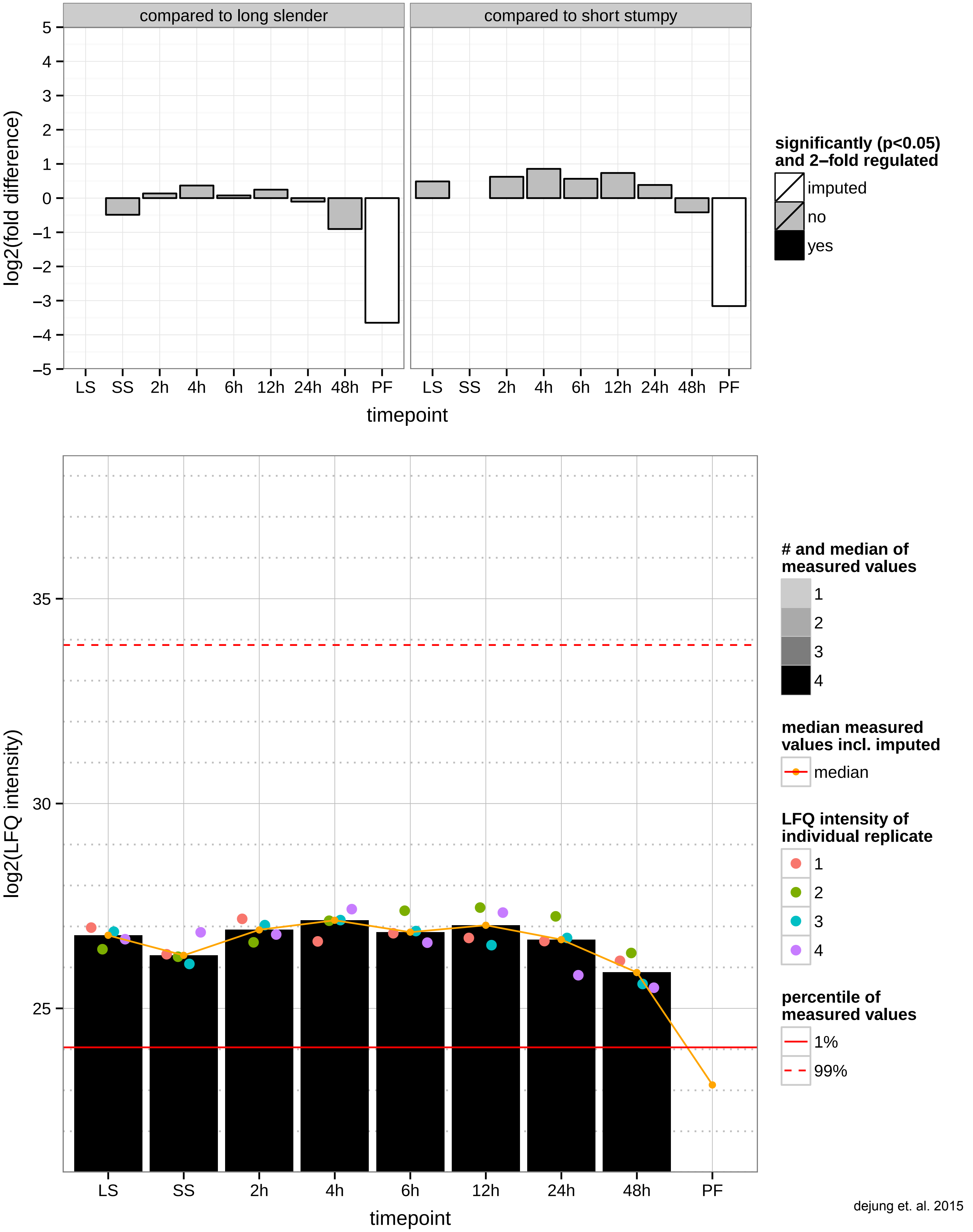

dejung et. al. 2015

hypothetical protein, conserved
Tb927.10.15640
AGOF: null
AGOC: null
AGOP: null
PGOF: protein binding
PGOC: null
PGOP: null

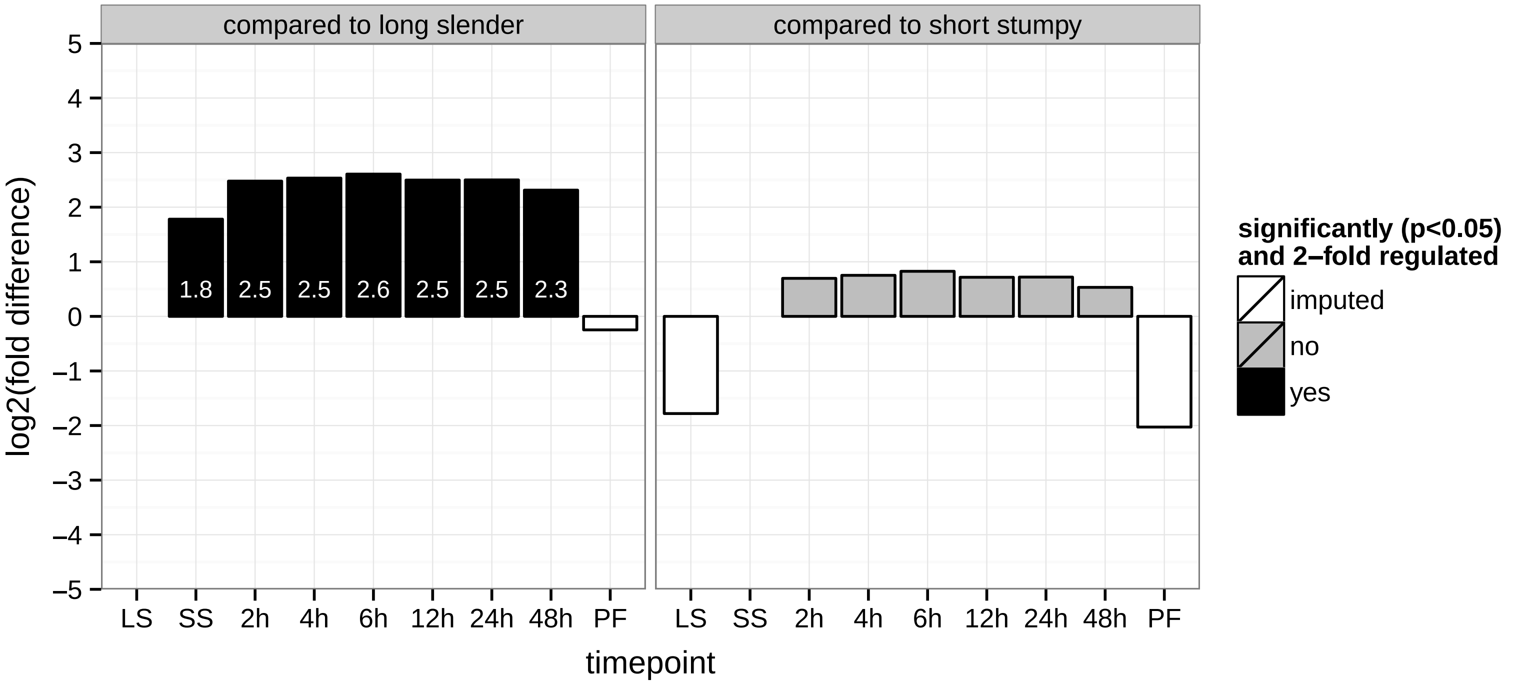

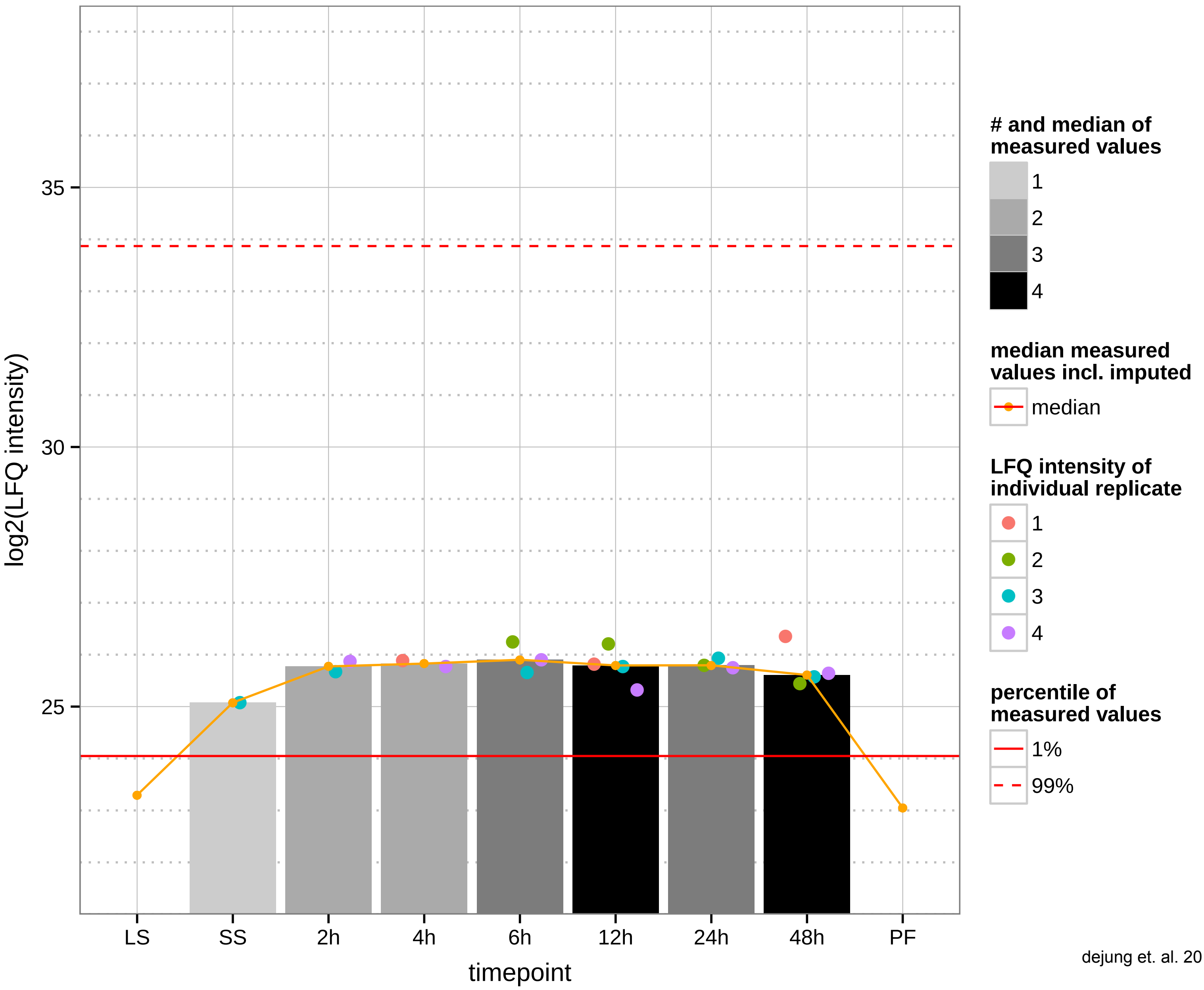

dejung et. al. 2015

mitogen activated protein kinase 4, putative, map kinase (MAPK2)
Tb927.10.16030
AGOF: ATP binding, MAP kinase activity, kinase activity, protein serine/threonine kinase activity, protein tyrosine kinase activit
AGOC: null
AGOP: protein phosphorylation
PGOF: ATP binding, protein kinase activity, transferase activity, transferring phosphorus–containing groups
PGOC: null
PGOP: protein phosphorylation

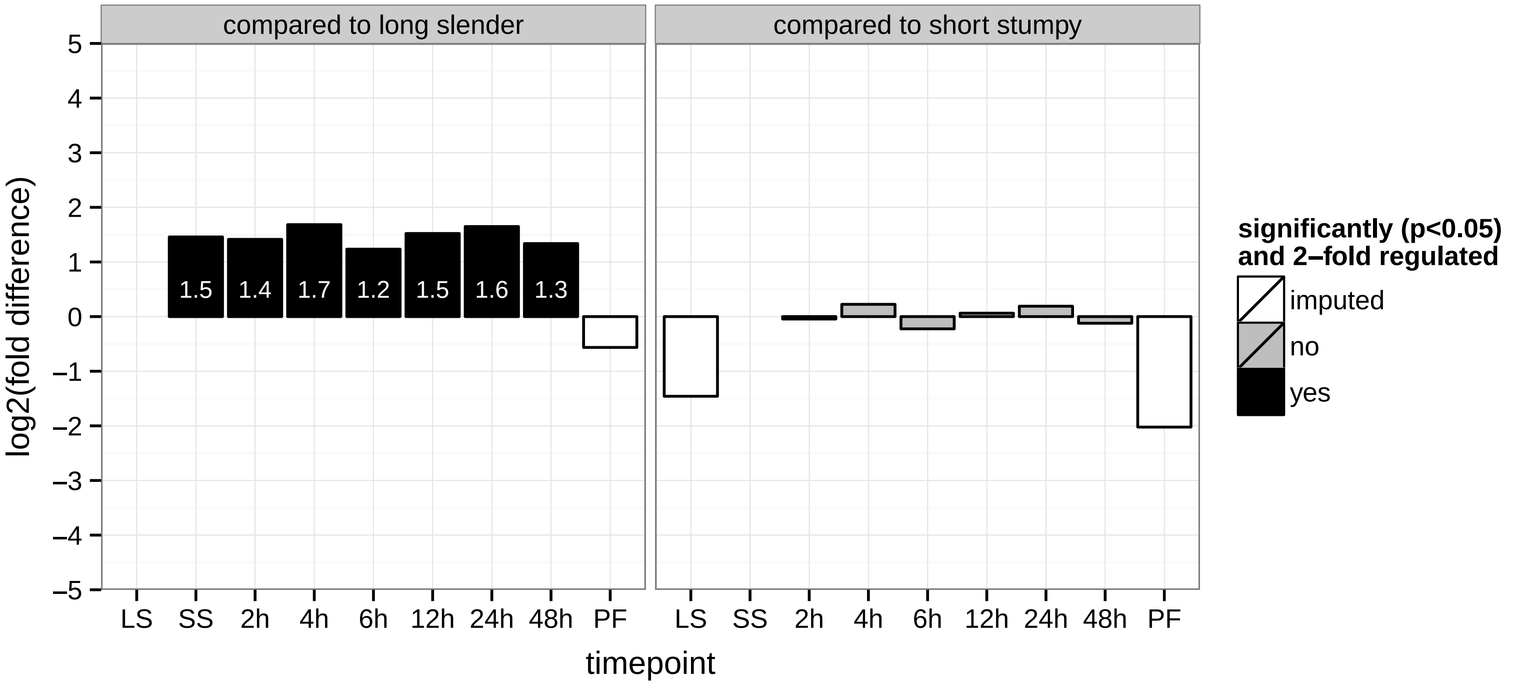

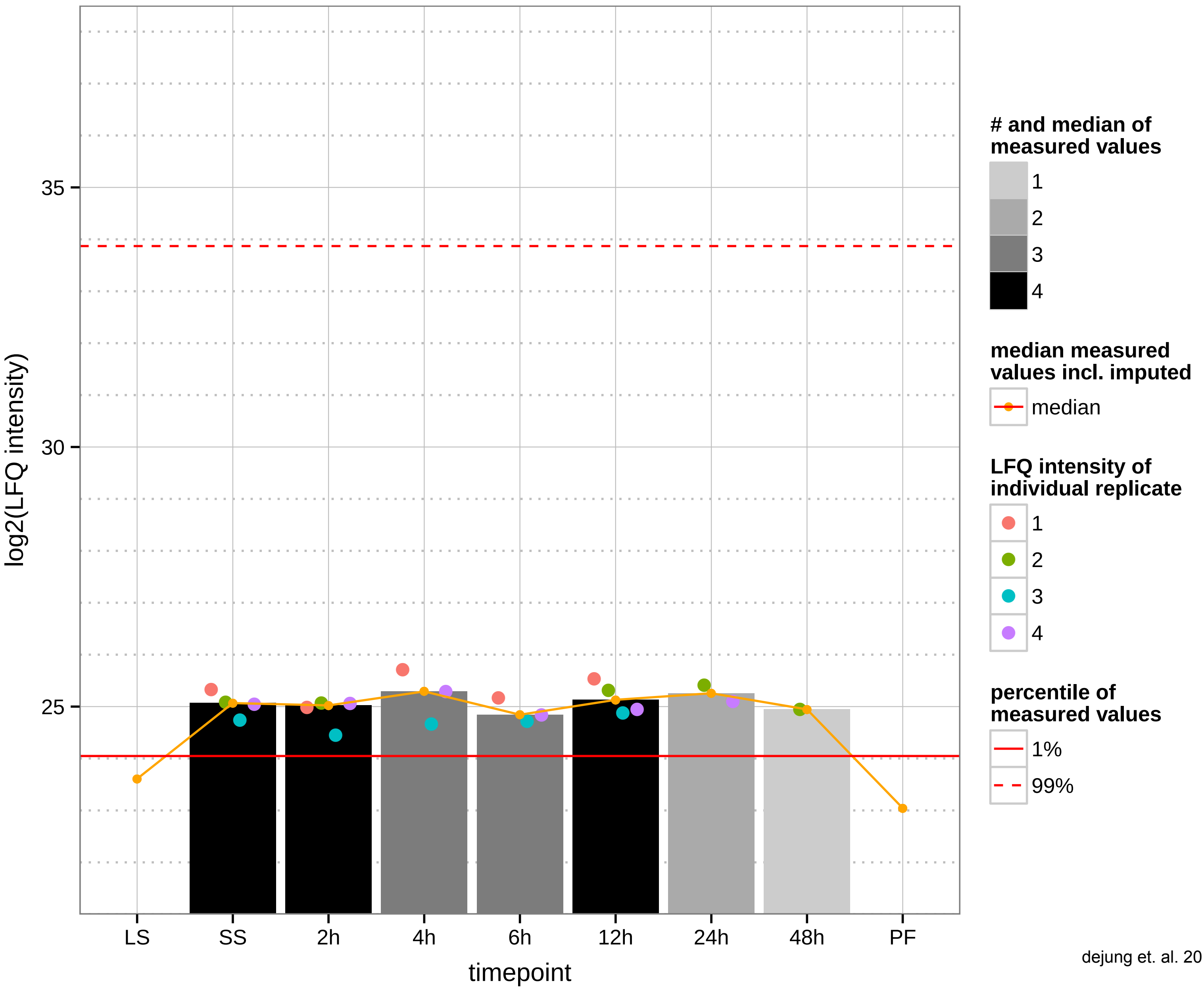

dejung et. al. 2015

peptidylprolyl isomerase–like protein, putative, FK506–binding protein (FKBP)–type peptidyl–prolyl isomerase
Tb927.10.16100;Tb11.v5.0411
AGOF: null, heat shock protein binding, peptidyl–prolyl cis–trans isomerase activity
AGOC: null
AGOP: null, protein folding
PGOF: protein binding
PGOC: null
PGOP: protein folding

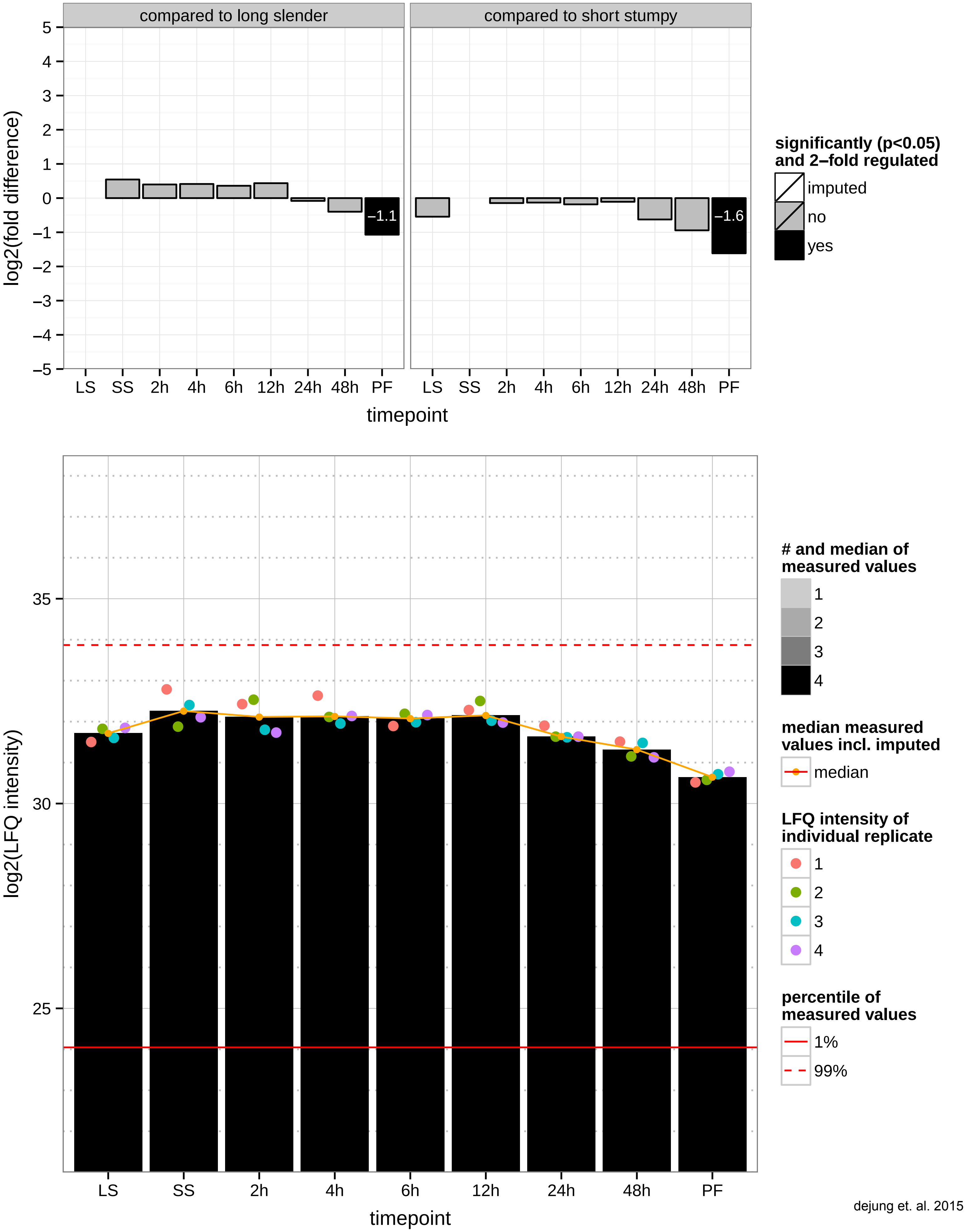

dejung et. al. 2015

syntaxin, putative
Tb927.10.1830
AGOF: transporter activity
AGOC: membrane
AGOP: vesicle–mediated transport
PGOF: protein binding
PGOC: null
PGOP: null

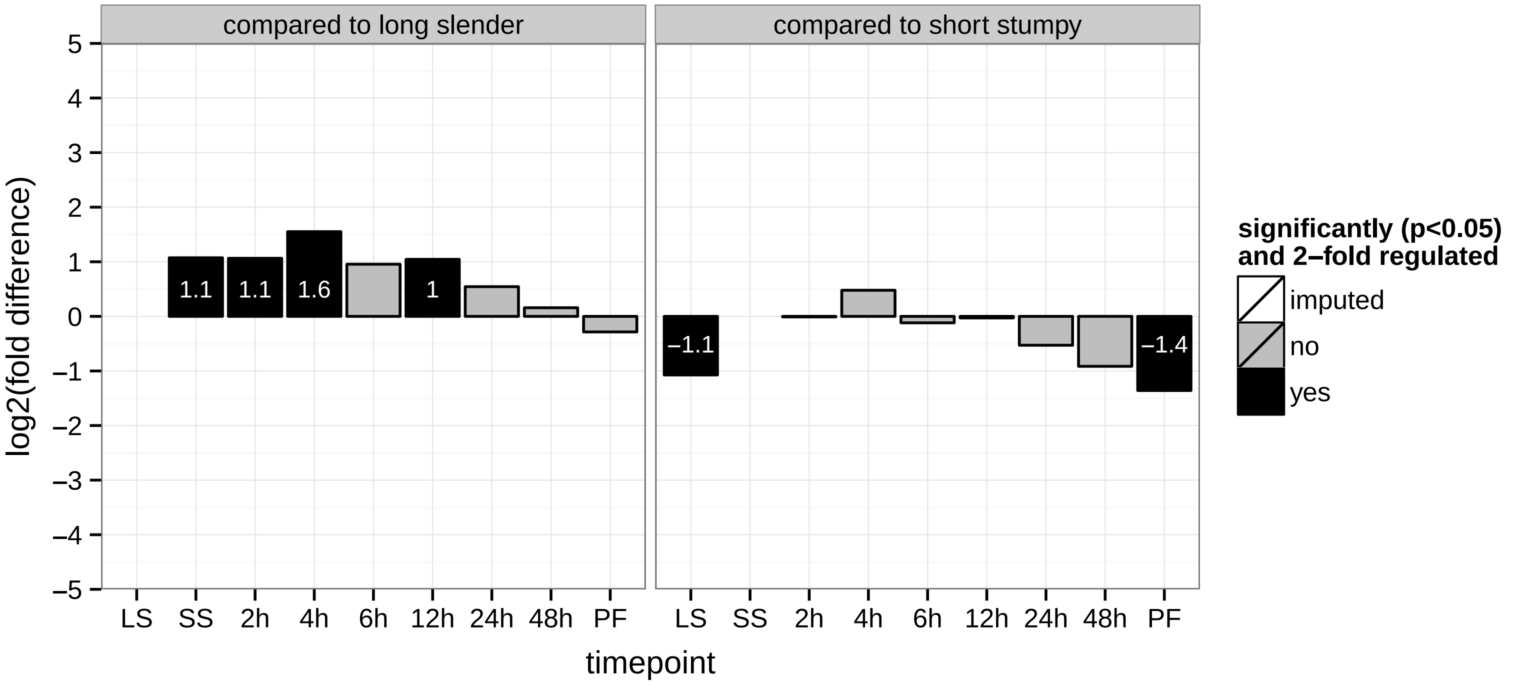

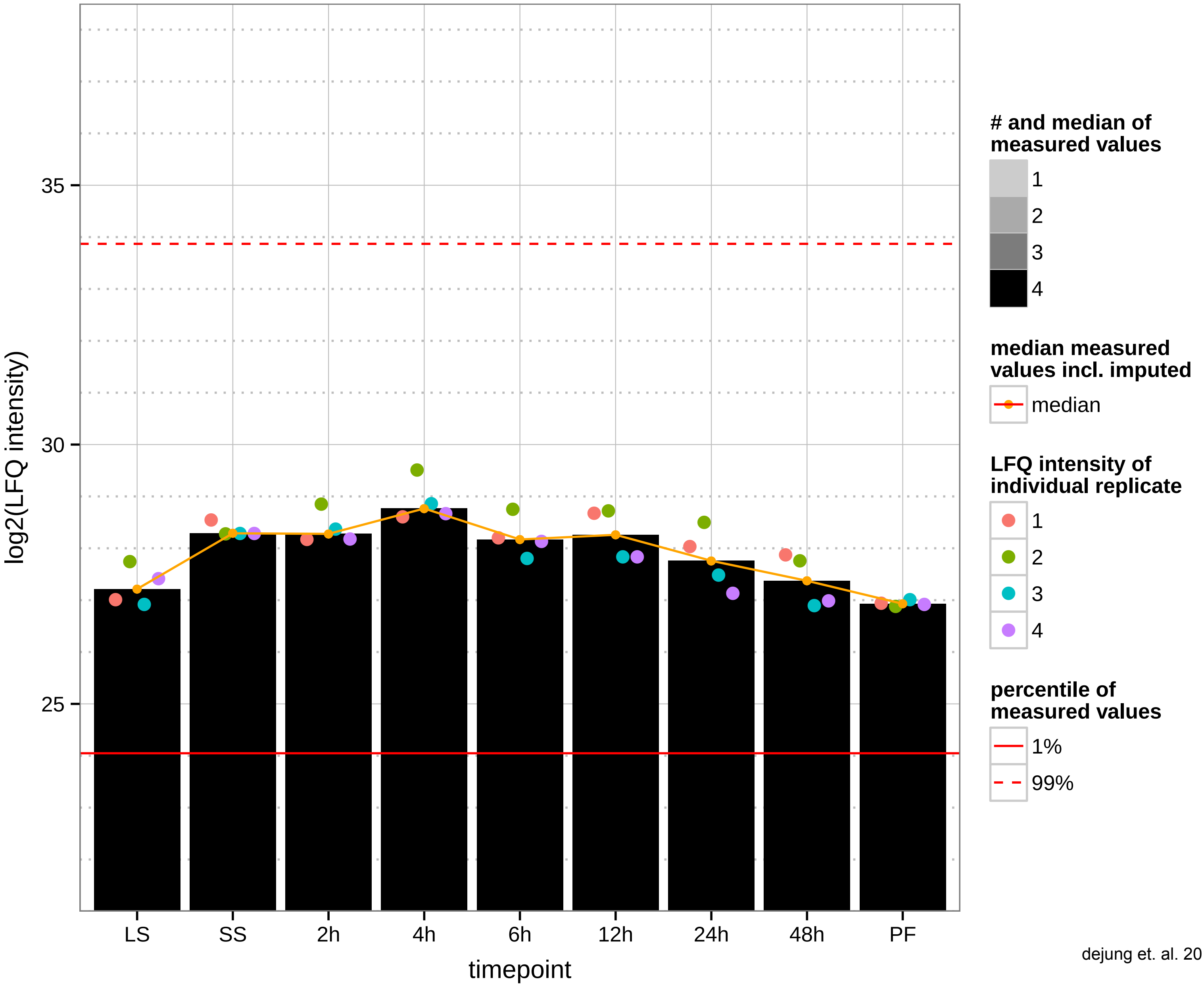

dejung et. al. 2015

leucine−rich repeat protein (LRRP), putative
Tb927.10.1950
AGOF: null
AGOC: mitochondrion
AGOP: null
PGOF: protein binding
PGOC: null
PGOP: null

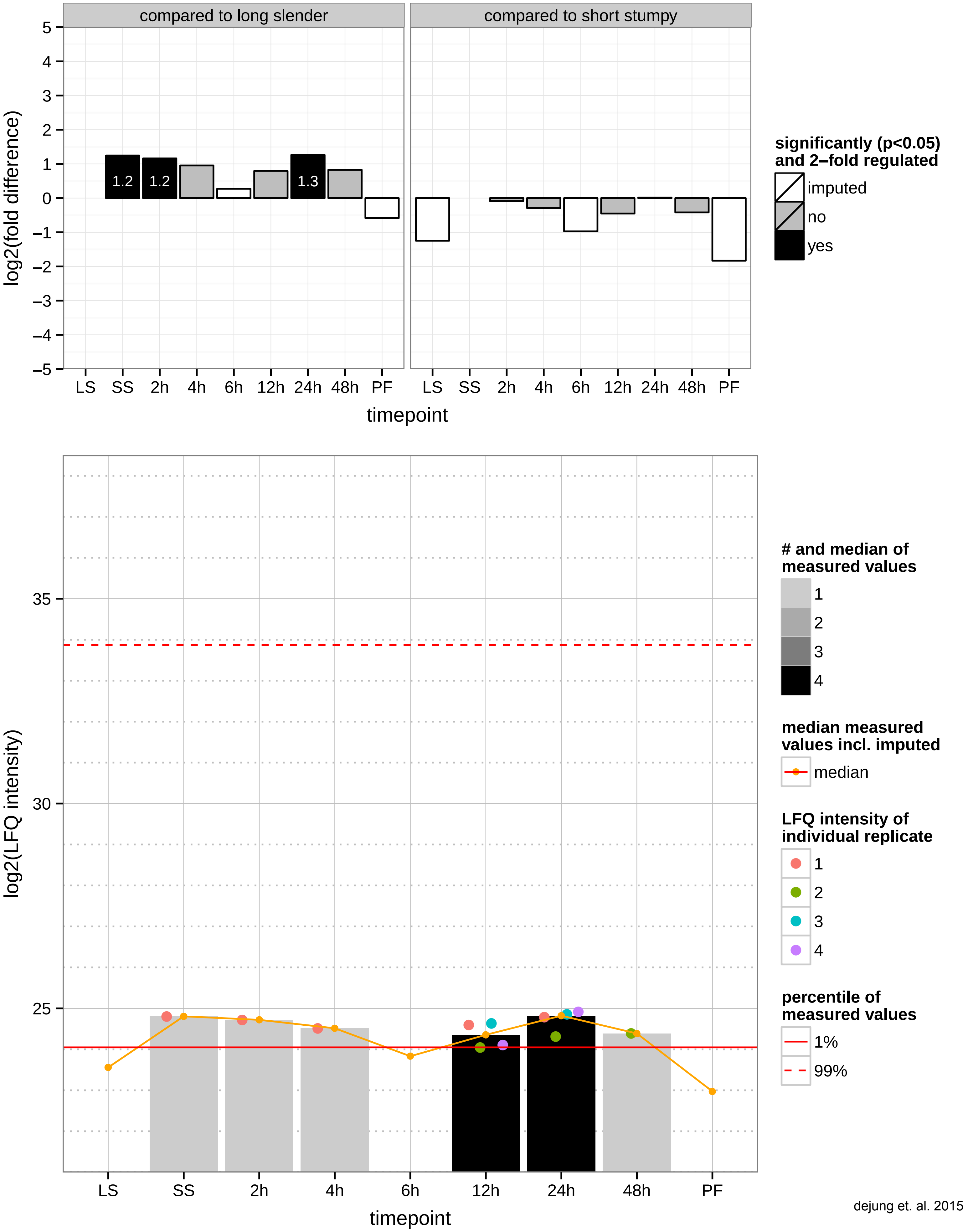

dejung et. al. 2015

hexokinase (HK1)
Tb927.10.2010
AGOF: ATP binding, hexokinase activity
AGOC: glycosome
AGOP: carbohydrate metabolic process, glycolysis, protein hexamerization
PGOF: ATP binding, phosphotransferase activity, alcohol group as acceptor
PGOC: null
PGOP: carbohydrate metabolic process

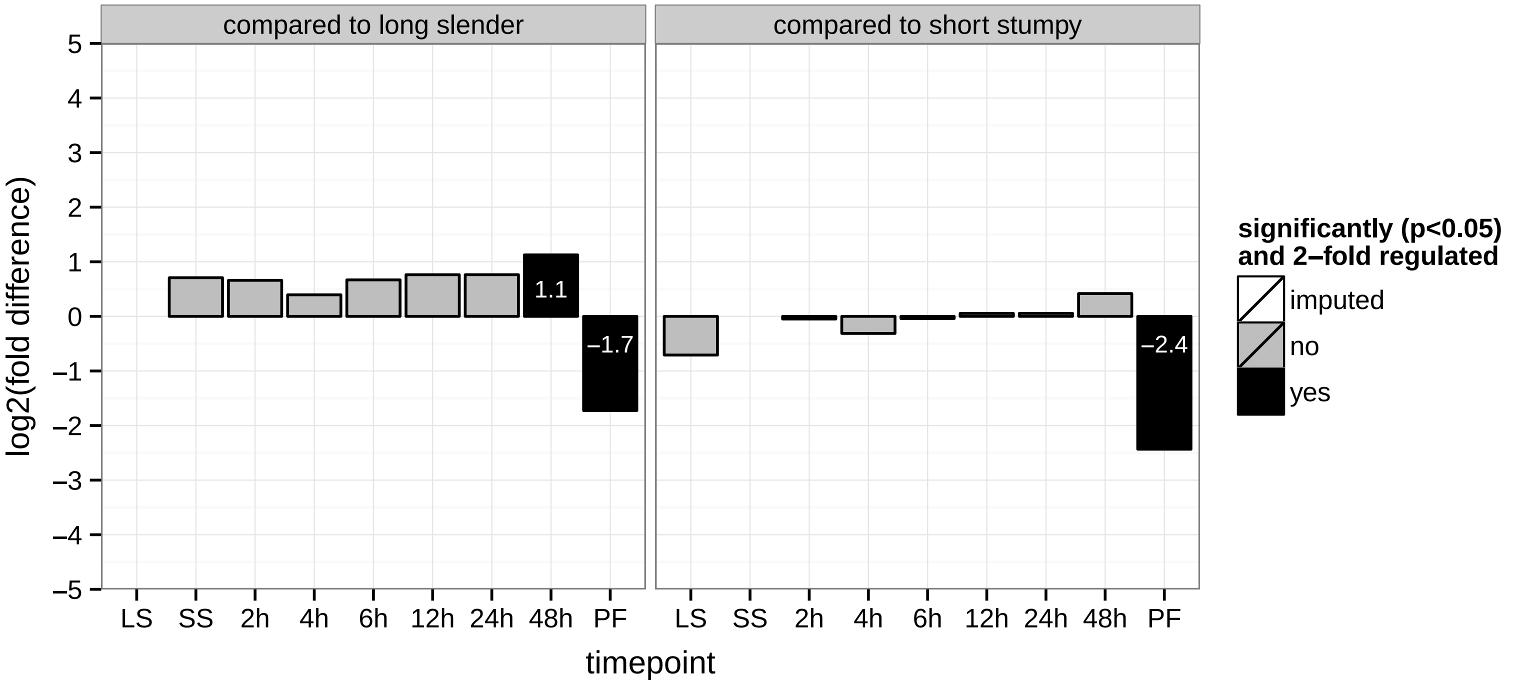

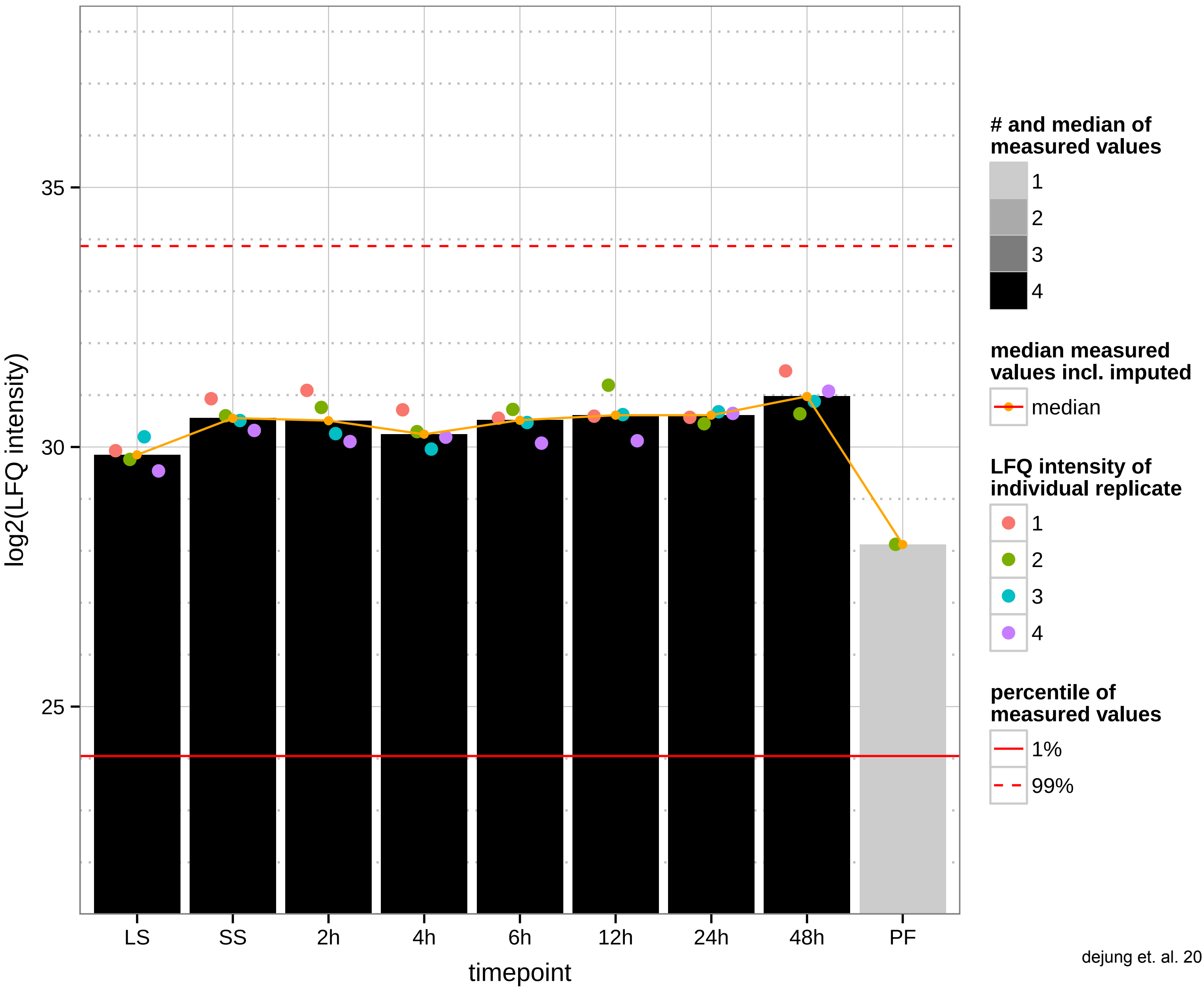

dejung et. al. 2015

hexokinase (HK2)
Tb927.10.2020
AGOF: ATP binding, hexokinase activity
AGOC: glycosome
AGOP: carbohydrate metabolic process, glycolysis
PGOF: ATP binding, phosphotransferase activity, alcohol group as acceptor
PGOC: null
PGOP: carbohydrate metabolic process

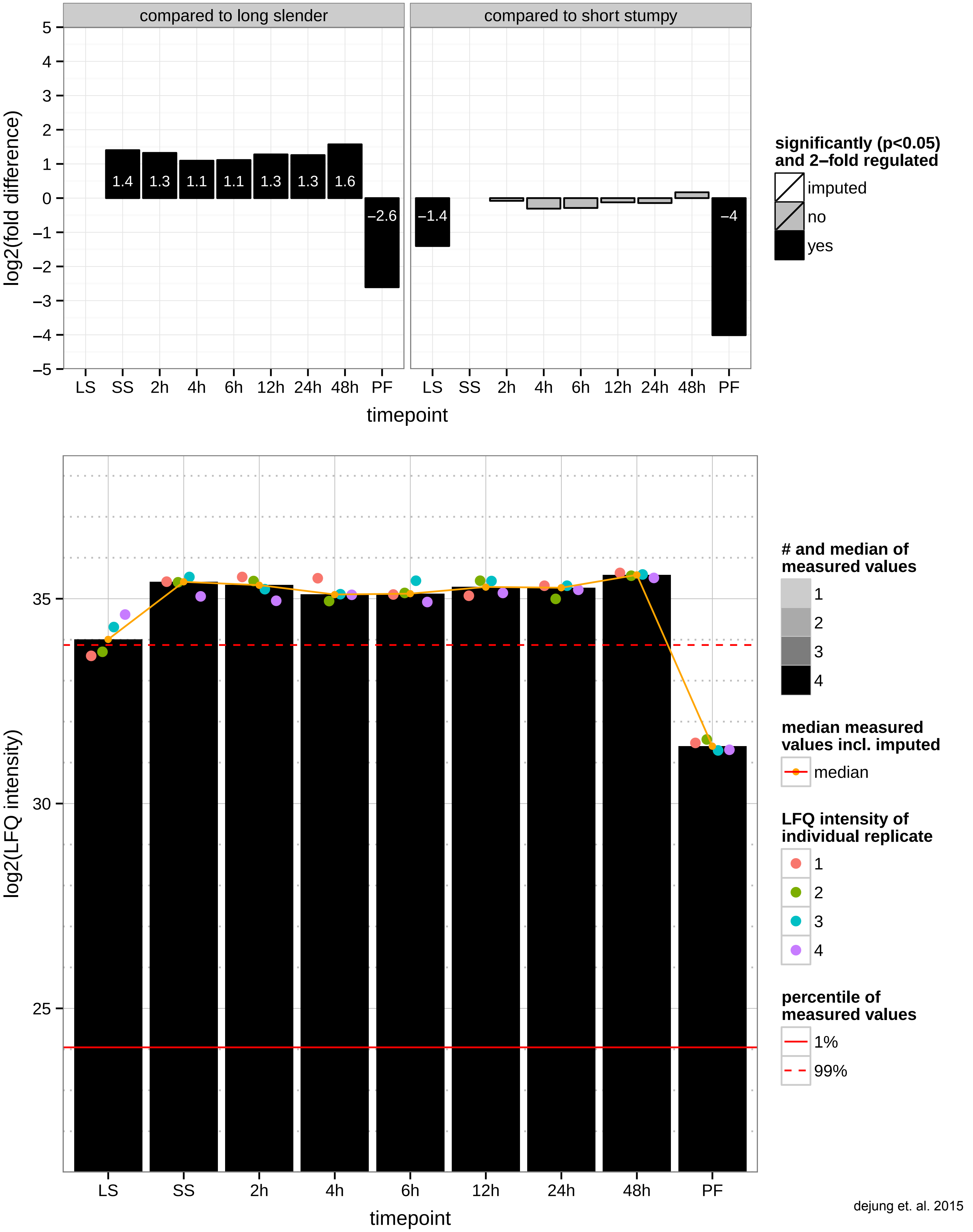

dejung et. al. 2015

hypothetical protein, conserved
Tb927.10.260
AGOF: null
AGOC: null
AGOP: null
PGOF: null
PGOC: null
PGOP: null

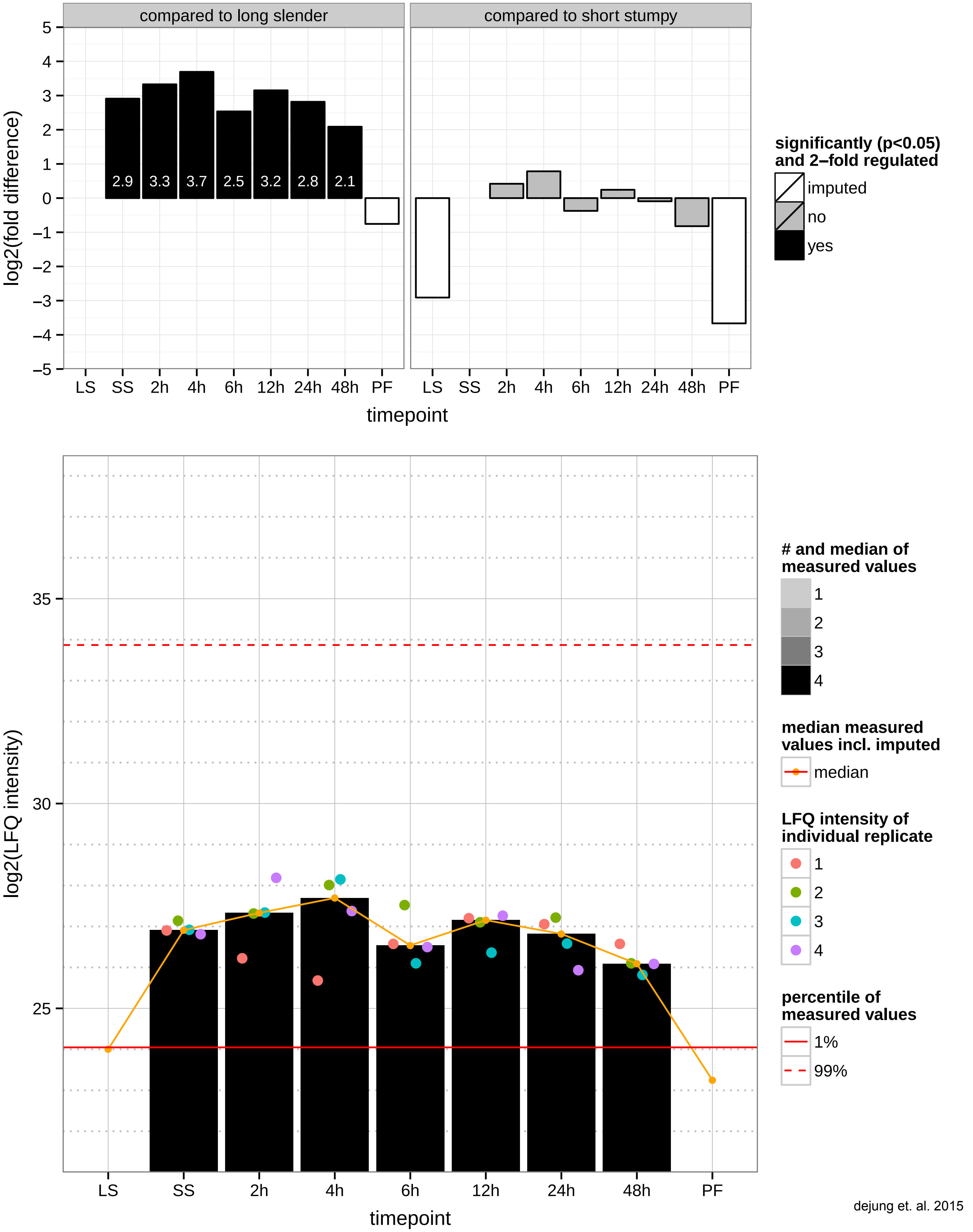

dejung et. al. 2015

hypothetical protein, conserved
Tb927.10.2940
AGOF: null
AGOC: Golgi apparatus, endoplasmic reticulum
AGOP: intracellular protein transport
PGOF: null
PGOC: null
PGOP: null

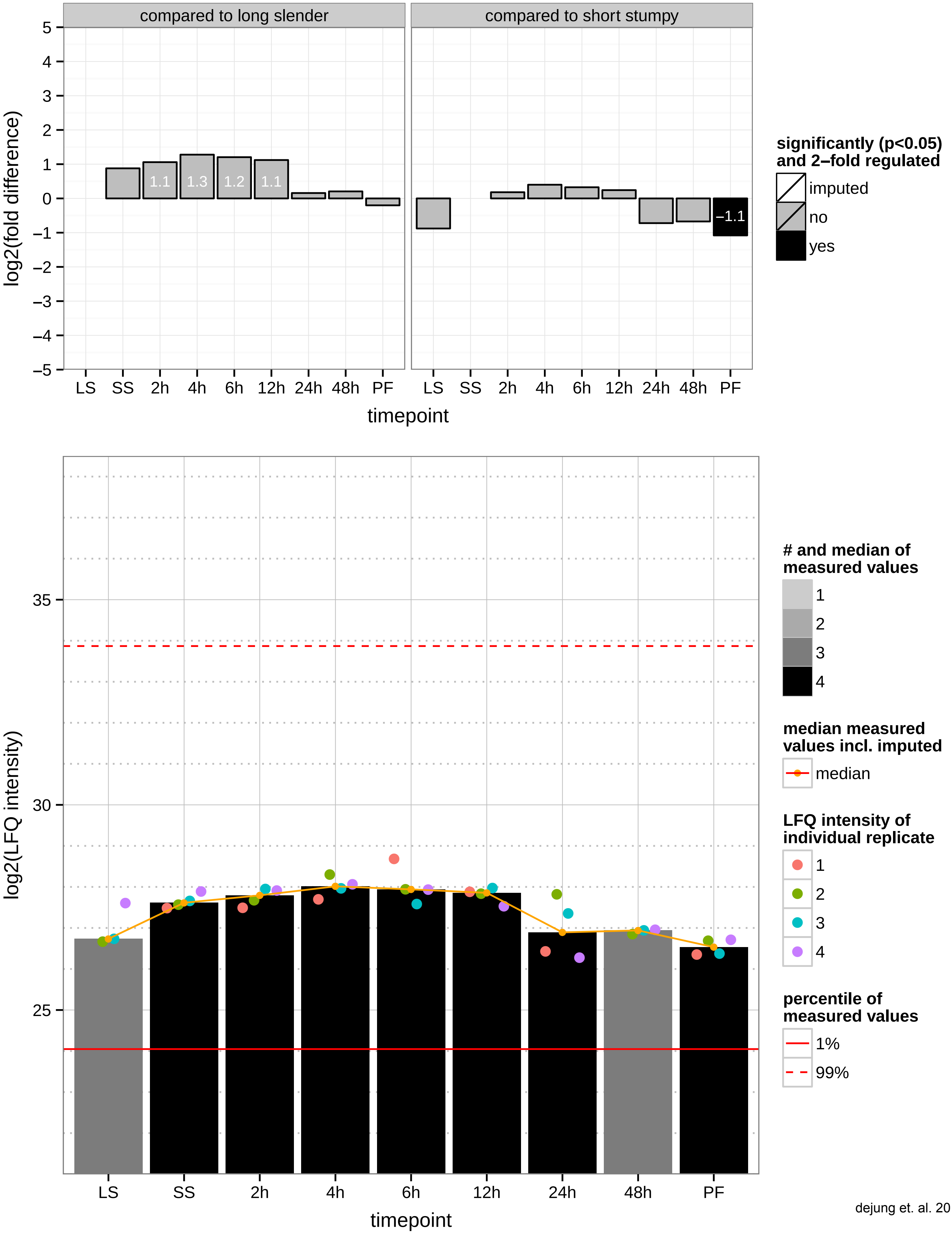

dejung et. al. 2015

glycerol–3–phosphate acyltransferase, putative
Tb927.10.3100
AGOF: acyl–phosphate glycerol–3–phosphate acyltransferase activity
AGOC: null
AGOP: metabolic process, phospholipid biosynthetic process
PGOF: transferase activity, transferring acyl groups
PGOC: null
PGOP: metabolic process

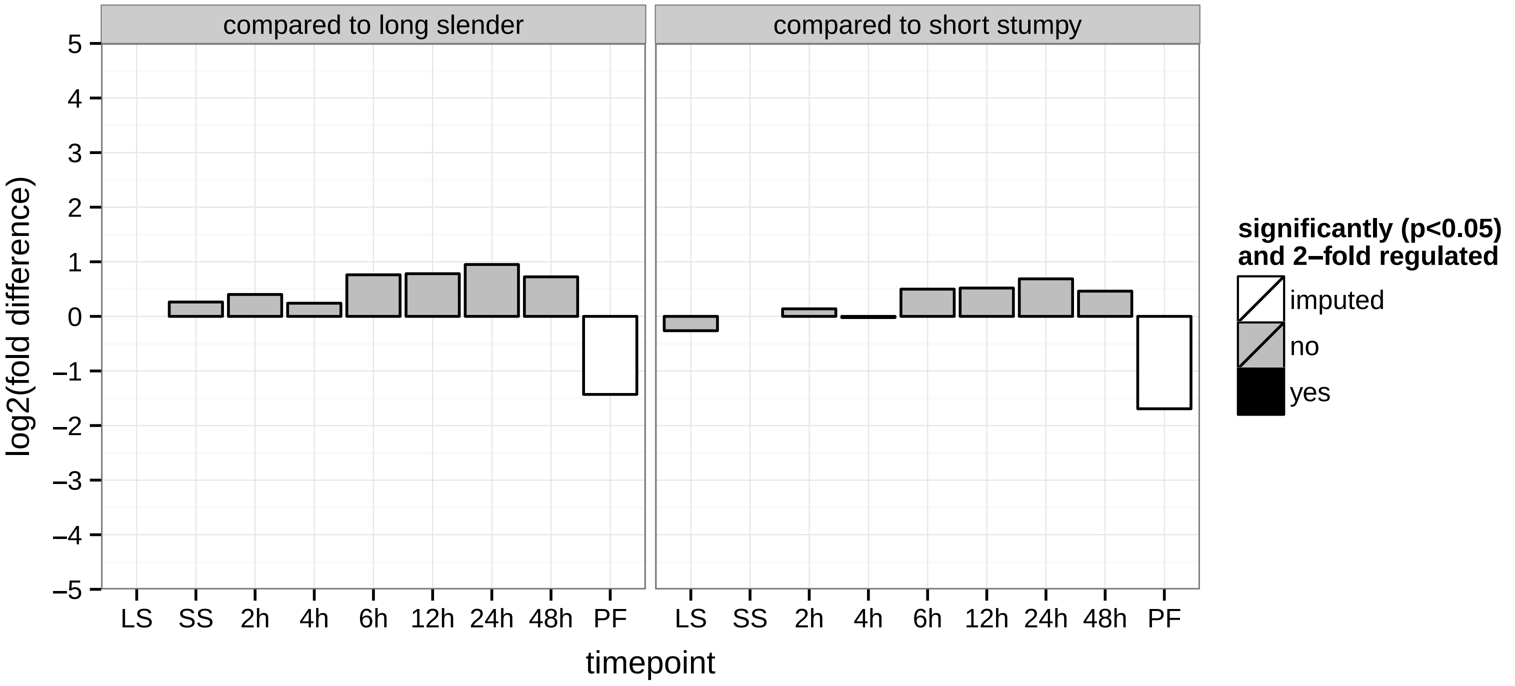

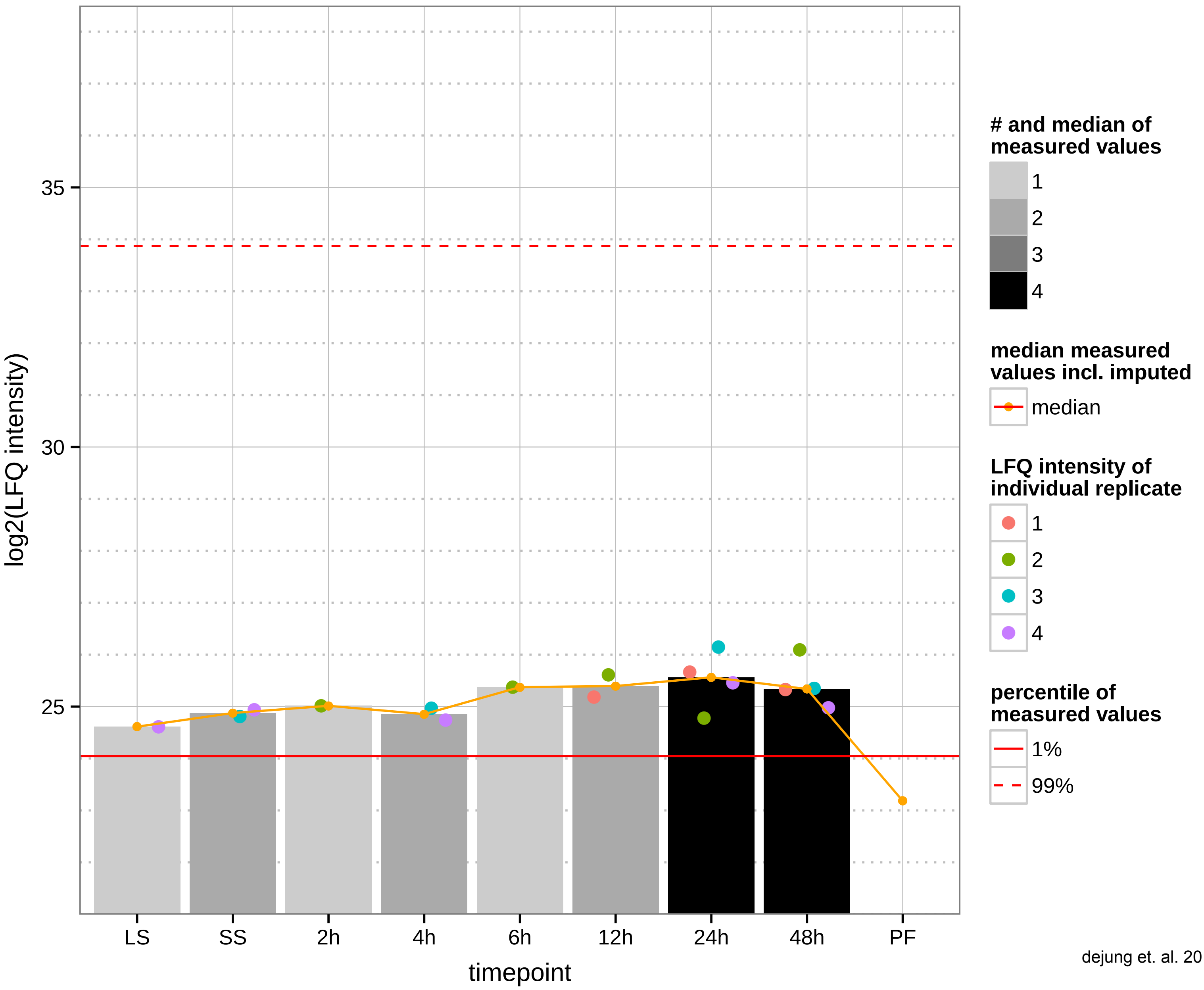

dejung et. al. 2015

hypothetical protein, conserved
Tb927.10.3330
AGOF: null
AGOC: null
AGOP: null
PGOF: null
PGOC: null
PGOP: null

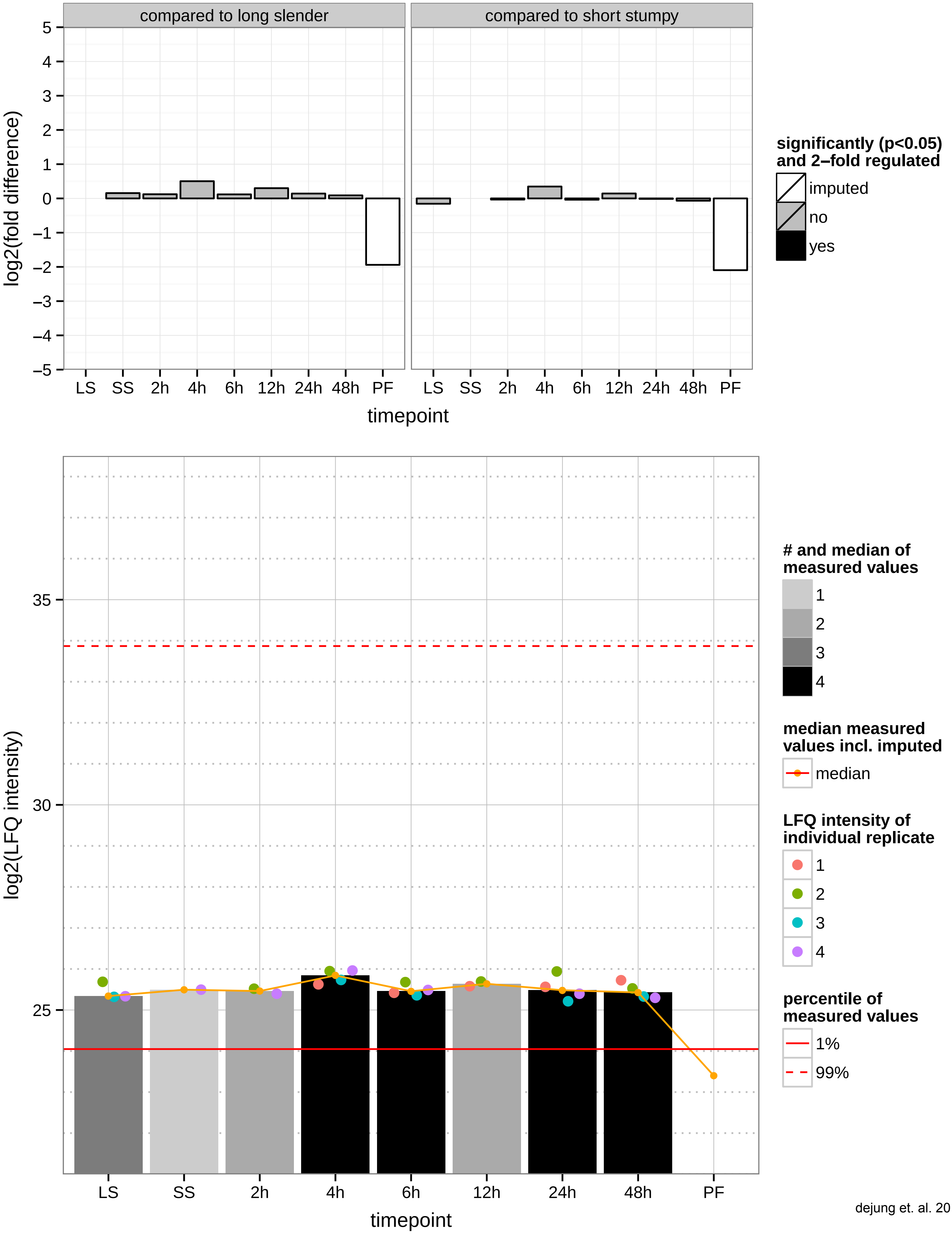

dejung et. al. 2015

hypothetical protein, conserved
Tb927.10.3880
AGOF: null
AGOC: null
AGOP: null
PGOF: null
PGOC: null
PGOP: null

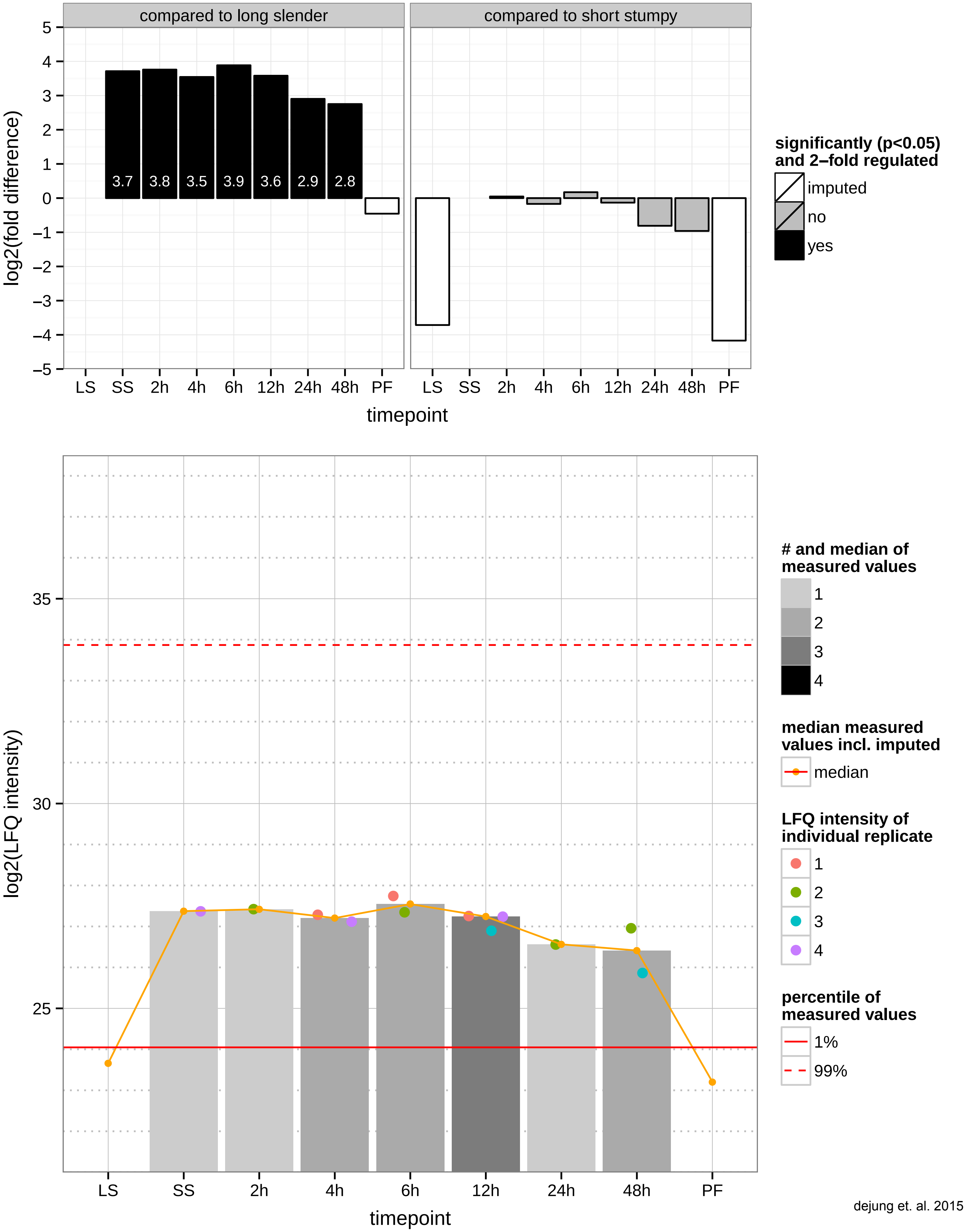

dejung et. al. 2015

protein kinase, putative
Tb927.10.3900
AGOF: ATP binding, protein kinase activity, protein serine/threonine kinase activity, protein tyrosine kinase activity
AGOC: null
AGOP: protein phosphorylation
PGOF: ATP binding, protein kinase activity, transferase activity, transferring phosphorus–containing groups
PGOC: null
PGOP: protein phosphorylation

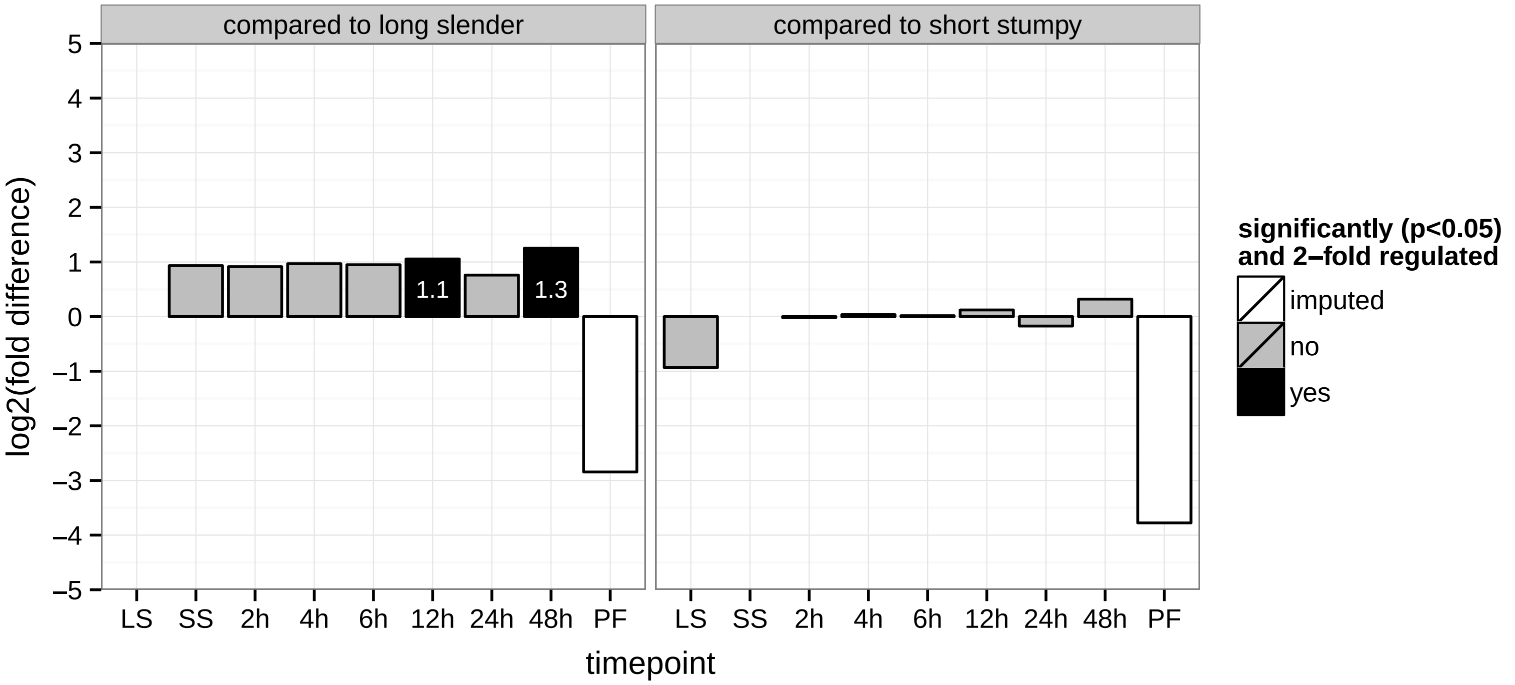

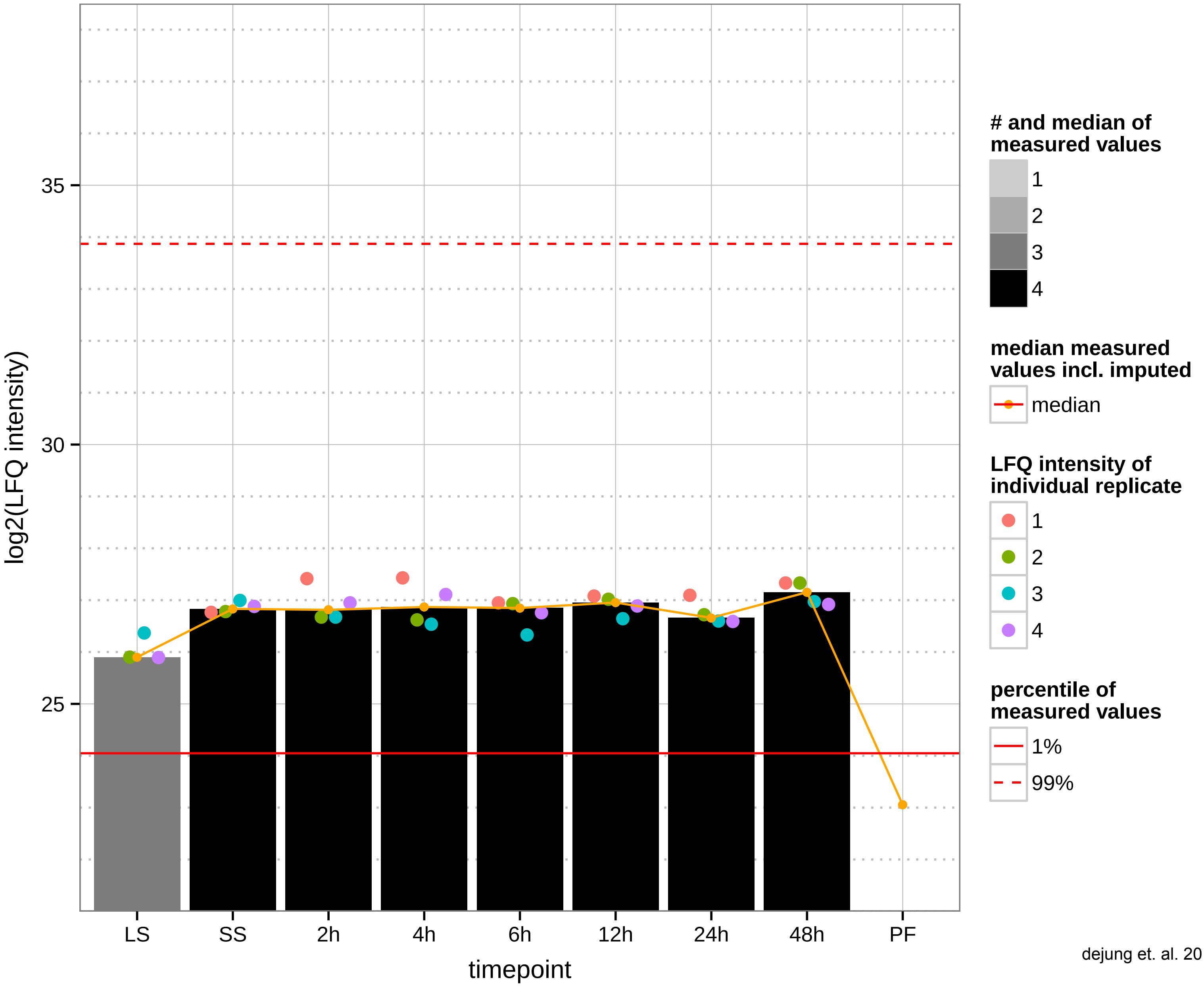

dejung et. al. 2015

hypothetical protein, conserved
Tb927.10.4100
AGOF: null
AGOC: null
AGOP: null
PGOF: null
PGOC: null
PGOP: null

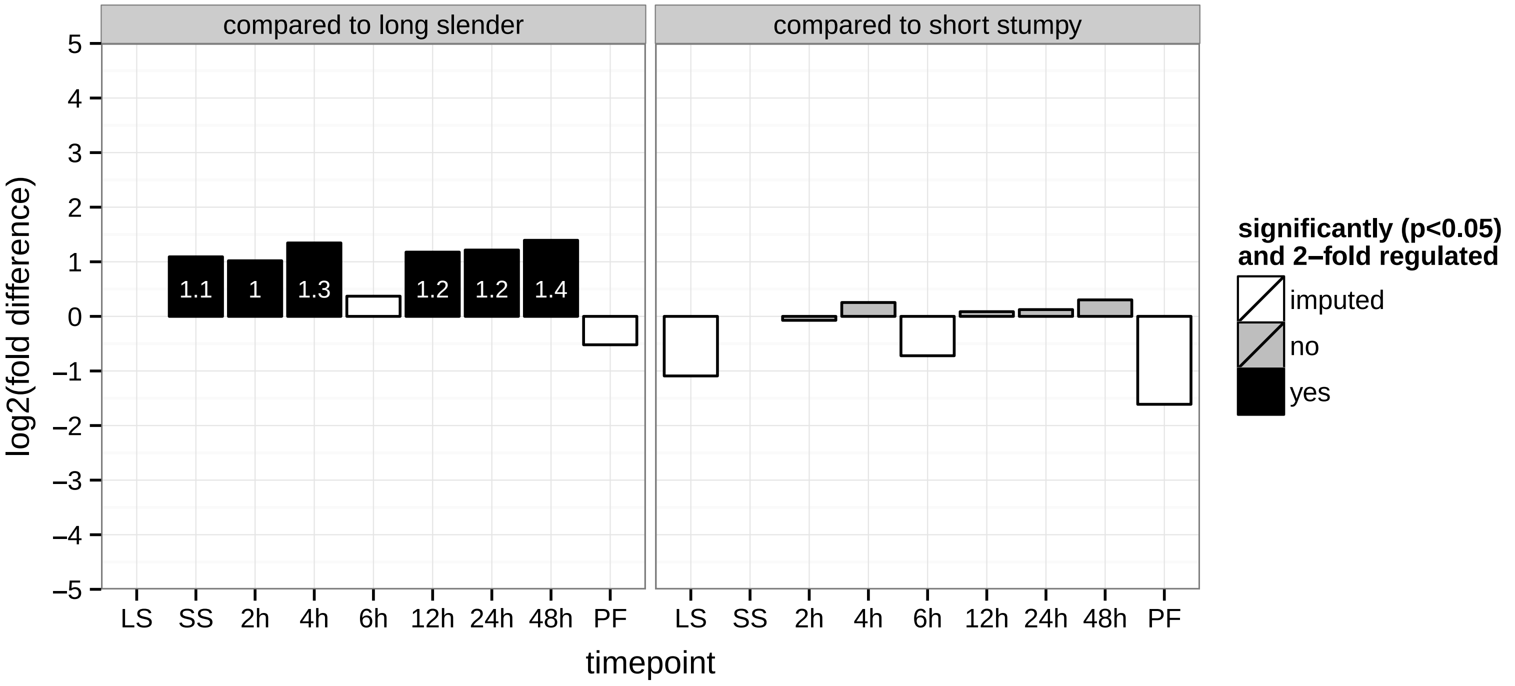

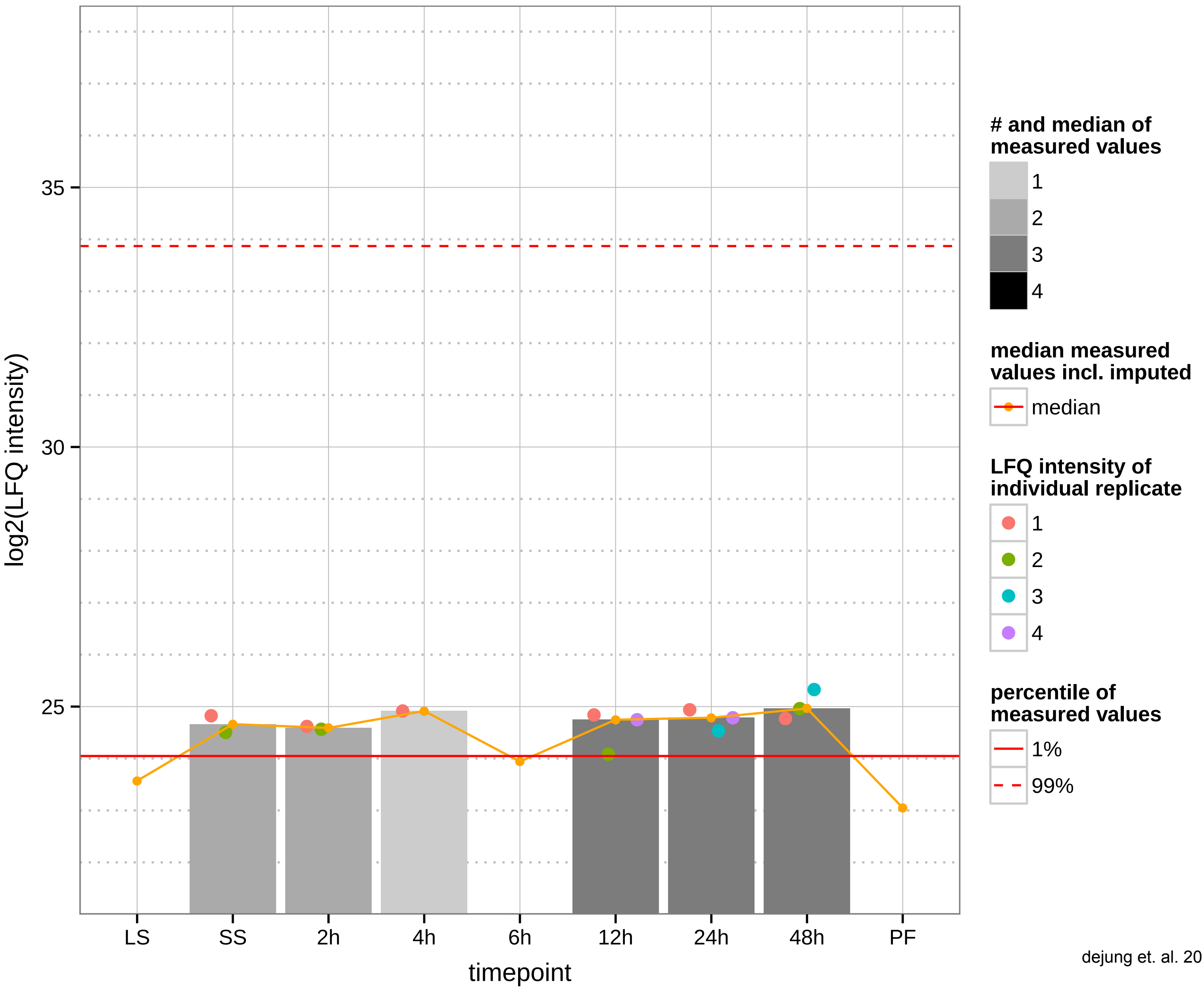

dejung et. al. 2015

hypothetical protein, conserved
Tb927.10.4260
AGOF: null
AGOC: null
AGOP: null
PGOF: null
PGOC: null
PGOP: null

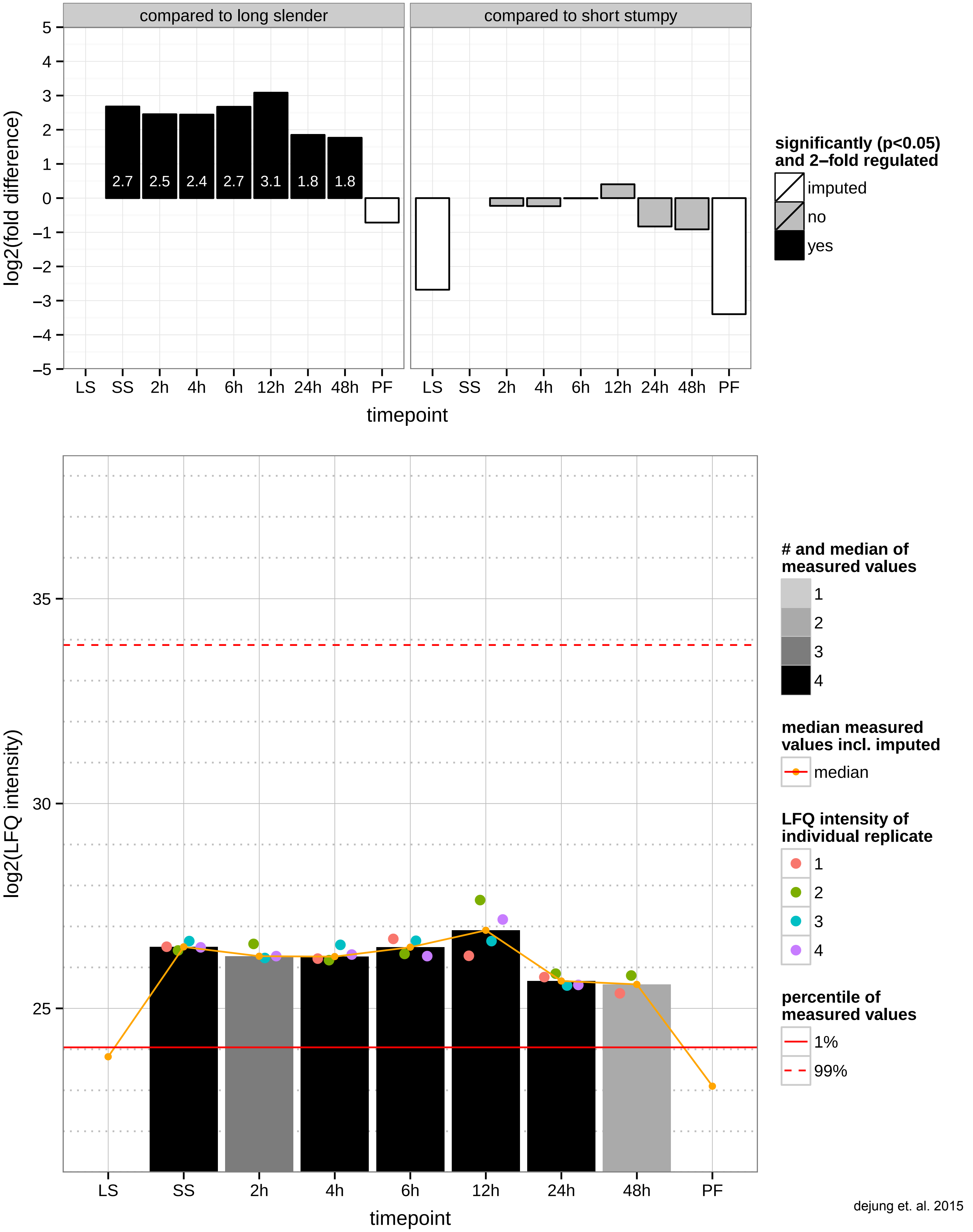

dejung et. al. 2015

hypothetical protein, conserved
Tb927.10.450
AGOF: null
AGOC: null
AGOP: ciliary or flagellar motility
PGOF: null
PGOC: null
PGOP: null

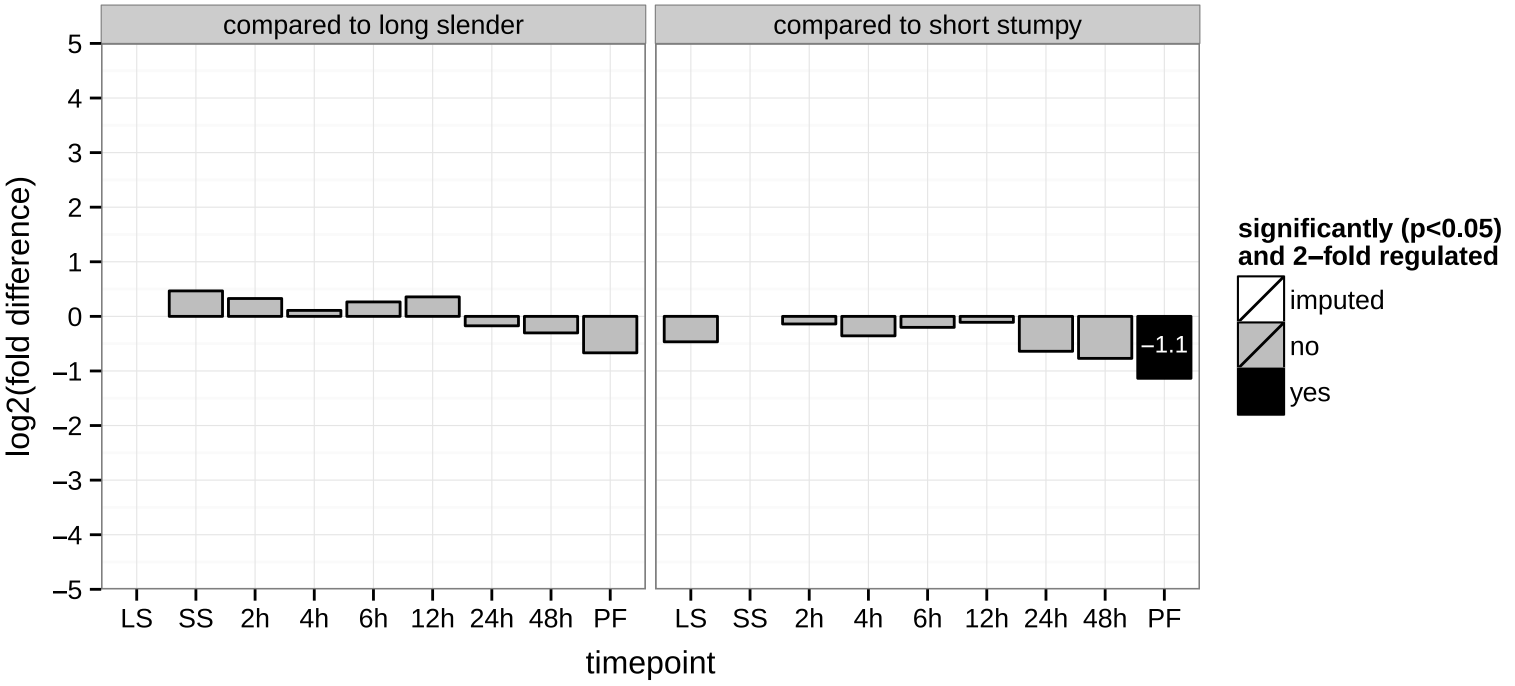

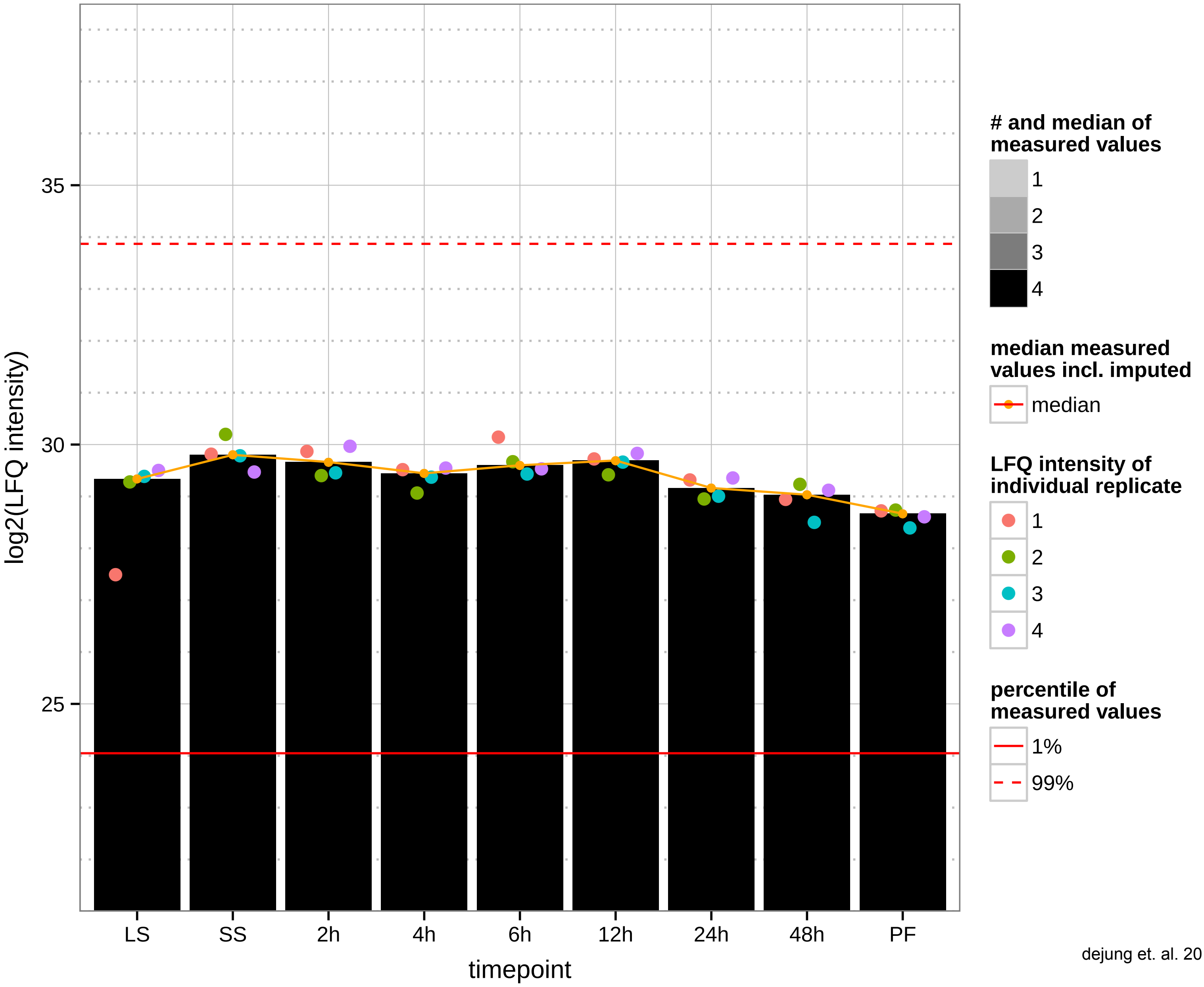

dejung et. al. 2015

U2 small nuclear ribonucleoprotein 16.5K
Tb927.10.4950
AGOF: RNA binding
AGOC: U2 snRNP, nucleus
AGOP: alternative nuclear mRNA splicing, via spliceosome
PGOF: null
PGOC: null
PGOP: null

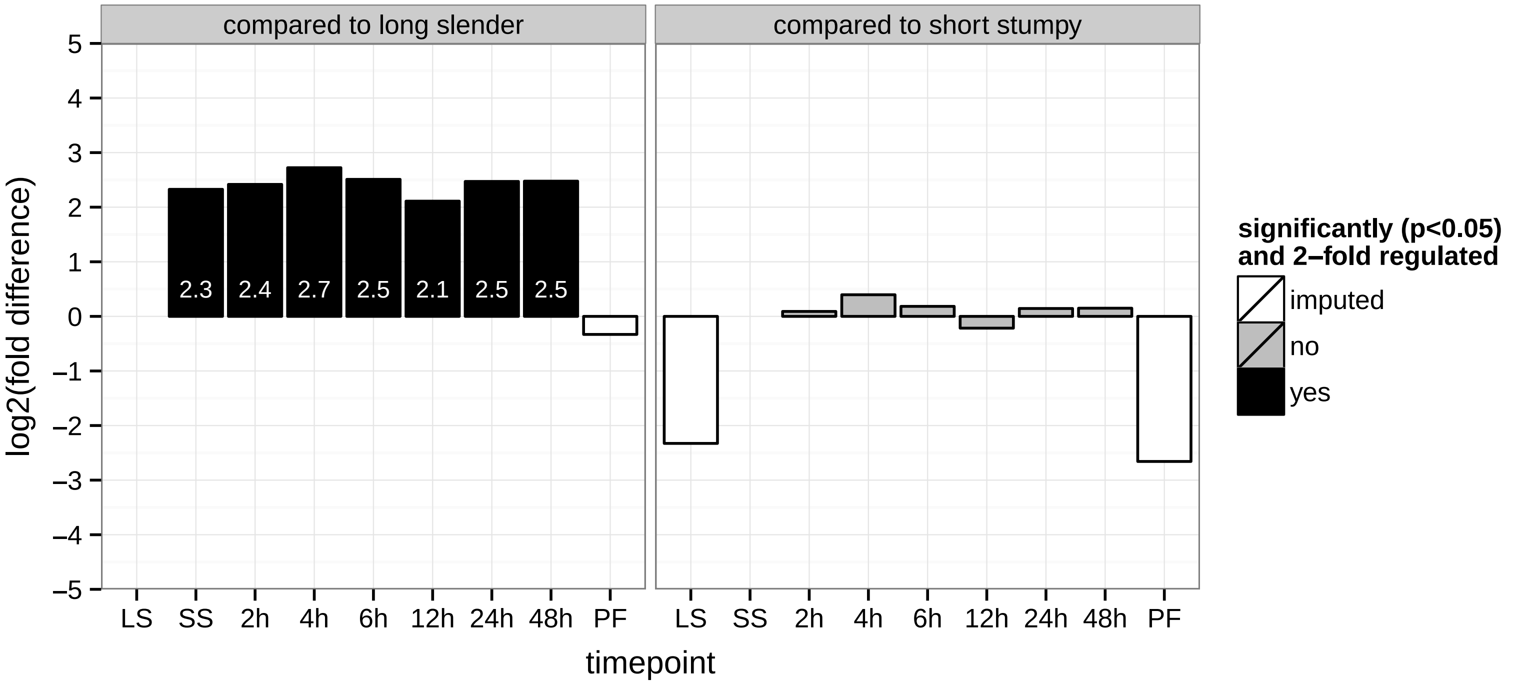

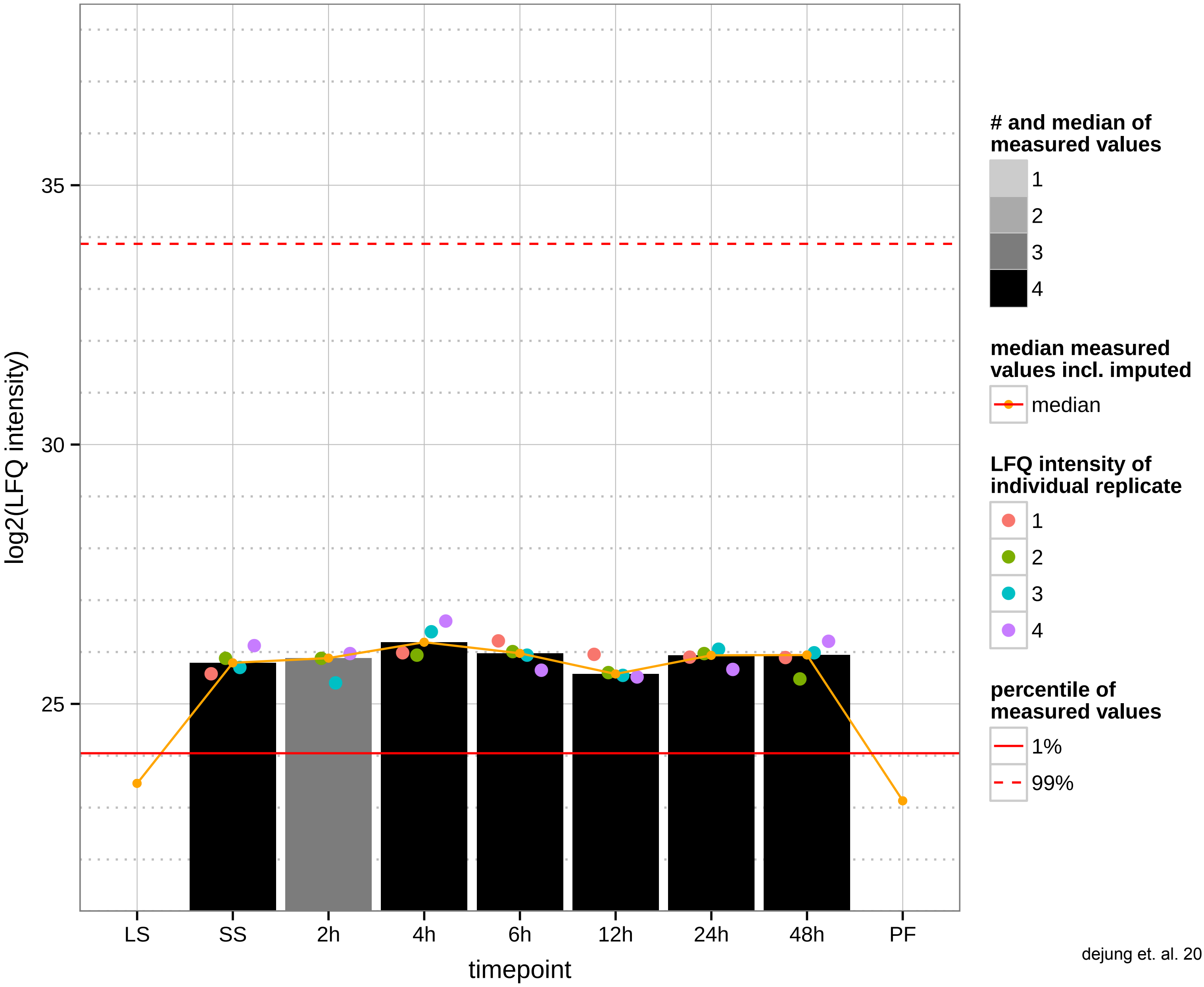

dejung et. al. 2015

KREPA6, RNA–editing complex protein (KREPA6)
Tb927.10.5120
AGOF: RNA binding, endonuclease activity
AGOC: mitochondrial mRNA editing complex, mitochondrion
AGOP: RNA modification
PGOF: single–stranded DNA binding
PGOC: null
PGOP: null

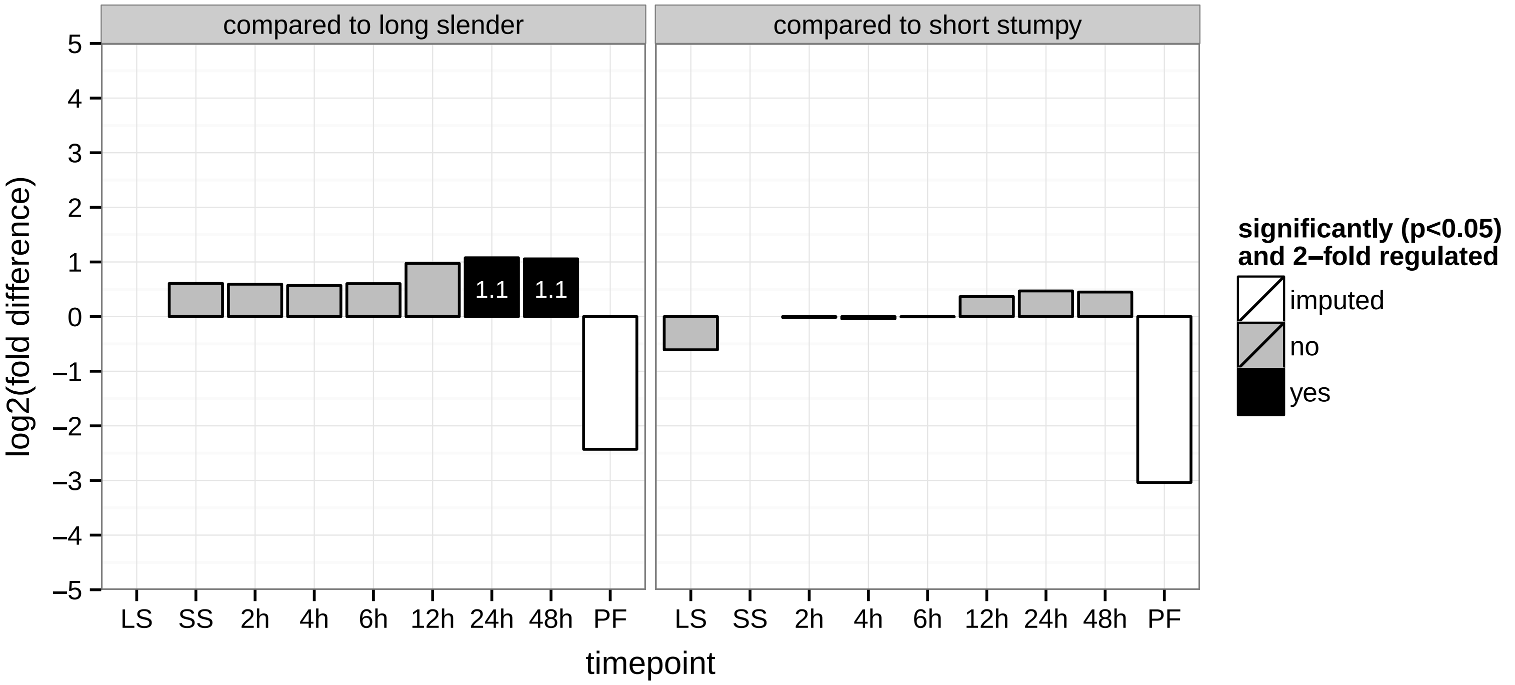

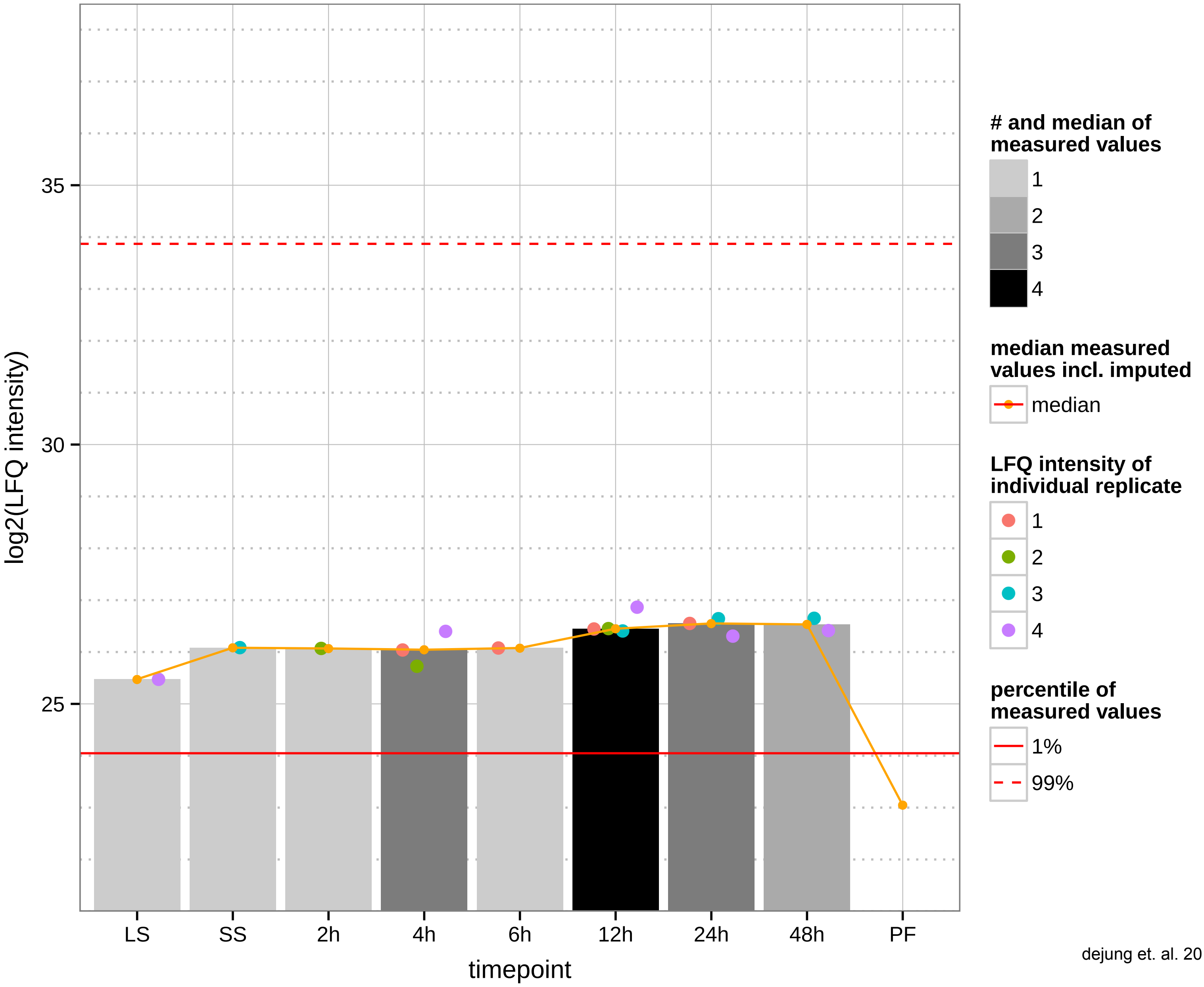

dejung et. al. 2015

hypothetical protein, conserved
Tb927.10.5400
AGOF: null
AGOC: mitochondrion
AGOP: null
PGOF: null
PGOC: null
PGOP: null

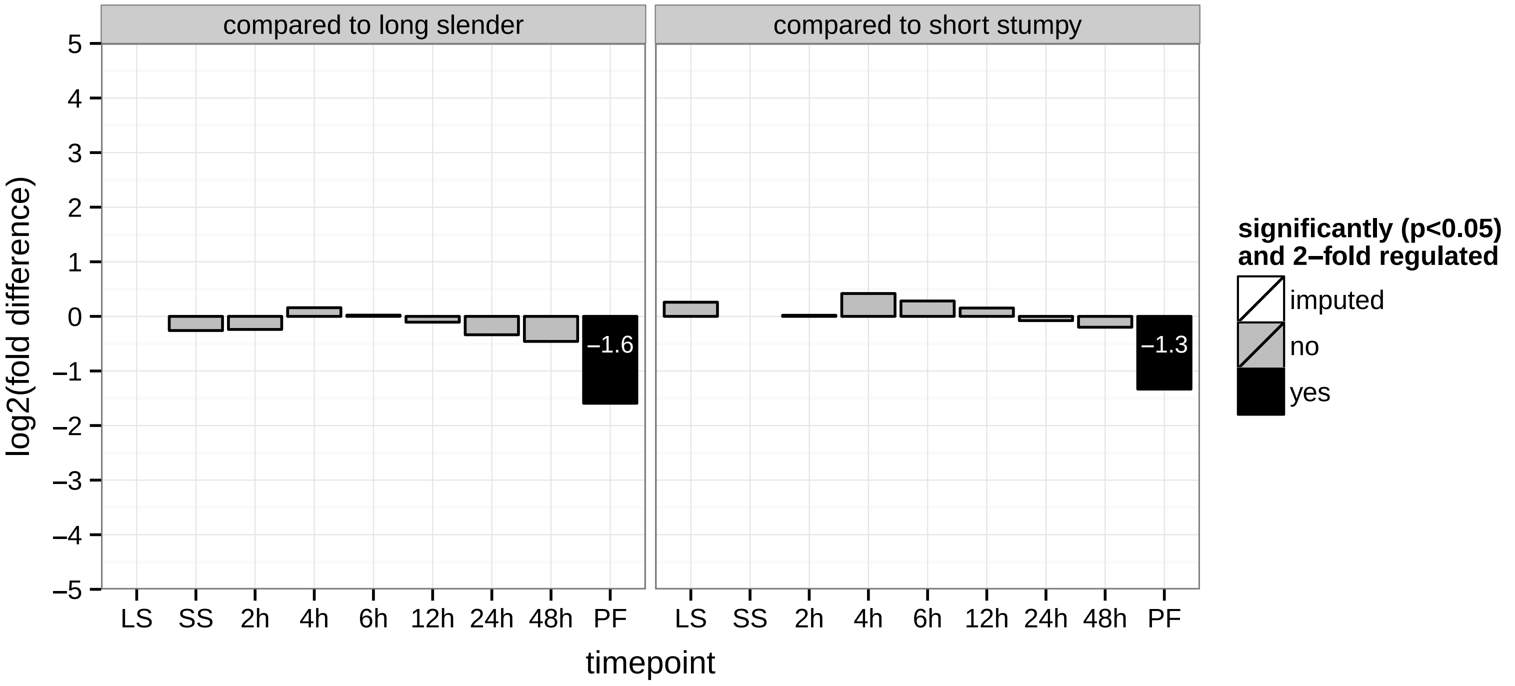

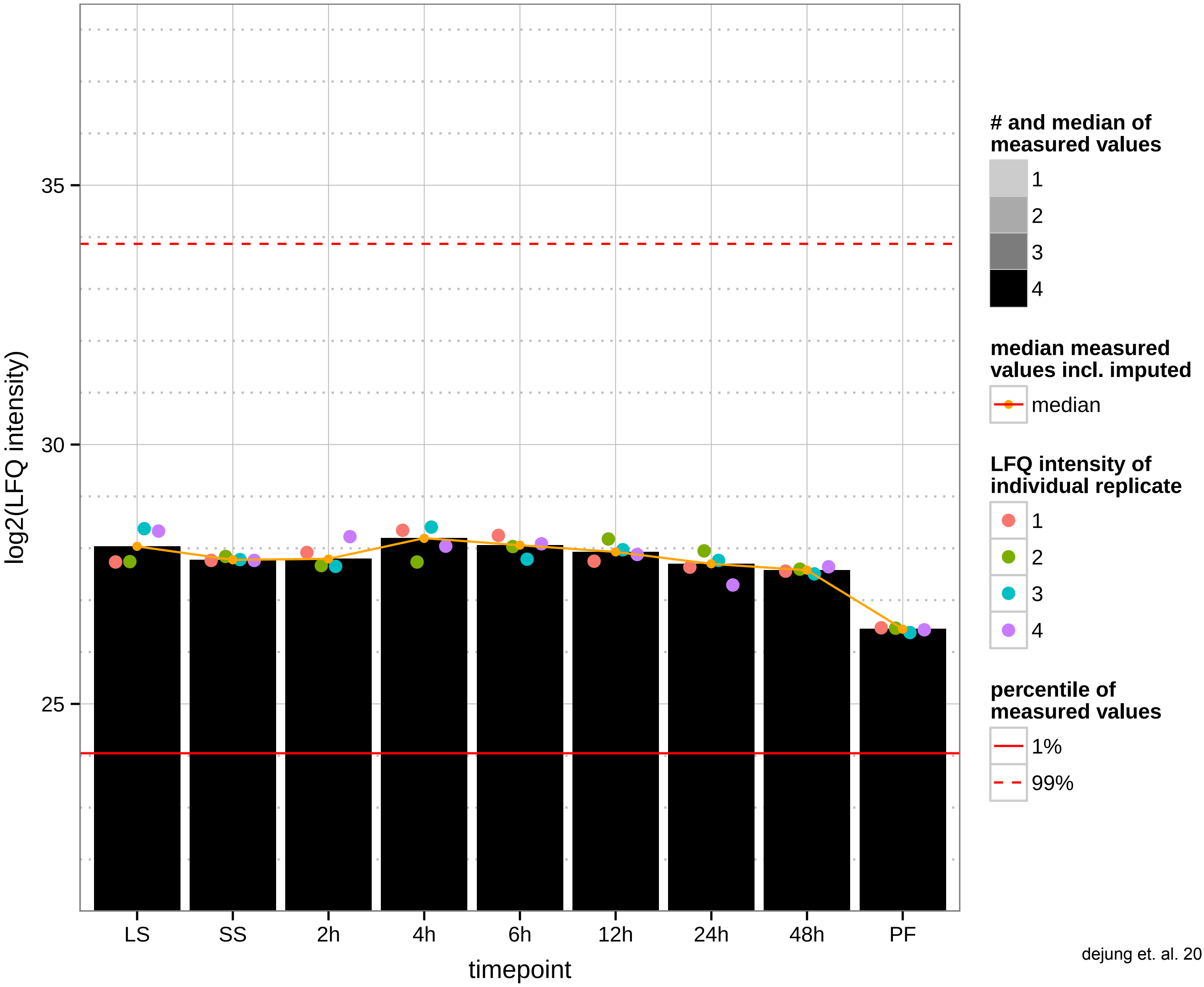

dejung et. al. 2015

neurobeachin/beige protein, putative
Tb927.10.6150;Tb11.v5.0207
AGOF: null
AGOC: null
AGOP: null
PGOF: protein binding
PGOC: null
PGOP: null

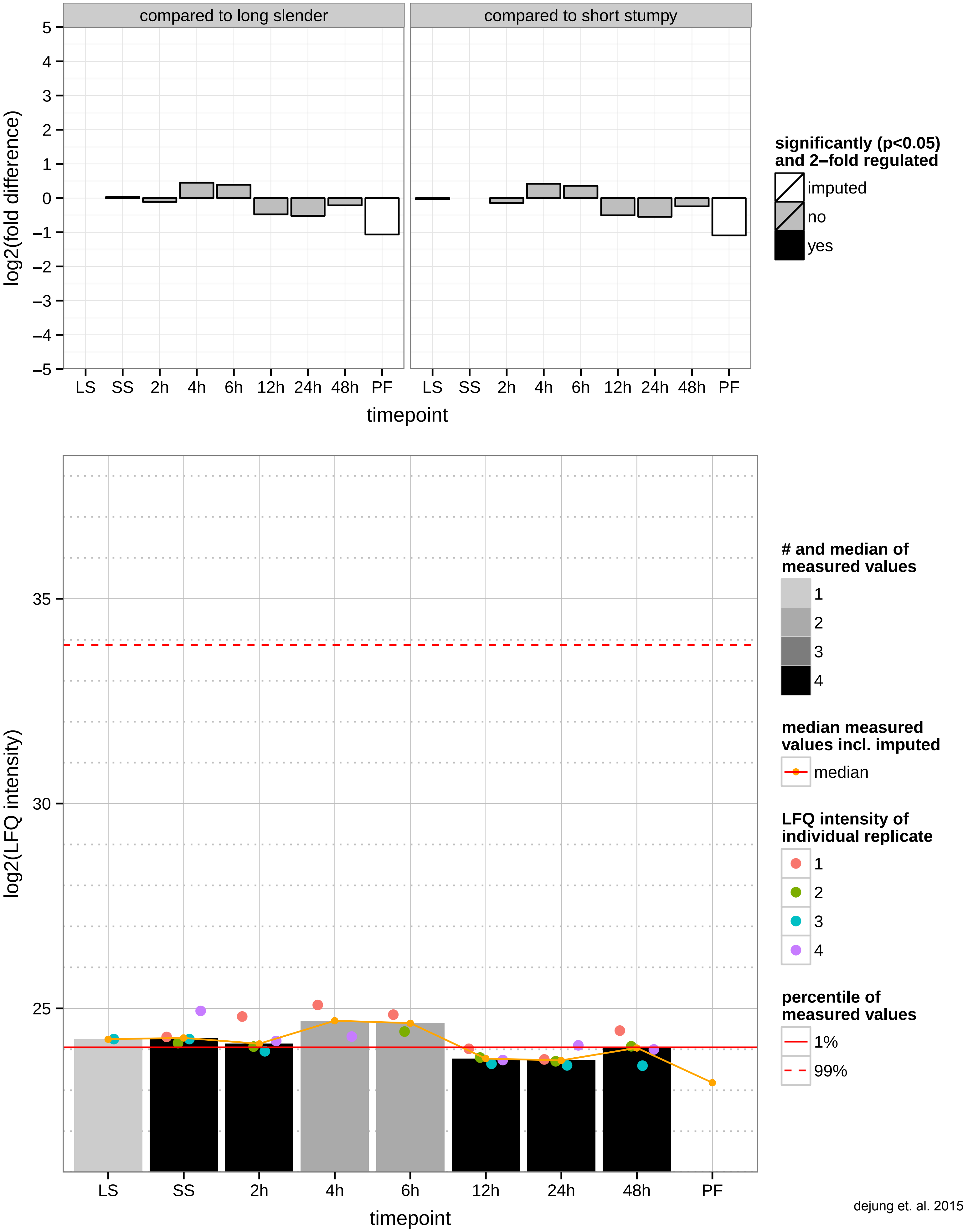

dejung et. al. 2015

MORN repeat containing protein
Tb927.10.6230
AGOF: null
AGOC: null
AGOP: null
PGOF: null
PGOC: null
PGOP: null

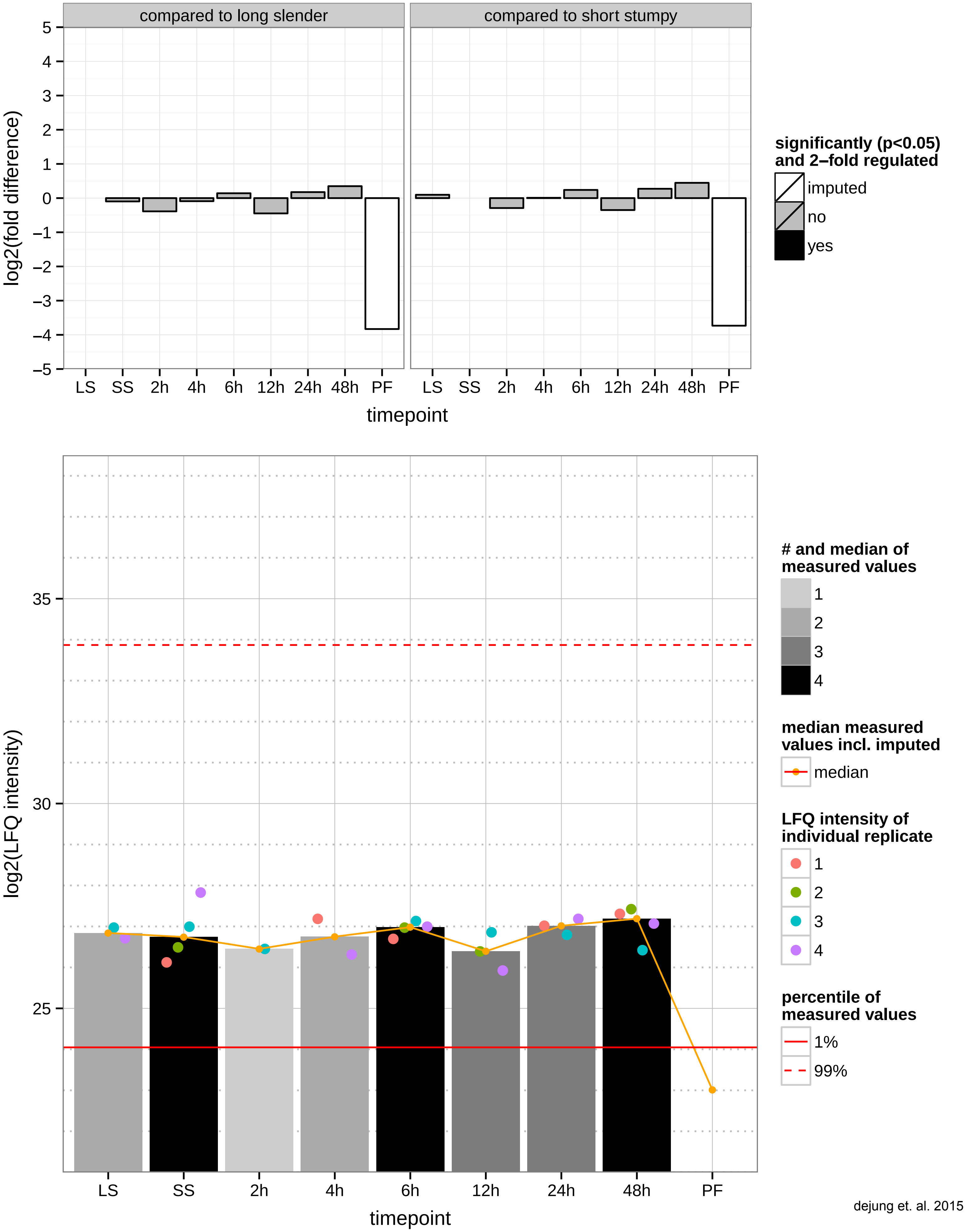

dejung et. al. 2015

hypothetical protein, conserved
Tb927.10.6590
AGOF: null
AGOC: null
AGOP: null
PGOF: null
PGOC: null
PGOP: null

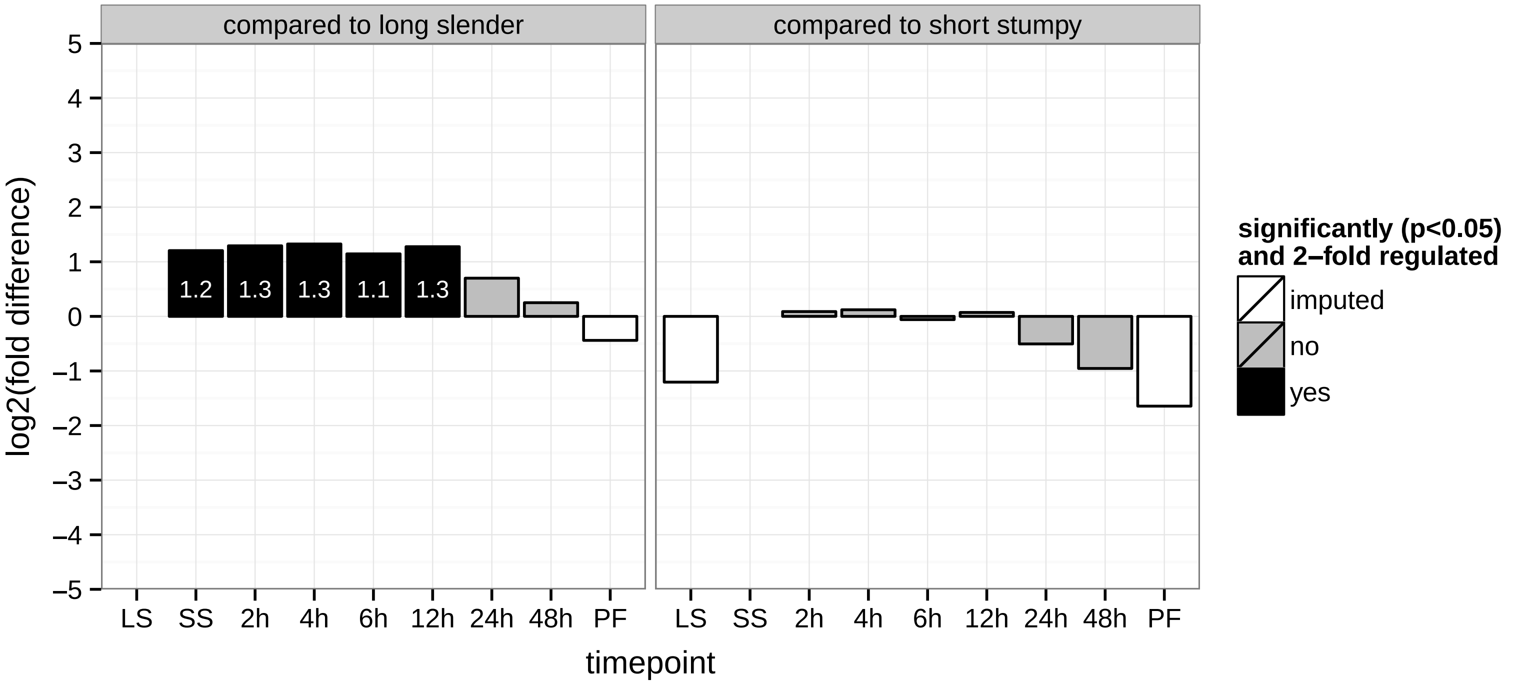

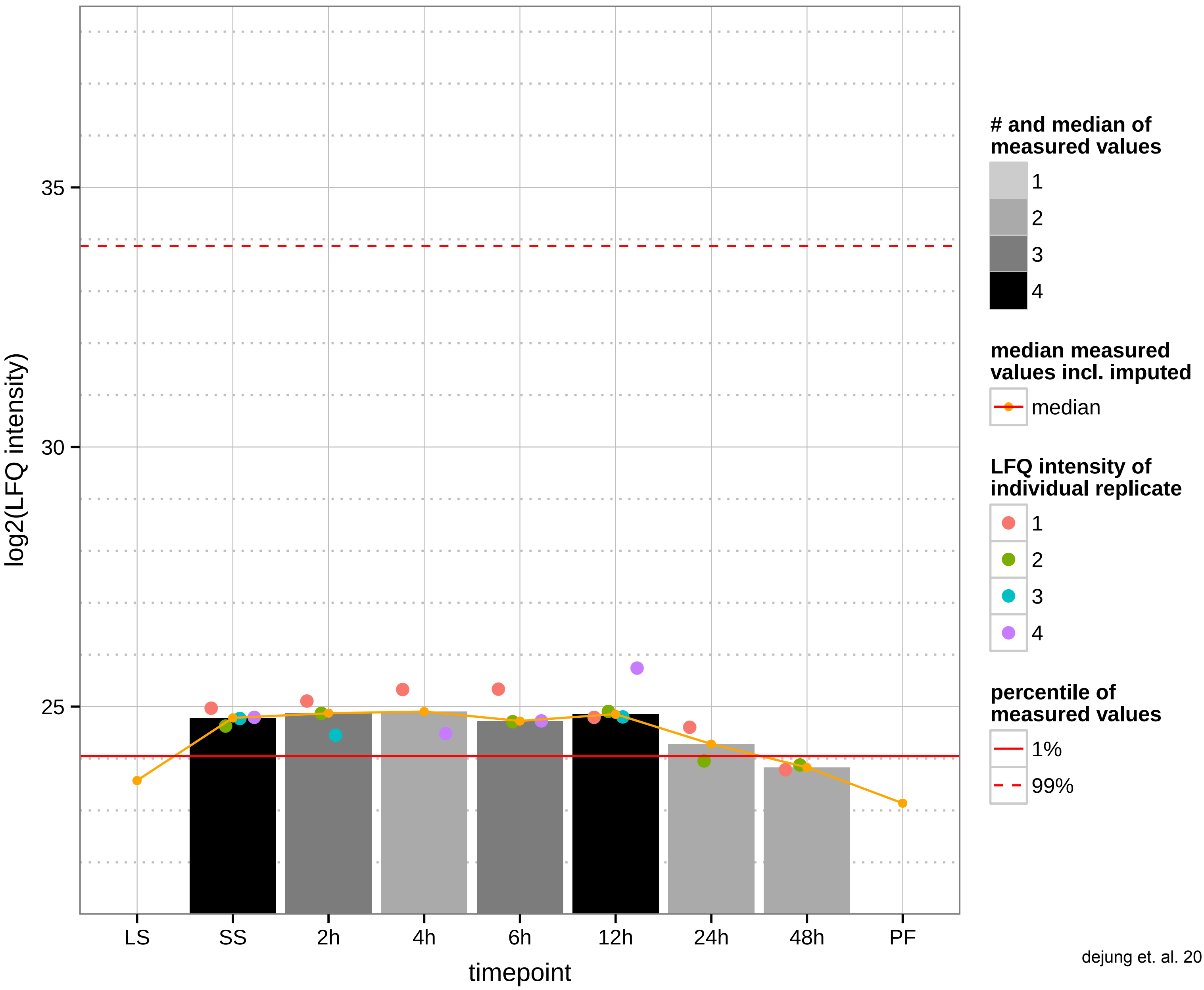

dejung et. al. 2015

protein tyrosine phosphatase, putative (TbPTP1)
Tb927.10.6690
AGOF: protein tyrosine phosphatase activity
AGOC: null
AGOP: protein dephosphorylation
PGOF: phosphatase activity, protein tyrosine phosphatase activity
PGOC: null
PGOP: dephosphorylation, protein dephosphorylation

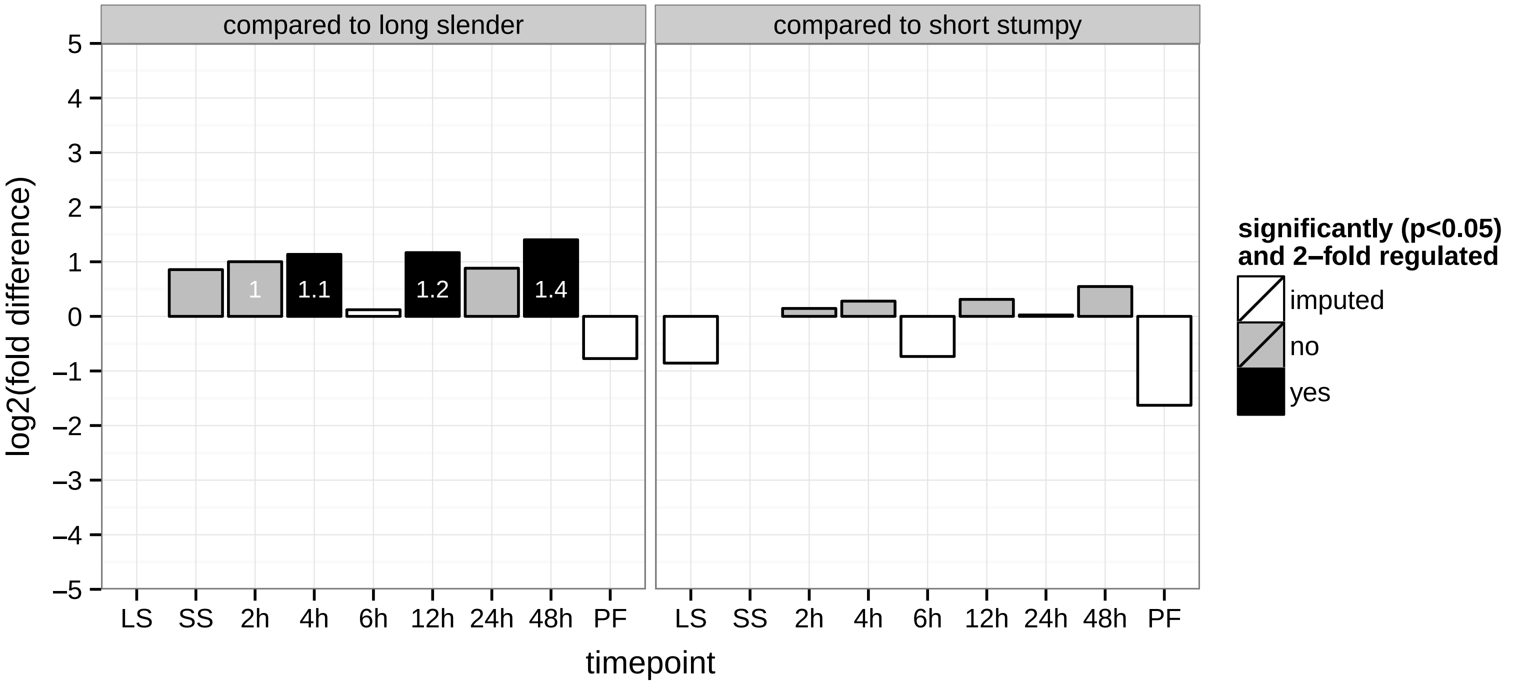

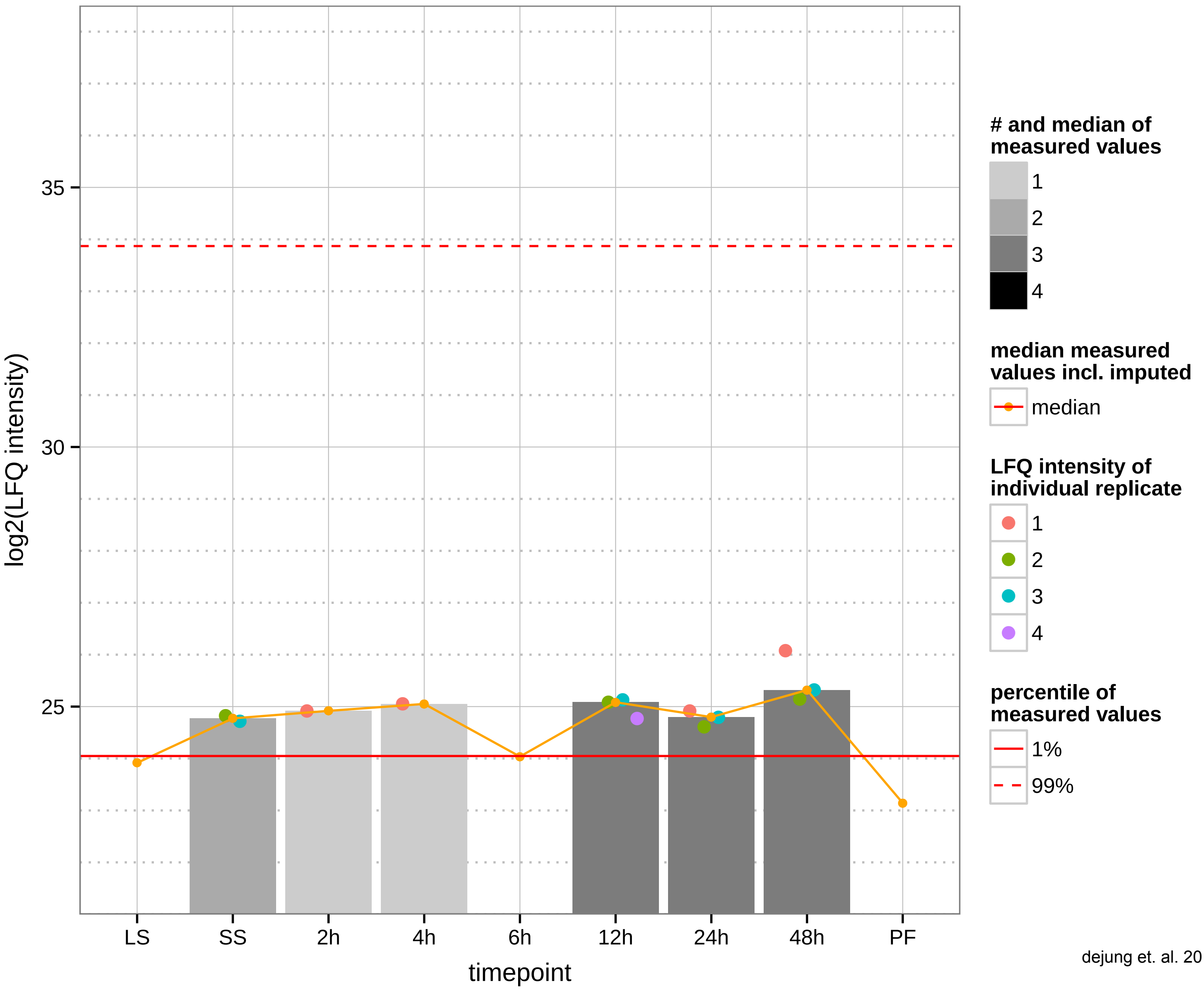

dejung et. al. 2015

hypothetical protein, conserved
Tb927.10.6700;Tb11.v5.0902
AGOF: null
AGOC: null
AGOP: null
PGOF: protein binding
PGOC: null
PGOP: null

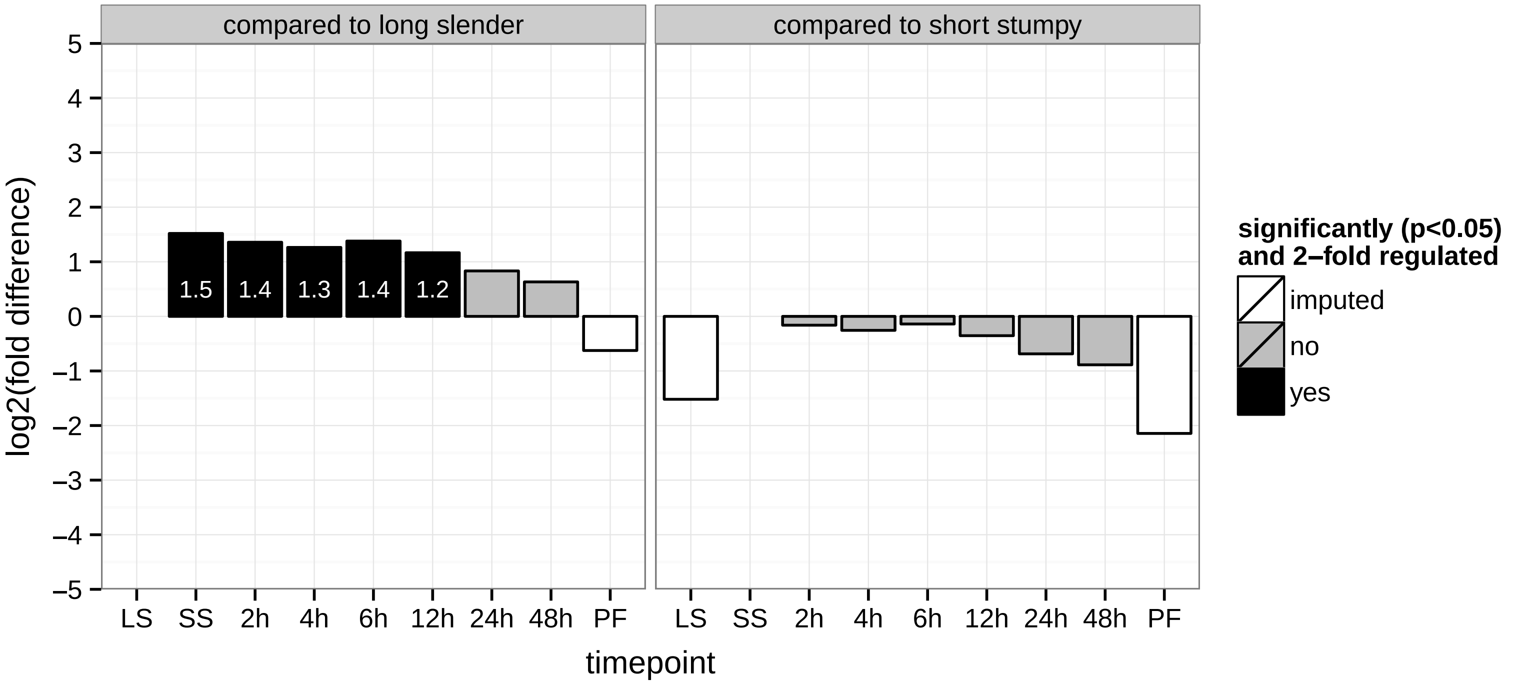

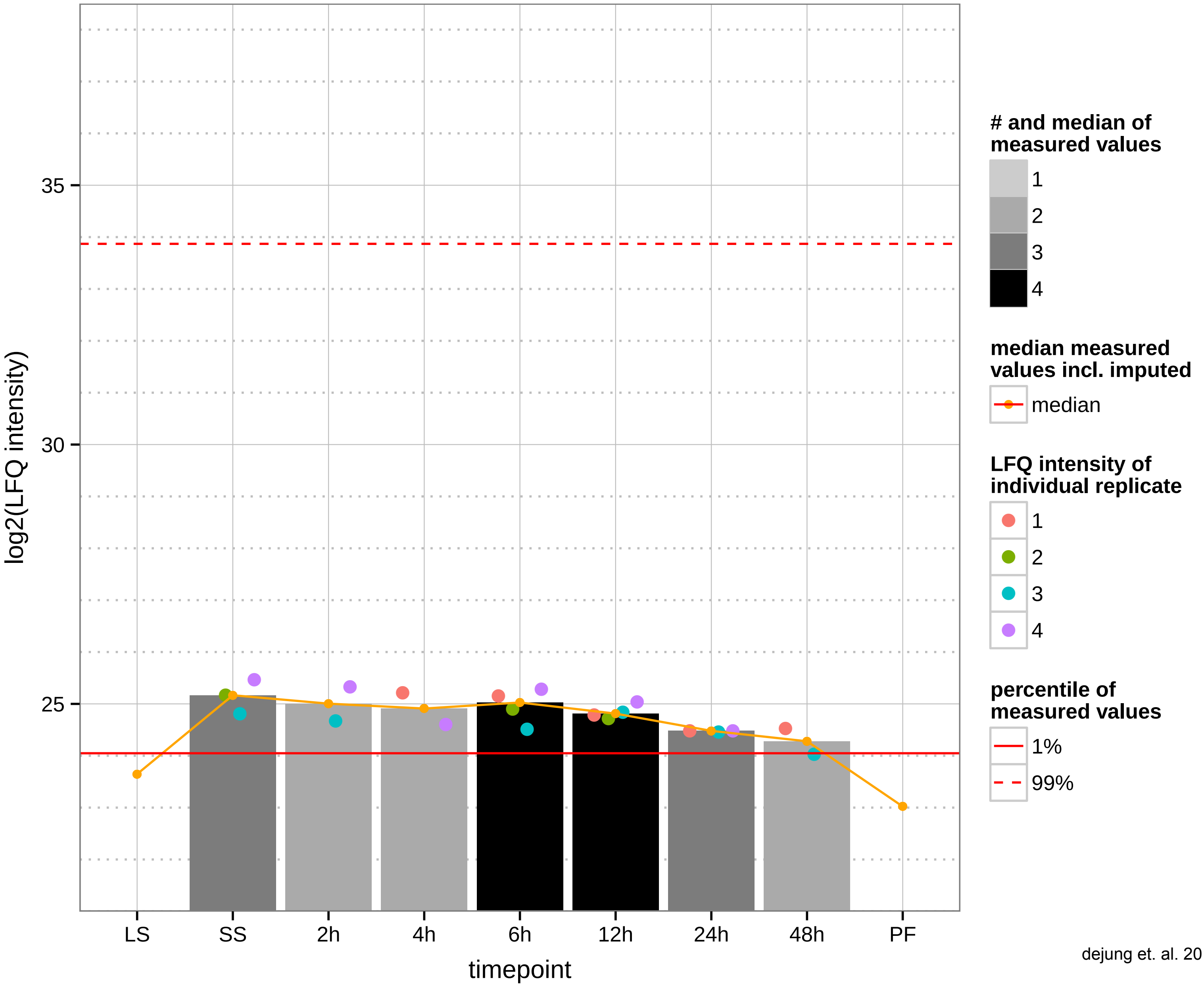

dejung et. al. 2015

hypothetical protein, conserved
Tb927.10.6990
AGOF: zinc ion binding
AGOC: null
AGOP: null
PGOF: null
PGOC: null
PGOP: null

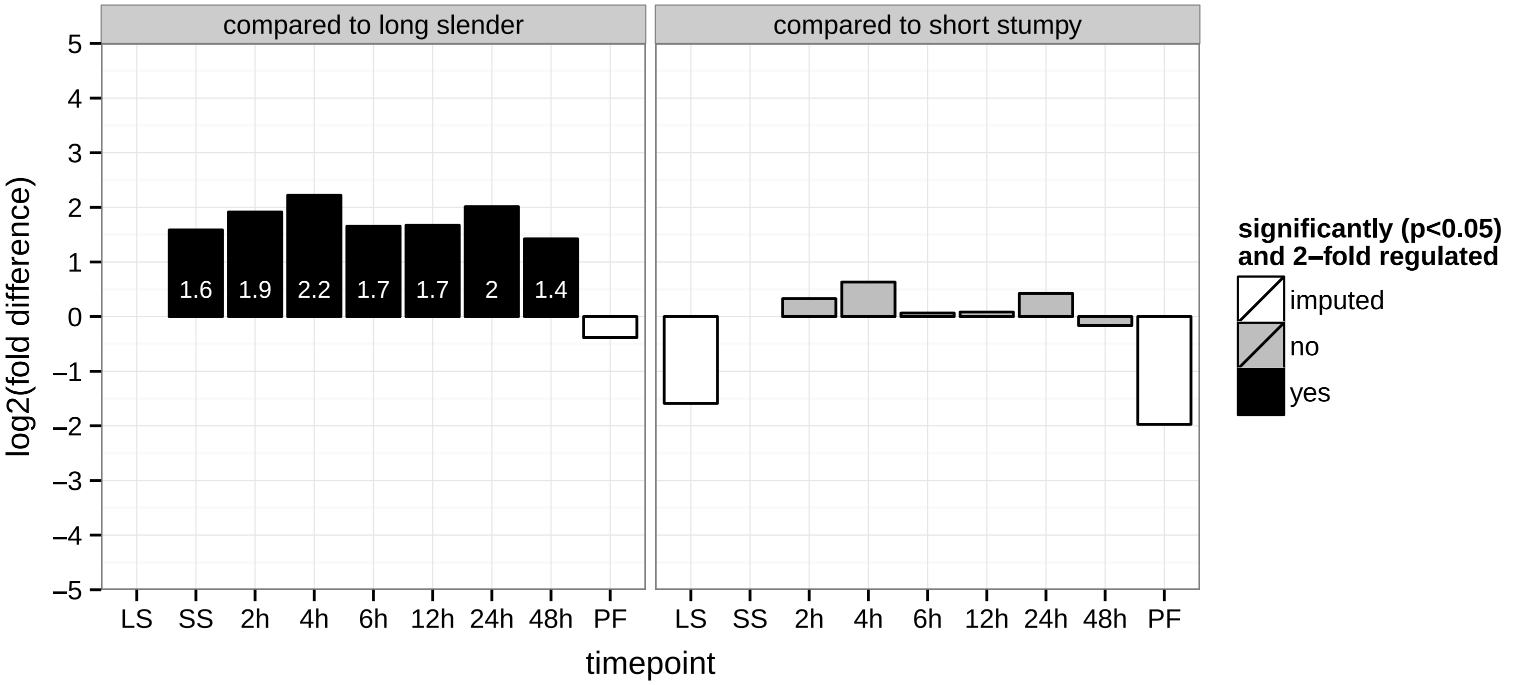

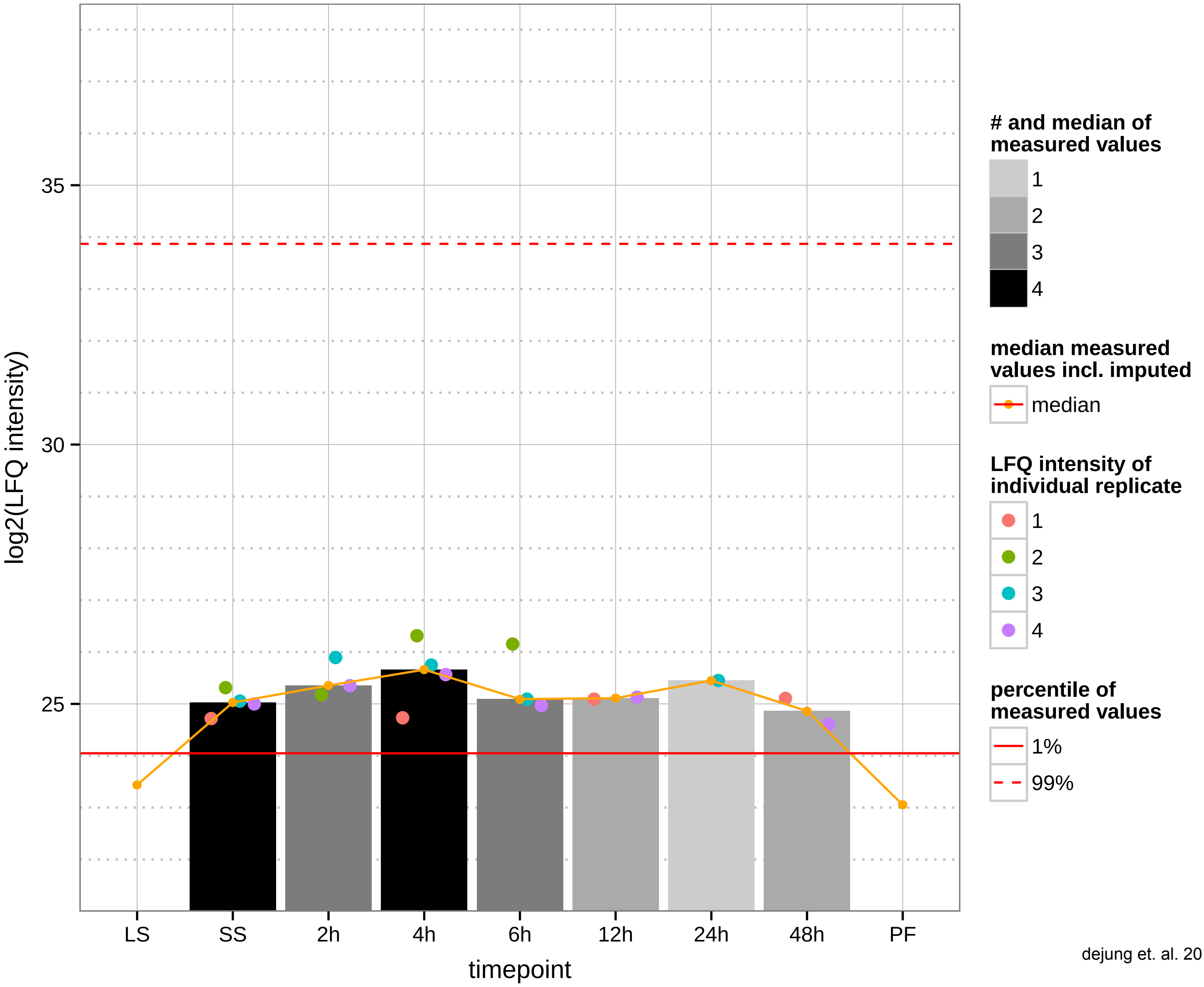

dejung et. al. 2015

hypothetical protein, conserved
Tb927.10.720
AGOF: null
AGOC: null
AGOP: null
PGOF: protein binding
PGOC: null
PGOP: null

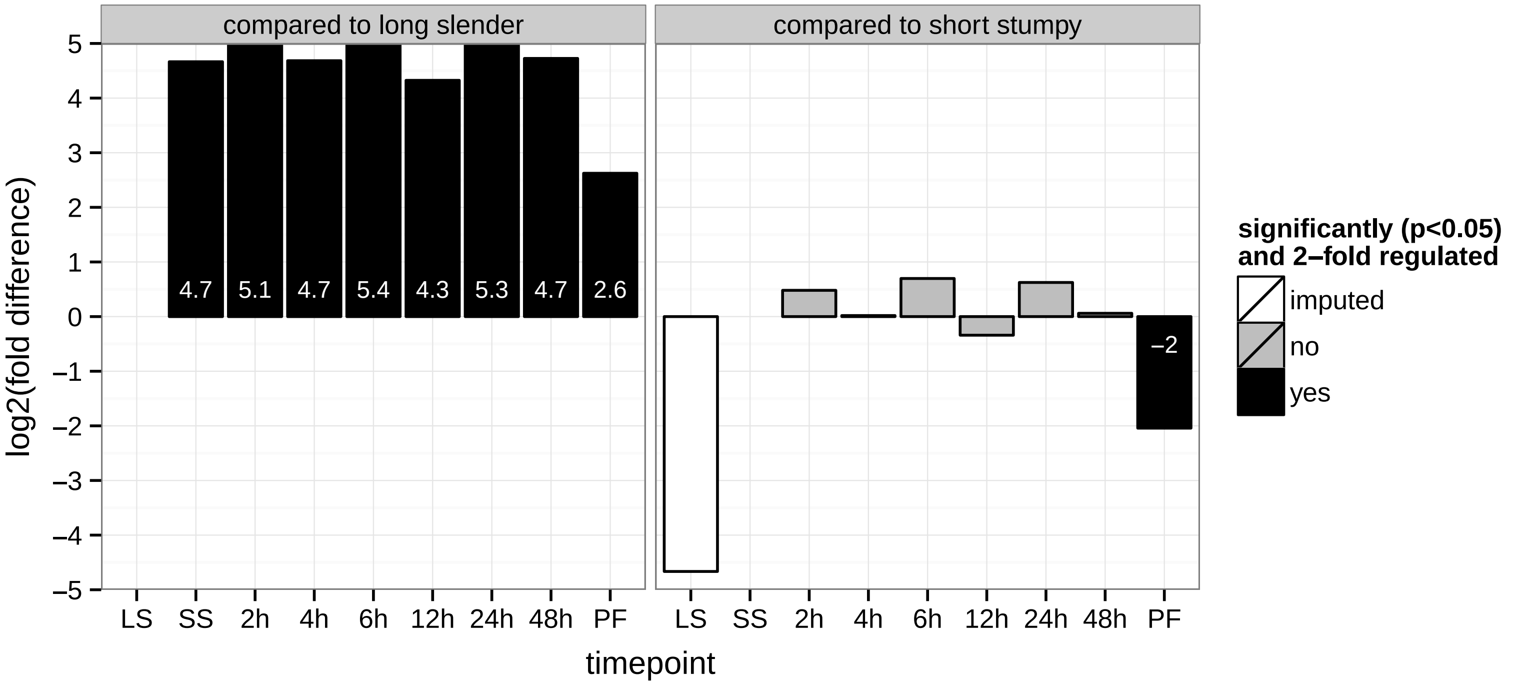

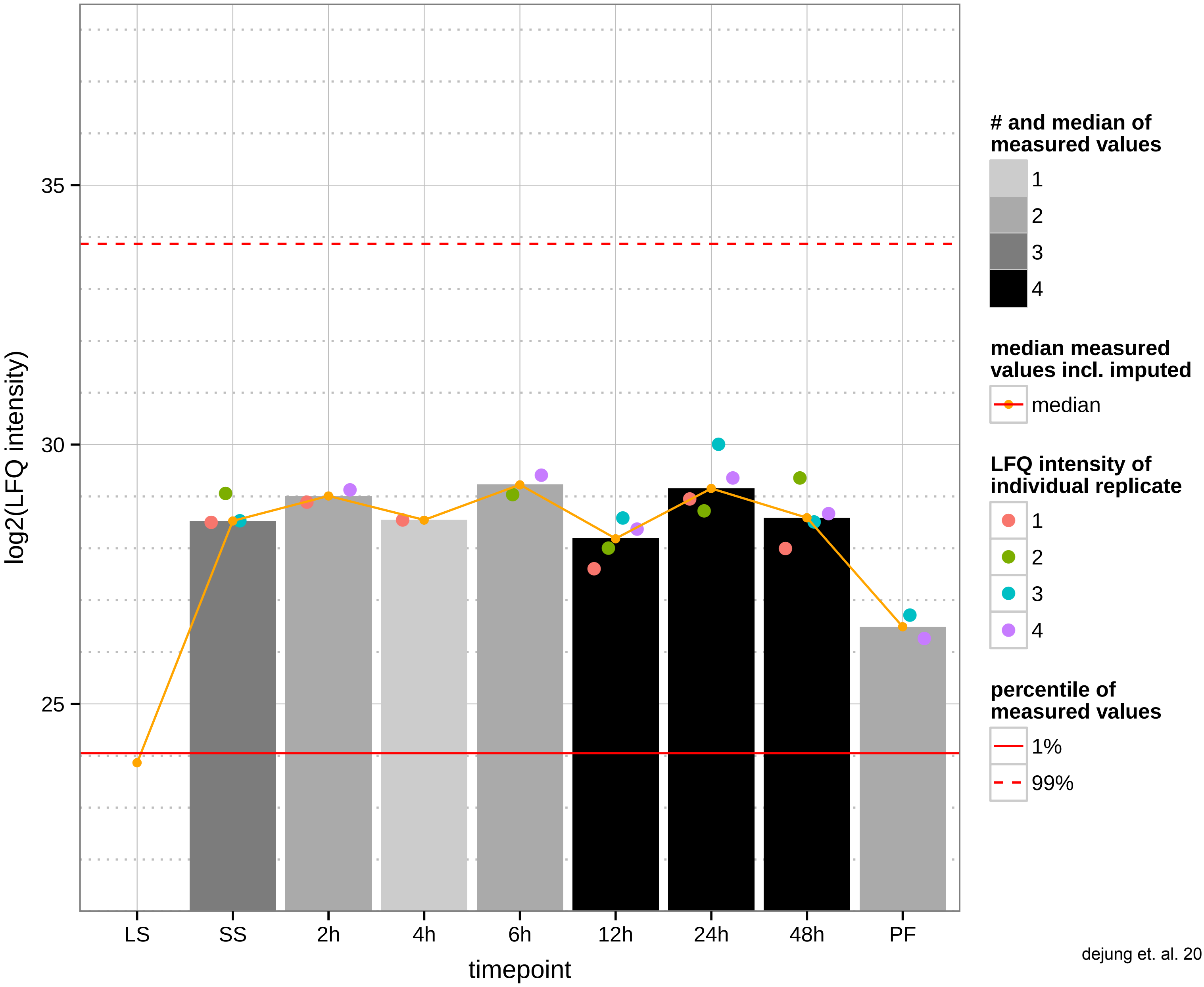

dejung et. al. 2015

hypothetical protein, conserved
Tb927.10.8140
AGOF: null
AGOC: null
AGOP: null
PGOF: null
PGOC: null
PGOP: null

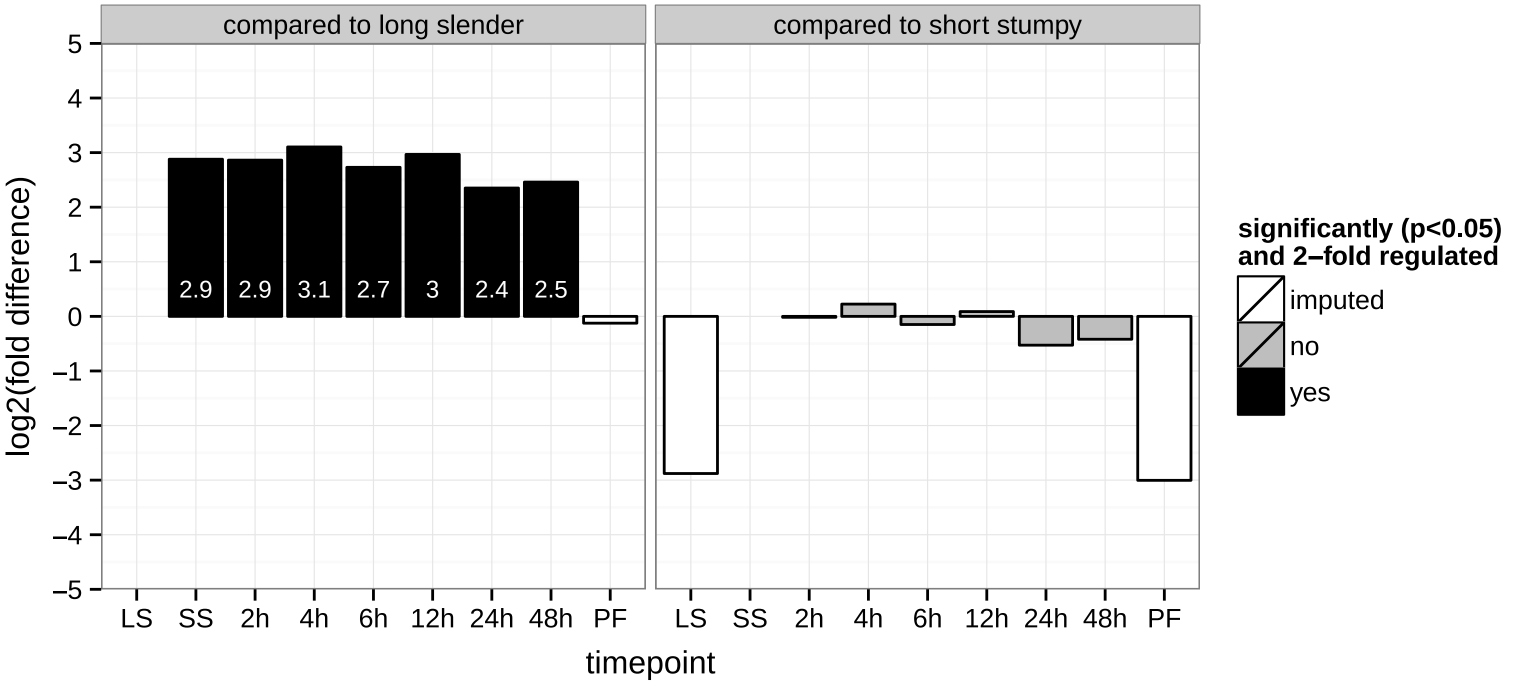

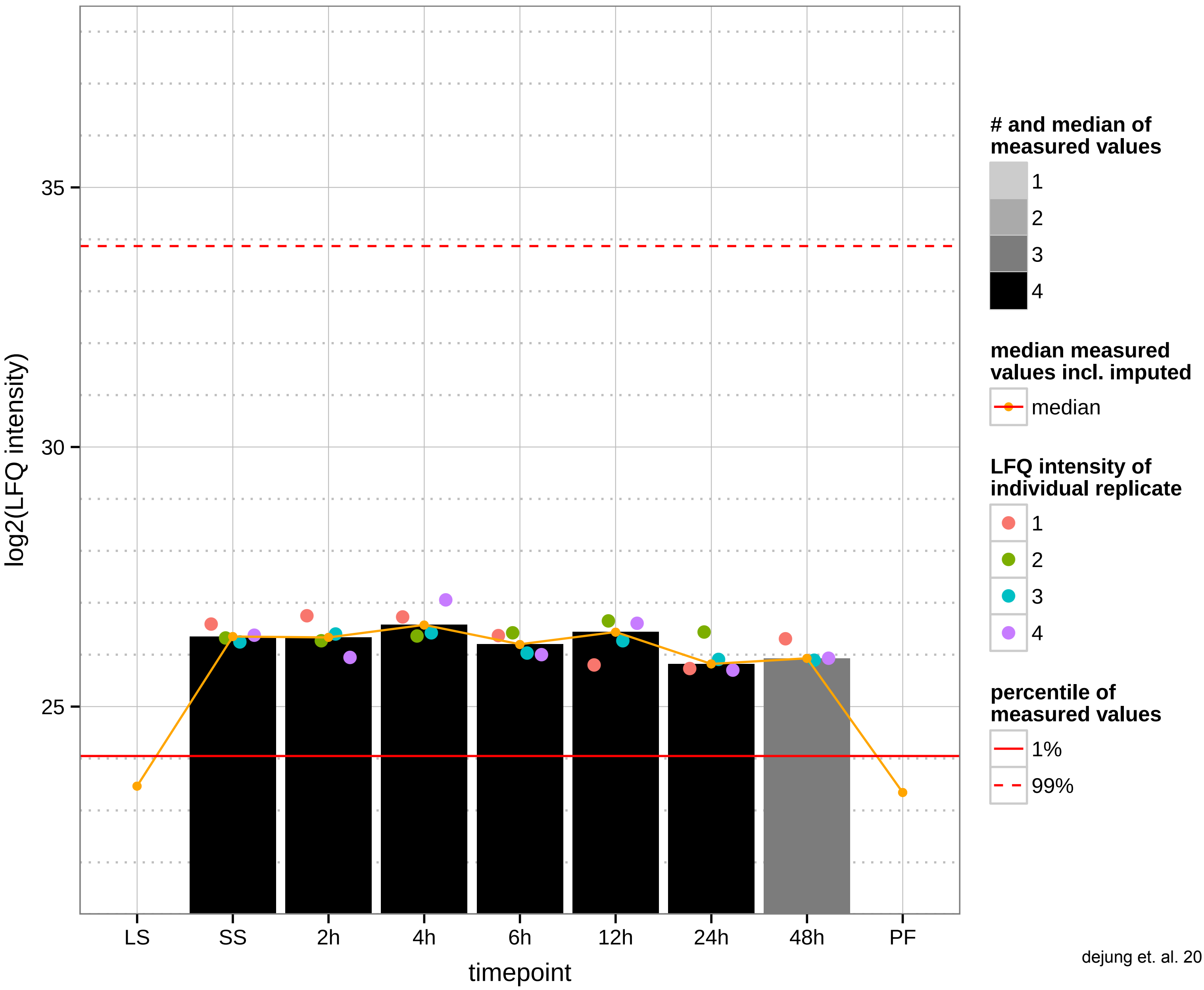

dejung et. al. 2015

hypothetical protein, conserved
Tb927.10.8160
AGOF: null
AGOC: mitochondrion
AGOP: null
PGOF: null
PGOC: null
PGOP: null

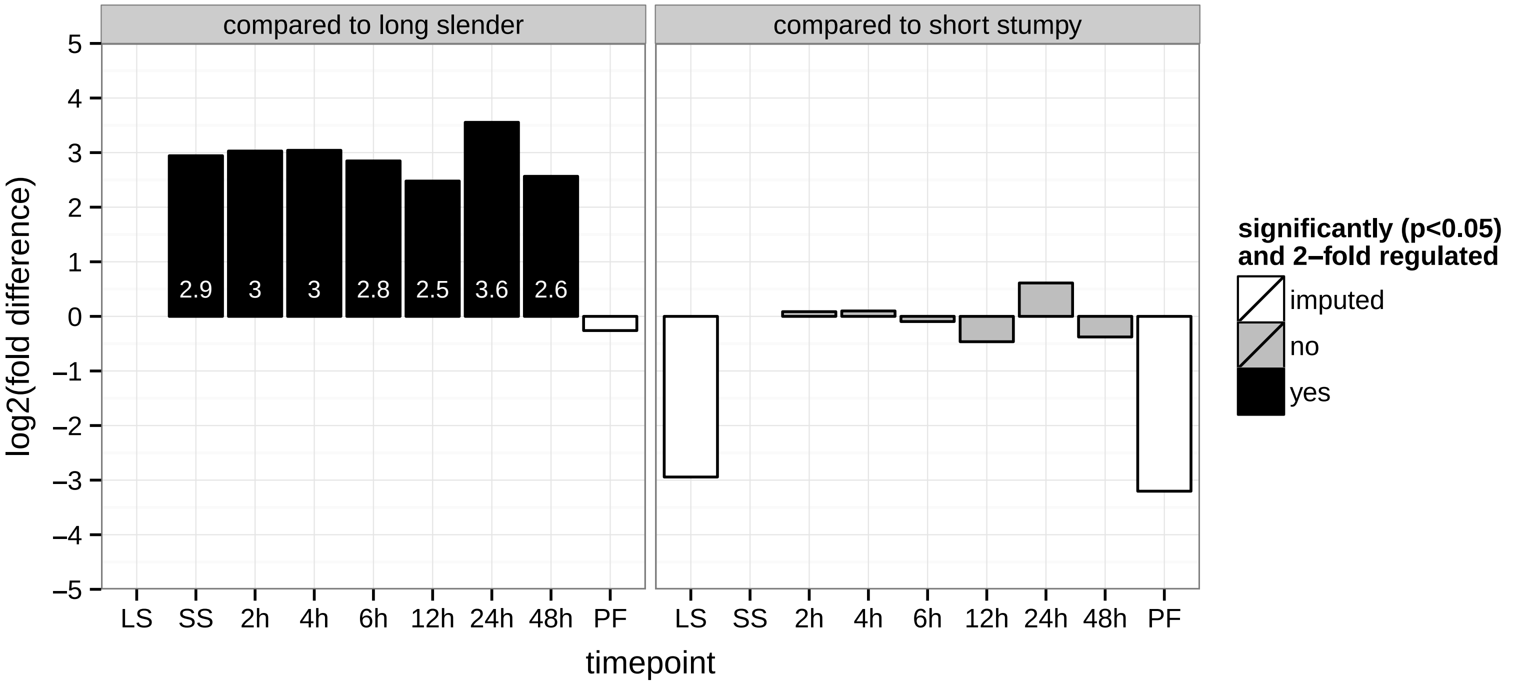

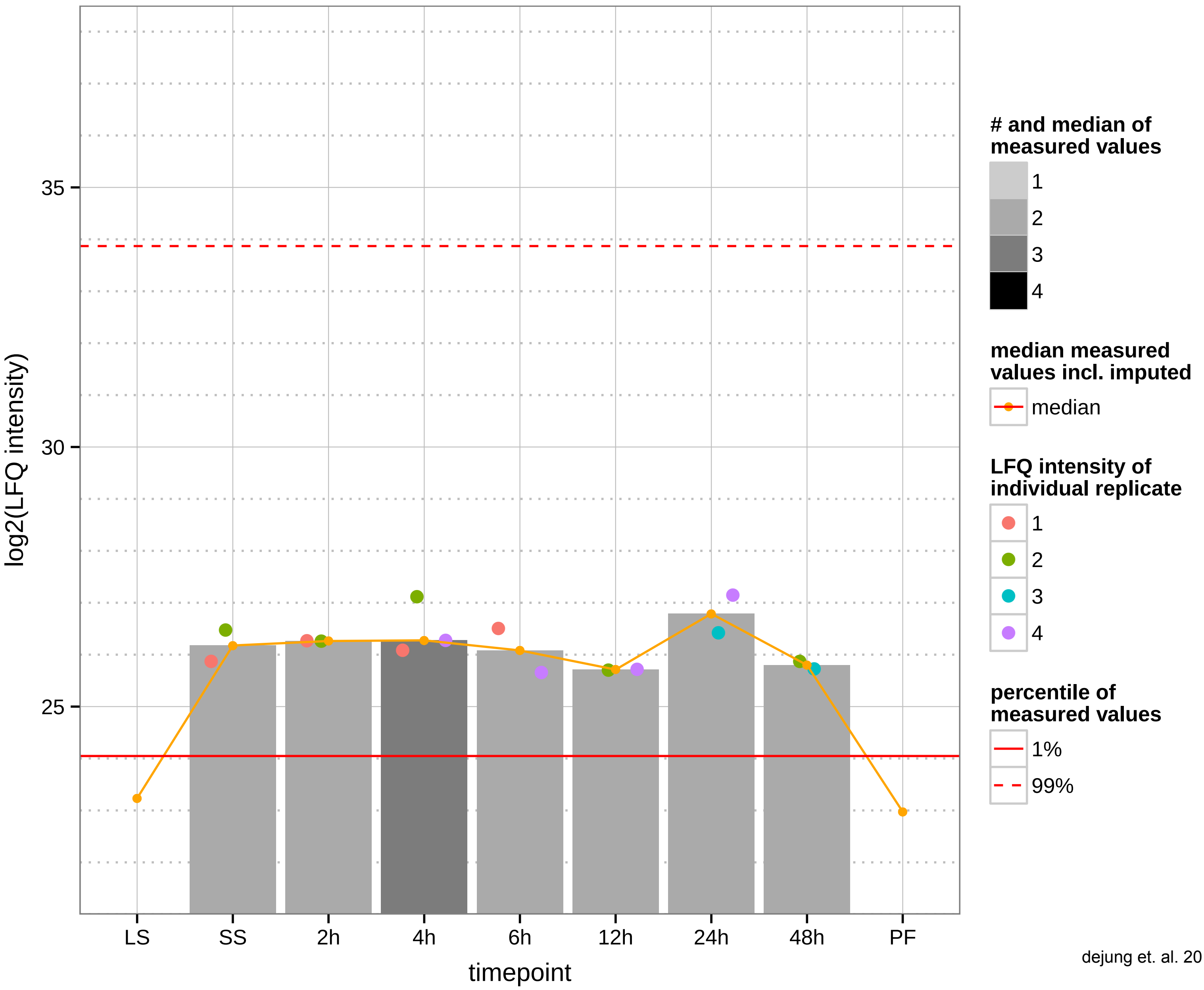

dejung et. al. 2015

chaperone protein DNAj, putative
Tb927.10.8540
AGOF: heat shock protein binding, unfolded protein binding
AGOC: null
AGOP: protein folding, protein refolding, response to unfolded protein
PGOF: heat shock protein binding, unfolded protein binding
PGOC: null
PGOP: protein folding

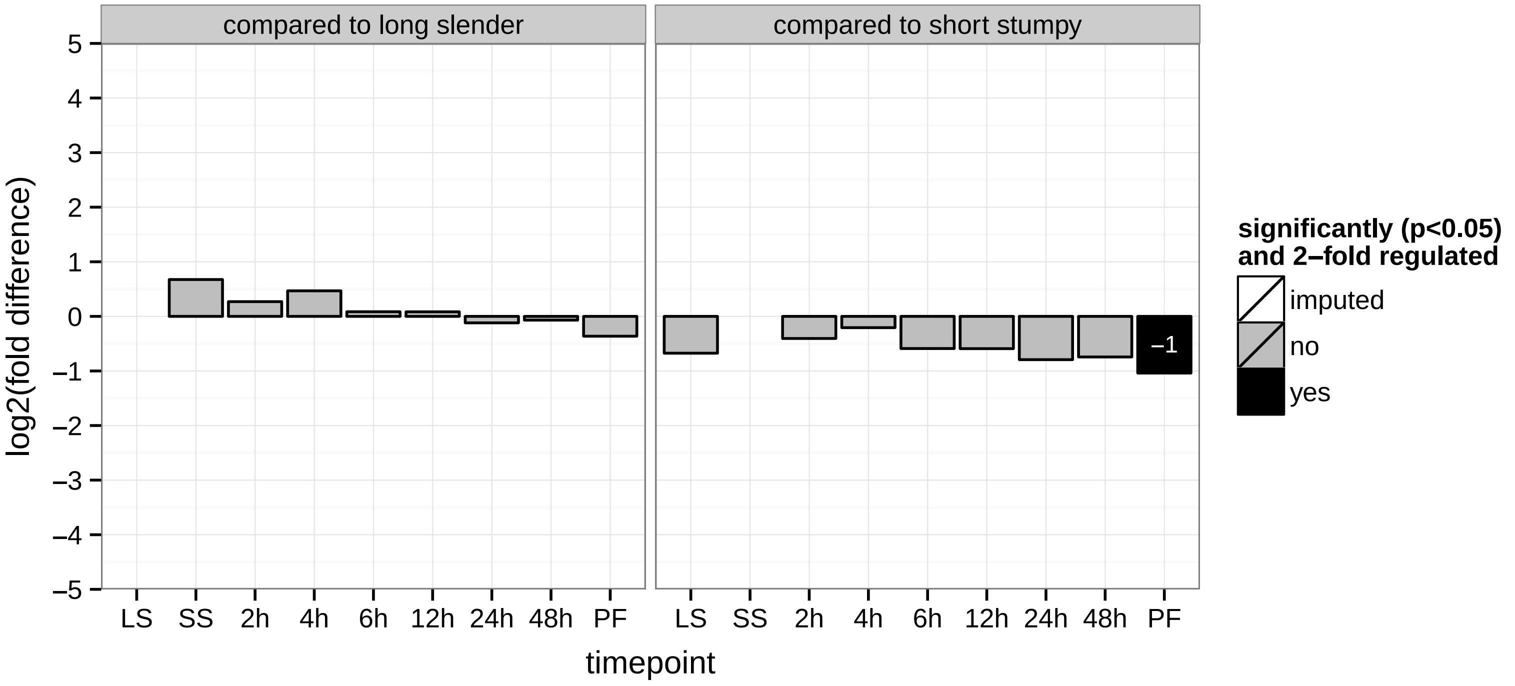

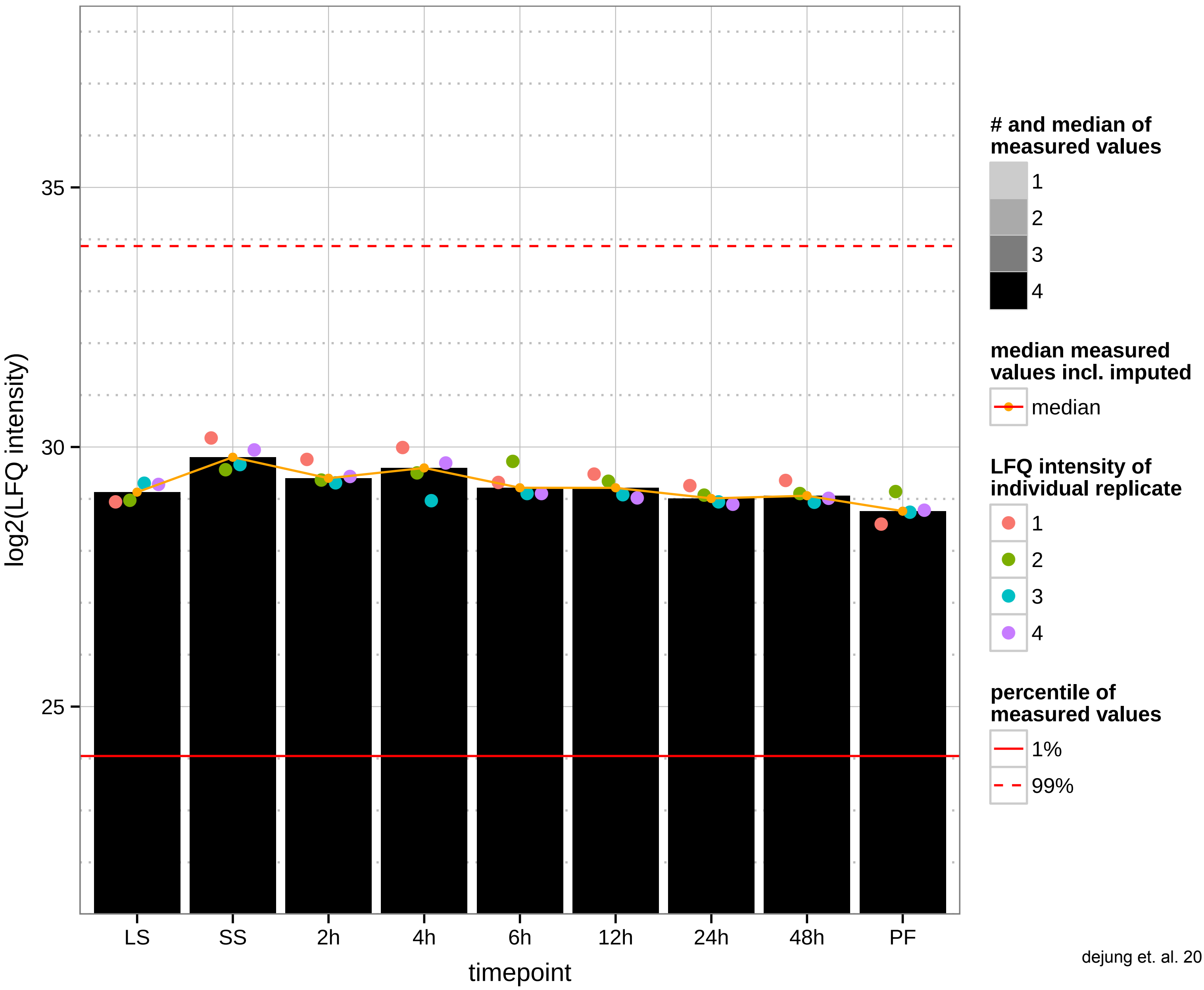

dejung et. al. 2015

hypothetical protein, conserved
Tb927.10.8600
AGOF: null
AGOC: null
AGOP: retrograde transport, endosome to Golgi
PGOF: null
PGOC: null
PGOP: retrograde transport, endosome to Golgi

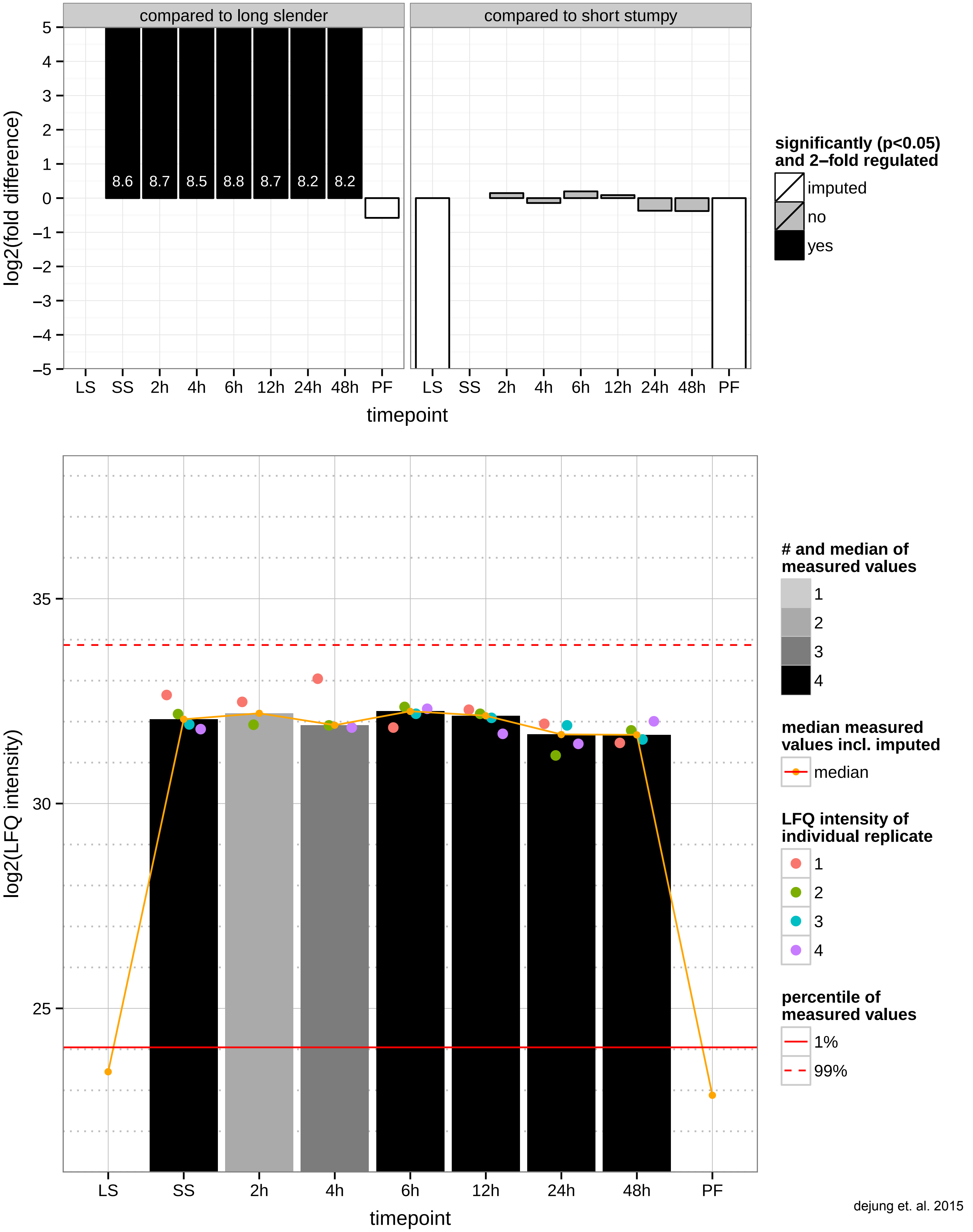

dejung et. al. 2015

RNA polymerase–associated protein CDC73, putative
Tb927.11.10230
AGOF: RNA polymerase II core binding
AGOC: Cdc73/Paf1 complex
AGOP: positive regulation of transcription elongation from RNA polymerase II promoter
PGOF: null
PGOC: null
PGOP: null

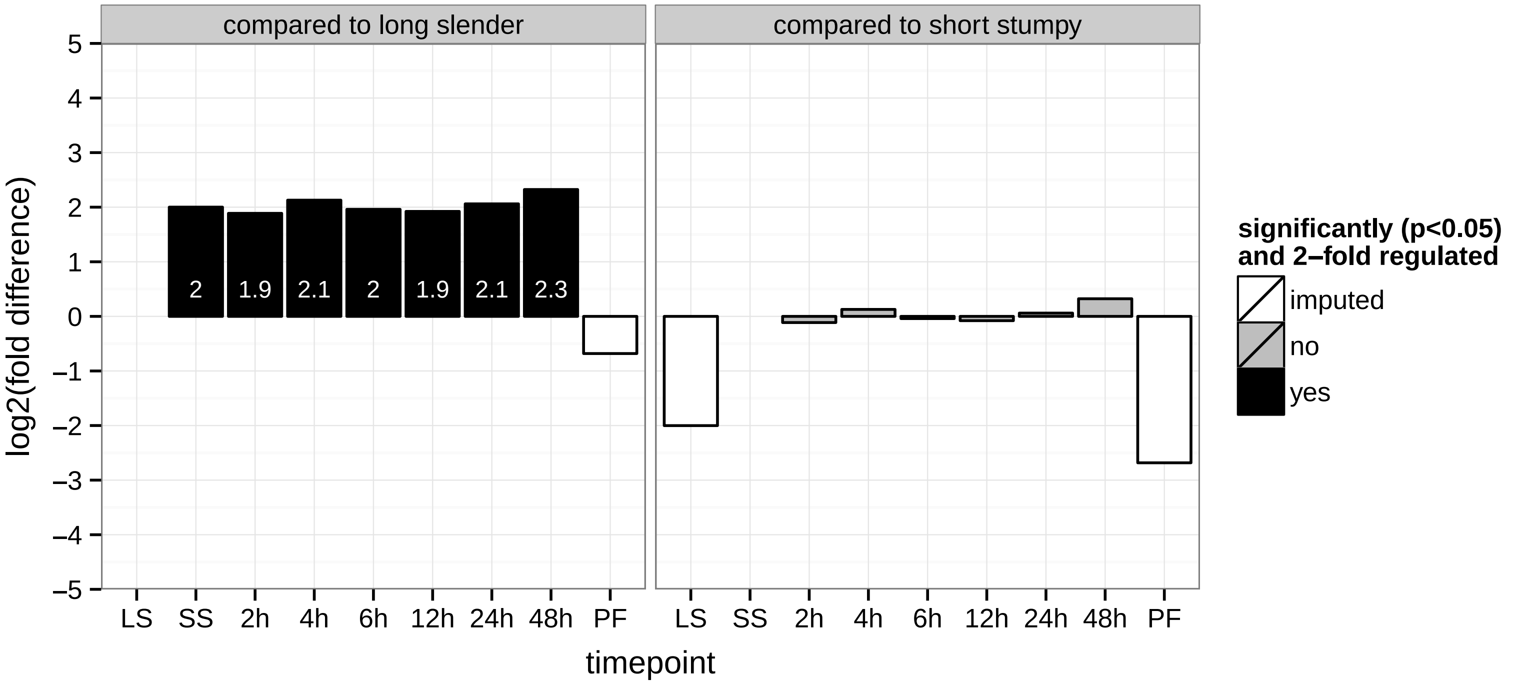

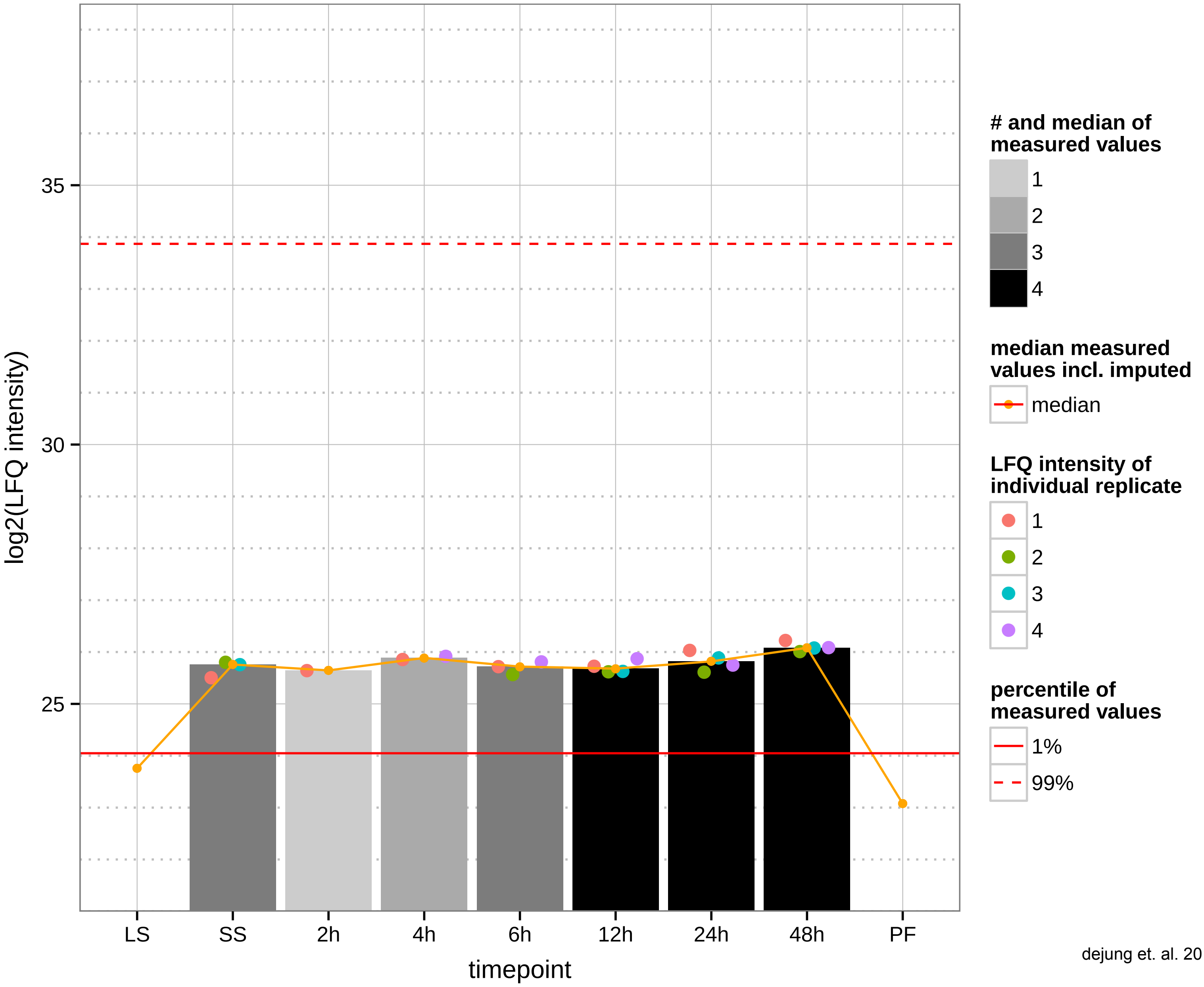

dejung et. al. 2015

golgi SNARE protein–like, GOLGI SNAP receptor complex member, putative
Tb927.11.10270
AGOF: null
AGOC: membrane
AGOP: intracellular protein transport
PGOF: null
PGOC: null
PGOP: null

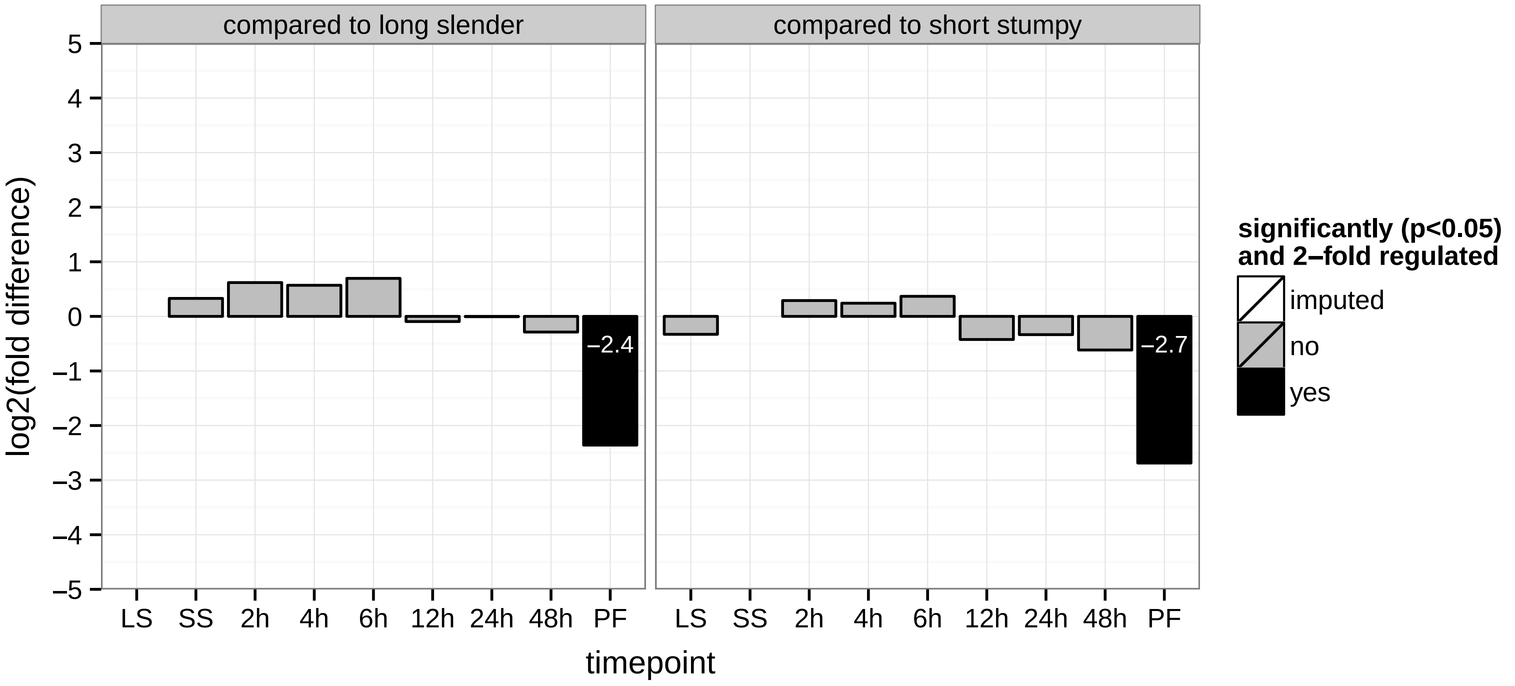

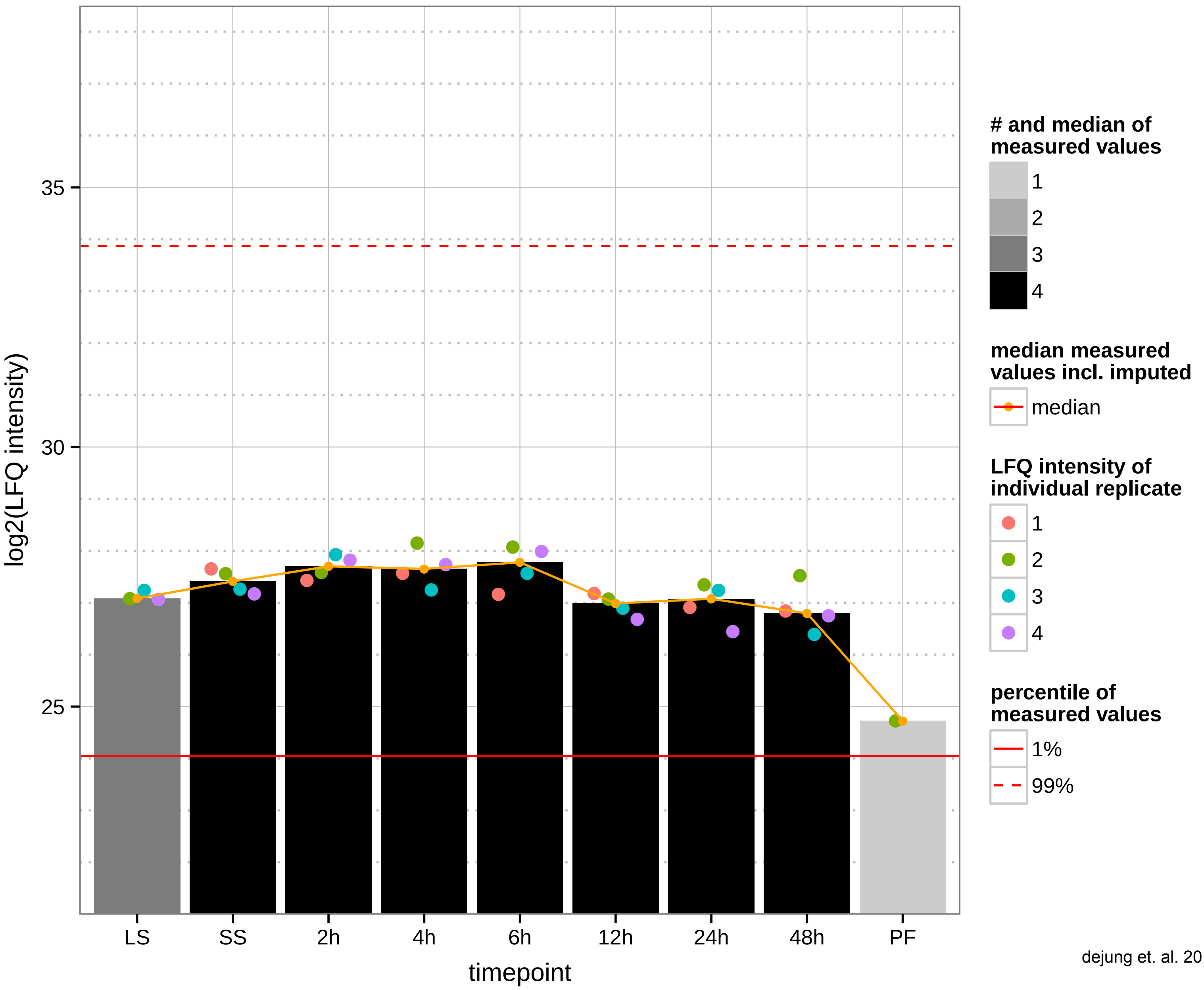

dejung et. al. 2015

hypothetical protein, conserved
Tb927.11.10620
AGOF: null
AGOC: null
AGOP: null
PGOF: null
PGOC: null
PGOP: null

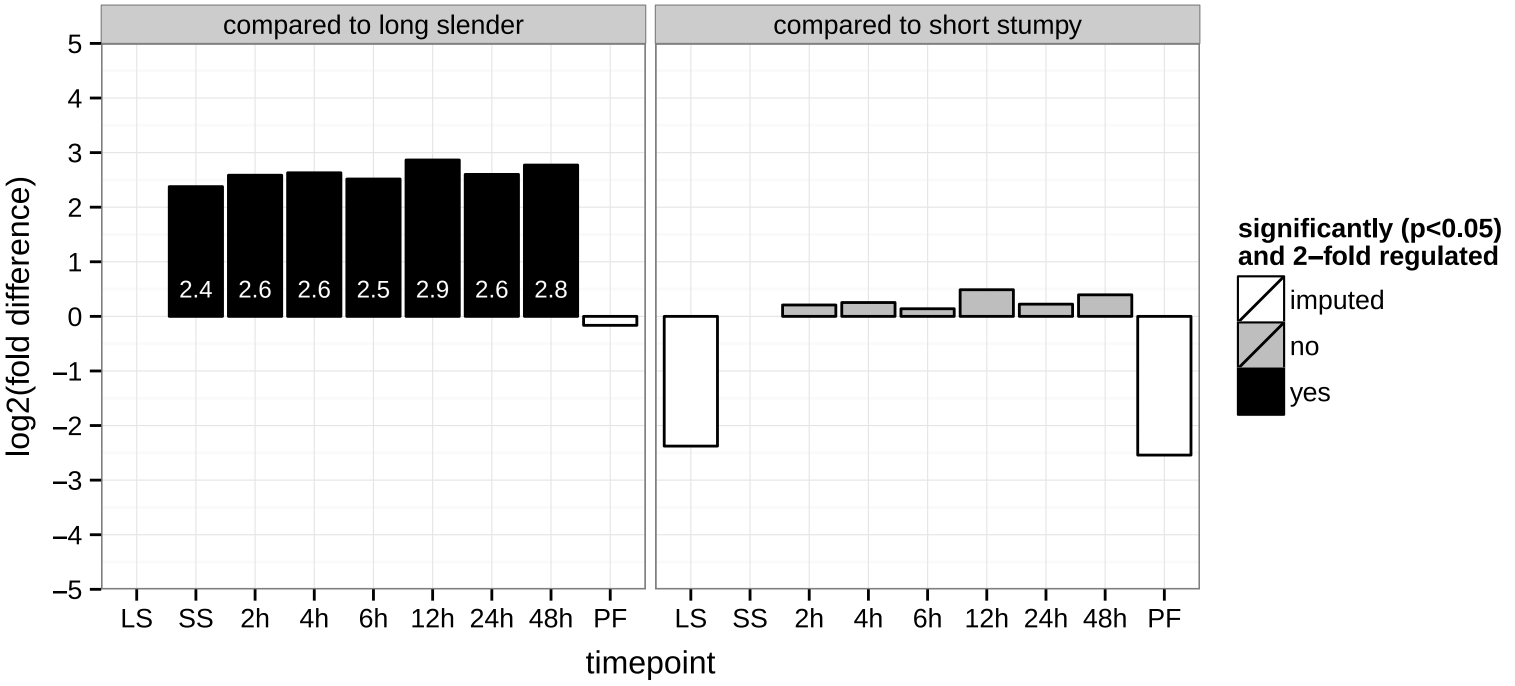

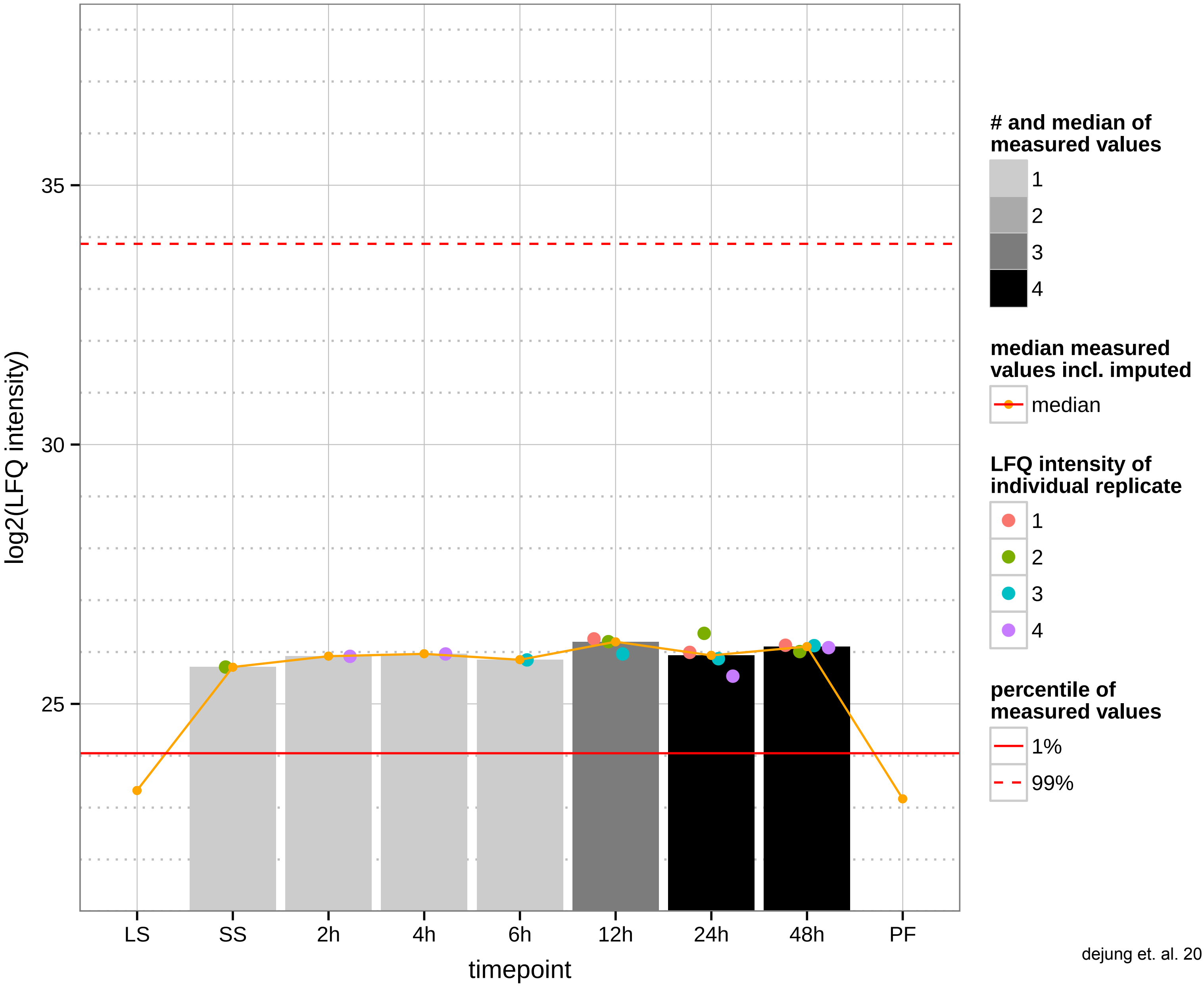

dejung et. al. 2015

SNF2 DNA repair protein, putative, ATP–dependent helicase, SNF2 family helicase
Tb927.11.10730
AGOF: ATP binding, ATPase activity, DNA binding, helicase activity
AGOC: null
AGOP: chromatin remodeling, meiosis
PGOF: ATP binding, DNA binding, helicase activity, nucleic acid binding
PGOC: null
PGOP: null

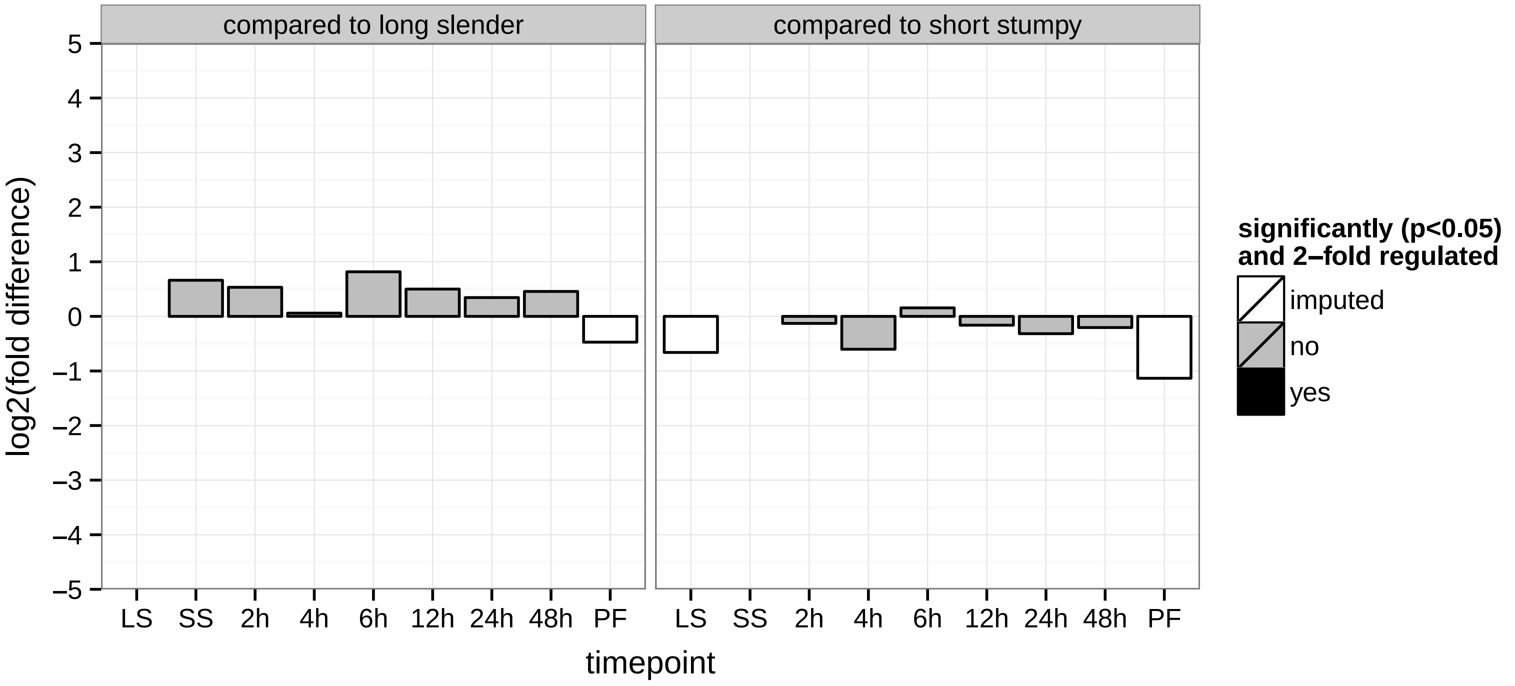

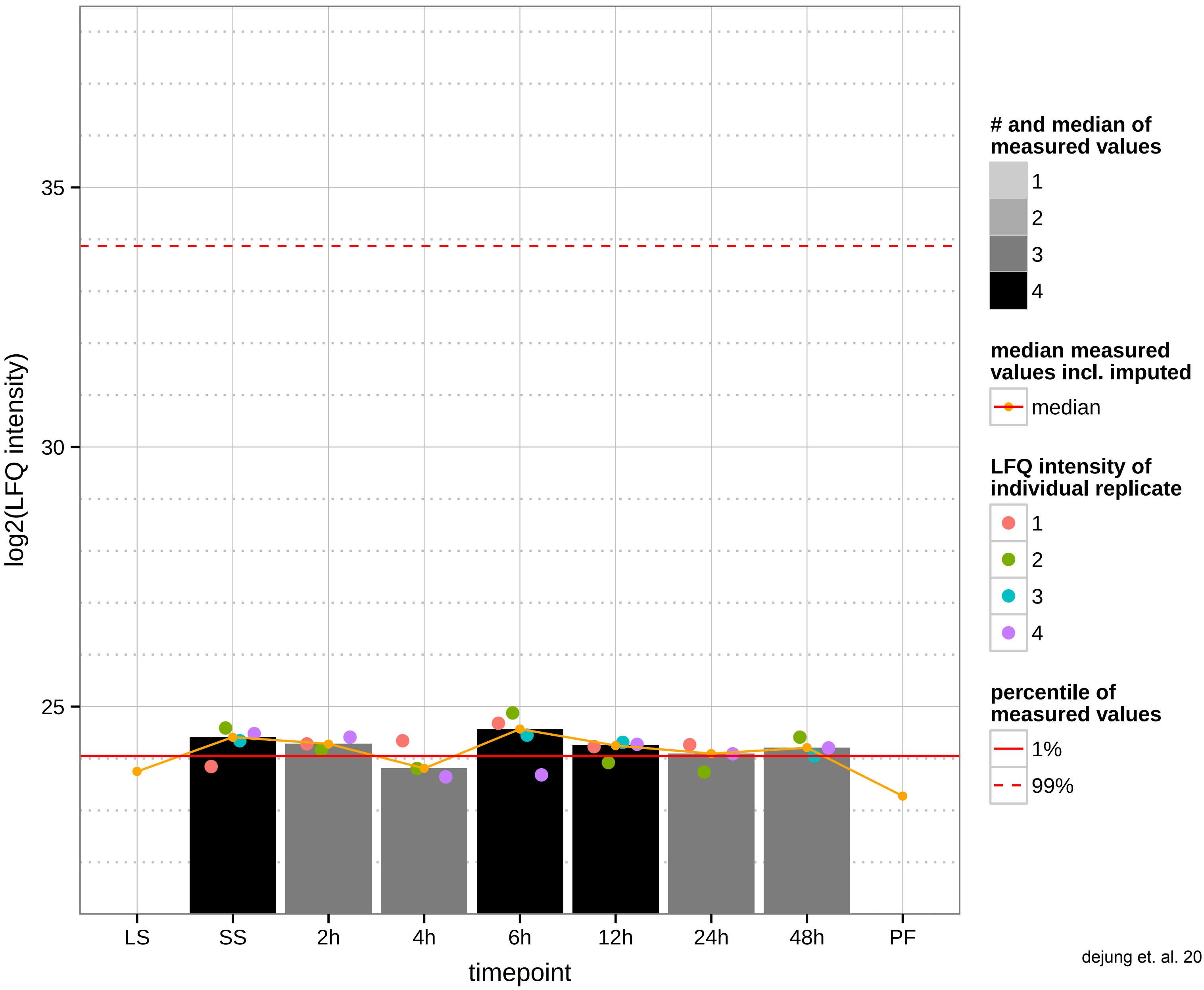

dejung et. al. 2015

MENG
Tb927.11.10900
AGOF: null
AGOC: null
AGOP: ciliary or flagellar motility
PGOF: null
PGOC: null
PGOP: null

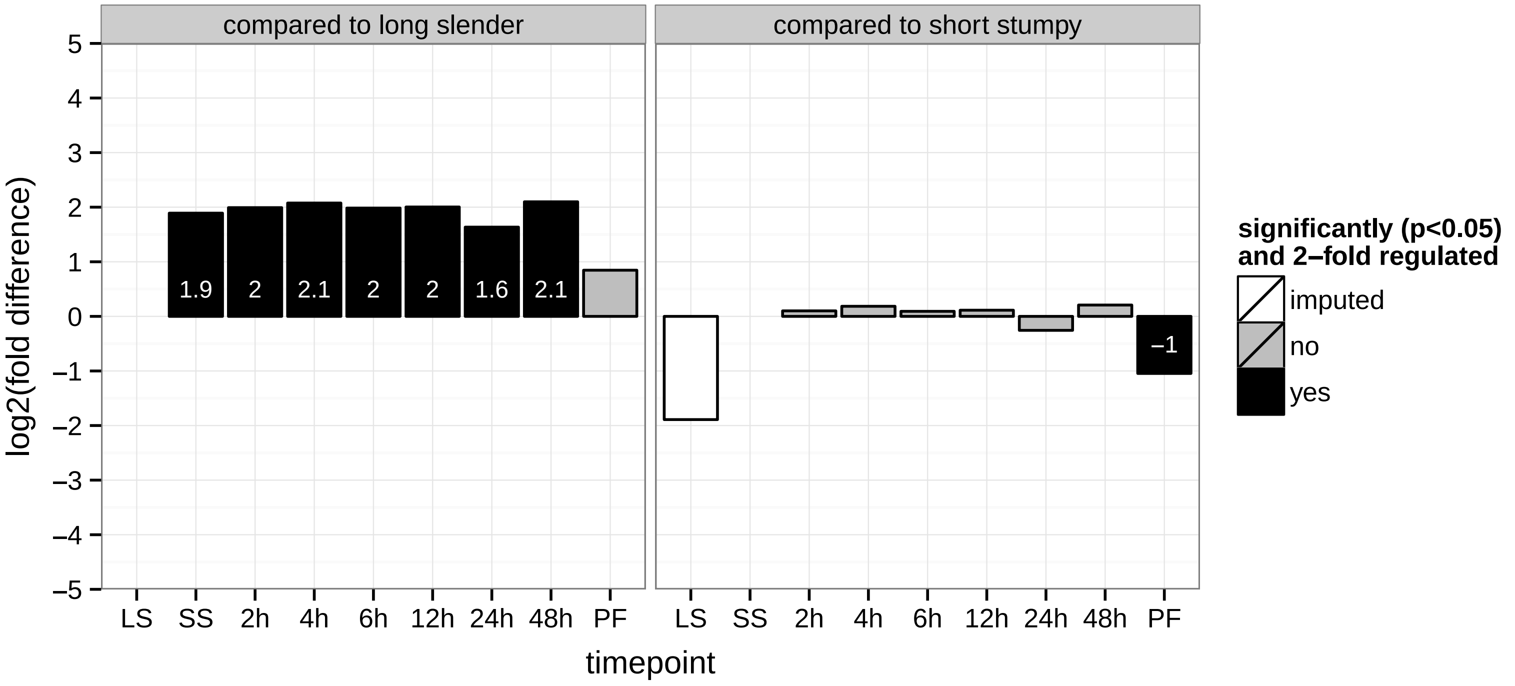

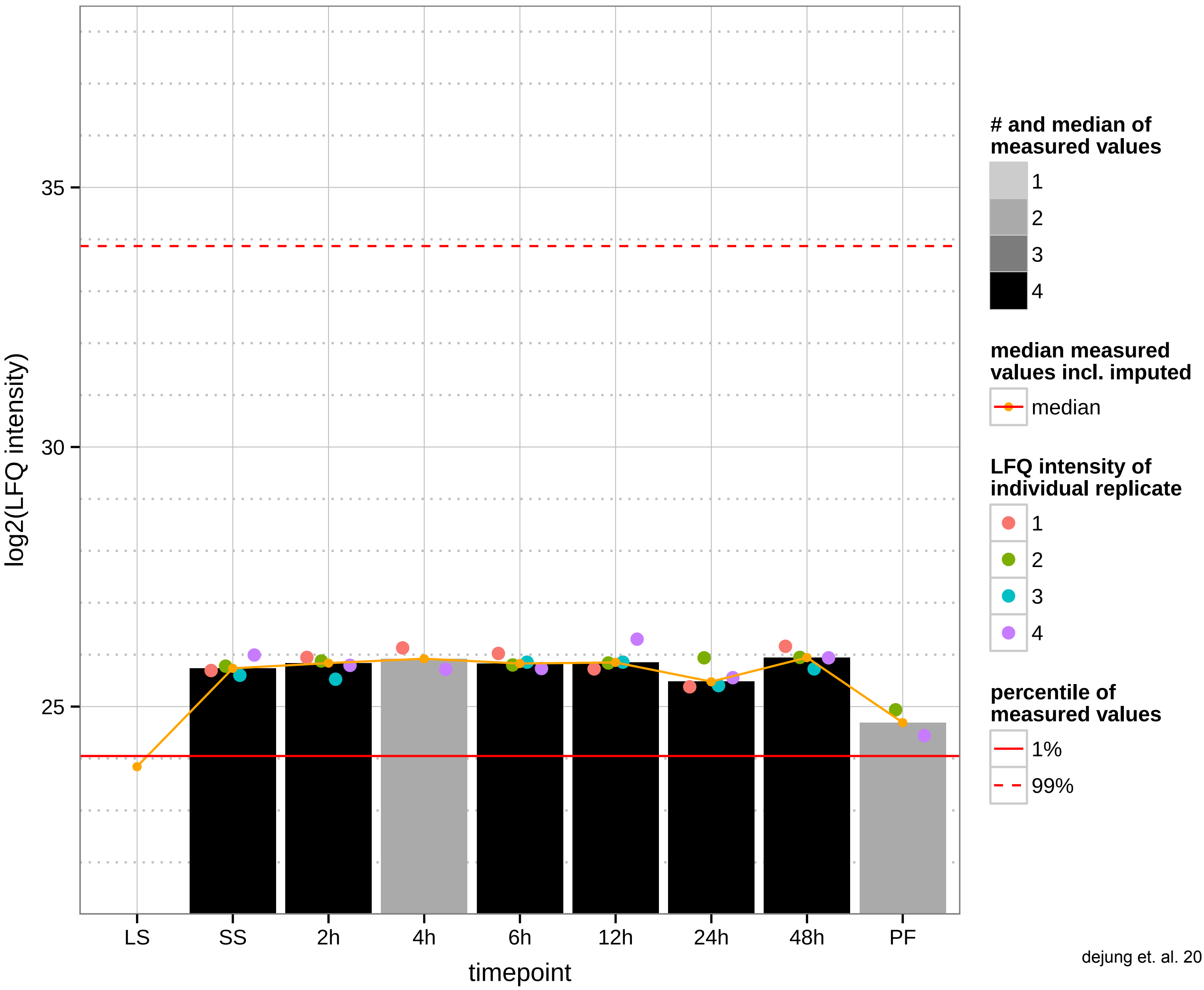

dejung et. al. 2015

hypothetical protein, conserved
Tb927.11.10970
AGOF: null
AGOC: null
AGOP: null
PGOF: protein binding
PGOC: null
PGOP: null

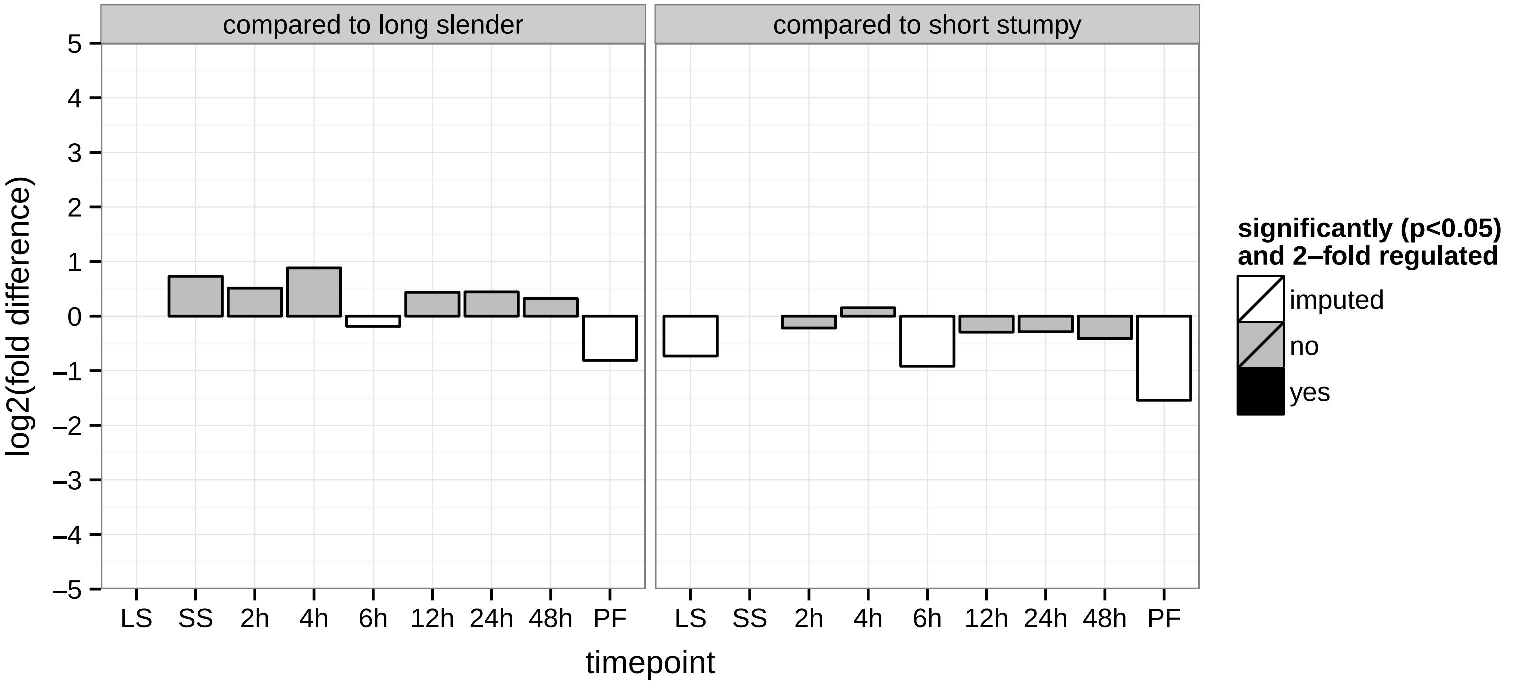

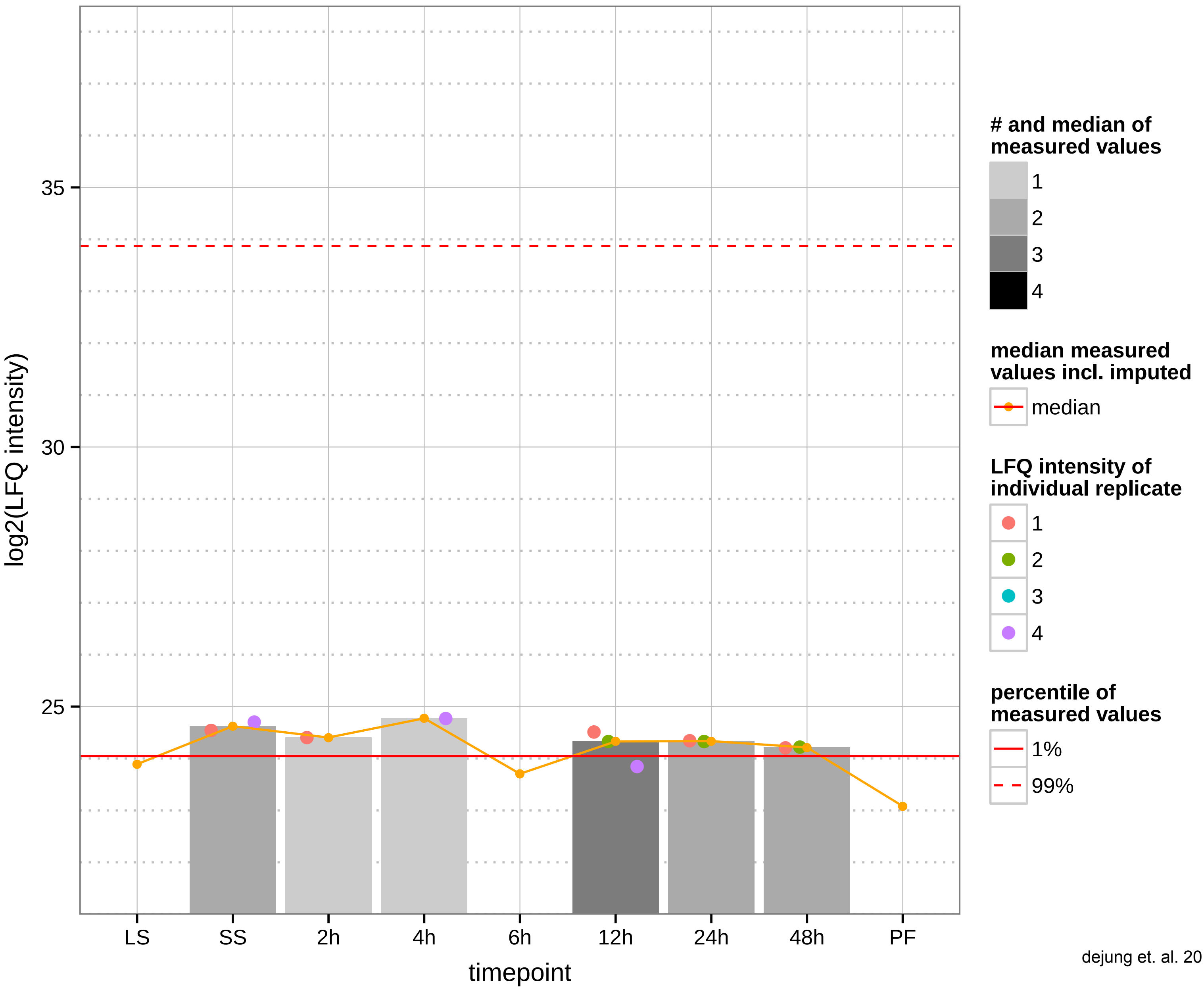

dejung et. al. 2015

glucosamine 6−phosphate n−acetyltransferase (GNA1)
Tb927.11.11100
AGOF: glucosamine 6−phosphate N−acetyltransferase activity
AGOC: glycosome
AGOP: N−acetylglucosamine metabolic process, metabolic process
PGOF: N−acetyltransferase activity
PGOC: null
PGOP: null

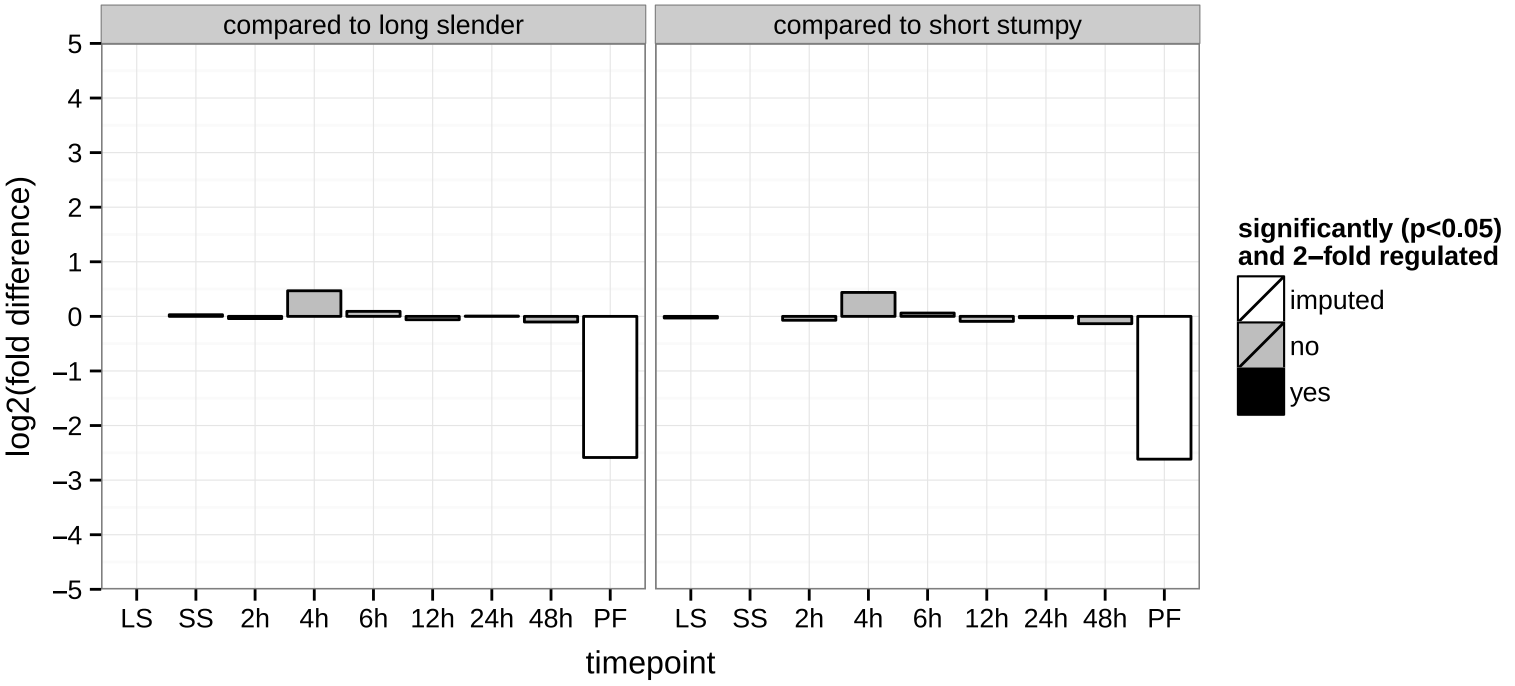

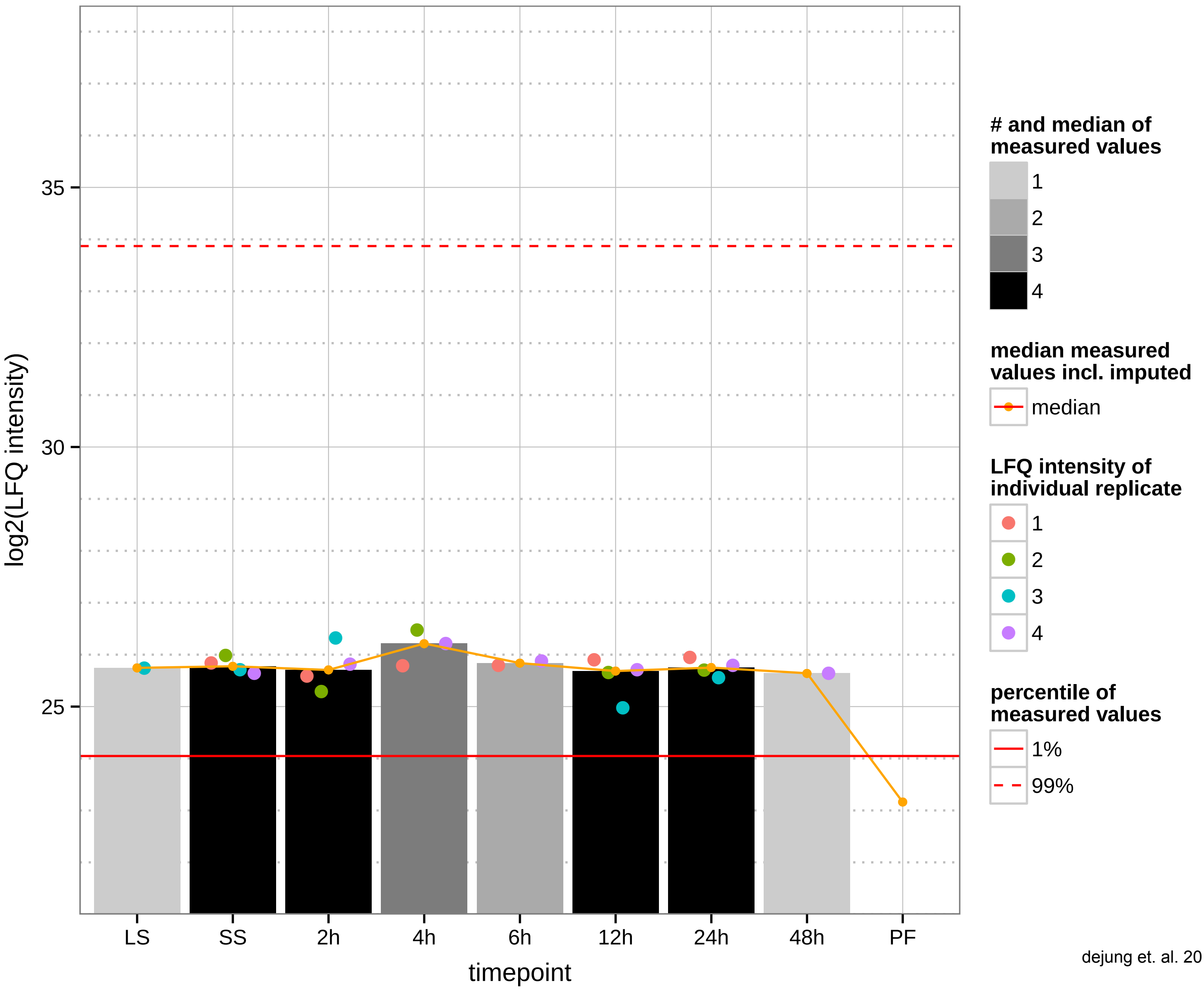

dejung et. al. 2015

hypothetical protein
Tb927.11.11140
AGOF: null
AGOC: null
AGOP: null
PGOF: protein binding
PGOC: null
PGOP: null

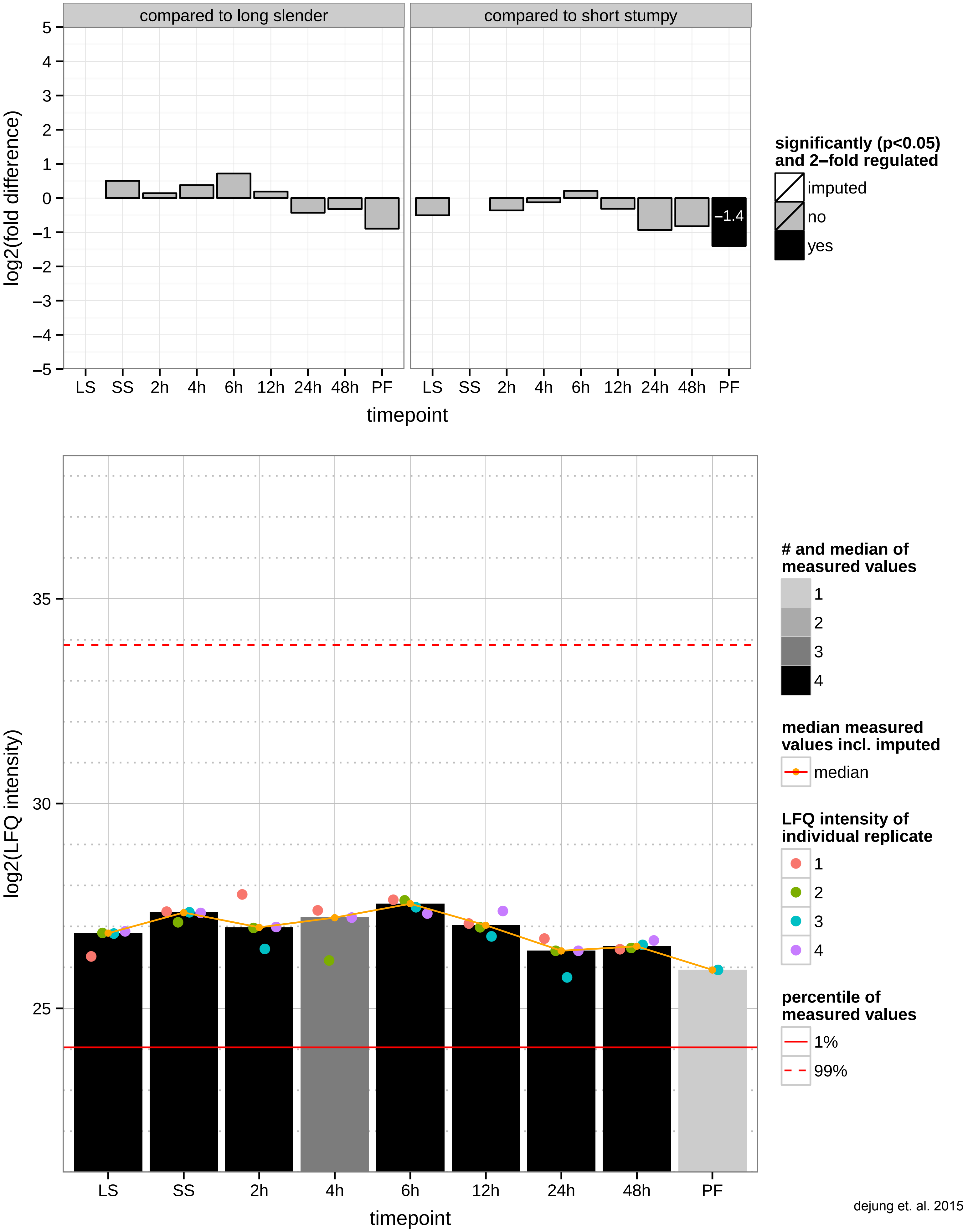

dejung et. al. 2015

hypothetical protein, conserved
Tb927.11.11610
AGOF: null
AGOC: null
AGOP: null
PGOF: protein binding
PGOC: null
PGOP: null

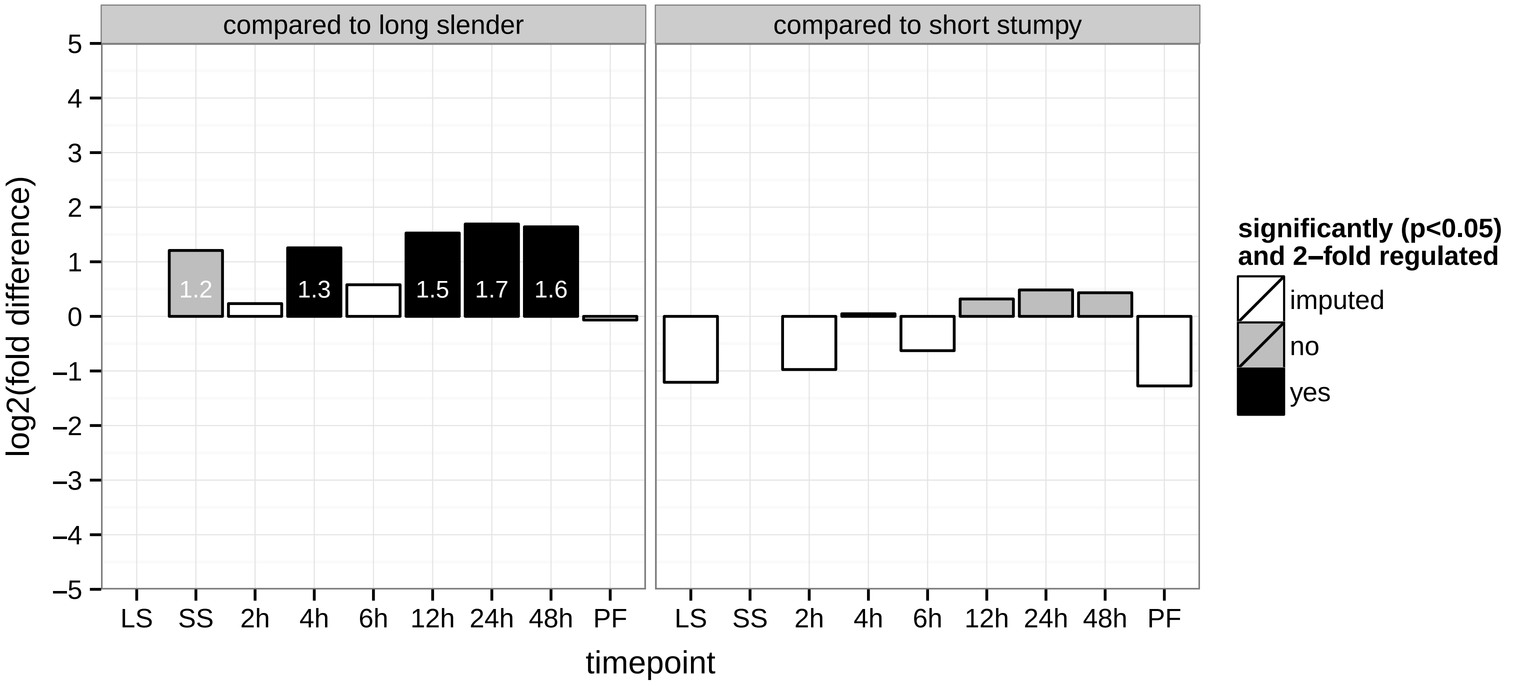

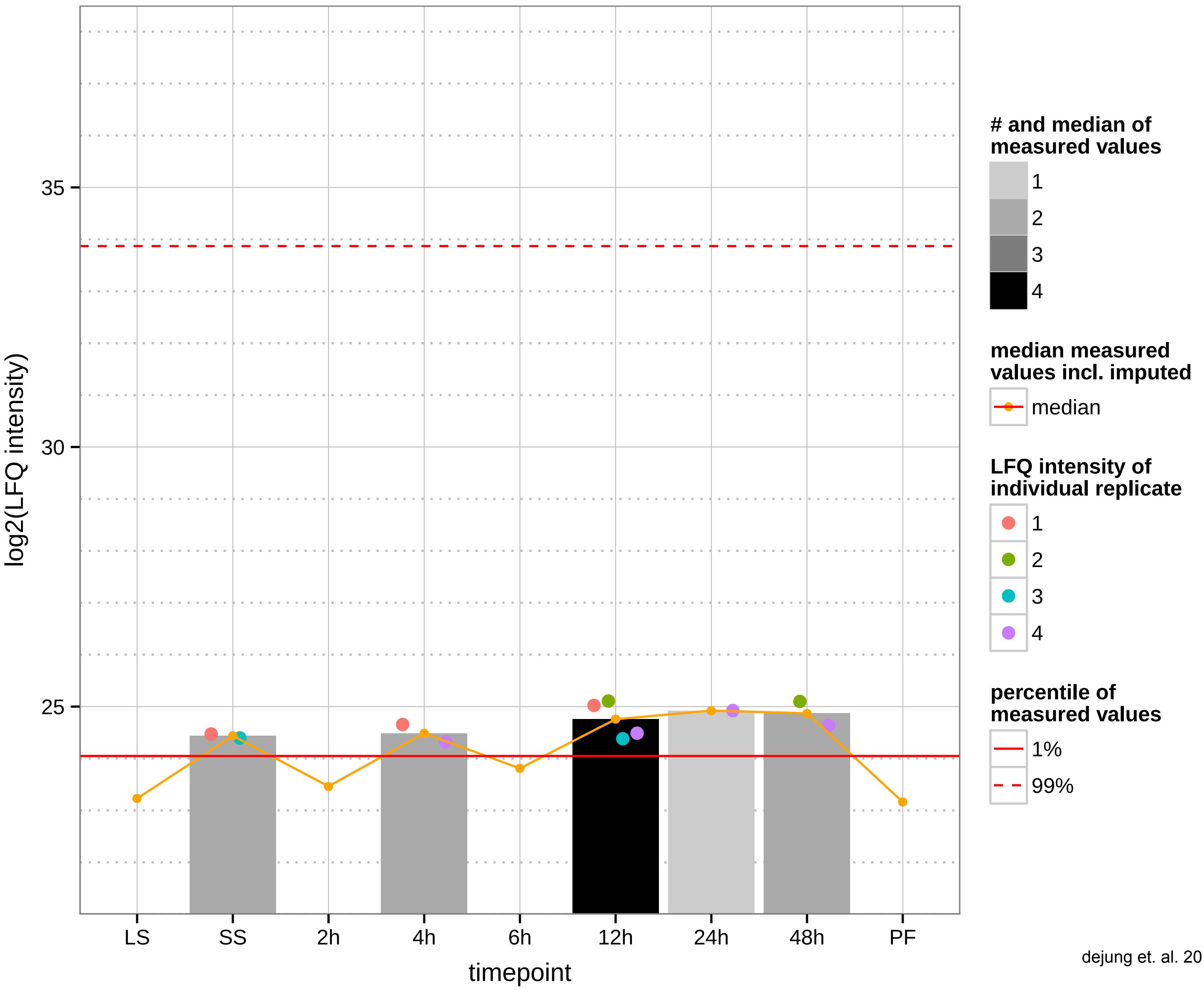

dejung et. al. 2015

leucine–rich repeat protein (LRRP), putative
Tb927.11.11860
AGOF: null
AGOC: null
AGOP: null
PGOF: protein binding
PGOC: null
PGOP: null

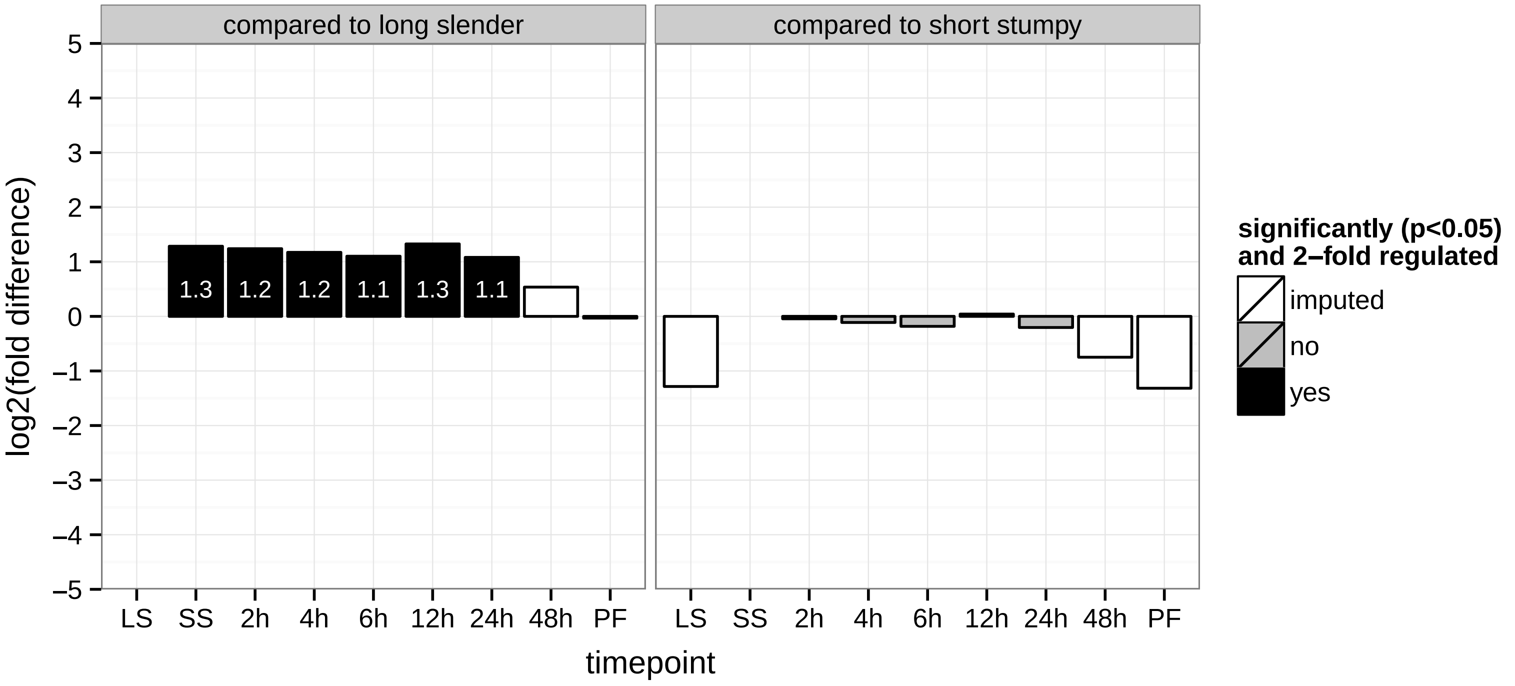

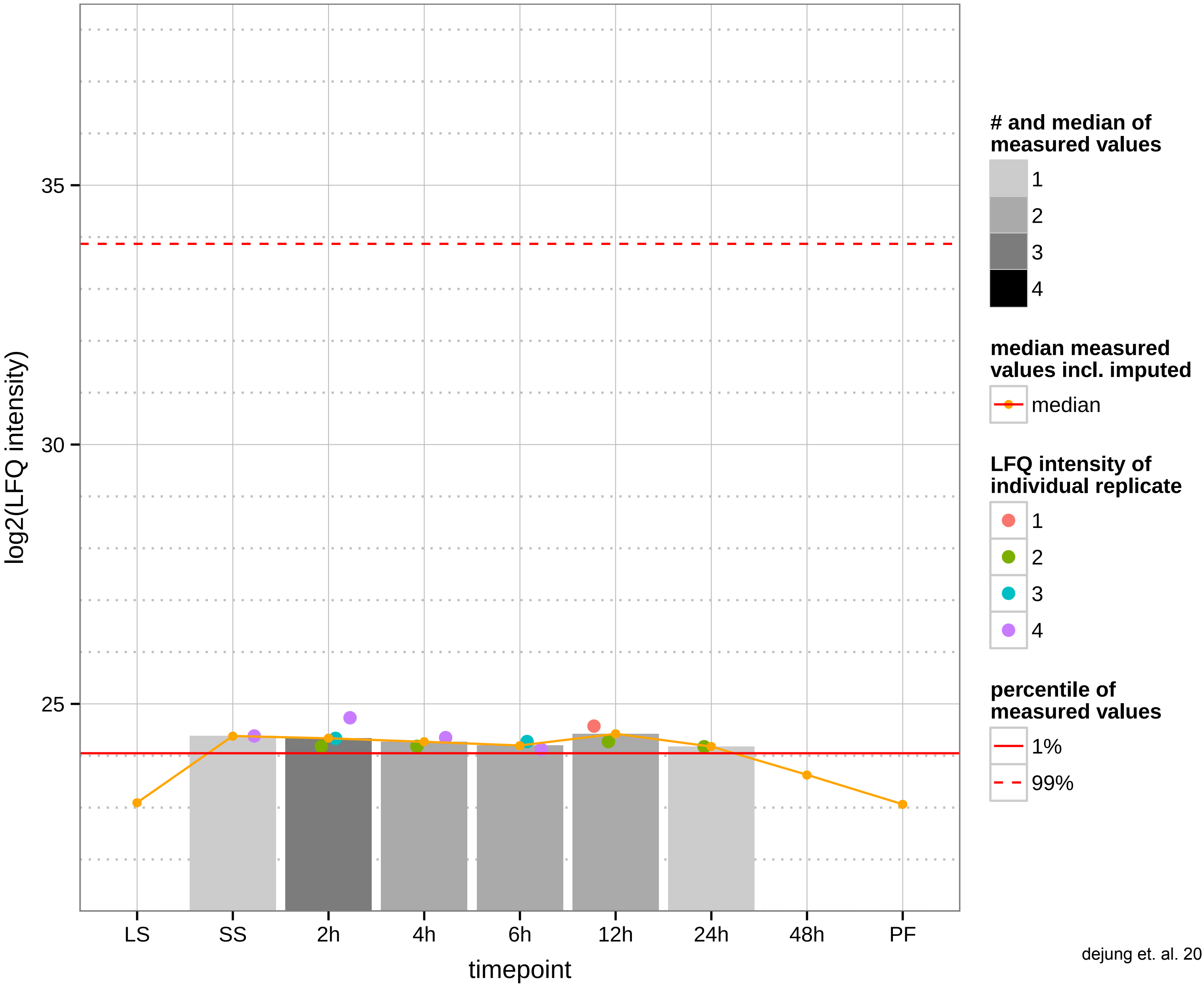

dejung et. al. 2015

copper−transporting ATPase−like protein, putative
Tb927.11.1260
AGOF: ATP binding, copper ion transmembrane transporter activity, copper−exporting ATPase activity, metal ion binding
AGOC: integral to membrane, membrane
AGOP: ATP biosynthetic process, copper ion transport, metabolic process
PGOF: ATP binding, ATPase activity, coupled to transmembrane movement of ions, phosphorylative mechanism, catalytic acti
PGOC: integral to membrane, membrane
PGOP: ATP biosynthetic process, cation transport, metabolic process, metal ion transport

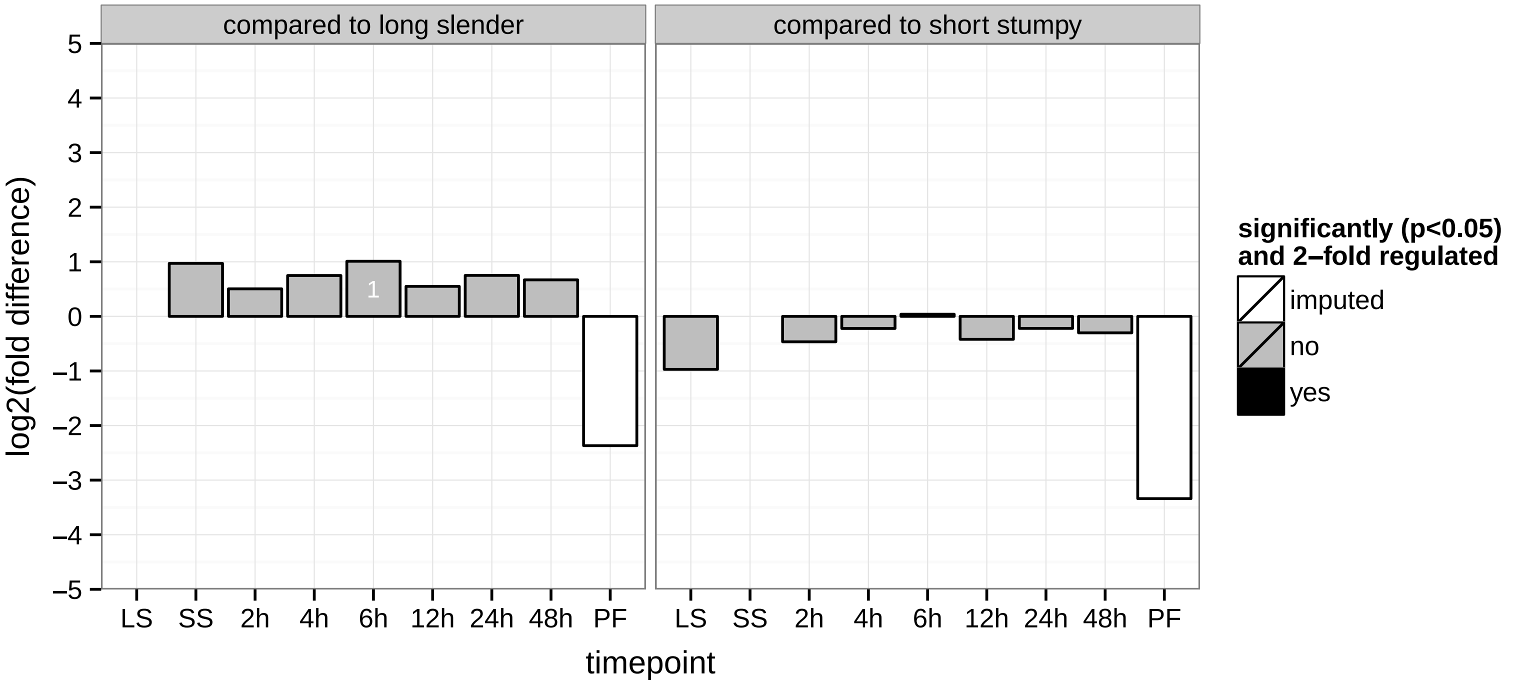

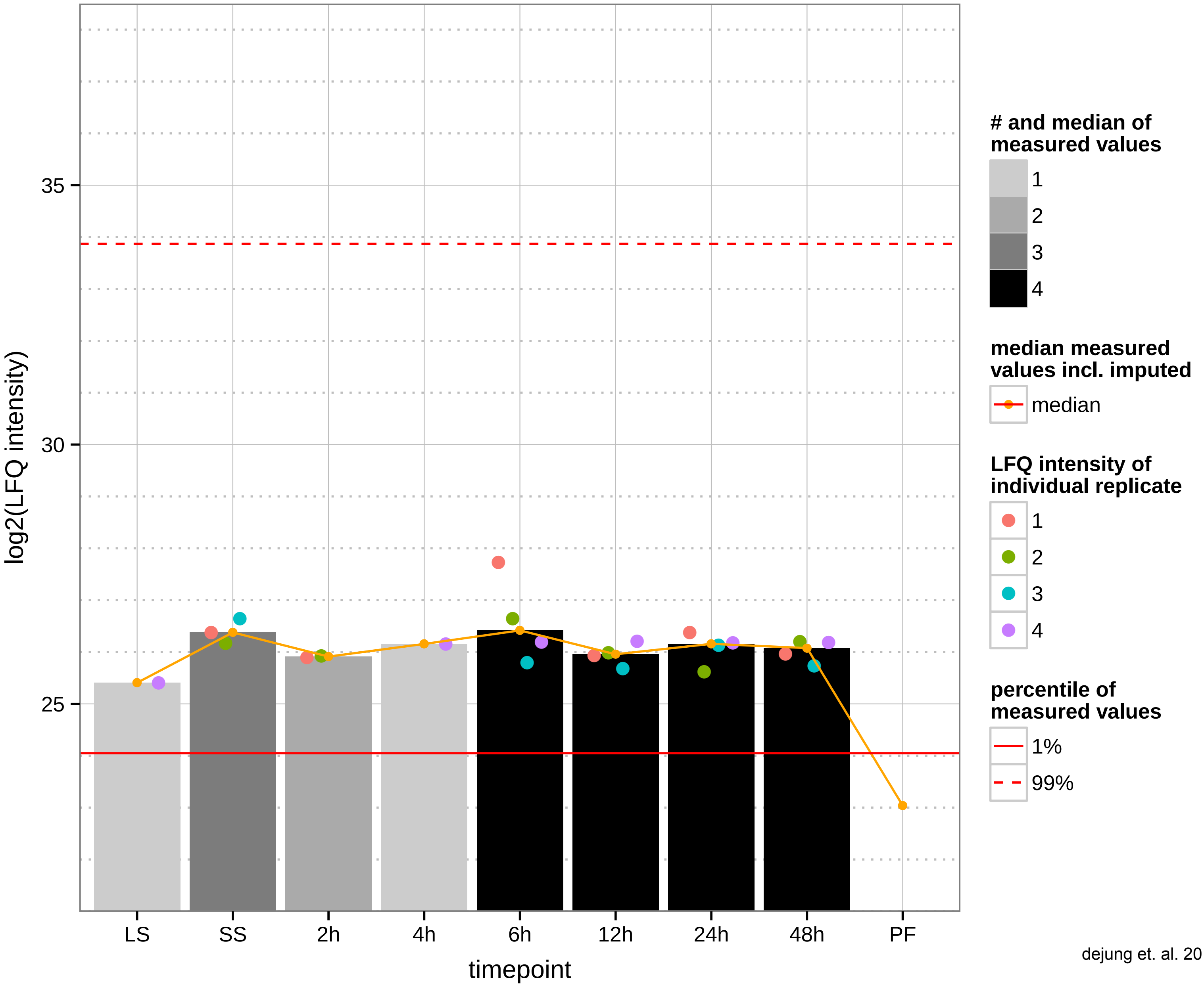

dejung et. al. 2015

hypothetical protein, conserved
Tb927.11.13230
AGOF: structural molecule activity
AGOC: null
AGOP: null
PGOF: structural molecule activity
PGOC: null
PGOP: null

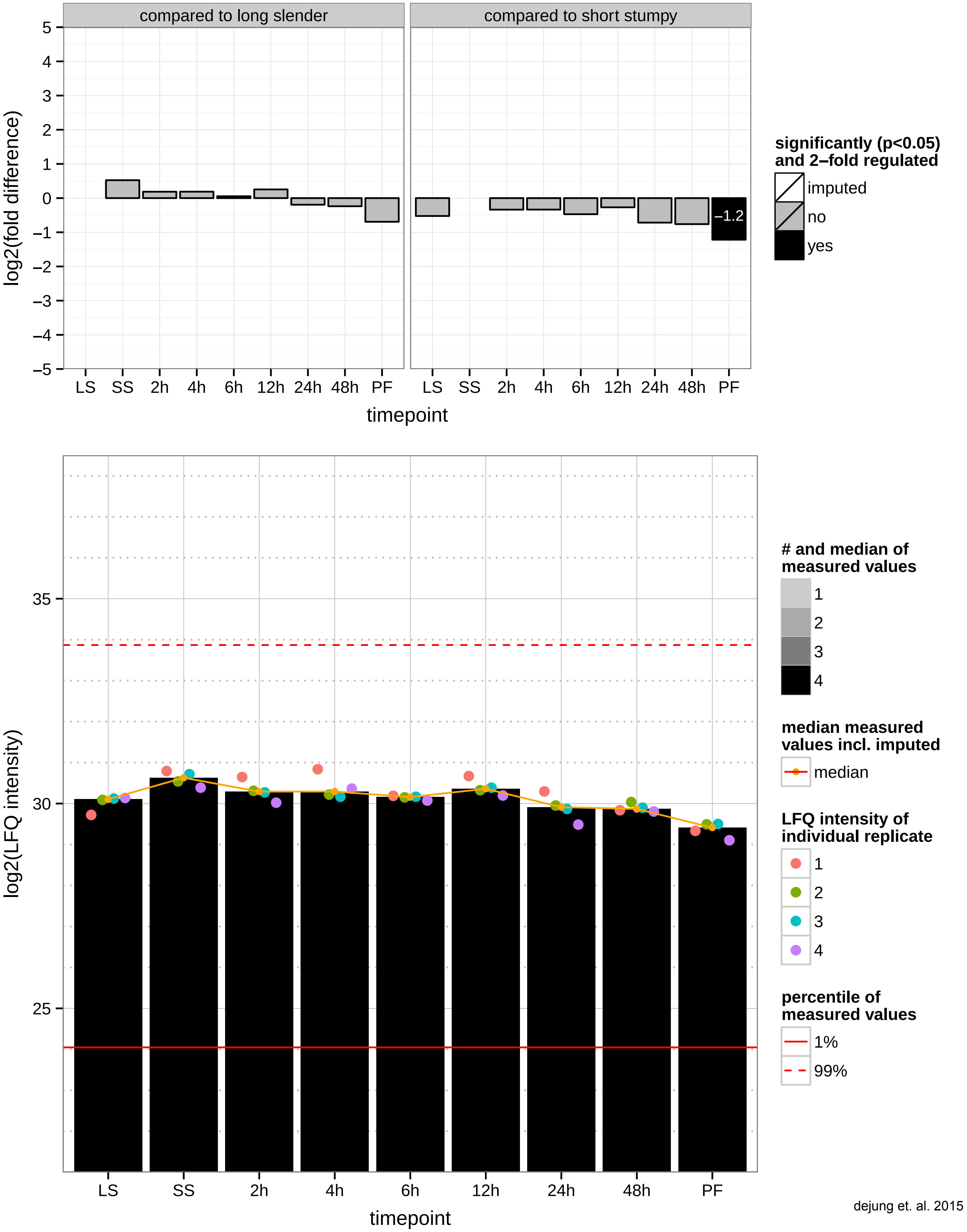

dejung et. al. 2015

hypothetical protein, conserved
Tb927.11.13910
AGOF: null
AGOC: mitochondrion
AGOP: null
PGOF: catalytic activity
PGOC: null
PGOP: null

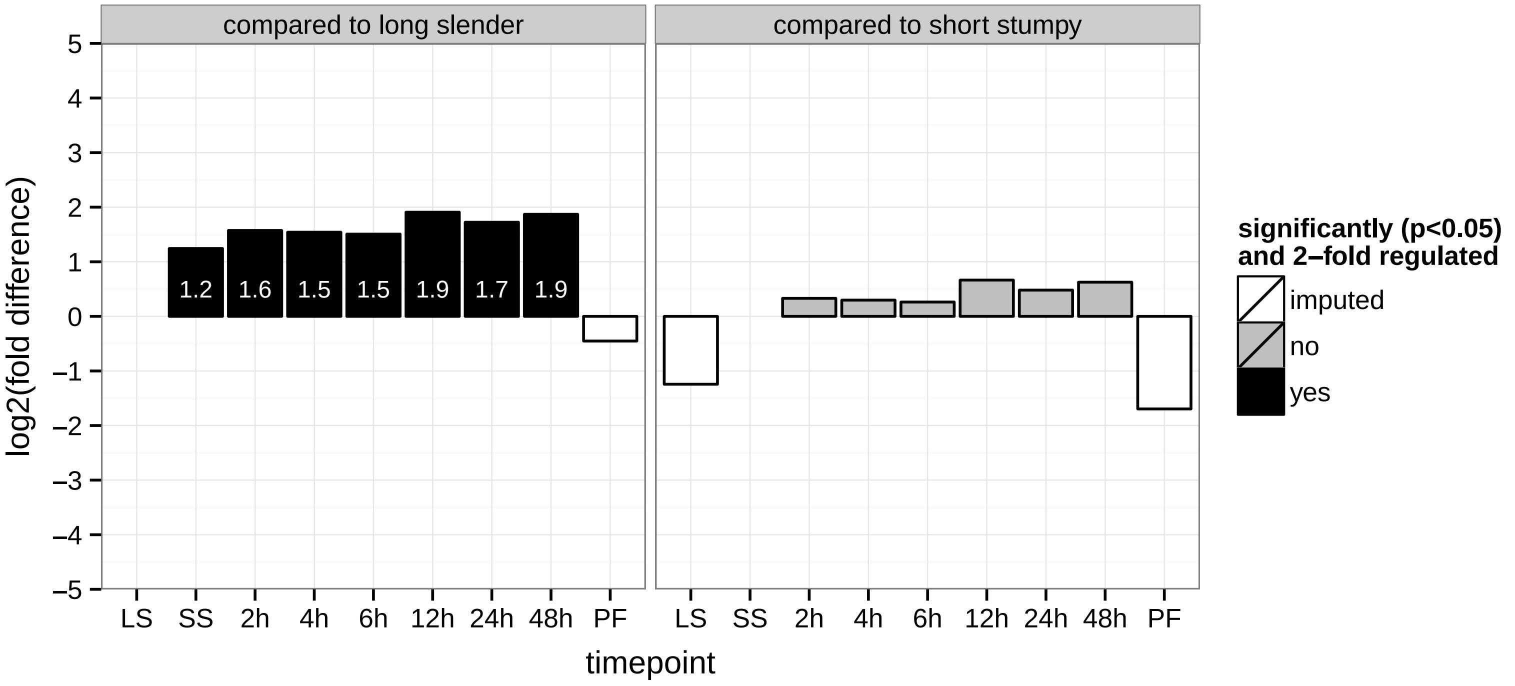

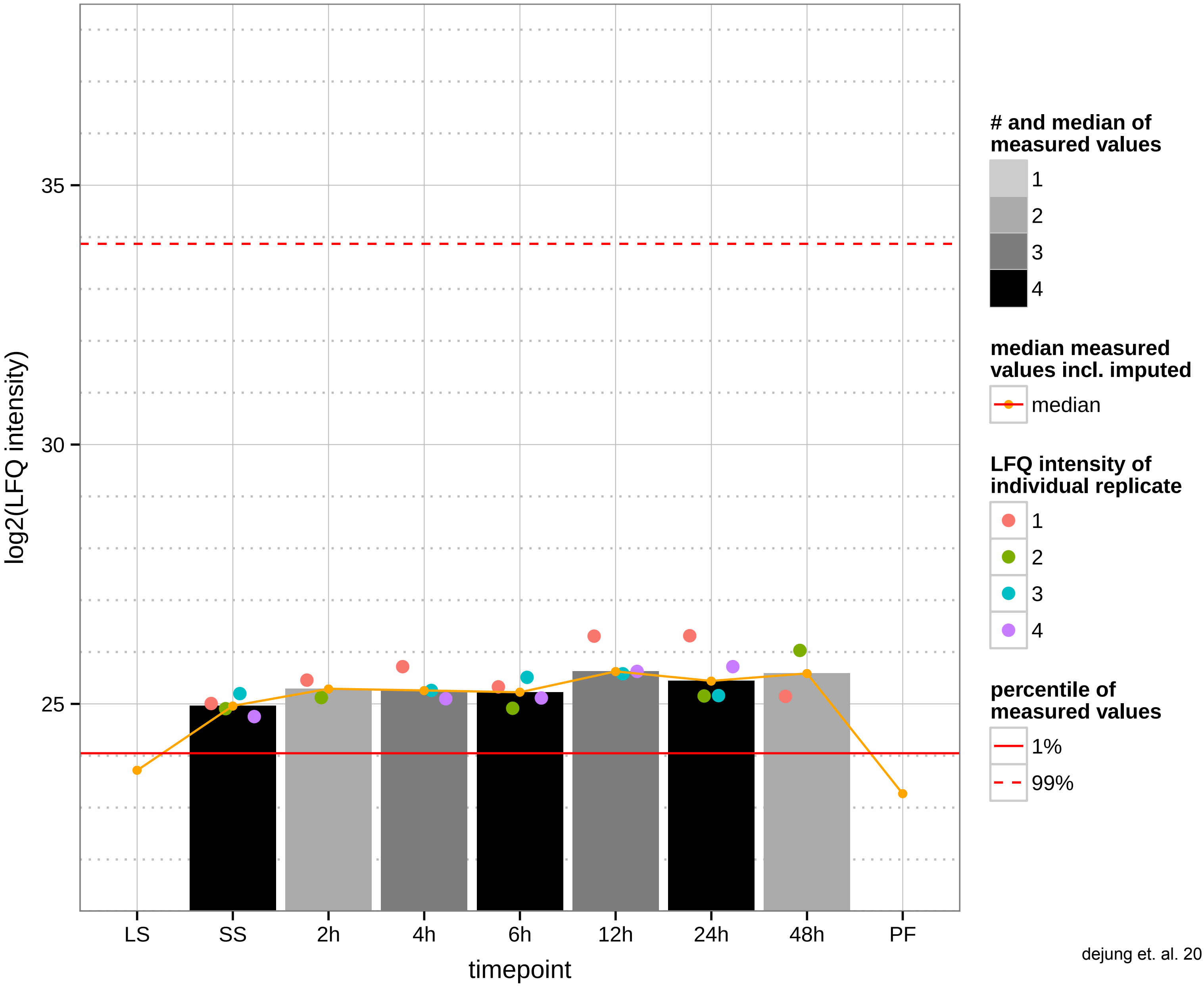

dejung et. al. 2015

kinetoplast polyadenylation/uridylation factor 2 (KPAF2)
Tb927.11.14380
AGOF: protein binding, ribosome binding
AGOC: mitochondrion
AGOP: mRNA 3'−end processing, mRNA polyadenylation, regulation of mRNA stability
PGOF: null
PGOC: null
PGOP: null

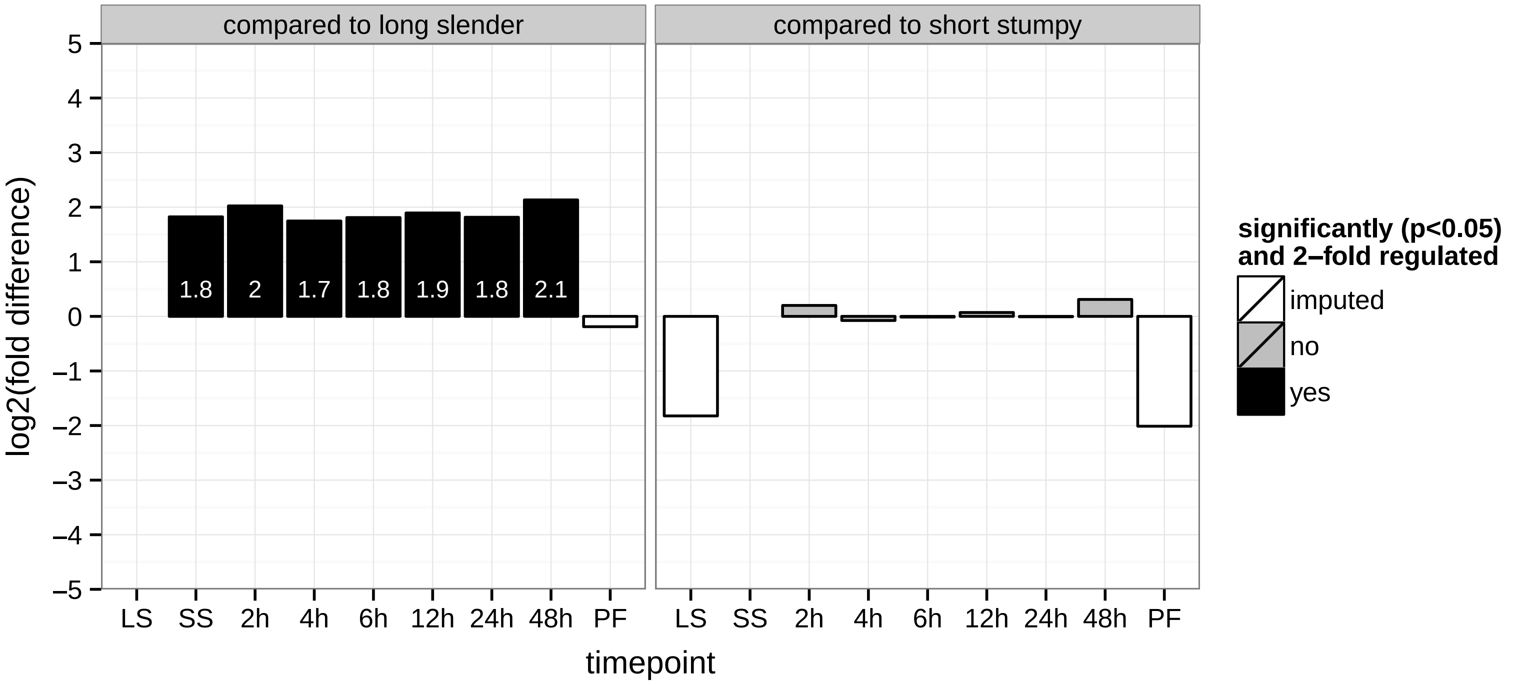

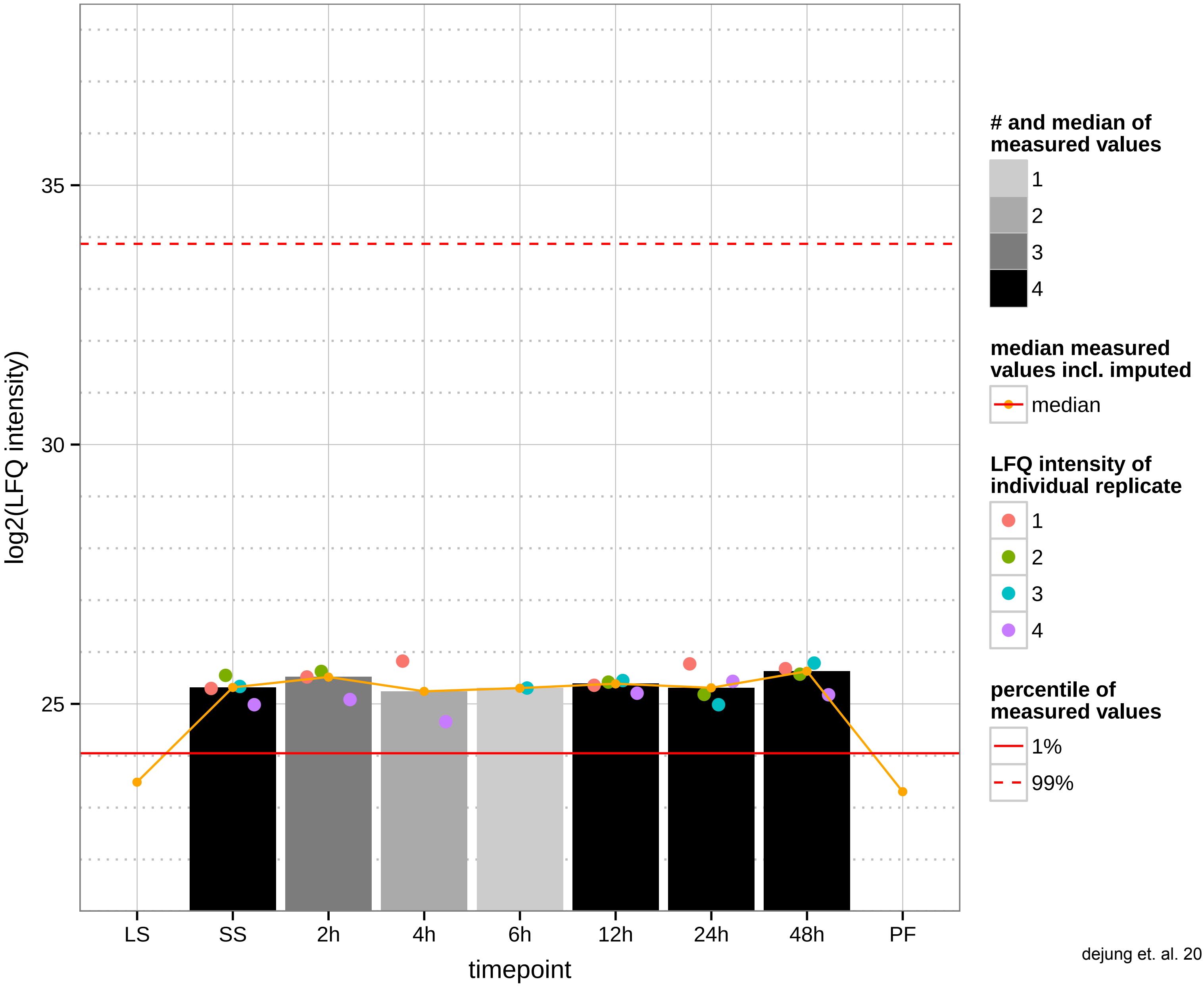

dejung et. al. 2015

hypothetical protein, conserved
Tb927.11.1440
AGOF: null
AGOC: null
AGOP: null
PGOF: null
PGOC: null
PGOP: null

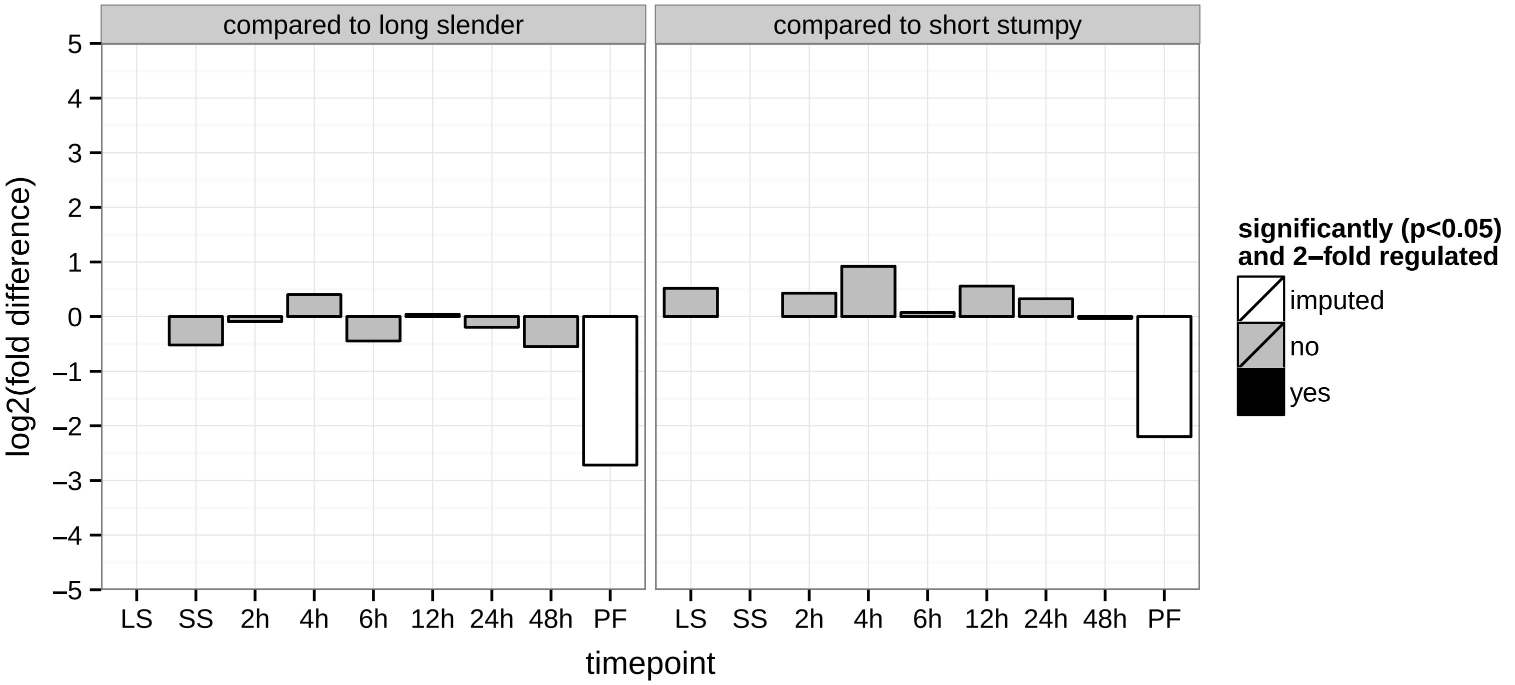

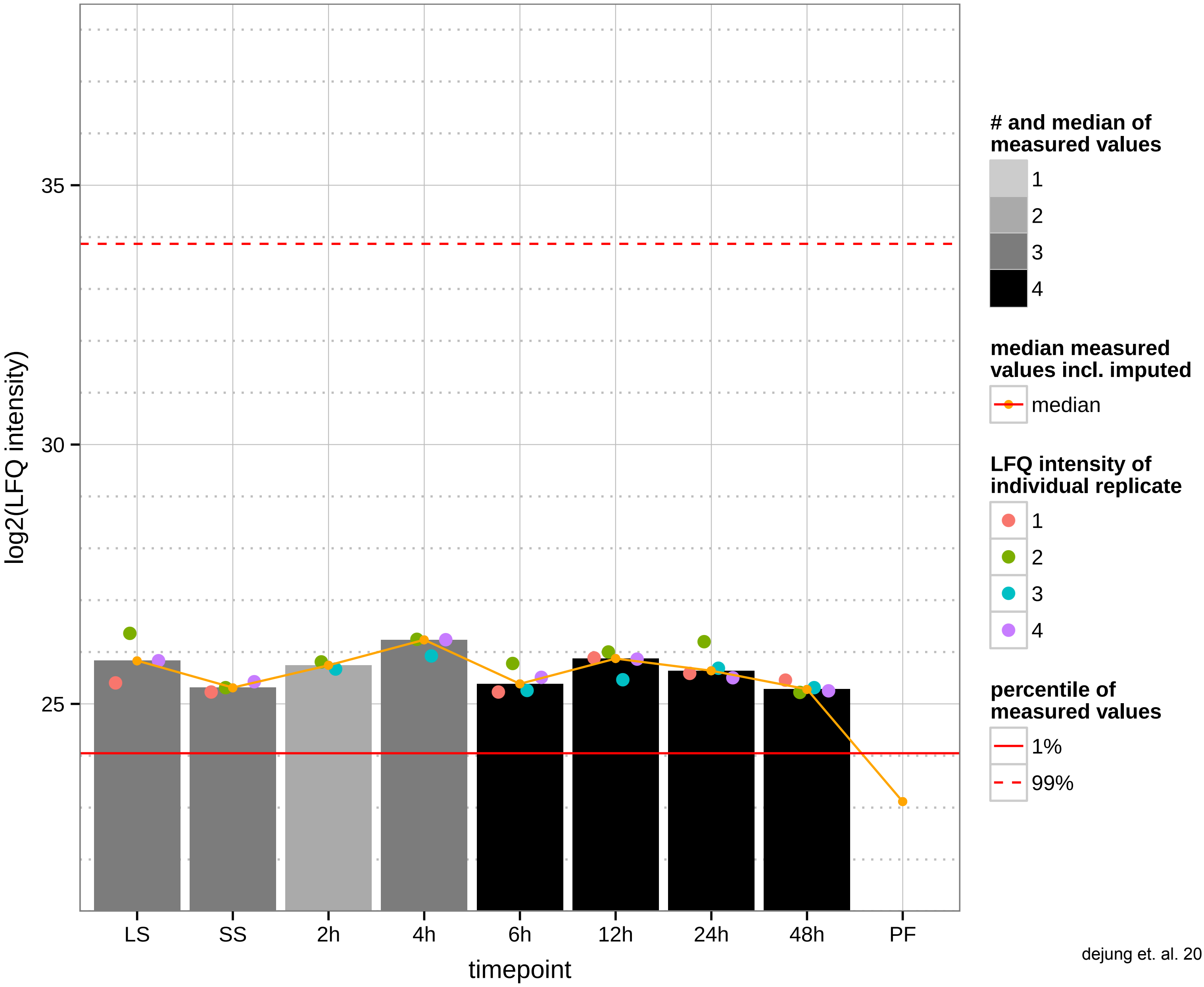

dejung et. al. 2015

hypothetical protein, conserved
Tb927.11.14570
AGOF: null
AGOC: mitochondrion
AGOP: null
PGOF: null
PGOC: null
PGOP: null

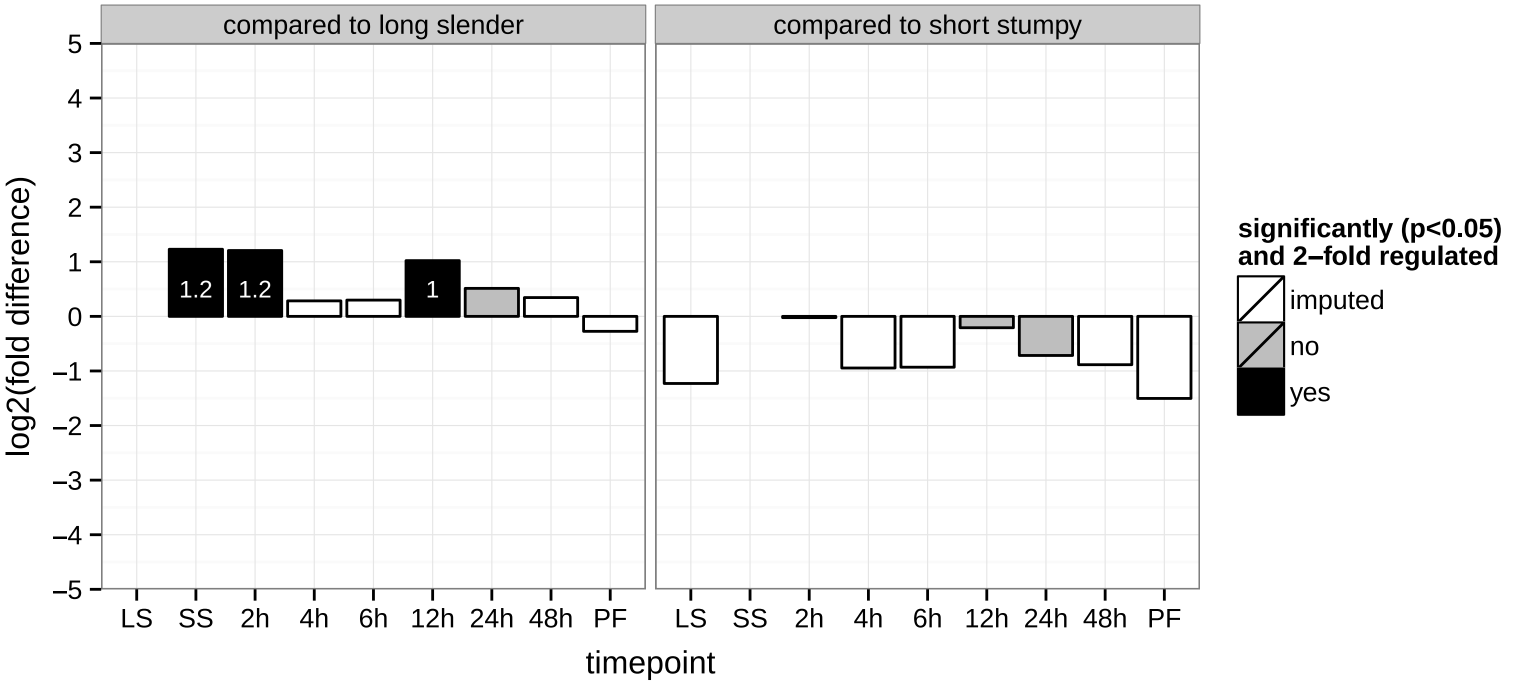

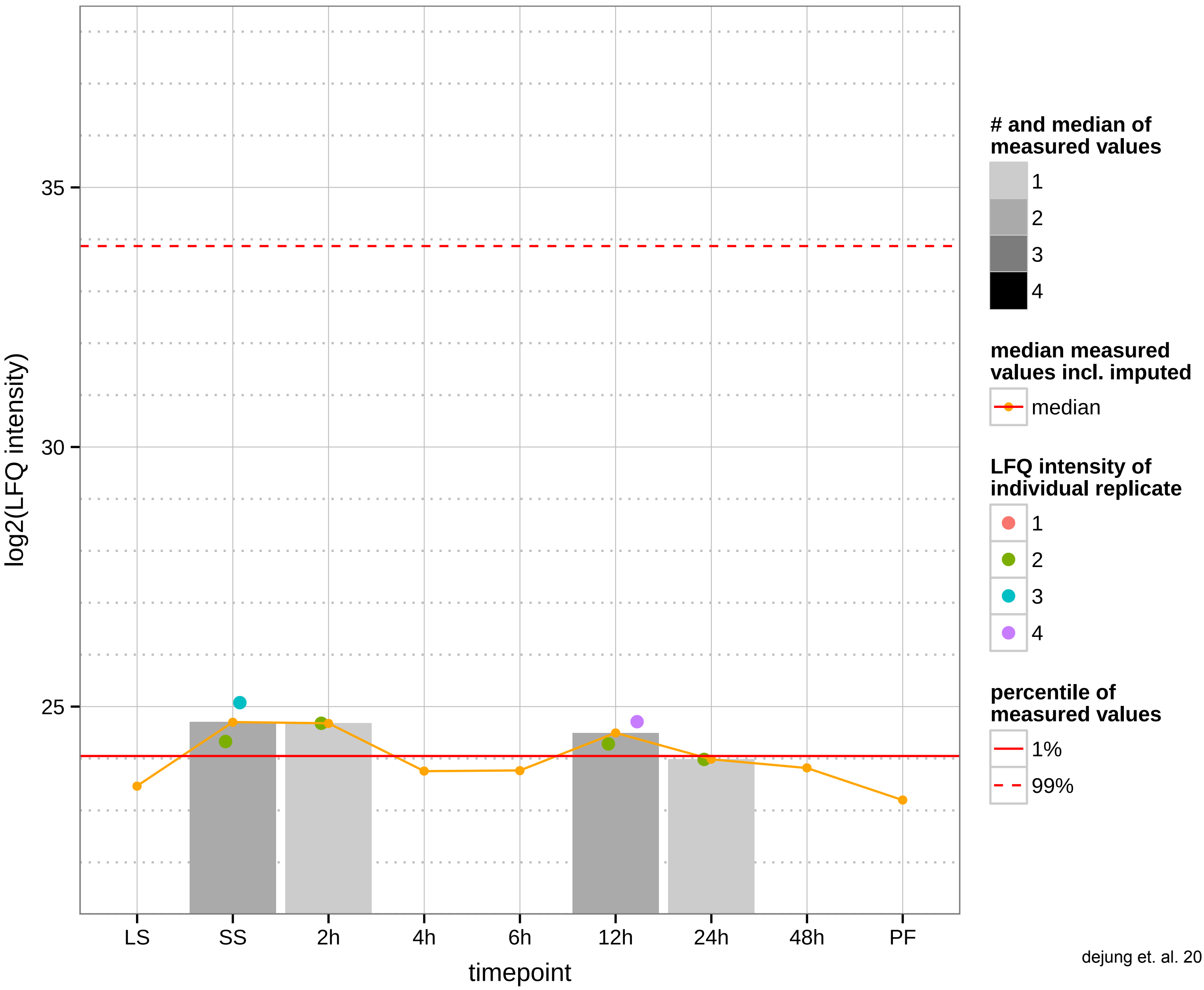

dejung et. al. 2015

hypothetical protein, conserved
Tb927.11.14690
AGOF: null
AGOC: null
AGOP: null
PGOF: null
PGOC: null
PGOP: null

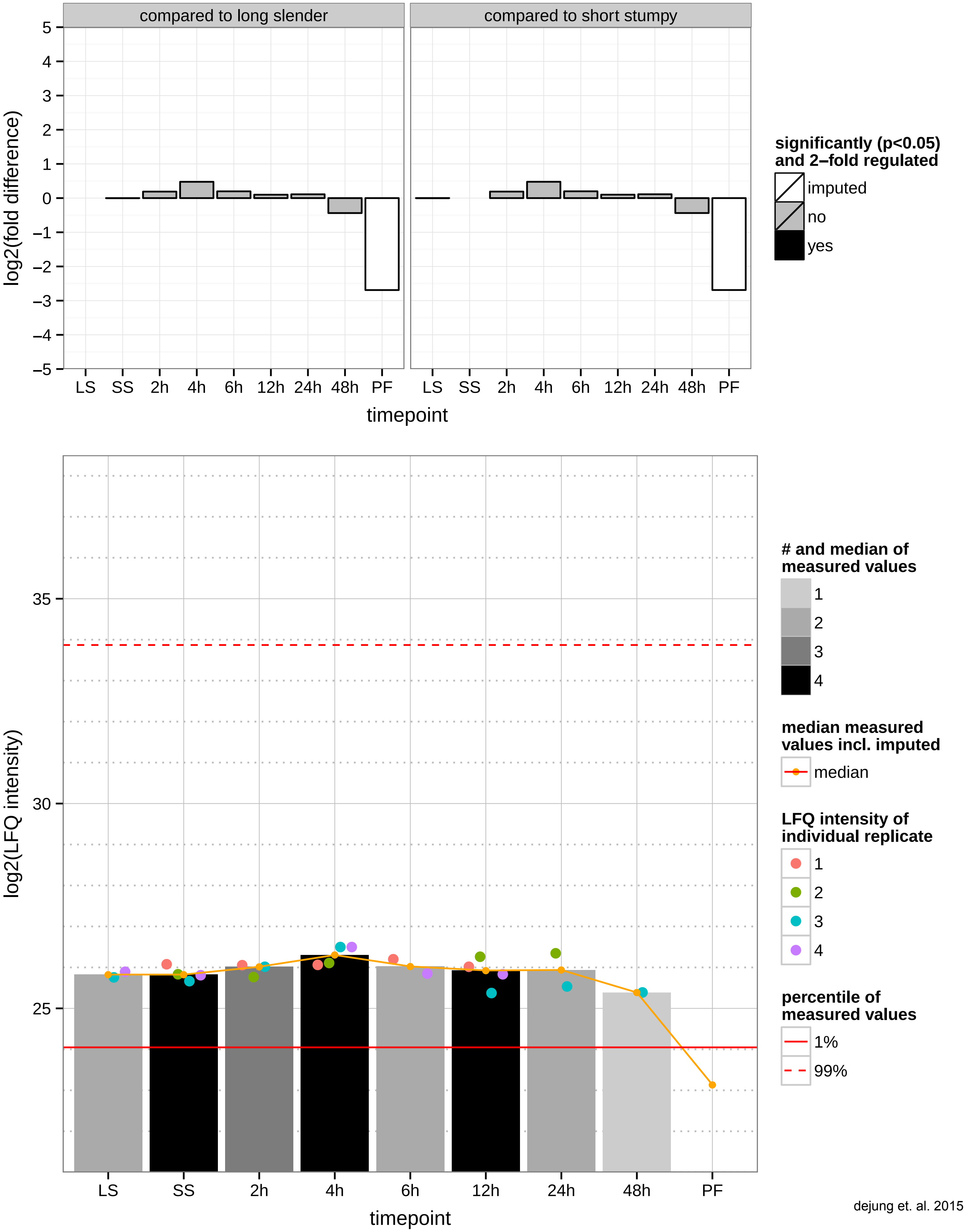

dejung et. al. 2015

small GTPase (X3)
Tb927.11.15030
AGOF: GTP binding, GTPase activity
AGOC: intracellular
AGOP: intracellular protein transport, nucleocytoplasmic transport, small GTPase mediated signal transduction
PGOF: GTP binding, GTPase activity
PGOC: intracellular, membrane
PGOP: GTP catabolic process, intracellular protein transport, nucleocytoplasmic transport, protein transport, signal transducti

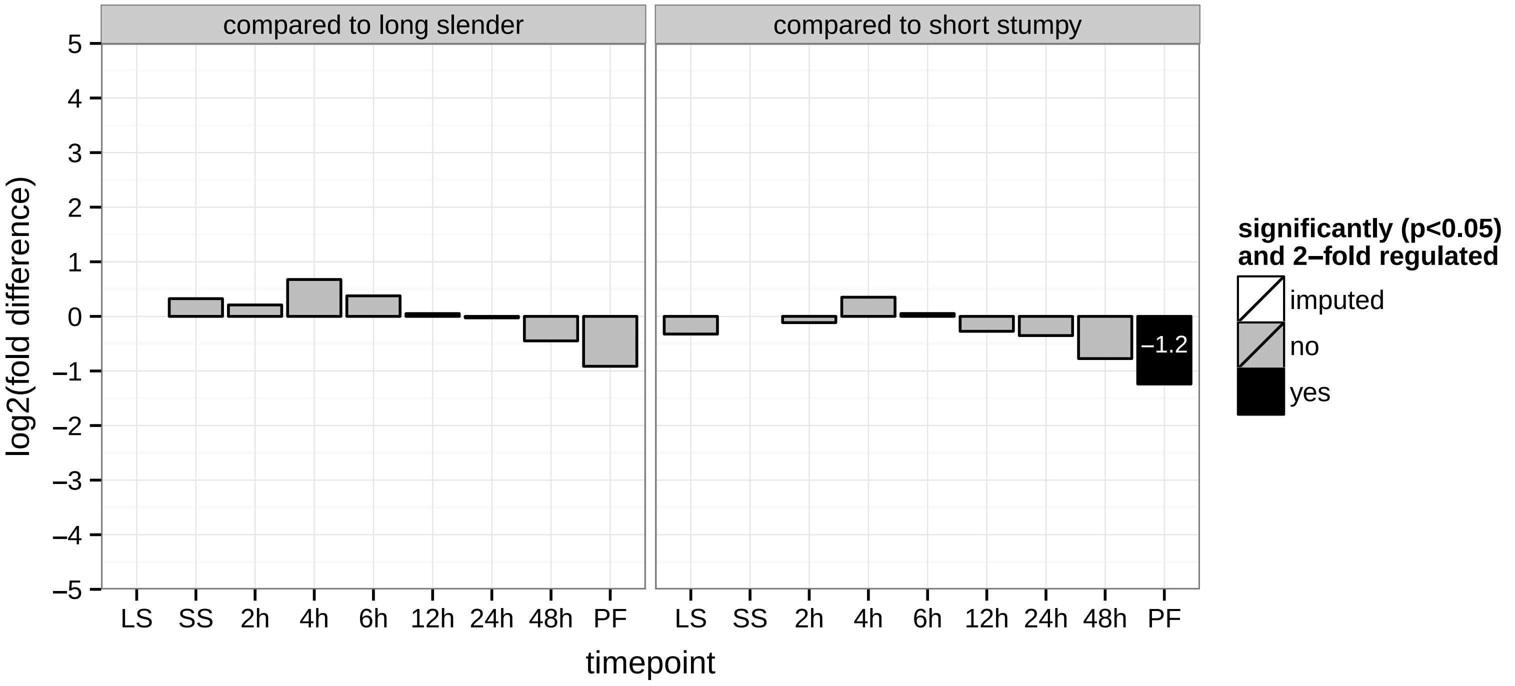

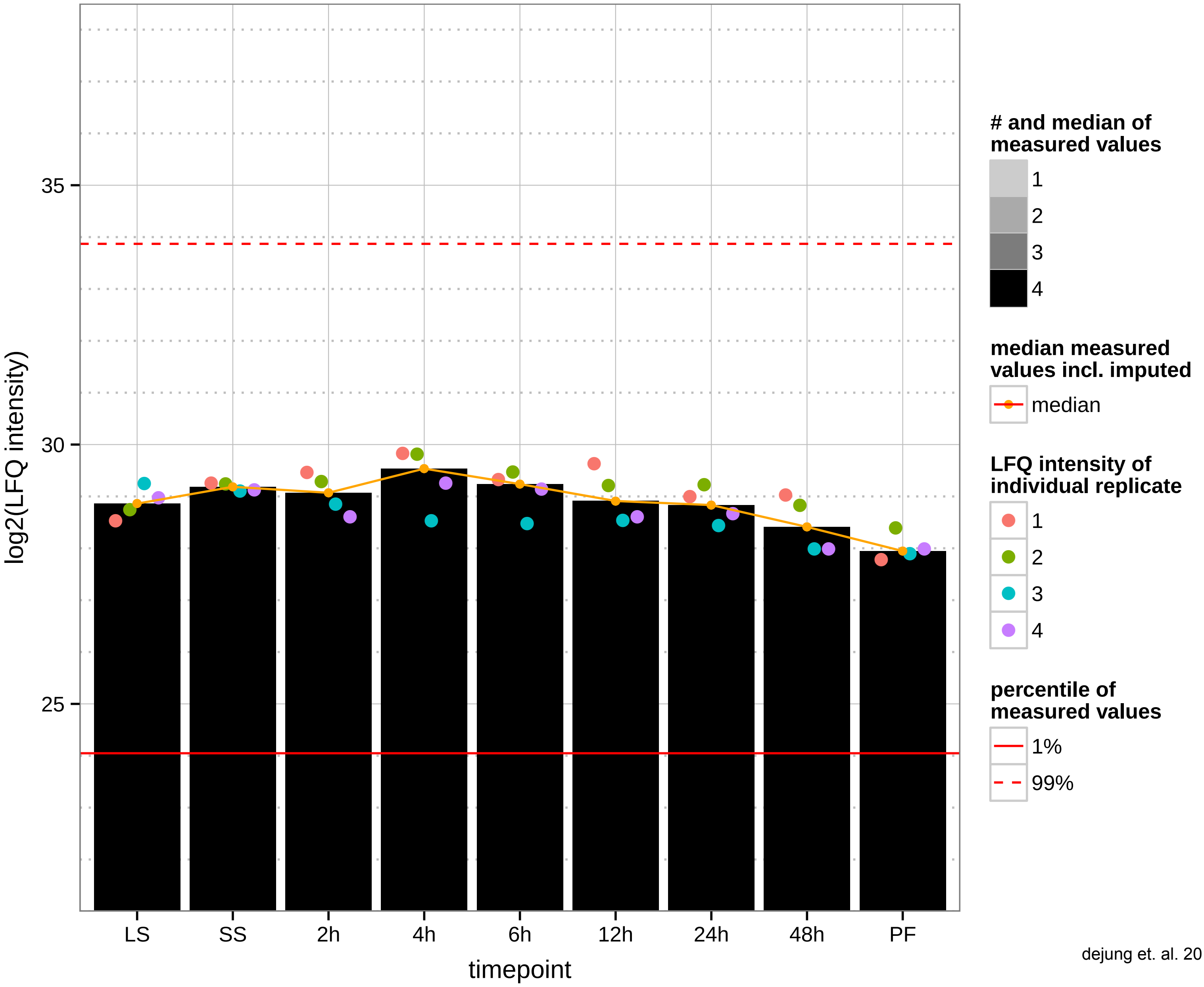

dejung et. al. 2015

hypothetical protein, conserved
Tb927.11.15120
AGOF: null
AGOC: null
AGOP: null
PGOF: null
PGOC: null
PGOP: null

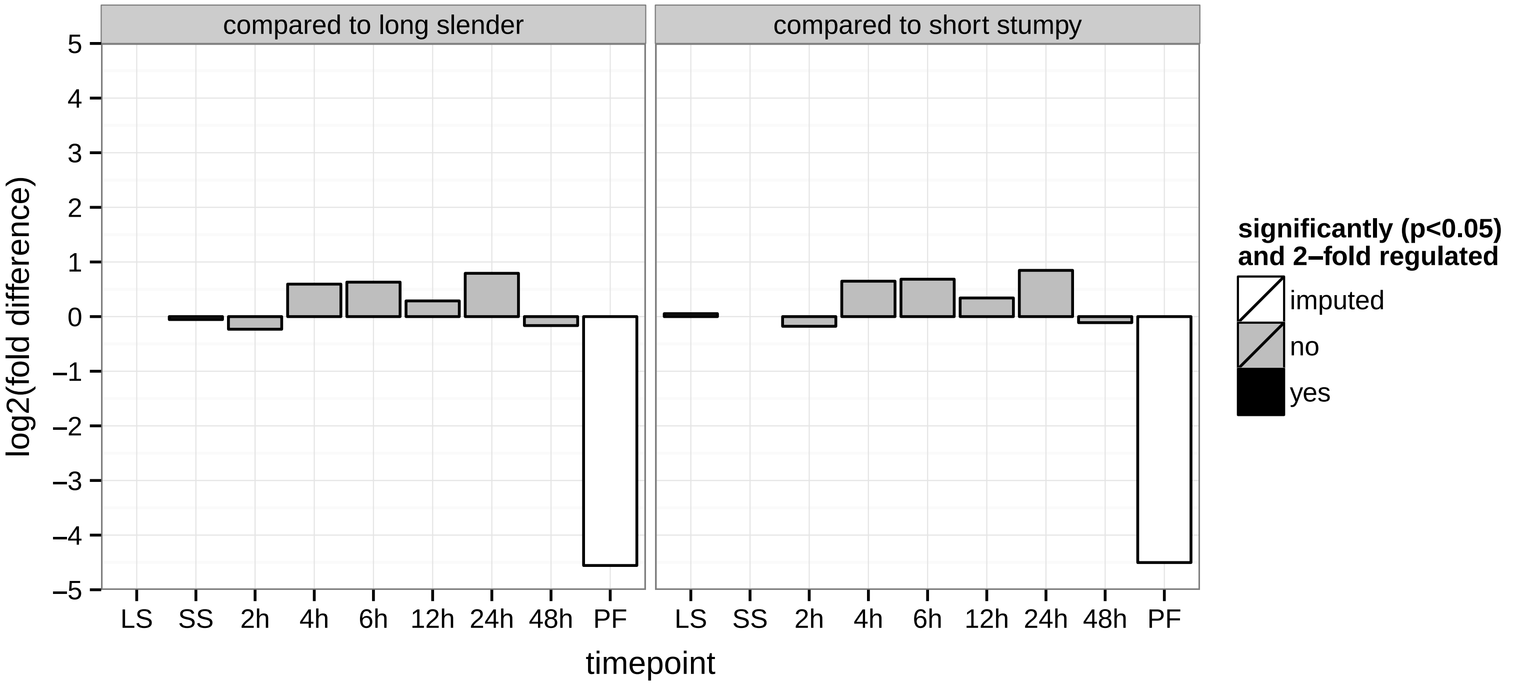

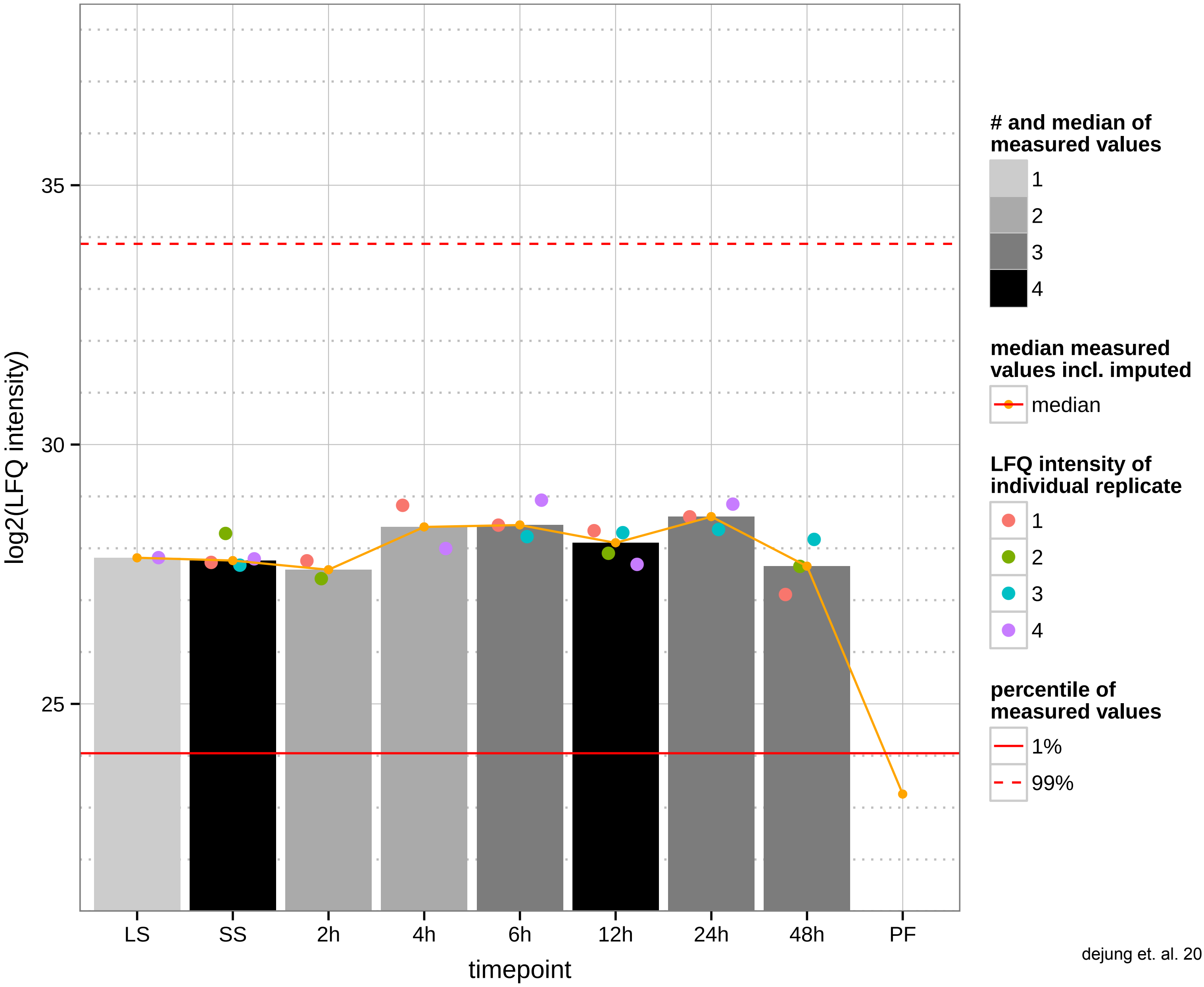

dejung et. al. 2015

hypothetical protein, conserved
Tb927.11.15400
AGOF: null
AGOC: null, integral to membrane
AGOP: null, vesicle–mediated transport
PGOF: null, protein binding
PGOC: null, integral to membrane
PGOP: null, vesicle–mediated transport

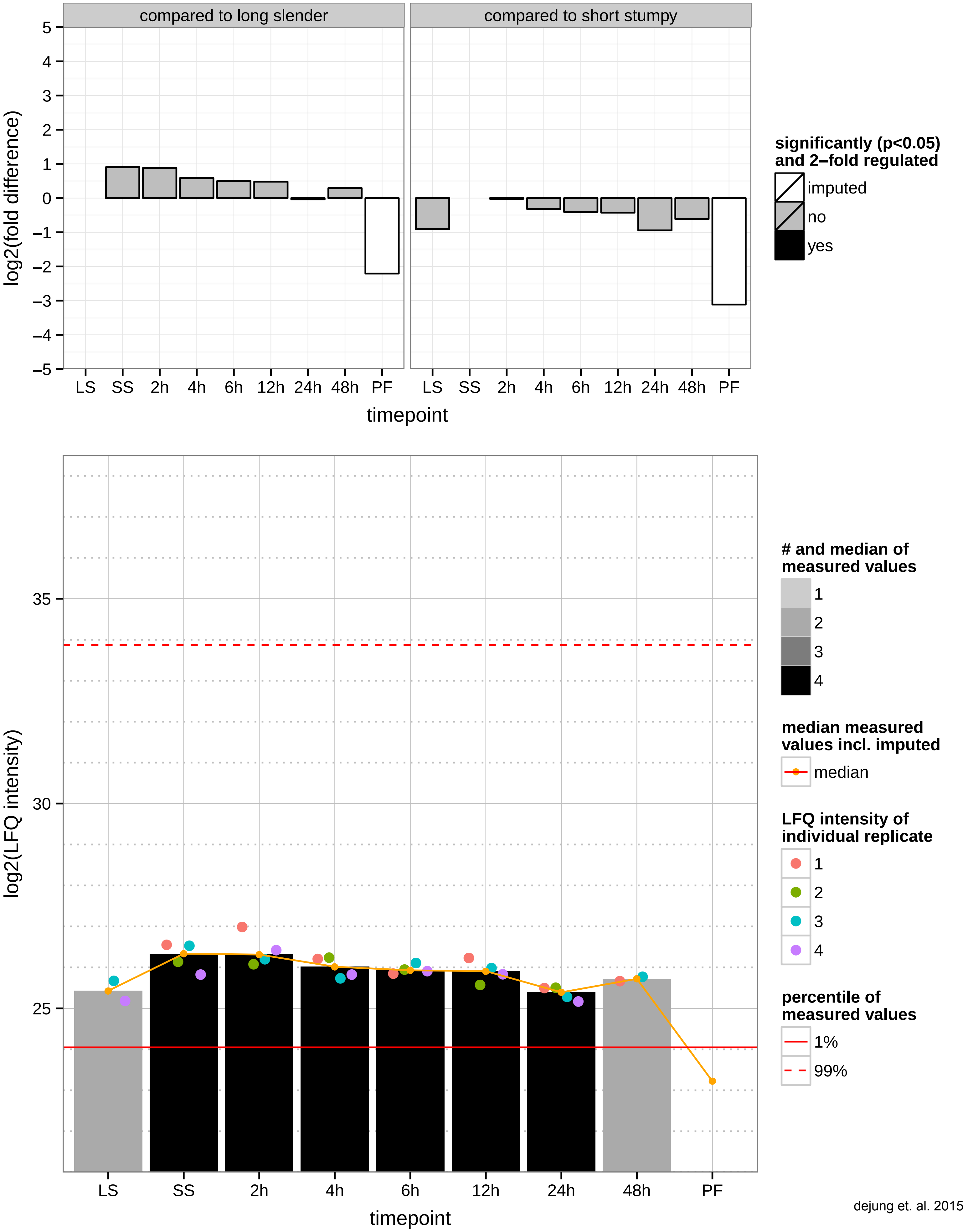

dejung et. al. 2015

hypothetical protein, conserved
Tb927.11.16320
AGOF: null
AGOC: null
AGOP: null
PGOF: protein binding
PGOC: null
PGOP: null

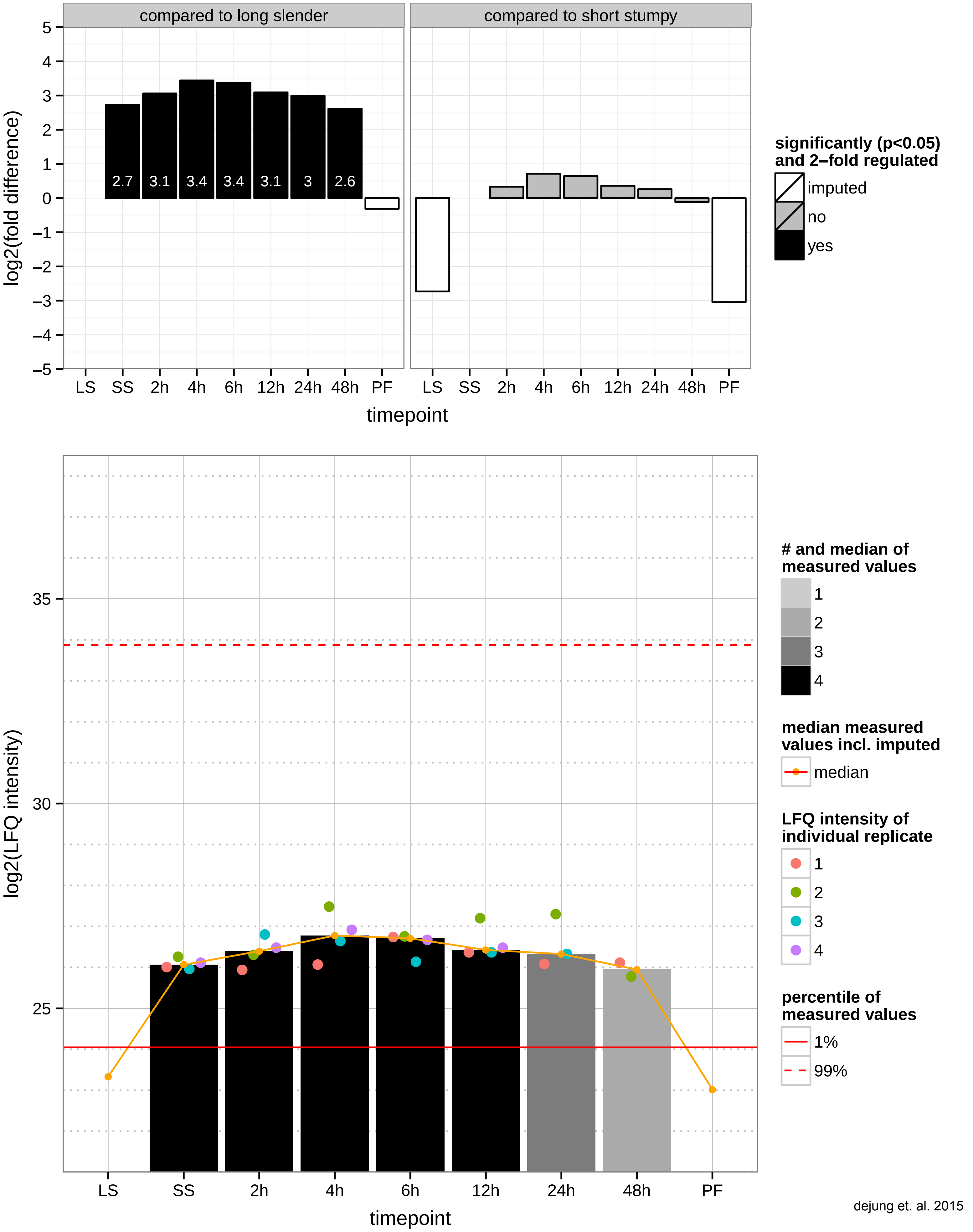

dejung et. al. 2015

hypothetical protein, conserved
Tb927.11.16540
AGOF: null
AGOC: null
AGOP: null
PGOF: null
PGOC: null
PGOP: null

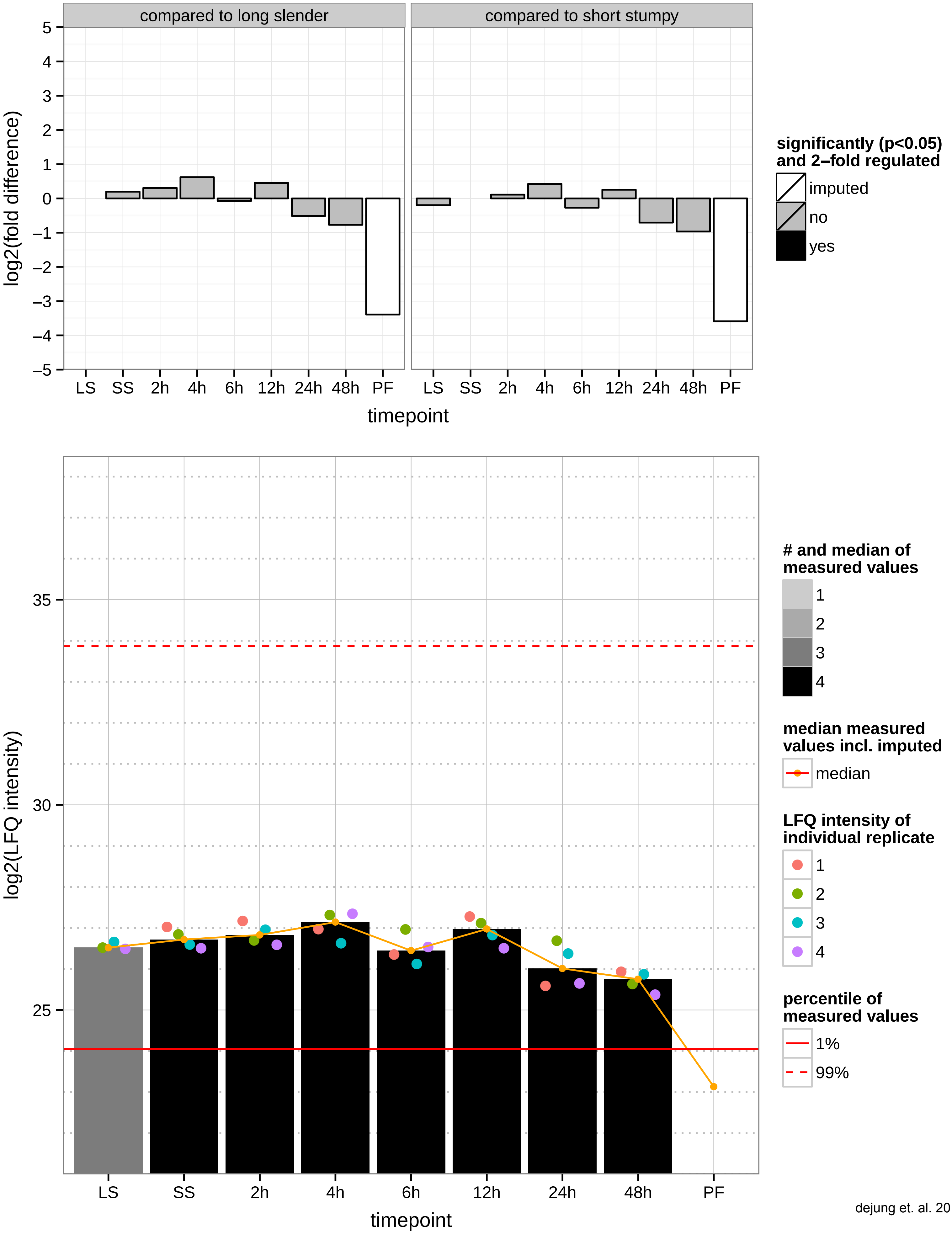

dejung et. al. 2015

hypothetical protein, conserved
Tb927.11.16610
AGOF: null
AGOC: intracellular
AGOP: null
PGOF: null
PGOC: null
PGOP: null

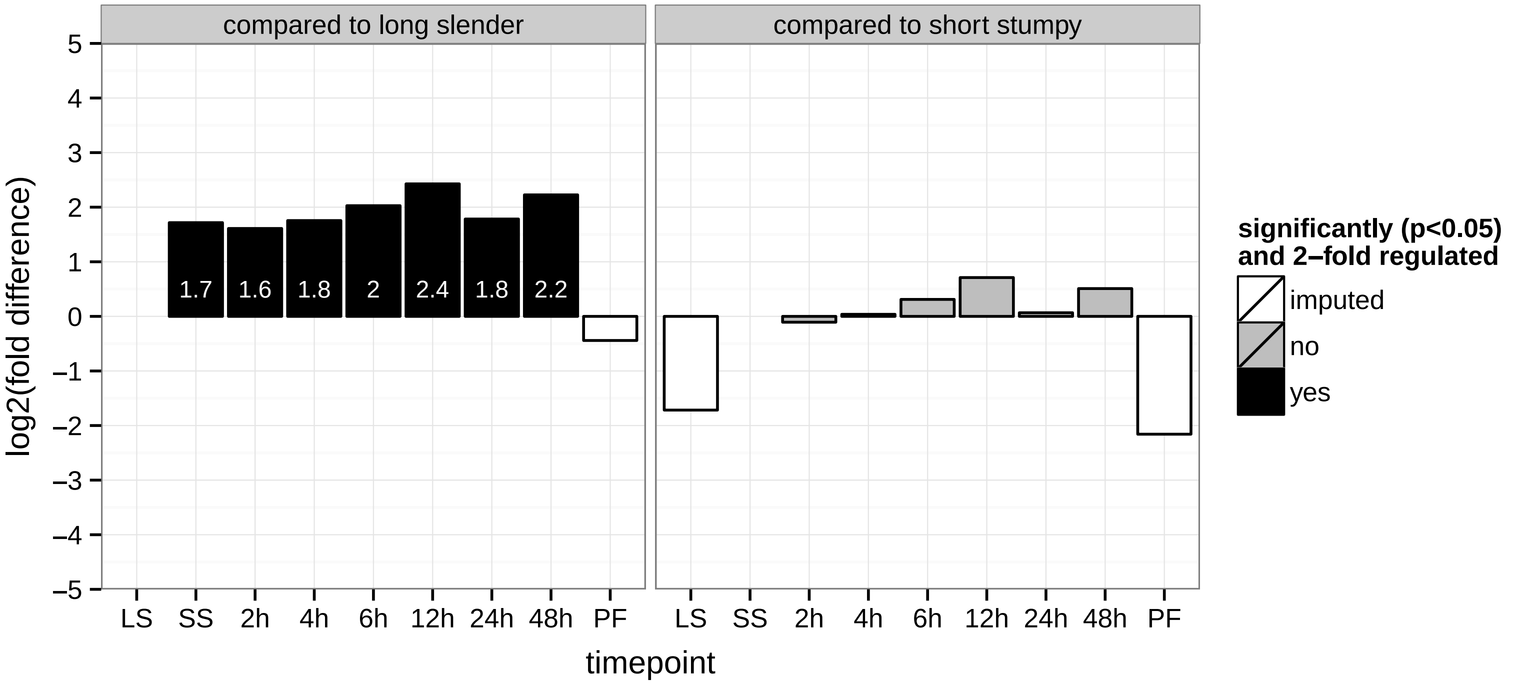

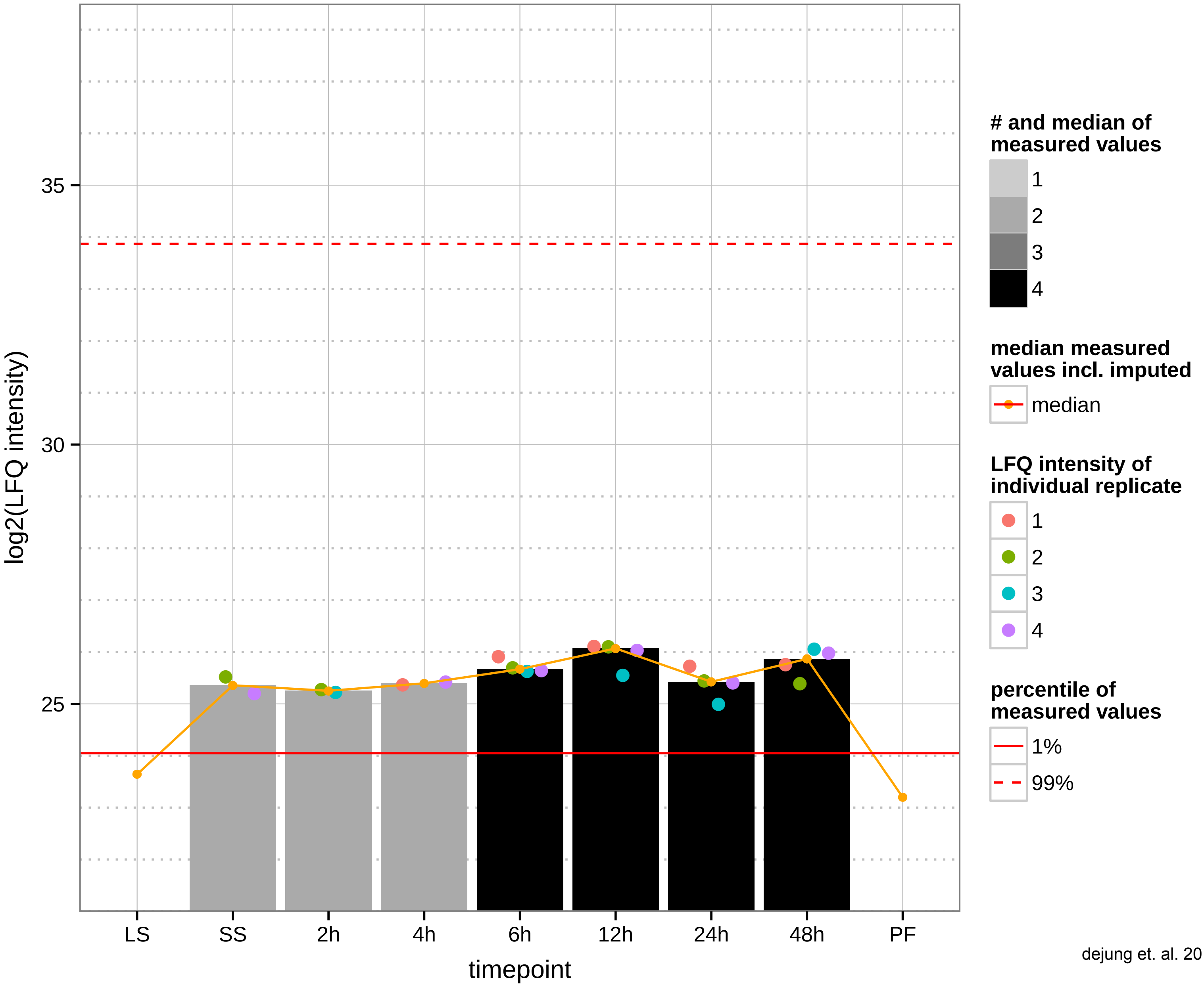

dejung et. al. 2015

chloride channel protein, putative
Tb927.11.16690
AGOF: voltage–gated chloride channel activity
AGOC: membrane
AGOP: chloride transport
PGOF: ion channel activity, protein binding, voltage–gated chloride channel activity
PGOC: membrane
PGOP: chloride transport, transmembrane transport

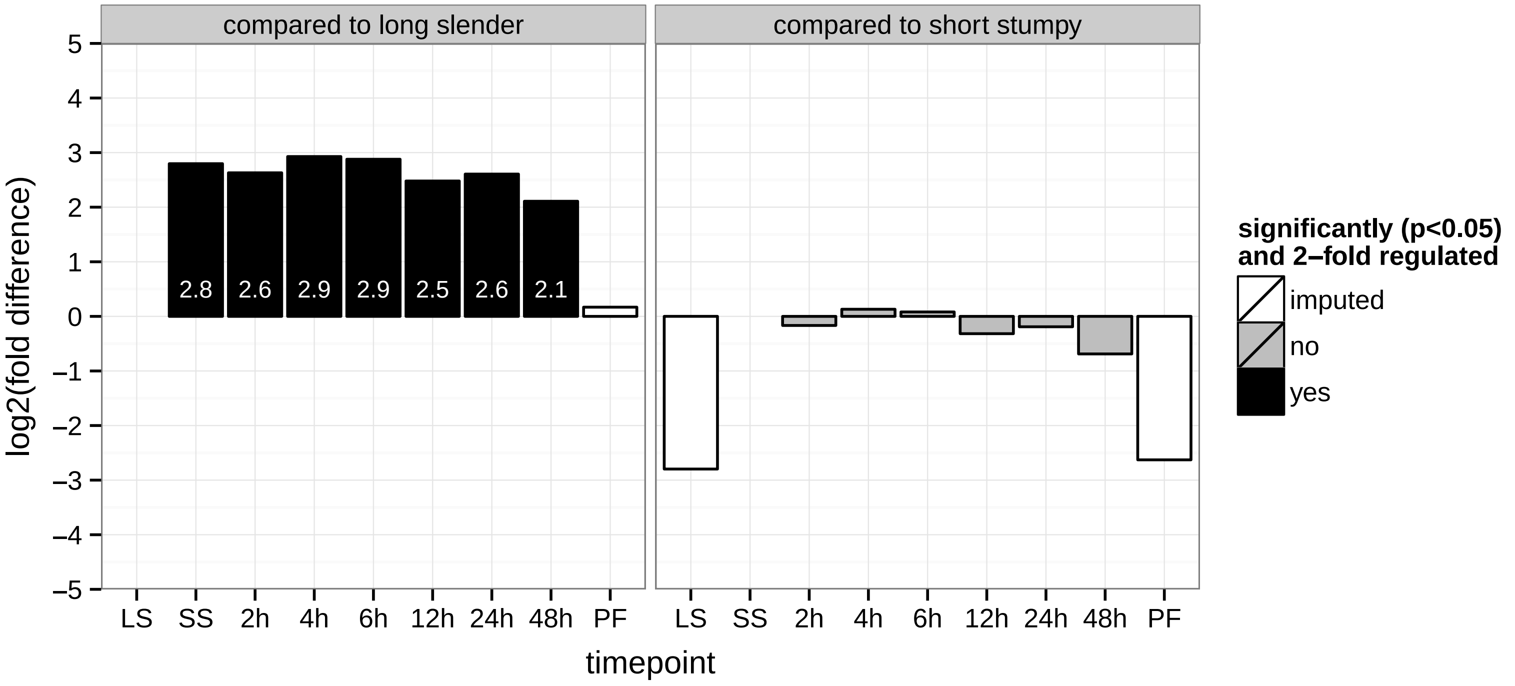

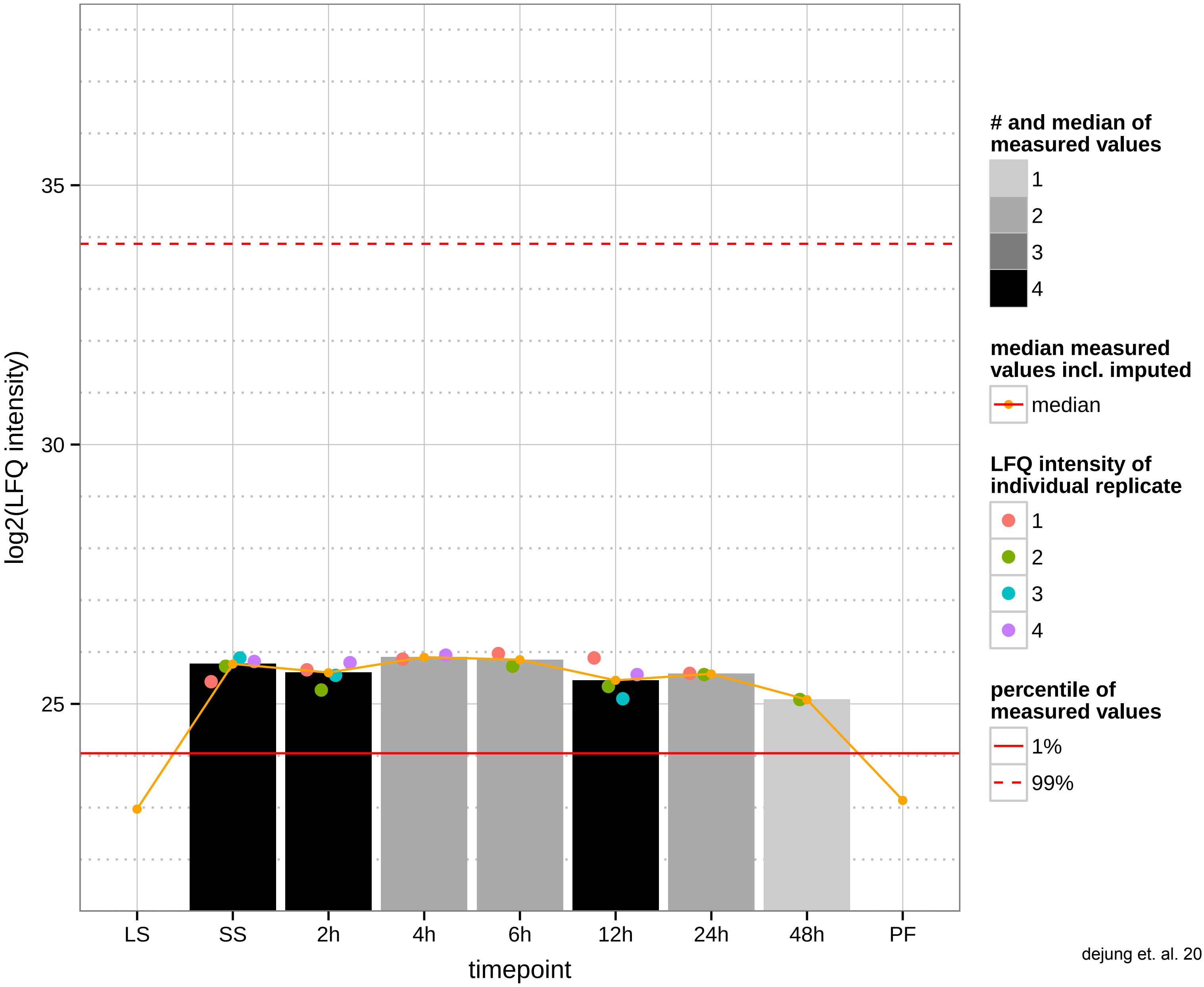

dejung et. al. 2015

cation transporter protein, putative
Tb927.11.1910
AGOF: cation transmembrane transporter activity, efflux transmembrane transporter activity
AGOC: integral to membrane, membrane
AGOP: cation transport
PGOF: cation transmembrane transporter activity
PGOC: integral to membrane
PGOP: cation transport, transmembrane transport

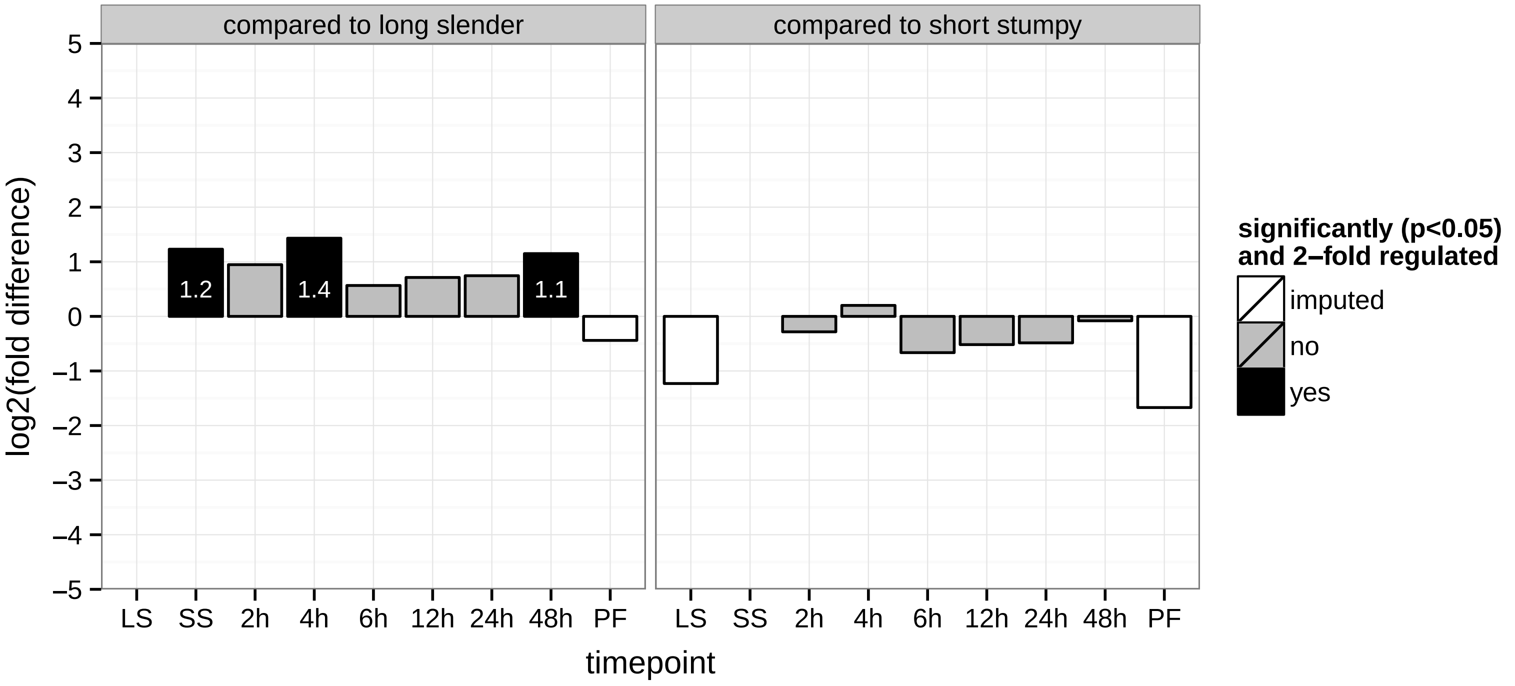

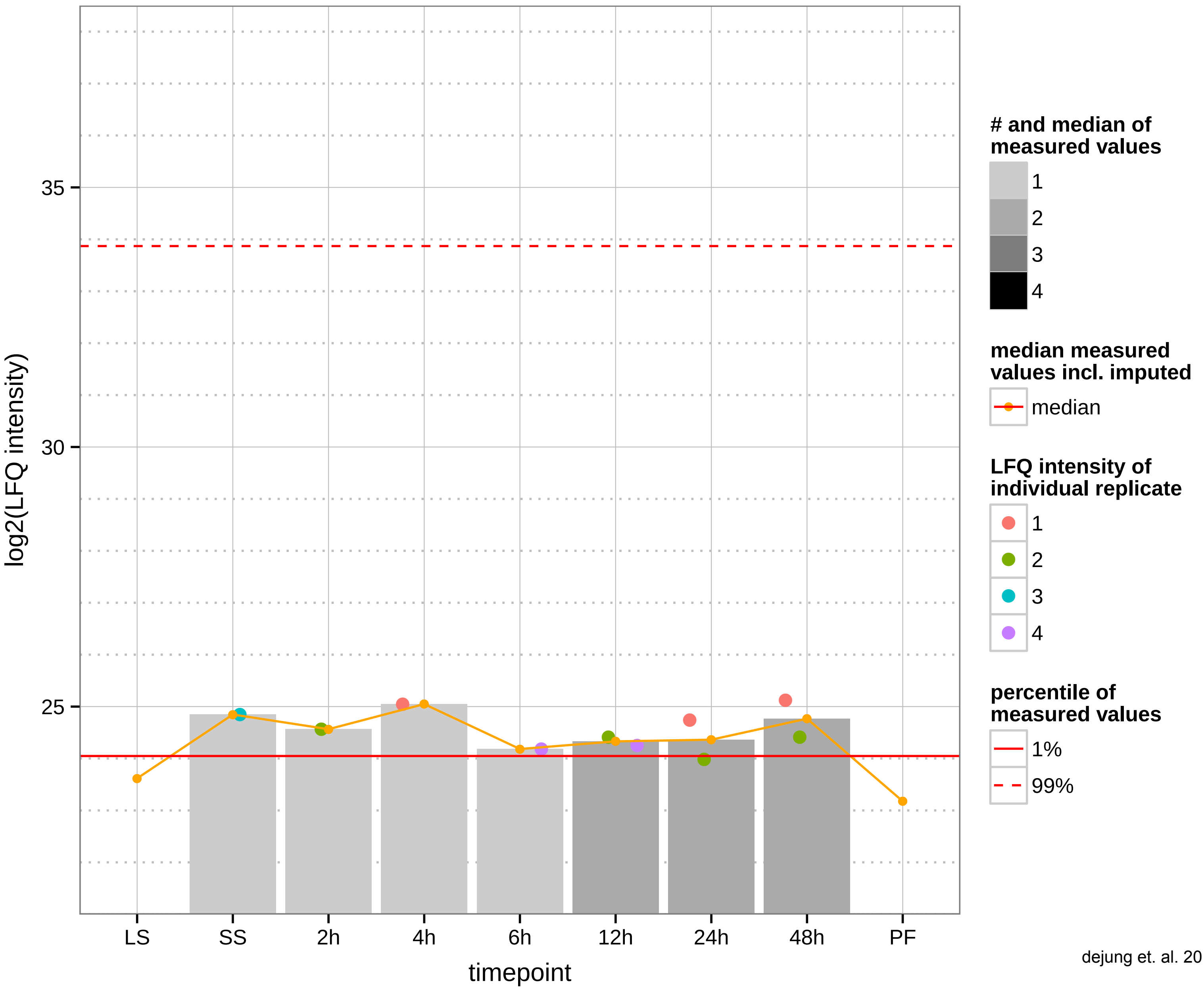

dejung et. al. 2015

Tubulin cofactor C domain−containing protein 1 (tbccd1)
Tb927.11.2440
AGOF: null
AGOC: Golgi apparatus, apical part of cell, centriole
AGOP: Golgi inheritance, establishment of nucleus localization, mitochondrial DNA inheritance
PGOF: null
PGOC: null
PGOP: null

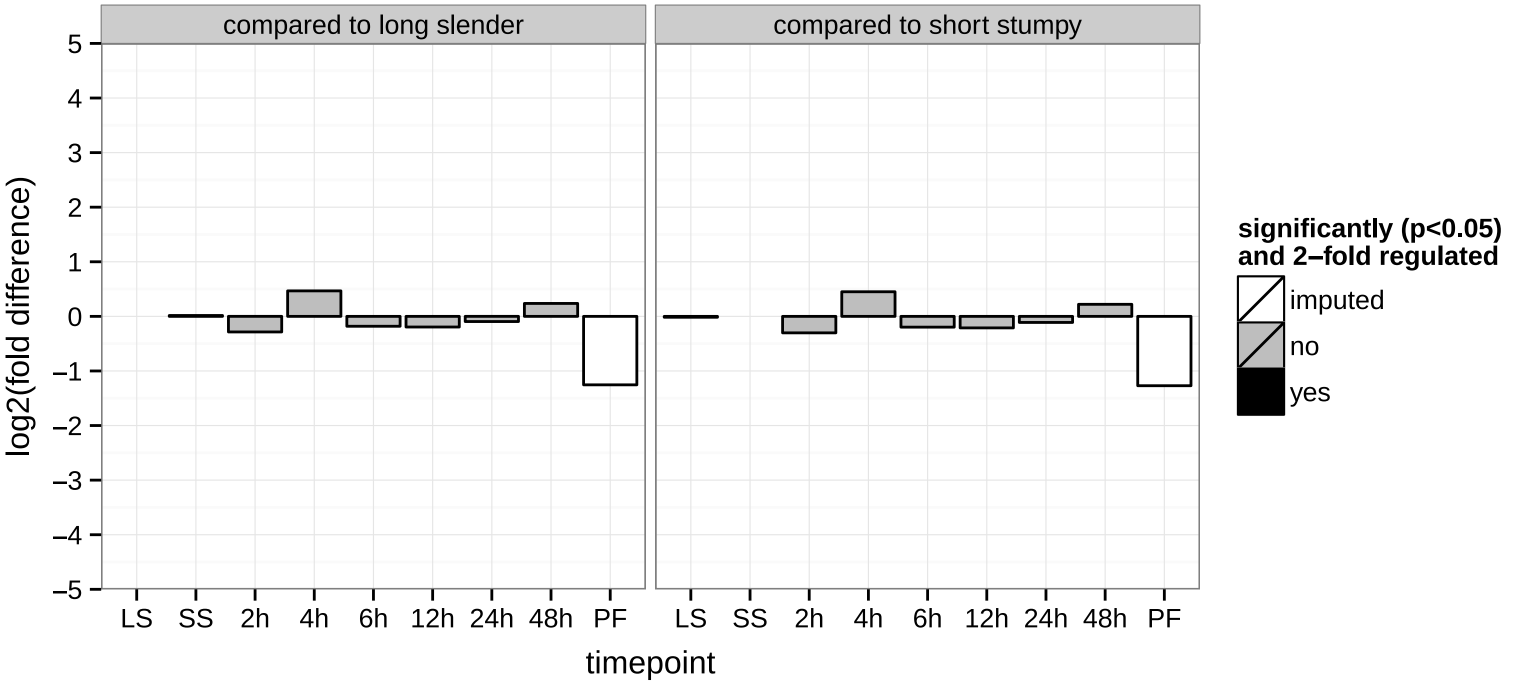

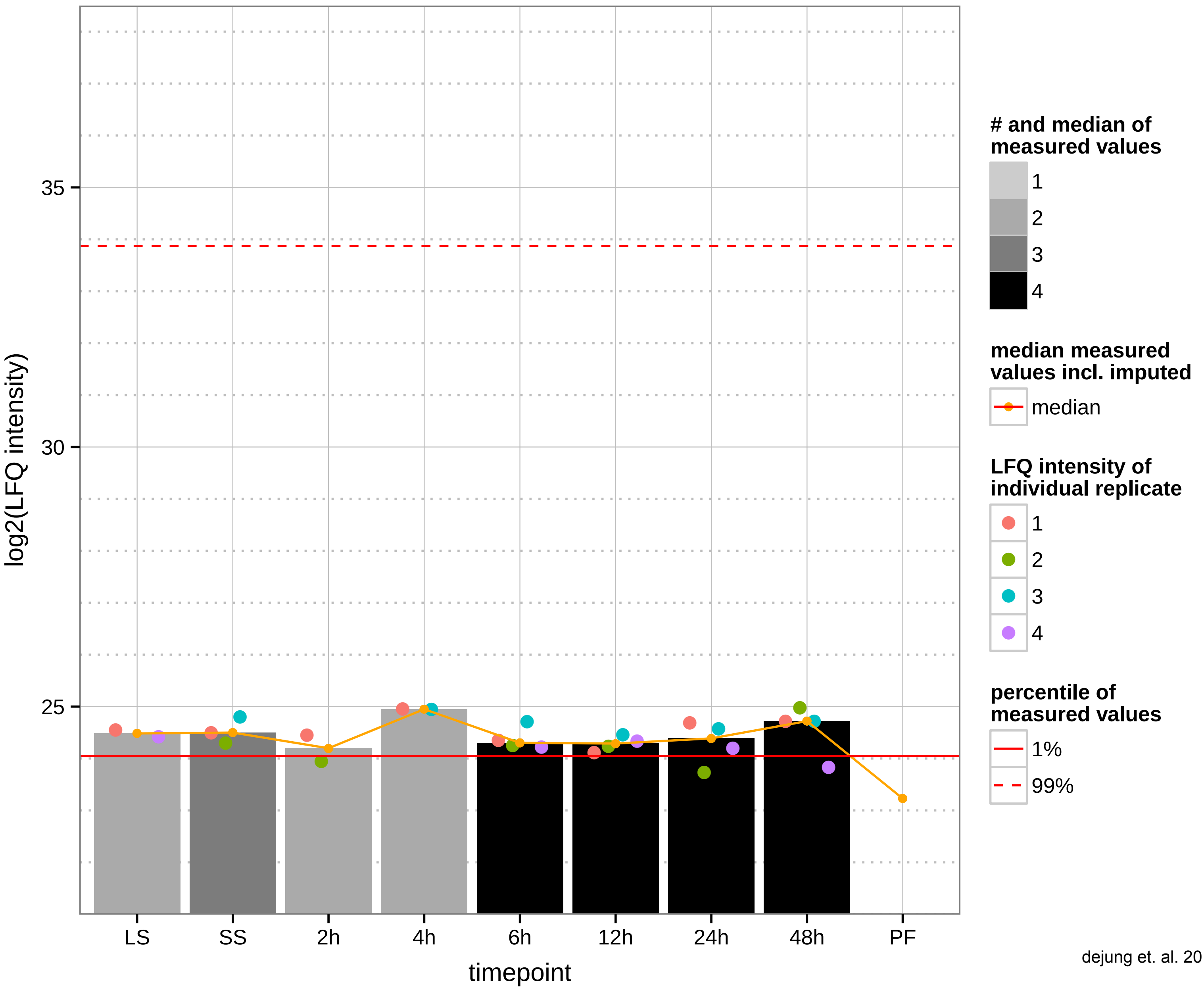

dejung et. al. 2015

ubiquitin carboxyl–terminal hydrolase, putative, cysteine peptidase, Clan CA, family C12
Tb927.11.250
AGOF: phospholipase C activity, ubiquitin thiolesterase activity
AGOC: cytoplasm, intracellular
AGOP: intracellular signal transduction, lipid metabolic process, ubiquitin–dependent protein catabolic process
PGOF: phospholipase C activity, ubiquitin thiolesterase activity
PGOC: intracellular
PGOP: intracellular signal transduction, lipid metabolic process, ubiquitin–dependent protein catabolic process

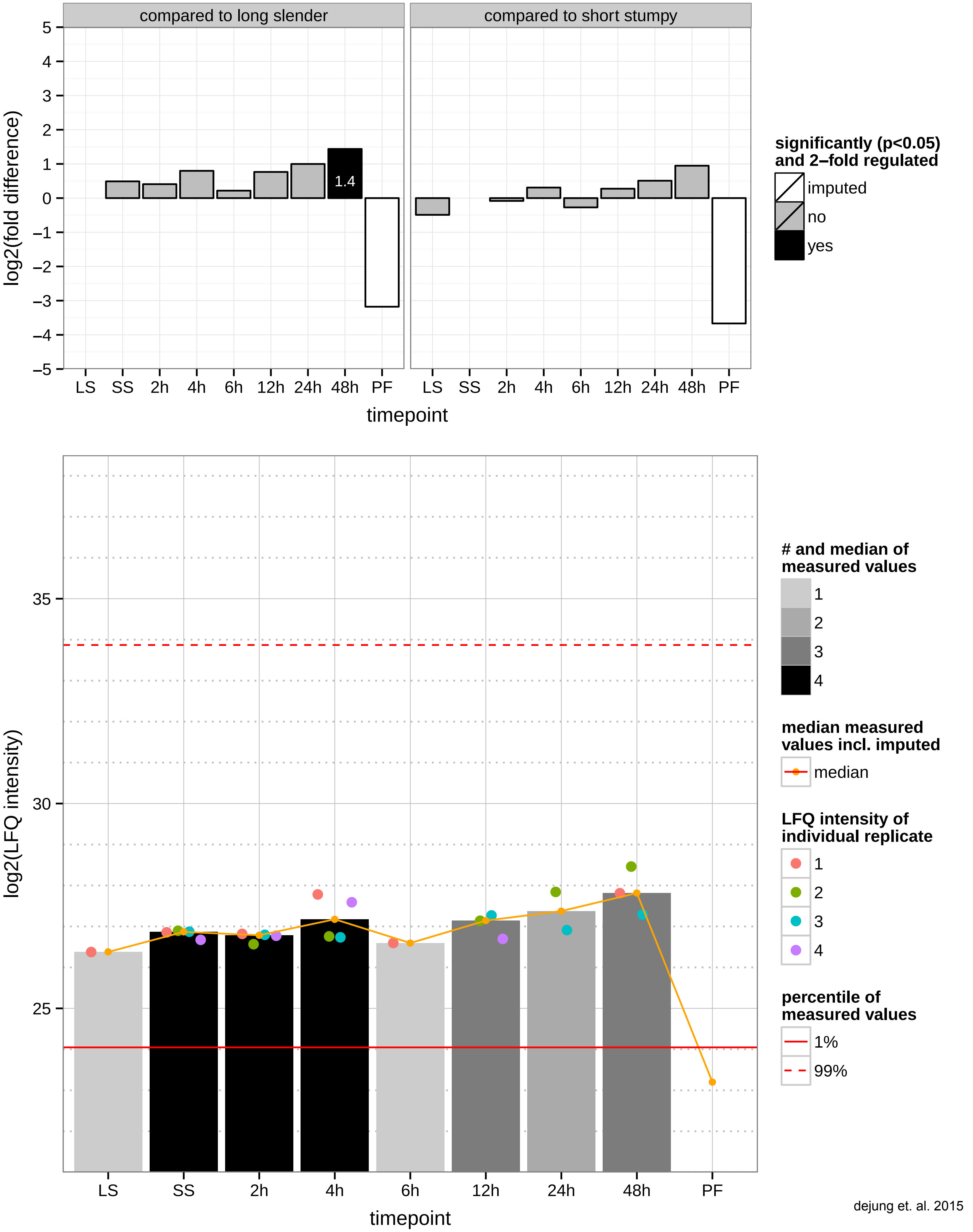

dejung et. al. 2015

succinyl–coA:3–ketoacid–coenzyme A transferase, mitochondrial precursor, putative
Tb927.11.2690
AGOF: CoA–transferase activity
AGOC: mitochondrion
AGOP: ketone body catabolic process, metabolic process
PGOF: CoA–transferase activity
PGOC: null
PGOP: ketone body catabolic process, metabolic process

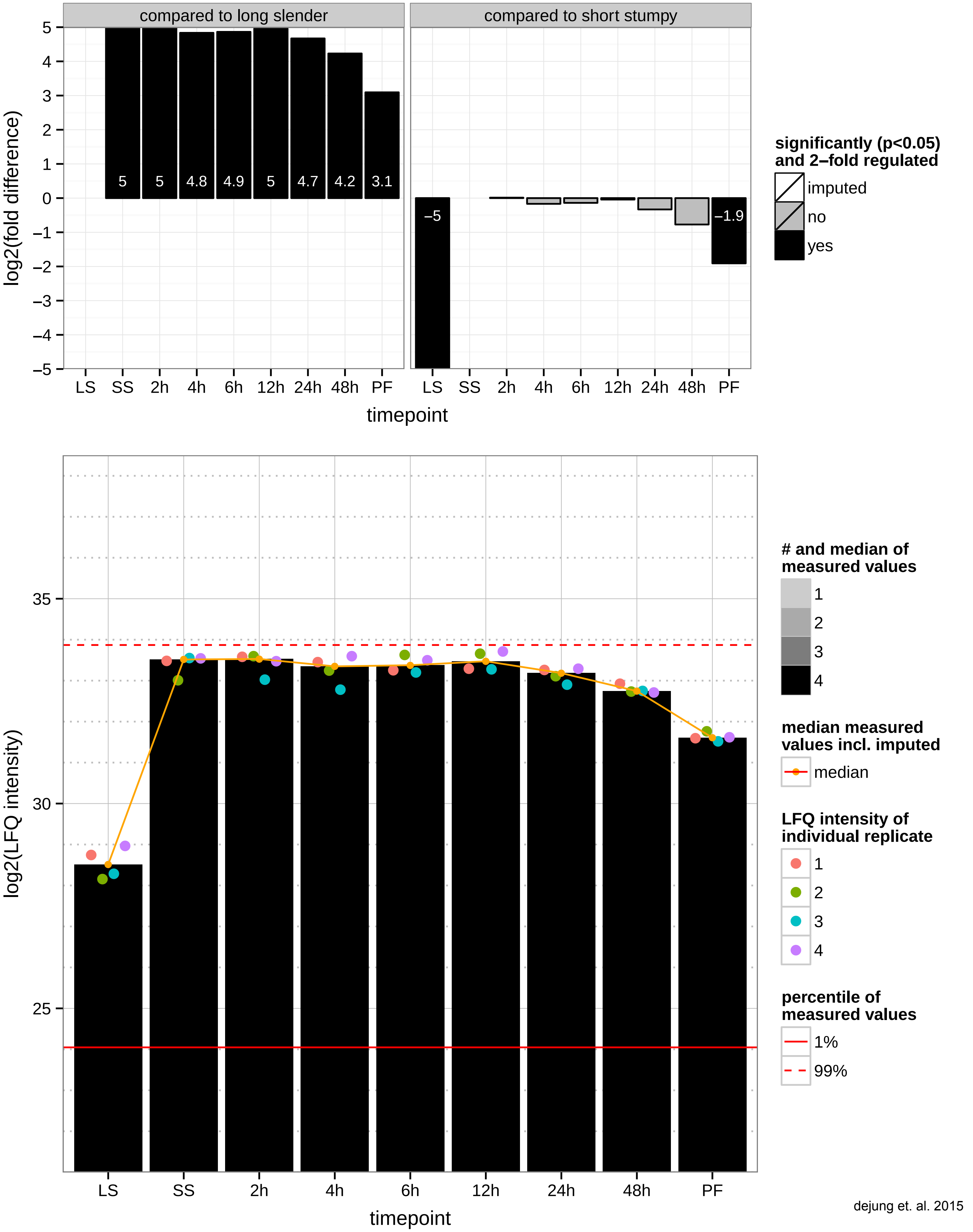

dejung et. al. 2015

hypothetical protein, conserved
Tb927.11.2750
AGOF: null
AGOC: null
AGOP: null
PGOF: null
PGOC: null
PGOP: null

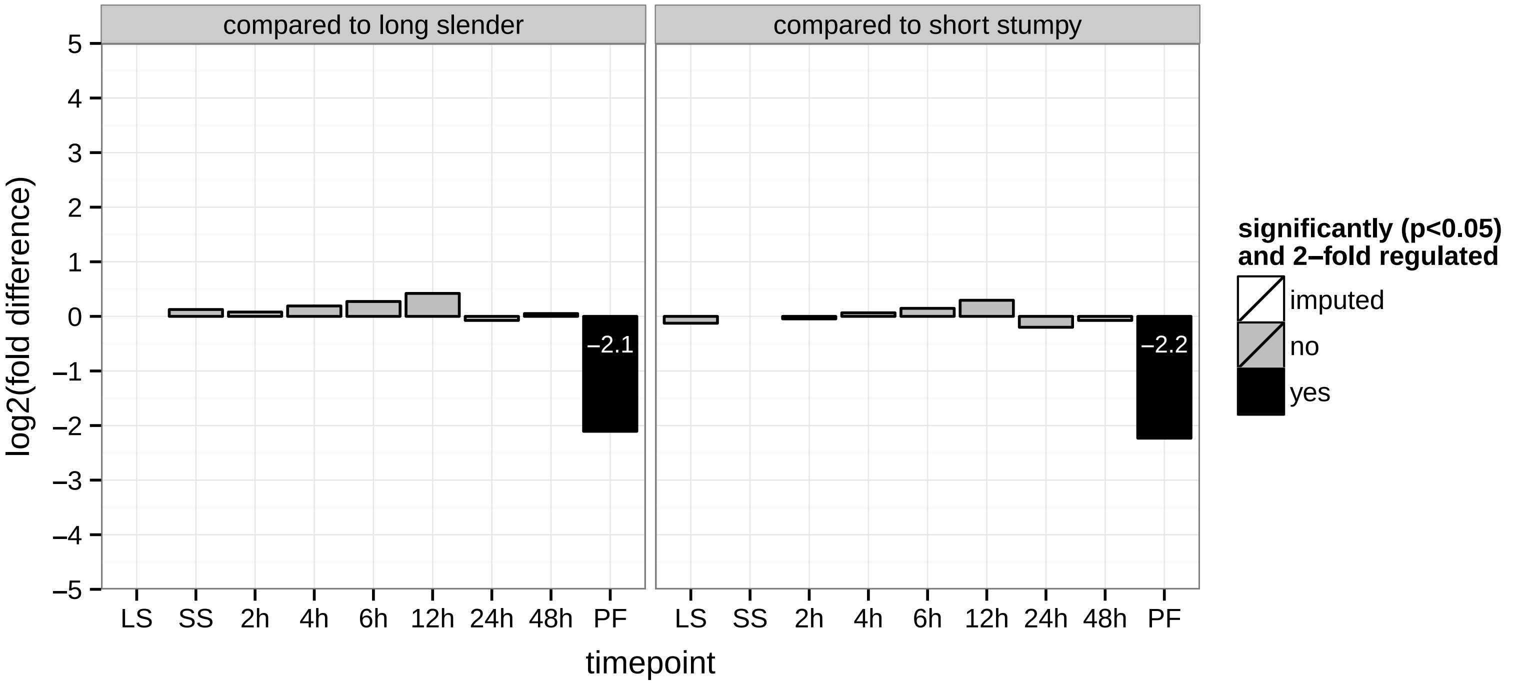

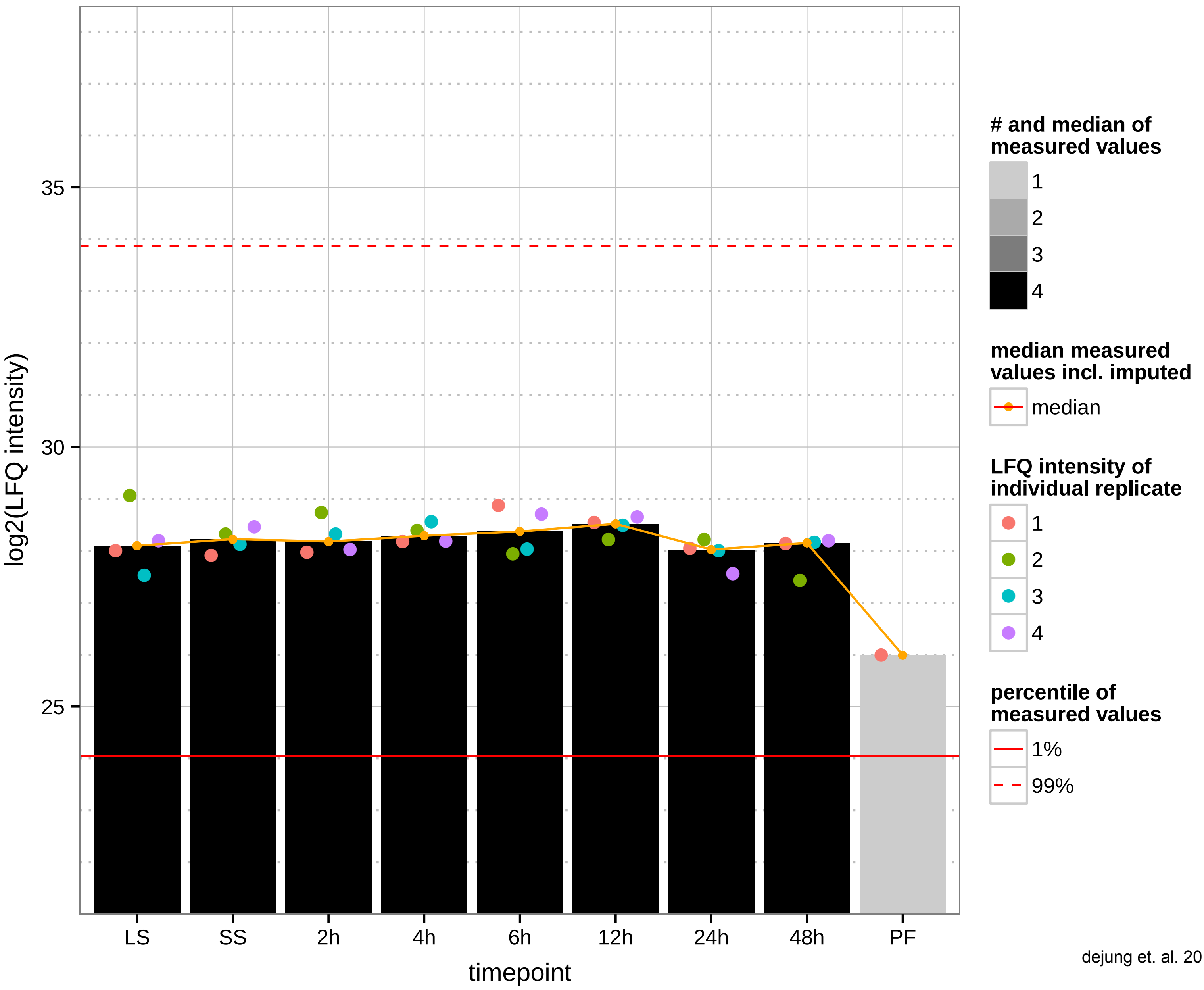

dejung et. al. 2015

kinesin, putative, MCAK–like kinesin, putative (Kif13–5)
Tb927.11.3280
AGOF: ATP binding, microtubule motor activity, motor activity
AGOC: cell, microtubule associated complex
AGOP: microtubule–based movement
PGOF: ATP binding, microtubule motor activity, protein binding
PGOC: null
PGOP: microtubule–based movement

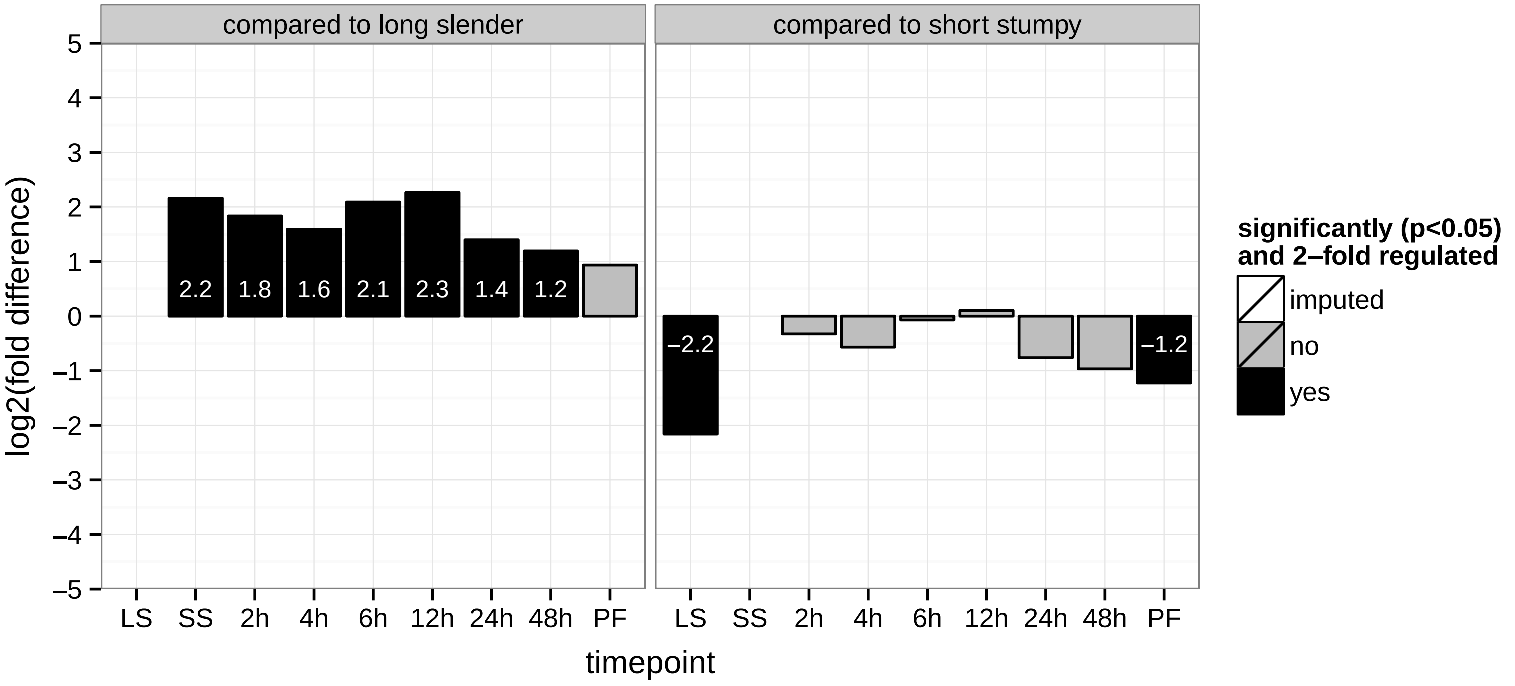

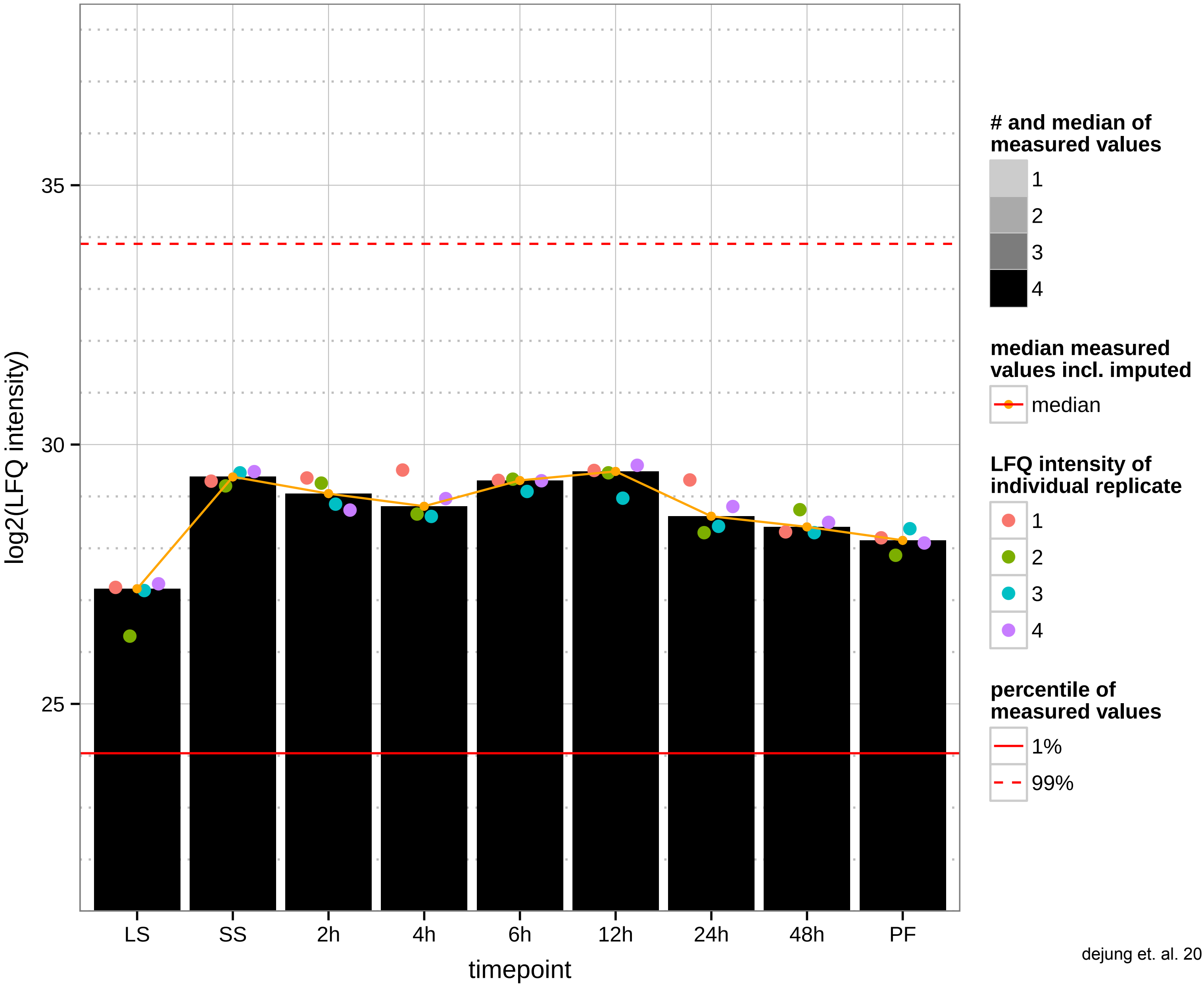

dejung et. al. 2015

hypothetical protein, conserved
Tb927.11.3690
AGOF: catalytic activity
AGOC: null
AGOP: null
PGOF: null
PGOC: null
PGOP: null

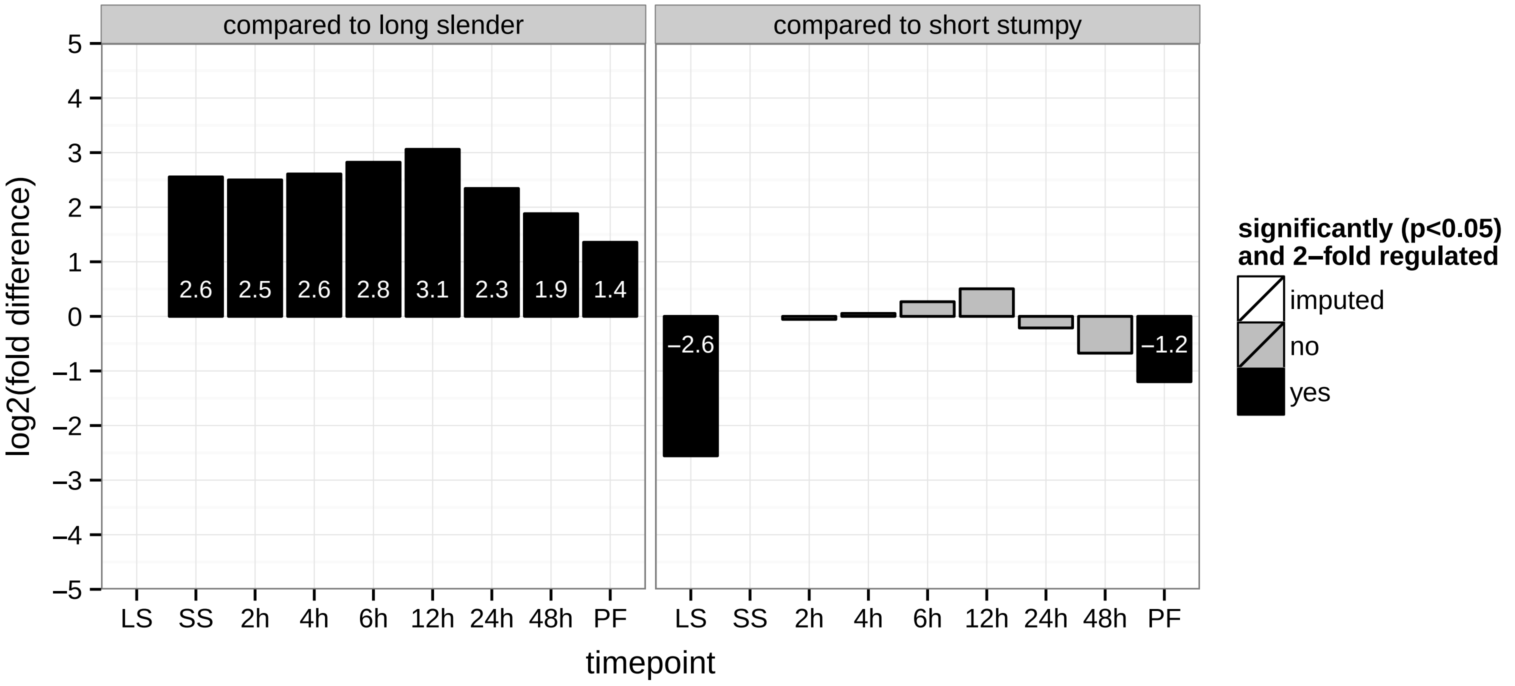

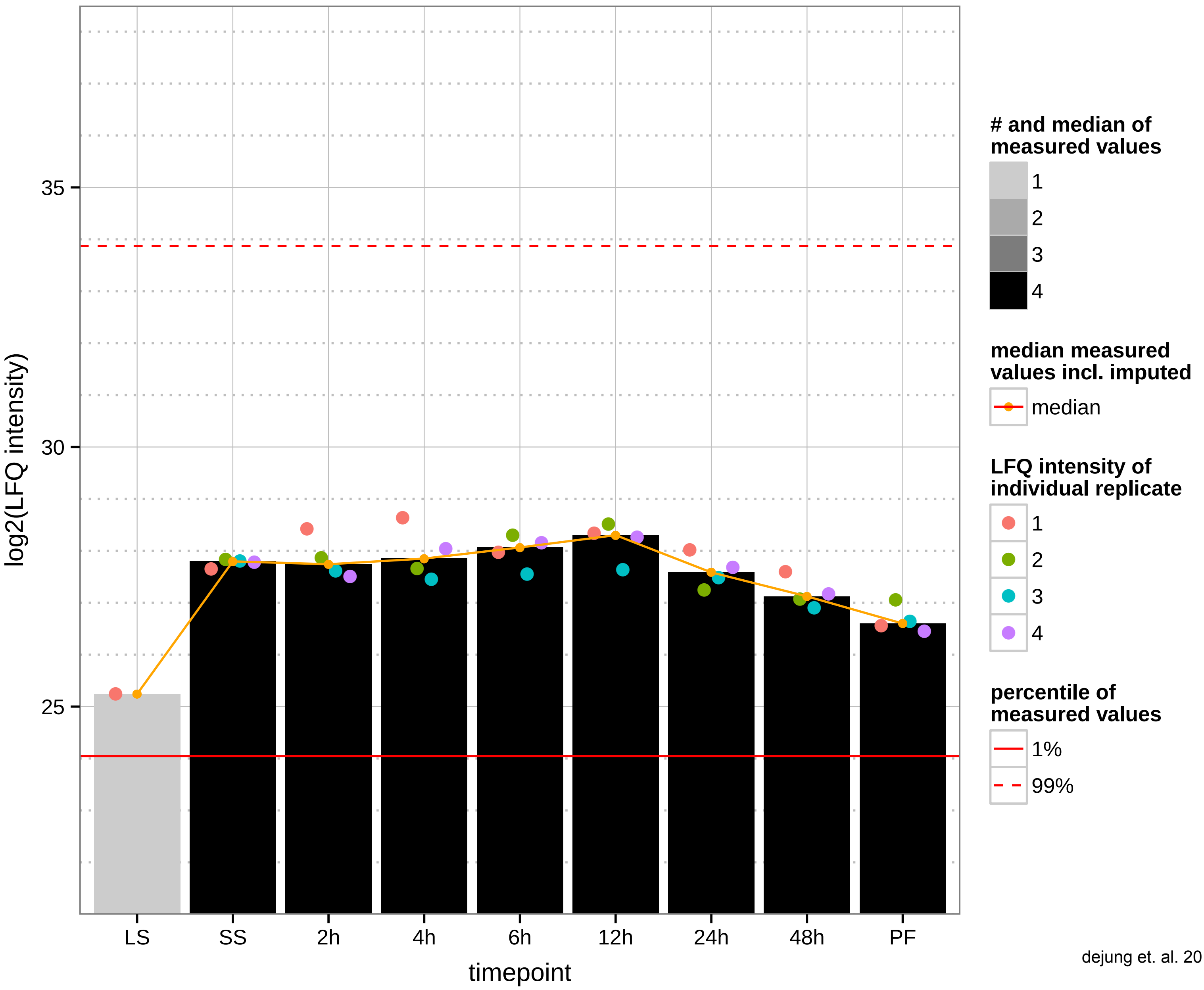

dejung et. al. 2015

hypothetical protein, conserved
Tb927.11.3840
AGOF: null
AGOC: null
AGOP: null
PGOF: null
PGOC: null
PGOP: null

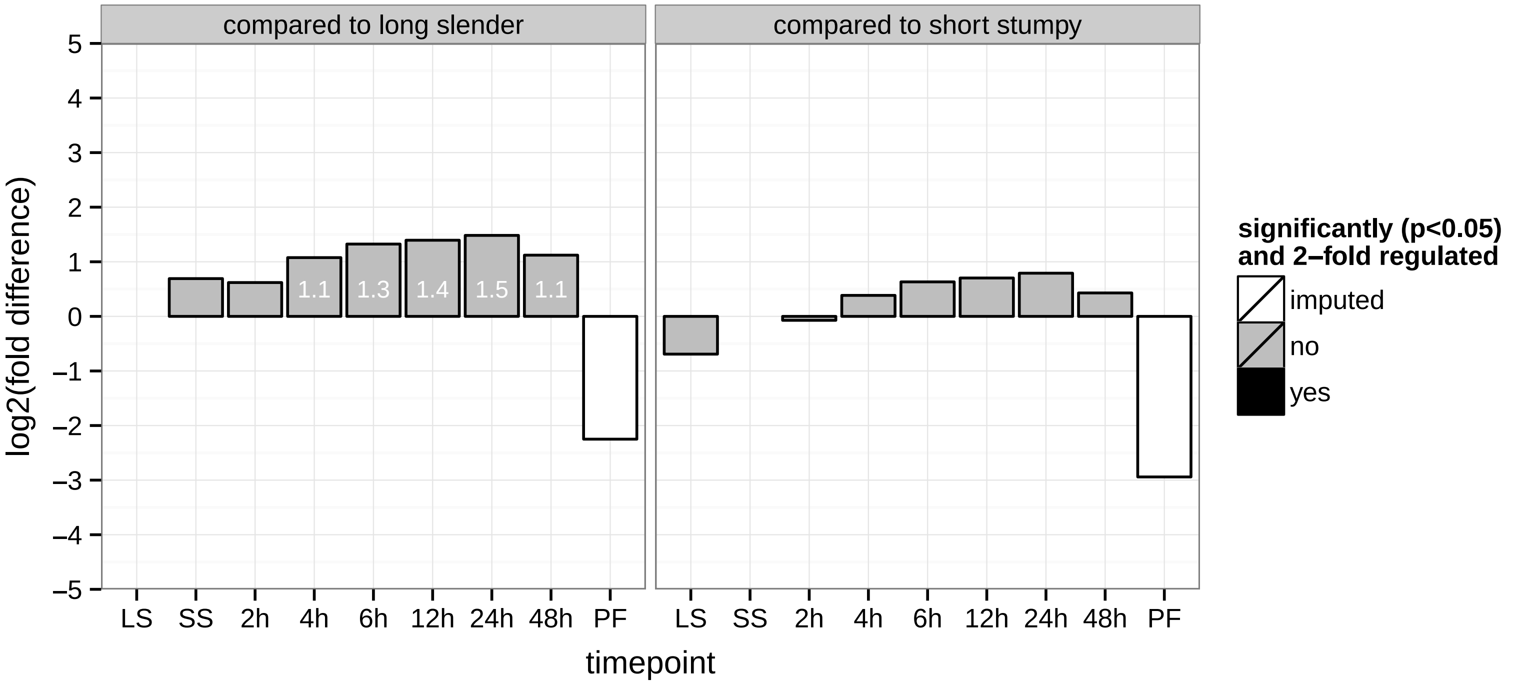

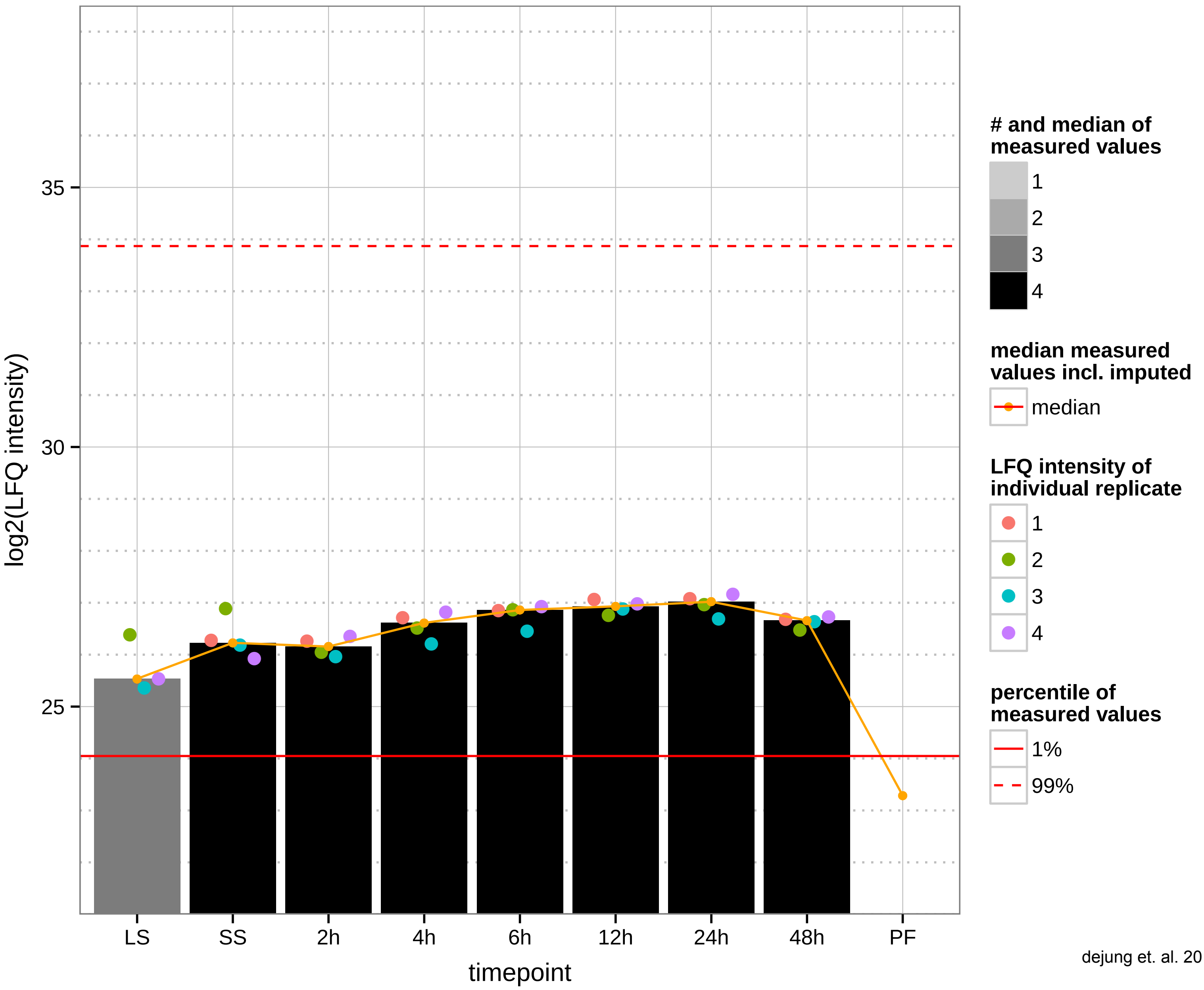

dejung et. al. 2015

hypothetical protein, conserved
Tb927.11.3920
AGOF: null
AGOC: null
AGOP: null
PGOF: null
PGOC: null
PGOP: null

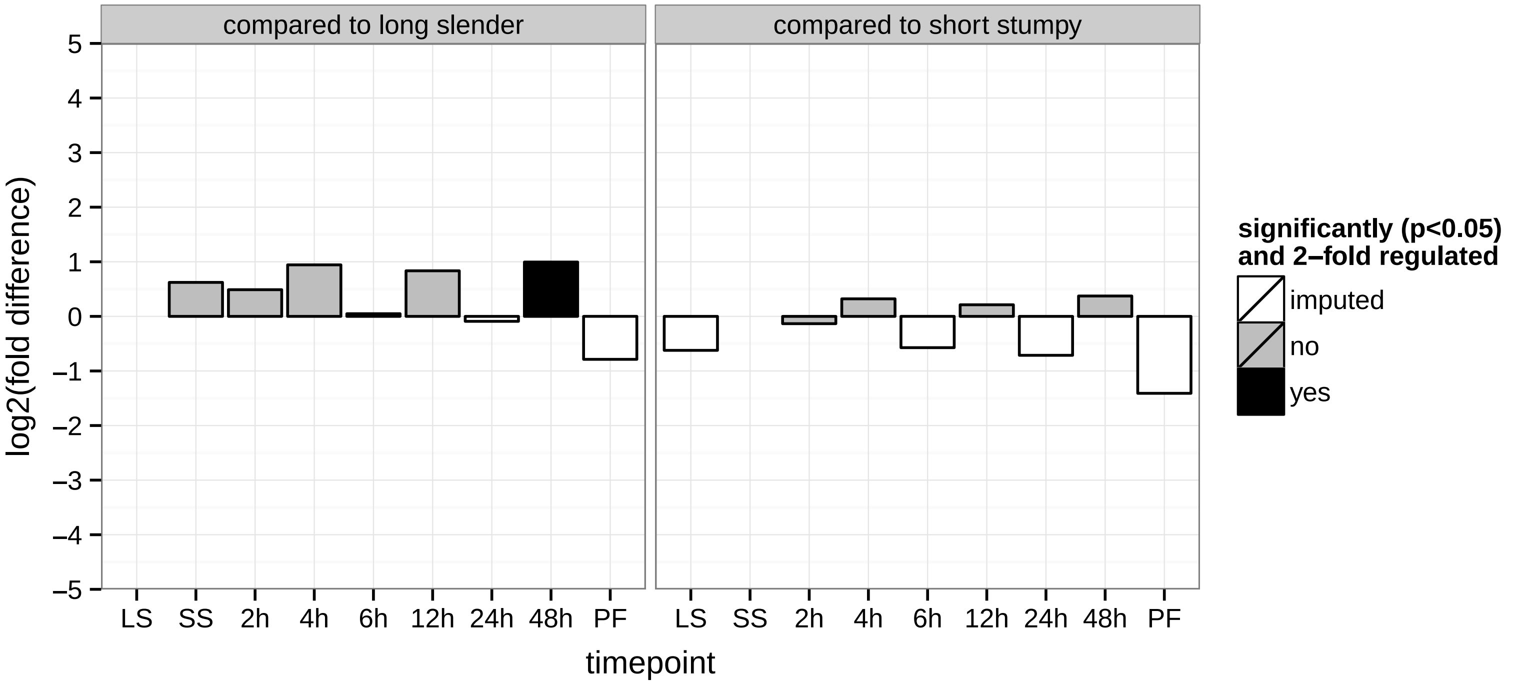

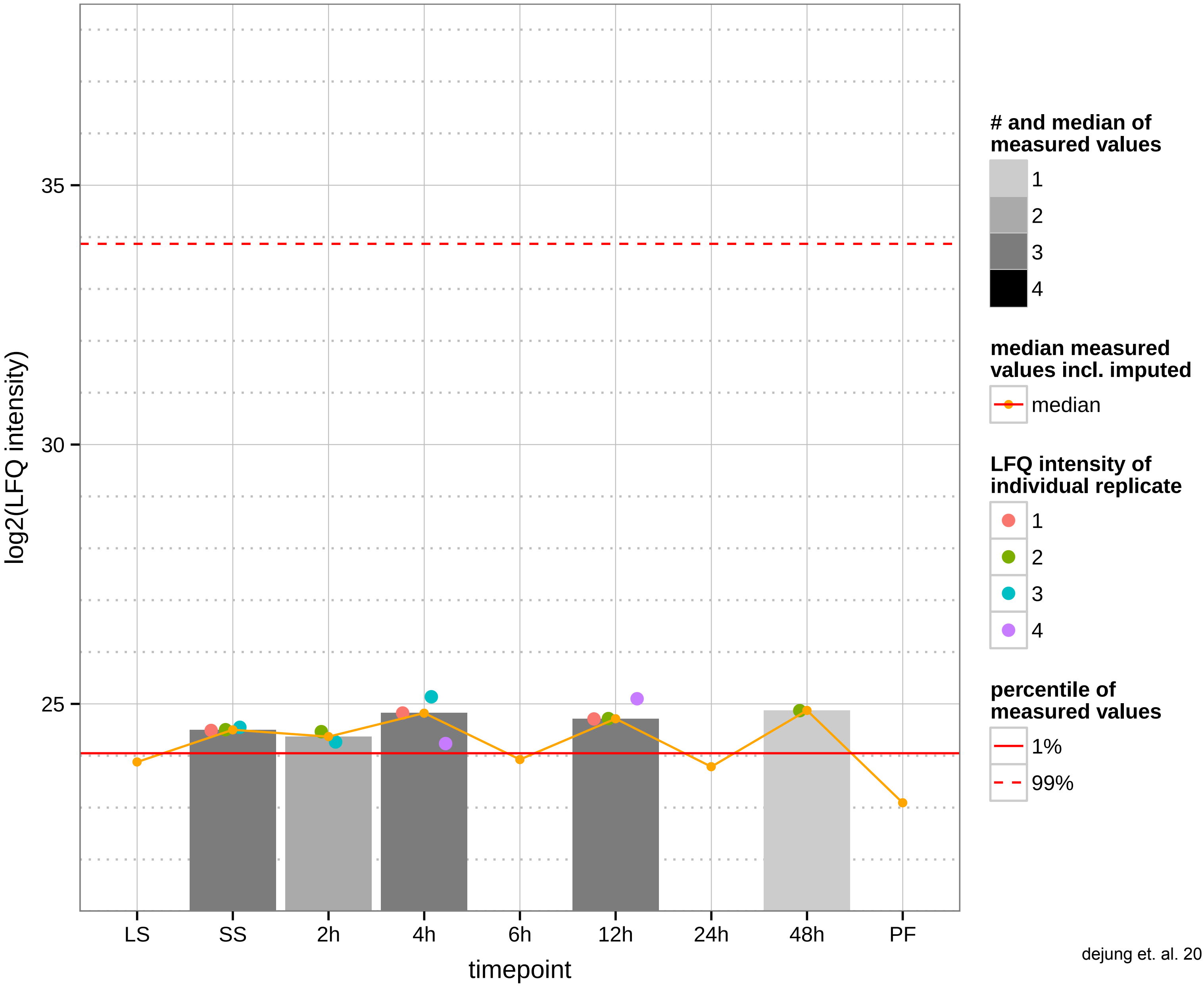

dejung et. al. 2015

hypothetical protein, conserved
Tb927.11.4030
AGOF: null
AGOC: null
AGOP: null
PGOF: null
PGOC: null
PGOP: null

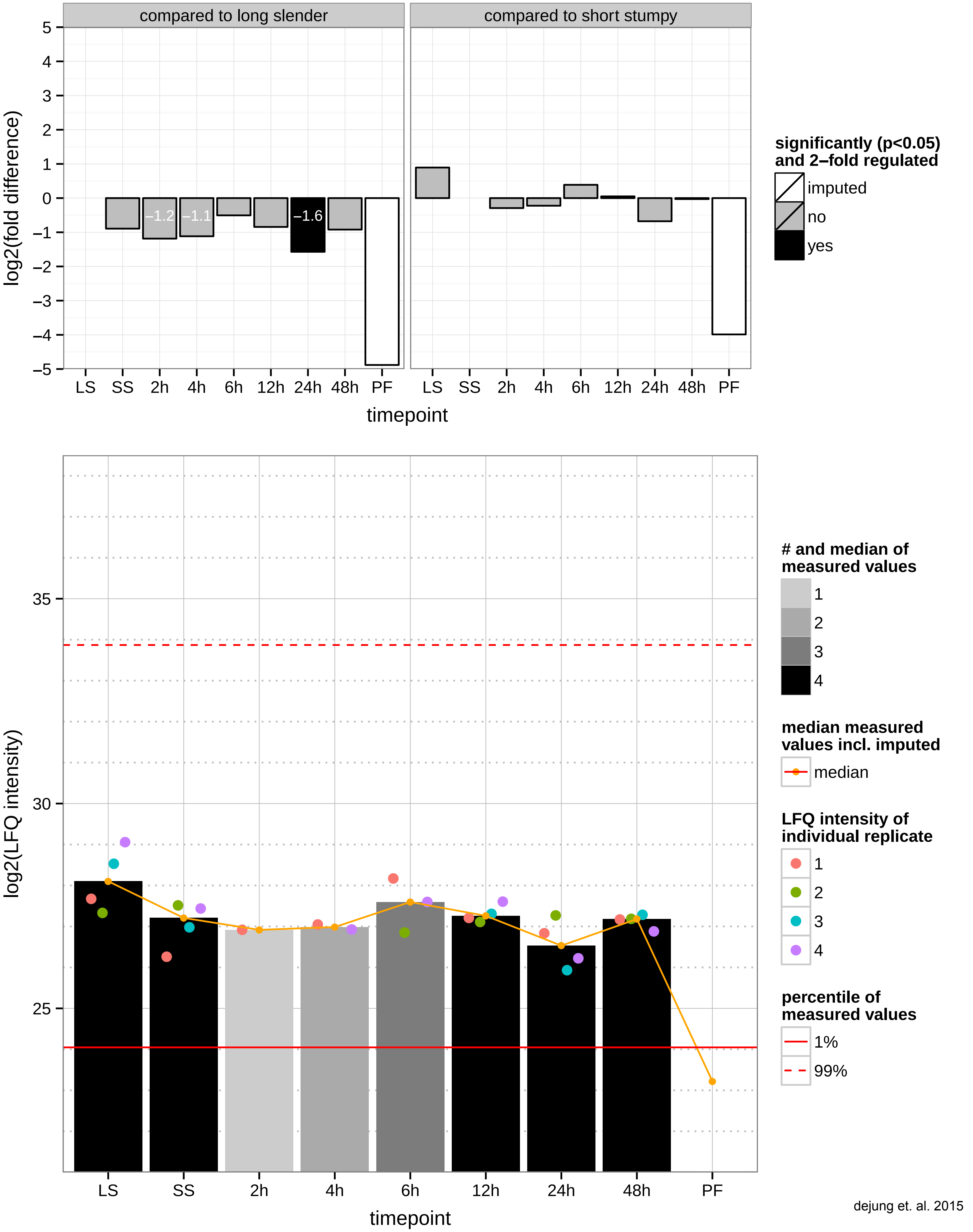

dejung et. al. 2015

hypothetical protein, conserved
Tb927.11.5150
AGOF: null
AGOC: null
AGOP: null
PGOF: null
PGOC: null
PGOP: null

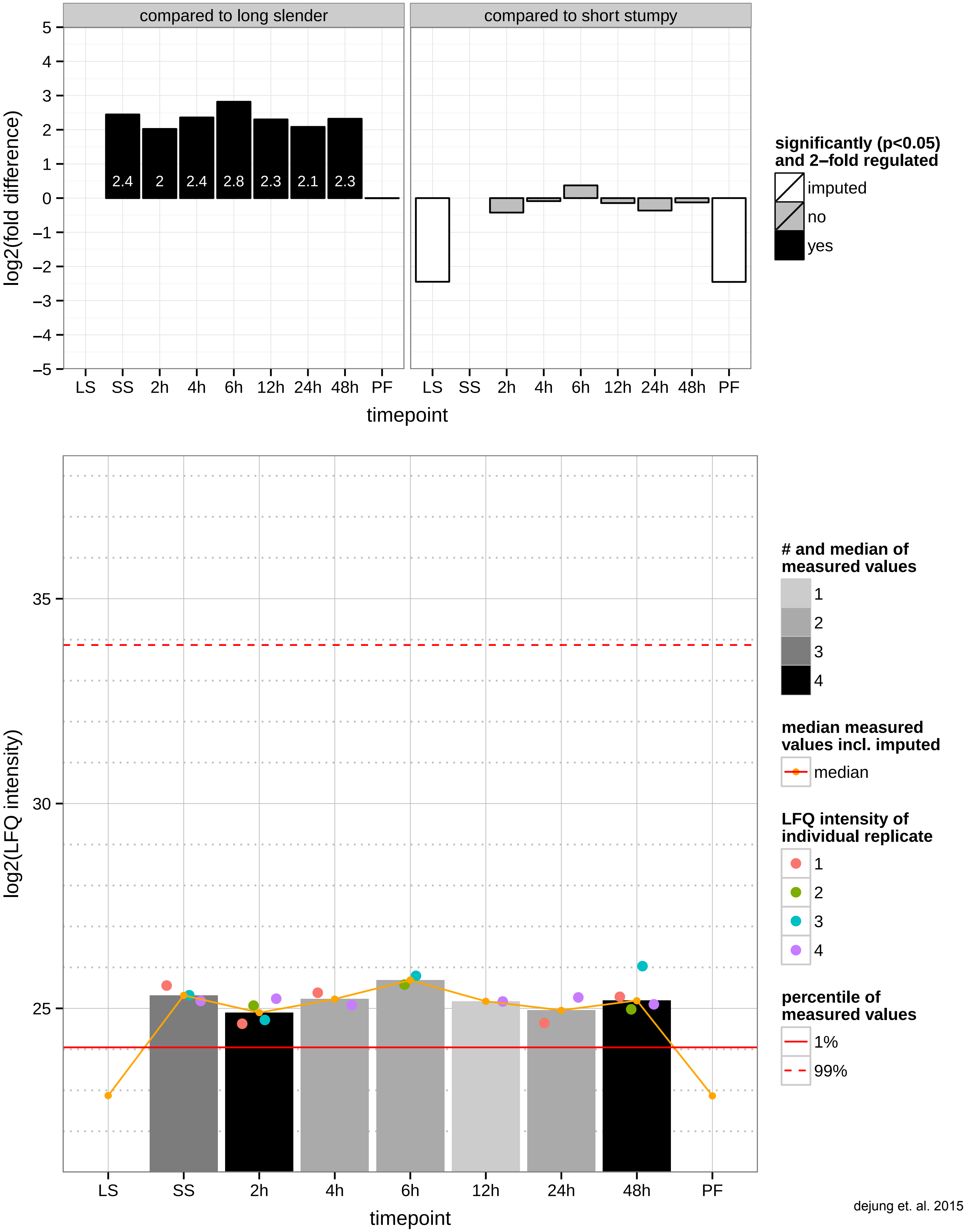

dejung et. al. 2015

pretranslocation protein, alpha subunit, putative, SEC61–like (pretranslocation process) protein
Tb927.11.6230
AGOF: P–P–bond–hydrolysis–driven protein transmembrane transporter activity
AGOC: endoplasmic reticulum, integral to membrane, membrane
AGOP: protein secretion, protein transport
PGOF: P–P–bond–hydrolysis–driven protein transmembrane transporter activity
PGOC: membrane
PGOP: protein transport

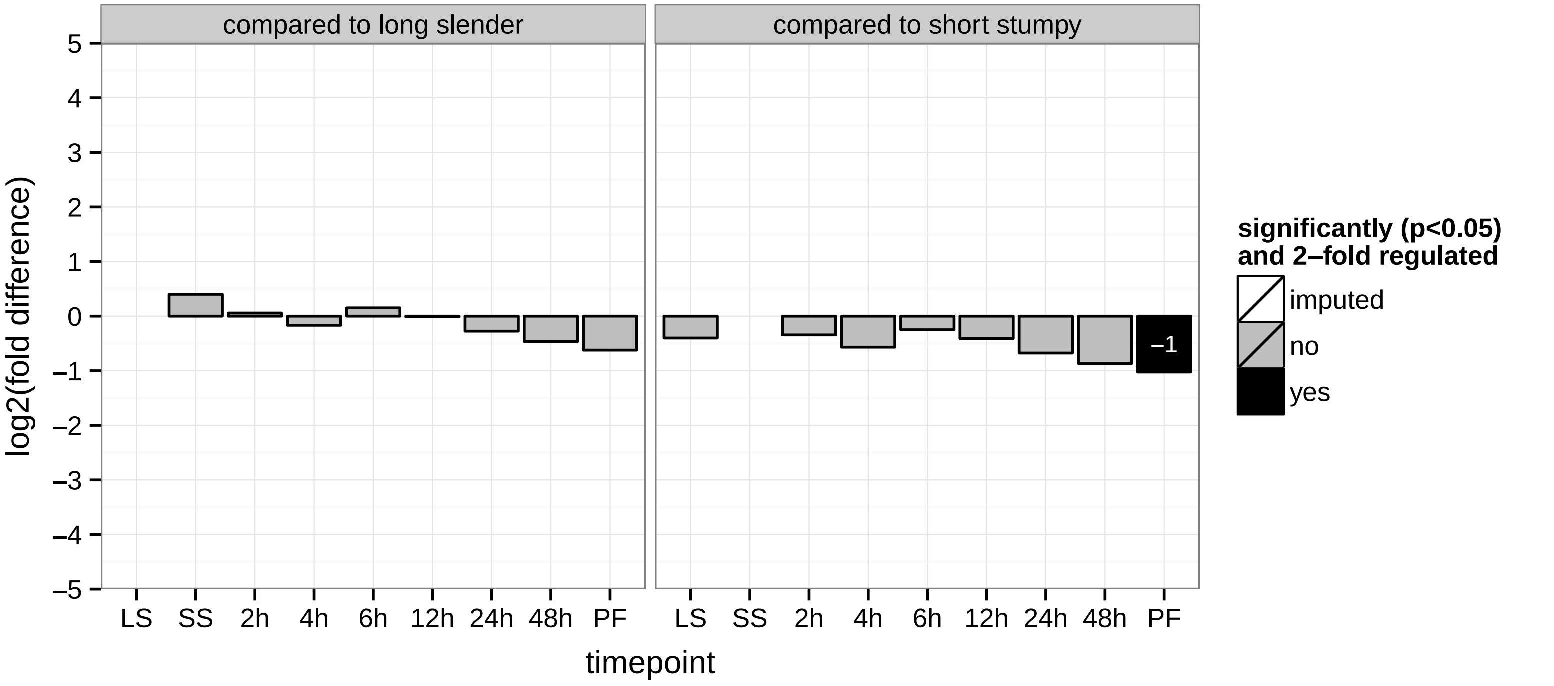

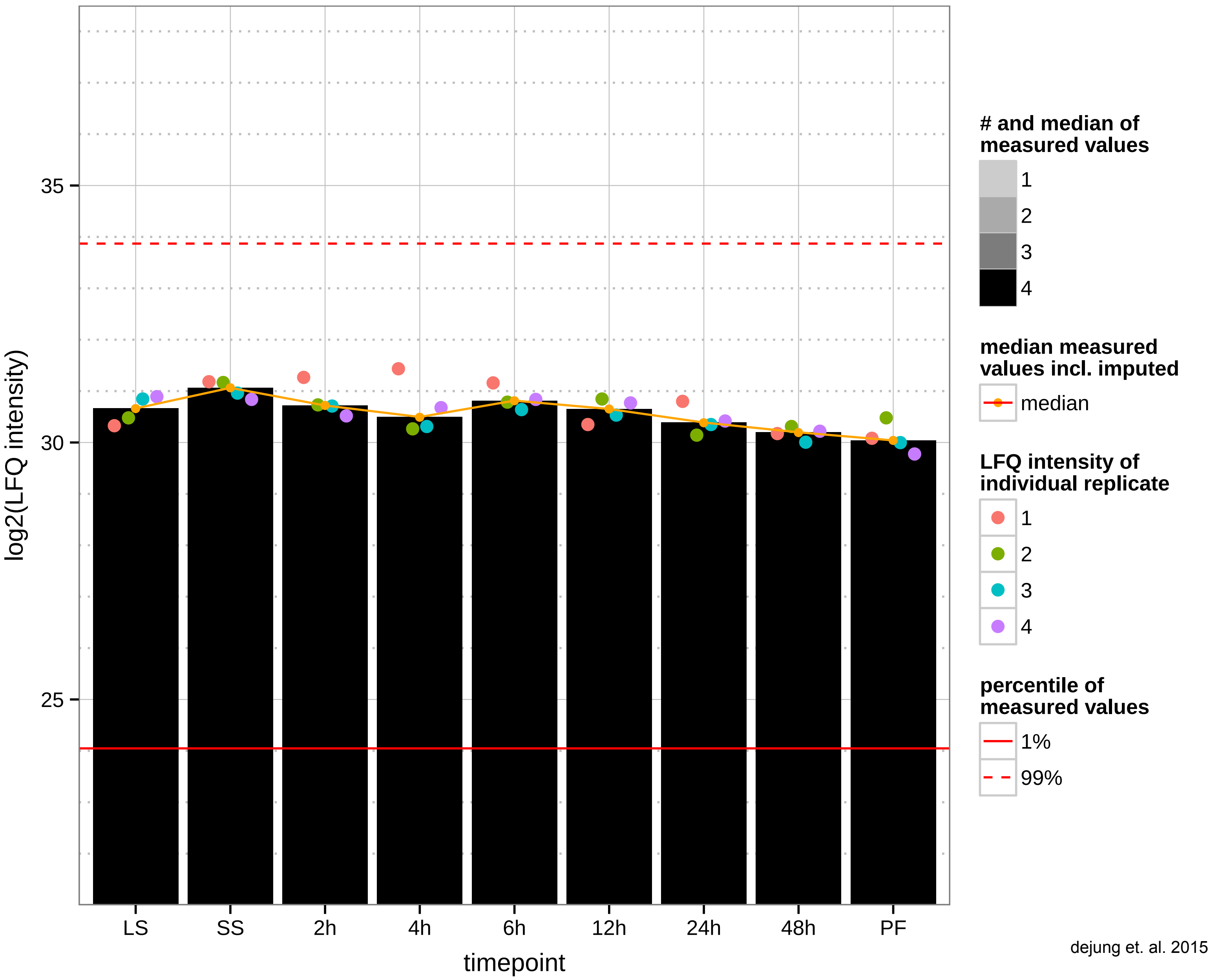

dejung et. al. 2015

hypothetical protein, conserved
Tb927.11.6520
AGOF: null
AGOC: null
AGOP: null
PGOF: null
PGOC: null
PGOP: null

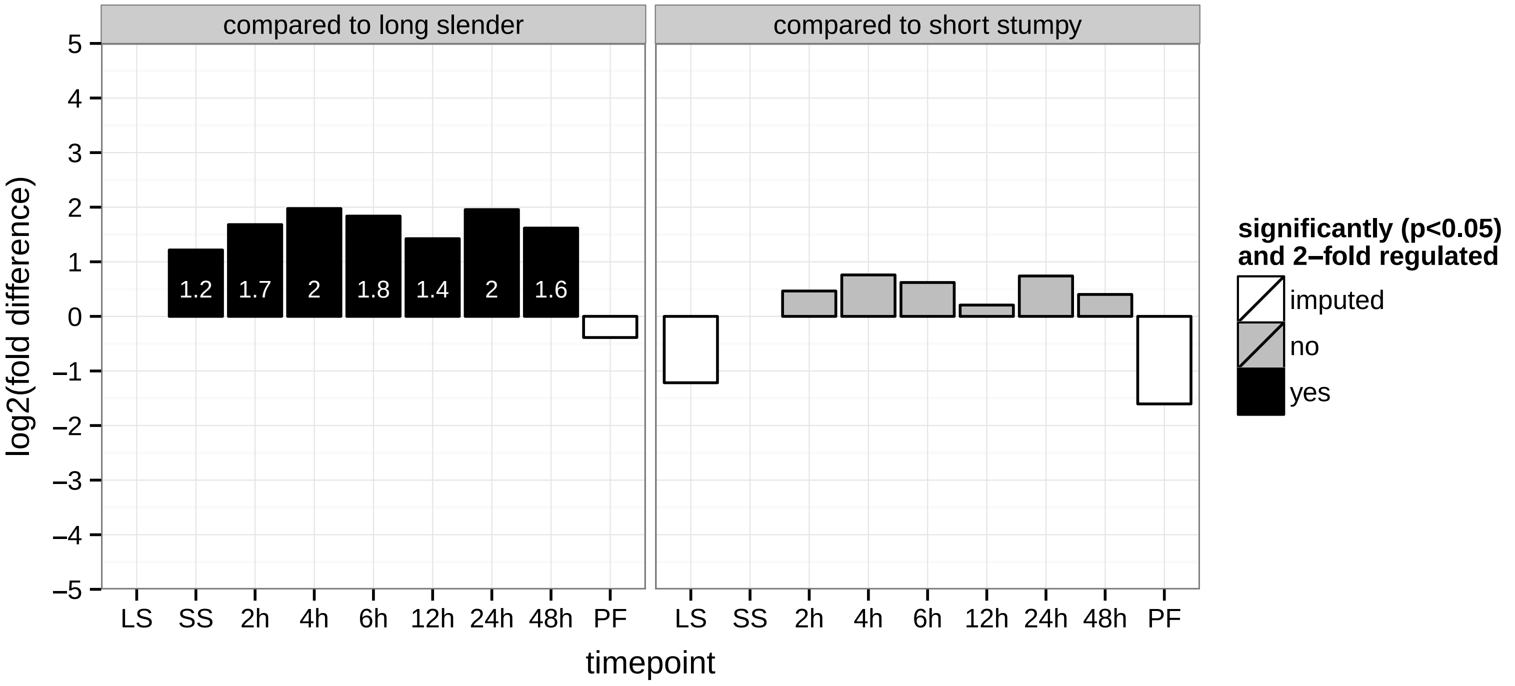

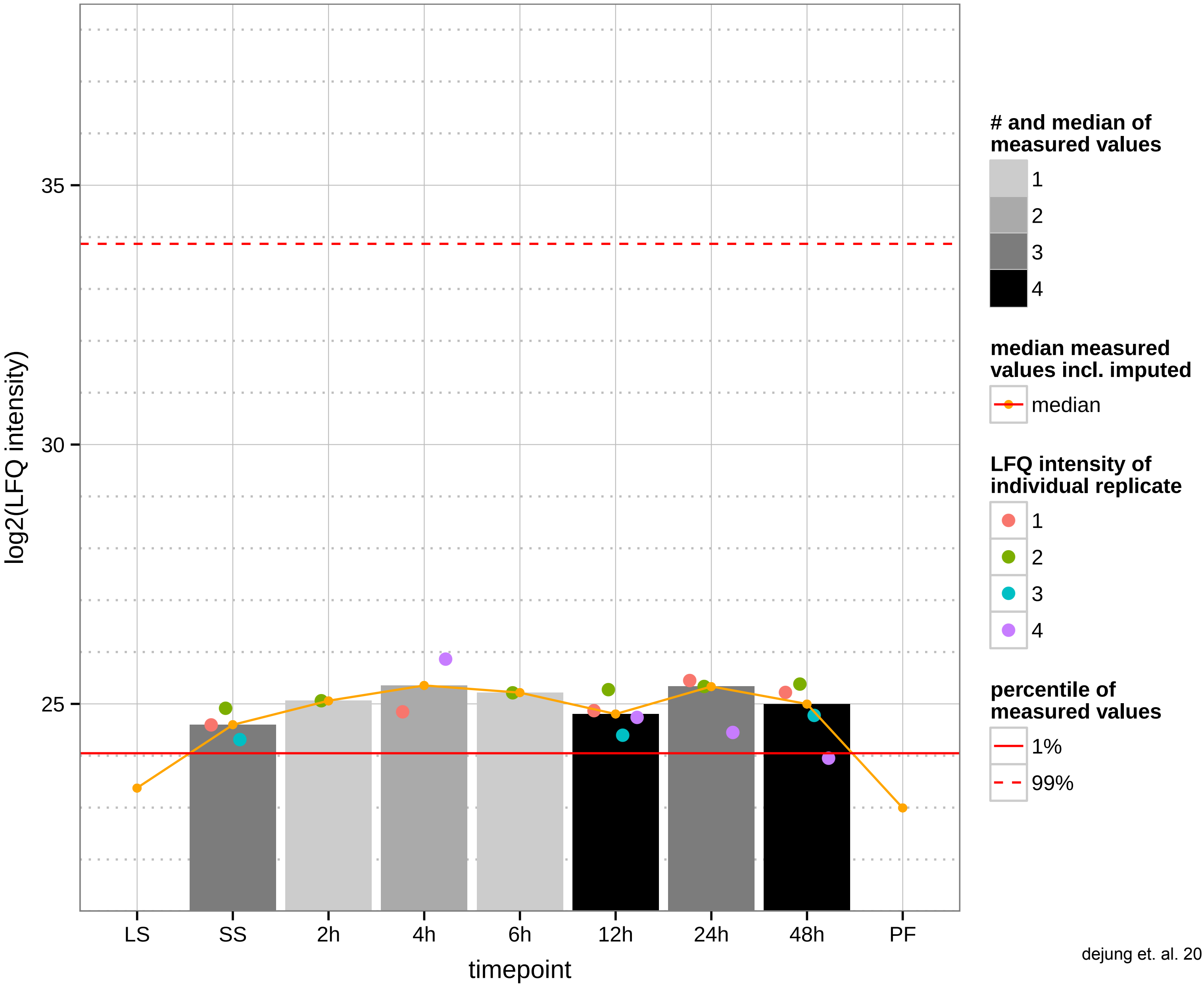

dejung et. al. 2015

hypothetical protein, conserved
Tb927.11.6920
AGOF: null
AGOC: null
AGOP: null
PGOF: null
PGOC: null
PGOP: null

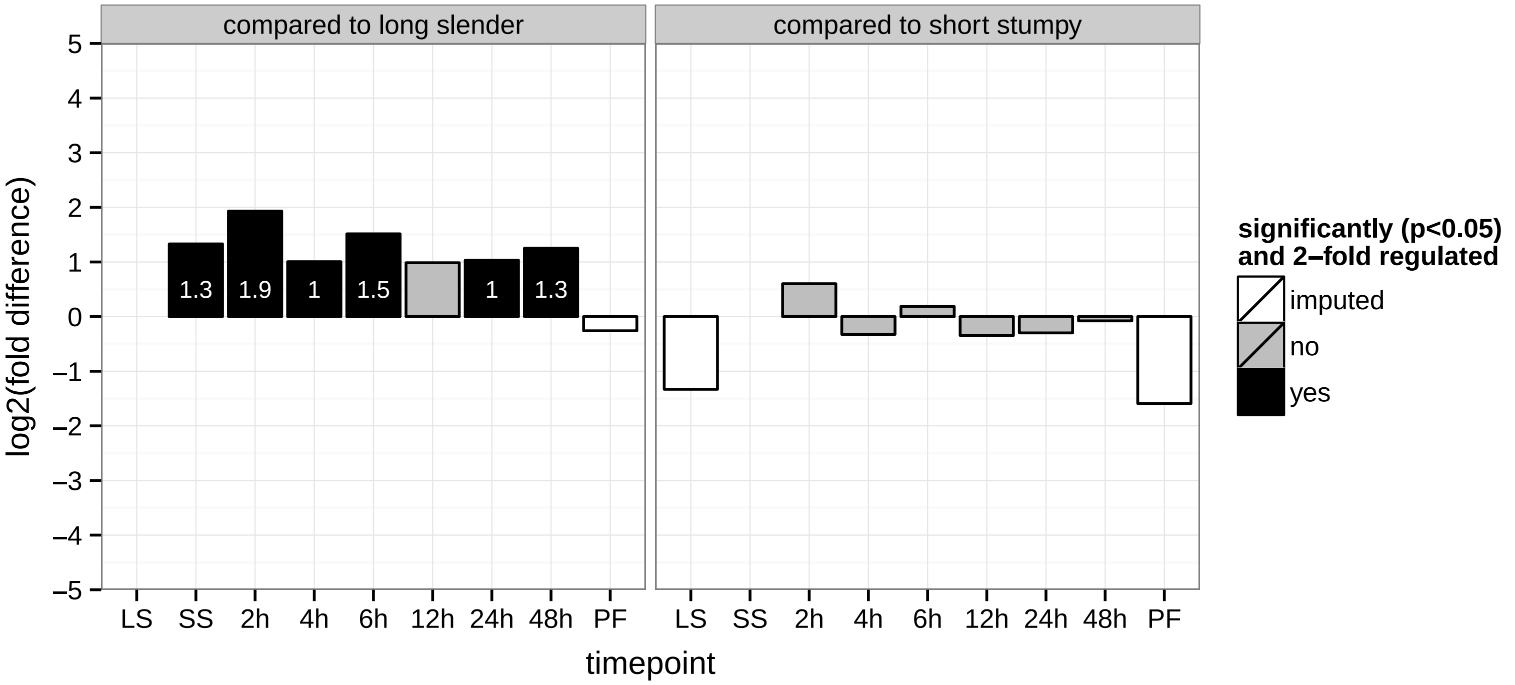

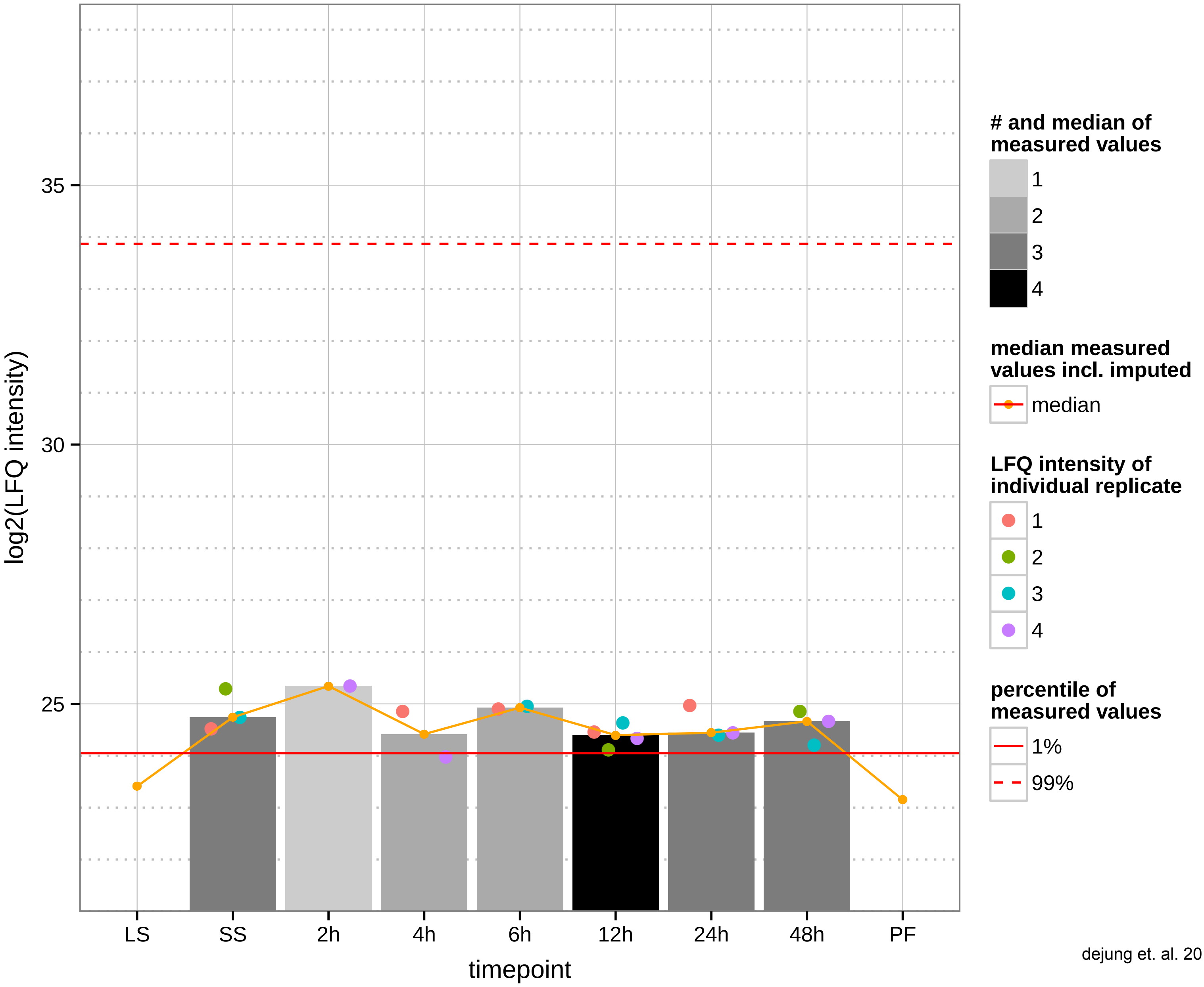

dejung et. al. 2015

pterin–4–alpha–carbinolamine dehydratase, putative
Tb927.11.7040
AGOF: 4–alpha–hydroxytetrahydrobiopterin dehydratase activity
AGOC: mitochondrion
AGOP: tetrahydrobiopterin biosynthetic process
PGOF: 4–alpha–hydroxytetrahydrobiopterin dehydratase activity
PGOC: null
PGOP: tetrahydrobiopterin biosynthetic process

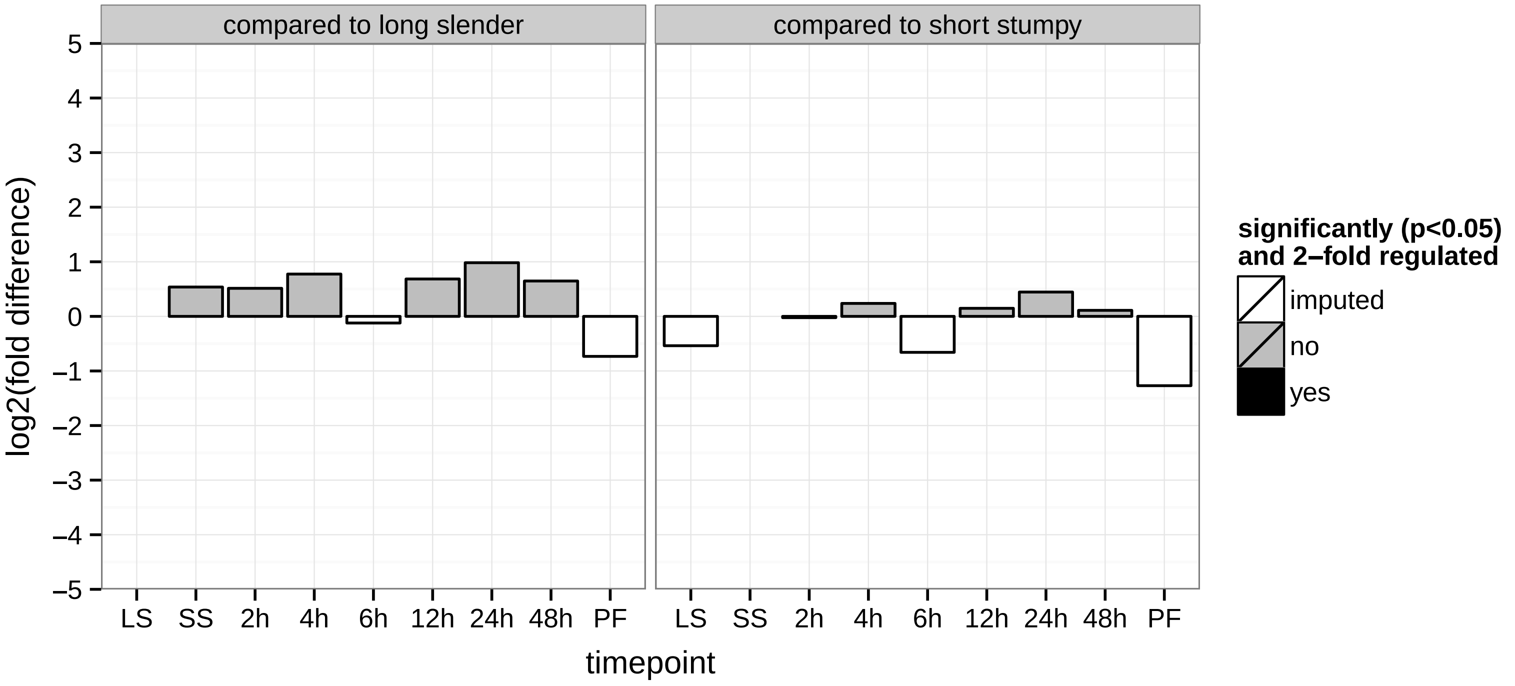

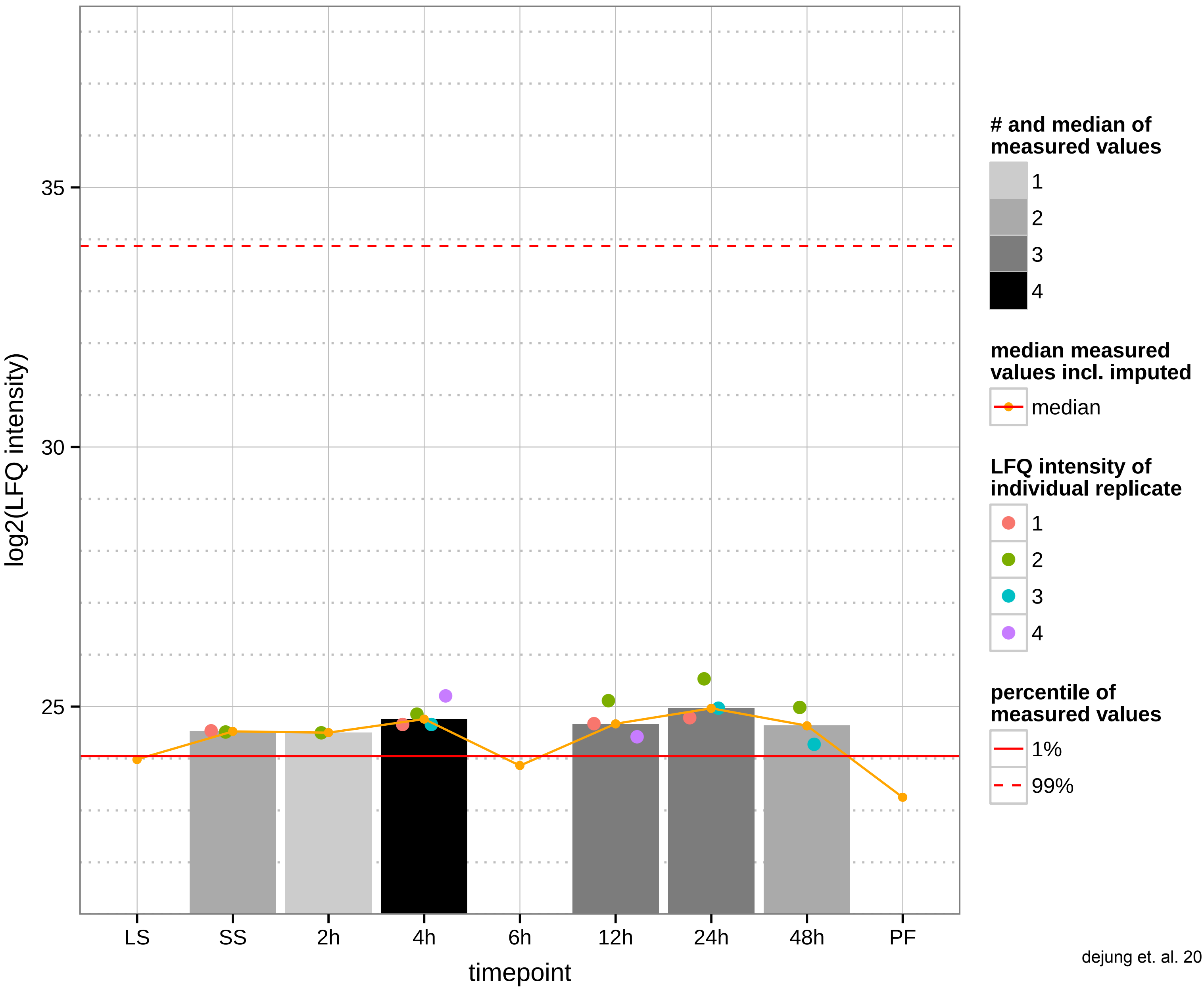

dejung et. al. 2015

glycerol–3–phosphate dehydrogenase (FAD–dependent), mitochondrial
Tb927.11.7380
AGOF: flavin adenine dinucleotide binding, glycerol–3–phosphate dehydrogenase activity
AGOC: glycerol–3–phosphate dehydrogenase complex, mitochondrion
AGOP: glycerol catabolic process, glycerol–3–phosphate metabolic process
PGOF: oxidoreductase activity
PGOC: null
PGOP: oxidation–reduction process

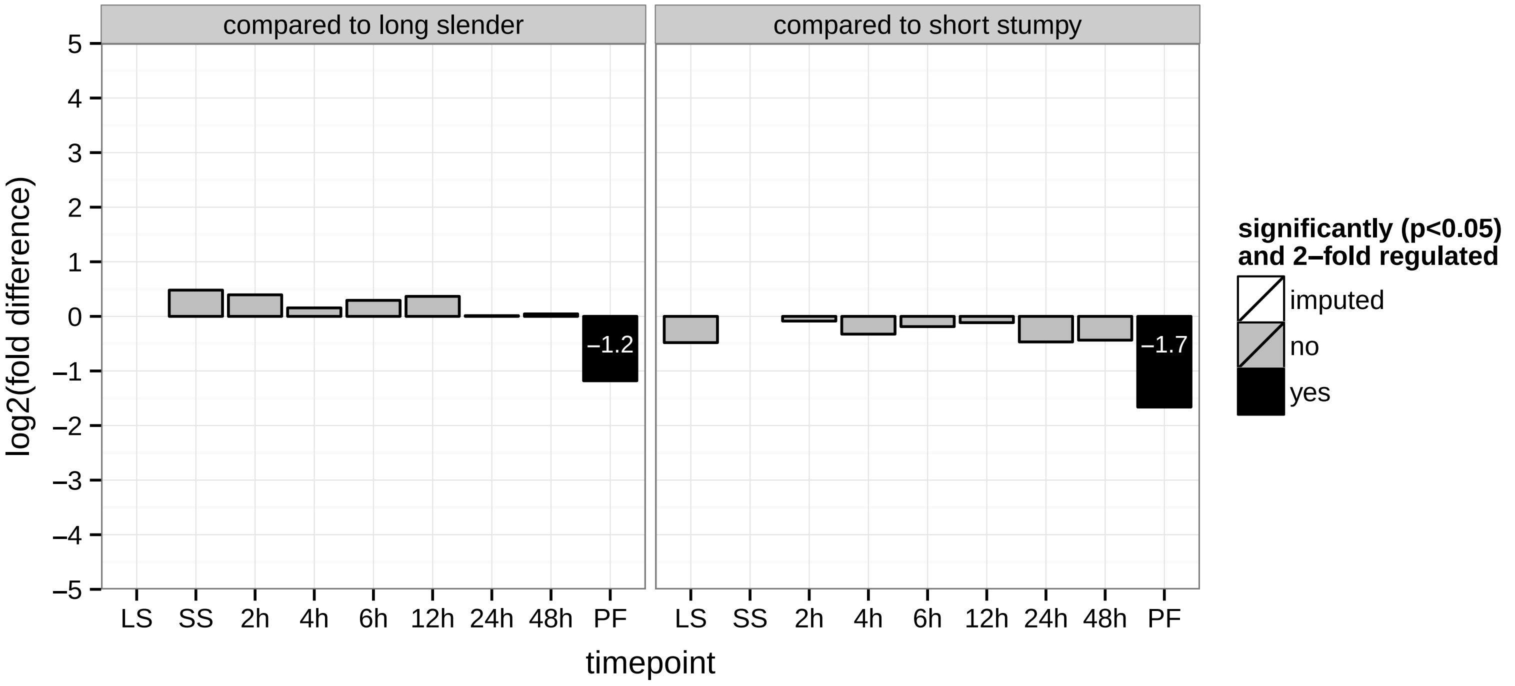

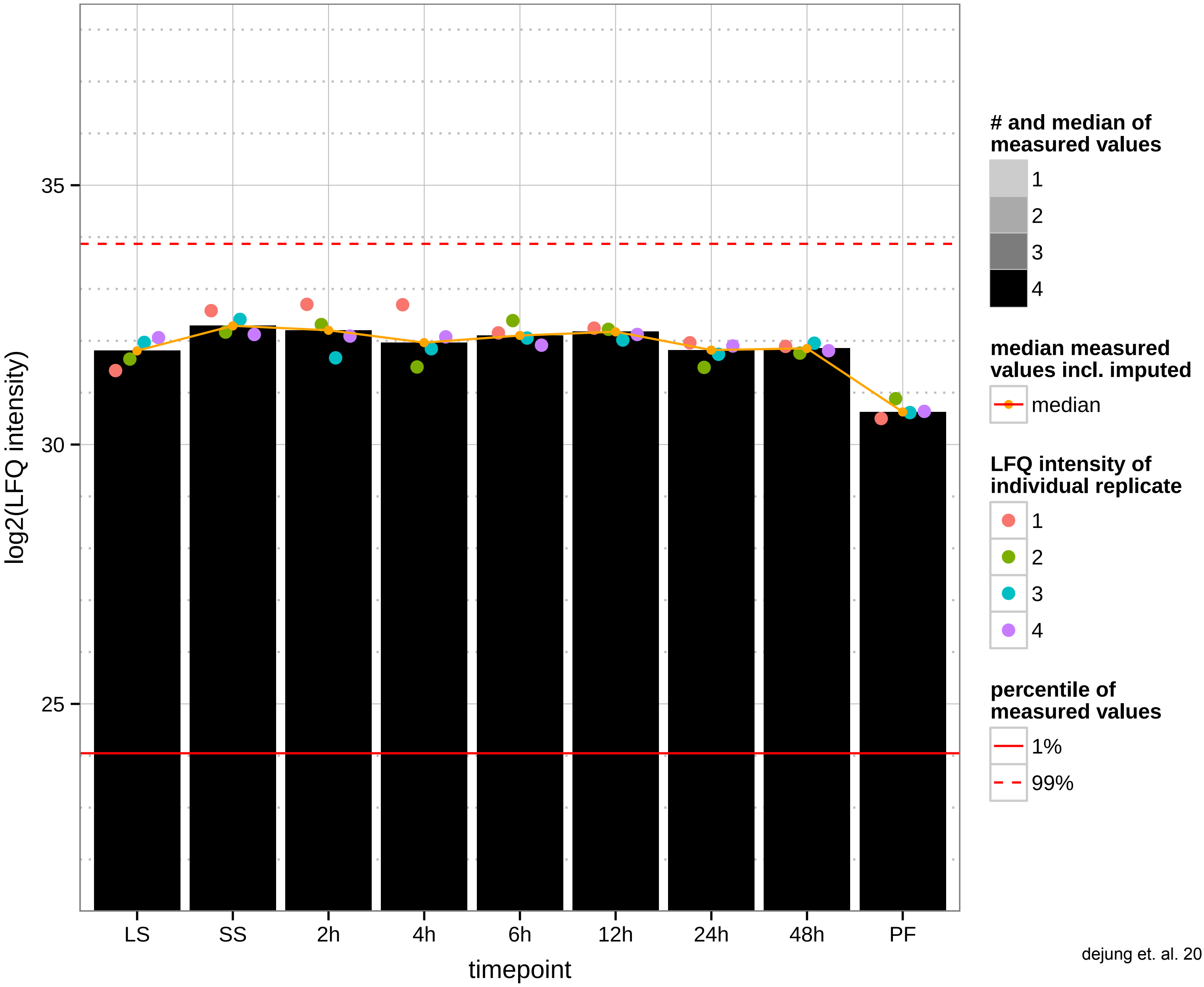

dejung et. al. 2015

NADPH--cytochrome p450 reductase, putative (CPR)
Tb927.11.8380
AGOF: FMN binding, electron carrier activity, iron ion binding, oxidoreductase activity
AGOC: integral to membrane
AGOP: oxidation–reduction process
PGOF: FMN binding, oxidoreductase activity
PGOC: null
PGOP: oxidation–reduction process

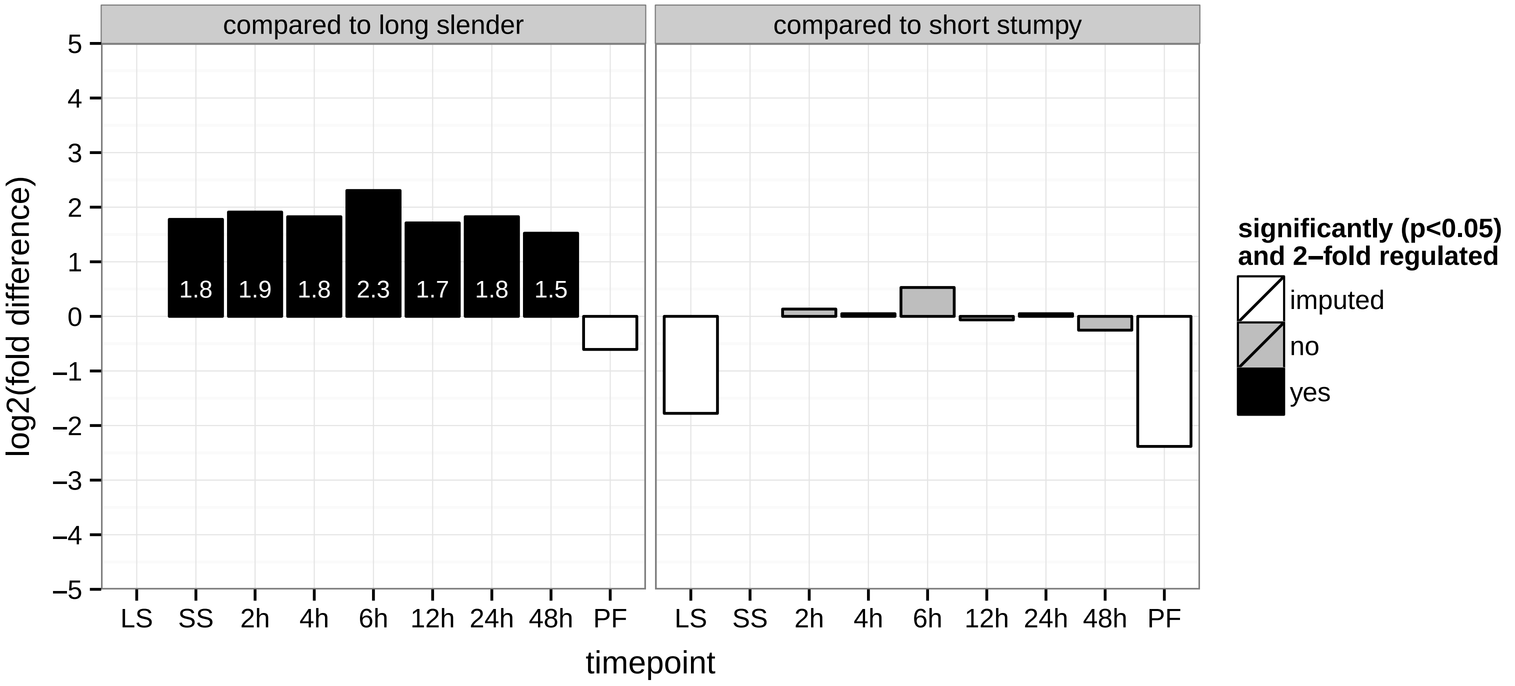

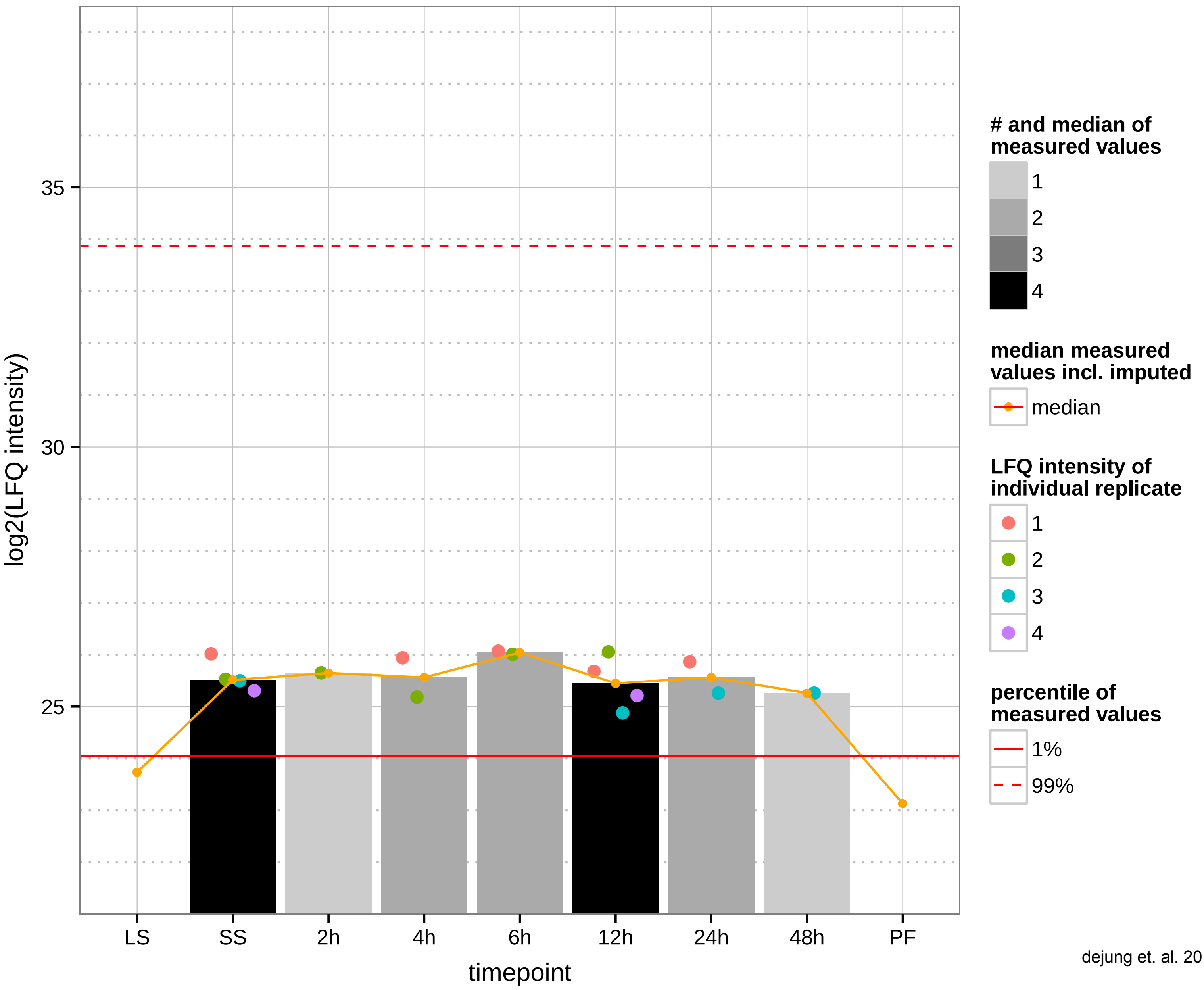

dejung et. al. 2015

protein kinase, putative
Tb927.11.850
AGOF: ATP binding, protein kinase activity
AGOC: null
AGOP: protein phosphorylation
PGOF: ATP binding, catalytic activity, protein kinase activity, transferase activity, transferring phosphorus–containing groups
PGOC: null
PGOP: protein phosphorylation

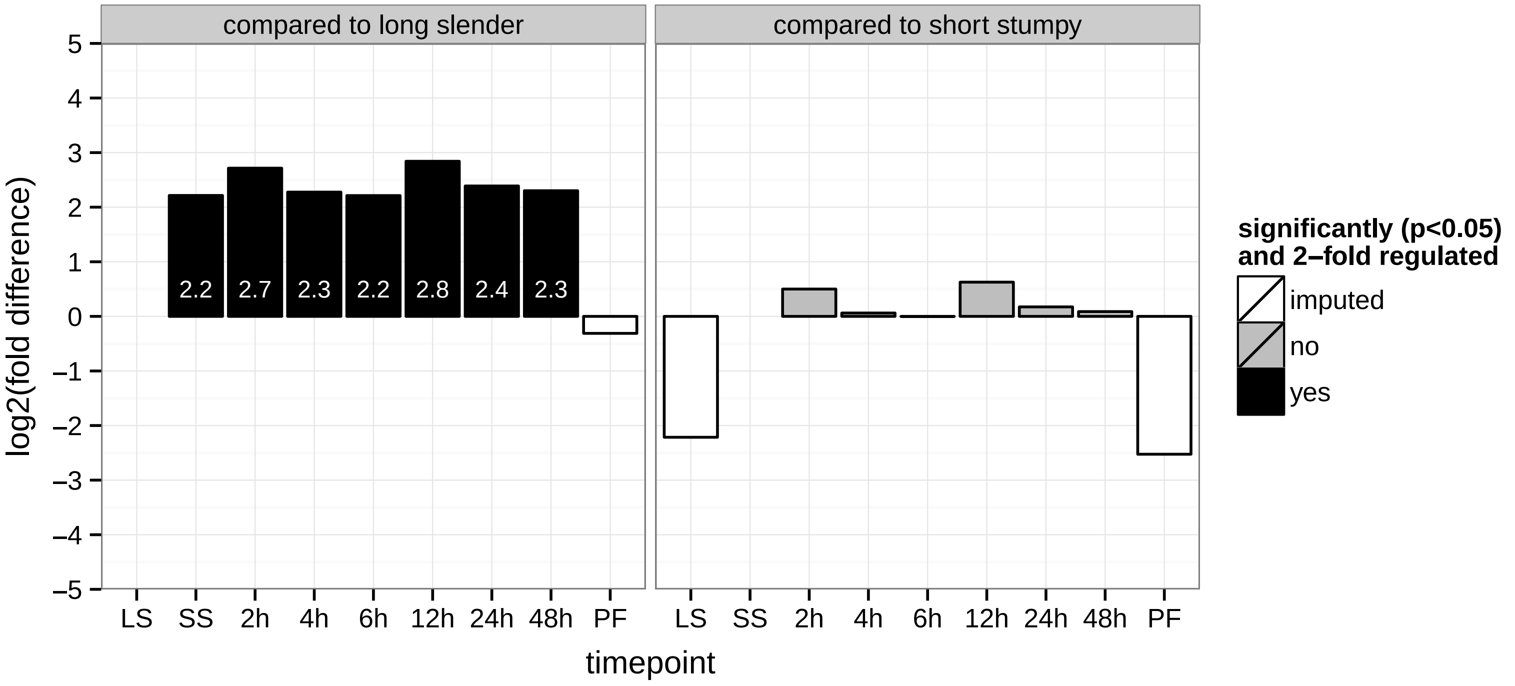

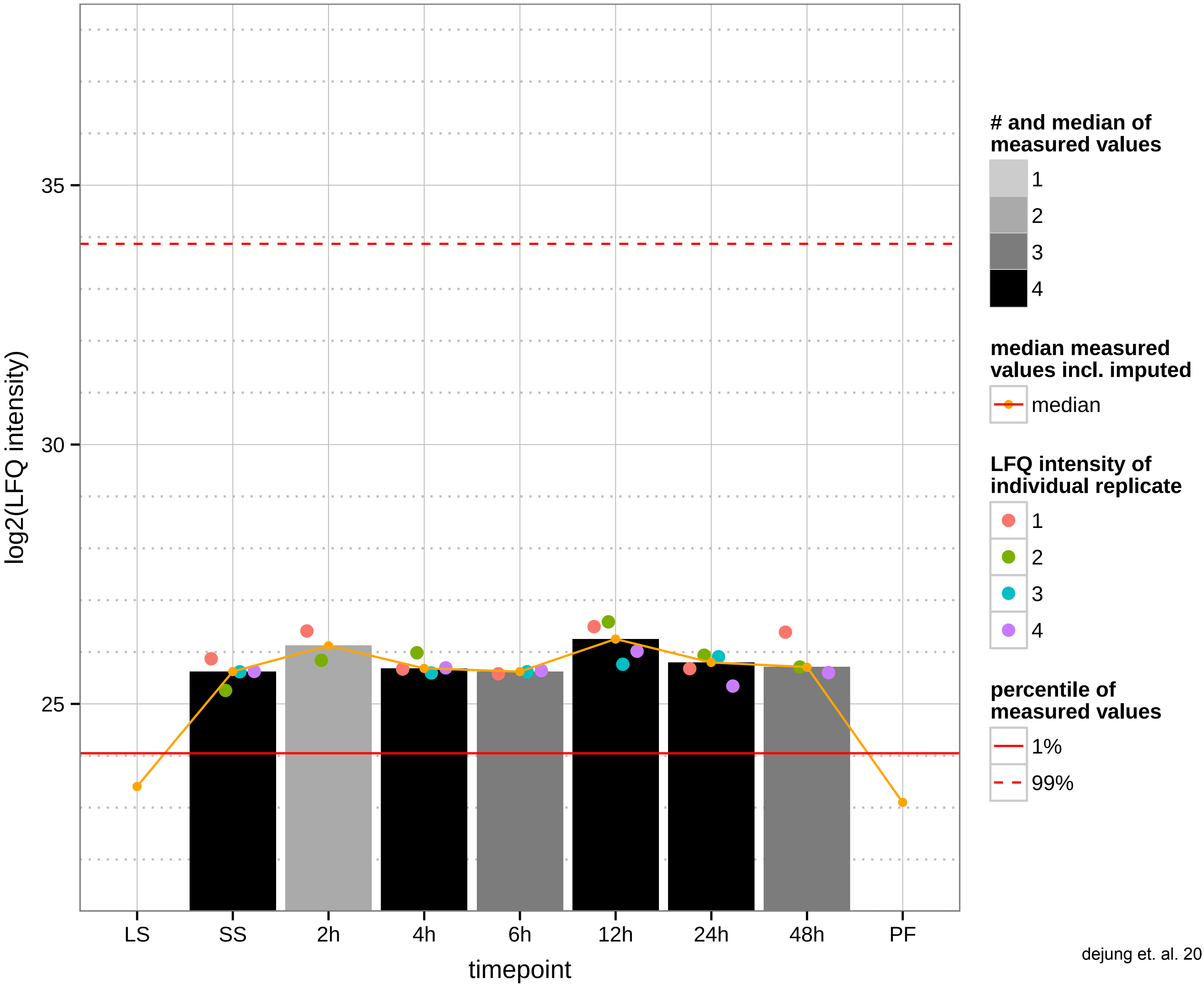

dejung et. al. 2015

hypothetical protein, conserved
Tb927.11.9100
AGOF: null
AGOC: null
AGOP: null
PGOF: null
PGOC: null
PGOP: null

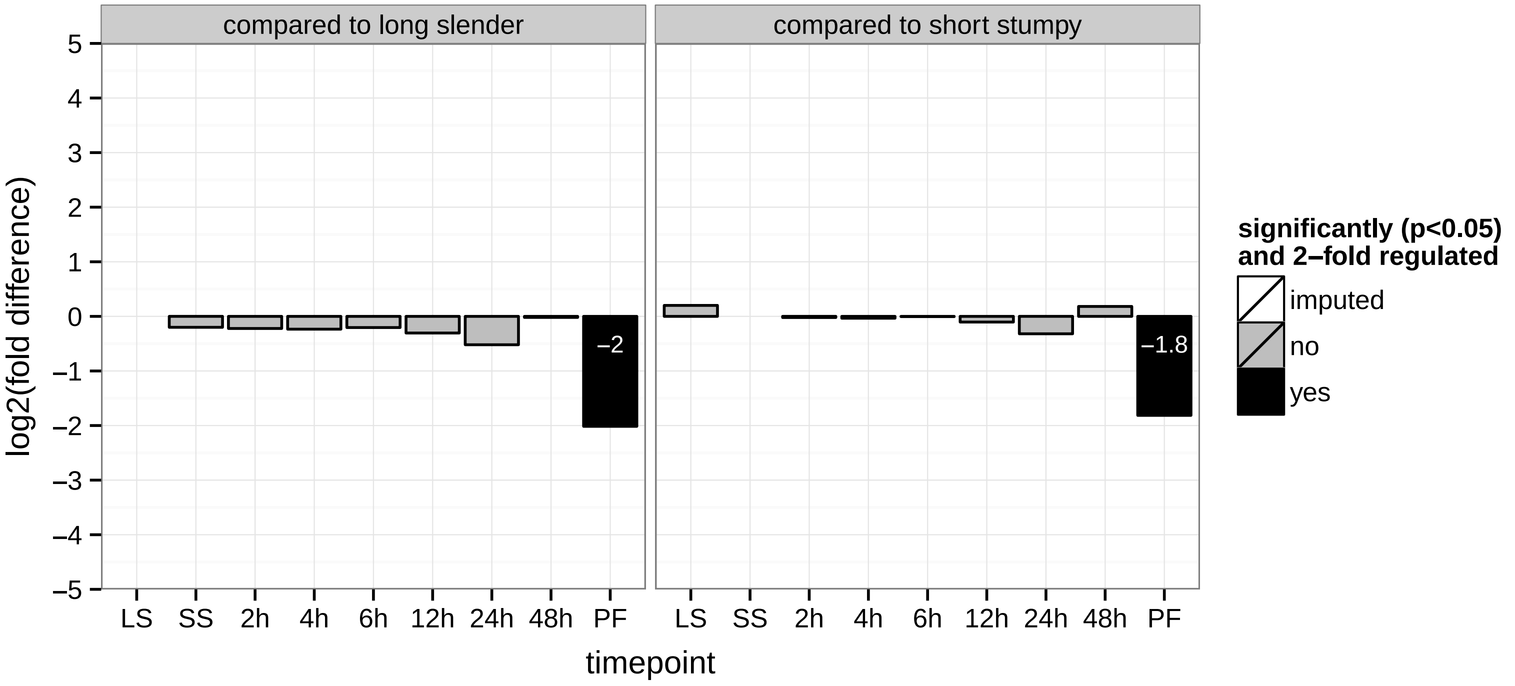

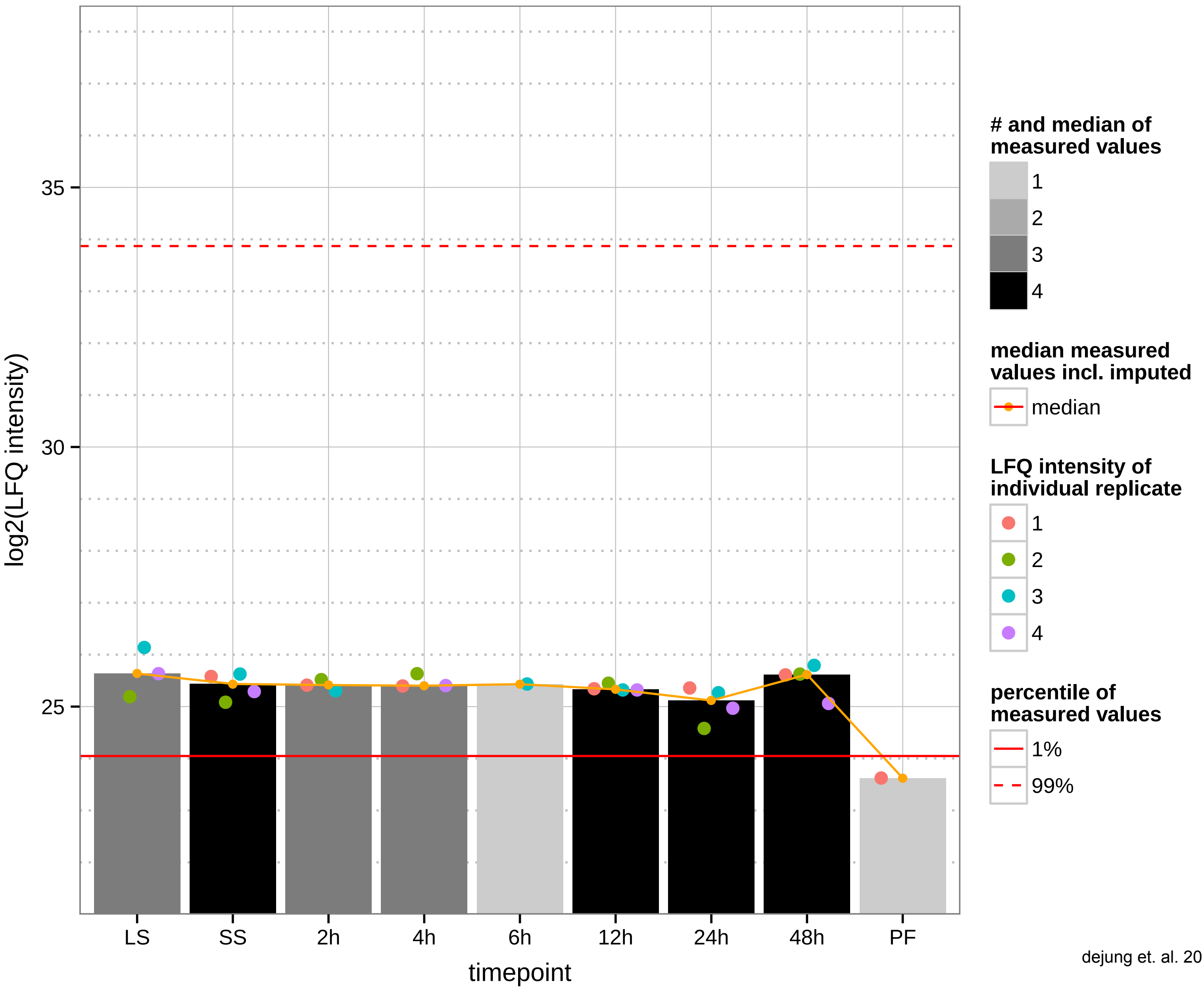

dejung et. al. 2015

oxidoreductase, putative
Tb927.11.9560
AGOF: flavin adenine dinucleotide binding, oxidoreductase activity, acting on CH–OH group of donors
AGOC: mitochondrion
AGOP: alcohol metabolic process, oxidation–reduction process
PGOF: choline dehydrogenase activity, flavin adenine dinucleotide binding, oxidoreductase activity, acting on CH–OH group o
PGOC: null
PGOP: alcohol metabolic process, oxidation–reduction process

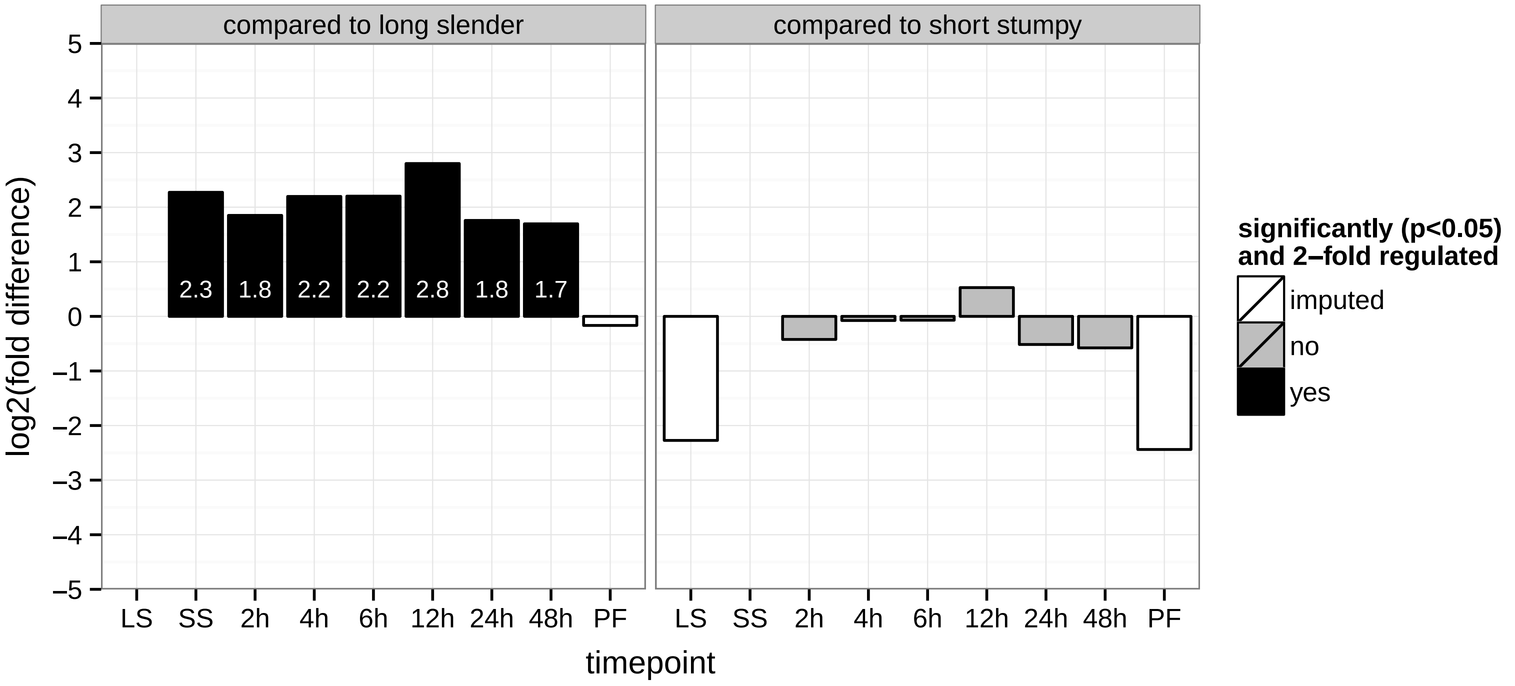

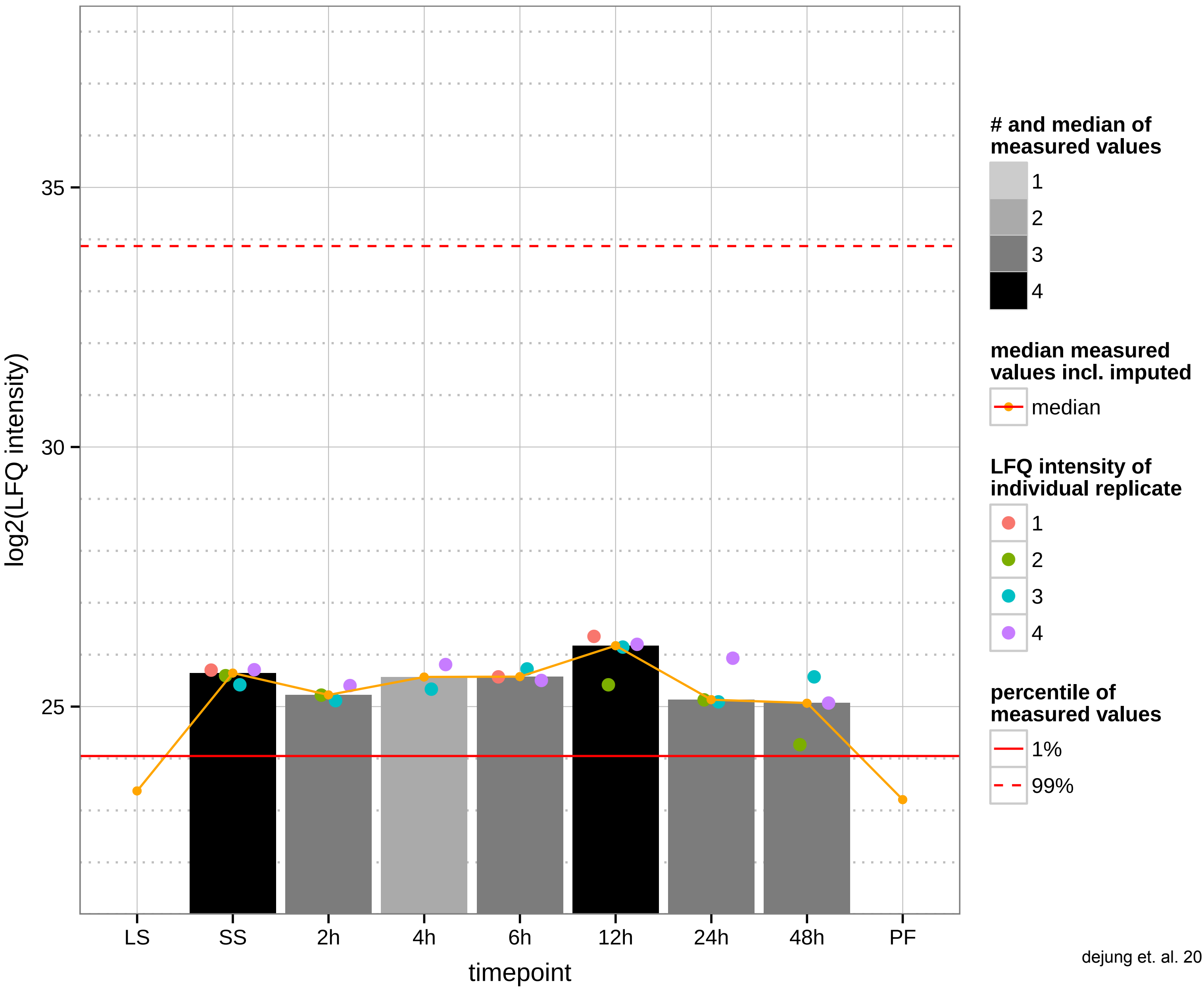

dejung et. al. 2015

hypothetical protein, conserved
Tb927.11.9960
AGOF: null
AGOC: null
AGOP: null
PGOF: null
PGOC: null
PGOP: null

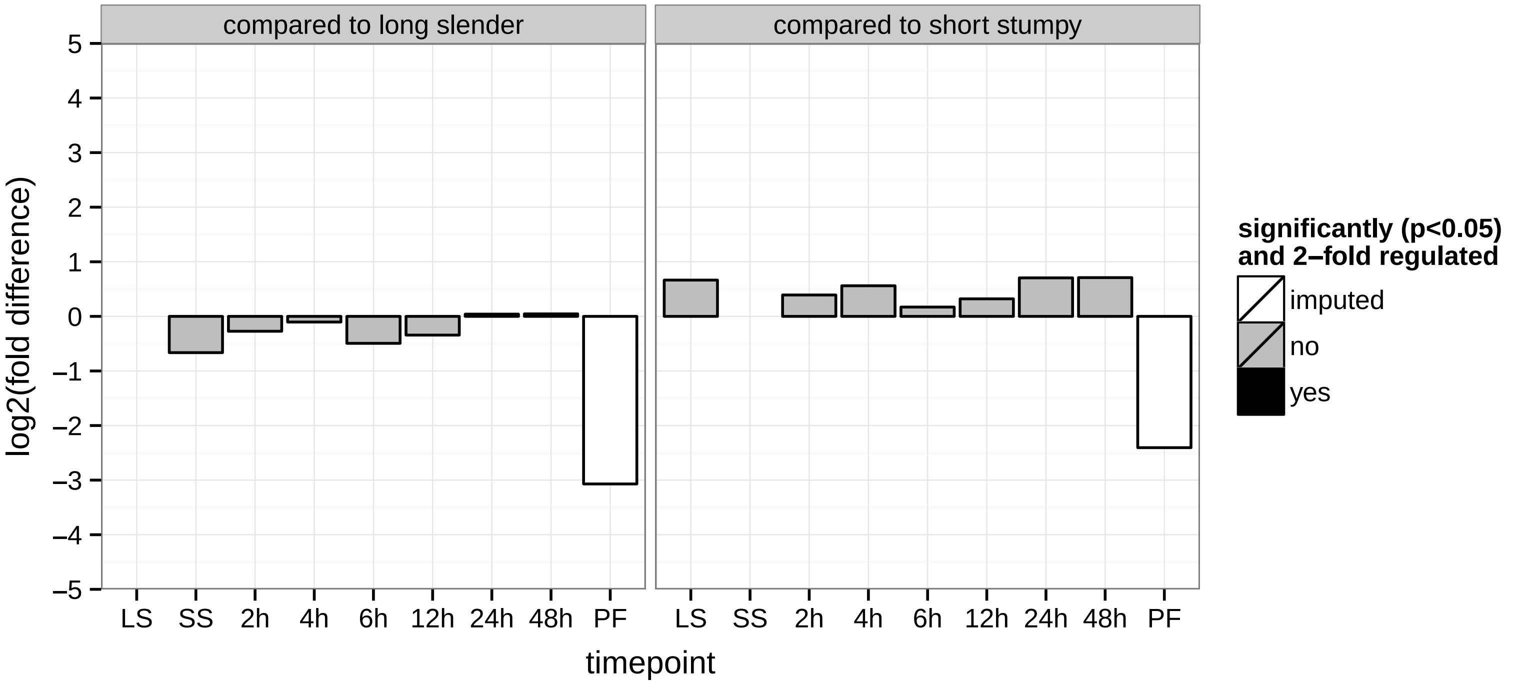

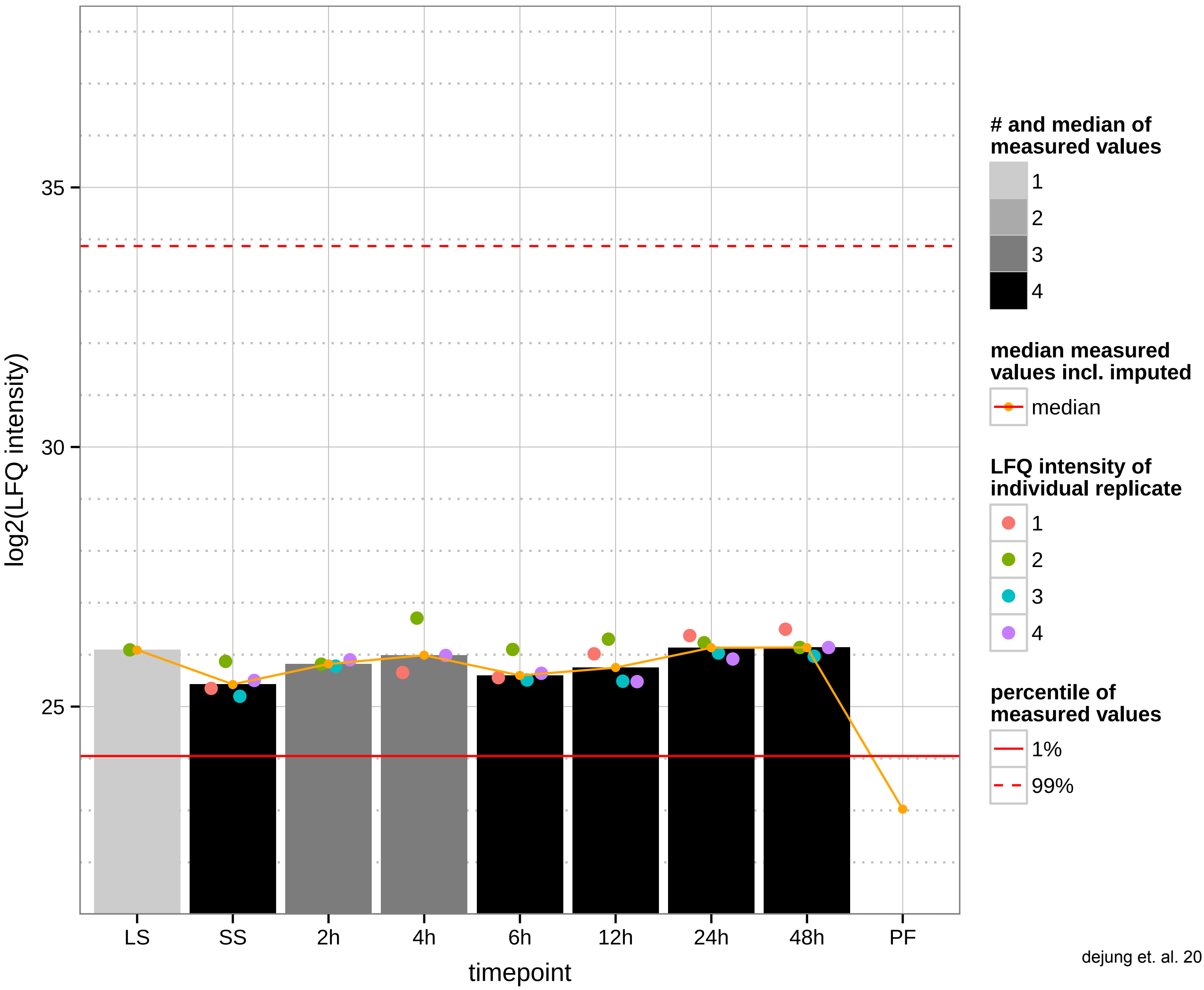

dejung et. al. 2015

2–oxoglutarate dehydrogenase E1 component, putative
Tb927.11.9980
AGOF: oxoglutarate dehydrogenase (succinyl–transferring) activity, thiamine pyrophosphate binding
AGOC: mitochondrion, oxoglutarate dehydrogenase complex
AGOP: glycolysis, lysine catabolic process, tricarboxylic acid cycle, tryptophan metabolic process
PGOF: oxidoreductase activity, acting on the aldehyde or oxo group of donors, disulfide as acceptor, oxoglutarate dehydrogen
PGOC: null
PGOP: metabolic process, oxidation–reduction process, tricarboxylic acid cycle

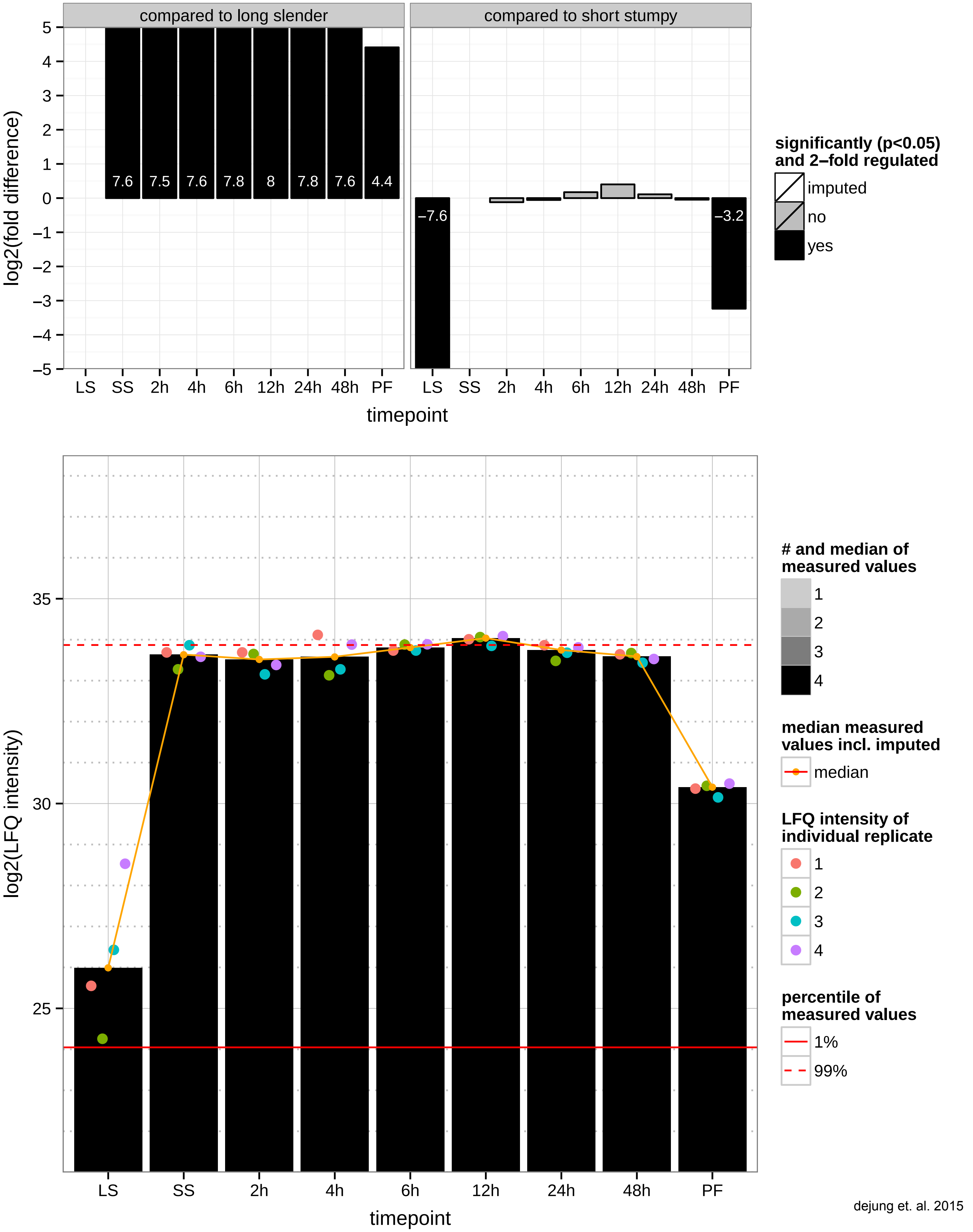

dejung et. al. 2015

hypothetical protein, conserved
Tb927.2.2390
AGOF: null
AGOC: null
AGOP: null
PGOF: null
PGOC: null
PGOP: null

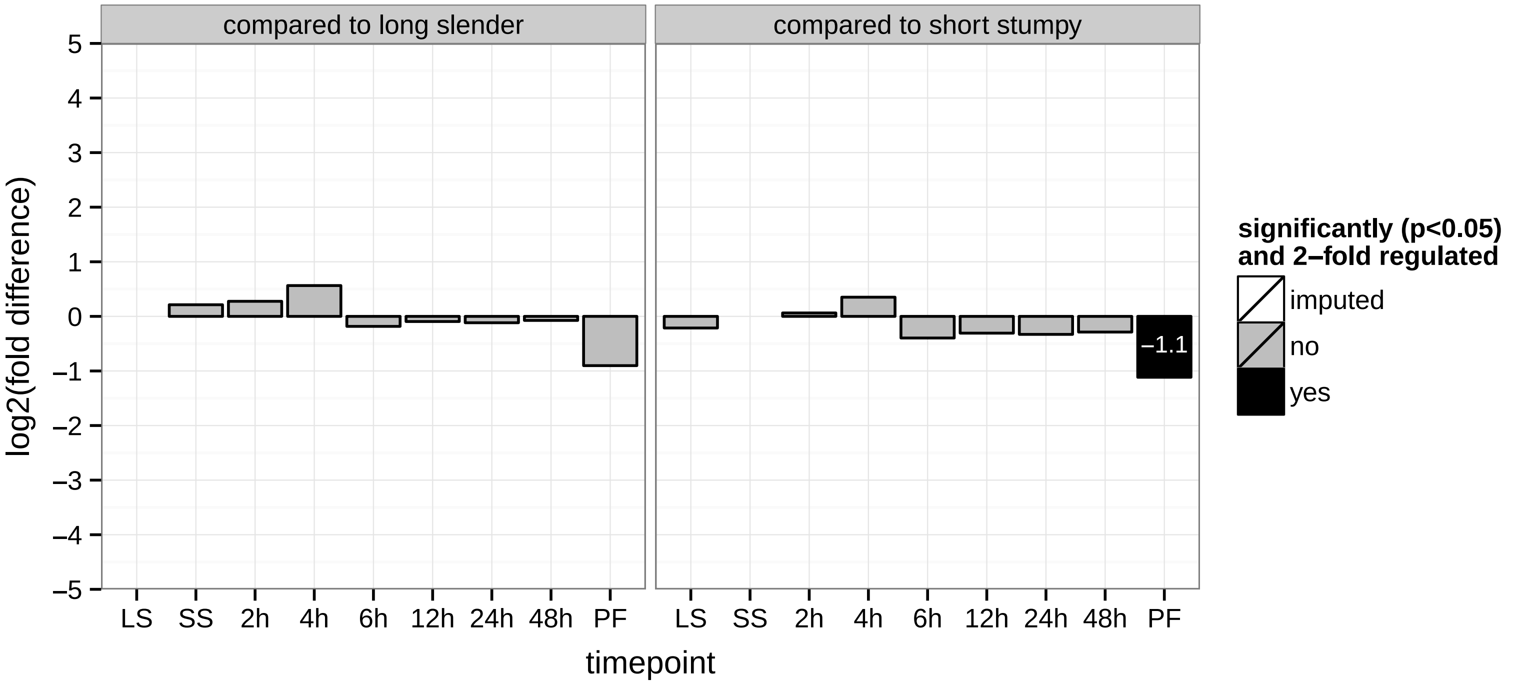

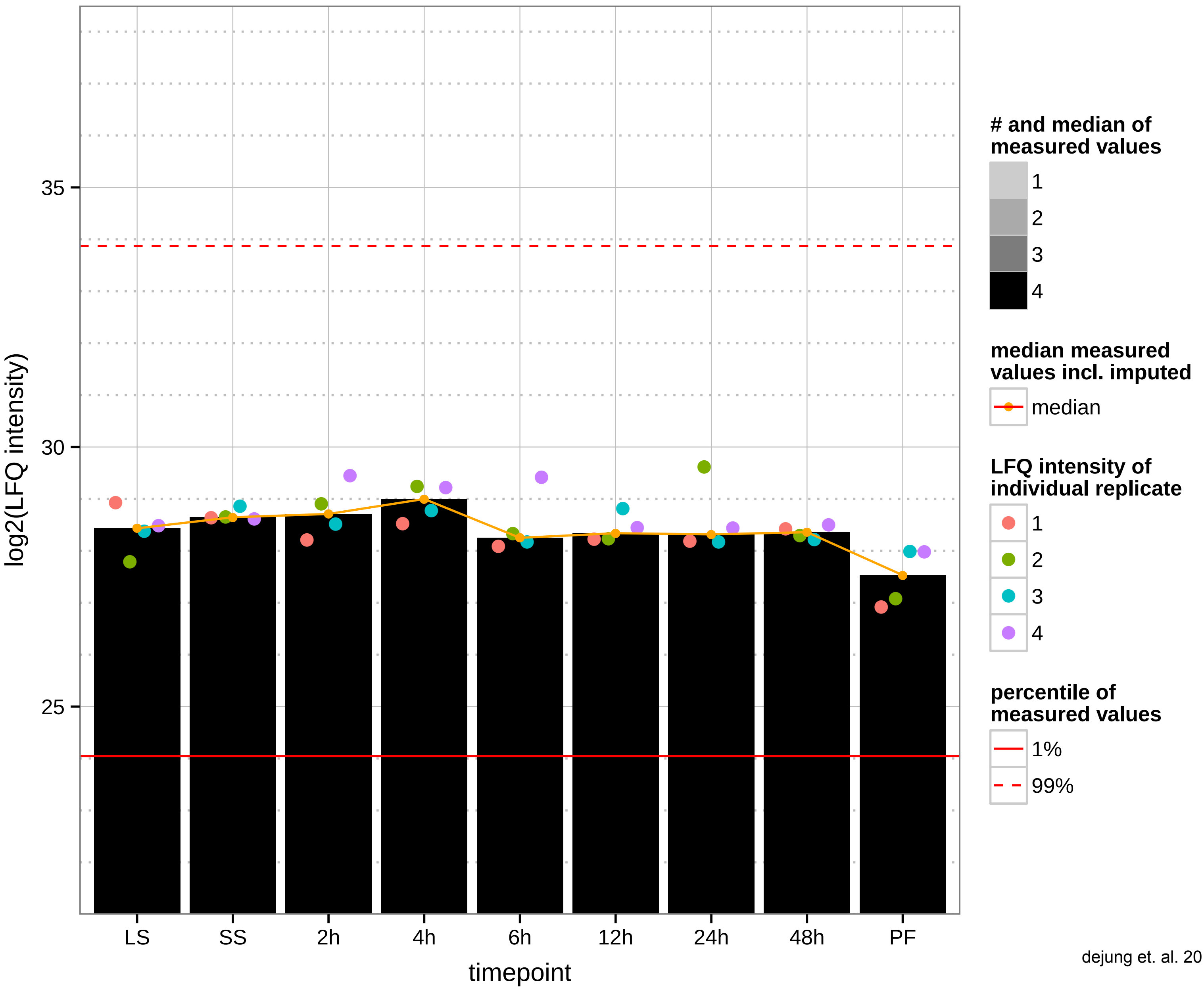

dejung et. al. 2015

retrotransposon hot spot protein (RHS, pseudogene), putative, retrotransposon hot spot protein 3 (RHS3), frameshift
Tb927.2.270
AGOF: null
AGOC: null
AGOP: null
PGOF: null
PGOC: null
PGOP: null

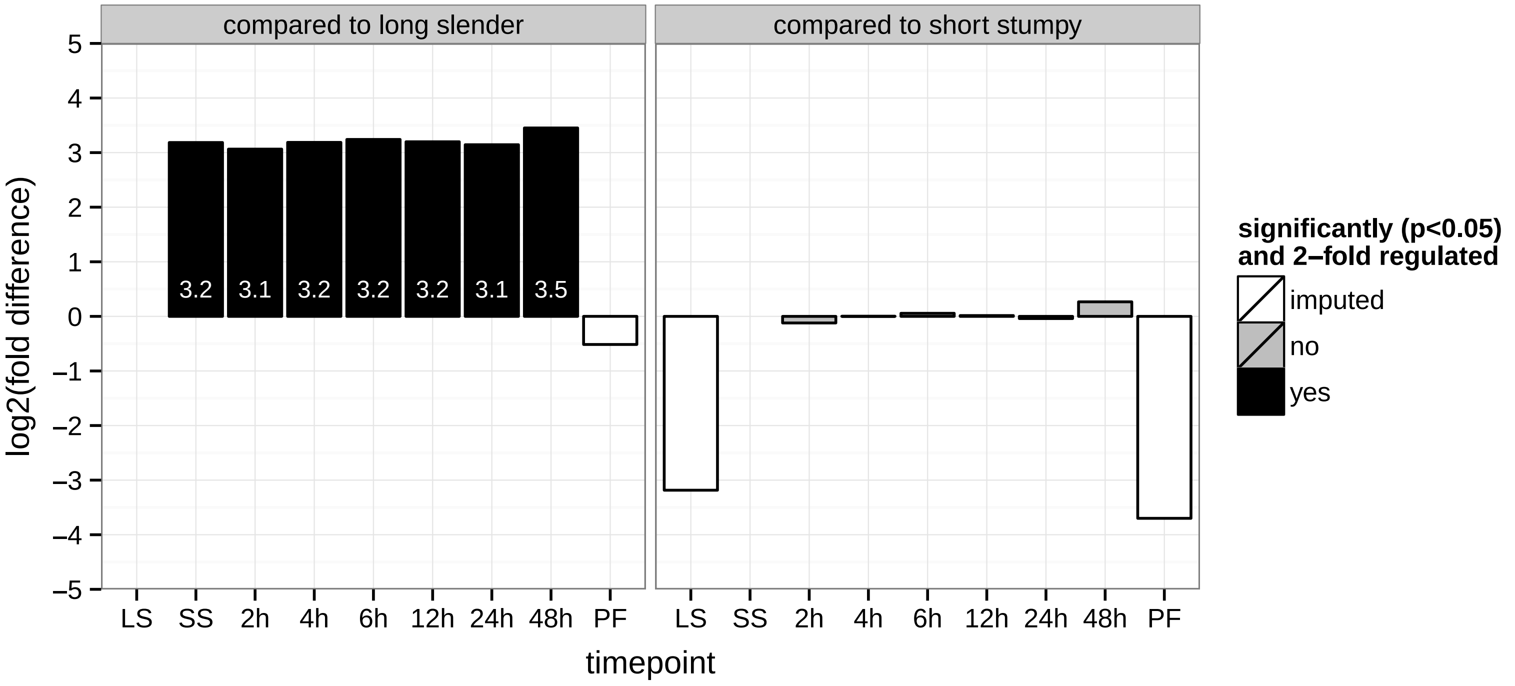

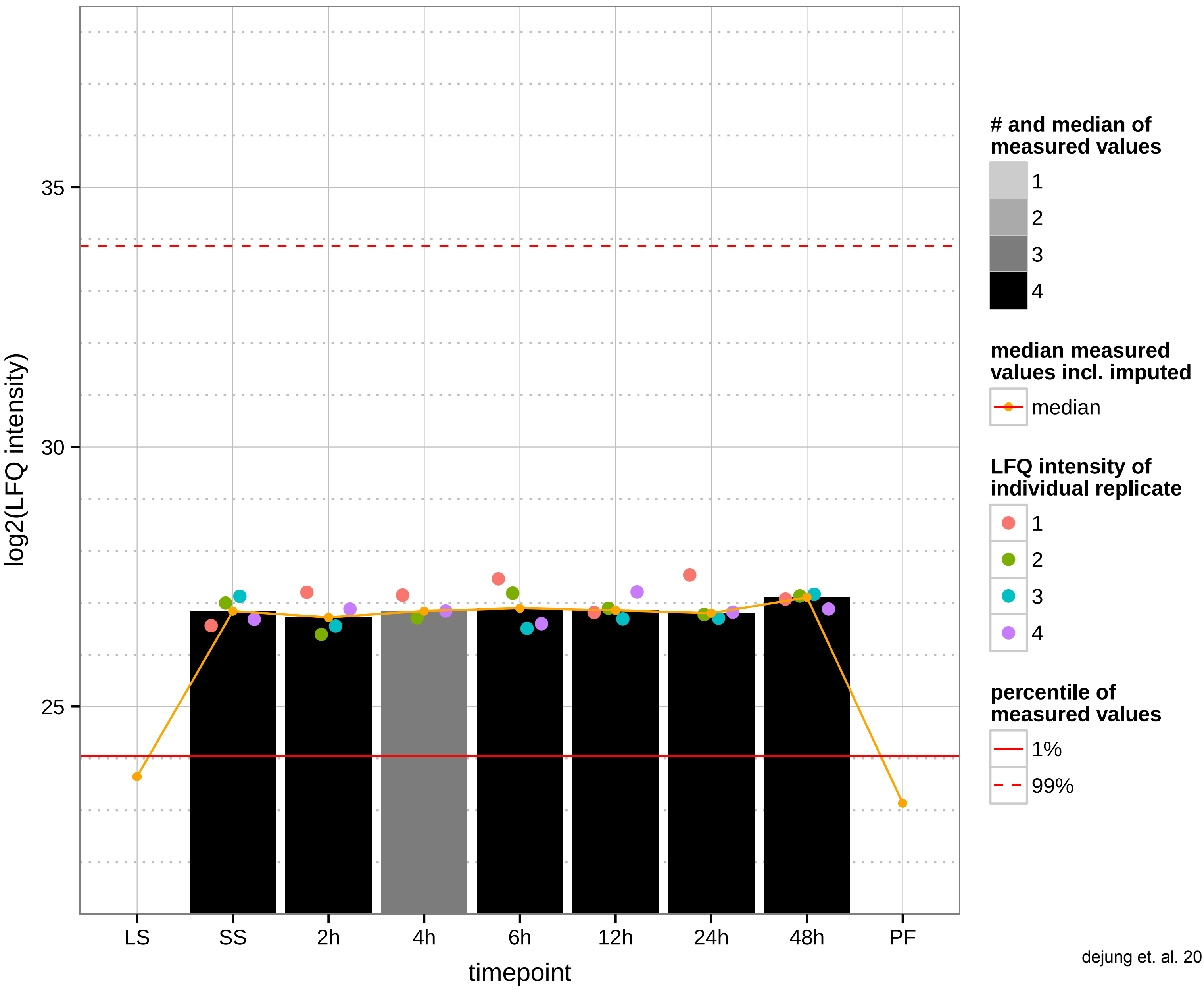

dejung et. al. 2015

retrotransposon hot spot protein 4 (RHS4), putative
Tb927.2.340
AGOF: null
AGOC: nucleus
AGOP: null
PGOF: null
PGOC: null
PGOP: null

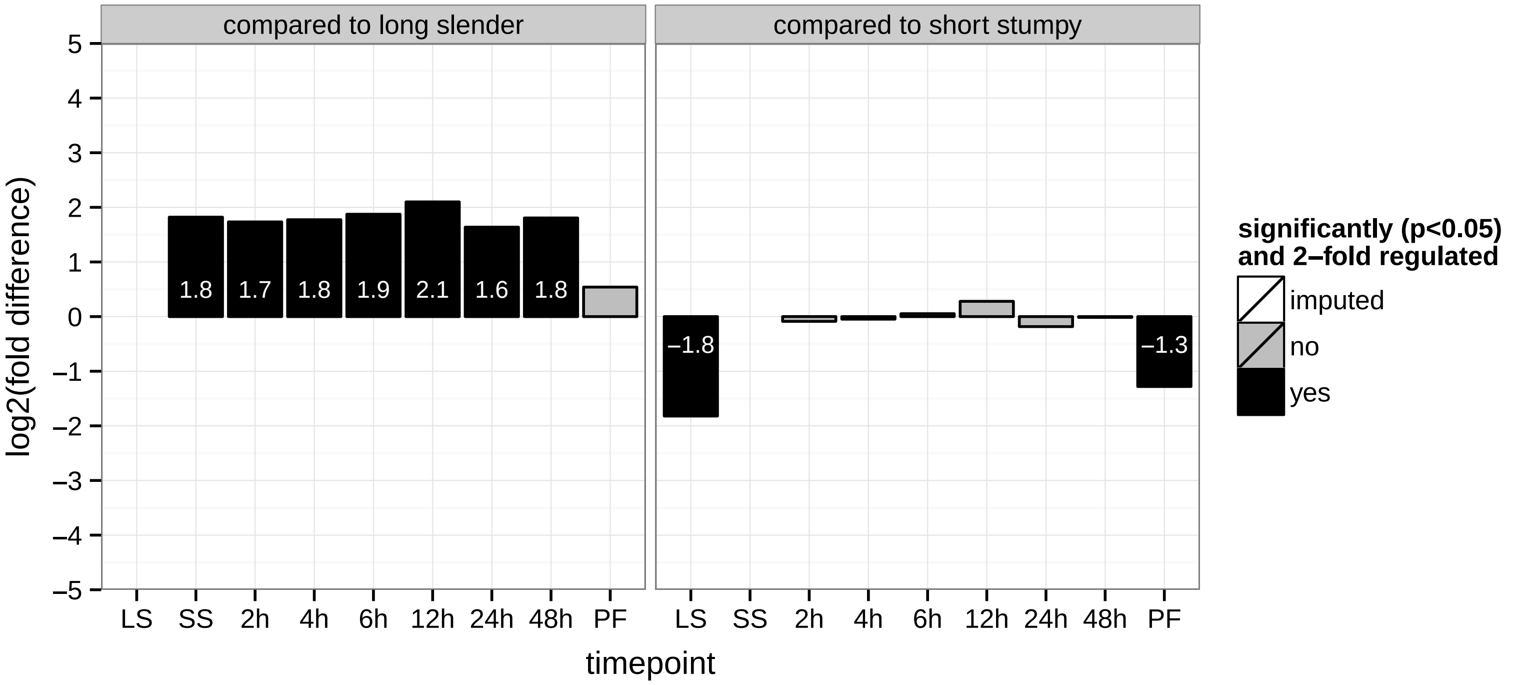

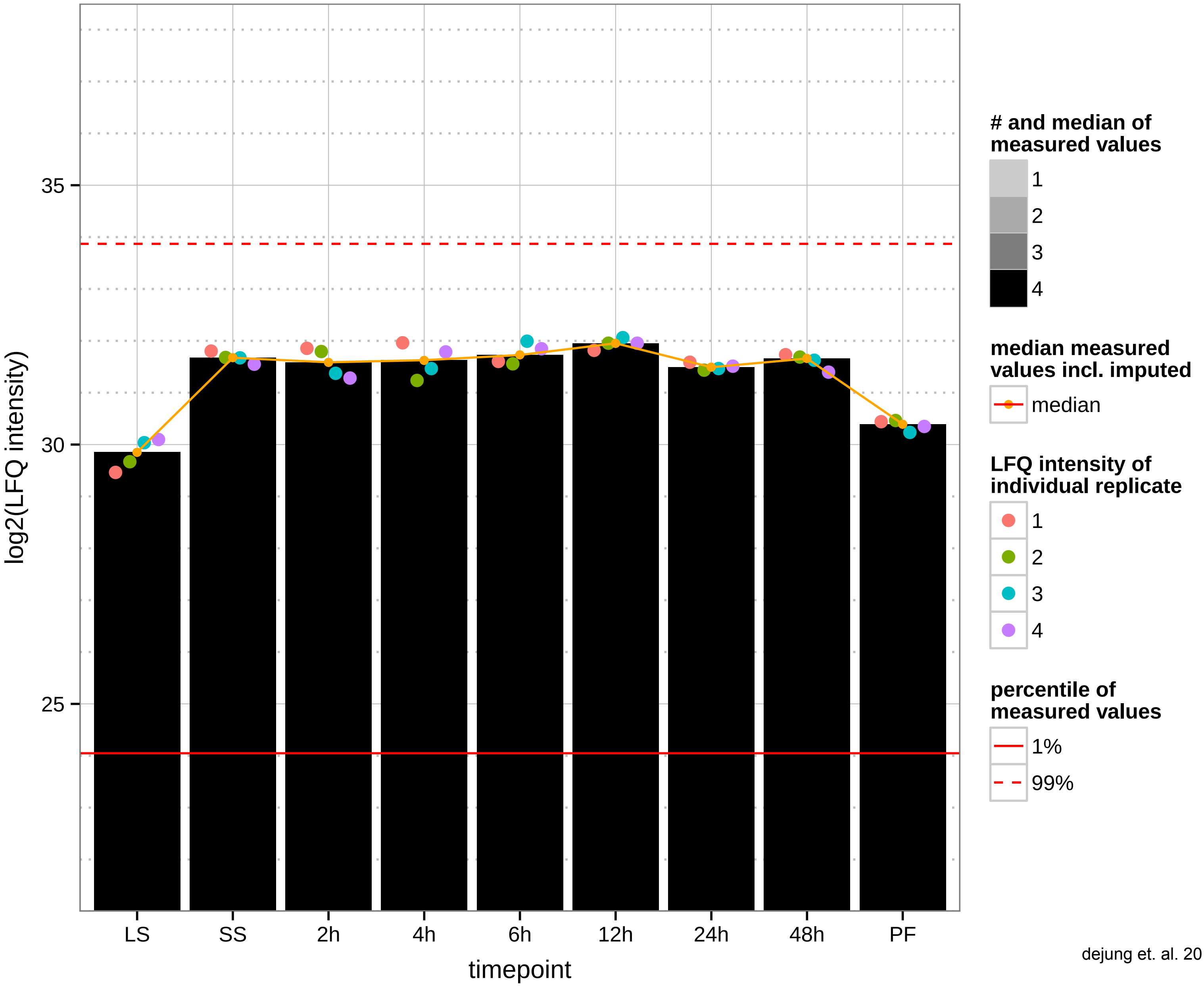

dejung et. al. 2015

hypothetical protein, conserved
Tb927.2.3990
AGOF: null
AGOC: null
AGOP: null
PGOF: null
PGOC: null
PGOP: null

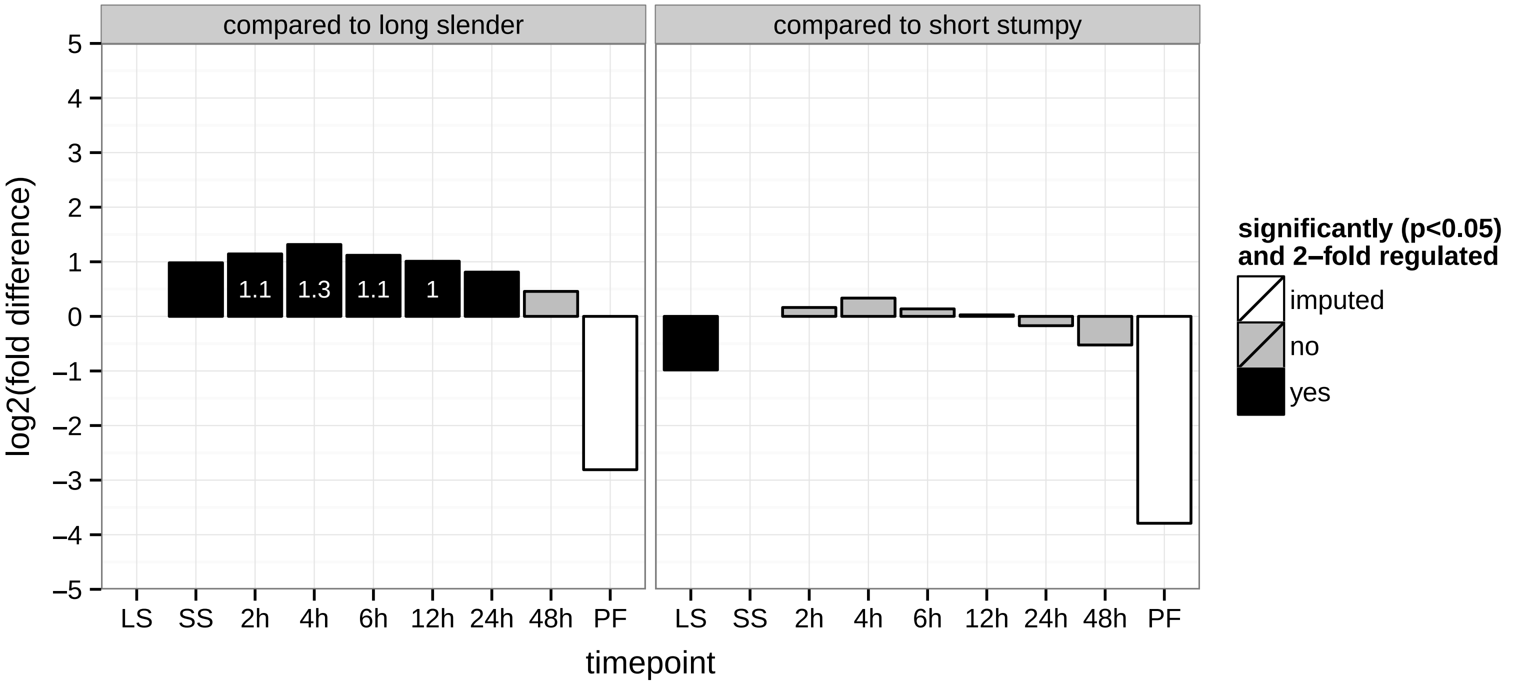

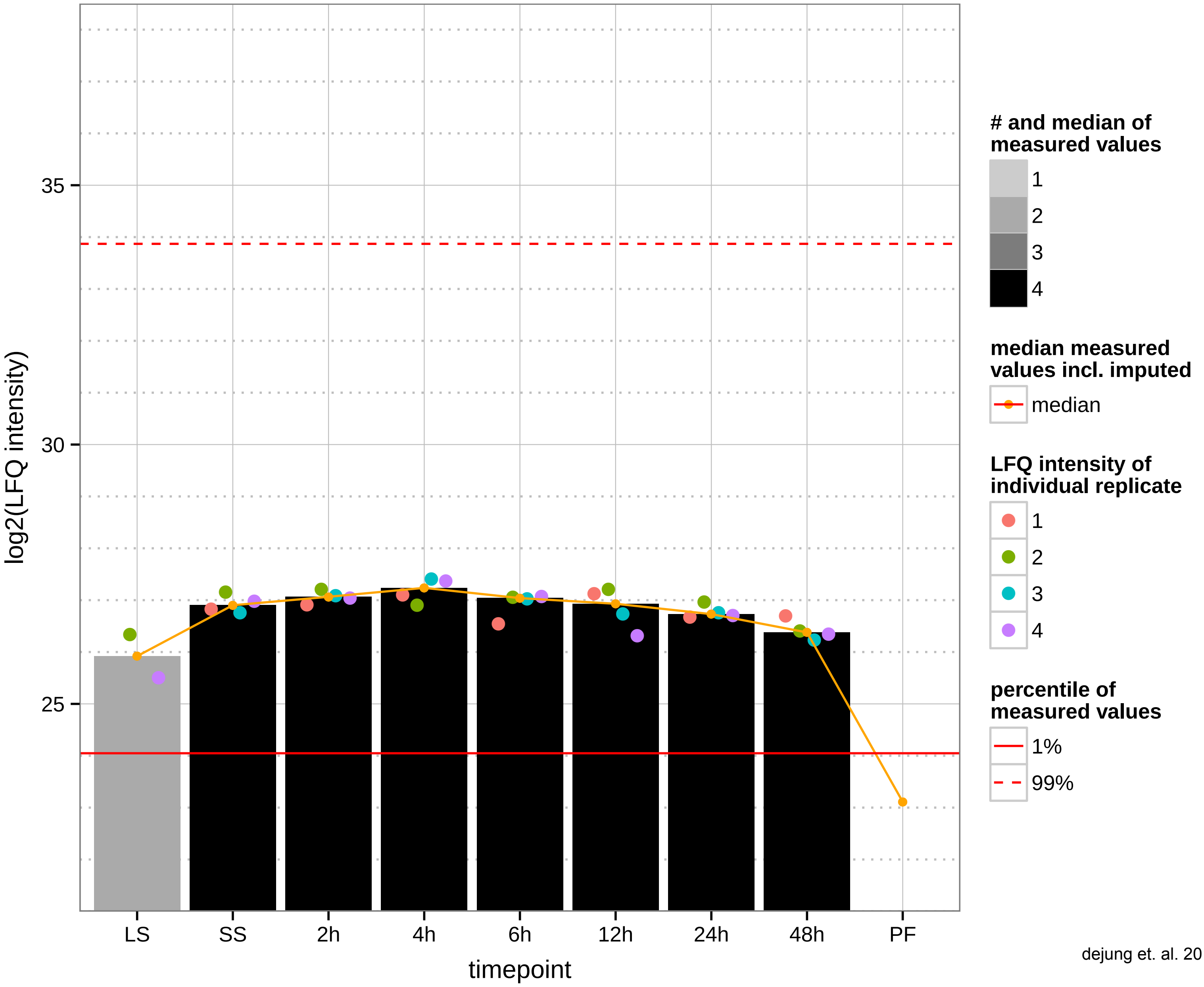

dejung et. al. 2015

hypothetical protein, conserved
Tb927.2.4380
AGOF: null
AGOC: mitochondrion
AGOP: null
PGOF: null
PGOC: null
PGOP: null

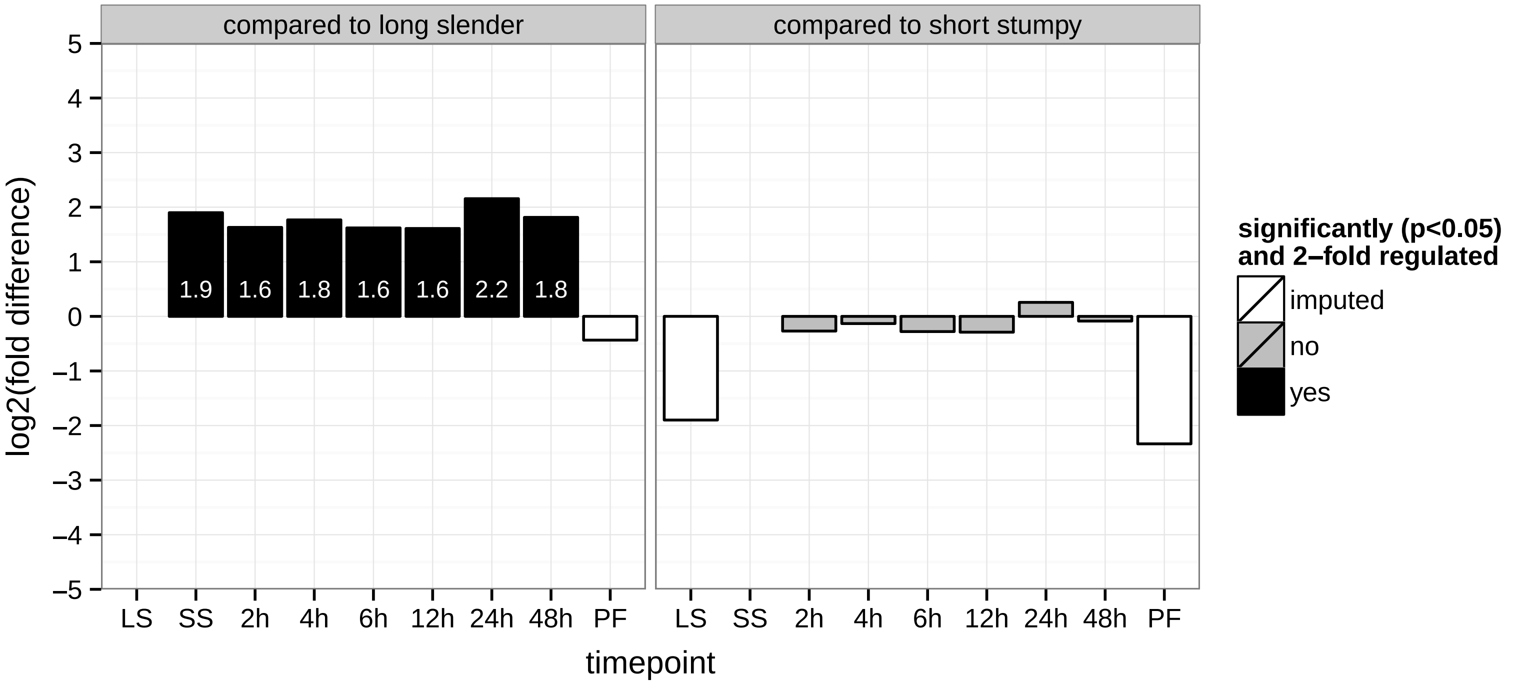

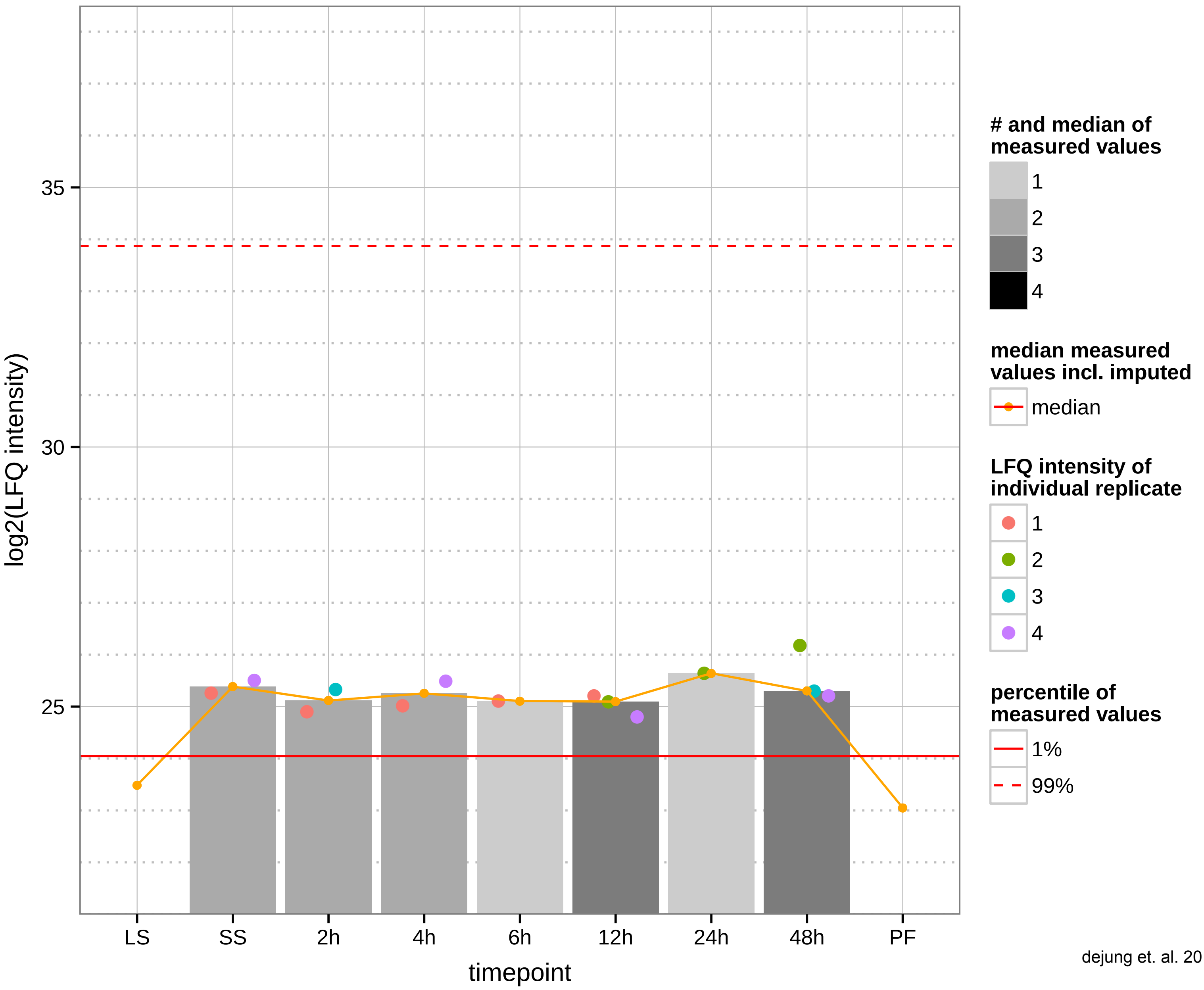

dejung et. al. 2015

retrotransposon hot spot (RHS), putative, retrotransposon hot spot protein 4 (RHS4), retrotransposon hot spot protein (RHS, p
Tb927.2.470
AGOF: null
AGOC: null, nucleus
AGOP: null
PGOF: null
PGOC: null
PGOP: null

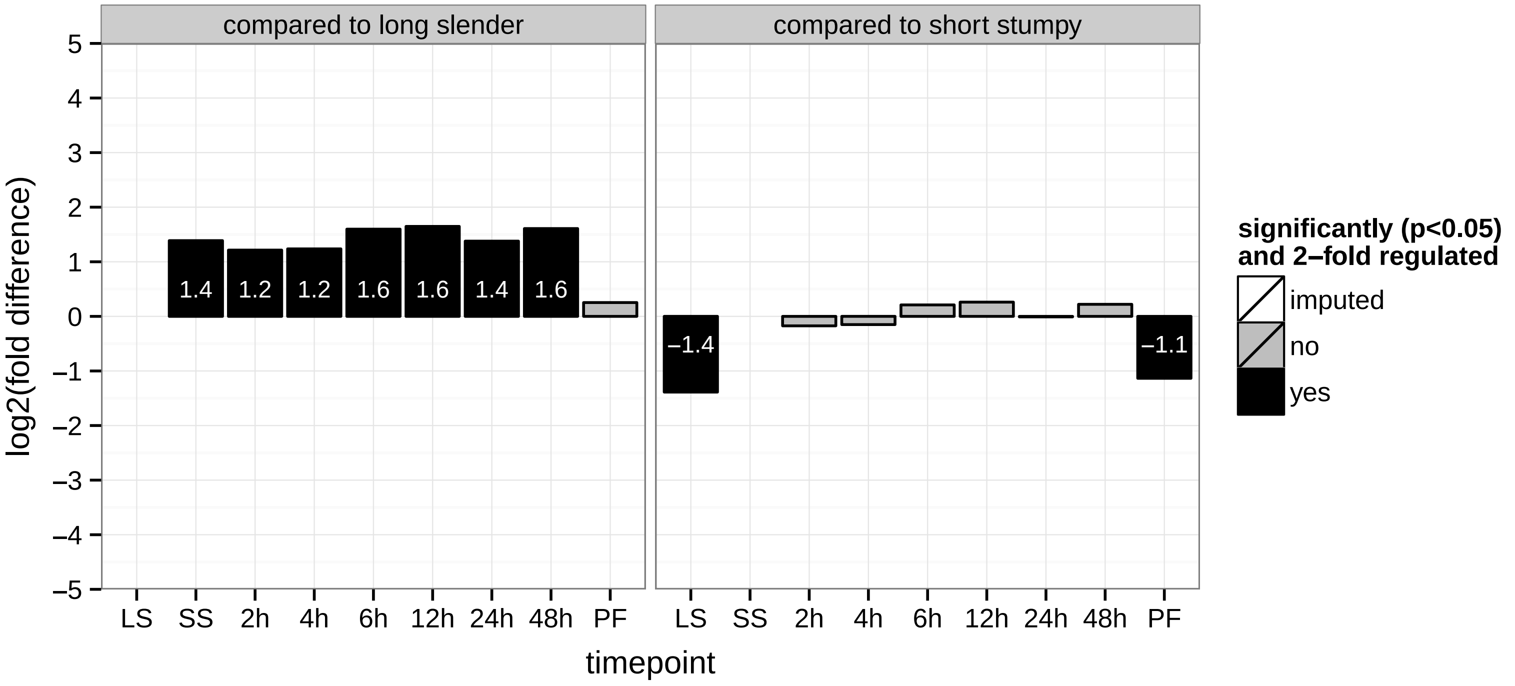

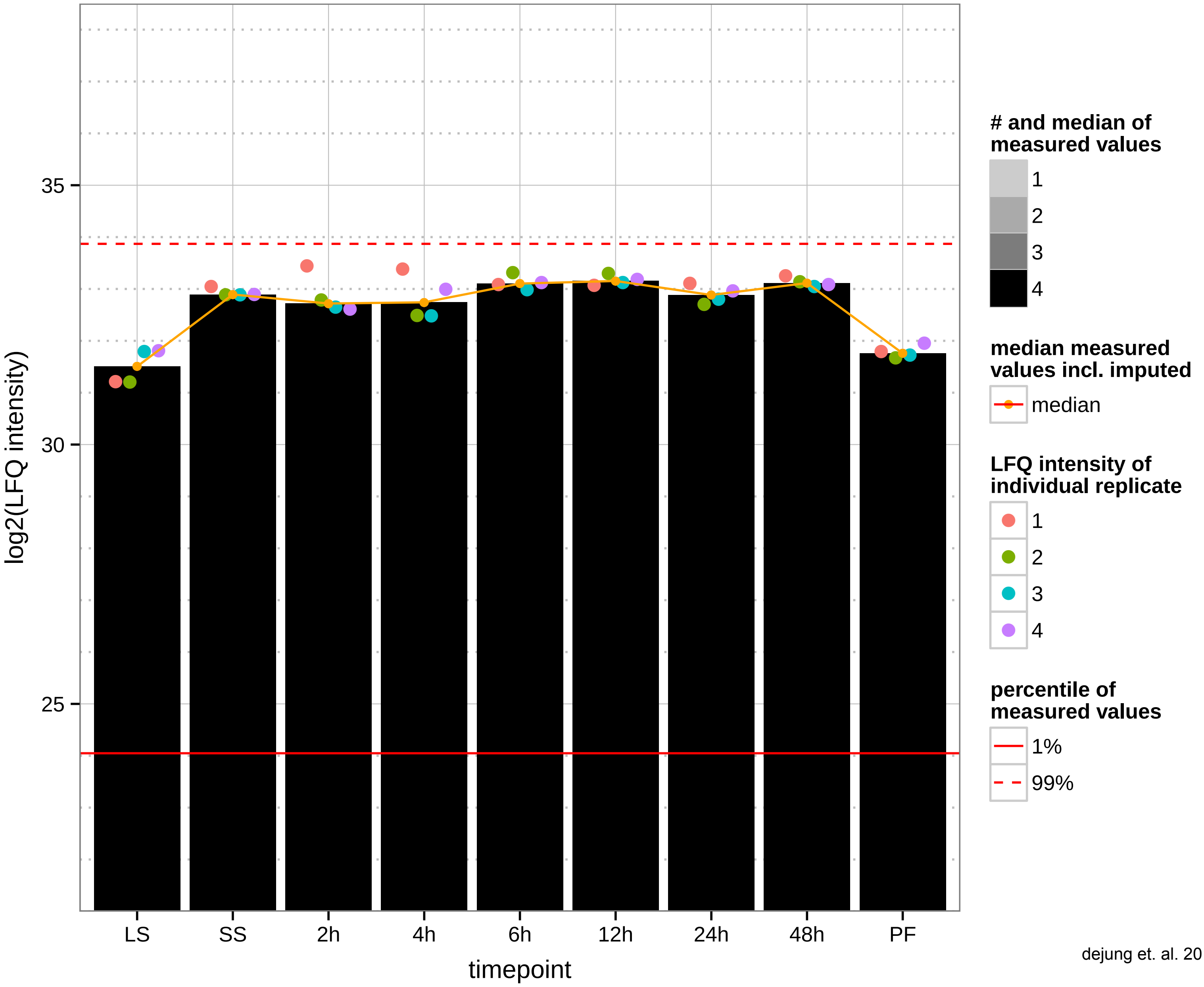

dejung et. al. 2015

hypothetical protein, conserved
Tb927.2.4780
AGOF: null
AGOC: null
AGOP: null
PGOF: null
PGOC: null
PGOP: null

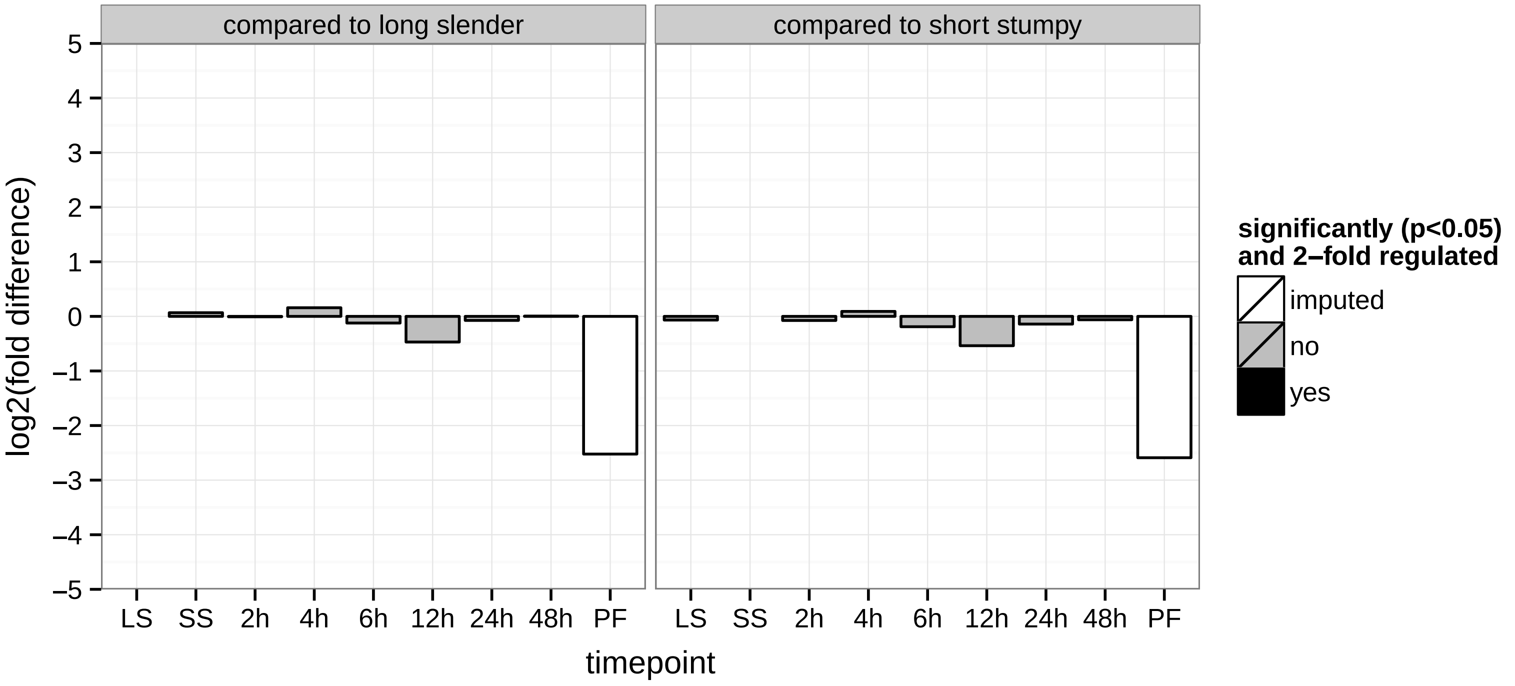

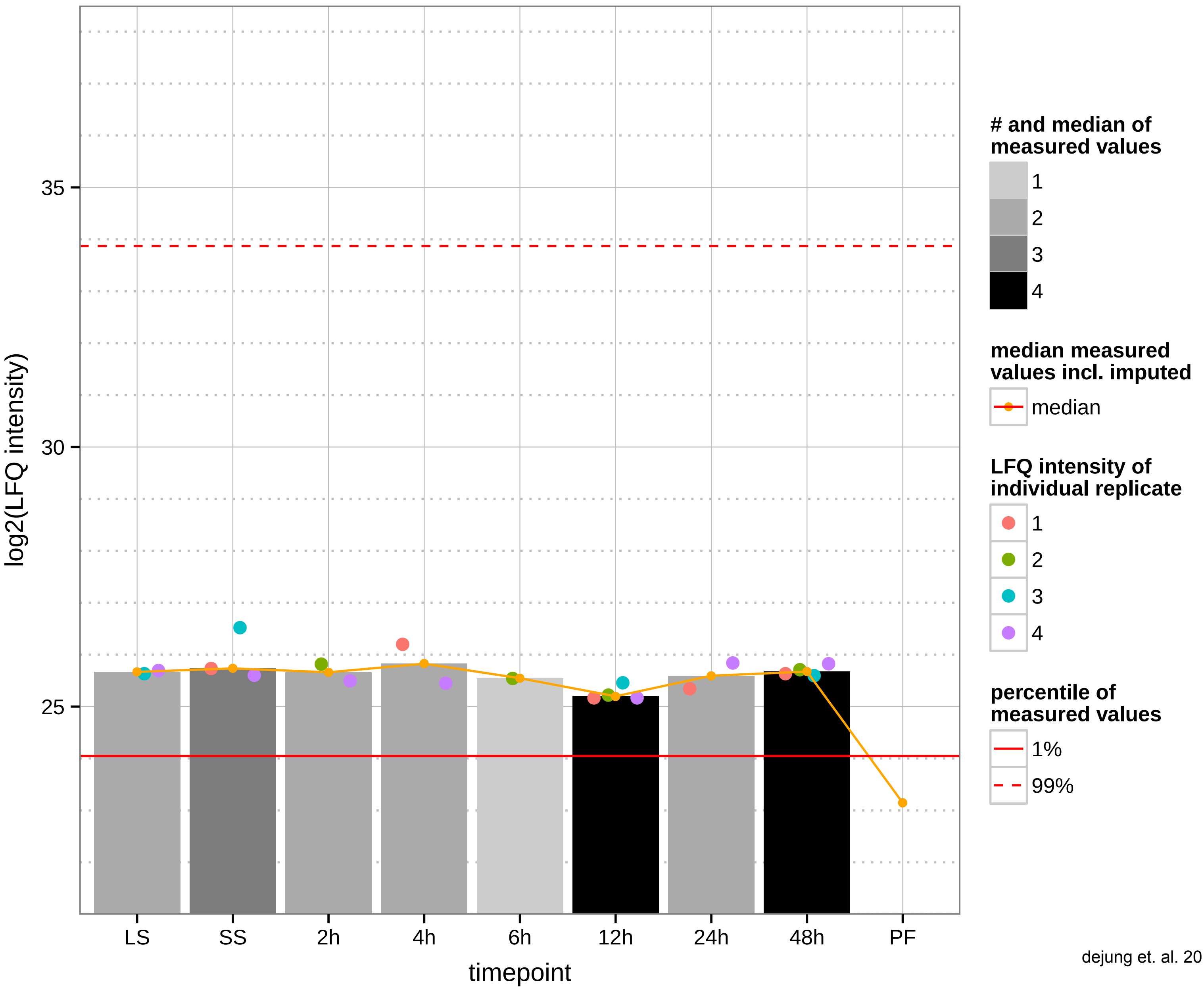

dejung et. al. 2015

retrotransposon hot spot protein 4 (RHS4), putative, retrotransposon hot spot protein 7 (RHS7)
Tb927.2.560
AGOF: null
AGOC: nucleus, null
AGOP: null
PGOF: null
PGOC: null
PGOP: null

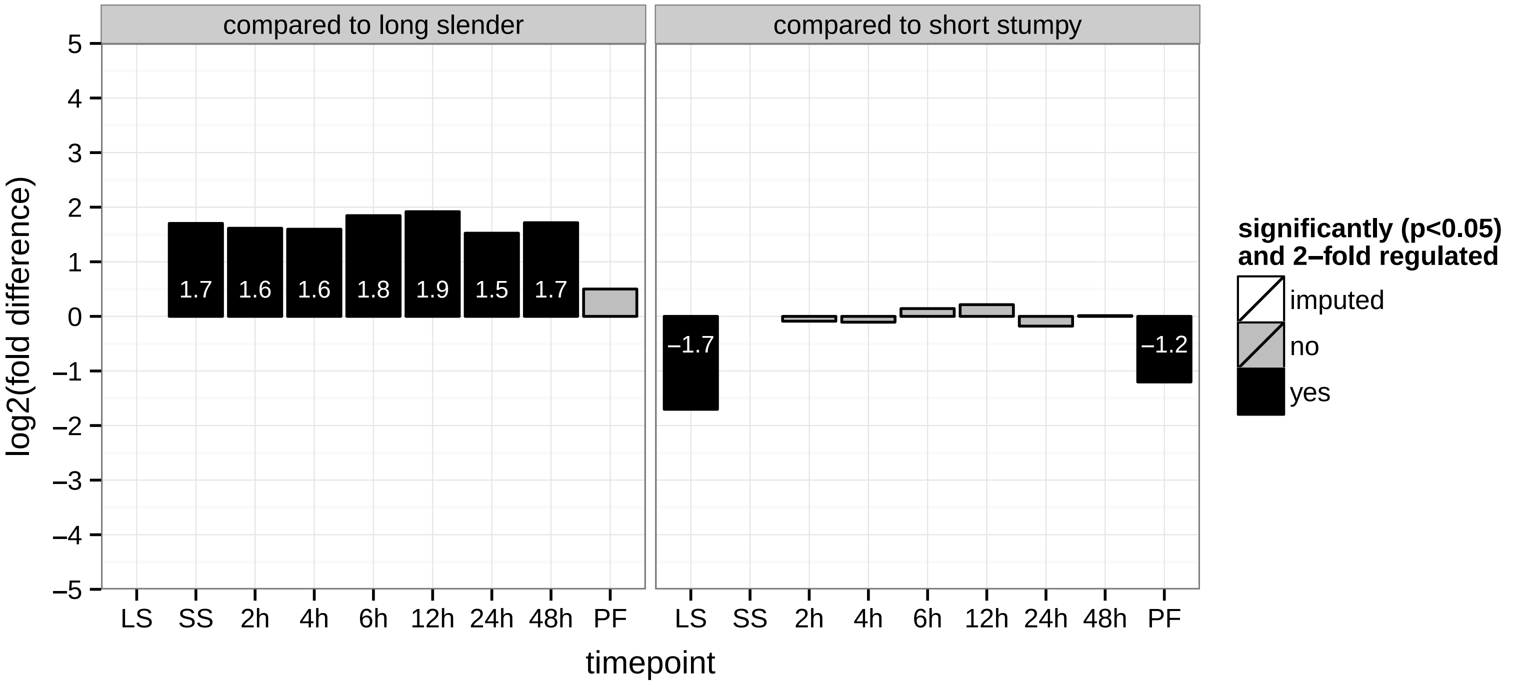

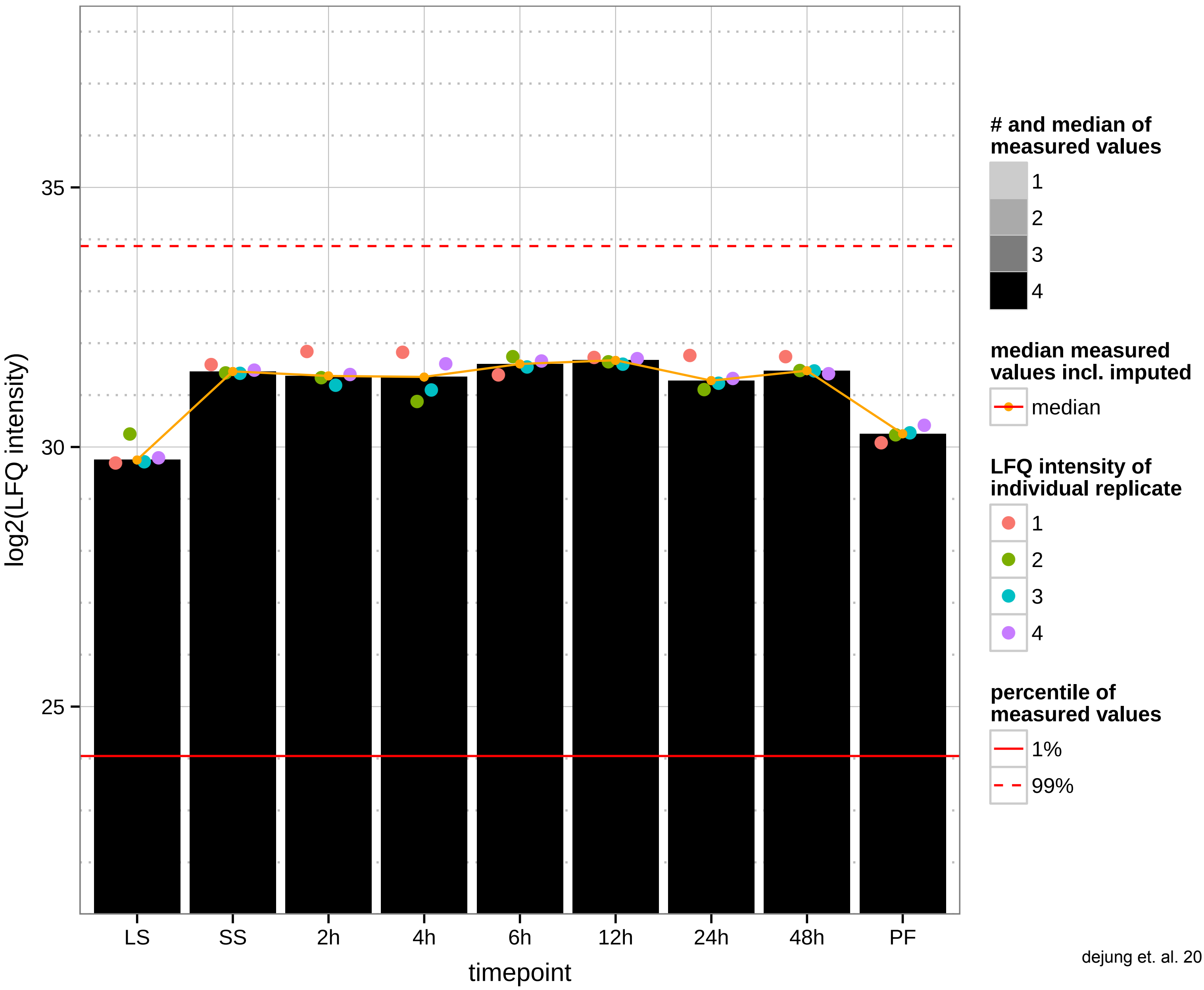

dejung et. al. 2015

hypothetical protein, conserved
Tb927.3.1140
AGOF: RNA binding
AGOC: null
AGOP: RNA processing
PGOF: RNA binding
PGOC: null
PGOP: RNA processing

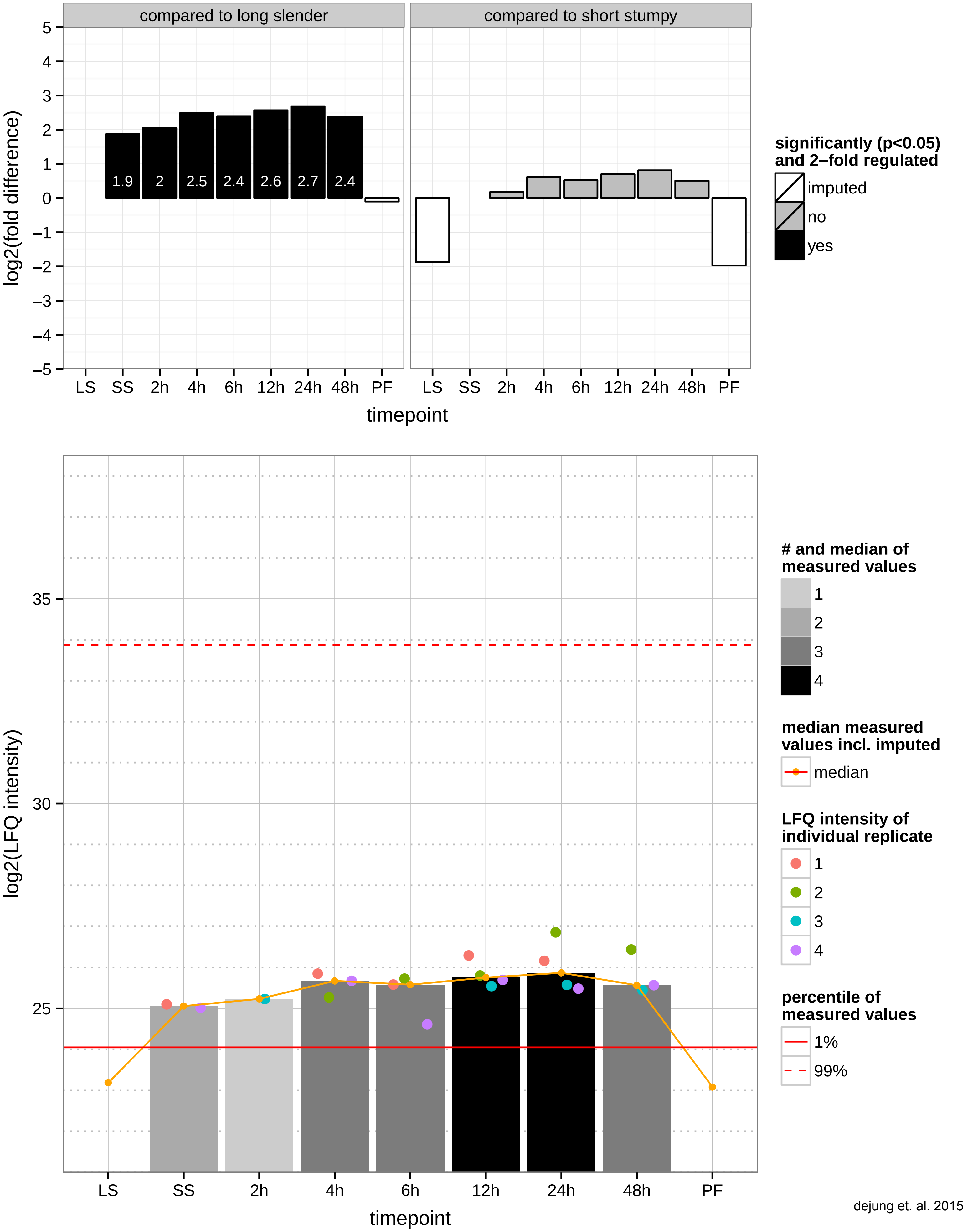

dejung et. al. 2015

hypothetical protein, conserved
Tb927.3.1800
AGOF: null
AGOC: null
AGOP: null
PGOF: null
PGOC: null
PGOP: null

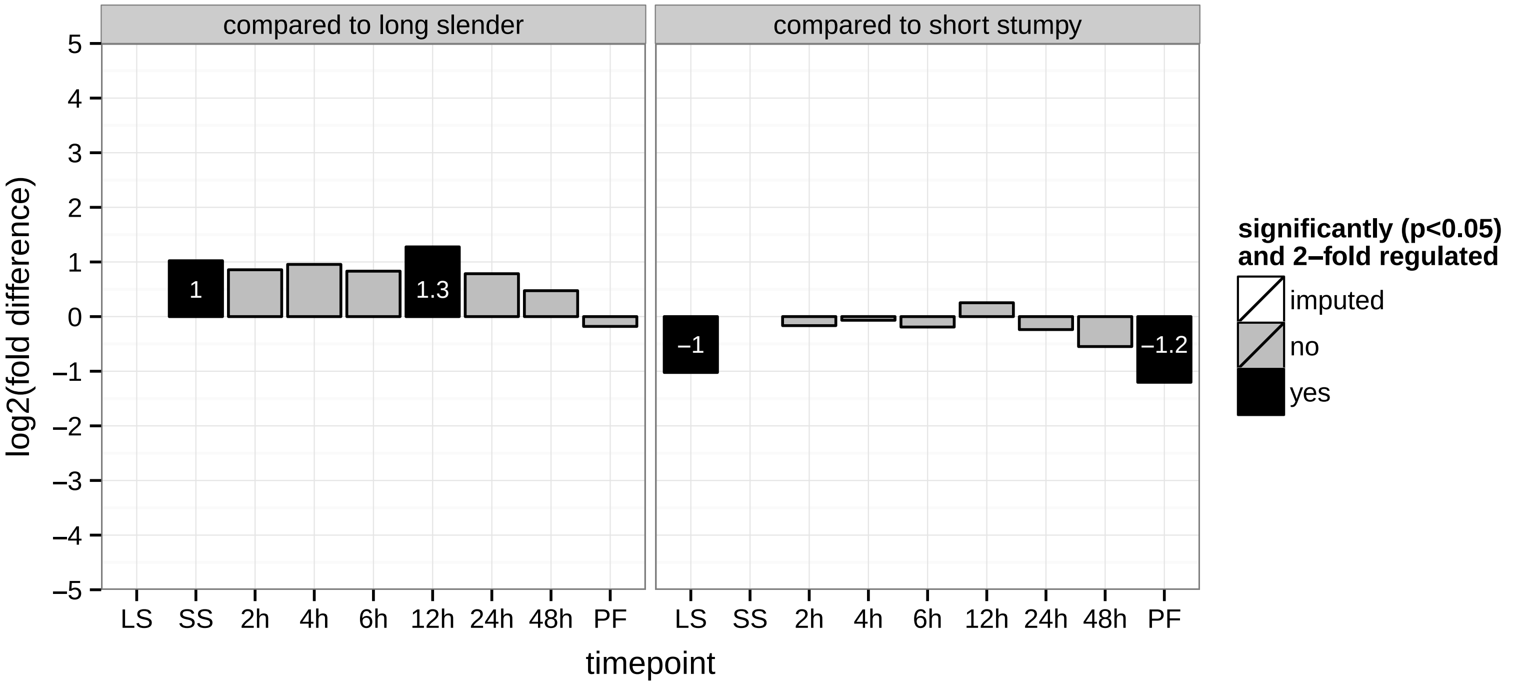

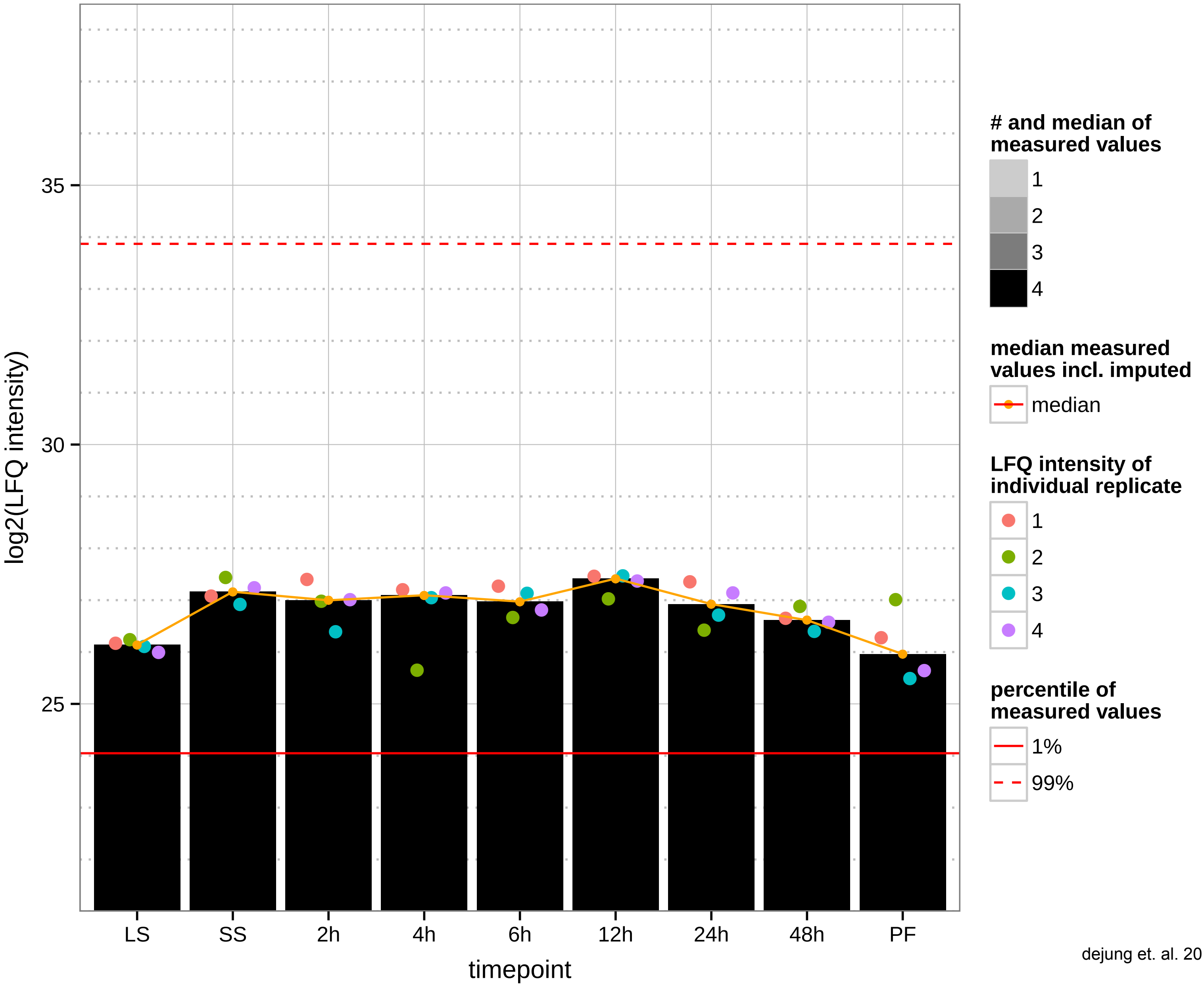

dejung et. al. 2015

hypothetical protein, conserved
Tb927.3.1980
AGOF: null
AGOC: null
AGOP: null
PGOF: null
PGOC: null
PGOP: null

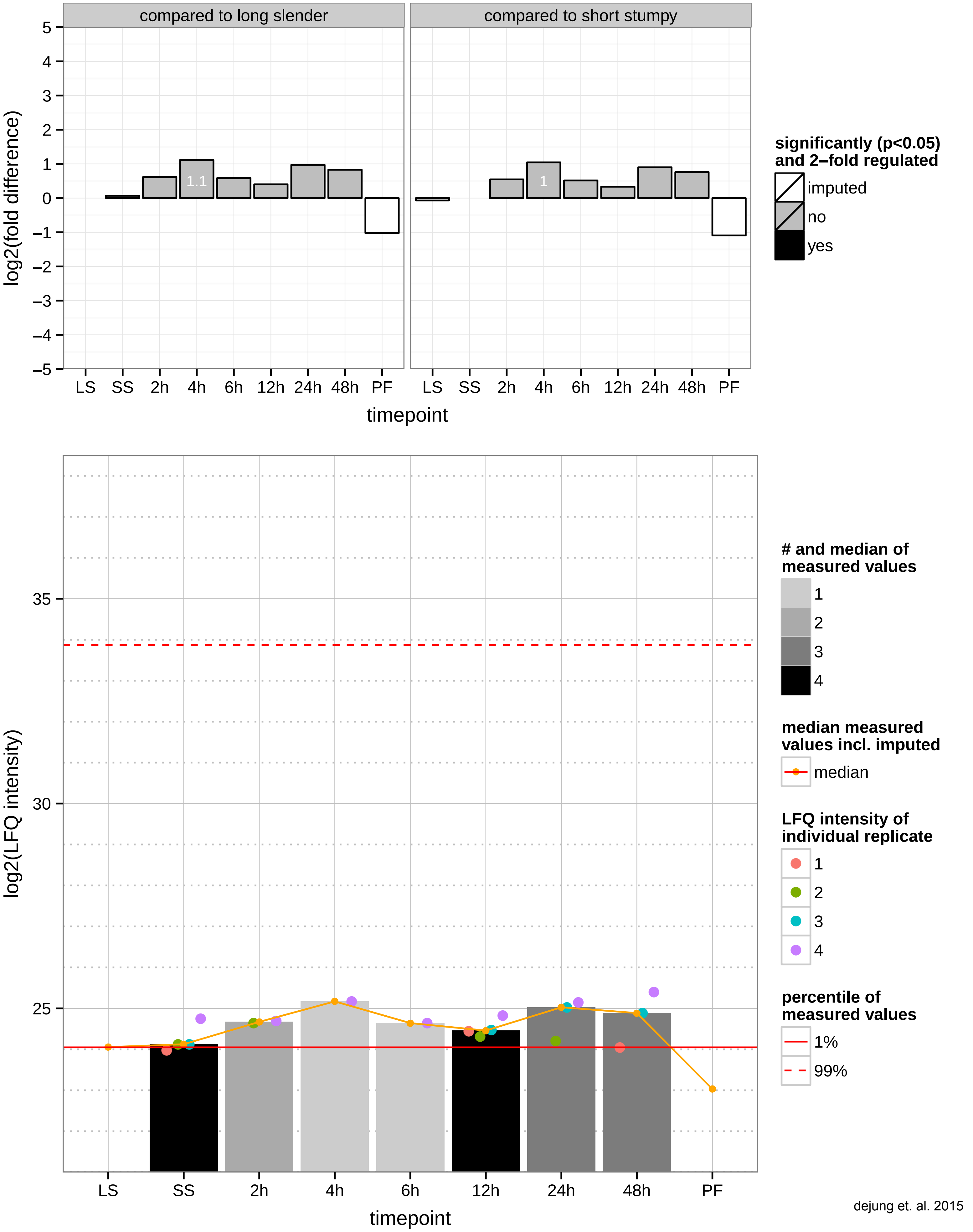

dejung et. al. 2015

RNA–binding protein, putative (RBP21)
Tb927.3.2000
AGOF: null
AGOC: null
AGOP: null
PGOF: null
PGOC: null
PGOP: null

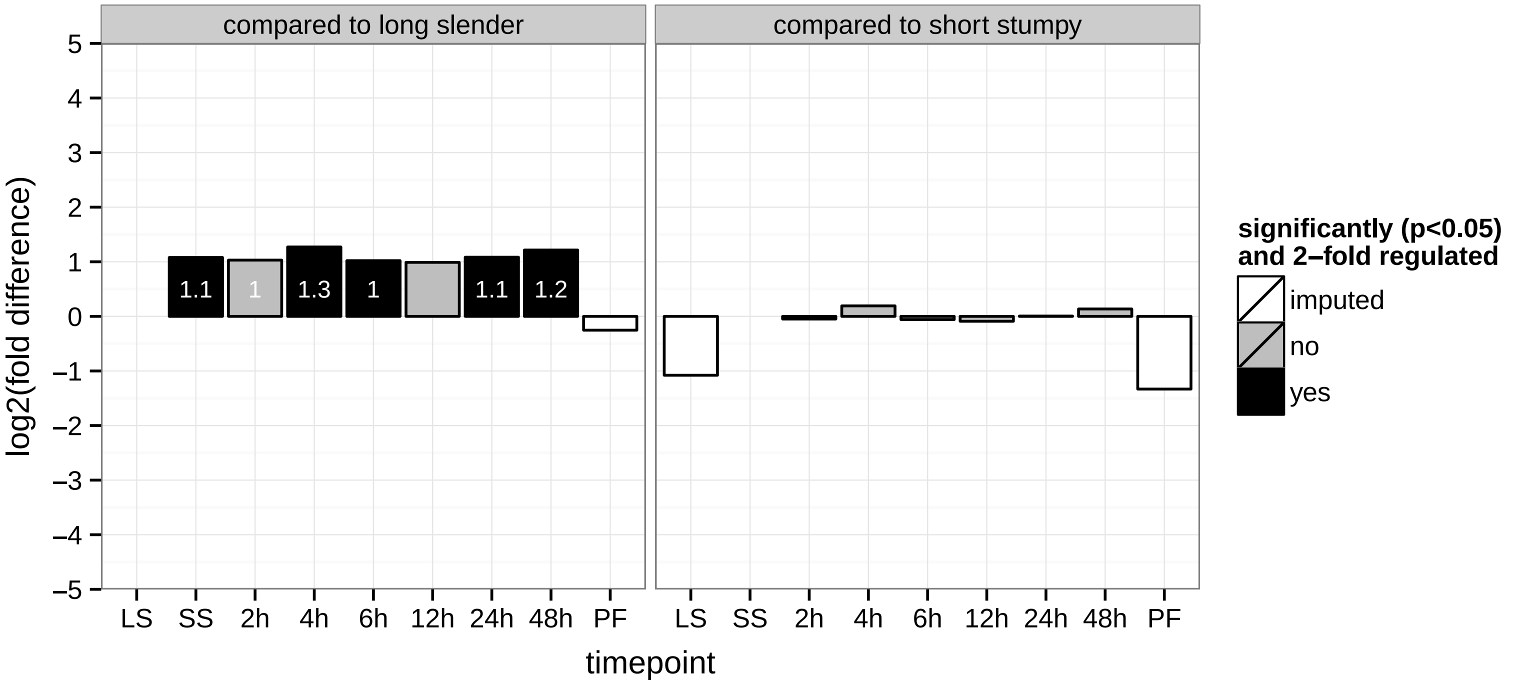

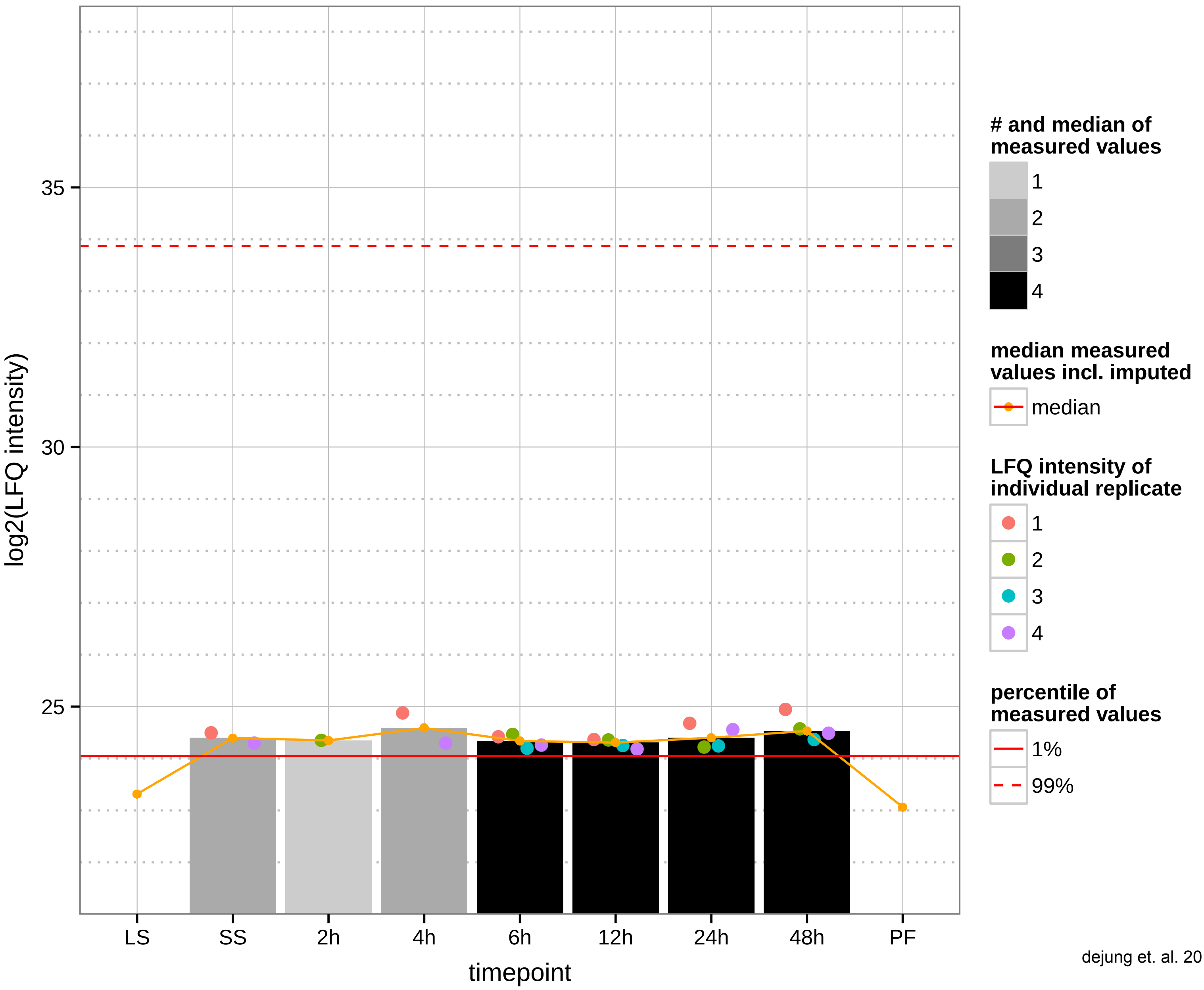

dejung et. al. 2015

vacuolar sorting protein 33, putative
Tb927.3.2280
AGOF: null
AGOC: null
AGOP: vesicle docking involved in exocytosis, vesicle–mediated transport
PGOF: null
PGOC: null
PGOP: vesicle docking involved in exocytosis, vesicle–mediated transport

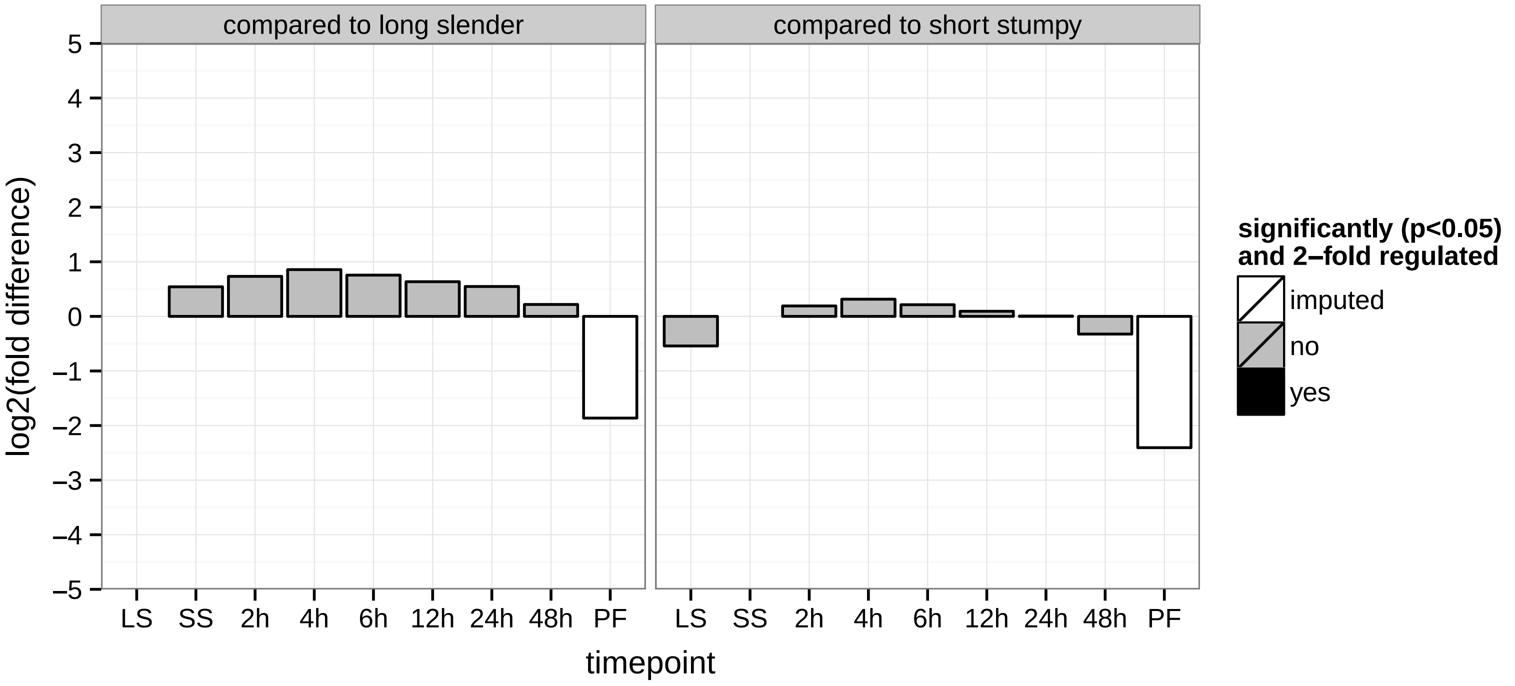

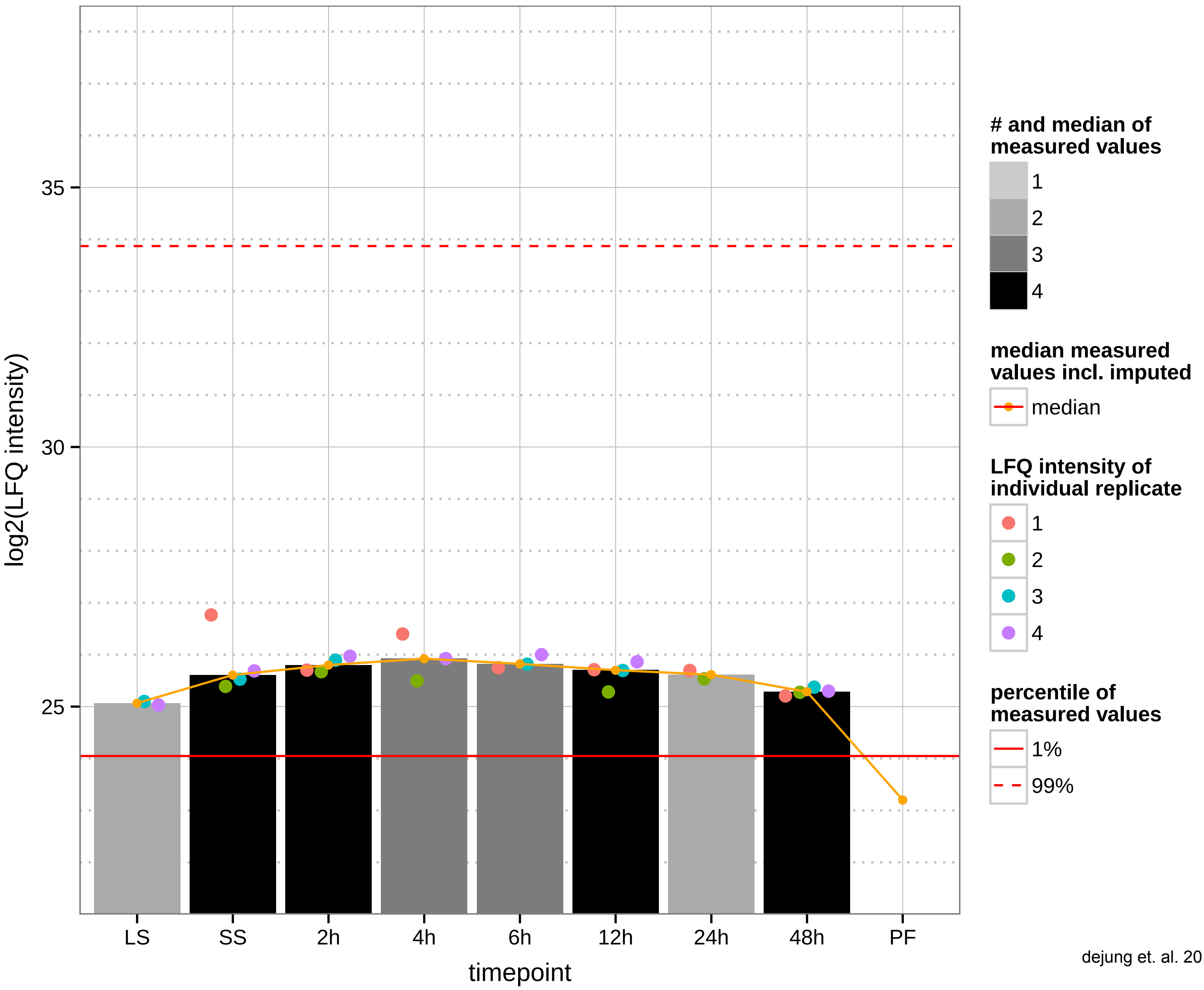

dejung et. al. 2015

hypothetical protein, conserved
Tb927.3.2870
AGOF: copper ion binding, oxidoreductase activity
AGOC: null
AGOP: null
PGOF: copper ion binding, oxidoreductase activity
PGOC: null
PGOP: oxidation−reduction process

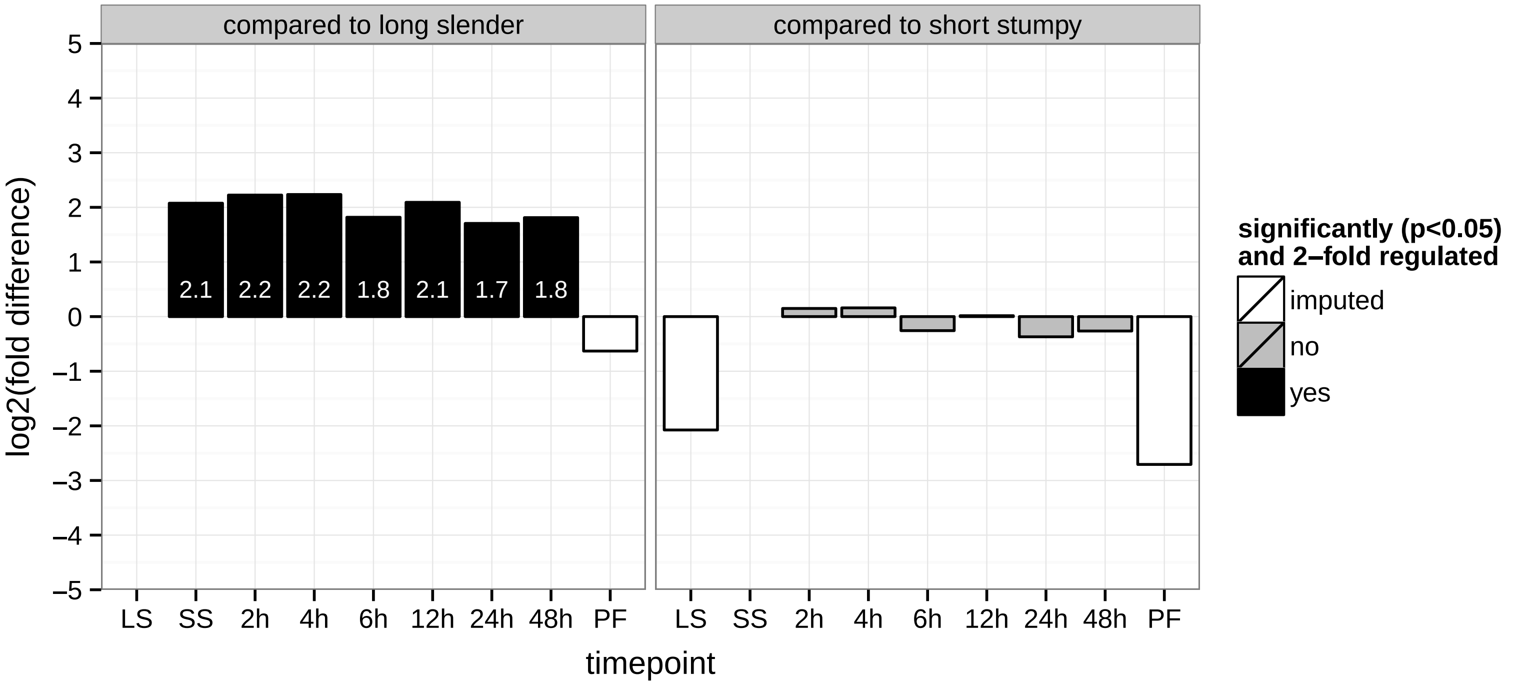

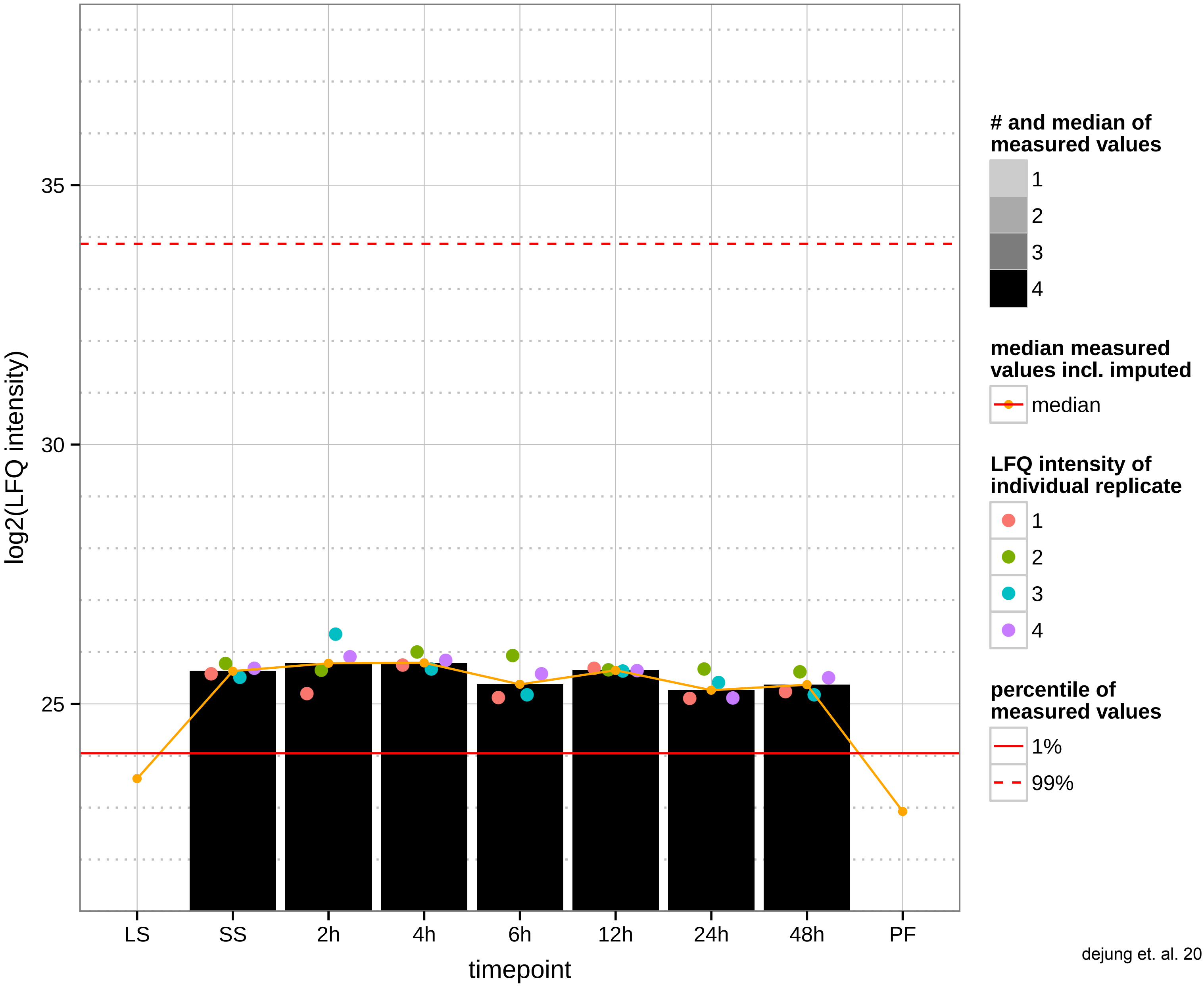

dejung et. al. 2015

serine/threonine–protein kinase, putative, protein kinase
Tb927.3.3190
AGOF: ATP binding, protein kinase activity, protein serine/threonine kinase activity, protein tyrosine kinase activity
AGOC: null
AGOP: protein phosphorylation
PGOF: ATP binding, protein kinase activity, transferase activity, transferring phosphorus–containing groups
PGOC: null
PGOP: protein phosphorylation

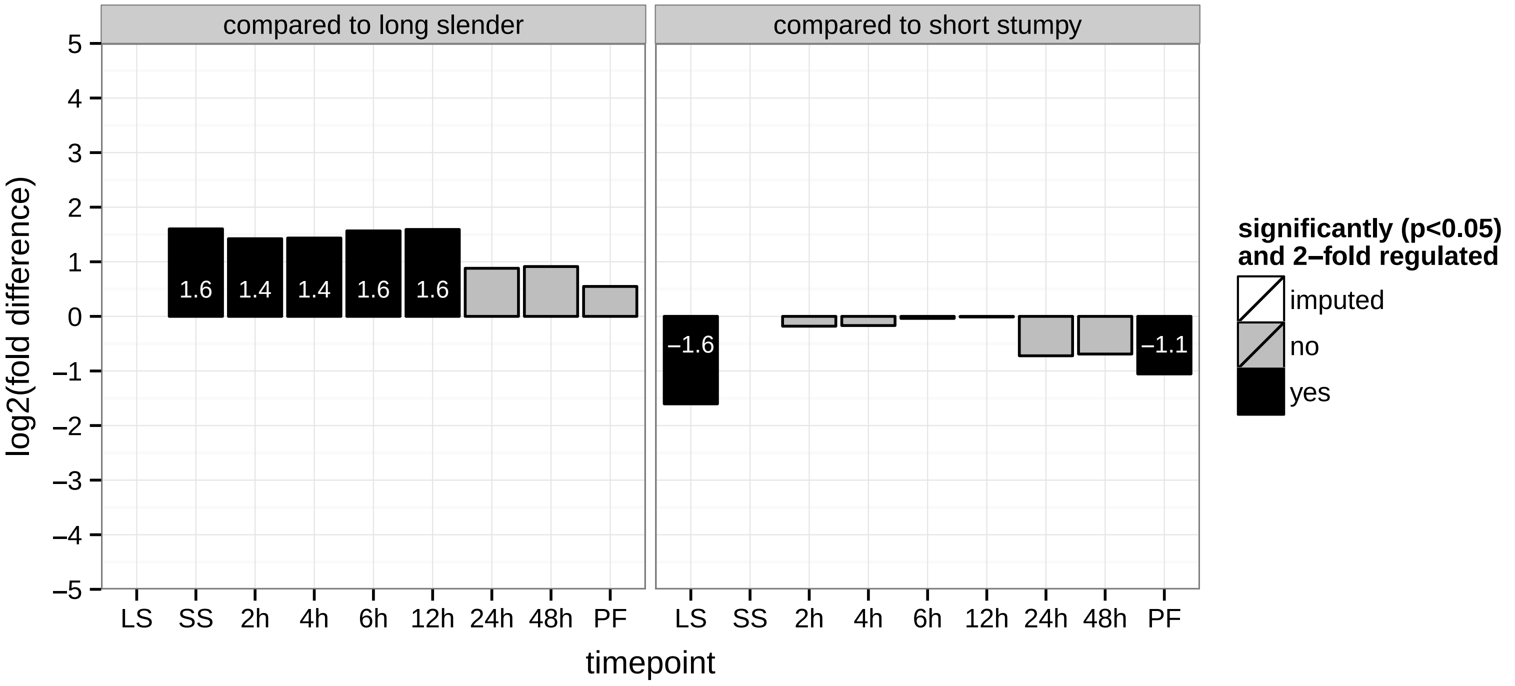

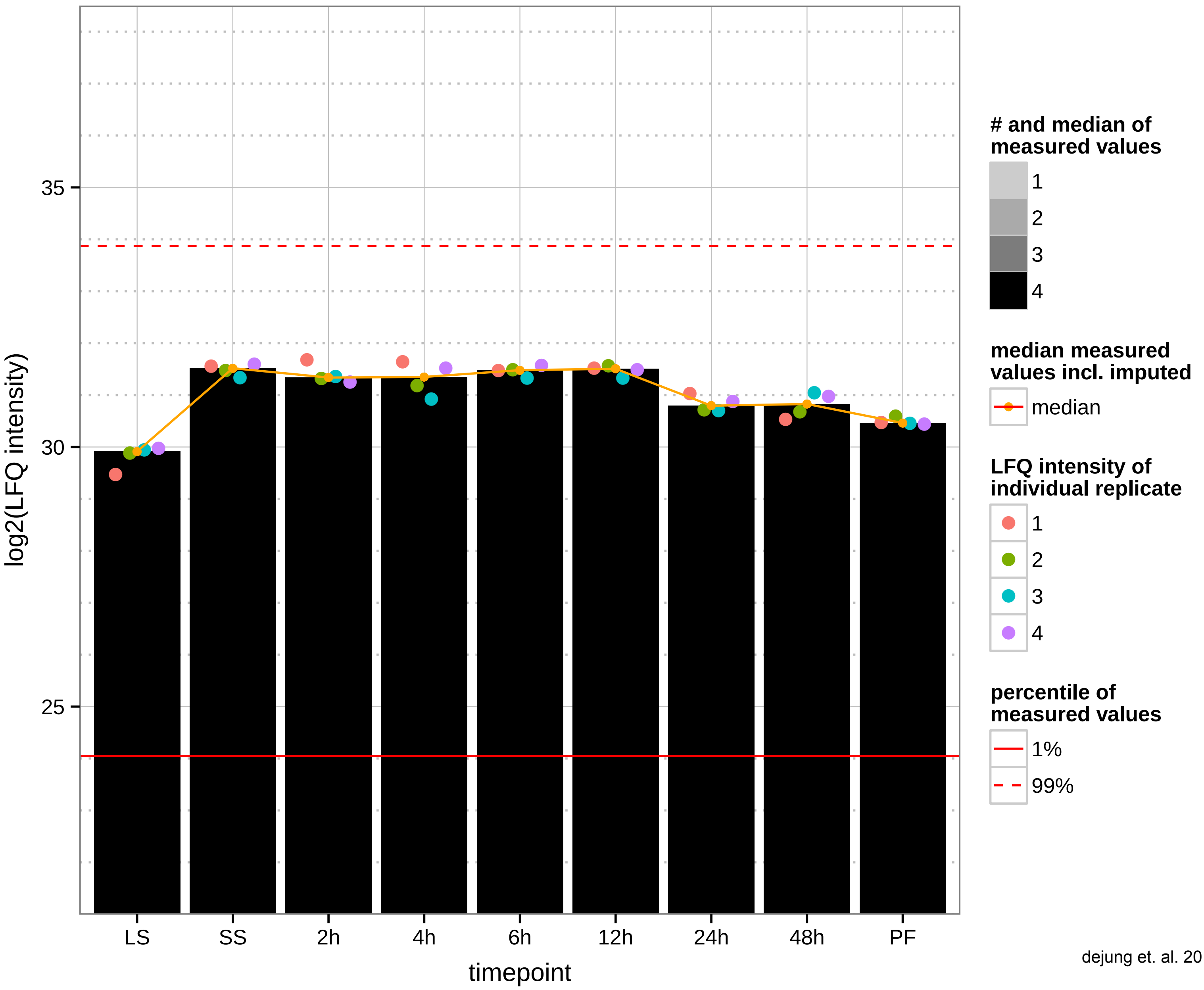

dejung et. al. 2015

acyltransferase, putative
Tb927.3.3360
AGOF: transferase activity, transferring acyl groups
AGOC: null
AGOP: metabolic process
PGOF: transferase activity, transferring acyl groups
PGOC: null
PGOP: metabolic process

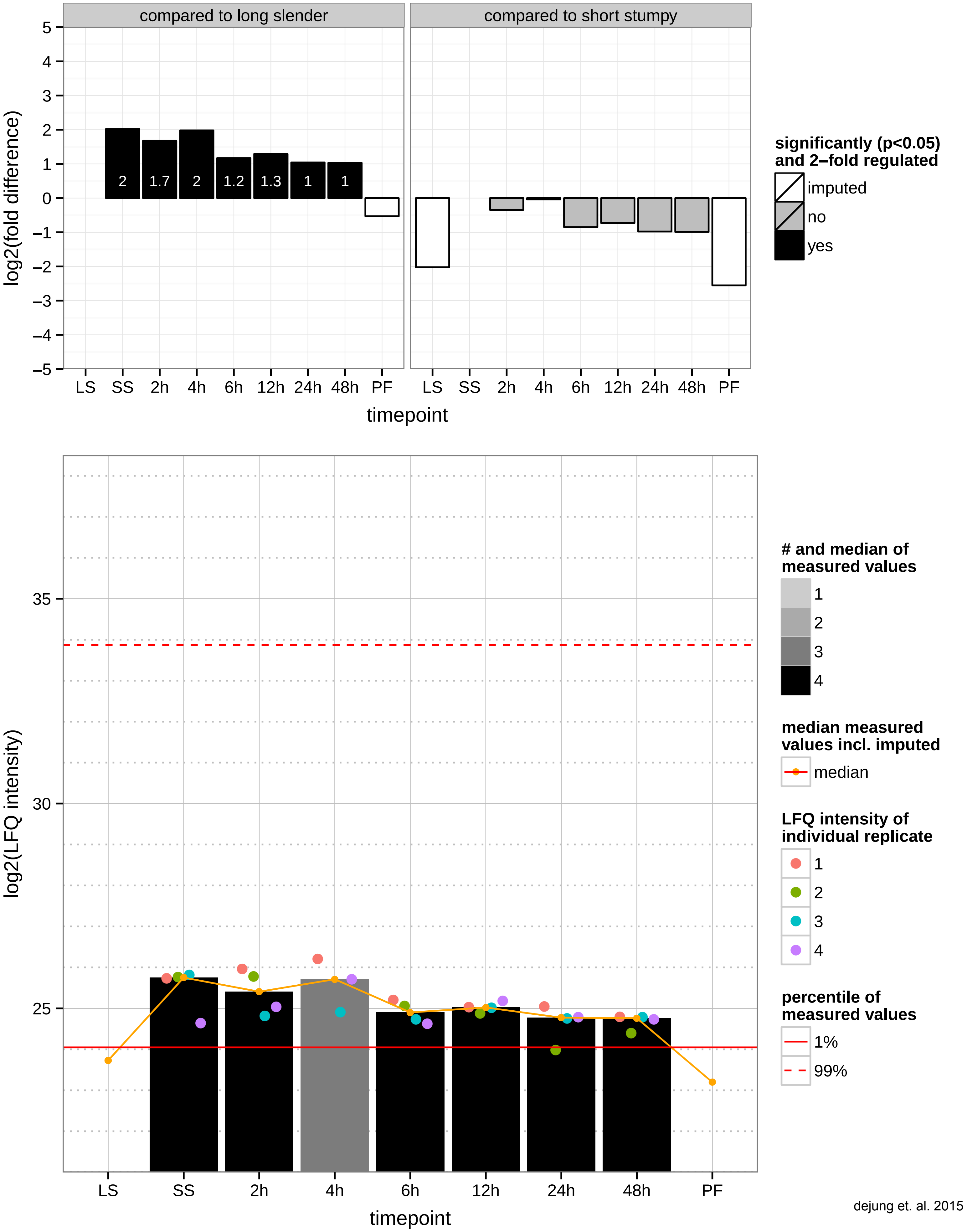

dejung et. al. 2015

lipase domain protein, putative
Tb927.3.3870
AGOF: triglyceride lipase activity
AGOC: null
AGOP: lipid metabolic process
PGOF: triglyceride lipase activity
PGOC: null
PGOP: lipid metabolic process

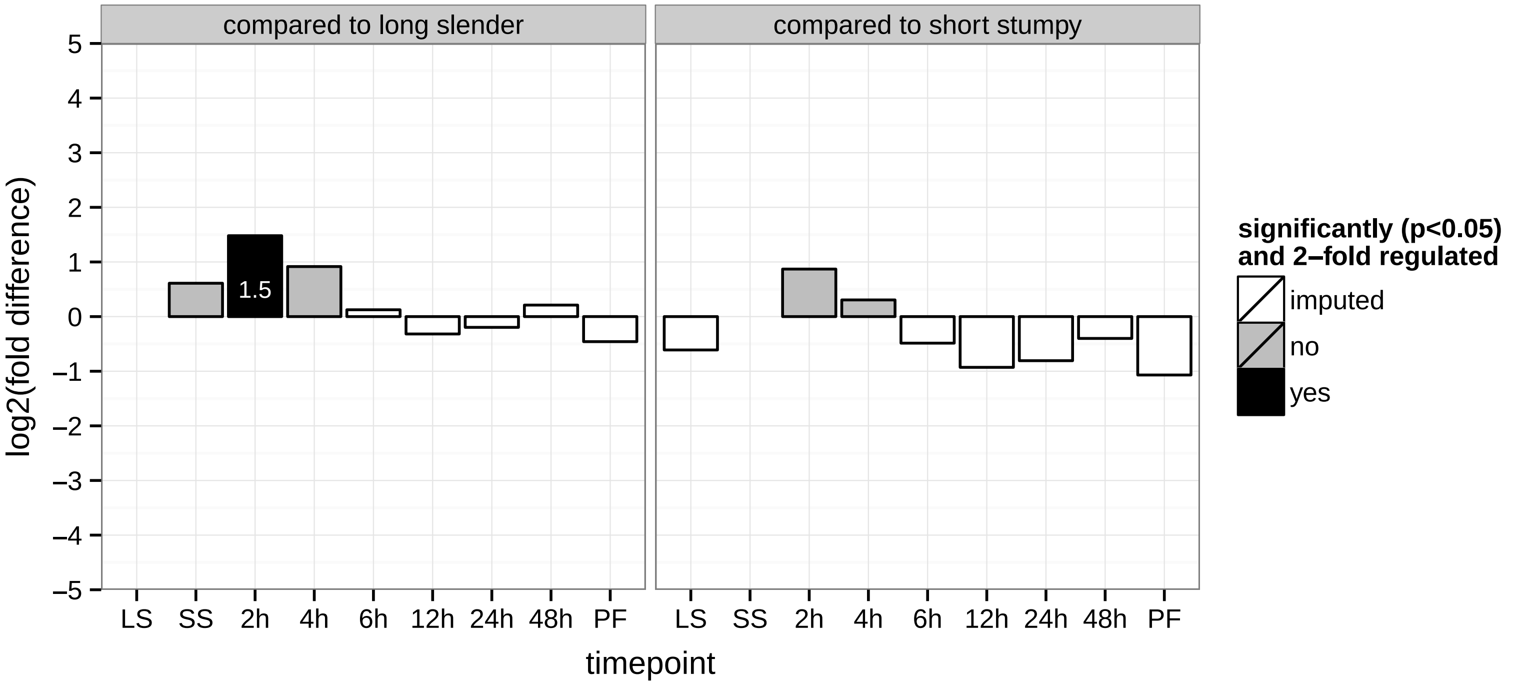

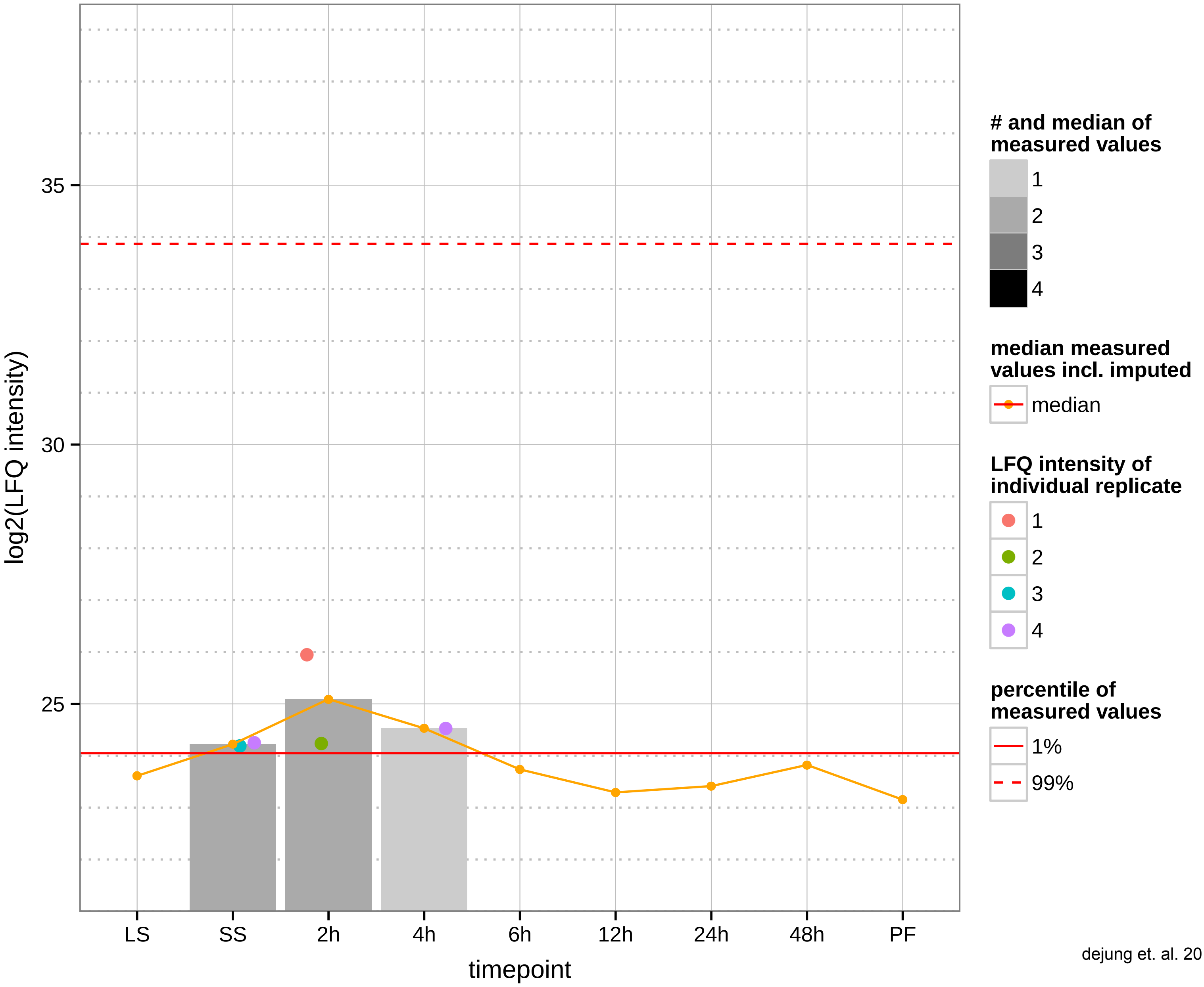

dejung et. al. 2015

hypothetical protein, conserved
Tb927.3.4260
AGOF: null
AGOC: mitochondrion
AGOP: null
PGOF: null
PGOC: null
PGOP: null

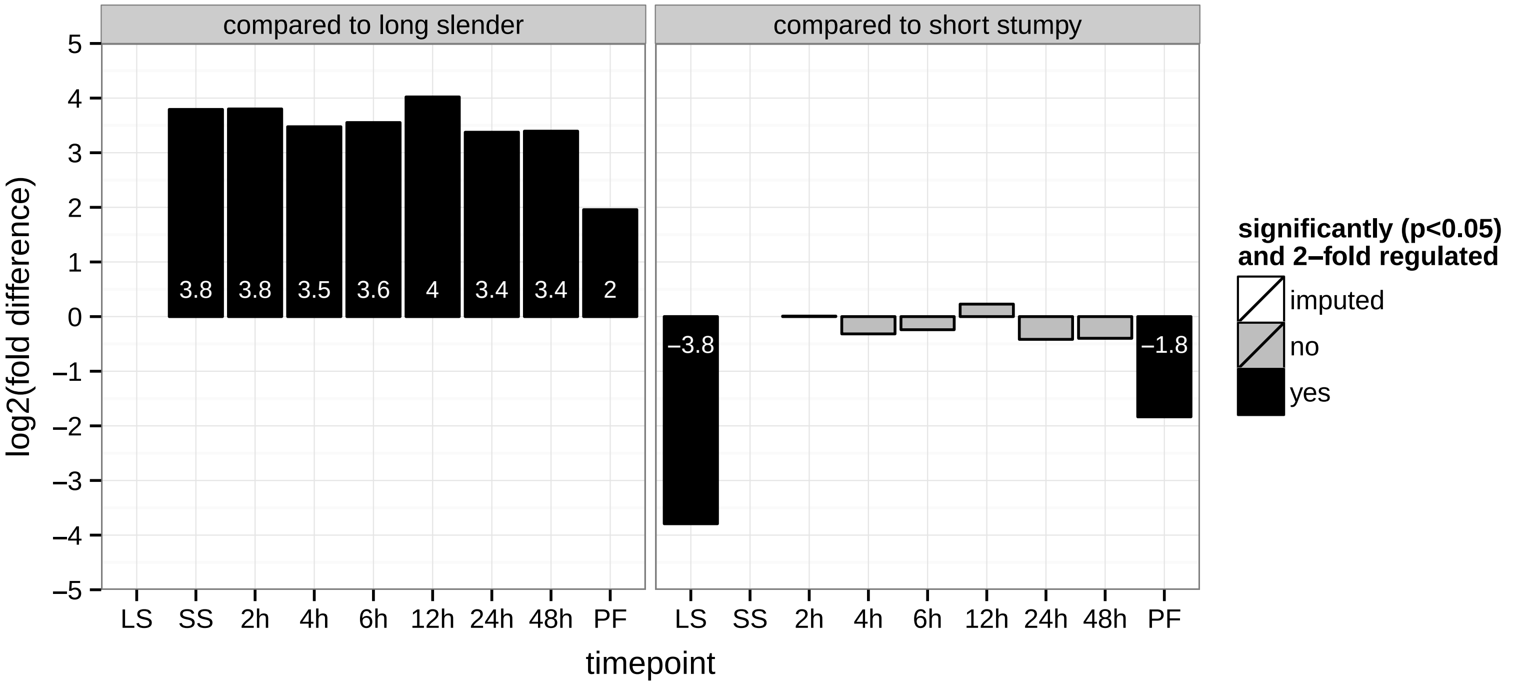

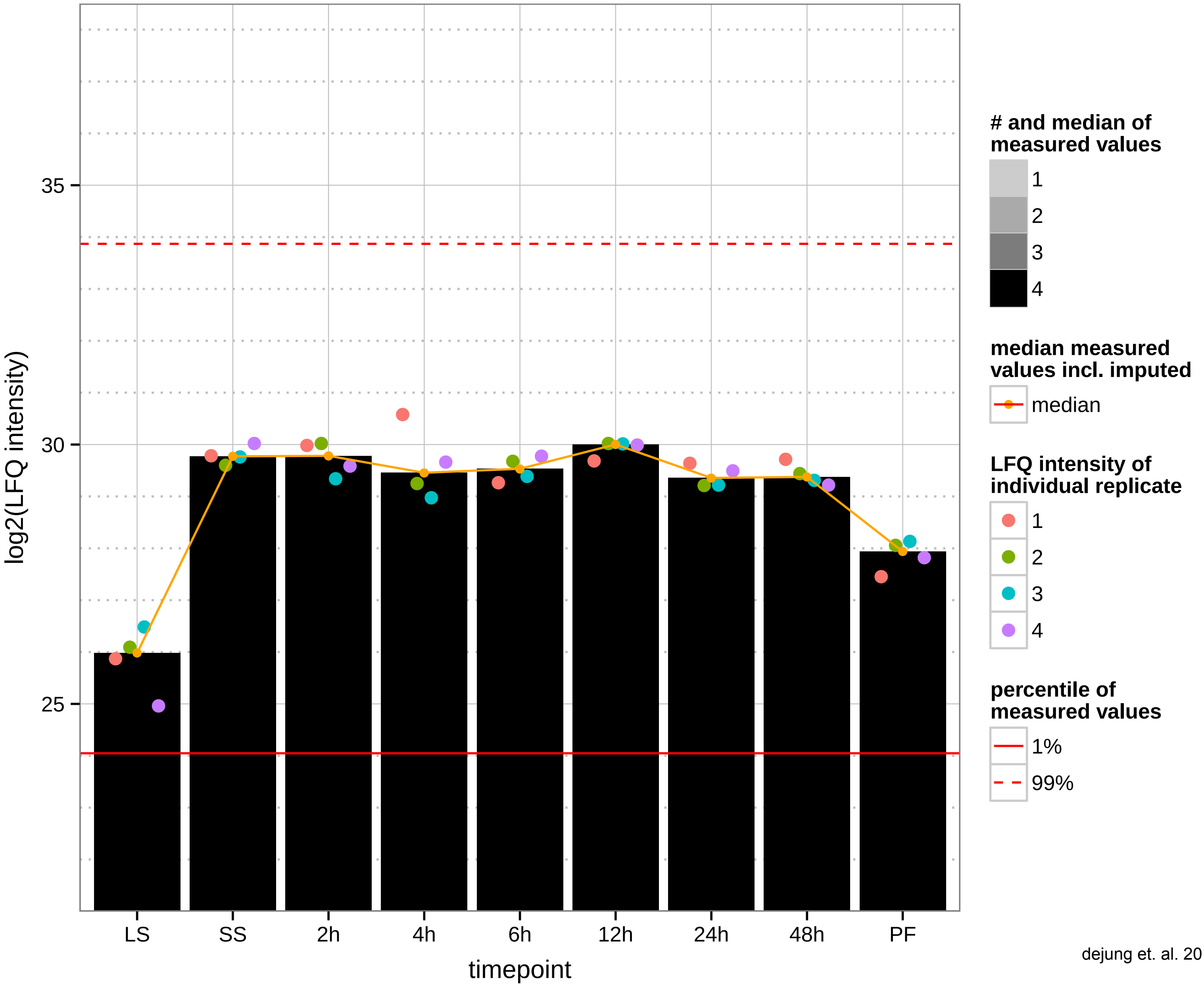

dejung et. al. 2015

hypothetical protein, conserved
Tb927.3.4420
AGOF: null
AGOC: mitochondrion
AGOP: null
PGOF: null
PGOC: null
PGOP: null

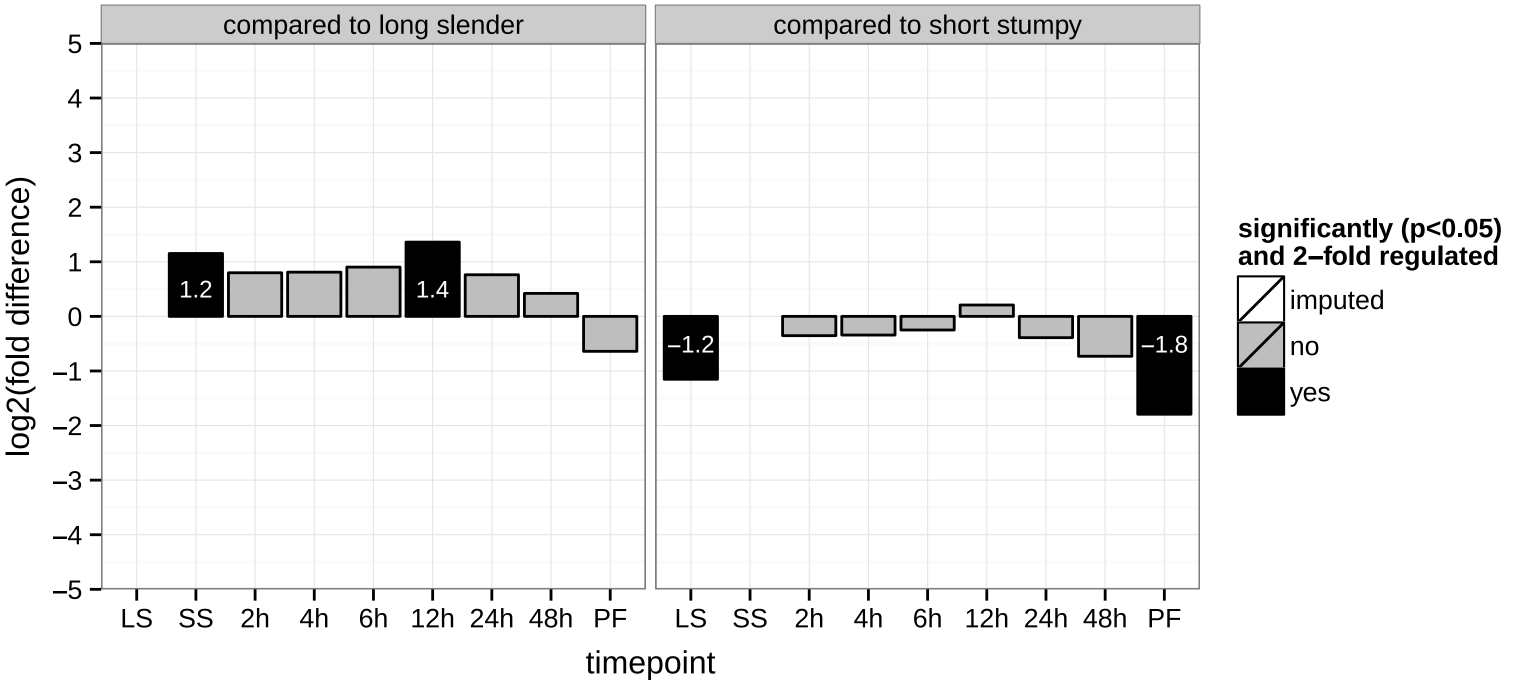

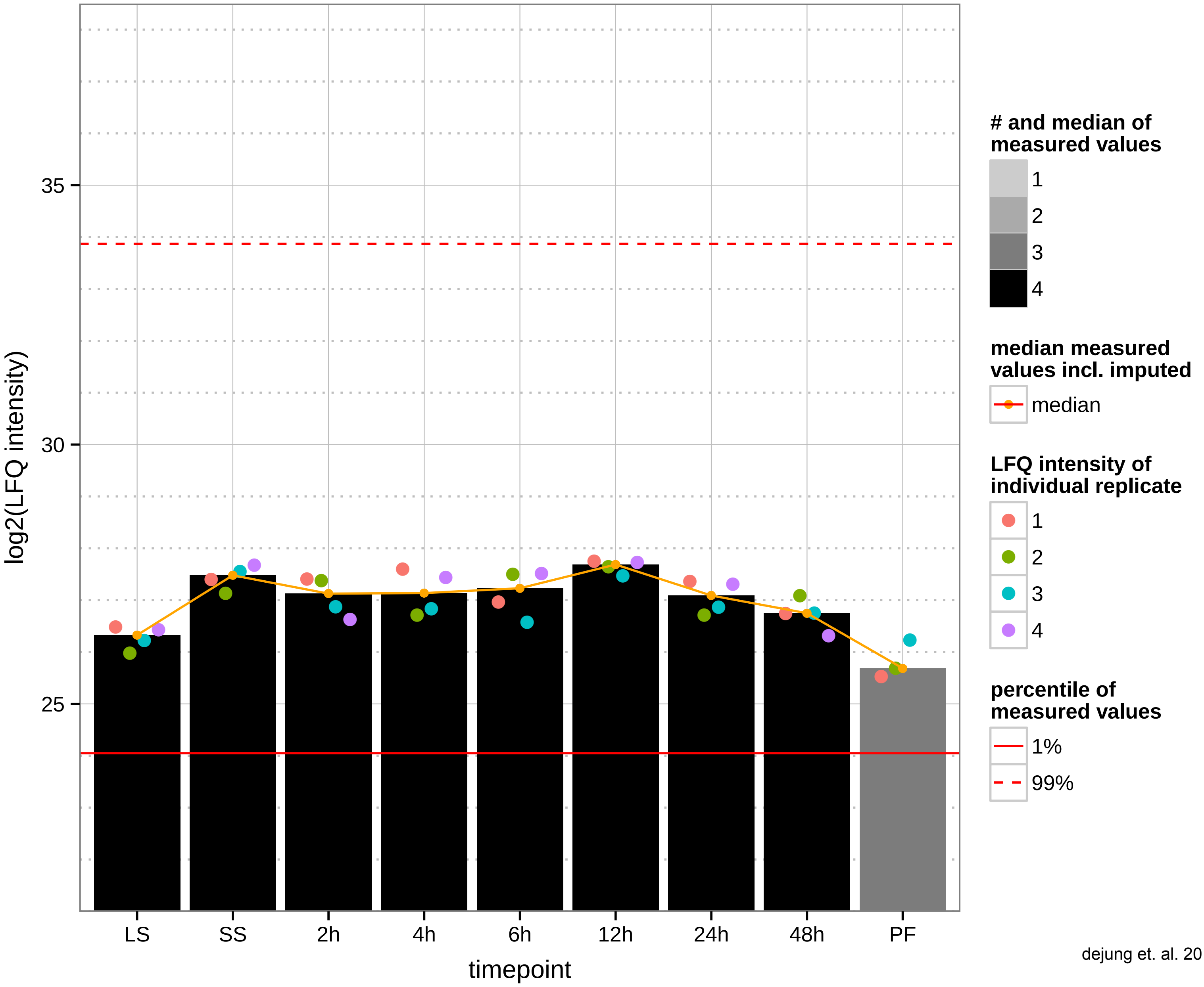

dejung et. al. 2015

RAB GDP dissociation inhibitor alpha, putative
Tb927.3.4680
AGOF: Rab GDP−dissociation inhibitor activity
AGOC: null
AGOP: protein transport, regulation of GTPase activity
PGOF: null
PGOC: null
PGOP: null

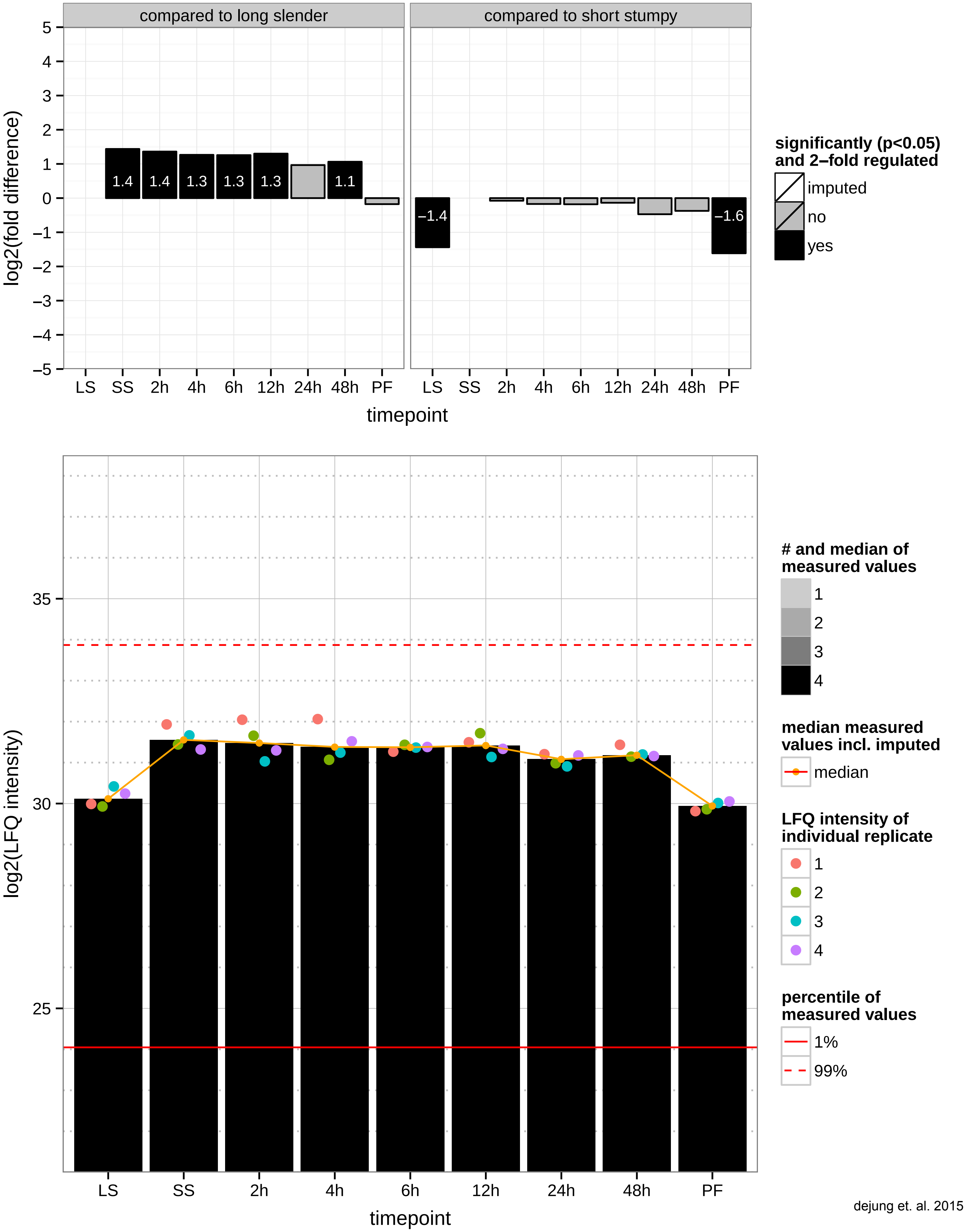

dejung et. al. 2015

hypothetical protein, conserved
Tb927.3.4800
AGOF: null
AGOC: null
AGOP: null
PGOF: null
PGOC: null
PGOP: null

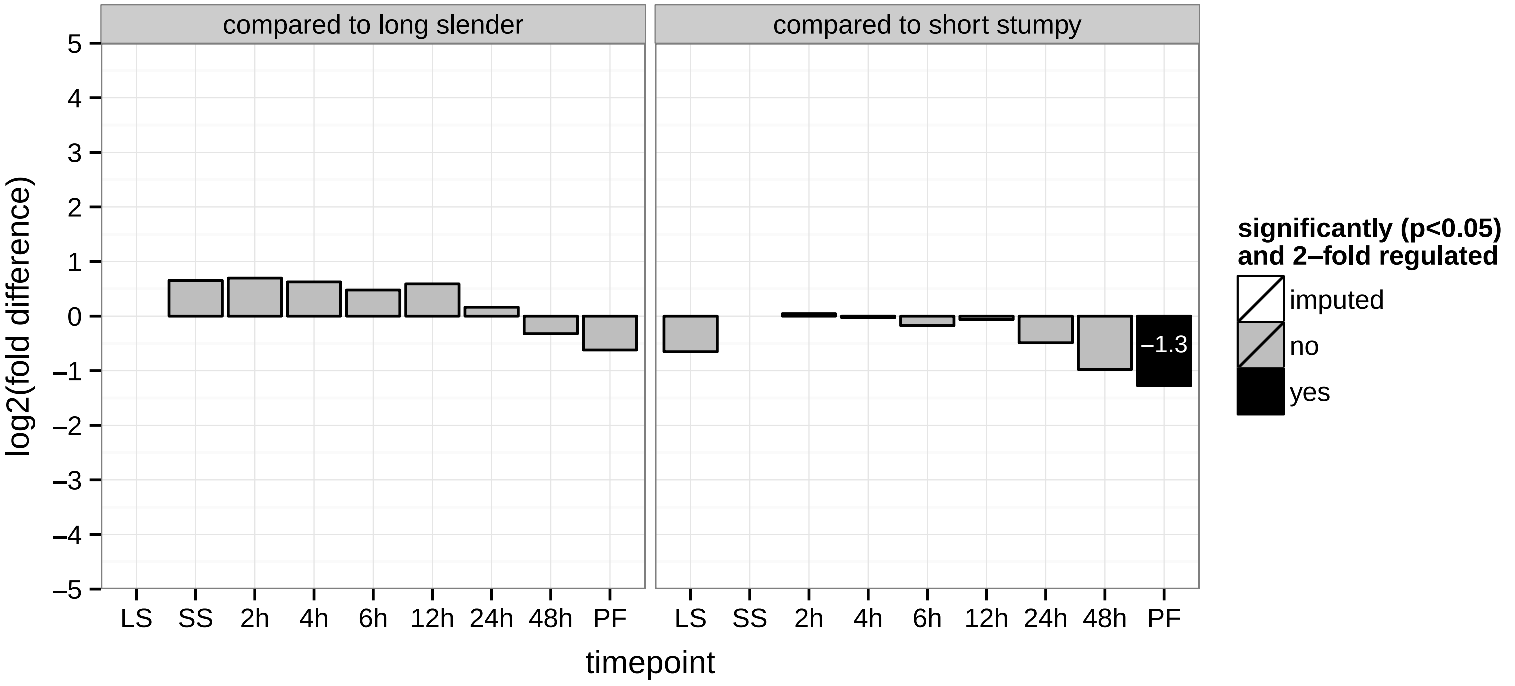

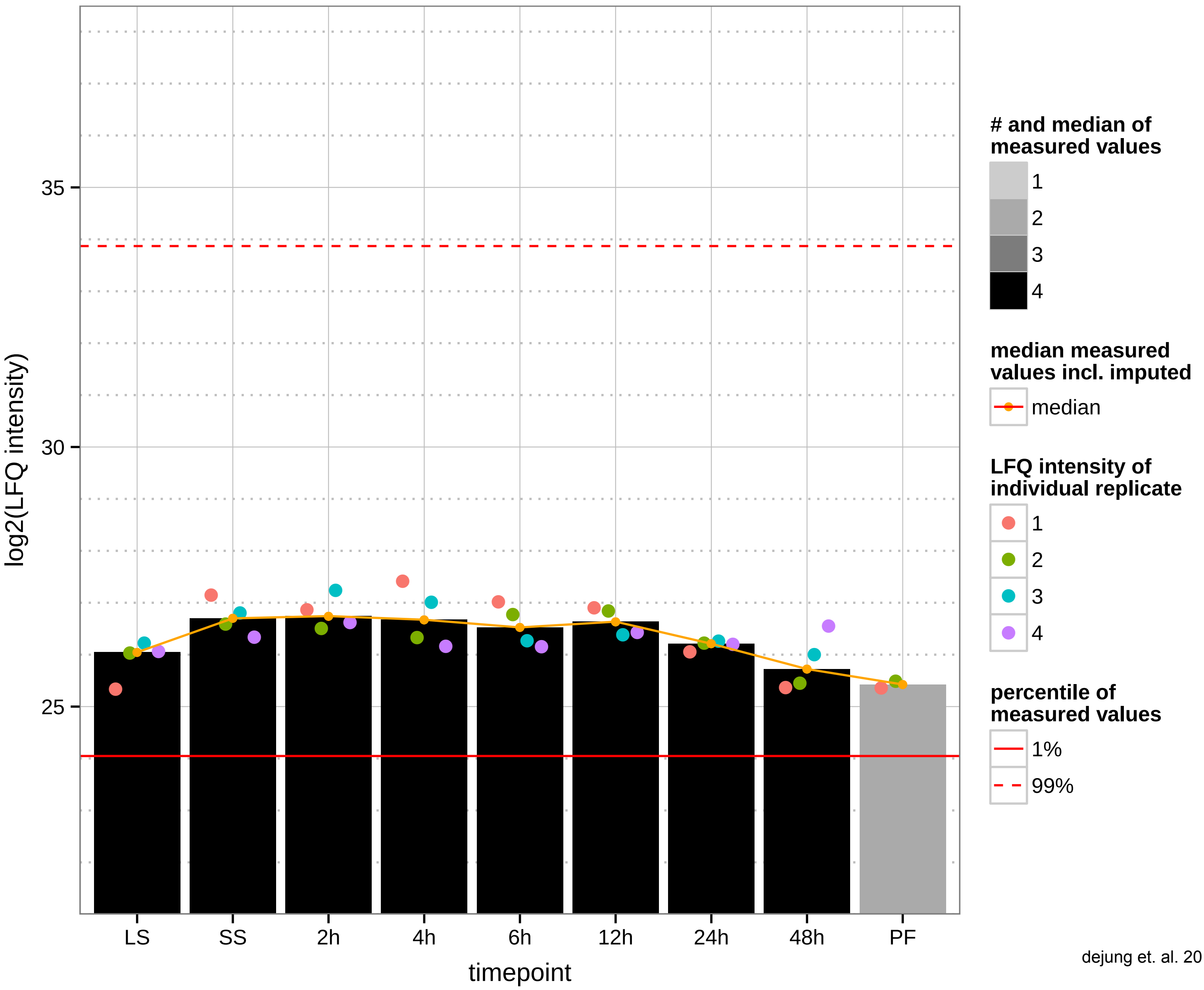

dejung et. al. 2015

ubiquitin hydrolase, putative, cysteine peptidase, Clan CA, family C19
Tb927.3.4840
AGOF: ubiquitin thiolesterase activity, zinc ion binding
AGOC: null
AGOP: ubiquitin–dependent protein catabolic process
PGOF: omega peptidase activity, protein binding, ubiquitin thiolesterase activity, zinc ion binding
PGOC: null
PGOP: ubiquitin–dependent protein catabolic process

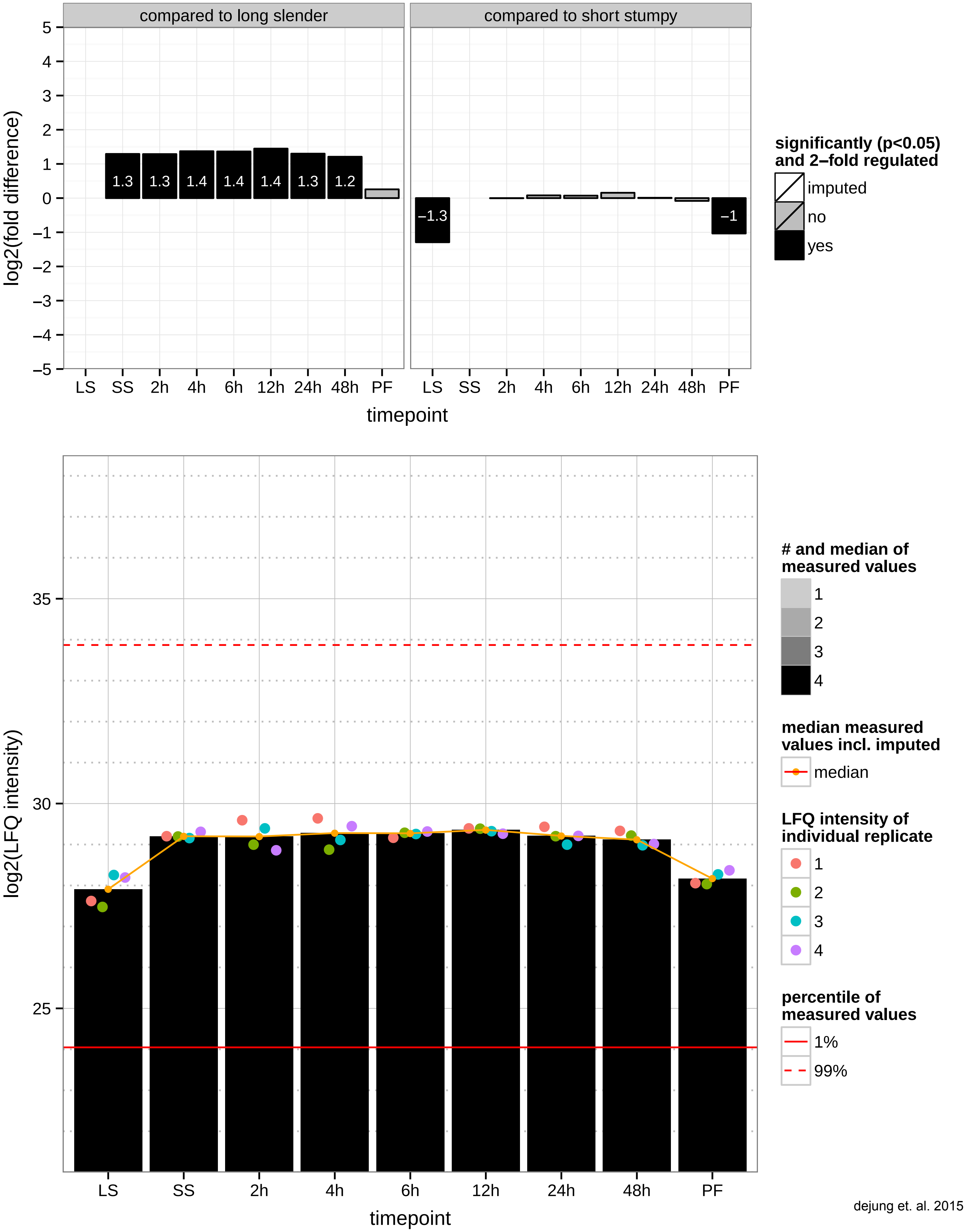

dejung et. al. 2015

spliceosome–associated protein 49, putative (DRBD1)
Tb927.3.5280
AGOF: nucleic acid binding, protein binding
AGOC: nucleus
AGOP: RNA processing
PGOF: nucleic acid binding
PGOC: null
PGOP: null

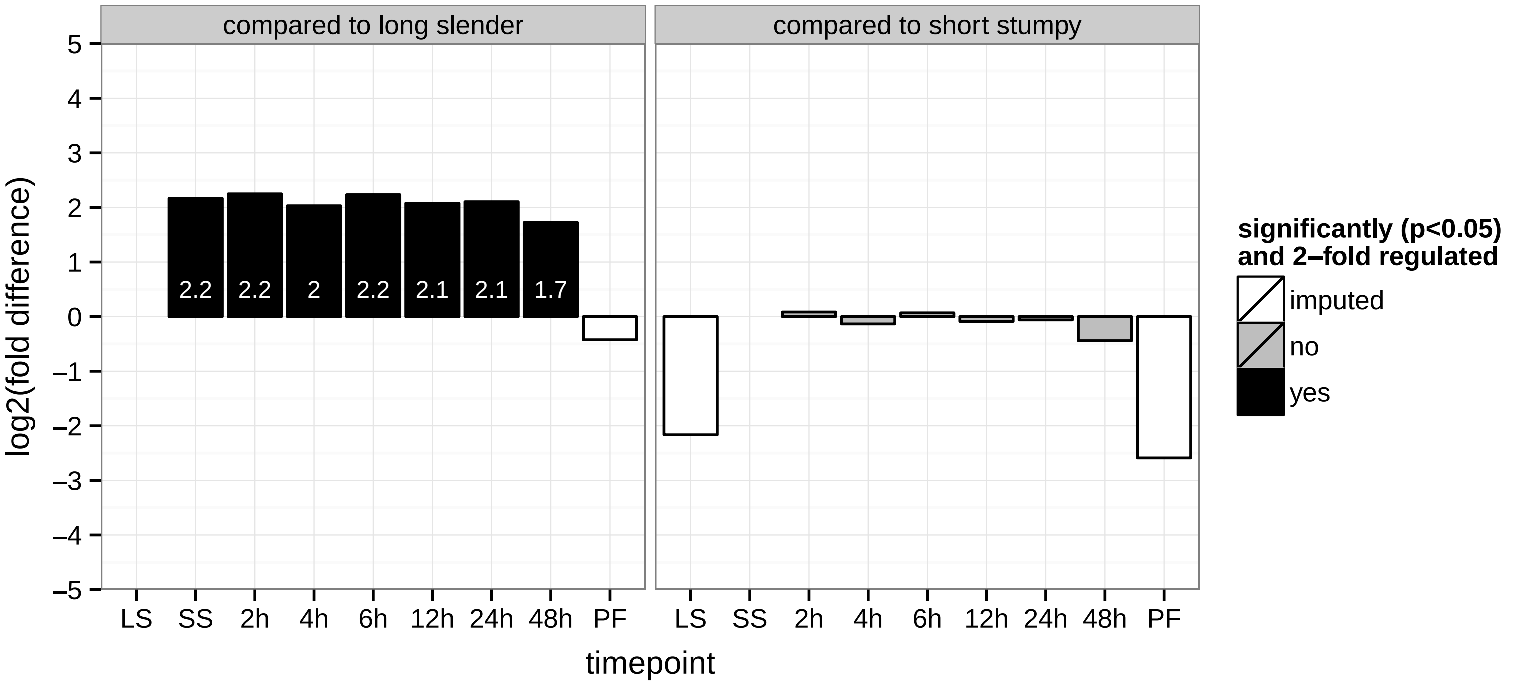

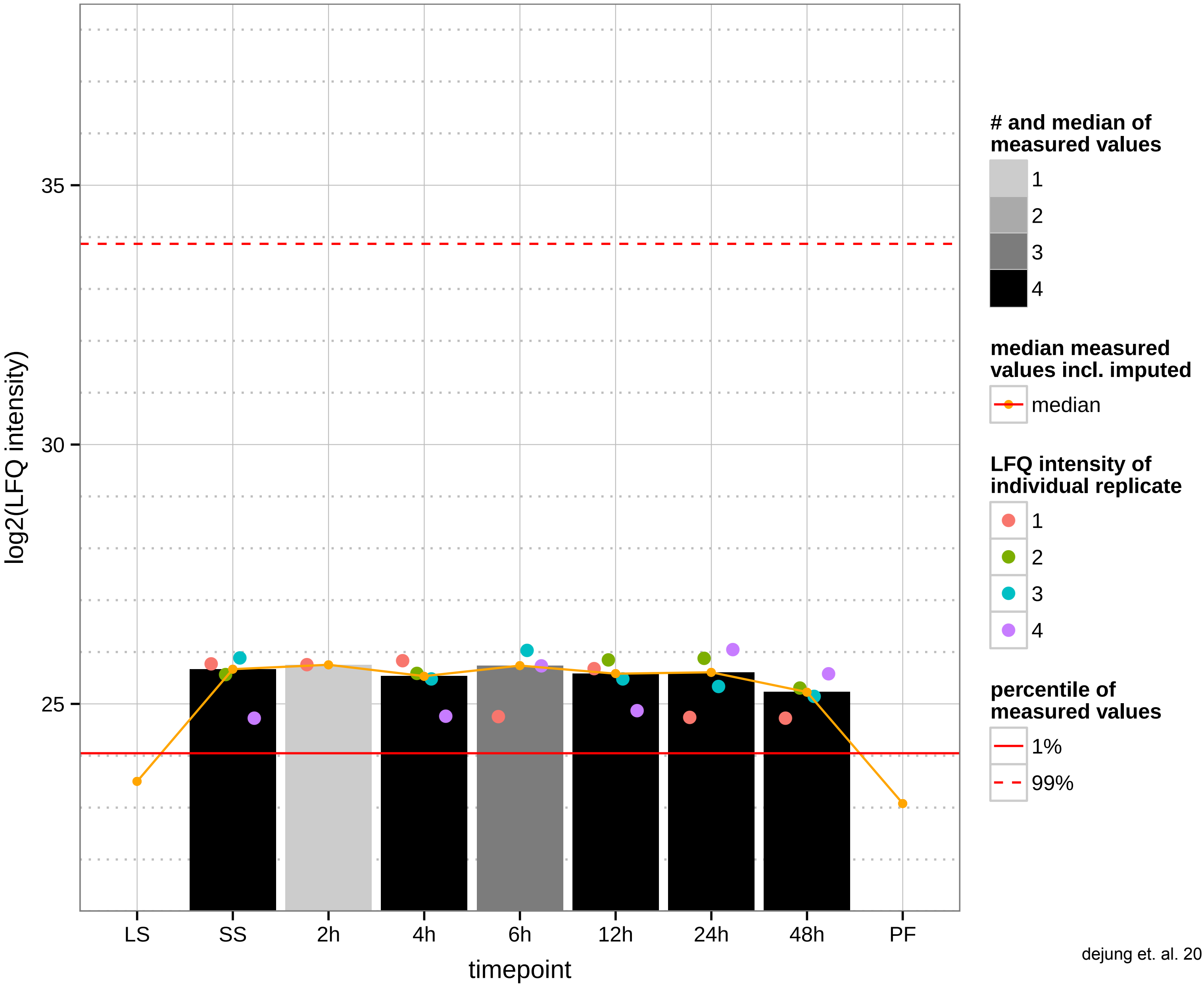

dejung et. al. 2015

hypothetical protein, conserved
Tb927.3.5360
AGOF: null
AGOC: mitochondrion
AGOP: null
PGOF: protein binding
PGOC: null
PGOP: null

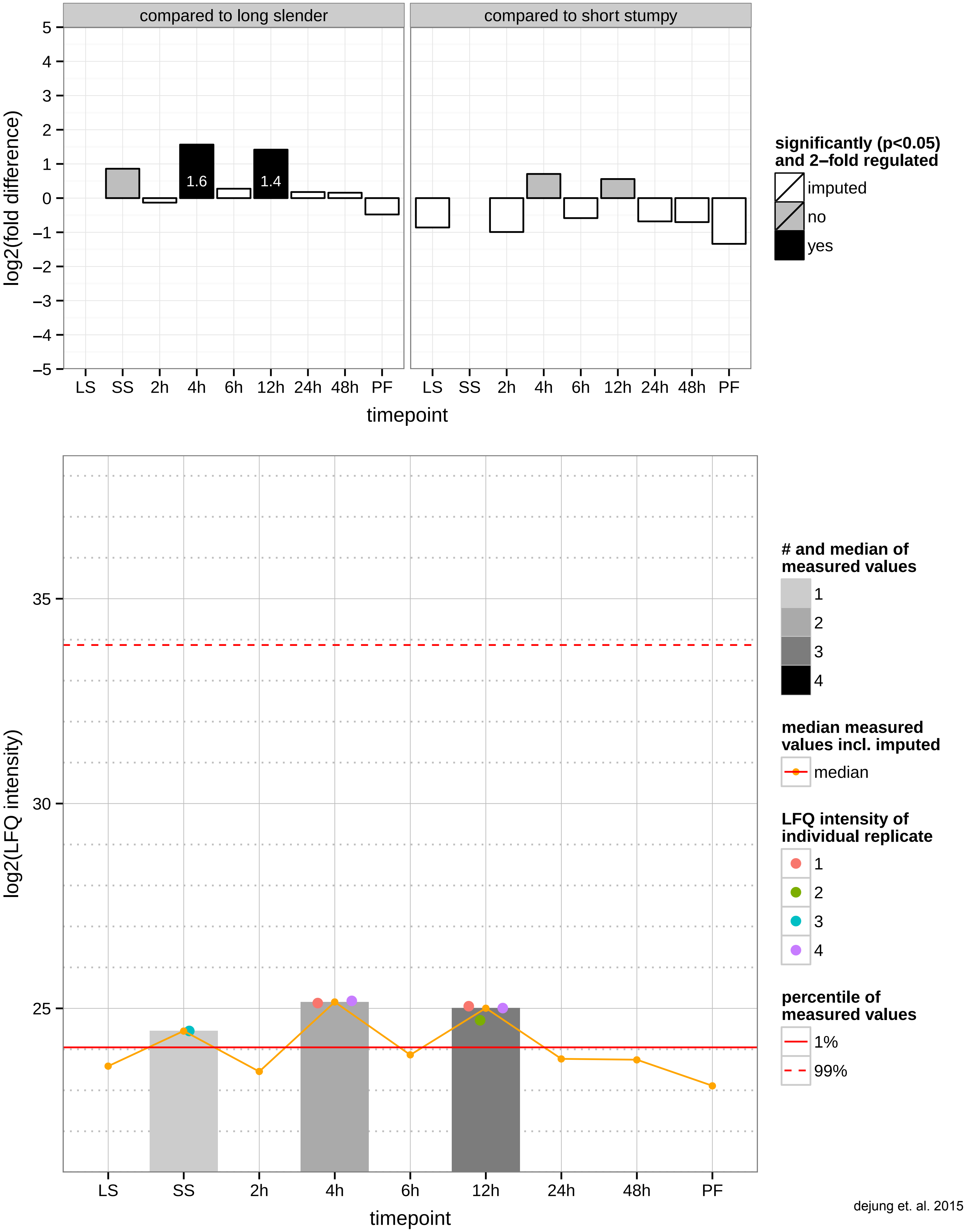

small GTP–binding protein, putative (TbRX1)
Tb927.3.5550
AGOF: GTP binding, GTPase activity
AGOC: intracellular
AGOP: protein transport, small GTPase mediated signal transduction
PGOF: GTP binding, GTPase activity
PGOC: intracellular, membrane
PGOP: GTP catabolic process, protein transport, signal transduction, small GTPase mediated signal transduction

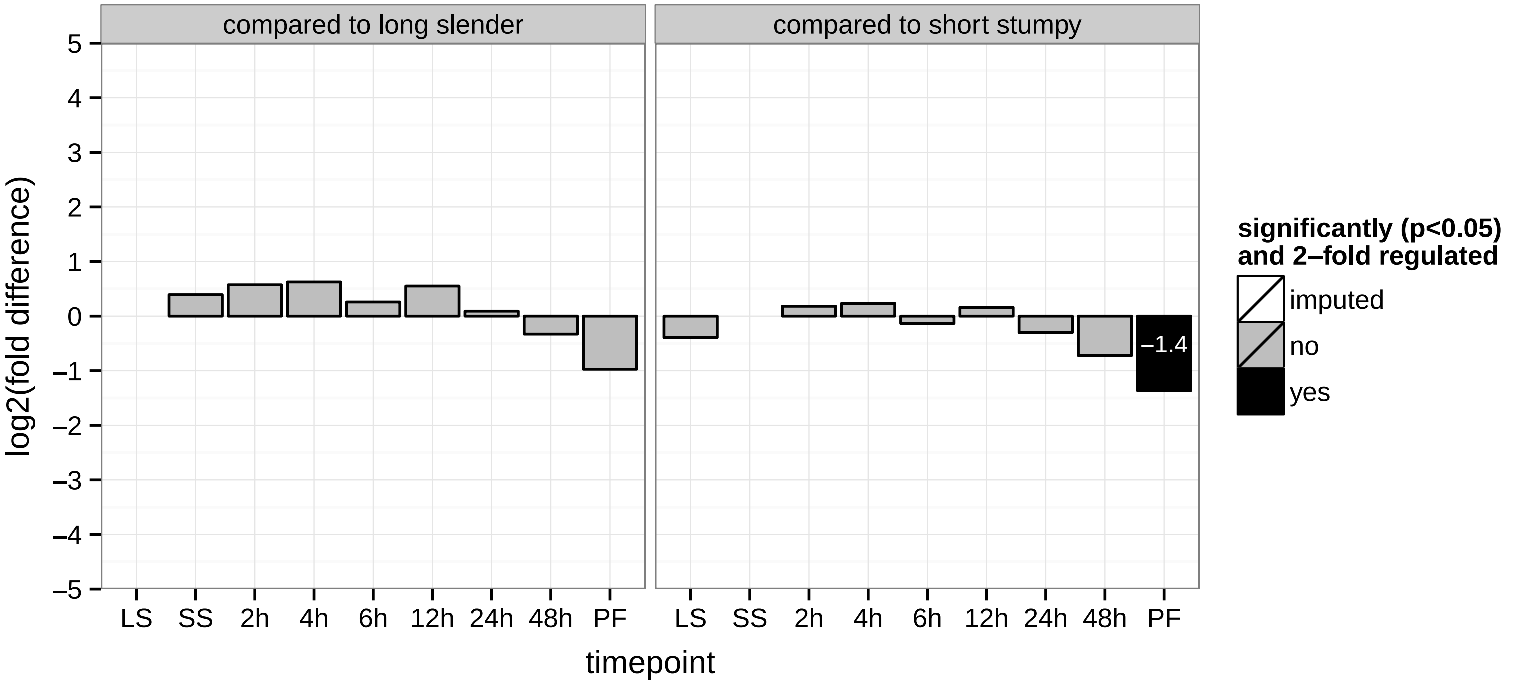

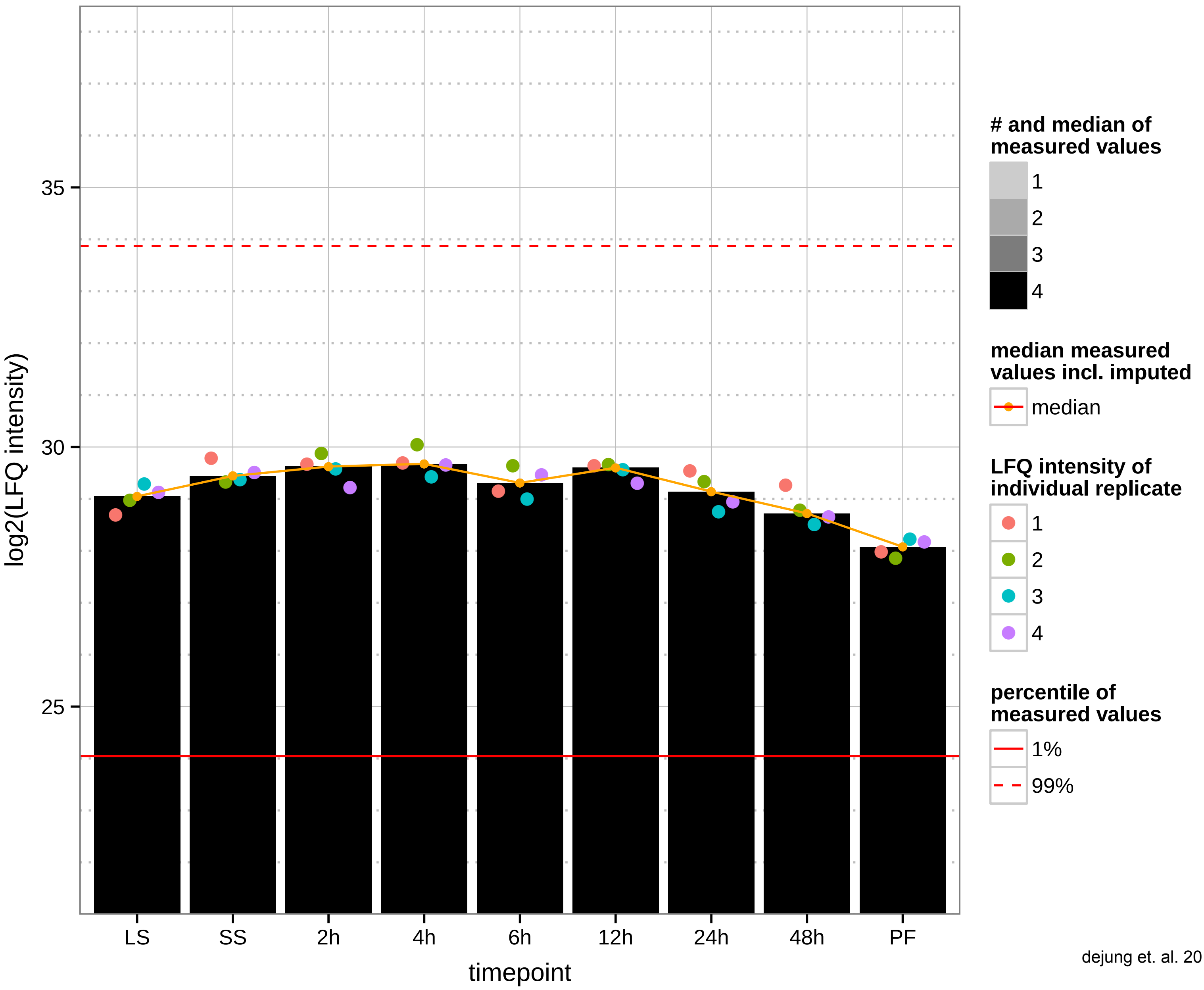

dejung et. al. 2015

syntaxin, putative
Tb927.3.5570
AGOF: null
AGOC: membrane
AGOP: vesicle−mediated transport
PGOF: protein binding
PGOC: membrane
PGOP: vesicle−mediated transport

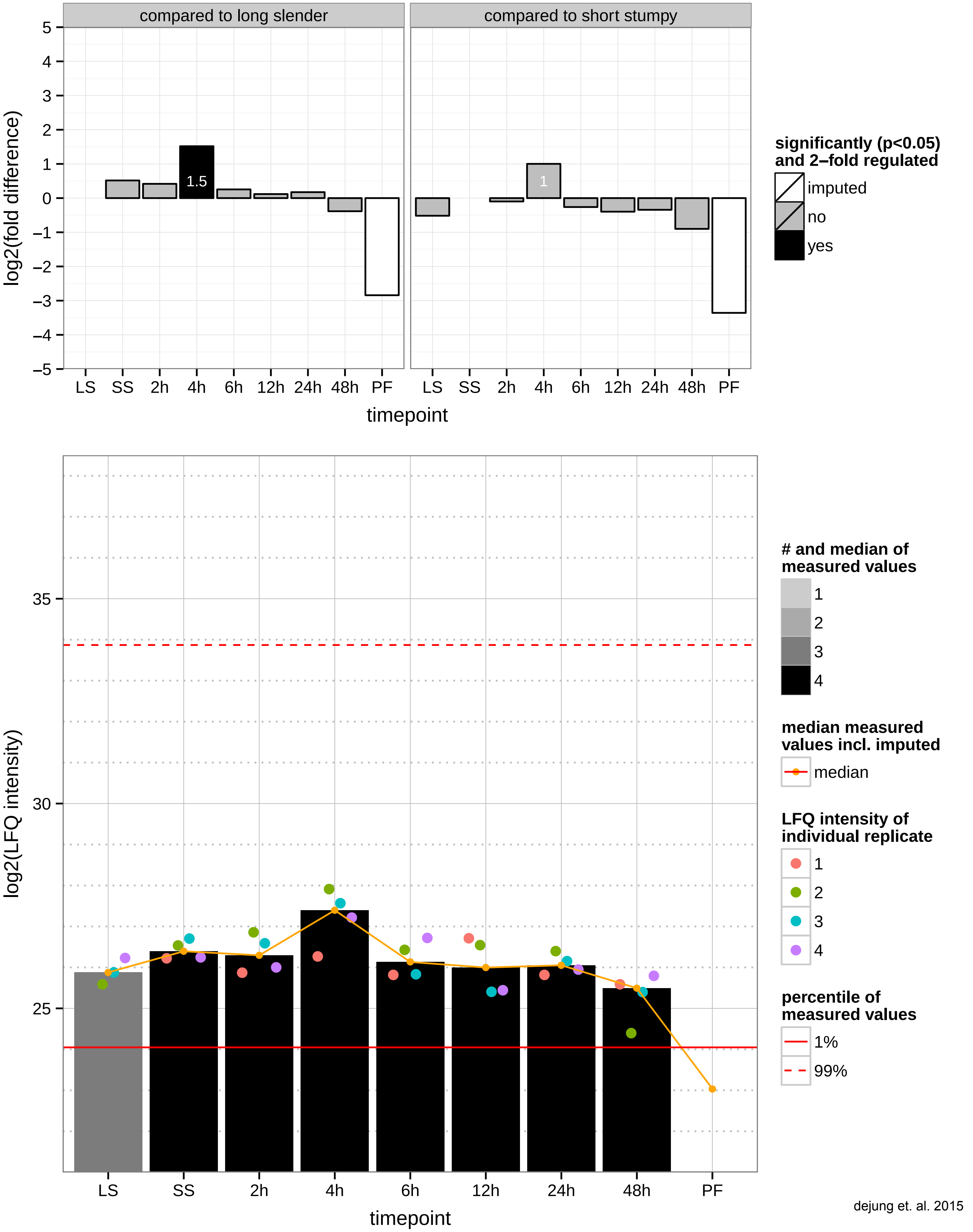

dejung et. al. 2015

hypothetical protein, conserved
Tb927.3.880
AGOF: null
AGOC: null
AGOP: null
PGOF: protein binding
PGOC: null
PGOP: null

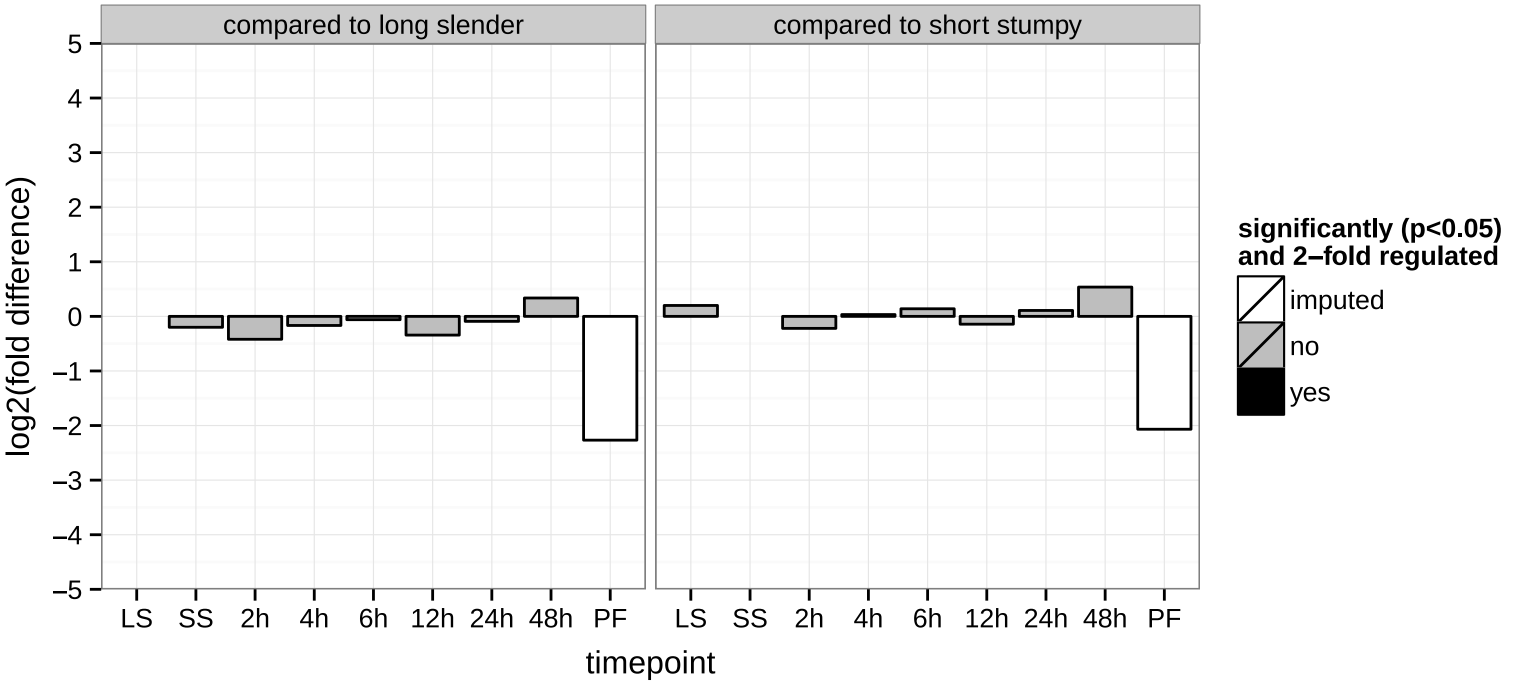

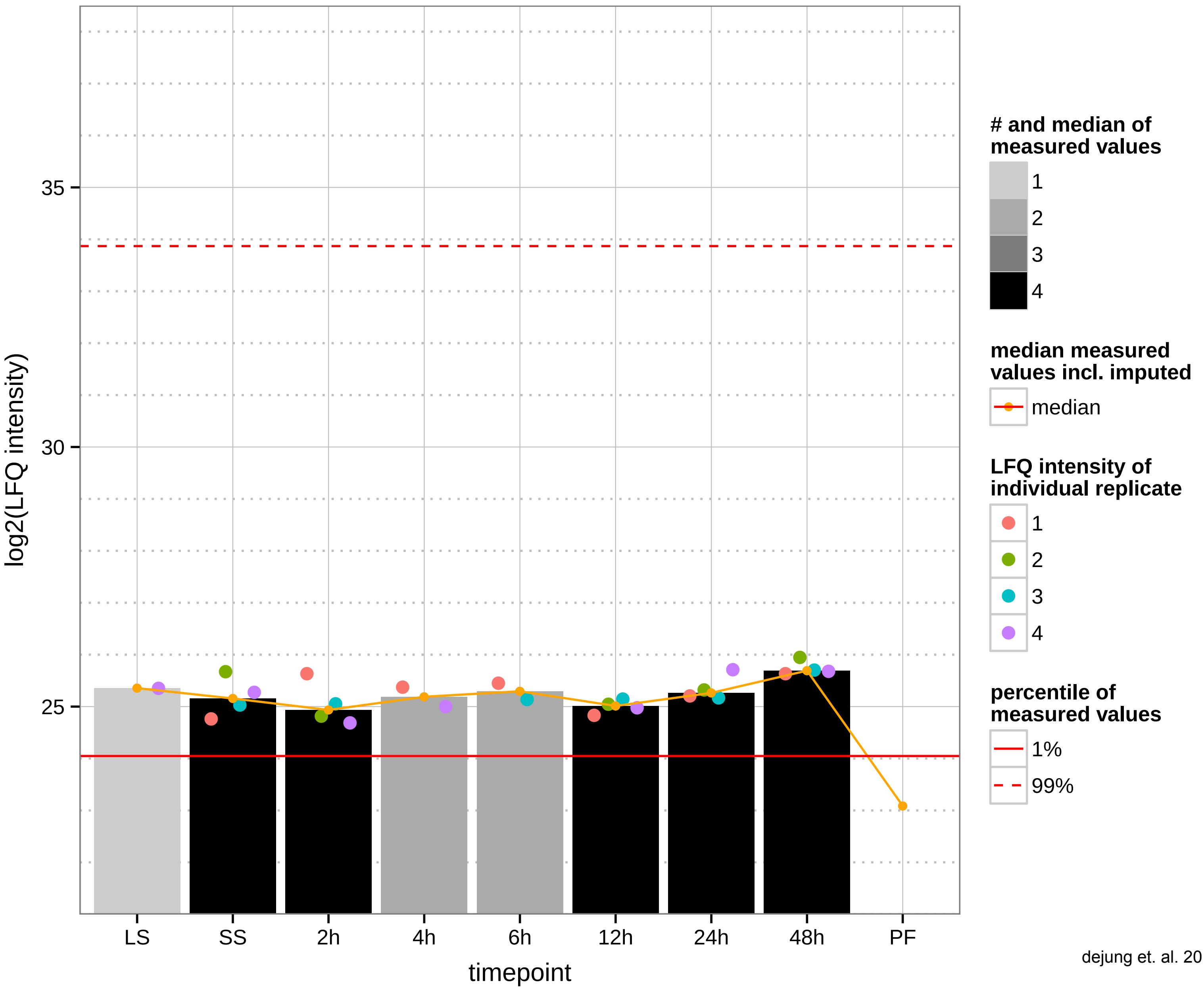

dejung et. al. 2015

DNA topoisomerase IB, large subunit
Tb927.4.1330
AGOF: DNA topoisomerase (ATP−hydrolyzing) activity, DNA topoisomerase type I activity
AGOC: chromosome, membrane, nucleus
AGOP: DNA topological change, regulation of transcription, DNA−dependent
PGOF: DNA binding, DNA topoisomerase (ATP−hydrolyzing) activity, DNA topoisomerase type I activity
PGOC: chromosome
PGOP: DNA topological change

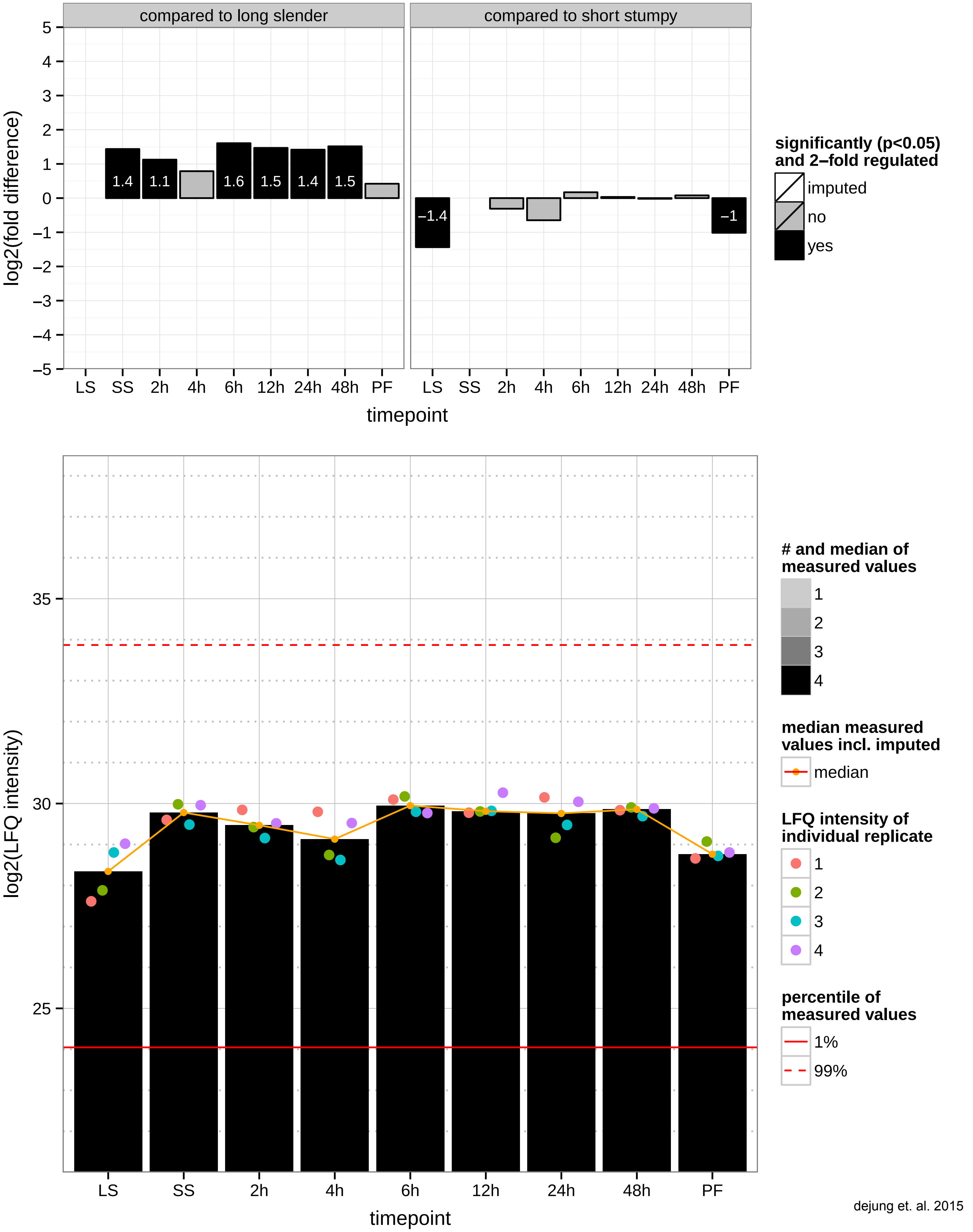

dejung et. al. 2015

hypothetical protein, conserved
Tb927.4.1610
AGOF: null
AGOC: null
AGOP: null
PGOF: null
PGOC: null
PGOP: null

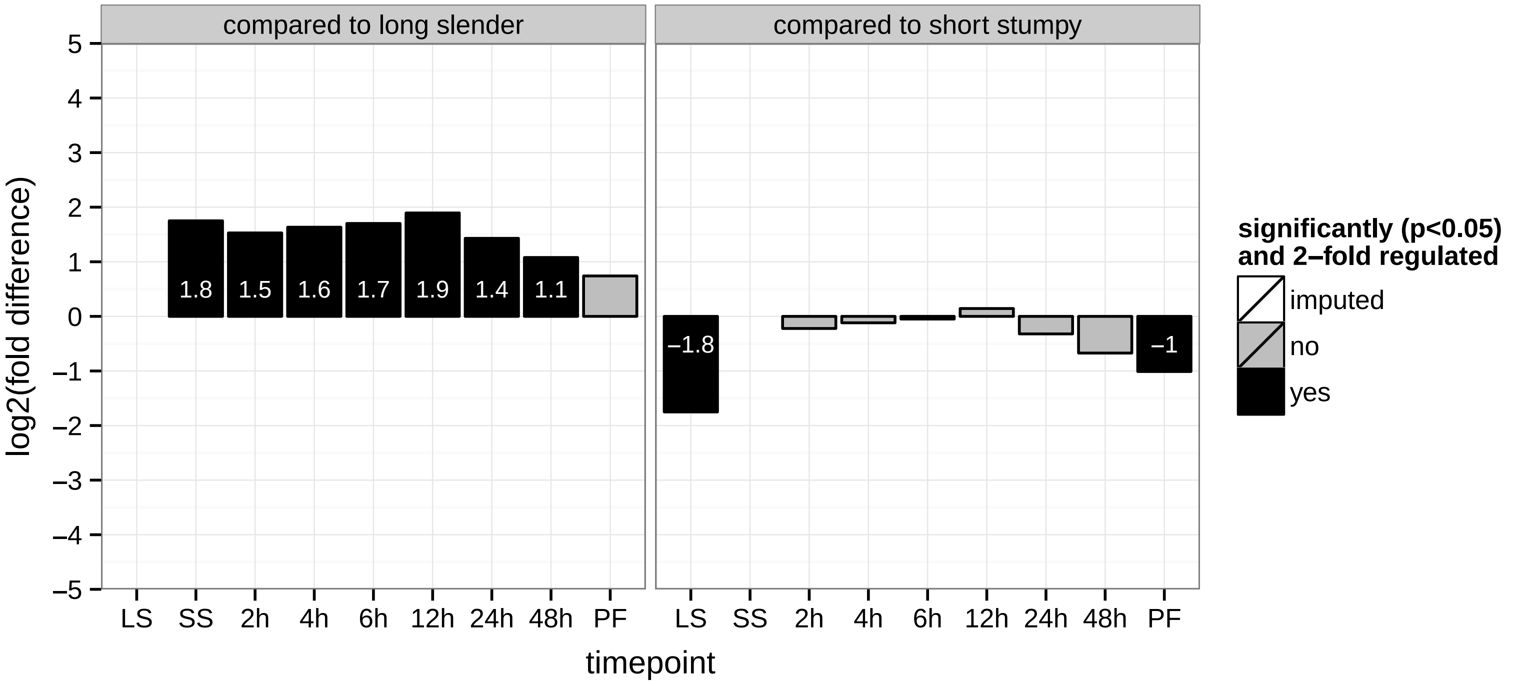

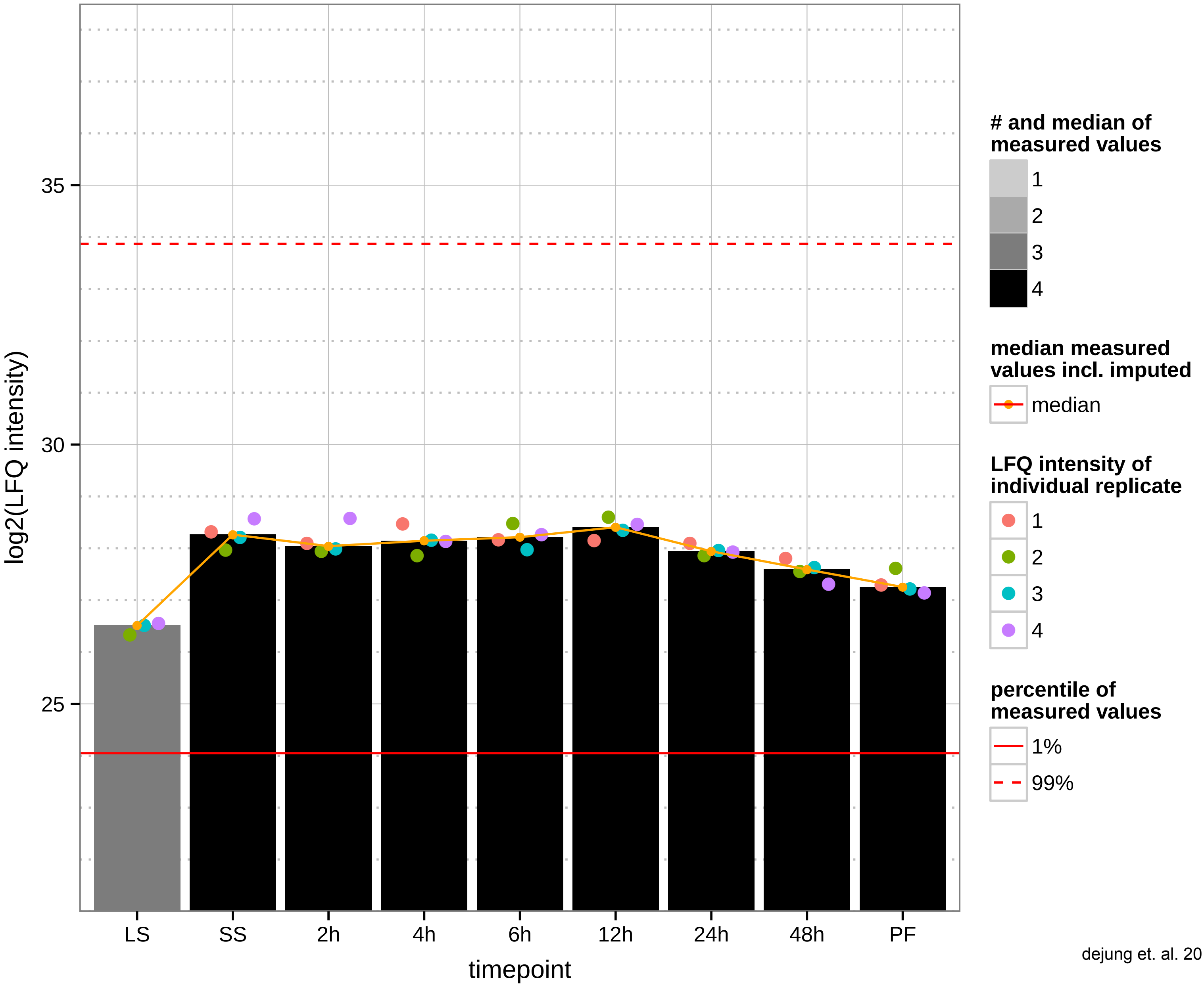

dejung et. al. 2015

antigenic protein, putative
Tb927.4.2070
AGOF: ATP binding, aminoacyl–tRNA ligase activity
AGOC: cytoplasm
AGOP: tRNA aminoacylation for protein translation, translation
PGOF: null
PGOC: null
PGOP: null

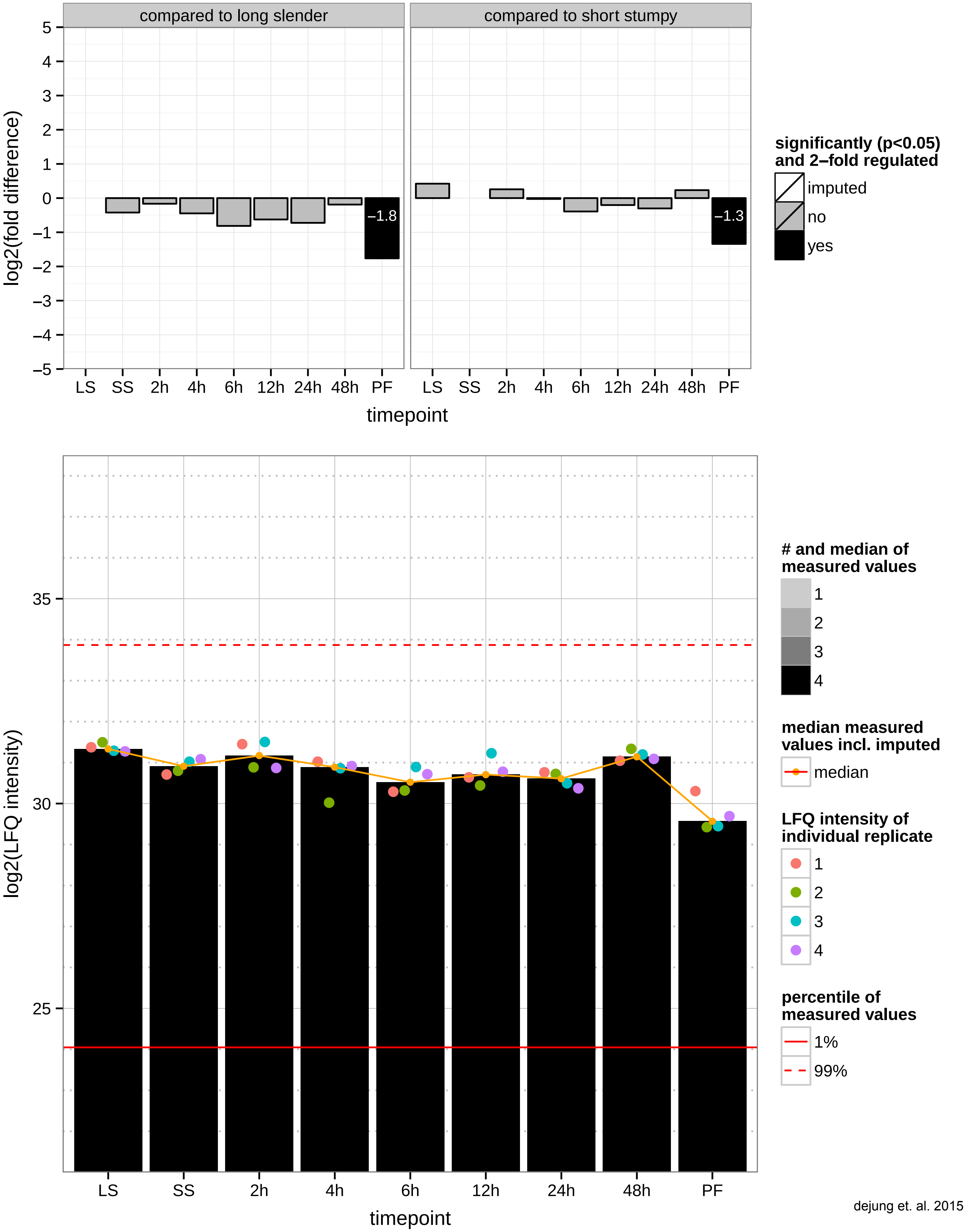

dejung et. al. 2015

MBO2, flagellar component
Tb927.4.2140
AGOF: null
AGOC: null
AGOP: ciliary or flagellar motility
PGOF: null
PGOC: null
PGOP: null

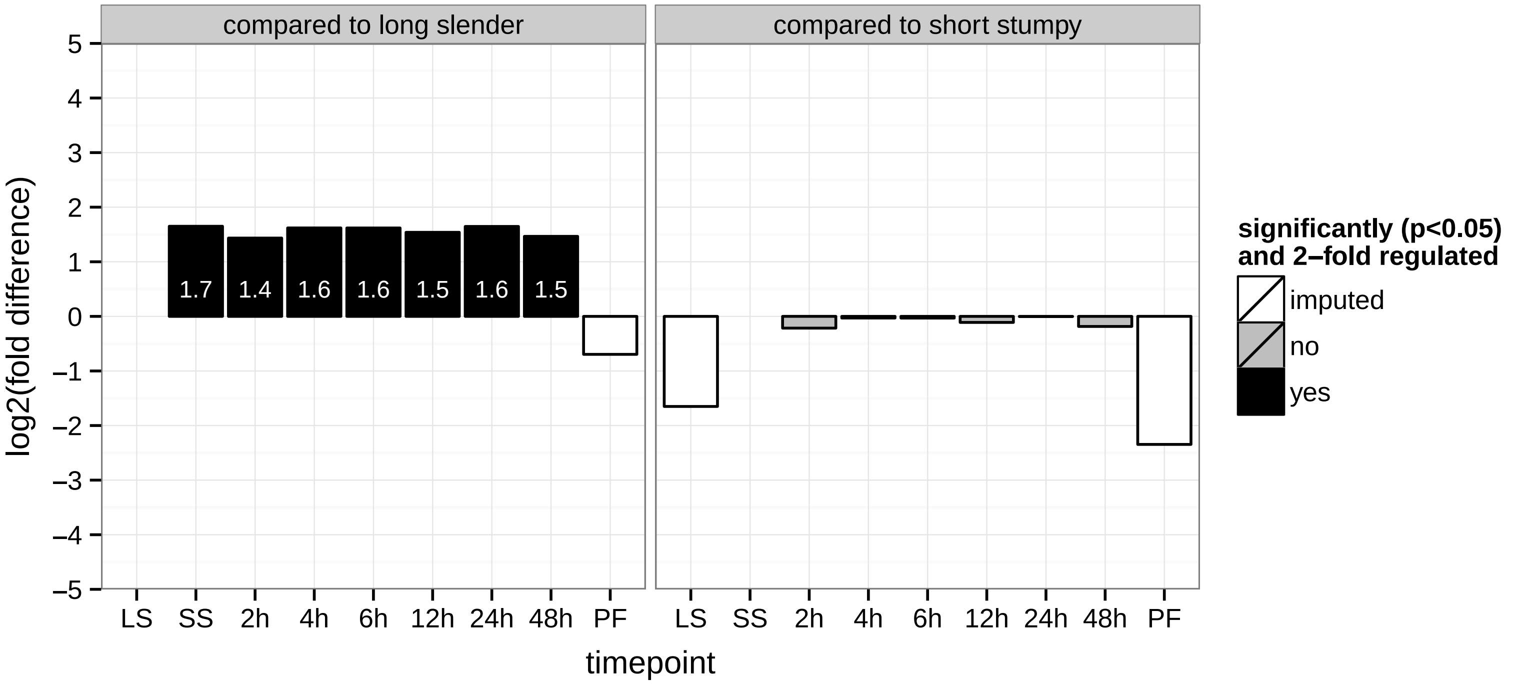

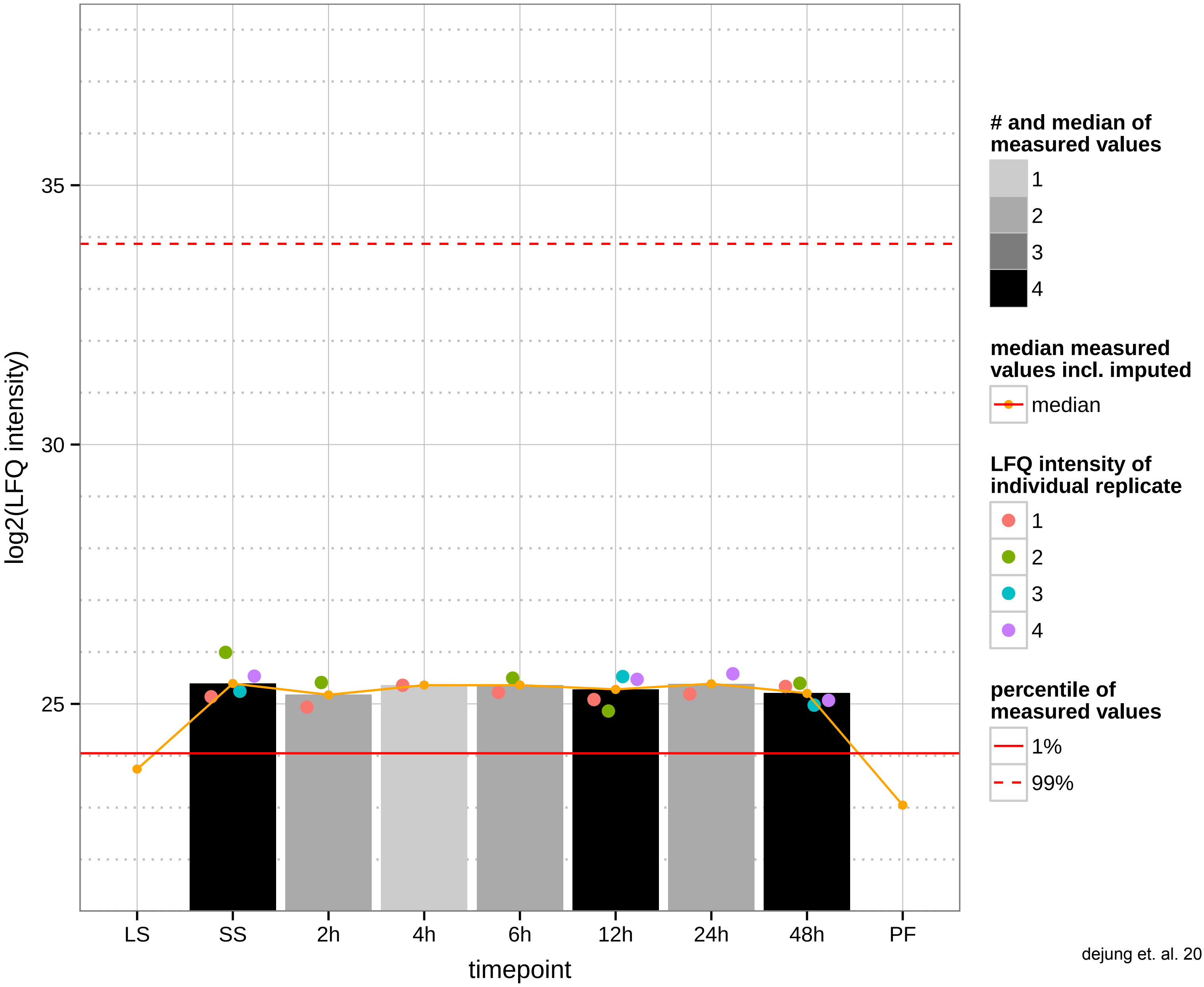

dejung et. al. 2015

hypothetical protein, conserved
Tb927.4.2930
AGOF: null
AGOC: null
AGOP: response to stress
PGOF: null
PGOC: null
PGOP: response to stress

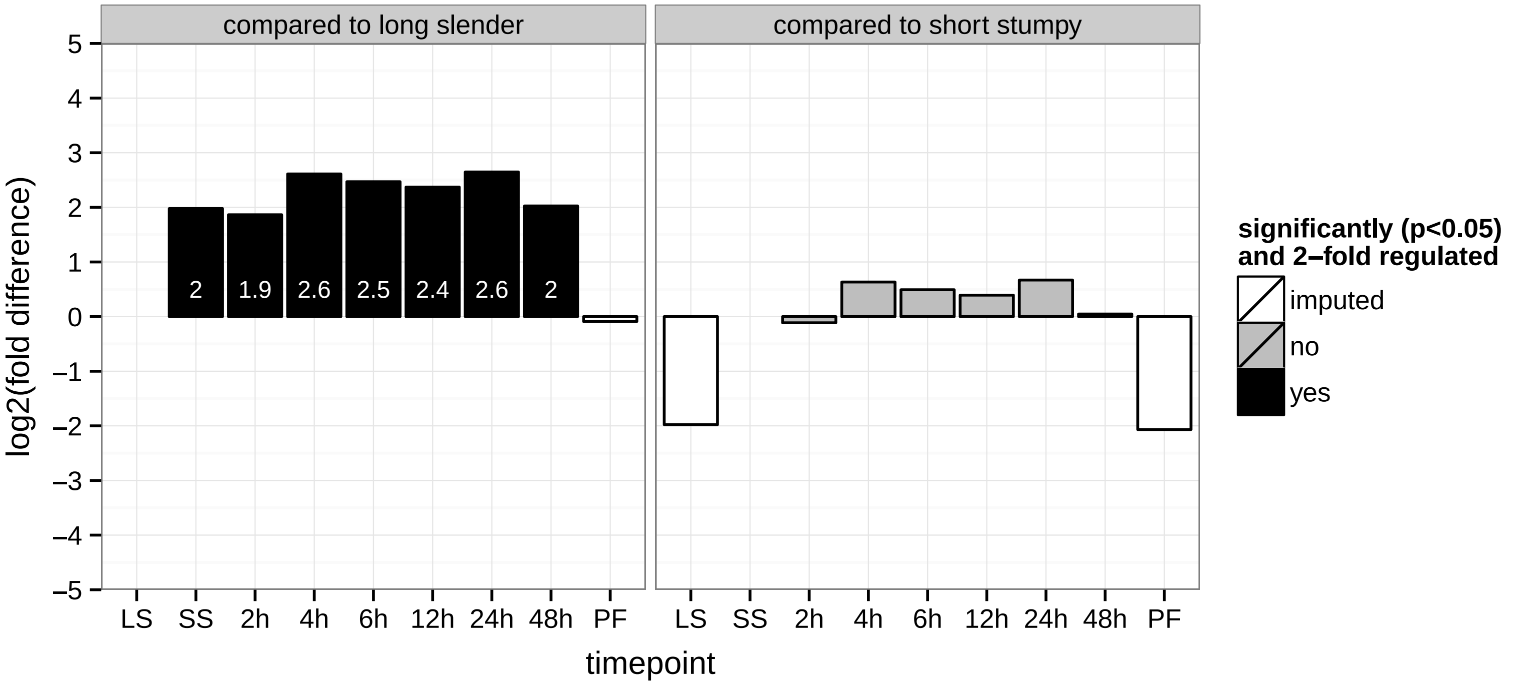

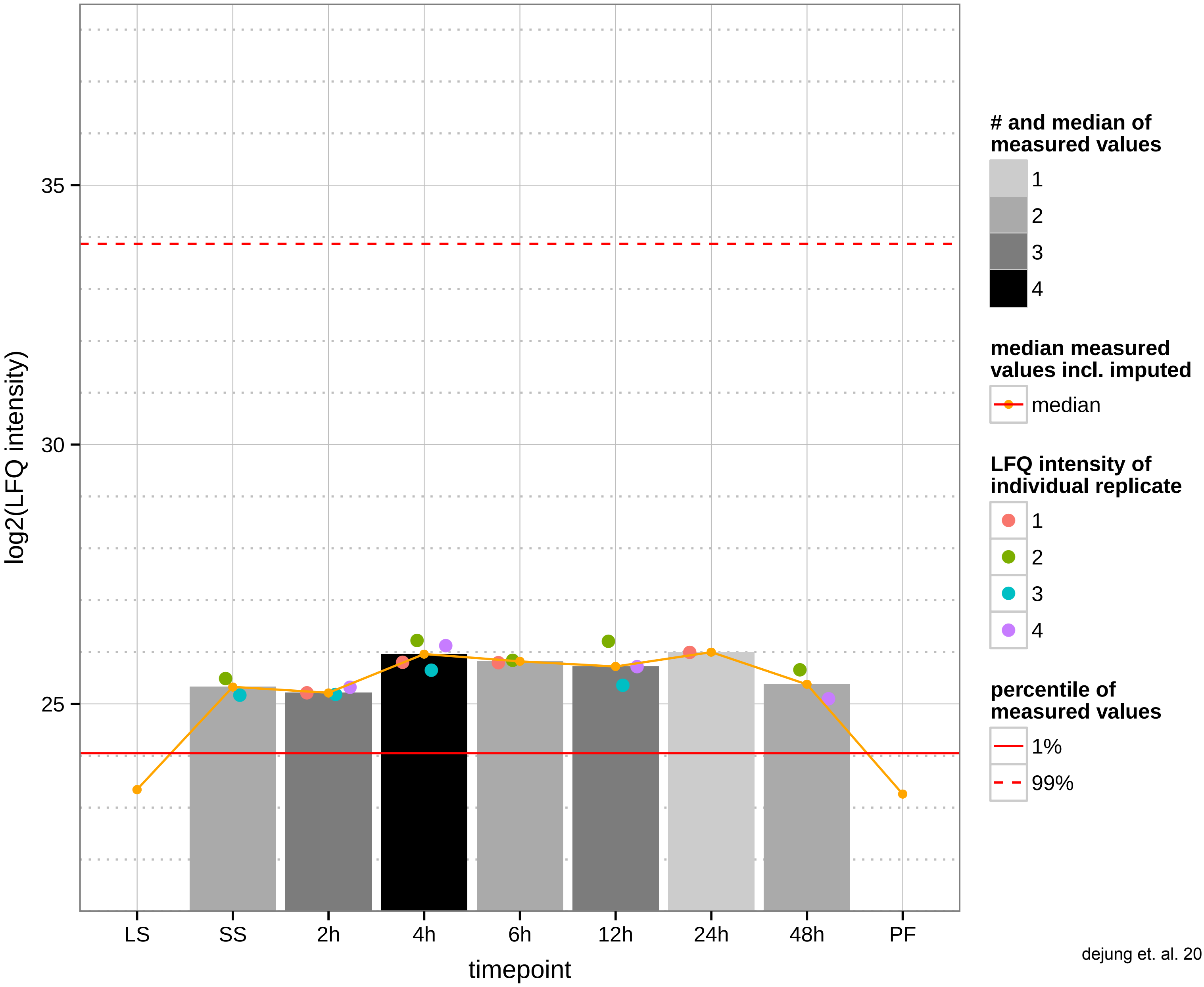

dejung et. al. 2015

hypothetical protein, conserved
Tb927.4.310
AGOF: acid–amino acid ligase activity
AGOC: intracellular
AGOP: cellular protein modification process
PGOF: acid–amino acid ligase activity, protein binding, transferase activity, transferring phosphorus–containing groups
PGOC: intracellular
PGOP: cellular protein modification process

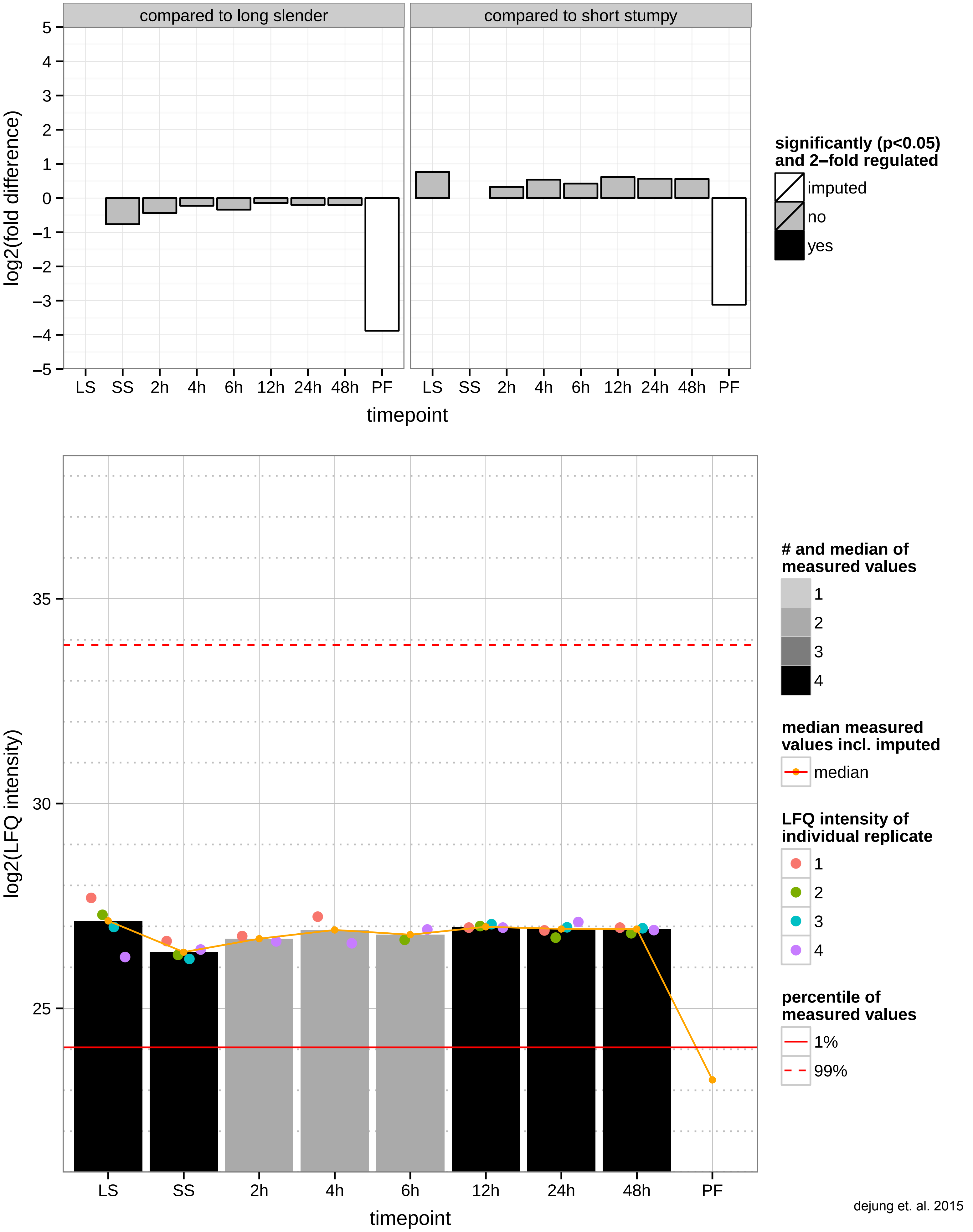

dejung et. al. 2015

tuzin, putative
Tb927.4.3390
AGOF: null
AGOC: mitochondrion
AGOP: null
PGOF: null
PGOC: null
PGOP: null

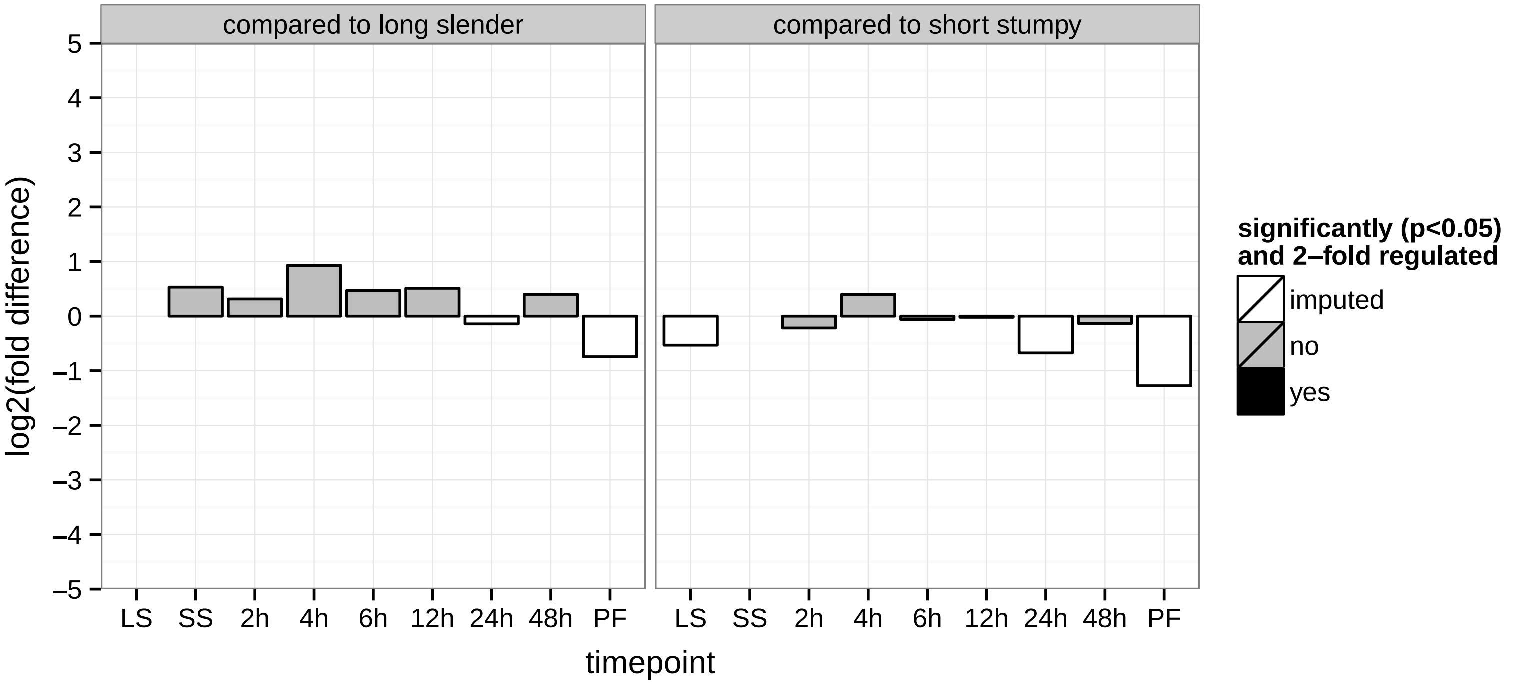

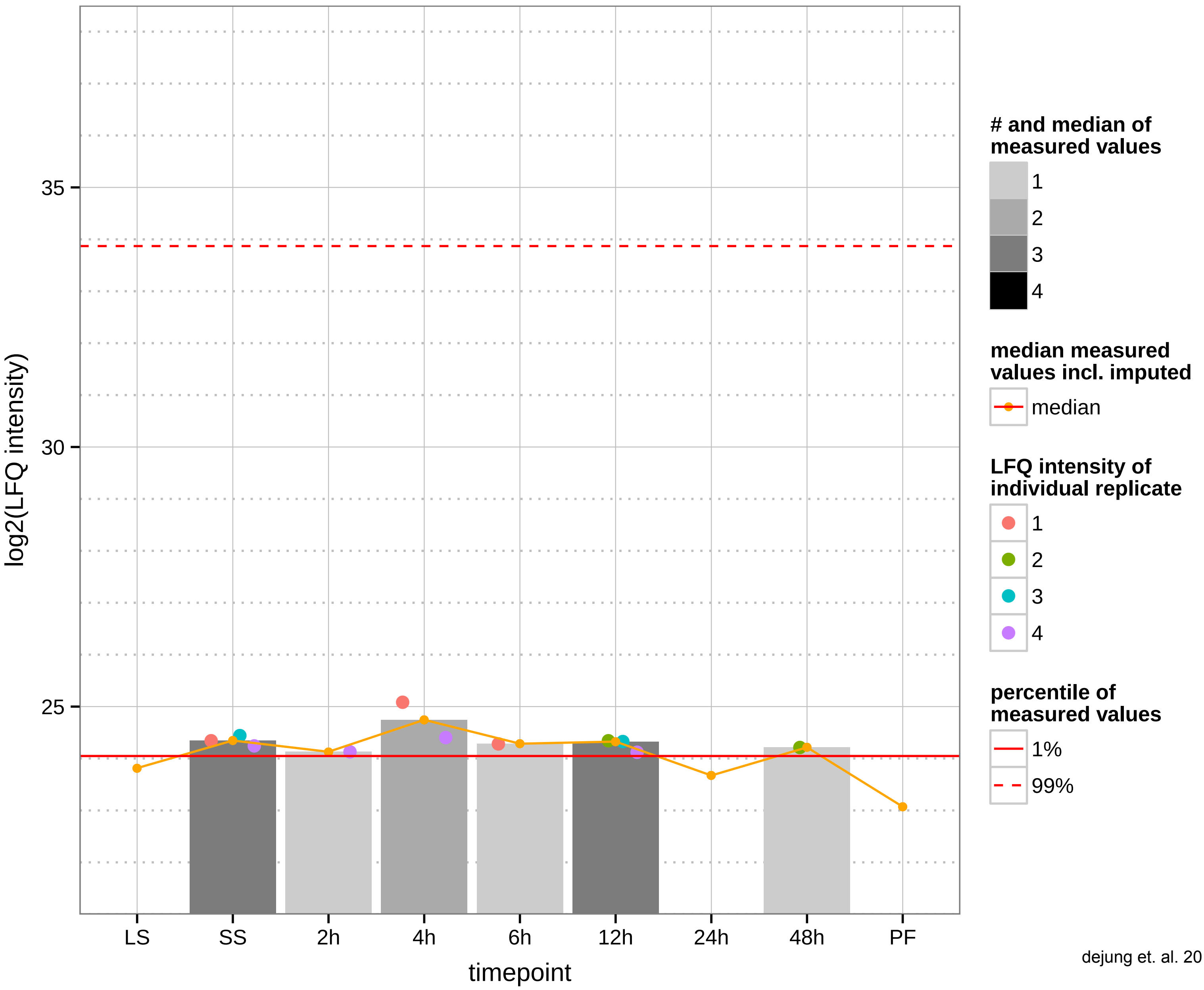

dejung et. al. 2015

phosphatidylinositol 3−kinase tor, putative, phosphatidylinositol 3−kinase (tor2) (TOR2)
Tb927.4.420
AGOF: null, phosphotransferase activity, alcohol group as acceptor, protein binding
AGOC: null, TORC2 complex, cytosol, endoplasmic reticulum, mitochondrion
AGOP: null, actin cytoskeleton organization
PGOF: phosphotransferase activity, alcohol group as acceptor, transferase activity, transferring phosphorus−containing groups
PGOC: null
PGOP: null

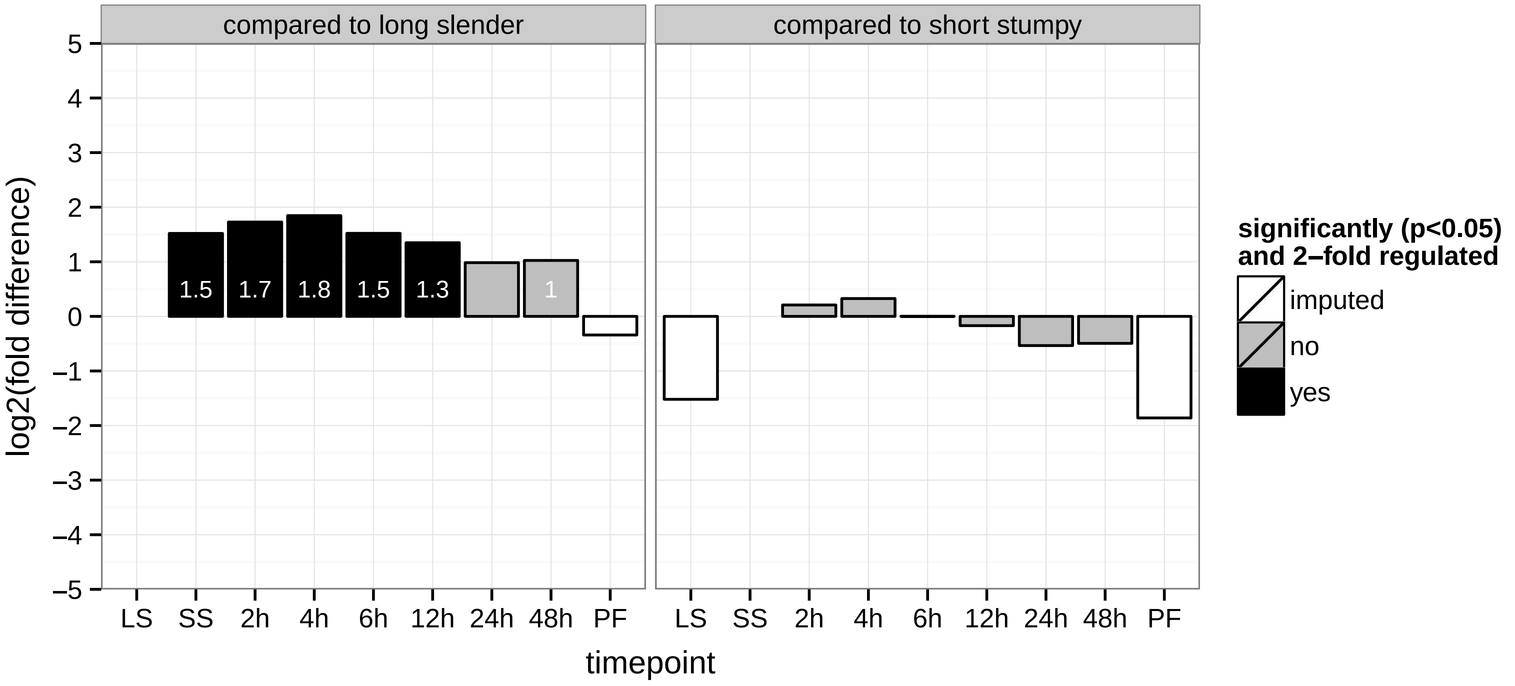

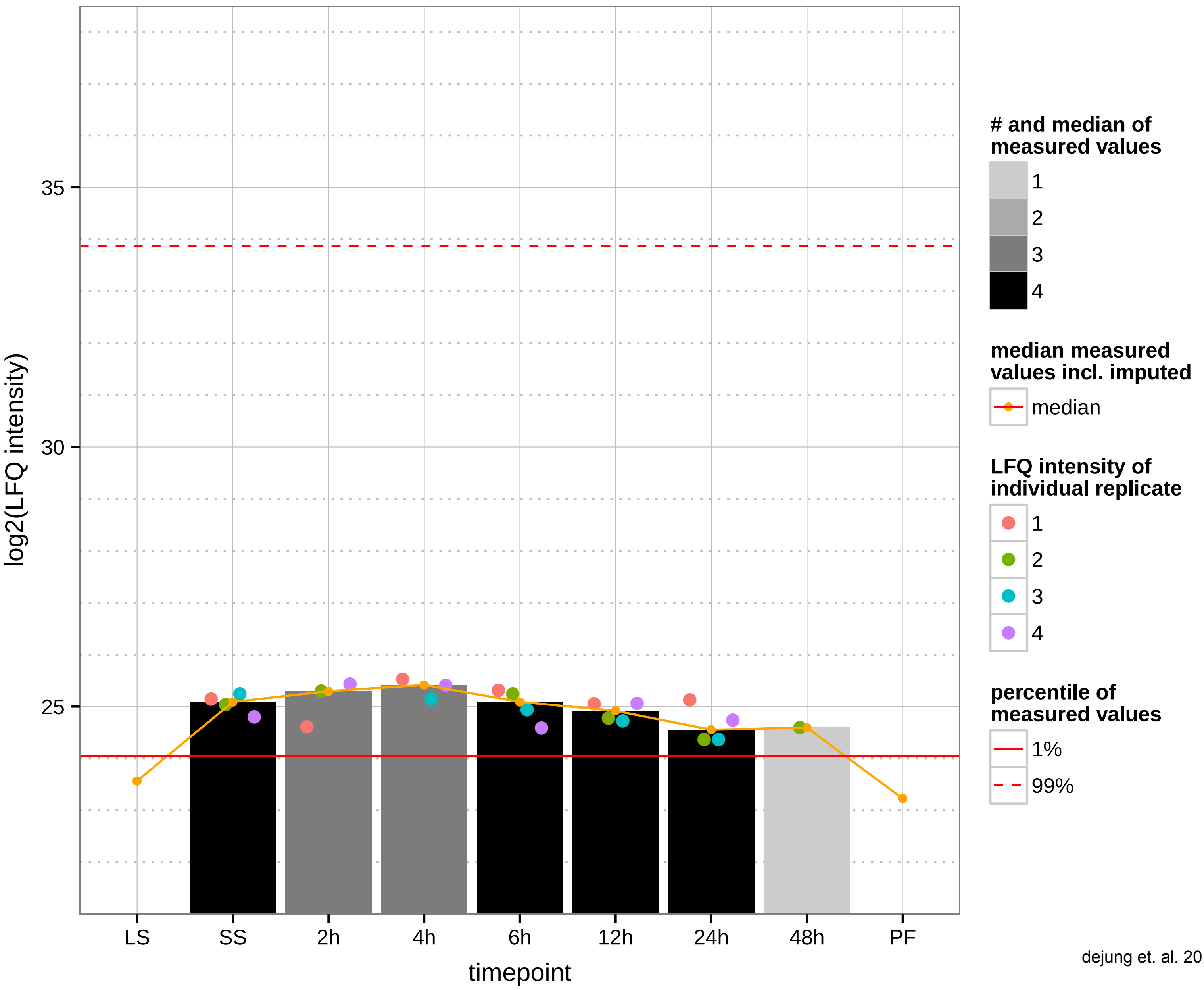

dejung et. al. 2015

adenylyl cyclase (GRESAG 4.4)
Tb927.4.4430;Tb927.4.4450
AGOF: ATP binding, adenylate cyclase activity
AGOC: integral to membrane
AGOP: cyclic nucleotide biosynthetic process, intracellular signal transduction
PGOF: phosphorus–oxygen lyase activity
PGOC: null
PGOP: cyclic nucleotide biosynthetic process, intracellular signal transduction

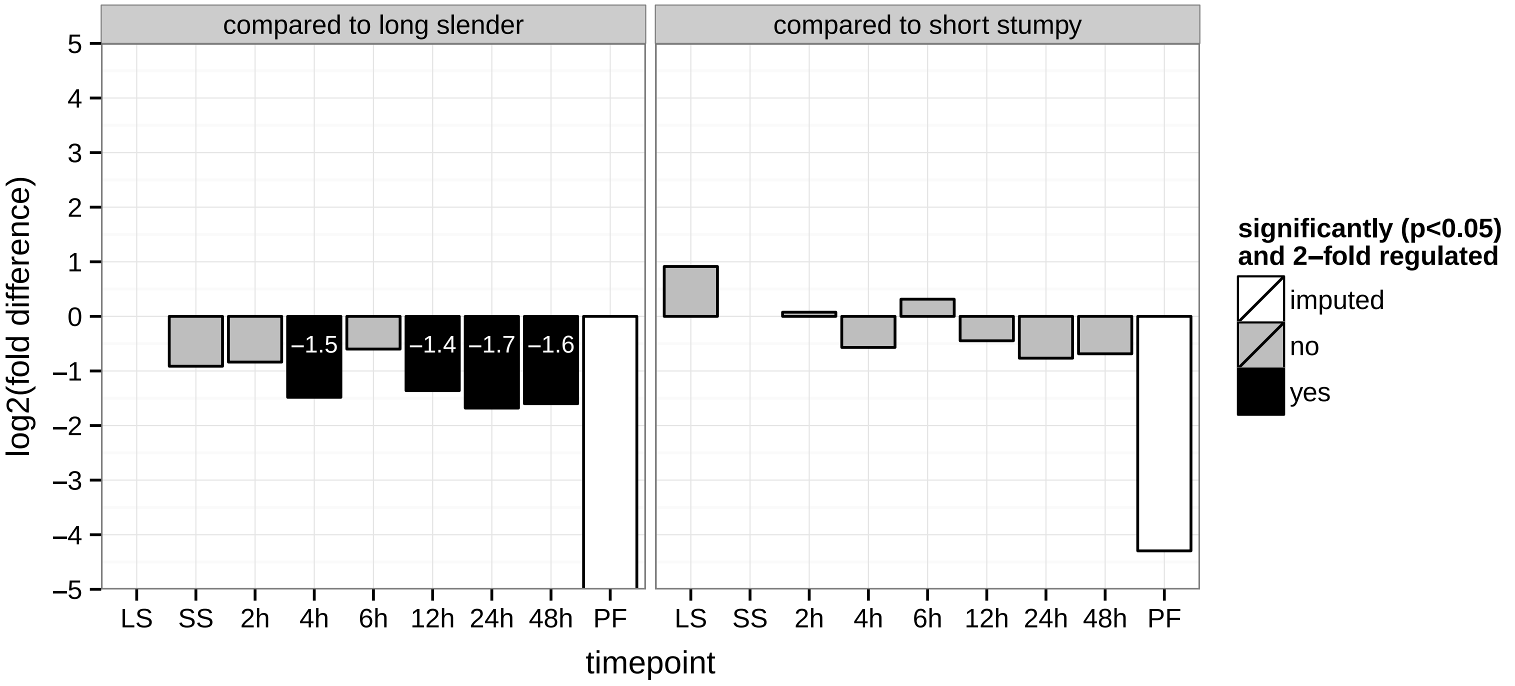

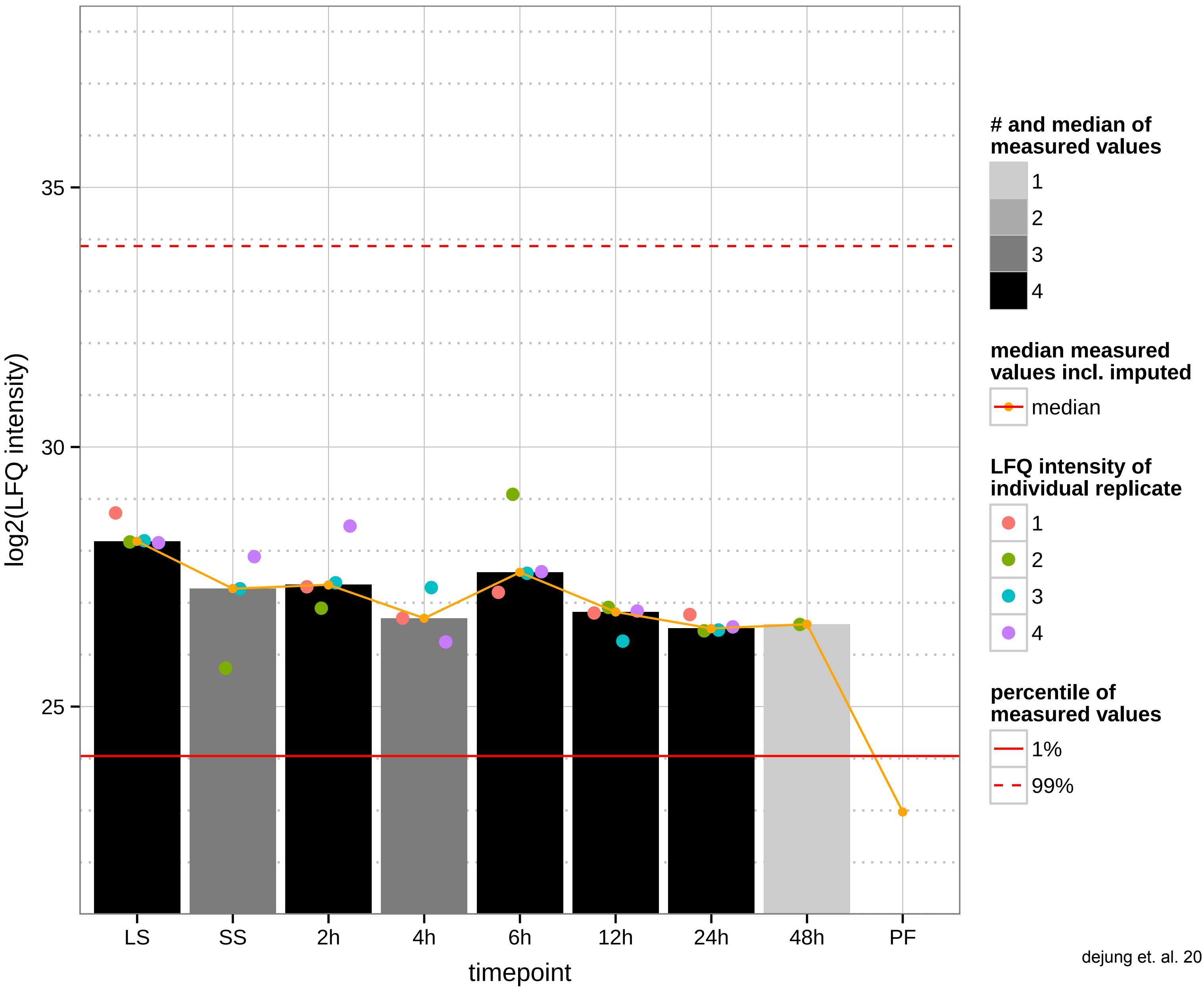

dejung et. al. 2015

hypothetical protein, conserved
Tb927.4.4750
AGOF: null
AGOC: null
AGOP: null
PGOF: null
PGOC: null
PGOP: null

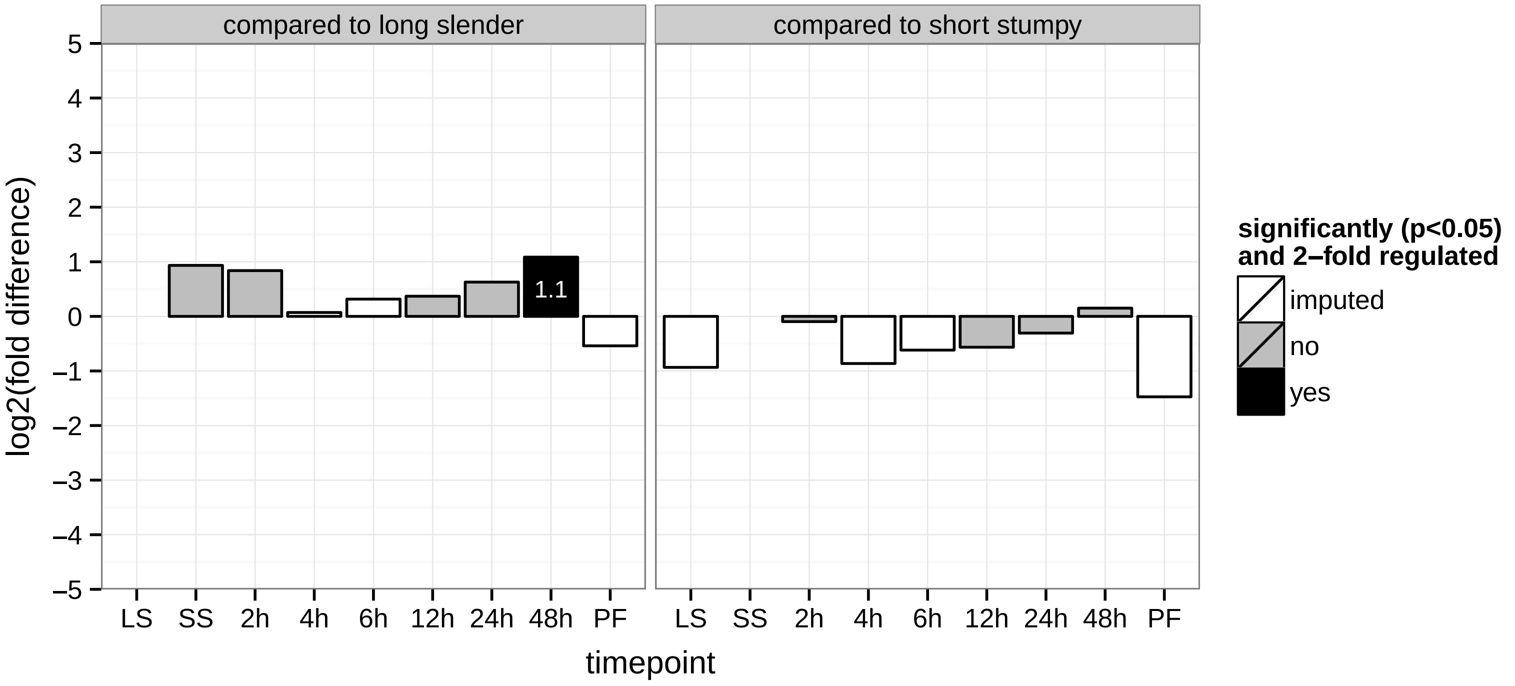

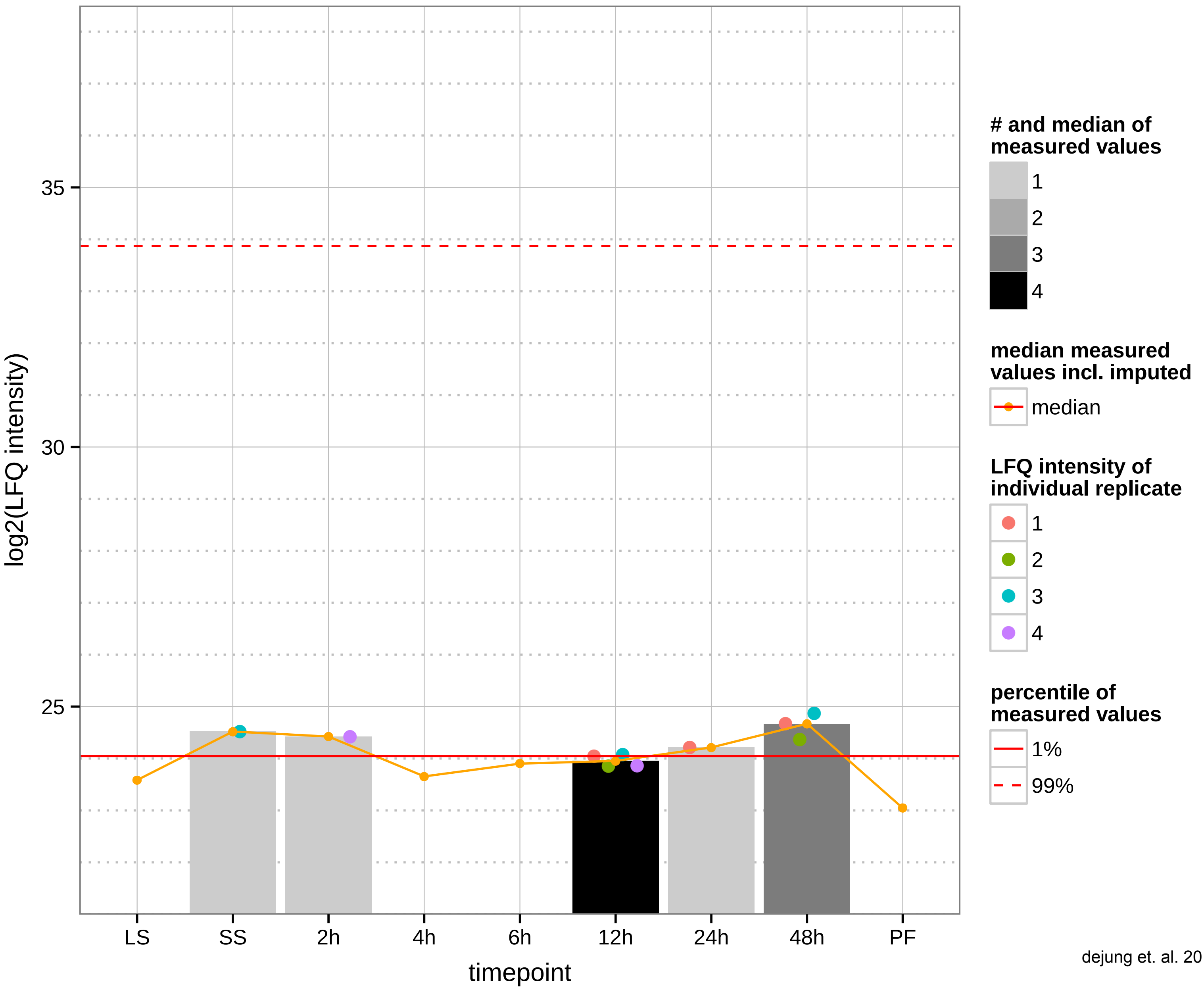

dejung et. al. 2015

hypothetical protein, conserved
Tb927.4.5080
AGOF: null
AGOC: null
AGOP: null
PGOF: null
PGOC: endoplasmic reticulum, integral to membrane
PGOP: intracellular protein transport

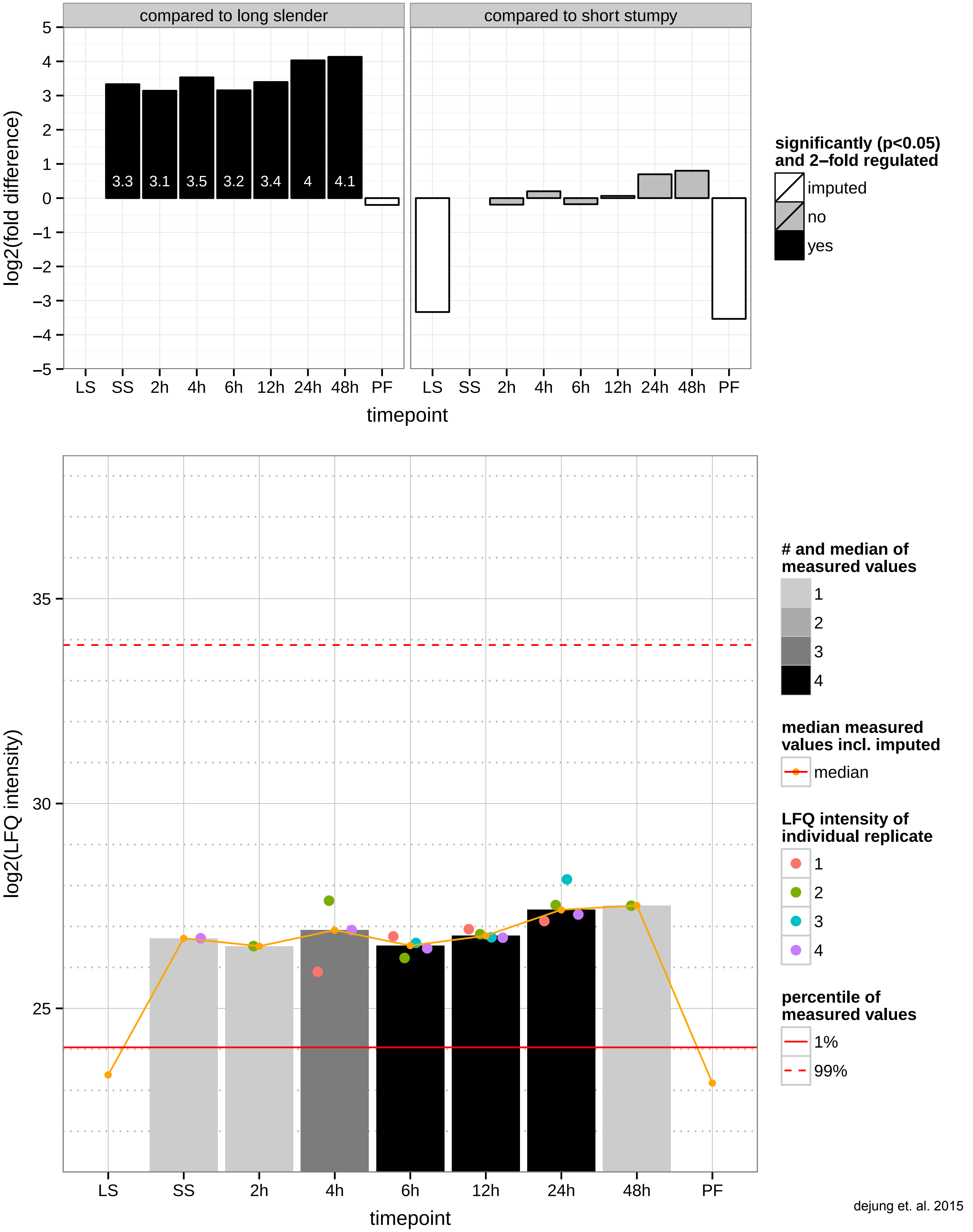

dejung et. al. 2015

hypothetical protein, conserved
Tb927.4.640
AGOF: null
AGOC: null
AGOP: null
PGOF: protein binding
PGOC: null
PGOP: protein targeting to Golgi

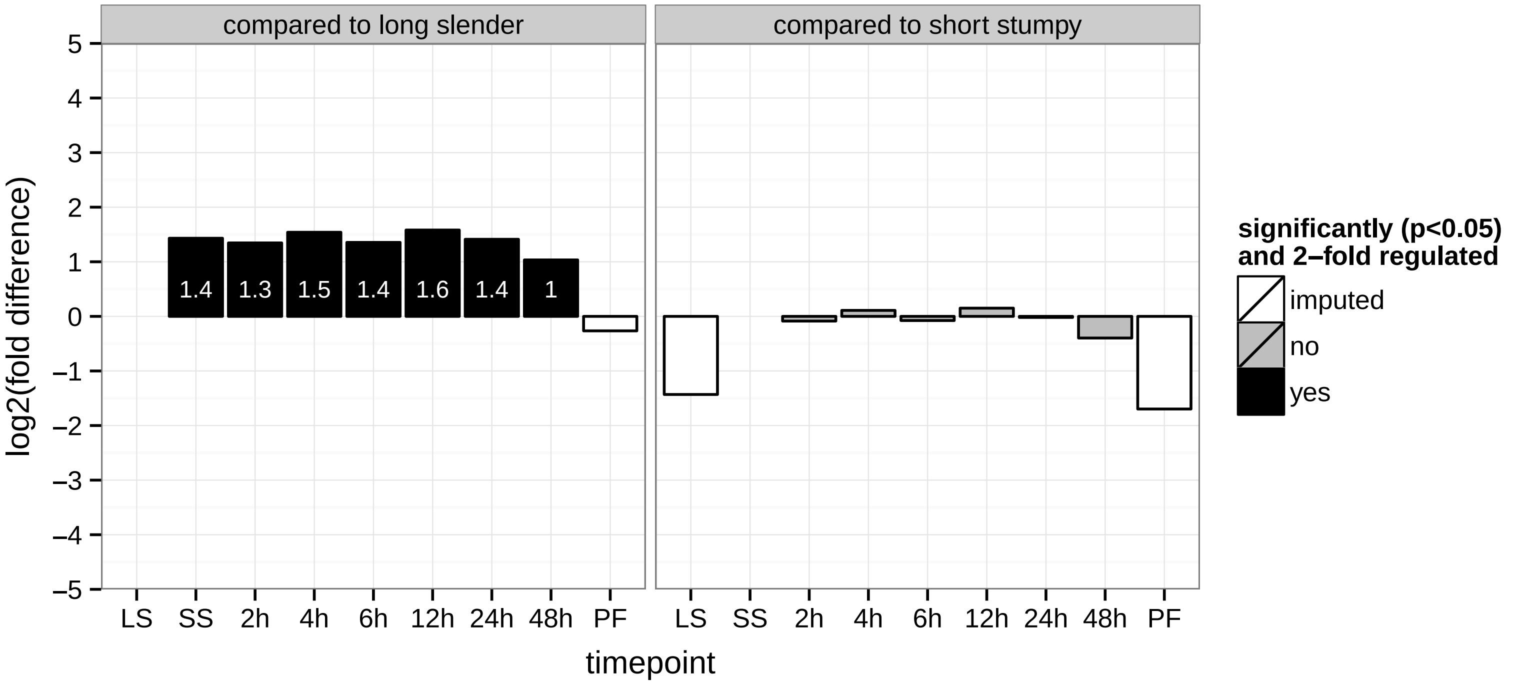

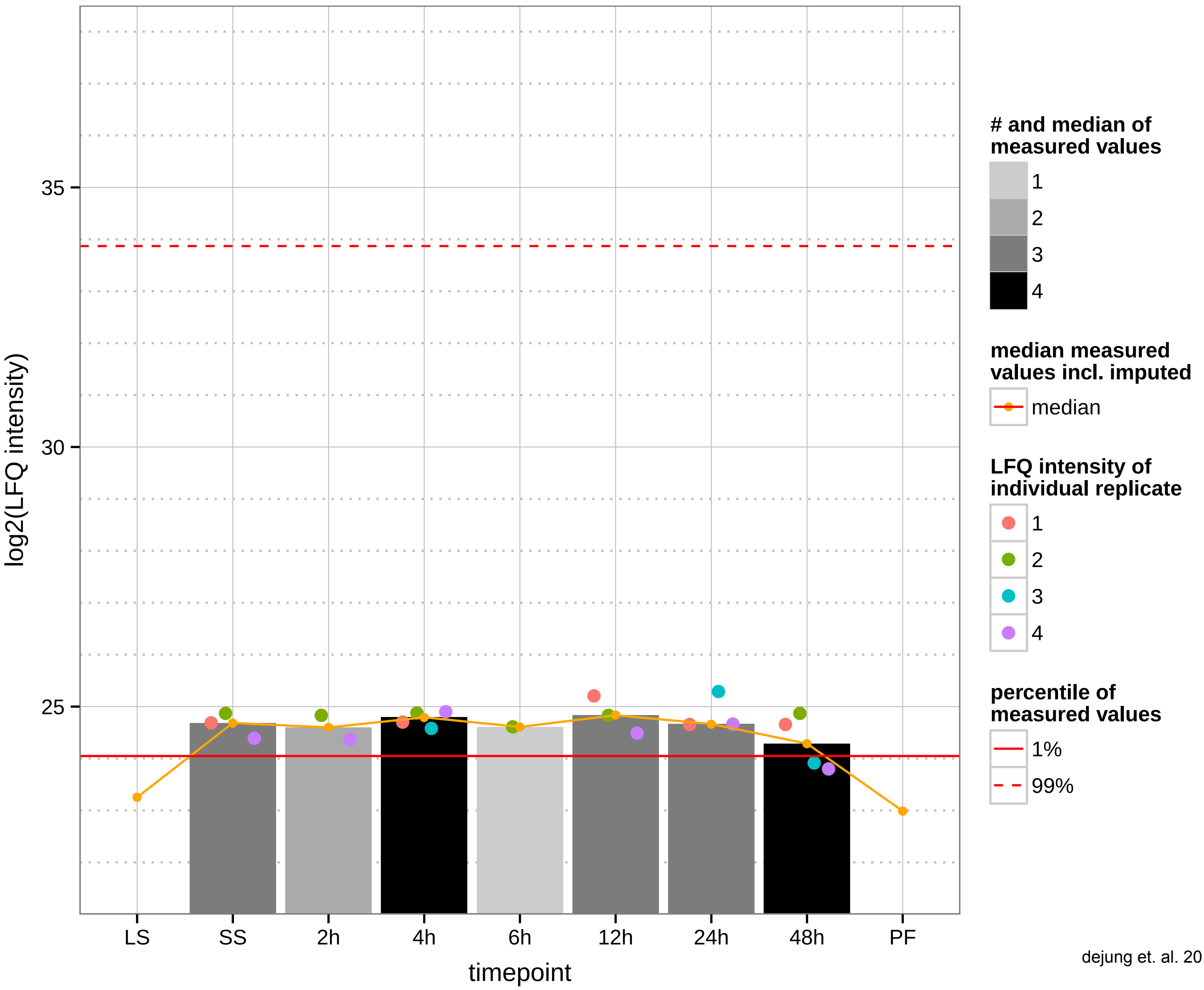

dejung et. al. 2015

Iron−sulfur assembly protein 2 (Isa2)
Tb927.5.1030
AGOF: null
AGOC: mitochondrion
AGOP: [4Fe−4S] cluster assembly, growth, regulation of reactive oxygen species metabolic process
PGOF: null
PGOC: null
PGOP: null

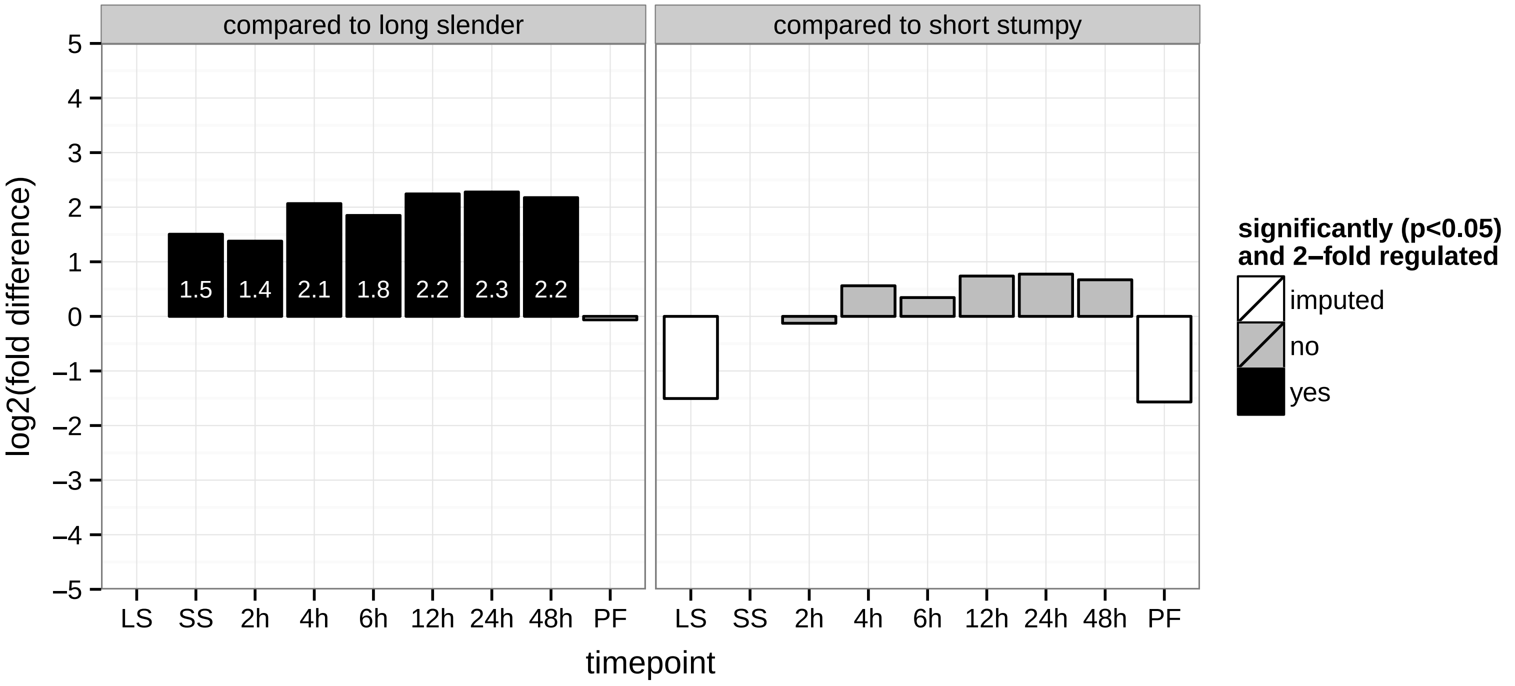

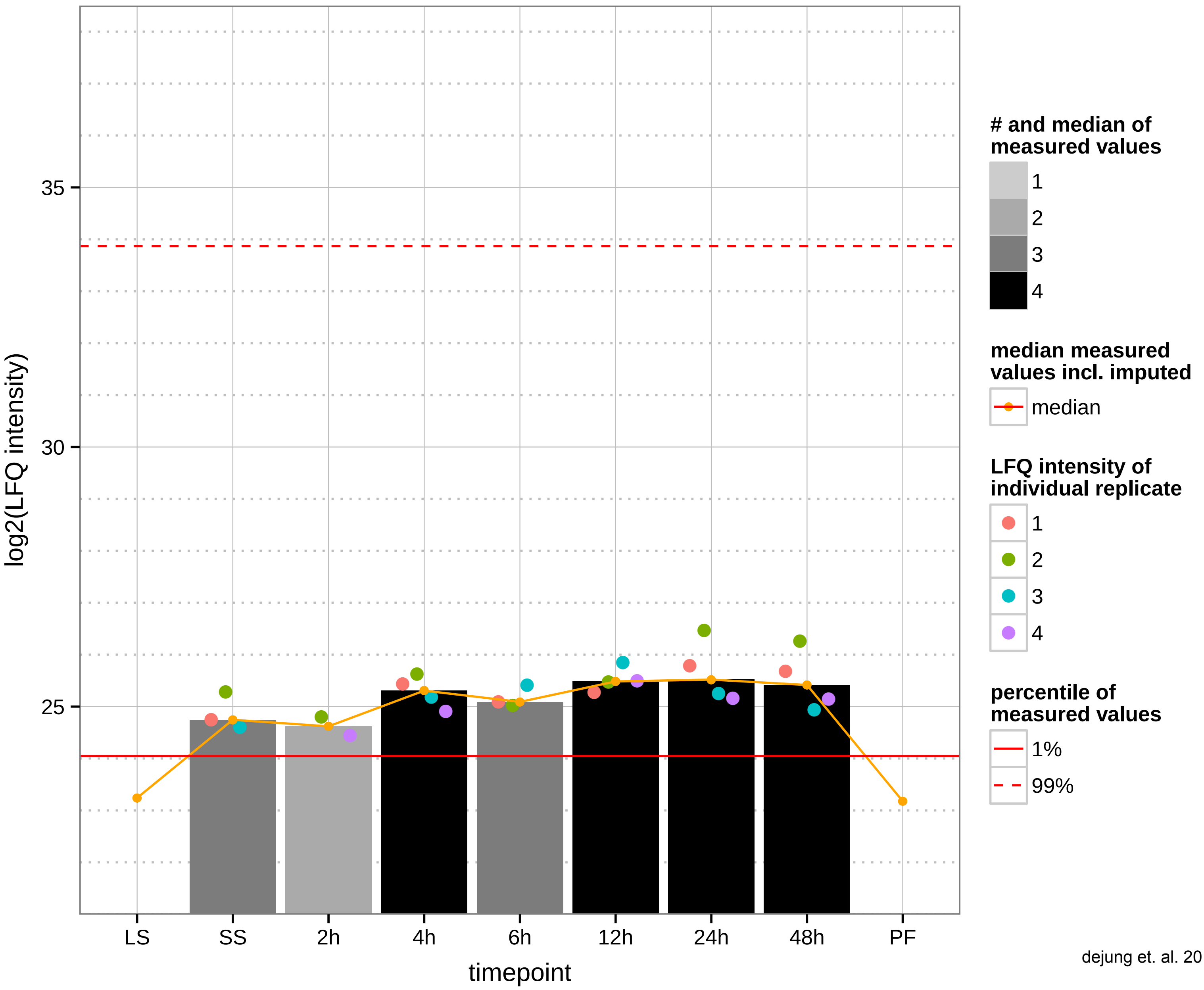

dejung et. al. 2015

alanine racemase, putative
Tb927.5.1280
AGOF: alanine racemase activity
AGOC: cytosol
AGOP: null
PGOF: null
PGOC: null
PGOP: null

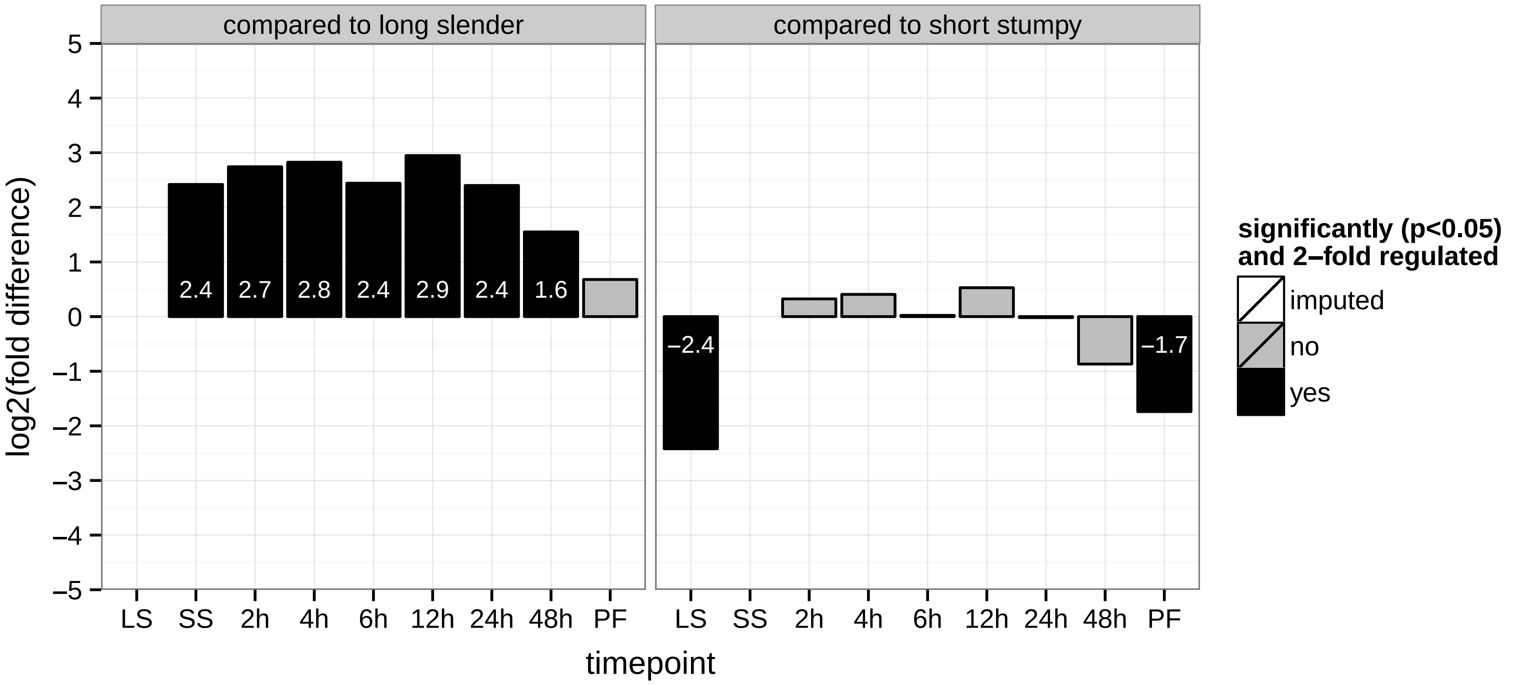

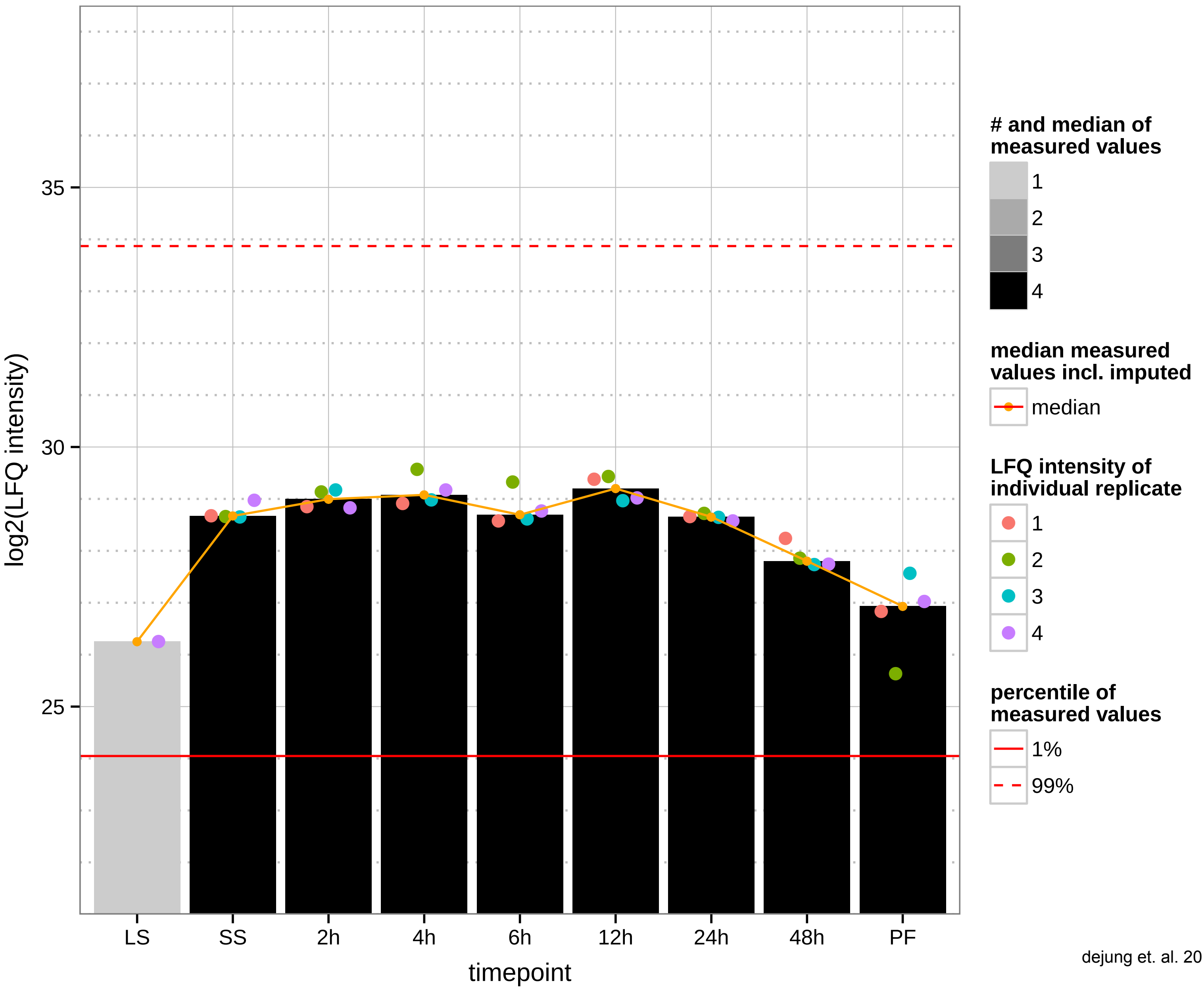

dejung et. al. 2015

hypothetical protein, conserved
Tb927.5.1320
AGOF: null
AGOC: null
AGOP: null
PGOF: null
PGOC: null
PGOP: null

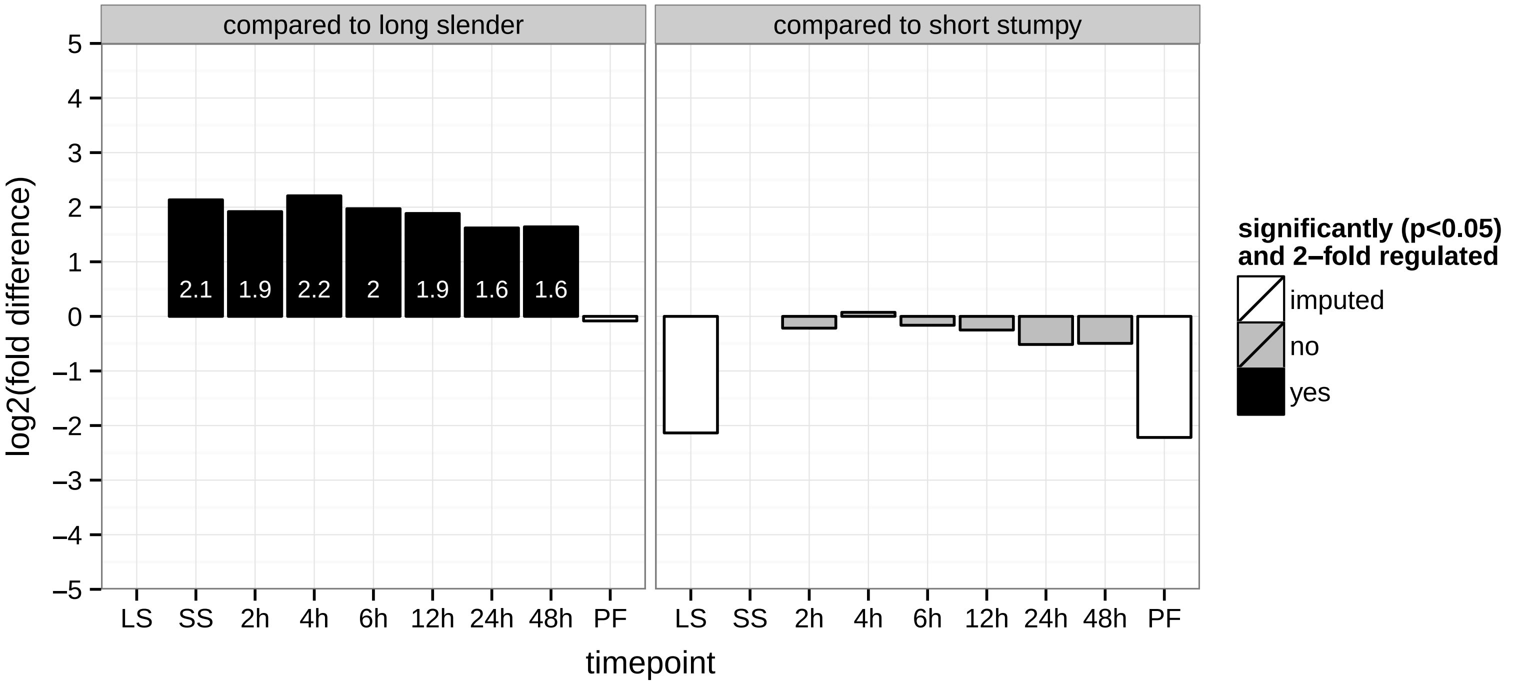

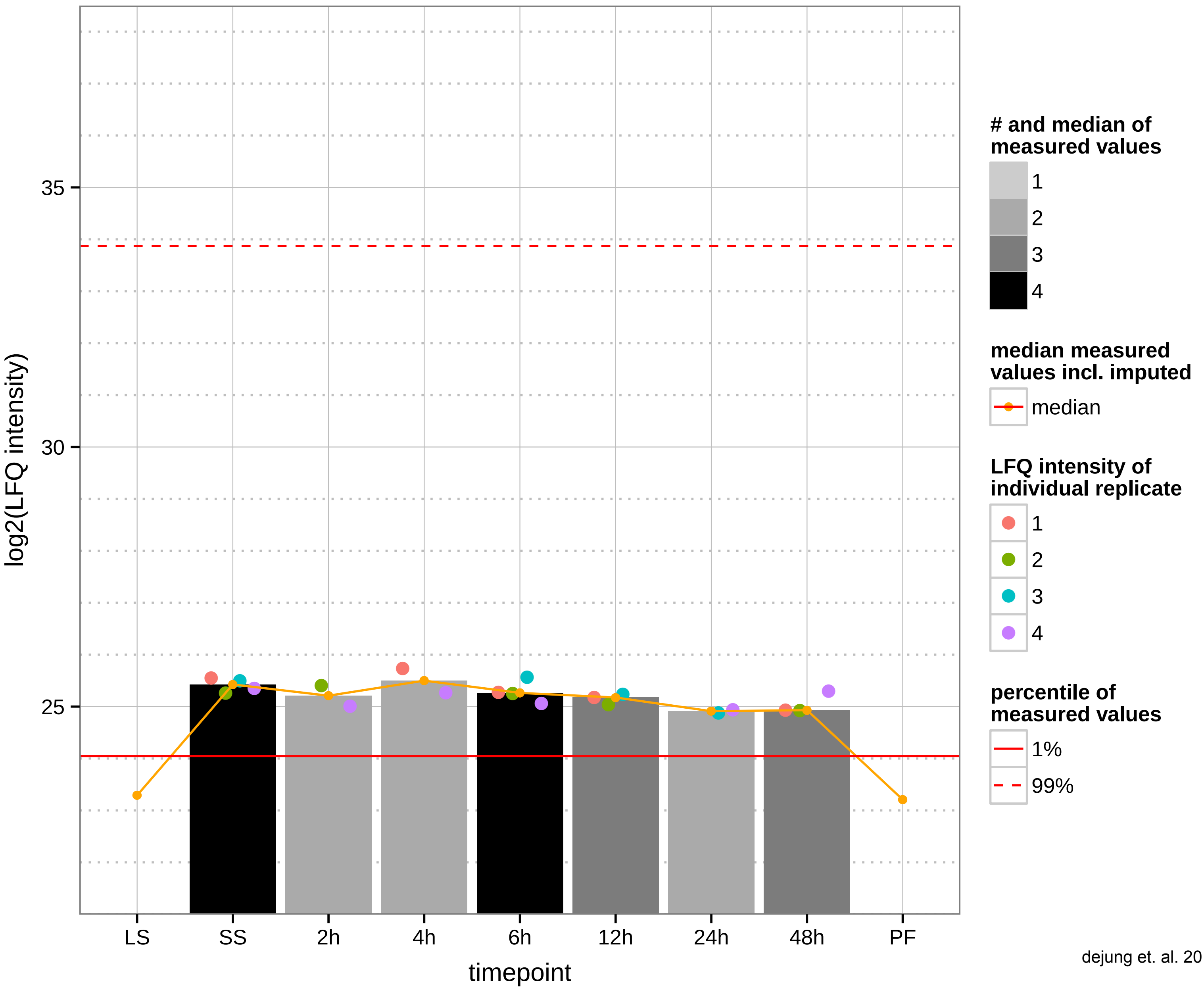

dejung et. al. 2015

hypothetical protein, conserved
Tb927.5.1540
AGOF: null
AGOC: null
AGOP: null
PGOF: null
PGOC: null
PGOP: null

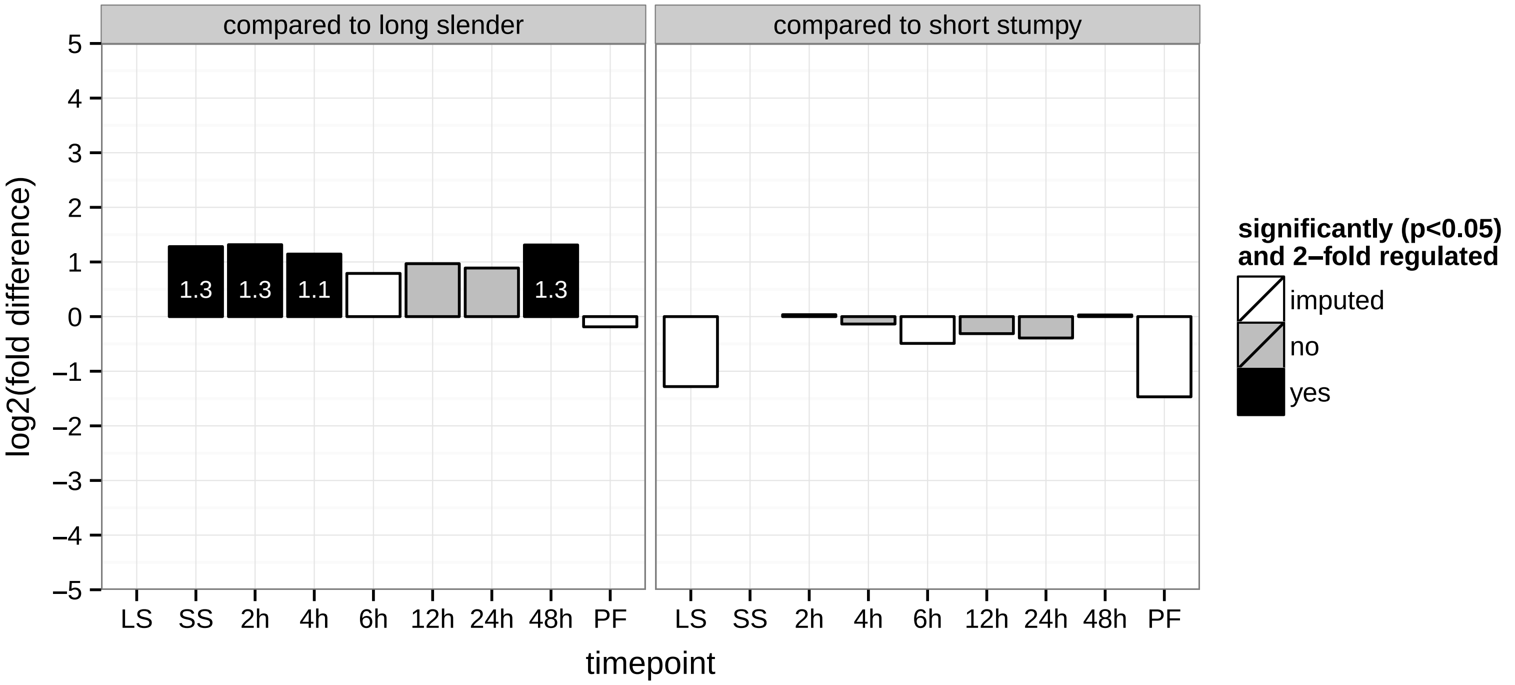

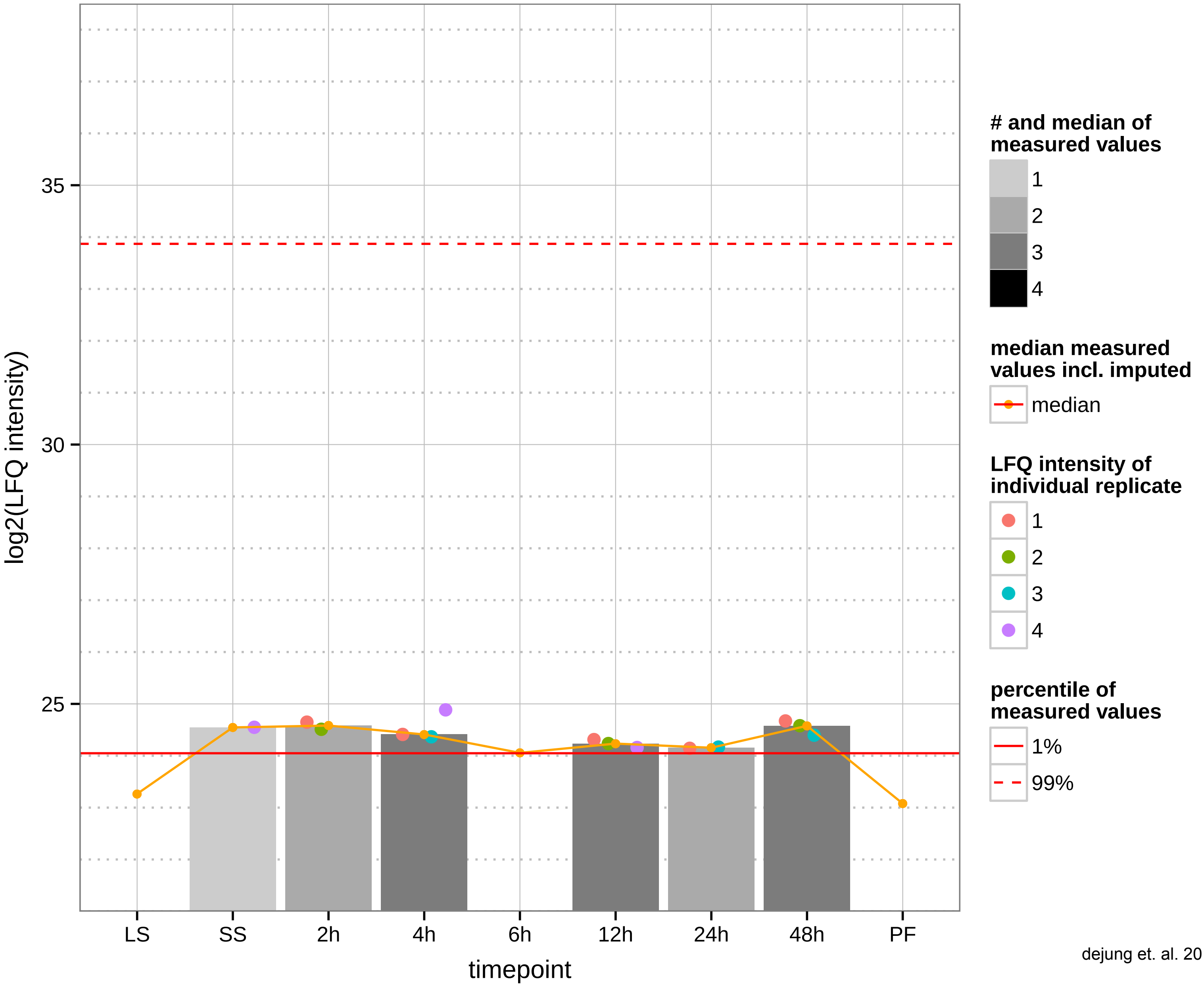

dejung et. al. 2015

zinc finger CCCH domain containing protein 13 (ZC3H13)
Tb927.5.1580
AGOF: RNA binding, zinc ion binding
AGOC: cytosol
AGOP: null
PGOF: nucleic acid binding, zinc ion binding
PGOC: null
PGOP: null

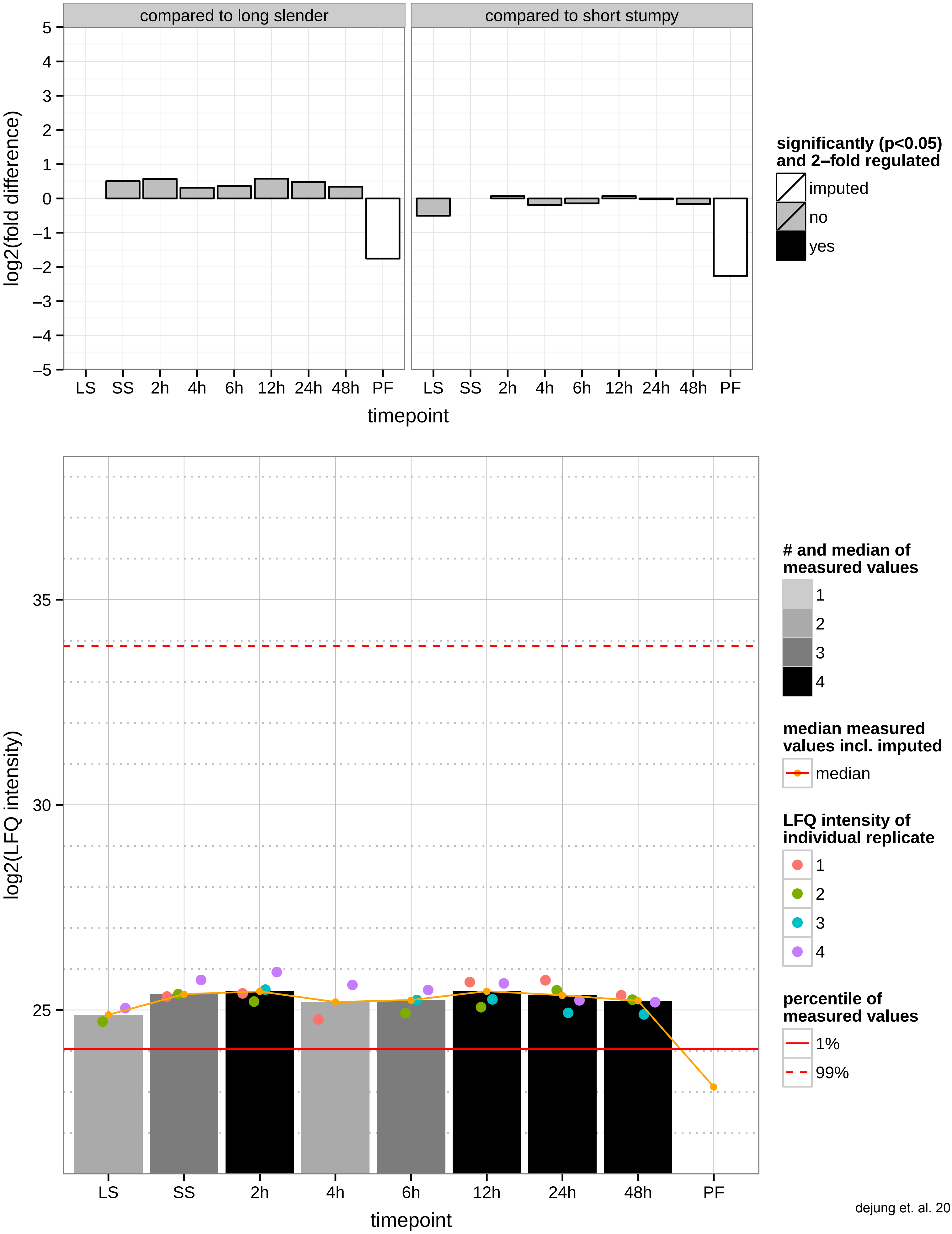

dejung et. al. 2015

hypothetical protein, conserved
Tb927.5.1590
AGOF: null
AGOC: null
AGOP: null
PGOF: null
PGOC: null
PGOP: null

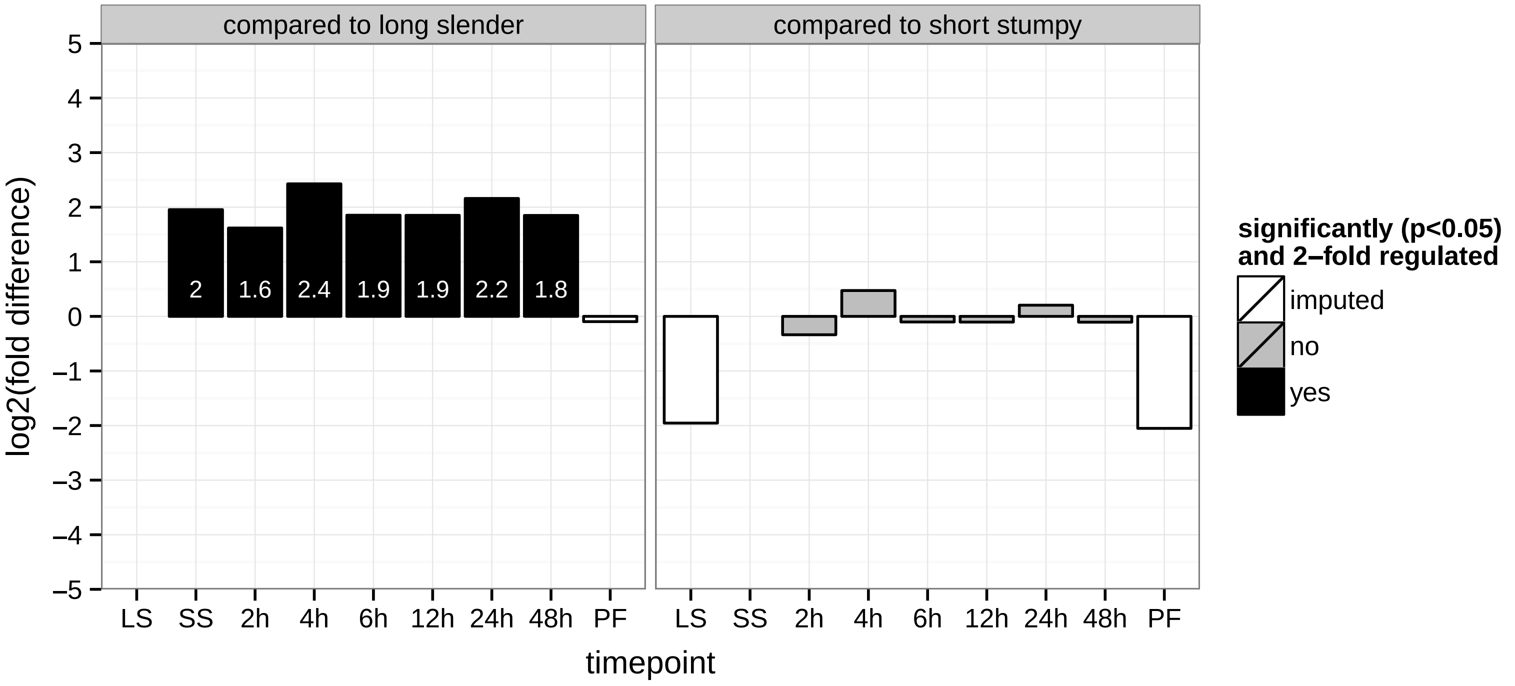

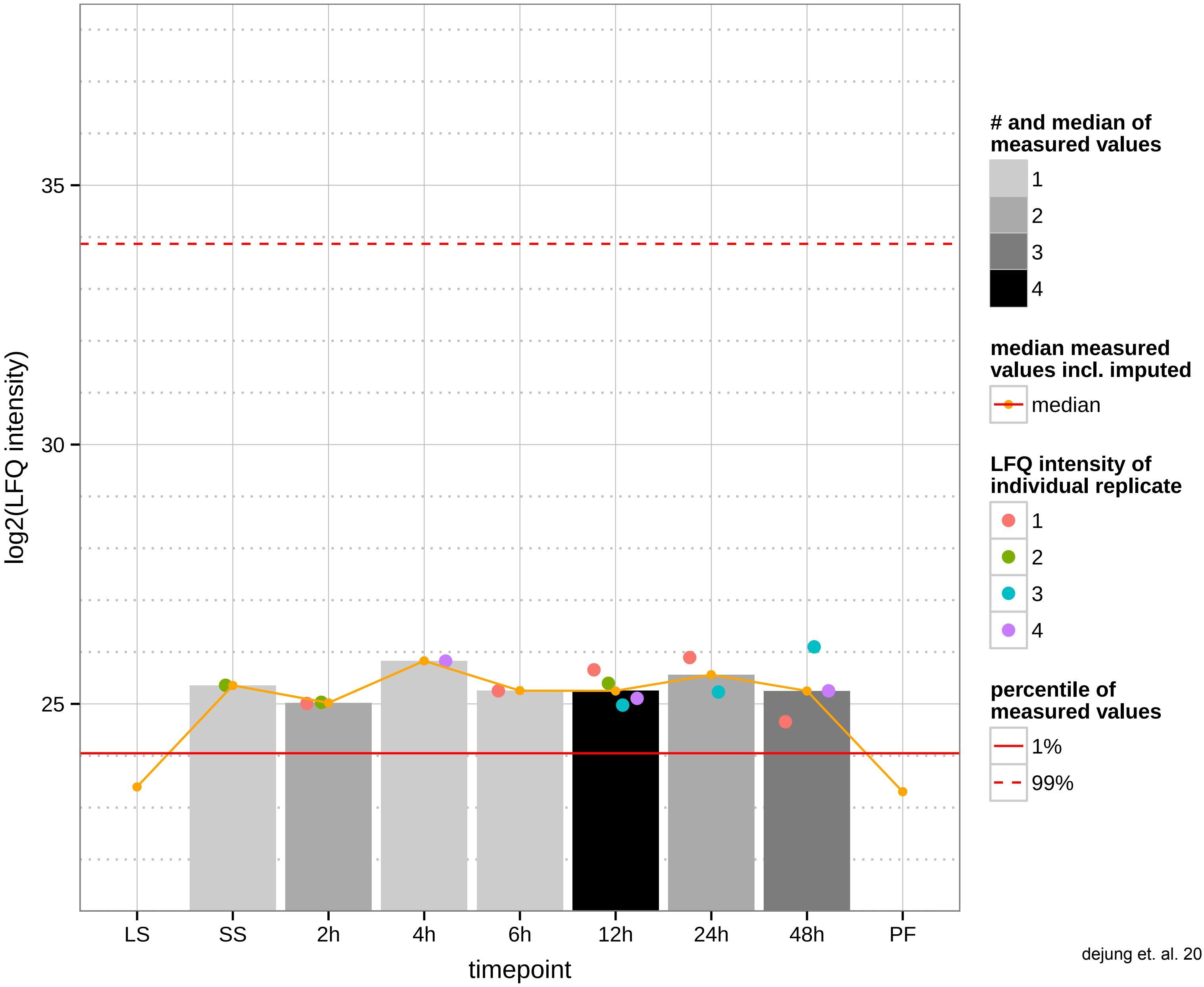

dejung et. al. 2015

Isoprenylcysteine alpha−carbonyl methylesterase, putative
Tb927.5.1760
AGOF: hydrolase activity
AGOC: null
AGOP: null
PGOF: hydrolase activity
PGOC: null
PGOP: metabolic process

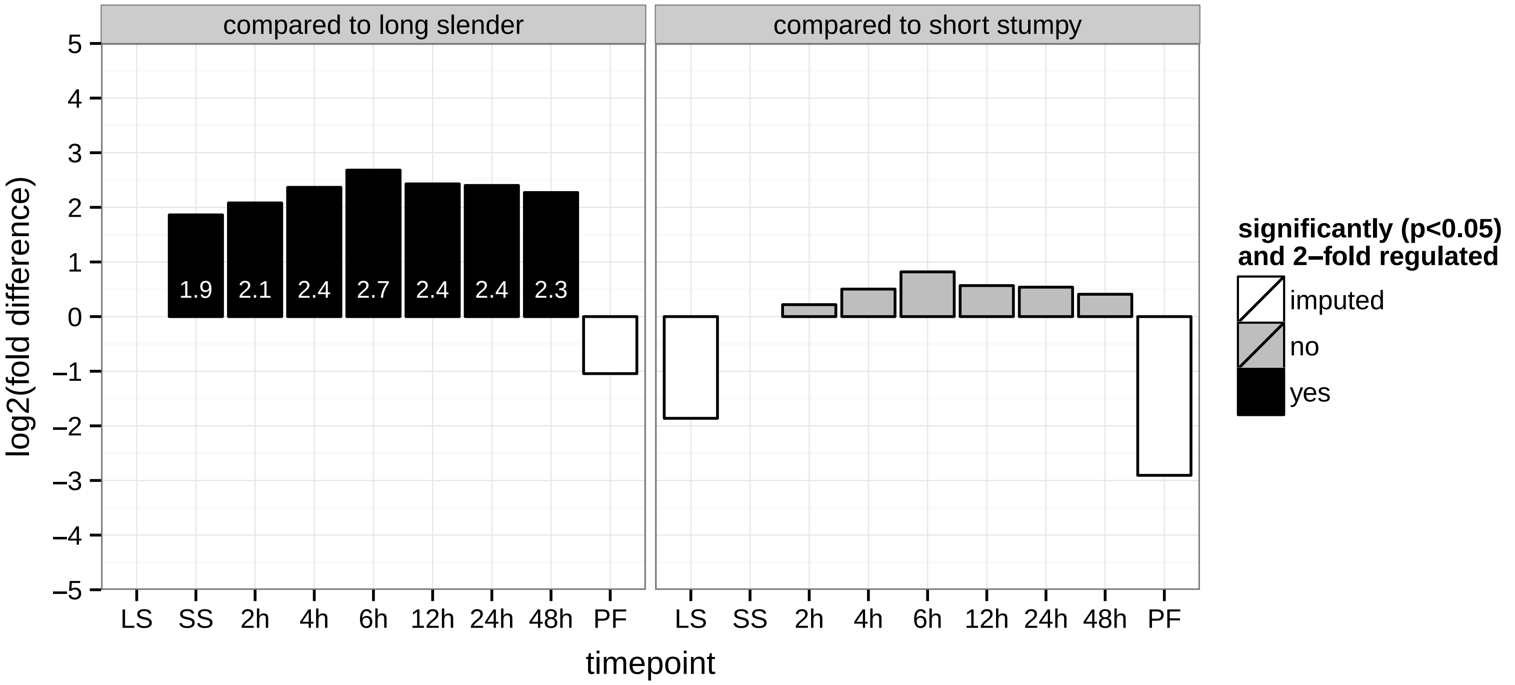

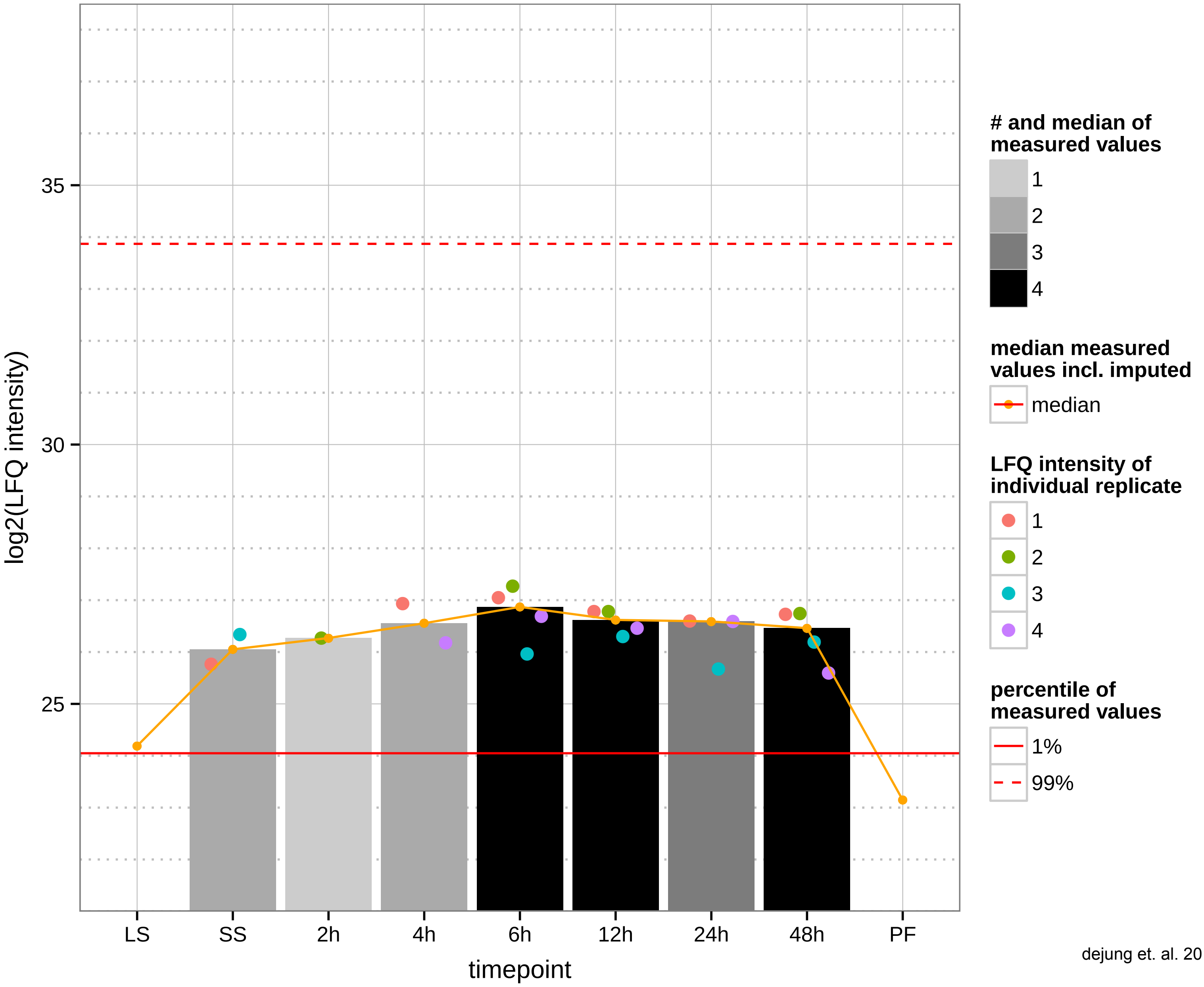

dejung et. al. 2015

hypothetical protein, conserved
Tb927.5.1910
AGOF: null
AGOC: null
AGOP: null
PGOF: null
PGOC: null
PGOP: null

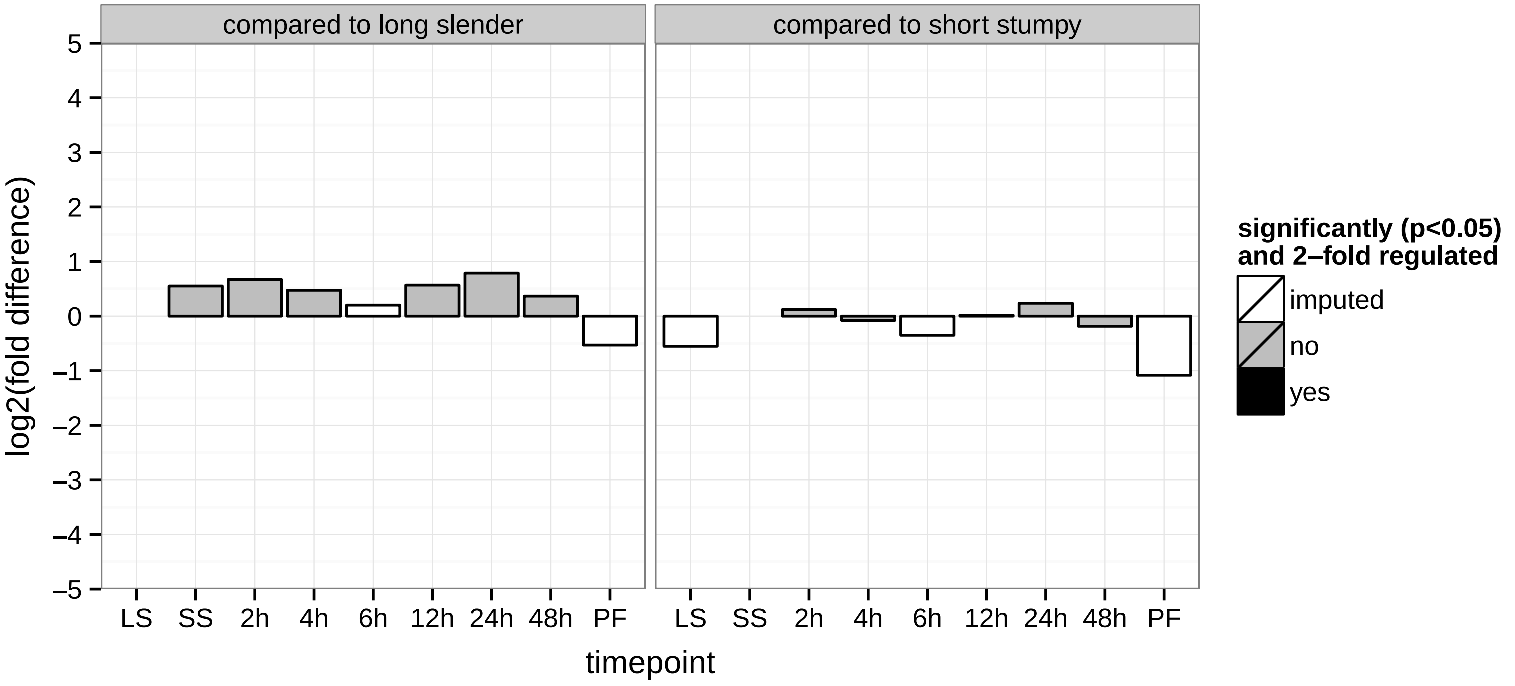

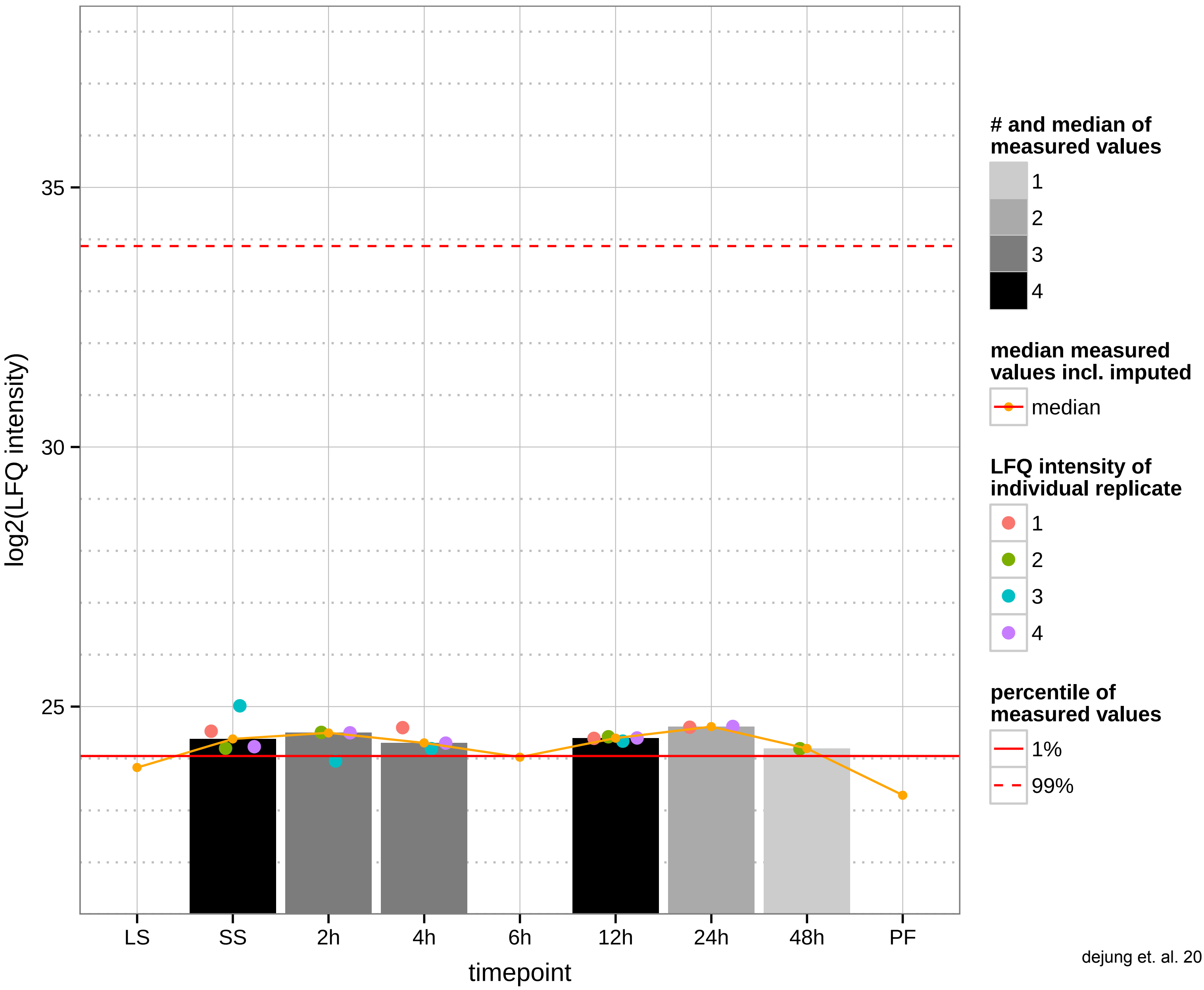

dejung et. al. 2015

cell division control protein, putative
Tb927.5.2060
AGOF: DNA binding
AGOC: null
AGOP: null
PGOF: DNA binding
PGOC: null
PGOP: null

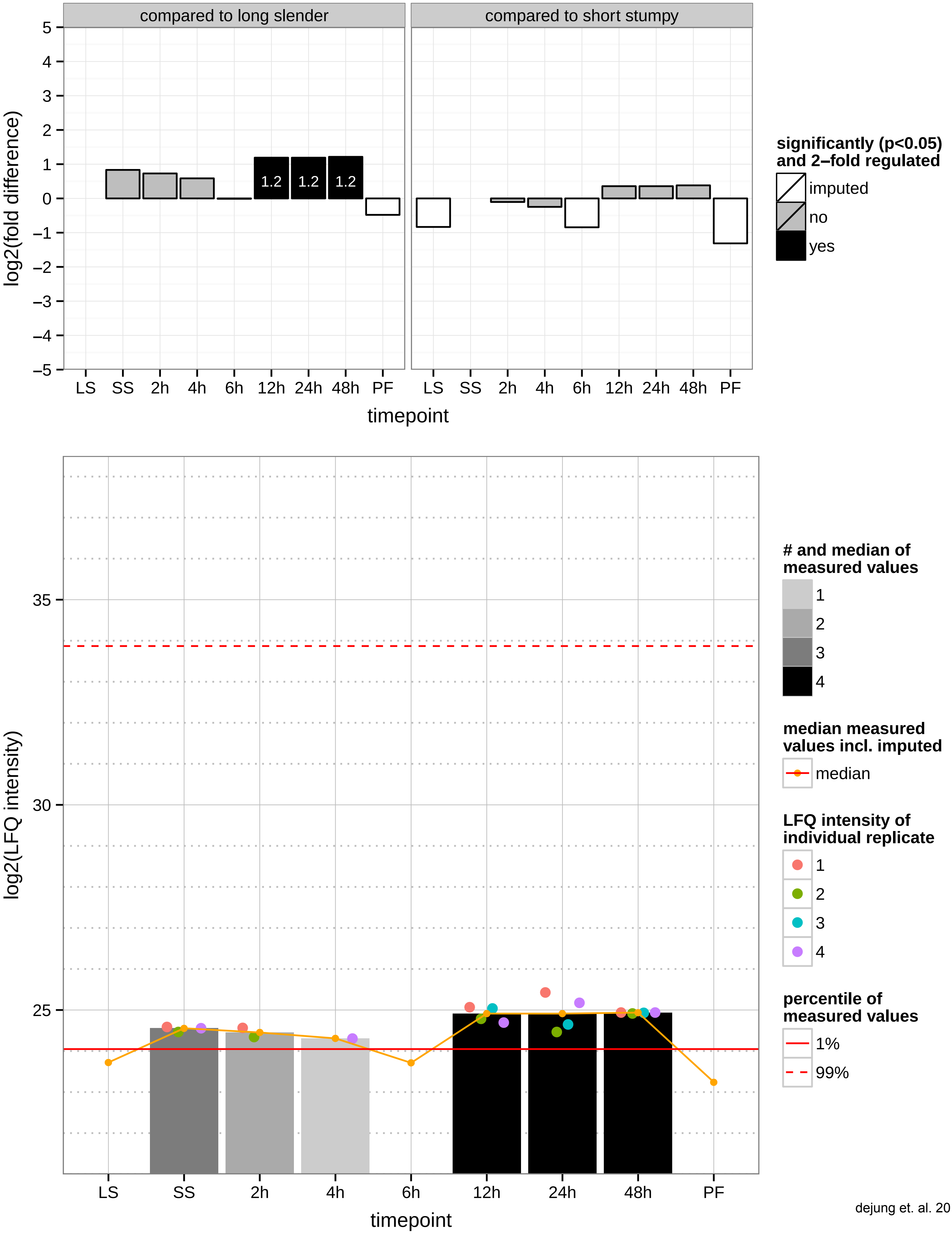

dejung et. al. 2015

hypothetical protein, conserved
Tb927.5.2250;Tb927.5.2220;Tb927.5.2190
AGOF: null
AGOC: null
AGOP: null
PGOF: null
PGOC: null
PGOP: null

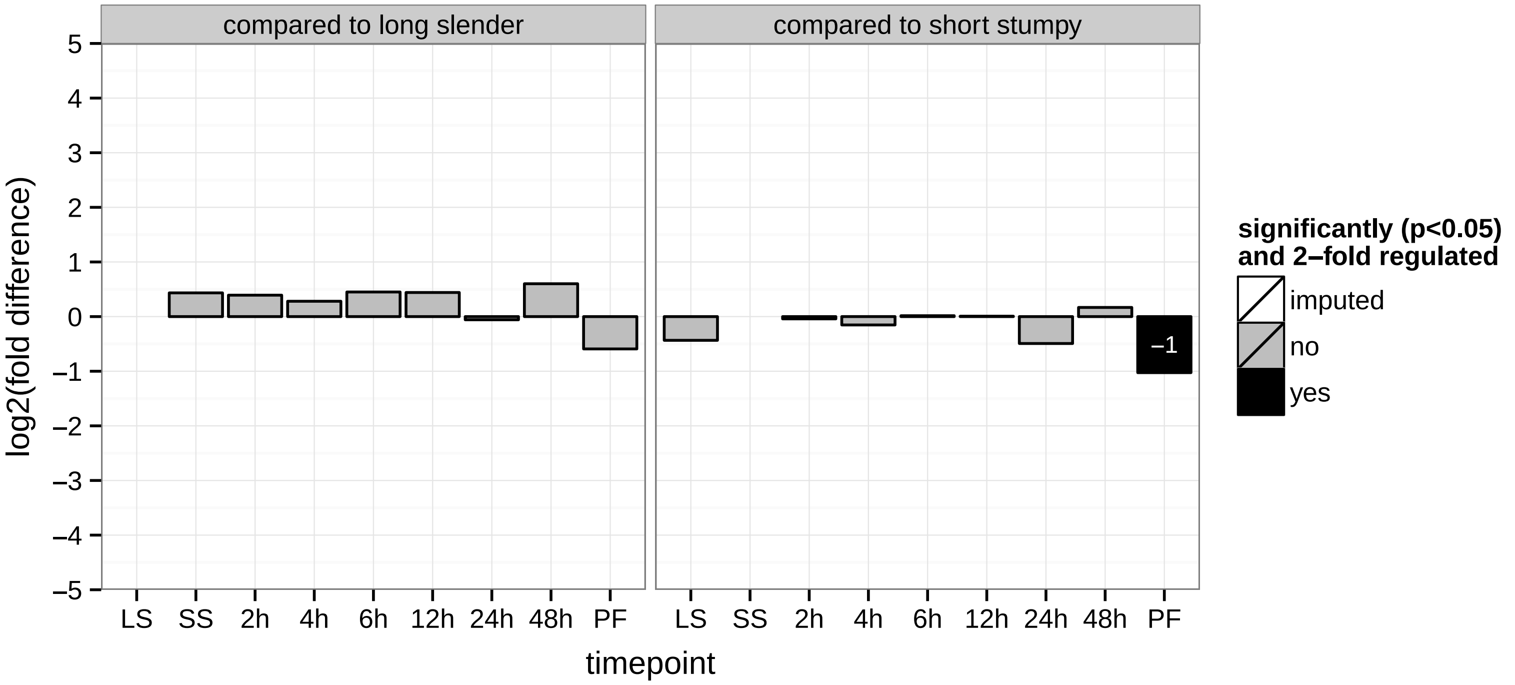

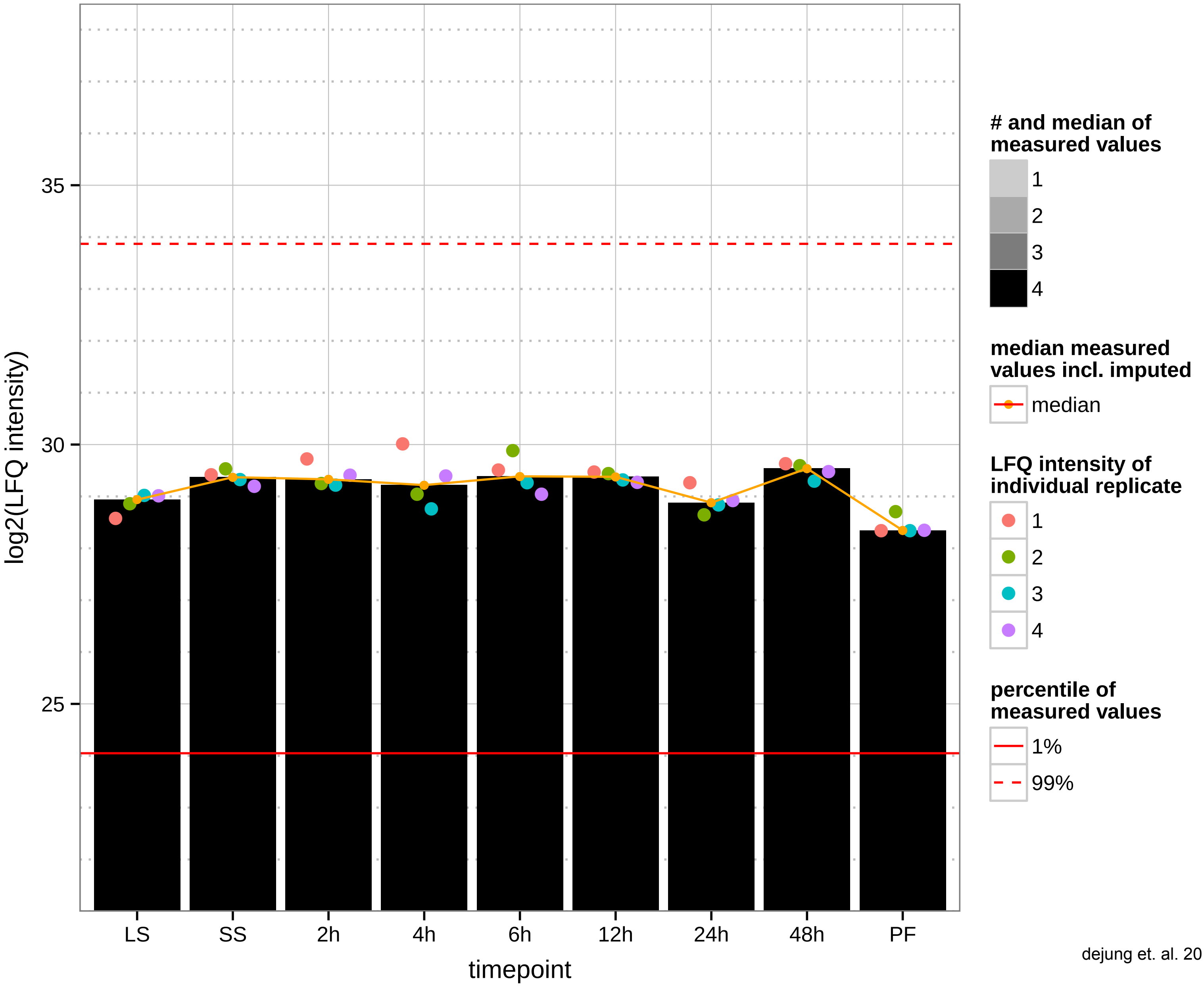

dejung et. al. 2015

hypothetical protein, conserved
Tb927.5.2980
AGOF: null
AGOC: null
AGOP: null
PGOF: null
PGOC: null
PGOP: null

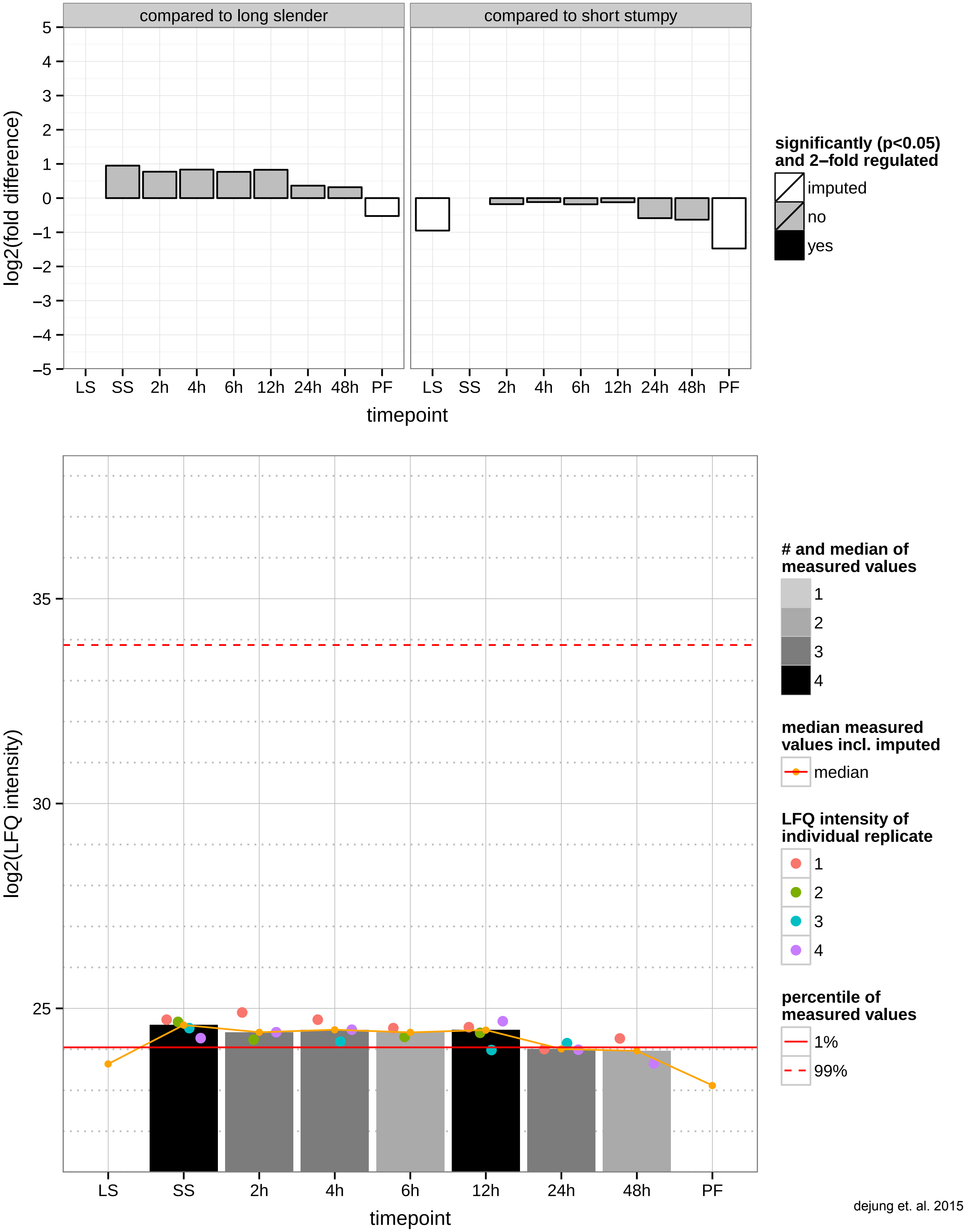

dejung et. al. 2015

ATP−dependent DEAD/H RNA helicase, putative
Tb927.5.3600
AGOF: ATP binding, ATP−dependent helicase activity, helicase activity, nucleic acid binding
AGOC: null
AGOP: nucleobase−containing compound metabolic process
PGOF: ATP binding, ATP−dependent helicase activity, helicase activity, nucleic acid binding
PGOC: null
PGOP: null

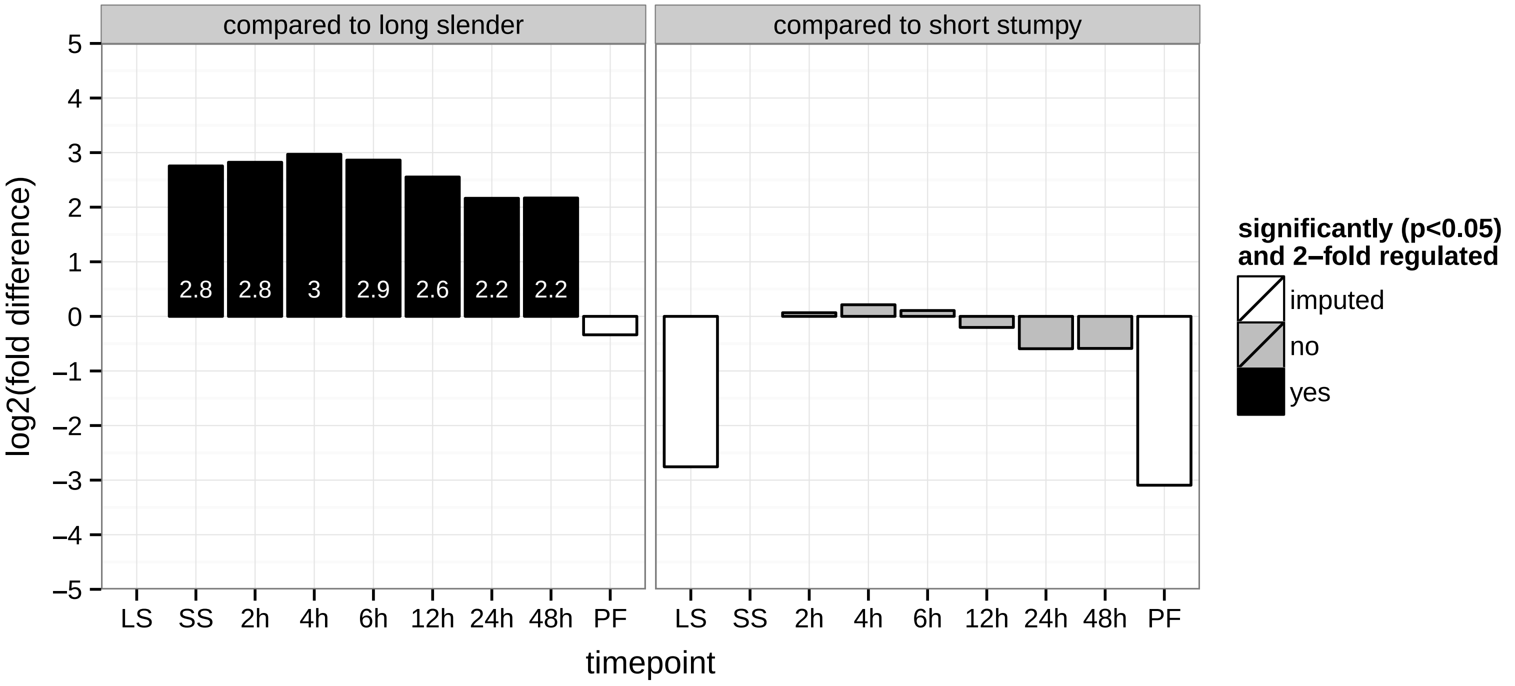

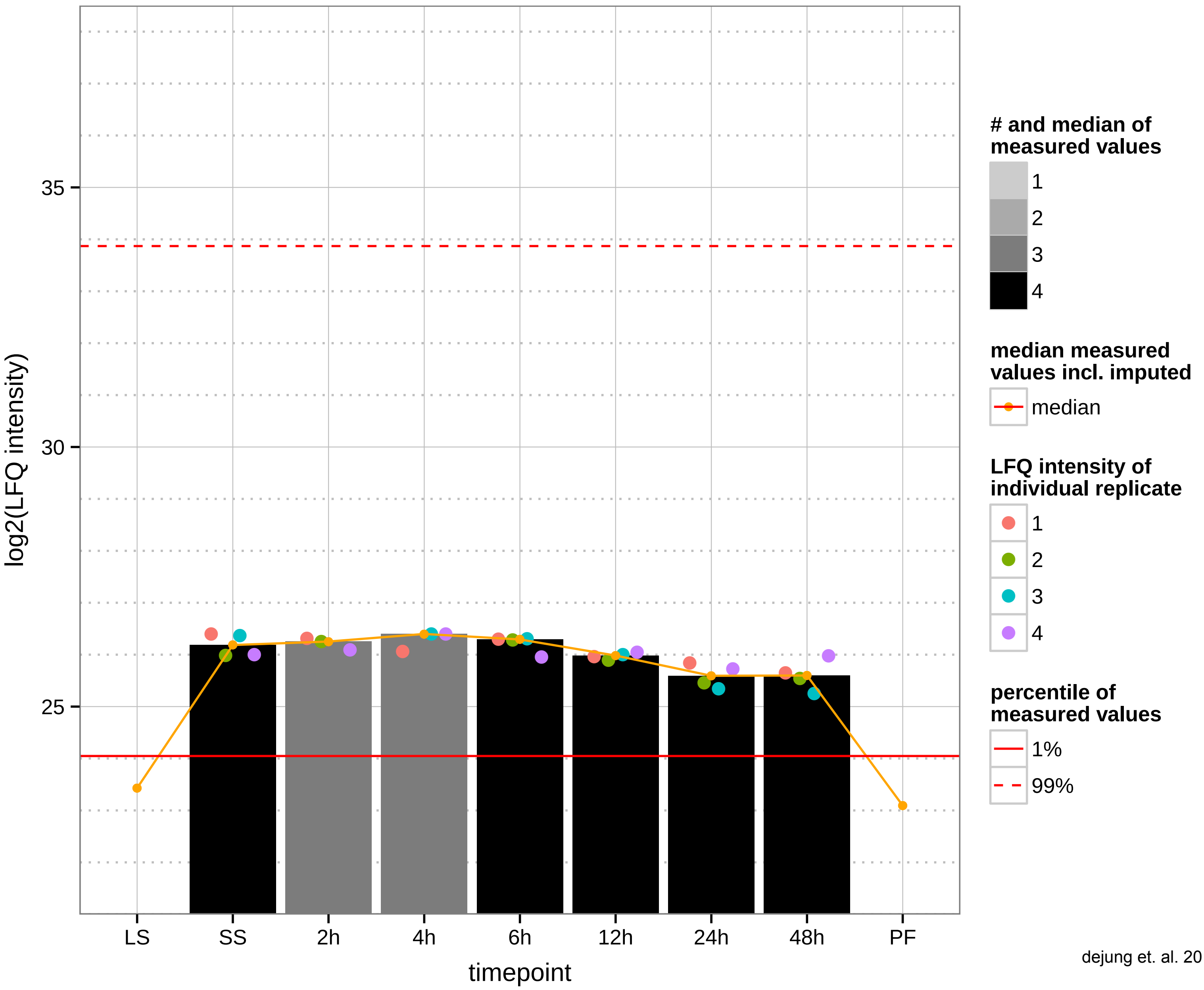

dejung et. al. 2015

RNA–binding protein, putative (PPCT1)
Tb927.5.3750
AGOF: RNA binding, peptidyl–prolyl cis–trans isomerase activity
AGOC: null
AGOP: RNA processing
PGOF: nucleic acid binding, peptidyl–prolyl cis–trans isomerase activity
PGOC: null
PGOP: protein folding

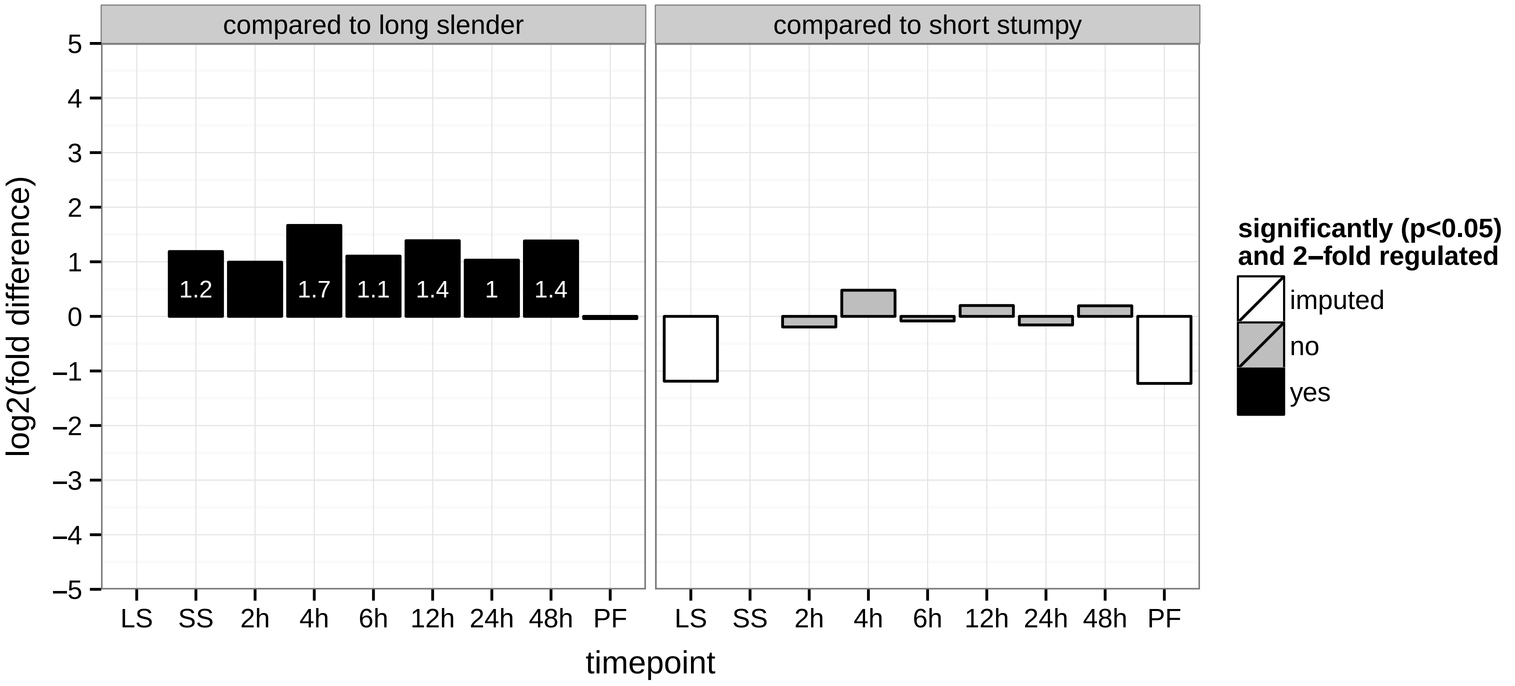

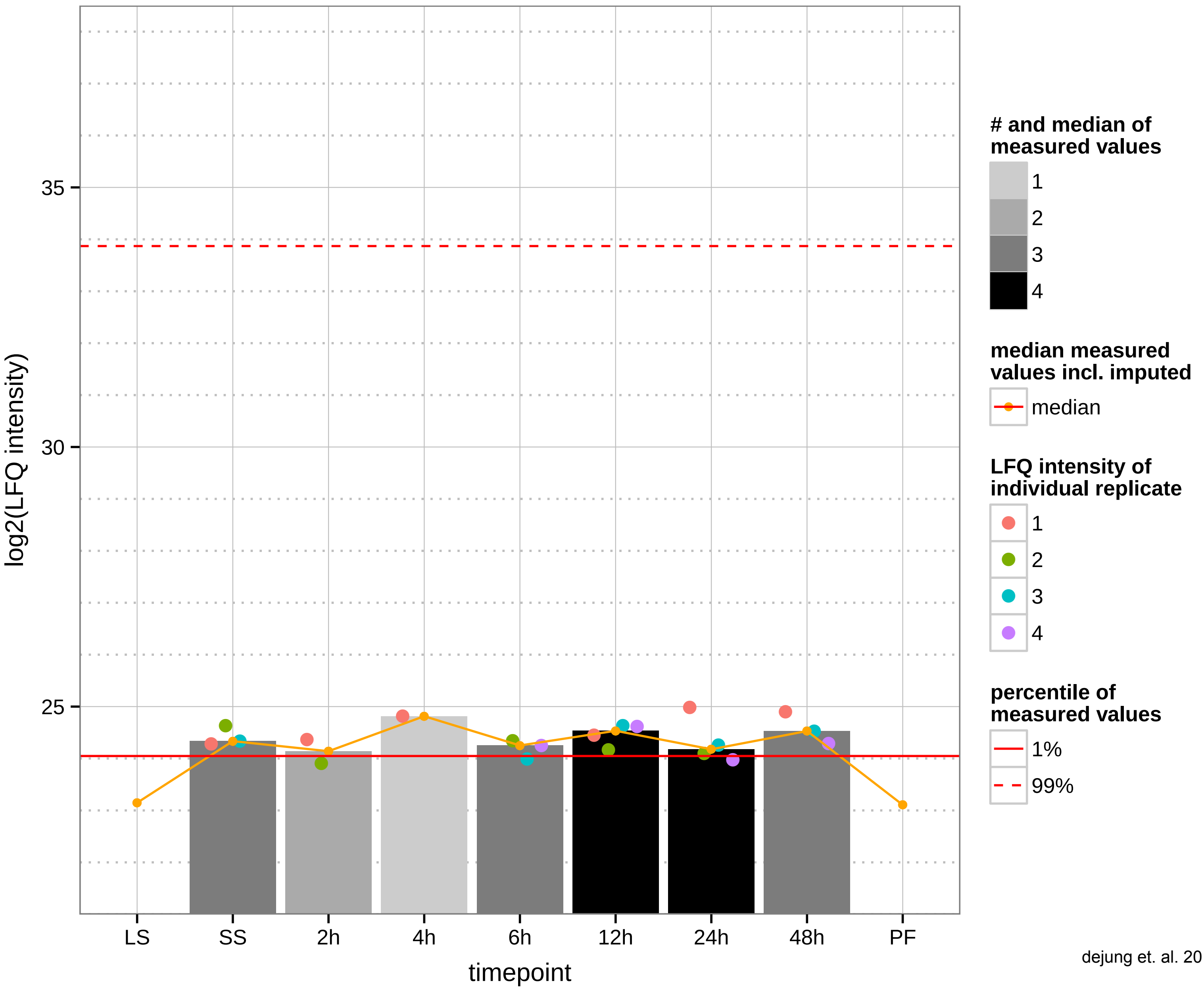

dejung et. al. 2015

Selenoprotein T, putative (SelT)
Tb927.5.860
AGOF: selenium binding
AGOC: null
AGOP: cell redox homeostasis
PGOF: selenium binding
PGOC: null
PGOP: cell redox homeostasis

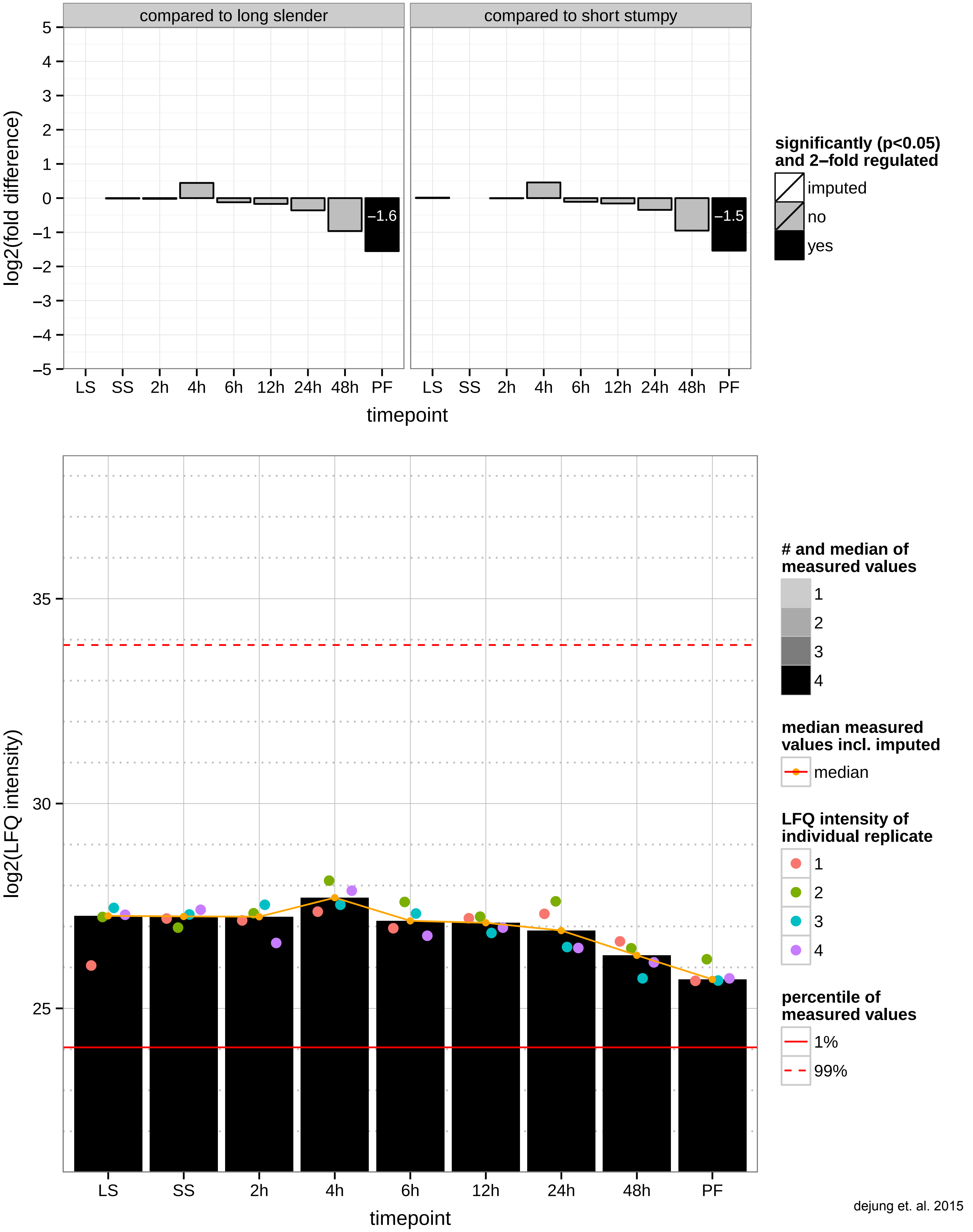

dejung et. al. 2015

hypothetical protein, conserved
Tb927.5.990
AGOF: null
AGOC: null
AGOP: null
PGOF: null
PGOC: null
PGOP: null

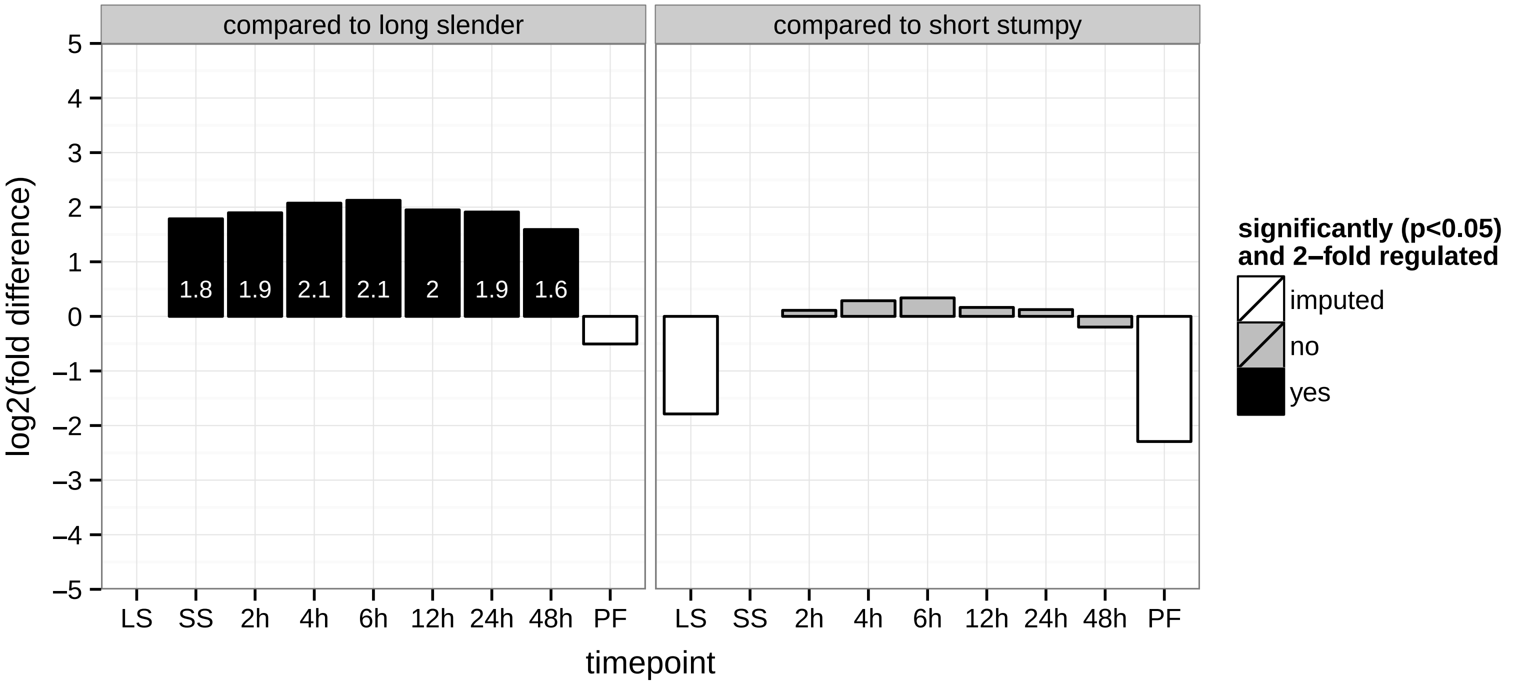

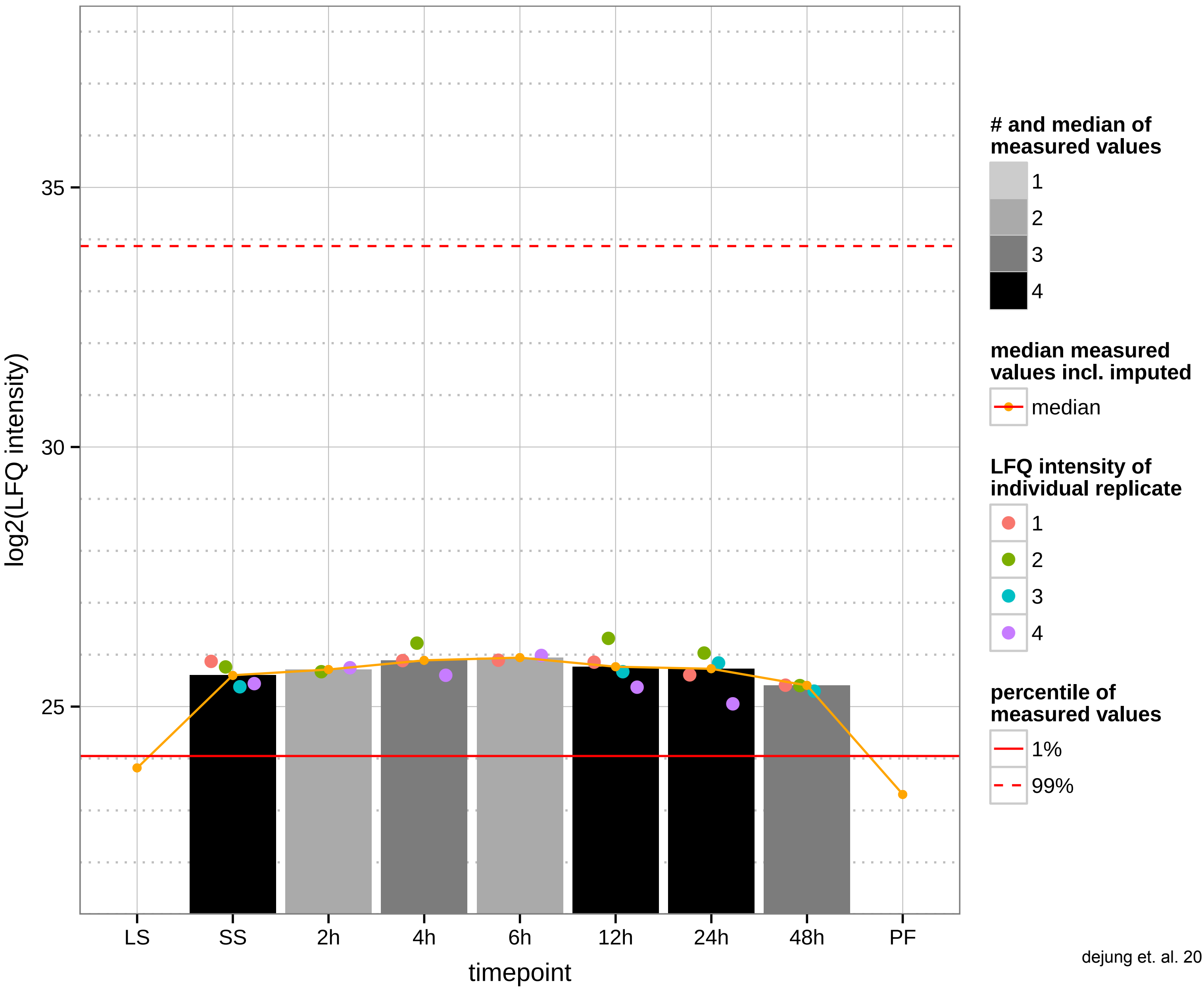

dejung et. al. 2015

hypothetical protein, conserved
Tb927.6.1470
AGOF: null
AGOC: null
AGOP: null
PGOF: null
PGOC: null
PGOP: null

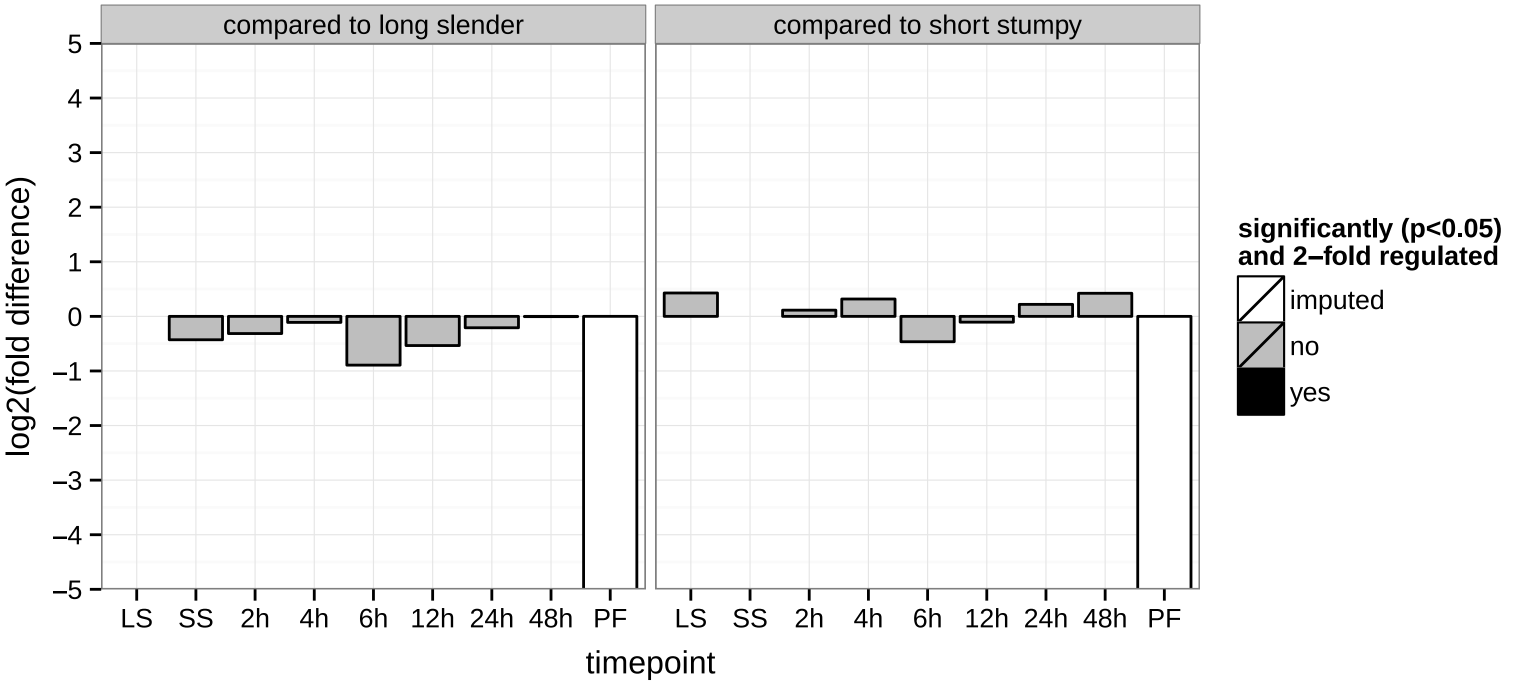

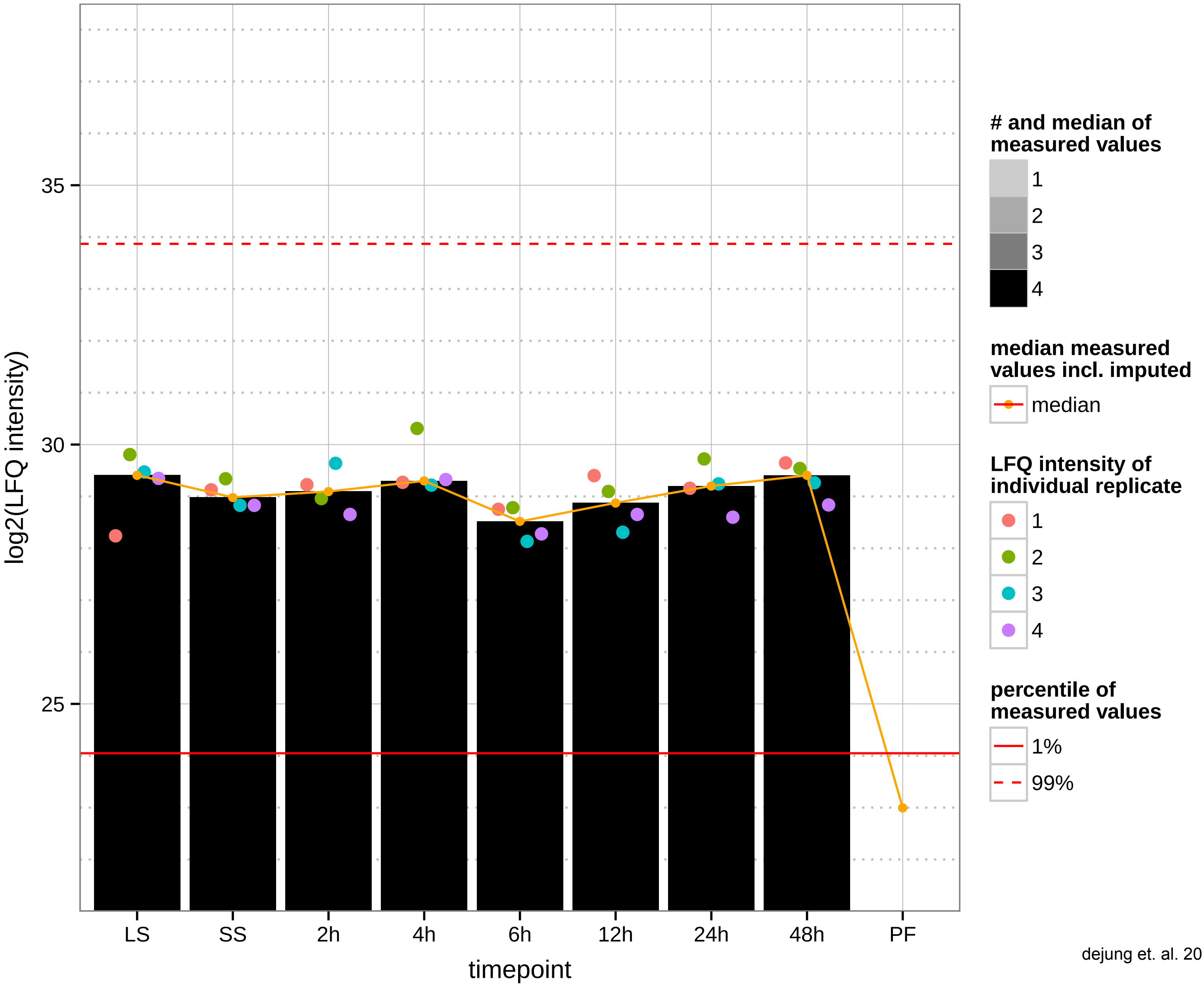

dejung et. al. 2015

hypothetical protein, conserved
Tb927.6.1480
AGOF: null
AGOC: null
AGOP: null
PGOF: protein binding
PGOC: null
PGOP: null

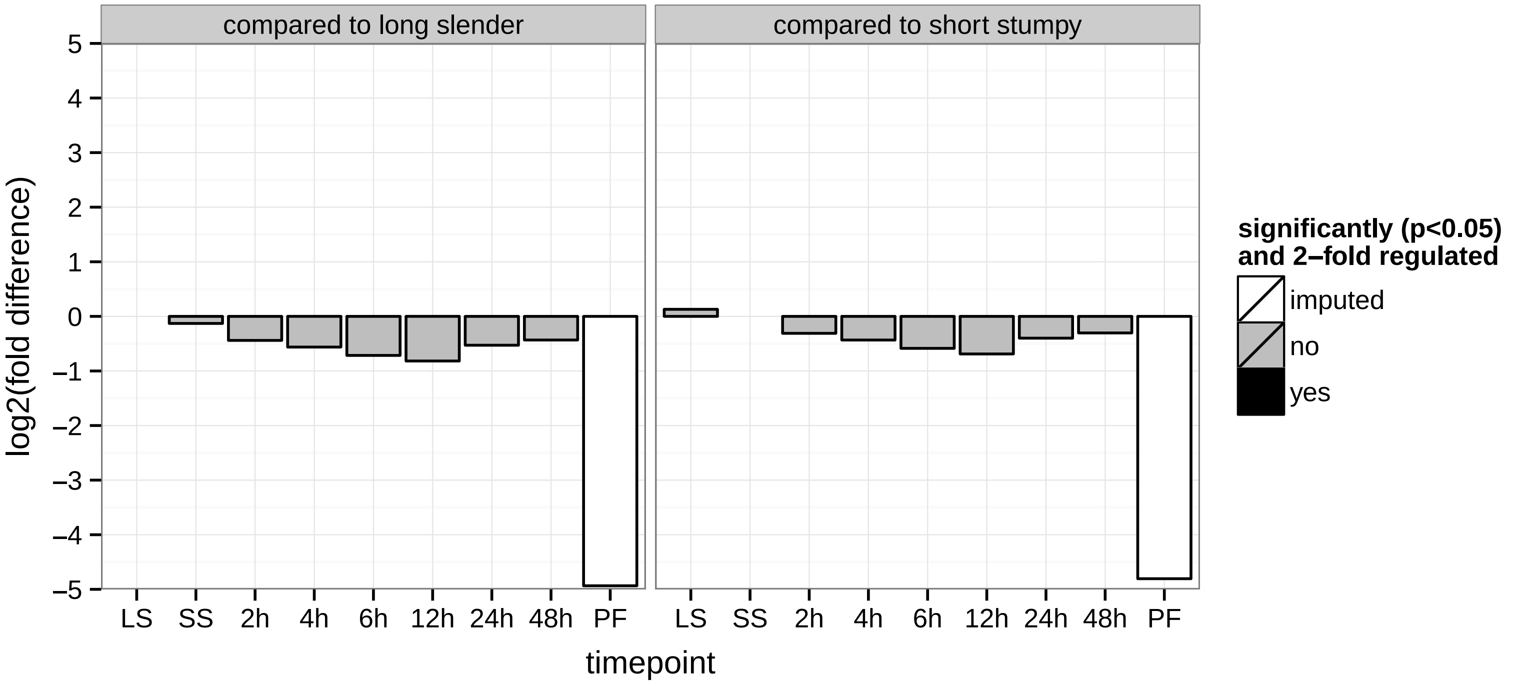

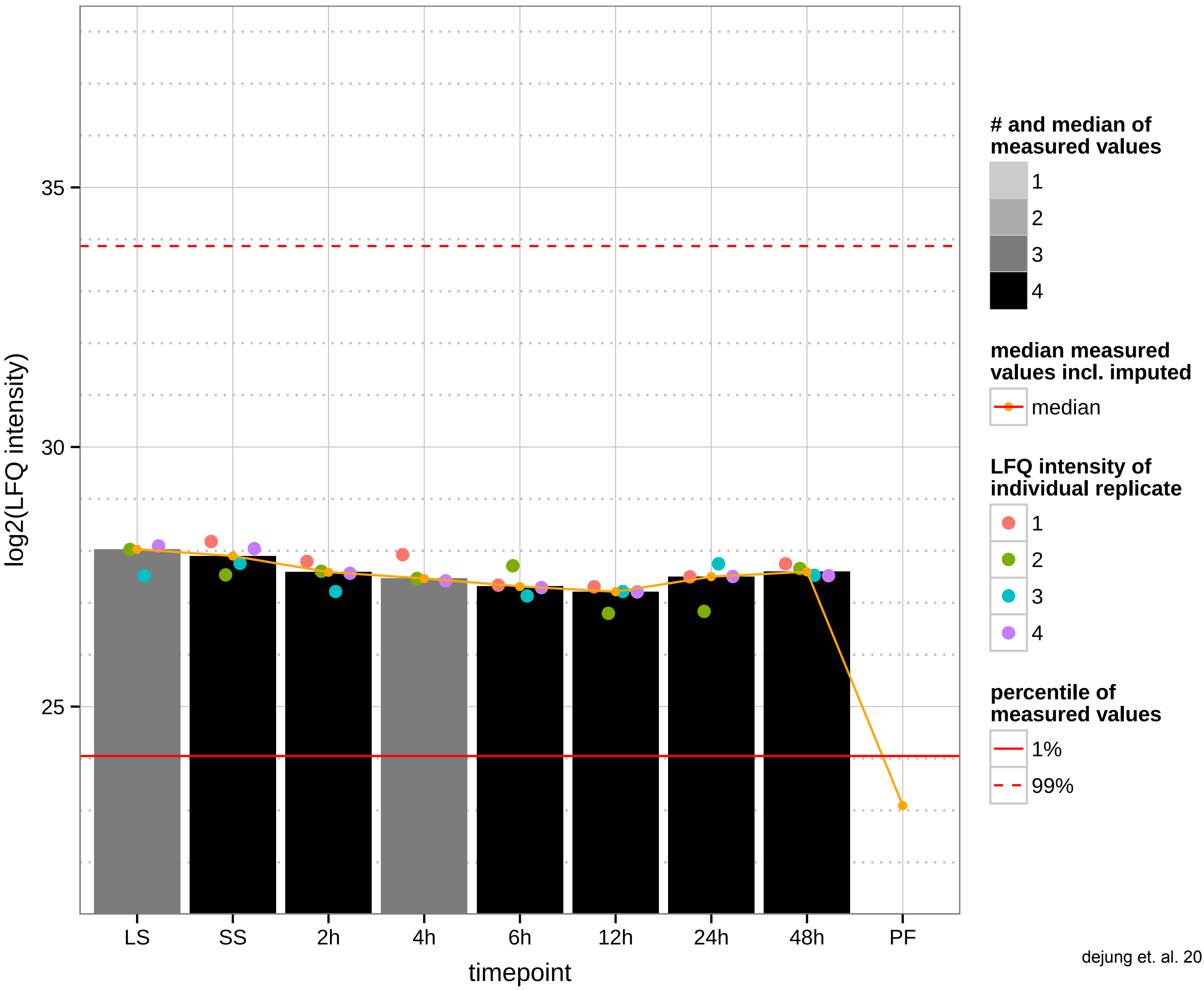

dejung et. al. 2015

Aquaglyceroporin 1 (AQP1)
Tb927.6.1520
AGOF: drug transmembrane transporter activity, glycerol channel activity, water channel activity
AGOC: membrane
AGOP: detection of osmotic stimulus, drug transmembrane transport, glycerol transport, hypotonic response, response to dru
PGOF: transporter activity
PGOC: integral to membrane, membrane
PGOP: transmembrane transport, transport, water transport

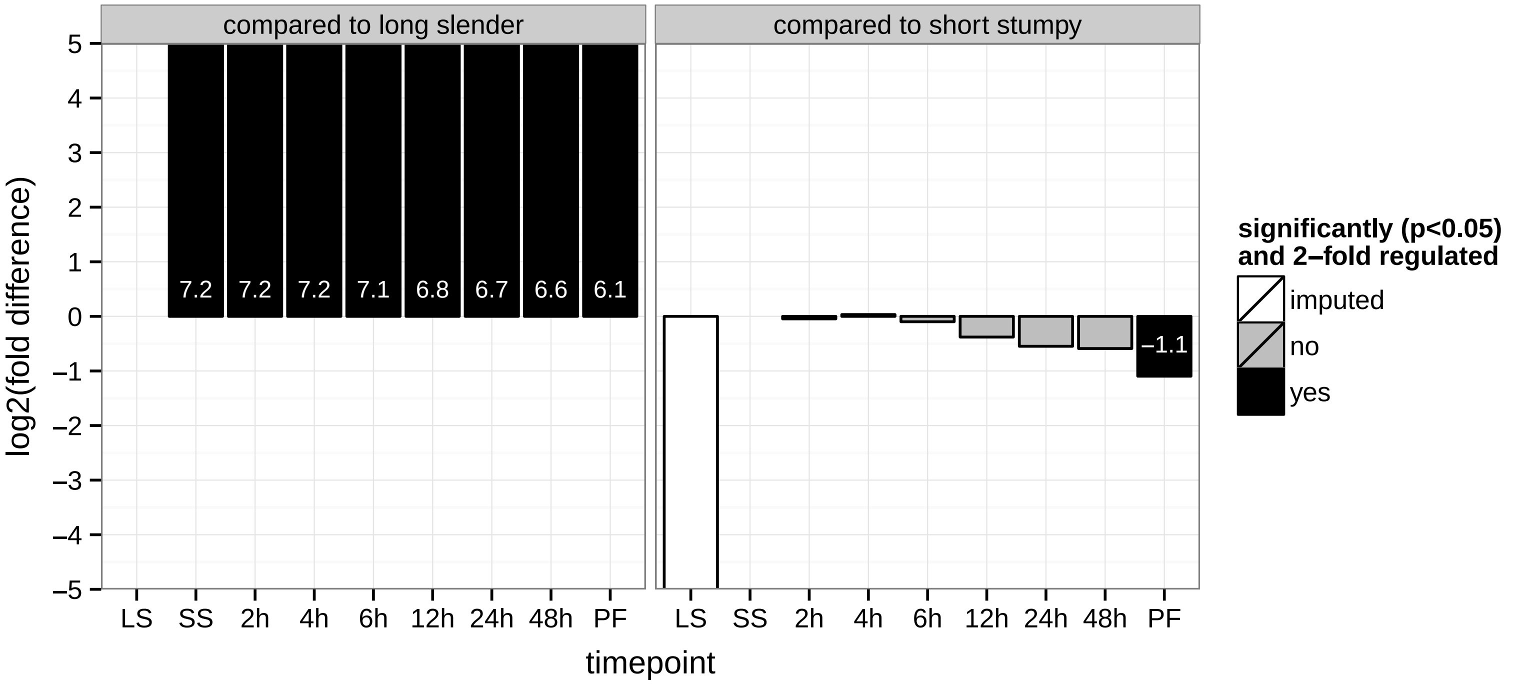

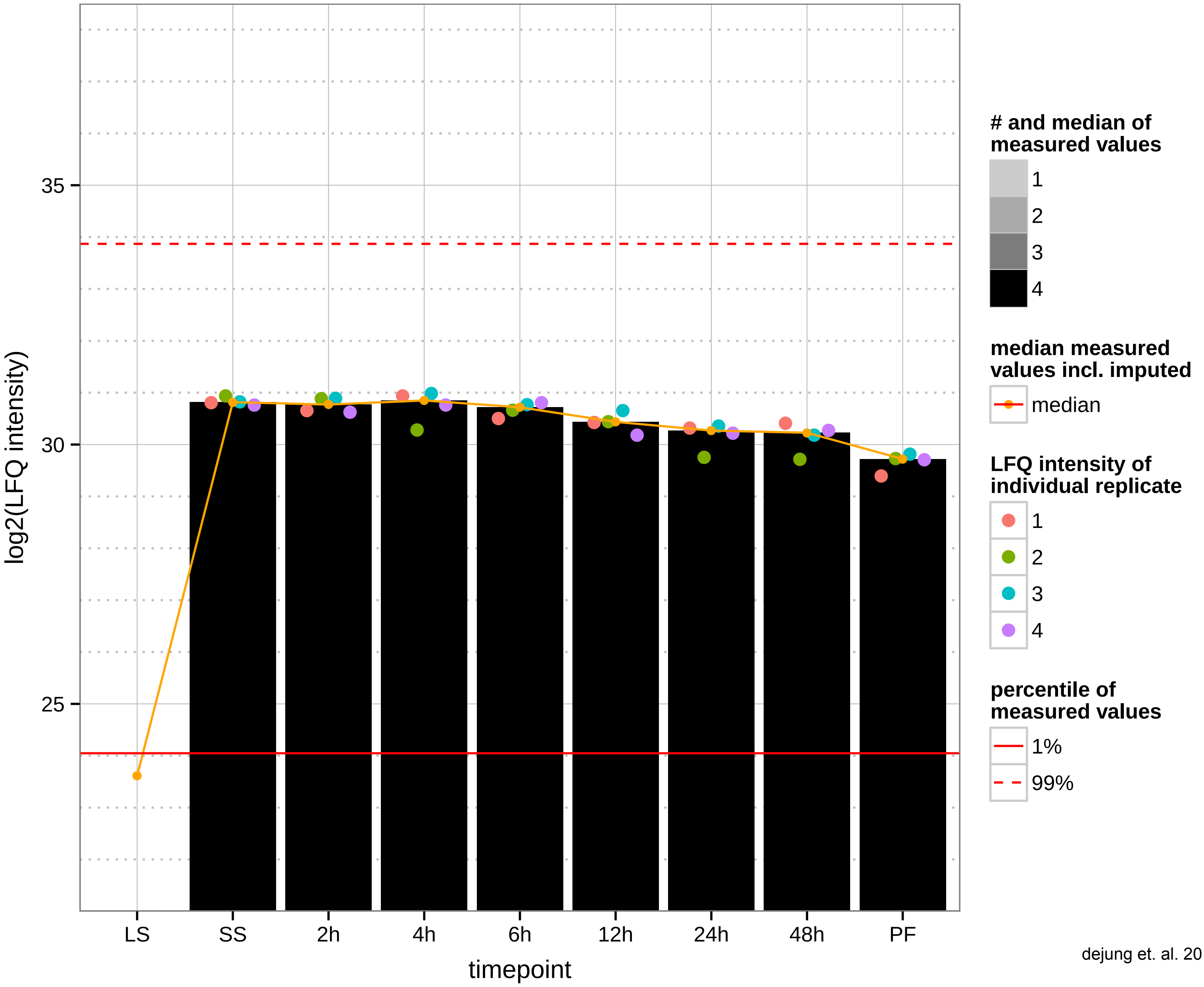

dejung et. al. 2015

hypothetical protein, conserved
Tb927.6.1540
AGOF: null
AGOC: null
AGOP: null
PGOF: null
PGOC: membrane
PGOP: null

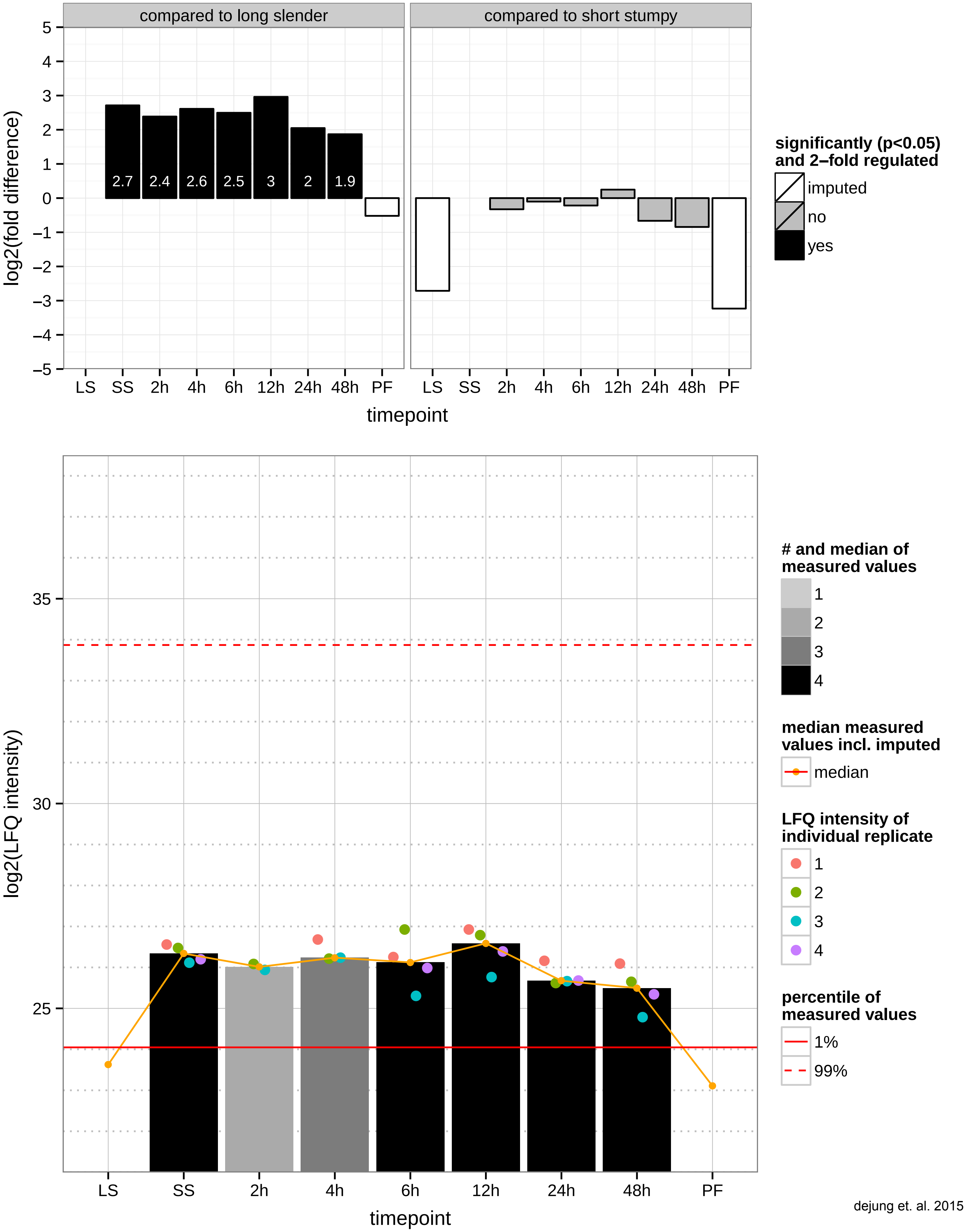

leucine−rich repeat protein (LRRP), putative
Tb927.6.1550
AGOF: null
AGOC: null
AGOP: null
PGOF: null
PGOC: null
PGOP: null

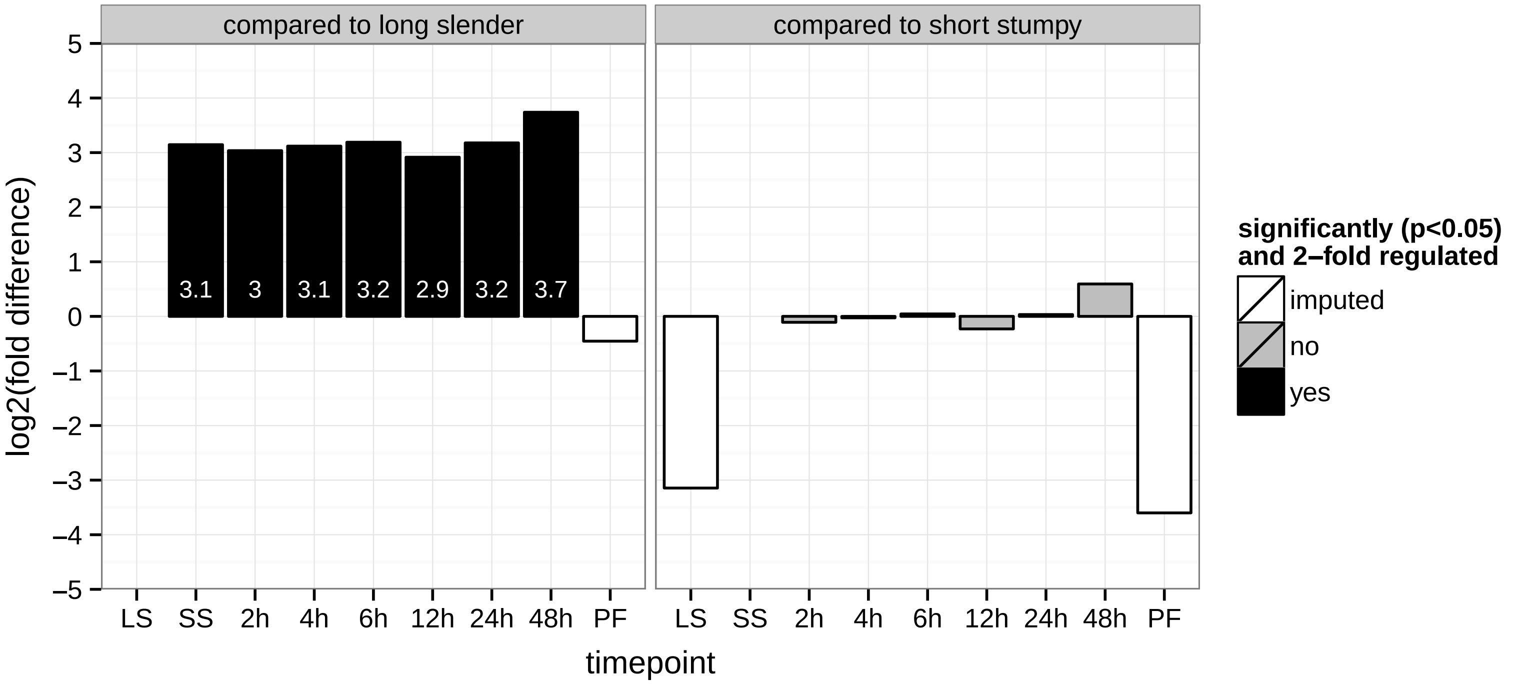

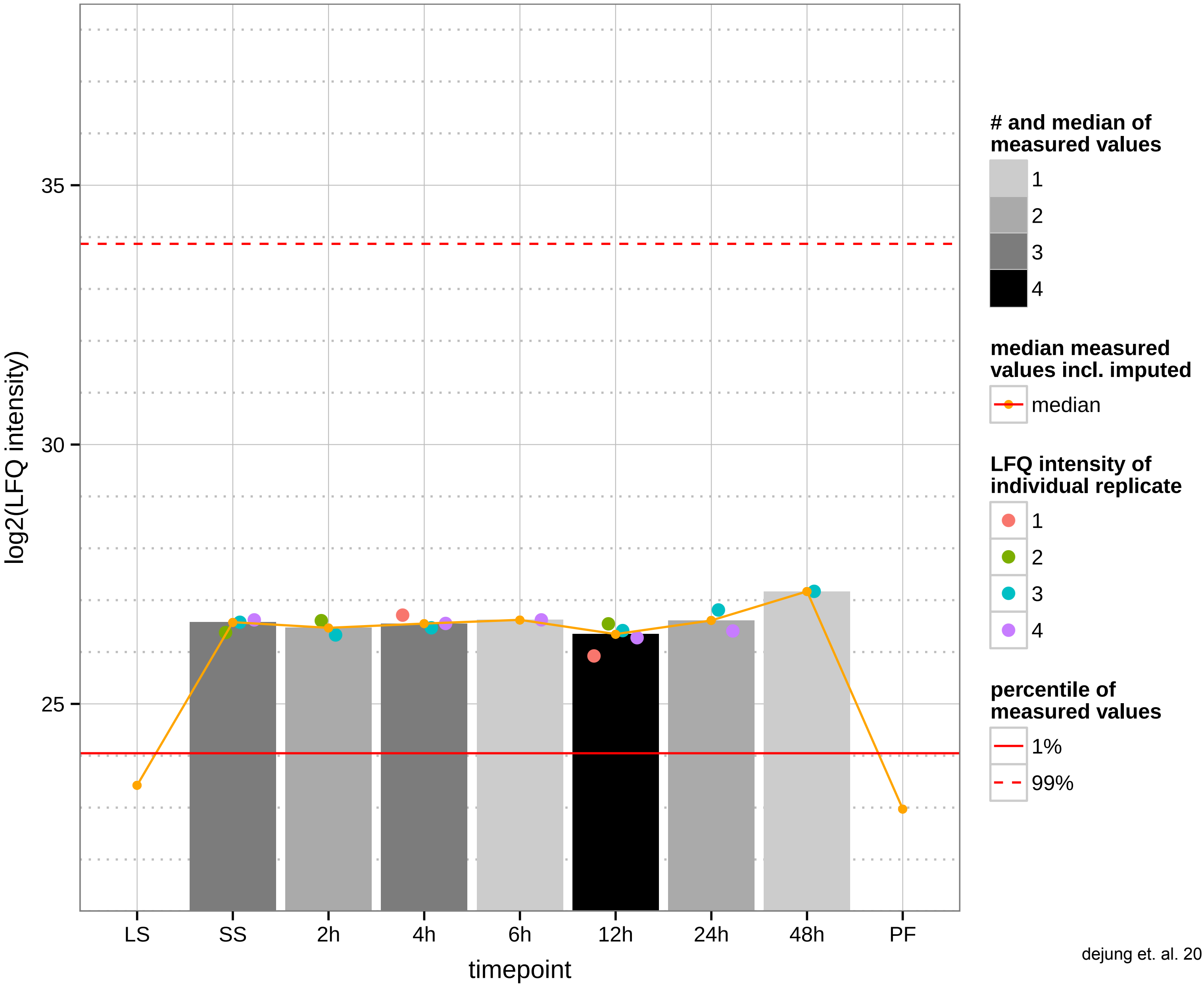

dejung et. al. 2015

polynucleotide kinase 3'−phosphatase, putative
Tb927.6.1580
AGOF: null
AGOC: null
AGOP: null
PGOF: null
PGOC: null
PGOP: null

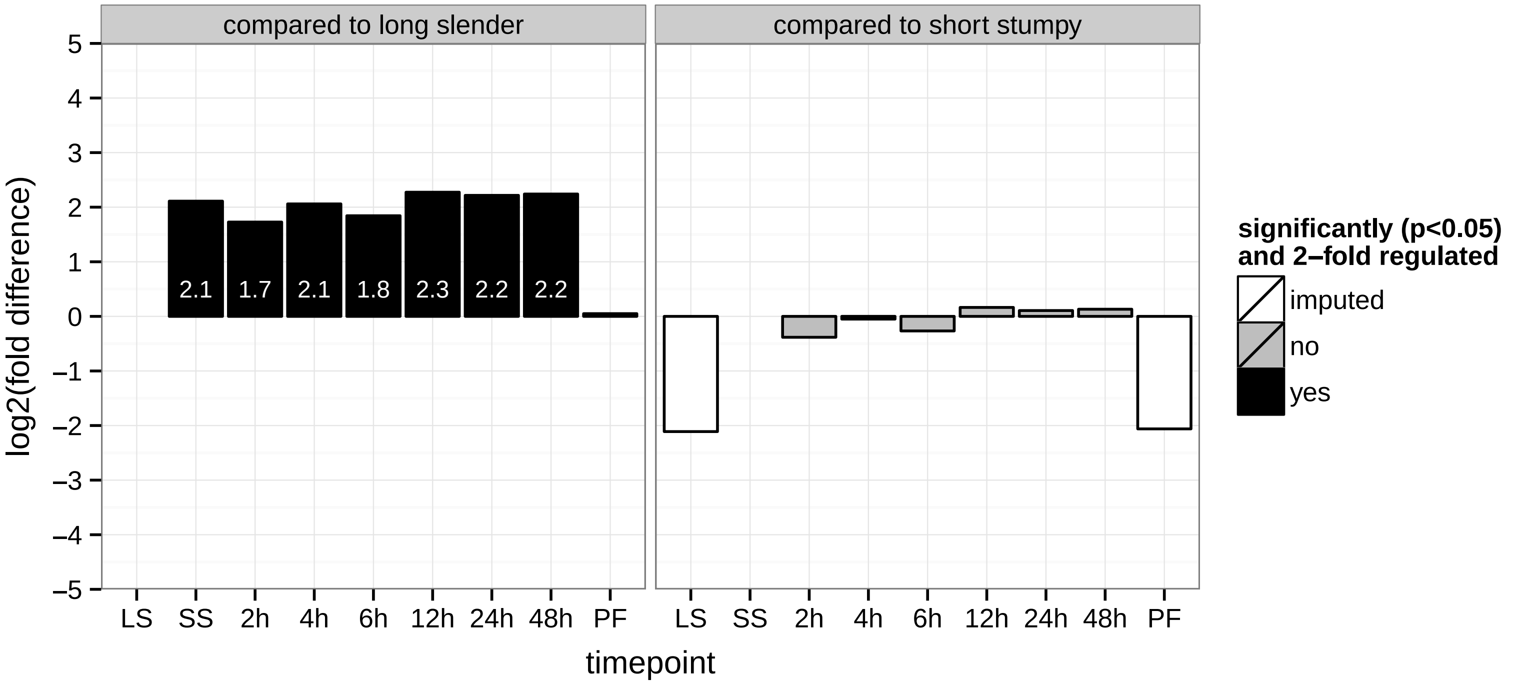

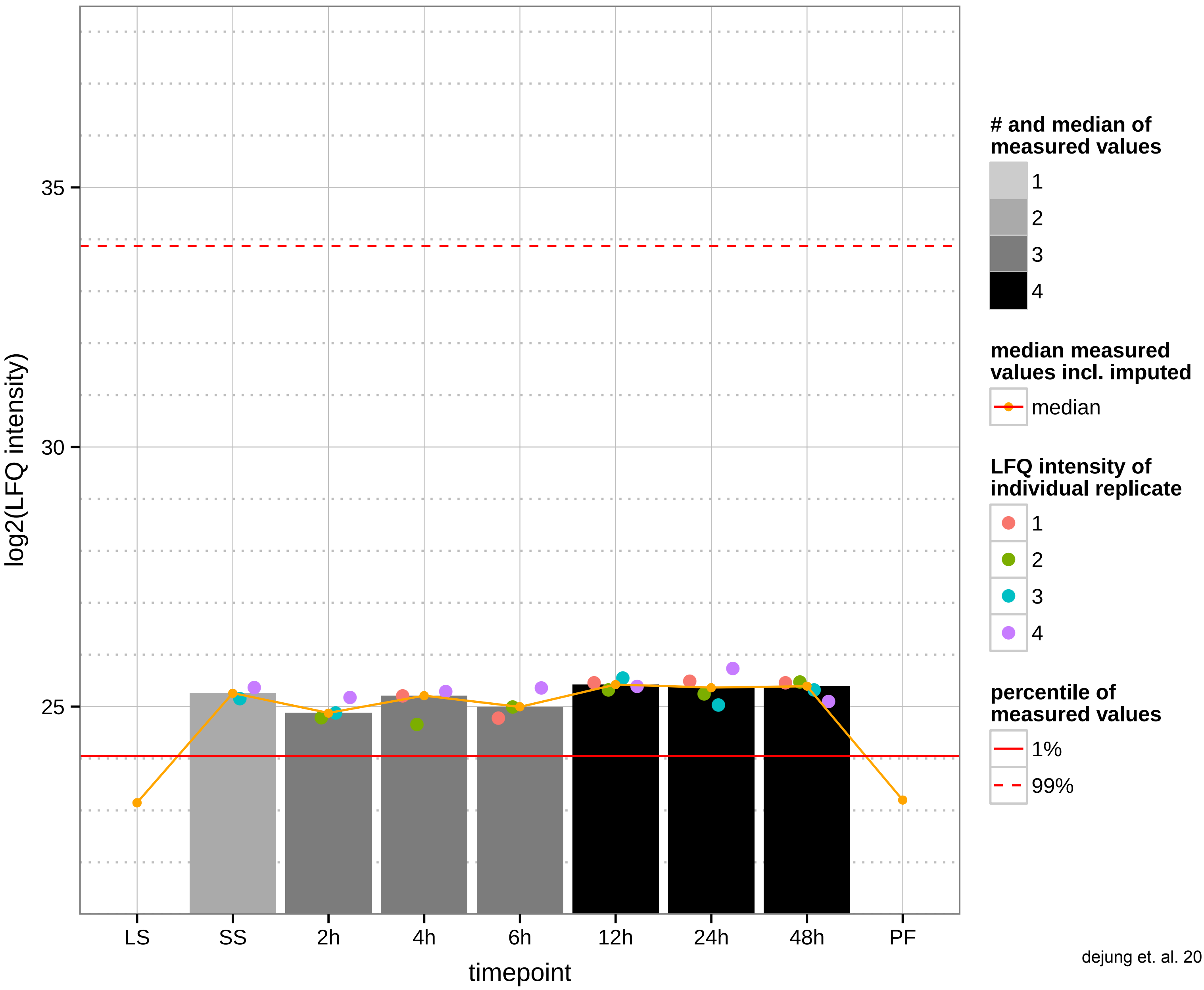

dejung et. al. 2015

kinesin, putative
Tb927.6.1770
AGOF: ATP binding, microtubule motor activity, motor activity
AGOC: microtubule associated complex
AGOP: microtubule−based movement
PGOF: ATP binding, microtubule motor activity
PGOC: null
PGOP: microtubule−based movement

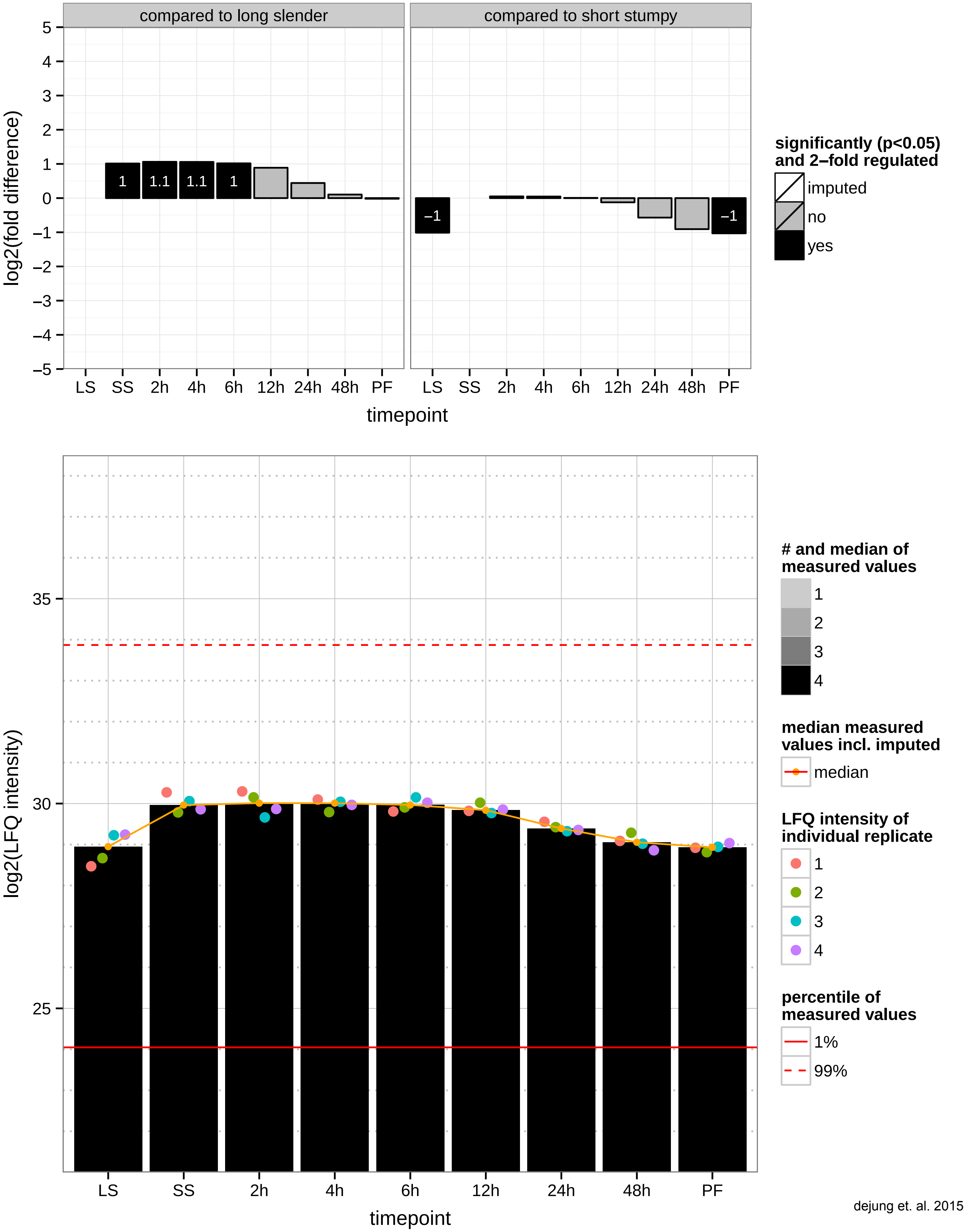

dejung et. al. 2015

protein phosphatase 2C, putative
Tb927.6.1800
AGOF: phosphatase activity
AGOC: null
AGOP: dephosphorylation
PGOF: catalytic activity
PGOC: null
PGOP: null

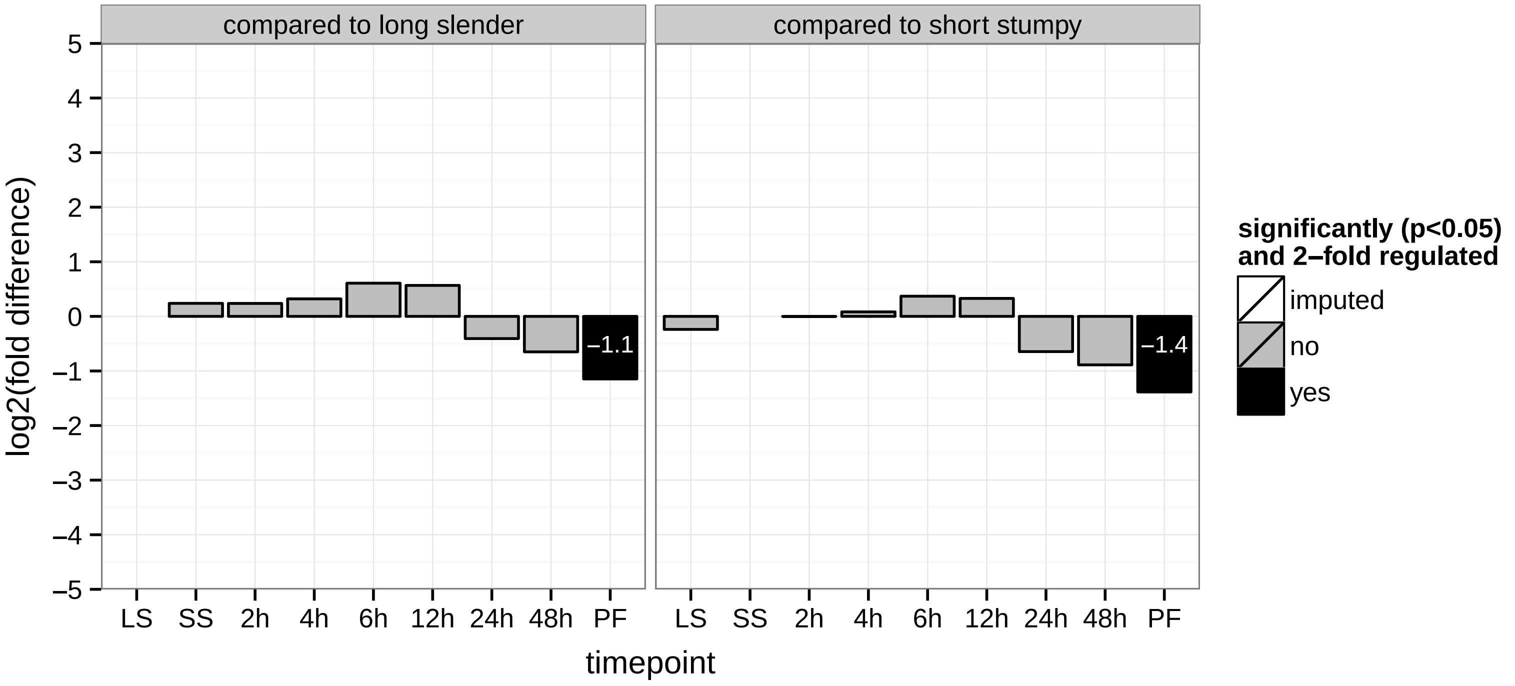

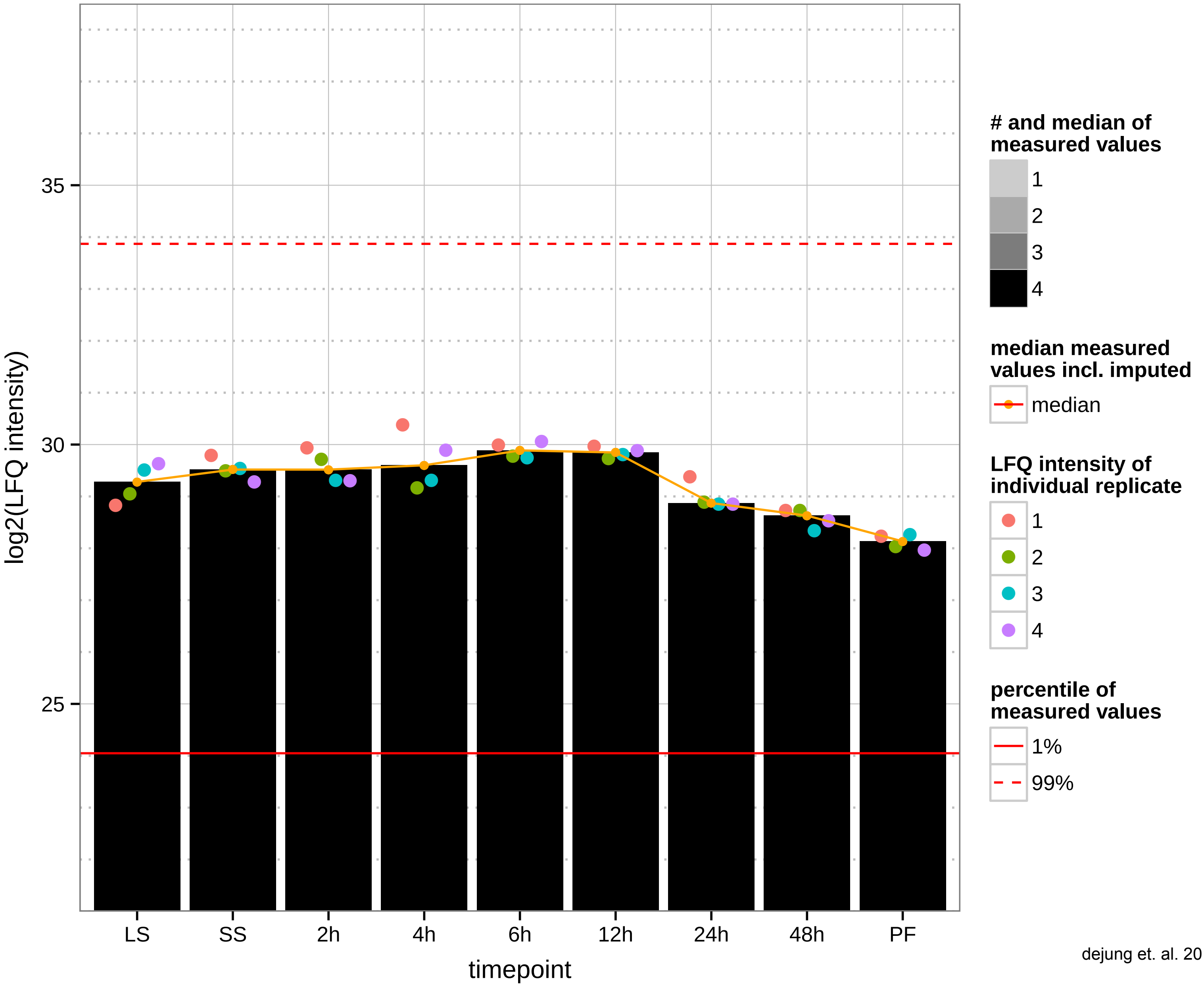

dejung et. al. 2015

hypothetical protein, conserved
Tb927.6.2510
AGOF: null
AGOC: mitochondrion
AGOP: null
PGOF: null
PGOC: null
PGOP: null

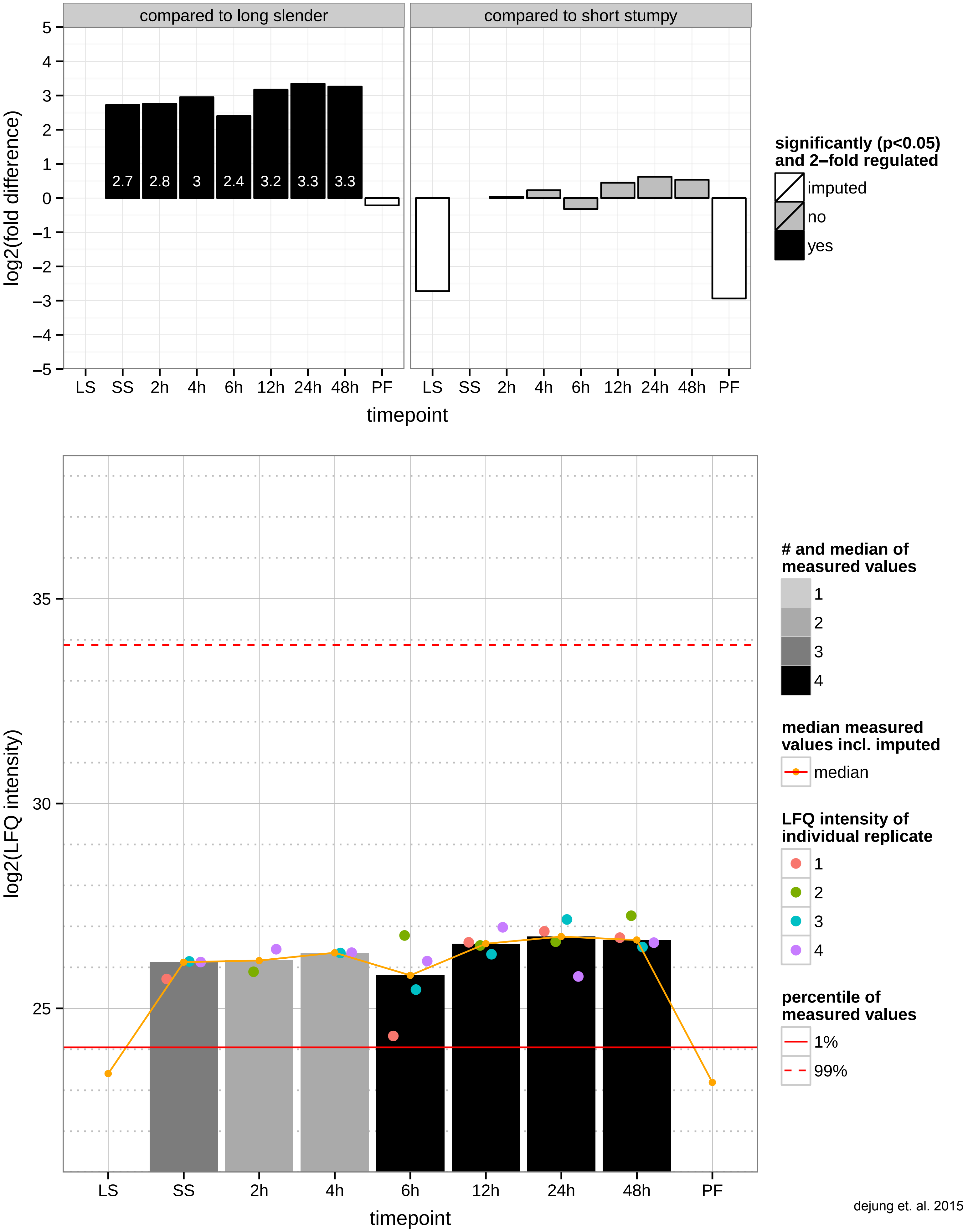

dejung et. al. 2015

hypothetical protein, conserved
Tb927.6.2600
AGOF: null
AGOC: null
AGOP: null
PGOF: null
PGOC: null
PGOP: null

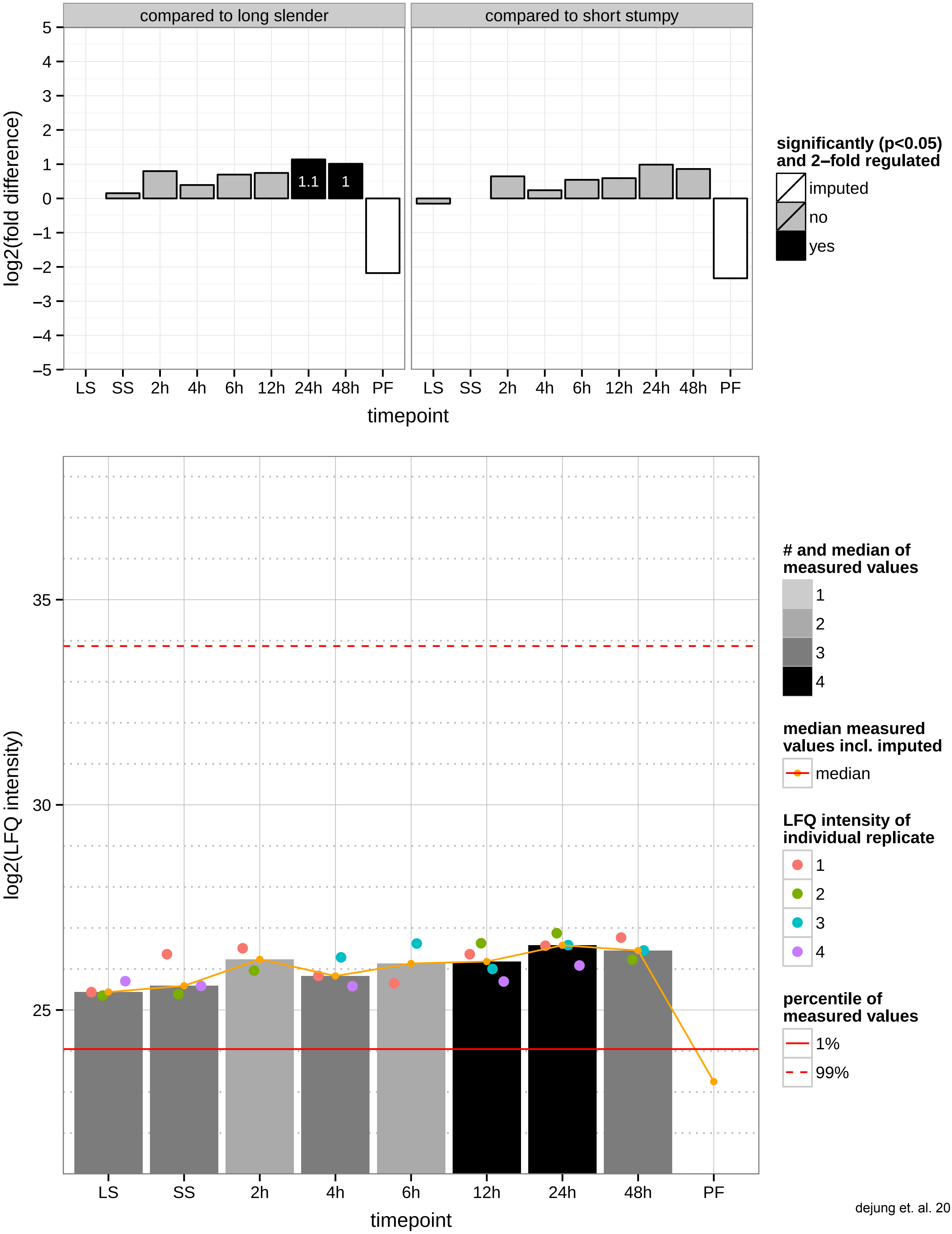

dejung et. al. 2015

GTPase activating protein, conserved
Tb927.6.2830
AGOF: Rab GTPase activator activity
AGOC: intracellular
AGOP: regulation of Rab GTPase activity
PGOF: Rab GTPase activator activity
PGOC: intracellular
PGOP: regulation of Rab GTPase activity

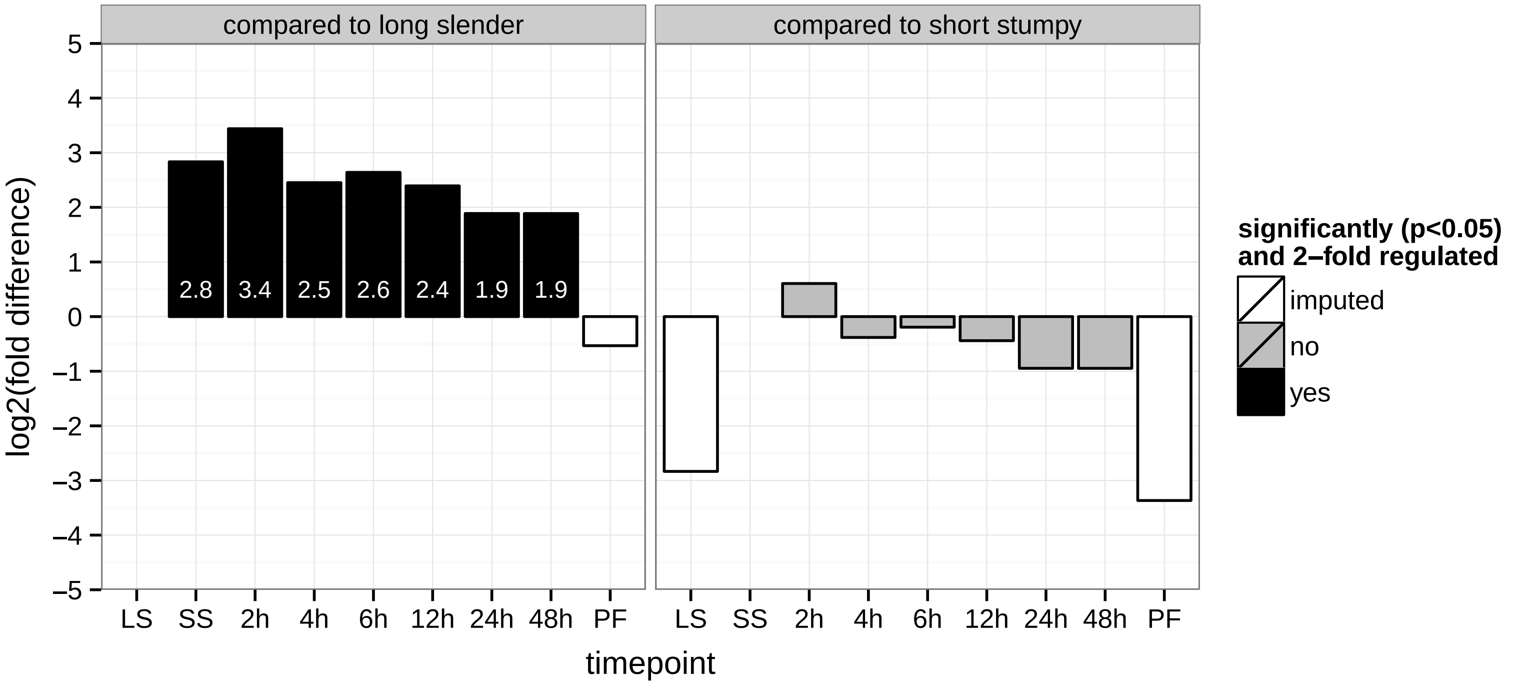

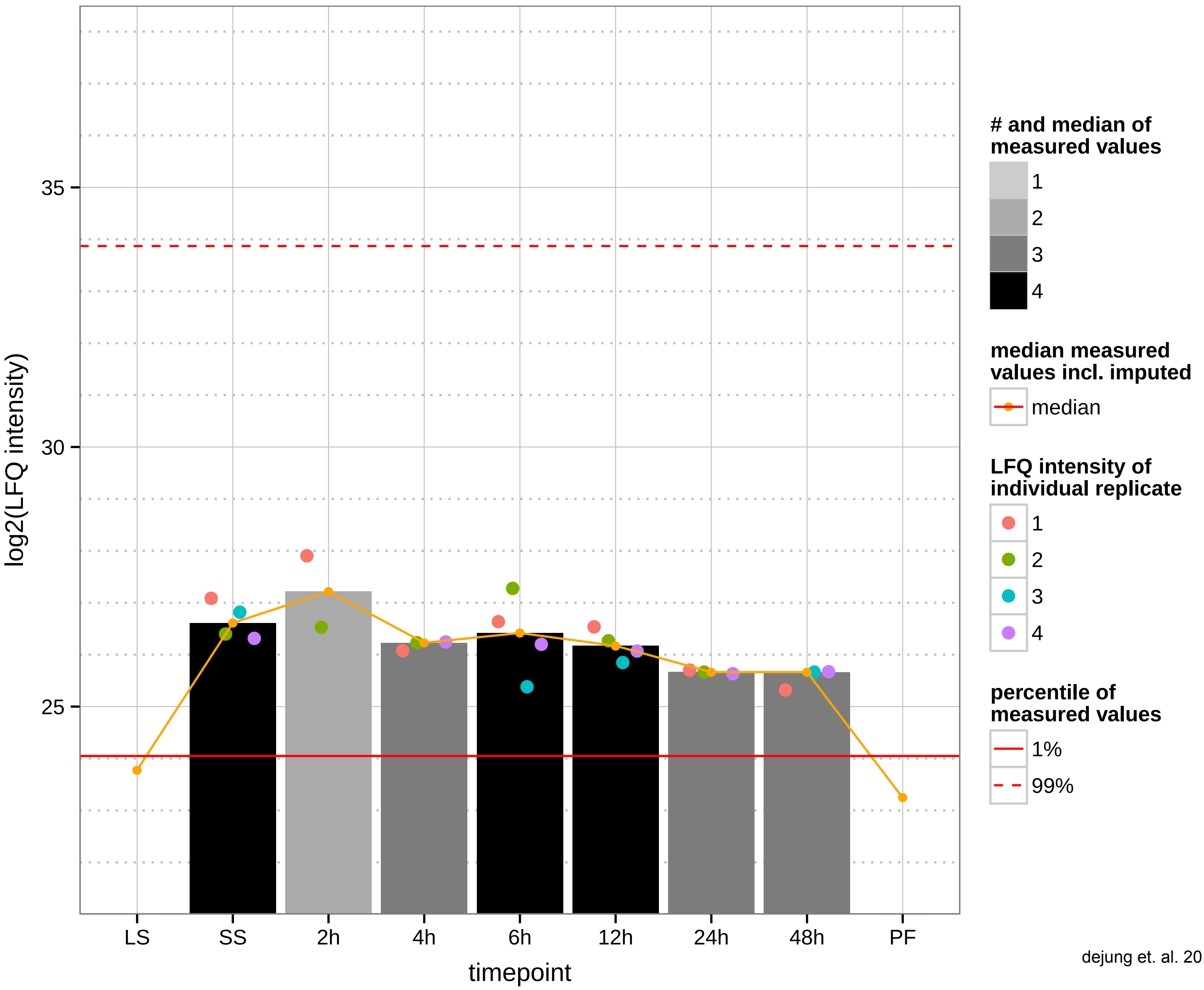

dejung et. al. 2015

hypothetical protein, conserved
Tb927.6.2930
AGOF: ATP binding, nucleoside−triphosphatase activity
AGOC: null
AGOP: null
PGOF: ATP binding, nucleoside−triphosphatase activity, nucleotide binding
PGOC: null
PGOP: null

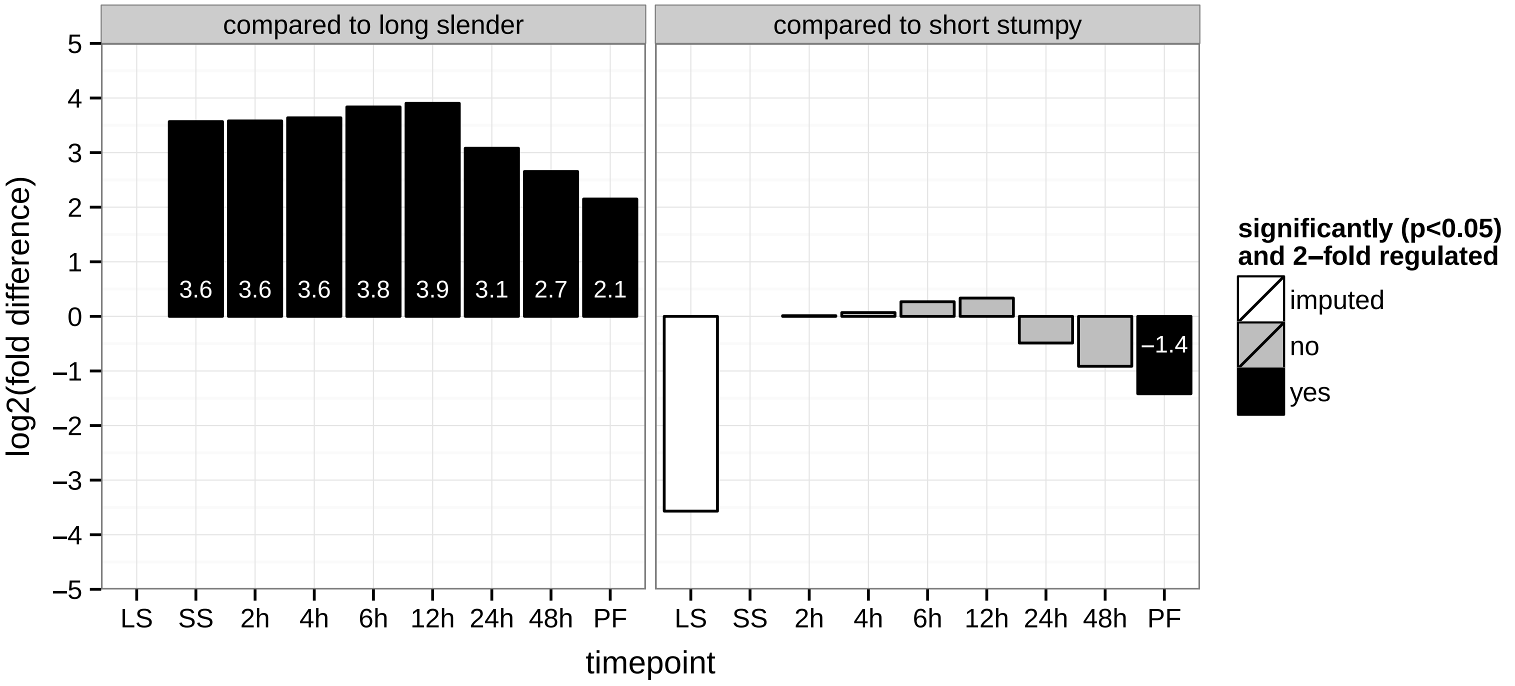

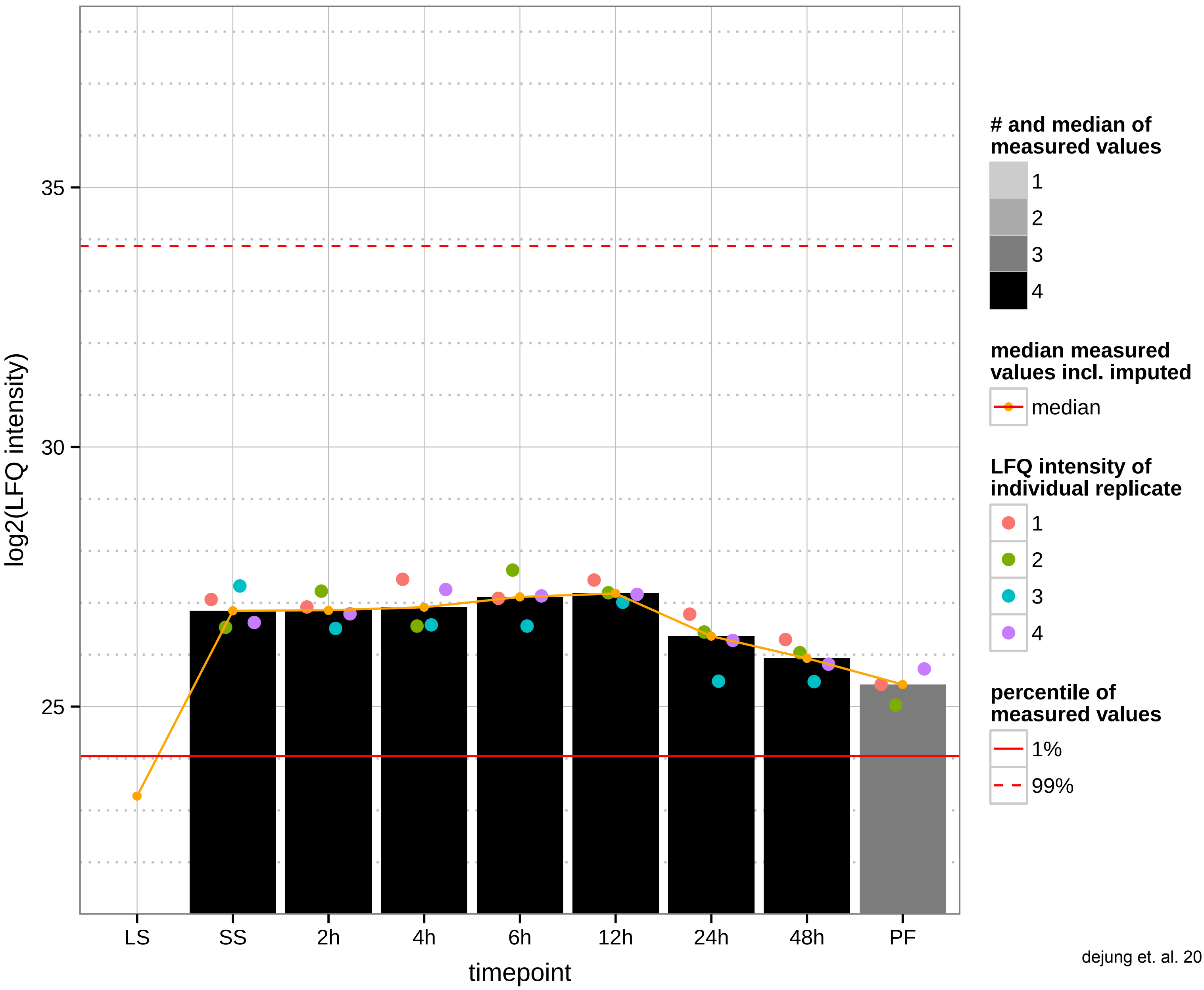

dejung et. al. 2015

protein kinase, putative, cdc2−related kinase, putative (CRK11)
Tb927.6.3110
AGOF: ATP binding, protein kinase activity, protein serine/threonine kinase activity, protein tyrosine kinase activity
AGOC: null
AGOP: protein phosphorylation
PGOF: ATP binding, protein kinase activity, transferase activity, transferring phosphorus−containing groups
PGOC: null
PGOP: protein phosphorylation

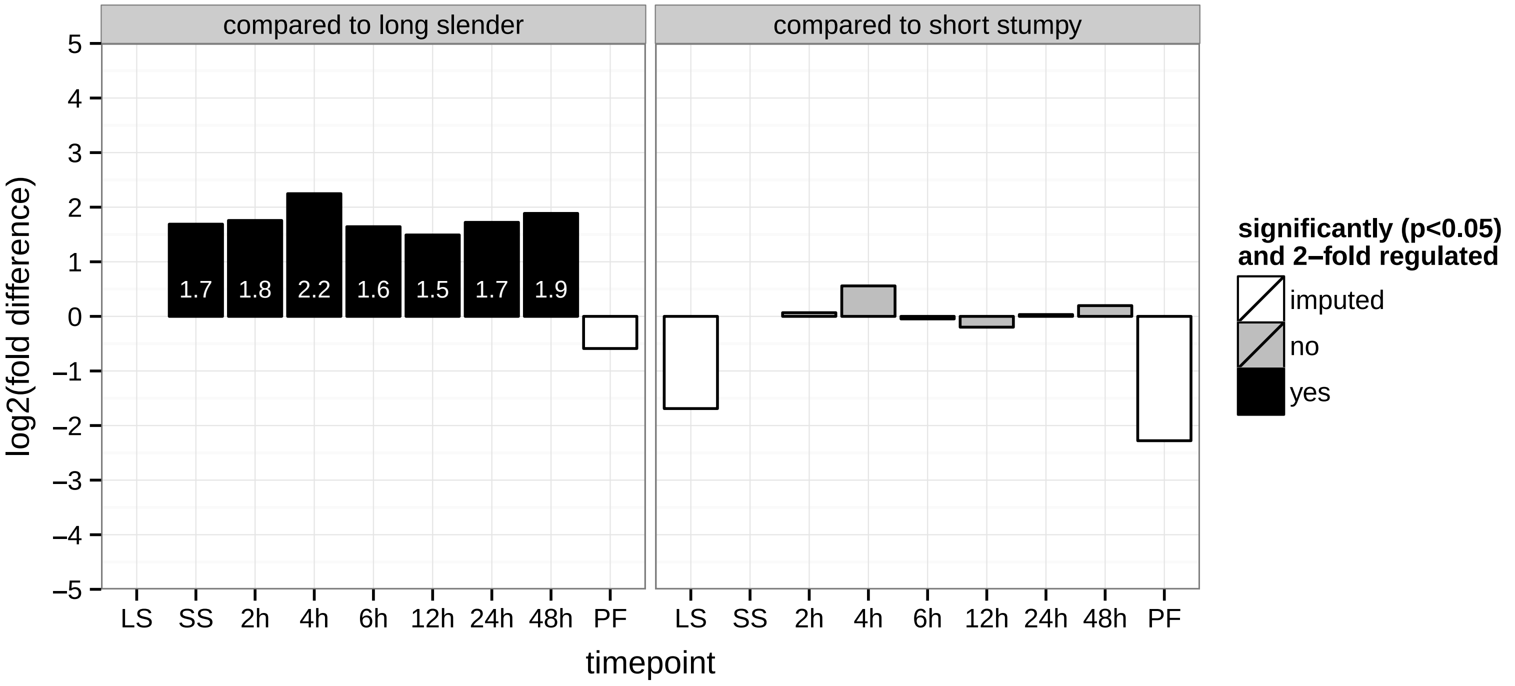

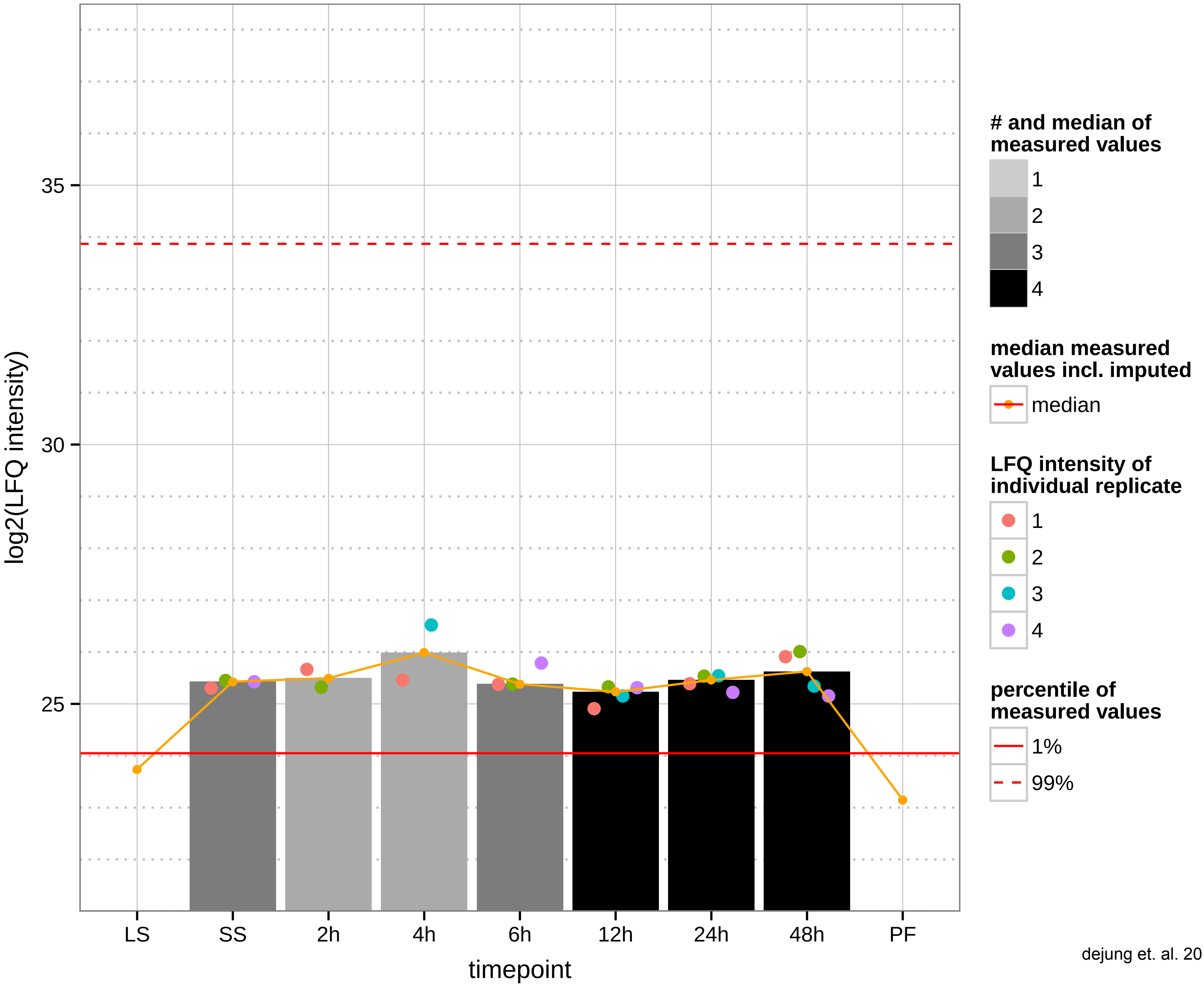

dejung et. al. 2015

hypothetical protein, conserved
Tb927.6.3140
AGOF: null
AGOC: null
AGOP: null
PGOF: protein binding
PGOC: null
PGOP: null

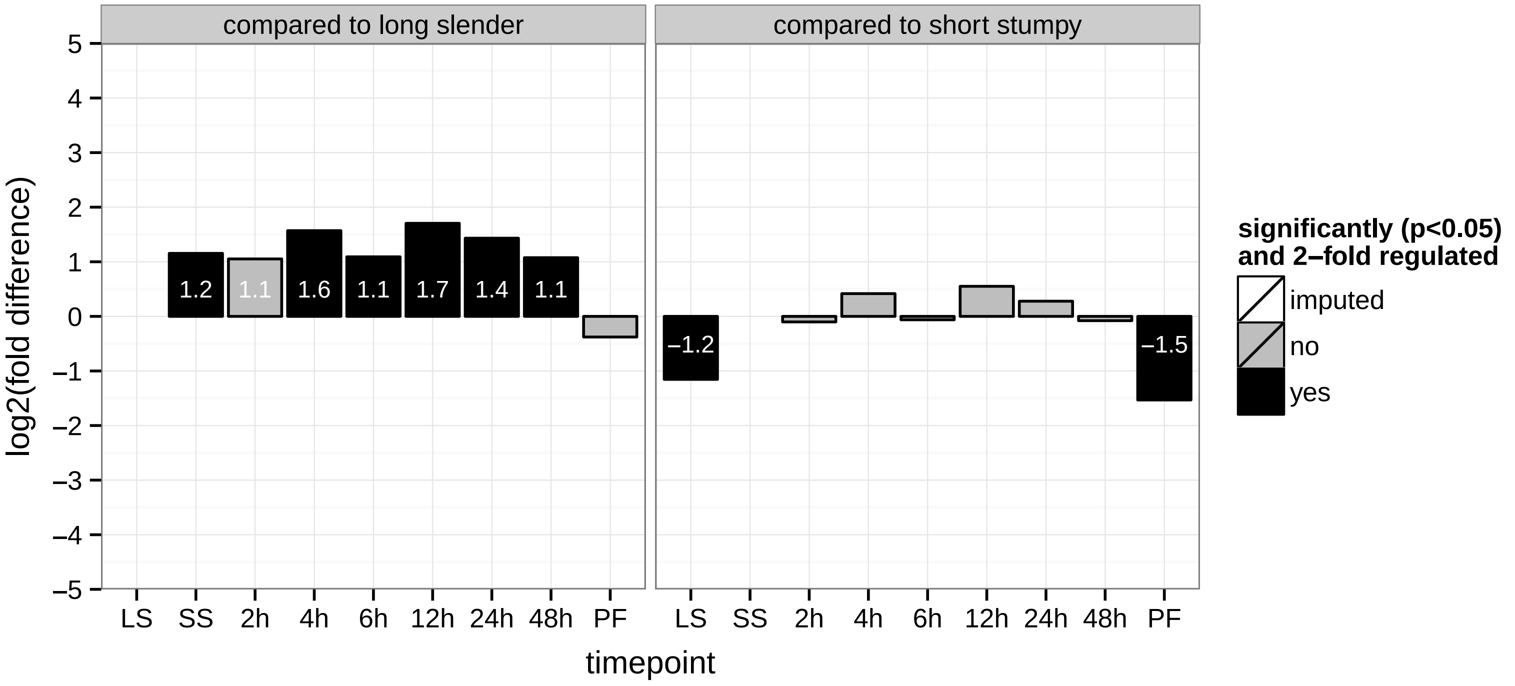

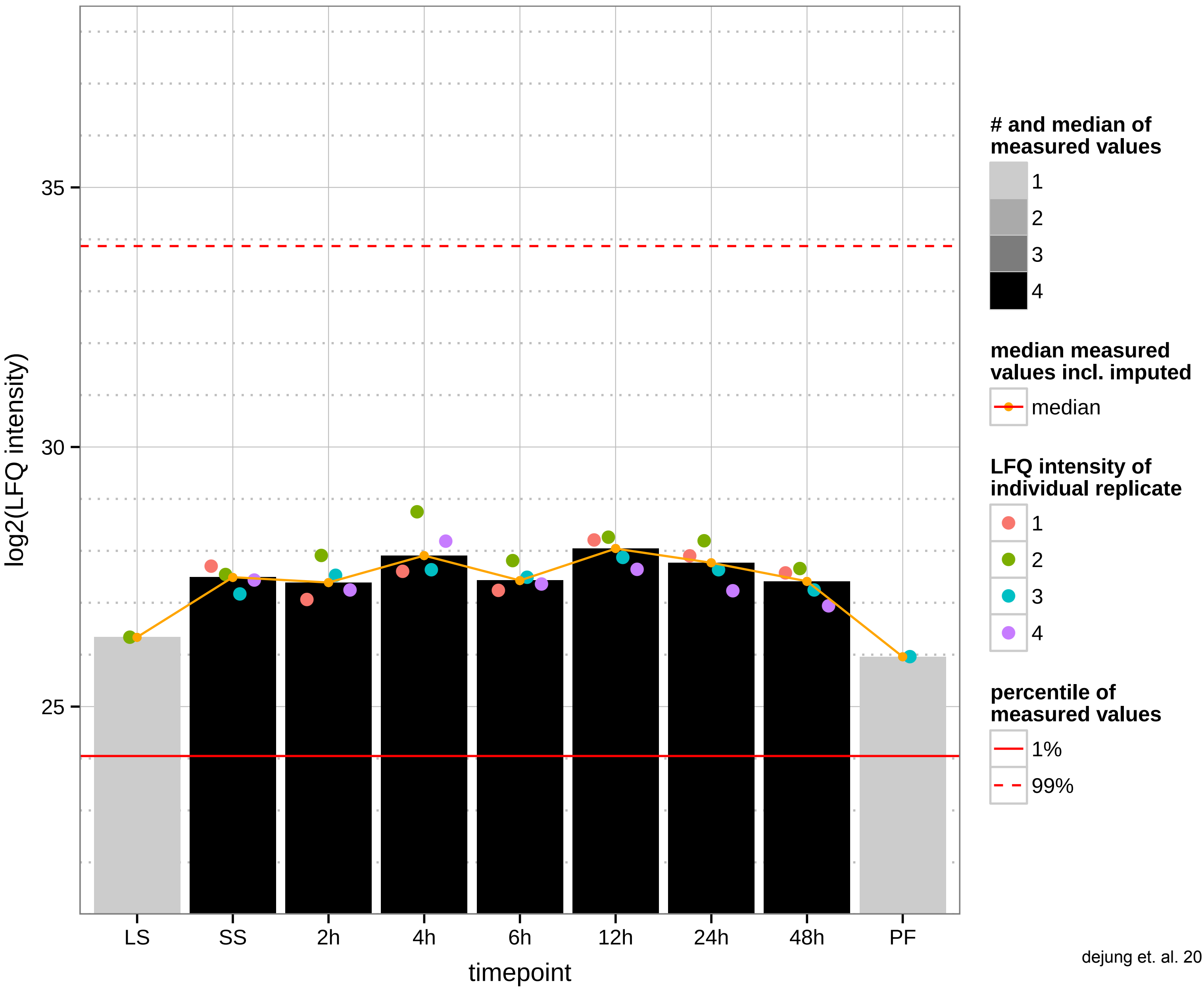

dejung et. al. 2015

valosin–containing protein homolog, putative, AAA ATPase
Tb927.6.3790
AGOF: ATP binding, nucleoside–triphosphatase activity
AGOC: null
AGOP: null
PGOF: ATP binding, nucleoside–triphosphatase activity, nucleotide binding
PGOC: null
PGOP: null

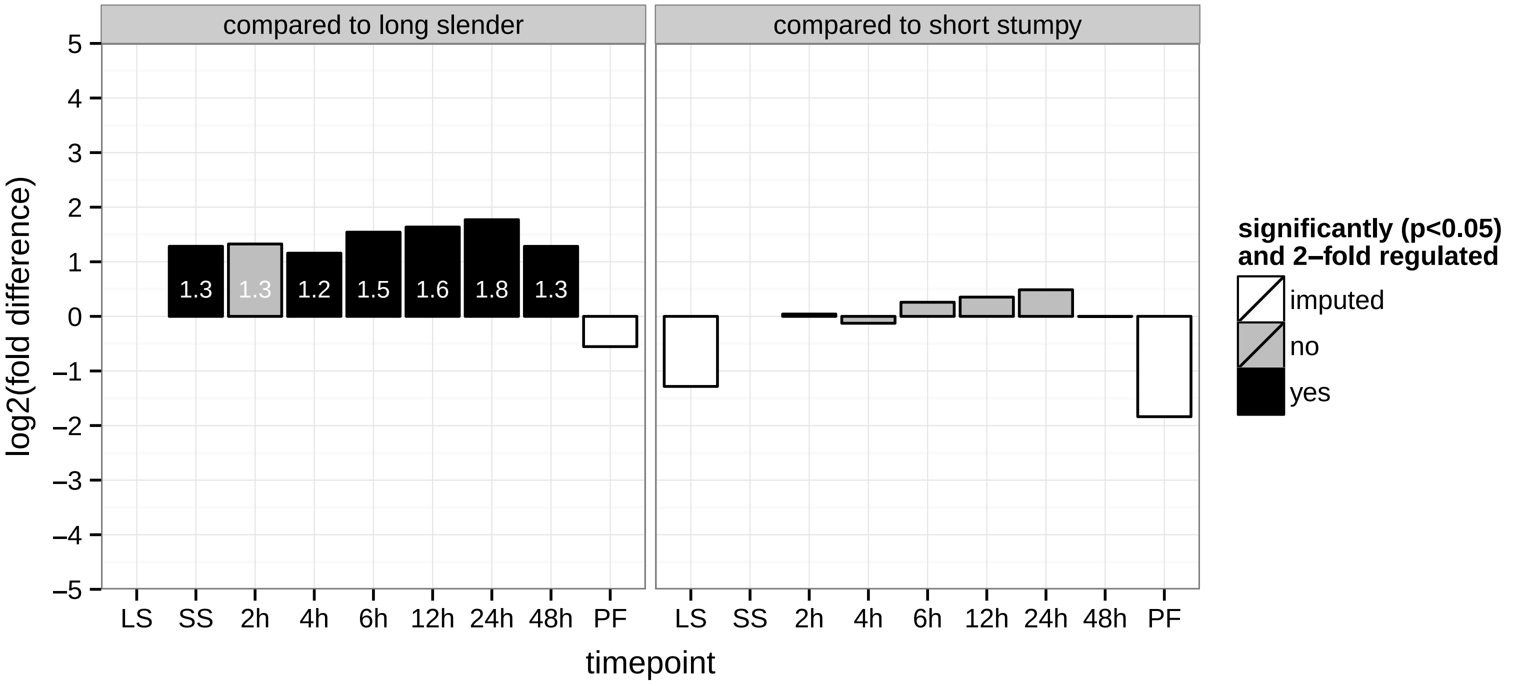

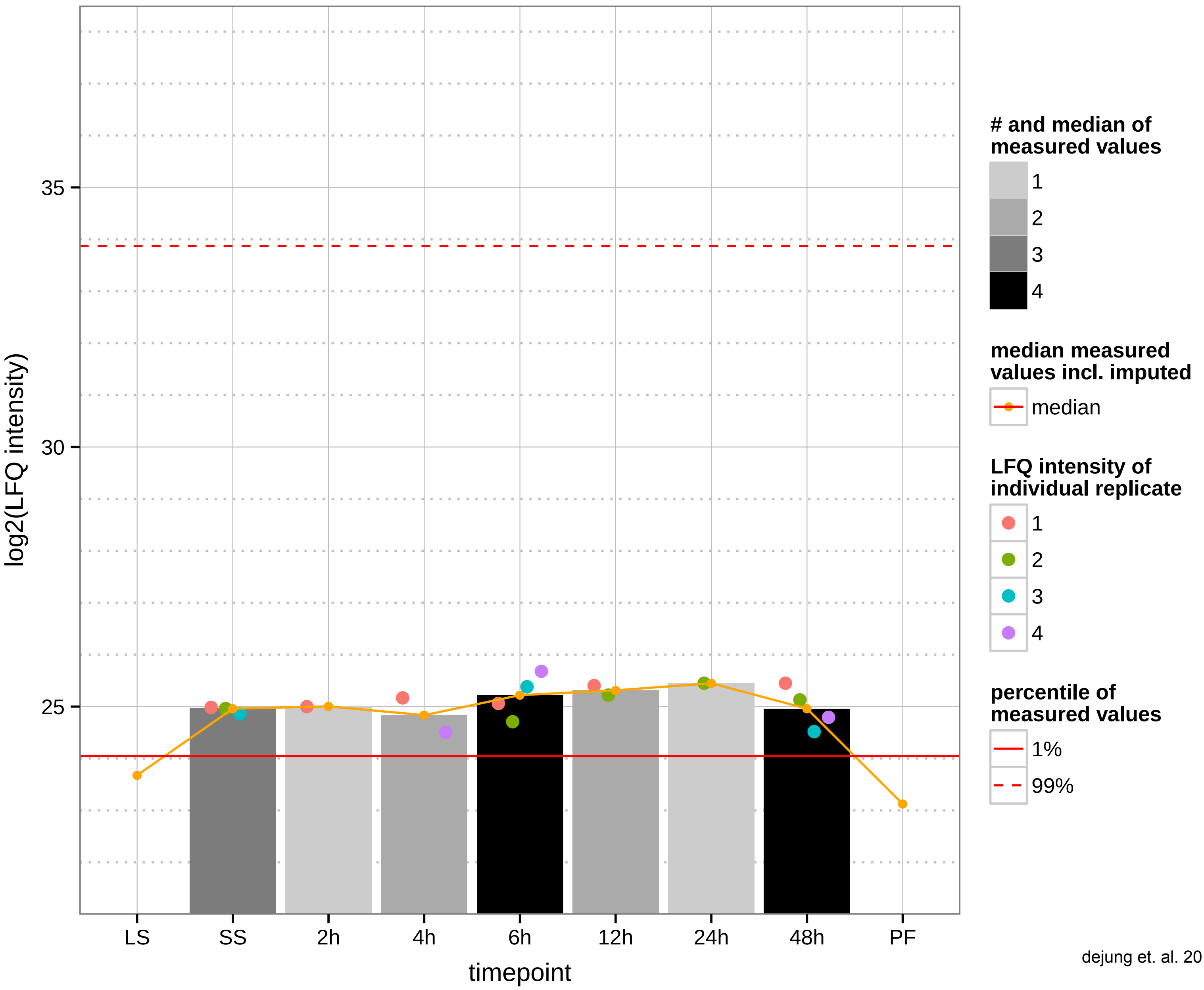

dejung et. al. 2015

hypothetical protein, conserved
Tb927.6.3820
AGOF: null
AGOC: null
AGOP: null
PGOF: null
PGOC: null
PGOP: null

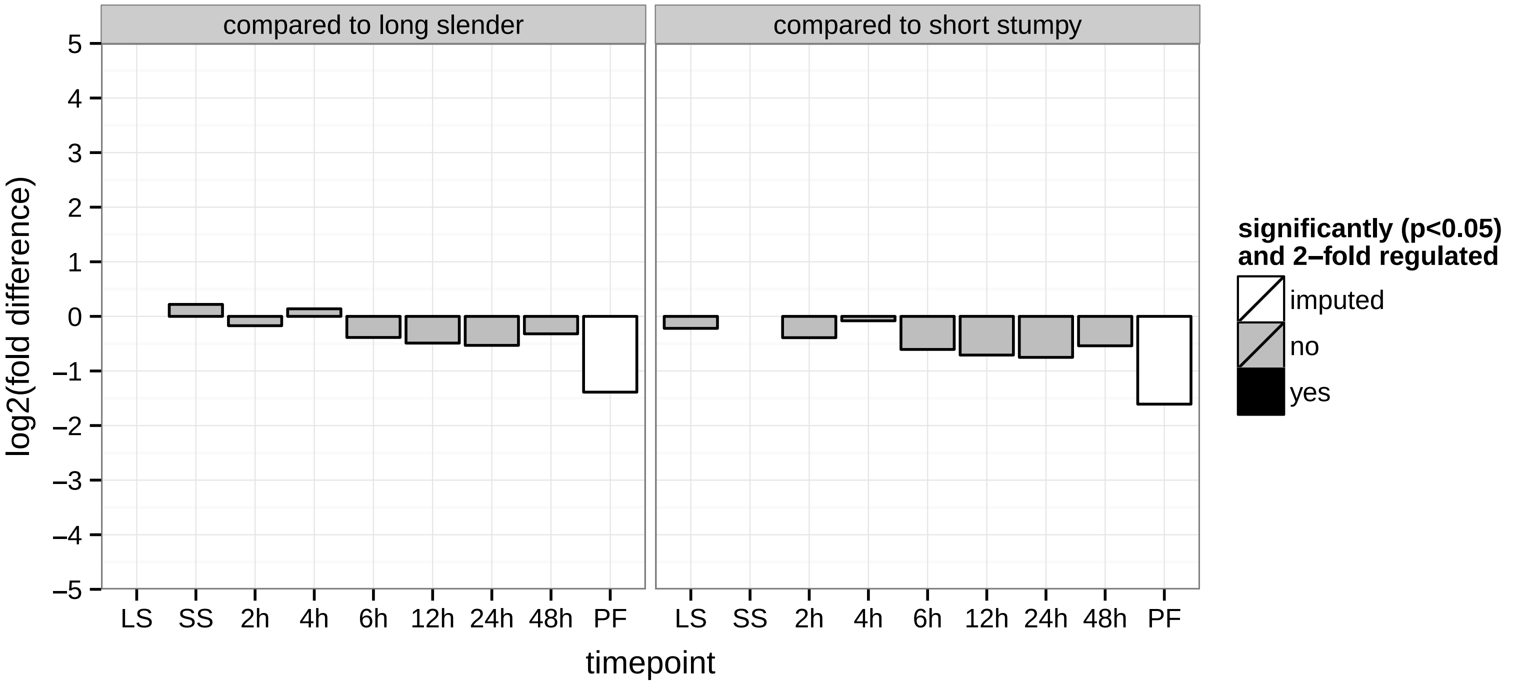

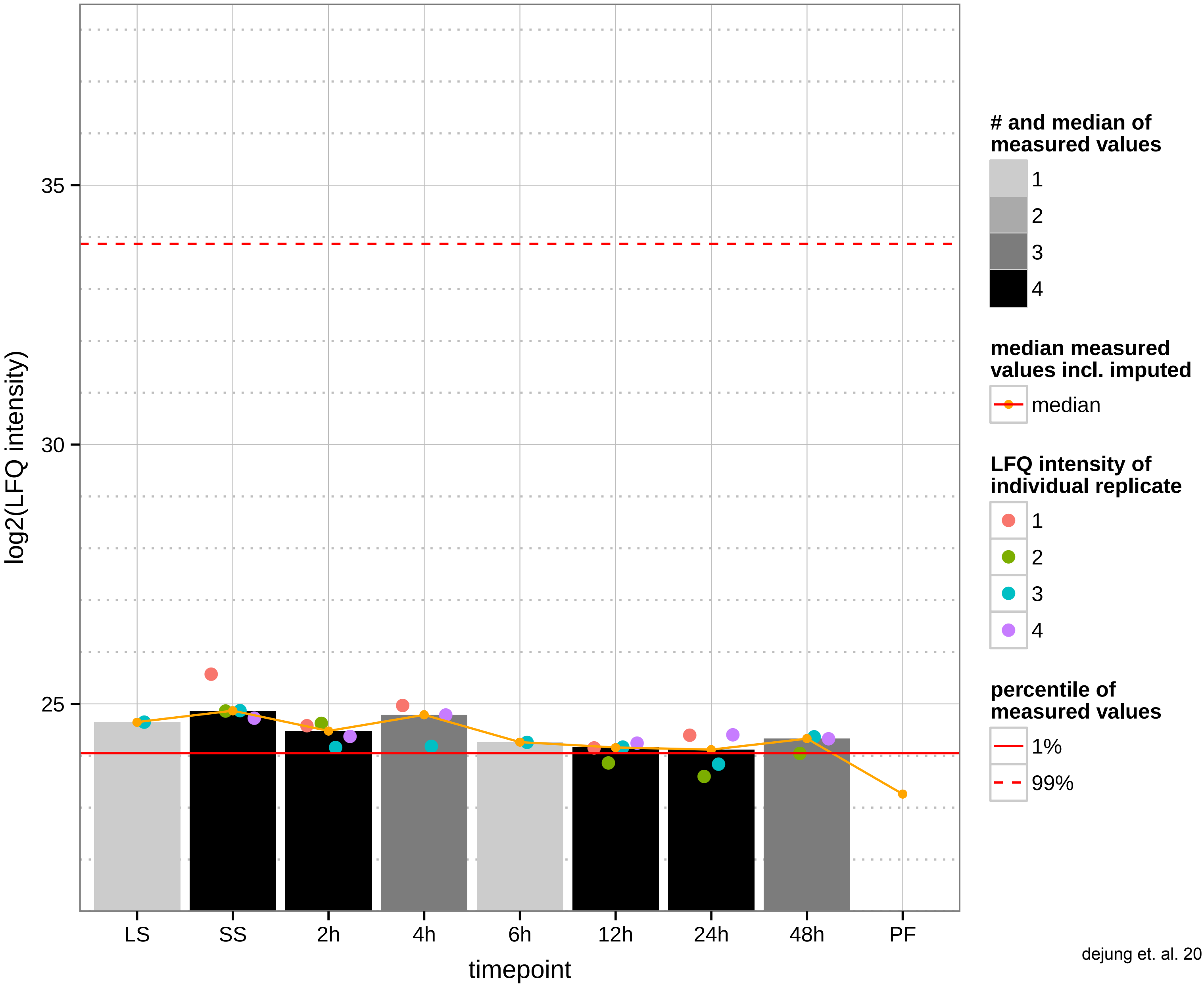

dejung et. al. 2015

hypothetical protein, conserved
Tb927.6.410
AGOF: null
AGOC: null
AGOP: null
PGOF: null
PGOC: null
PGOP: null

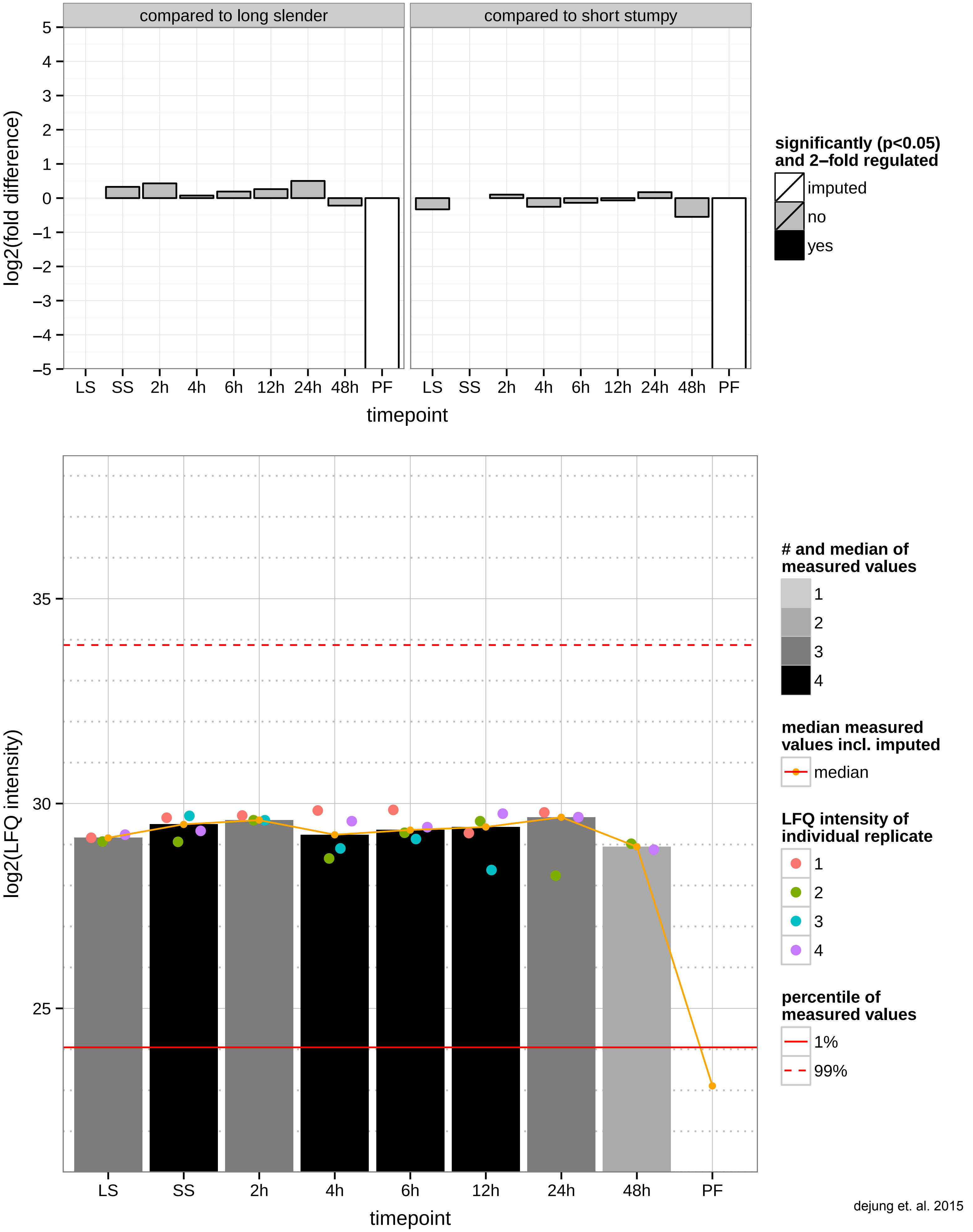

dejung et. al. 2015

hypothetical protein, conserved
Tb927.6.4180
AGOF: null
AGOC: null
AGOP: null
PGOF: null
PGOC: null
PGOP: null

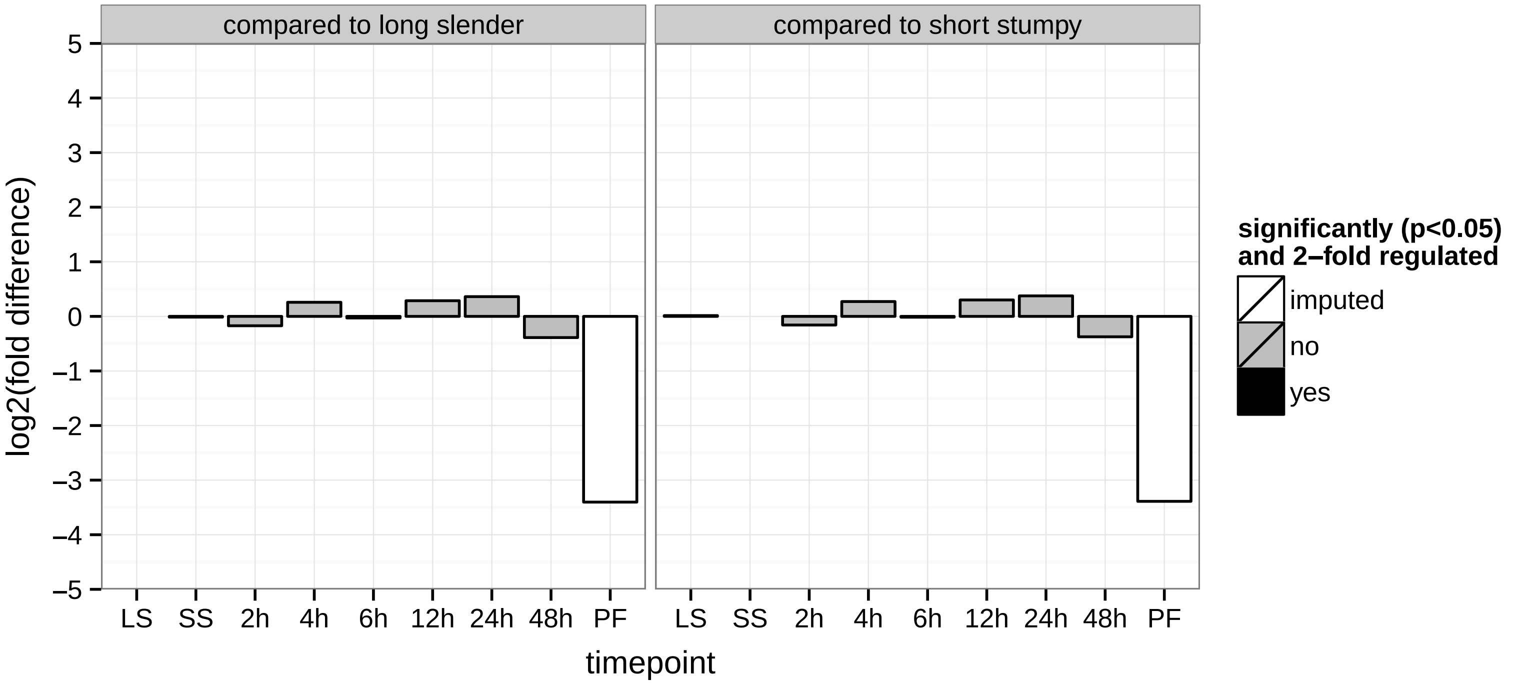

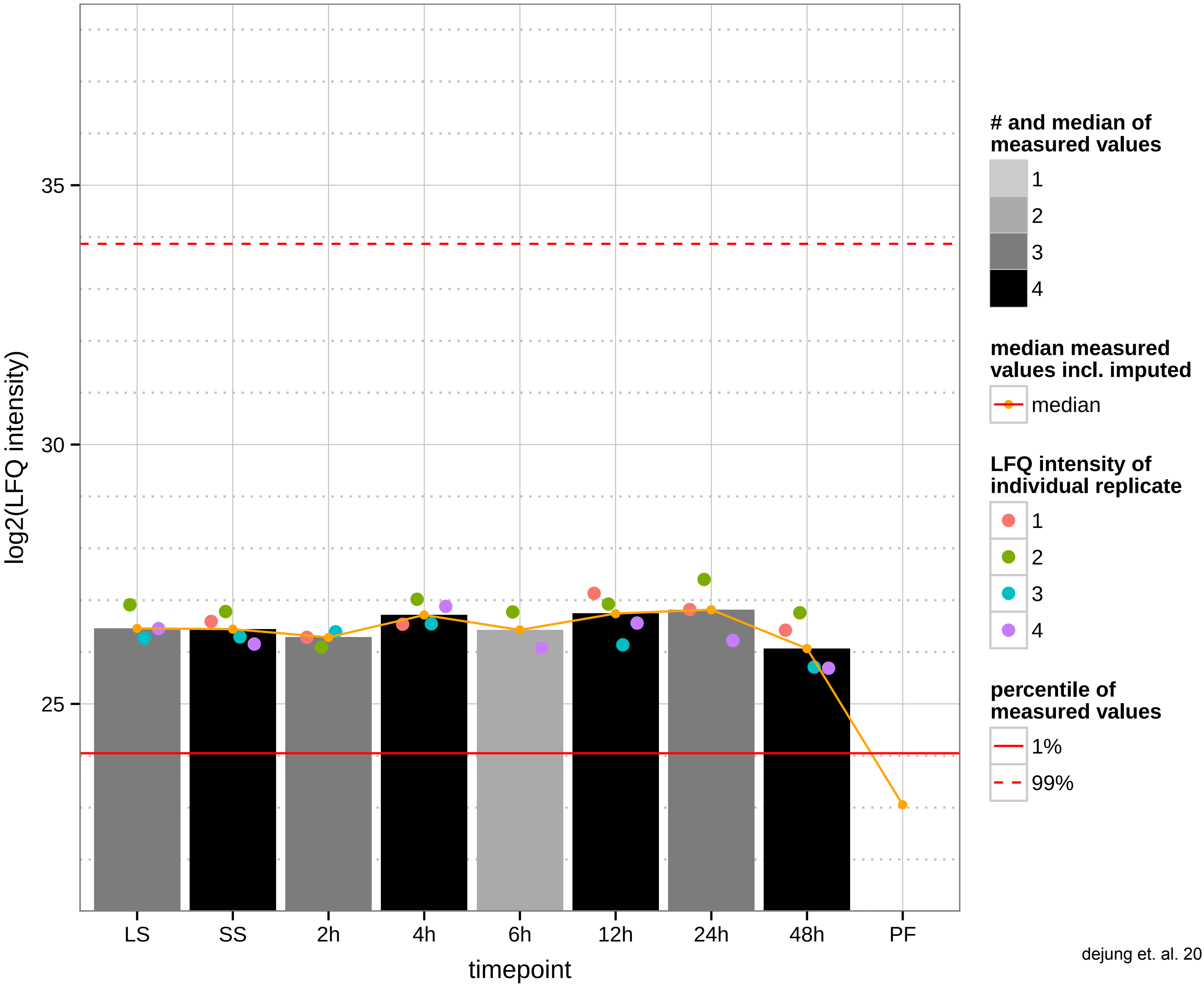

dejung et. al. 2015

mitogen–activated protein kinase 5 (MAPK5)
Tb927.6.4220
AGOF: ATP binding, protein serine/threonine kinase activity, protein tyrosine kinase activity
AGOC: null
AGOP: protein phosphorylation
PGOF: ATP binding, protein kinase activity, transferase activity, transferring phosphorus–containing groups
PGOC: null
PGOP: protein phosphorylation

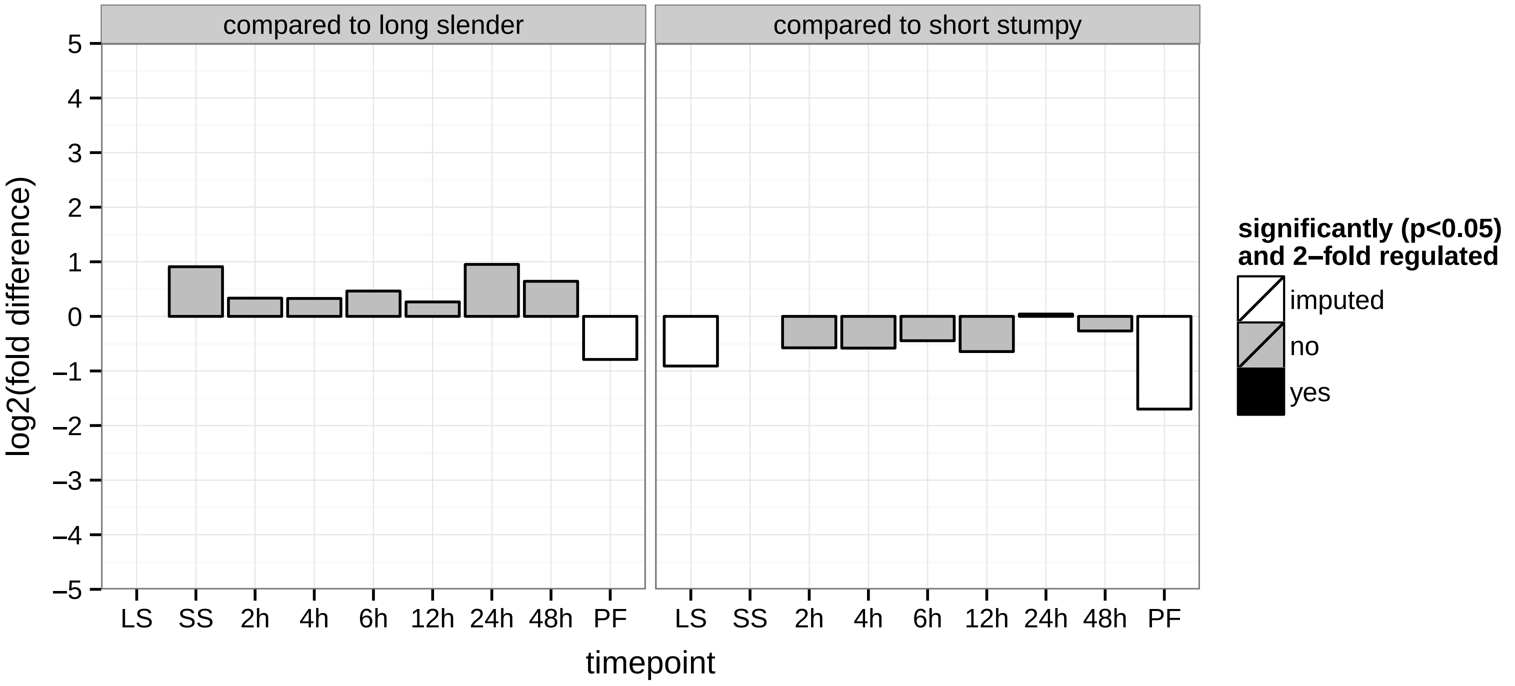

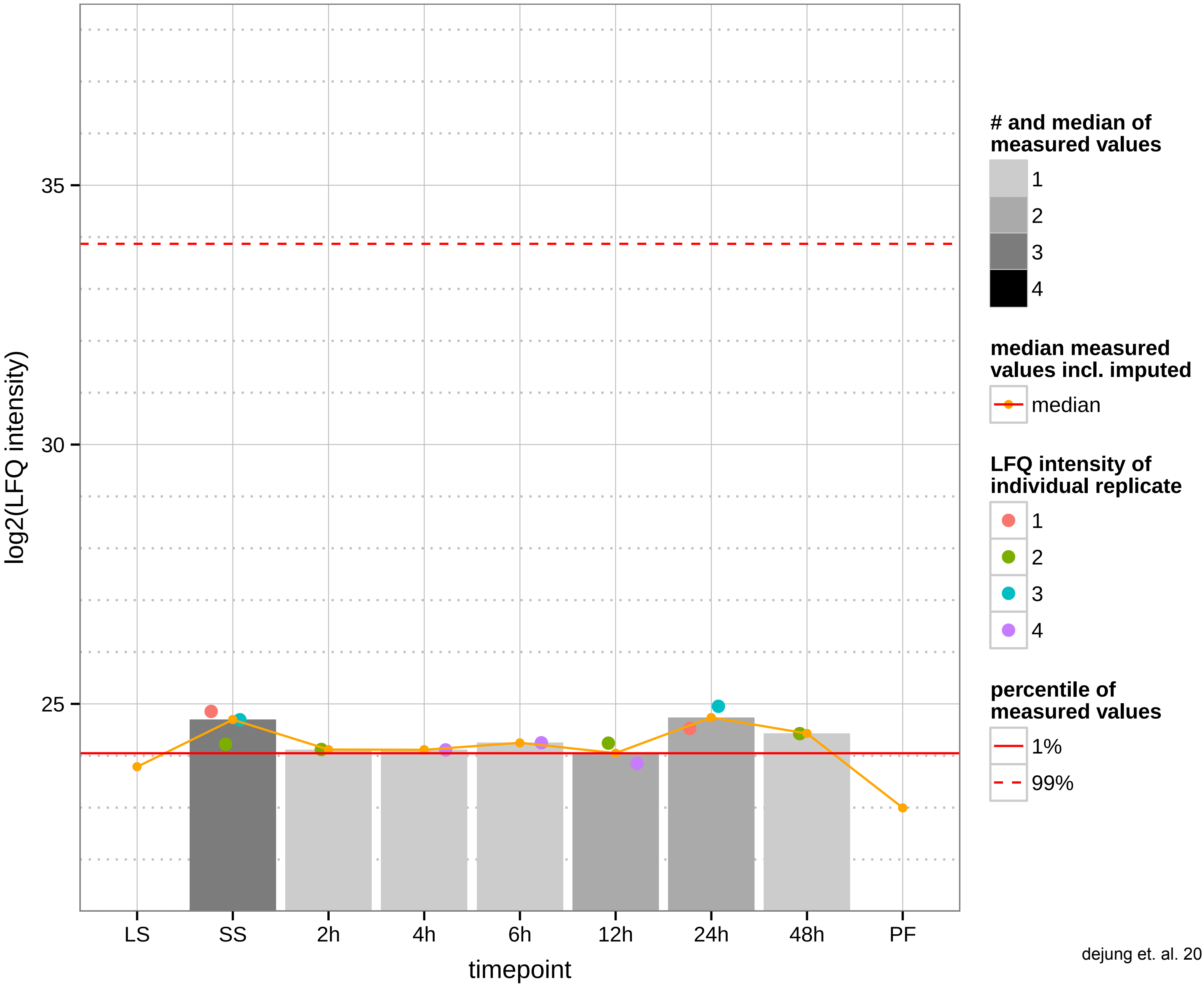

dejung et. al. 2015

hypothetical protein, conserved
Tb927.6.4250
AGOF: null
AGOC: null
AGOP: null
PGOF: null
PGOC: null
PGOP: null

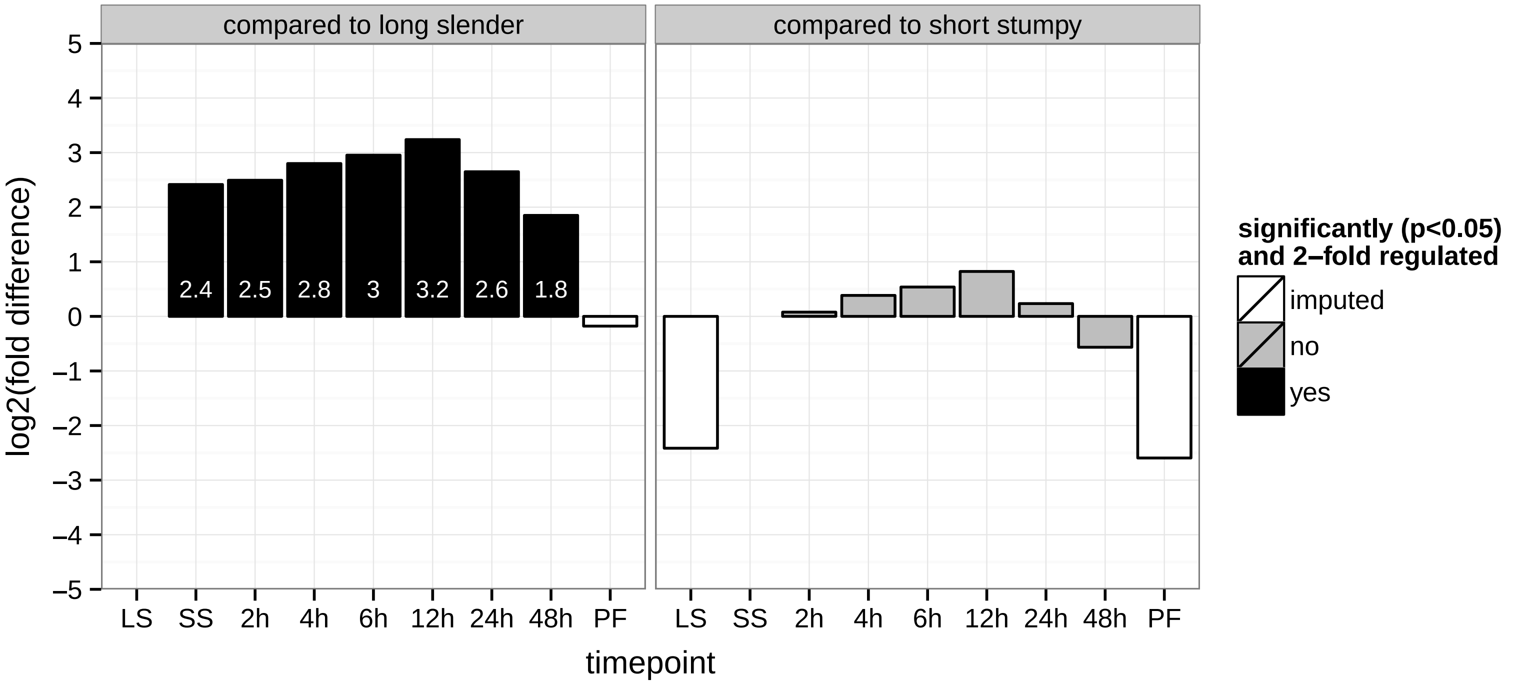

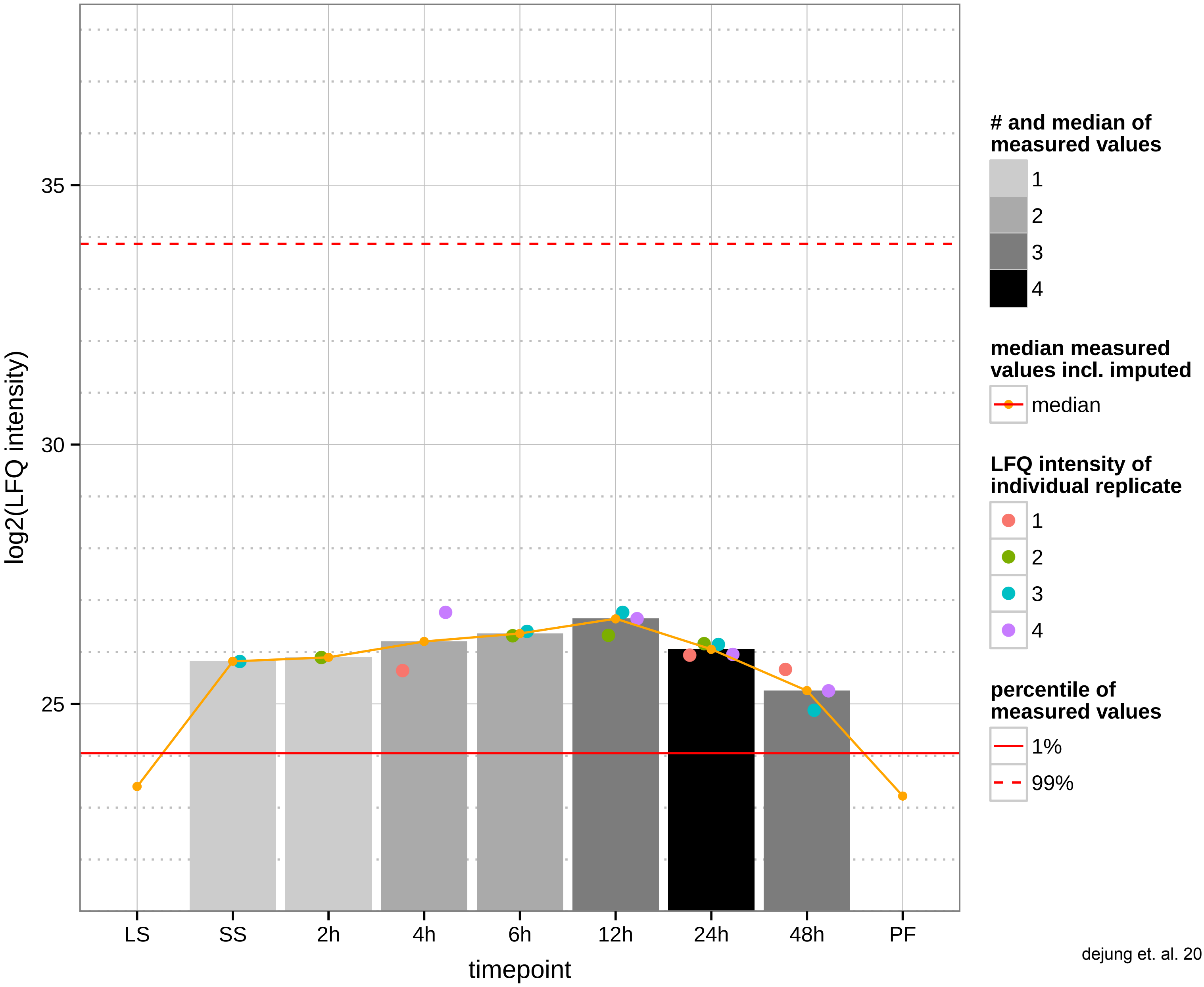

dejung et. al. 2015

glyceraldehyde 3–phosphate dehydrogenase, glycosomal (GAPDH)
Tb927.6.4300;Tb927.6.4280
AGOF: glyceraldehyde–3–phosphate dehydrogenase (NAD+) (phosphorylating) activity
AGOC: glycosome
AGOP: glycolysis
PGOF: NAD binding, NADP binding, oxidoreductase activity, acting on the aldehyde or oxo group of donors, NAD or NADP as
PGOC: null
PGOP: glucose metabolic process, oxidation–reduction process, null

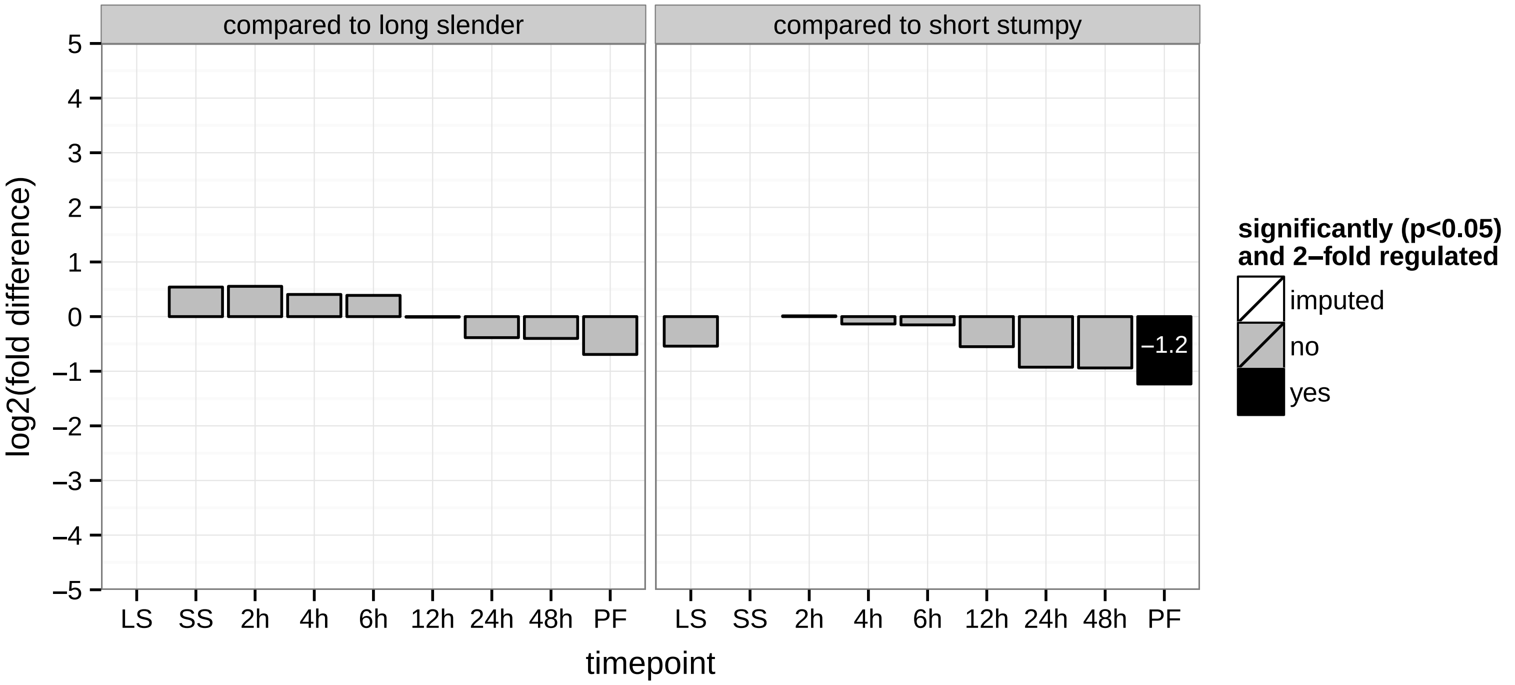

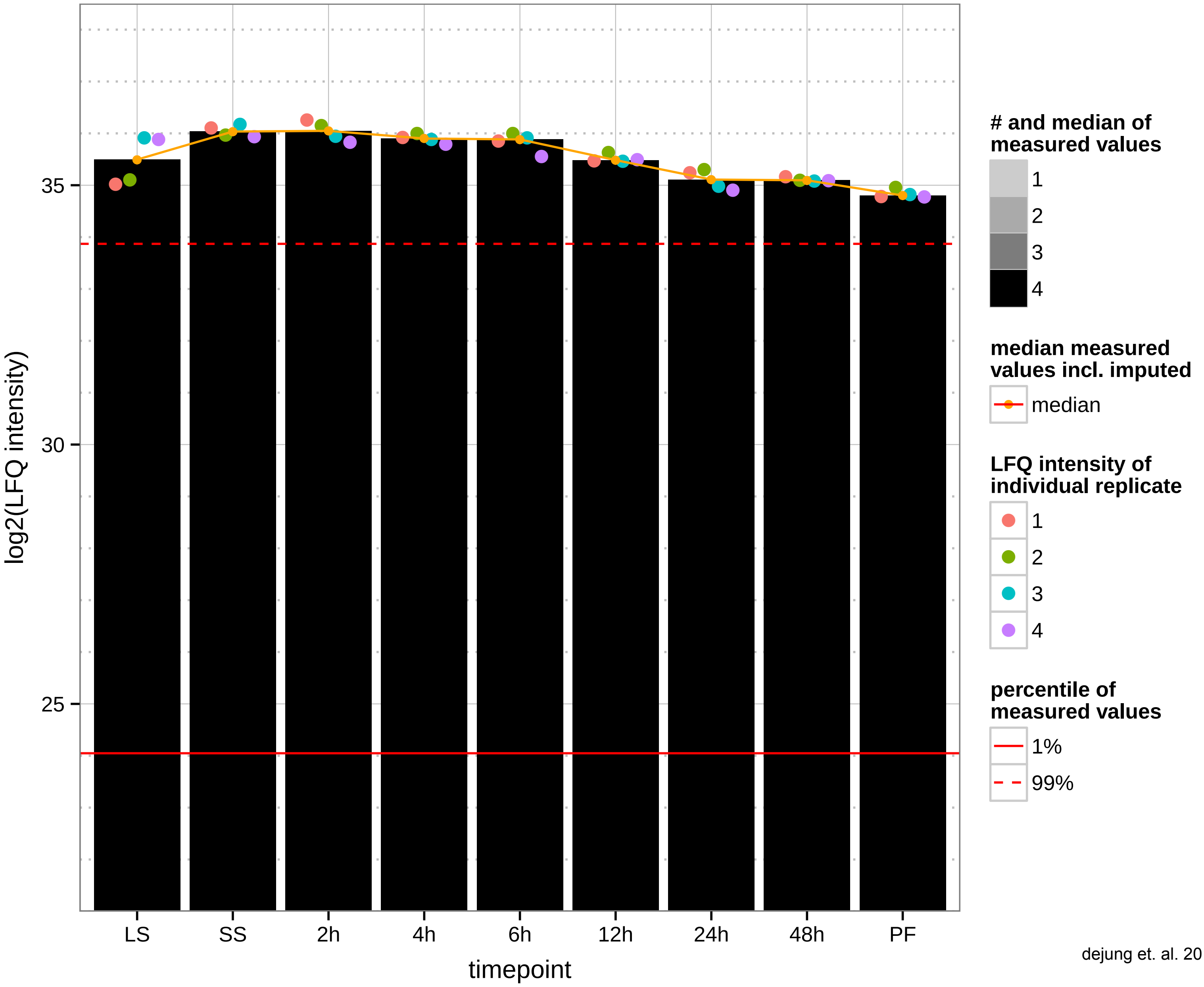

dejung et. al. 2015

hypothetical protein, conserved
Tb927.6.4330
AGOF: null
AGOC: null
AGOP: null
PGOF: null
PGOC: null
PGOP: null

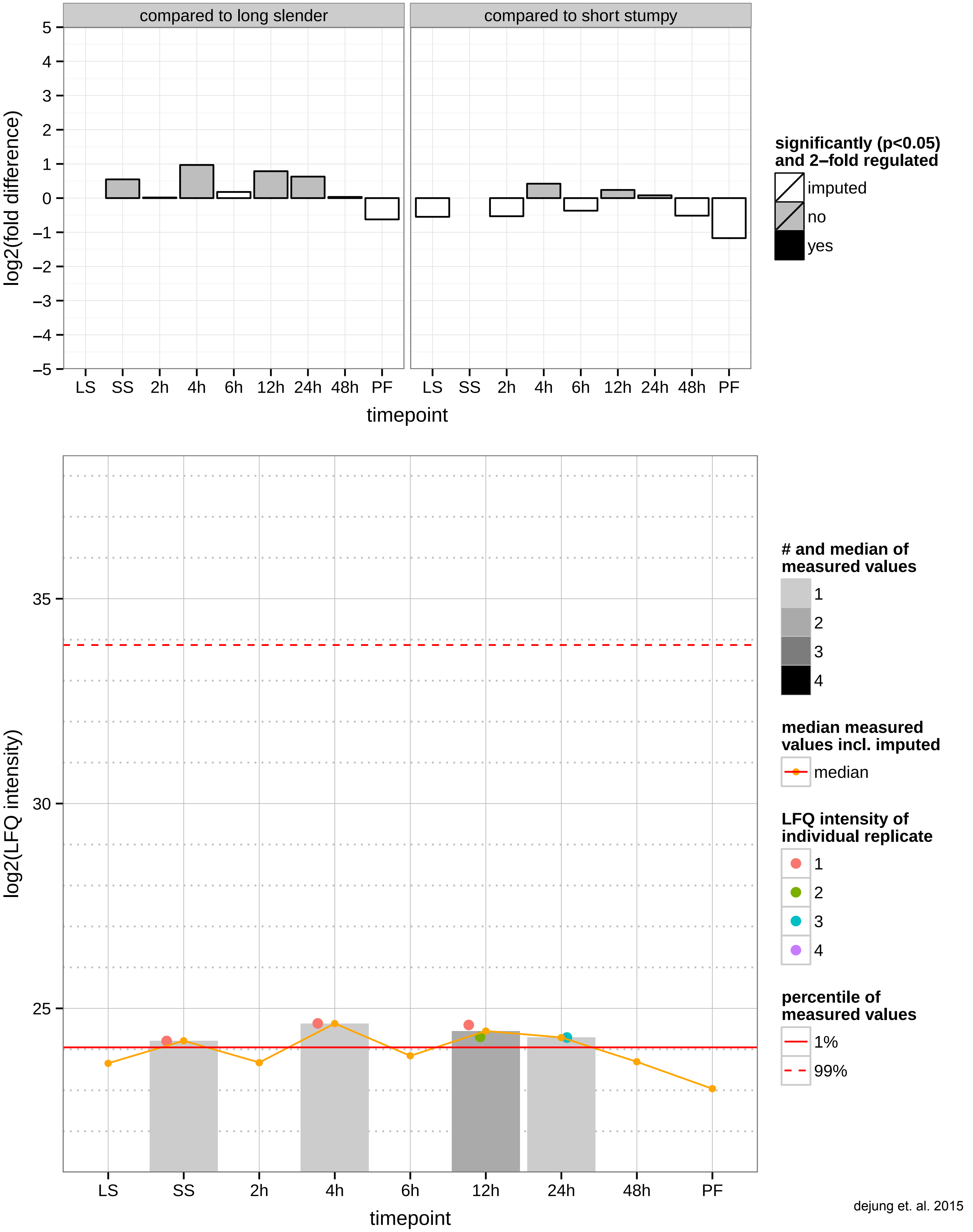

dejung et. al. 2015

pumillio RNA binding protein 4, putative, pumilio RNA binding protein, putative (PUF4)
Tb927.6.820
AGOF: null, RNA binding
AGOC: null
AGOP: null
PGOF: RNA binding, binding
PGOC: null
PGOP: null

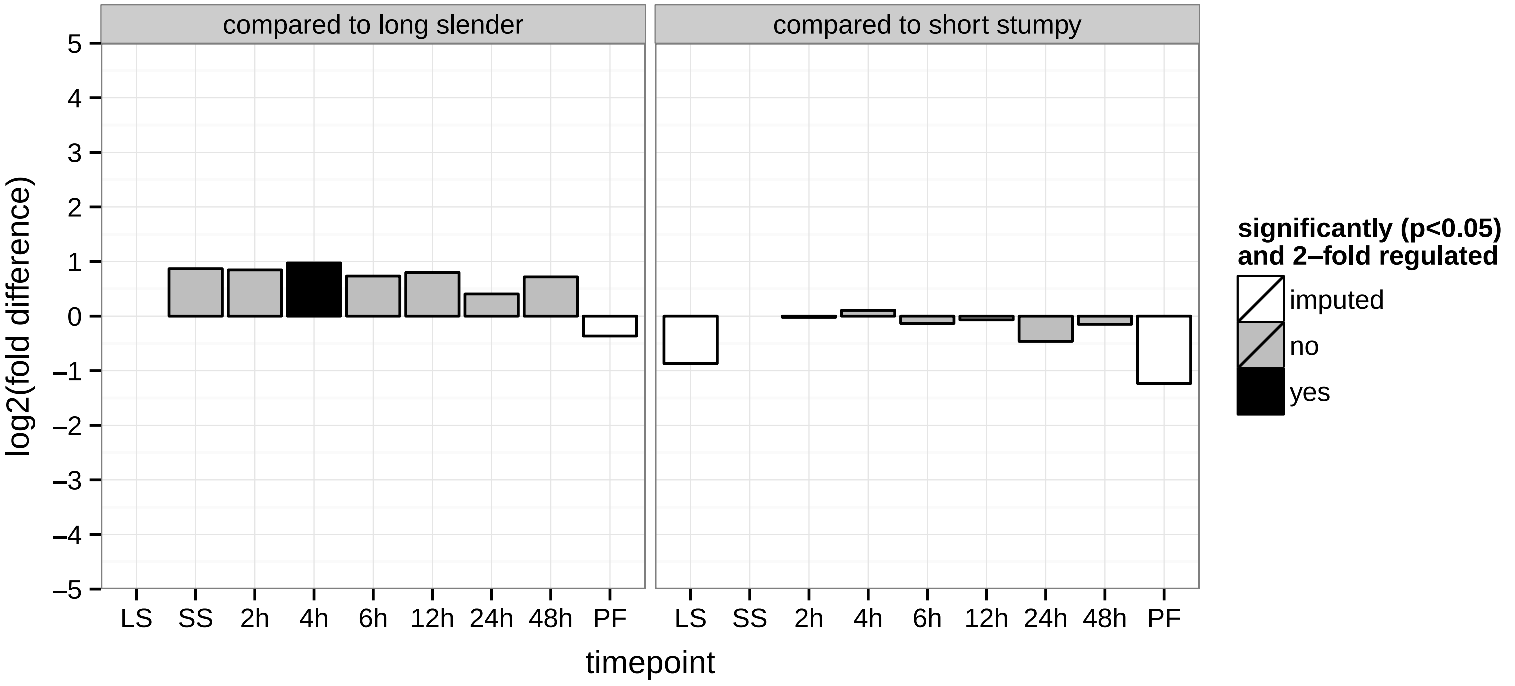

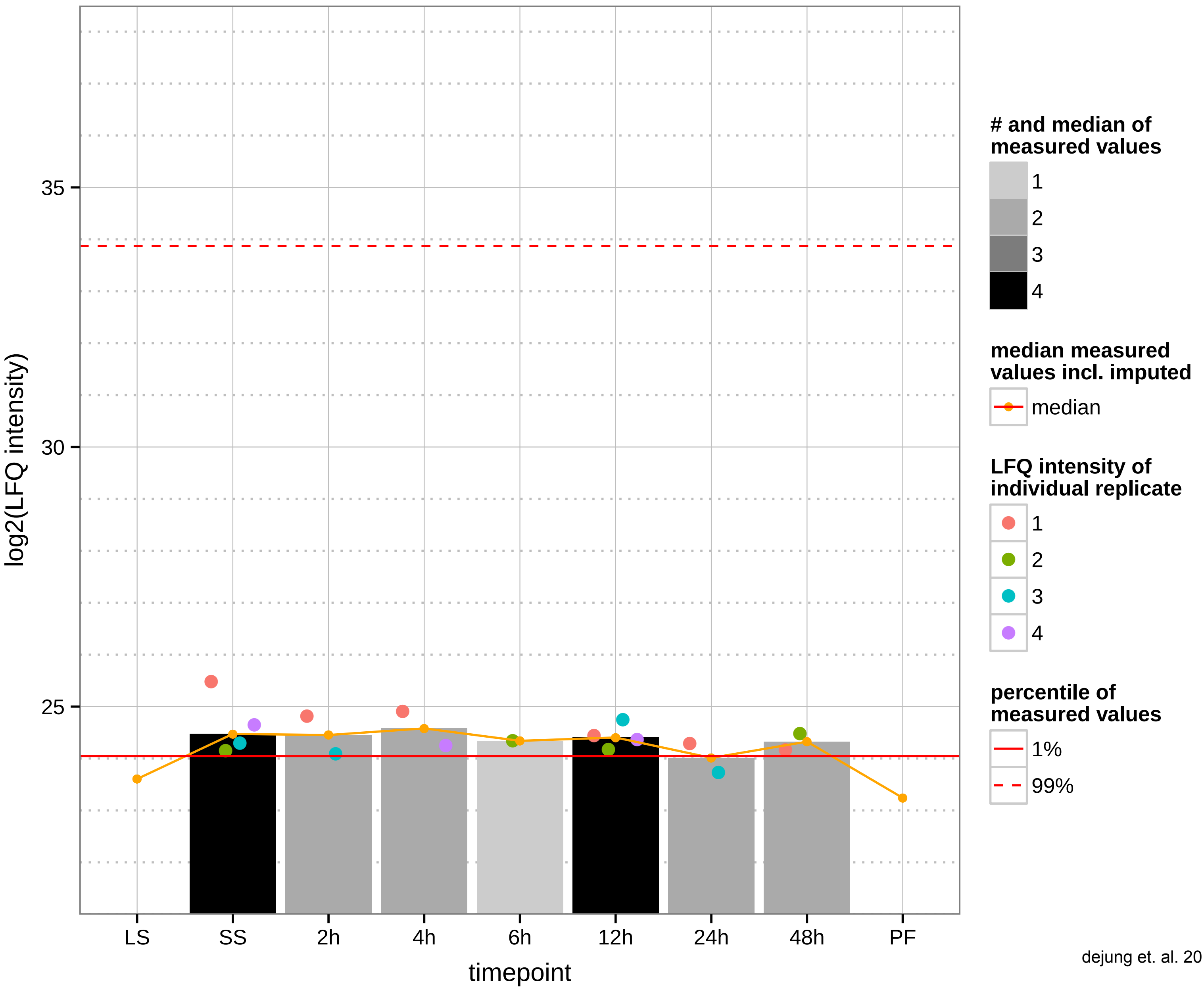

dejung et. al. 2015

asparagine synthetase a, putative
Tb927.7.1110
AGOF: ATP binding, aminoacyl–tRNA ligase activity, aspartate–ammonia ligase activity
AGOC: cytoplasm
AGOP: asparagine biosynthetic process, tRNA aminoacylation for protein translation, translation
PGOF: ATP binding, aminoacyl–tRNA ligase activity, aspartate–ammonia ligase activity, nucleotide binding
PGOC: cytoplasm
PGOP: asparagine biosynthetic process, tRNA aminoacylation for protein translation

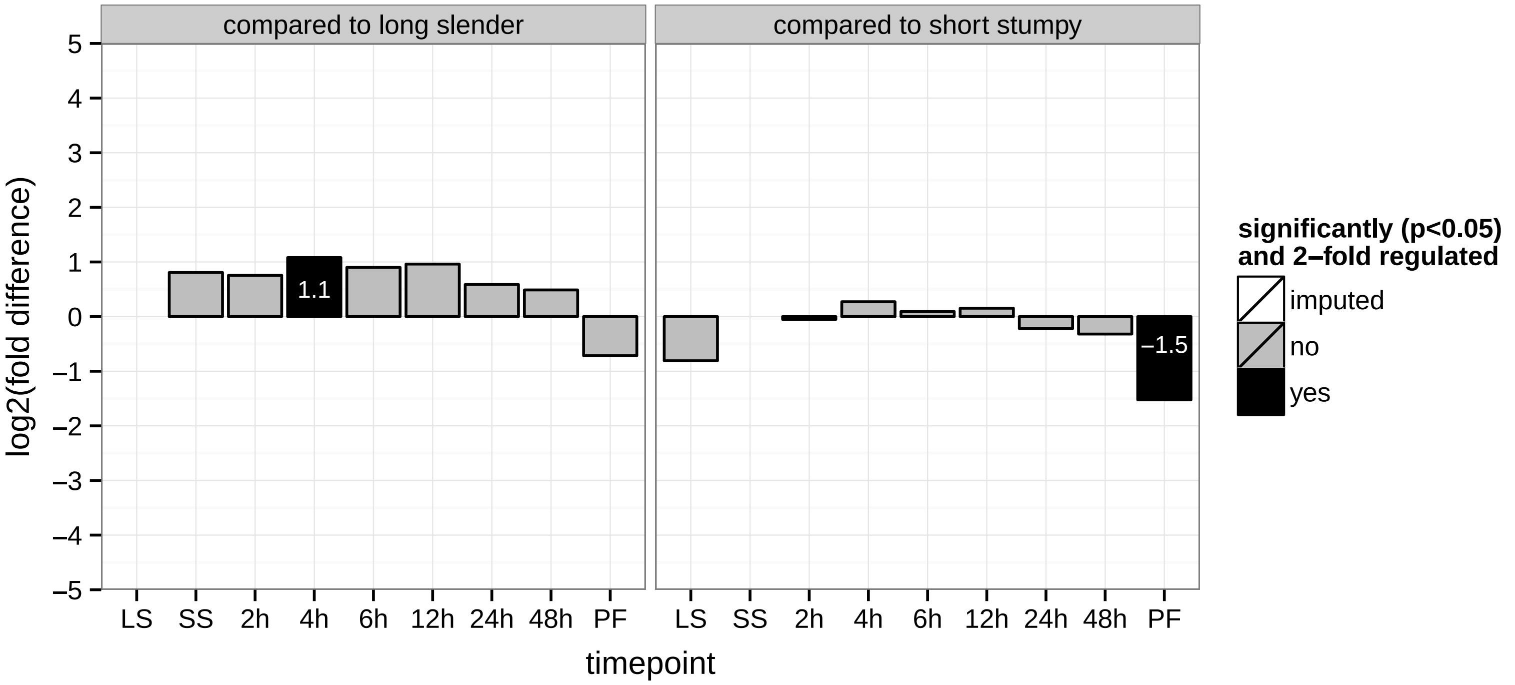

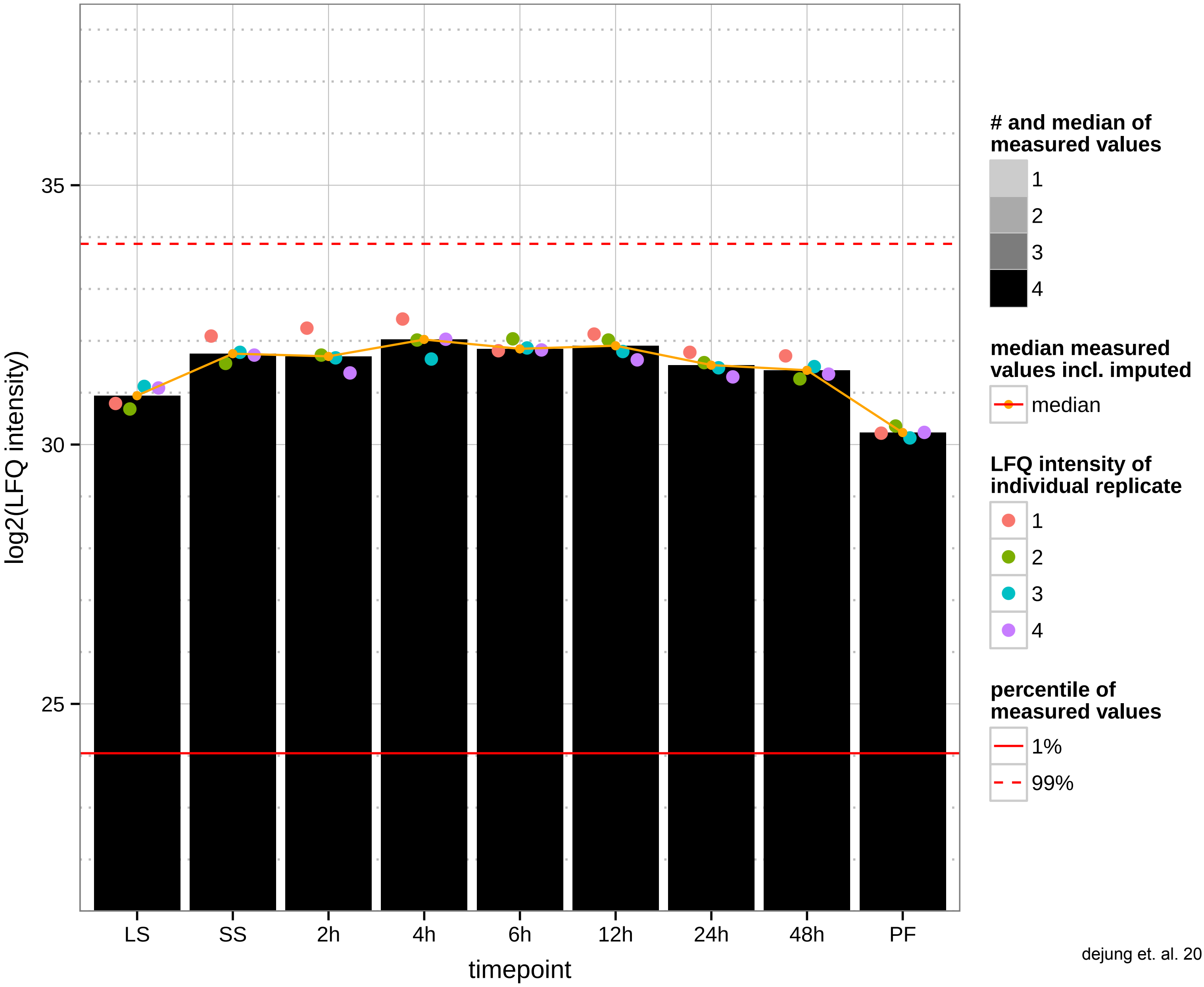

dejung et. al. 2015

hypothetical protein, conserved
Tb927.7.1280;Tb927.7.1260
AGOF: null
AGOC: null
AGOP: null
PGOF: null
PGOC: null
PGOP: null

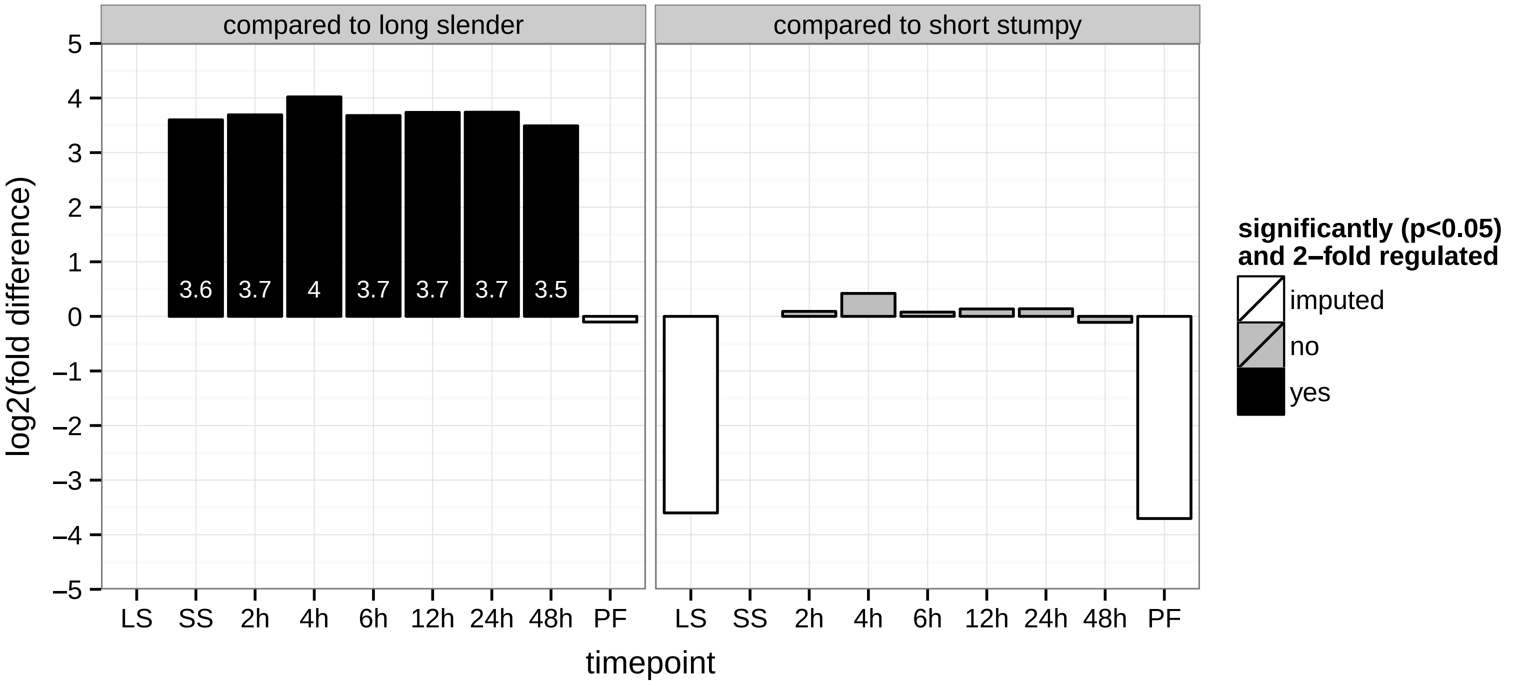

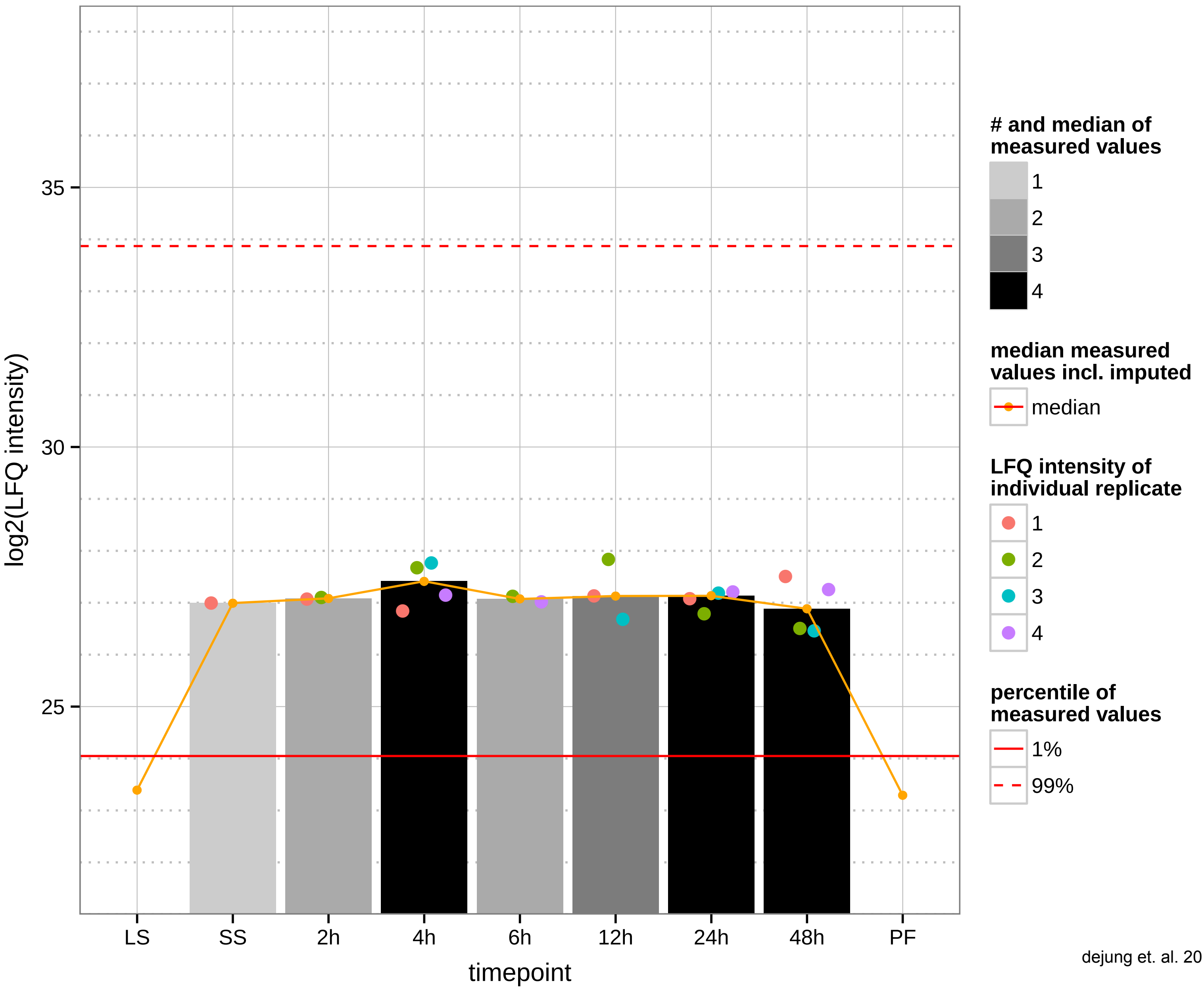

dejung et. al. 2015

hypothetical protein, conserved
Tb927.7.1330
AGOF: null
AGOC: null
AGOP: null
PGOF: null
PGOC: null
PGOP: null

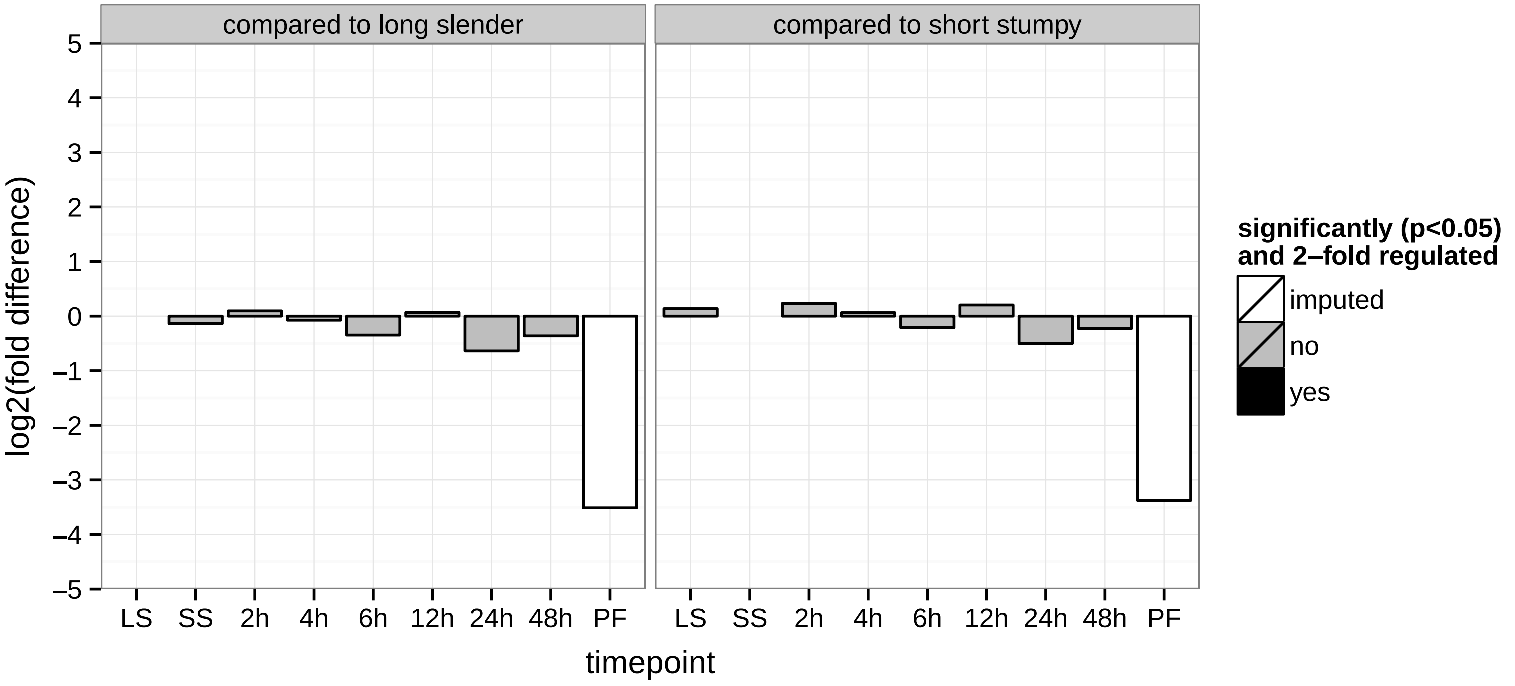

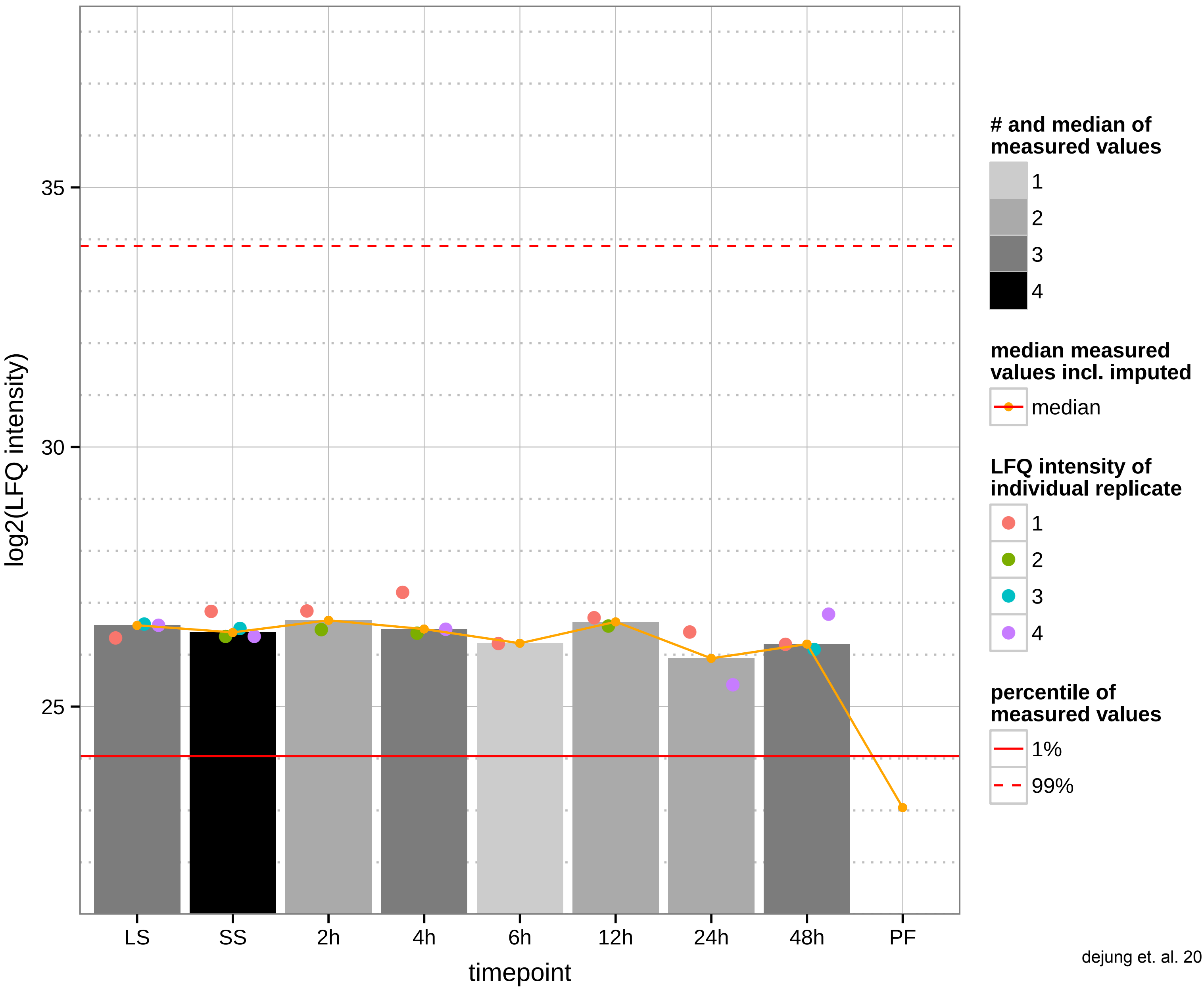

dejung et. al. 2015

spliced leader RNA PSE–promoter transcription factor, putative (PPB1)
Tb927.7.1370
AGOF: null
AGOC: mitochondrion
AGOP: null
PGOF: null
PGOC: null
PGOP: null

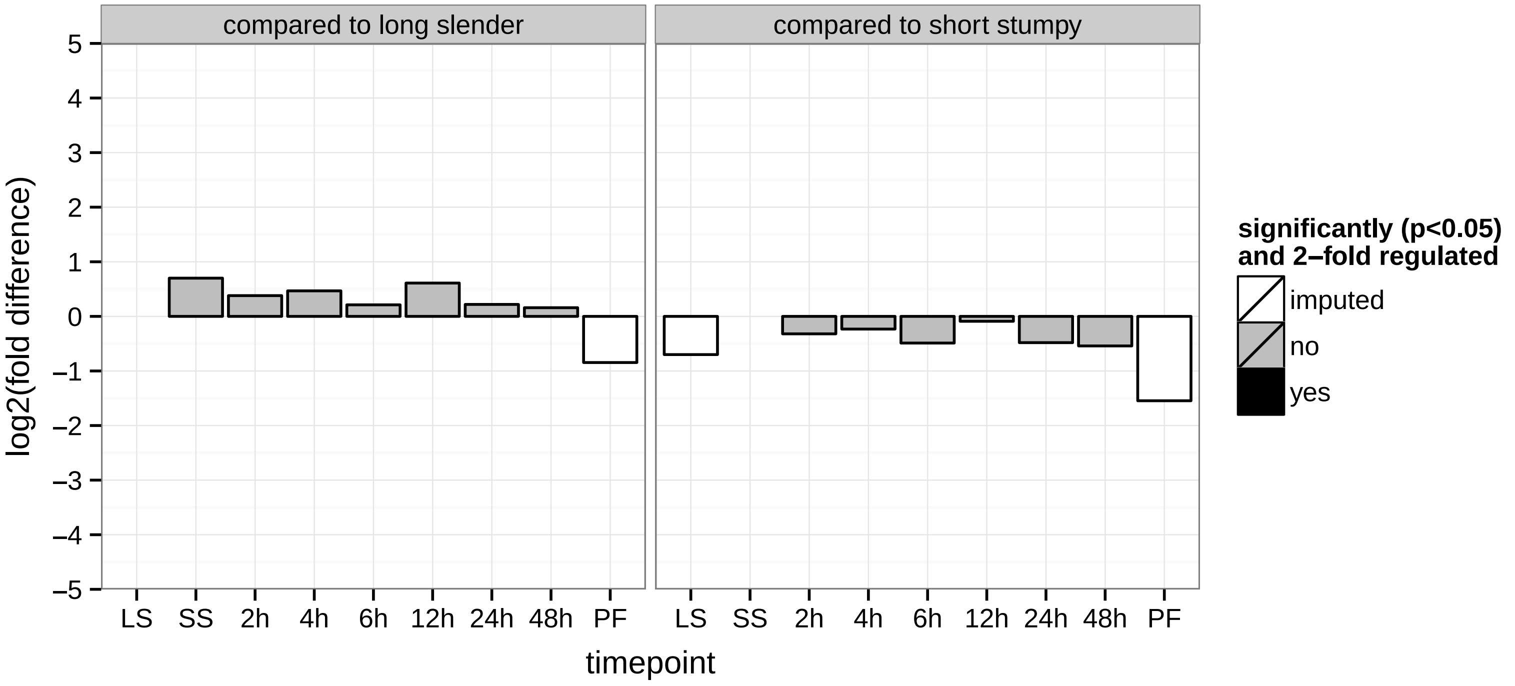

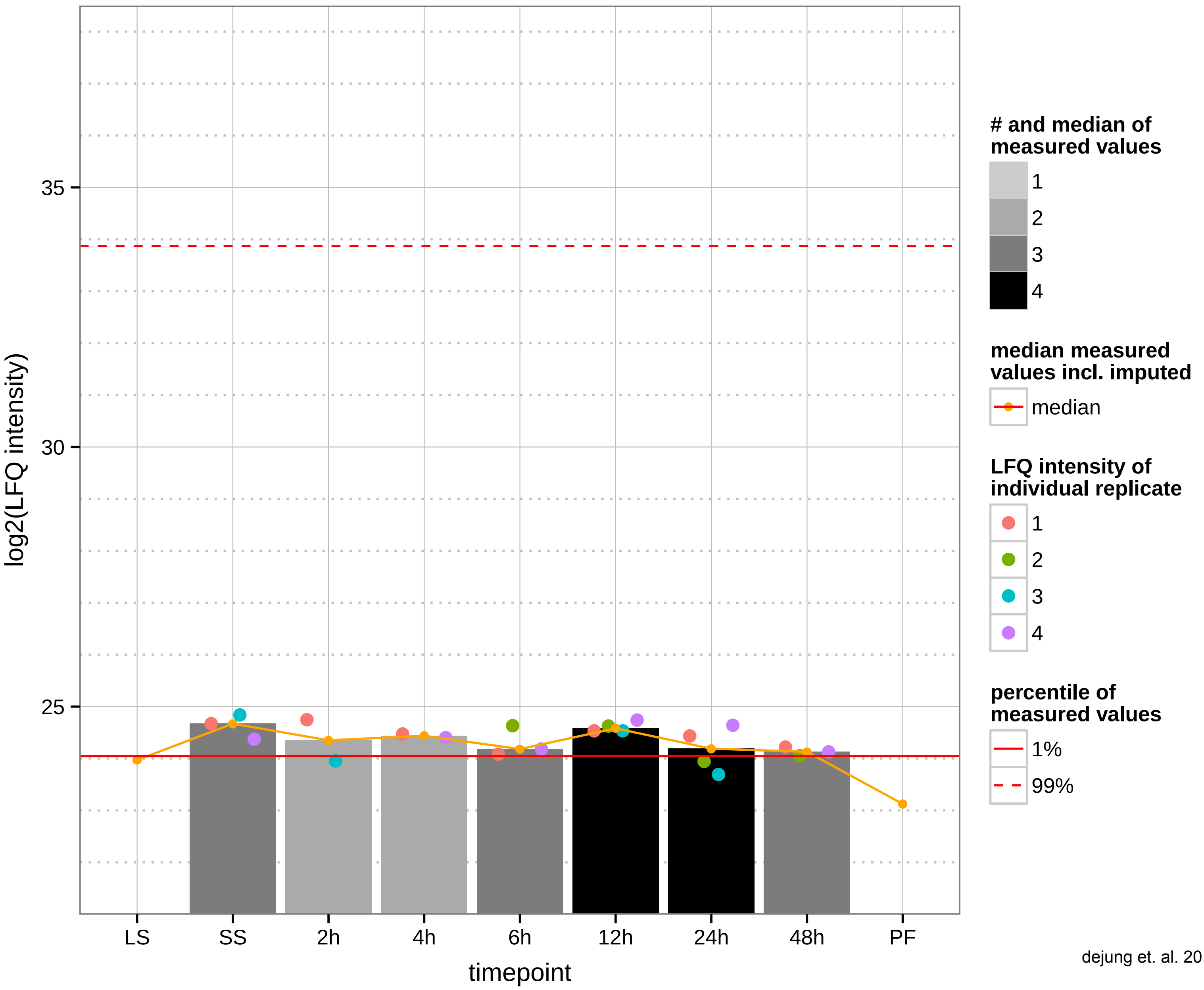

dejung et. al. 2015

hypothetical protein, conserved
Tb927.7.1400
AGOF: null
AGOC: null
AGOP: null
PGOF: null
PGOC: null
PGOP: null

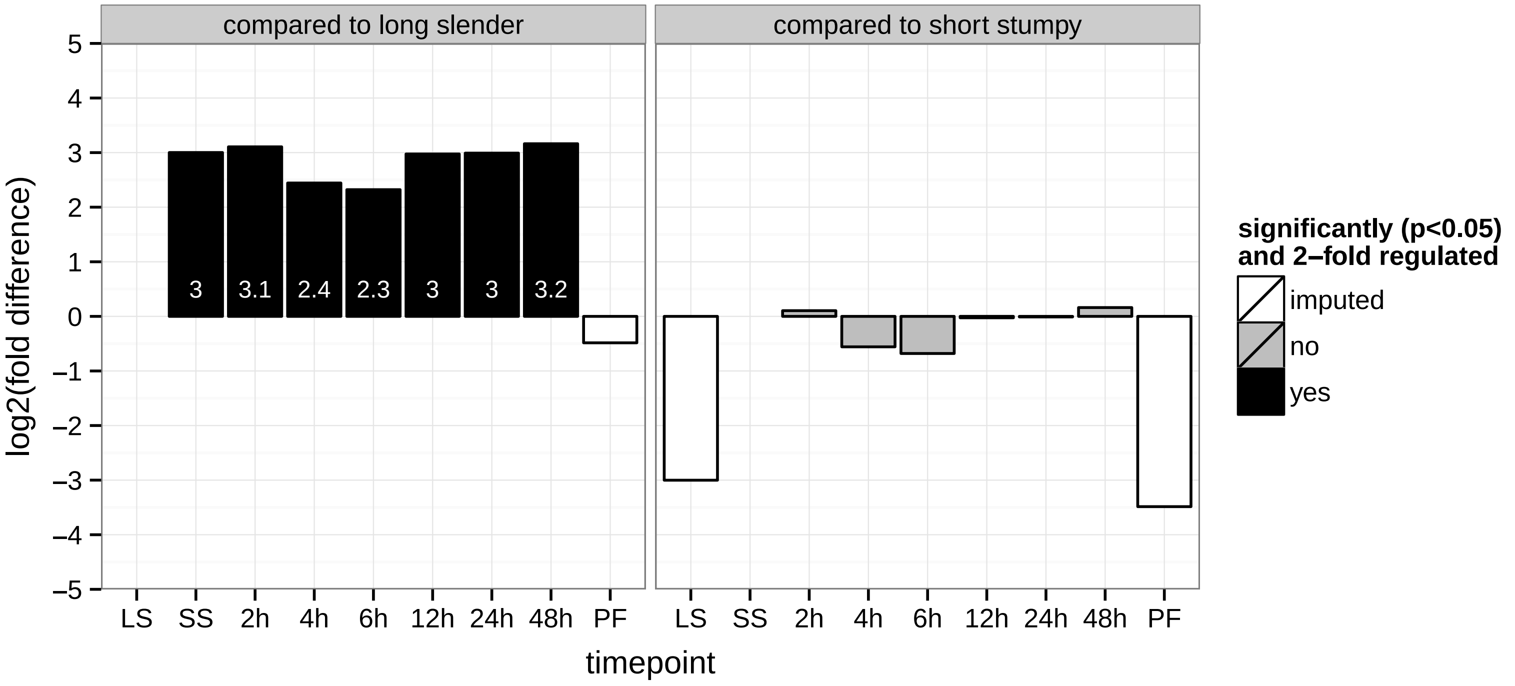

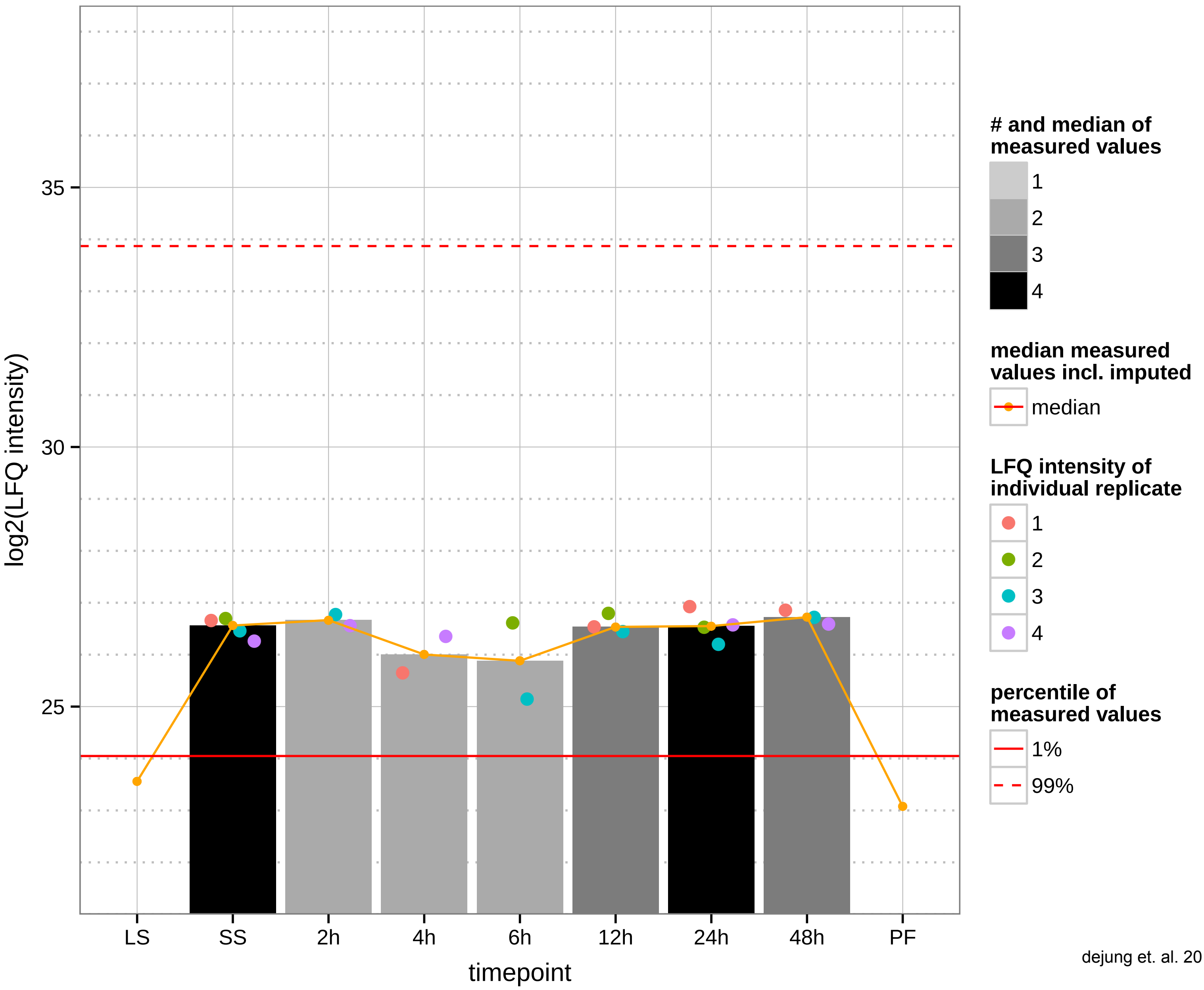

dejung et. al. 2015

hypothetical protein, conserved
Tb927.7.1580
AGOF: null
AGOC: null
AGOP: null
PGOF: null
PGOC: null
PGOP: null

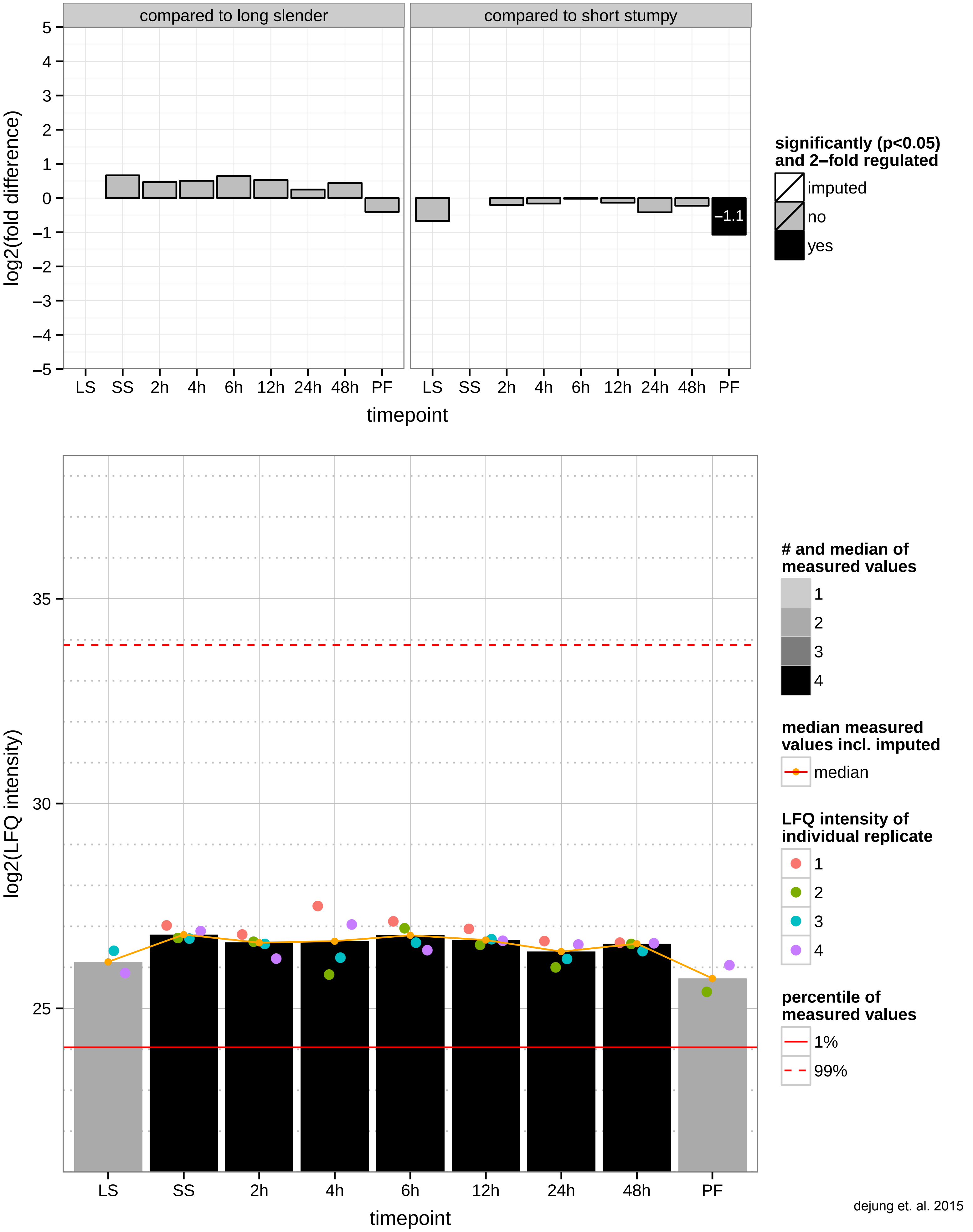

dejung et. al. 2015

hypothetical protein, conserved
Tb927.7.1620
AGOF: null
AGOC: null
AGOP: null
PGOF: mRNA binding
PGOC: mRNA cleavage factor complex
PGOP: mRNA polyadenylation

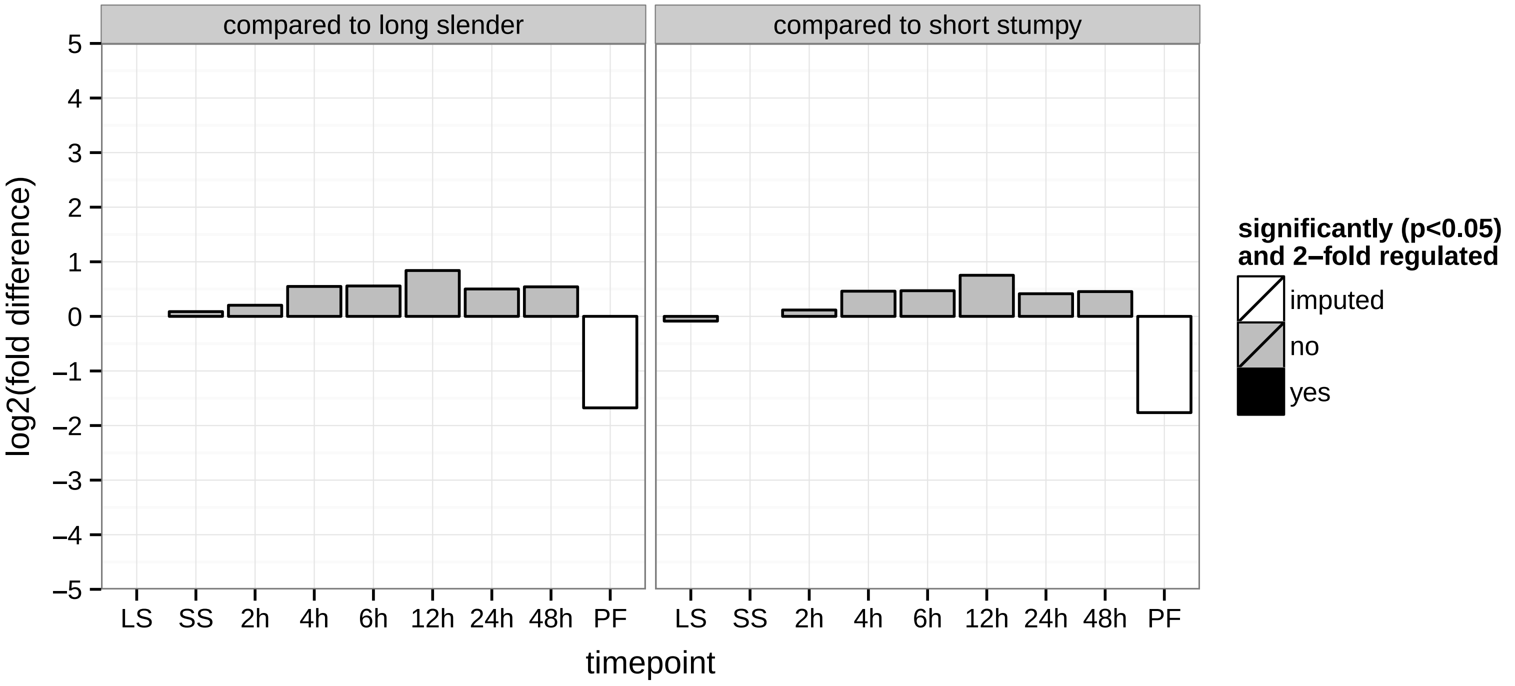

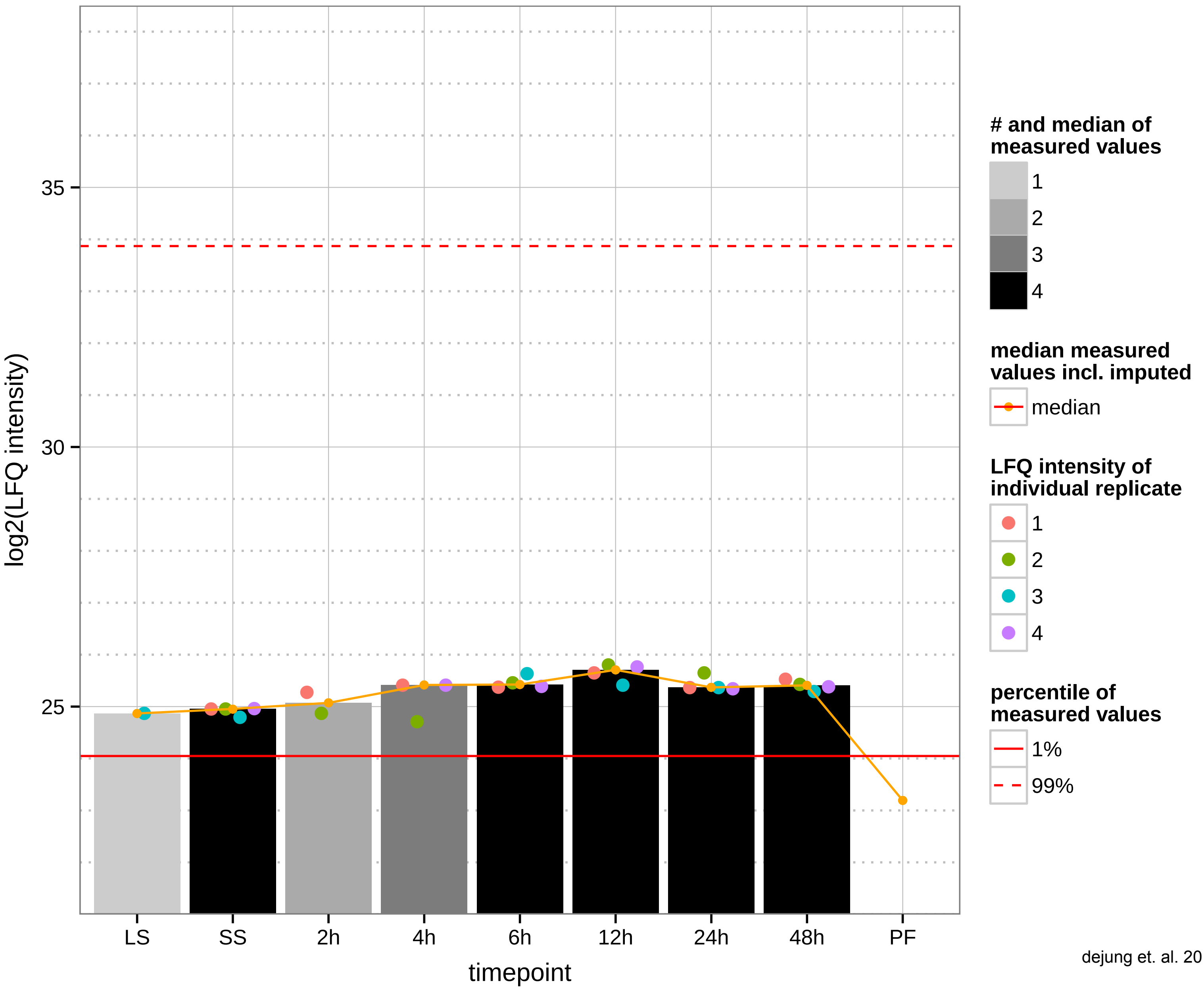

dejung et. al. 2015

GTPase activating protein, putative
Tb927.7.2470
AGOF: Rab GTPase activator activity
AGOC: intracellular
AGOP: regulation of Rab GTPase activity
PGOF: Rab GTPase activator activity
PGOC: intracellular
PGOP: regulation of Rab GTPase activity

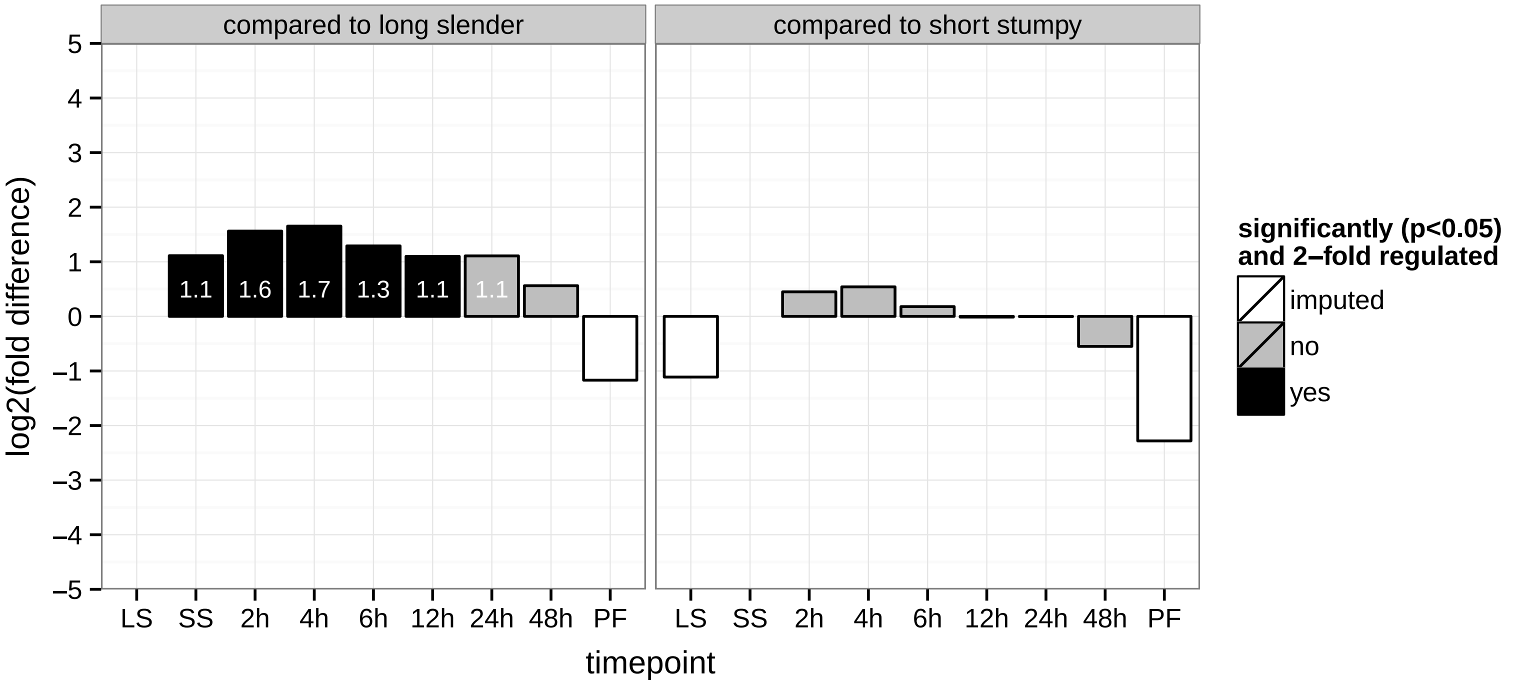

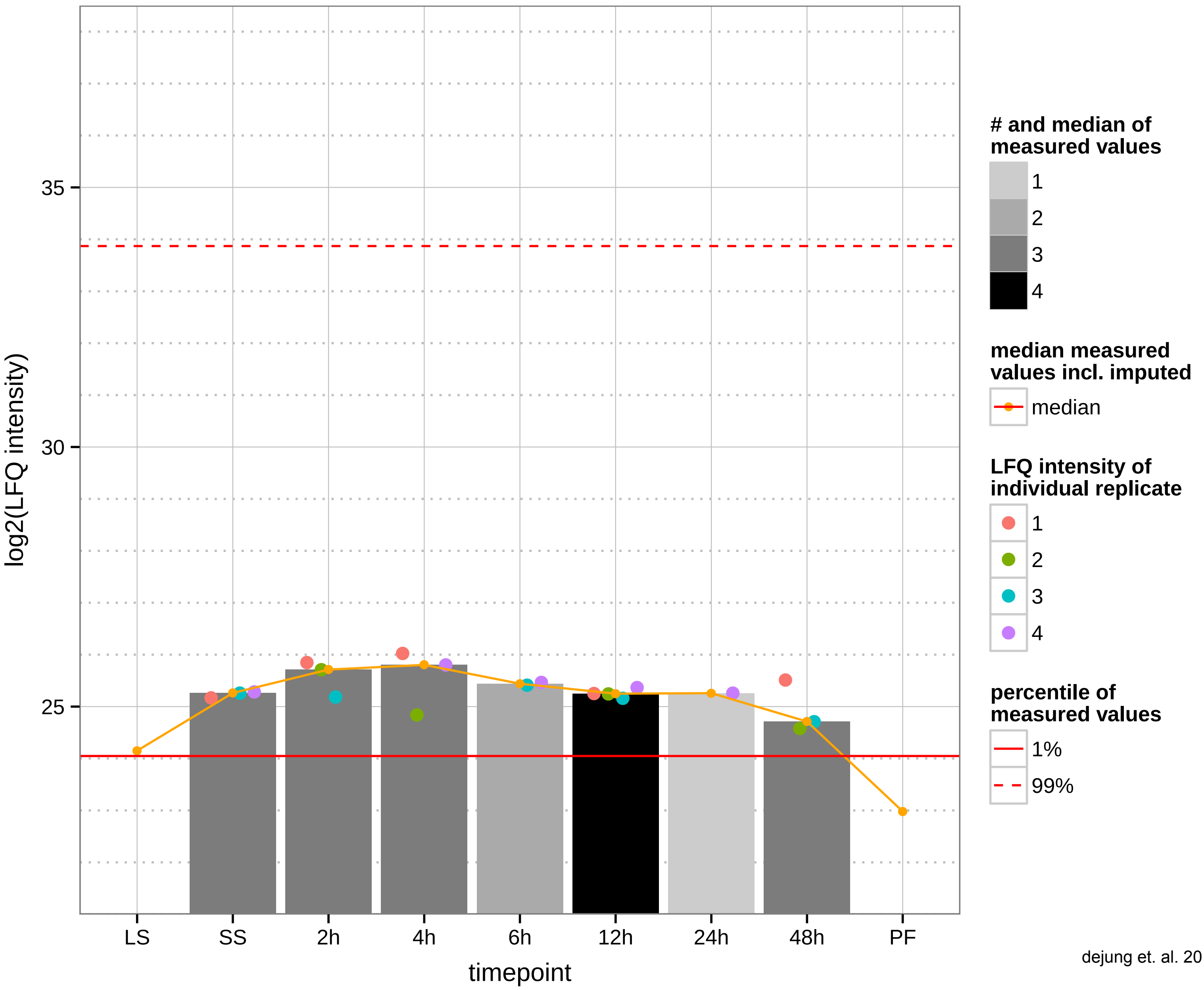

dejung et. al. 2015

endoplasmatic reticulum retrieval protein, putative
Tb927.7.2510
AGOF: null
AGOC: integral to membrane
AGOP: null
PGOF: null
PGOC: integral to membrane
PGOP: null

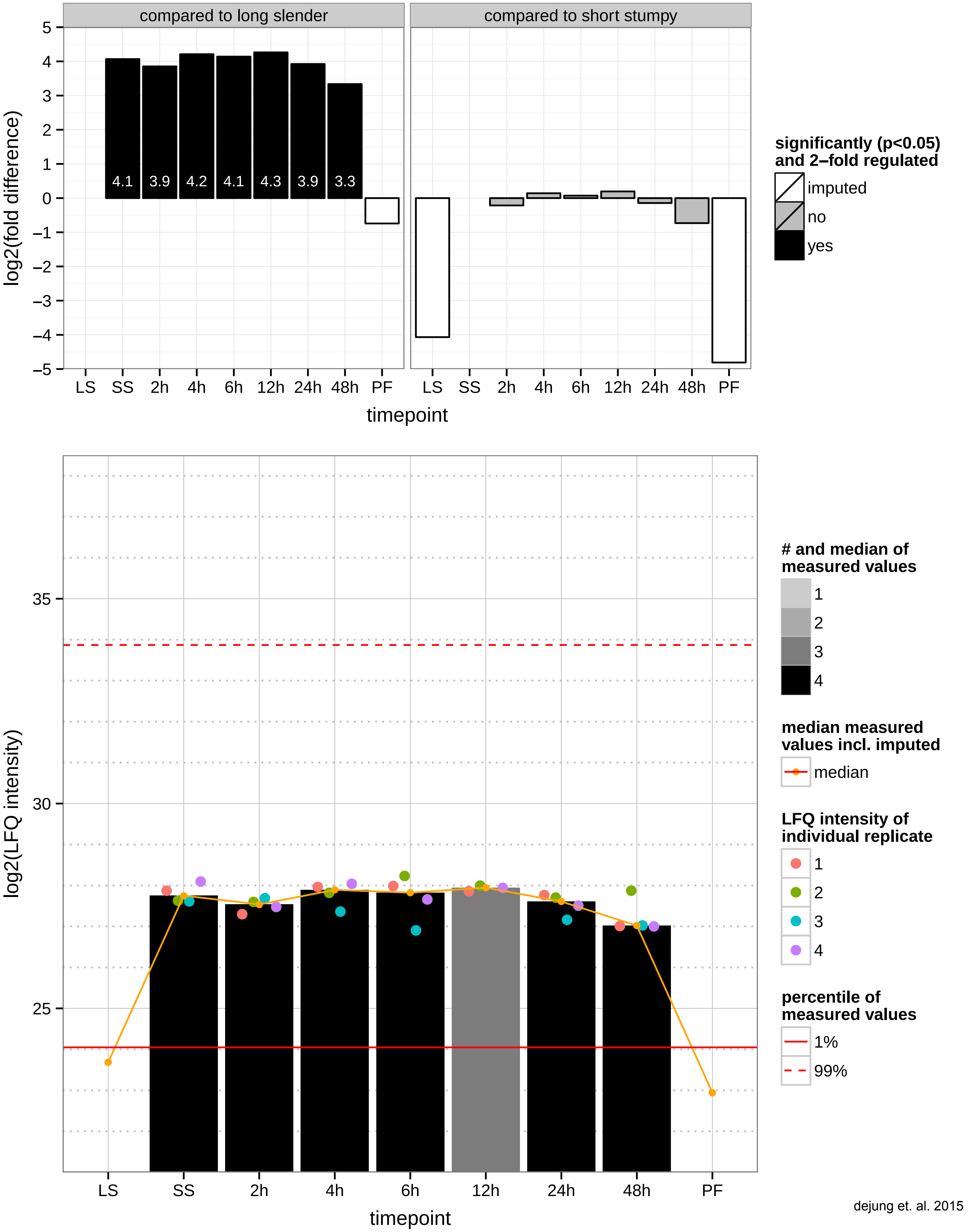

dejung et. al. 2015

hypothetical protein, conserved
Tb927.7.3070
AGOF: null
AGOC: null
AGOP: null
PGOF: null
PGOC: null
PGOP: transmembrane transport

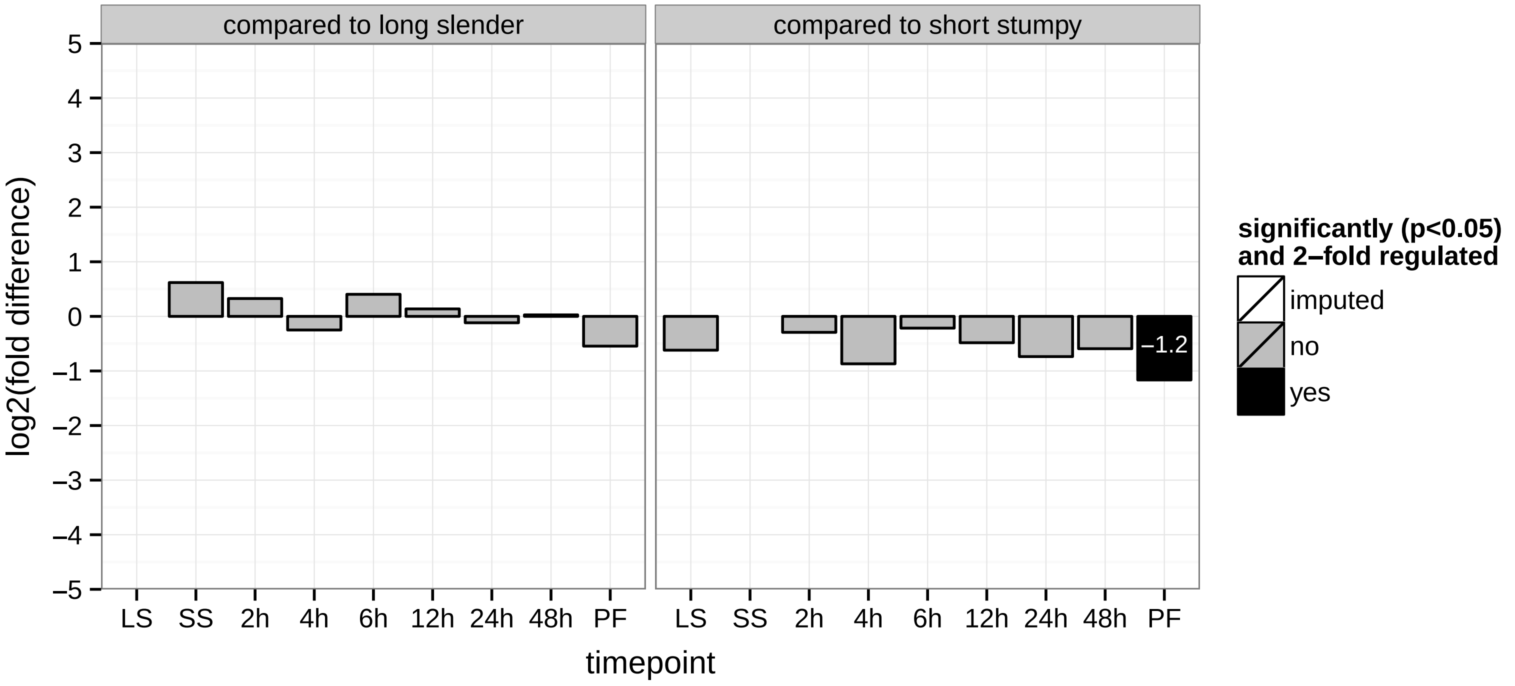

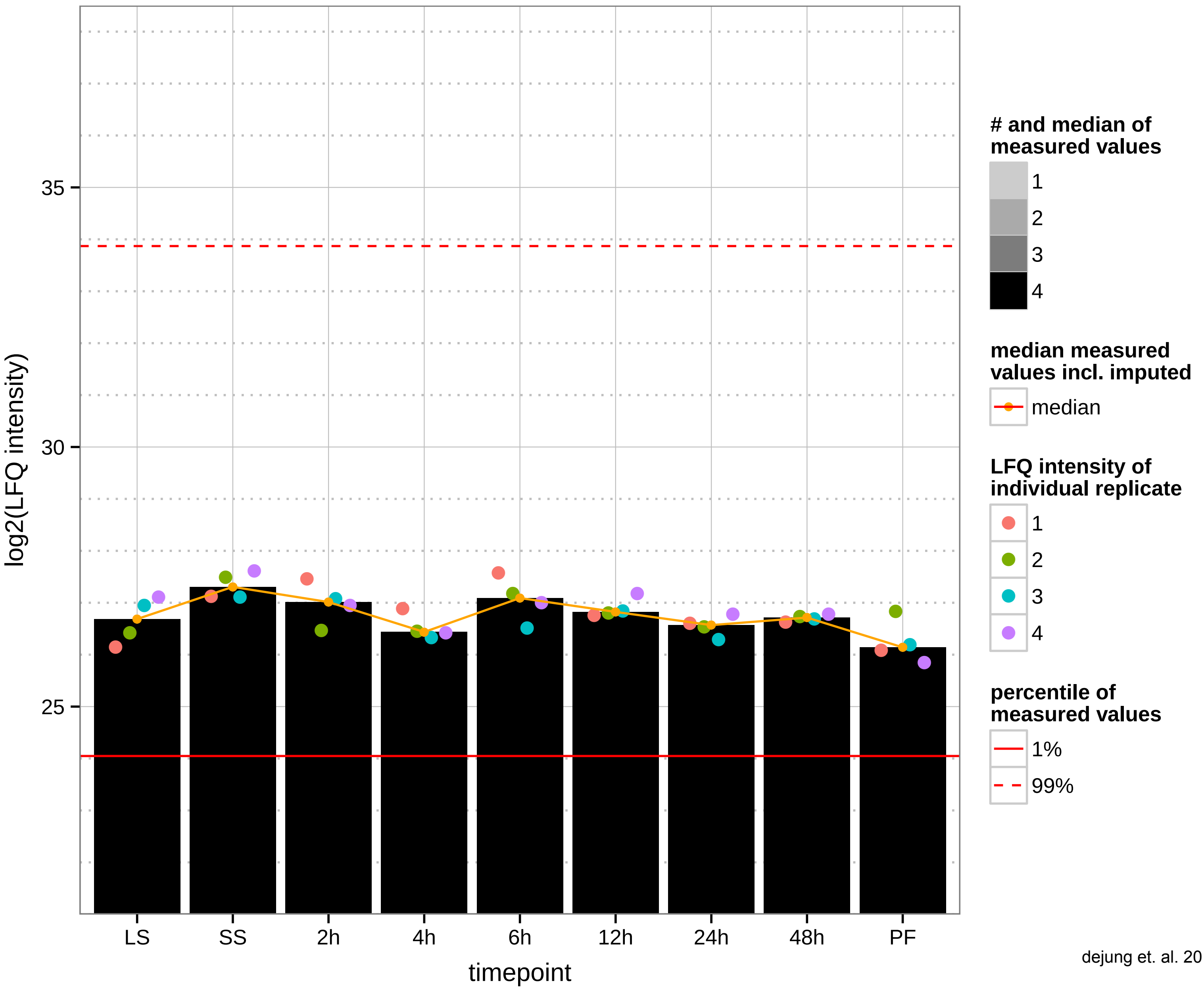

dejung et. al. 2015

peptidyl−prolyl cis−trans isomerase, putative (PPIase)
Tb927.7.3420
AGOF: null
AGOC: null
AGOP: protein folding
PGOF: null
PGOC: null
PGOP: protein folding

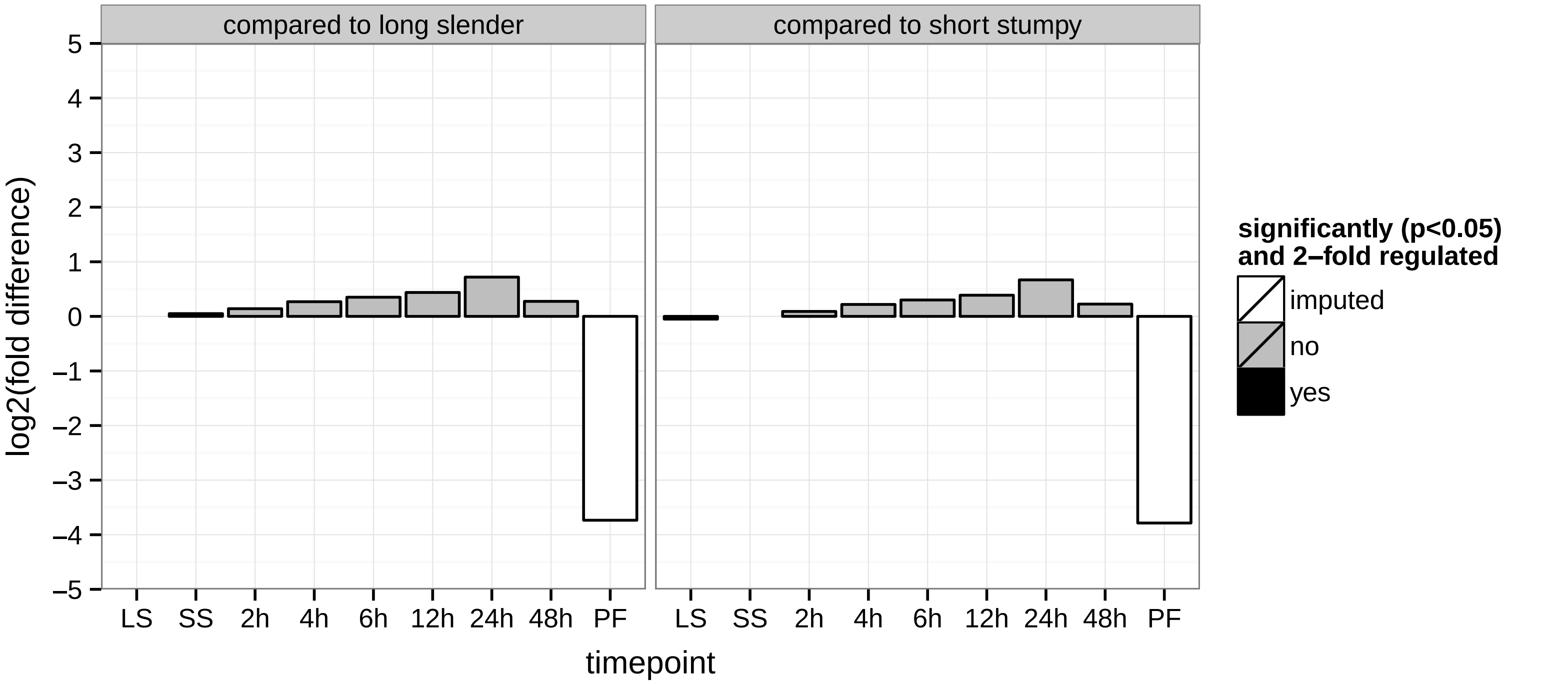

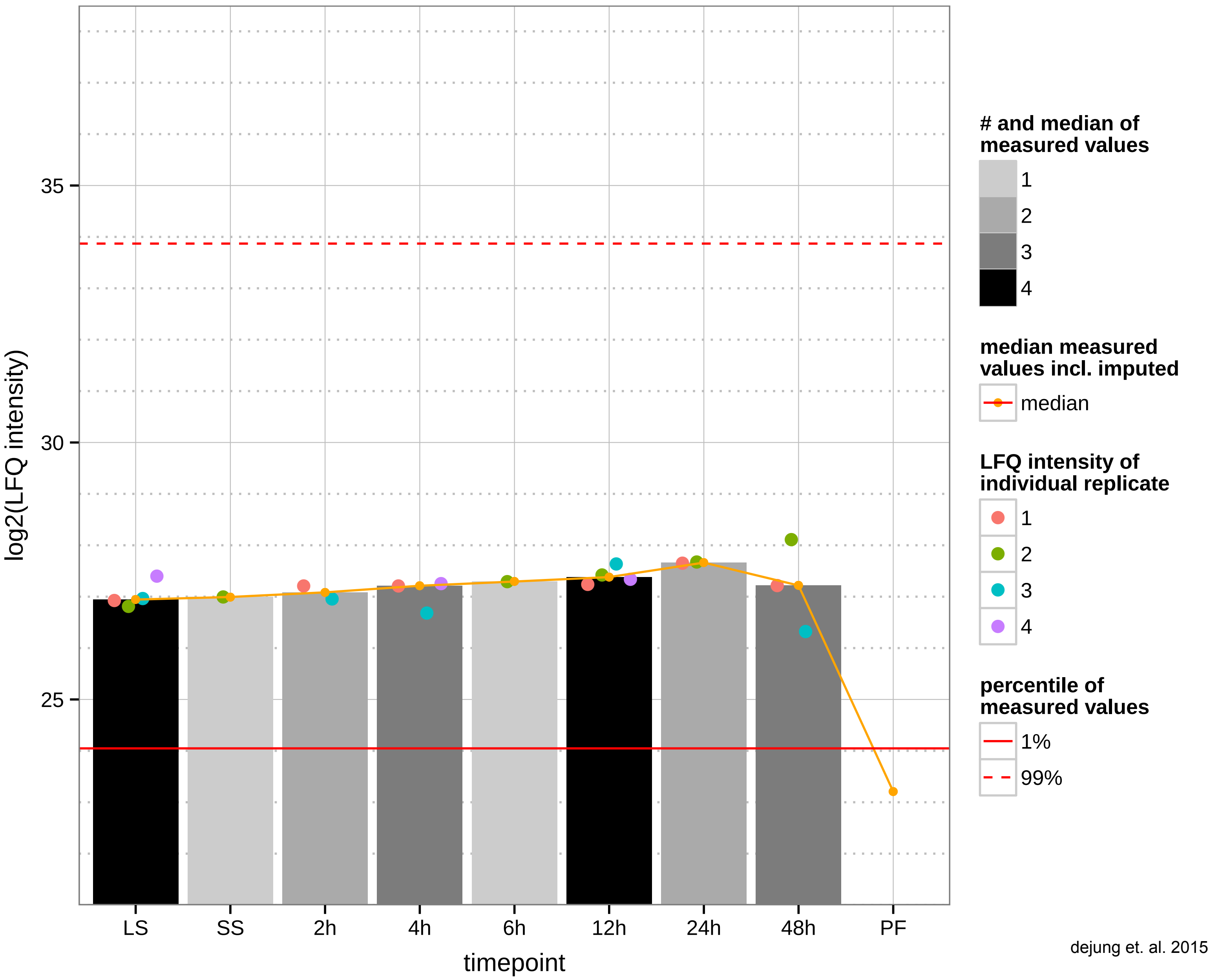

dejung et. al. 2015

hypothetical protein, conserved
Tb927.7.3510
AGOF: null
AGOC: mitochondrion
AGOP: null
PGOF: null
PGOC: null
PGOP: null

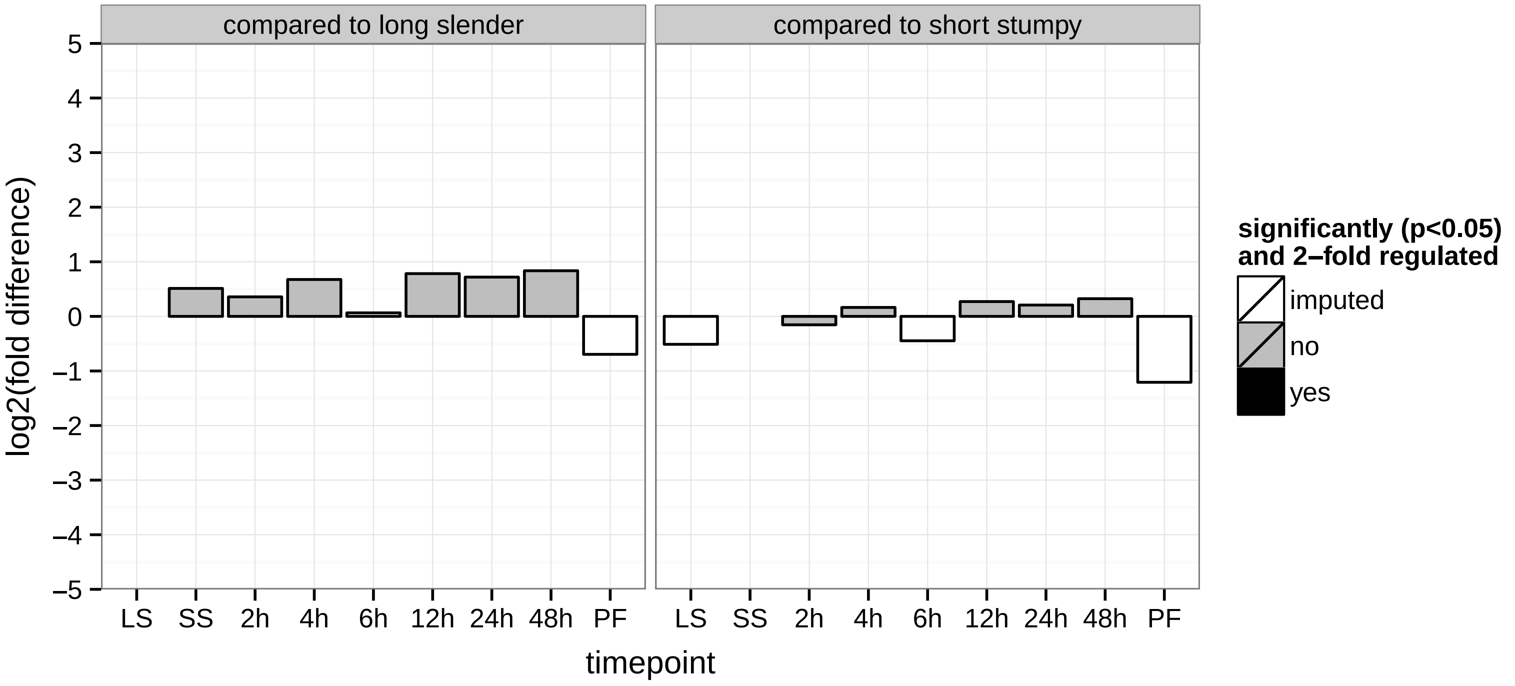

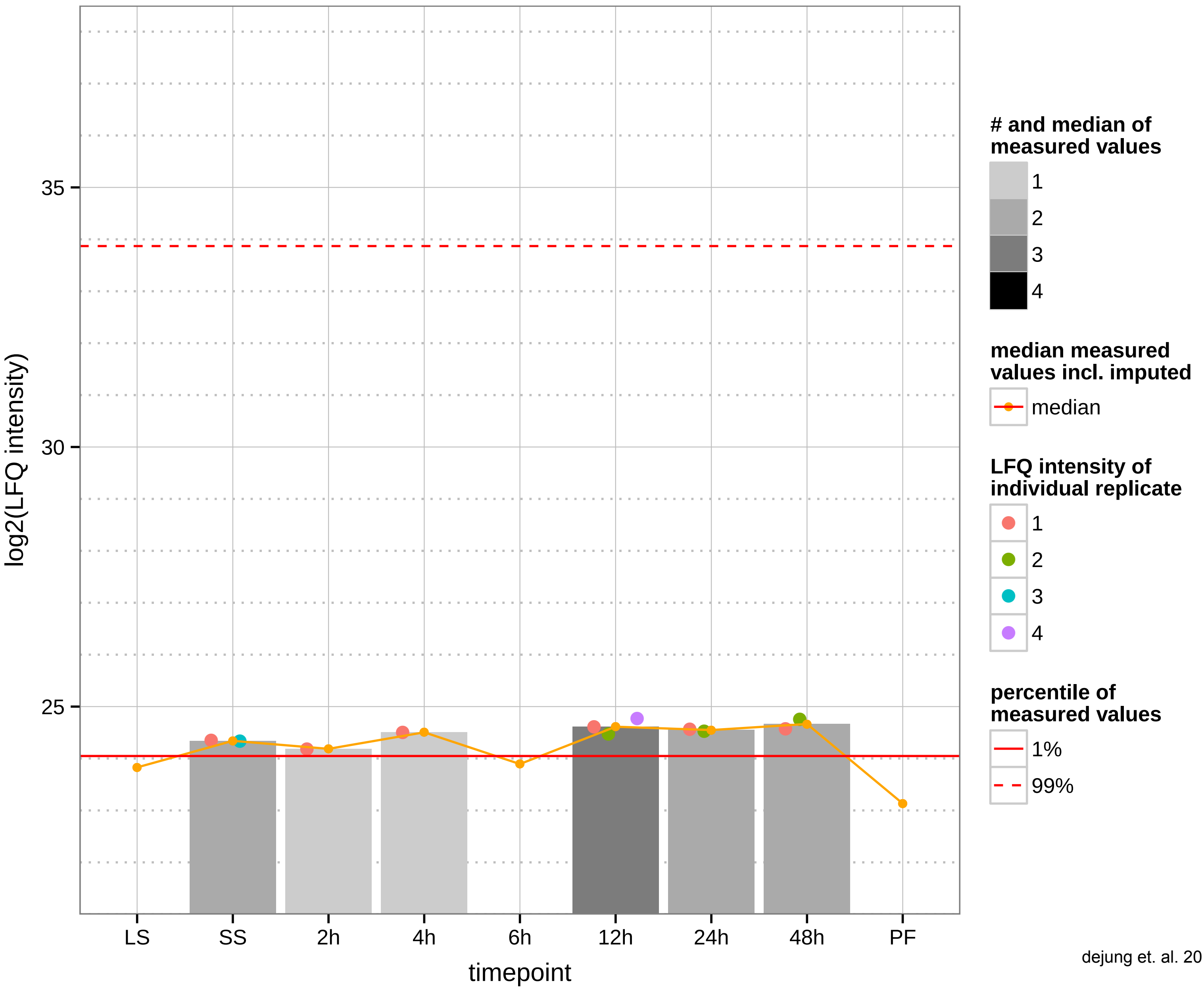

dejung et. al. 2015

hypothetical protein, conserved
Tb927.7.3530
AGOF: null
AGOC: null
AGOP: null
PGOF: binding
PGOC: null
PGOP: null

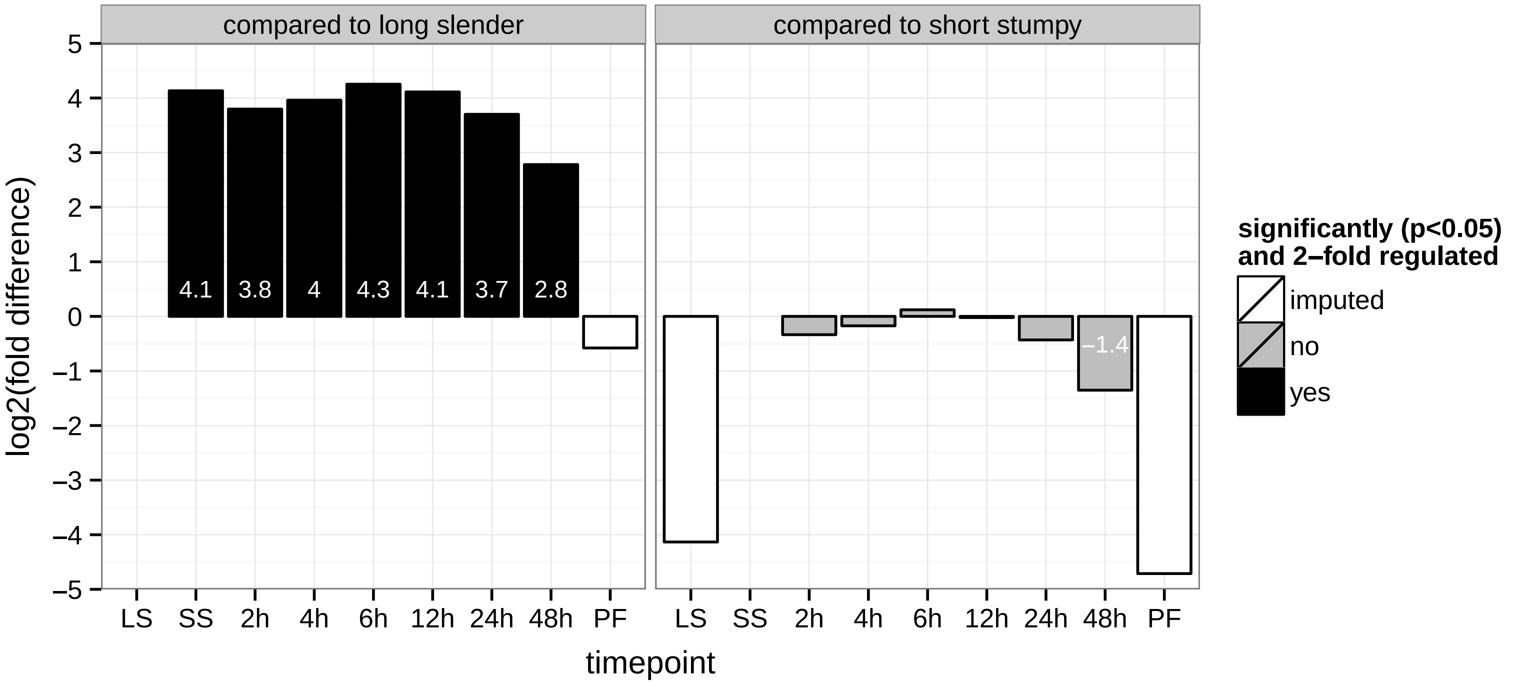

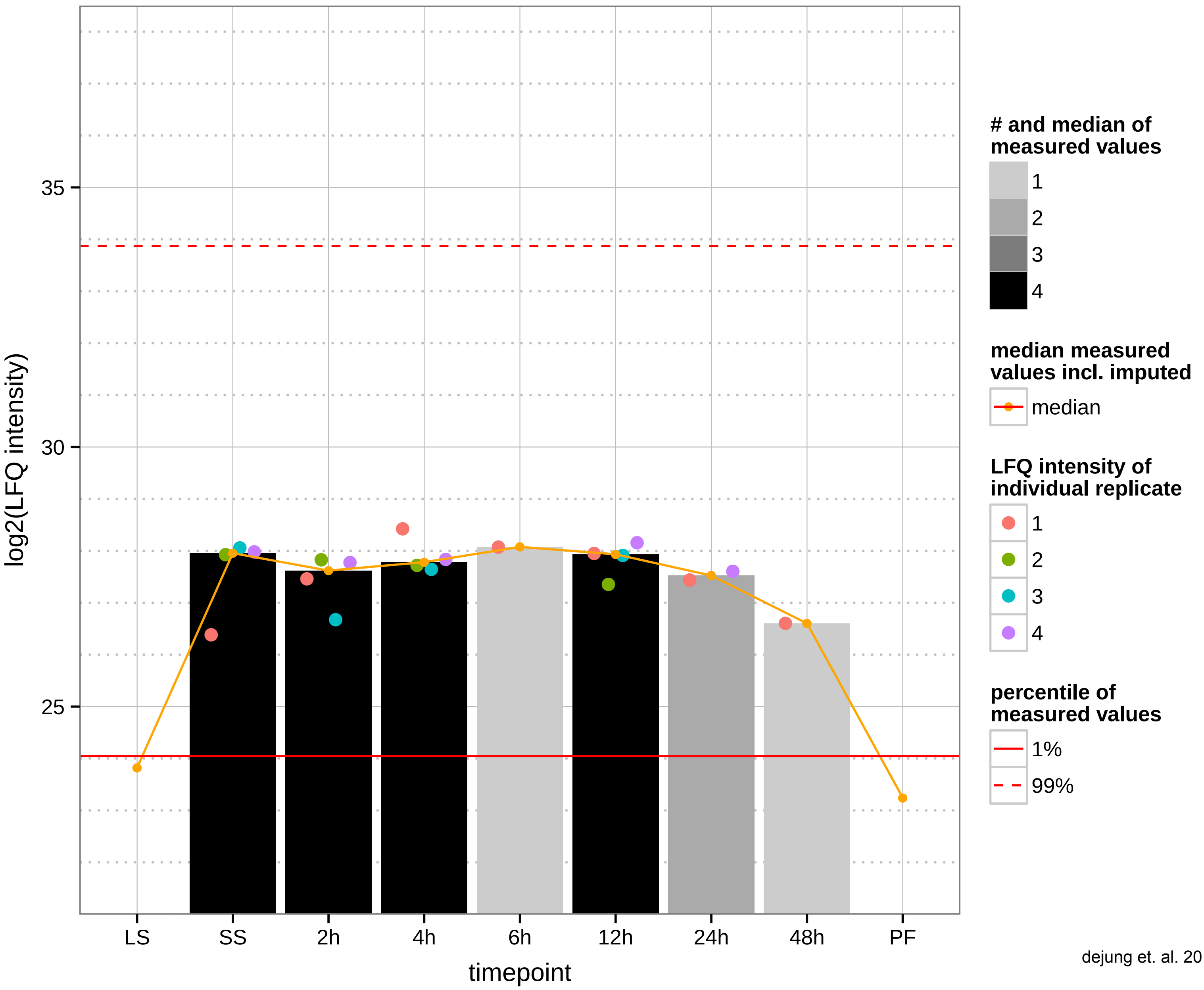

dejung et. al. 2015

TPR–repeat–containing chaperone protein DNAJ, putative
Tb927.7.3630
AGOF: heat shock protein binding
AGOC: null
AGOP: null
PGOF: heat shock protein binding, protein binding
PGOC: null
PGOP: null

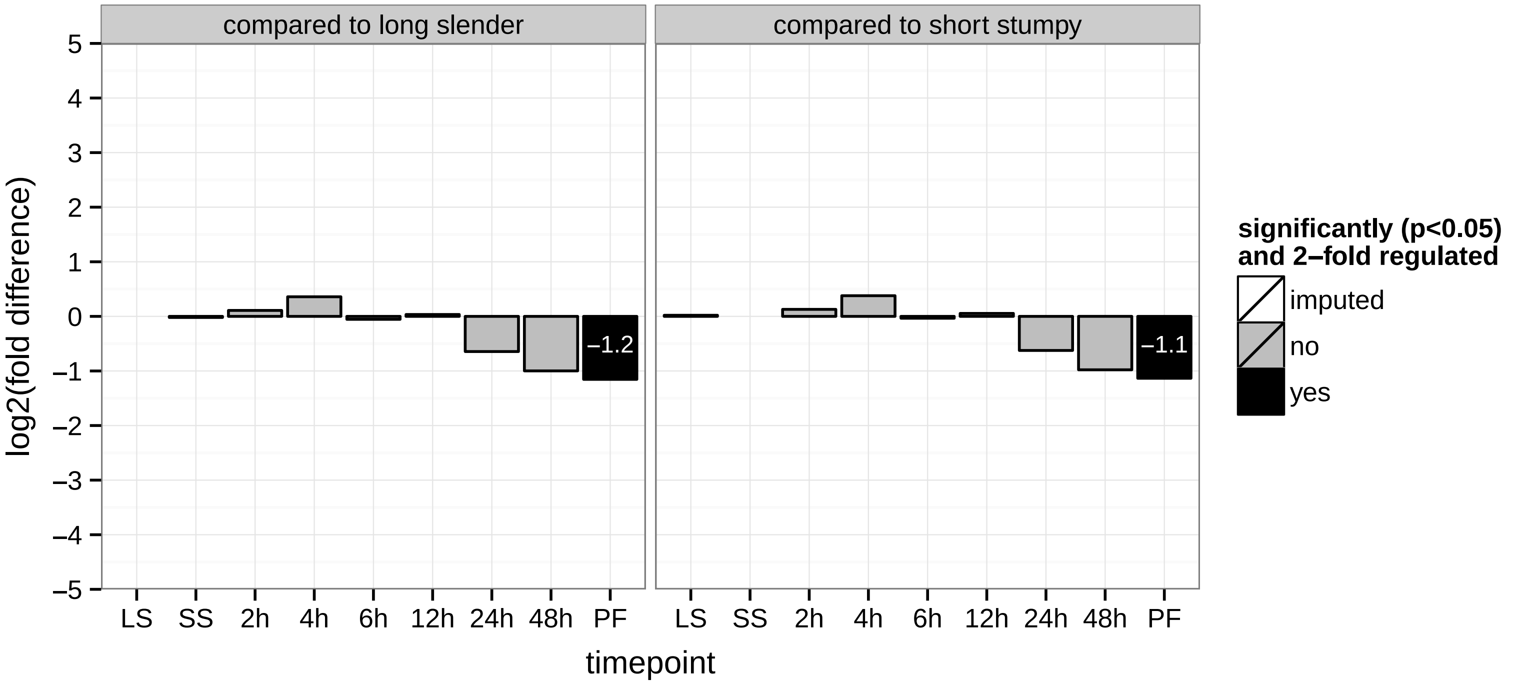

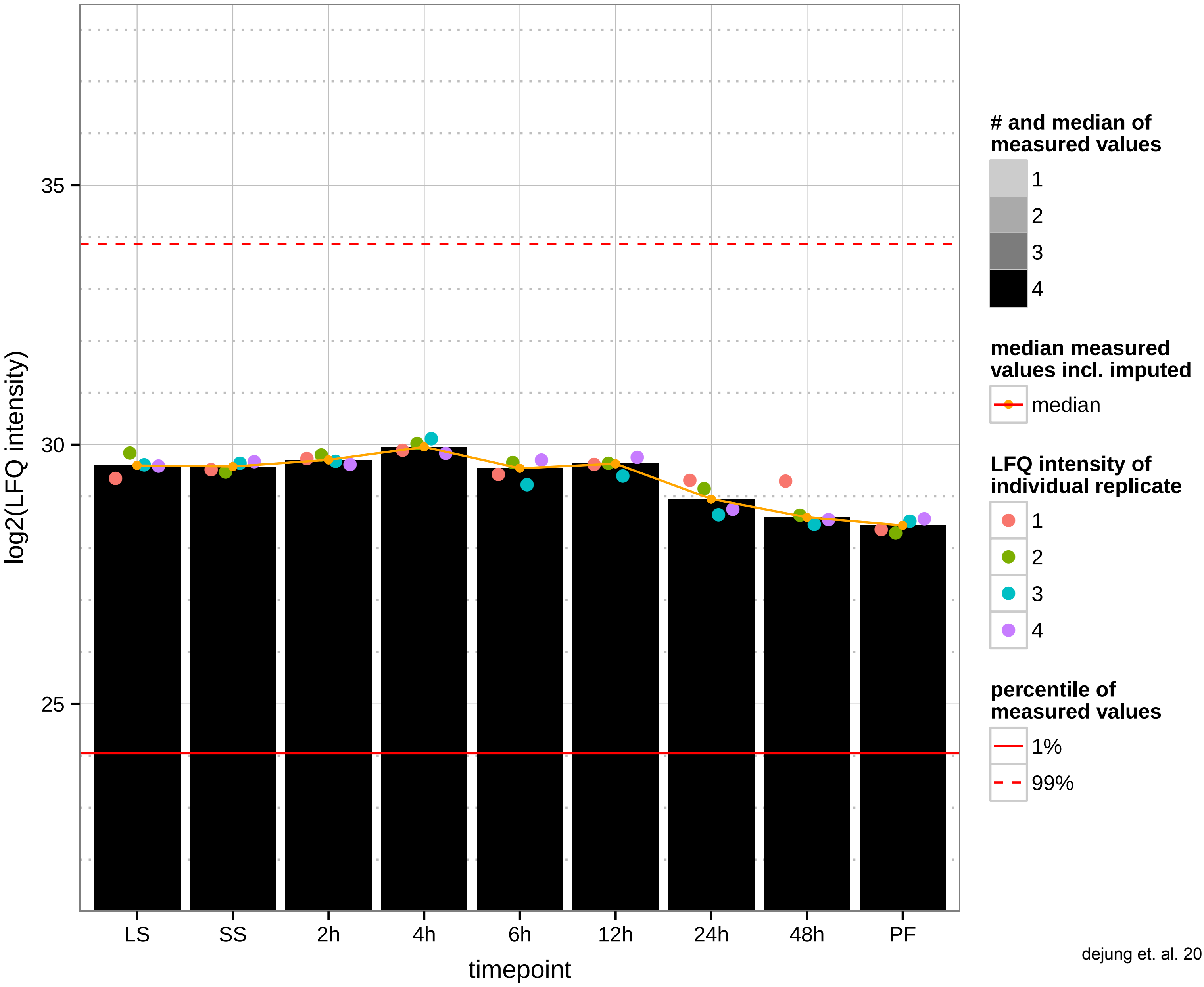

dejung et. al. 2015

hypothetical protein, conserved
Tb927.7.3870
AGOF: null
AGOC: null
AGOP: null
PGOF: null
PGOC: null
PGOP: null

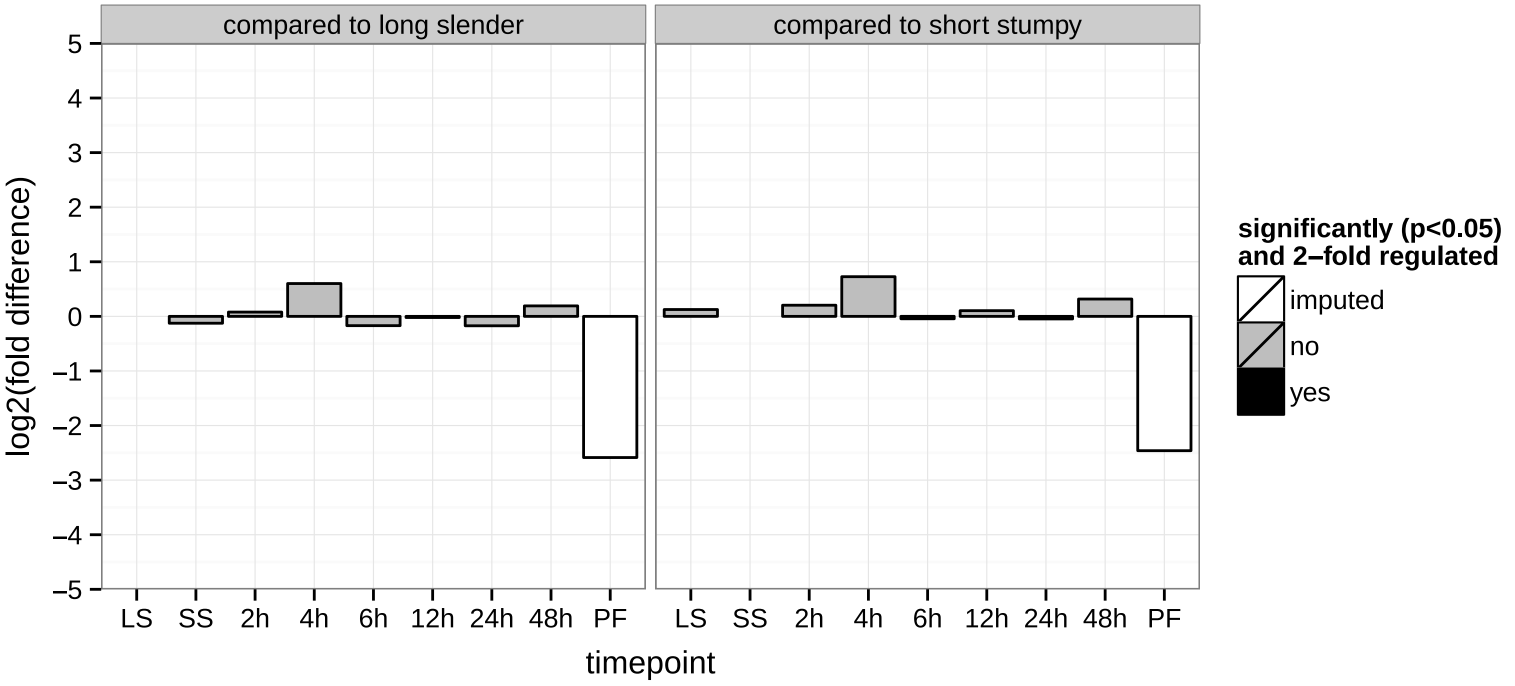

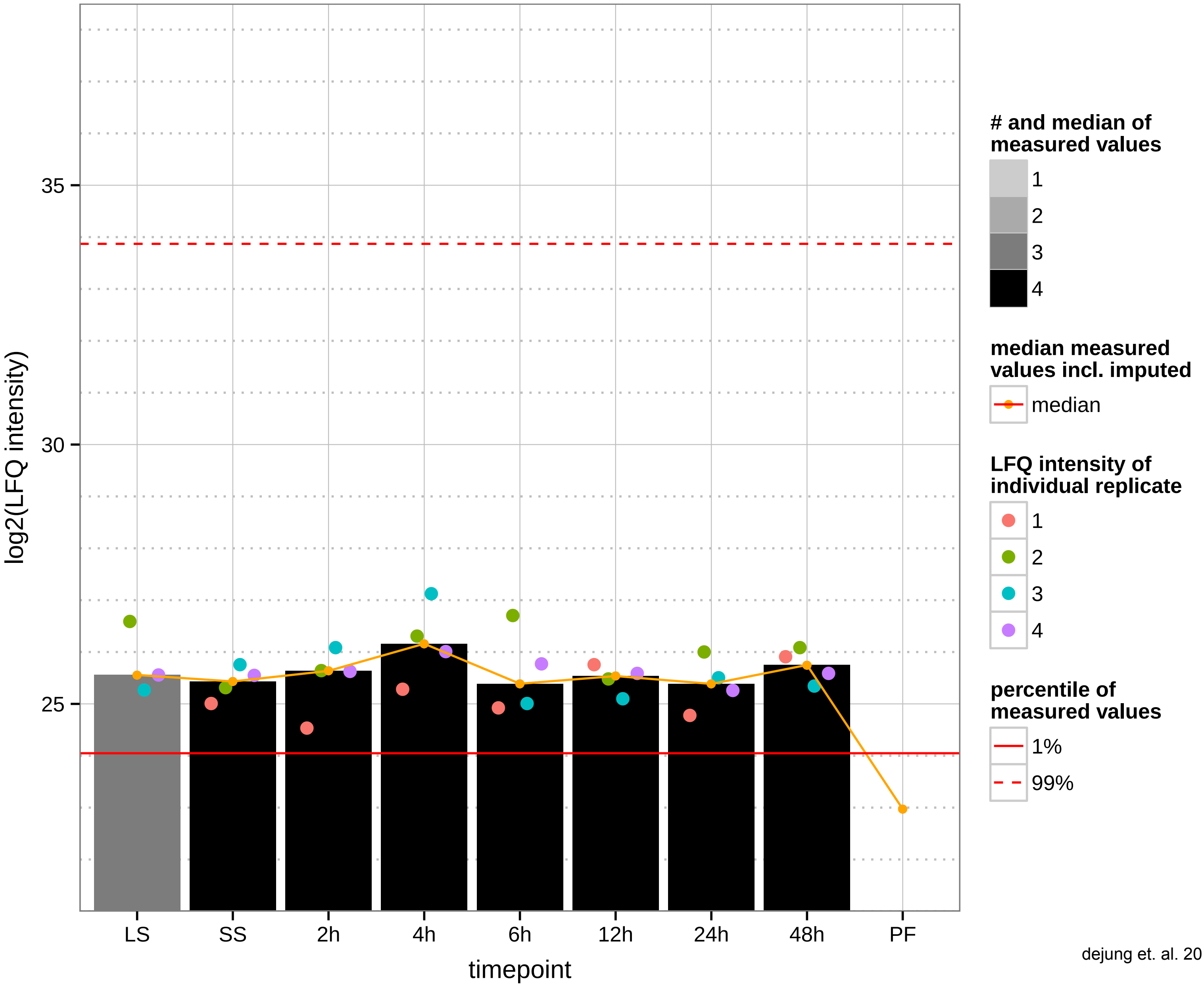

dejung et. al. 2015

hypothetical protein, conserved
Tb927.7.4100
AGOF: null
AGOC: null
AGOP: null
PGOF: null
PGOC: null
PGOP: null

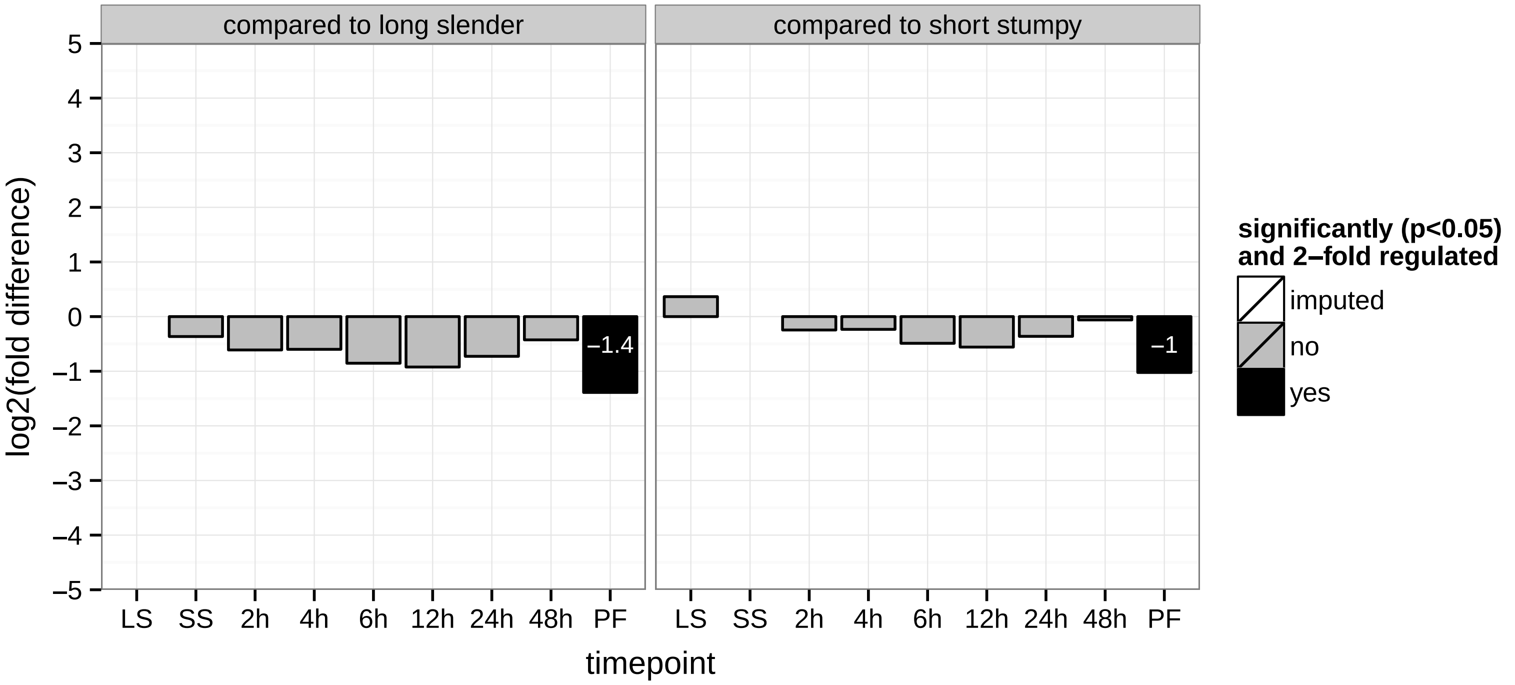

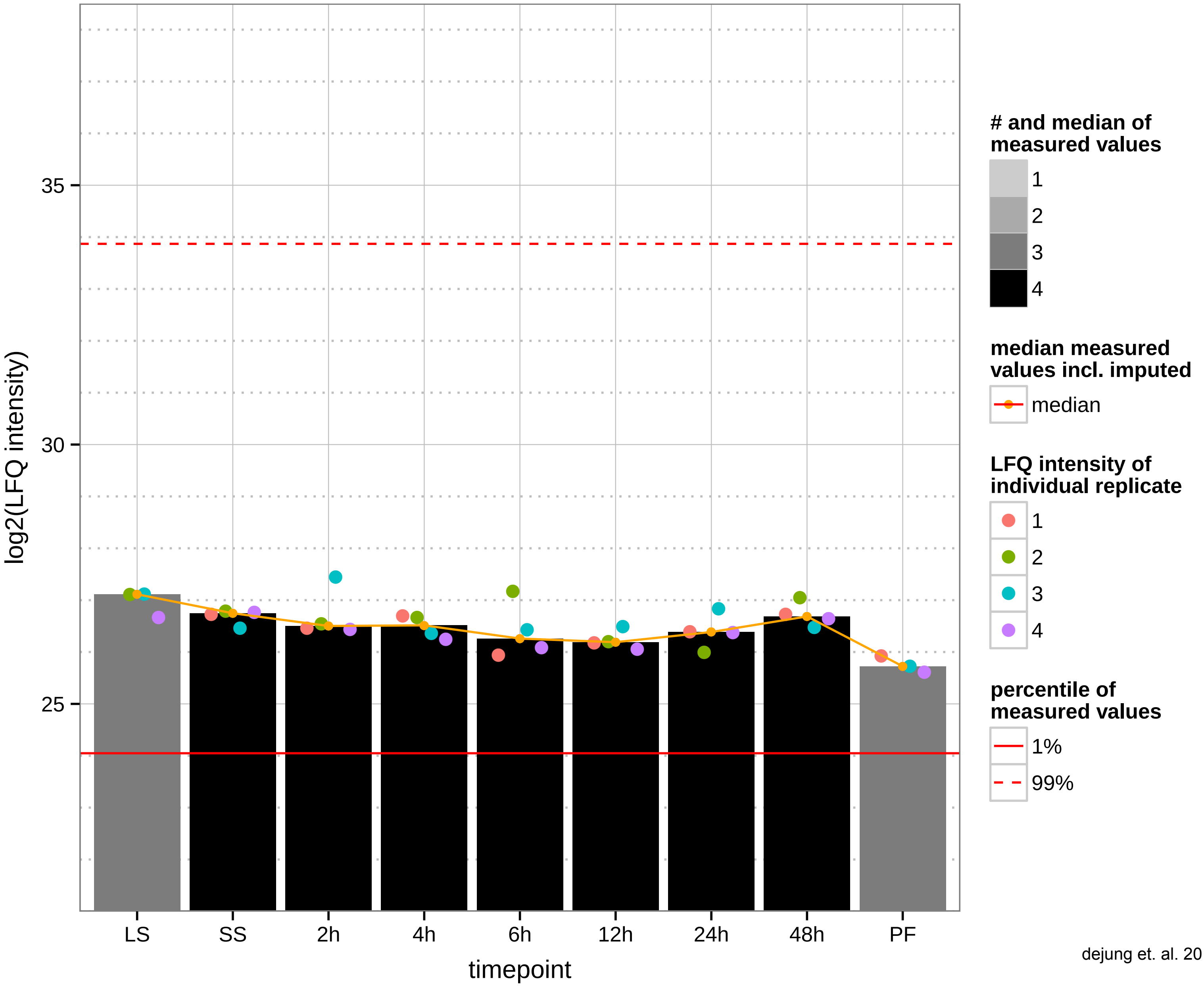

dejung et. al. 2015

fatty acid elongase, putative
Tb927.7.4170
AGOF: fatty acid elongase activity
AGOC: integral to endoplasmic reticulum membrane
AGOP: long−chain fatty acid biosynthetic process
PGOF: null
PGOC: integral to membrane
PGOP: null

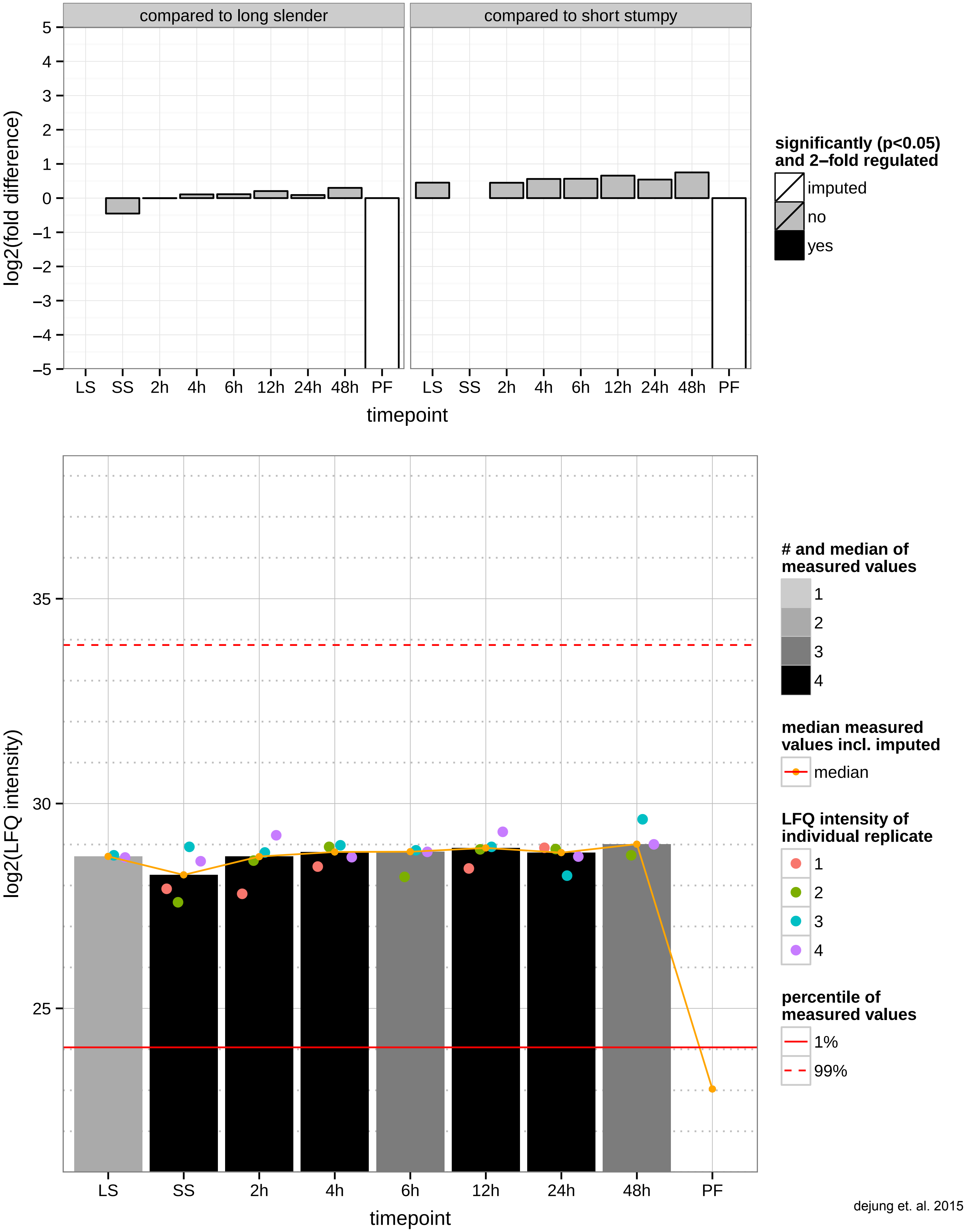

dejung et. al. 2015

hypothetical protein, conserved
Tb927.7.450;Tb927.7.390
AGOF: null
AGOC: null
AGOP: null
PGOF: null
PGOC: null
PGOP: null

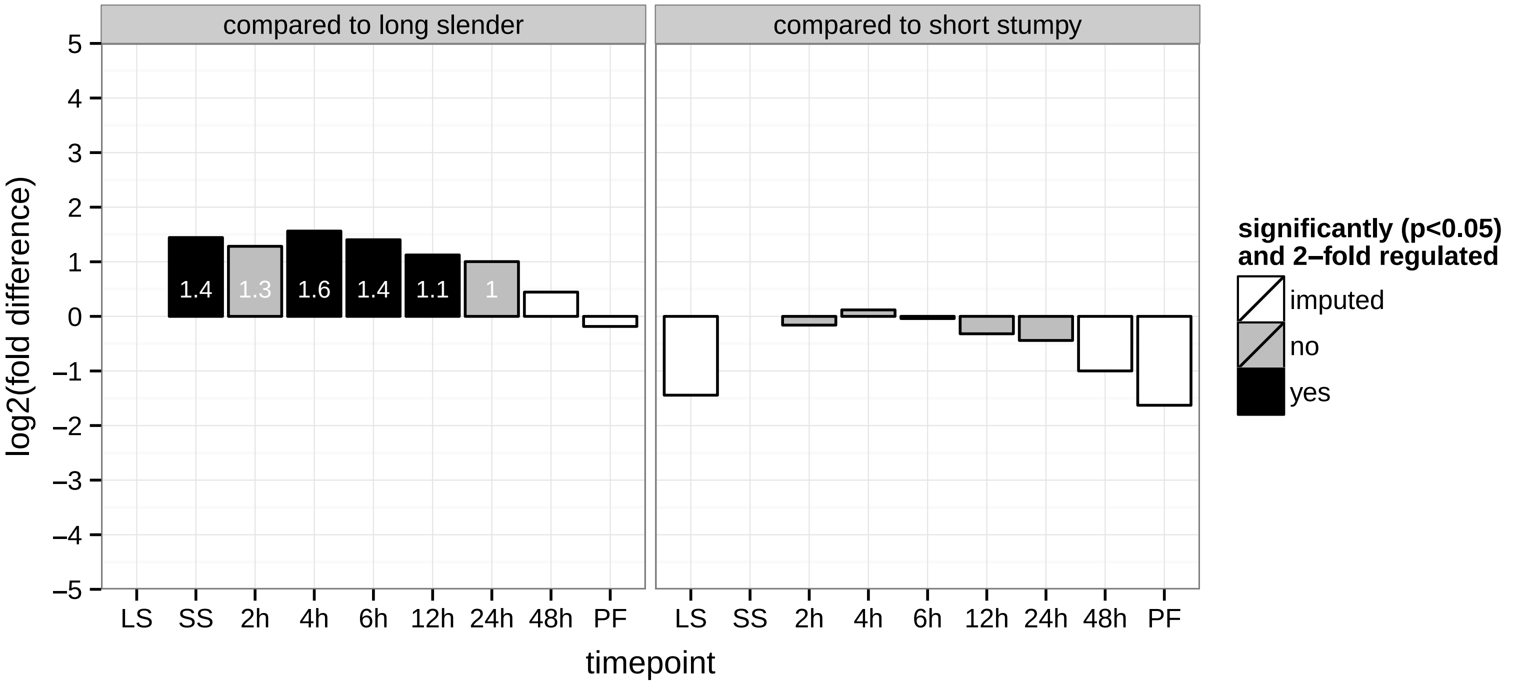

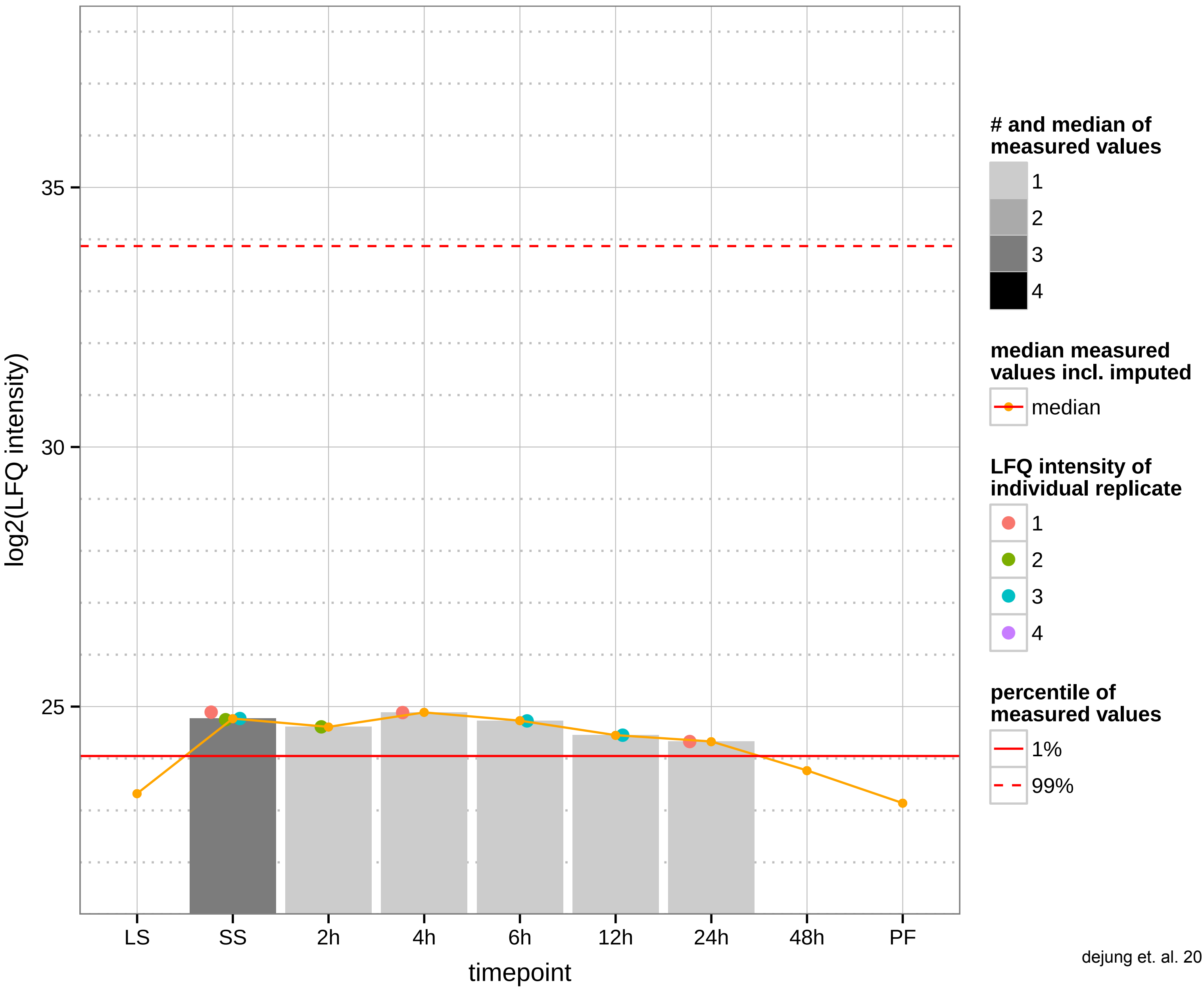

dejung et. al. 2015

hypothetical protein, conserved
Tb927.7.5310
AGOF: null
AGOC: nucleus
AGOP: regulation of transcription, DNA–dependent
PGOF: null
PGOC: nucleus
PGOP: regulation of transcription, DNA–dependent

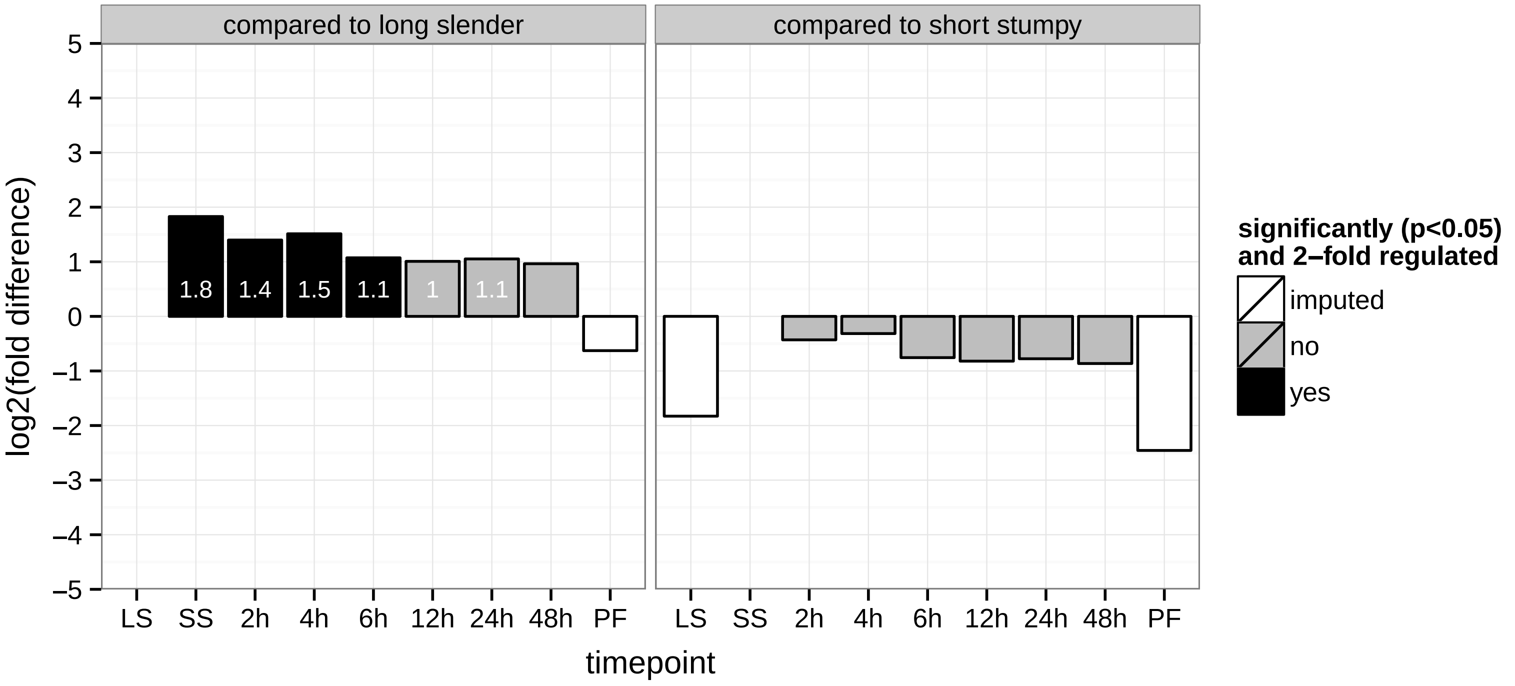

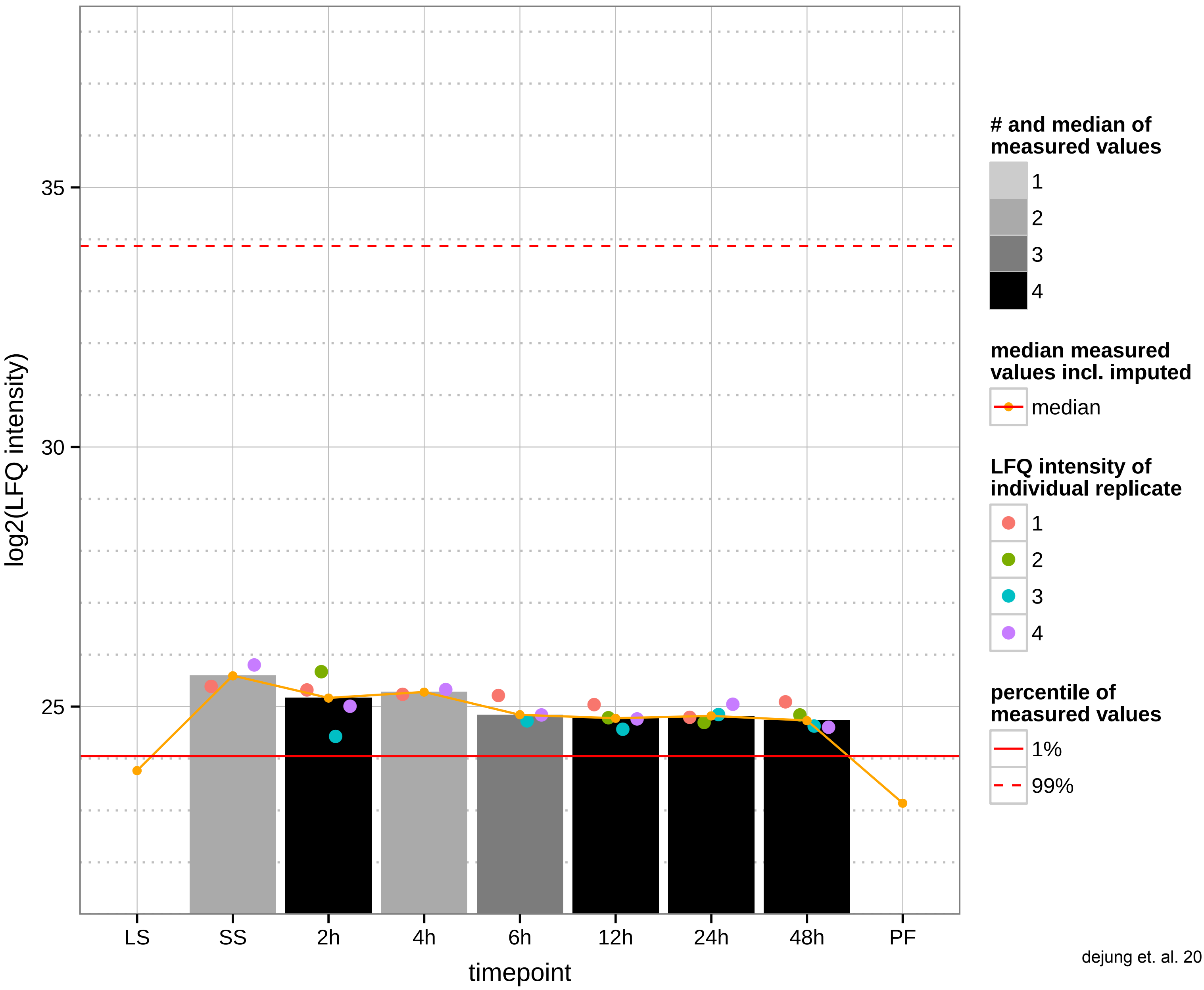

dejung et. al. 2015

hypothetical protein, conserved
Tb927.7.5330
AGOF: null
AGOC: null
AGOP: null
PGOF: null
PGOC: null
PGOP: null

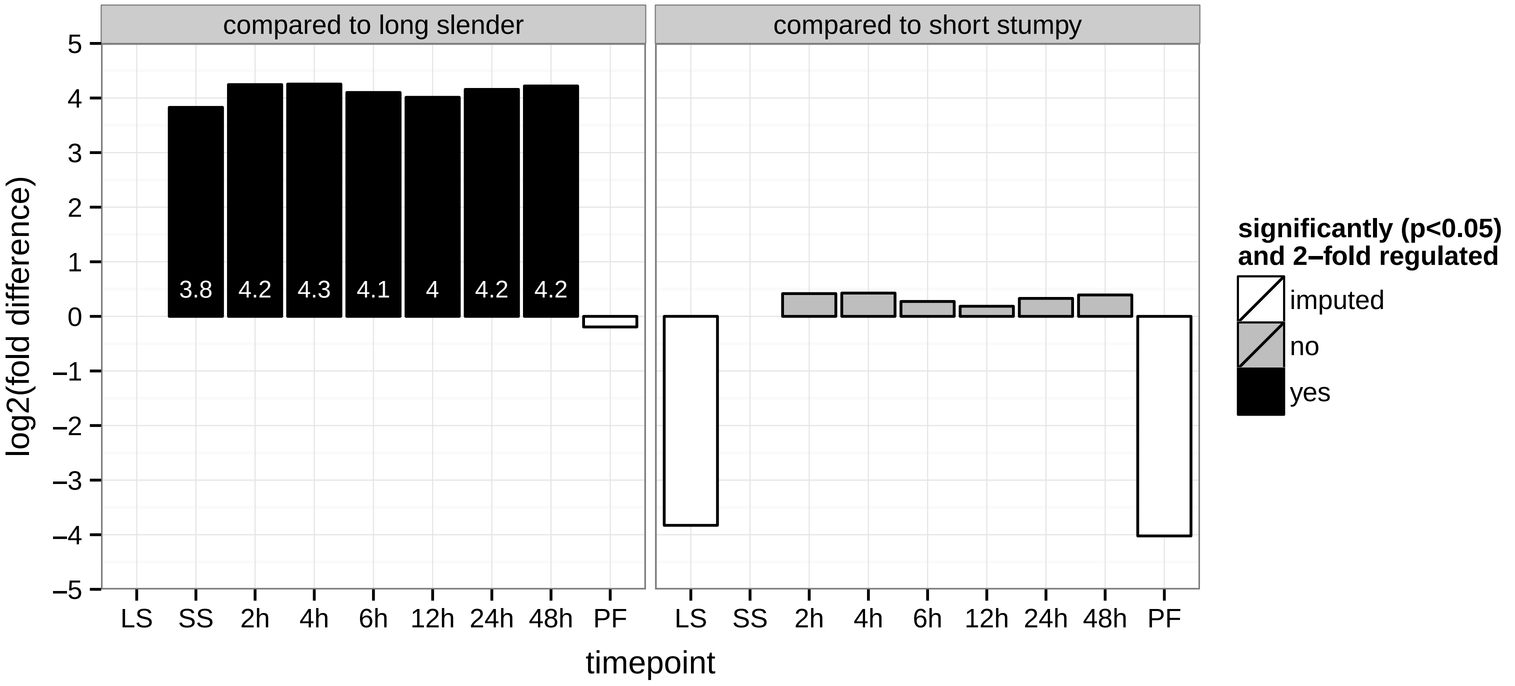

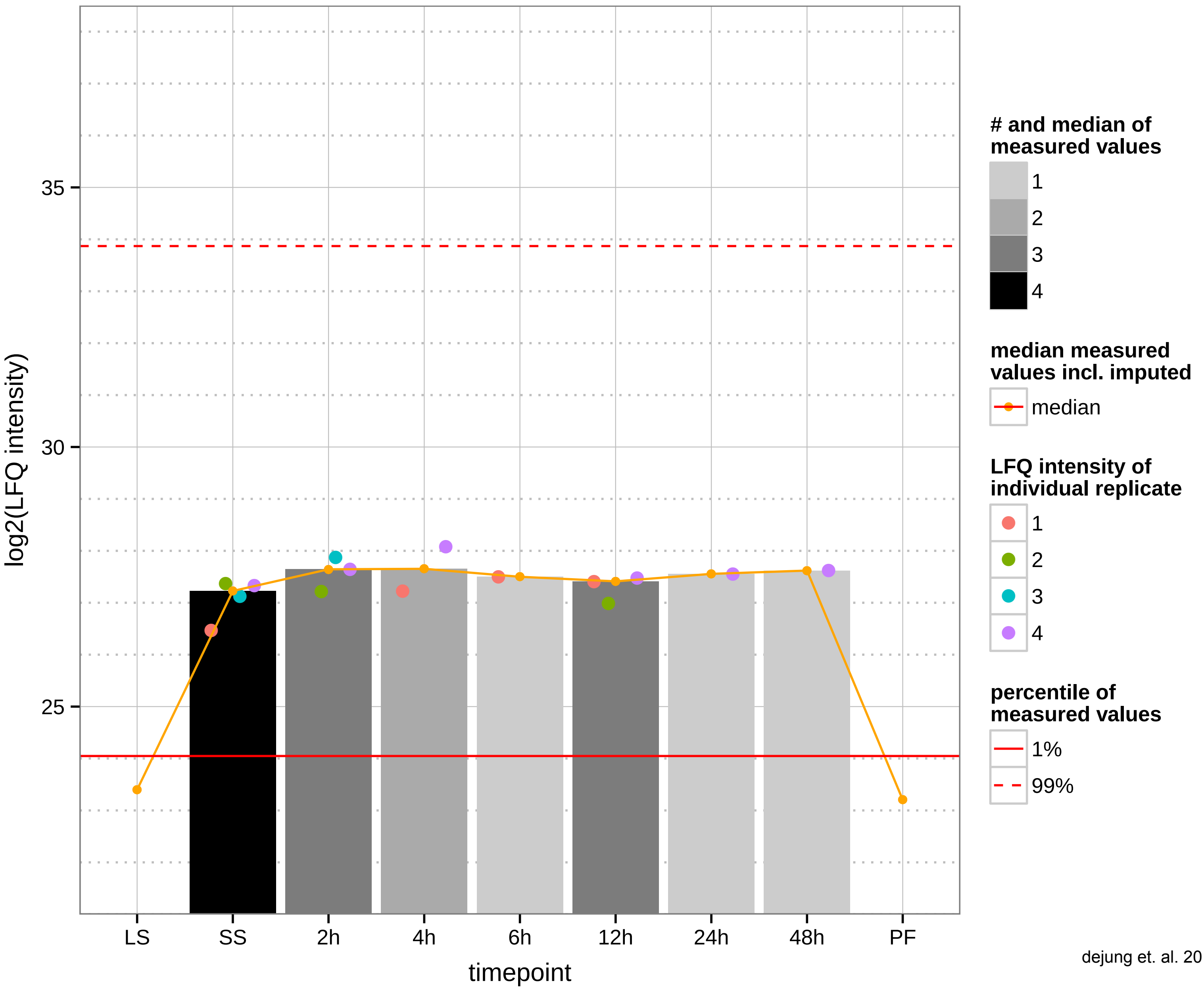

dejung et. al. 2015

chaperone protein DNAj, putative
Tb927.7.540
AGOF: heat shock protein binding, unfolded protein binding
AGOC: null
AGOP: protein folding
PGOF: heat shock protein binding
PGOC: null
PGOP: null

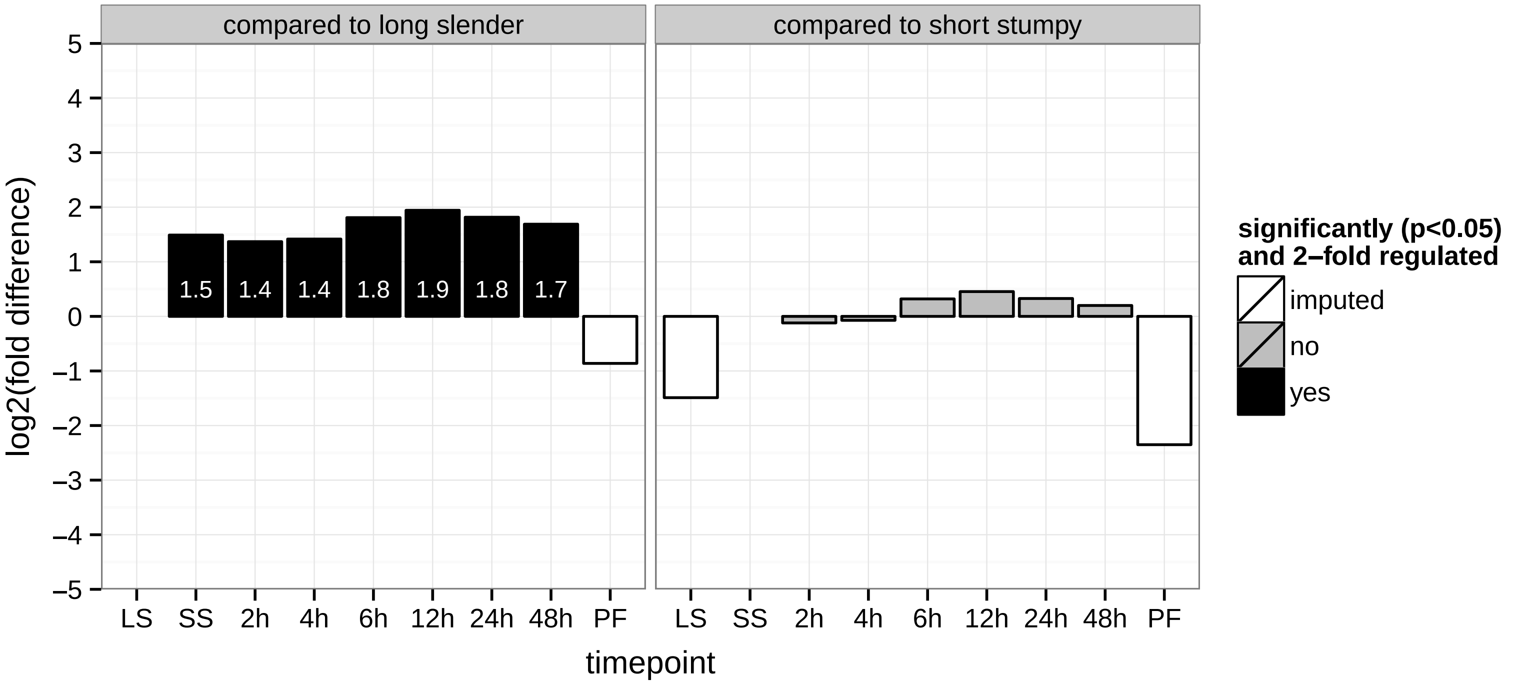

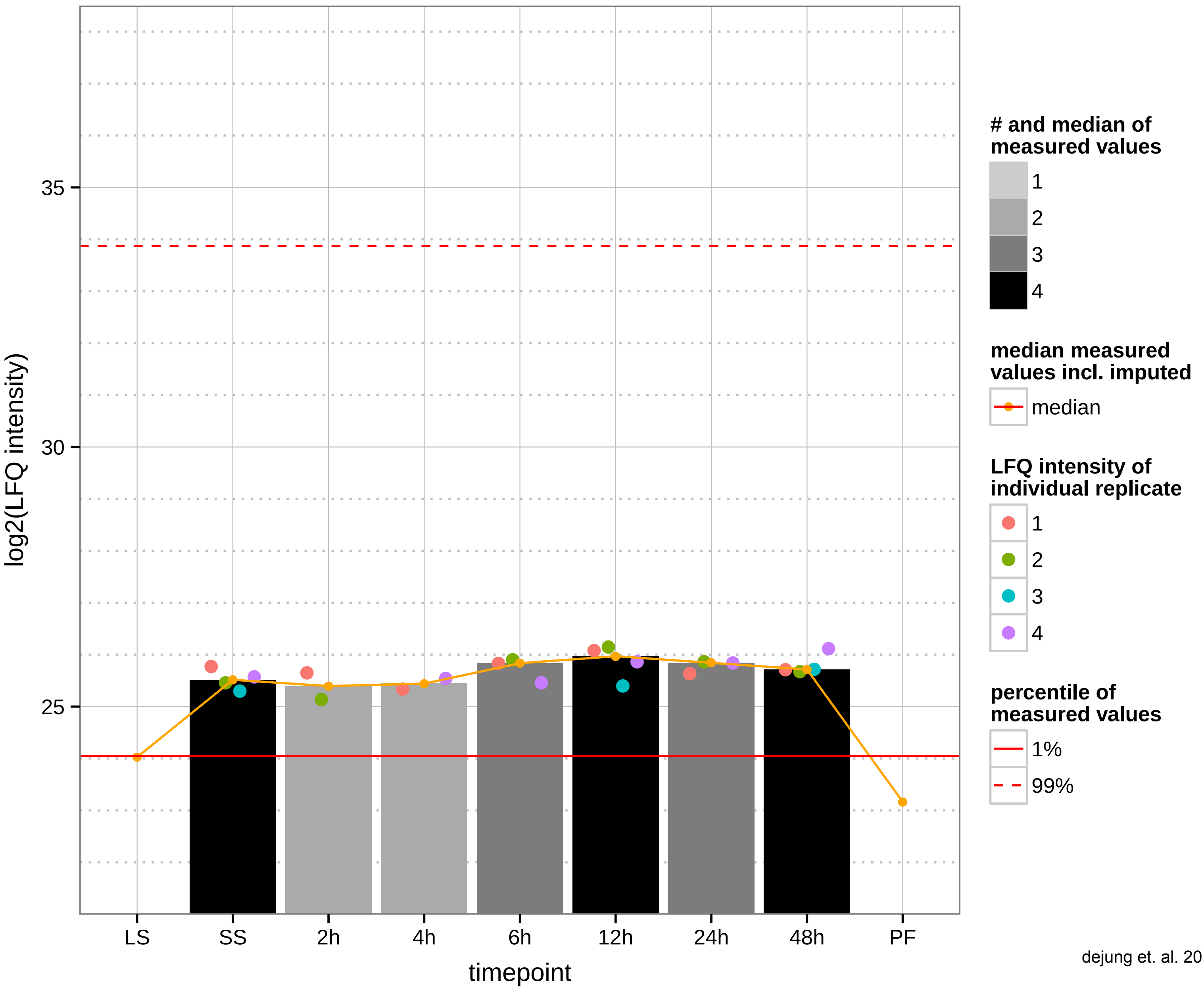

dejung et. al. 2015

protein disulfide isomerase, putative
Tb927.7.5790
AGOF: null
AGOC: endoplasmic reticulum
AGOP: cell redox homeostasis
PGOF: electron carrier activity, isomerase activity, protein disulfide oxidoreductase activity
PGOC: null
PGOP: cell redox homeostasis, glycerol ether metabolic process

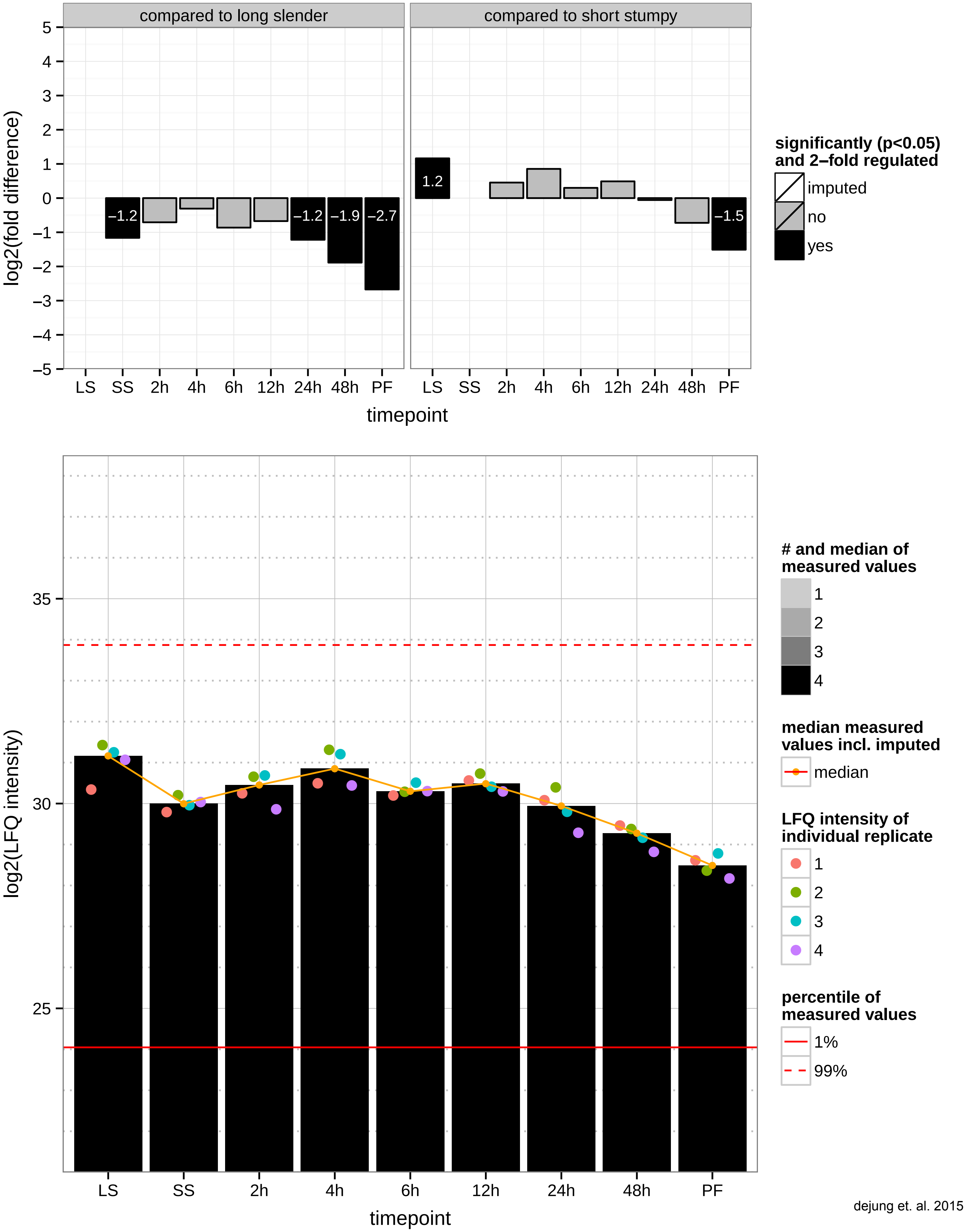

dejung et. al. 2015

hypothetical protein, conserved
Tb927.7.5850
AGOF: null
AGOC: null
AGOP: null
PGOF: null
PGOC: null
PGOP: null

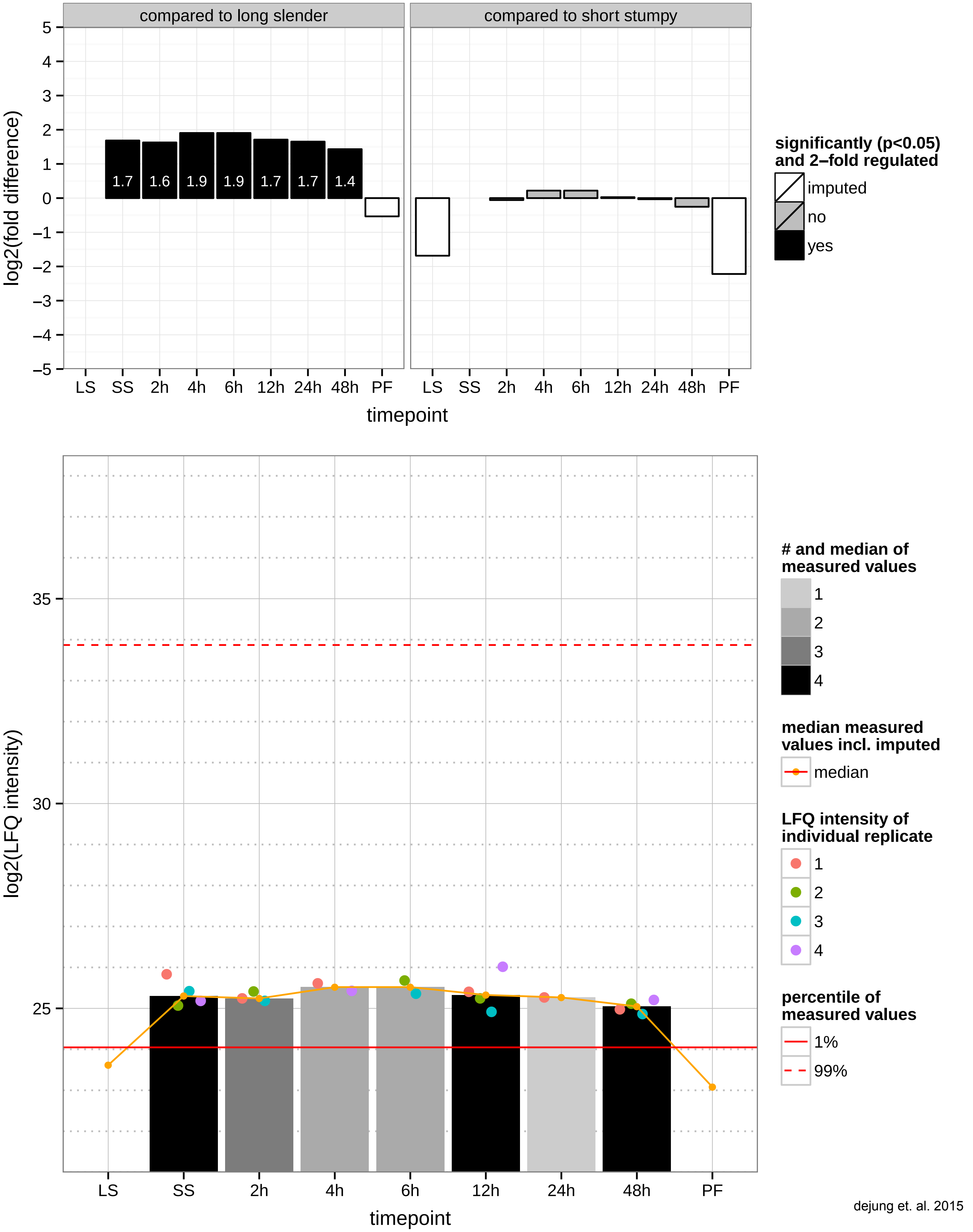

dejung et. al. 2015

hypothetical protein, conserved
Tb927.7.6340
AGOF: null
AGOC: null
AGOP: null
PGOF: null
PGOC: null
PGOP: null

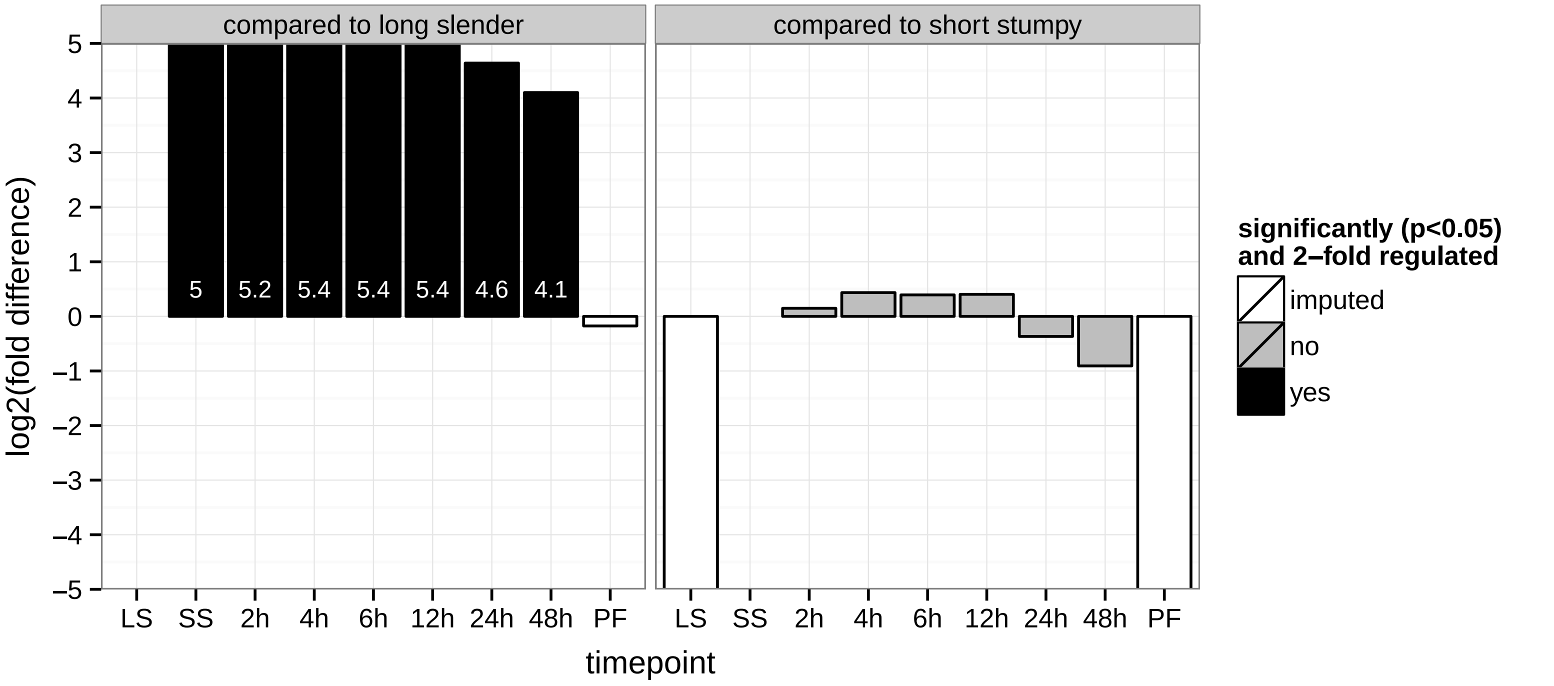

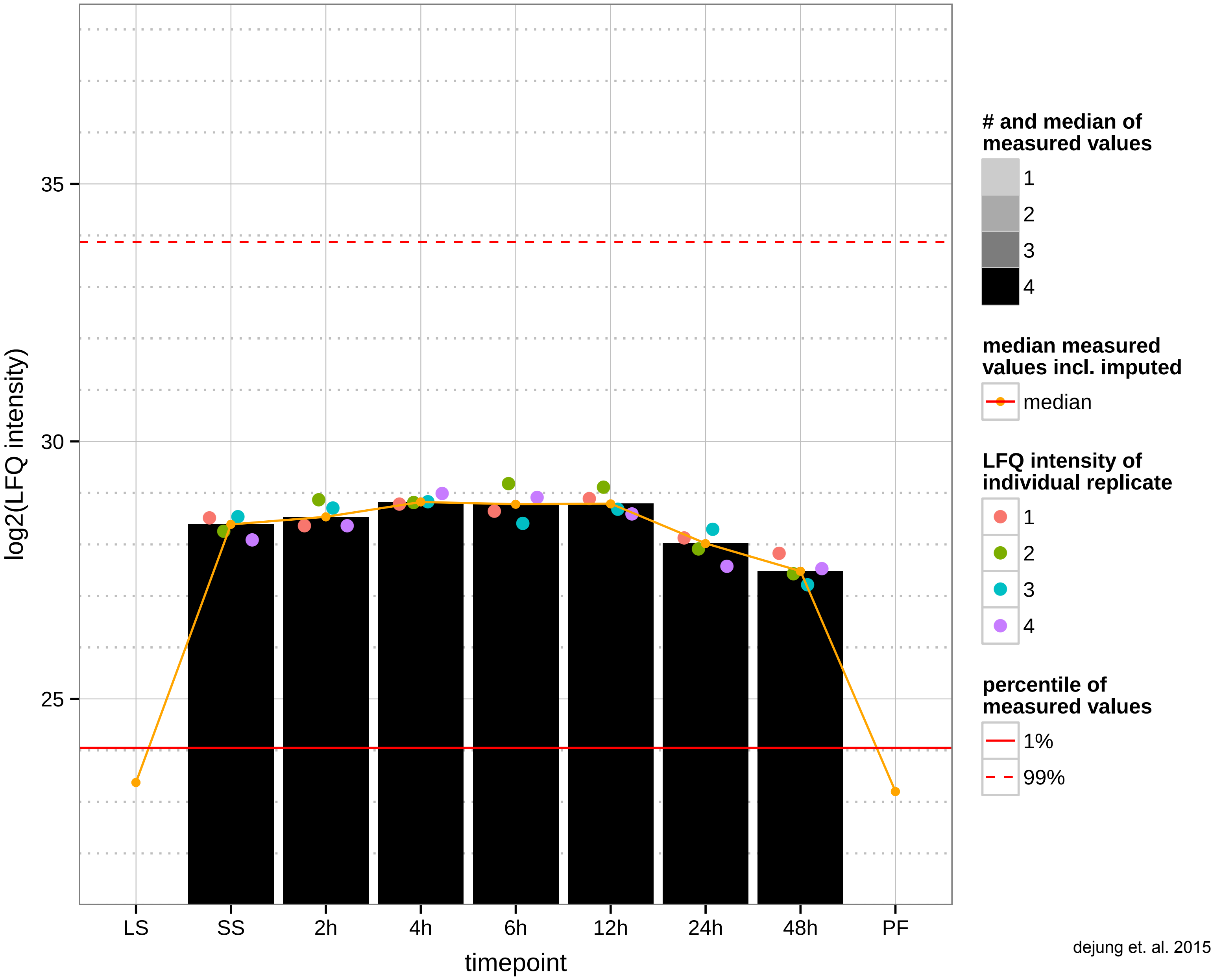

dejung et. al. 2015

hypothetical protein, conserved
Tb927.7.7400
AGOF: null
AGOC: null
AGOP: null
PGOF: null
PGOC: null
PGOP: null

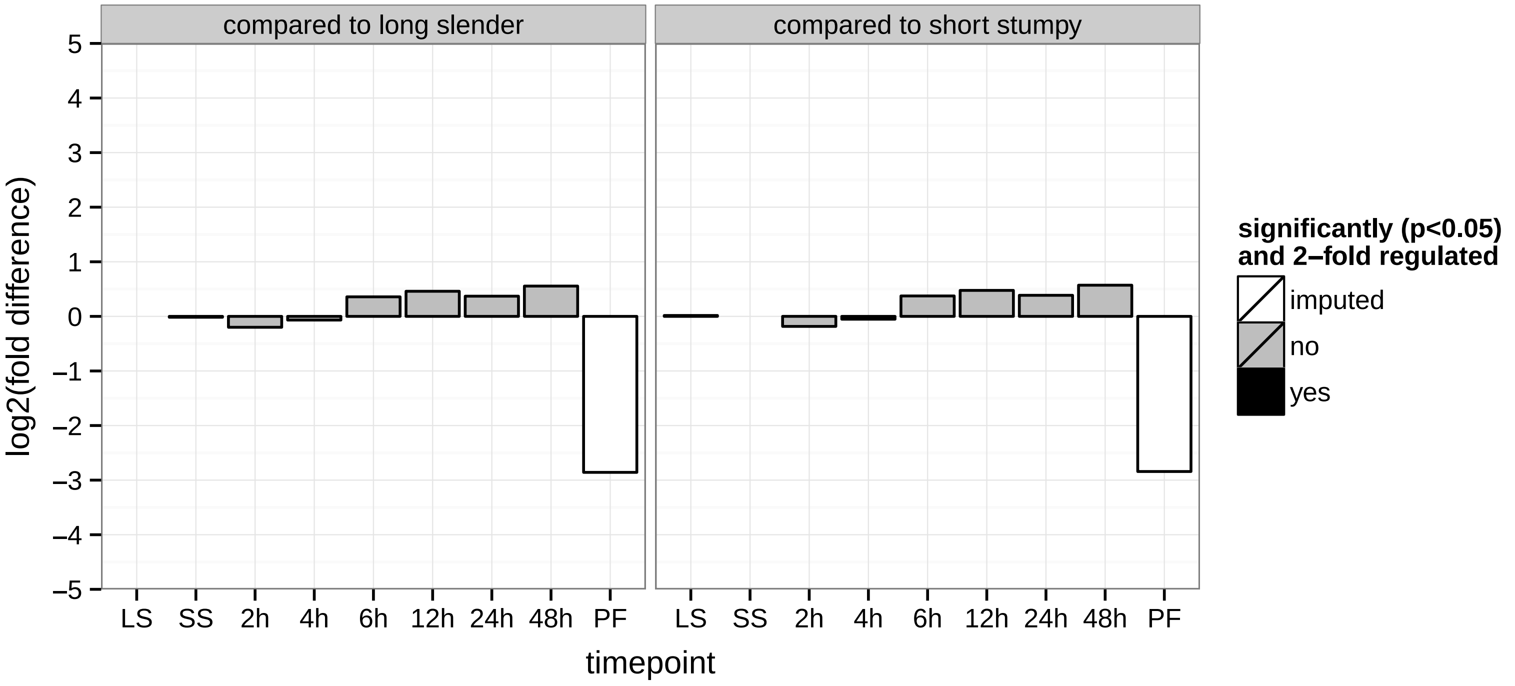

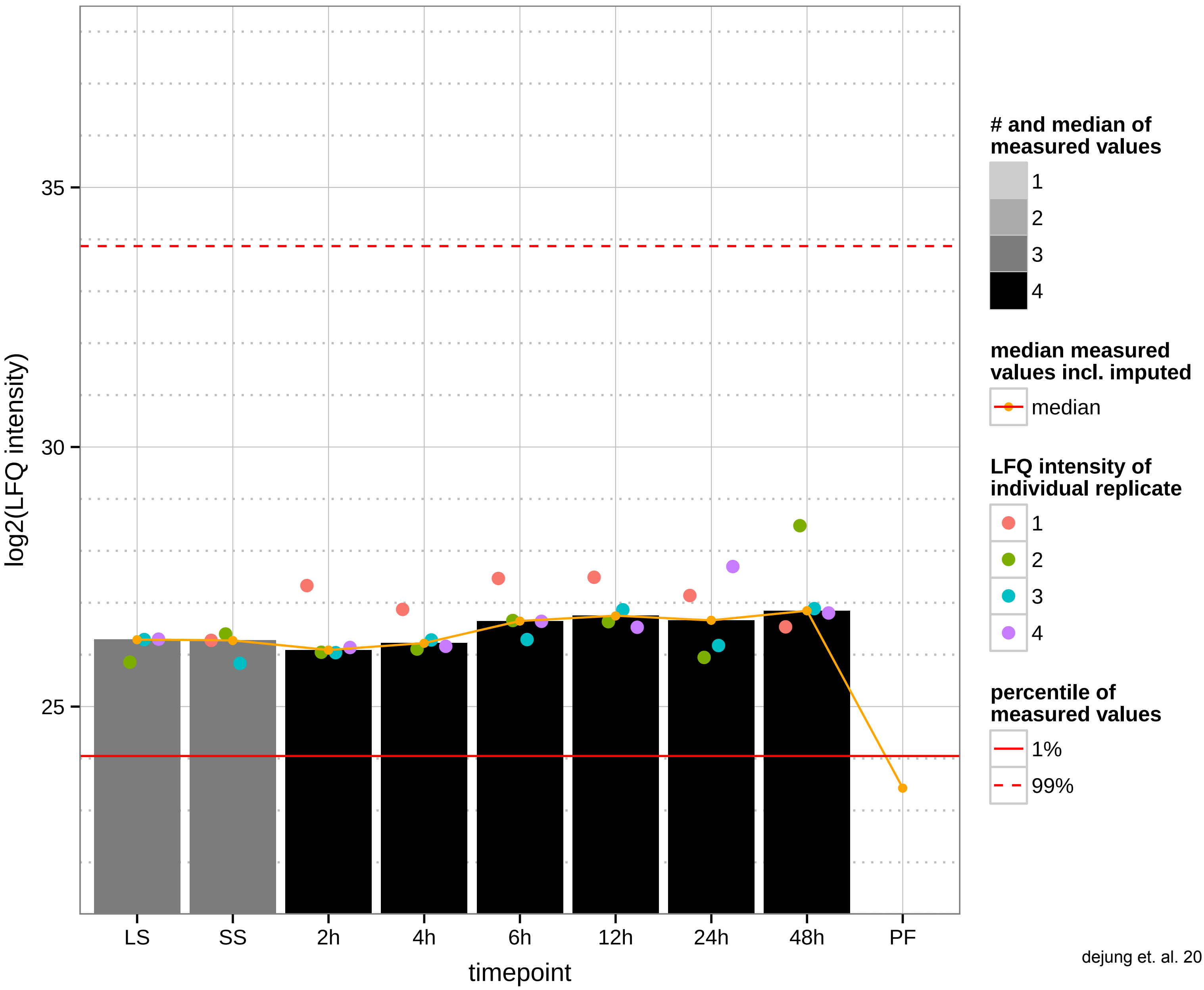

dejung et. al. 2015

hypothetical protein, conserved
Tb927.8.1140
AGOF: null
AGOC: null
AGOP: null
PGOF: null
PGOC: null
PGOP: null

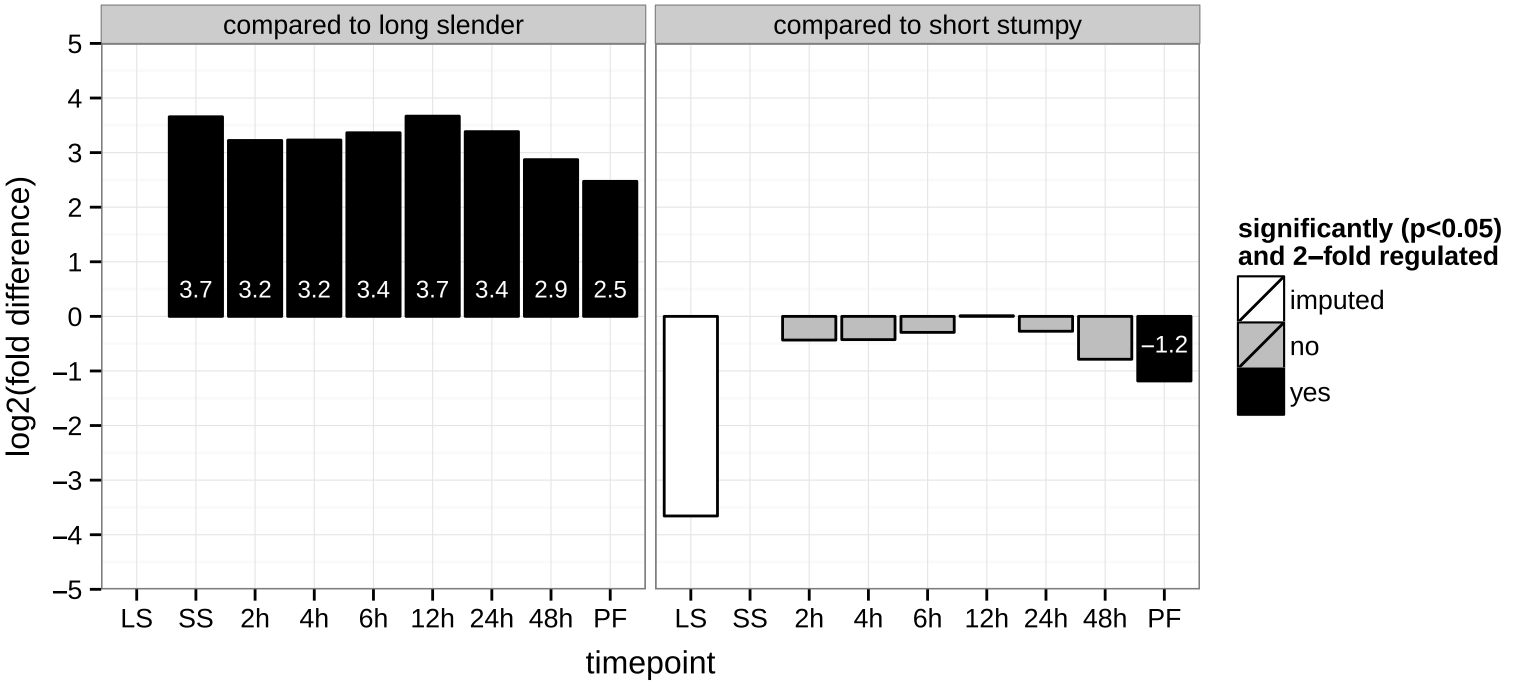

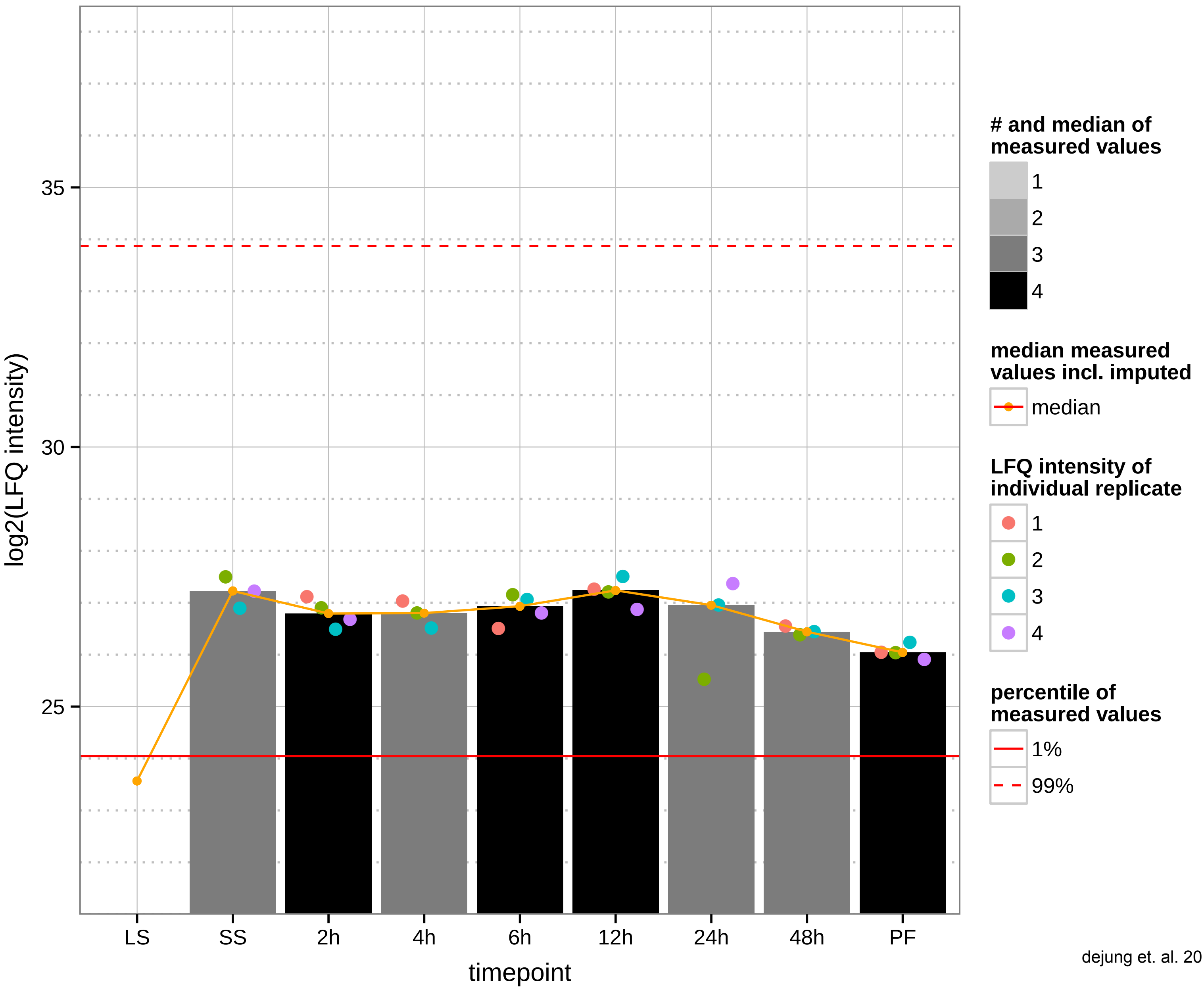

dejung et. al. 2015

hypothetical protein, conserved
Tb927.8.1290
AGOF: null
AGOC: null
AGOP: null
PGOF: null
PGOC: null
PGOP: null

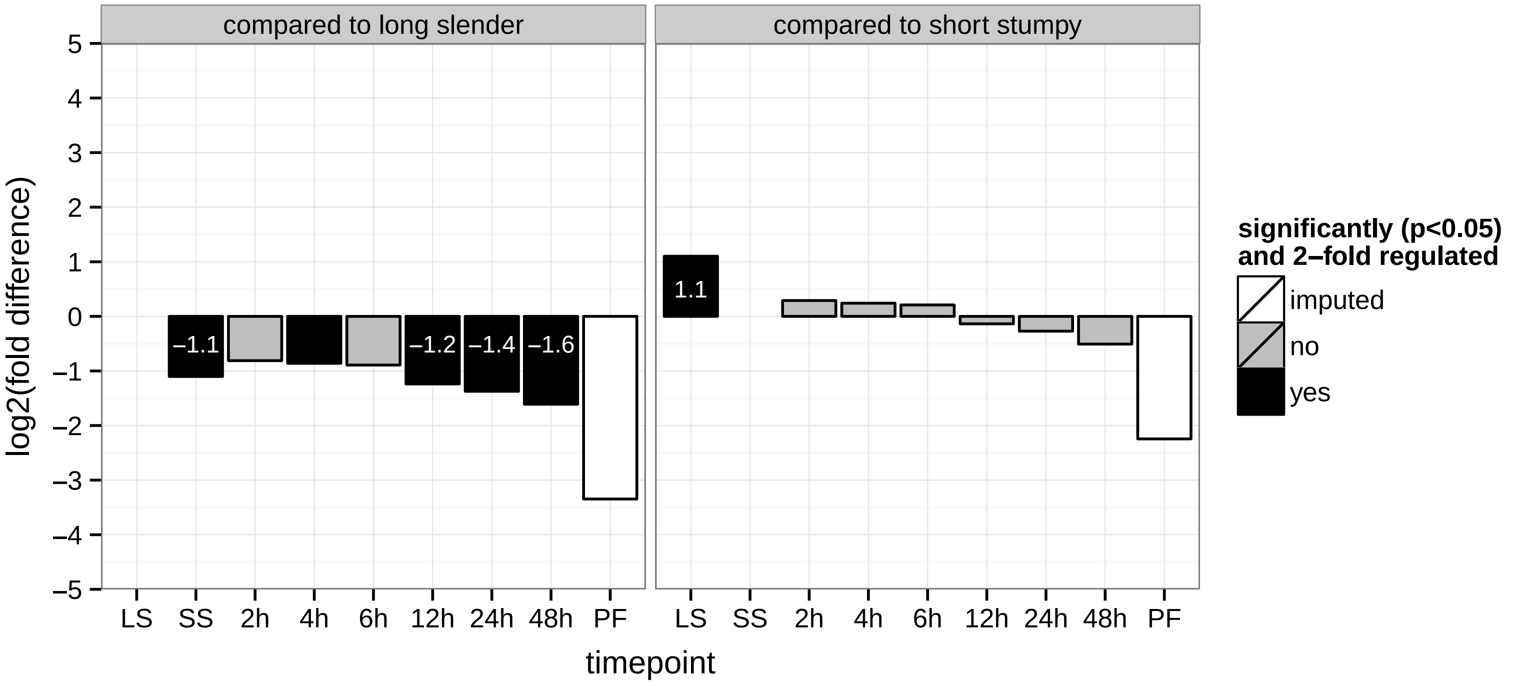

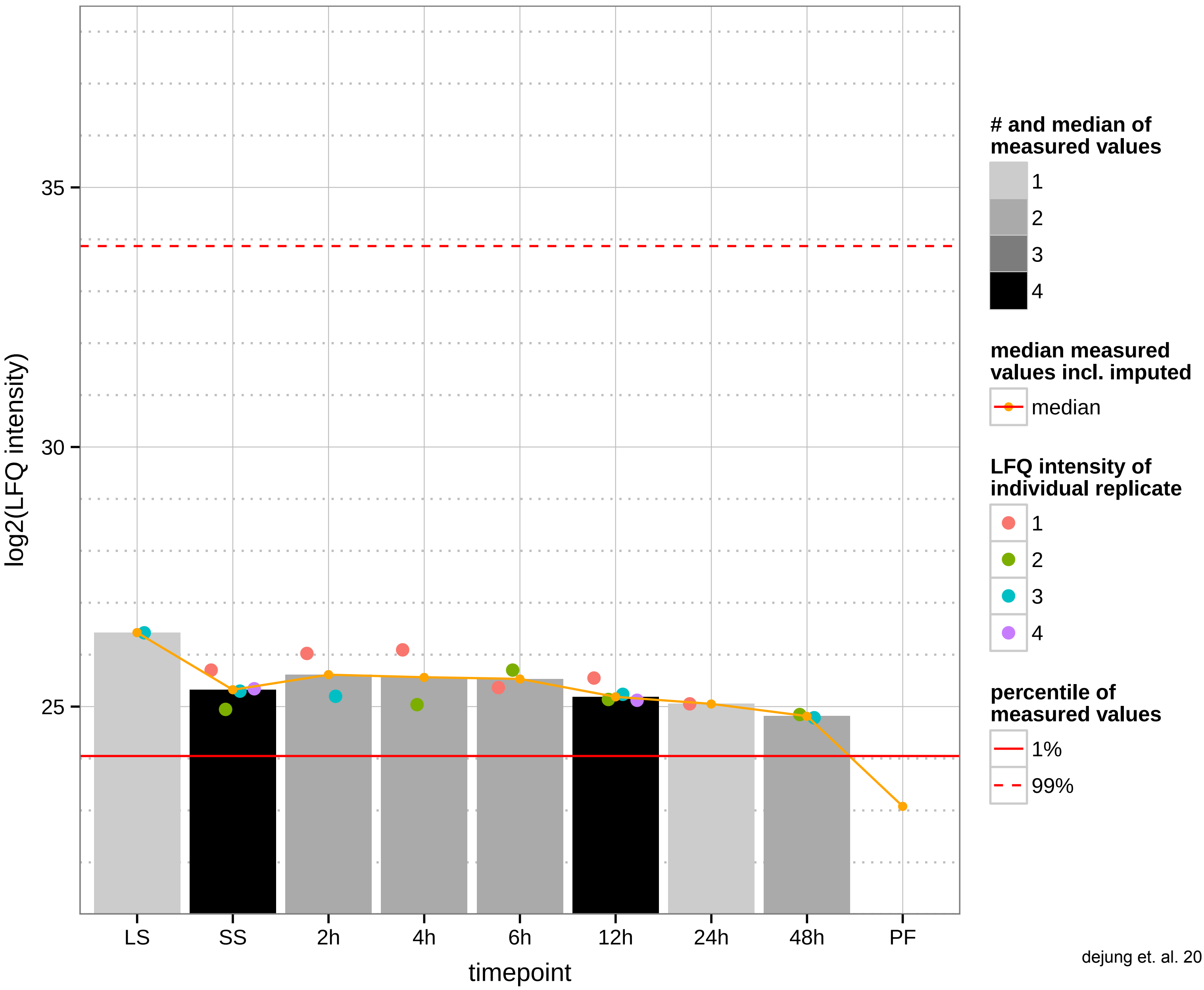

dejung et. al. 2015

hypothetical protein, conserved
Tb927.8.1320
AGOF: null
AGOC: null
AGOP: null
PGOF: null
PGOC: null
PGOP: null

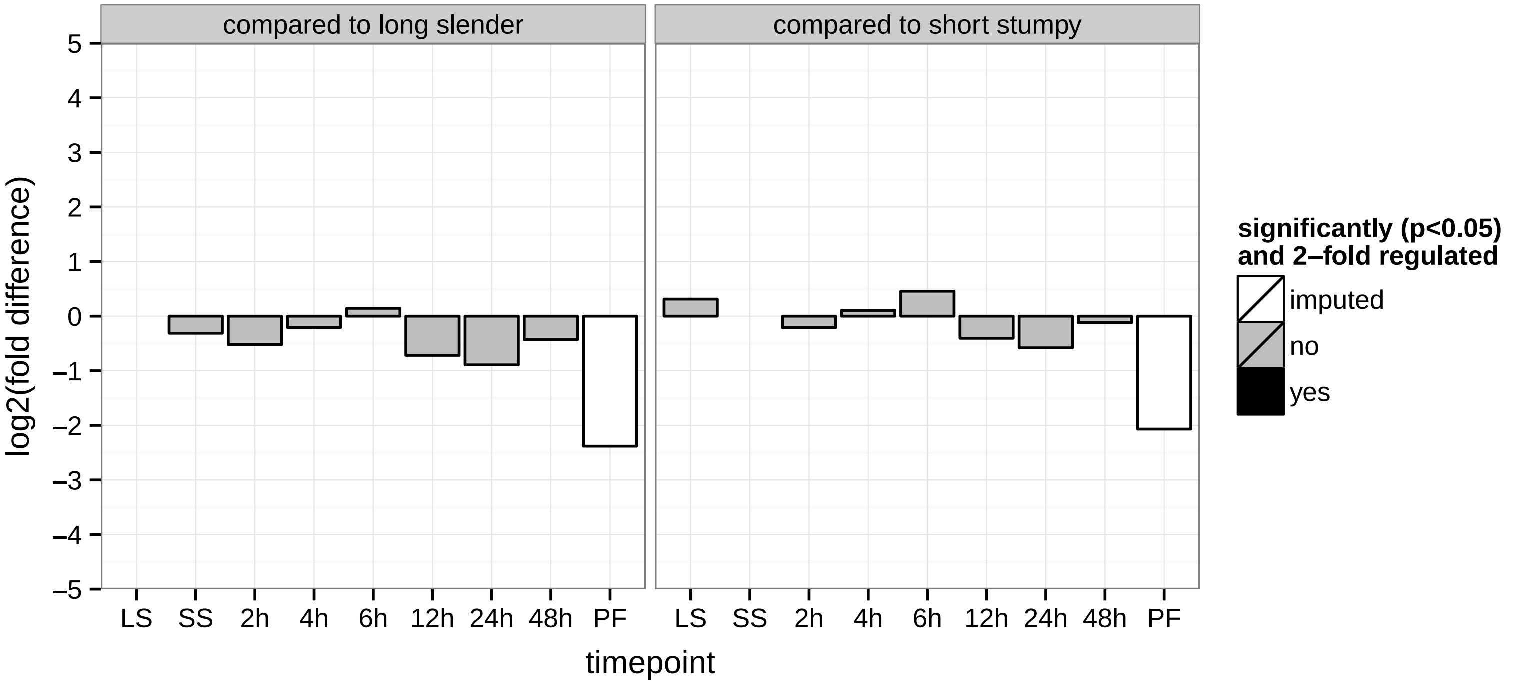

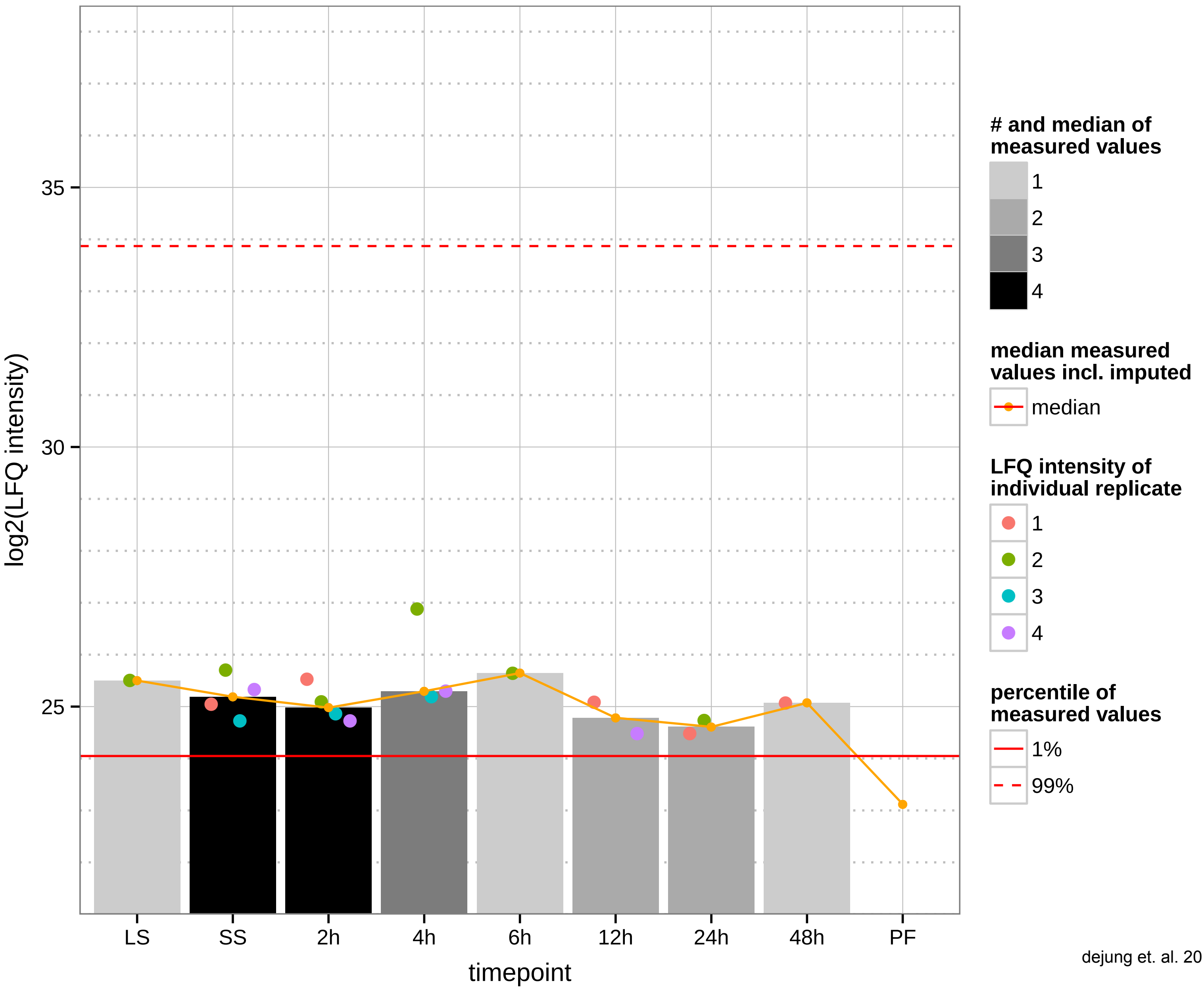

dejung et. al. 2015

hypothetical protein, conserved
Tb927.8.1360
AGOF: null
AGOC: null
AGOP: null
PGOF: null
PGOC: null
PGOP: null

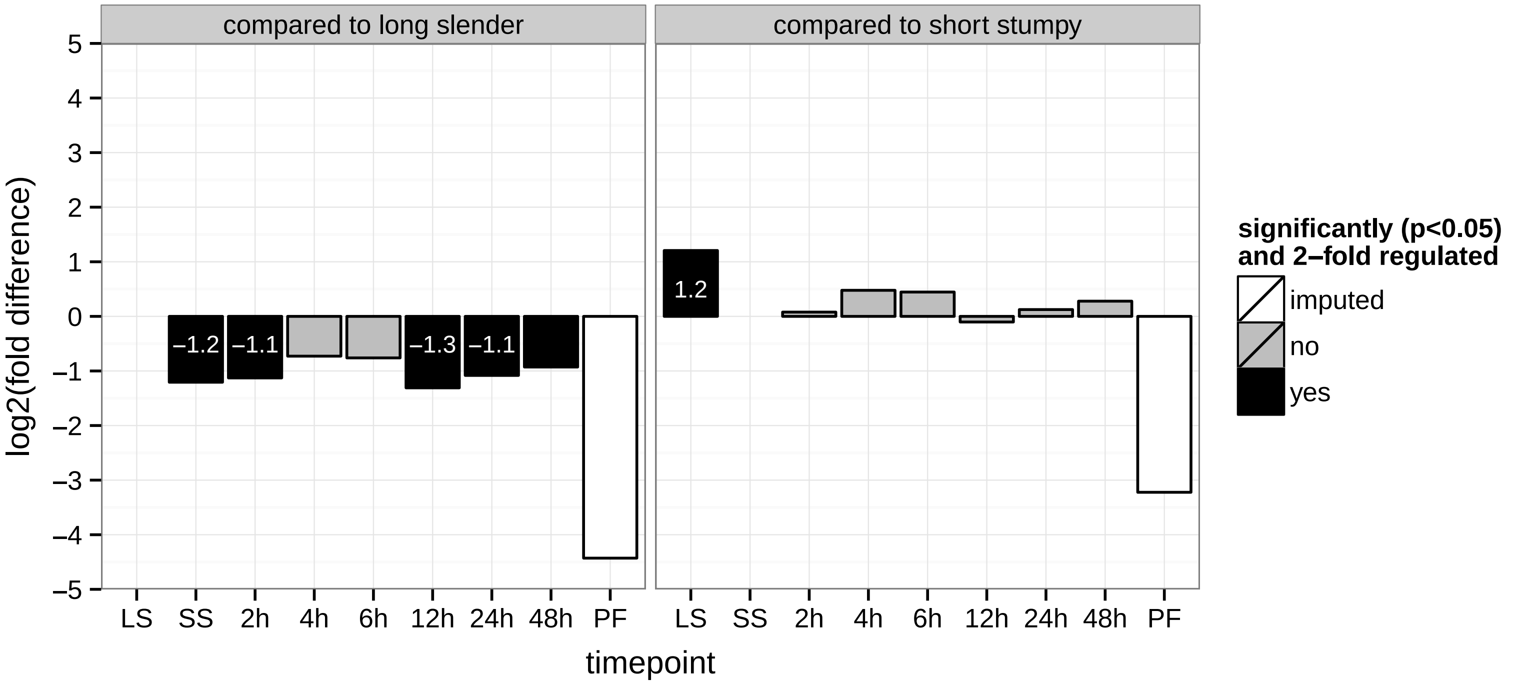

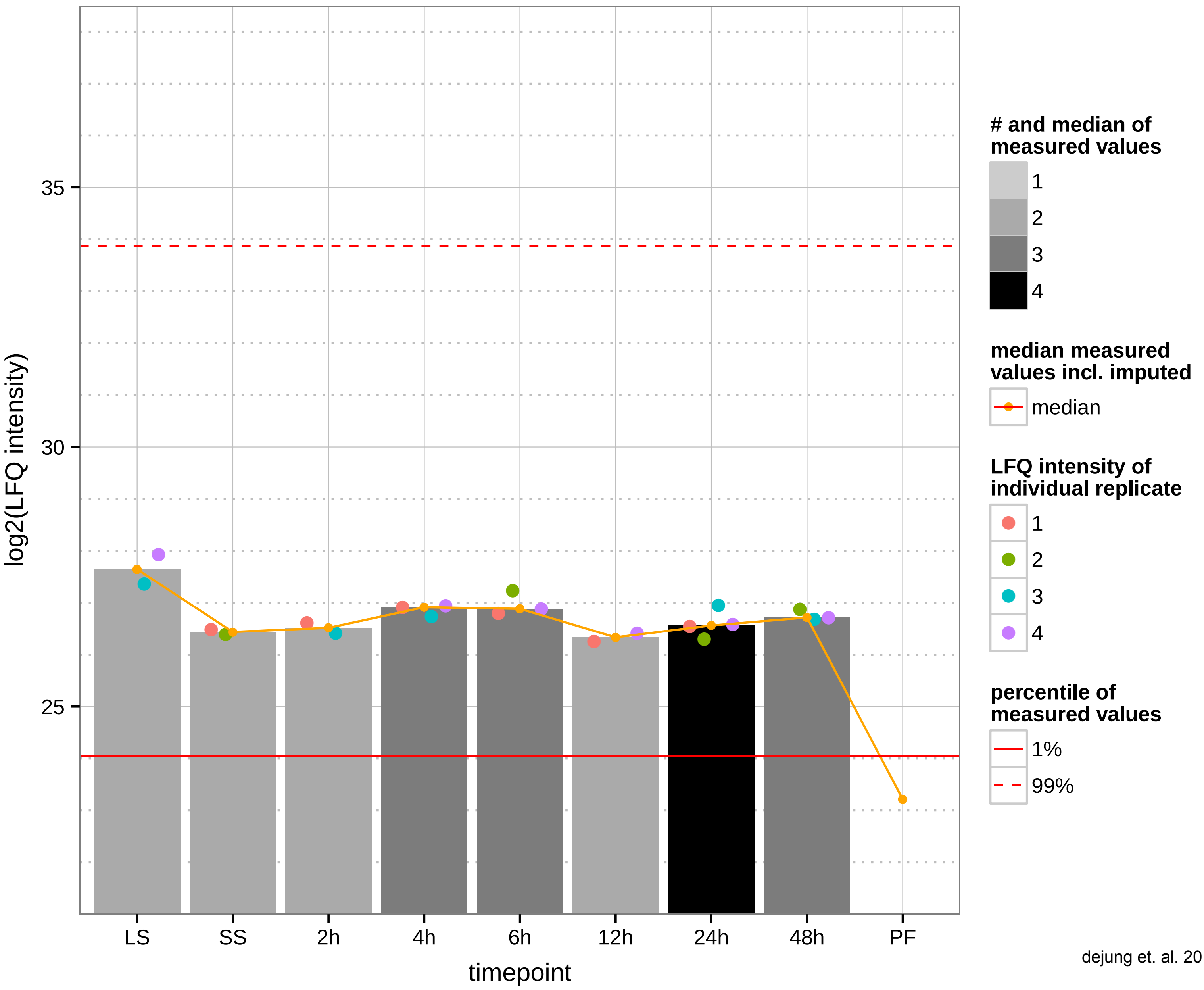

dejung et. al. 2015

hypothetical protein, conserved
Tb927.8.1450
AGOF: null
AGOC: null
AGOP: null
PGOF: null
PGOC: null
PGOP: null

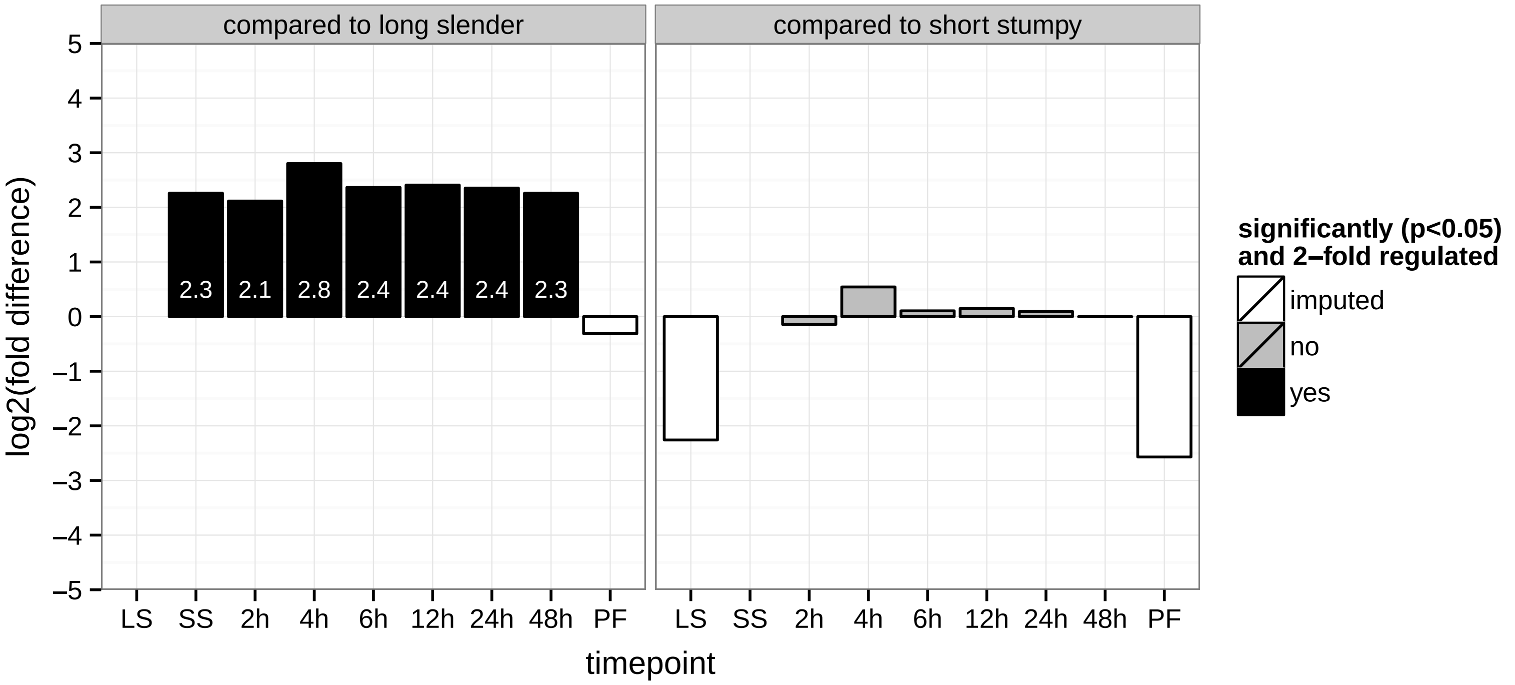

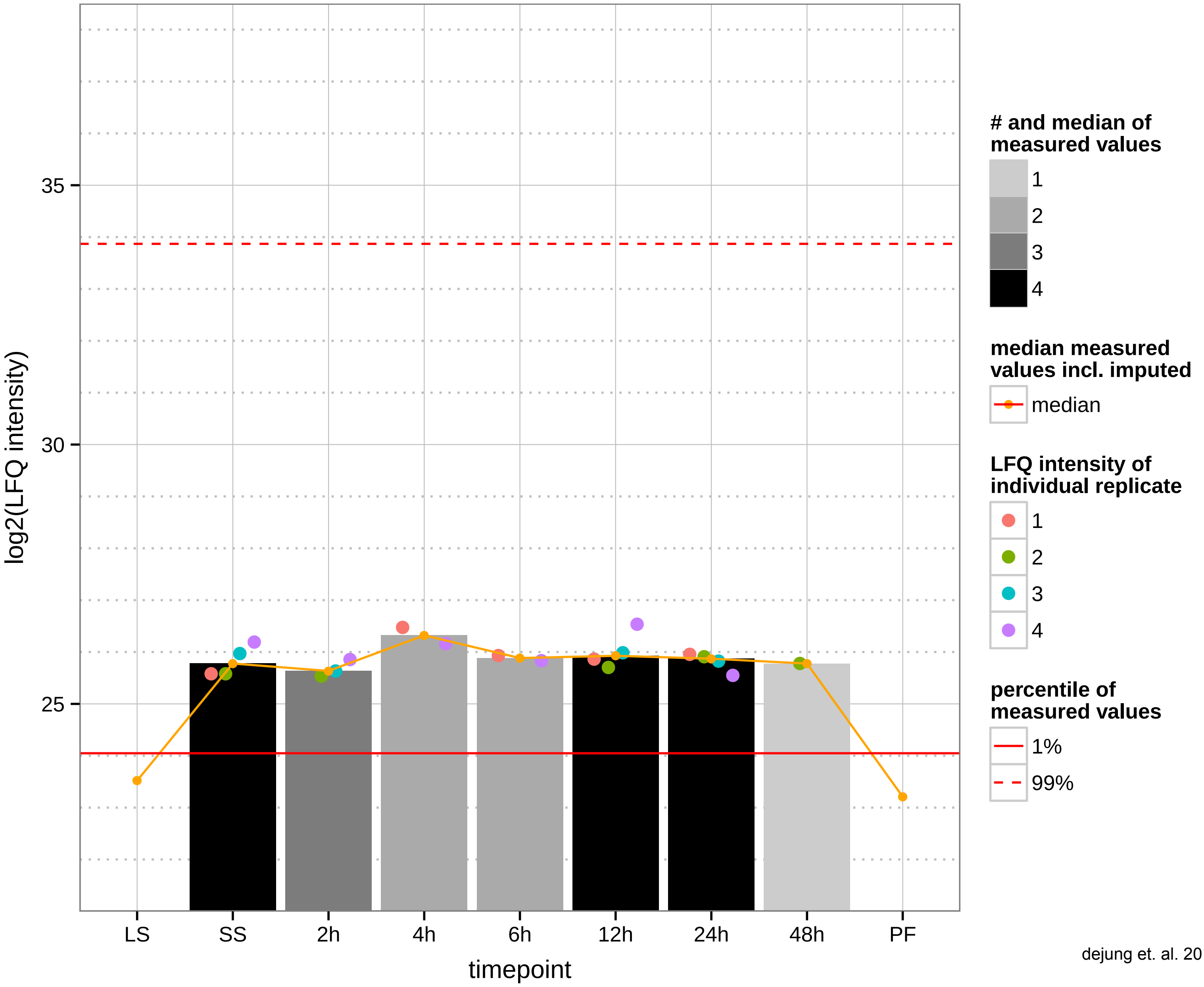

dejung et. al. 2015

hypothetical protein, conserved
Tb927.8.1560
AGOF: null
AGOC: null
AGOP: null
PGOF: protein binding
PGOC: null
PGOP: null

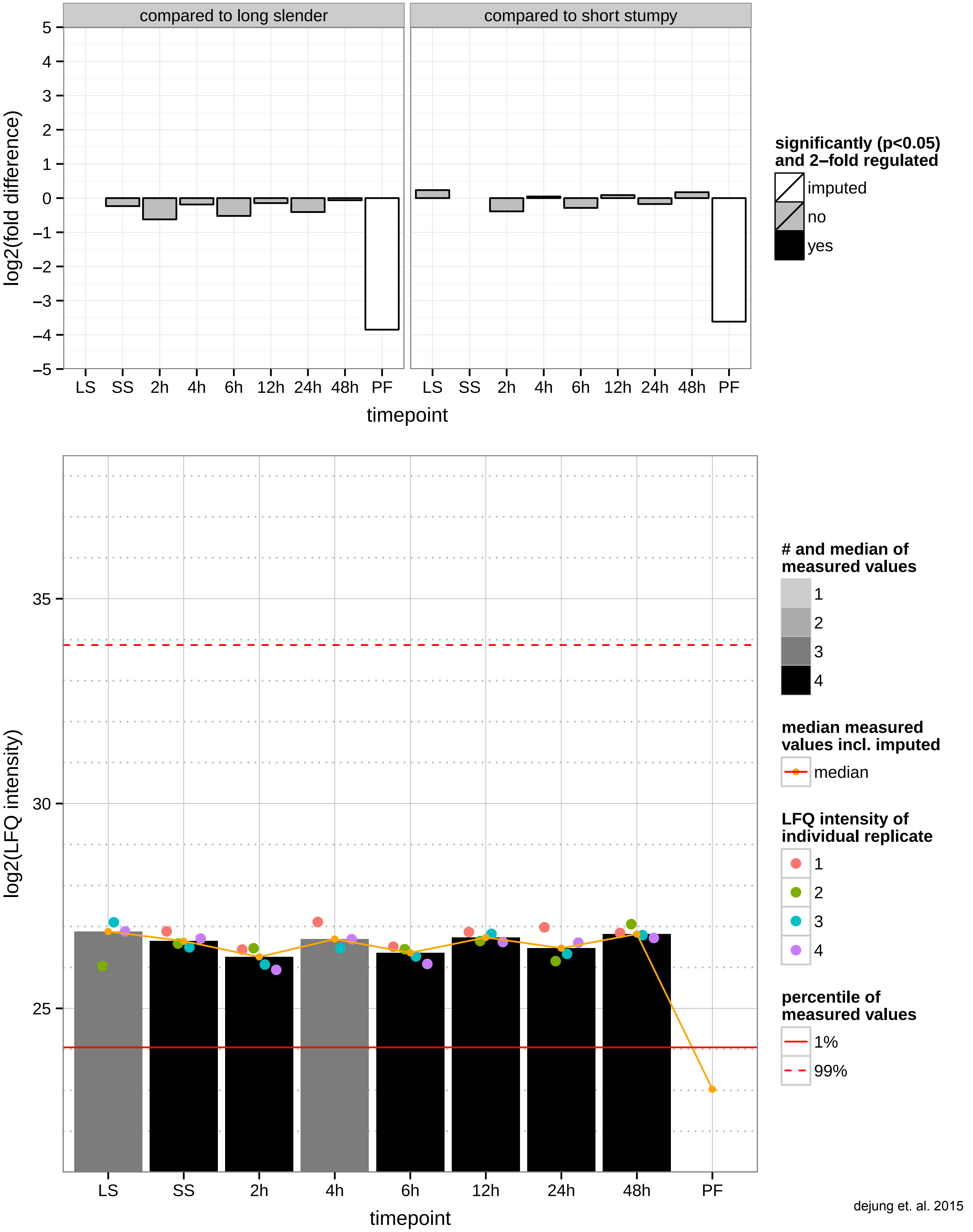

dejung et. al. 2015

rhomboid–like protein, serine peptidase, Clan S–, family S54, putative
Tb927.8.1810
AGOF: null
AGOC: integral to membrane
AGOP: null
PGOF: null
PGOC: null
PGOP: null

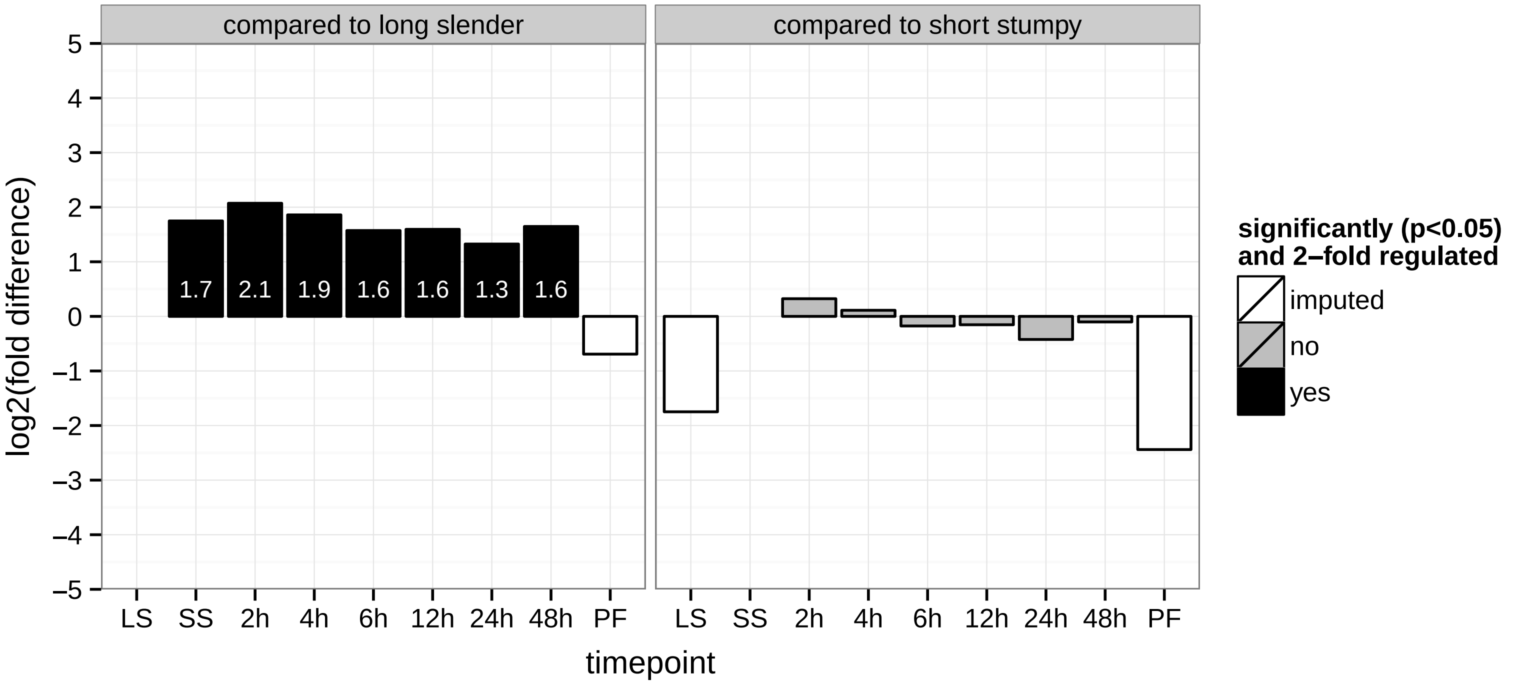

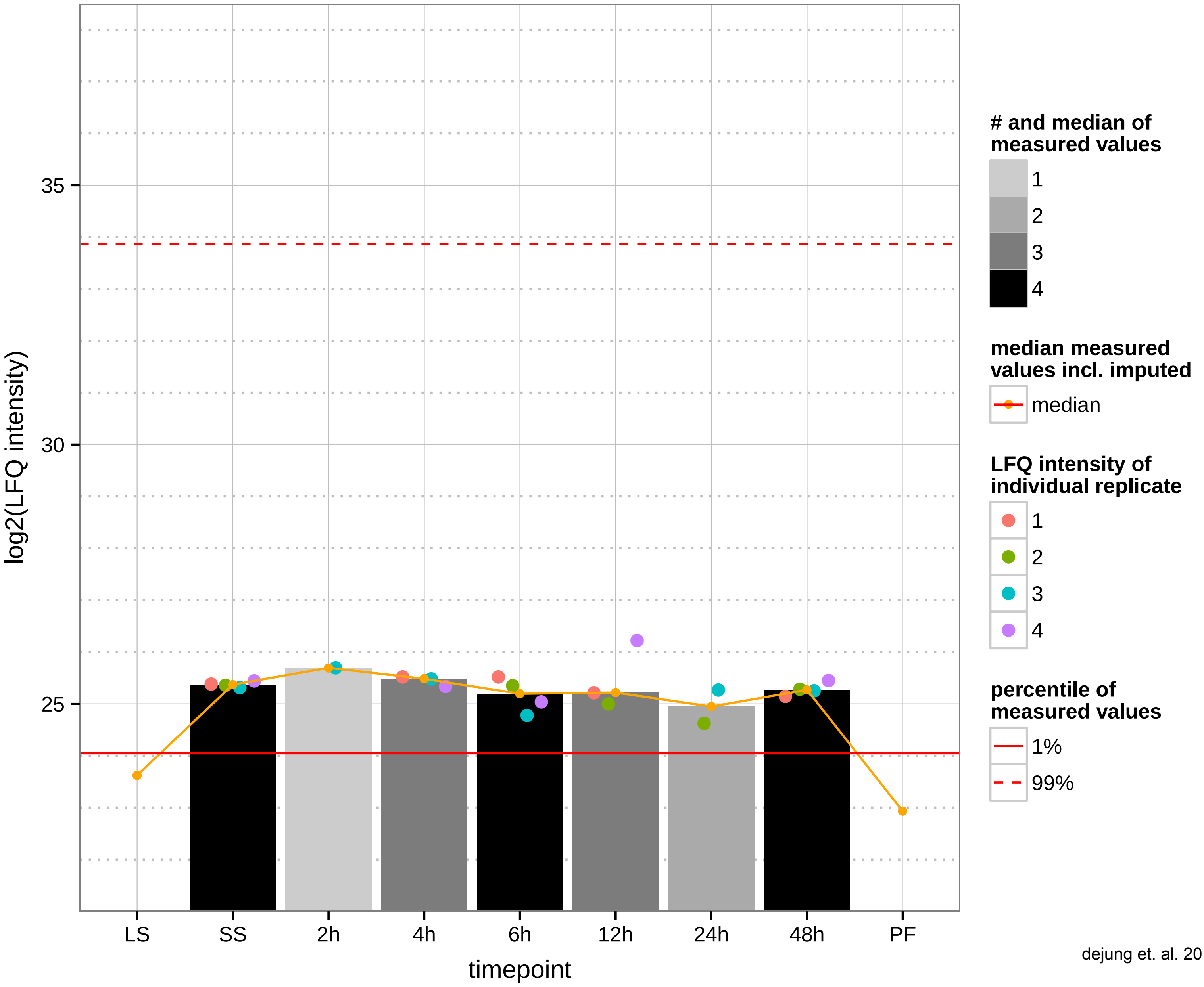

dejung et. al. 2015

hypothetical protein, conserved
Tb927.8.2490
AGOF: null
AGOC: null
AGOP: null
PGOF: protein binding
PGOC: null
PGOP: null

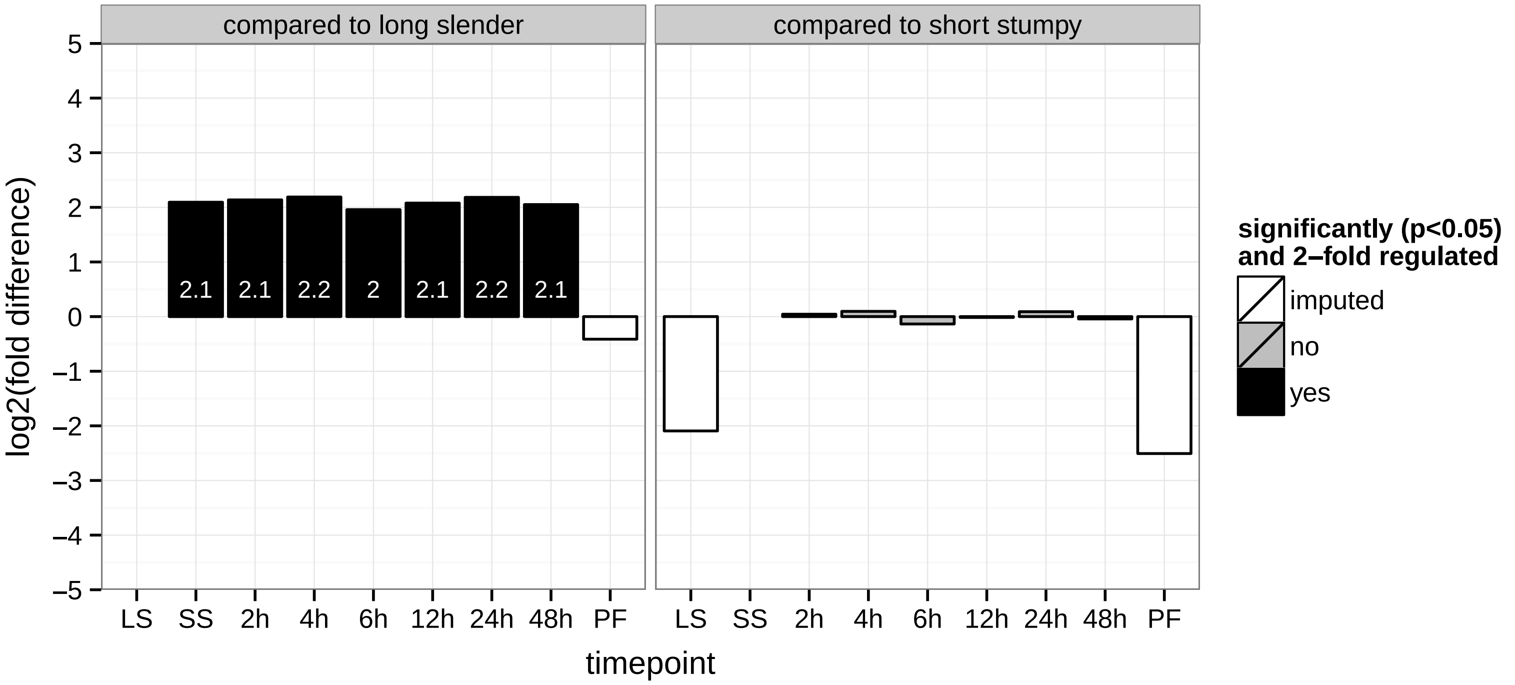

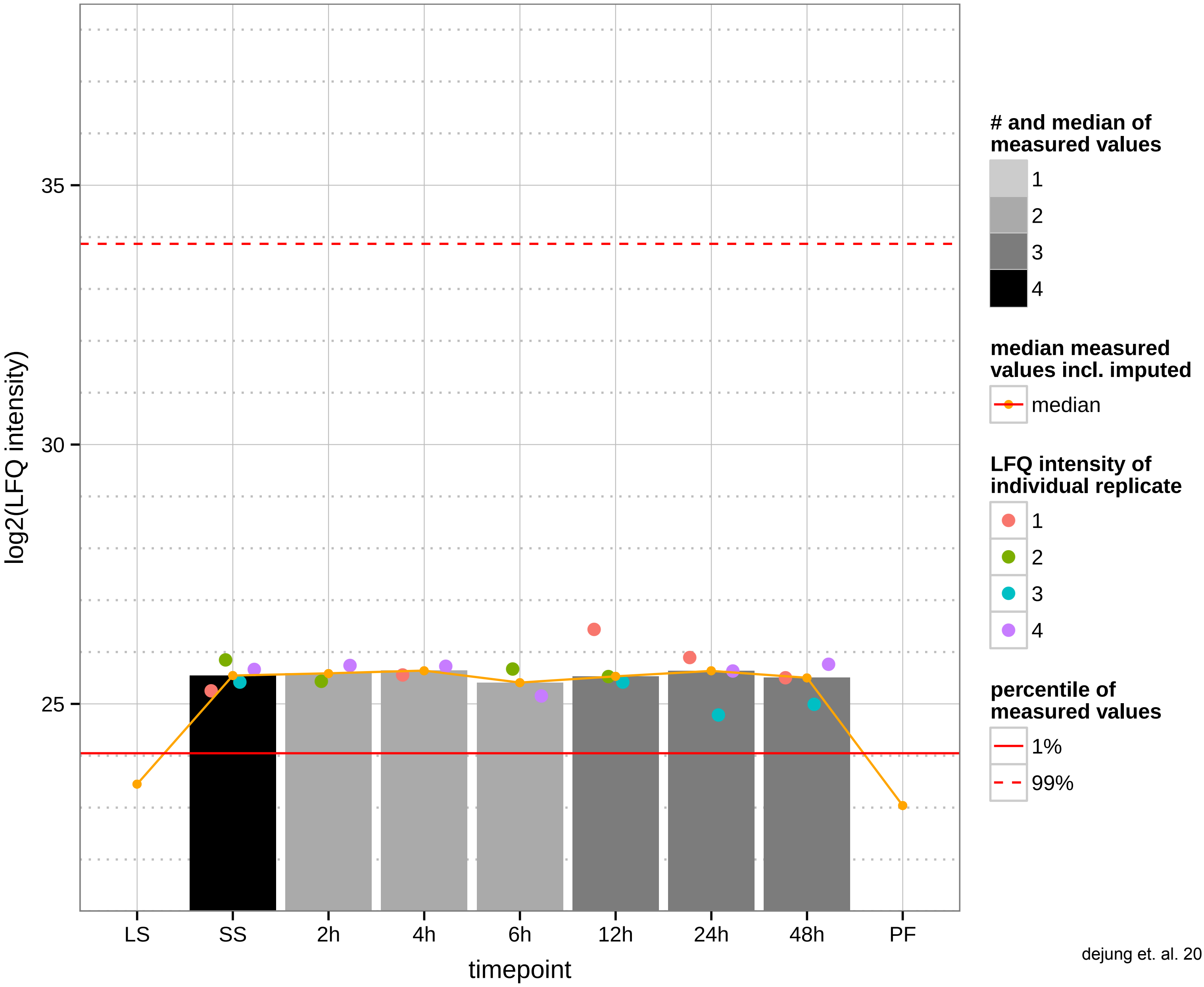

dejung et. al. 2015

spliceosomal U5 snRNP−specific protein, putative
Tb927.8.2560
AGOF: null
AGOC: nucleus, spliceosomal complex
AGOP: RNA splicing, mitosis, nuclear migration along microfilament
PGOF: null
PGOC: spliceosomal complex
PGOP: mitosis

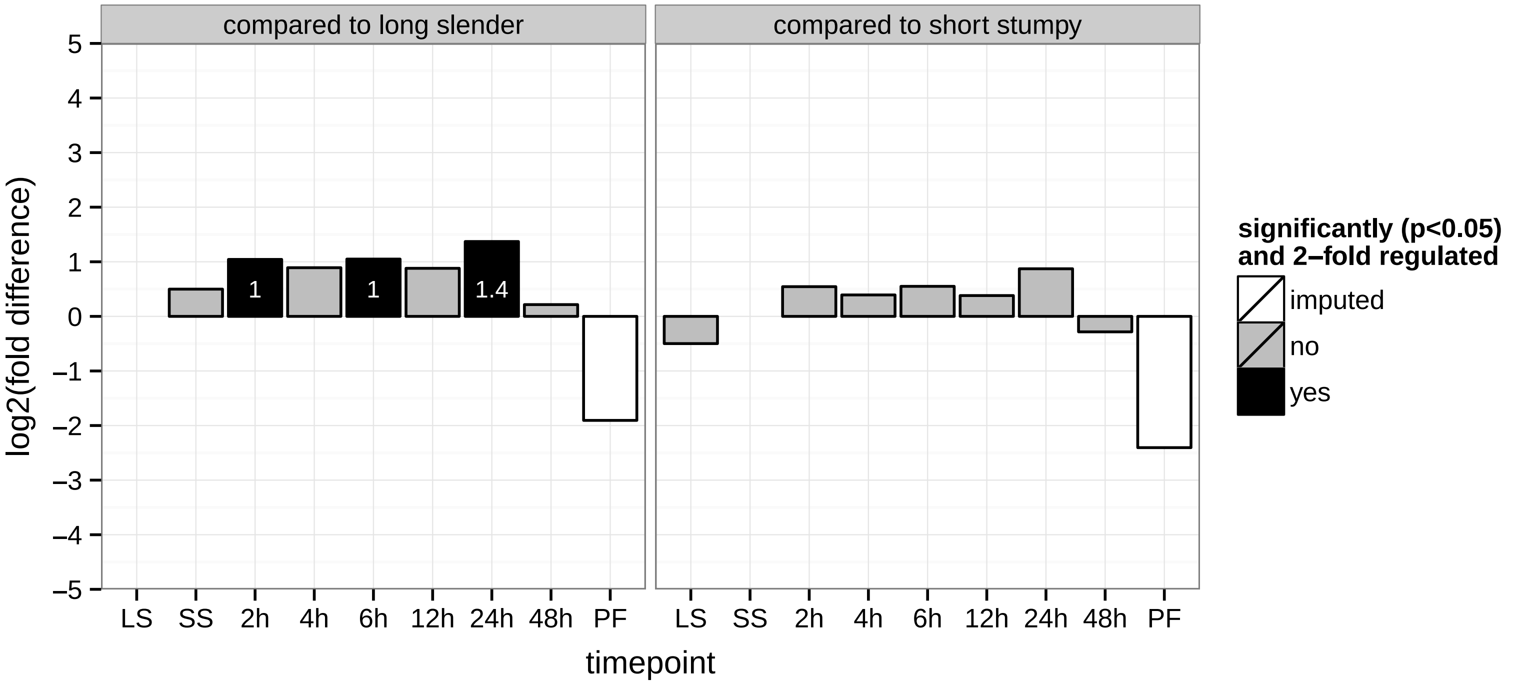

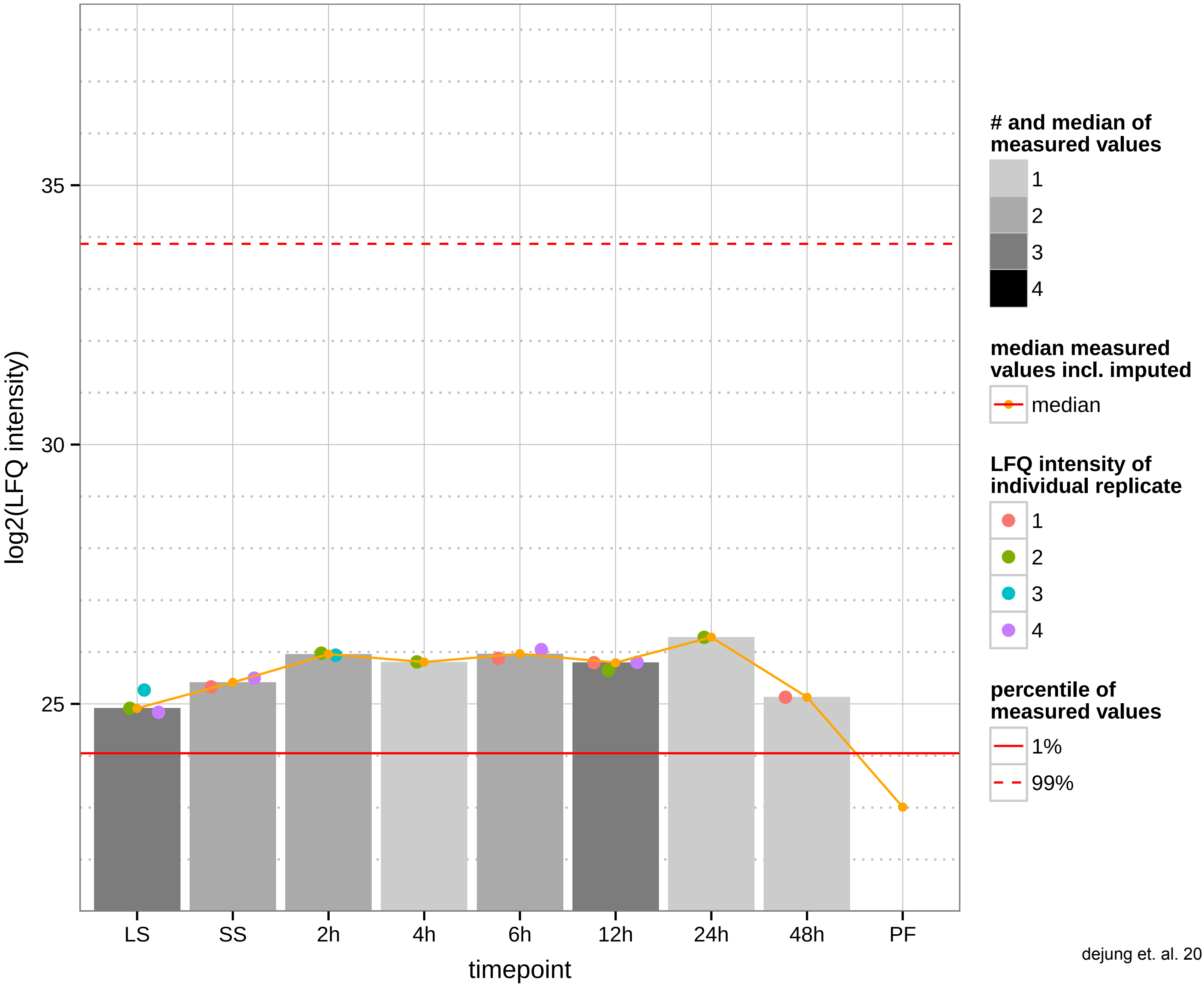

dejung et. al. 2015

hypothetical protein, conserved
Tb927.8.2660
AGOF: null
AGOC: null
AGOP: null
PGOF: null
PGOC: null
PGOP: null

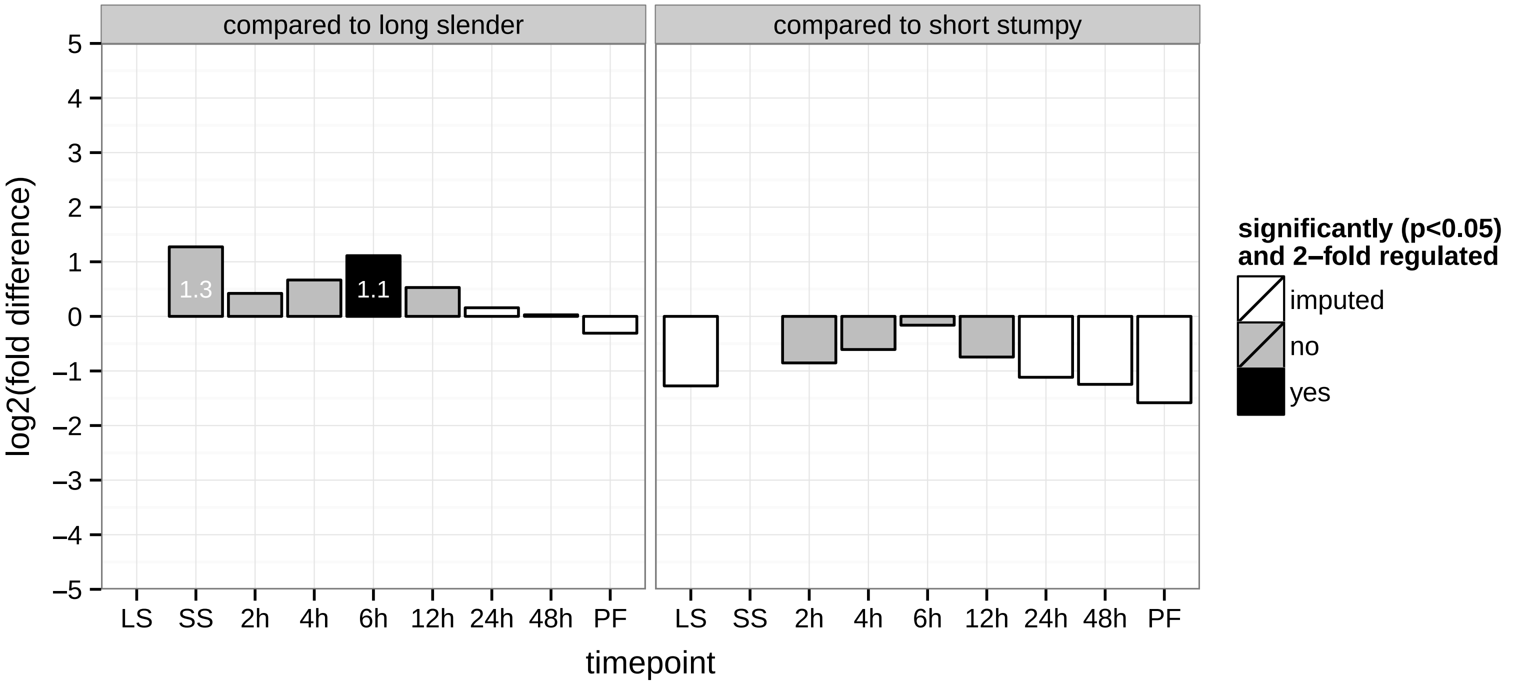

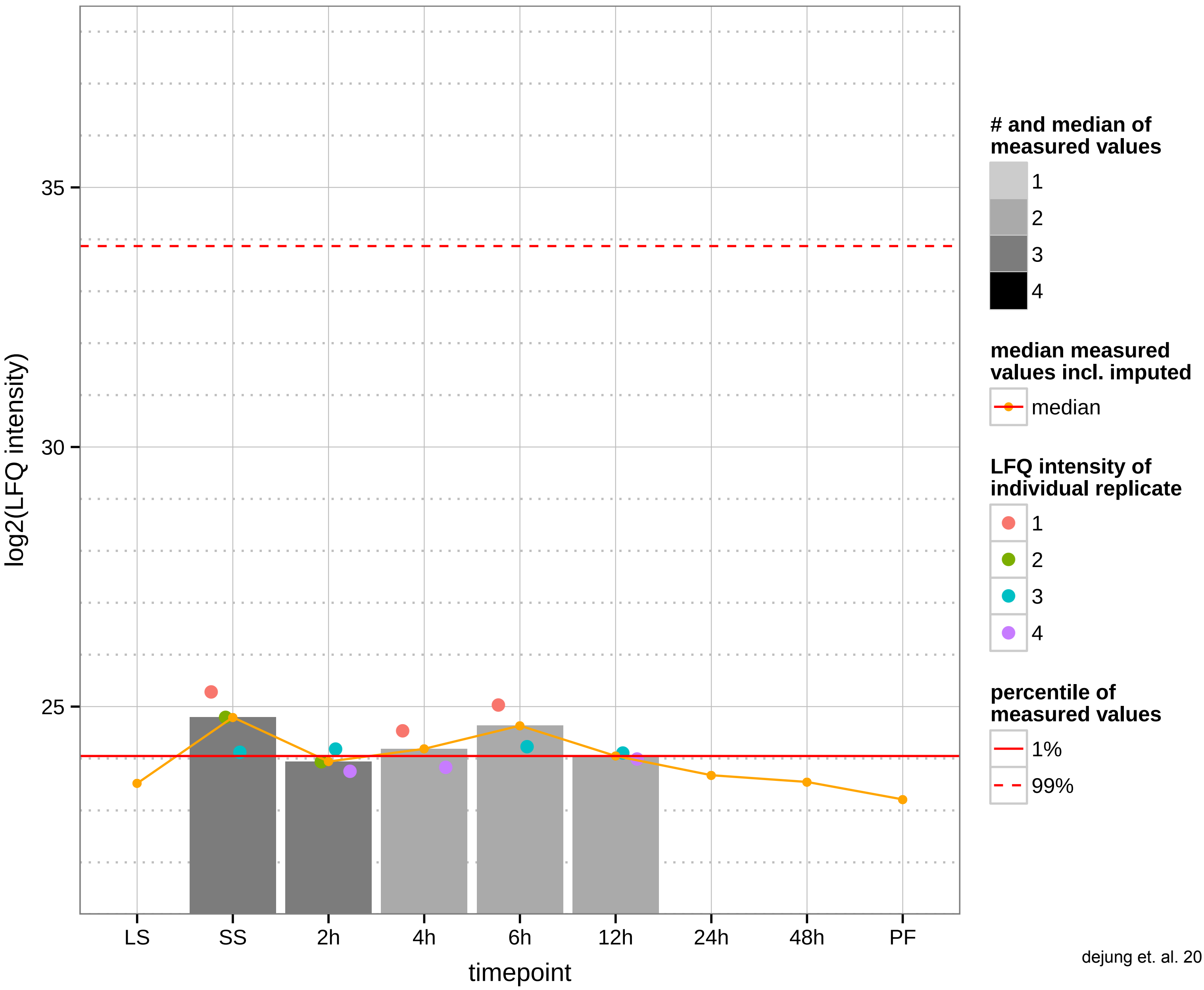

dejung et. al. 2015

hypothetical protein, conserved
Tb927.8.2690
AGOF: null
AGOC: null
AGOP: null
PGOF: protein binding
PGOC: null
PGOP: null

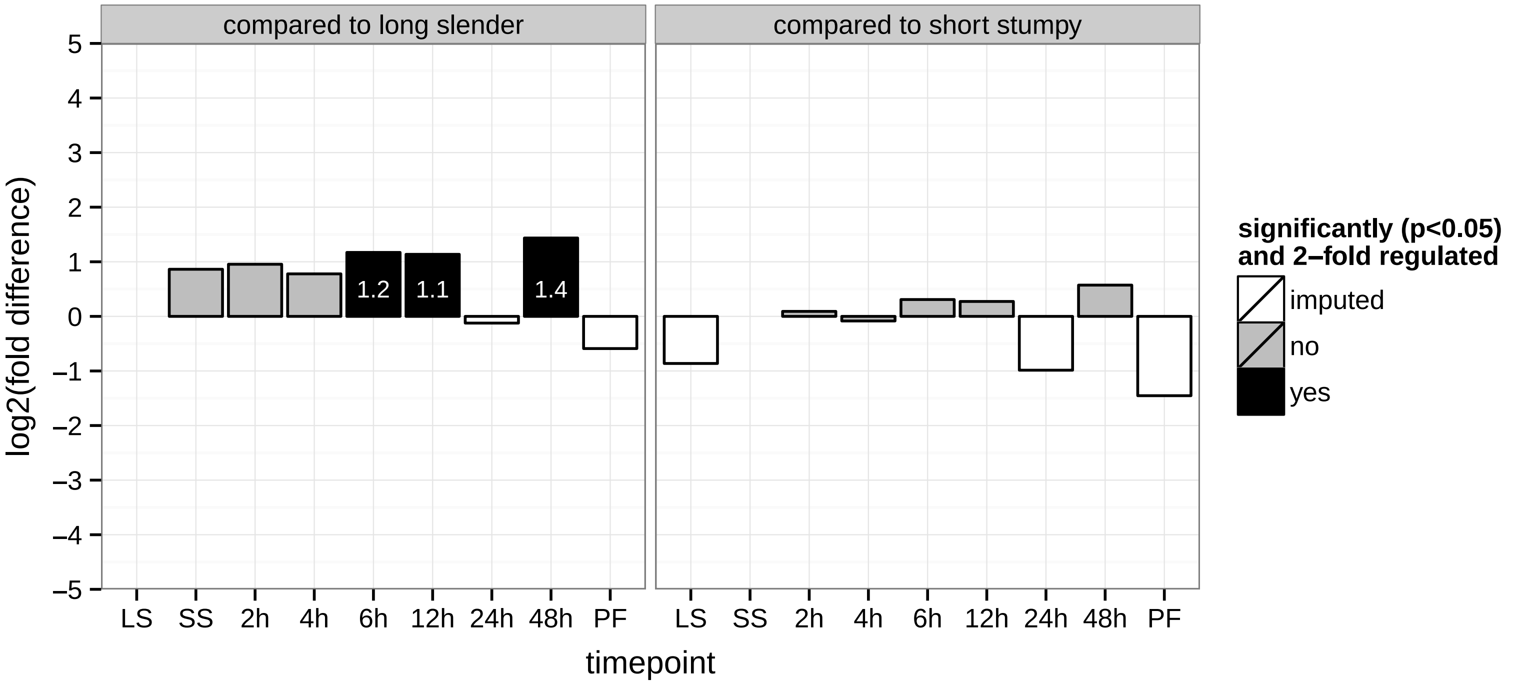

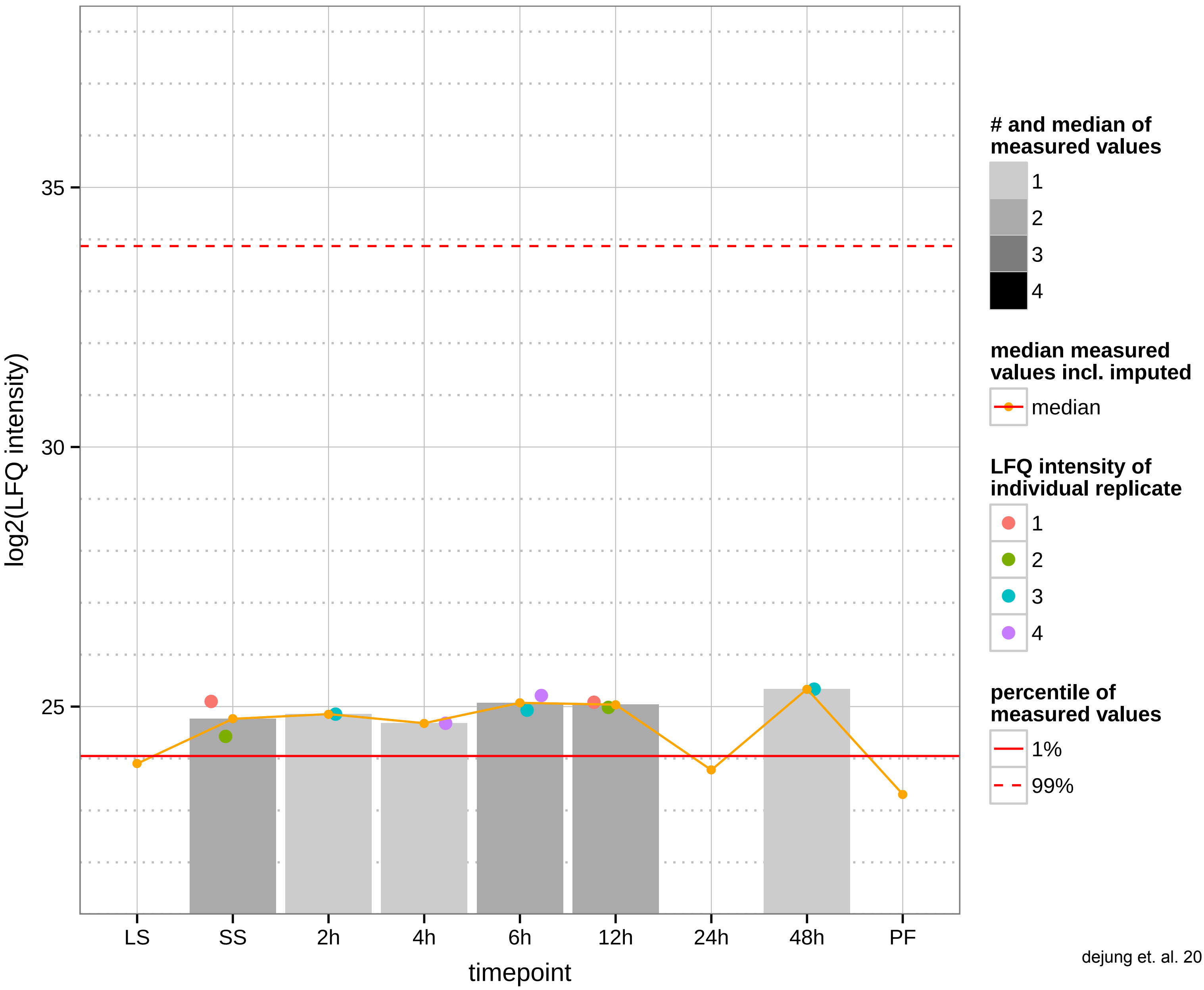

dejung et. al. 2015

hypothetical protein, conserved
Tb927.8.3410
AGOF: null
AGOC: null
AGOP: null
PGOF: null
PGOC: null
PGOP: null

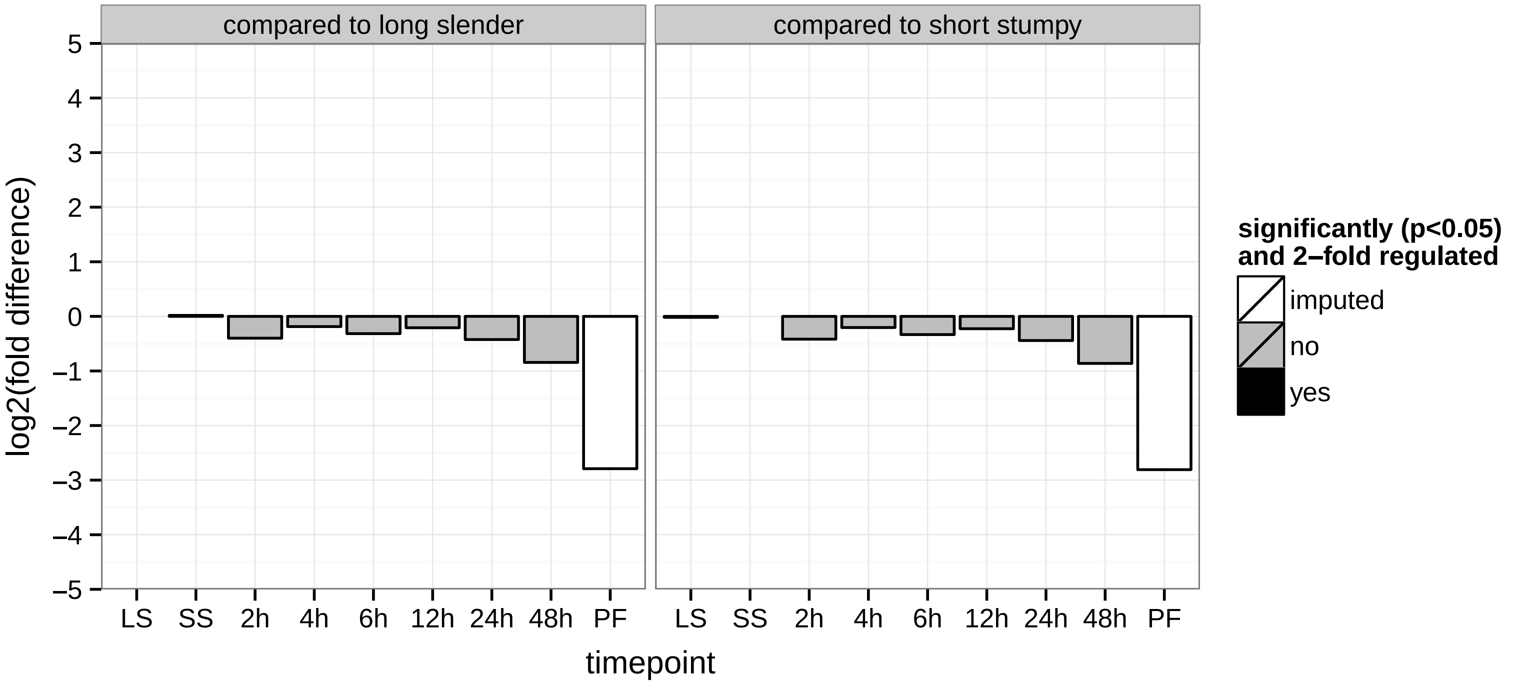

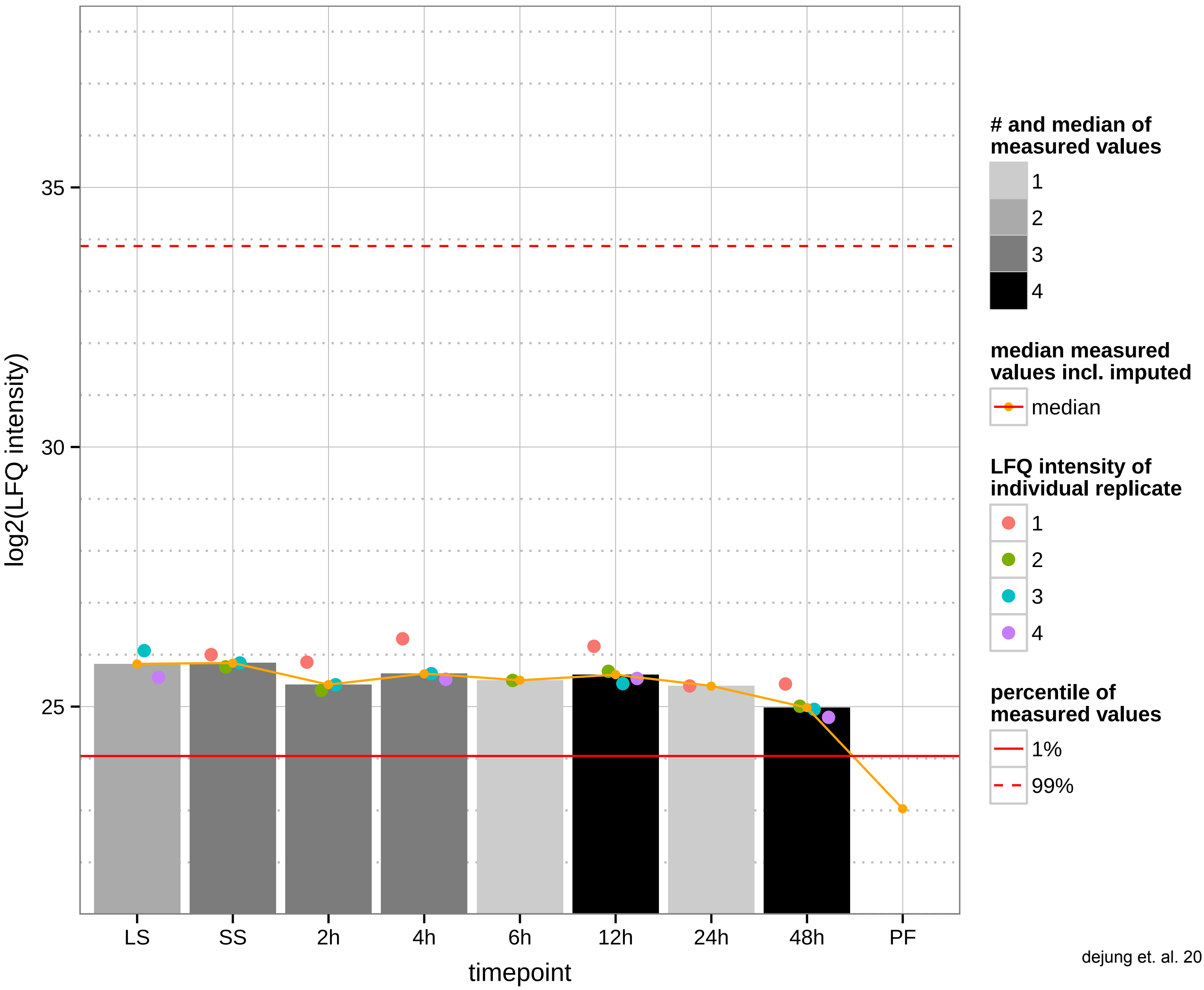

dejung et. al. 2015

hypothetical protein, conserved
Tb927.8.3710
AGOF: exonuclease activity, nucleic acid binding
AGOC: intracellular
AGOP: null
PGOF: exonuclease activity, nucleic acid binding
PGOC: intracellular
PGOP: null

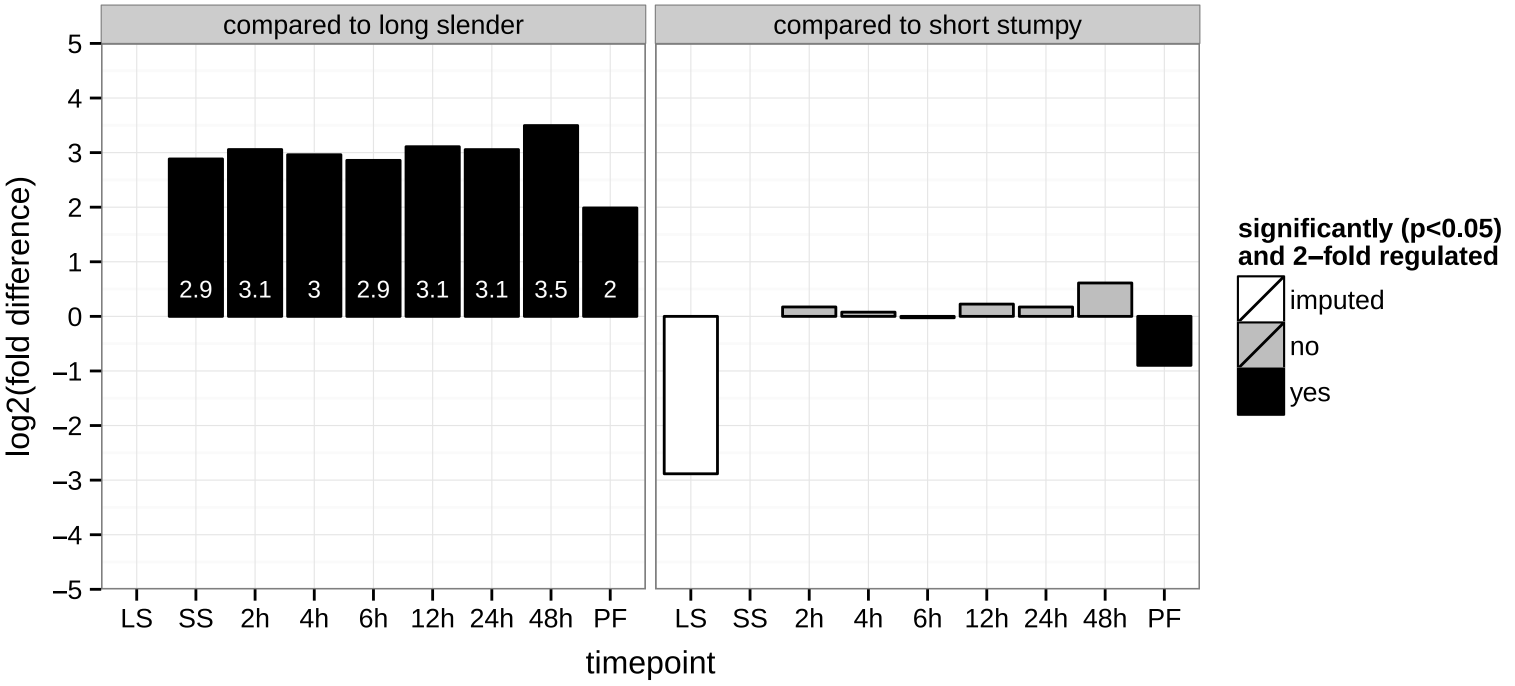

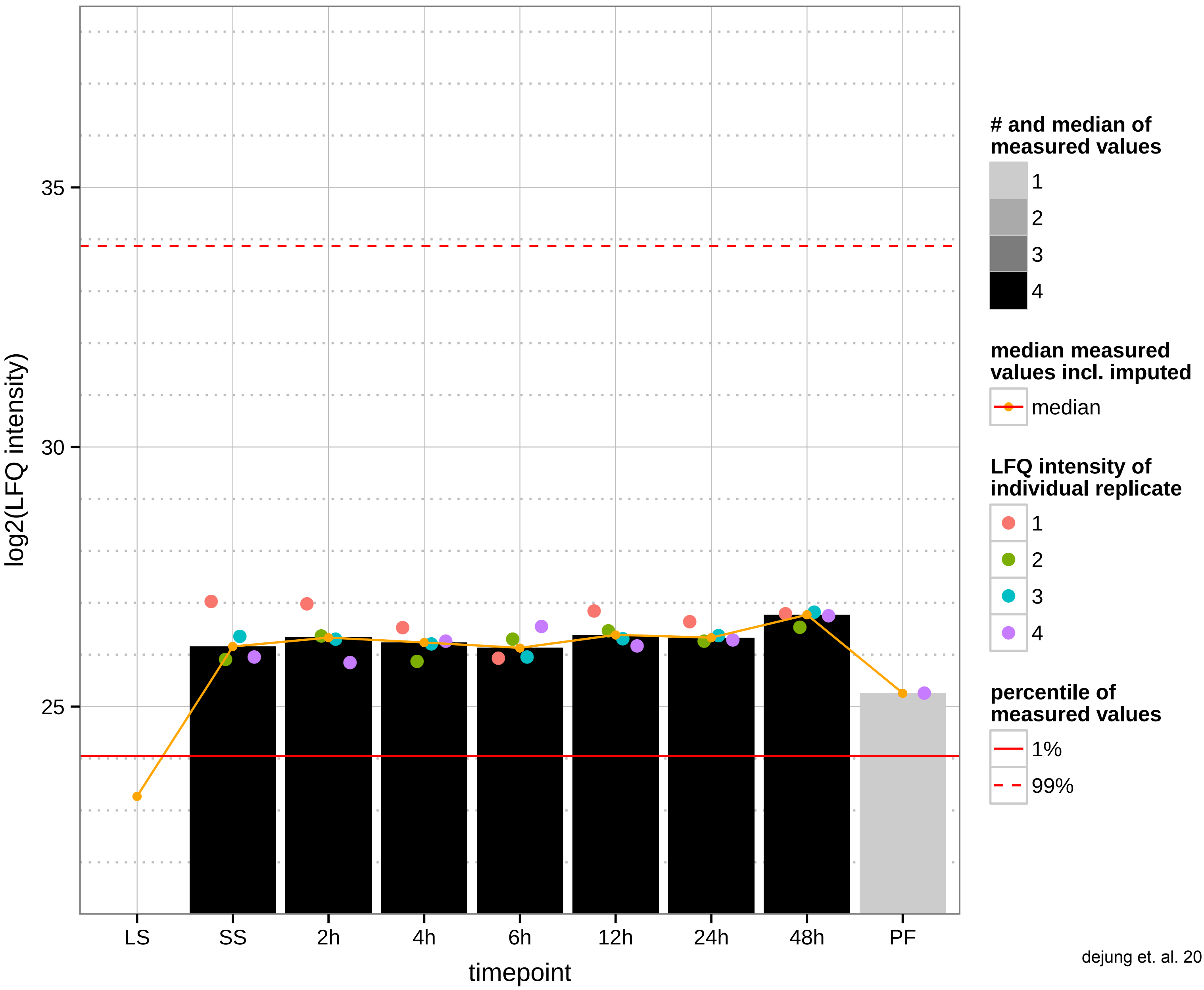

dejung et. al. 2015

hypothetical protein, conserved
Tb927.8.4250
AGOF: null
AGOC: mitochondrion
AGOP: null
PGOF: protein binding
PGOC: null
PGOP: null

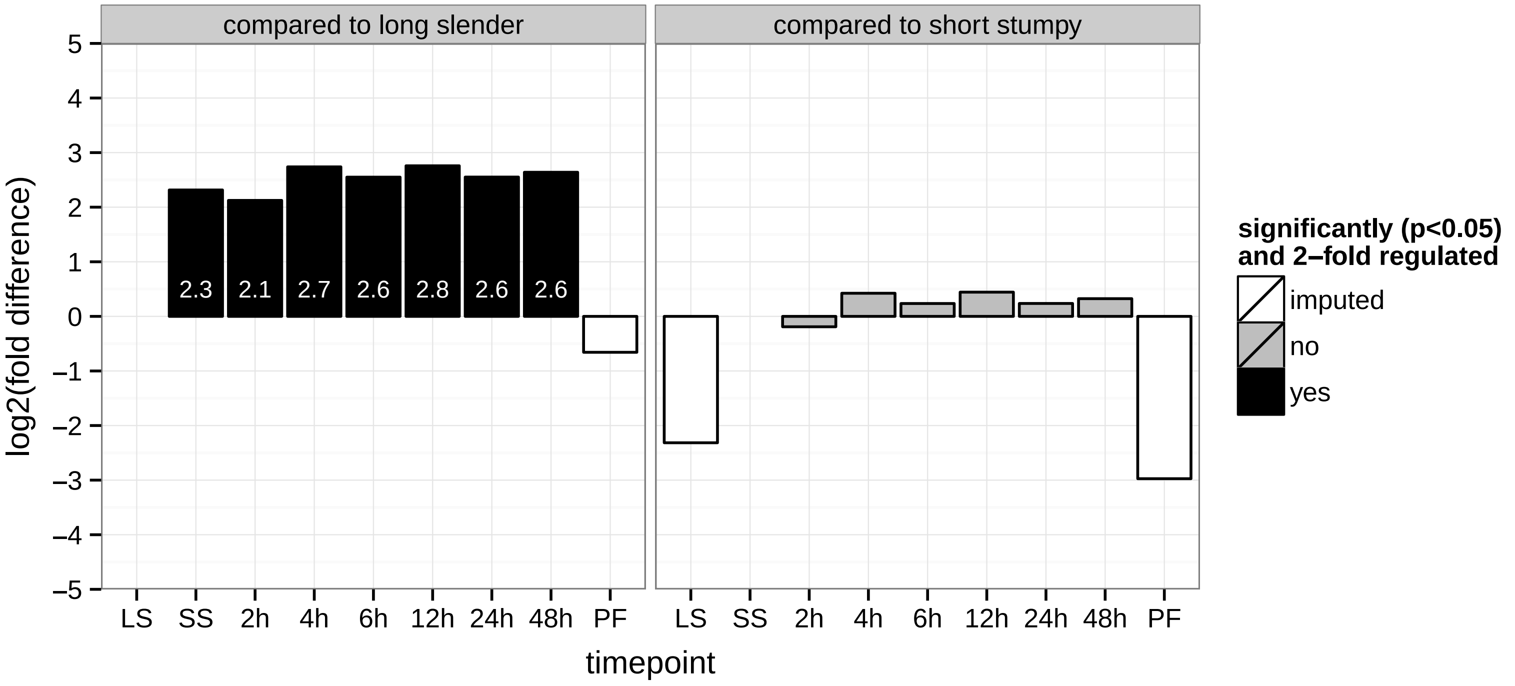

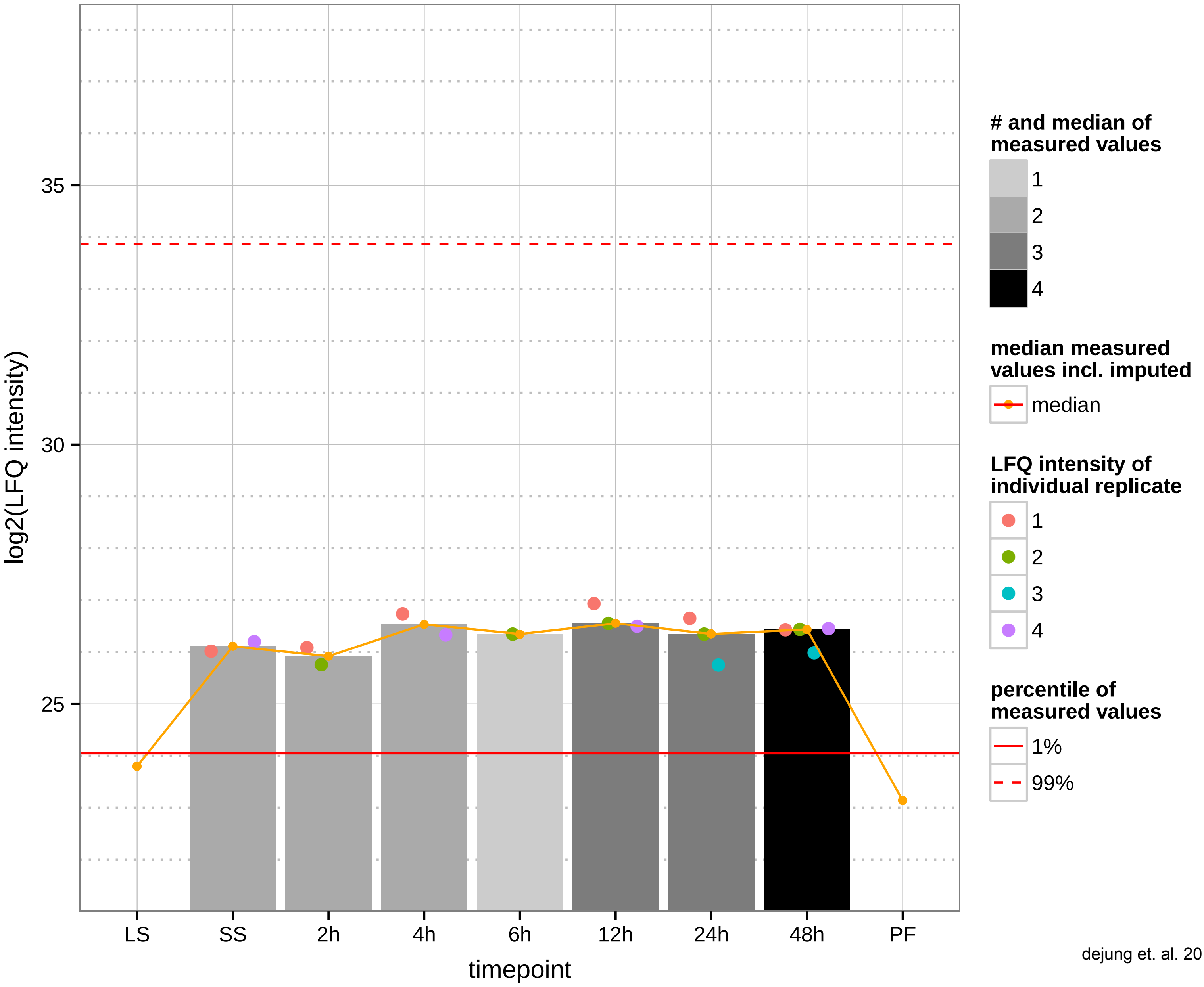

dejung et. al. 2015

trafficking protein particle complex subunit 3, putative, TRAPP, transport protein particle (TRAPP) component, Bet3
Tb927.8.5030
AGOF: null
AGOC: TRAPP complex, integral to membrane
AGOP: ER to Golgi vesicle−mediated transport
PGOF: null
PGOC: null
PGOP: null

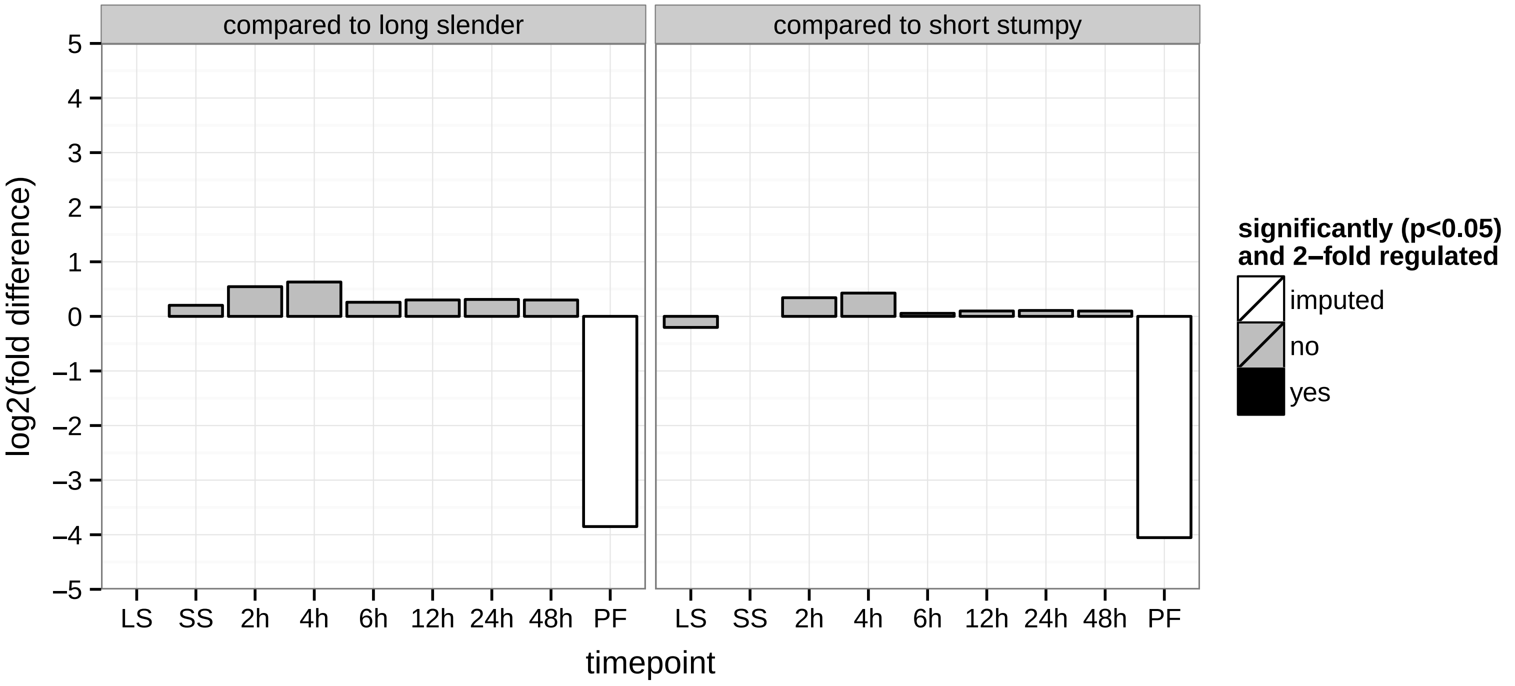

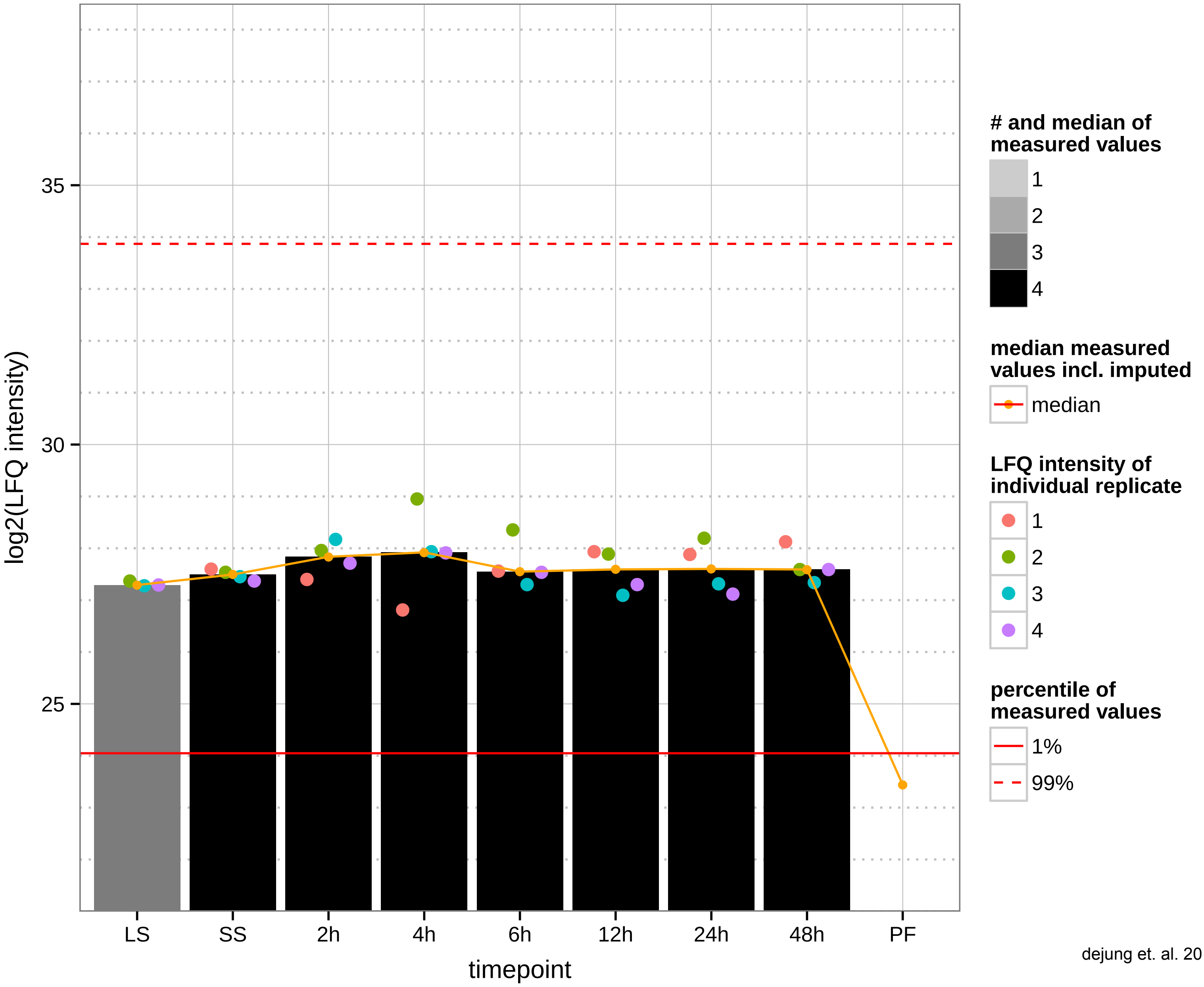

dejung et. al. 2015

recombination initiation protein NBS1, putative
Tb927.8.5710
AGOF: null
AGOC: null
AGOP: null
PGOF: protein binding
PGOC: null
PGOP: null

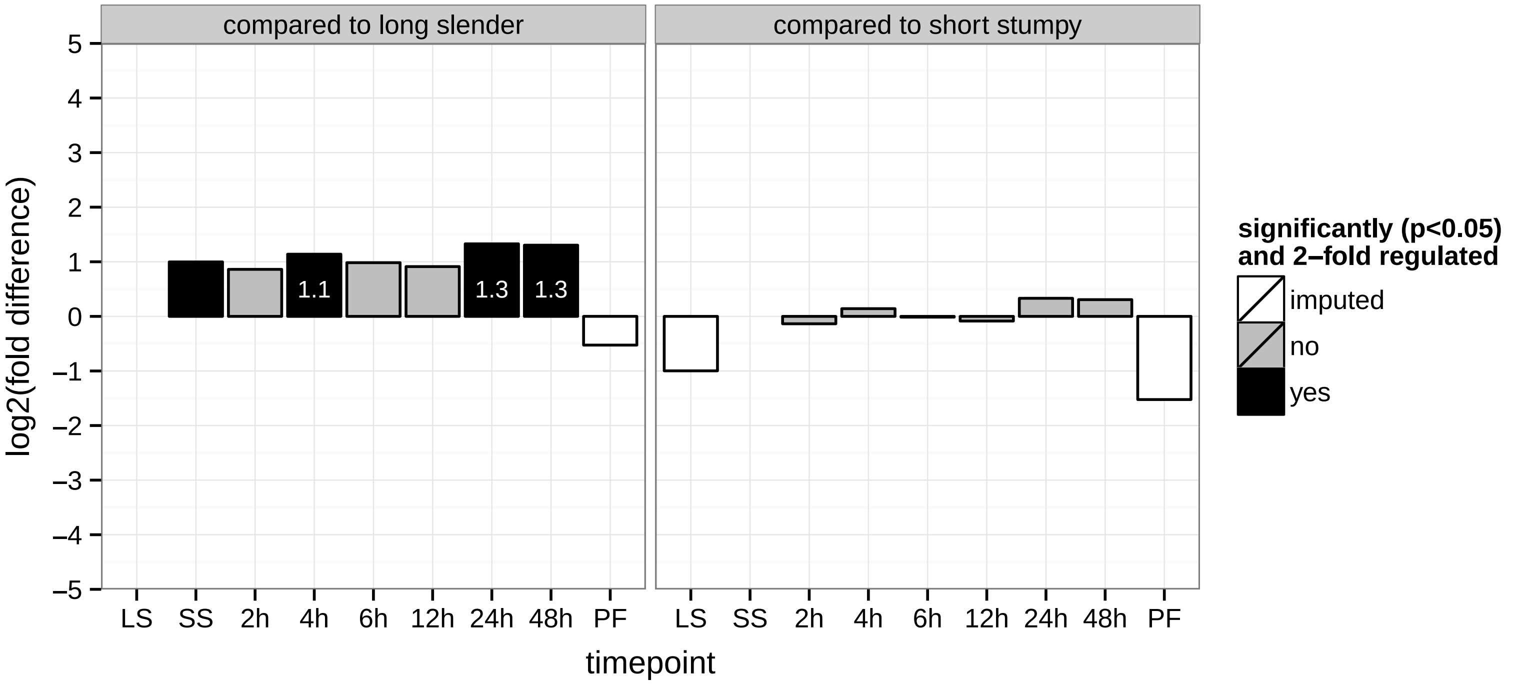

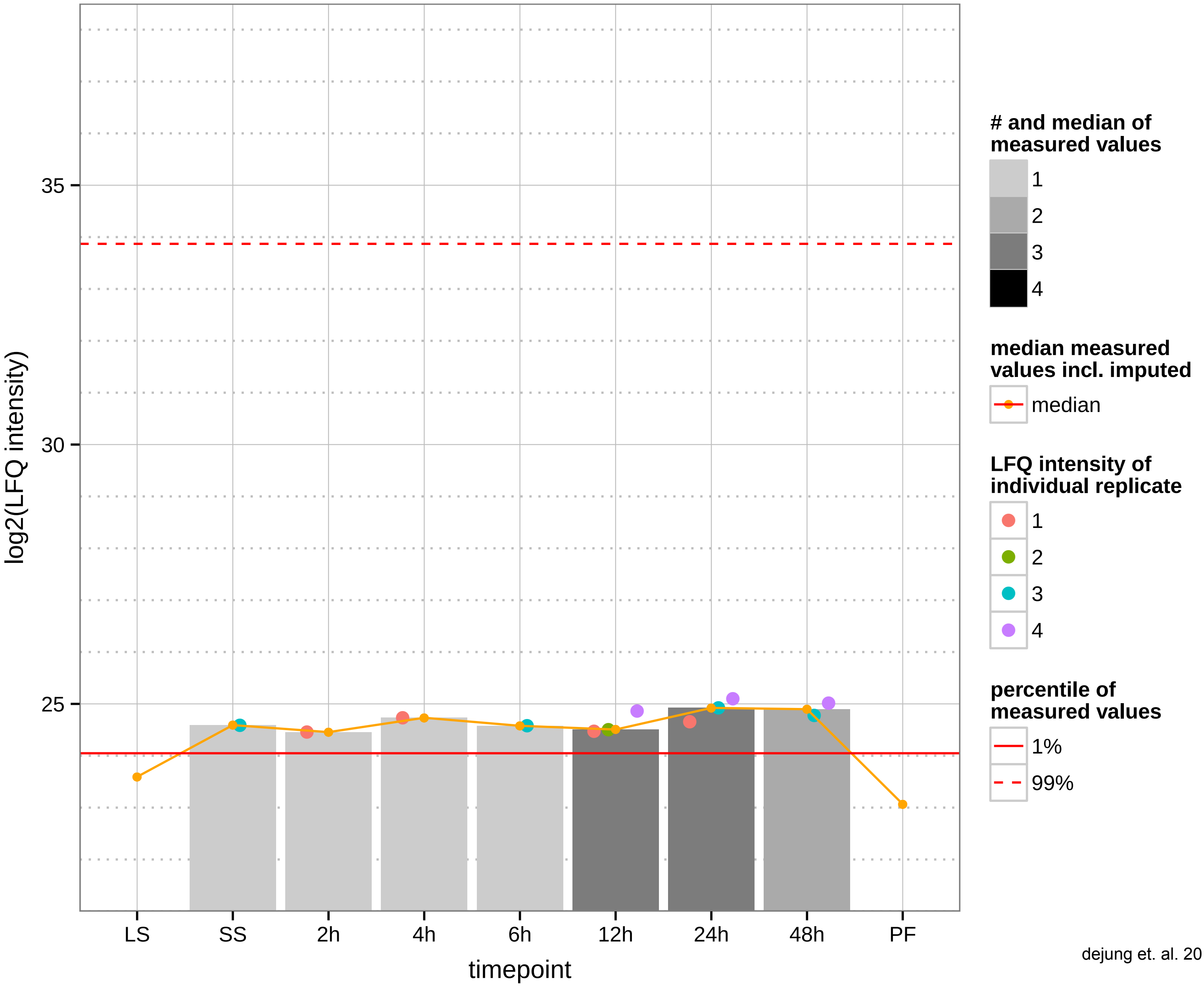

dejung et. al. 2015

basal body component, ML protein (TBBC)
Tb927.8.6070
AGOF: null
AGOC: cilium, cilium basal body, nucleus
AGOP: cilium assembly
PGOF: null
PGOC: null
PGOP: null

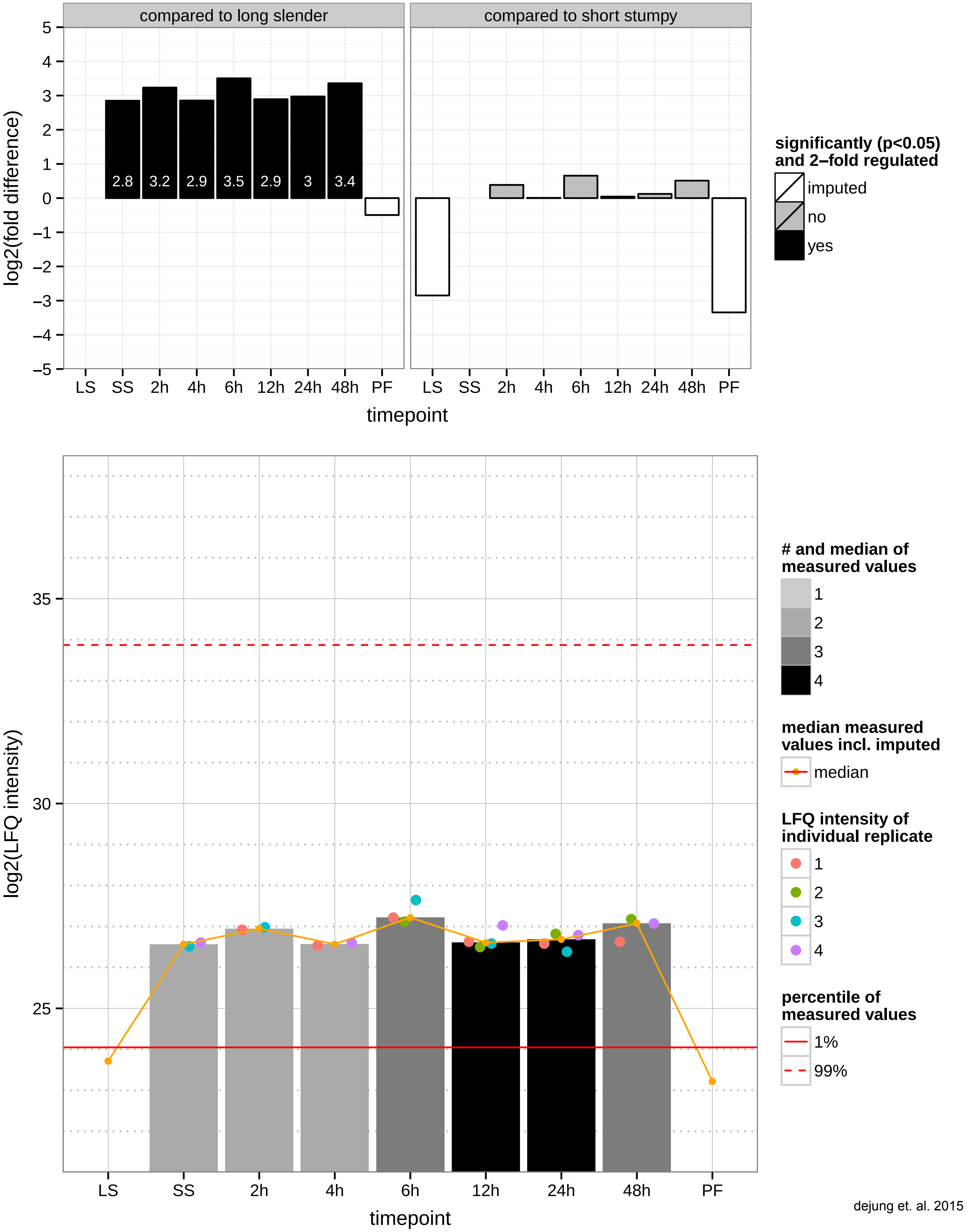

dejung et. al. 2015

hypothetical protein, conserved
Tb927.8.6190
AGOF: null
AGOC: null
AGOP: null
PGOF: null
PGOC: null
PGOP: null

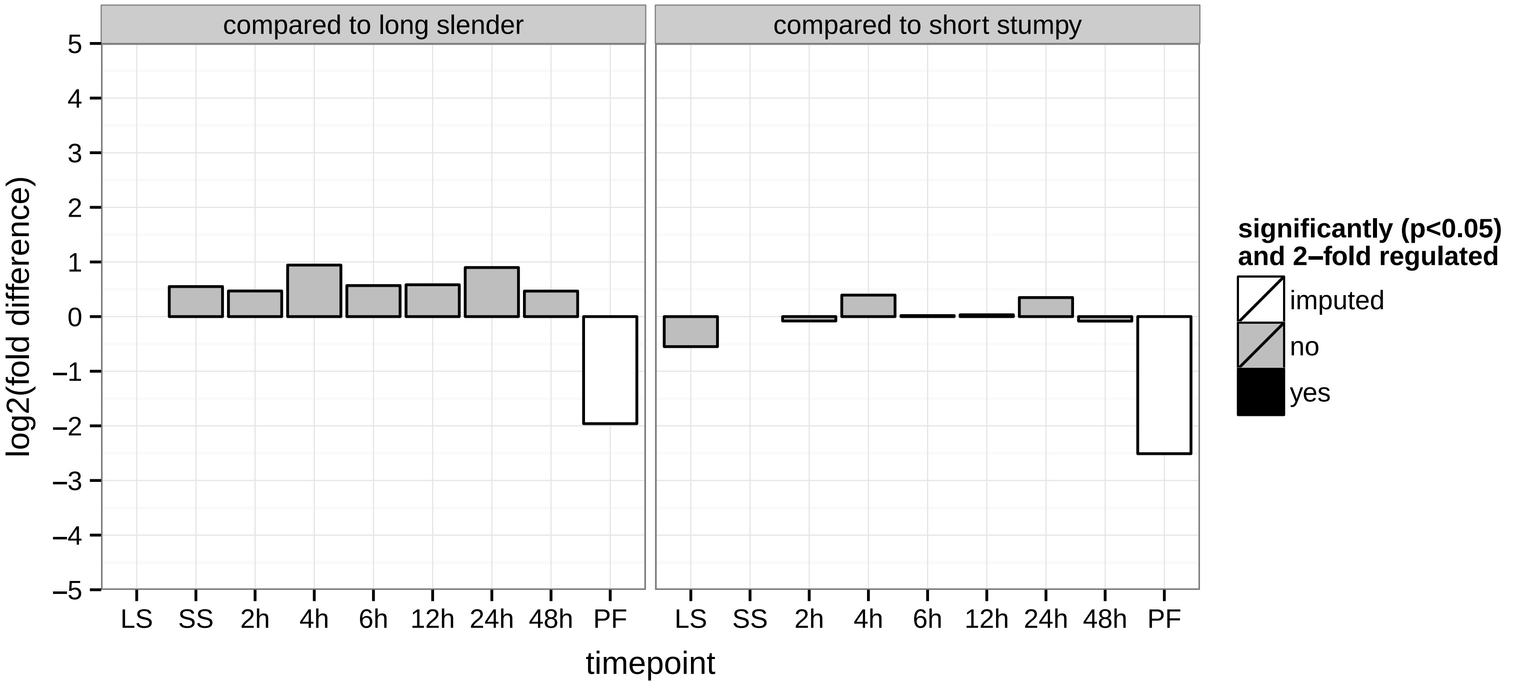

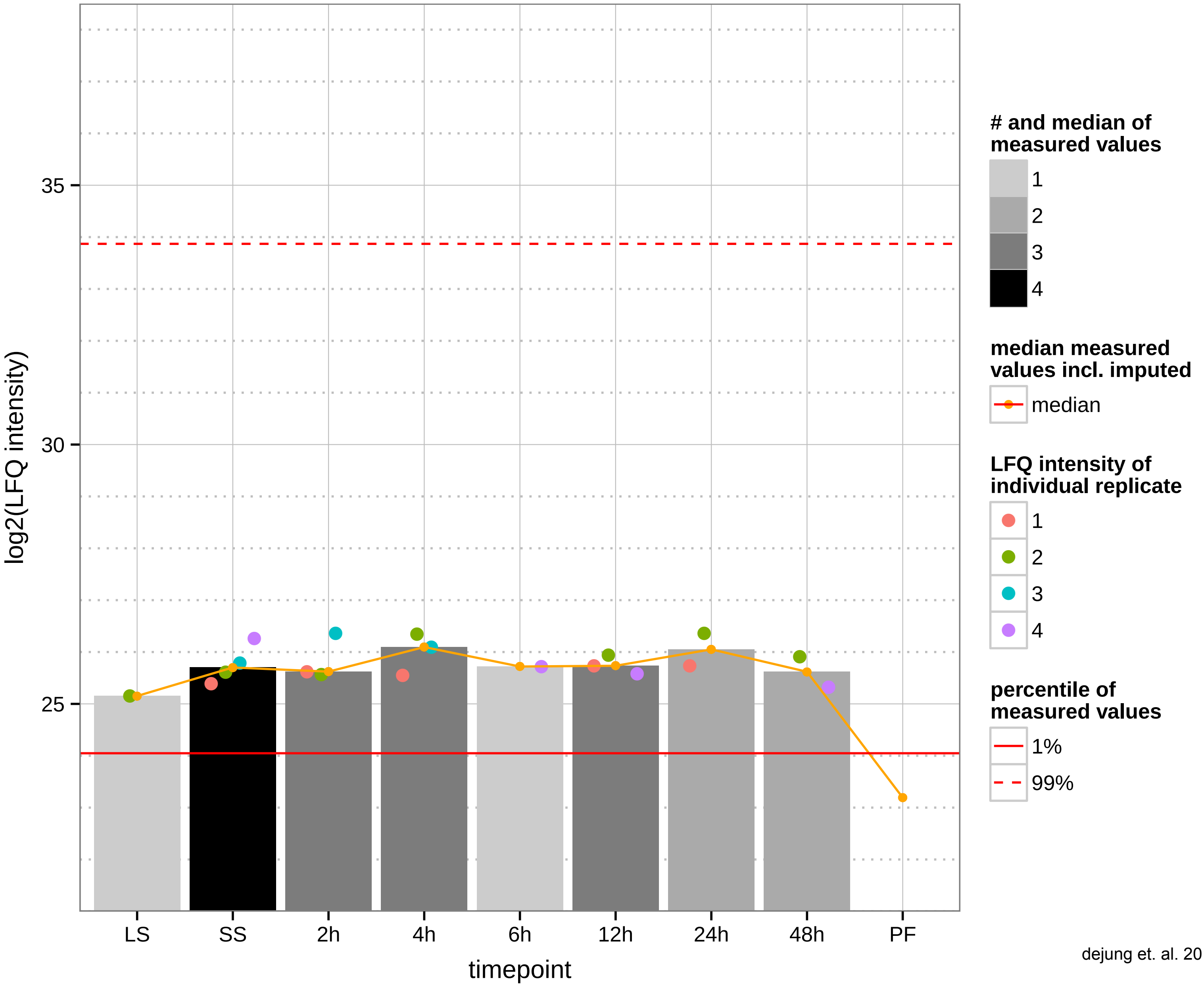

dejung et. al. 2015

hypothetical protein, conserved
Tb927.8.7210
AGOF: null
AGOC: null
AGOP: null
PGOF: null
PGOC: null
PGOP: null

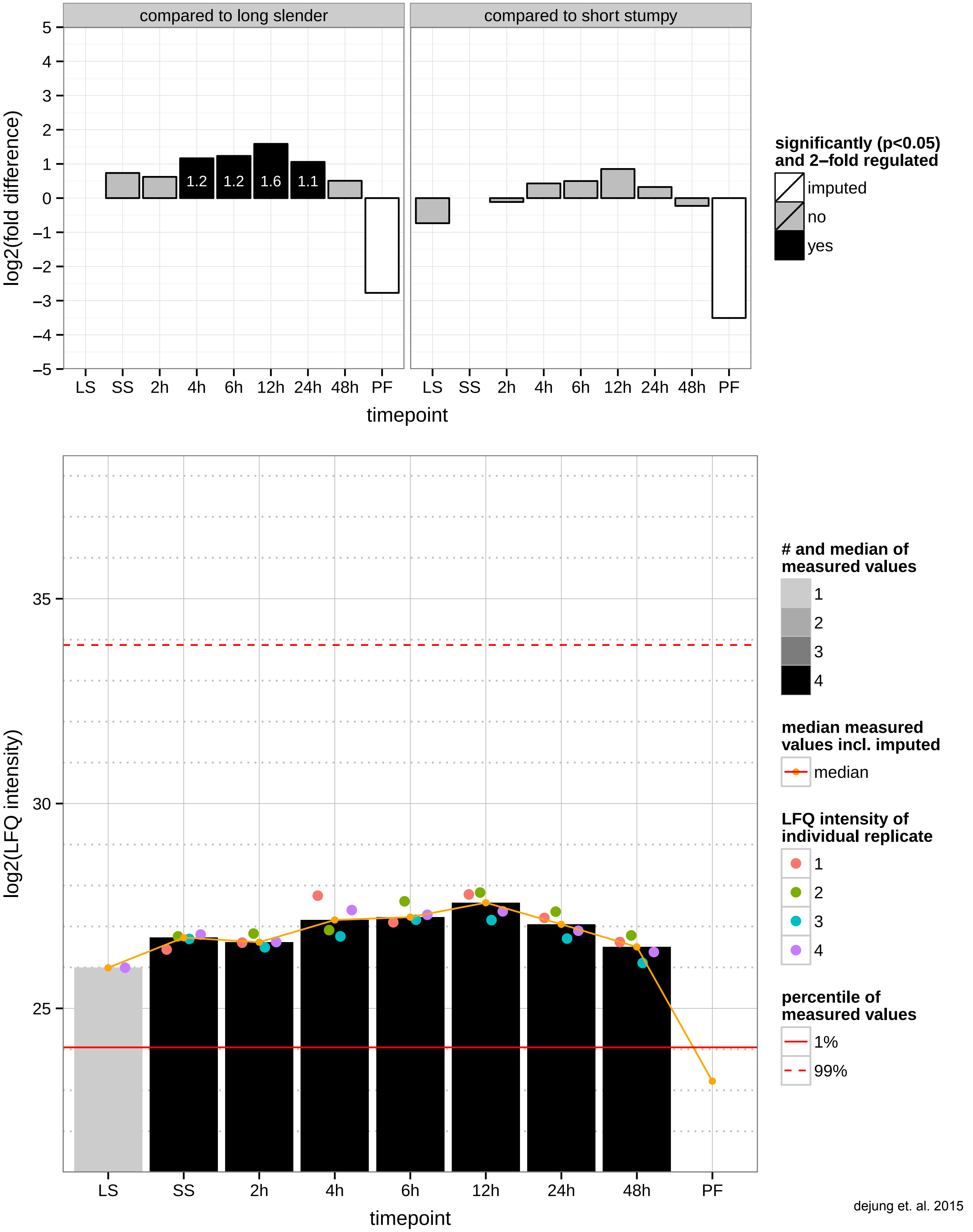

dejung et. al. 2015

hypothetical protein, conserved
Tb927.8.7790
AGOF: null
AGOC: null
AGOP: null
PGOF: null
PGOC: null
PGOP: null

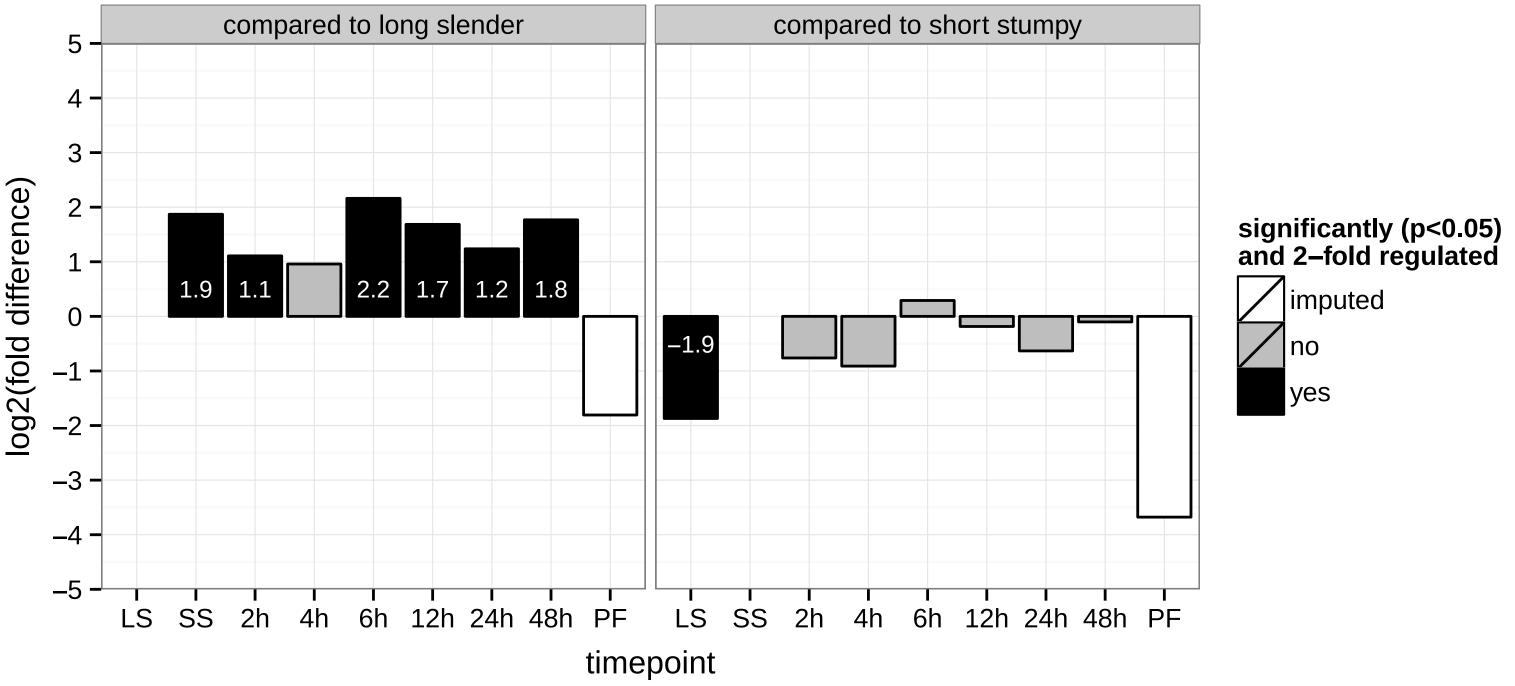

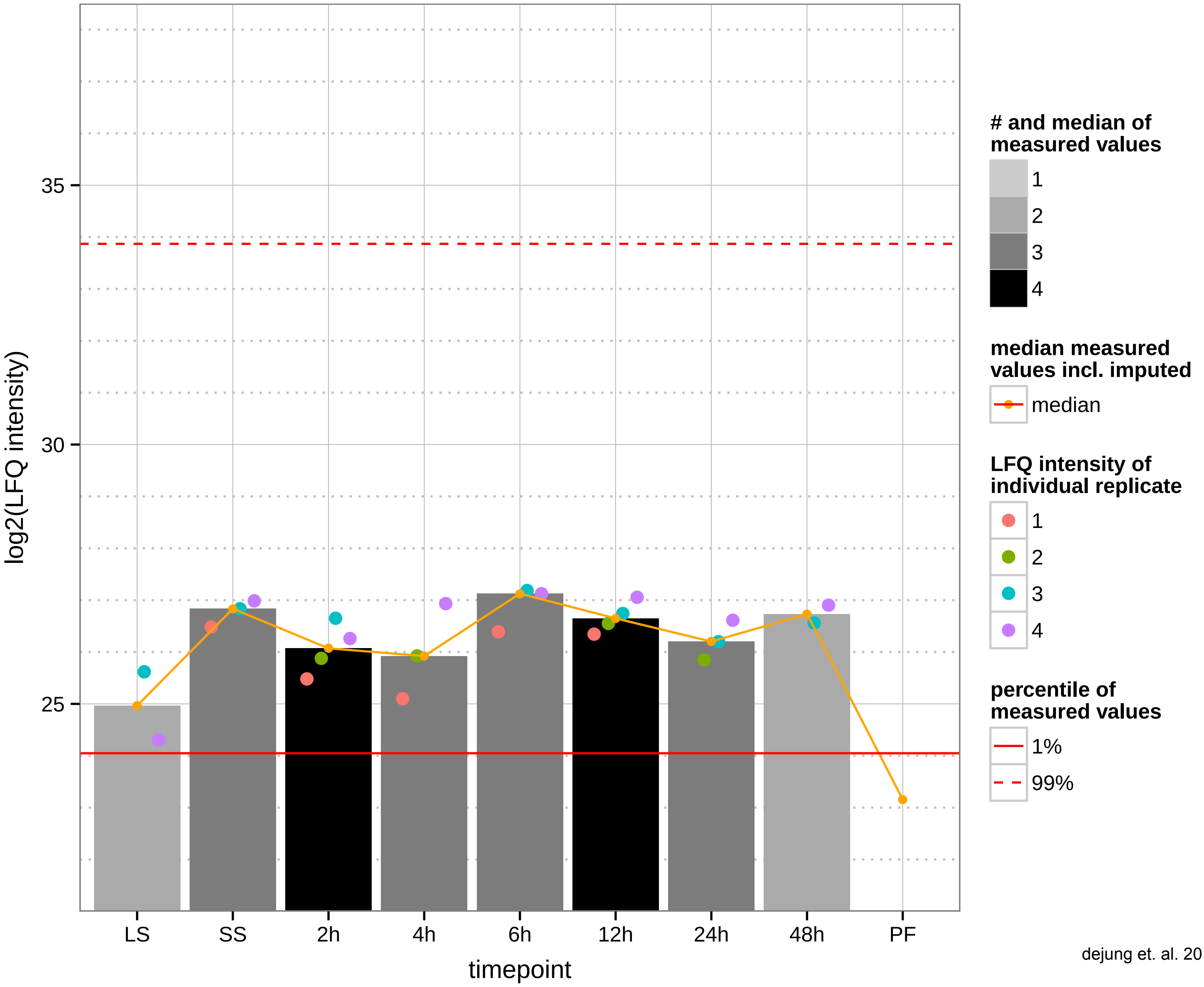

dejung et. al. 2015

hypothetical protein, conserved
Tb927.8.7990
AGOF: zinc ion binding
AGOC: intracellular
AGOP: null
PGOF: zinc ion binding
PGOC: intracellular
PGOP: null

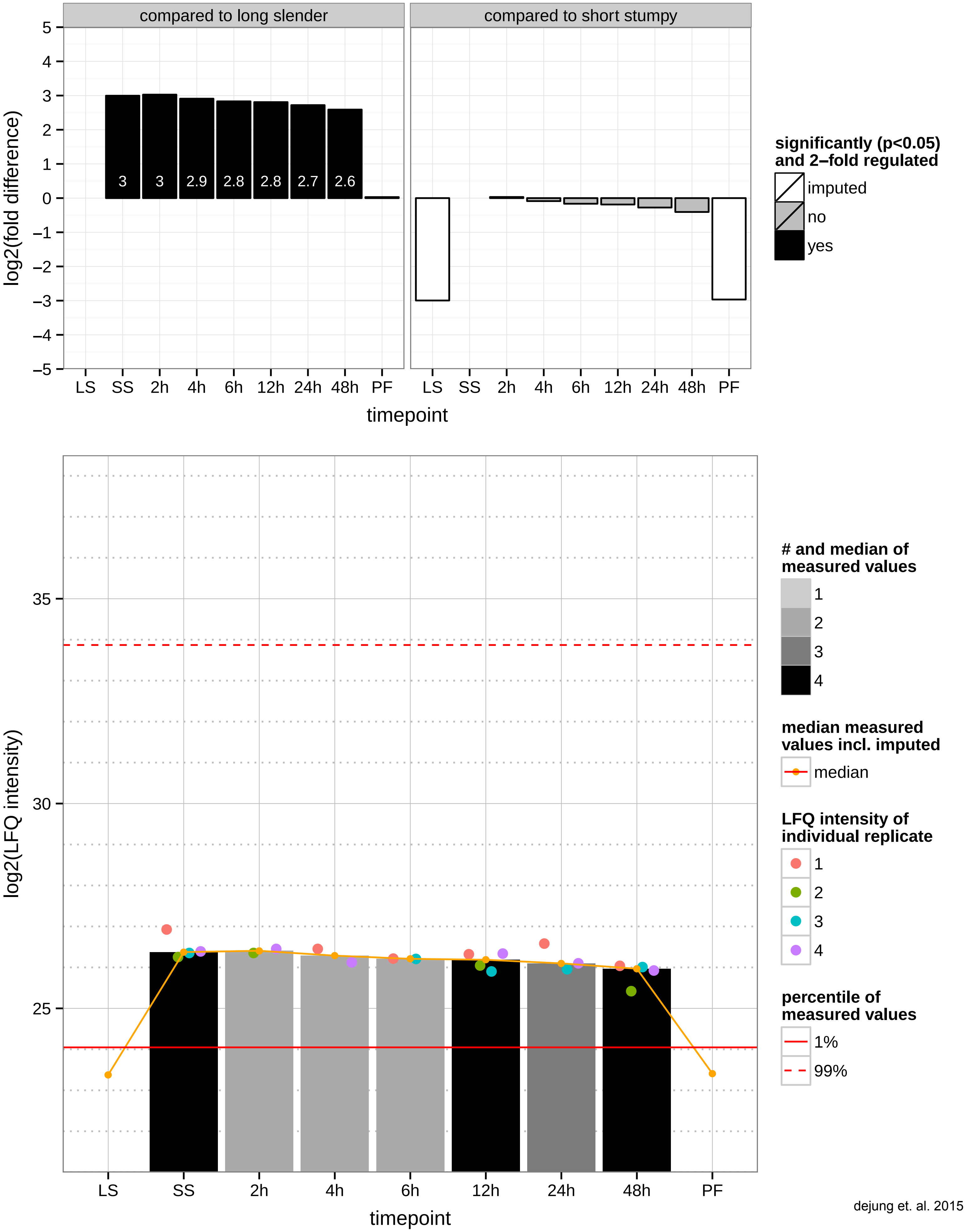

dejung et. al. 2015

hypothetical protein, conserved
Tb927.8.8120
AGOF: null
AGOC: null
AGOP: null
PGOF: null
PGOC: null
PGOP: null

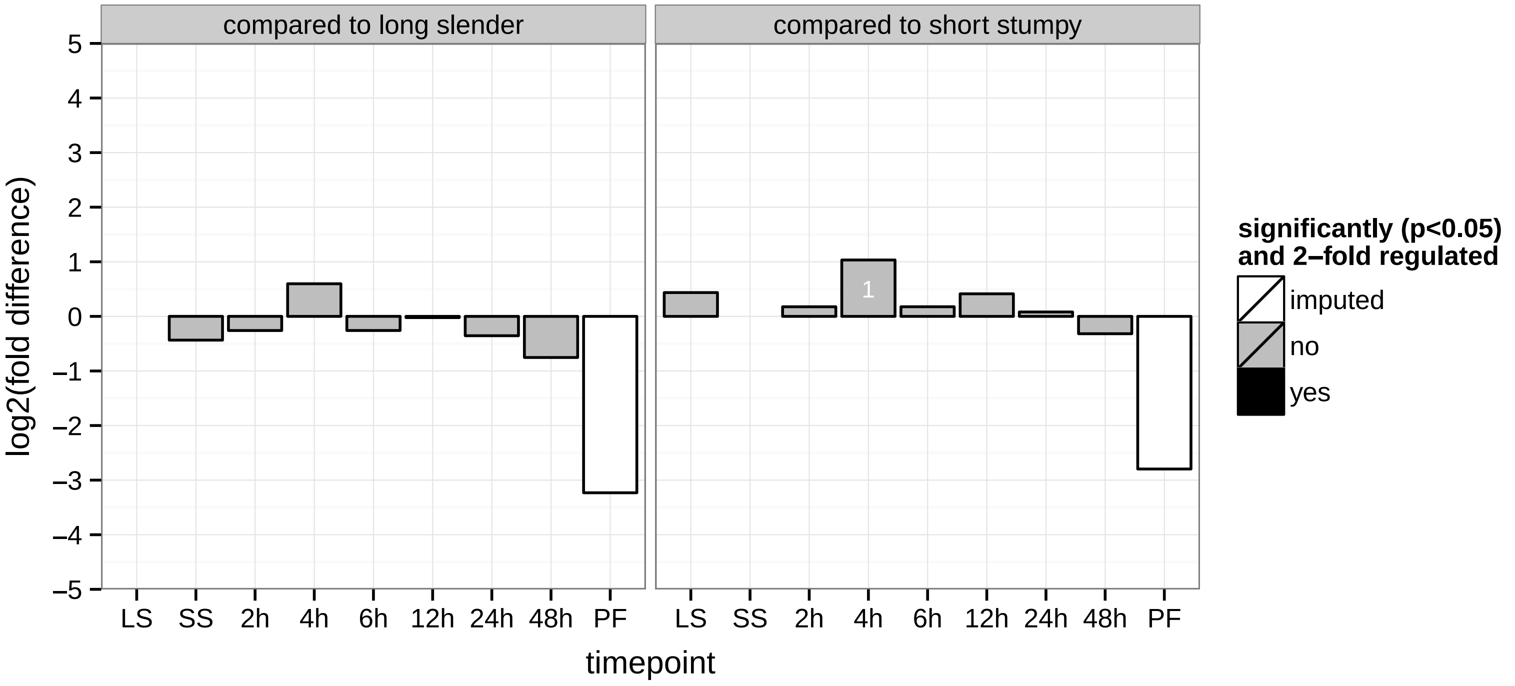

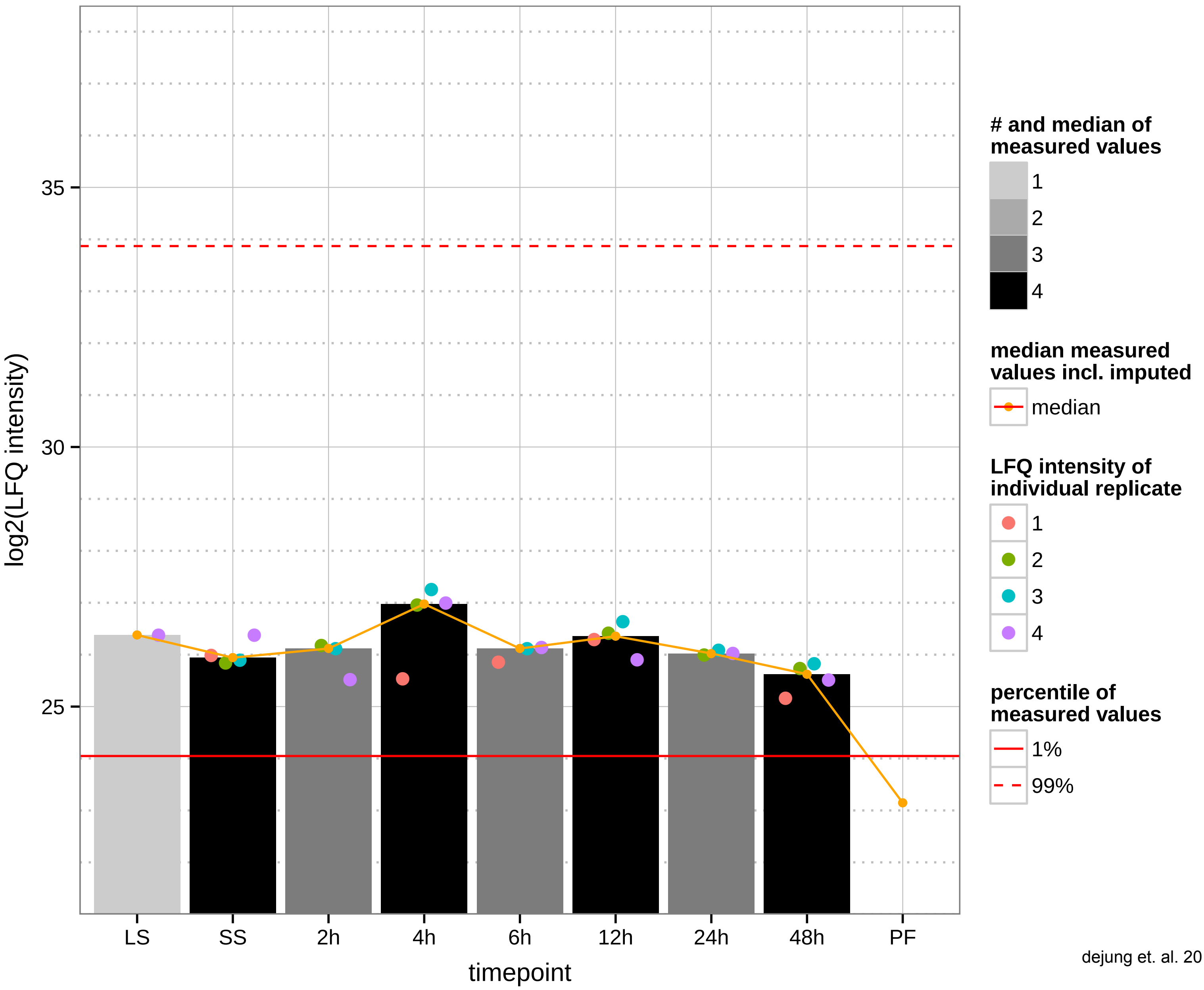

dejung et. al. 2015

chaperone protein DNAj, putative
Tb927.8.8310
AGOF: heat shock protein binding, unfolded protein binding
AGOC: null
AGOP: protein folding
PGOF: heat shock protein binding
PGOC: null
PGOP: null

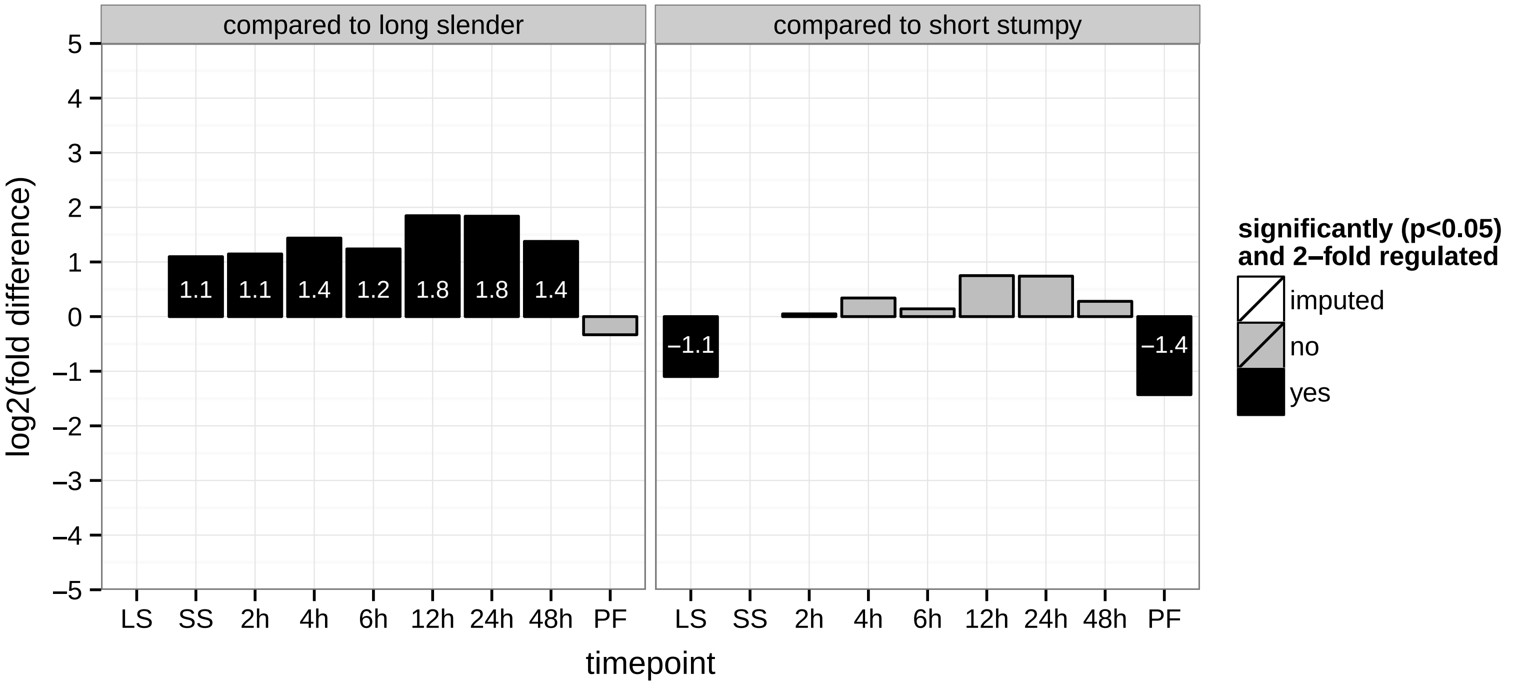

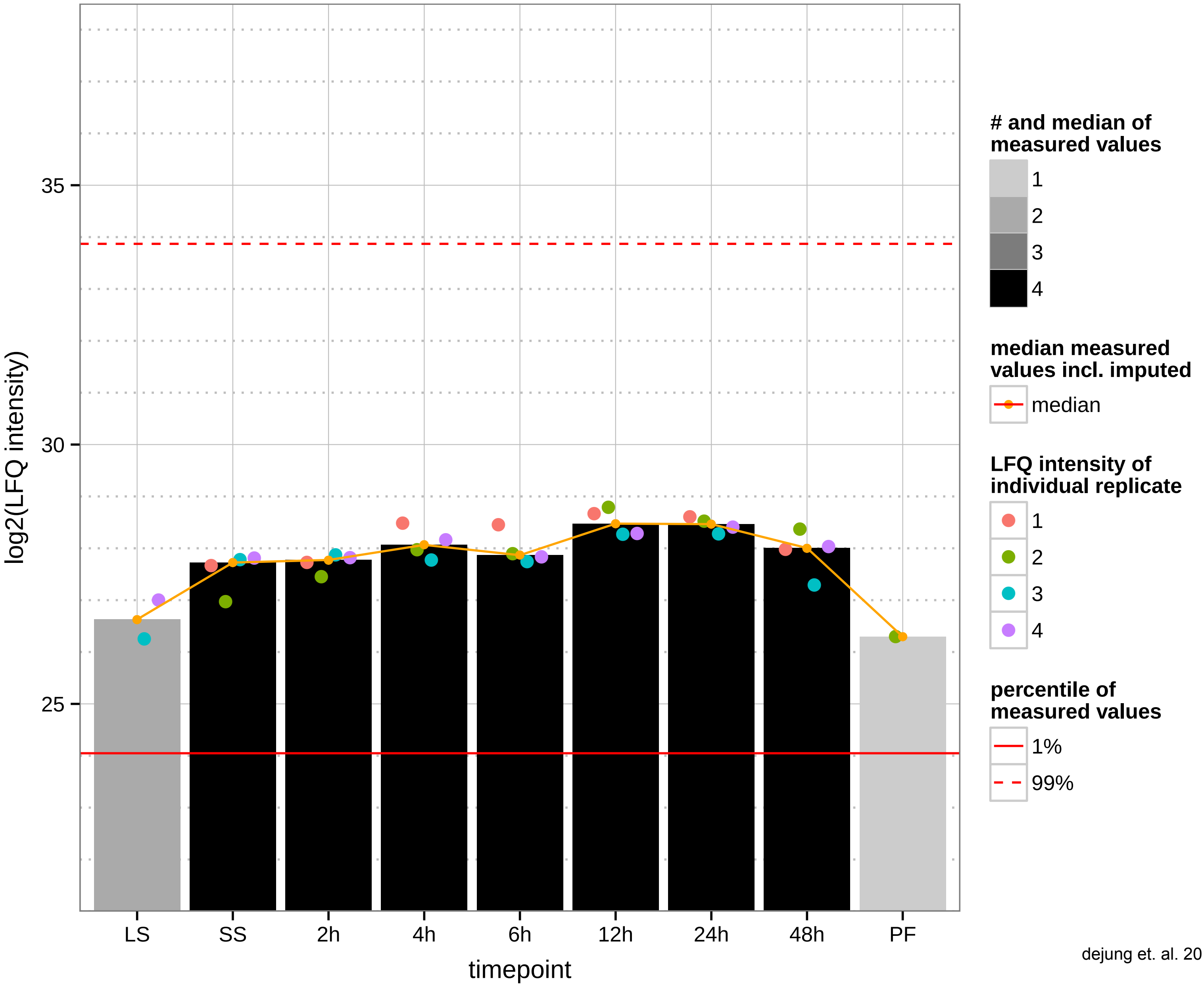

dejung et. al. 2015

hypothetical protein, conserved
Tb927.8.8340
AGOF: null
AGOC: null
AGOP: null
PGOF: null
PGOC: null
PGOP: null

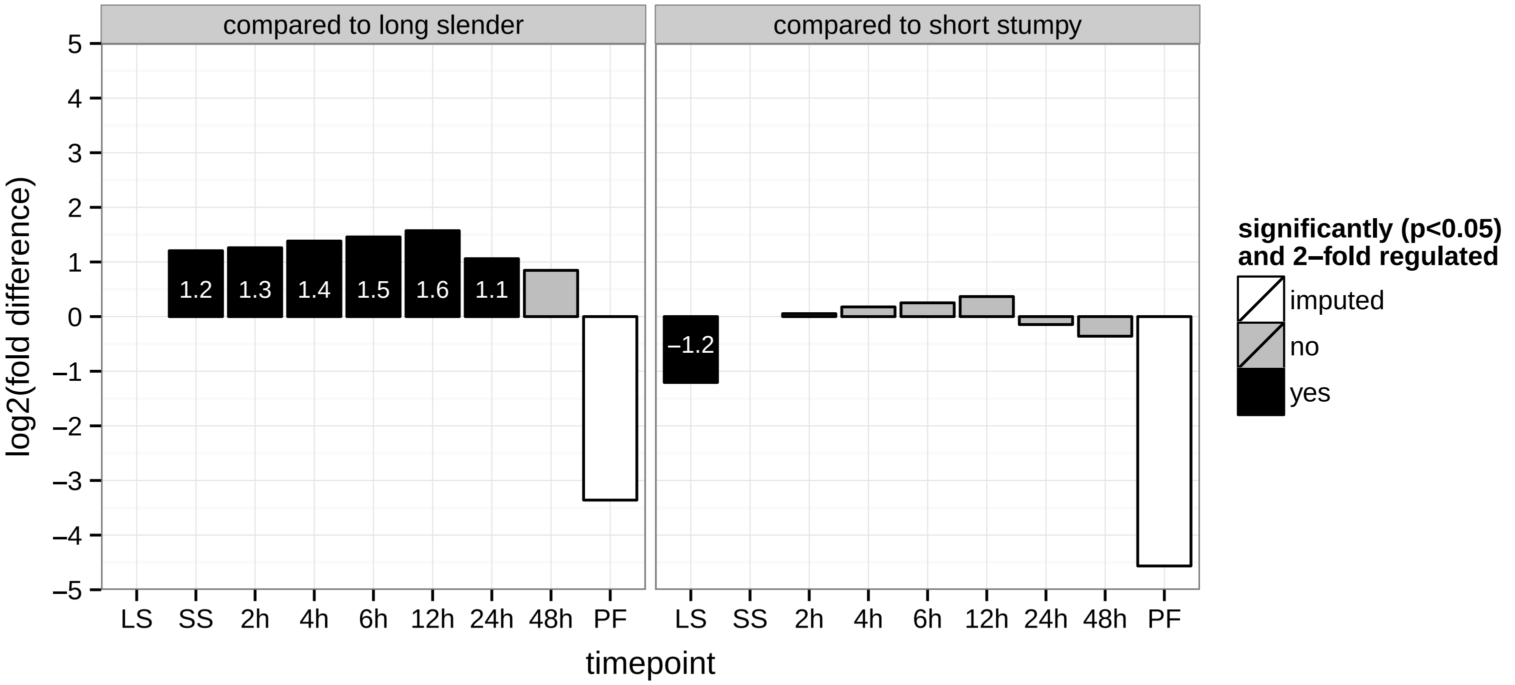

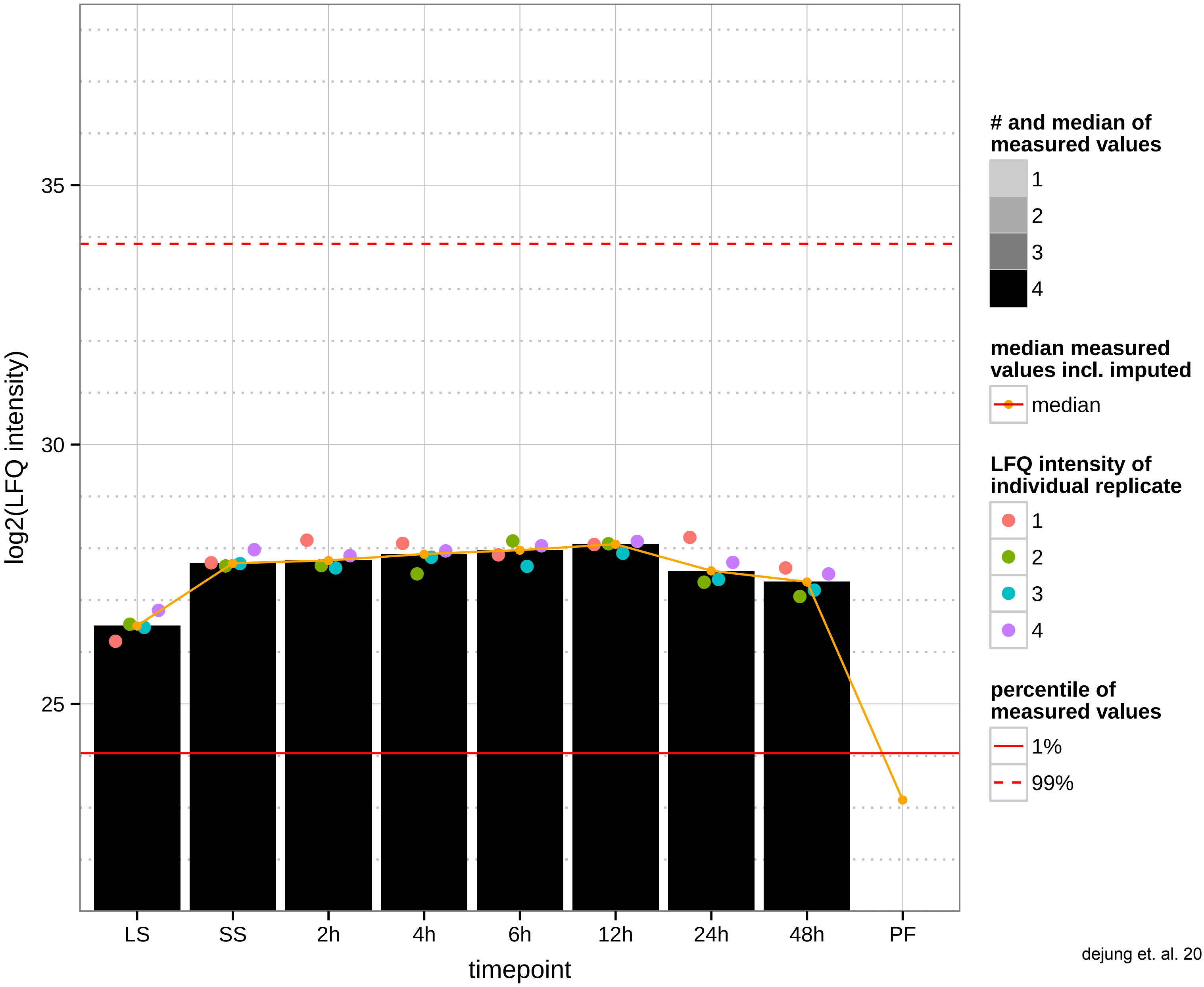

dejung et. al. 2015

serine/threonine kinase, putative, protein kinase
Tb927.8.870
AGOF: ATP binding, protein serine/threonine kinase activity, protein tyrosine kinase activity
AGOC: null
AGOP: protein phosphorylation
PGOF: ATP binding, protein kinase activity, transferase activity, transferring phosphorus–containing groups
PGOC: null
PGOP: protein phosphorylation

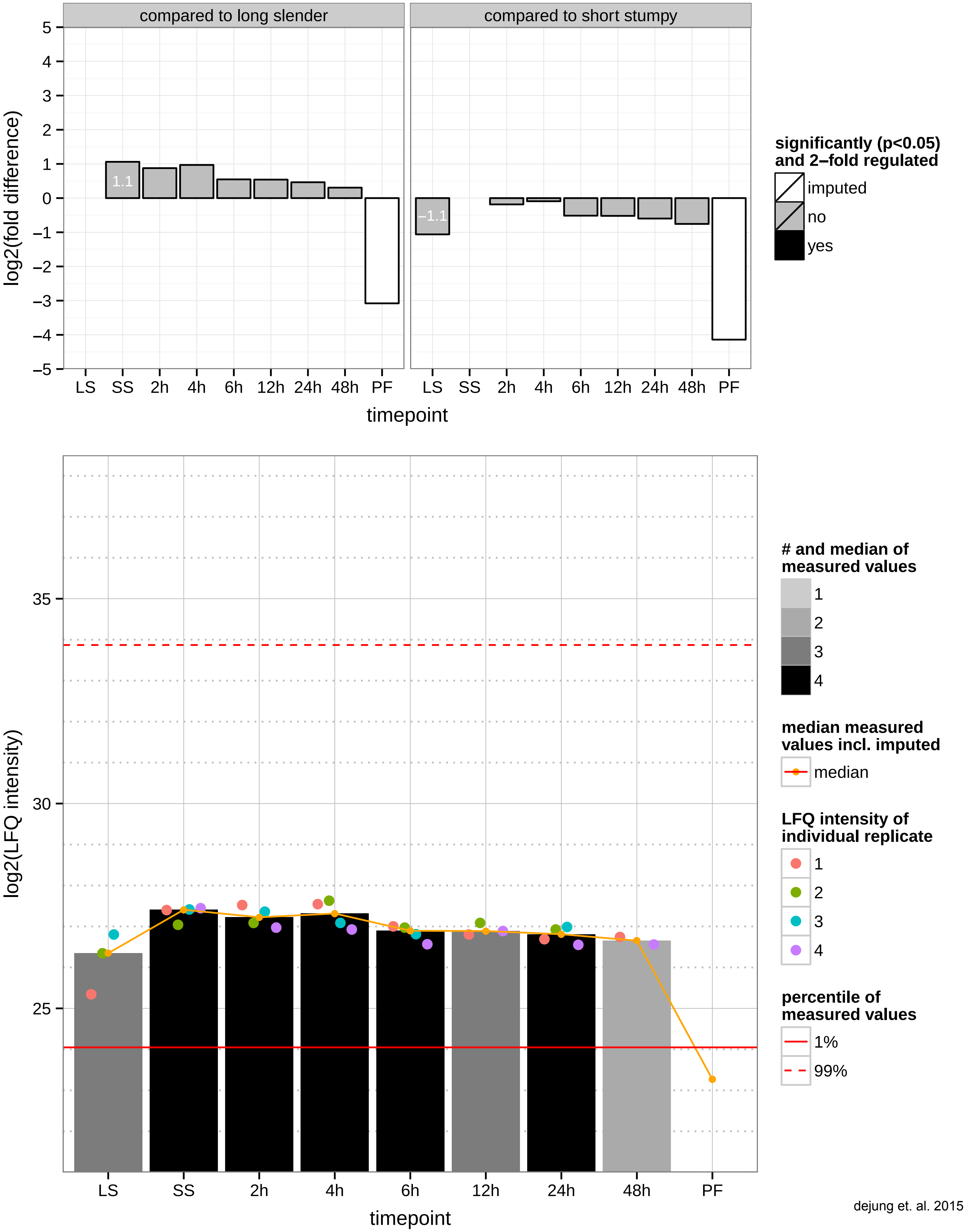

dejung et. al. 2015

Ras–related protein Rab1A (Rab1A)
Tb927.8.890
AGOF: GTP binding, GTPase activity
AGOC: intracellular
AGOP: intracellular protein transport, nucleocytoplasmic transport, small GTPase mediated signal transduction
PGOF: GTP binding, GTPase activity
PGOC: intracellular, membrane
PGOP: GTP catabolic process, intracellular protein transport, nucleocytoplasmic transport, protein transport, signal transducti

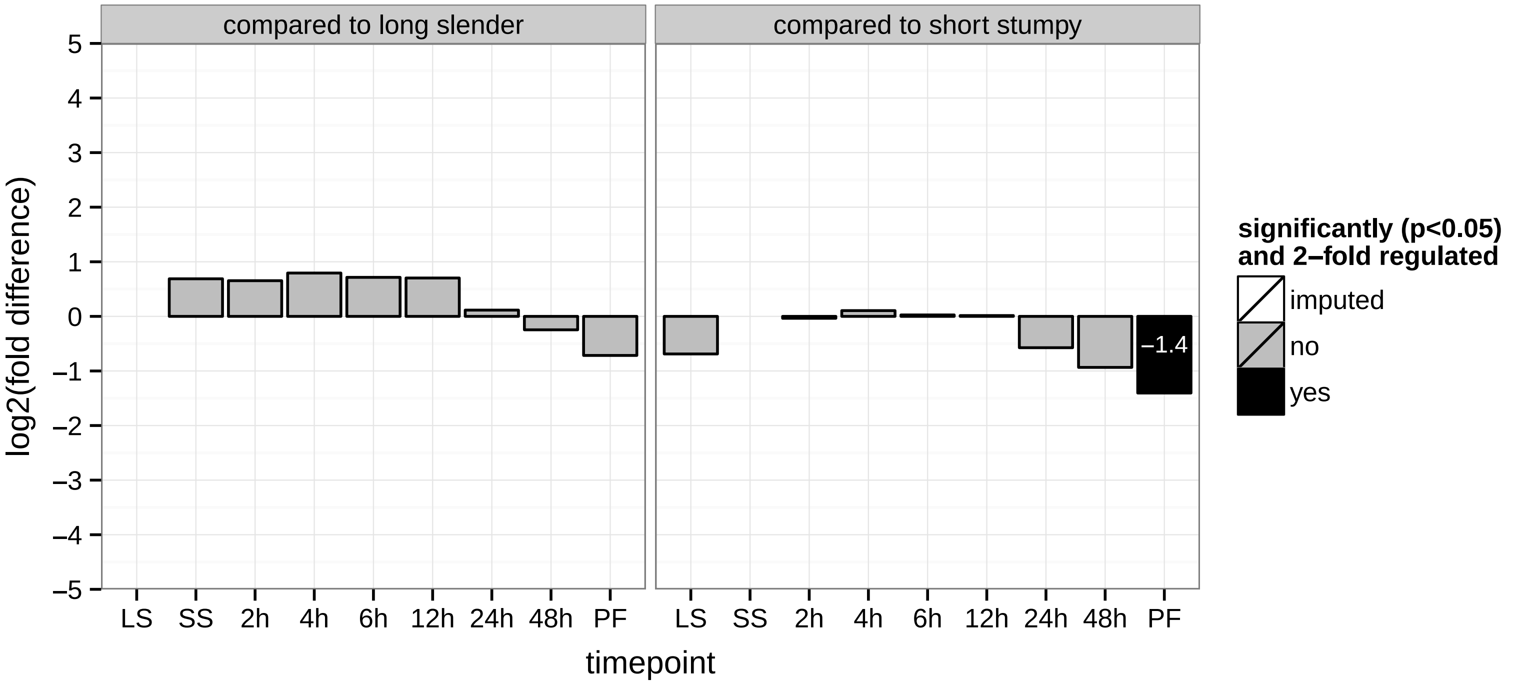

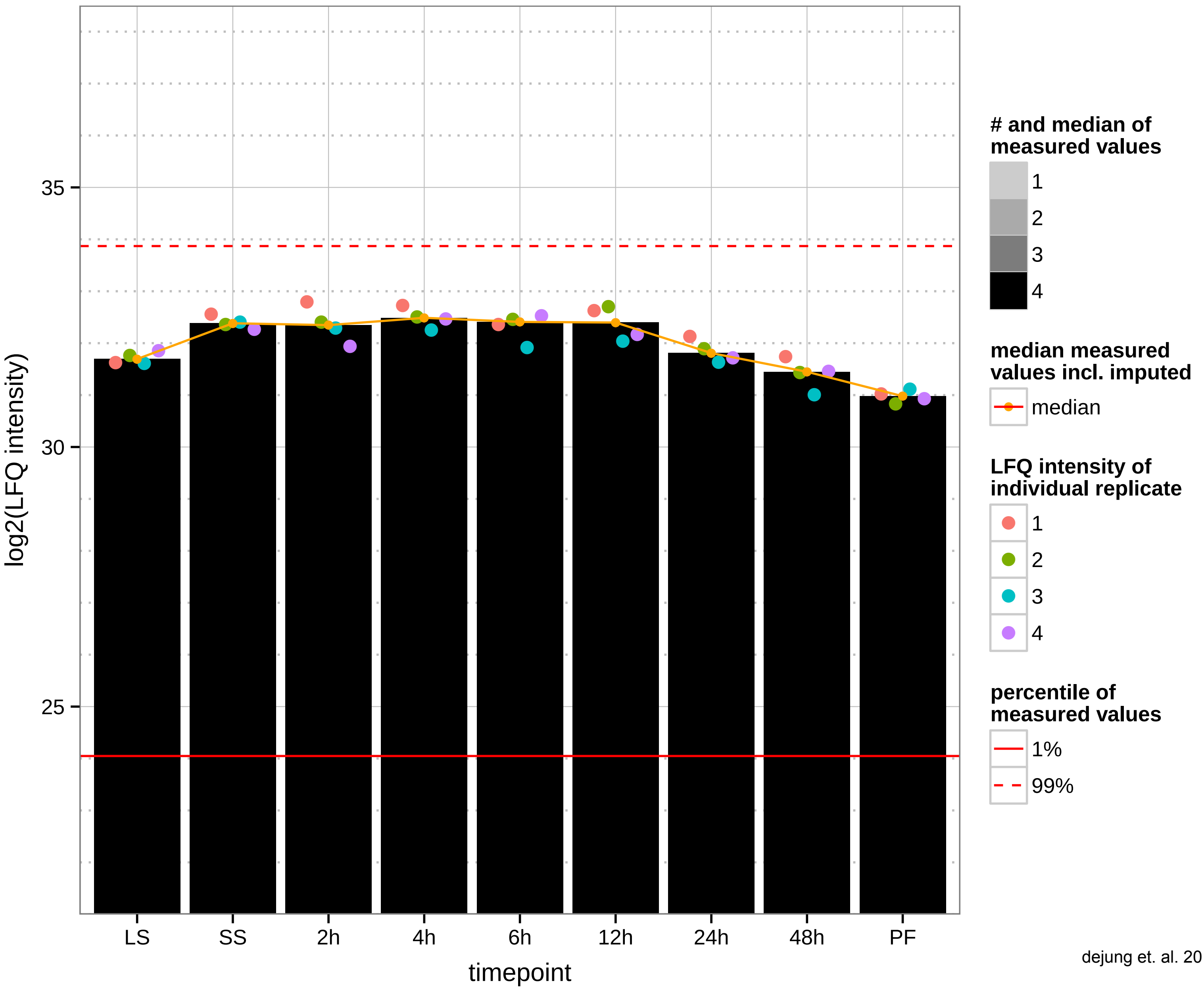

dejung et. al. 2015

splicing factor TSR1 (TSR1)
Tb927.8.900
AGOF: RNA binding
AGOC: nucleus
AGOP: RNA processing
PGOF: nucleic acid binding
PGOC: null
PGOP: null

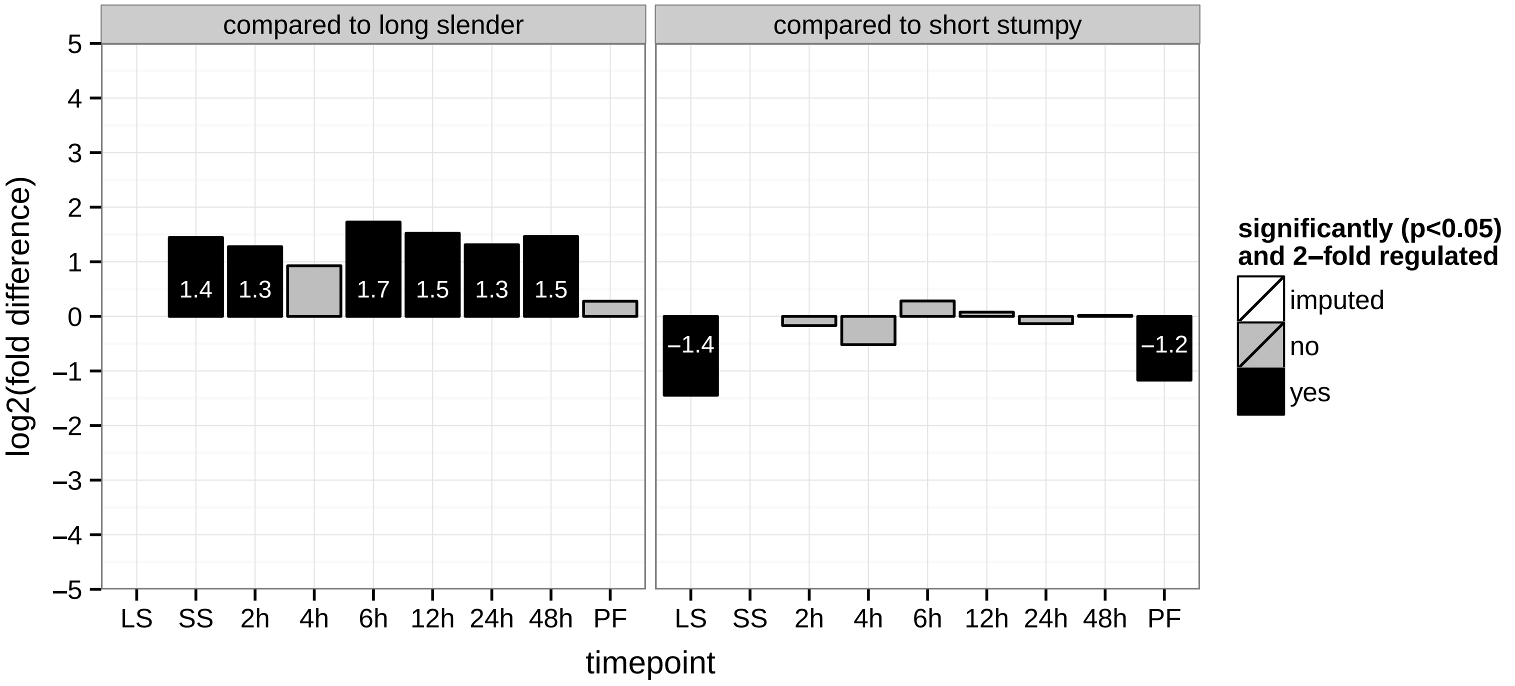

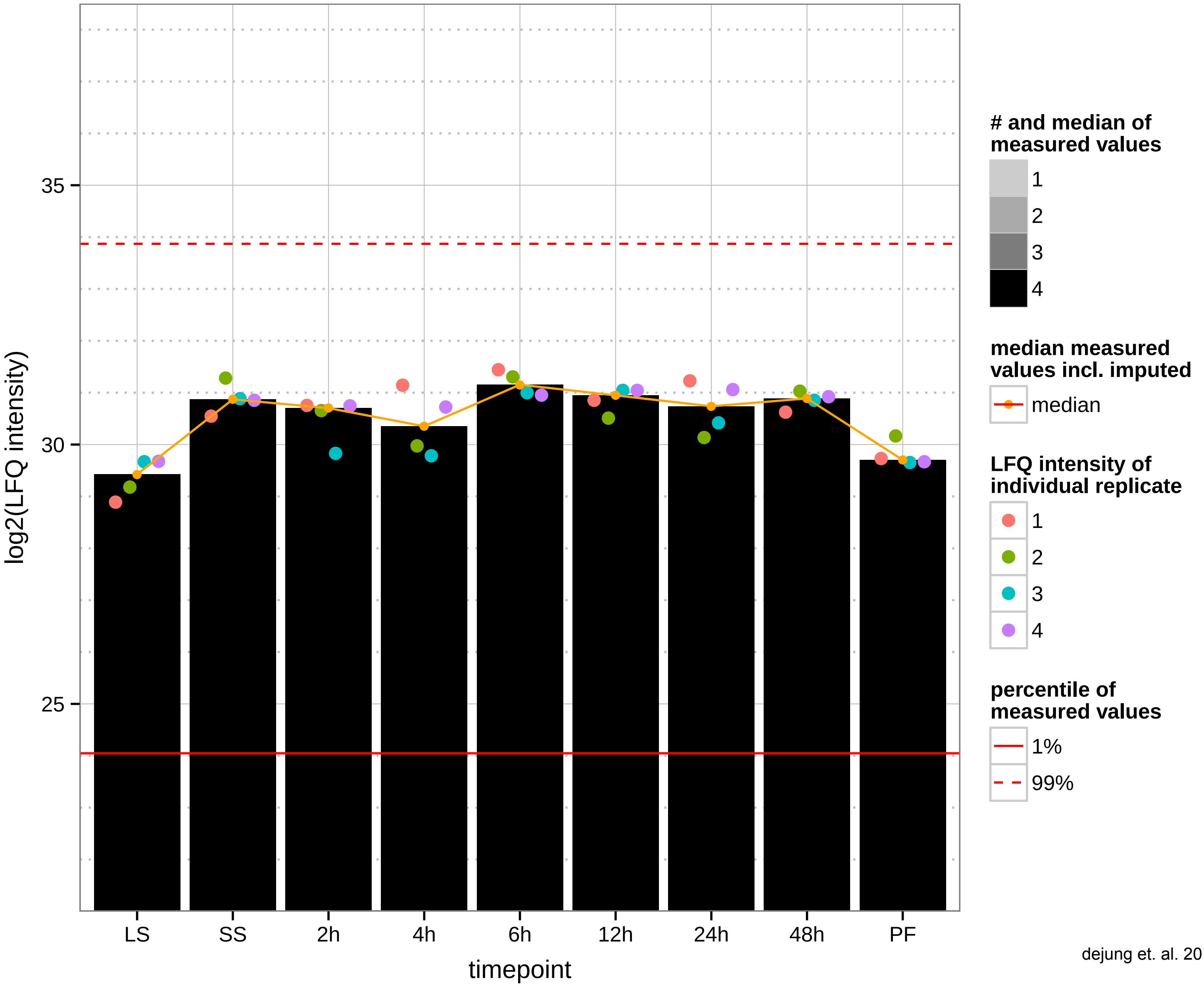

dejung et. al. 2015

hypothetical protein, conserved
Tb927.9.10350
AGOF: null
AGOC: null
AGOP: protein transport, vesicle–mediated transport
PGOF: null
PGOC: null
PGOP: protein transport

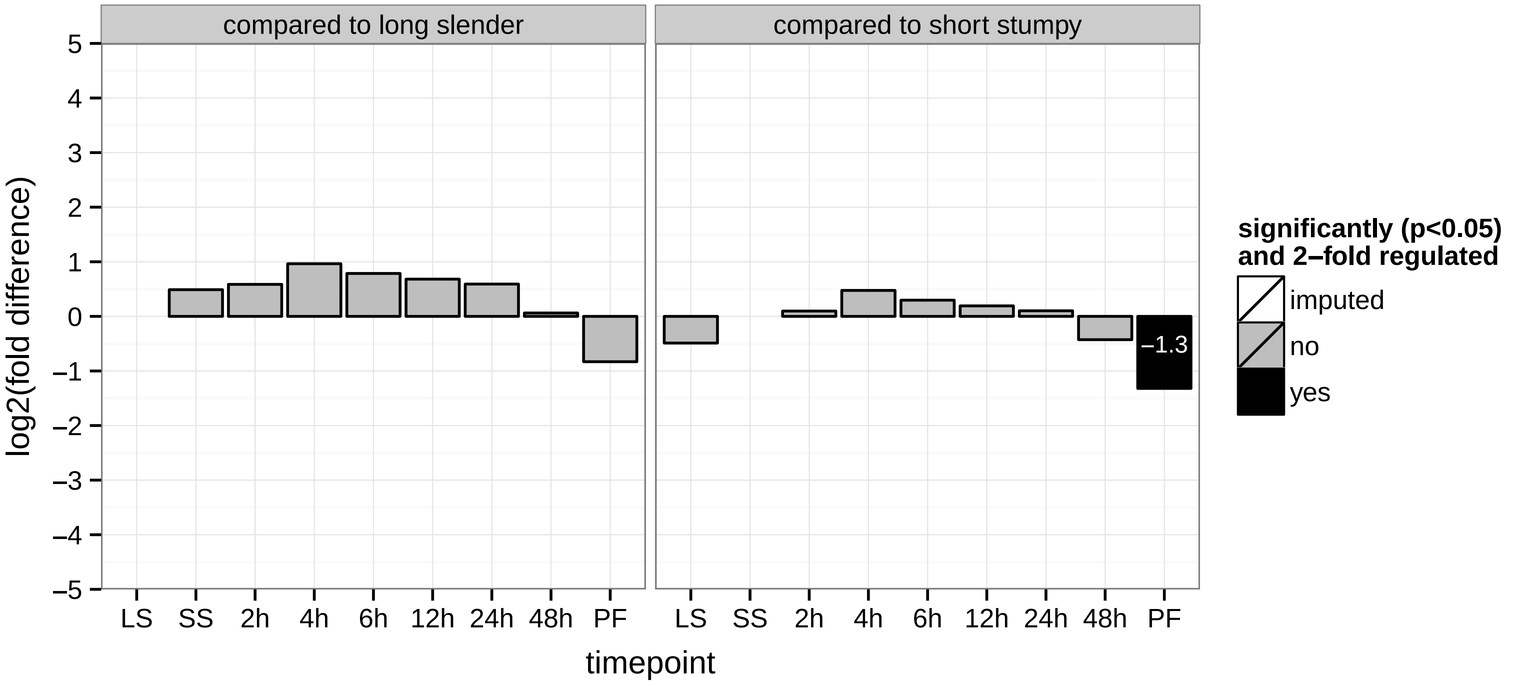

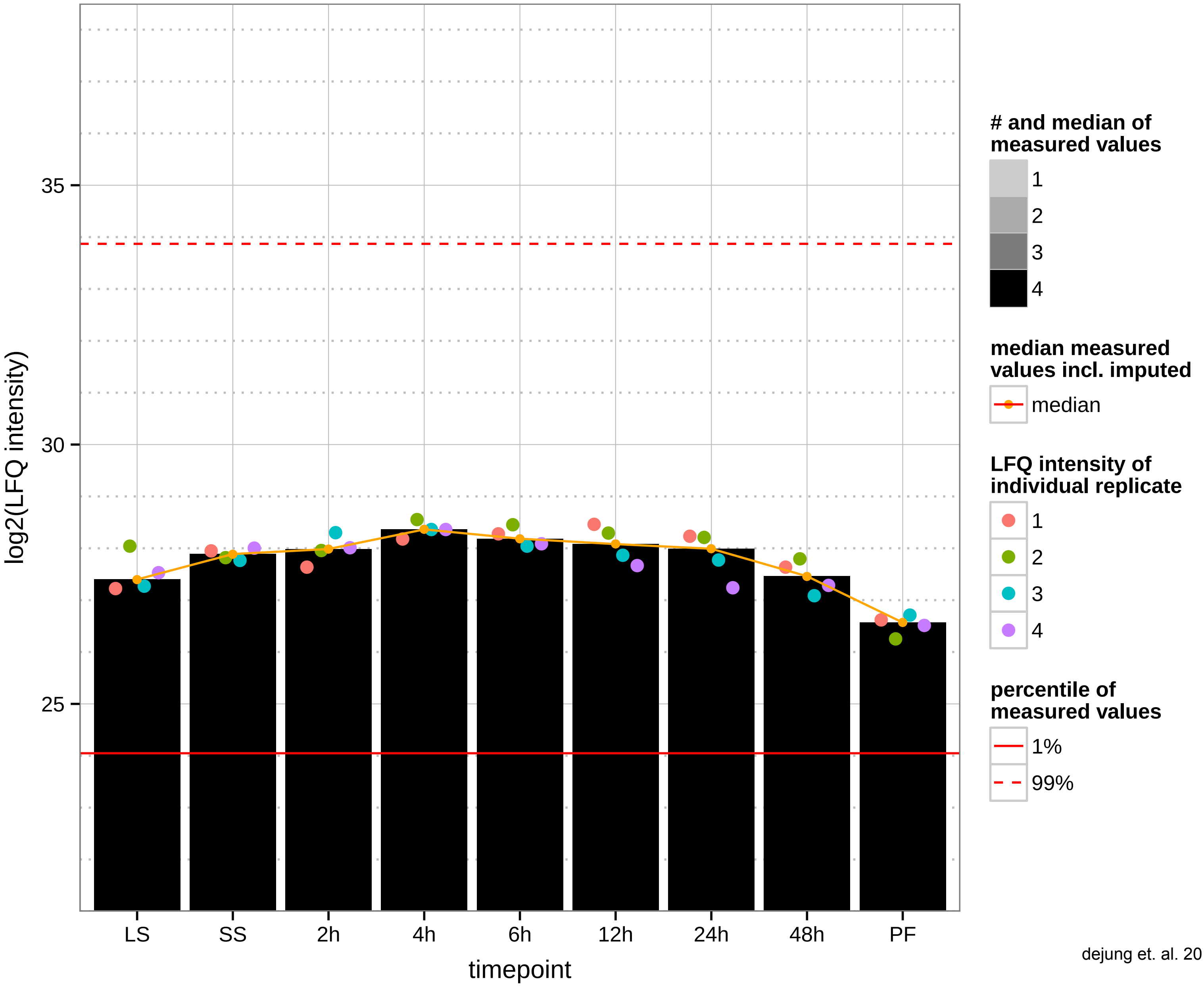

dejung et. al. 2015

hypothetical protein, conserved
Tb927.9.10450
AGOF: hydrolase activity, acting on ester bonds
AGOC: intrinsic to endoplasmic reticulum membrane
AGOP: GPI anchor metabolic process, intracellular protein transport
PGOF: hydrolase activity, acting on ester bonds
PGOC: intrinsic to endoplasmic reticulum membrane
PGOP: GPI anchor metabolic process, intracellular protein transport

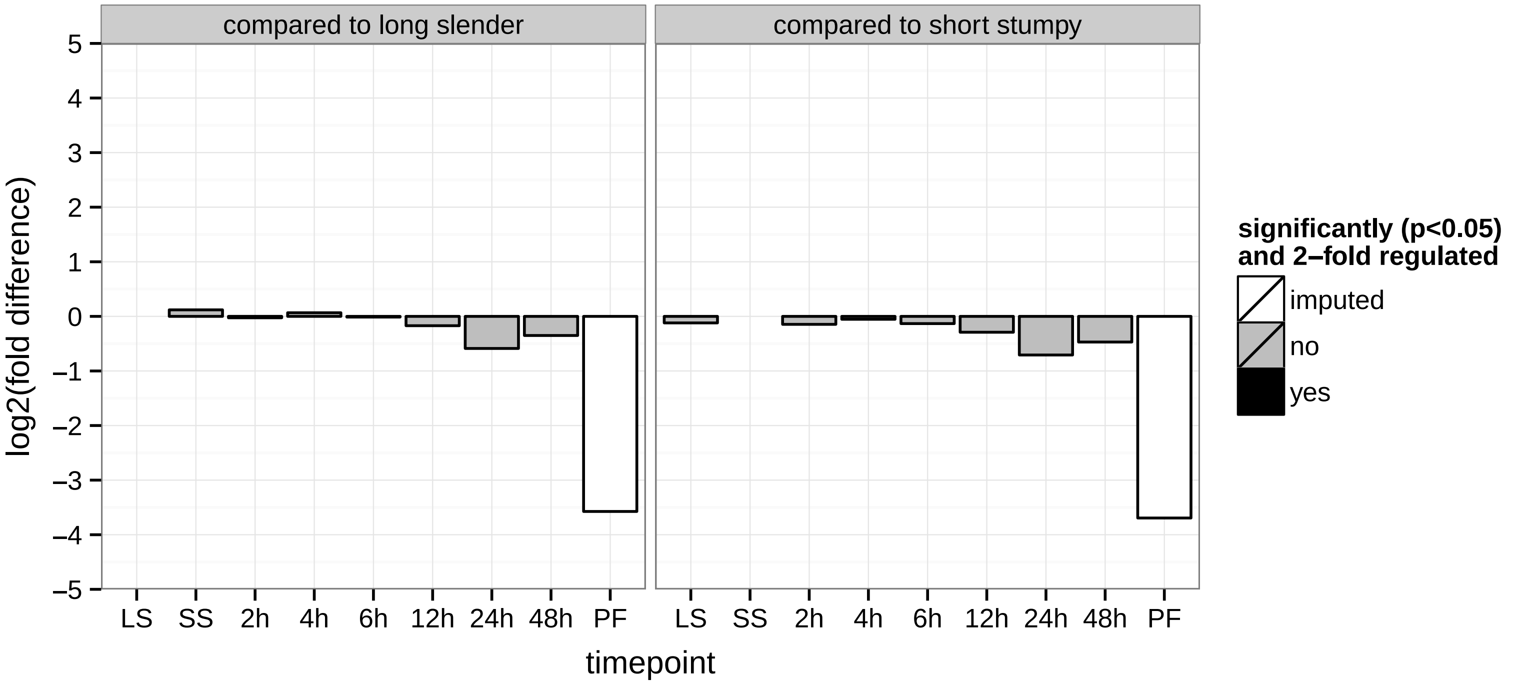

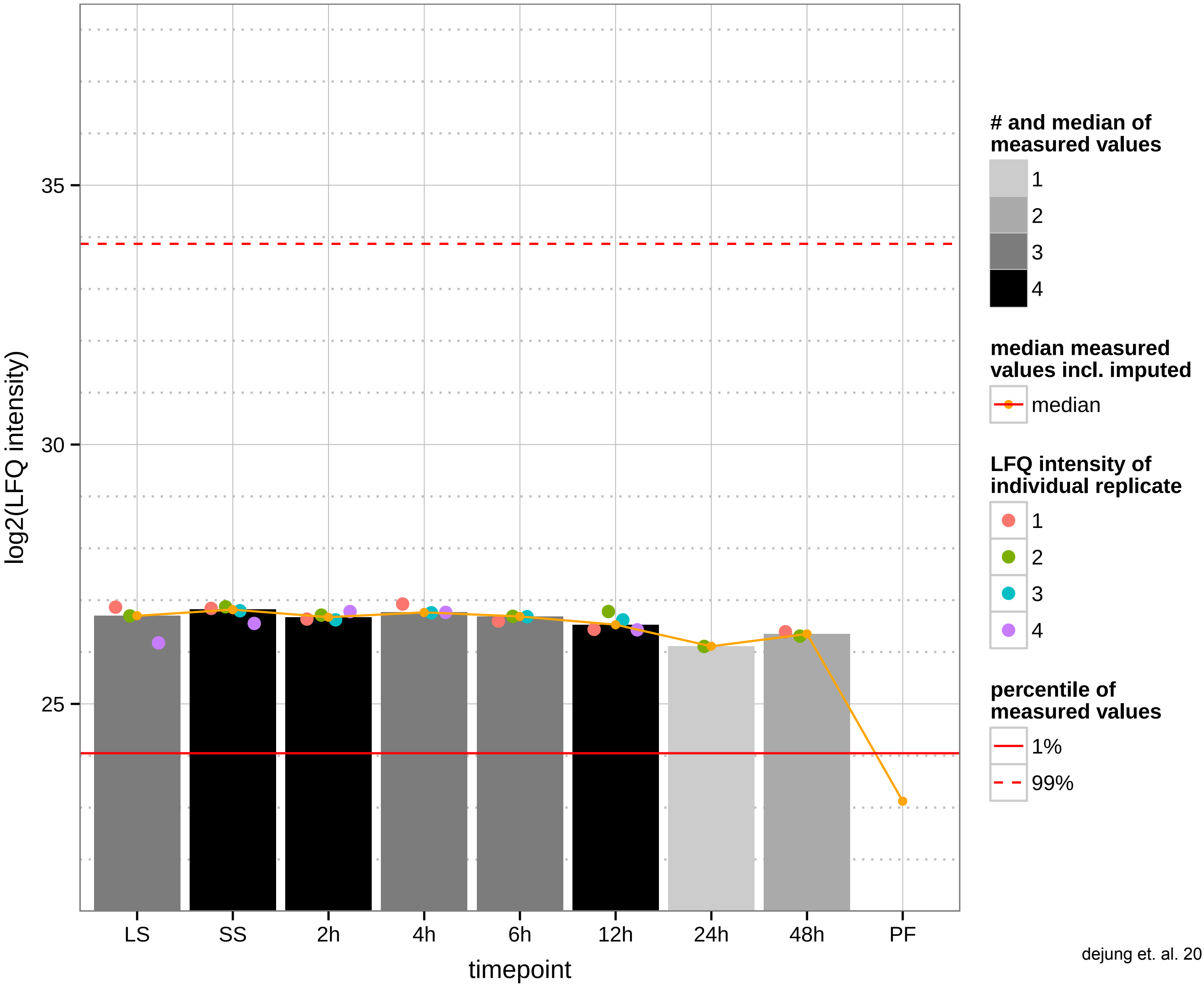

dejung et. al. 2015

hypothetical protein, conserved
Tb927.9.10550
AGOF: null
AGOC: null
AGOP: null
PGOF: null
PGOC: null
PGOP: null

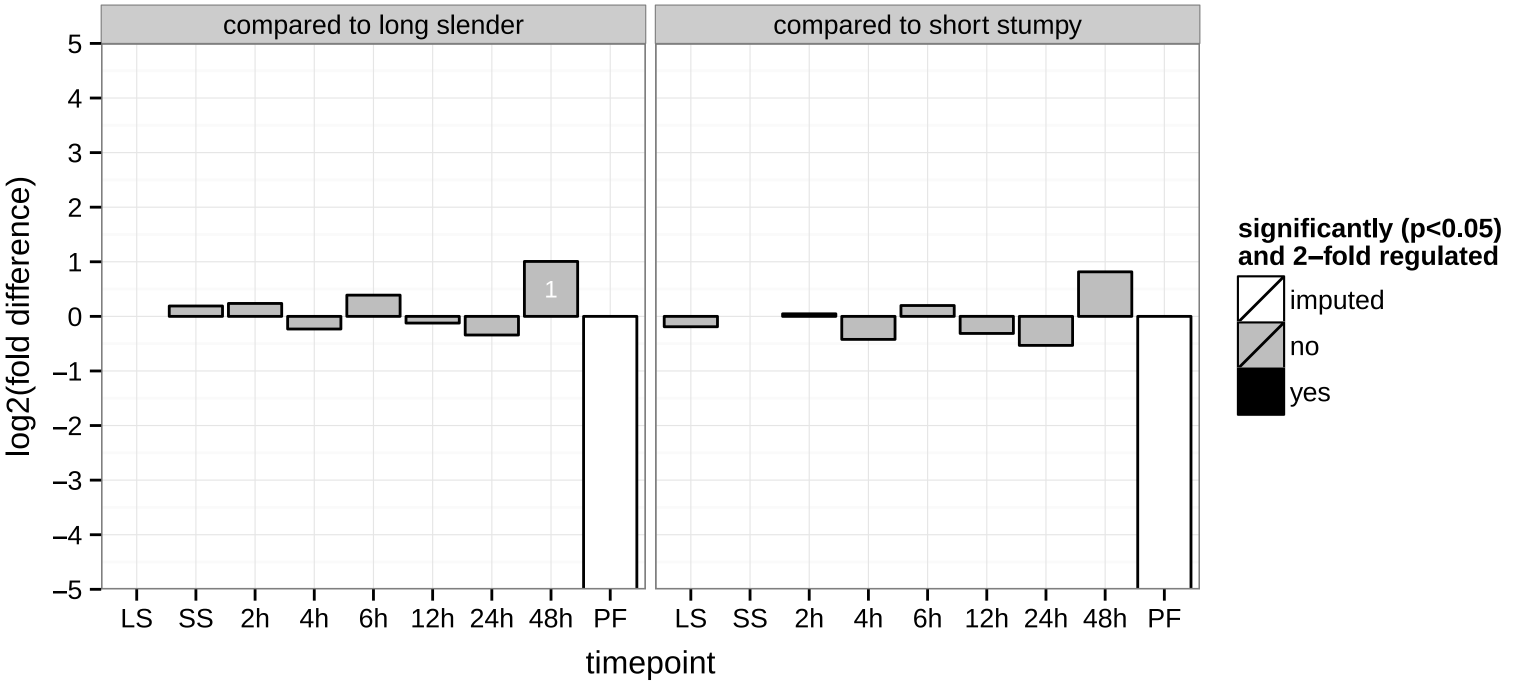

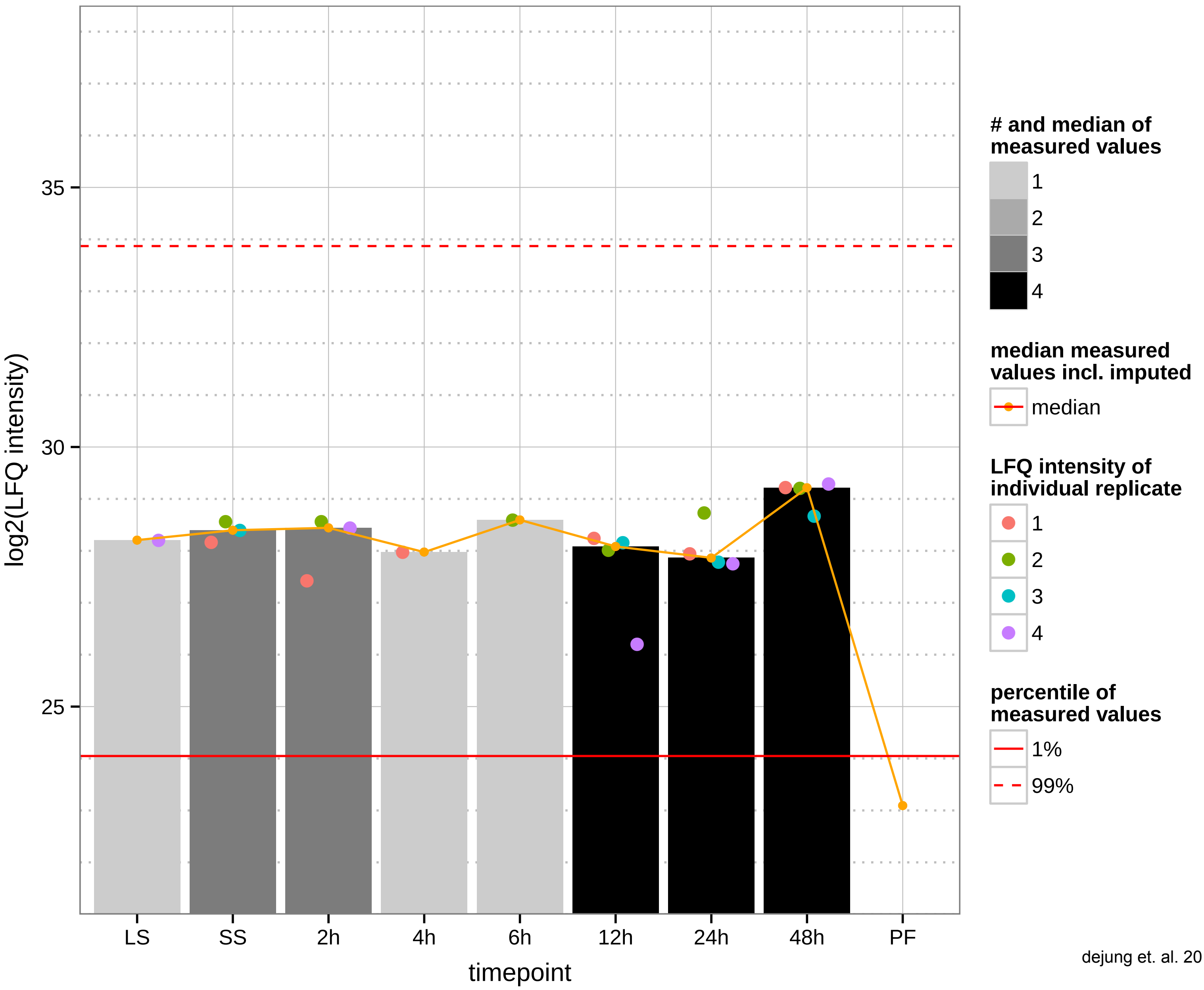

dejung et. al. 2015

4E–interacting protein, putative
Tb927.9.11050
AGOF: translation regulator activity
AGOC: cytoplasm
AGOP: regulation of translation
PGOF: null
PGOC: null
PGOP: null

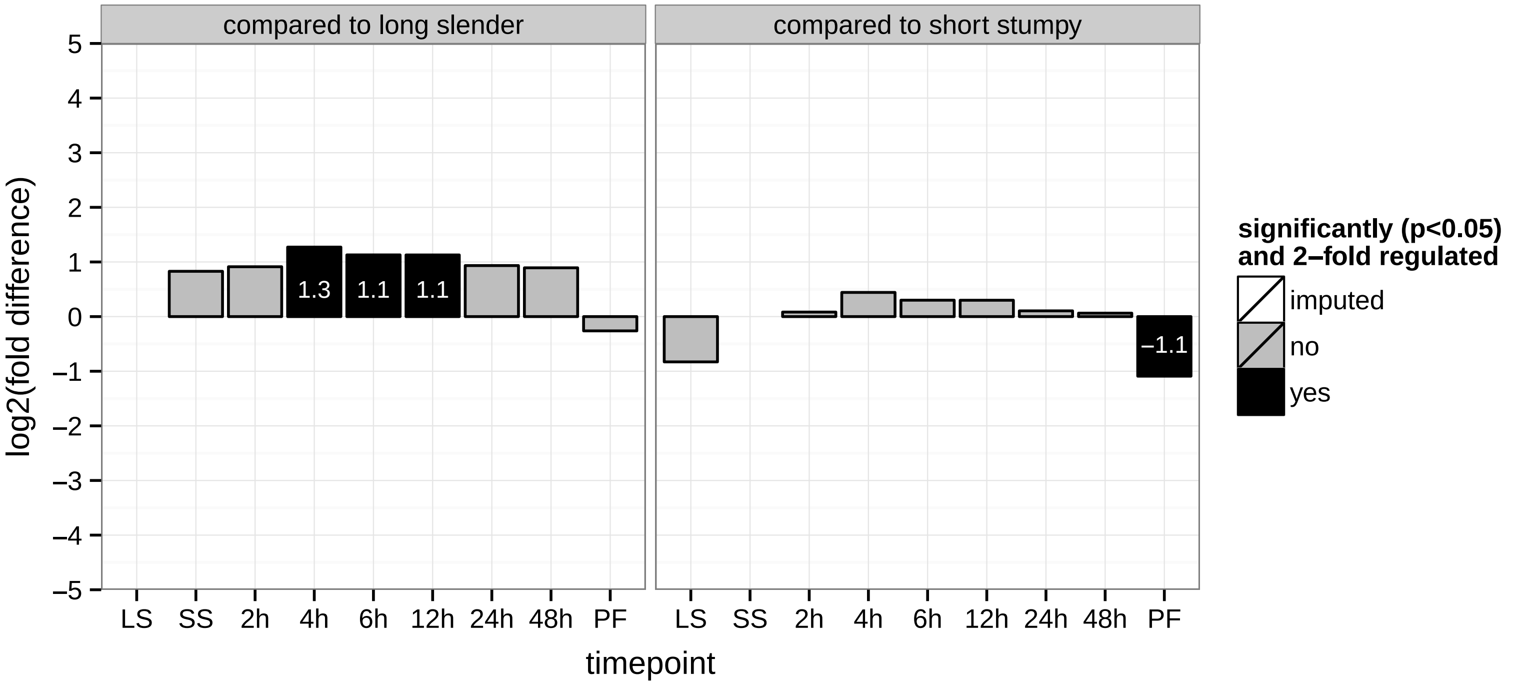

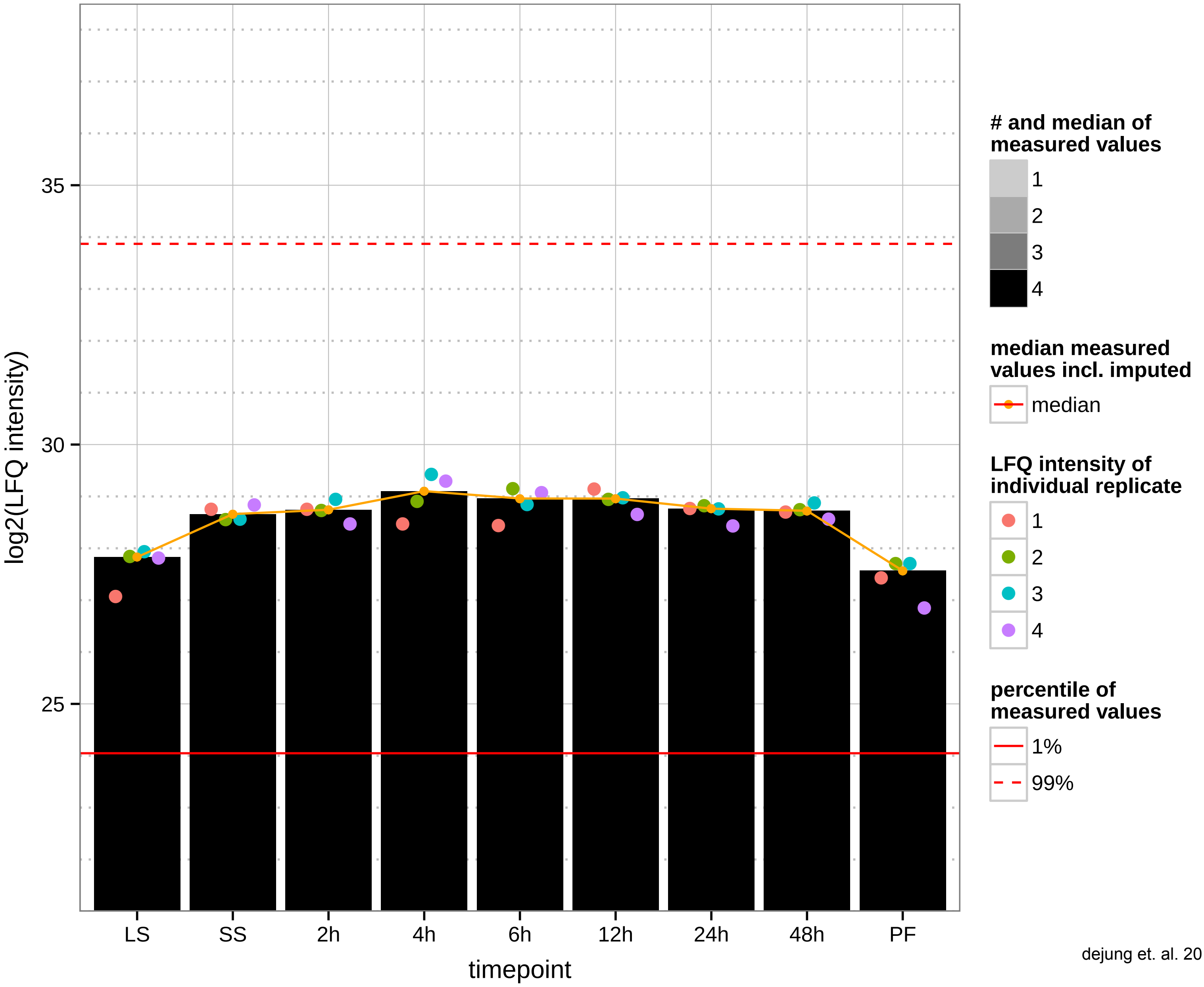

dejung et. al. 2015

hypothetical protein, conserved
Tb927.9.12000
AGOF: null
AGOC: membrane
AGOP: null
PGOF: null
PGOC: membrane
PGOP: null

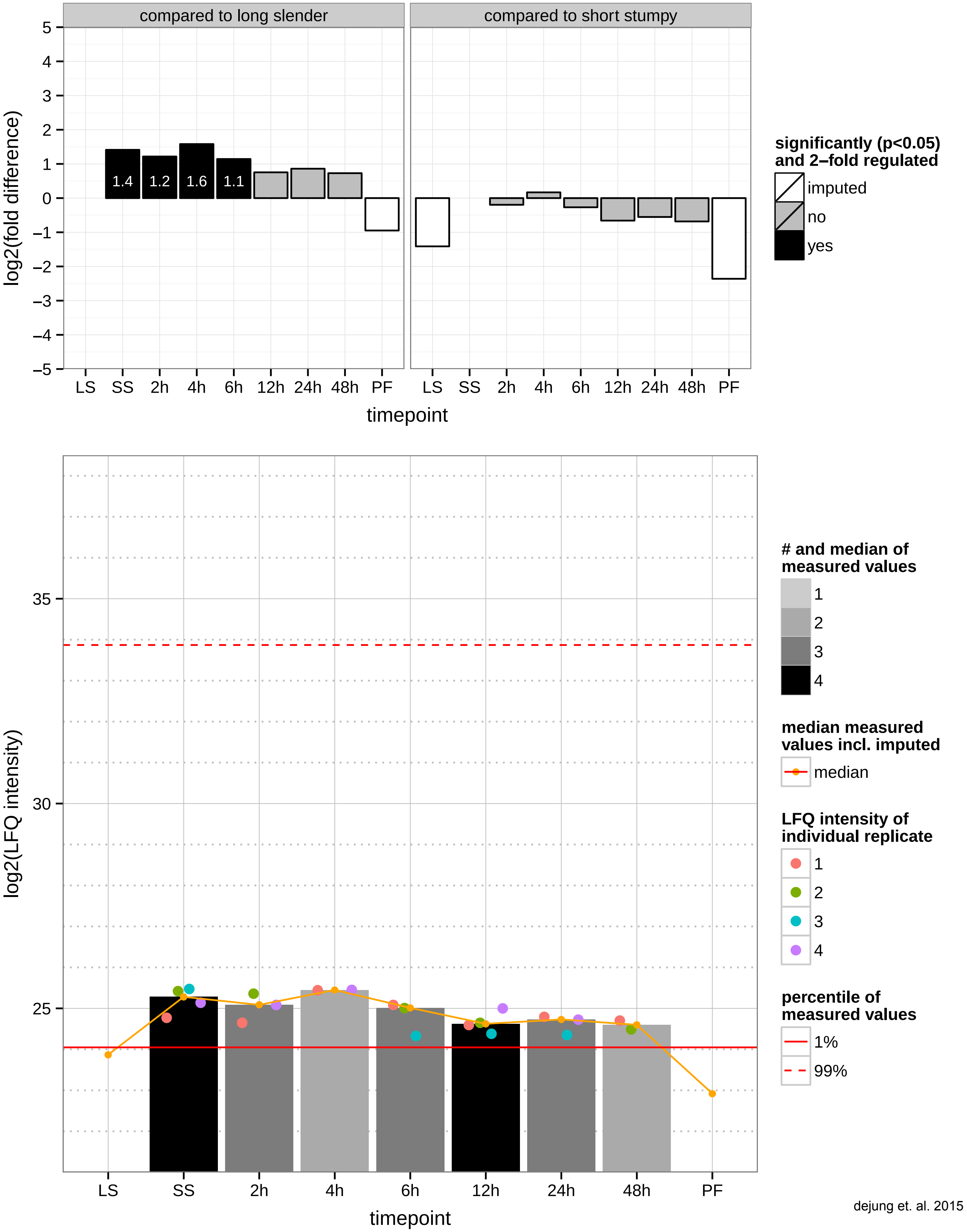

dejung et. al. 2015

hypothetical protein, conserved
Tb927.9.12040
AGOF: mRNA (nucleoside−2'−O−)−methyltransferase activity
AGOC: null
AGOP: 7−methylguanosine mRNA capping
PGOF: null
PGOC: null
PGOP: null

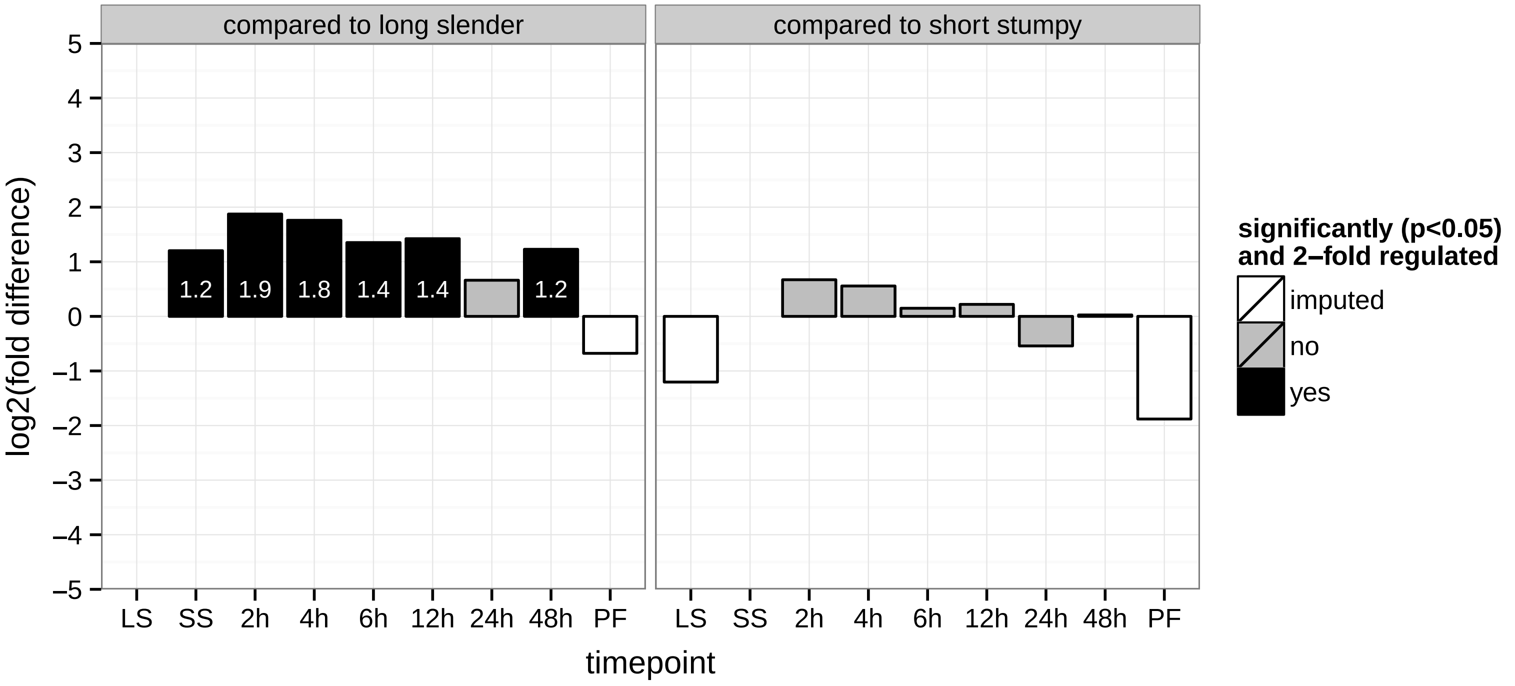

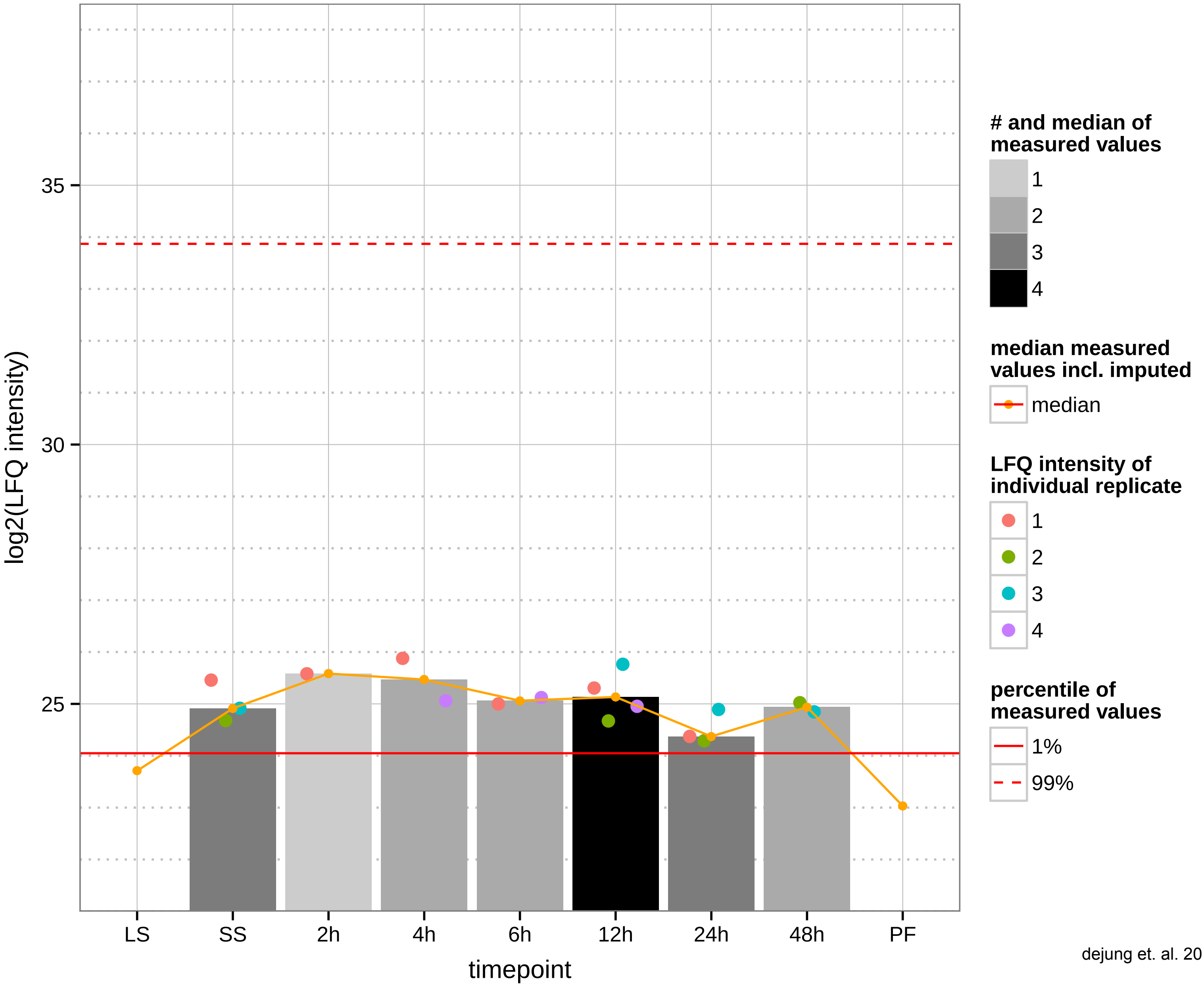

dejung et. al. 2015

hypothetical protein, conserved
Tb927.9.12870
AGOF: null
AGOC: cis–Golgi network
AGOP: ER to Golgi vesicle–mediated transport
PGOF: null
PGOC: cis–Golgi network
PGOP: ER to Golgi vesicle–mediated transport, transport

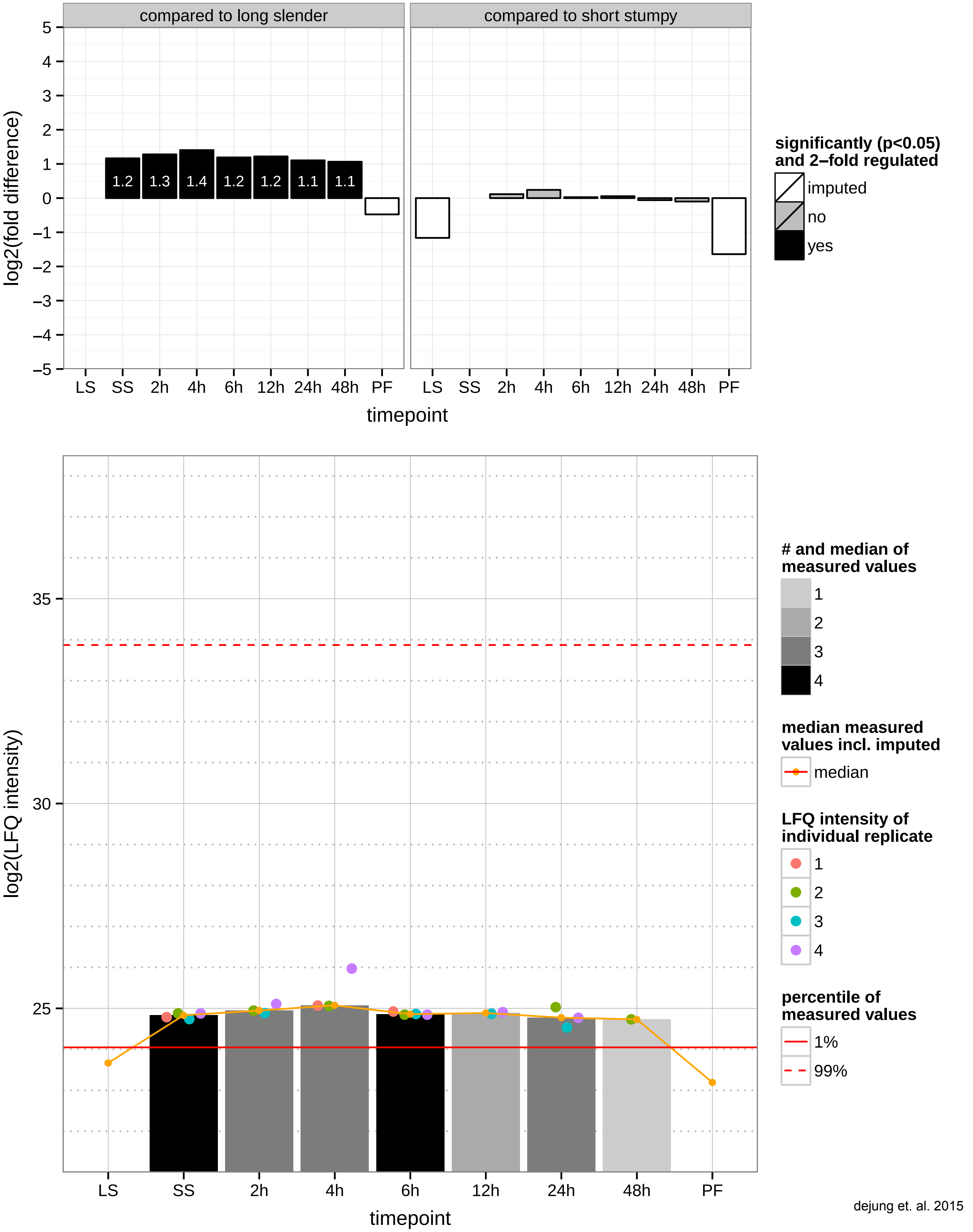

dejung et. al. 2015

hypothetical protein, conserved
Tb927.9.13050
AGOF: null
AGOC: null
AGOP: null
PGOF: null
PGOC: null
PGOP: null

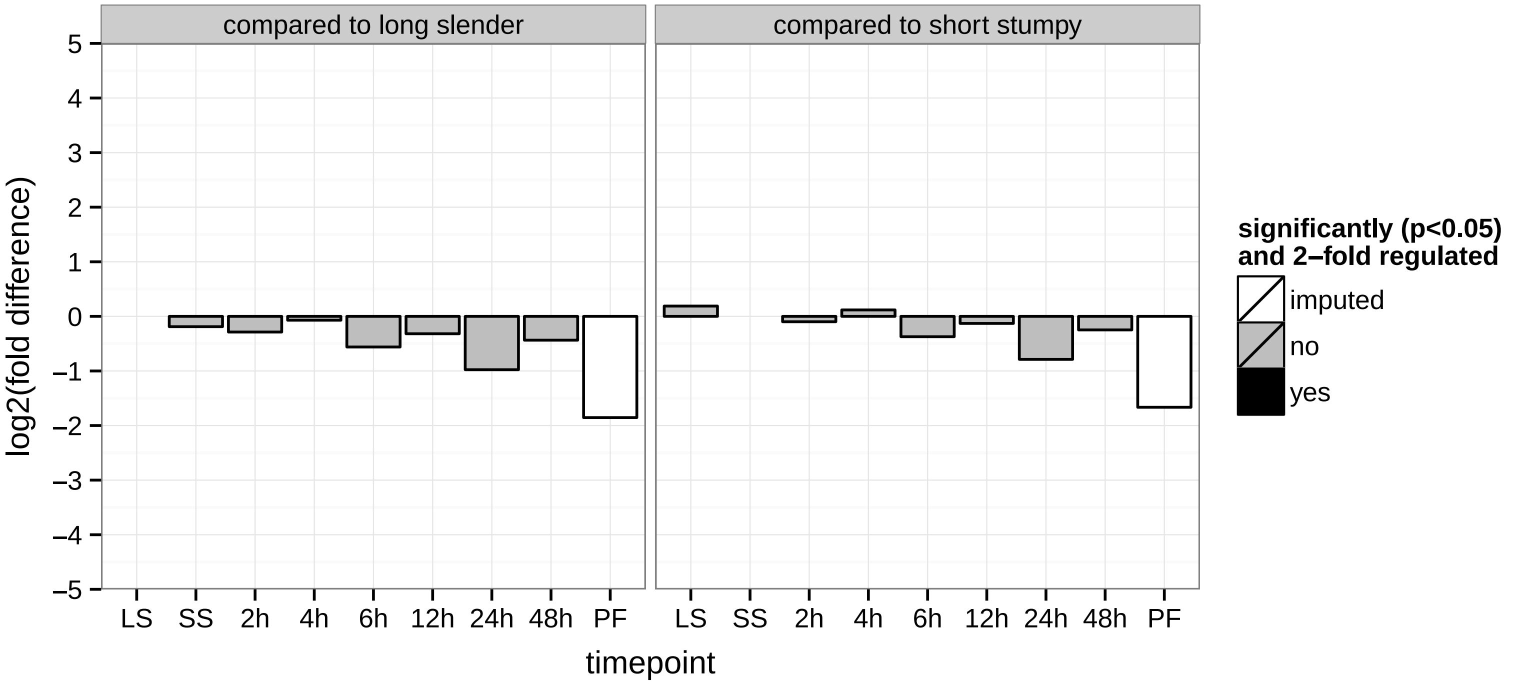

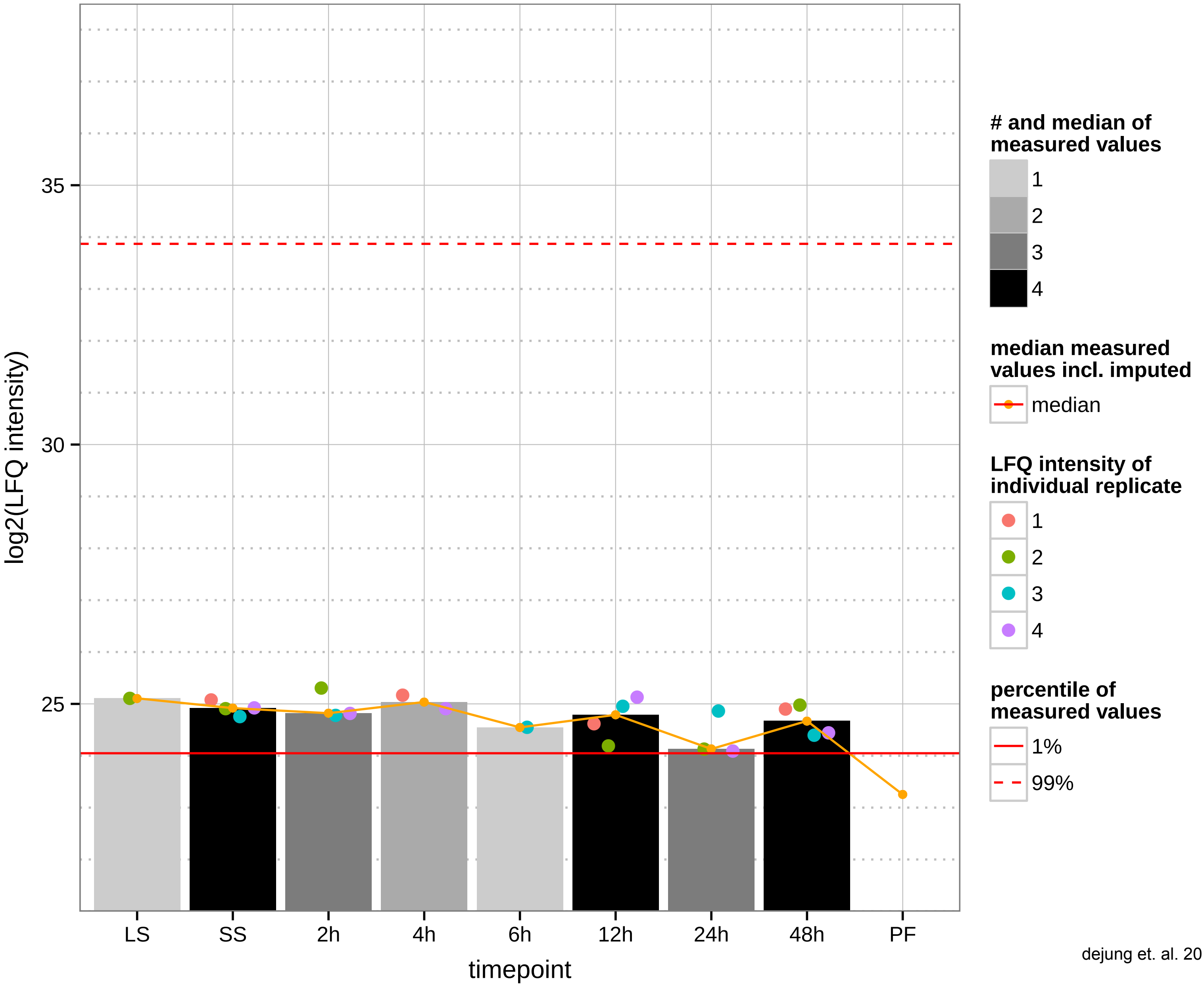

dejung et. al. 2015

hypothetical protein, conserved
Tb927.9.13340
AGOF: null
AGOC: null
AGOP: null
PGOF: null
PGOC: null
PGOP: null

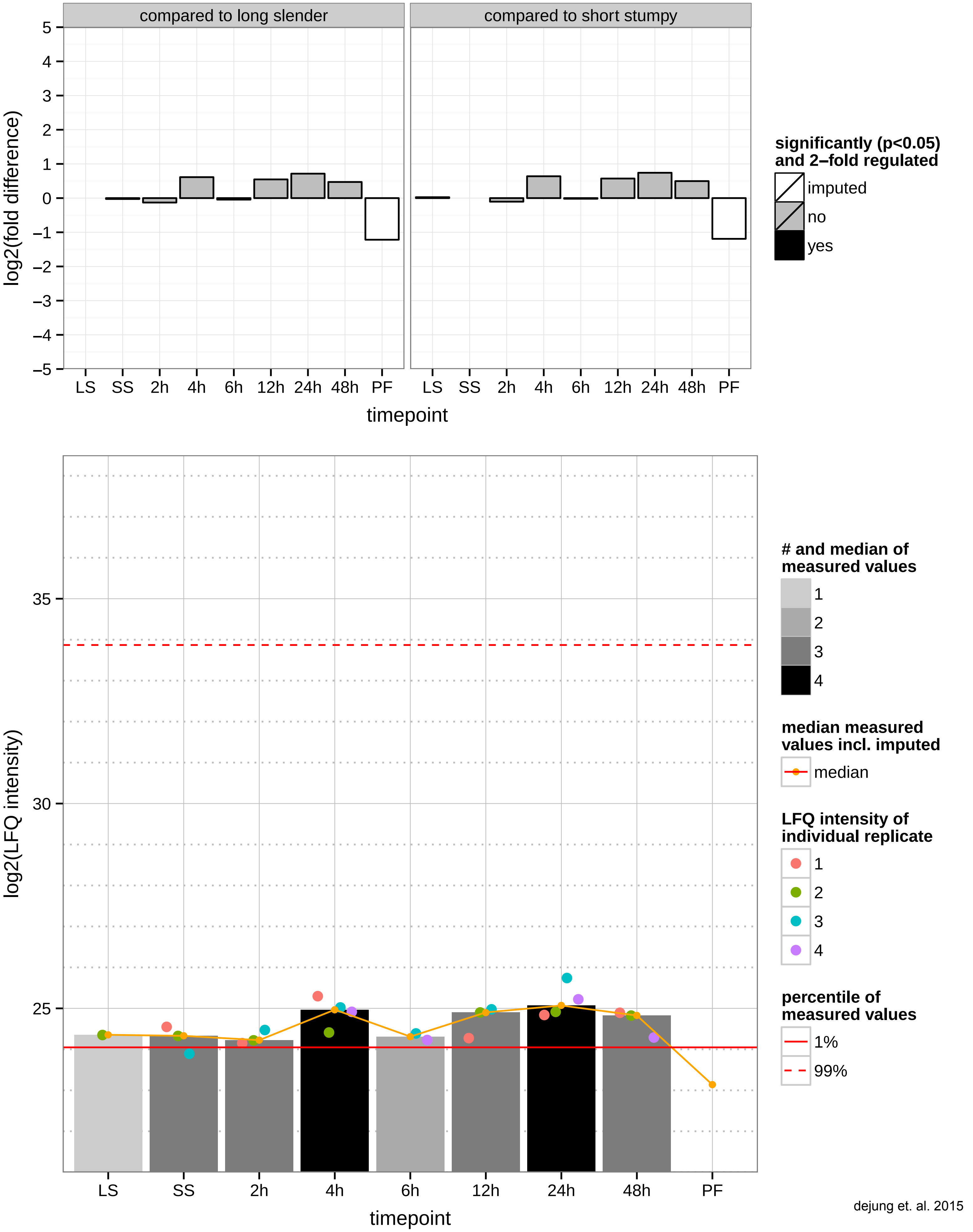

dejung et. al. 2015

hypothetical protein, conserved
Tb927.9.2400
AGOF: null
AGOC: null
AGOP: null
PGOF: null
PGOC: null
PGOP: null

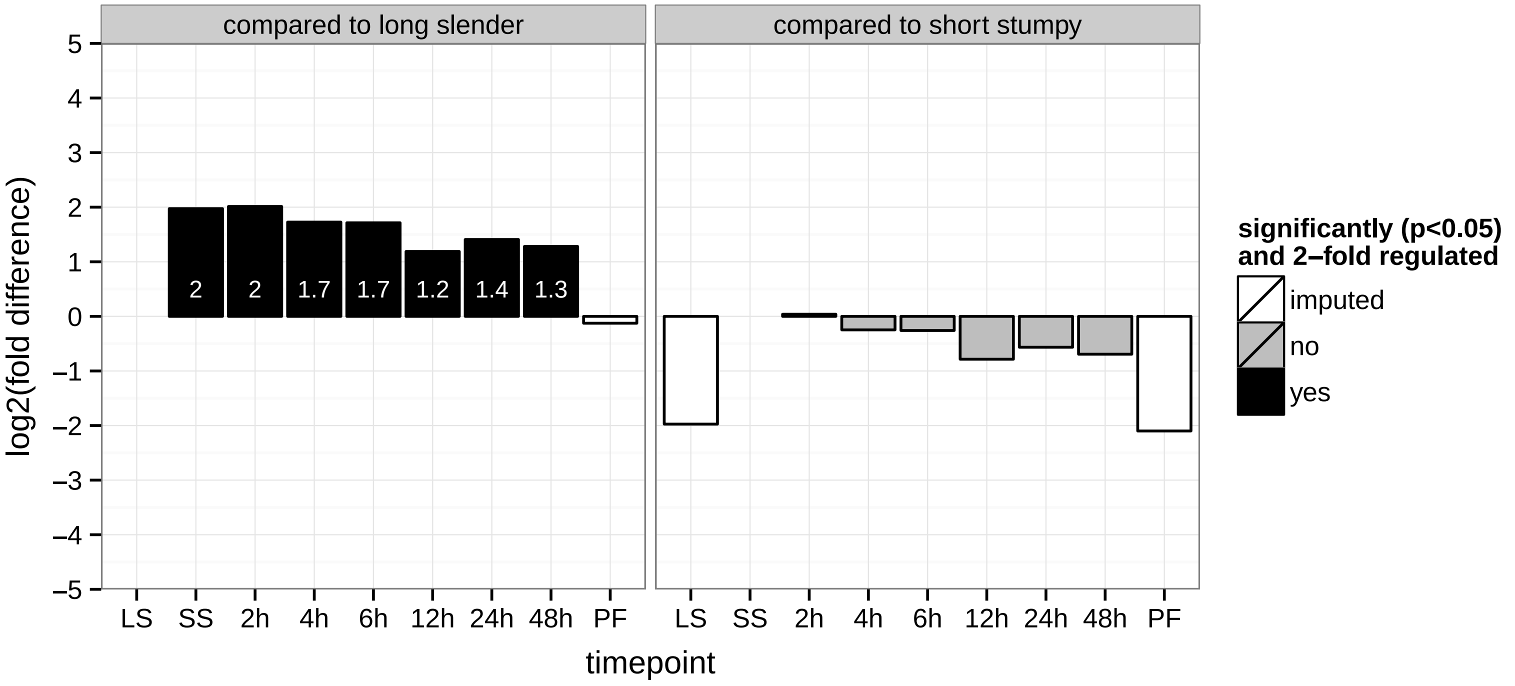

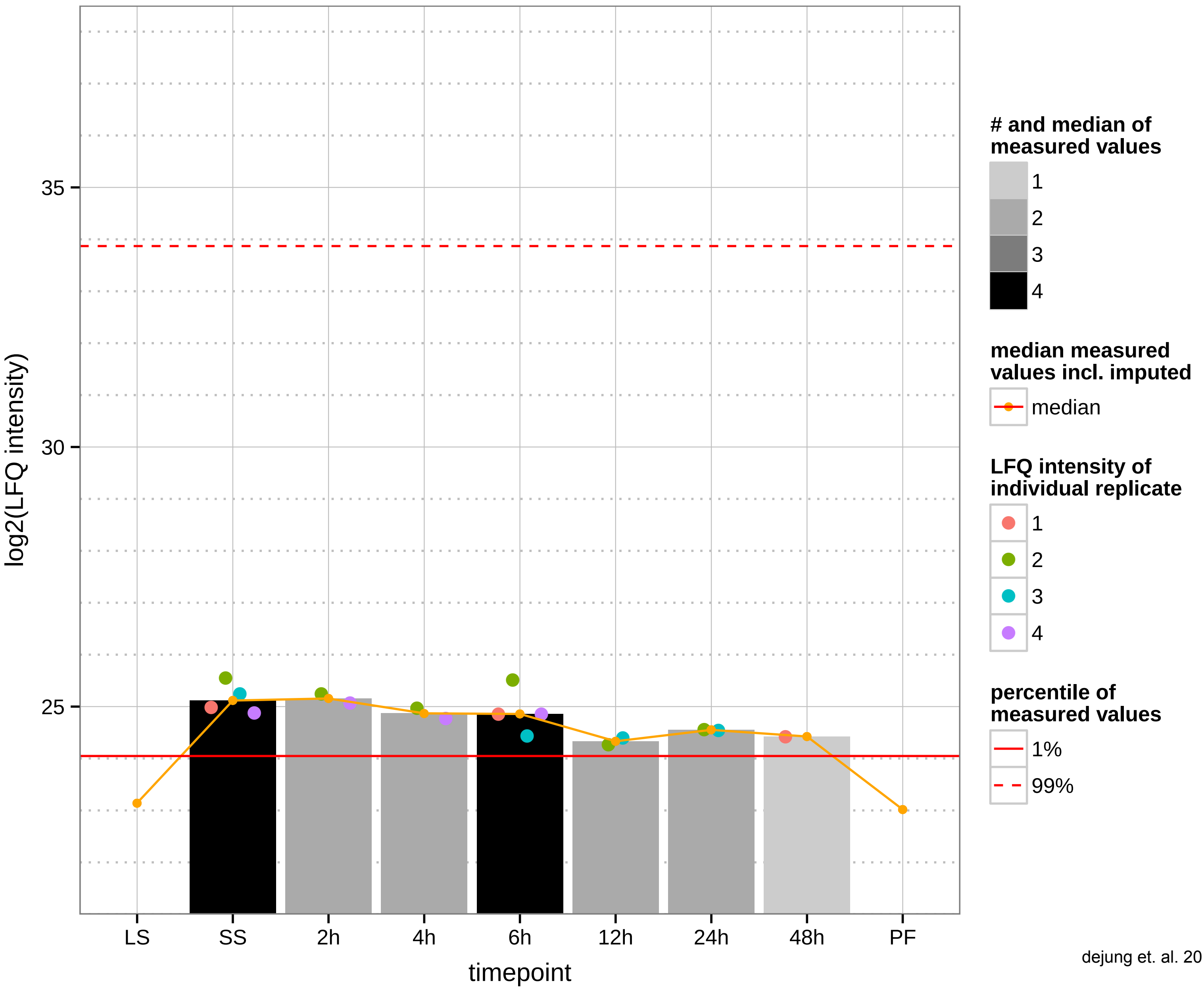

dejung et. al. 2015

hypothetical protein, conserved
Tb927.9.3640
AGOF: null
AGOC: mitochondrion
AGOP: null
PGOF: null
PGOC: null
PGOP: null

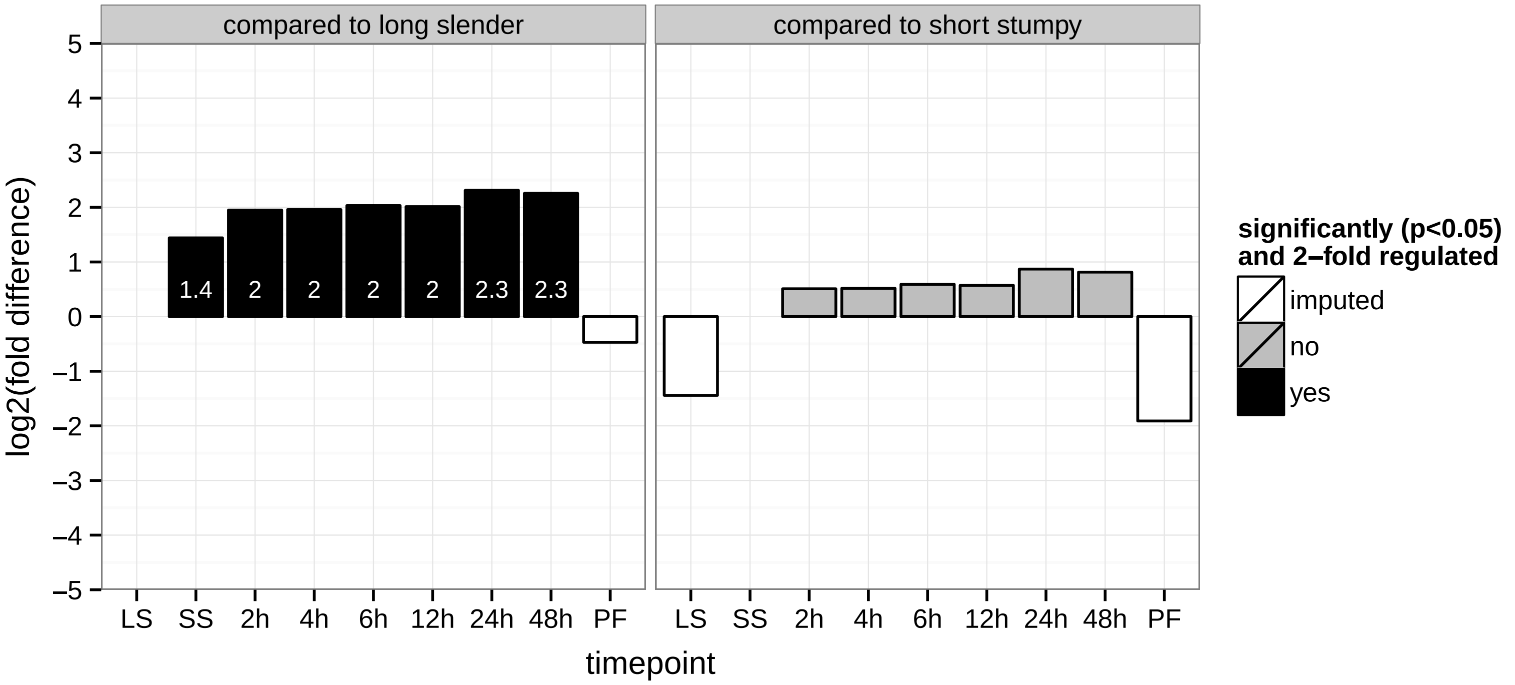

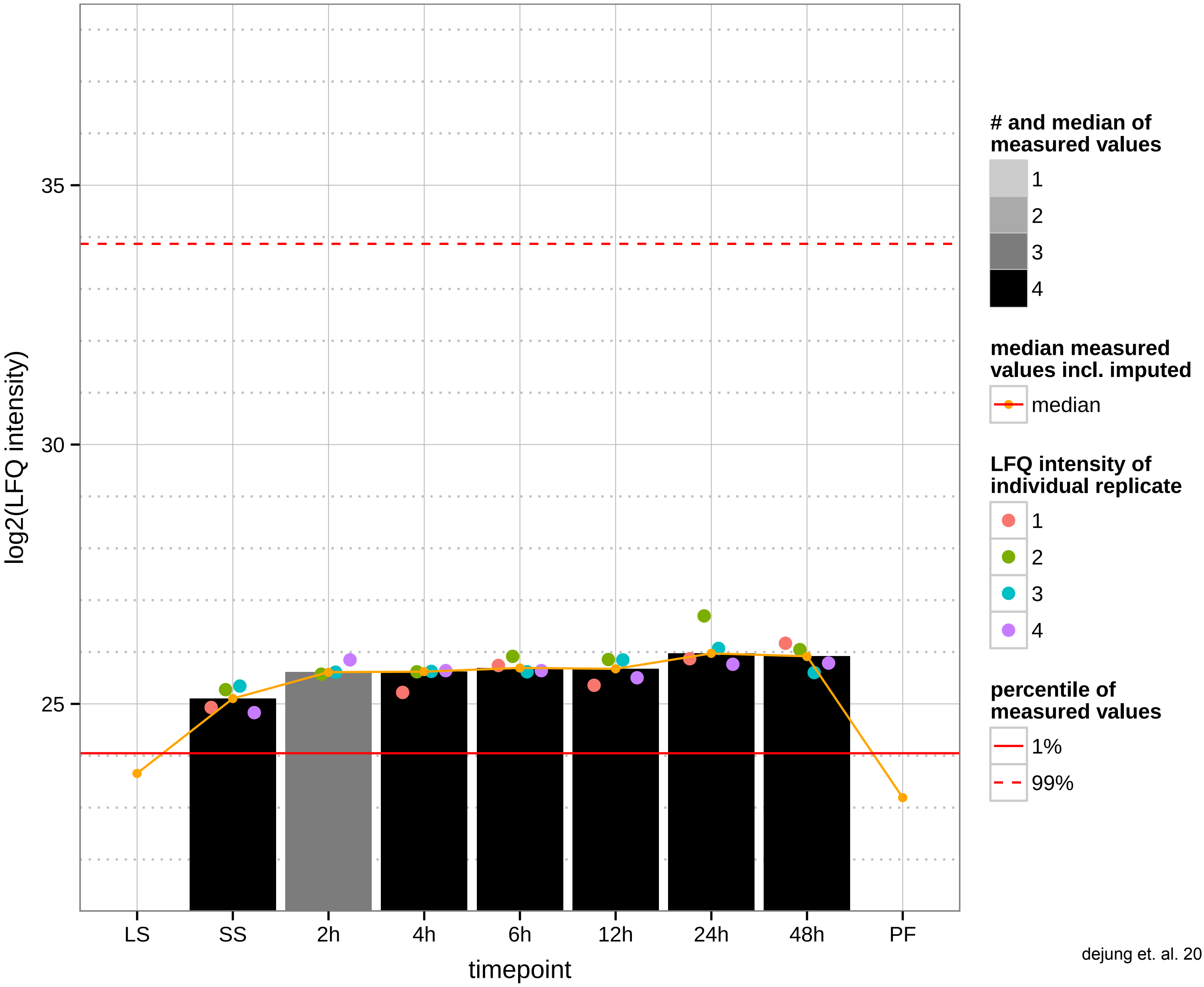

dejung et. al. 2015

hypothetical protein, conserved
Tb927.9.3750
AGOF: null
AGOC: null
AGOP: null
PGOF: null
PGOC: null
PGOP: null

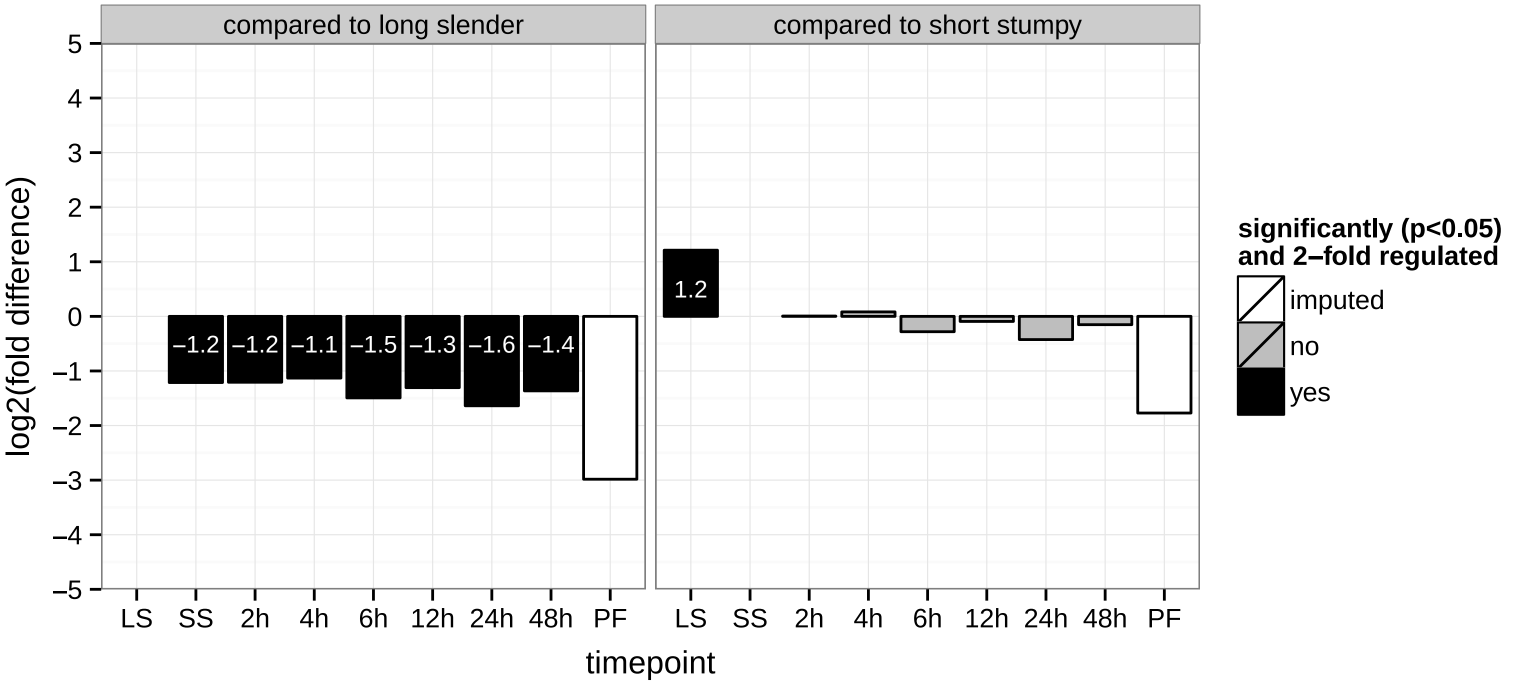

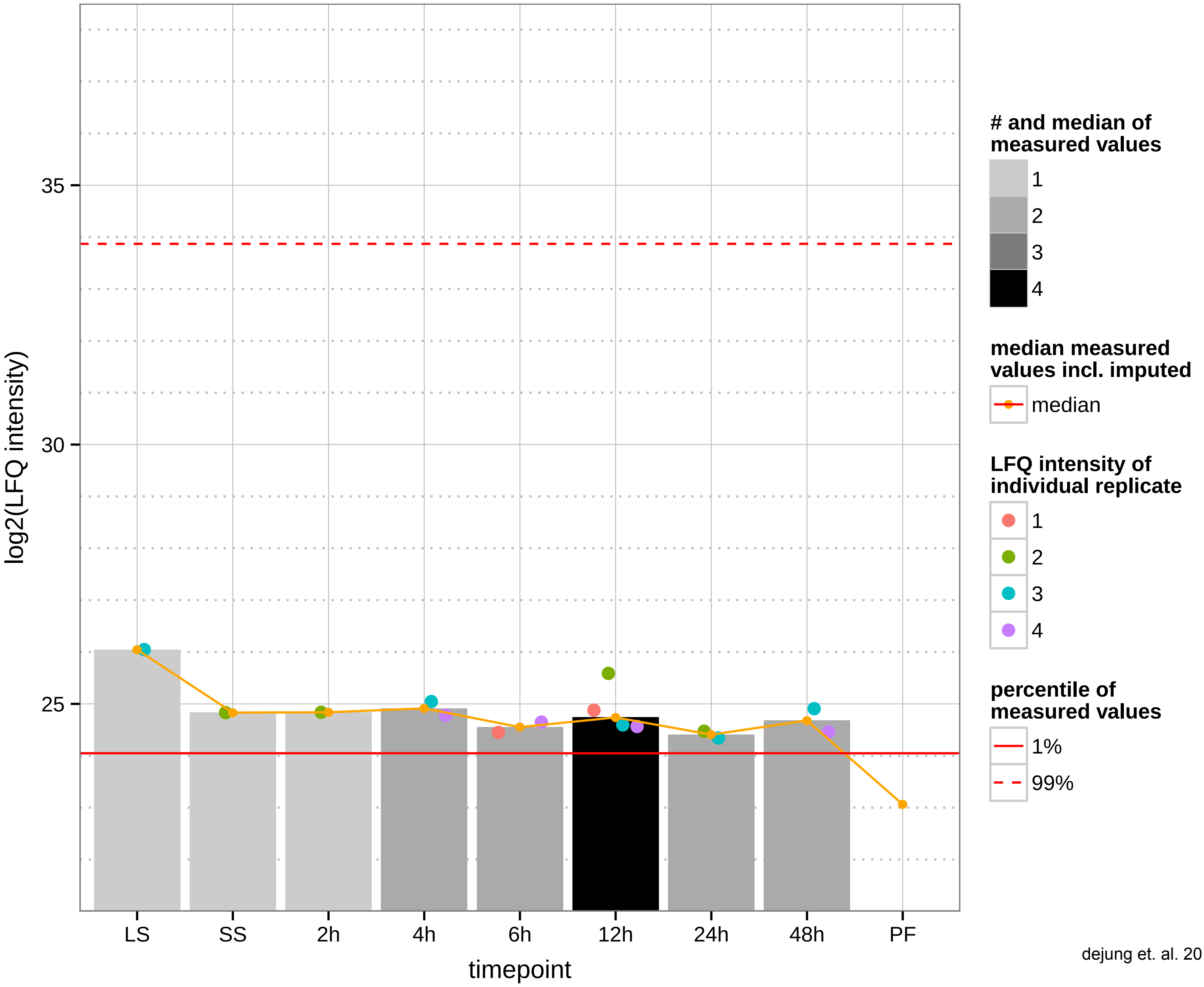

dejung et. al. 2015

fatty acyl CoA synthetase, putative, fatty acyl CoA synthetase 3 (ACS3)
Tb927.9.4210;Tb11.v5.0561
AGOF: null, fatty–acyl–CoA synthase activity
AGOC: null, membrane
AGOP: null, fatty acid metabolic process
PGOF: catalytic activity
PGOC: null
PGOP: metabolic process

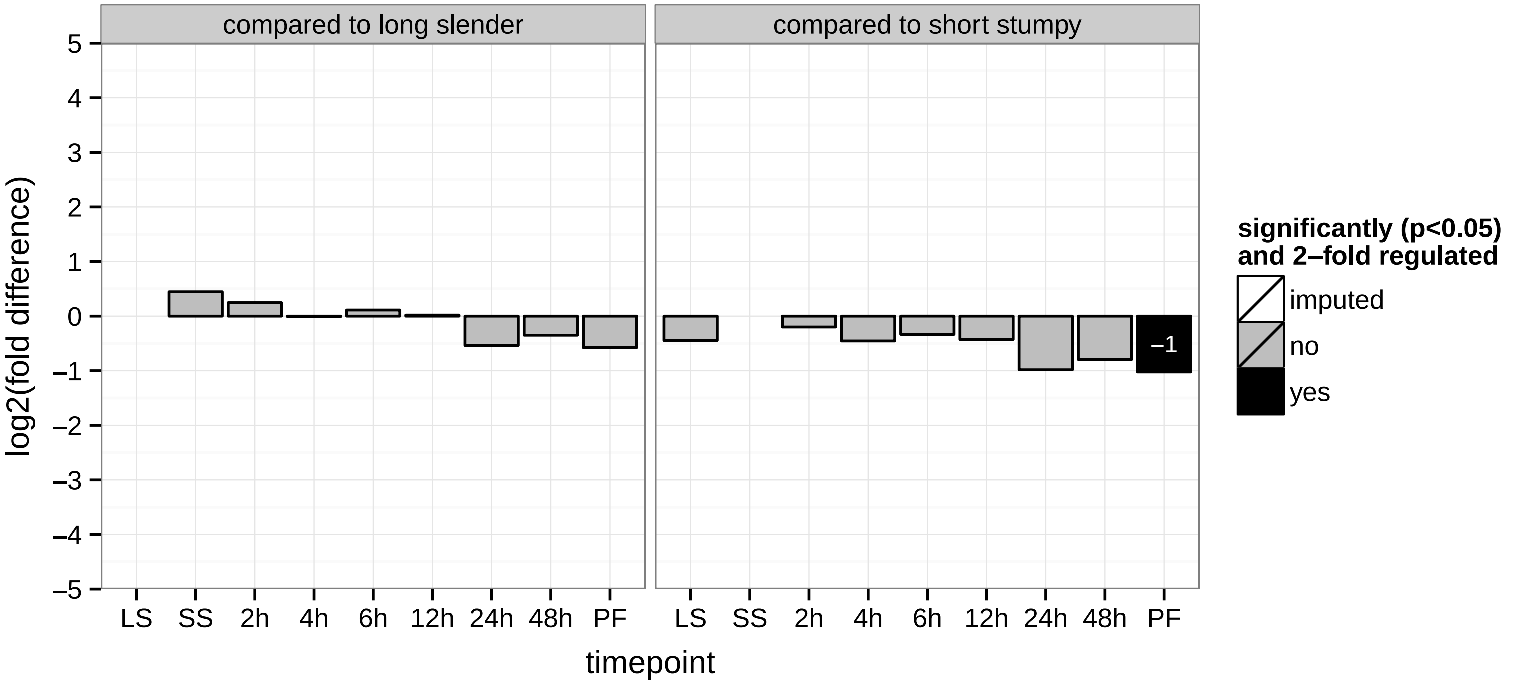

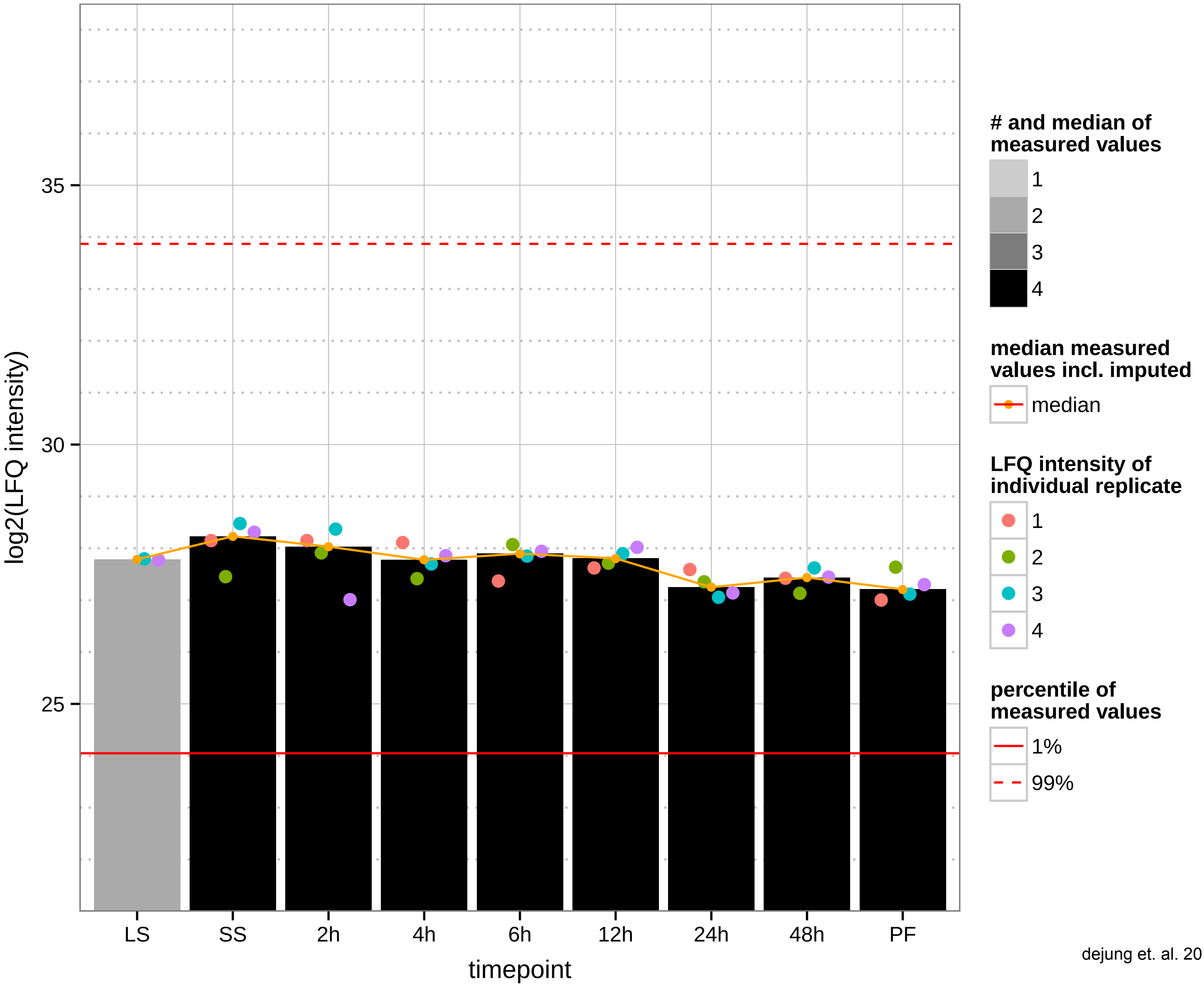

dejung et. al. 2015

cAMP–specific phosphodiesterase (PDEB2)
Tb927.9.5100
AGOF: 3', 5'–cyclic–nucleotide phosphodiesterase activity
AGOC: cytoplasm
AGOP: cell proliferation, signal transduction
PGOF: 3', 5'–cyclic–nucleotide phosphodiesterase activity, catalytic activity, protein binding
PGOC: null
PGOP: signal transduction

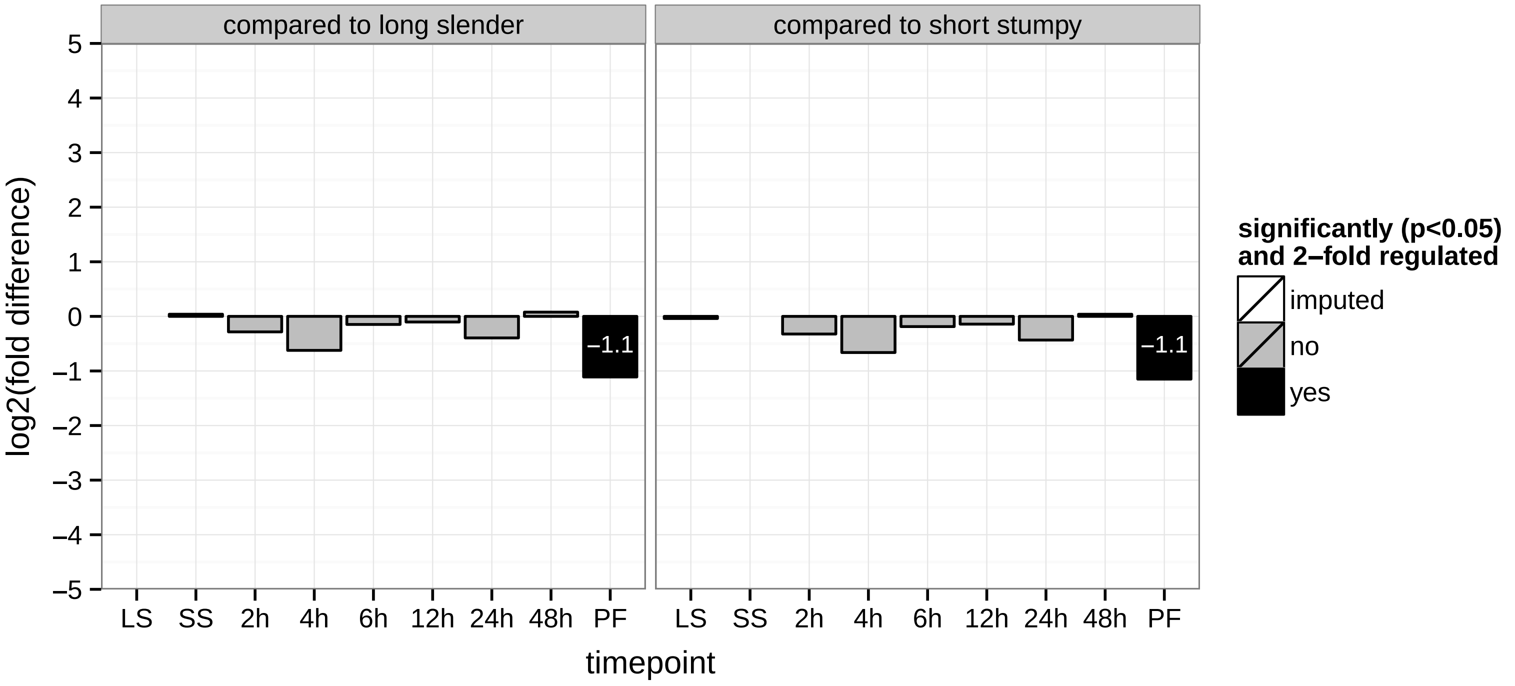

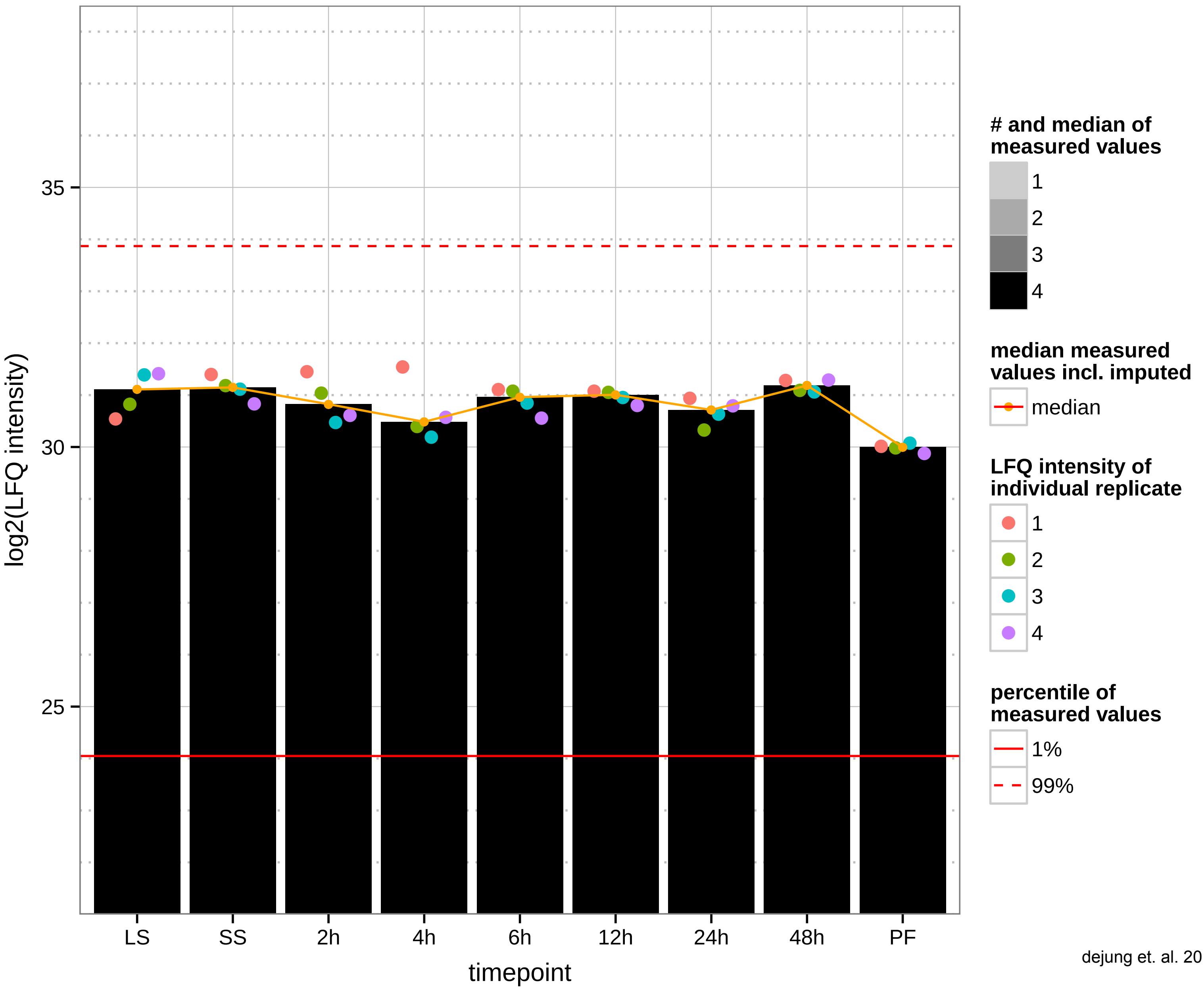

dejung et. al. 2015

ubiquitin carboxyl–terminal hydrolase, putative, cysteine peptidase, Clan CA, family C19
Tb927.9.5520
AGOF: cysteine–type endopeptidase activity, ubiquitin thiolesterase activity
AGOC: null
AGOP: ubiquitin–dependent protein catabolic process
PGOF: ubiquitin thiolesterase activity
PGOC: null
PGOP: ubiquitin–dependent protein catabolic process

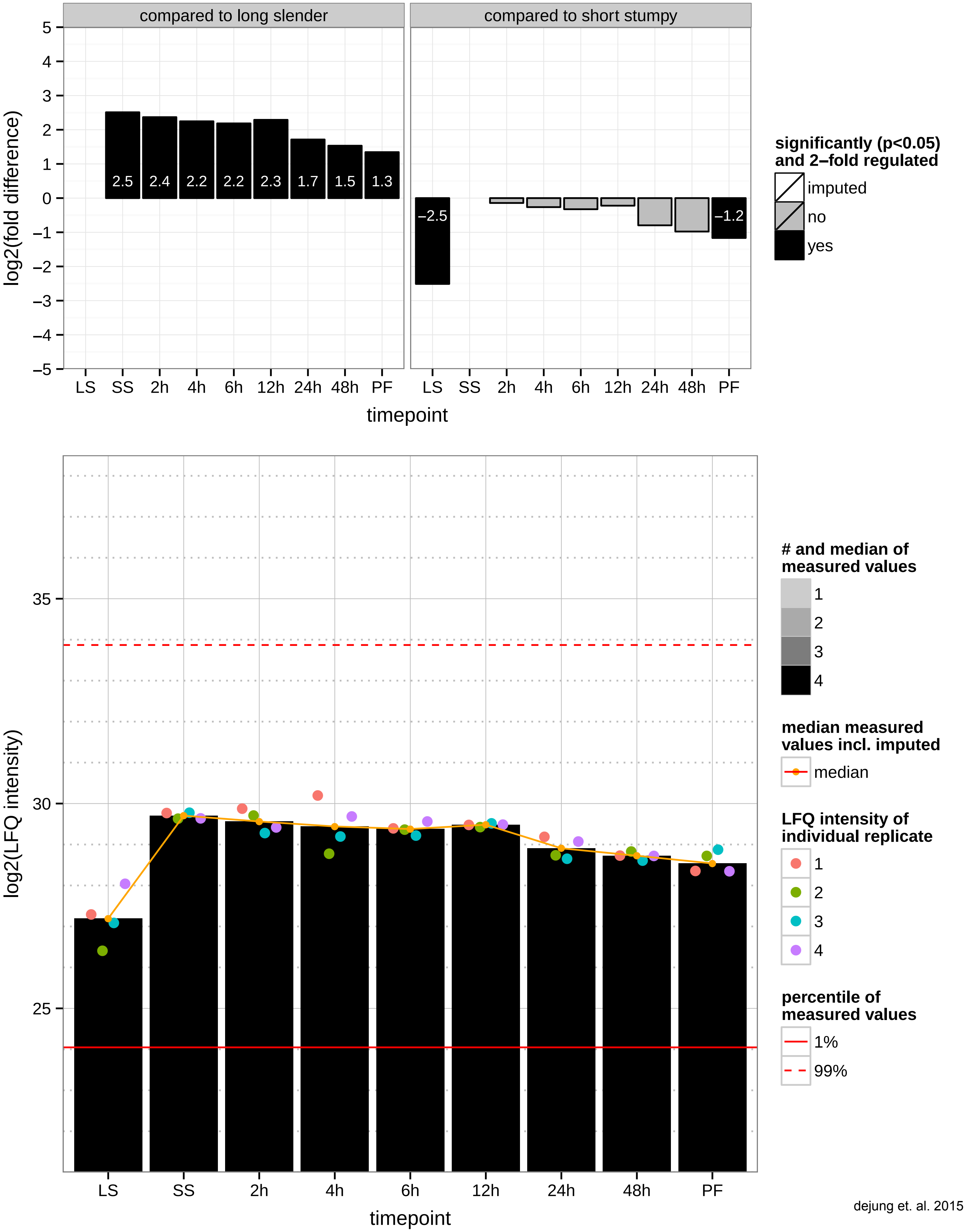

dejung et. al. 2015

arginine kinase, AK1 (AK)
Tb927.9.6290
AGOF: ATP binding, arginine kinase activity, transferase activity, transferring phosphorus−containing groups
AGOC: cytosol
AGOP: biological_process
PGOF: kinase activity, transferase activity, transferring phosphorus−containing groups
PGOC: null
PGOP: null

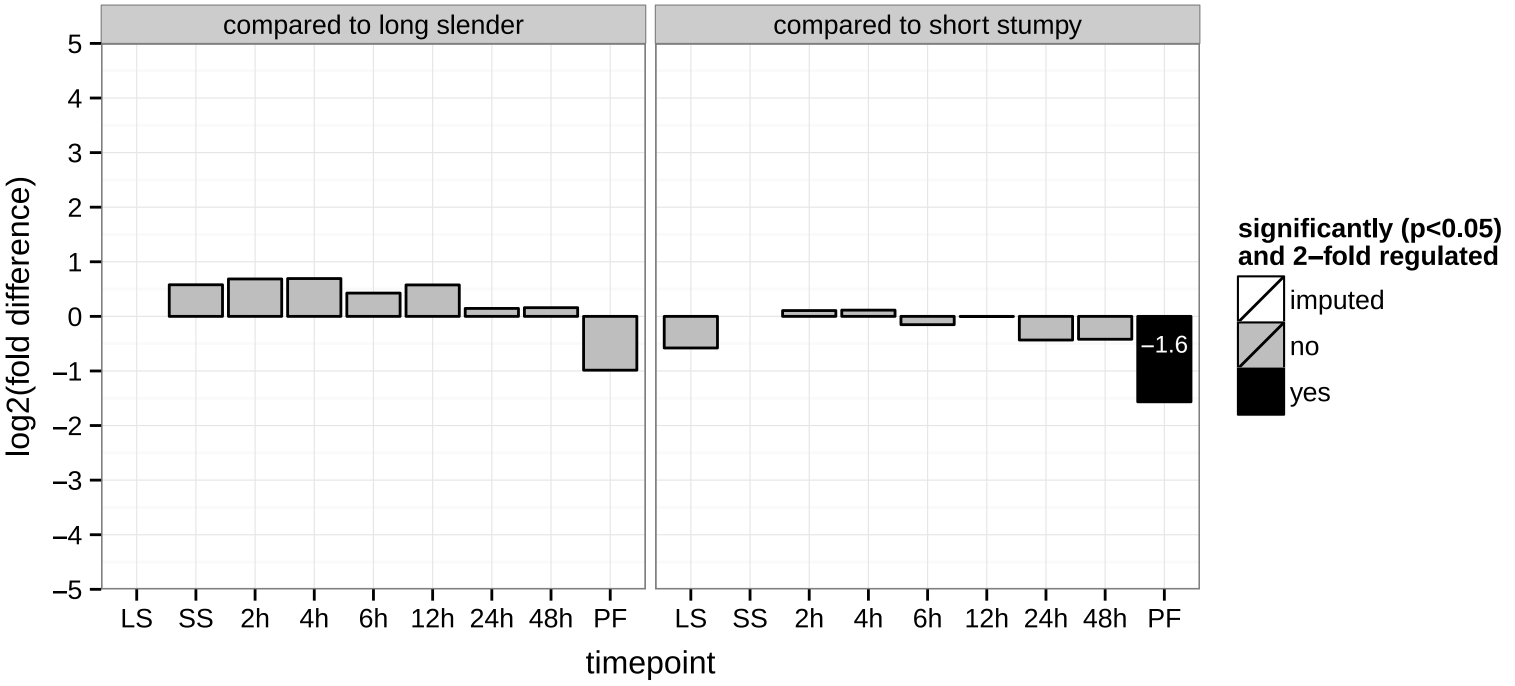

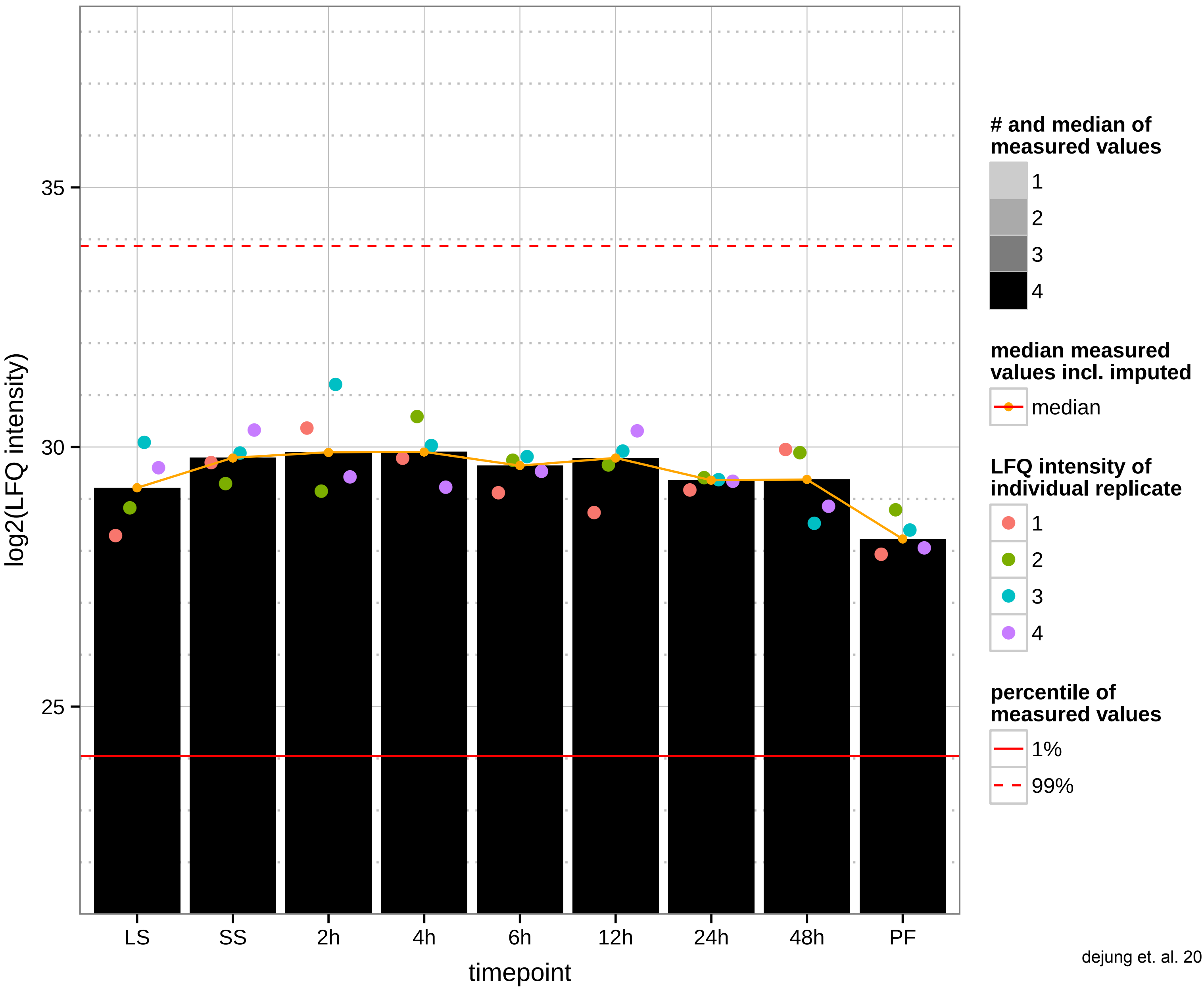

dejung et. al. 2015

hypothetical protein, conserved
Tb927.9.7690
AGOF: nucleotidyltransferase activity
AGOC: null
AGOP: null
PGOF: null
PGOC: null
PGOP: null

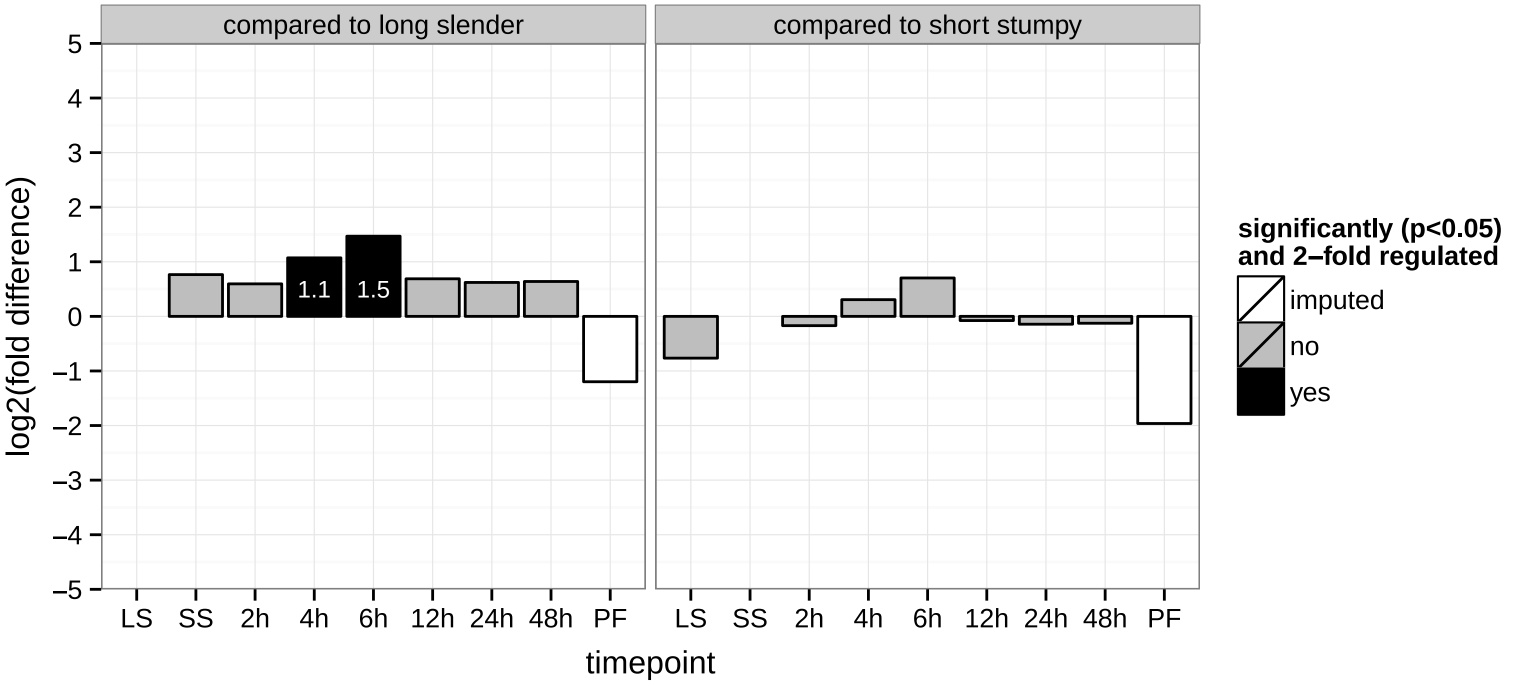

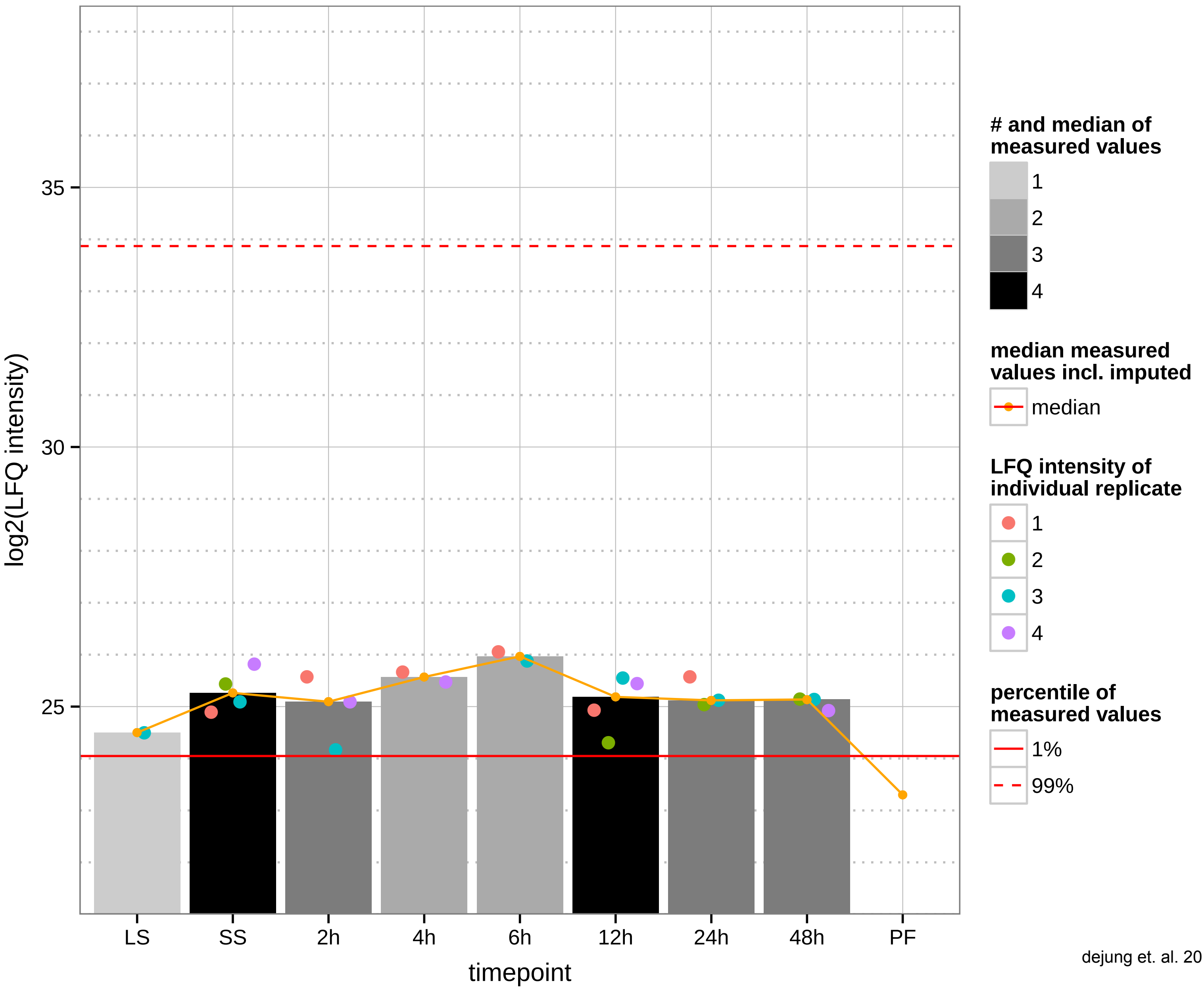

dejung et. al. 2015

hypothetical protein, conserved
Tb927.9.9230
AGOF: null
AGOC: null
AGOP: null
PGOF: null
PGOC: null
PGOP: null

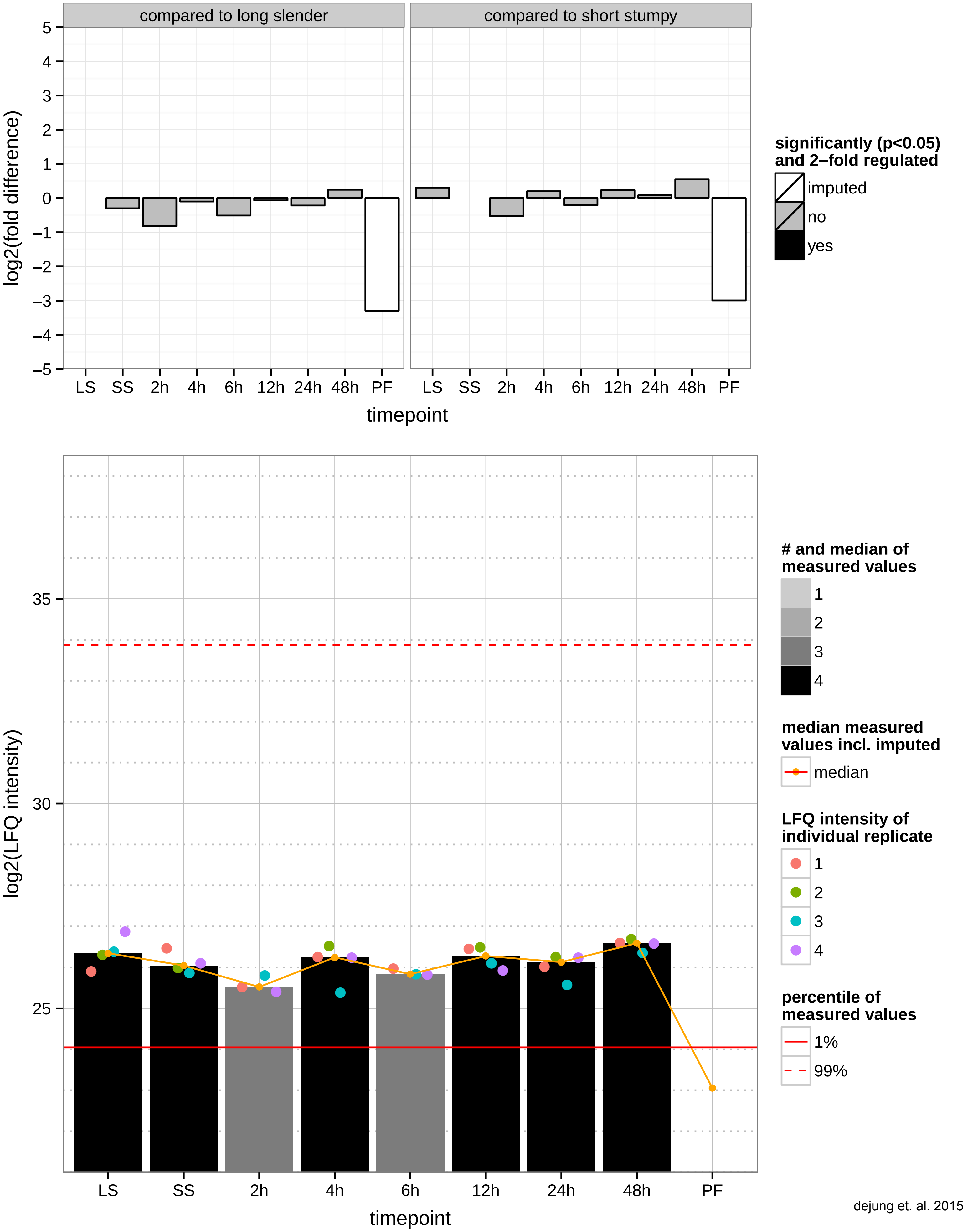

dejung et. al. 2015

sphingomyelin/ceramide phosphorylethanolamine synthase, bifunctional, sphingomyelin synthase (SLS4), sphingomyelin synt
Tb927.9.9410;Tb927.9.9390;Tb927.9.9380;Tb927.9.9400
AGOF: ceramide cholinephosphotransferase activity, ethanolaminephosphotransferase activity, sphingomyelin synthase activi
AGOC: Golgi apparatus, membrane, membrane
AGOP: ceramide biosynthetic process, sphingomyelin biosynthetic process, ethanolamine biosynthetic process, sphingomyeli
PGOF: null
PGOC: null
PGOP: null

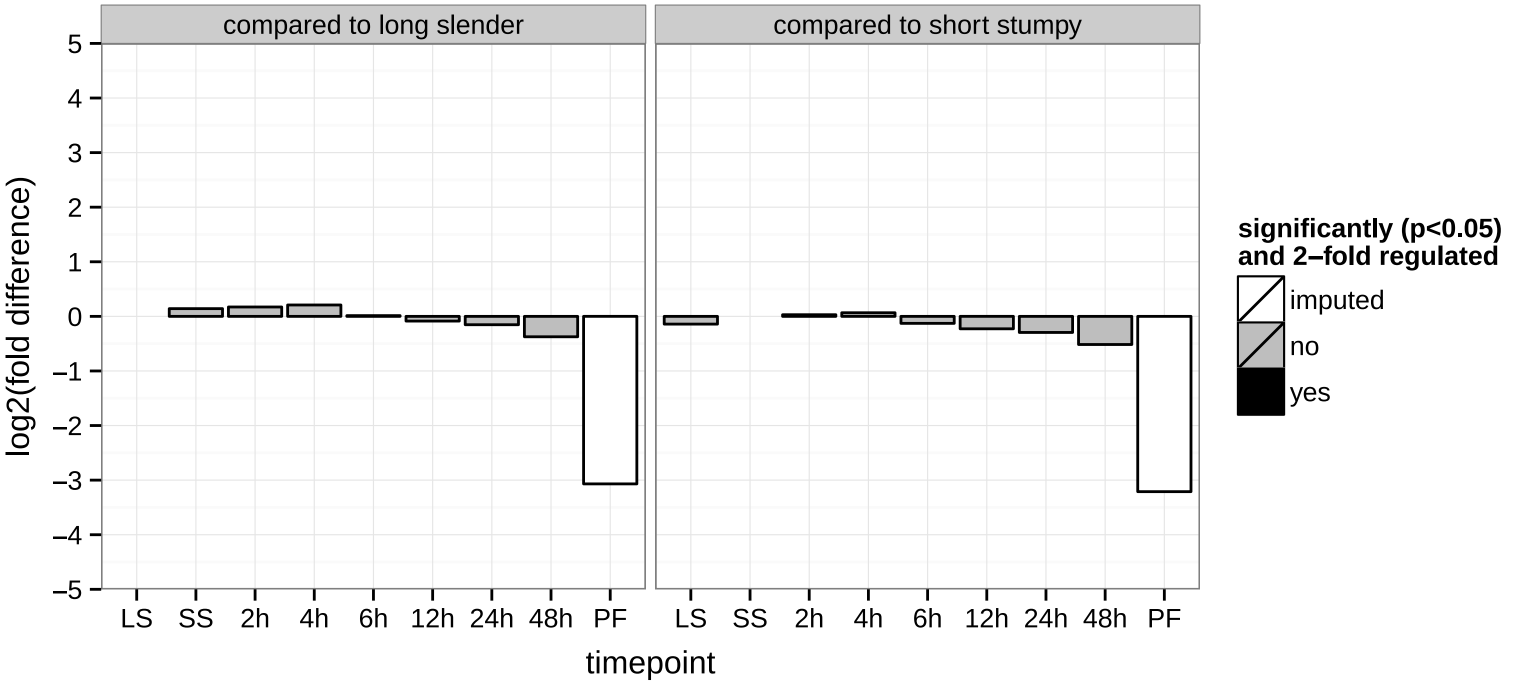

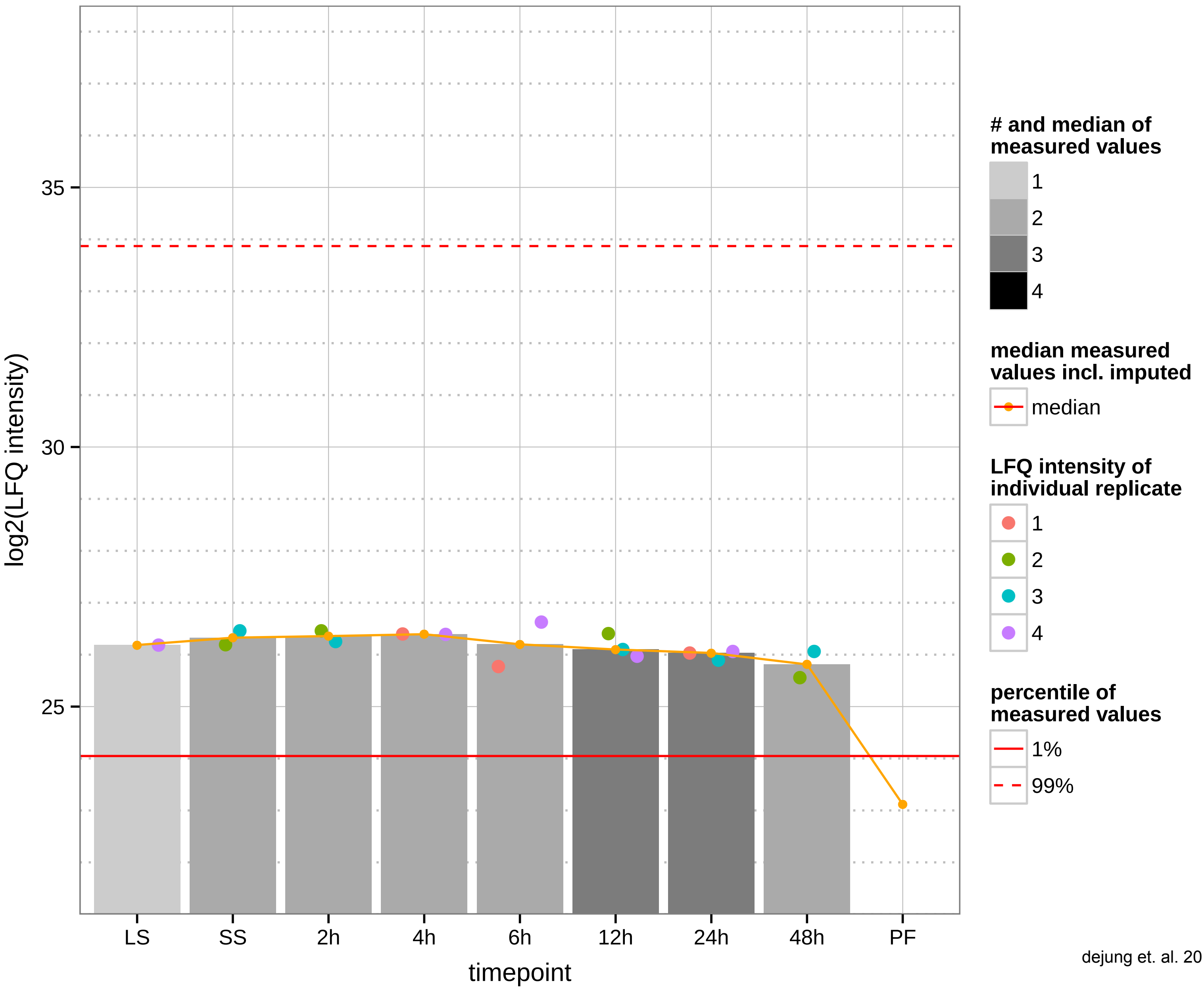

dejung et. al. 2015

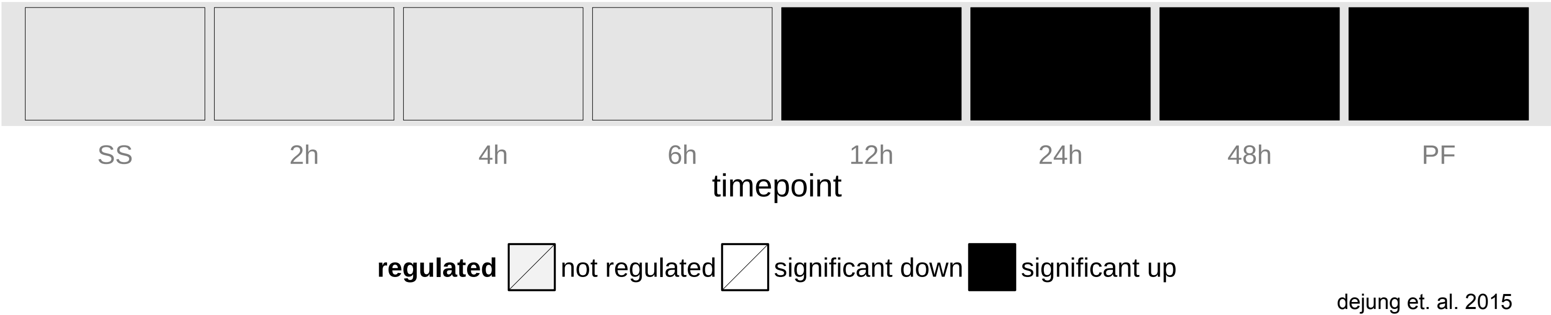
SS
2h
4h
6h
12h
24h
48h
PF
timepoint
regulated
not regulated
significant down
significant up
dejung et. al. 2015

hypothetical protein, conserved
Tb11.02.5105b;Tb11.02.5105
AGOF: null
AGOC: null
AGOP: null
PGOF: null
PGOC: null
PGOP: null

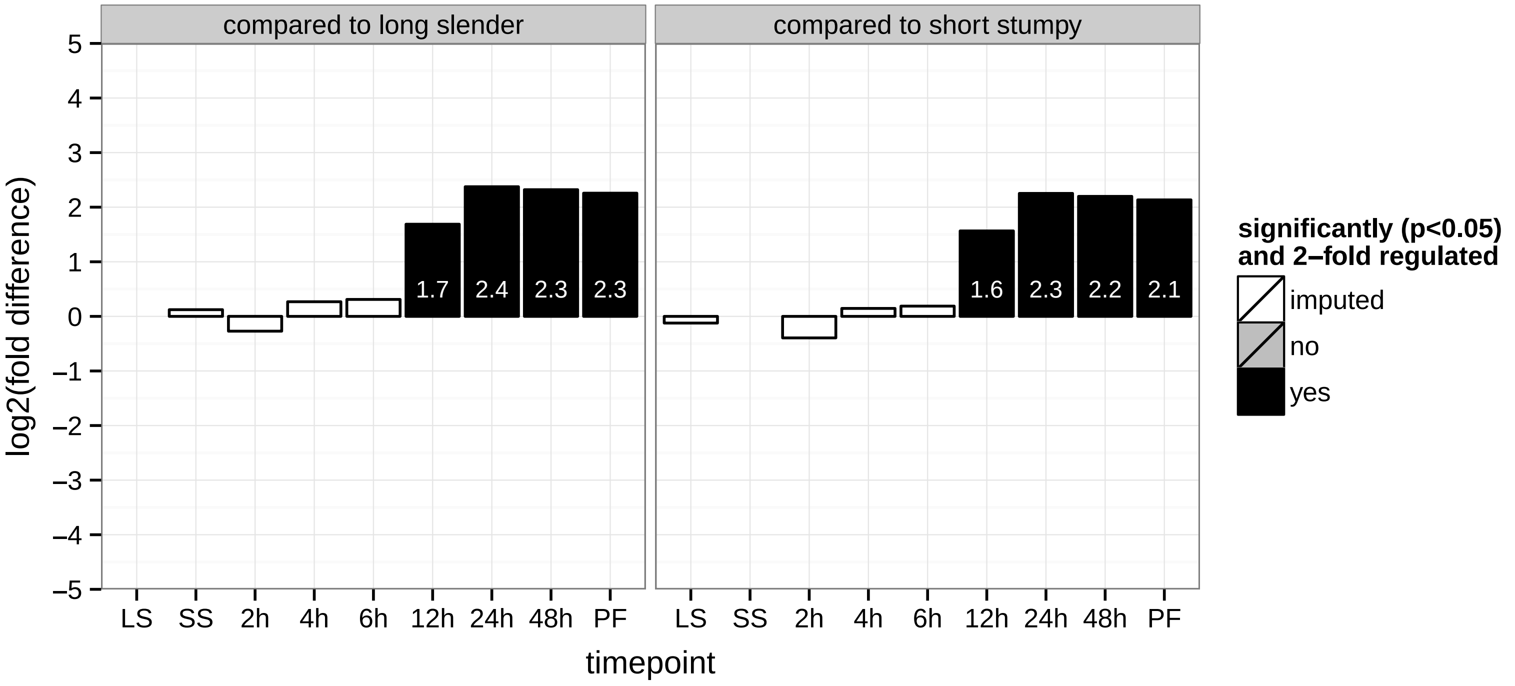

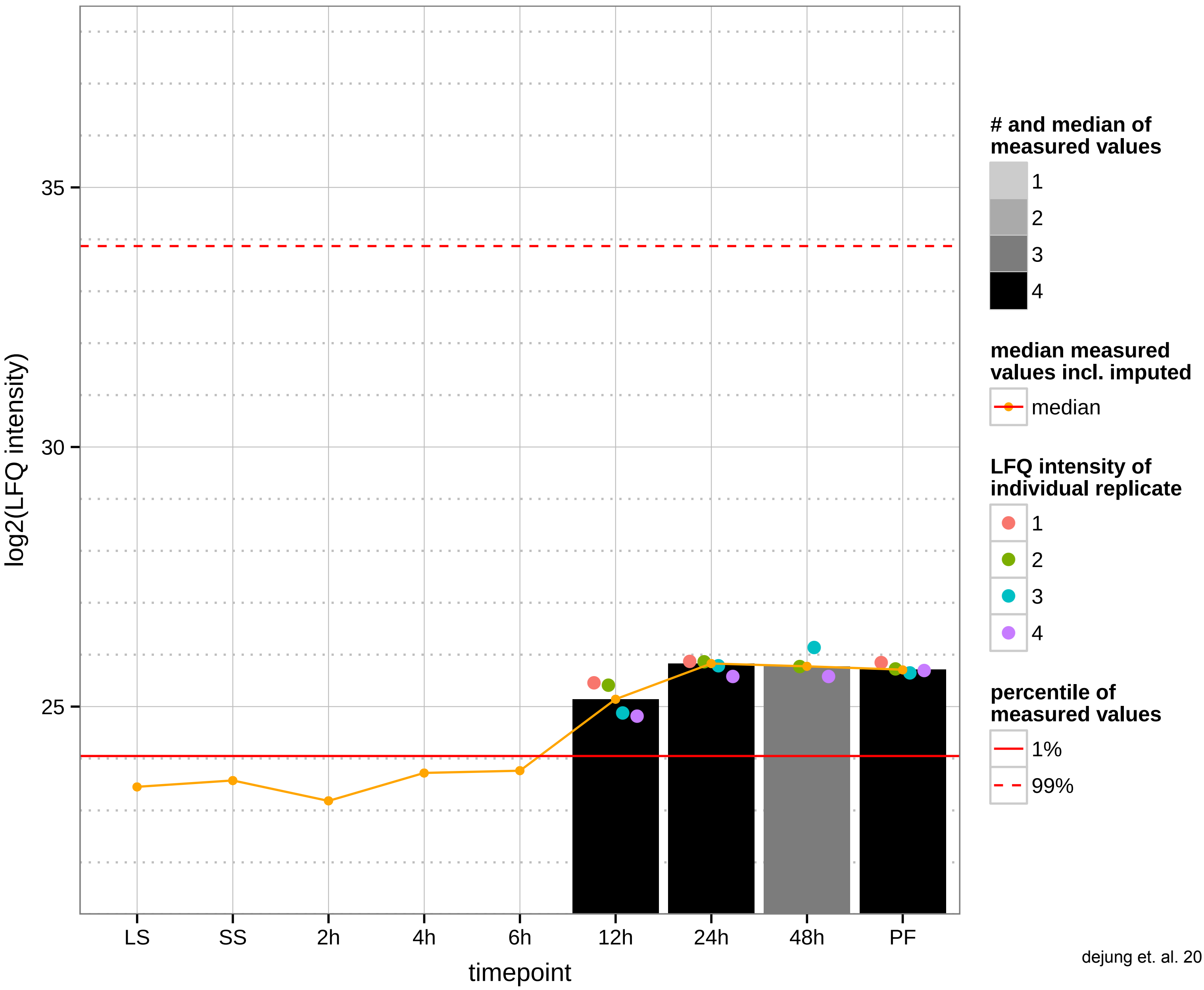

dejung et. al. 2015

hypothetical protein, chrXI additional, unordered contigs, P27 protein, putative (P27)
Tb11.0400;Tb927.11.7700;Tb11.0300
AGOF: null
AGOC: null, cytochrome complex, mitochondrial inner membrane
AGOP: null, regulation of oxidative phosphorylation
PGOF: null
PGOC: null
PGOP: null

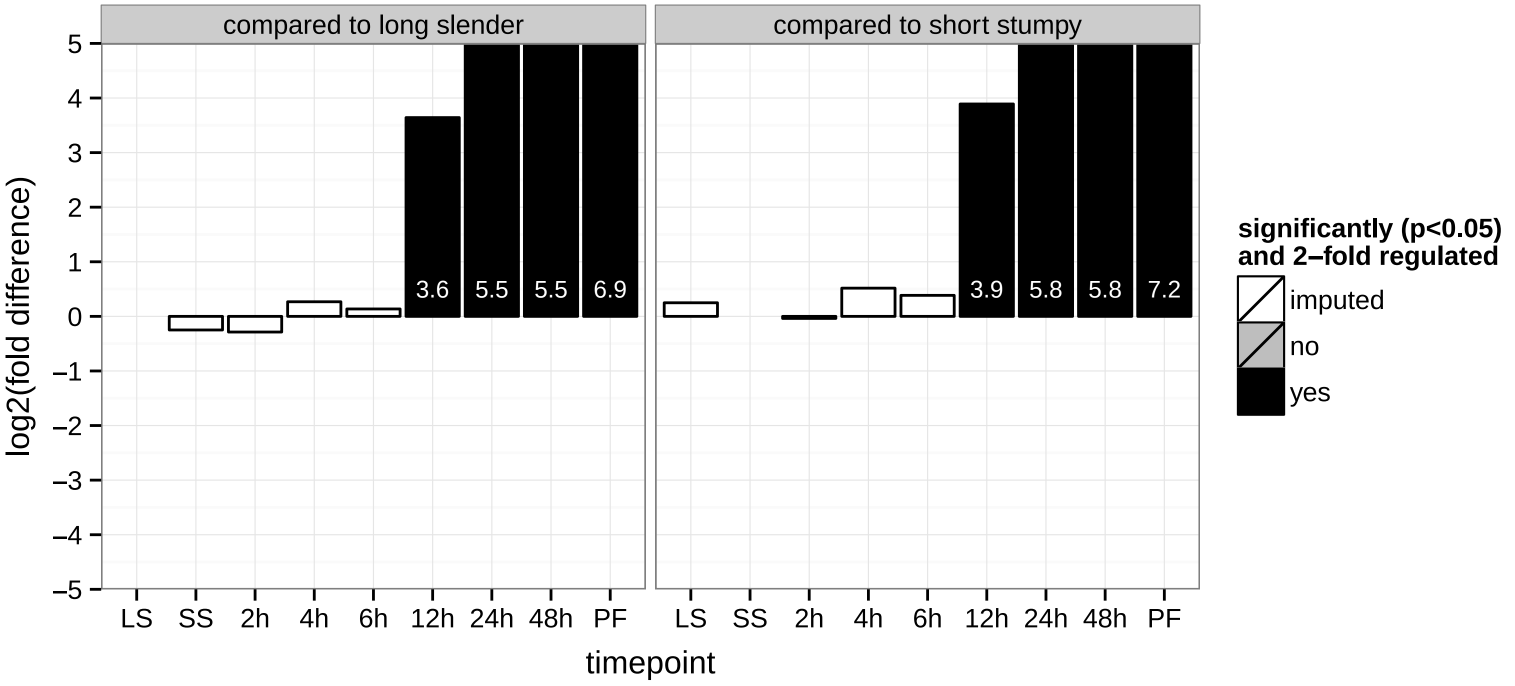

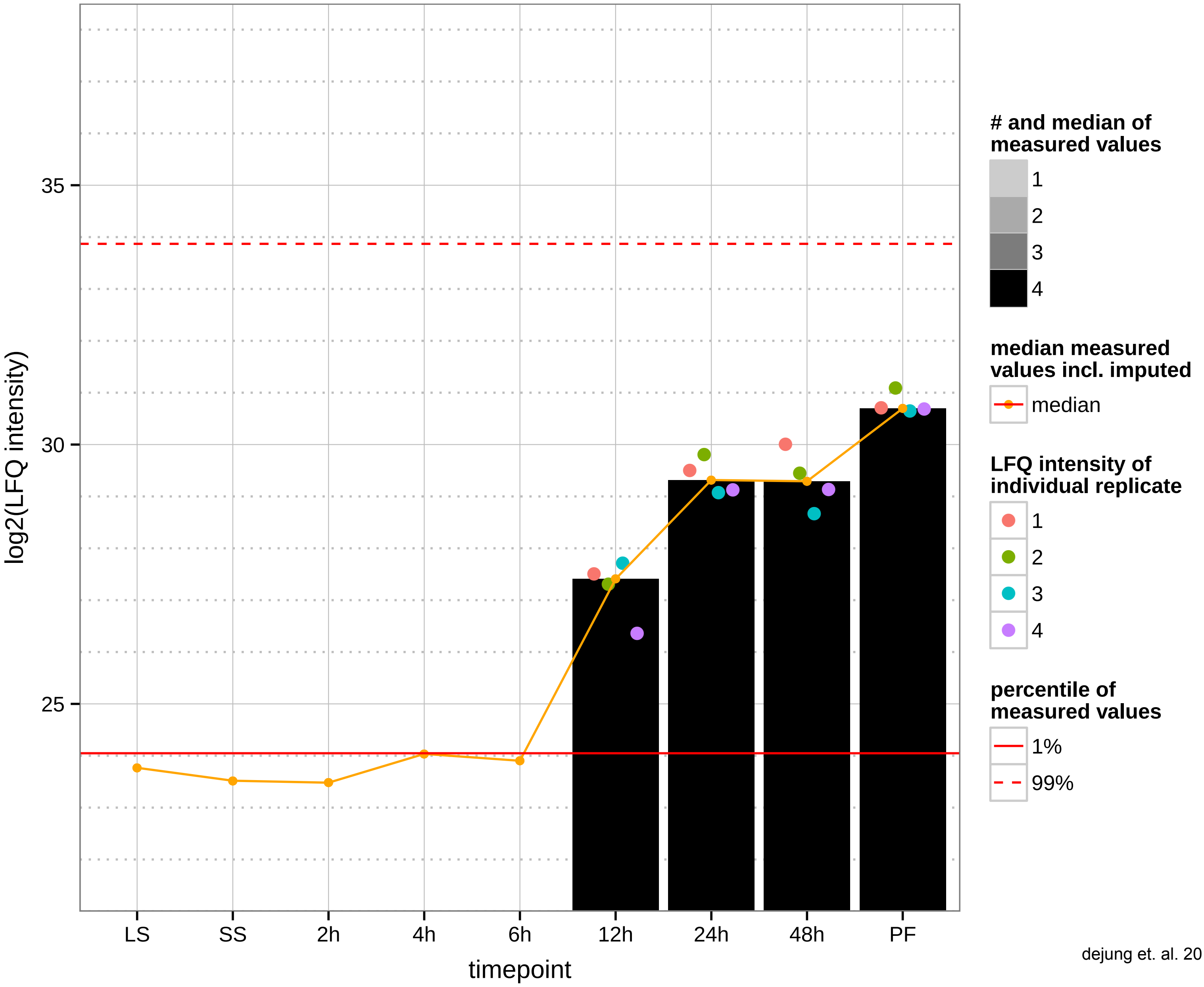

dejung et. al. 2015

hypothetical protein, conserved
Tb927.7.4820;Tb11.v5.0164
AGOF: null
AGOC: null
AGOP: null
PGOF: null
PGOC: null
PGOP: null

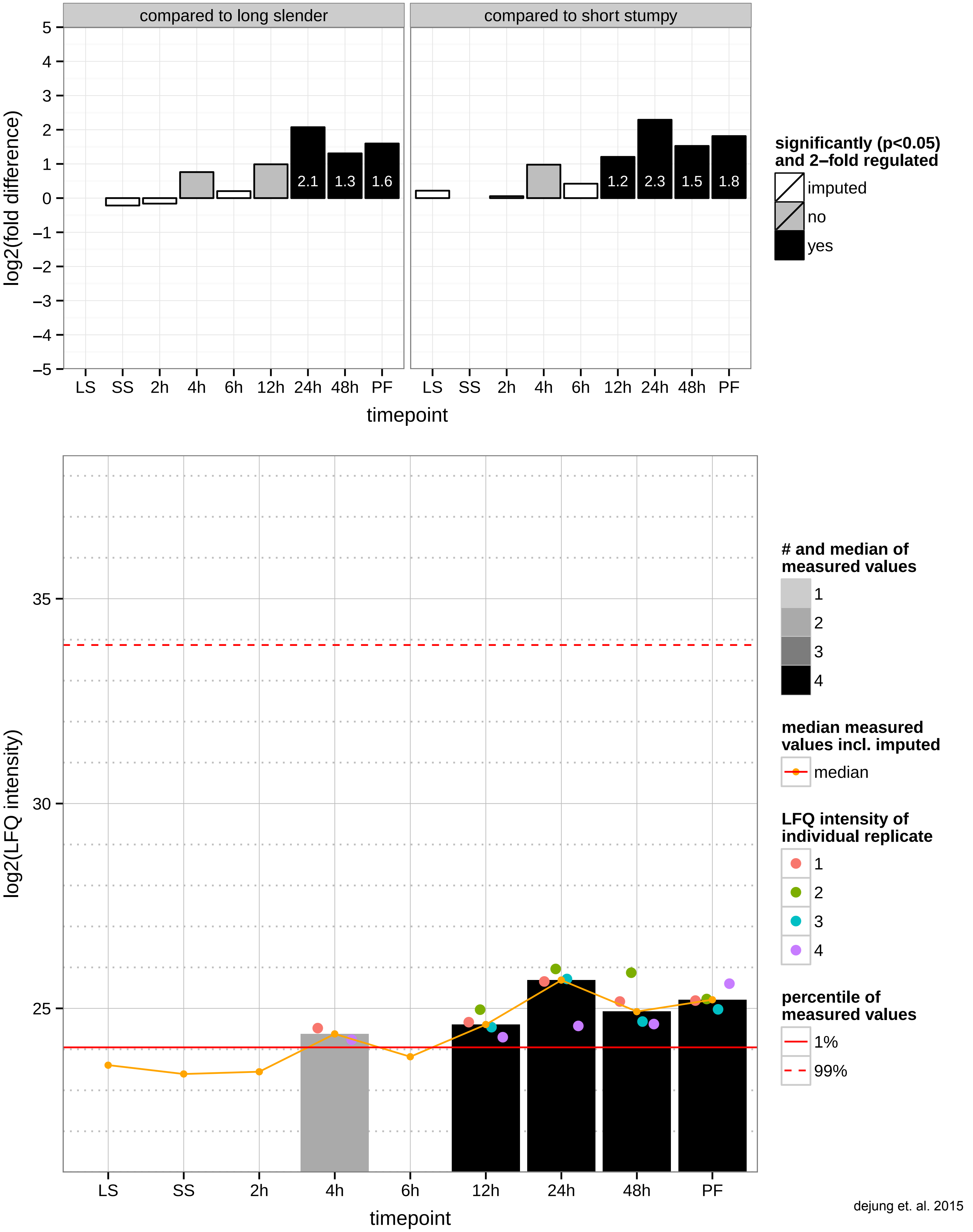

dejung et. al. 2015

oligopeptidase B protein, putative, serine peptidase, clan SC, family S9A–like protein
Tb927.7.4940;Tb11.v5.0175
AGOF: null, serine–type endopeptidase activity
AGOC: null, mitochondrion
AGOP: null, proteolysis
PGOF: serine–type endopeptidase activity, serine–type peptidase activity
PGOC: null
PGOP: proteolysis

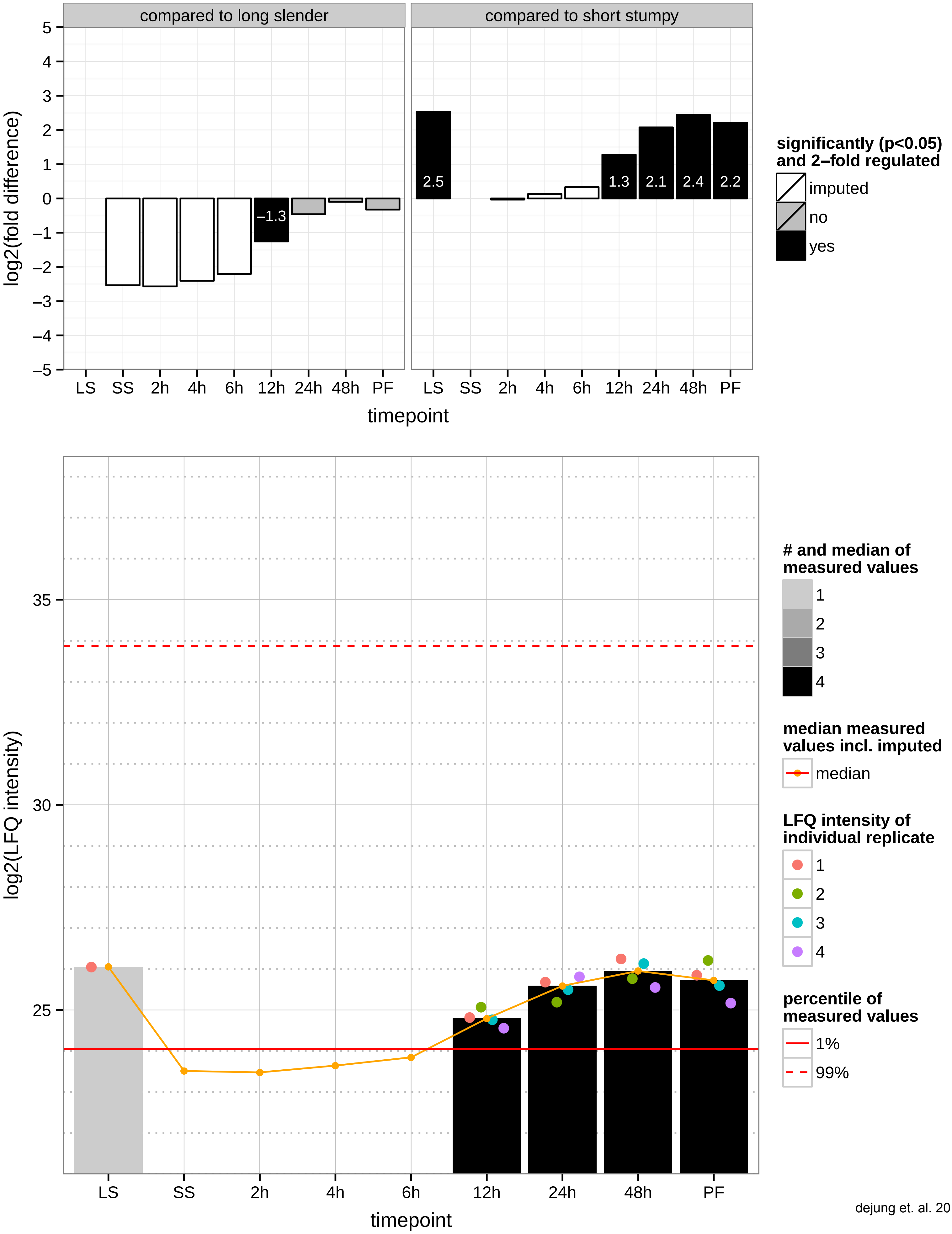

dejung et. al. 2015

universal minicircle sequence binding protein, putative, universal minicircle sequence binding protein (UMSBP), DNA–binding
Tb927.10.6060;Tb11.v5.0199
AGOF: null, nucleic acid binding, zinc ion binding
AGOC: null
AGOP: null
PGOF: nucleic acid binding, zinc ion binding
PGOC: null
PGOP: null

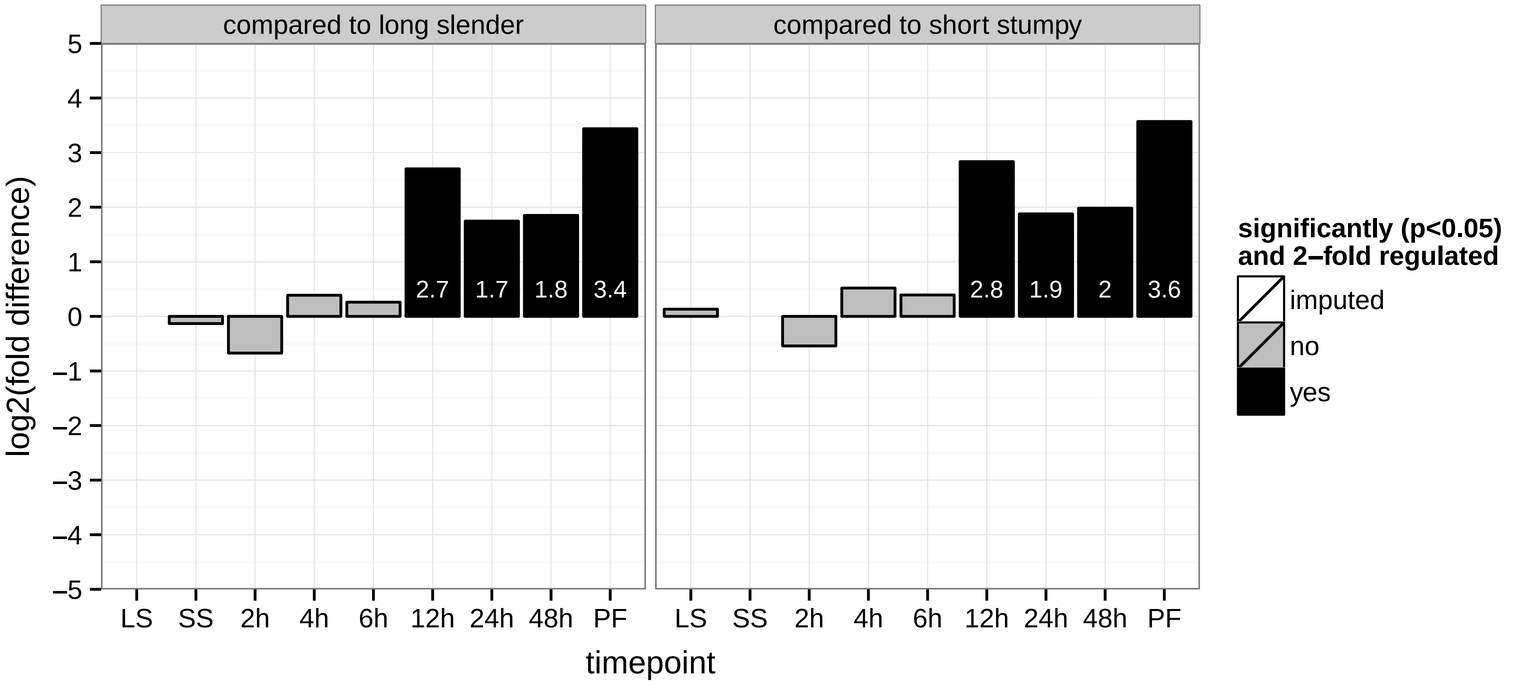

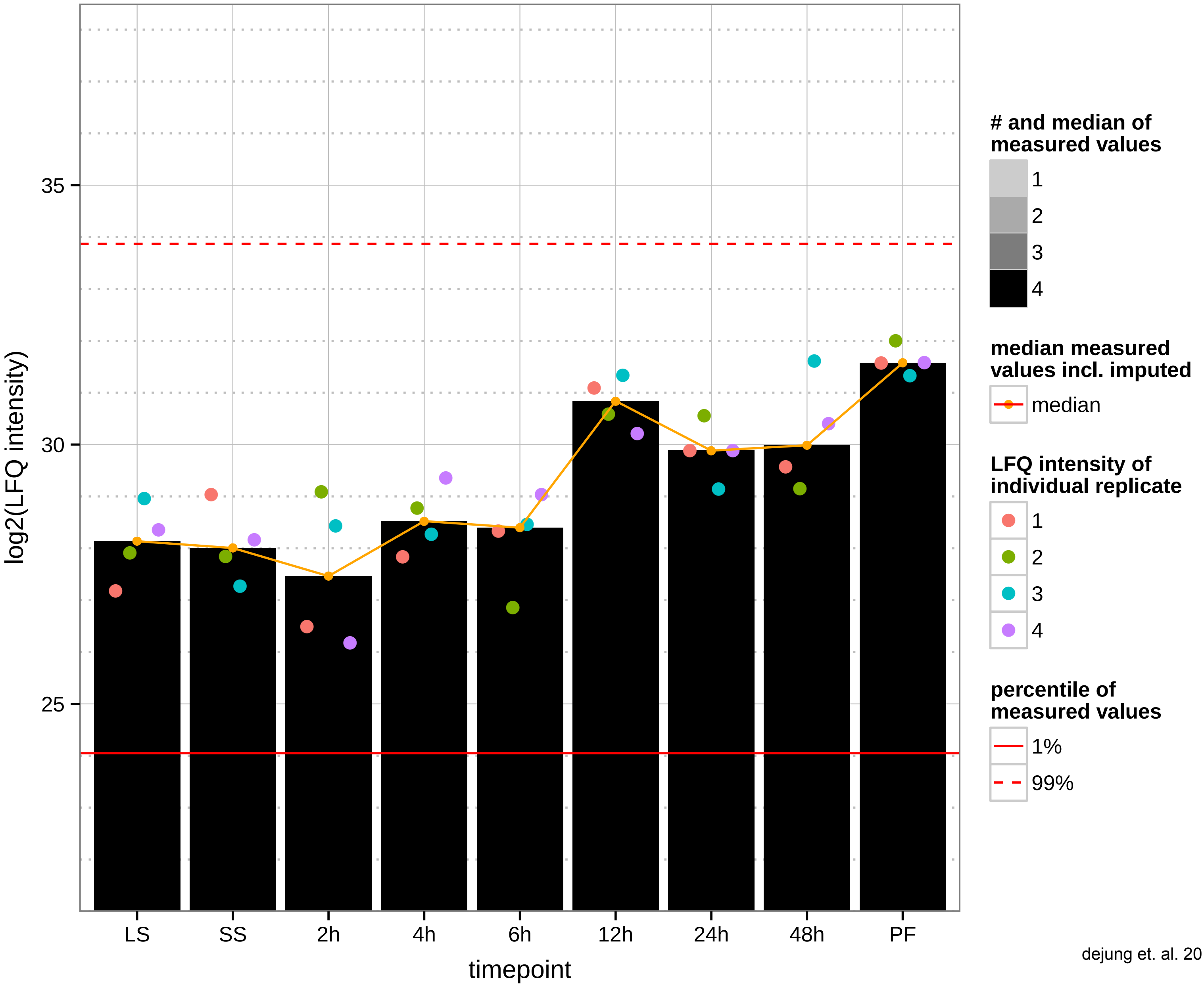

dejung et. al. 2015

eukaryotic translation initiation factor 5, putative
Tb927.10.2770;Tb11.v5.0684
AGOF: null, translation initiation factor activity
AGOC: null
AGOP: null, translational initiation
PGOF: protein binding, translation initiation factor activity
PGOC: null
PGOP: translational initiation

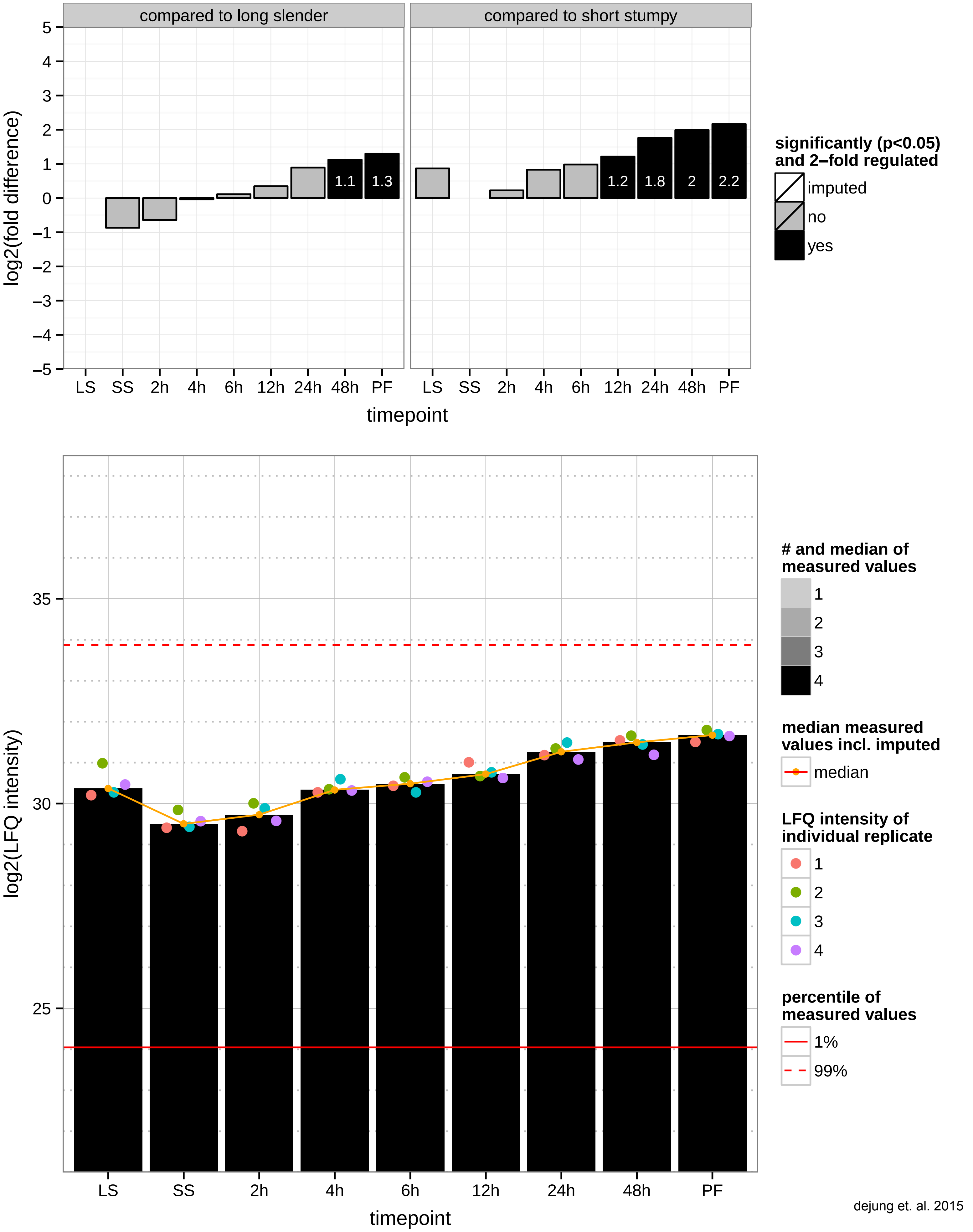

dejung et. al. 2015

conserved protein, unknown function
Tb927.1.1210
AGOF: null
AGOC: mitochondrion
AGOP: null
PGOF: null
PGOC: null
PGOP: null

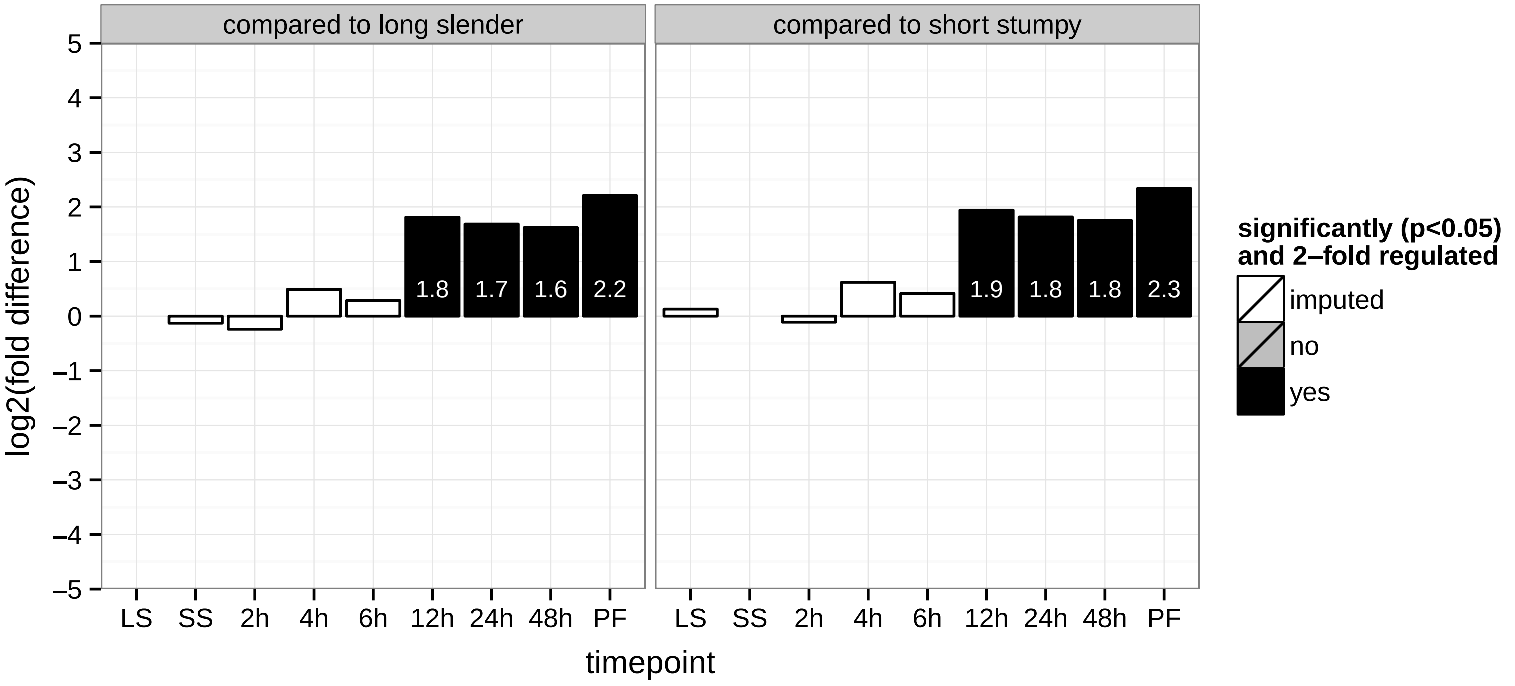

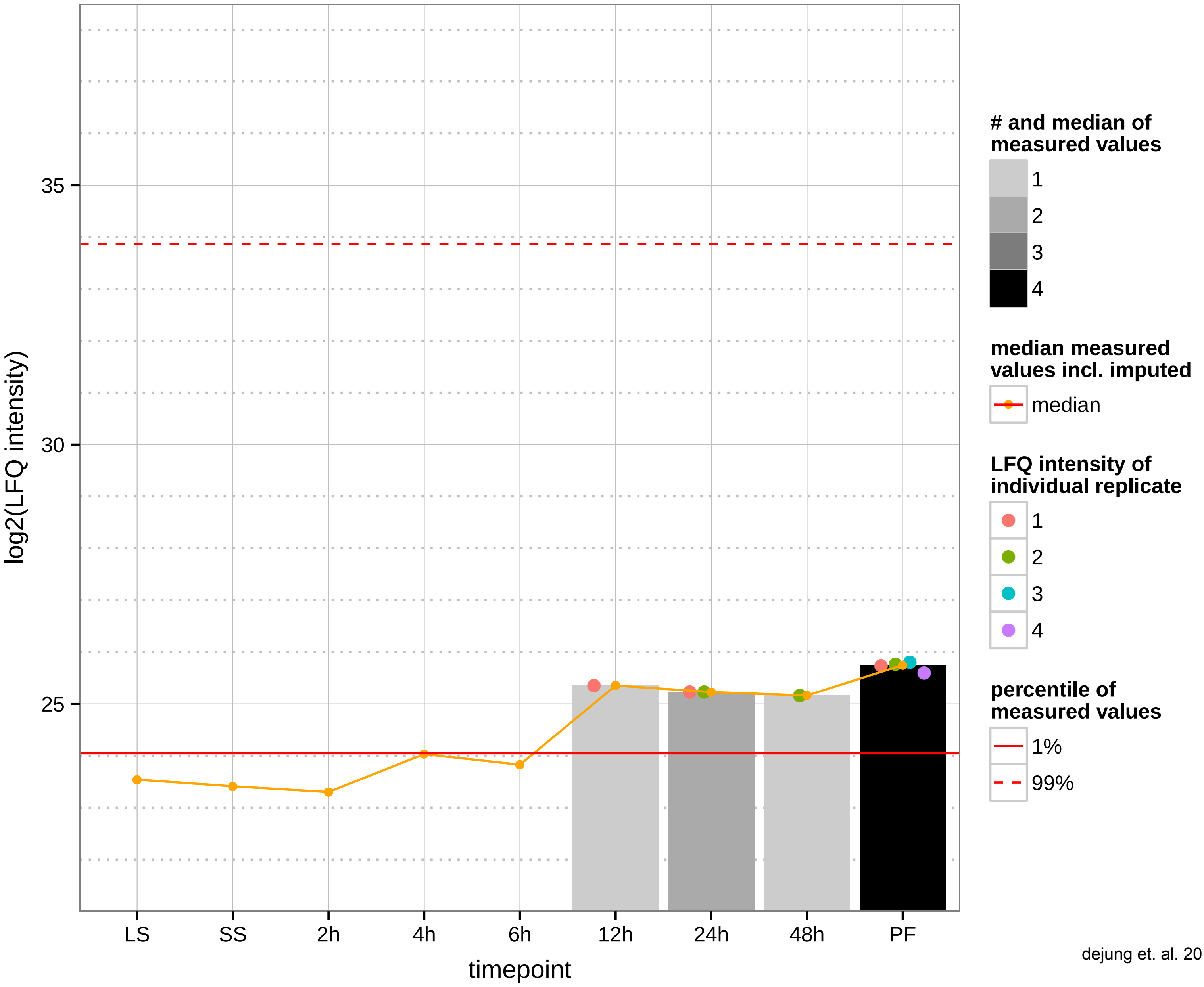

dejung et. al. 2015

RWD domain−containing protein
Tb927.1.1220
AGOF: null
AGOC: null
AGOP: null
PGOF: protein binding
PGOC: null
PGOP: null

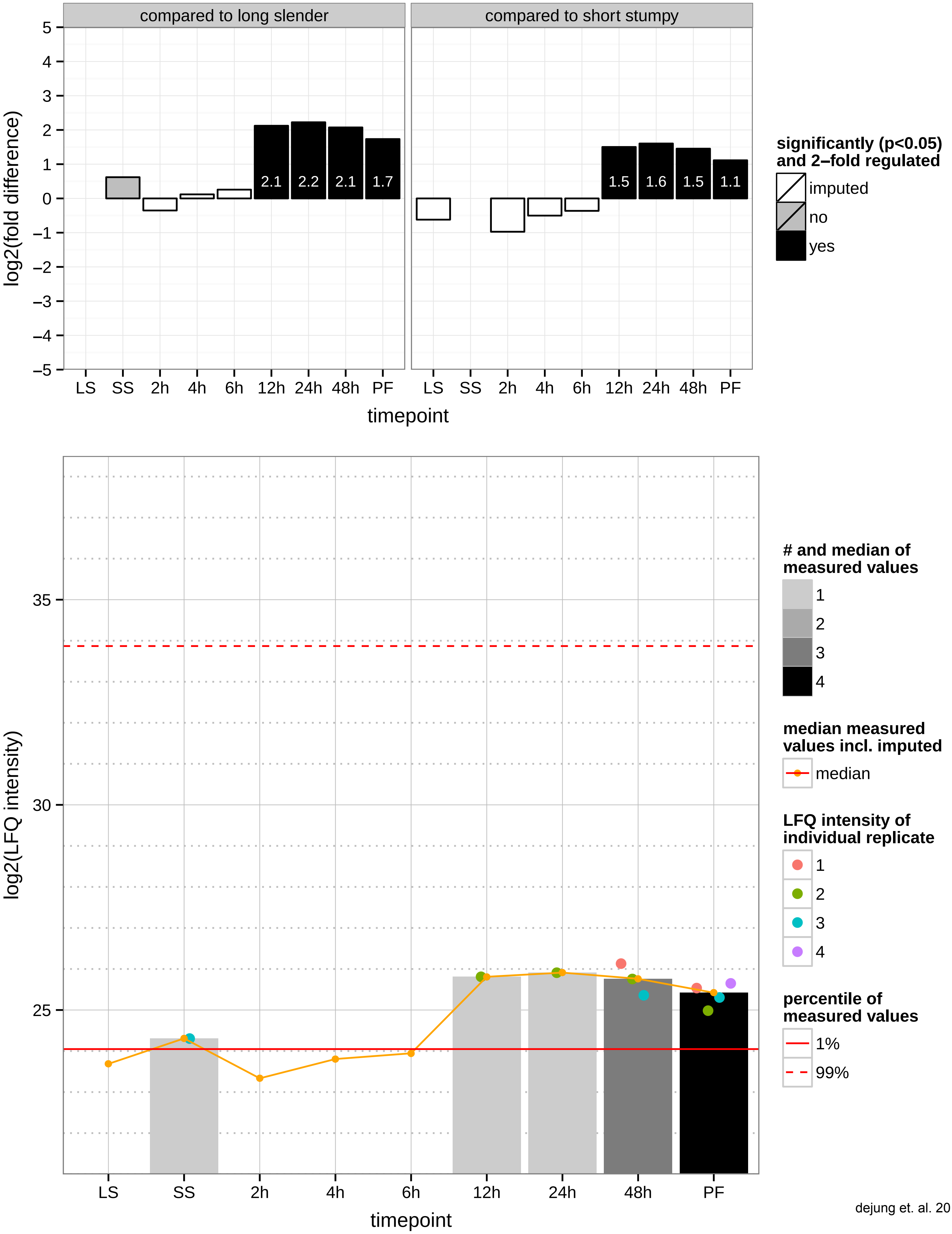

dejung et. al. 2015

hypothetical protein, conserved
Tb927.1.3310
AGOF: null
AGOC: null
AGOP: null
PGOF: null
PGOC: null
PGOP: null

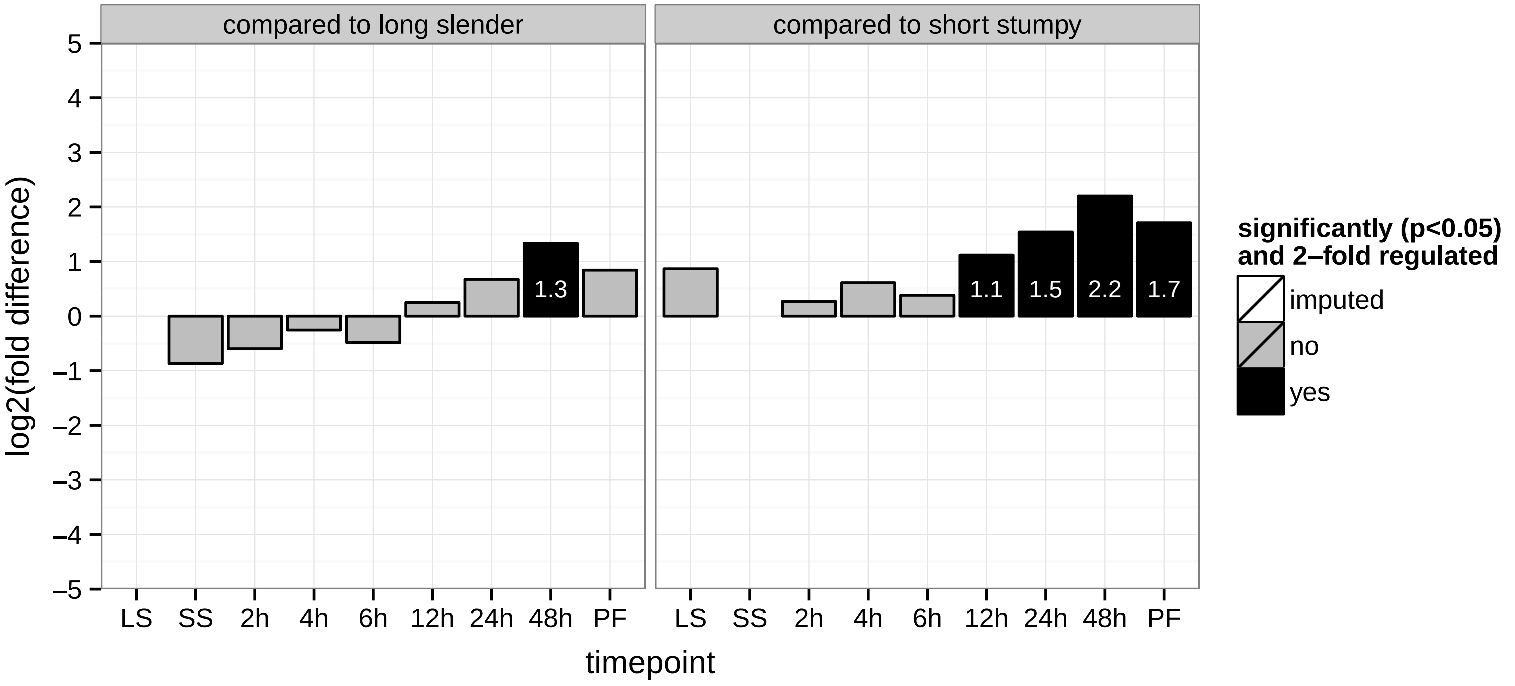

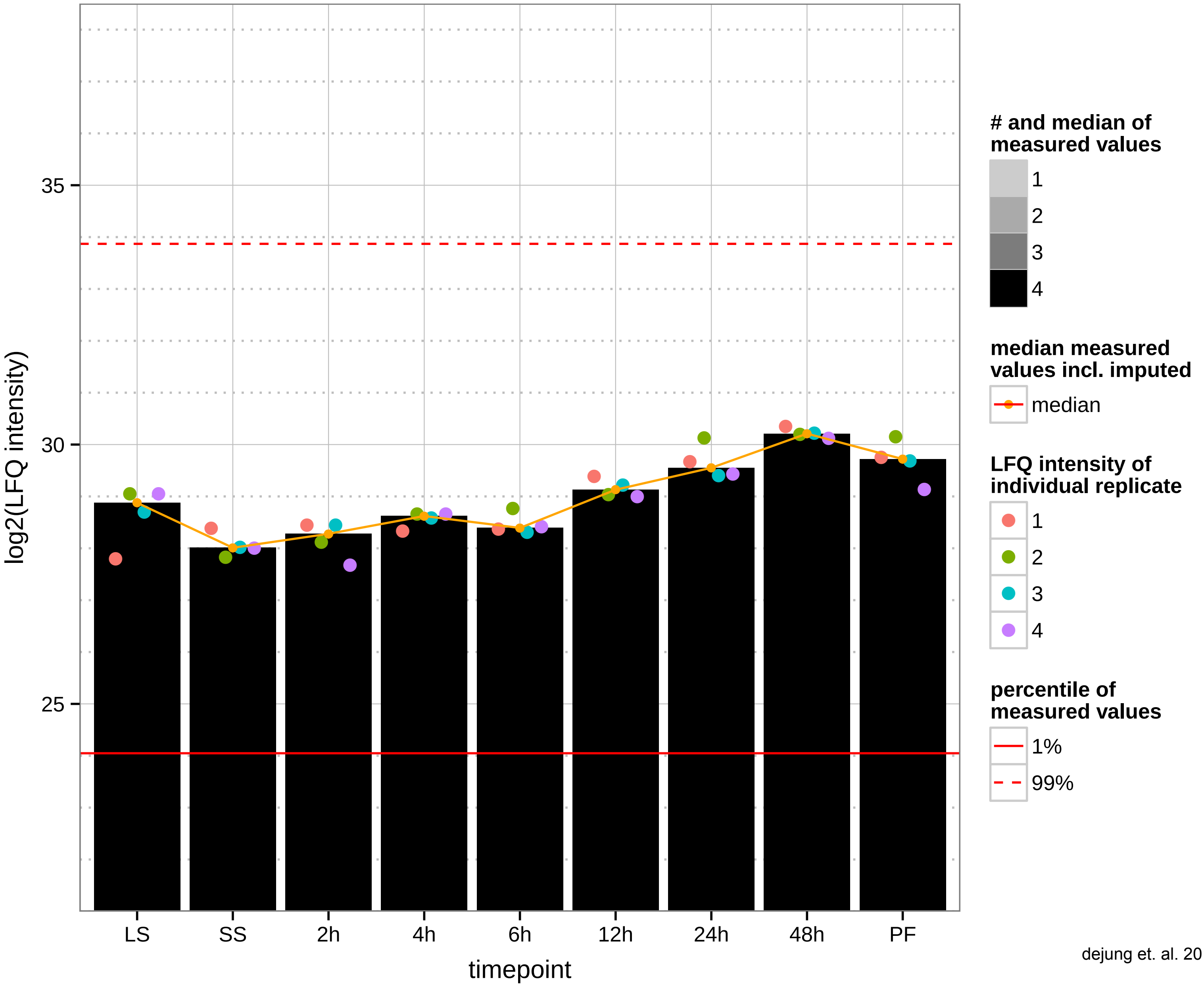

dejung et. al. 2015

hypothetical protein, conserved
Tb927.1.4230
AGOF: null
AGOC: mitochondrion
AGOP: null
PGOF: null
PGOC: null
PGOP: null

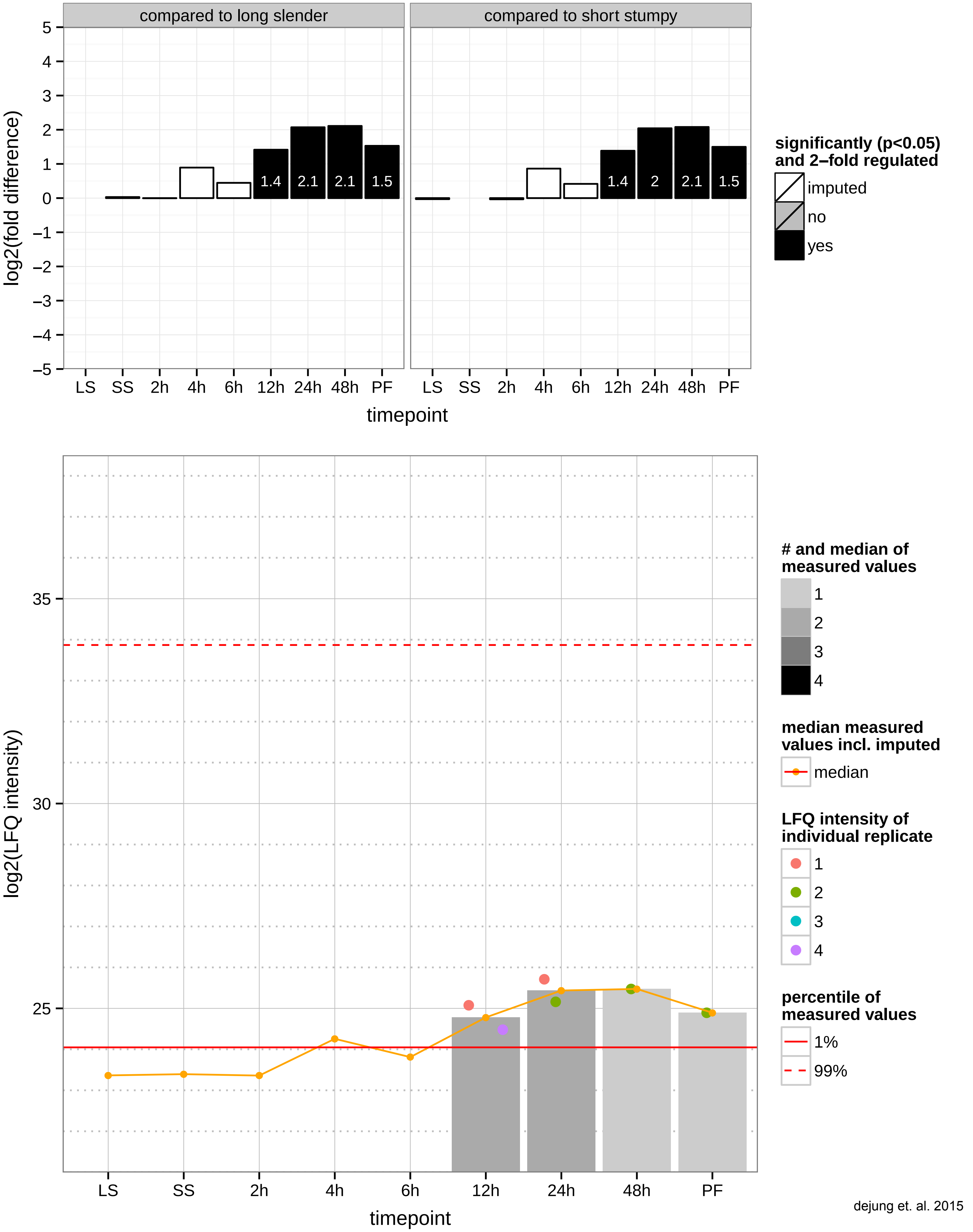

dejung et. al. 2015

structural maintenance of chromosome 2, putative, putative (SMC2)
Tb927.10.10340;Tb11.v5.0811
AGOF: null, ATP binding
AGOC: null, chromosome, cohesin complex, nucleus
AGOP: null, chromosome organization, mitosis, mitotic sister chromatid segregation
PGOF: ATP binding, protein binding
PGOC: chromosome
PGOP: chromosome organization

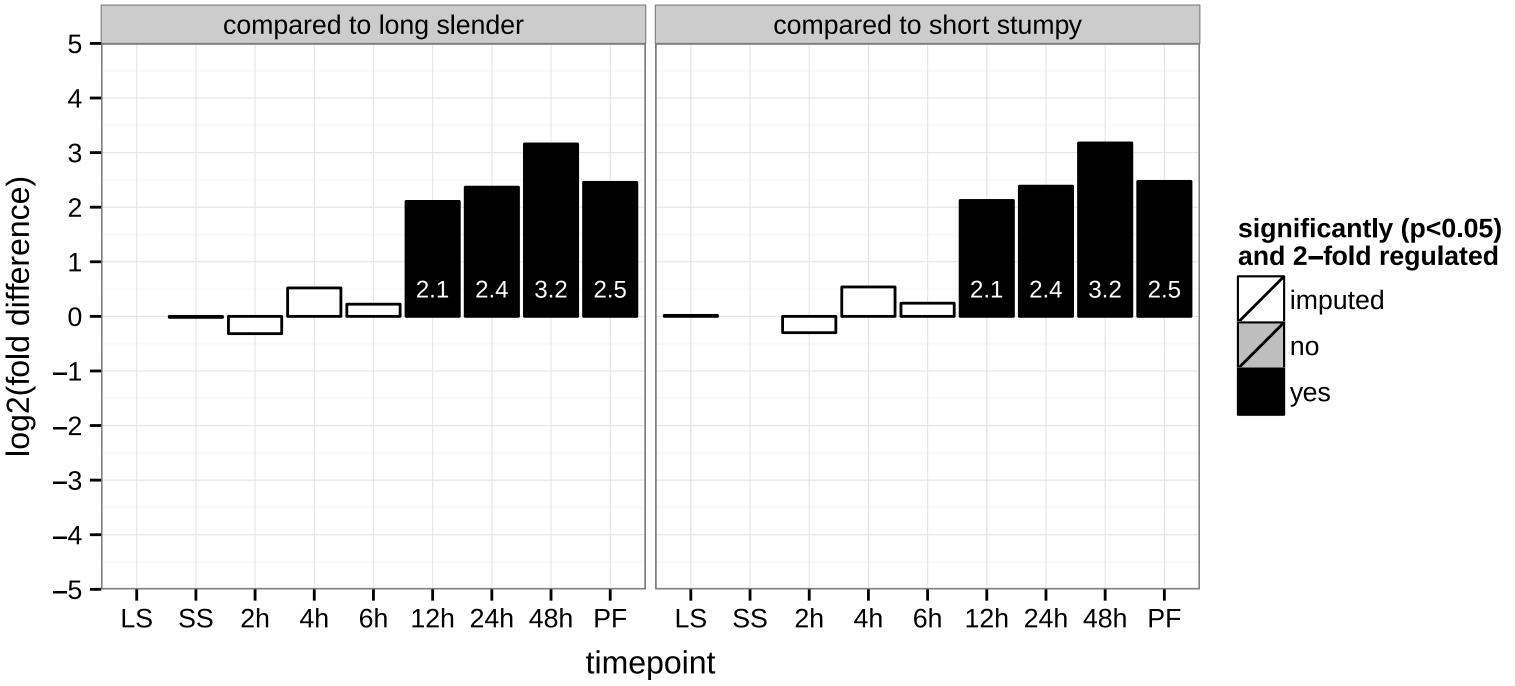

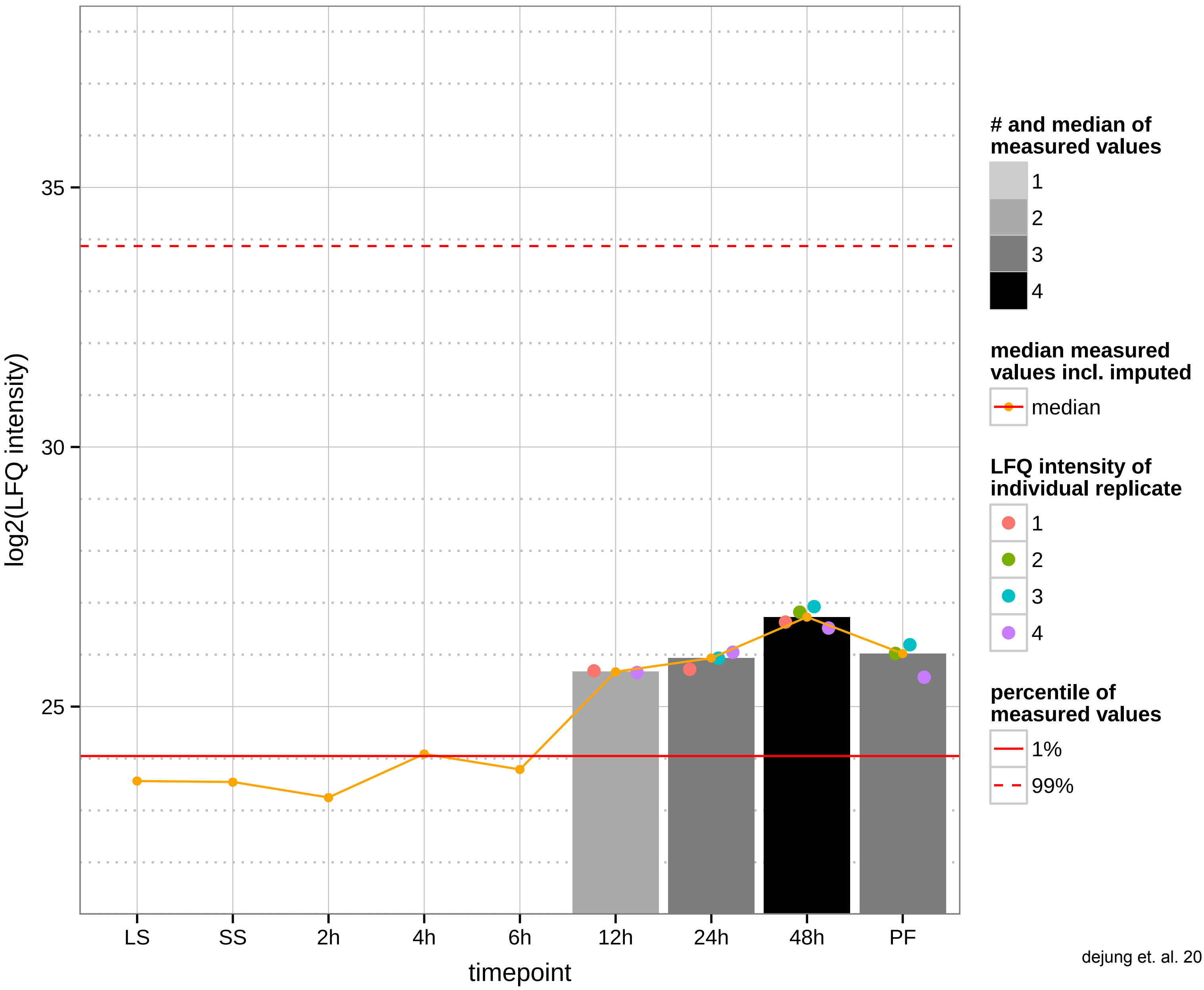

dejung et. al. 2015

RNA–binding protein, putative (RBP23)
Tb927.10.11270
AGOF: RNA binding
AGOC: null
AGOP: RNA processing
PGOF: nucleic acid binding
PGOC: null
PGOP: null

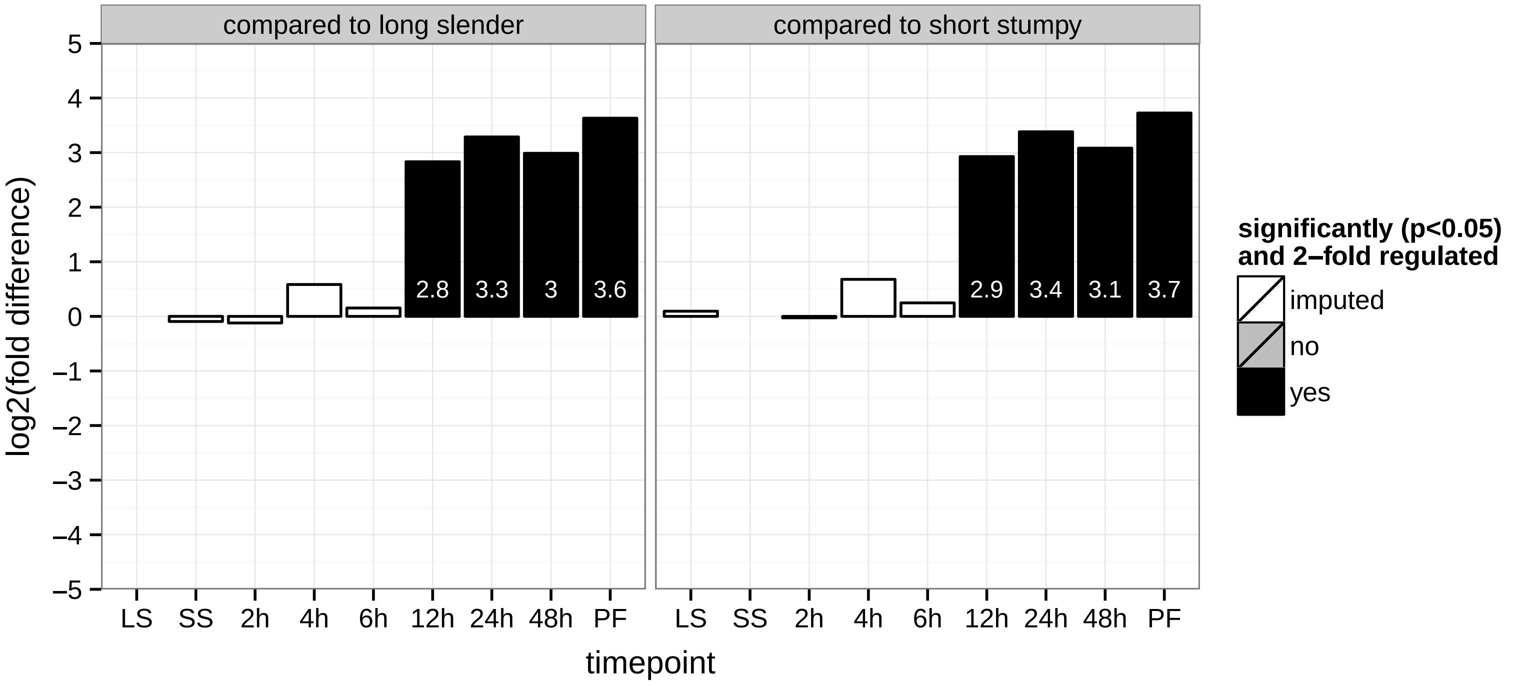

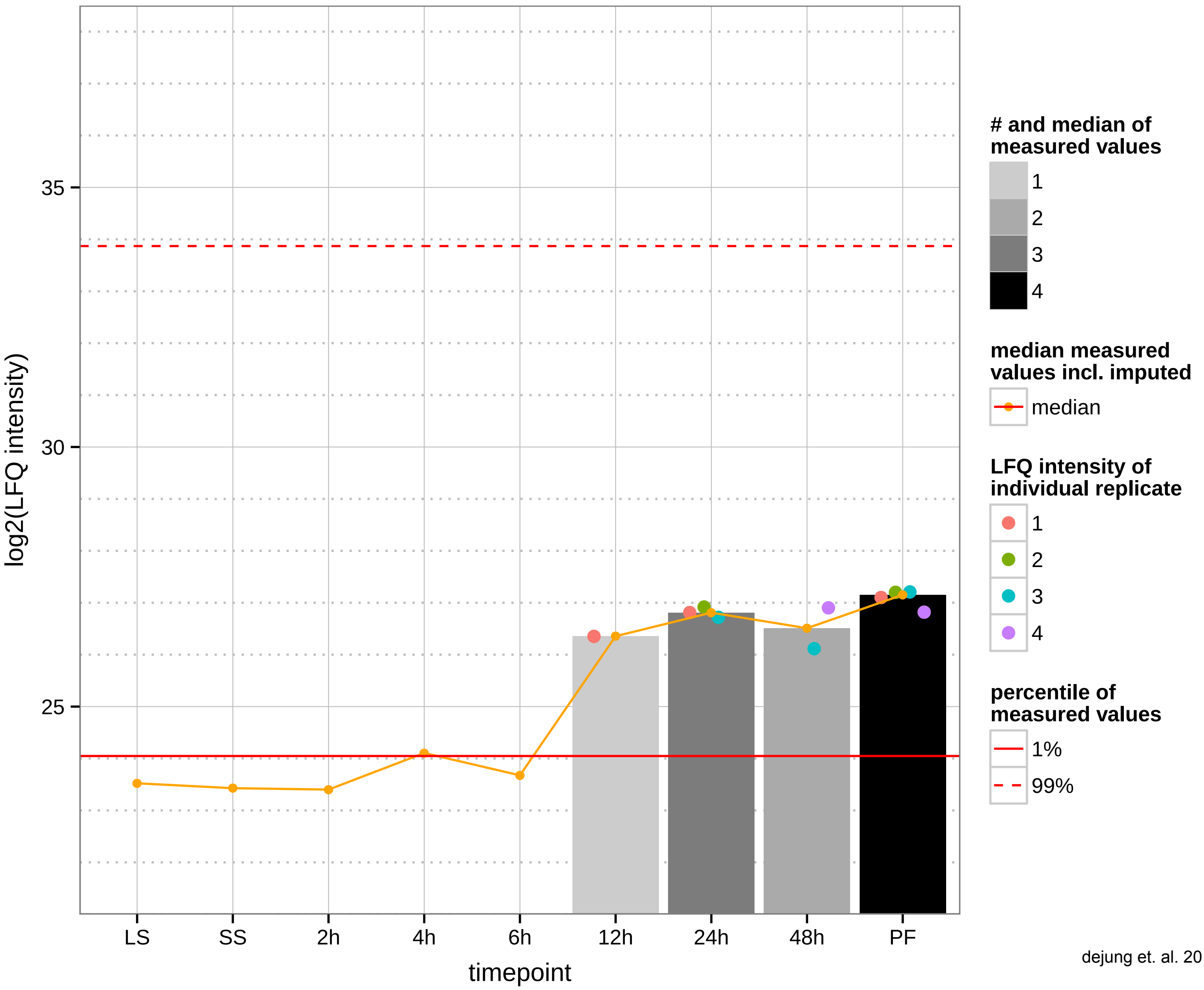

dejung et. al. 2015

UDP−Gal or UDP−GlcNAc−dependent glycosyltransferase, putative
Tb927.10.12290
AGOF: UDP−glycosyltransferase activity, galactosyltransferase activity
AGOC: membrane
AGOP: GPI anchor biosynthetic process, protein glycosylation
PGOF: null
PGOC: null
PGOP: null

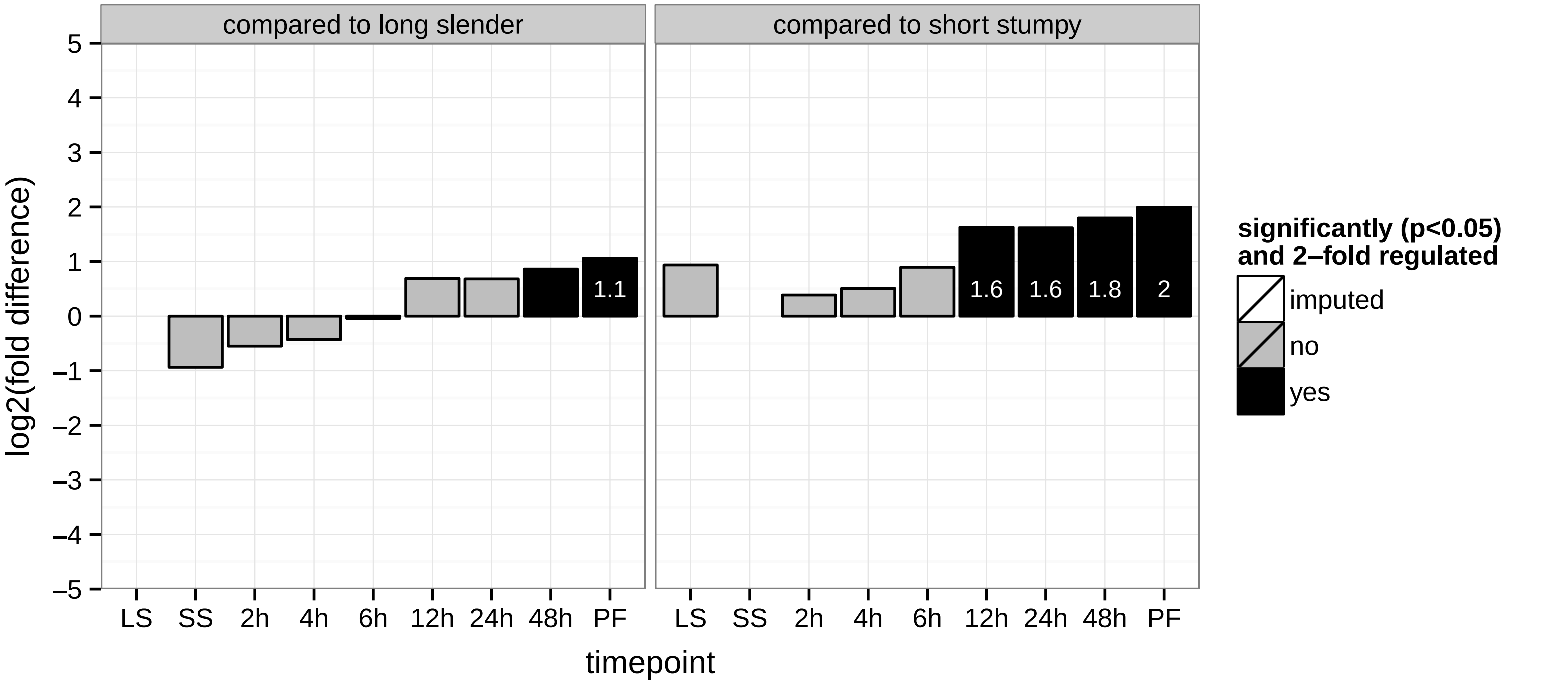

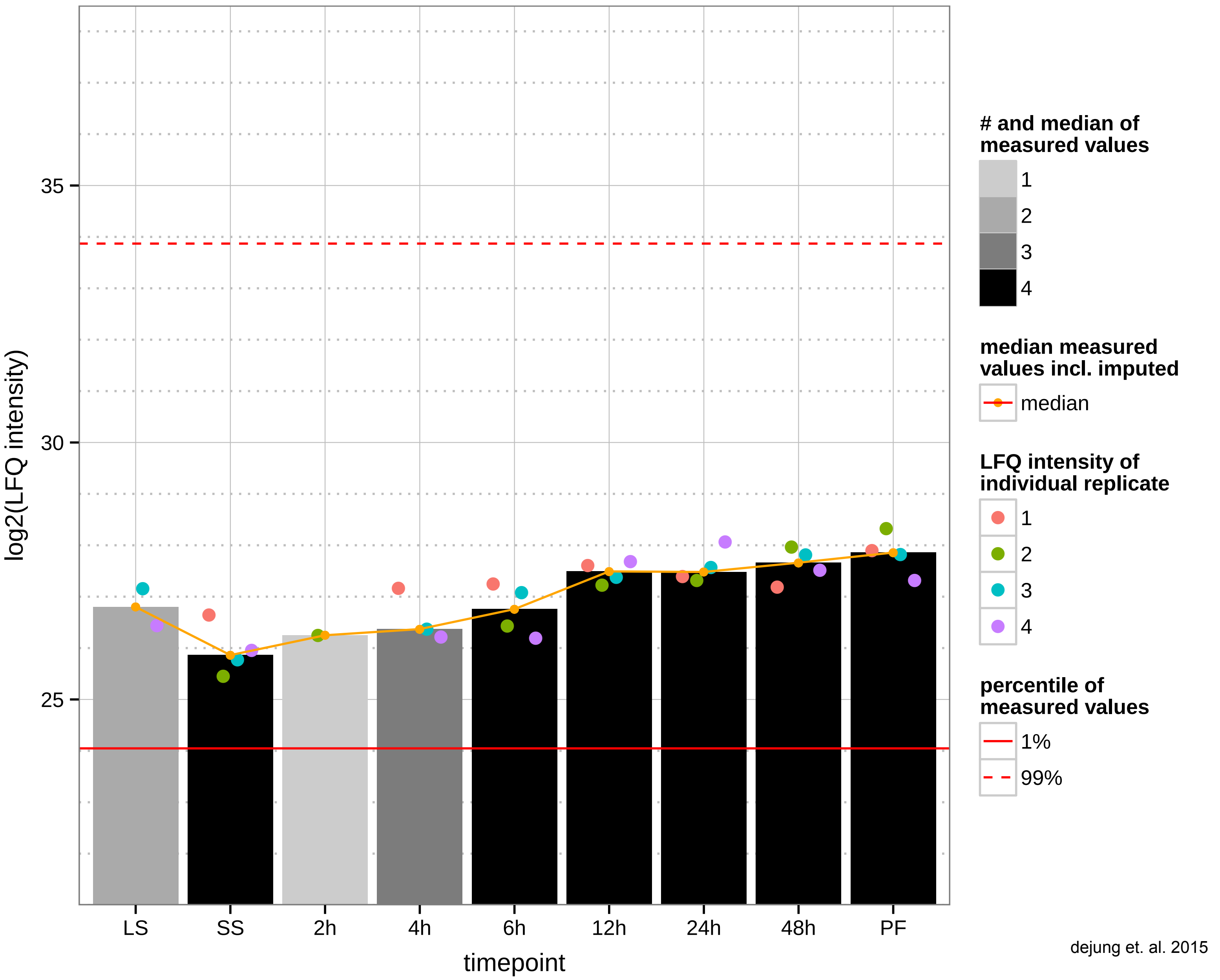

dejung et. al. 2015

zinc finger protein family member, putative (ZC3H34)
Tb927.10.12330
AGOF: RNA binding, zinc ion binding
AGOC: null
AGOP: null
PGOF: nucleic acid binding, zinc ion binding
PGOC: null
PGOP: null

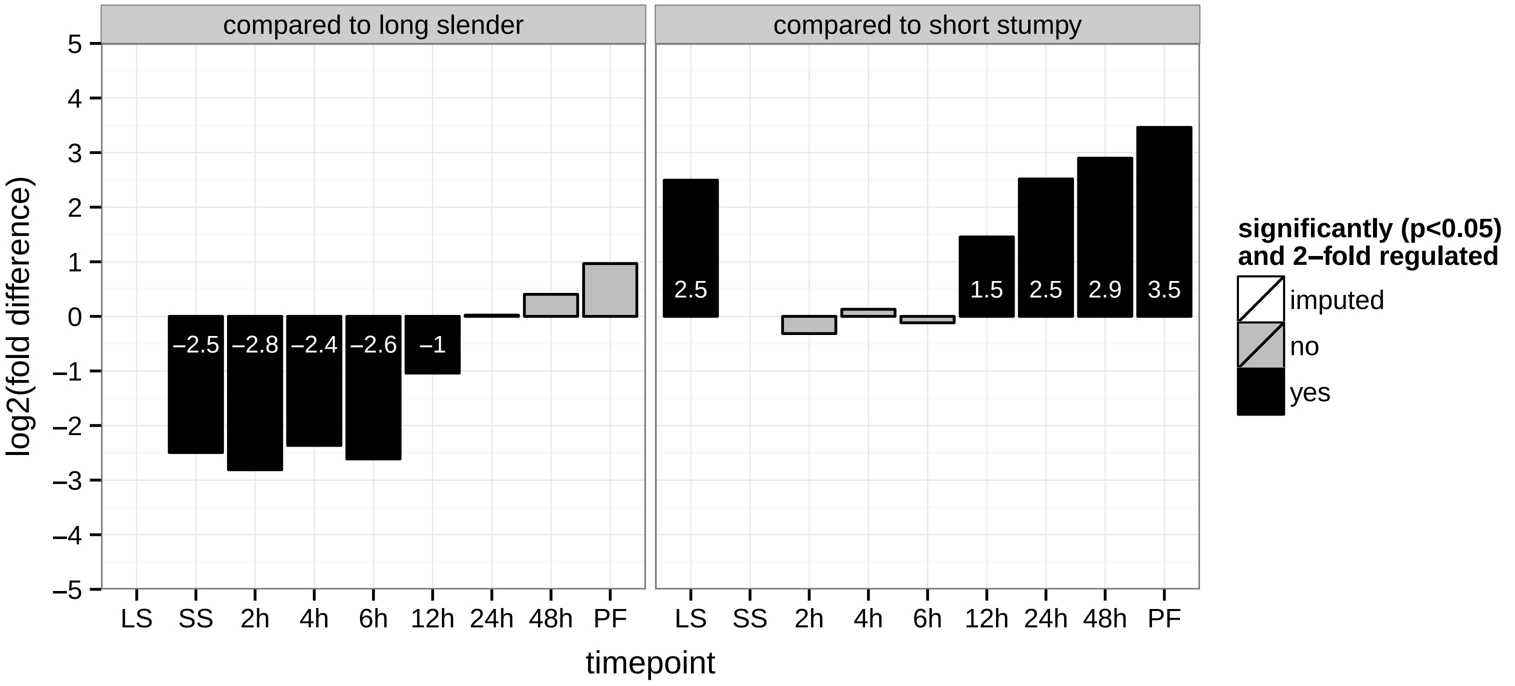

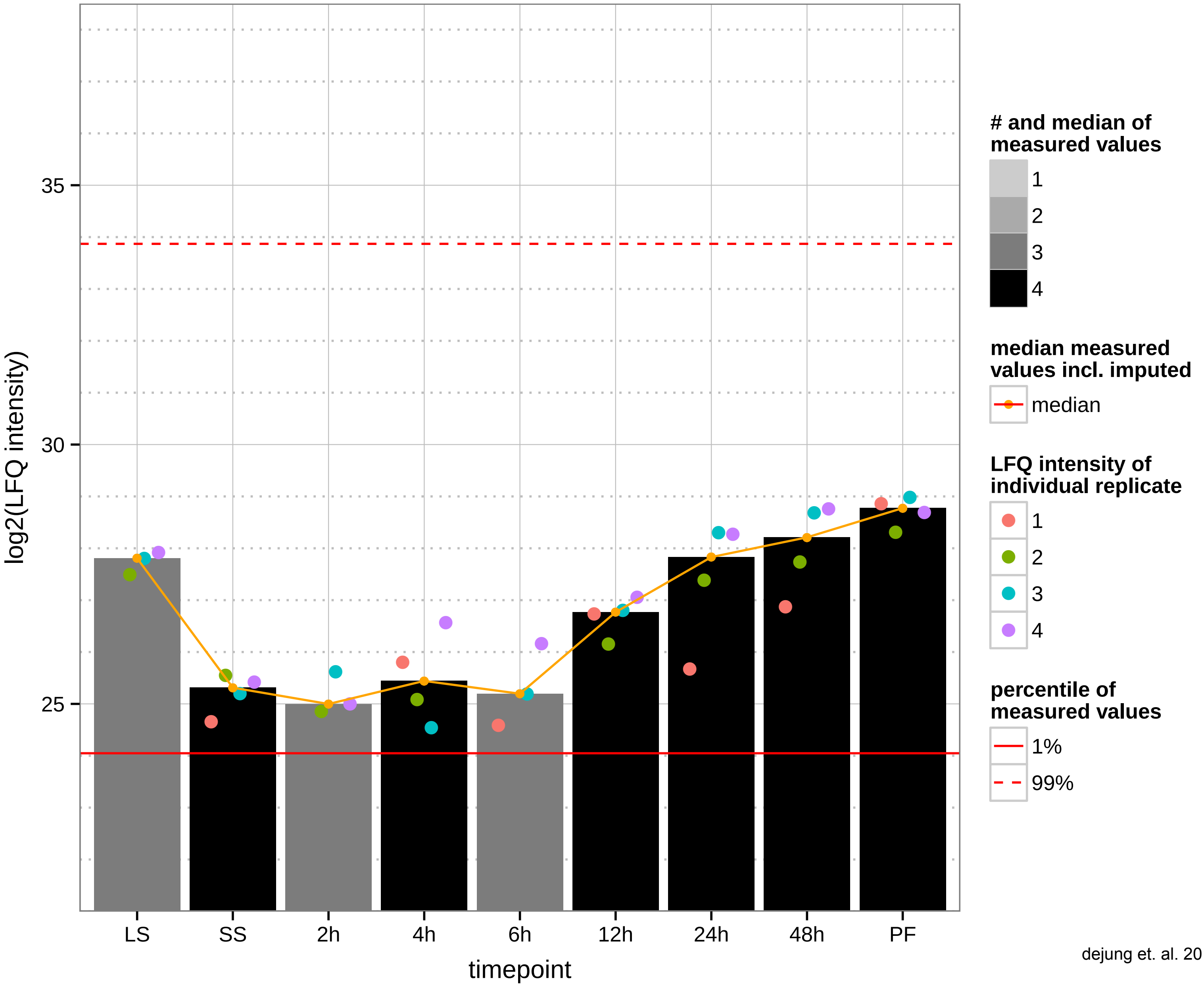

dejung et. al. 2015

hypothetical protein, conserved
Tb927.10.1250
AGOF: null
AGOC: null
AGOP: null
PGOF: null
PGOC: null
PGOP: null

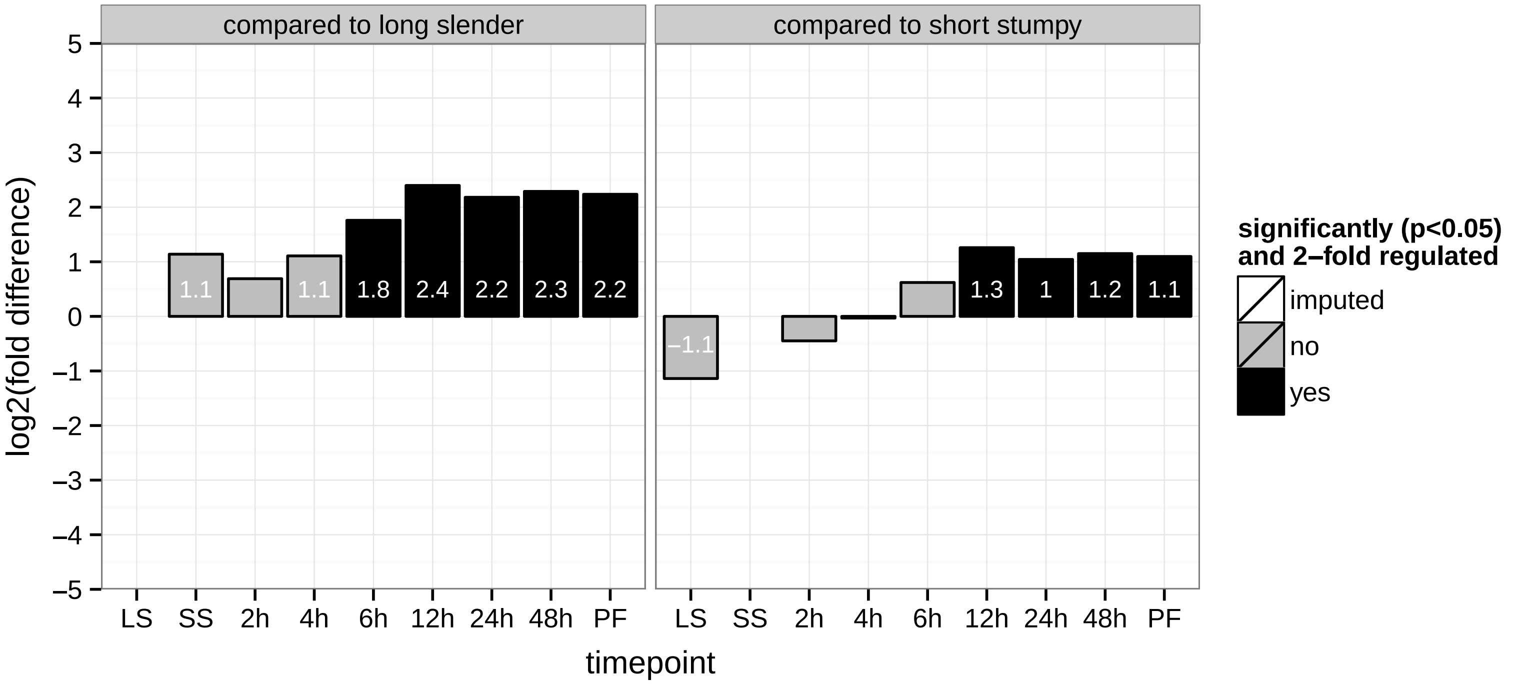

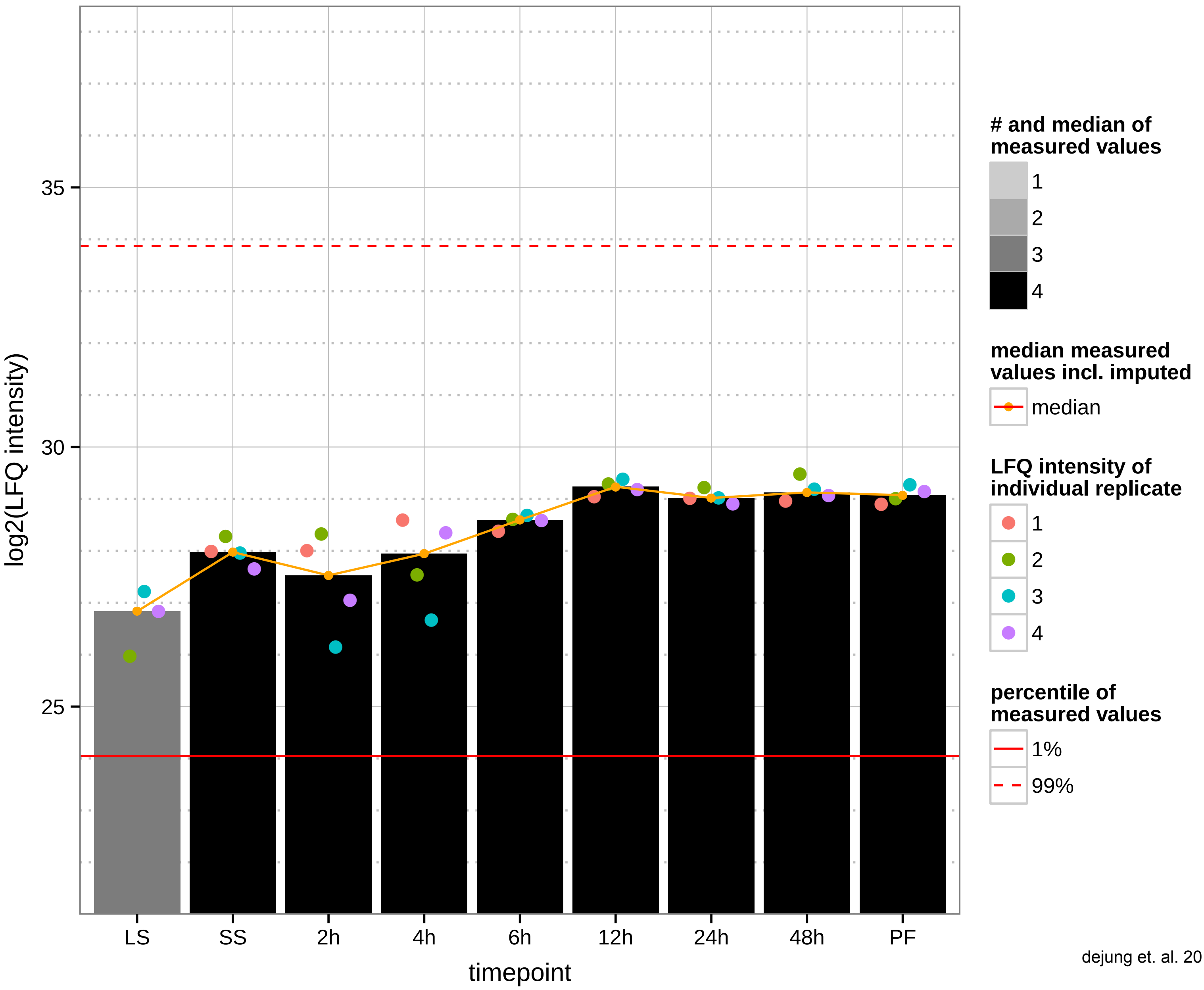

dejung et. al. 2015

hypothetical protein, conserved
Tb927.10.1280
AGOF: null
AGOC: null
AGOP: null
PGOF: null
PGOC: null
PGOP: null

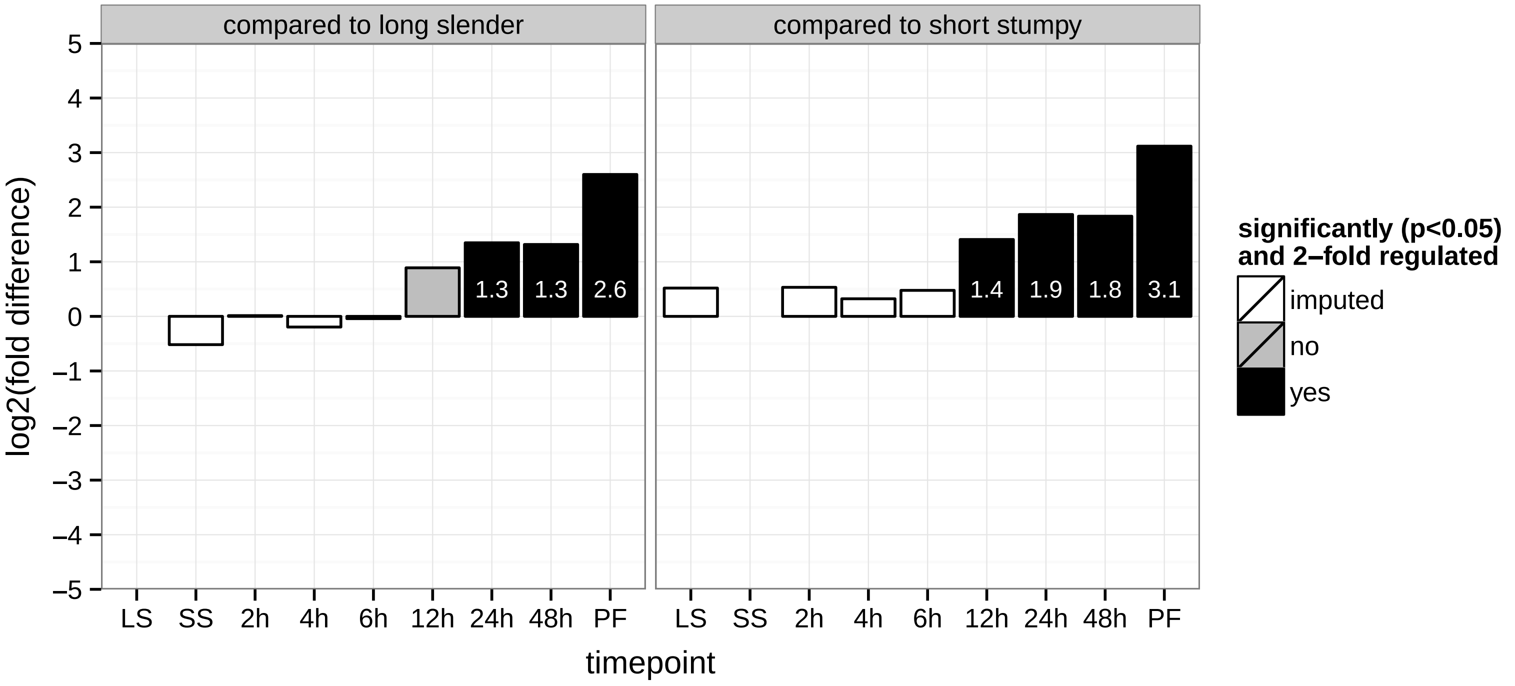

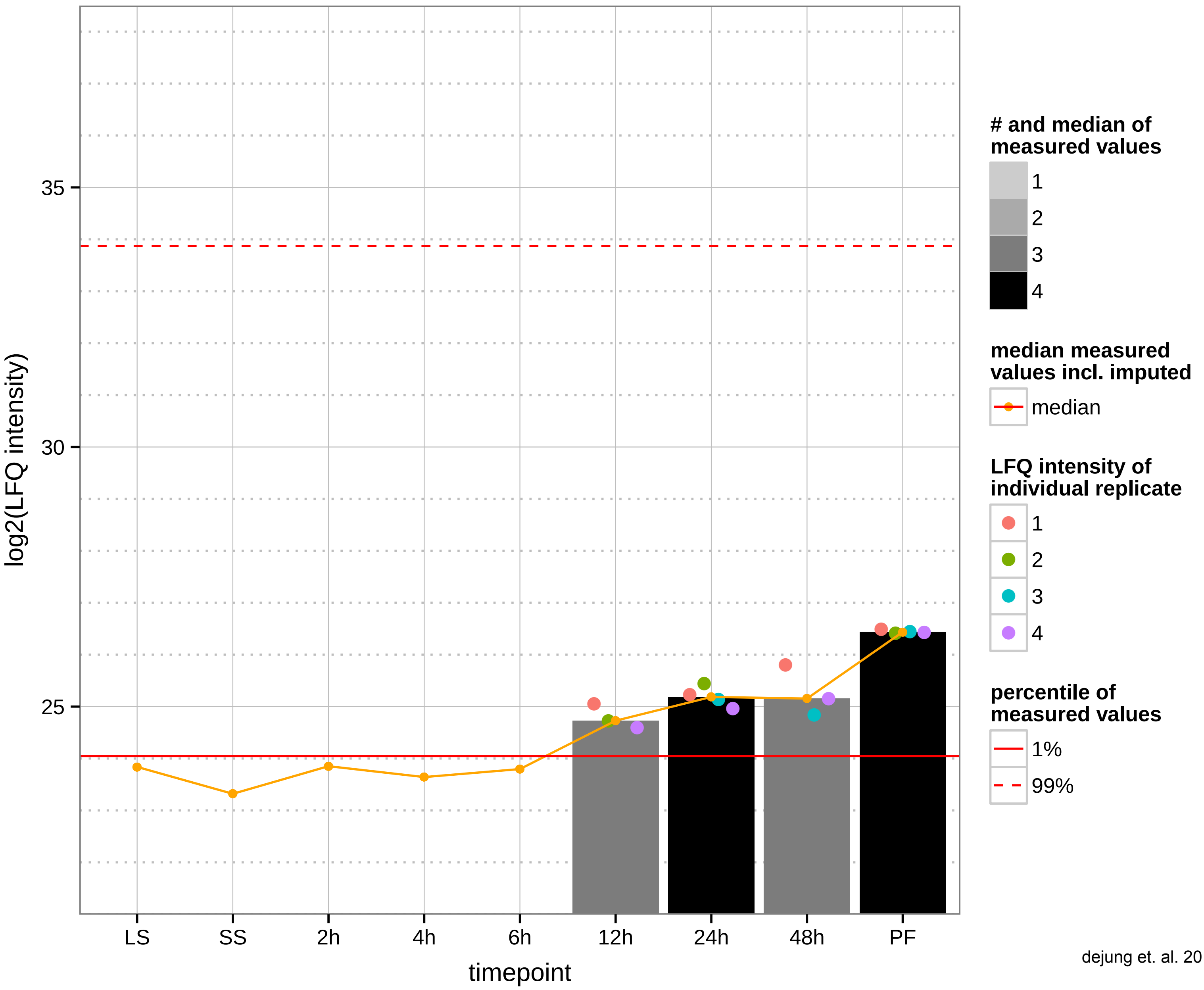

dejung et. al. 2015

hypothetical protein, conserved
Tb927.10.12860
AGOF: null
AGOC: null
AGOP: null
PGOF: null
PGOC: null
PGOP: null

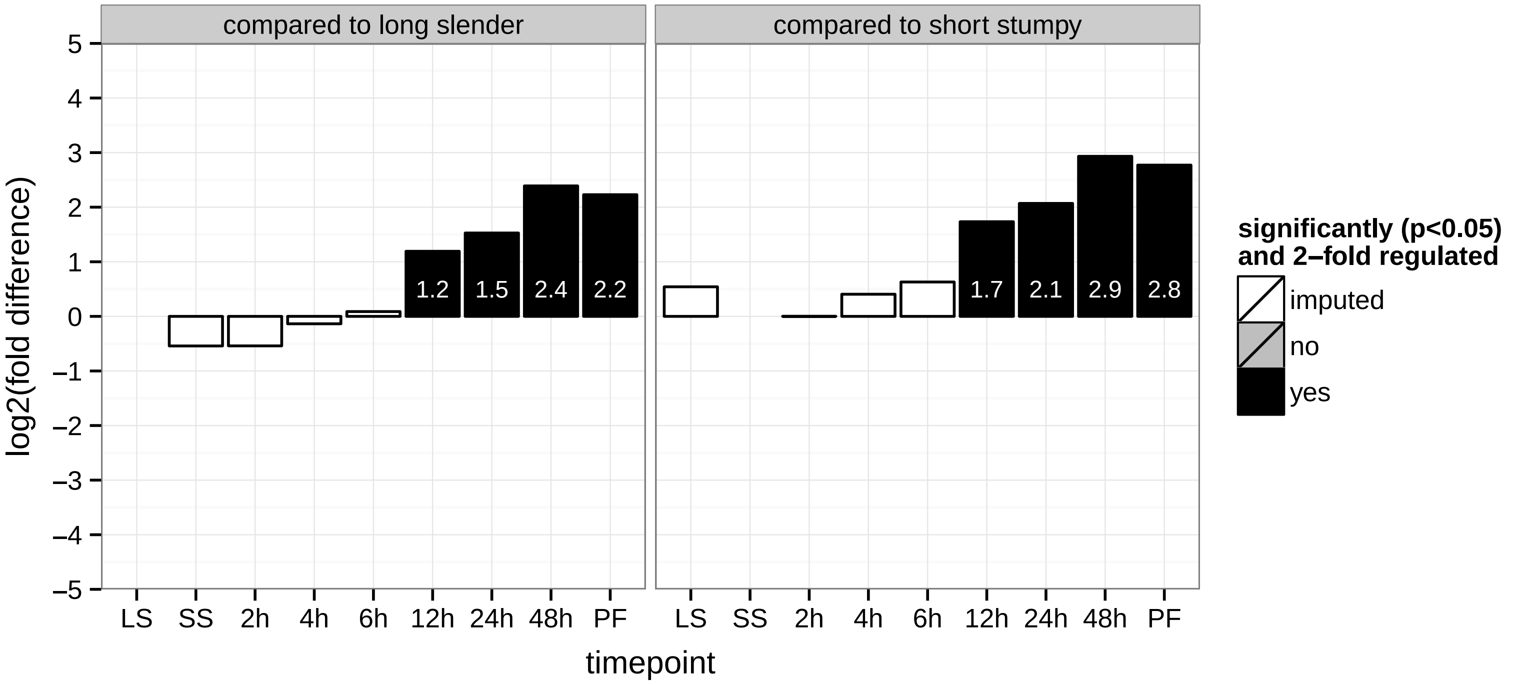

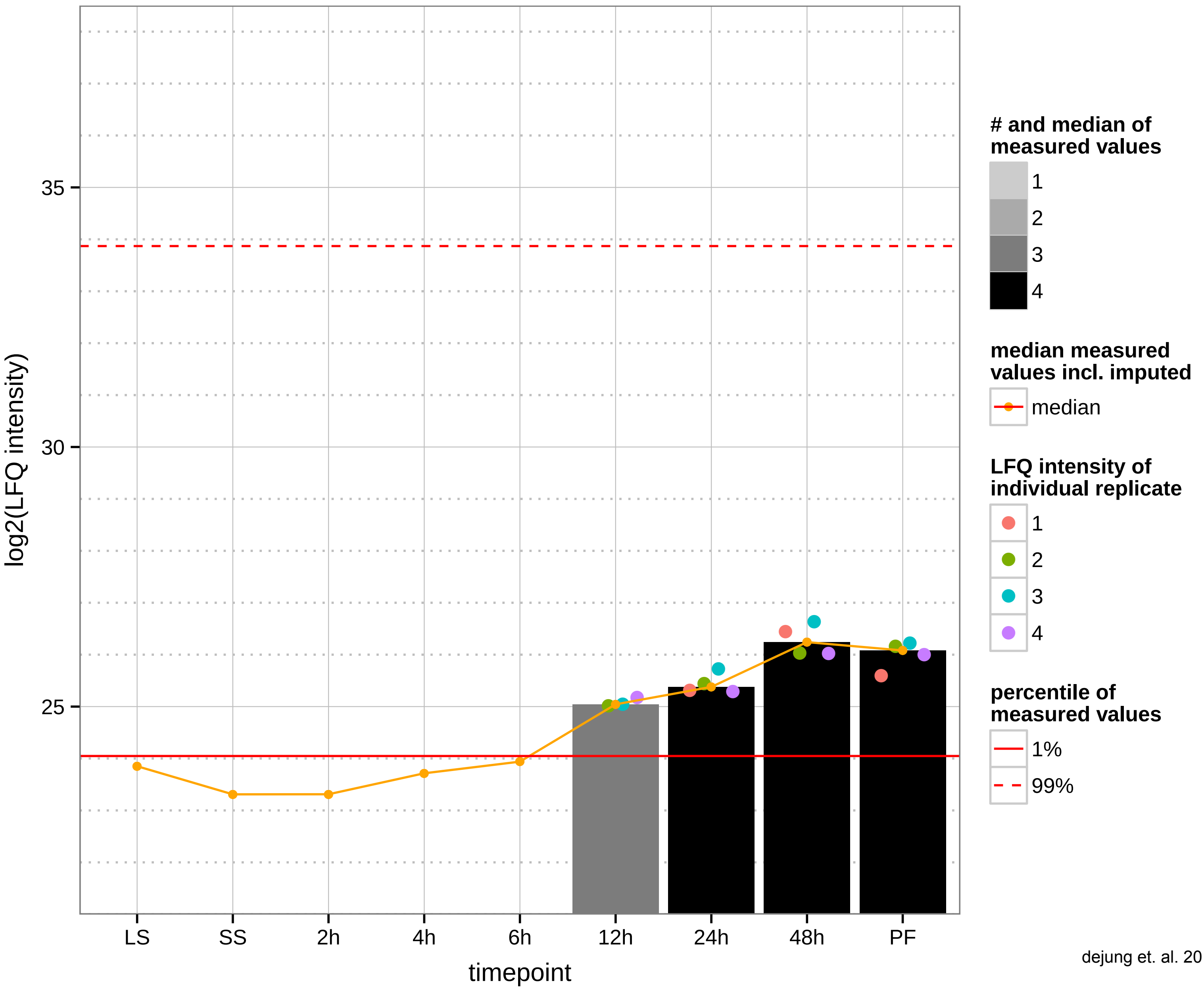

dejung et. al. 2015

hypothetical protein, conserved
Tb927.10.13140
AGOF: null
AGOC: null
AGOP: null
PGOF: null
PGOC: null
PGOP: null

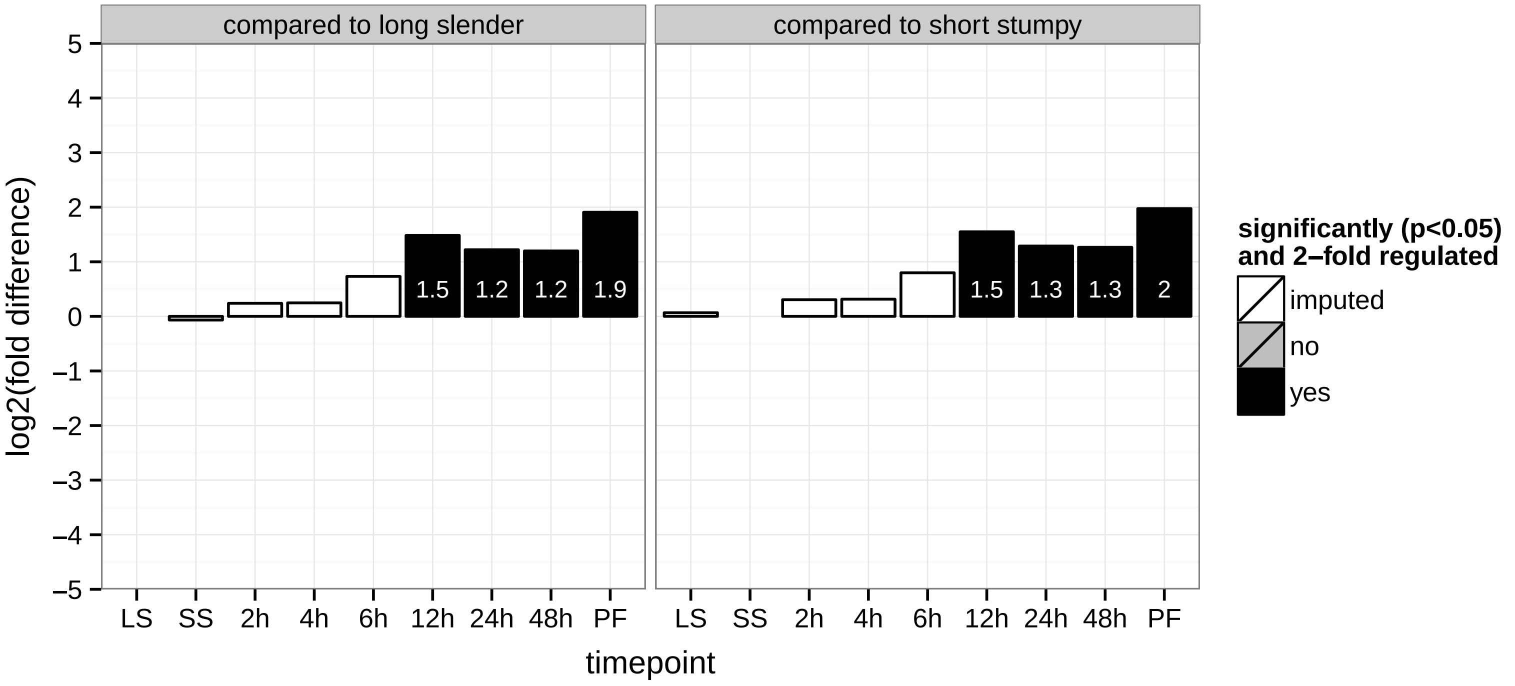

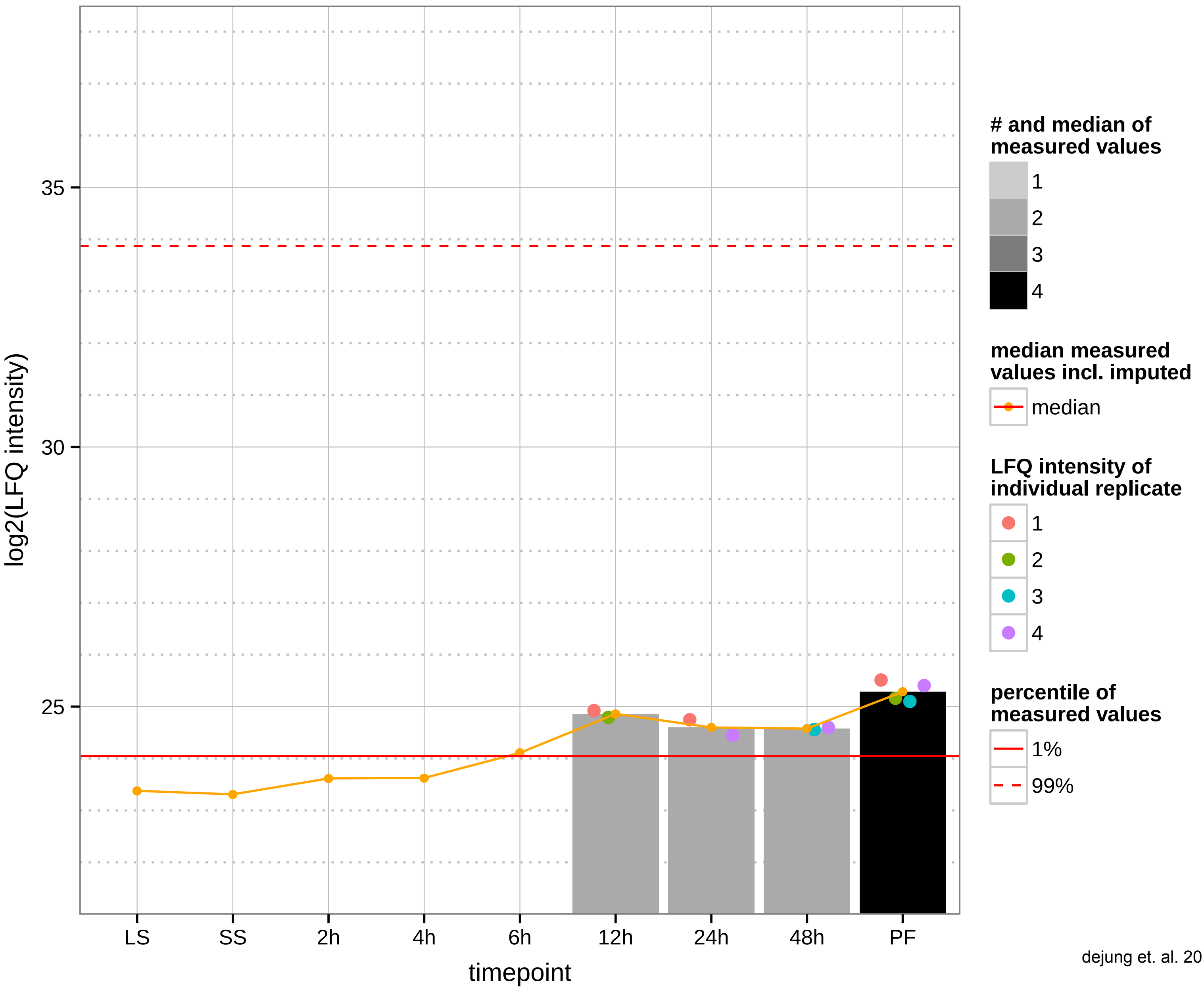

dejung et. al. 2015

DNA–directed RNA polymerase I subunit RPB5z, putative (RPB5z)
Tb927.10.13310
AGOF: DNA binding, DNA–directed RNA polymerase activity
AGOC: nucleus
AGOP: transcription, DNA–dependent
PGOF: DNA binding, DNA–directed RNA polymerase activity
PGOC: nucleus
PGOP: transcription, DNA–dependent

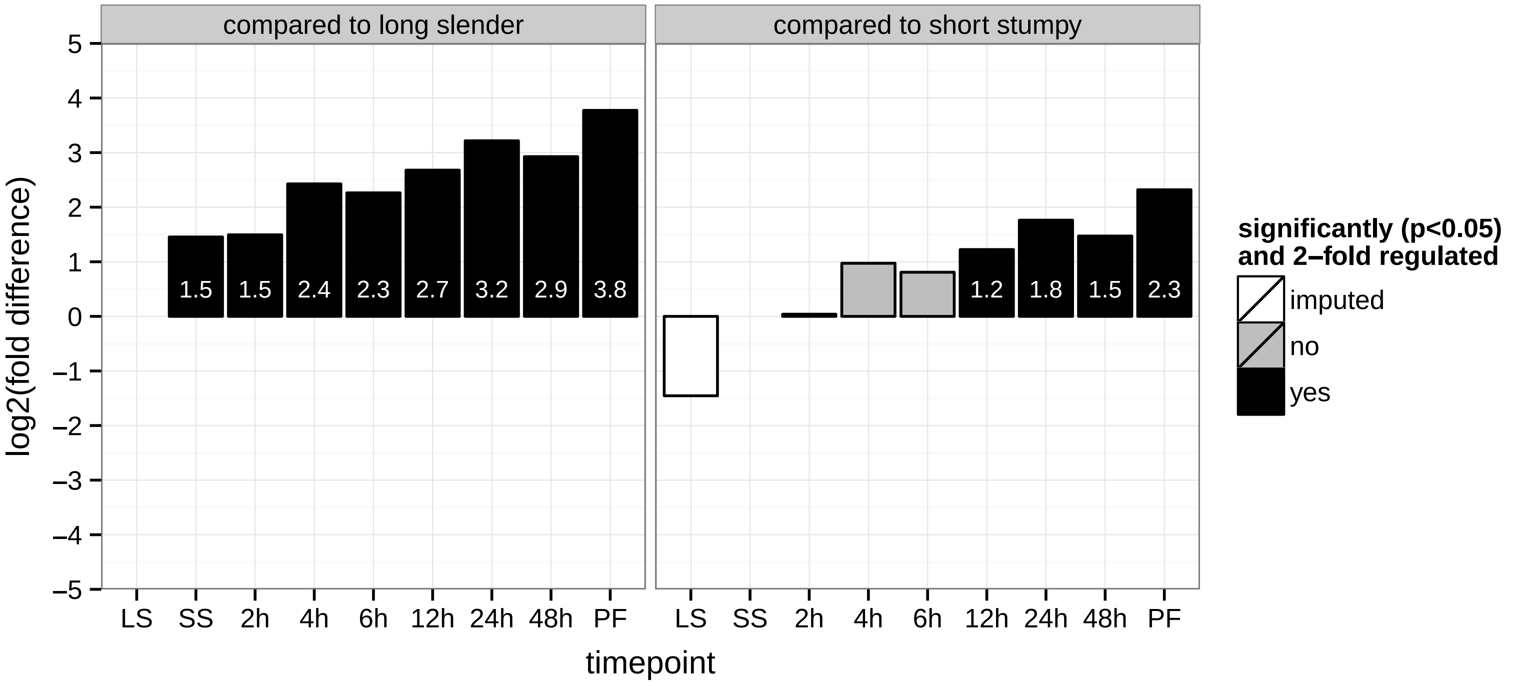

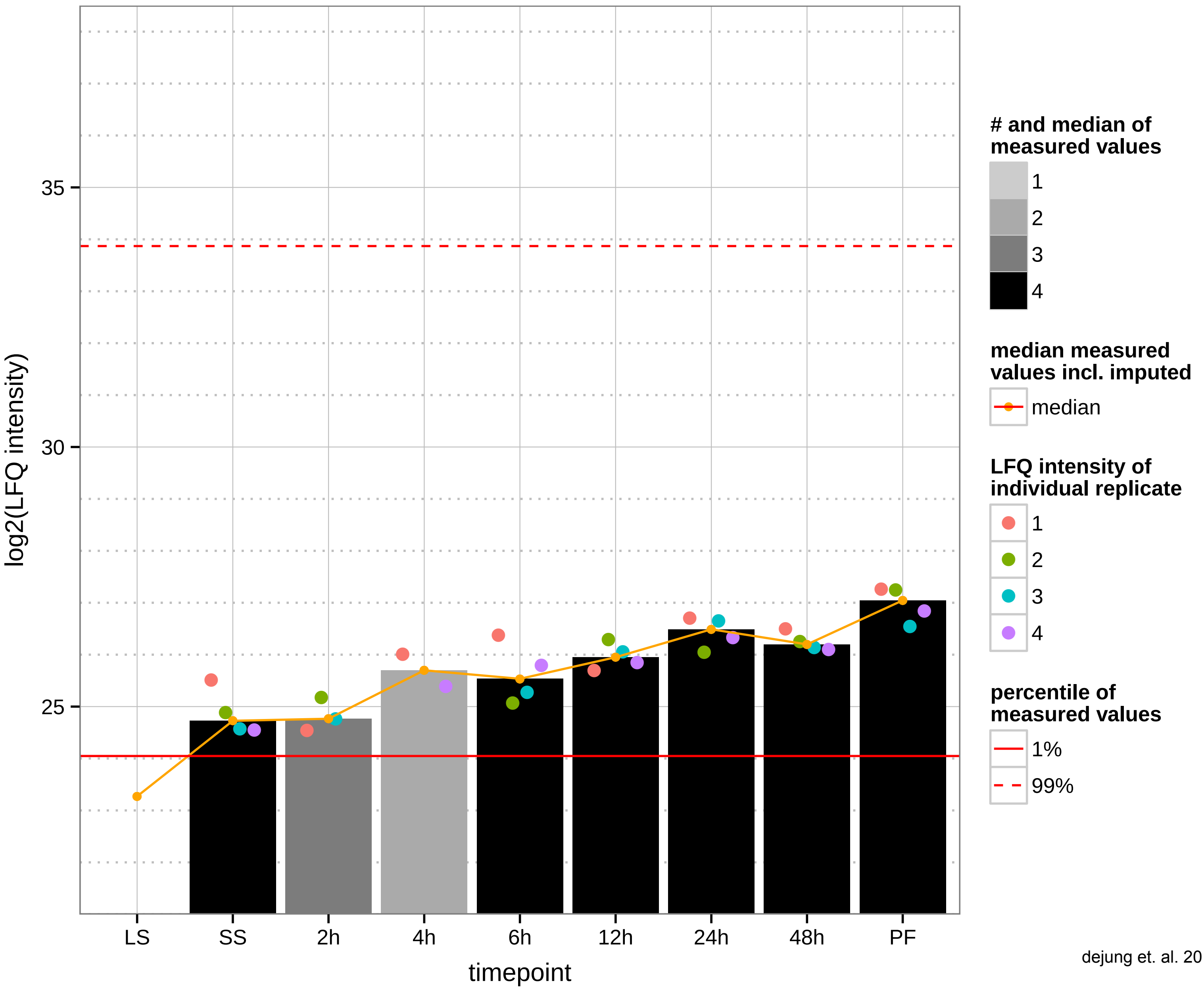

dejung et. al. 2015

hypothetical protein, conserved
Tb927.10.13800
AGOF: null
AGOC: null
AGOP: null
PGOF: null
PGOC: null
PGOP: null

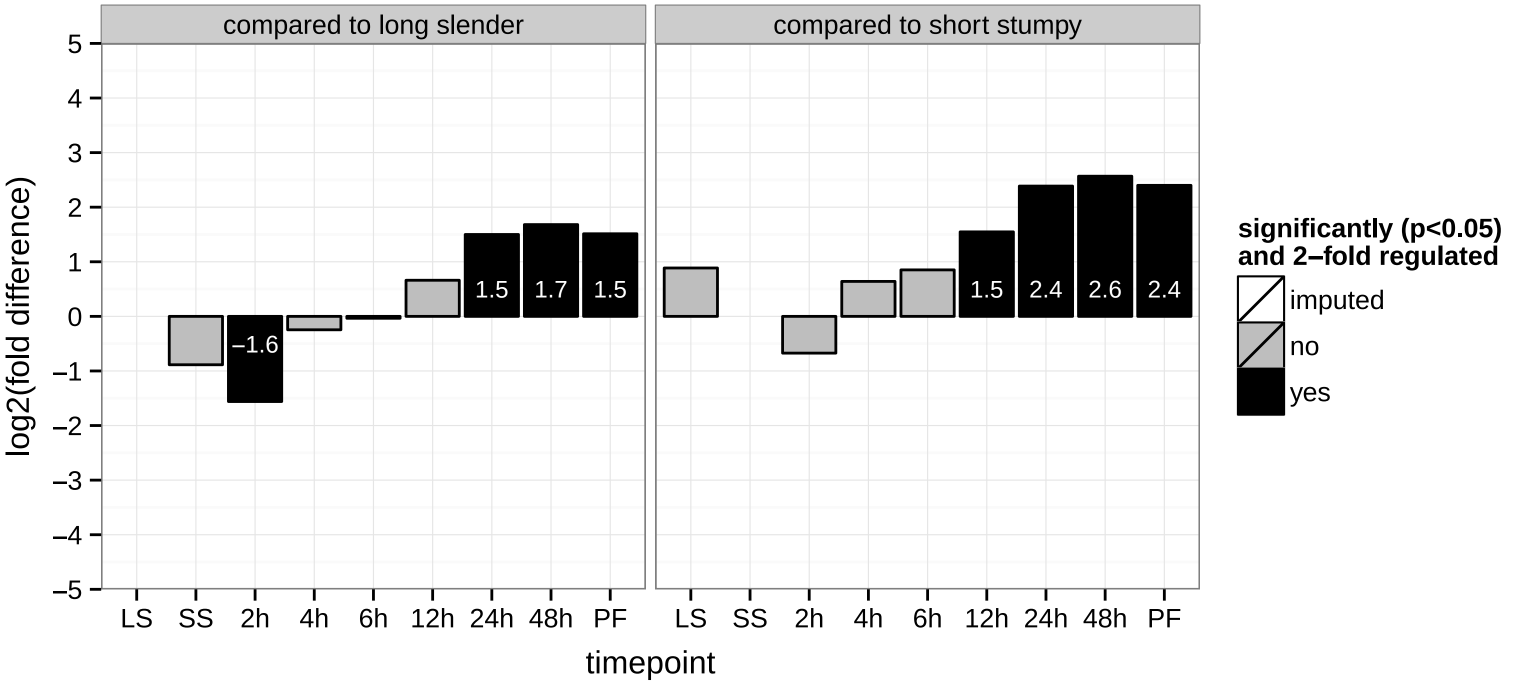

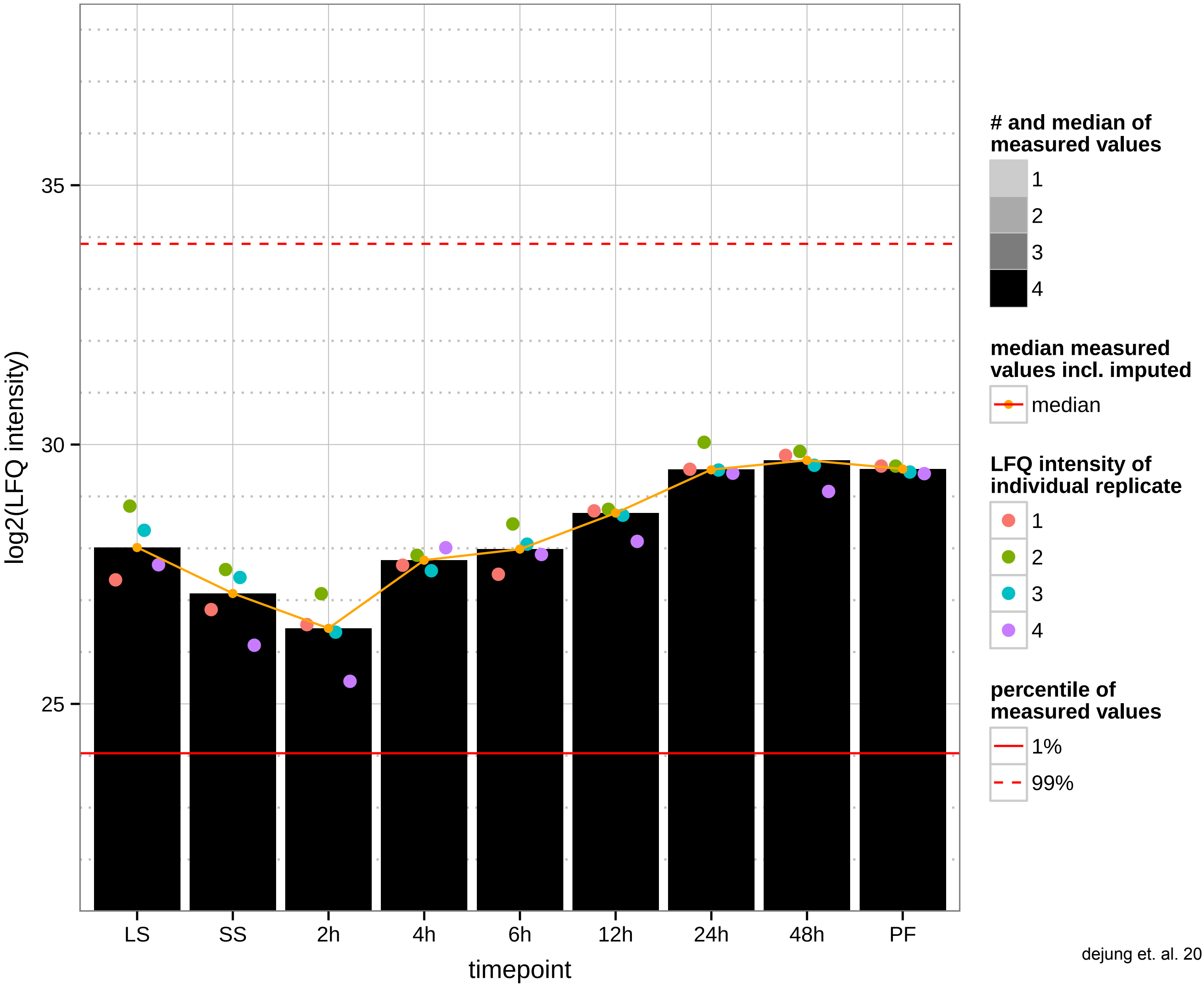

dejung et. al. 2015

aquaglyceroporin 2 (AQP2)
Tb927.10.14170
AGOF: glycerol channel activity, water channel activity, water transmembrane transporter activity
AGOC: flagellar pocket, plasma membrane
AGOP: glycerol transport
PGOF: transporter activity
PGOC: membrane
PGOP: transport

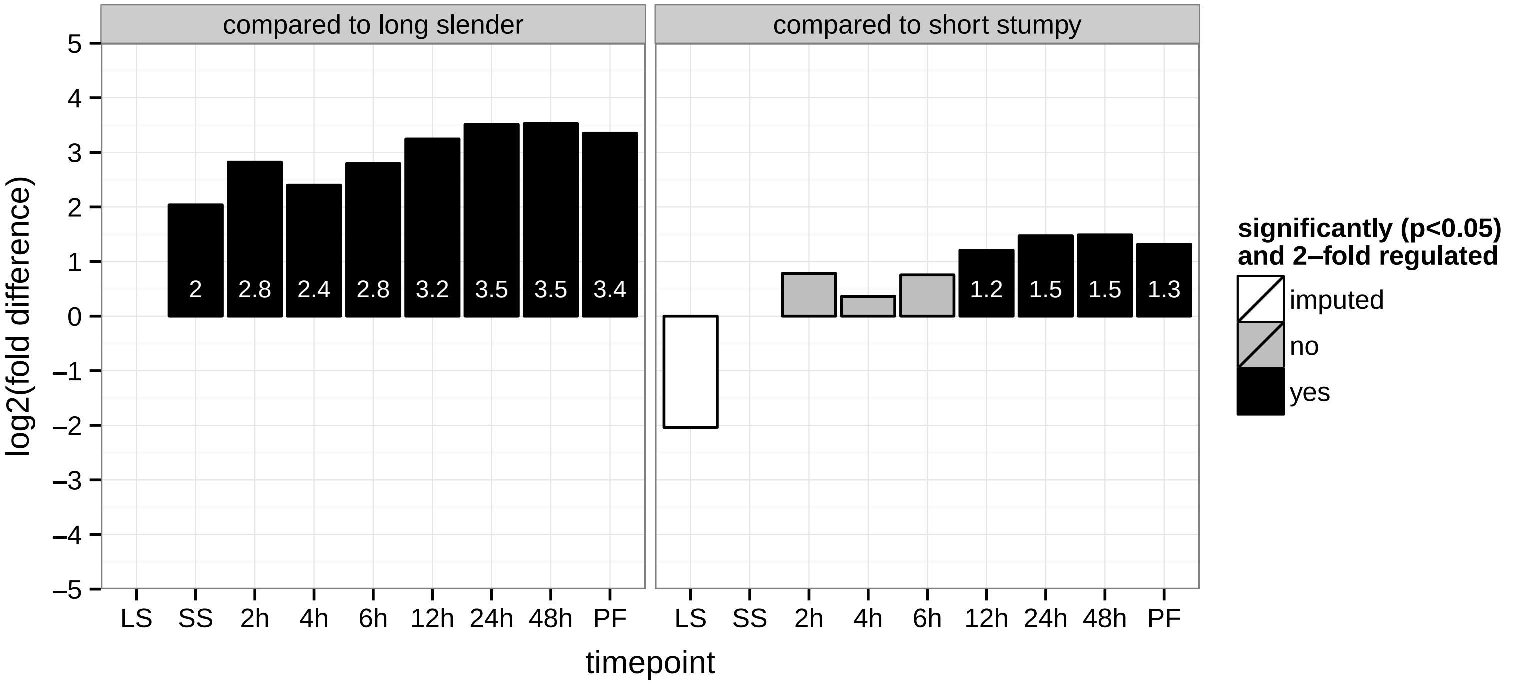

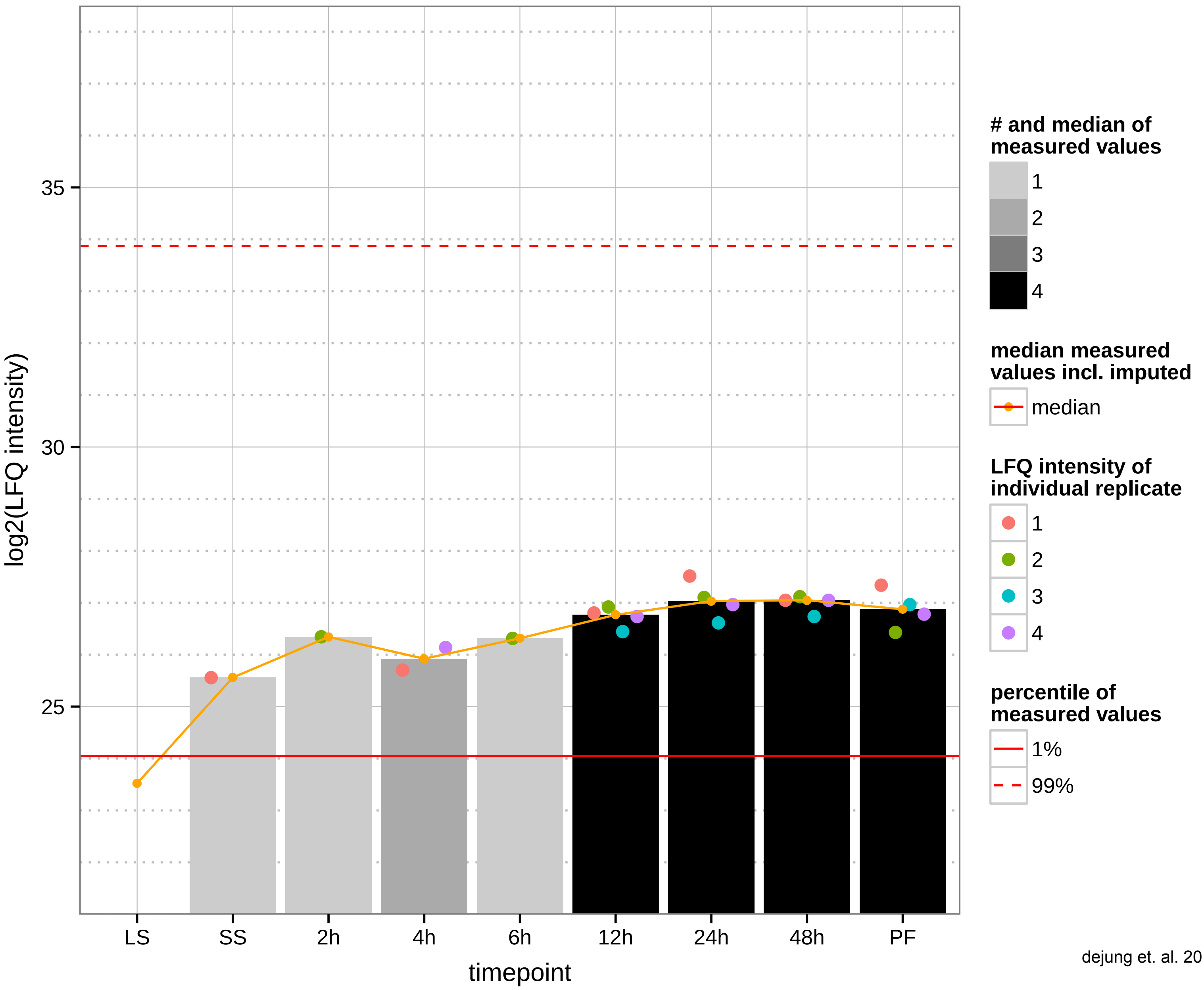

dejung et. al. 2015

peroxin 13, SH3 domain protein, conserved (PEX13)
Tb927.10.14720
AGOF: null
AGOC: glycosome membrane
AGOP: peroxisomal membrane transport, peroxisome organization
PGOF: protein binding
PGOC: null
PGOP: null

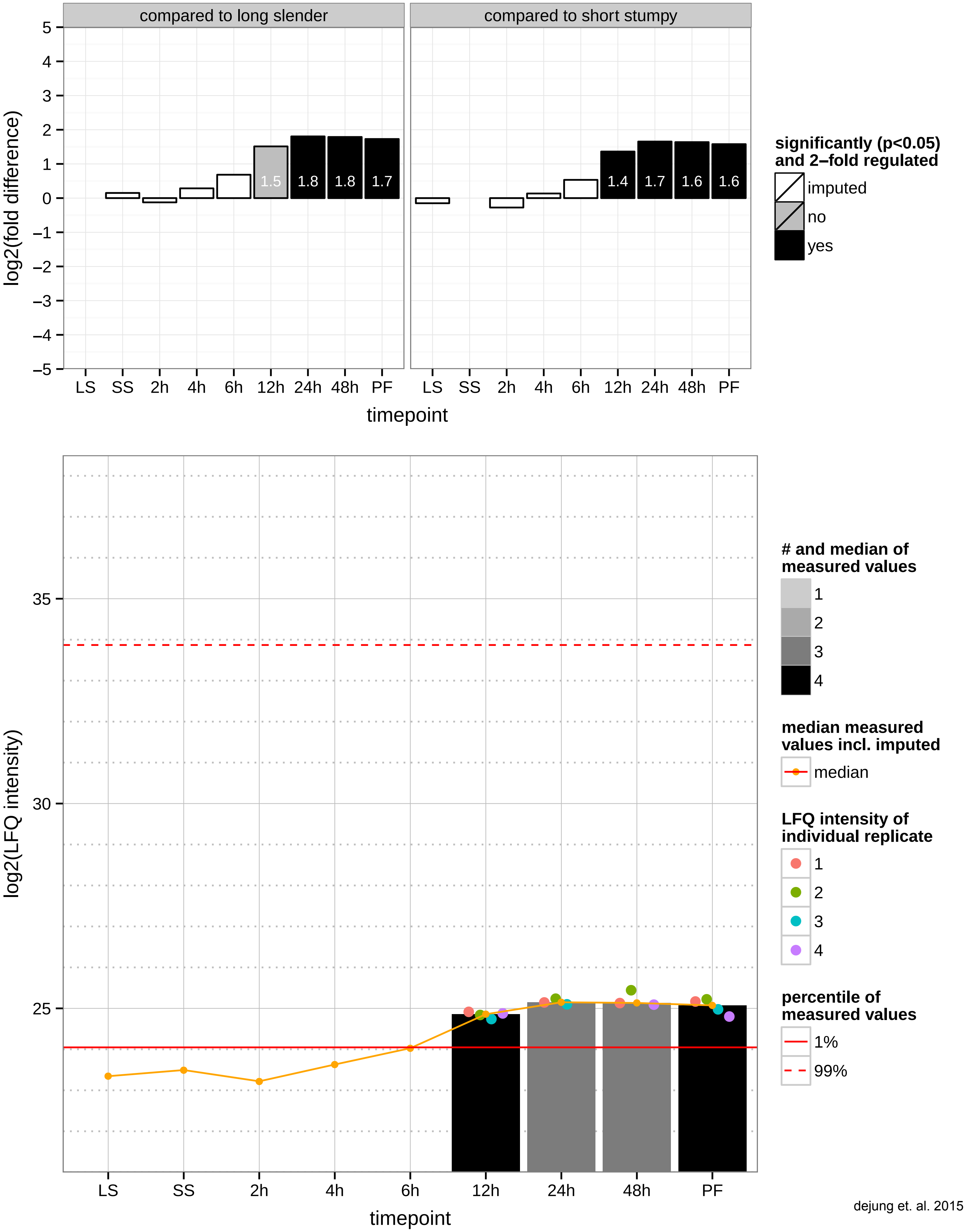

dejung et. al. 2015

Multiprotein–bridging factor 1, putative (mbf1)
Tb927.10.14810
AGOF: DNA binding, sequence–specific DNA binding, transcription coactivator activity
AGOC: null
AGOP: positive regulation of transcription from RNA polymerase II promoter
PGOF: DNA binding, sequence–specific DNA binding
PGOC: null
PGOP: null

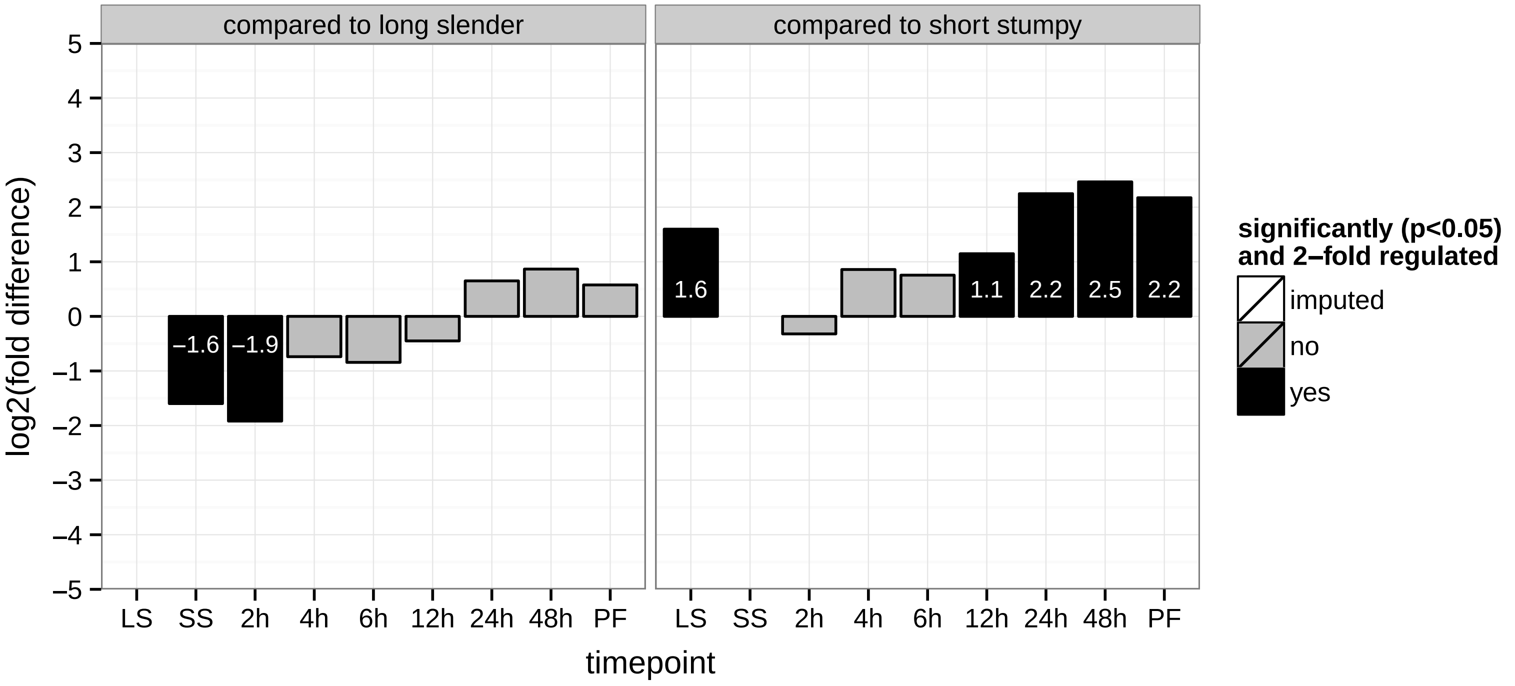

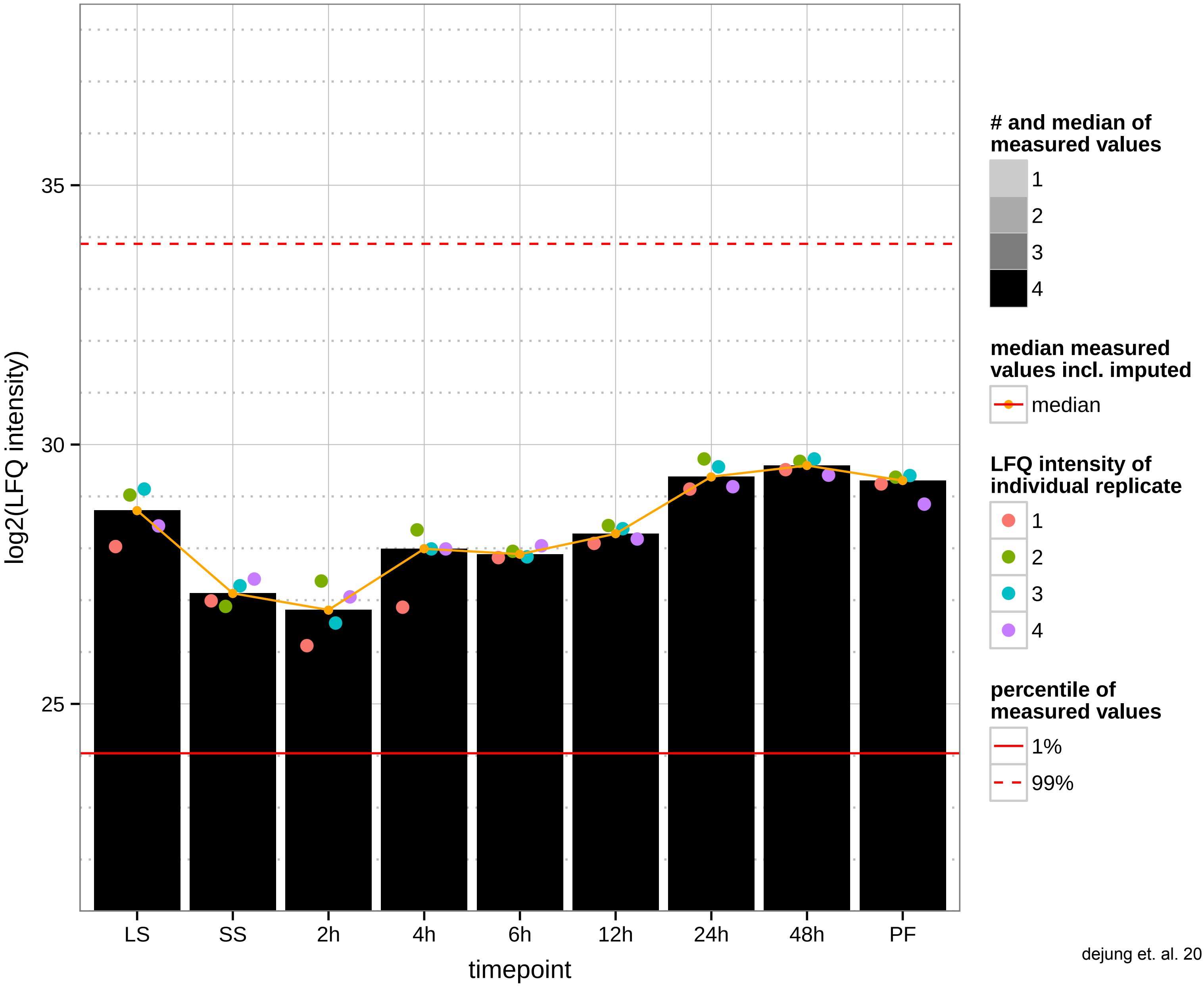

dejung et. al. 2015

Zinc finger CCCH domain–containing protein 39 (ZC3H39)
Tb927.10.14930
AGOF: RNA binding, ubiquitin–protein ligase activity
AGOC: ubiquitin ligase complex
AGOP: protein ubiquitination
PGOF: nucleic acid binding, zinc ion binding
PGOC: null
PGOP: null

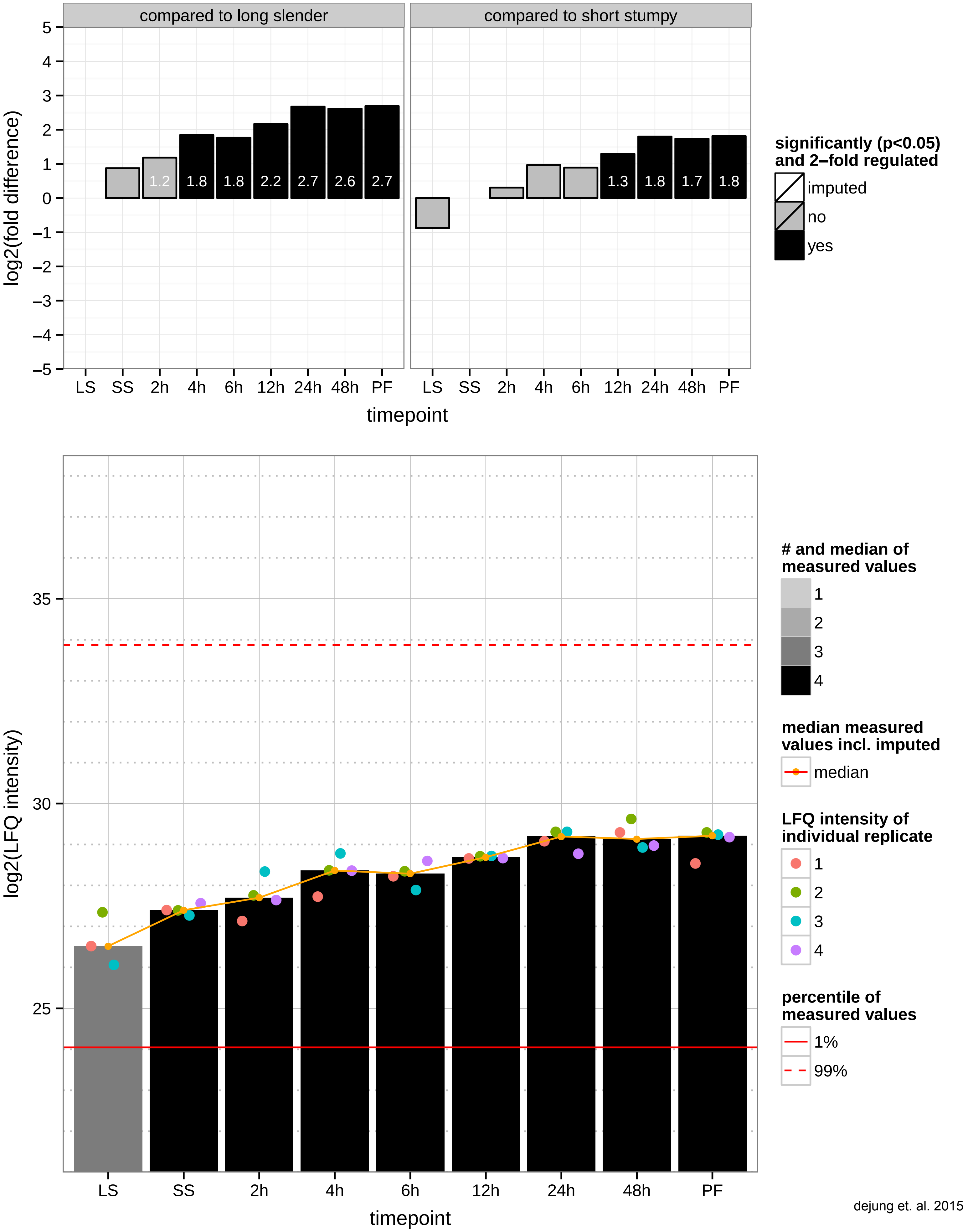

dejung et. al. 2015

zinc finger protein family member, putative (ZC3H30)
Tb927.10.1540
AGOF: RNA binding
AGOC: null
AGOP: null
PGOF: null
PGOC: null
PGOP: null

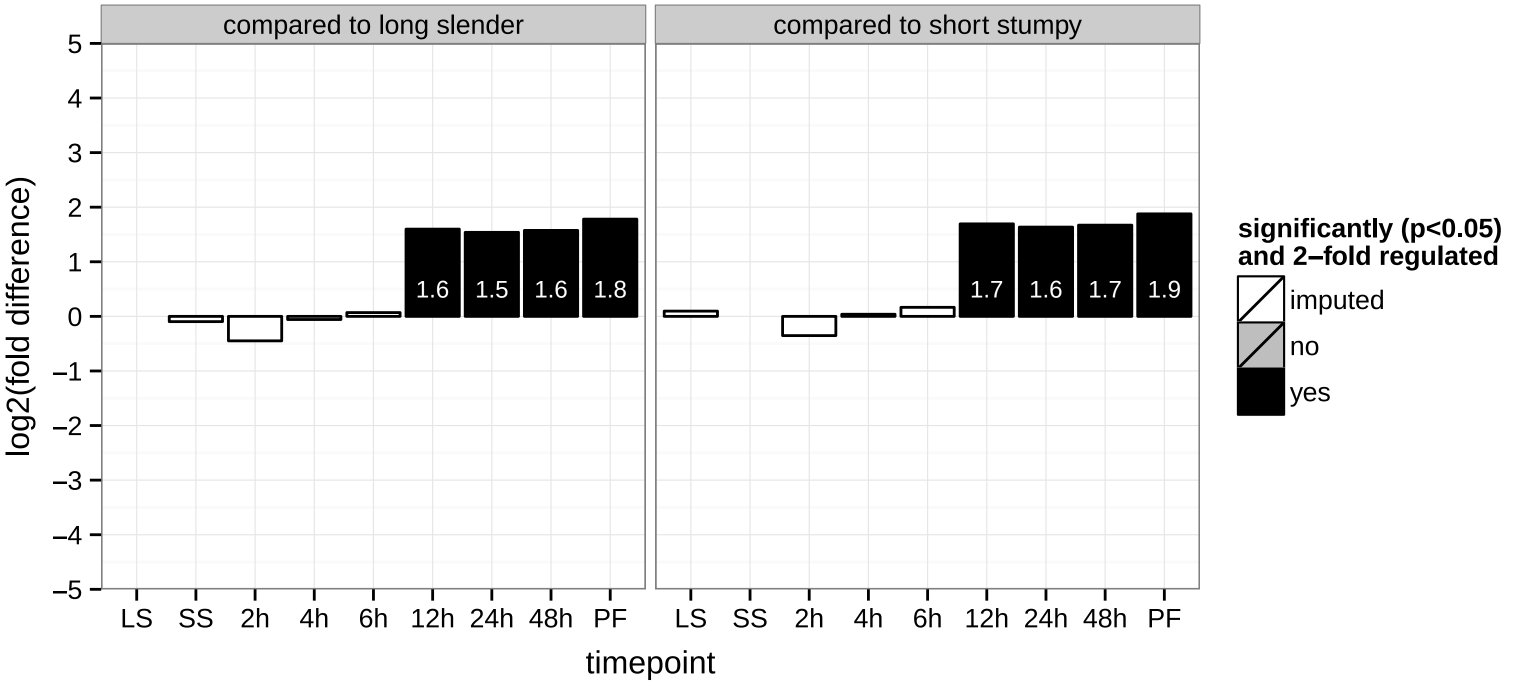

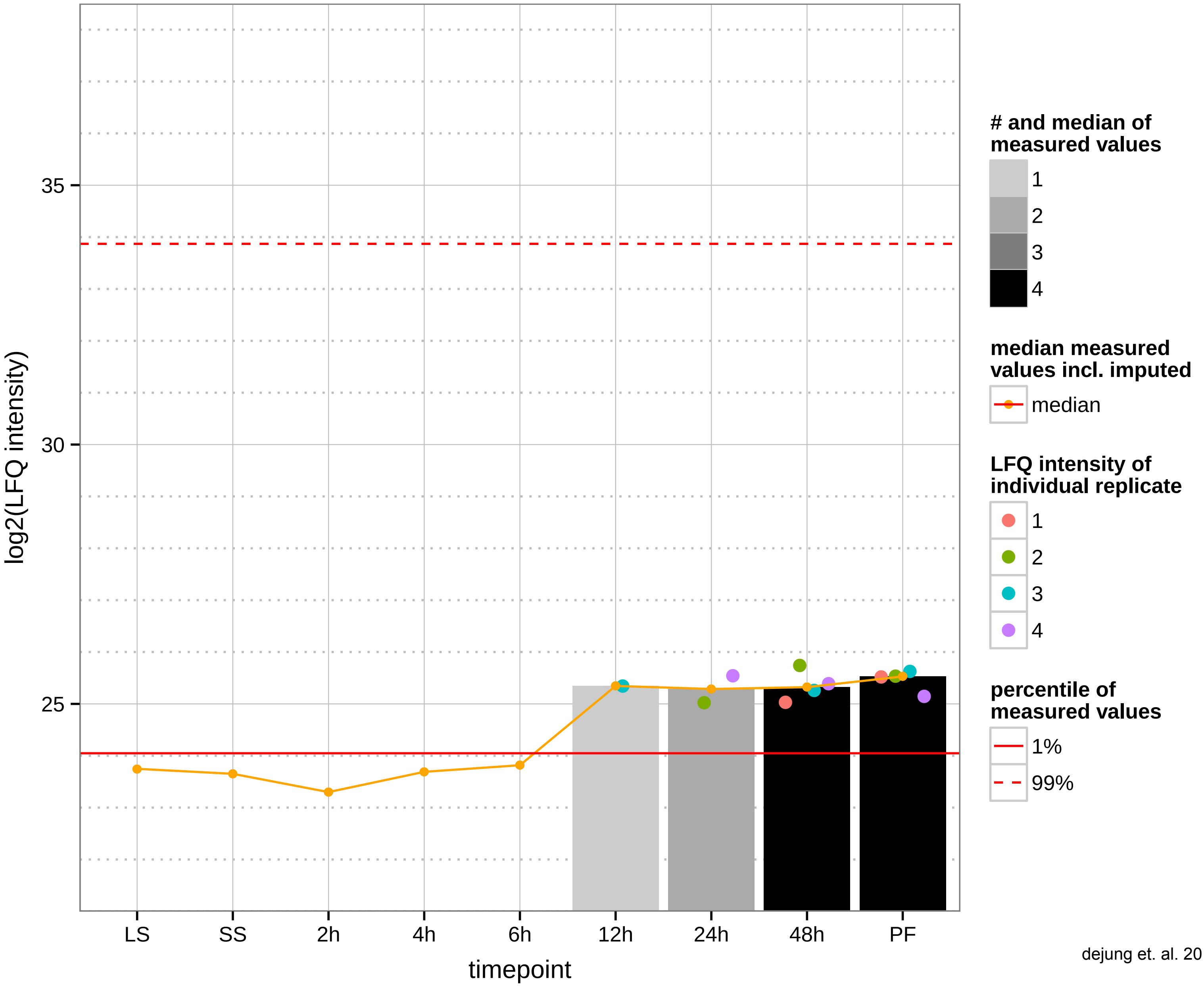

dejung et. al. 2015

hypothetical protein, conserved
Tb927.10.2060
AGOF: null
AGOC: mitochondrion
AGOP: null
PGOF: null
PGOC: null
PGOP: null

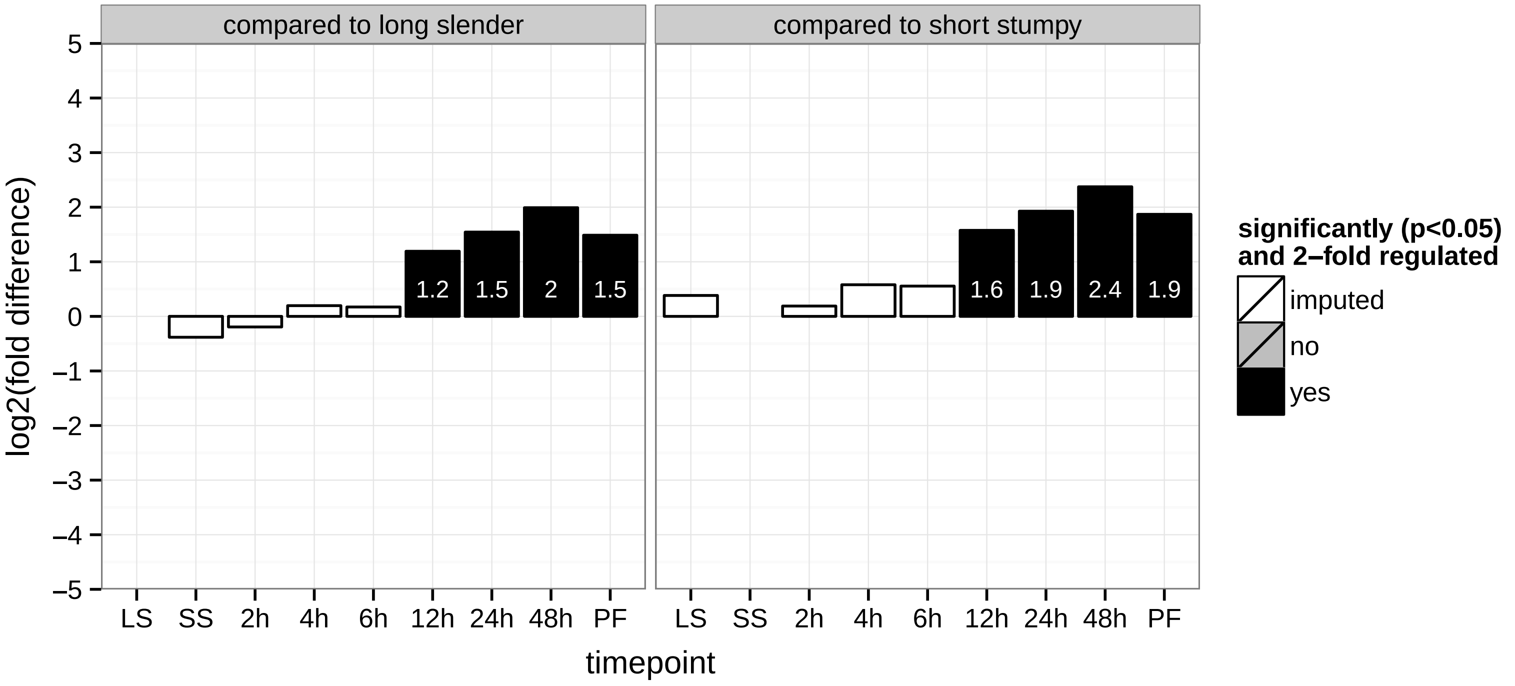

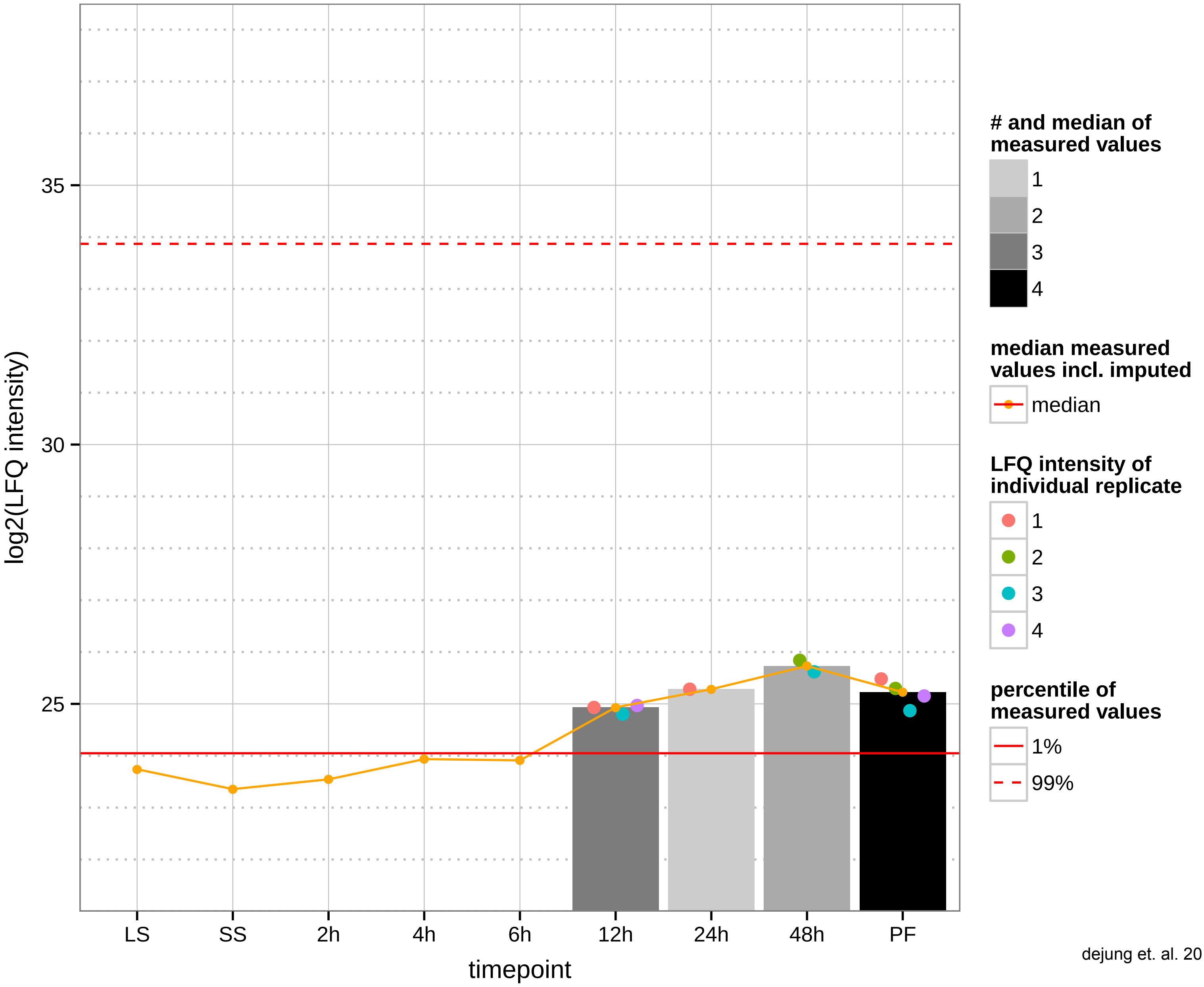

dejung et. al. 2015

pumillio/PUF RNA binding protein 3 (PUF3)
Tb927.10.310
AGOF: RNA binding
AGOC: null
AGOP: mRNA metabolic process
PGOF: RNA binding, binding
PGOC: null
PGOP: null

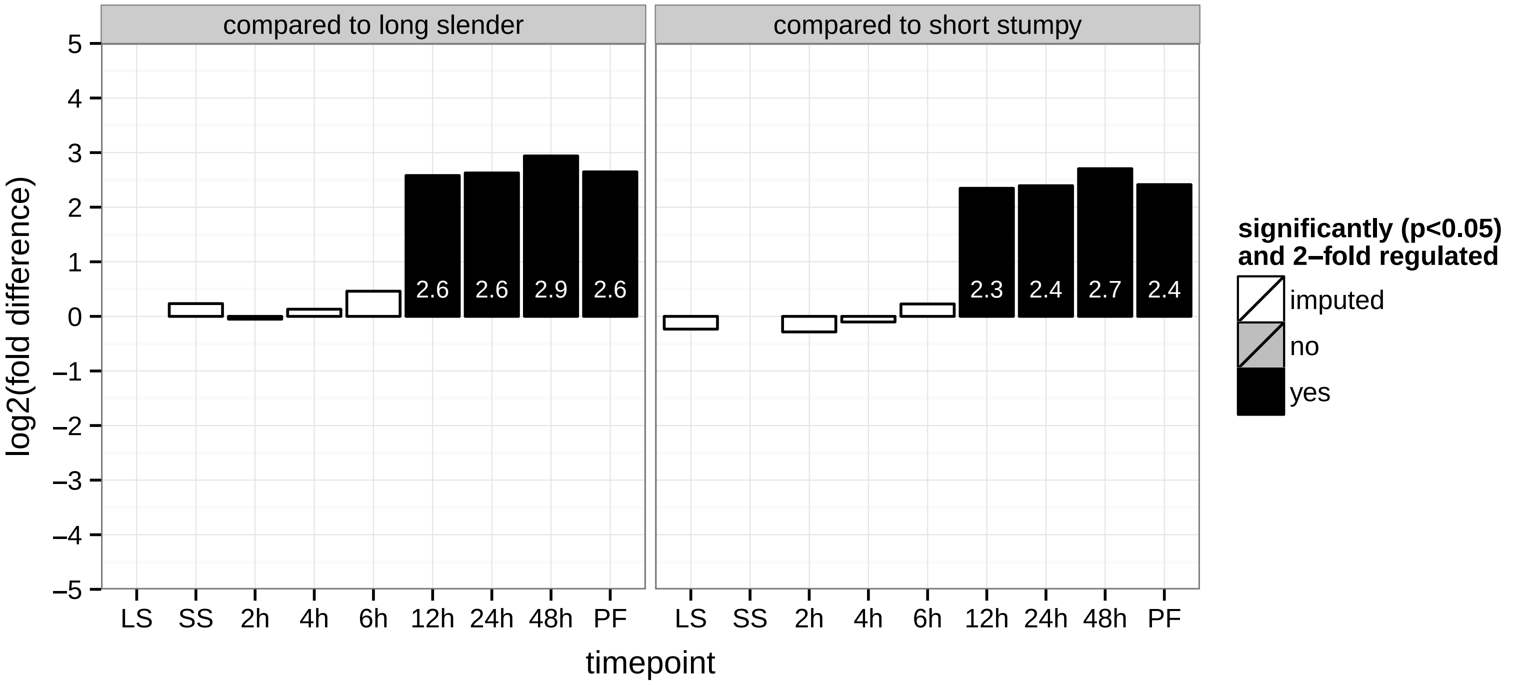

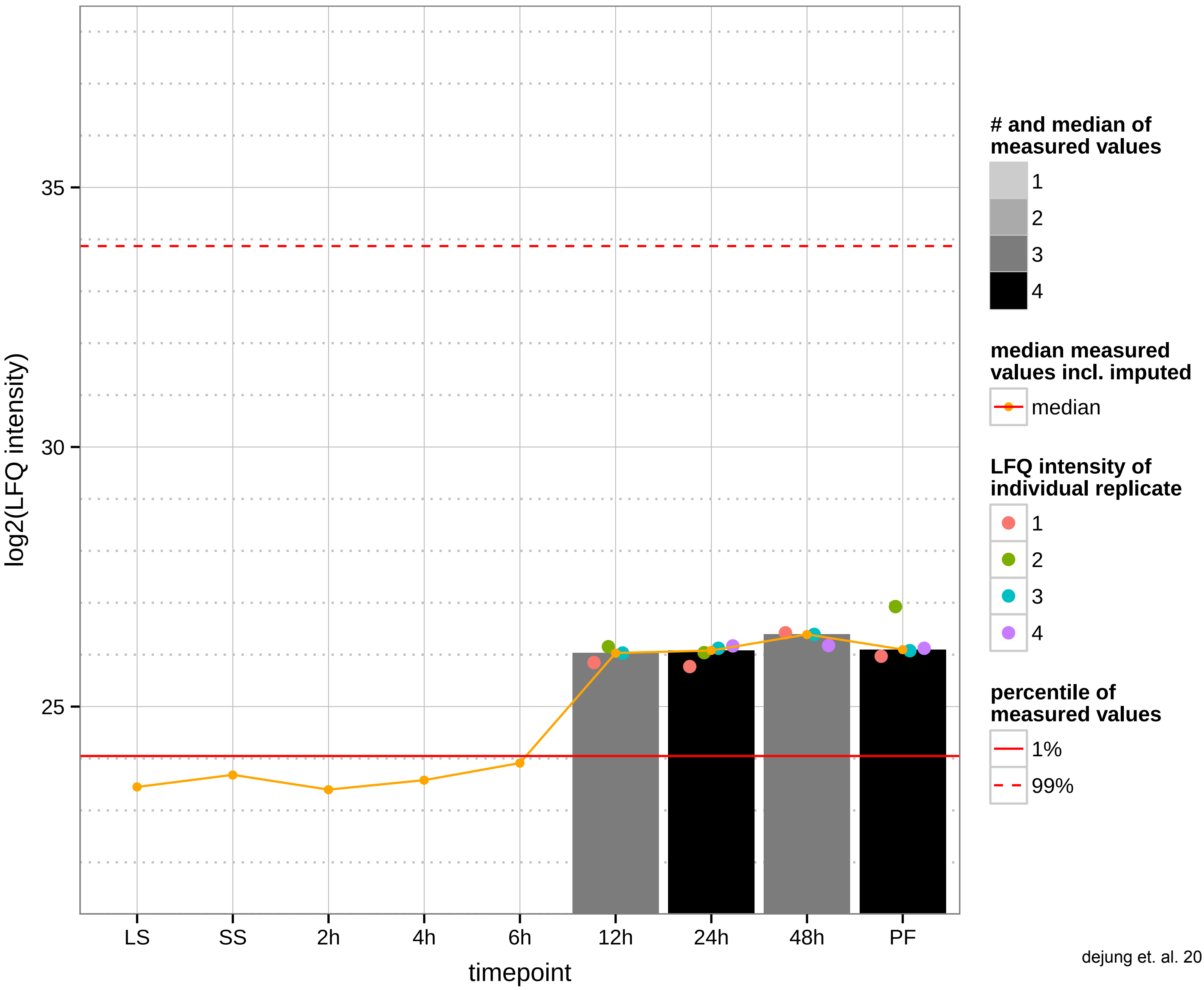

dejung et. al. 2015

hypothetical protein, conserved
Tb927.10.3250
AGOF: null
AGOC: null, mitochondrion
AGOP: null
PGOF: null
PGOC: null
PGOP: null

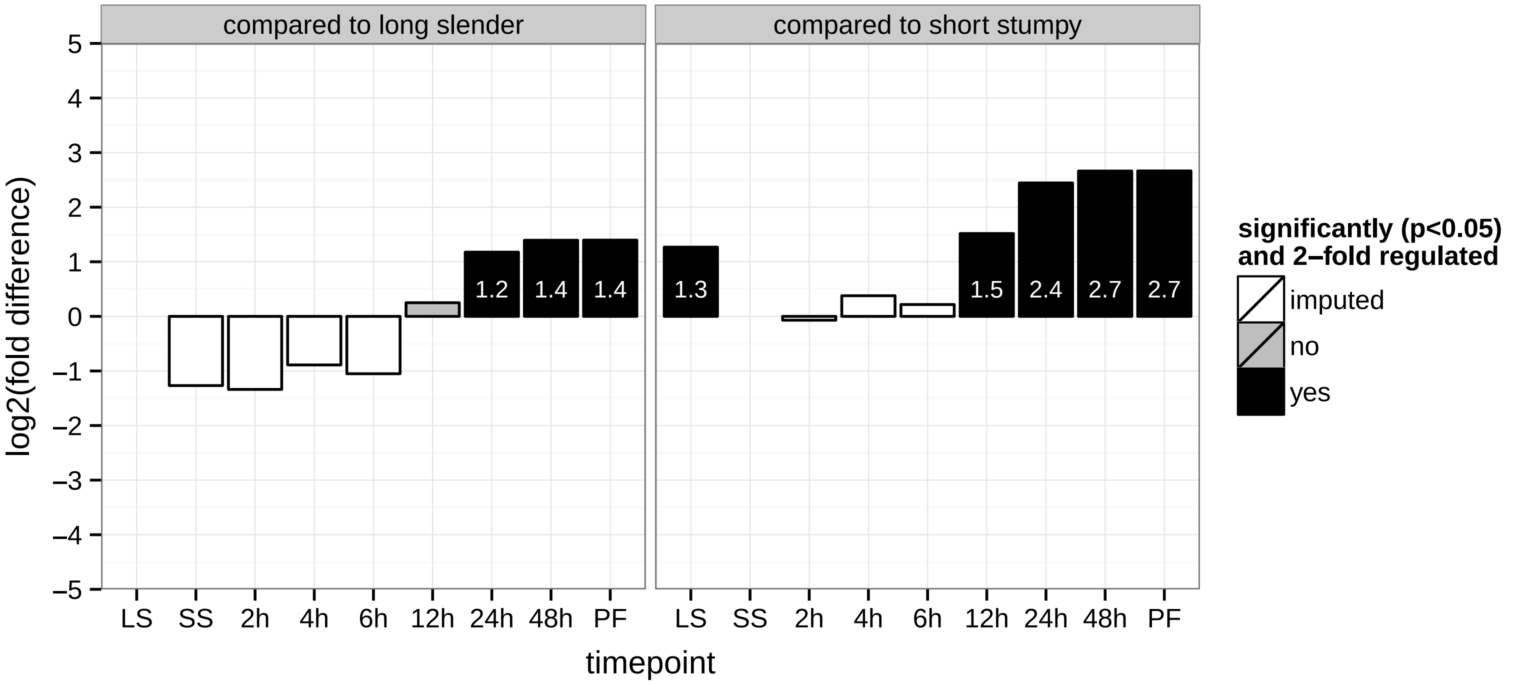

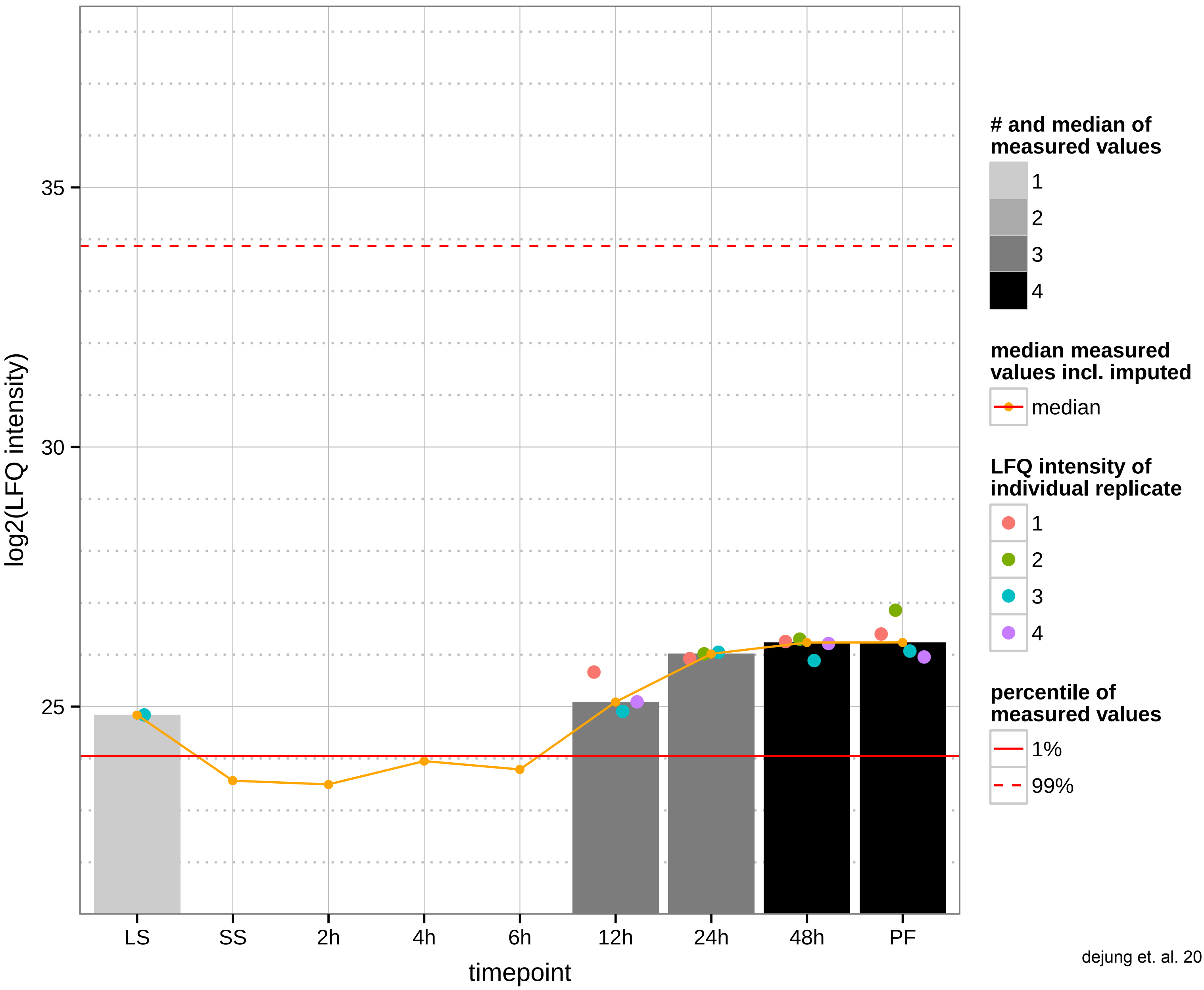

dejung et. al. 2015

hypothetical protein, conserved
Tb927.10.4240
AGOF: null
AGOC: mitochondrial inner membrane, mitochondrion
AGOP: null
PGOF: null
PGOC: null
PGOP: null

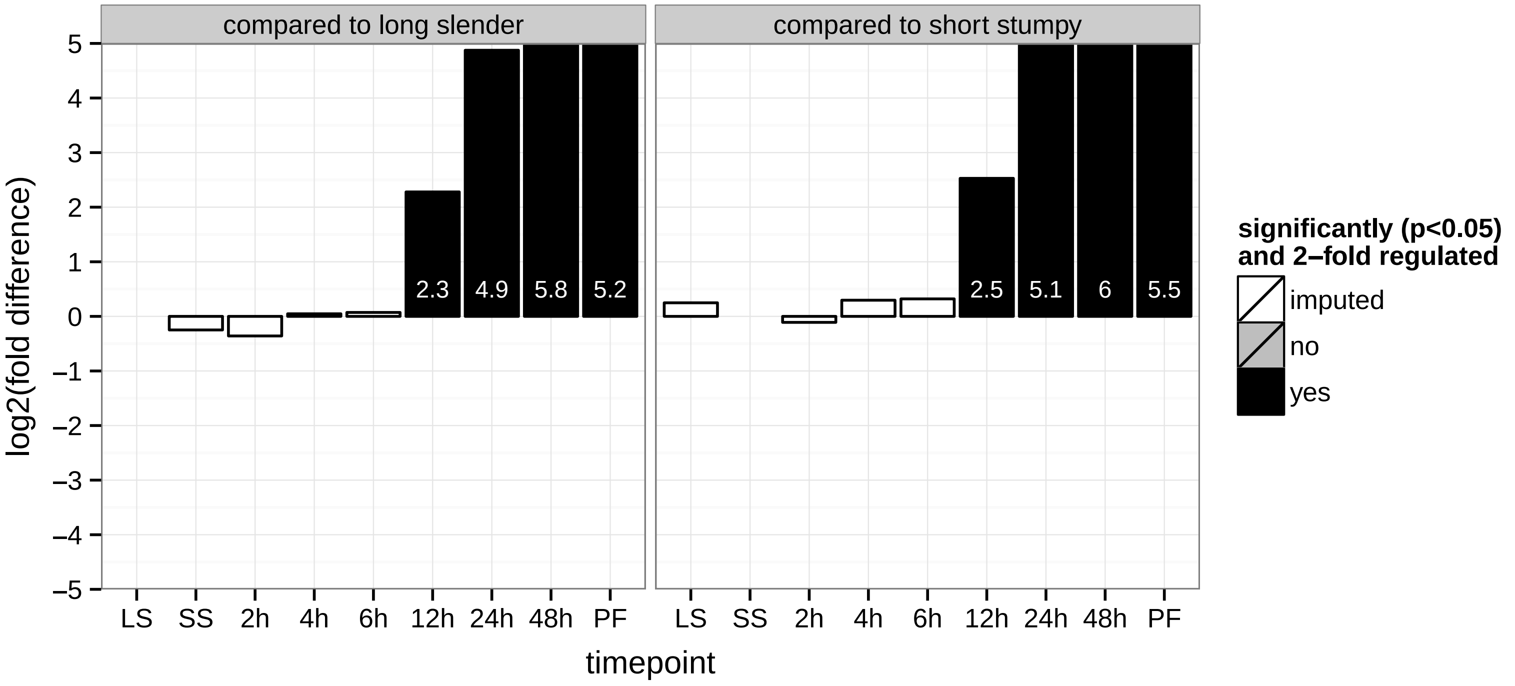

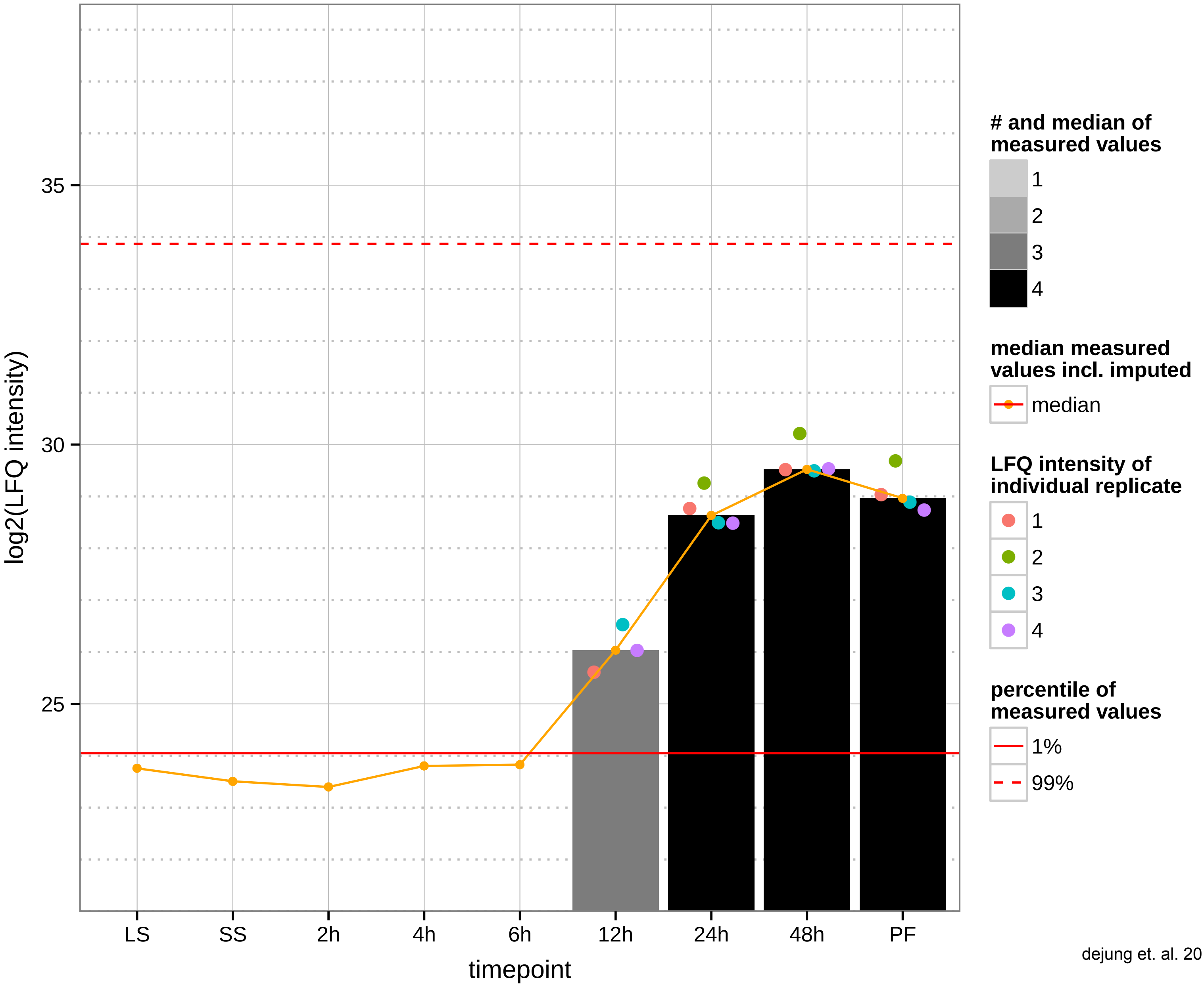

dejung et. al. 2015

hypothetical protein, conserved
Tb927.10.4280
AGOF: ubiquinol–cytochrome–c reductase activity
AGOC: mitochondrion
AGOP: mitochondrial electron transport, ubiquinol to cytochrome c
PGOF: ubiquinol–cytochrome–c reductase activity
PGOC: null
PGOP: mitochondrial electron transport, ubiquinol to cytochrome c

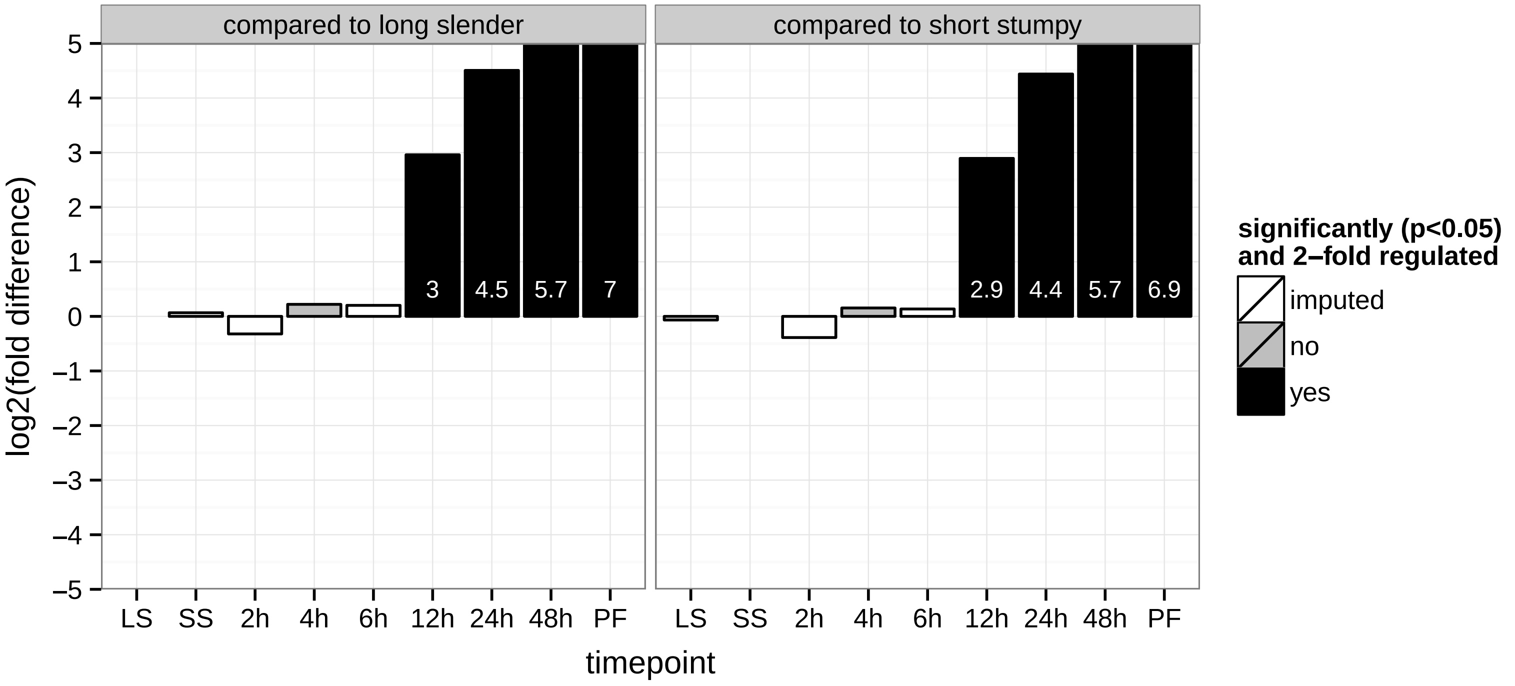

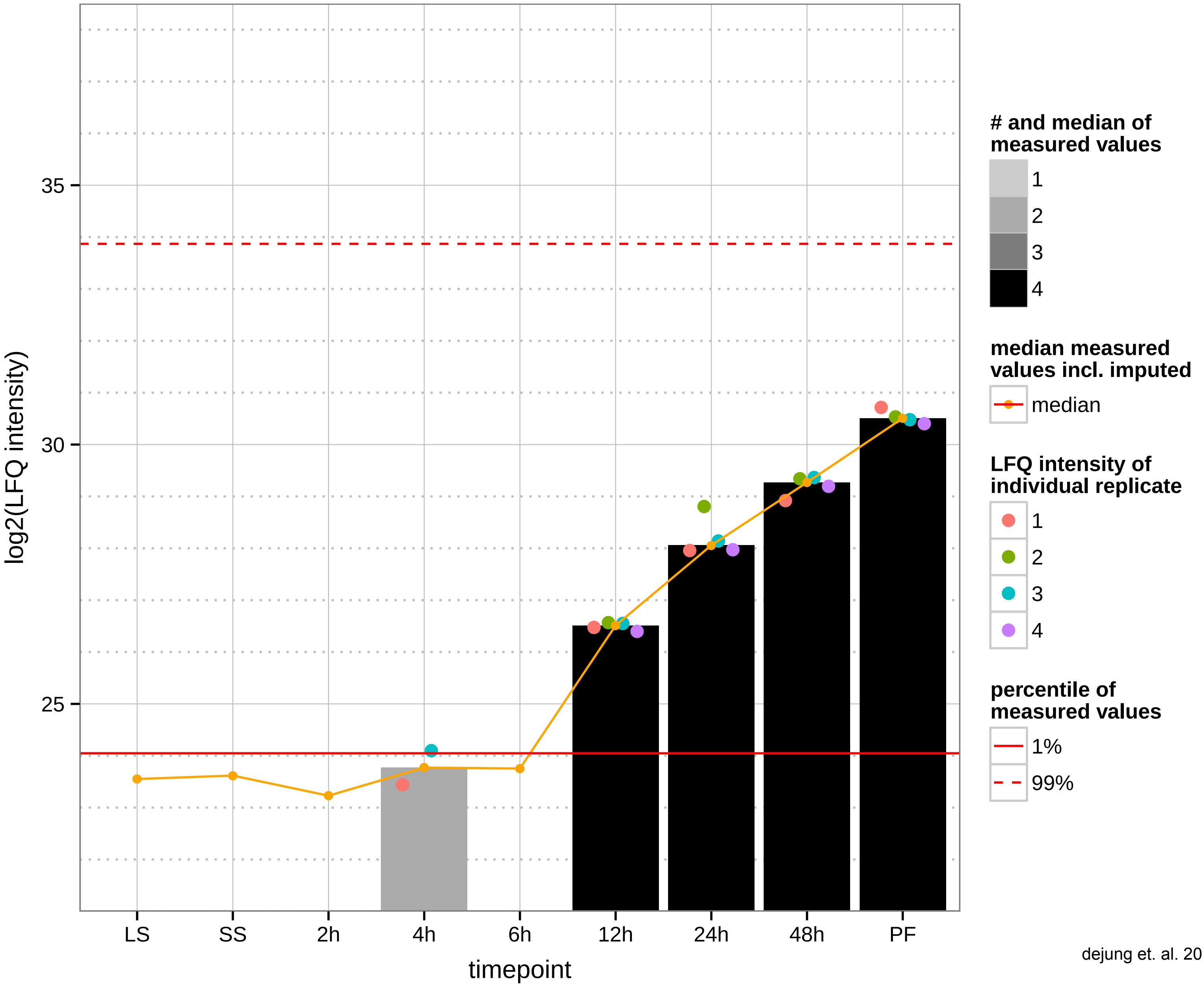

dejung et. al. 2015

hypothetical protein, conserved
Tb927.10.430
AGOF: heme binding, peroxidase activity
AGOC: mitochondrion
AGOP: response to oxidative stress
PGOF: heme binding, peroxidase activity
PGOC: null
PGOP: oxidation–reduction process, response to oxidative stress

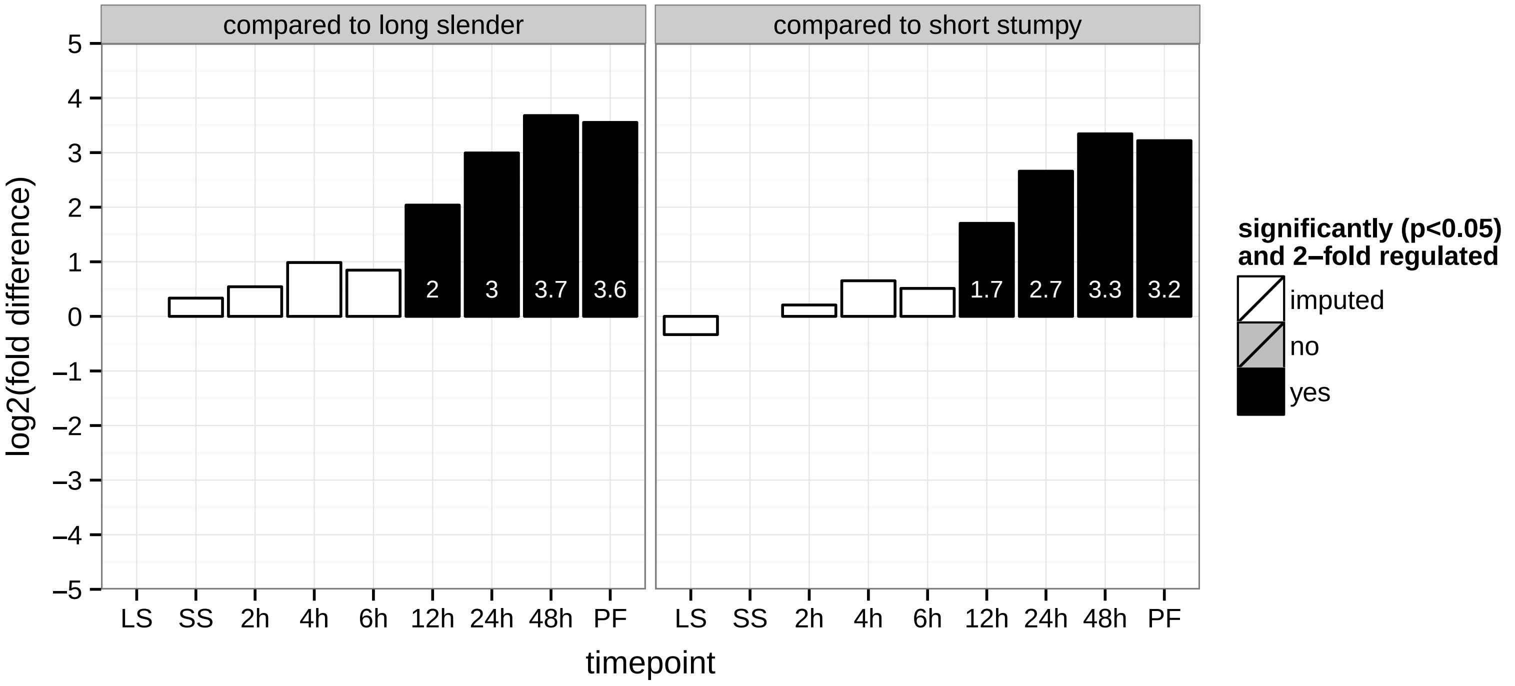

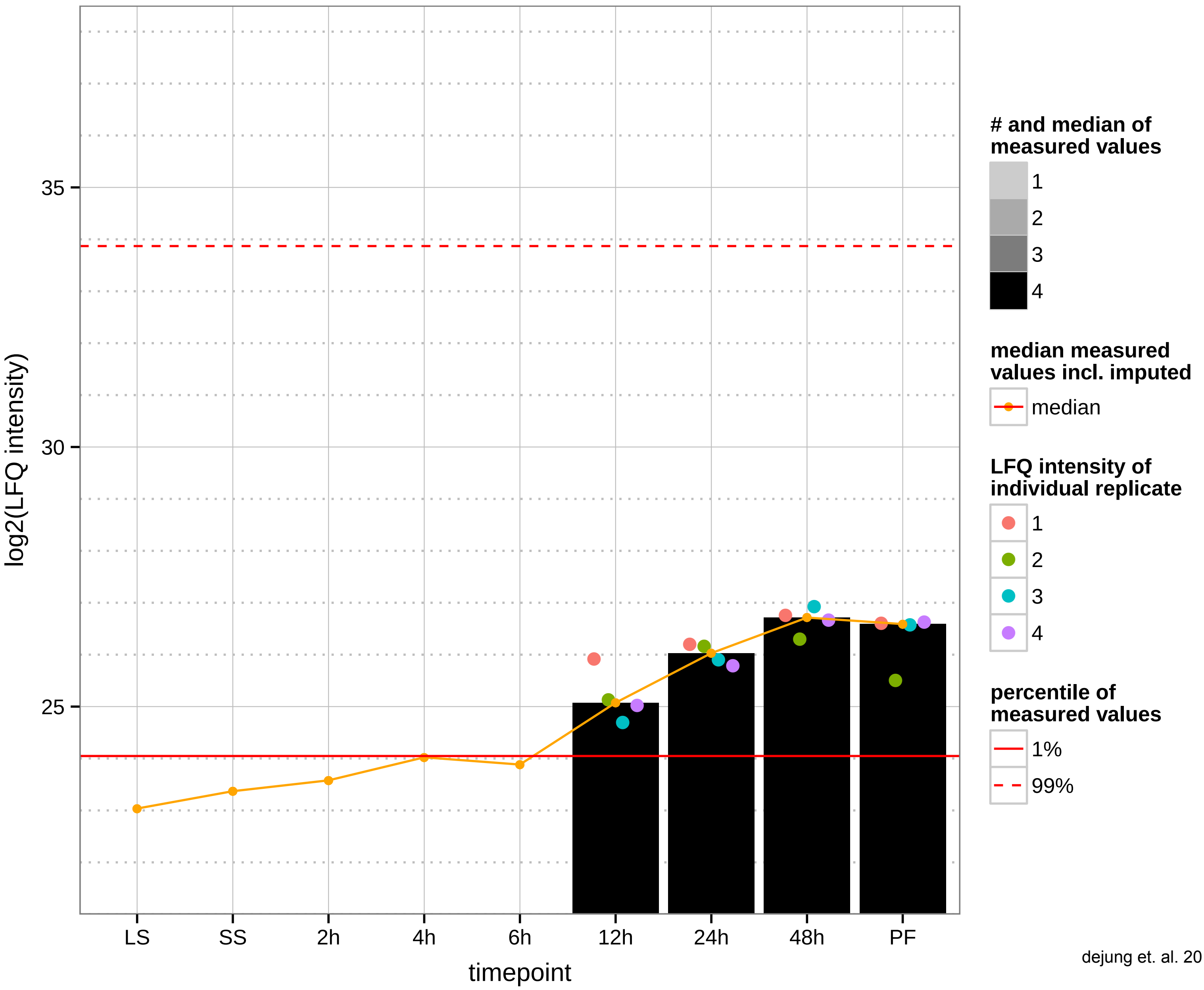

dejung et. al. 2015

hypothetical protein, conserved
Tb927.10.4880
AGOF: null
AGOC: mitochondrion
AGOP: null
PGOF: null
PGOC: null
PGOP: null

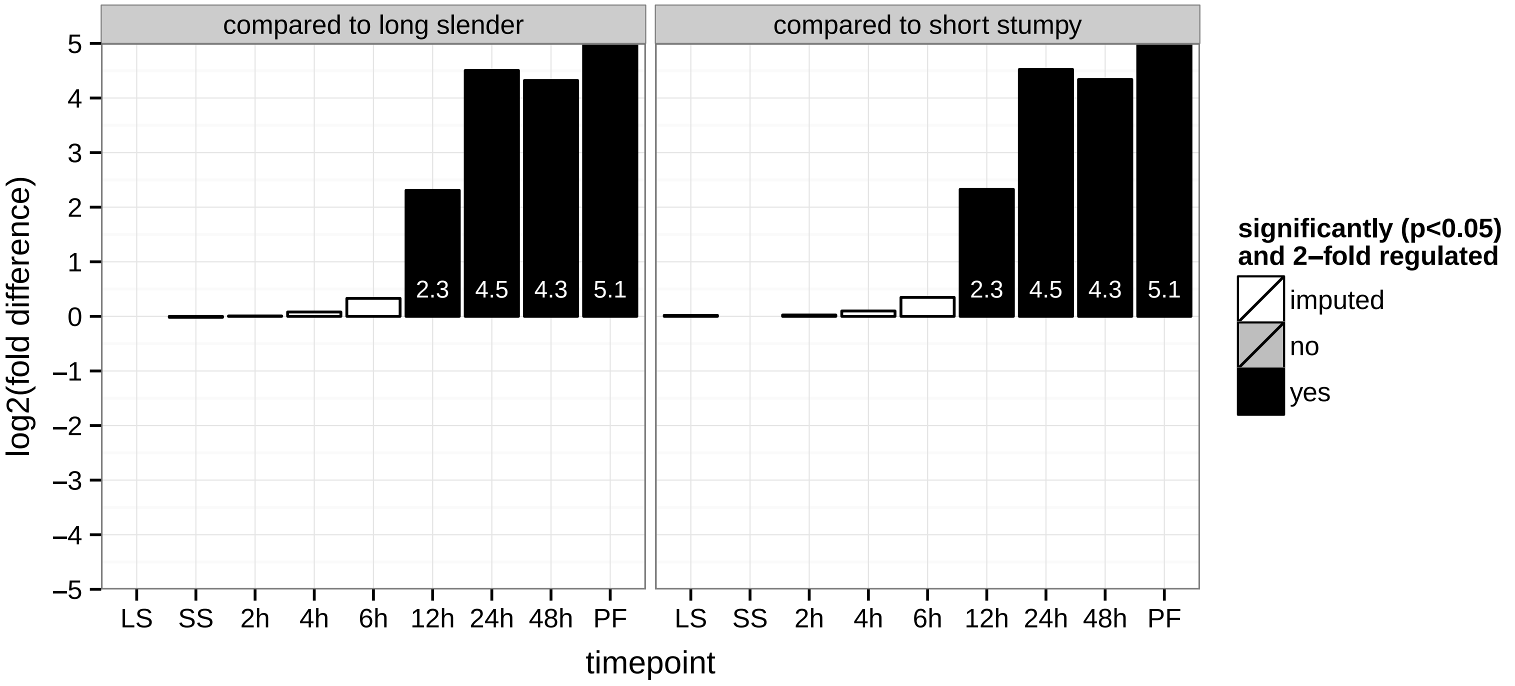

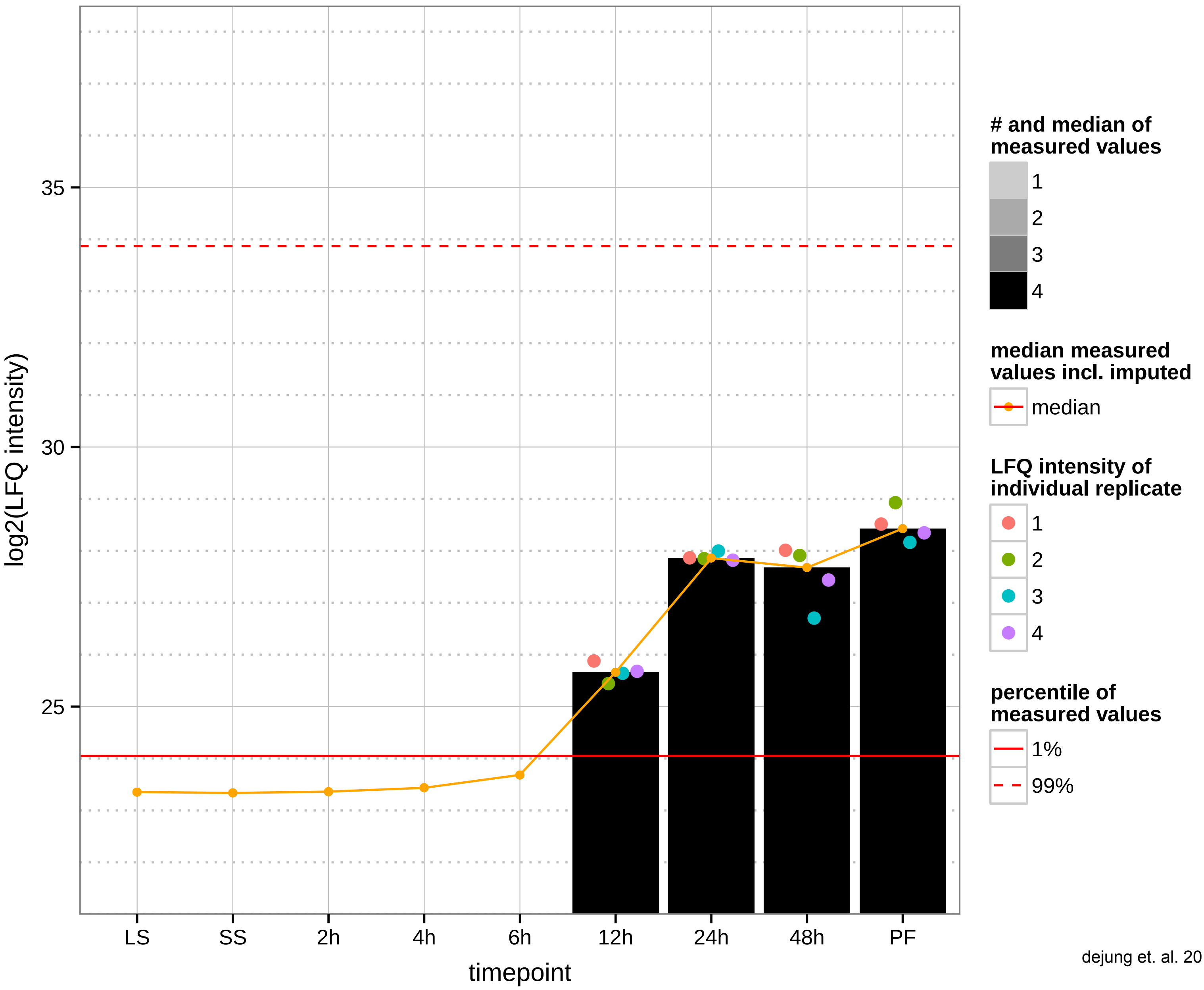

dejung et. al. 2015

Elongation factor G1, mitochondrial, putative (EF−G1)
Tb927.10.5010
AGOF: GTP binding, GTPase activity, translation elongation factor activity
AGOC: intracellular, mitochondrion
AGOP: growth, oxidative phosphorylation, translational elongation
PGOF: GTP binding, GTPase activity, translation elongation factor activity
PGOC: intracellular
PGOP: translational elongation

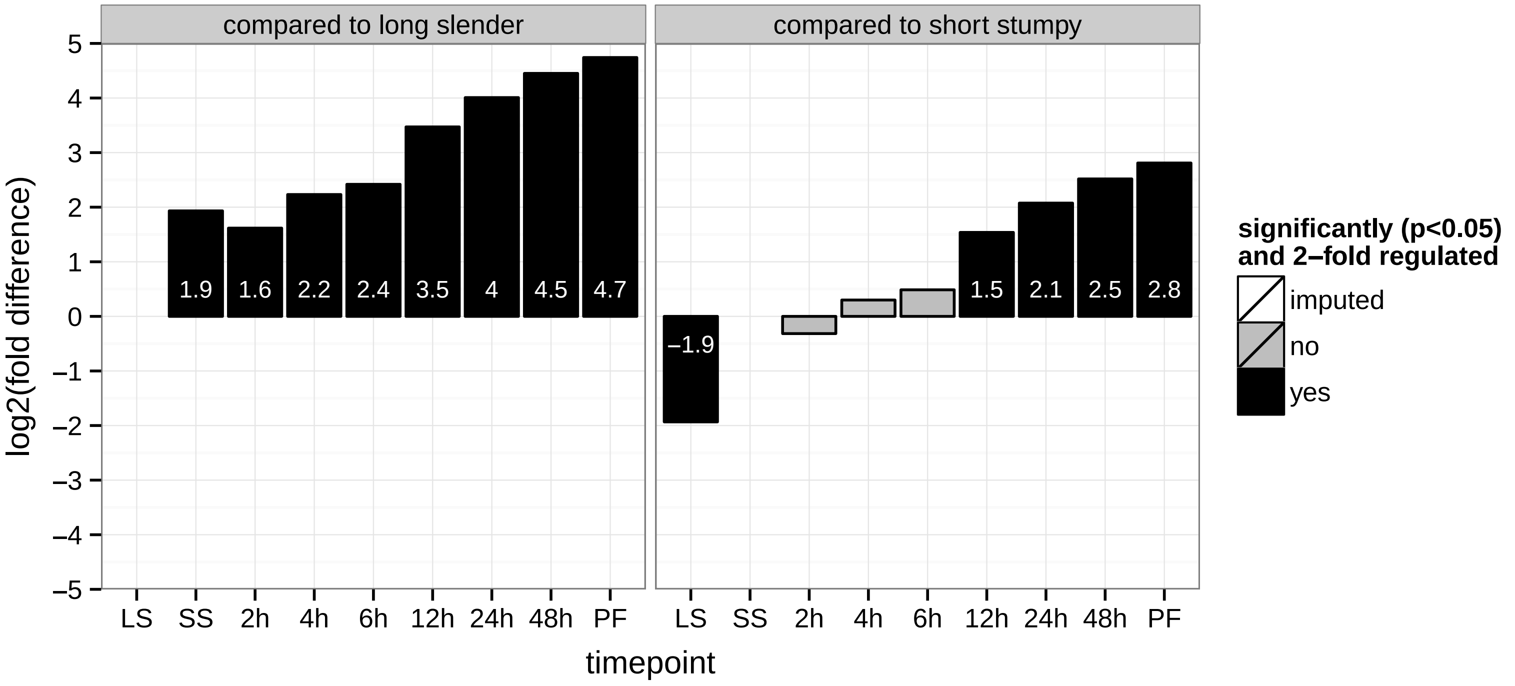

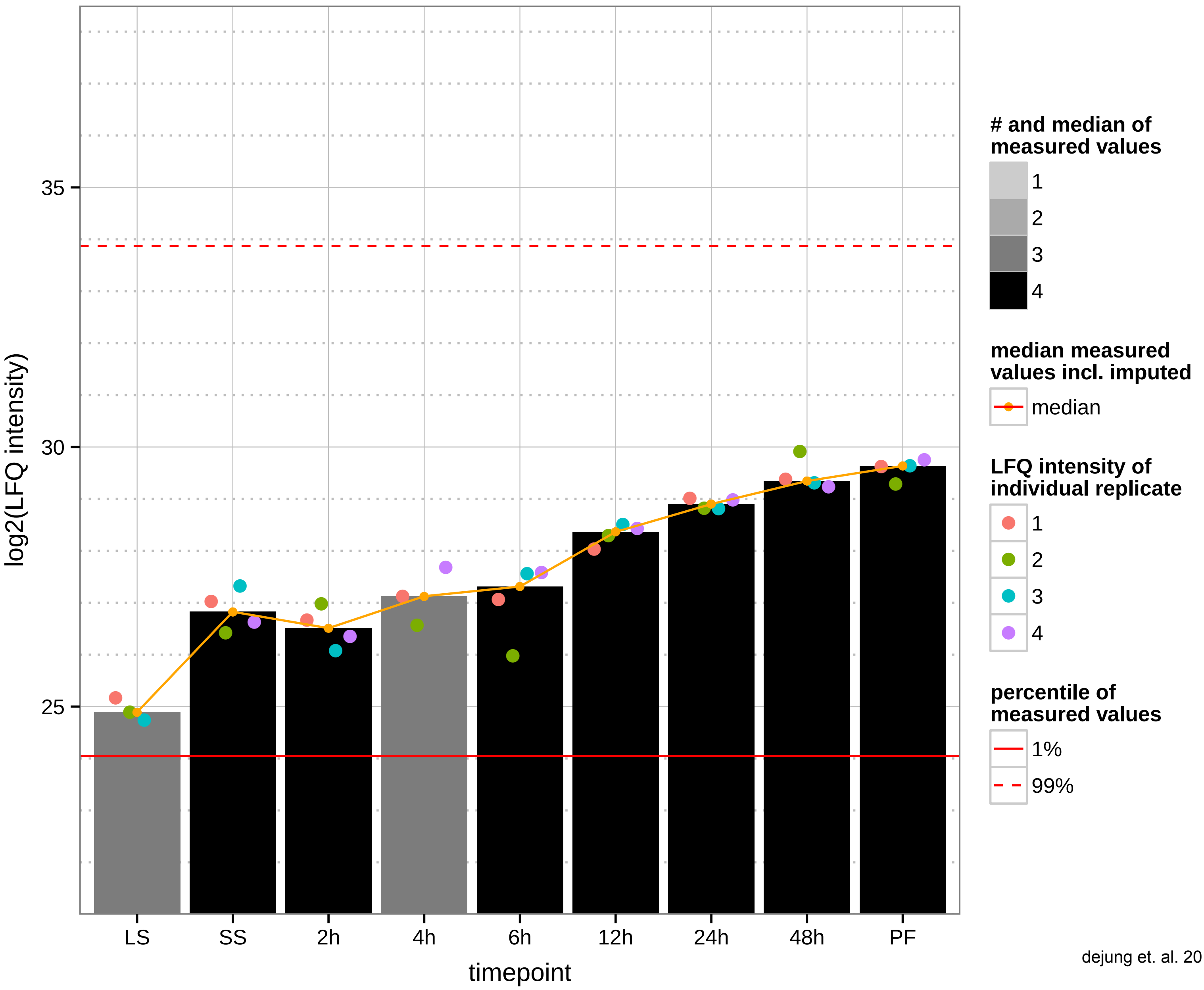

dejung et. al. 2015

translation elongation factor 1–beta, putative
Tb927.10.5840
AGOF: translation elongation factor activity
AGOC: eukaryotic translation elongation factor 1 complex
AGOP: translational elongation
PGOF: translation elongation factor activity
PGOC: eukaryotic translation elongation factor 1 complex
PGOP: translational elongation

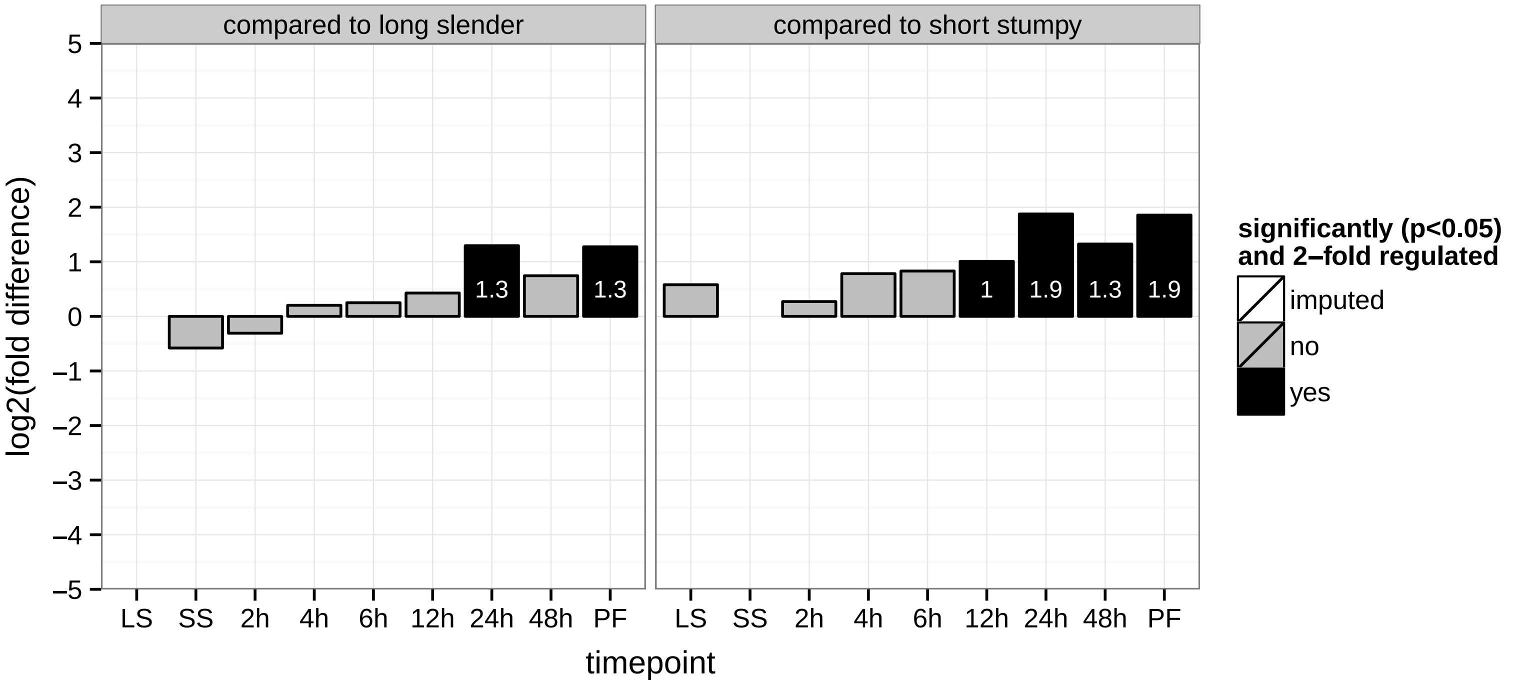

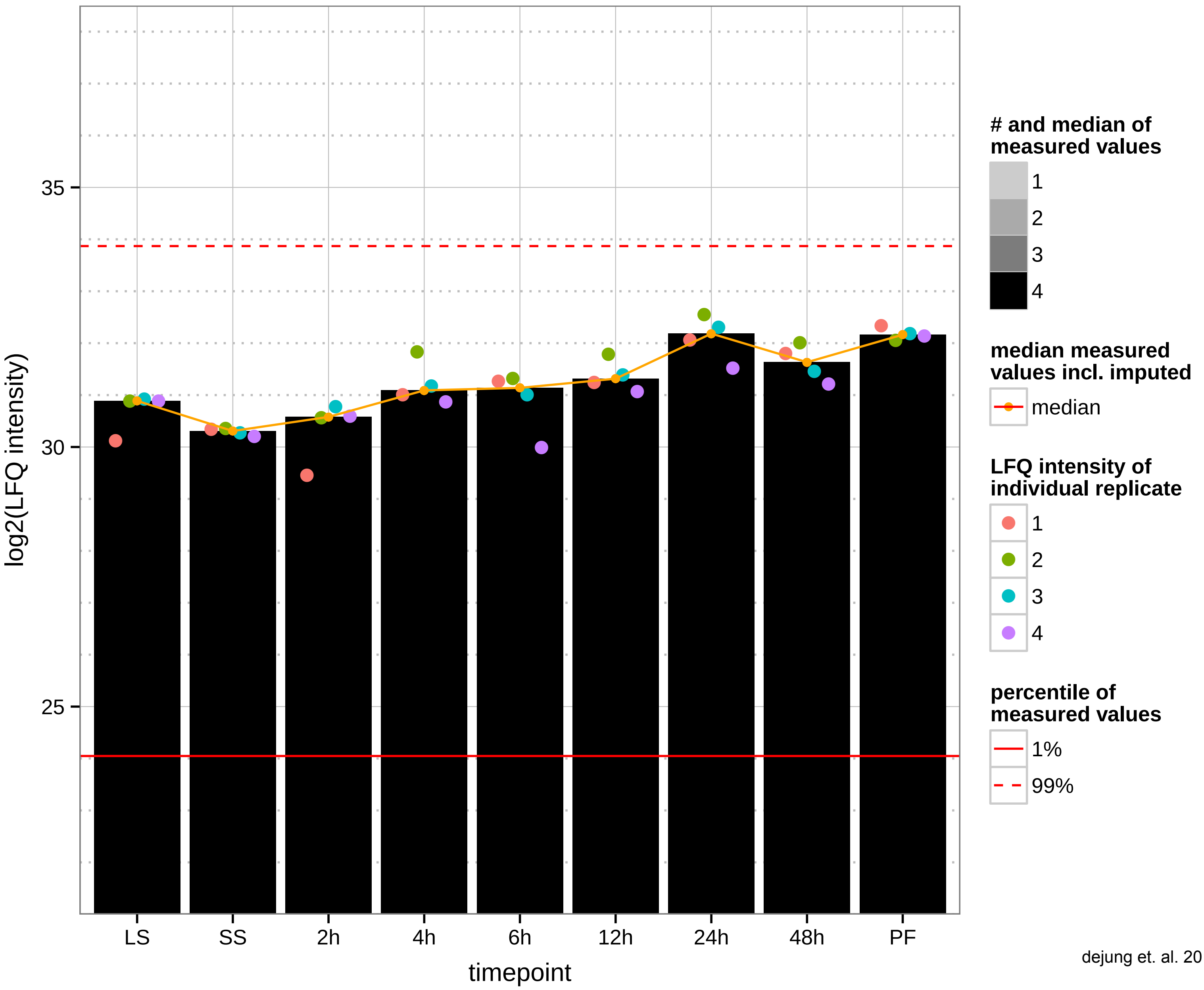

dejung et. al. 2015

hypothetical protein, conserved
Tb927.10.6020
AGOF: null
AGOC: null
AGOP: null
PGOF: null
PGOC: null
PGOP: null

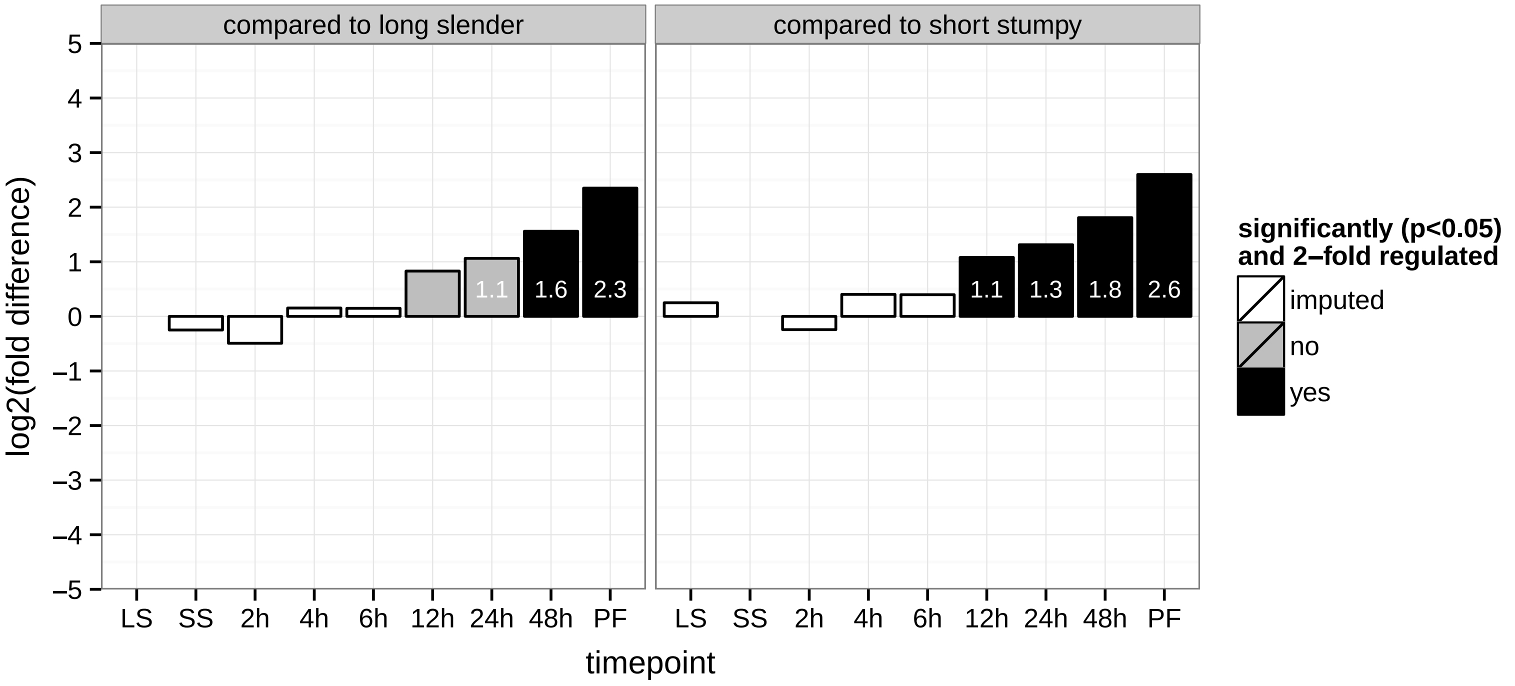

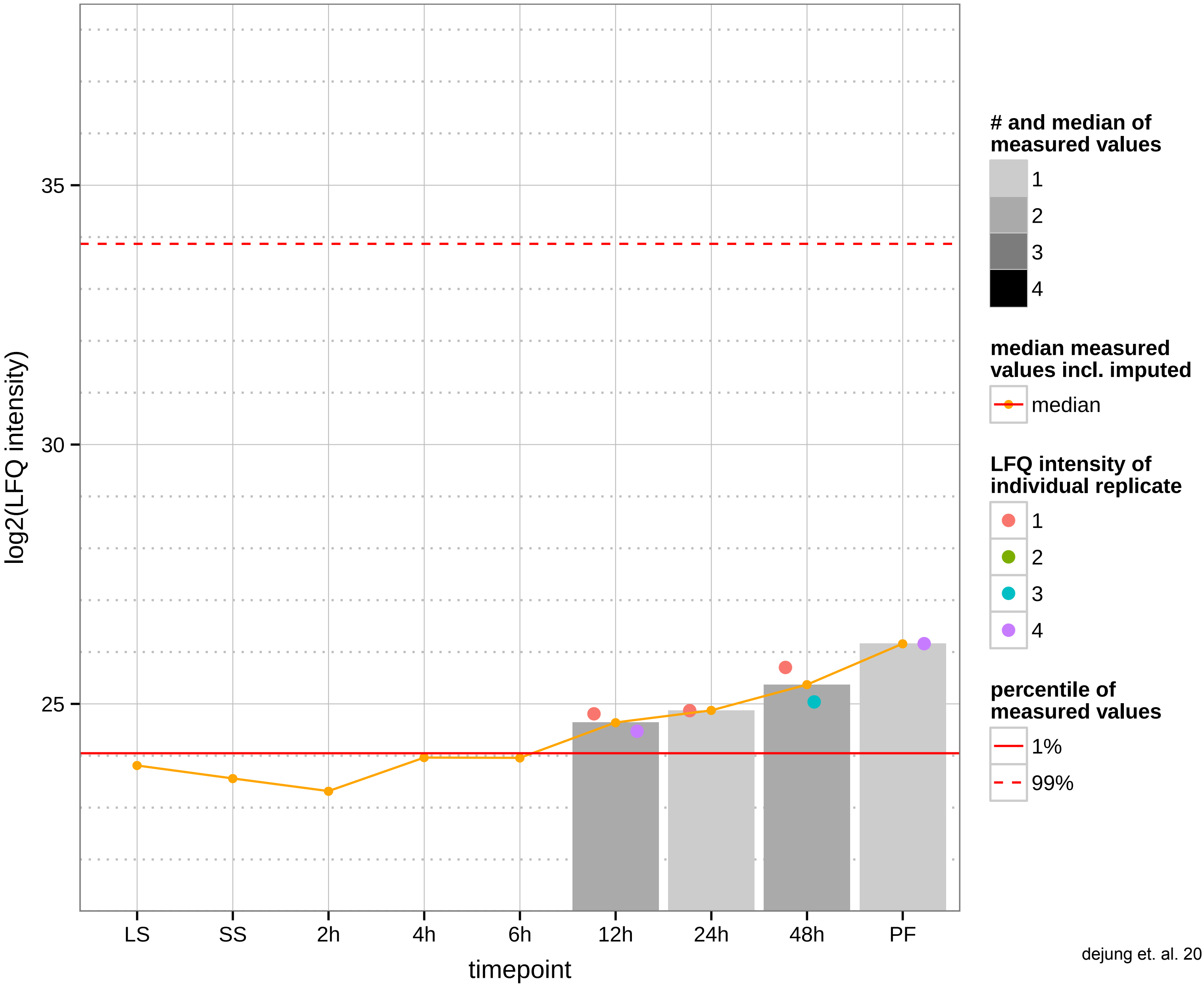

dejung et. al. 2015

universal minicircle sequence binding protein (UMSBP), putative, predicted zinc finger protein
Tb927.10.6070
AGOF: nucleic acid binding, zinc ion binding
AGOC: null
AGOP: null
PGOF: nucleic acid binding, zinc ion binding
PGOC: null
PGOP: null

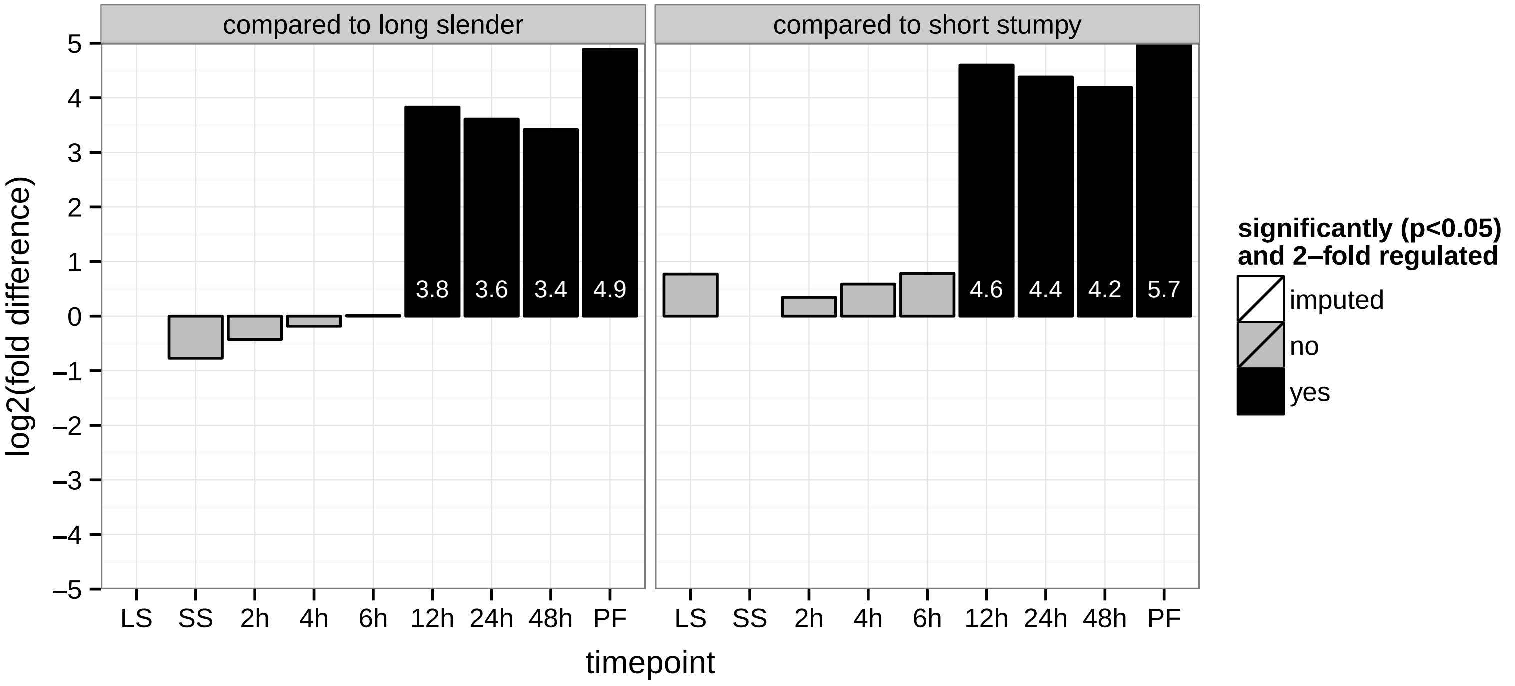

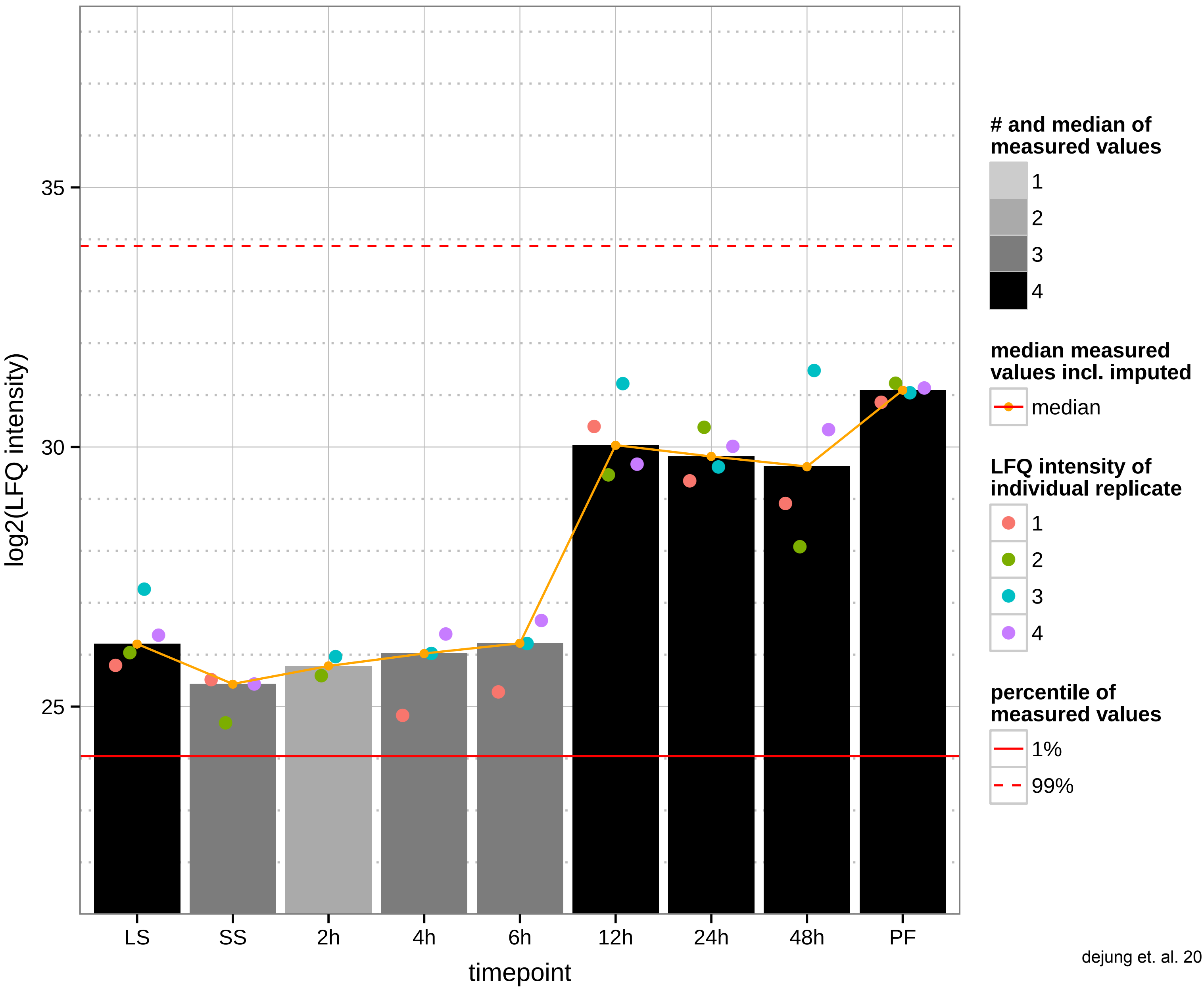

dejung et. al. 2015

aldehyde dehydrogenase, putative (ALDH)
Tb927.10.6190
AGOF: glutathione–disulfide reductase activity
AGOC: null
AGOP: gamma–aminobutyric acid catabolic process
PGOF: oxidoreductase activity
PGOC: null
PGOP: metabolic process, oxidation–reduction process

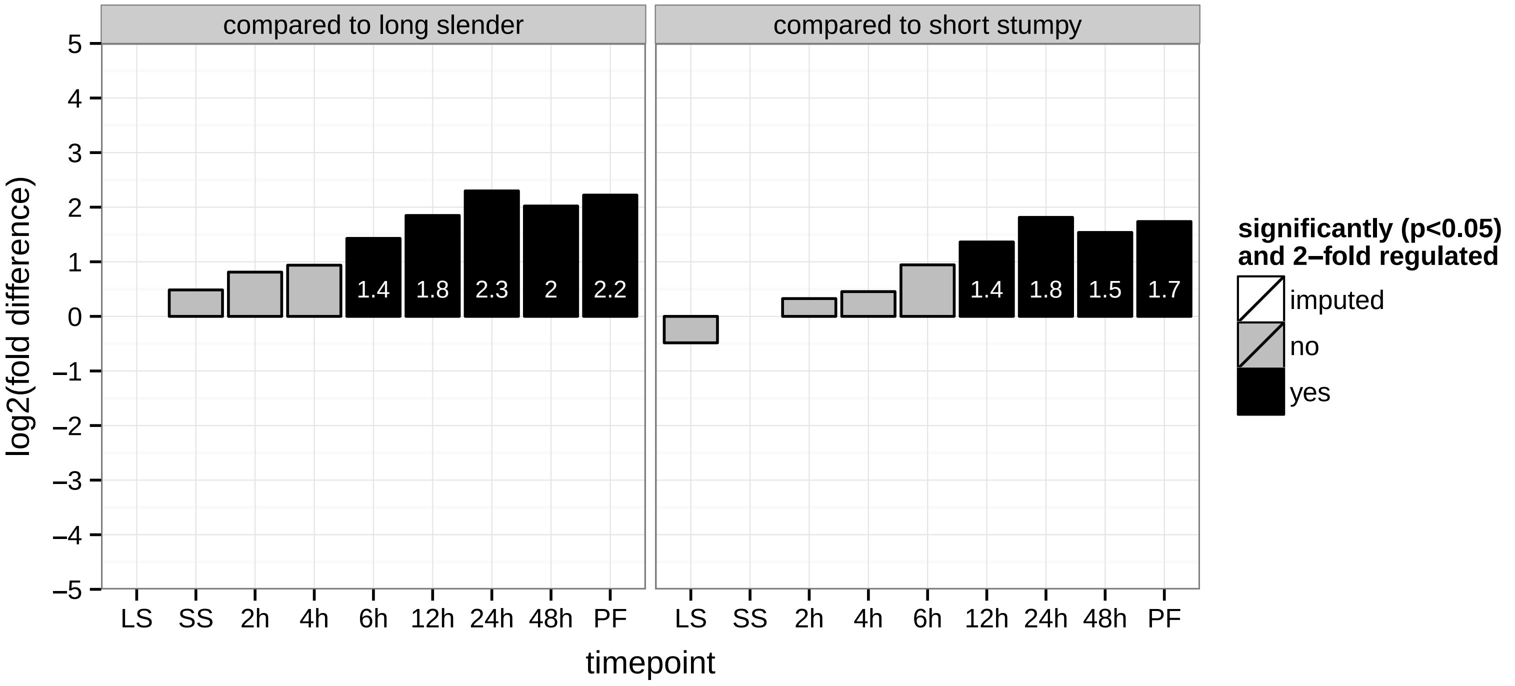

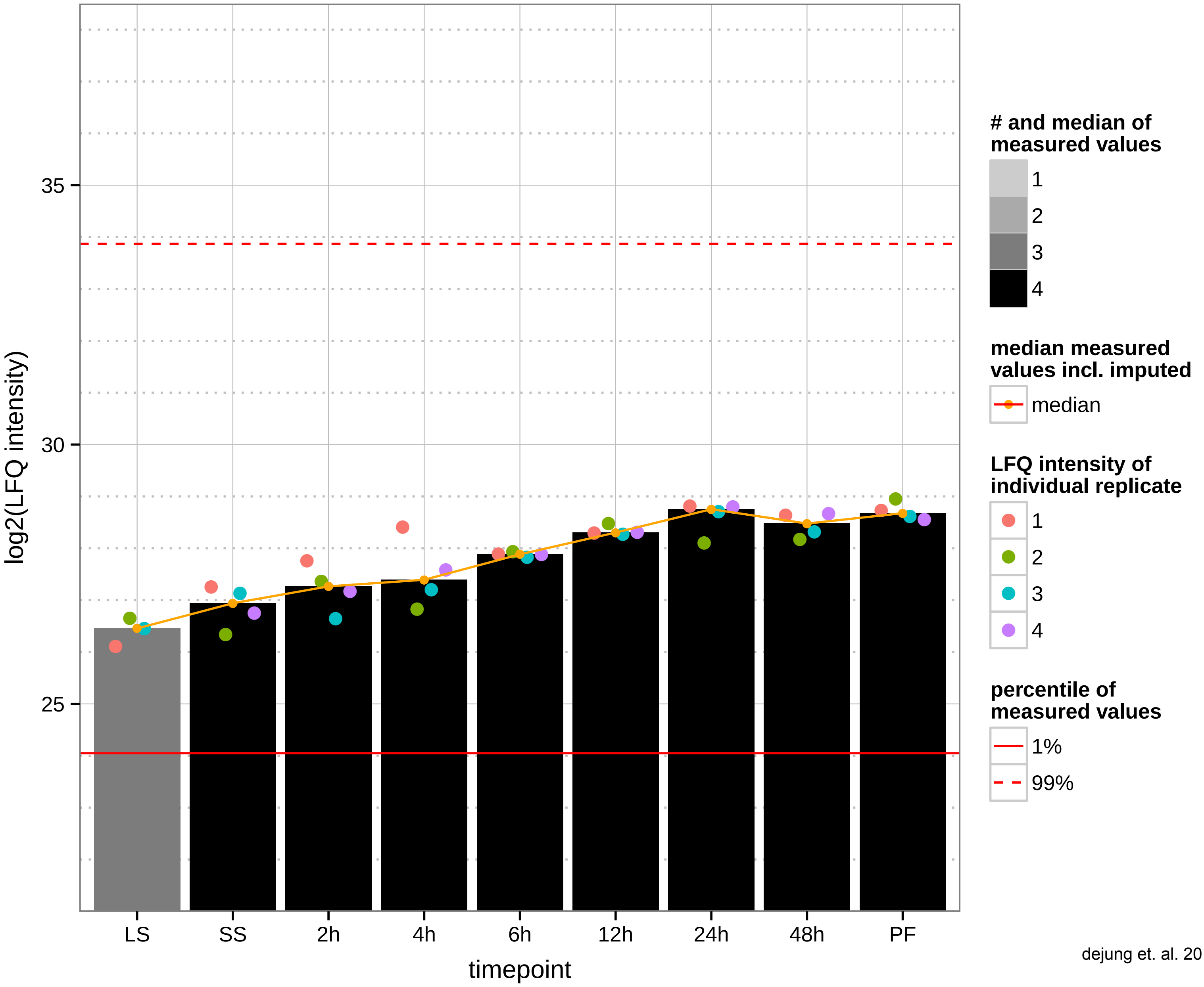

dejung et. al. 2015

hypothetical protein, conserved
Tb927.10.760
AGOF: null
AGOC: mitochondrion
AGOP: null
PGOF: null
PGOC: null
PGOP: null

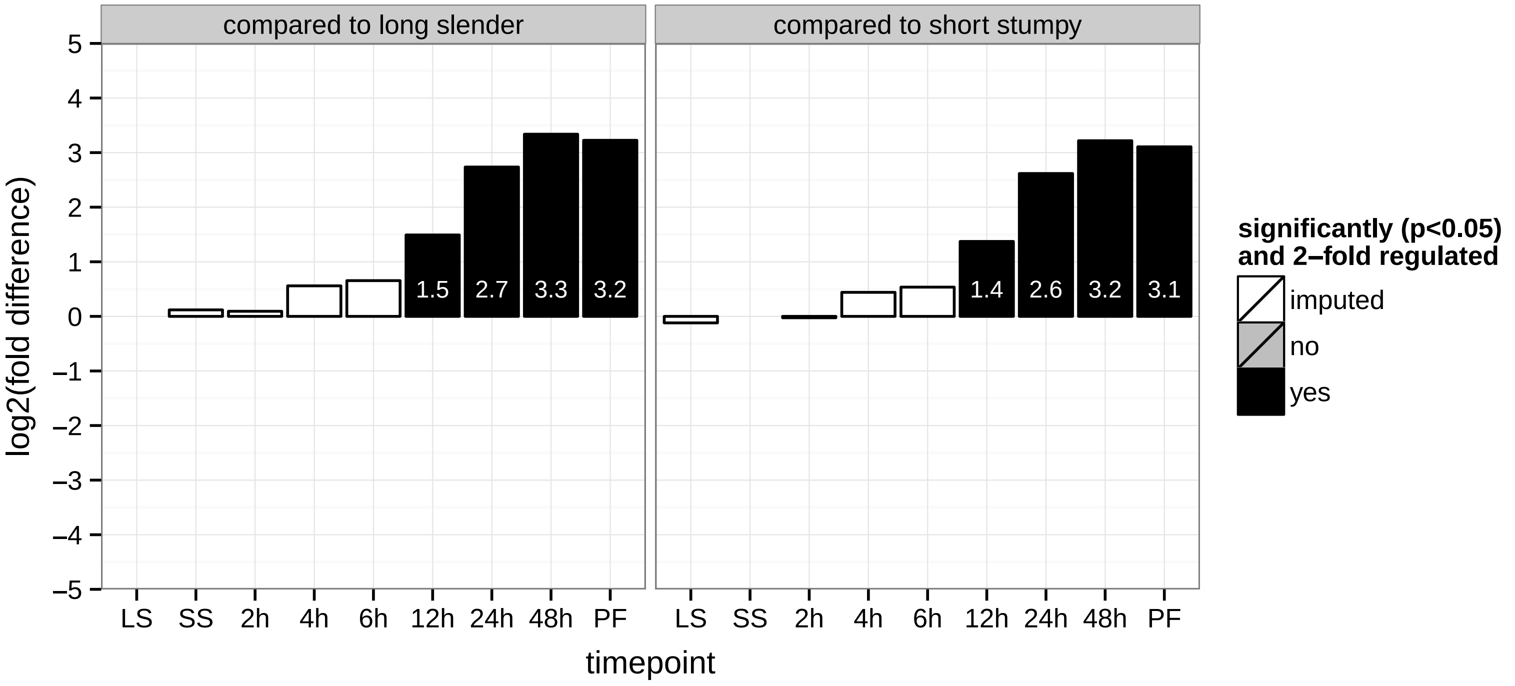

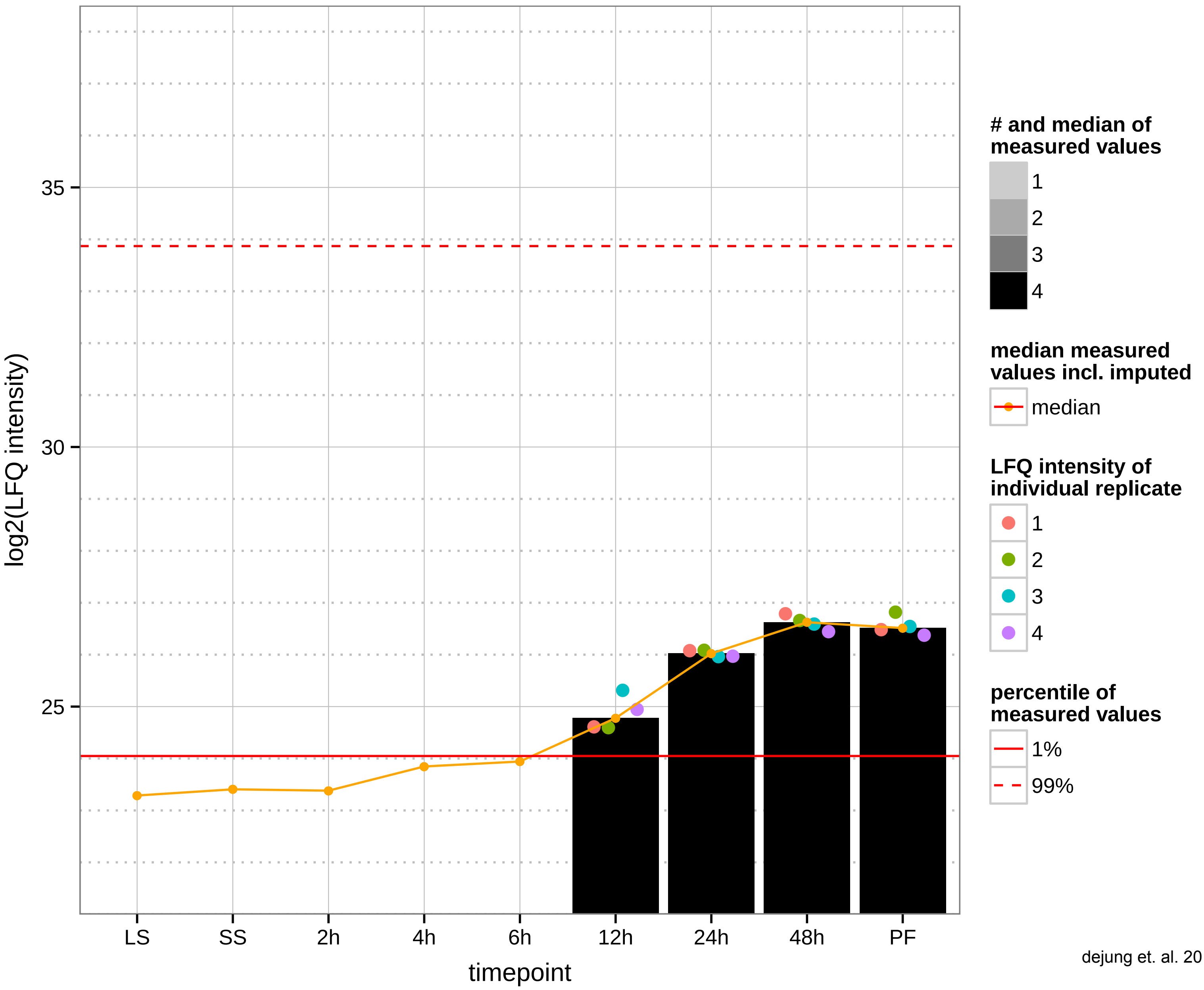

dejung et. al. 2015

cytochrome oxidase subunit IX (COXIX)
Tb927.10.8320
AGOF: null
AGOC: mitochondrion
AGOP: null
PGOF: null
PGOC: null
PGOP: null

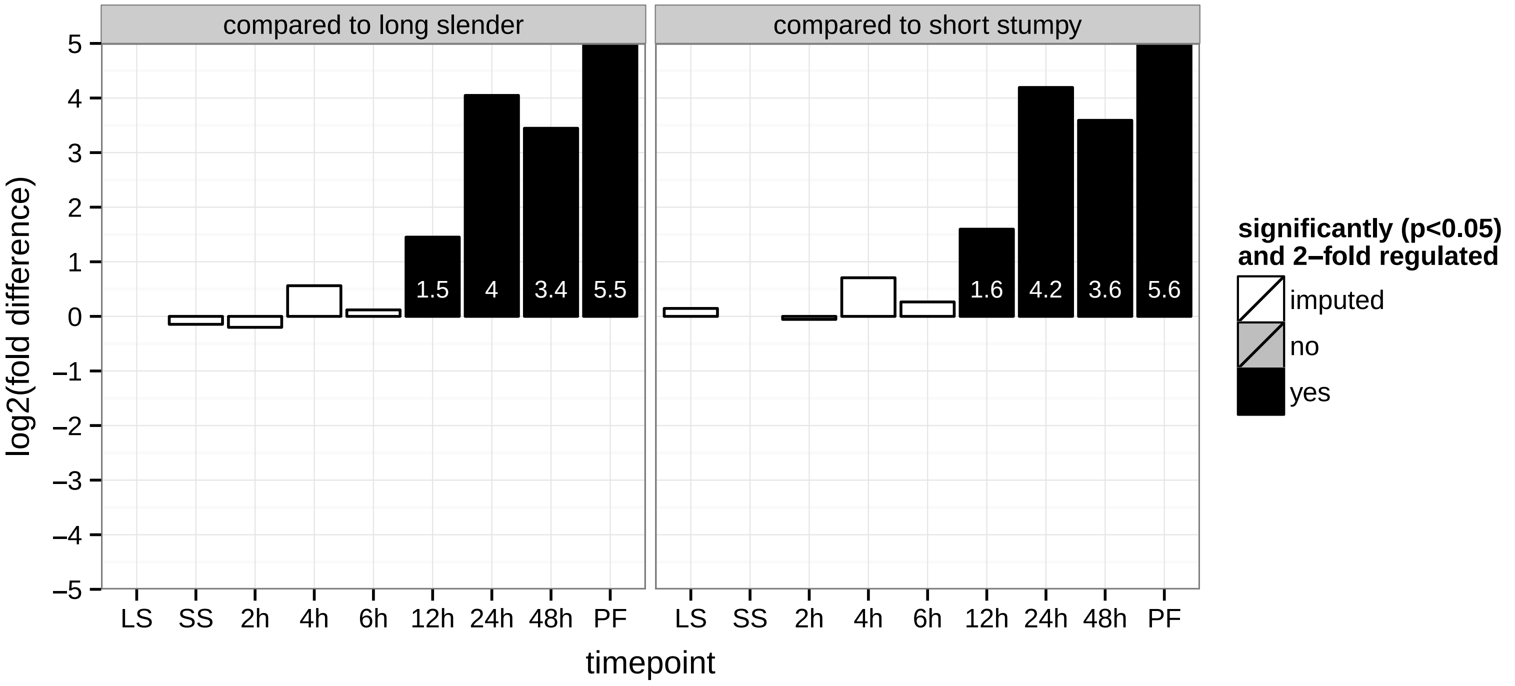

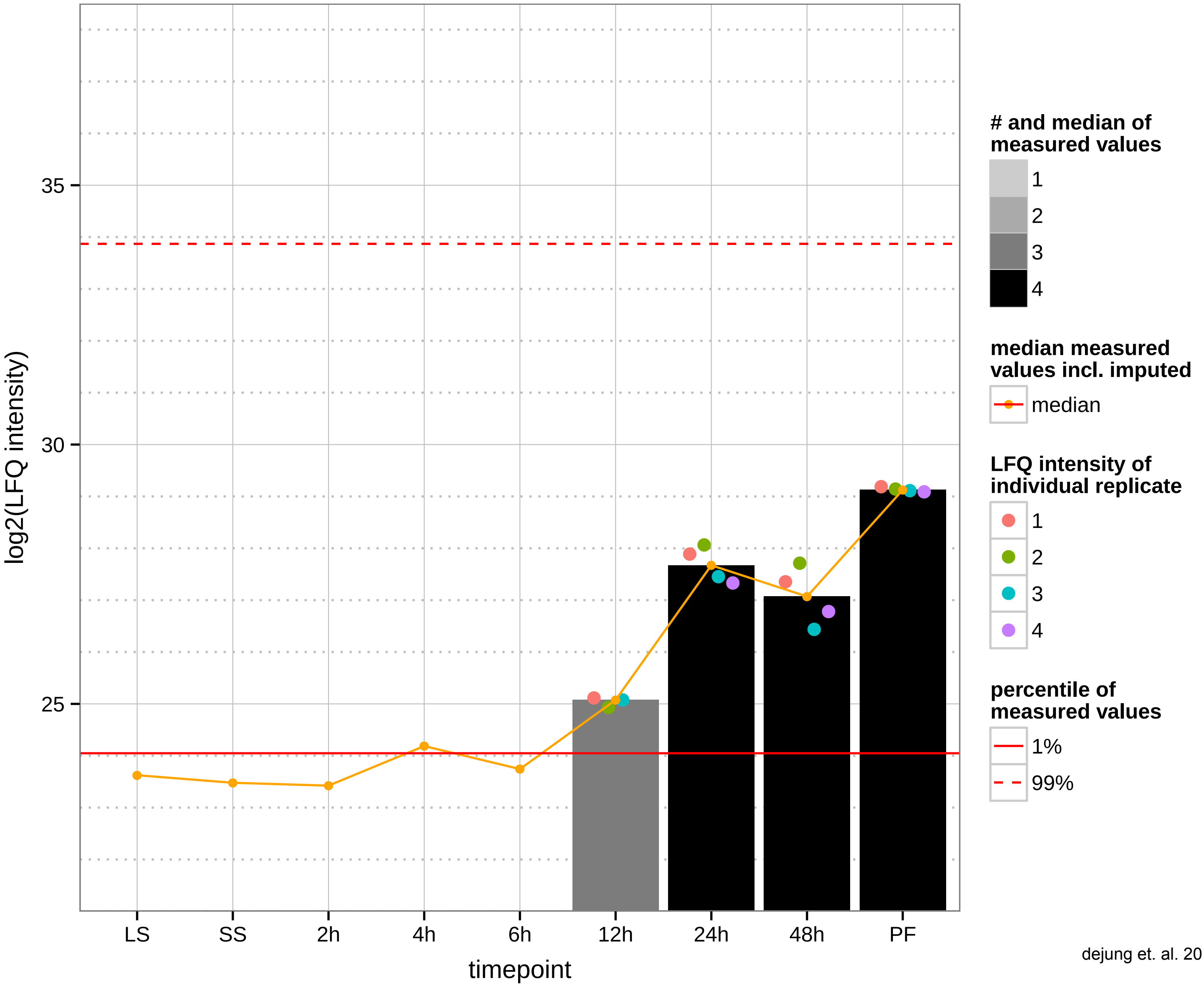

dejung et. al. 2015

hypothetical protein, conserved
Tb927.10.8620
AGOF: null
AGOC: mitochondrion
AGOP: null
PGOF: null
PGOC: null
PGOP: null

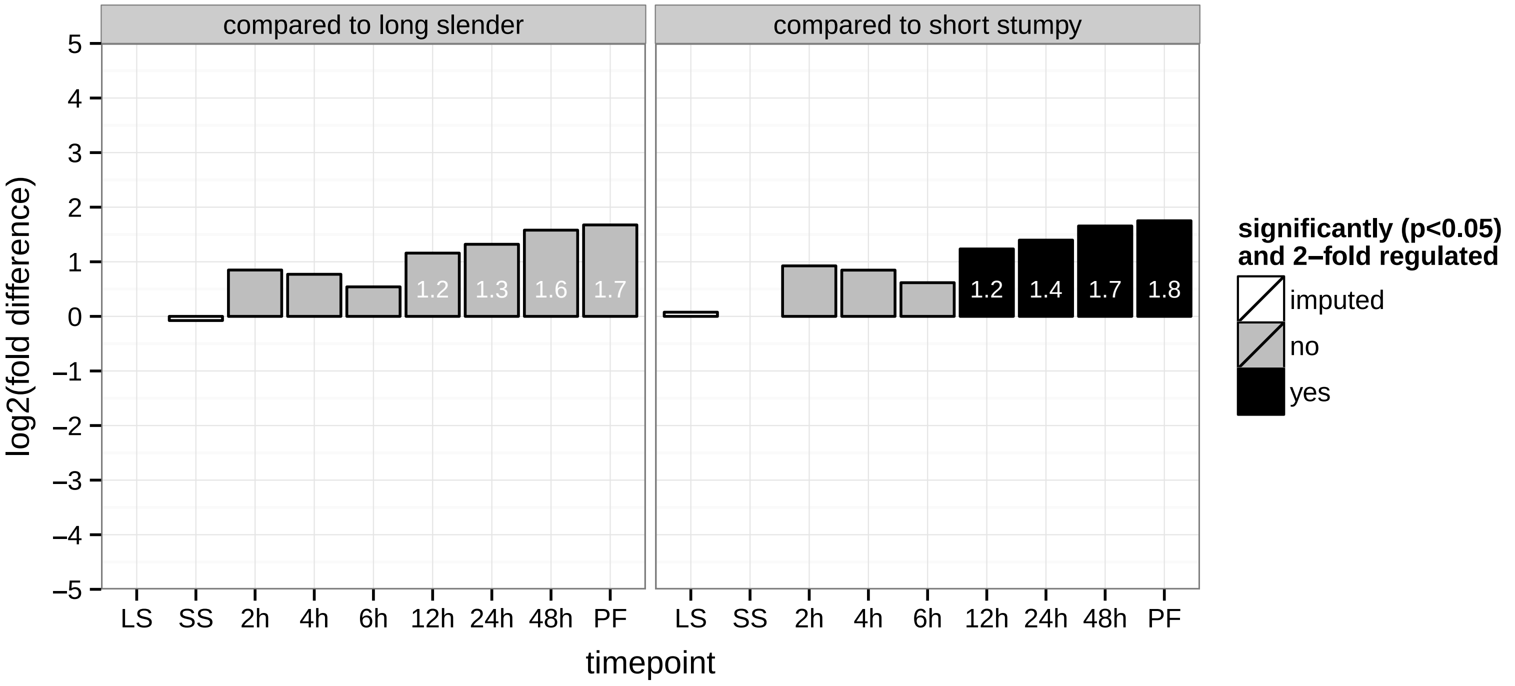

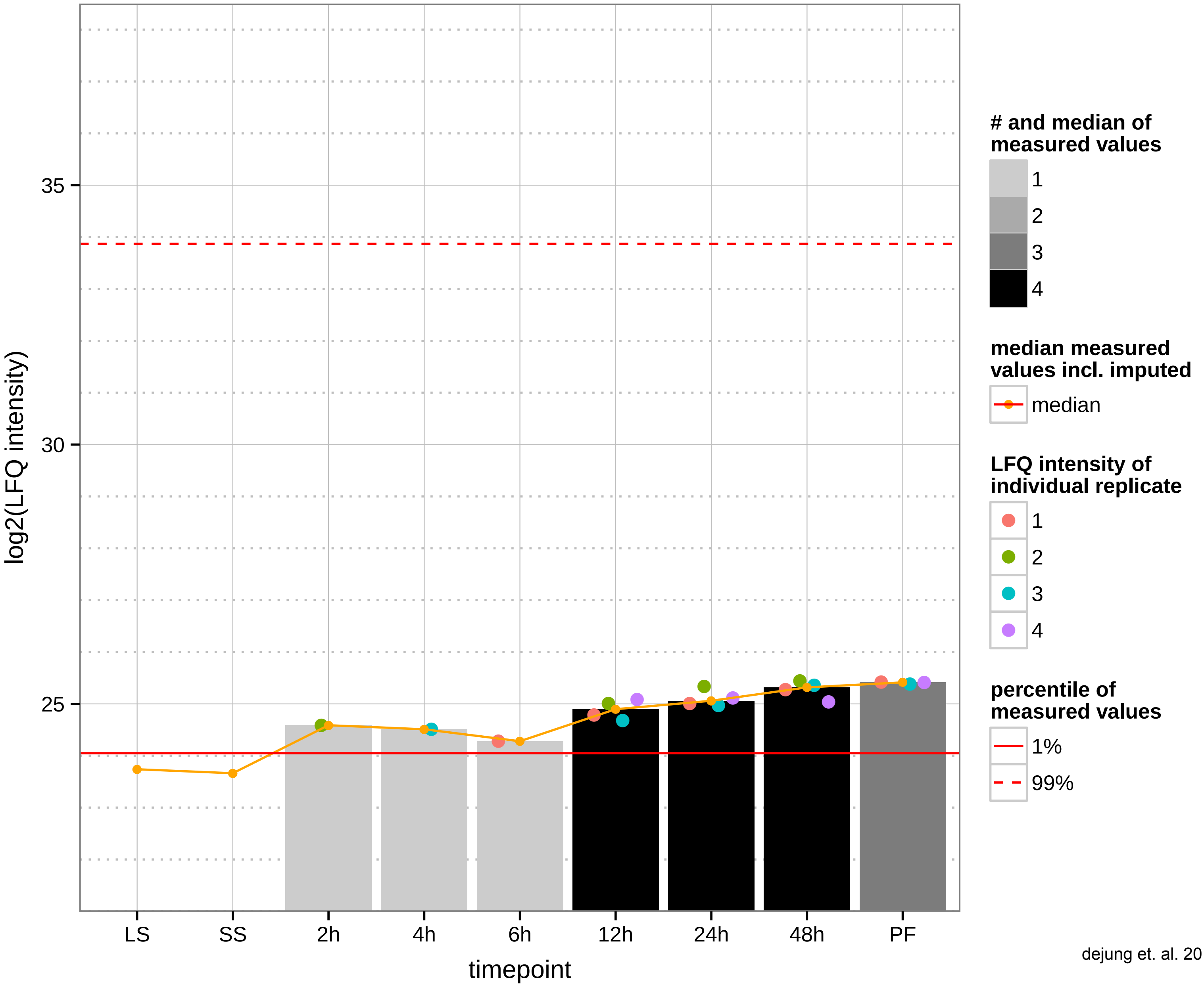

dejung et. al. 2015

hypothetical protein, conserved
Tb927.10.8870
AGOF: null
AGOC: null
AGOP: null
PGOF: null
PGOC: null
PGOP: null

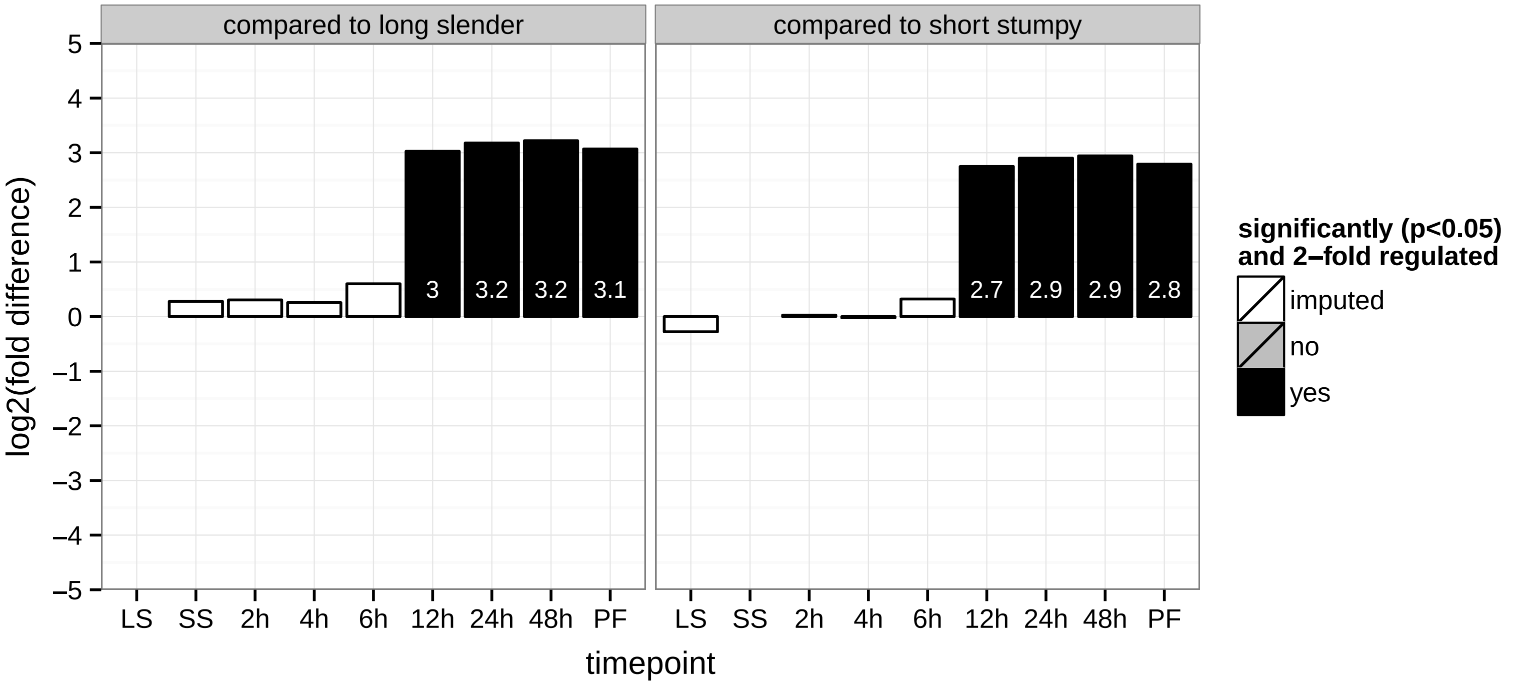

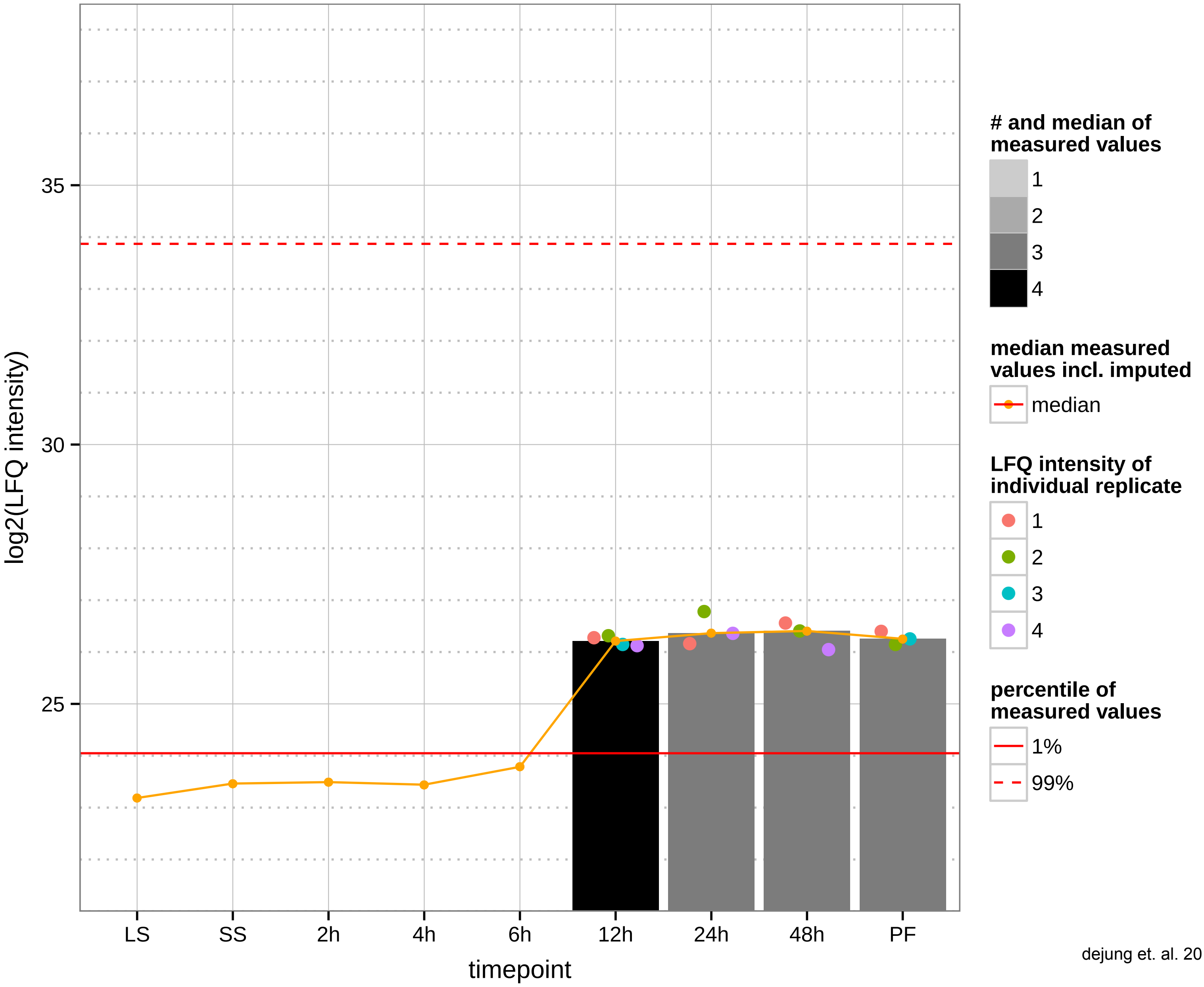

dejung et. al. 2015

hypothetical protein, conserved
Tb927.11.10140
AGOF: null
AGOC: mitochondrial inner membrane, mitochondrion
AGOP: null
PGOF: null
PGOC: null
PGOP: null

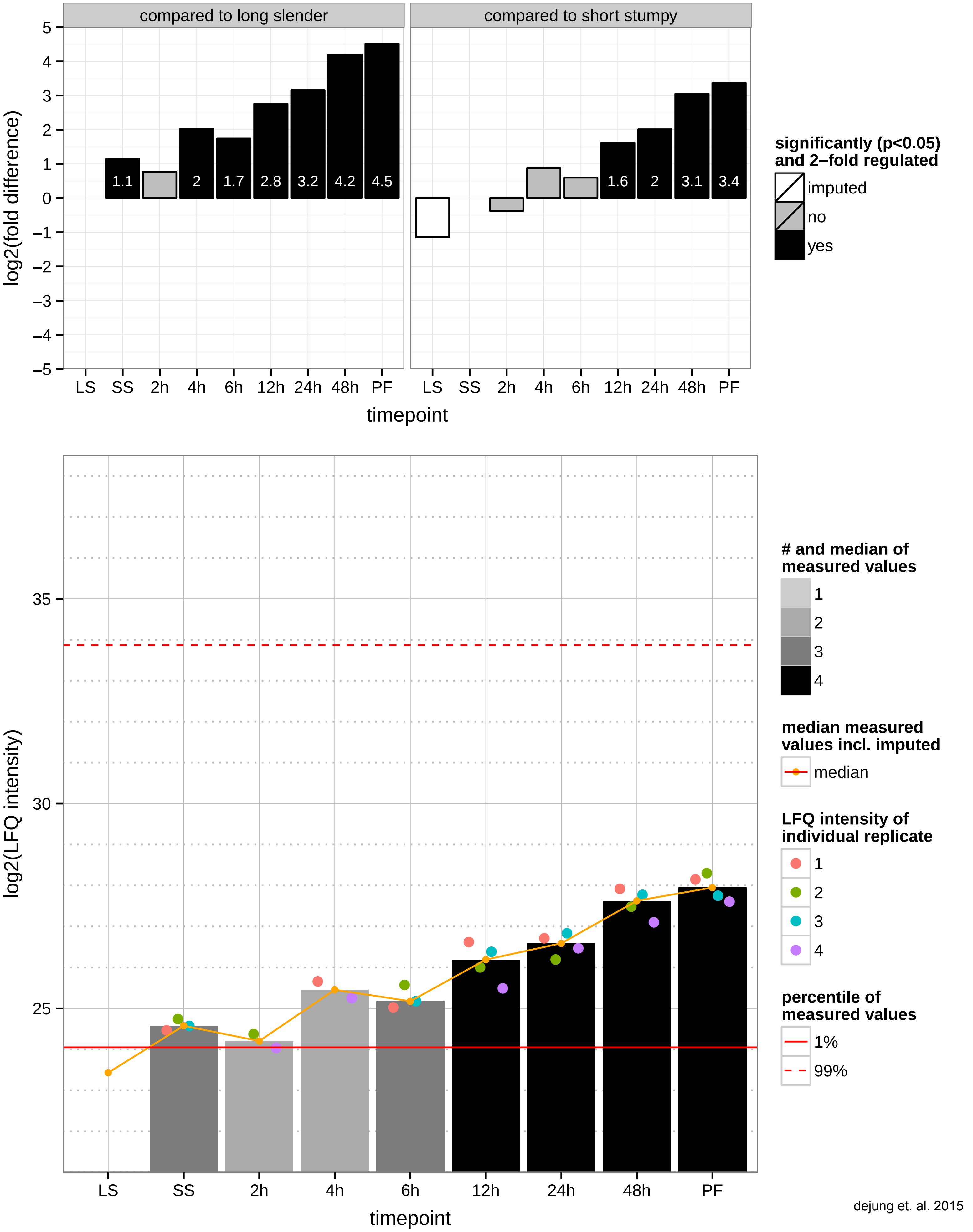

dejung et. al. 2015

eukaryotic translation initation factor 4 gamma, putative
Tb927.11.10560
AGOF: RNA binding
AGOC: cytoplasm
AGOP: RNA metabolic process, nucleobase–containing compound metabolic process, translation
PGOF: DNA binding, RNA binding, binding, protein binding
PGOC: null
PGOP: null

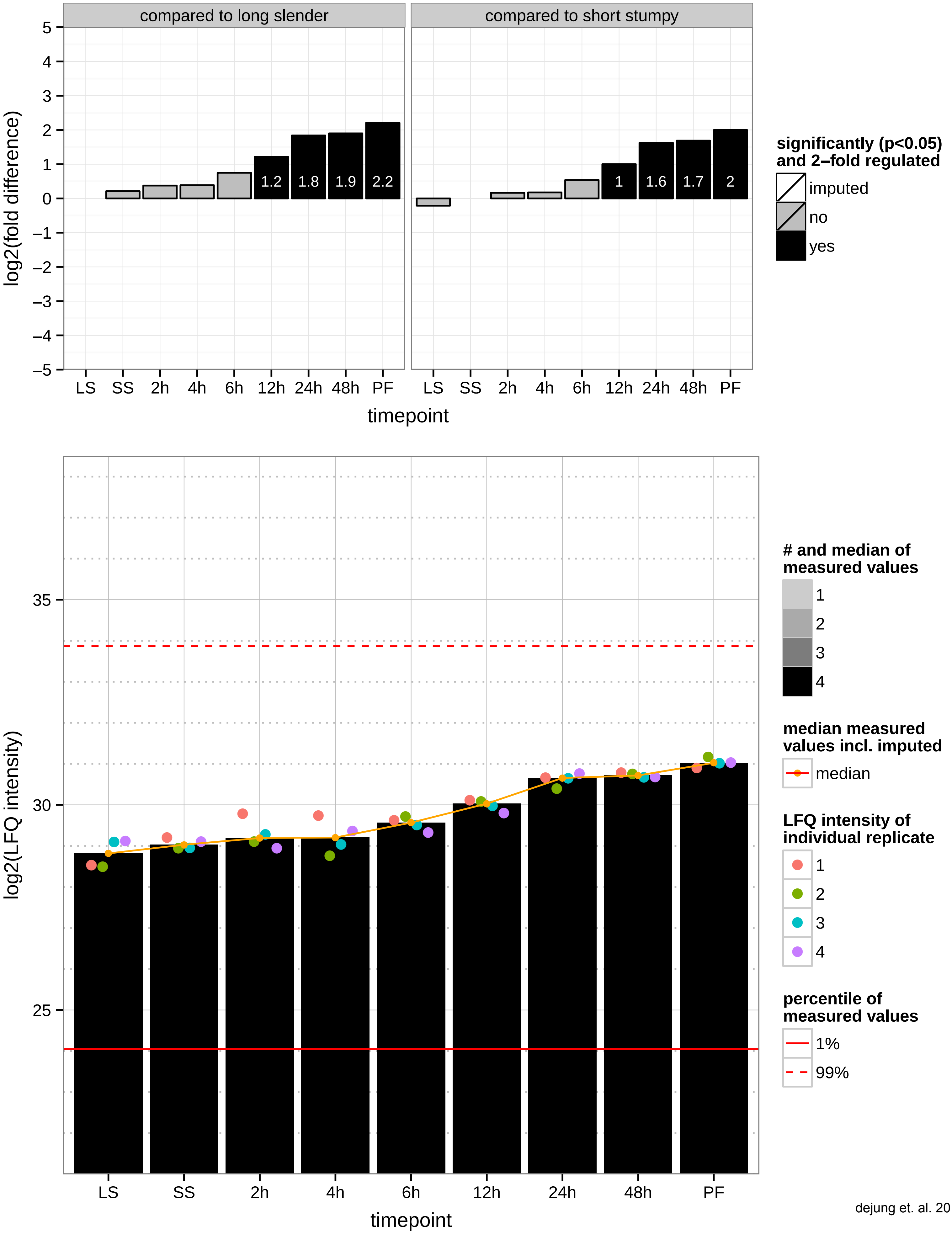

dejung et. al. 2015

hypothetical protein, conserved
Tb927.11.11630
AGOF: null
AGOC: mitochondrion
AGOP: null
PGOF: null
PGOC: null
PGOP: null

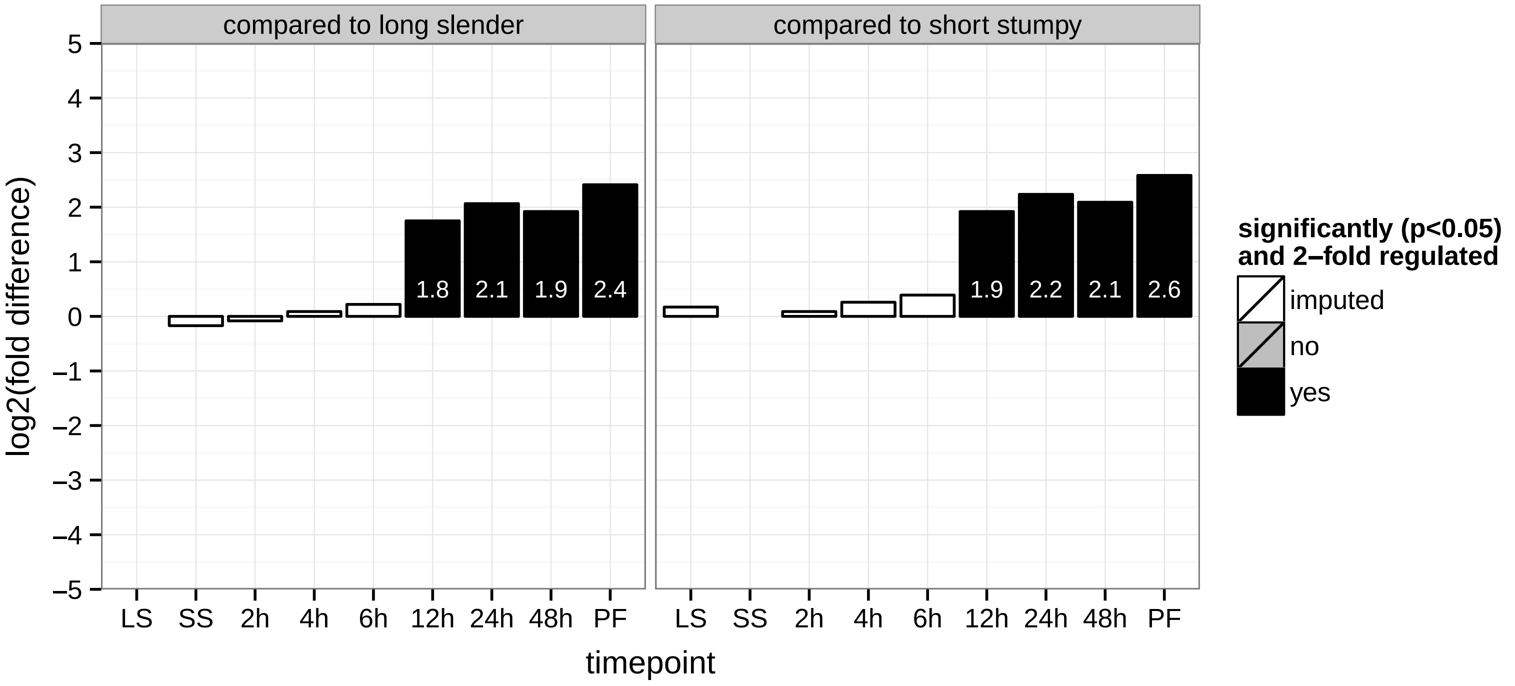

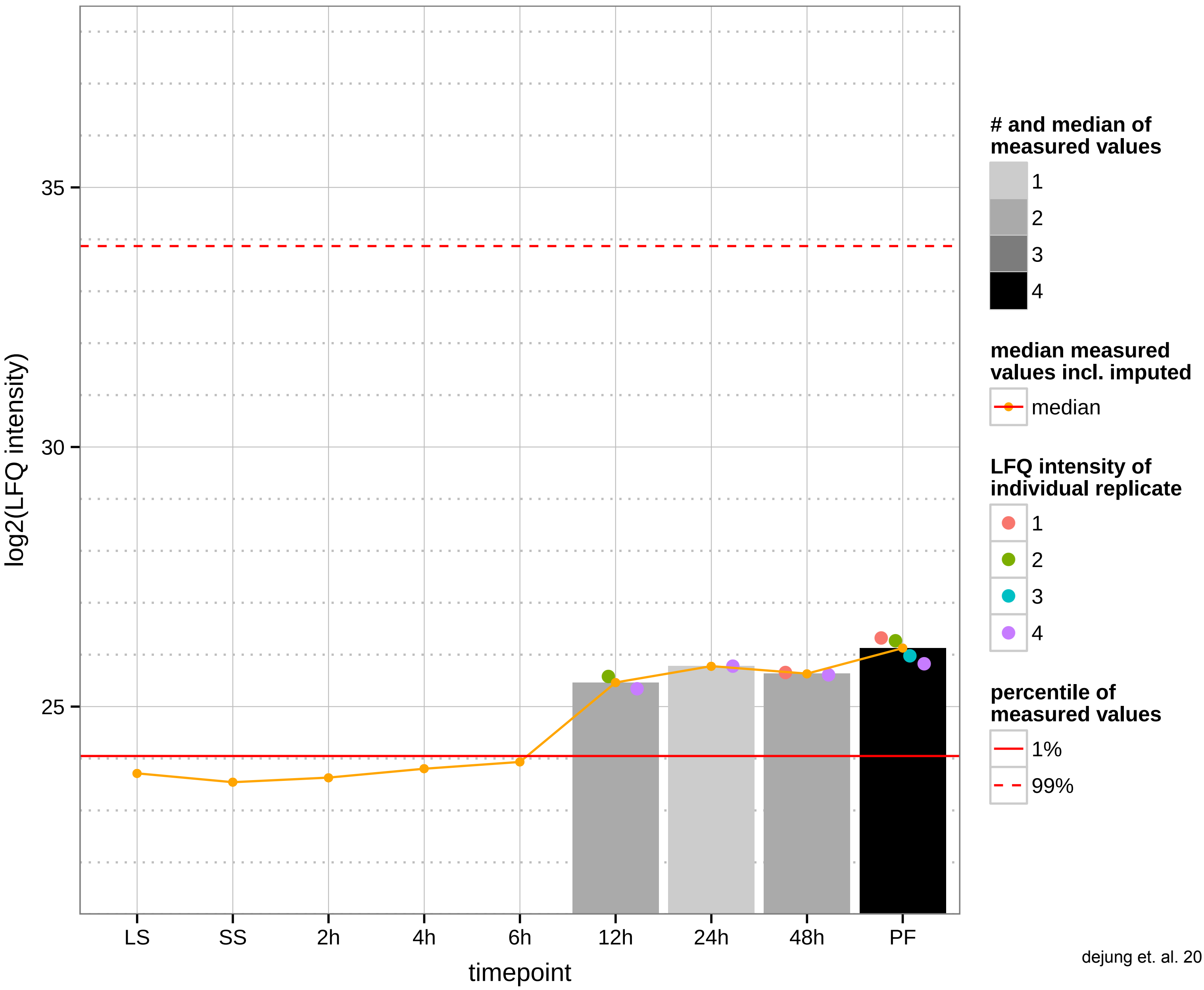

dejung et. al. 2015

eukaryotic translation initiation factor 4e, putative
Tb927.11.11770
AGOF: RNA binding, RNA cap binding, translation initiation factor activity
AGOC: cytoplasm
AGOP: translational initiation
PGOF: RNA binding, translation initiation factor activity
PGOC: cytoplasm
PGOP: translational initiation

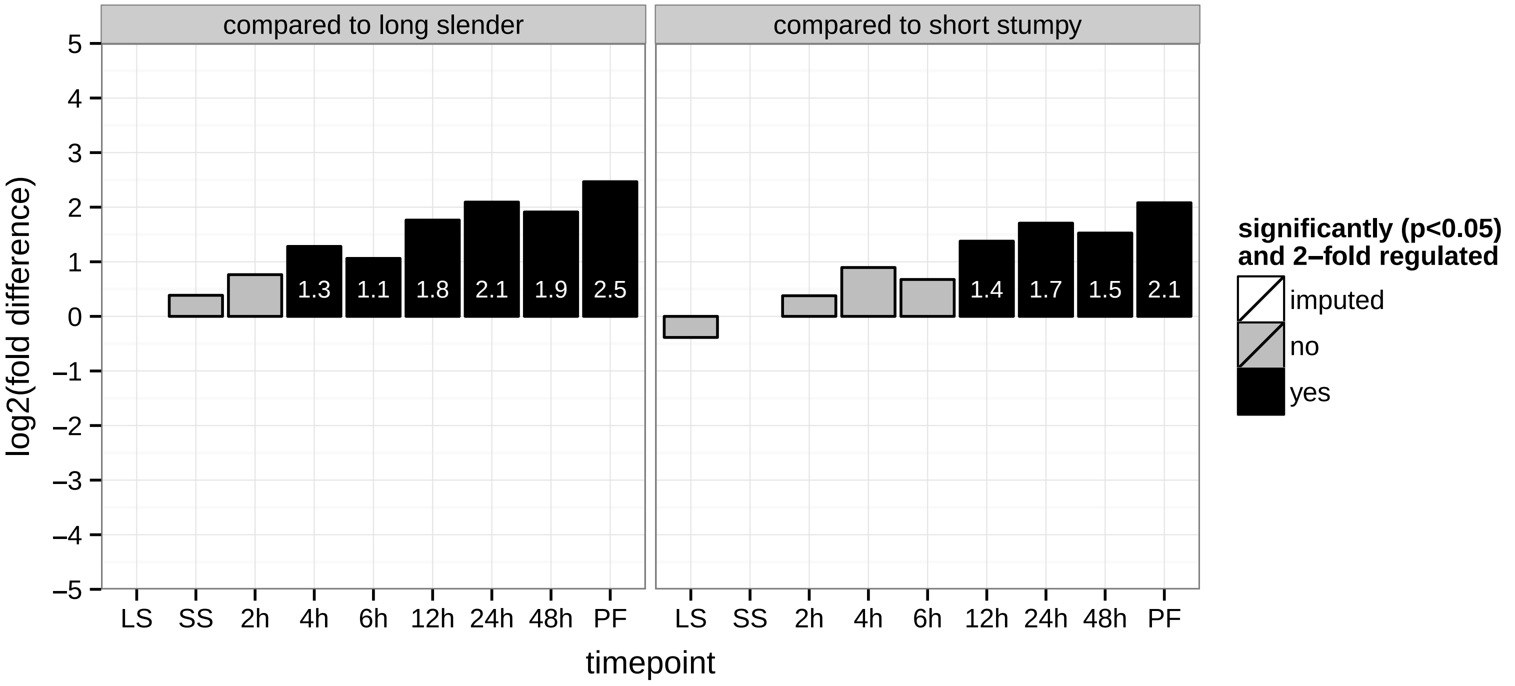

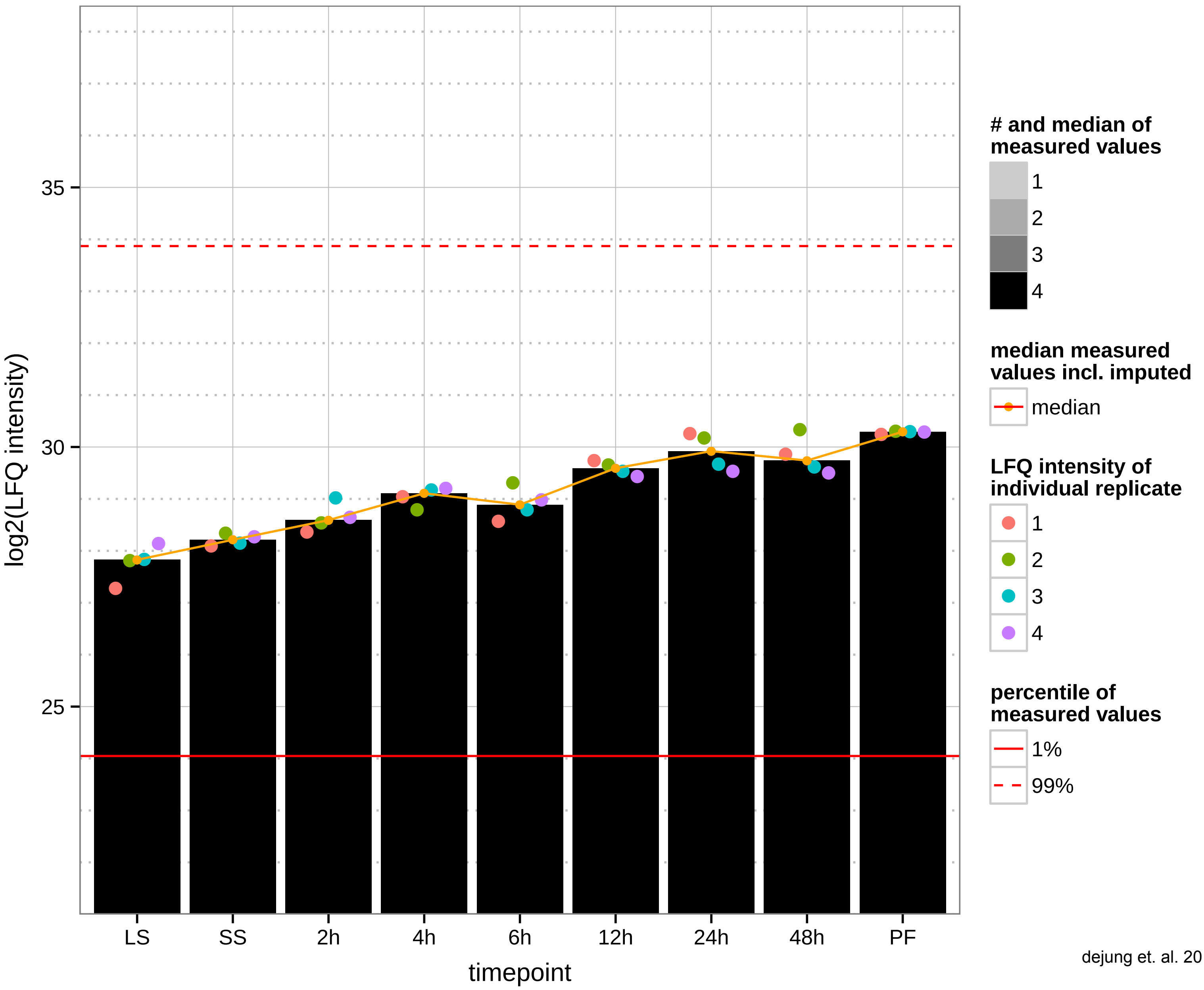

dejung et. al. 2015

ubiquitin carboxyl–terminal hydrolase, putative, cysteine peptidase, Clan CA, family C19
Tb927.11.12240
AGOF: ubiquitin thiolesterase activity
AGOC: null
AGOP: ubiquitin–dependent protein catabolic process
PGOF: ubiquitin thiolesterase activity
PGOC: null
PGOP: ubiquitin–dependent protein catabolic process

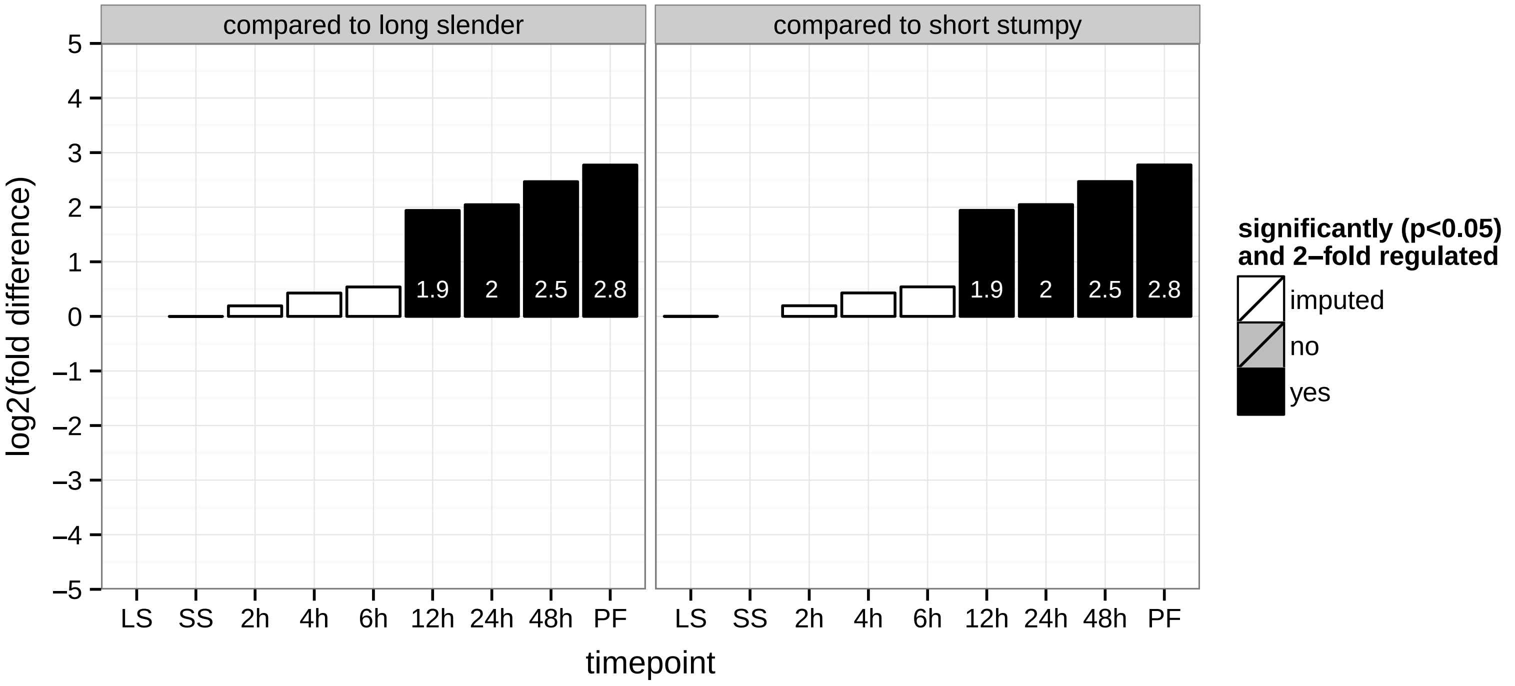

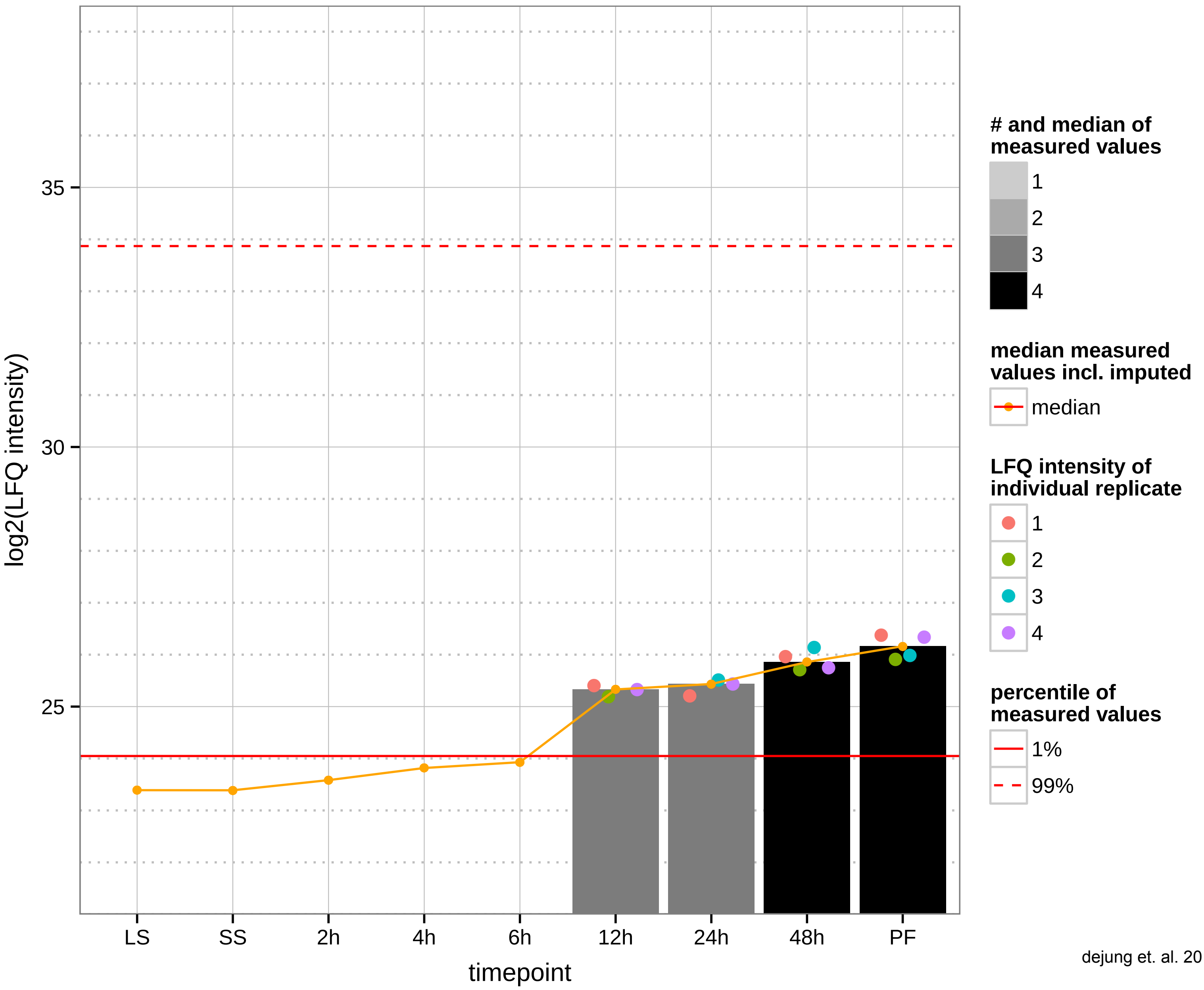

dejung et. al. 2015

Mitochondrial SSU ribosomal protein, putative, mitochondrial edited mRNA stability factor 1 subunit, kinteoplast poly(A) polym
Tb927.11.1250
AGOF: RNA binding
AGOC: mitochondrion
AGOP: RNA polyadenylation, mRNA stabilization
PGOF: null
PGOC: null
PGOP: null

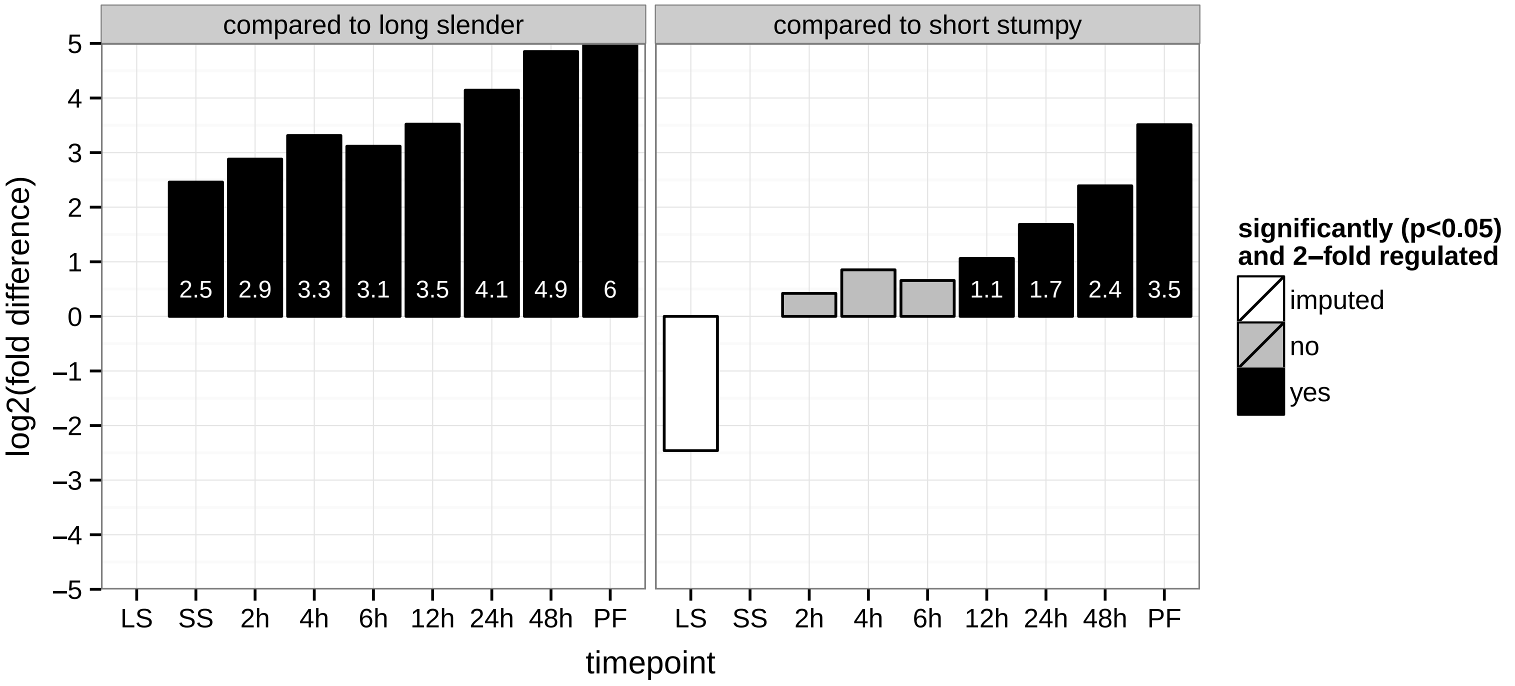

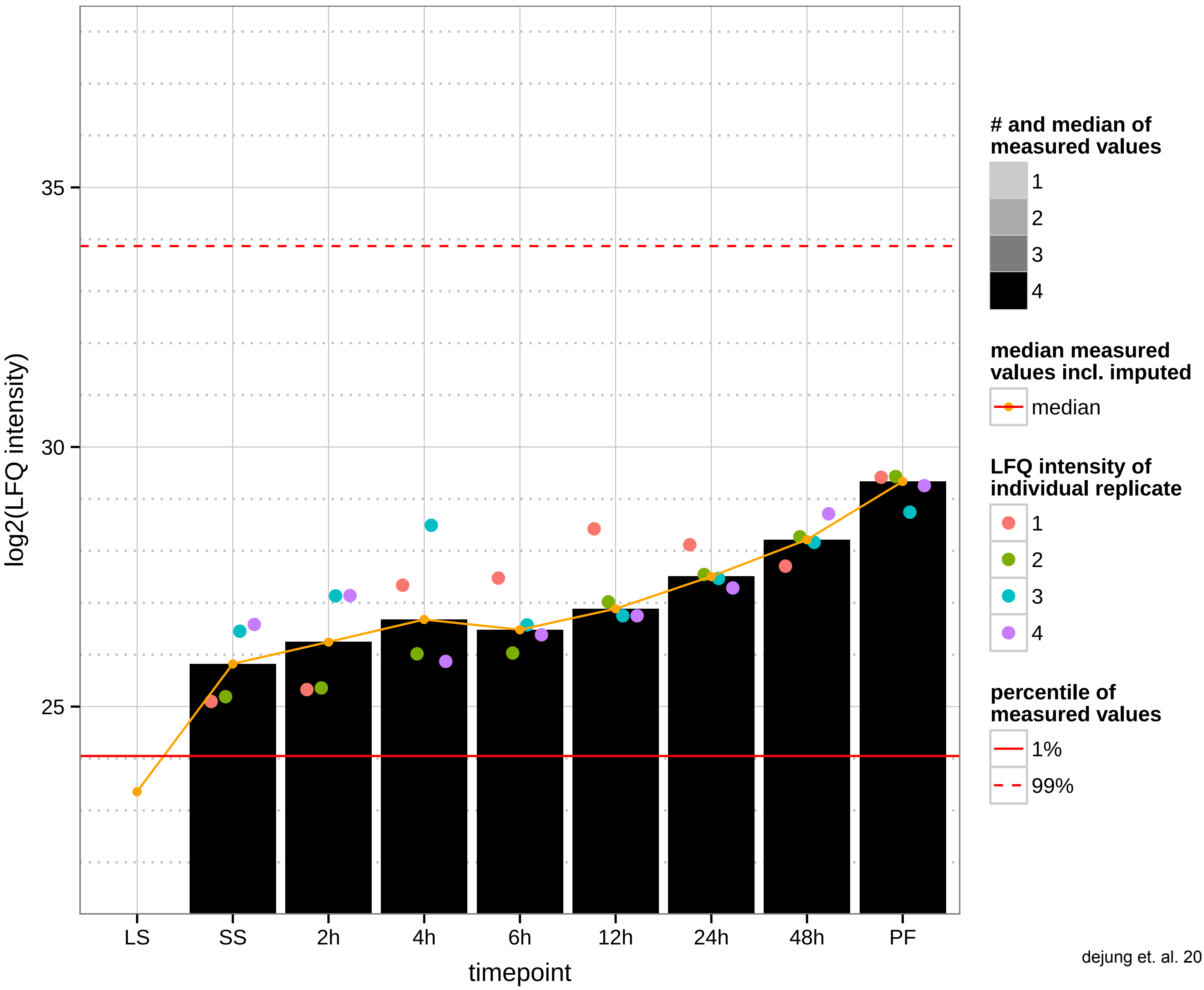

dejung et. al. 2015

prefoldin subunit 2, putative
Tb927.11.12680
AGOF: unfolded protein binding
AGOC: prefoldin complex
AGOP: protein folding
PGOF: unfolded protein binding
PGOC: prefoldin complex
PGOP: protein folding

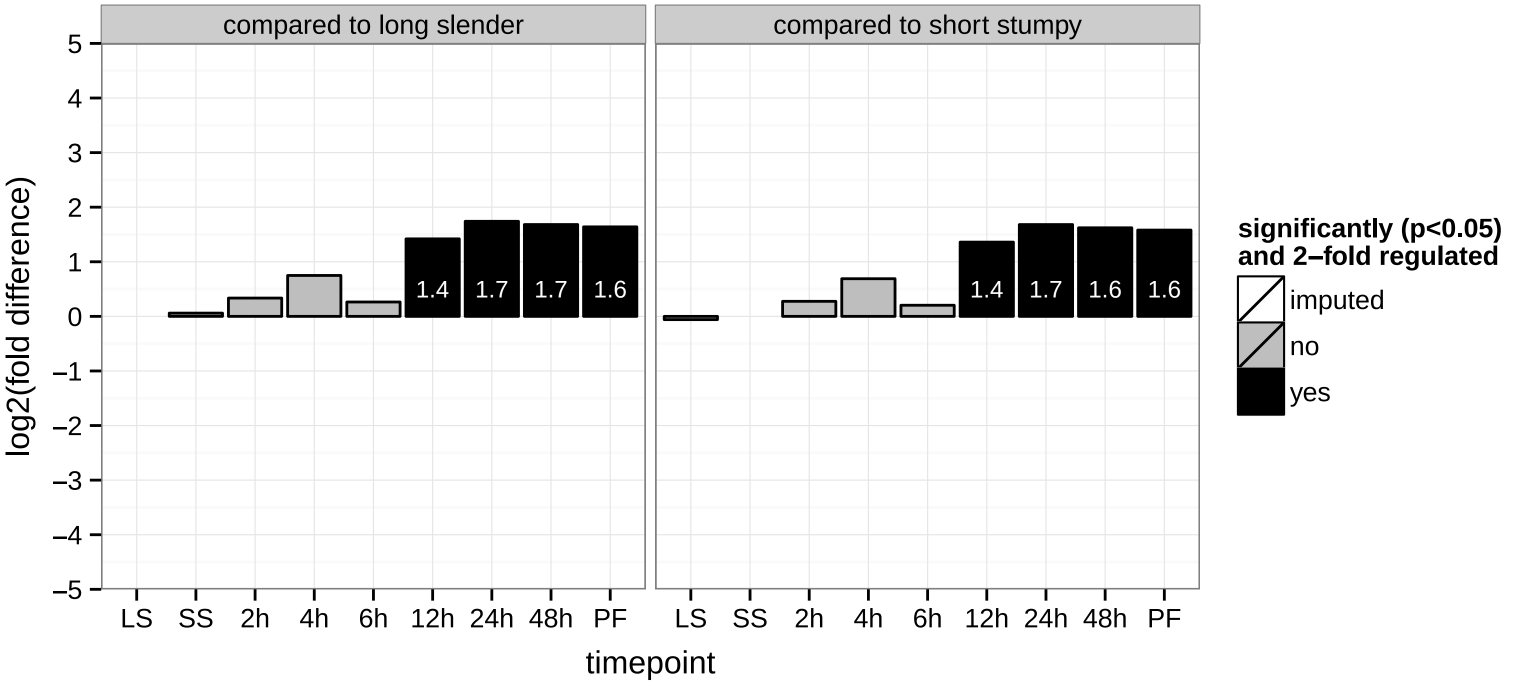

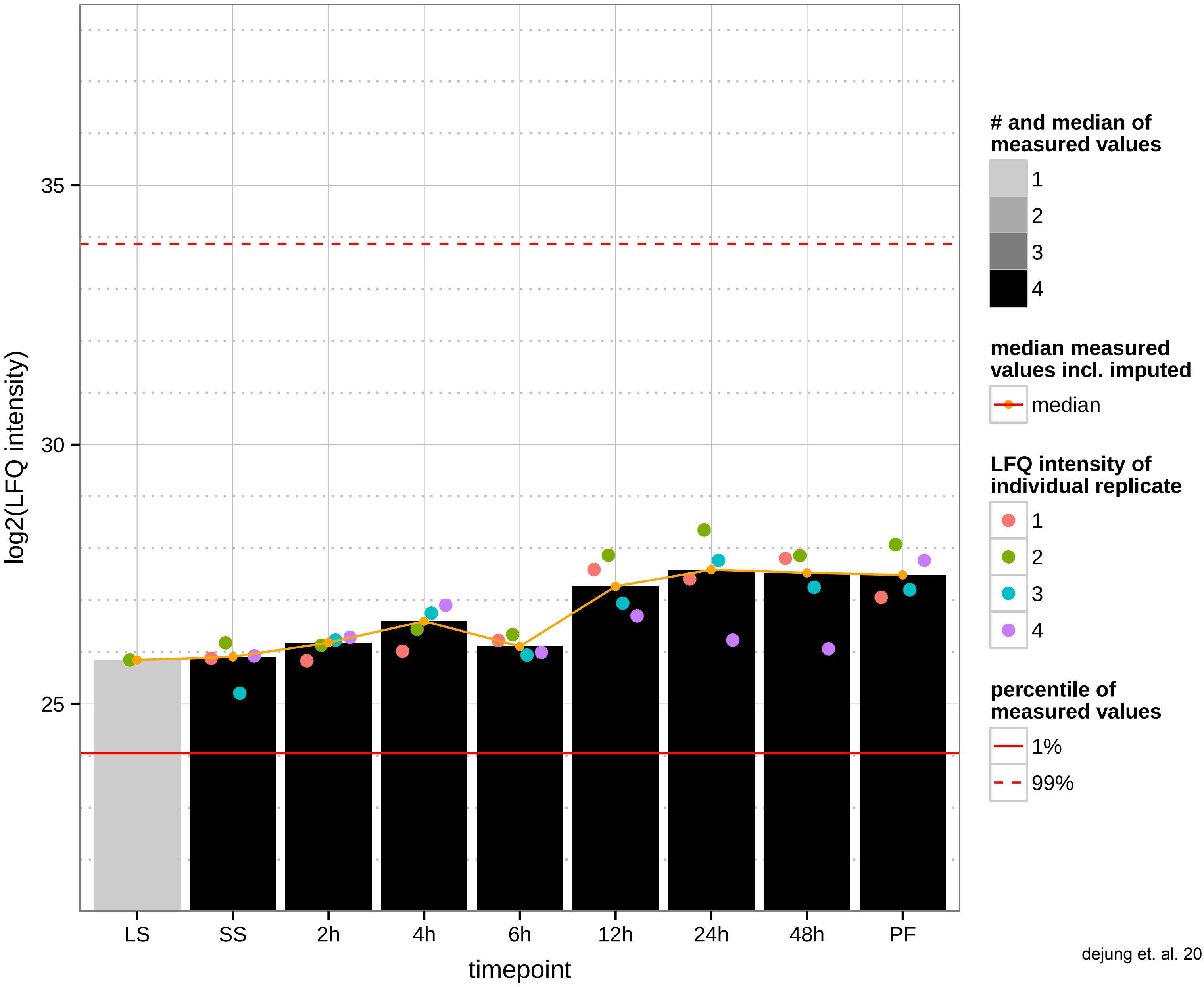

dejung et. al. 2015

ribonucleoside–diphosphate reductase small chain, putative, ribonucleoside–diphosphate reductase small chain (RNR2)
Tb927.11.12800;Tb927.11.12790;Tb927.11.12780;Tb927.11.12820;Tb11.v5.0288;Tb927.11.12810
AGOF: null, ribonucleoside–diphosphate reductase activity, thioredoxin disulfide as acceptor, transition metal ion binding
AGOC: null
AGOP: null, deoxyribonucleoside diphosphate metabolic process
PGOF: oxidoreductase activity, ribonucleoside–diphosphate reductase activity, thioredoxin disulfide as acceptor, transition me
PGOC: null
PGOP: deoxyribonucleoside diphosphate metabolic process, oxidation–reduction process

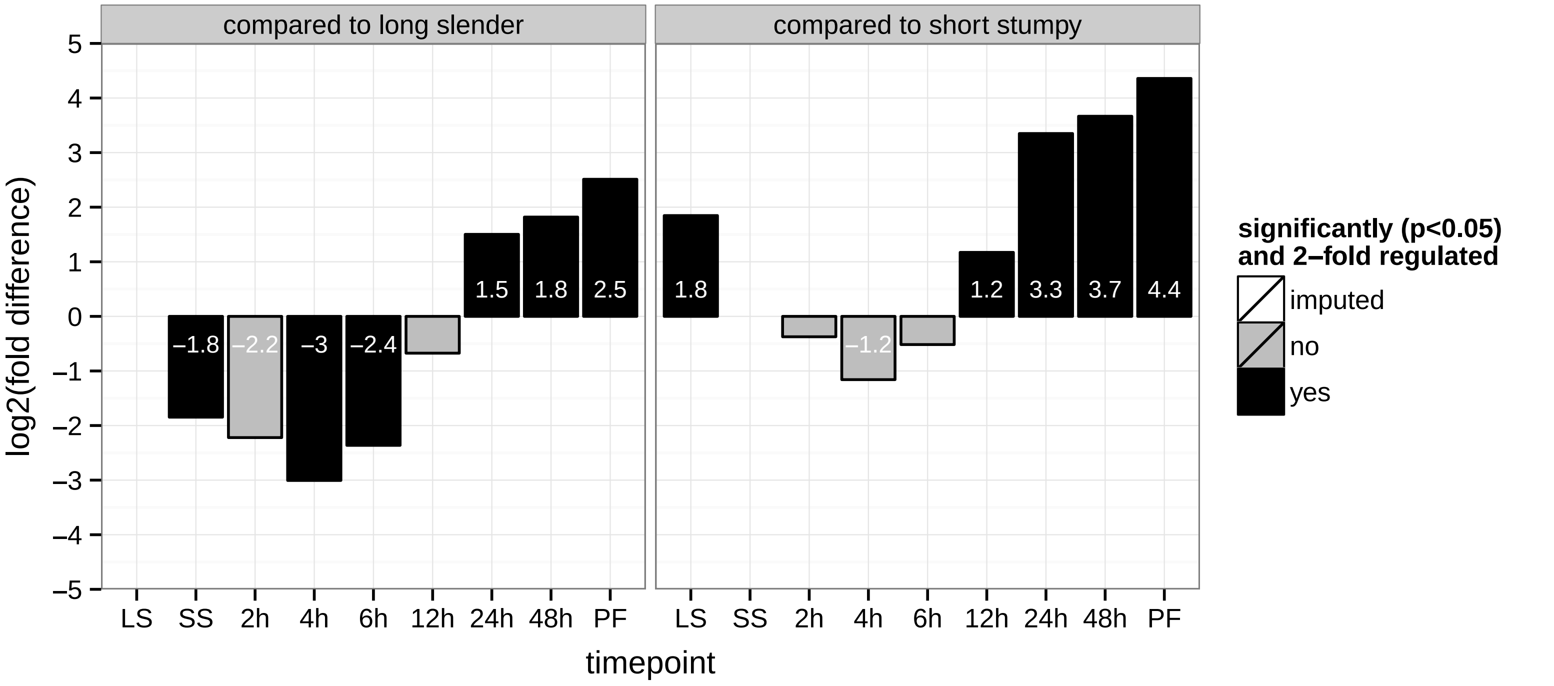

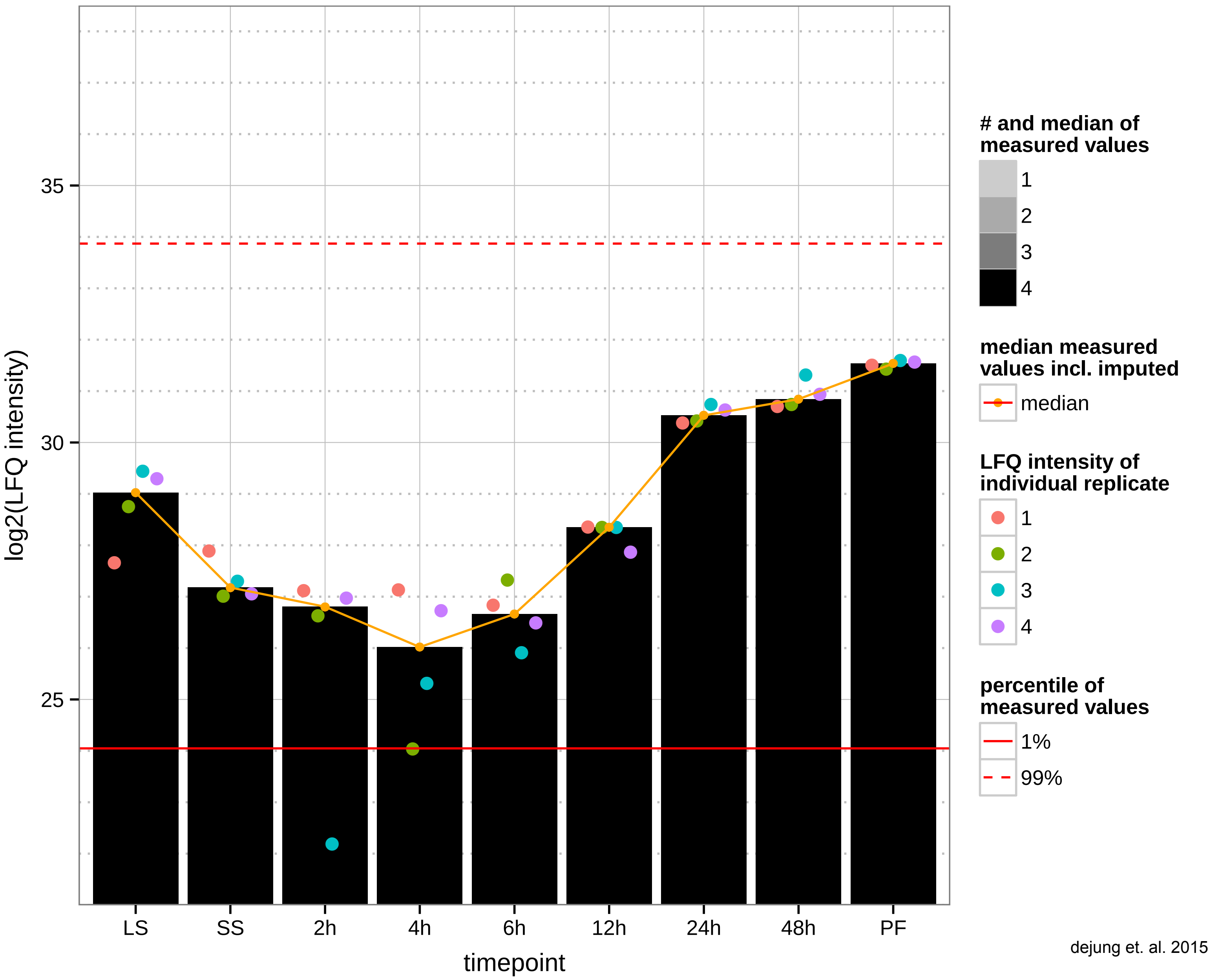

dejung et. al. 2015

cytochrome oxidase subunit X (COXX)
Tb927.11.13140
AGOF: null
AGOC: mitochondrion
AGOP: null
PGOF: null
PGOC: null
PGOP: null

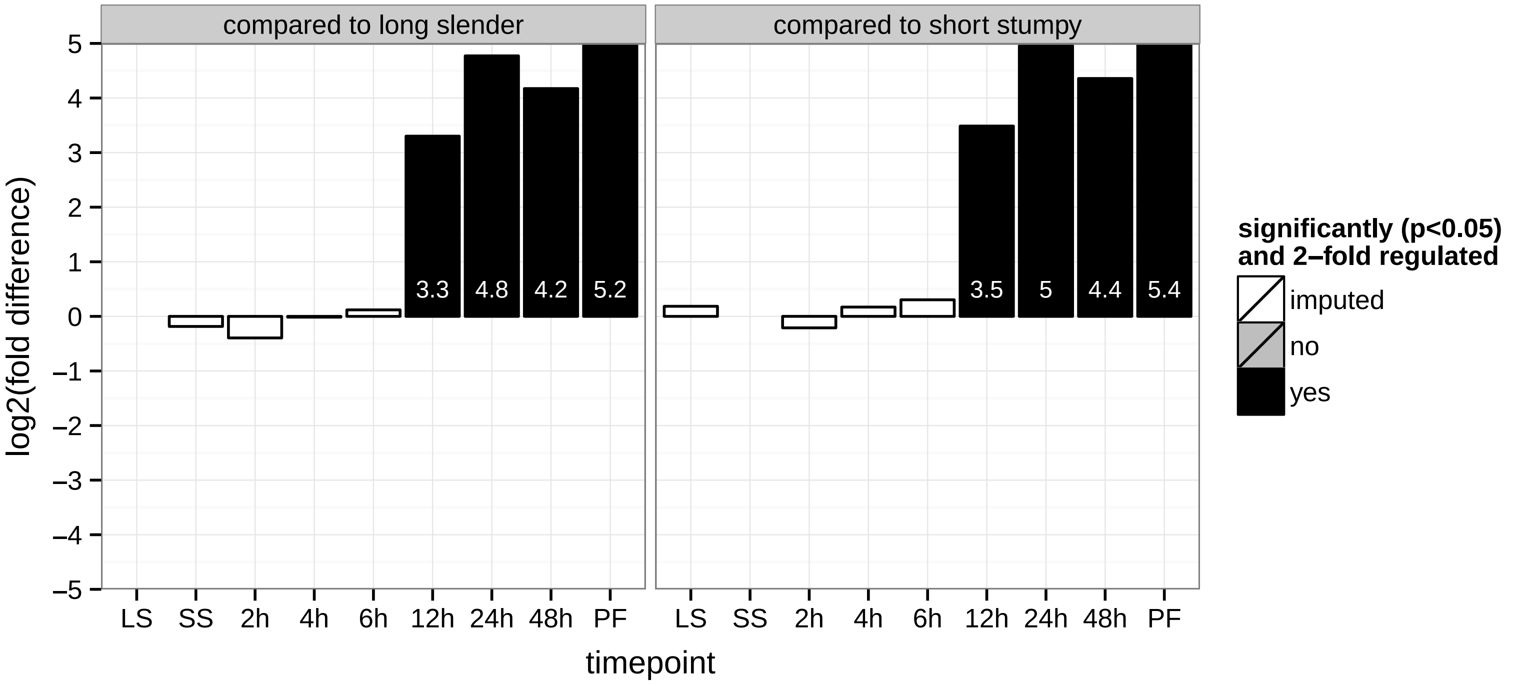

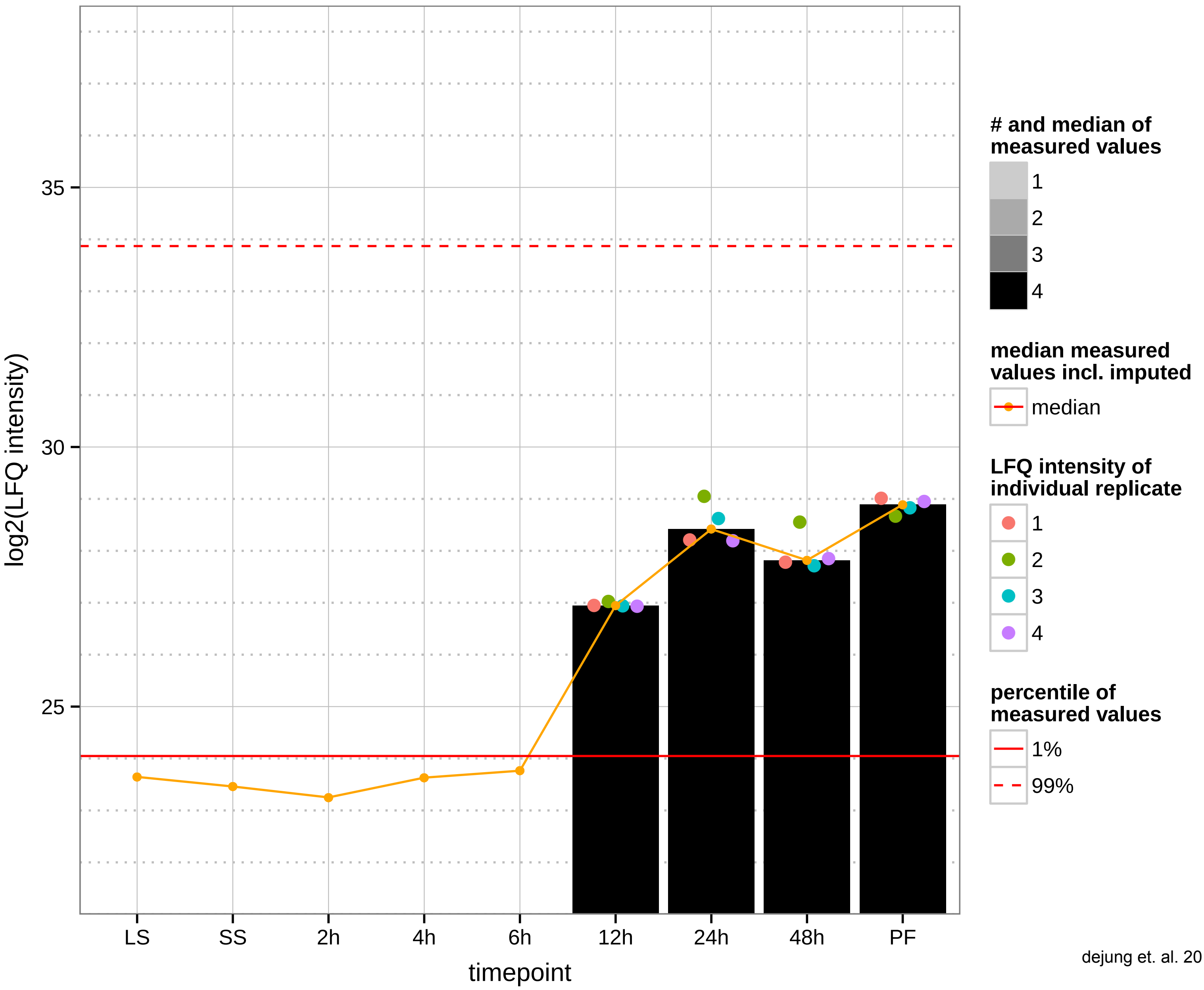

dejung et. al. 2015

hypothetical protein, conserved
Tb927.11.13510
AGOF: null
AGOC: mitochondrion
AGOP: null
PGOF: null
PGOC: null
PGOP: null

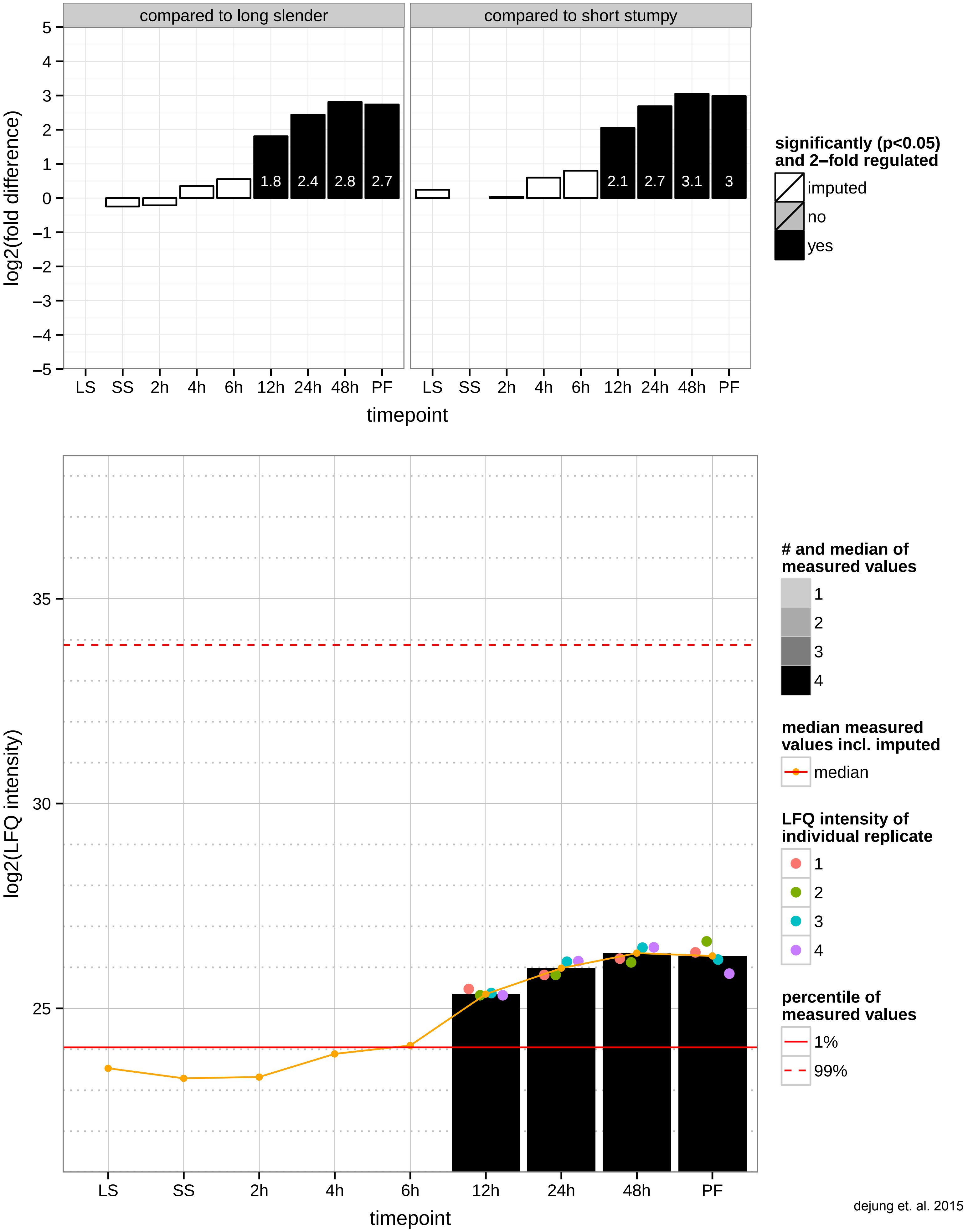

dejung et. al. 2015

hypothetical protein, conserved
Tb927.11.13610
AGOF: null
AGOC: null
AGOP: null
PGOF: null
PGOC: null
PGOP: null

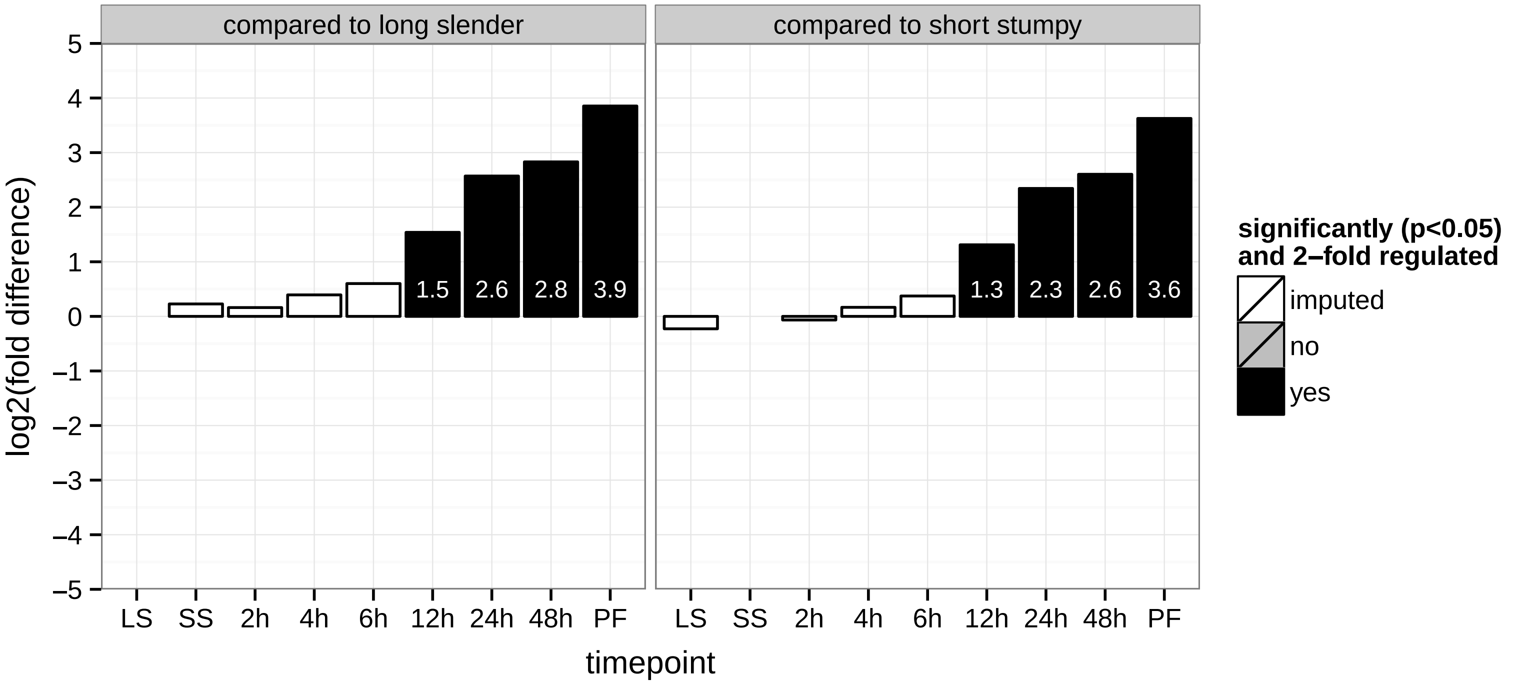

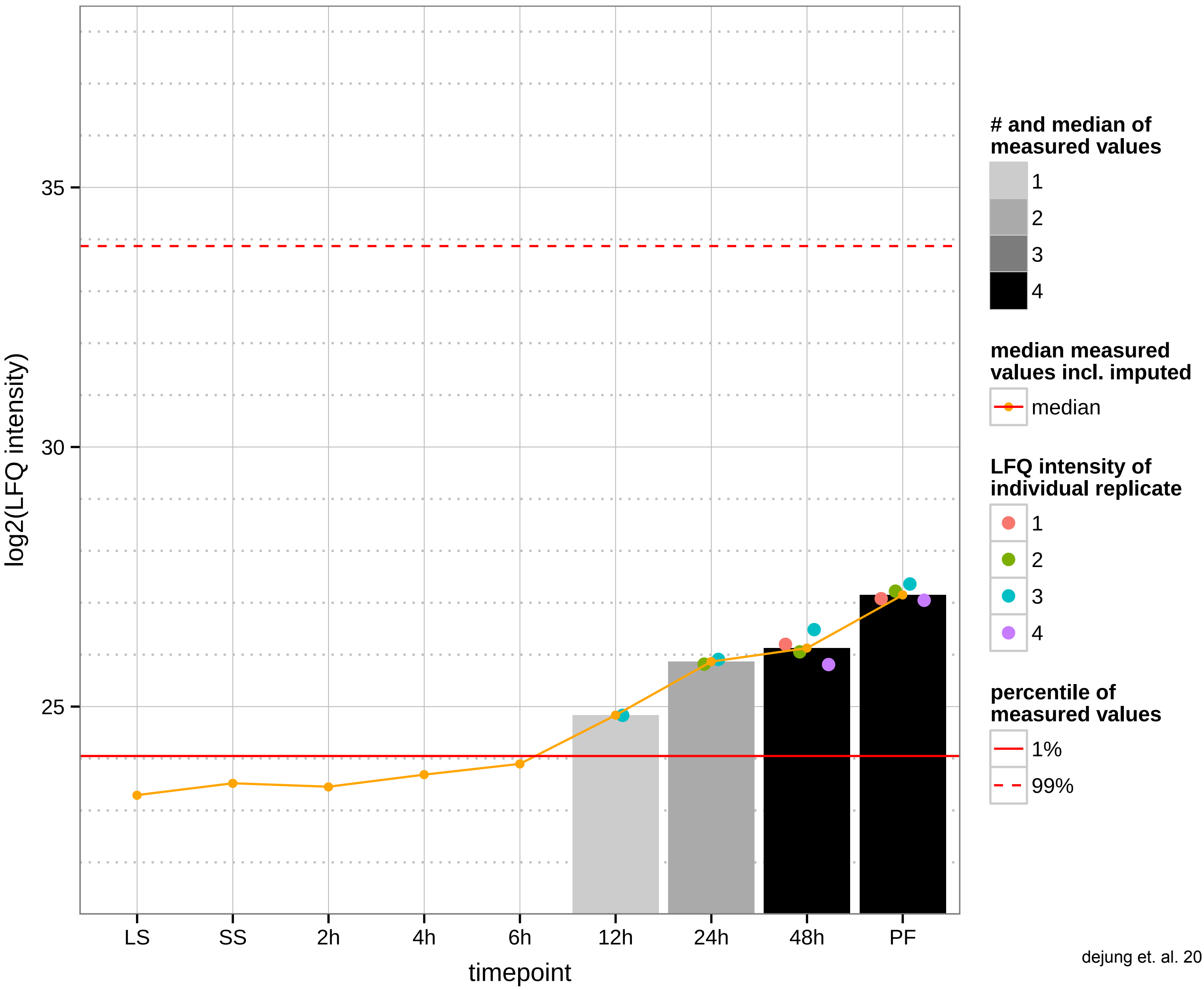

dejung et. al. 2015

hypothetical protein, conserved
Tb927.11.13620
AGOF: null
AGOC: mitochondrion
AGOP: null
PGOF: null
PGOC: null
PGOP: null

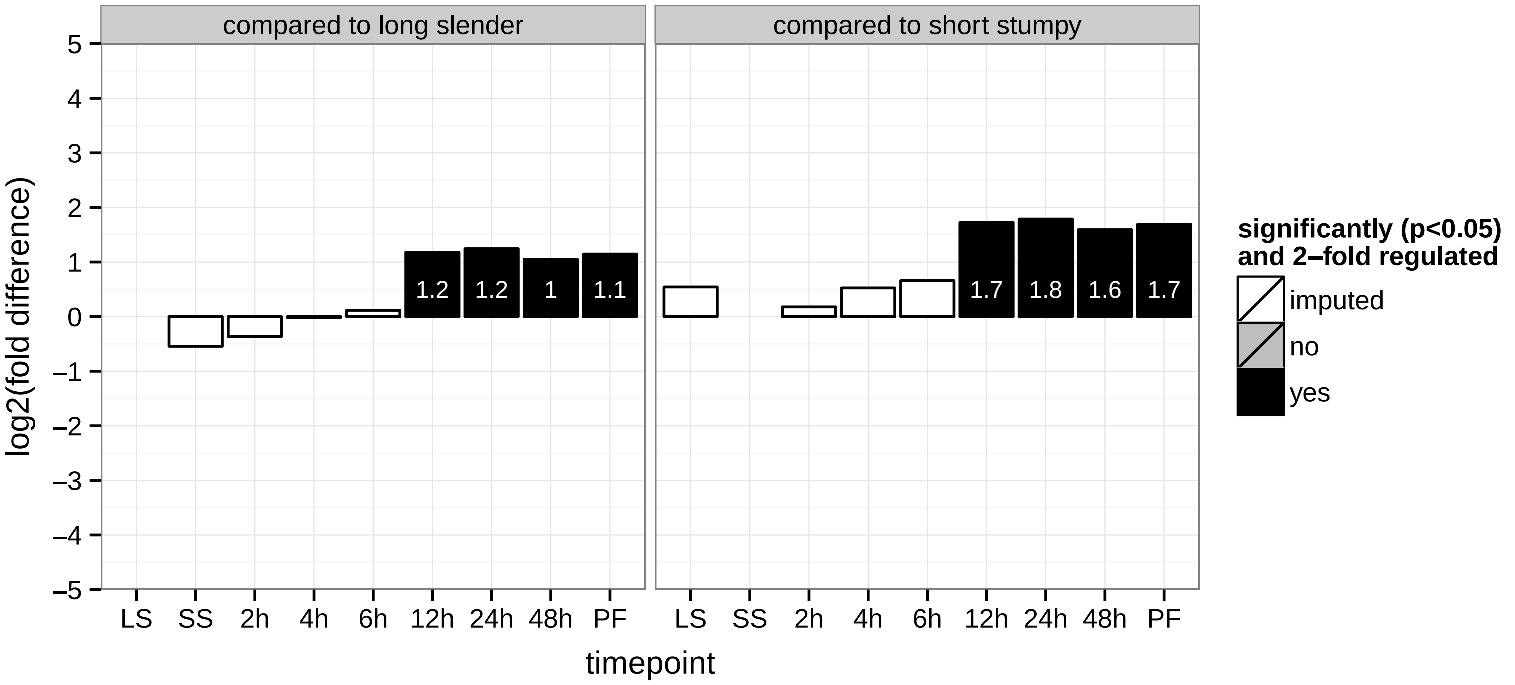

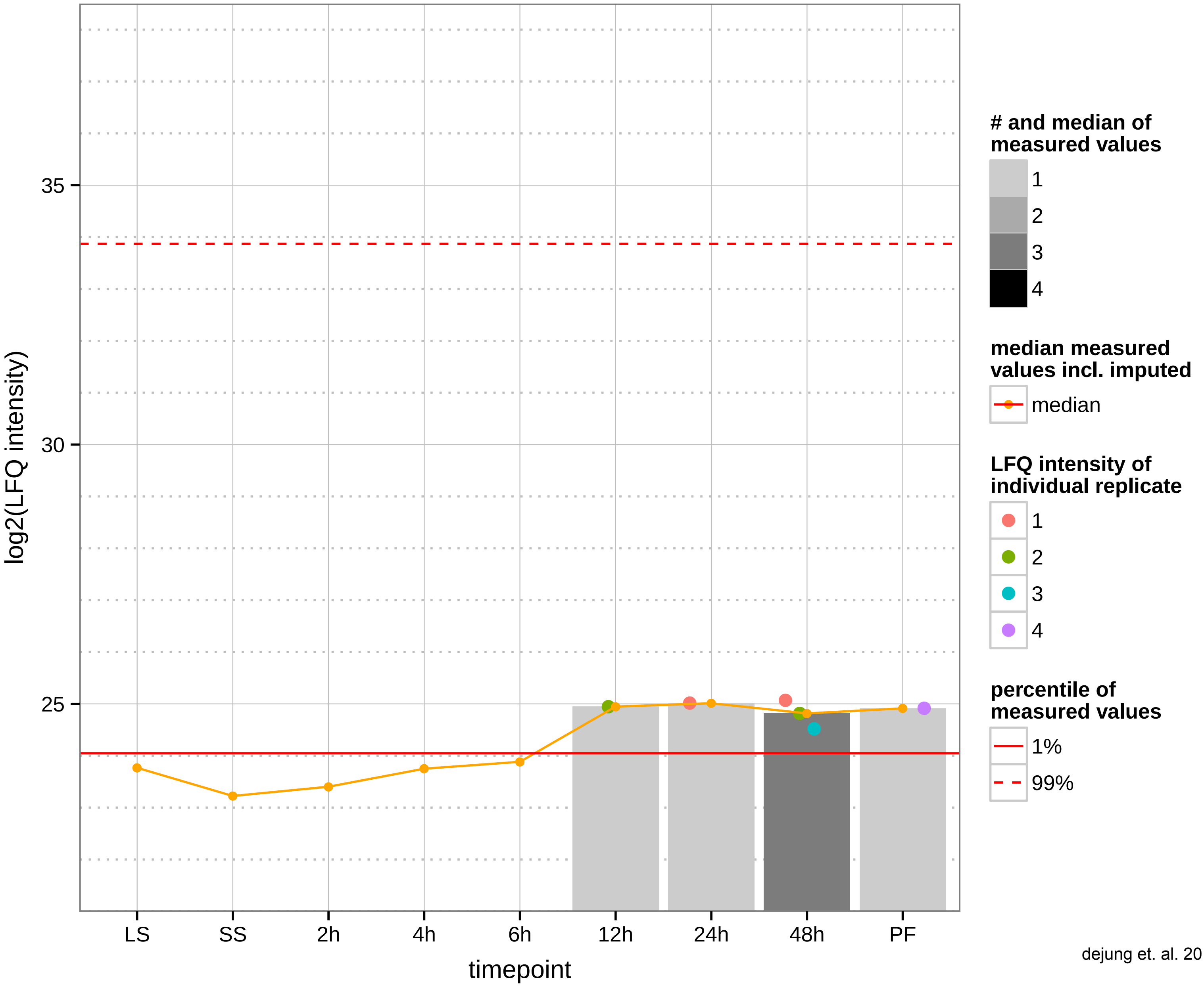

dejung et. al. 2015

receptor–type adenylate cyclase GRESAG 4, putative
Tb927.11.13740
AGOF: adenylate cyclase activity
AGOC: integral to membrane
AGOP: cyclic nucleotide biosynthetic process, intracellular signal transduction
PGOF: phosphorus–oxygen lyase activity
PGOC: null
PGOP: cyclic nucleotide biosynthetic process, intracellular signal transduction

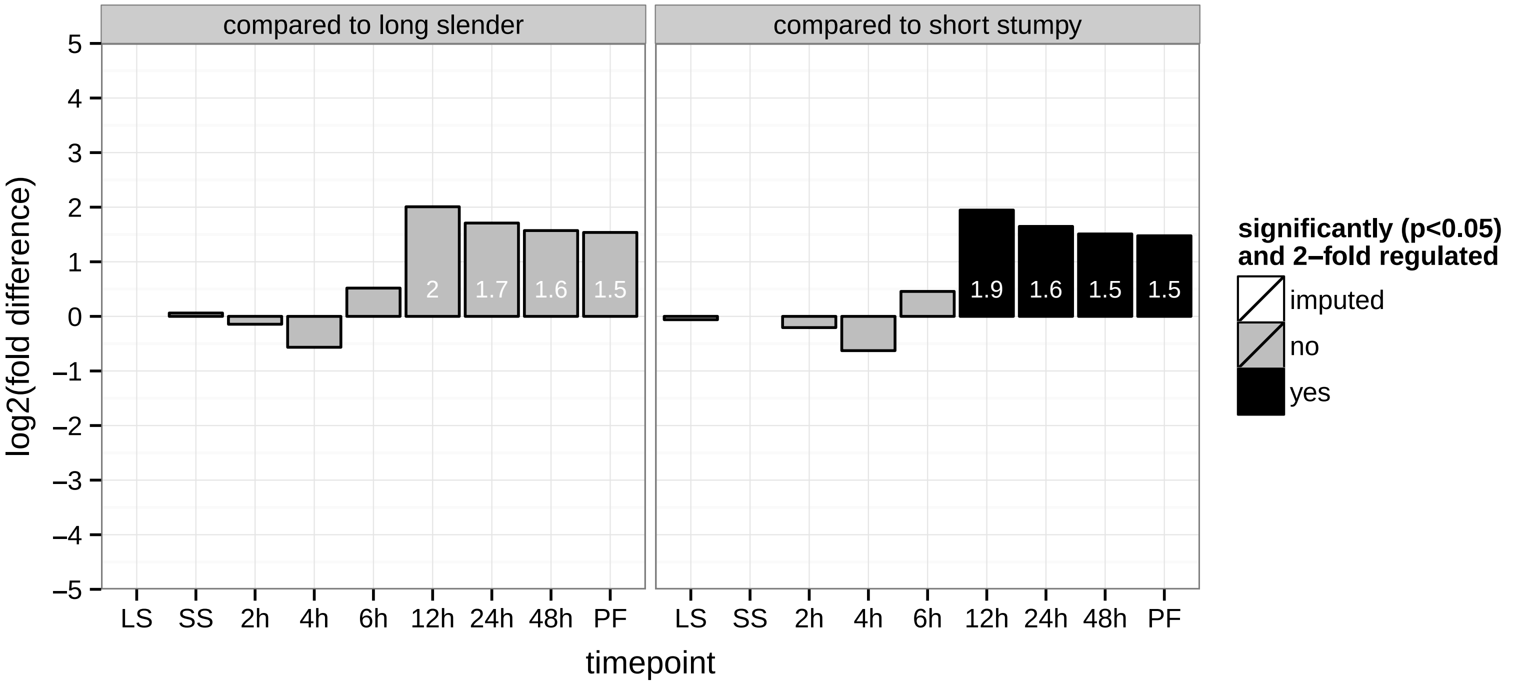

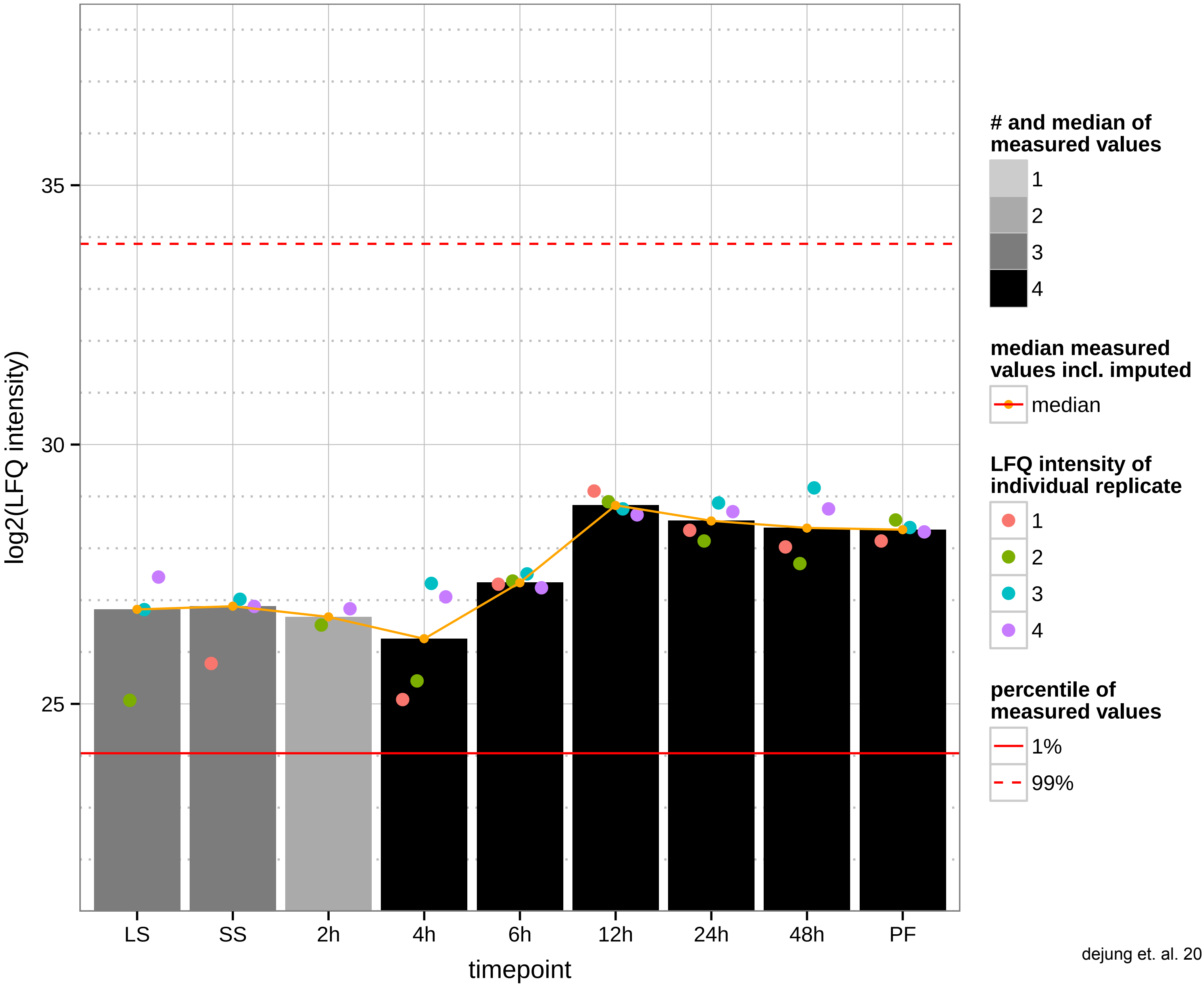

dejung et. al. 2015

hypothetical protein, conserved
Tb927.11.13830
AGOF: heat shock protein binding
AGOC: integral to membrane, mitochondrial membrane, mitochondrion
AGOP: null
PGOF: heat shock protein binding
PGOC: null
PGOP: null

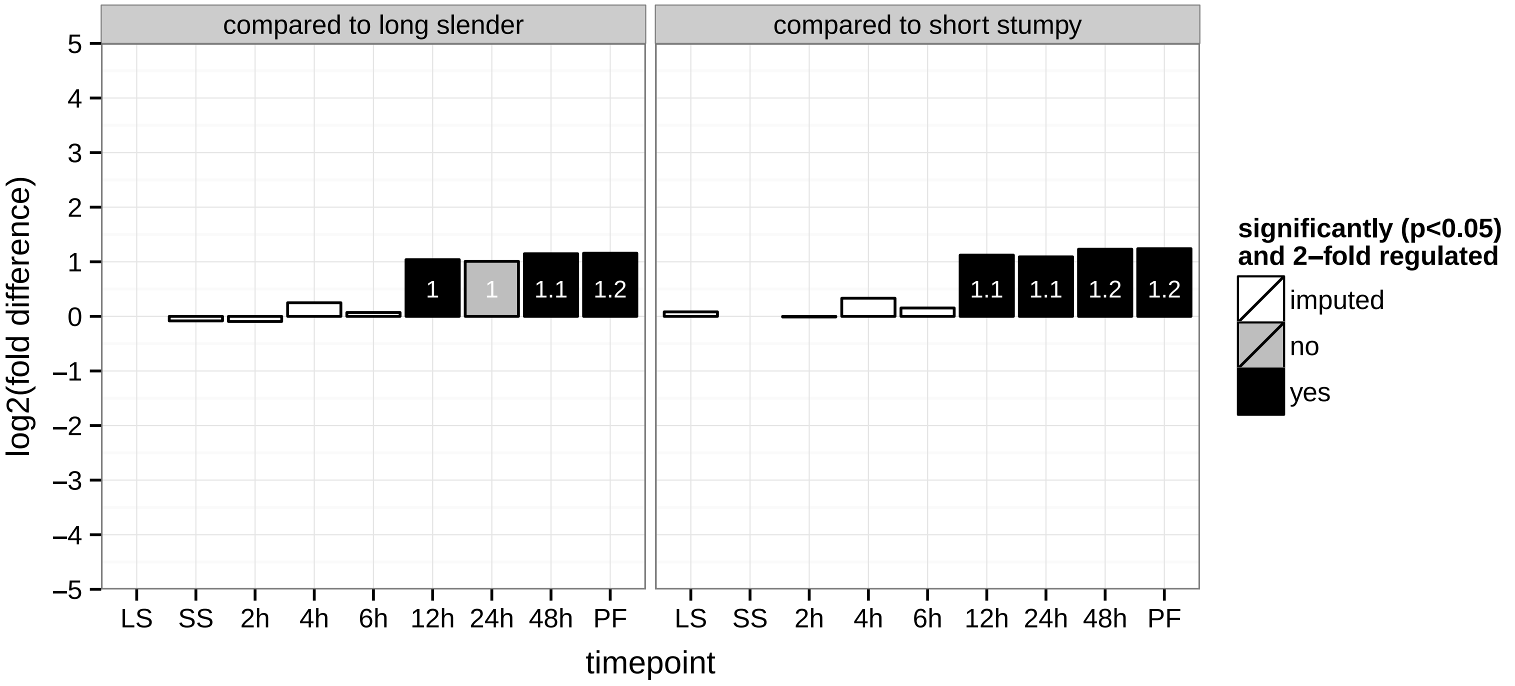

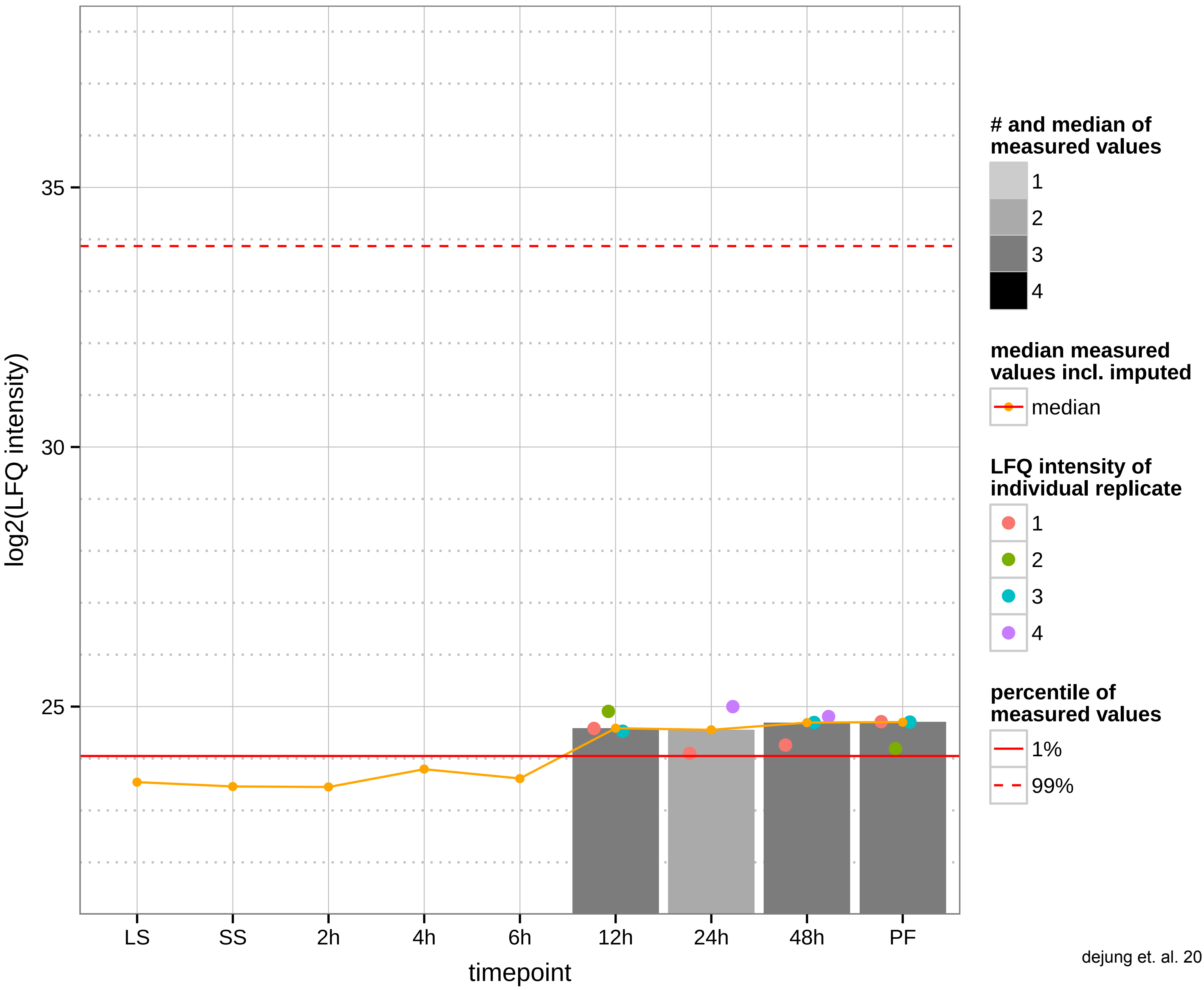

dejung et. al. 2015

hypothetical protein, conserved
Tb927.11.13890
AGOF: catalytic activity
AGOC: intracellular, mitochondrion
AGOP: RNA metabolic process
PGOF: catalytic activity
PGOC: intracellular
PGOP: RNA metabolic process

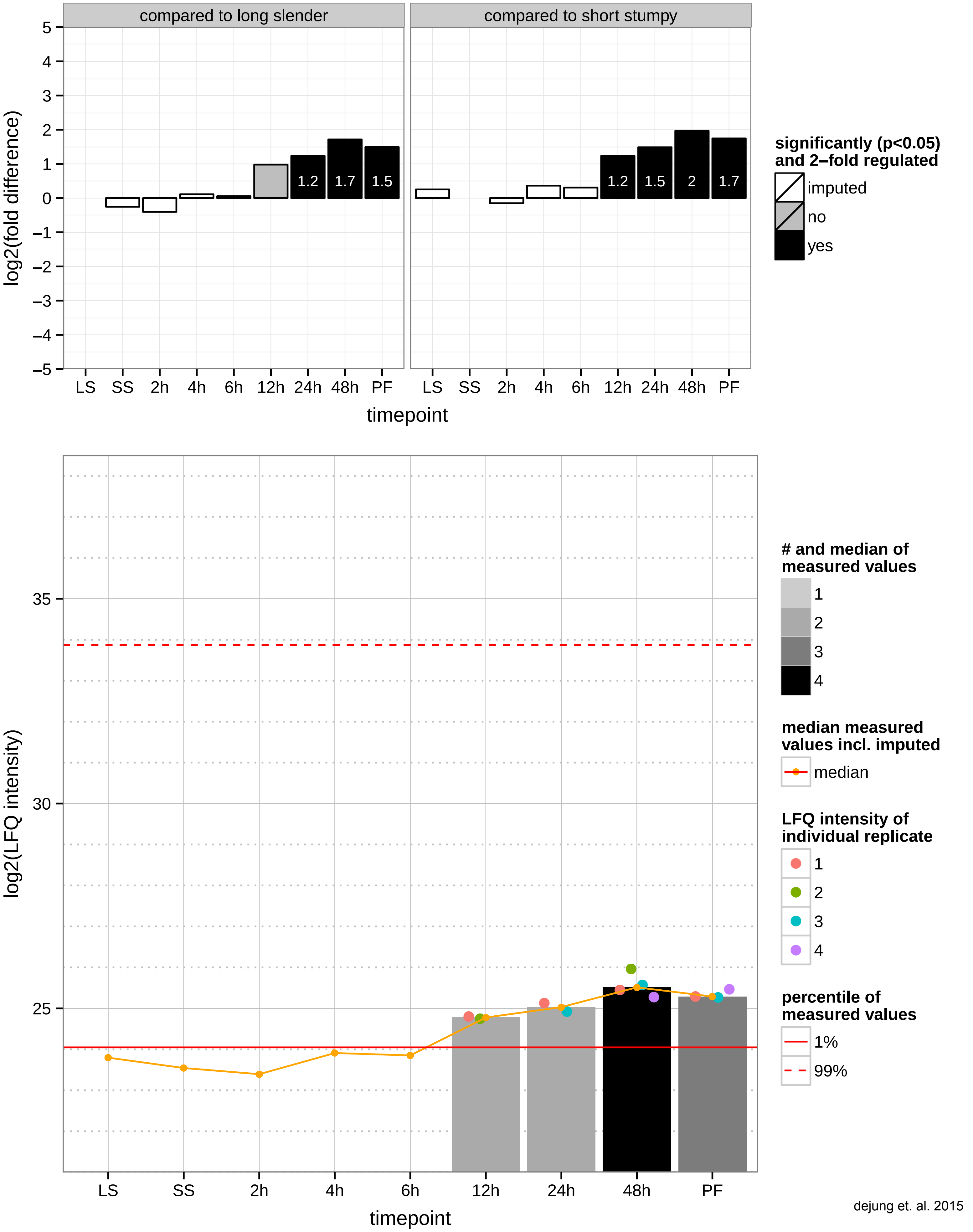

dejung et. al. 2015

hypothetical protein, conserved
Tb927.11.14220
AGOF: null
AGOC: null
AGOP: null
PGOF: null
PGOC: null
PGOP: null

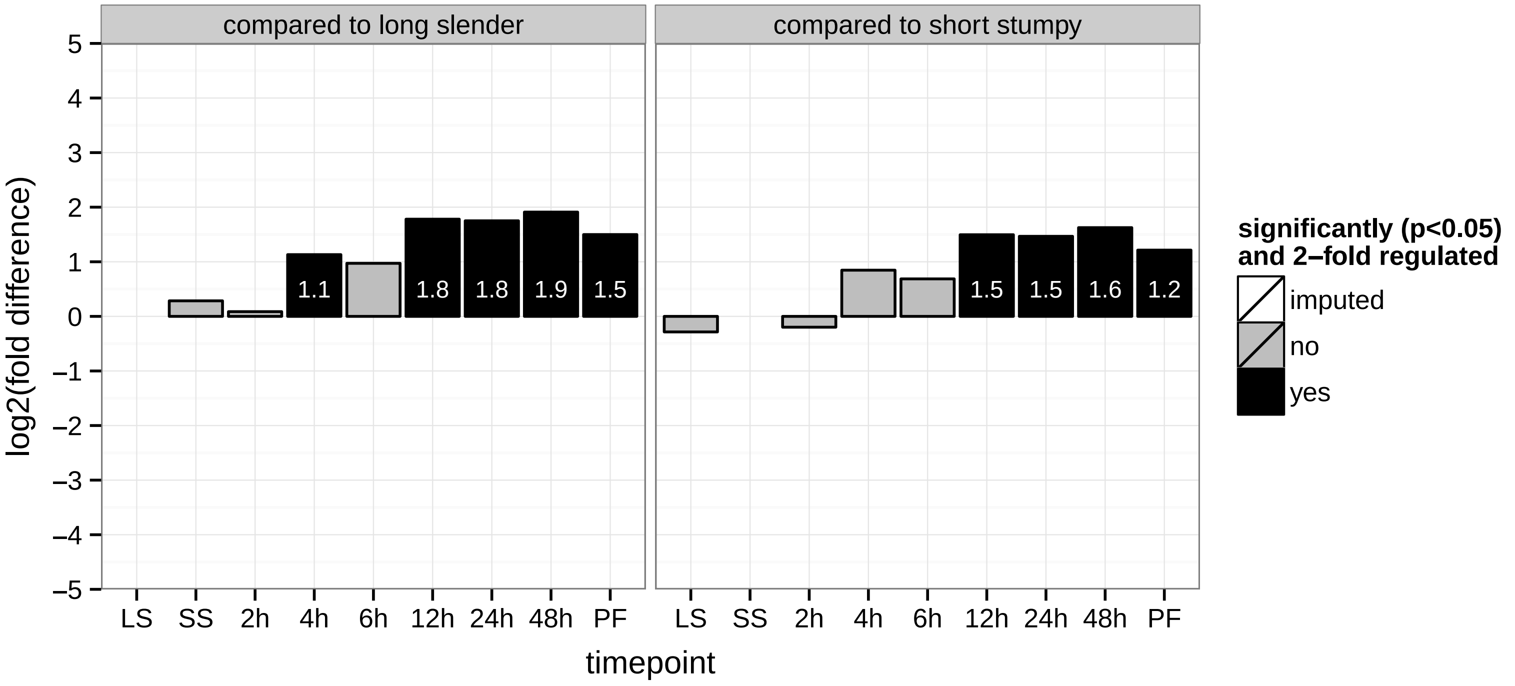

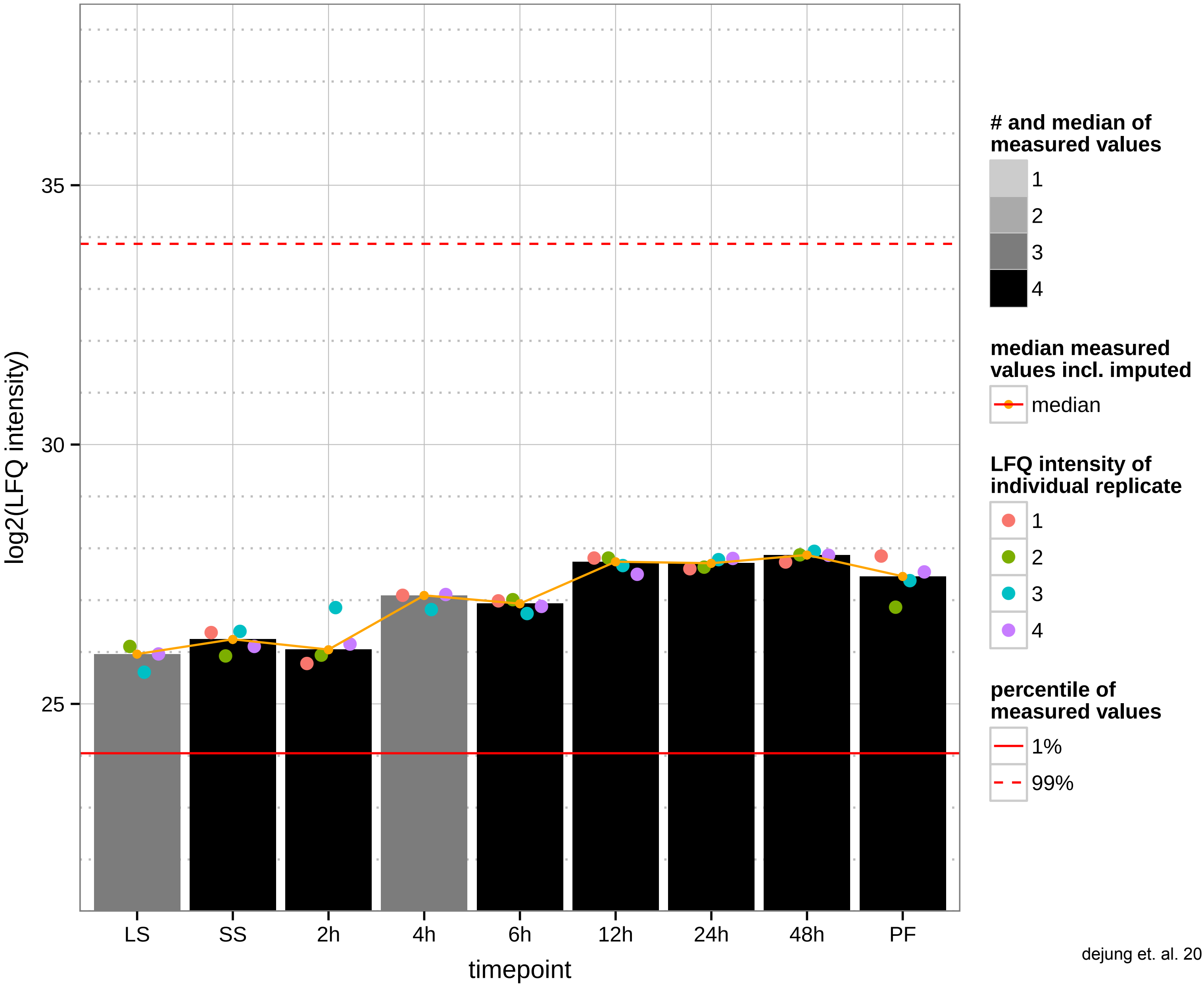

dejung et. al. 2015

hypothetical protein, conserved
Tb927.11.14760
AGOF: null
AGOC: null
AGOP: null
PGOF: null
PGOC: null
PGOP: null

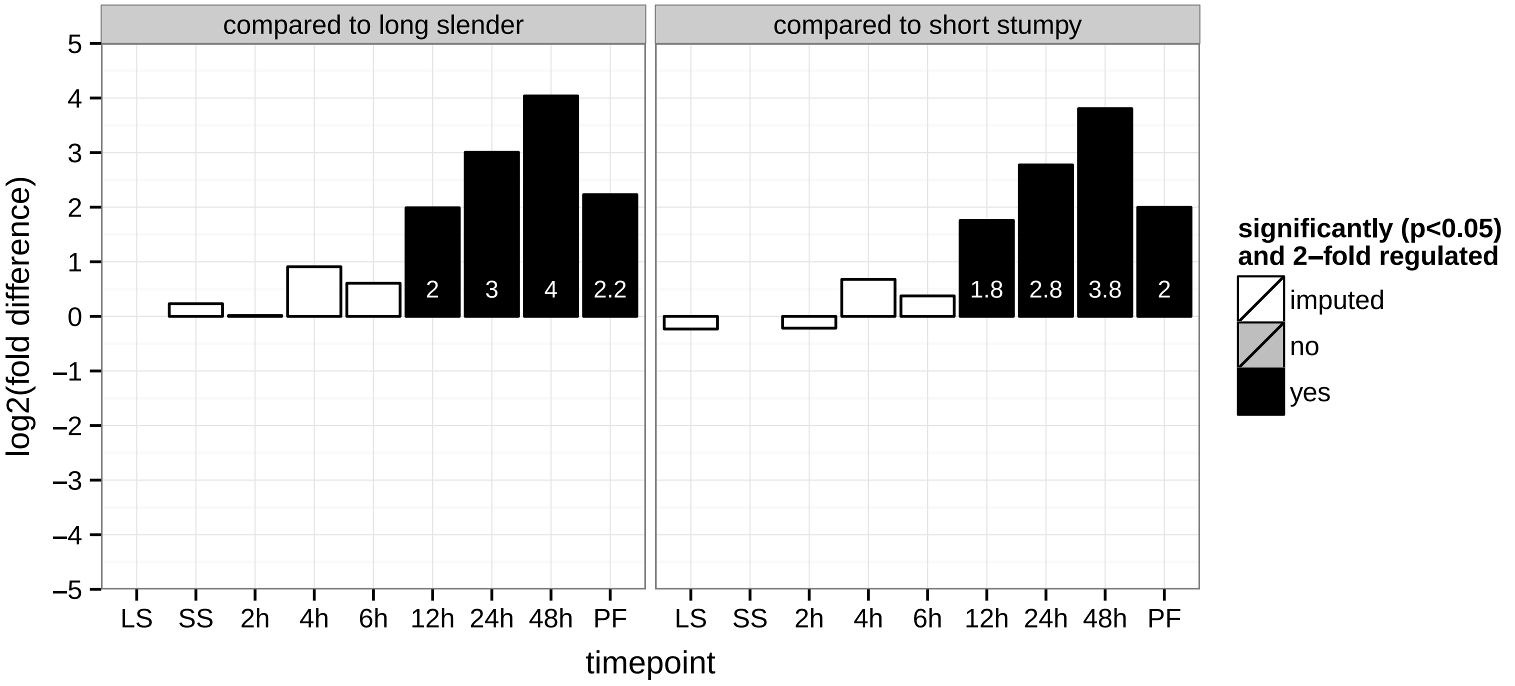

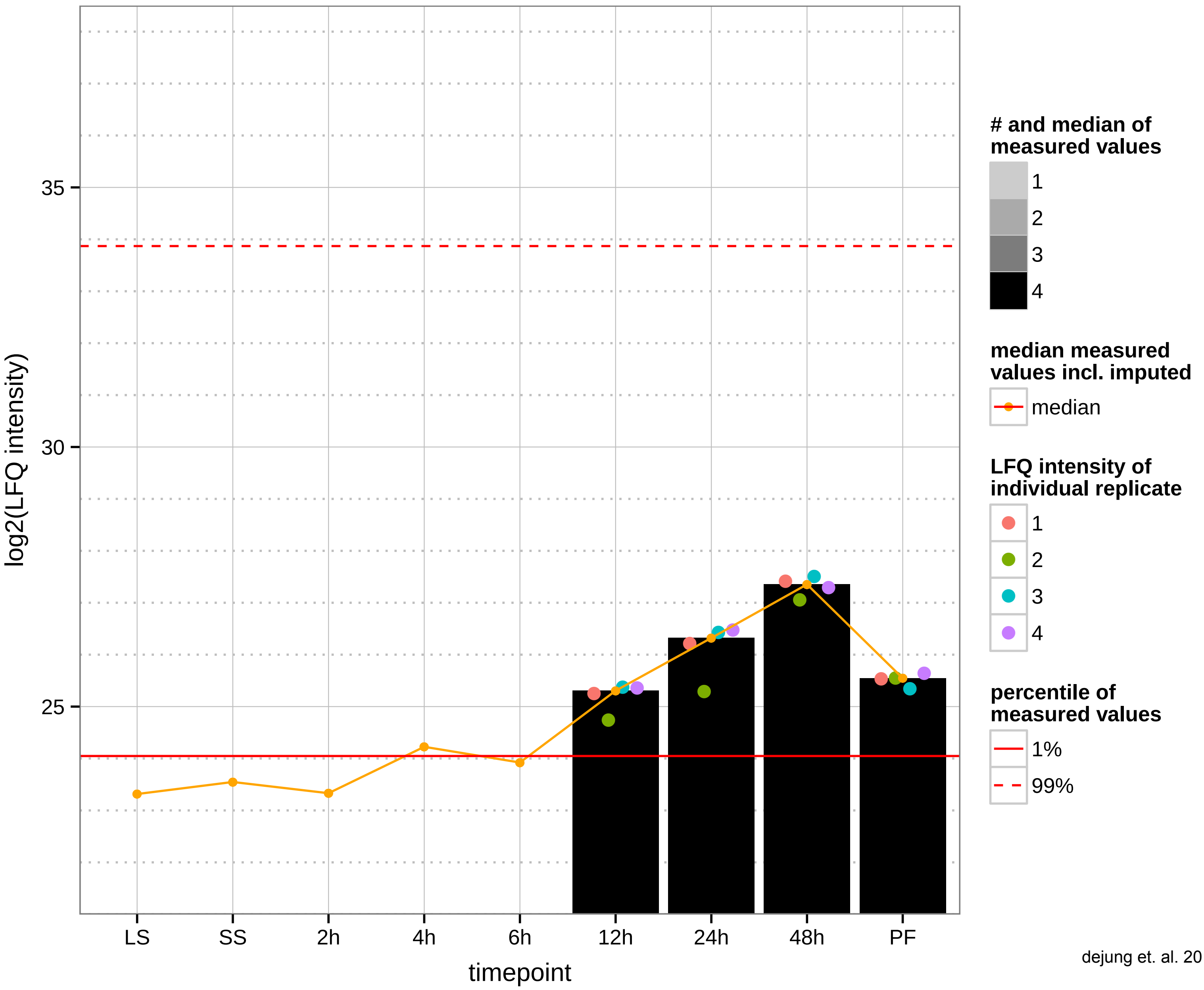

dejung et. al. 2015

phosphomannose isomerase, putative
Tb927.11.14780
AGOF: mannose−6−phosphate isomerase activity, zinc ion binding
AGOC: cytoplasm
AGOP: carbohydrate metabolic process
PGOF: mannose−6−phosphate isomerase activity, zinc ion binding
PGOC: null
PGOP: carbohydrate metabolic process

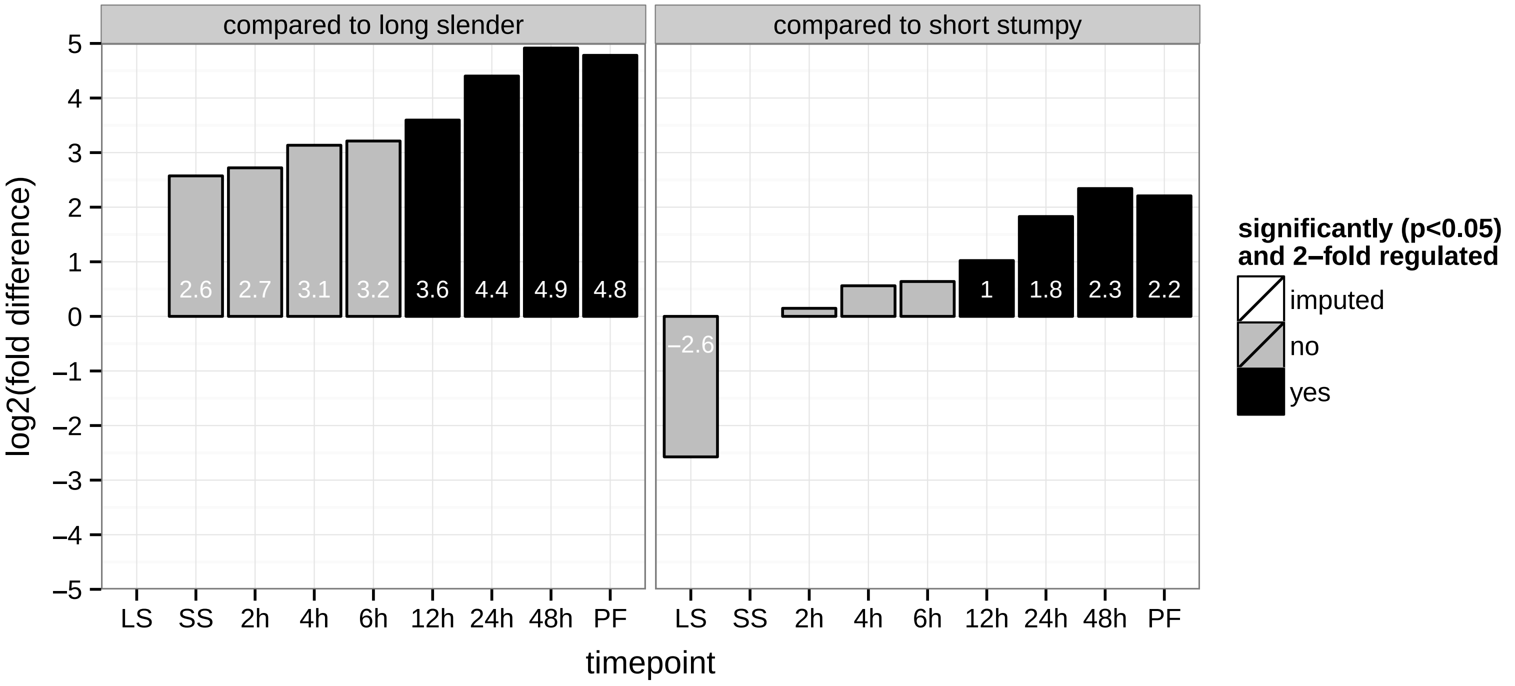

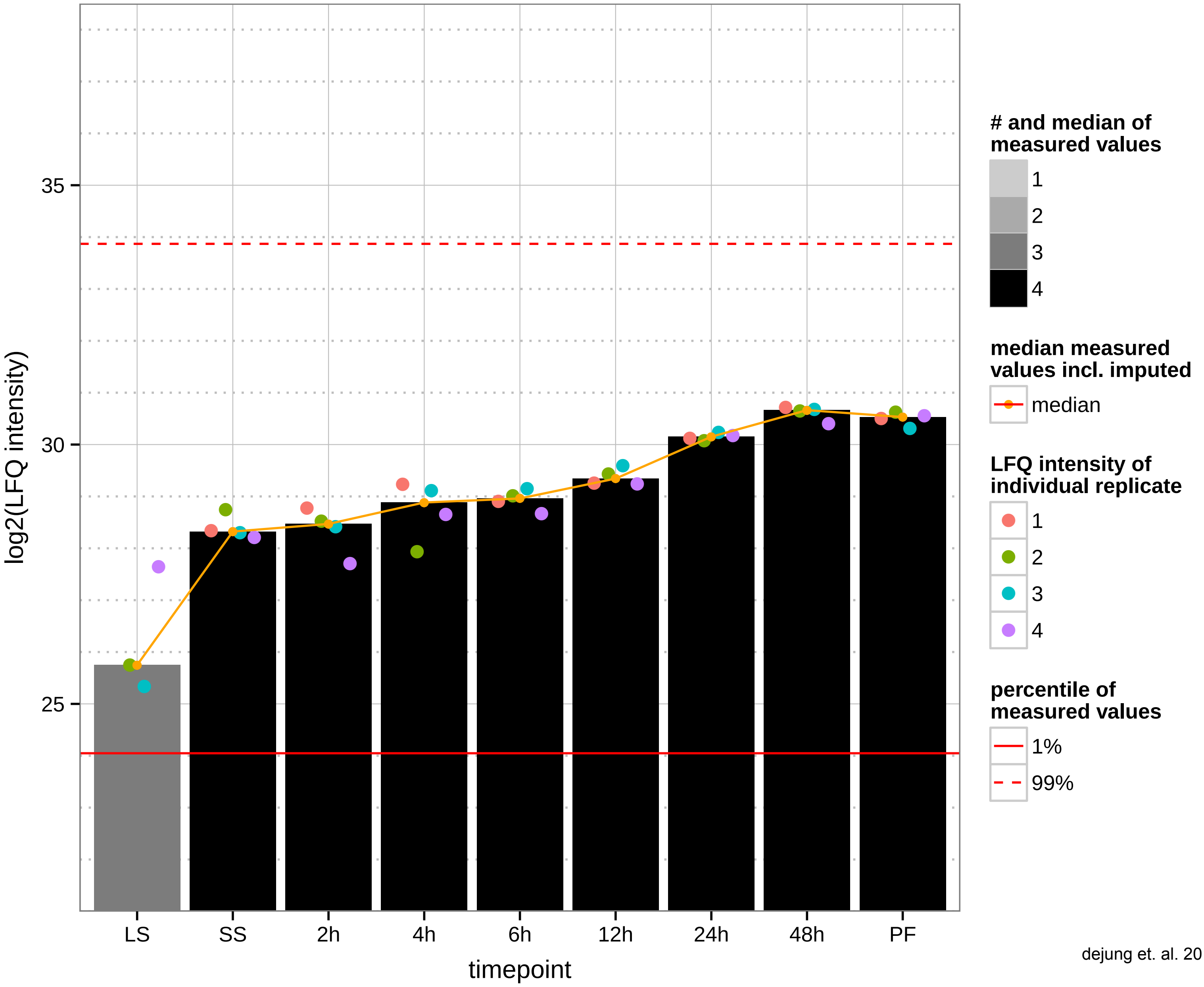

dejung et. al. 2015

iron superoxide dismutase (TbSODB)
Tb927.11.15020
AGOF: metal ion binding, superoxide dismutase activity
AGOC: glycosome
AGOP: oxidation–reduction process, superoxide metabolic process
PGOF: metal ion binding, superoxide dismutase activity
PGOC: null
PGOP: oxidation–reduction process, superoxide metabolic process

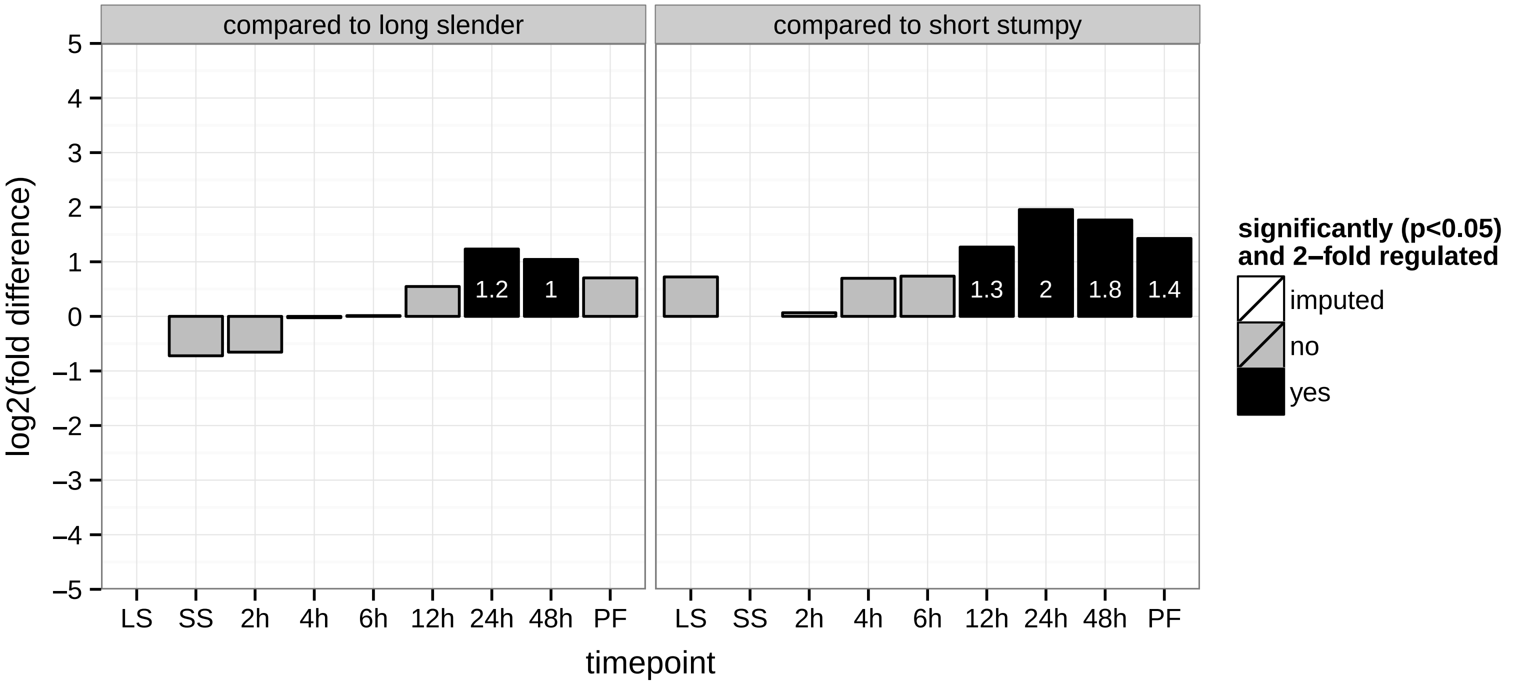

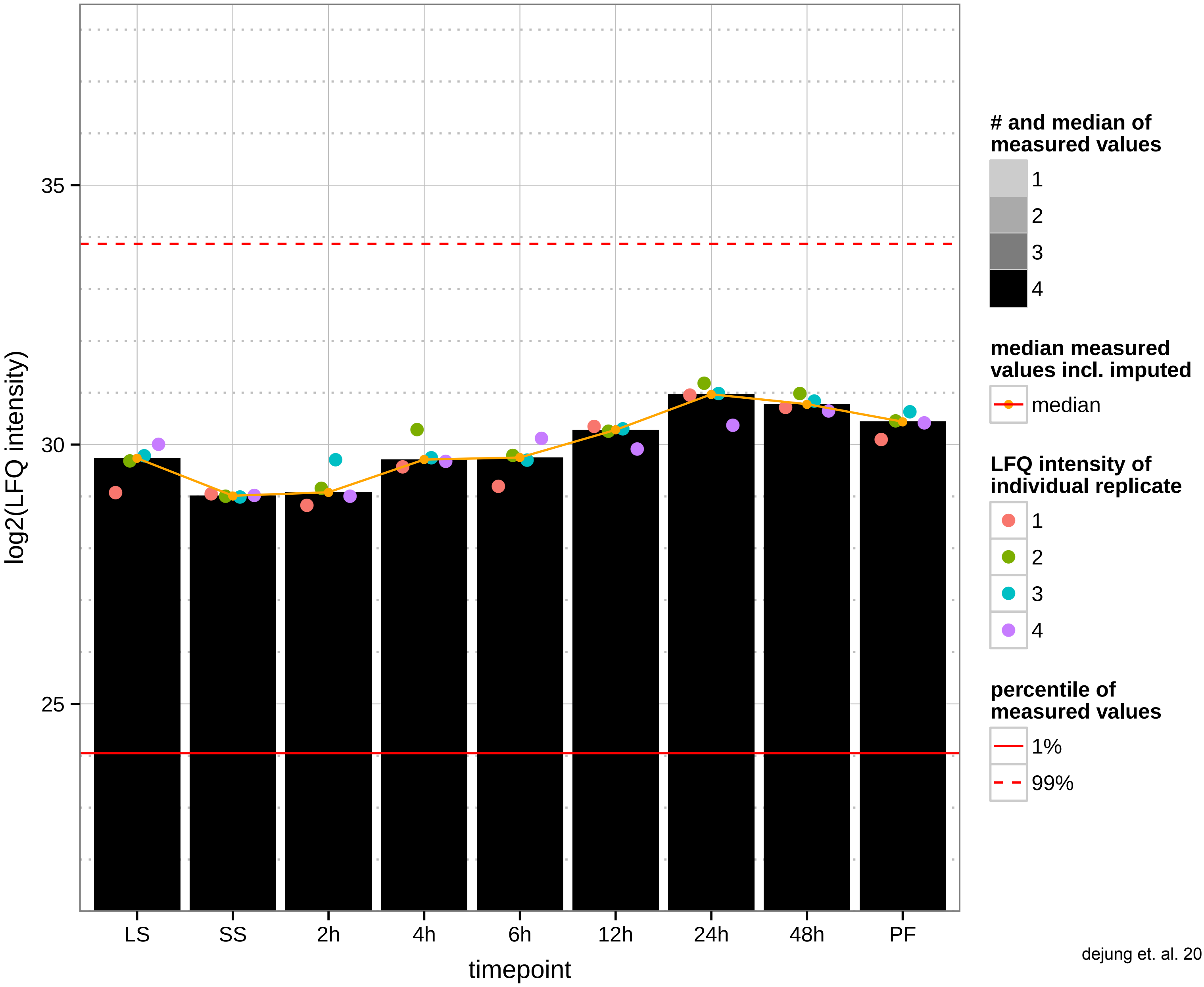

dejung et. al. 2015

hypothetical protein
Tb927.11.15450
AGOF: null
AGOC: null
AGOP: null
PGOF: null
PGOC: null
PGOP: null

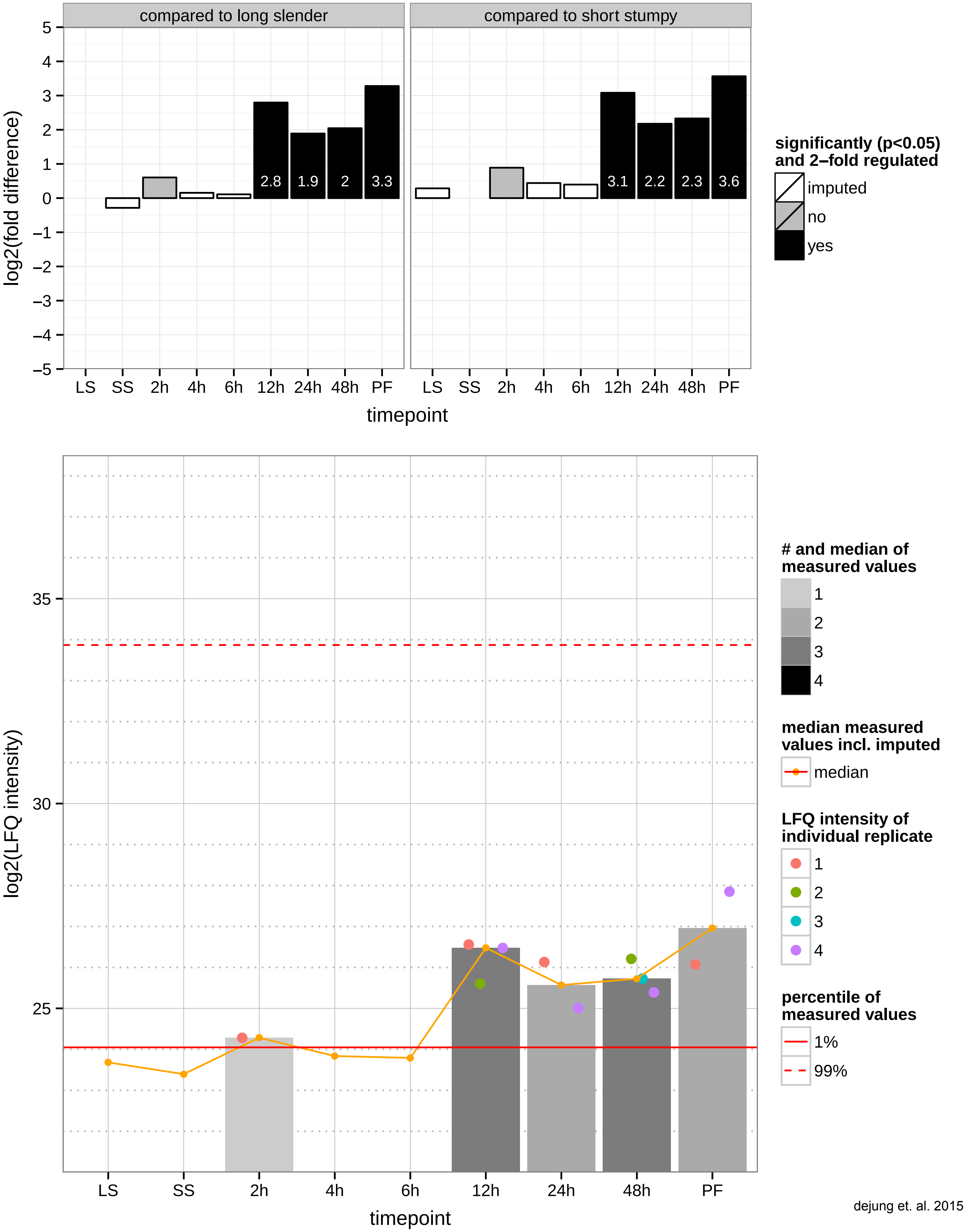

dejung et. al. 2015

NADH−cytochrome b5 reductase, putative (B5R)
Tb927.11.15550
AGOF: cytochrome−b5 reductase activity, electron carrier activity
AGOC: null
AGOP: oxidation−reduction process
PGOF: oxidoreductase activity
PGOC: null
PGOP: oxidation−reduction process

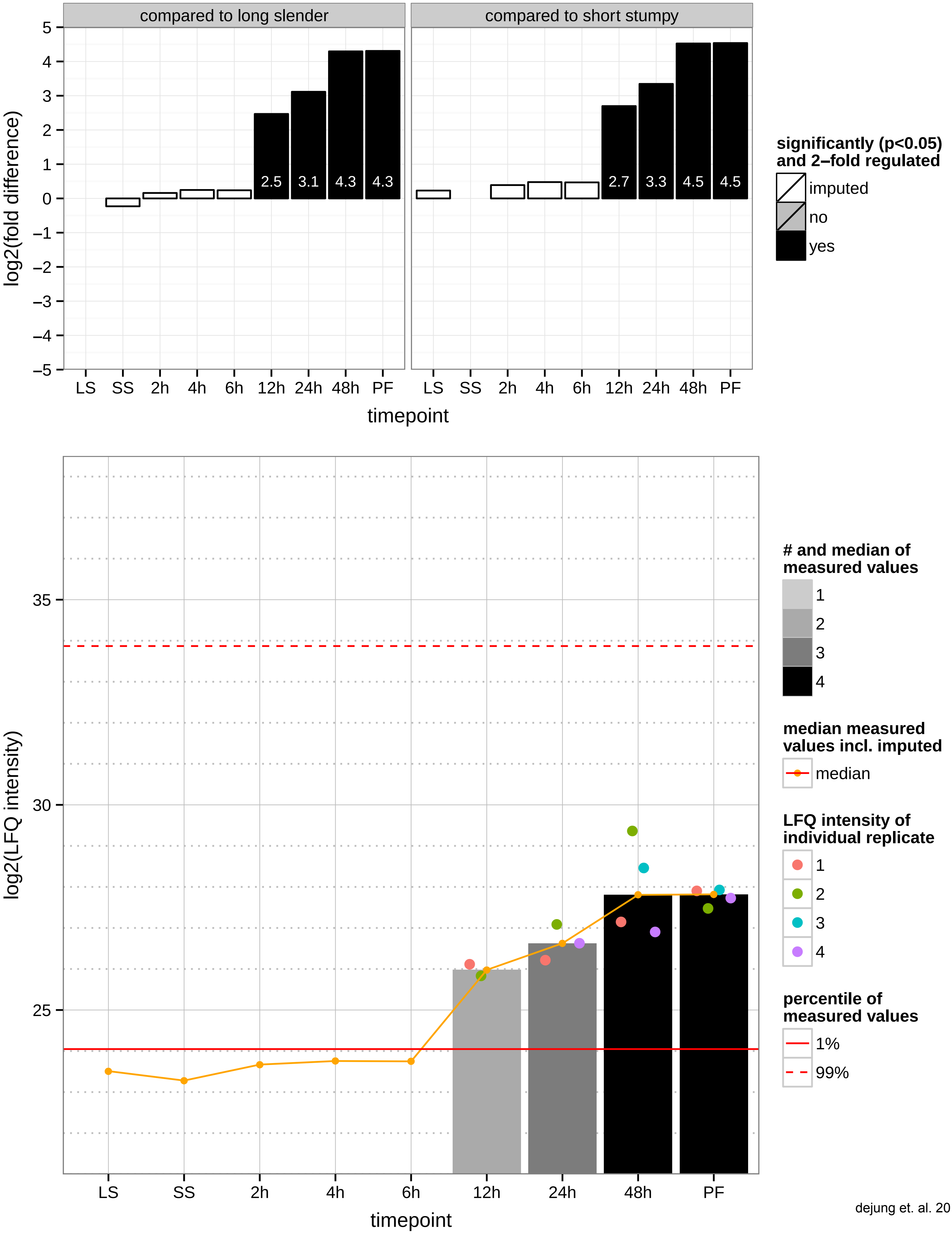

dejung et. al. 2015

superoxide dismutase, putative
Tb927.11.15820
AGOF: metal ion binding, superoxide dismutase activity
AGOC: mitochondrion
AGOP: oxidation–reduction process, superoxide metabolic process
PGOF: metal ion binding, superoxide dismutase activity
PGOC: null
PGOP: oxidation–reduction process, superoxide metabolic process

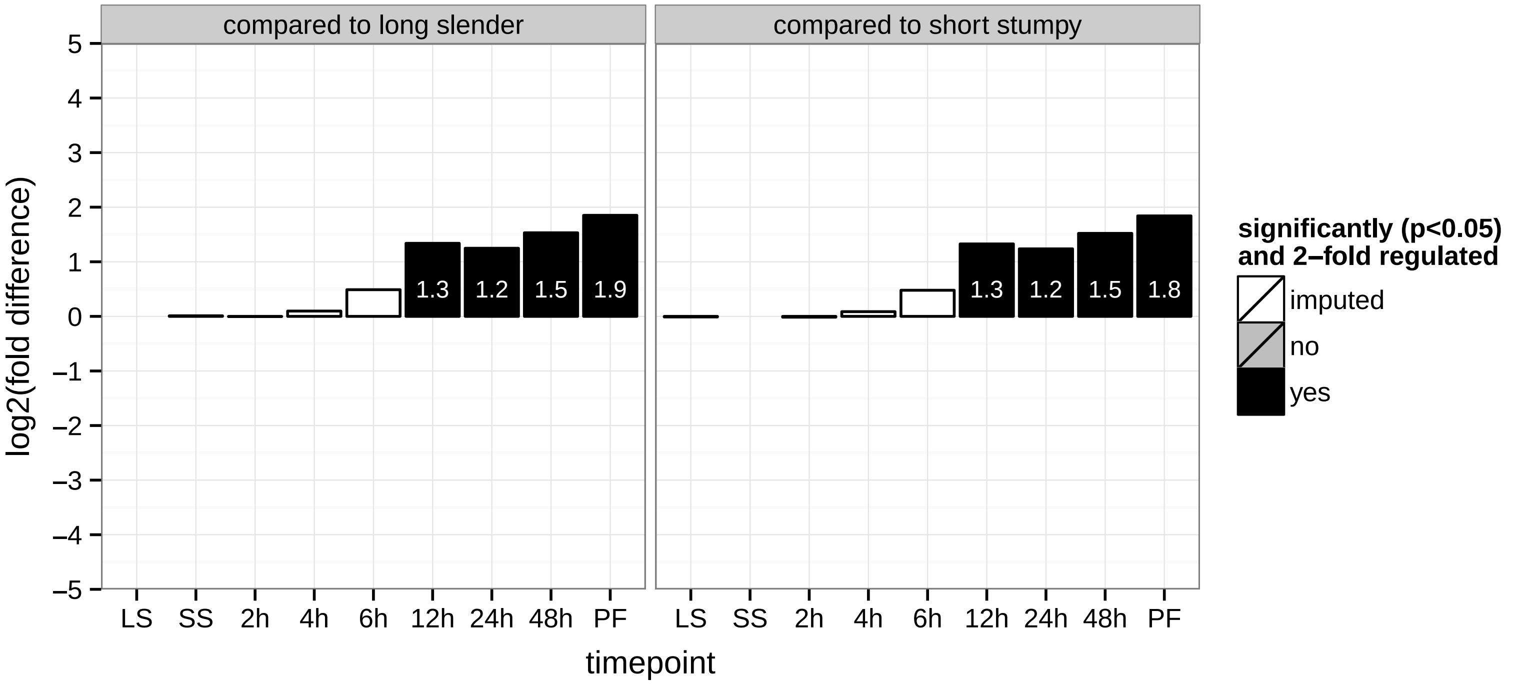

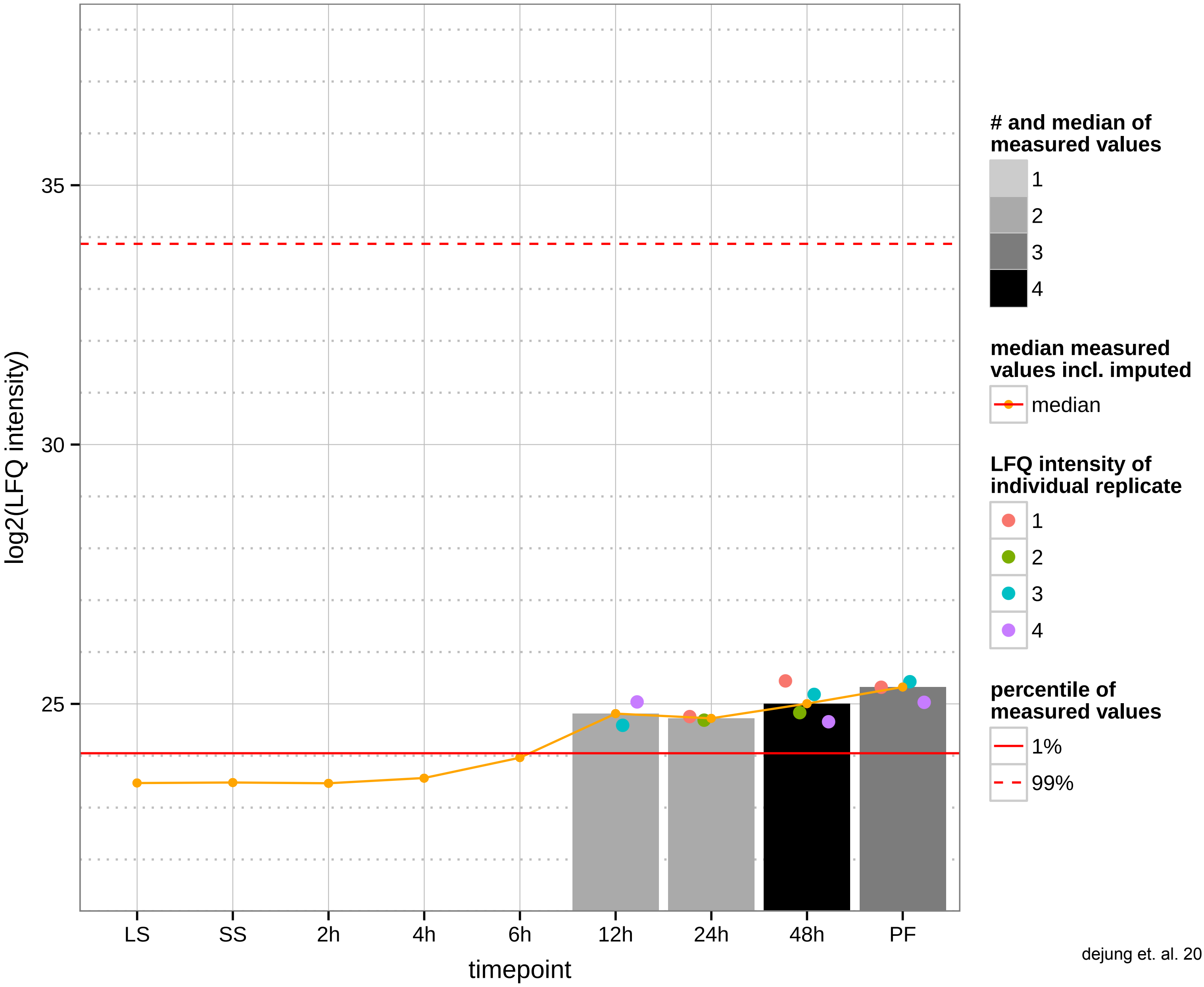

dejung et. al. 2015

RNA–binding protein, putative (DRBD10)
Tb927.11.16020
AGOF: RNA binding
AGOC: null
AGOP: RNA processing
PGOF: nucleic acid binding
PGOC: null
PGOP: null

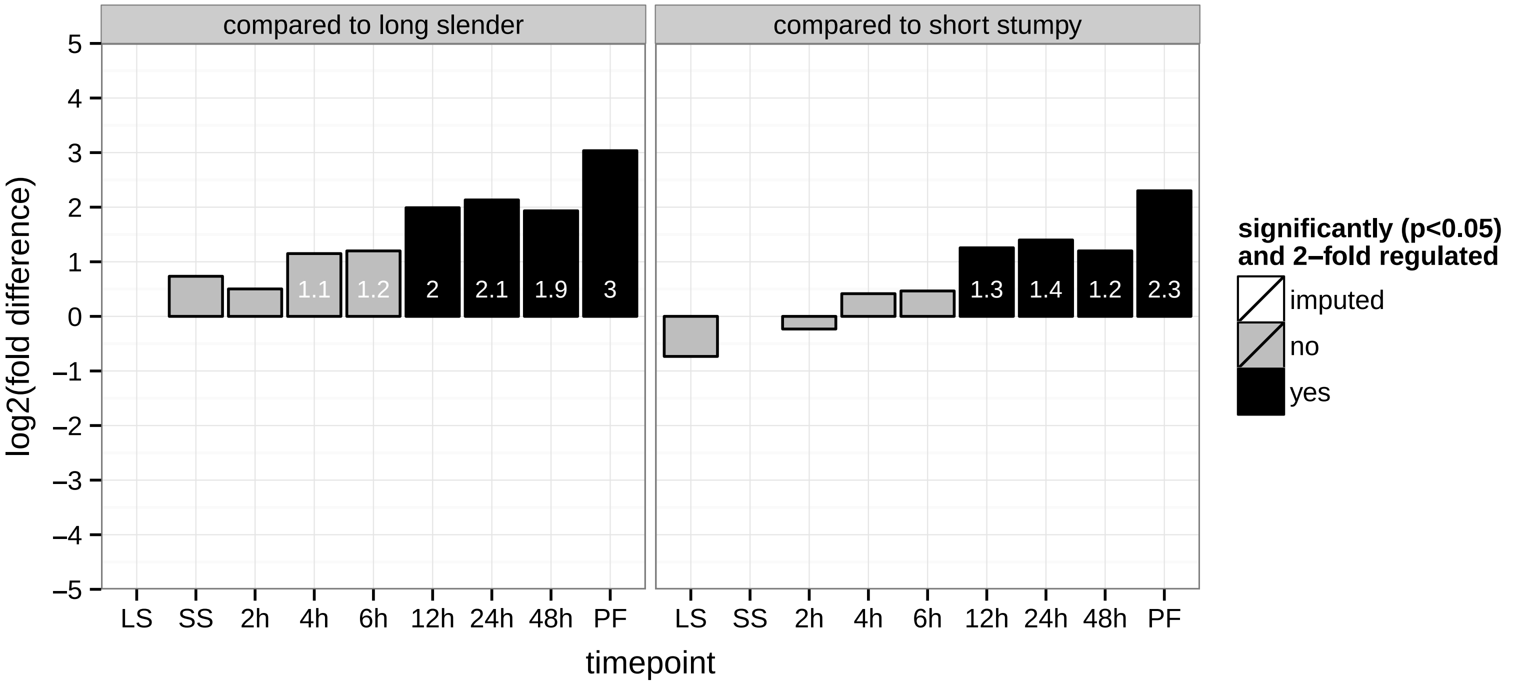

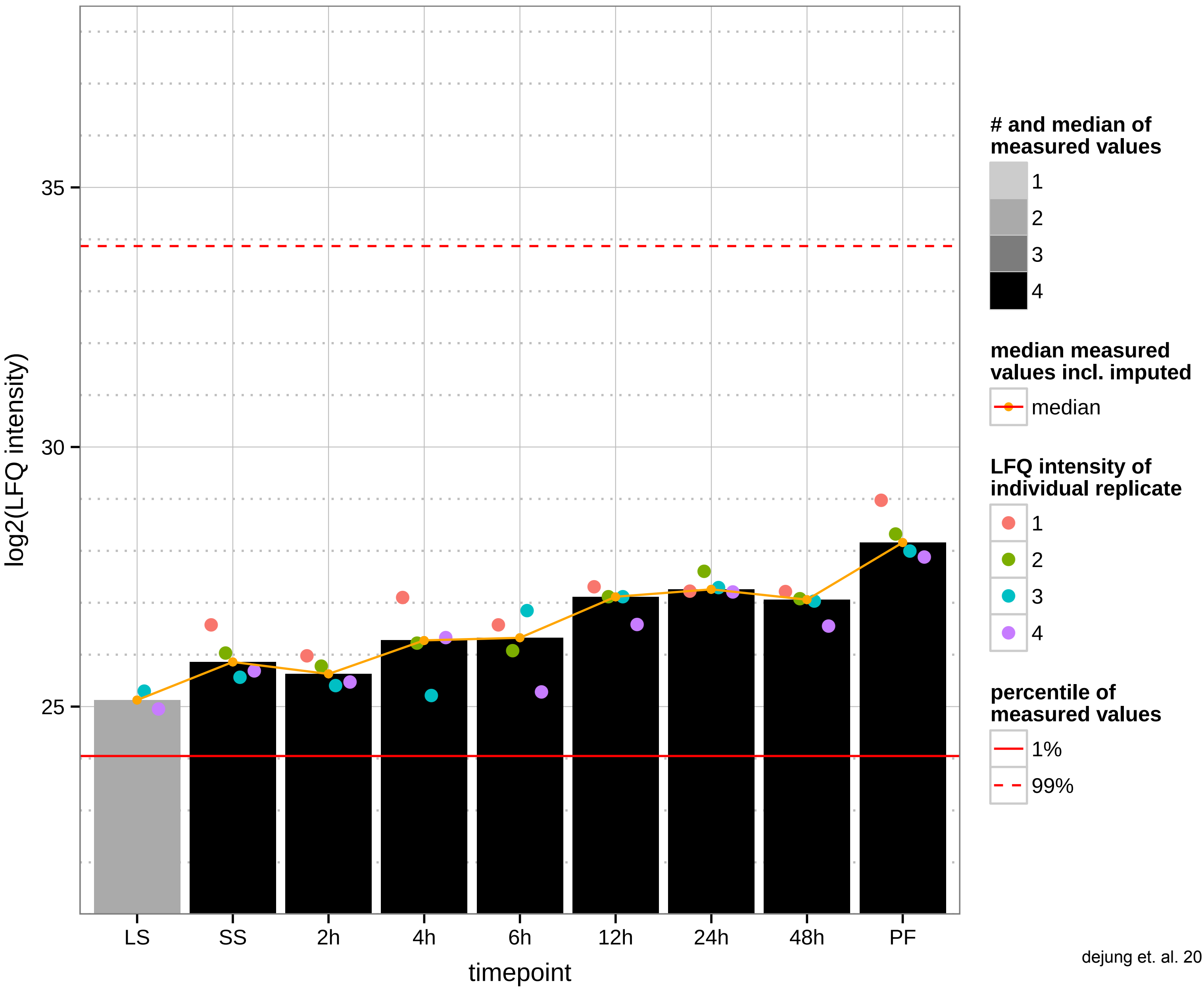

dejung et. al. 2015

hypothetical protein, conserved
Tb927.11.16220
AGOF: null
AGOC: mitochondrion
AGOP: null
PGOF: null
PGOC: null
PGOP: null

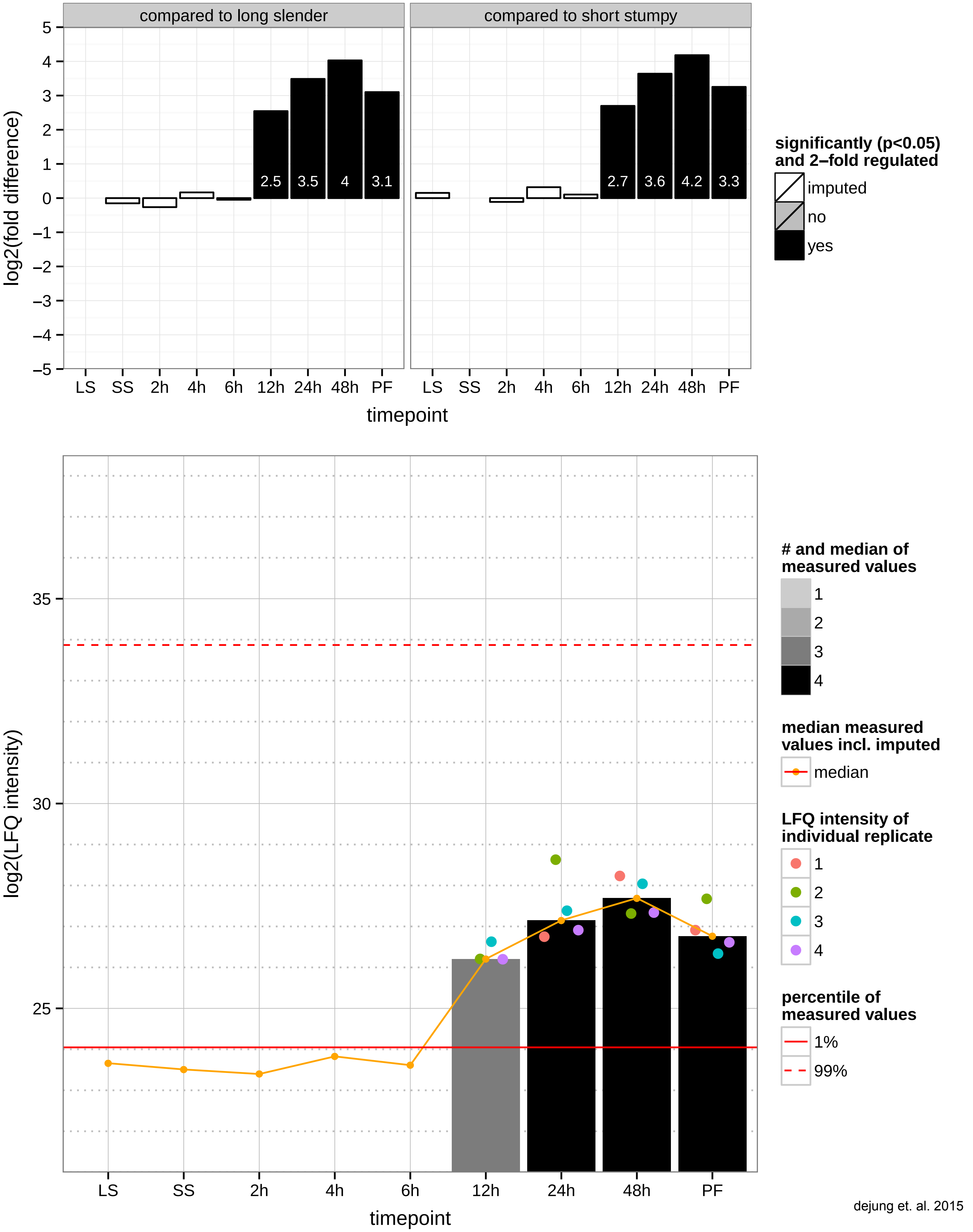

dejung et. al. 2015

hypothetical protein, conserved
Tb927.11.16340
AGOF: protein transporter activity
AGOC: cytoplasm, nuclear pore, nucleus
AGOP: intracellular protein transport, protein import into nucleus, docking
PGOF: binding, protein transporter activity
PGOC: null
PGOP: intracellular protein transport

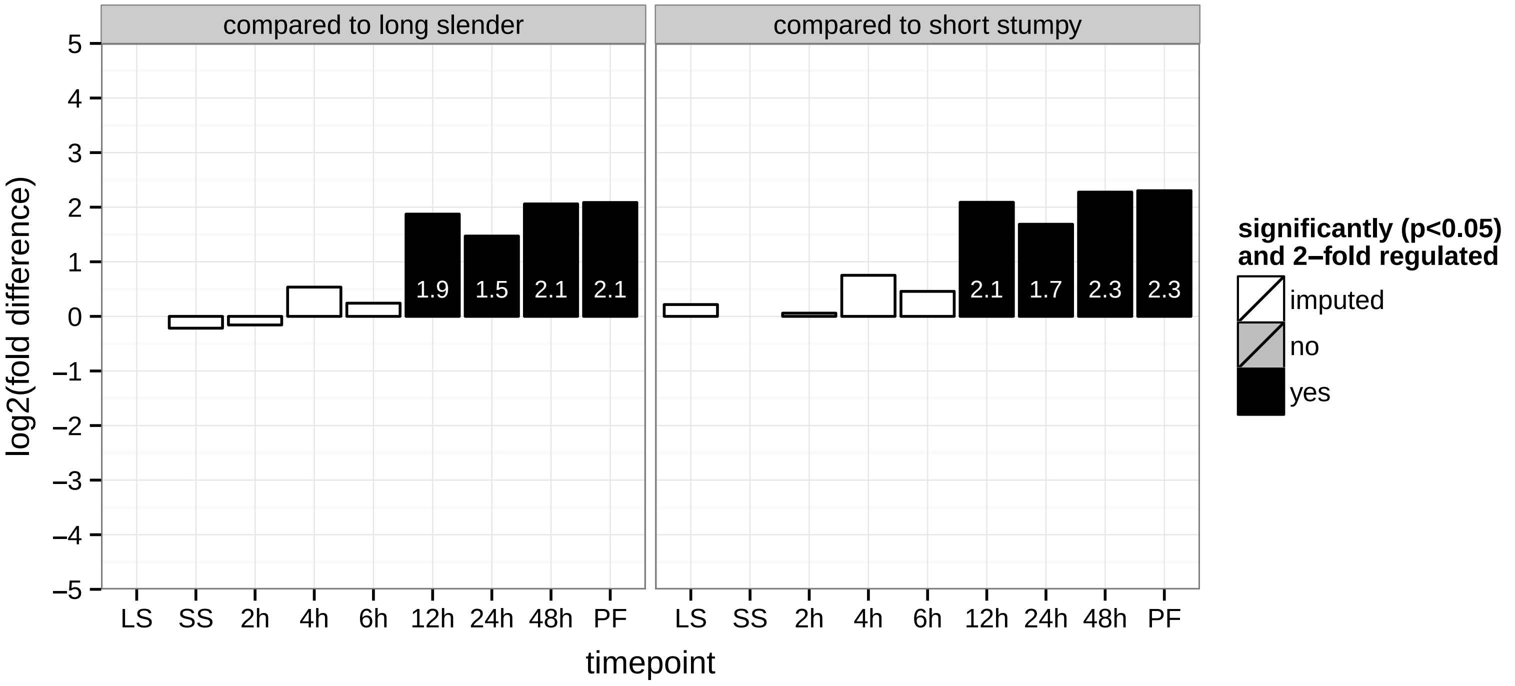

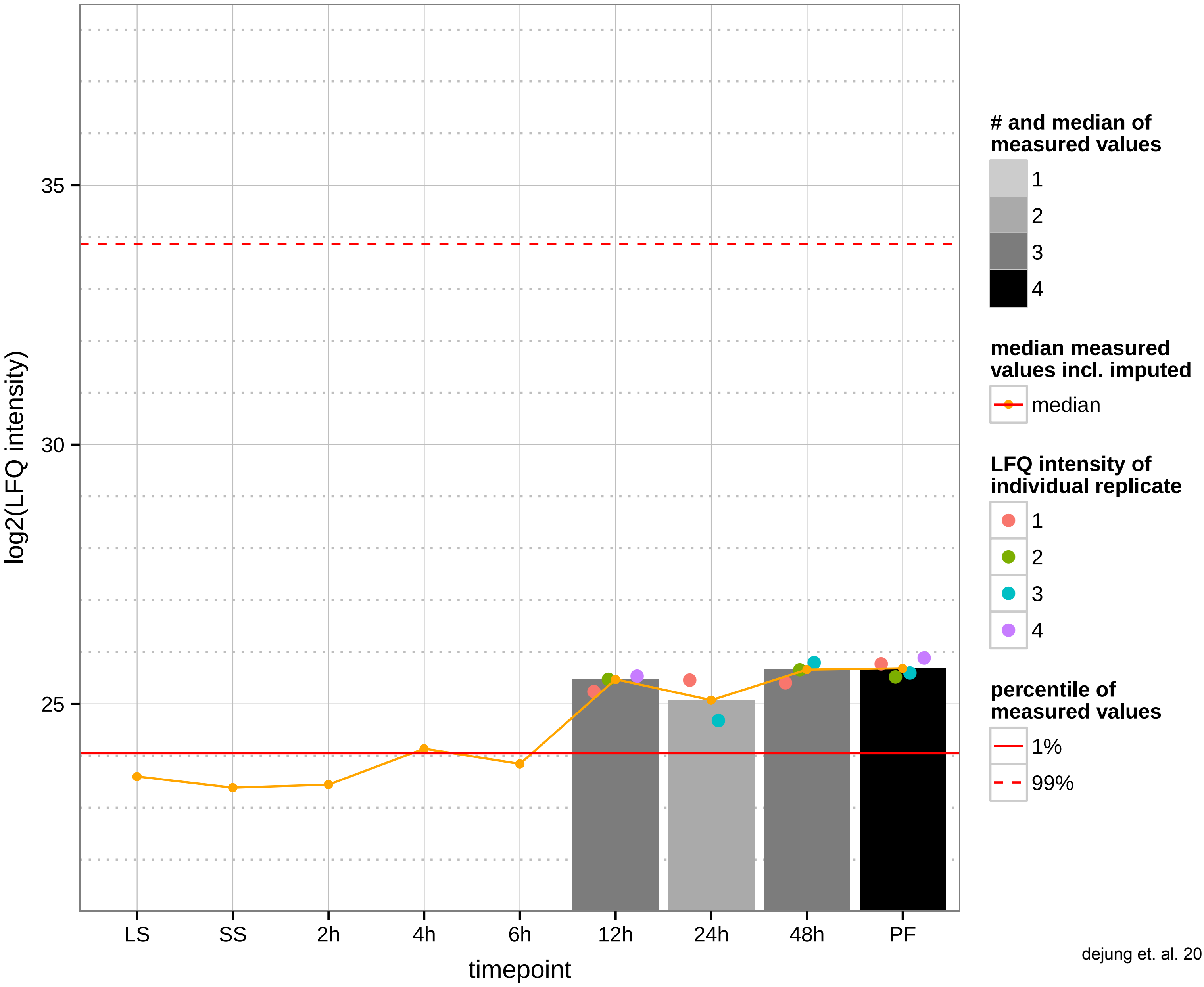

dejung et. al. 2015

hypothetical protein, conserved
Tb927.11.16450
AGOF: null
AGOC: null
AGOP: null
PGOF: null
PGOC: null
PGOP: null

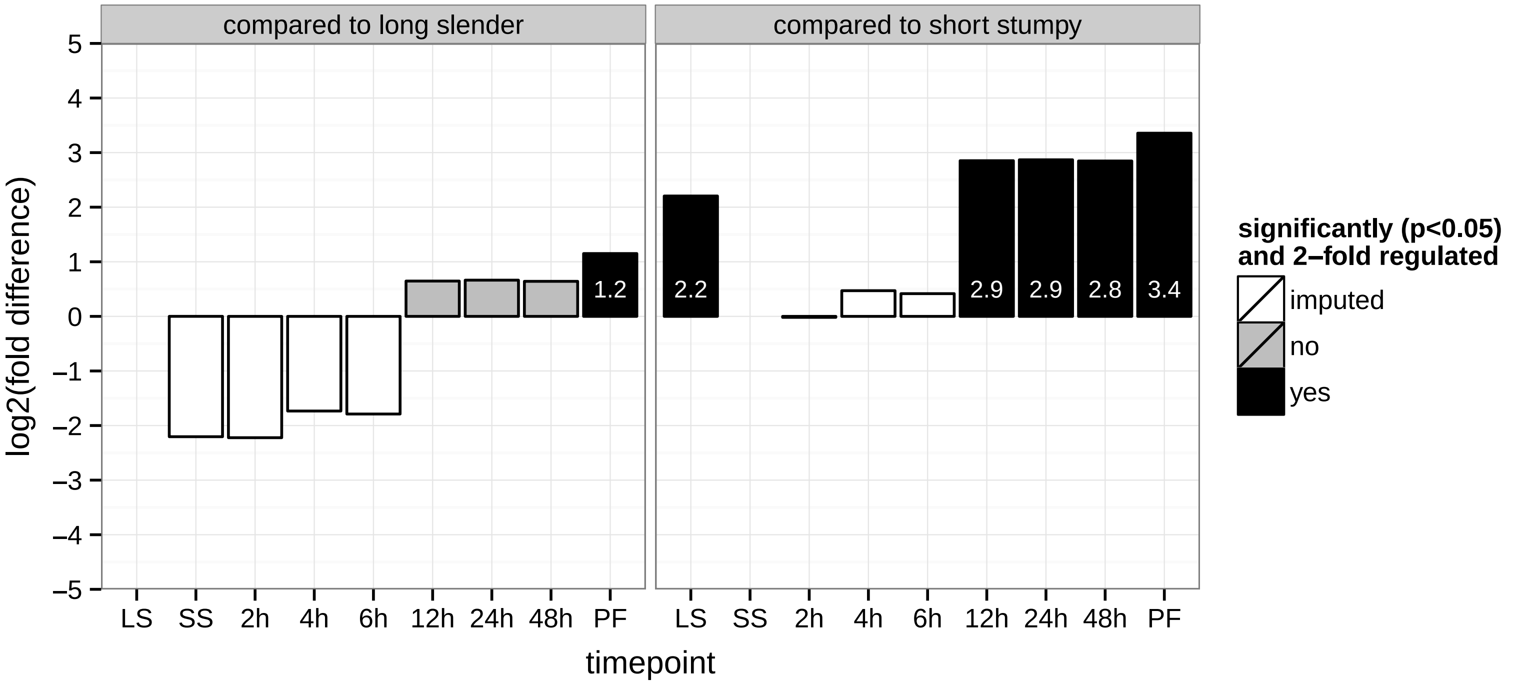

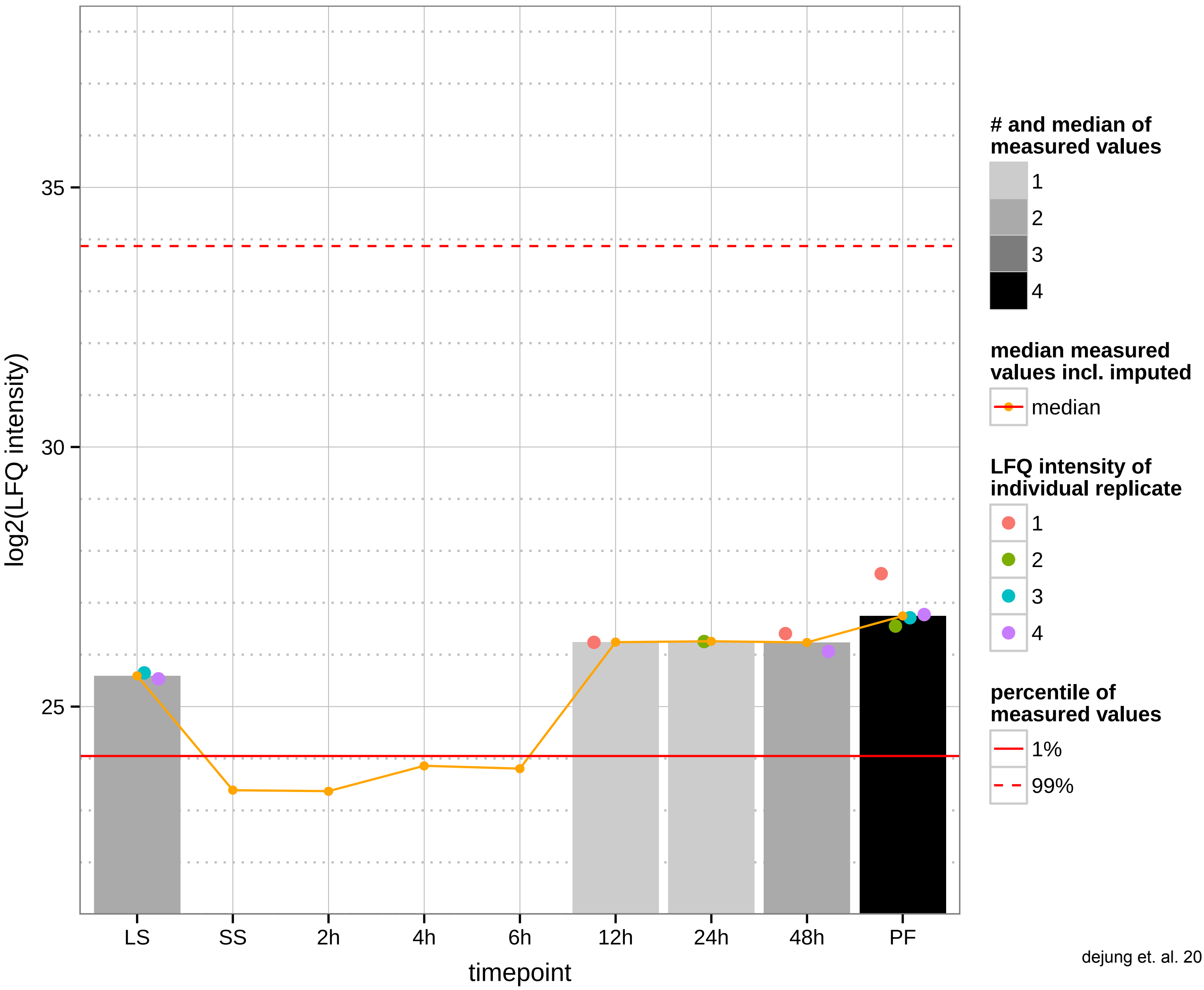

dejung et. al. 2015

hypothetical protein, conserved
Tb927.11.16510
AGOF: null
AGOC: mitochondrion
AGOP: null
PGOF: null
PGOC: null
PGOP: null

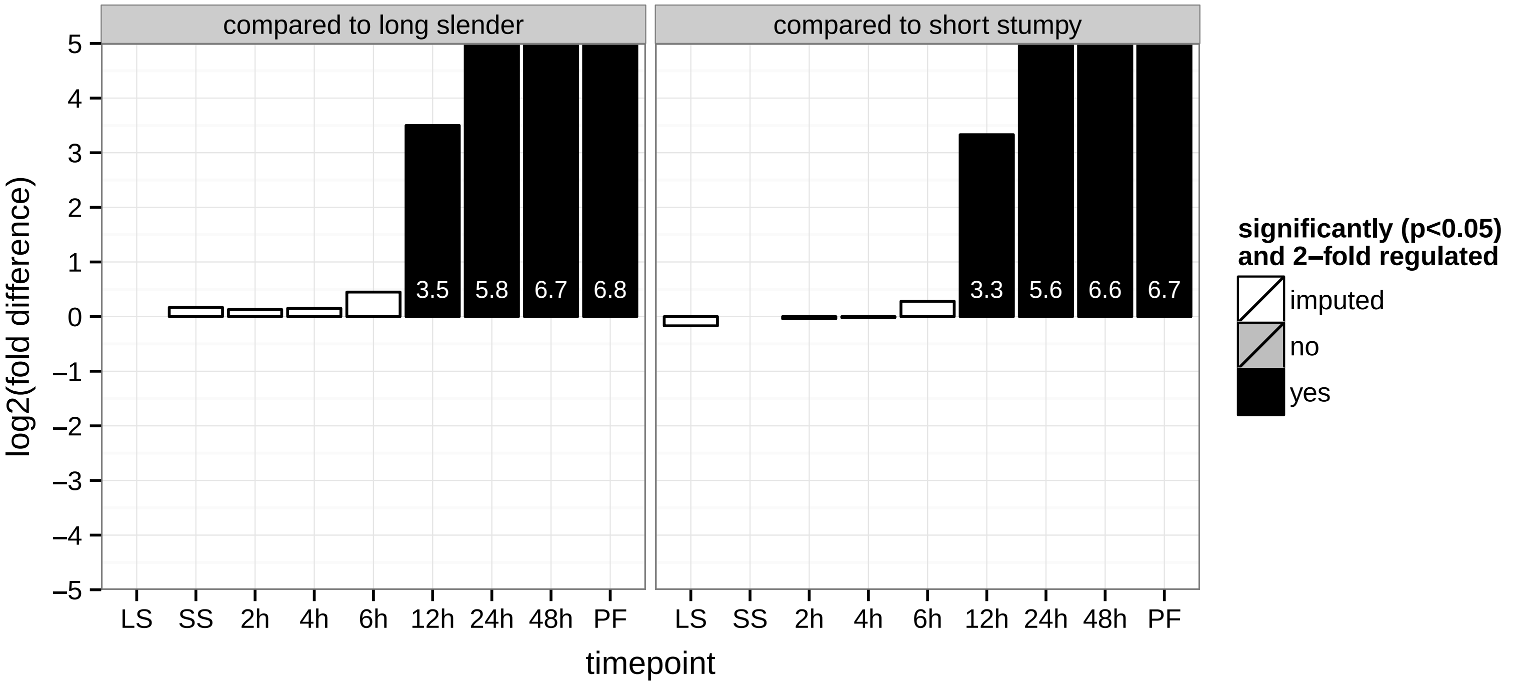

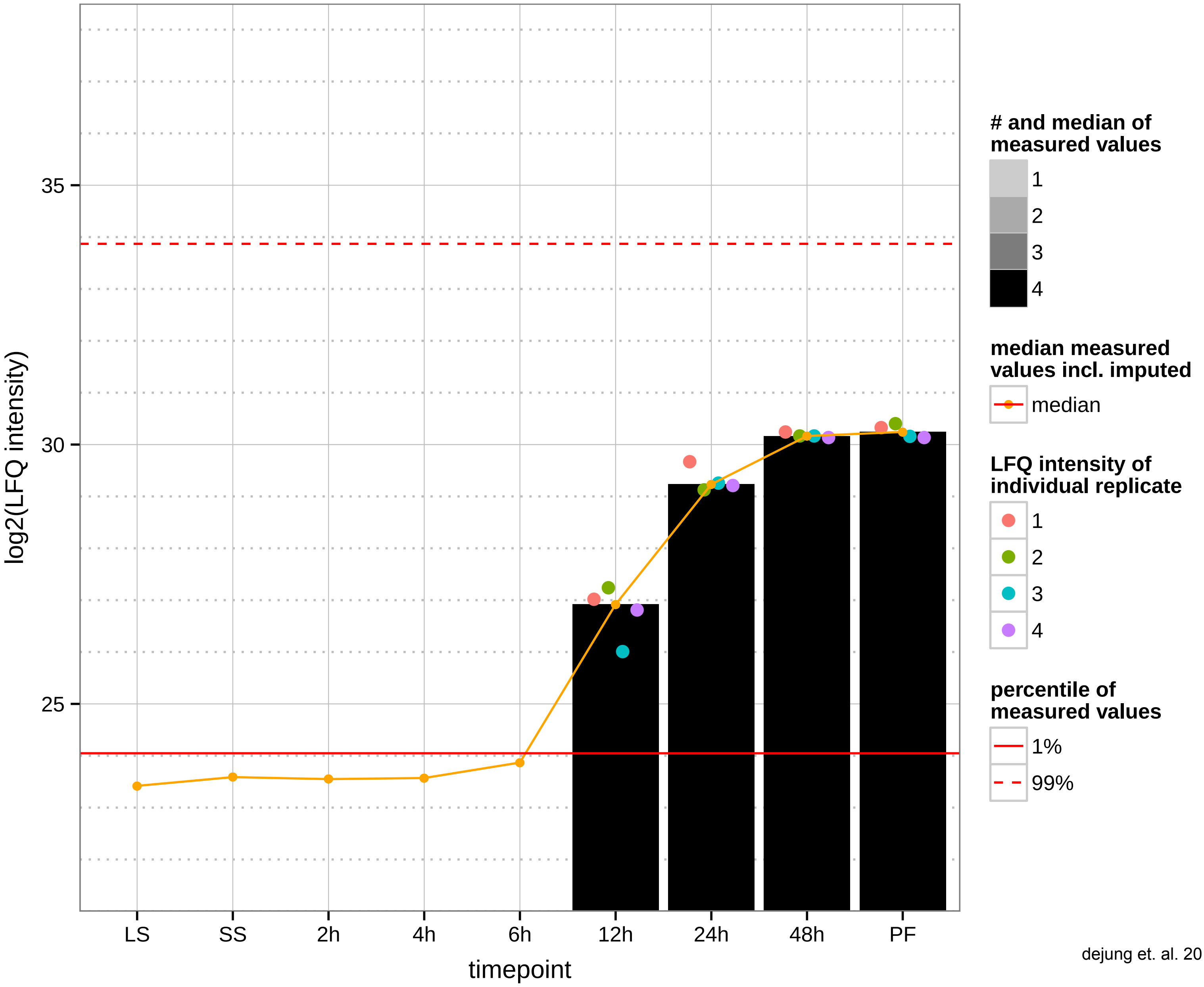

dejung et. al. 2015

electron transfer flavoprotein, putative
Tb927.11.180
AGOF: electron carrier activity
AGOC: mitochondrion
AGOP: oxidation−reduction process
PGOF: electron carrier activity
PGOC: null
PGOP: null

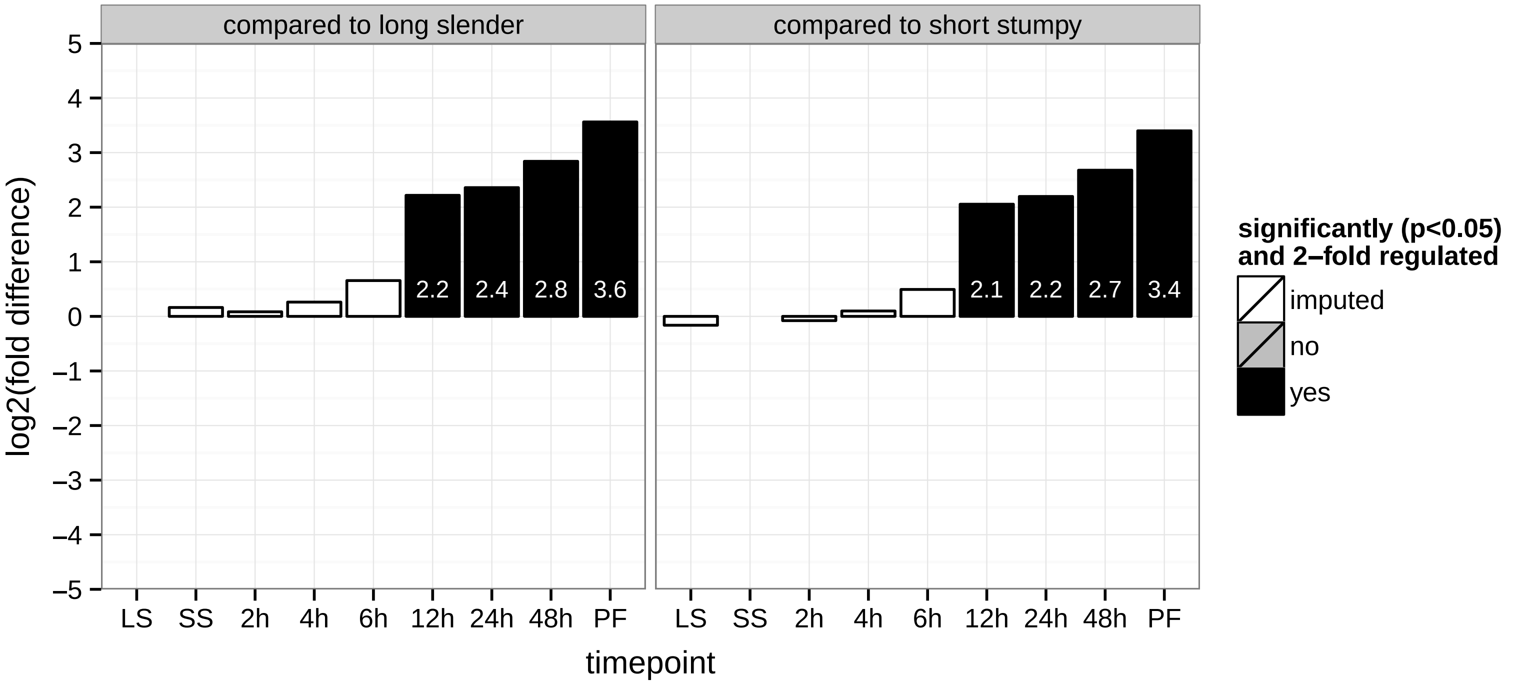

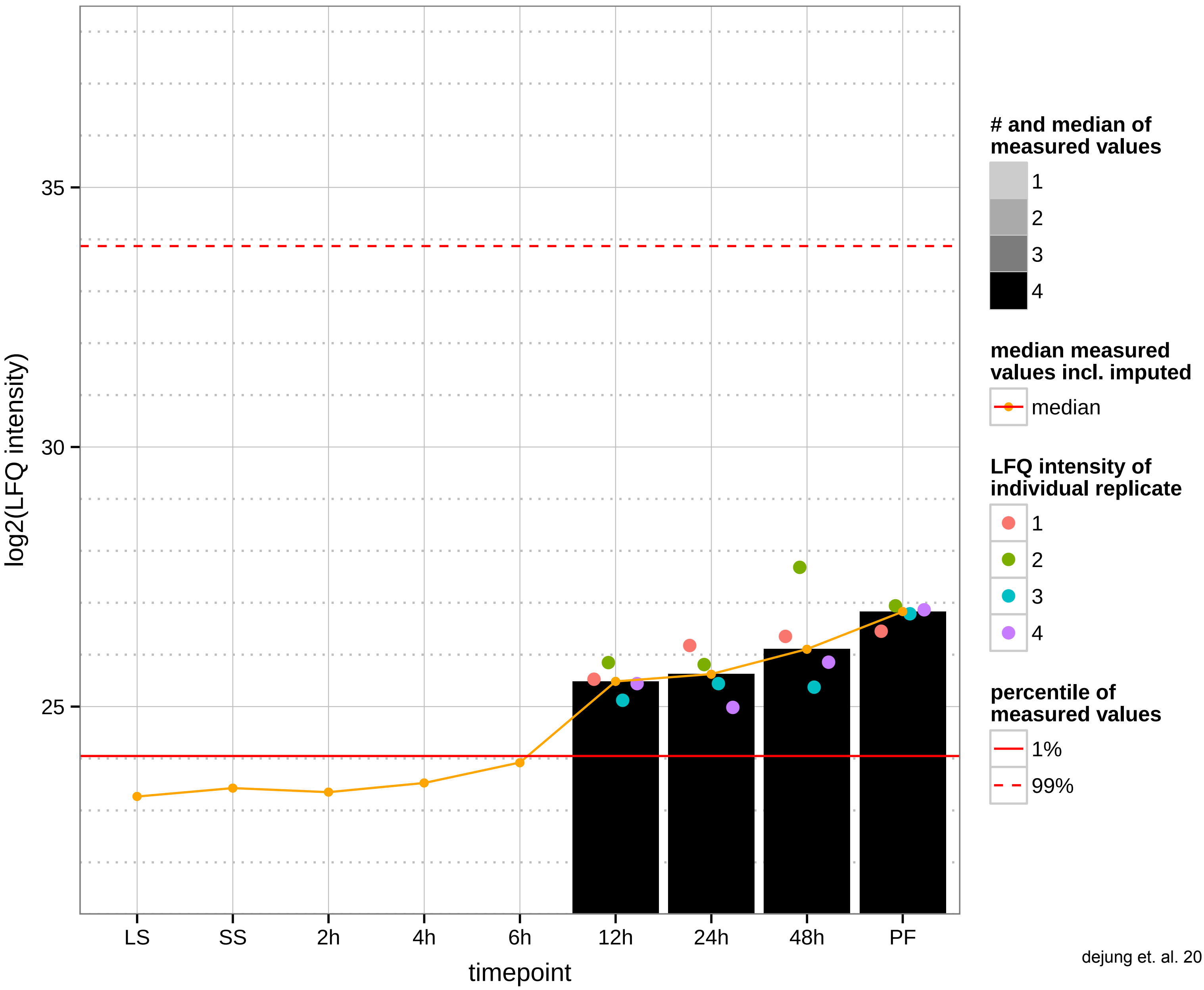

dejung et. al. 2015

elongation factor, putative
Tb927.11.190
AGOF: GTP binding, GTPase activity
AGOC: null
AGOP: null
PGOF: GTP binding, GTPase activity
PGOC: null
PGOP: null

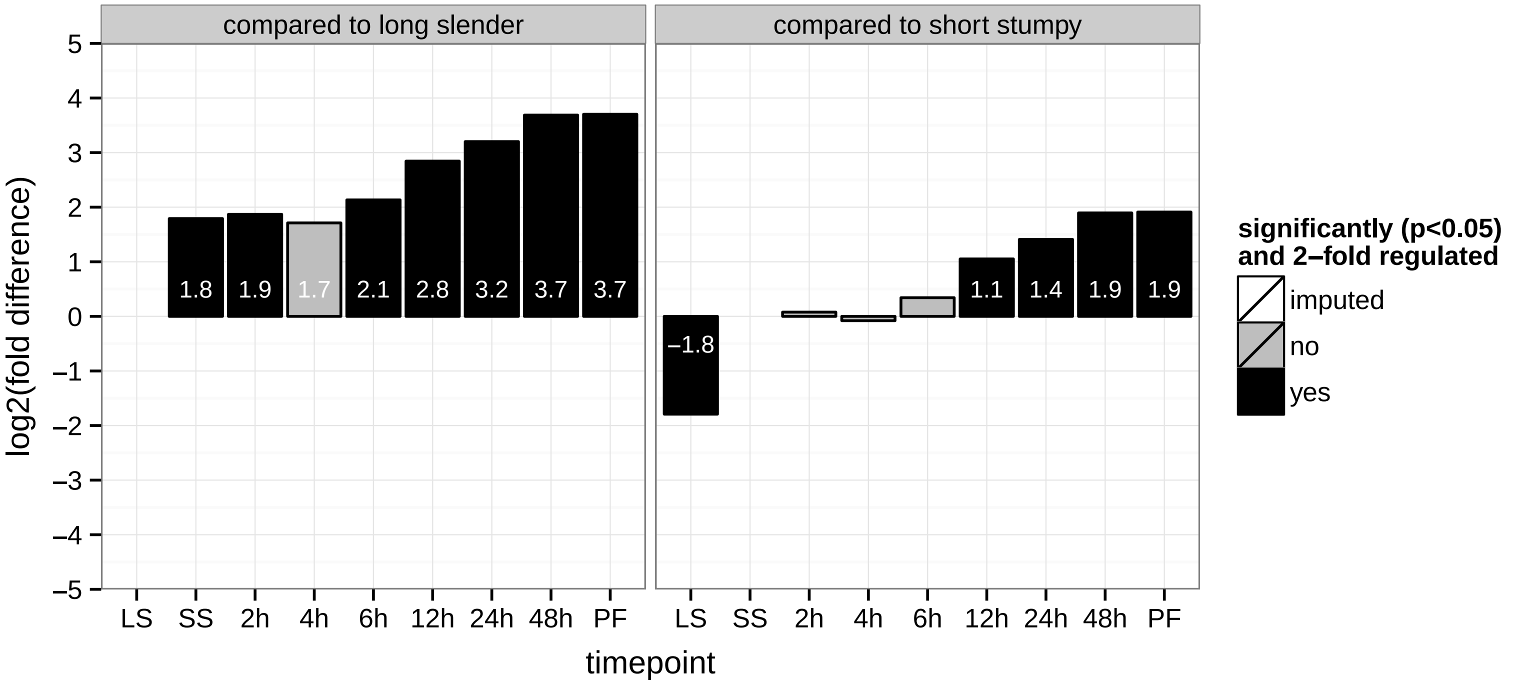

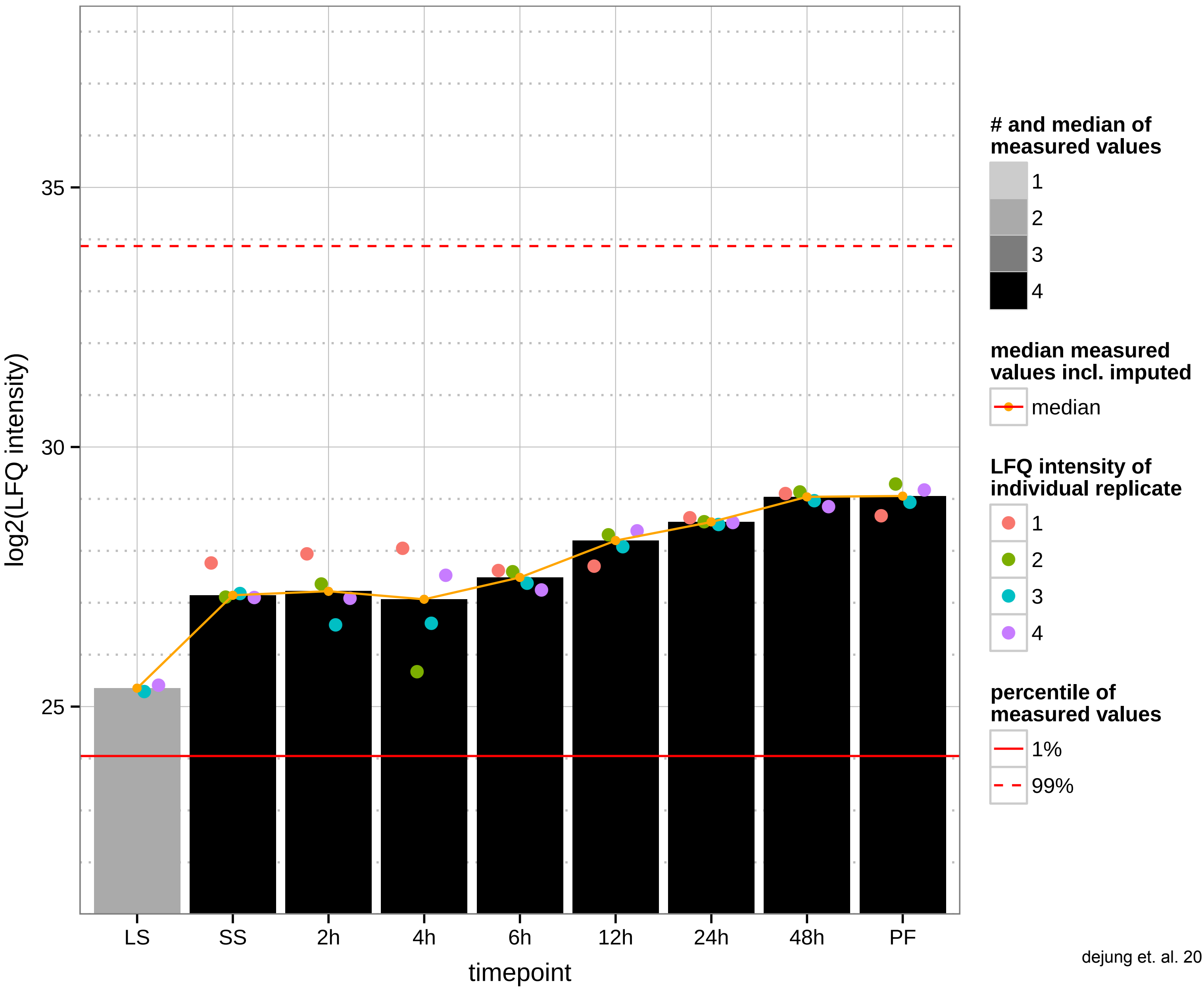

dejung et. al. 2015

60S ribosomal protein L37, putative
Tb927.9.9210;Tb927.11.3000
AGOF: structural constituent of ribosome
AGOC: intracellular, ribosome
AGOP: translation
PGOF: structural constituent of ribosome
PGOC: intracellular, ribosome
PGOP: translation

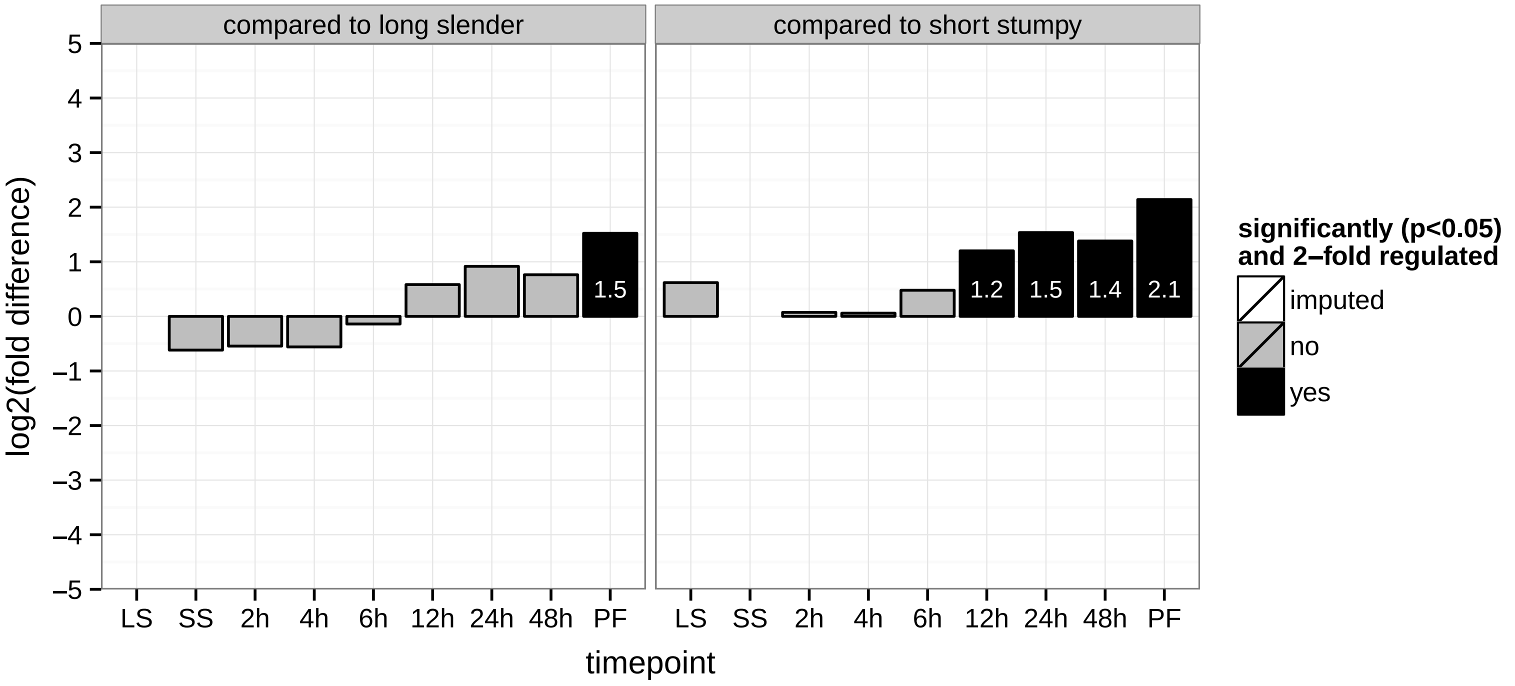

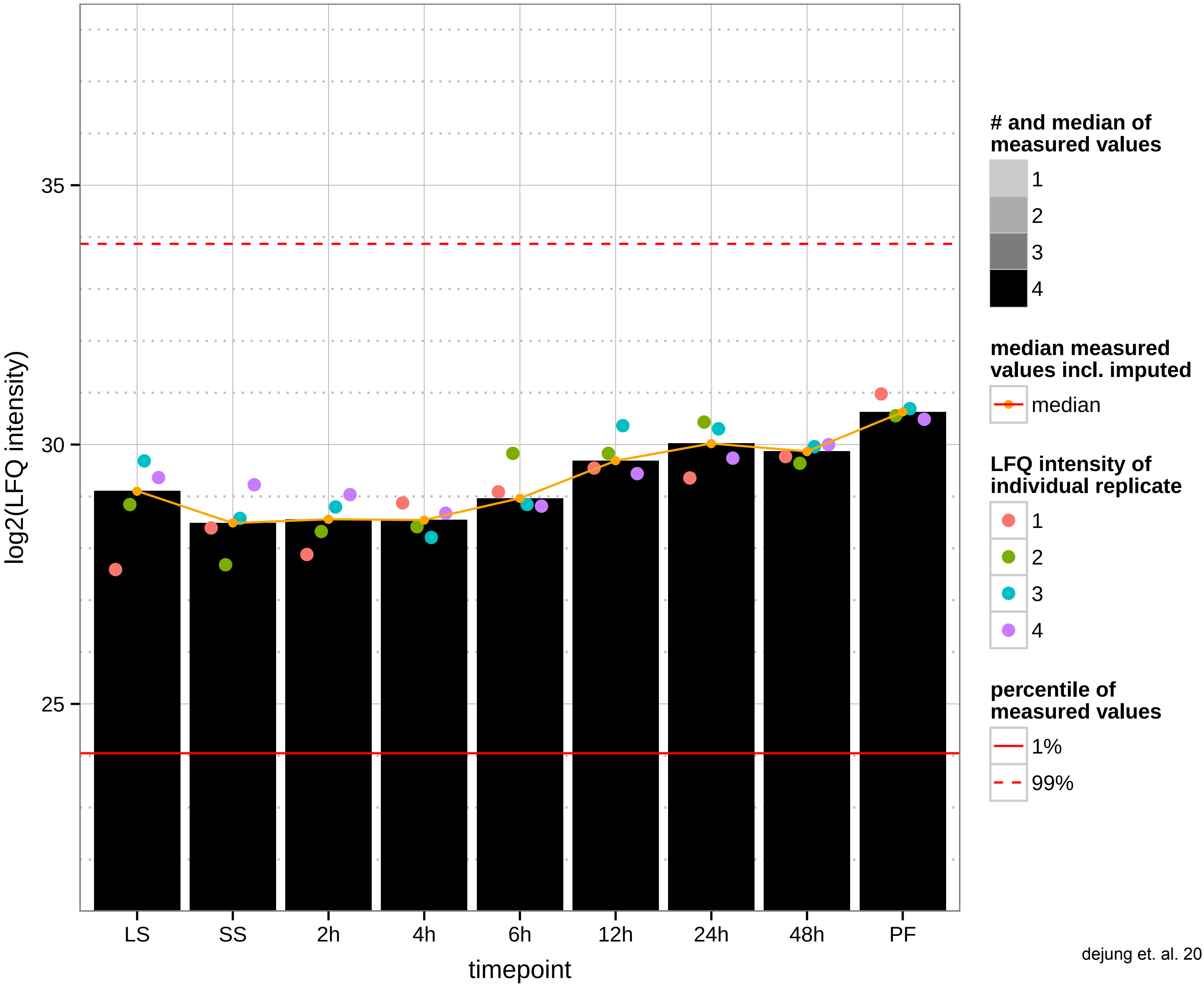

dejung et. al. 2015

mitochondrial DNA polymerase I protein D, putative (POLID)
Tb927.11.3260
AGOF: DNA binding, DNA−directed DNA polymerase activity
AGOC: mitochondrion
AGOP: DNA replication
PGOF: 3'−5' exonuclease activity, DNA binding, DNA−directed DNA polymerase activity, nucleic acid binding
PGOC: intracellular
PGOP: DNA replication, nucleobase−containing compound metabolic process

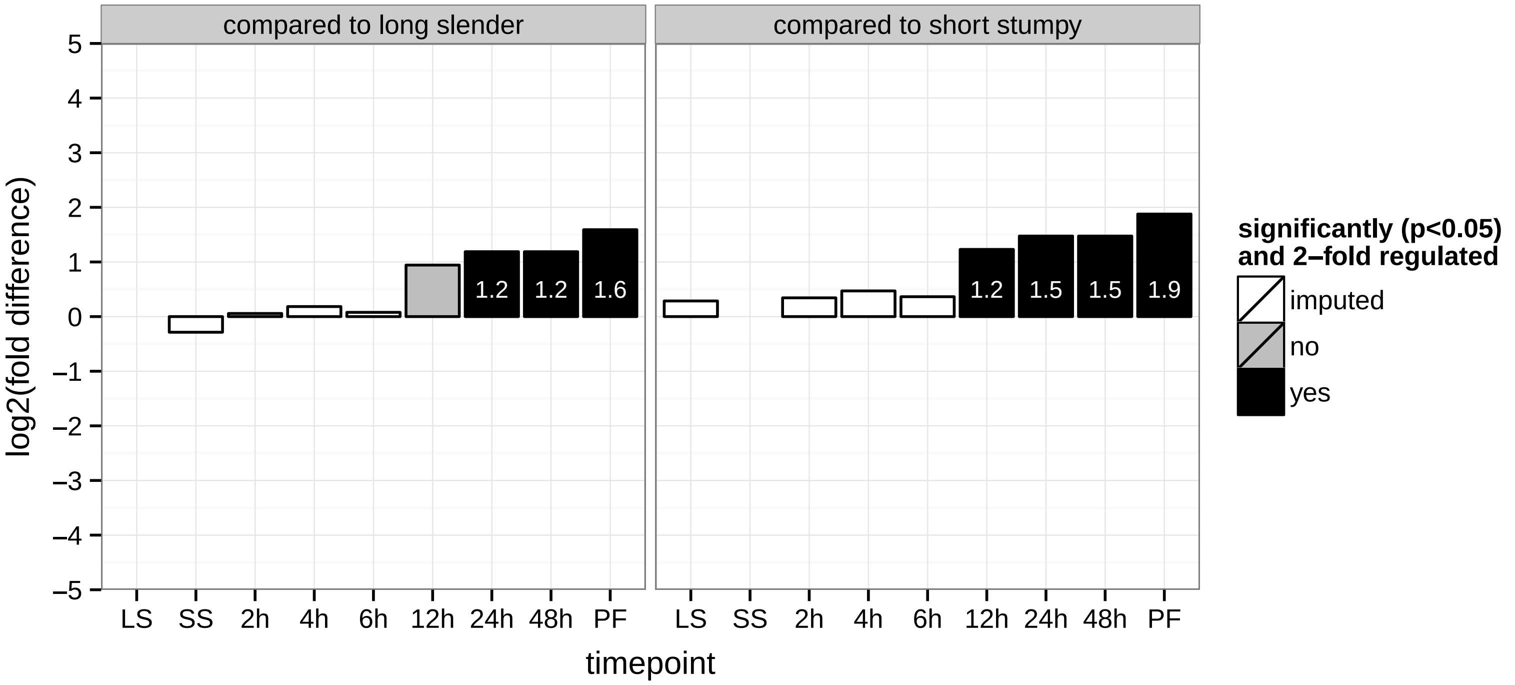

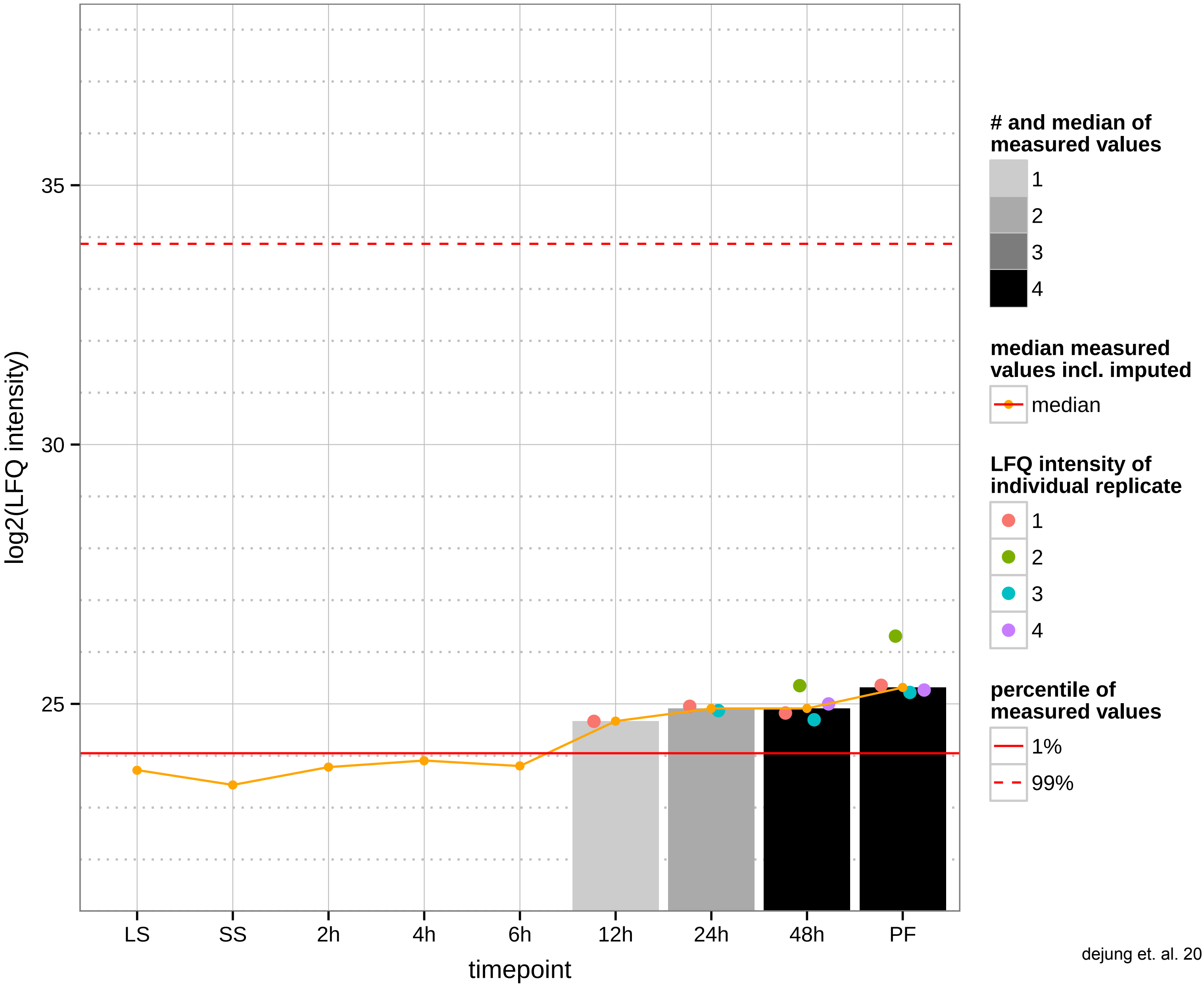

dejung et. al. 2015

squalene monooxygenase, putative
Tb927.11.3270
AGOF: flavin adenine dinucleotide binding, monooxygenase activity, squalene monooxygenase activity
AGOC: integral to membrane
AGOP: cellular aromatic compound metabolic process, oxidation–reduction process
PGOF: flavin adenine dinucleotide binding, squalene monooxygenase activity
PGOC: integral to membrane
PGOP: oxidation–reduction process

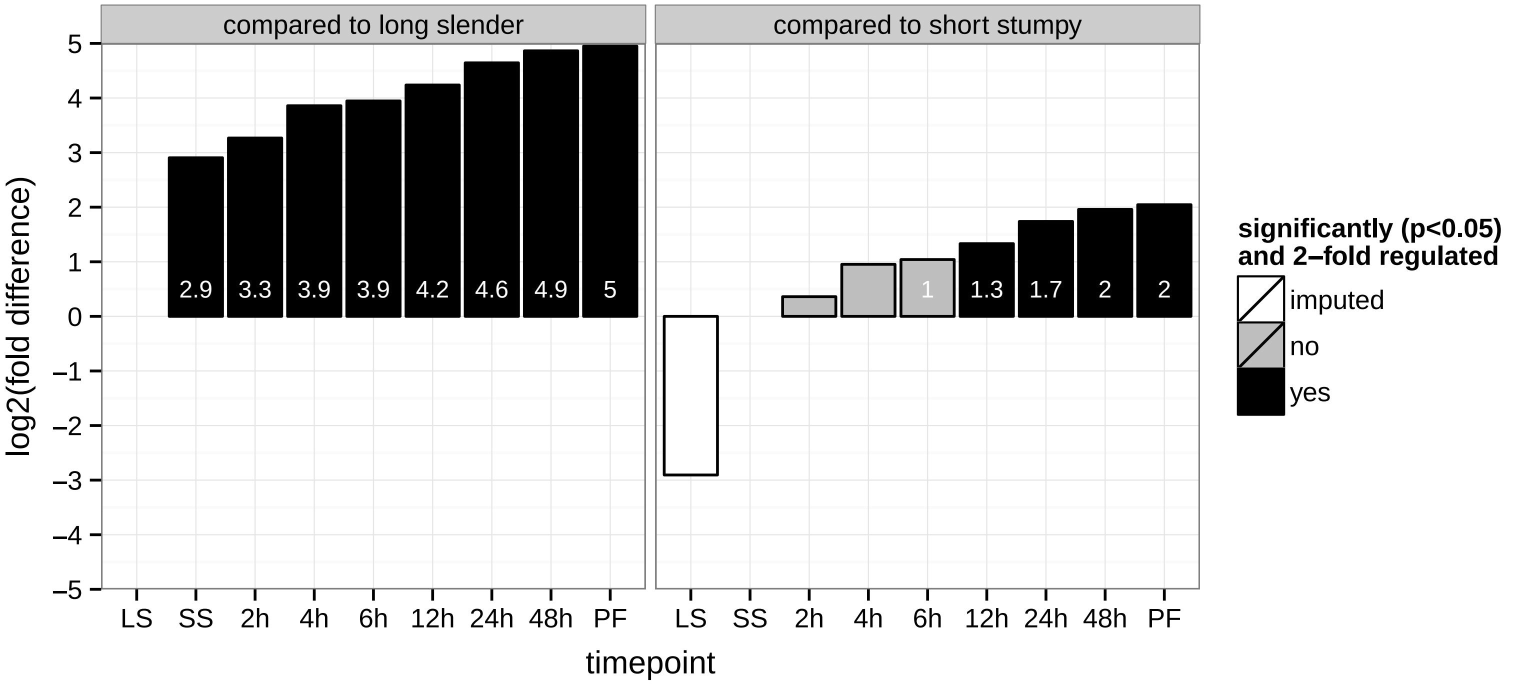

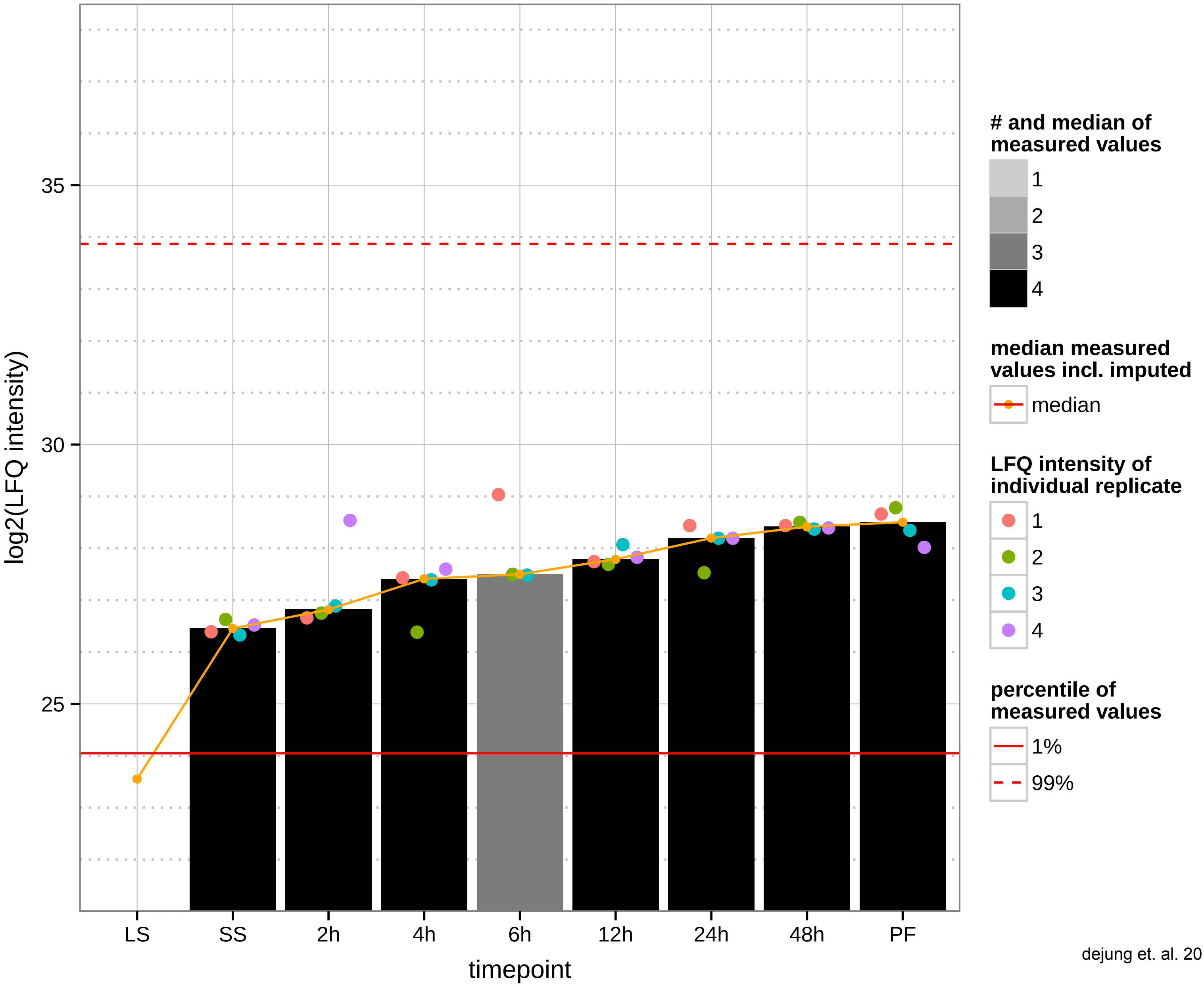

dejung et. al. 2015

hypothetical protein, conserved
Tb927.11.3400
AGOF: null
AGOC: null
AGOP: null
PGOF: null
PGOC: null
PGOP: null

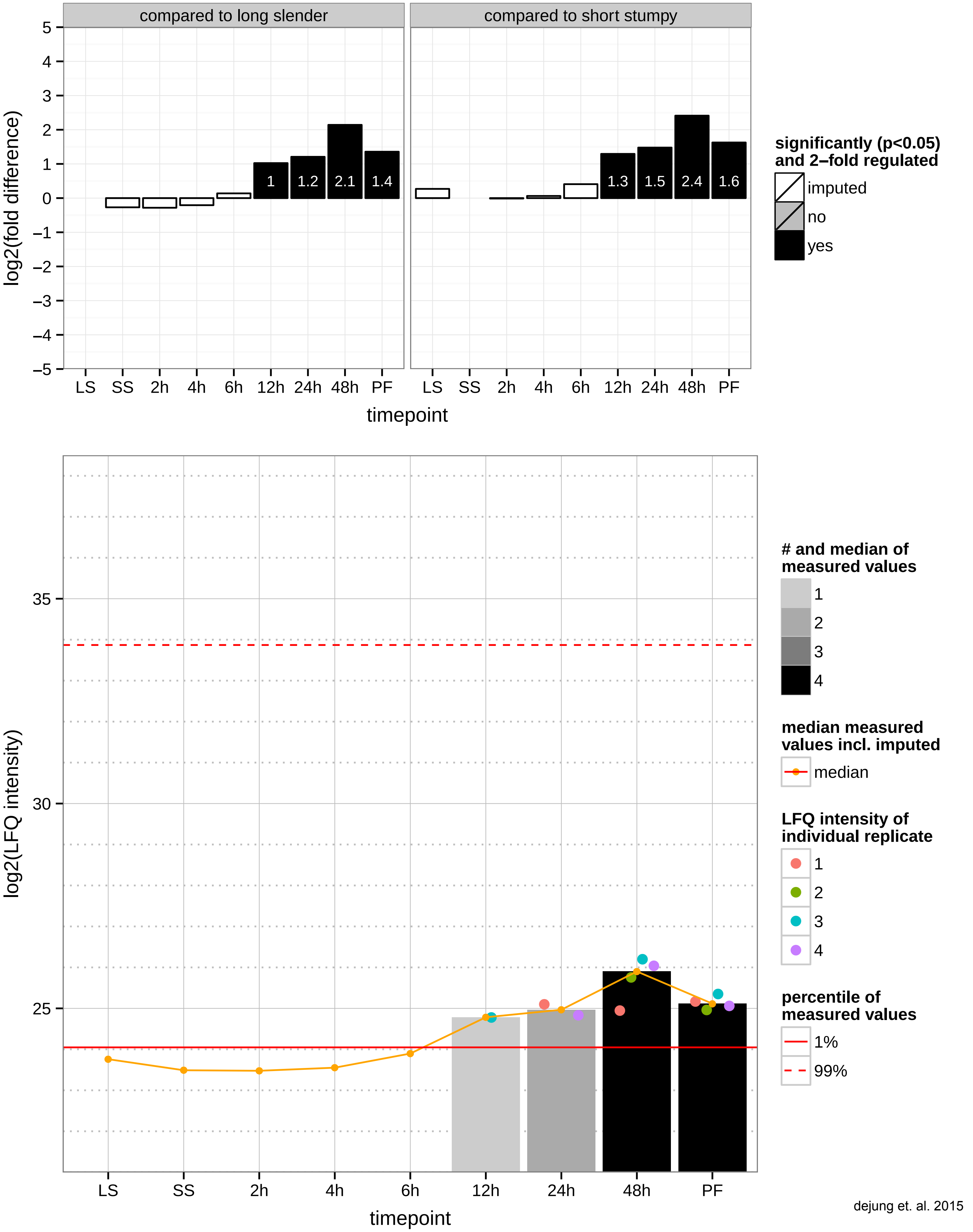

dejung et. al. 2015

hypothetical protein, conserved
Tb927.11.5060
AGOF: methyltransferase activity
AGOC: mitochondrion
AGOP: translation
PGOF: methyltransferase activity
PGOC: null
PGOP: translation

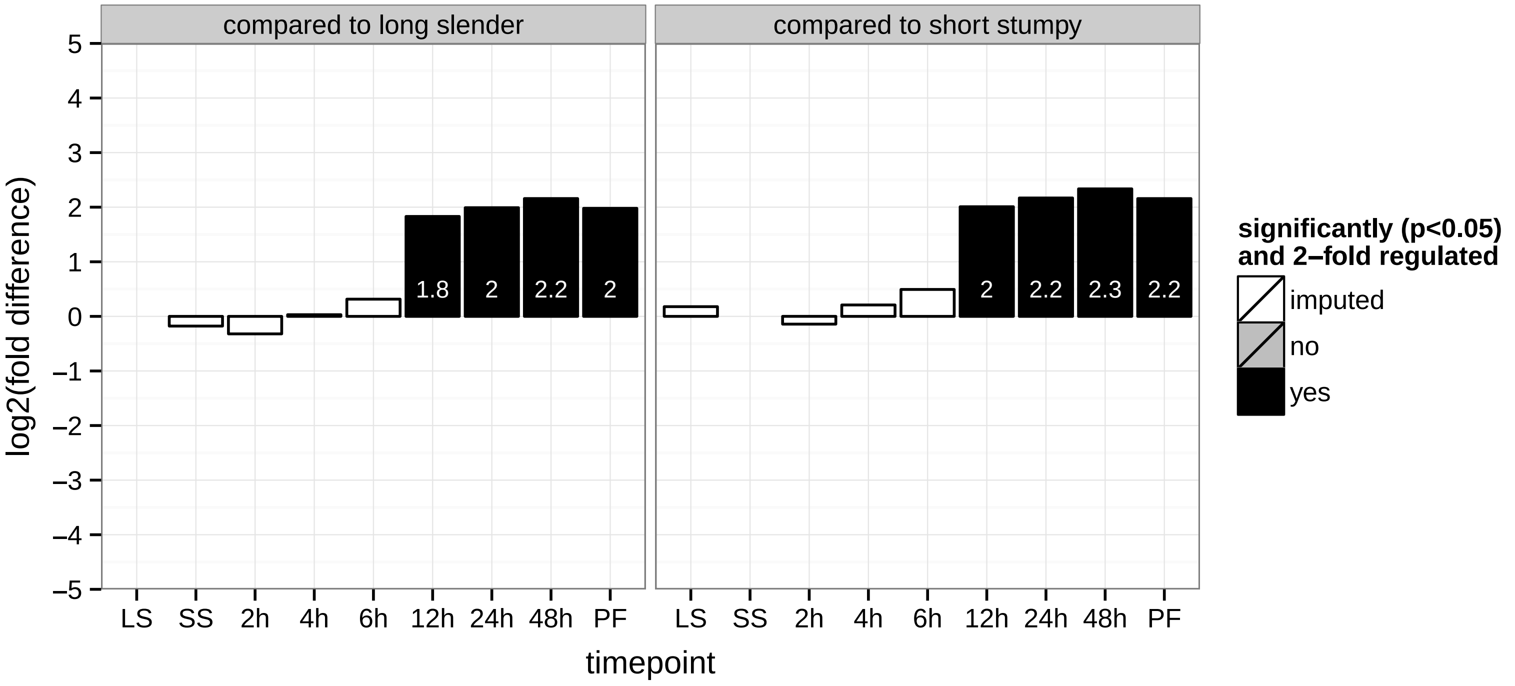

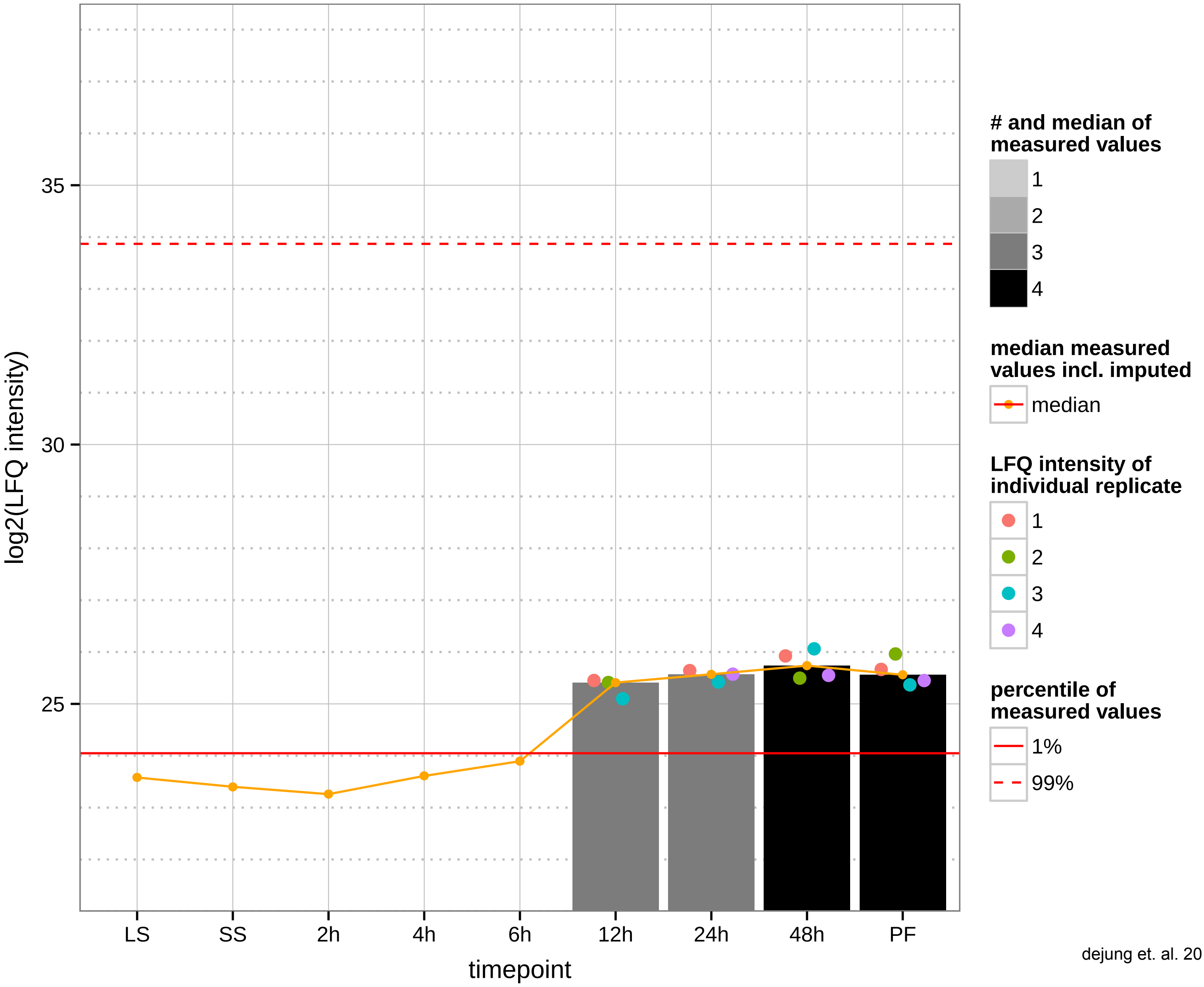

dejung et. al. 2015

pyruvate phosphate dikinase (PPDK)
Tb927.11.6280
AGOF: ATP binding, kinase activity, pyruvate, phosphate dikinase activity
AGOC: glycosome
AGOP: phosphorylation
PGOF: ATP binding, catalytic activity, kinase activity, pyruvate, phosphate dikinase activity, transferase activity, transferring ph
PGOC: null
PGOP: phosphorylation

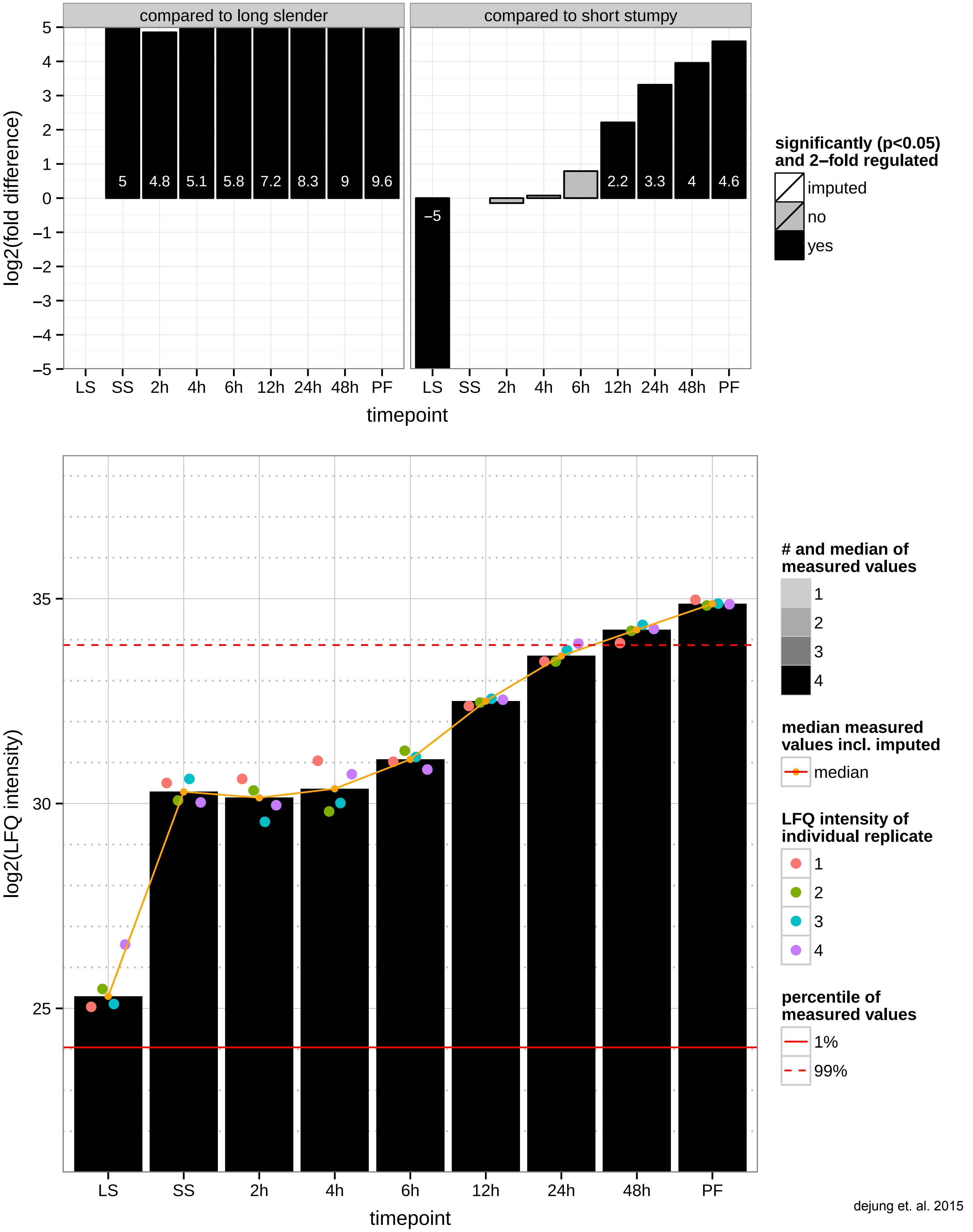

hypothetical protein, conserved
Tb927.11.6440
AGOF: null
AGOC: null
AGOP: null
PGOF: null
PGOC: null
PGOP: null

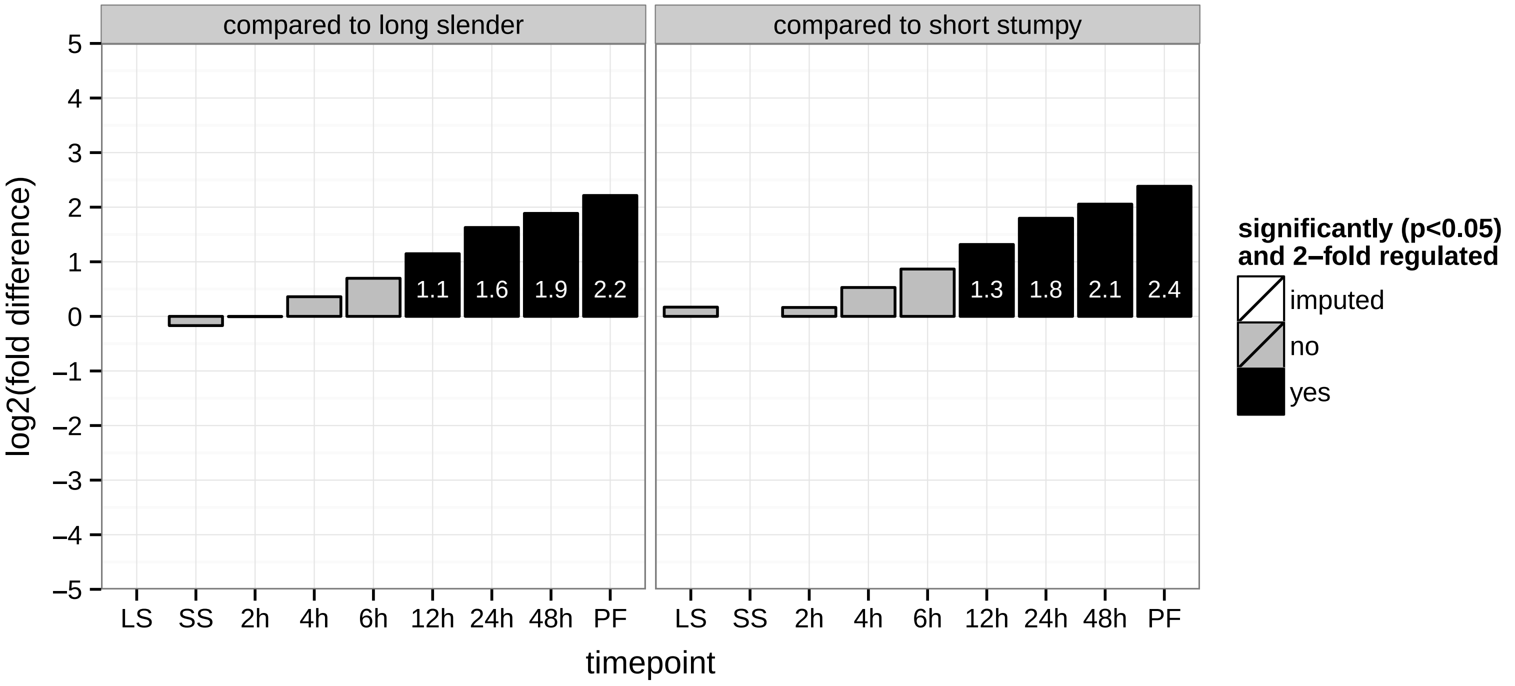

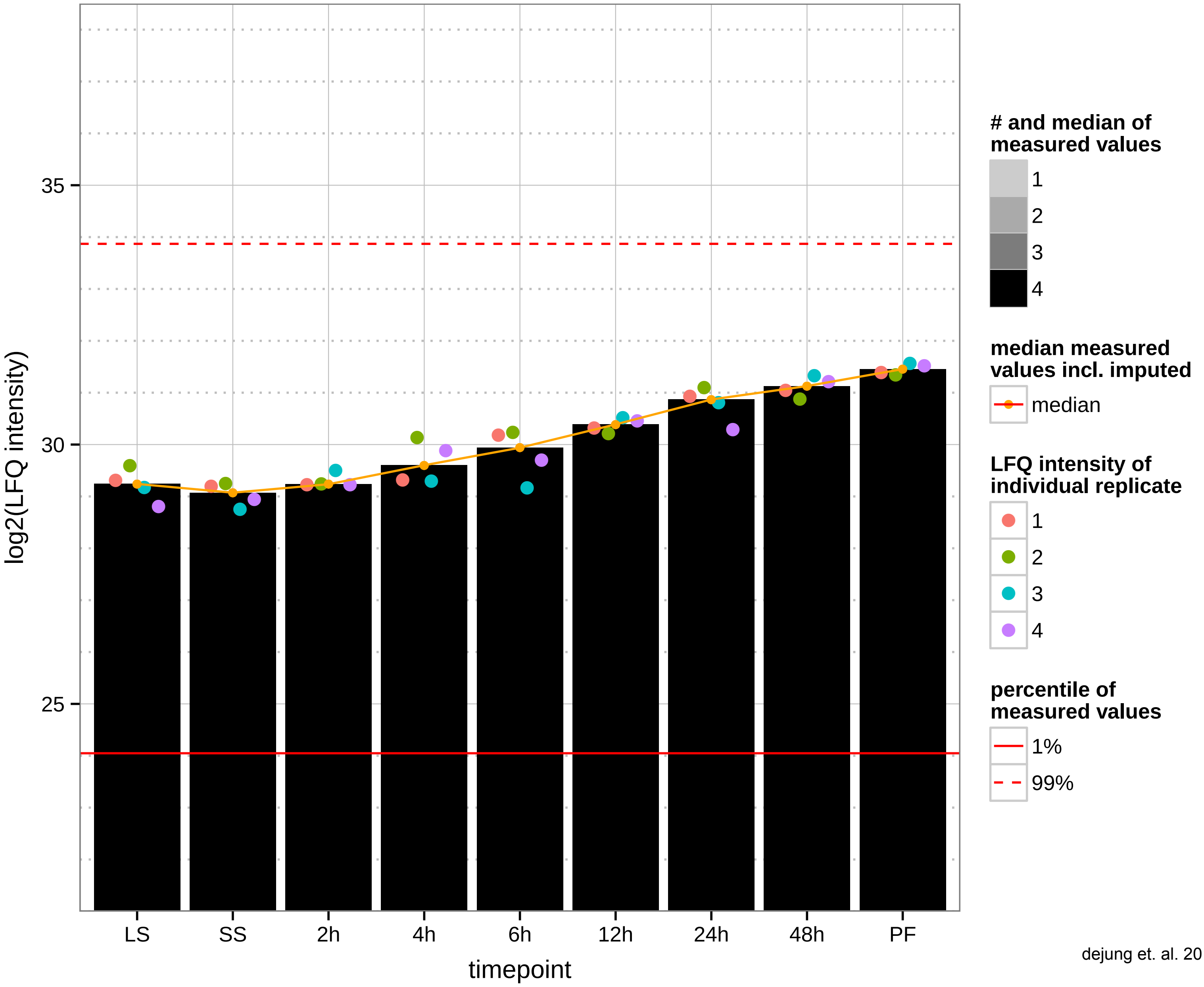

dejung et. al. 2015

mRNA cap guanine−N7 methyltransferase, putative
Tb927.11.6720
AGOF: mRNA (guanine−N7−)−methyltransferase activity, protein binding
AGOC: eukaryotic translation initiation factor 4F complex
AGOP: null
PGOF: null
PGOC: null
PGOP: 7−methylguanosine mRNA capping

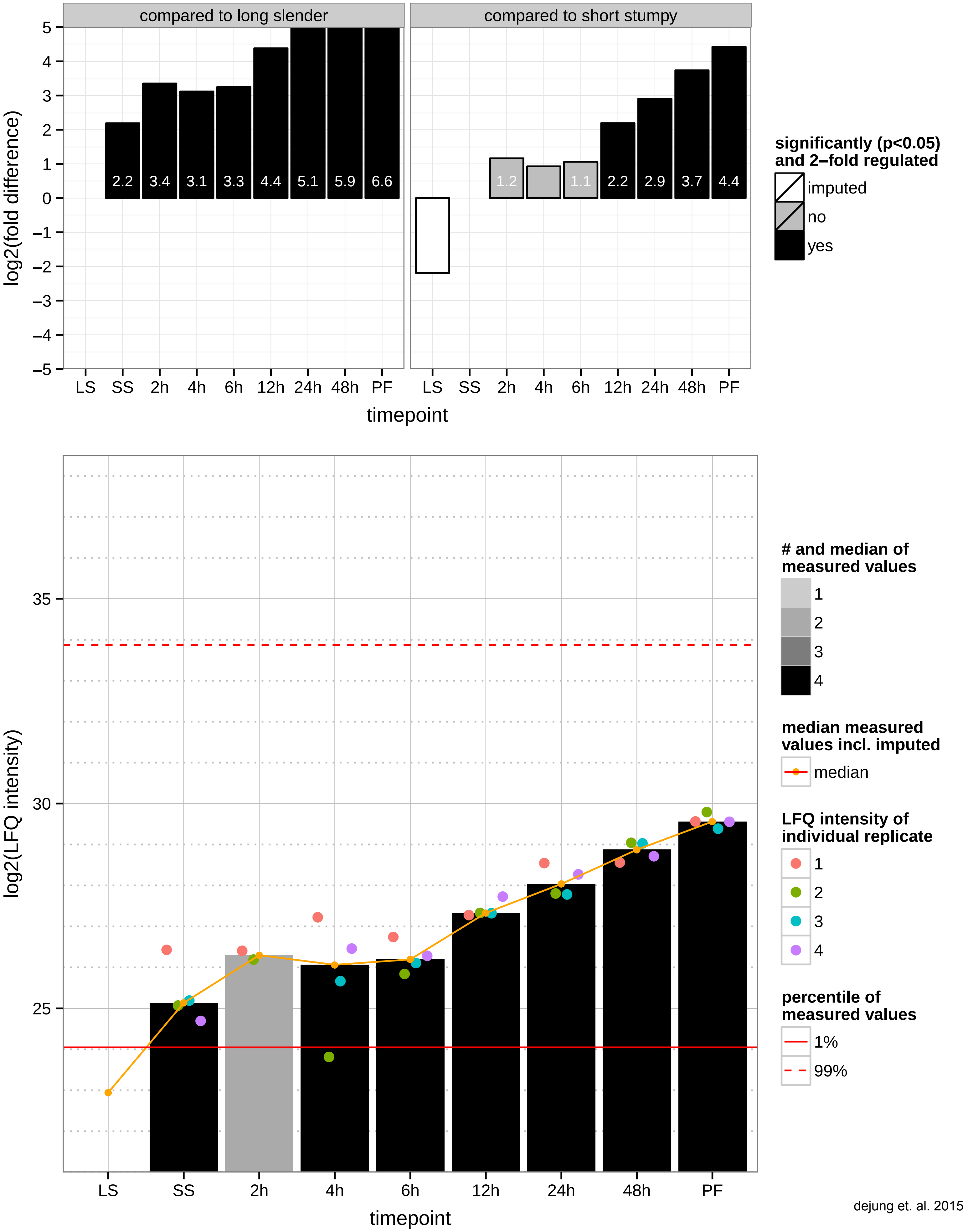

dejung et. al. 2015

hypothetical protein, conserved
Tb927.11.6850
AGOF: null
AGOC: null
AGOP: null
PGOF: null
PGOC: null
PGOP: null

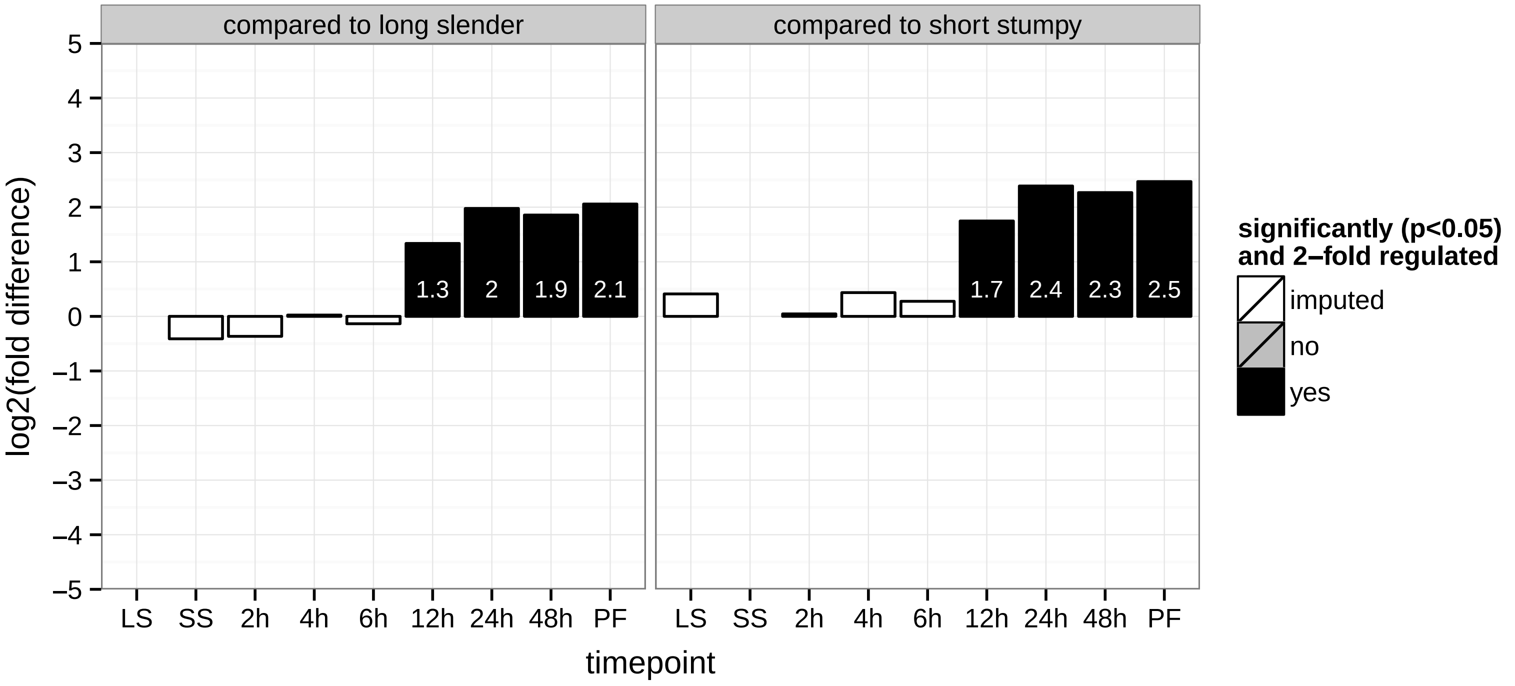

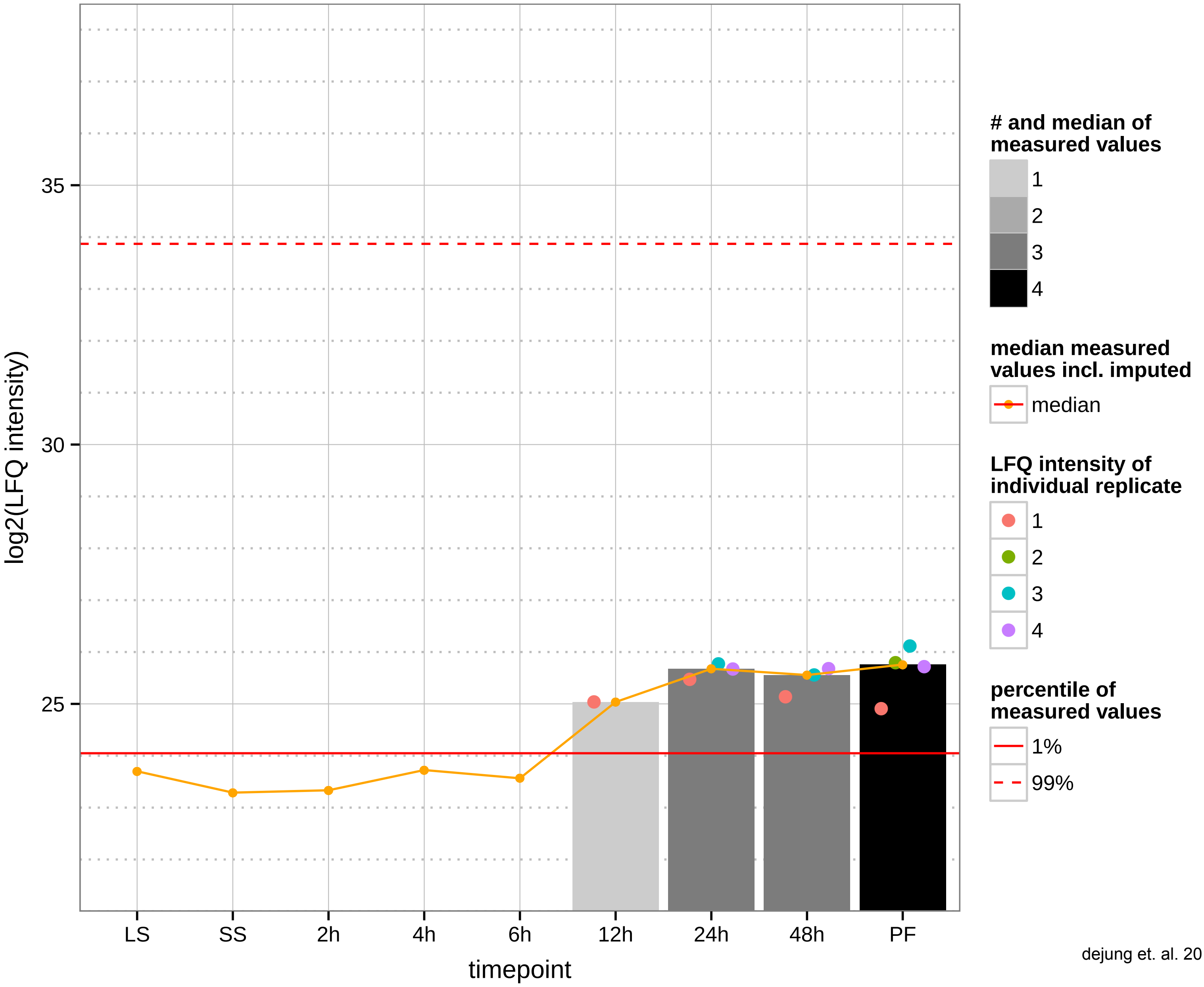

dejung et. al. 2015

hypothetical protein, conserved
Tb927.11.7520
AGOF: null
AGOC: null
AGOP: null
PGOF: null
PGOC: null
PGOP: null

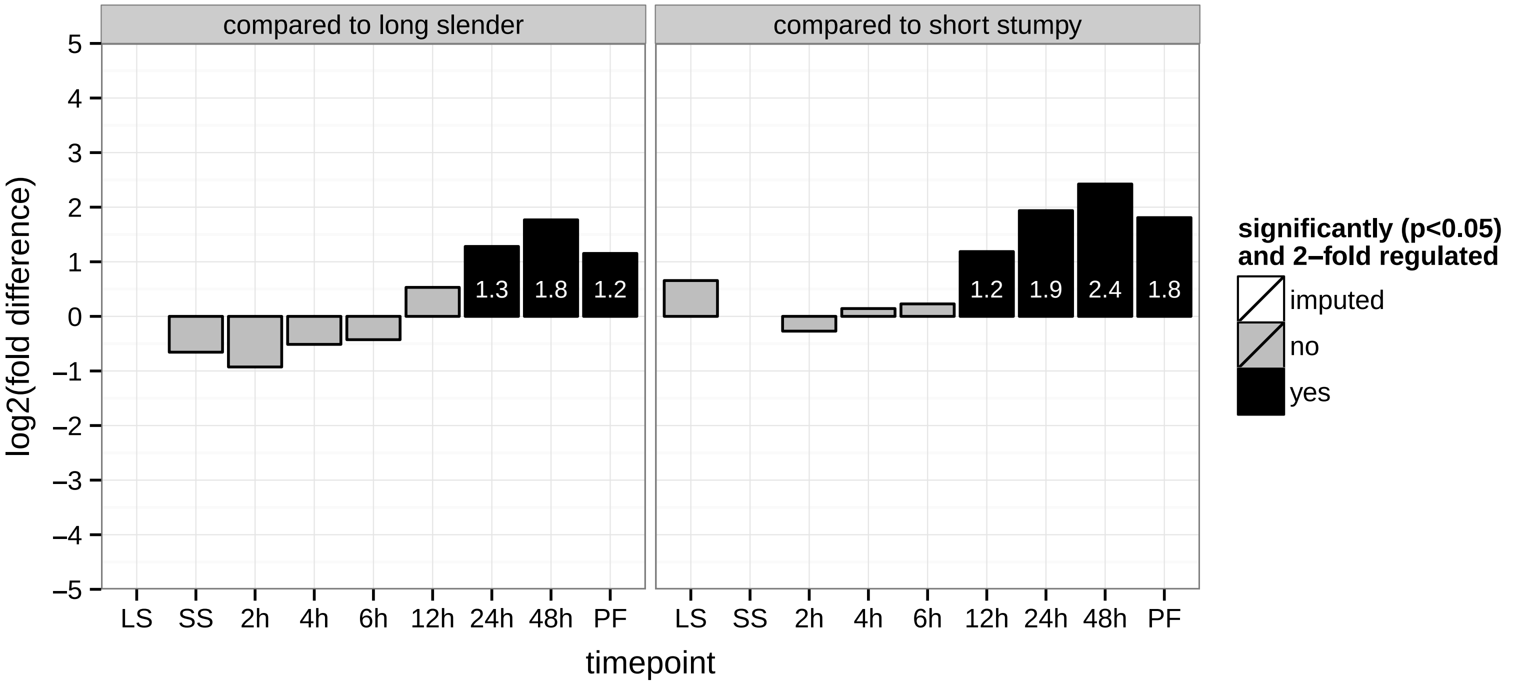

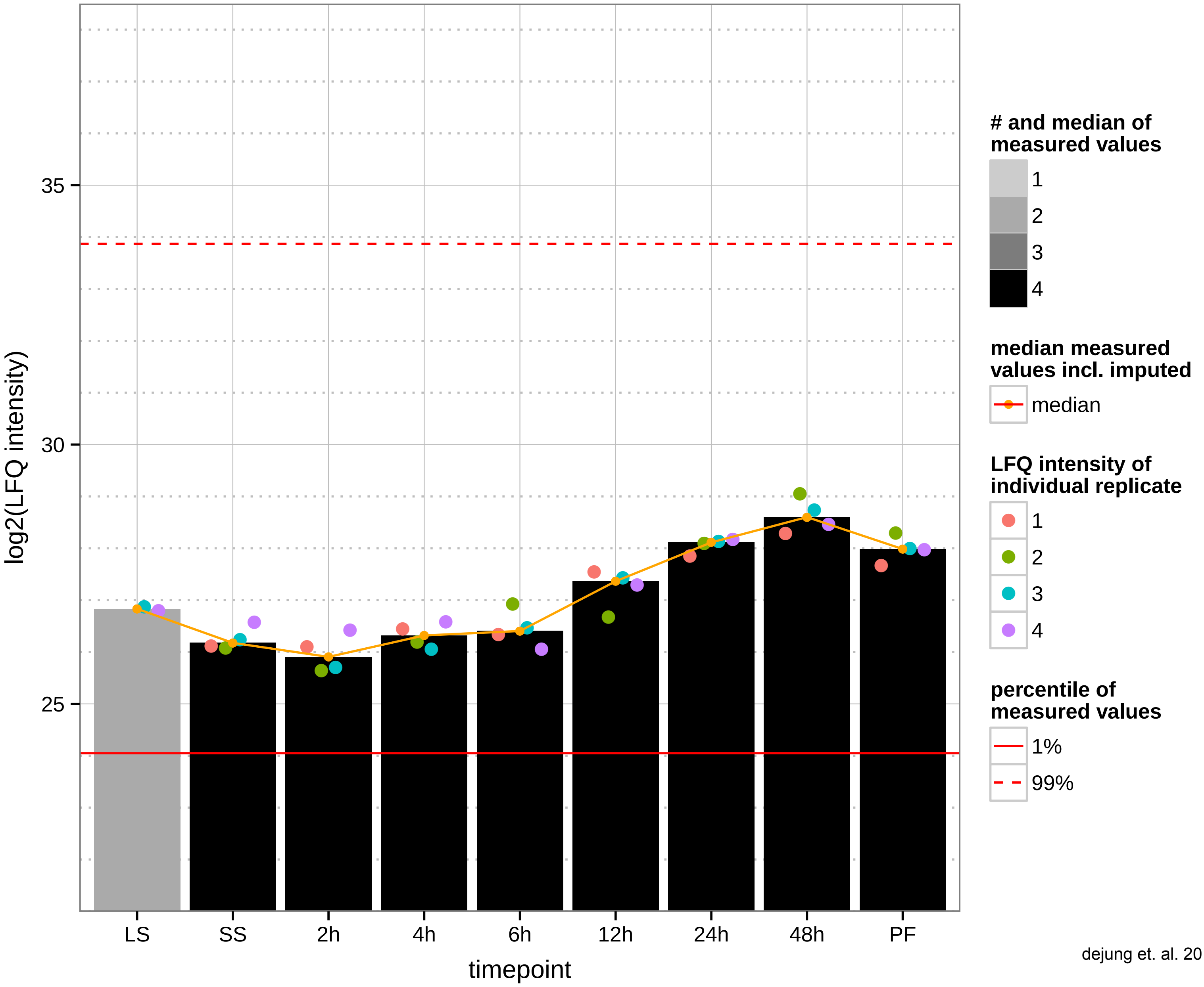

dejung et. al. 2015

electron–transfer–flavoprotein, alpha polypeptide, putative
Tb927.11.7540
AGOF: electron carrier activity, flavin adenine dinucleotide binding
AGOC: mitochondrion
AGOP: oxidation–reduction process
PGOF: electron carrier activity, flavin adenine dinucleotide binding
PGOC: null
PGOP: null

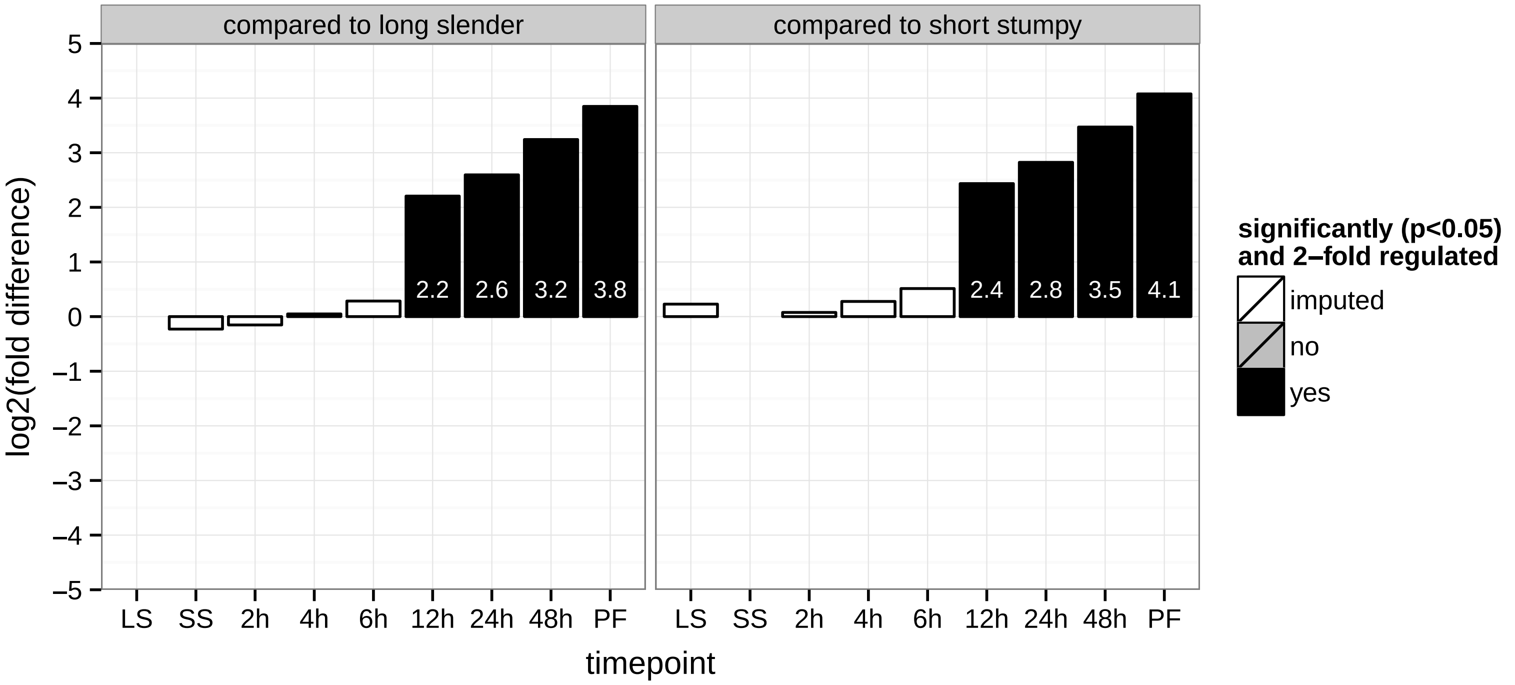

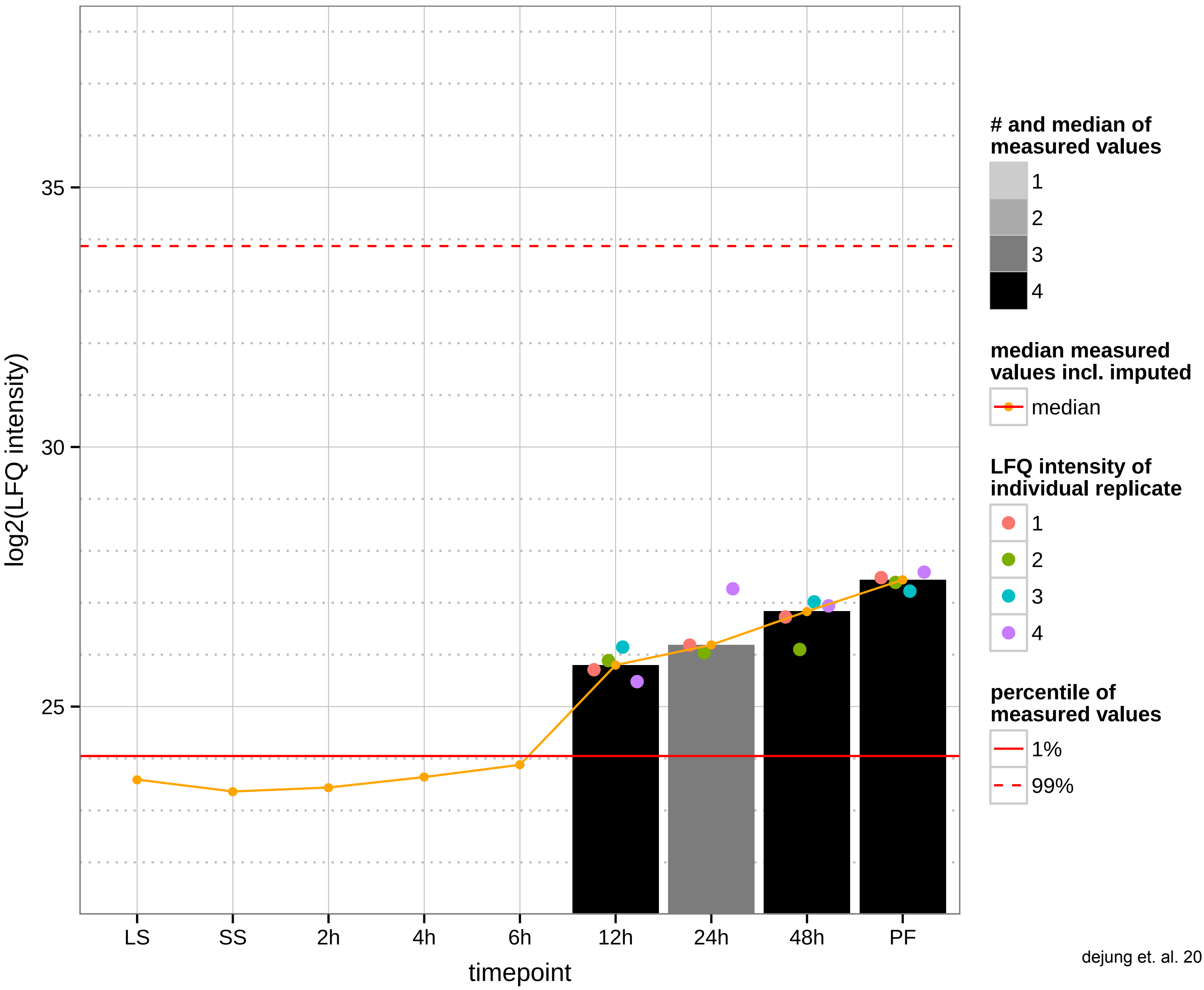

dejung et. al. 2015

hypothetical protein, conserved
Tb927.11.7600
AGOF: null
AGOC: null
AGOP: null
PGOF: null
PGOC: null
PGOP: null

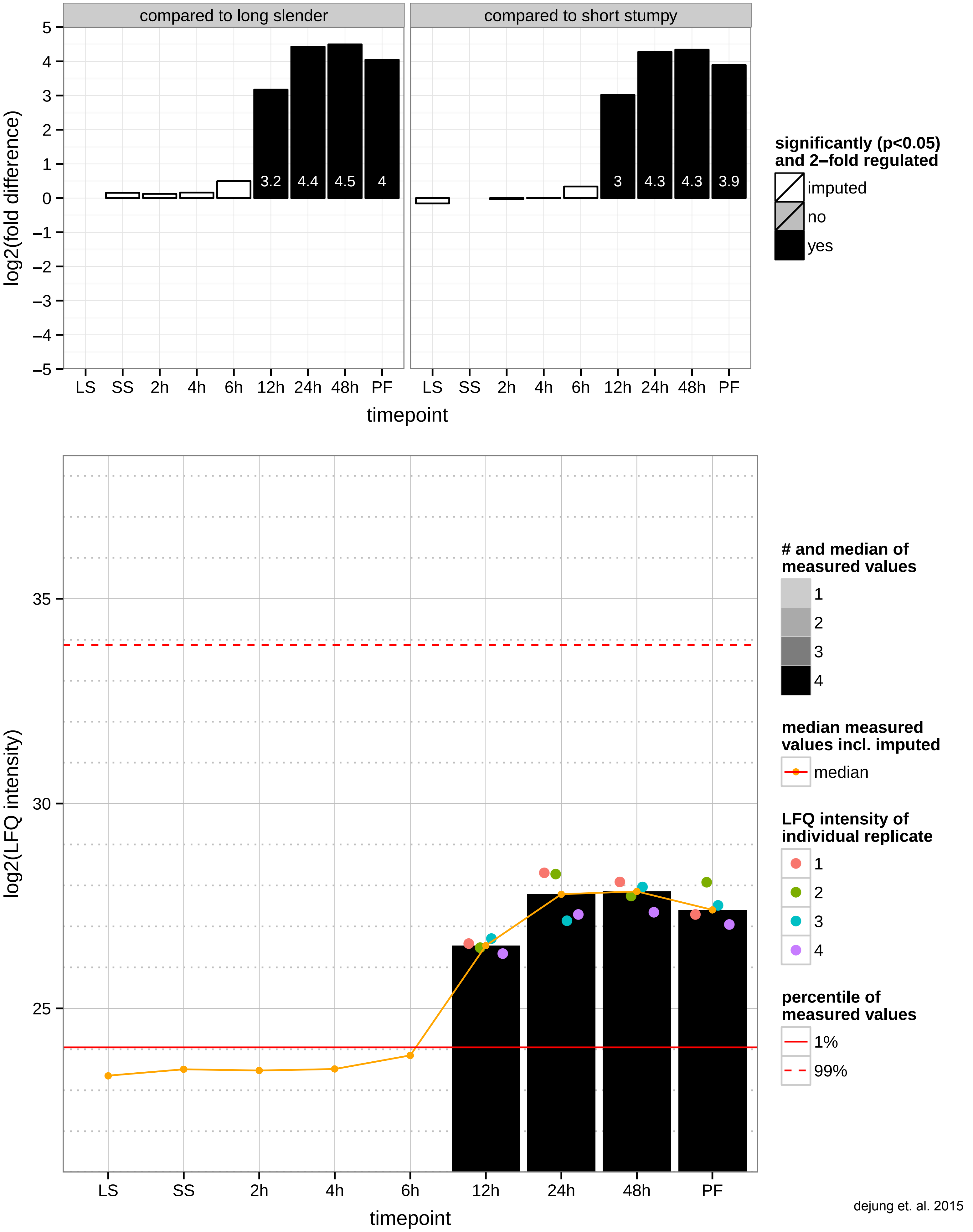

dejung et. al. 2015

DIS3–like exonuclease, putative
Tb927.11.8290
AGOF: null, RNA binding, ribonuclease activity
AGOC: null
AGOP: null
PGOF: RNA binding, ribonuclease activity
PGOC: null
PGOP: null

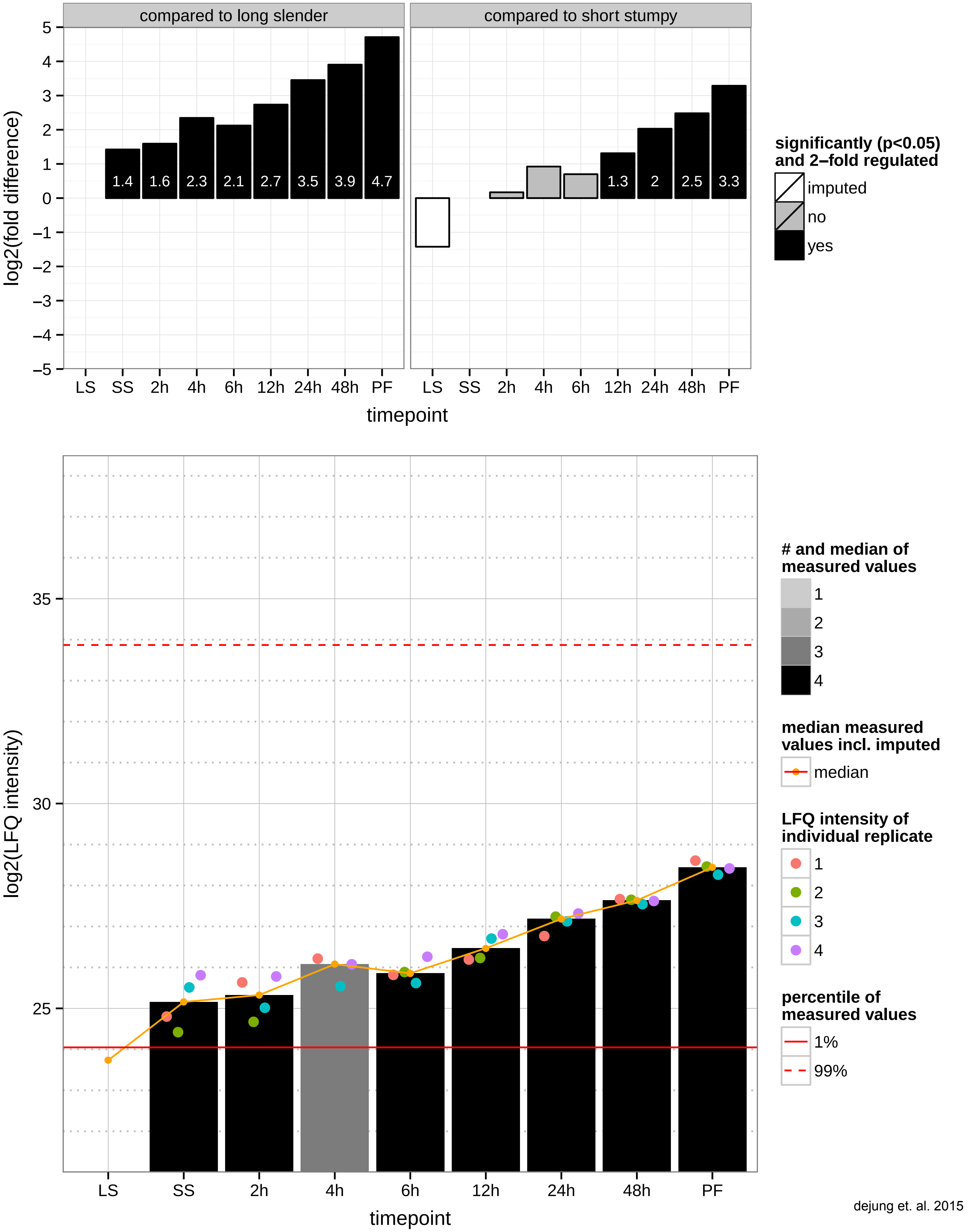

dejung et. al. 2015

mitochondrial DEAD box protein, KREH1 (KREH1)
Tb927.11.8870
AGOF: ATP binding, ATP−dependent helicase activity, helicase activity, nucleic acid binding
AGOC: mitochondrion
AGOP: nucleobase−containing compound metabolic process
PGOF: ATP binding, ATP−dependent helicase activity, helicase activity, nucleic acid binding
PGOC: null
PGOP: null

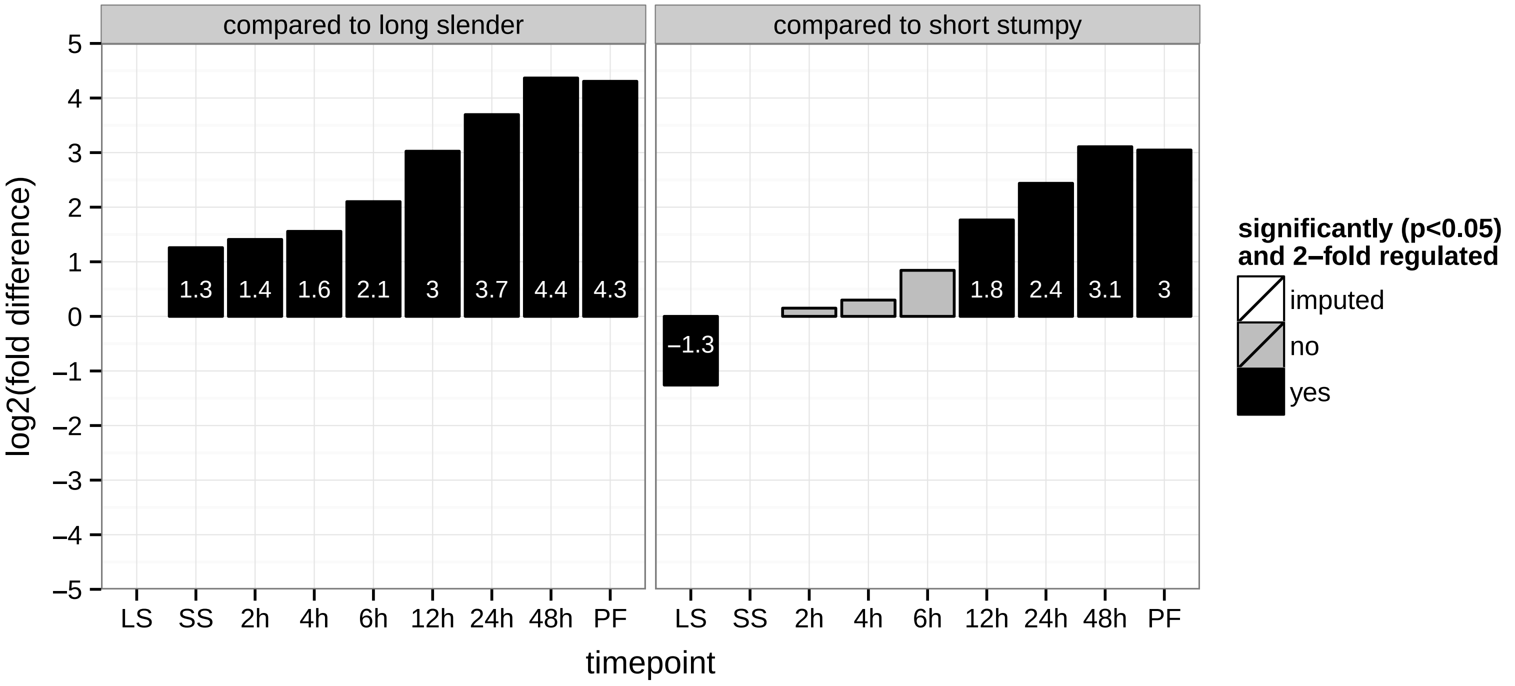

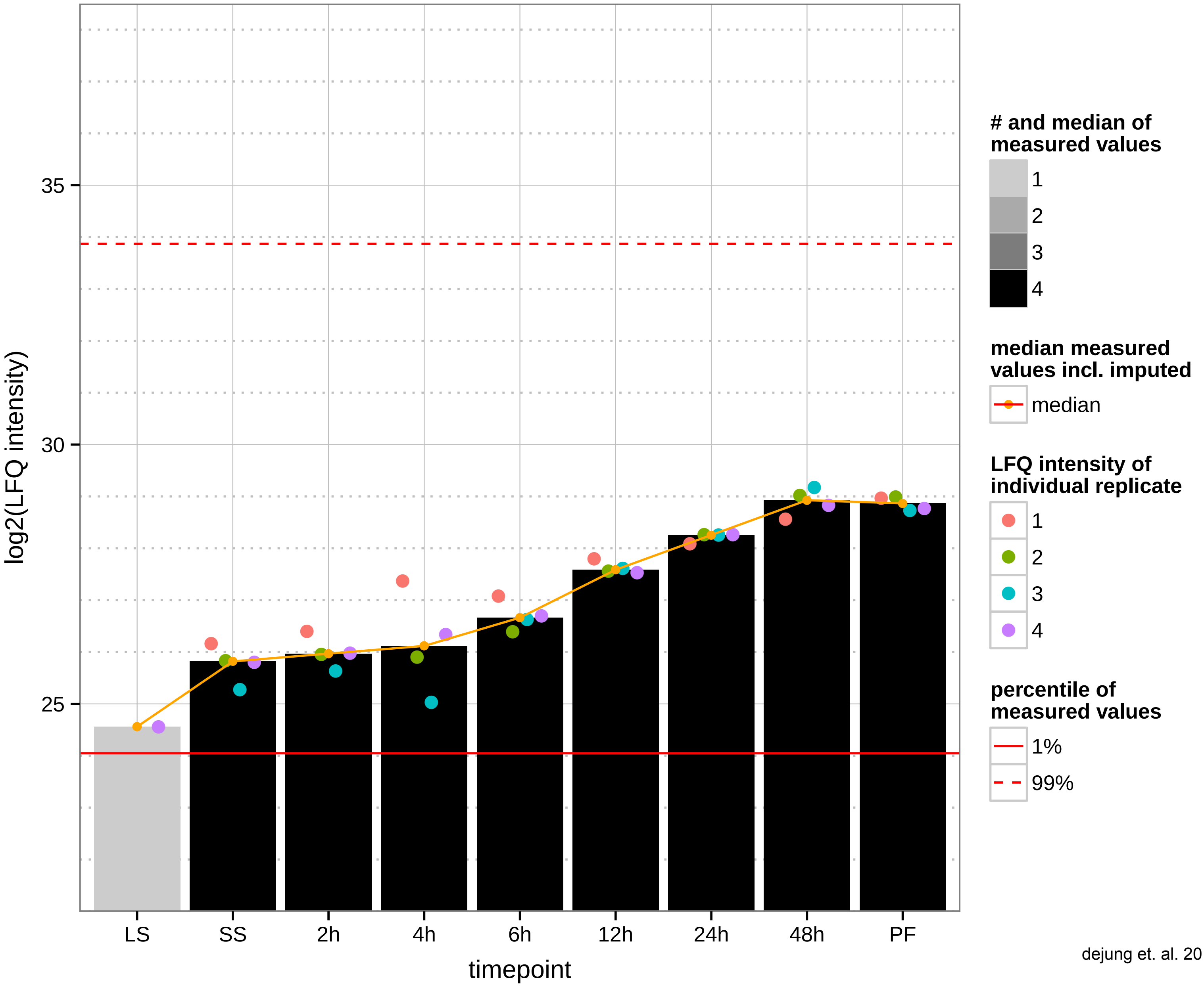

dejung et. al. 2015

conserved protein
Tb927.11.9040
AGOF: DNA binding
AGOC: null
AGOP: null
PGOF: DNA binding
PGOC: null
PGOP: null

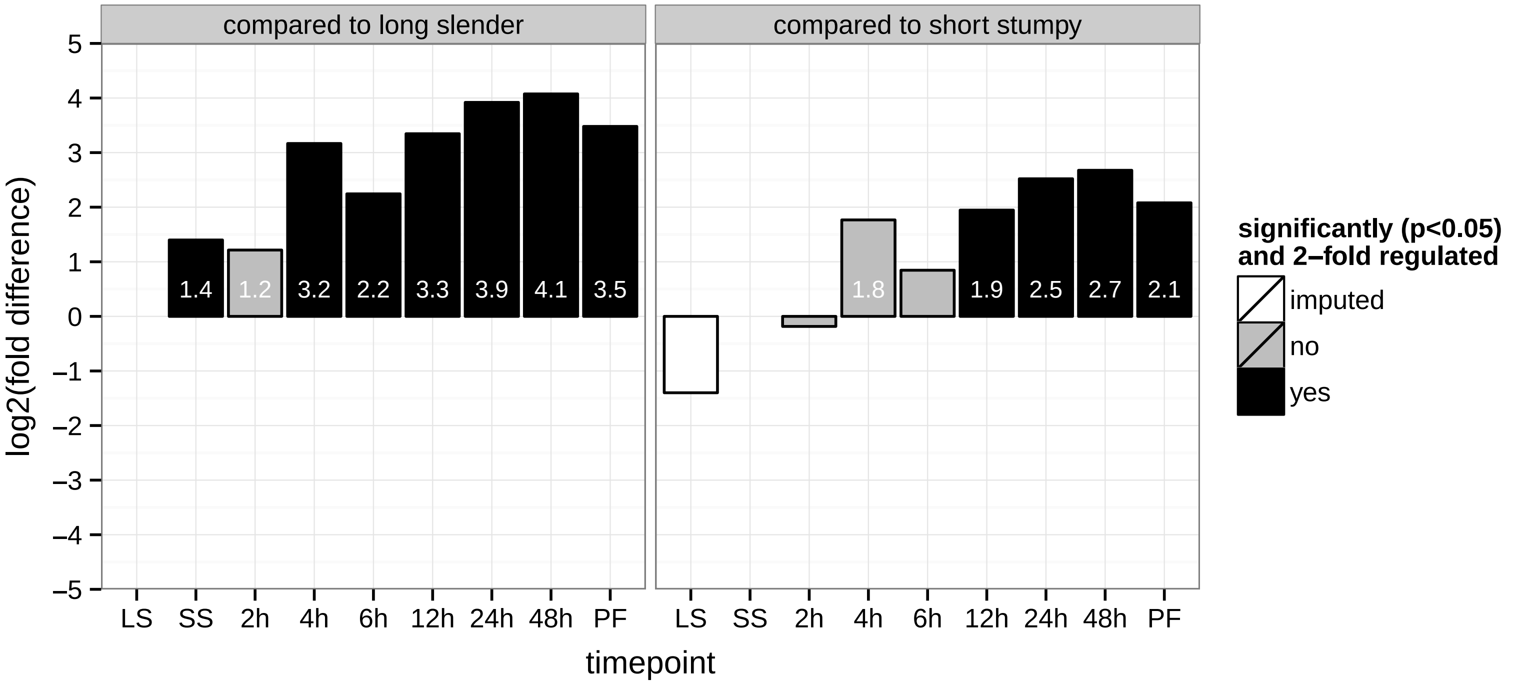

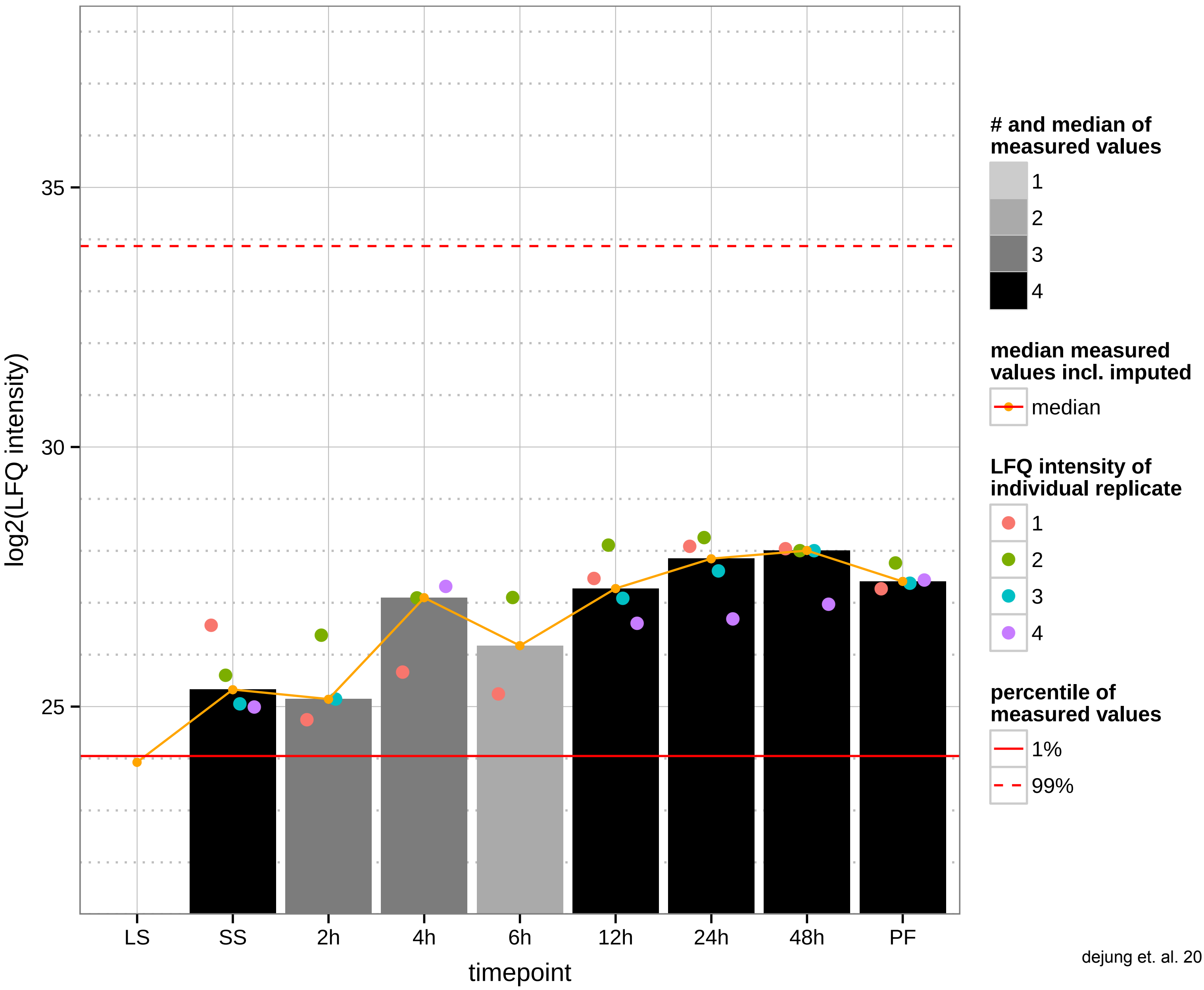

dejung et. al. 2015

hypothetical protein, conserved
Tb927.11.9280
AGOF: null
AGOC: mitochondrion
AGOP: null
PGOF: null
PGOC: null
PGOP: null

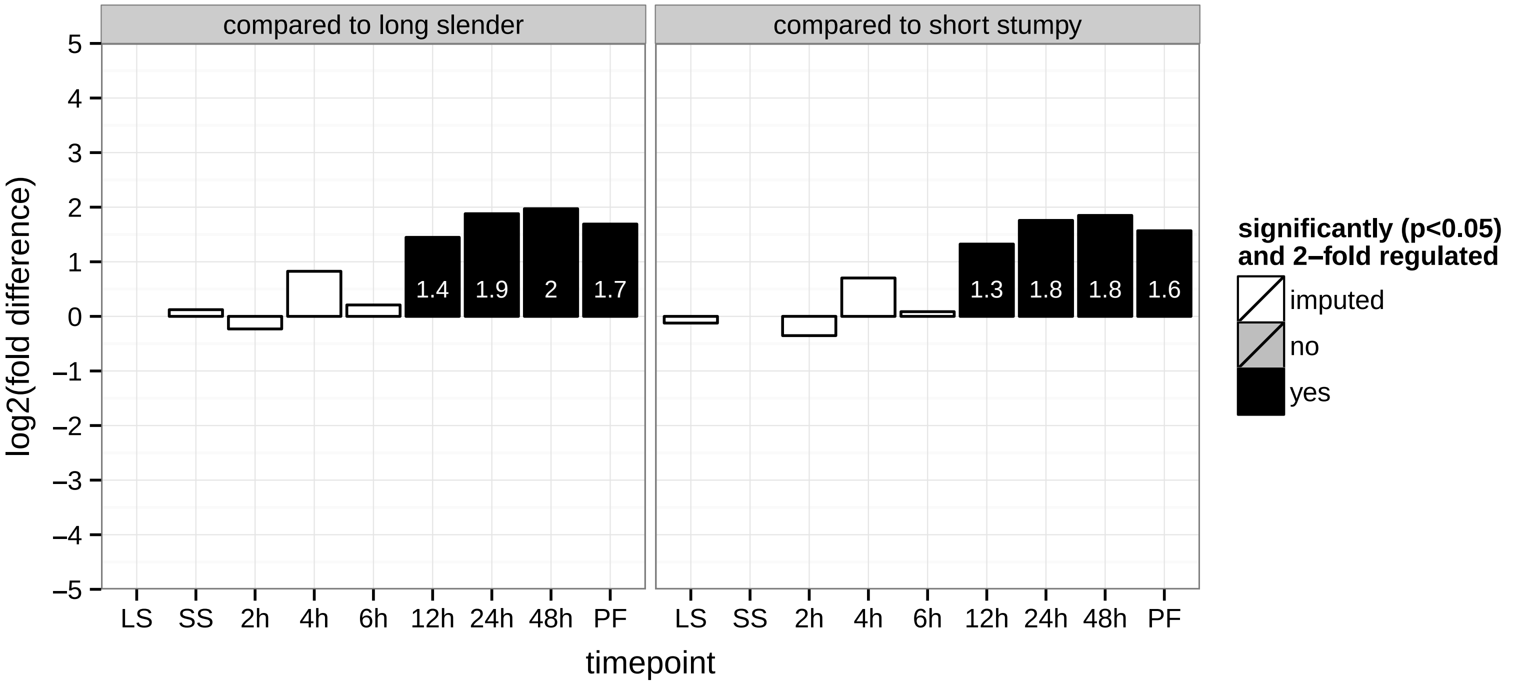

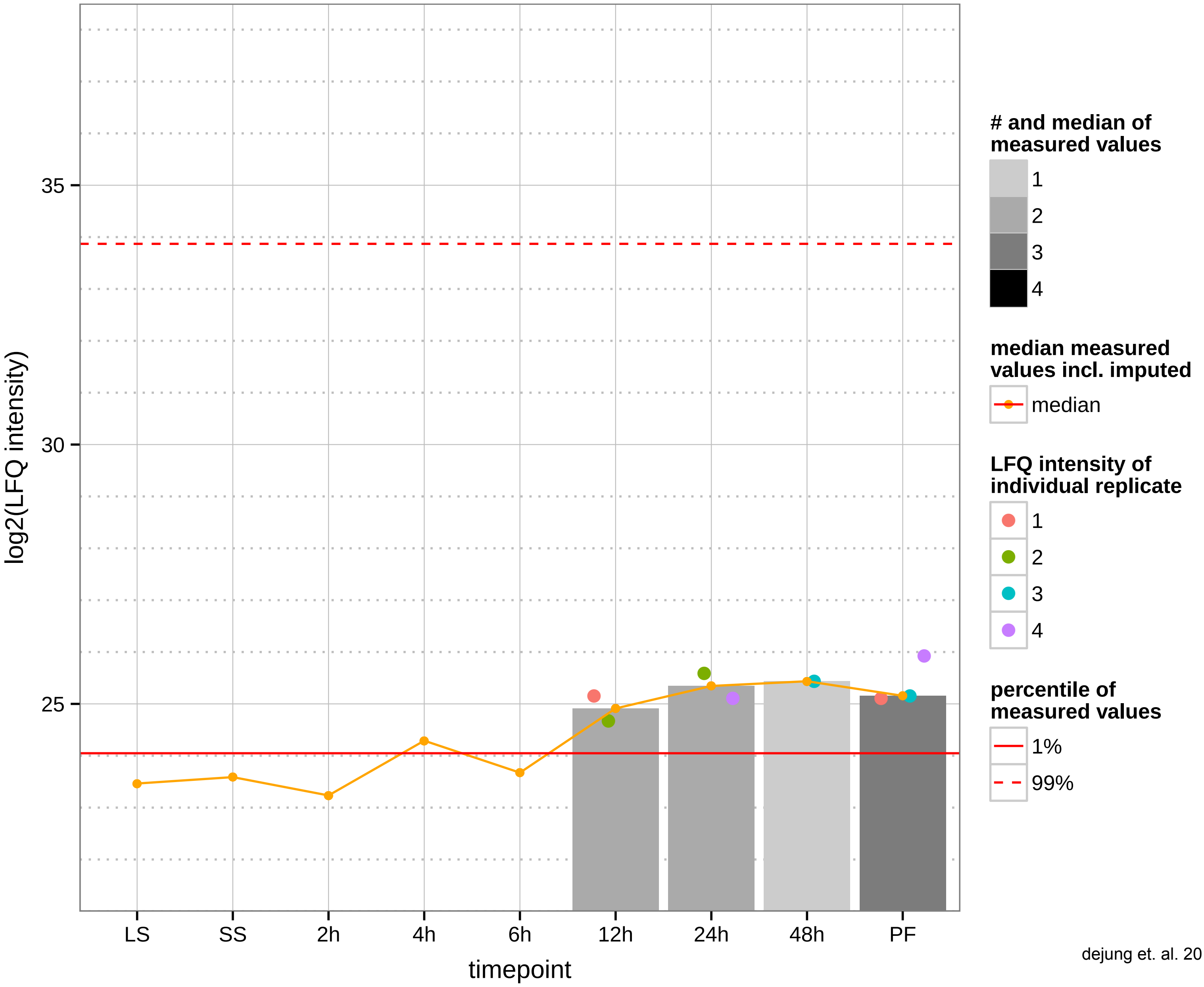

dejung et. al. 2015

casein kinase II, alpha chain (CK2A2)
Tb927.2.2430
AGOF: ATP binding, protein kinase activity, protein serine/threonine kinase activity, protein tyrosine kinase activity
AGOC: null
AGOP: protein phosphorylation
PGOF: ATP binding, protein kinase activity, transferase activity, transferring phosphorus−containing groups
PGOC: null
PGOP: protein phosphorylation

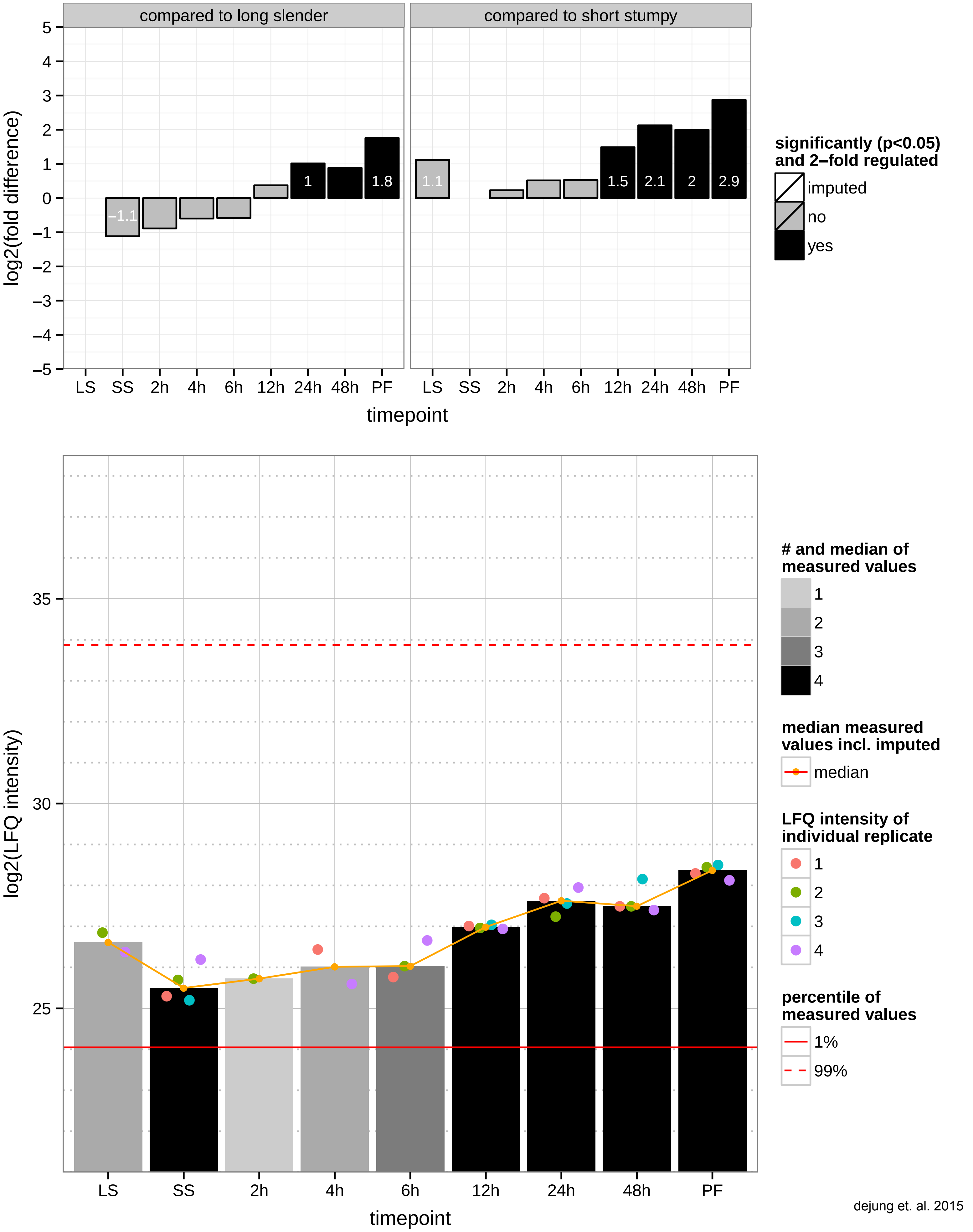

dejung et. al. 2015

UDP–Gal or UDP–GlcNAc–dependent glycosyltransferase, putative
Tb927.2.3370
AGOF: UDP–glycosyltransferase activity, galactosyltransferase activity
AGOC: integral to membrane, membrane
AGOP: GPI anchor biosynthetic process, protein glycosylation
PGOF: null
PGOC: null
PGOP: null

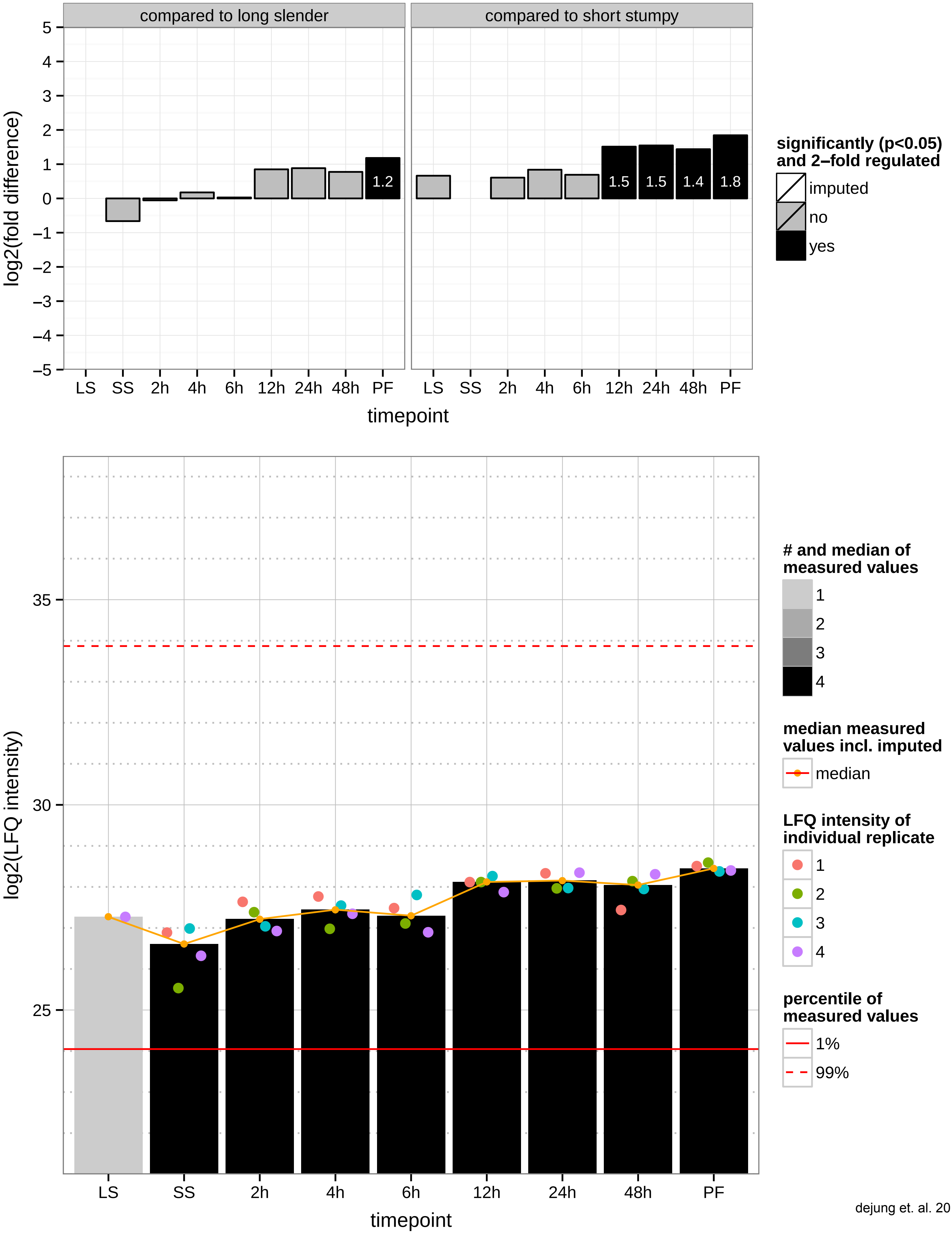

dejung et. al. 2015

GTP binding protein, putative
Tb927.2.4240
AGOF: GTP binding
AGOC: intracellular
AGOP: null
PGOF: GTP binding
PGOC: null
PGOP: null

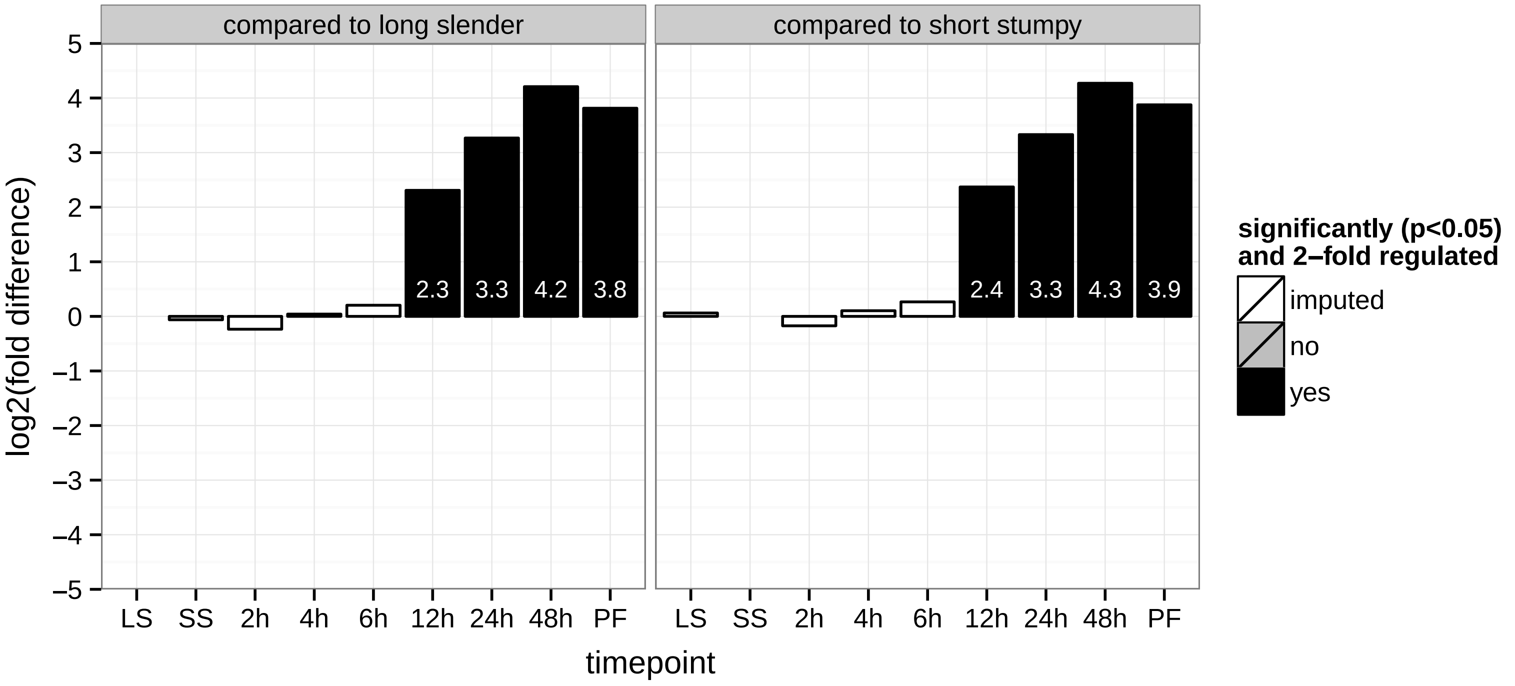

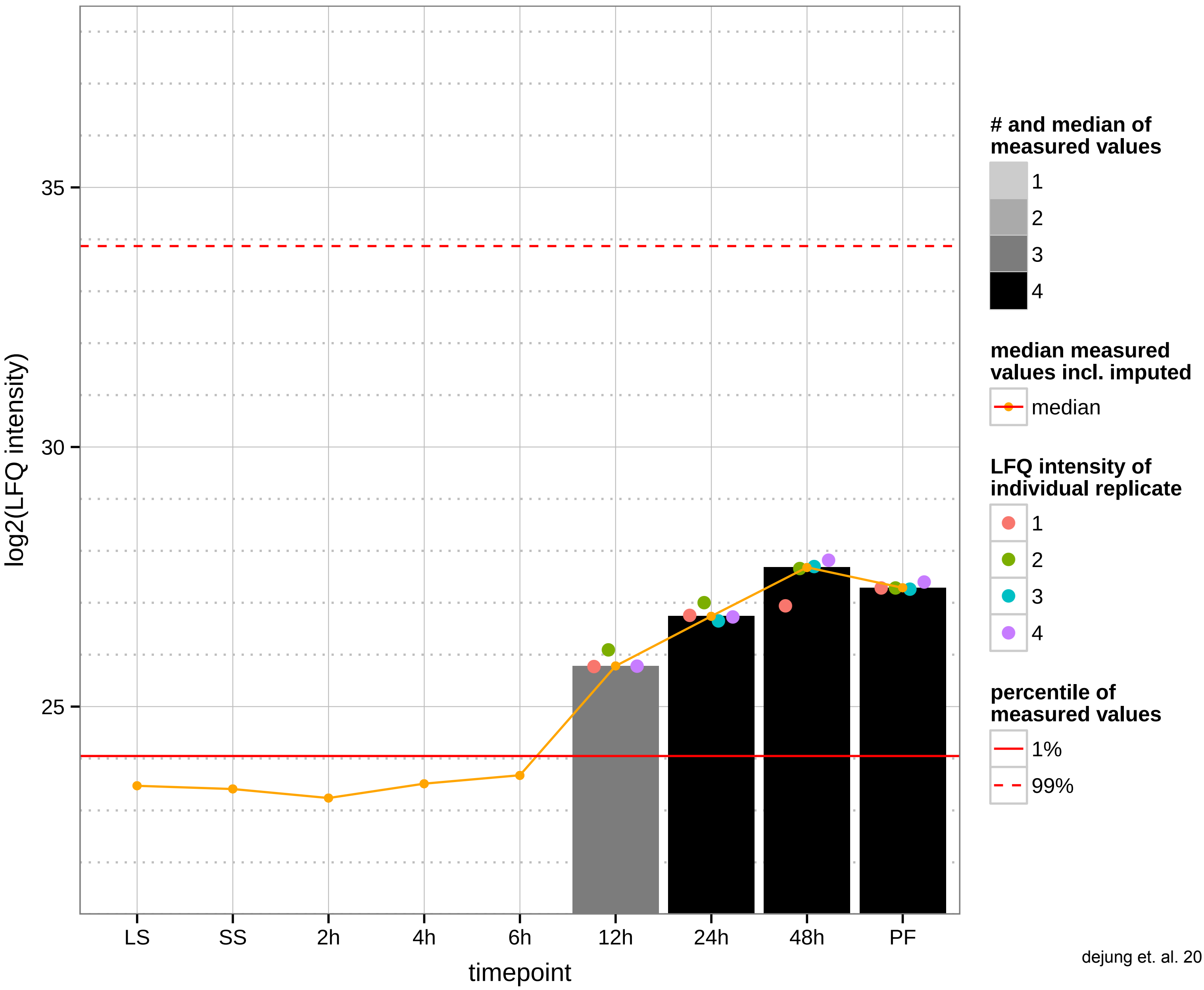

dejung et. al. 2015

hypothetical protein, conserved
Tb927.2.4400
AGOF: null
AGOC: mitochondrion
AGOP: null
PGOF: null
PGOC: null
PGOP: null

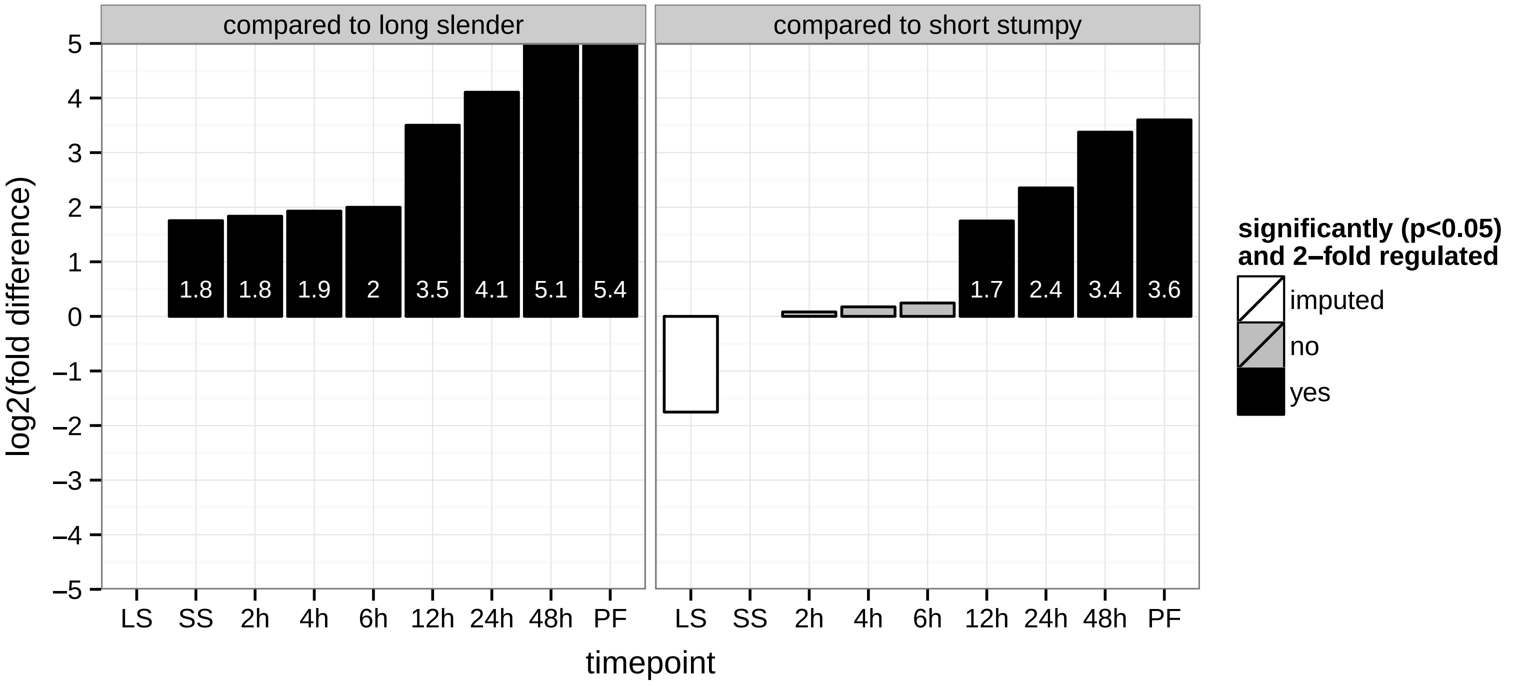

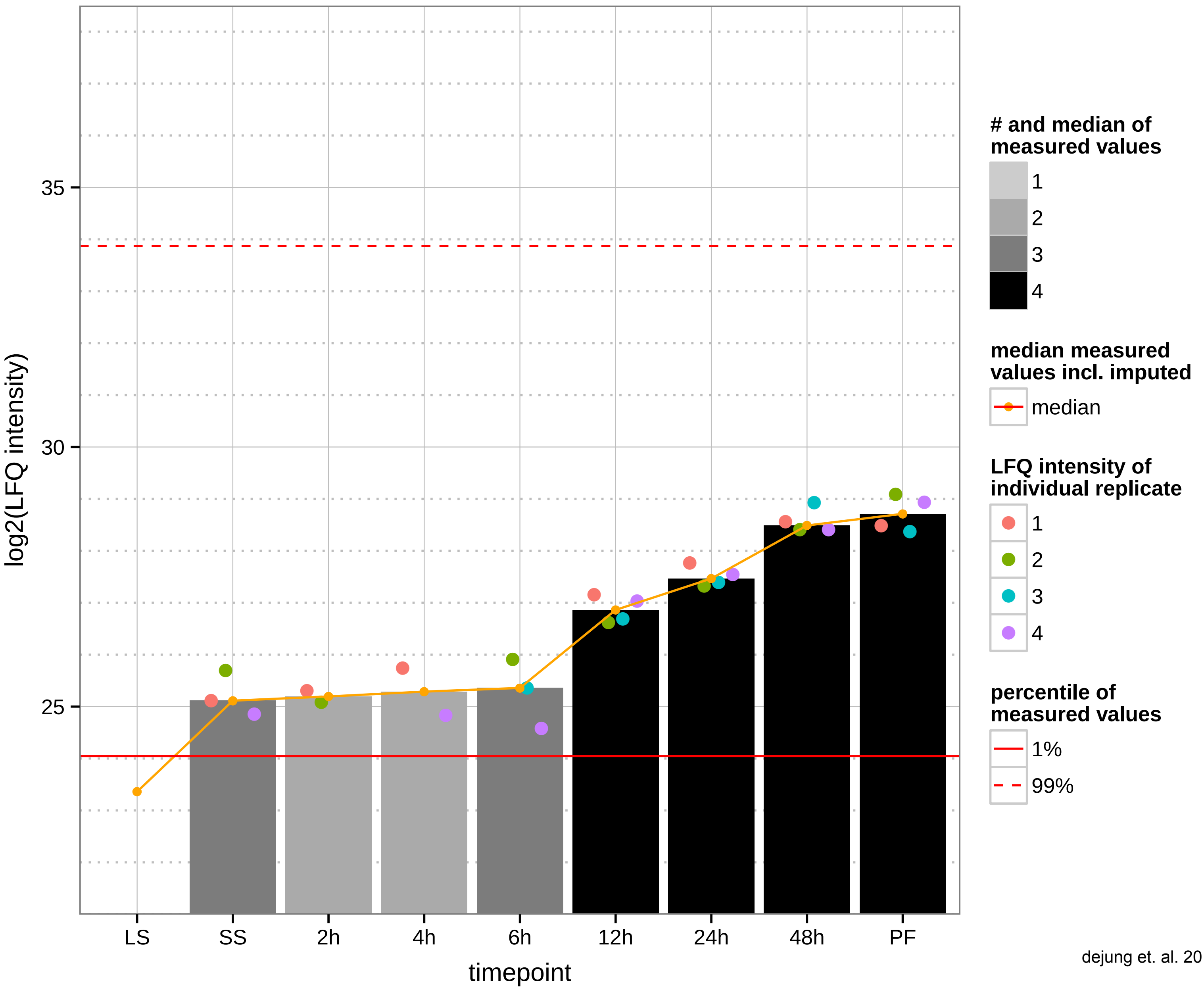

dejung et. al. 2015

hypothetical protein, conserved
Tb927.2.4460
AGOF: null
AGOC: null
AGOP: null
PGOF: null
PGOC: null
PGOP: null

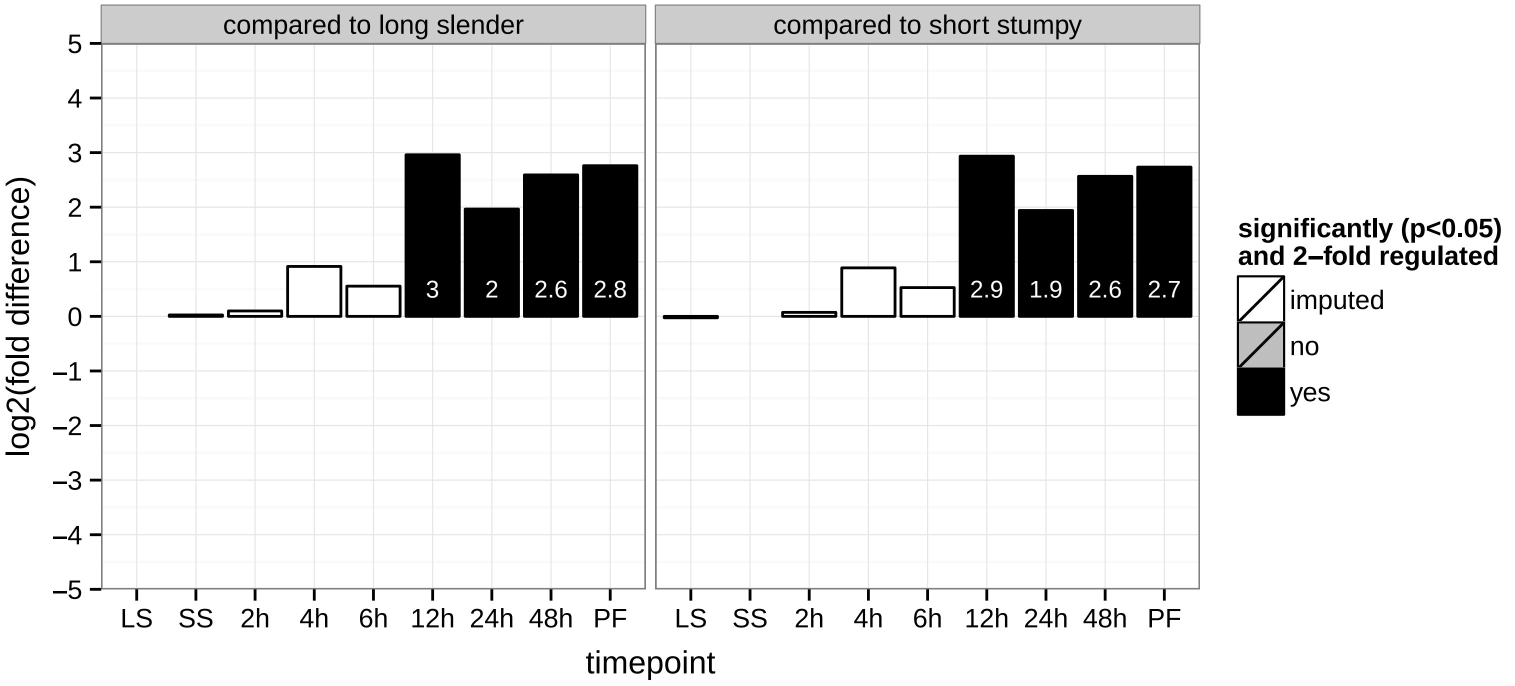

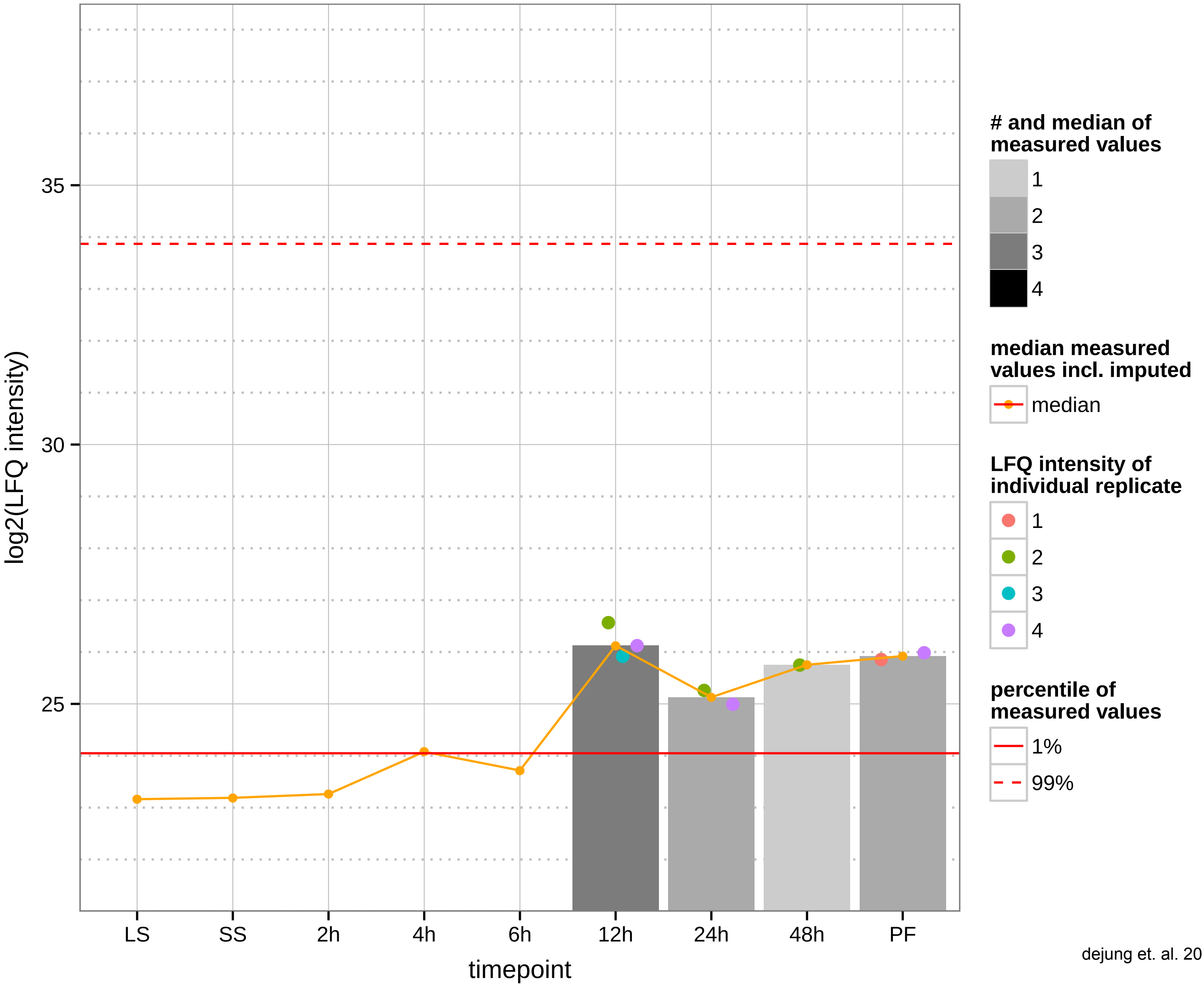

dejung et. al. 2015

hypothetical protein, conserved
Tb927.2.4650
AGOF: null
AGOC: null
AGOP: null
PGOF: null
PGOC: null
PGOP: null

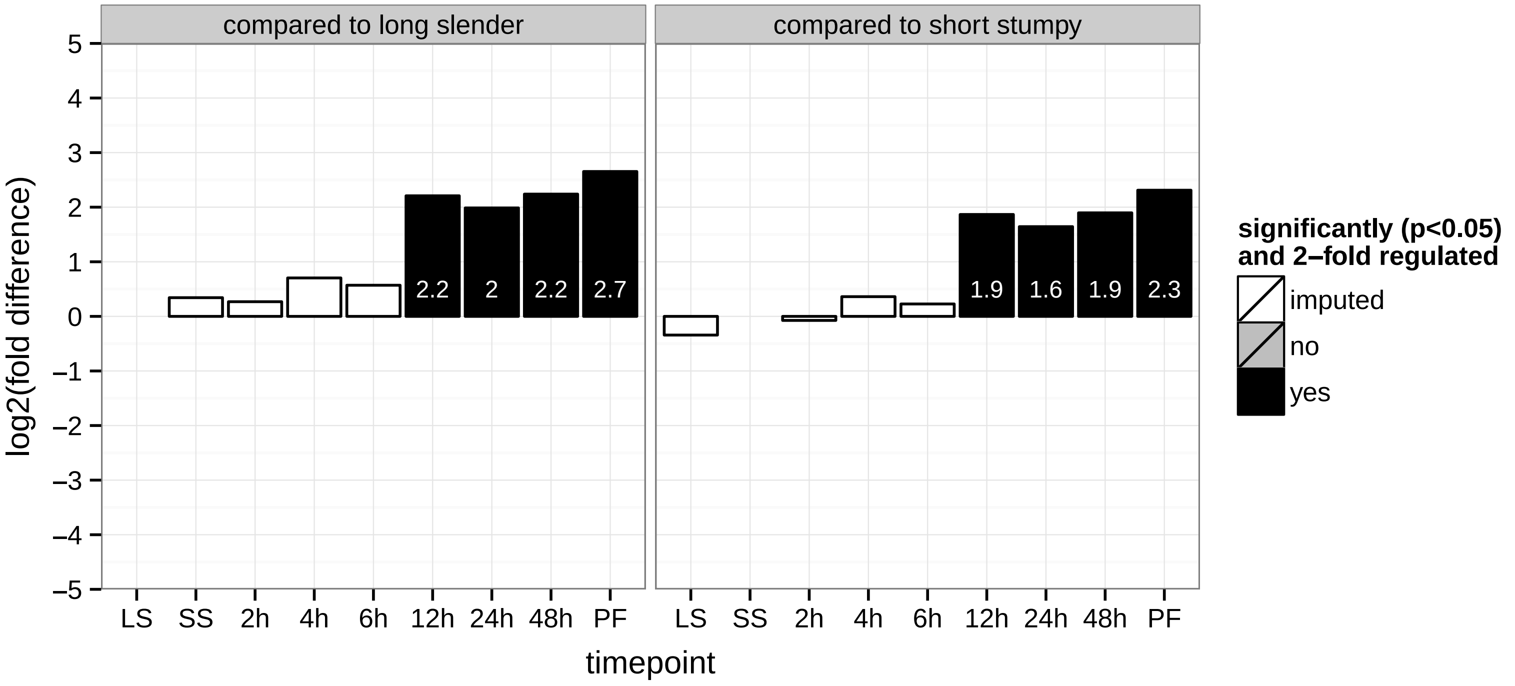

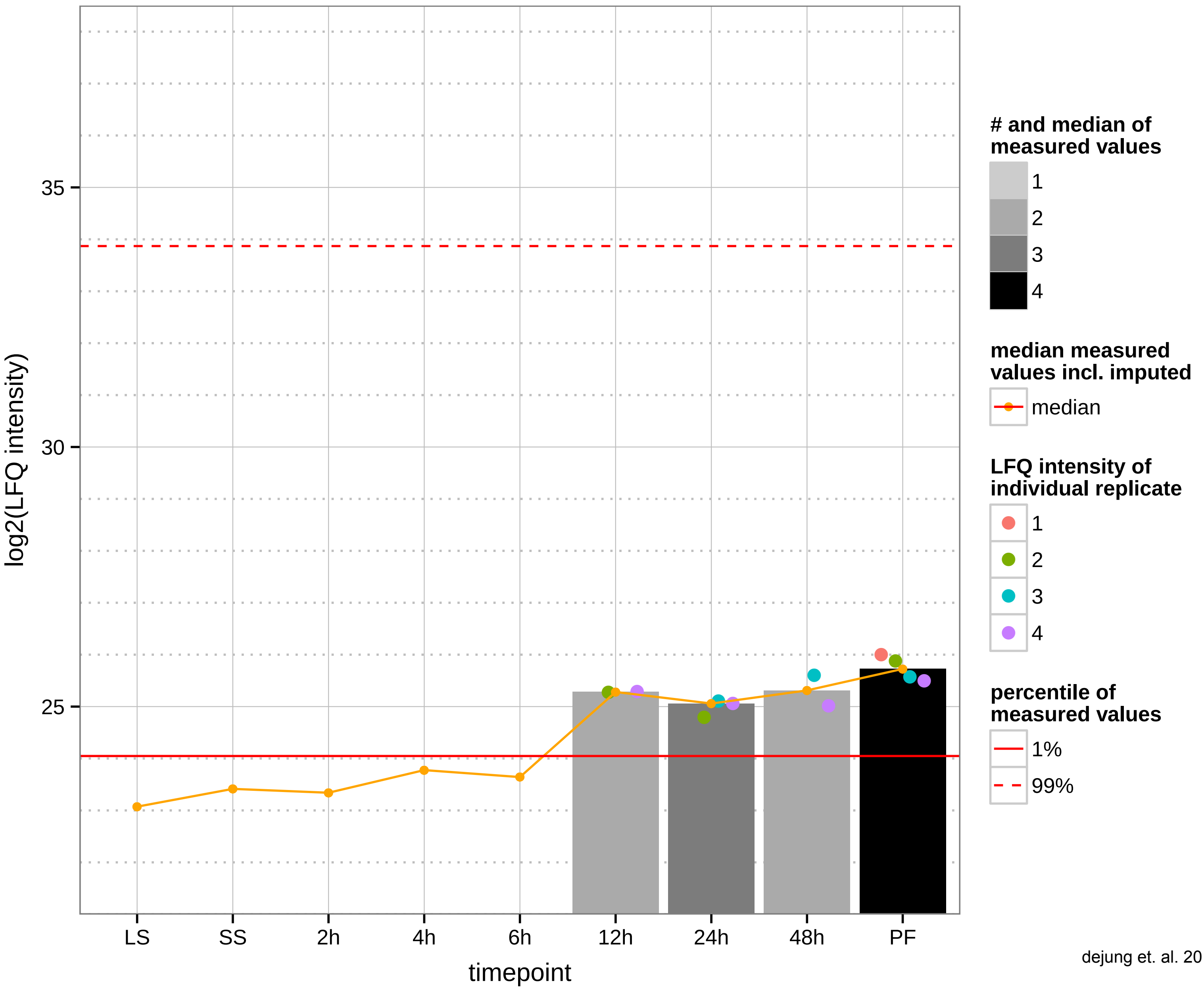

dejung et. al. 2015

acyl–CoA oxidase, putative
Tb927.2.5020
AGOF: acyl–CoA oxidase activity
AGOC: mitochondrion, peroxisome
AGOP: fatty acid beta–oxidation
PGOF: acyl–CoA oxidase activity, oxidoreductase activity, acting on the CH–CH group of donors
PGOC: peroxisome
PGOP: fatty acid beta–oxidation, metabolic process, oxidation–reduction process

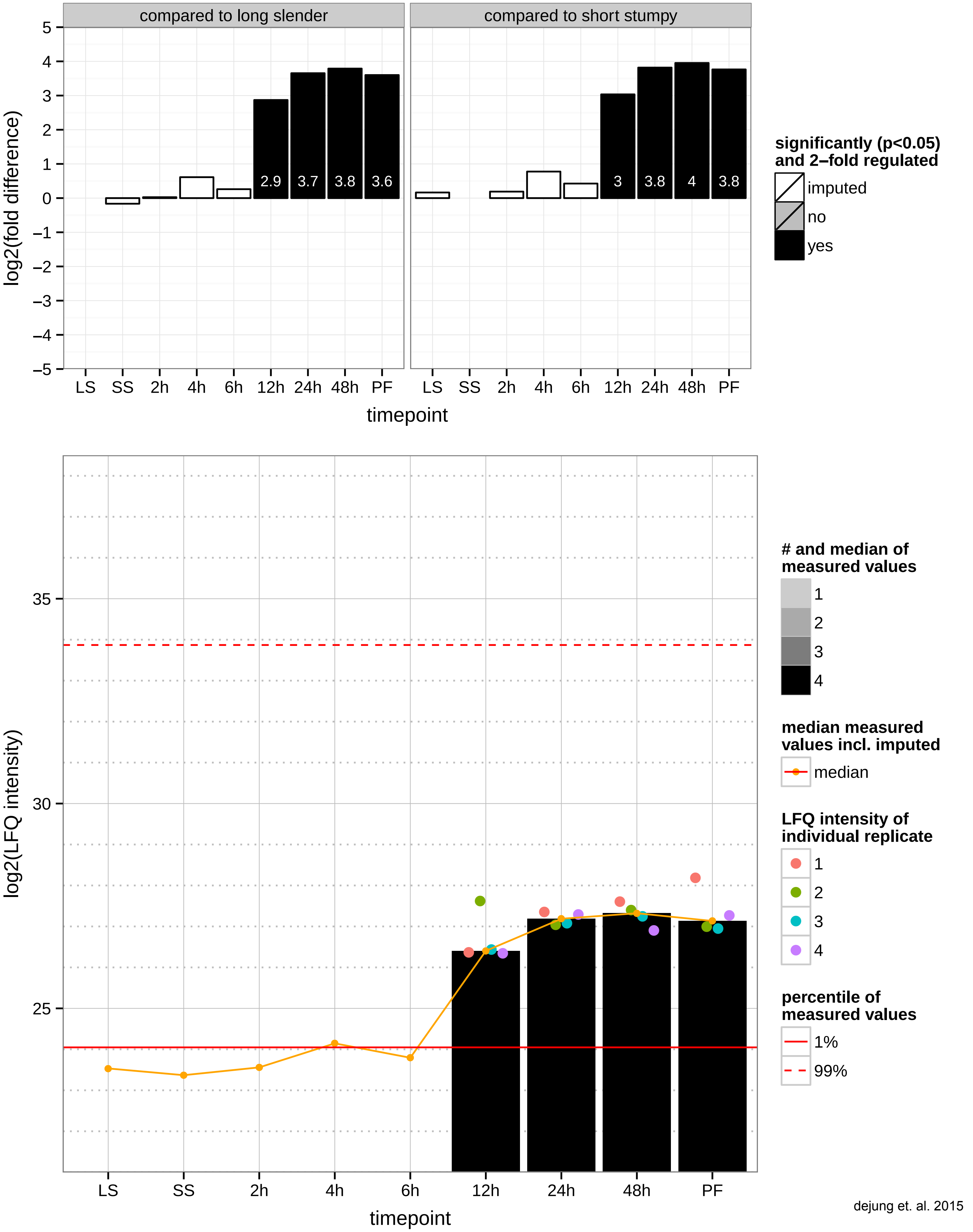

dejung et. al. 2015

hypothetical protein, conserved
Tb927.2.5610;Tb927.2.5530
AGOF: null
AGOC: null
AGOP: null
PGOF: null
PGOC: null
PGOP: null

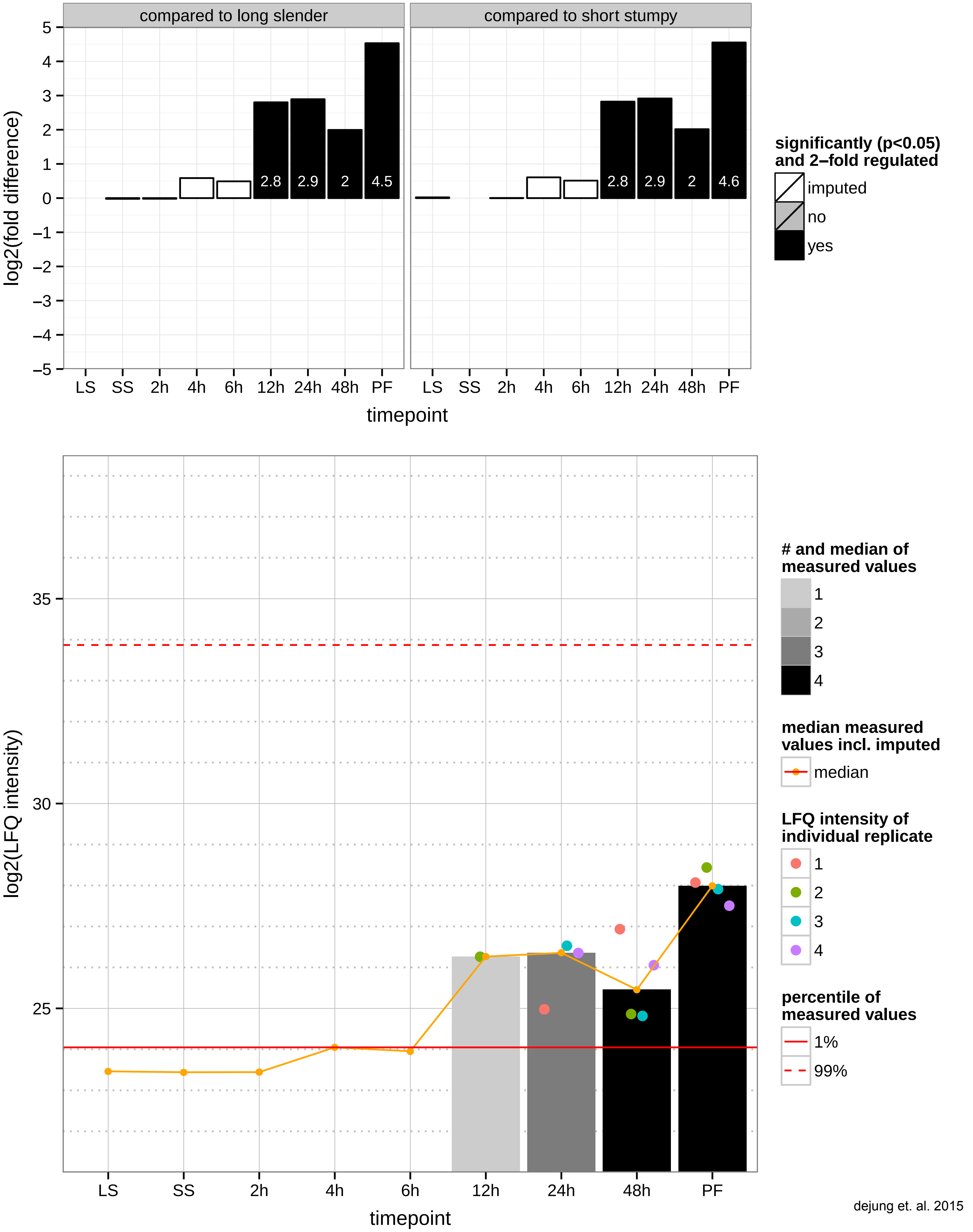

dejung et. al. 2015

cytochrome oxidase subunit VII (COXVII)
Tb927.3.1410
AGOF: null
AGOC: mitochondrion
AGOP: null
PGOF: null
PGOC: null
PGOP: null

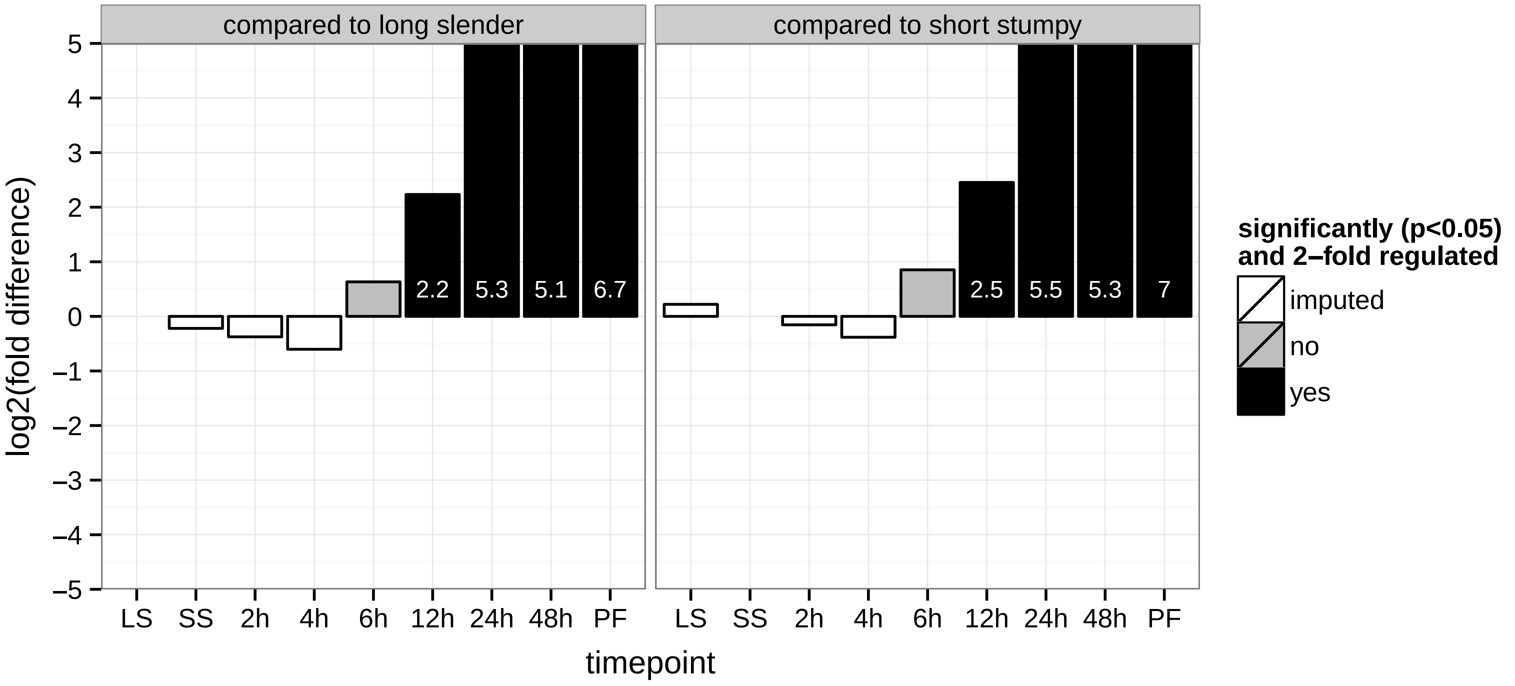

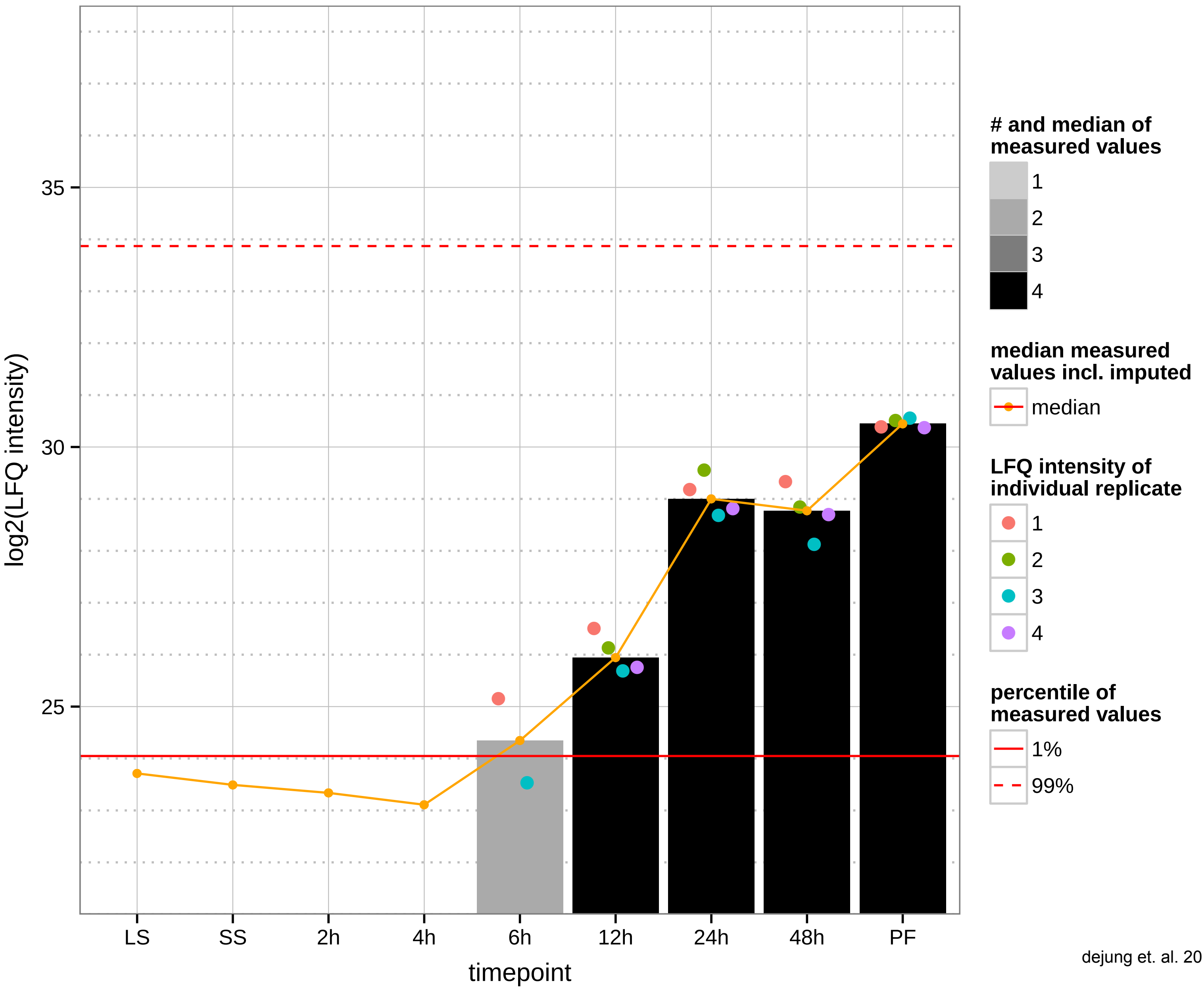

dejung et. al. 2015

hypothetical protein, conserved
Tb927.3.1680
AGOF: null
AGOC: null
AGOP: null
PGOF: null
PGOC: null
PGOP: null

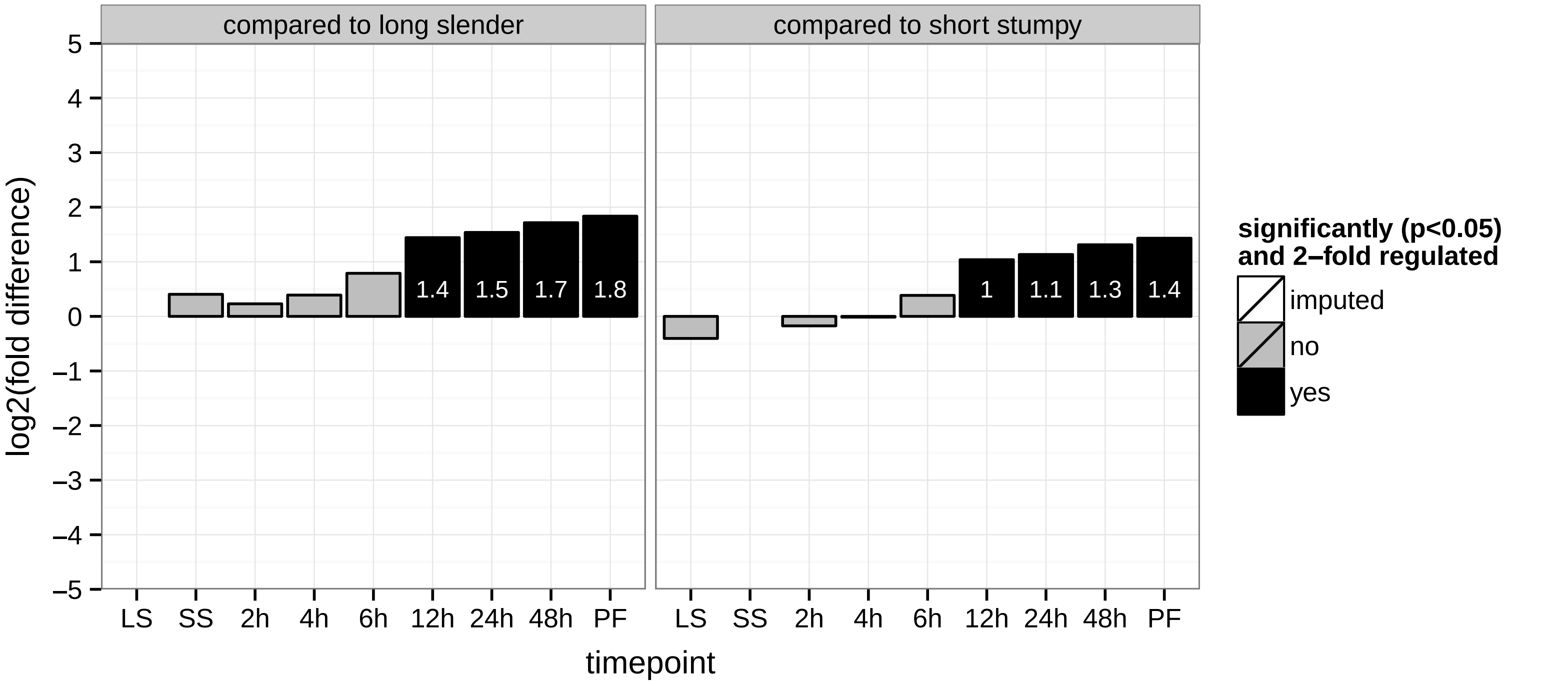

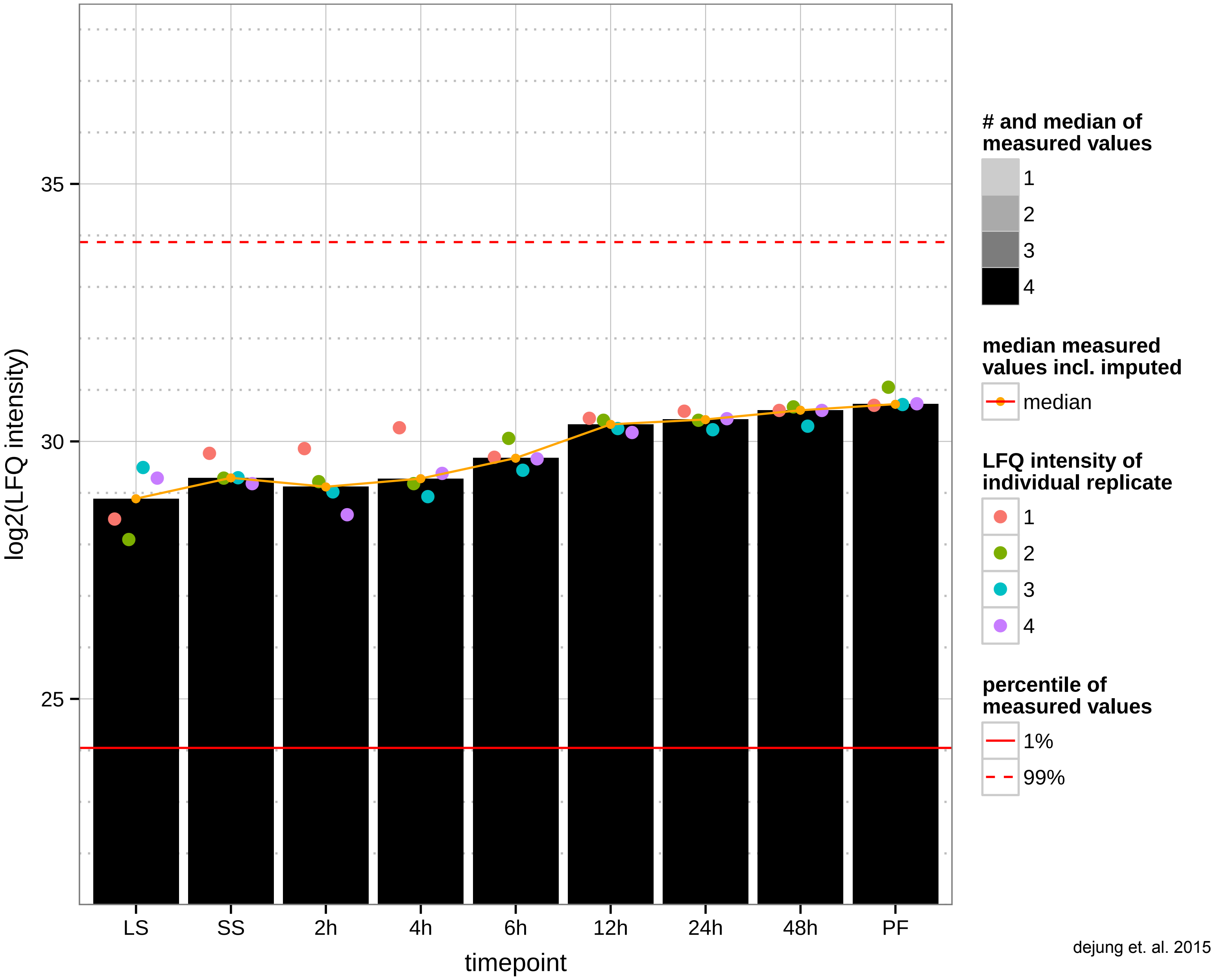

dejung et. al. 2015

hypothetical protein, conserved
Tb927.3.1730
AGOF: null
AGOC: mitochondrion
AGOP: null
PGOF: null
PGOC: null
PGOP: null

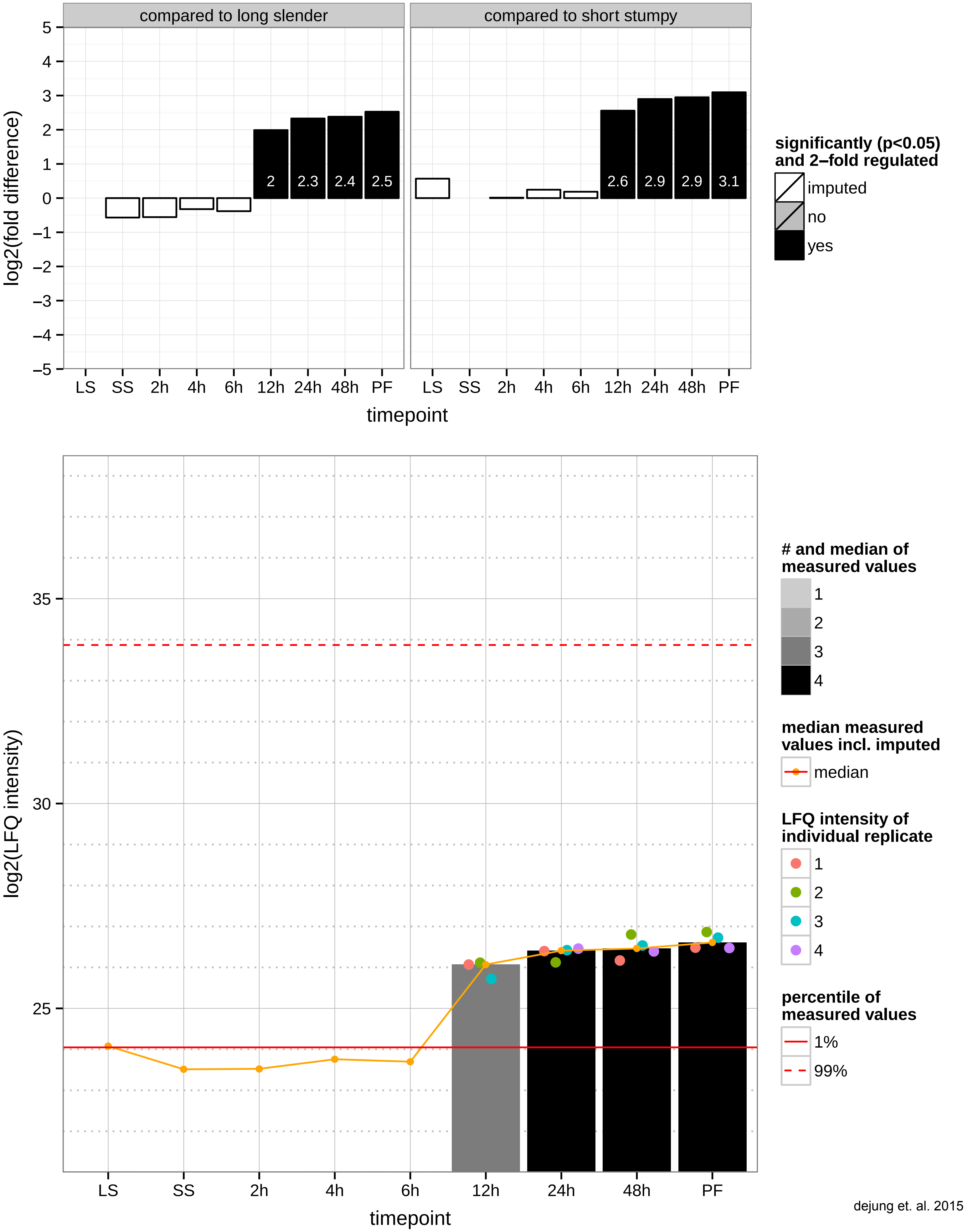

dejung et. al. 2015

hypothetical protein, conserved
Tb927.3.1940
AGOF: null
AGOC: mitochondrion
AGOP: null
PGOF: null
PGOC: null
PGOP: null

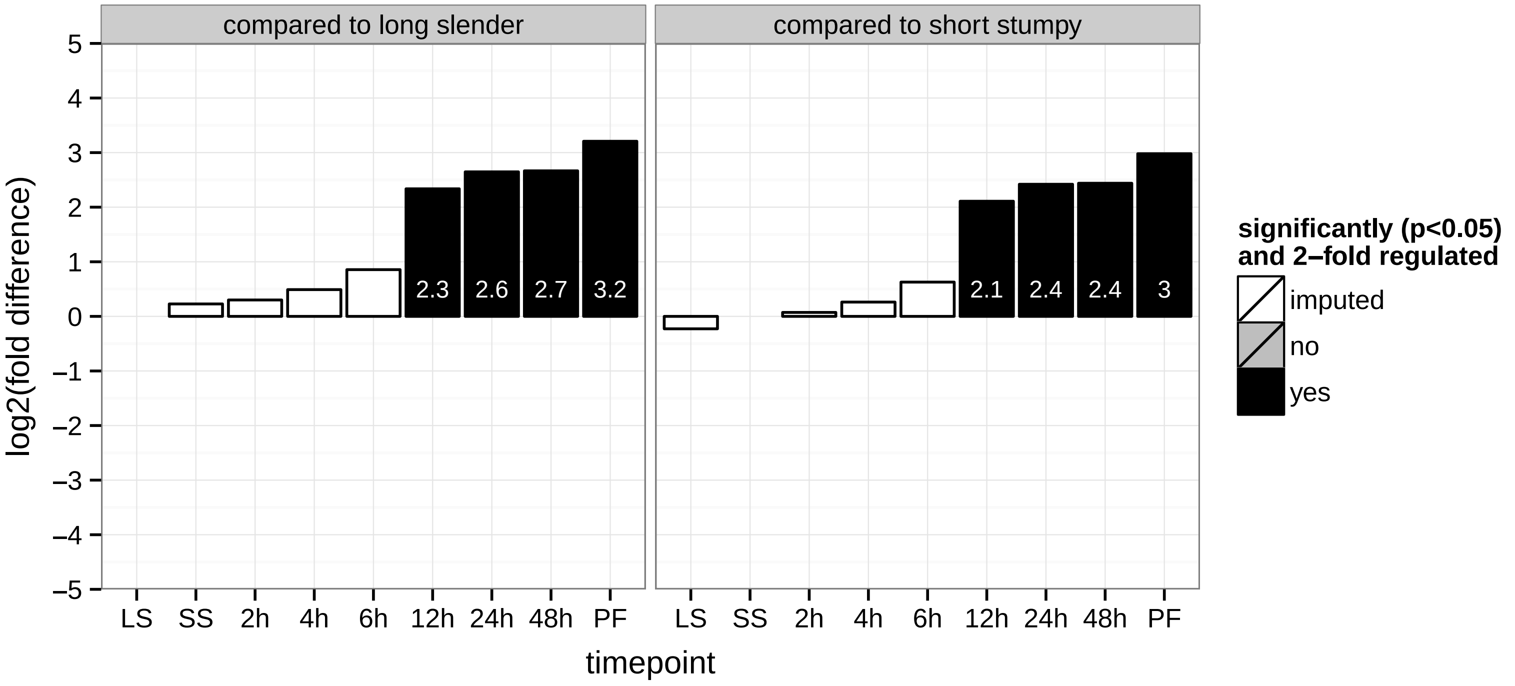

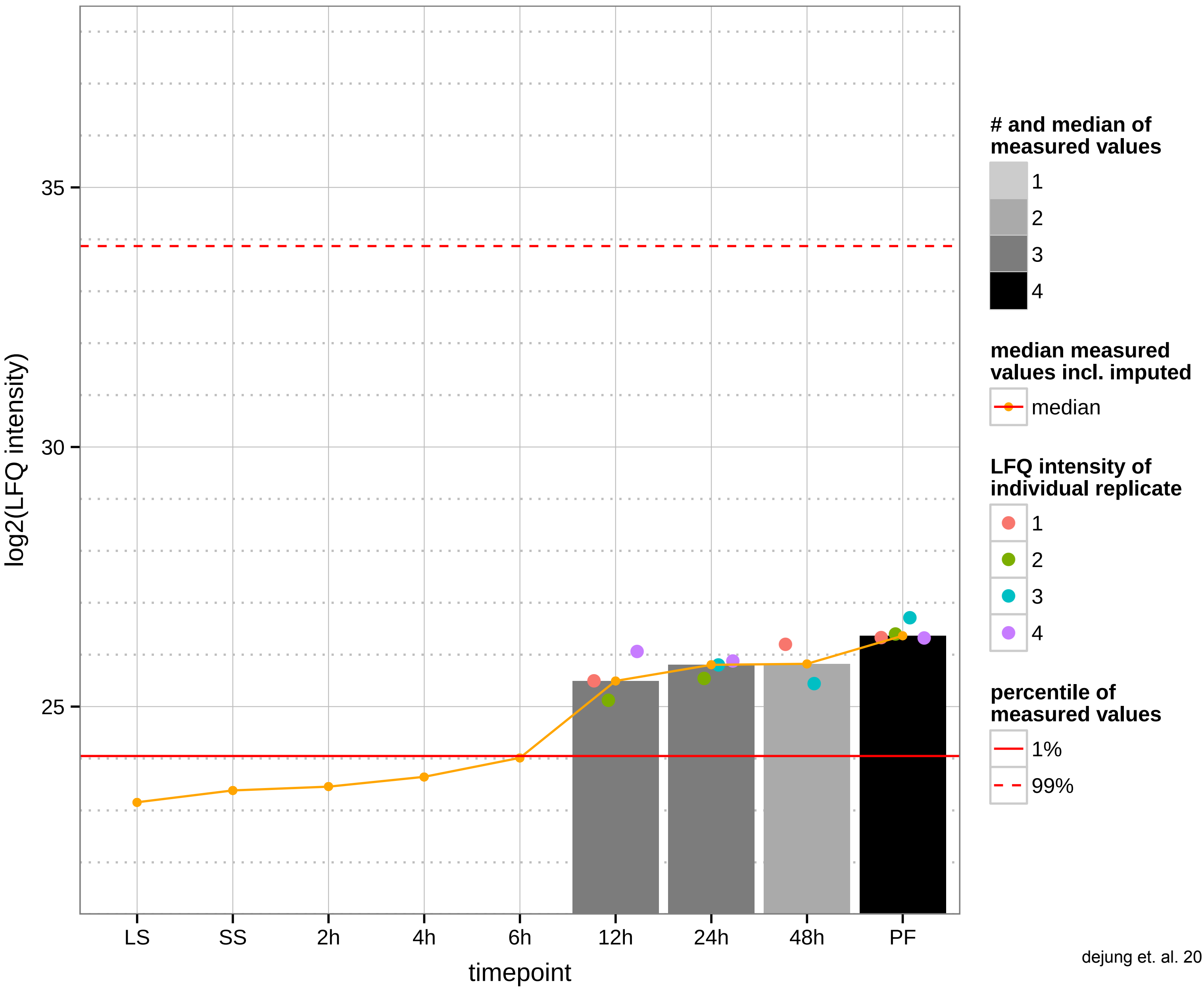

dejung et. al. 2015

hypothetical protein, conserved
Tb927.3.2180
AGOF: null
AGOC: mitochondrion
AGOP: null
PGOF: null
PGOC: null
PGOP: null

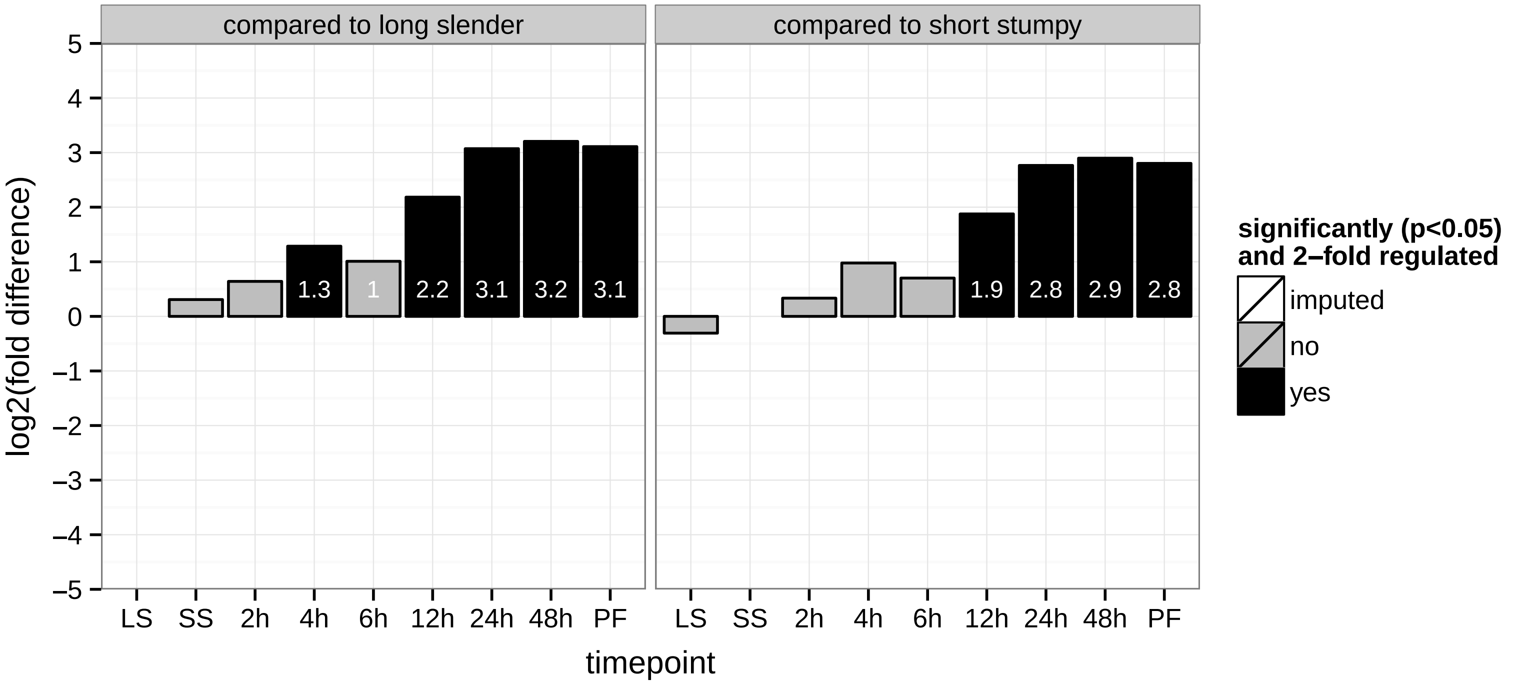

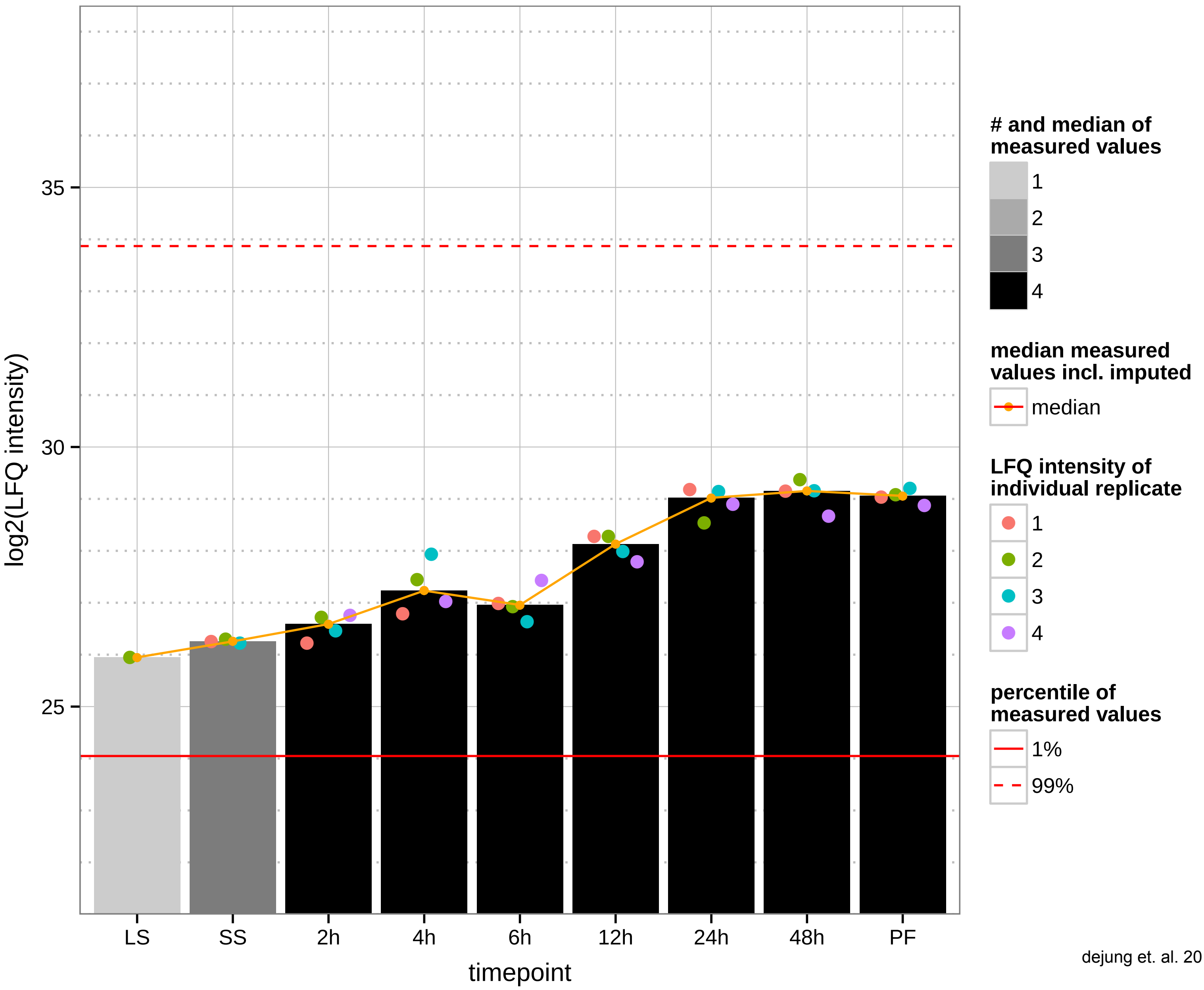

dejung et. al. 2015

hypothetical protein, conserved
Tb927.3.2880
AGOF: null
AGOC: mitochondrion
AGOP: null
PGOF: null
PGOC: null
PGOP: null

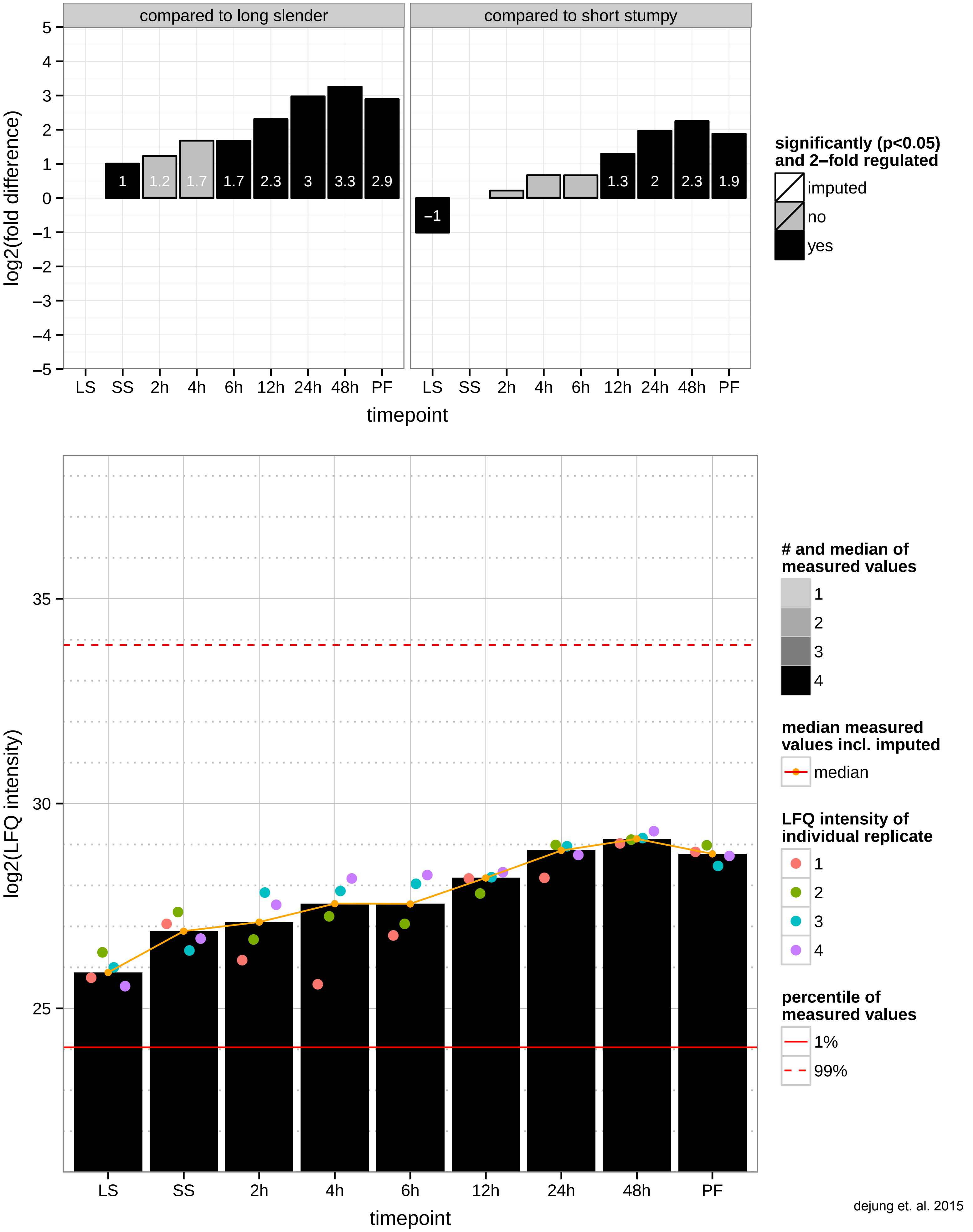

dejung et. al. 2015

peptidyl–prolyl cis–trans isomerase NIMA–interacting 4, putative
Tb927.3.3100
AGOF: isomerase activity
AGOC: null
AGOP: null
PGOF: isomerase activity
PGOC: null
PGOP: null

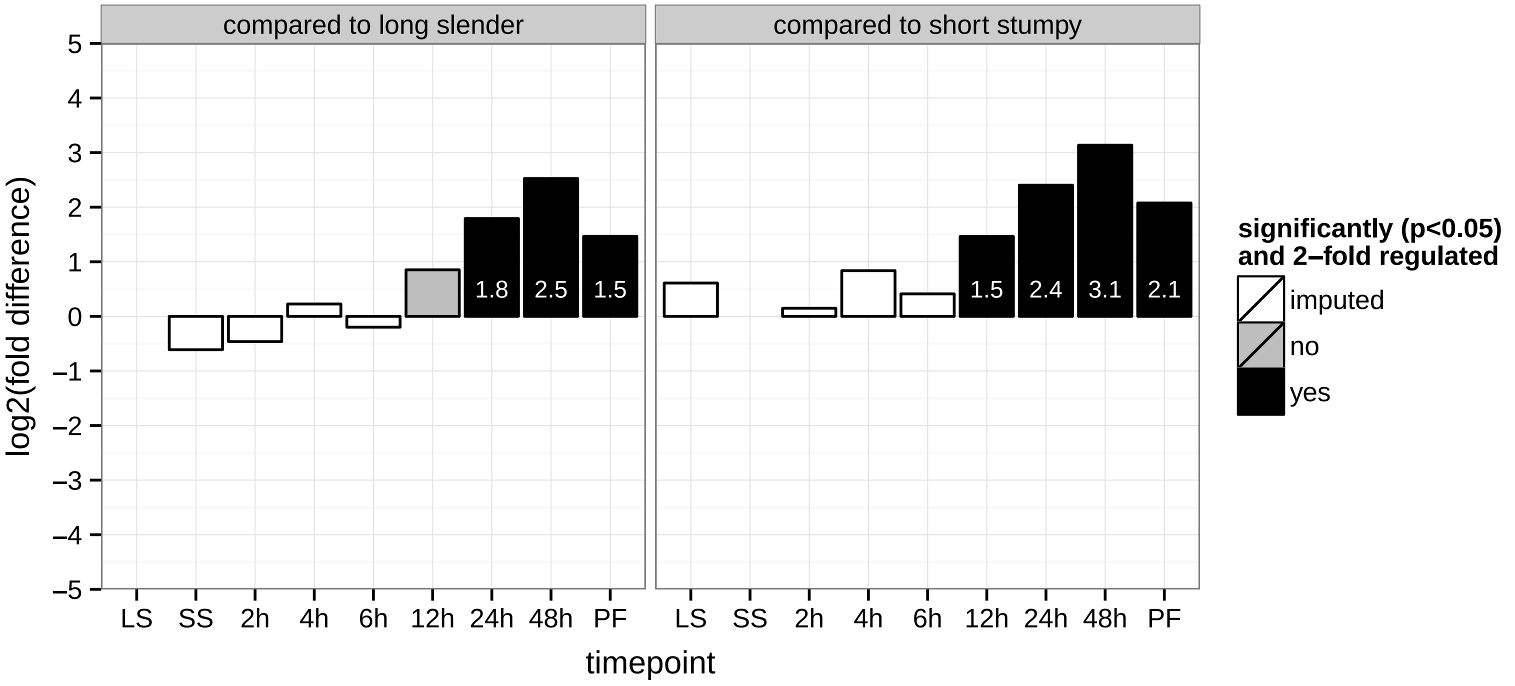

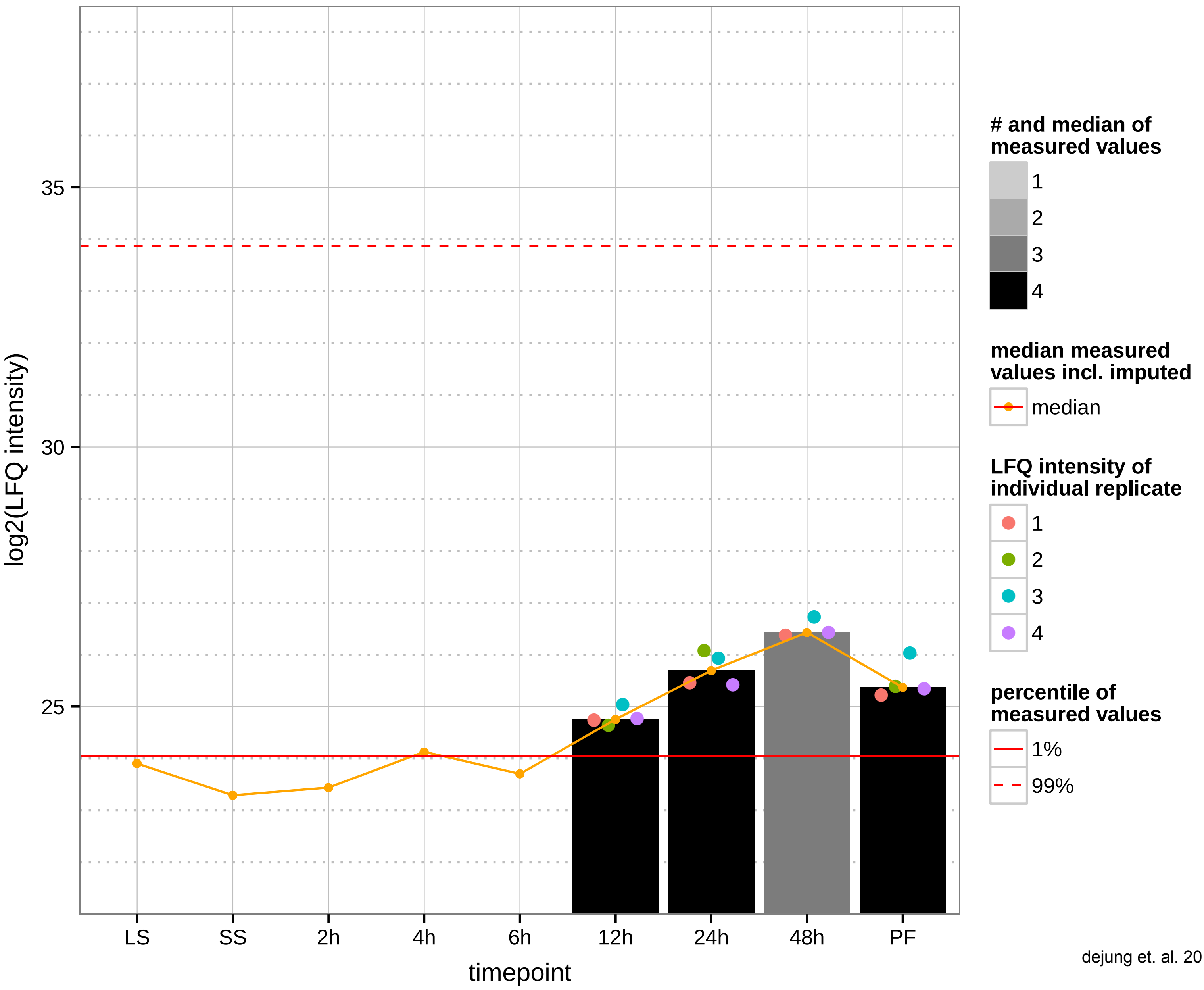

dejung et. al. 2015

hypothetical protein, conserved
Tb927.3.3660
AGOF: null
AGOC: mitochondrion
AGOP: null
PGOF: null
PGOC: null
PGOP: null

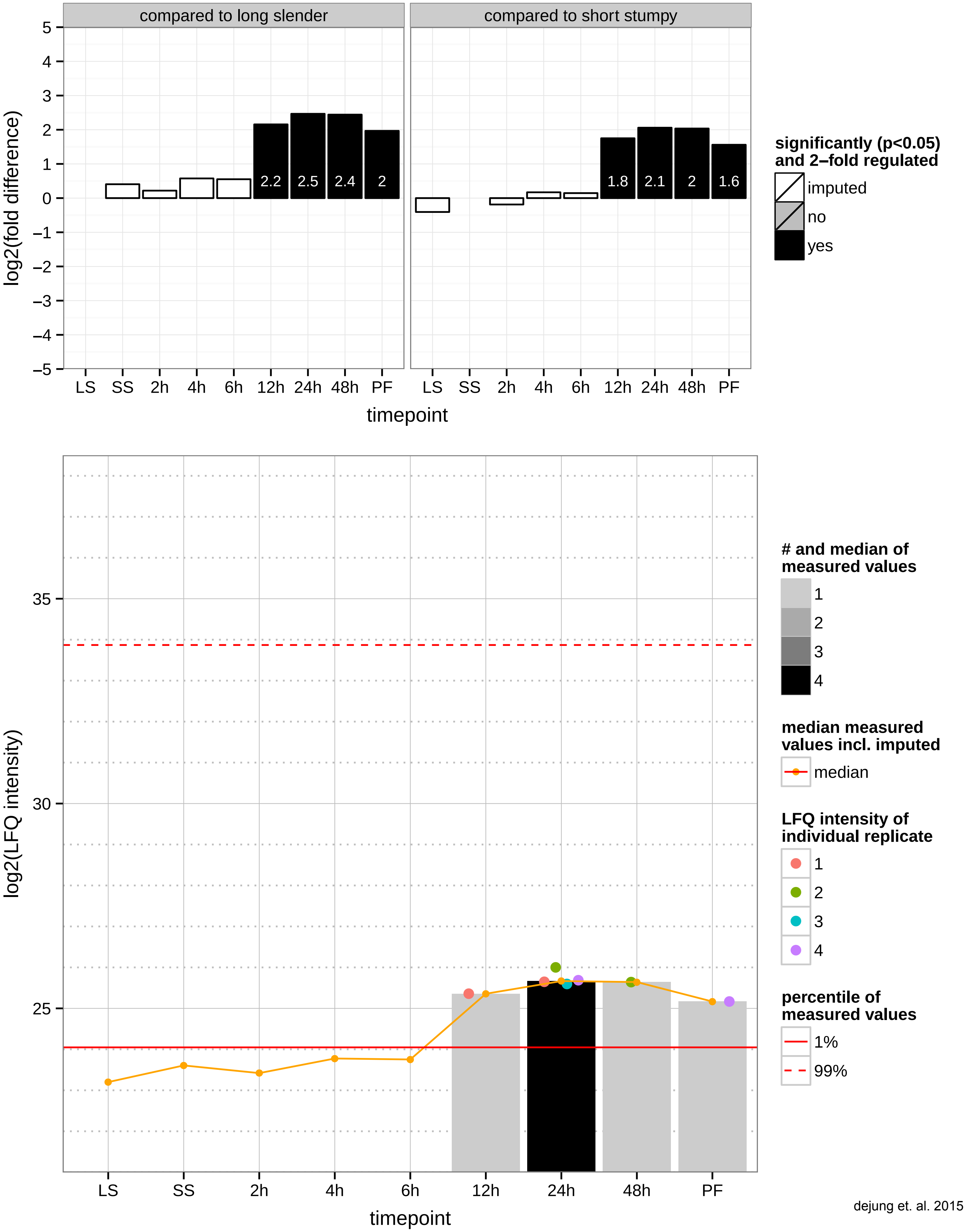

dejung et. al. 2015

hypothetical protein, conserved
Tb927.3.3890
AGOF: null
AGOC: mitochondrion
AGOP: null
PGOF: null
PGOC: null
PGOP: null

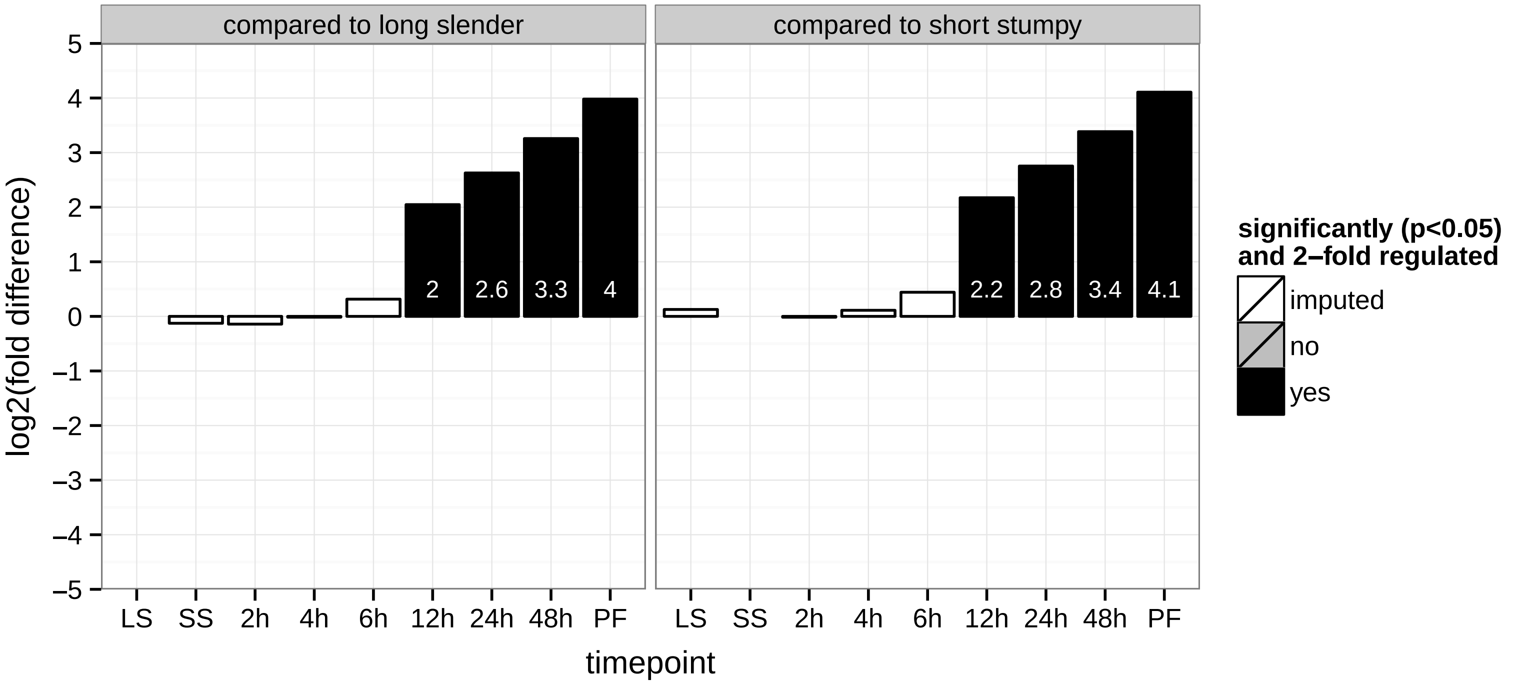

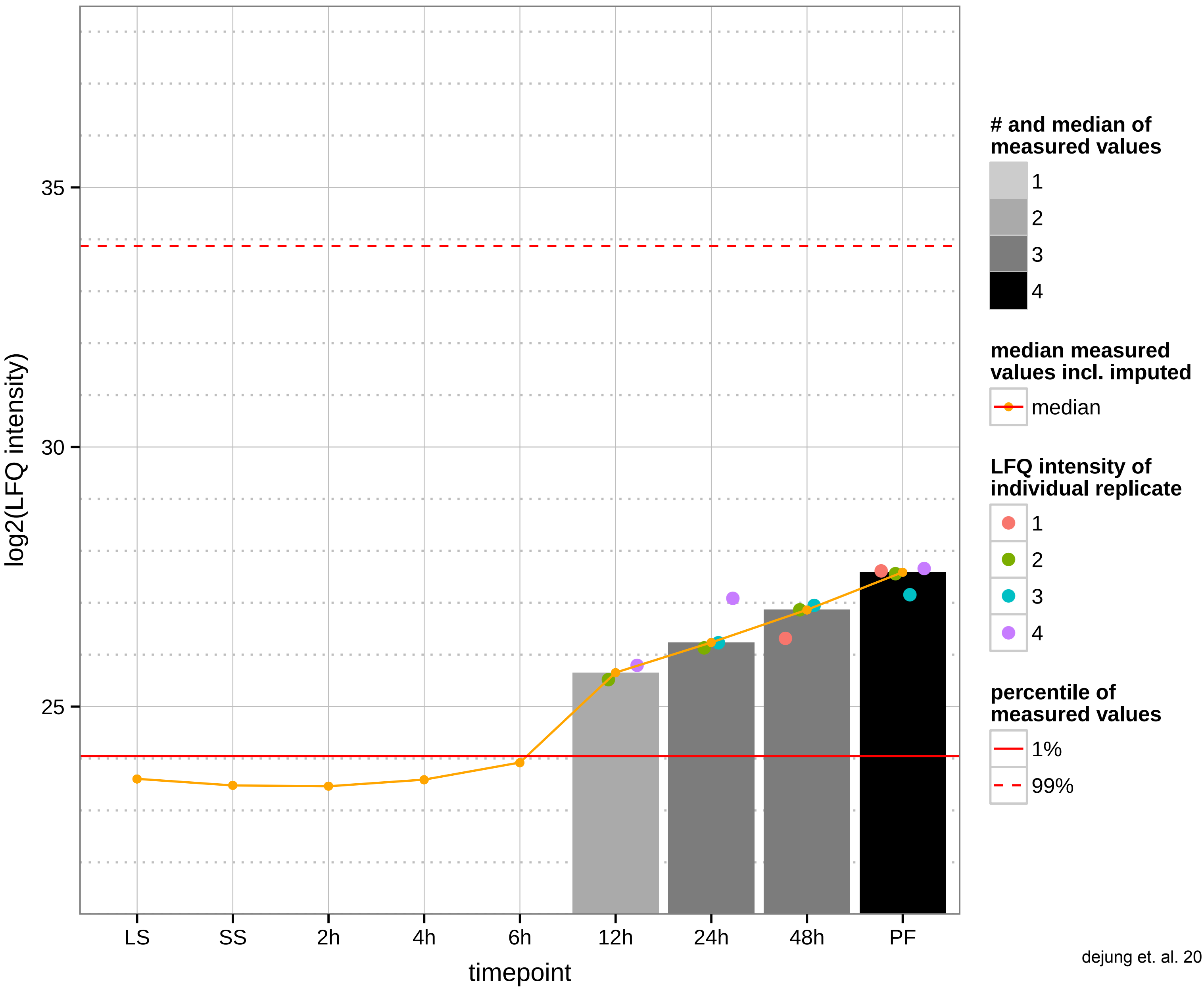

dejung et. al. 2015

RNA–binding protein (DRBD6B), RNA–binding protein, putative (DRBD11), RNA–binding protein (DRBD6A)
Tb927.3.3940
AGOF: nucleic acid binding, RNA binding
AGOC: null
AGOP: RNA processing
PGOF: nucleic acid binding
PGOC: null
PGOP: null

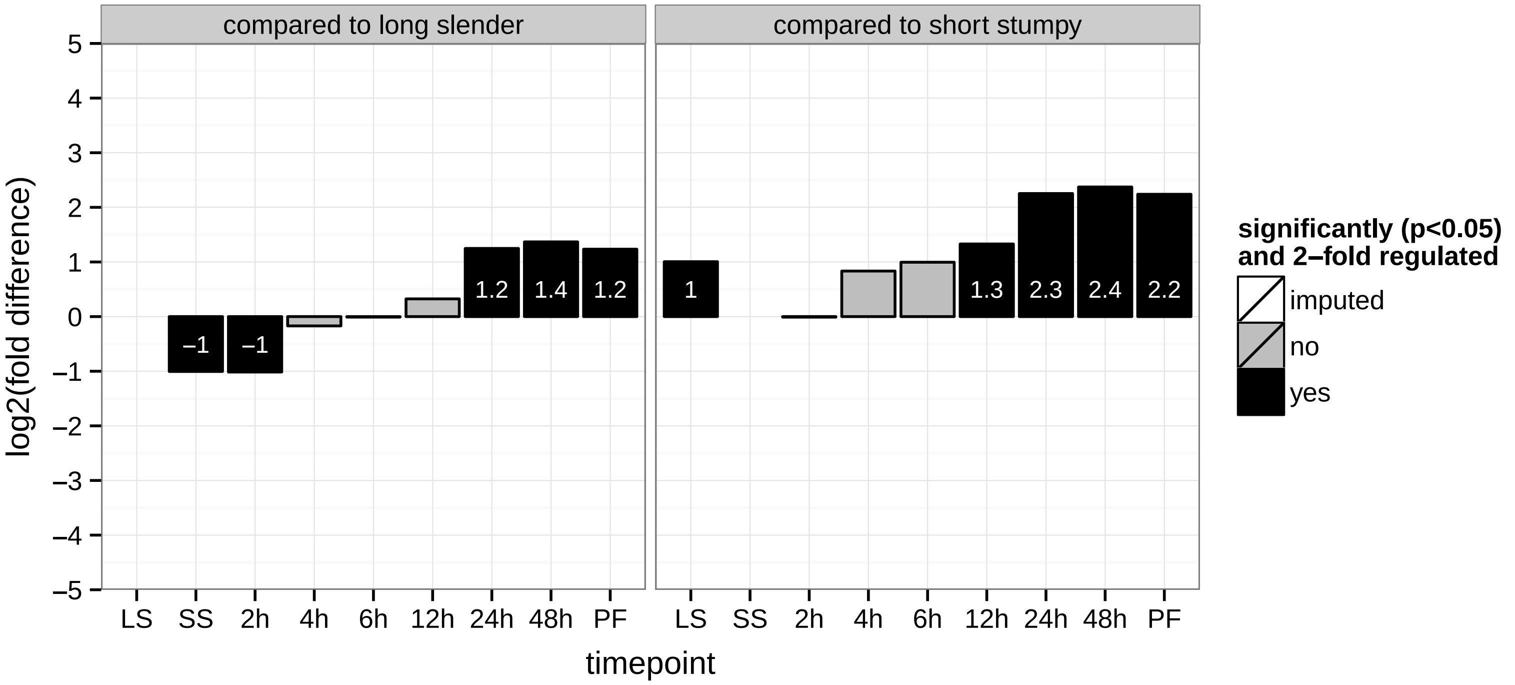

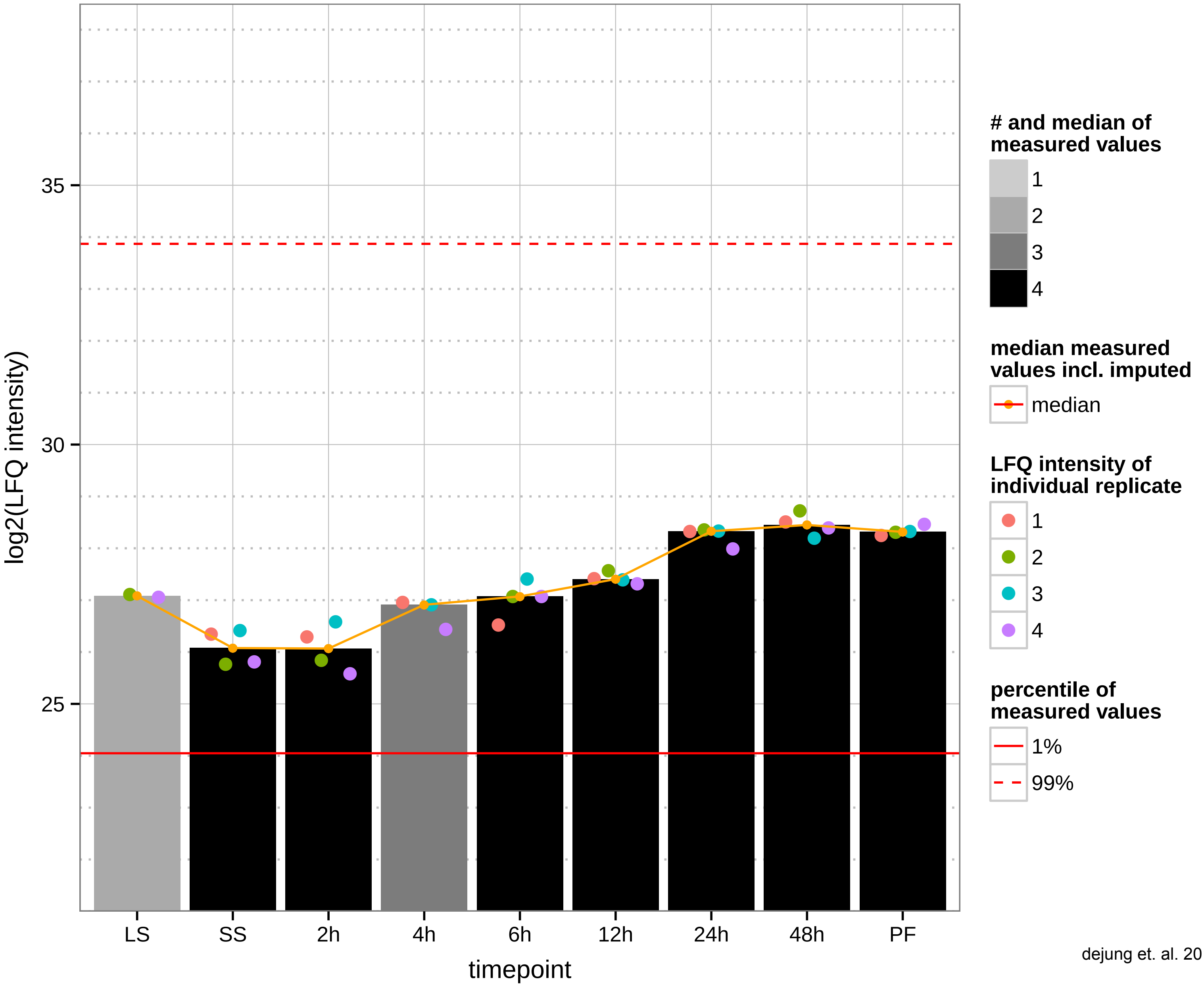

dejung et. al. 2015

Zinc finger CCCH domain–containing protein 8 (ZC3H8)
Tb927.3.5250
AGOF: RNA binding
AGOC: null
AGOP: null
PGOF: nucleic acid binding, zinc ion binding
PGOC: null
PGOP: null

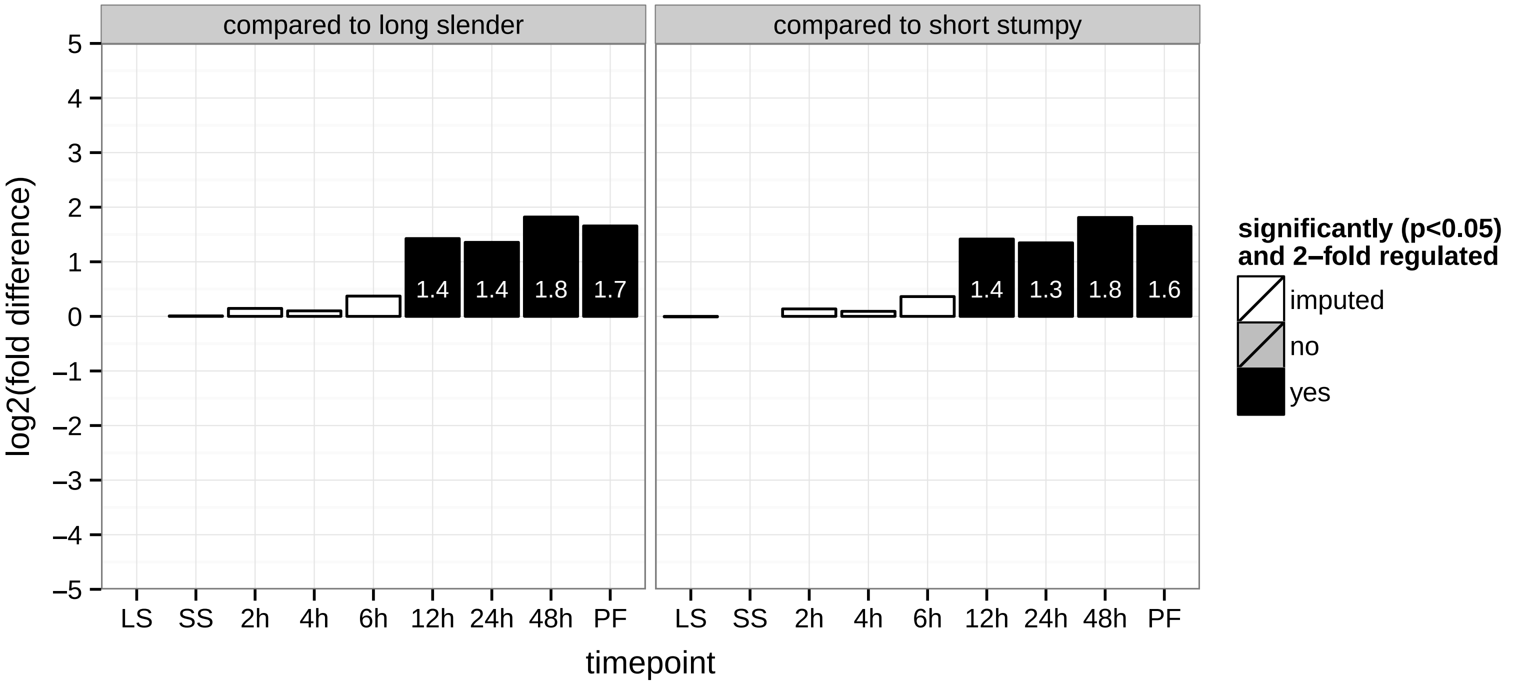

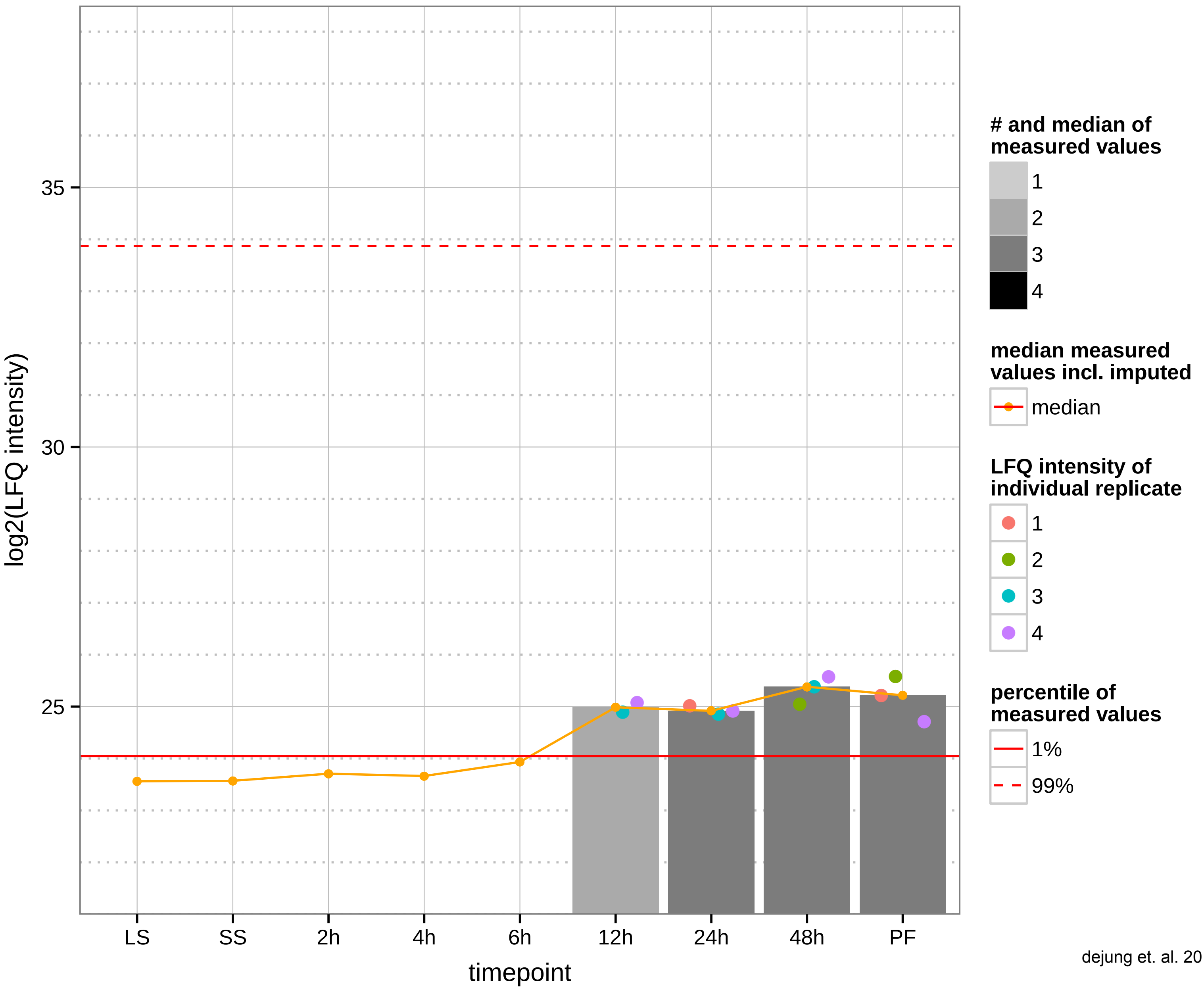

dejung et. al. 2015

N–acetyltransferase complex ARD1 subunit, putative
Tb927.3.610
AGOF: N–acetyltransferase activity
AGOC: null
AGOP: metabolic process
PGOF: N–acetyltransferase activity
PGOC: null
PGOP: null

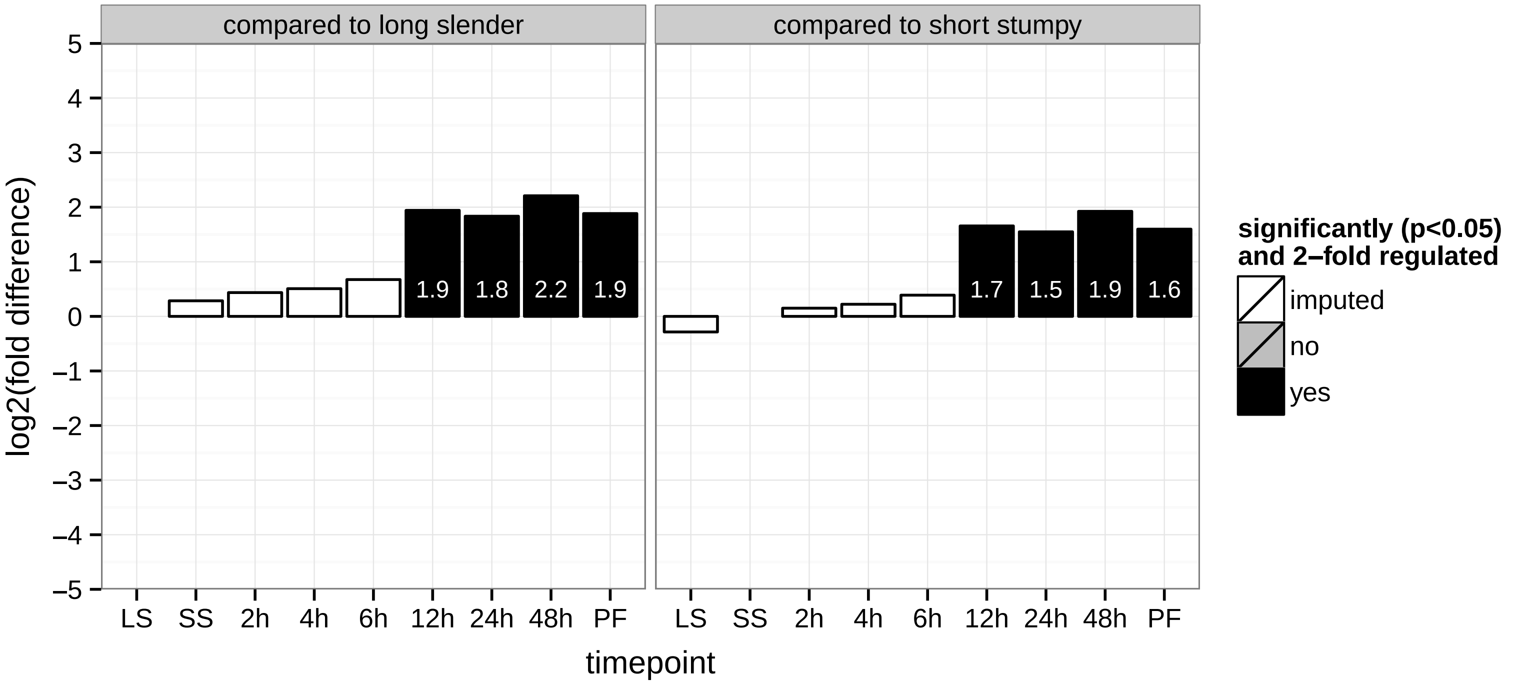

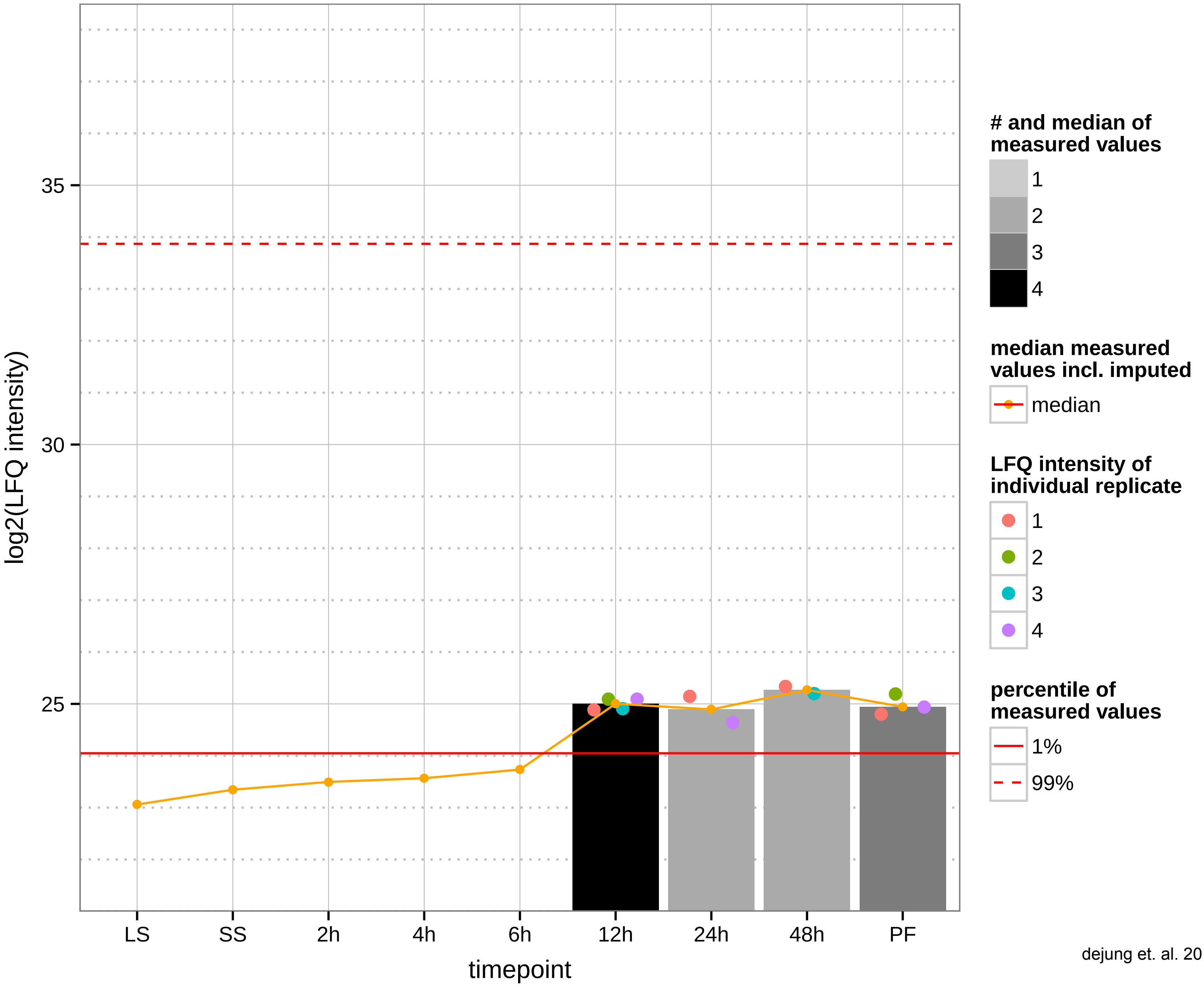

dejung et. al. 2015

zinc finger protein 3, ZFP family member, putative (ZFP3)
Tb927.3.720
AGOF: RNA binding, zinc ion binding
AGOC: ribonucleoprotein complex
AGOP: null
PGOF: nucleic acid binding, protein binding, zinc ion binding
PGOC: null
PGOP: null

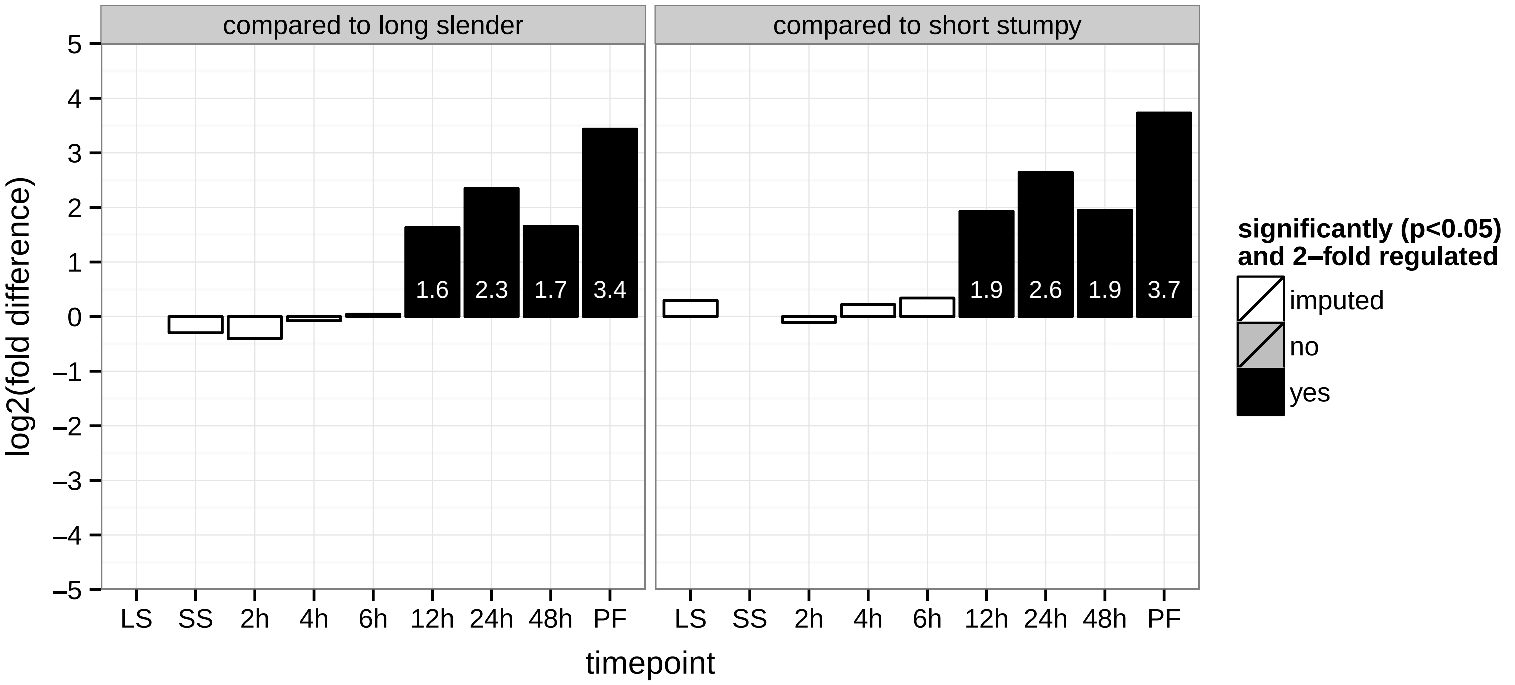

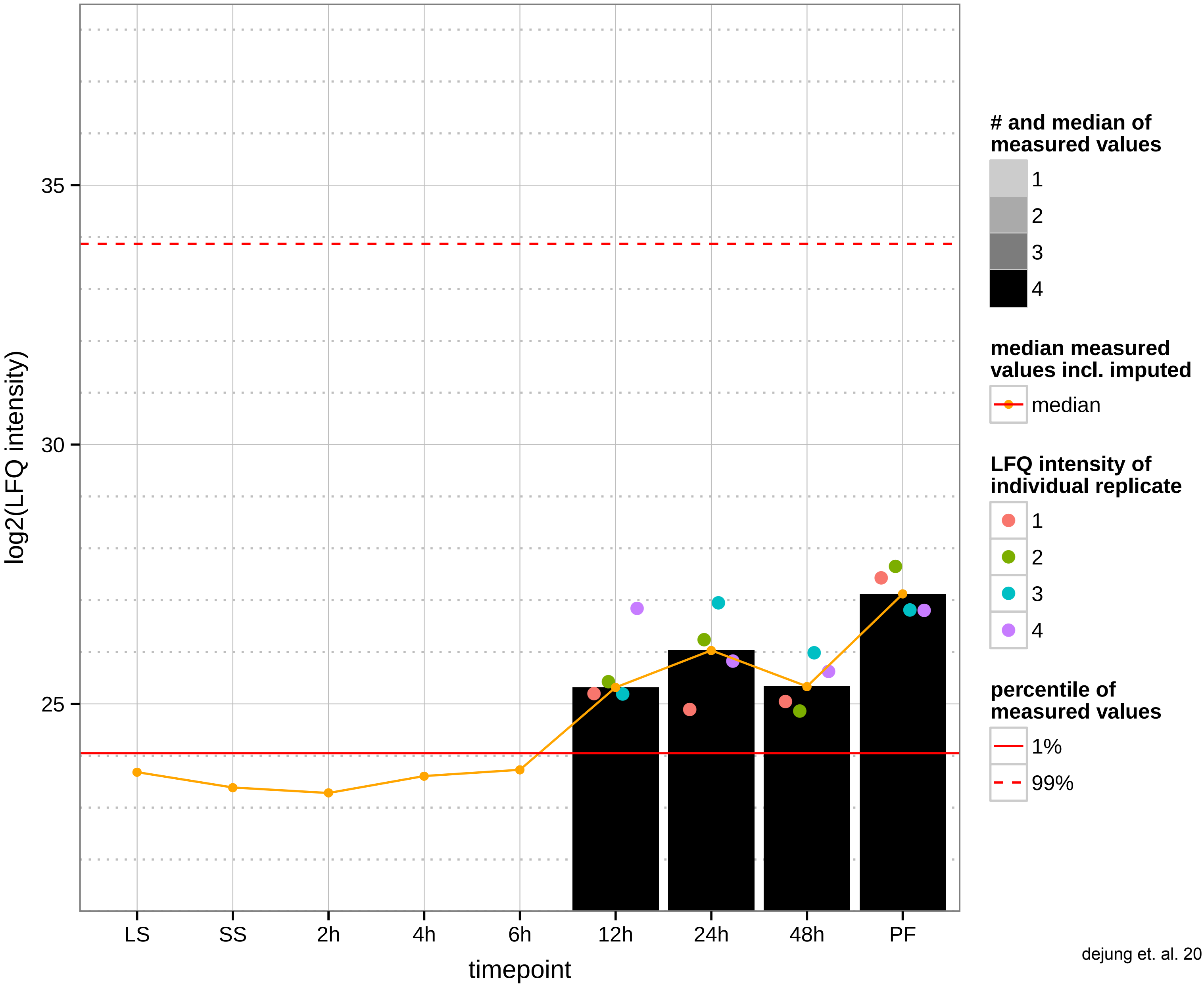

dejung et. al. 2015

hypothetical protein, conserved
Tb927.3.950
AGOF: oxidoreductase activity
AGOC: mitochondrion
AGOP: null
PGOF: oxidoreductase activity, oxidoreductase activity, acting on paired donors, with incorporation or reduction of molecular o
PGOC: null
PGOP: oxidation–reduction process

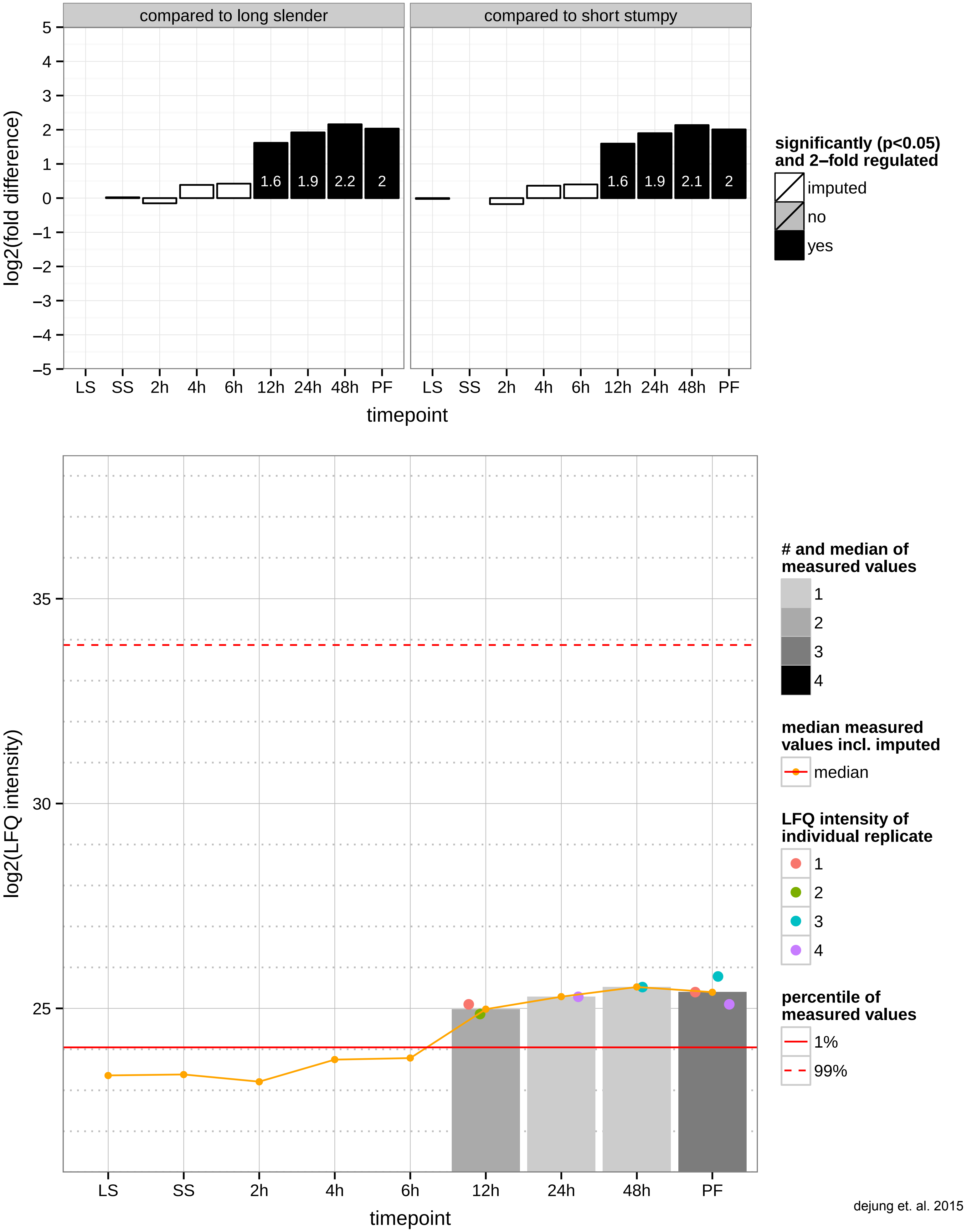

dejung et. al. 2015

ankyrin, putative
Tb927.4.1170;Tb11.v5.0430
AGOF: null
AGOC: null
AGOP: null
PGOF: null, protein binding
PGOC: null
PGOP: null

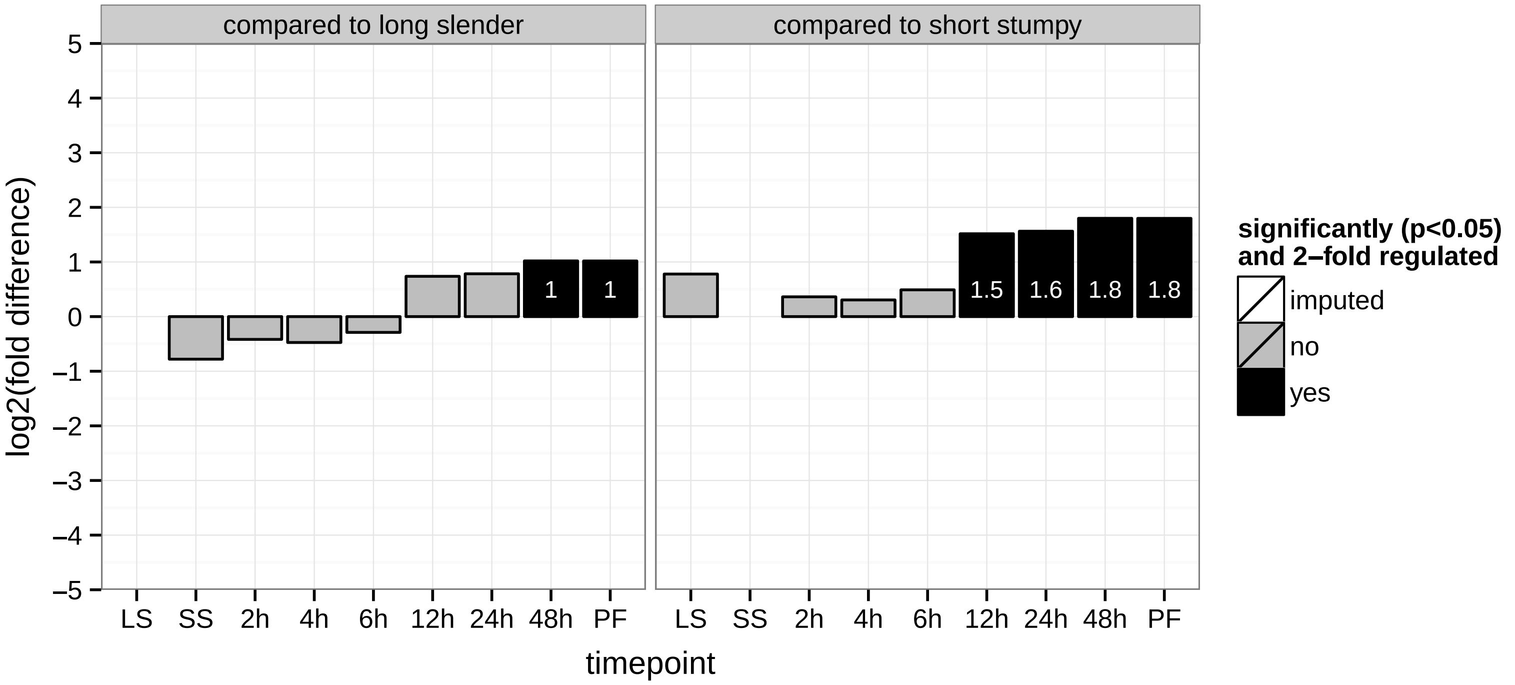

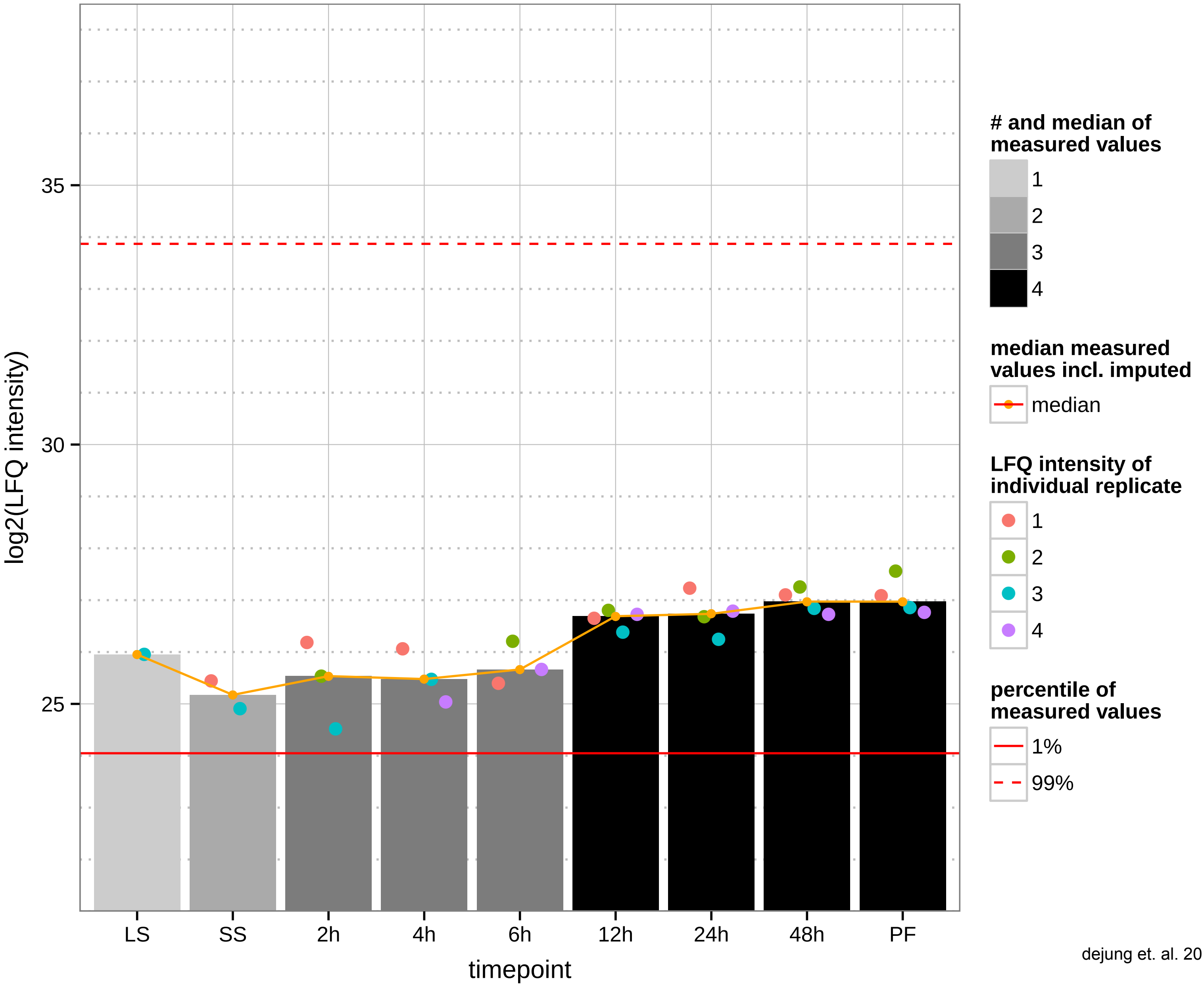

dejung et. al. 2015

expression site–associated gene (ESAG, pseudogene), putative, expression site–associated gene 4 (ESAG4), pseudogene, r
Tb927.4.130
AGOF: phosphorus–oxygen lyase activity, null
AGOC: null
AGOP: cyclic nucleotide biosynthetic process, intracellular signal transduction, null
PGOF: phosphorus–oxygen lyase activity, null
PGOC: null
PGOP: cyclic nucleotide biosynthetic process, intracellular signal transduction, null

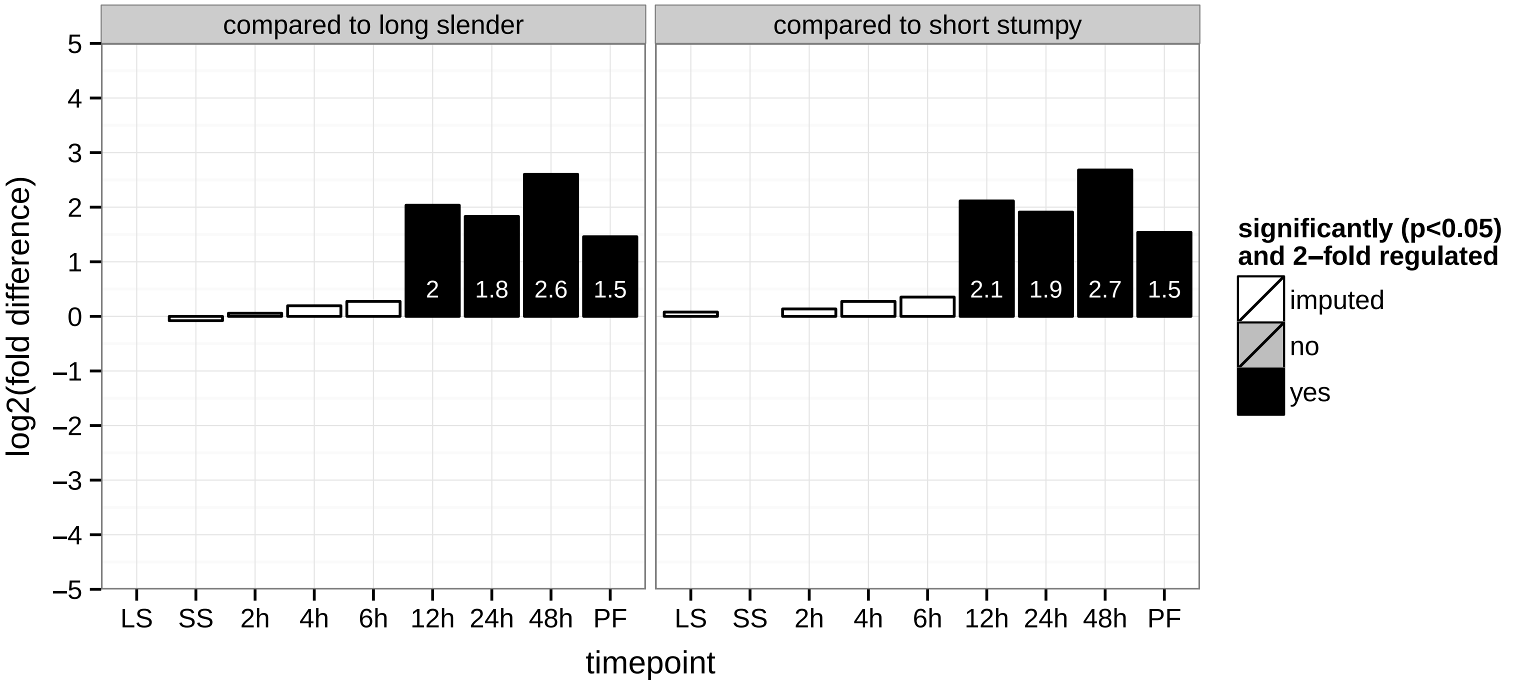

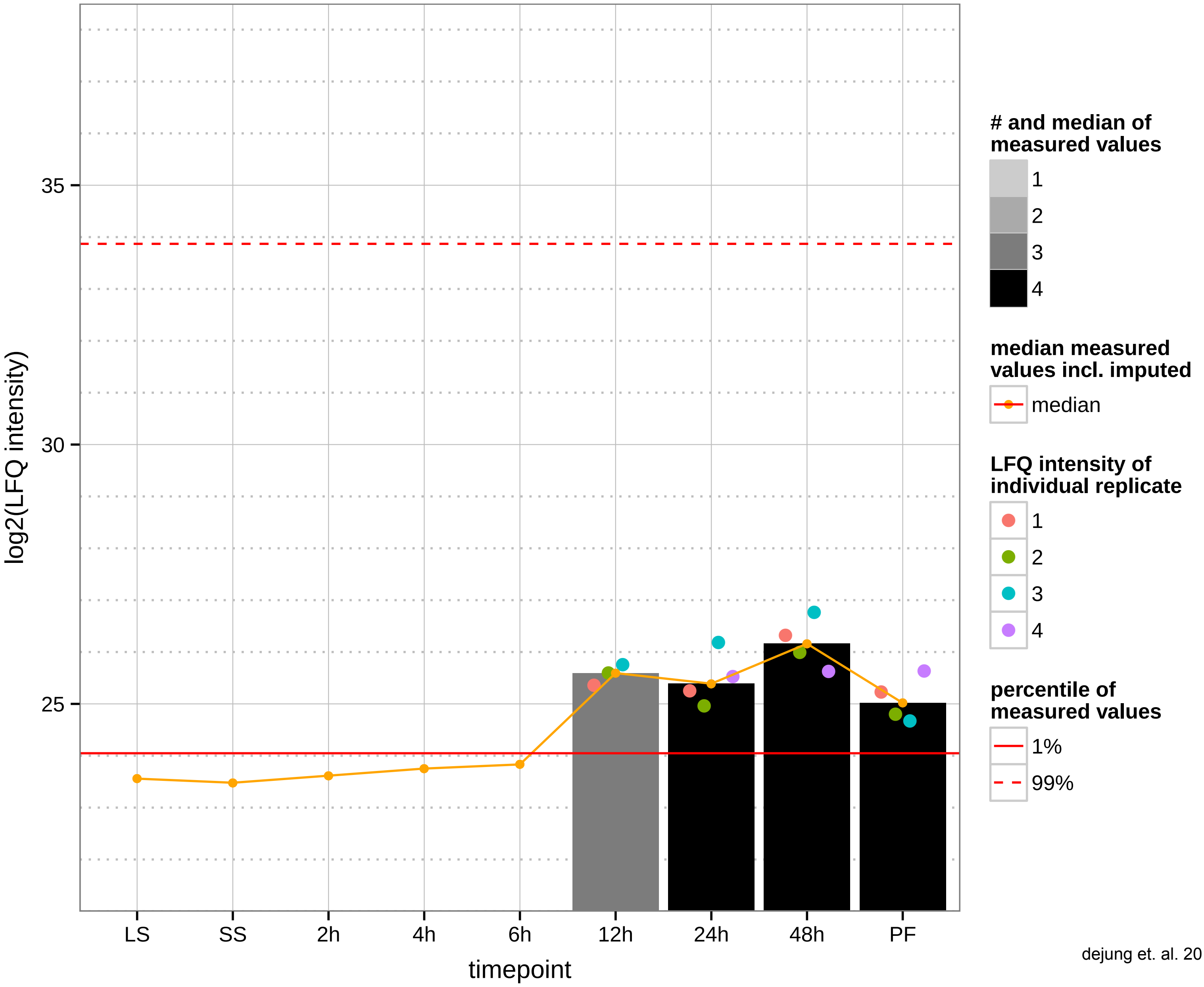

dejung et. al. 2015

ATP−dependent DEAD/H RNA helicase, putative
Tb927.4.2630
AGOF: ATP binding, ATP−dependent helicase activity, RNA binding, helicase activity
AGOC: nucleus
AGOP: nucleobase−containing compound metabolic process
PGOF: ATP binding, ATP−dependent helicase activity, RNA binding, helicase activity, hydrolase activity, acting on acid anhydr
PGOC: nucleus
PGOP: null

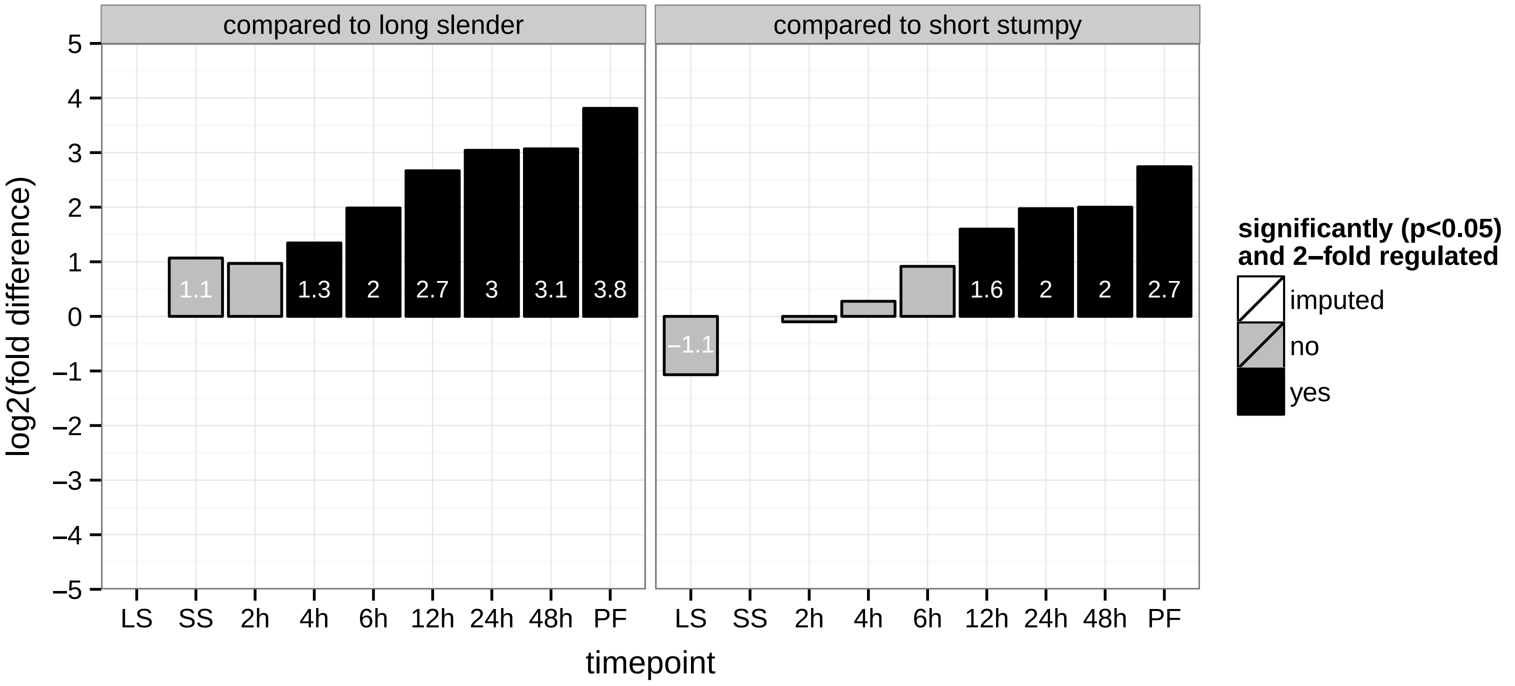

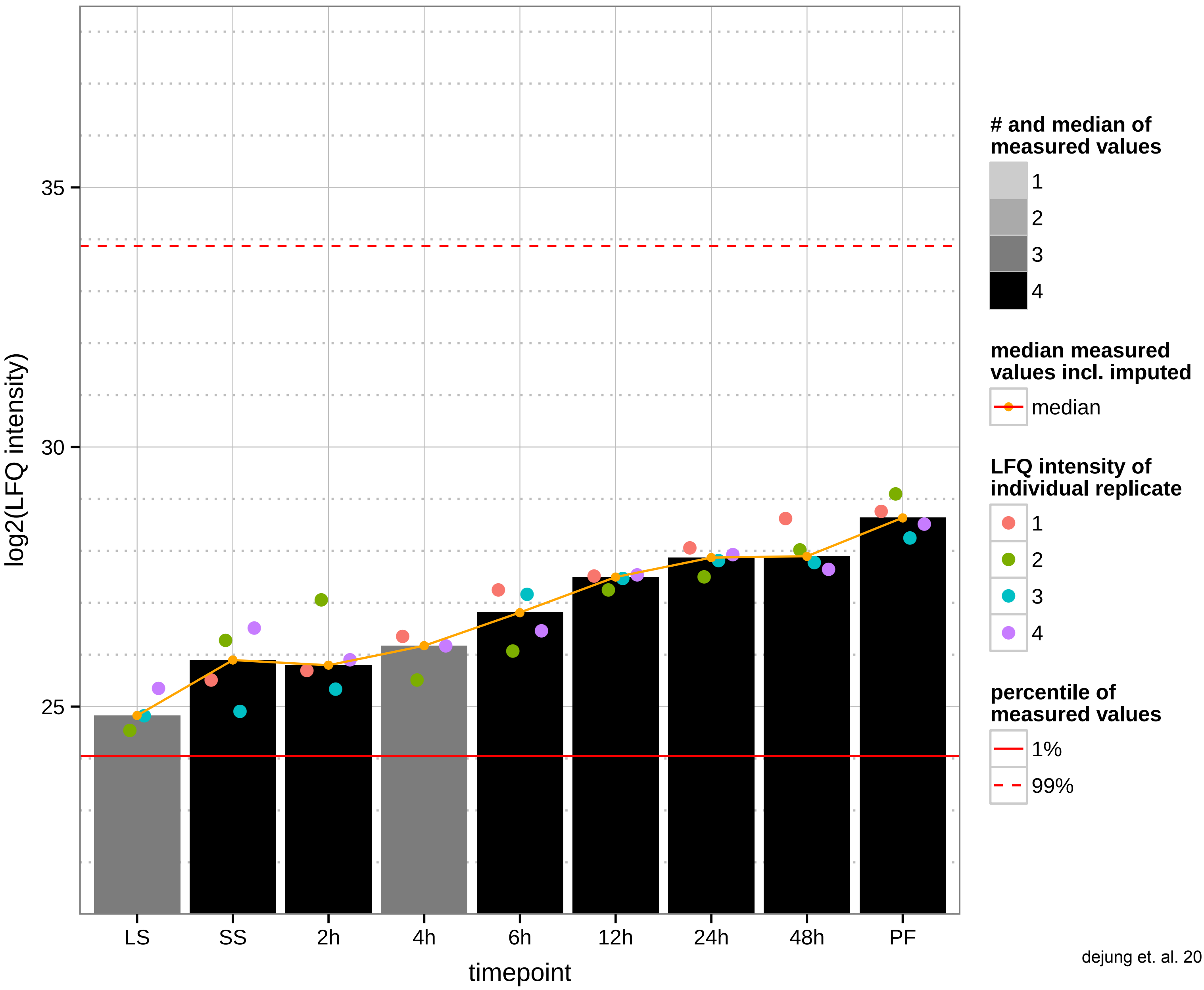

dejung et. al. 2015

3, 2–trans–enoyl–CoA isomerase, mitochondrial precursor, putative
Tb927.4.4910
AGOF: catalytic activity
AGOC: mitochondrion
AGOP: metabolic process
PGOF: catalytic activity
PGOC: null
PGOP: metabolic process

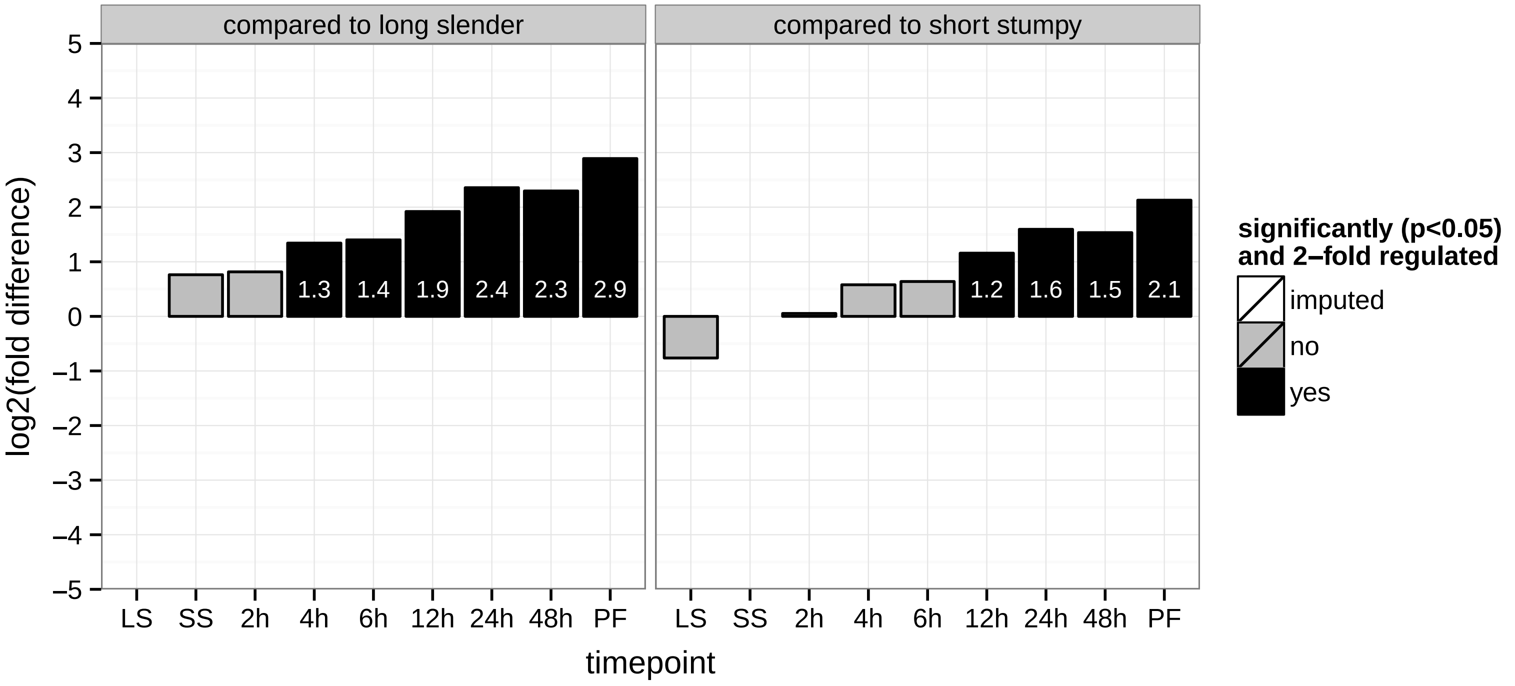

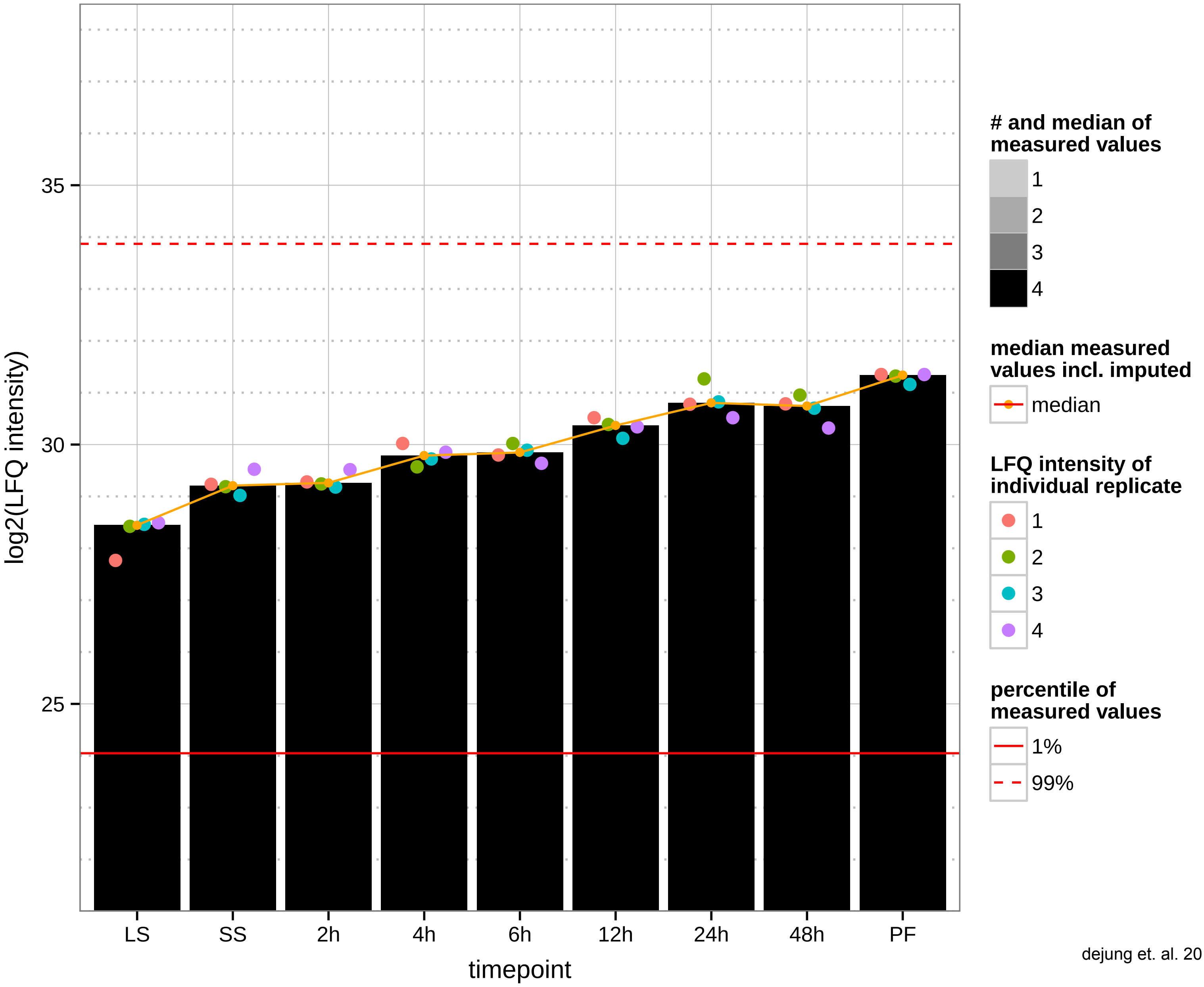

dejung et. al. 2015

hypothetical protein, conserved
Tb927.4.5120
AGOF: null
AGOC: null
AGOP: null
PGOF: null
PGOC: null
PGOP: null

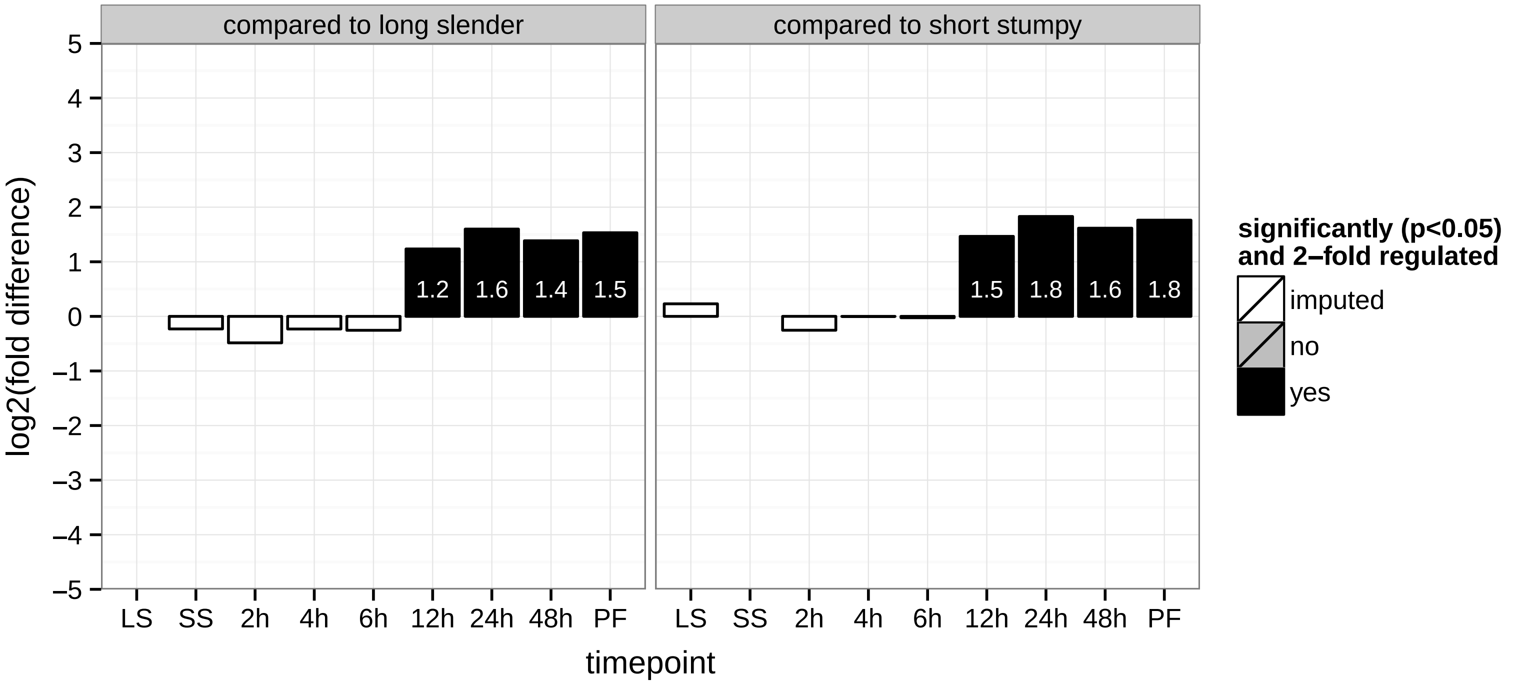

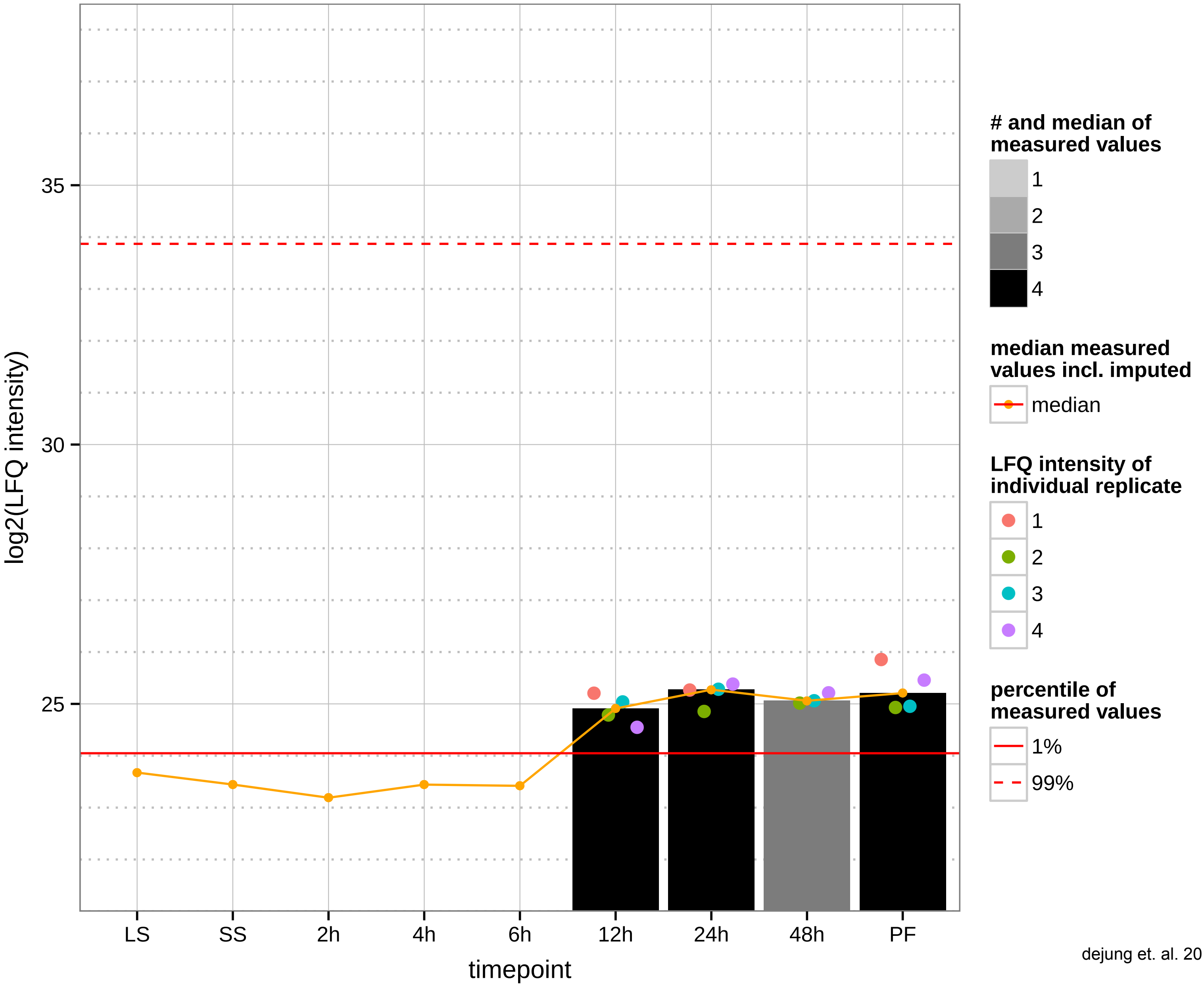

dejung et. al. 2015

ATP−dependent DEAD/H RNA helicase, putative
Tb927.5.1560
AGOF: ATP binding, ATP−dependent helicase activity, helicase activity, nucleic acid binding
AGOC: null
AGOP: nucleobase−containing compound metabolic process
PGOF: ATP binding, ATP−dependent helicase activity, helicase activity, nucleic acid binding
PGOC: null
PGOP: null

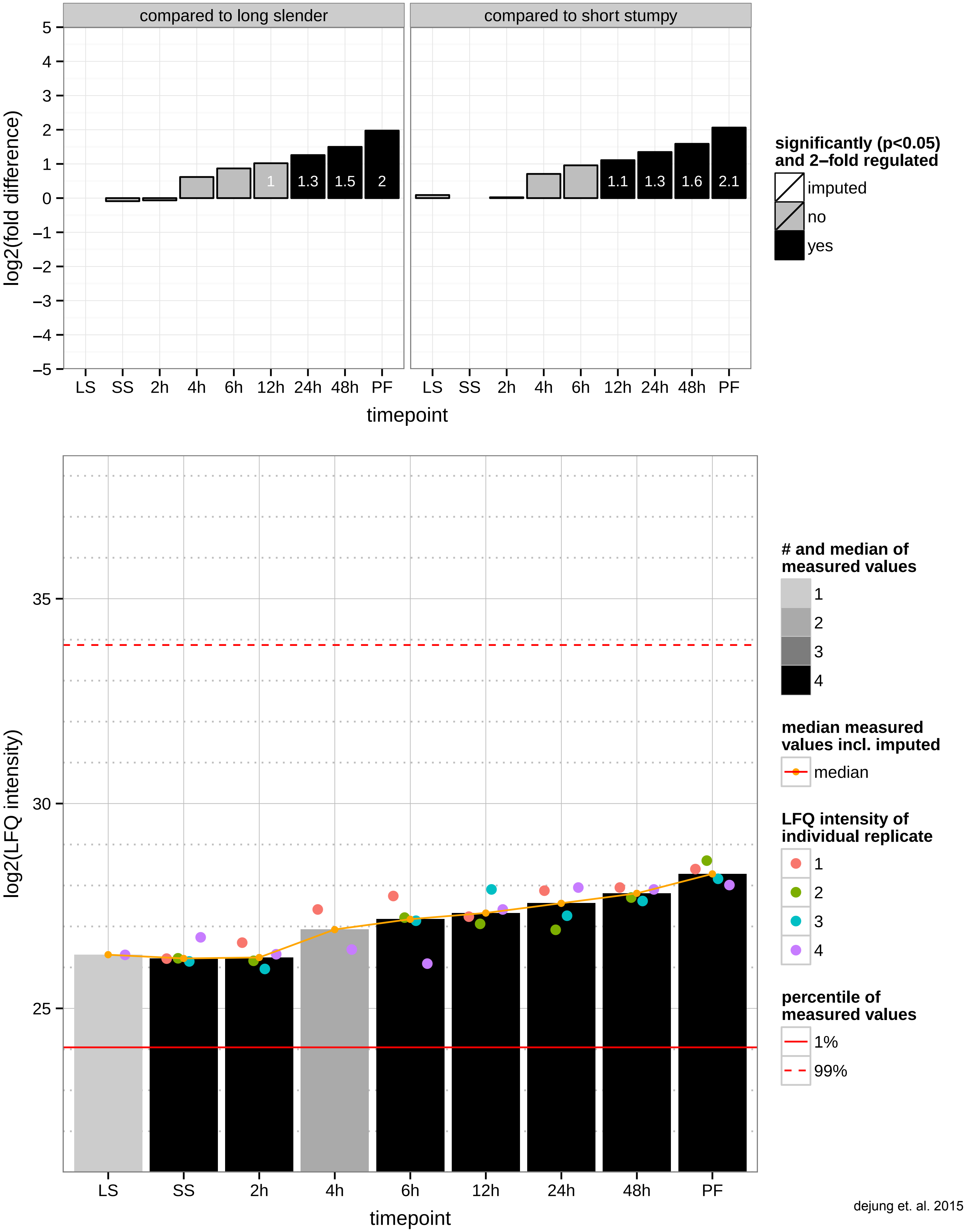

dejung et. al. 2015

ribonucleoprotein p18, mitochondrial precursor, putative
Tb927.5.1710
AGOF: null
AGOC: mitochondrion
AGOP: null
PGOF: null
PGOC: null
PGOP: null

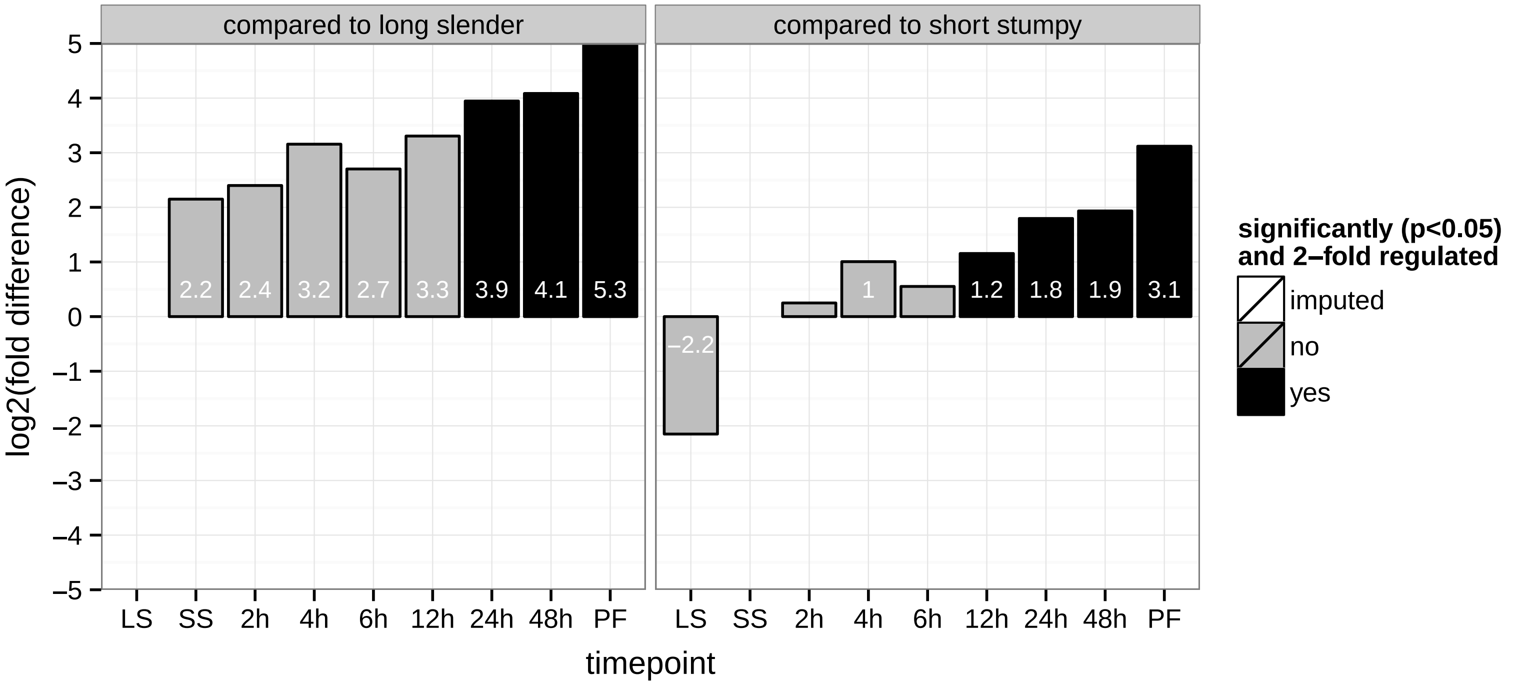

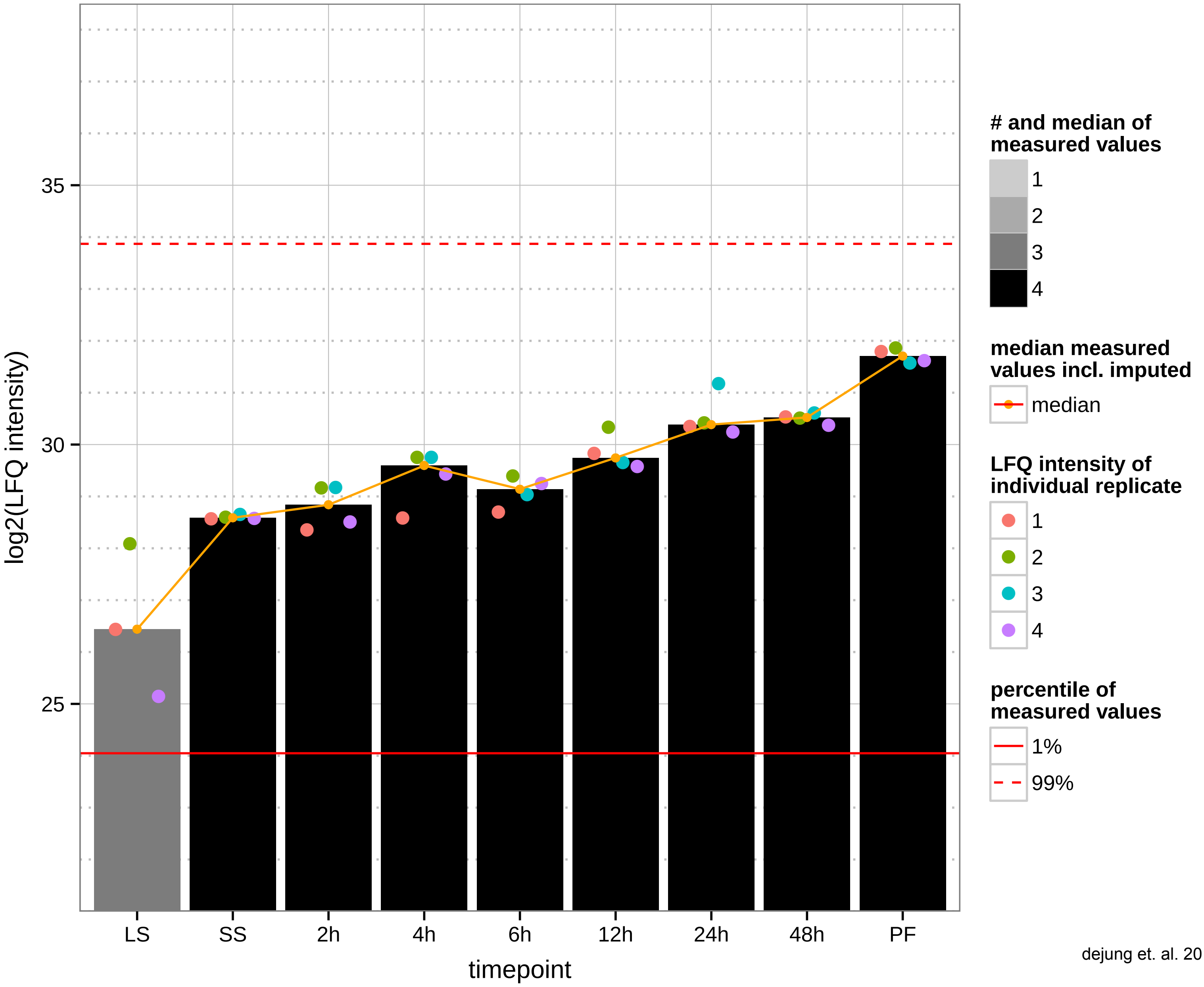

dejung et. al. 2015

60S acidic ribosomal protein, putative
Tb927.5.1820
AGOF: structural constituent of ribosome
AGOC: intracellular, ribosome
AGOP: translation, translational elongation
PGOF: structural constituent of ribosome
PGOC: intracellular, ribosome
PGOP: translational elongation

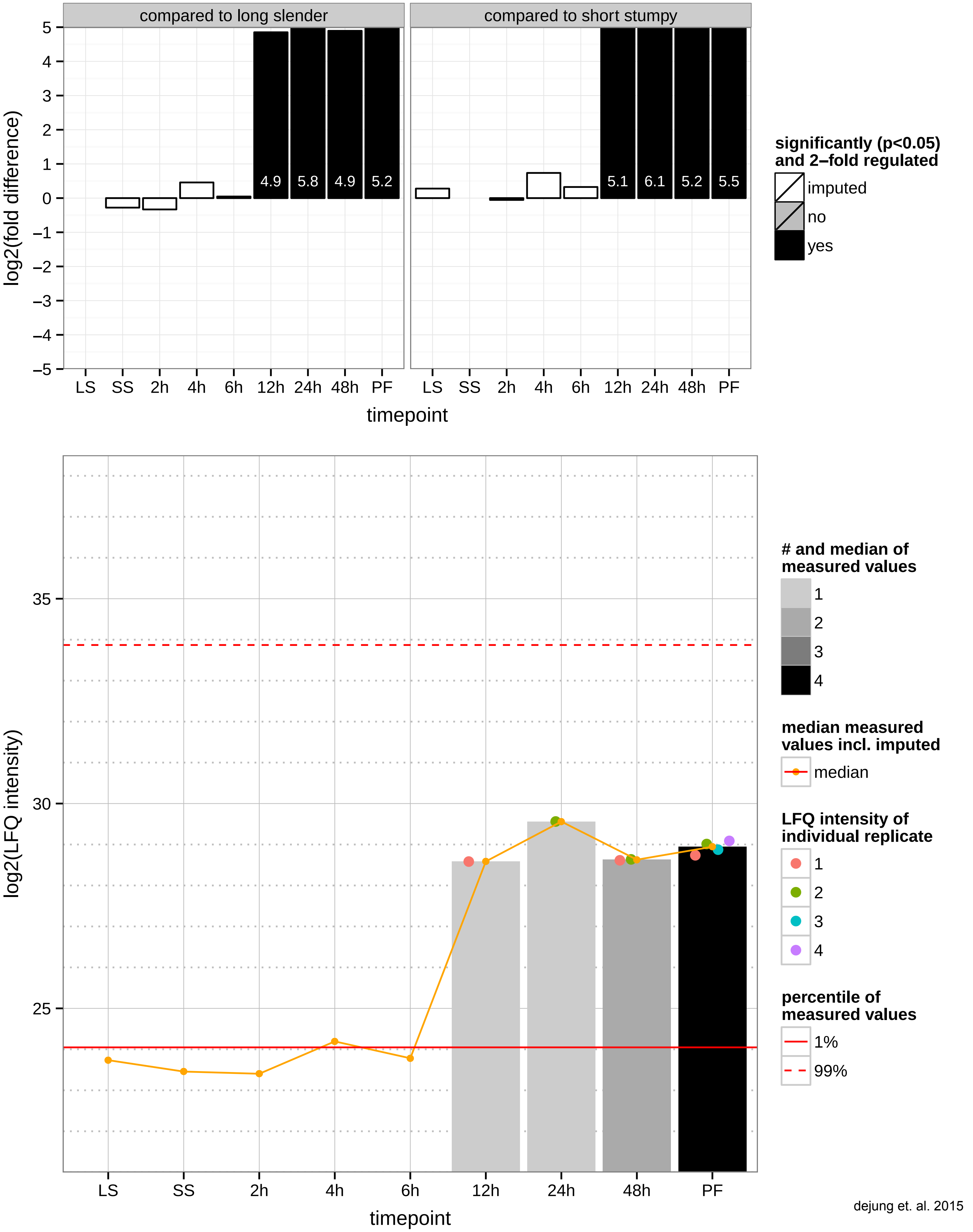

dejung et. al. 2015

conserved protein
Tb927.5.2160
AGOF: null
AGOC: null
AGOP: null
PGOF: null
PGOC: null
PGOP: null

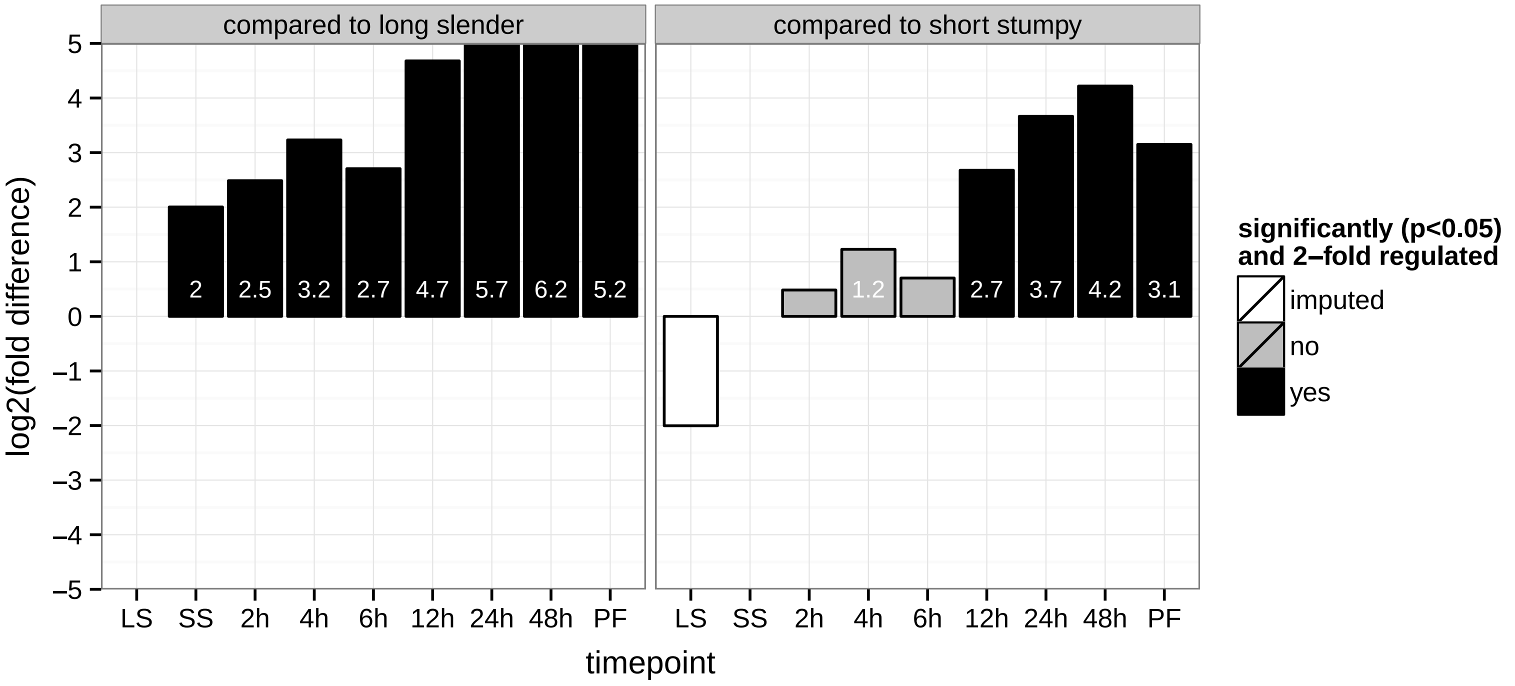

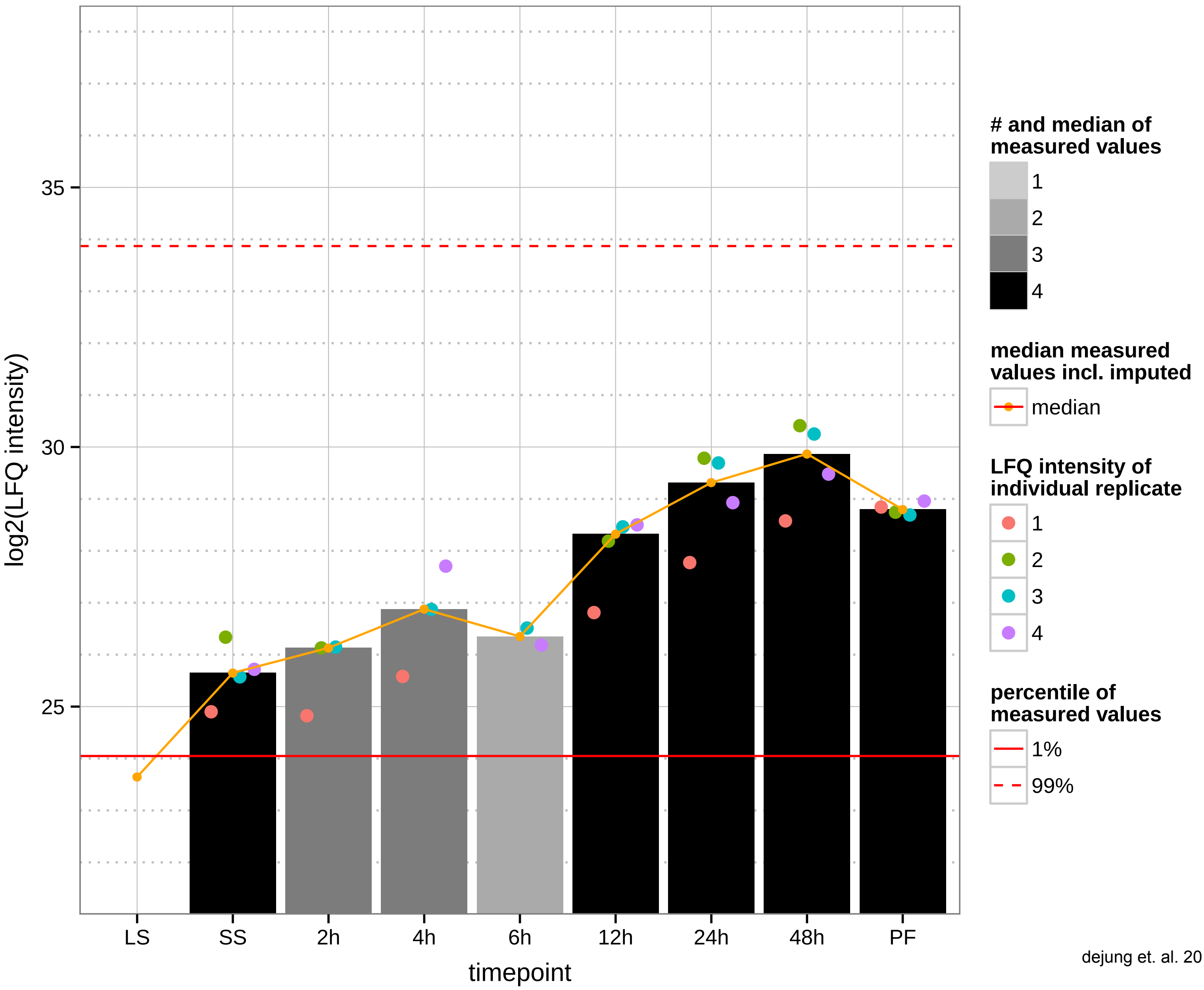

dejung et. al. 2015

hypothetical protein, conserved
Tb927.5.2560
AGOF: null
AGOC: null
AGOP: null
PGOF: null
PGOC: null
PGOP: null

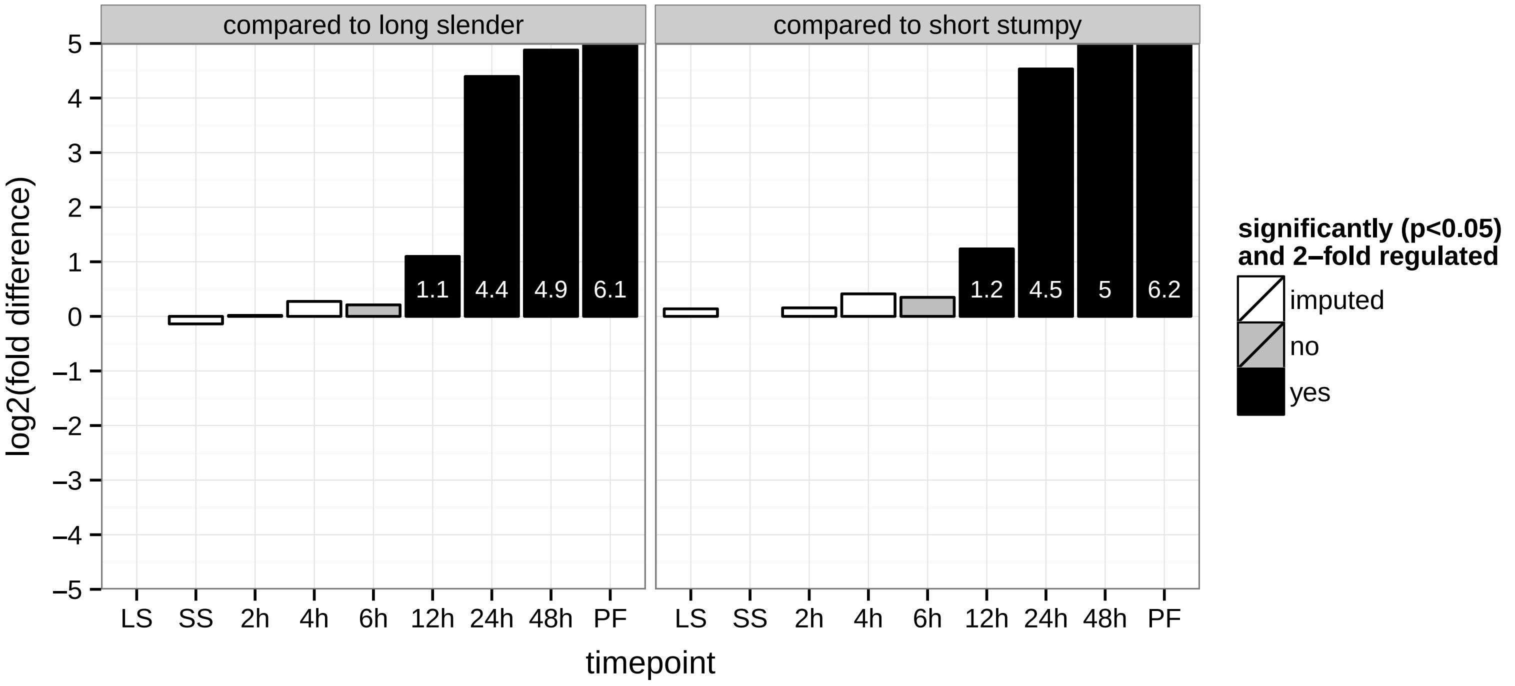

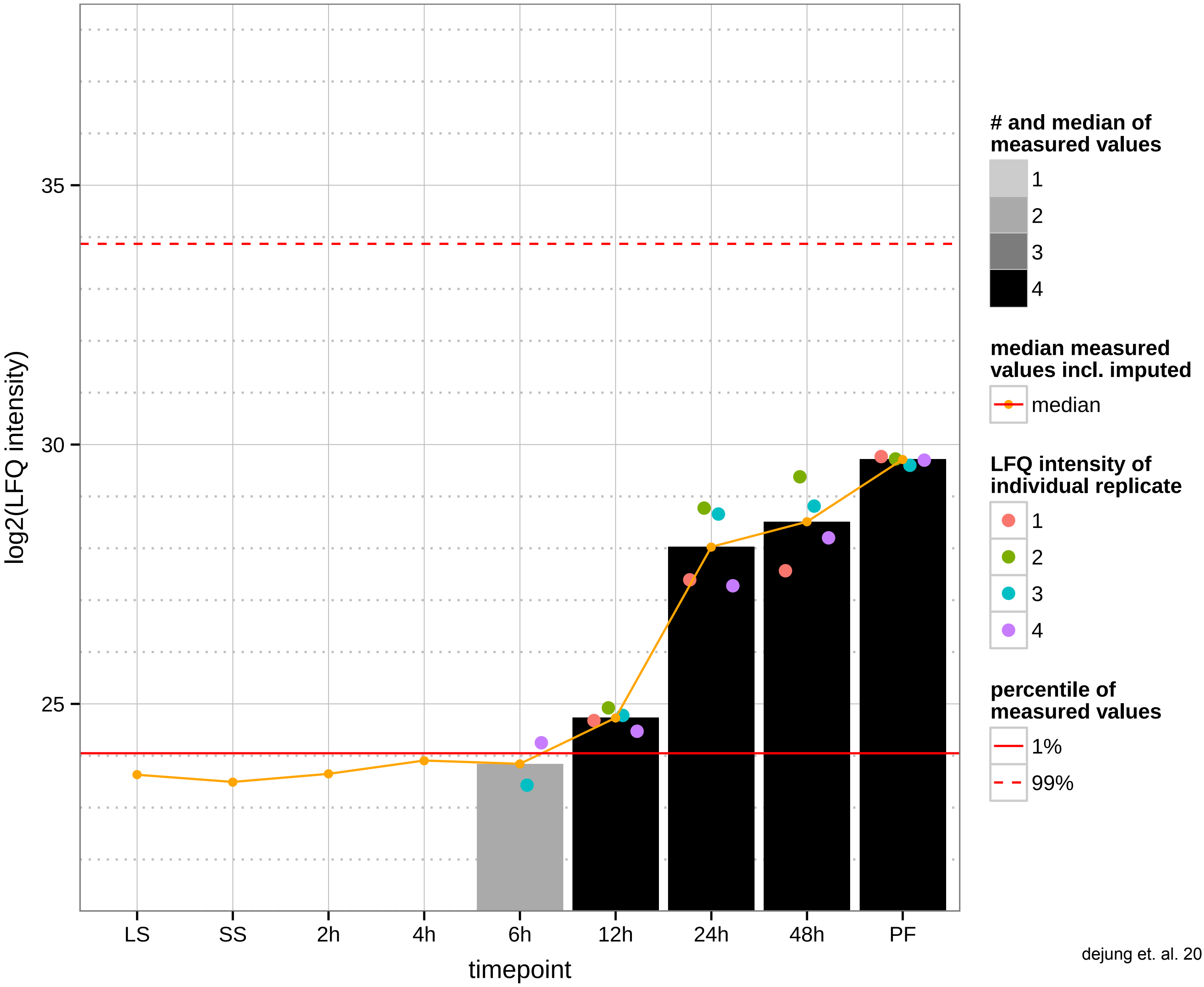

dejung et. al. 2015

hypothetical protein, conserved
Tb927.5.3090
AGOF: null
AGOC: mitochondrion
AGOP: null
PGOF: null
PGOC: null
PGOP: null

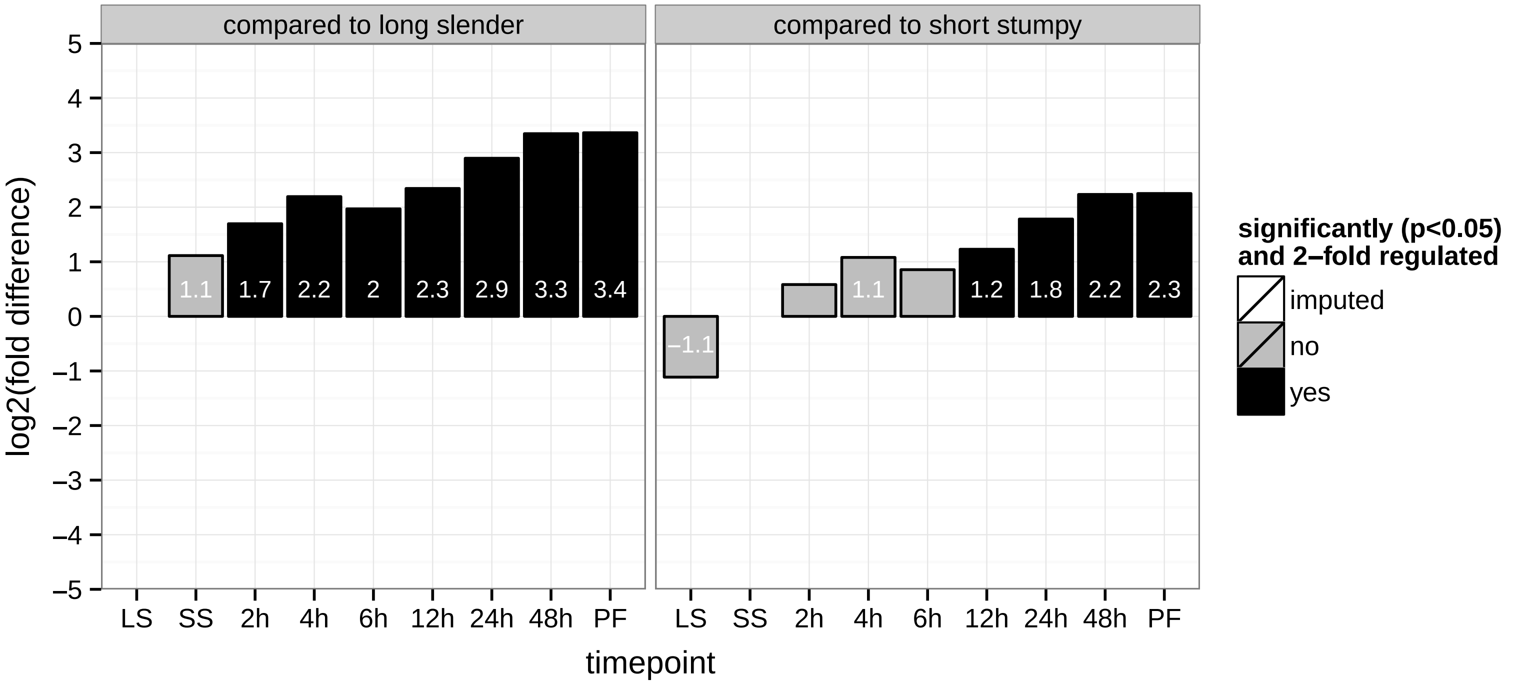

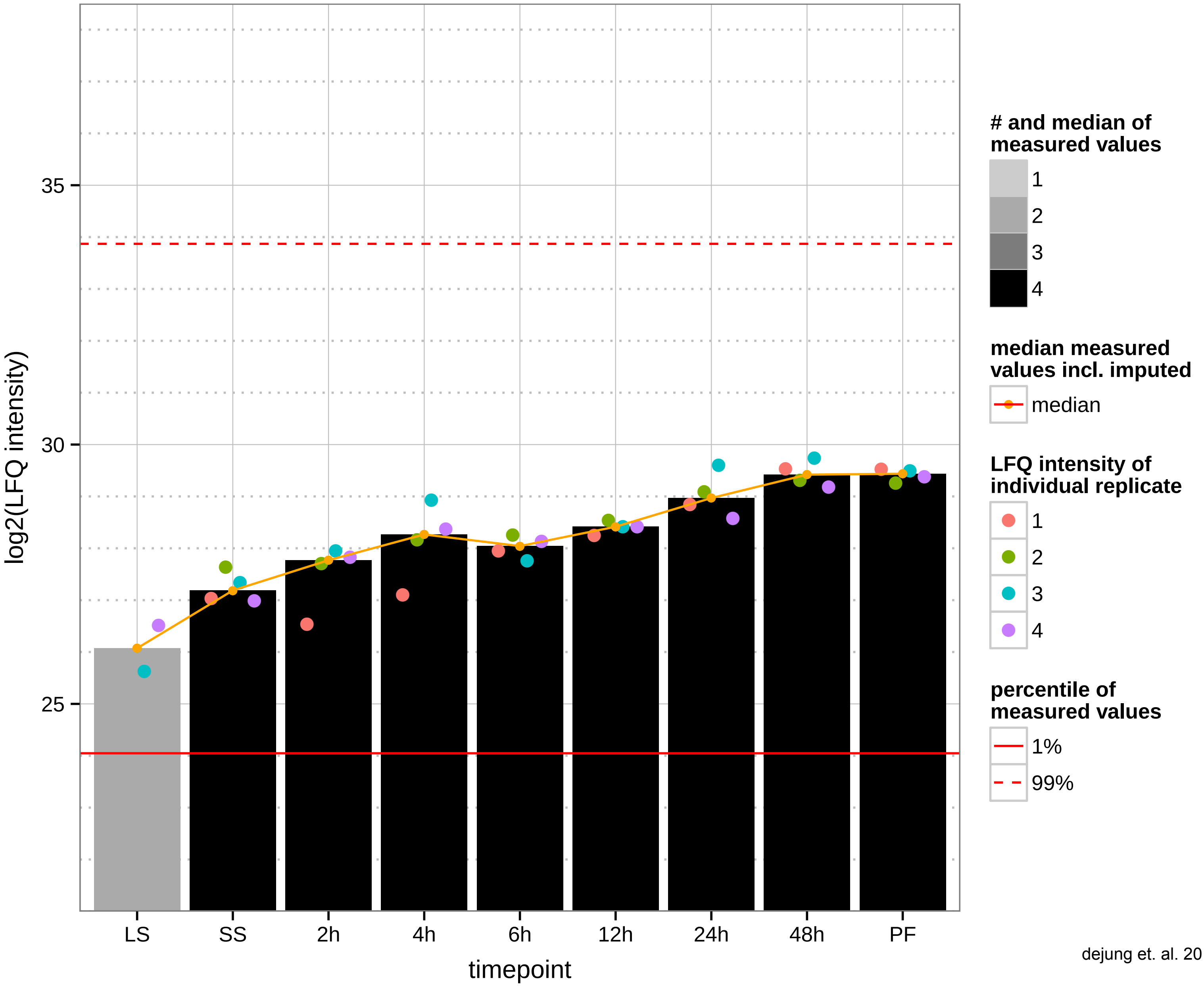

dejung et. al. 2015

hypothetical protein, conserved
Tb927.5.3110
AGOF: null
AGOC: mitochondrion
AGOP: null
PGOF: null
PGOC: null
PGOP: null

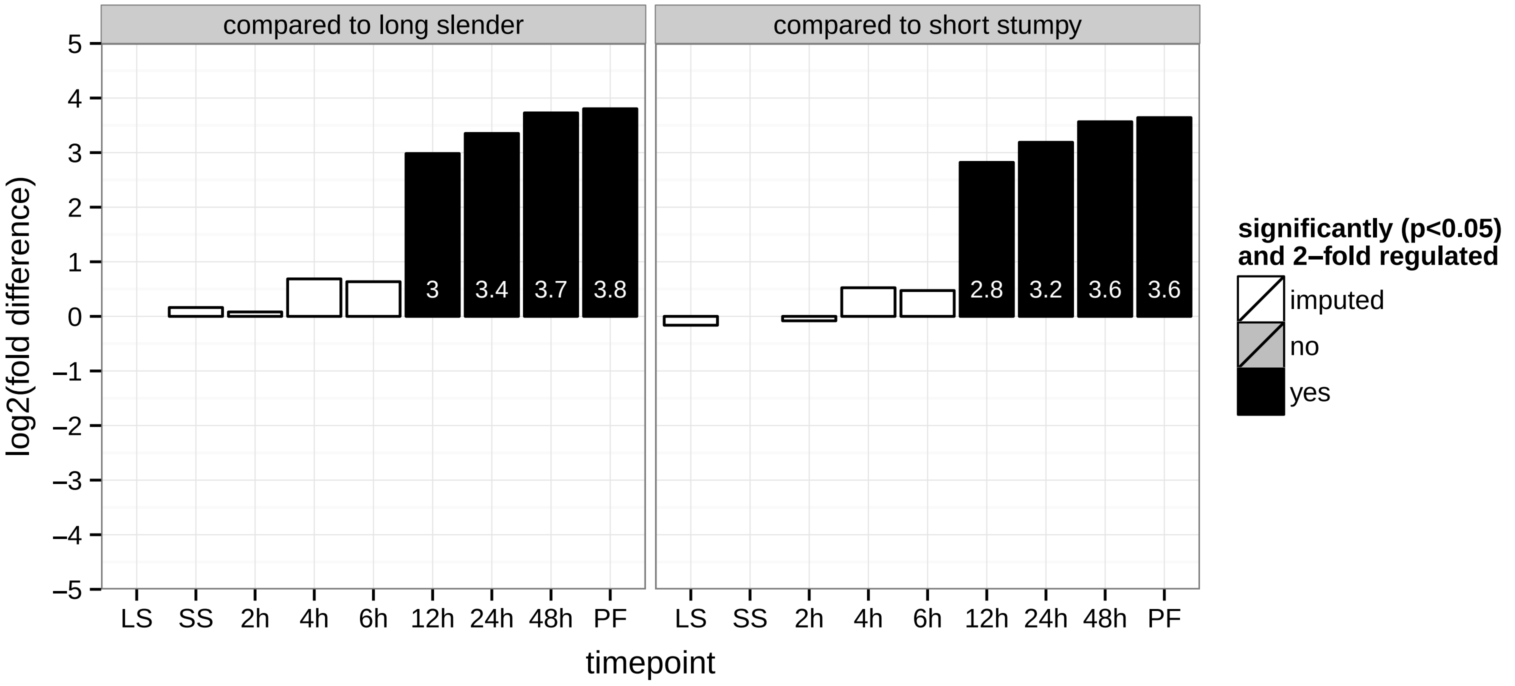

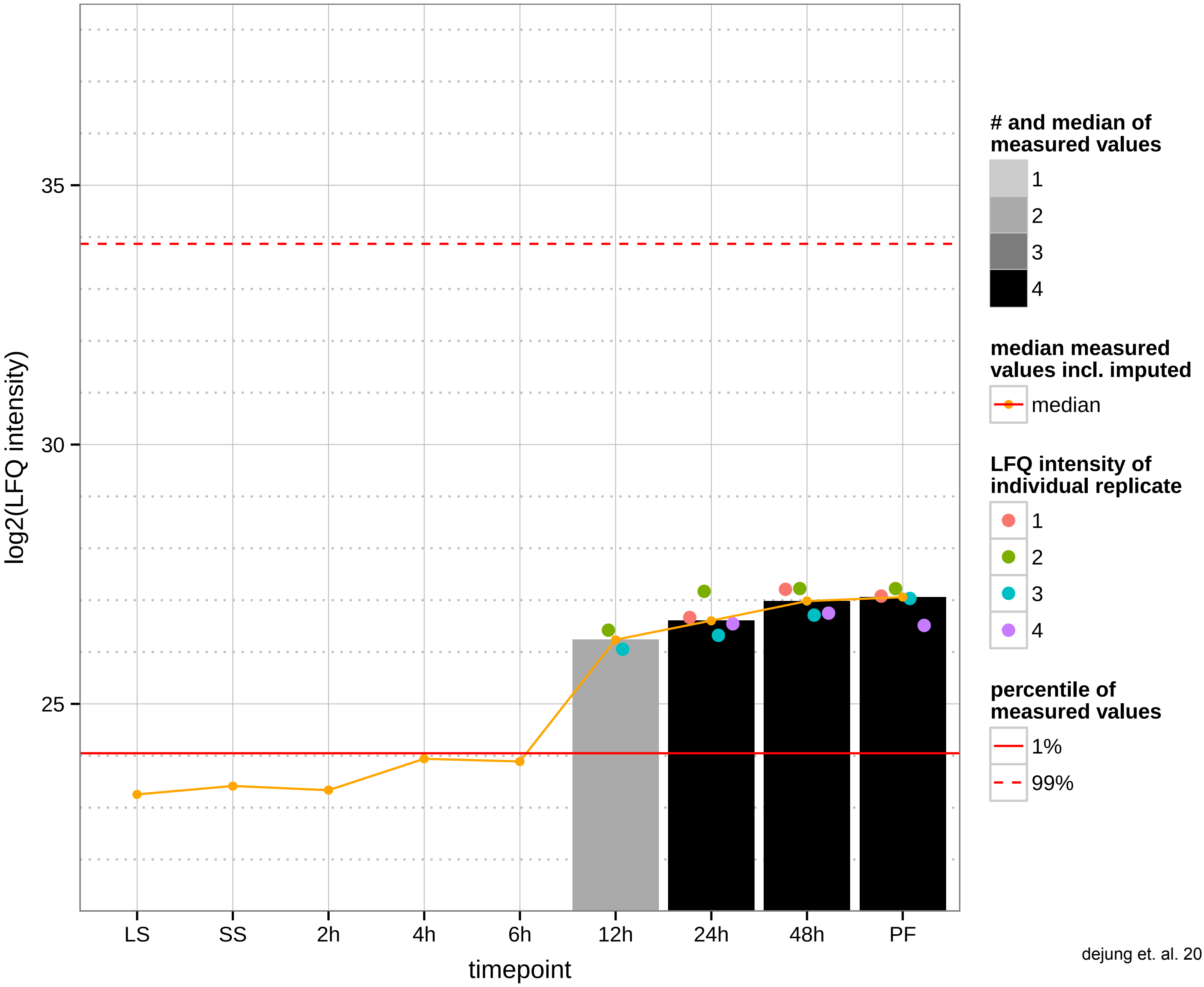

dejung et. al. 2015

eukaryotic translation initiation factor 2 beta subunit, putative (EIF2B)
Tb927.5.3120
AGOF: translation initiation factor activity, translation initiation factor binding
AGOC: eukaryotic translation initiation factor 2 complex
AGOP: formation of translation preinitiation complex, translational initiation
PGOF: translation initiation factor activity
PGOC: null
PGOP: translational initiation

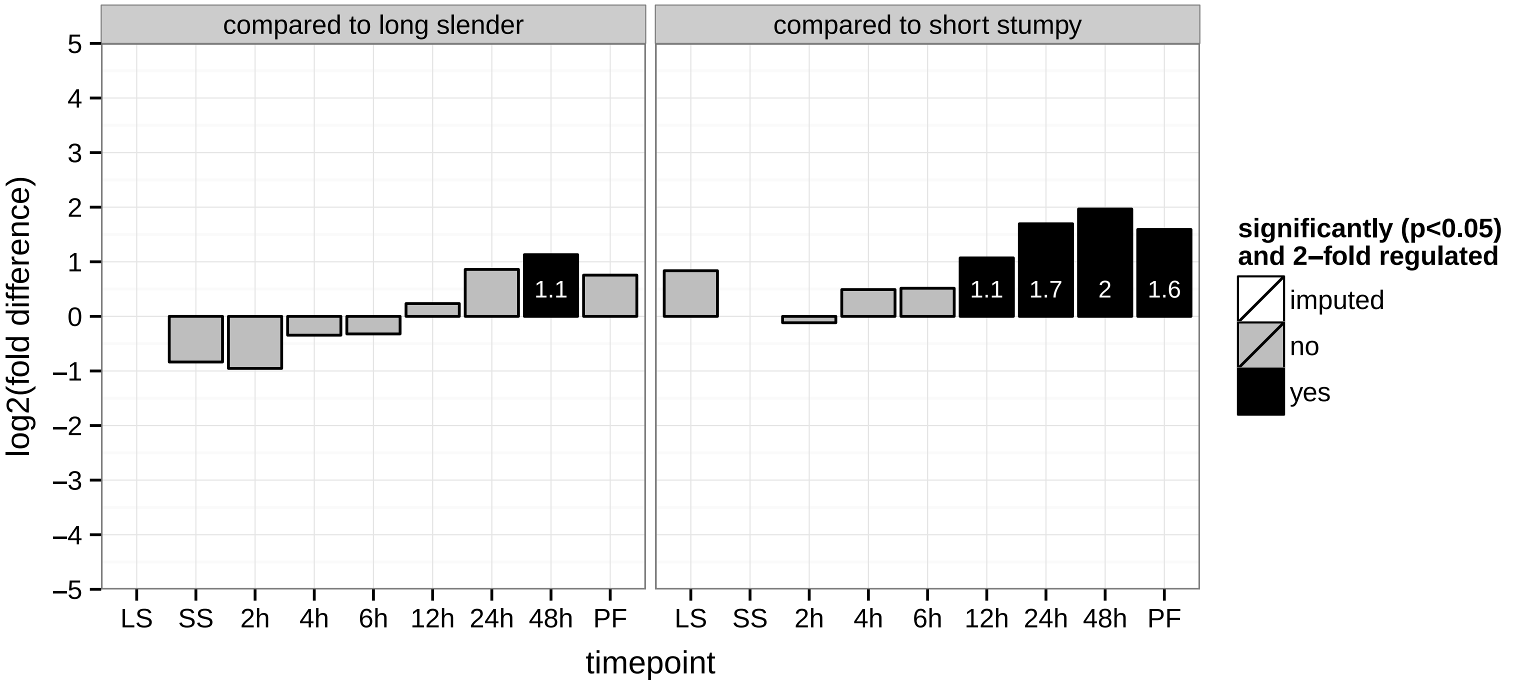

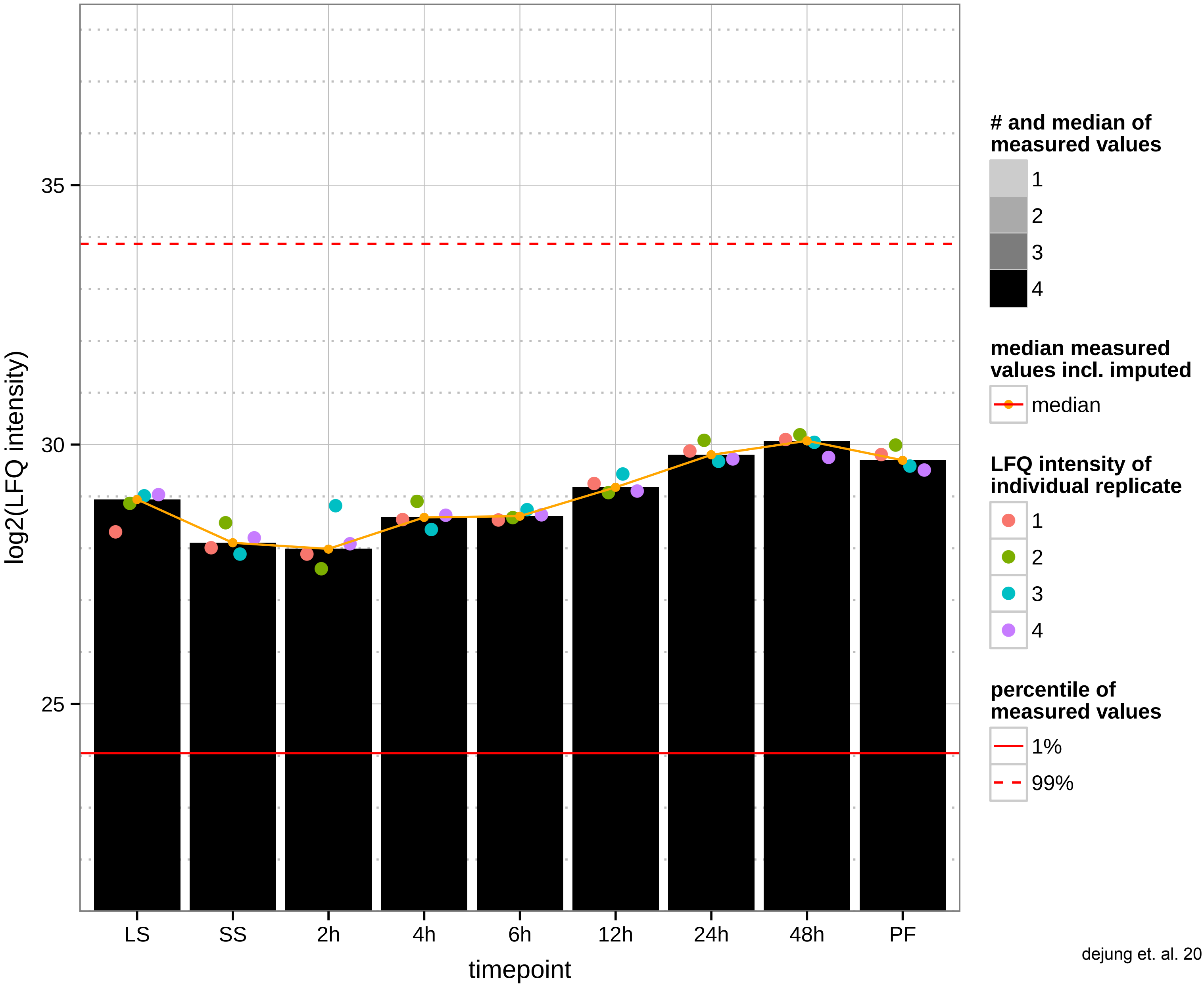

dejung et. al. 2015

hypothetical protein, conserved
Tb927.5.3980
AGOF: structural constituent of ribosome
AGOC: large ribosomal subunit, mitochondrion
AGOP: translation
PGOF: null
PGOC: null
PGOP: null

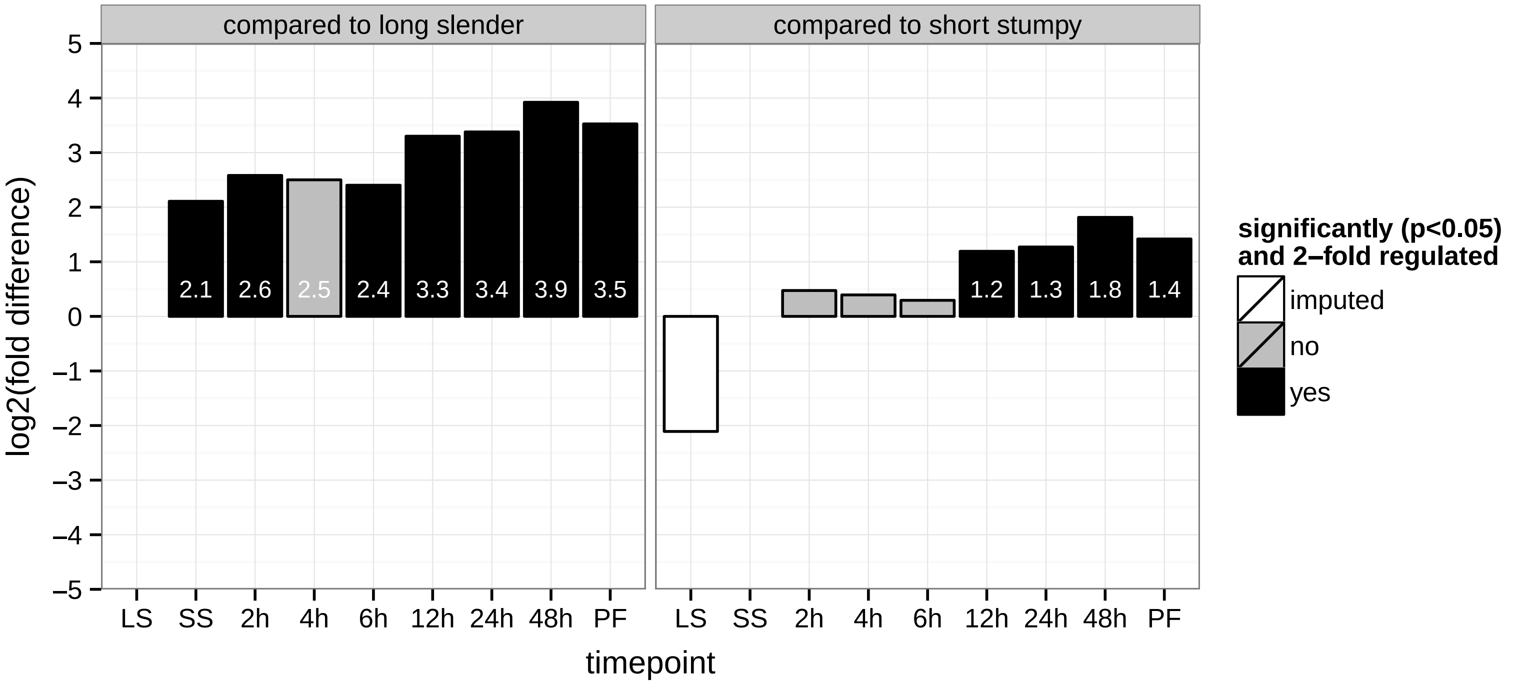

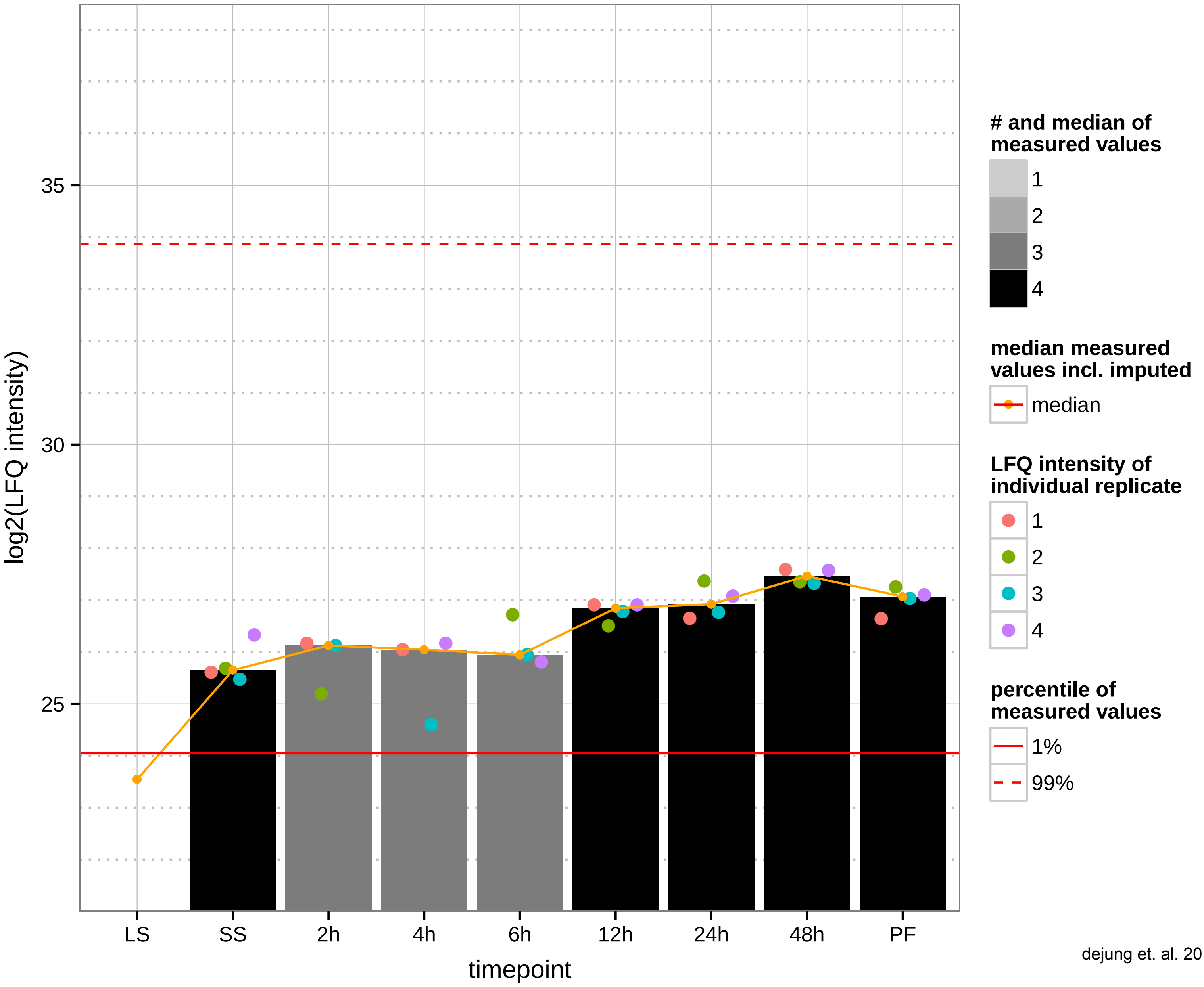

dejung et. al. 2015

hypothetical protein, conserved
Tb927.5.4090
AGOF: null
AGOC: null
AGOP: null
PGOF: protein binding
PGOC: null
PGOP: null

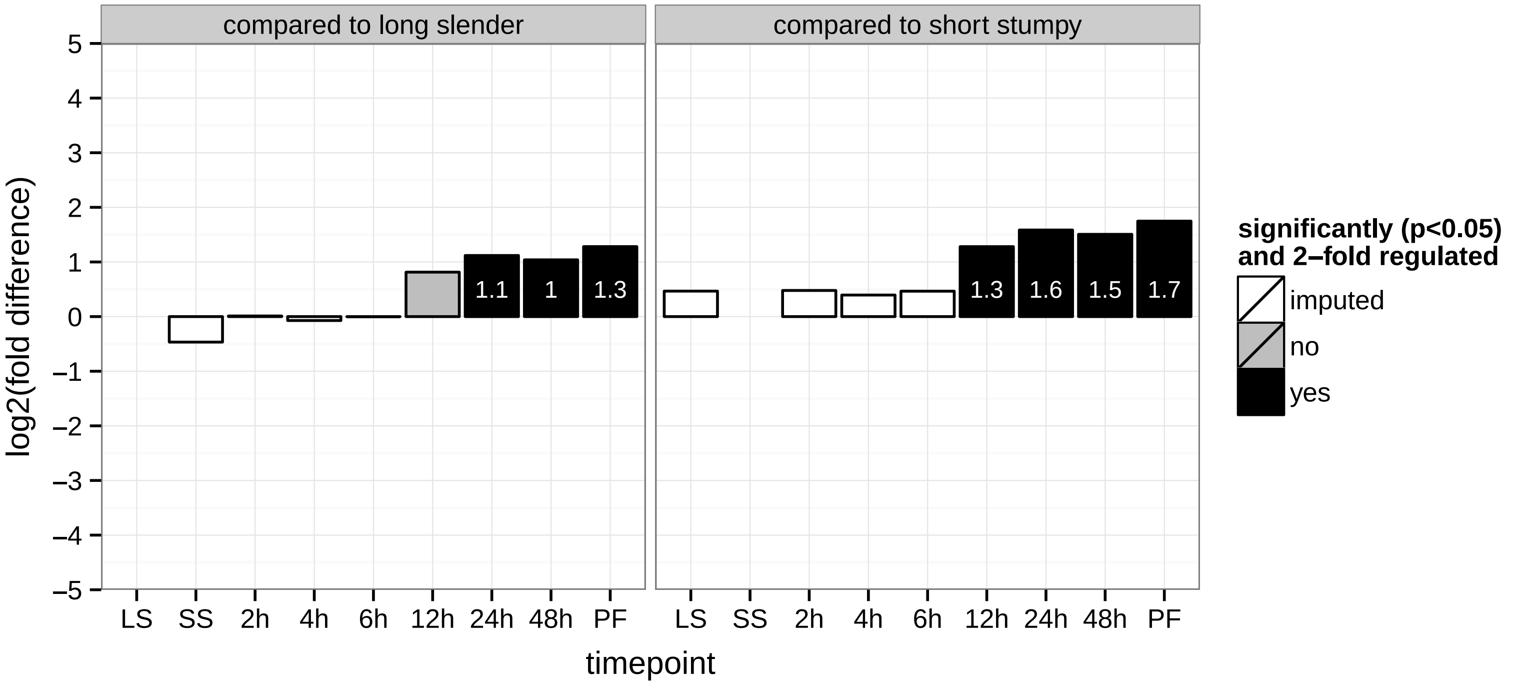

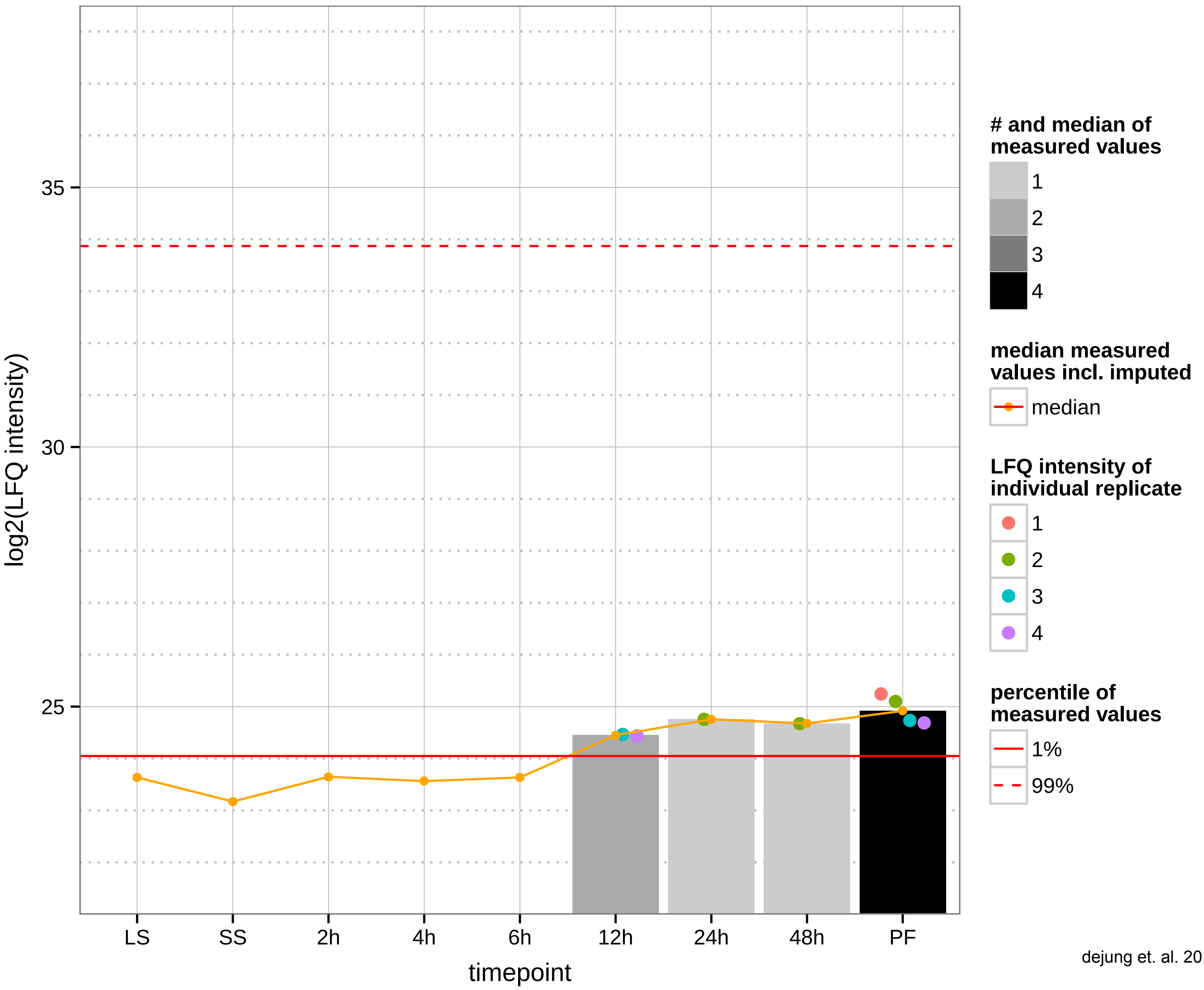

dejung et. al. 2015

hypothetical protein, conserved
Tb927.6.1890
AGOF: null
AGOC: mitochondrion
AGOP: null
PGOF: null
PGOC: null
PGOP: null

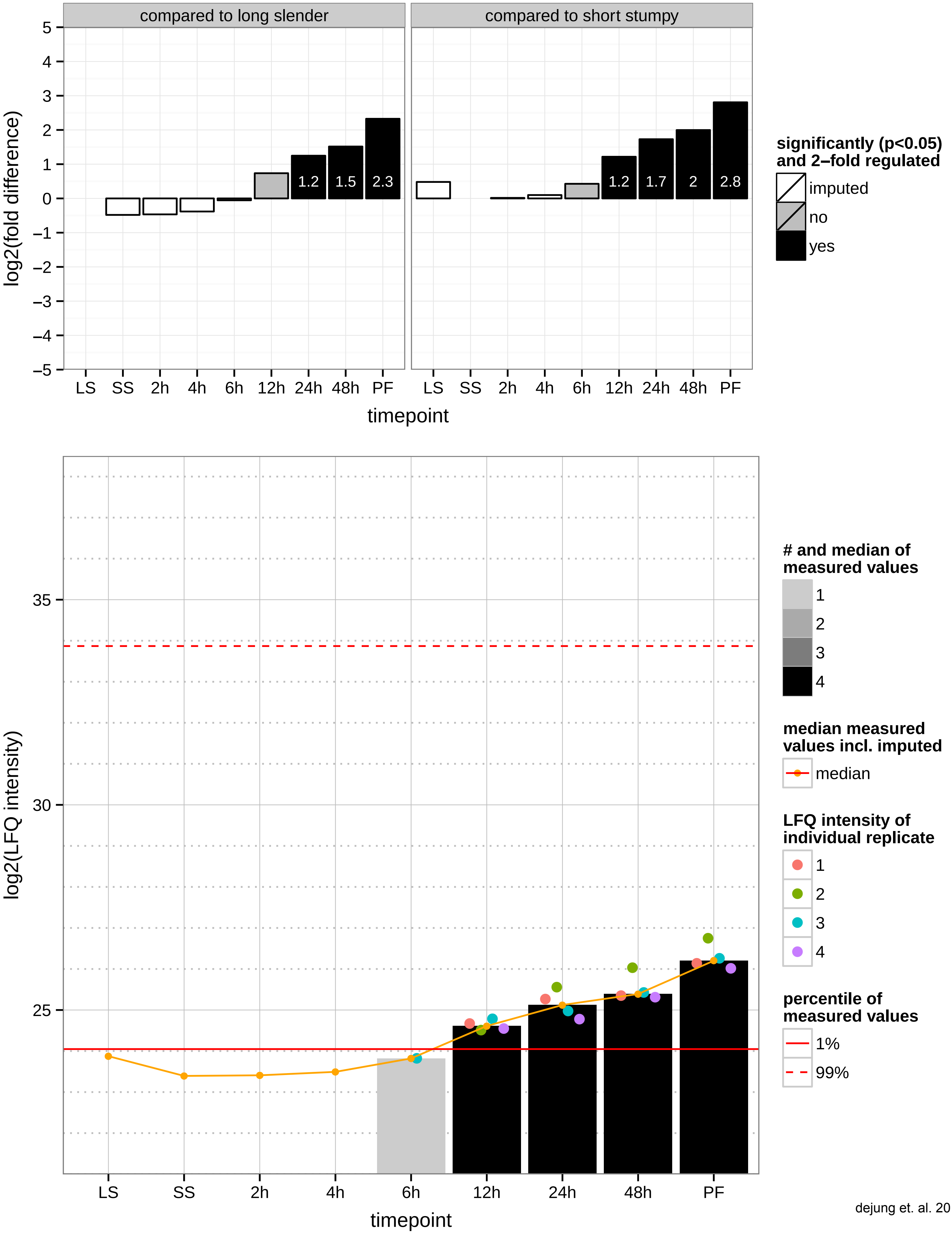

dejung et. al. 2015

hypothetical protein, conserved
Tb927.6.2080
AGOF: null
AGOC: mitochondrion
AGOP: null
PGOF: null
PGOC: null
PGOP: null

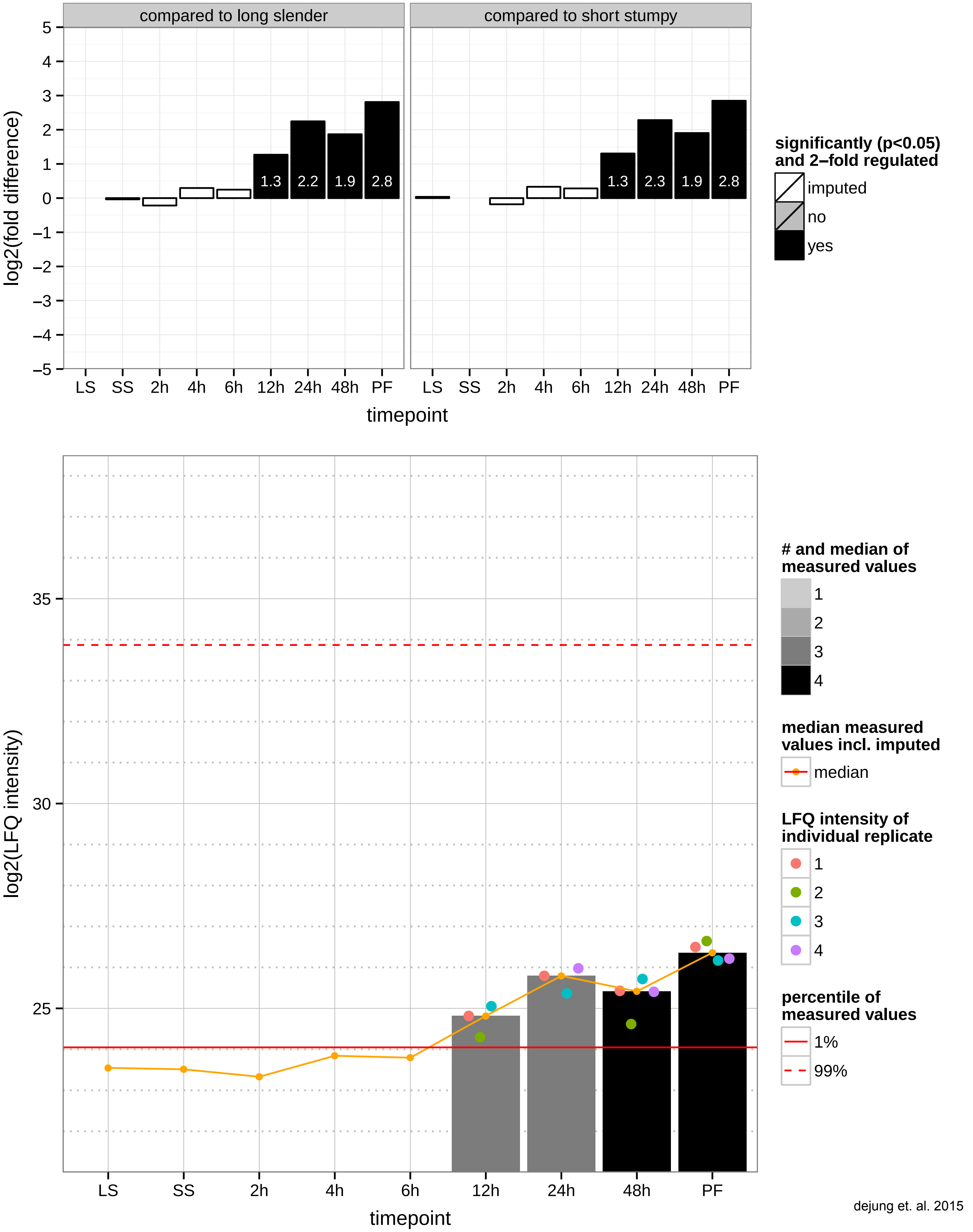

dejung et. al. 2015

protein kinase, putative, protein kinase B kinase
Tb927.6.2450
AGOF: ATP binding, protein kinase activity, protein serine/threonine kinase activity, protein tyrosine kinase activity
AGOC: null
AGOP: protein phosphorylation
PGOF: ATP binding, protein kinase activity, transferase activity, transferring phosphorus–containing groups
PGOC: null
PGOP: protein phosphorylation

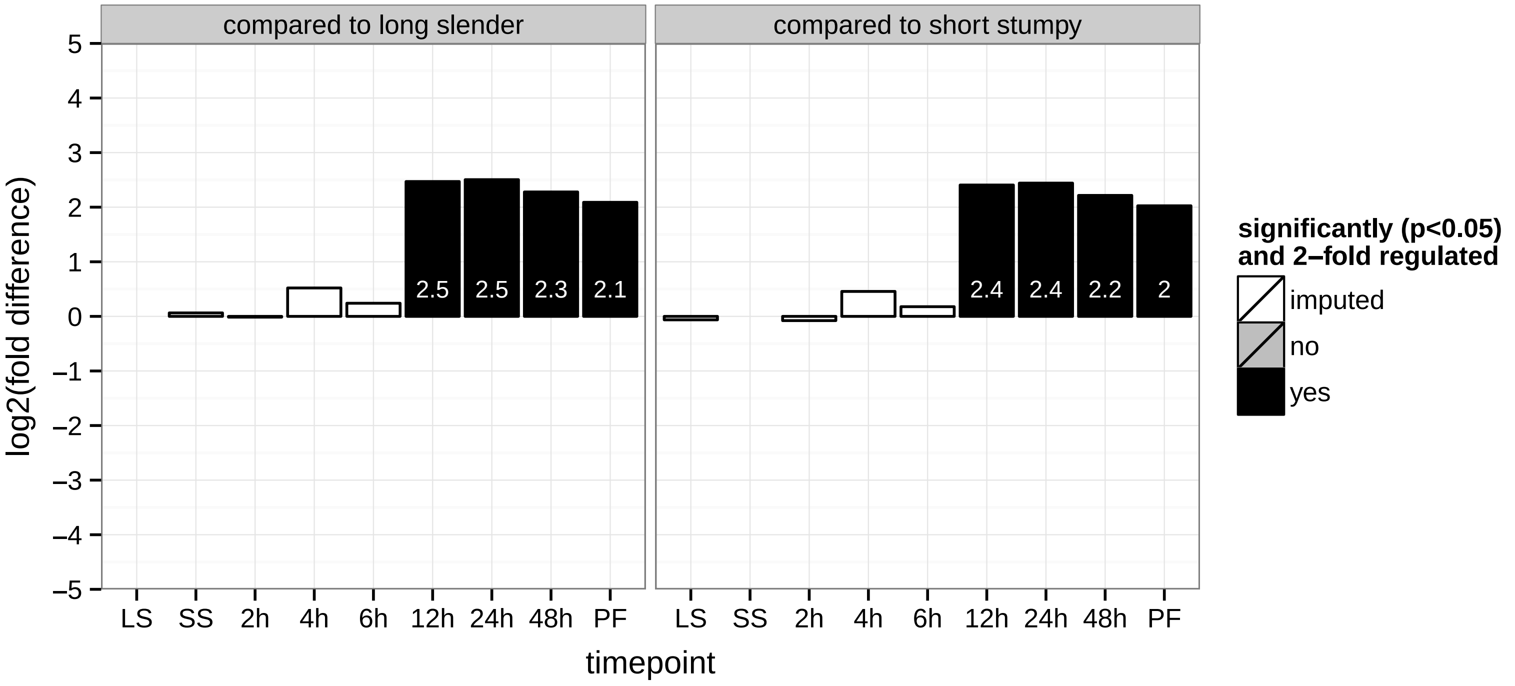

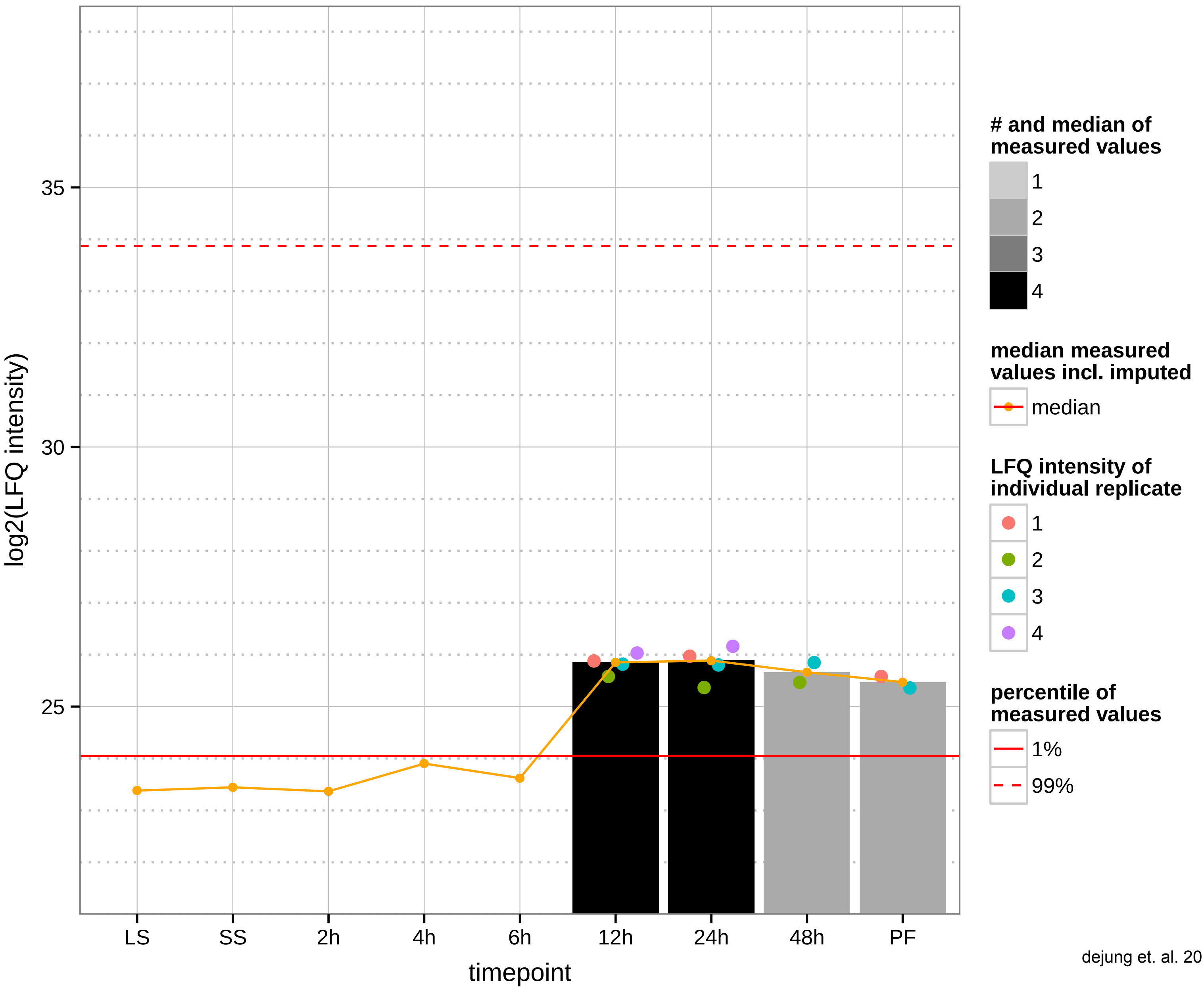

dejung et. al. 2015

receptor–type adenylate cyclase GRESAG 4, pseudogene, putative, degenerate
Tb927.6.320
AGOF: phosphorus–oxygen lyase activity
AGOC: null
AGOP: cyclic nucleotide biosynthetic process, intracellular signal transduction
PGOF: phosphorus–oxygen lyase activity
PGOC: null
PGOP: cyclic nucleotide biosynthetic process, intracellular signal transduction

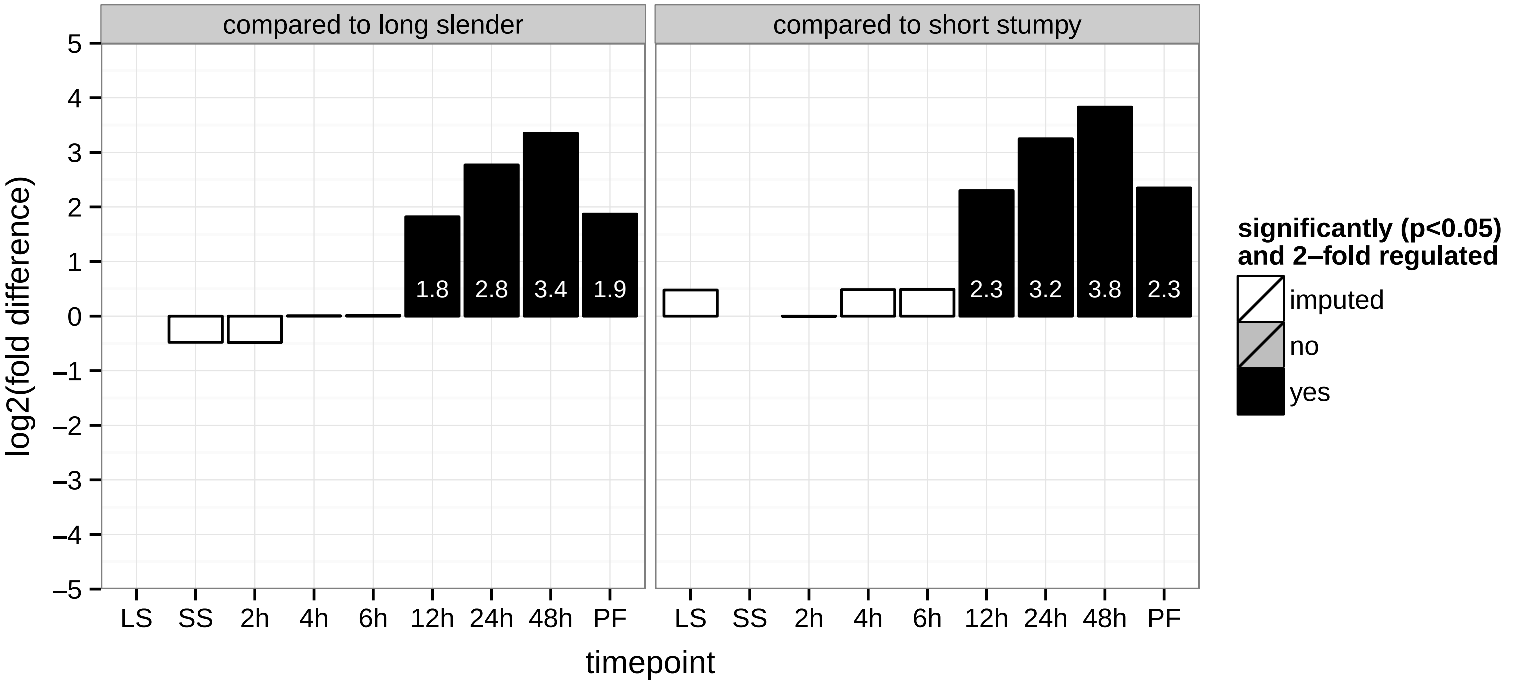

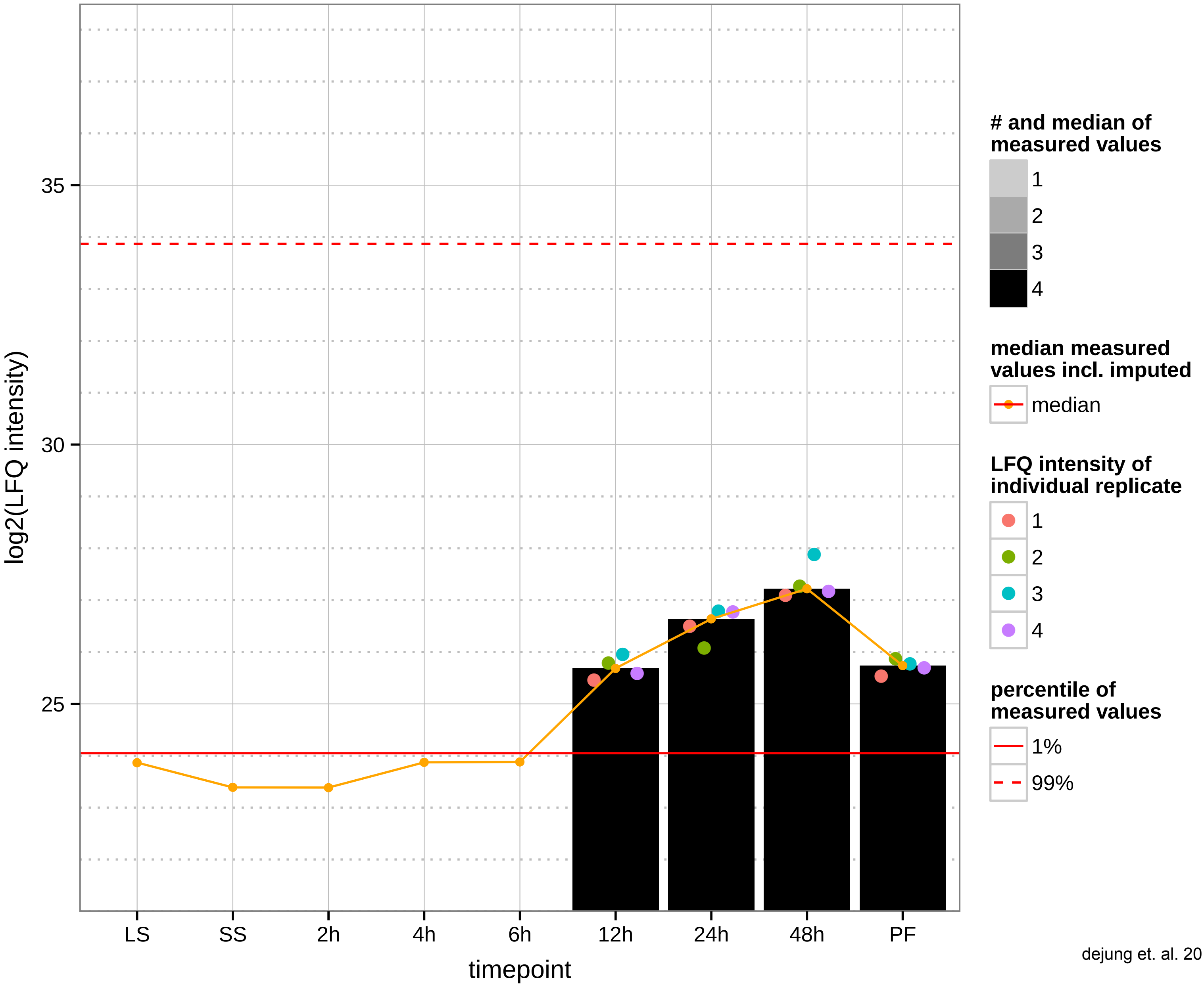

dejung et. al. 2015

hypothetical protein, conserved
Tb927.6.3250
AGOF: zinc ion binding
AGOC: intracellular
AGOP: null
PGOF: zinc ion binding
PGOC: intracellular
PGOP: null

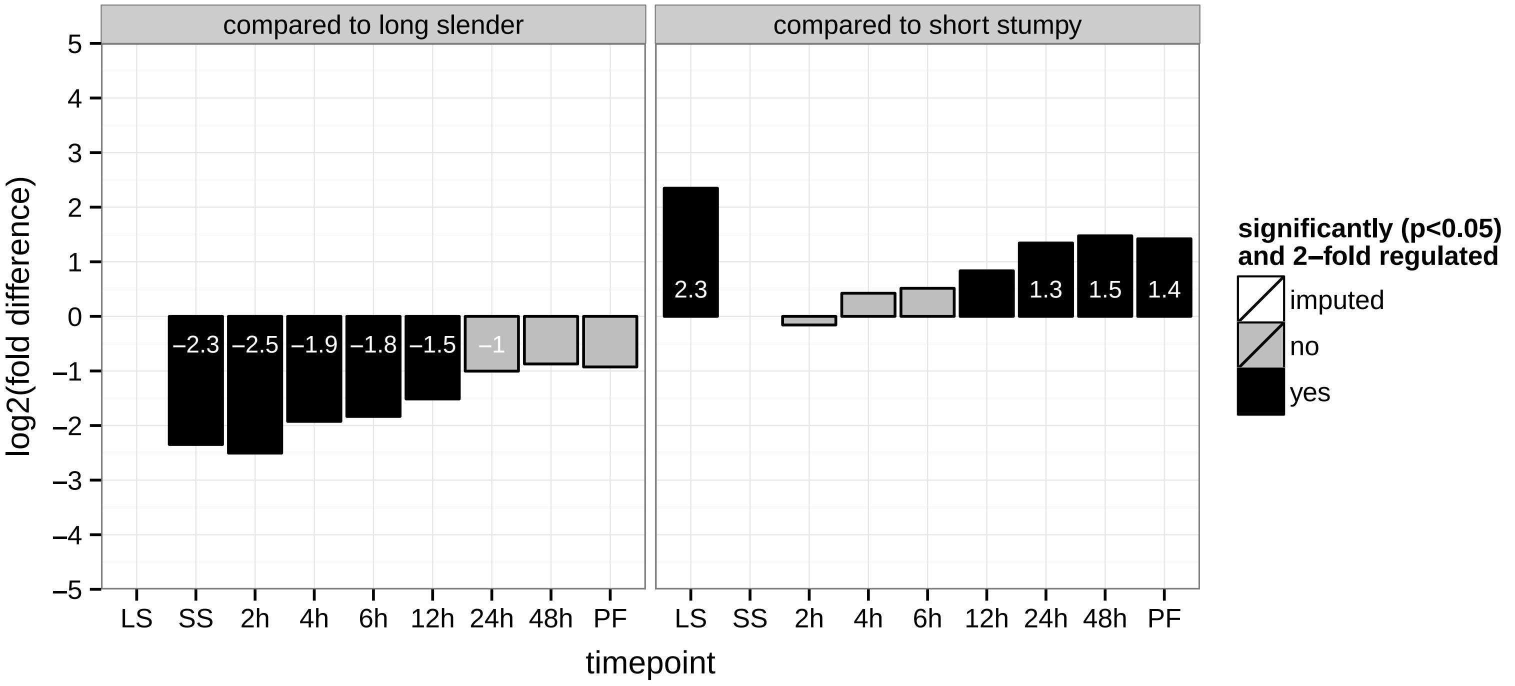

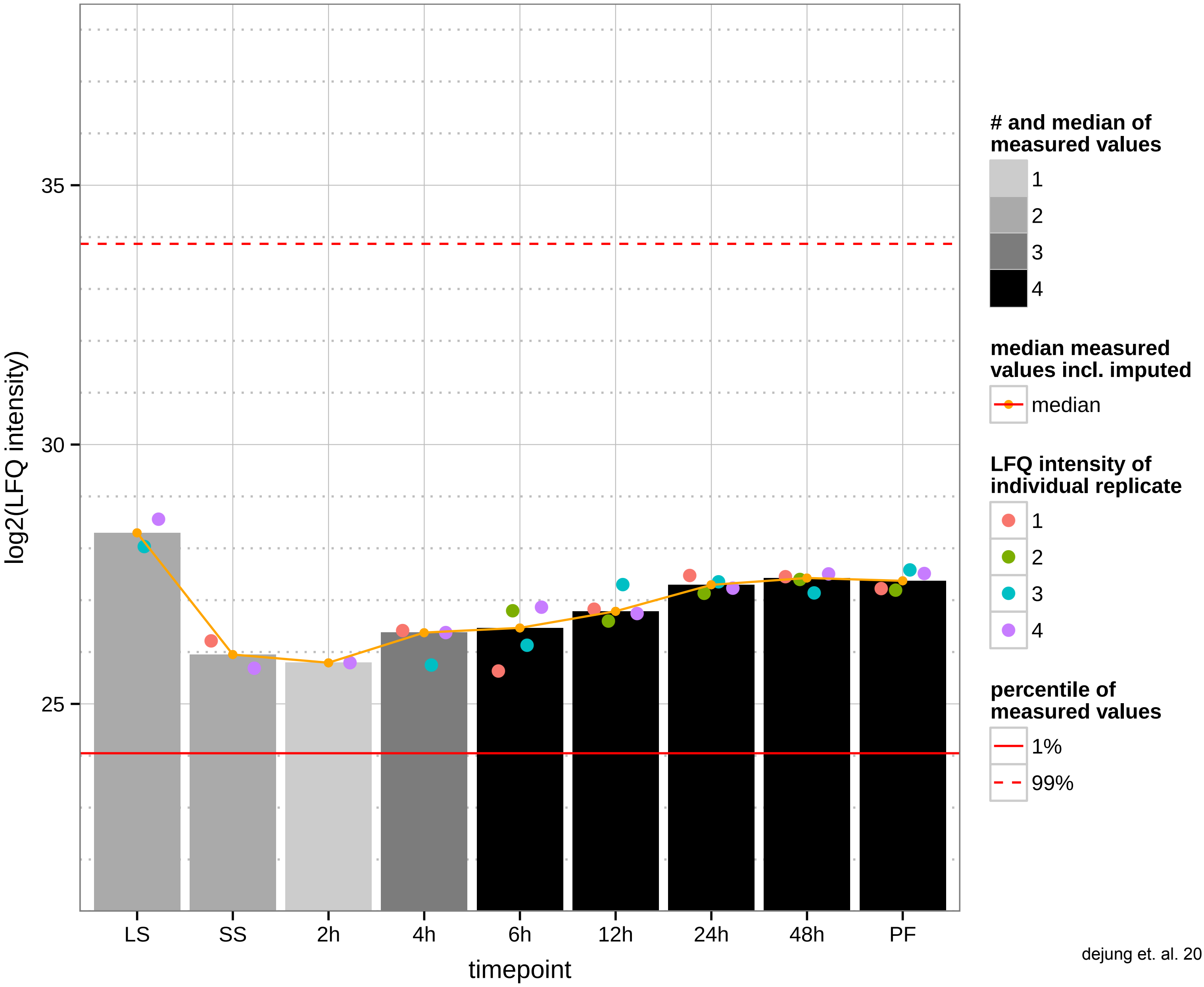

dejung et. al. 2015

RNA–binding protein 42 (RNA–binding motif protein 42) (RBP42)
Tb927.6.4440
AGOF: RNA polymerase binding, mRNA binding
AGOC: polysome
AGOP: null
PGOF: null
PGOC: null
PGOP: null

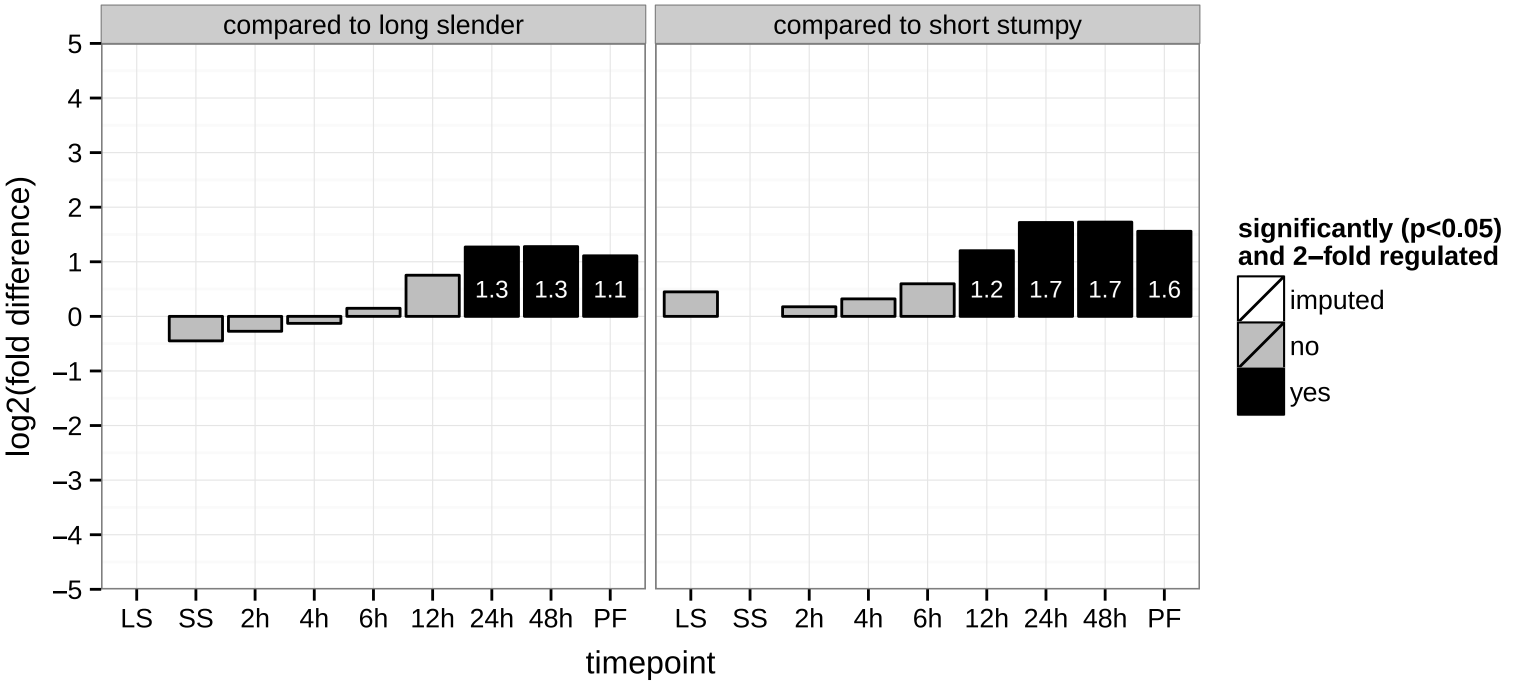

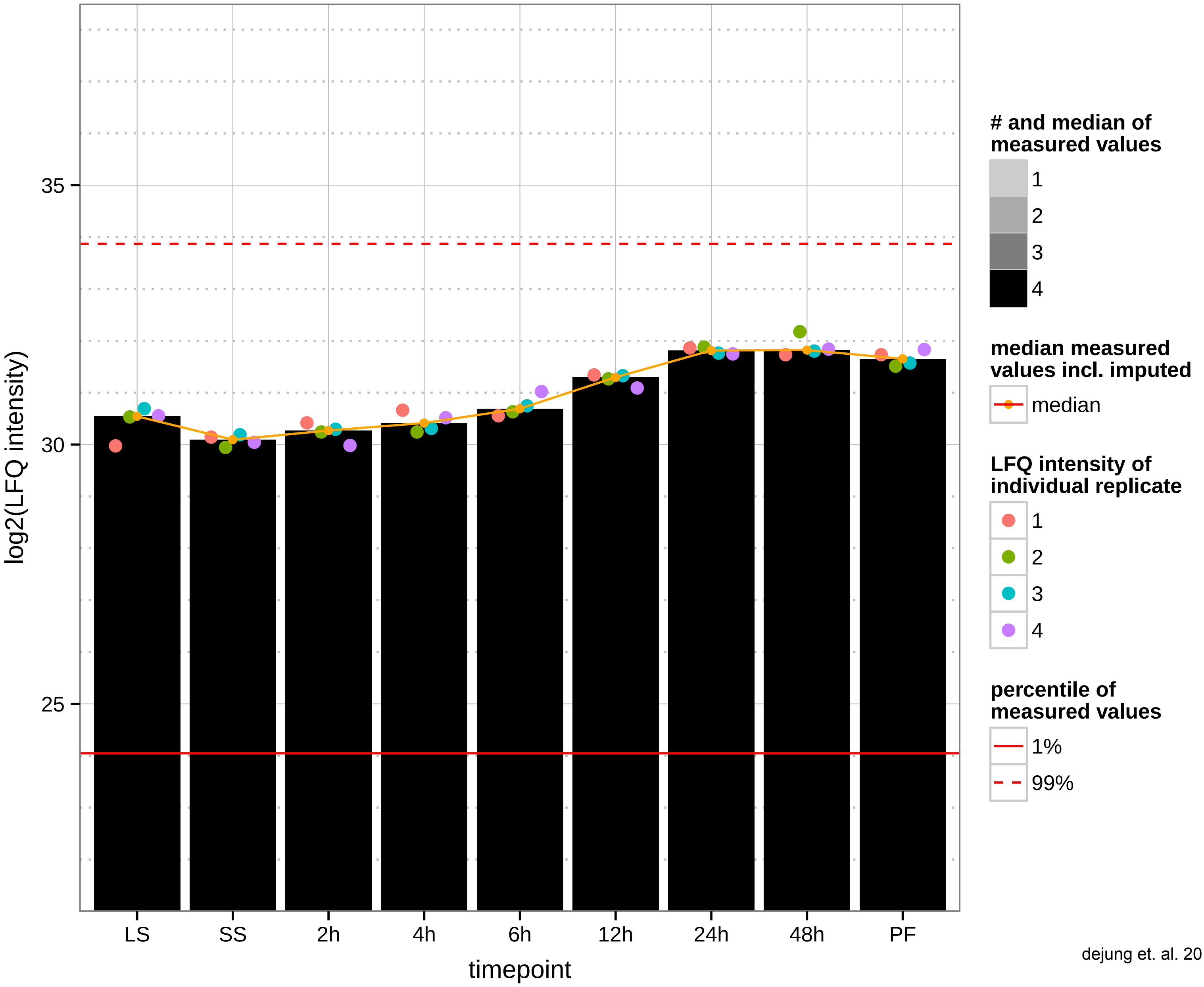

dejung et. al. 2015

3–hydroxy–3–methylglutaryl–CoA reductase, putative
Tb927.6.4540
AGOF: NADP binding
AGOC: integral to membrane, mitochondrial inner membrane, mitochondrion
AGOP: coenzyme A metabolic process, oxidation–reduction process
PGOF: NADP binding, coenzyme binding, hydroxymethylglutaryl–CoA reductase (NADPH) activity, oxidoreductase activity, ac
PGOC: integral to membrane
PGOP: coenzyme A metabolic process, isoprenoid biosynthetic process, oxidation–reduction process

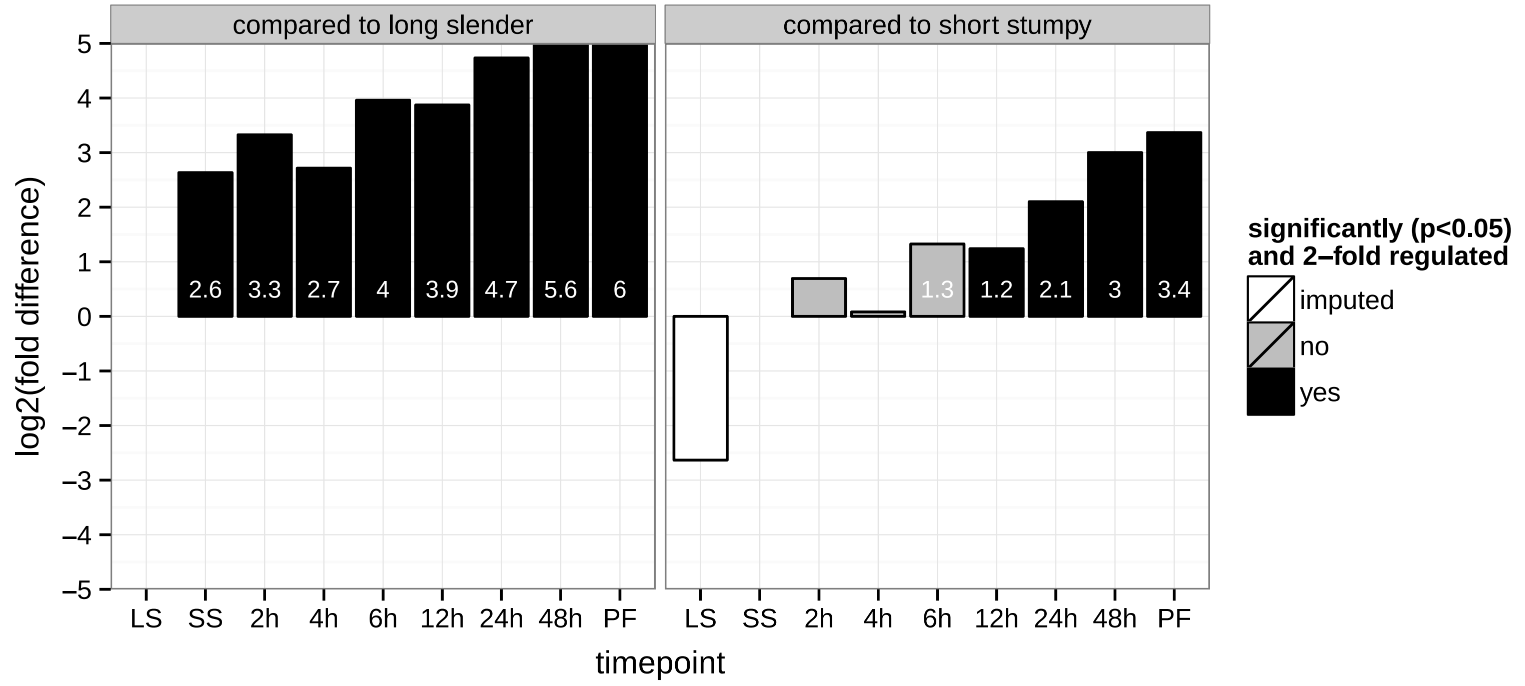

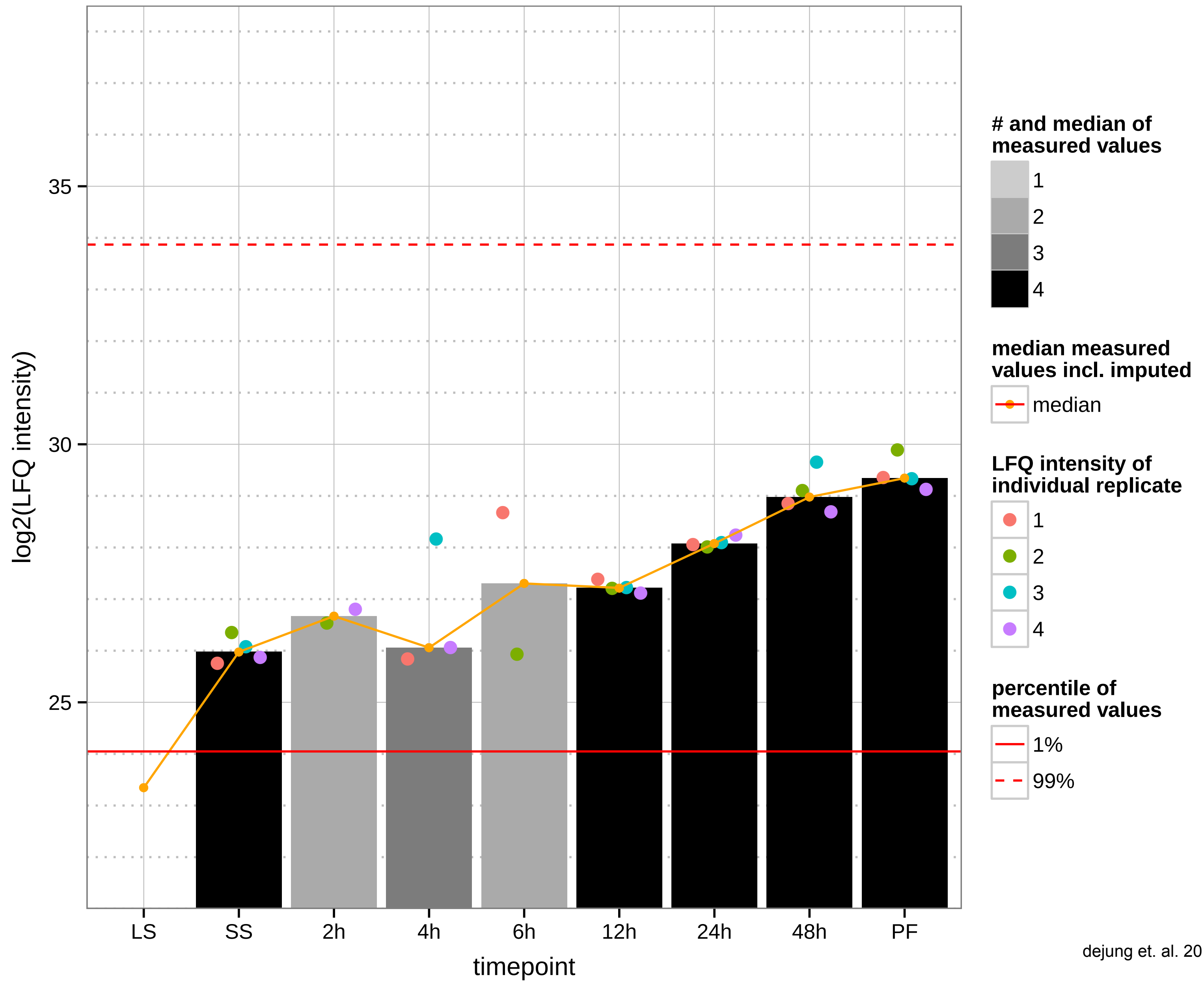

dejung et. al. 2015

hypothetical protein, conserved
Tb927.6.4560
AGOF: null
AGOC: mitochondrion
AGOP: null
PGOF: null
PGOC: null
PGOP: null

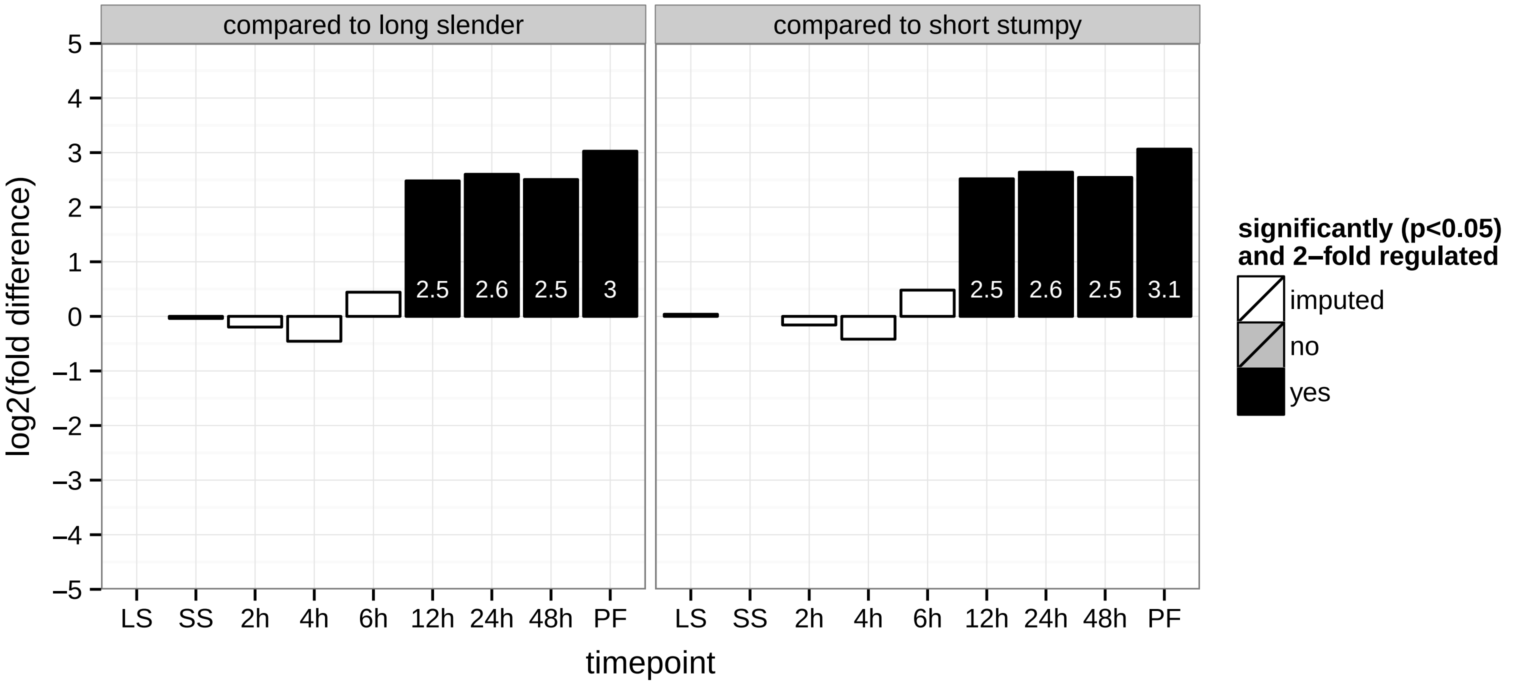

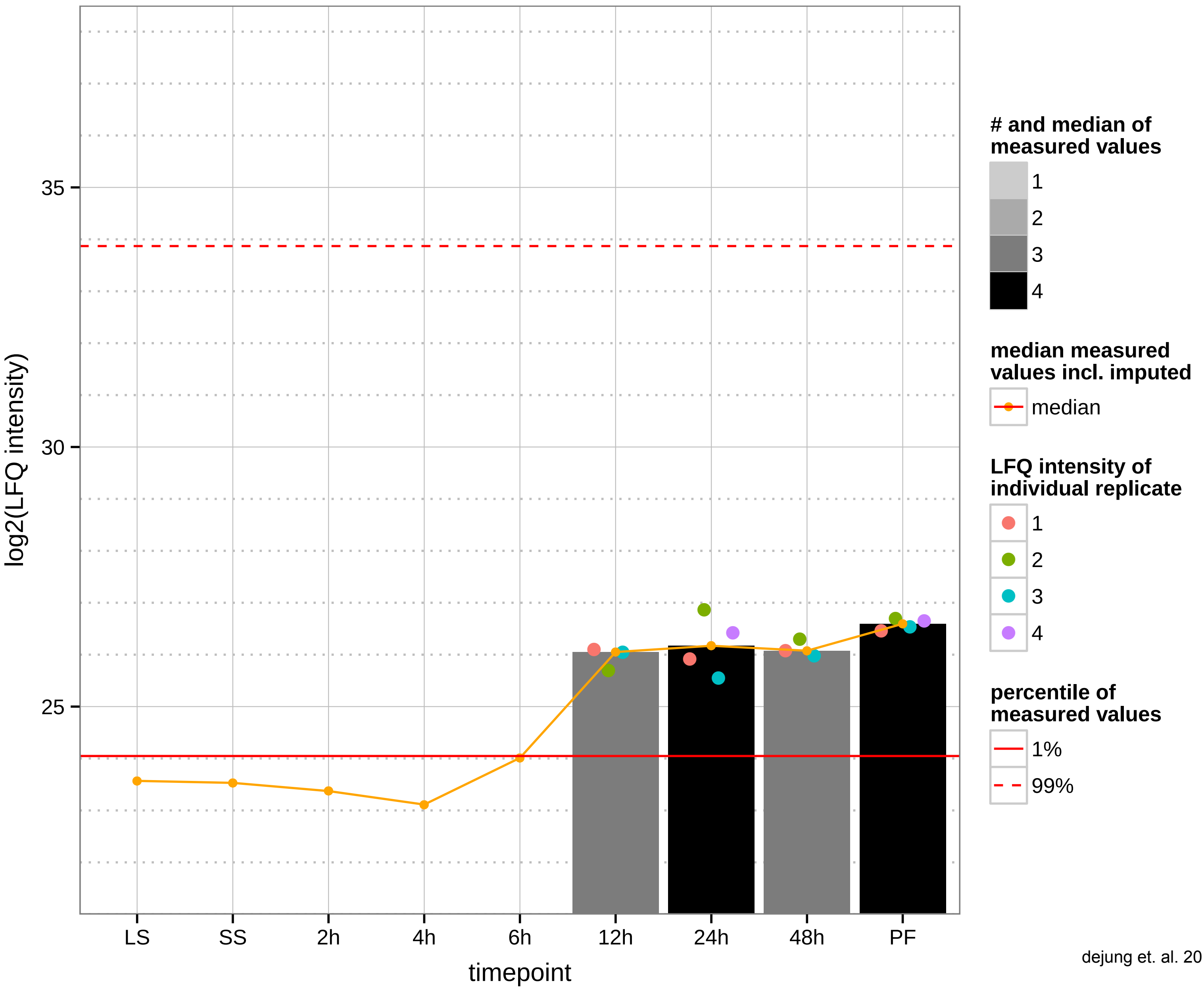

dejung et. al. 2015

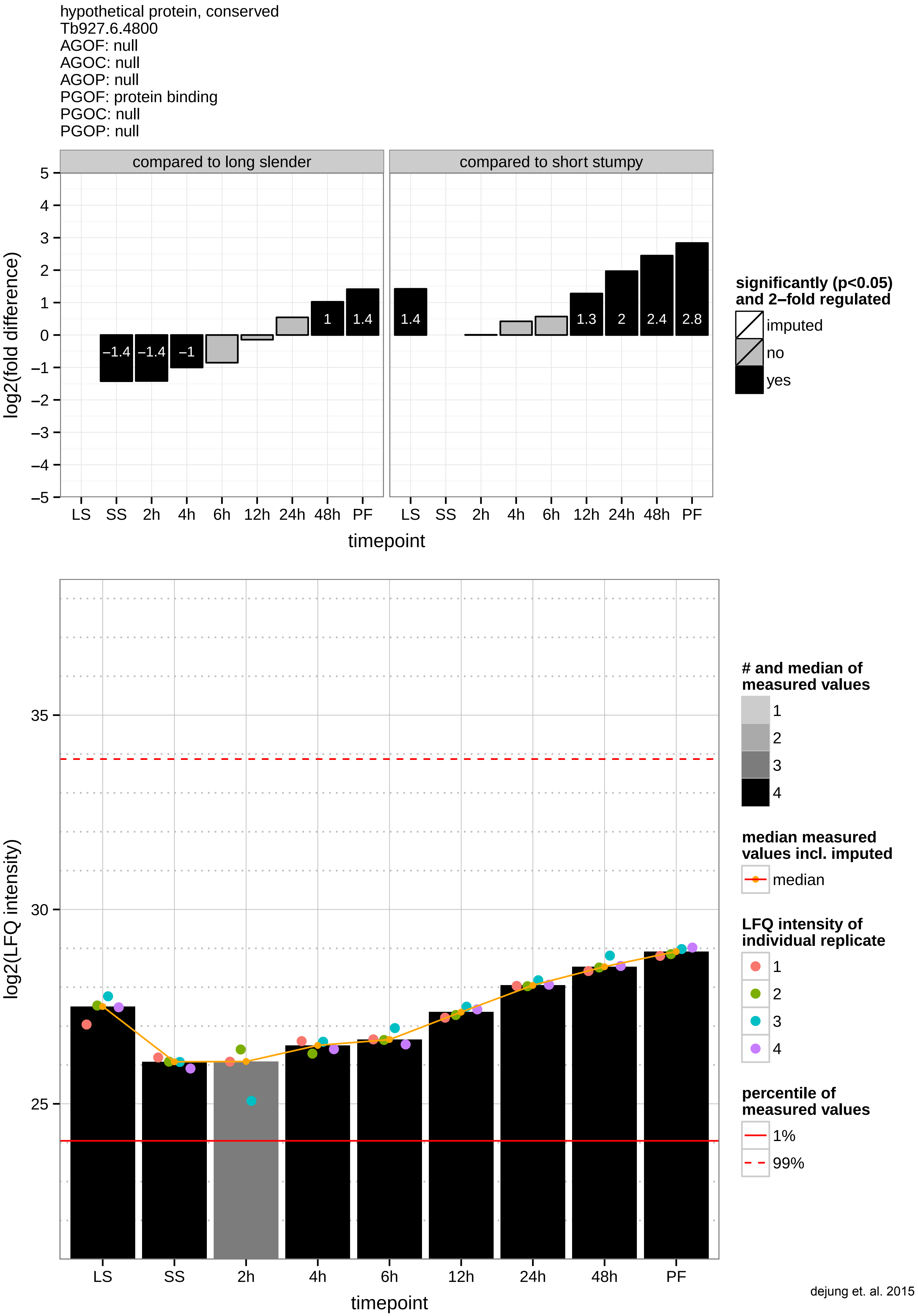

hypothetical protein, conserved
Tb927.6.4800
AGOF: null
AGOC: null
AGOP: null
PGOF: protein binding
PGOC: null
PGOP: null
compared to long slender
compared to short stumpy
log2(fold difference)
−1.4
−1.4
−1
1
1.4
1.4
1.3
2
2.4
2.8
LS SS 2h 4h 6h 12h 24h 48h PF
timepoint
significantly (p<0.05) and 2−fold regulated
imputed
no
yes
log2(LFQ intensity)
# and median of measured values
1
2
3
4
median measured values incl. imputed
median
LFQ intensity of individual replicate
1
2
3
4
percentile of measured values
1%
99%
dejung et. al. 2015

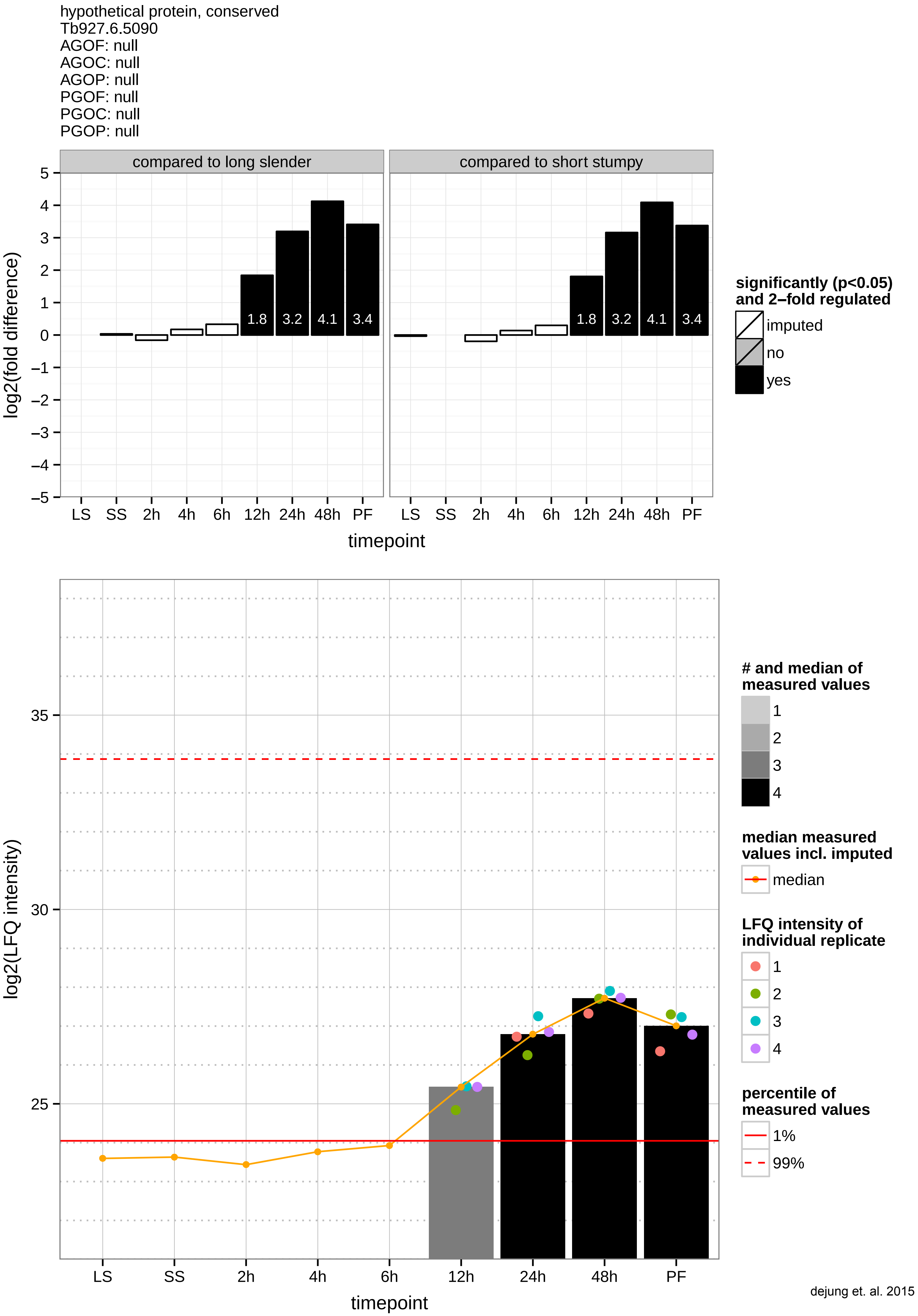

hypothetical protein, conserved
Tb927.6.5090
AGOF: null
AGOC: null
AGOP: null
PGOF: null
PGOC: null
PGOP: null
compared to long slender
compared to short stumpy
log2(fold difference)
1.8
3.2
4.1
3.4
LS SS 2h 4h 6h 12h 24h 48h PF
timepoint
significantly (p<0.05) and 2–fold regulated
imputed
no
yes
log2(LFQ intensity)
# and median of measured values
median measured values incl. imputed
median
LFQ intensity of individual replicate
percentile of measured values
1%
99%
dejung et. al. 2015

thimet oligopeptidase, putative (THOP1)
Tb927.7.190
AGOF: metalloendopeptidase activity
AGOC: null
AGOP: intracellular signal transduction, proteolysis
PGOF: metalloendopeptidase activity
PGOC: null
PGOP: proteolysis

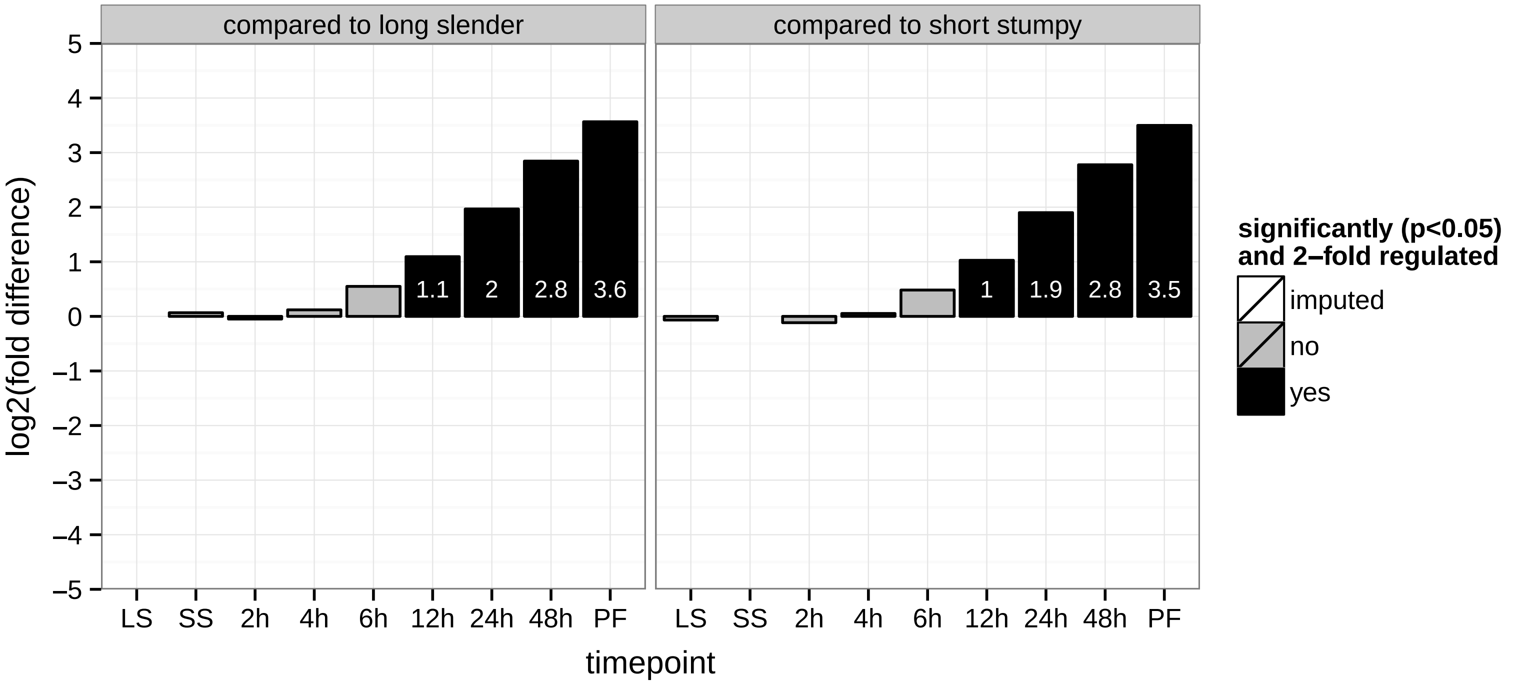

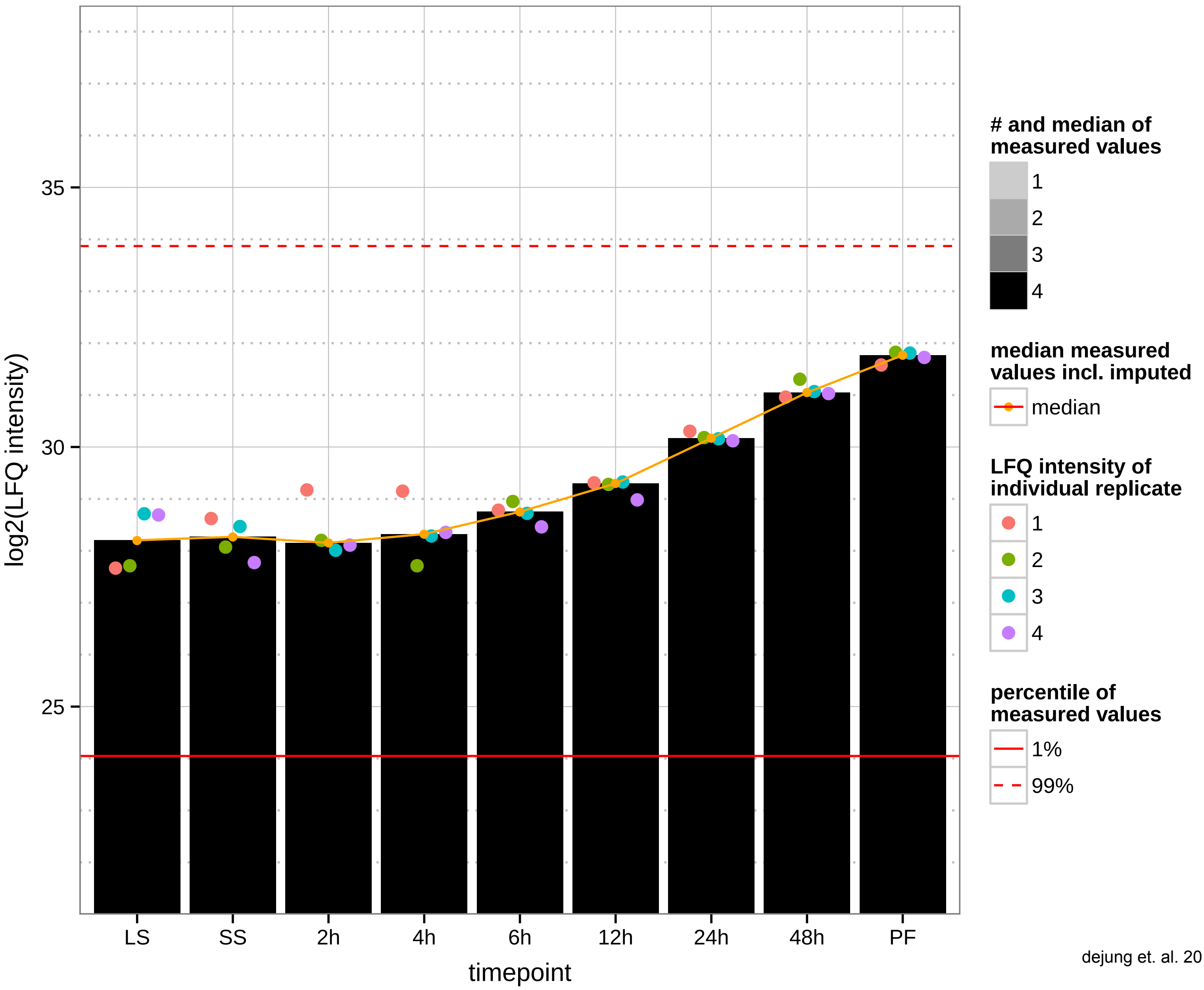

dejung et. al. 2015

glutathione−S−transferase/glutaredoxin, putative
Tb927.7.3500
AGOF: electron carrier activity, protein disulfide oxidoreductase activity
AGOC: null
AGOP: cell redox homeostasis
PGOF: electron carrier activity, protein disulfide oxidoreductase activity
PGOC: null
PGOP: cell redox homeostasis

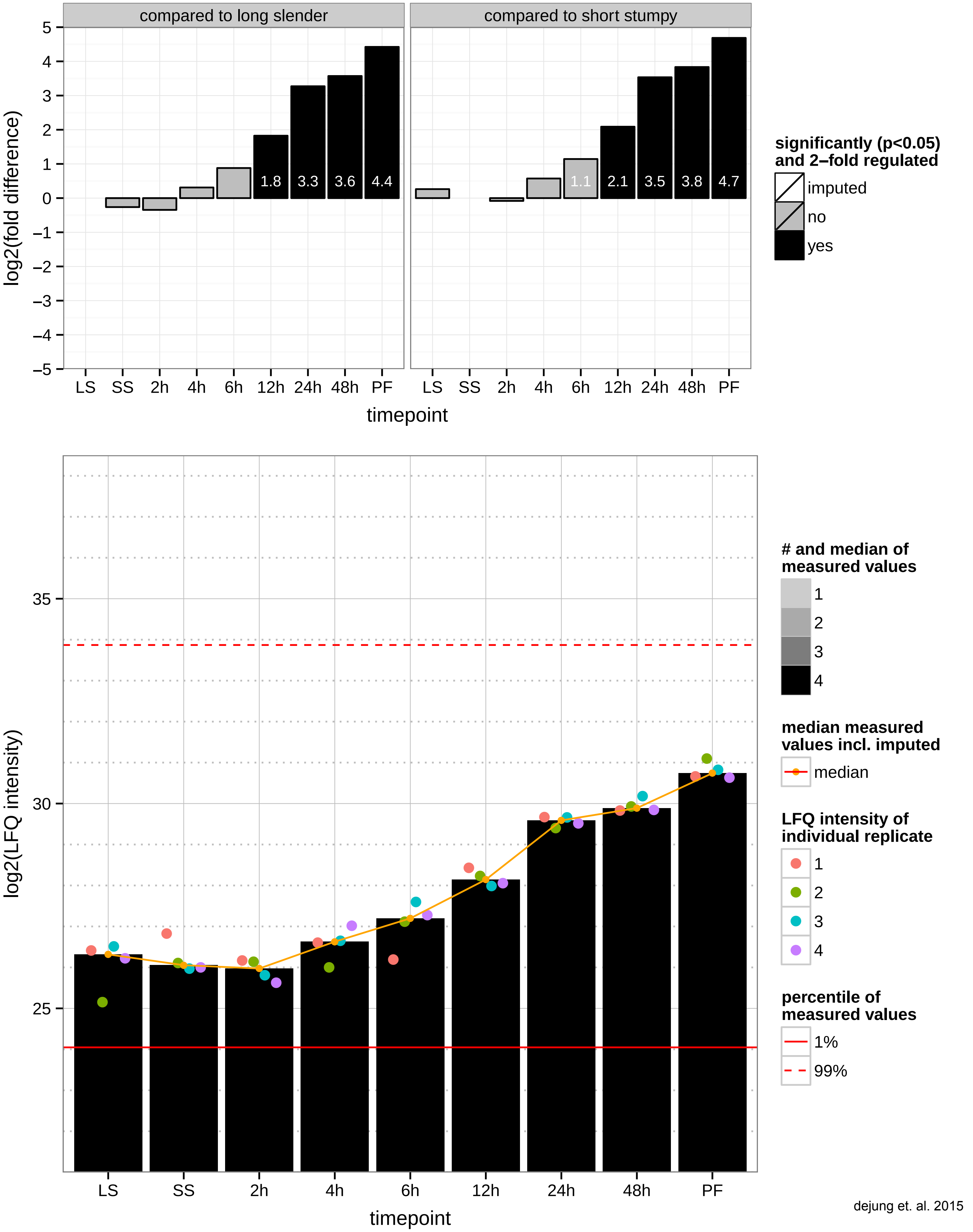

dejung et. al. 2015

fatty acid elongase, putative
Tb927.7.4160
AGOF: fatty acid elongase activity
AGOC: integral to endoplasmic reticulum membrane
AGOP: long−chain fatty acid biosynthetic process
PGOF: null
PGOC: integral to membrane
PGOP: null

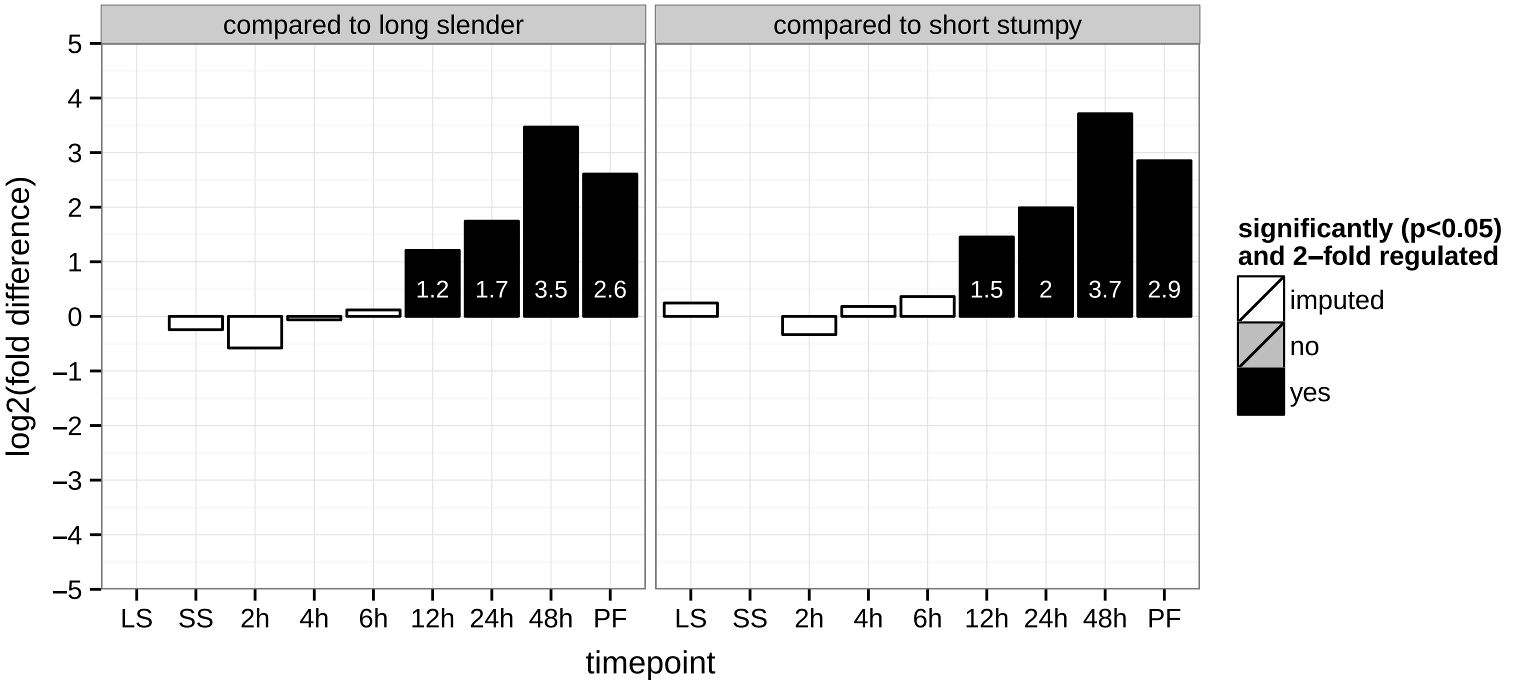

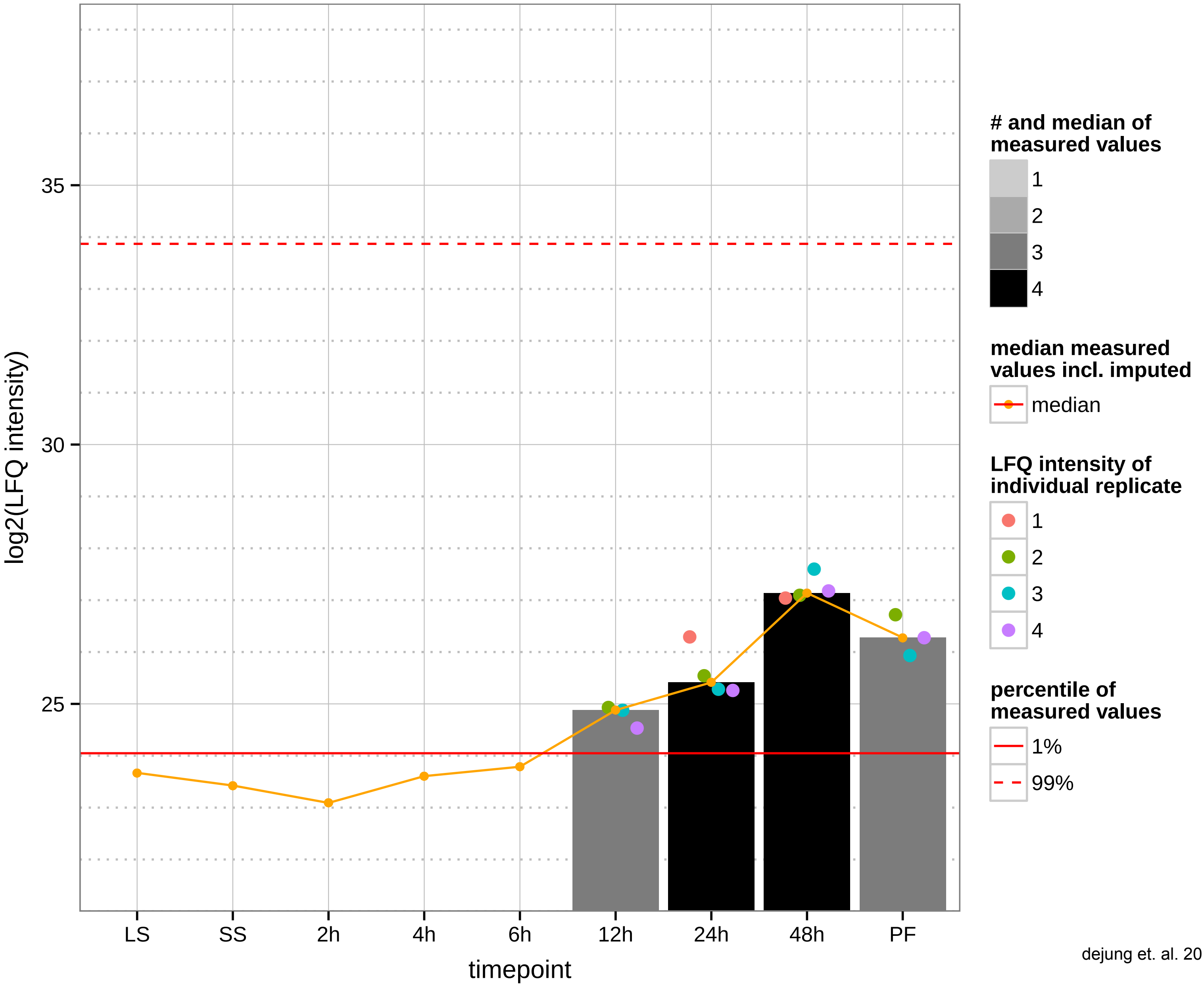

dejung et. al. 2015

hypothetical protein, conserved
Tb927.7.5140
AGOF: null
AGOC: null
AGOP: null
PGOF: null
PGOC: null
PGOP: null

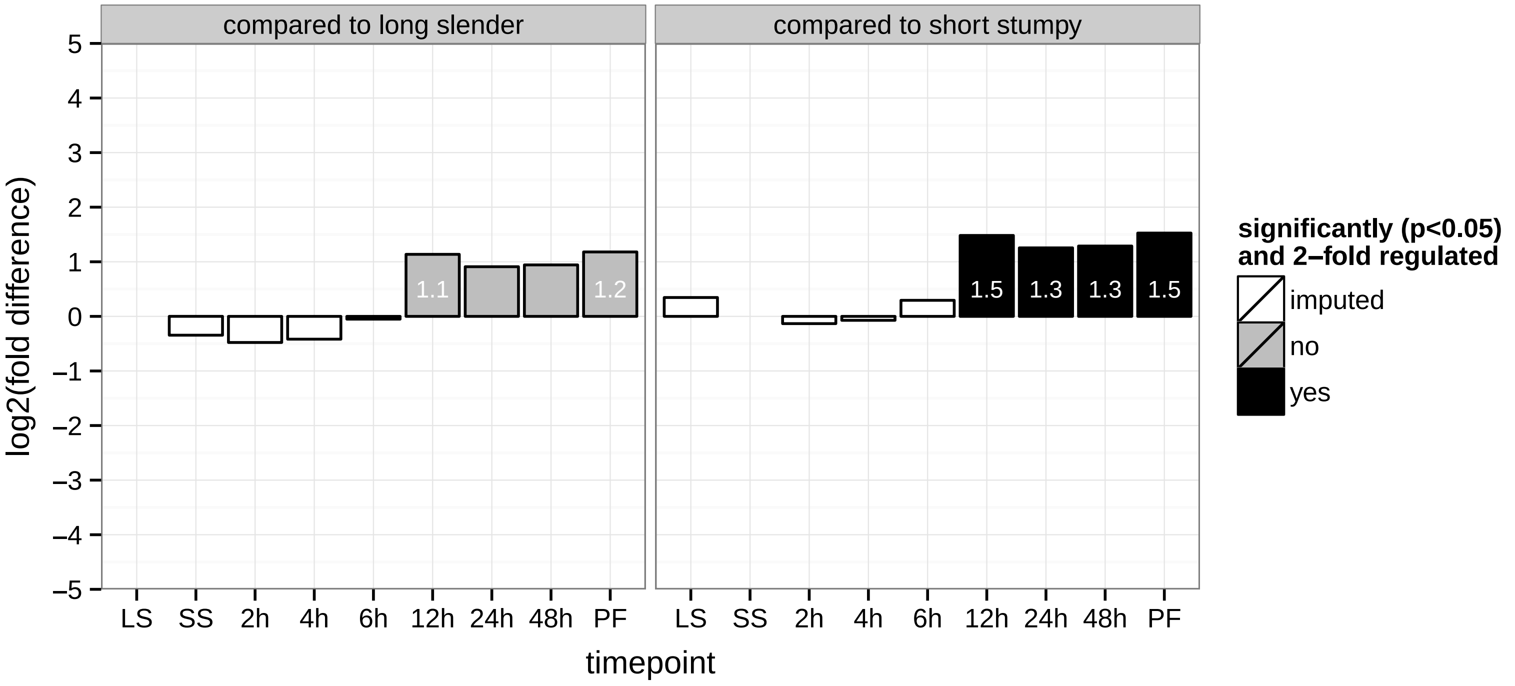

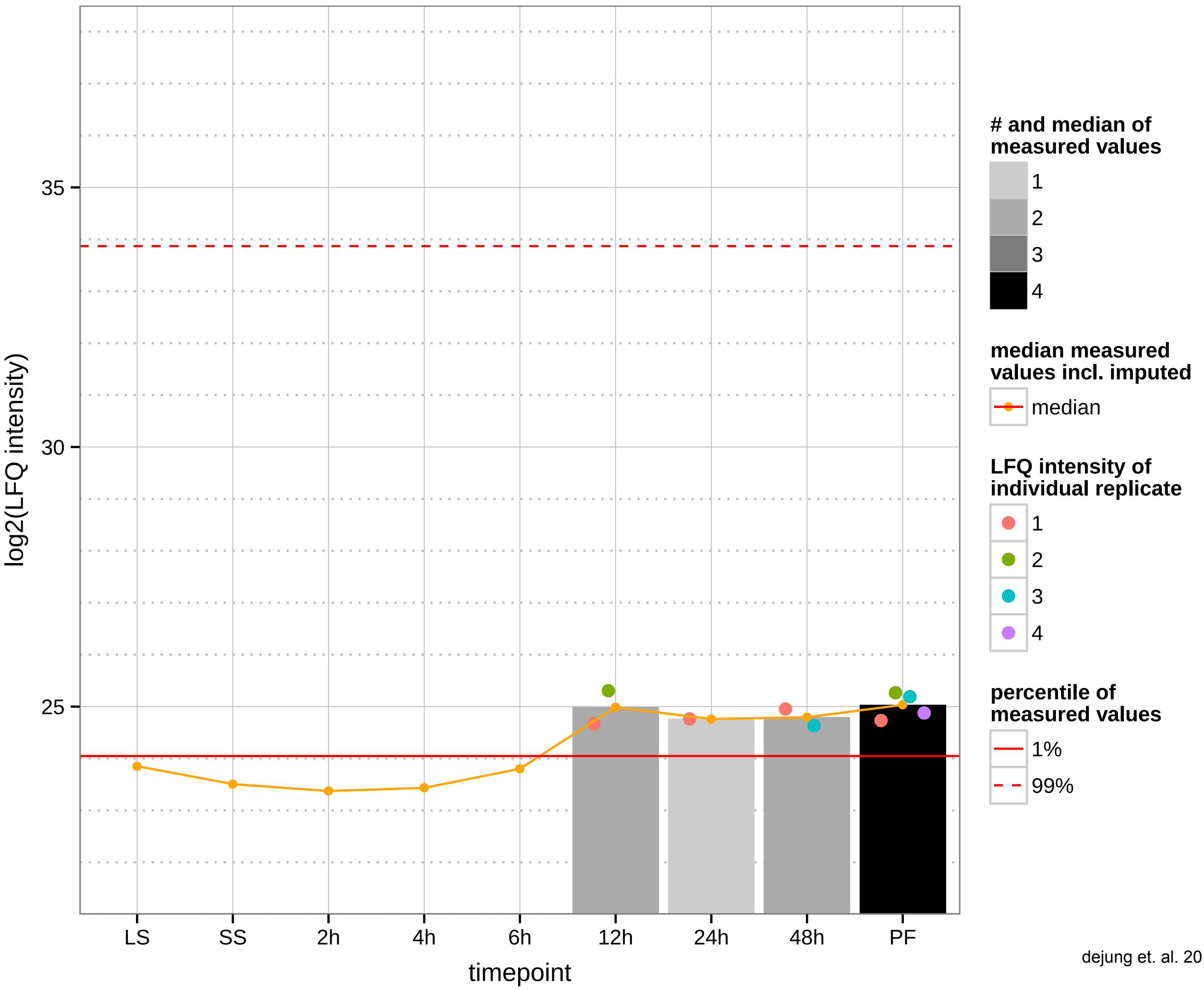

dejung et. al. 2015

chaperone protein DNAj, putative
Tb927.7.6200
AGOF: heat shock protein binding, unfolded protein binding
AGOC: integral to membrane, mitochondrial inner membrane, mitochondrion
AGOP: protein folding
PGOF: heat shock protein binding
PGOC: null
PGOP: null

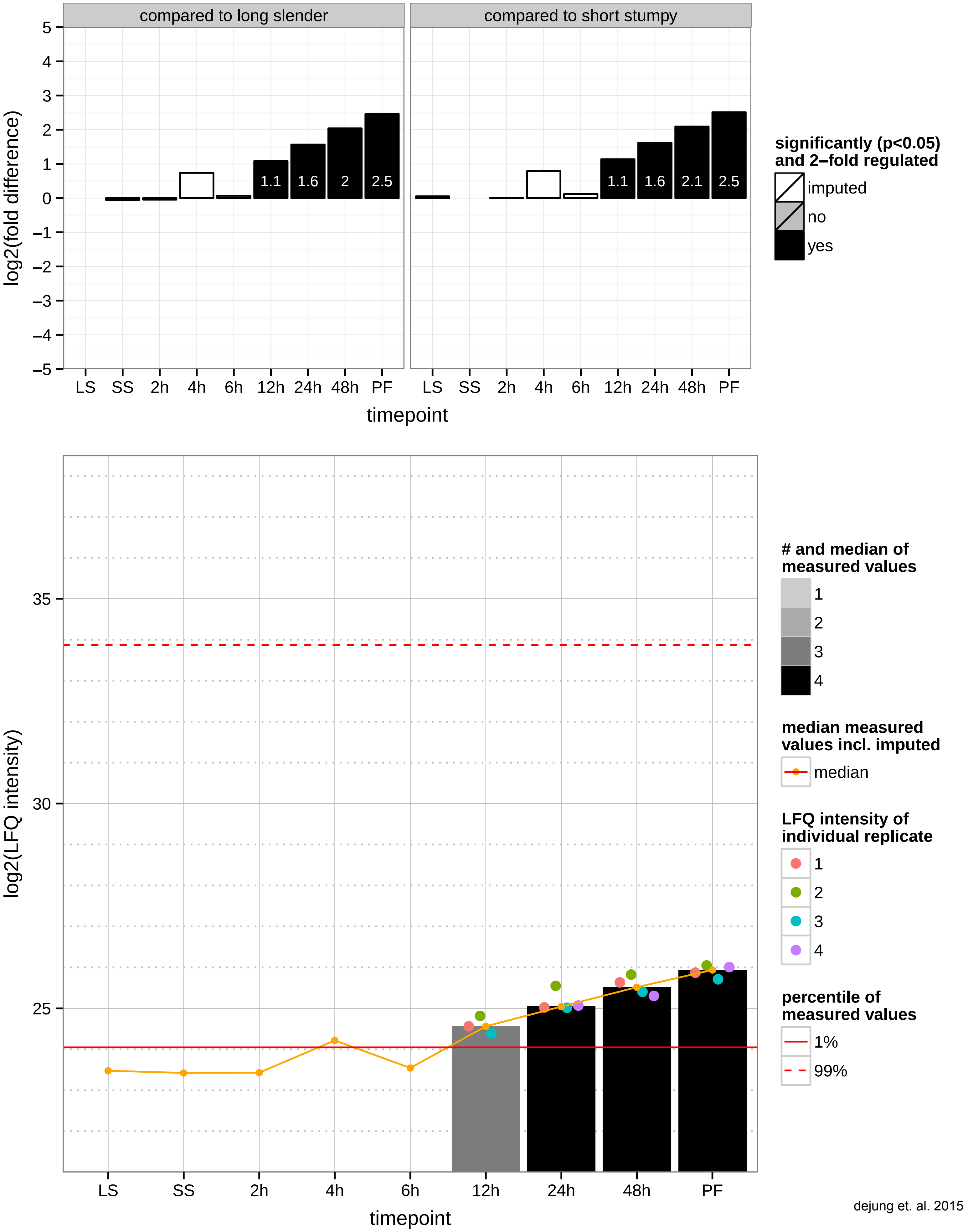

dejung et. al. 2015

peptidase t, putative, aminotripeptidase
Tb927.7.6270
AGOF: hydrolase activity, metallopeptidase activity, protein dimerization activity, tripeptide aminopeptidase activity, zinc ion bir
AGOC: cytoplasm
AGOP: peptide metabolic process, proteolysis
PGOF: hydrolase activity, tripeptide aminopeptidase activity, zinc ion binding
PGOC: cytoplasm
PGOP: metabolic process, peptide metabolic process

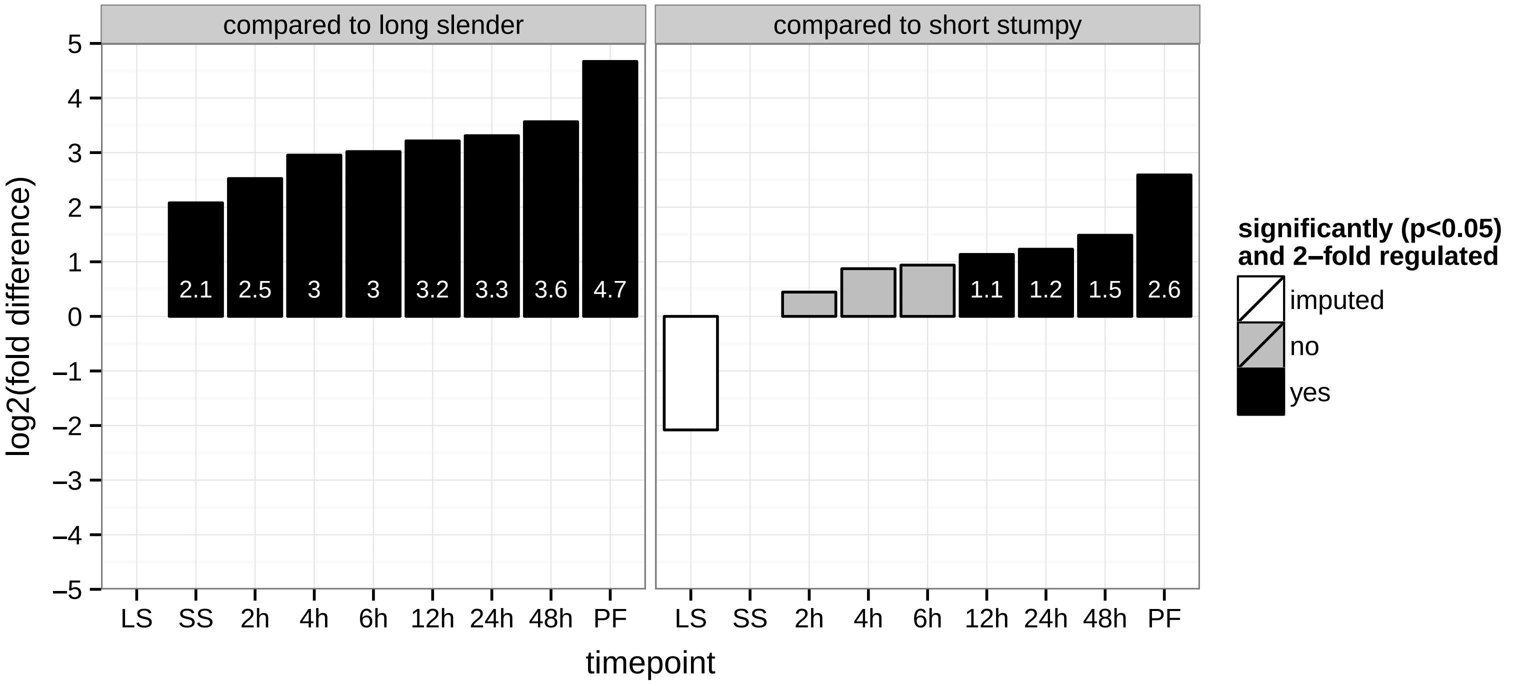

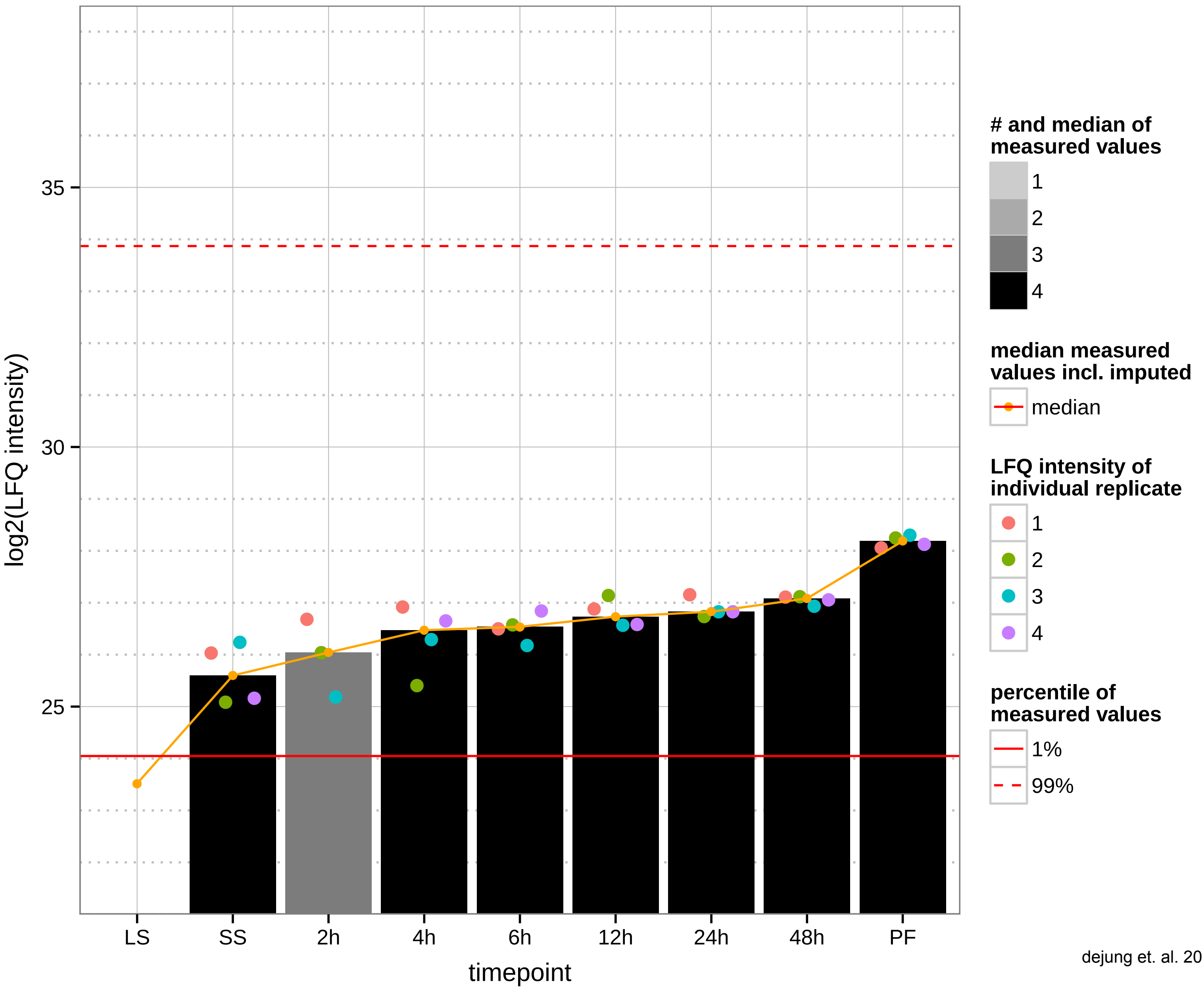

dejung et. al. 2015

O–sialoglycoprotein endopeptidase, putative, metallo–peptidase, Clan MK, Family M67
Tb927.7.6470
AGOF: metalloendopeptidase activity, zinc ion binding
AGOC: null
AGOP: proteolysis
PGOF: metalloendopeptidase activity
PGOC: null
PGOP: proteolysis

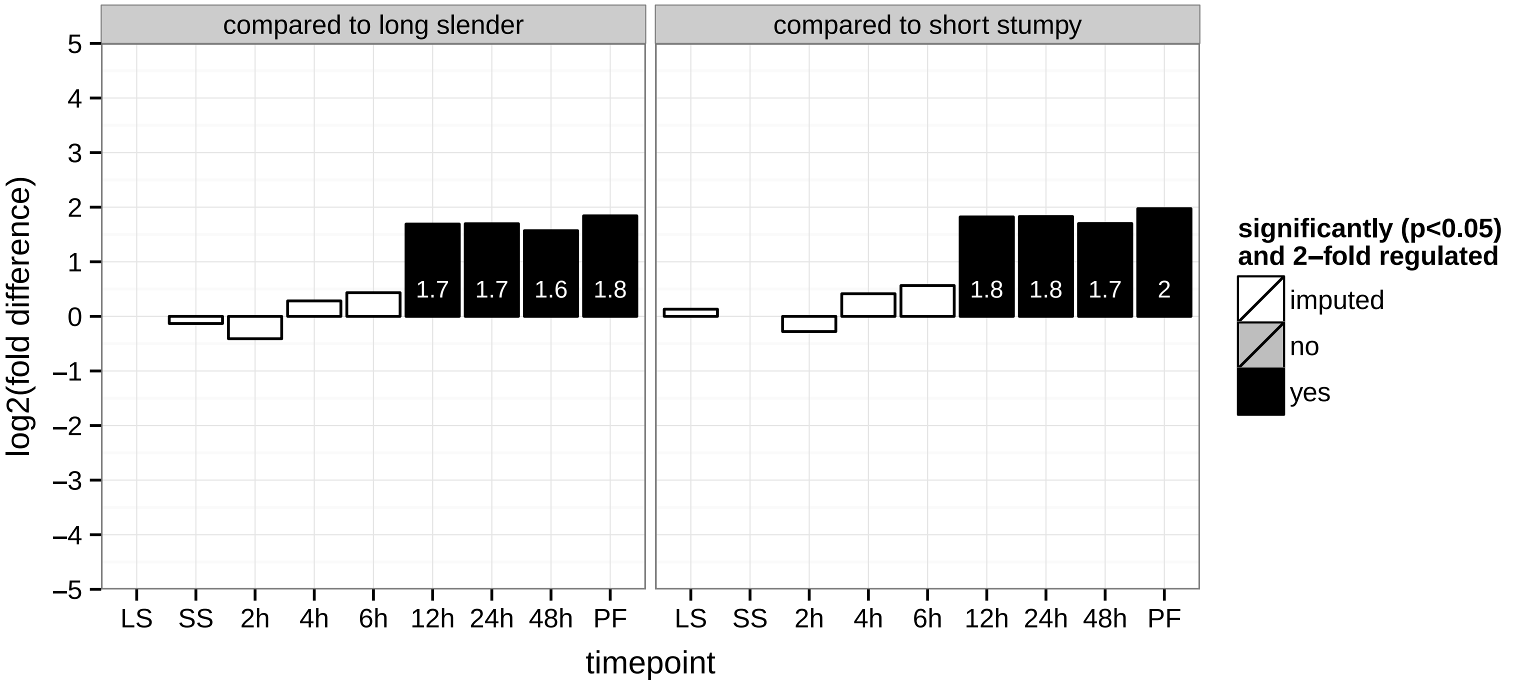

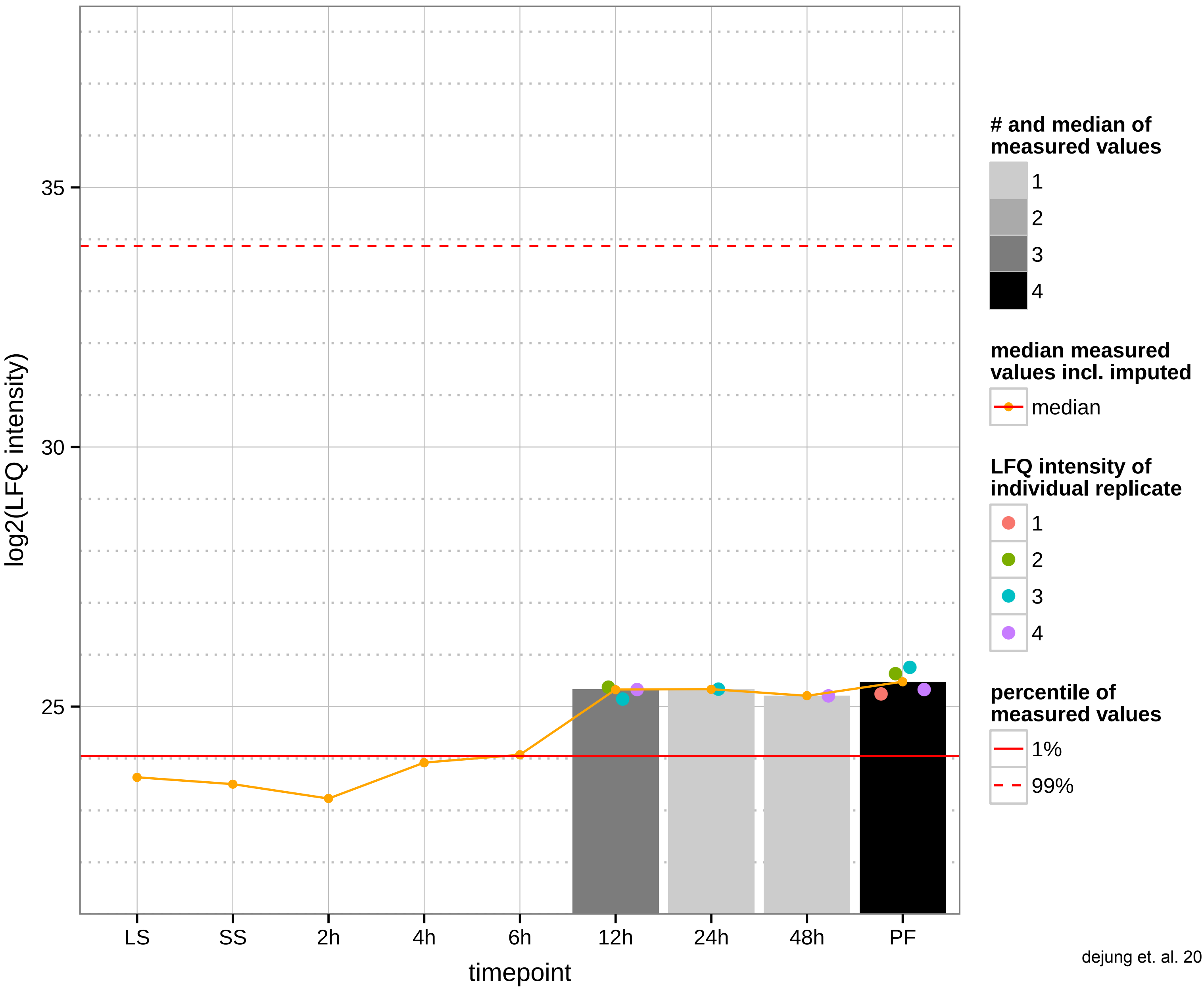

dejung et. al. 2015

hypothetical protein, conserved
Tb927.7.6790
AGOF: null
AGOC: null
AGOP: null
PGOF: null
PGOC: null
PGOP: null

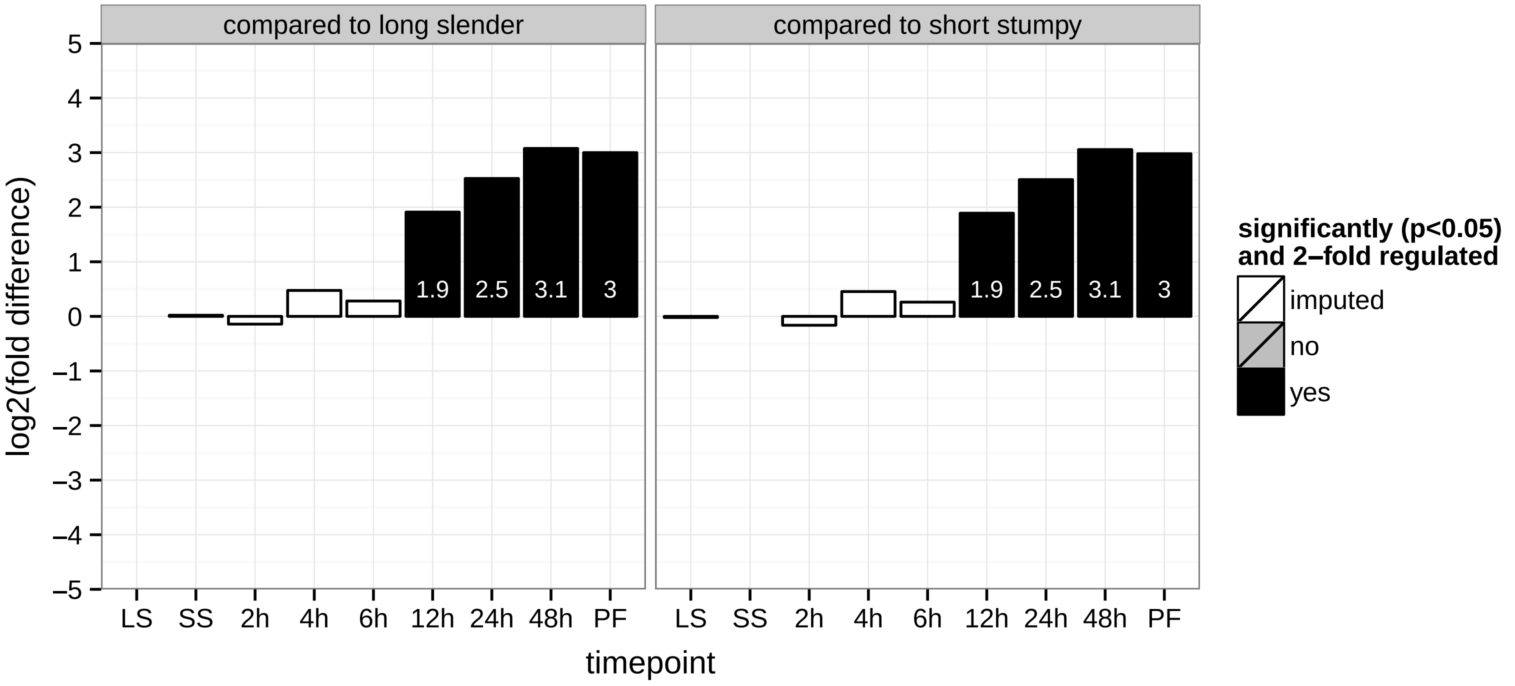

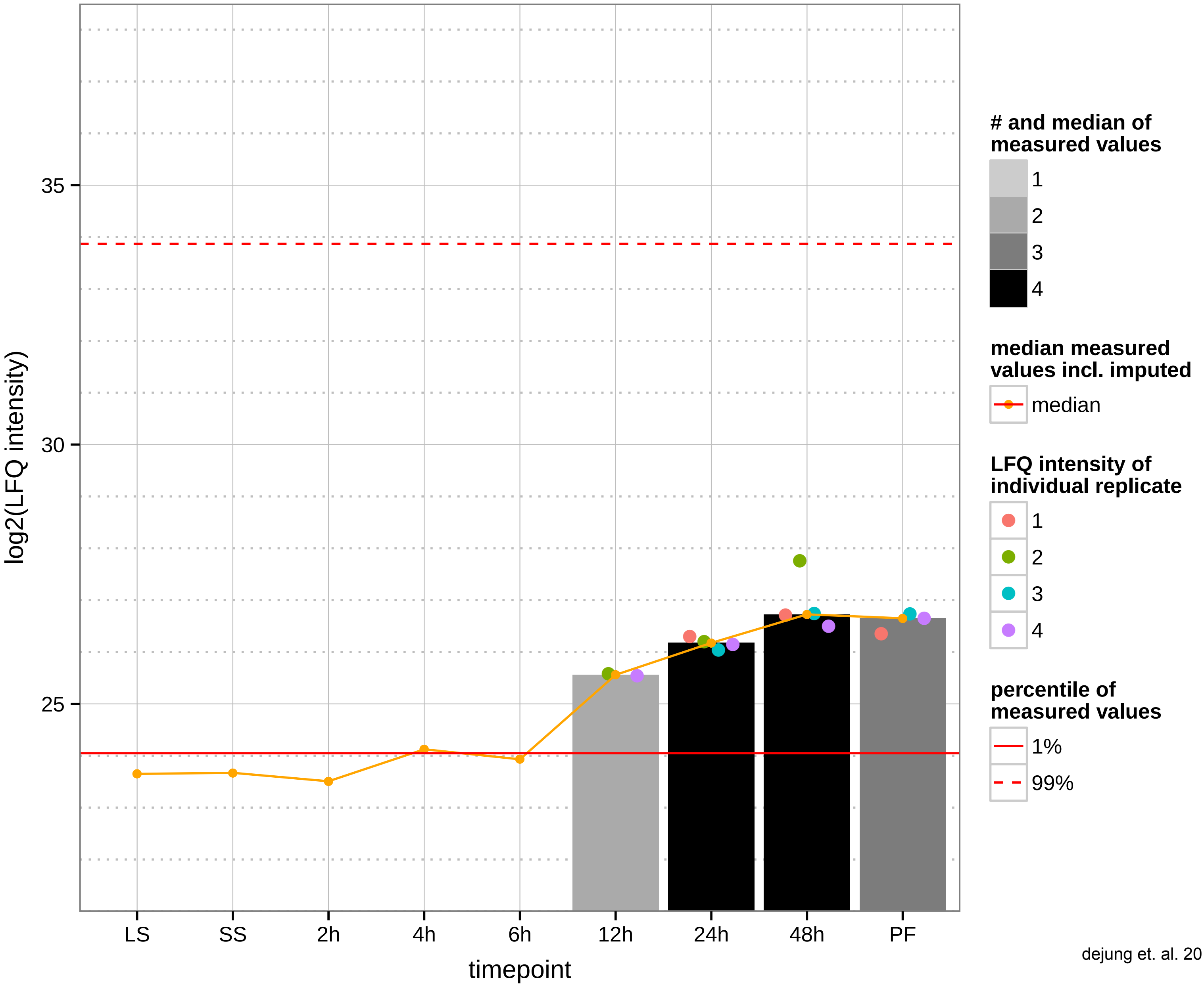

dejung et. al. 2015

leucine−rich repeat protein (LRRP), putative, leucine−rich repeat protein 1 (LRRP1)
Tb927.7.7180;Tb927.7.7110
AGOF: null
AGOC: null
AGOP: null
PGOF: null
PGOC: null
PGOP: null

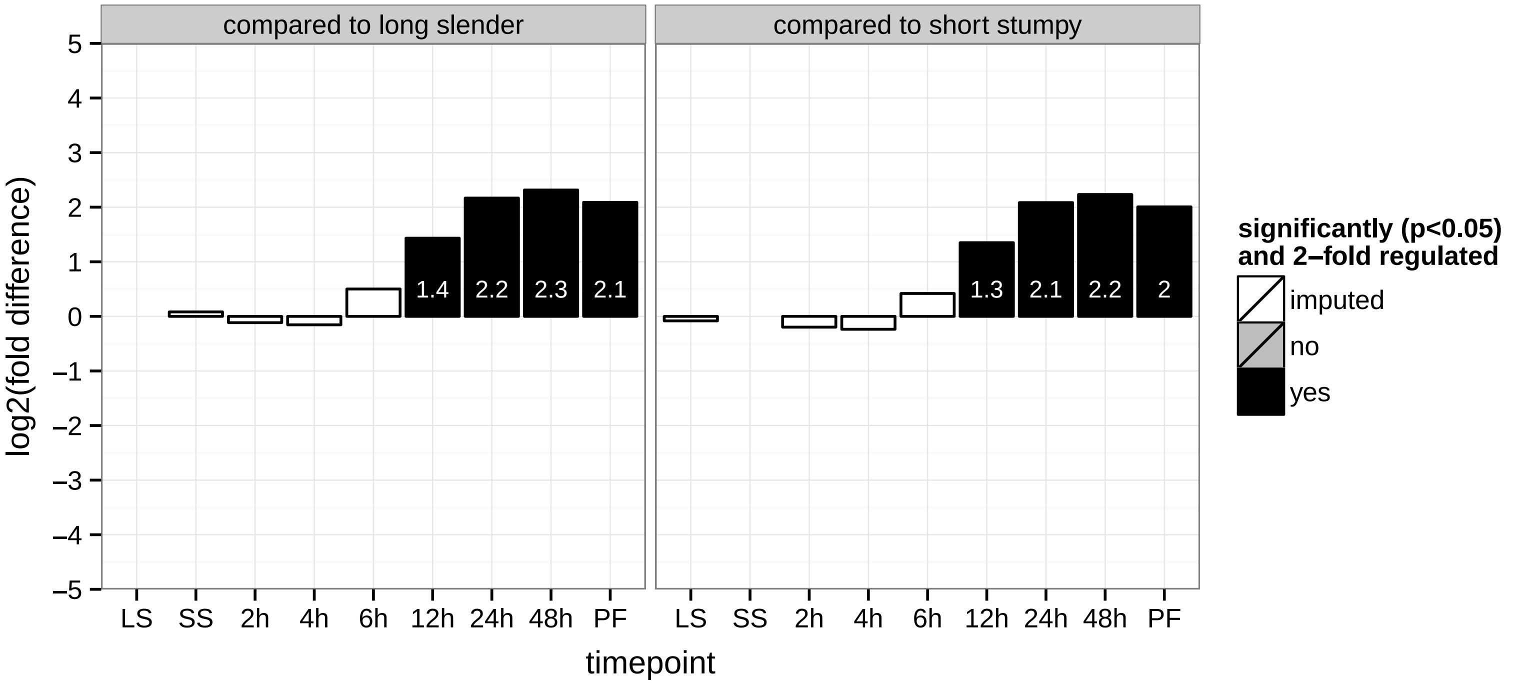

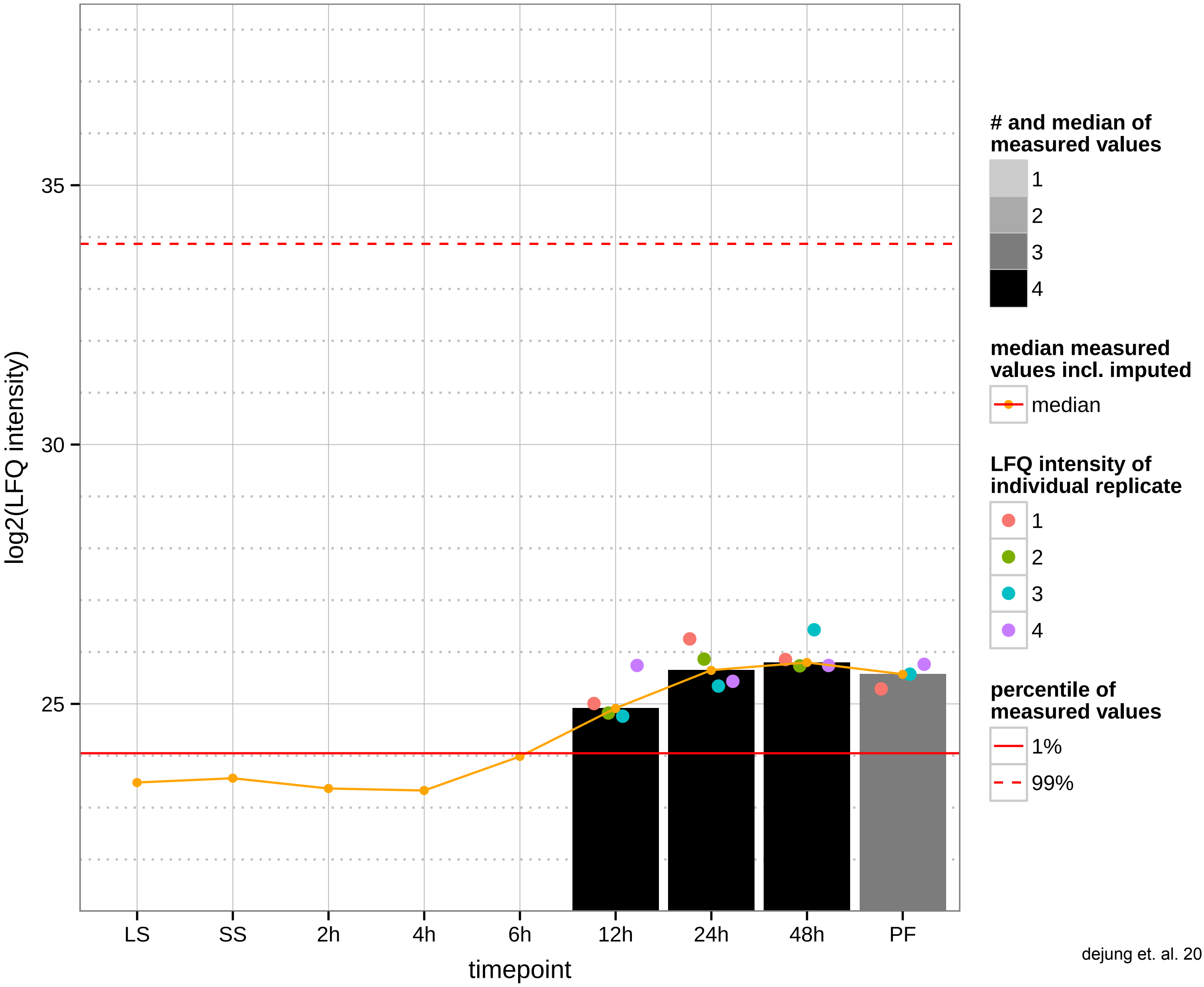

dejung et. al. 2015

hypothetical protein, conserved
Tb927.7.7440
AGOF: null
AGOC: mitochondrion
AGOP: null
PGOF: null
PGOC: null
PGOP: null

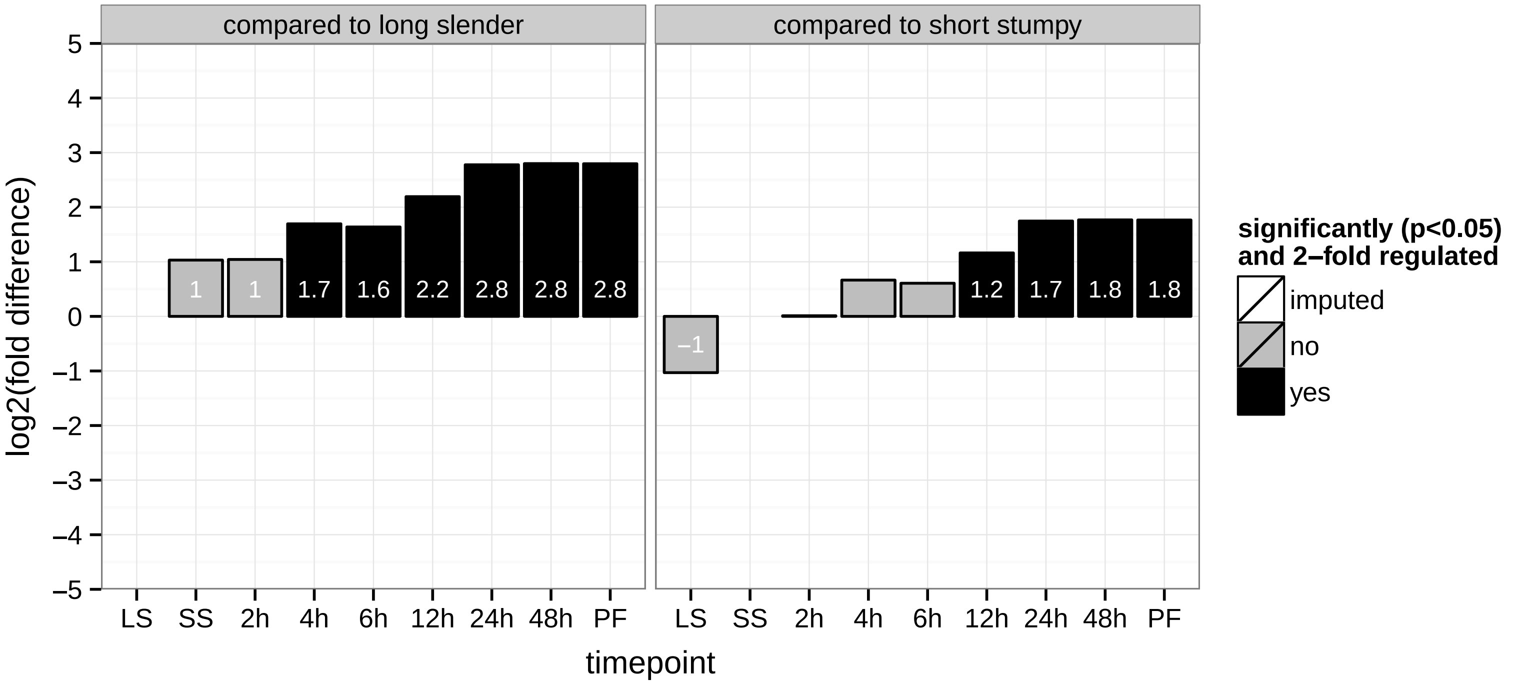

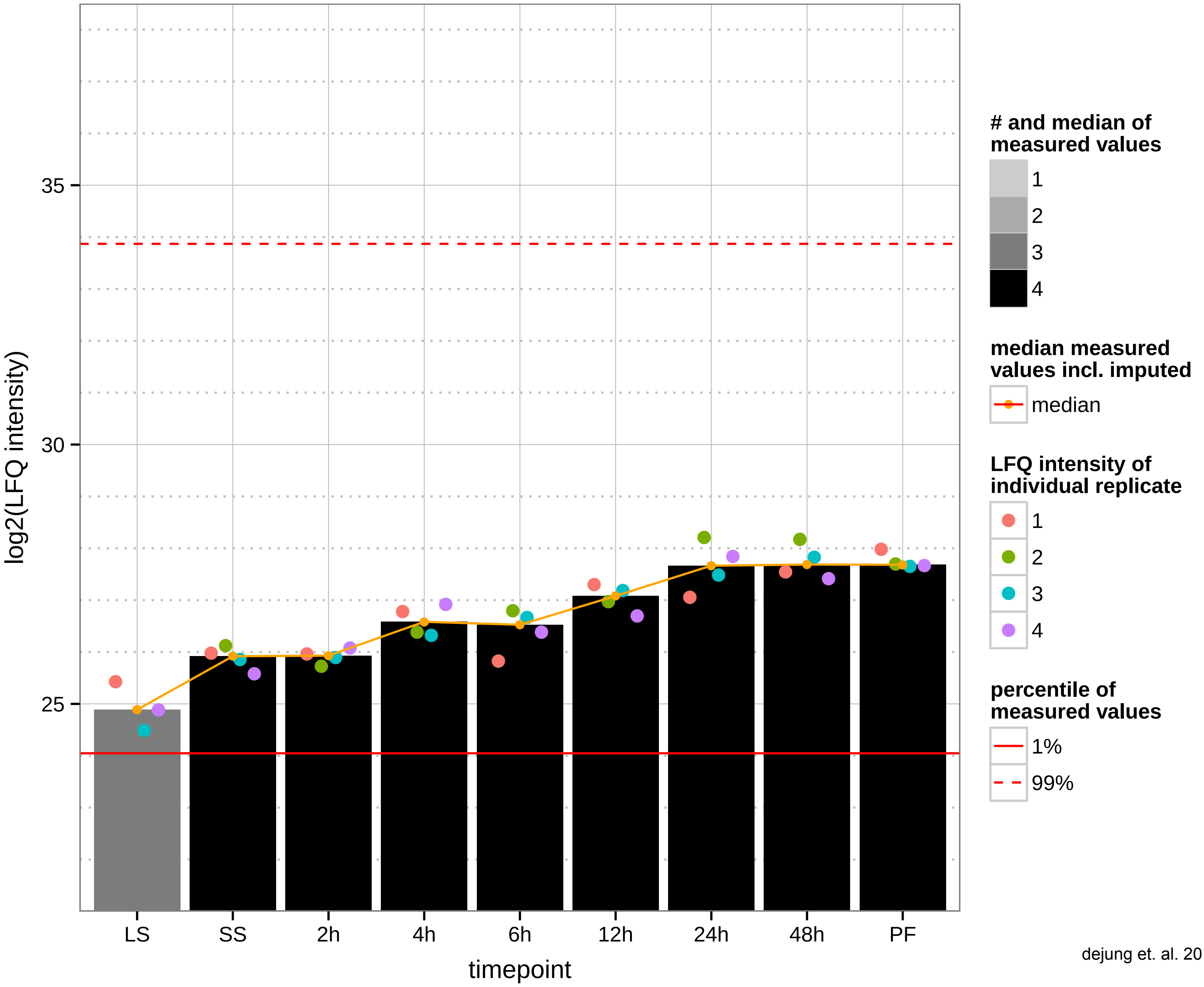

dejung et. al. 2015

hypothetical protein, conserved
Tb927.8.1880
AGOF: null
AGOC: mitochondrion
AGOP: null
PGOF: null
PGOC: null
PGOP: null

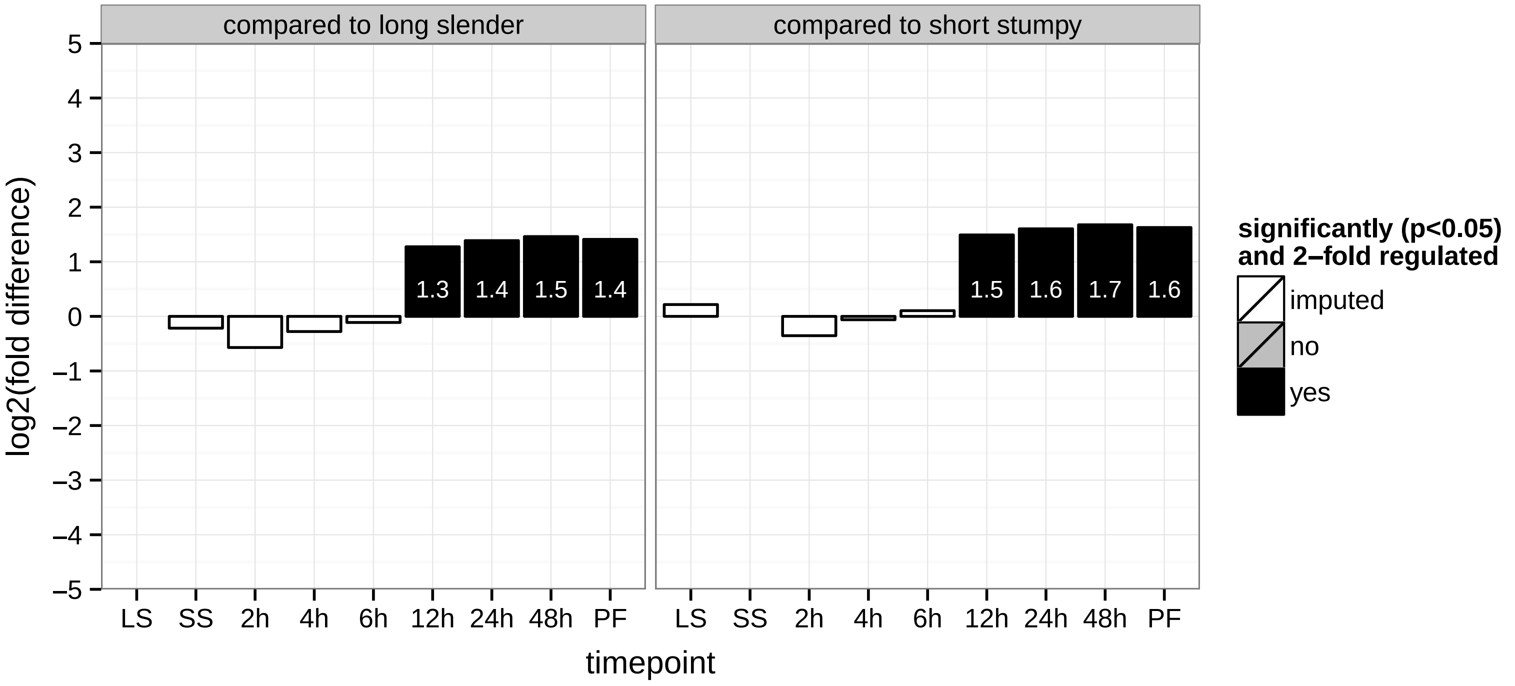

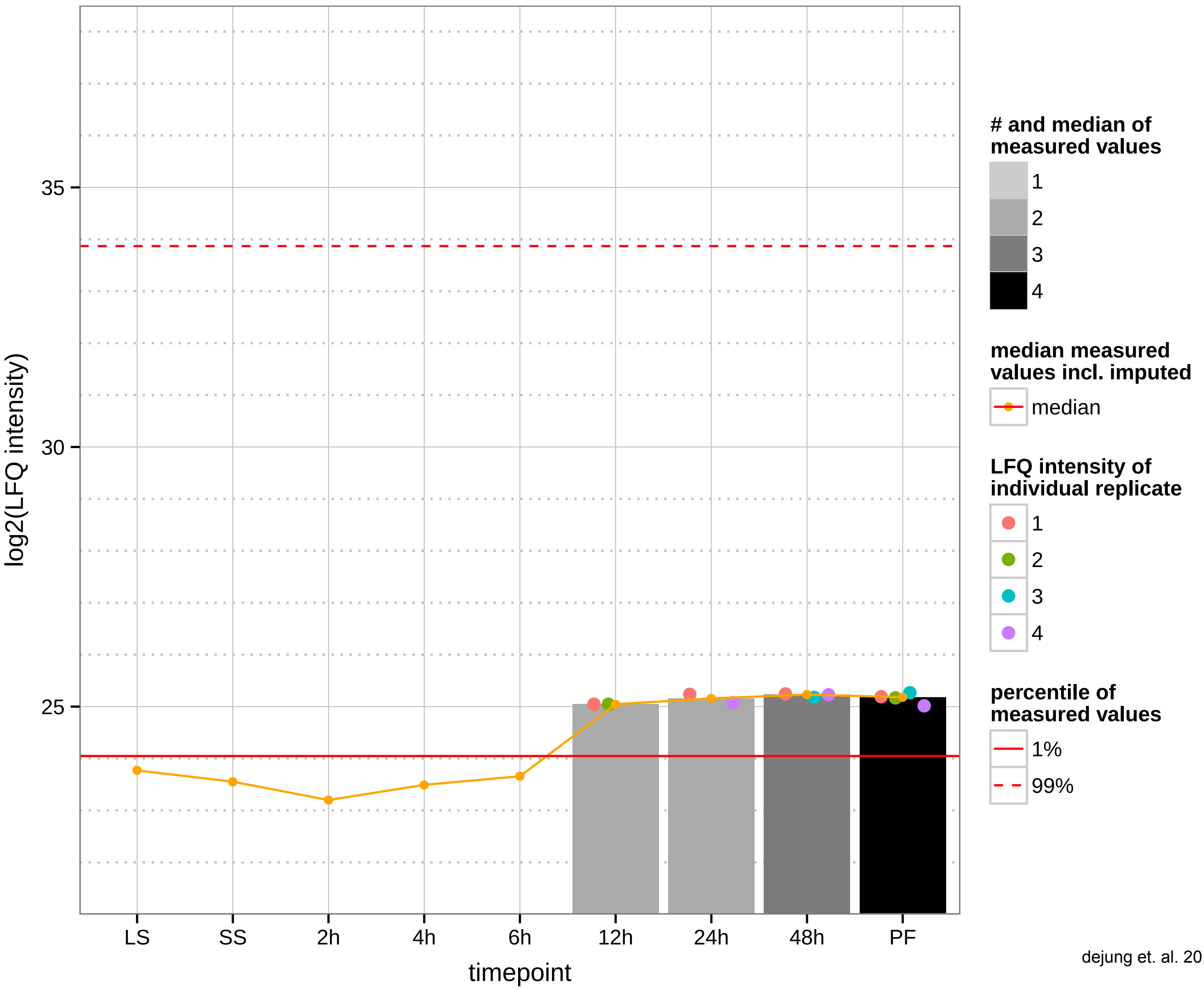

dejung et. al. 2015

cytochrome c1, heme protein, mitochondrial precursor
Tb927.8.1890
AGOF: electron carrier activity, heme binding, iron ion binding
AGOC: mitochondrial respiratory chain, mitochondrion
AGOP: oxidation–reduction process
PGOF: electron carrier activity, heme binding, iron ion binding
PGOC: null
PGOP: null

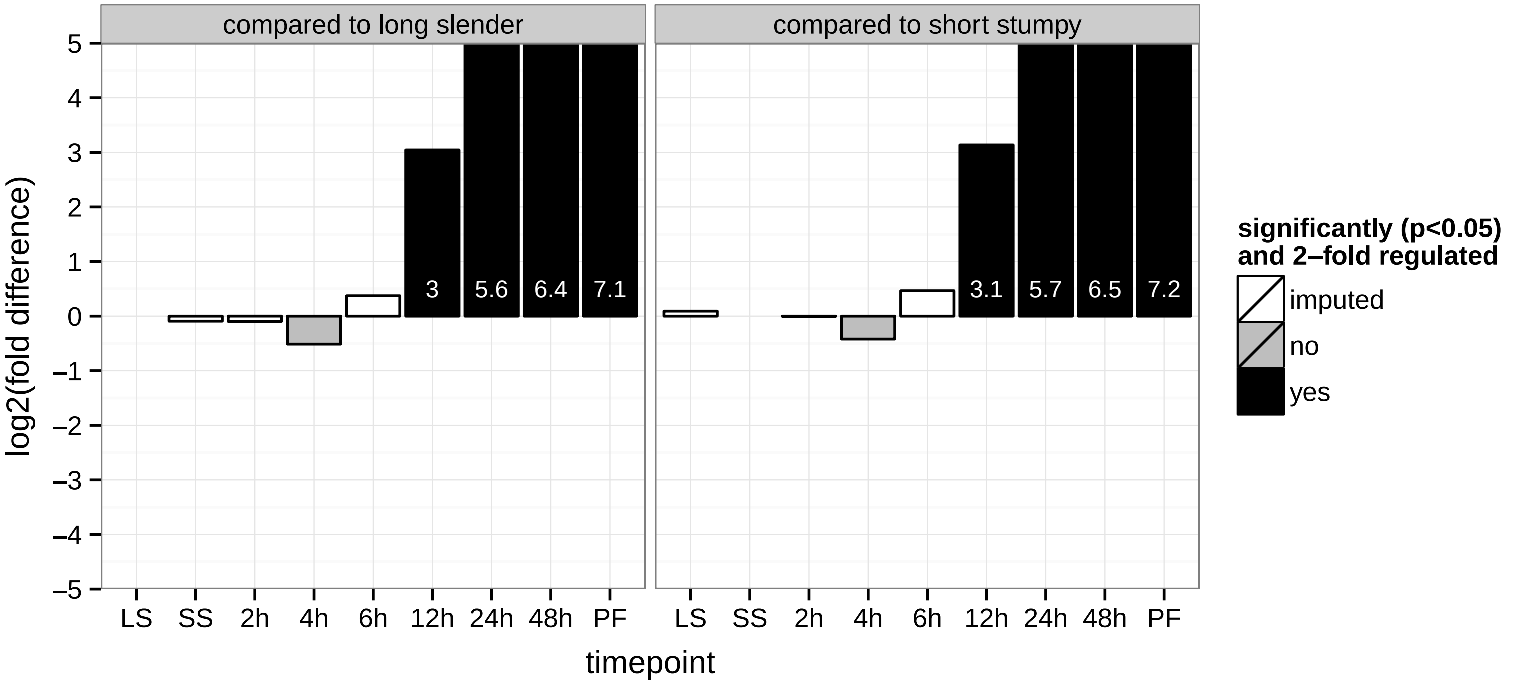

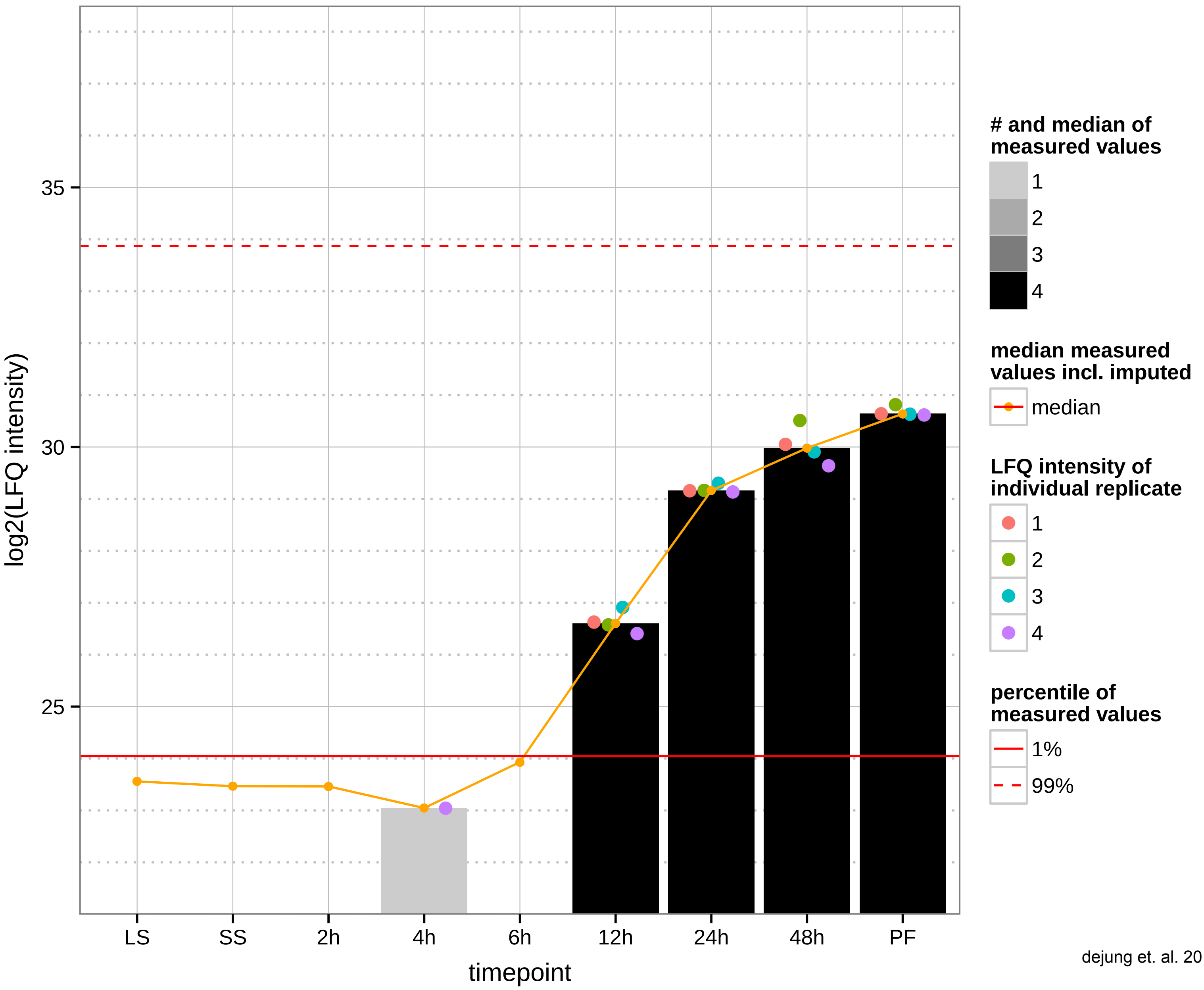

dejung et. al. 2015

cyclophilin, putative, cyclophilin type peptidyl−prolyl cis−trans isomerase, peptidyl−prolyl cis−trans isomerase, putative (NCP1
Tb927.8.2000
AGOF: peptidyl−prolyl cis−trans isomerase activity, protein binding, rRNA binding
AGOC: nucleus
AGOP: protein folding, rRNA processing
PGOF: peptidyl−prolyl cis−trans isomerase activity
PGOC: null
PGOP: protein folding

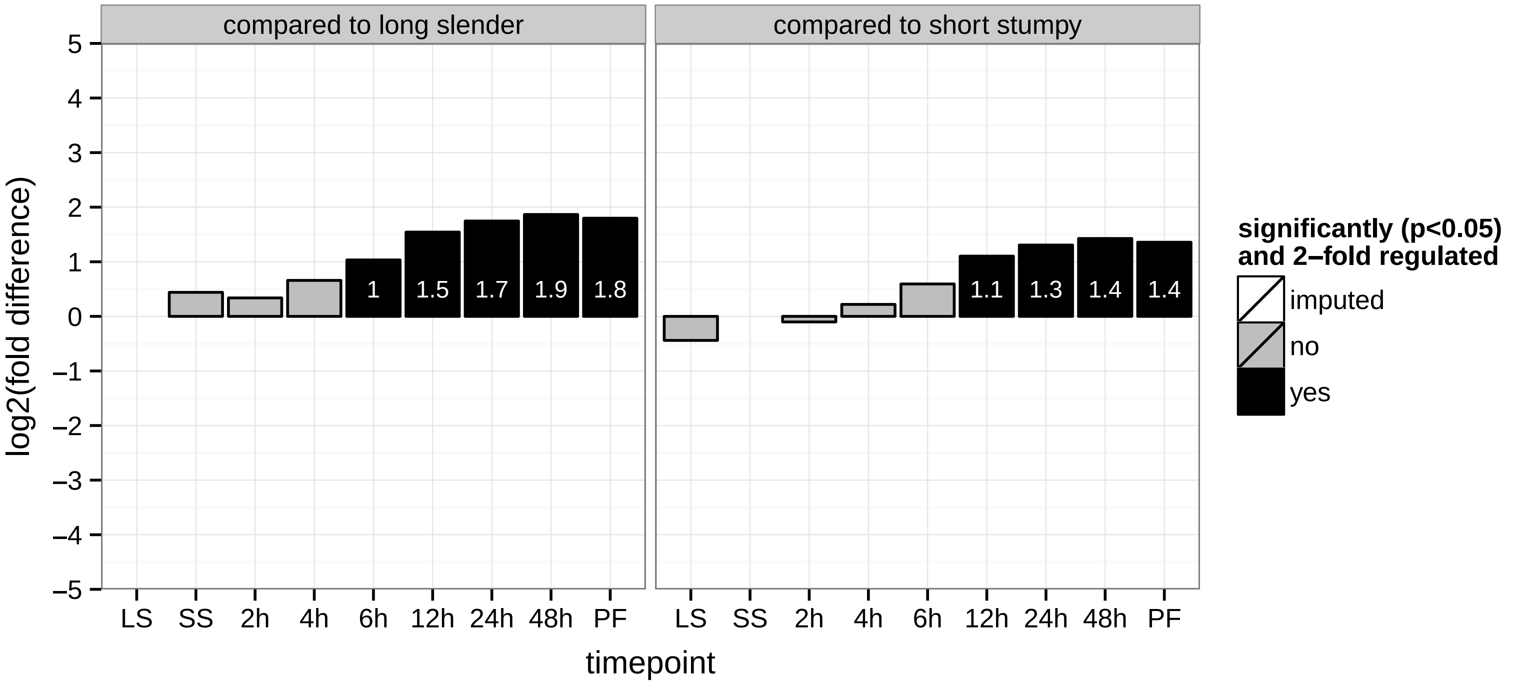

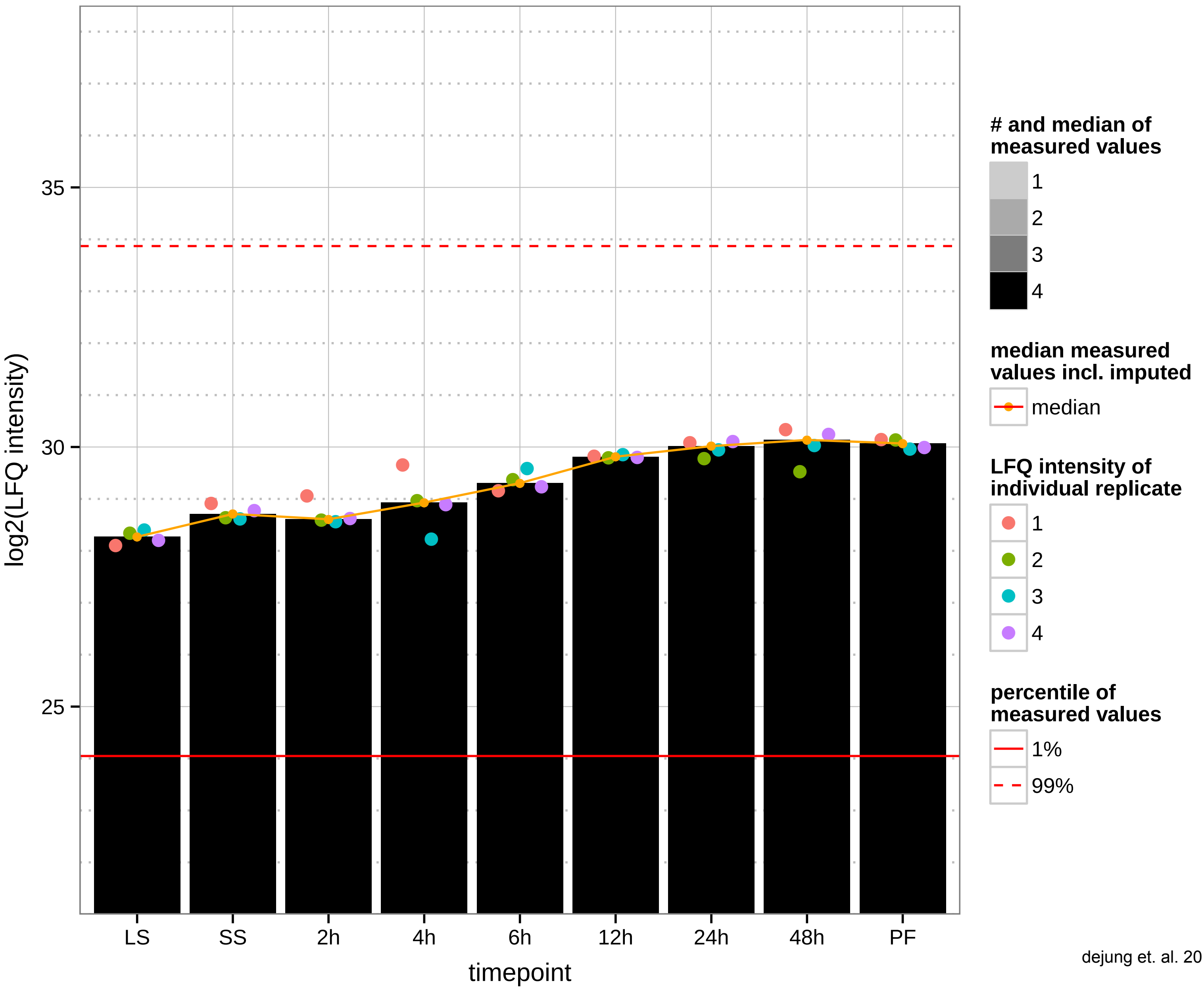

dejung et. al. 2015

multidrug resistance protein A, p–glycoprotein (PGPA)
Tb927.8.2160
AGOF: ATP binding, ATPase activity, coupled to transmembrane movement of substances
AGOC: ATP–binding cassette (ABC) transporter complex, integral to membrane
AGOP: response to drug, transport
PGOF: ATP binding, ATPase activity, coupled to transmembrane movement of substances, nucleoside–triphosphatase activity
PGOC: integral to membrane
PGOP: transmembrane transport, transport

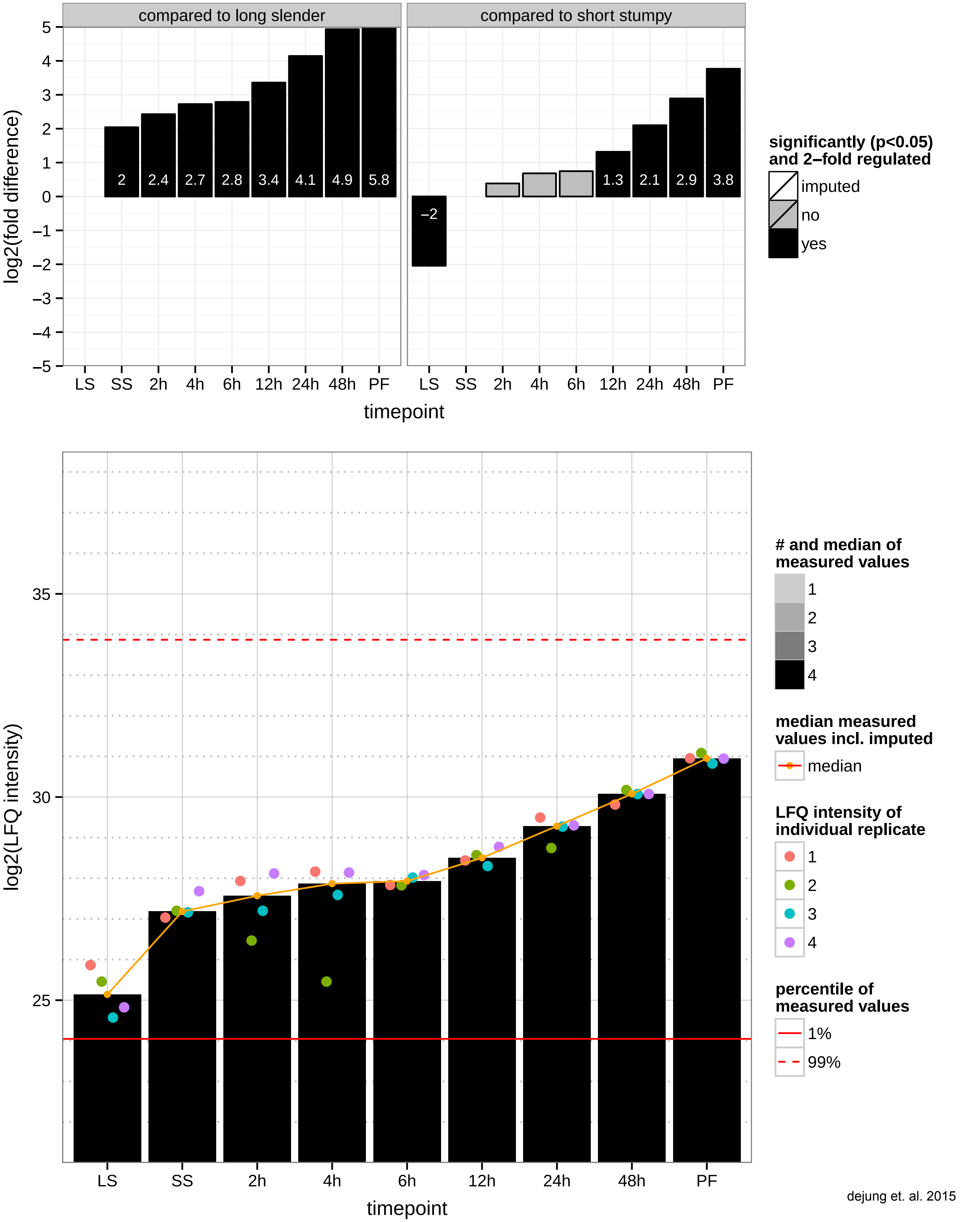

dejung et. al. 2015

3–ketoacyl–CoA thiolase, putative, ERG10
Tb927.8.2540
AGOF: acetyl–CoA C–acyltransferase activity
AGOC: mitochondrion
AGOP: metabolic process
PGOF: catalytic activity, transferase activity, transferring acyl groups other than amino–acyl groups
PGOC: null
PGOP: metabolic process

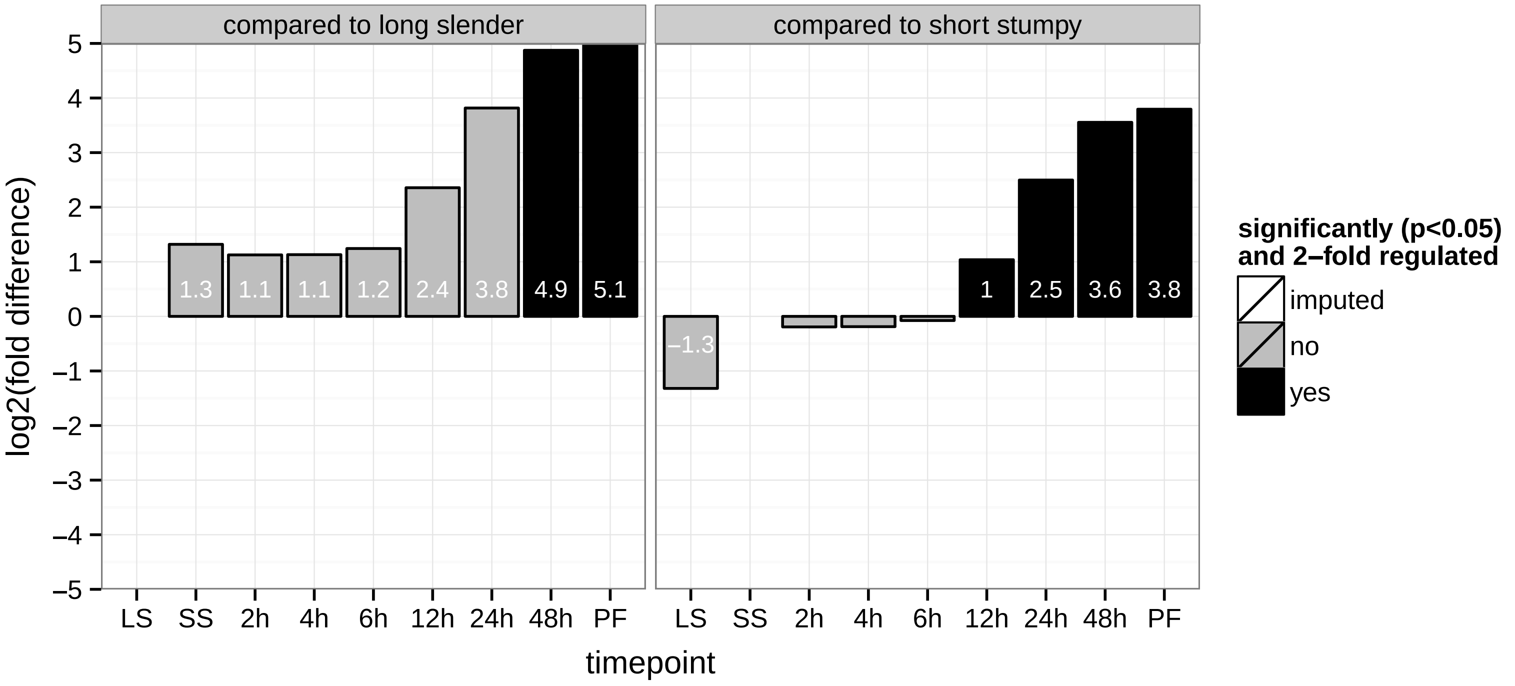

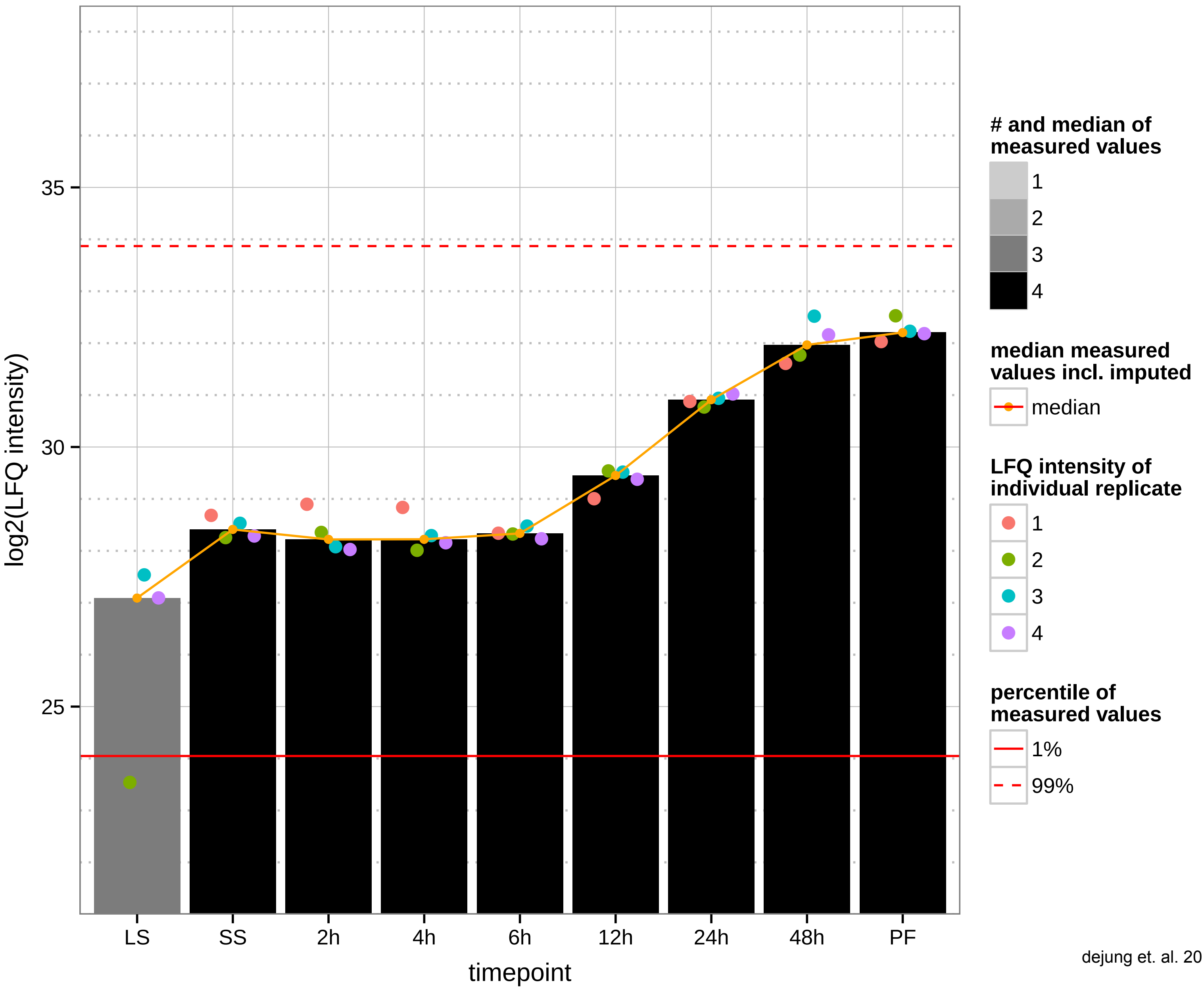

dejung et. al. 2015

mitochondrial DNA primase (PRI1)
Tb927.8.2550
AGOF: DNA primase activity
AGOC: kinetoplast, mitochondrial matrix
AGOP: mitochondrial DNA replication
PGOF: hydrolase activity, acting on acid anhydrides
PGOC: null
PGOP: null

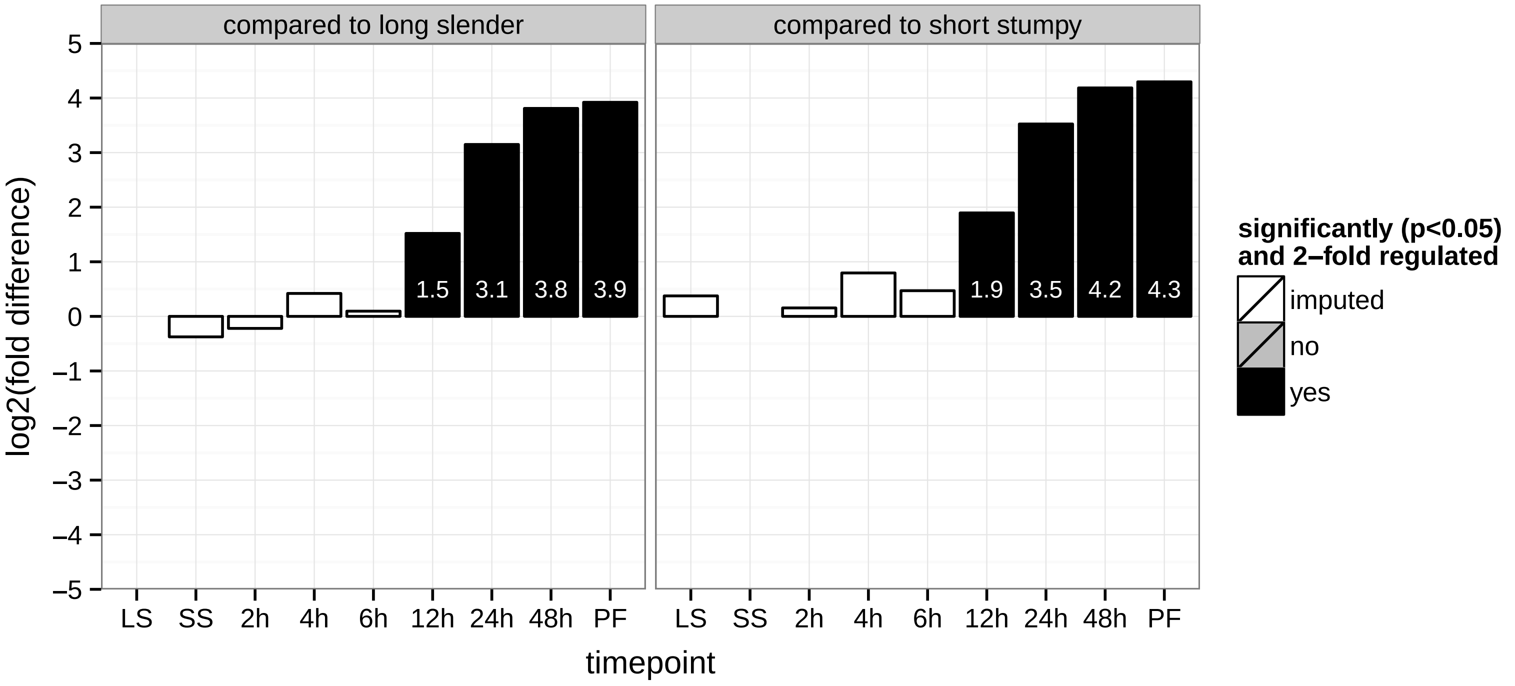

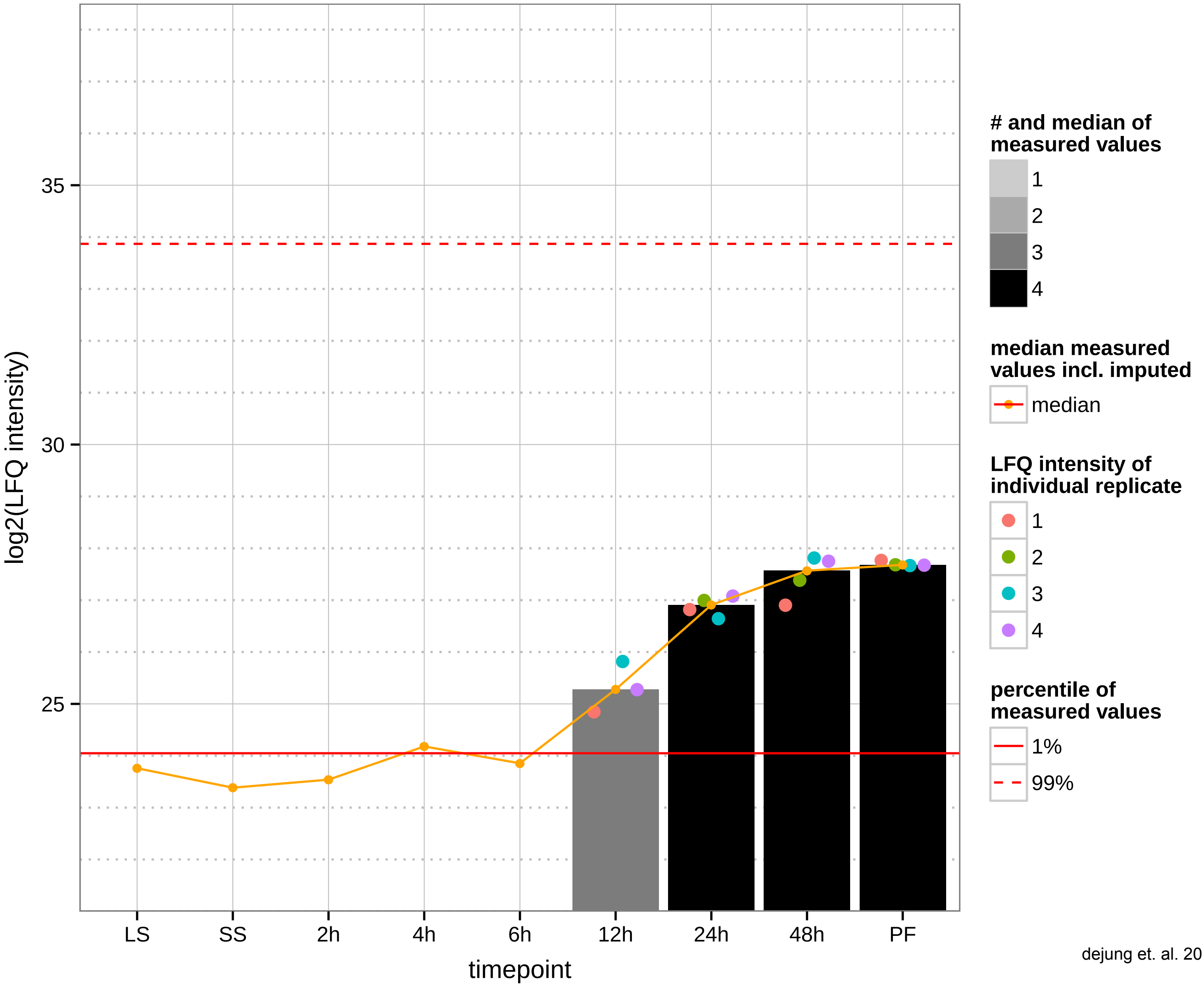

dejung et. al. 2015

hypothetical protein, conserved
Tb927.8.2880
AGOF: null
AGOC: mitochondrion
AGOP: null
PGOF: null
PGOC: null
PGOP: null

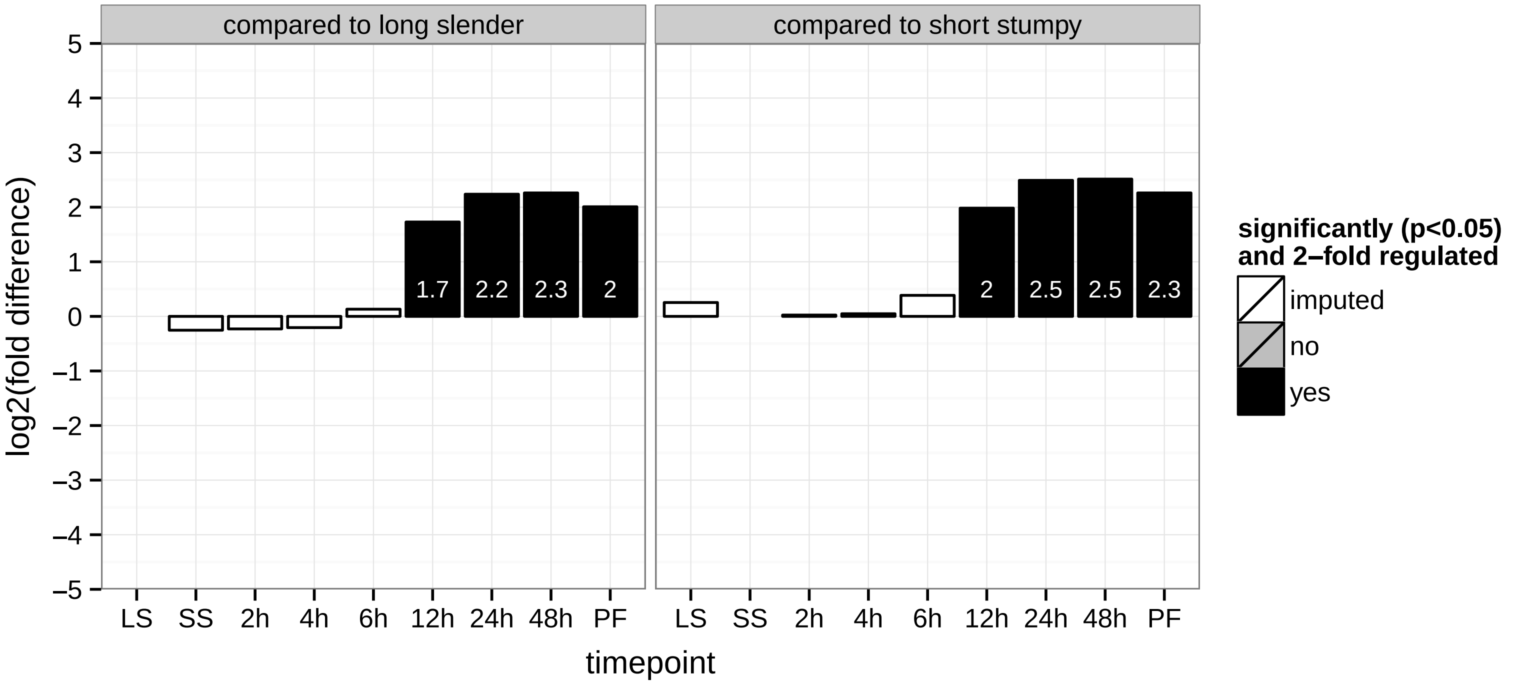

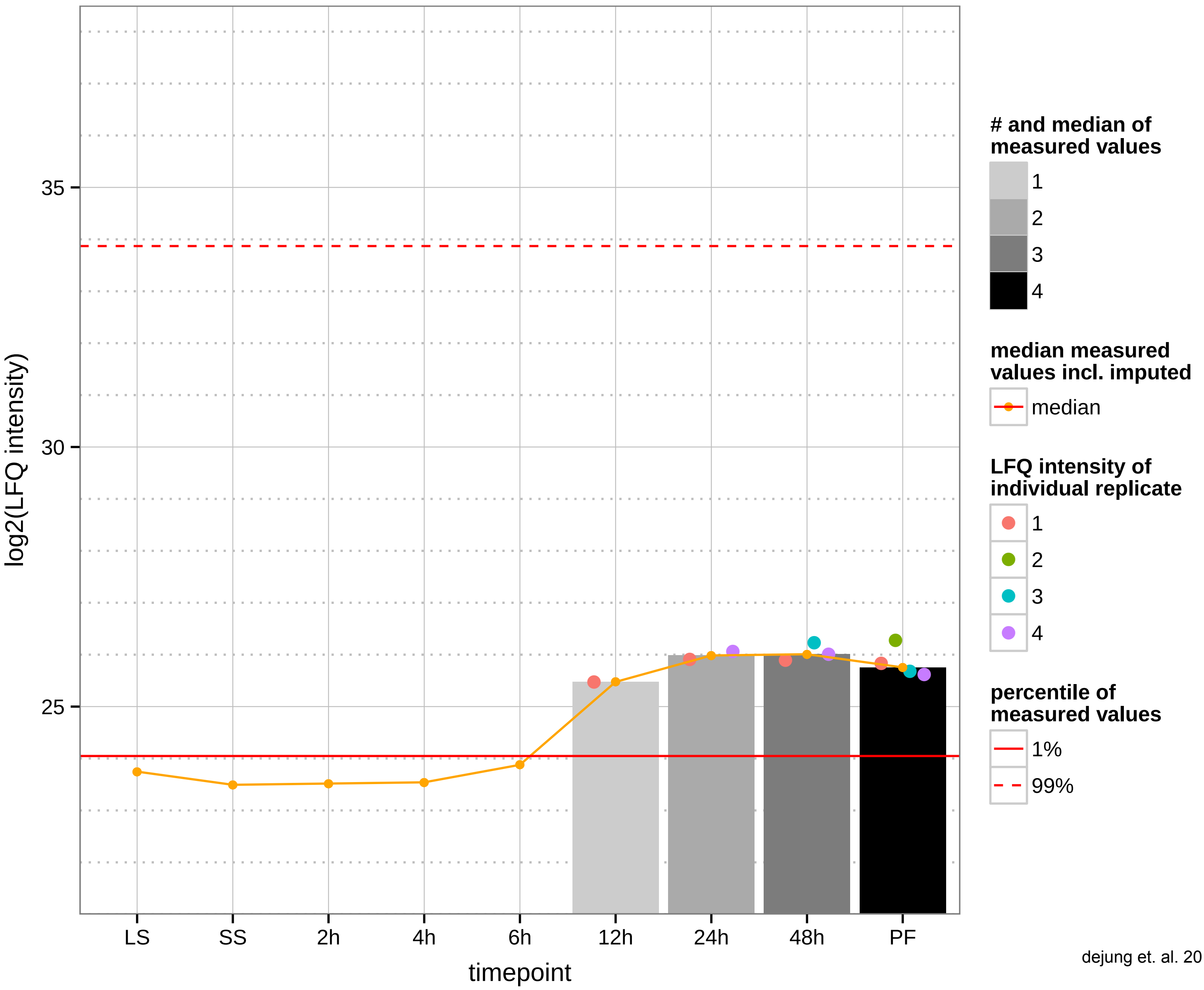

dejung et. al. 2015

hypothetical protein, conserved
Tb927.8.3010
AGOF: null
AGOC: null
AGOP: null
PGOF: null
PGOC: null
PGOP: null

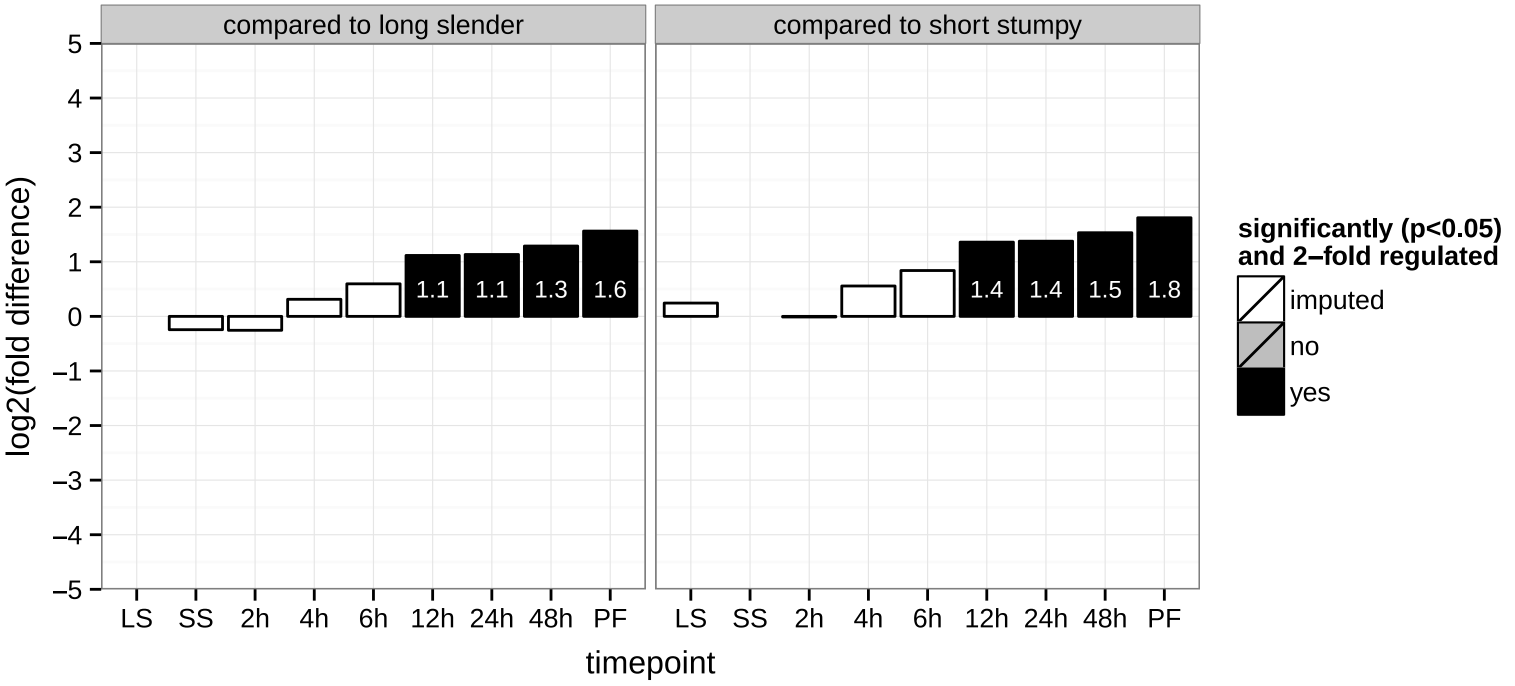

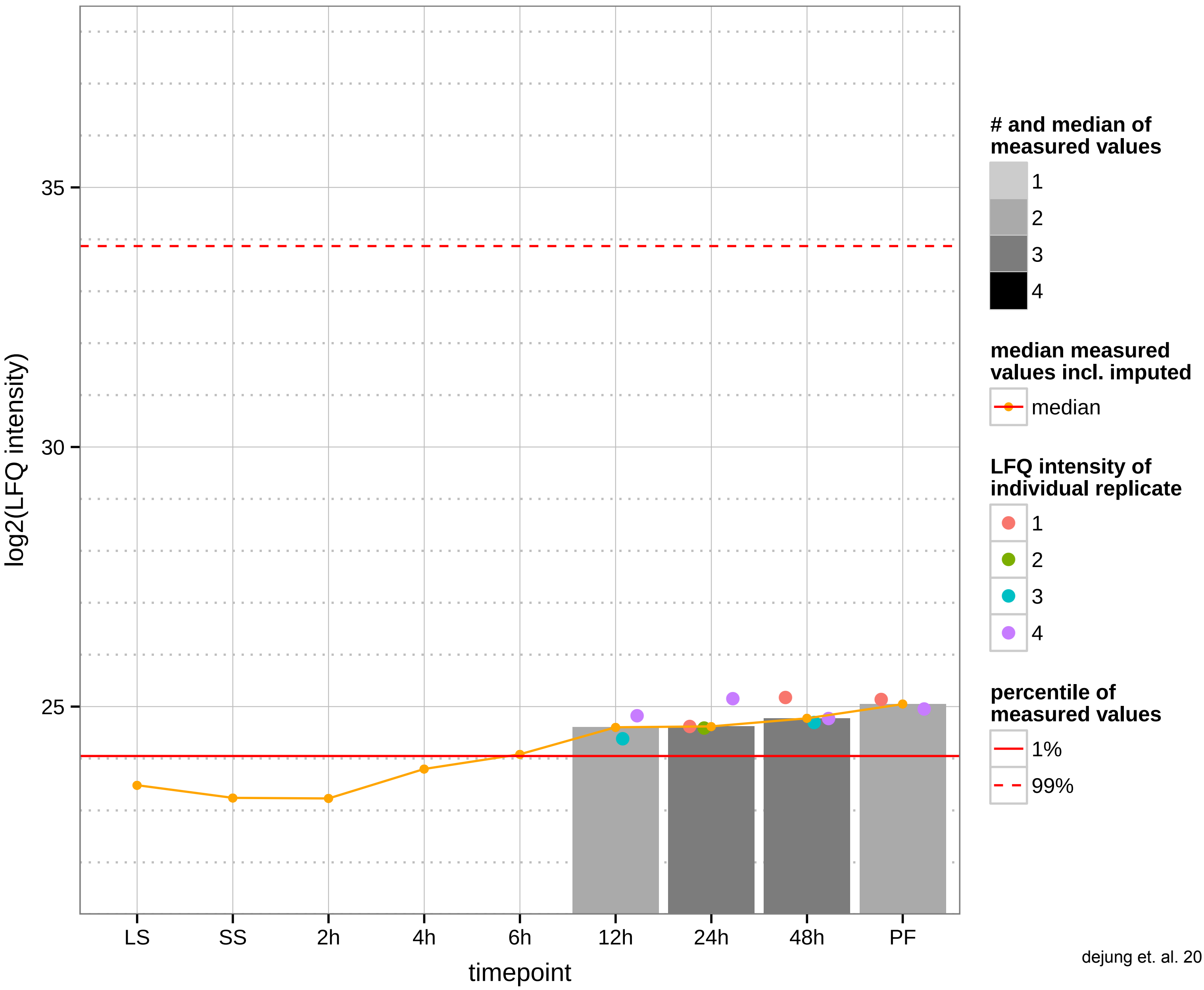

dejung et. al. 2015

hypothetical protein, conserved
Tb927.8.3340
AGOF: null
AGOC: null
AGOP: null
PGOF: null
PGOC: null
PGOP: null

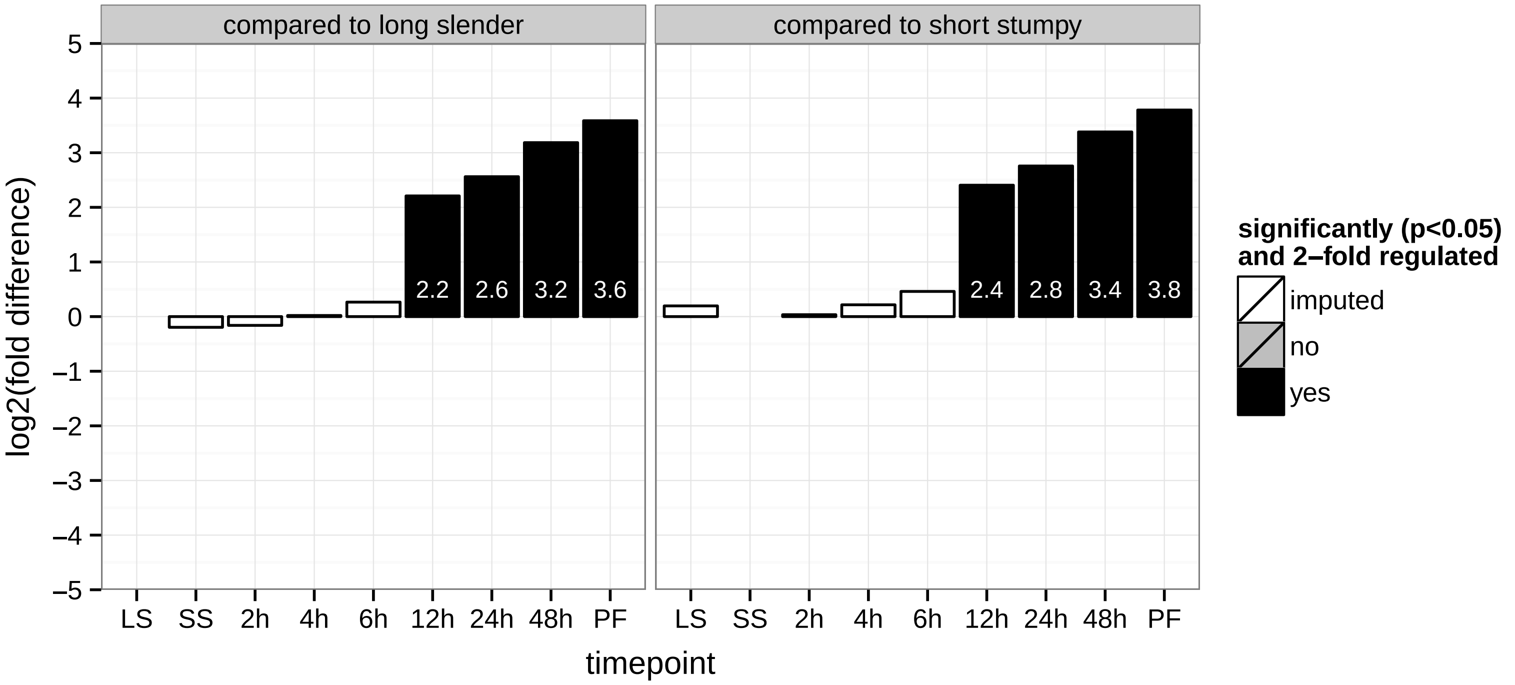

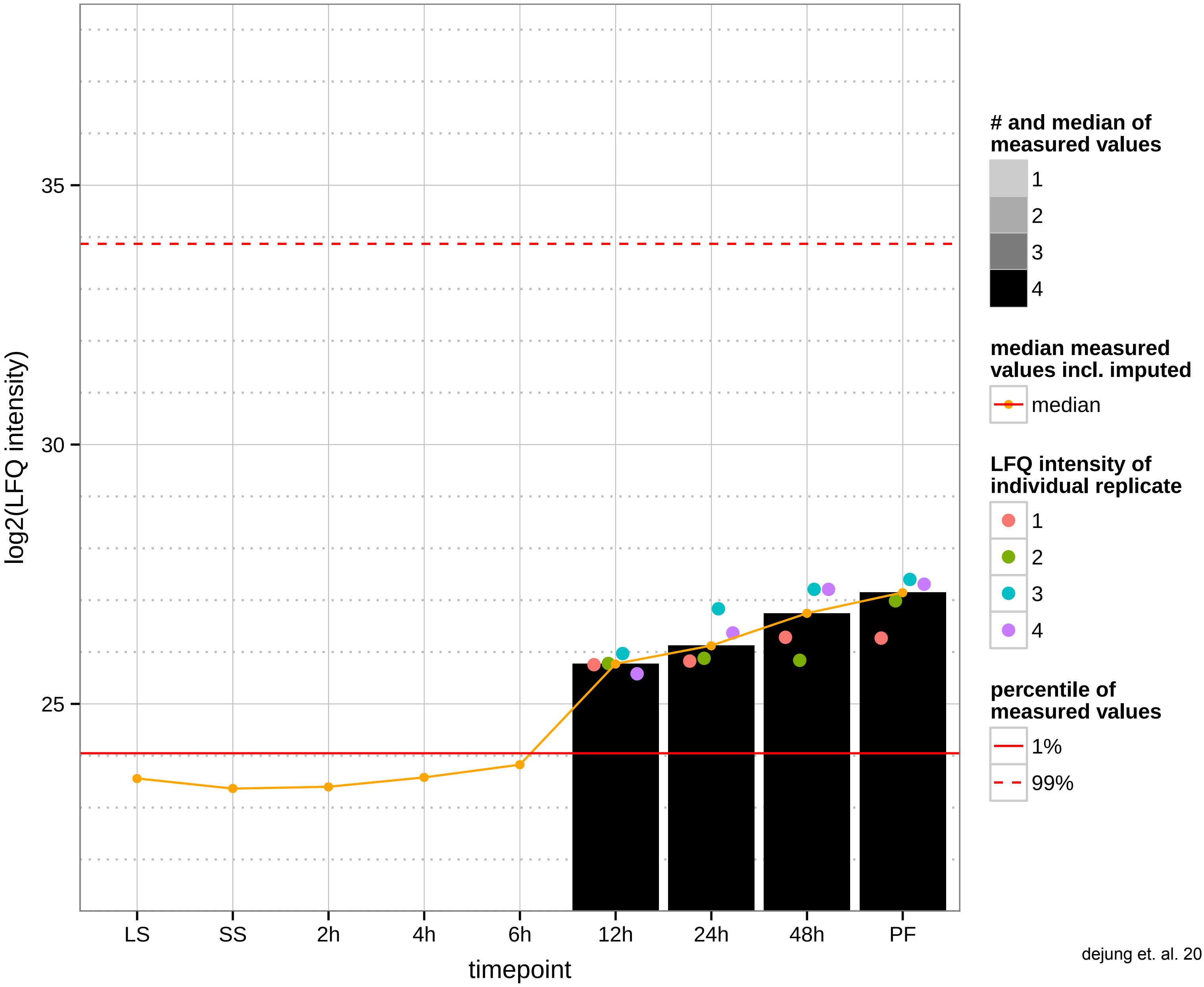

dejung et. al. 2015

hypothetical protein, conserved
Tb927.8.5070
AGOF: null
AGOC: null
AGOP: null
PGOF: null
PGOC: null
PGOP: null

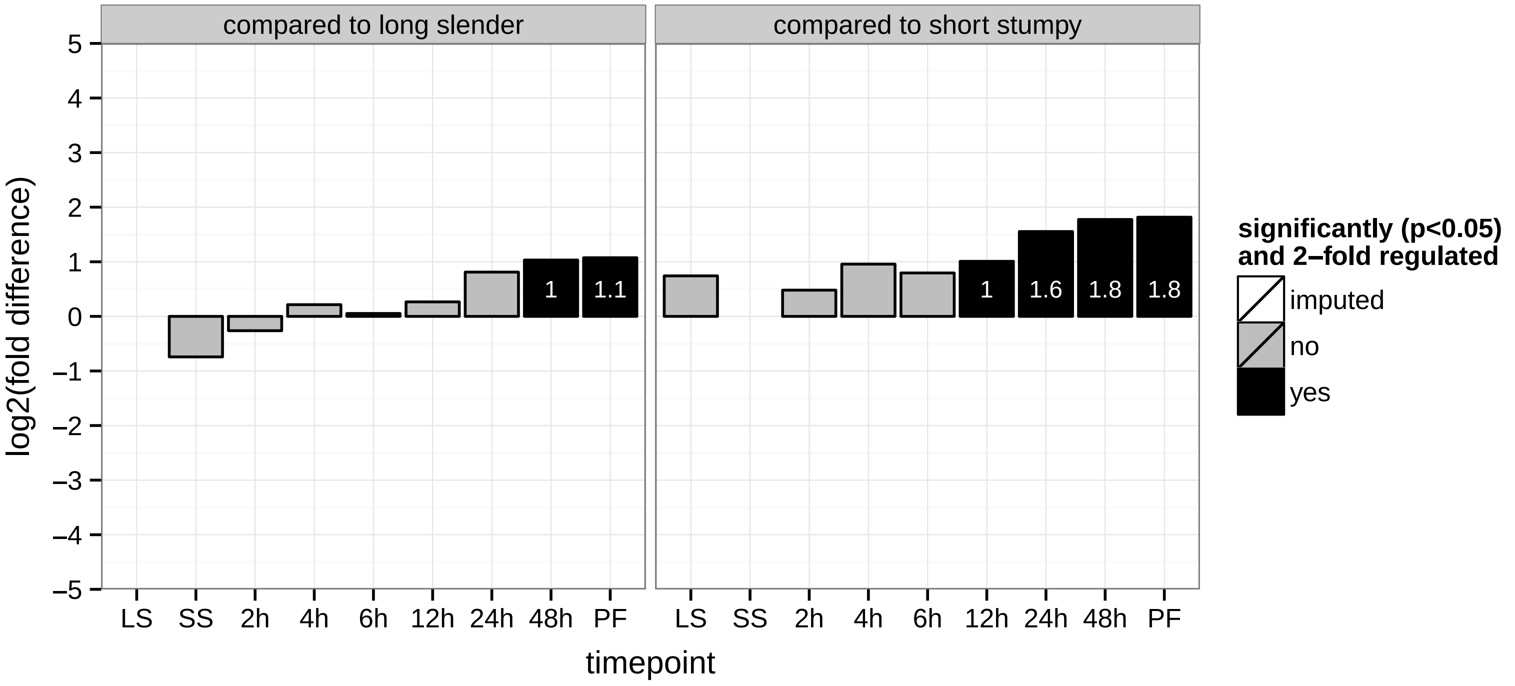

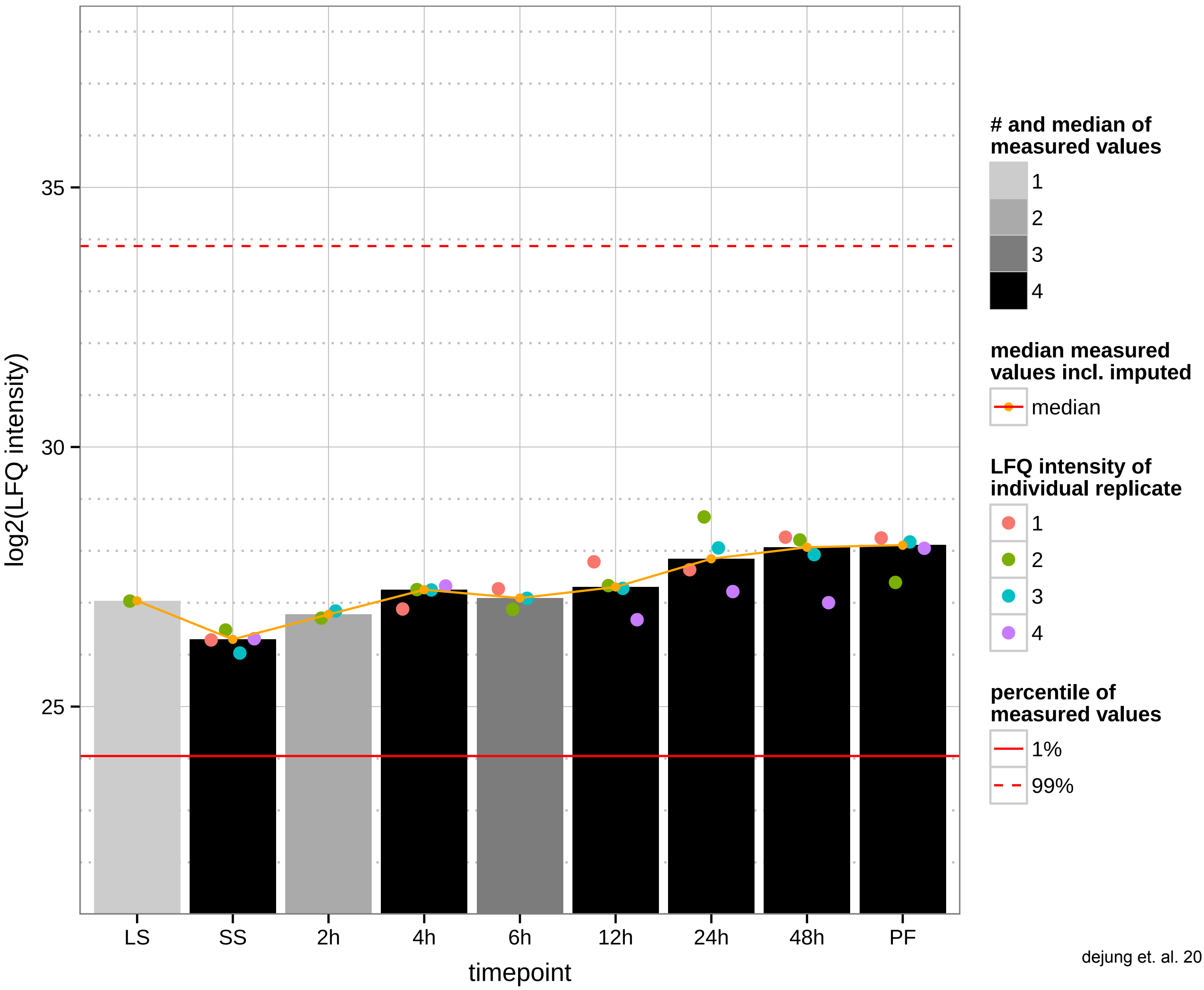

dejung et. al. 2015

hypothetical protein, conserved
Tb927.8.5490
AGOF: null
AGOC: preribosome, small subunit precursor
AGOP: rRNA processing
PGOF: null
PGOC: preribosome, small subunit precursor
PGOP: rRNA processing

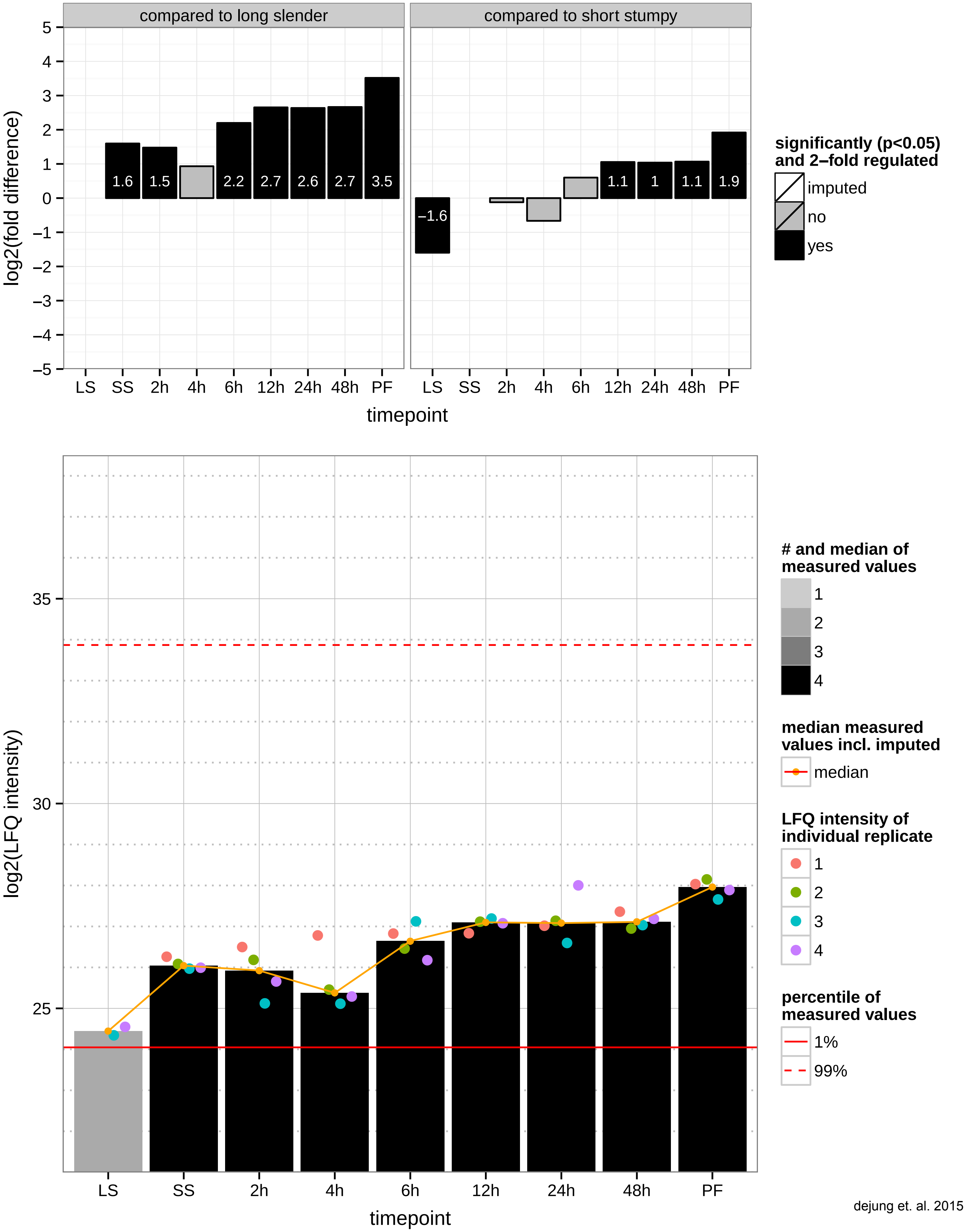

dejung et. al. 2015

eukaryotic translation initiation factor 1A, putative
Tb927.8.5880
AGOF: RNA binding, translation initiation factor activity
AGOC: cytoplasm
AGOP: translational initiation
PGOF: RNA binding, translation initiation factor activity
PGOC: null
PGOP: translational initiation

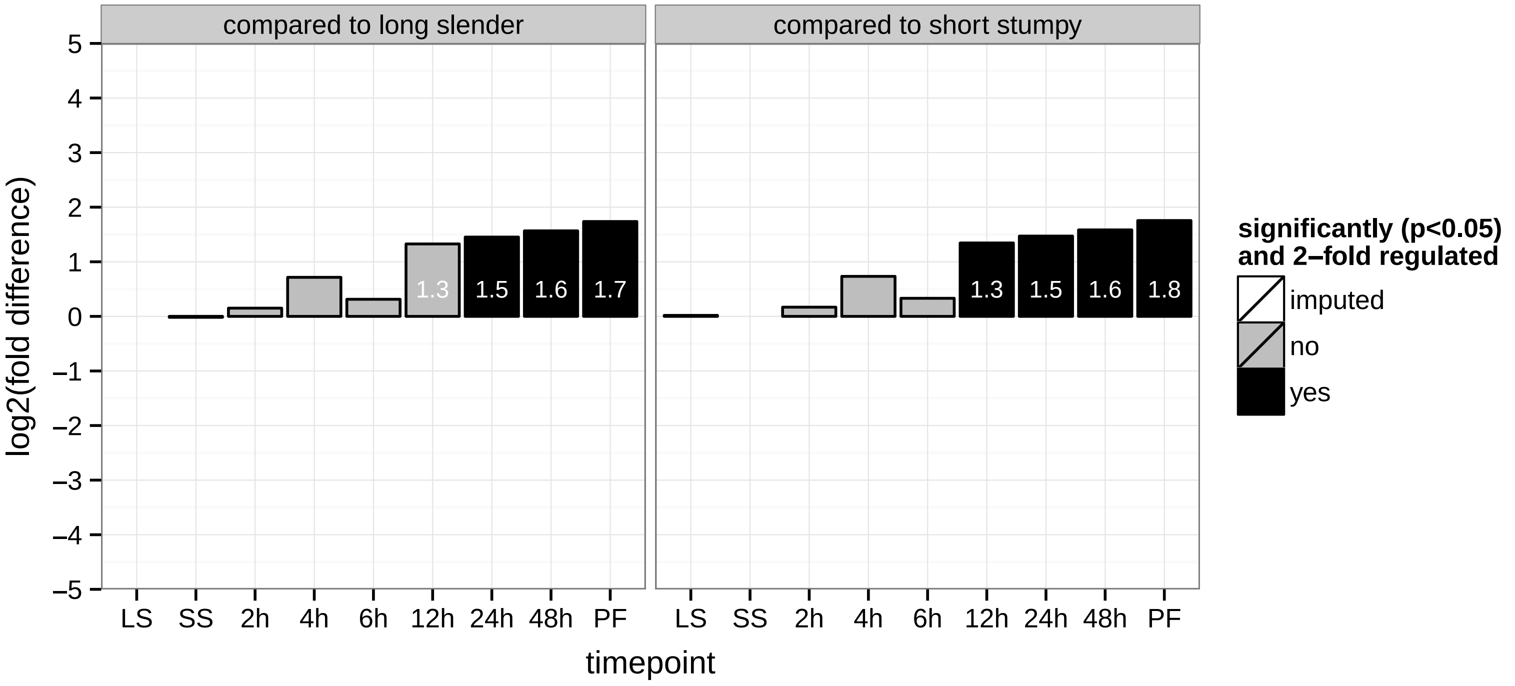

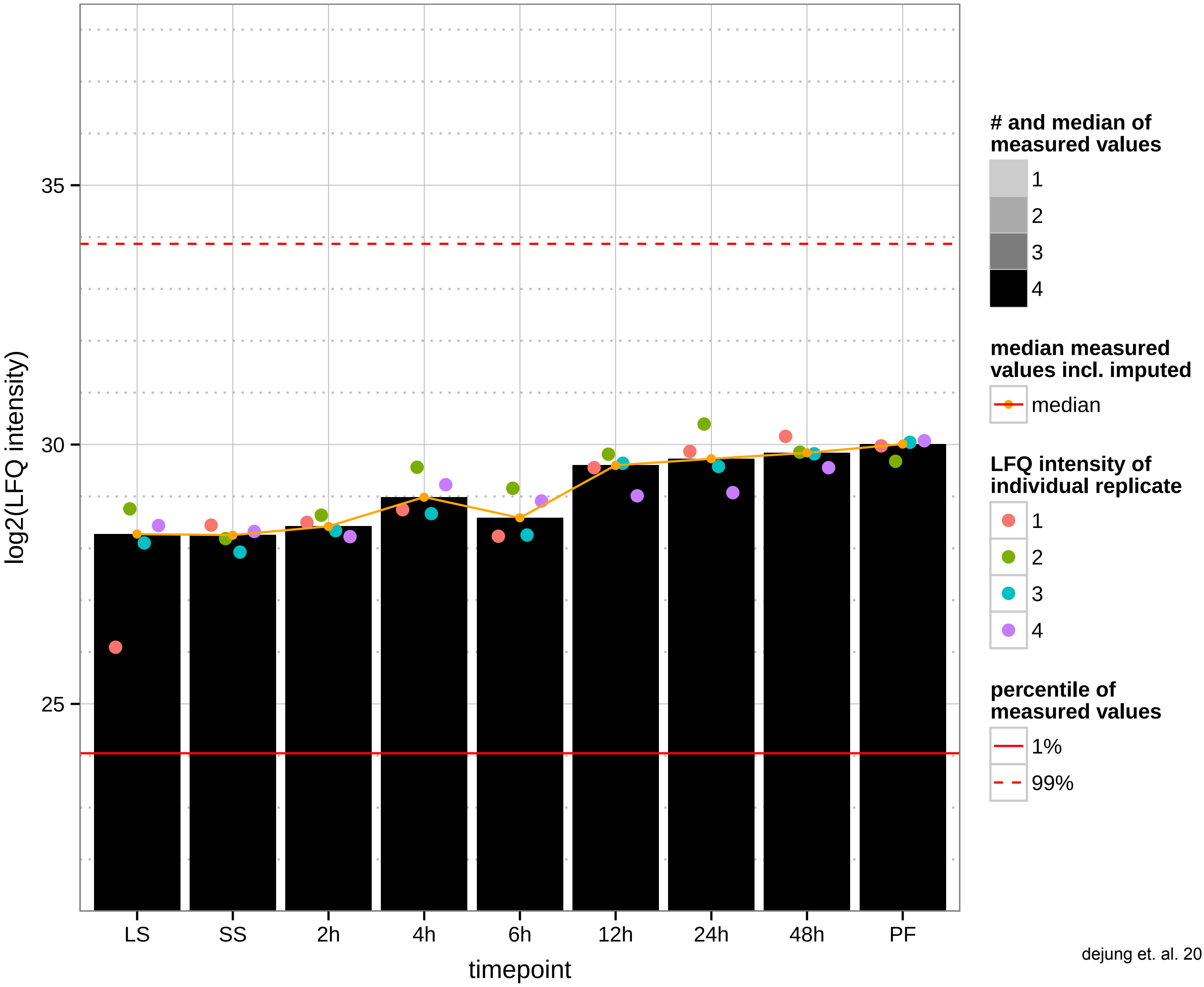

dejung et. al. 2015

hypothetical protein, conserved
Tb927.8.6080
AGOF: glycerophosphodiester phosphodiesterase activity
AGOC: mitochondrion
AGOP: glycerol metabolic process, lipid metabolic process, phospholipid catabolic process
PGOF: glycerophosphodiester phosphodiesterase activity, phosphoric diester hydrolase activity
PGOC: null
PGOP: glycerol metabolic process, lipid metabolic process

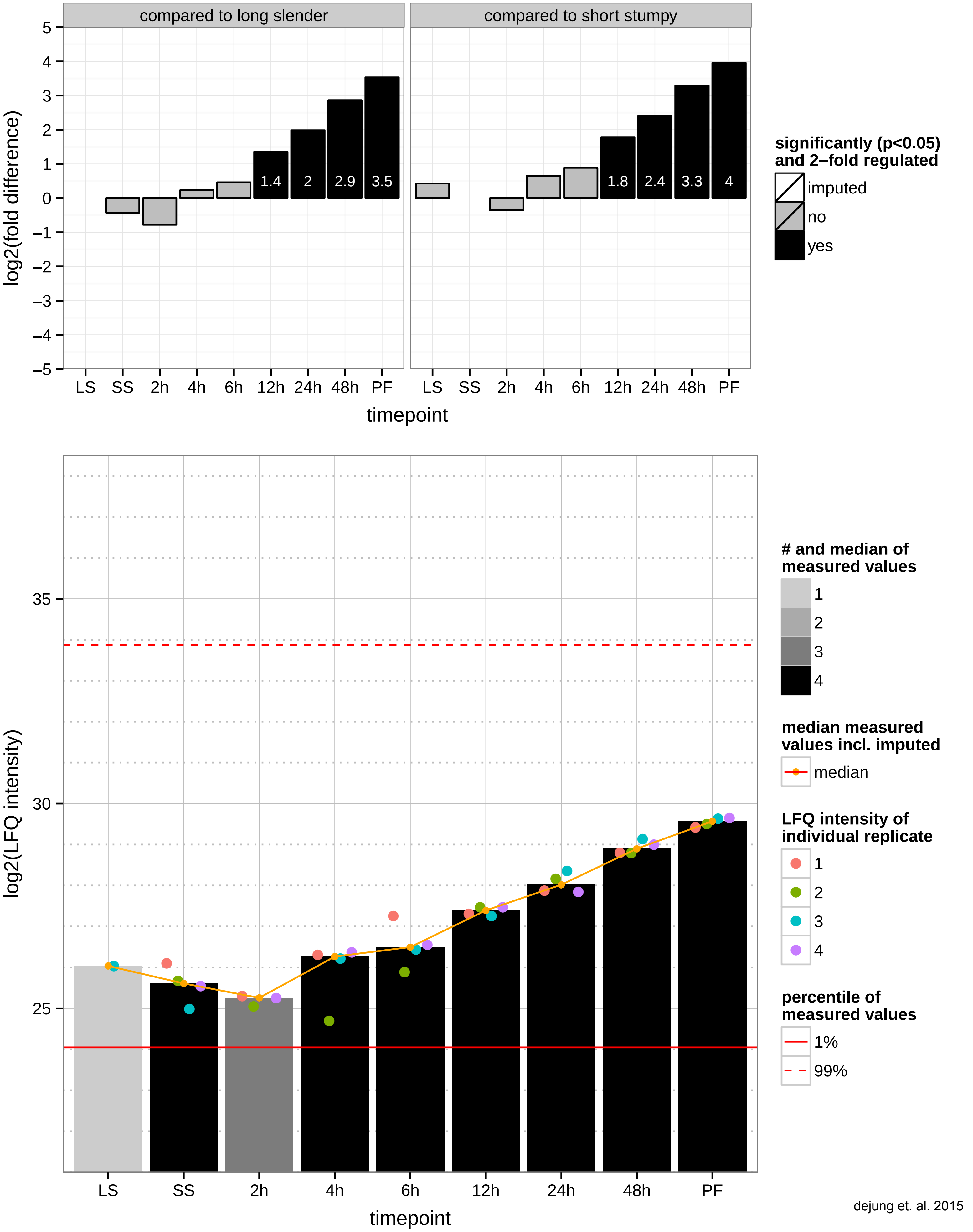

dejung et. al. 2015

ubiquitin–conjugating enzyme E2, putative, ubiquitin–protein ligase, ubiquitin carrier protein
Tb927.8.6090
AGOF: ATP binding, ubiquitin–protein ligase activity
AGOC: null
AGOP: cellular protein modification process, post–translational protein modification, protein ubiquitination, regulation of protei
PGOF: acid–amino acid ligase activity
PGOC: null
PGOP: null

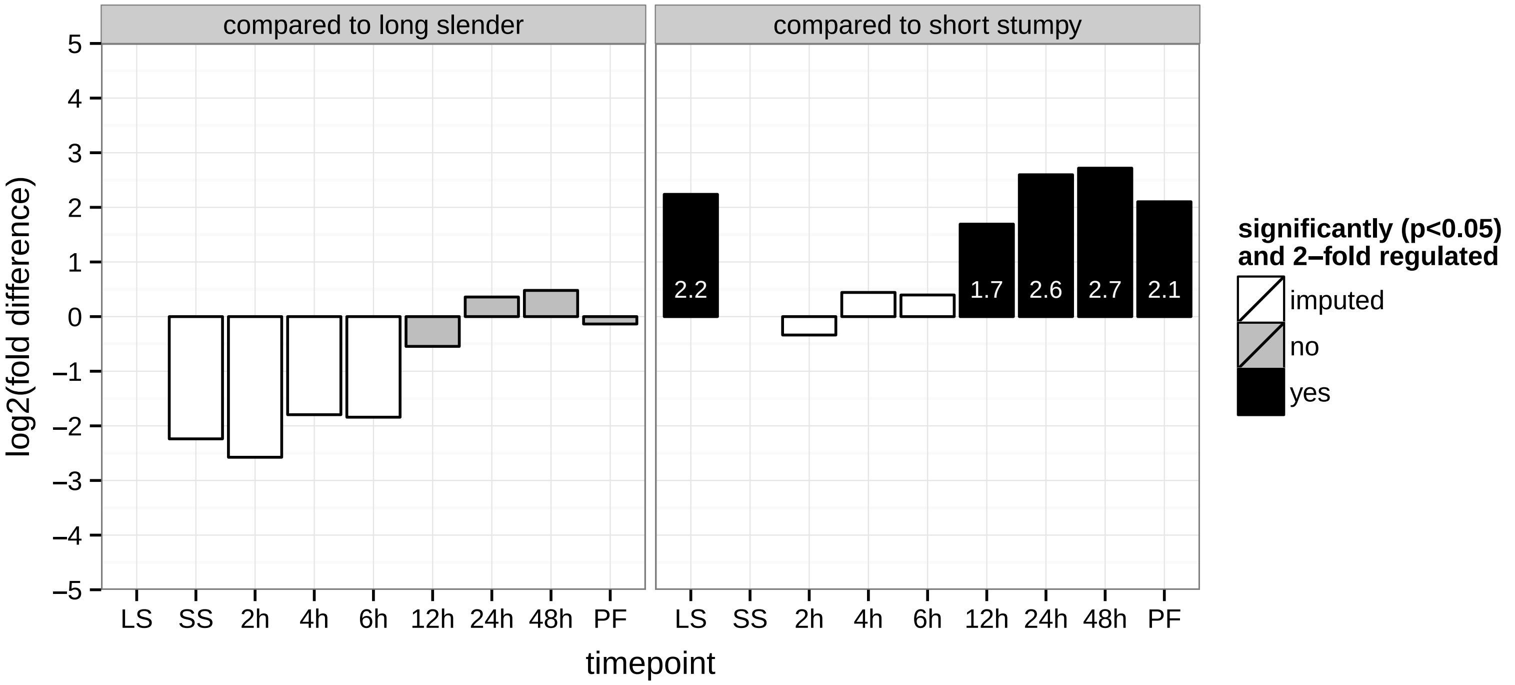

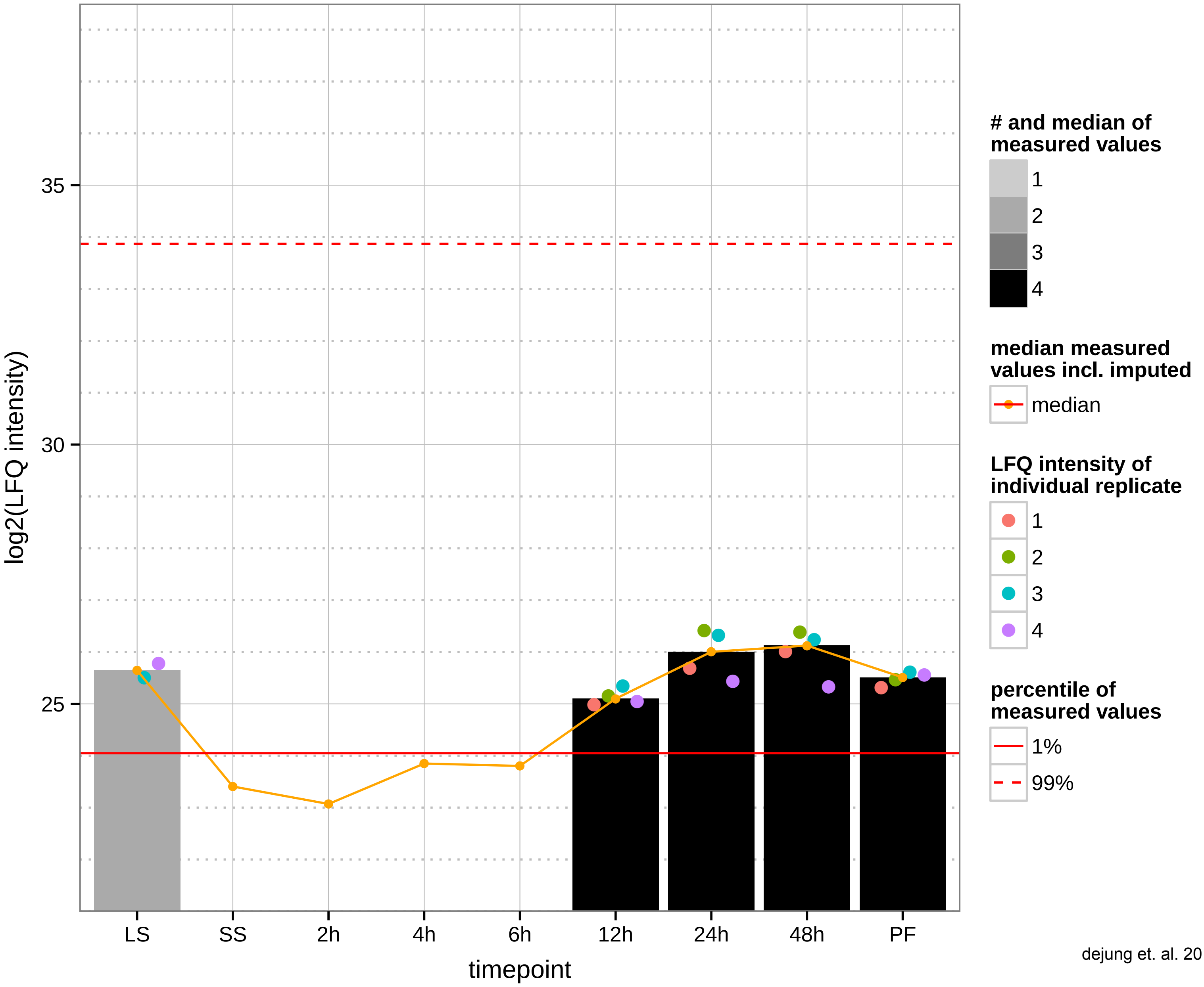

dejung et. al. 2015

transketolase, putative (TK)
Tb927.8.6170
AGOF: transketolase activity
AGOC: cytoplasm, glycosome
AGOP: pentose−phosphate shunt
PGOF: catalytic activity, transketolase activity
PGOC: null
PGOP: metabolic process

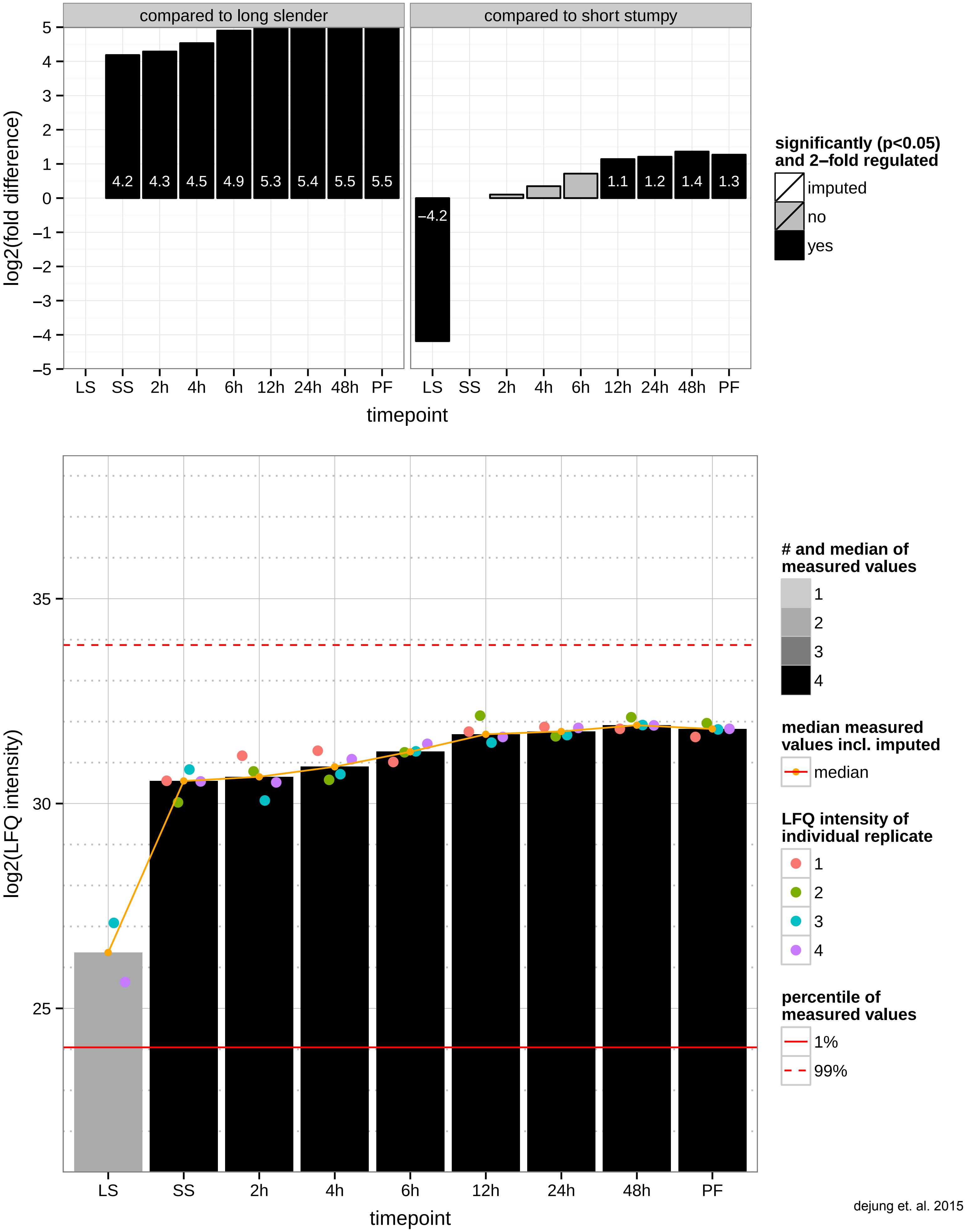

dejung et. al. 2015

short–chain dehydrogenase, putative
Tb927.8.6410
AGOF: oxidoreductase activity
AGOC: integral to membrane, mitochondrial inner membrane, mitochondrion
AGOP: metabolic process
PGOF: oxidoreductase activity
PGOC: null
PGOP: metabolic process

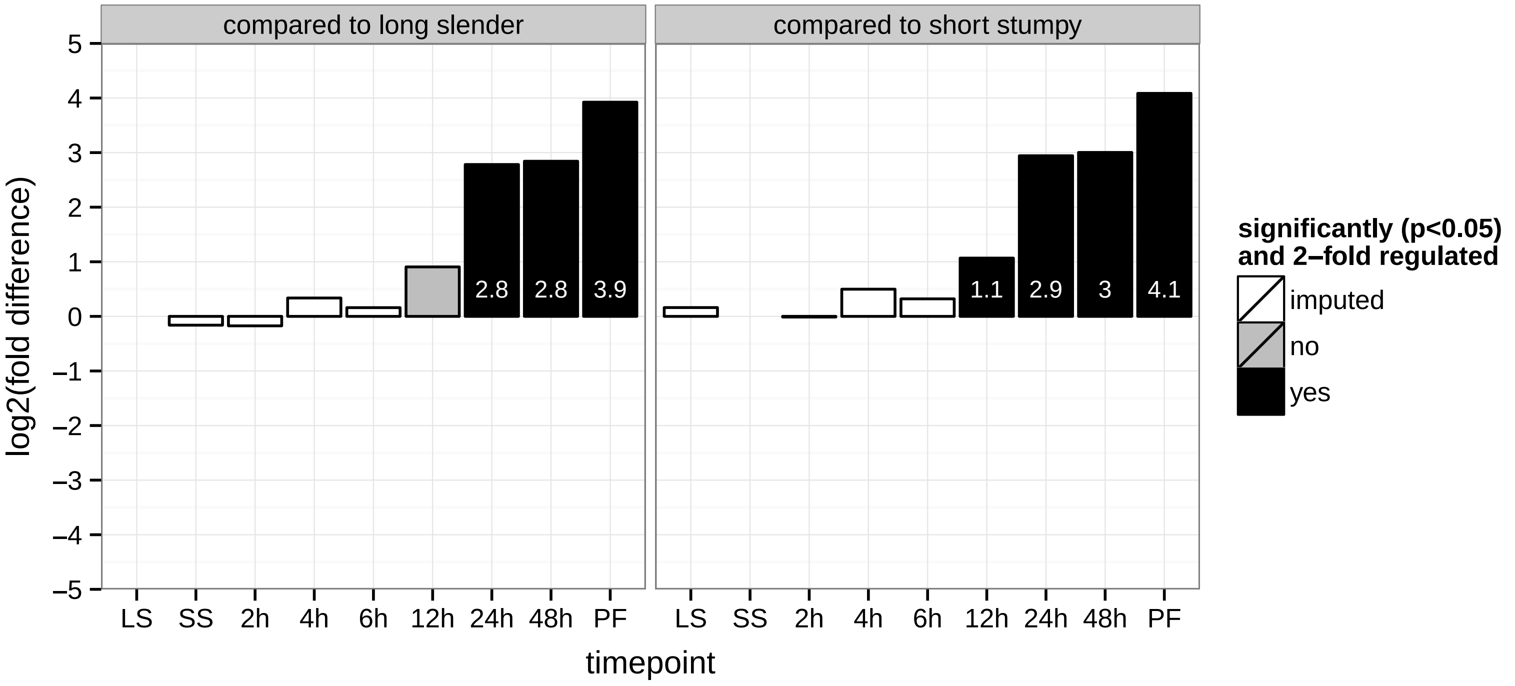

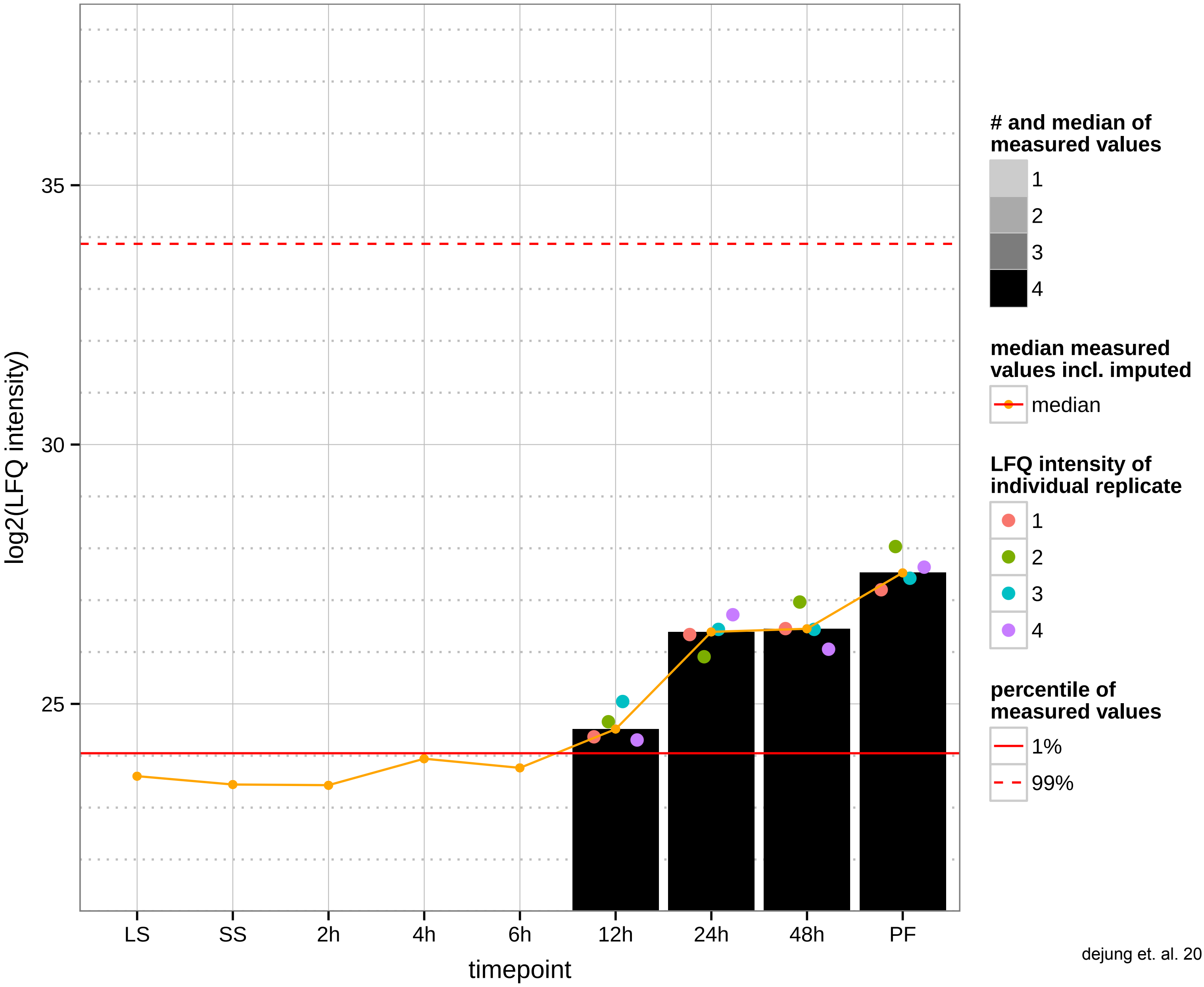

dejung et. al. 2015

translationally controlled tumor protein (TCTP), putative
Tb927.8.6750
AGOF: null
AGOC: cytoplasm, mitochondrion
AGOP: translation
PGOF: null
PGOC: null
PGOP: null

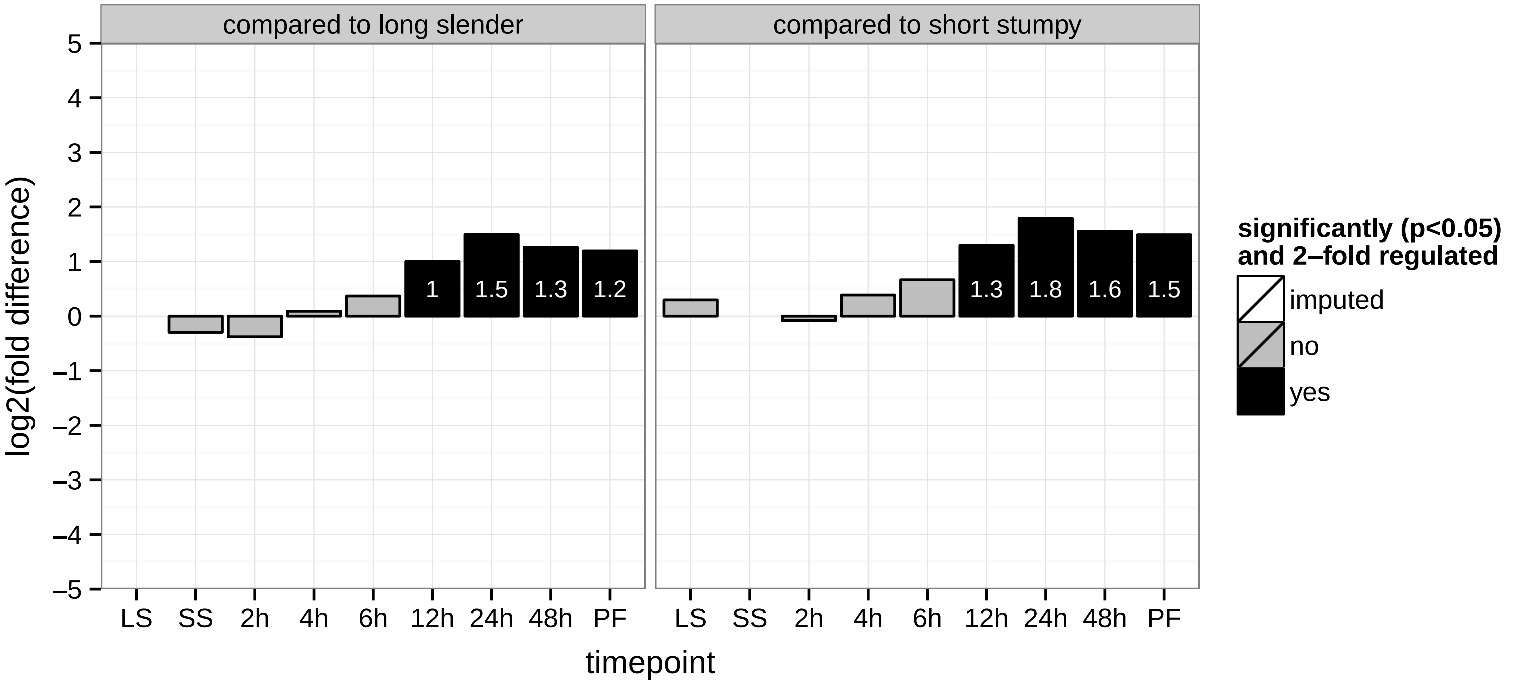

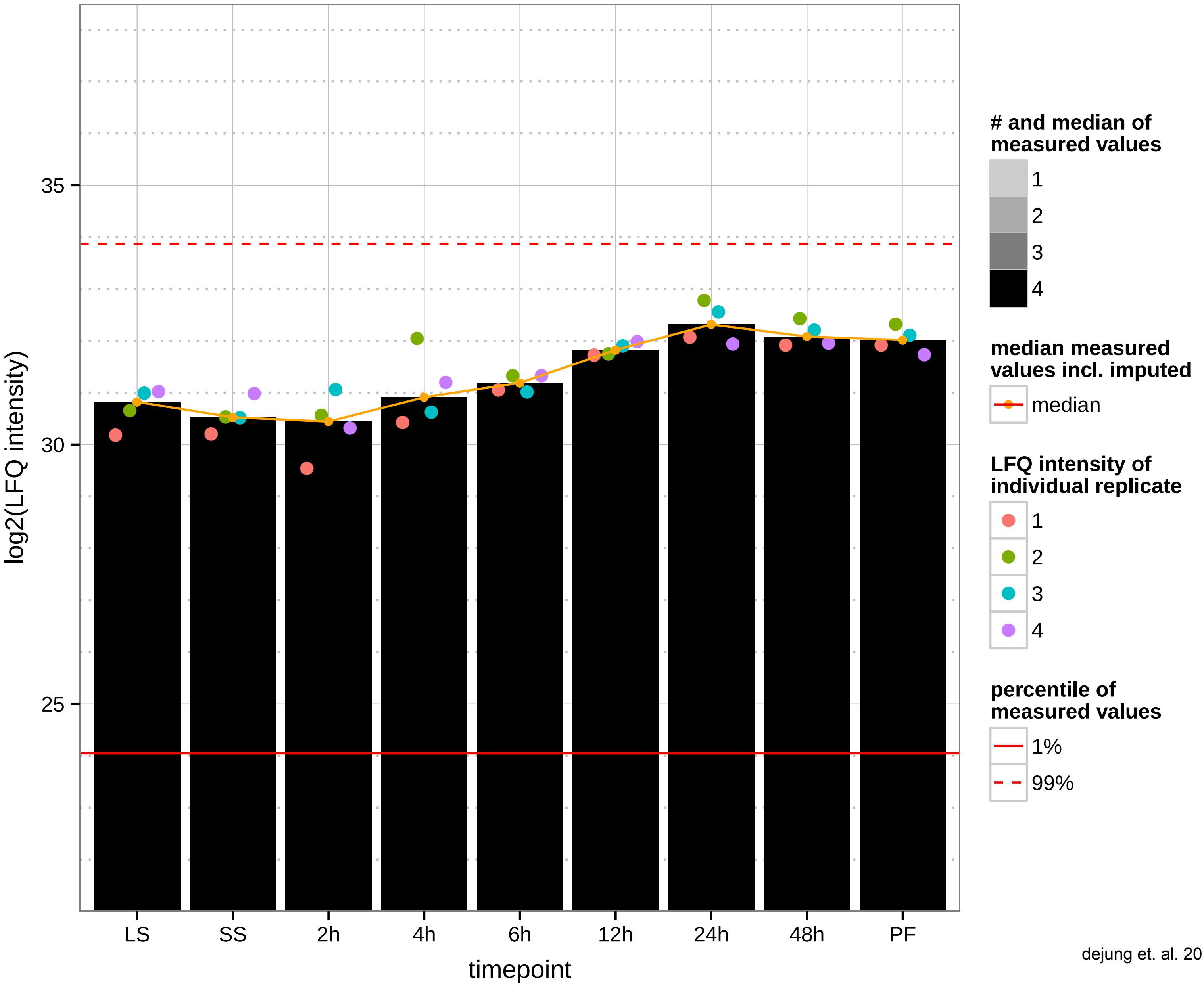

dejung et. al. 2015

hypothetical protein, conserved
Tb927.8.6890
AGOF: null
AGOC: mitochondrion
AGOP: null
PGOF: null
PGOC: null
PGOP: null

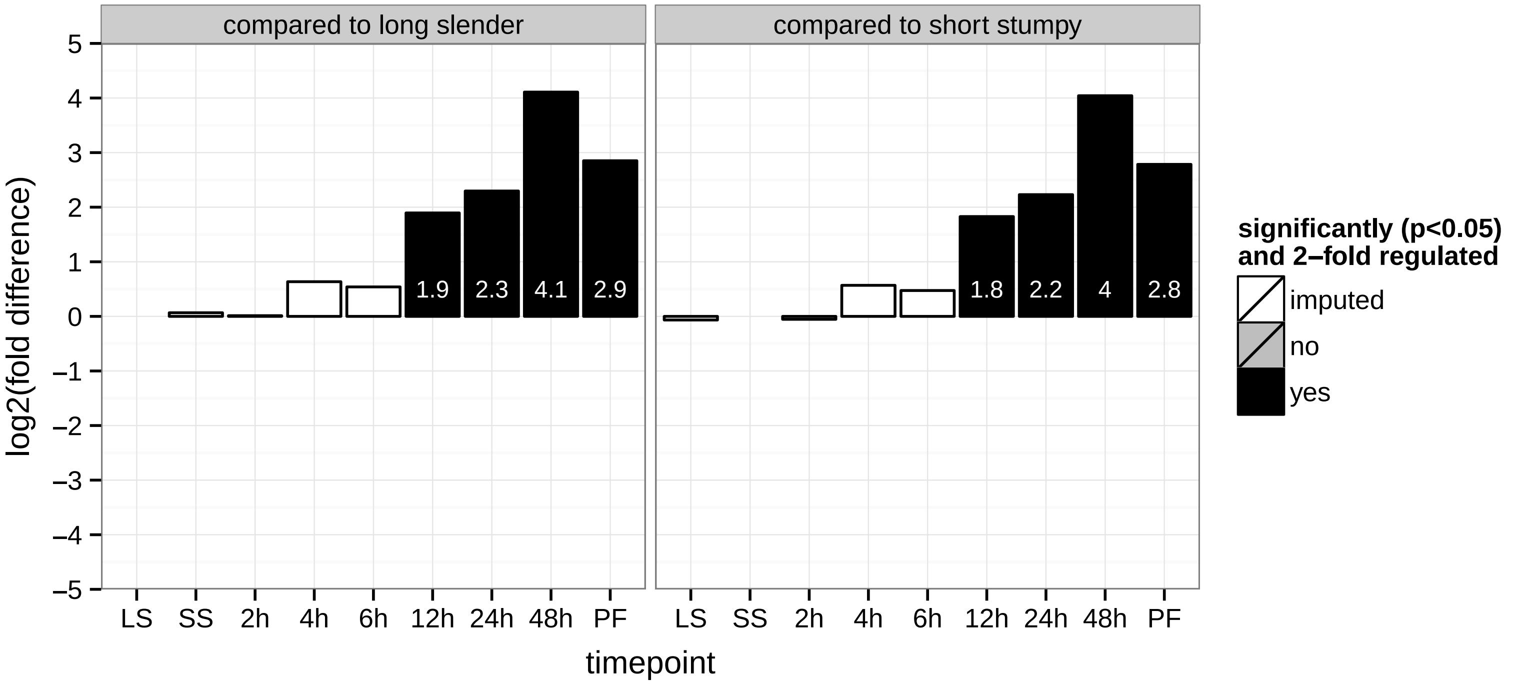

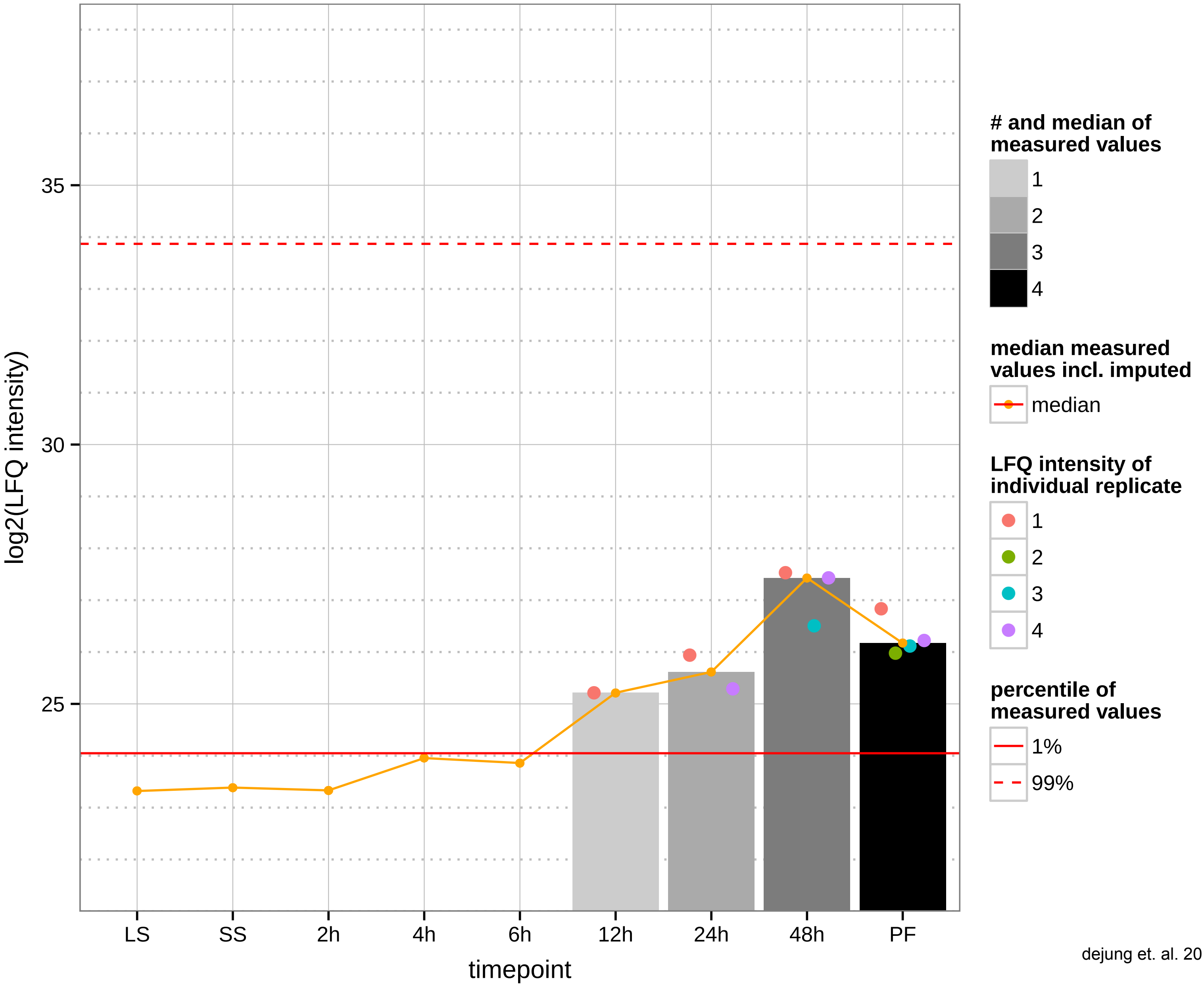

dejung et. al. 2015

hypothetical protein, conserved
Tb927.8.6960
AGOF: null
AGOC: mitochondrion
AGOP: null
PGOF: null
PGOC: null
PGOP: null

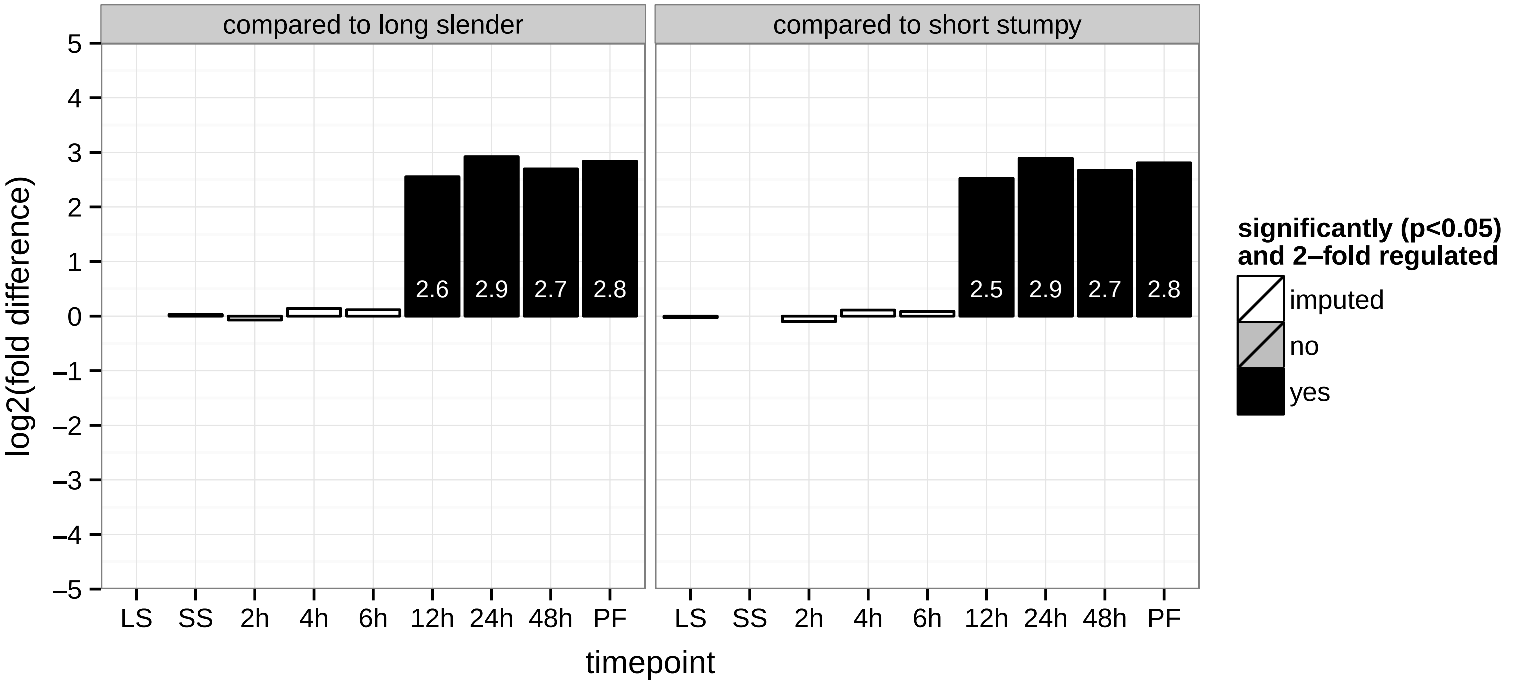

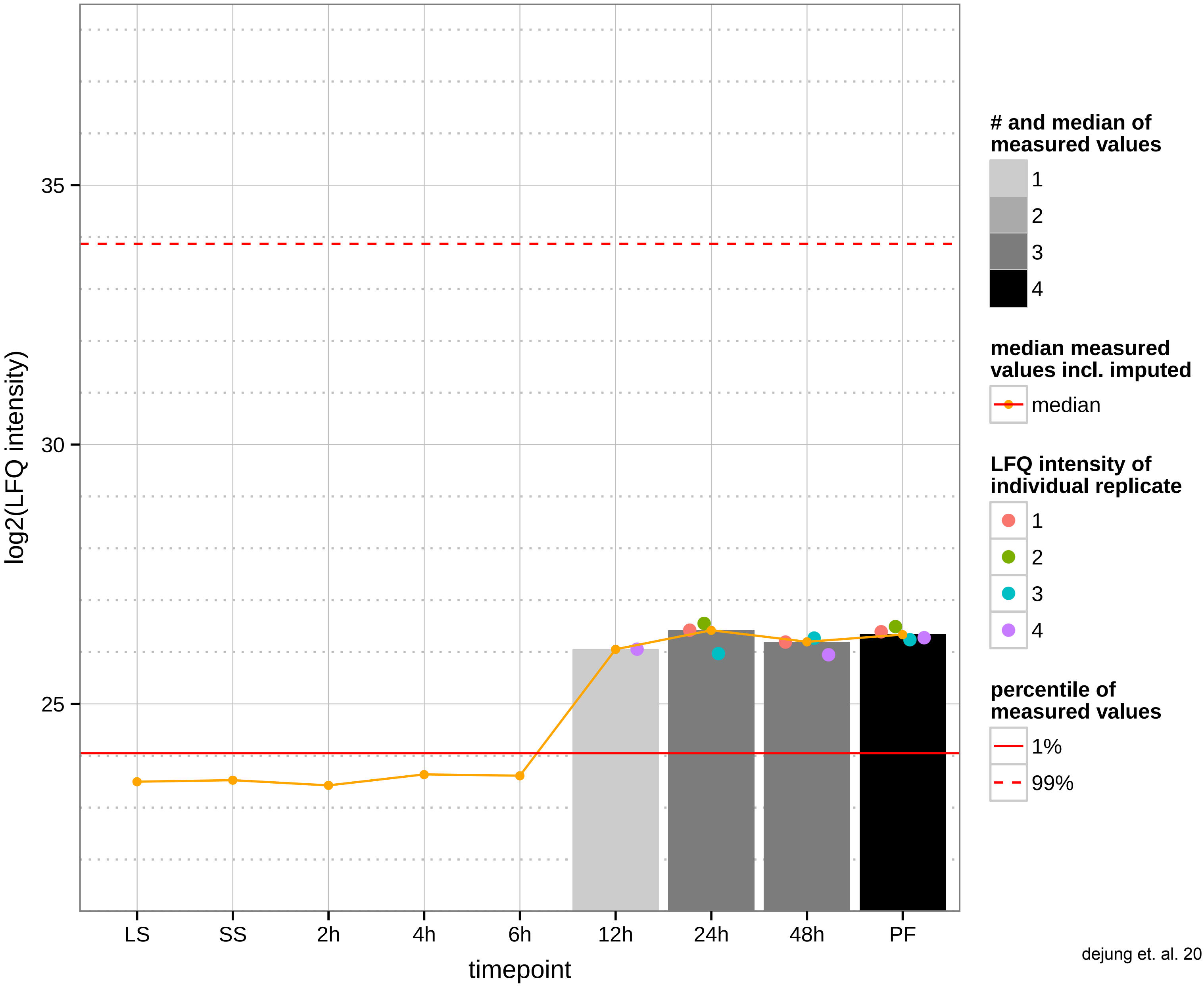

dejung et. al. 2015

3, 2–trans–enoyl–CoA isomerase, mitochondrial precursor, putative
Tb927.8.7530
AGOF: catalytic activity
AGOC: mitochondrion
AGOP: metabolic process
PGOF: catalytic activity
PGOC: null
PGOP: metabolic process

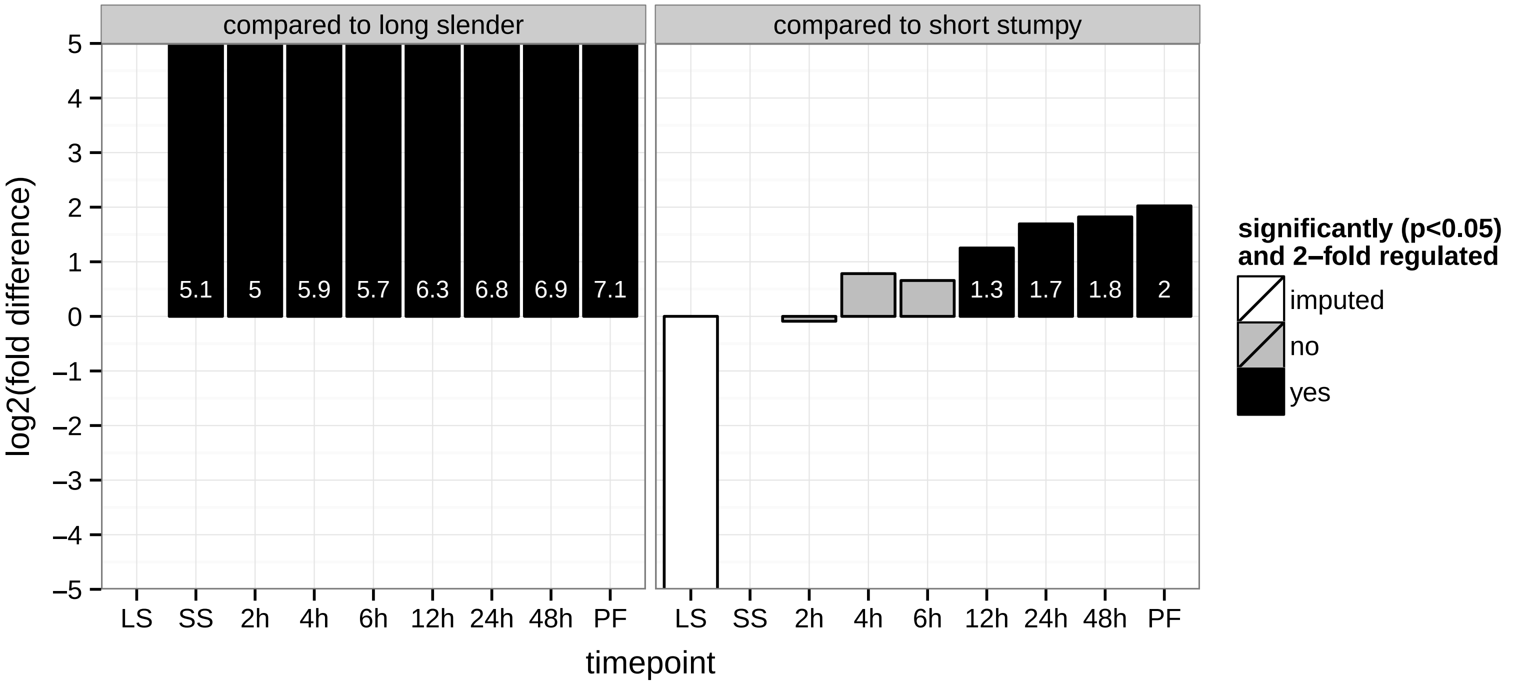

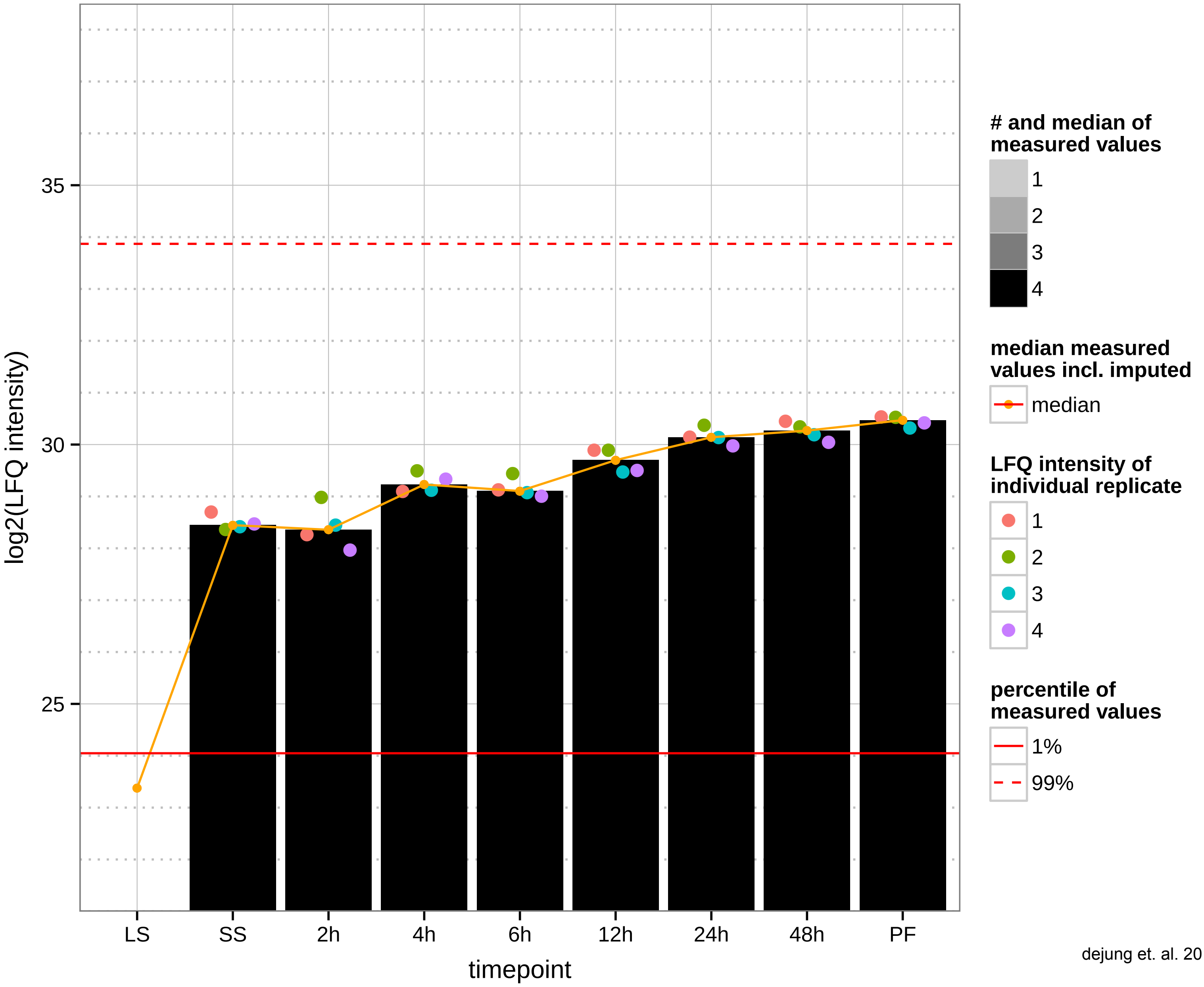

dejung et. al. 2015

hypothetical protein, conserved
Tb927.8.960;Tb927.8.940
AGOF: null
AGOC: null
AGOP: null
PGOF: null
PGOC: null
PGOP: null

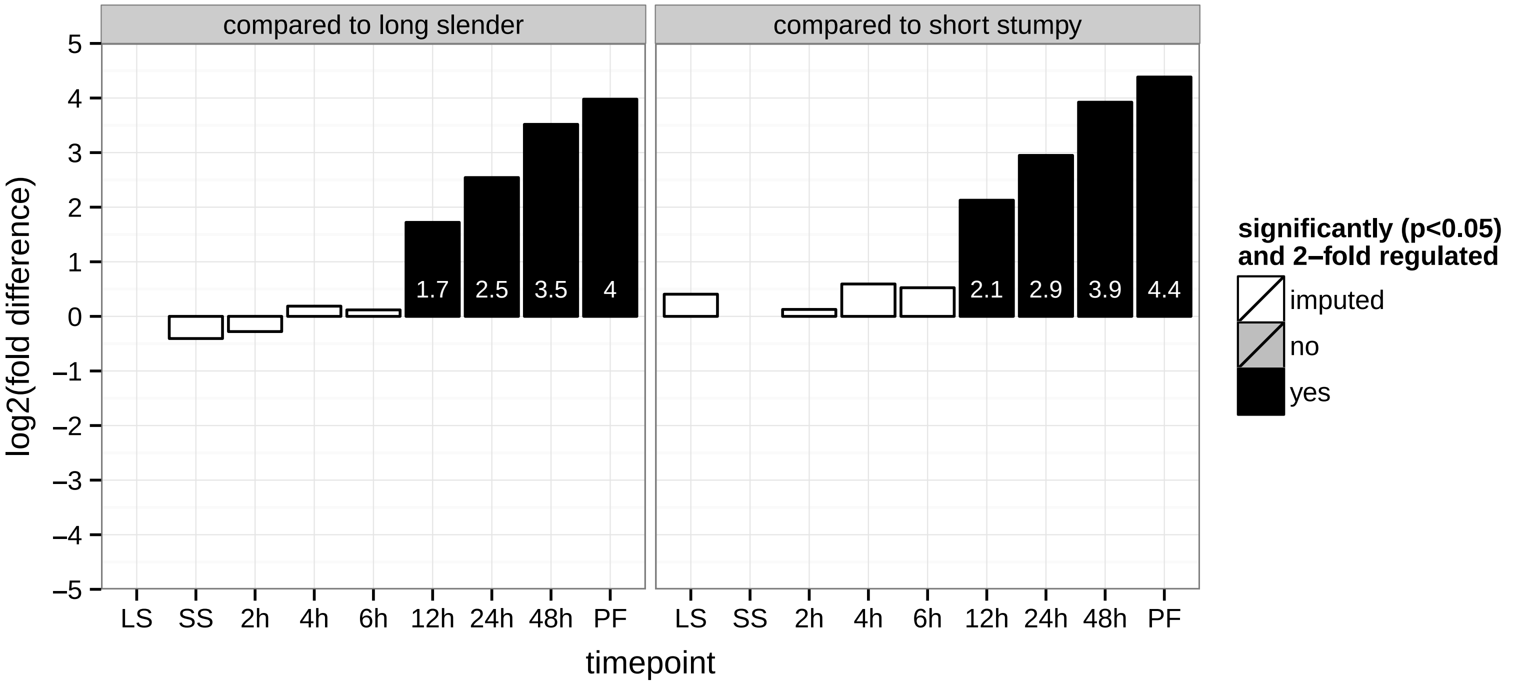

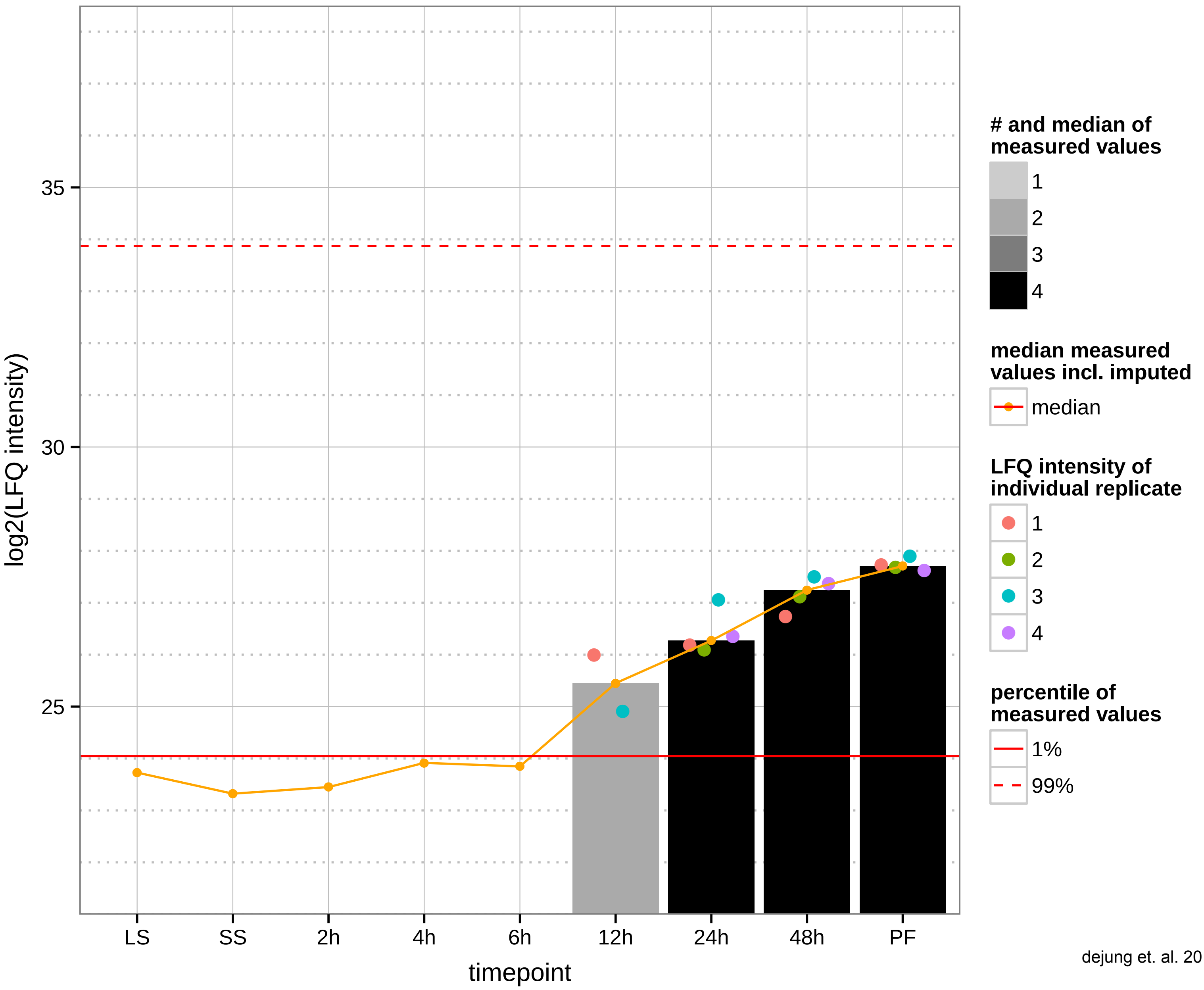

dejung et. al. 2015

hypothetical protein, conserved
Tb927.9.10490
AGOF: null
AGOC: null
AGOP: ciliary or flagellar motility
PGOF: null
PGOC: null
PGOP: null

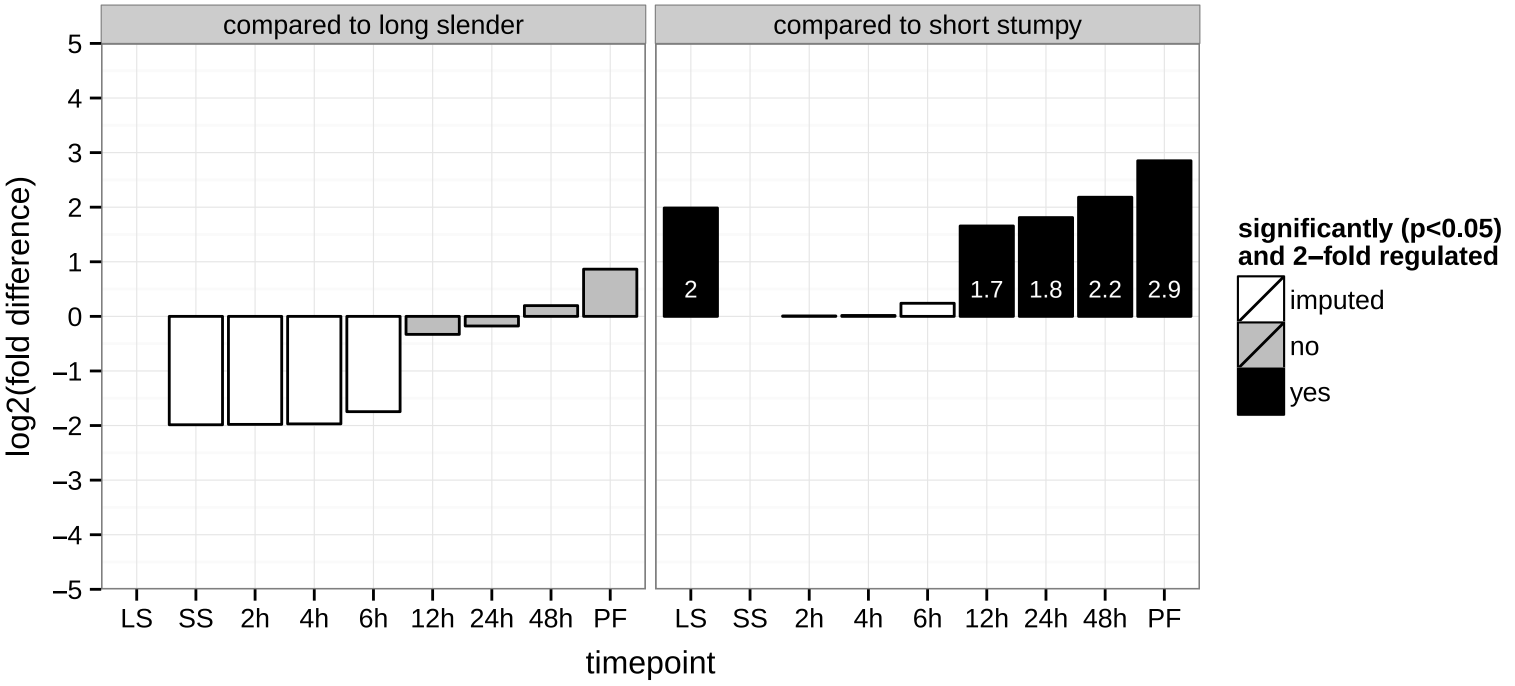

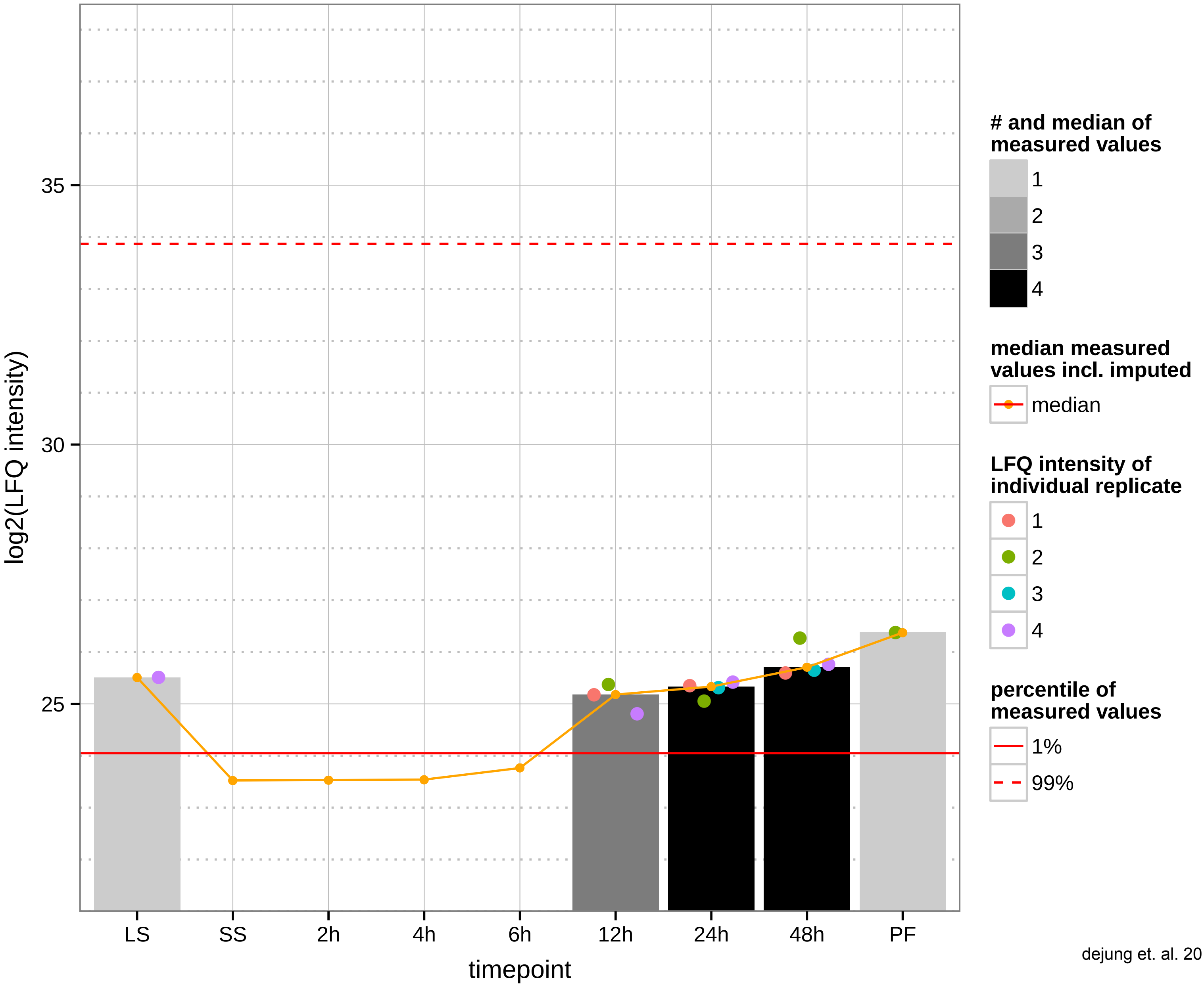

dejung et. al. 2015

hypothetical protein, conserved
Tb927.9.10880
AGOF: null
AGOC: null
AGOP: null
PGOF: null
PGOC: null
PGOP: null

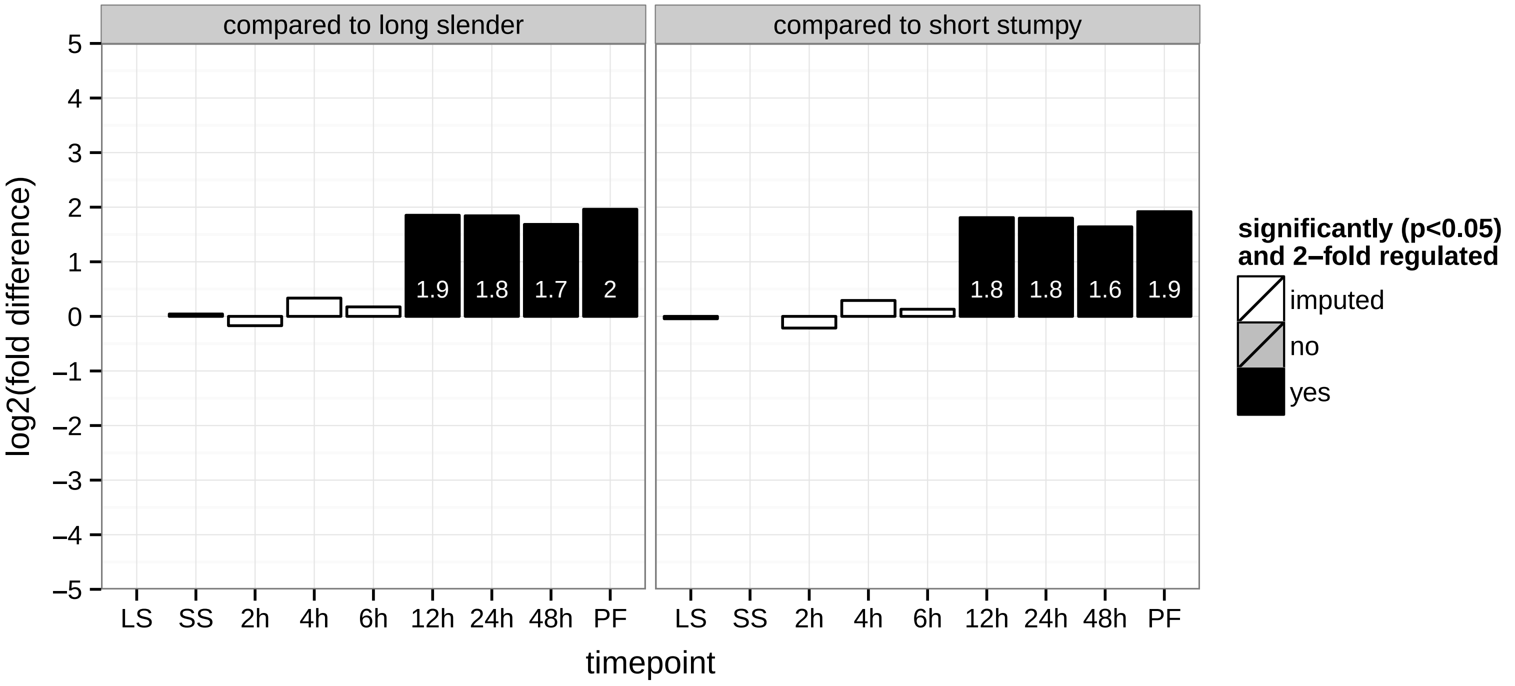

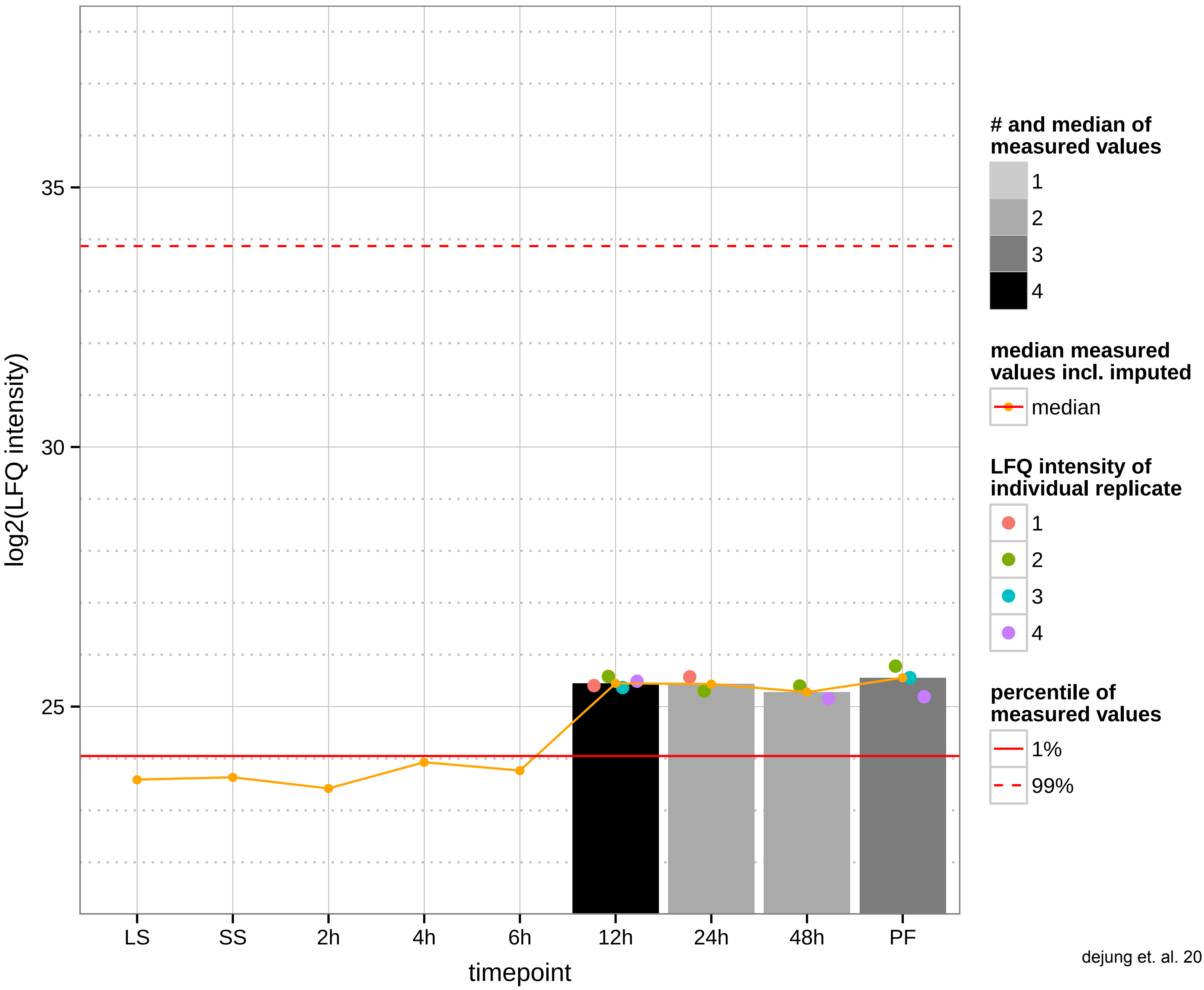

dejung et. al. 2015

RNA–binding protein, putative (RBP35)
Tb927.9.12360
AGOF: null
AGOC: null
AGOP: null
PGOF: null
PGOC: null
PGOP: null

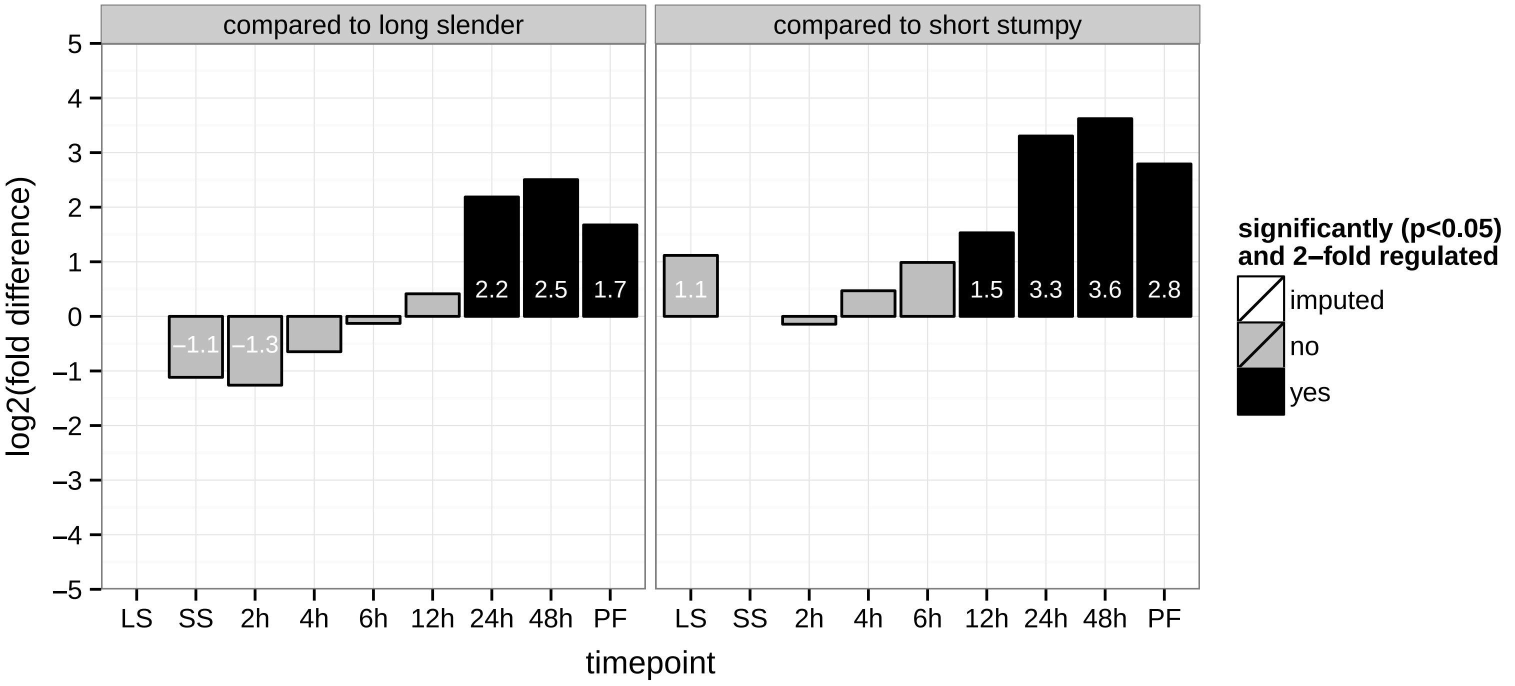

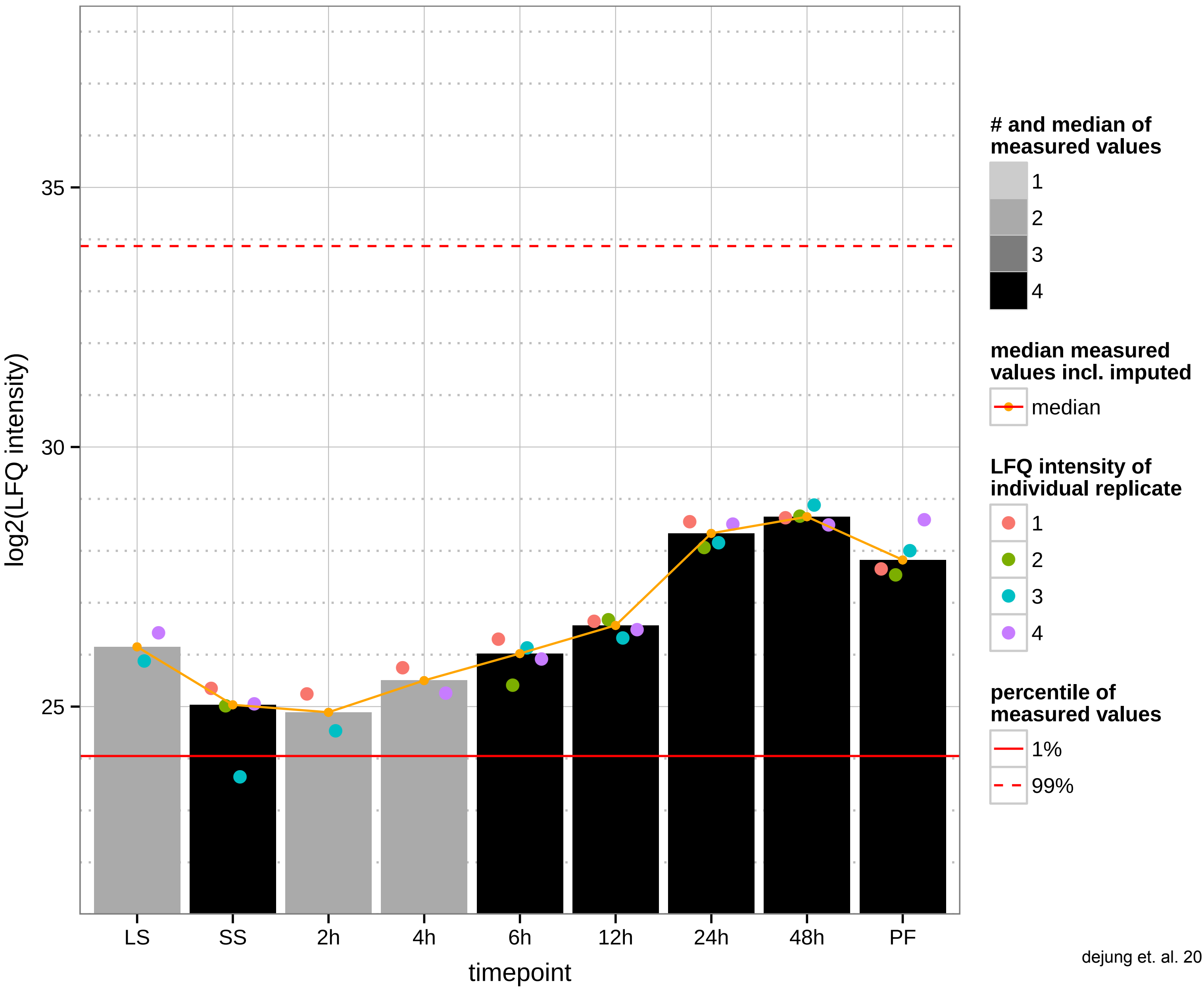

dejung et. al. 2015

Double RNA binding domain protein 9 (DRBD9)
Tb927.9.13280
AGOF: RNA binding
AGOC: nucleus
AGOP: RNA processing
PGOF: nucleic acid binding
PGOC: null
PGOP: null

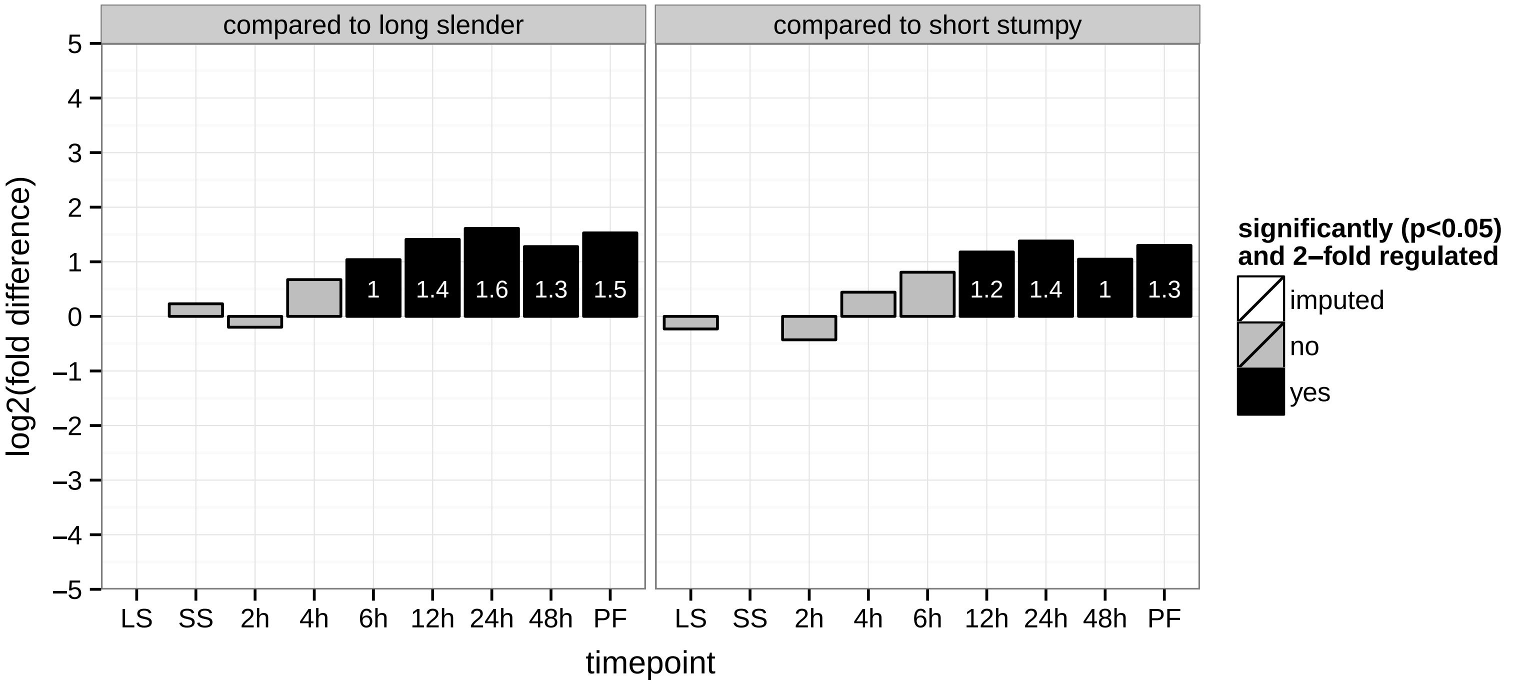

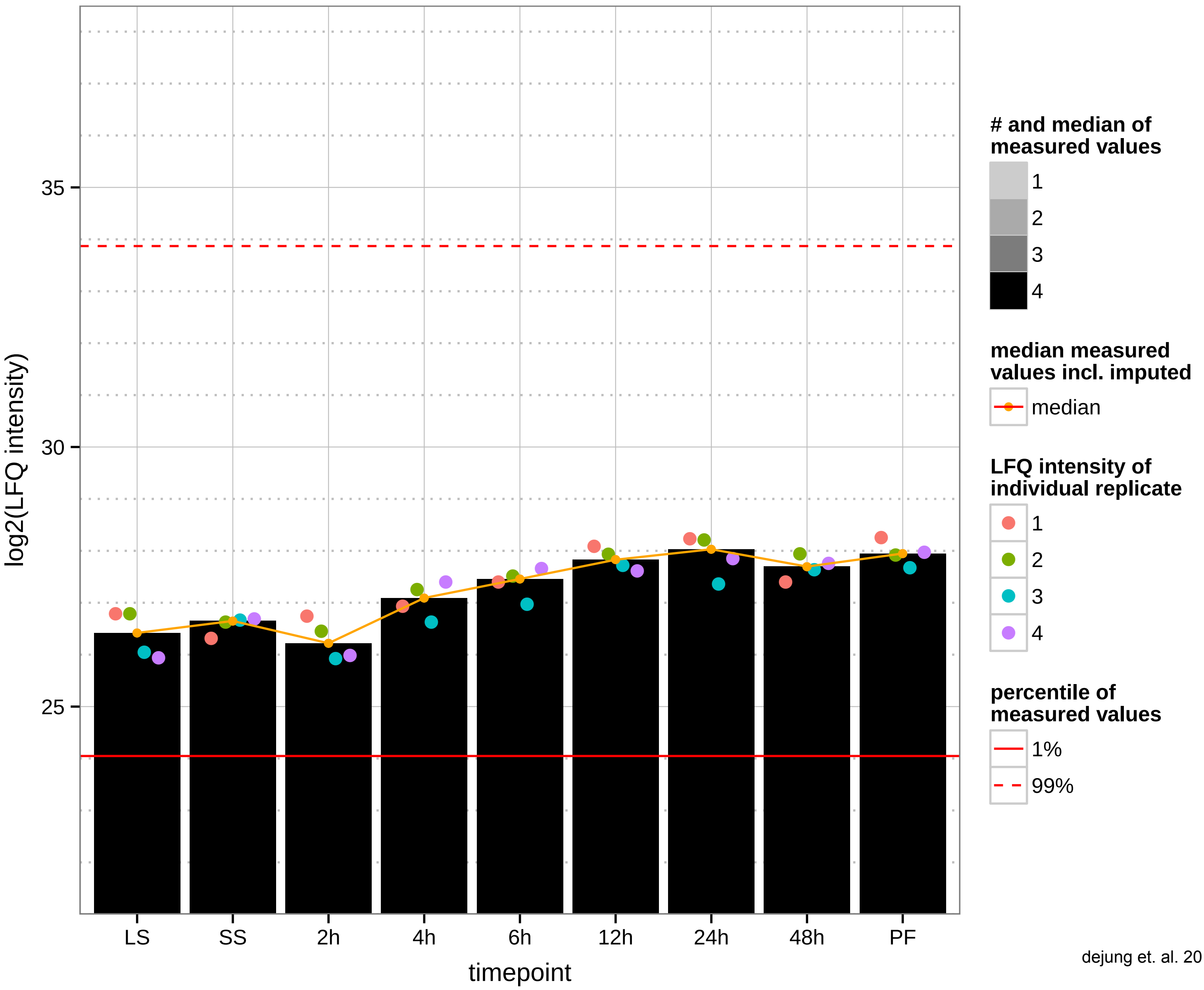

dejung et. al. 2015

hypothetical protein, conserved
Tb927.9.14200
AGOF: methyltransferase activity
AGOC: mitochondrial inner membrane, mitochondrion
AGOP: metabolic process
PGOF: null
PGOC: null
PGOP: null

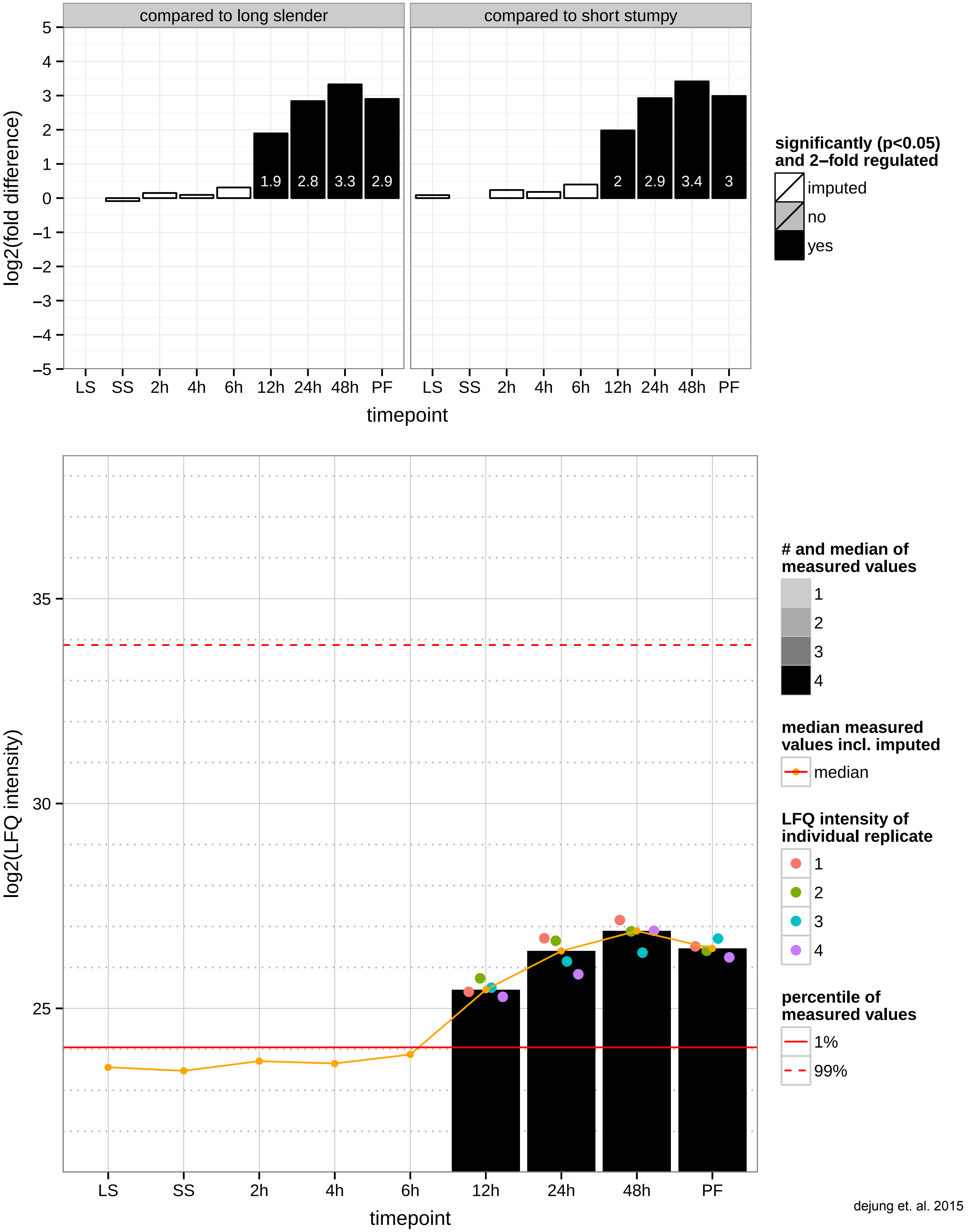

dejung et. al. 2015

unspecified product
Tb927.9.14550
AGOF: null
AGOC: null
AGOP: null
PGOF: null
PGOC: null
PGOP: null

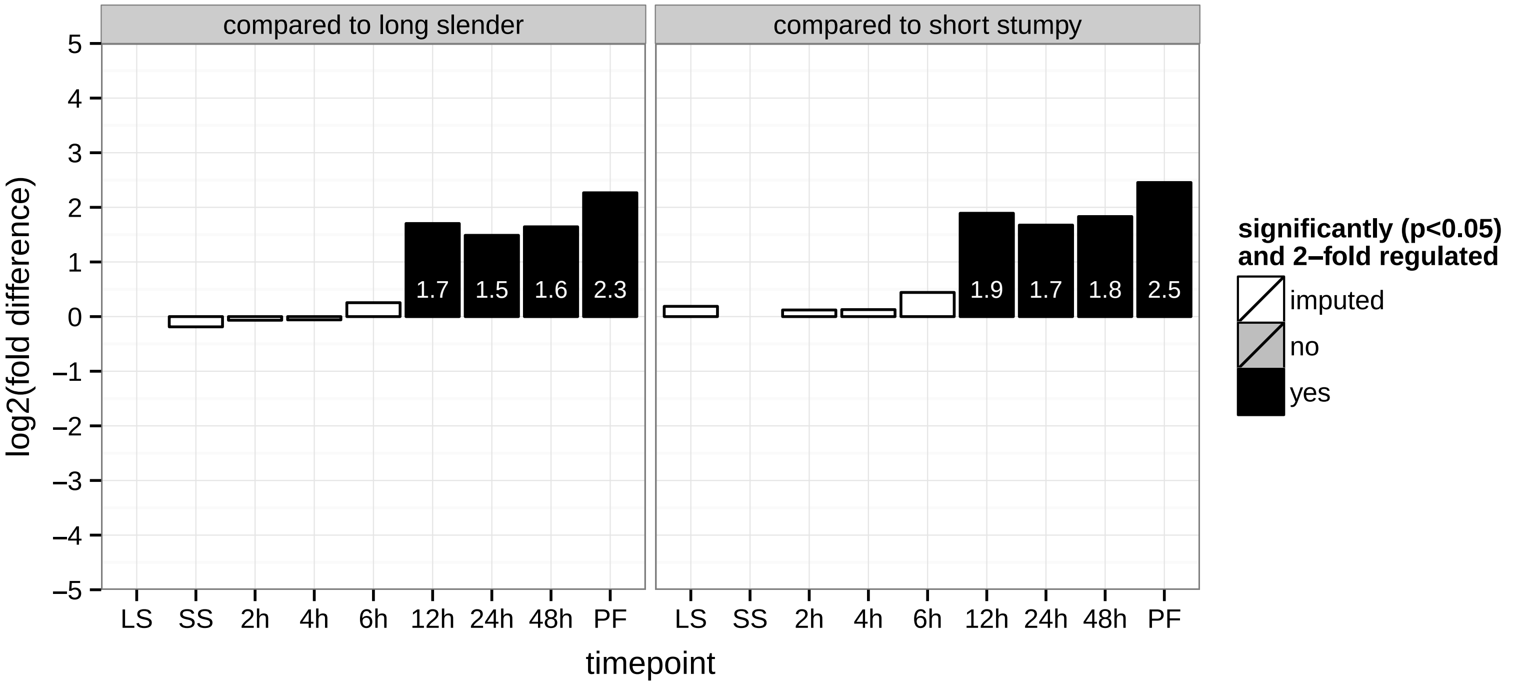

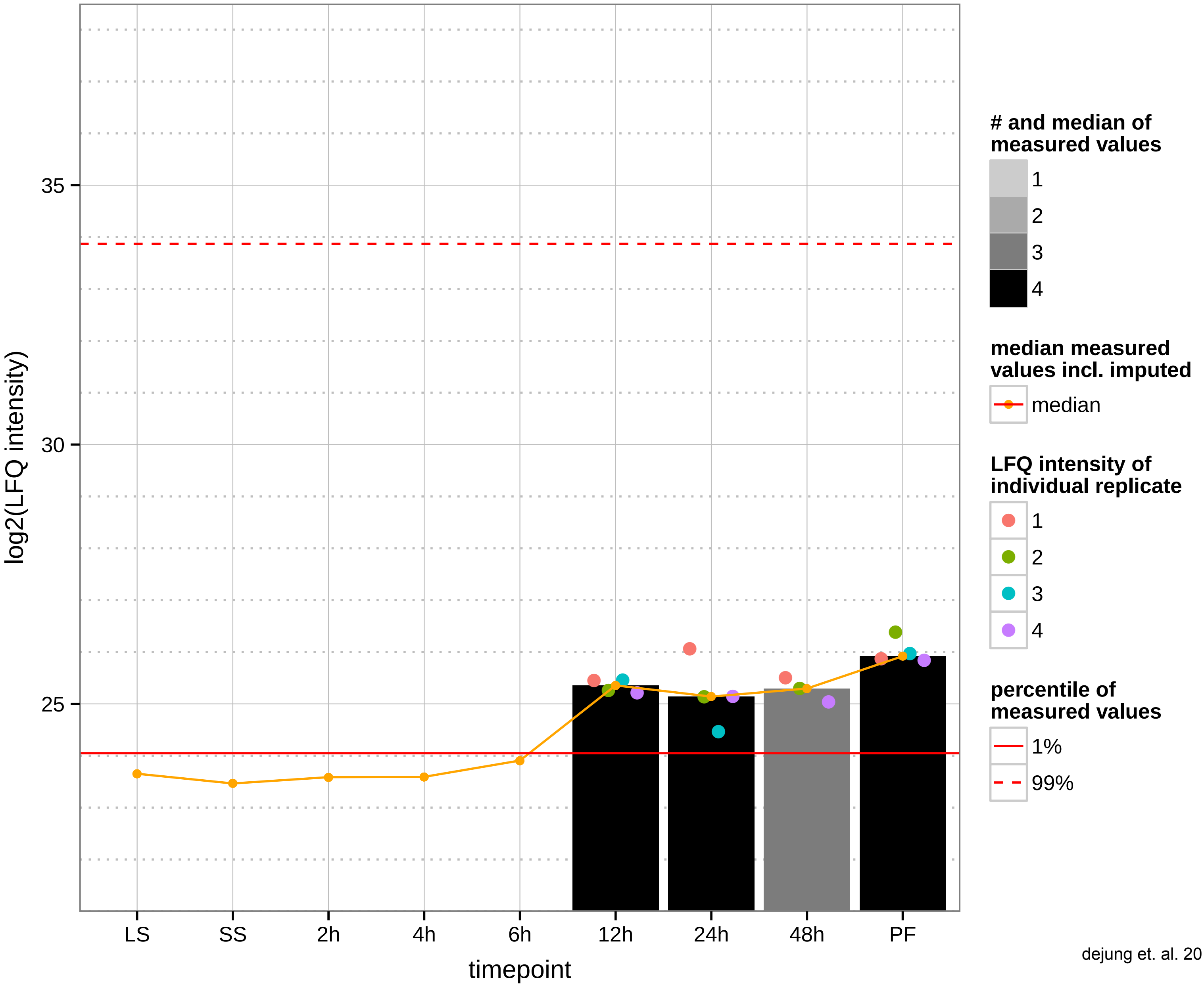

dejung et. al. 2015

hypothetical protein, conserved
Tb927.9.15330
AGOF: null
AGOC: null
AGOP: null
PGOF: null
PGOC: null
PGOP: null

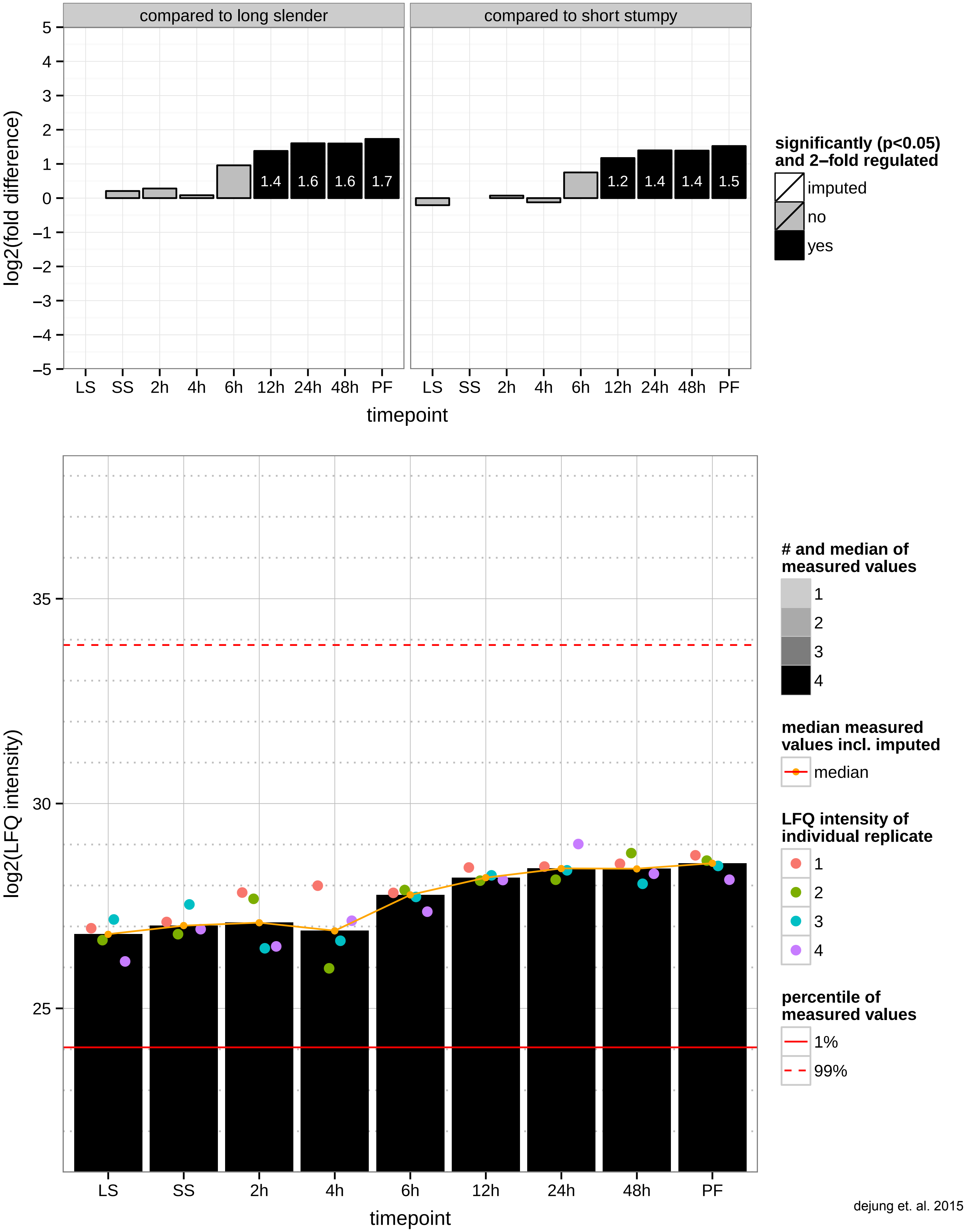

dejung et. al. 2015

hypothetical protein, conserved
Tb927.9.2320
AGOF: methyltransferase activity
AGOC: null
AGOP: metabolic process
PGOF: null
PGOC: null
PGOP: null

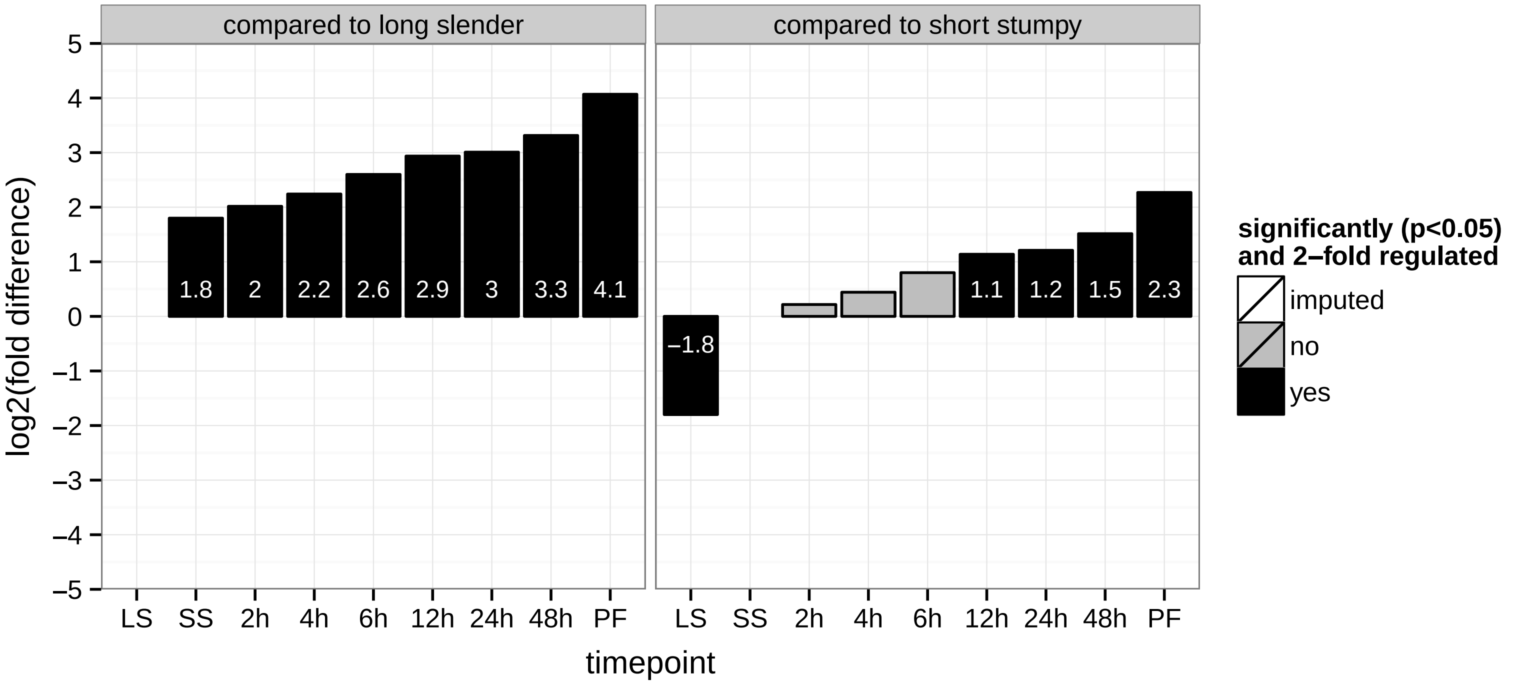

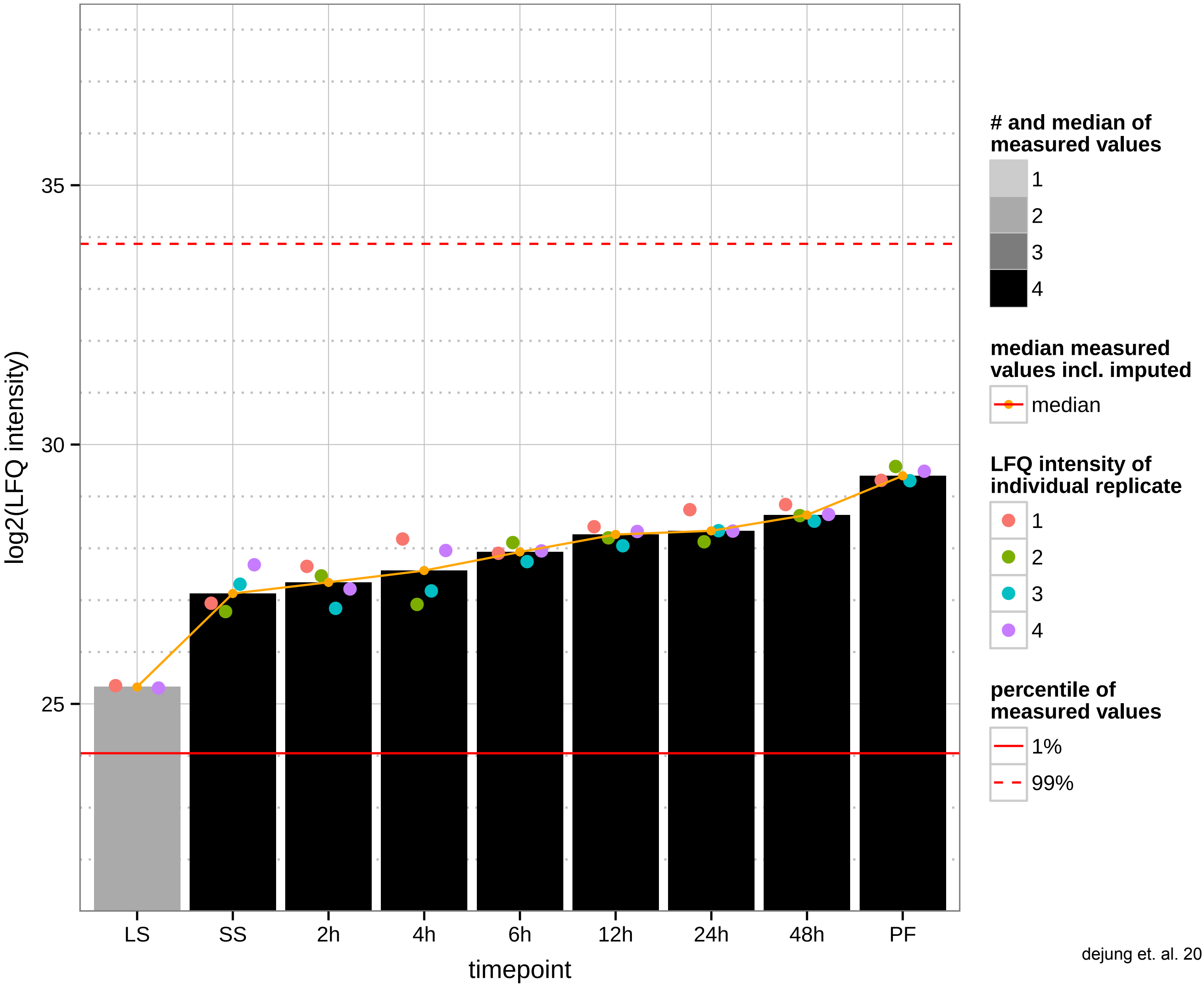

dejung et. al. 2015

hypothetical protein, conserved
Tb927.9.3080
AGOF: null
AGOC: null
AGOP: null
PGOF: null
PGOC: null
PGOP: null

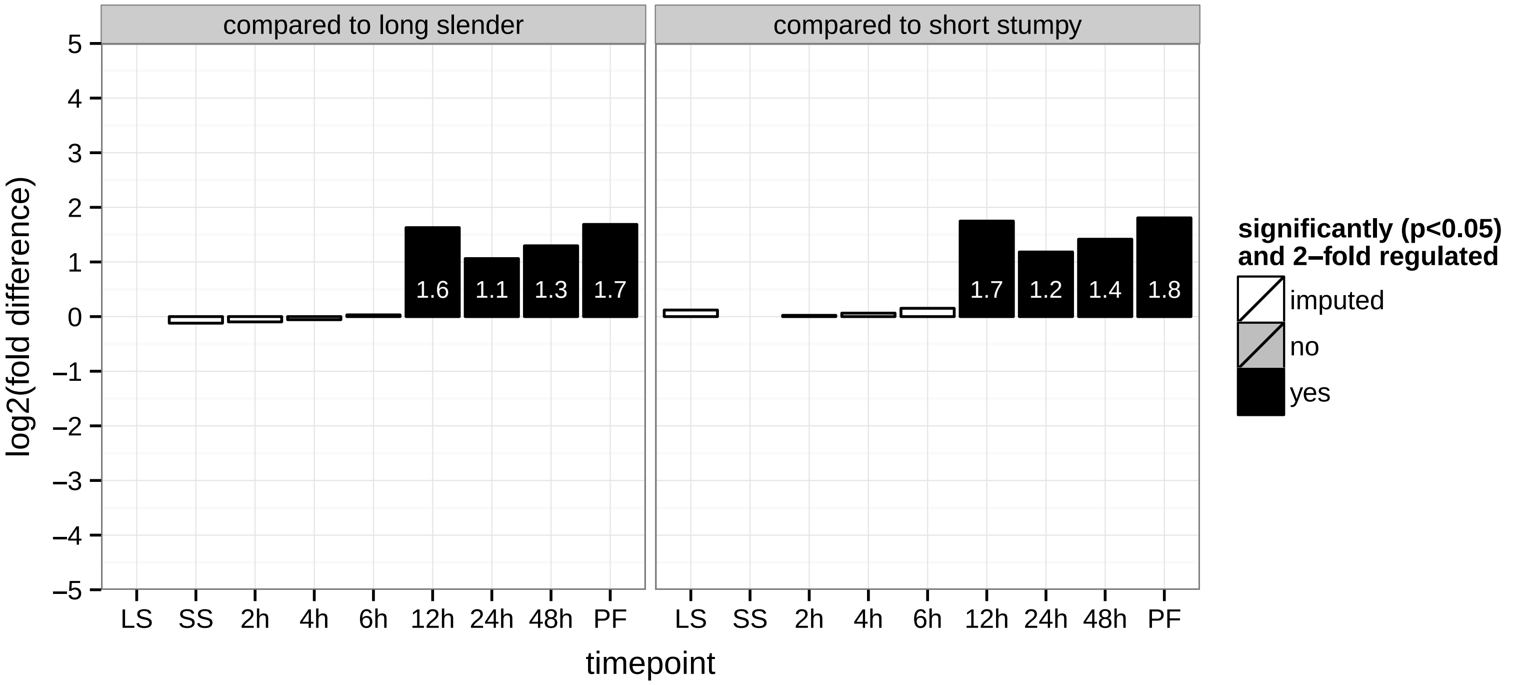

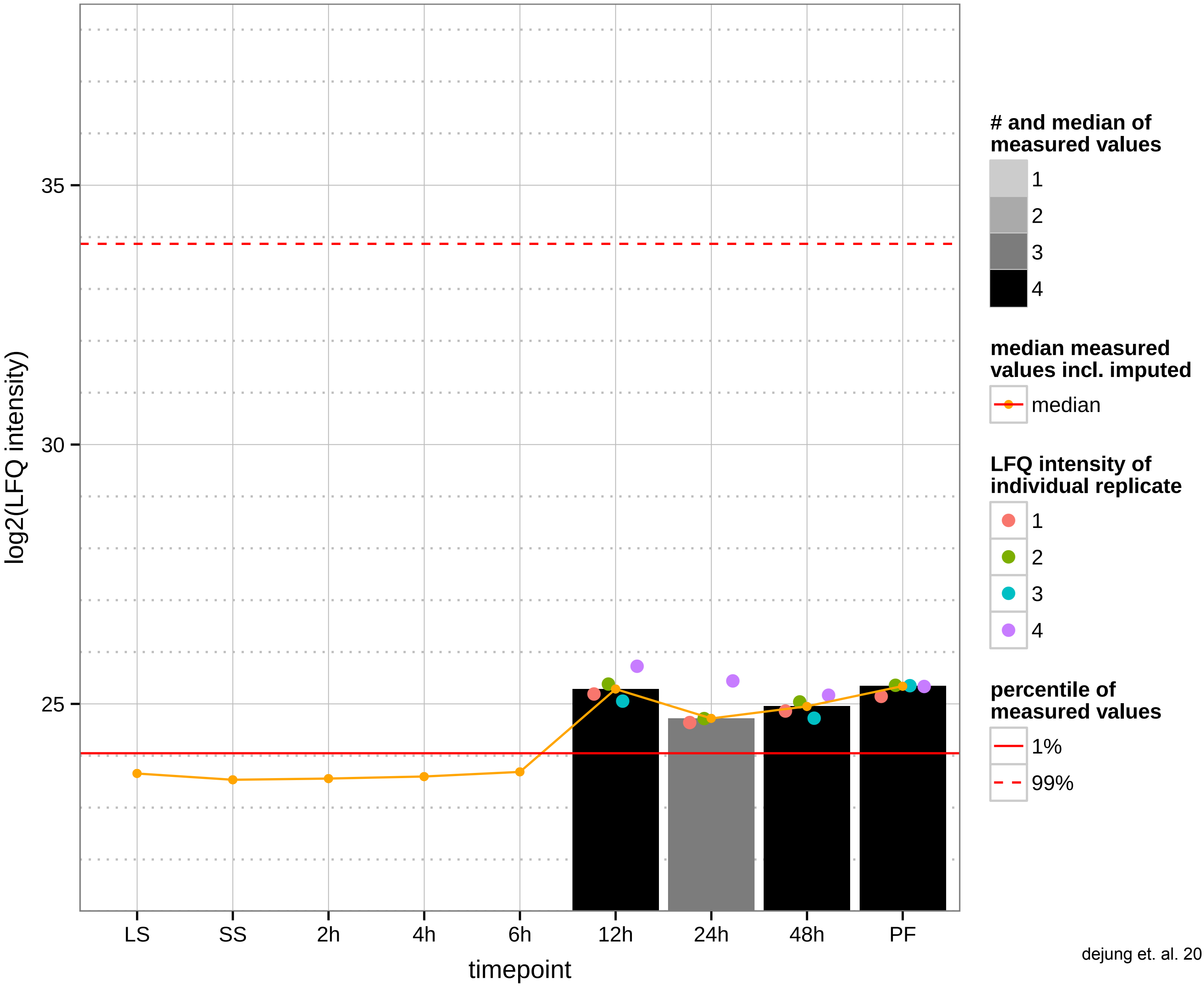

dejung et. al. 2015

solanesyl–diphosphate synthase, putative, trans–octaprenyltranstransferase
Tb927.9.5890
AGOF: trans–hexaprenyltranstransferase activity, trans–octaprenyltranstransferase activity
AGOC: mitochondrion
AGOP: isoprenoid biosynthetic process, ubiquinone metabolic process
PGOF: null
PGOC: null
PGOP: isoprenoid biosynthetic process

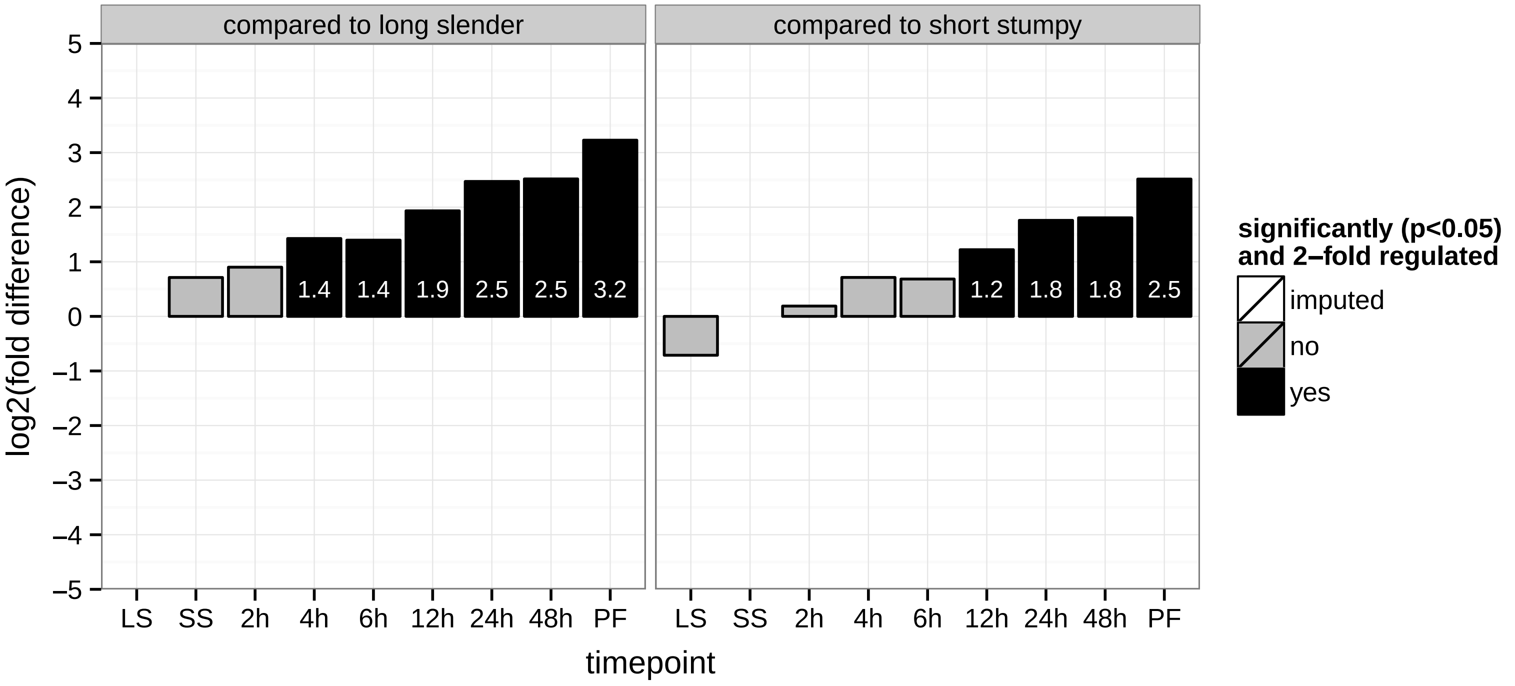

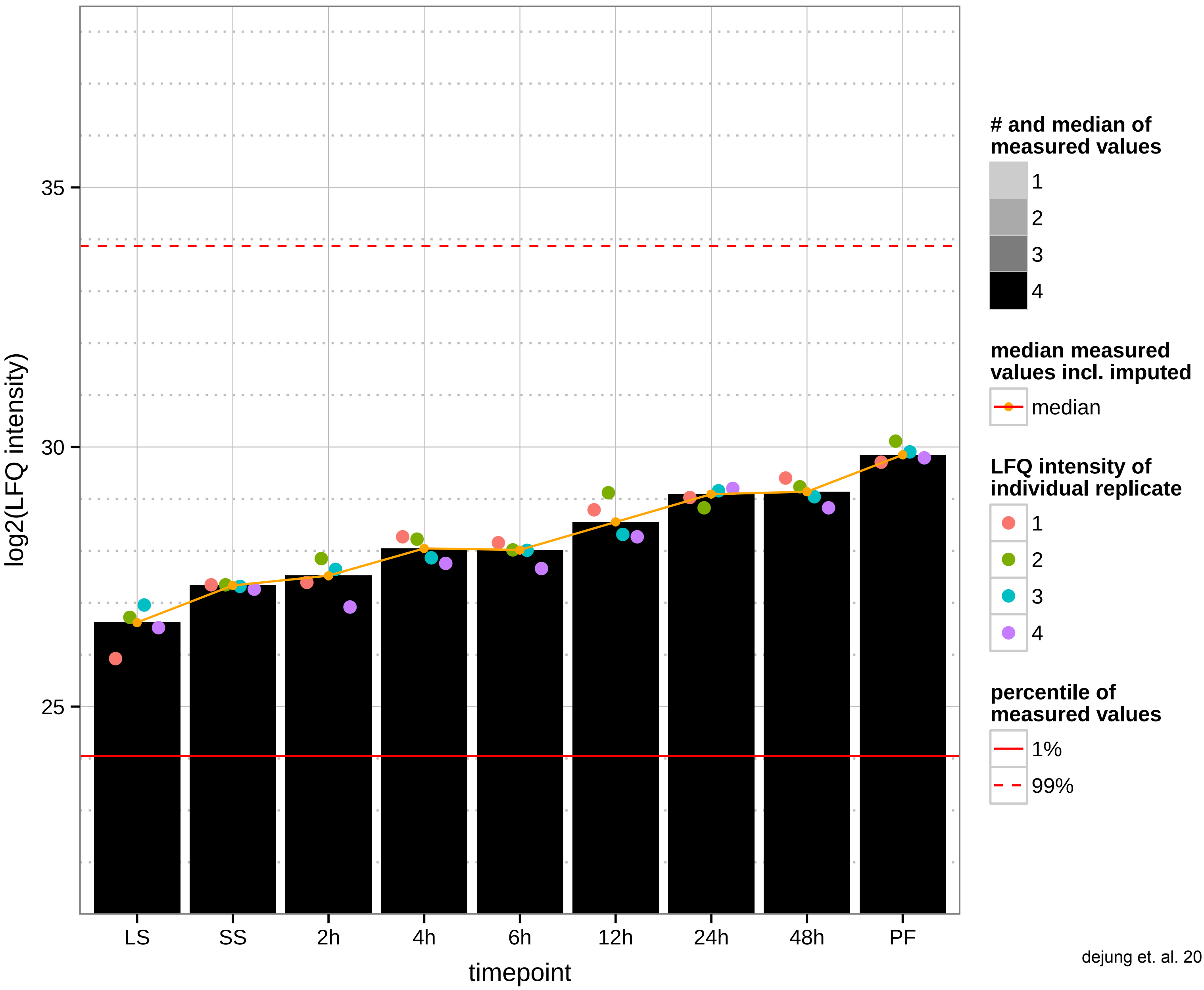

dejung et. al. 2015

hypothetical protein, conserved
Tb927.9.6920
AGOF: null
AGOC: null
AGOP: null
PGOF: null
PGOC: null
PGOP: null

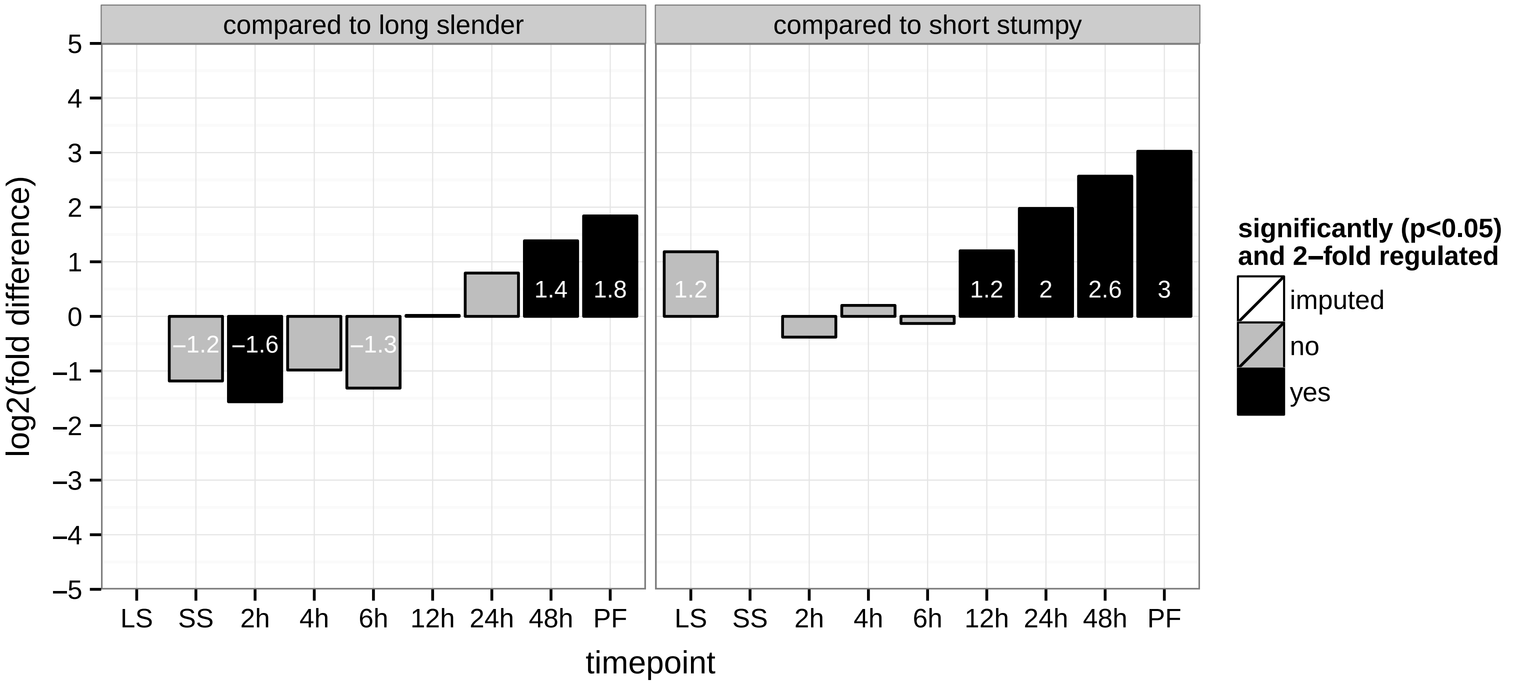

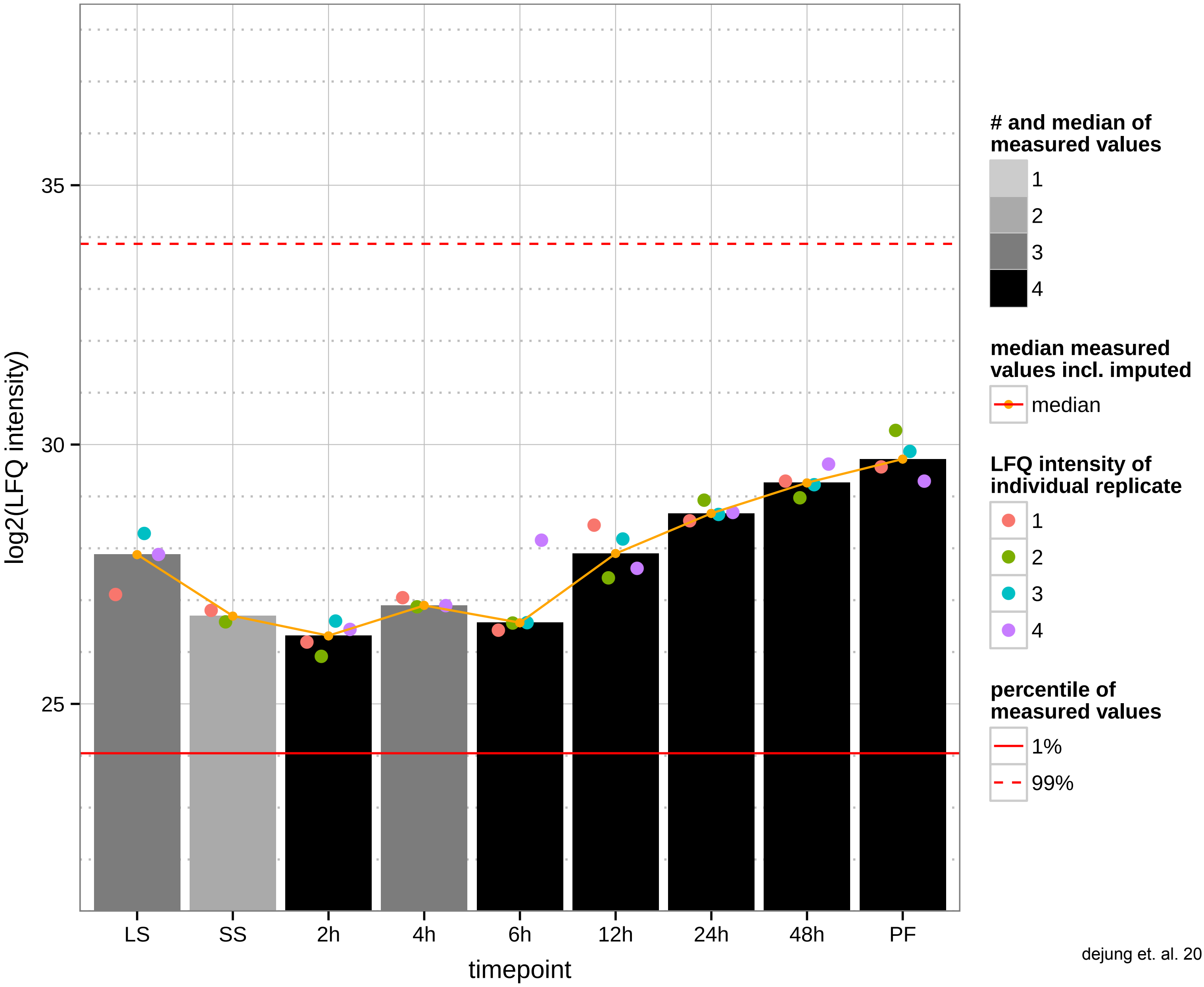

dejung et. al. 2015

adenosine transporter 2 (TbNT2/927), adenosine transporter 2, putative (TbNT3), adenosine transporter 2 (TbNT5), putative (
Tb927.9.7470
AGOF: purine nucleoside transmembrane transporter activity, nucleoside transmembrane transporter activity, nucleoside:hydr
AGOC: integral to membrane, membrane
AGOP: purine ribonucleoside salvage, transport, nucleoside transport, adenosine transport, inosine transport, nucleoside tran
PGOF: nucleoside transmembrane transporter activity
PGOC: integral to membrane
PGOP: transport

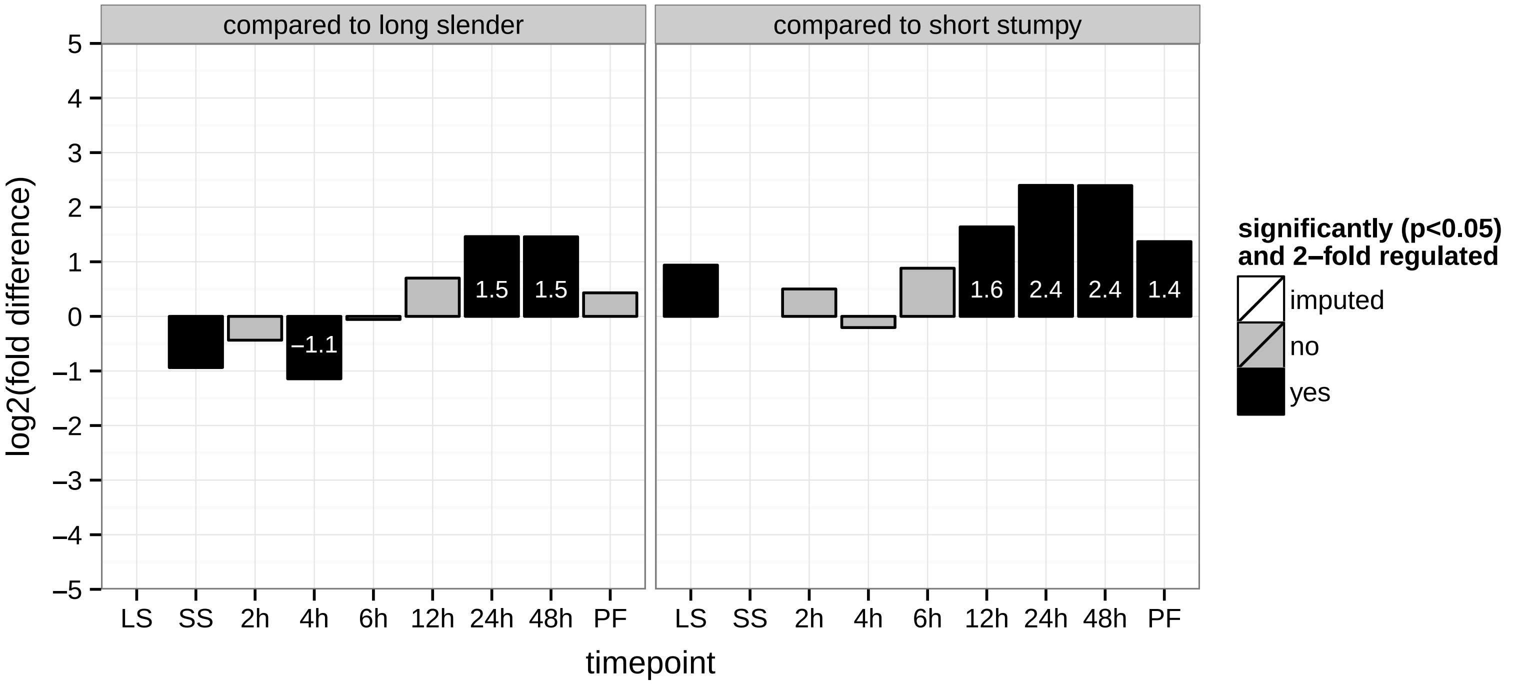

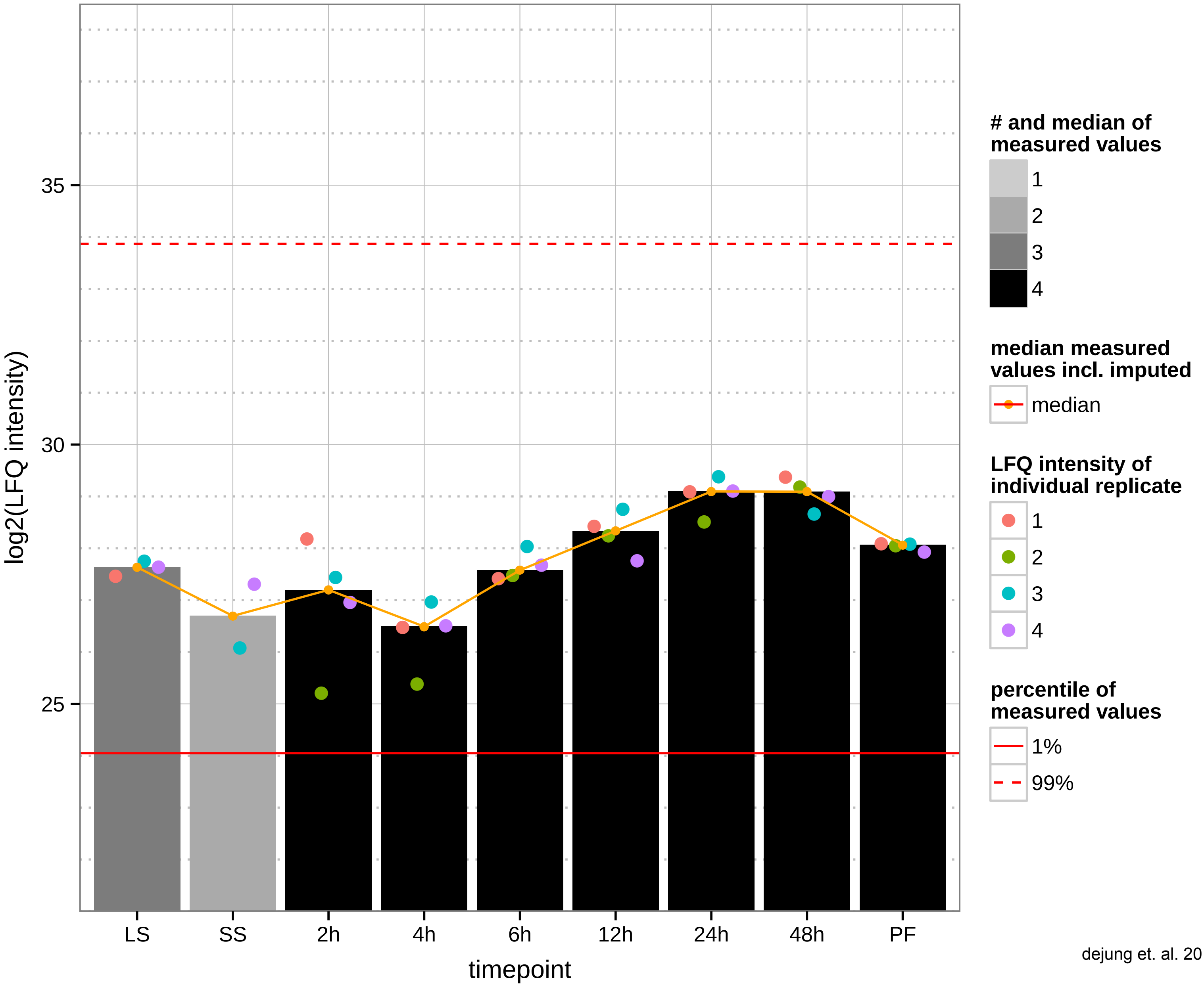

dejung et. al. 2015

Peptide deformylase 2
Tb927.9.8210
AGOF: iron ion binding, peptide deformylase activity
AGOC: mitochondrion
AGOP: translation
PGOF: null
PGOC: null
PGOP: null

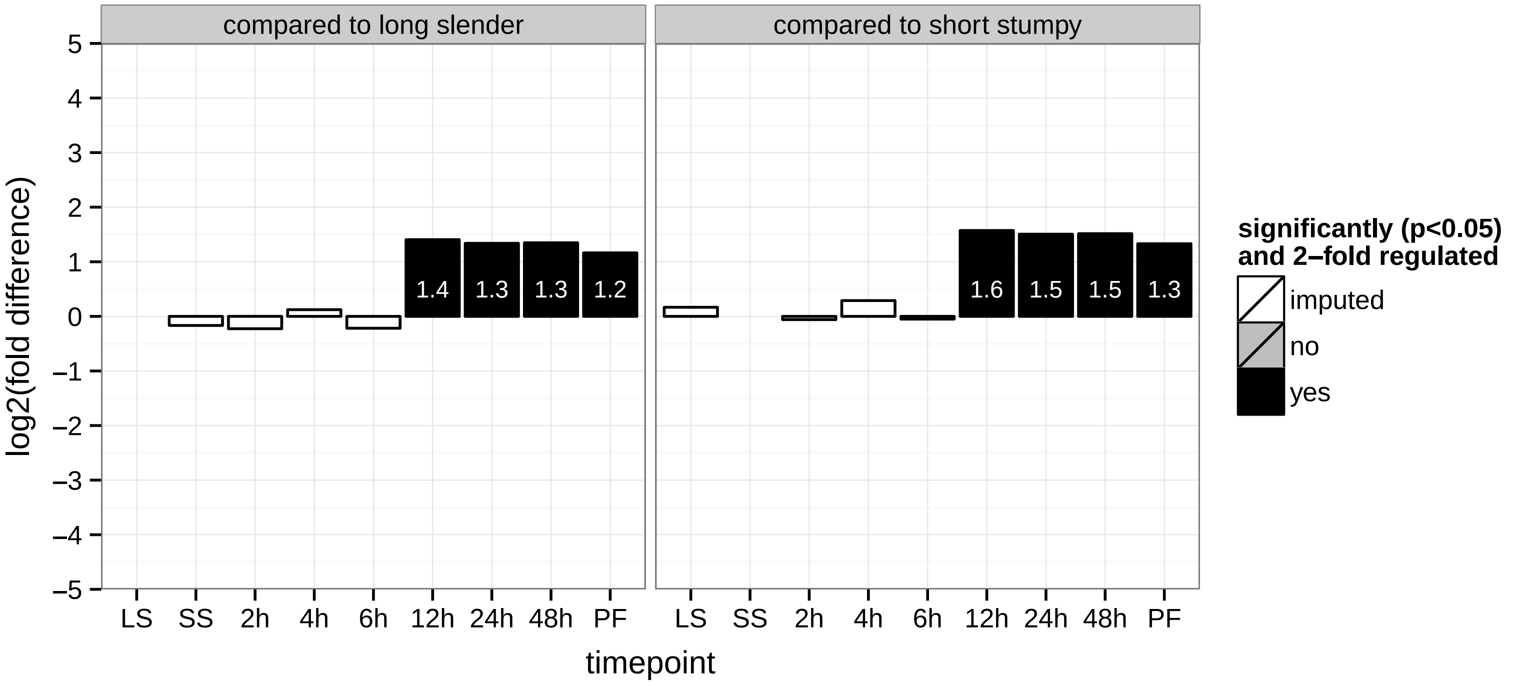

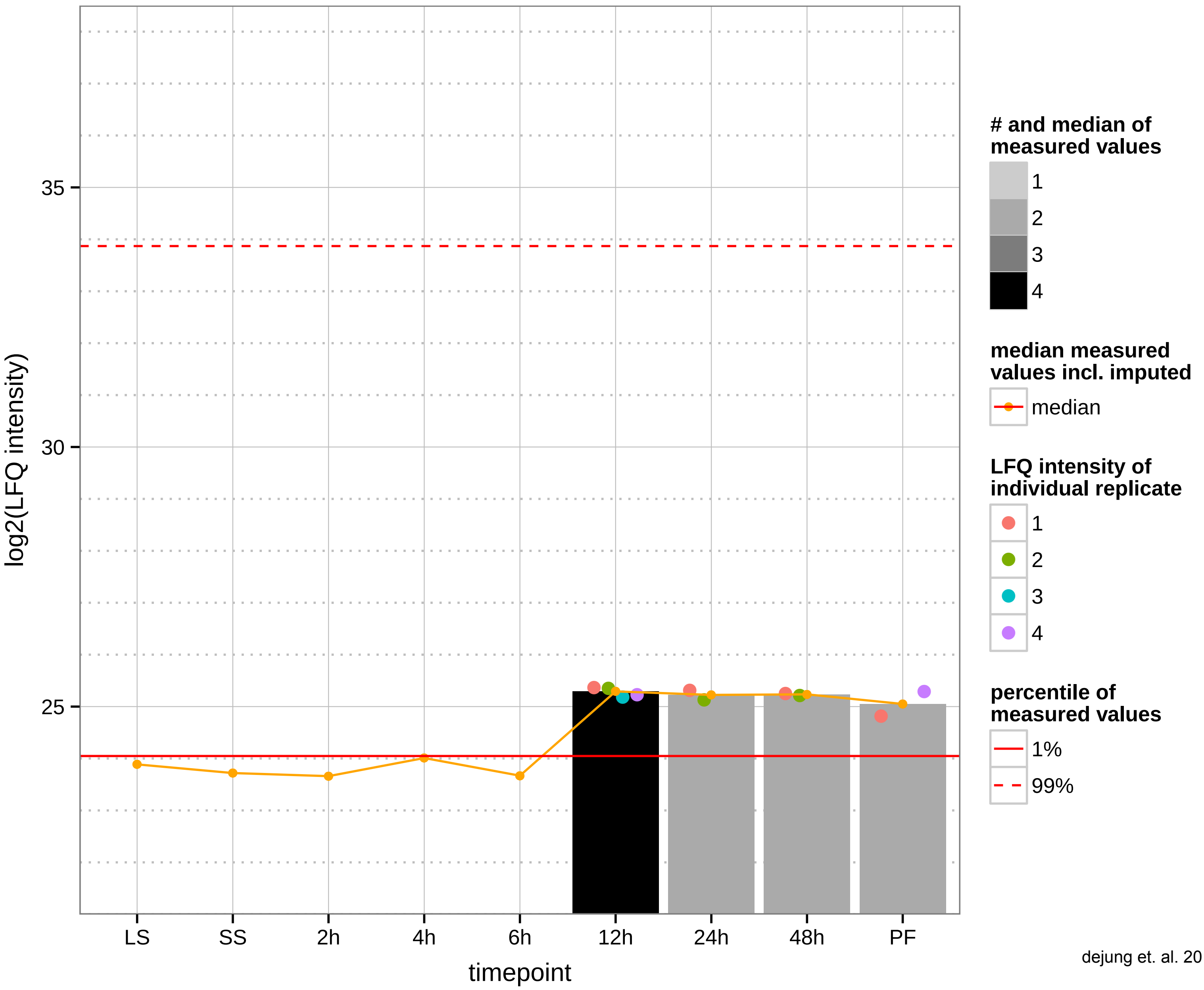

dejung et. al. 2015

hypothetical protein, conserved
Tb927.9.8380
AGOF: null
AGOC: null
AGOP: null
PGOF: null
PGOC: null
PGOP: null

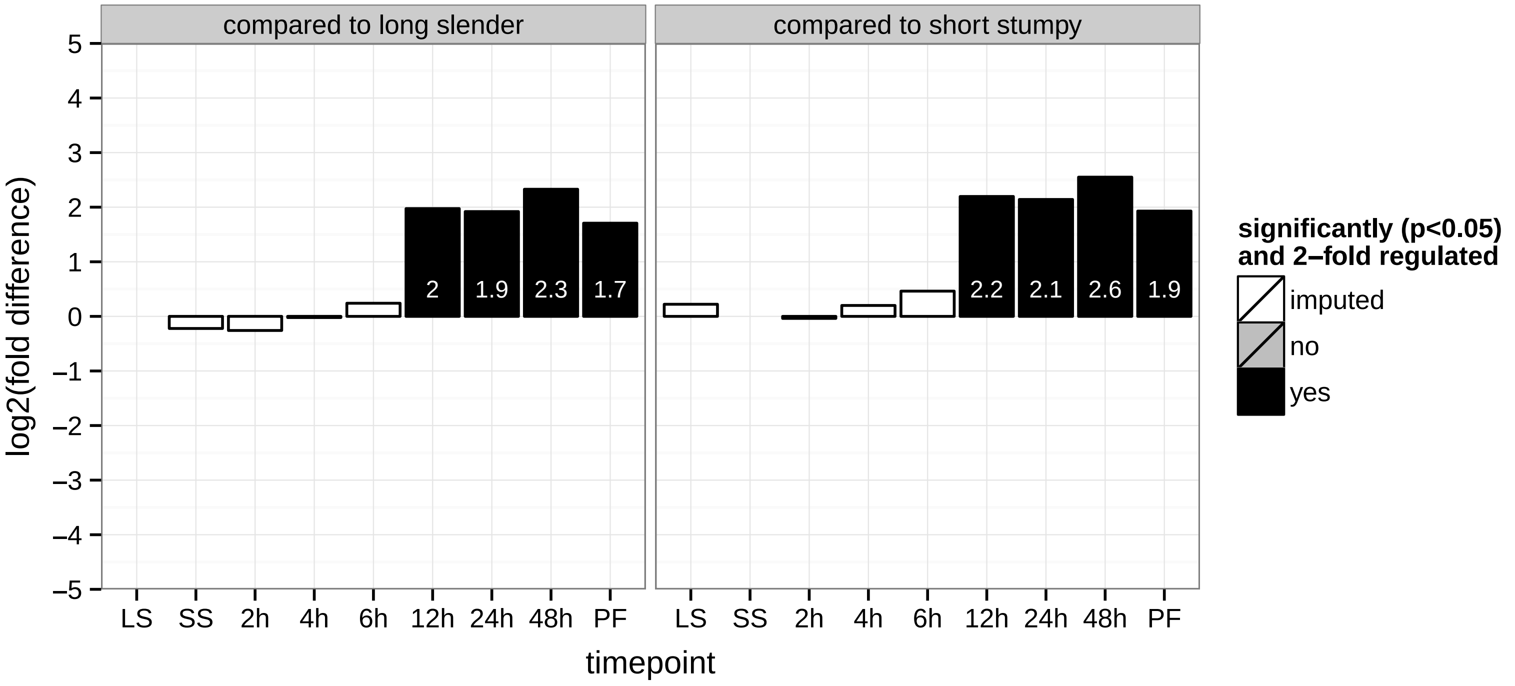

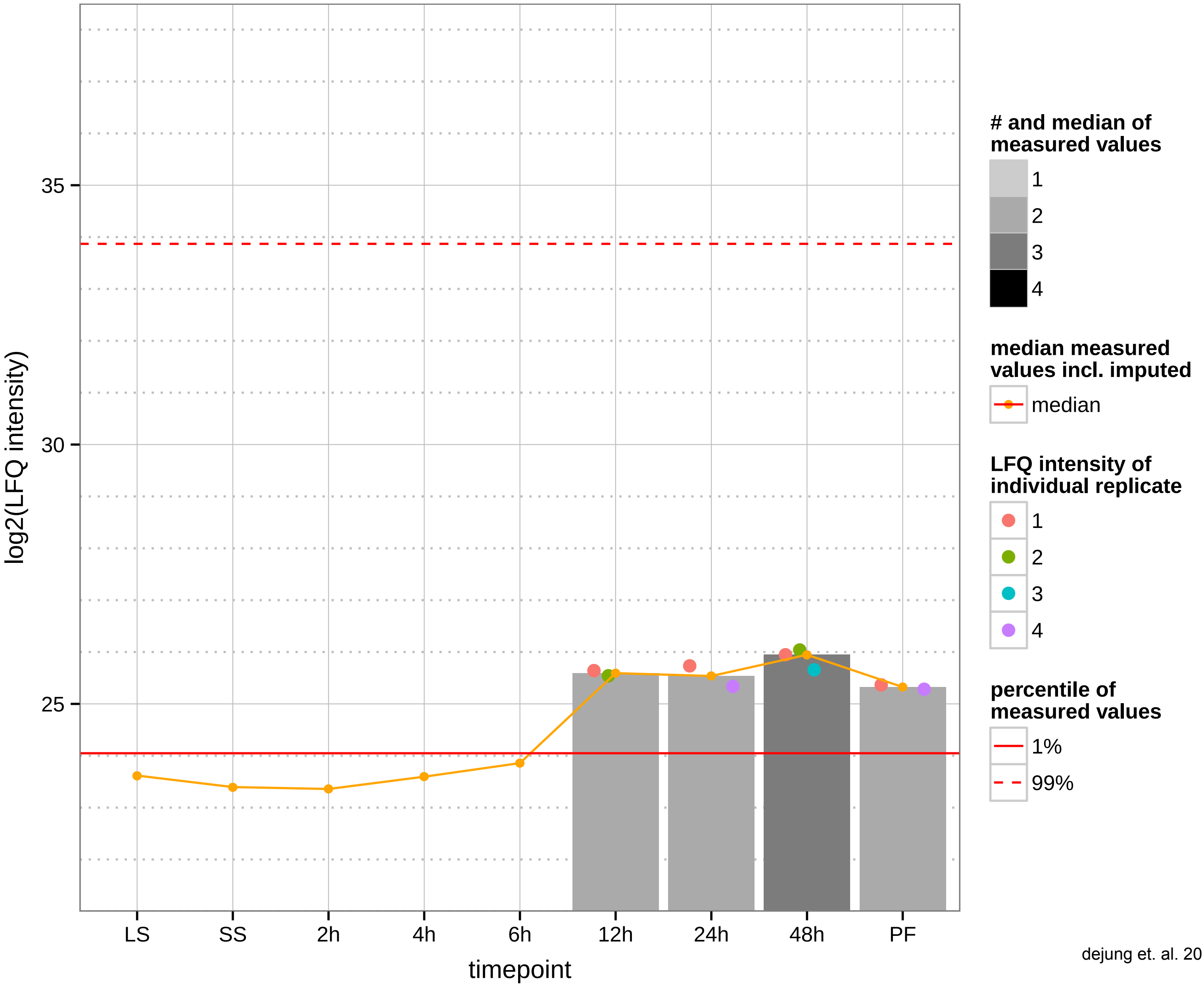

dejung et. al. 2015

hypothetical protein, conserved
Tb927.9.9310
AGOF: null
AGOC: mitochondrion
AGOP: null
PGOF: null
PGOC: null
PGOP: null

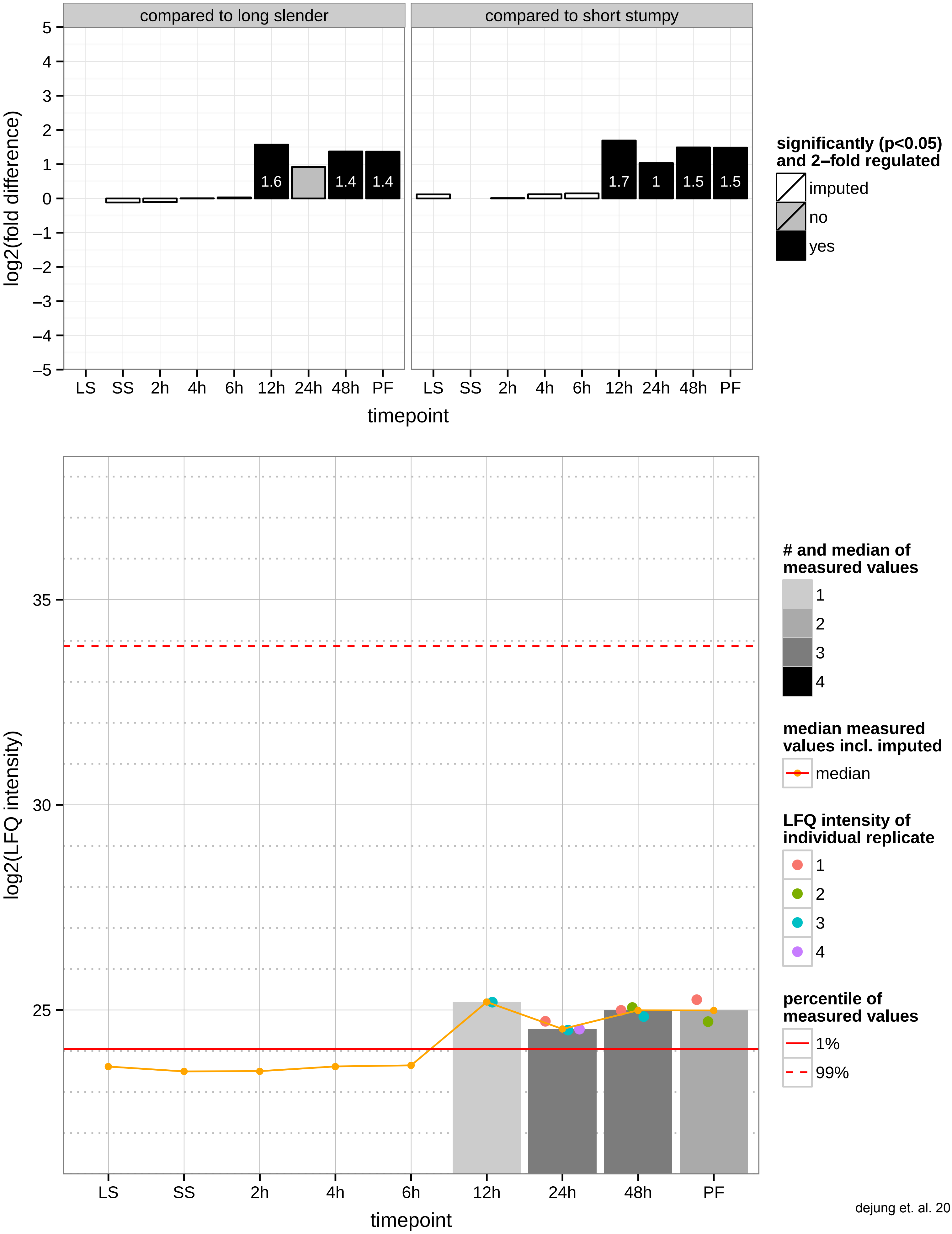

dejung et. al. 2015

zinc finger protein family member, putative (ZC3H29)
Tb927.9.9520
AGOF: RNA binding, zinc ion binding
AGOC: null
AGOP: null
PGOF: nucleic acid binding, zinc ion binding
PGOC: null
PGOP: null

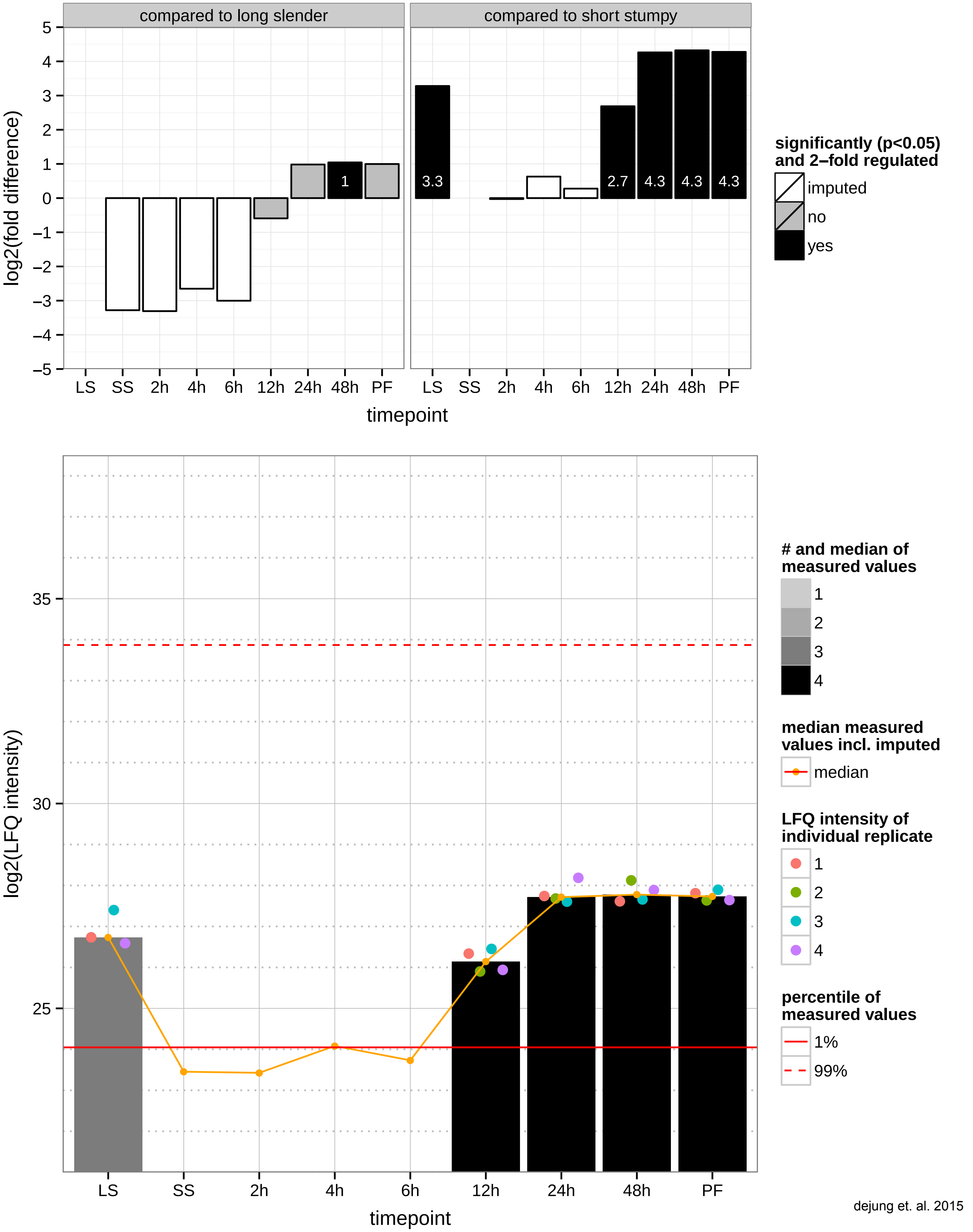

dejung et. al. 2015

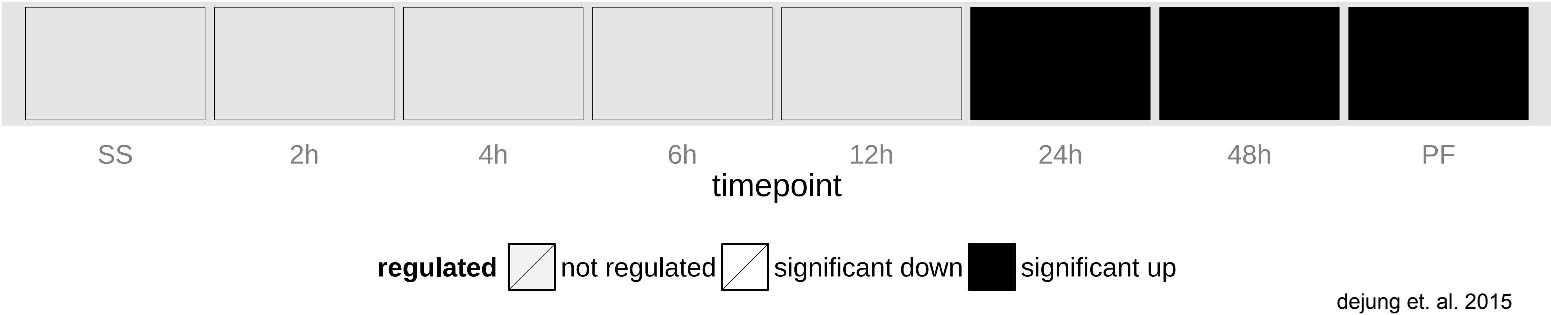
SS
2h
4h
6h
12h
24h
48h
PF
timepoint
regulated
not regulated
significant down
significant up
dejung et. al. 2015

nucleolar RNA helicase II, putative, nucleolar RNA helicase Gu, BAC from homologous region on chr5
Tb927.5.4420;Tb05.5K5.70
AGOF: ATP binding, ATP−dependent helicase activity, RNA binding, helicase activity
AGOC: nucleus, nucleolus, nucleus
AGOP: null, nucleobase−containing compound metabolic process
PGOF: ATP binding, ATP−dependent helicase activity, RNA binding, helicase activity, nucleic acid binding
PGOC: nucleus
PGOP: null

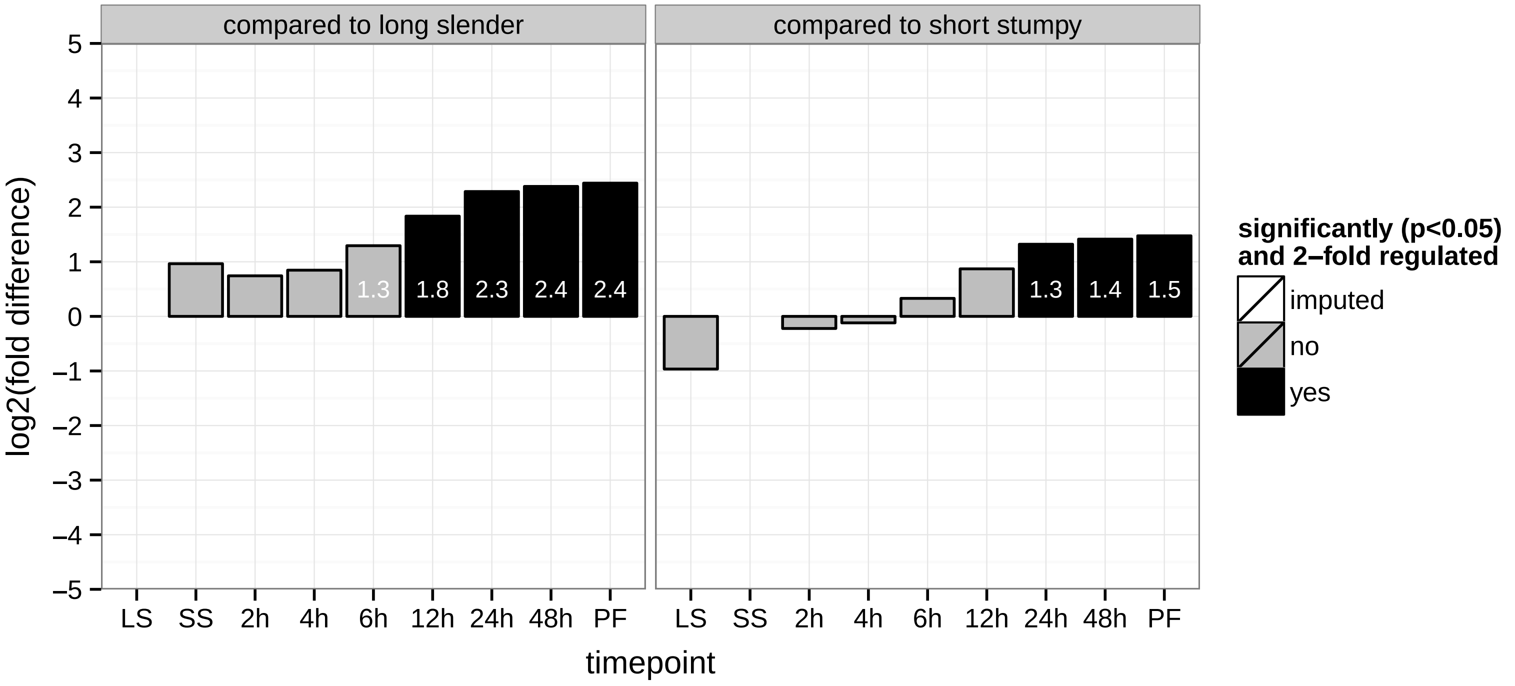

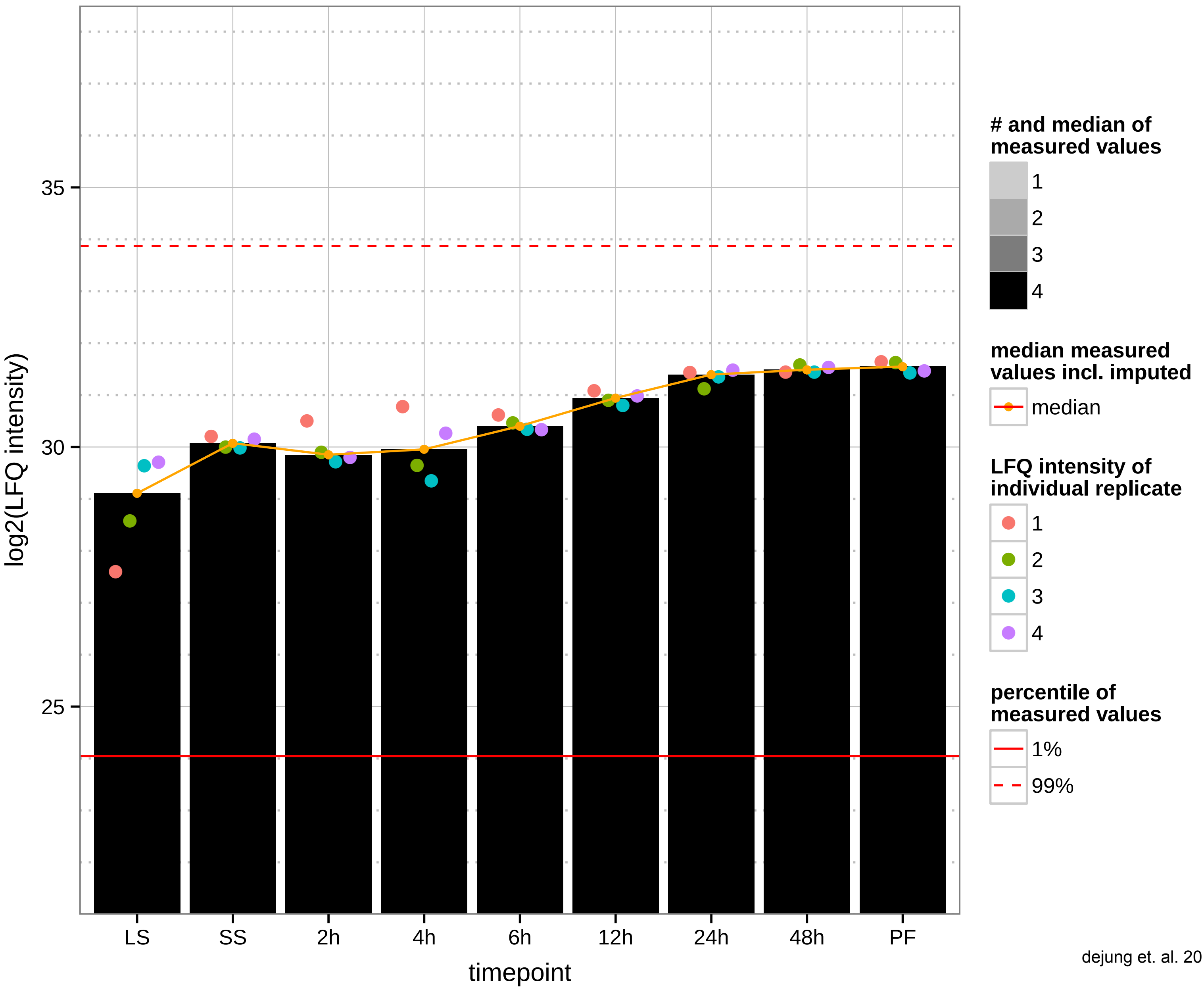

dejung et. al. 2015

hypothetical protein, conserved, chrXI additional, unordered contigs
Tb11.1420;Tb11.1370;Tb927.11.1850
AGOF: null
AGOC: null
AGOP: null
PGOF: null
PGOC: null
PGOP: null

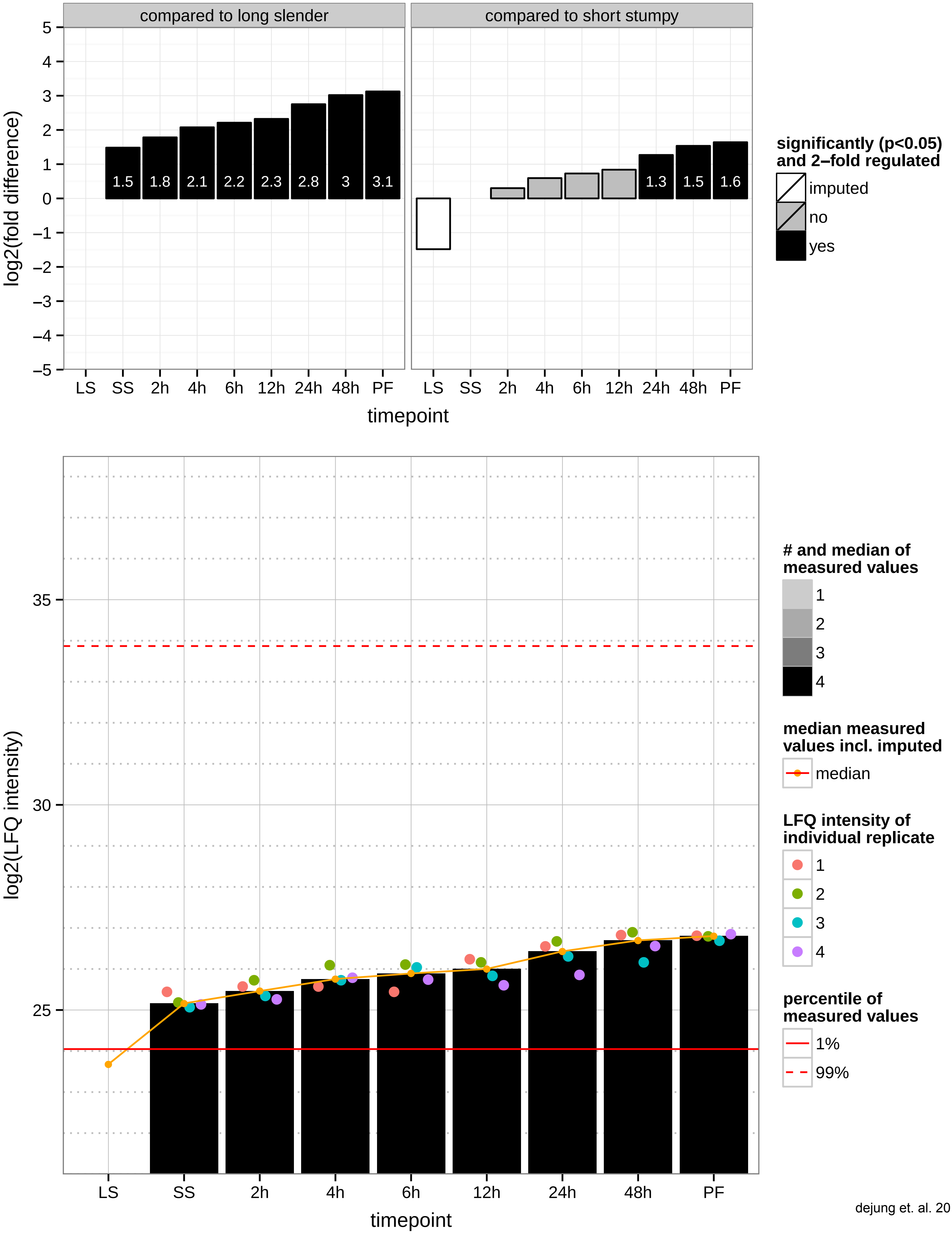

dejung et. al. 2015

2–oxoisovalerate dehydrogenase alpha subunit, putative
Tb927.10.660;Tb11.v5.0500
AGOF: null, 2–oxoisovalerate dehydrogenase (acylating) activity, oxidoreductase activity, acting on the aldehyde or oxo group
AGOC: null, mitochondrion
AGOP: null, metabolic process
PGOF: oxidoreductase activity, acting on the aldehyde or oxo group of donors, disulfide as acceptor
PGOC: null
PGOP: metabolic process

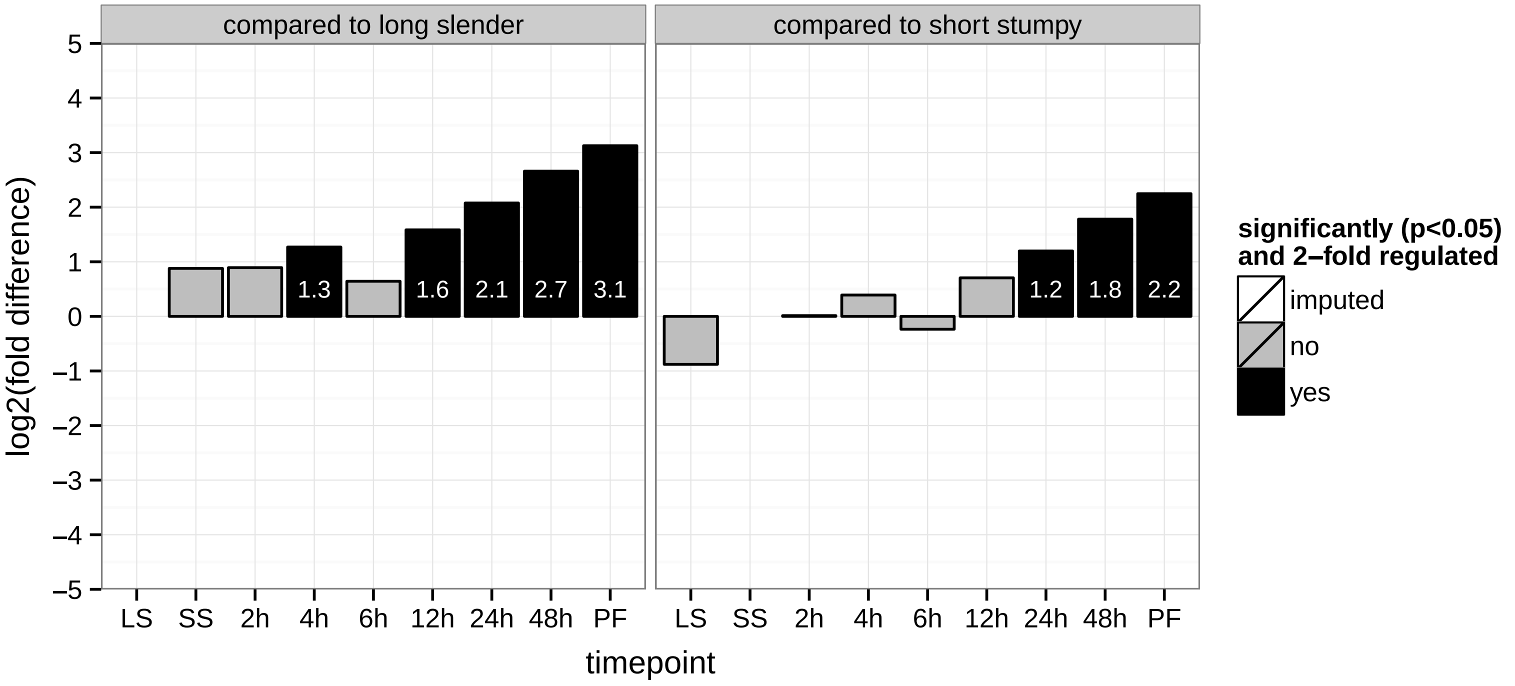

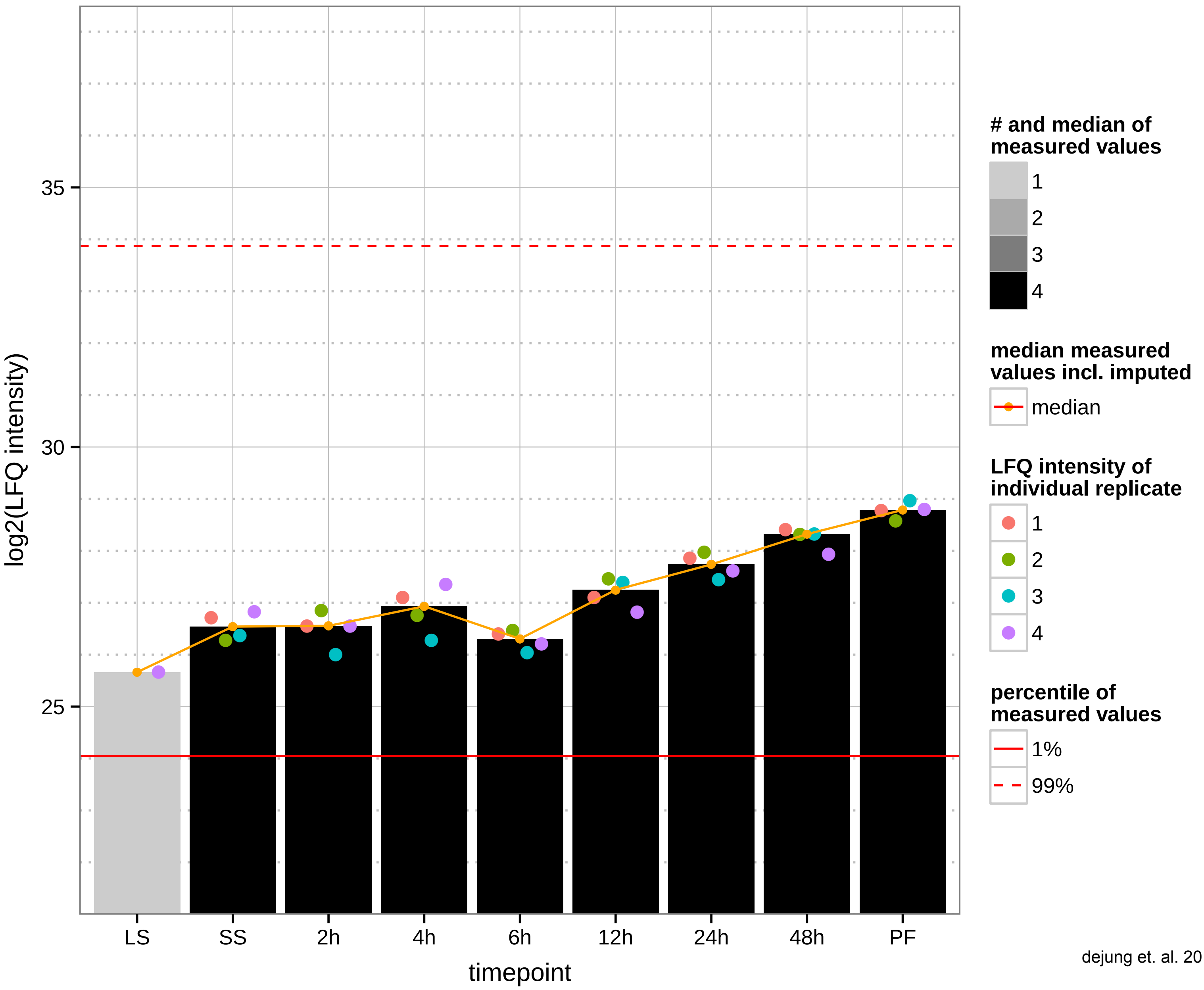

dejung et. al. 2015

calpain–like protein fragment, putative
Tb927.1.2260
AGOF: null
AGOC: null
AGOP: null
PGOF: null
PGOC: null
PGOP: null

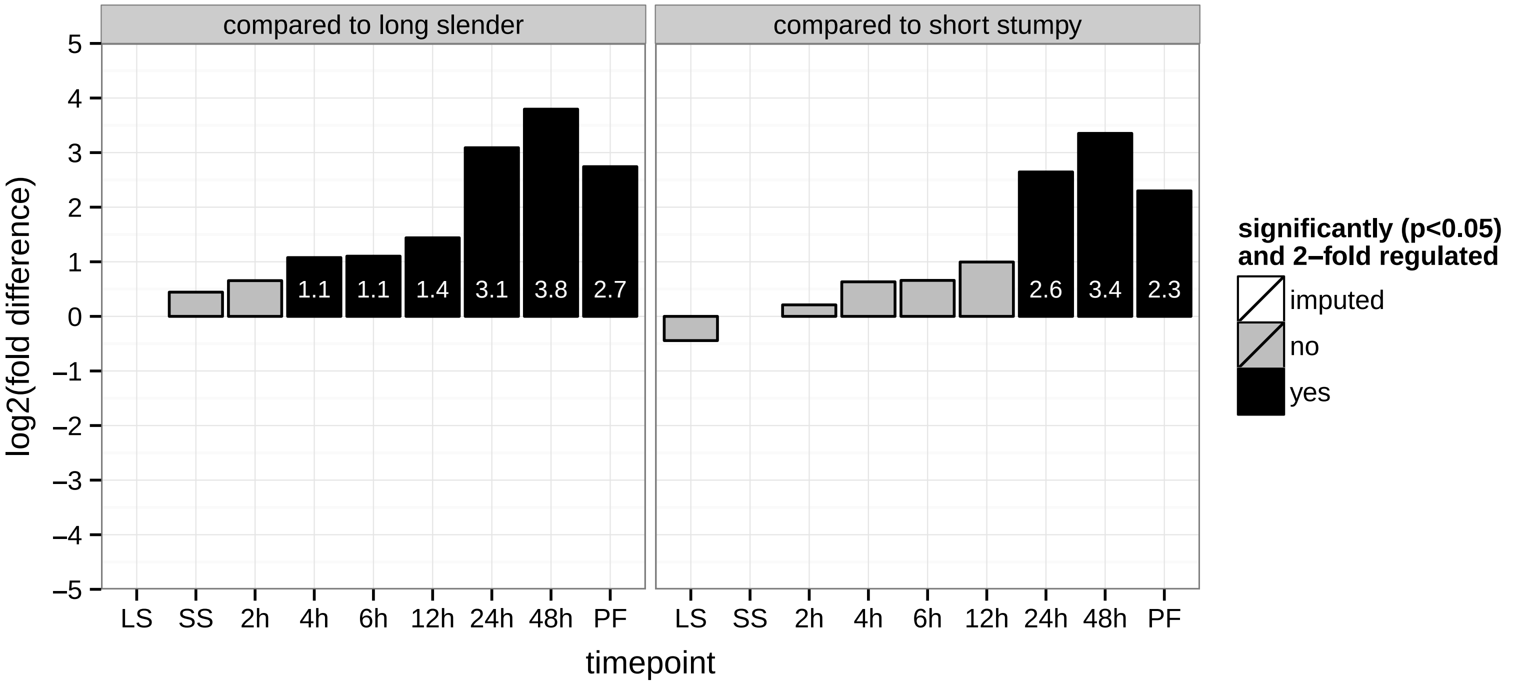

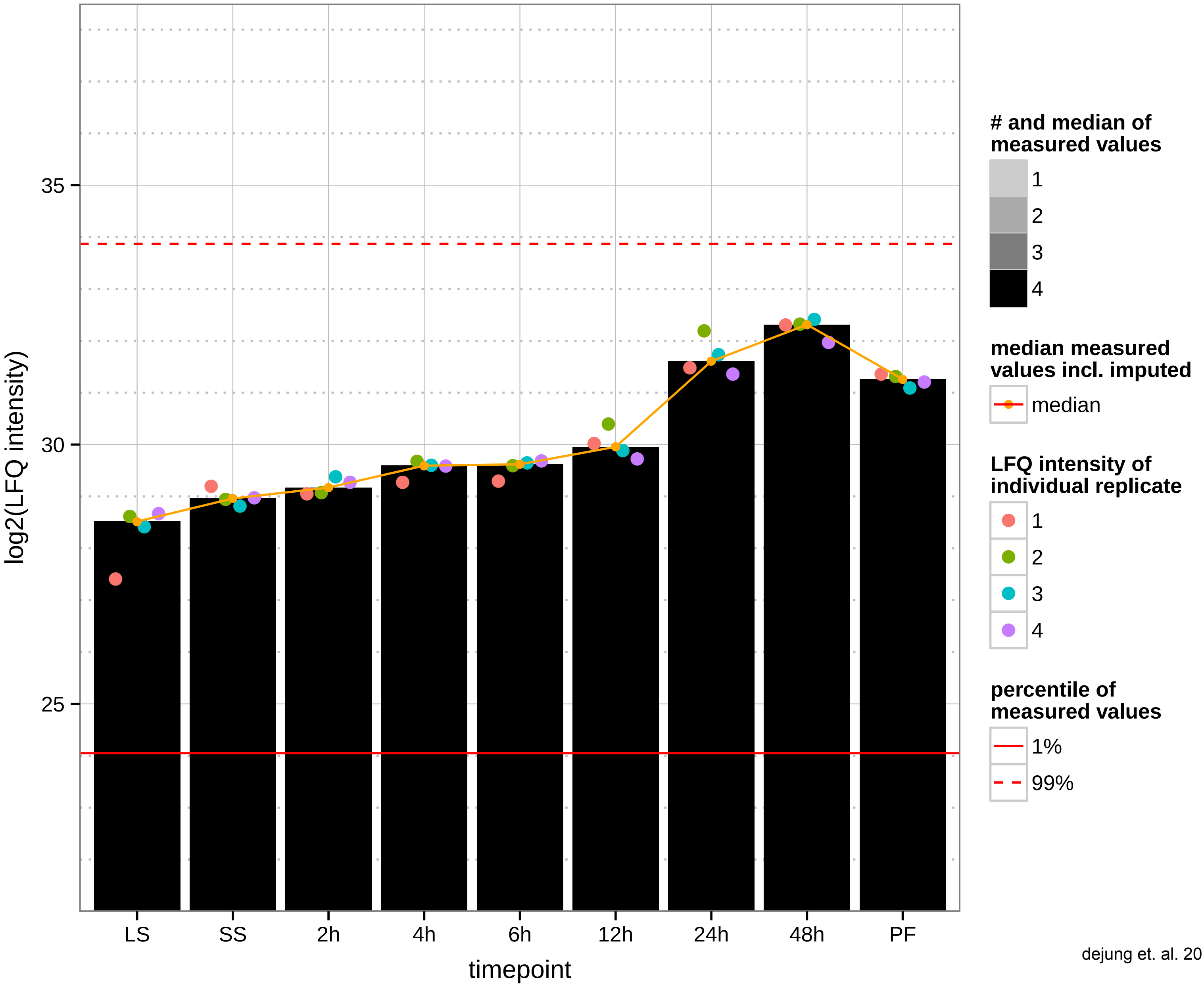

dejung et. al. 2015

pumilio/PUF RNA binding protein 9 (PUF9)
Tb927.1.2600
AGOF: mRNA binding
AGOC: null
AGOP: regulation of mRNA stability
PGOF: RNA binding, binding
PGOC: null
PGOP: null

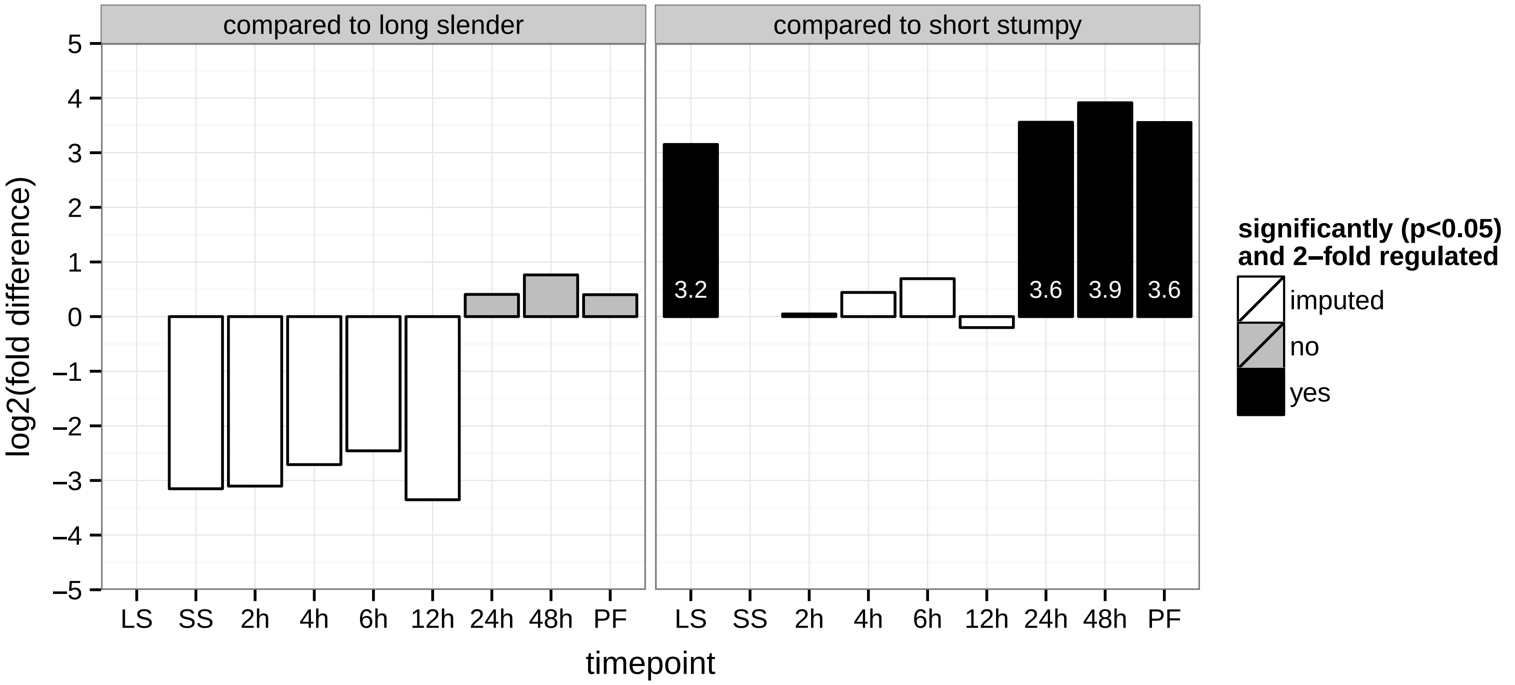

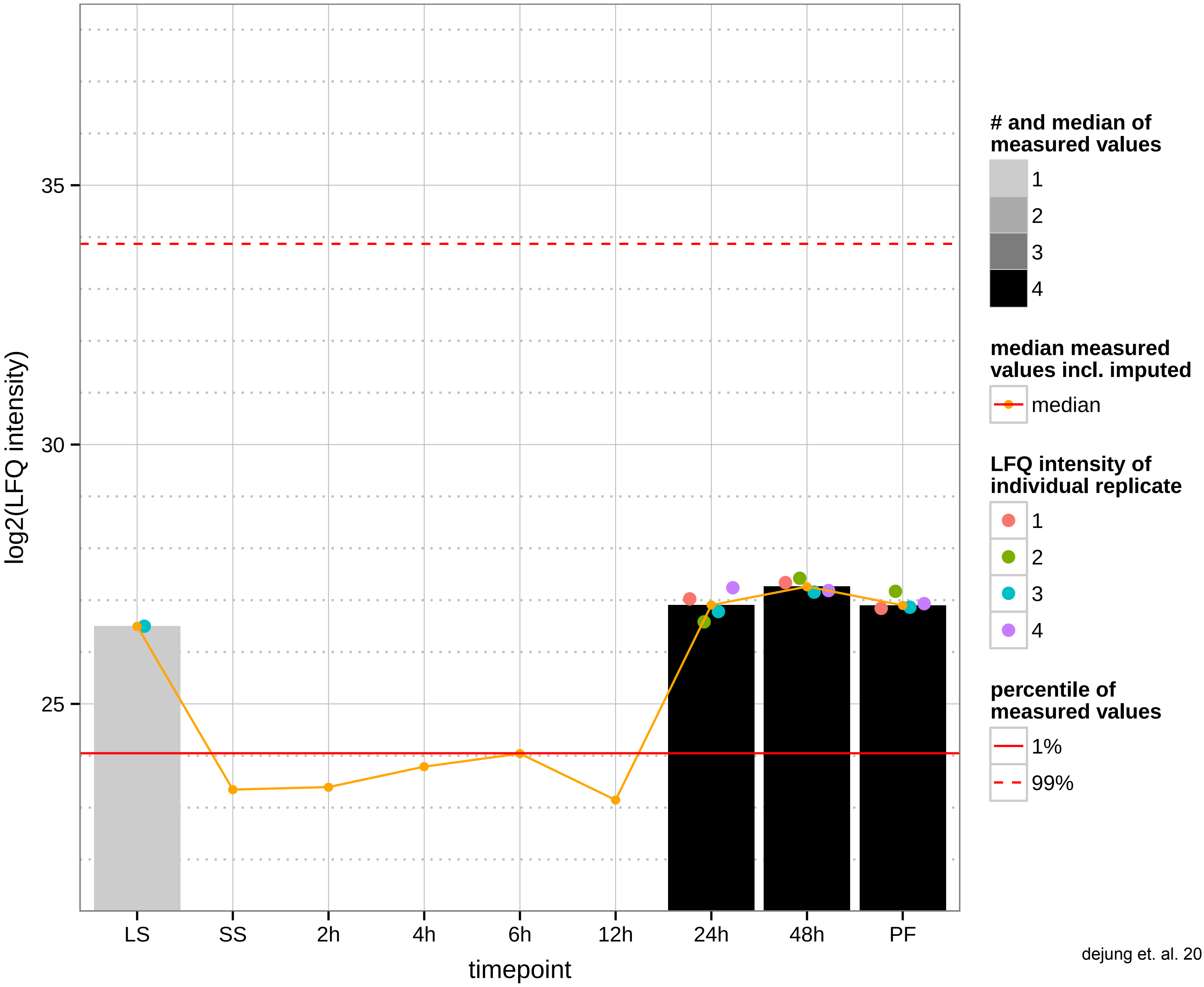

dejung et. al. 2015

monothiol glutaredoxin, putative
Tb927.10.10420
AGOF: electron carrier activity, protein disulfide isomerase activity, protein disulfide oxidoreductase activity
AGOC: mitochondrion
AGOP: cell redox homeostasis, oxidation–reduction process
PGOF: electron carrier activity, protein disulfide oxidoreductase activity
PGOC: null
PGOP: cell redox homeostasis

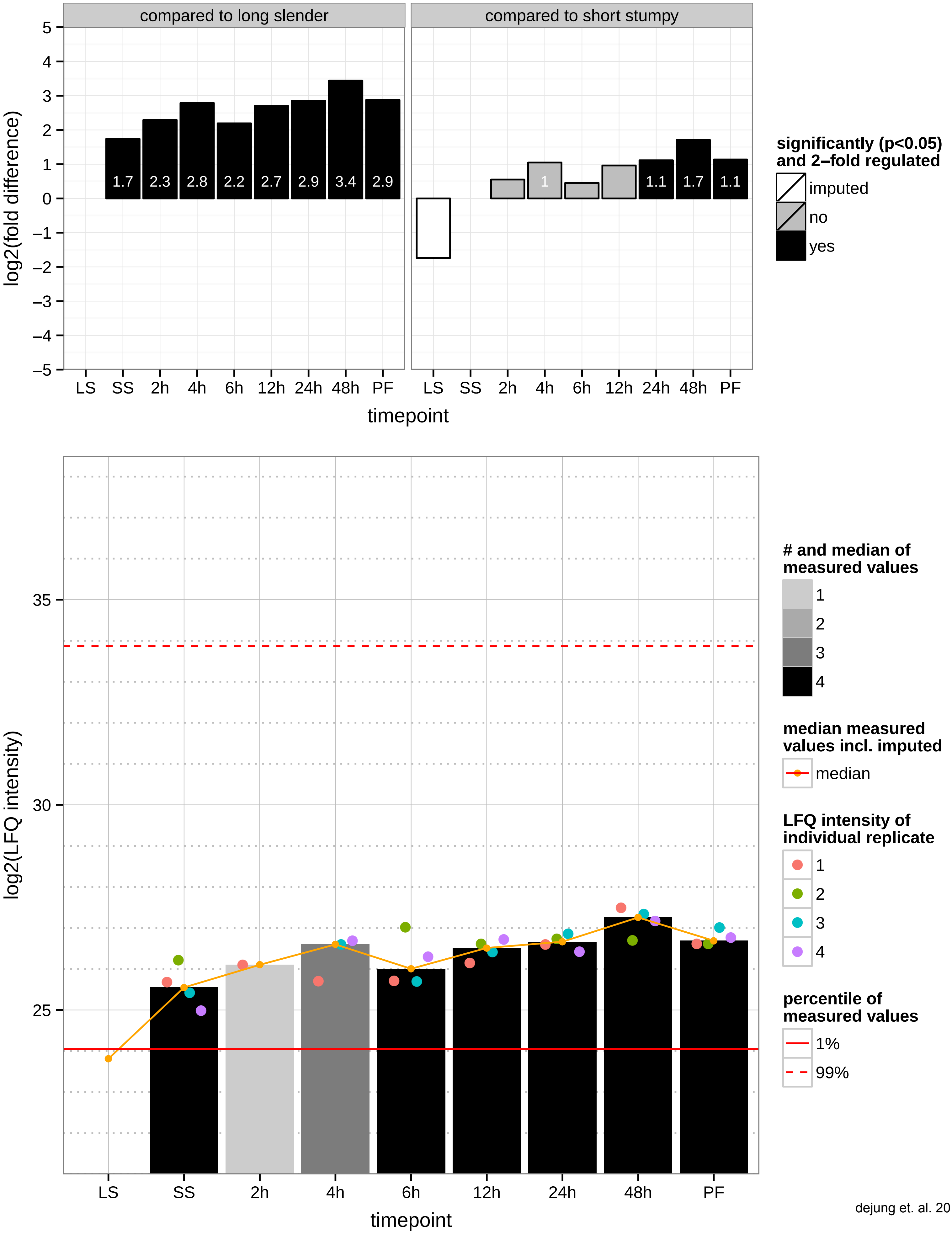

dejung et. al. 2015

RGG−containing protein 2, MRB1−associated protein, RGGm (RGG2)
Tb927.10.10830
AGOF: mRNA binding
AGOC: external side of mitochondrial outer membrane, mitochondrion
AGOP: RNA processing
PGOF: nucleic acid binding
PGOC: null
PGOP: null

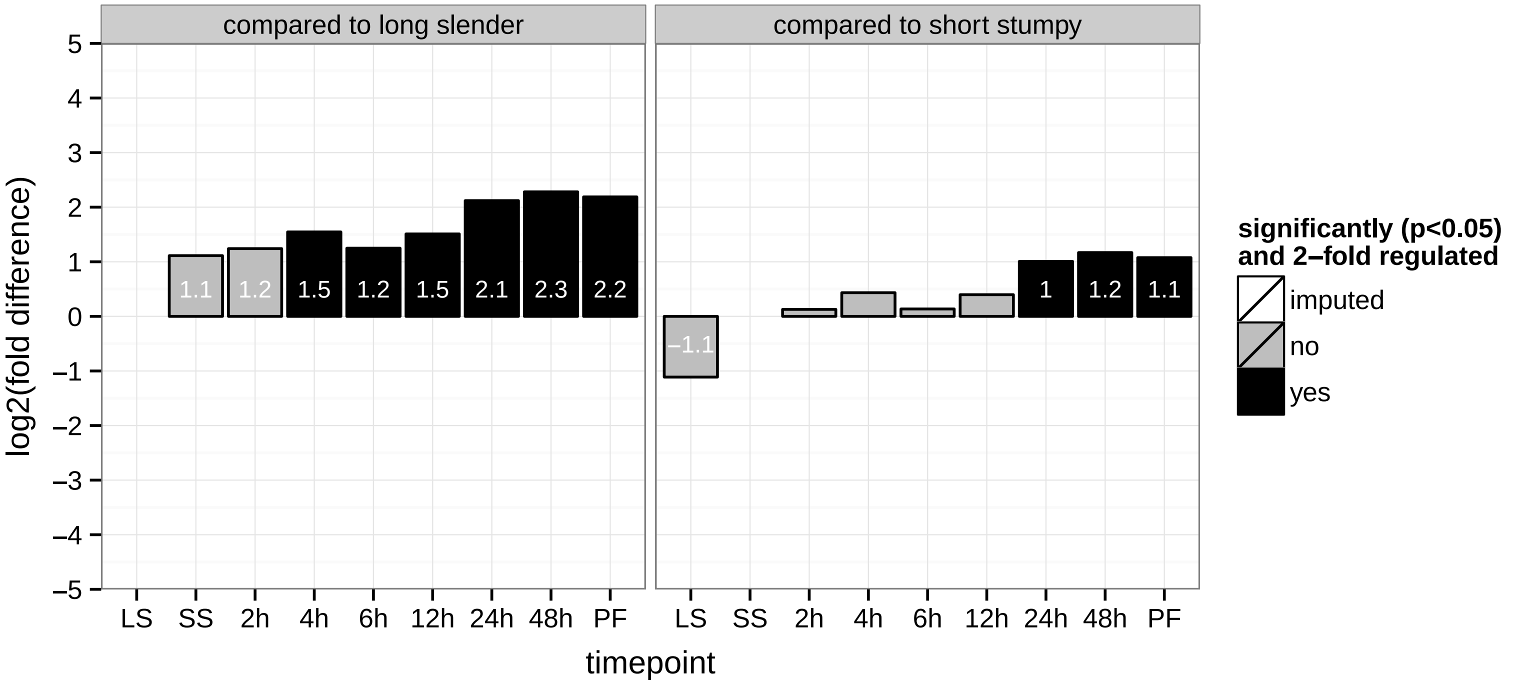

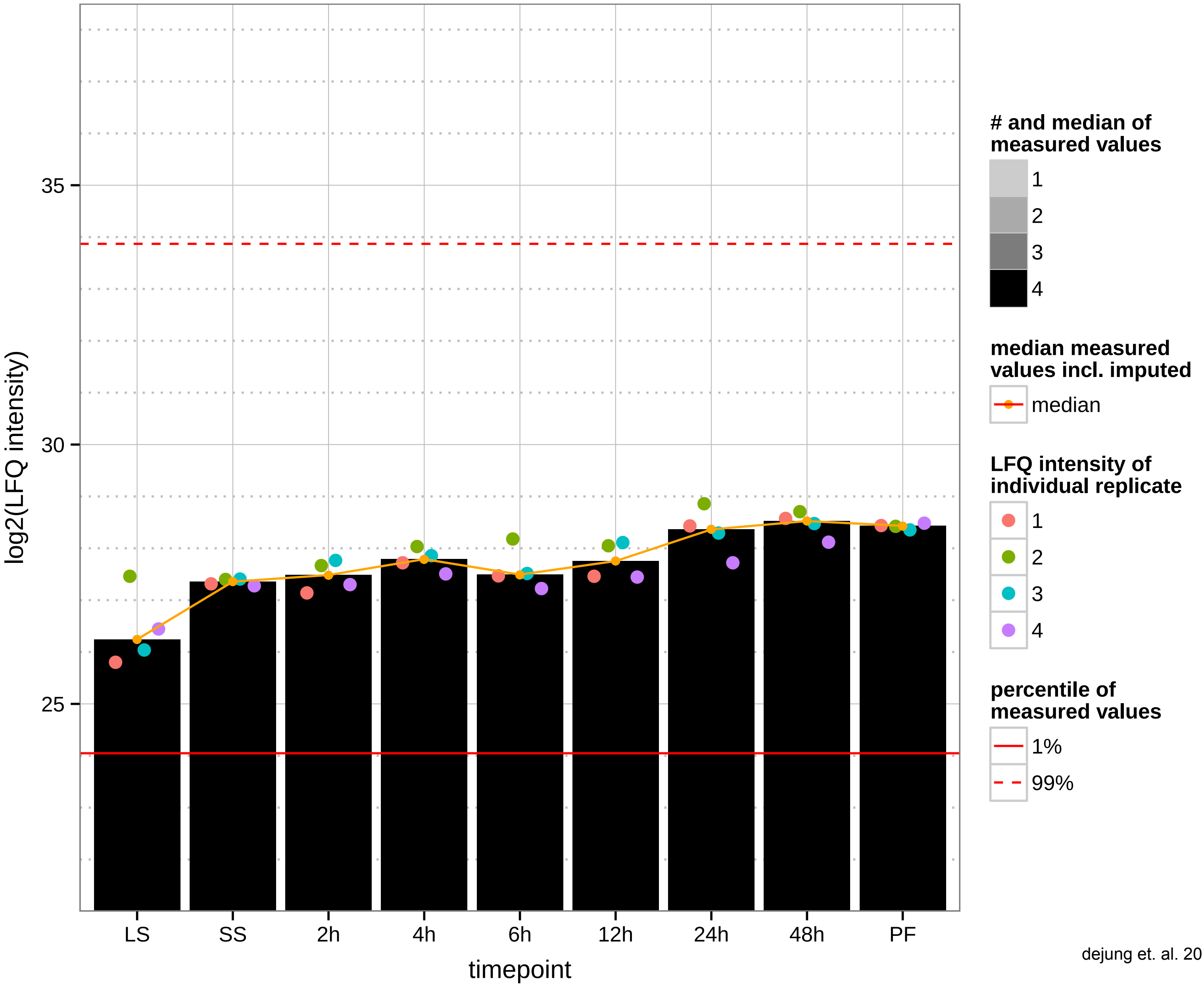

dejung et. al. 2015

hypothetical protein, conserved
Tb927.10.11030
AGOF: null
AGOC: mitochondrion
AGOP: null
PGOF: null
PGOC: null
PGOP: null

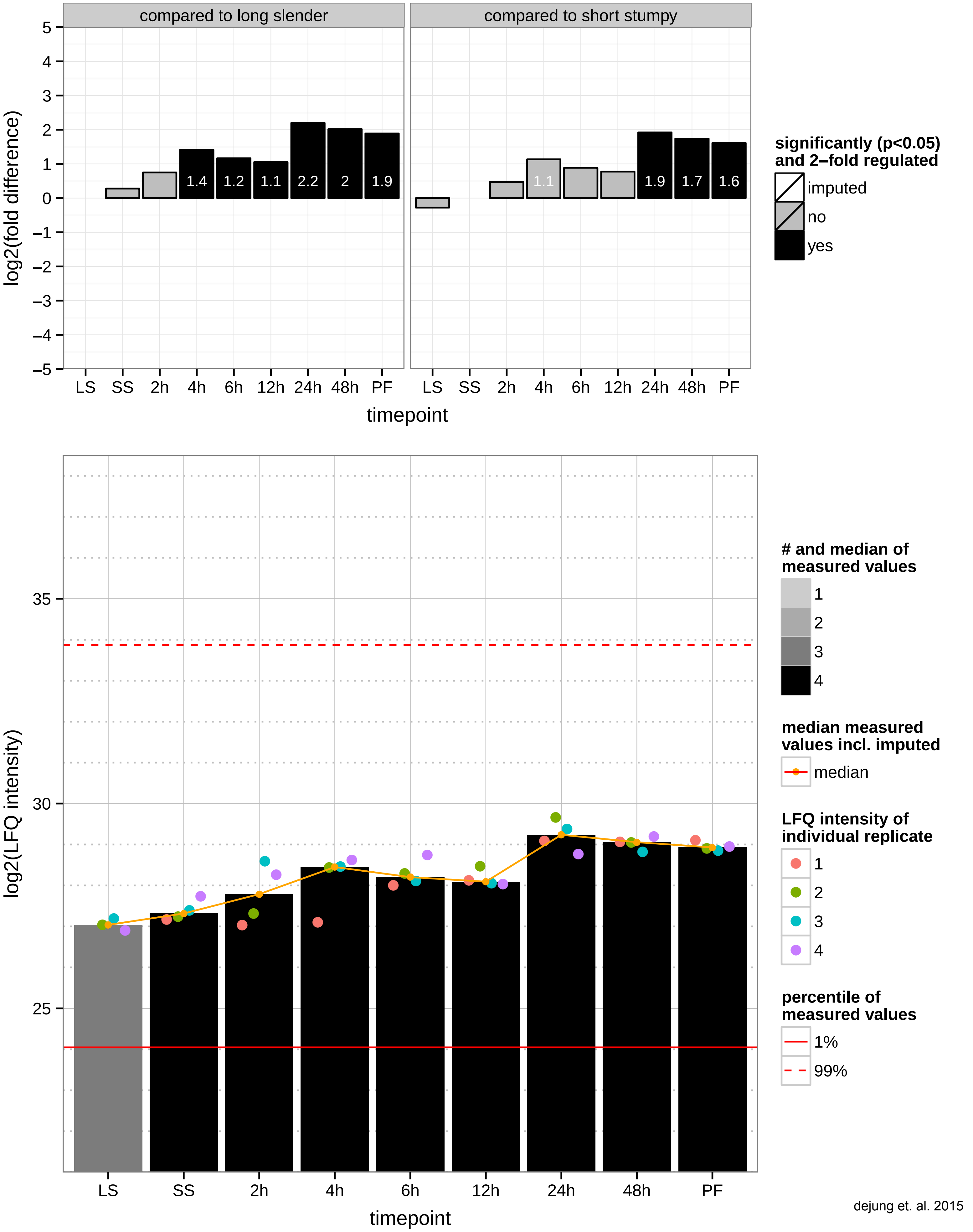

dejung et. al. 2015

pumilio/PUF RNA binding protein 6 (PUF6)
Tb927.10.11760
AGOF: RNA binding
AGOC: null
AGOP: mRNA metabolic process
PGOF: RNA binding, binding
PGOC: null
PGOP: null

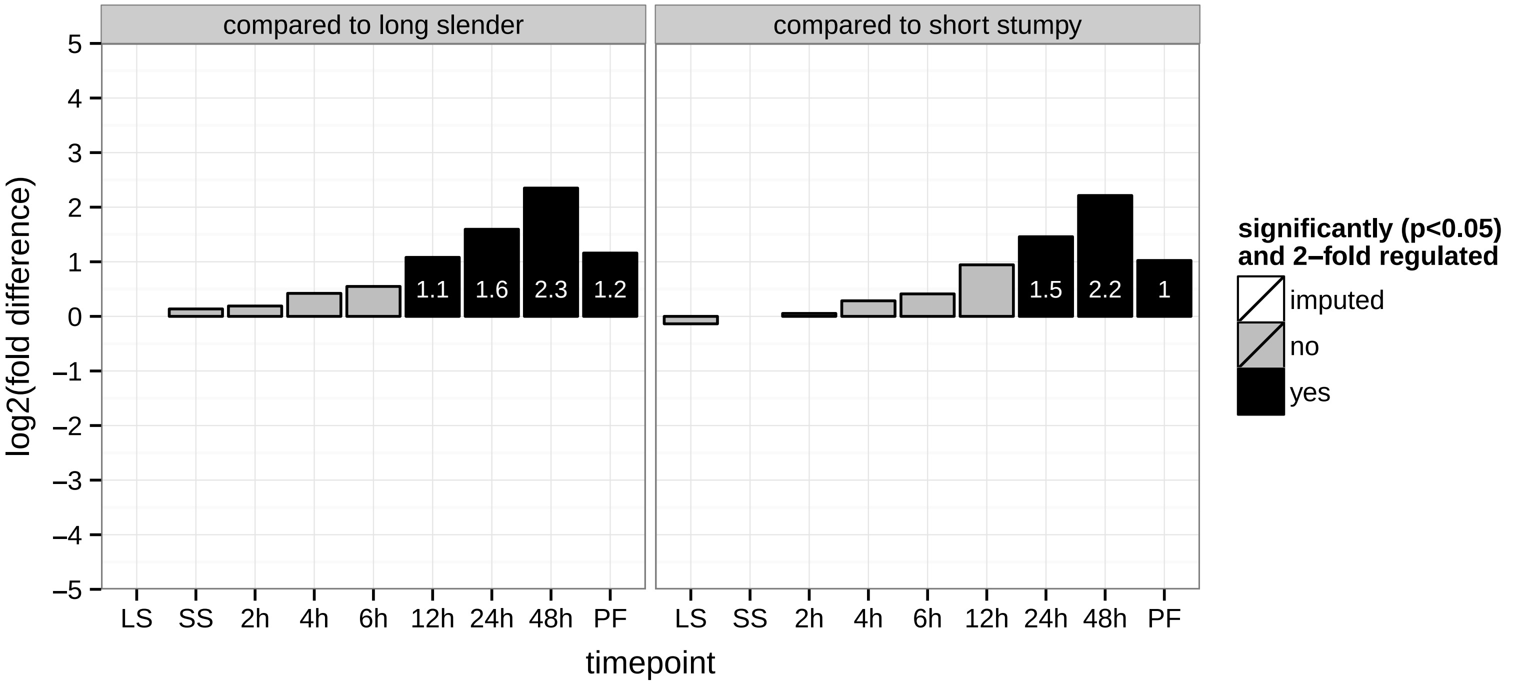

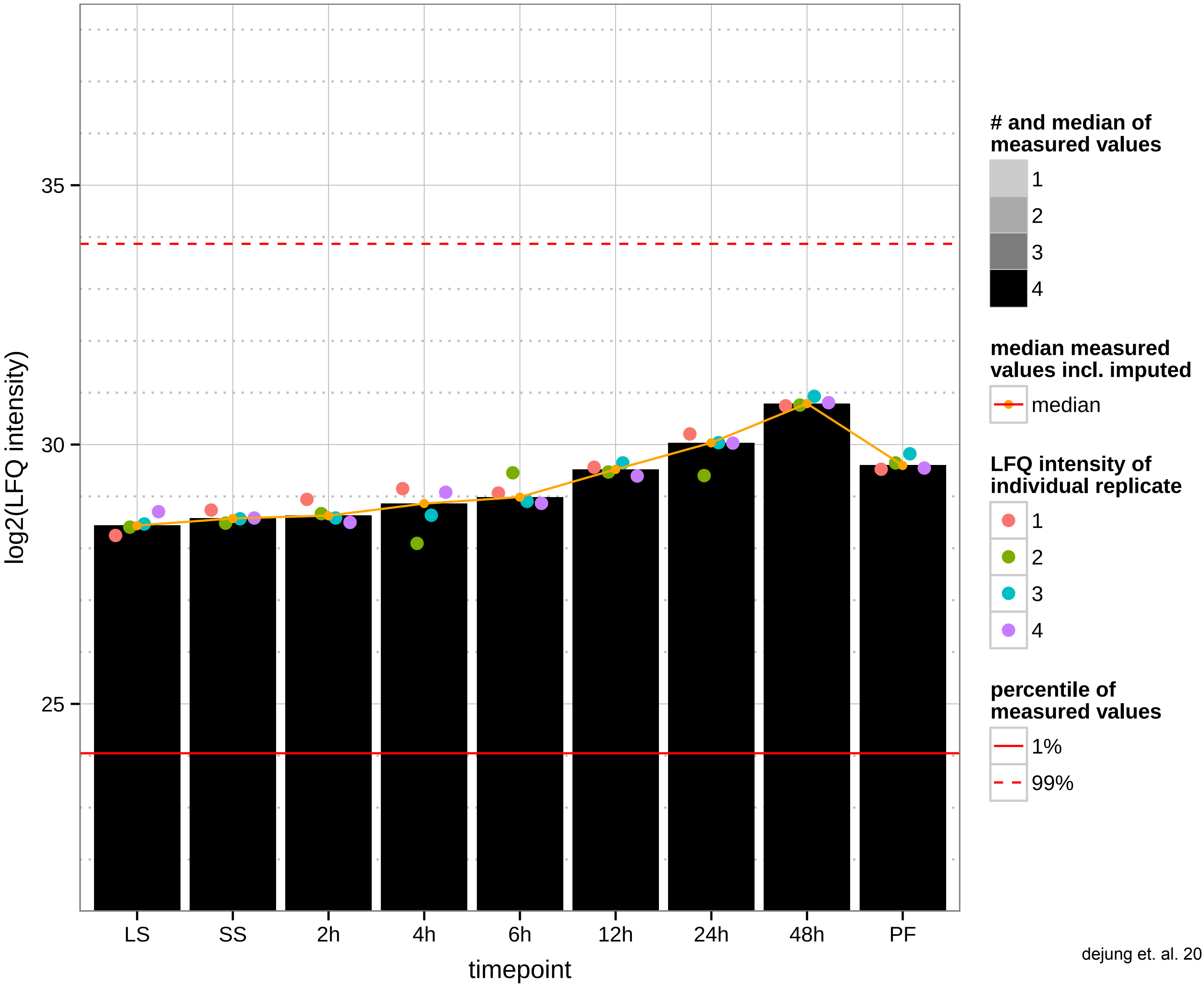

dejung et. al. 2015

Elongation factor Tu, mitochondrial (EF−Tu)
Tb927.10.13360
AGOF: GTP binding, GTPase activity, translation elongation factor activity
AGOC: intracellular, mitochondrion
AGOP: growth, mitochondrial translation, oxidative phosphorylation, translational elongation
PGOF: GTP binding, GTPase activity
PGOC: null
PGOP: null

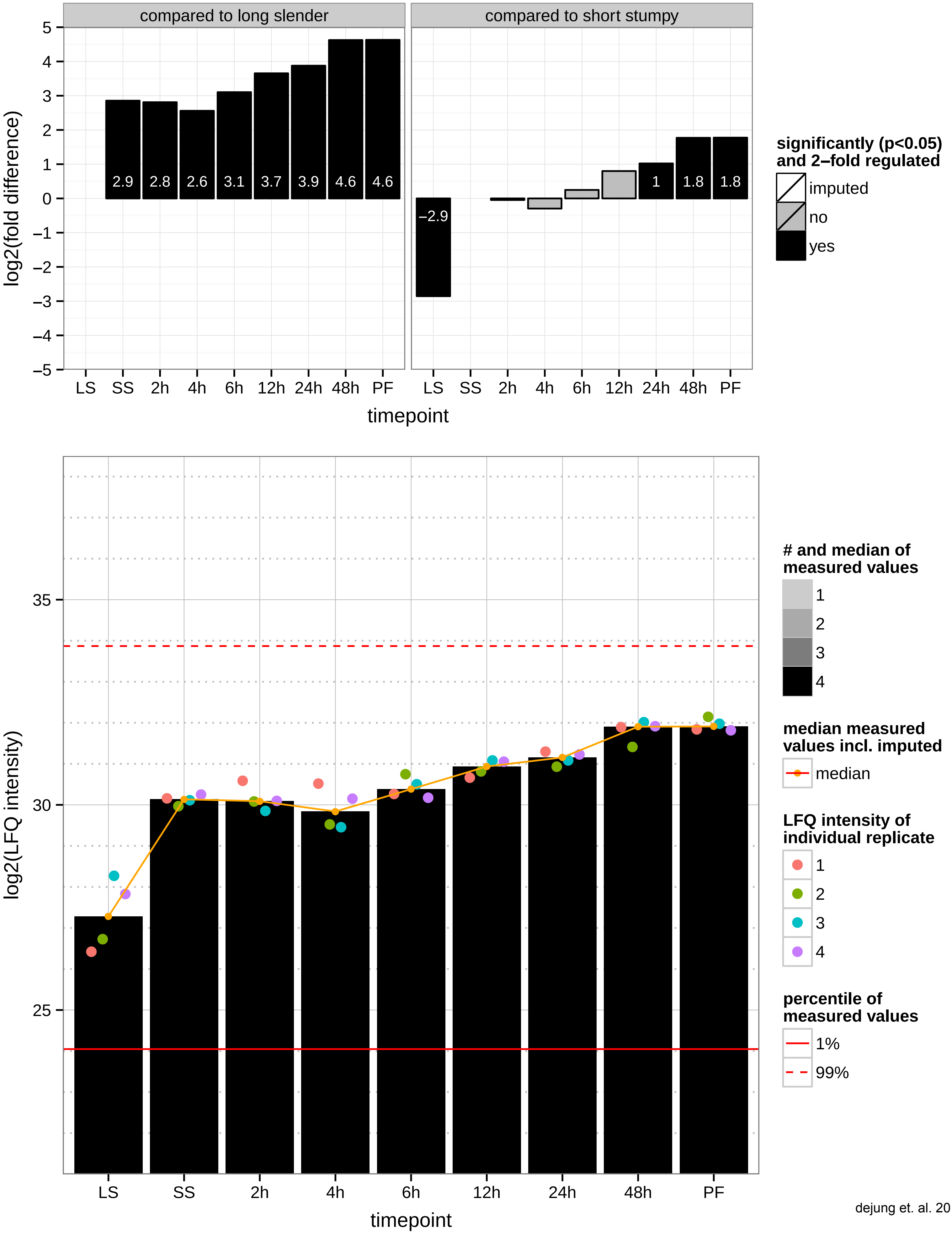

dejung et. al. 2015

hypoxanthine–guanine phosphoribosyltransferase, putative
Tb927.10.1390
AGOF: hypoxanthine phosphoribosyltransferase activity
AGOC: cytoplasm
AGOP: purine ribonucleoside salvage
PGOF: null
PGOC: null
PGOP: nucleoside metabolic process

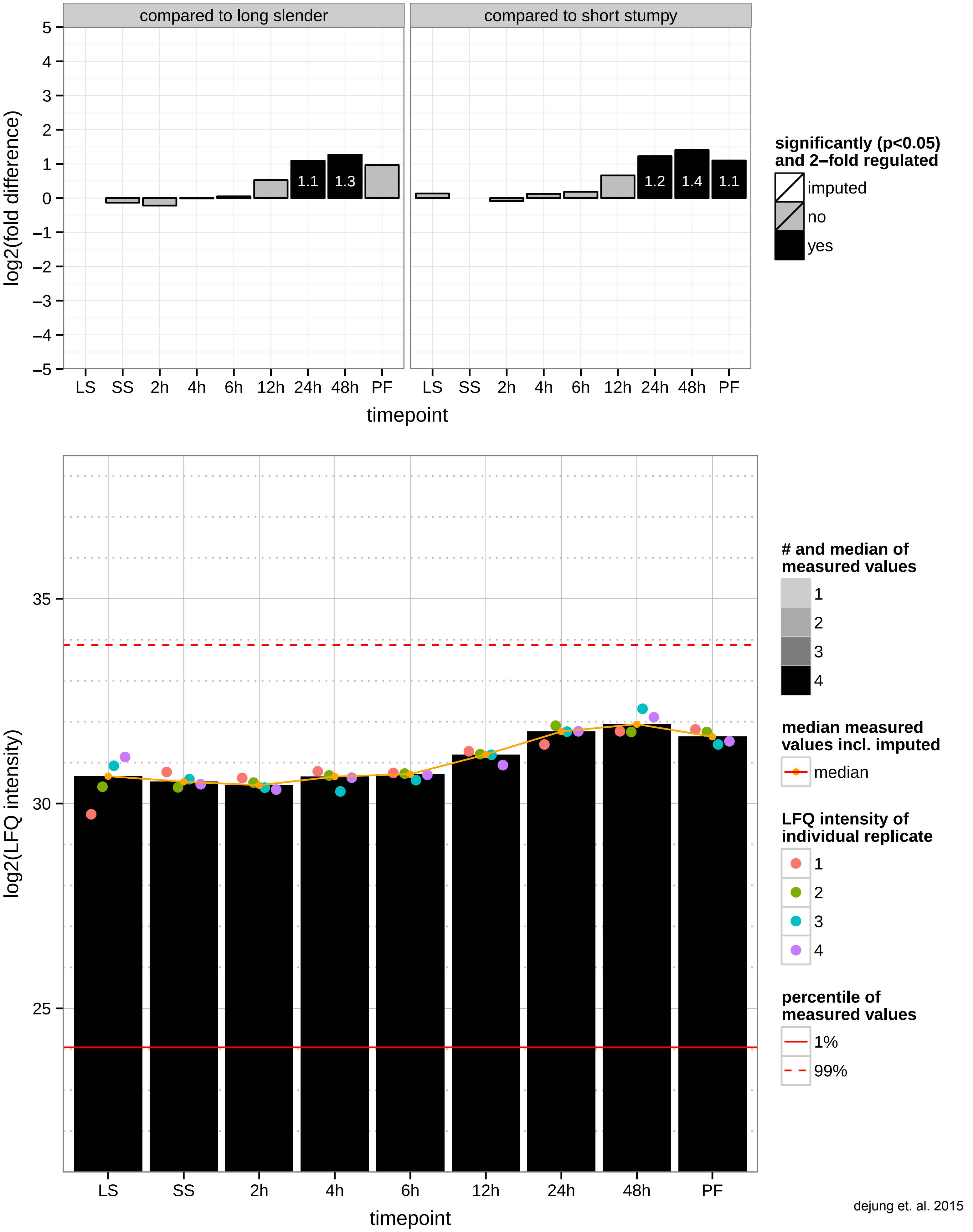

dejung et. al. 2015

hypothetical protein, conserved
Tb927.10.14860
AGOF: null
AGOC: mitochondrion
AGOP: null
PGOF: null
PGOC: null
PGOP: null

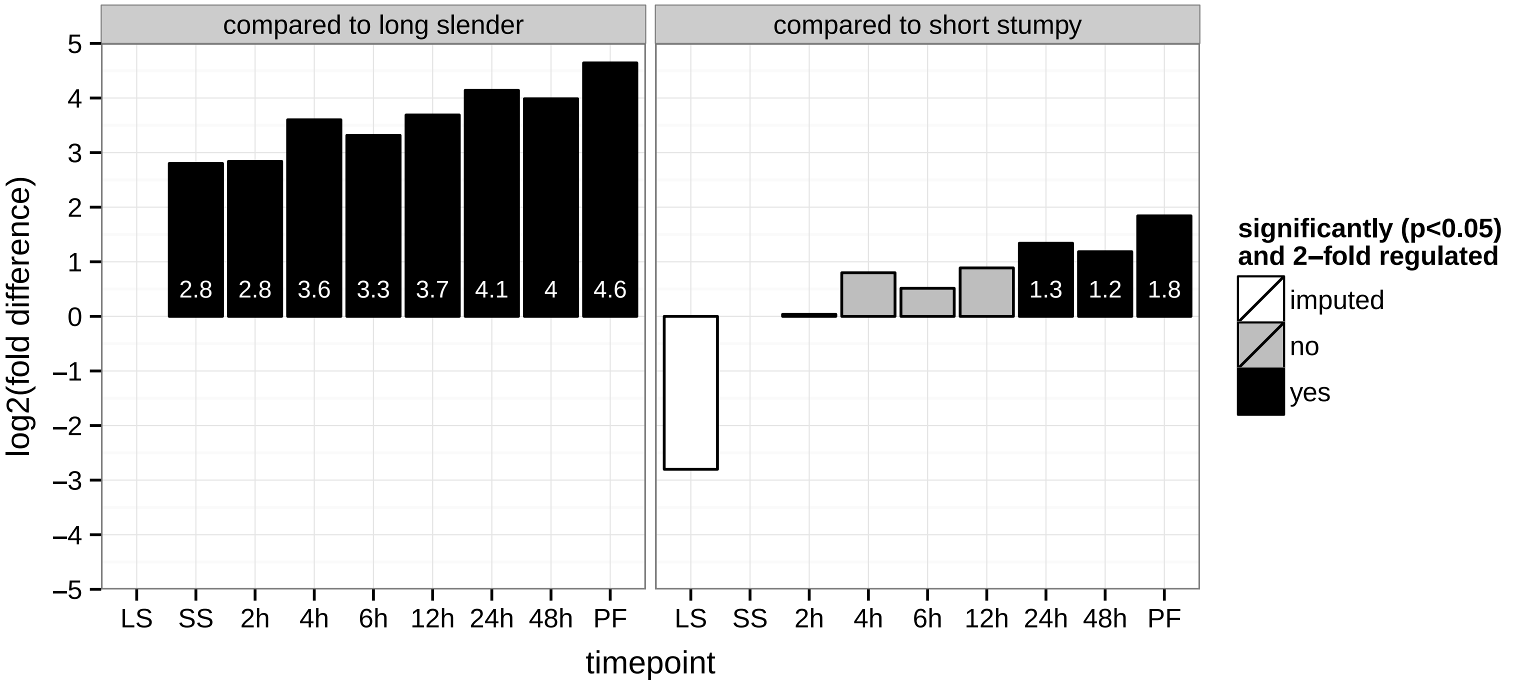

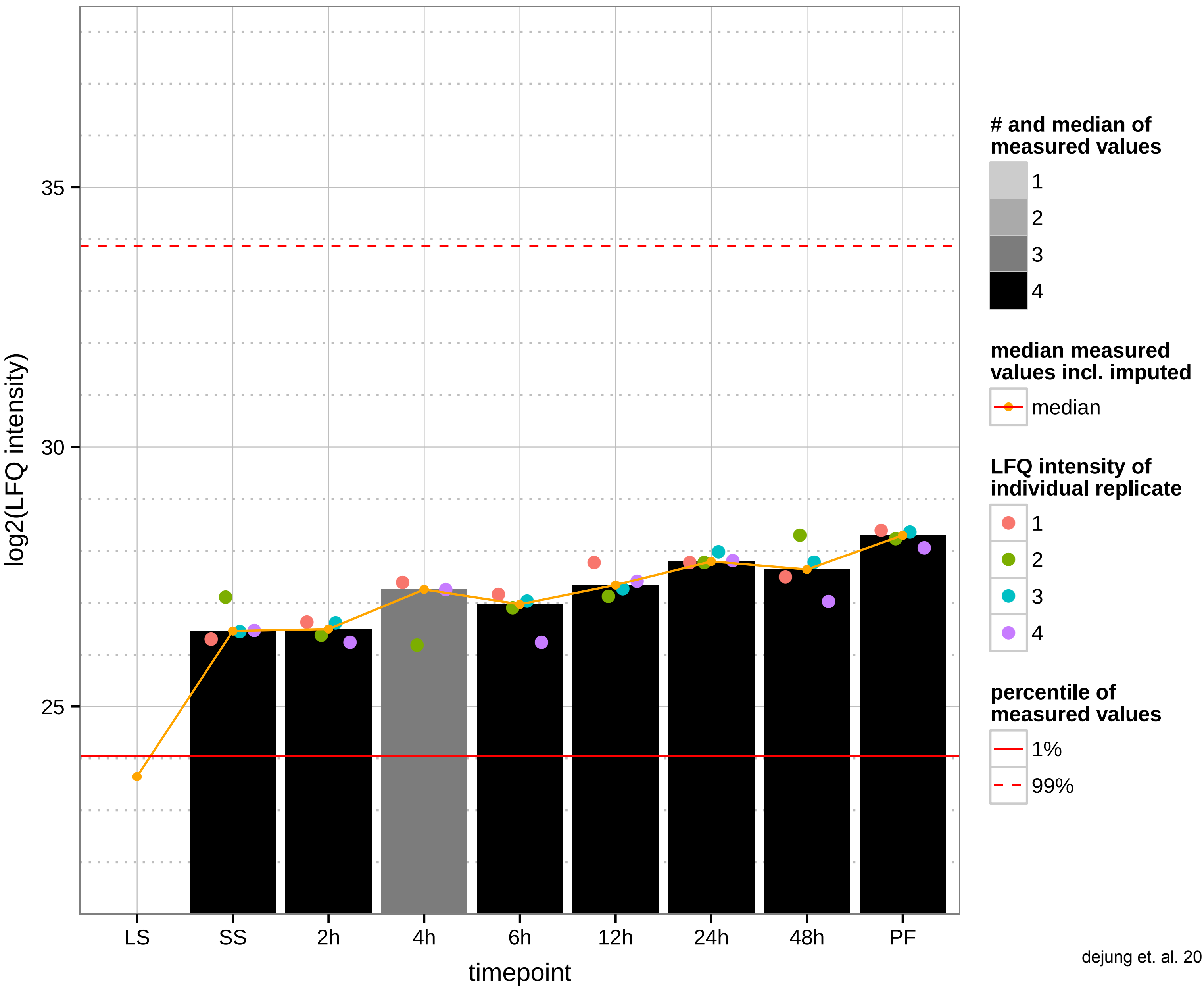

dejung et. al. 2015

methionyl–tRNA synthetase, putative (MetRS)
Tb927.10.1500
AGOF: ATP binding, methionine–tRNA ligase activity
AGOC: cytoplasm
AGOP: methionyl–tRNA aminoacylation, translation
PGOF: ATP binding, aminoacyl–tRNA ligase activity, methionine–tRNA ligase activity, nucleotide binding
PGOC: cytoplasm
PGOP: methionyl–tRNA aminoacylation, tRNA aminoacylation for protein translation

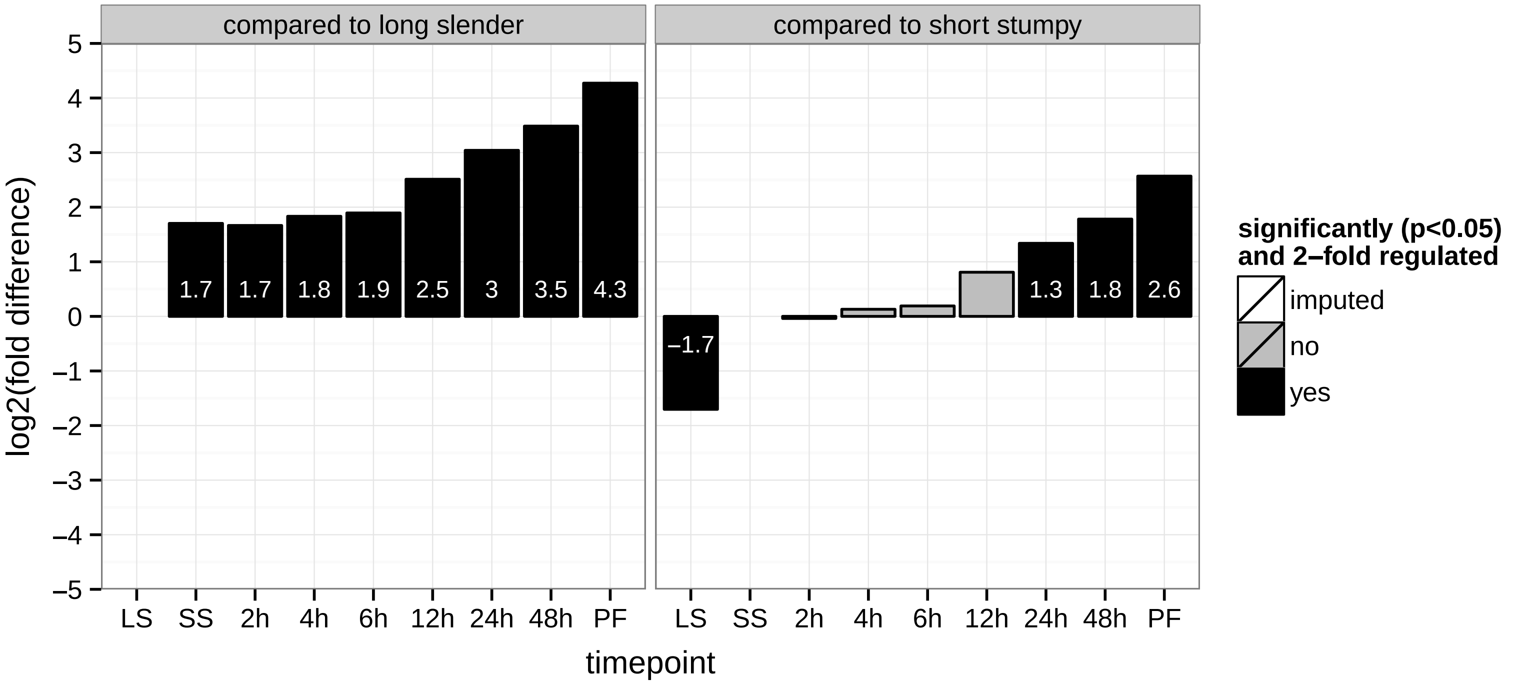

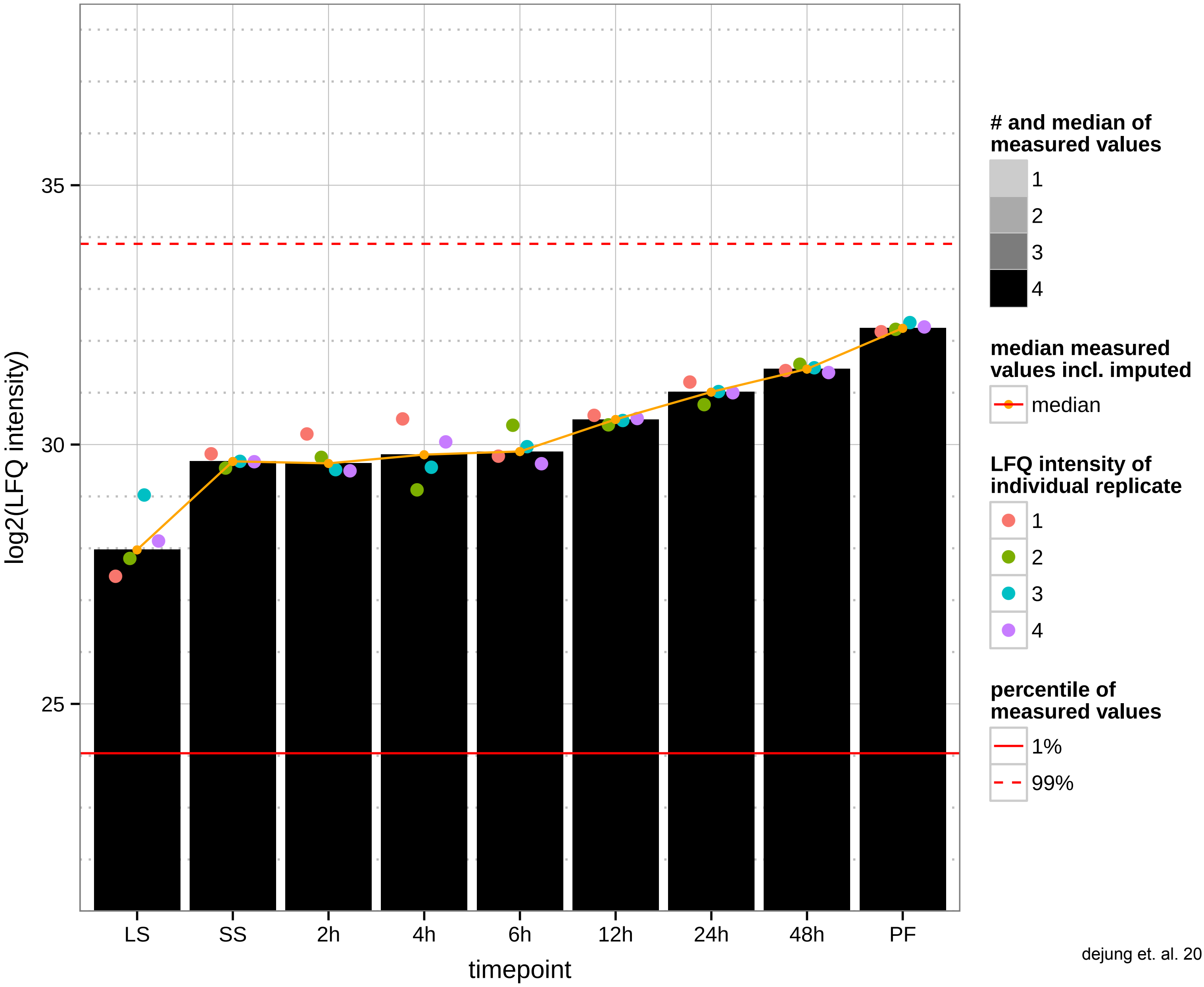

dejung et. al. 2015

hypothetical protein, conserved
Tb927.10.15170
AGOF: null
AGOC: null
AGOP: null
PGOF: null
PGOC: null
PGOP: null

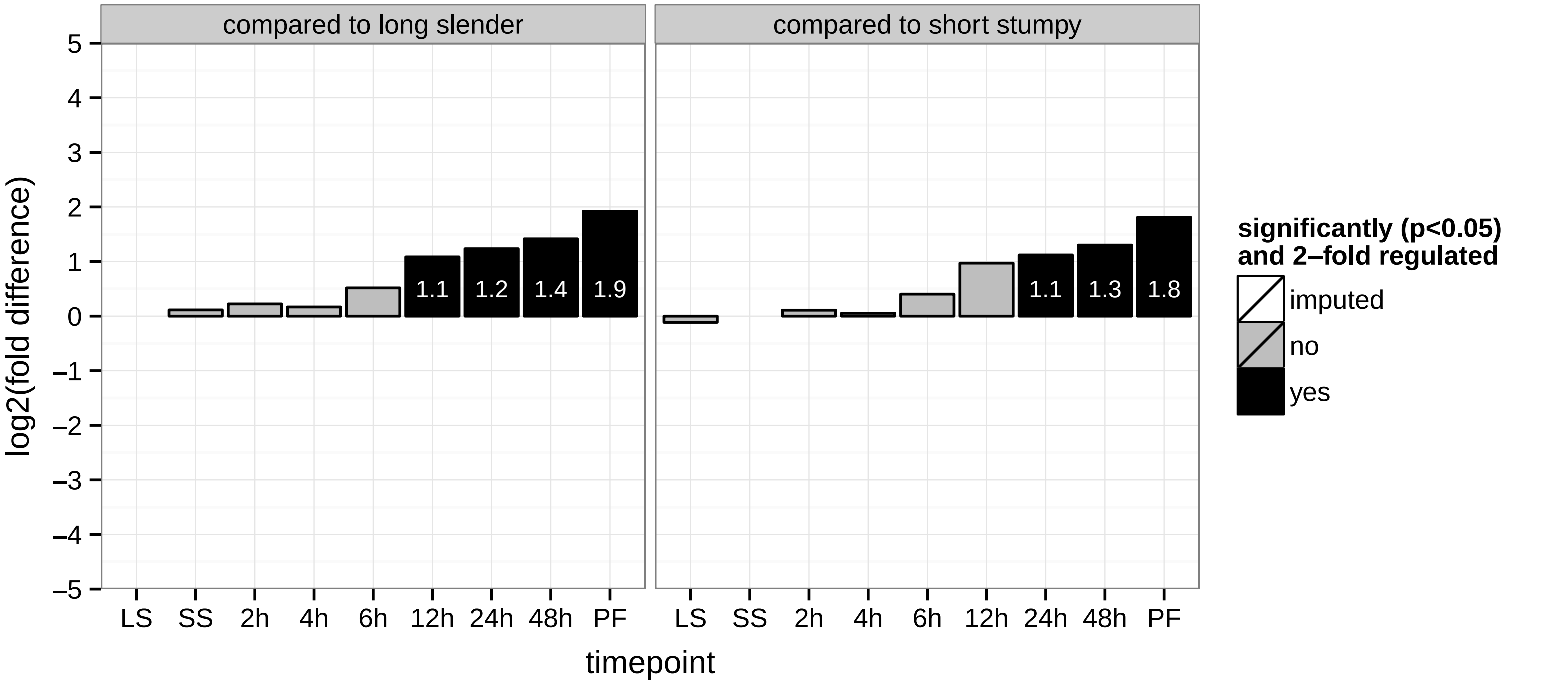

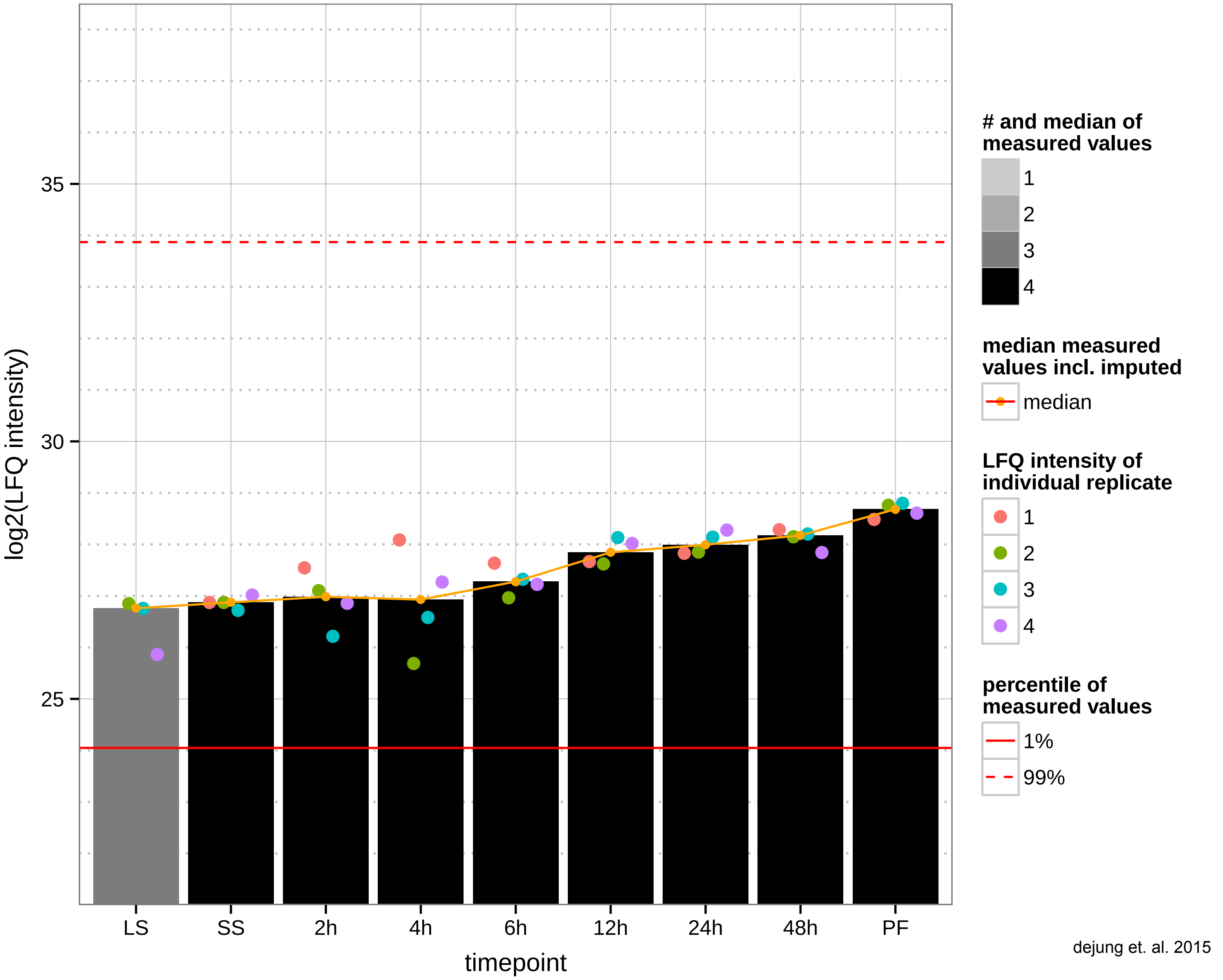

dejung et. al. 2015

hypothetical protein, conserved
Tb927.10.15790
AGOF: null
AGOC: mitochondrion
AGOP: null
PGOF: null
PGOC: null
PGOP: null

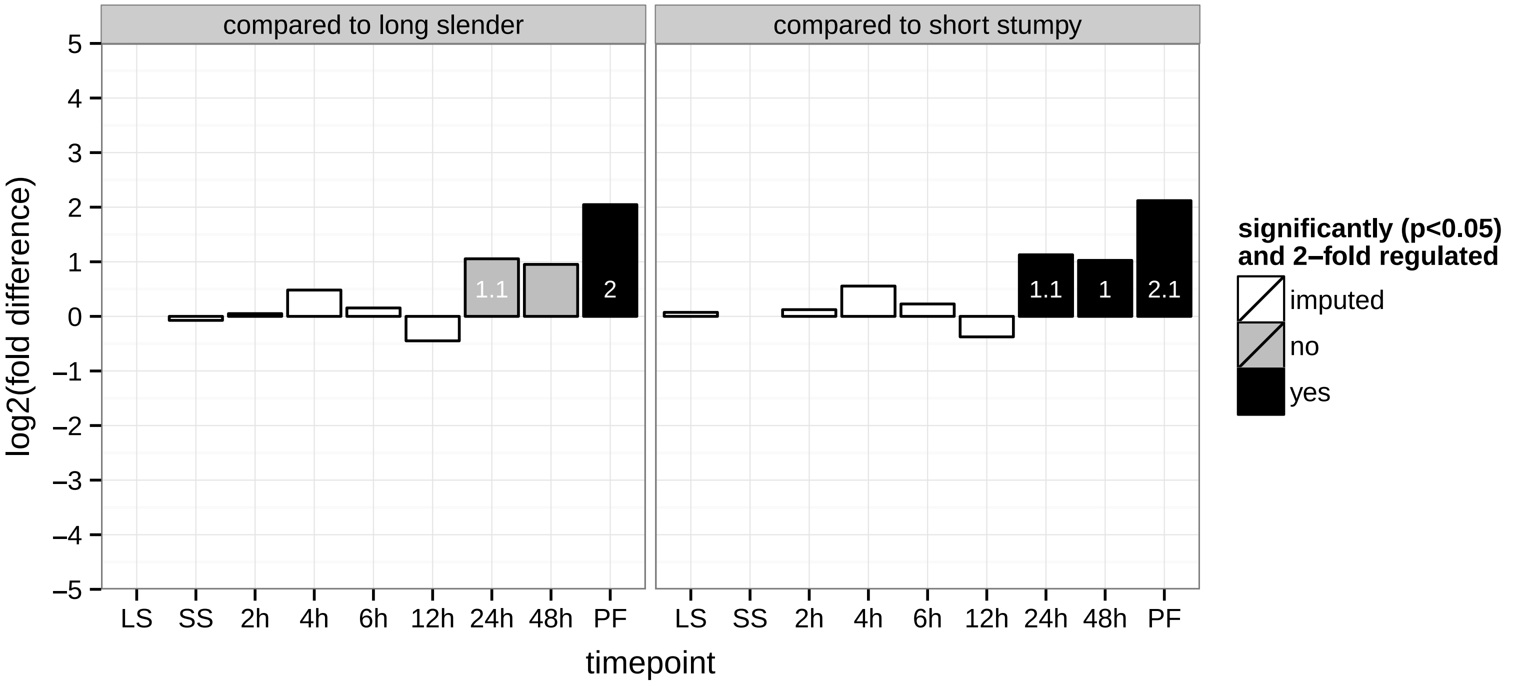

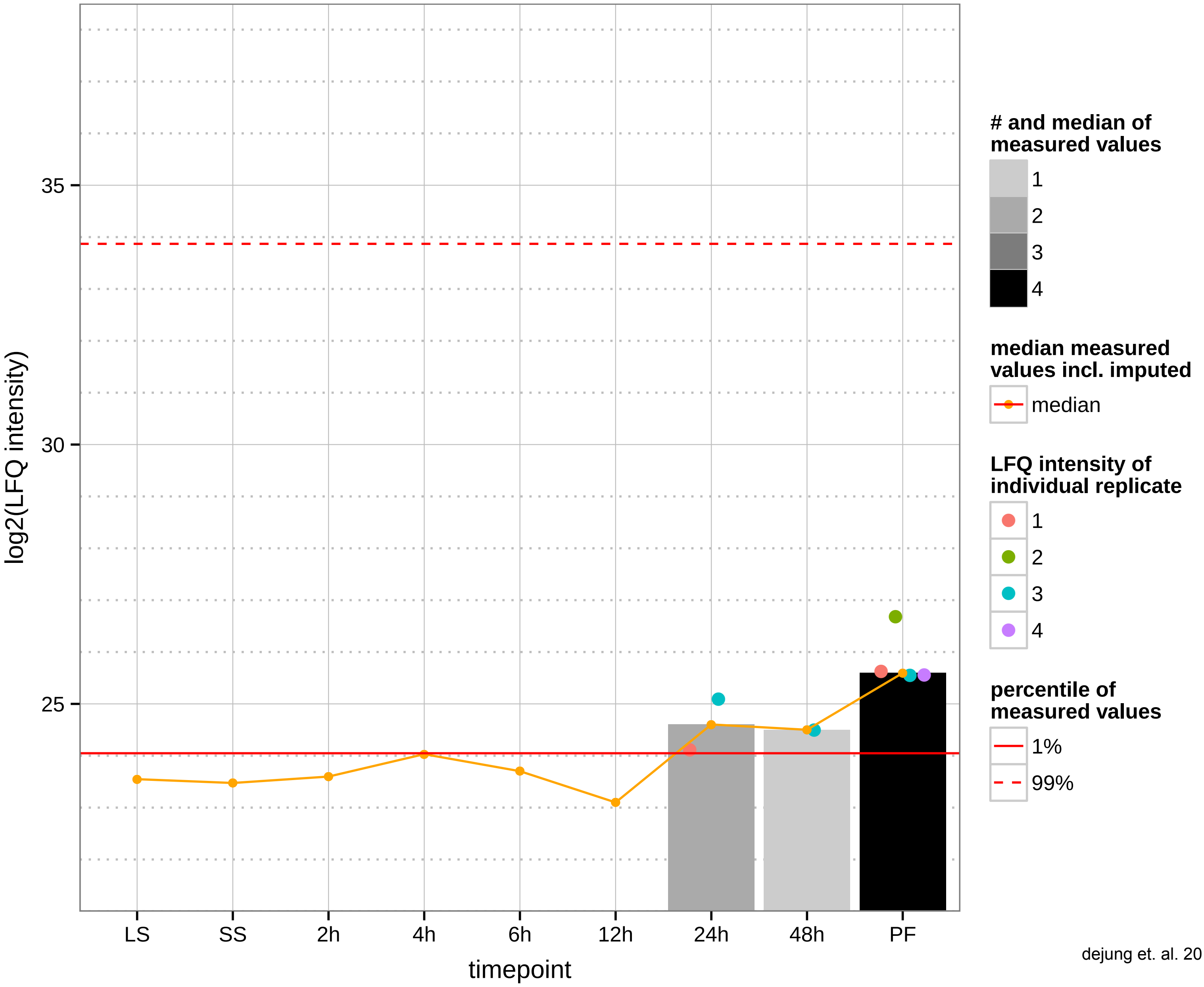

dejung et. al. 2015

ATP synthase F1 subunit gamma protein, putative
Tb927.10.180
AGOF: hydrogen ion transporting ATP synthase activity, rotational mechanism, proton−transporting ATPase activity
AGOC: mitochondrion, proton−transporting ATP synthase complex, catalytic core F(1), proton−transporting two−sector ATPas
AGOP: ATP synthesis coupled proton transport
PGOF: hydrogen ion transporting ATP synthase activity, rotational mechanism, proton−transporting ATPase activity
PGOC: proton−transporting ATP synthase complex, catalytic core F(1)
PGOP: ATP synthesis coupled proton transport

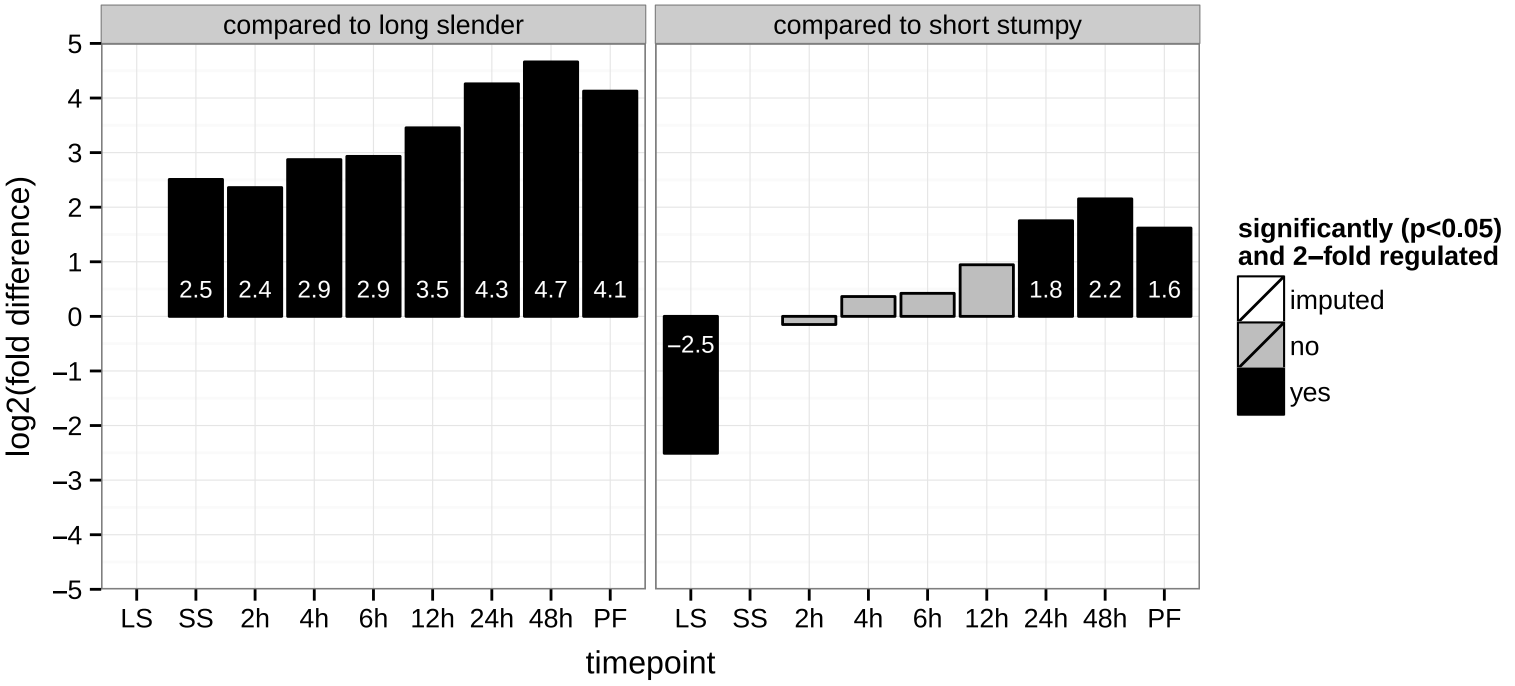

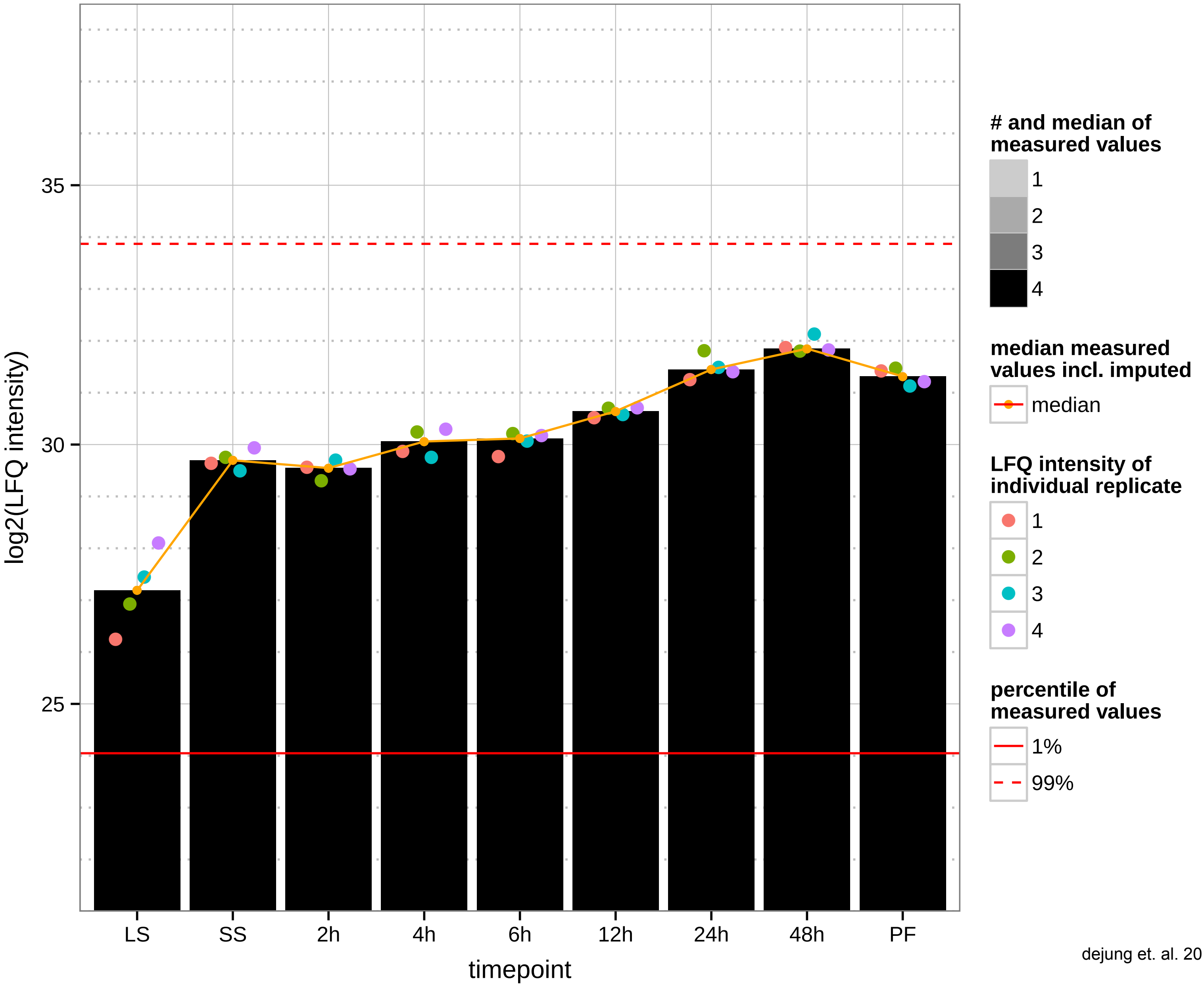

dejung et. al. 2015

NA, 40S ribosomal protein S6, putative
Tb927.10.190
AGOF: NA, structural constituent of ribosome
AGOC: NA, intracellular, ribosome
AGOP: NA, regulation of cell growth, regulation of cell proliferation, translation
PGOF: NA, structural constituent of ribosome
PGOC: NA, intracellular, ribosome
PGOP: NA, translation

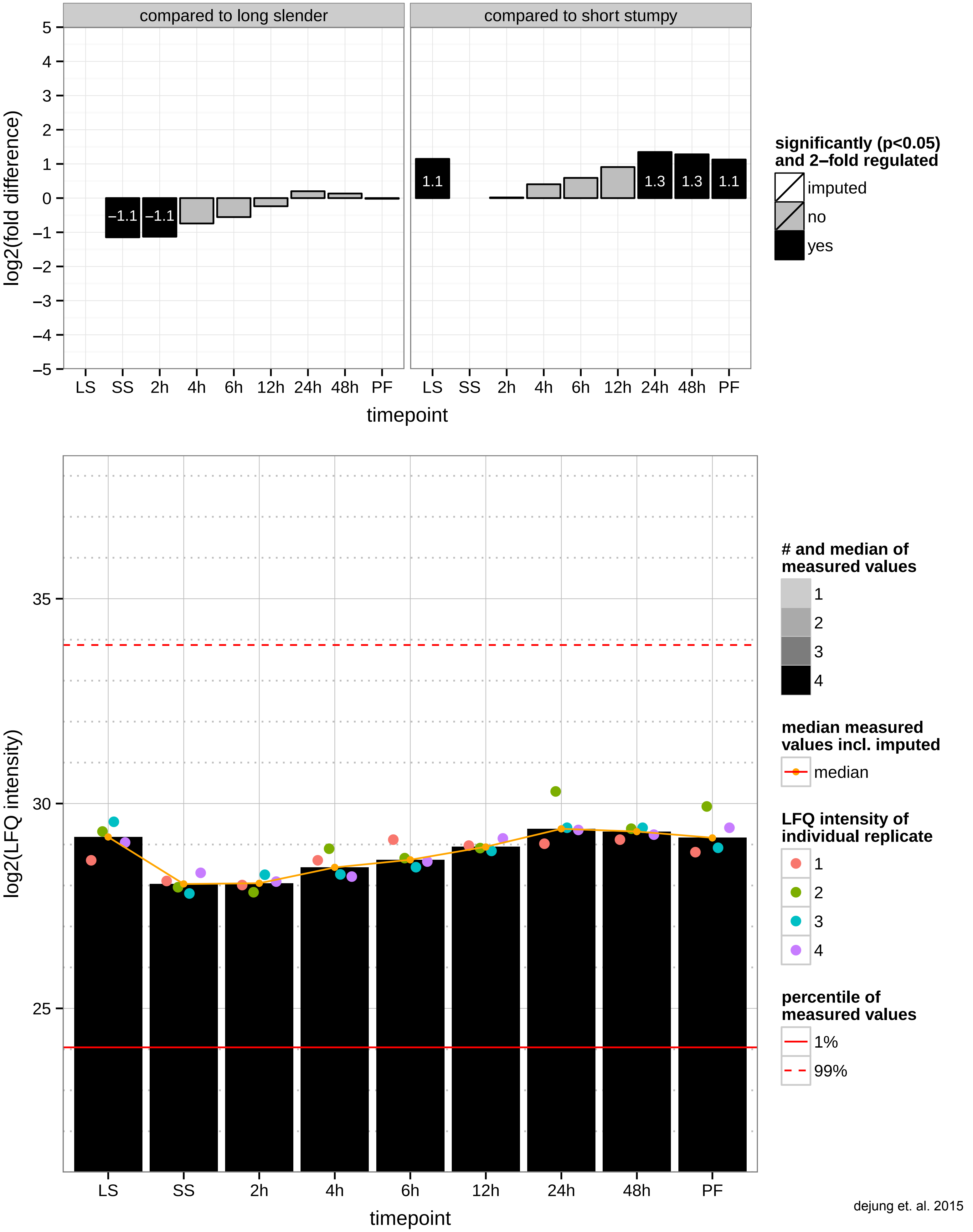

dejung et. al. 2015

DNA topoisomerase IA, putative
Tb927.10.1900
AGOF: DNA binding, DNA topoisomerase type I activity
AGOC: chromosome
AGOP: DNA modification, DNA topological change
PGOF: DNA binding, DNA topoisomerase activity
PGOC: chromosome
PGOP: DNA topological change

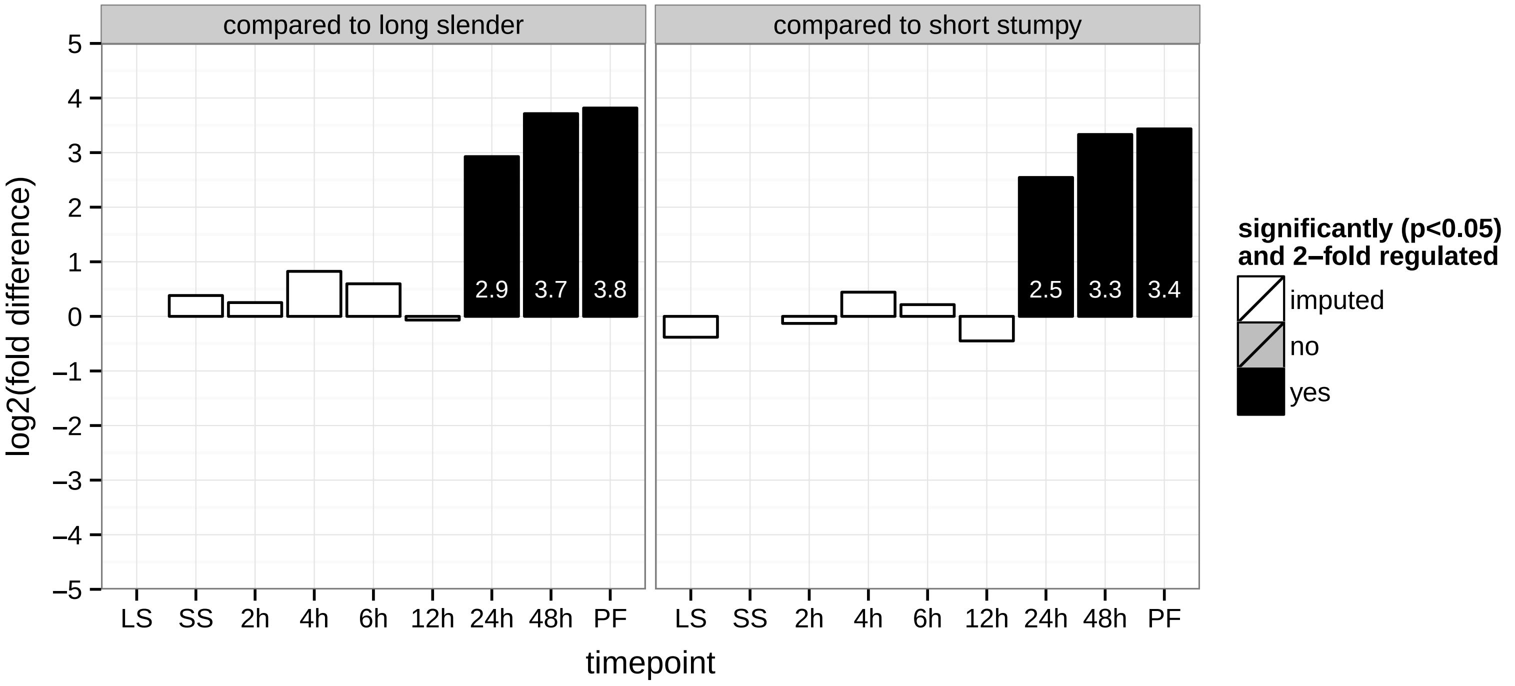

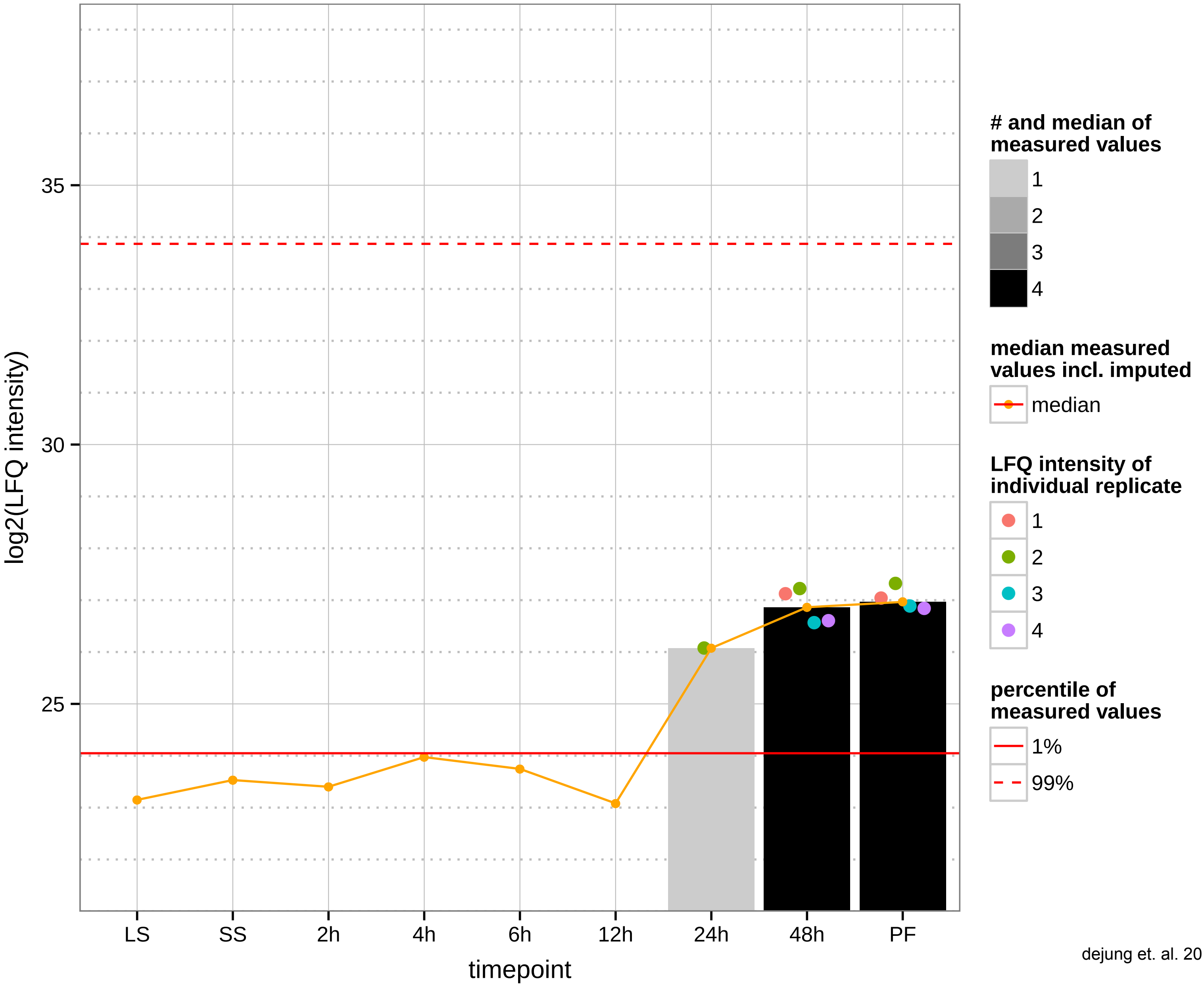

dejung et. al. 2015

hypothetical protein, conserved
Tb927.10.2070
AGOF: null
AGOC: null
AGOP: null
PGOF: null
PGOC: null
PGOP: null

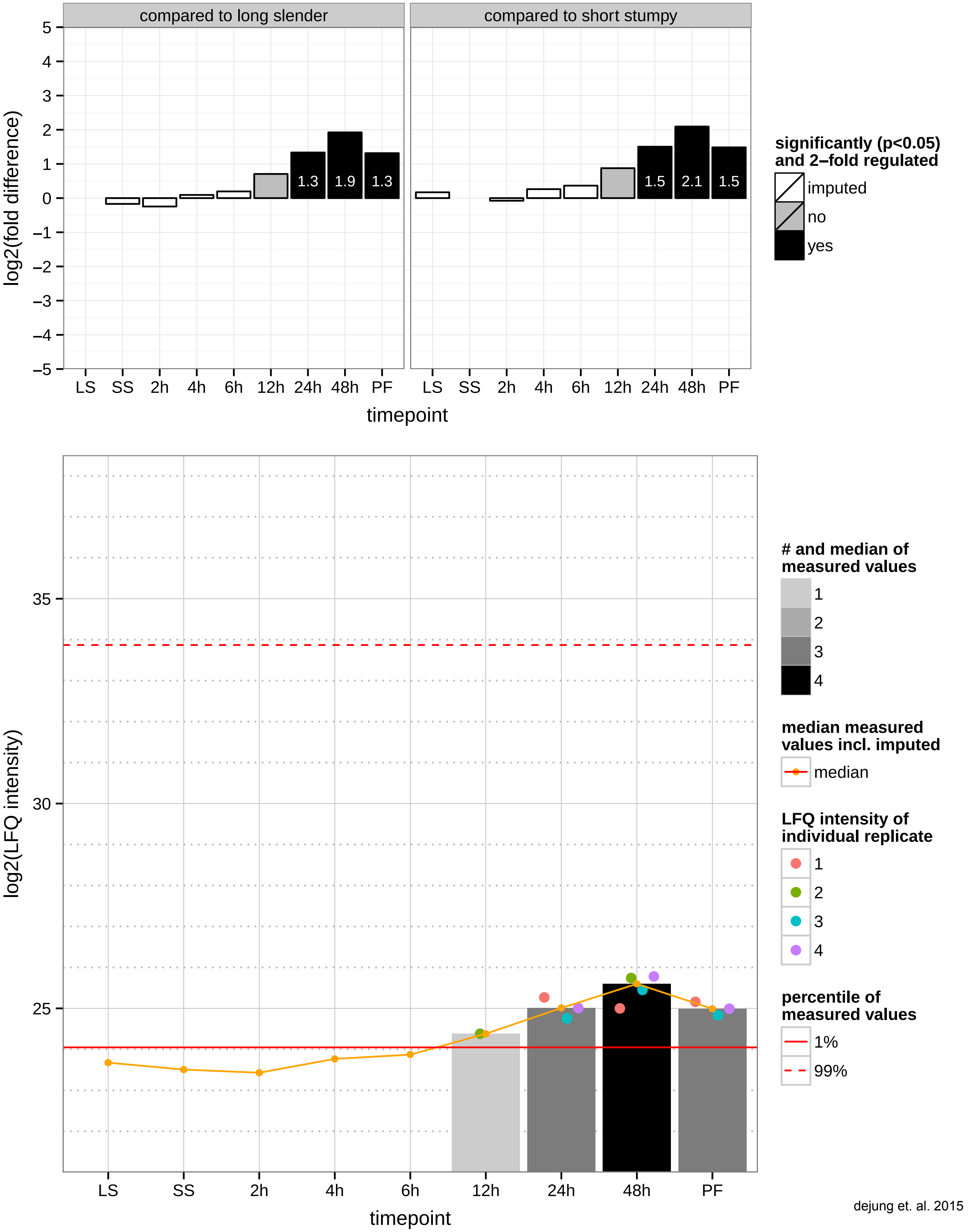

dejung et. al. 2015

hypothetical protein, conserved
Tb927.10.2240
AGOF: null
AGOC: intracellular
AGOP: transport
PGOF: null
PGOC: null
PGOP: null

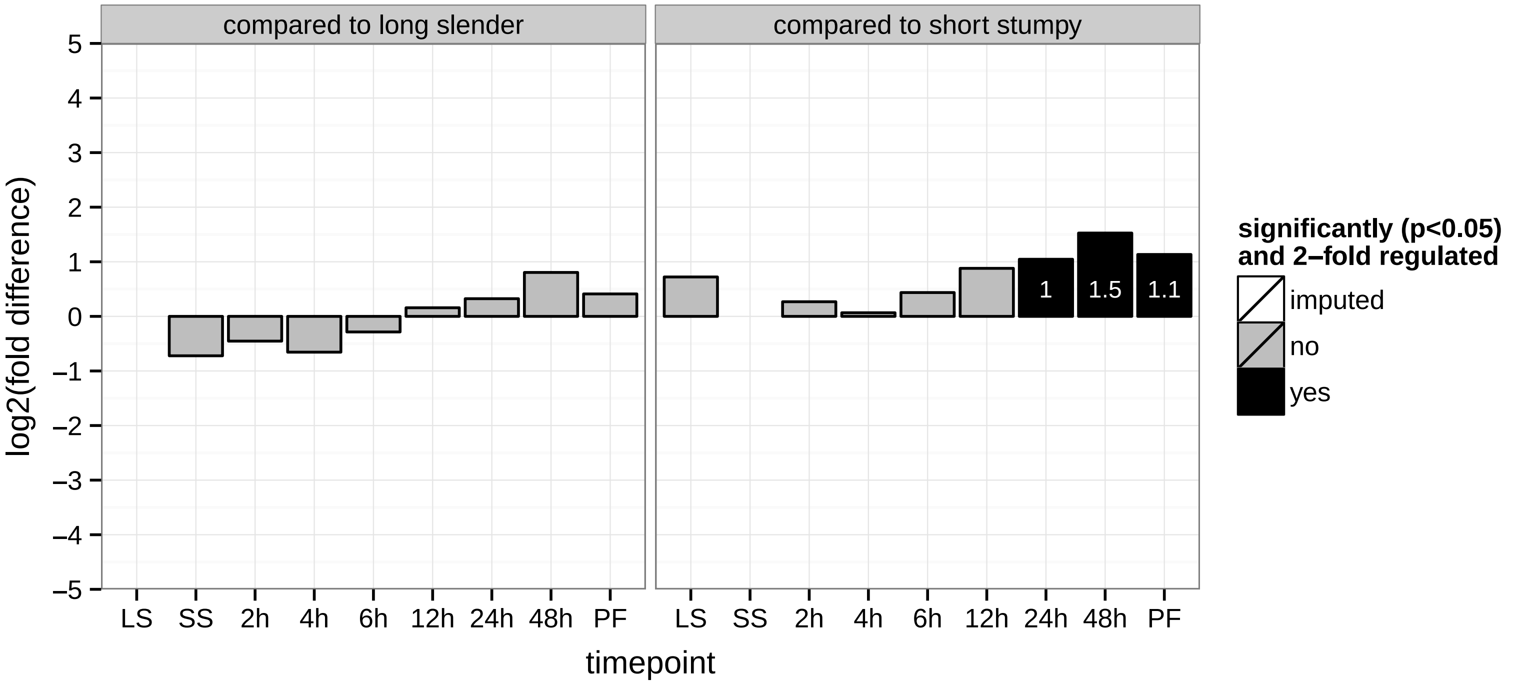

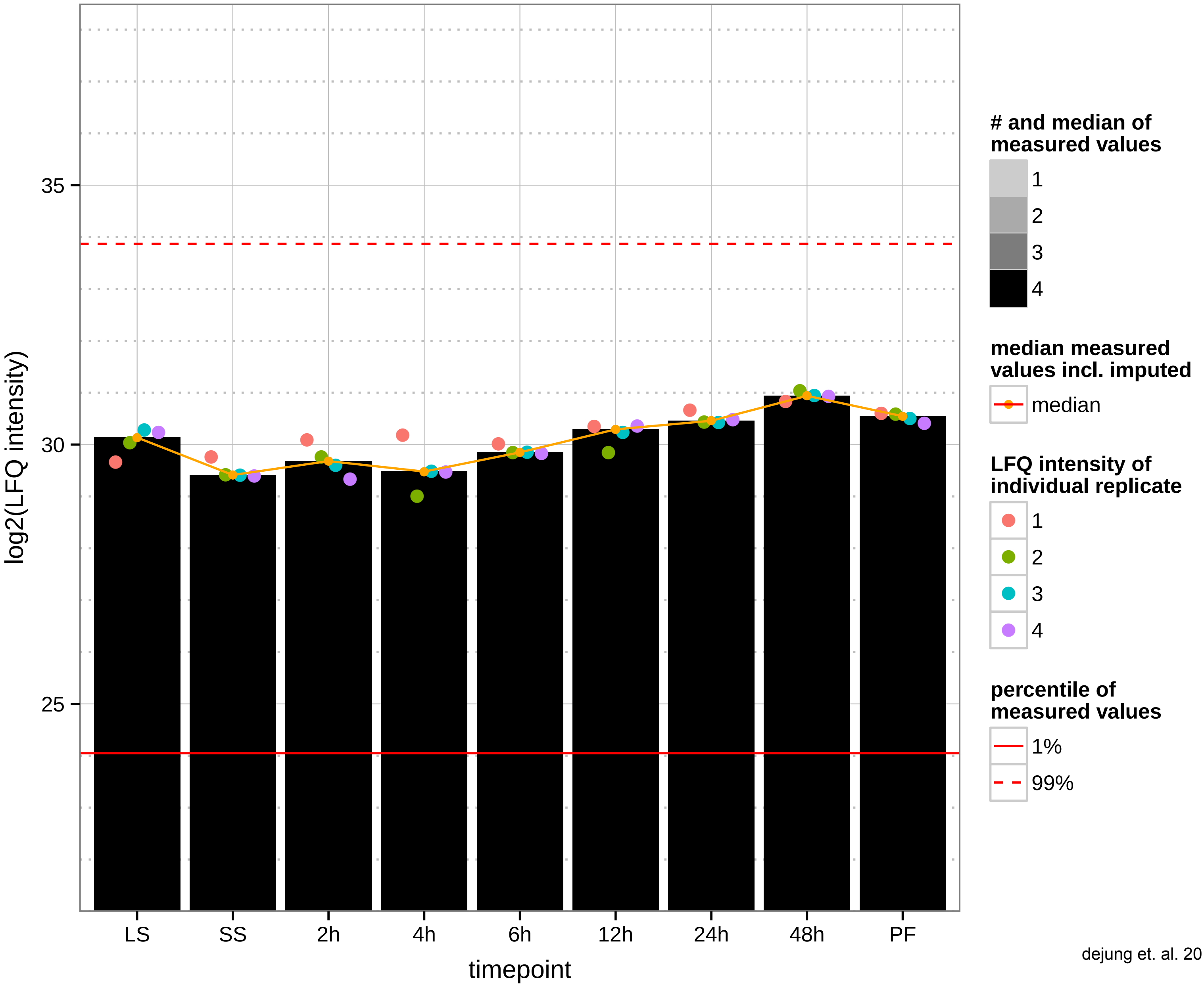

dejung et. al. 2015

60S ribosomal protein L32, putative, unspecified product
Tb927.9.15420;Tb927.10.270
AGOF: structural constituent of ribosome
AGOC: intracellular, ribosome
AGOP: null, translation
PGOF: structural constituent of ribosome
PGOC: intracellular, ribosome
PGOP: translation

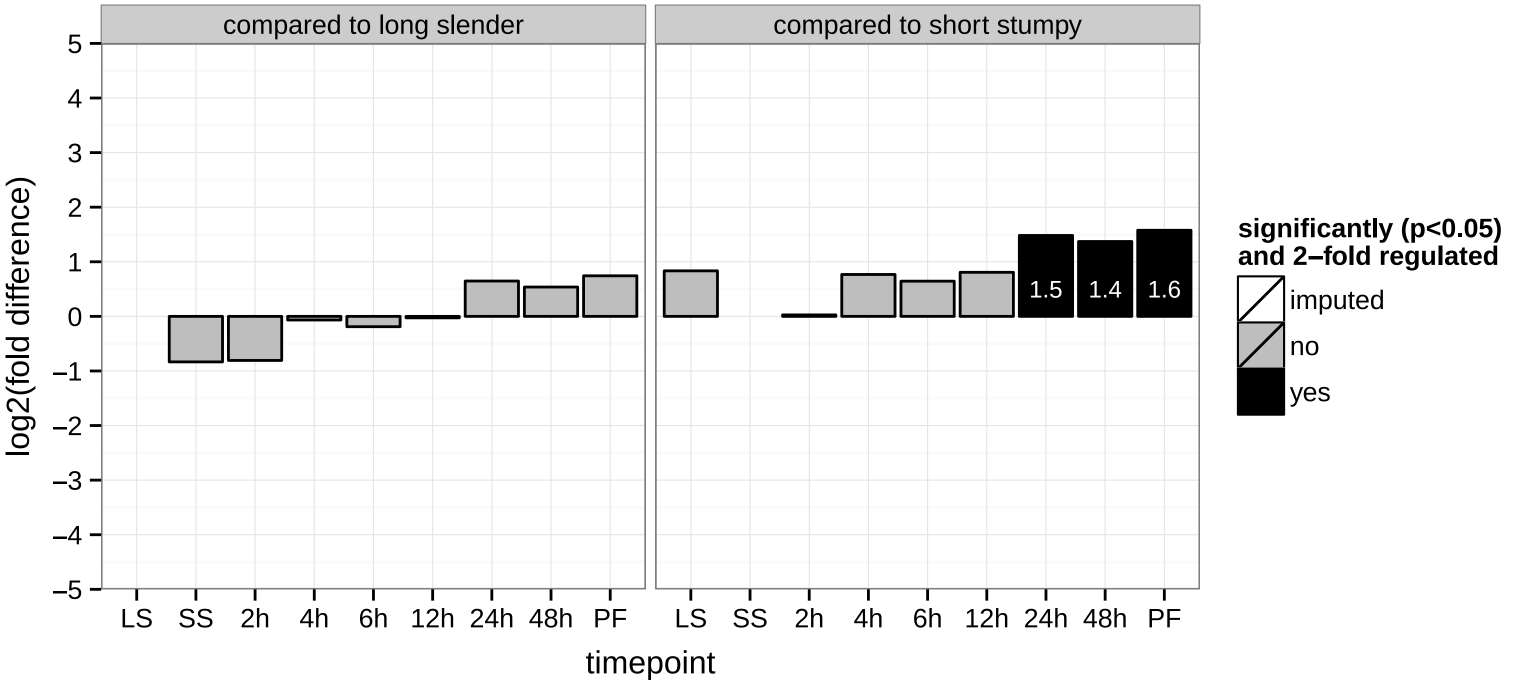

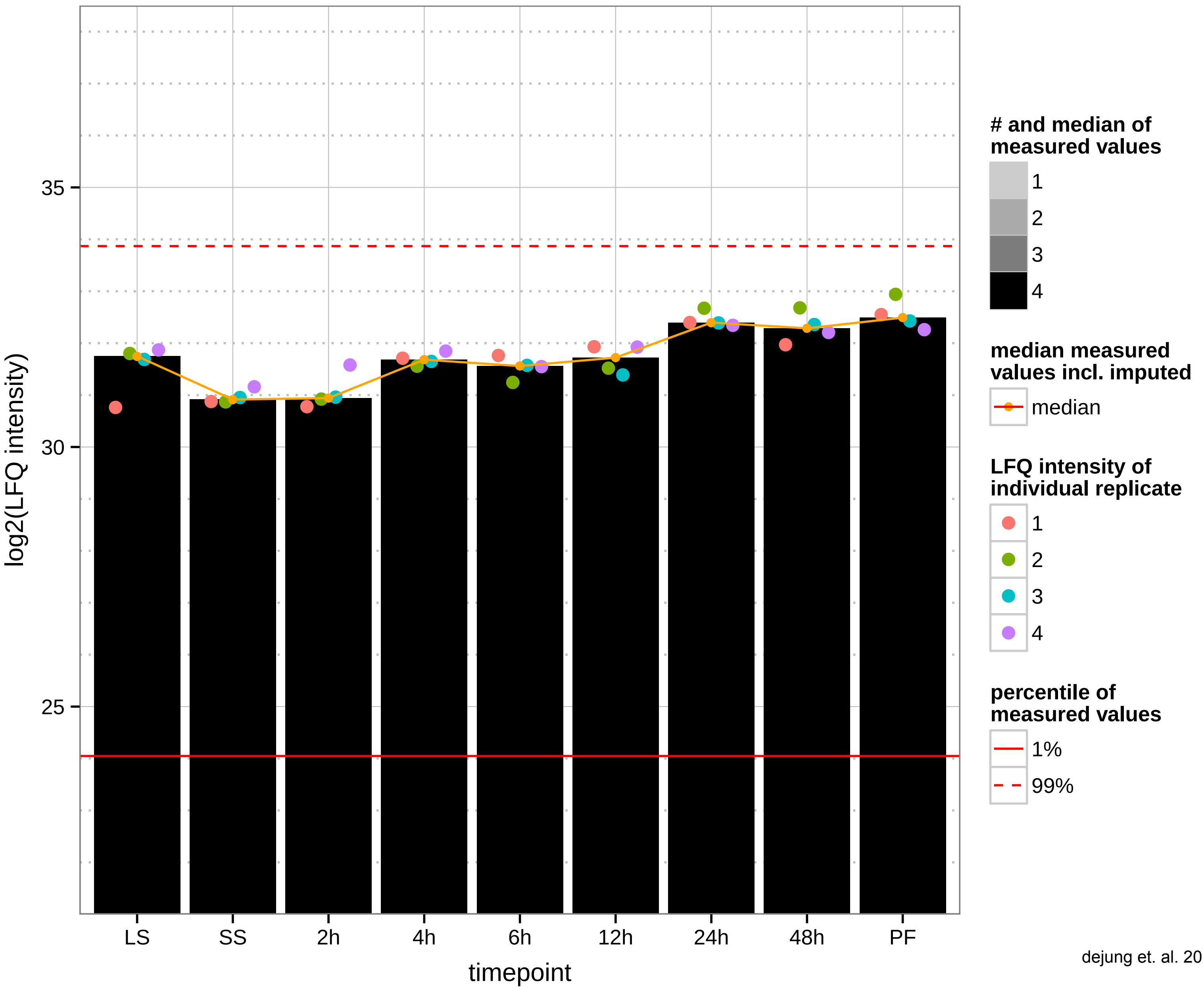

dejung et. al. 2015

60S ribosomal proteins L38, putative
Tb927.10.3280
AGOF: structural constituent of ribosome
AGOC: intracellular, ribosome
AGOP: translation
PGOF: structural constituent of ribosome
PGOC: intracellular, ribosome
PGOP: translation

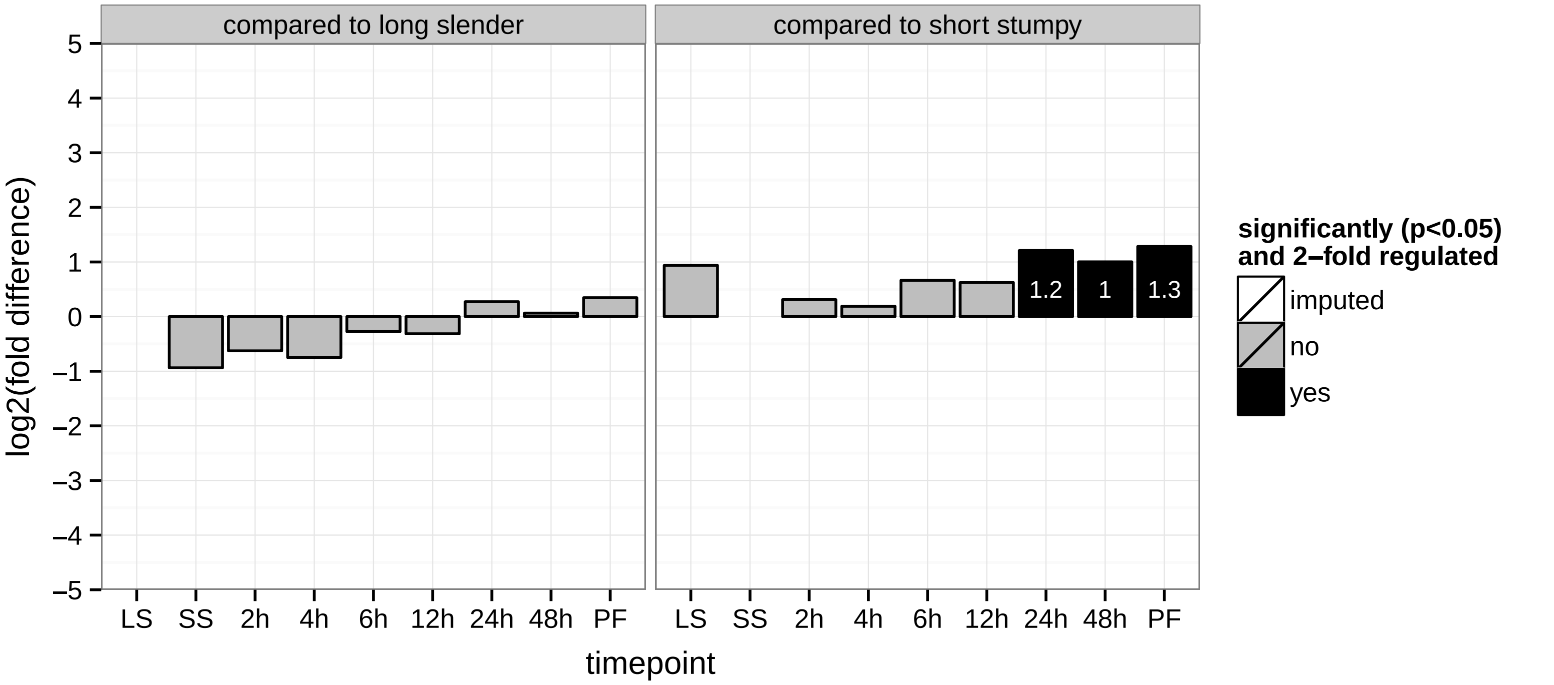

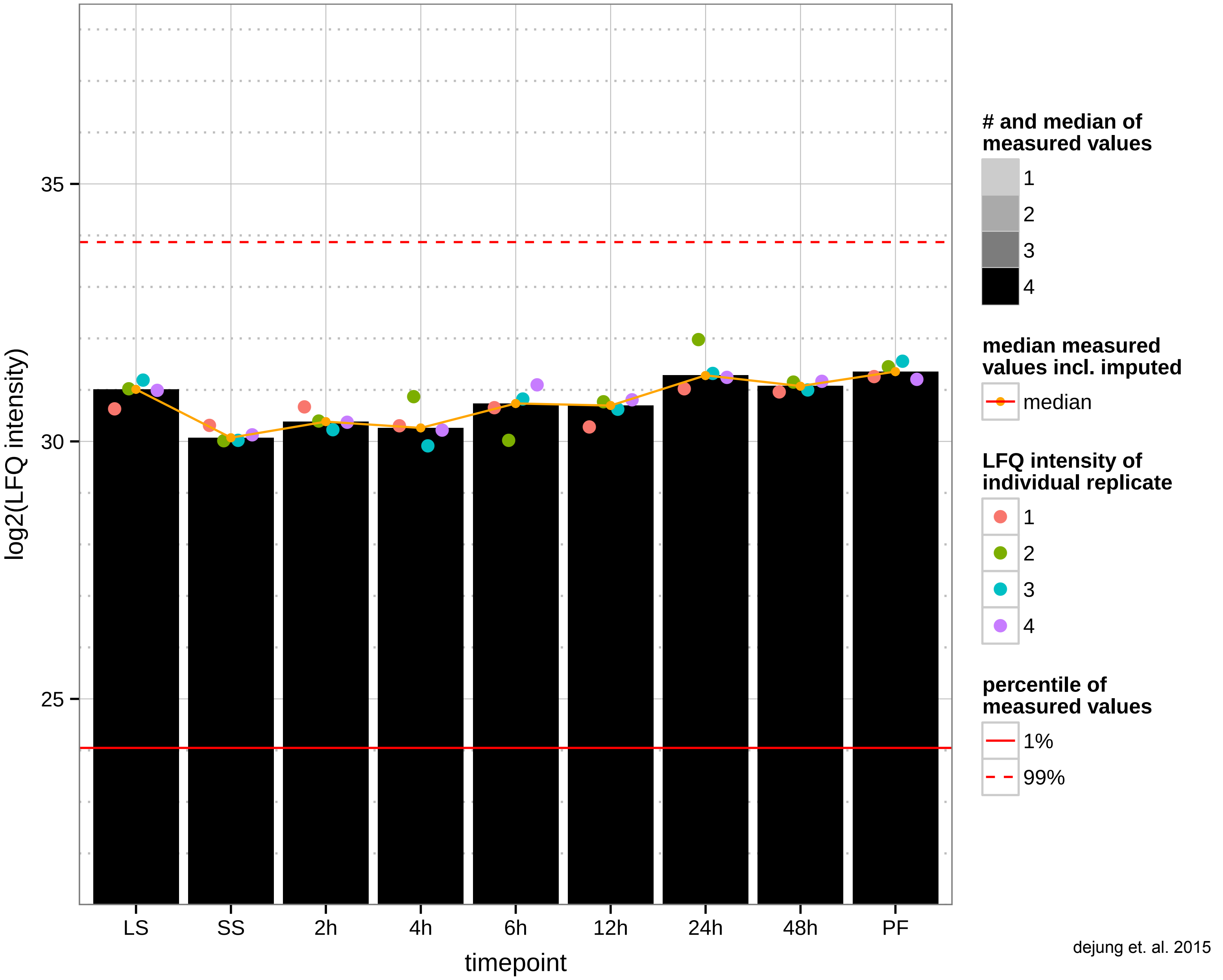

dejung et. al. 2015

mitochondrial structure specific endonuclease I (SSE−1), putative
Tb927.10.340
AGOF: 5'−3' exonuclease activity, DNA binding
AGOC: mitochondrion
AGOP: DNA catabolic process
PGOF: 5'−3' exonuclease activity, DNA binding, catalytic activity
PGOC: null
PGOP: null

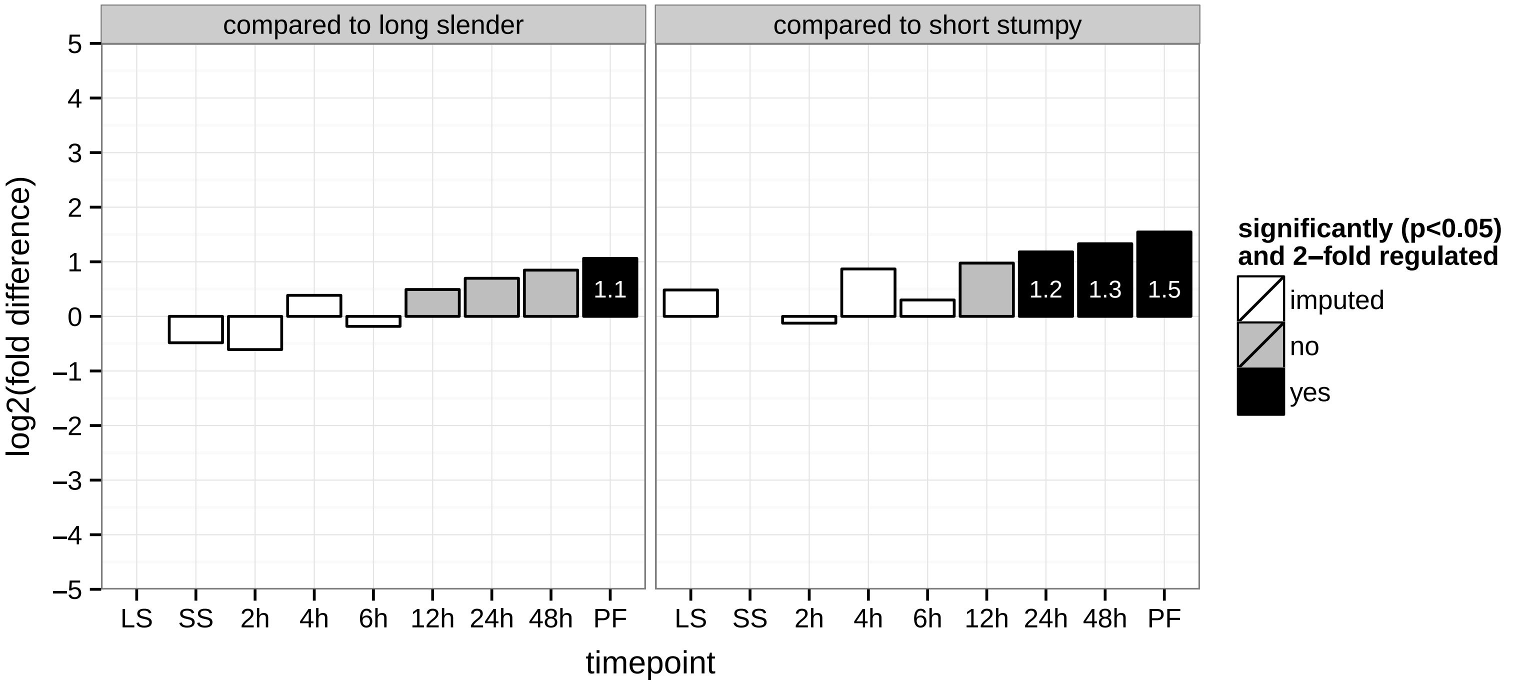

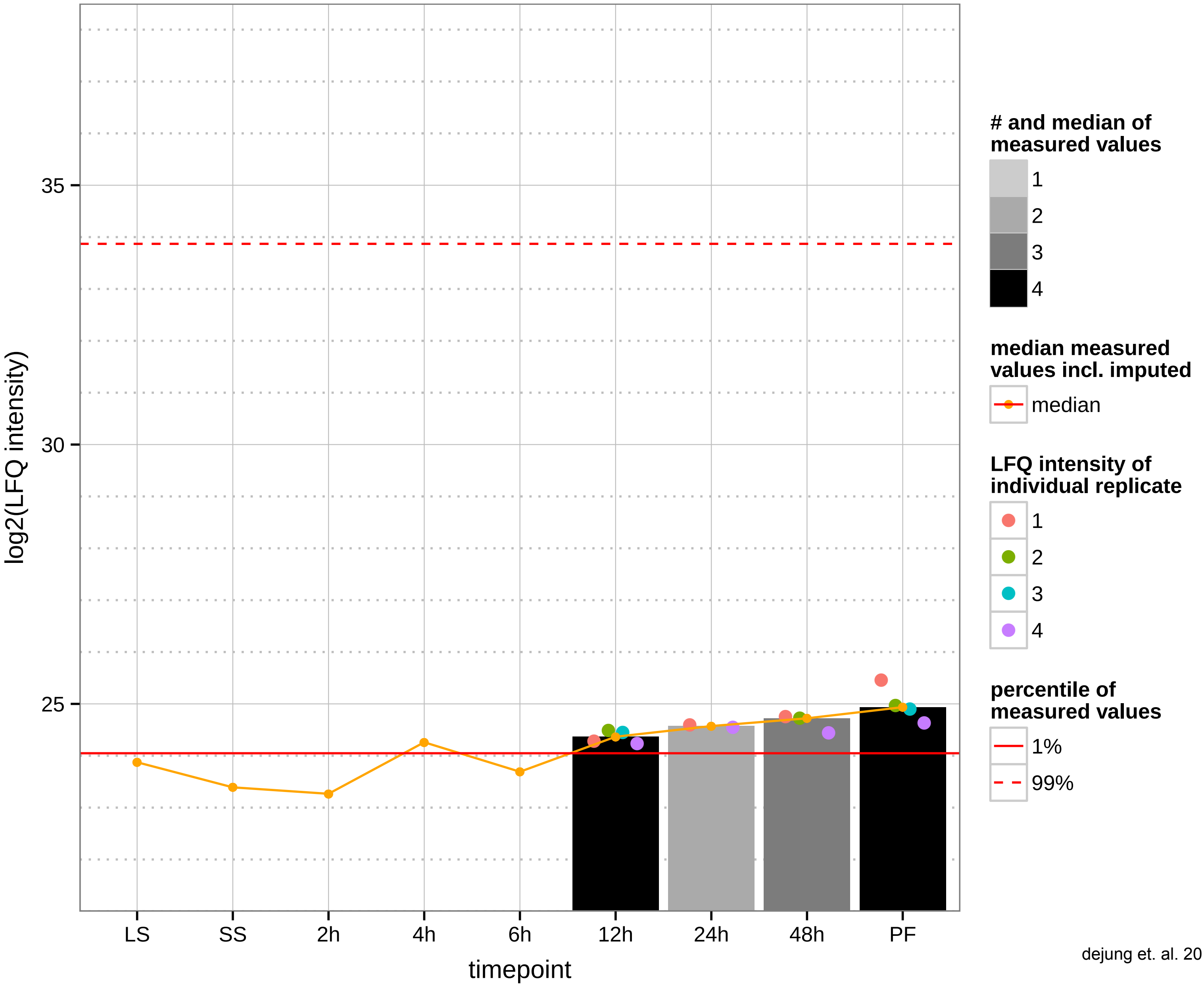

dejung et. al. 2015

NADH−dependent fumarate reductase, putative
Tb927.10.3650
AGOF: electron carrier activity, succinate dehydrogenase activity
AGOC: null
AGOP: thiamine biosynthetic process
PGOF: oxidoreductase activity, succinate dehydrogenase activity
PGOC: null
PGOP: oxidation−reduction process

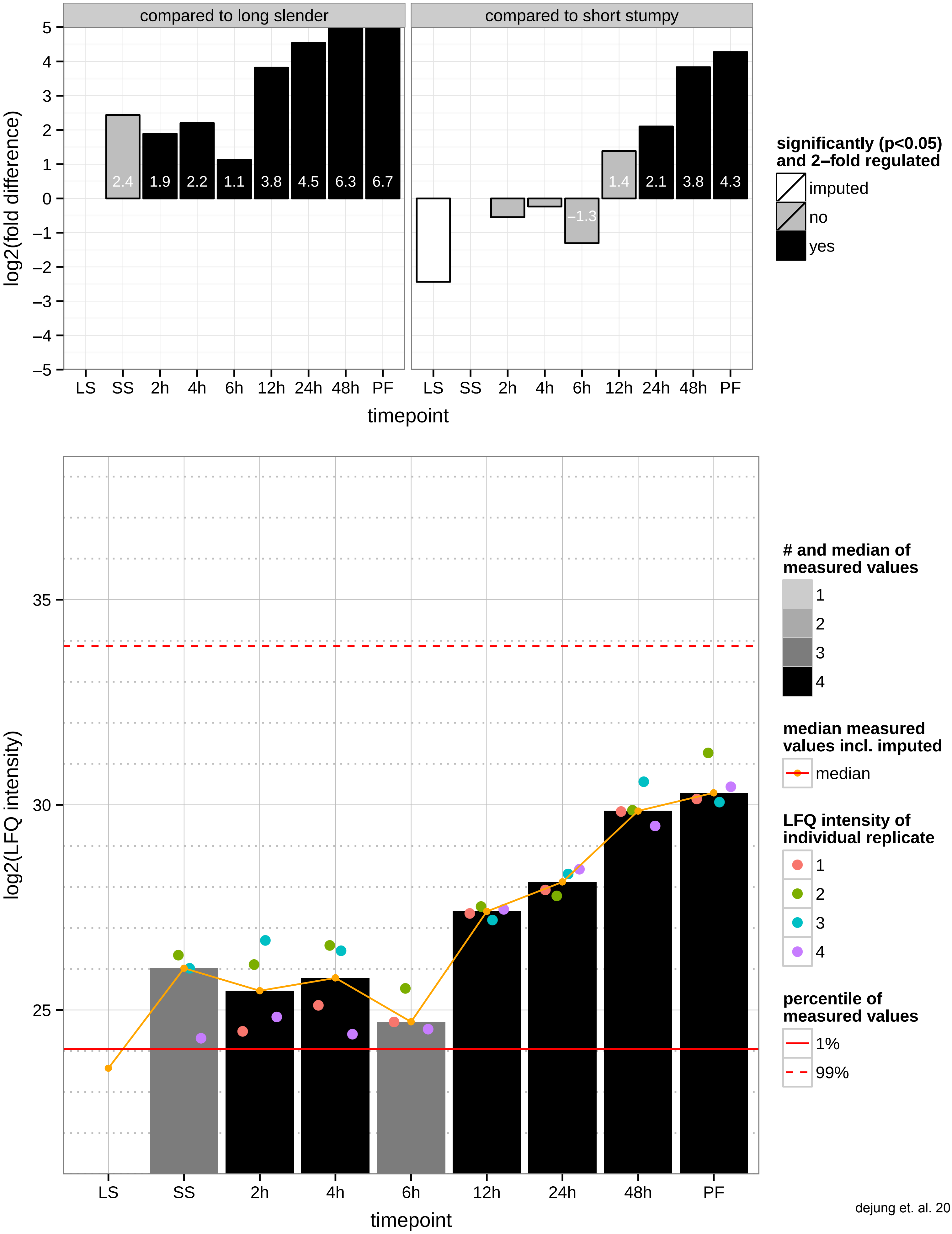

dejung et. al. 2015

hypothetical protein, conserved
Tb927.10.3790
AGOF: null
AGOC: null
AGOP: null
PGOF: binding, null
PGOC: null
PGOP: null

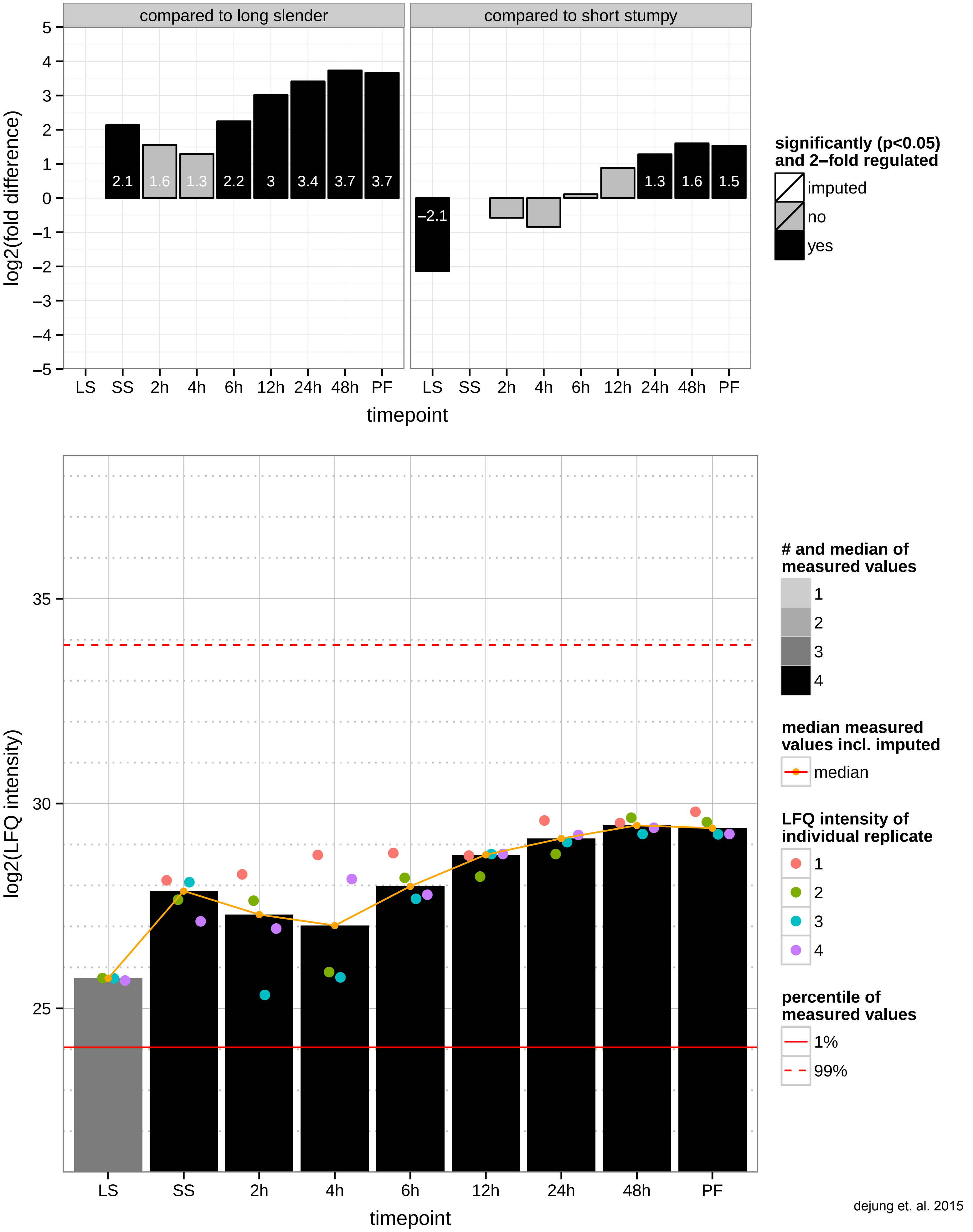

dejung et. al. 2015

hypothetical protein, conserved
Tb927.10.4080
AGOF: oxidoreductase activity
AGOC: mitochondrion
AGOP: fatty acid metabolic process
PGOF: null
PGOC: null
PGOP: oxidation–reduction process

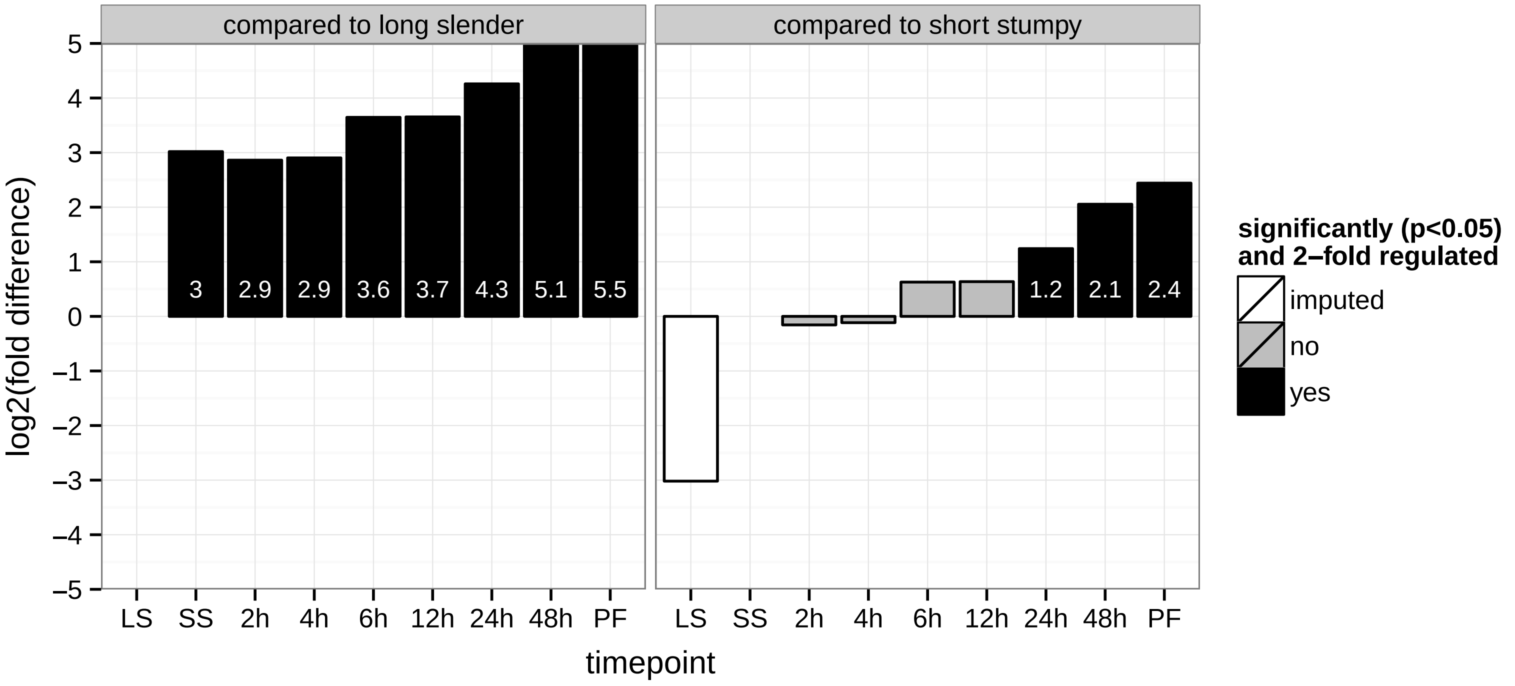

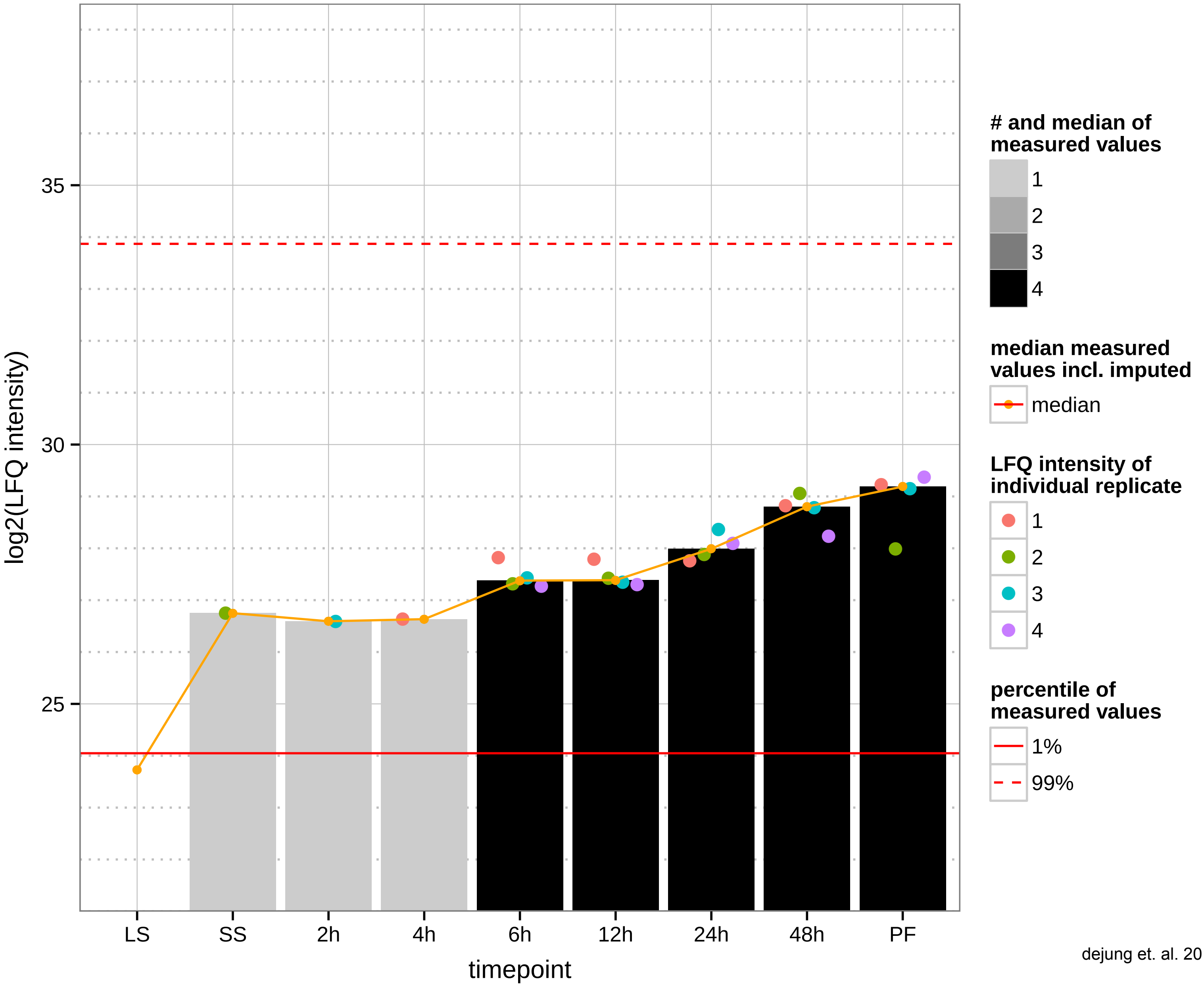

dejung et. al. 2015

hypothetical protein, conserved
Tb927.10.520
AGOF: null
AGOC: mitochondrial inner membrane, mitochondrion
AGOP: null
PGOF: null
PGOC: null
PGOP: null

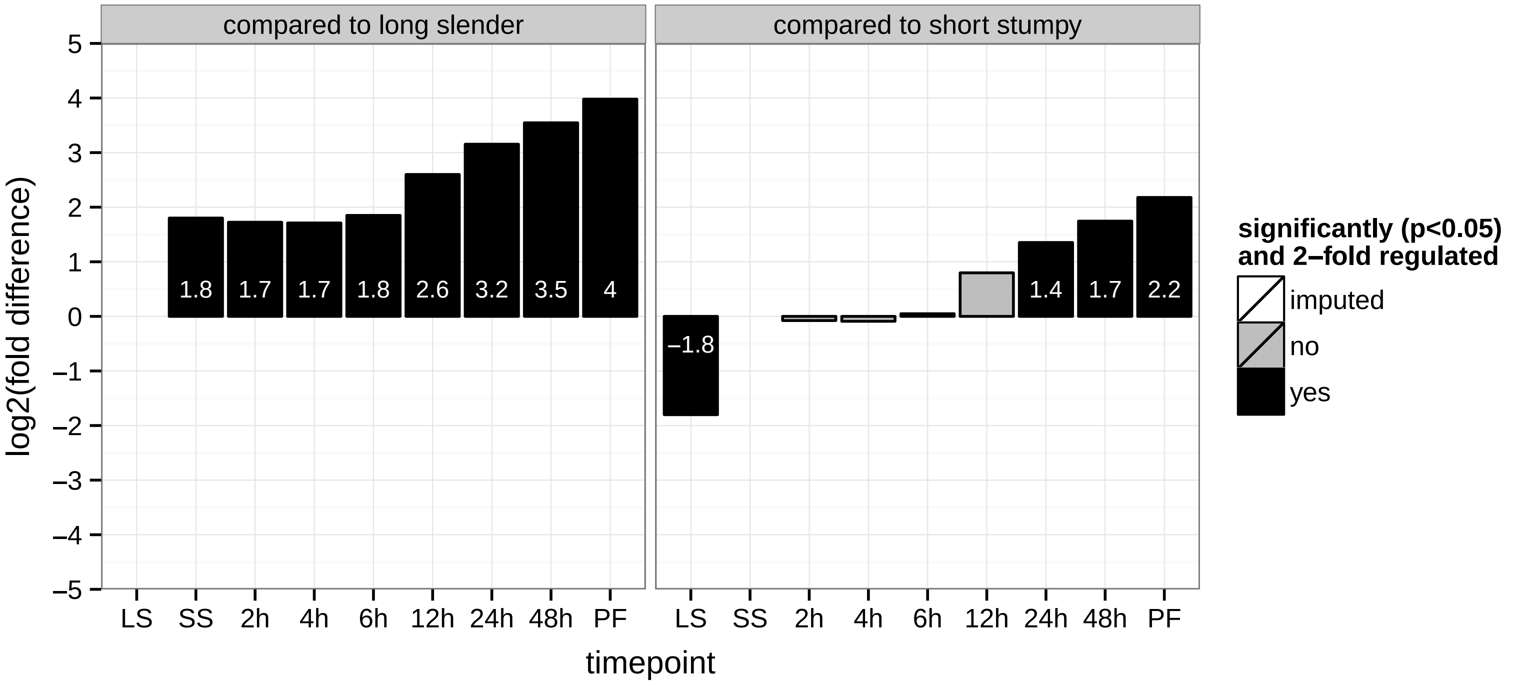

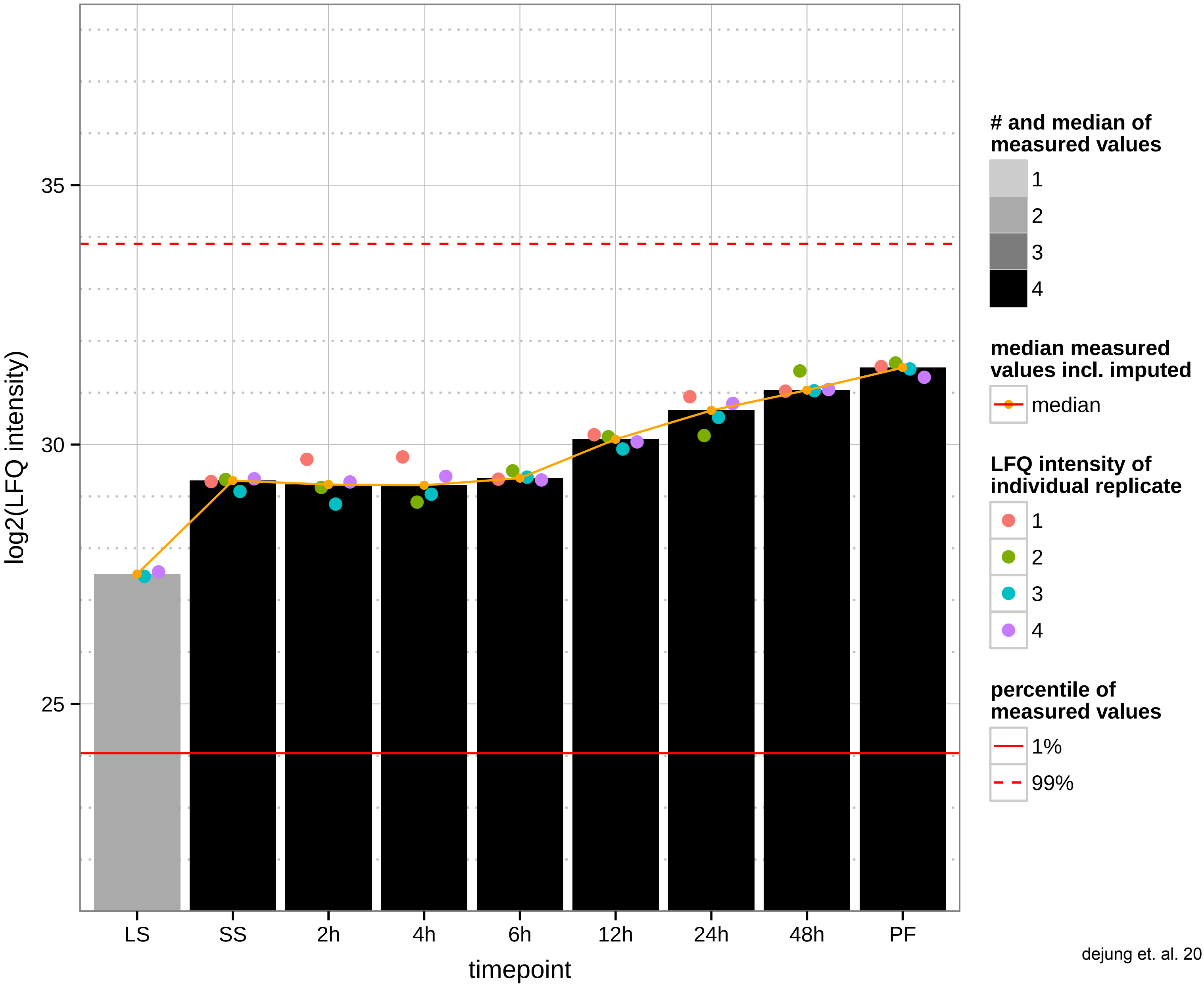

dejung et. al. 2015

40S ribosomal proteins S11, putative
Tb927.10.560
AGOF: structural constituent of ribosome
AGOC: intracellular, organellar small ribosomal subunit, ribosome
AGOP: translation
PGOF: structural constituent of ribosome
PGOC: intracellular, ribosome
PGOP: translation

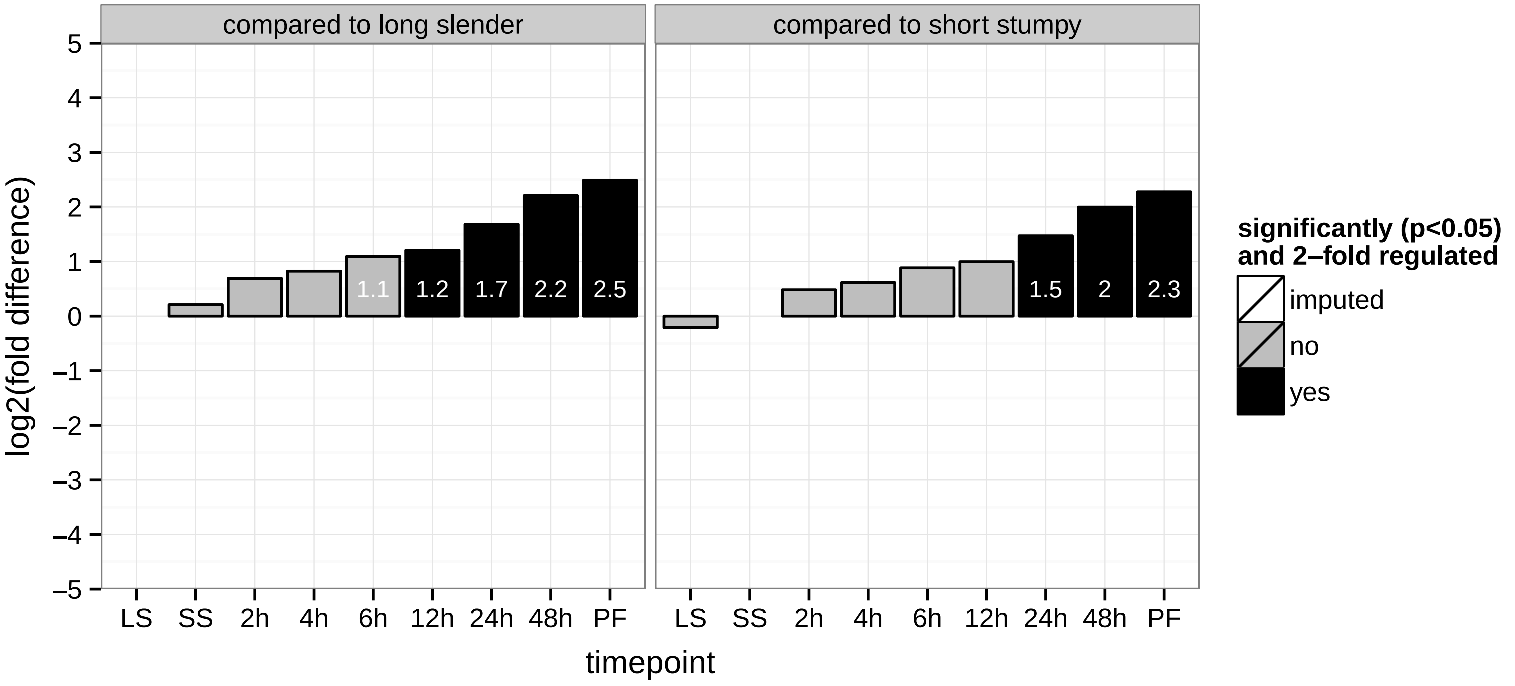

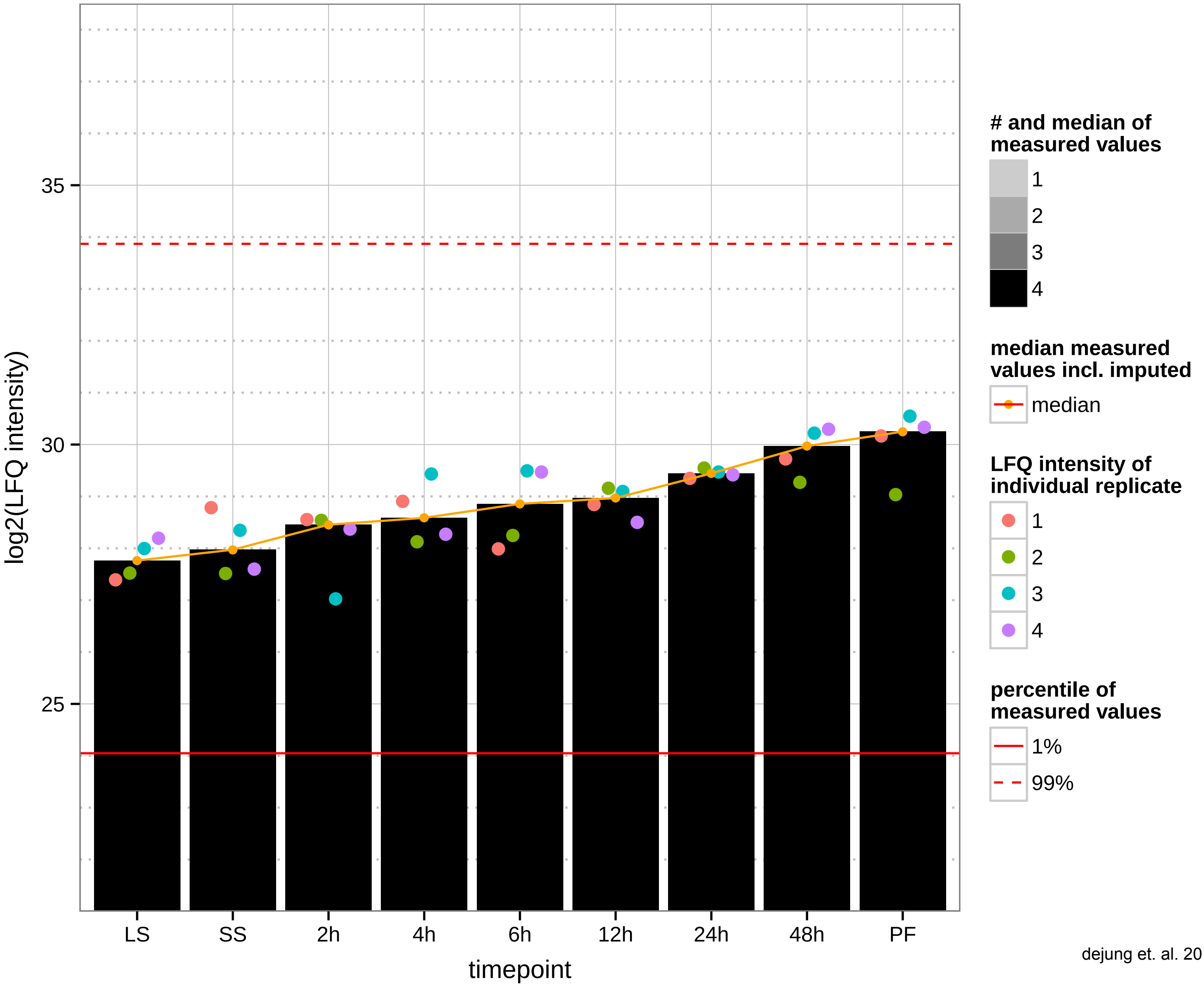

dejung et. al. 2015

adenylate kinase, putative
Tb927.10.5760
AGOF: ATP binding, adenylate kinase activity
AGOC: null
AGOP: nucleobase−containing compound metabolic process, nucleobase−containing small molecule interconversion
PGOF: ATP binding, nucleobase−containing compound kinase activity, nucleotide kinase activity, phosphotransferase activity,
PGOC: null
PGOP: nucleobase−containing compound metabolic process, nucleotide phosphorylation

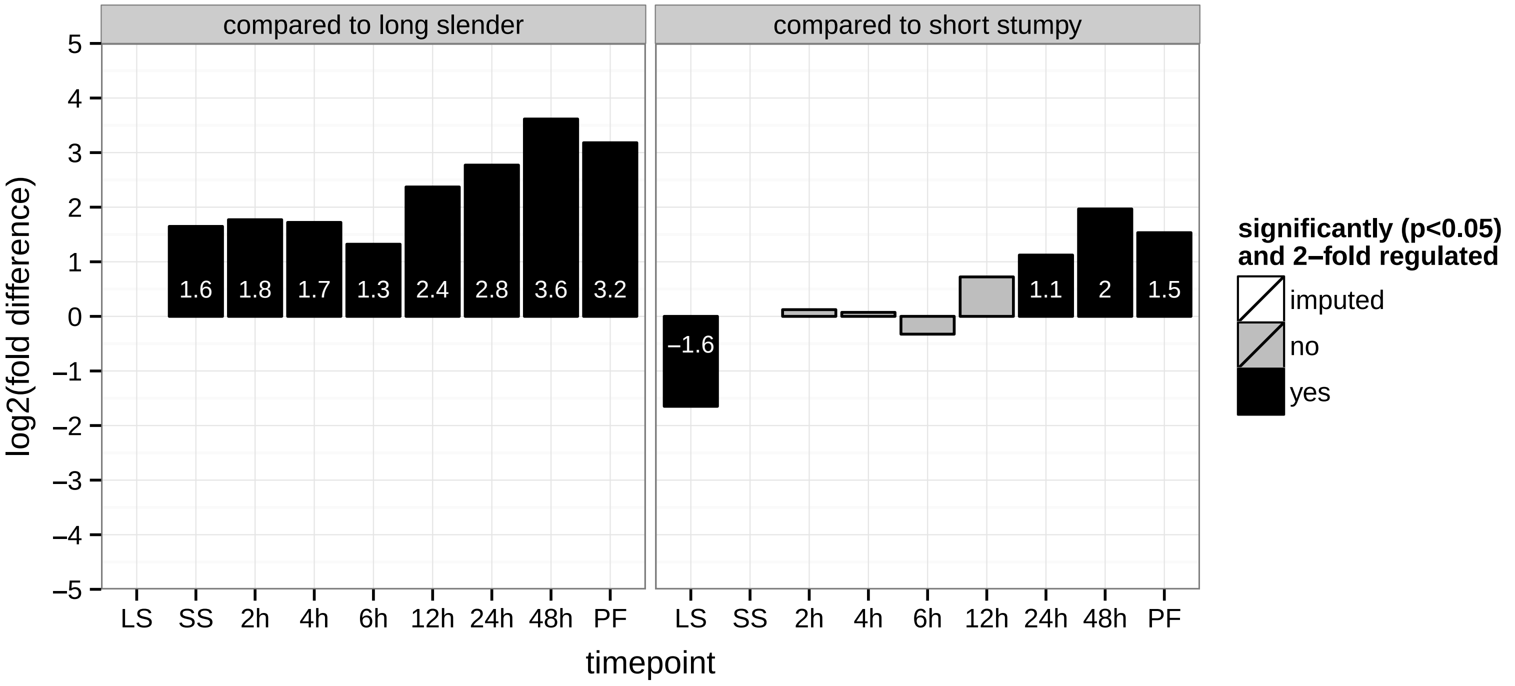

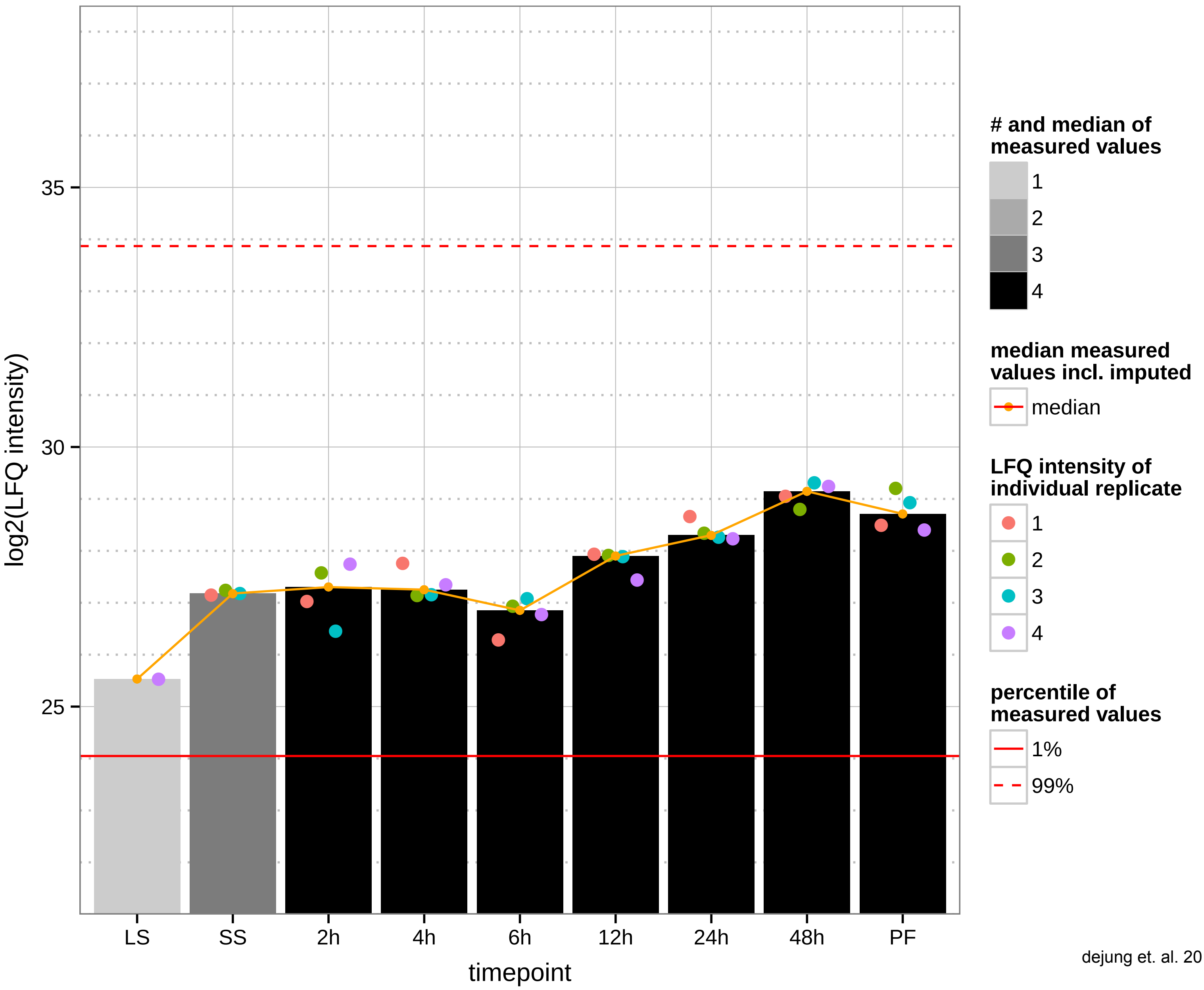

dejung et. al. 2015

hypothetical protein, conserved
Tb927.10.600
AGOF: null
AGOC: mitochondrion
AGOP: null
PGOF: null
PGOC: null
PGOP: null

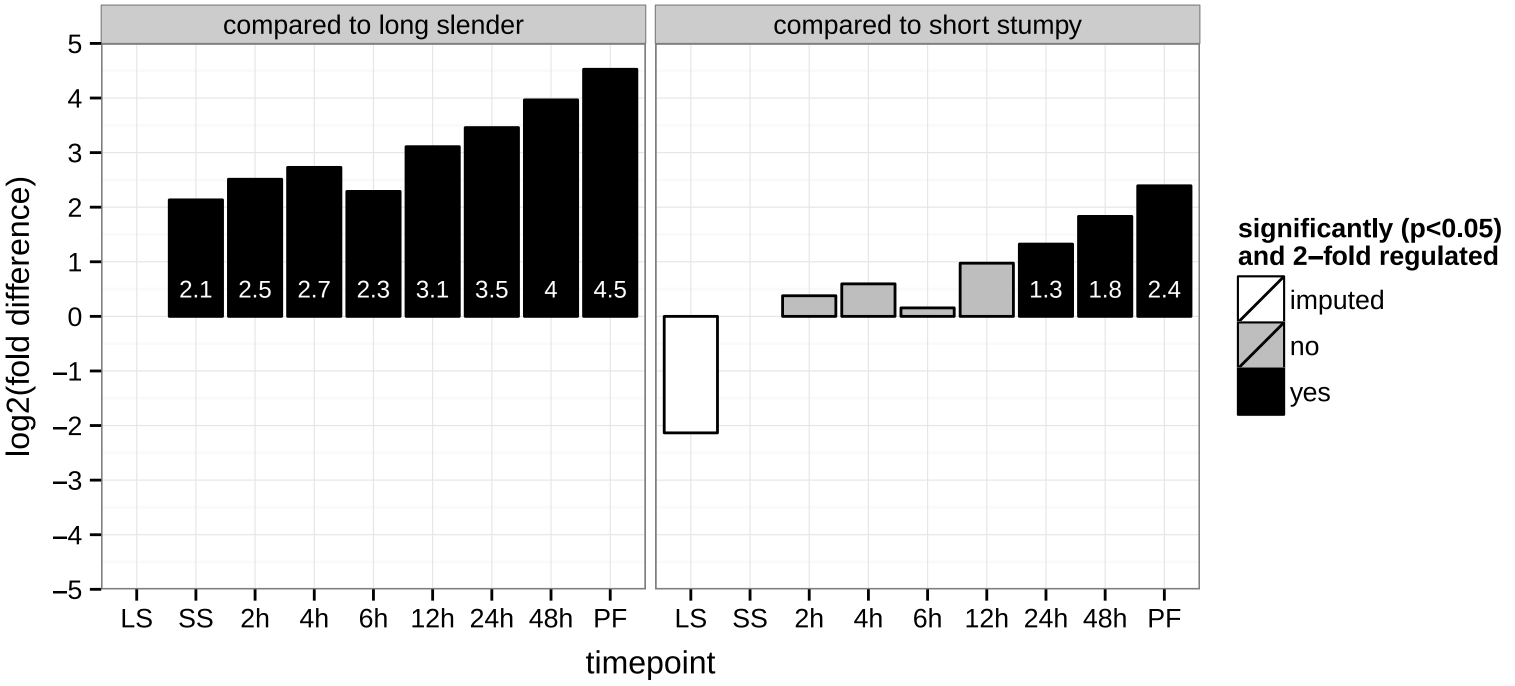

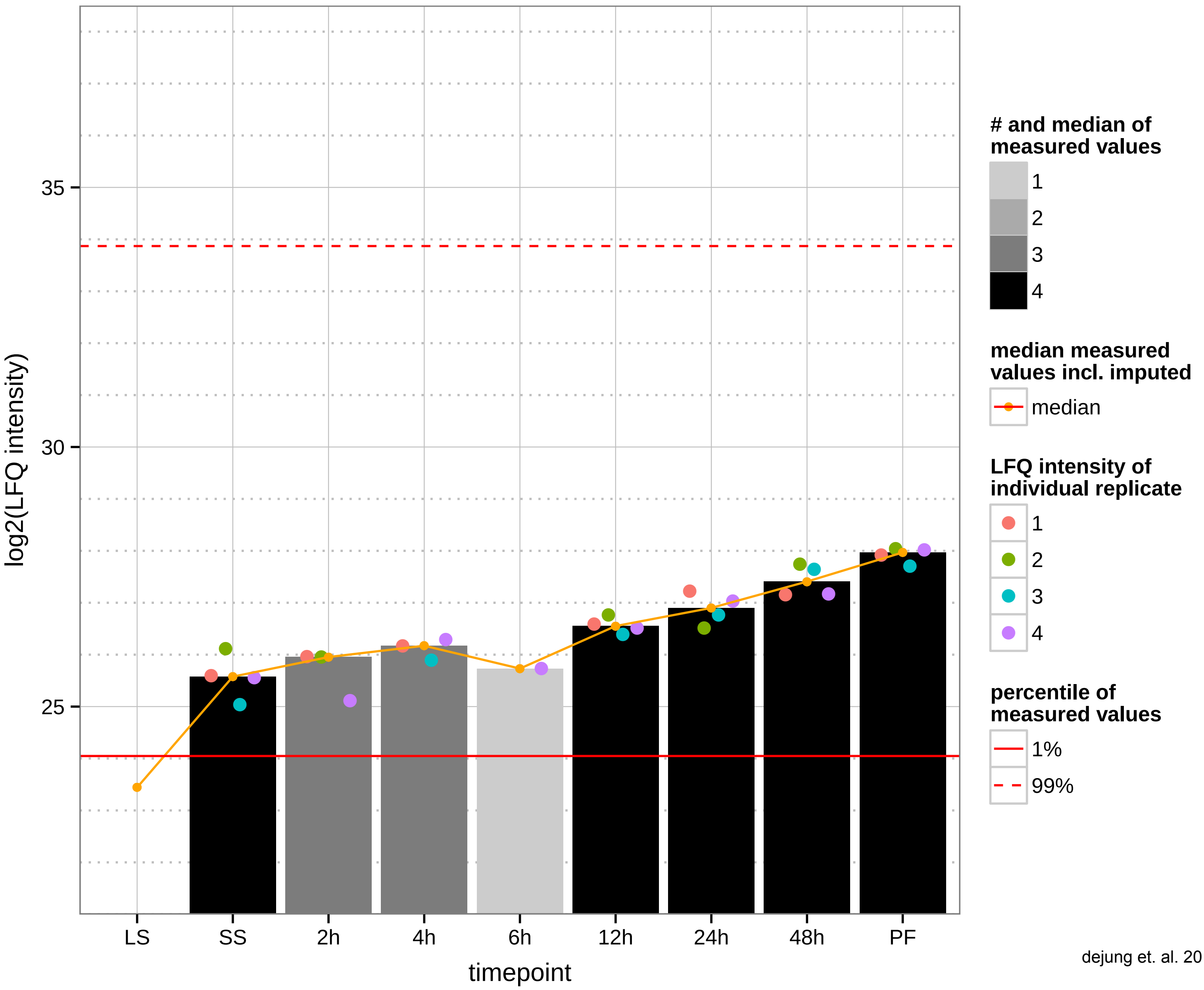

dejung et. al. 2015

hypothetical protein, conserved
Tb927.10.6200
AGOF: null
AGOC: mitochondrion
AGOP: null
PGOF: null
PGOC: null
PGOP: null

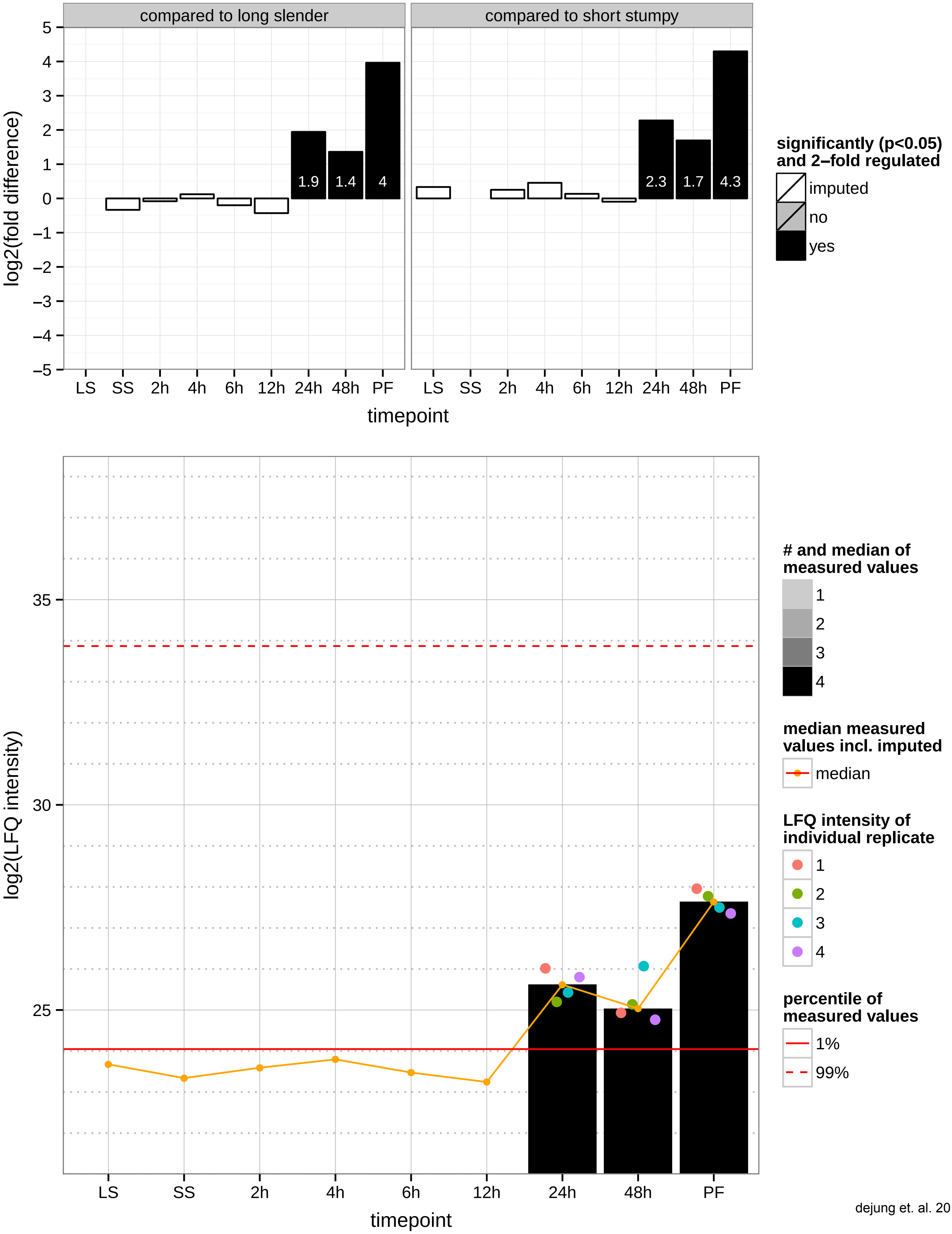

dejung et. al. 2015

thymidine kinase, putative
Tb927.10.880
AGOF: ATP binding, thymidine kinase activity
AGOC: mitochondrion
AGOP: nucleobase–containing small molecule interconversion
PGOF: ATP binding, thymidine kinase activity
PGOC: null
PGOP: null

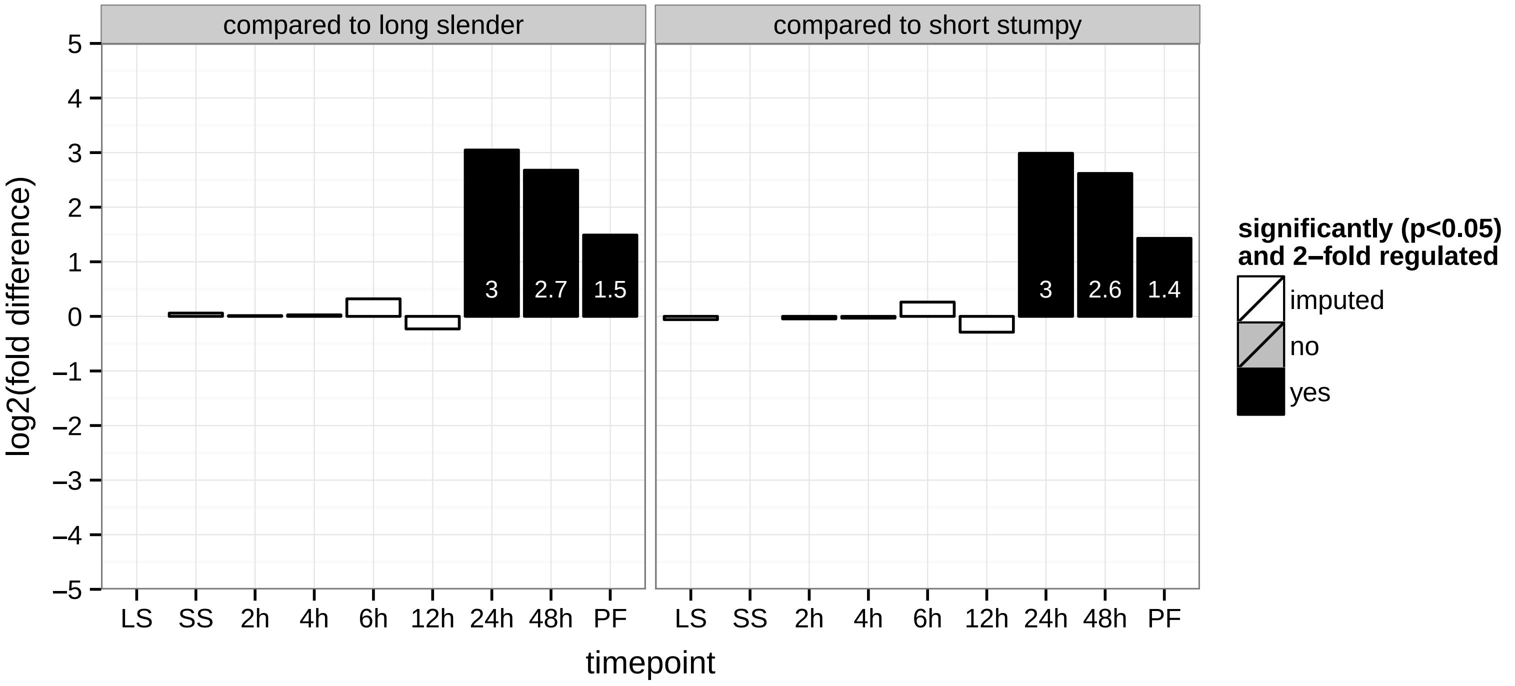

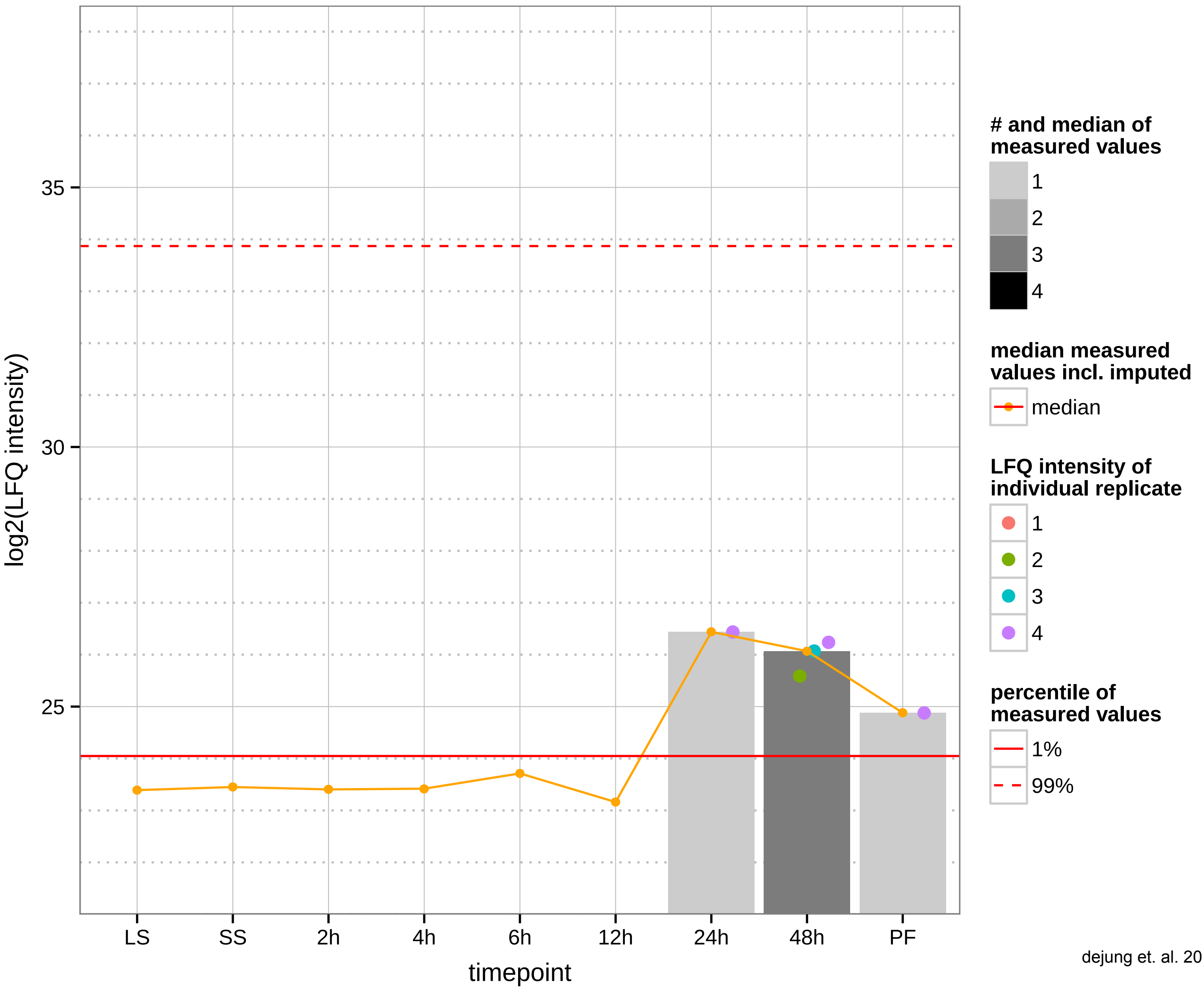

dejung et. al. 2015

ras–like small GTPase, putative (TbGRP)
Tb927.10.8920
AGOF: GTP binding
AGOC: cytoplasm, nucleus
AGOP: null
PGOF: GTP binding
PGOC: cytoplasm, nucleus
PGOP: null

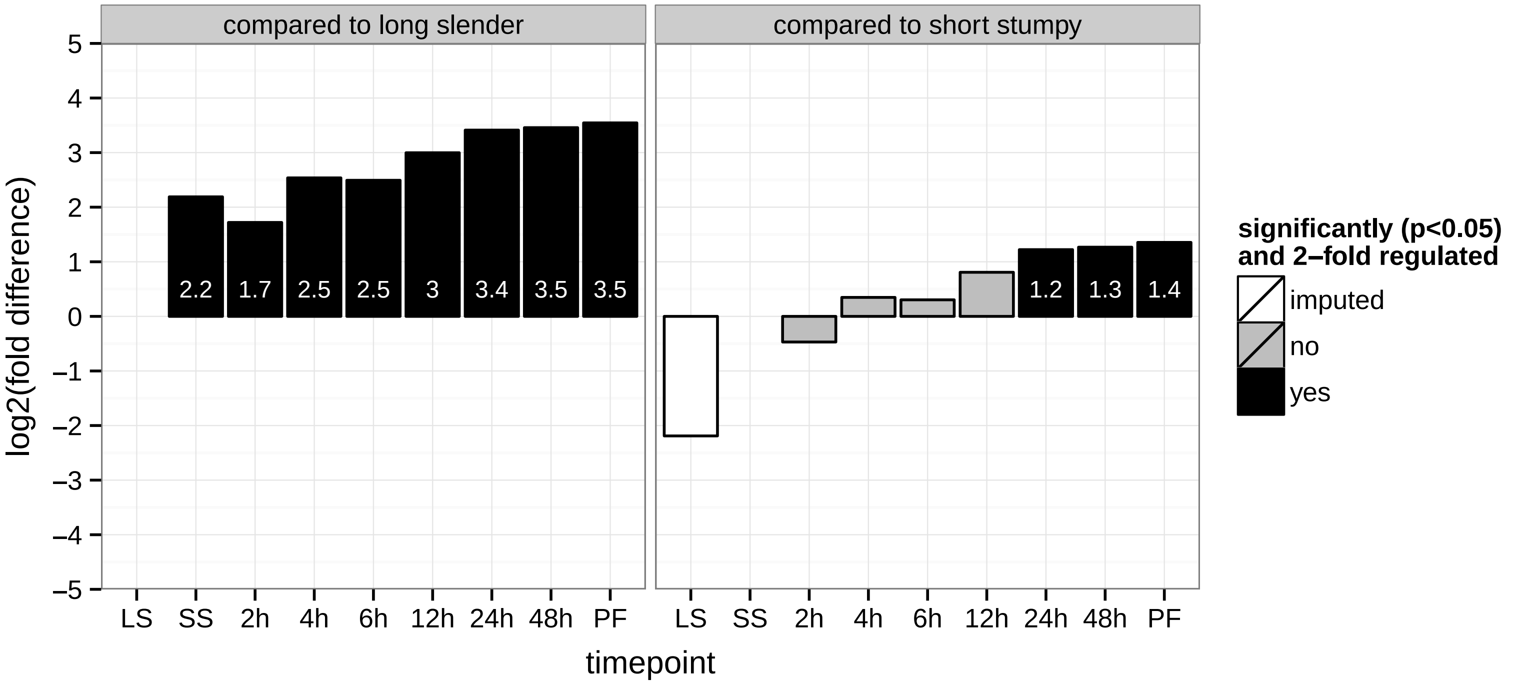

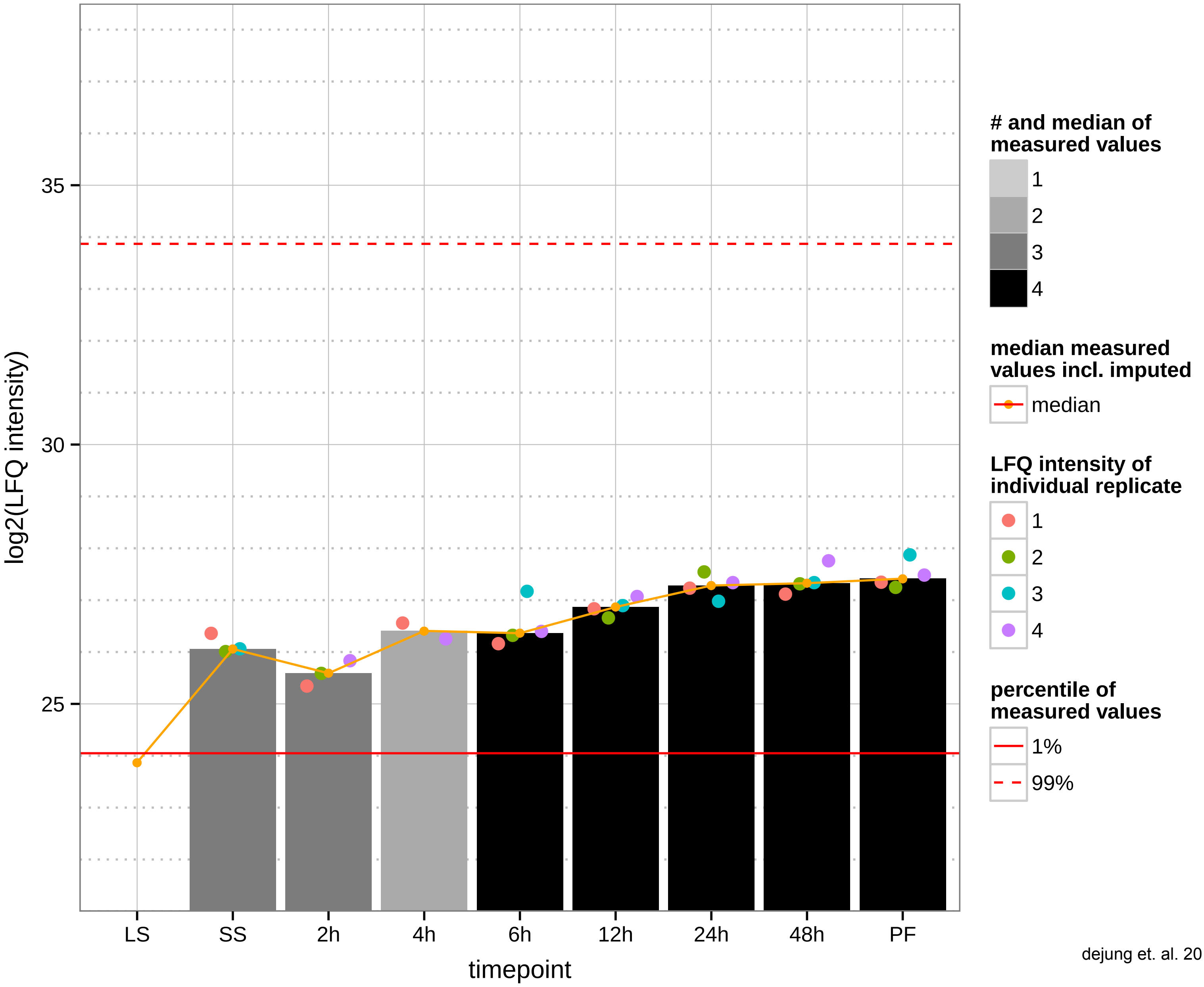

dejung et. al. 2015

hypothetical protein, conserved
Tb927.10.8980
AGOF: null
AGOC: mitochondrion
AGOP: null
PGOF: null
PGOC: null
PGOP: null

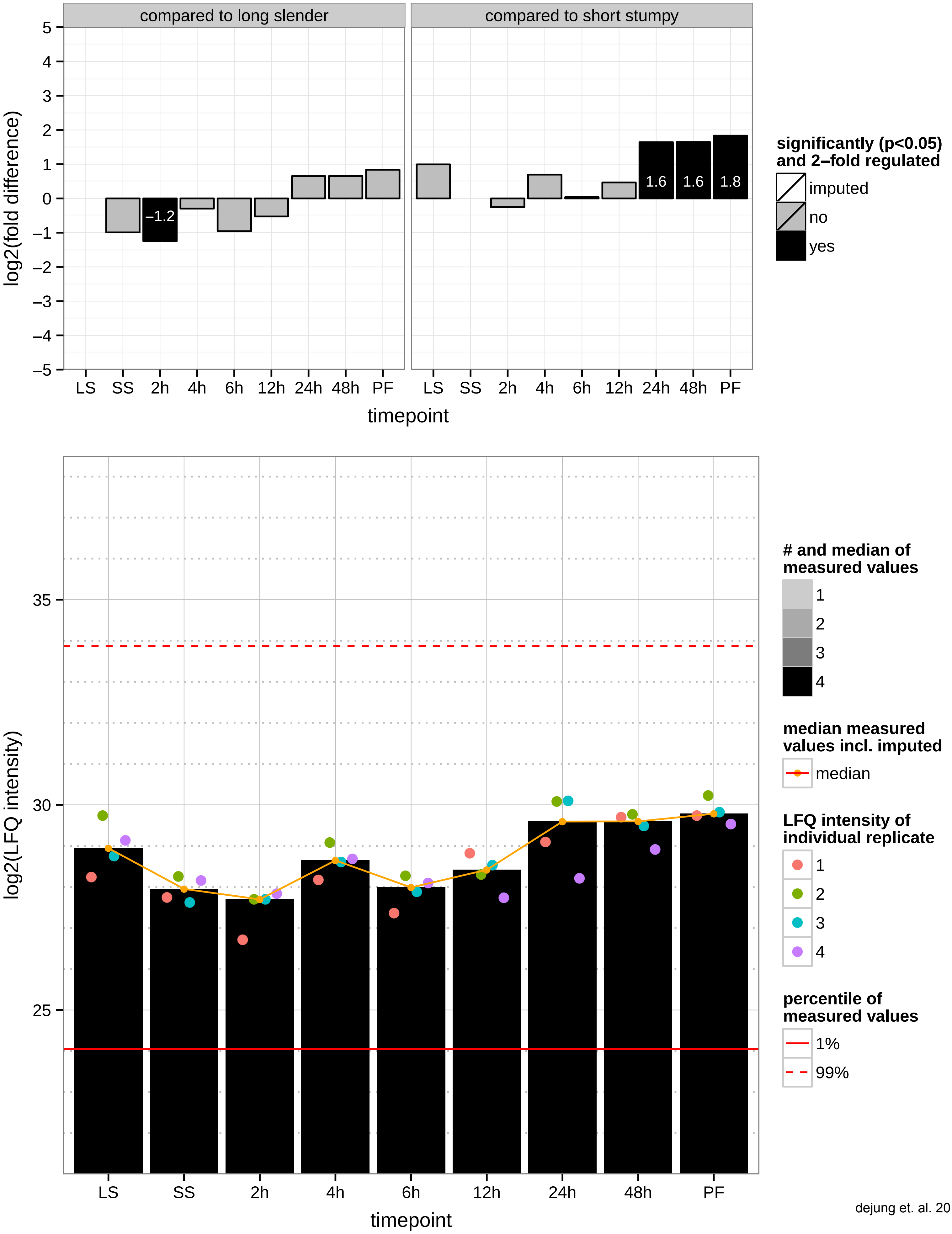

dejung et. al. 2015

hypothetical protein, conserved
Tb927.10.920
AGOF: RNA binding
AGOC: null
AGOP: RNA processing
PGOF: RNA binding, structural constituent of ribosome
PGOC: large ribosomal subunit
PGOP: translation

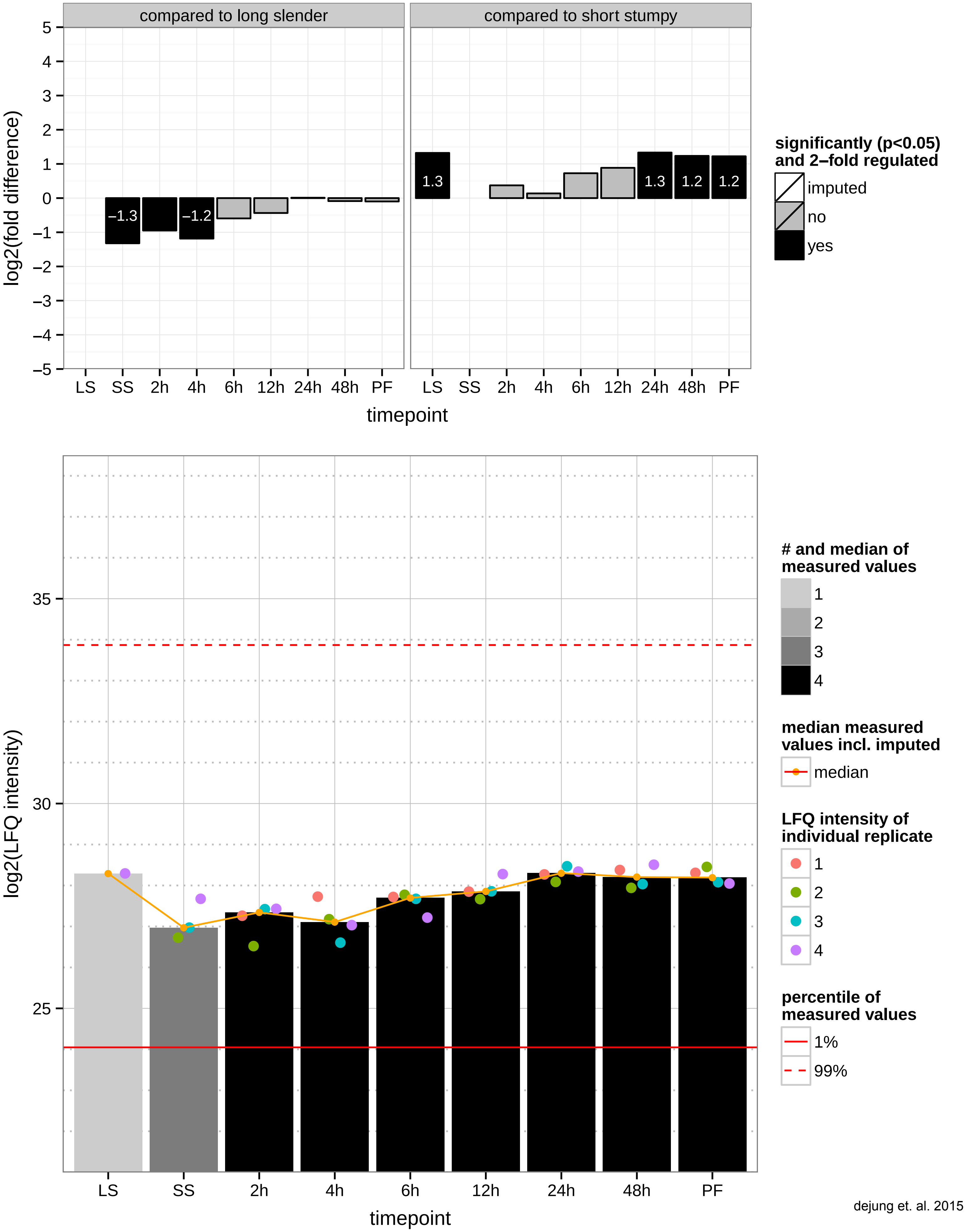

ATP−dependent DEAD/H RNA helicase, putative, ATP− dependent RNA helicase
Tb927.10.9780
AGOF: ATP binding, ATP−dependent helicase activity, helicase activity, nucleic acid binding
AGOC: null
AGOP: nucleobase−containing compound metabolic process
PGOF: ATP binding, ATP−dependent helicase activity, helicase activity, nucleic acid binding
PGOC: null
PGOP: null

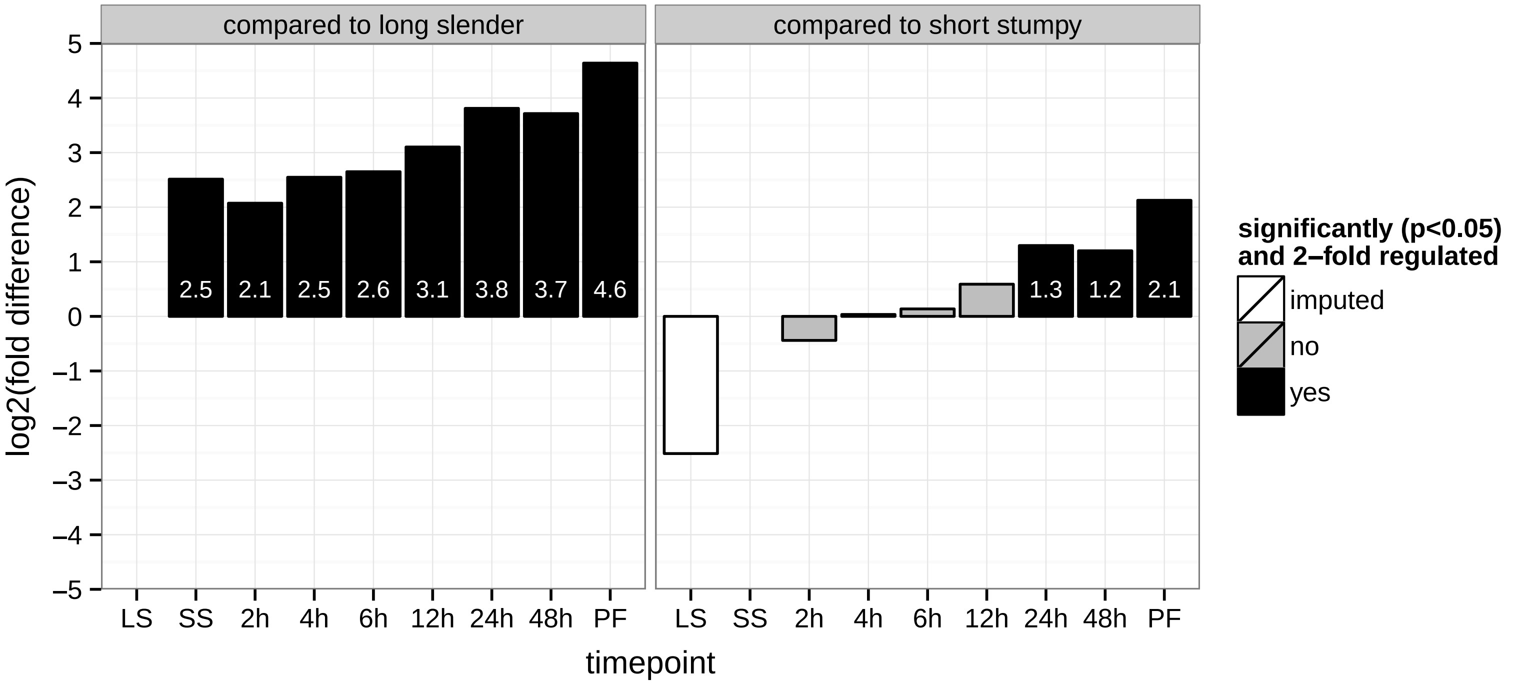

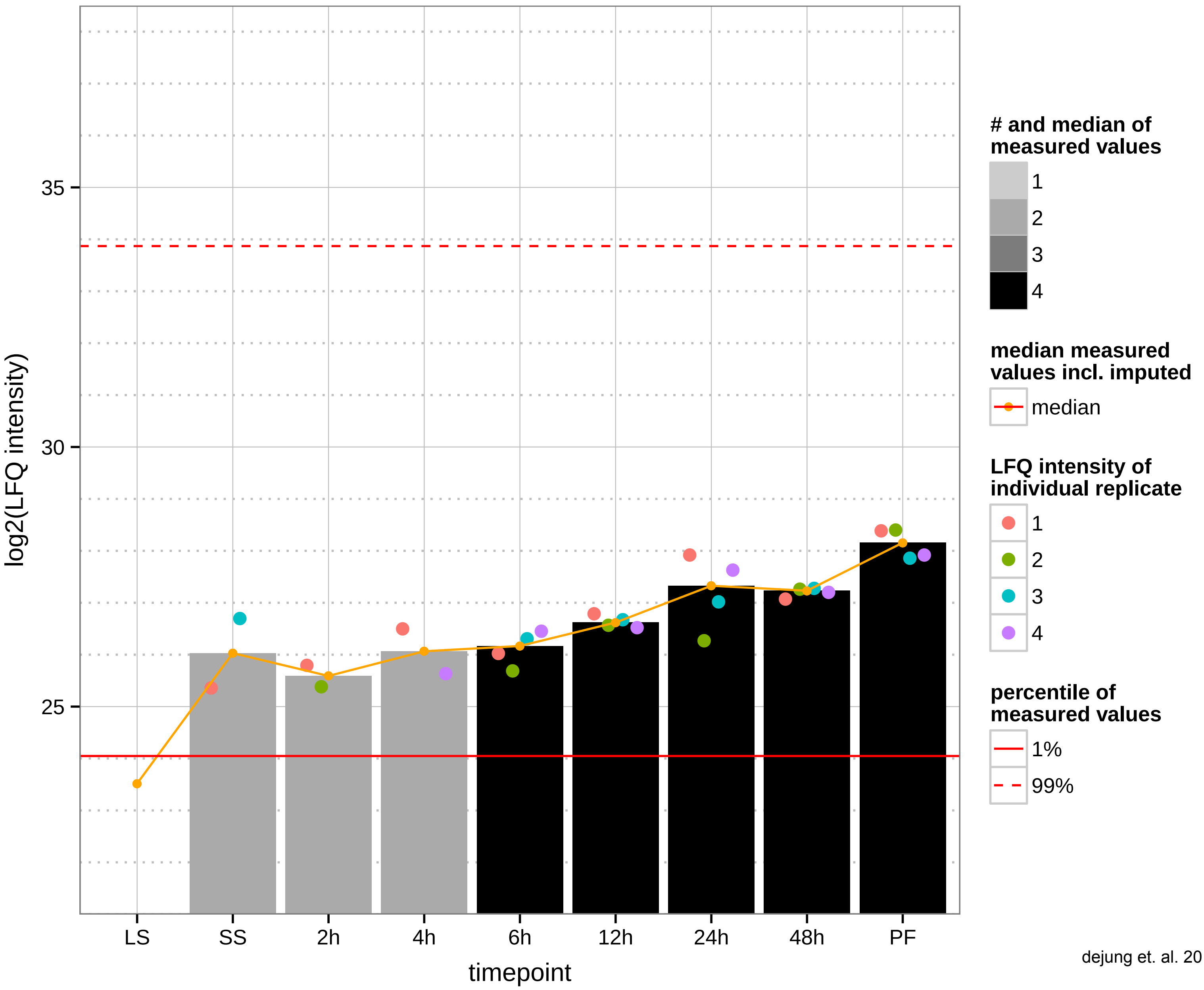

dejung et. al. 2015

hypothetical protein, conserved
Tb927.10.9810
AGOF: null
AGOC: mitochondrion
AGOP: null
PGOF: null
PGOC: null
PGOP: null

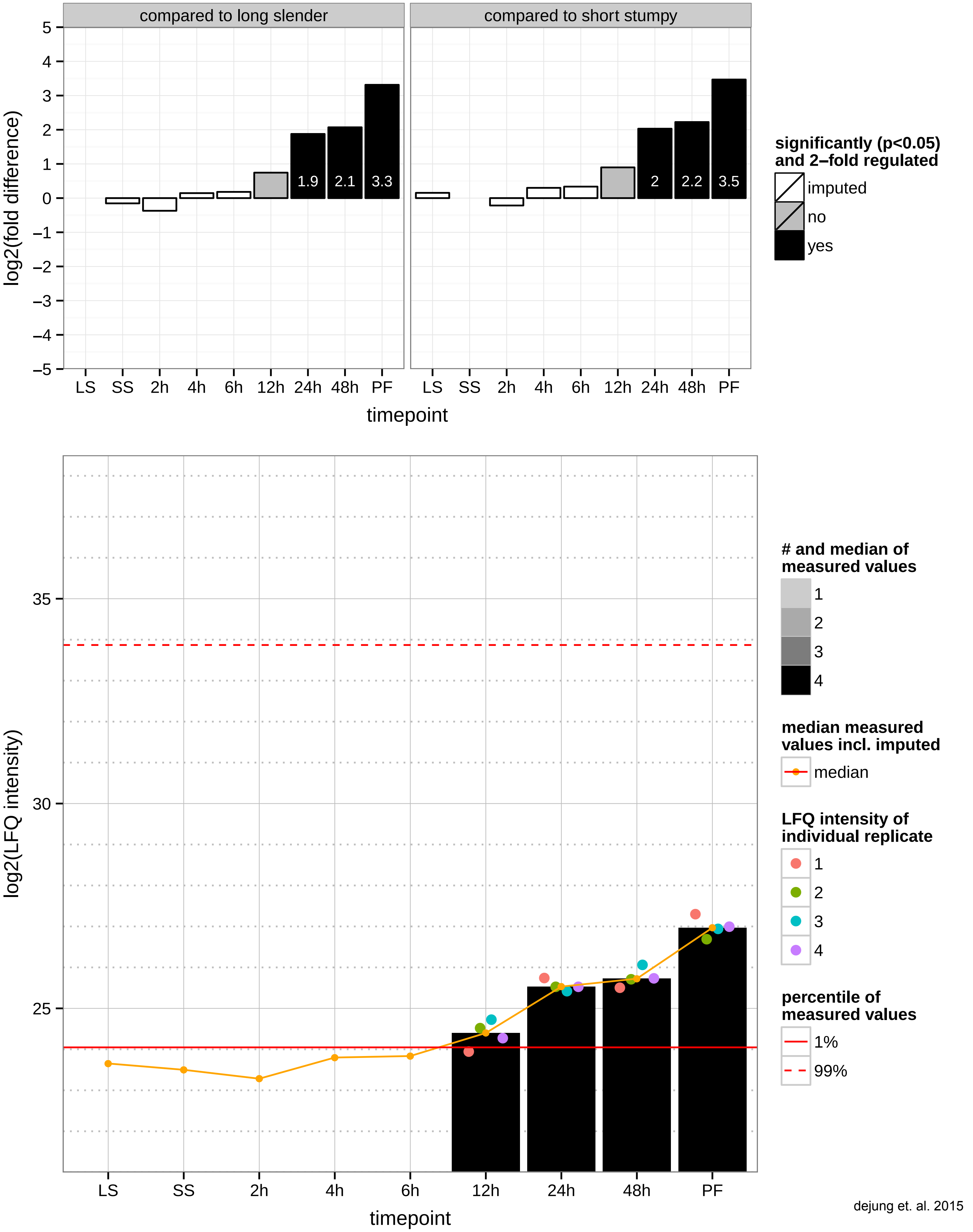

dejung et. al. 2015

mitochondrial intermediate peptidase, putative, metallo–peptidase, Clan MA(E) Family M3
Tb927.10.9820;Tb11.v5.0565
AGOF: null, metalloendopeptidase activity
AGOC: null, mitochondrion
AGOP: null, proteolysis
PGOF: metalloendopeptidase activity
PGOC: null
PGOP: proteolysis

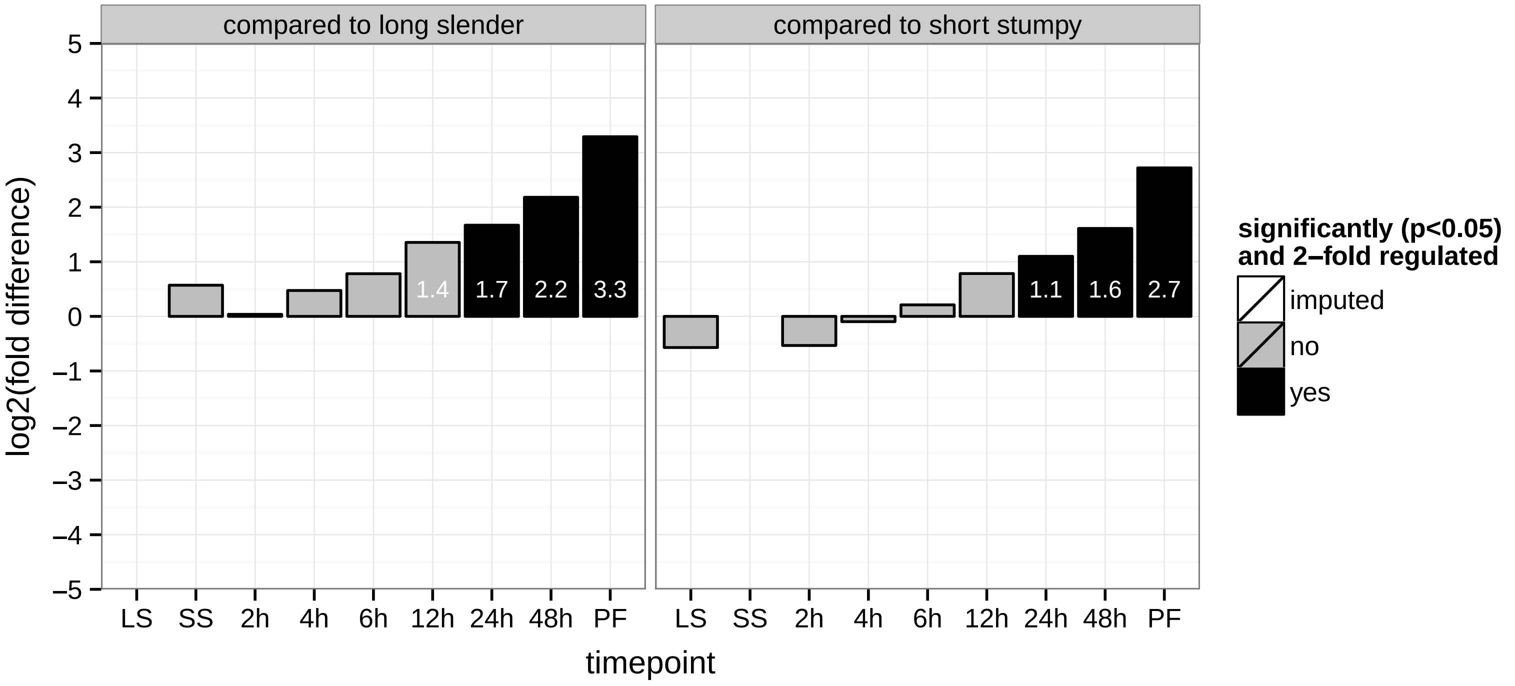

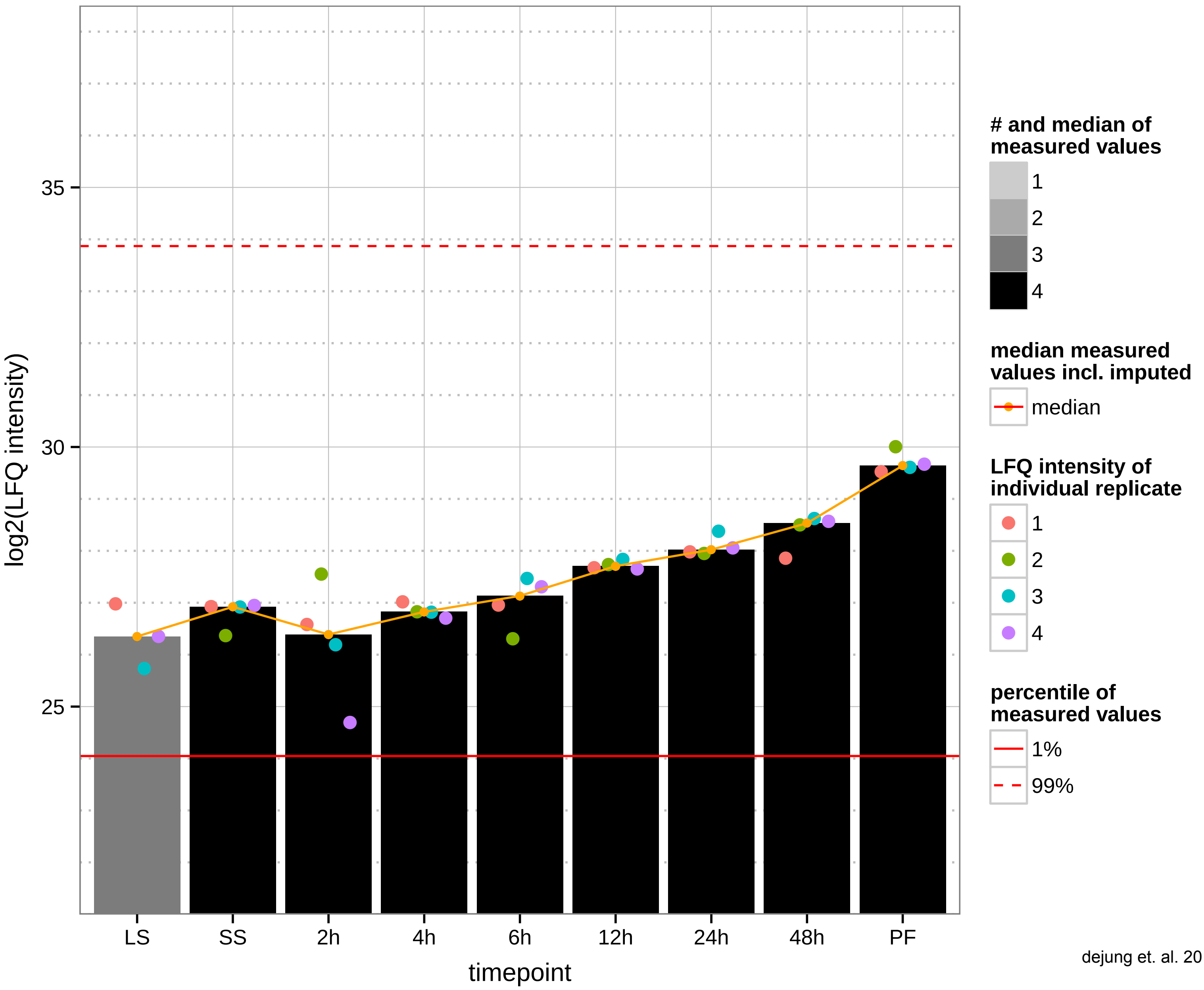

dejung et. al. 2015

hypothetical protein, conserved
Tb927.10.9830
AGOF: null
AGOC: mitochondrion
AGOP: null
PGOF: null
PGOC: null
PGOP: null

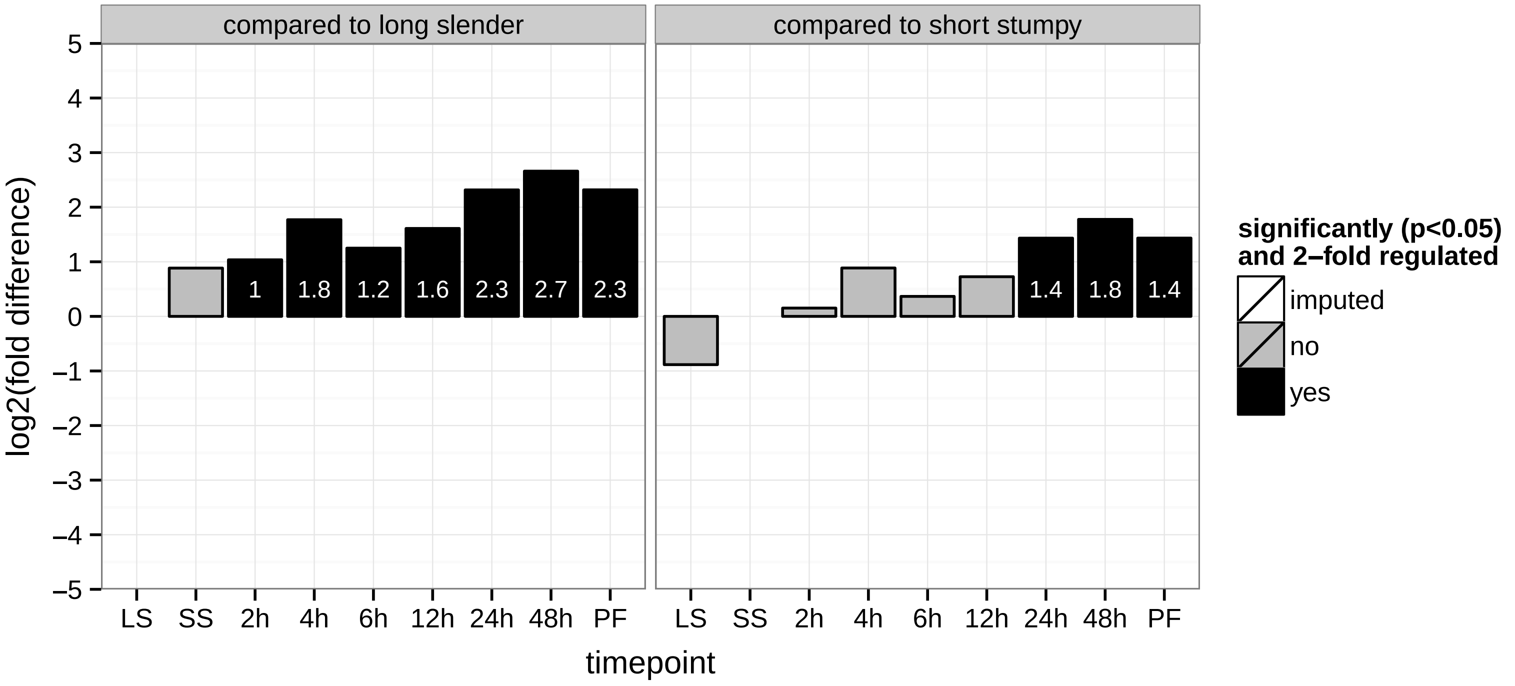

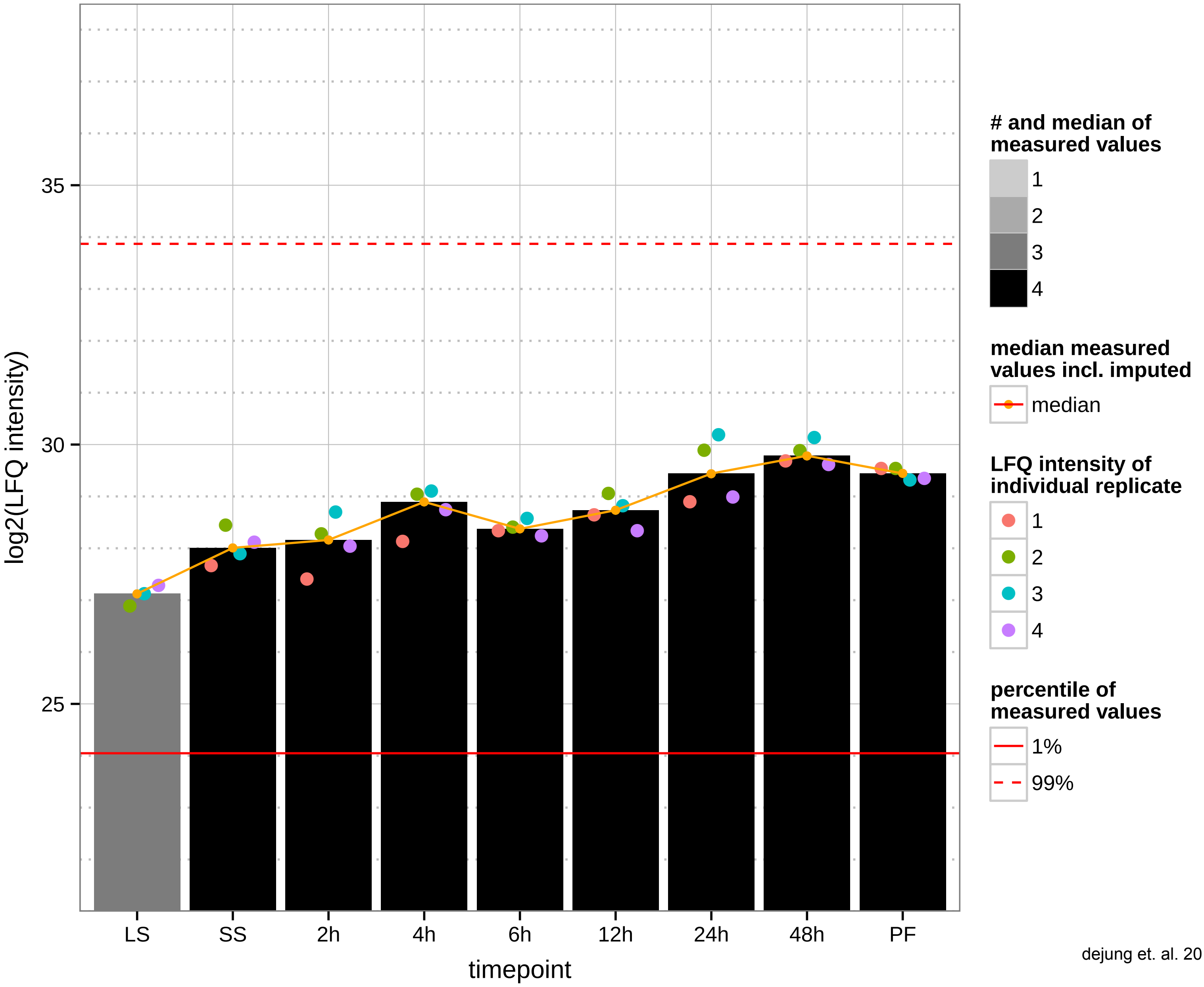

dejung et. al. 2015

hypothetical protein, conserved
Tb927.10.9990
AGOF: hydrolase activity
AGOC: null
AGOP: null
PGOF: hydrolase activity
PGOC: null
PGOP: null

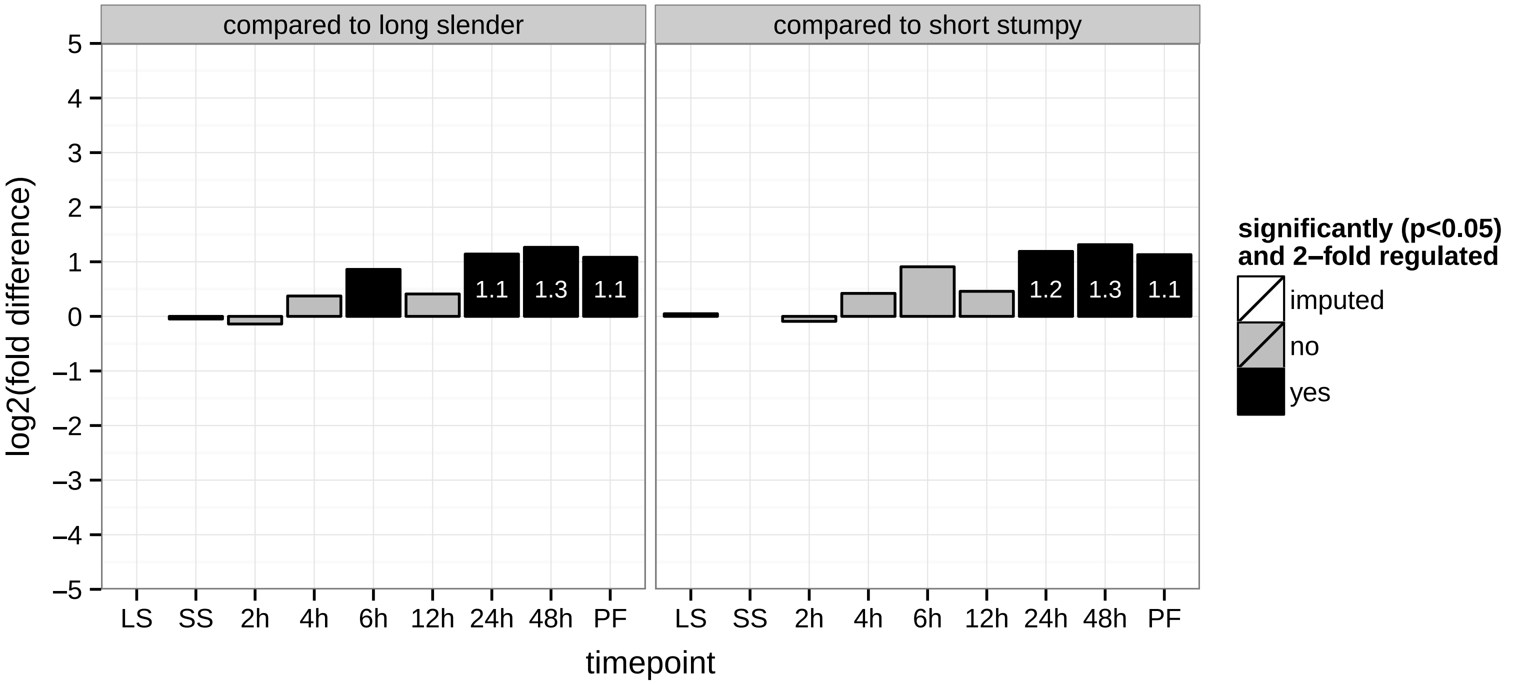

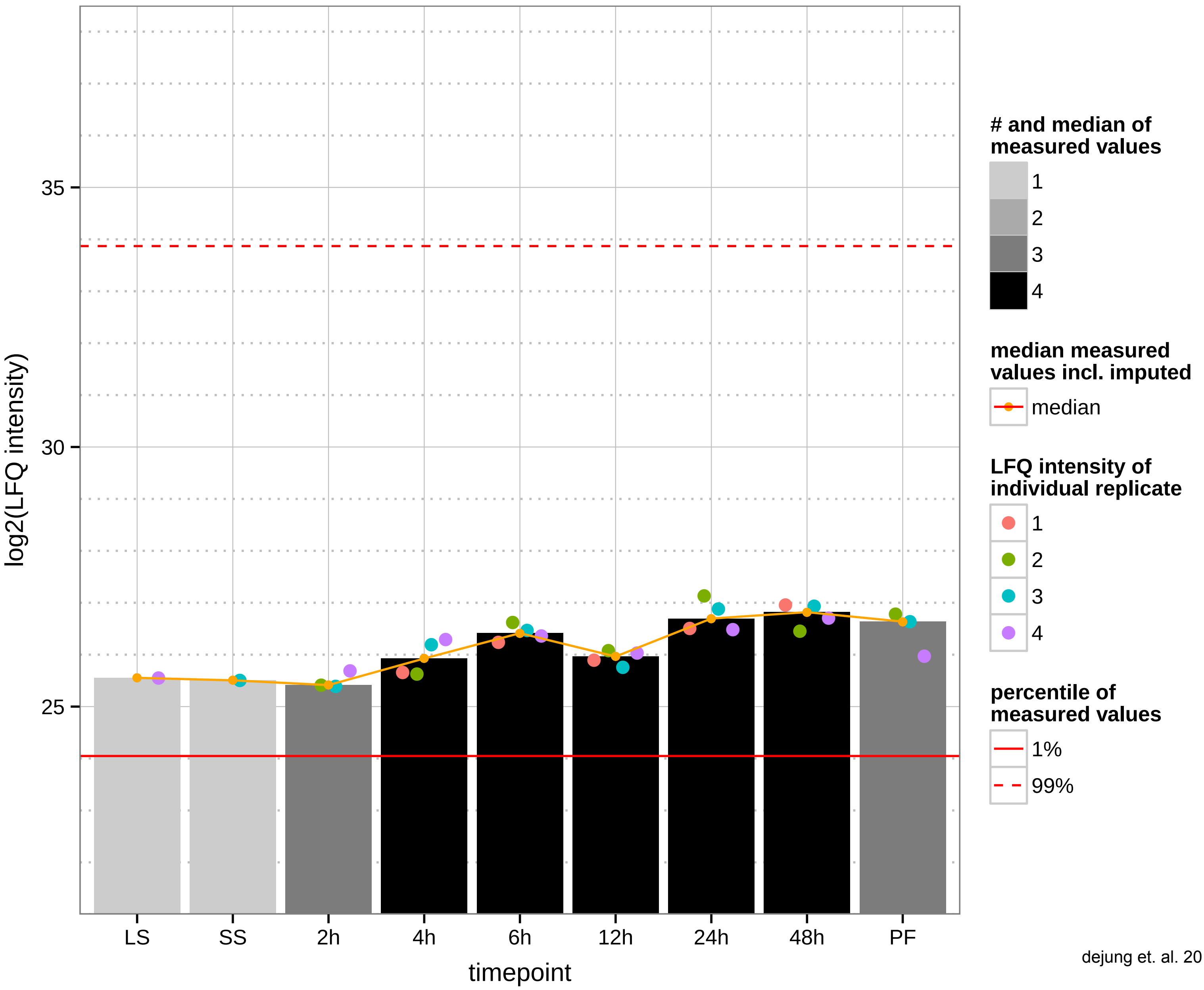

dejung et. al. 2015

hypothetical protein, conserved
Tb927.11.10400
AGOF: null
AGOC: mitochondrion
AGOP: null
PGOF: null
PGOC: null
PGOP: null

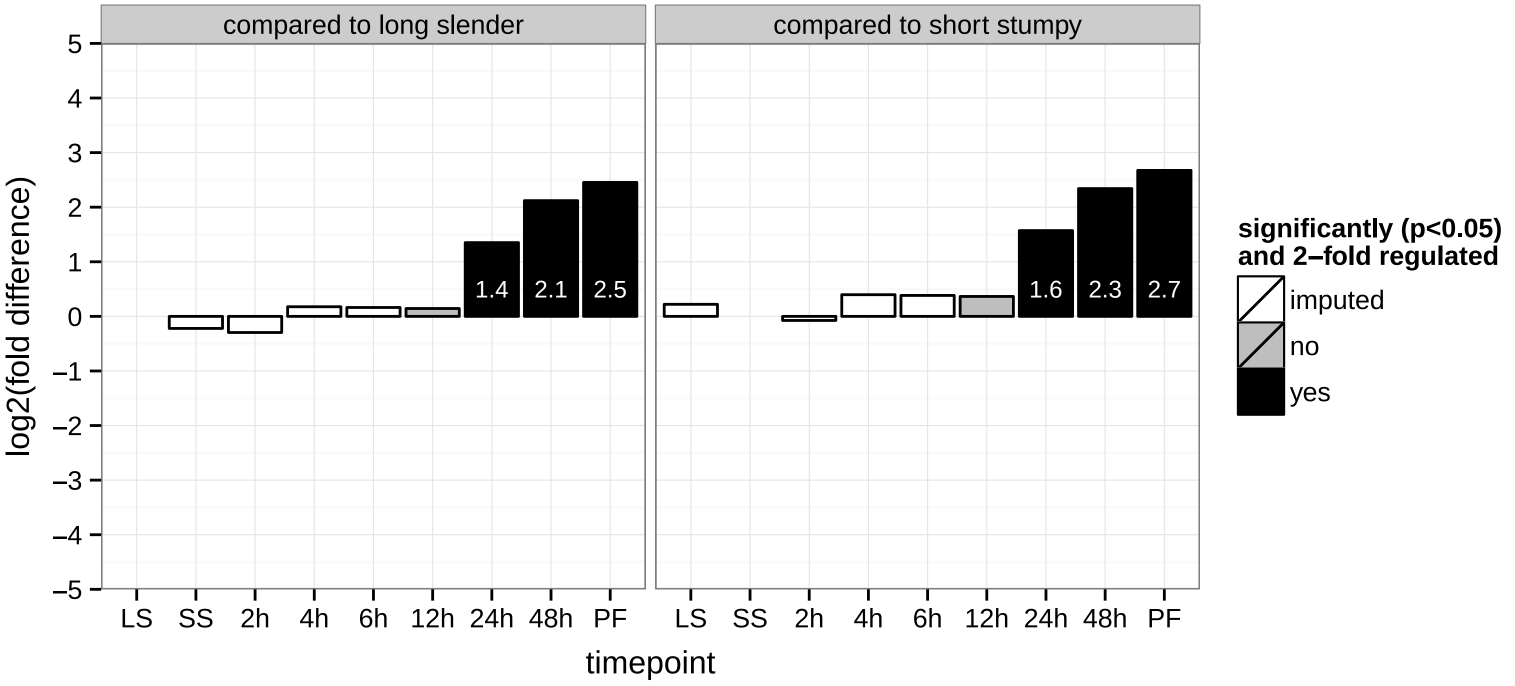

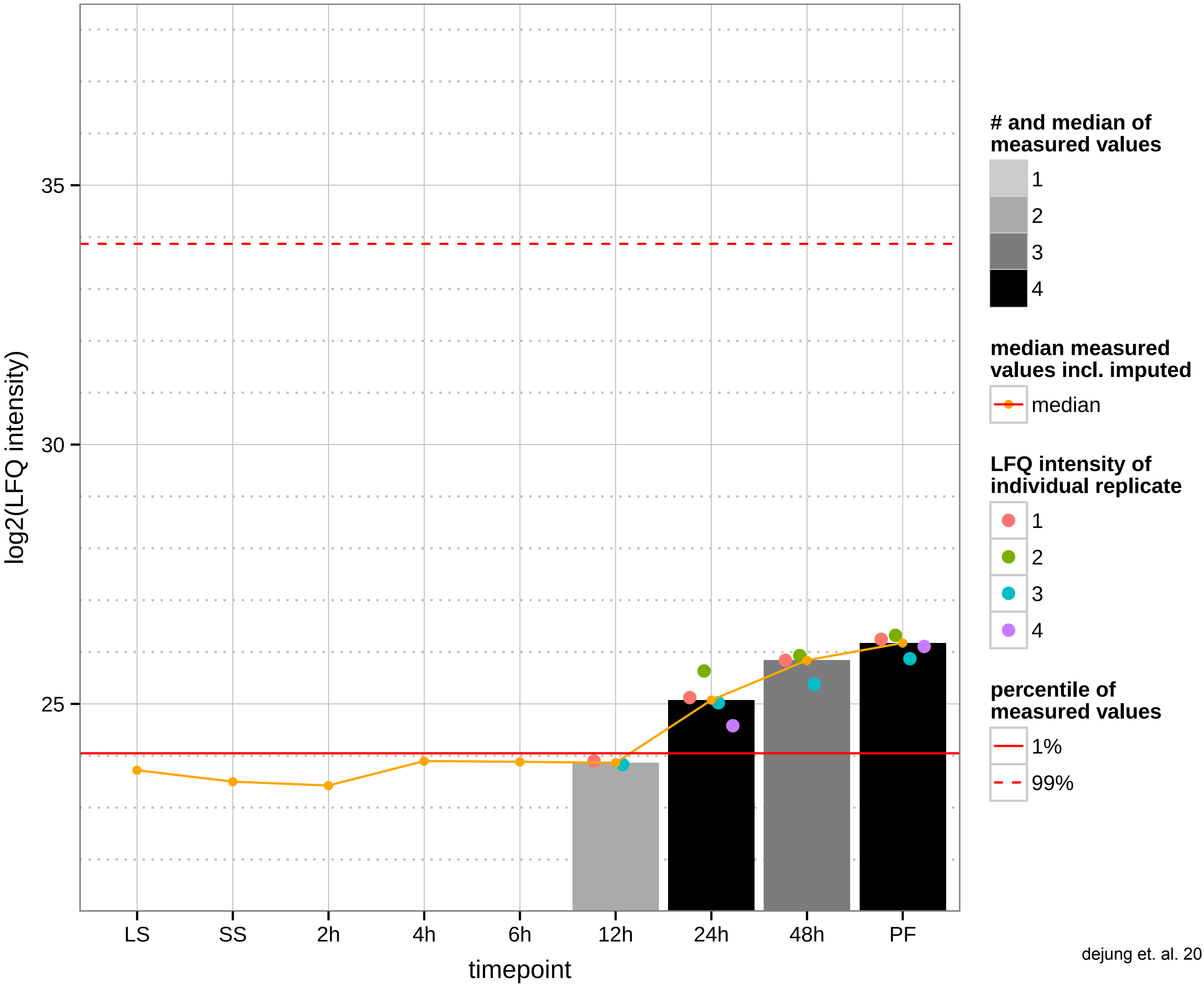

dejung et. al. 2015

glycosomal transporter (GAT3), putative (gat3)
Tb927.11.1070
AGOF: ATP binding, ATPase activity
AGOC: ATP−binding cassette (ABC) transporter complex, integral to membrane, membrane
AGOP: transport
PGOF: ATP binding, ATPase activity, coupled to transmembrane movement of substances, nucleoside−triphosphatase activity
PGOC: integral to membrane, membrane
PGOP: transmembrane transport, transport

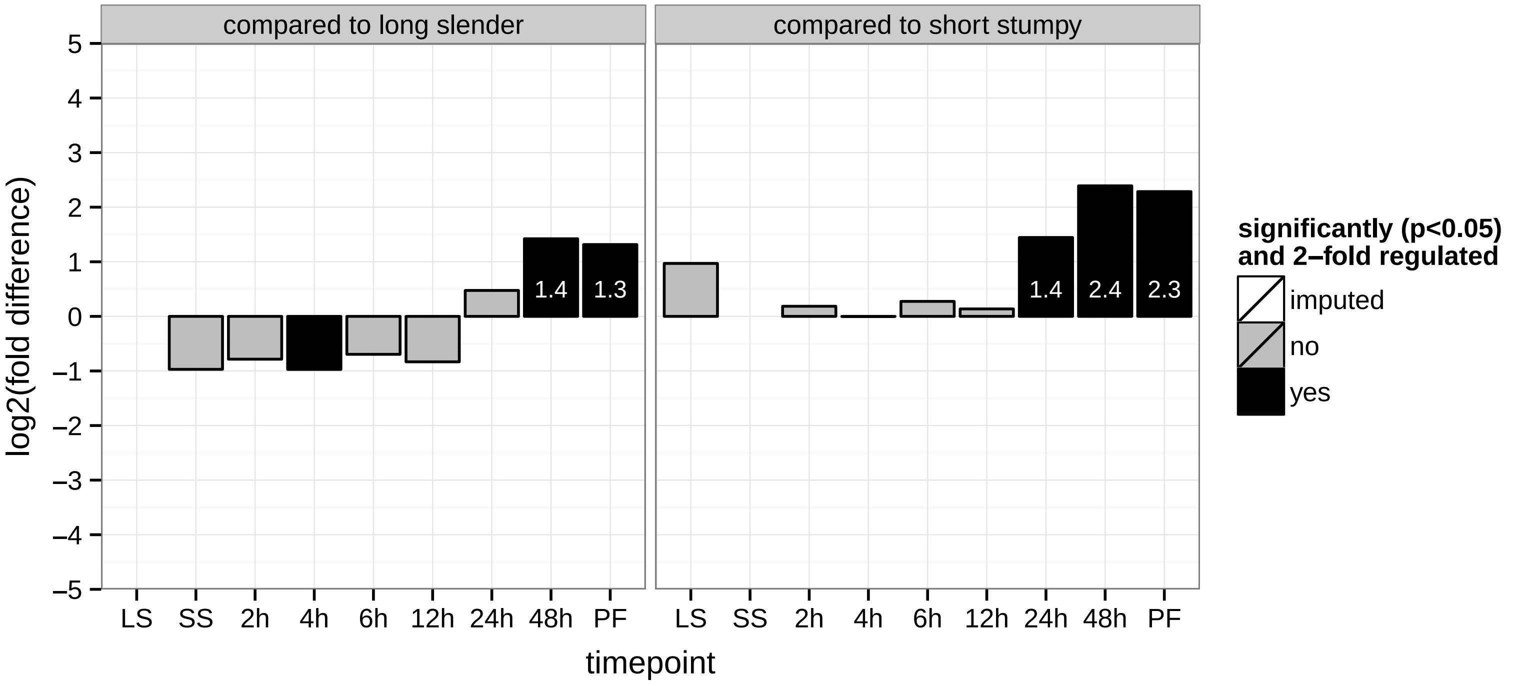

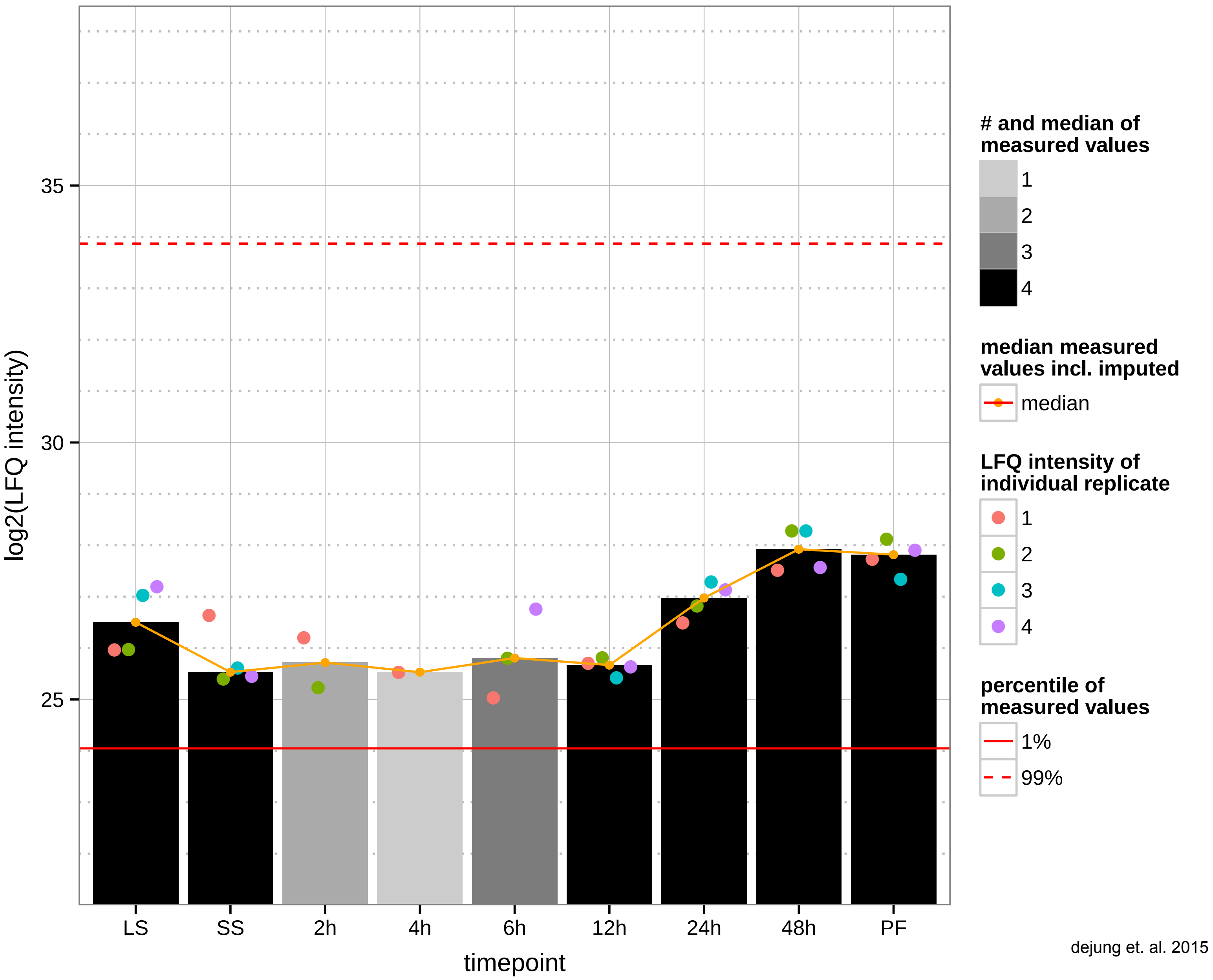

dejung et. al. 2015

kinesin–like protein, putative
Tb927.11.10760
AGOF: ATP binding, microtubule motor activity, motor activity
AGOC: microtubule associated complex
AGOP: microtubule–based movement
PGOF: ATP binding, microtubule motor activity
PGOC: null
PGOP: microtubule–based movement

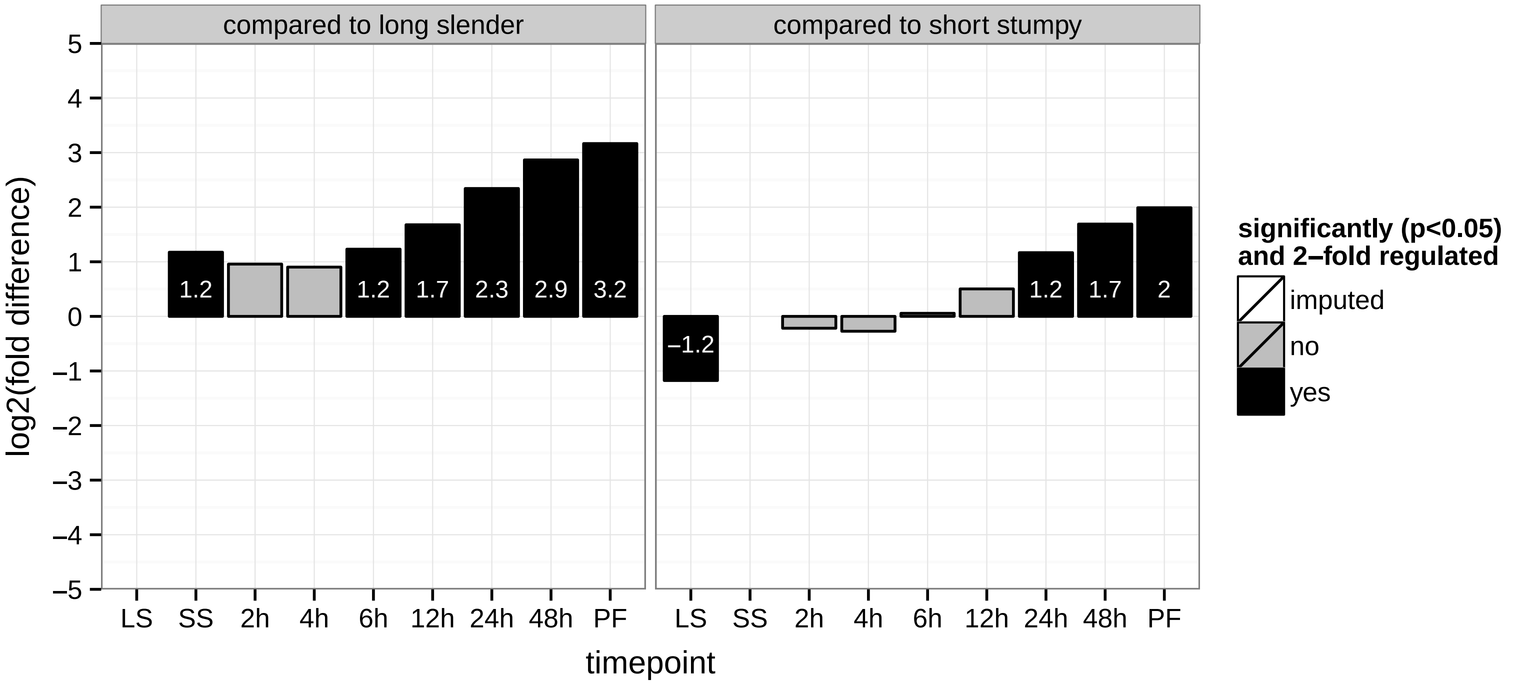

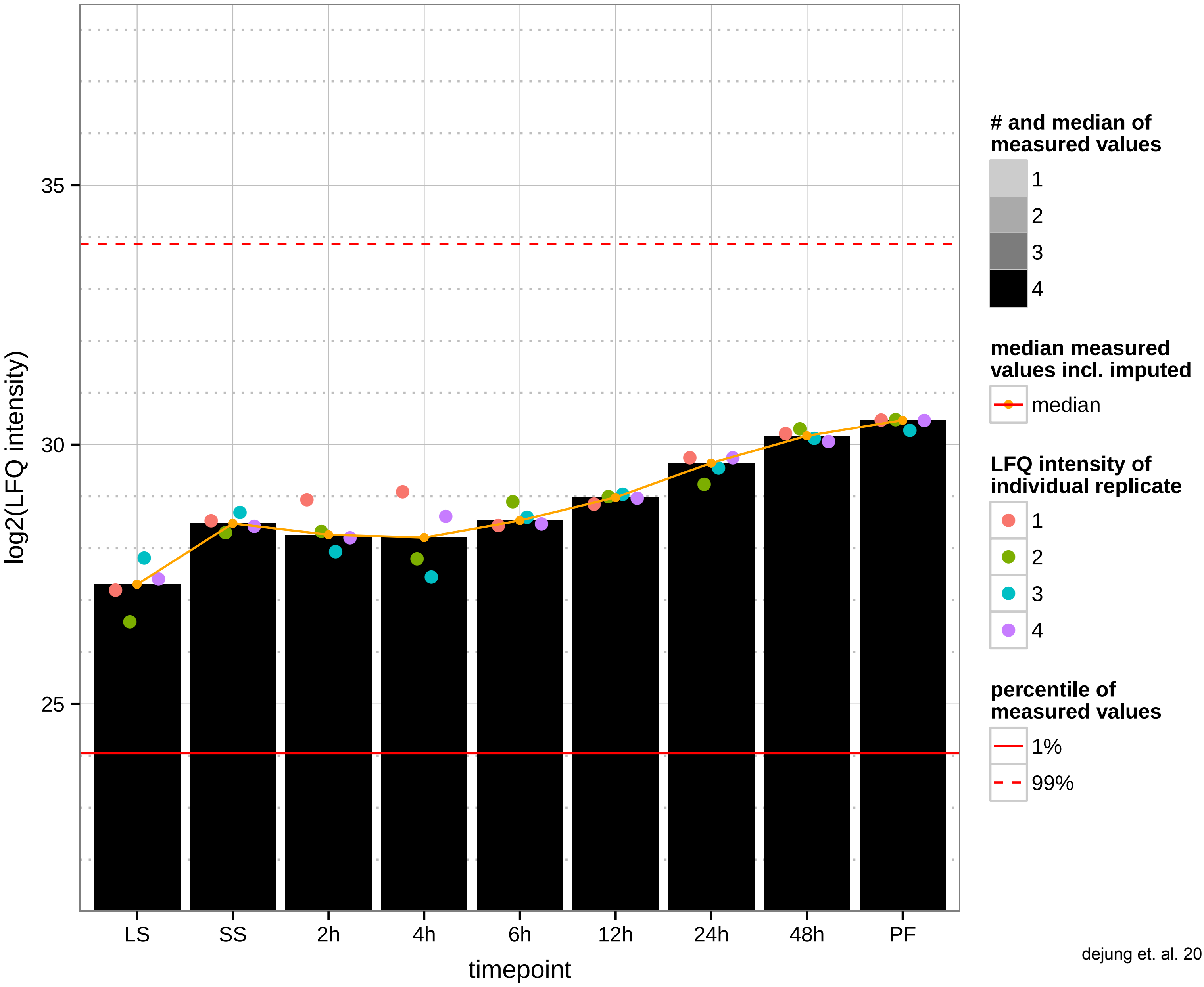

dejung et. al. 2015

hypothetical protein, conserved
Tb927.11.12040
AGOF: null
AGOC: mitochondrion
AGOP: null
PGOF: null
PGOC: null
PGOP: null

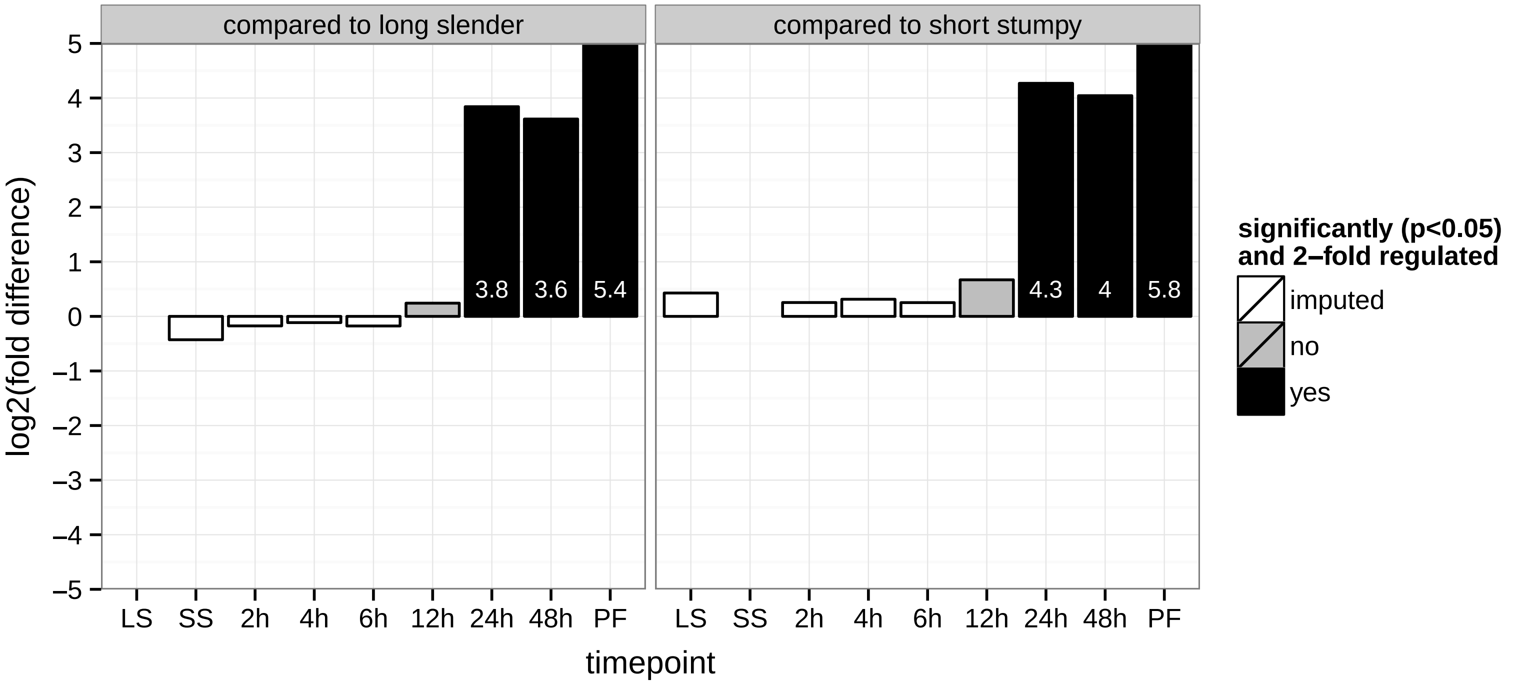

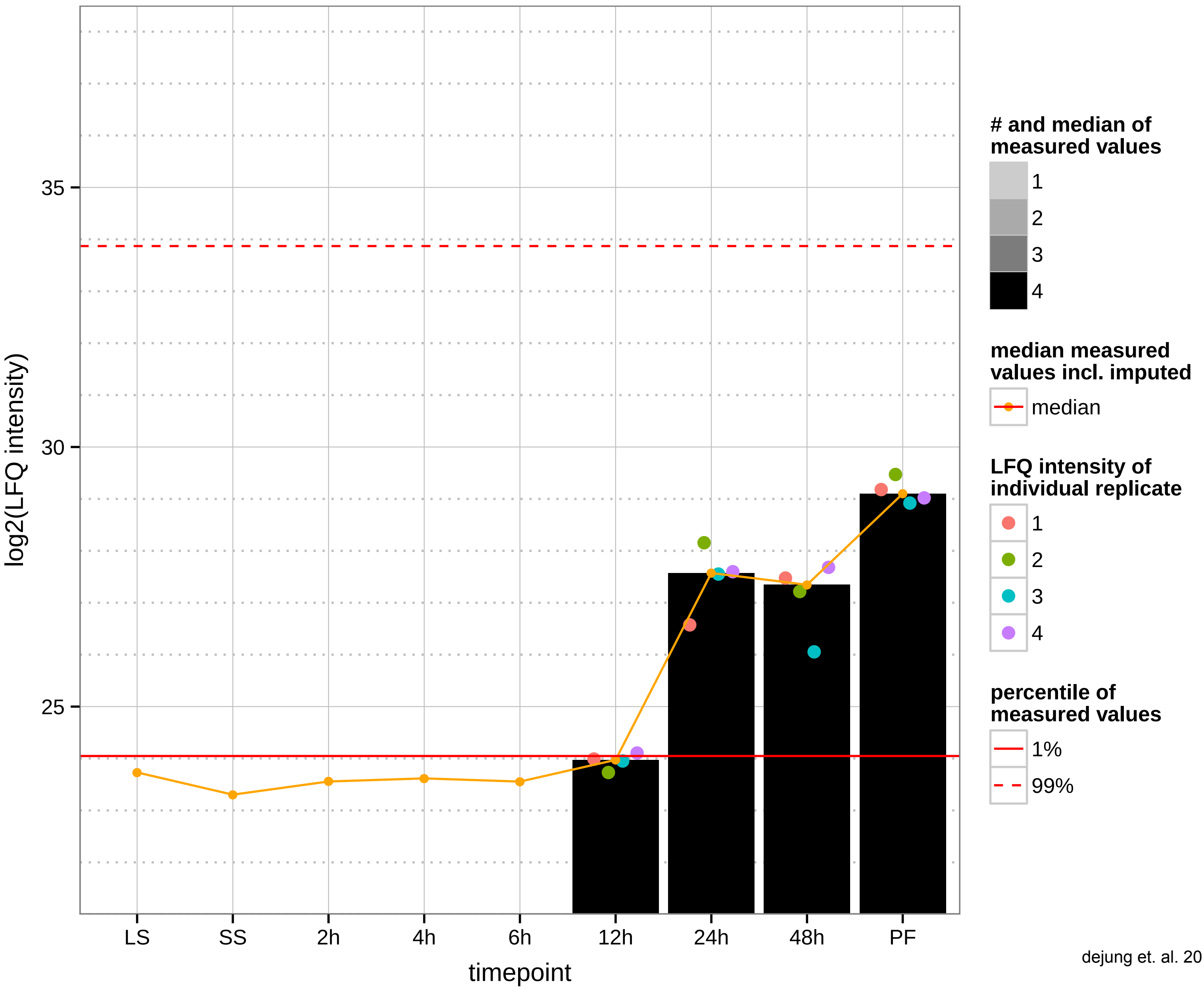

dejung et. al. 2015

hypothetical protein, conserved
Tb927.11.1270
AGOF: null
AGOC: mitochondrial inner membrane, mitochondrion
AGOP: null
PGOF: null
PGOC: null
PGOP: null

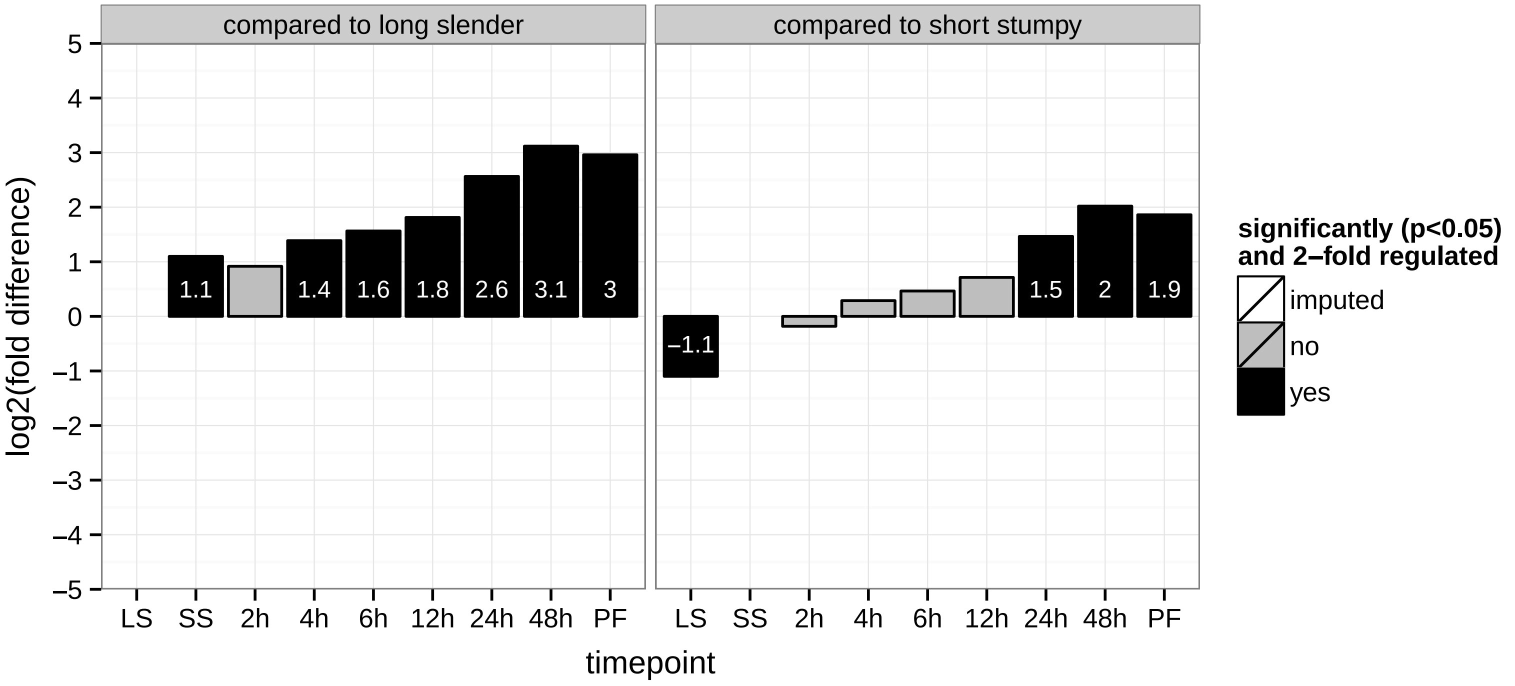

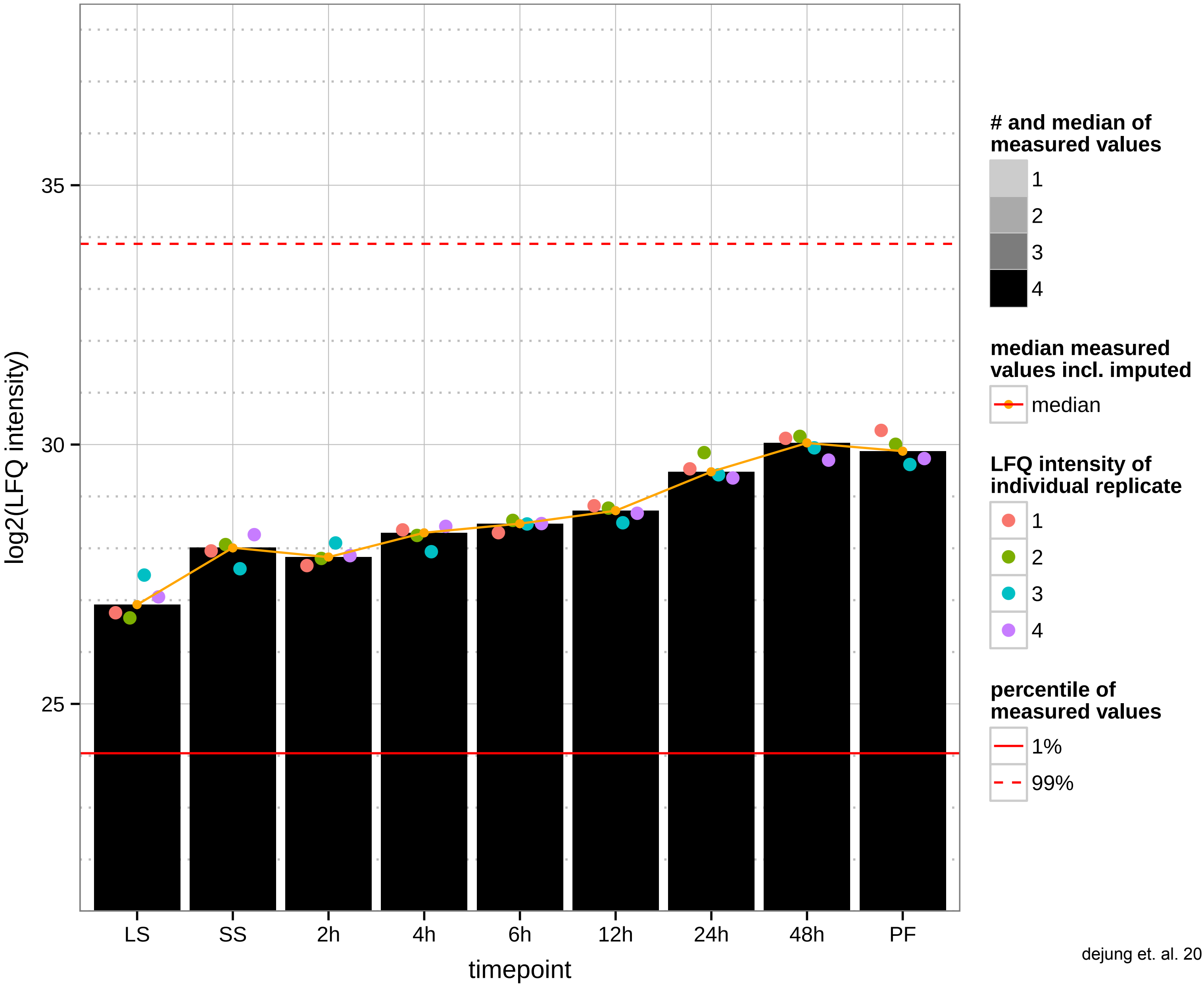

dejung et. al. 2015

hypothetical protein conserved
Tb927.11.13180
AGOF: null
AGOC: mitochondrial inner membrane, mitochondrion
AGOP: null
PGOF: null
PGOC: null
PGOP: null

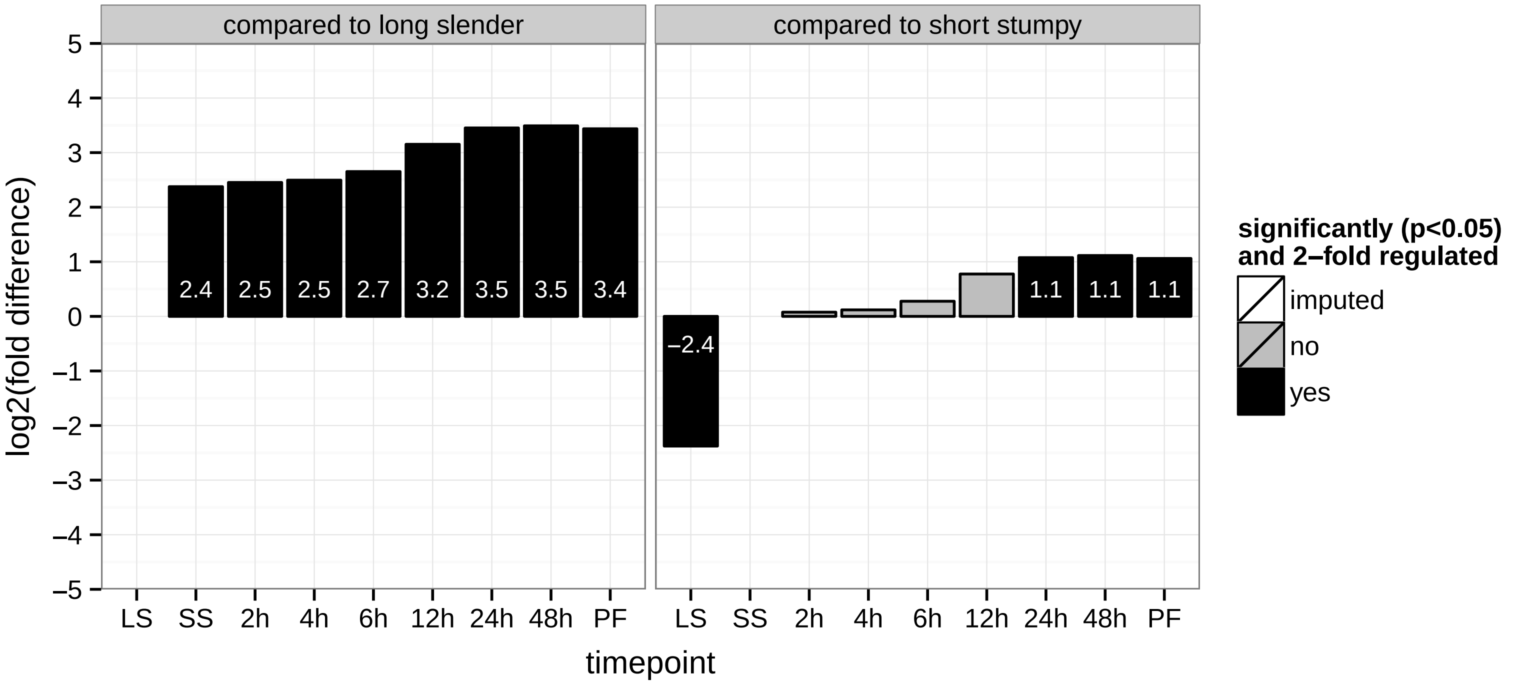

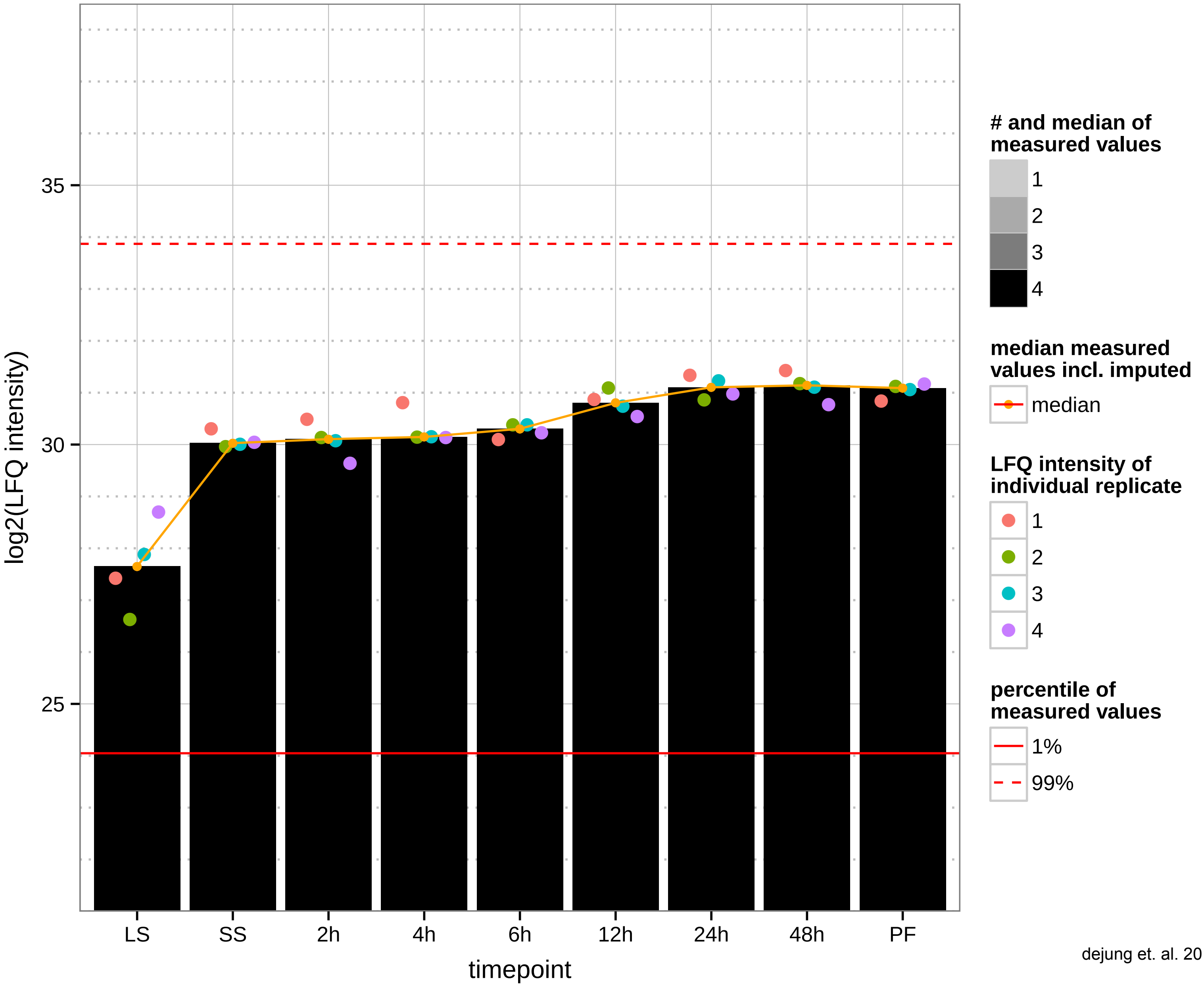

dejung et. al. 2015

eukaryotic translation initiation factor 2 gamma, putative (eIF−2−gamma)
Tb927.11.13250
AGOF: GTP binding, GTPase activity, histone methyltransferase activity, translation initiation factor activity
AGOC: eukaryotic 43S preinitiation complex
AGOP: translation, translational initiation
PGOF: GTP binding, GTPase activity
PGOC: null
PGOP: null

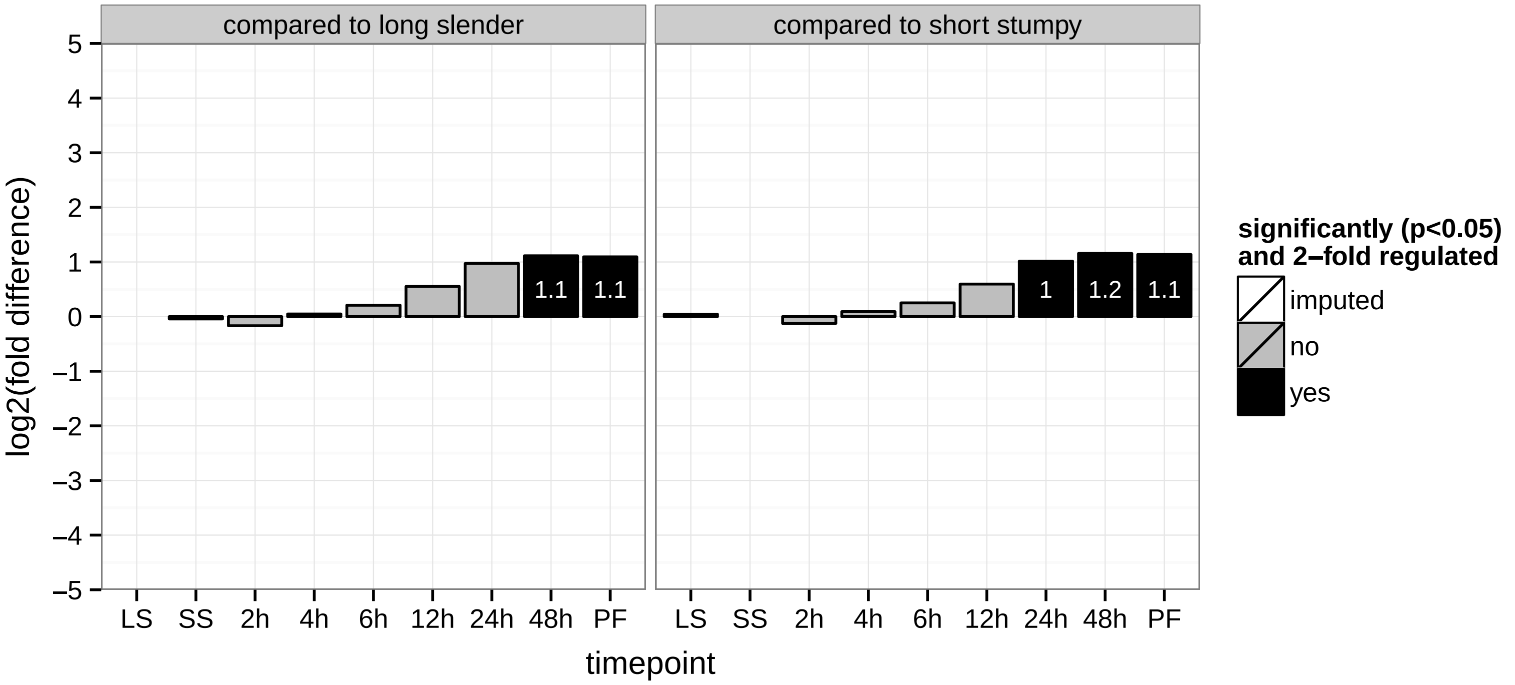

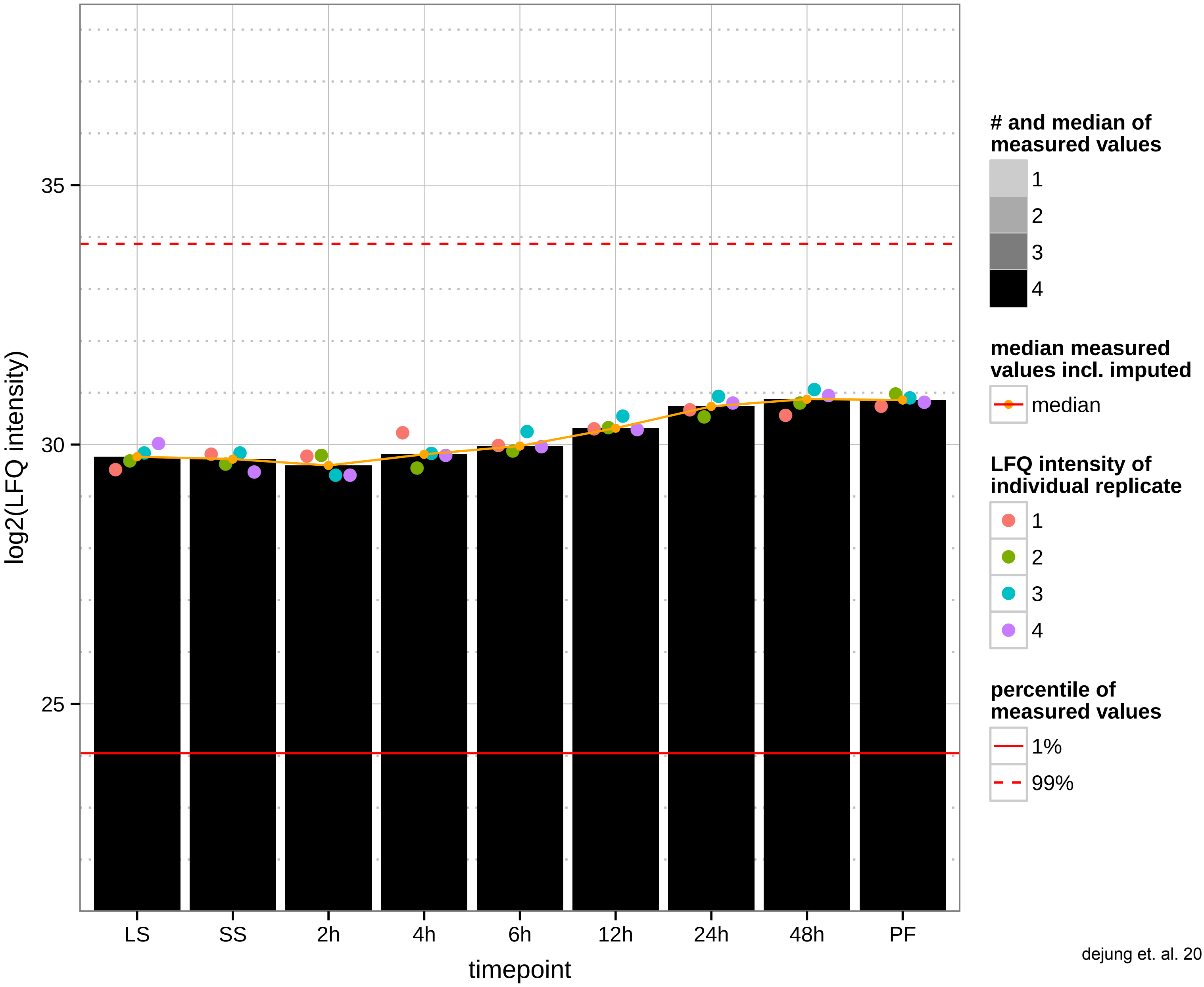

dejung et. al. 2015

hypothetical protein, conserved
Tb927.11.1340
AGOF: null
AGOC: null
AGOP: null
PGOF: transferase activity, transferring phosphorus–containing groups
PGOC: null
PGOP: null

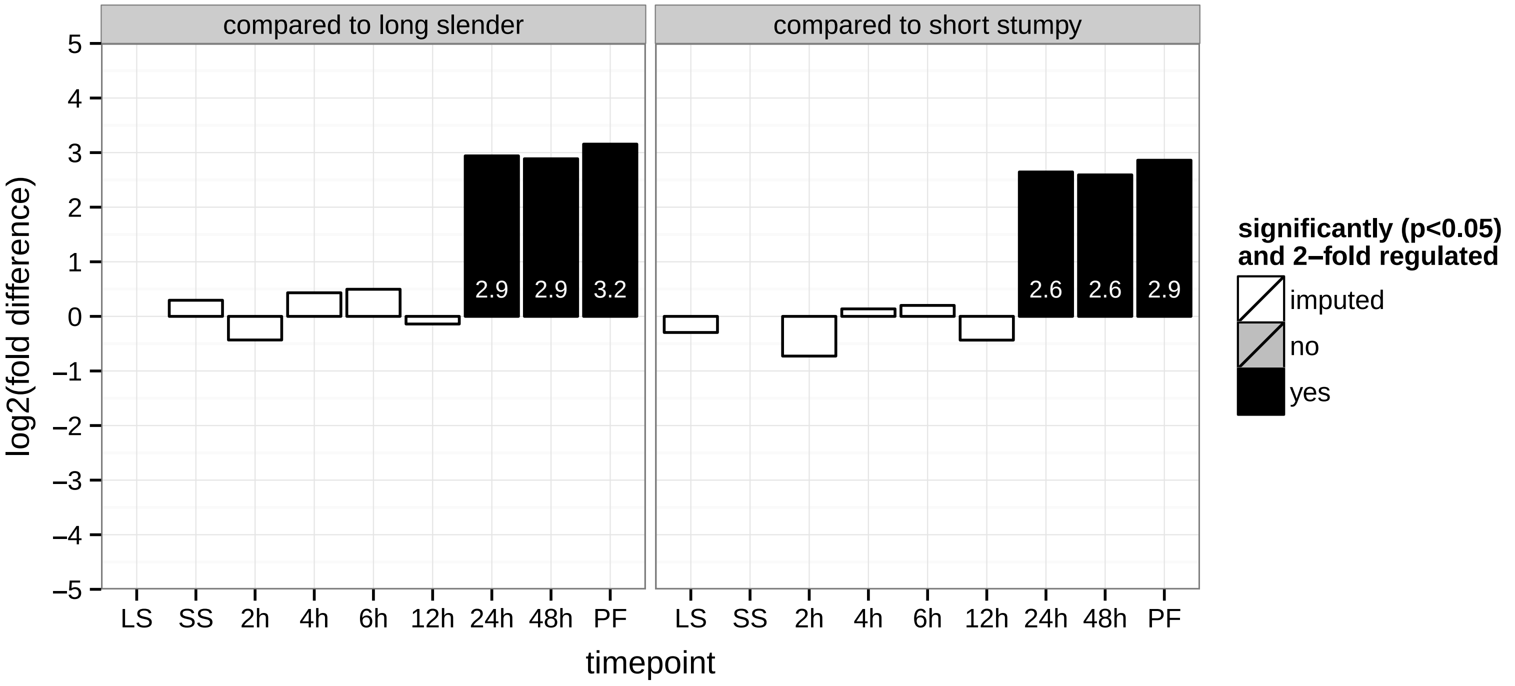

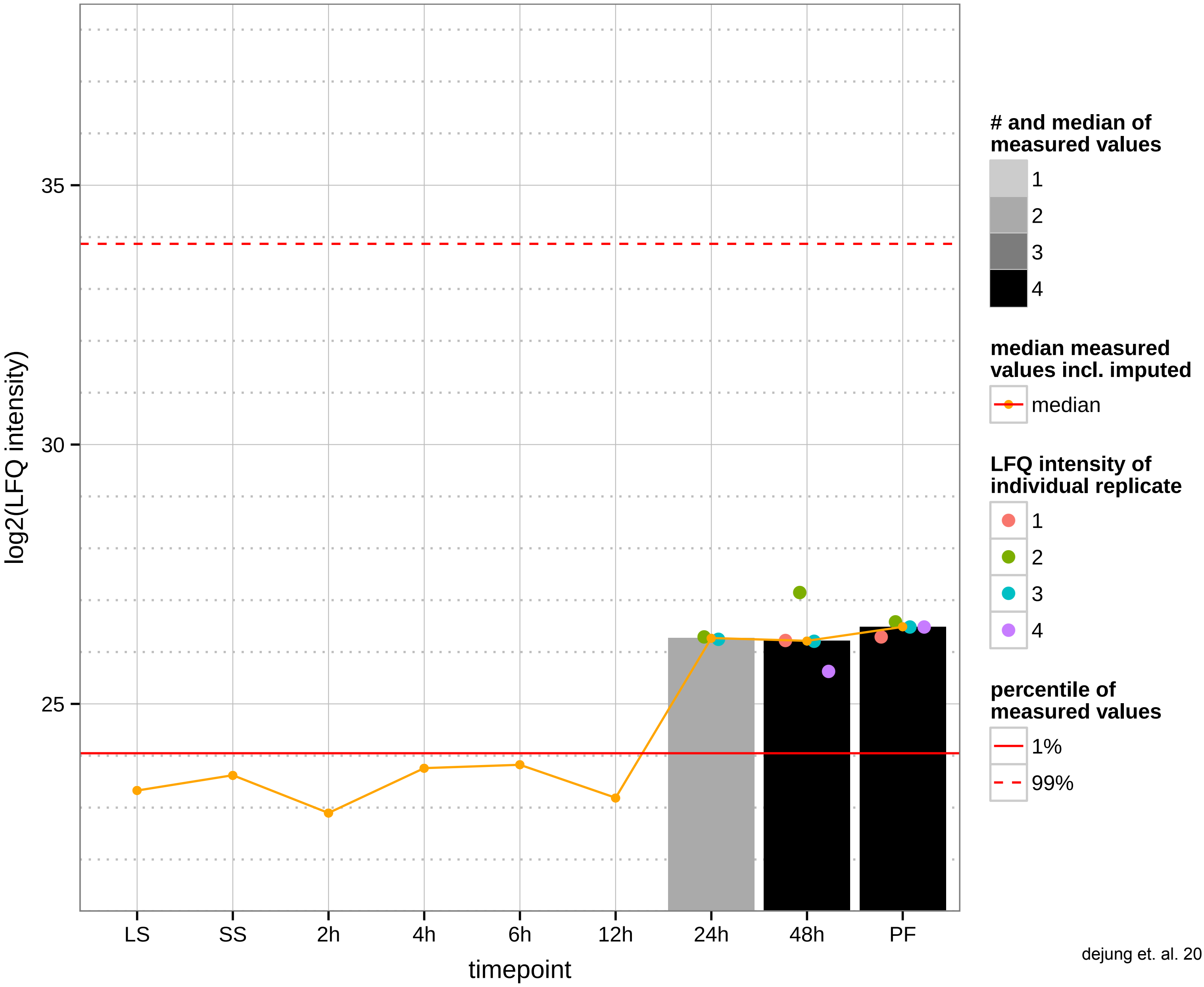

dejung et. al. 2015

G5–interacting protein (G5–IP)
Tb927.11.14590
AGOF: protein binding
AGOC: cytoplasm, eukaryotic translation initiation factor 4F complex
AGOP: RNA capping
PGOF: null
PGOC: null
PGOP: null

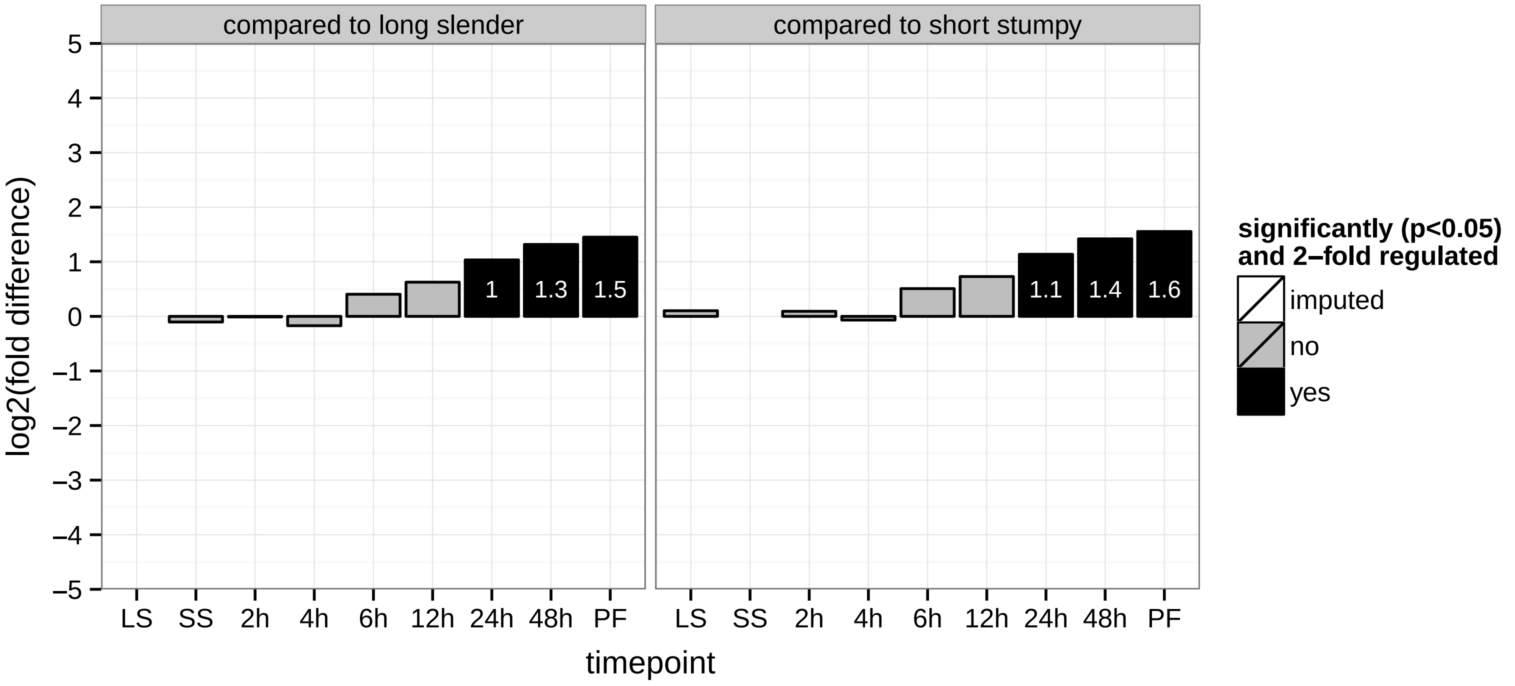

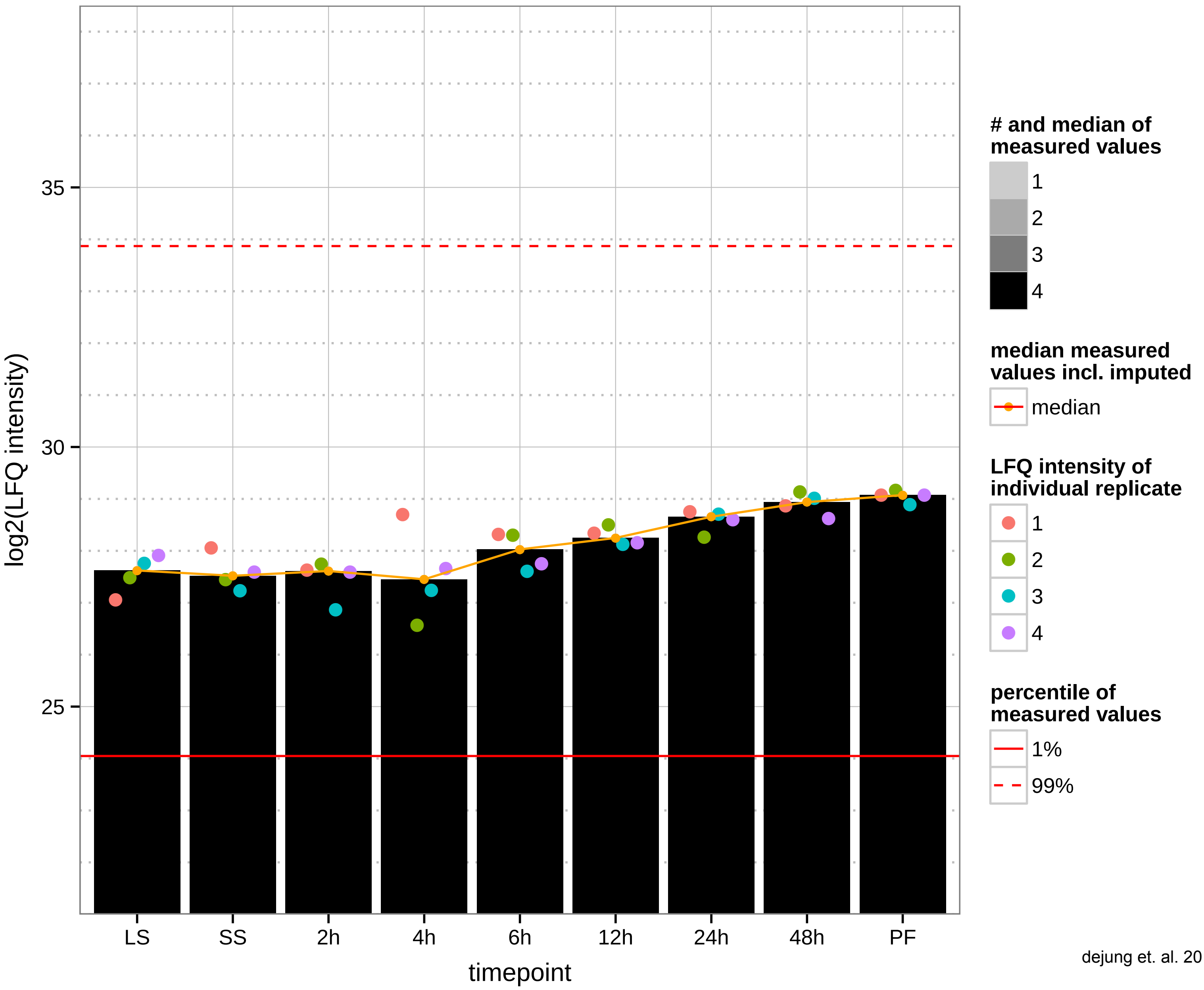

dejung et. al. 2015

zinc finger protein 2 (ZFP2)
Tb927.11.14950
AGOF: RNA binding, nucleic acid binding, zinc ion binding
AGOC: cytoplasm
AGOP: cell differentiation, microtubule polymerization
PGOF: nucleic acid binding, protein binding, zinc ion binding
PGOC: null
PGOP: null

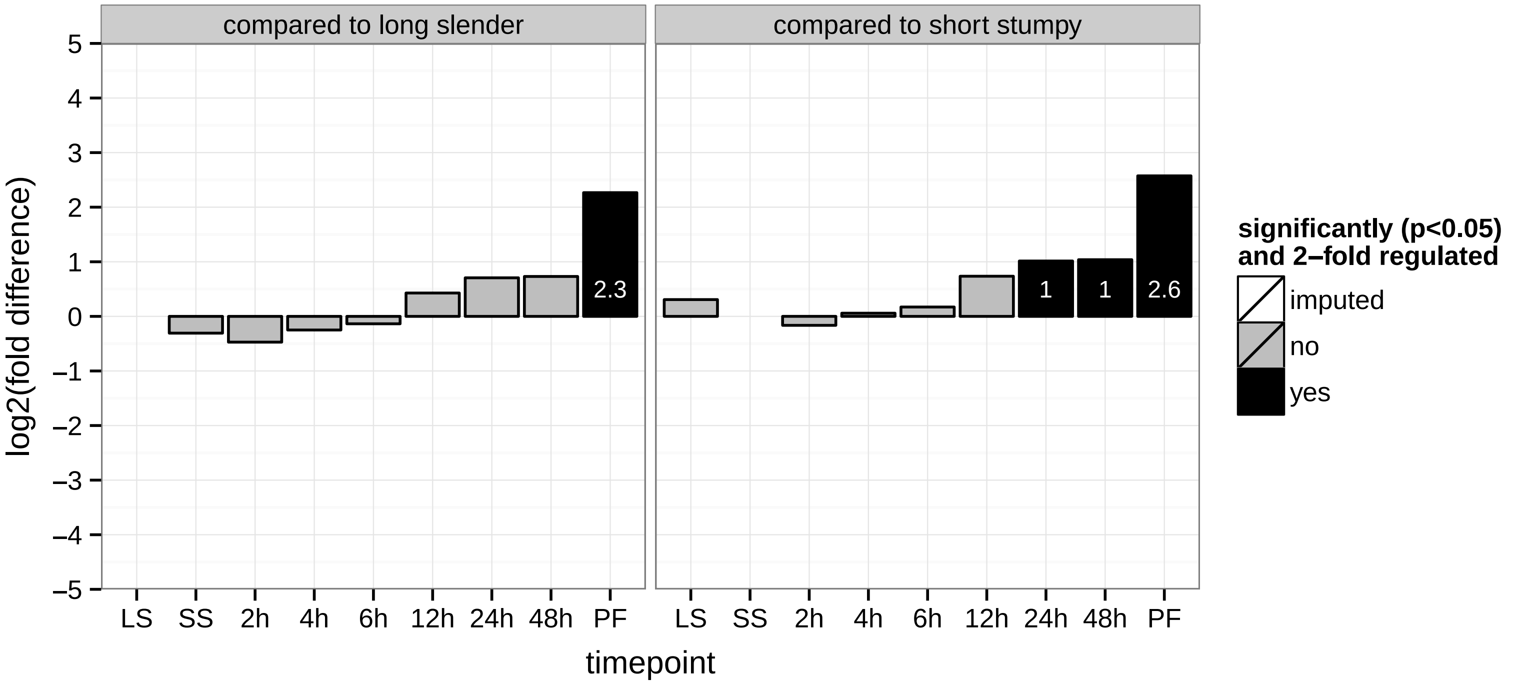

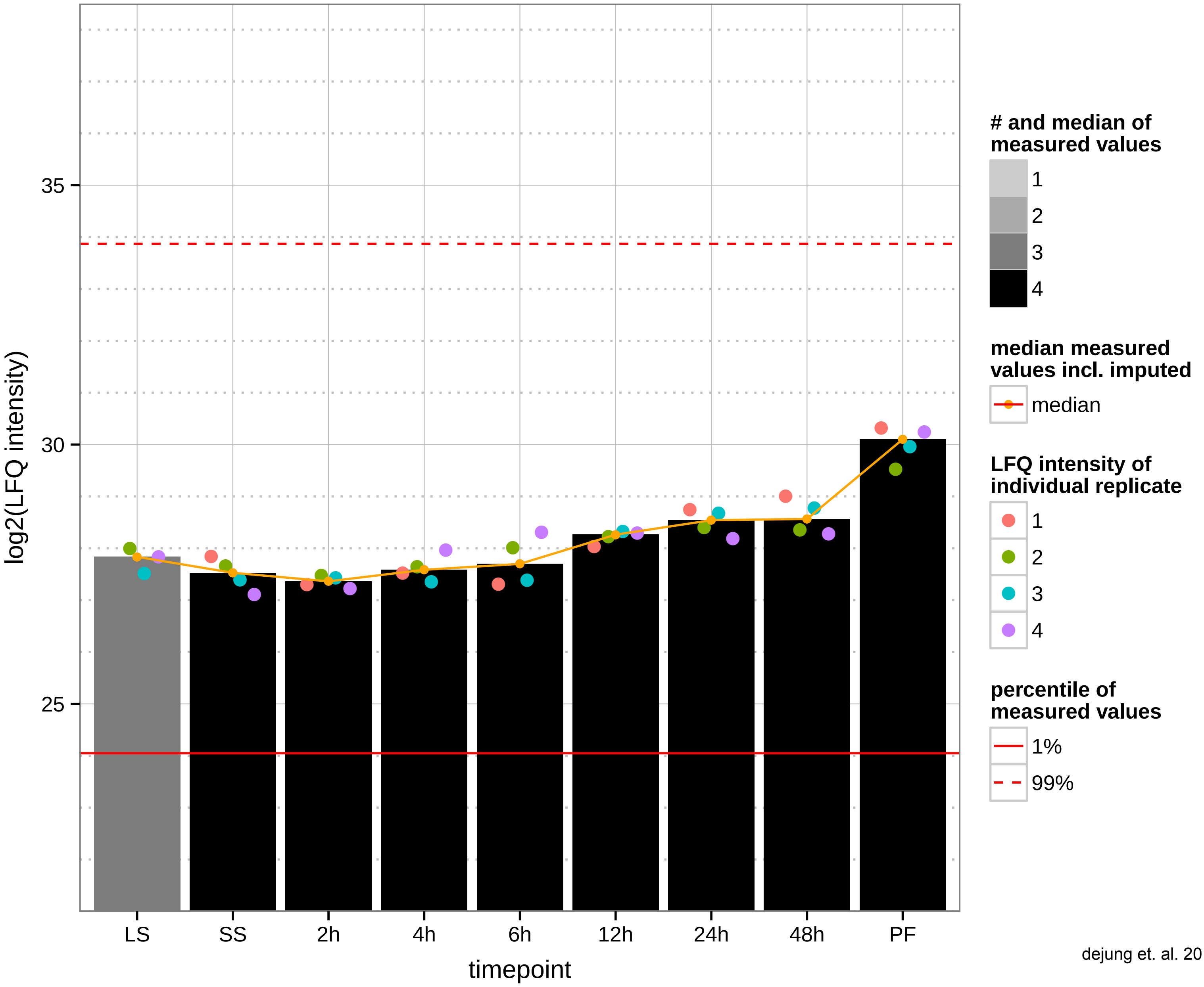

dejung et. al. 2015

hypothetical protein, conserved
Tb927.11.15500
AGOF: null
AGOC: mitochondrion
AGOP: null
PGOF: null
PGOC: null
PGOP: null

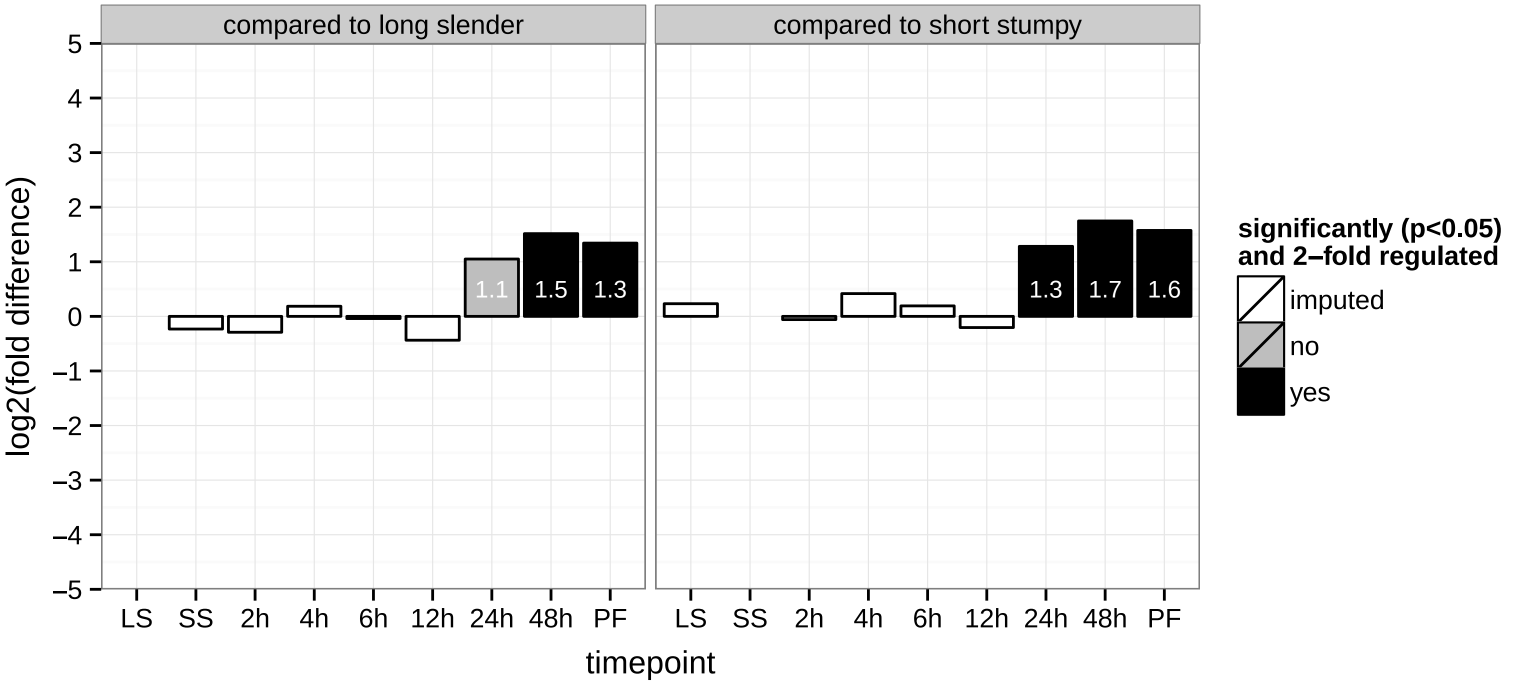

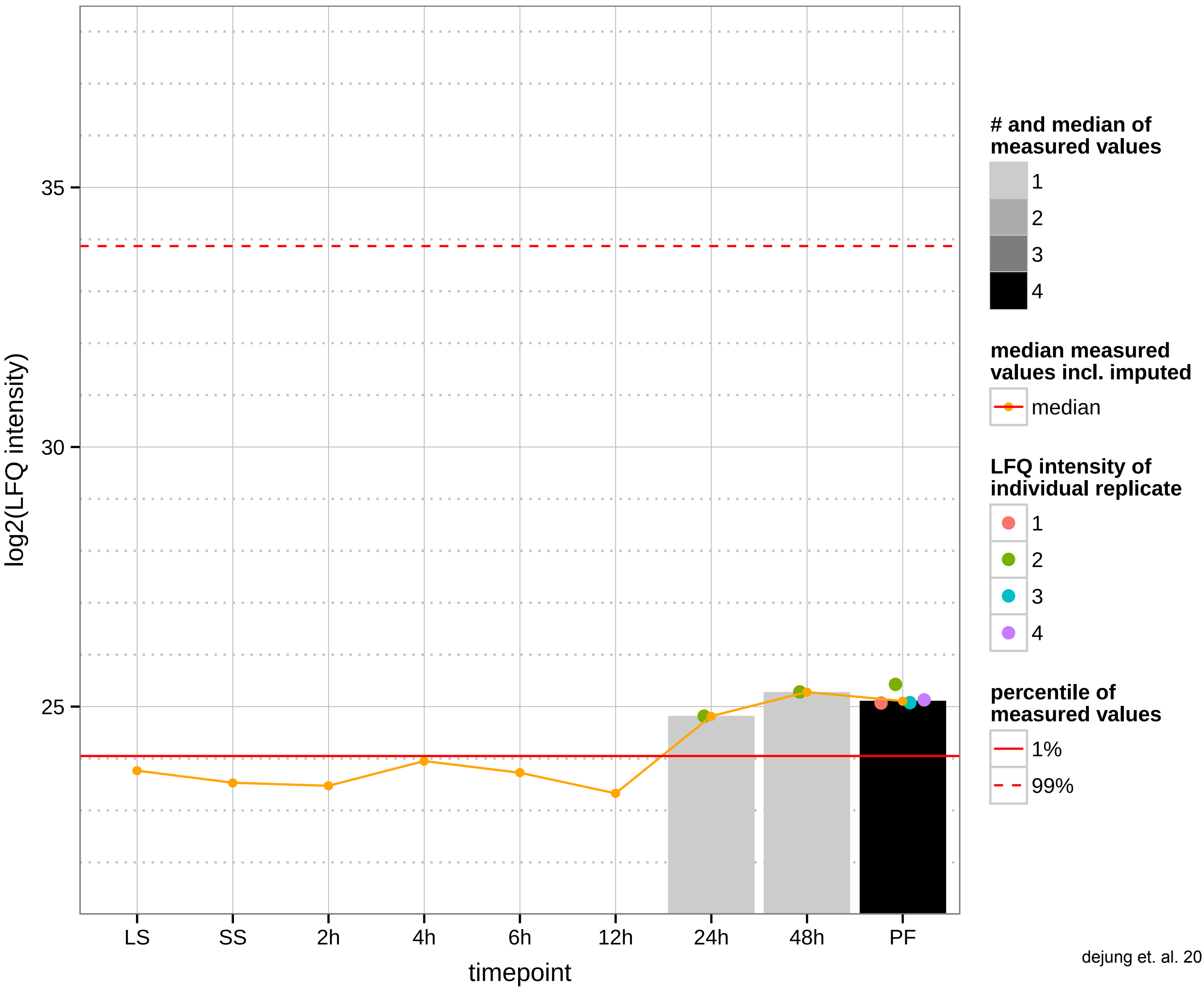

dejung et. al. 2015

hypothetical protein, conserved
Tb927.11.15800
AGOF: null
AGOC: null
AGOP: null
PGOF: protein binding
PGOC: null
PGOP: null

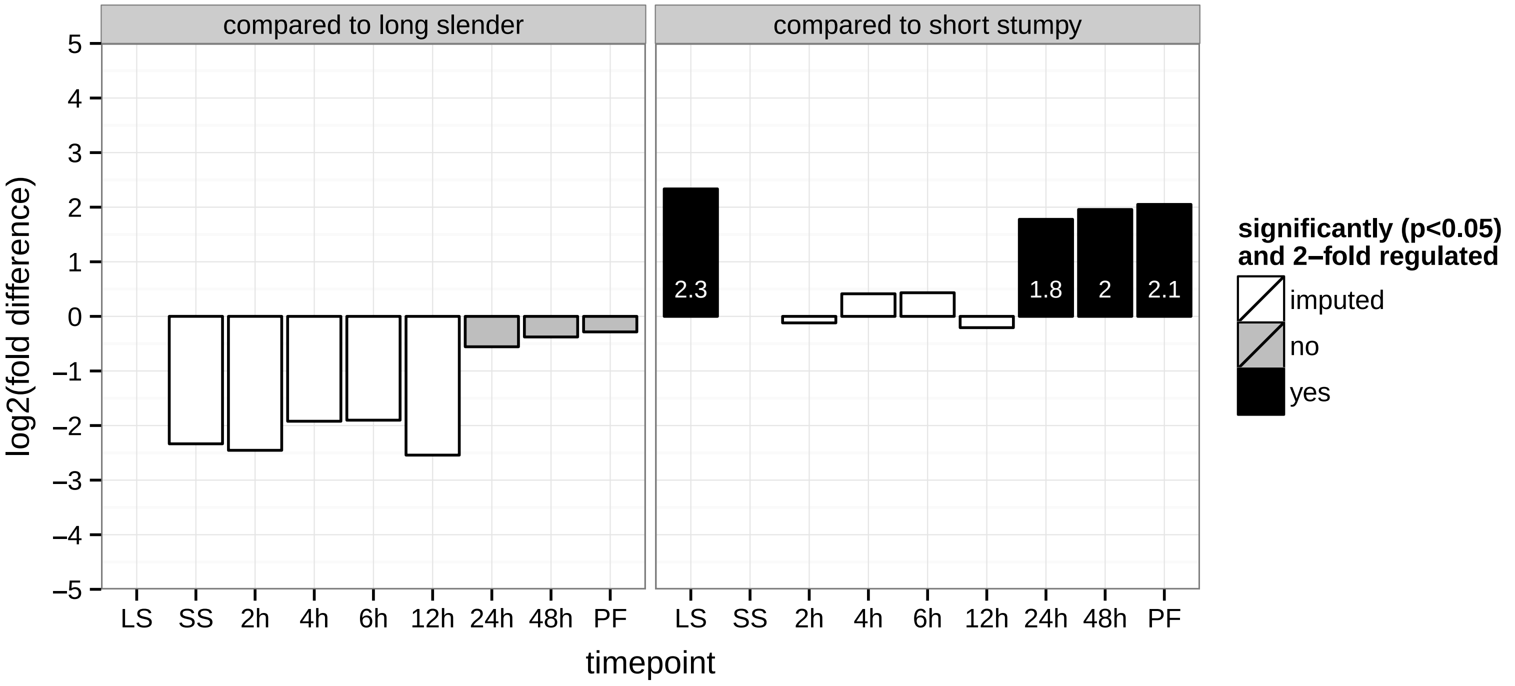

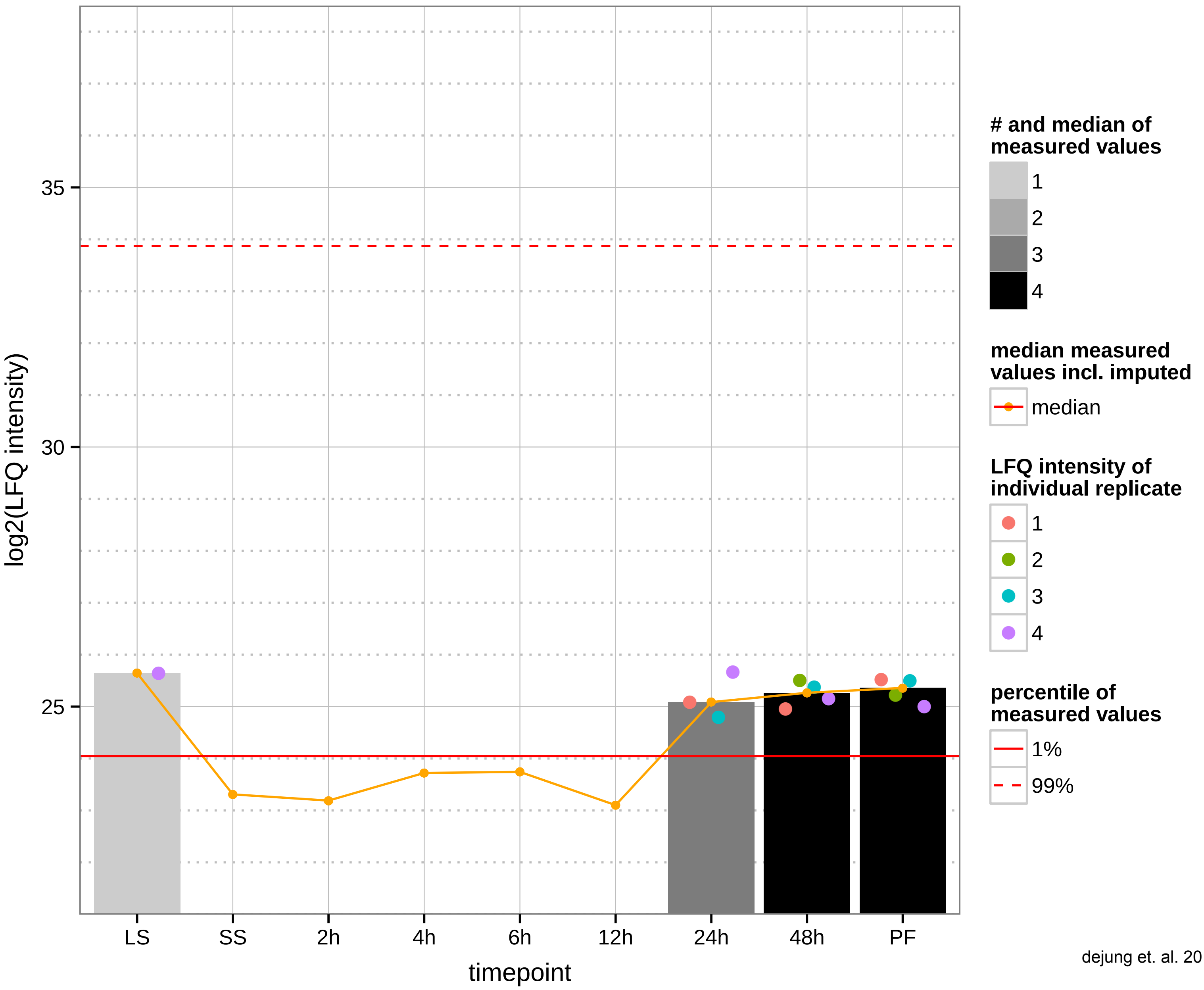

dejung et. al. 2015

ATP binding protein–like protein
Tb927.11.16160
AGOF: glutathione–disulfide reductase activity
AGOC: null
AGOP: oxidation–reduction process
PGOF: null
PGOC: null
PGOP: null

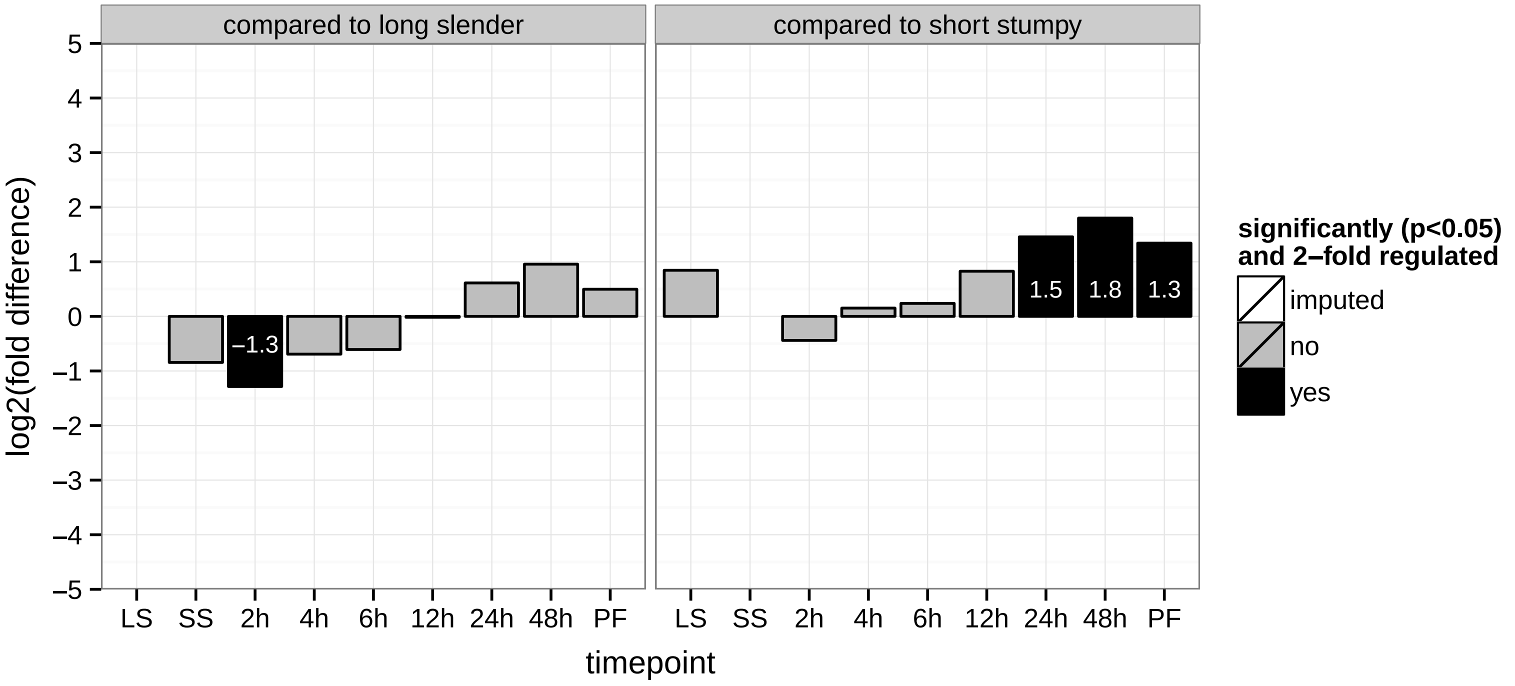

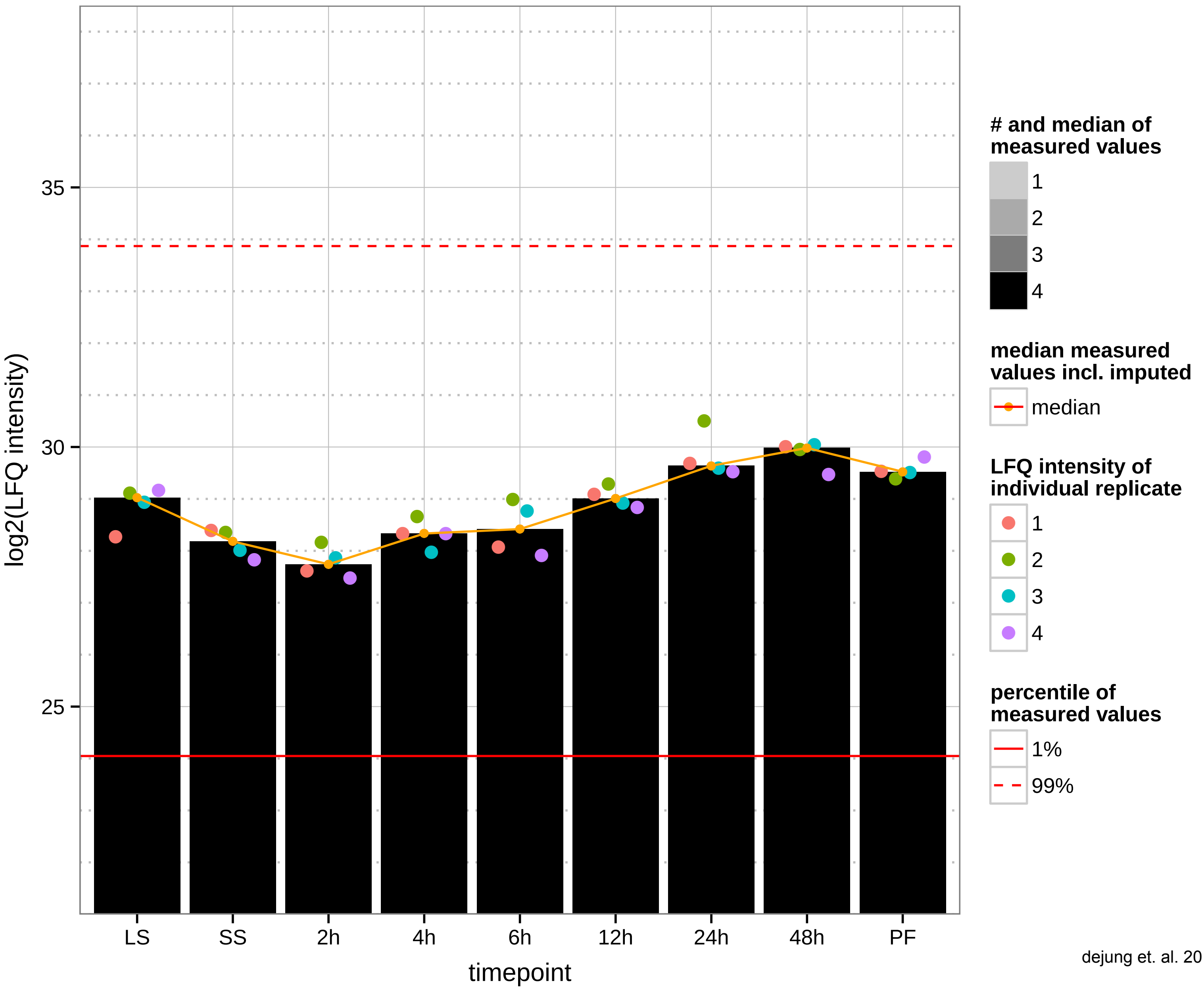

dejung et. al. 2015

enoyl–CoA hydratase/isomerase family protein, putative
Tb927.11.16480
AGOF: catalytic activity
AGOC: mitochondrion
AGOP: metabolic process
PGOF: catalytic activity
PGOC: null
PGOP: metabolic process

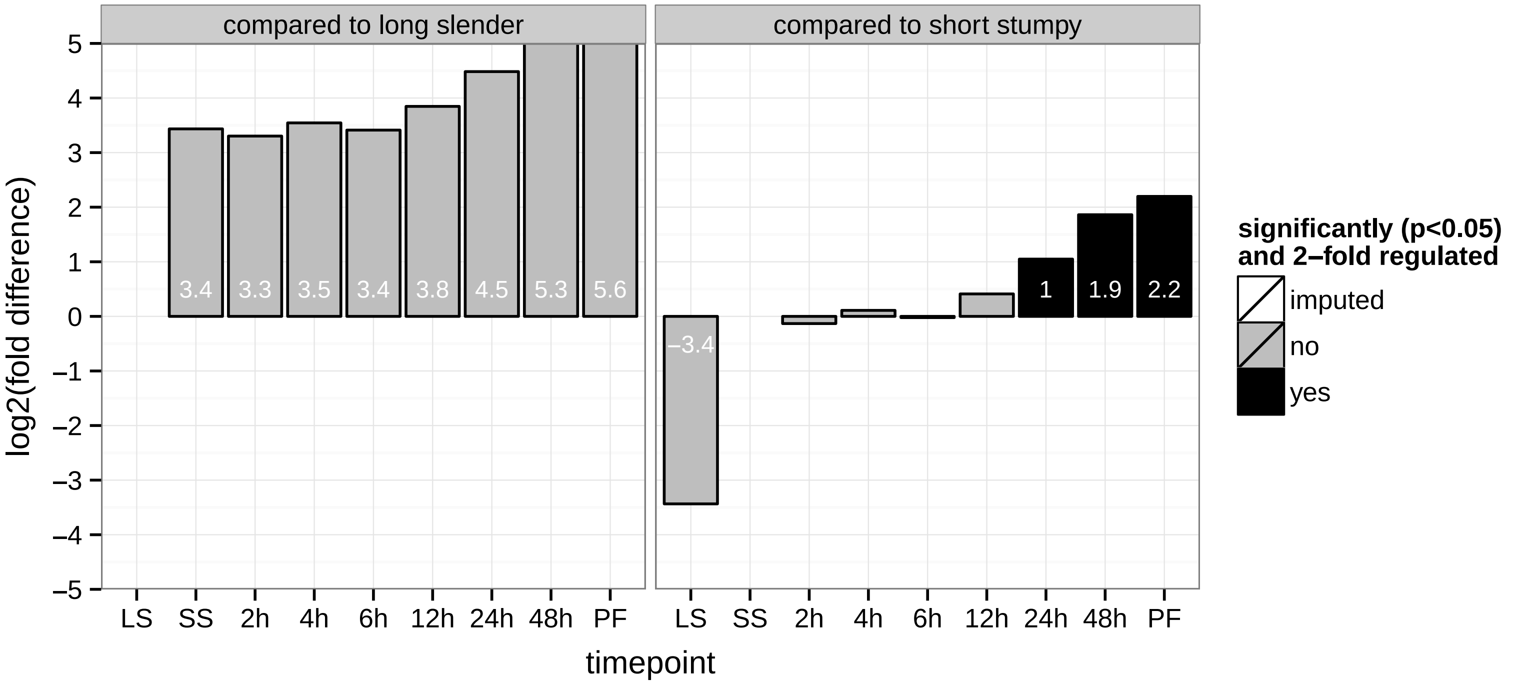

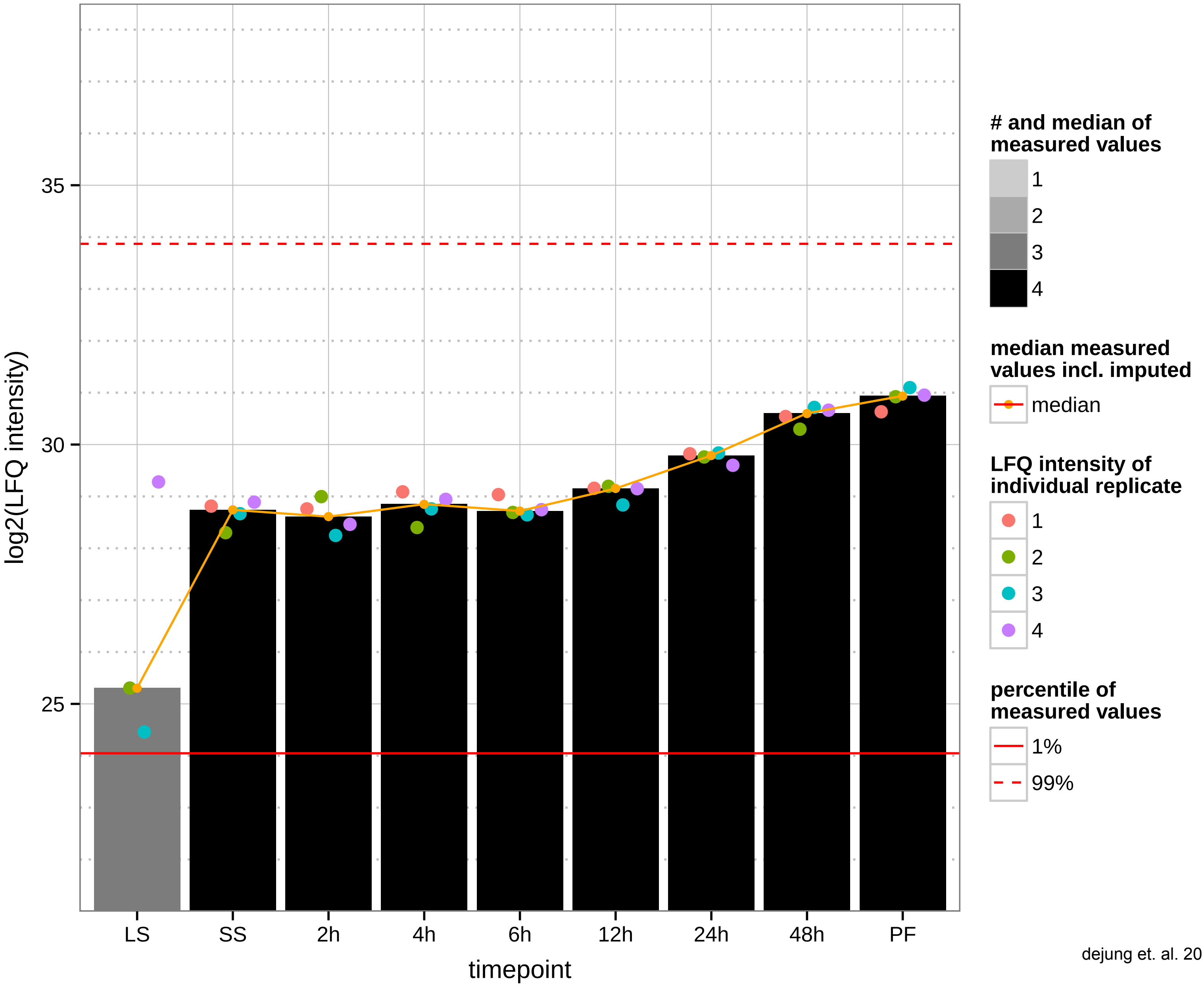

dejung et. al. 2015

NADH dehydrogenase subunit NI8M, putative, NADH–ubiquinone oxidoreductase chain NI8M
Tb927.11.16870
AGOF: null
AGOC: mitochondrion
AGOP: mitochondrial electron transport, NADH to ubiquinone
PGOF: null
PGOC: null
PGOP: null

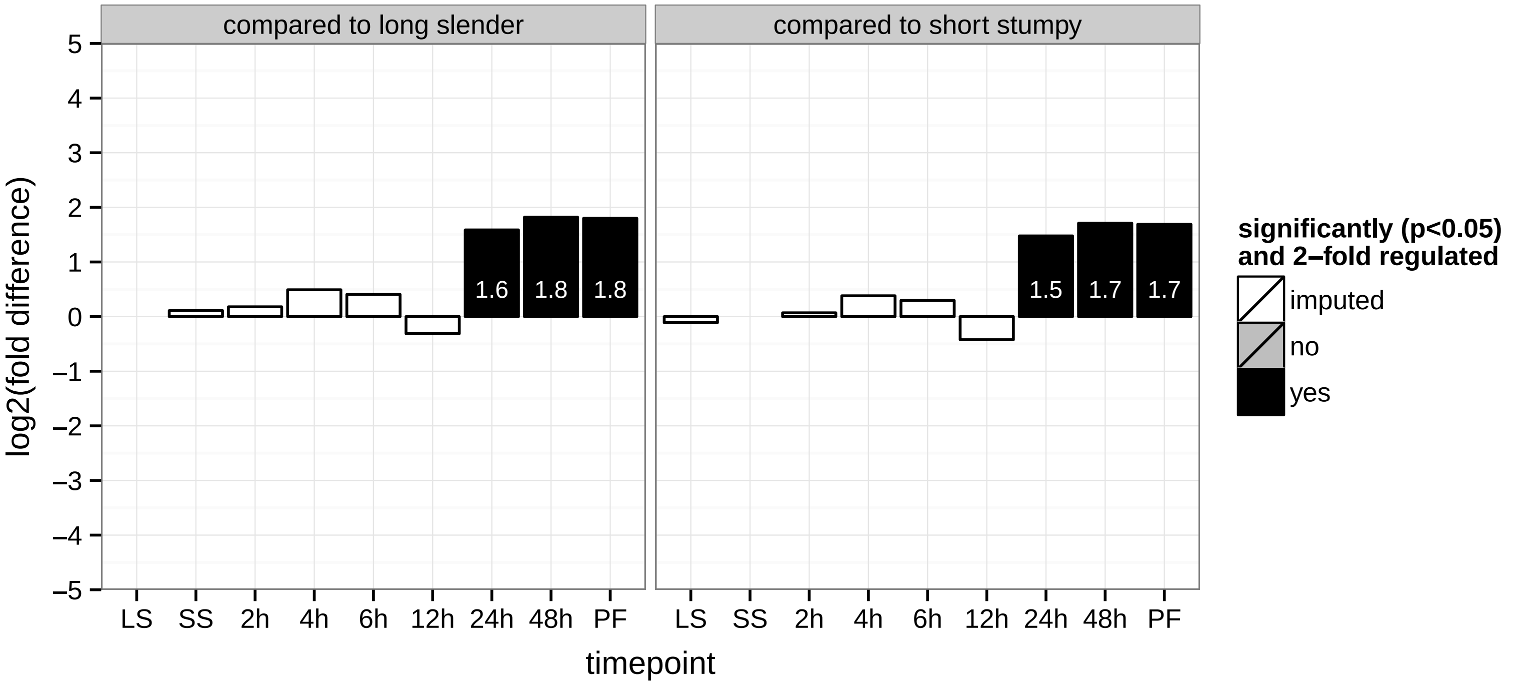

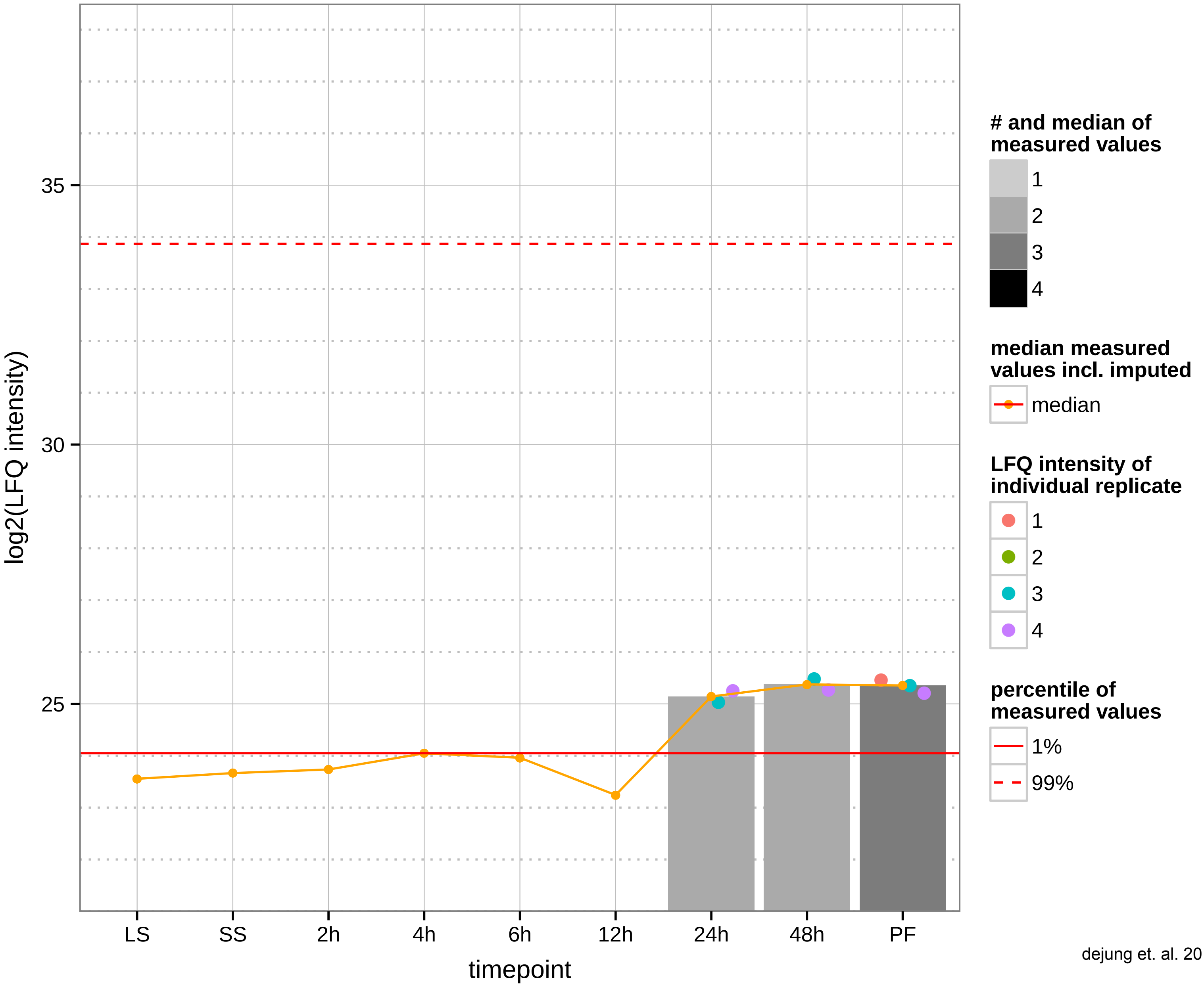

dejung et. al. 2015

hypothetical protein, conserved
Tb927.11.16910
AGOF: null, GTP binding
AGOC: null, intracellular
AGOP: null
PGOF: GTP binding, protein binding
PGOC: null
PGOP: null

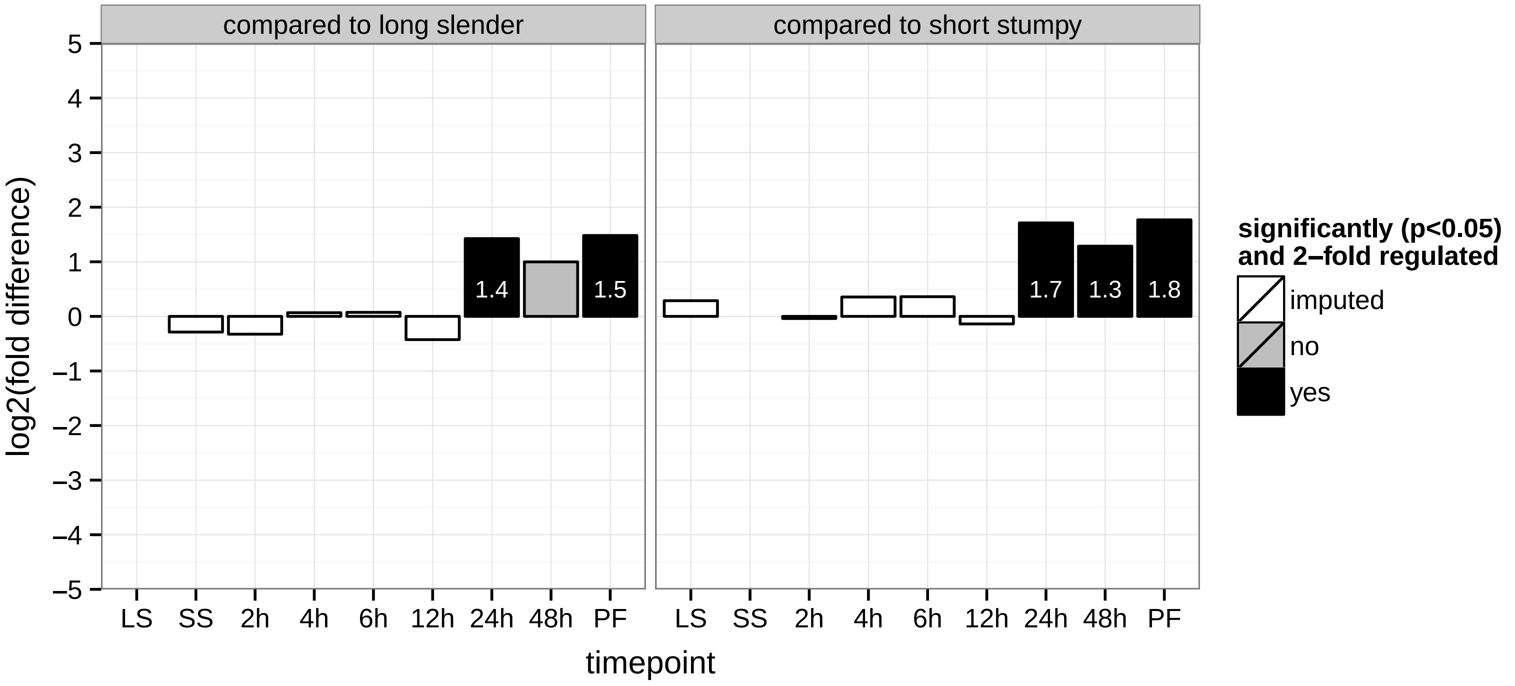

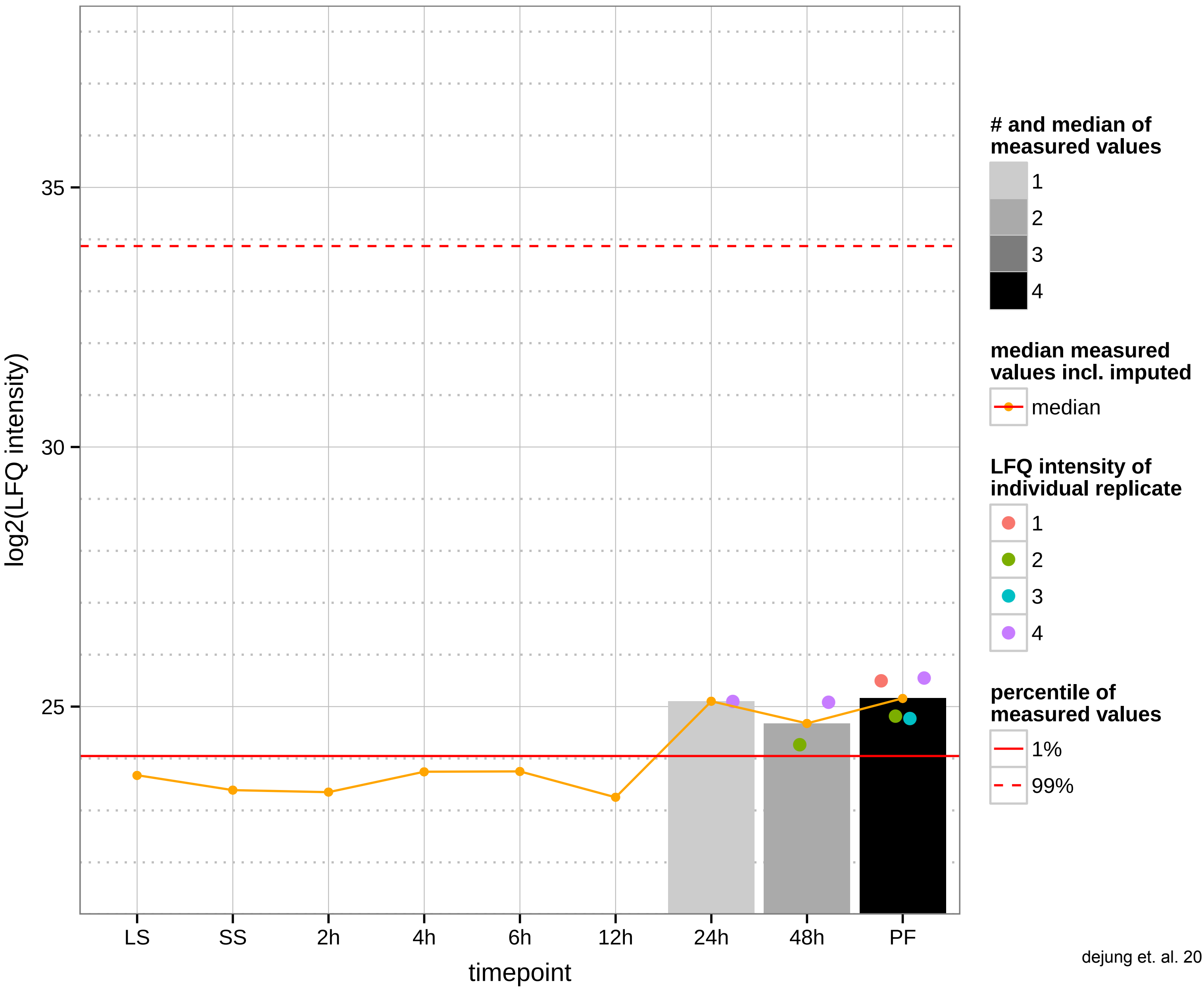

dejung et. al. 2015

zinc finger protein family member, putative (ZC3H41)
Tb927.11.1980
AGOF: ATP binding, RNA binding, helicase activity, zinc ion binding
AGOC: null
AGOP: null
PGOF: ATP binding, helicase activity, nucleic acid binding, zinc ion binding
PGOC: null
PGOP: null

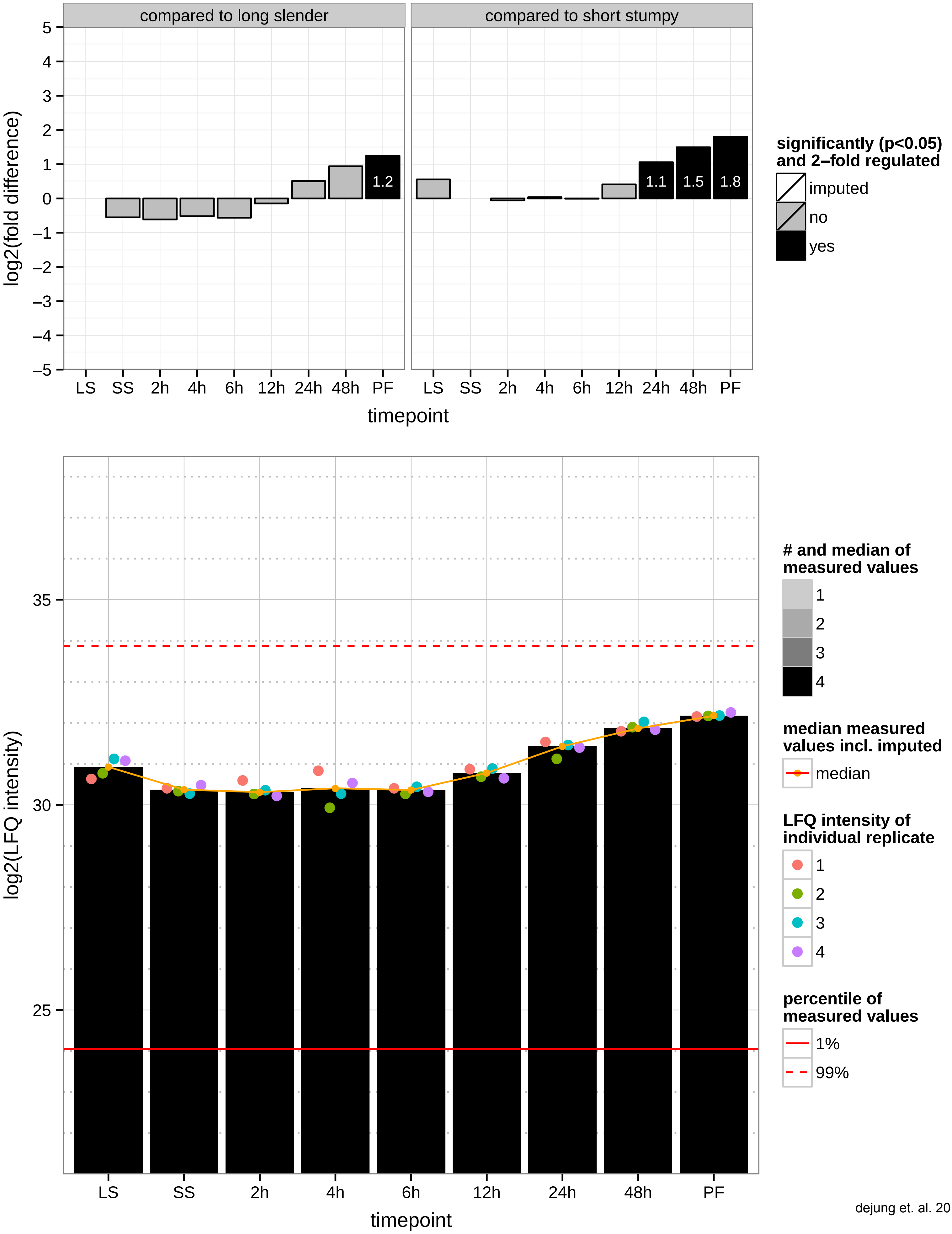

dejung et. al. 2015

mitochondrial RNA binding complex 1 subunit, kinteoplast poly(A) polymerase complex 1 subunit, mitochondrial edited mRNA
Tb927.11.2530
AGOF: RNA binding
AGOC: mitochondrion
AGOP: RNA polyadenylation, mRNA stabilization
PGOF: null
PGOC: null
PGOP: null

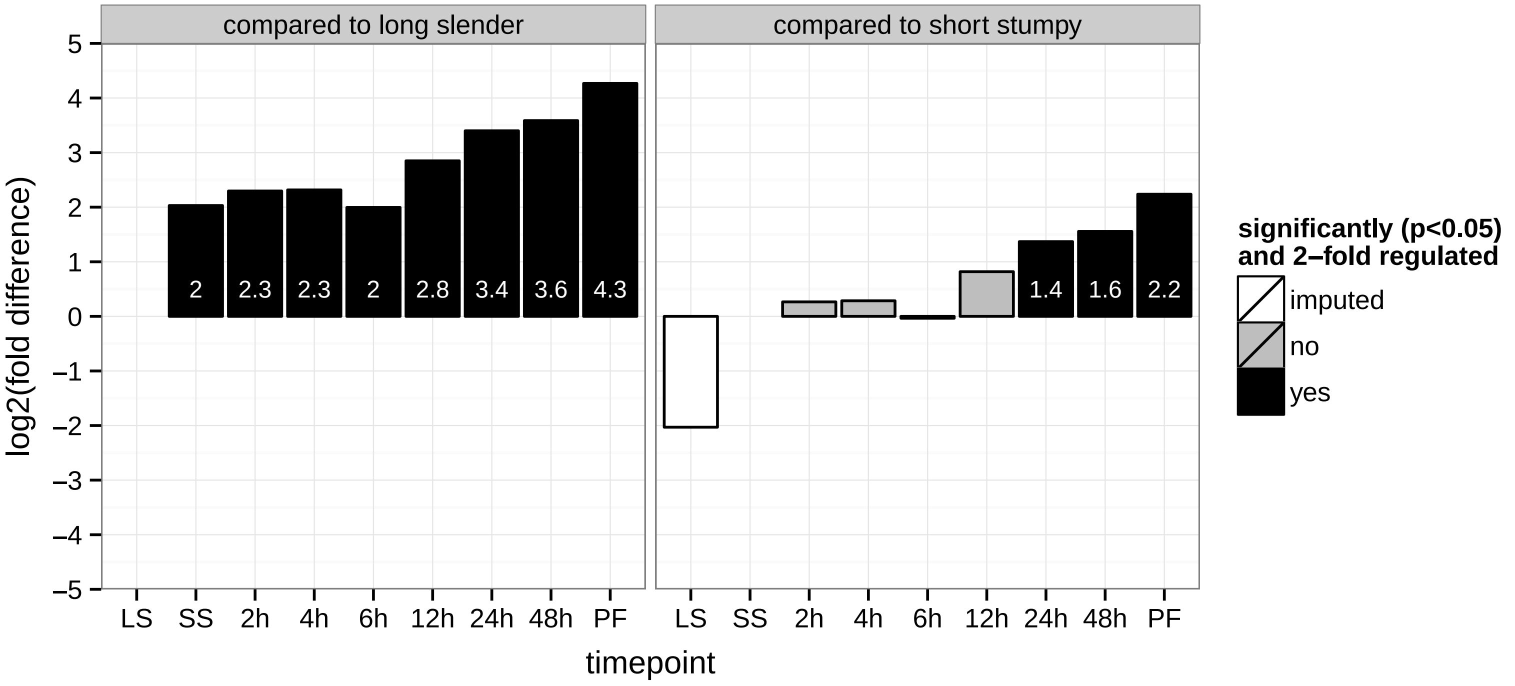

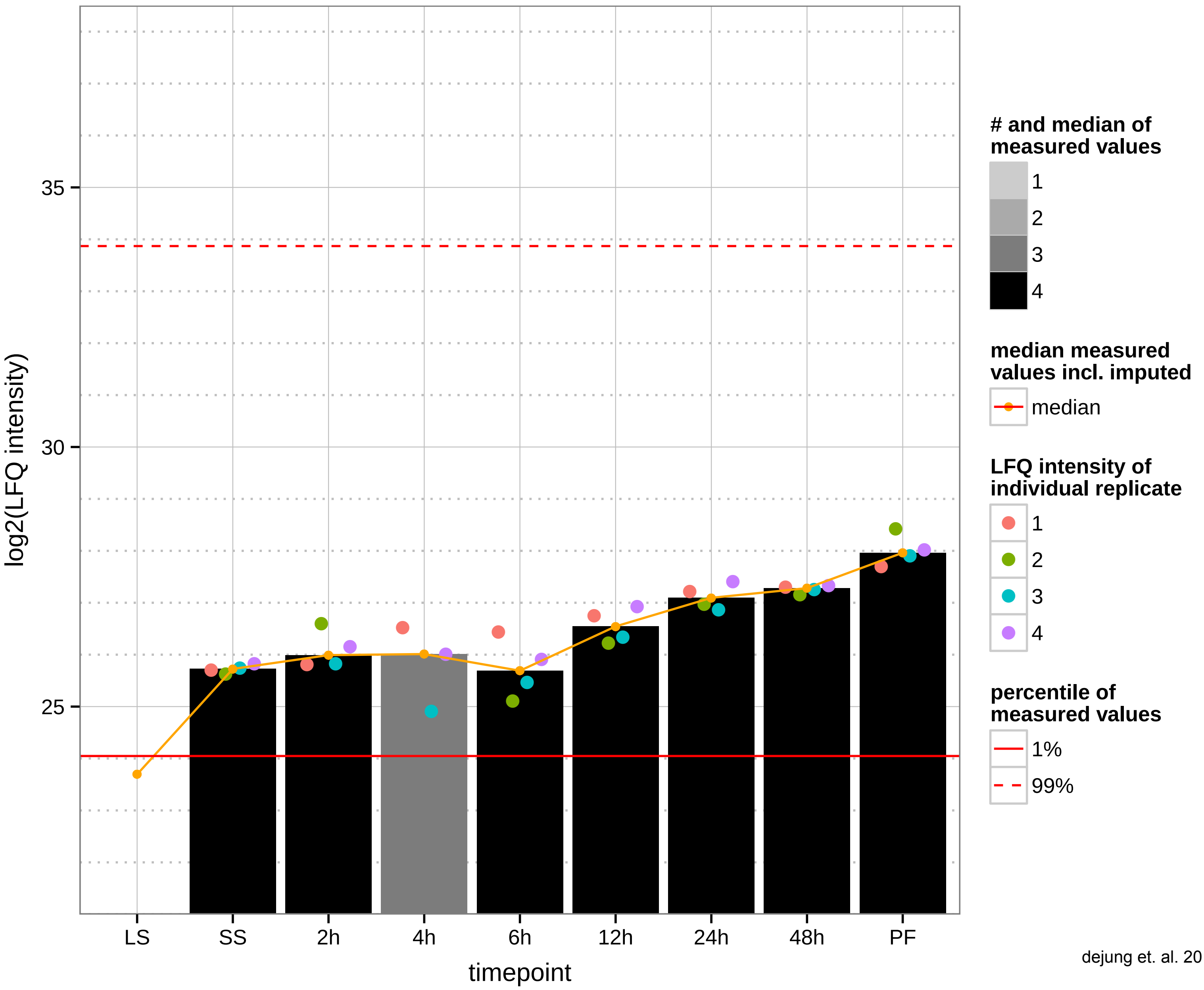

dejung et. al. 2015

hypothetical protein, conserved
Tb927.11.2930
AGOF: null
AGOC: mitochondrion
AGOP: null
PGOF: null
PGOC: null
PGOP: null

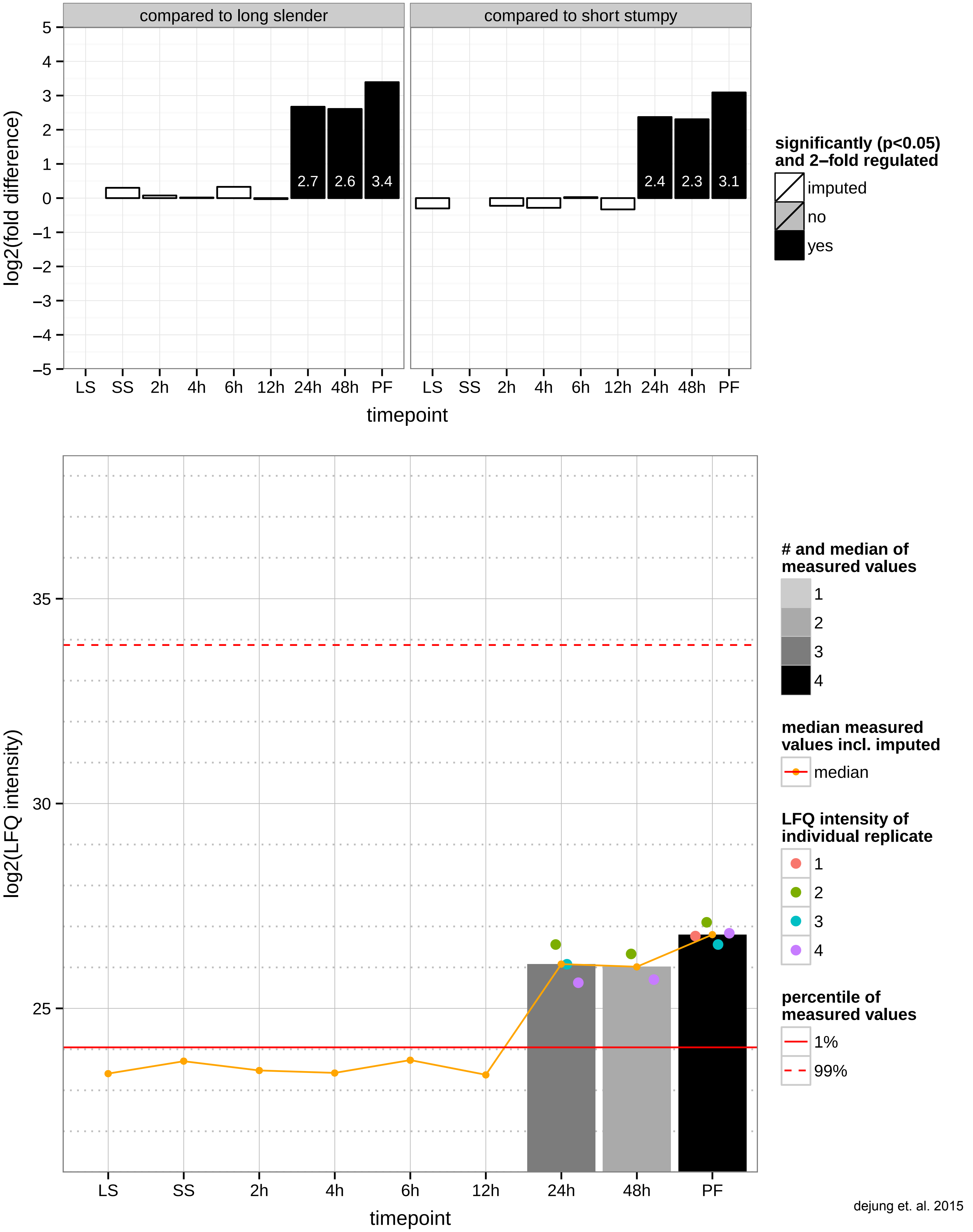

dejung et. al. 2015

hypothetical protein, conserved
Tb927.11.4180
AGOF: null
AGOC: null
AGOP: null
PGOF: null
PGOC: null
PGOP: null

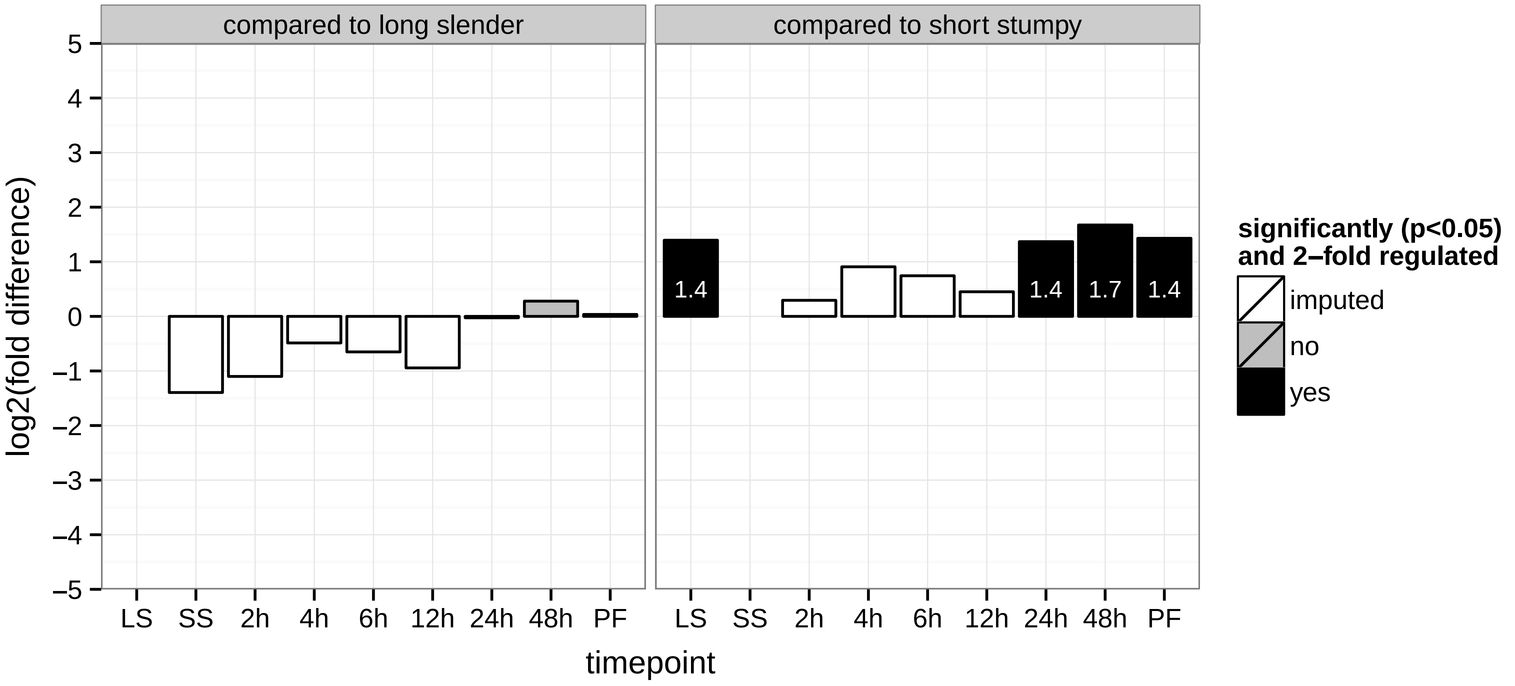

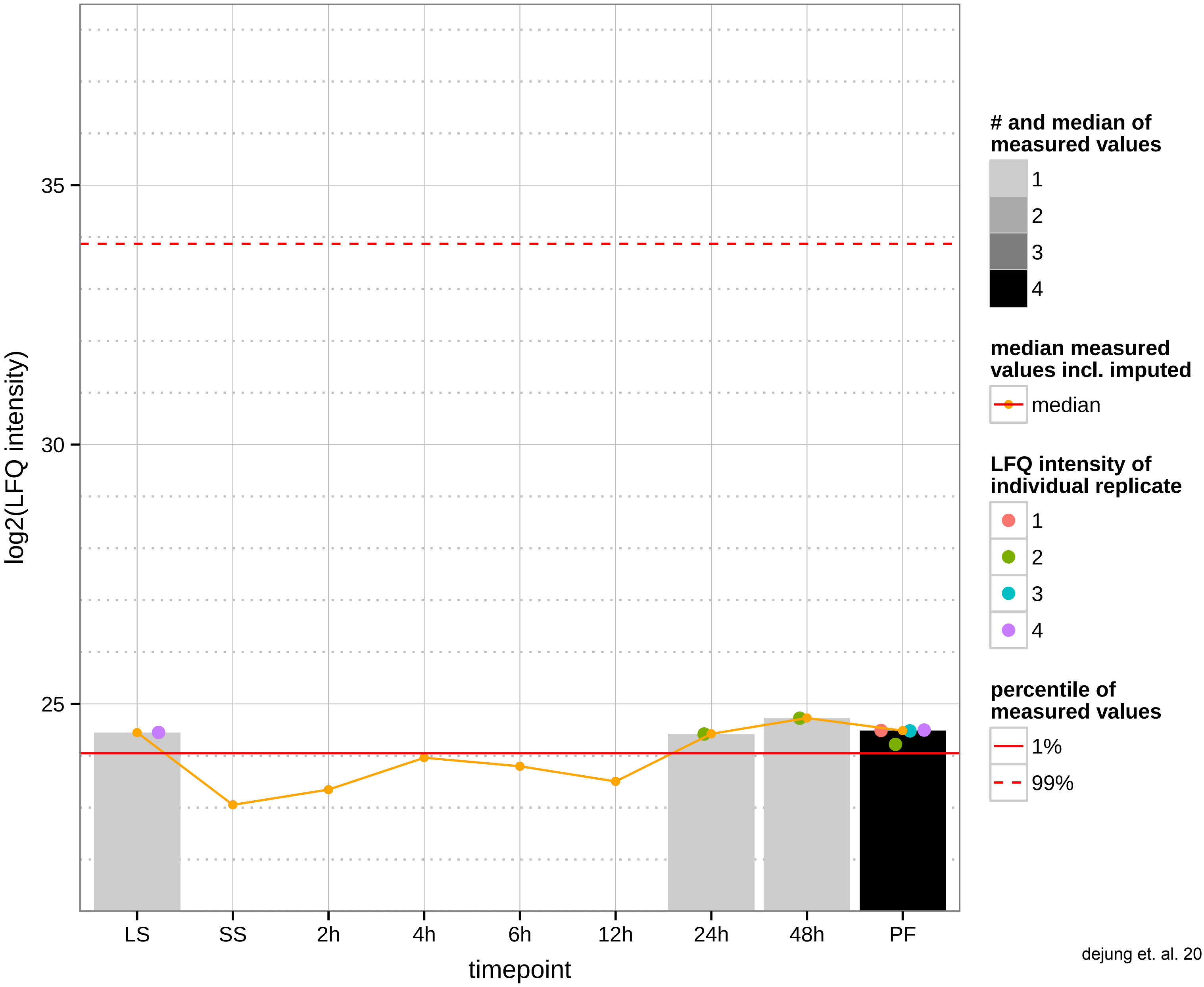

dejung et. al. 2015

prostaglandin f synthase
Tb927.11.4700
AGOF: D–arabinose 1–dehydrogenase [NAD(P)+] activity, aldo–keto reductase (NADP) activity
AGOC: null
AGOP: D–arabinose catabolic process, oxidation–reduction process
PGOF: oxidoreductase activity
PGOC: null
PGOP: oxidation–reduction process

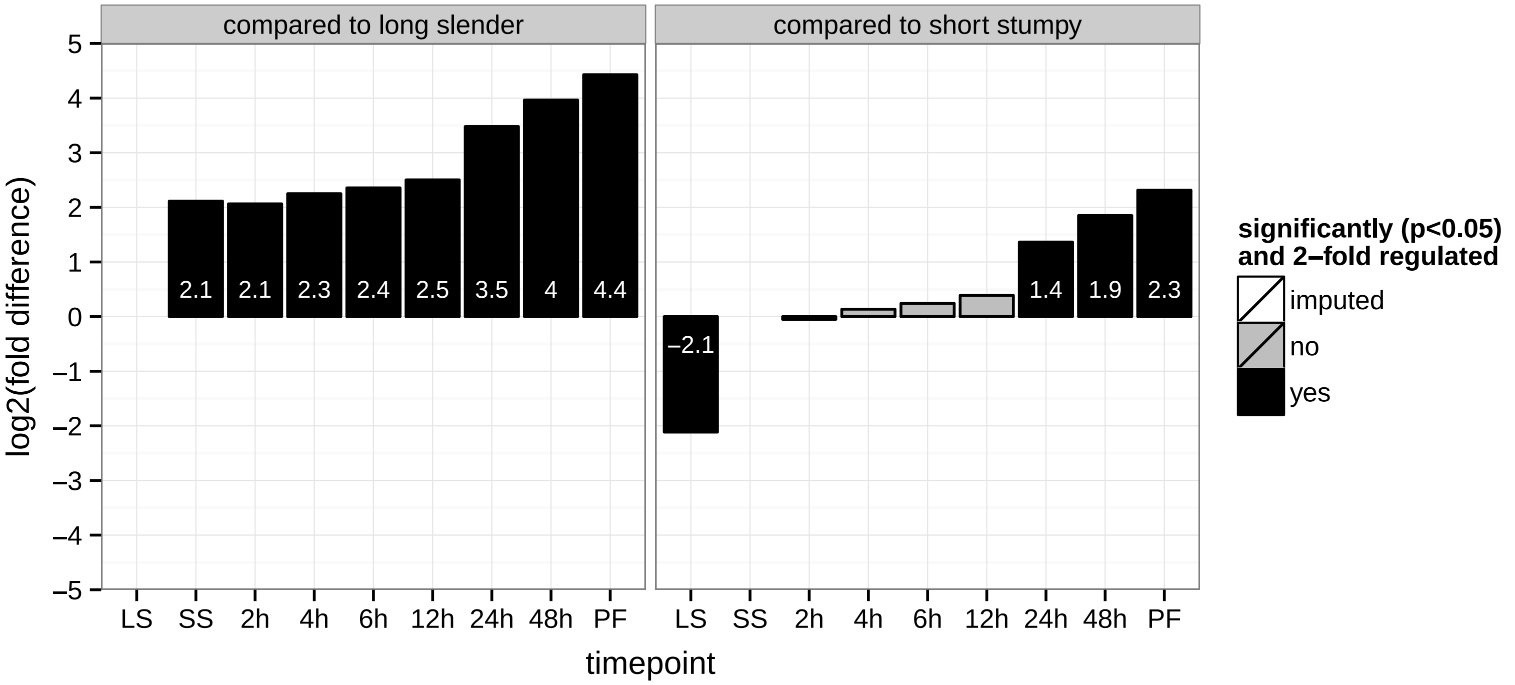

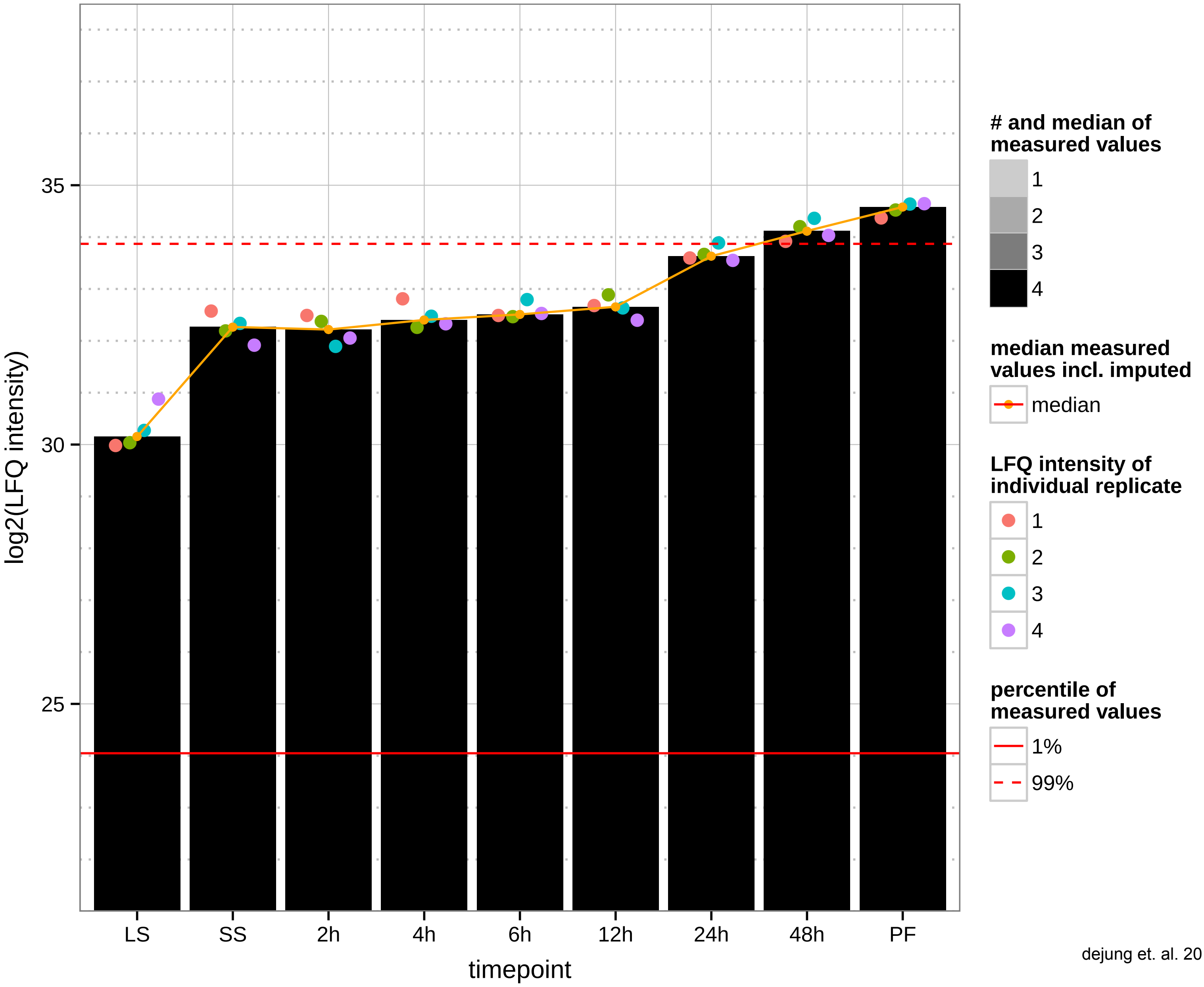

dejung et. al. 2015

predicted ankyrin repeat family protein
Tb927.11.4910
AGOF: null
AGOC: null
AGOP: null
PGOF: protein binding
PGOC: null
PGOP: null

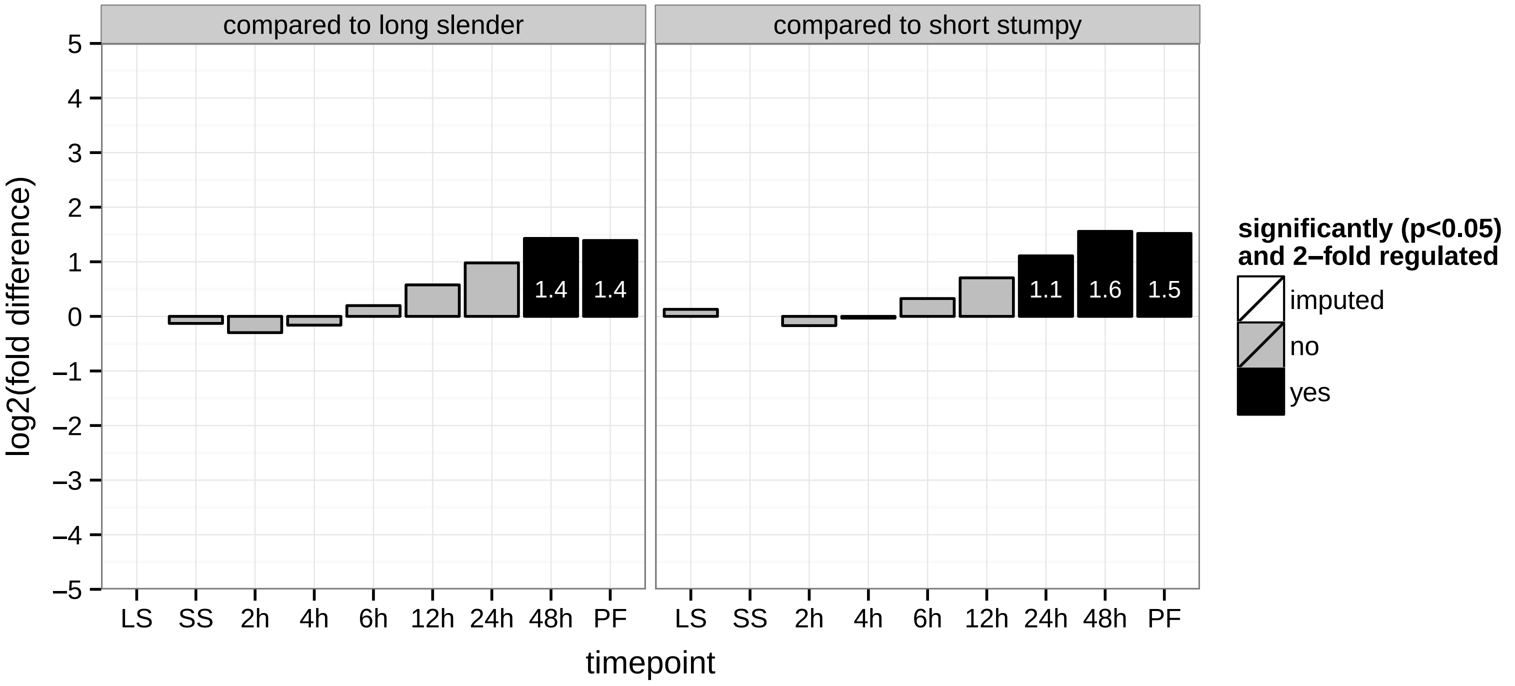

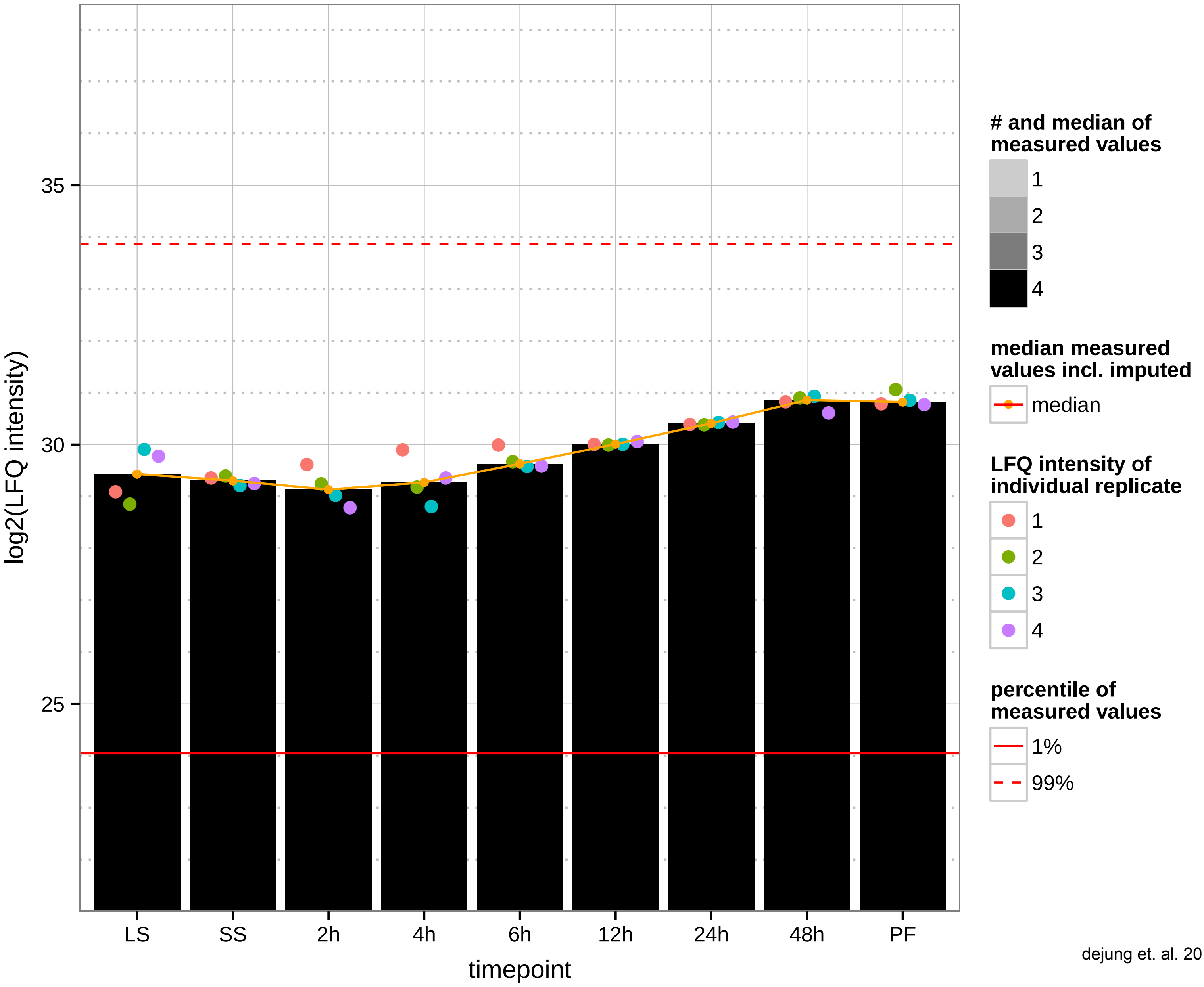

dejung et. al. 2015

RNA–binding protein, UBP2 (UBP2)
Tb927.11.510
AGOF: RNA binding, mRNA binding
AGOC: cytoplasm, nucleus
AGOP: RNA processing
PGOF: nucleic acid binding
PGOC: null
PGOP: null

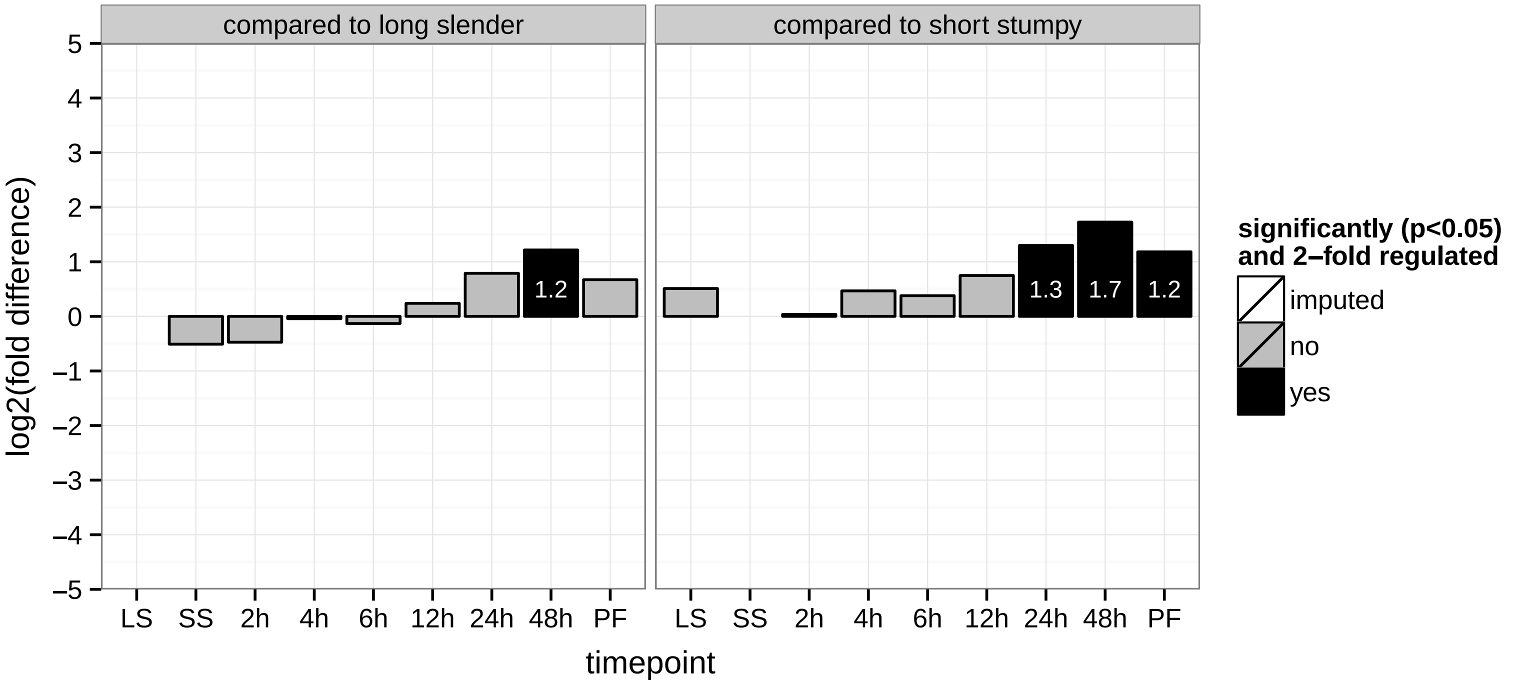

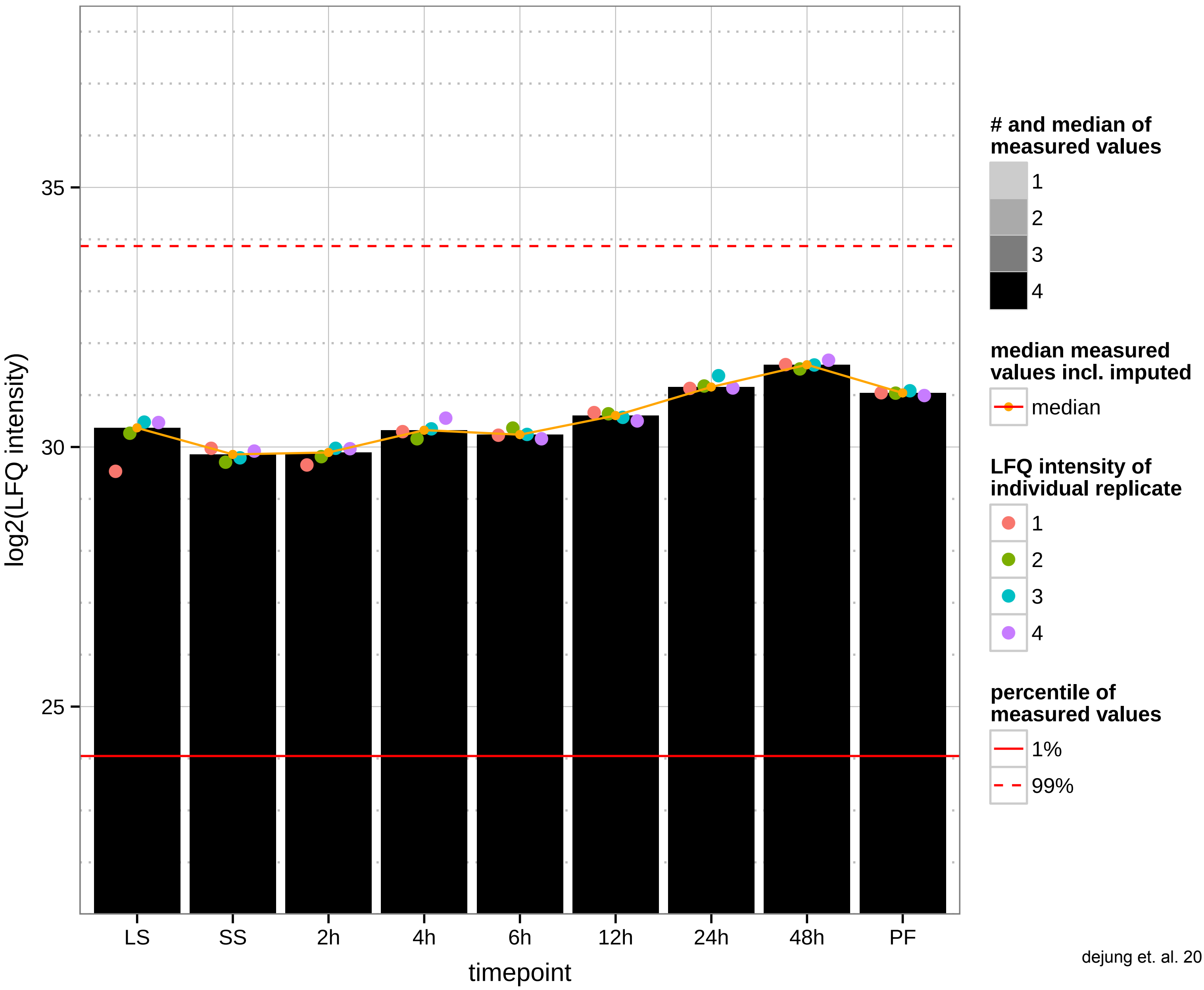

dejung et. al. 2015

hypothetical protein, conserved
Tb927.11.5600
AGOF: null
AGOC: mitochondrion
AGOP: null
PGOF: null
PGOC: null
PGOP: null

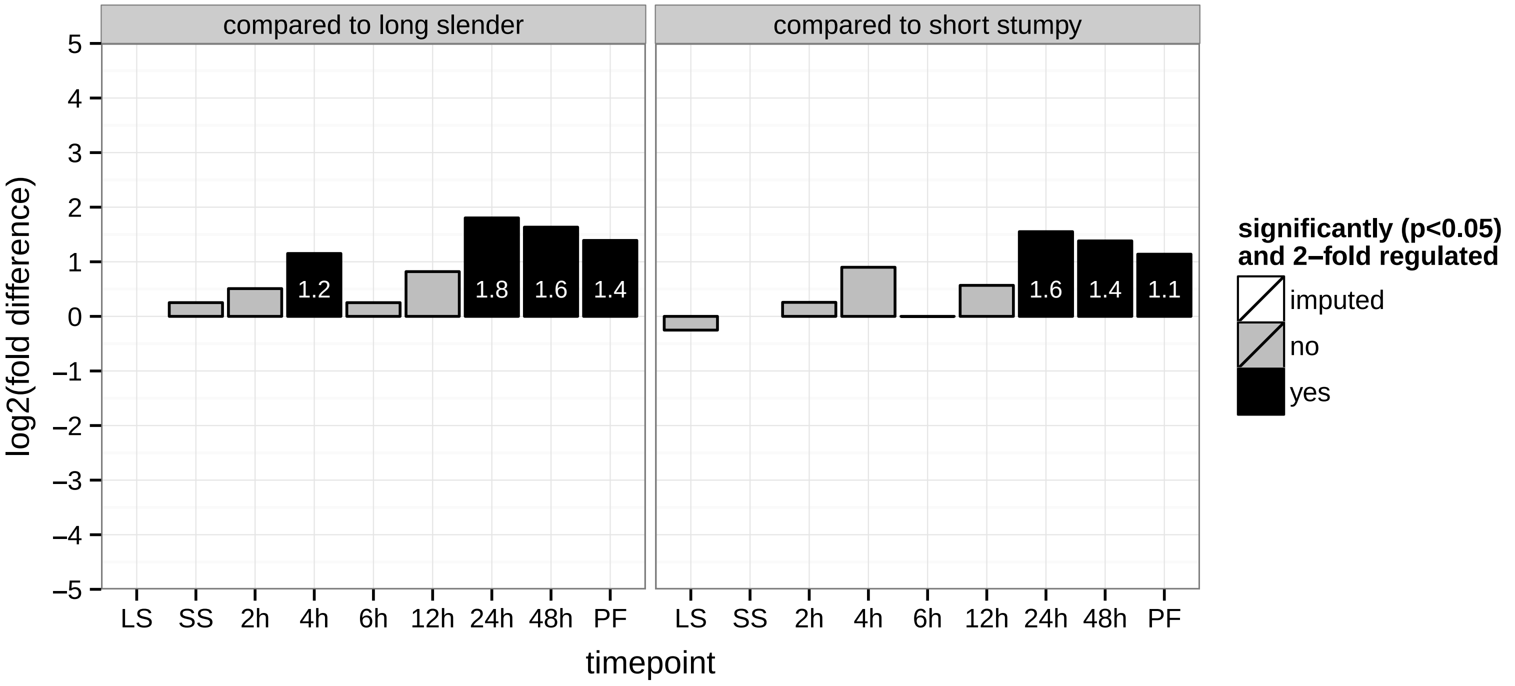

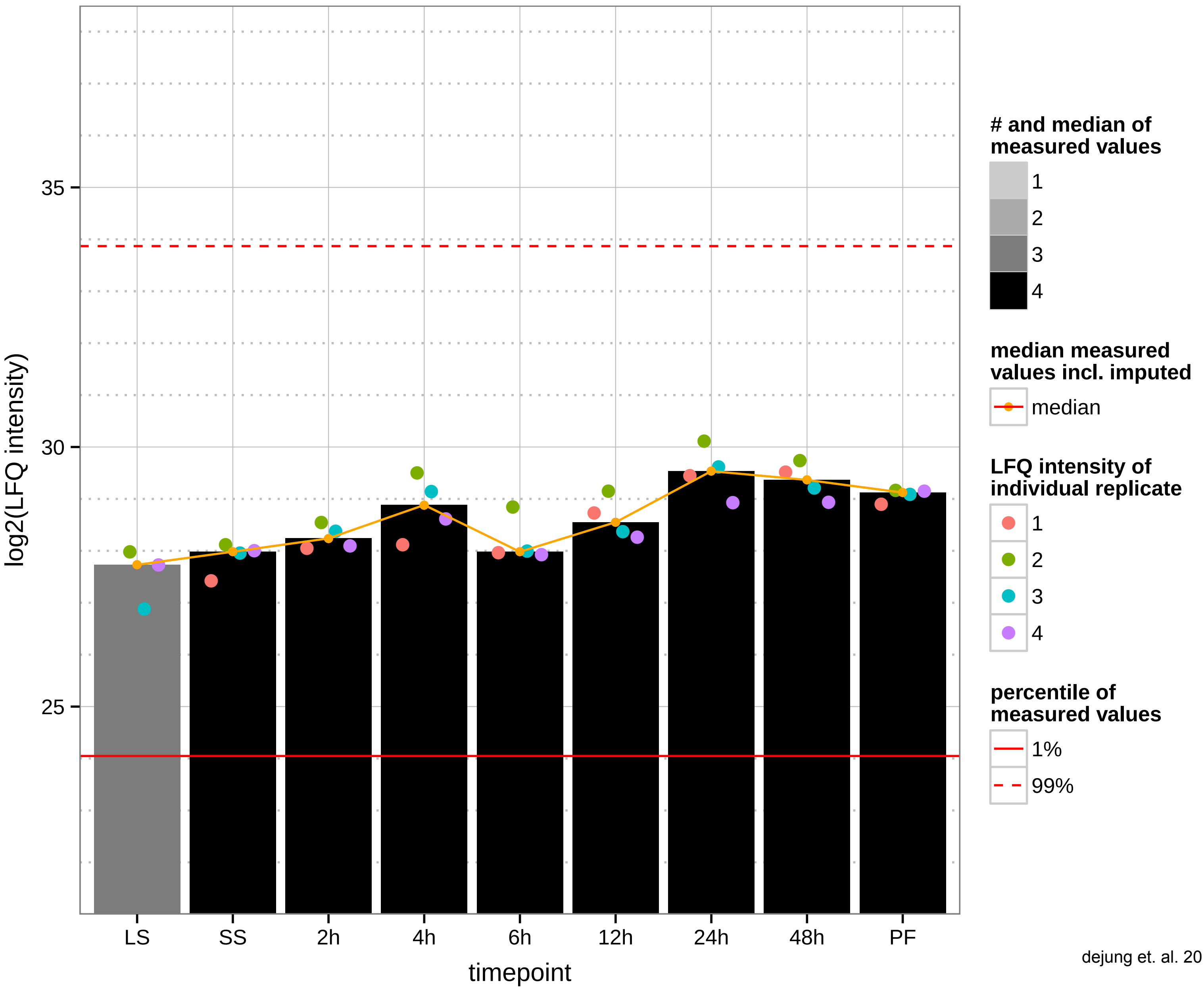

dejung et. al. 2015

Protein translation factor SUI1 homolog, putative
Tb927.11.5840
AGOF: translation initiation factor activity
AGOC: null
AGOP: translation, translational initiation
PGOF: translation initiation factor activity
PGOC: null
PGOP: translational initiation

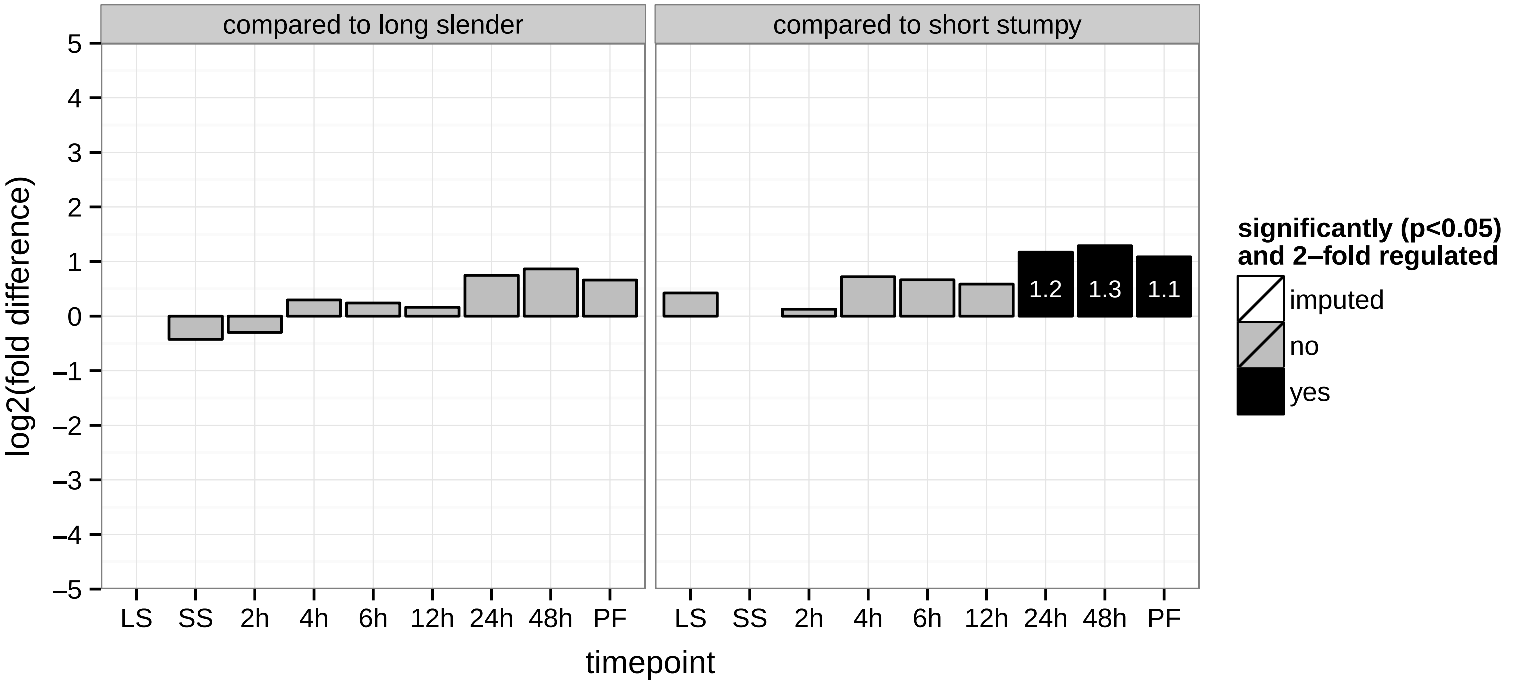

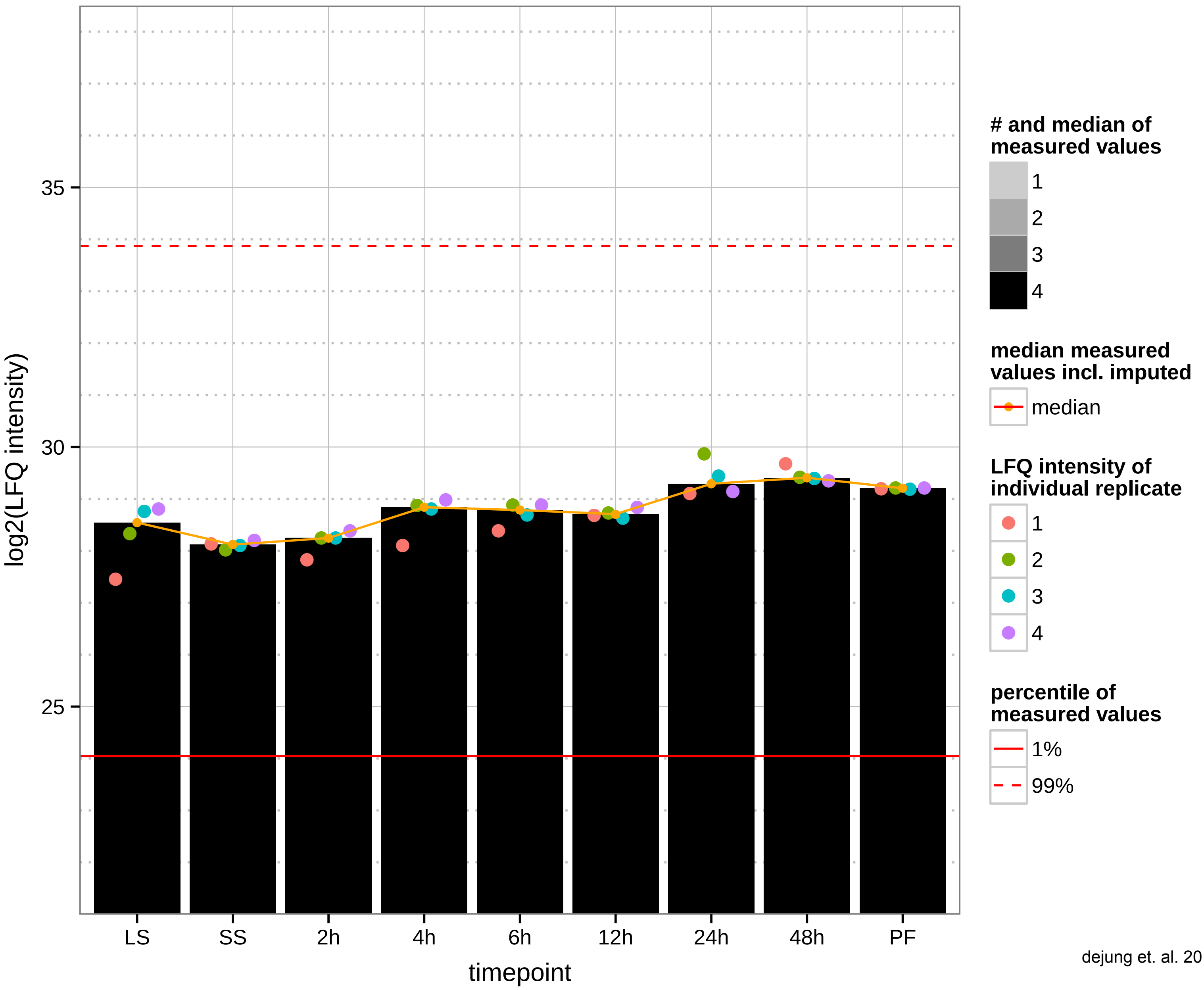

dejung et. al. 2015

60S ribosomal protein L28, putative
Tb927.11.6200;Tb927.11.6180
AGOF: structural constituent of ribosome
AGOC: intracellular, ribosome
AGOP: translation
PGOF: null
PGOC: null
PGOP: null

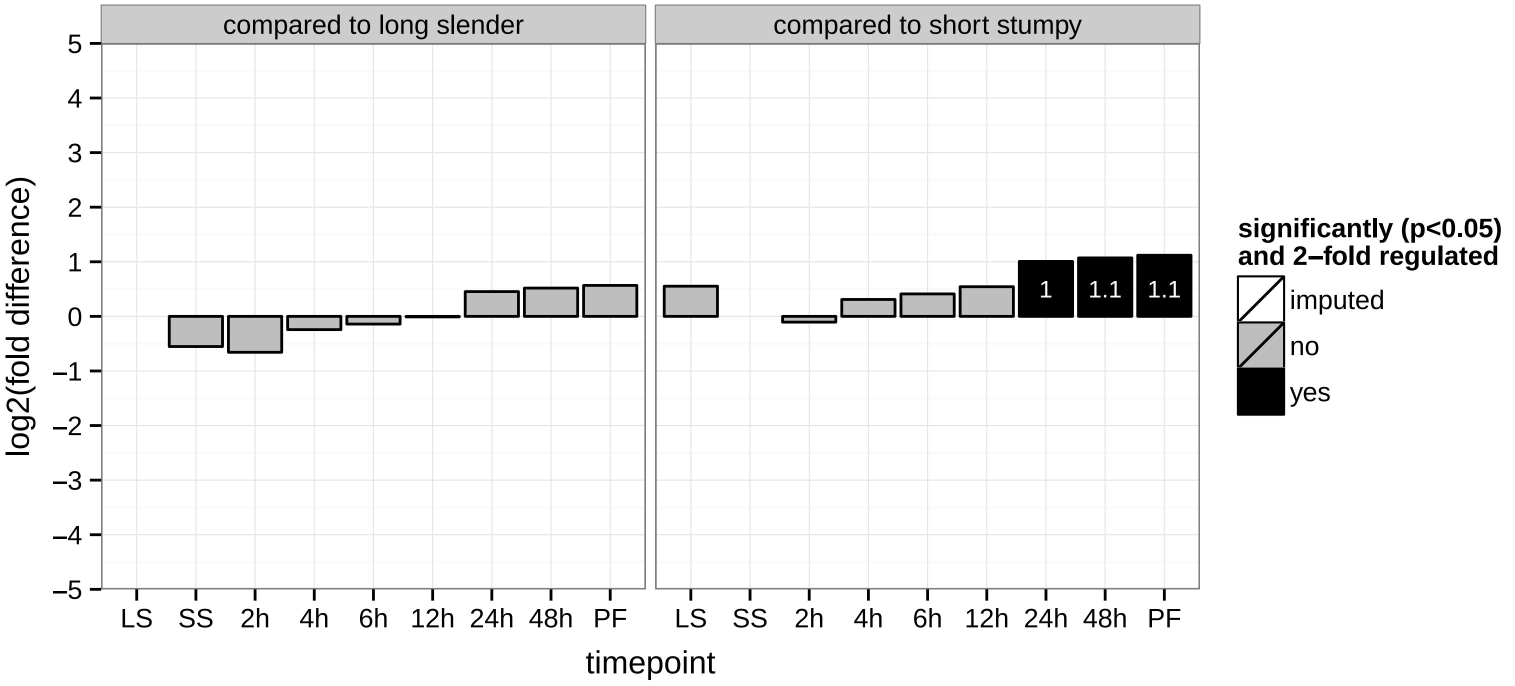

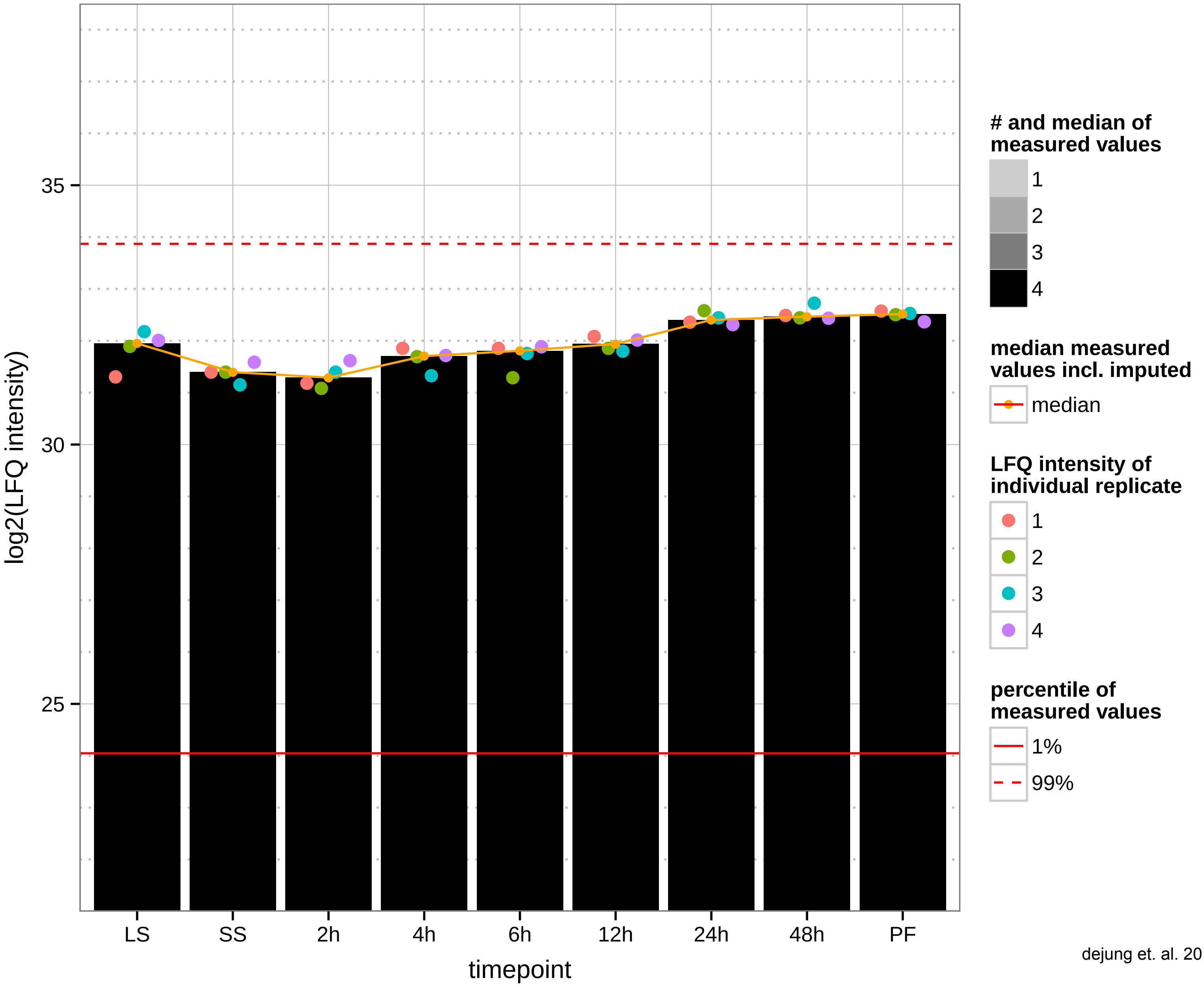

dejung et. al. 2015

hypothetical protein, conserved
Tb927.11.6250
AGOF: null
AGOC: mitochondrial inner membrane, mitochondrion
AGOP: null
PGOF: null
PGOC: null
PGOP: null

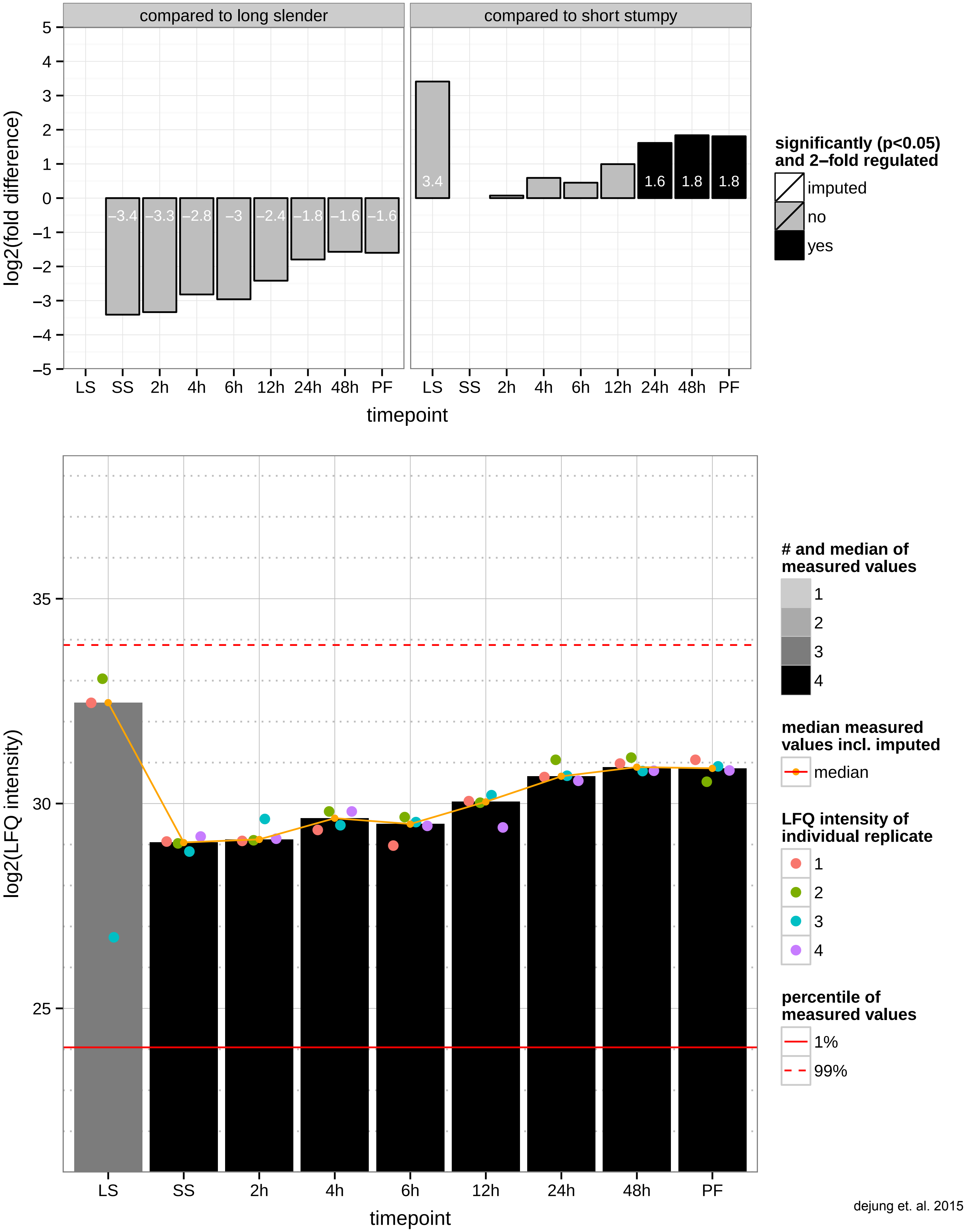

dejung et. al. 2015

pumilio/PUF RNA binding protein 10, putative
Tb927.11.6740
AGOF: RNA binding
AGOC: null
AGOP: mRNA metabolic process
PGOF: RNA binding, binding
PGOC: null
PGOP: null

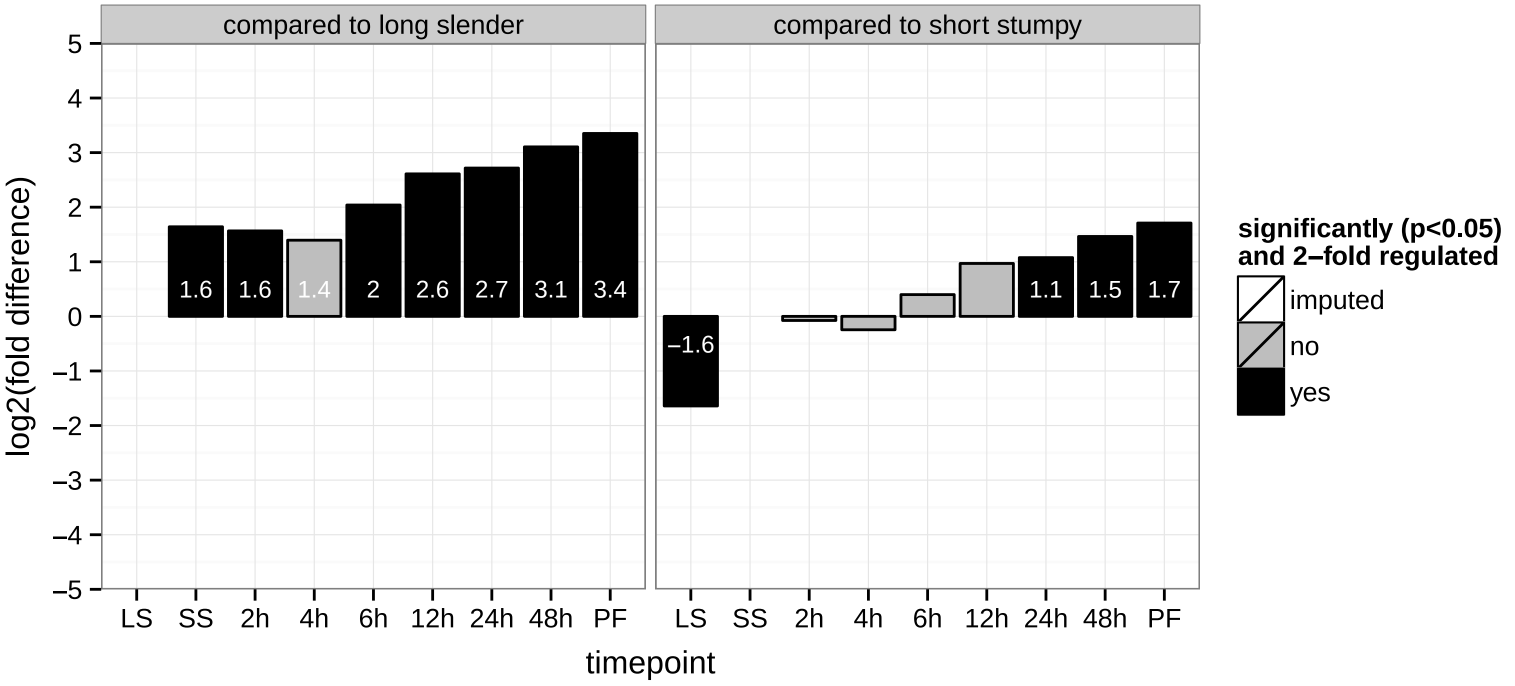

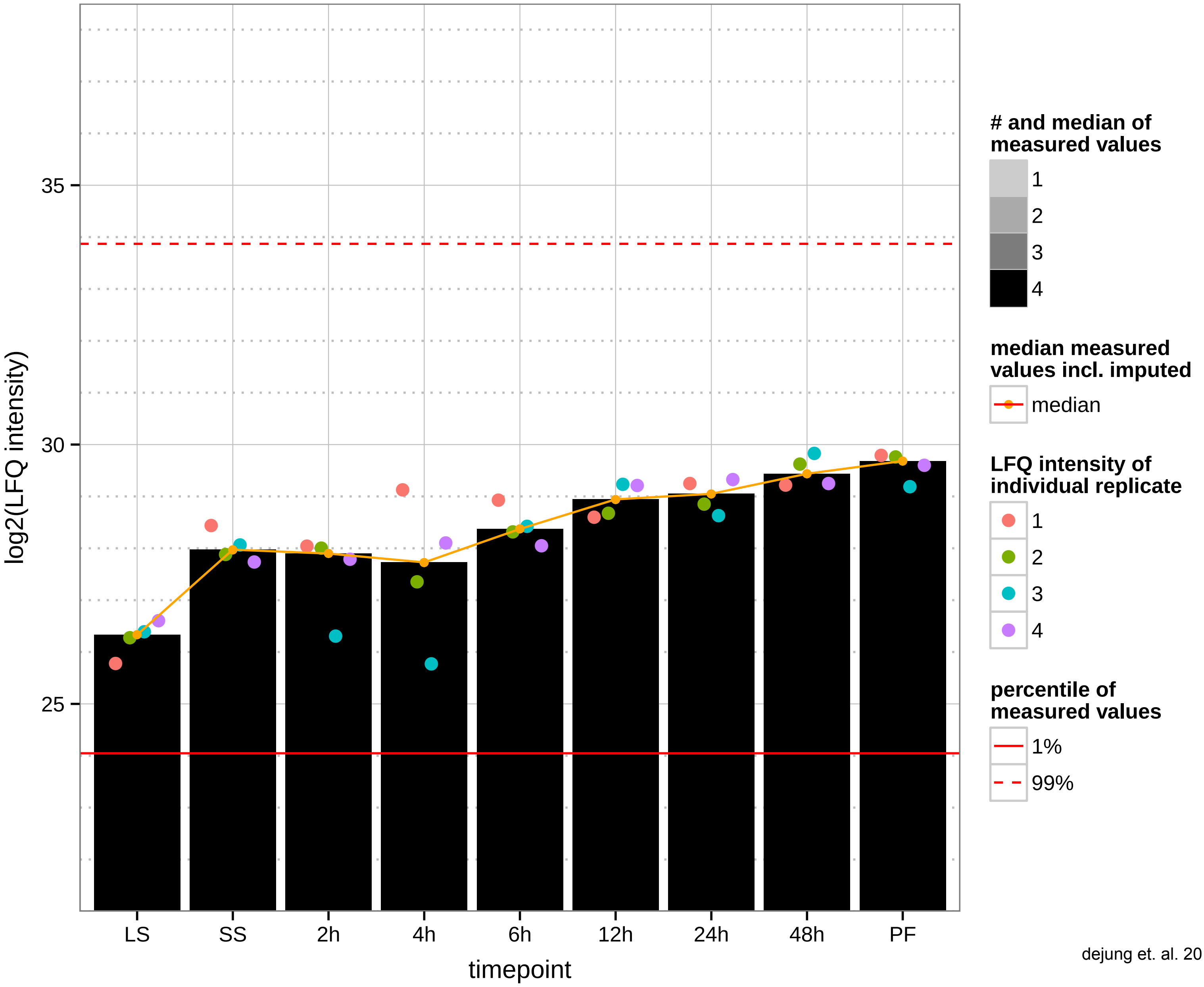

dejung et. al. 2015

eukaryotic translation initiation factor 5A, putative (eIF−5A)
Tb927.11.740
AGOF: RNA binding, ribosome binding, translation elongation factor activity, translation initiation factor activity
AGOC: cytoplasm
AGOP: peptidyl−lysine modification to hypusine, positive regulation of translational elongation, positive regulation of translatio
PGOF: RNA binding, ribosome binding, translation elongation factor activity
PGOC: null
PGOP: peptidyl−lysine modification to hypusine, positive regulation of translational elongation, positive regulation of translatio

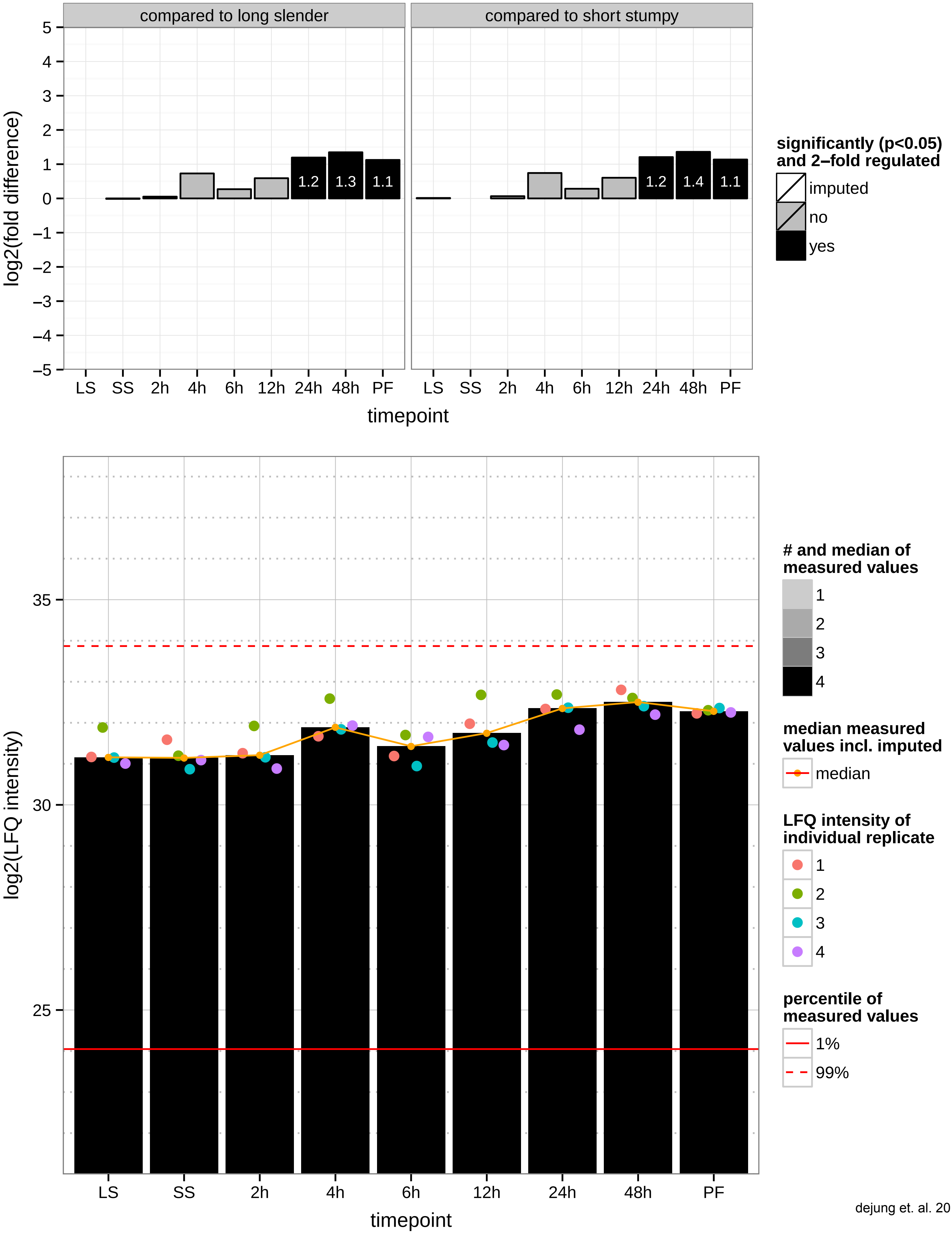

dejung et. al. 2015

hypothetical protein, conserved
Tb927.11.7790
AGOF: null
AGOC: mitochondrion
AGOP: null
PGOF: null
PGOC: null
PGOP: null

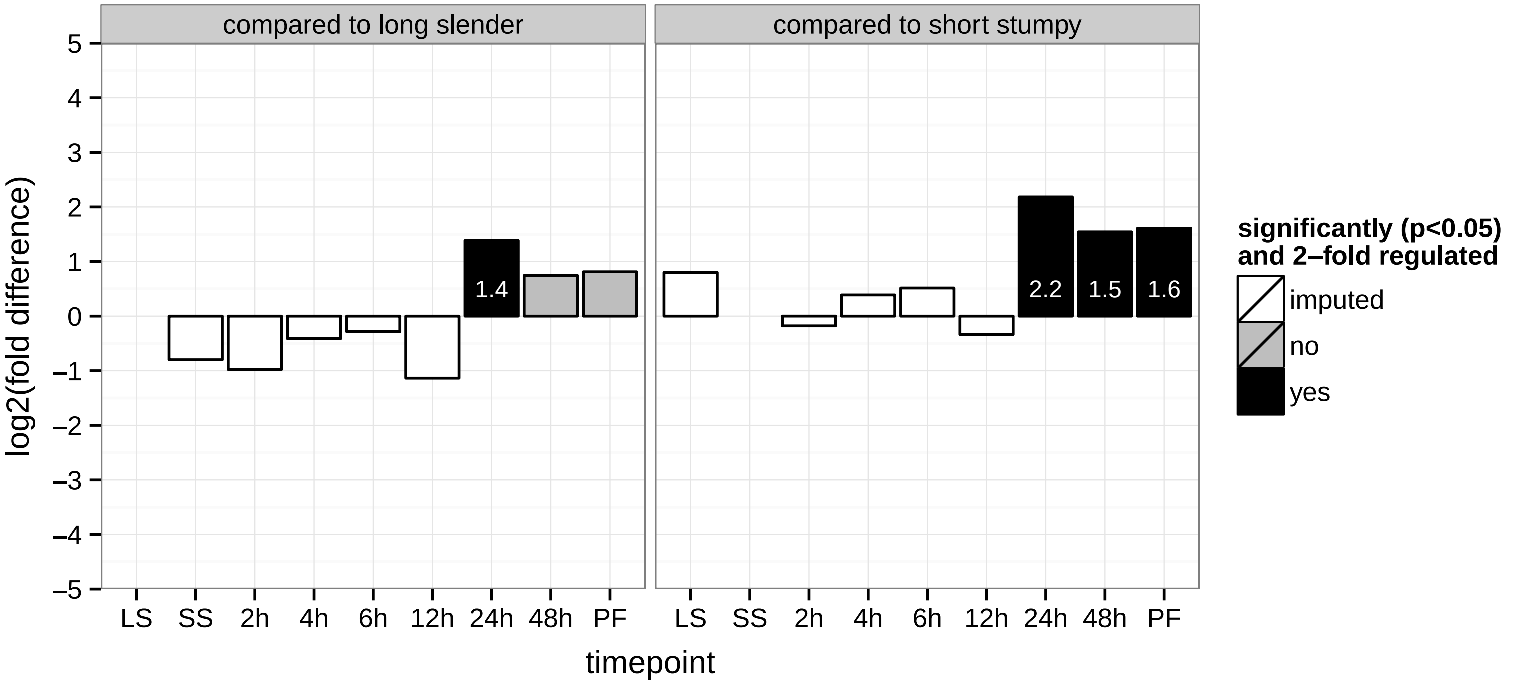

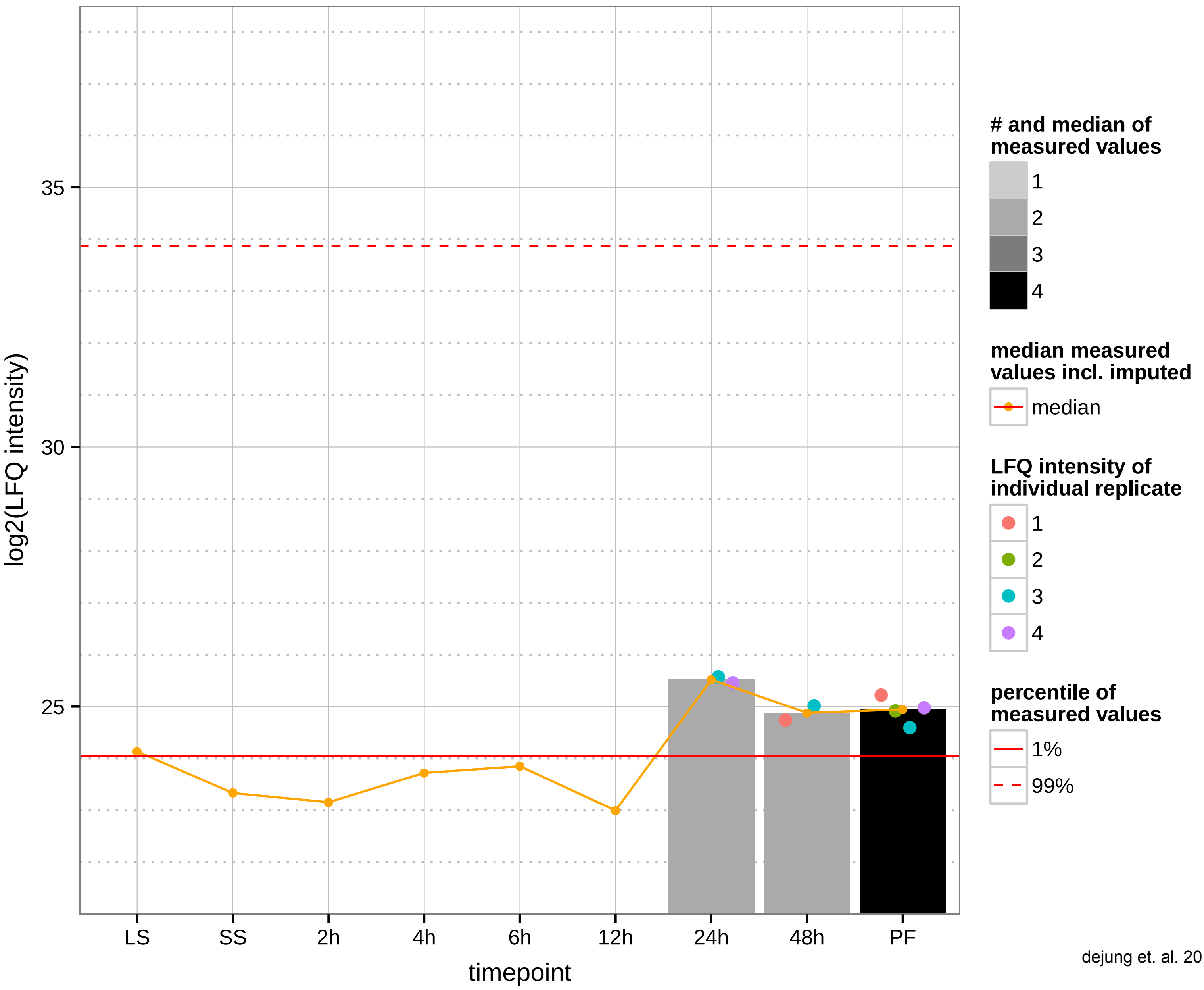

dejung et. al. 2015

hypothetical protein, conserved
Tb927.11.9570
AGOF: null
AGOC: null
AGOP: null
PGOF: null
PGOC: null
PGOP: null

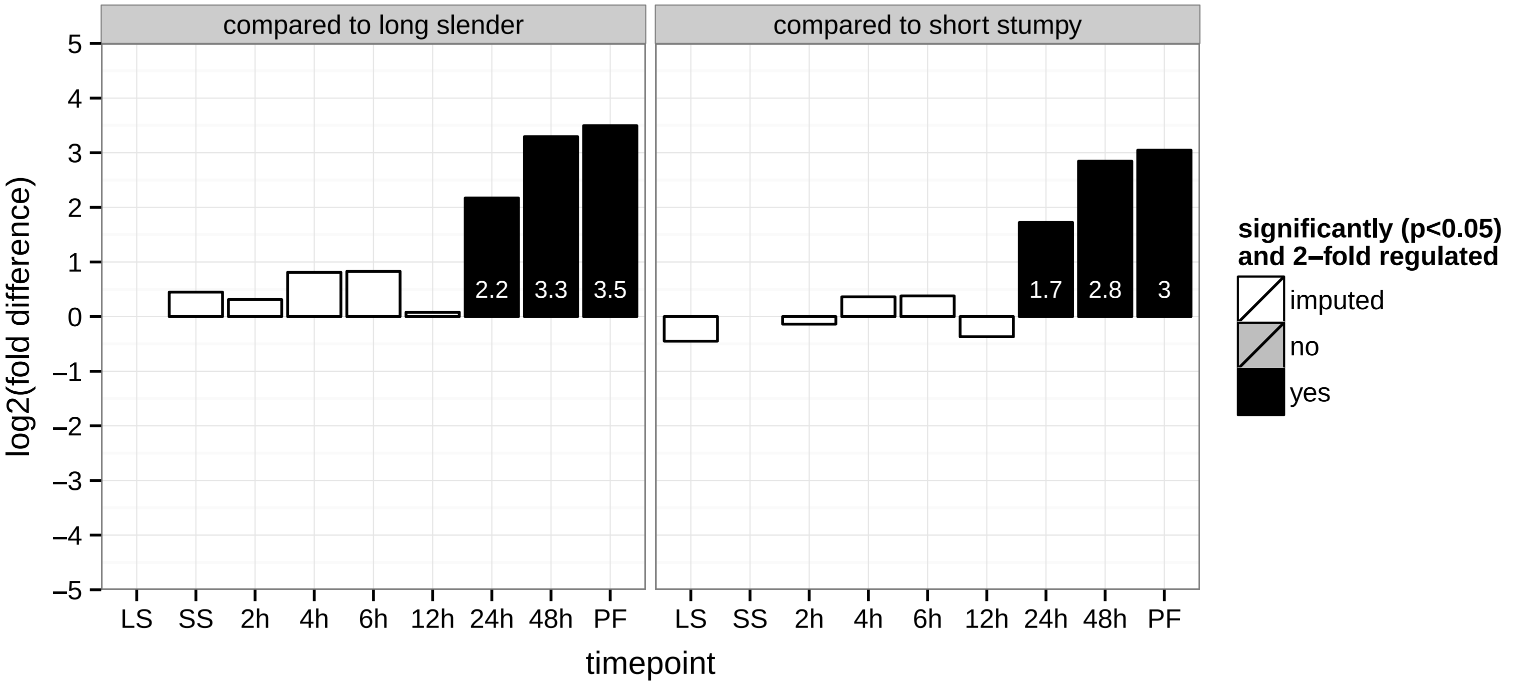

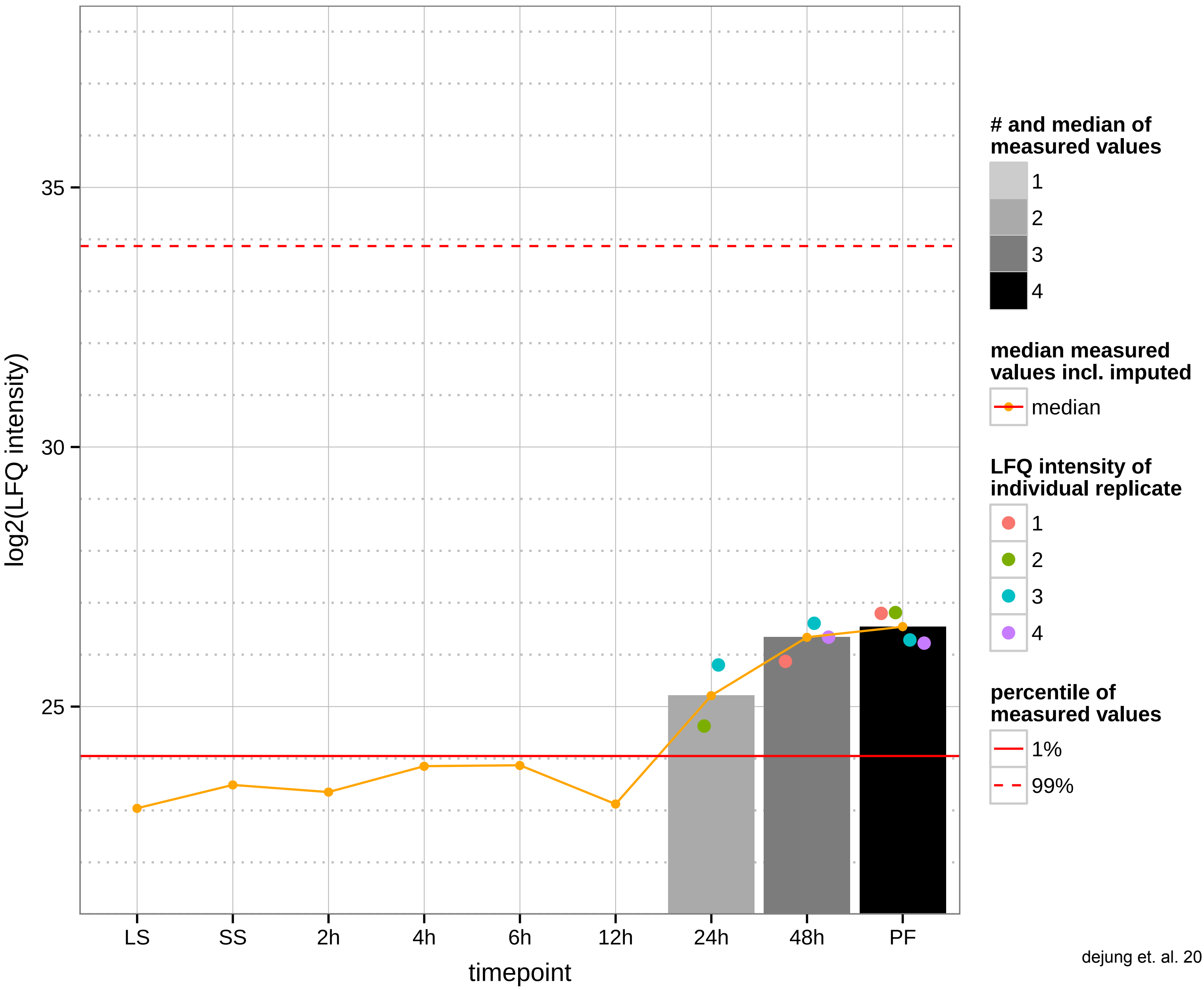

dejung et. al. 2015

hypothetical protein, conserved
Tb927.11.9970
AGOF: null
AGOC: null
AGOP: null
PGOF: null
PGOC: null
PGOP: null

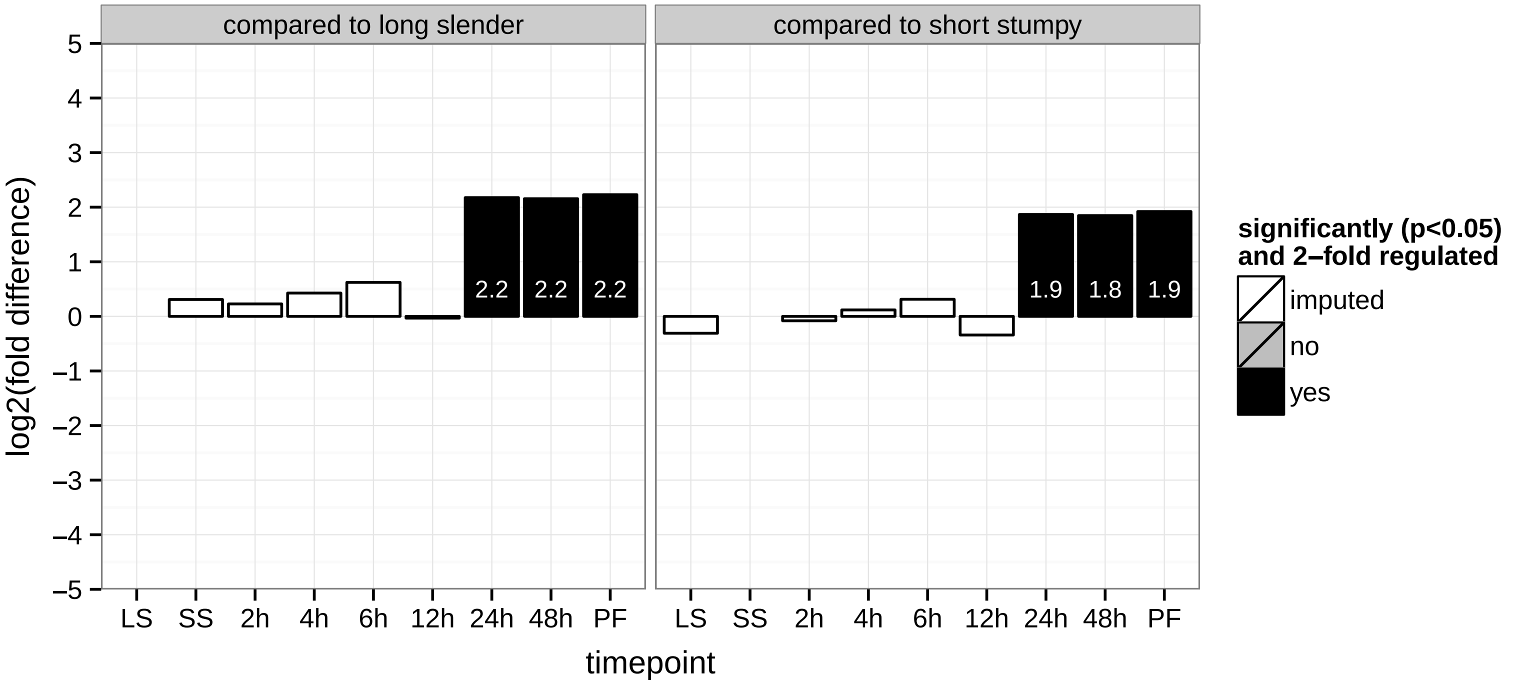

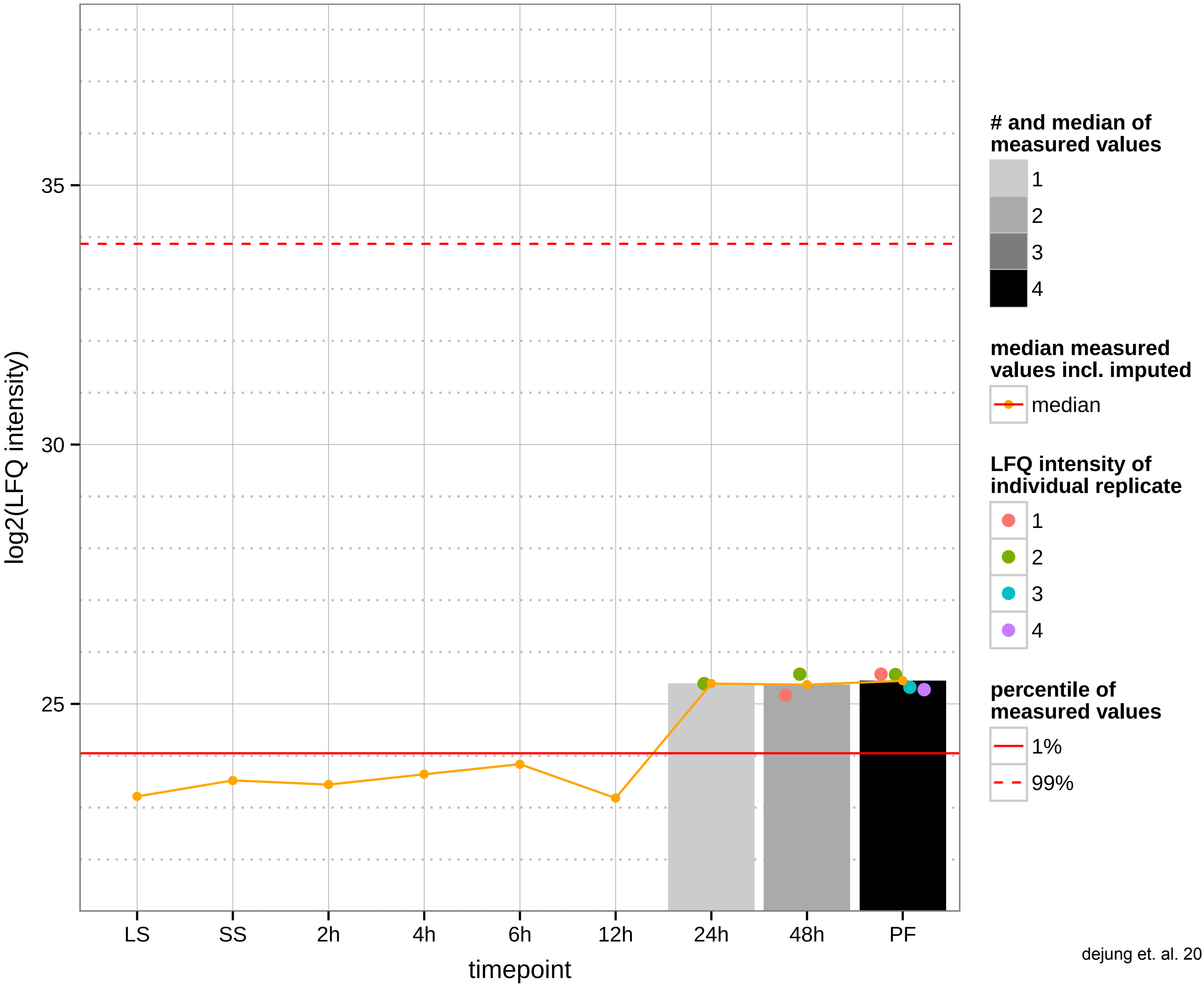

dejung et. al. 2015

hypothetical protein, conserved
Tb927.2.3610
AGOF: null
AGOC: mitochondrion
AGOP: null
PGOF: null
PGOC: null
PGOP: null

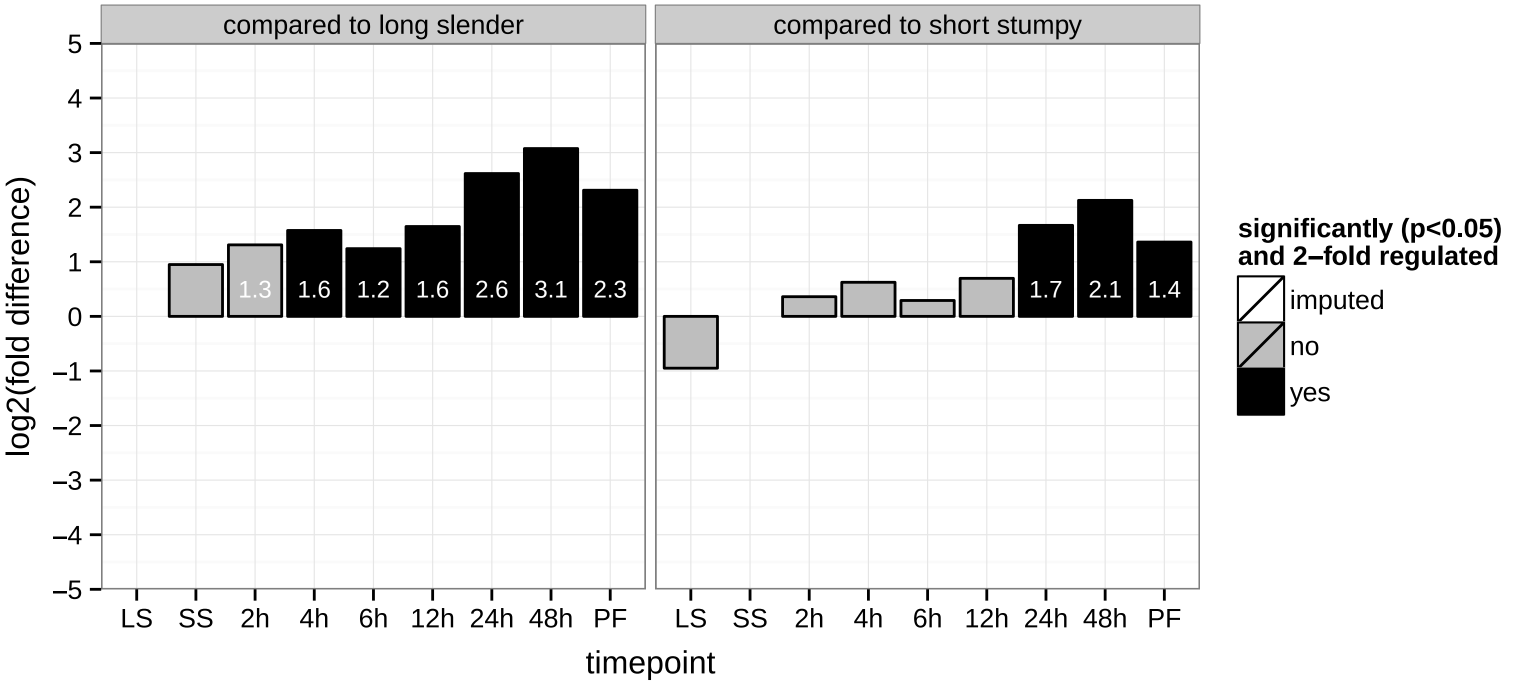

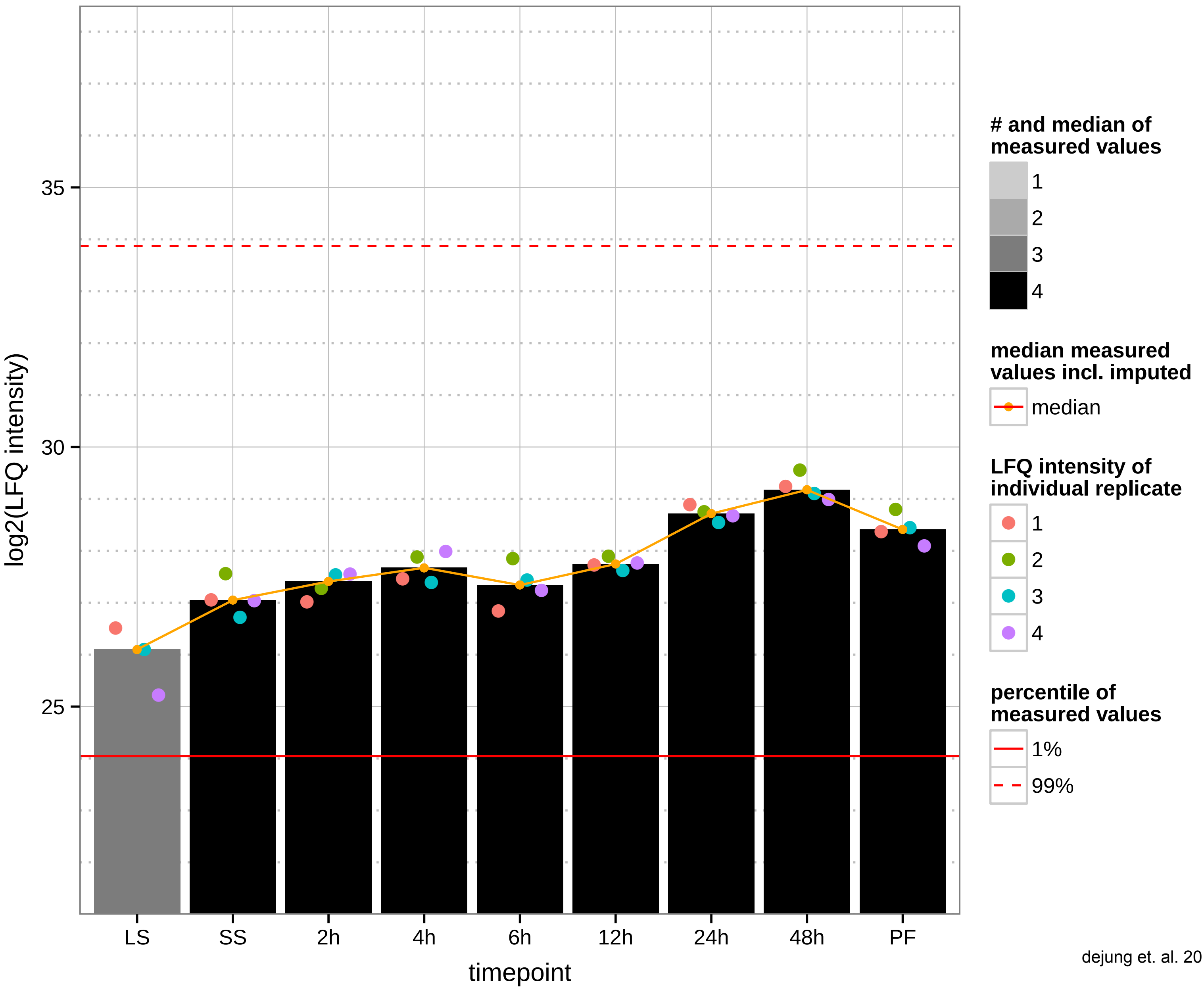

dejung et. al. 2015

ubiquitin–conjugating enzyme, putative
Tb927.2.3720
AGOF: ubiquitin–protein ligase activity
AGOC: nucleus
AGOP: cellular protein modification process, post–translational protein modification, regulation of protein metabolic process
PGOF: acid–amino acid ligase activity
PGOC: null
PGOP: null

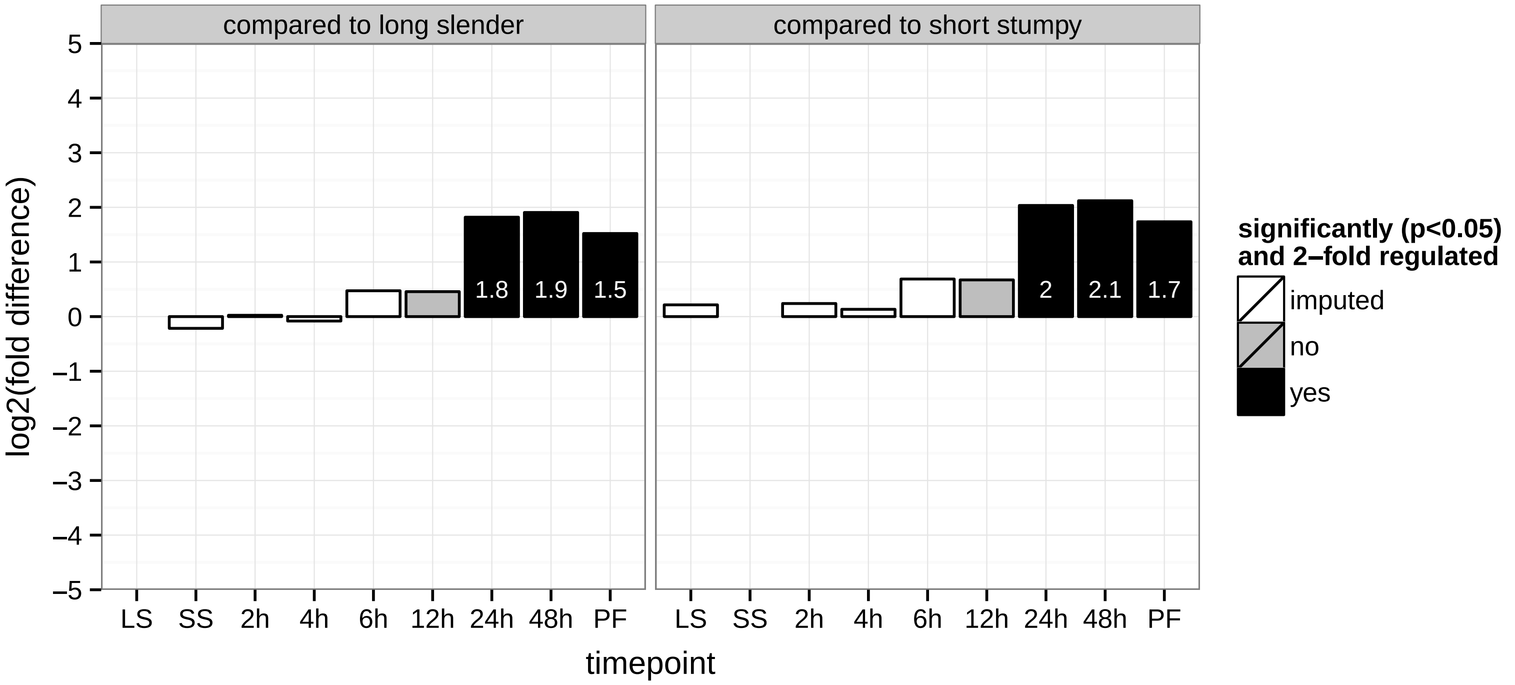

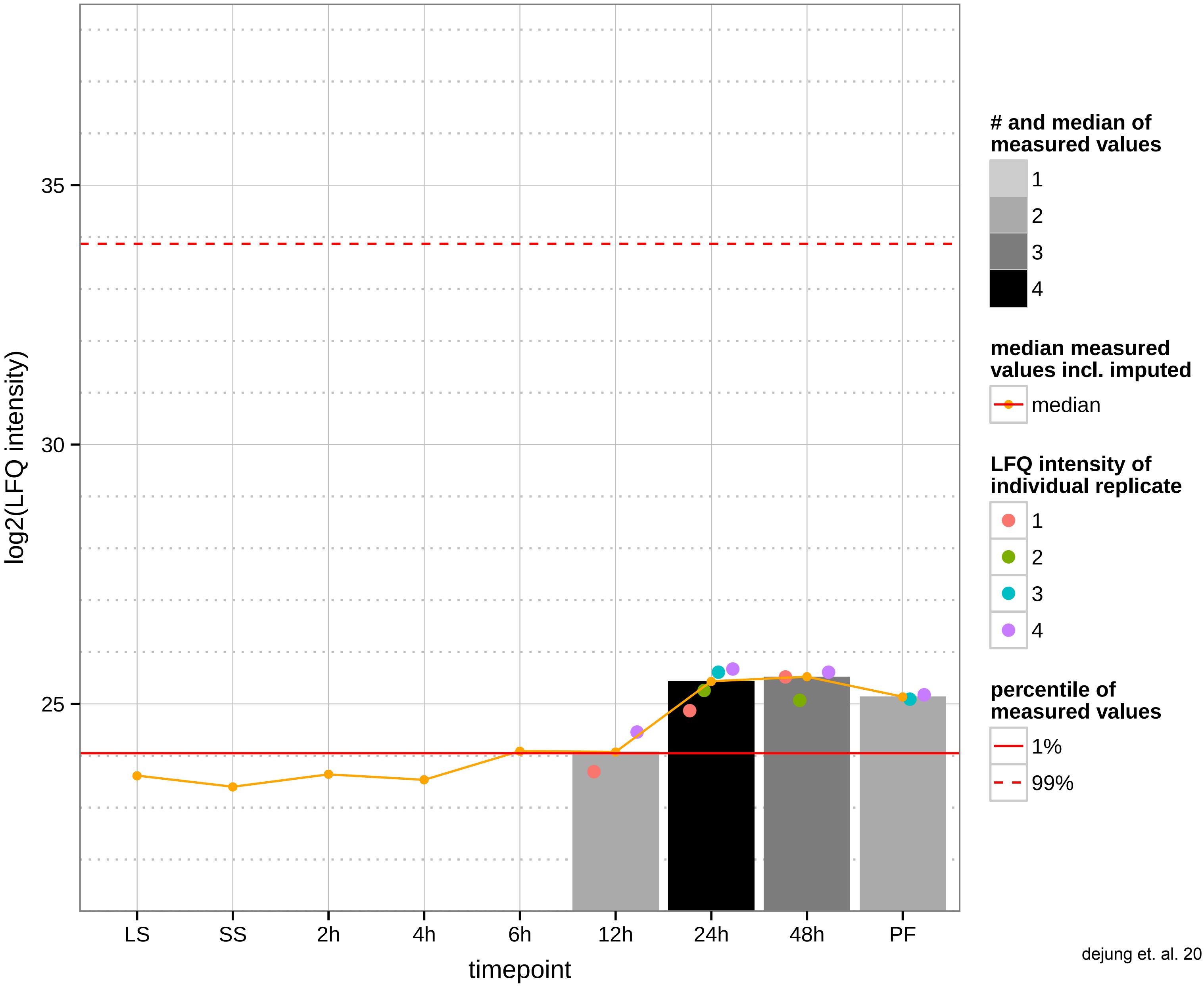

dejung et. al. 2015

glycosomal phosphoenolpyruvate carboxykinase (PEPCK)
Tb927.2.4210
AGOF: ATP binding, microtubule binding, phosphoenolpyruvate carboxykinase (ATP) activity
AGOC: glycosome, vesicle membrane
AGOP: gluconeogenesis
PGOF: ATP binding, phosphoenolpyruvate carboxykinase (ATP) activity, phosphoenolpyruvate carboxykinase activity, purine r
PGOC: null
PGOP: gluconeogenesis

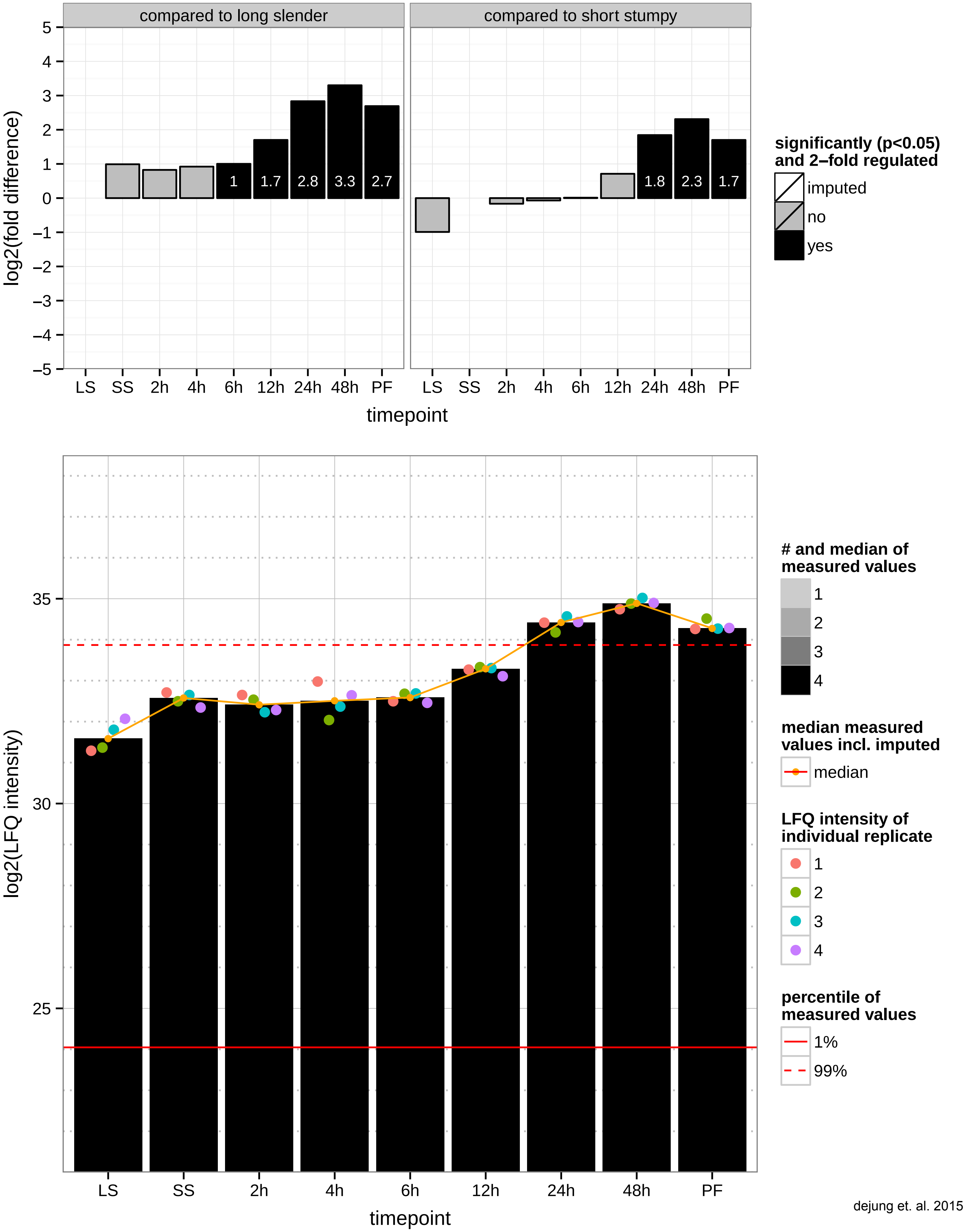

dejung et. al. 2015

hypothetical protein, conserved
Tb927.2.4980
AGOF: null
AGOC: nucleus
AGOP: null
PGOF: methyltransferase activity
PGOC: null
PGOP: null

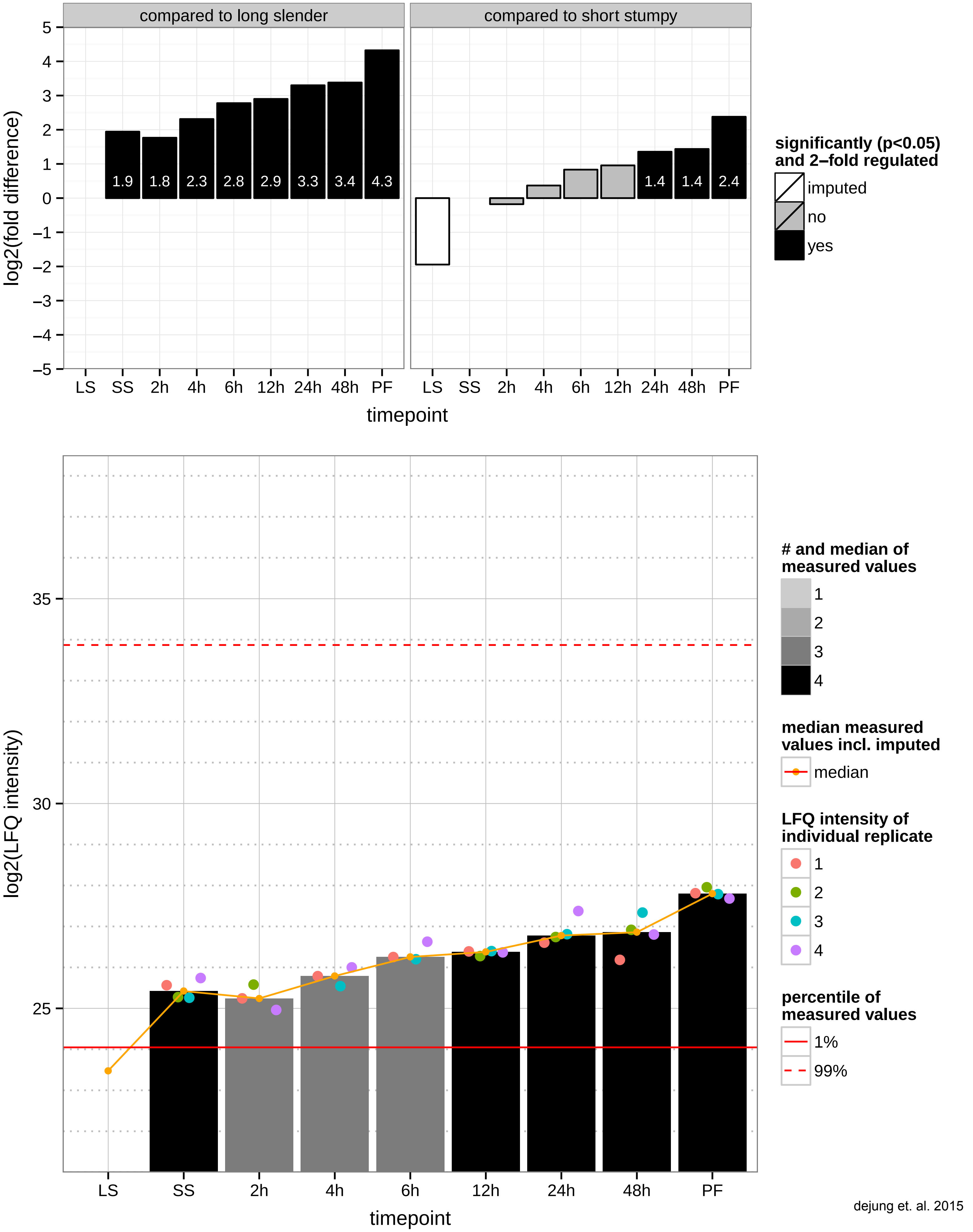

dejung et. al. 2015

hypothetical protein, conserved
Tb927.2.6100
AGOF: null
AGOC: null
AGOP: null
PGOF: null
PGOC: null
PGOP: null

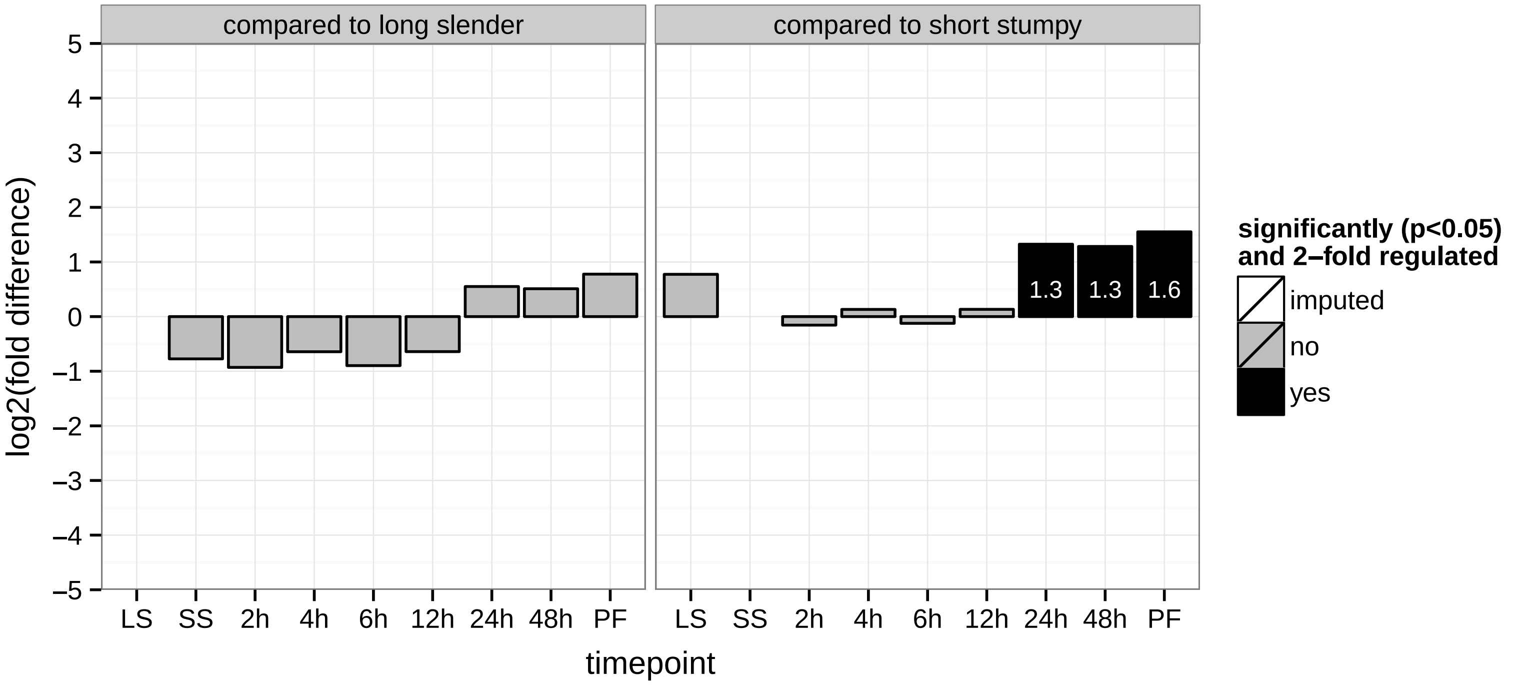

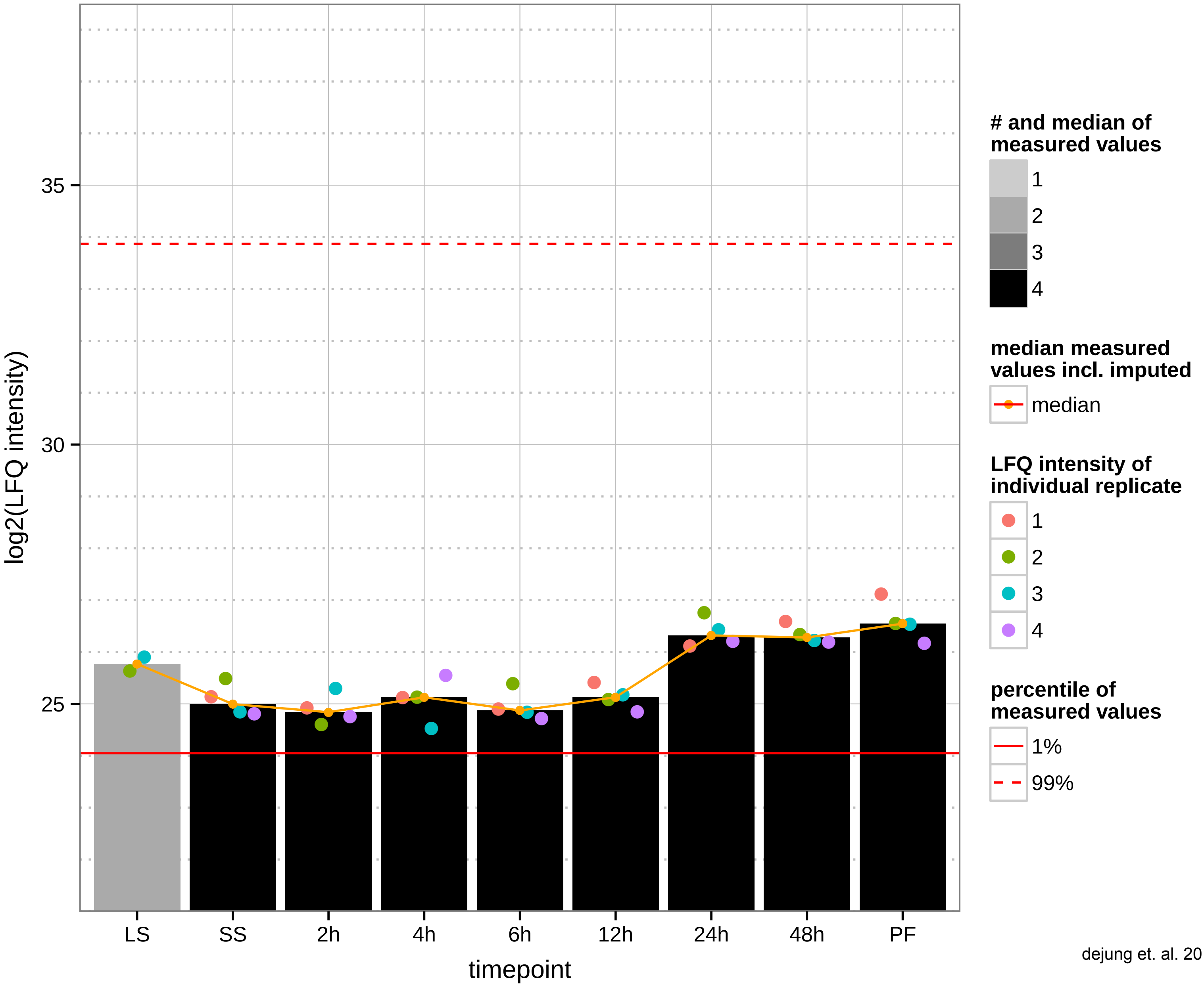

dejung et. al. 2015

ATP synthase beta chain, mitochondrial precursor, ATP synthase F1, beta subunit
Tb927.3.1380
AGOF: ATP binding, GTP binding, hydrogen ion transporting ATP synthase activity, rotational mechanism, hydrogen–exporting
AGOC: mitochondrial inner membrane, mitochondrial proton–transporting ATP synthase complex, catalytic core F(1), mitocho
AGOP: ATP synthesis coupled proton transport, proton transport
PGOF: ATP binding, hydrogen ion transporting ATP synthase activity, rotational mechanism, hydrogen–exporting ATPase activ
PGOC: proton–transporting ATP synthase complex, catalytic core F(1), proton–transporting two–sector ATPase complex, cata
PGOP: ATP hydrolysis coupled proton transport, ATP metabolic process, ATP synthesis coupled proton transport, proton trans

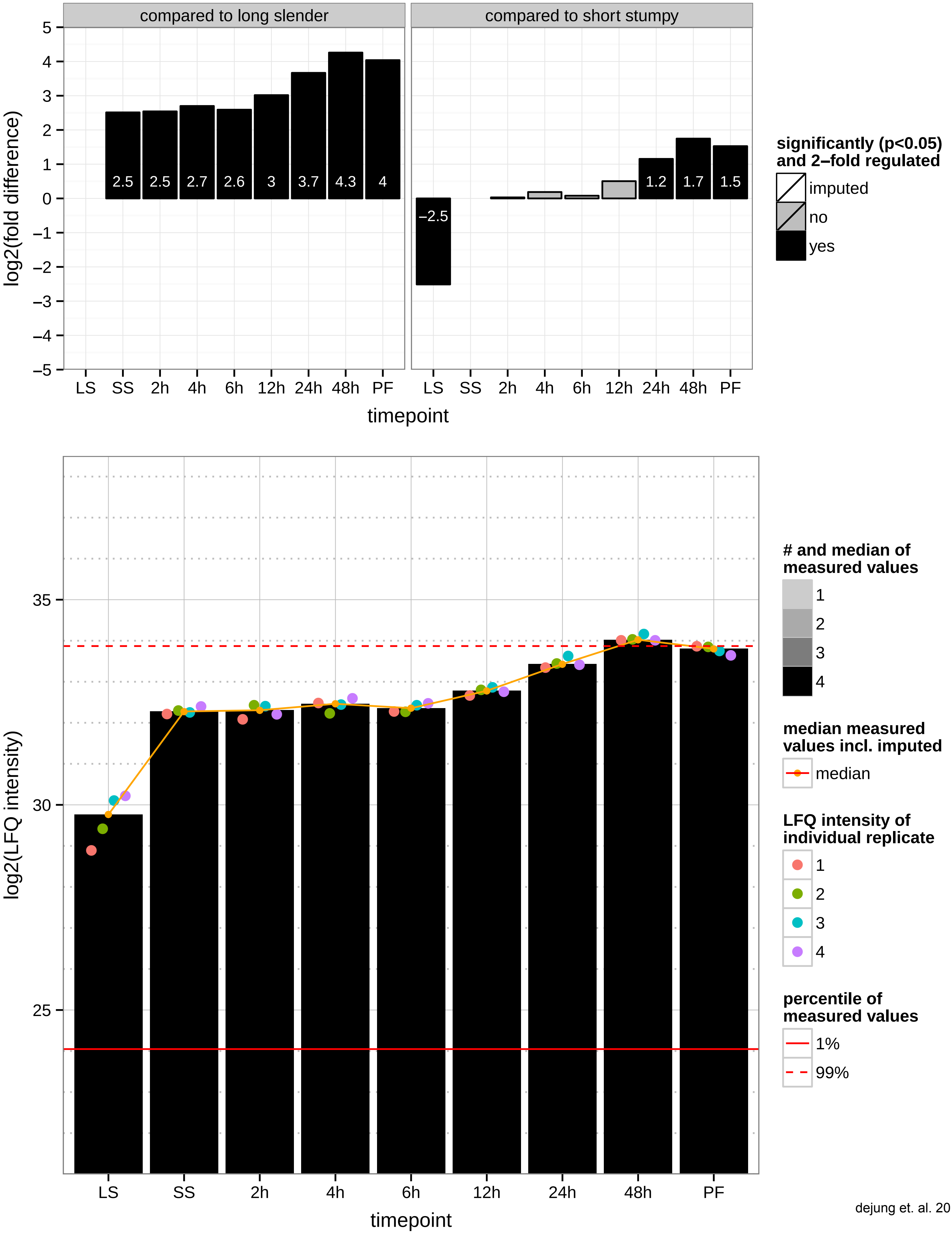

dejung et. al. 2015

hypothetical protein, conserved
Tb927.3.1690
AGOF: null
AGOC: mitochondrion
AGOP: null
PGOF: null
PGOC: null
PGOP: null

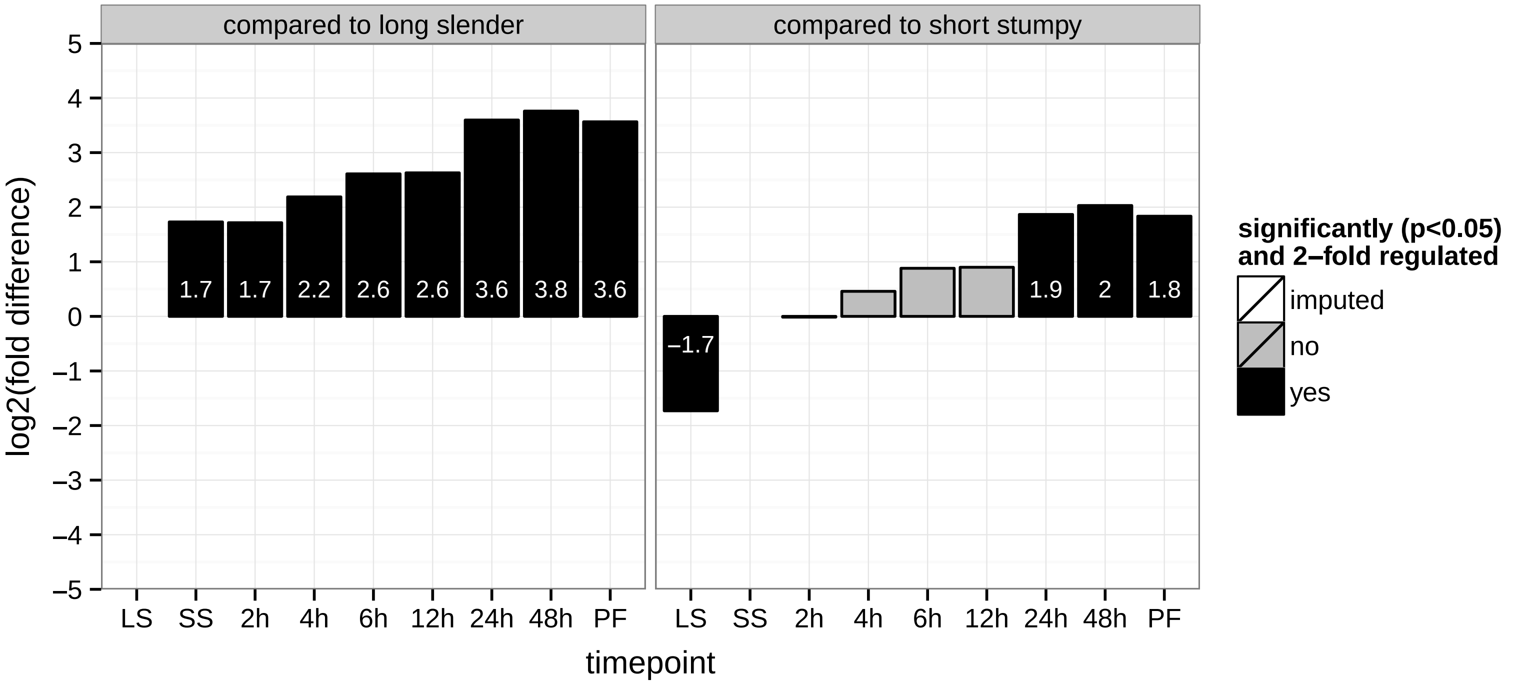

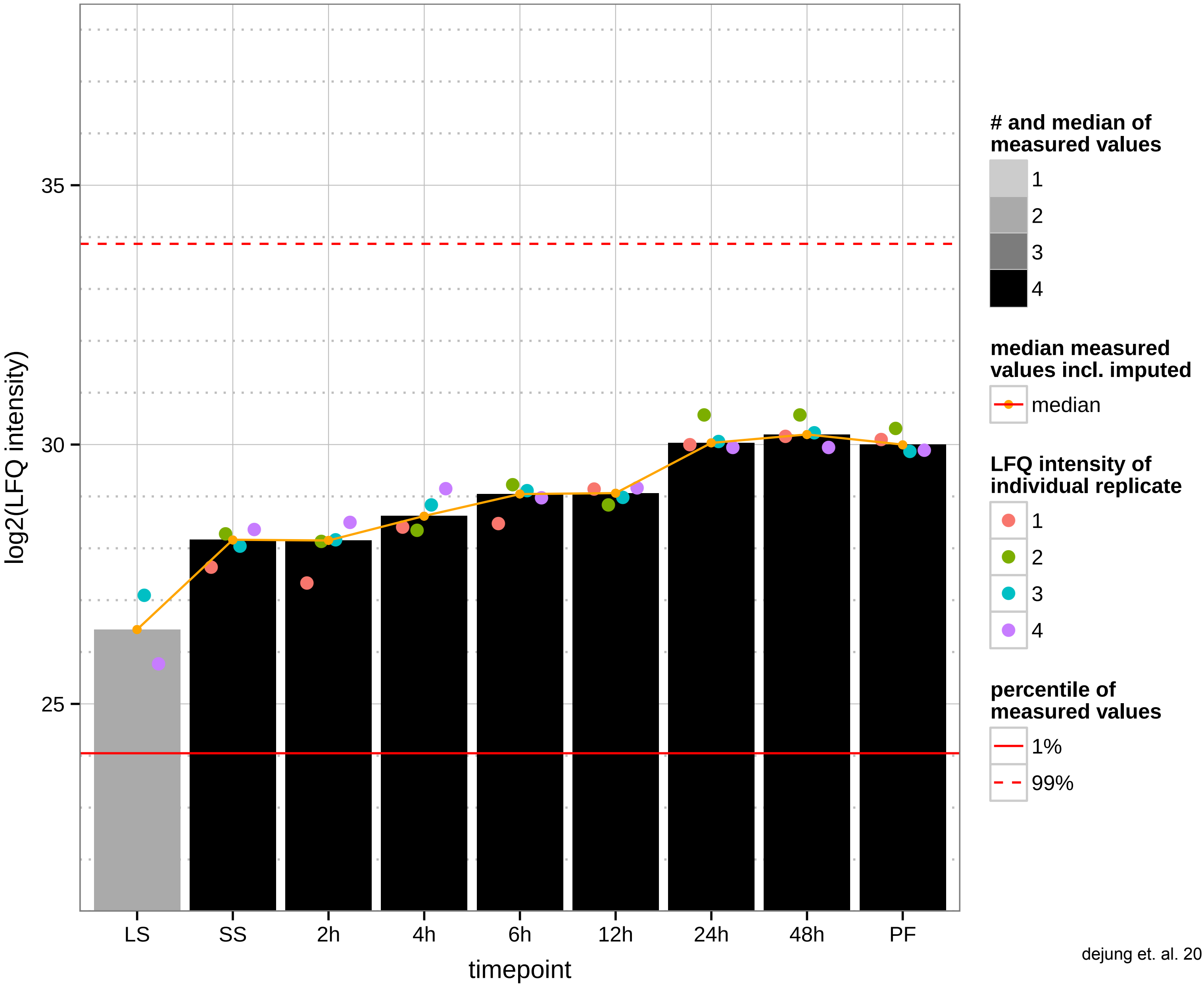

dejung et. al. 2015

hypothetical protein, conserved
Tb927.3.1810
AGOF: null
AGOC: mitochondrion
AGOP: null
PGOF: null
PGOC: null
PGOP: null

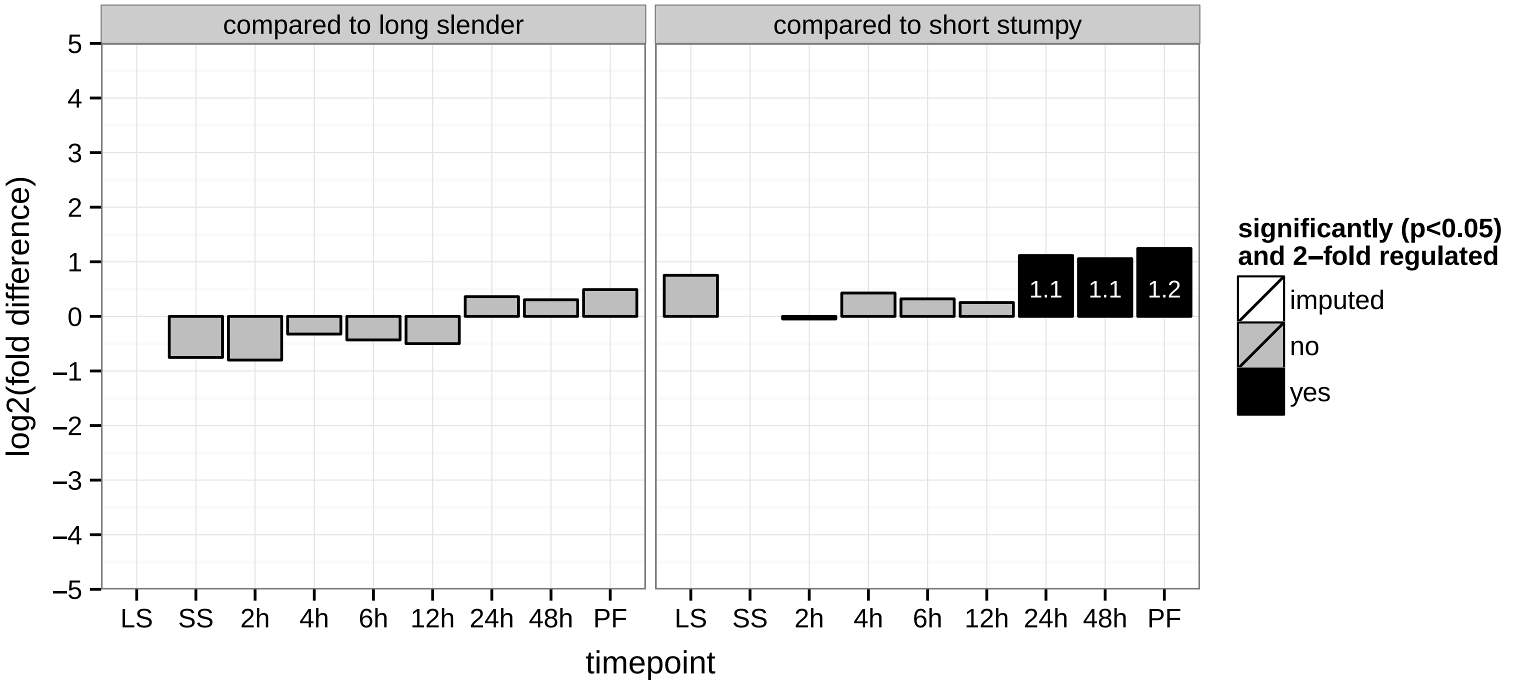

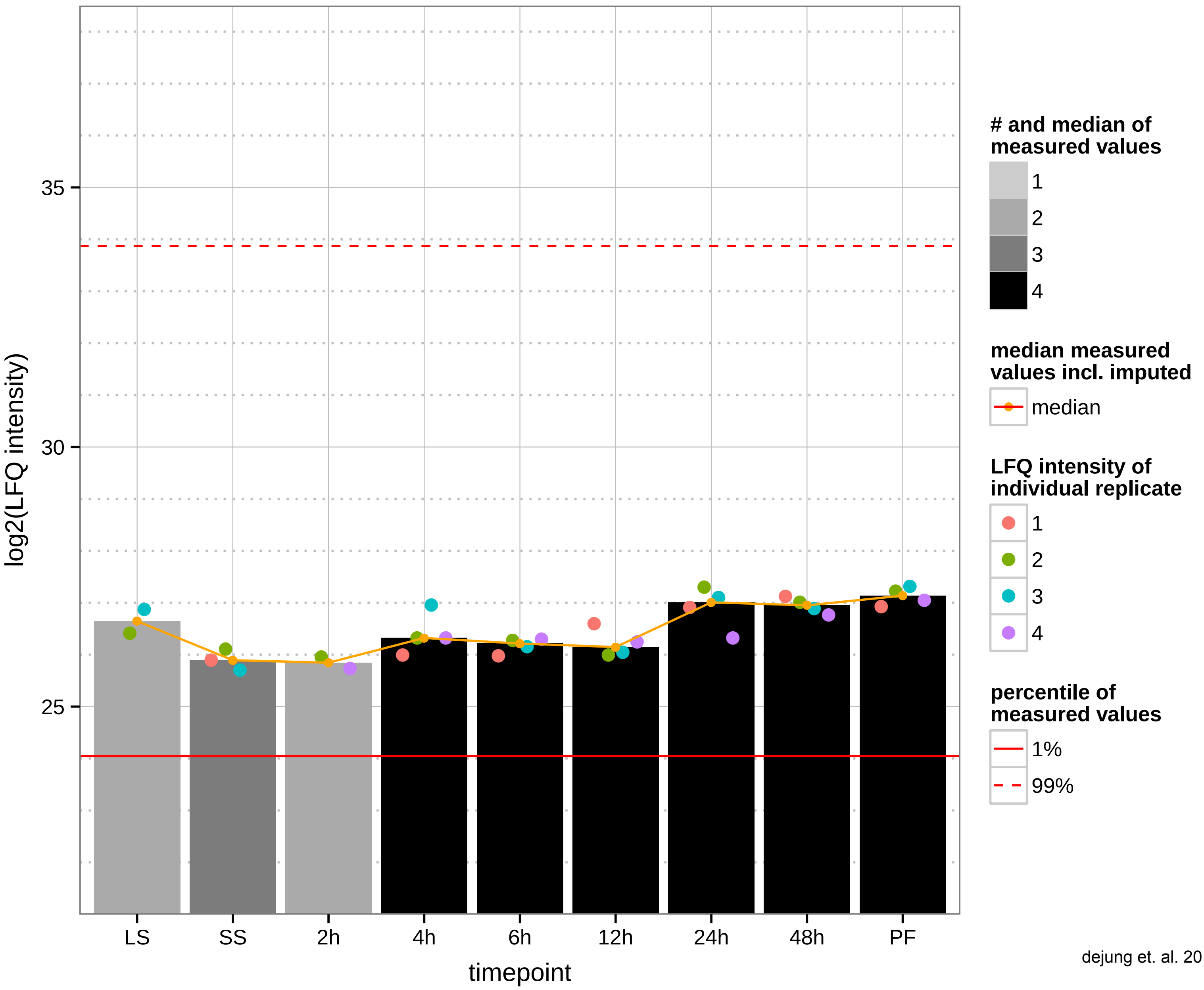

dejung et. al. 2015

protein phosphatase 2C, putative
Tb927.3.2150
AGOF: phosphoprotein phosphatase activity
AGOC: null
AGOP: null
PGOF: catalytic activity
PGOC: null
PGOP: null

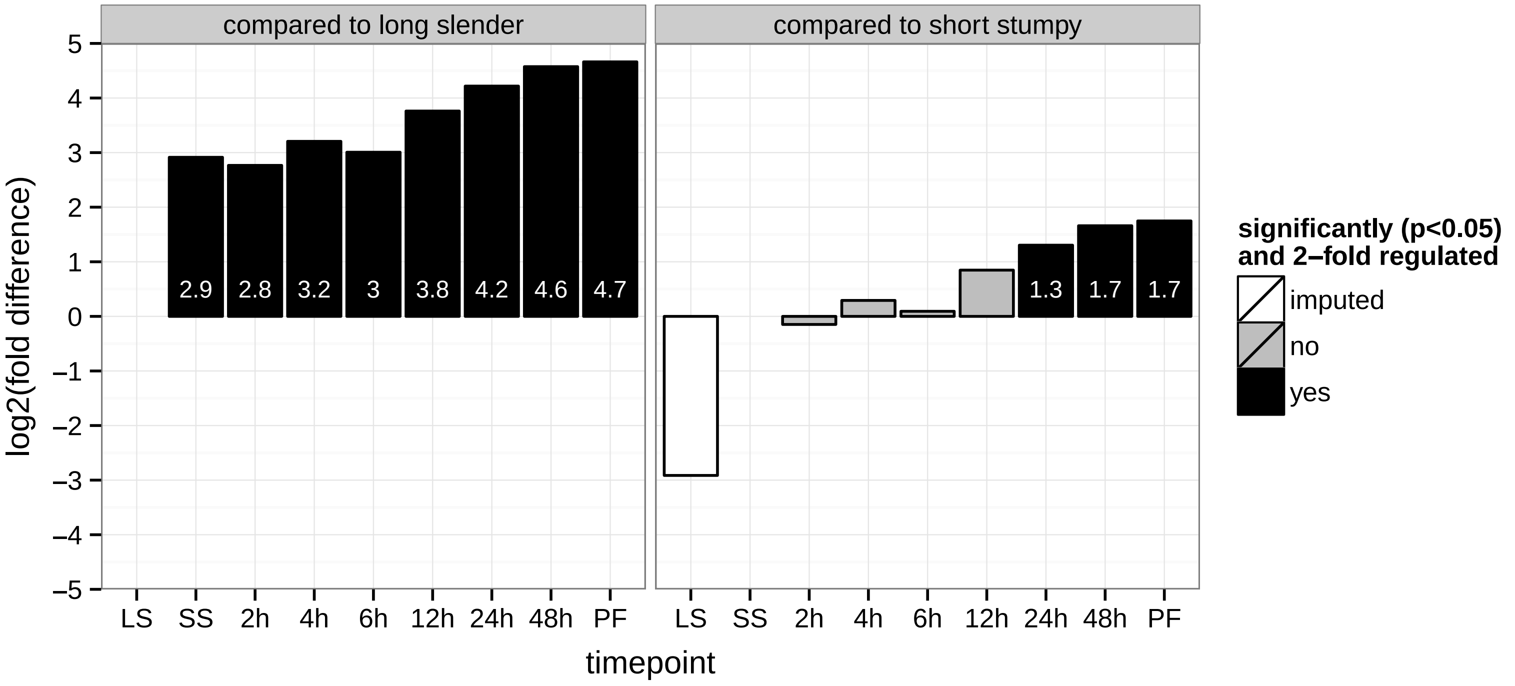

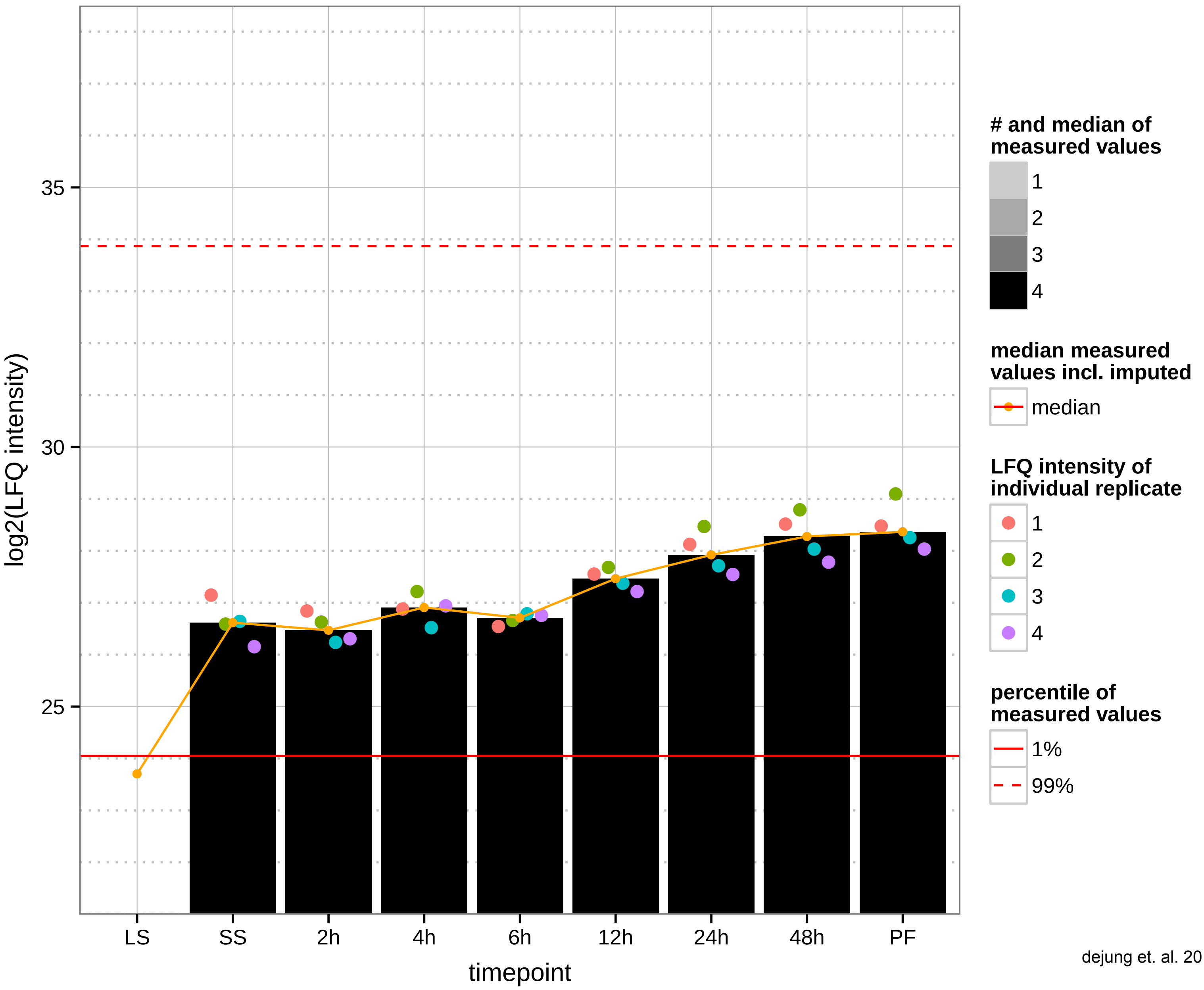

dejung et. al. 2015

hypothetical protein, conserved
Tb927.3.2750
AGOF: null
AGOC: null
AGOP: null
PGOF: null
PGOC: null
PGOP: null

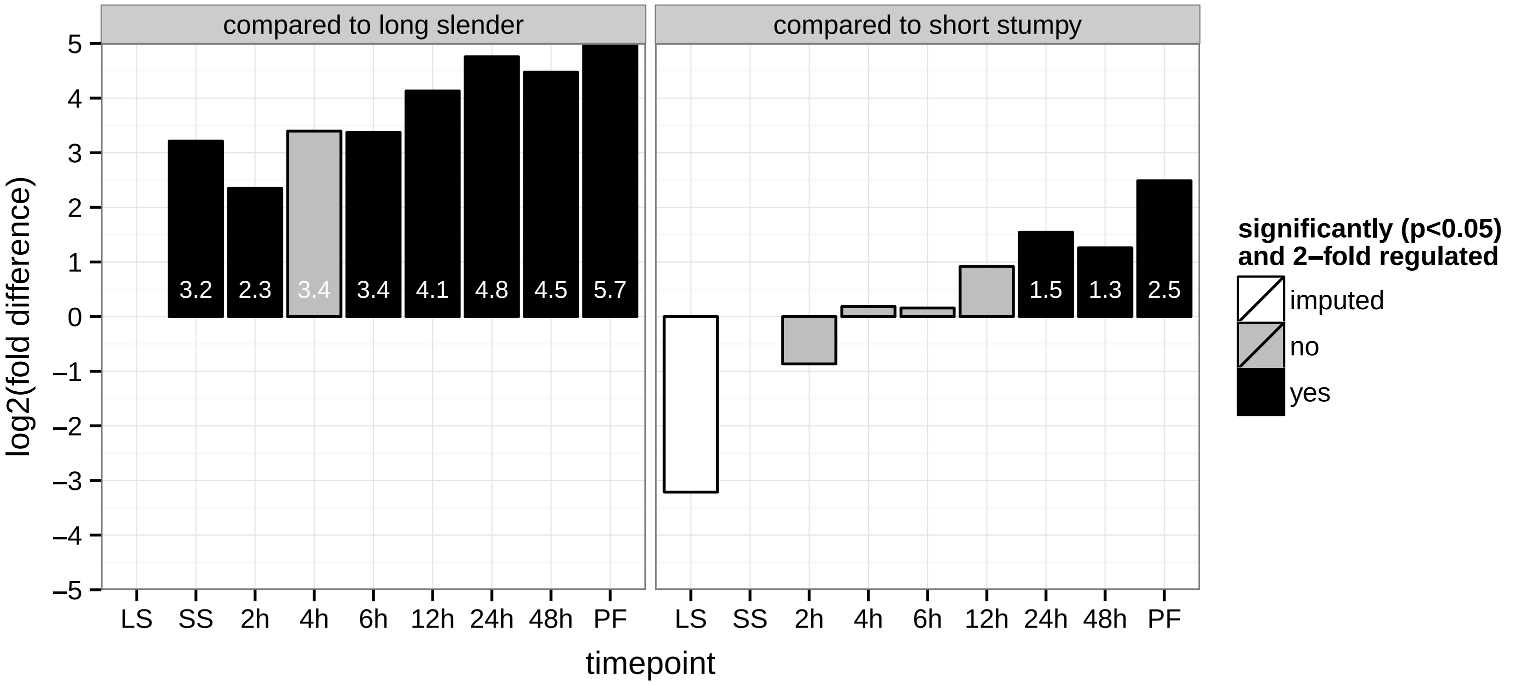

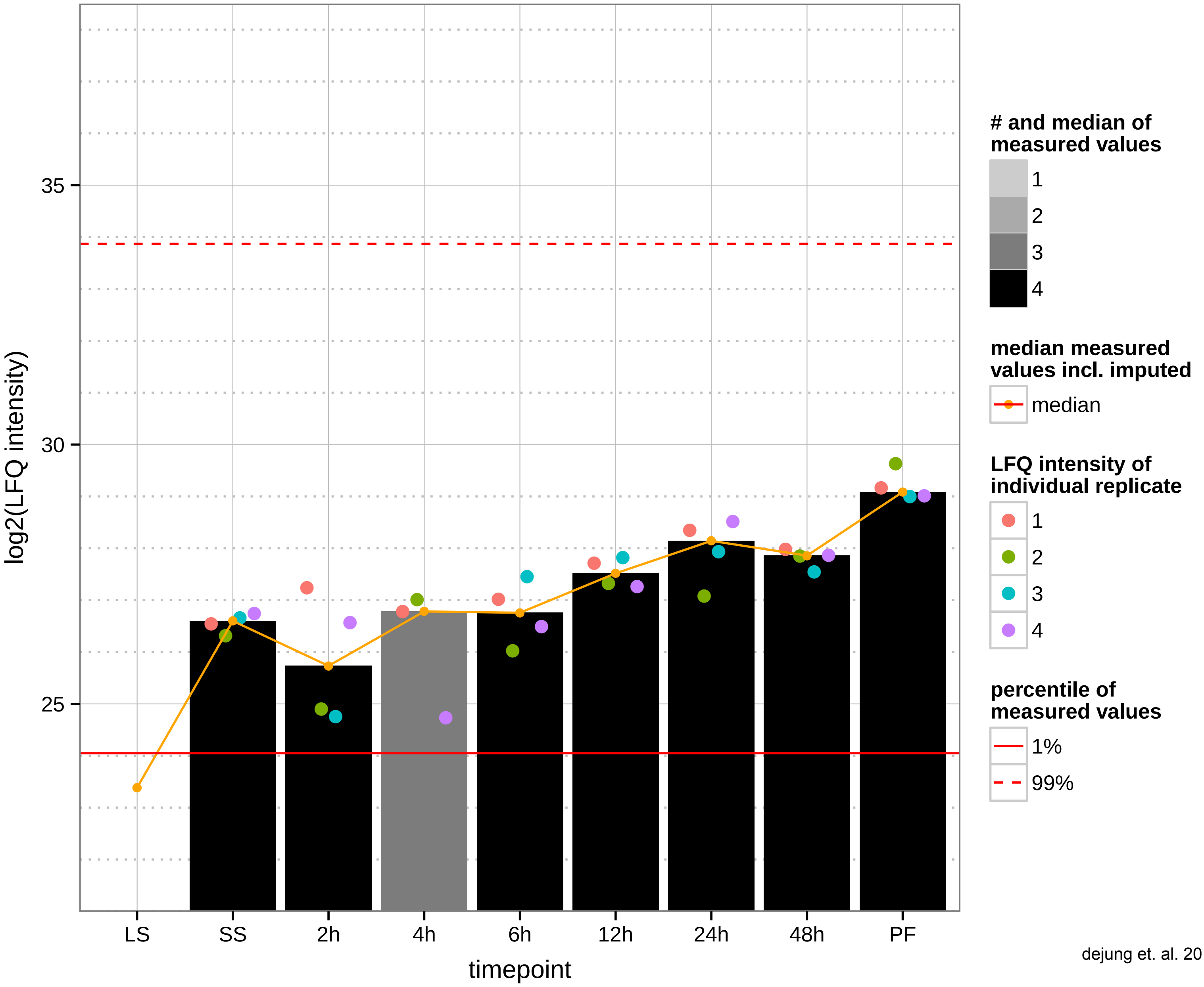

dejung et. al. 2015

eukaryotic initiation factor 2a, putative
Tb927.3.2900
AGOF: RNA binding, translation initiation factor activity
AGOC: eukaryotic translation initiation factor 2 complex
AGOP: translation
PGOF: RNA binding, translation initiation factor activity
PGOC: eukaryotic translation initiation factor 2 complex
PGOP: null

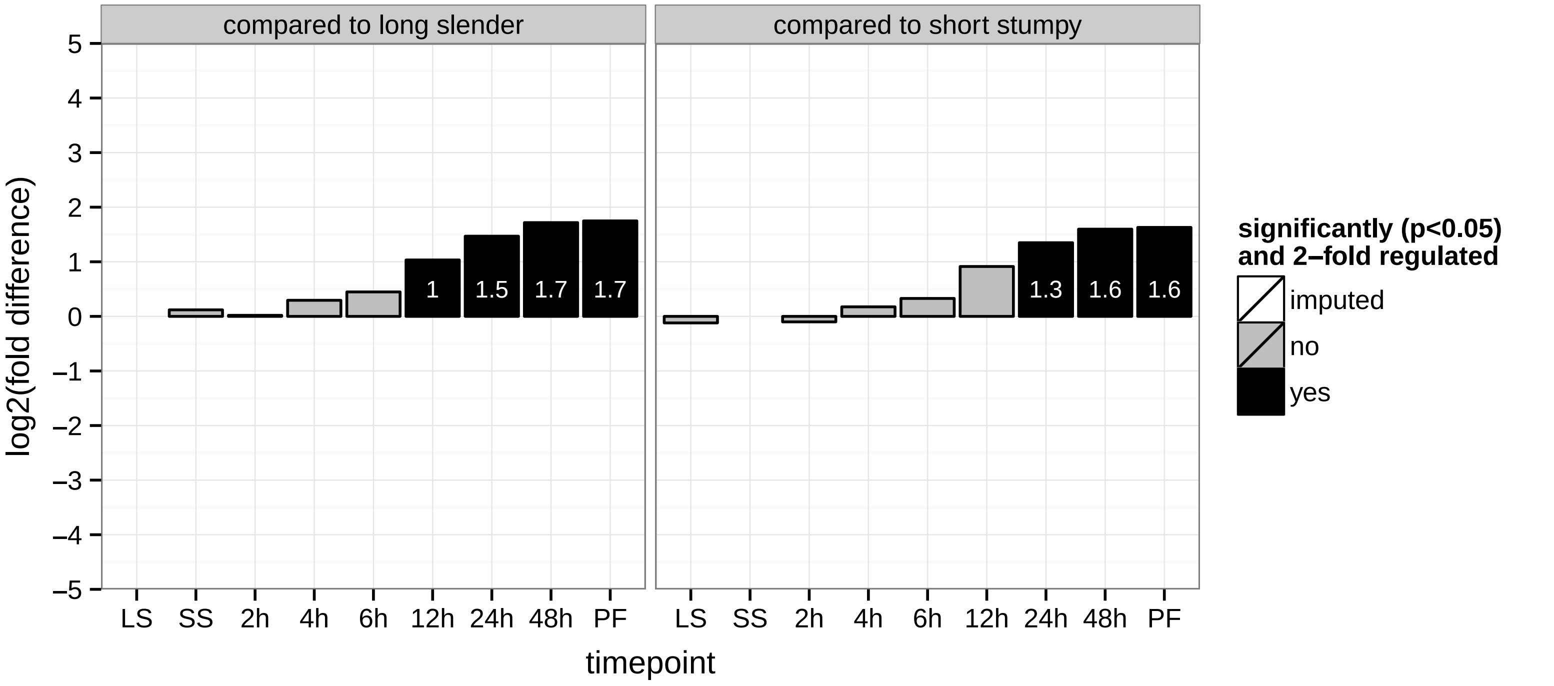

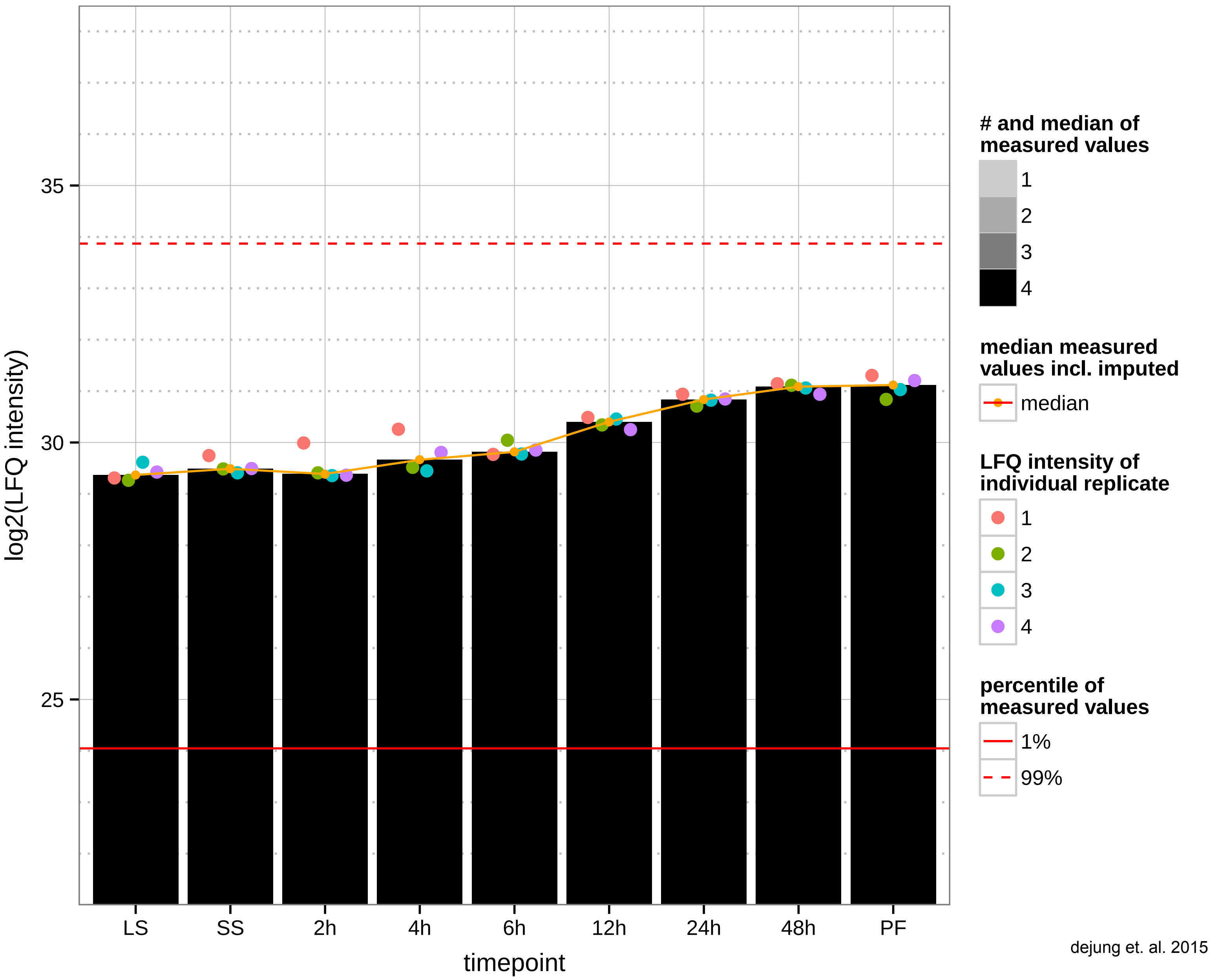

dejung et. al. 2015

hypothetical protein, conserved
Tb927.3.3170
AGOF: null
AGOC: null
AGOP: intracellular signal transduction
PGOF: null
PGOC: null
PGOP: intracellular signal transduction

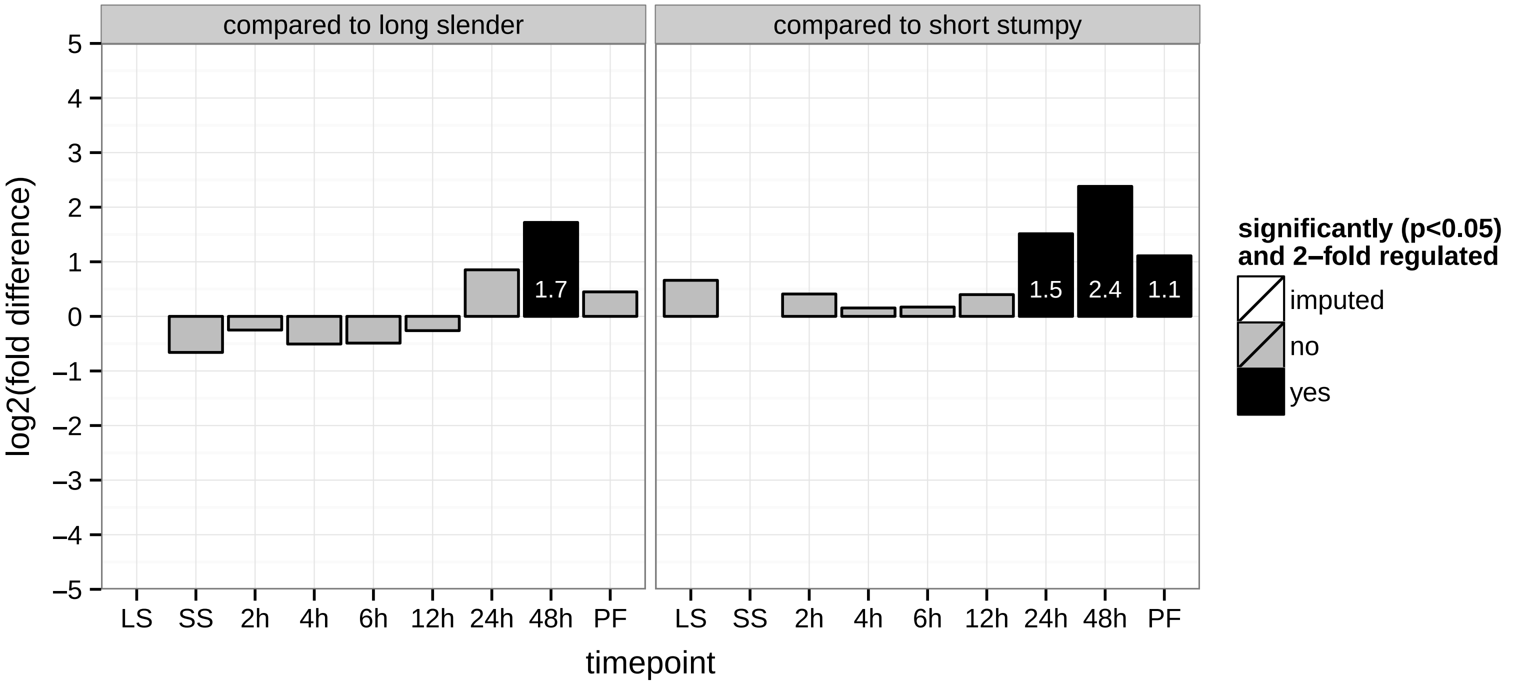

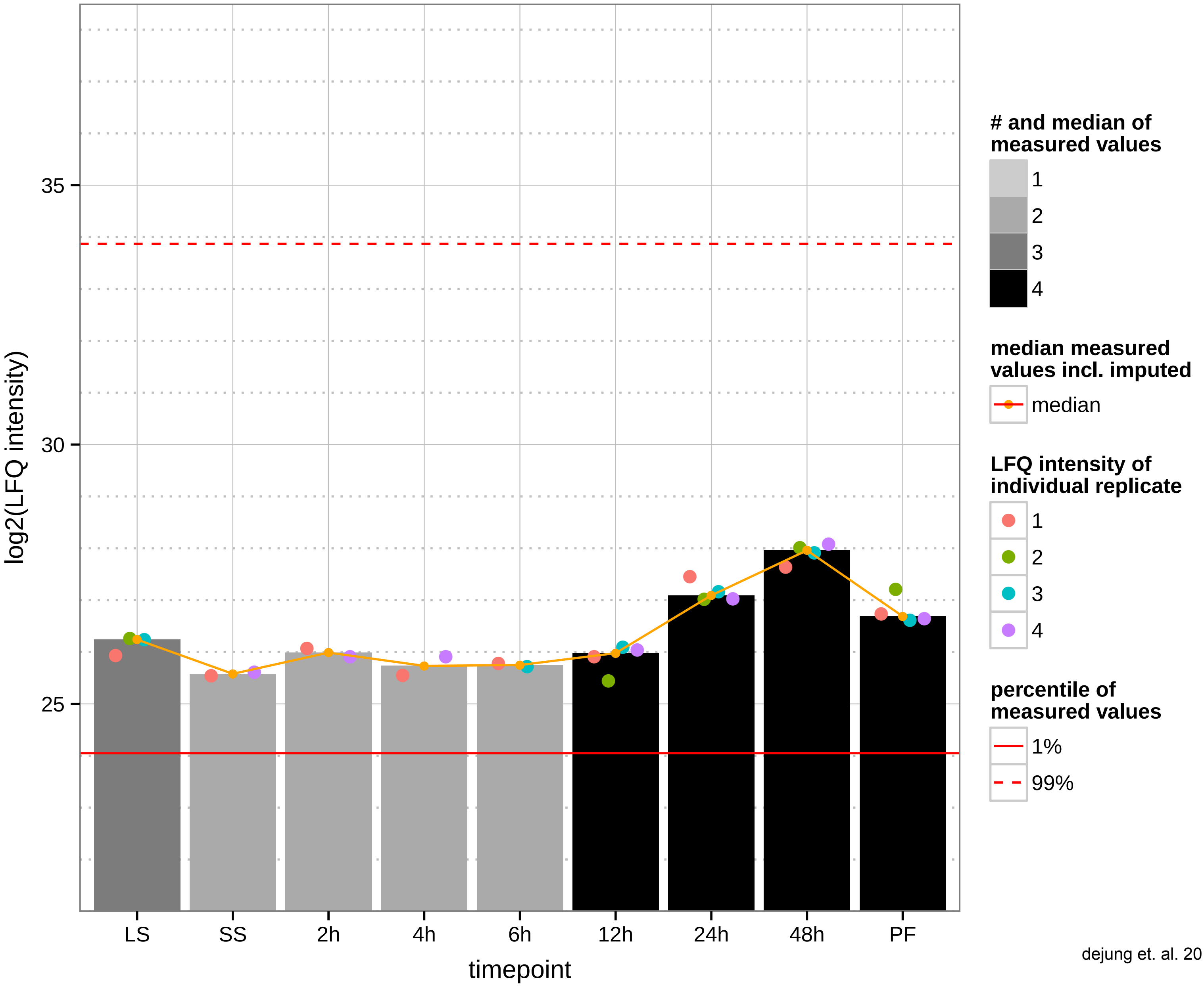

dejung et. al. 2015

hypothetical protein, conserved
Tb927.3.3460
AGOF: null
AGOC: null
AGOP: null
PGOF: null
PGOC: null
PGOP: null

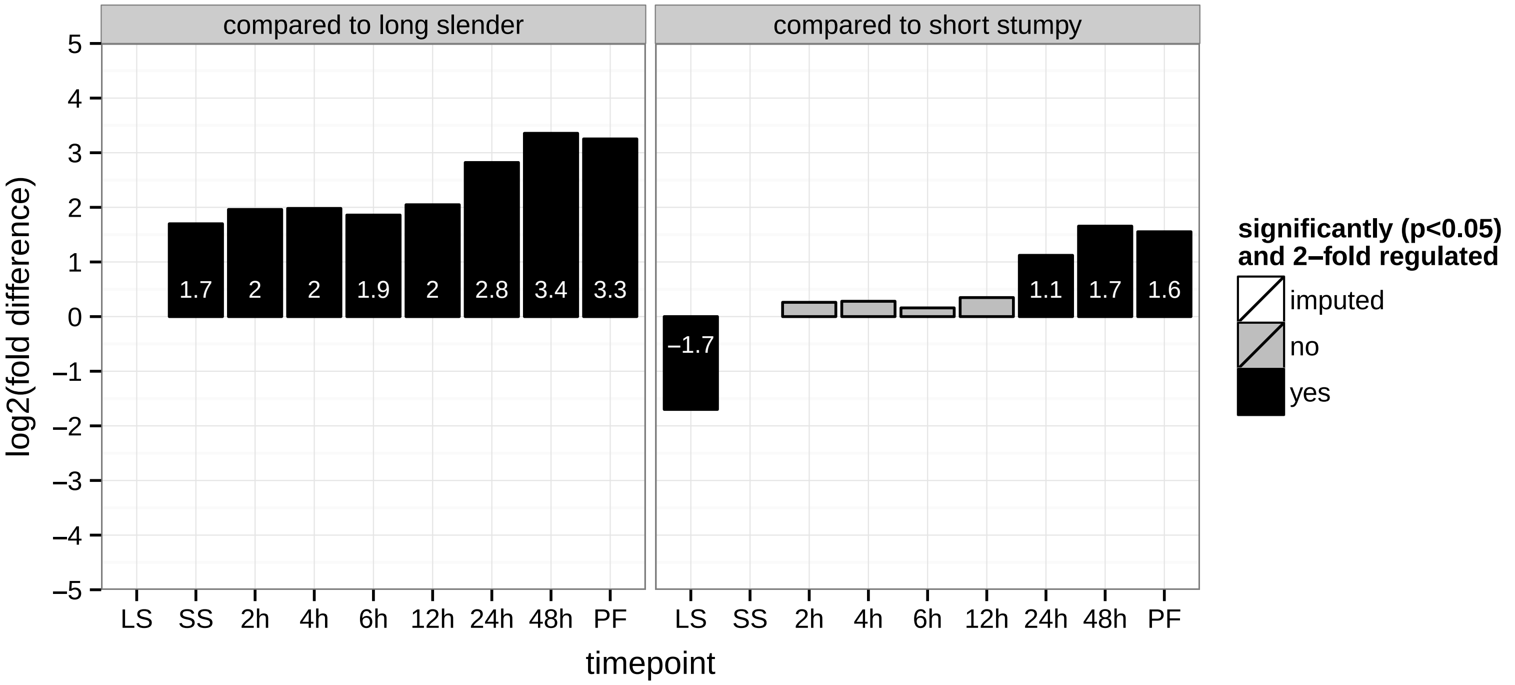

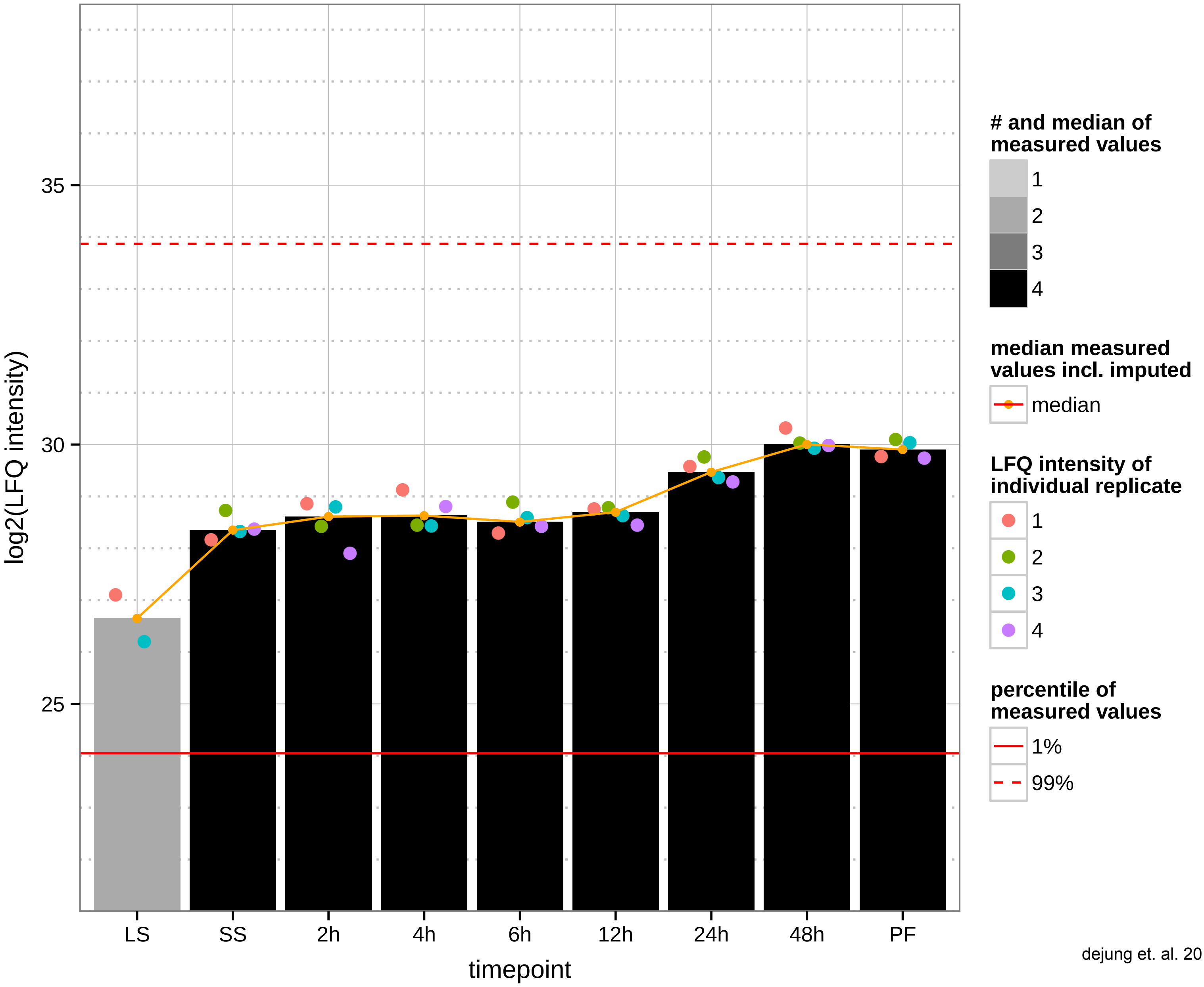

dejung et. al. 2015

peroxisomal targeting signal type 2 receptor, putative, peroxin 7, peroxin 7 (PEX7)
Tb927.3.3610
AGOF: peroxisome matrix targeting signal−2 binding
AGOC: cytoplasm, peroxisome
AGOP: null
PGOF: protein binding
PGOC: null
PGOP: null

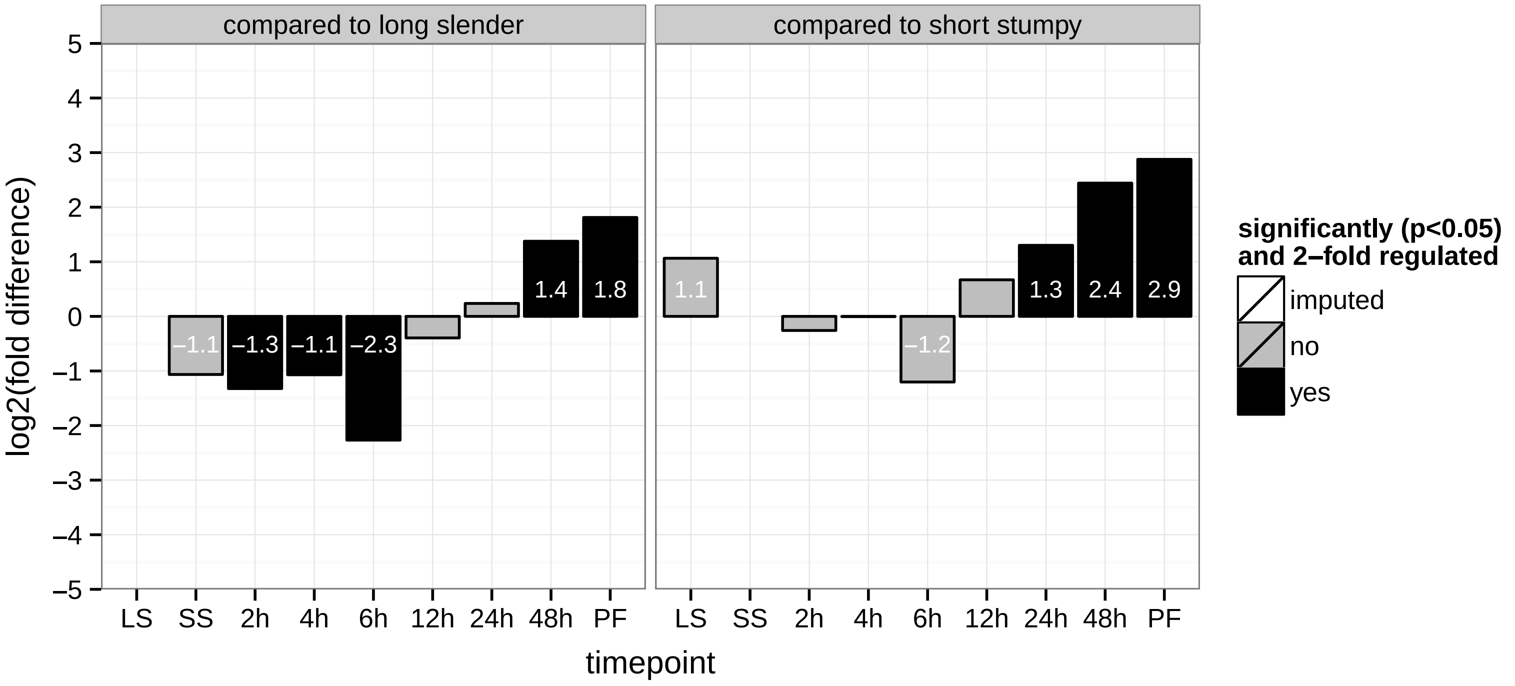

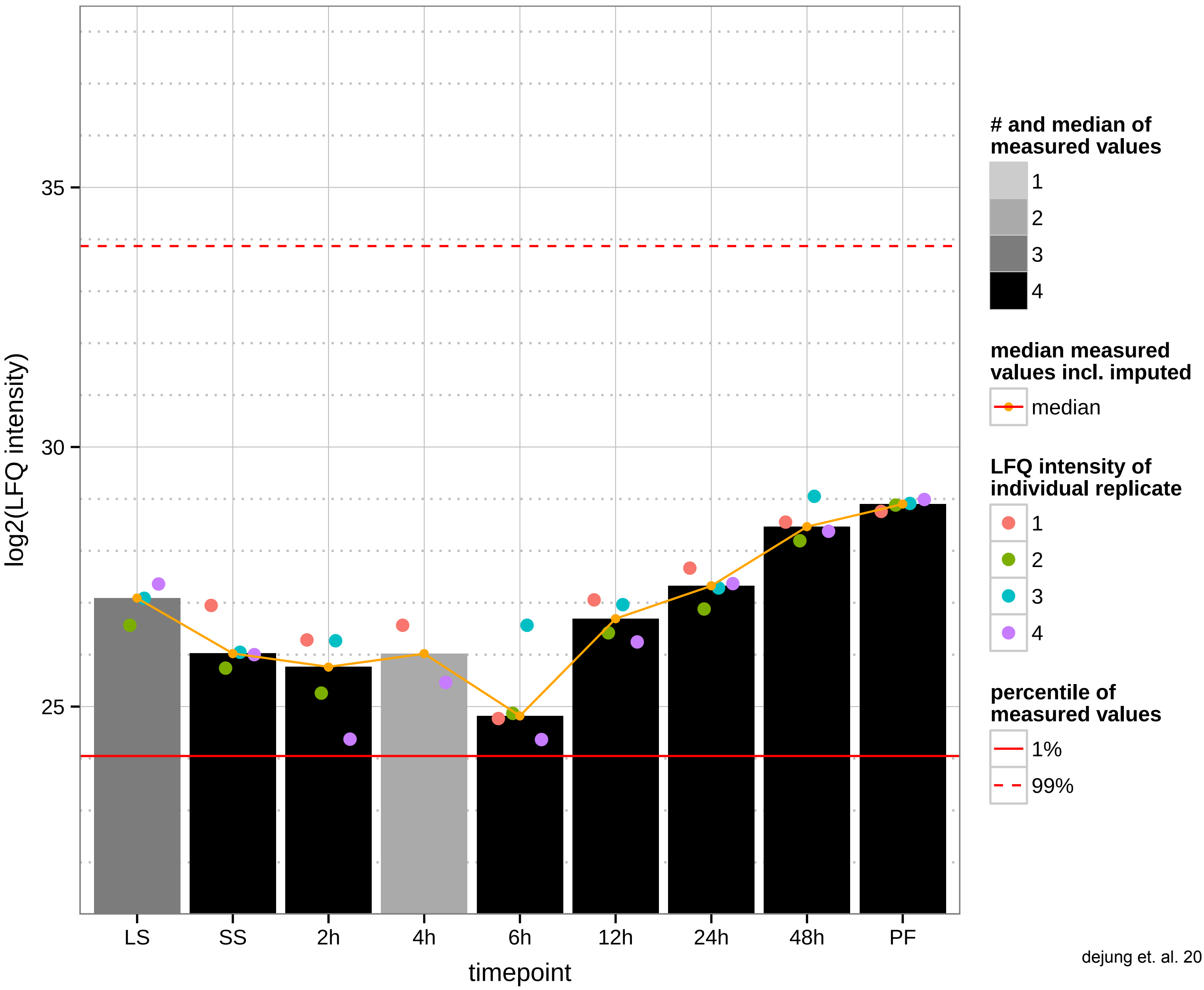

dejung et. al. 2015

SNF1/AMP–activated kinase catalytic subunit, putative
Tb927.3.4560
AGOF: ATP binding, protein serine/threonine kinase activity, protein tyrosine kinase activity
AGOC: null
AGOP: modulation of development of symbiont involved in interaction with host, protein phosphorylation, quorum sensing invo
PGOF: ATP binding, protein kinase activity, protein serine/threonine kinase activity, transferase activity, transferring phosphoru
PGOC: null
PGOP: protein phosphorylation

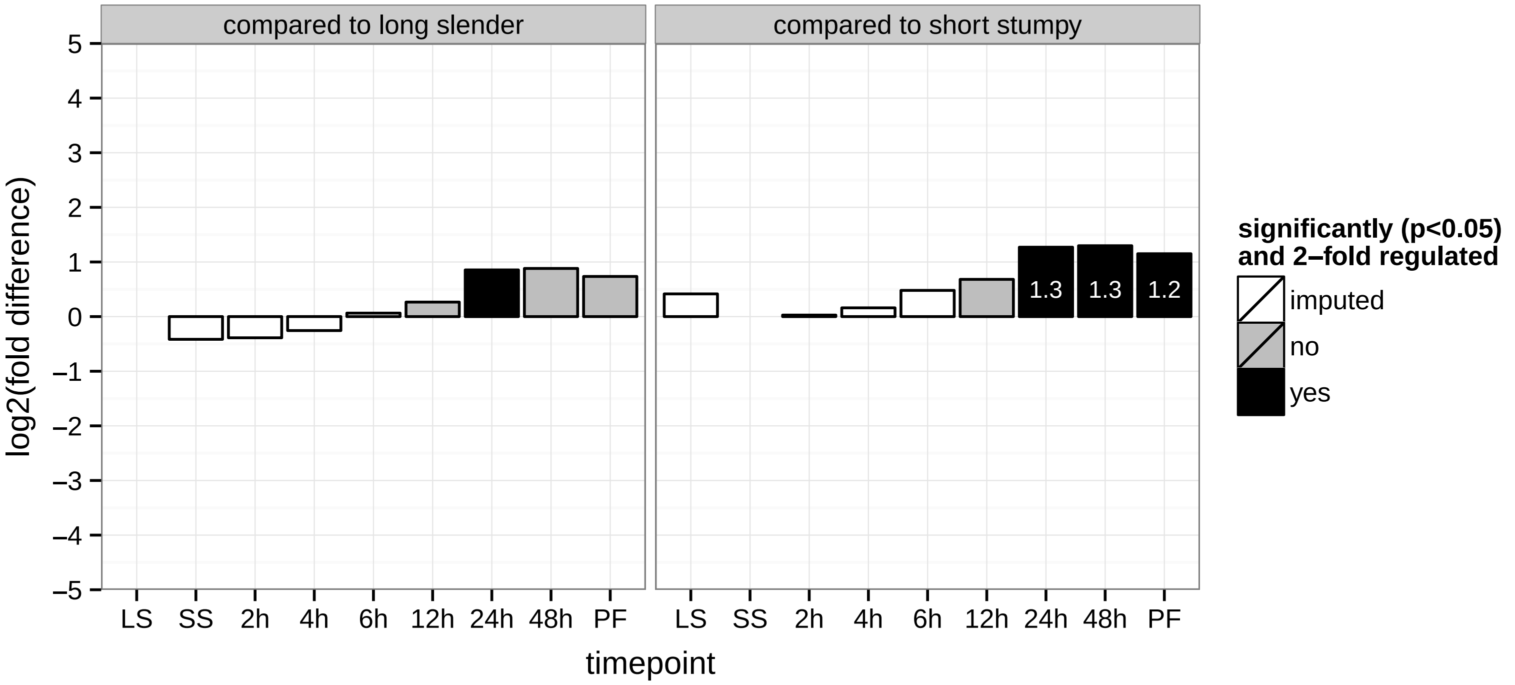

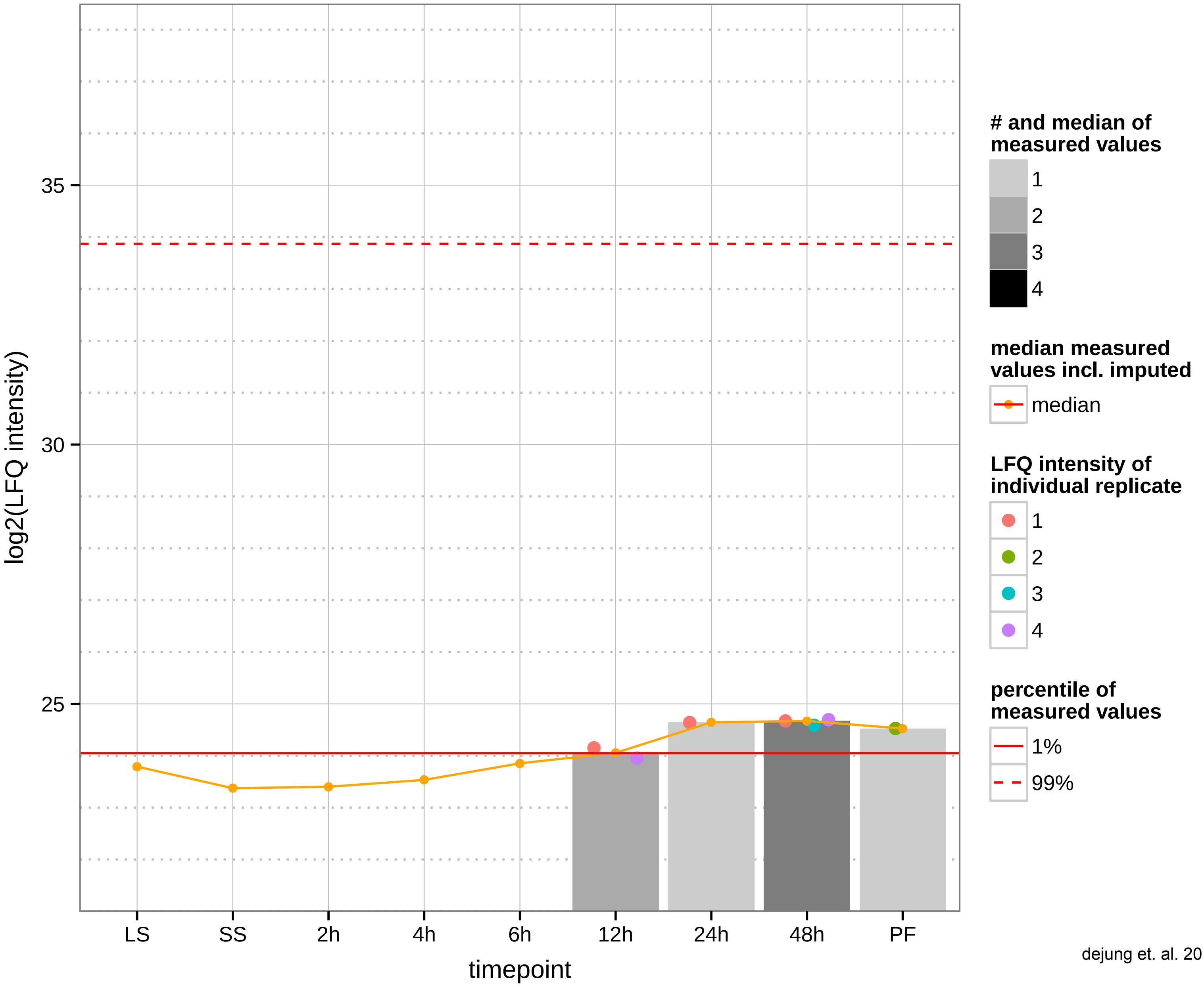

dejung et. al. 2015

hypothetical protein, conserved
Tb927.4.1280
AGOF: null
AGOC: null
AGOP: null
PGOF: protein binding
PGOC: null
PGOP: null

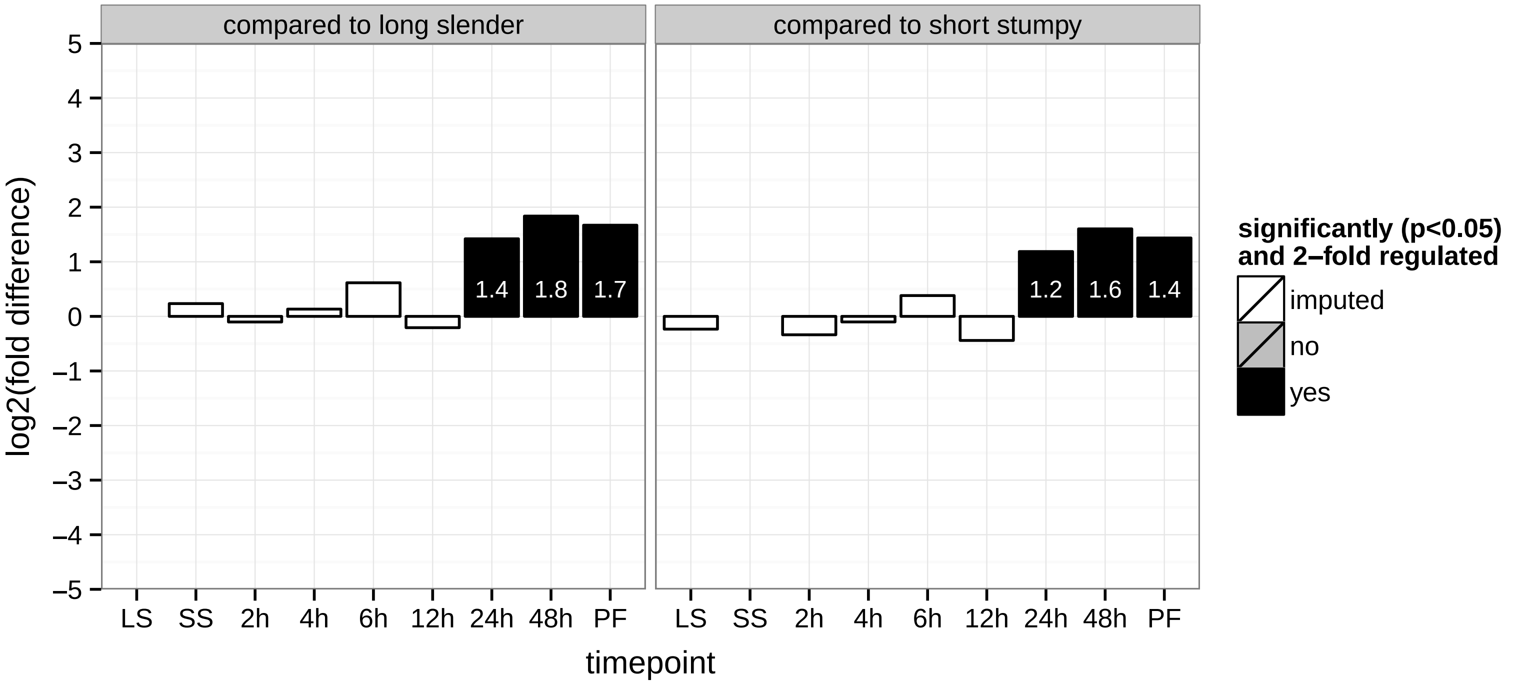

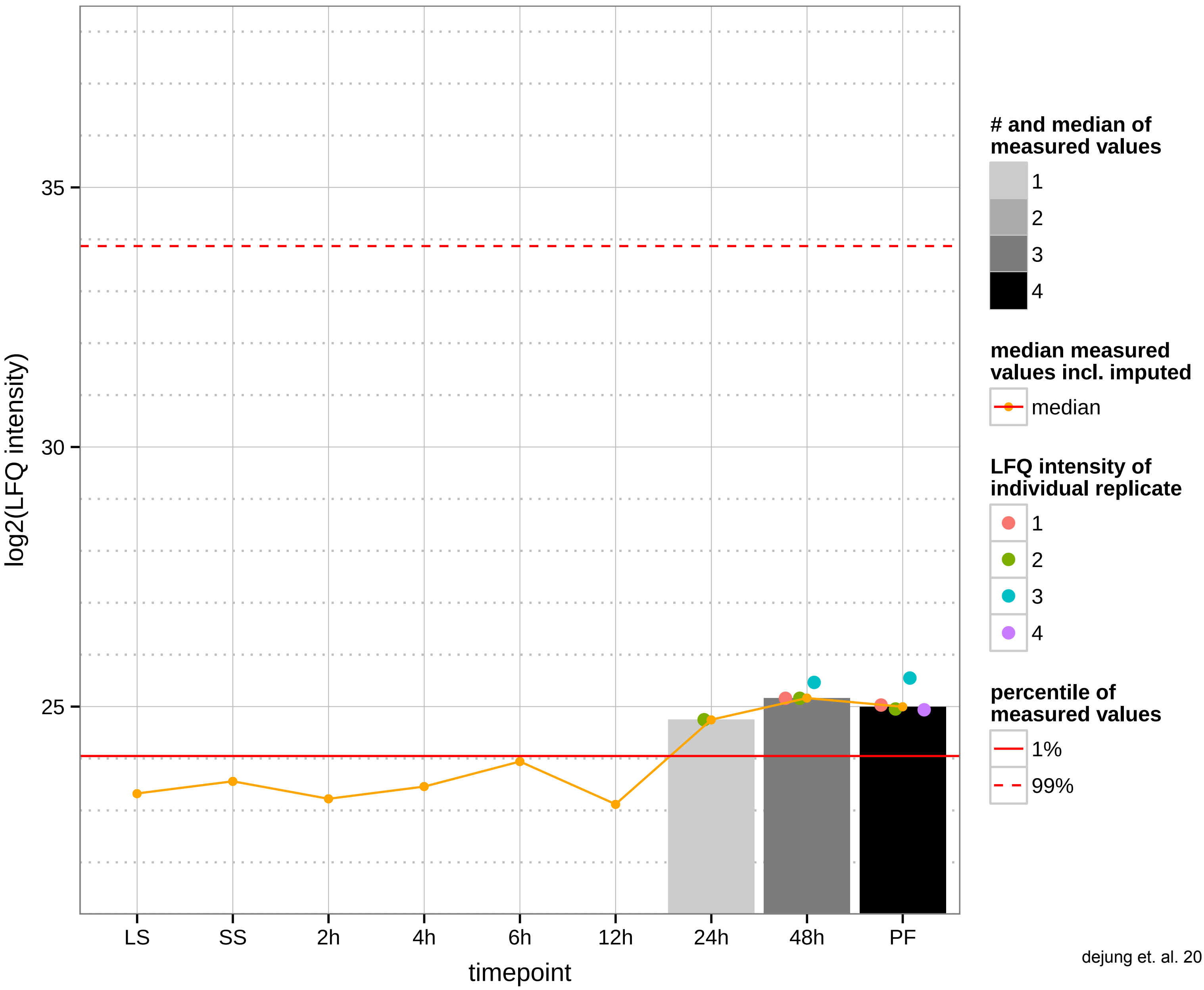

dejung et. al. 2015

RNA editing associated helicase 2, mitochondrial RNA binding complex 1 subunit (REH2)
Tb927.4.1500
AGOF: 3'−5' RNA helicase activity, ATP binding, RNA binding, double−stranded RNA binding, helicase activity, mRNA binding
AGOC: intracellular, mitochondrial mRNA editing complex, mitochondrion
AGOP: cytidine to uridine editing, mRNA modification, mRNA stabilization
PGOF: ATP binding, ATP−dependent helicase activity, double−stranded RNA binding, helicase activity, nucleic acid binding
PGOC: intracellular
PGOP: null

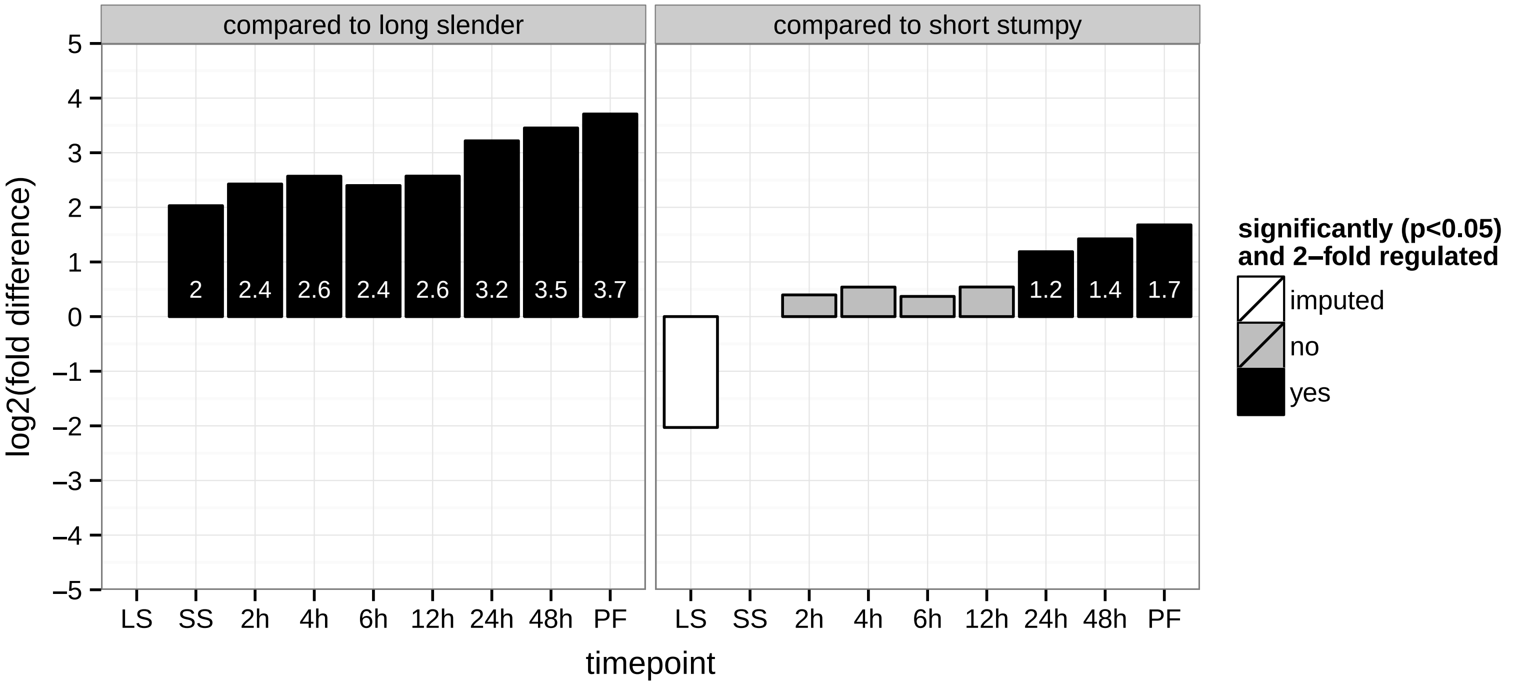

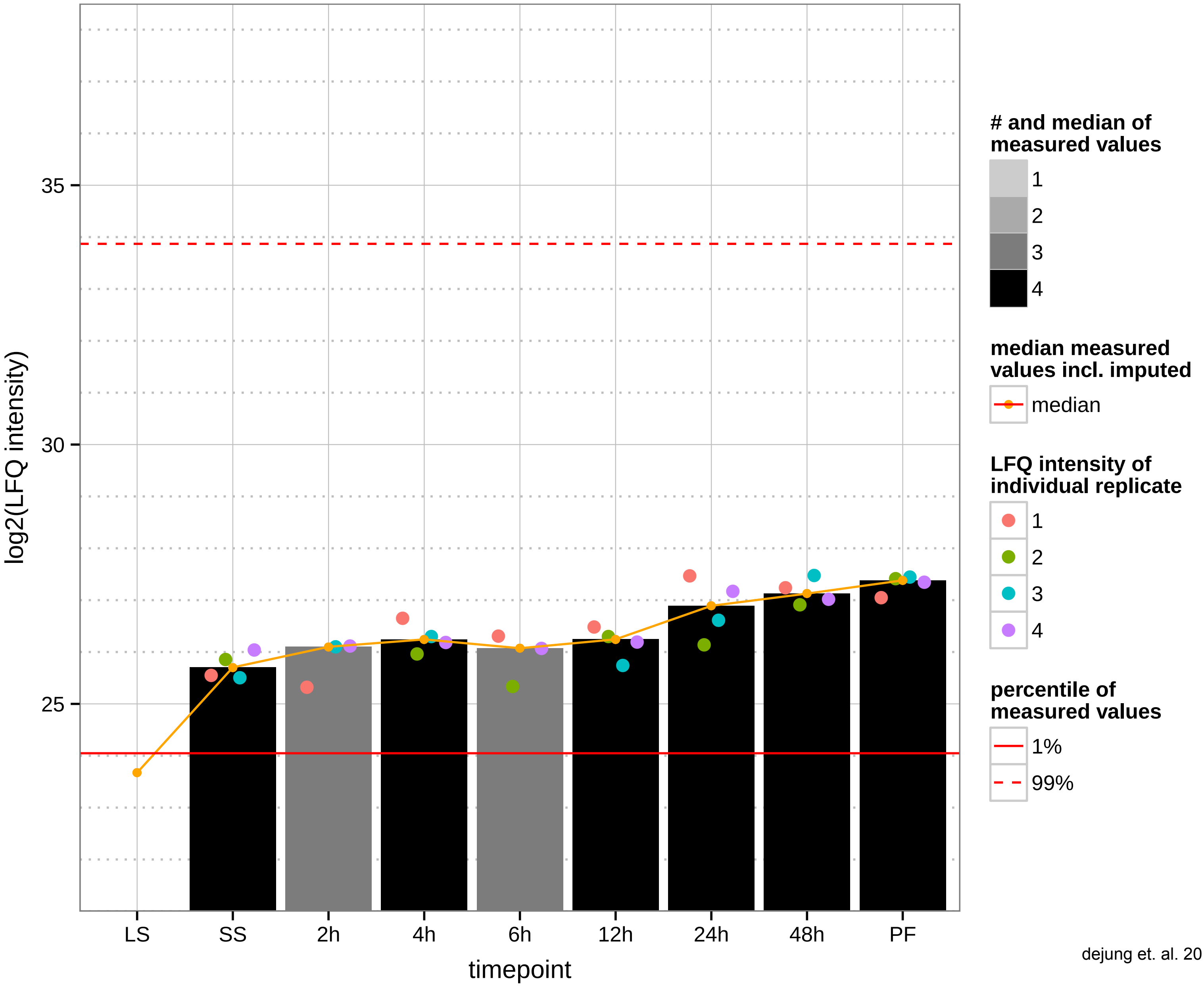

dejung et. al. 2015

hypothetical protein, conserved
Tb927.4.2680
AGOF: null
AGOC: null
AGOP: null
PGOF: ATP binding, kinase activity
PGOC: null
PGOP: null

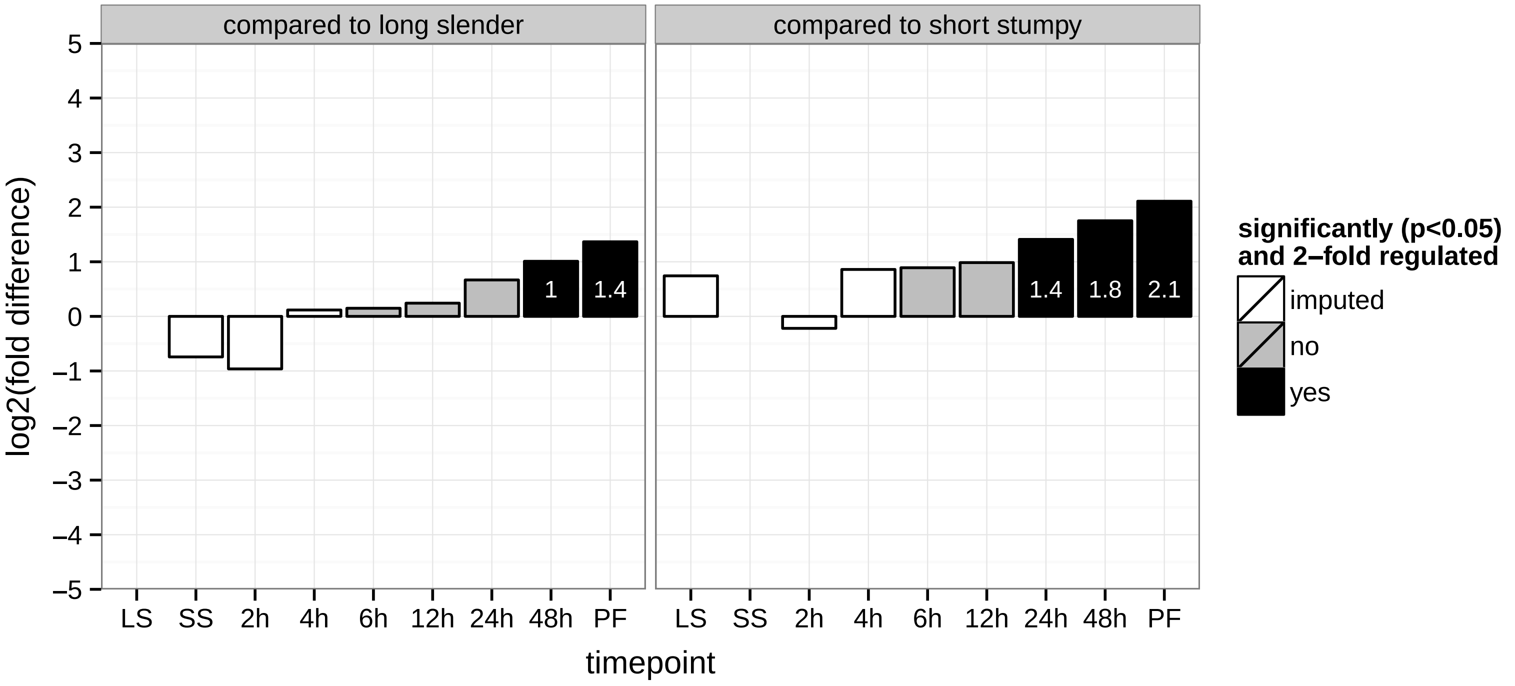

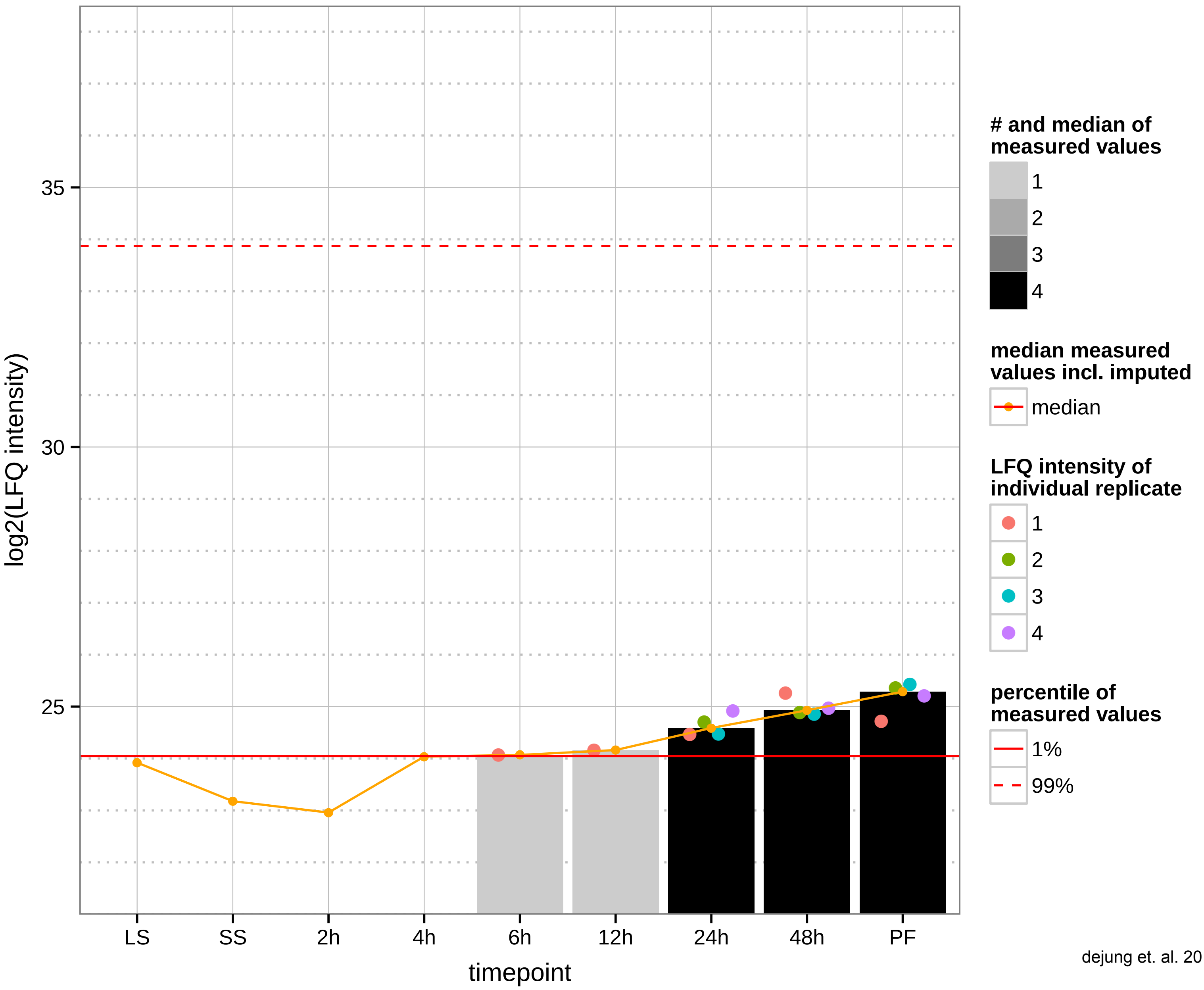

dejung et. al. 2015

hypothetical protein, conserved
Tb927.4.3450
AGOF: null
AGOC: mitochondrion
AGOP: null
PGOF: null
PGOC: null
PGOP: null

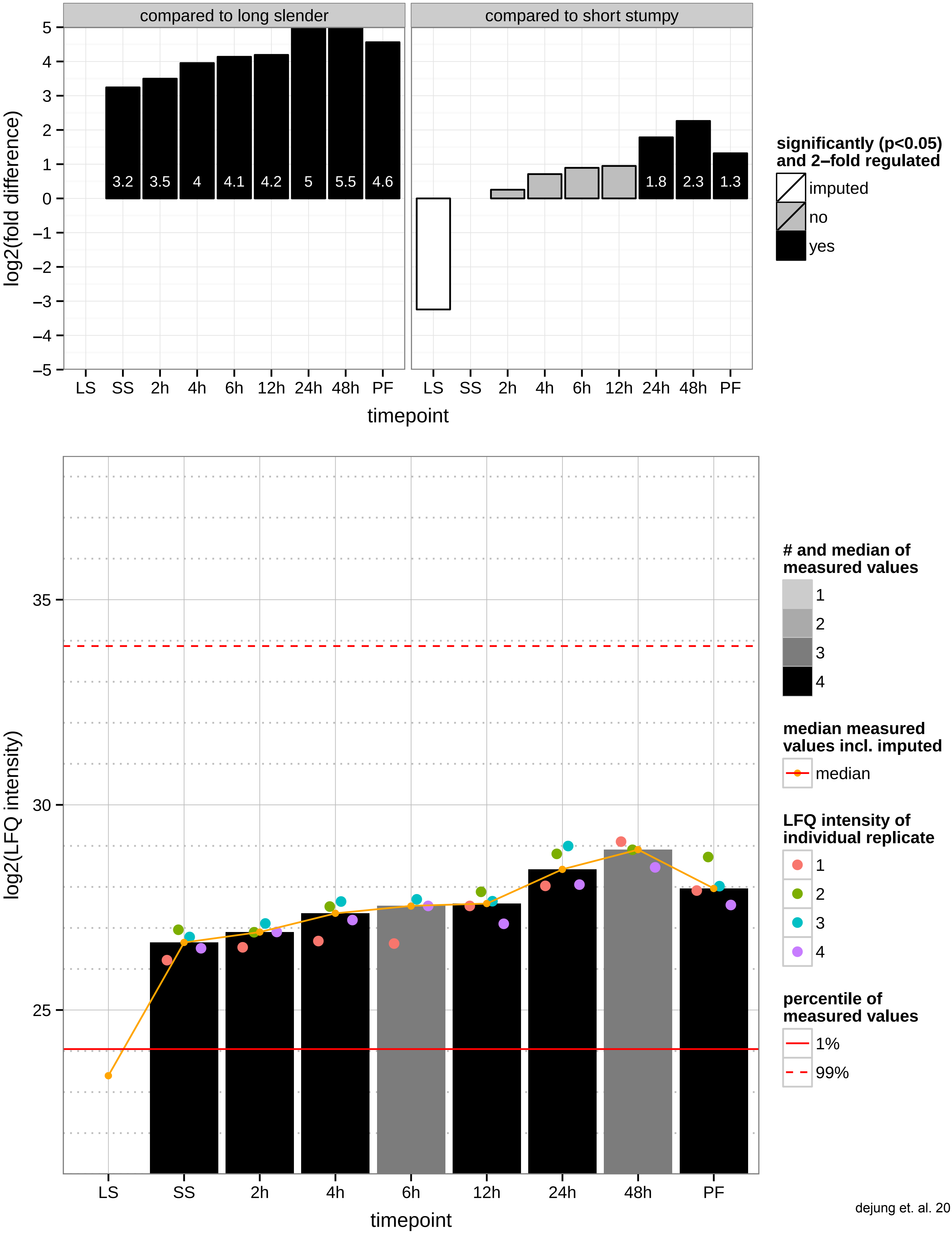

dejung et. al. 2015

Double RNA binding domain protein 7 (DRBD7)
Tb927.4.400
AGOF: RNA binding
AGOC: cytoplasm
AGOP: RNA processing
PGOF: nucleic acid binding
PGOC: null
PGOP: null

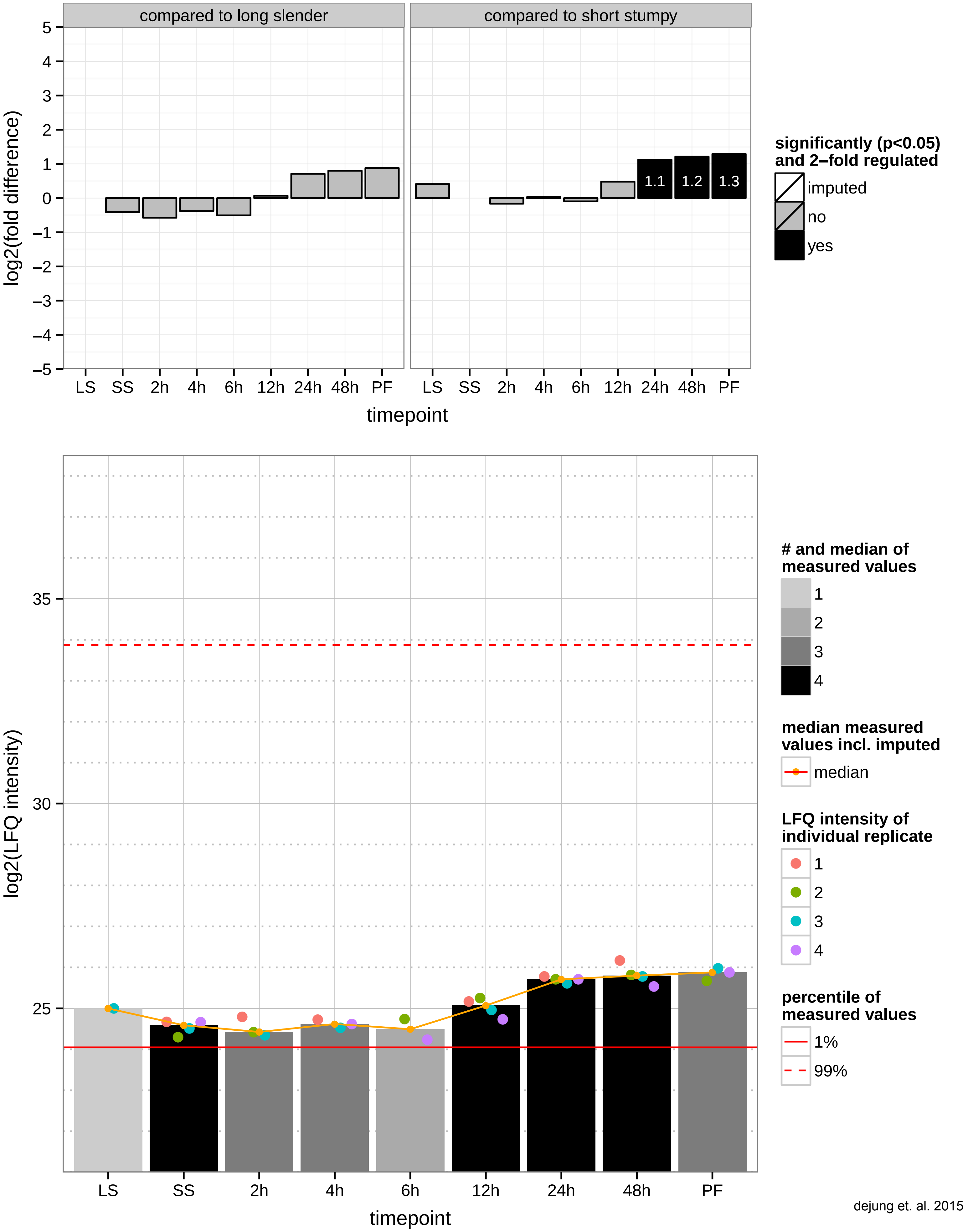

dejung et. al. 2015

hypothetical protein, conserved
Tb927.4.4570
AGOF: sequence−specific DNA binding transcription factor activity
AGOC: null
AGOP: regulation of transcription, DNA−dependent
PGOF: null
PGOC: null
PGOP: null

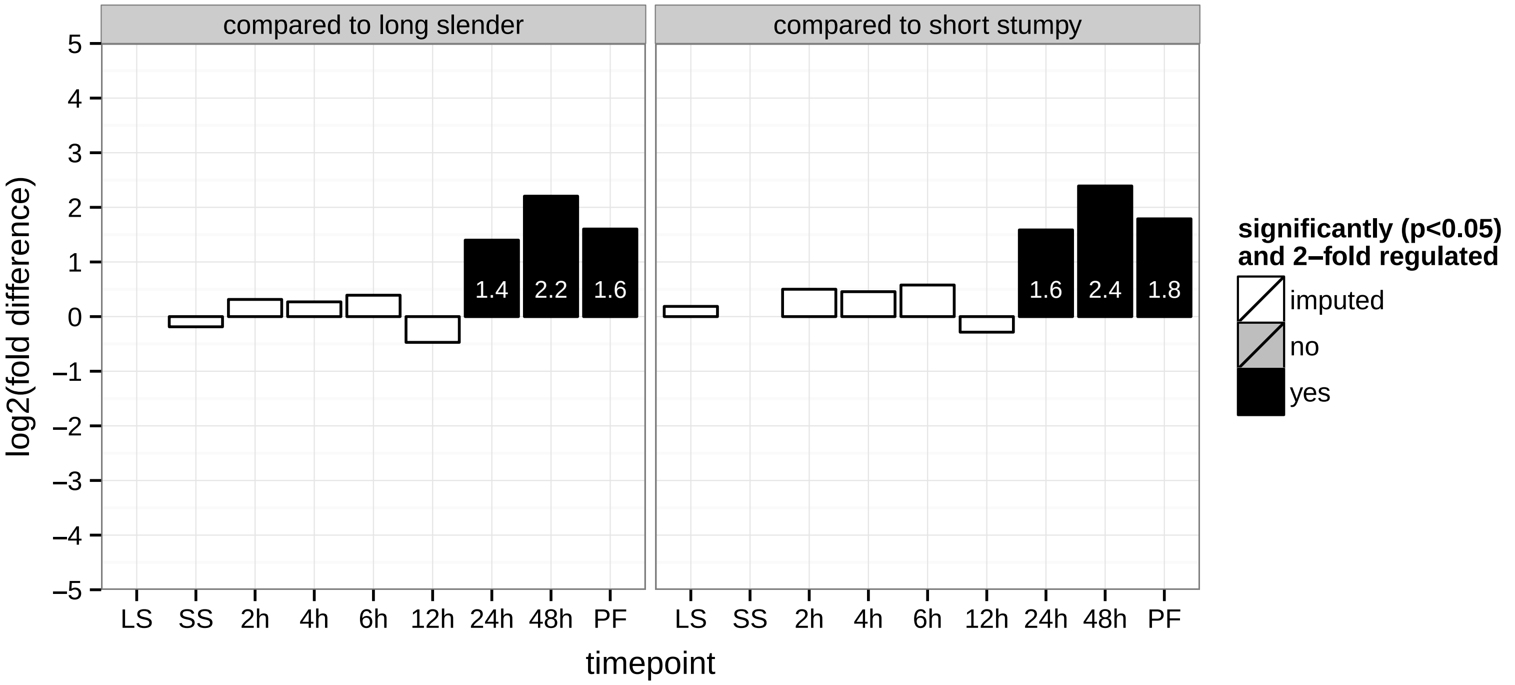

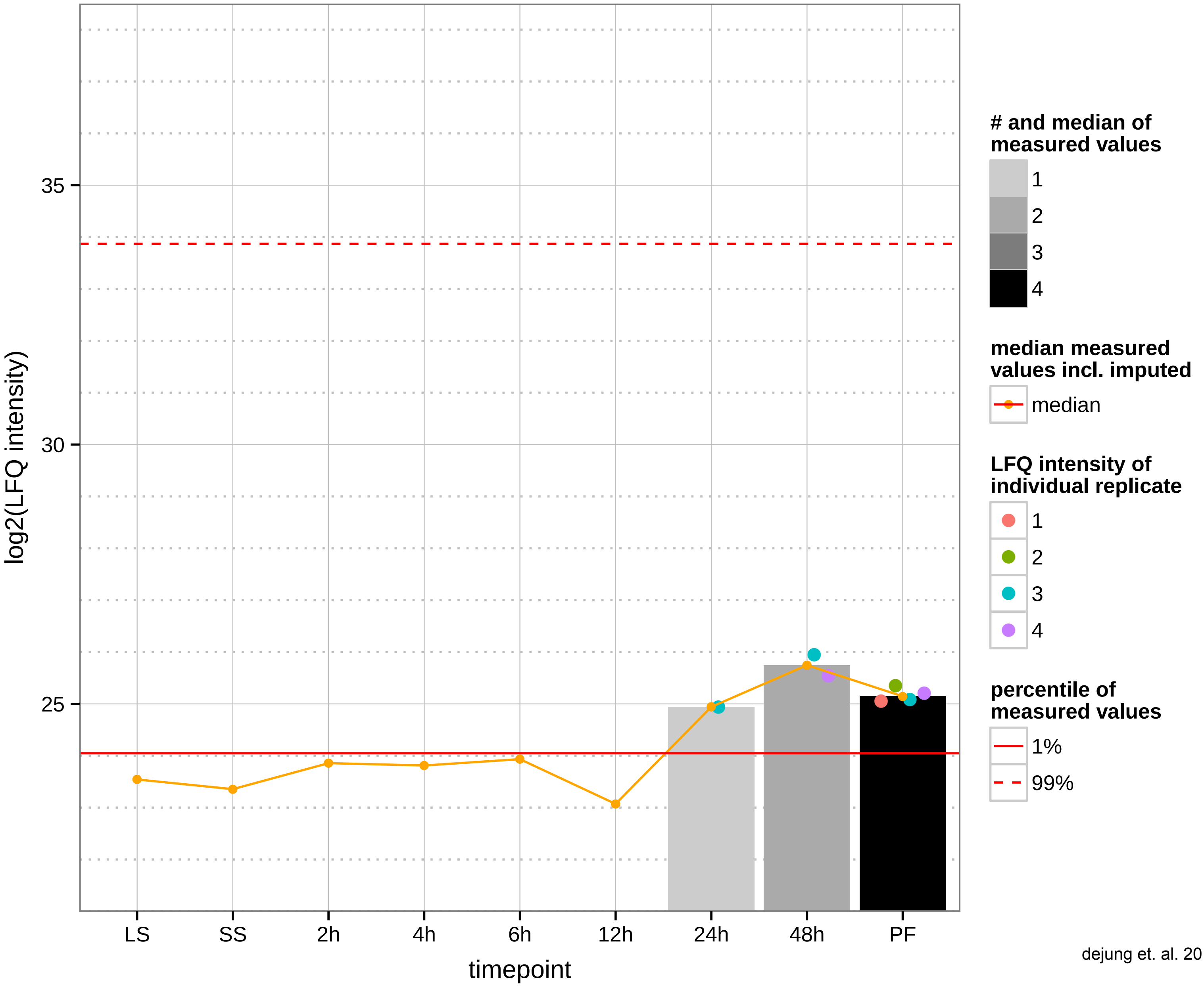

dejung et. al. 2015

cytochrome oxidase subunit VIII (COXVIII)
Tb927.4.4620
AGOF: null
AGOC: mitochondrion
AGOP: null
PGOF: null
PGOC: null
PGOP: null

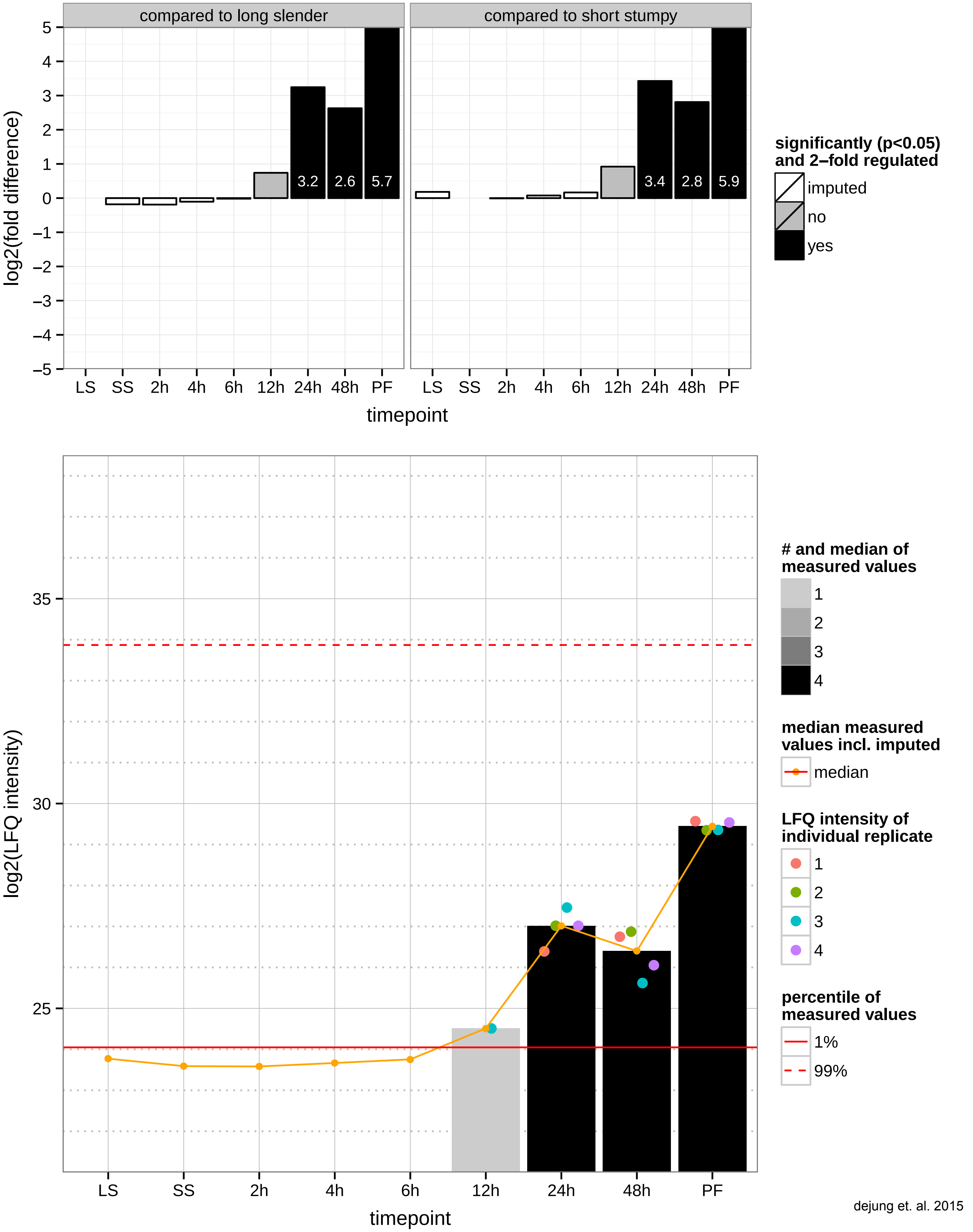

dejung et. al. 2015

hypothetical protein, conserved
Tb927.4.4940
AGOF: null
AGOC: null
AGOP: null
PGOF: null
PGOC: null
PGOP: null

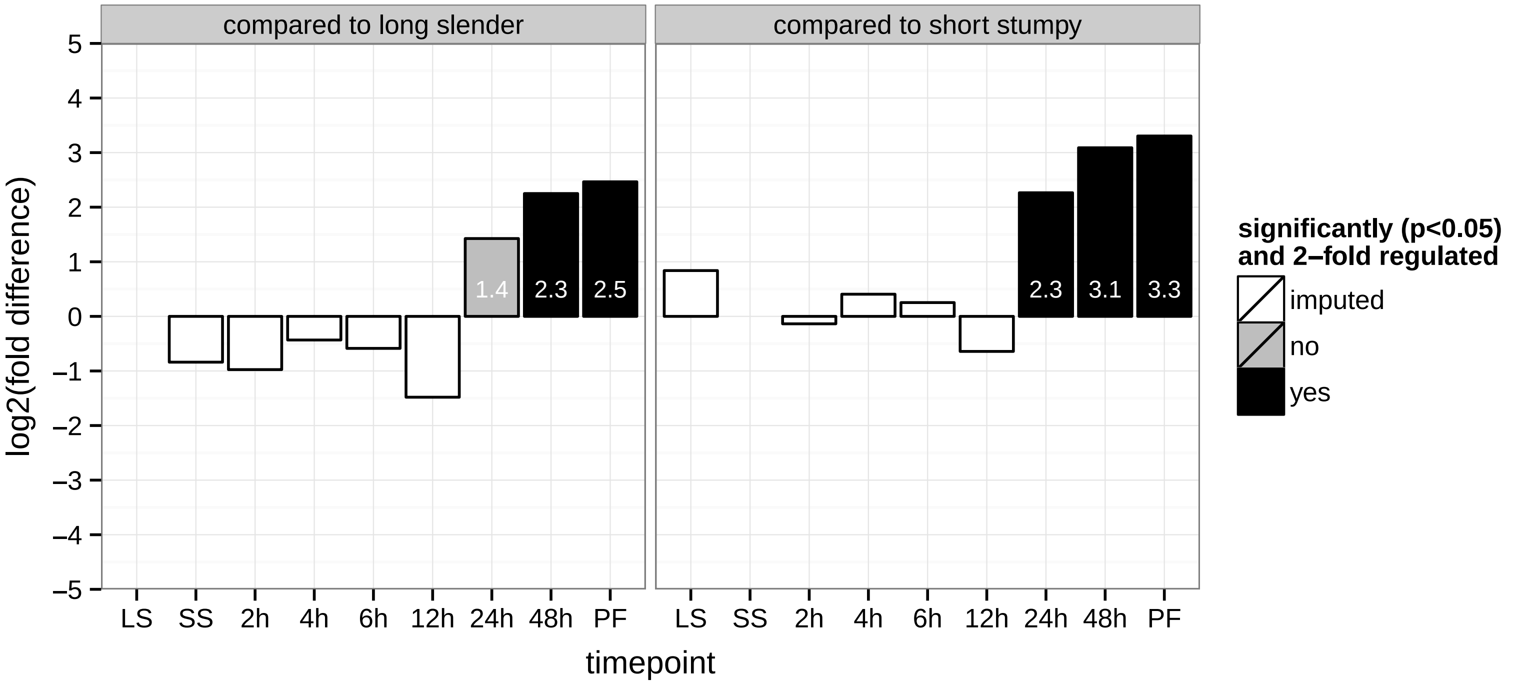

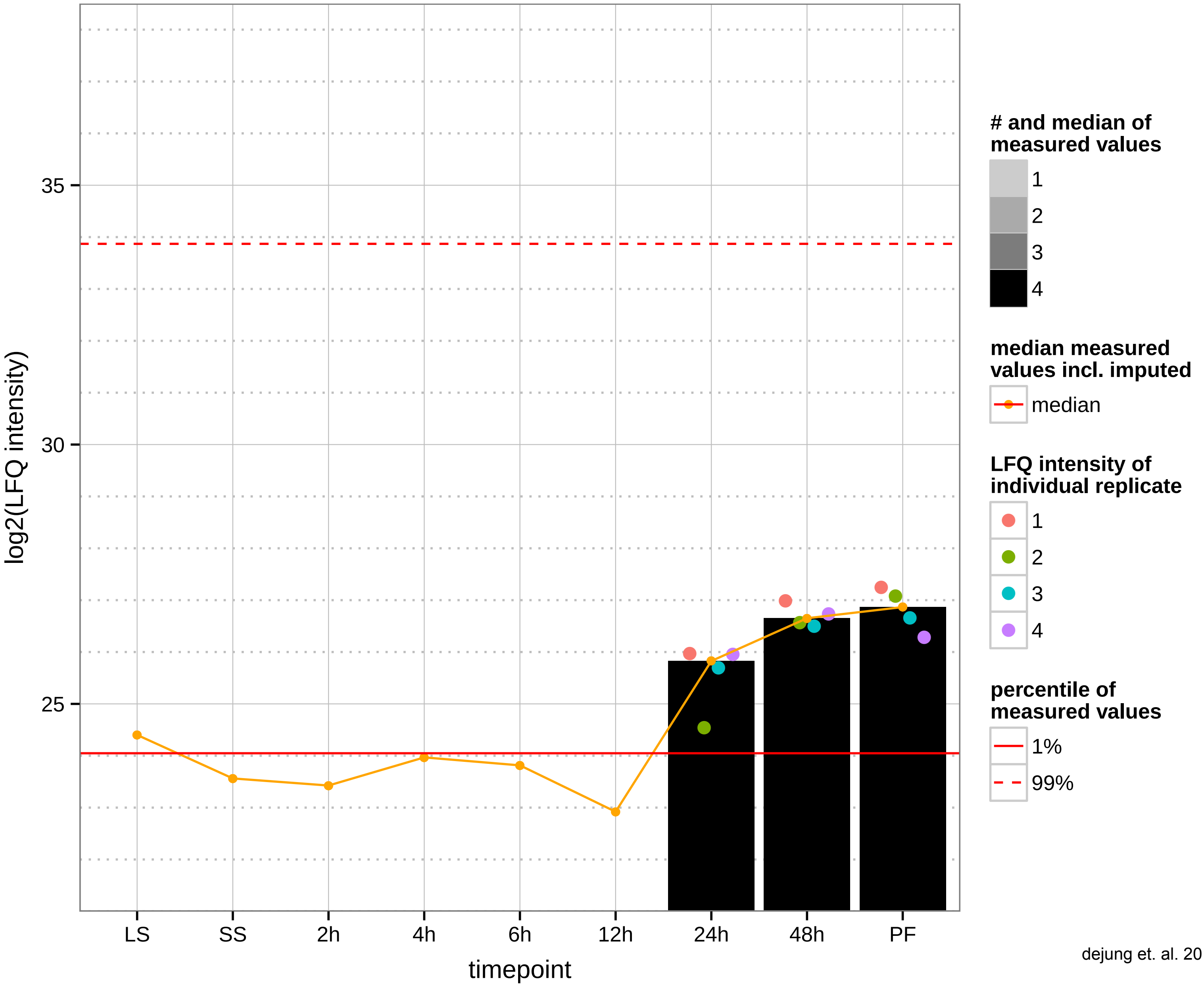

dejung et. al. 2015

50S ribosomal protein L14, putative
Tb927.4.930
AGOF: structural constituent of ribosome
AGOC: intracellular, large ribosomal subunit, ribosome
AGOP: translation
PGOF: structural constituent of ribosome
PGOC: ribosome
PGOP: translation

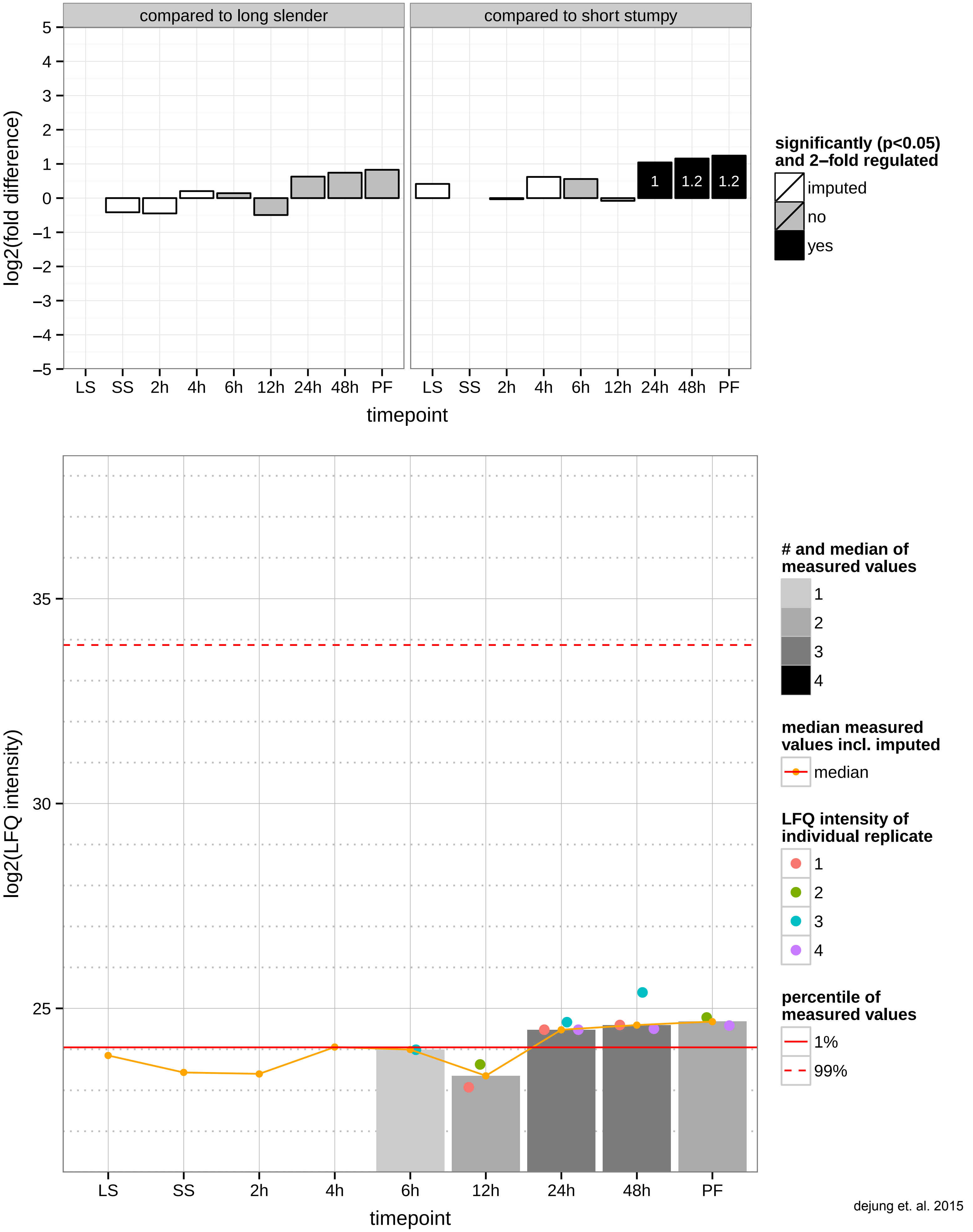

dejung et. al. 2015

peroxisome targeting signal 1 receptor (TbPEX5)
Tb927.5.1100
AGOF: null
AGOC: null
AGOP: null
PGOF: protein binding
PGOC: null
PGOP: null

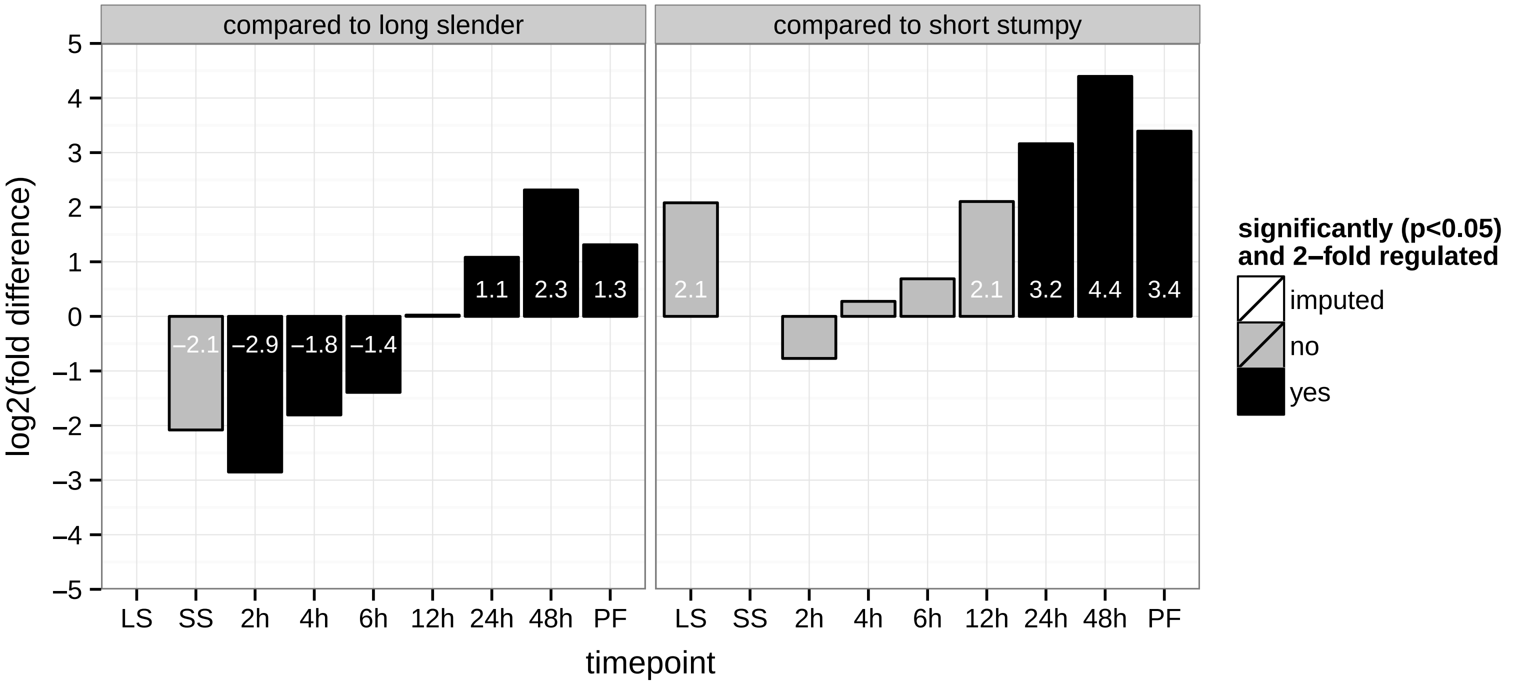

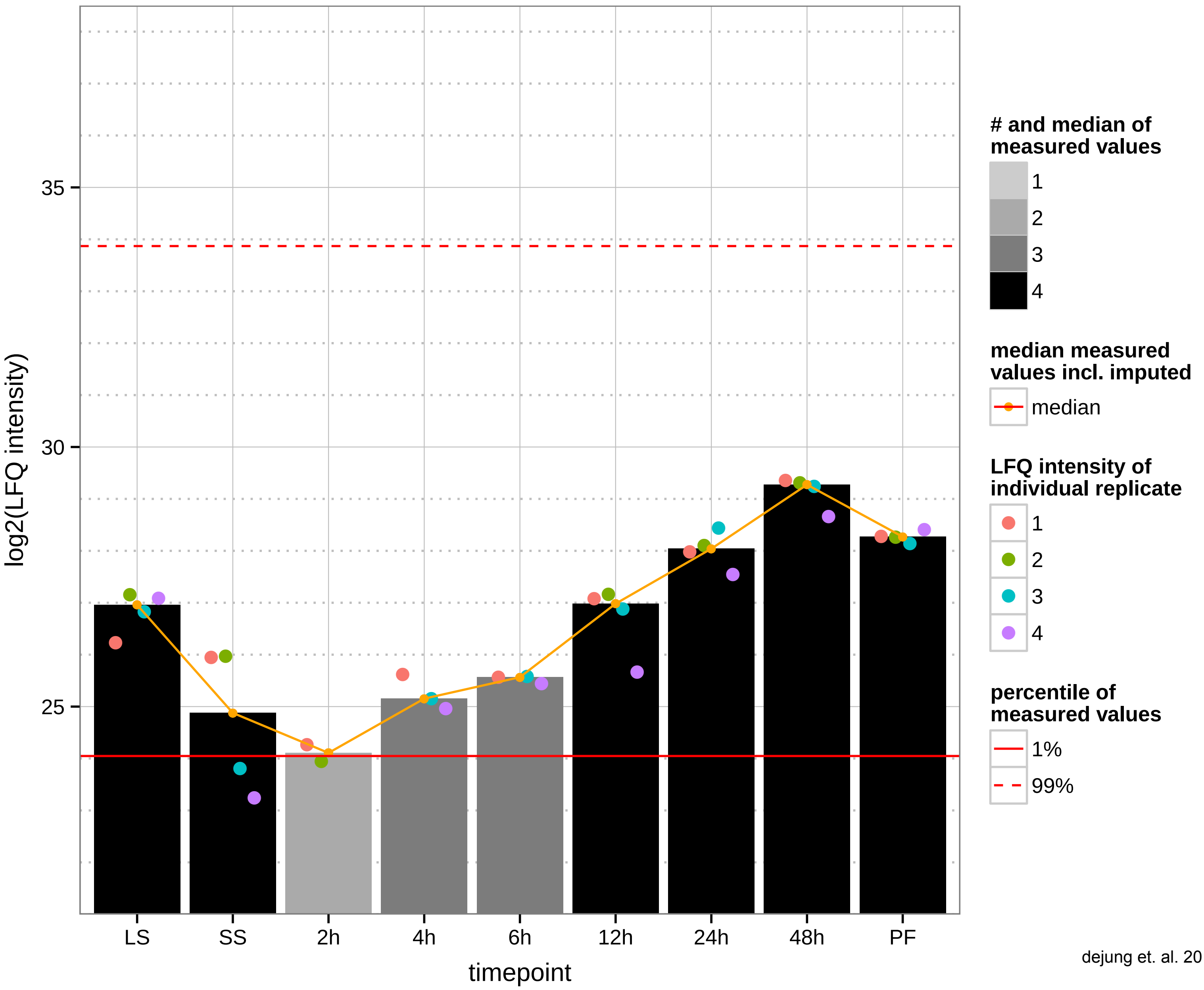

dejung et. al. 2015

RNA helicase Prp43
Tb927.5.1150
AGOF: ATP binding, helicase activity, nucleic acid binding, nucleoside−triphosphatase activity
AGOC: null
AGOP: RNA splicing
PGOF: ATP binding, ATP−dependent helicase activity, helicase activity, nucleic acid binding, nucleoside−triphosphatase activi
PGOC: null
PGOP: null

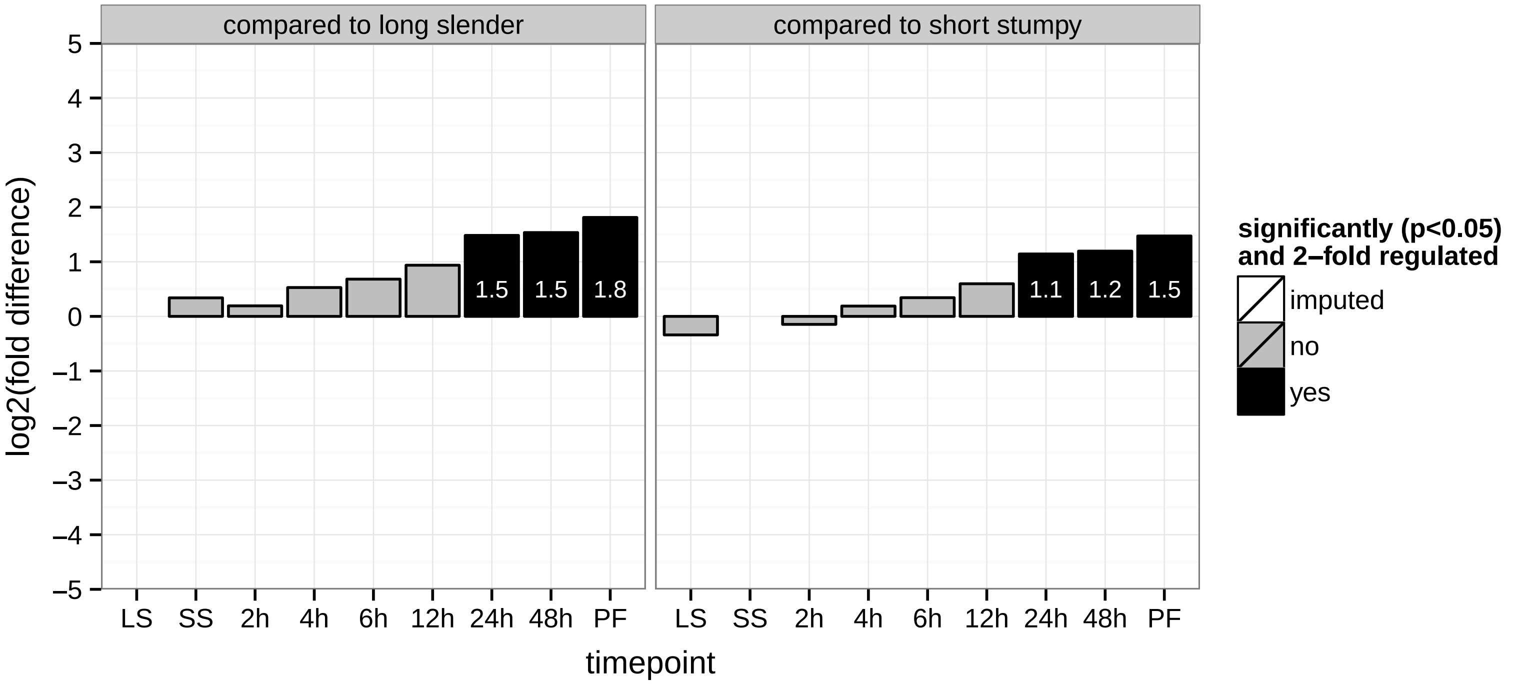

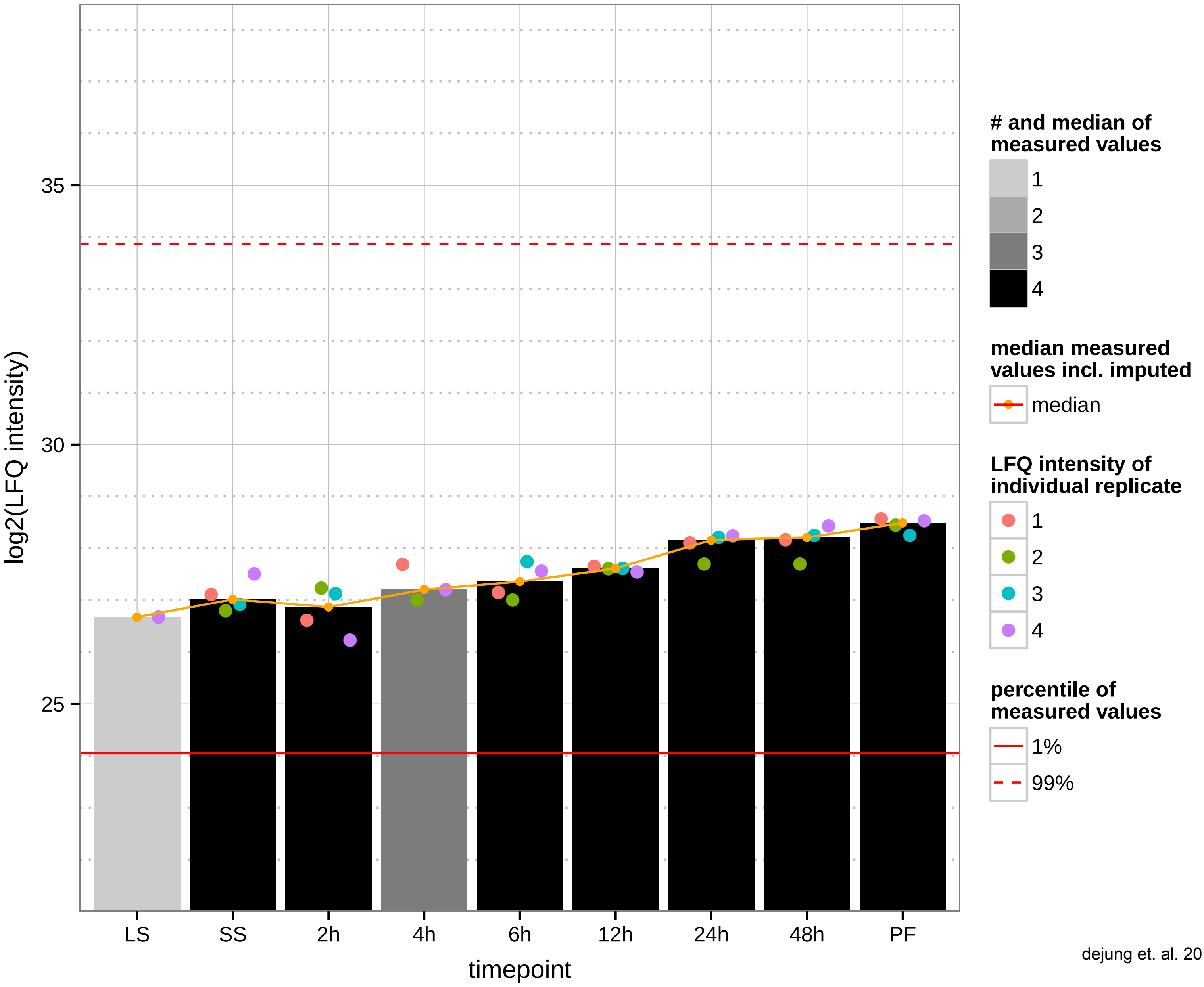

dejung et. al. 2015

hypothetical protein, conserved
Tb927.5.2800
AGOF: null
AGOC: null
AGOP: null
PGOF: null
PGOC: null
PGOP: null

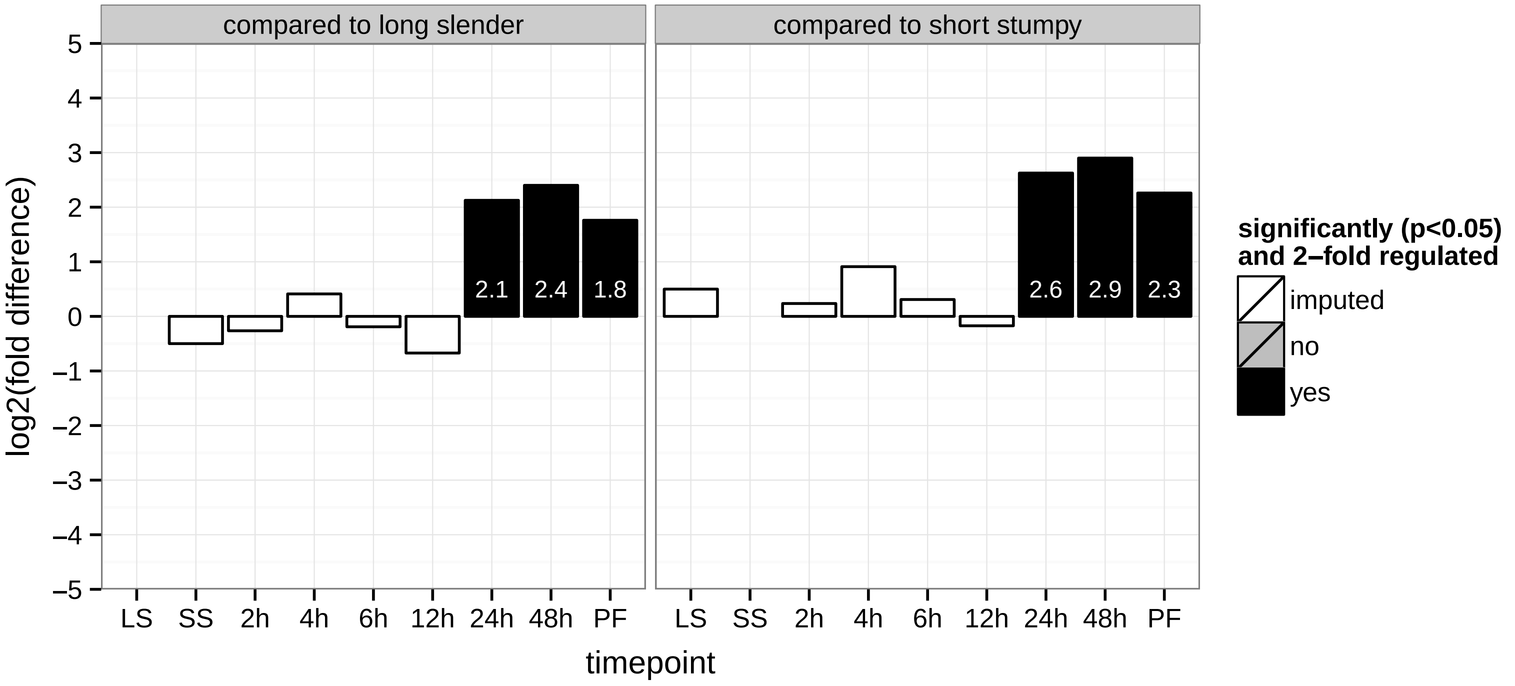

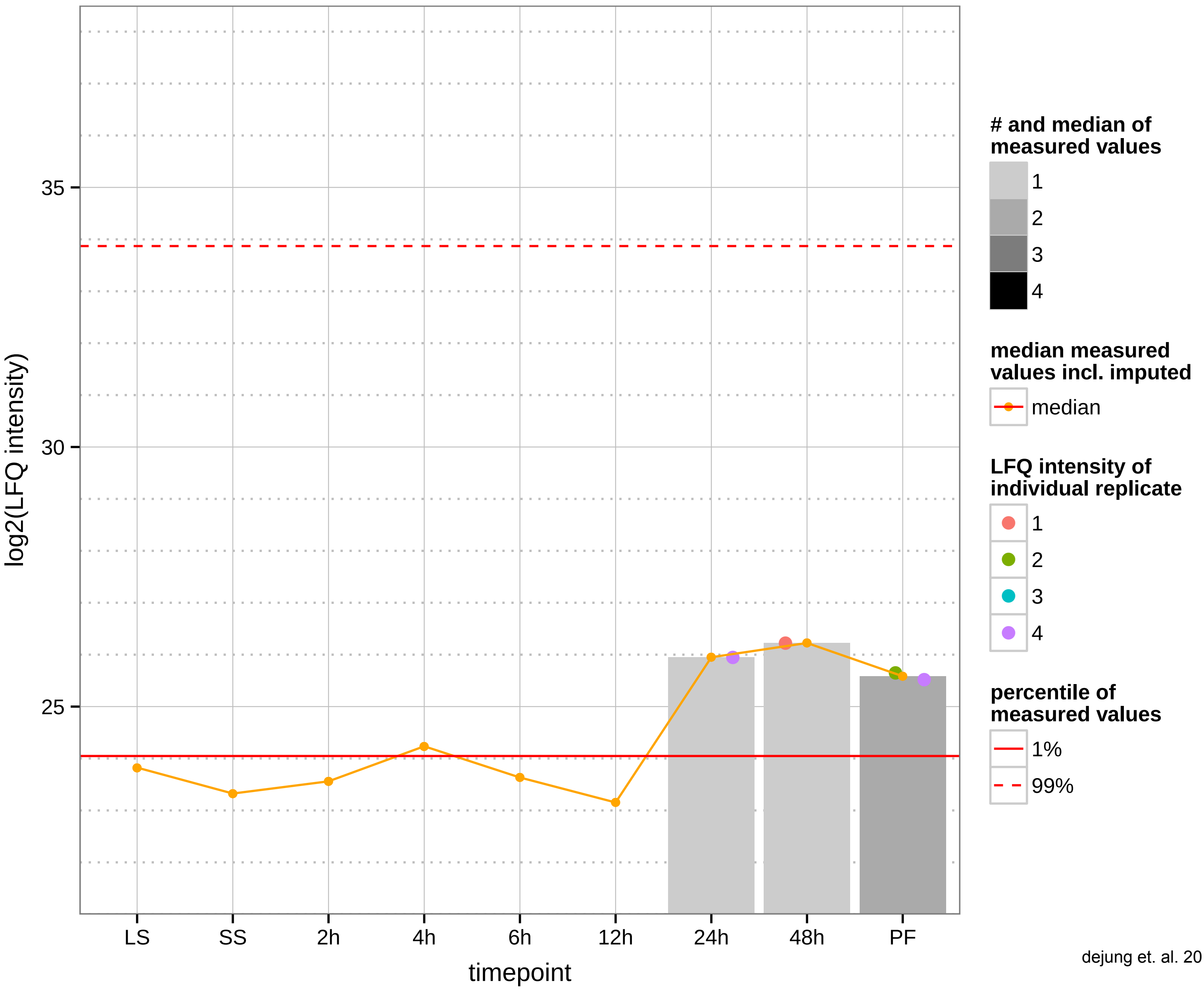

dejung et. al. 2015

hypothetical protein, conserved
Tb927.5.2930
AGOF: null
AGOC: mitochondrion
AGOP: null
PGOF: null
PGOC: null
PGOP: null

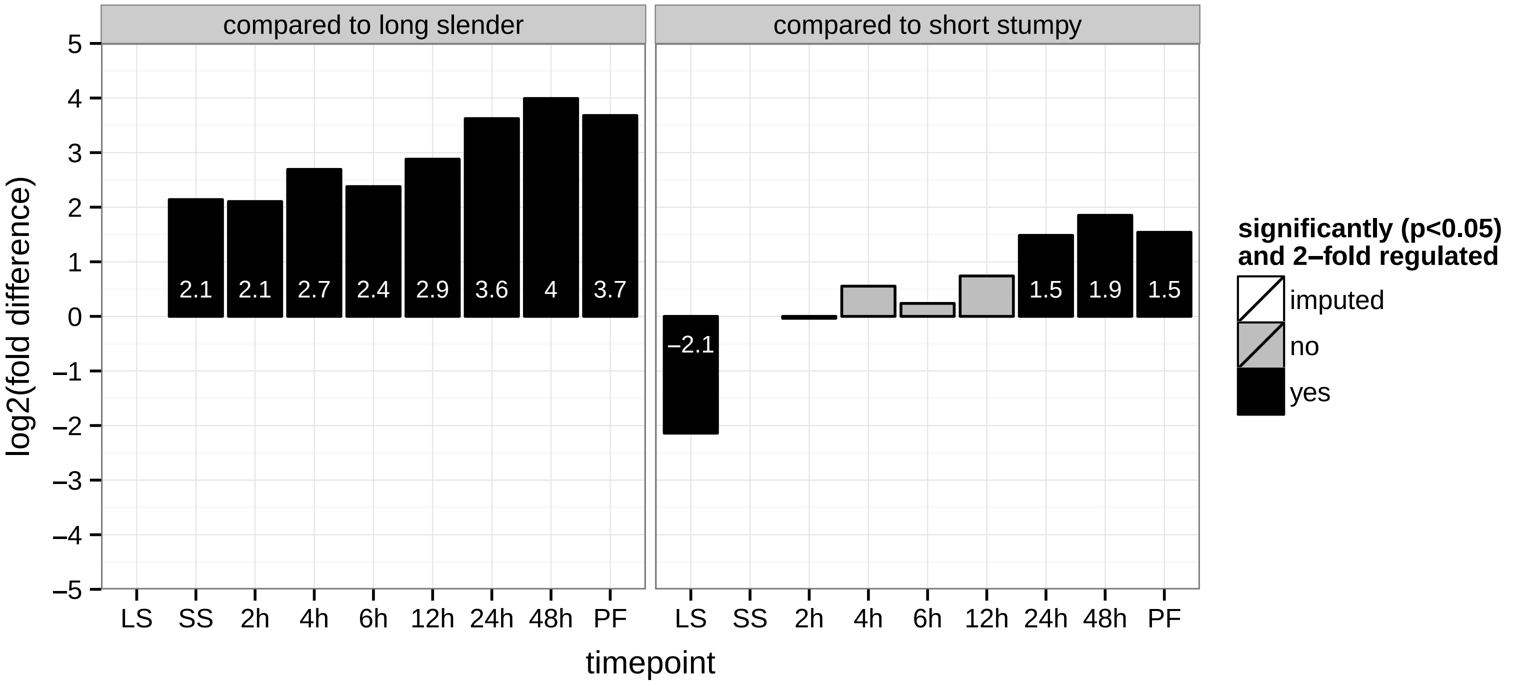

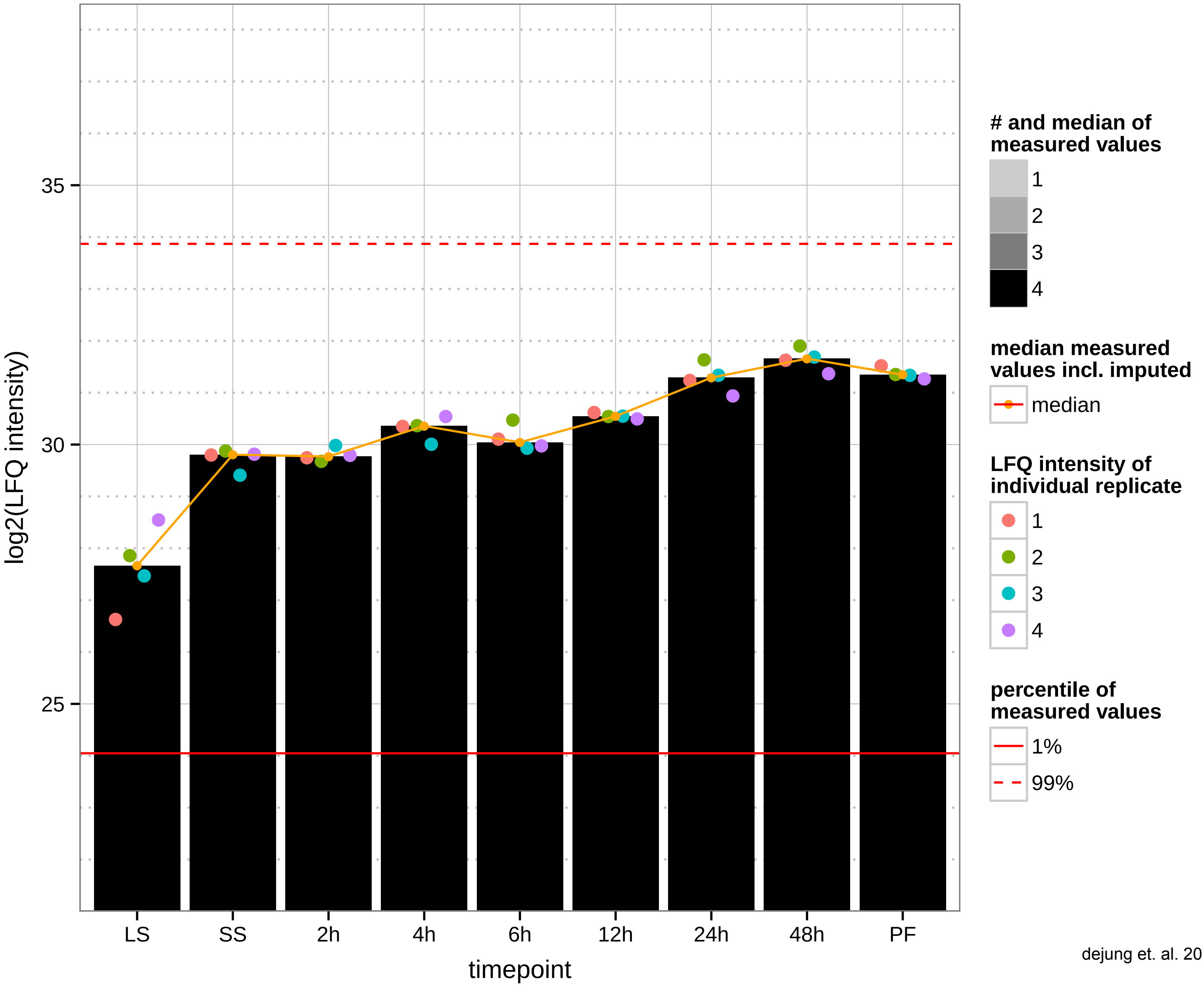

dejung et. al. 2015

poly(ADP−ribose) polymerase, putative
Tb927.5.3050
AGOF: NAD+ ADP−ribosyltransferase activity
AGOC: nucleus
AGOP: protein ADP−ribosylation
PGOF: NAD+ ADP−ribosyltransferase activity
PGOC: null
PGOP: protein ADP−ribosylation

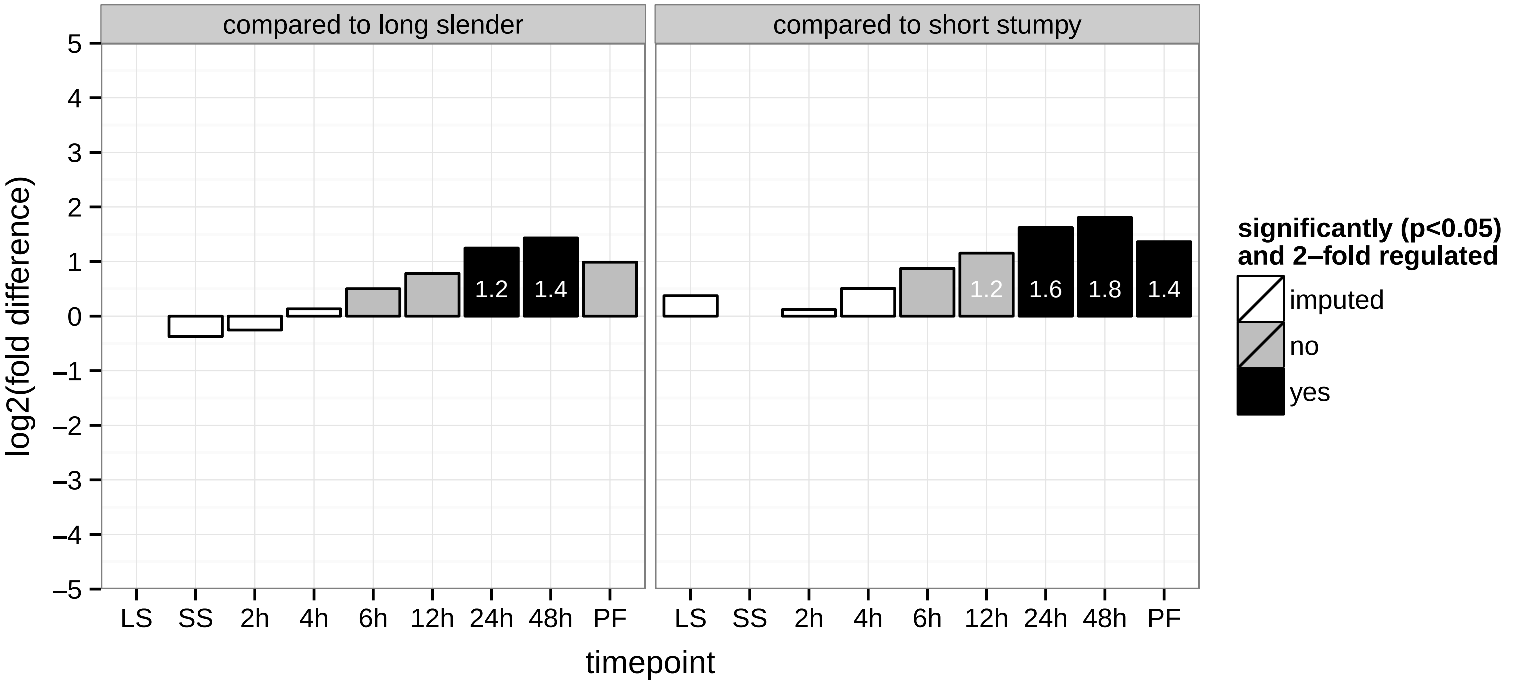

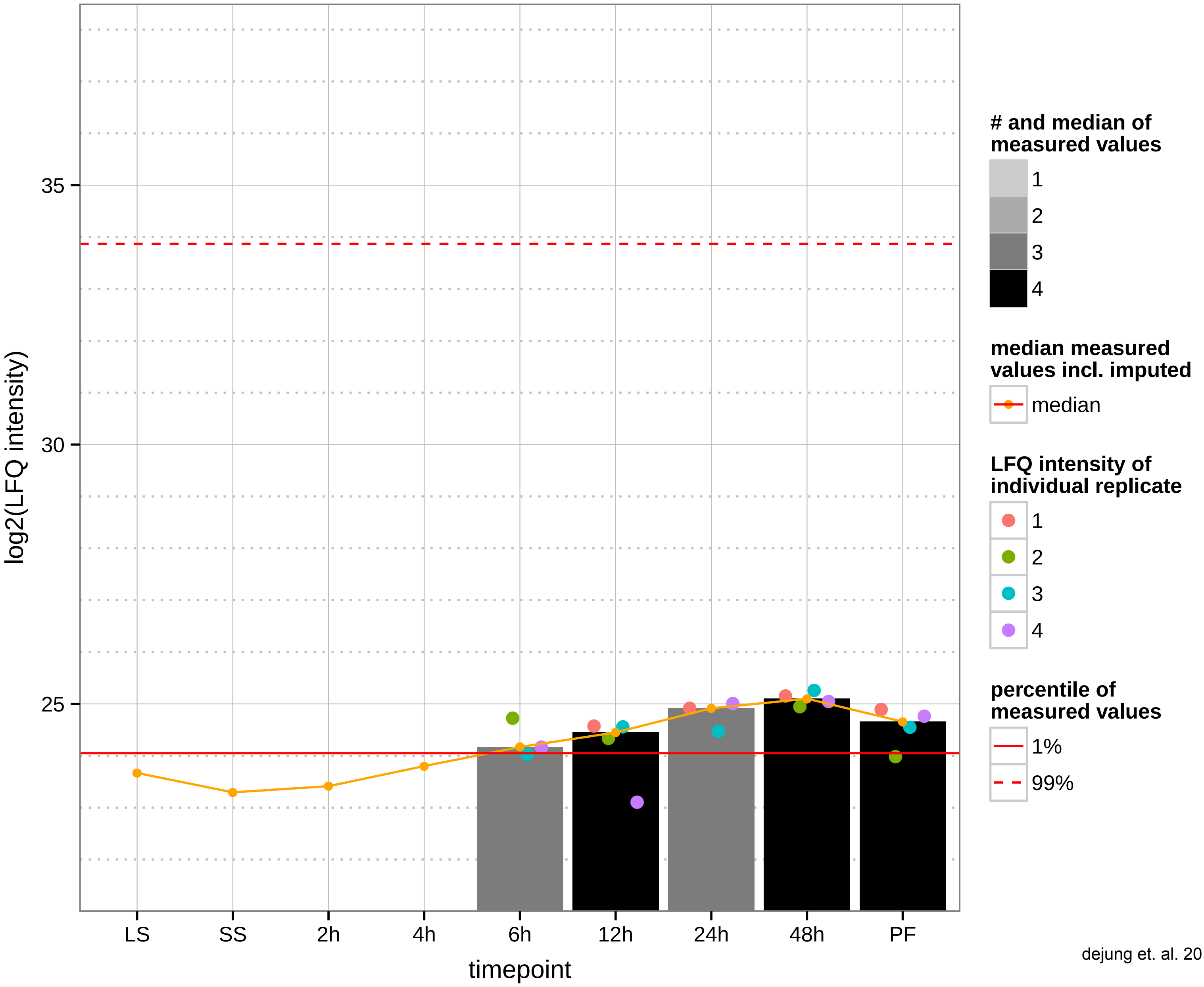

dejung et. al. 2015

hypothetical protein, conserved
Tb927.5.4040
AGOF: null
AGOC: mitochondrion
AGOP: cell wall macromolecule catabolic process
PGOF: null
PGOC: null
PGOP: cell wall macromolecule catabolic process

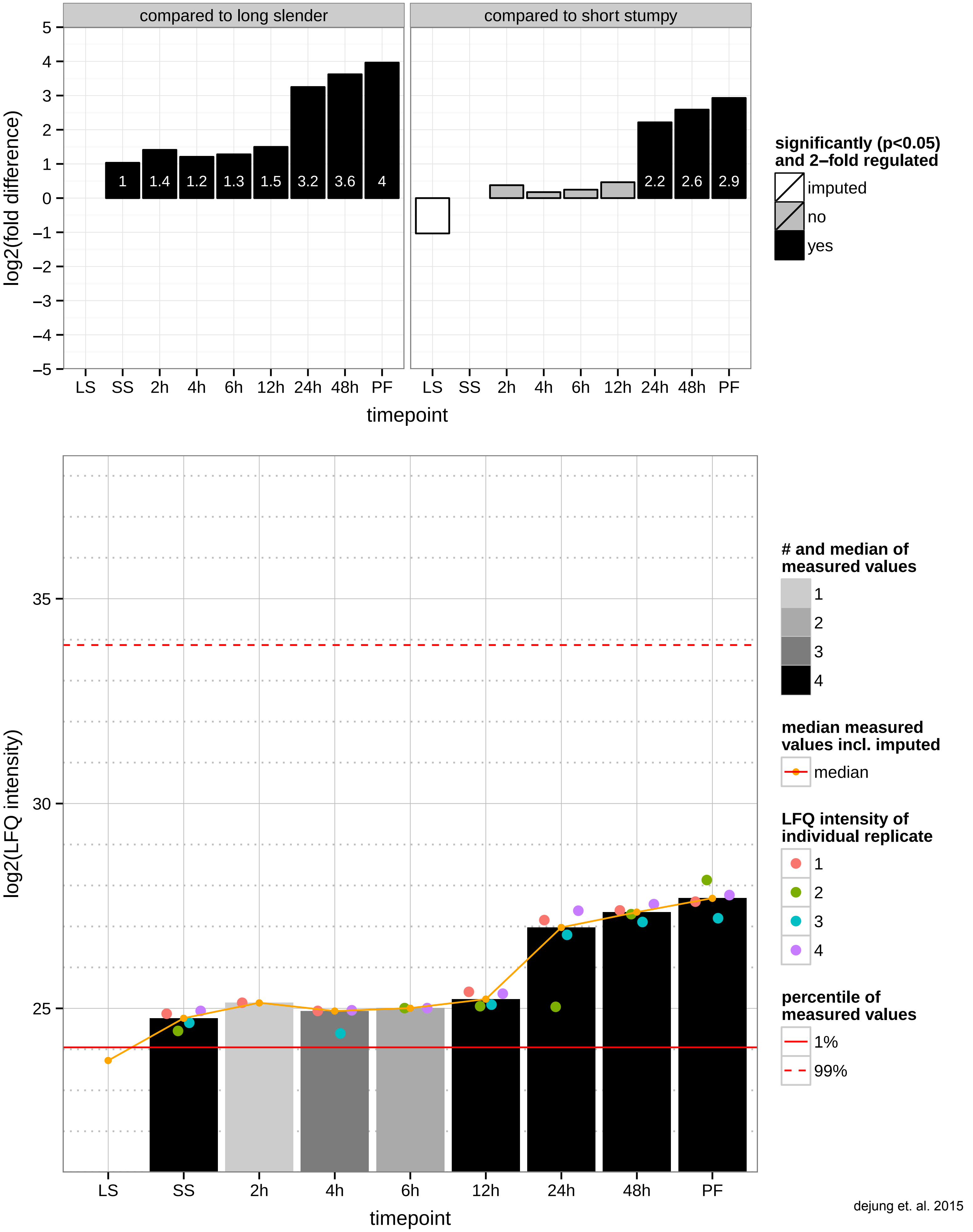

dejung et. al. 2015

dihydrolipoamide branched chain transacylase, putative
Tb927.5.4330
AGOF: cofactor binding, dihydrolipoyllysine−residue (2−methylpropanoyl)transferase activity, transferase activity, transferring a
AGOC: mitochondrion
AGOP: fatty−acyl−CoA biosynthetic process
PGOF: transferase activity, transferring acyl groups
PGOC: null
PGOP: metabolic process

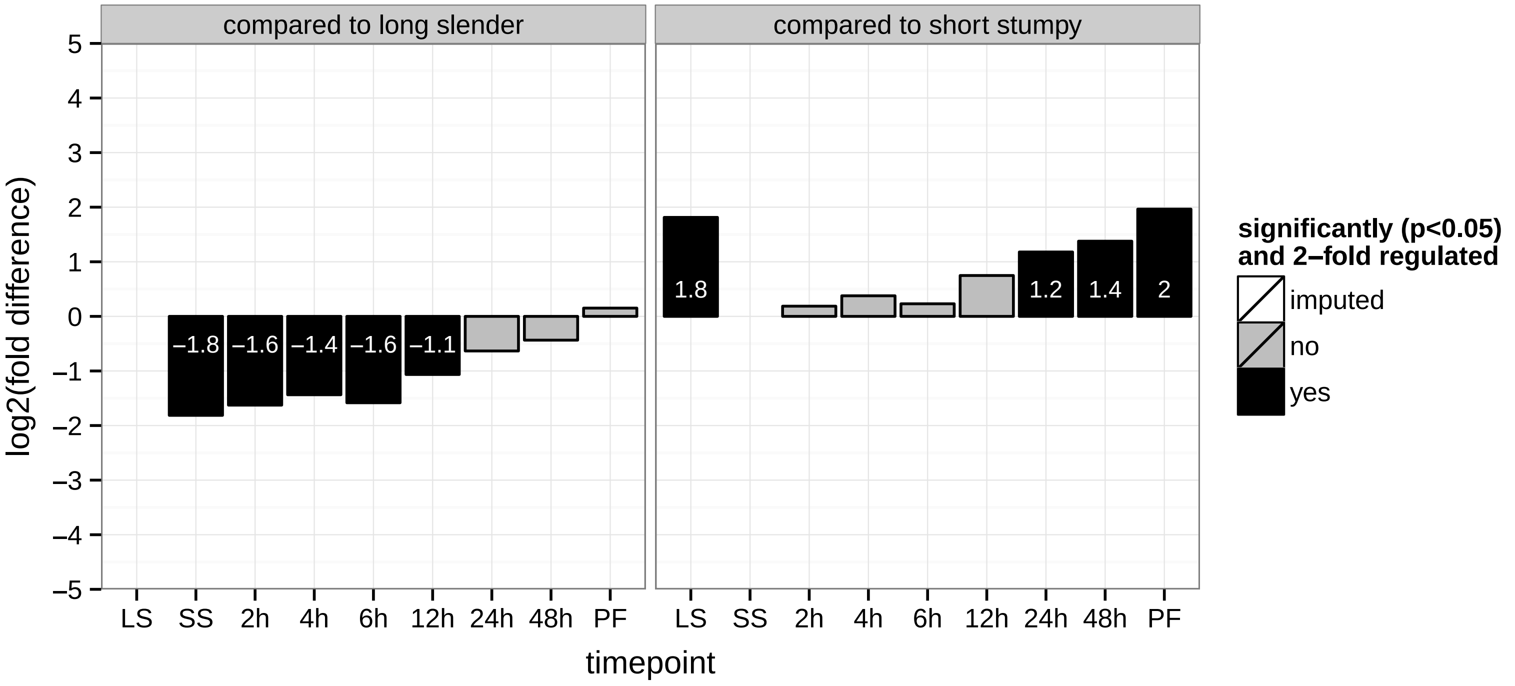

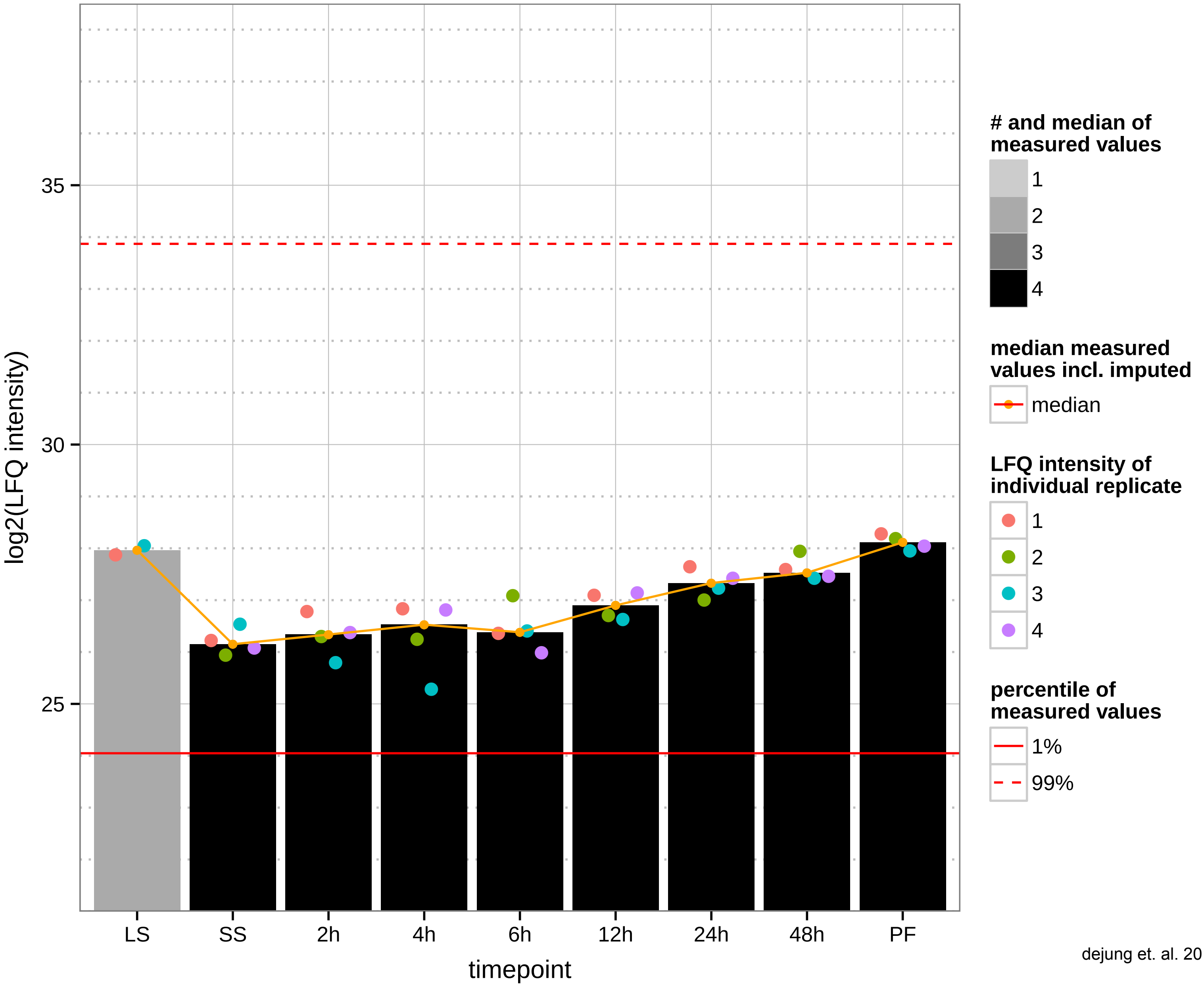

dejung et. al. 2015

NADH−dependent fumarate reductase, putative, hypothetical protein, NADH−dependent fumarate reductase (FRDg)
Tb927.5.930;Tb11.v5.0613
AGOF: null, electron carrier activity, fumarate reductase (NADH) activity, succinate dehydrogenase activity
AGOC: null, glycosome
AGOP: null, metabolic process, thiamine biosynthetic process
PGOF: oxidoreductase activity, null, succinate dehydrogenase activity
PGOC: null
PGOP: oxidation−reduction process, null

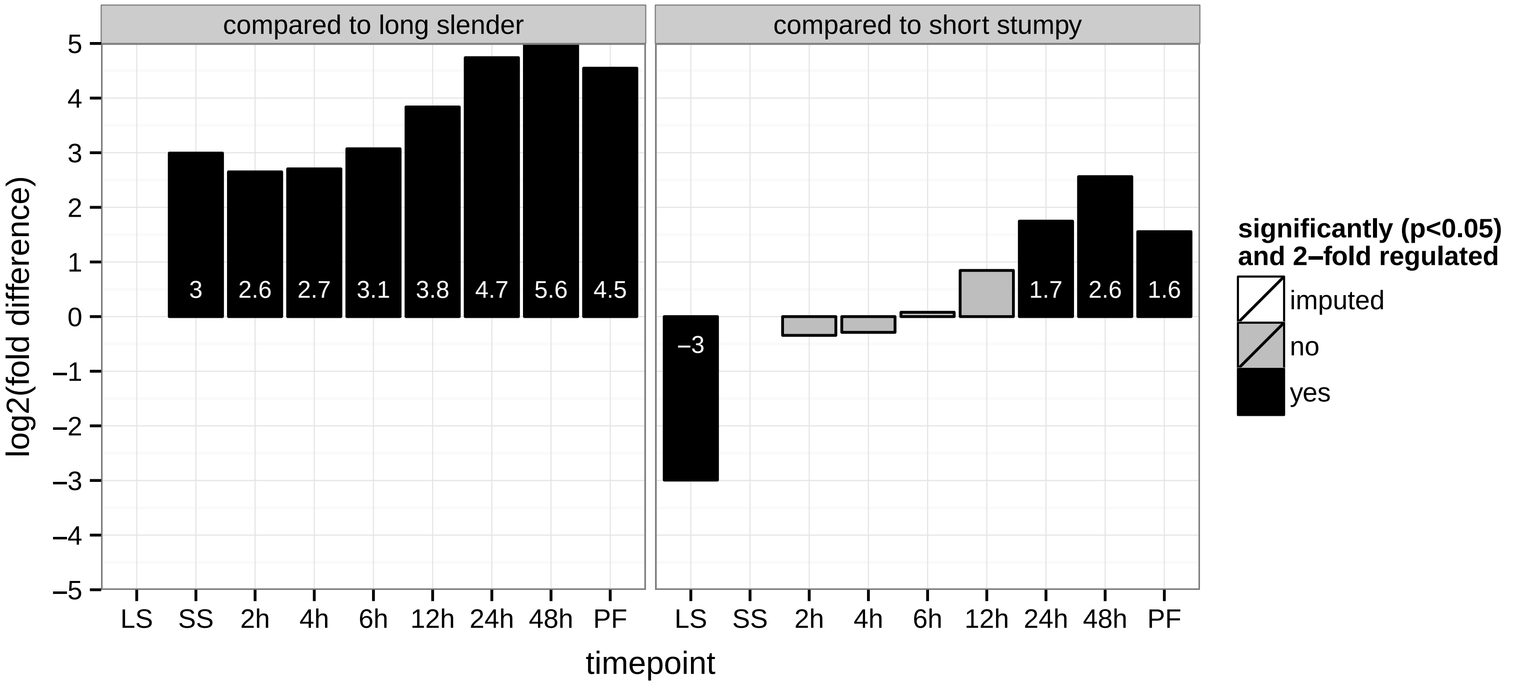

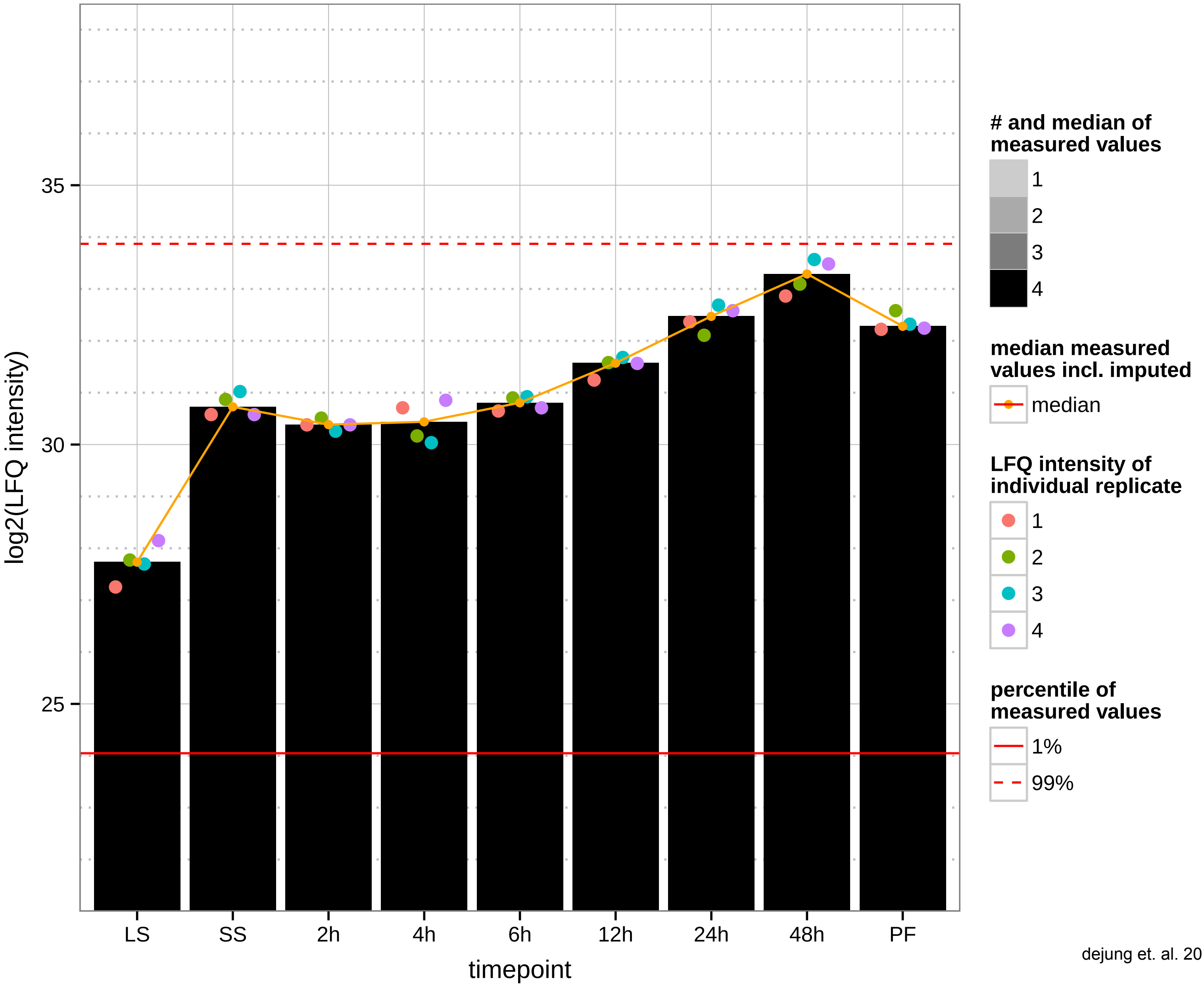

dejung et. al. 2015

hypothetical protein, conserved
Tb927.6.2180
AGOF: null
AGOC: mitochondrion
AGOP: null
PGOF: null
PGOC: null
PGOP: null

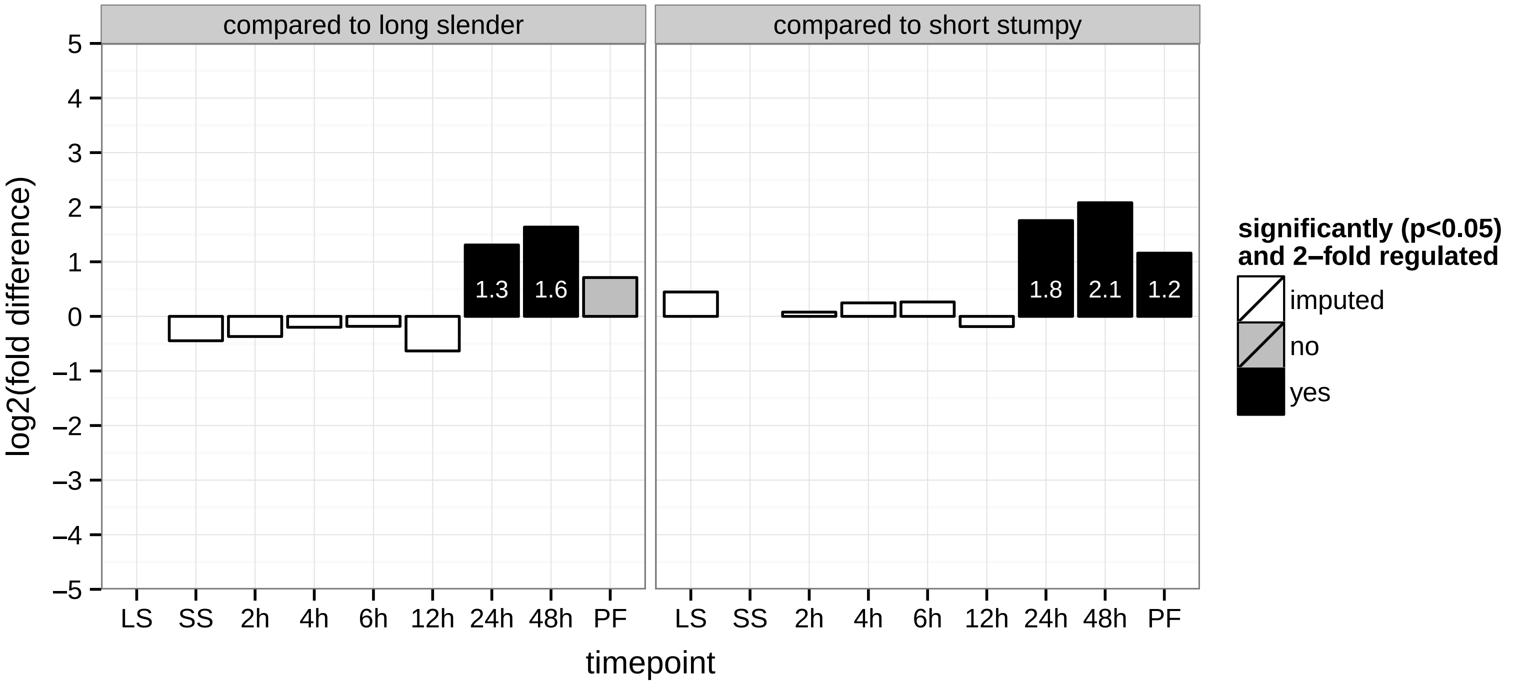

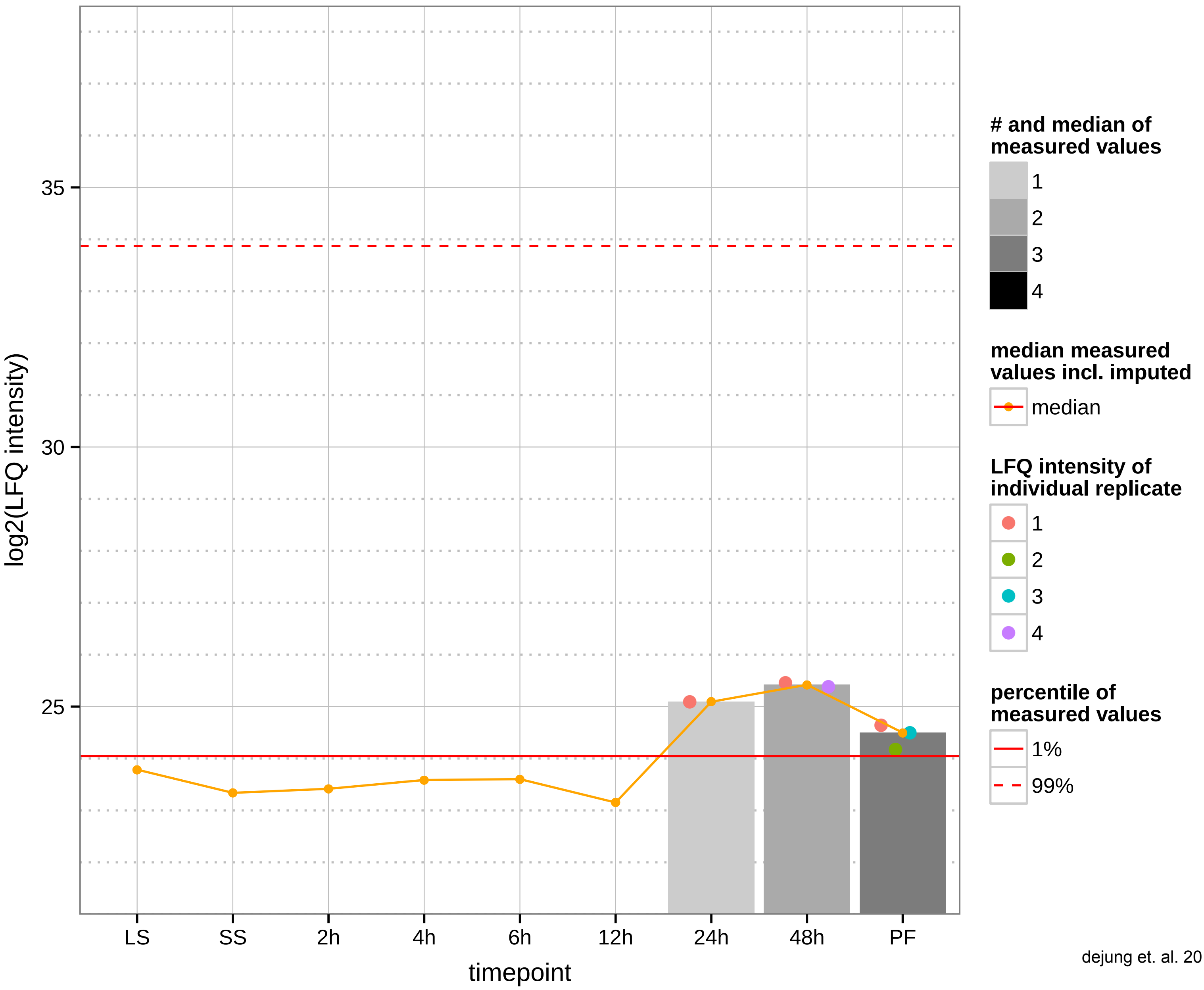

dejung et. al. 2015

hypothetical protein, conserved
Tb927.6.2320;Tb927.6.2260
AGOF: null
AGOC: null
AGOP: null
PGOF: null
PGOC: null
PGOP: null

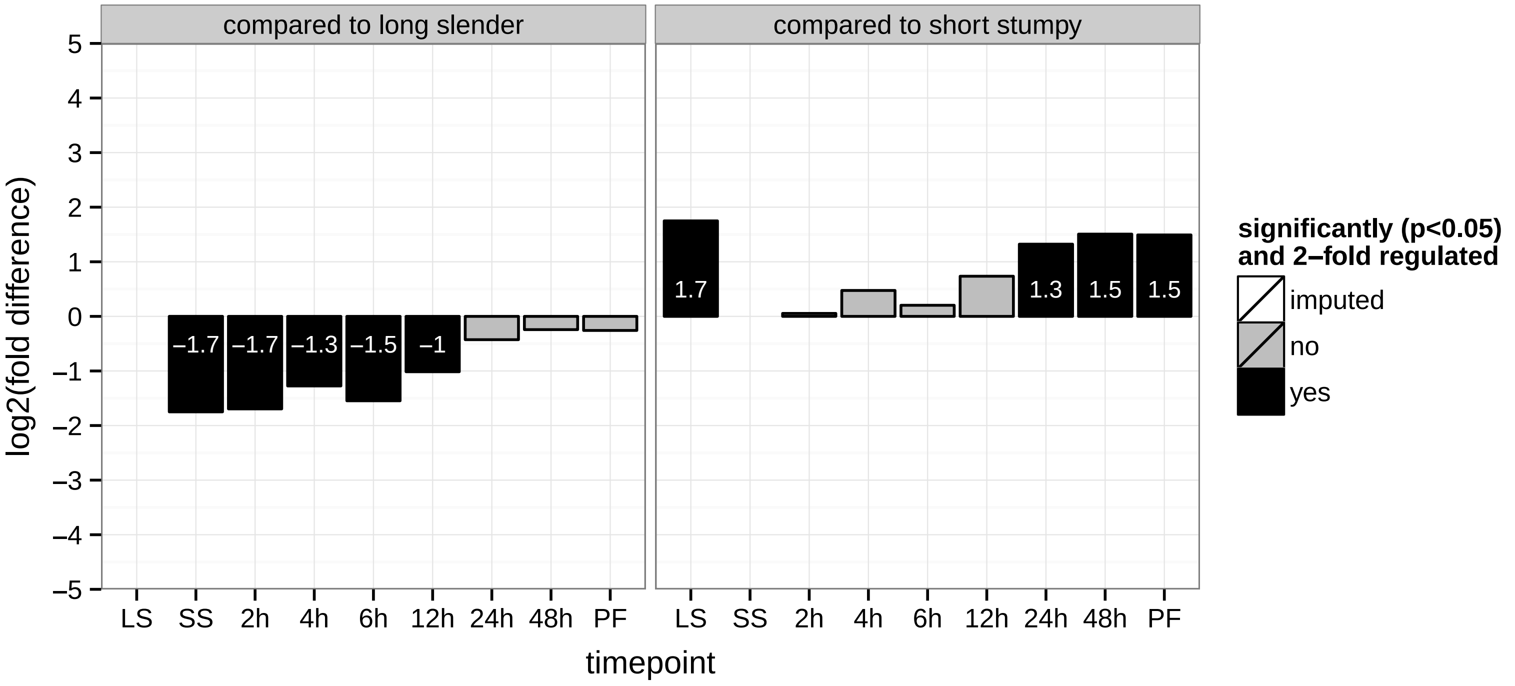

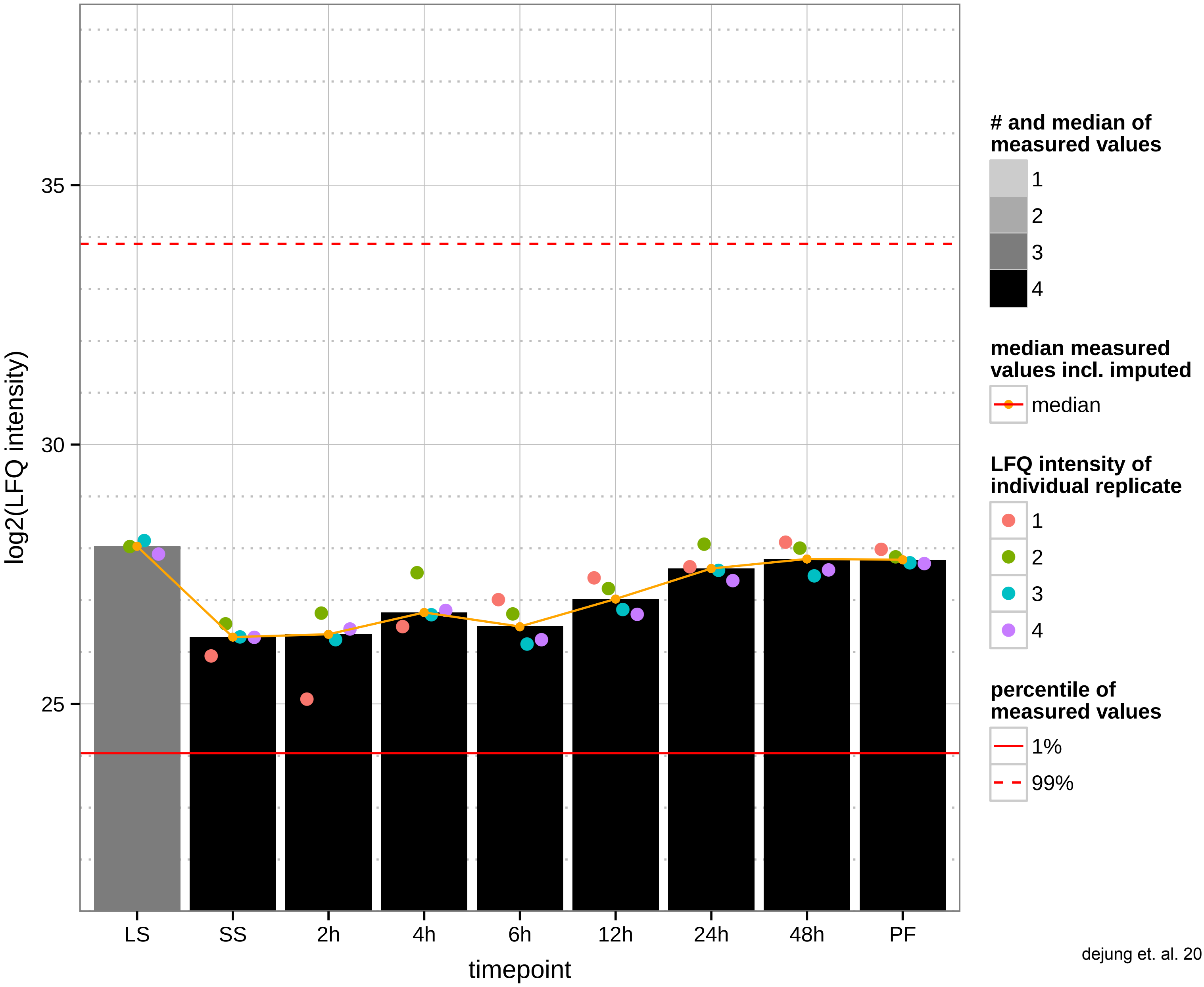

dejung et. al. 2015

hypothetical protein, conserved
Tb927.6.2490
AGOF: null
AGOC: mitochondrion
AGOP: null
PGOF: null
PGOC: null
PGOP: null

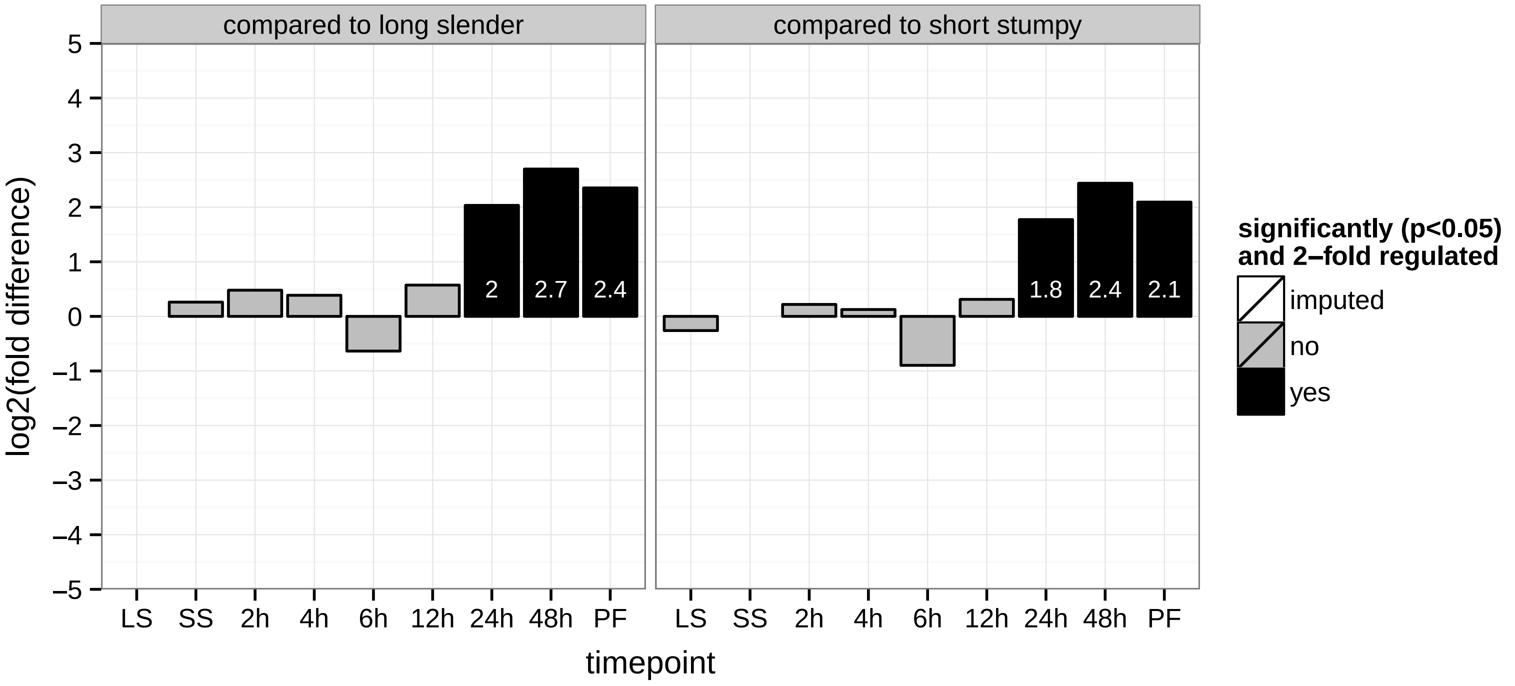

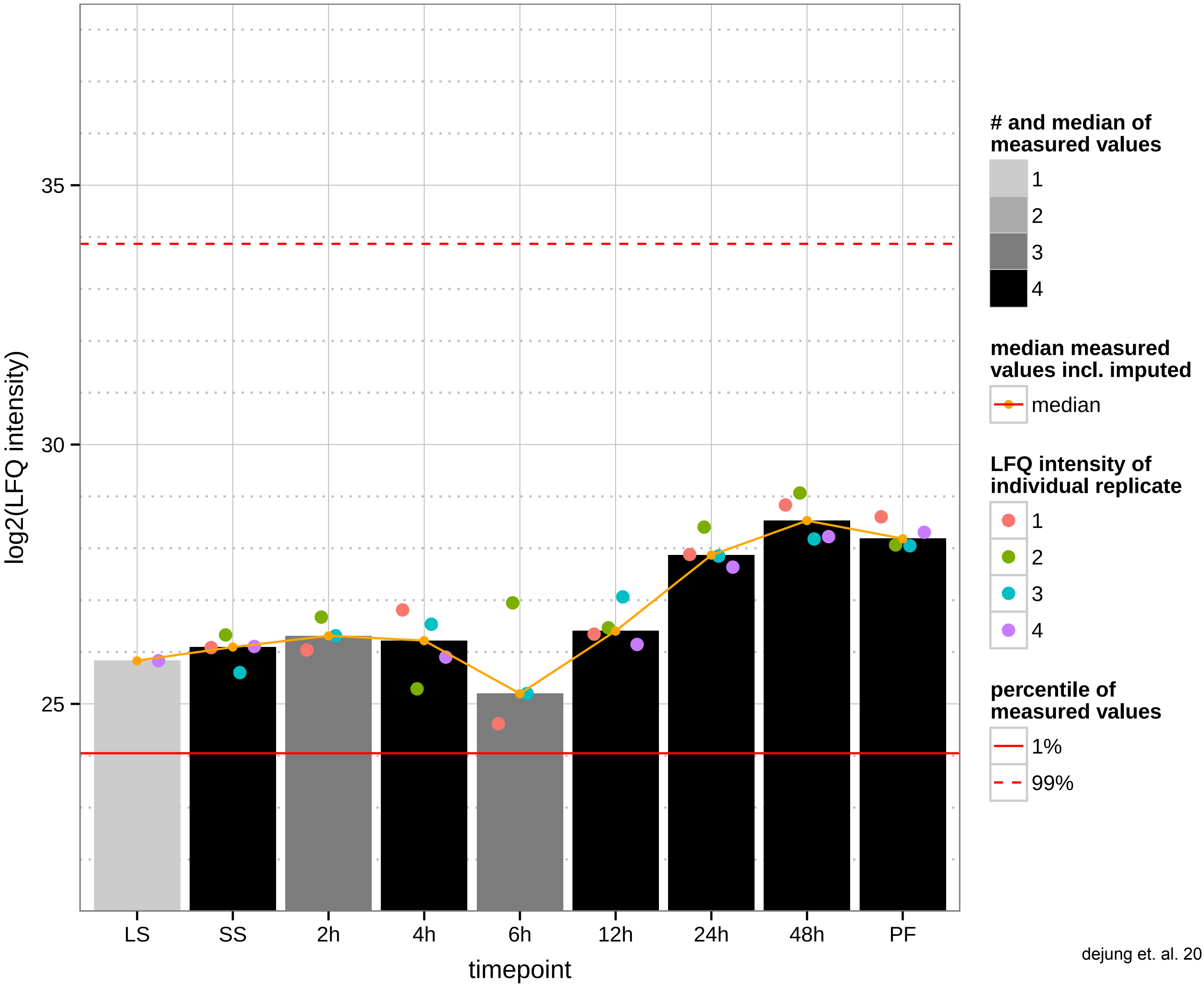

dejung et. al. 2015

hypothetical protein, conserved
Tb927.6.2570
AGOF: null
AGOC: null
AGOP: null
PGOF: null
PGOC: null
PGOP: null

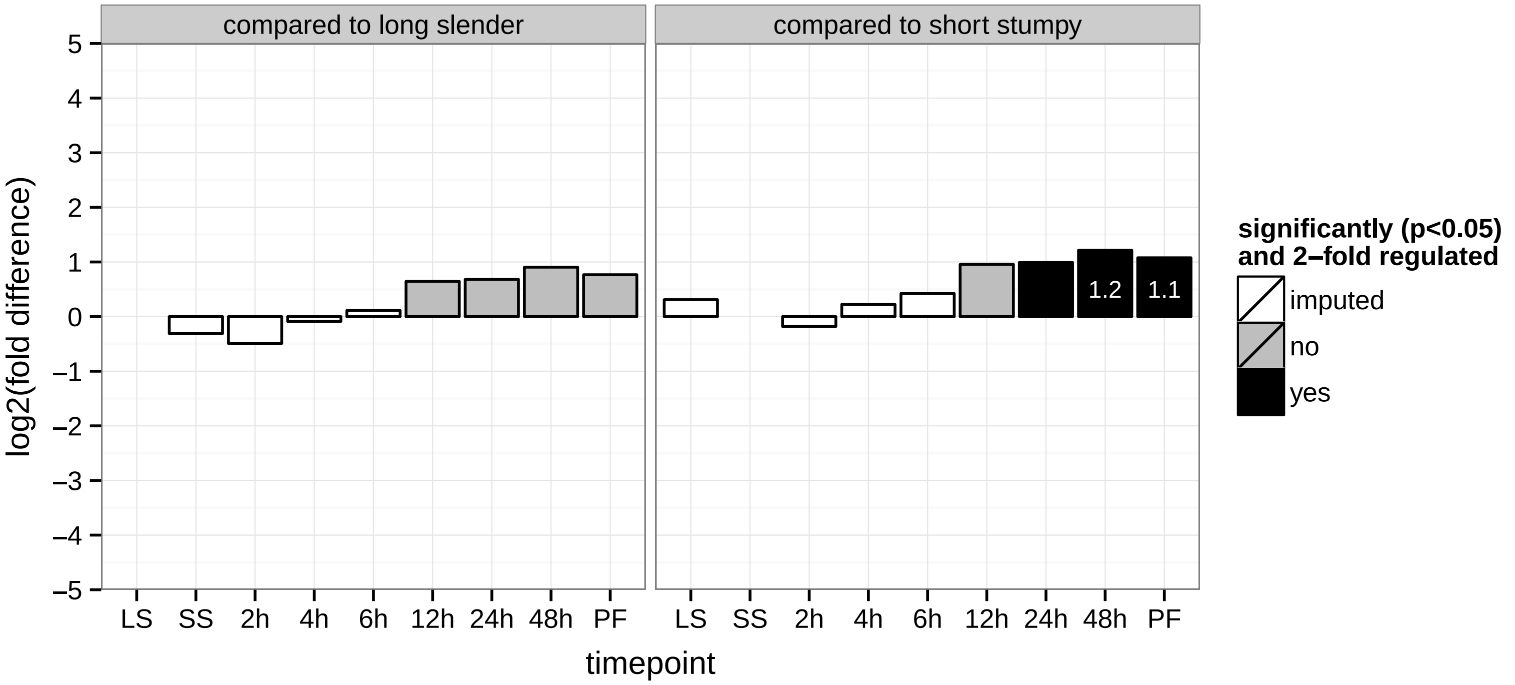

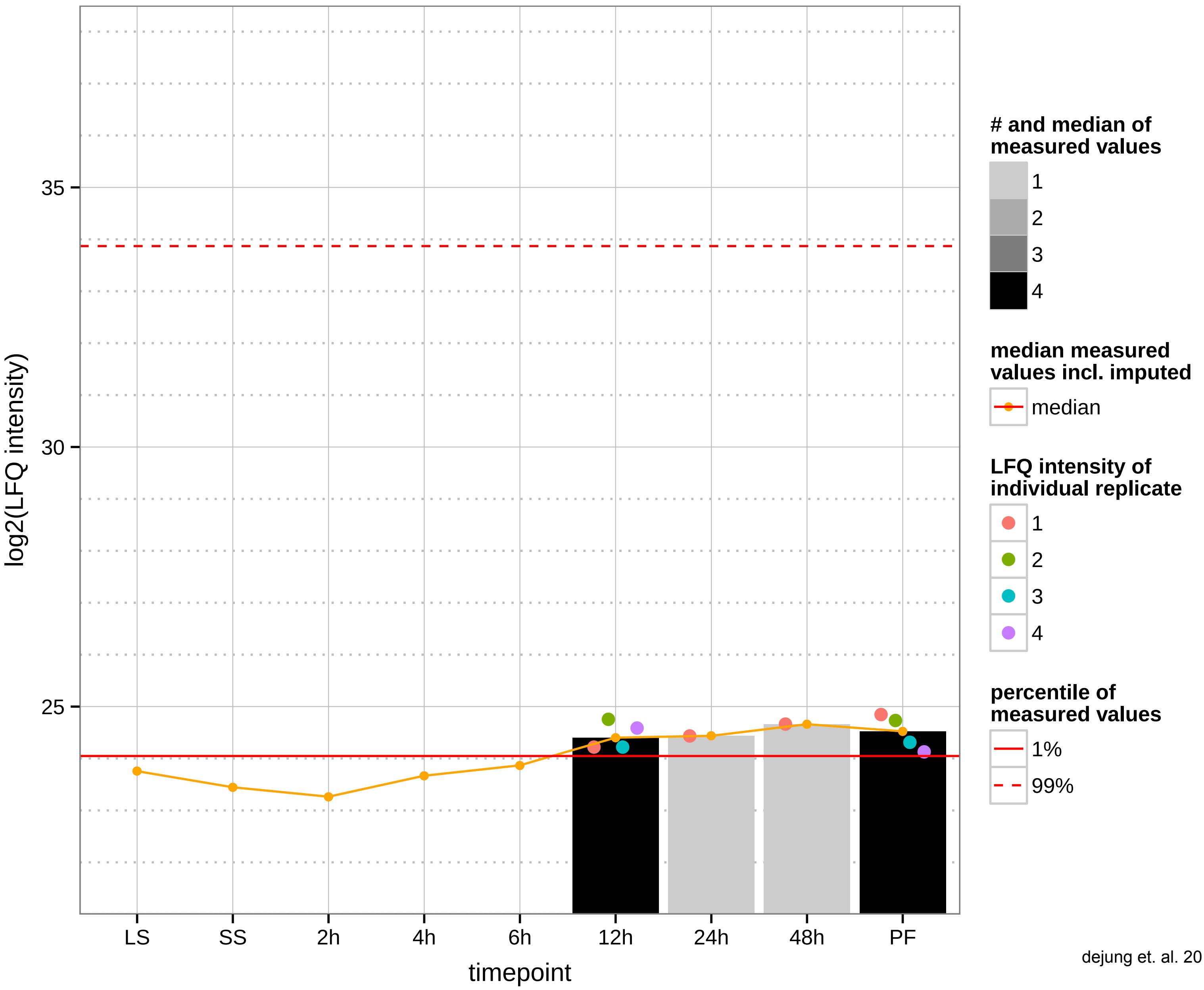

dejung et. al. 2015

superoxide dismutase, putative
Tb927.6.4030
AGOF: metal ion binding, superoxide dismutase activity
AGOC: mitochondrion
AGOP: oxidation–reduction process, superoxide metabolic process
PGOF: metal ion binding, superoxide dismutase activity
PGOC: null
PGOP: oxidation–reduction process, superoxide metabolic process

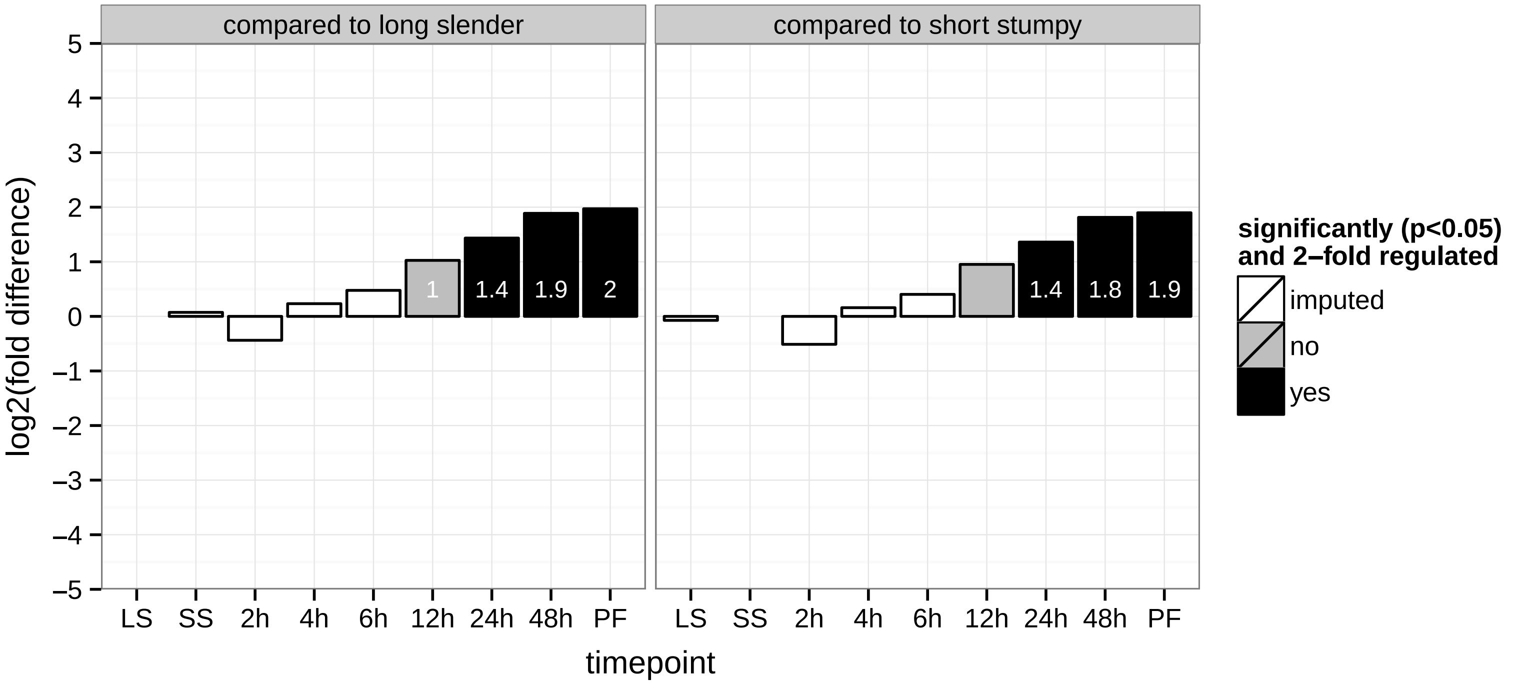

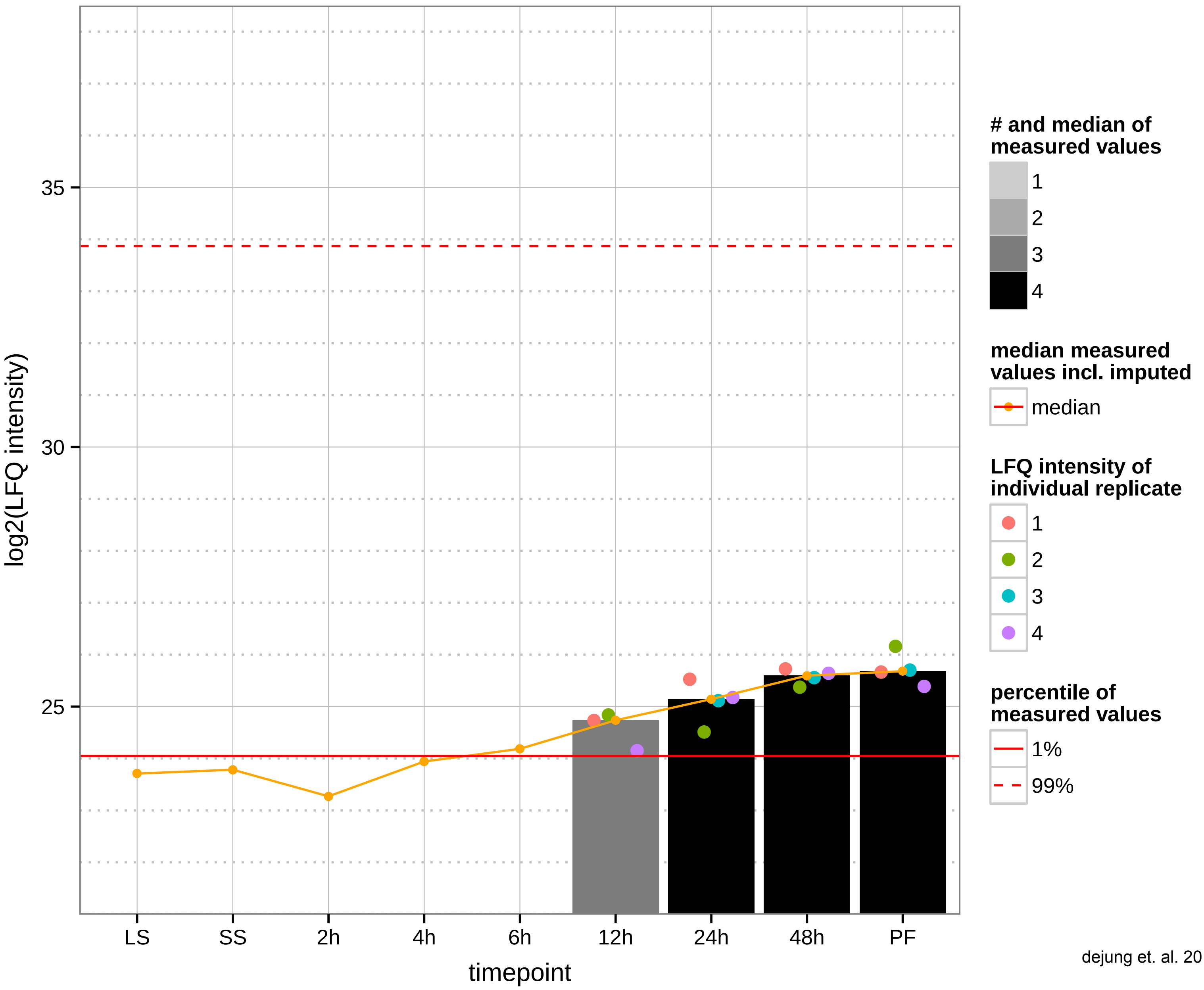

dejung et. al. 2015

hypothetical protein, conserved
Tb927.6.4070
AGOF: null
AGOC: mitochondrion
AGOP: null
PGOF: null
PGOC: null
PGOP: null

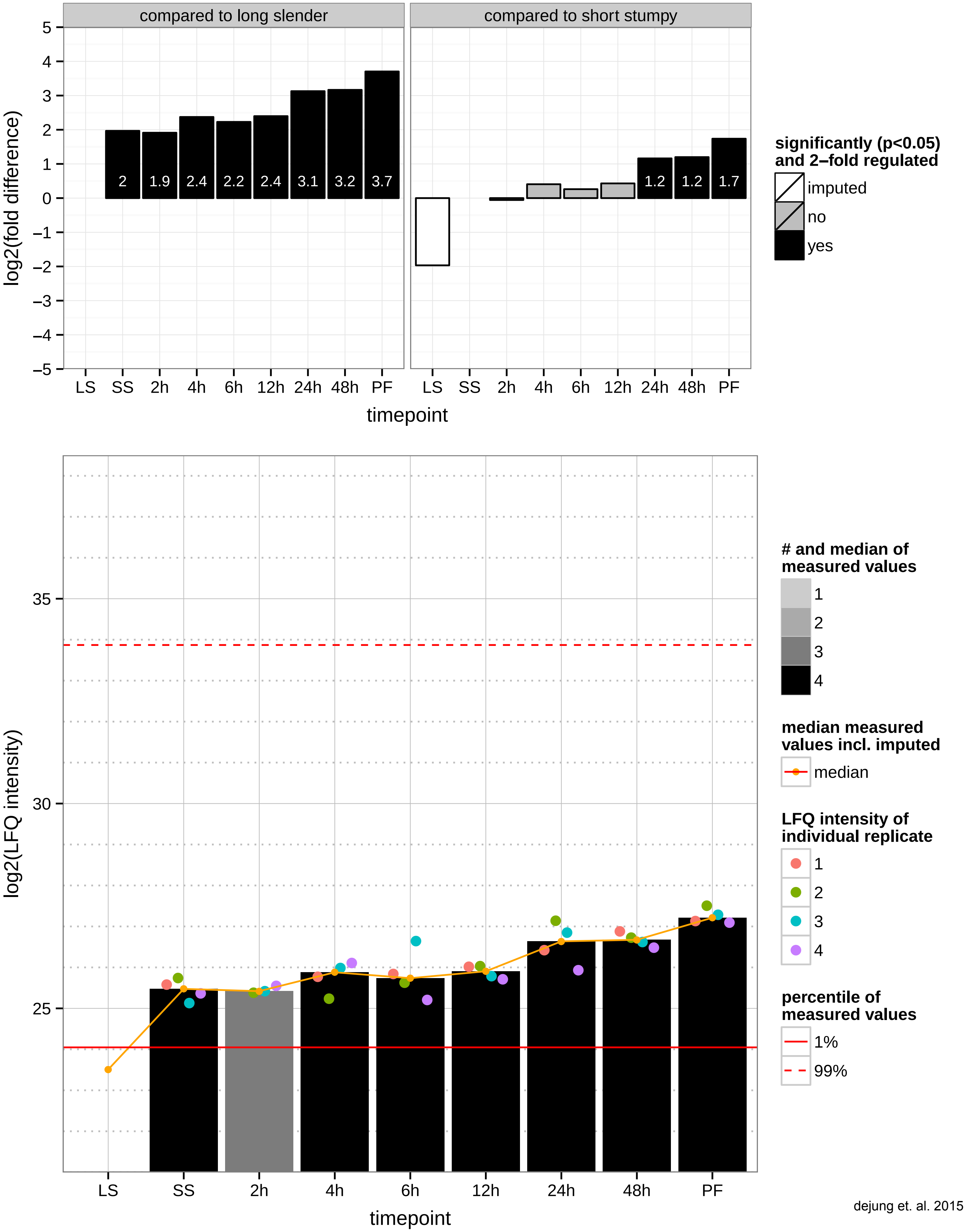

dejung et. al. 2015

RNA–binding protein, putative (RBP17)
Tb927.6.4530
AGOF: RNA binding
AGOC: null
AGOP: RNA processing
PGOF: nucleic acid binding
PGOC: null
PGOP: null

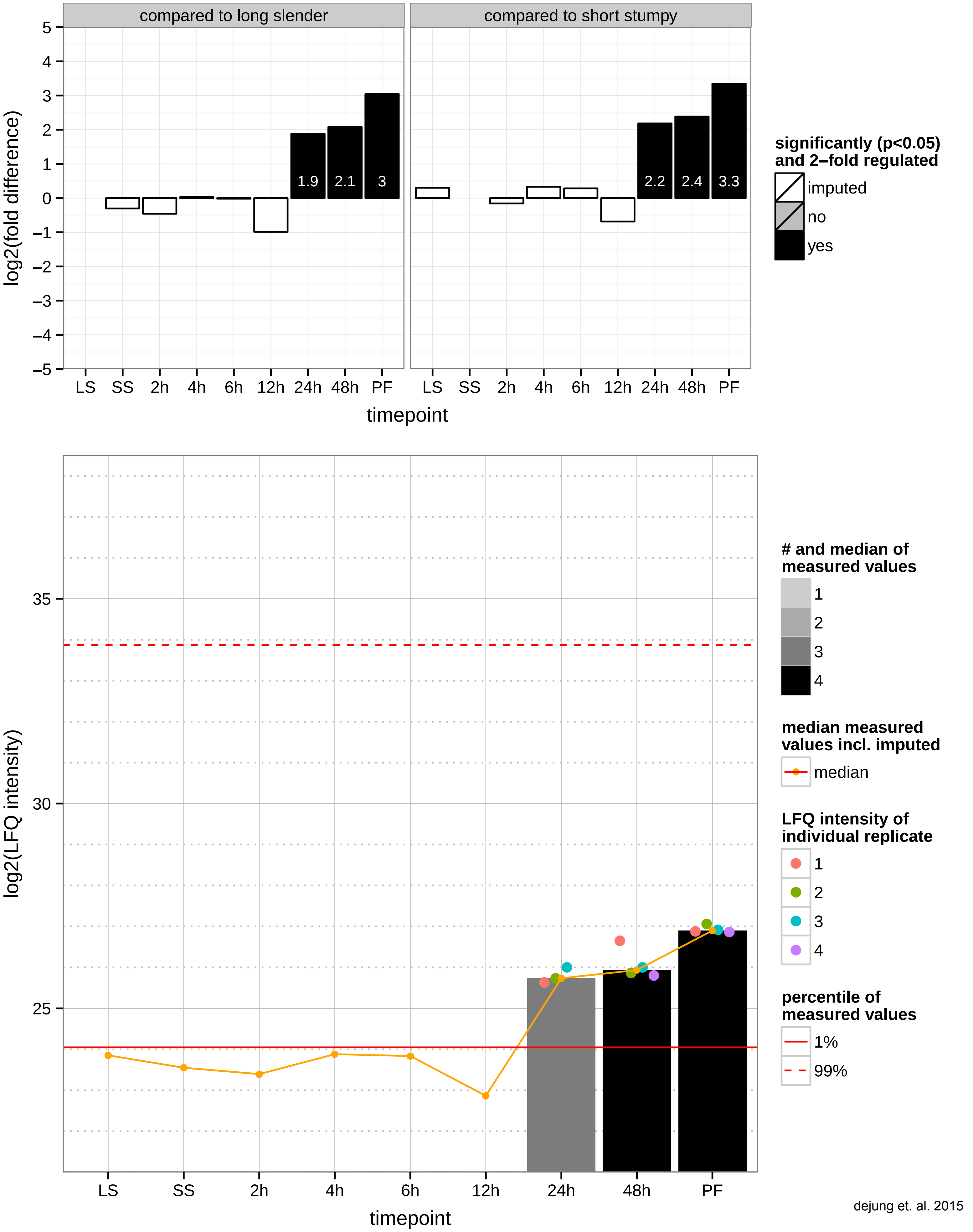

dejung et. al. 2015

hypothetical protein, conserved
Tb927.6.590
AGOF: null
AGOC: mitochondrion
AGOP: null
PGOF: null
PGOC: null
PGOP: null

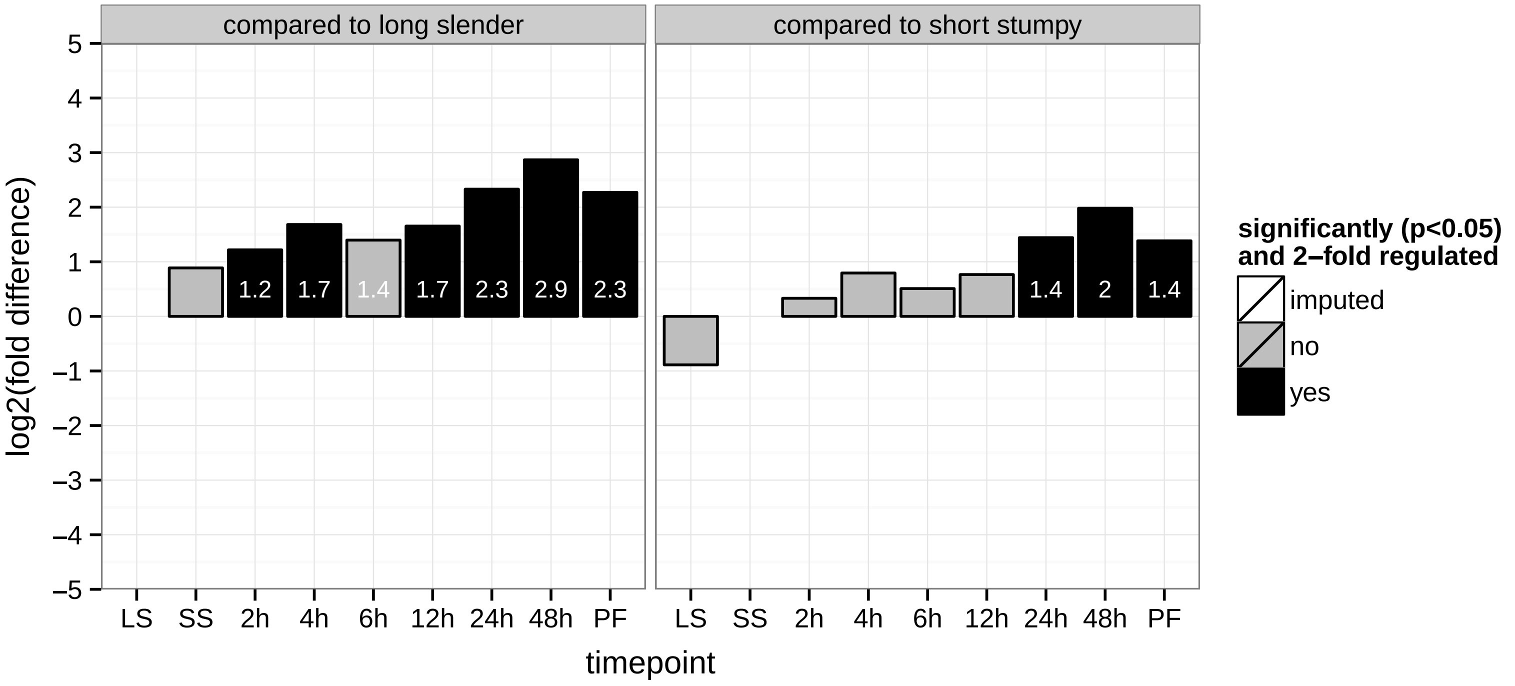

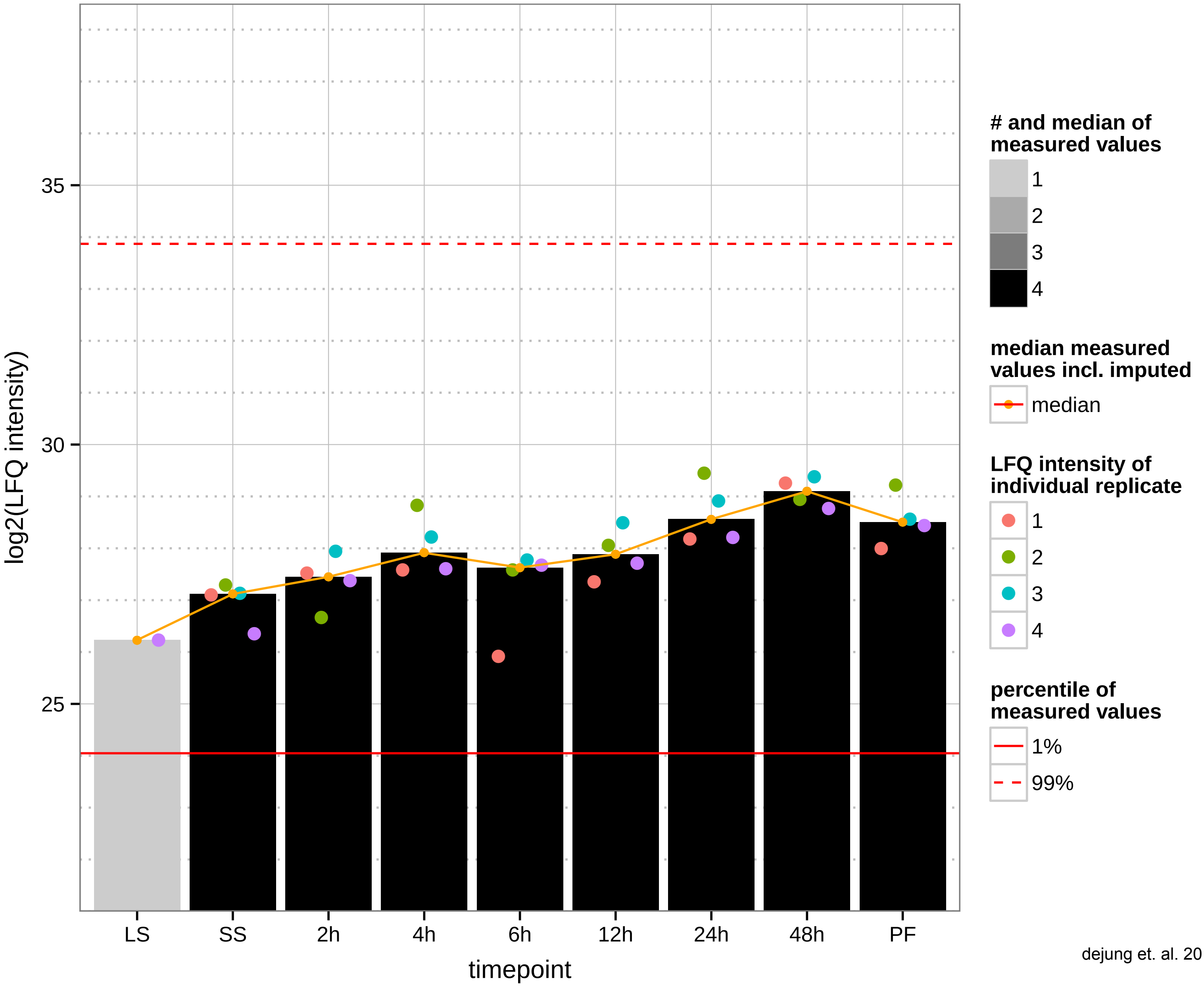

dejung et. al. 2015

60S ribosomal protein L7, putative
Tb927.7.1730;Tb927.7.1750;Tb927.7.1740
AGOF: structural constituent of ribosome
AGOC: intracellular, large ribosomal subunit, ribosome
AGOP: ribosome biogenesis, translation
PGOF: null
PGOC: null
PGOP: null

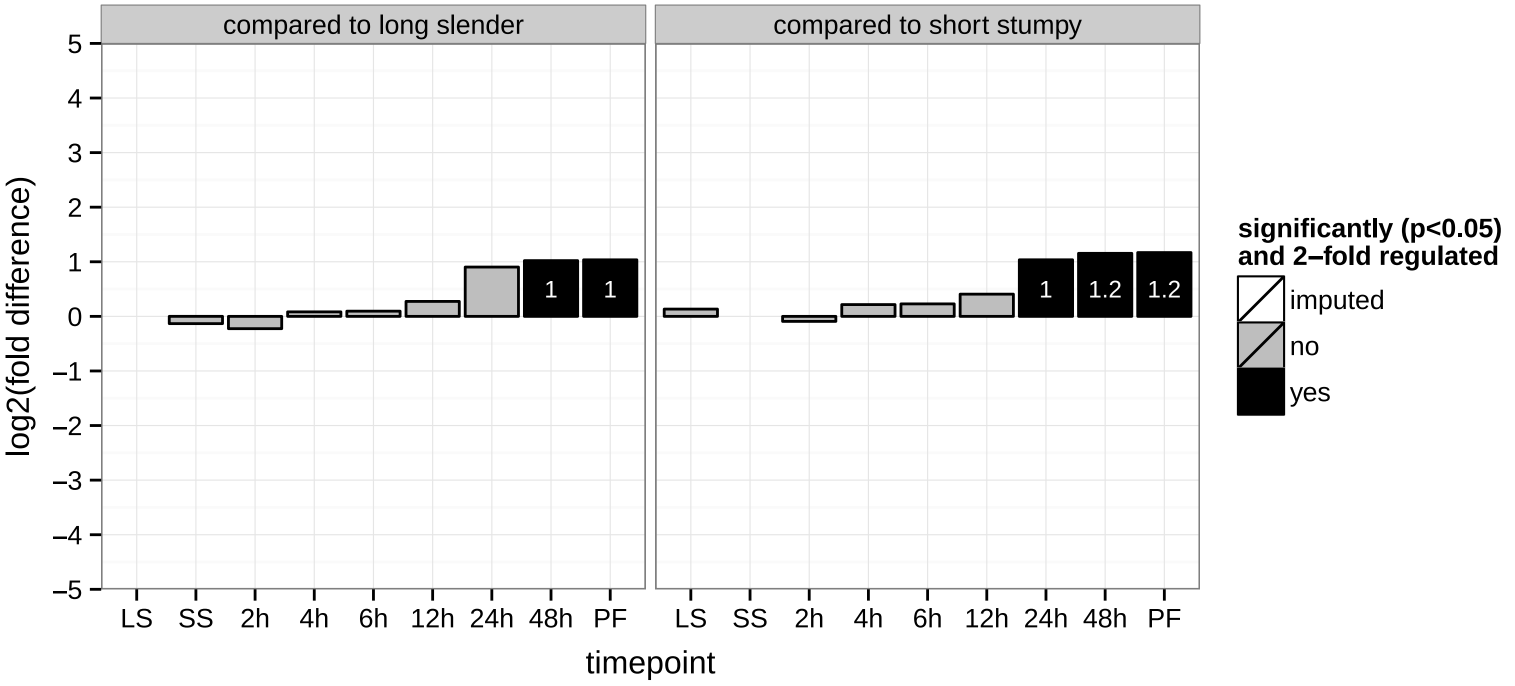

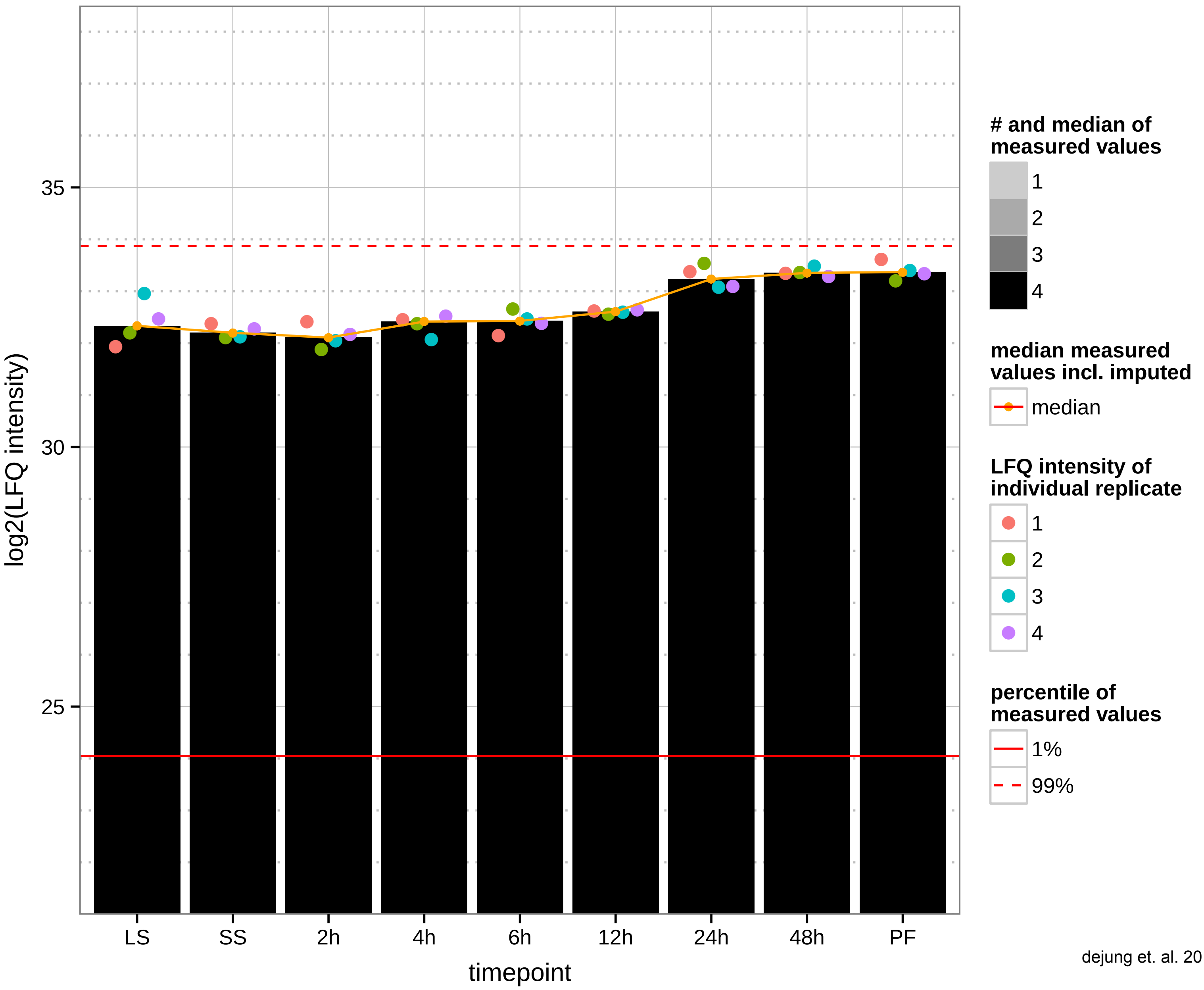

dejung et. al. 2015

Adenine phosphoribosyltransferase, putative
Tb927.7.1780
AGOF: null
AGOC: null
AGOP: purine ribonucleoside salvage
PGOF: null
PGOC: null
PGOP: nucleoside metabolic process

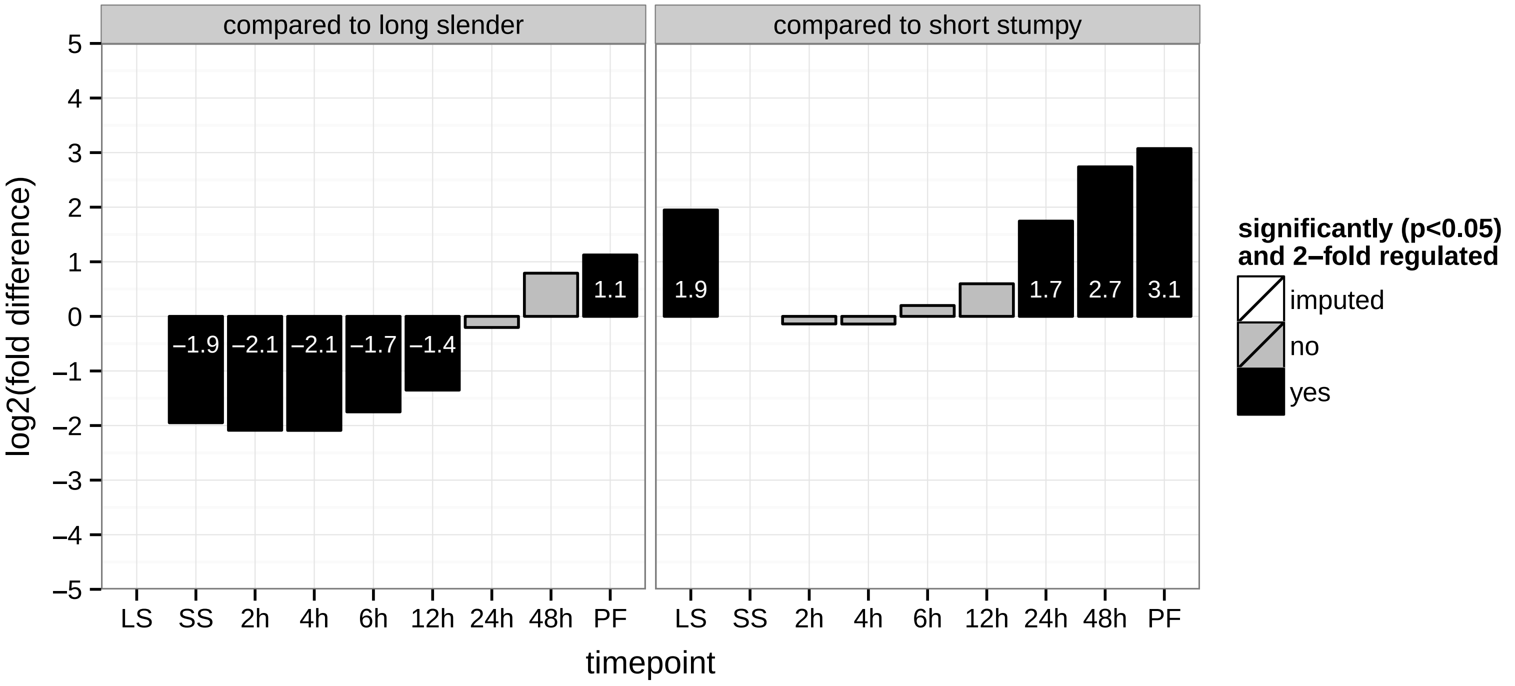

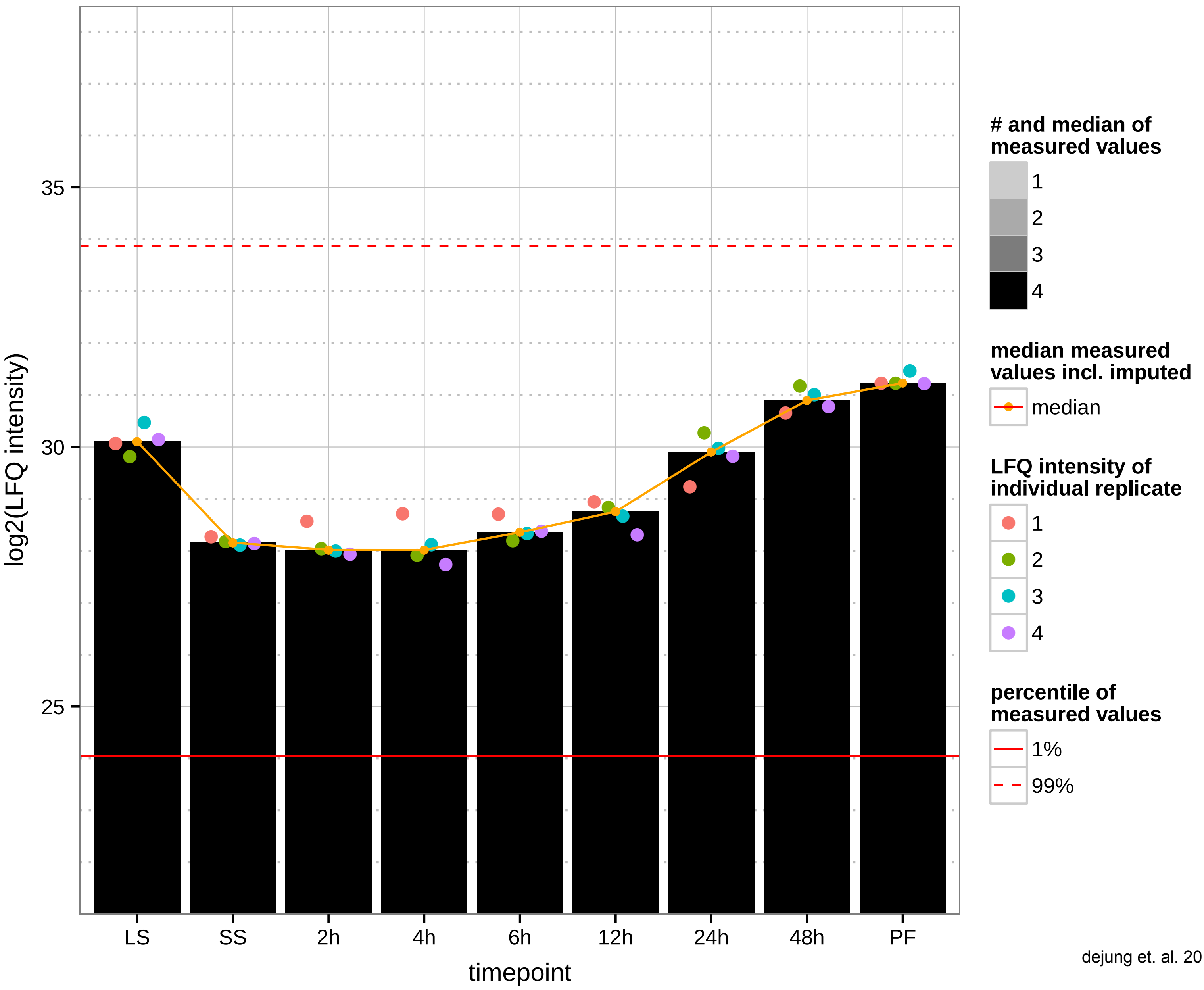

dejung et. al. 2015

hypothetical protein, conserved
Tb927.7.2220
AGOF: null
AGOC: mitochondrion
AGOP: null
PGOF: null
PGOC: null
PGOP: null

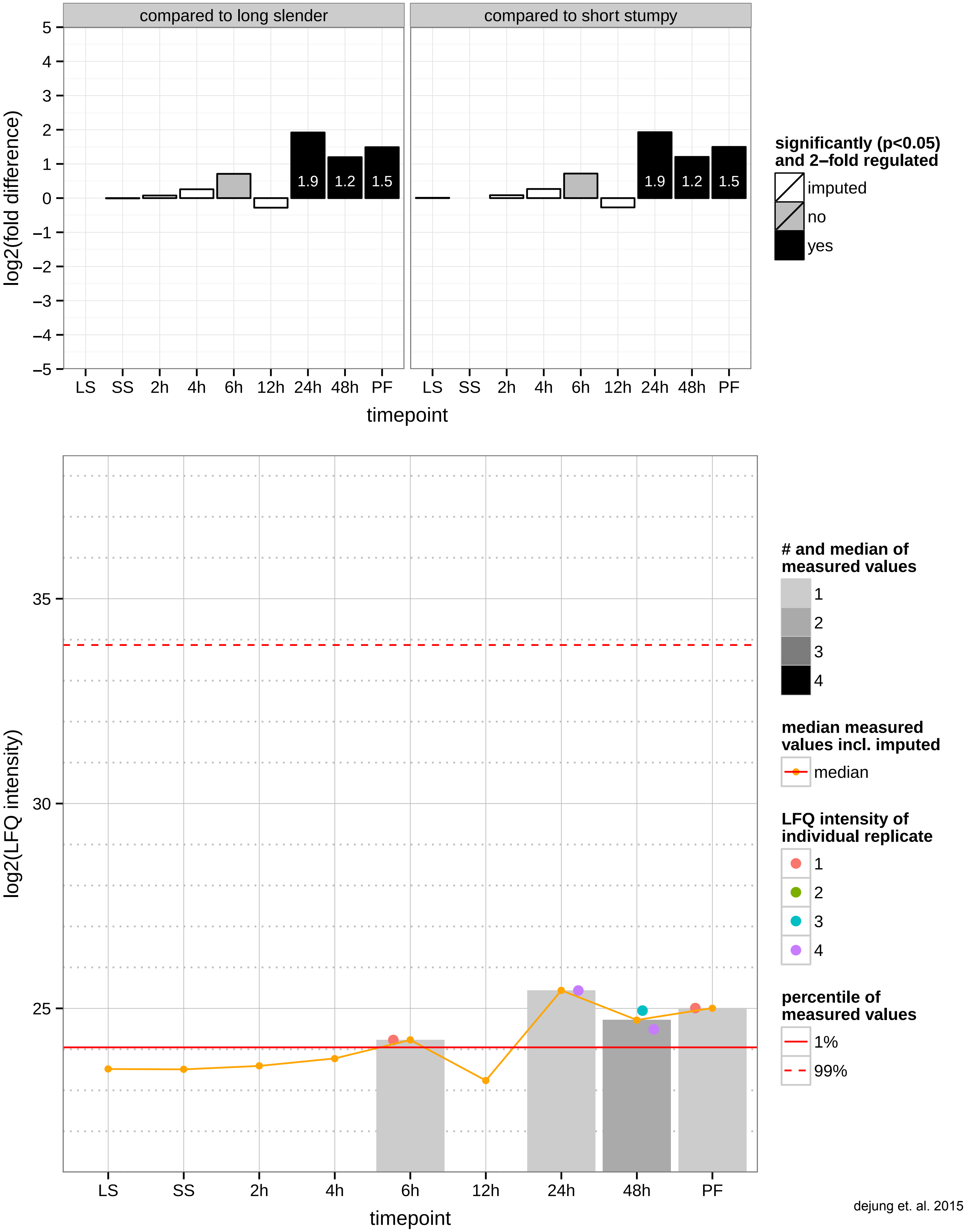

dejung et. al. 2015

tyrosyl–tRNA synthetase, putative
Tb927.7.2400
AGOF: tRNA binding
AGOC: null
AGOP: null
PGOF: tRNA binding
PGOC: null
PGOP: null

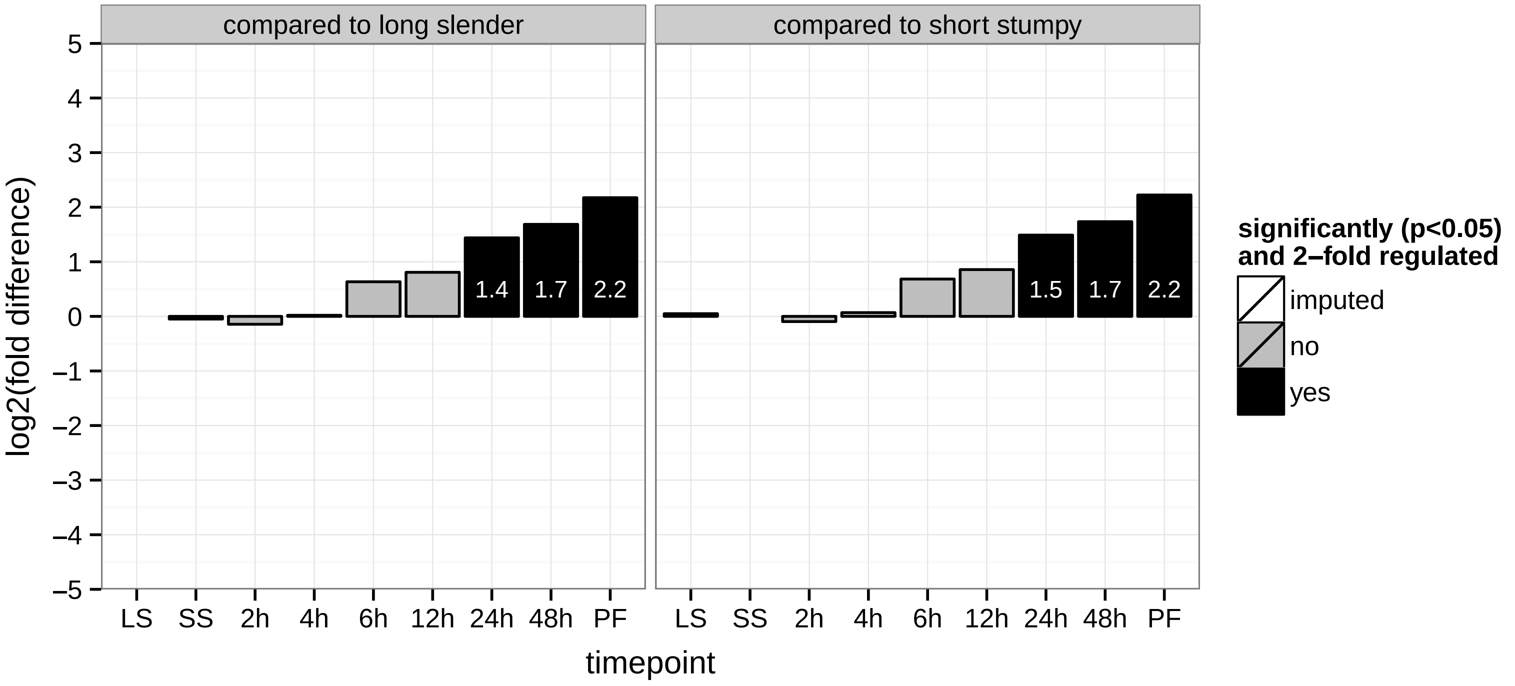

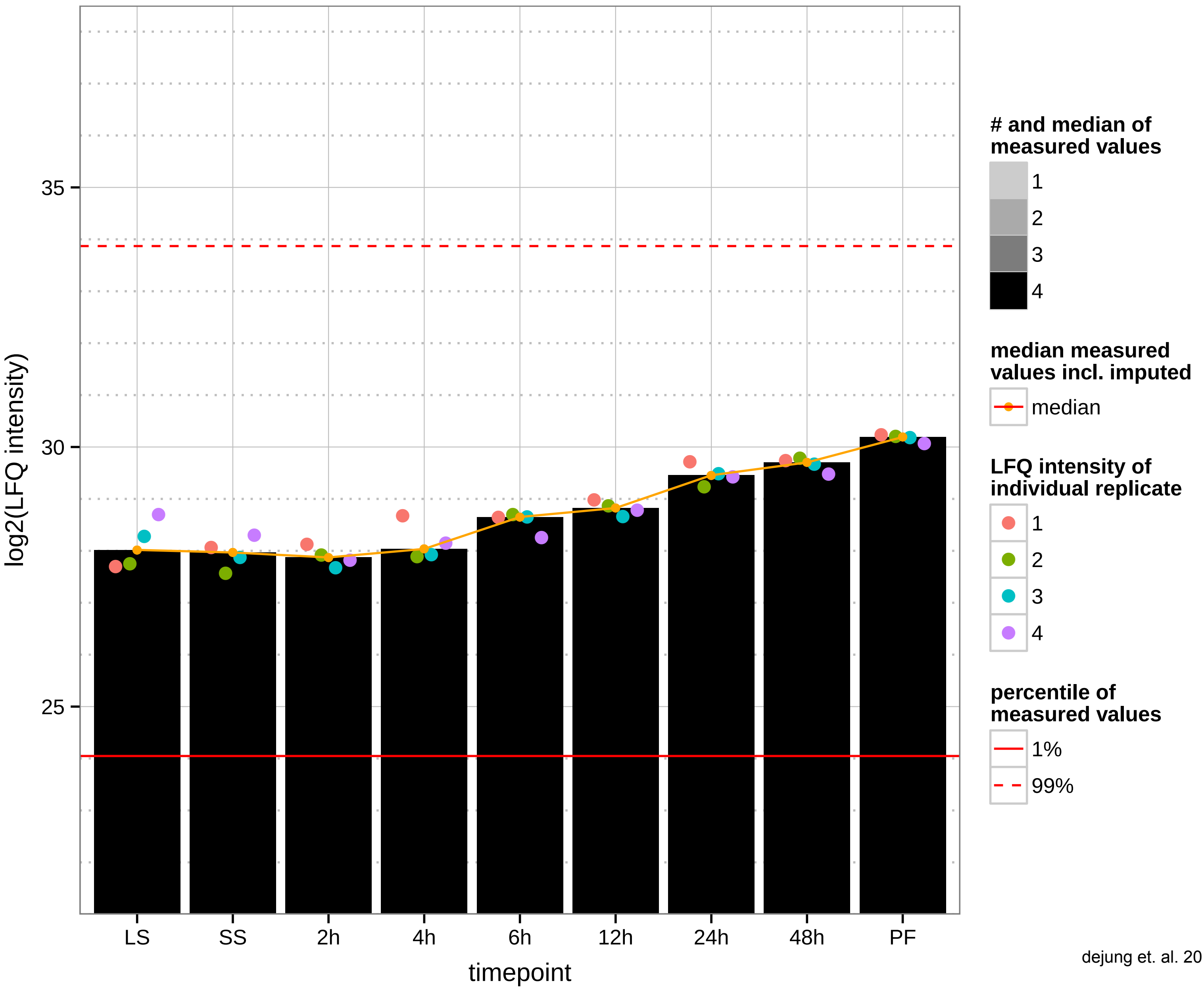

dejung et. al. 2015

NADH–cytochrome b5 reductase, putative (B5R)
Tb927.7.2700
AGOF: electron carrier activity, oxidoreductase activity
AGOC: mitochondrion
AGOP: null
PGOF: oxidoreductase activity
PGOC: null
PGOP: oxidation–reduction process

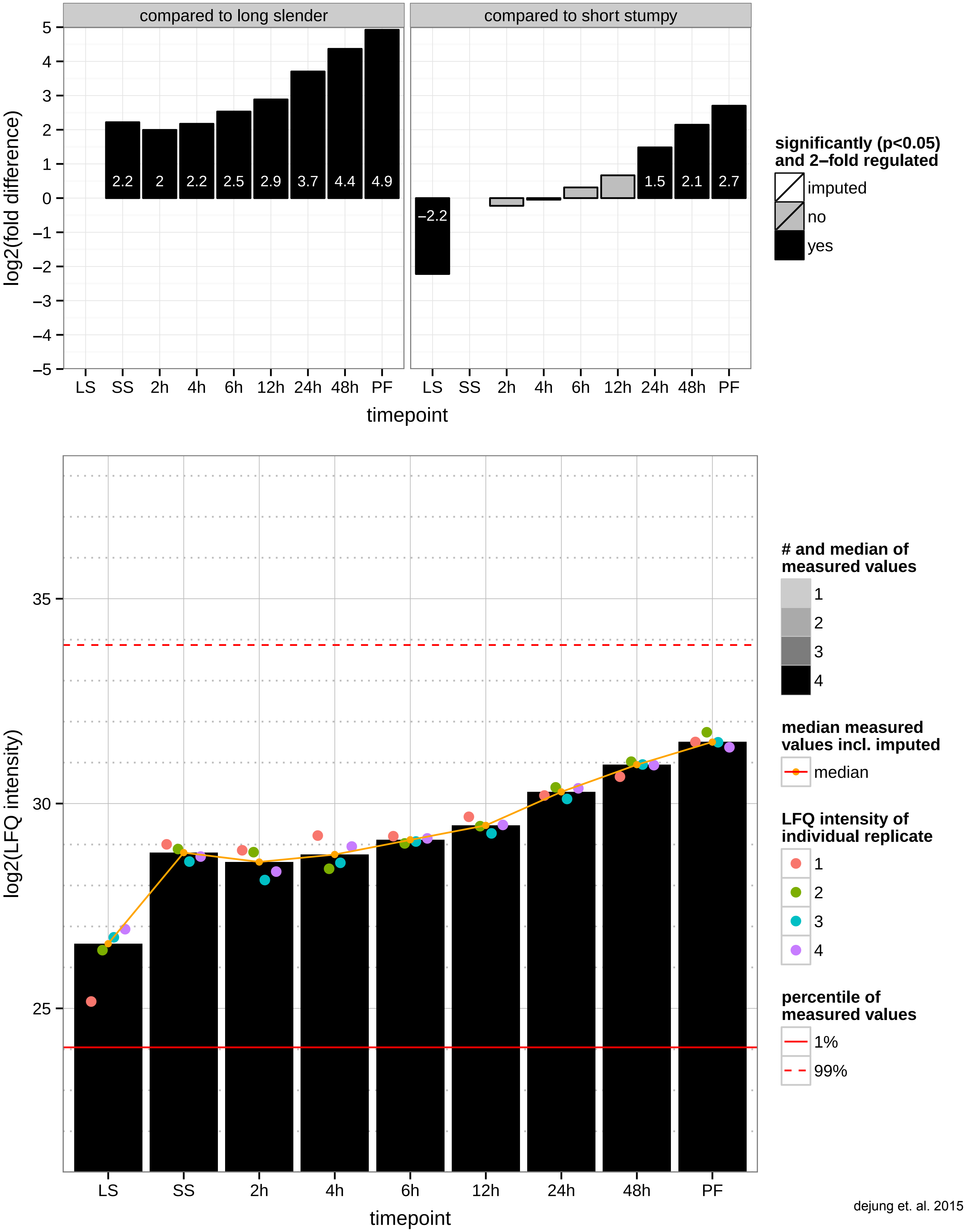

dejung et. al. 2015

NADH–cytochrome b5 reductase, putative
Tb927.7.2710
AGOF: electron carrier activity, oxidoreductase activity
AGOC: null
AGOP: null
PGOF: oxidoreductase activity
PGOC: null
PGOP: oxidation–reduction process

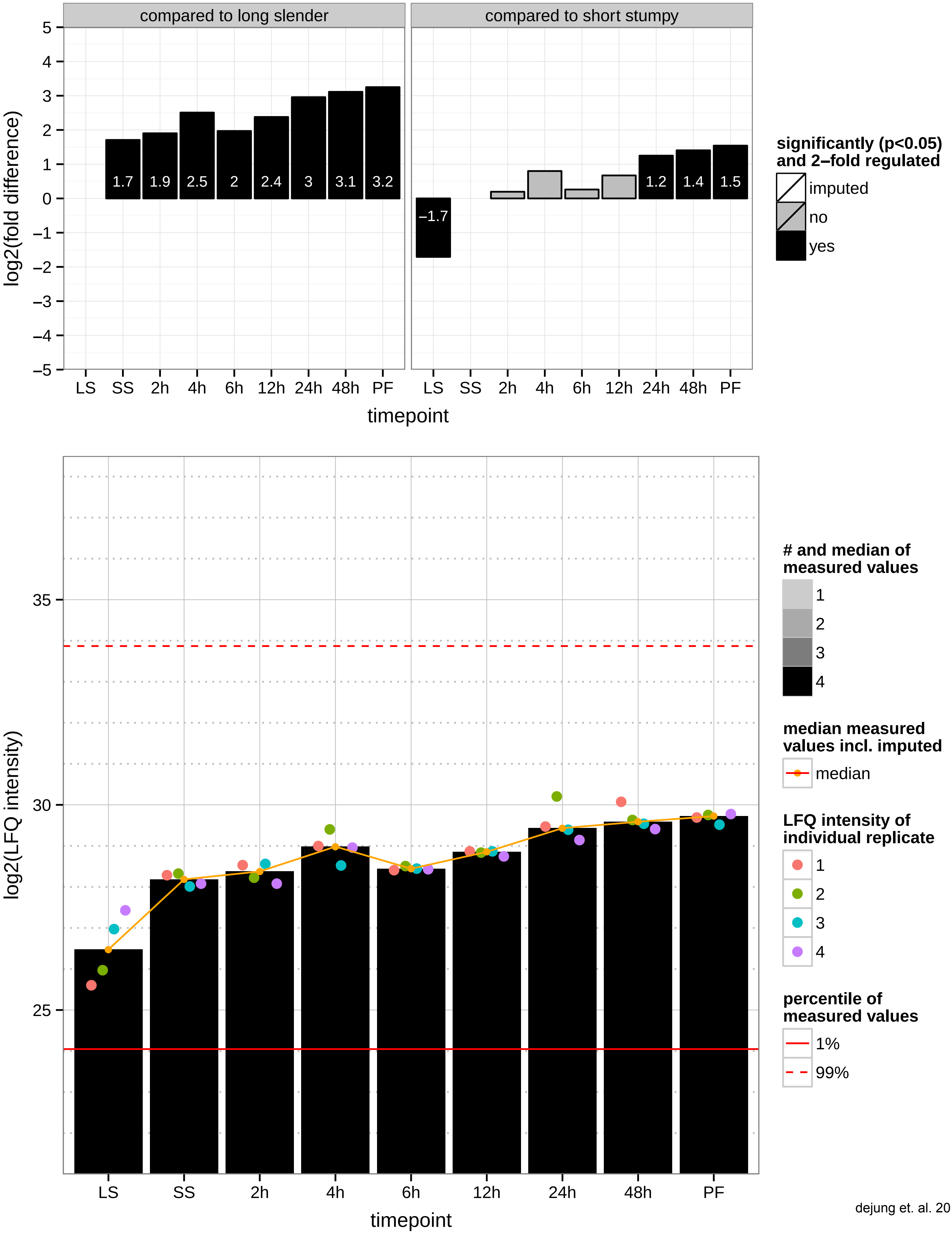

dejung et. al. 2015

cyclophilin–type peptidyl–prolyl cis–trans isomerase, putative (PPIase)
Tb927.7.280
AGOF: peptidyl–prolyl cis–trans isomerase activity
AGOC: null
AGOP: protein folding
PGOF: peptidyl–prolyl cis–trans isomerase activity
PGOC: null
PGOP: protein folding

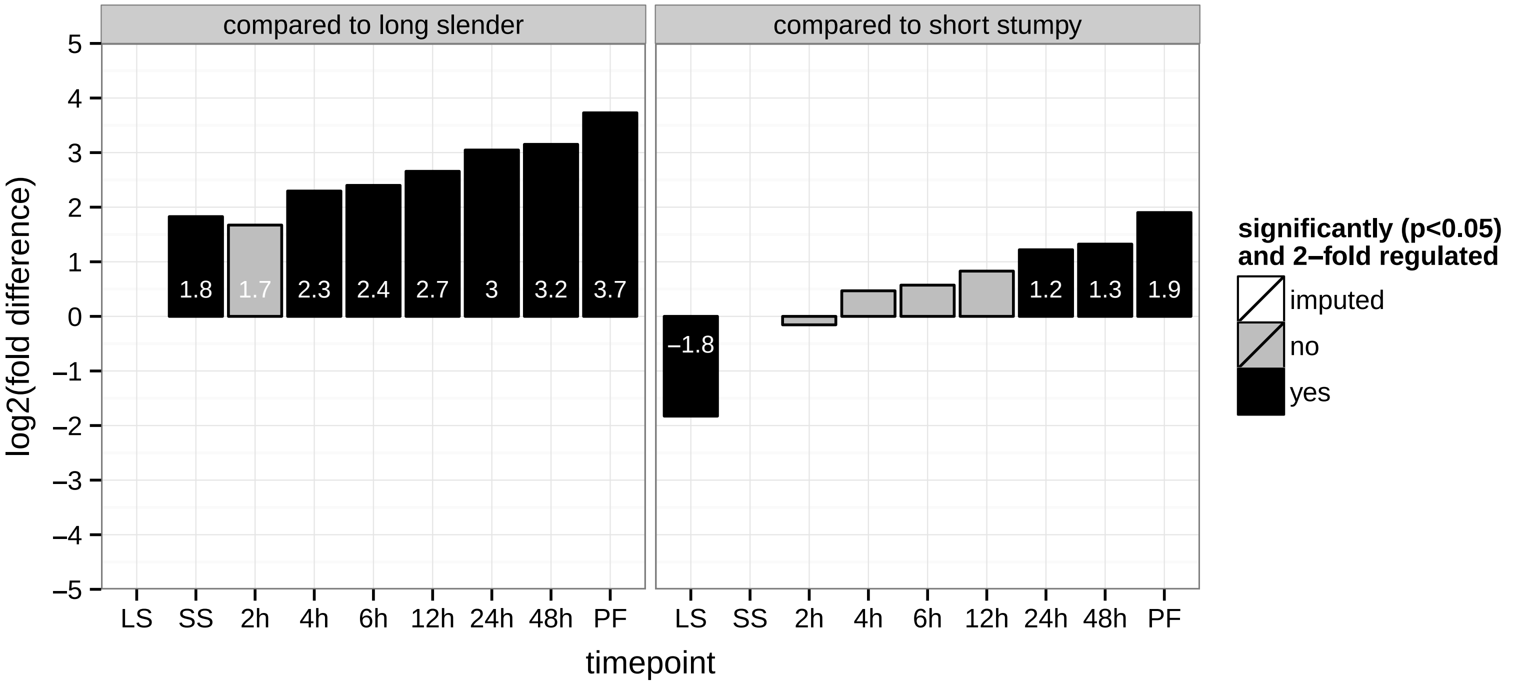

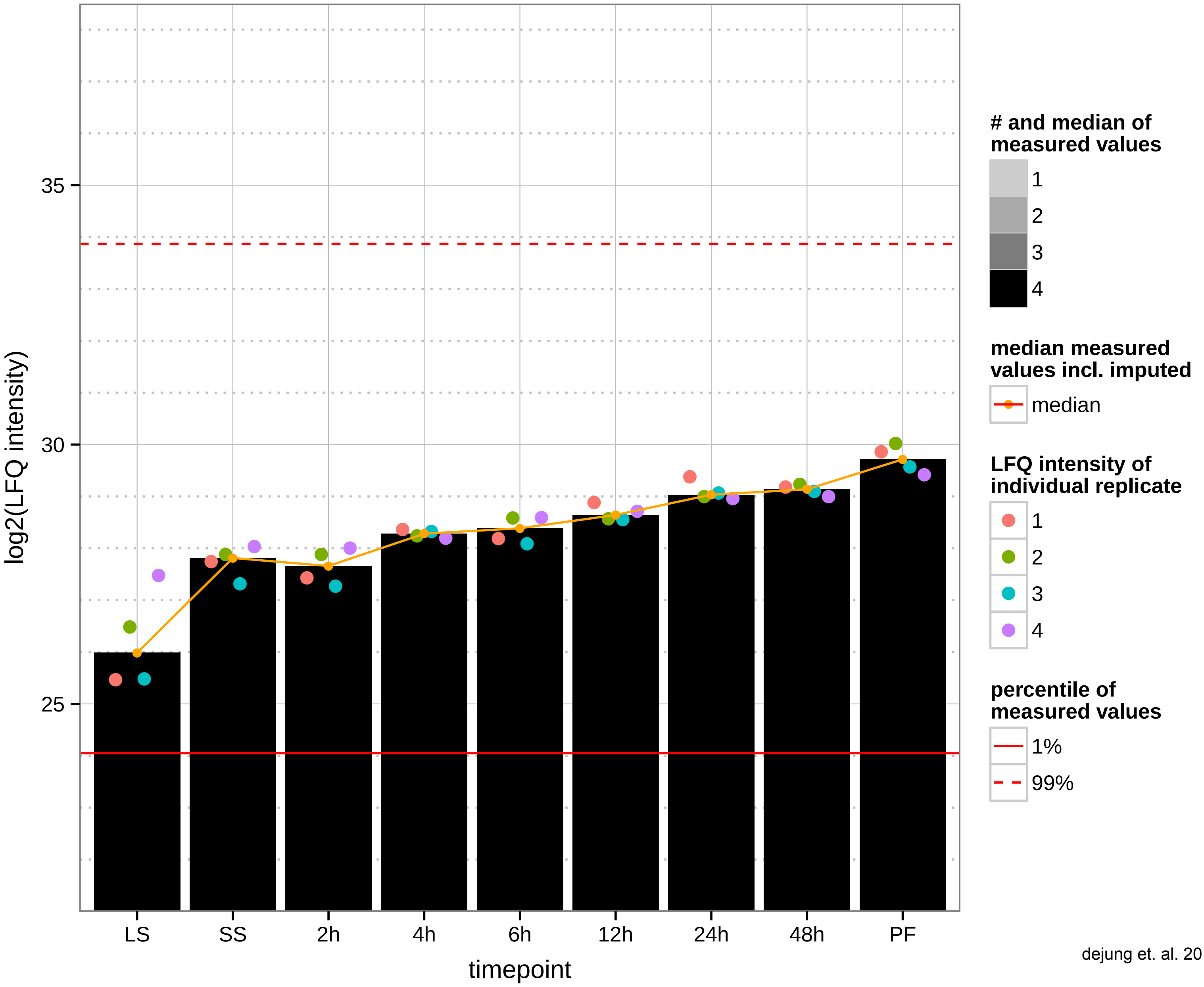

dejung et. al. 2015

hypothetical protein, conserved
Tb927.7.3590
AGOF: null
AGOC: mitochondrion
AGOP: null
PGOF: null
PGOC: null
PGOP: null

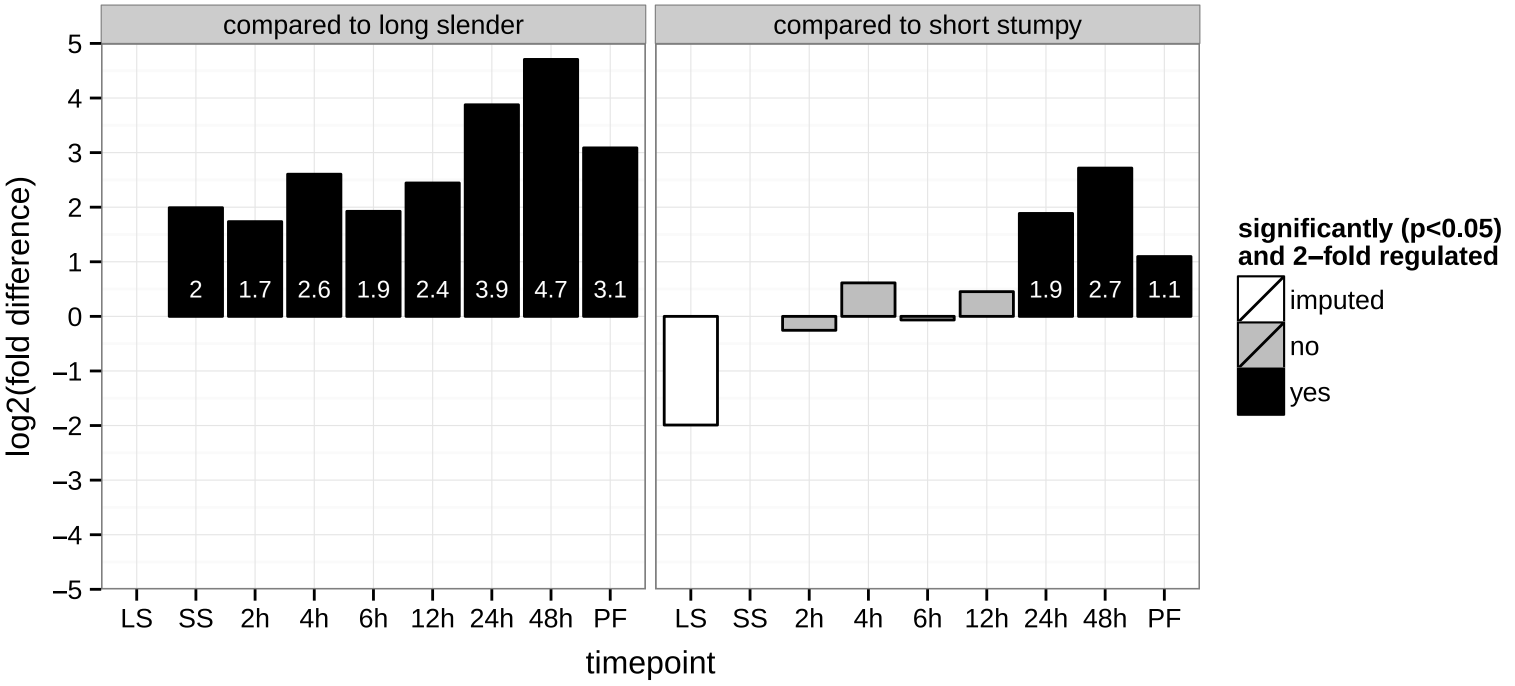

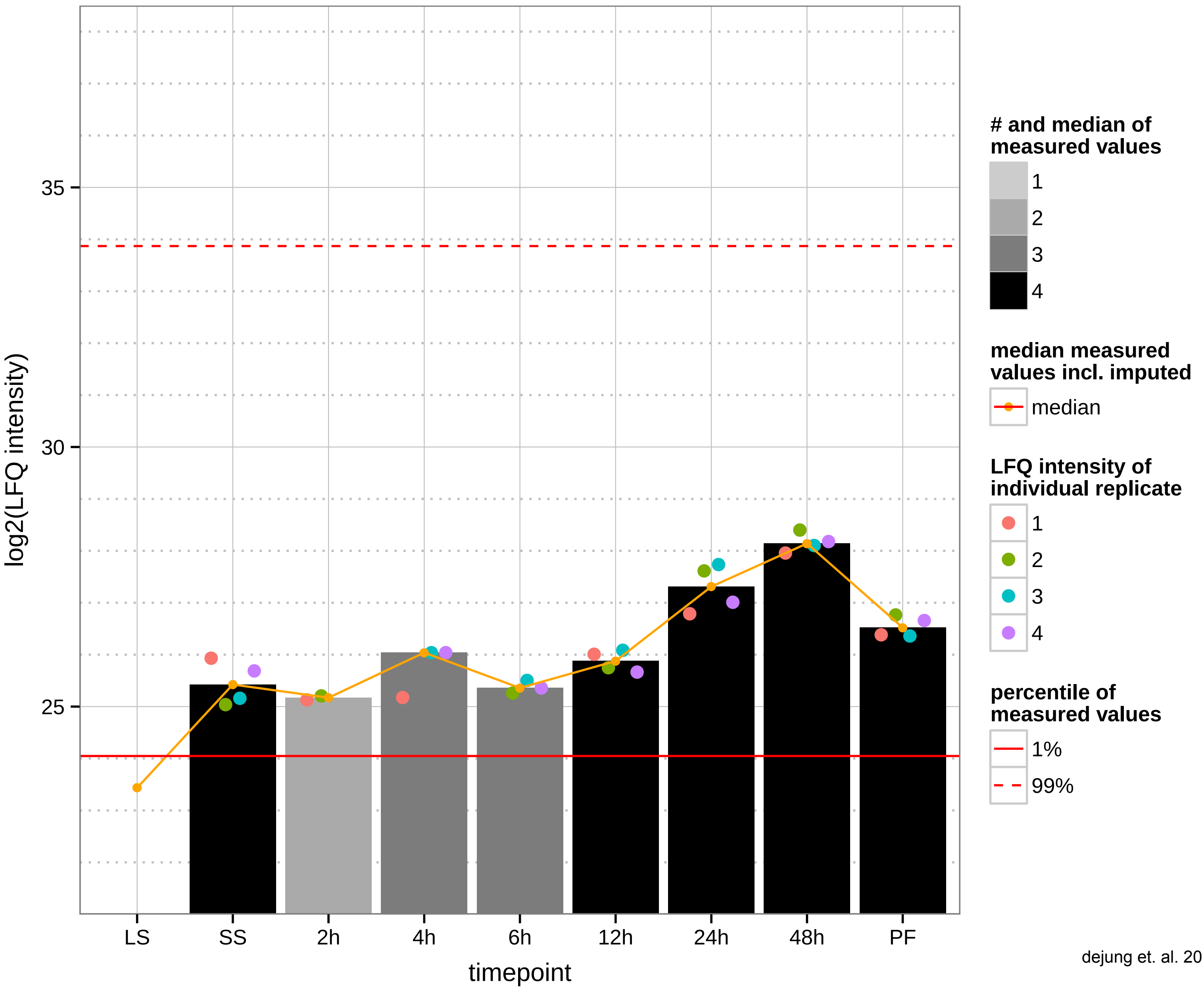

dejung et. al. 2015

mitochondrial carrier protein, ADP/ATP mitochondrial translocase, putative, adenine nucleotide mitochondrial translocator, put
Tb927.7.3940
AGOF: null
AGOC: mitochondrial inner membrane, mitochondrial part
AGOP: transport
PGOF: null
PGOC: null
PGOP: null

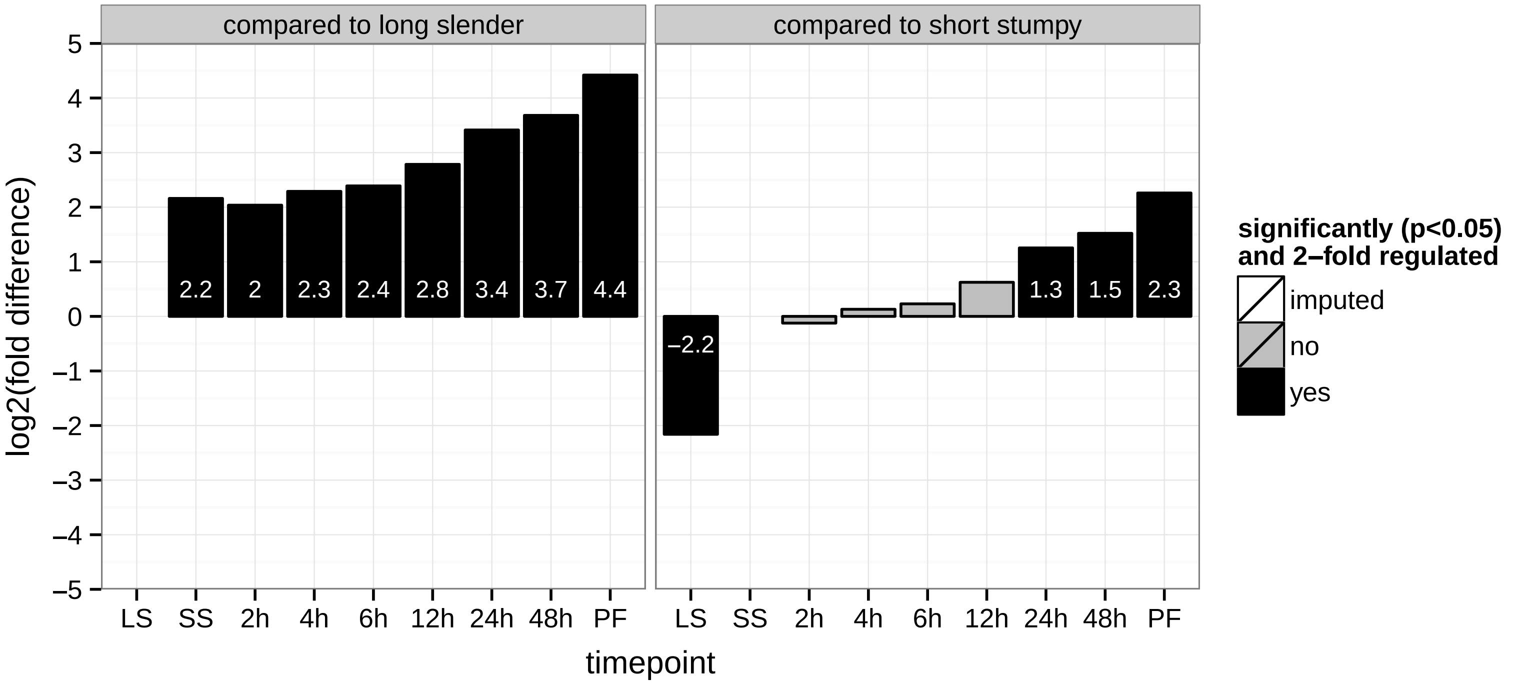

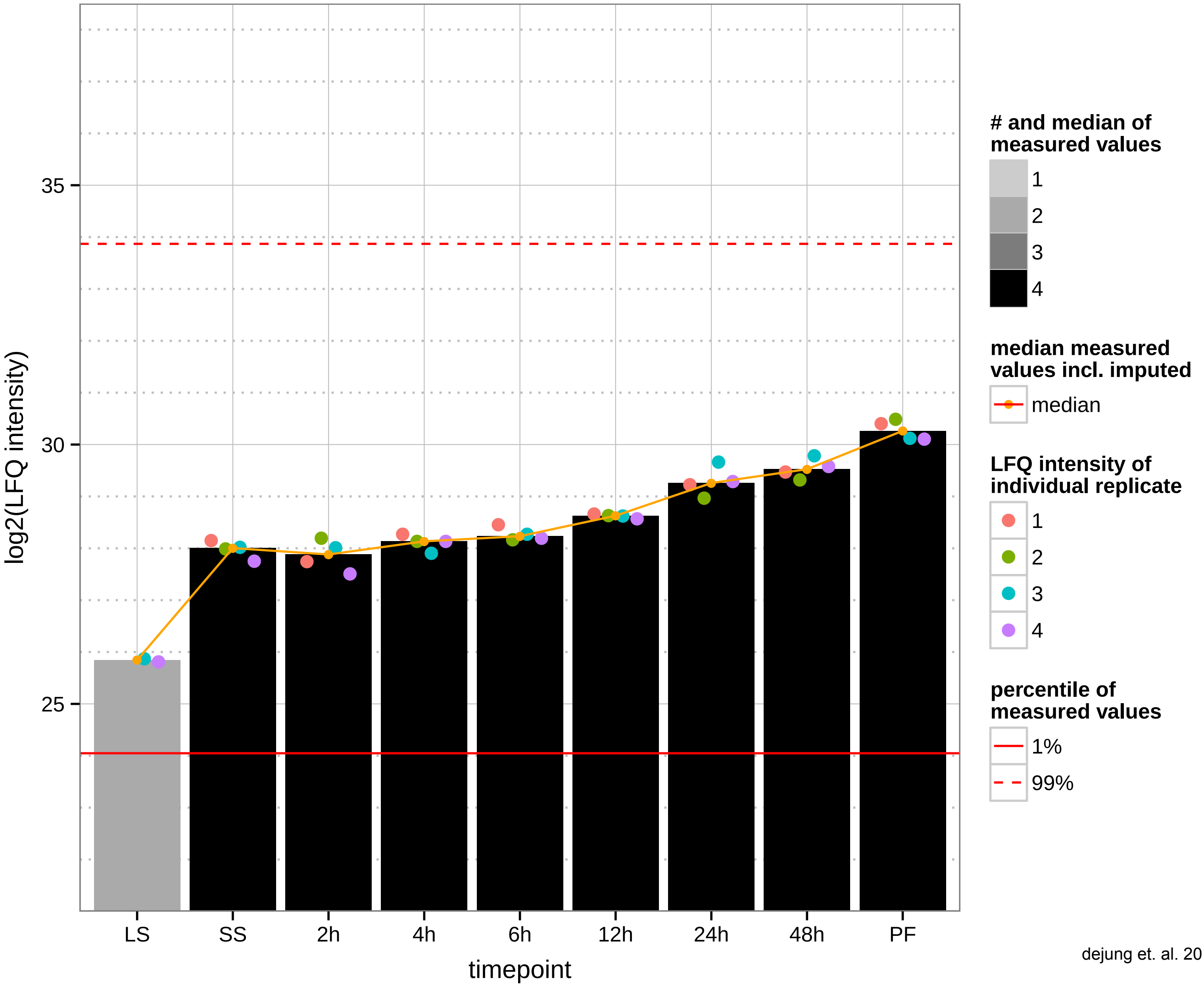

dejung et. al. 2015

deoxyuridine triphosphatase, putative, dUTP diphosphatase
Tb927.7.5160
AGOF: null
AGOC: null
AGOP: null
PGOF: null
PGOC: null
PGOP: null

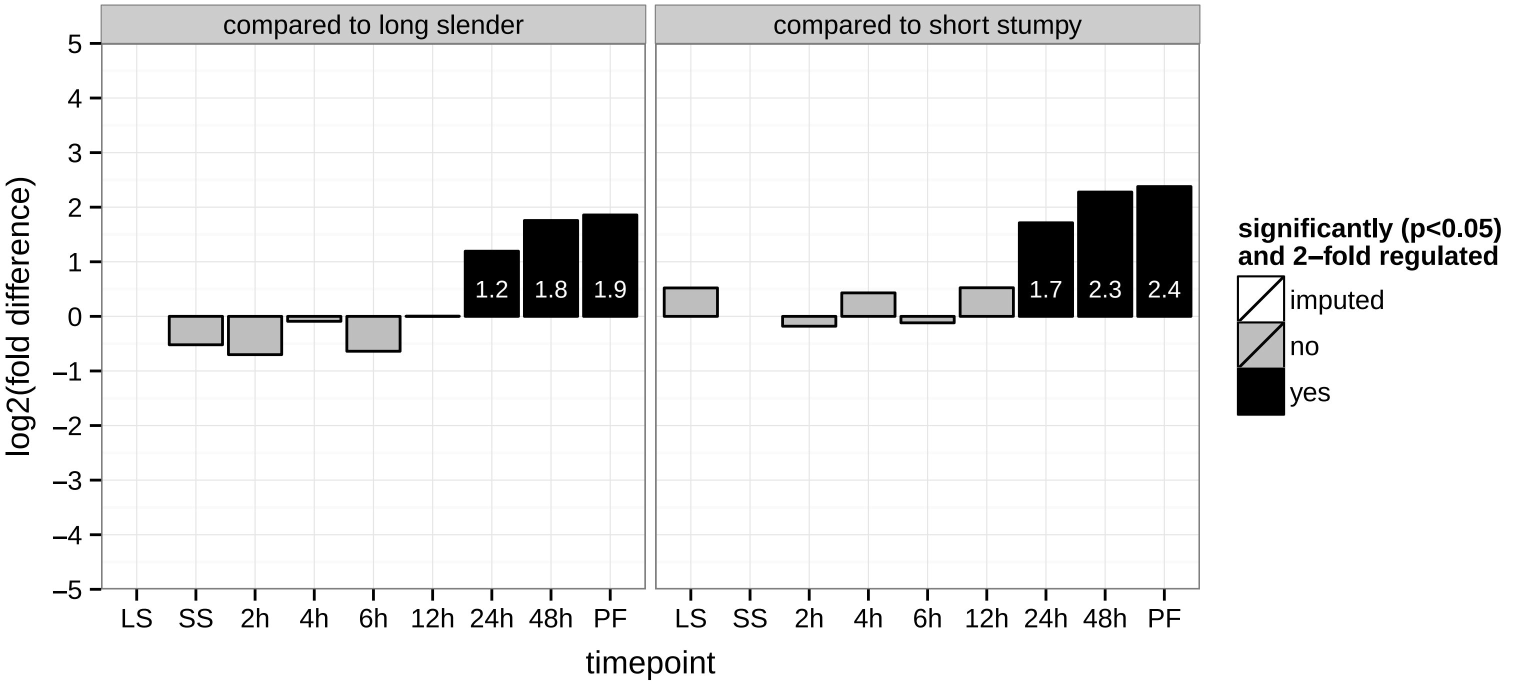

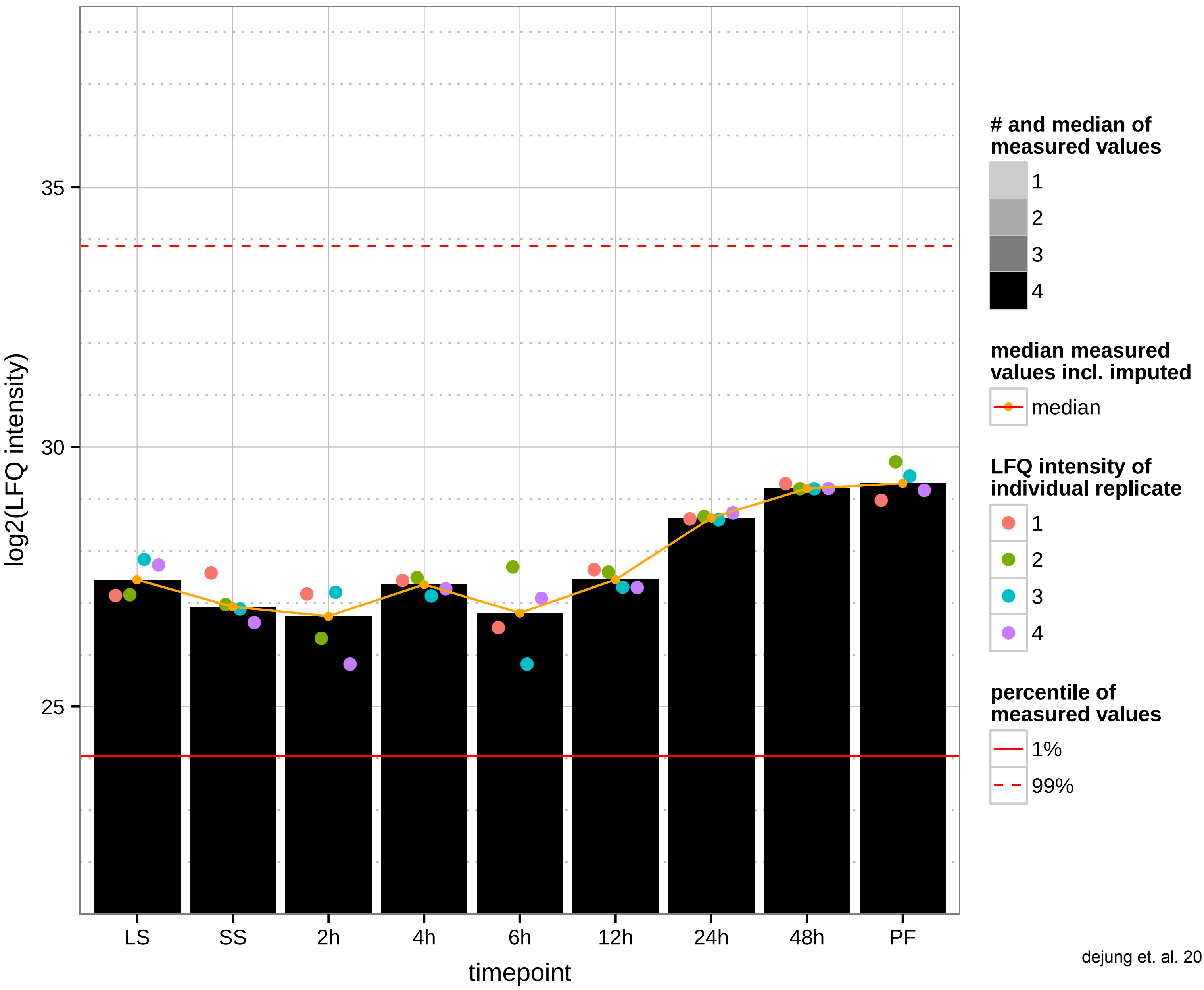

dejung et. al. 2015

hypothetical protein, conserved
Tb927.7.6670
AGOF: null
AGOC: null
AGOP: null
PGOF: null
PGOC: null
PGOP: null

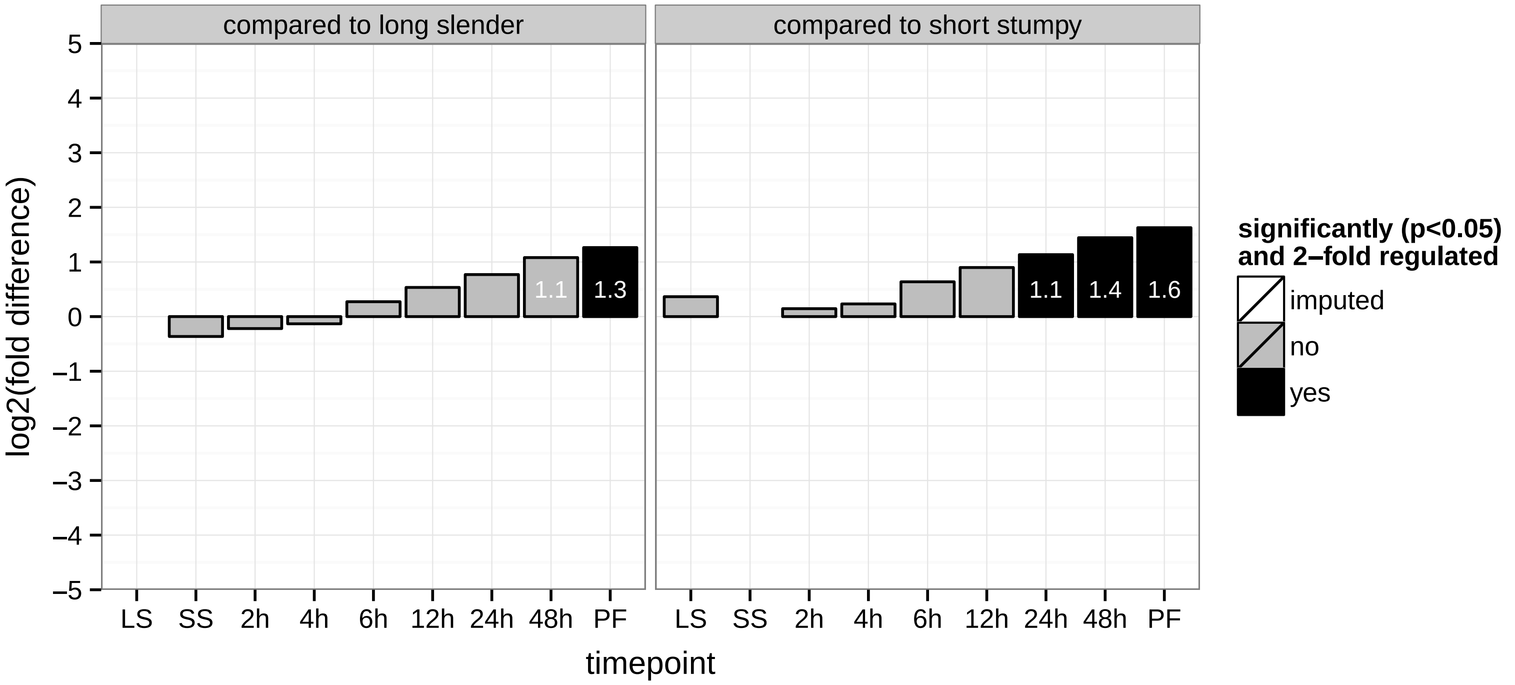

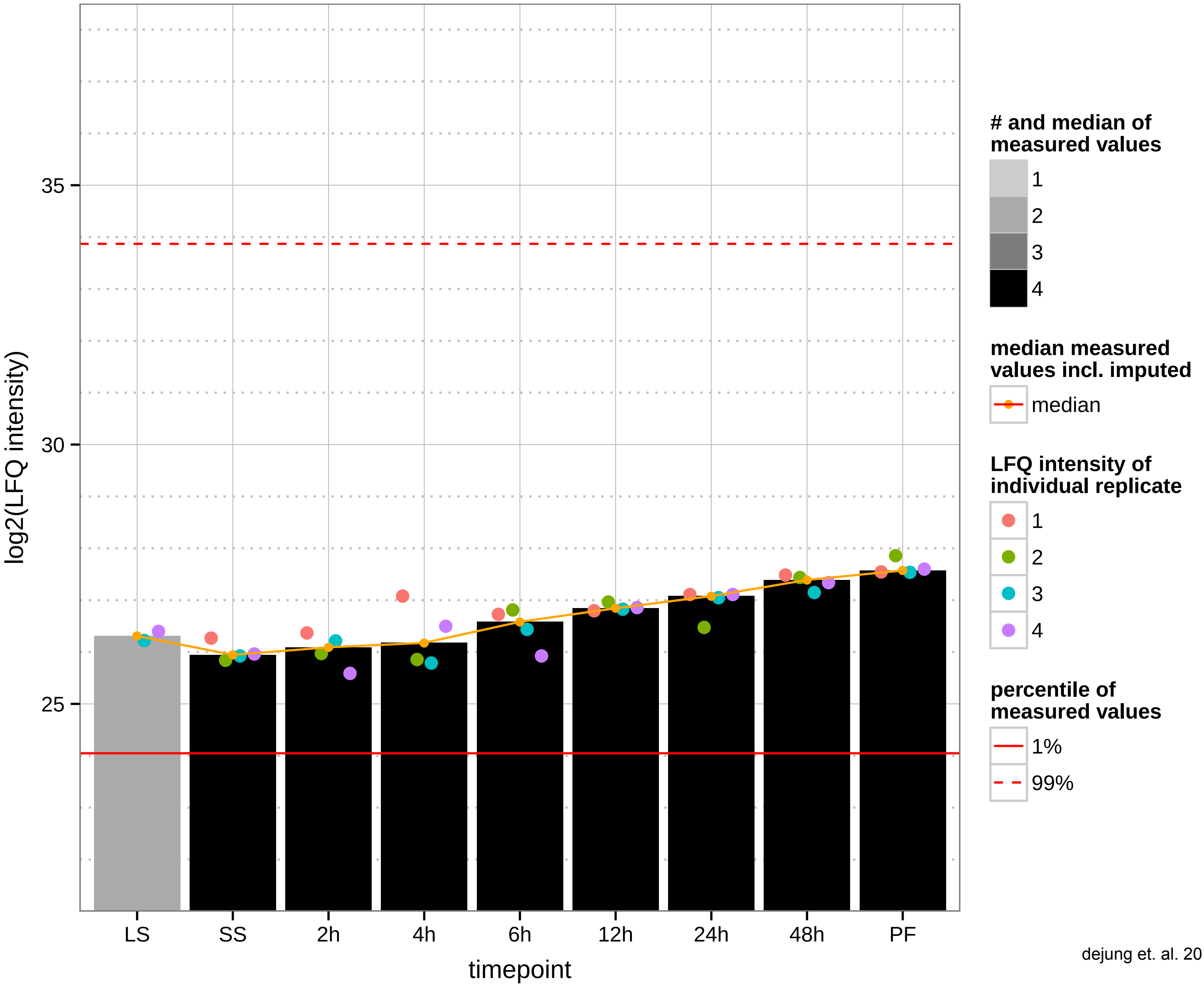

dejung et. al. 2015

hypothetical protein, conserved
Tb927.7.6800
AGOF: null
AGOC: mitochondrion
AGOP: null
PGOF: null
PGOC: null
PGOP: null

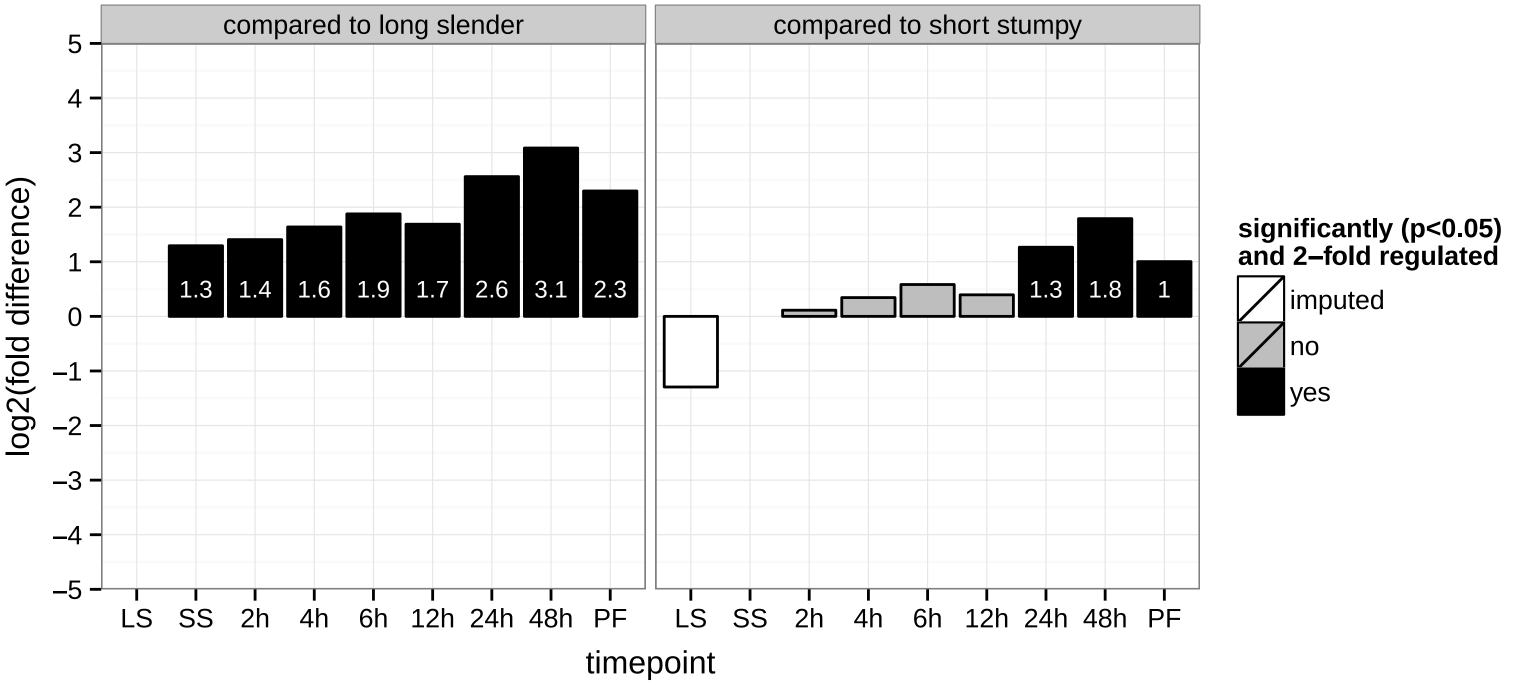

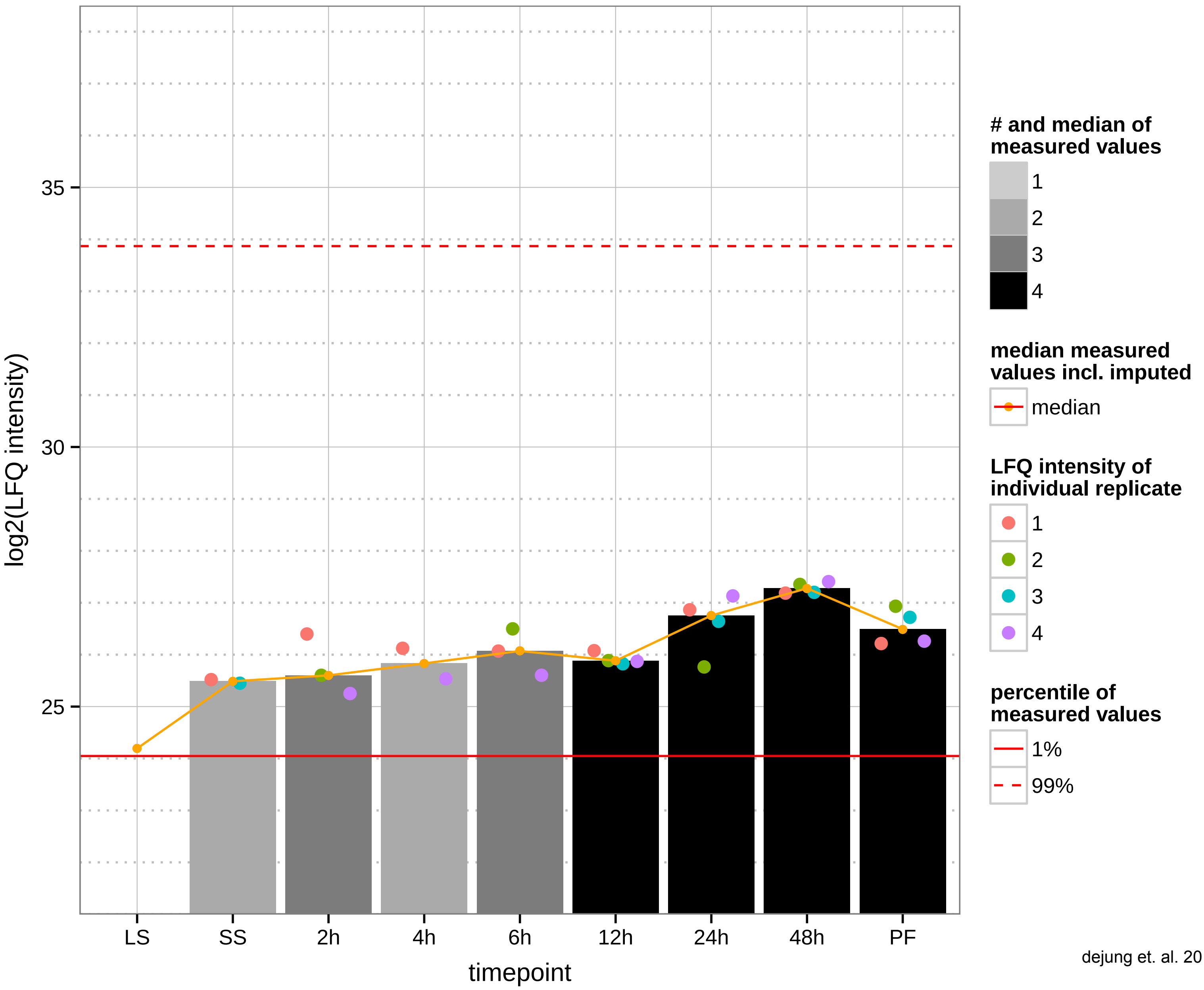

dejung et. al. 2015

hypothetical protein, conserved
Tb927.7.7090
AGOF: null
AGOC: mitochondrion
AGOP: null
PGOF: null
PGOC: null
PGOP: null

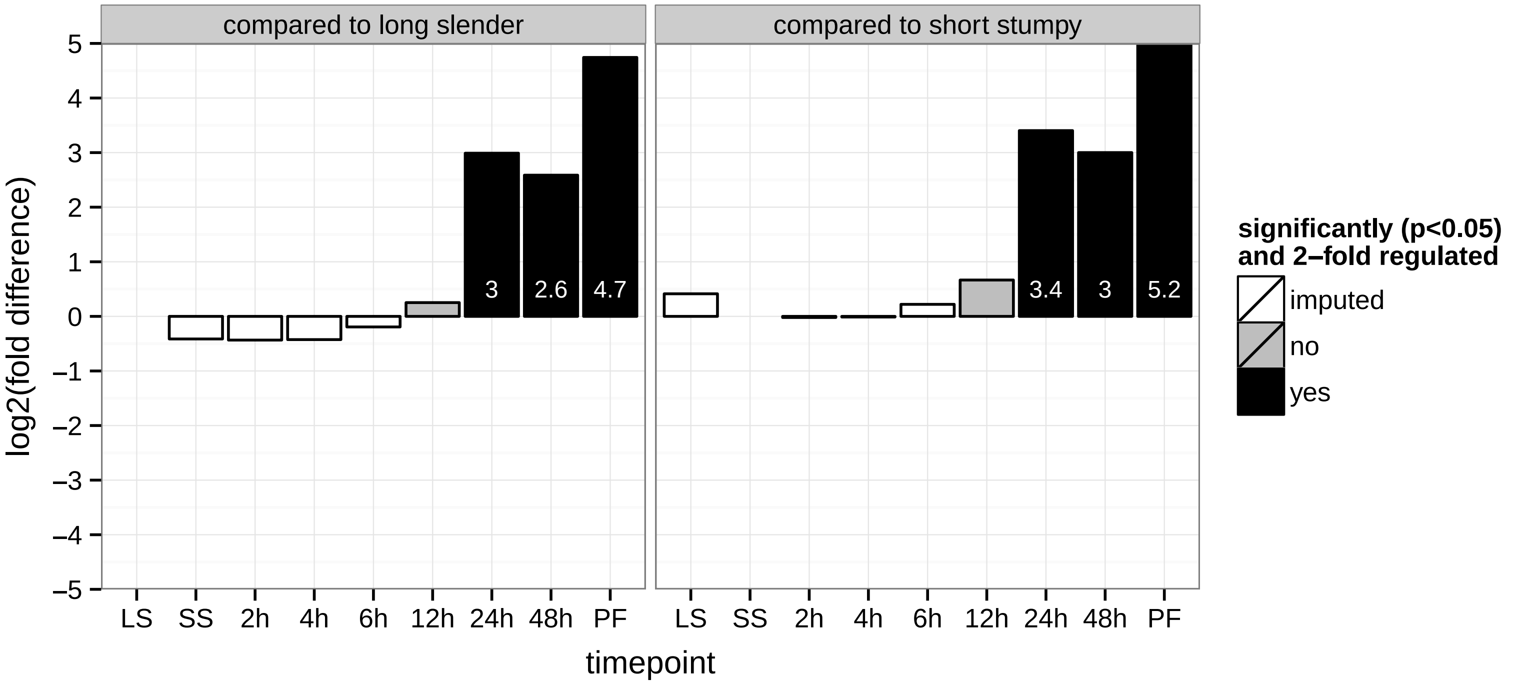

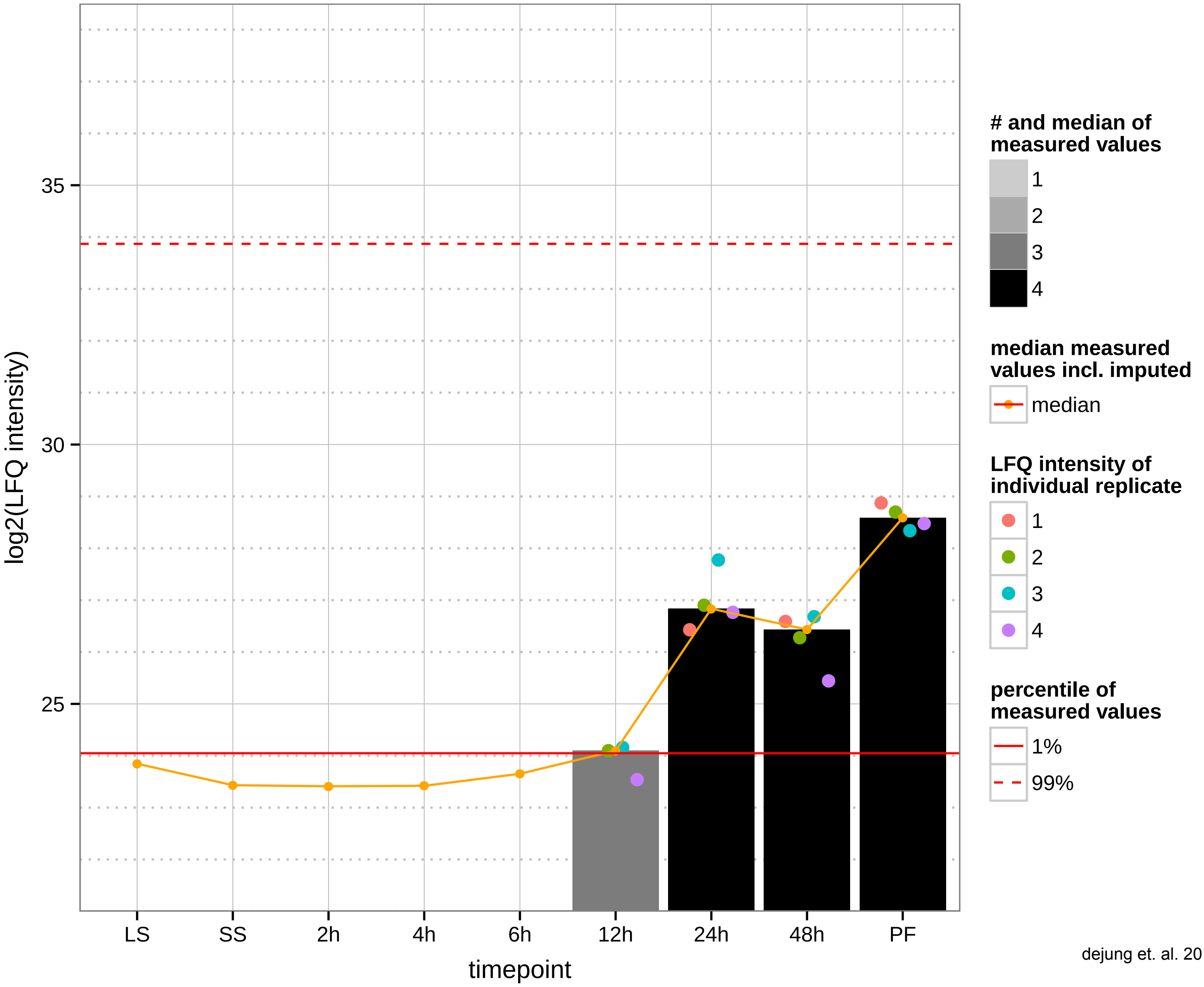

dejung et. al. 2015

hypothetical protein, conserved
Tb927.7.730
AGOF: null
AGOC: null
AGOP: ciliary or flagellar motility
PGOF: protein binding
PGOC: null
PGOP: null

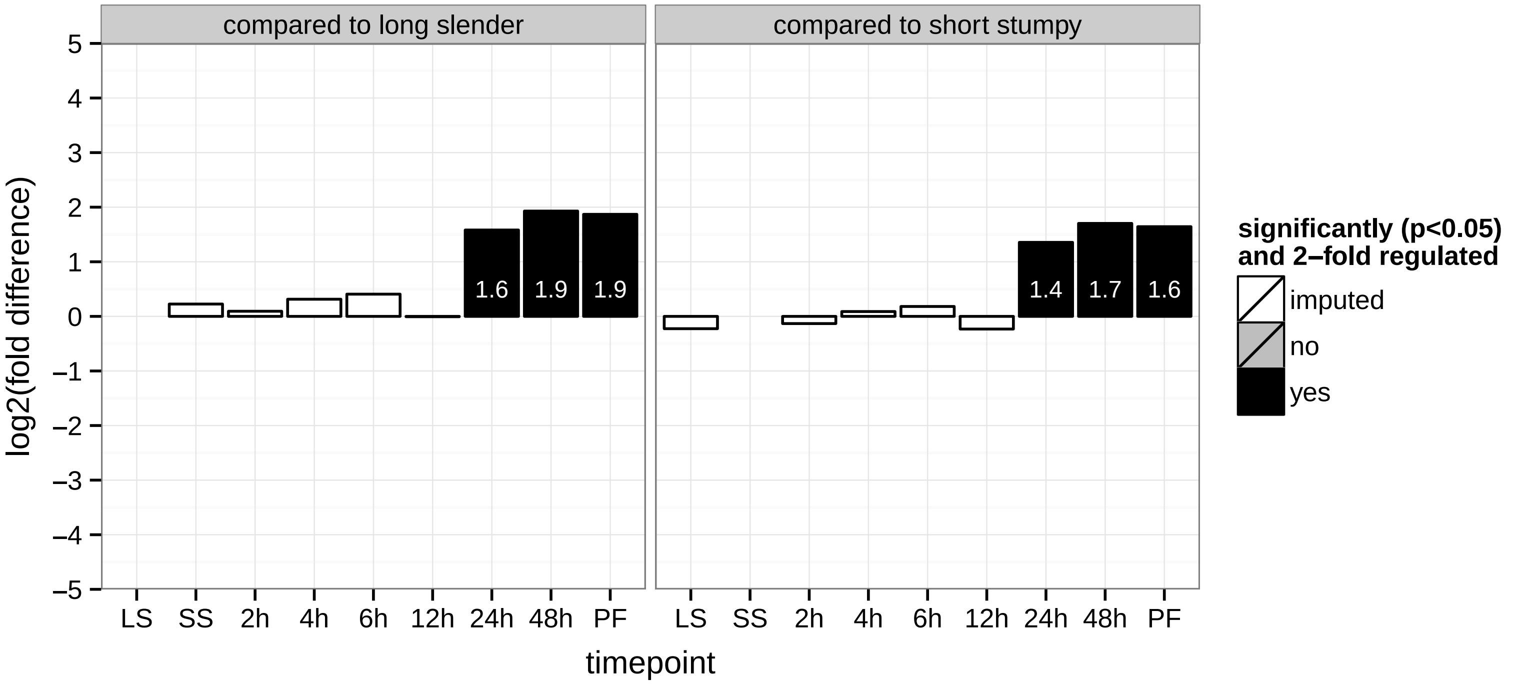

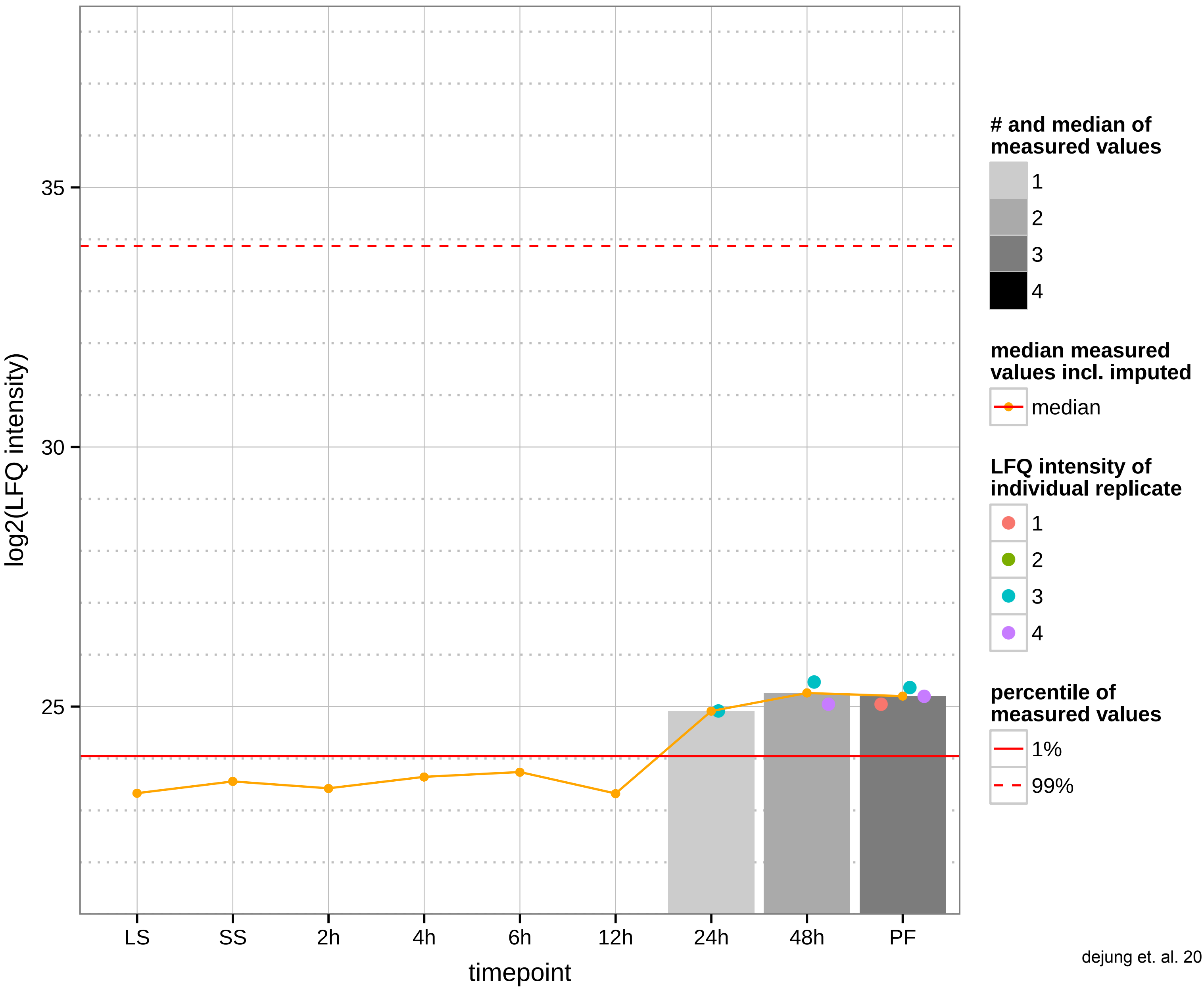

dejung et. al. 2015

ATP synthase alpha chain, mitochondrial precursor, ATP synthase F1, alpha subunit
Tb927.7.7430;Tb927.7.7420
AGOF: hydrogen ion transporting ATP synthase activity, rotational mechanism, proton−transporting ATPase activity
AGOC: mitochondrial inner membrane, mitochondrion, proton−transporting ATP synthase complex, catalytic core F(1), proton
AGOP: ATP synthesis coupled proton transport
PGOF: ATP binding, hydrogen ion transporting ATP synthase activity, rotational mechanism, hydrolase activity, acting on acid
PGOC: proton−transporting ATP synthase complex, catalytic core F(1), proton−transporting two−sector ATPase complex, cata
PGOP: ATP hydrolysis coupled proton transport, ATP synthesis coupled proton transport

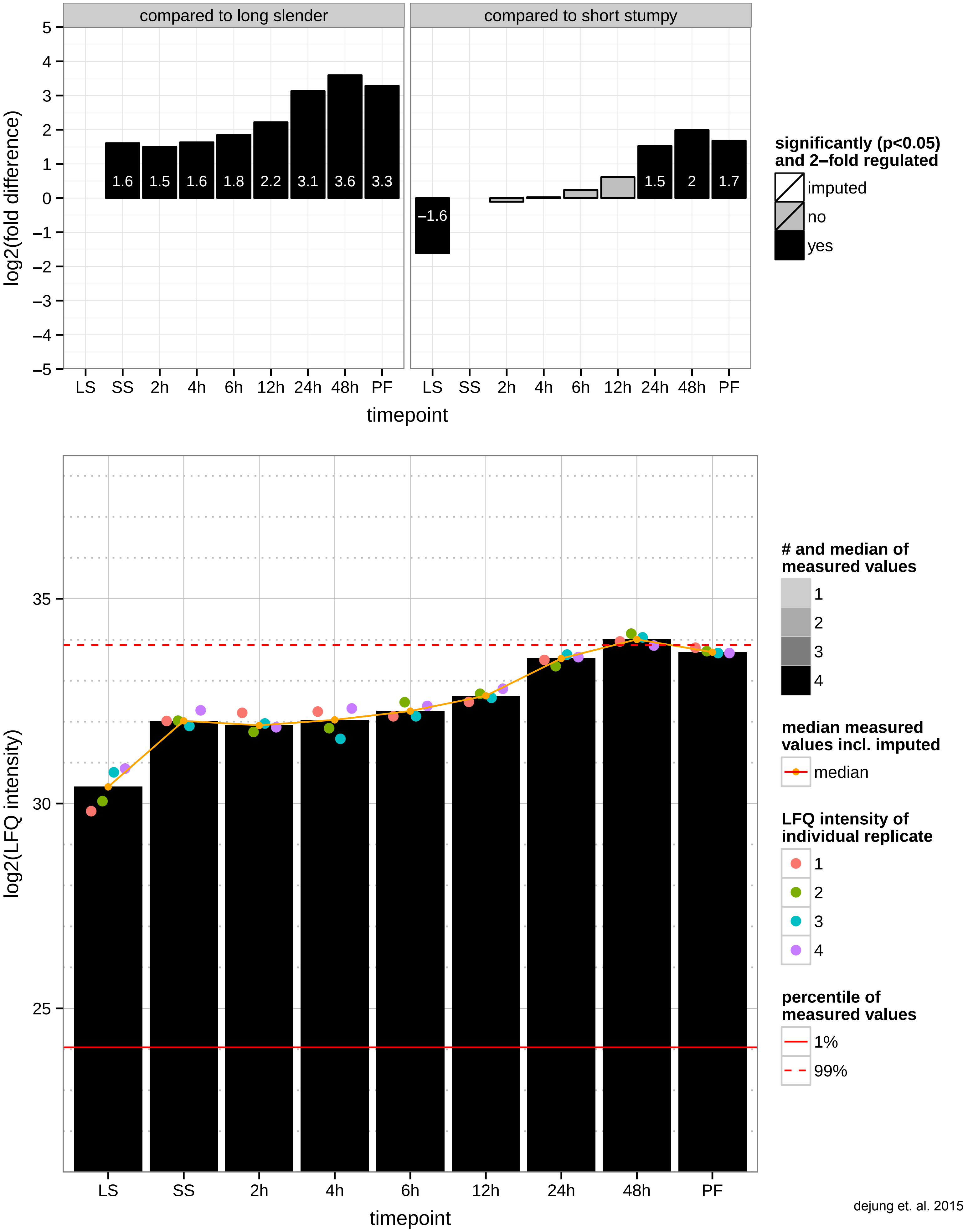

dejung et. al. 2015

hypothetical protein, conserved
Tb927.7.7460
AGOF: null
AGOC: null
AGOP: null
PGOF: null
PGOC: null
PGOP: null

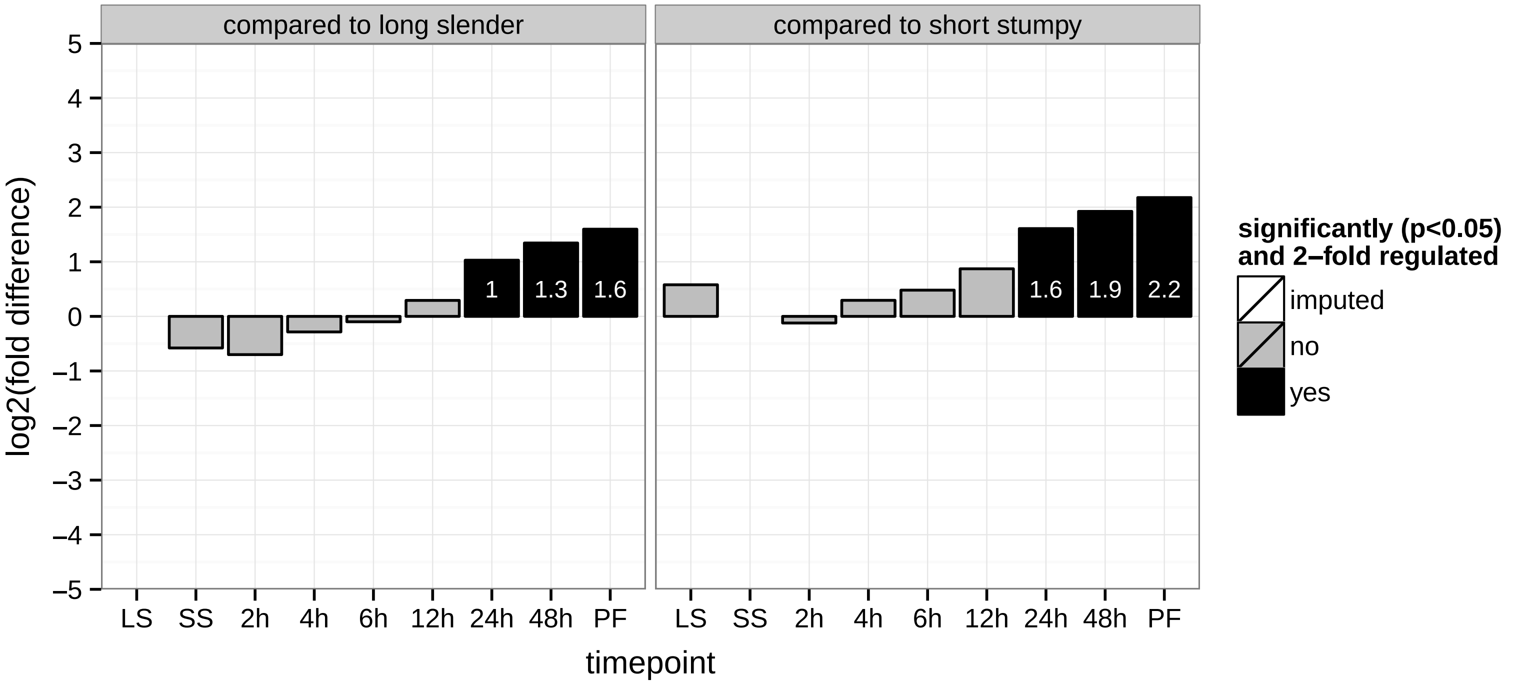

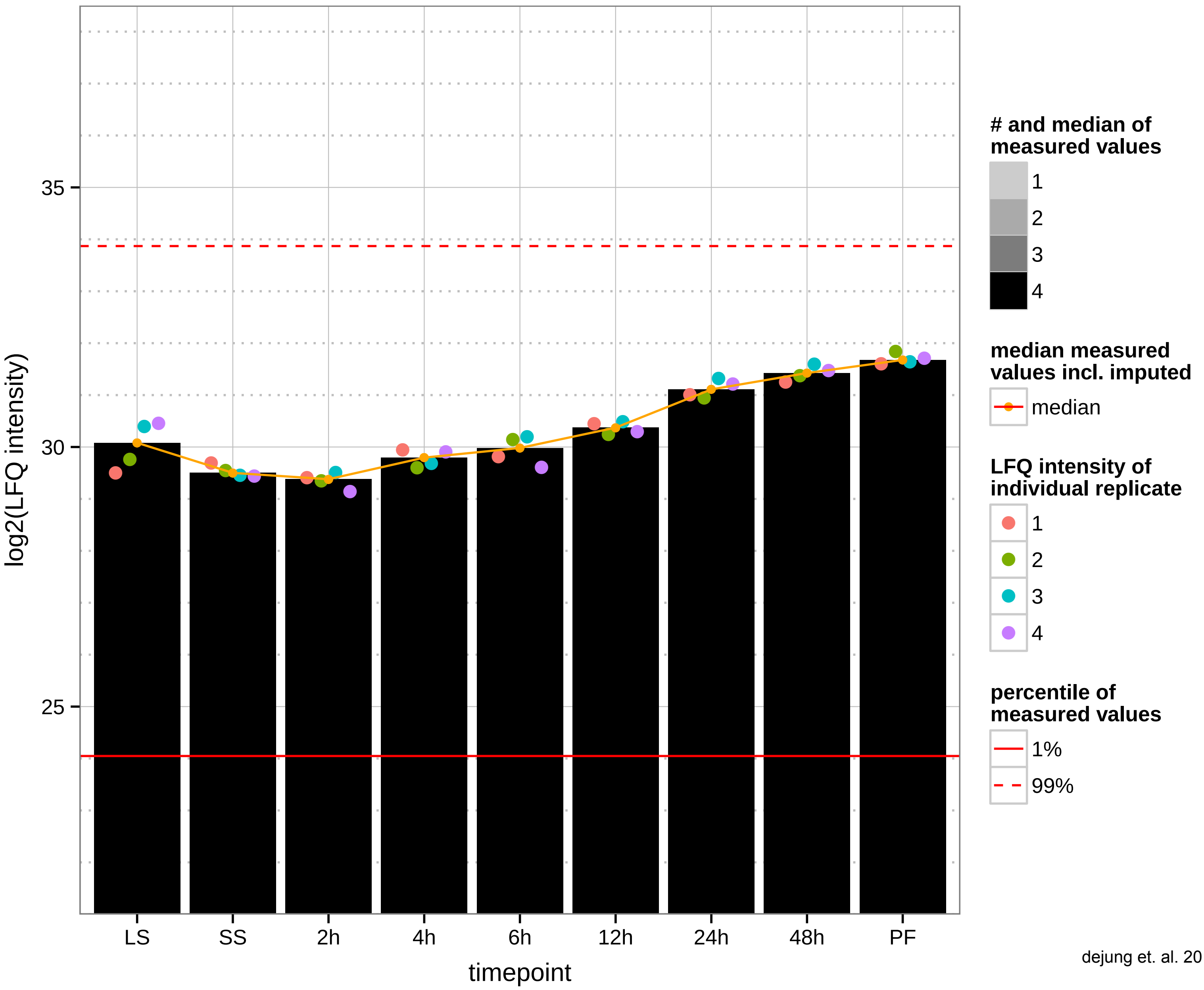

dejung et. al. 2015

hypothetical protein, conserved
Tb927.7.840
AGOF: null
AGOC: mitochondrial inner membrane, mitochondrion
AGOP: null
PGOF: null
PGOC: null
PGOP: null

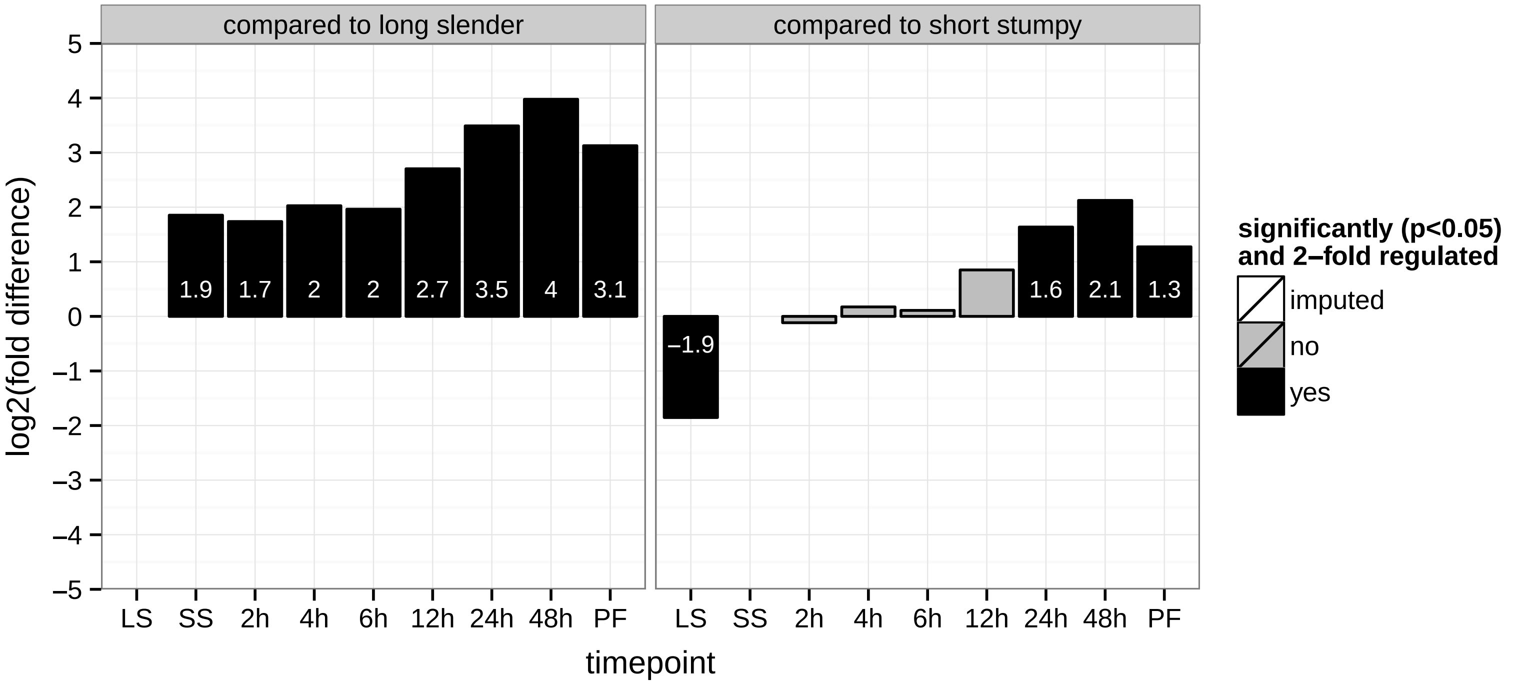

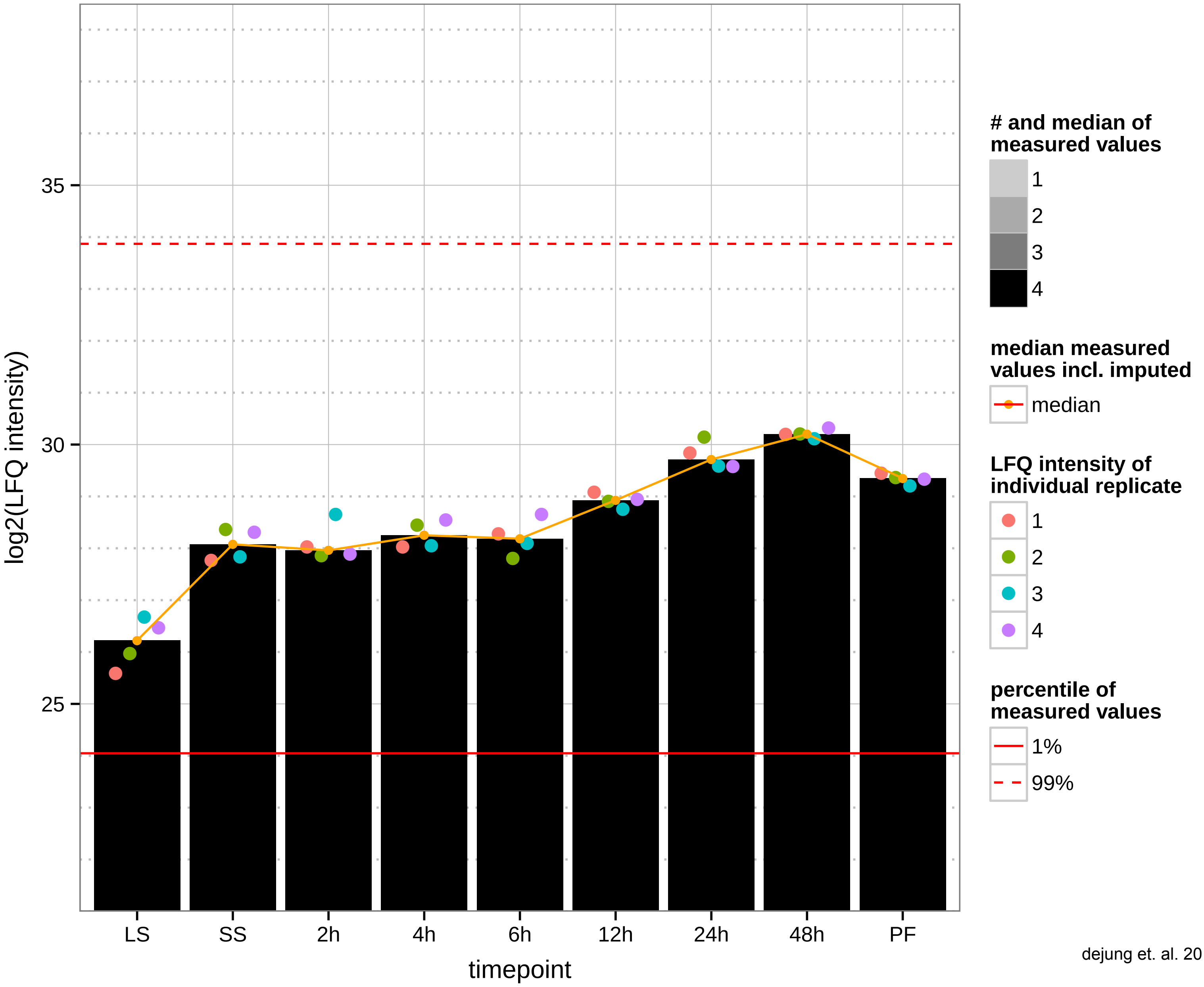

dejung et. al. 2015

hypothetical protein, conserved
Tb927.7.900
AGOF: null
AGOC: null
AGOP: null
PGOF: null
PGOC: null
PGOP: null

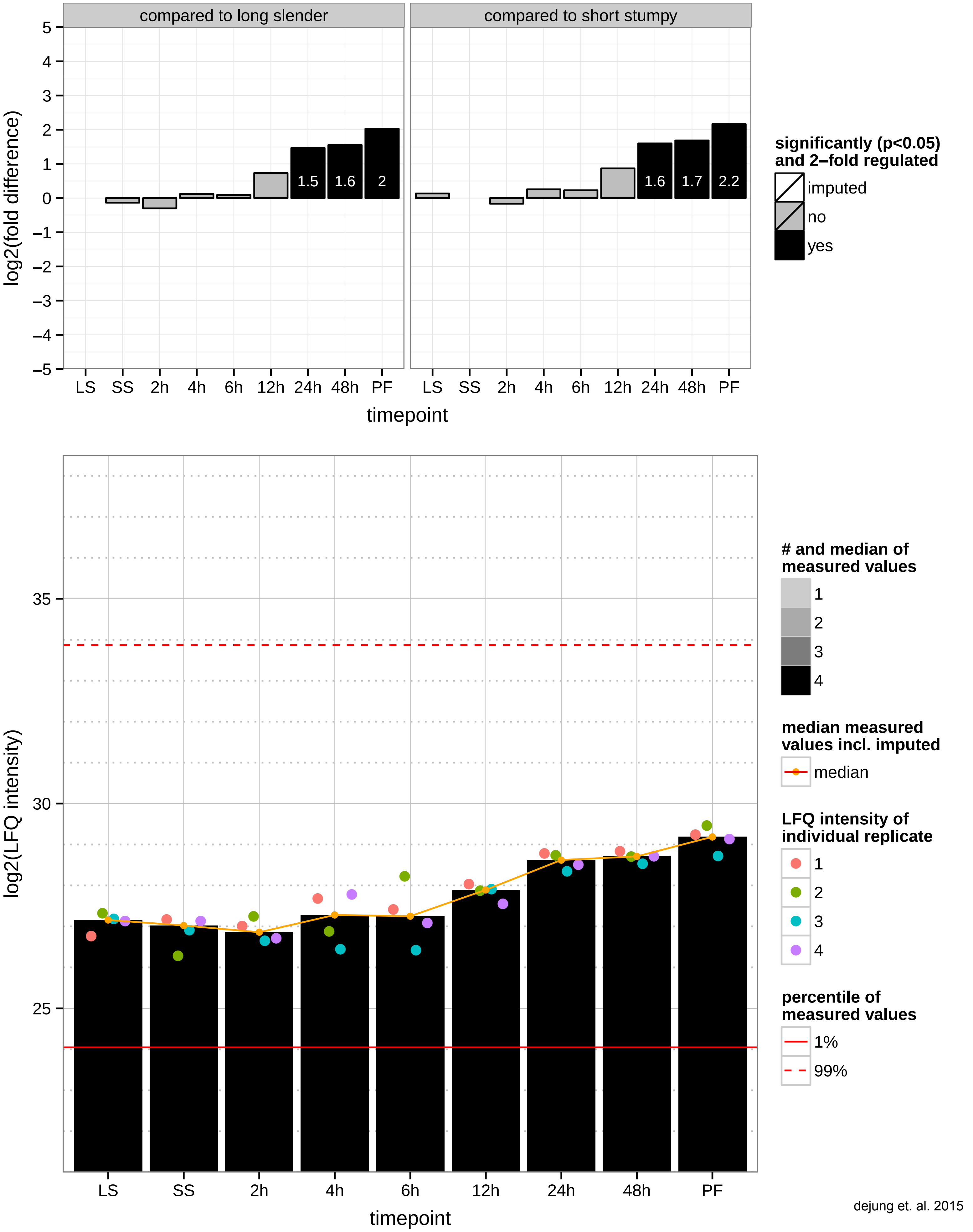

dejung et. al. 2015

hypothetical protein, conserved
Tb927.8.1500
AGOF: null
AGOC: null
AGOP: null
PGOF: null
PGOC: null
PGOP: null

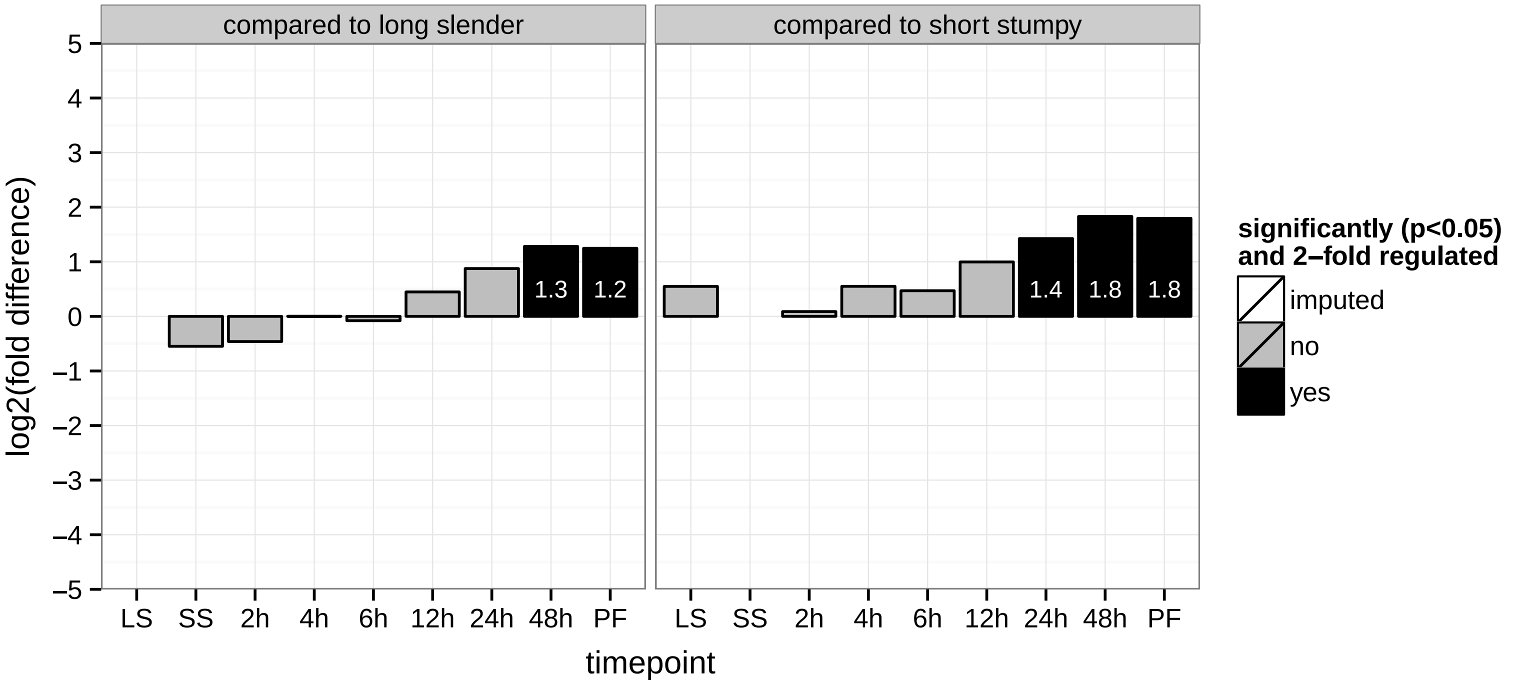

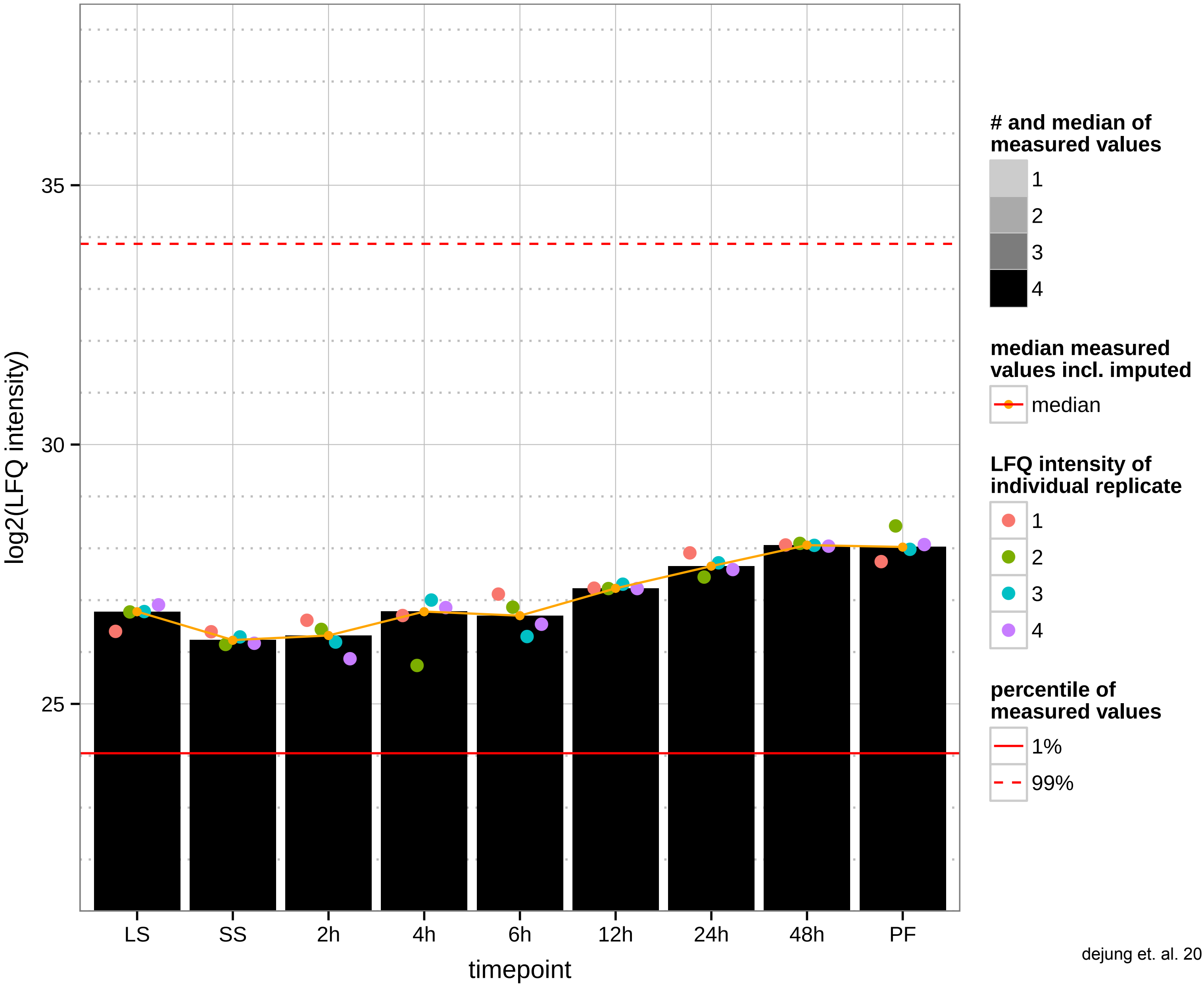

dejung et. al. 2015

hypothetical protein, conserved
Tb927.8.1790
AGOF: null
AGOC: null
AGOP: null
PGOF: null
PGOC: null
PGOP: null

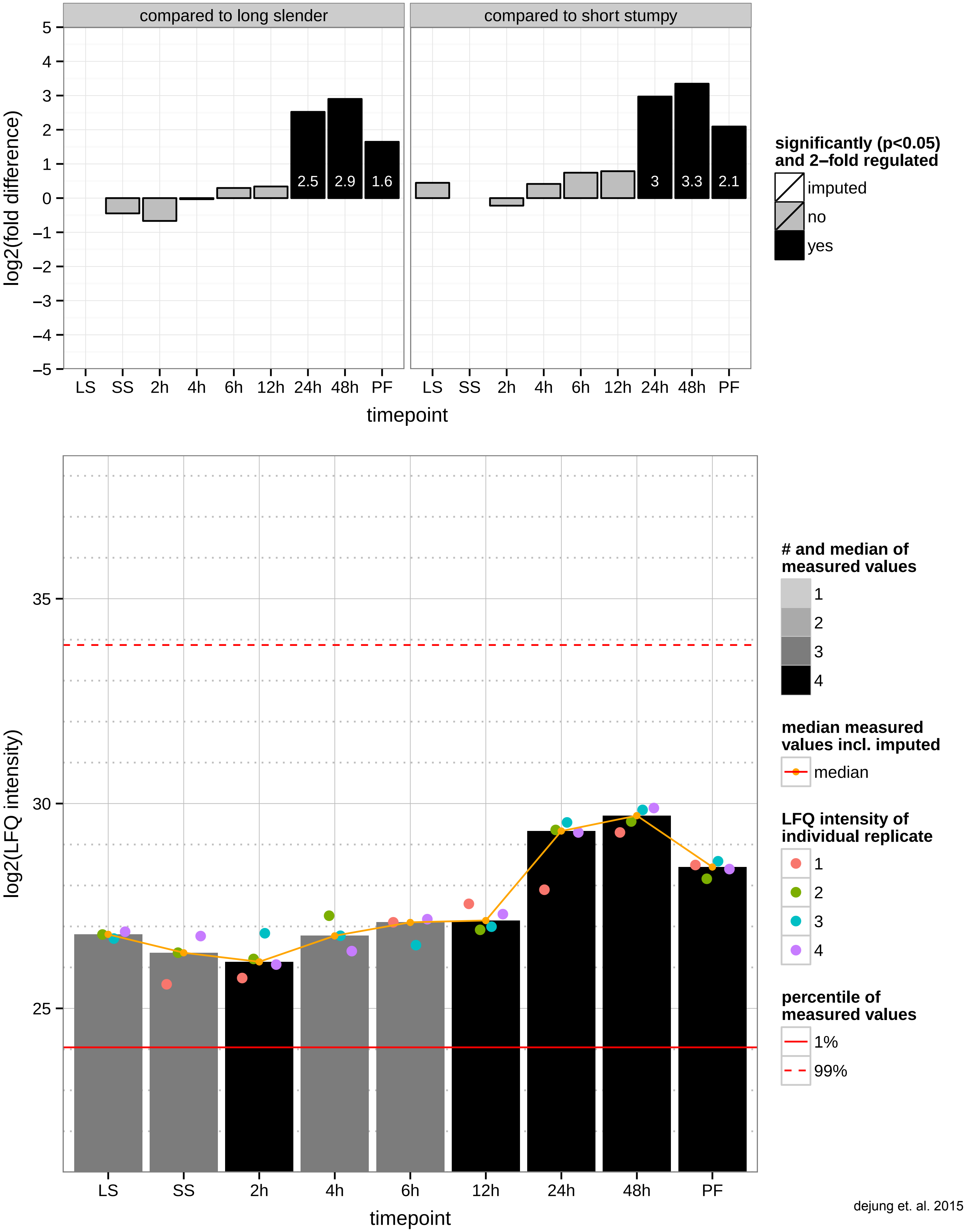

dejung et. al. 2015

hypothetical protein, conserved
Tb927.8.2030
AGOF: null
AGOC: null
AGOP: null
PGOF: null
PGOC: null
PGOP: null

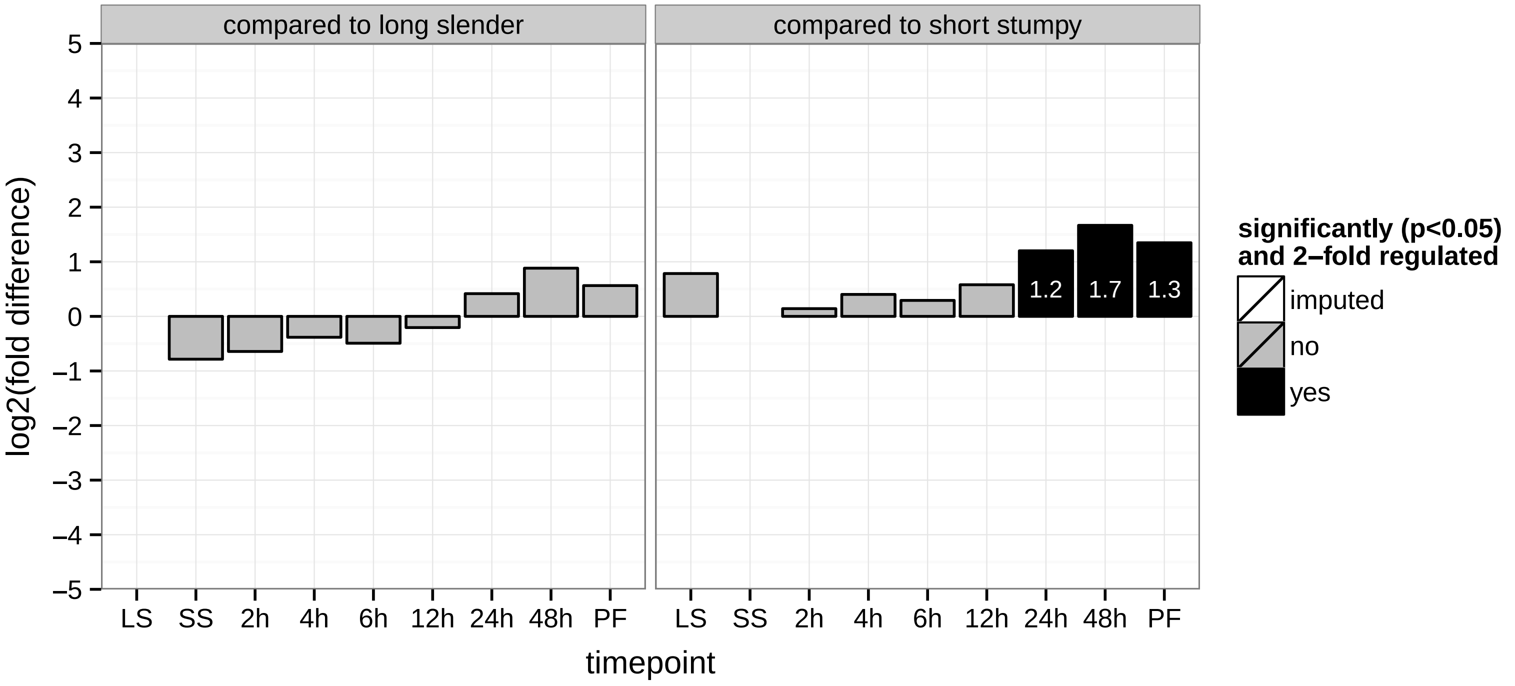

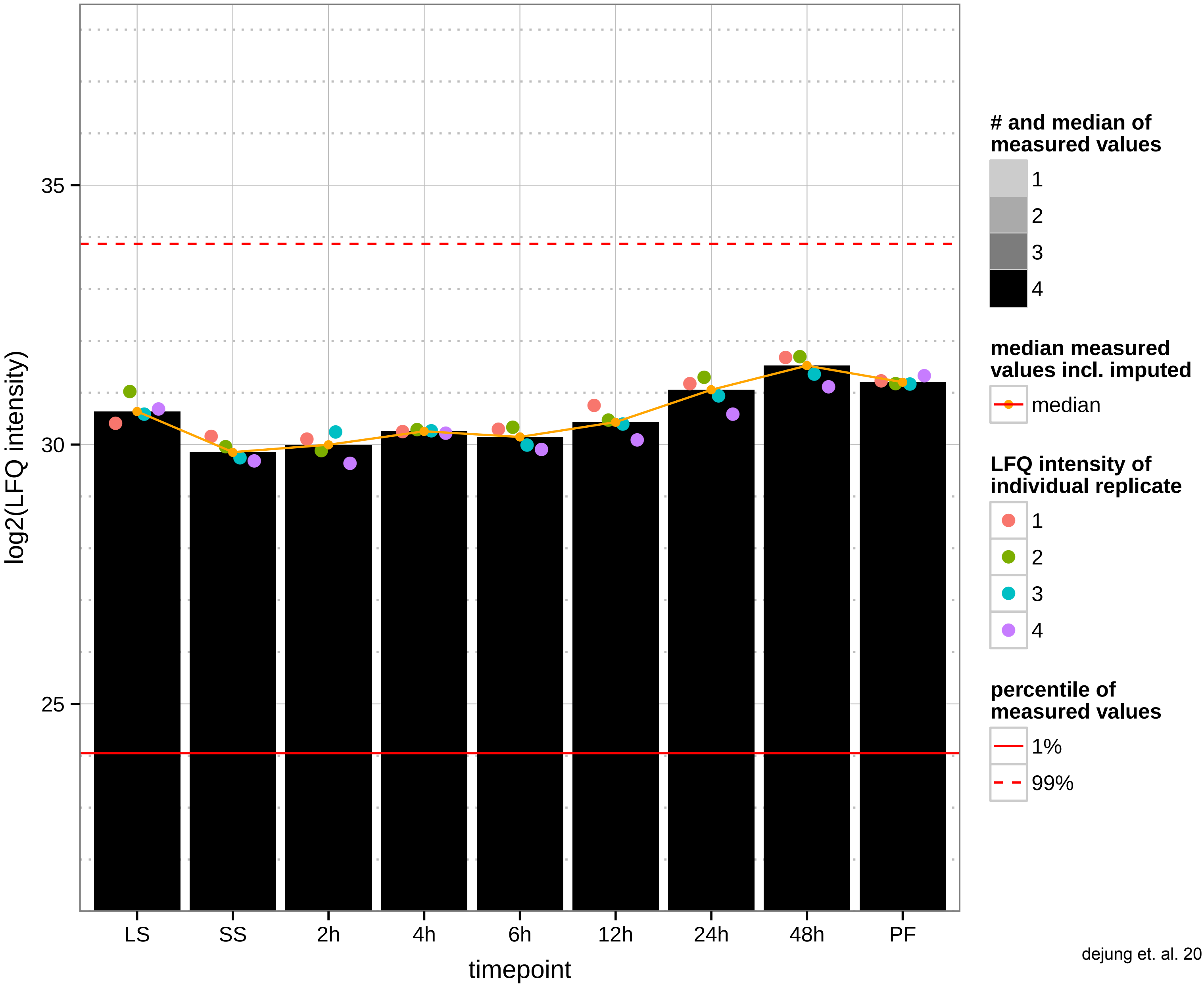

dejung et. al. 2015

kinesin, putative
Tb927.8.2630
AGOF: ATP binding, microtubule motor activity, motor activity
AGOC: kinesin complex, microtubule associated complex
AGOP: microtubule–based movement
PGOF: ATP binding, microtubule motor activity
PGOC: null
PGOP: microtubule–based movement

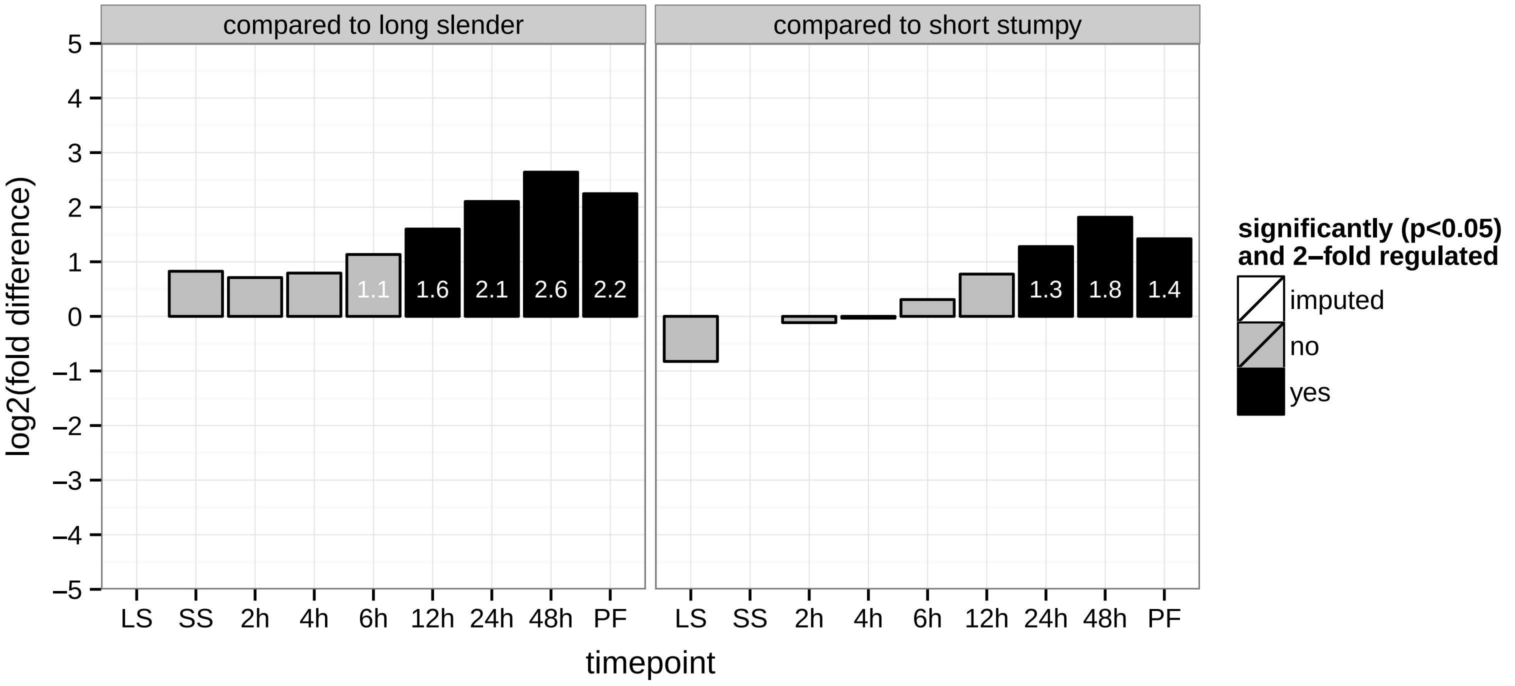

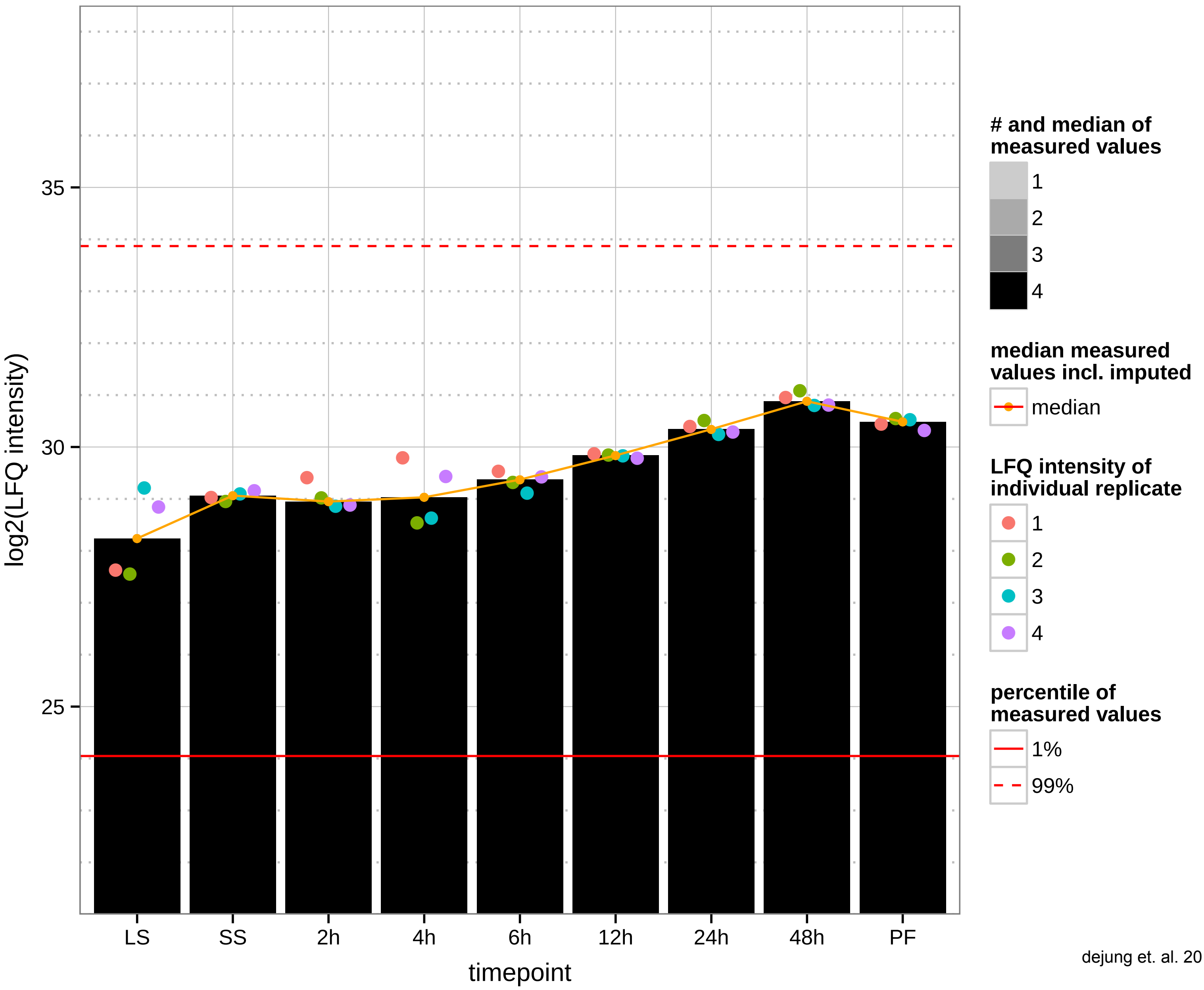

dejung et. al. 2015

DNA repair and recombination helicase protein PIF5 (PIF5)
Tb927.8.3560
AGOF: 5'–3' DNA helicase activity, nucleoside–triphosphatase activity, nucleotide binding
AGOC: mitochondrion
AGOP: DNA replication, Okazaki fragment processing, DNA replication, removal of RNA primer
PGOF: nucleoside–triphosphatase activity, nucleotide binding
PGOC: null
PGOP: null

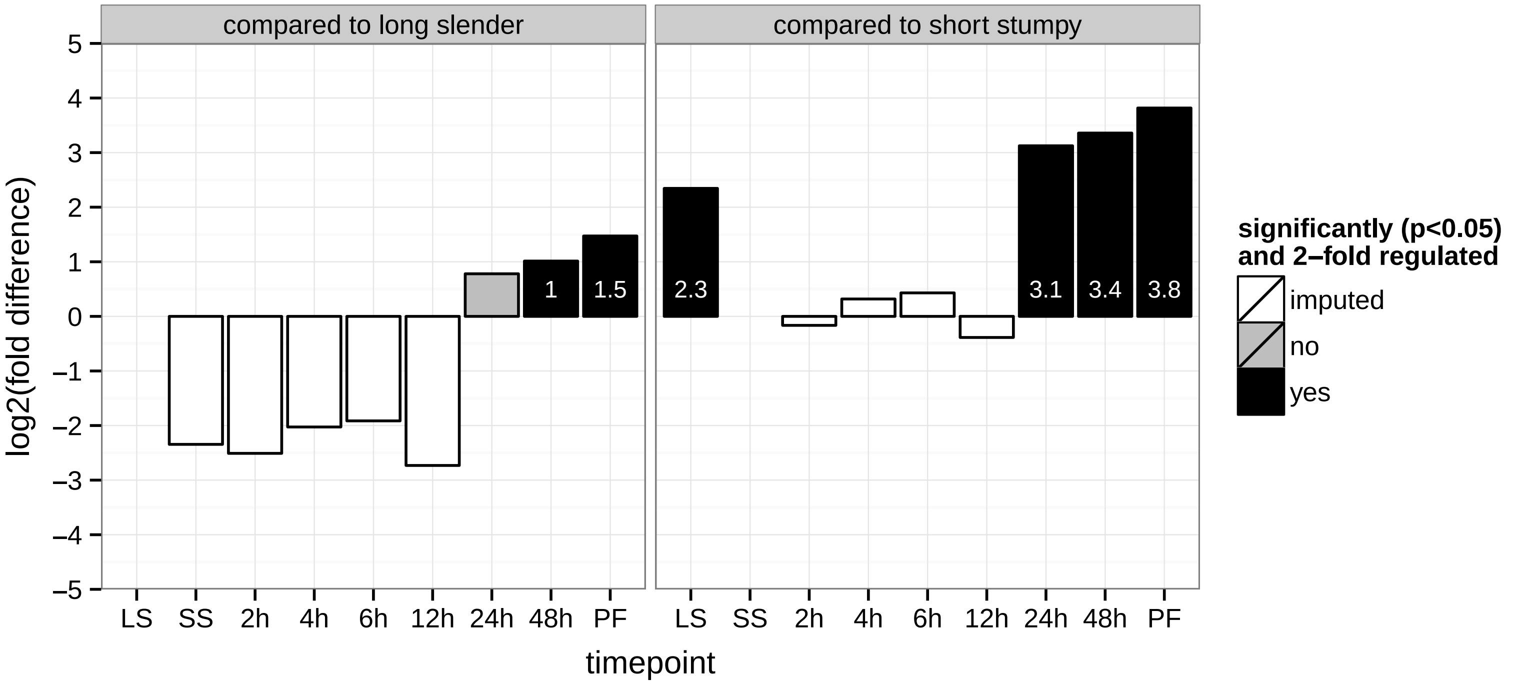

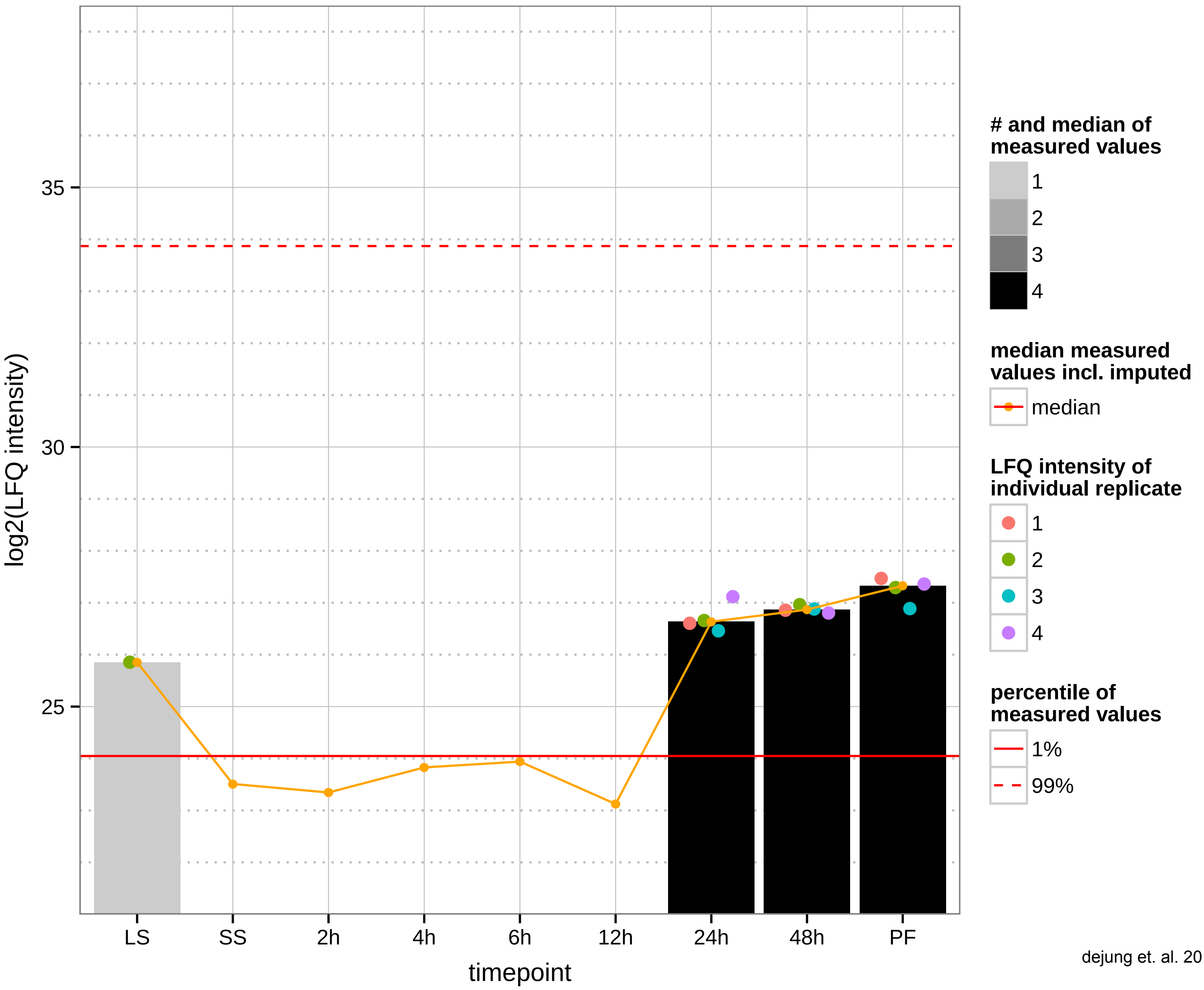

dejung et. al. 2015

hypothetical protein, conserved
Tb927.8.4230
AGOF: null
AGOC: null
AGOP: null
PGOF: null
PGOC: null
PGOP: null

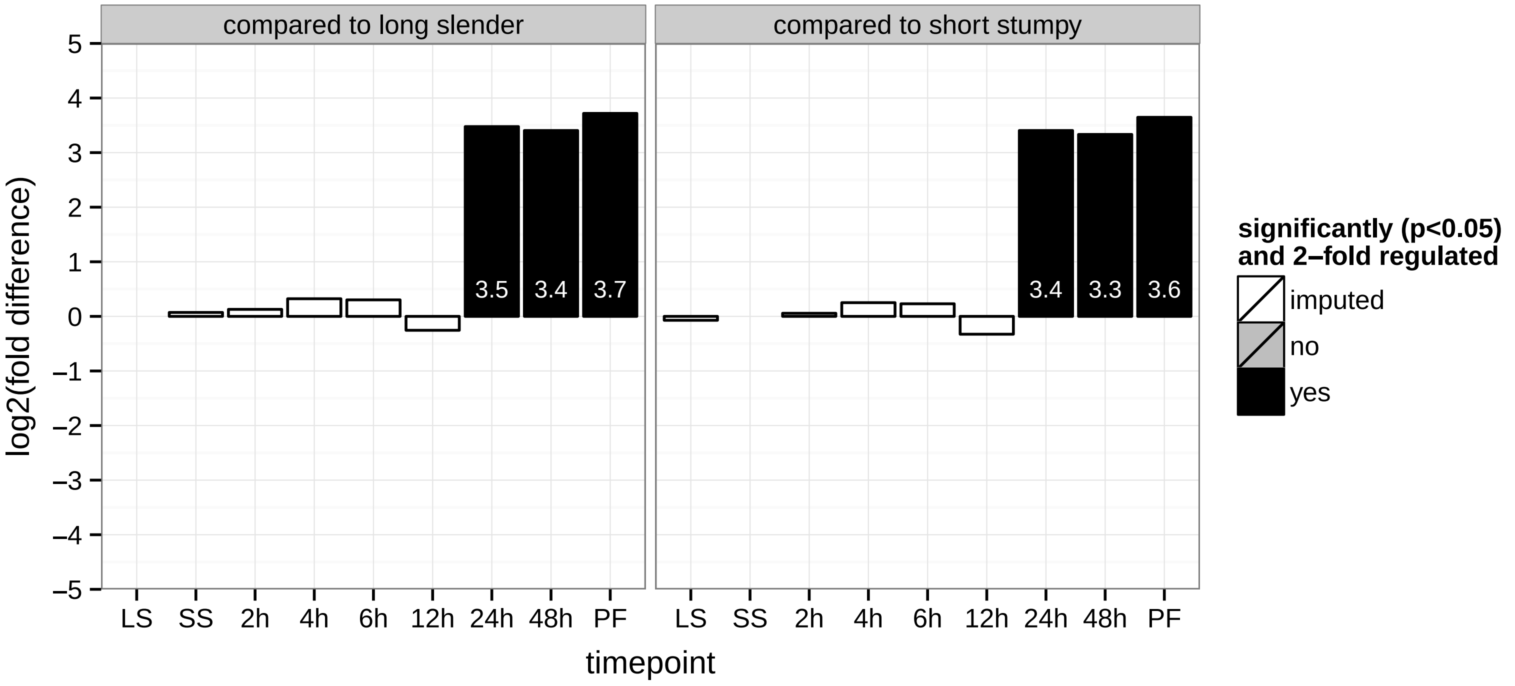

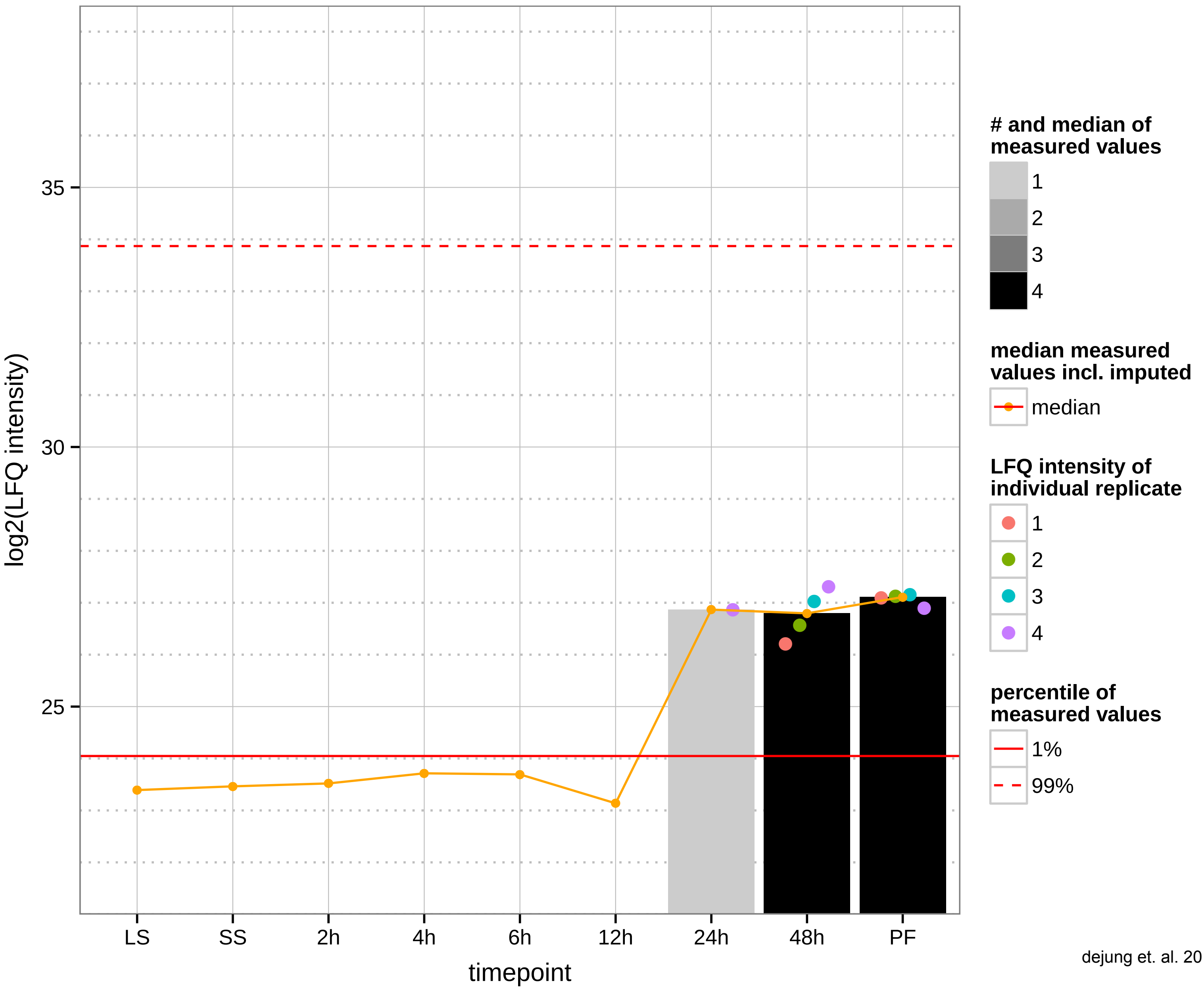

dejung et. al. 2015

hypothetical protein, conserved
Tb927.8.4380
AGOF: null
AGOC: mitochondrion
AGOP: null
PGOF: null
PGOC: null
PGOP: null

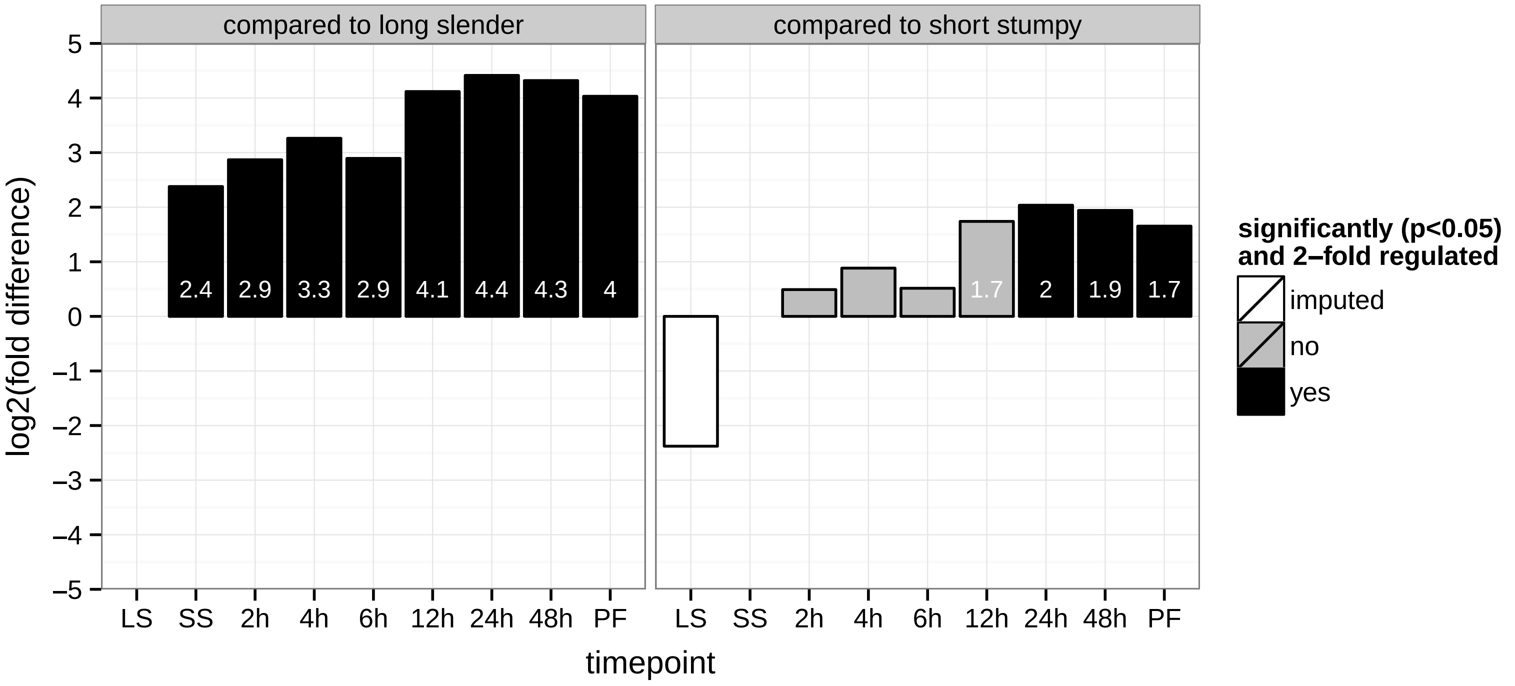

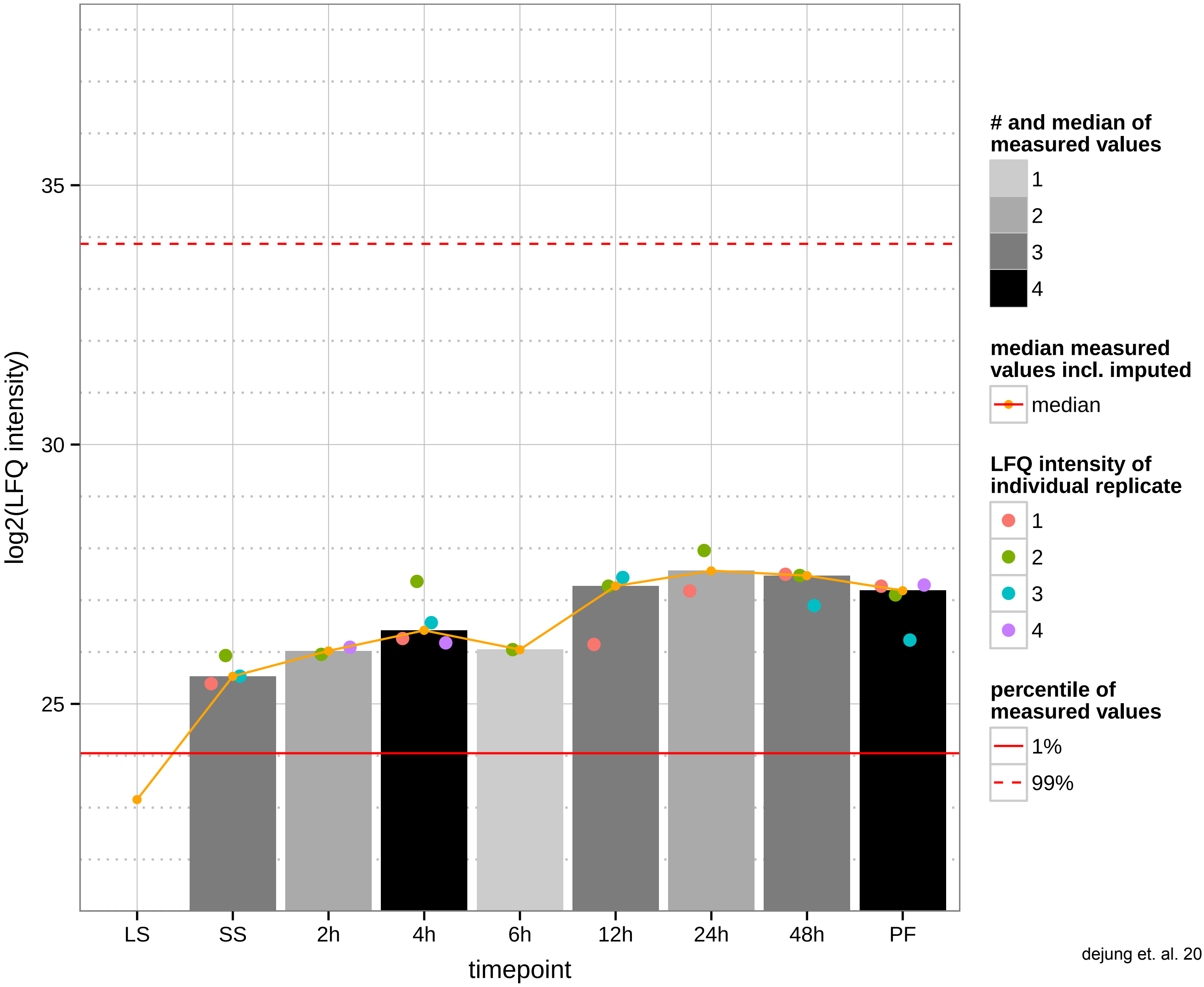

dejung et. al. 2015

hypothetical protein, conserved, eukaryotic translation initation factor 4 gamma, putative (eIF4G3)
Tb927.8.4820;Tb11.v5.0659
AGOF: null
AGOC: null
AGOP: null, RNA metabolic process, translation
PGOF: DNA binding, RNA binding, binding, protein binding
PGOC: null
PGOP: null

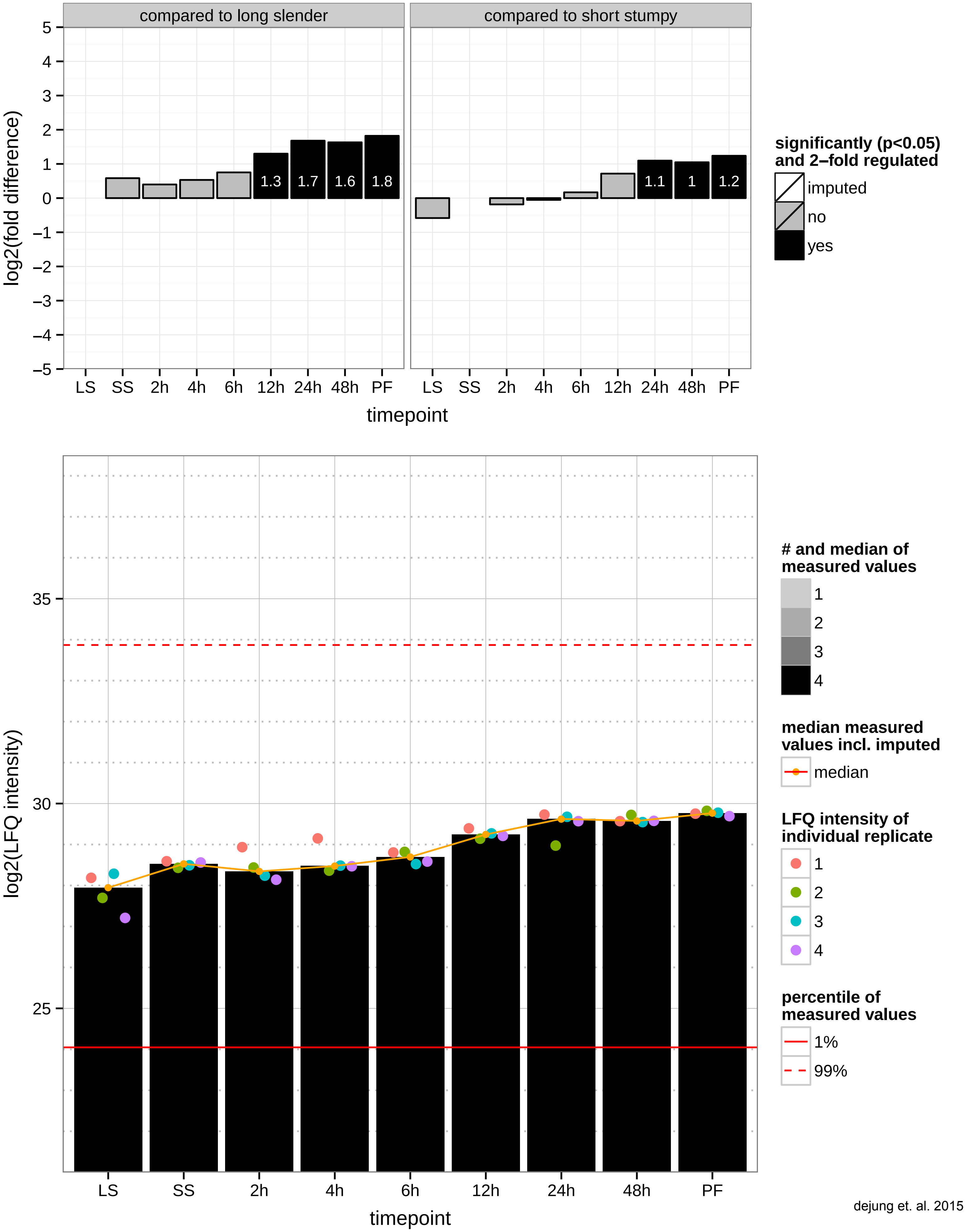

dejung et. al. 2015

DNA polymerase alpha catalytic subunit, DNA polymerase I
Tb927.8.4880
AGOF: DNA binding, DNA–directed DNA polymerase activity, nucleotide binding
AGOC: DNA–directed RNA polymerase I complex, nucleus
AGOP: DNA replication, lagging strand elongation
PGOF: DNA binding, DNA–directed DNA polymerase activity, nucleic acid binding, nucleotide binding
PGOC: null
PGOP: DNA replication, nucleobase–containing compound metabolic process

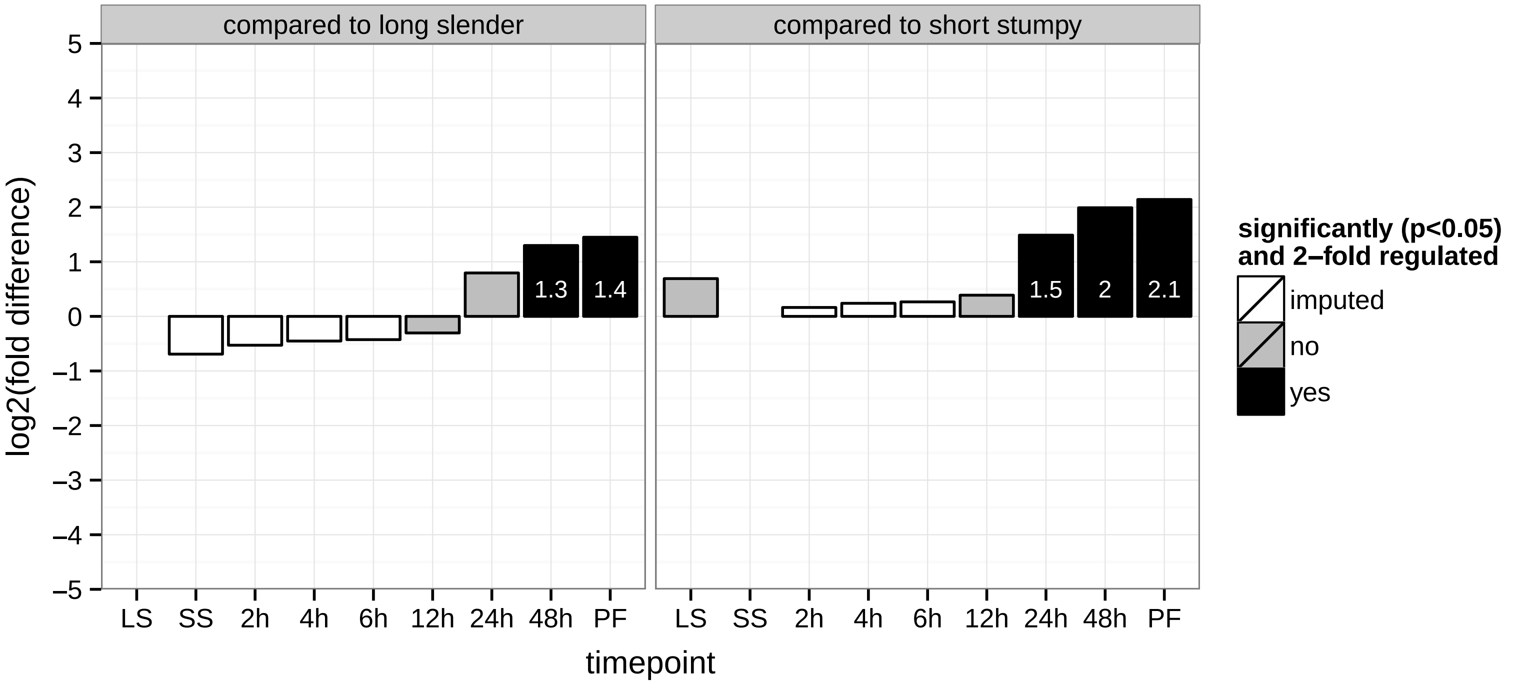

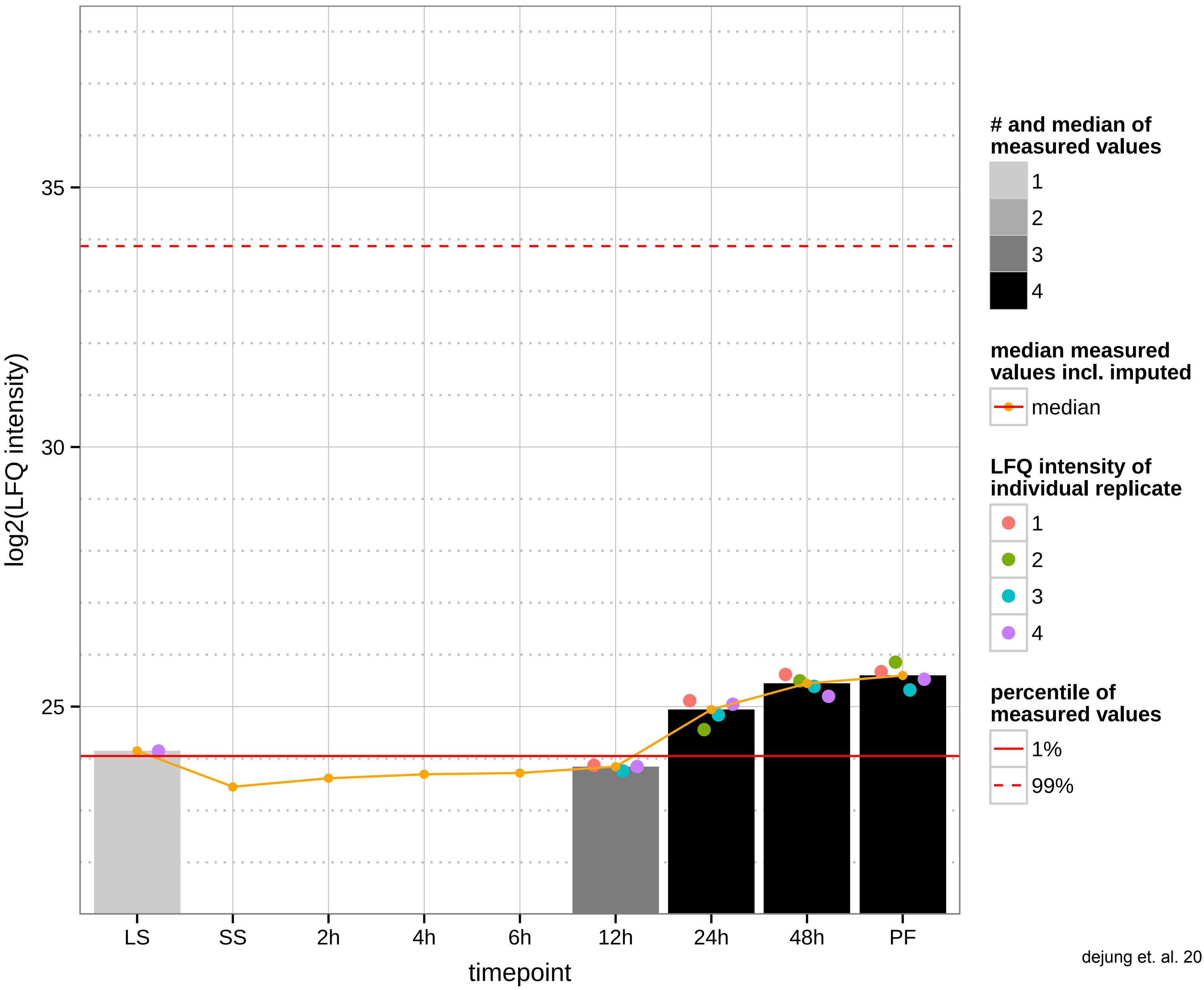

dejung et. al. 2015

hypothetical protein, conserved
Tb927.8.4920
AGOF: null
AGOC: mitochondrion
AGOP: null
PGOF: null
PGOC: null
PGOP: null

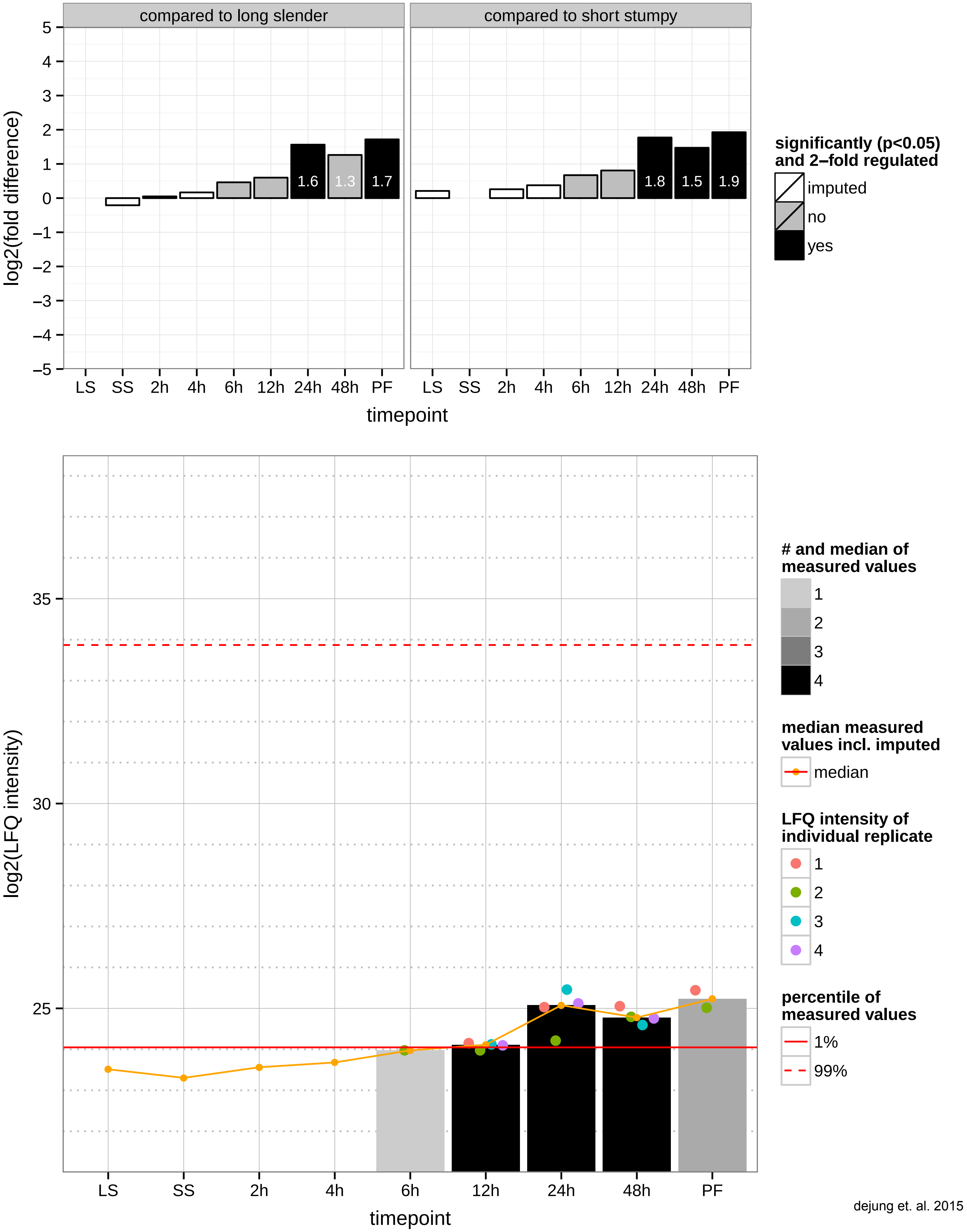

dejung et. al. 2015

DNA–directed rna polymerase I largest subunit, putative, DNA–directed RNA polymerase I largest subunit (RPA190)
Tb927.8.5090
AGOF: null, DNA binding, DNA–directed RNA polymerase activity, zinc ion binding
AGOC: null, nucleus
AGOP: null, transcription, DNA–dependent
PGOF: DNA binding, DNA–directed RNA polymerase activity
PGOC: null
PGOP: transcription, DNA–dependent

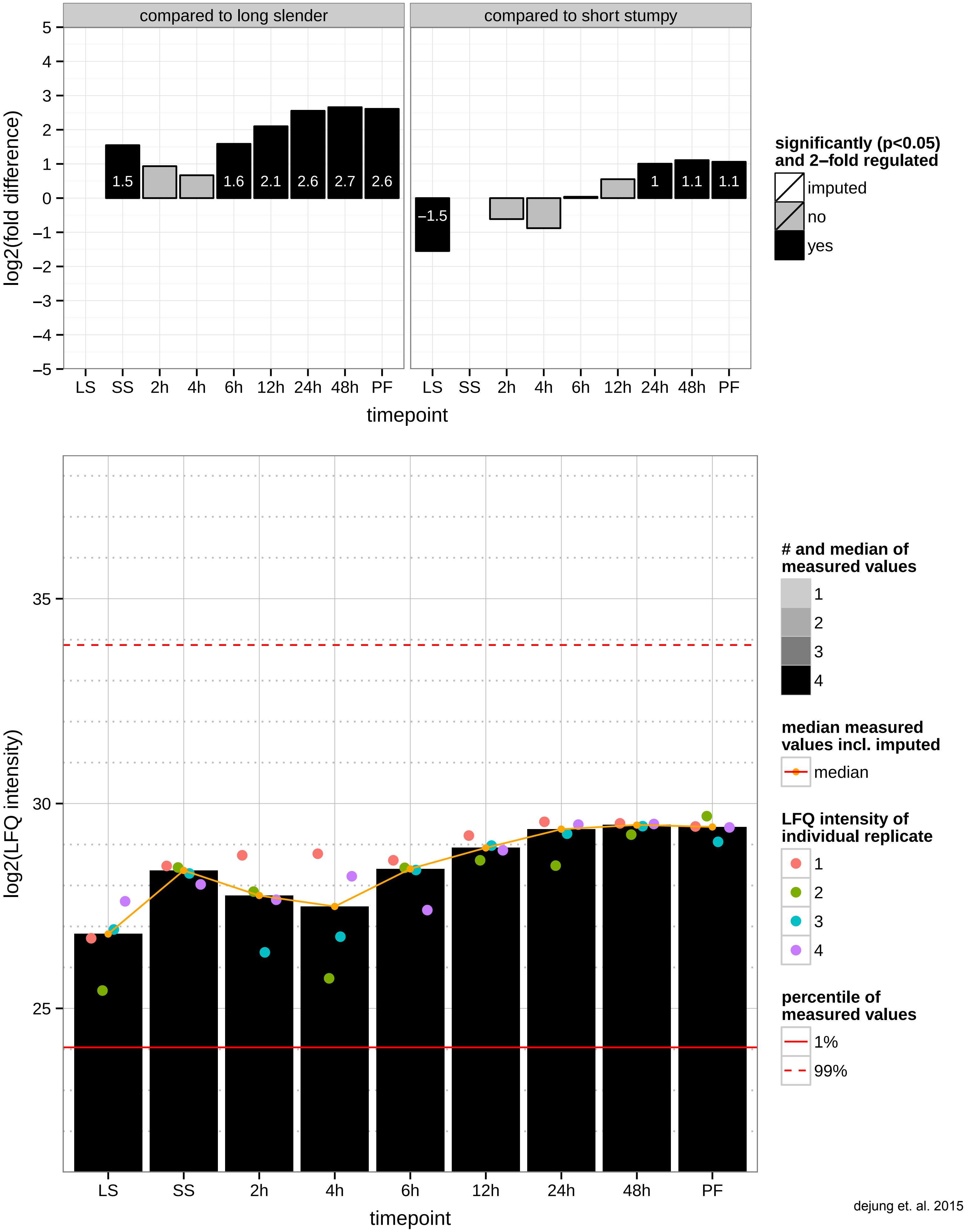

dejung et. al. 2015

hypothetical protein, conserved
Tb927.8.5420
AGOF: methyltransferase activity
AGOC: integral to membrane, mitochondrion
AGOP: metabolic process
PGOF: methyltransferase activity
PGOC: null
PGOP: metabolic process

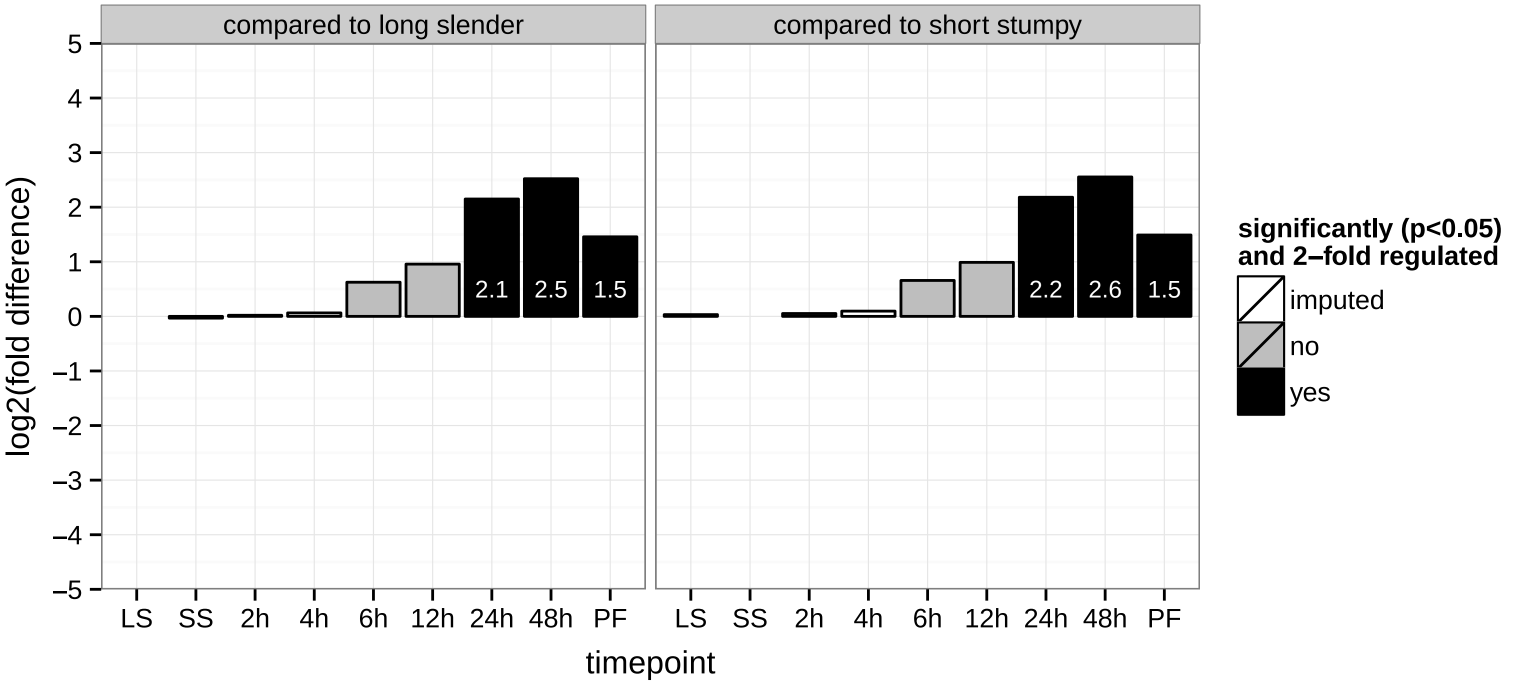

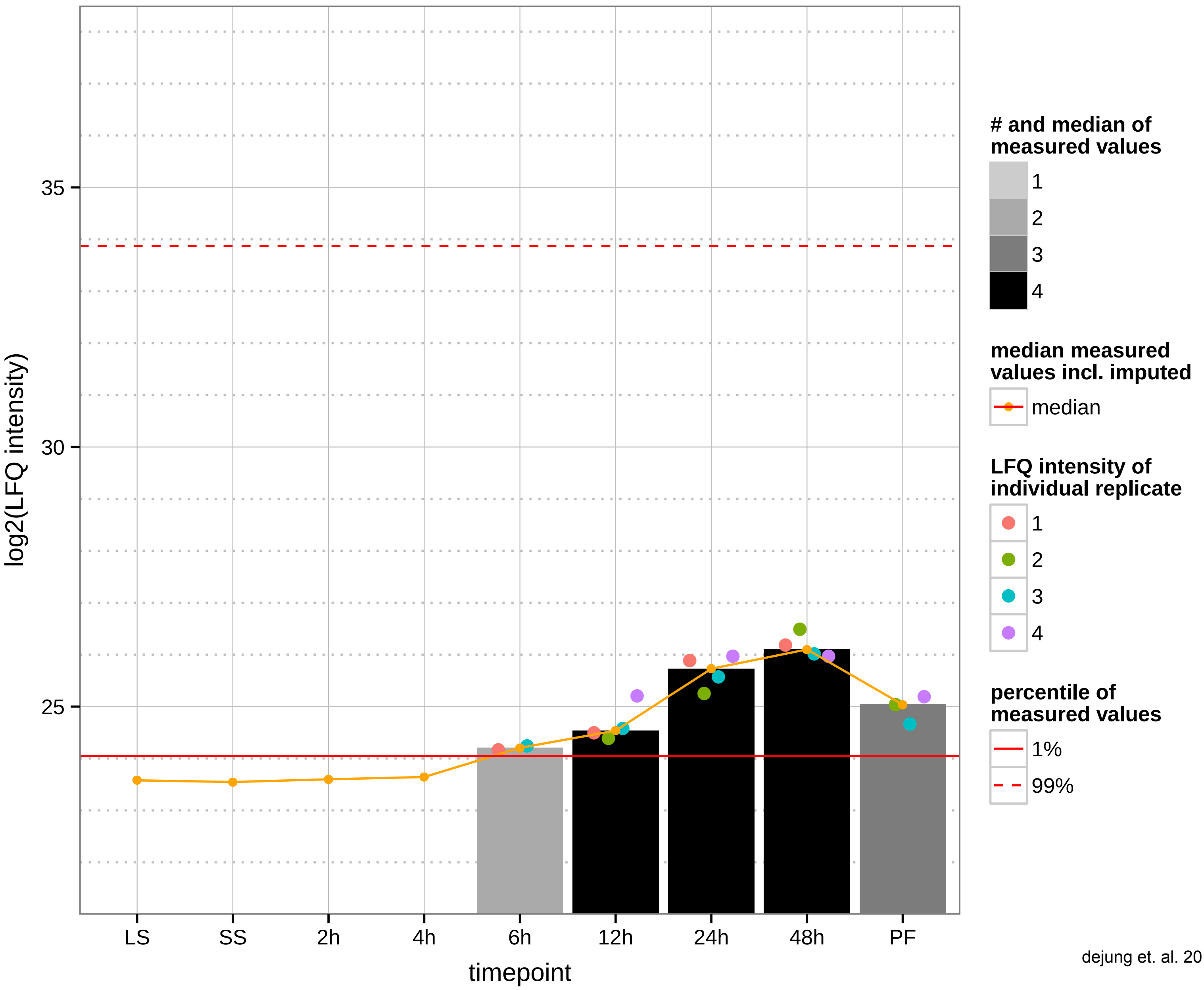

dejung et. al. 2015

hypothetical protein, conserved
Tb927.8.5640
AGOF: sequence−specific DNA binding transcription factor activity
AGOC: mitochondrion
AGOP: null
PGOF: null
PGOC: null
PGOP: null

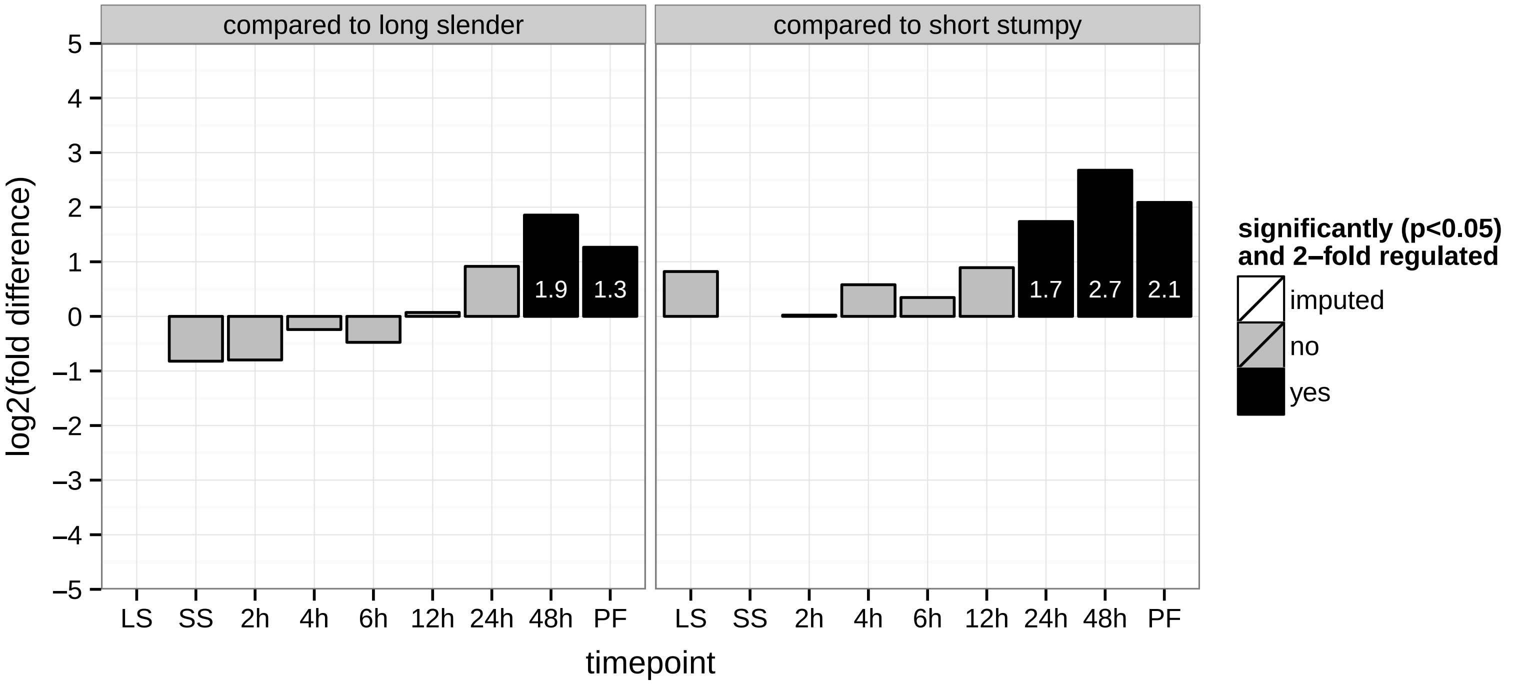

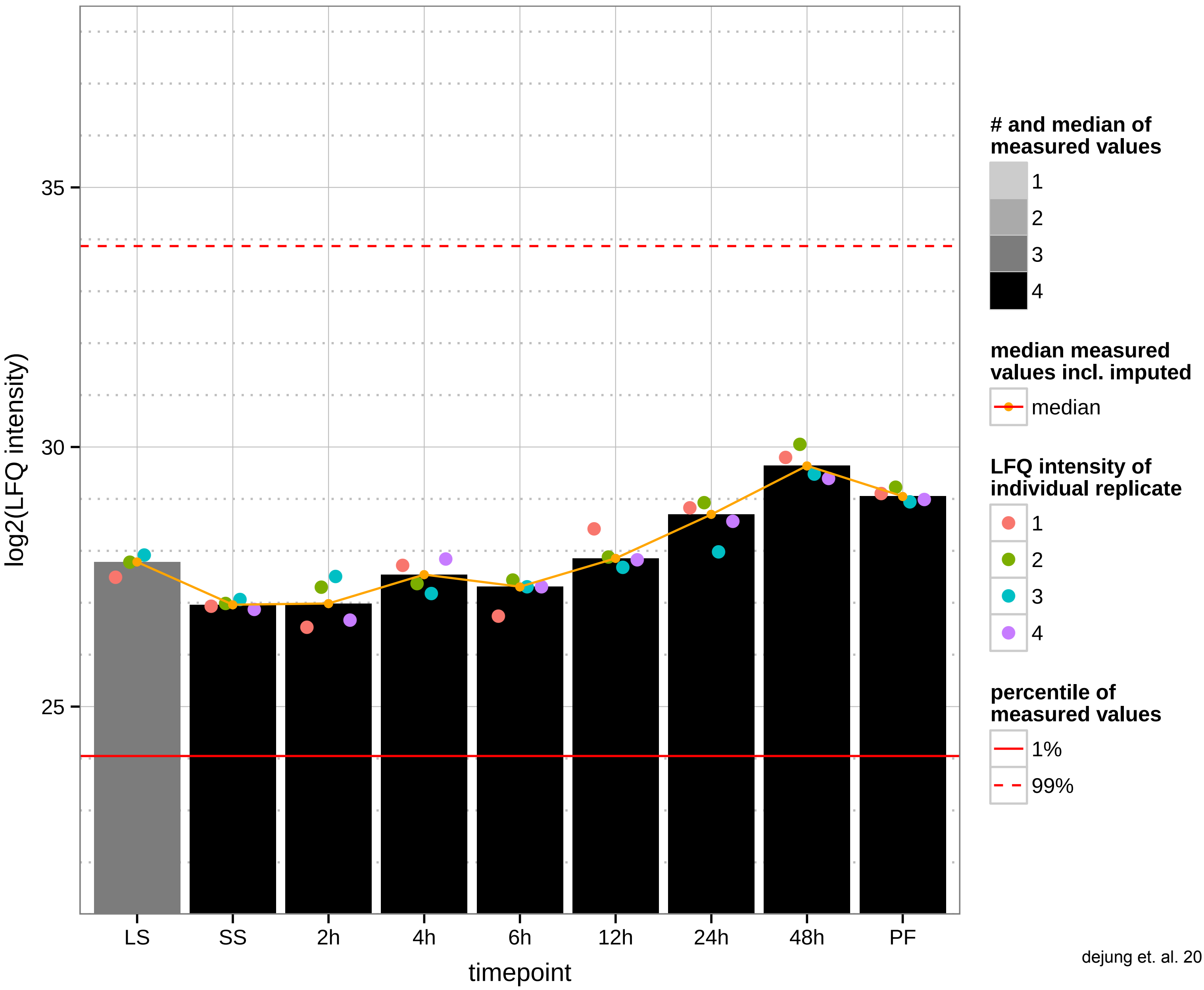

dejung et. al. 2015

succinate dehydrogenase flavoprotein, putative
Tb927.8.6580
AGOF: electron carrier activity, flavin adenine dinucleotide binding, succinate dehydrogenase activity
AGOC: mitochondrion
AGOP: electron transport chain, oxidation–reduction process, tricarboxylic acid cycle
PGOF: flavin adenine dinucleotide binding, oxidoreductase activity, acting on the CH–CH group of donors
PGOC: null
PGOP: electron transport chain, oxidation–reduction process, tricarboxylic acid cycle

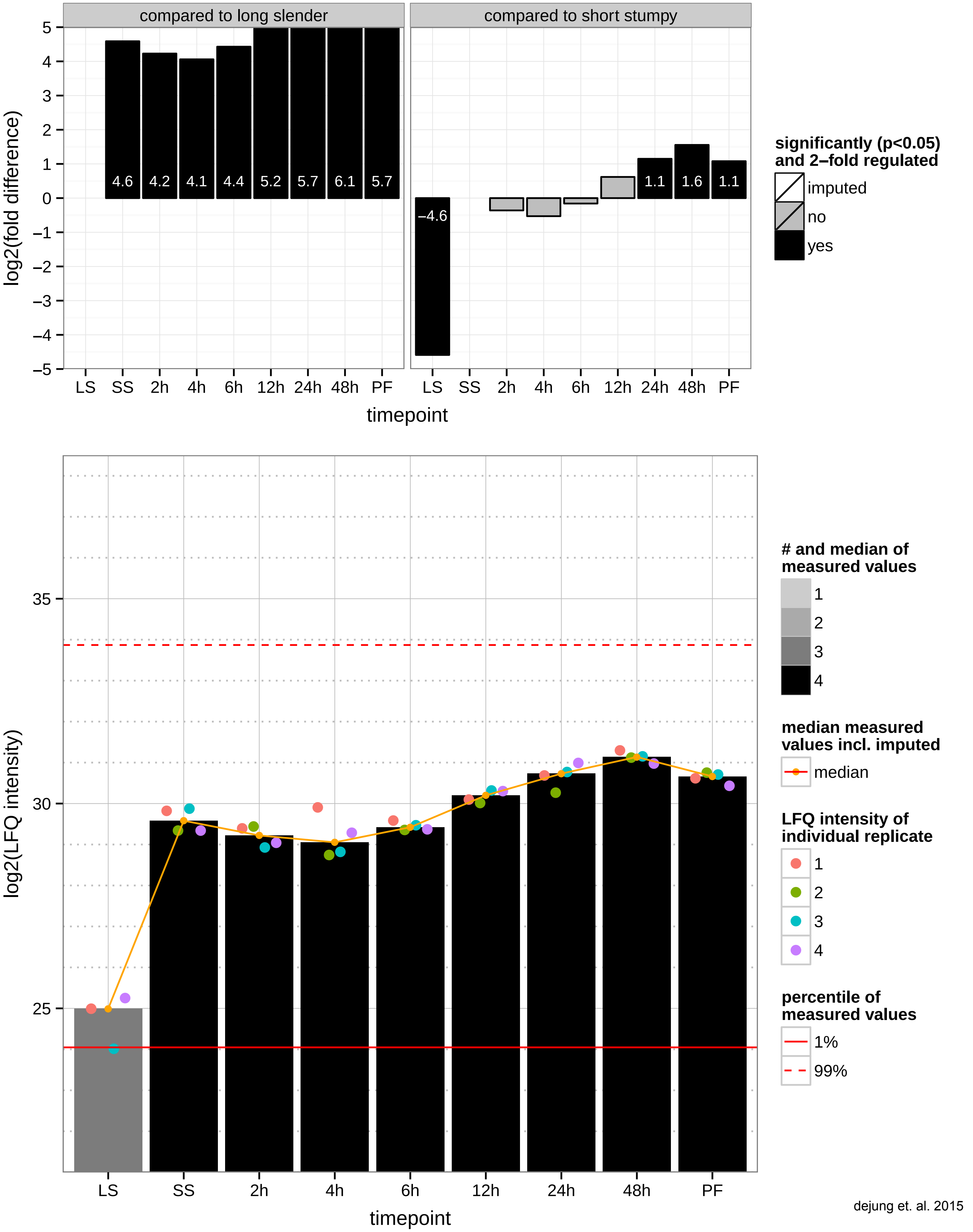

dejung et. al. 2015

dihydroceramide synthase, putative
Tb927.8.7730
AGOF: very long−chain fatty acid−CoA ligase activity
AGOC: cytosol, integral to membrane
AGOP: ceramide biosynthetic process
PGOF: null
PGOC: integral to membrane
PGOP: null

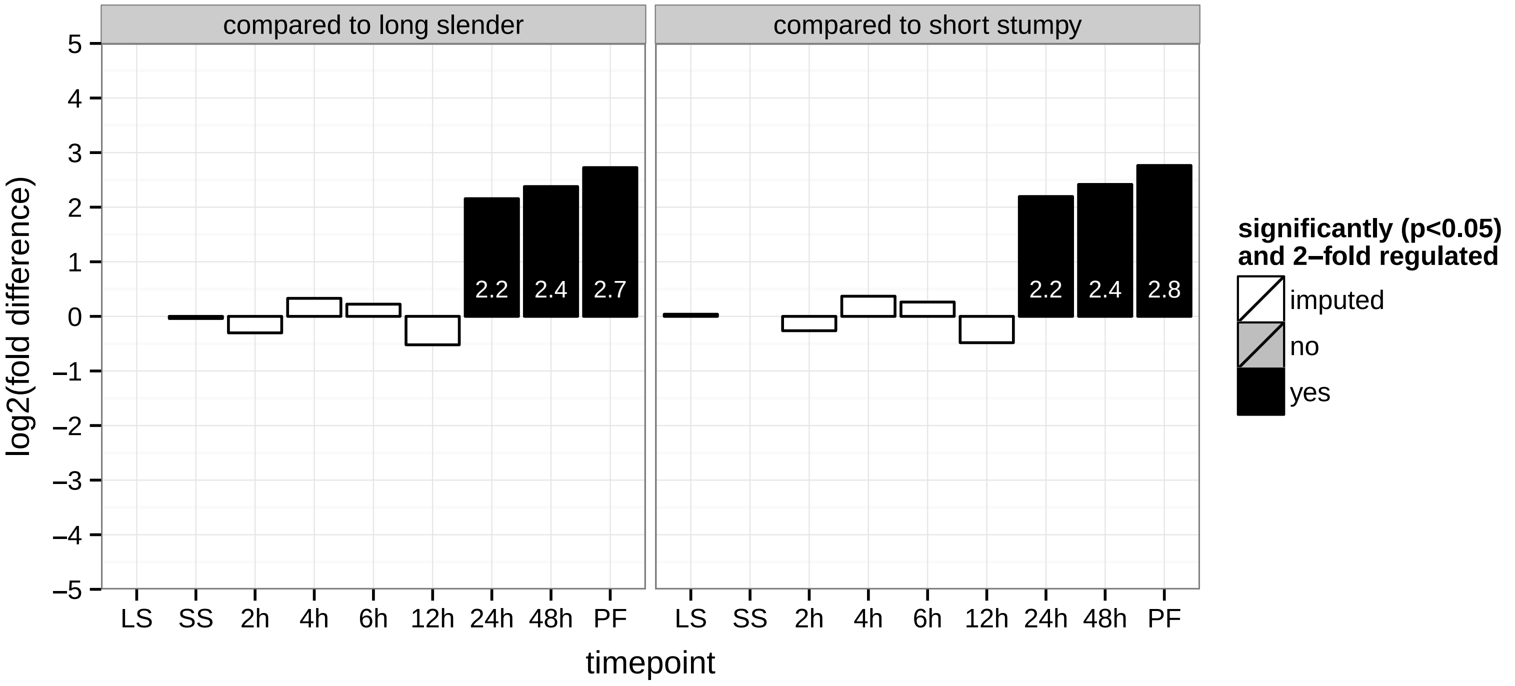

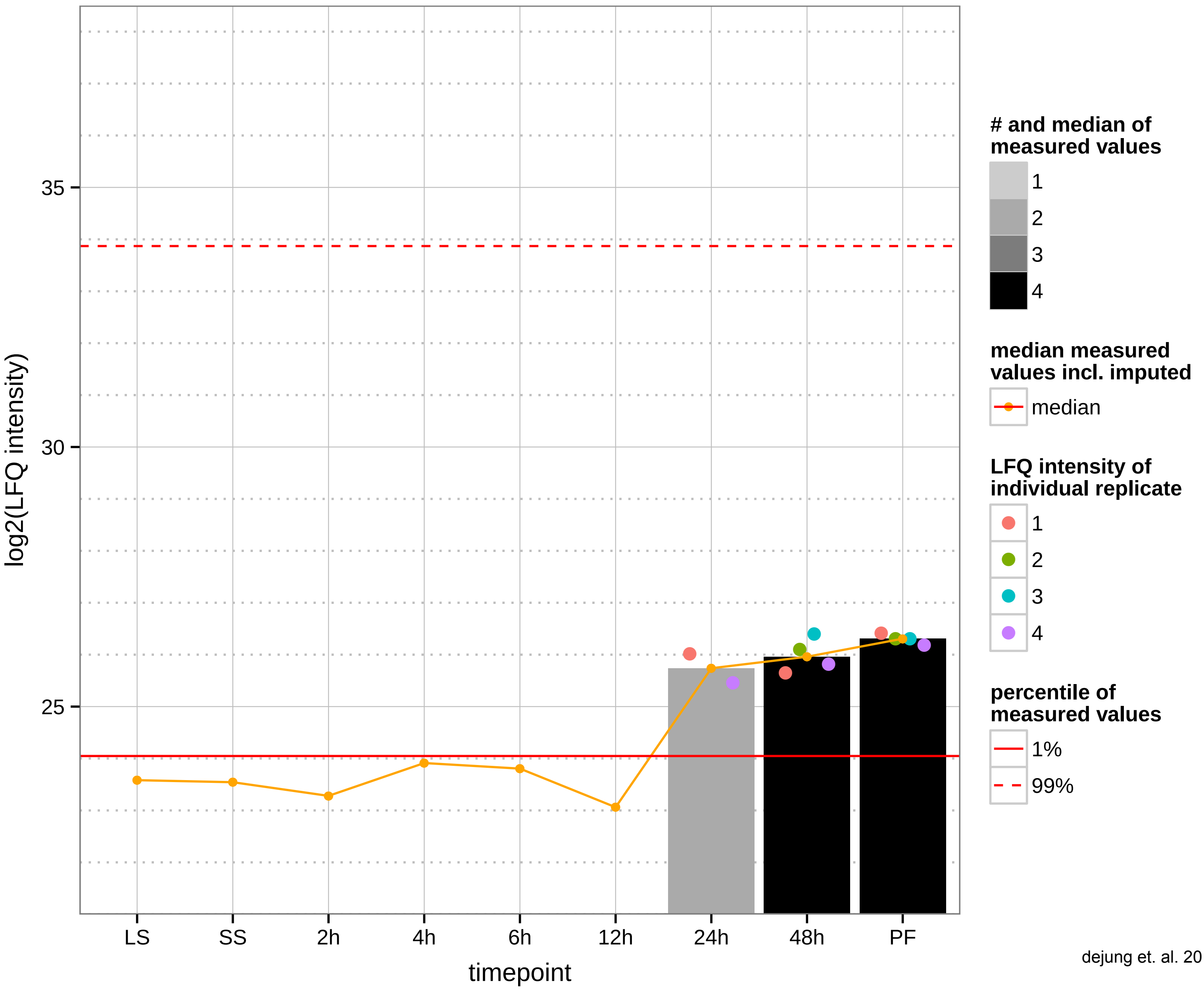

dejung et. al. 2015

60S ribosomal protein L27a, 60S ribosomal protein L28, 60S ribosomal protein L29, 60S ribosomal protein L29 (RPL27A)
Tb927.9.11490;Tb927.9.11470
AGOF: RNA binding, structural constituent of ribosome
AGOC: intracellular, ribosome
AGOP: translation, null
PGOF: null
PGOC: null
PGOP: null

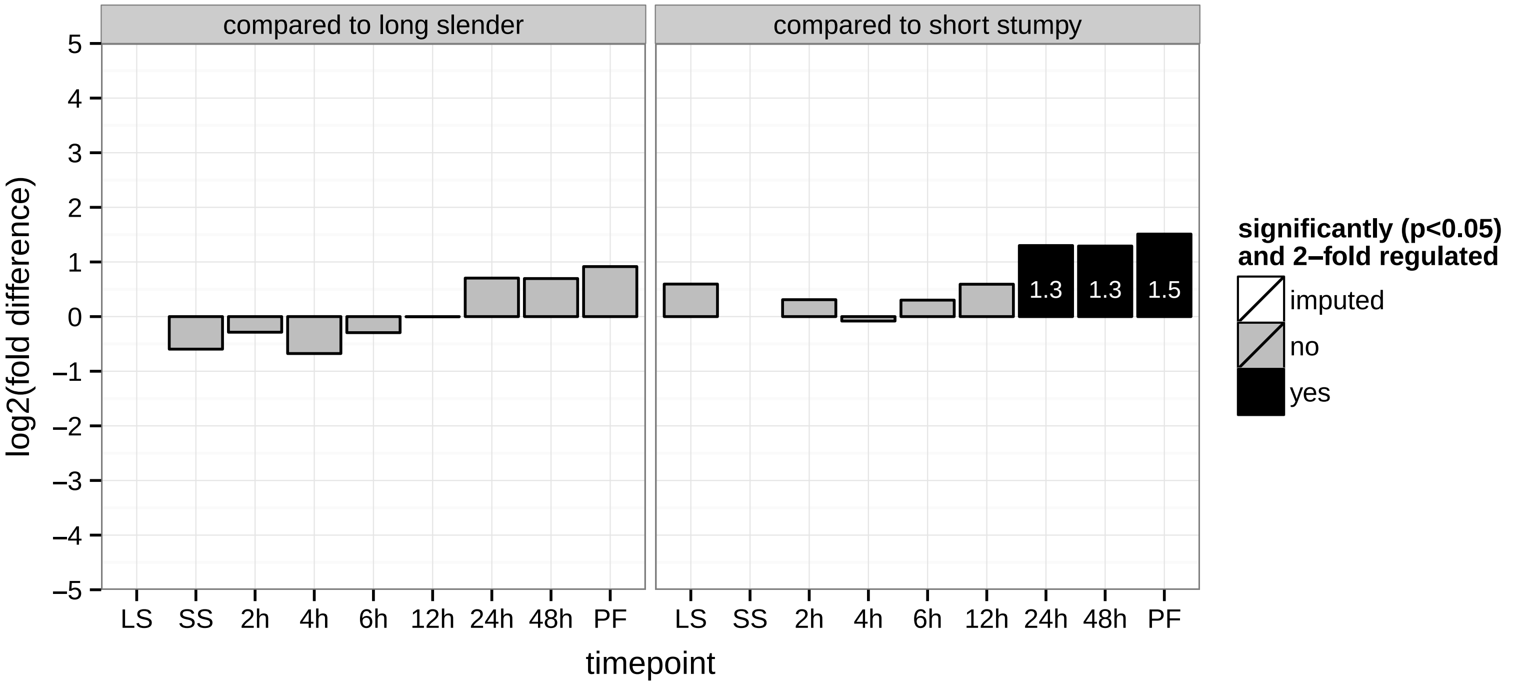

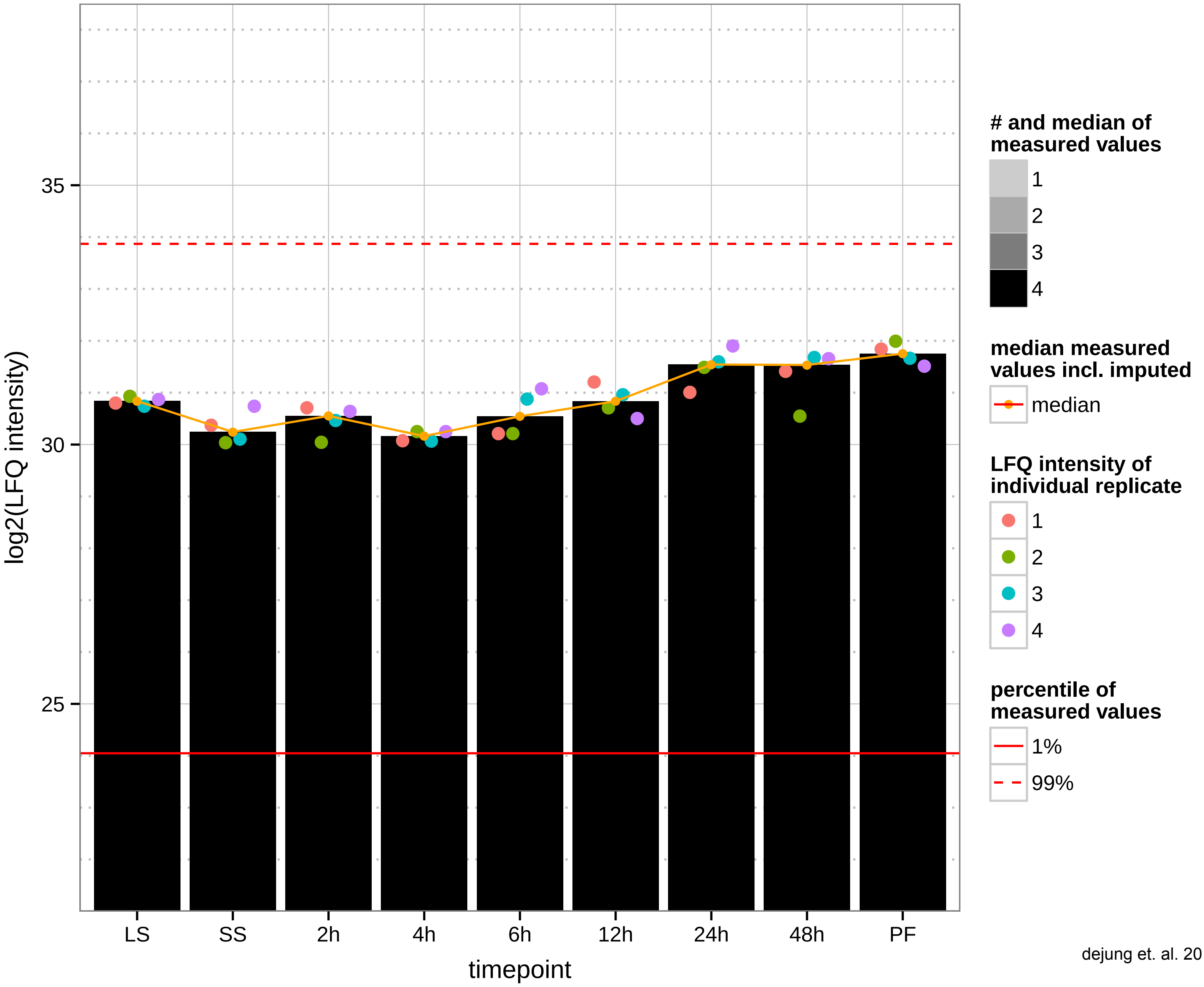

dejung et. al. 2015

hypothetical protein, conserved
Tb927.9.12160
AGOF: null
AGOC: mitochondrion
AGOP: null
PGOF: null
PGOC: null
PGOP: null

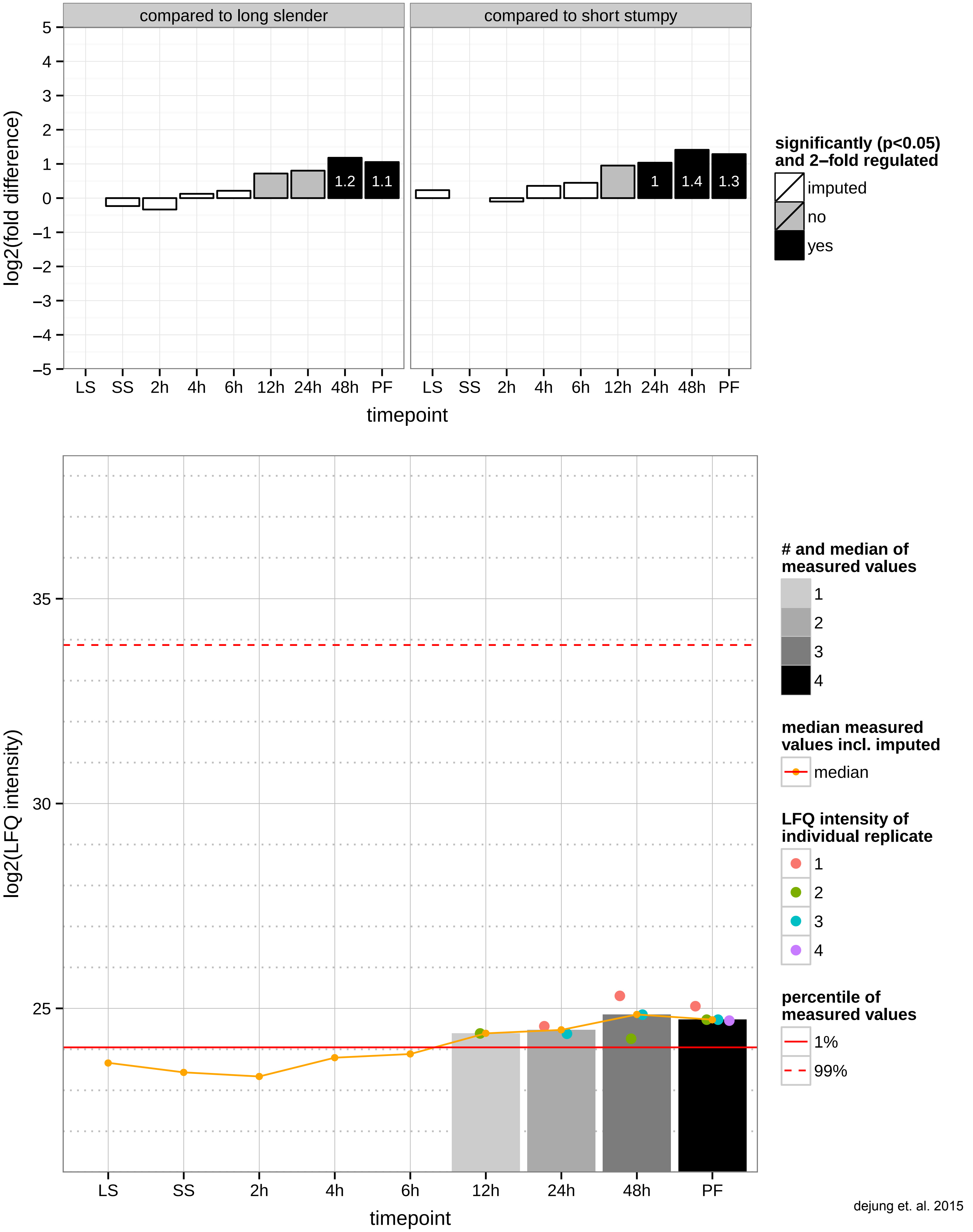

dejung et. al. 2015

hypothetical protein, conserved
Tb927.9.12770
AGOF: null
AGOC: mitochondrion
AGOP: null
PGOF: null
PGOC: null
PGOP: null

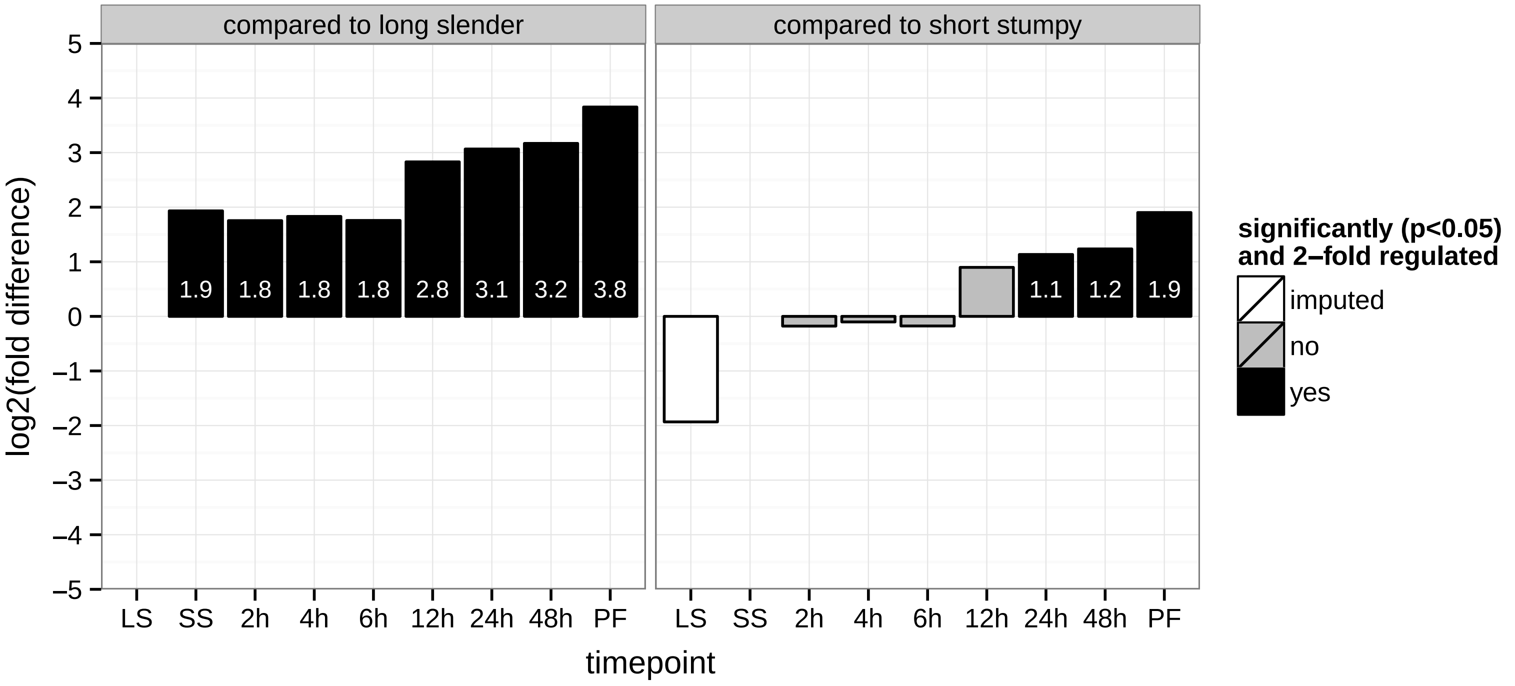

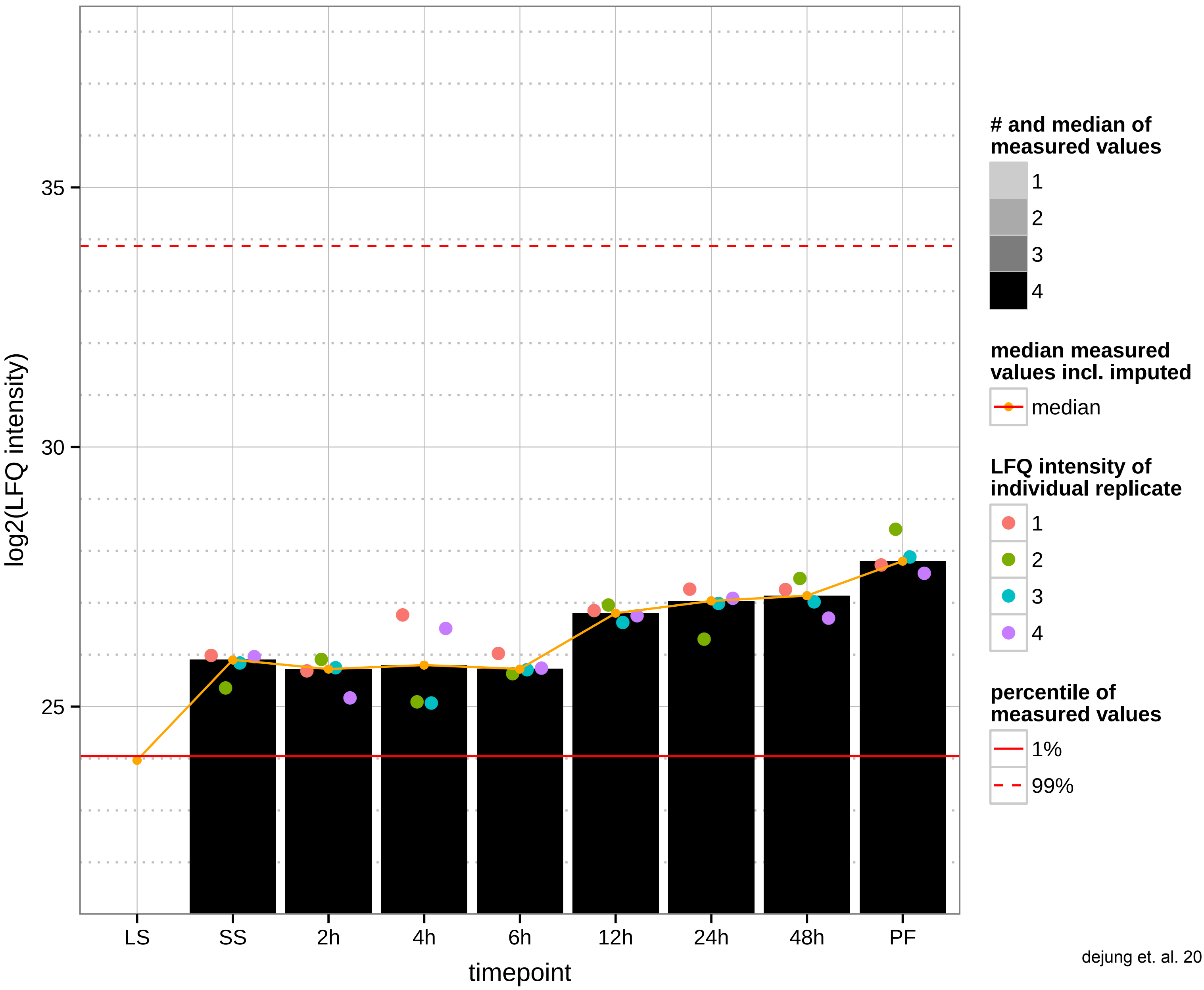

dejung et. al. 2015

aminopeptidase P, putative, metallo–peptidase, Clan MG, Family M24
Tb927.9.13490
AGOF: aminopeptidase activity, manganese ion binding
AGOC: null
AGOP: cellular process
PGOF: aminopeptidase activity, manganese ion binding
PGOC: null
PGOP: cellular process

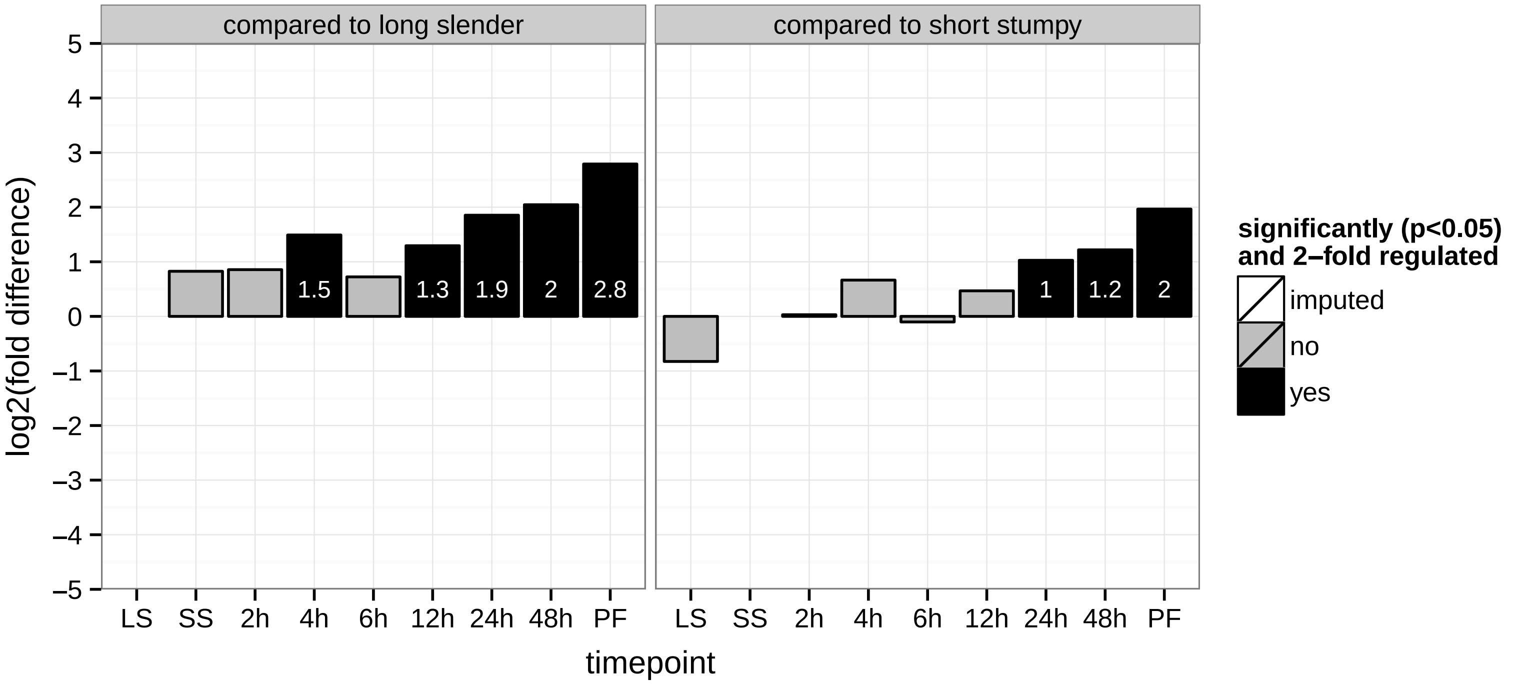

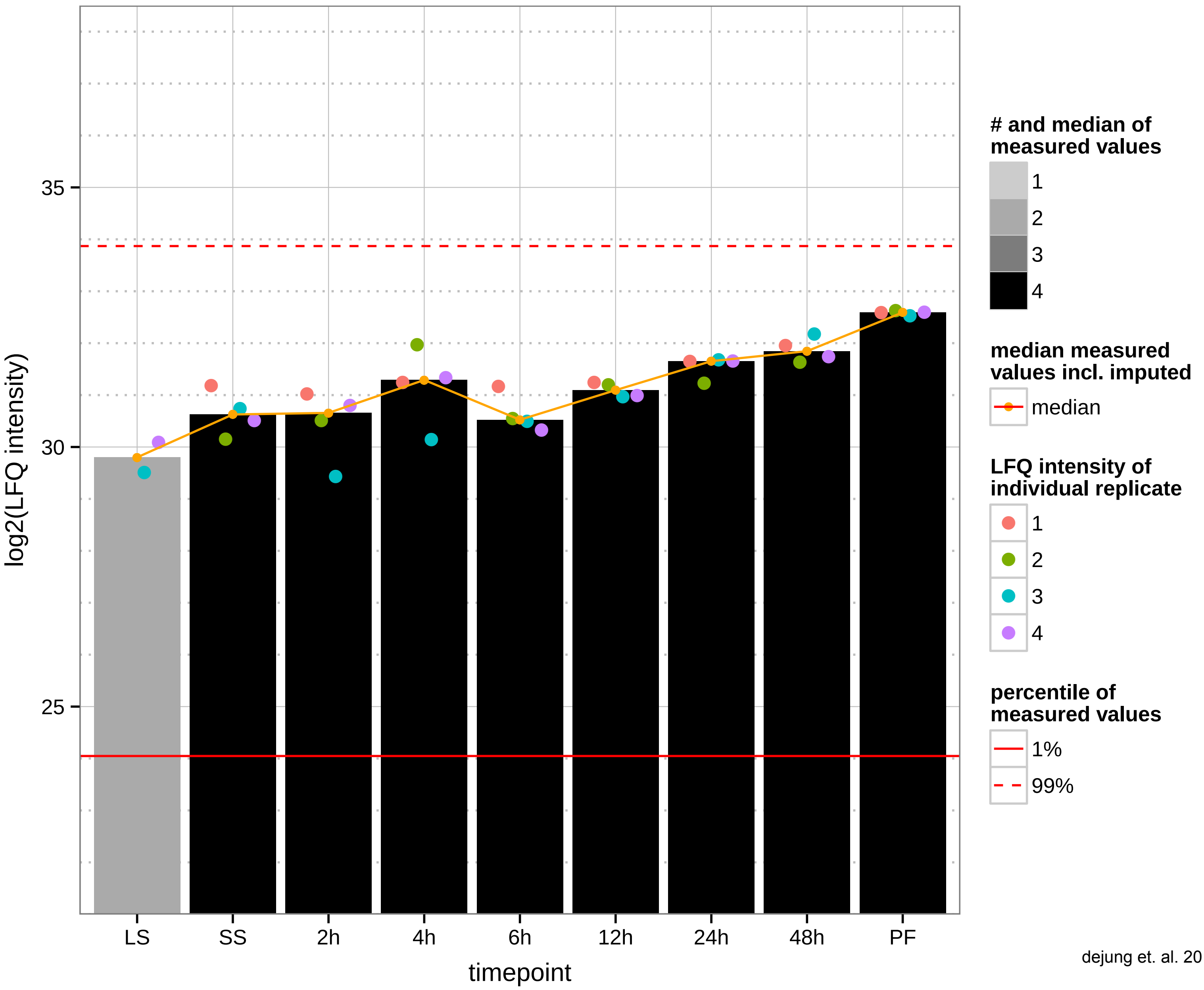

dejung et. al. 2015

hypothetical protein, conserved
Tb927.9.13580
AGOF: null
AGOC: mitochondrion
AGOP: null
PGOF: null
PGOC: null
PGOP: null

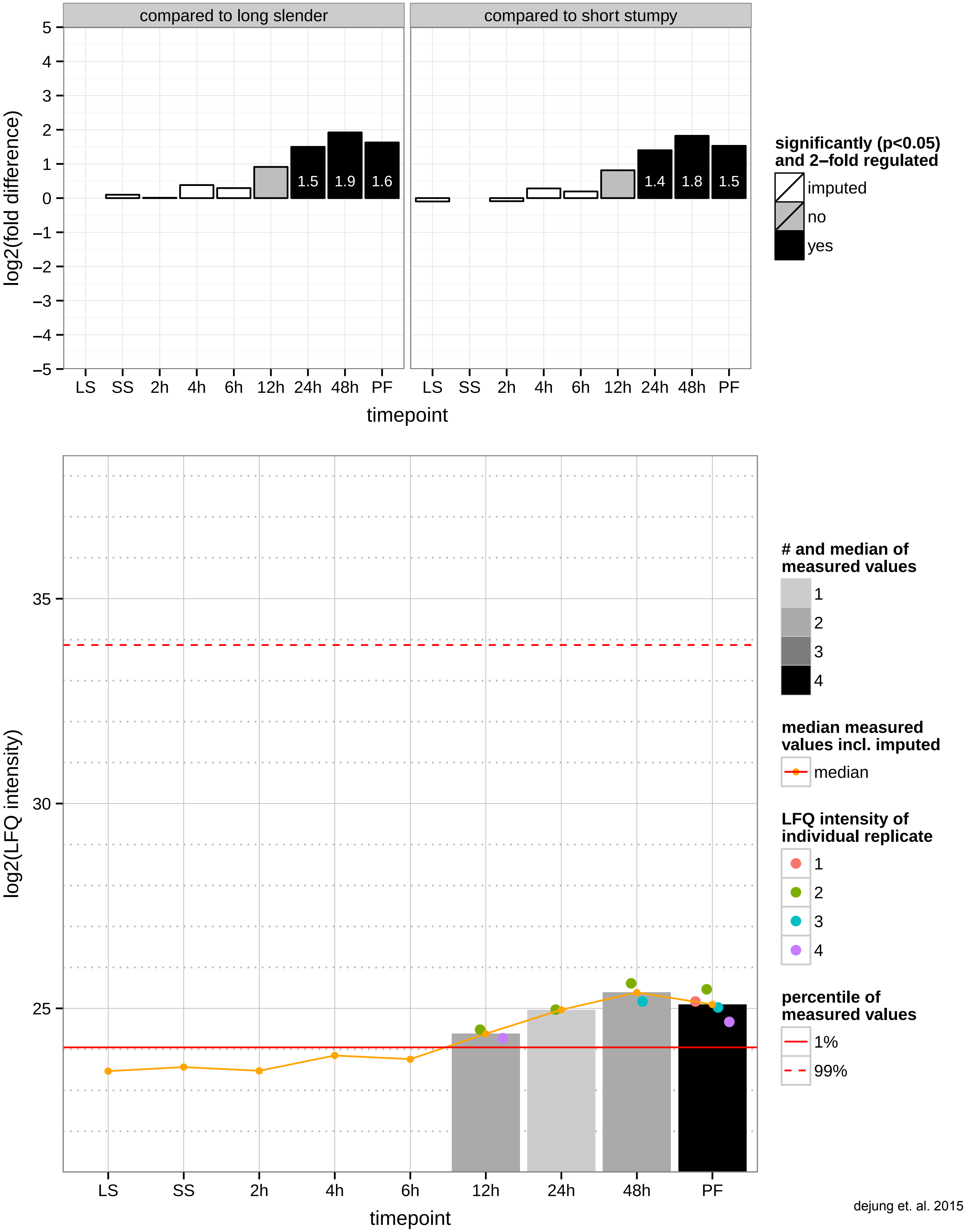

dejung et. al. 2015

RNA 3'–terminal phosphate cyclase–like protein
Tb927.9.14410
AGOF: RNA–3'–phosphate cyclase activity
AGOC: nucleus
AGOP: null
PGOF: catalytic activity
PGOC: nucleolus
PGOP: RNA processing, ribosome biogenesis

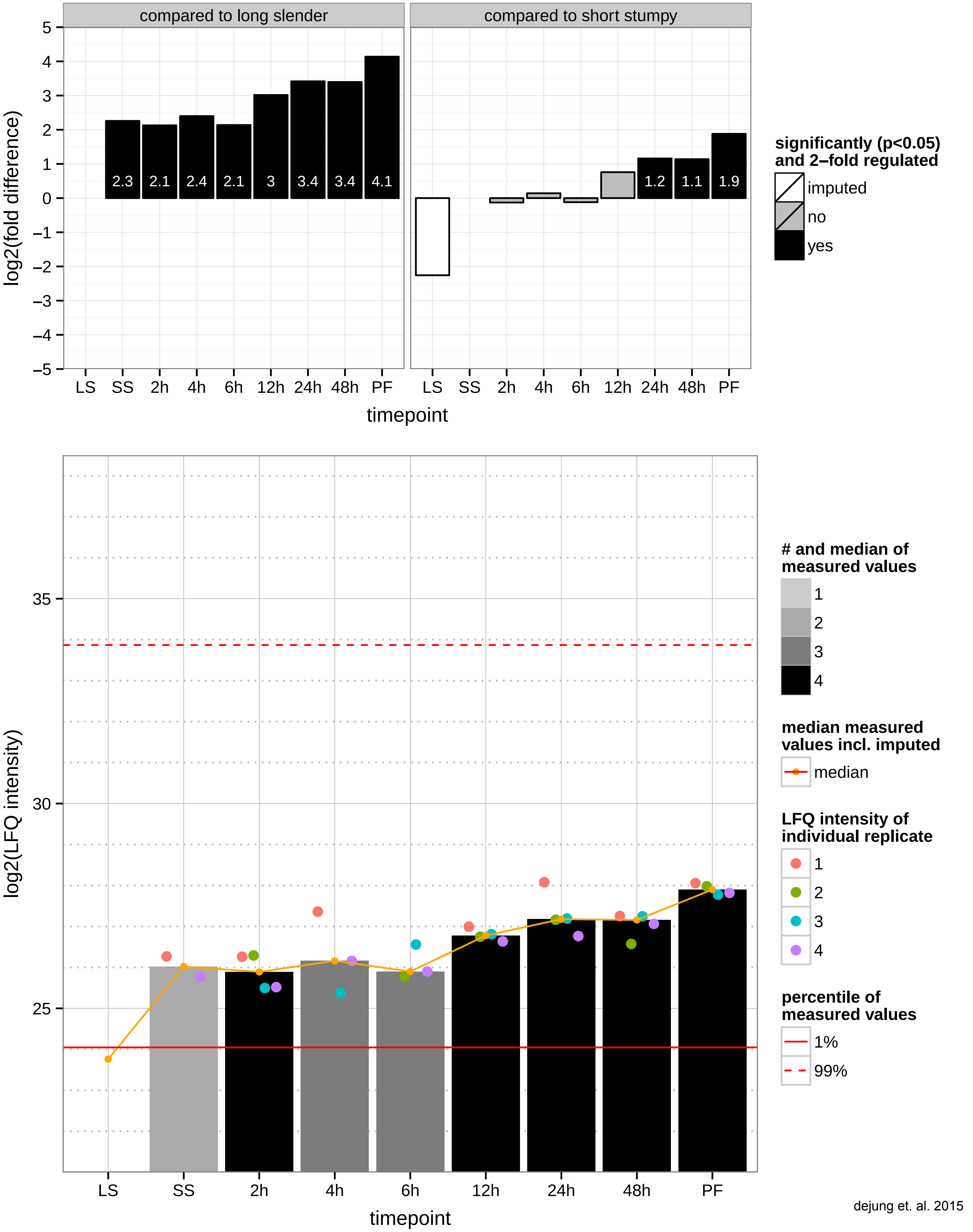

dejung et. al. 2015

cyclophilin–like protein, putative (PPIase)
Tb927.9.14420
AGOF: peptidyl–prolyl cis–trans isomerase activity
AGOC: integral to membrane, mitochondrion
AGOP: protein folding
PGOF: peptidyl–prolyl cis–trans isomerase activity
PGOC: null
PGOP: protein folding

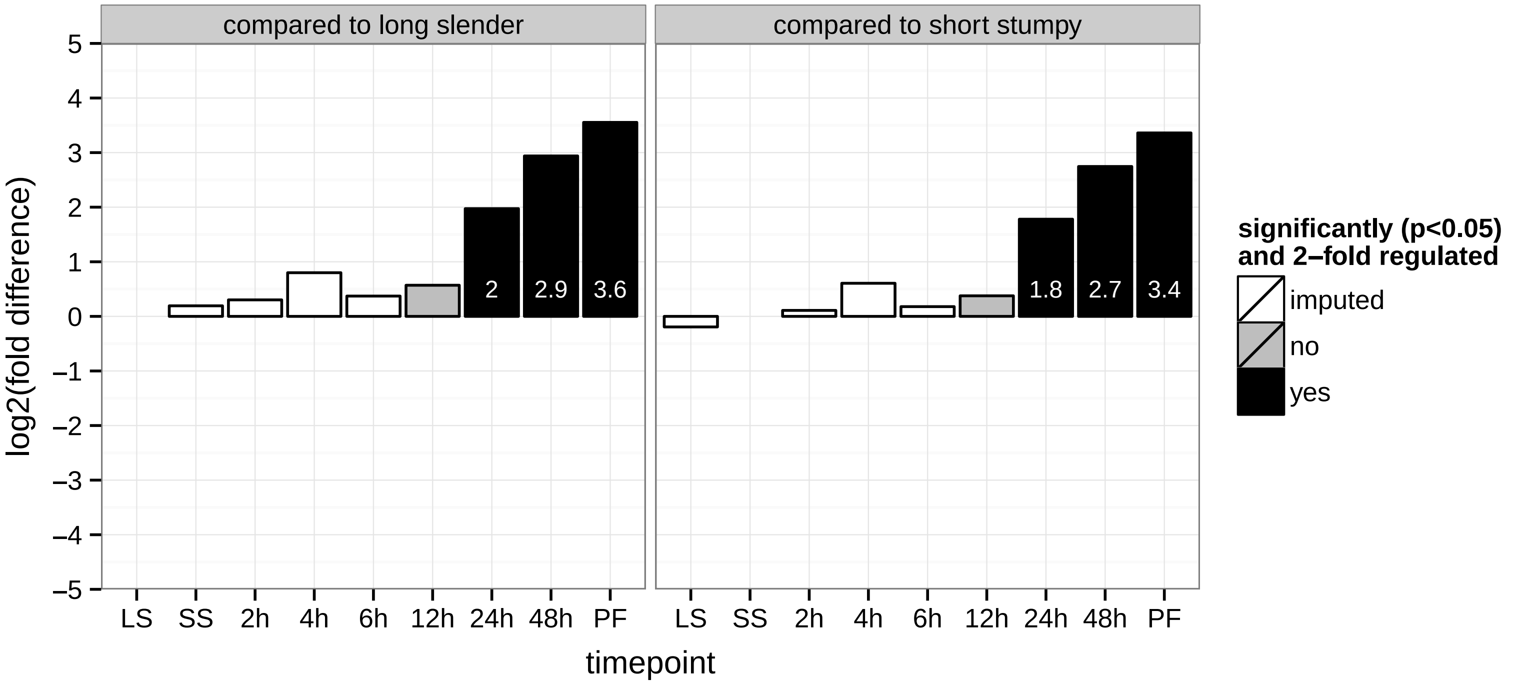

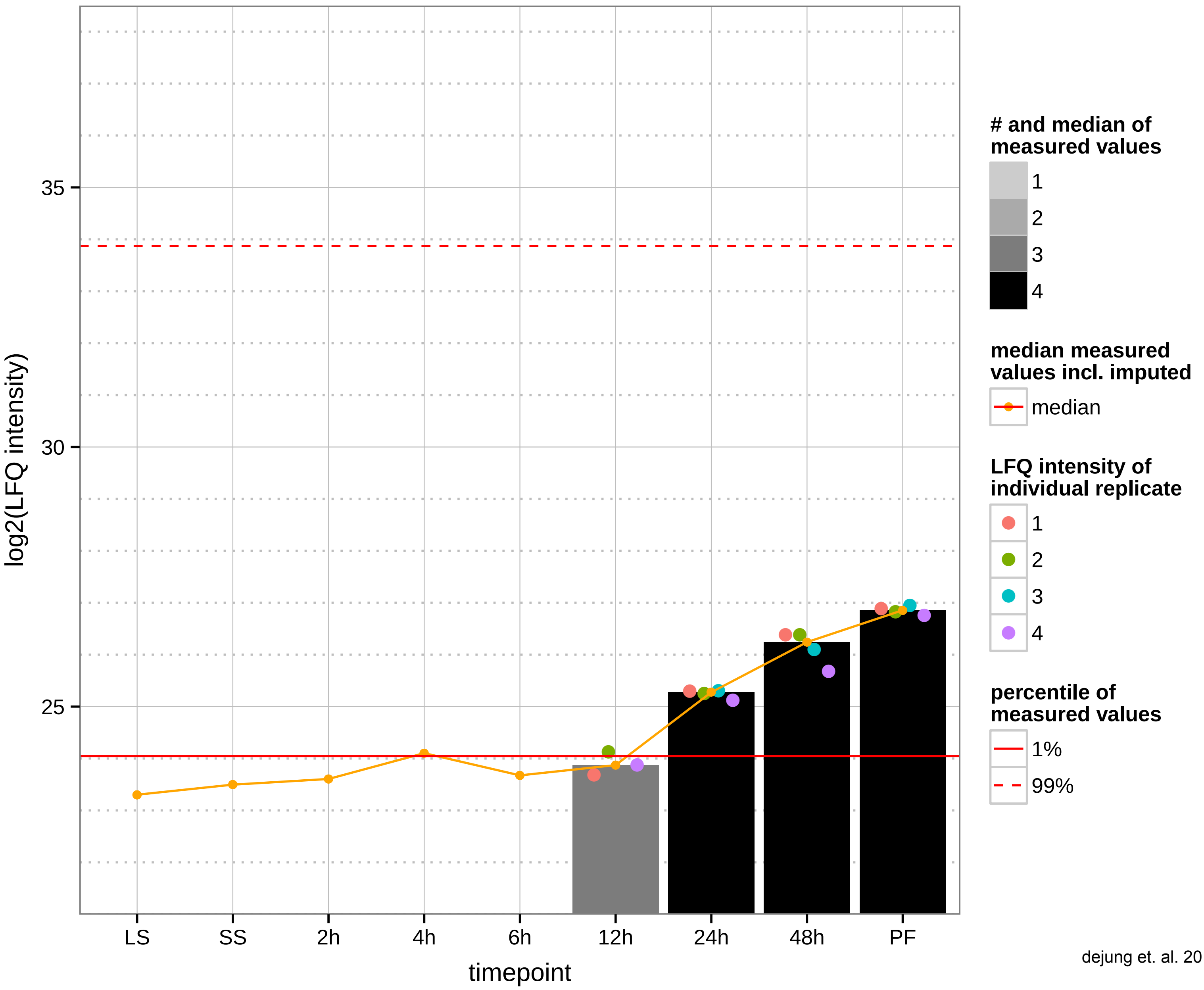

dejung et. al. 2015

hypothetical protein, conserved
Tb927.9.1510
AGOF: ATP binding, catalytic activity
AGOC: null
AGOP: null
PGOF: ATP binding, D–alanine–D–alanine ligase activity, metal ion binding, protein binding
PGOC: null
PGOP: null

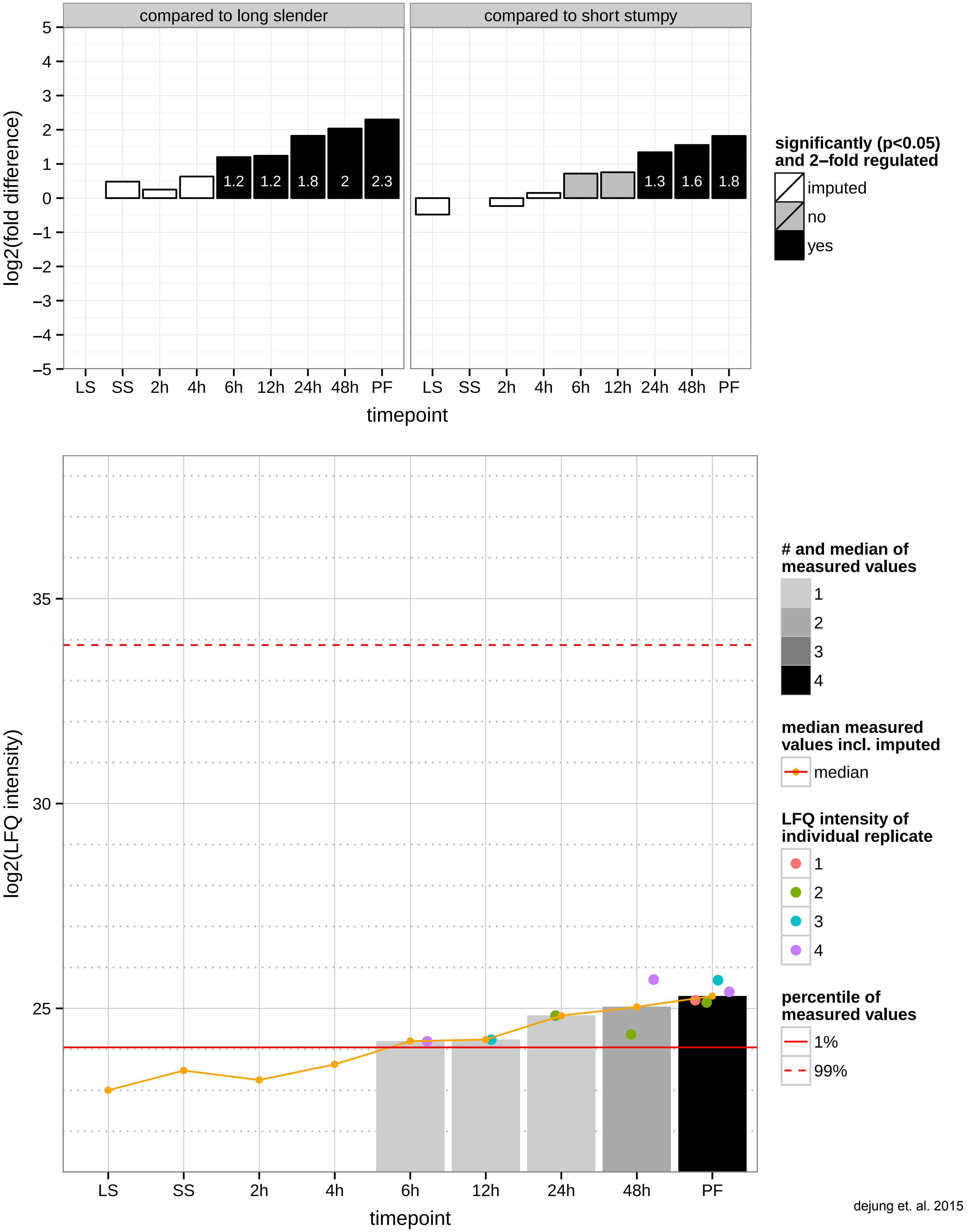

dejung et. al. 2015

60S ribosomal protein L35, putative
Tb927.9.1850;Tb927.9.1810
AGOF: structural constituent of ribosome
AGOC: intracellular, ribosome
AGOP: translation
PGOF: null, structural constituent of ribosome
PGOC: null, intracellular, ribosome
PGOP: null, translation

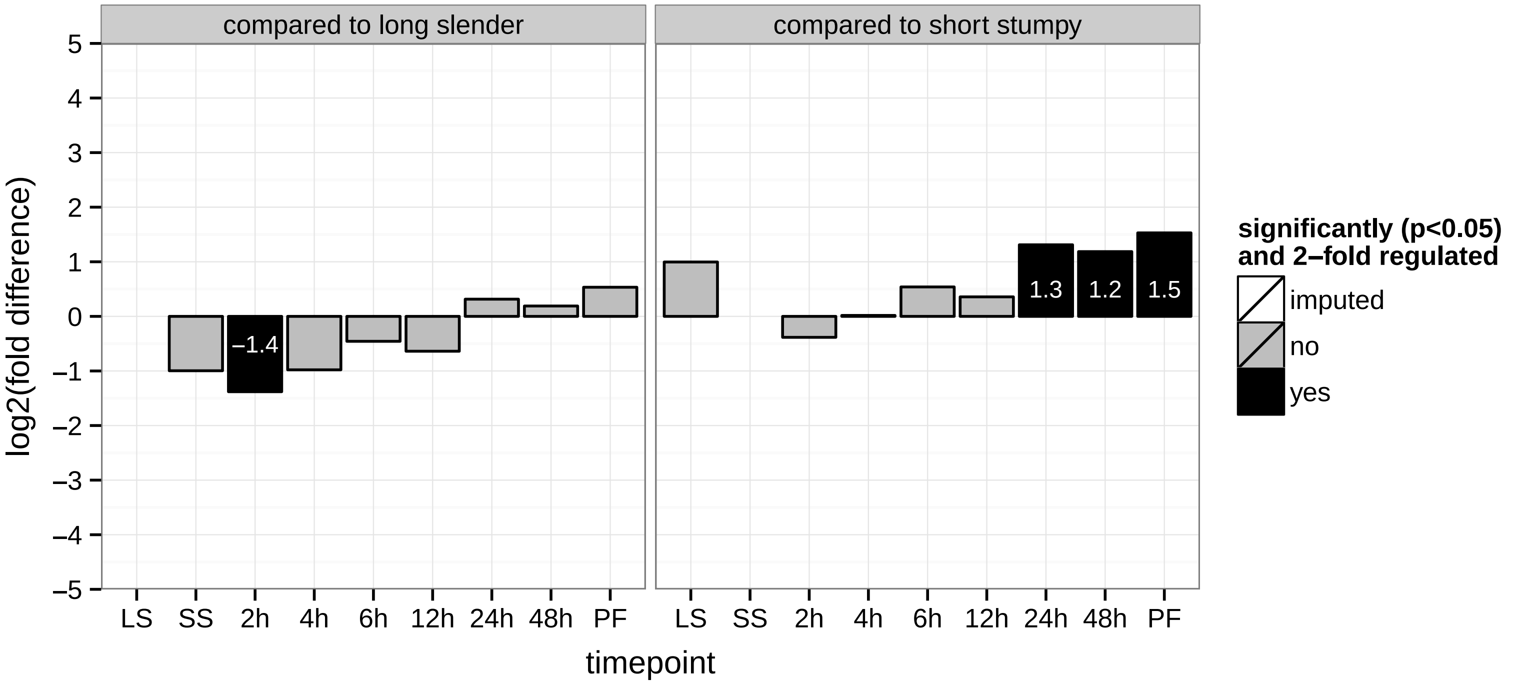

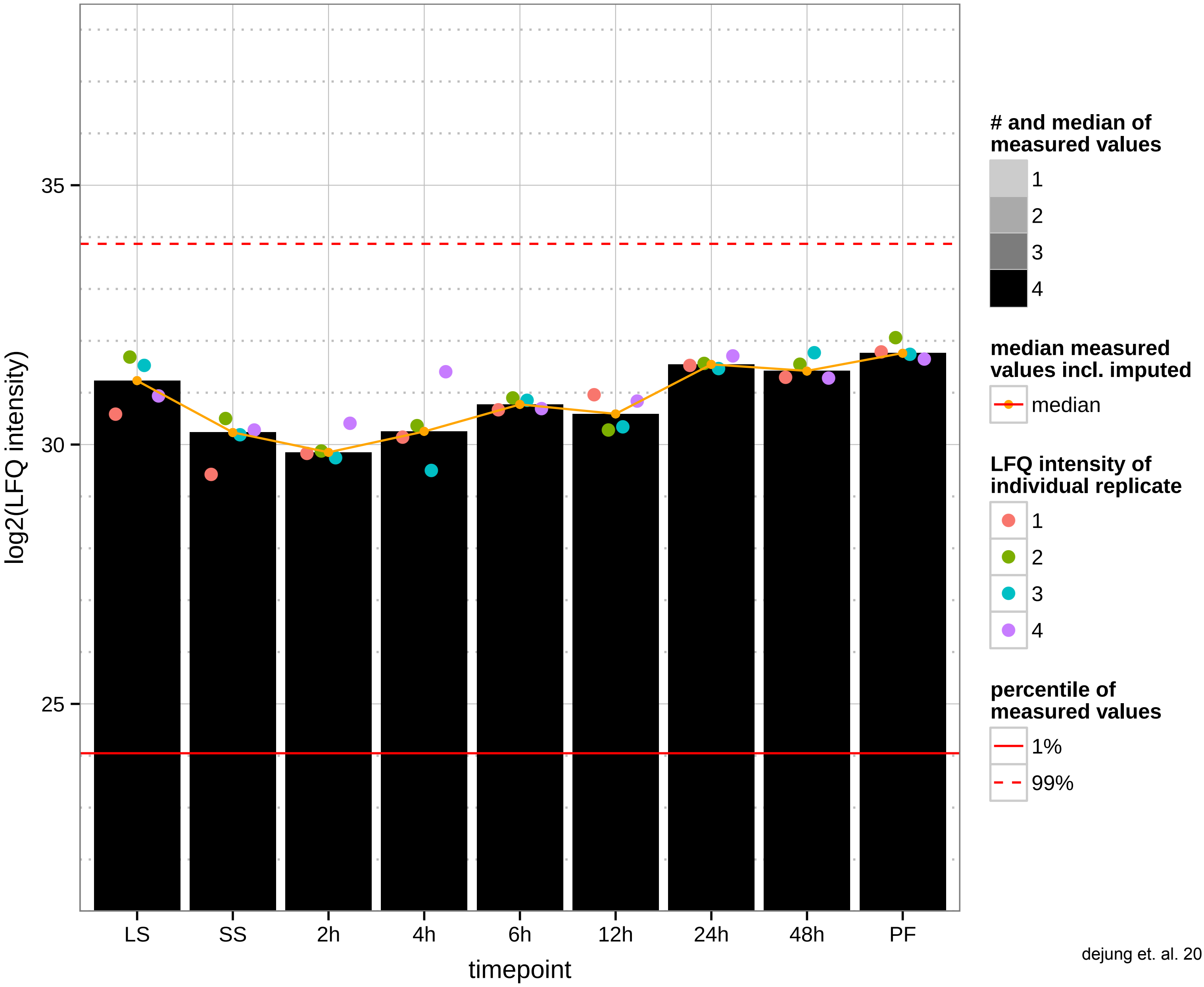

dejung et. al. 2015

hypothetical protein, conserved
Tb927.9.2120
AGOF: null
AGOC: null
AGOP: null
PGOF: null
PGOC: null
PGOP: null

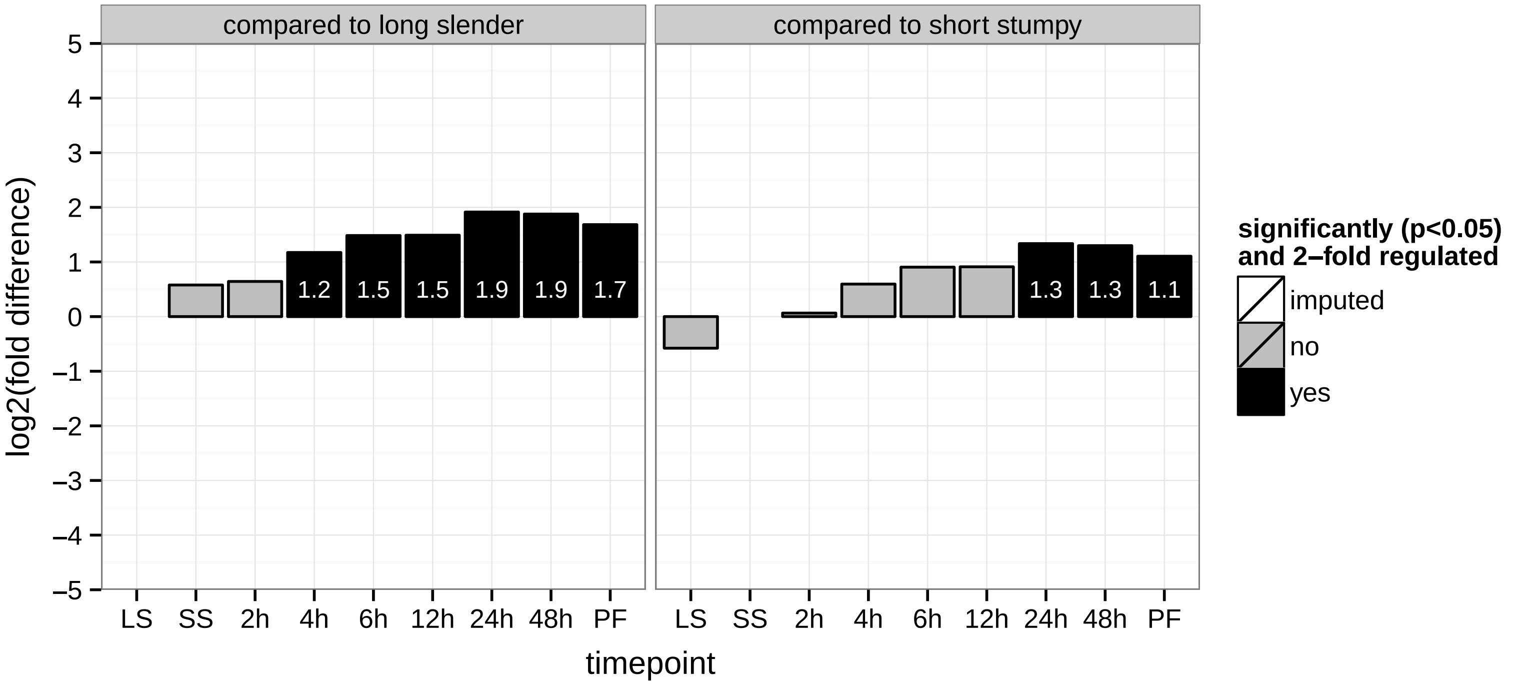

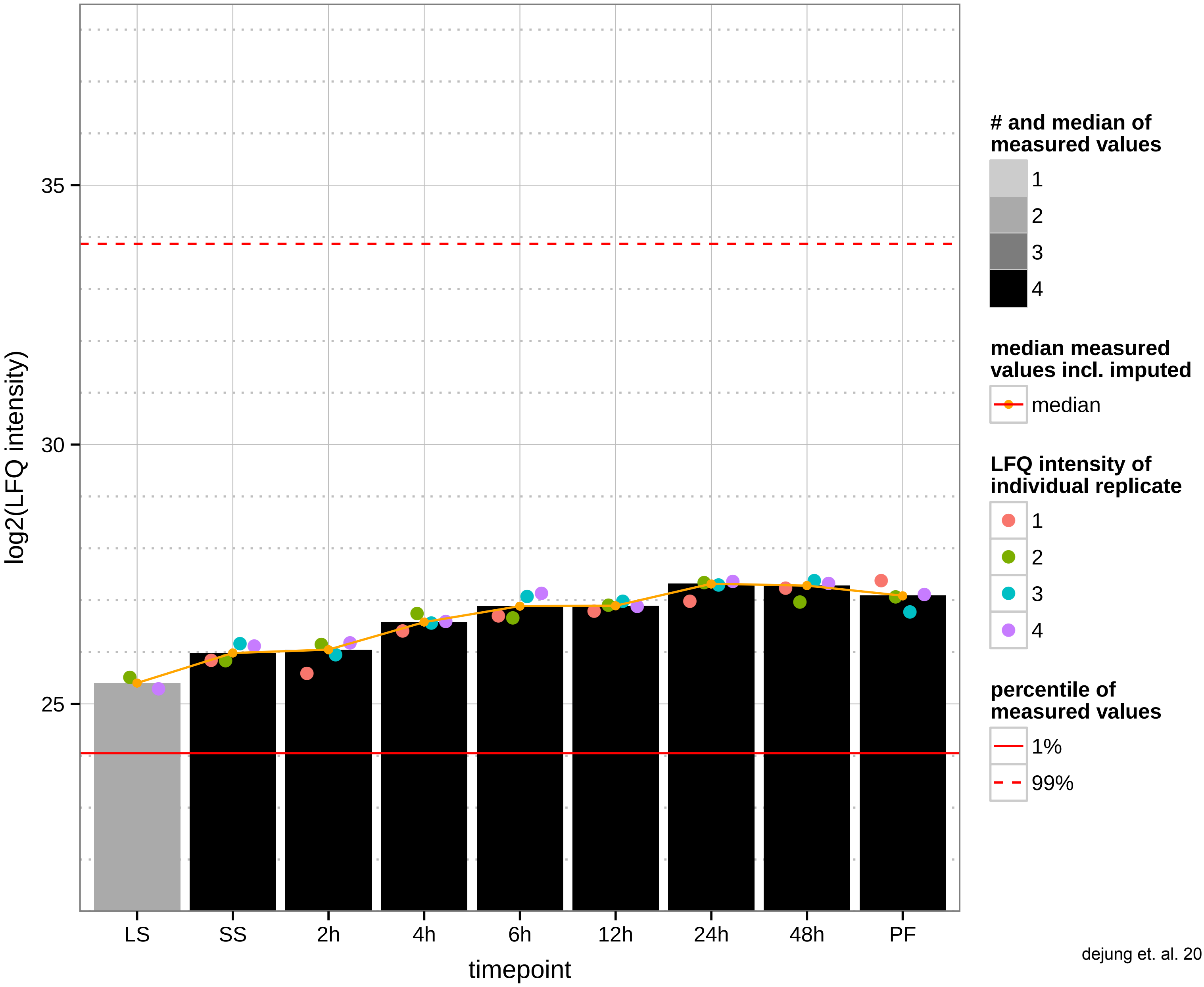

dejung et. al. 2015

electon transport protein SCO1/SCO2, putative
Tb927.9.2450
AGOF: copper ion binding
AGOC: mitochondrial inner membrane
AGOP: oxidation−reduction process
PGOF: null
PGOC: null
PGOP: null

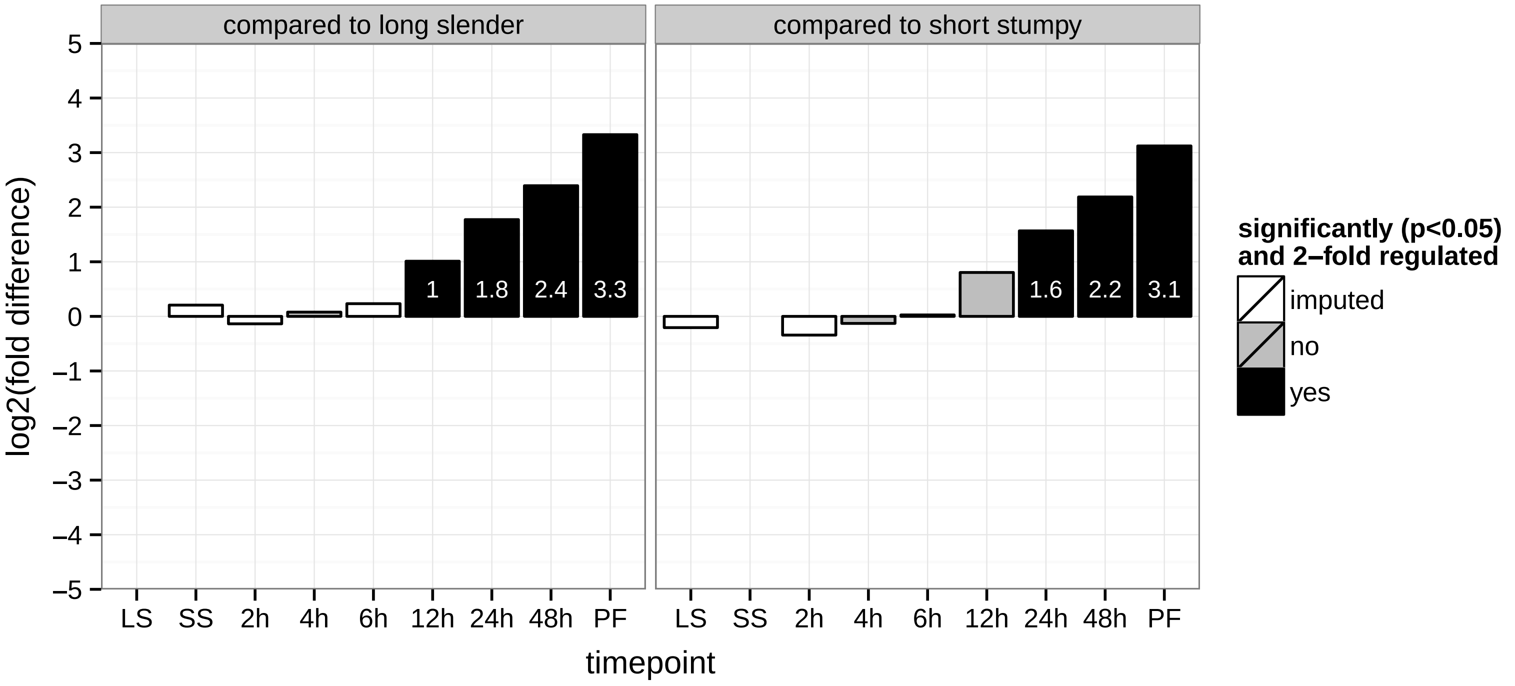

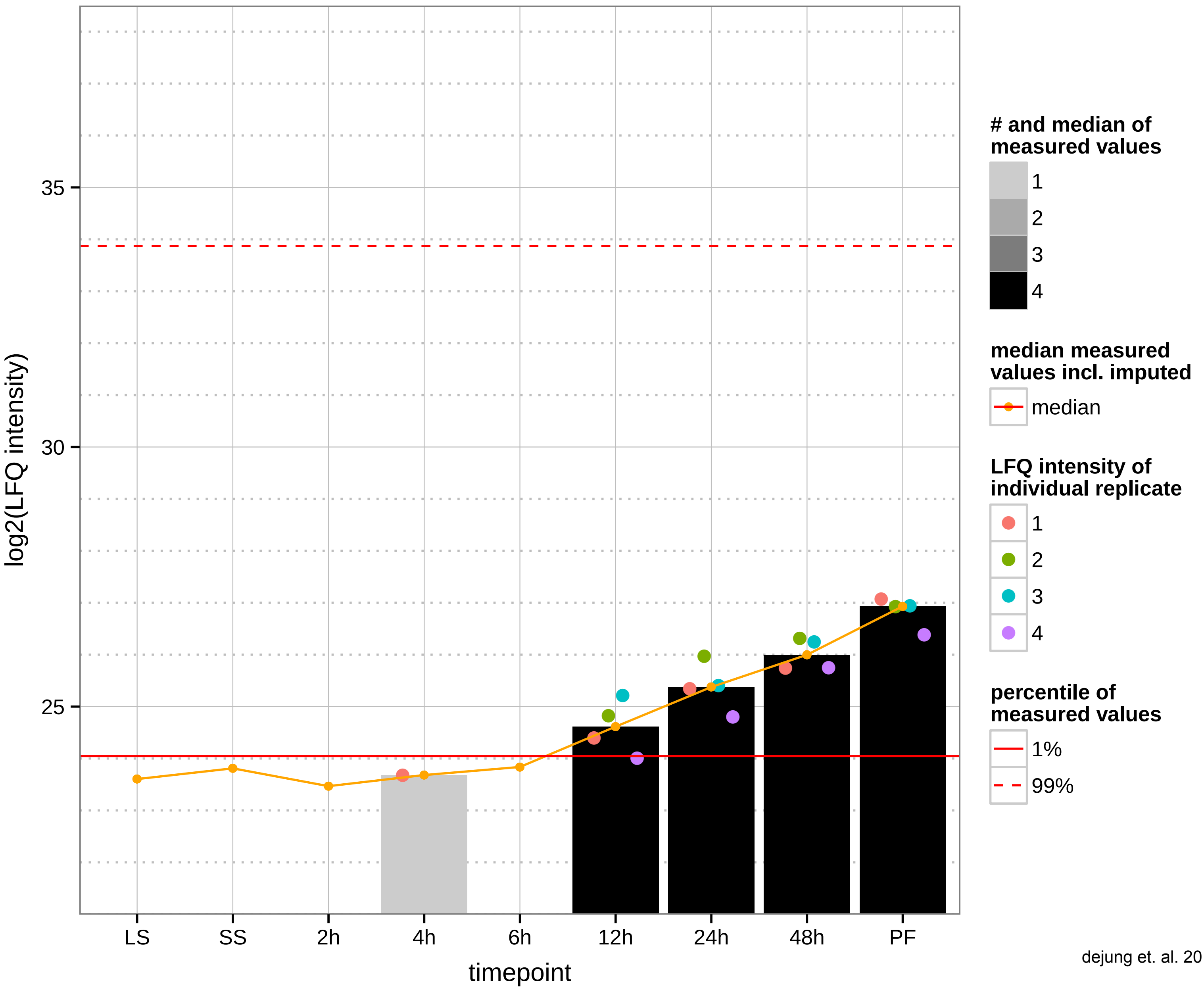

dejung et. al. 2015

fatty acyl CoA synthetase 2 (ACS2)
Tb927.9.4200
AGOF: fatty−acyl−CoA synthase activity
AGOC: membrane
AGOP: fatty acid metabolic process
PGOF: catalytic activity
PGOC: null
PGOP: metabolic process

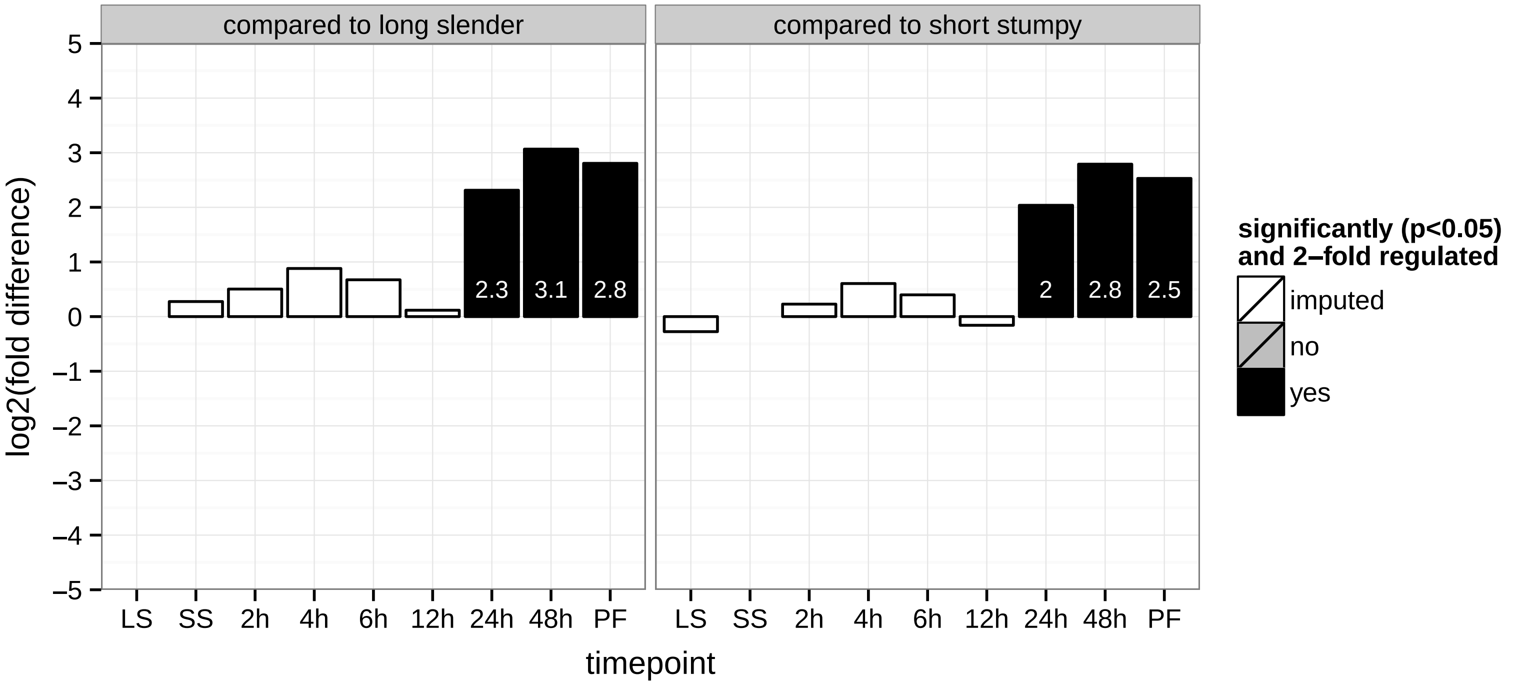

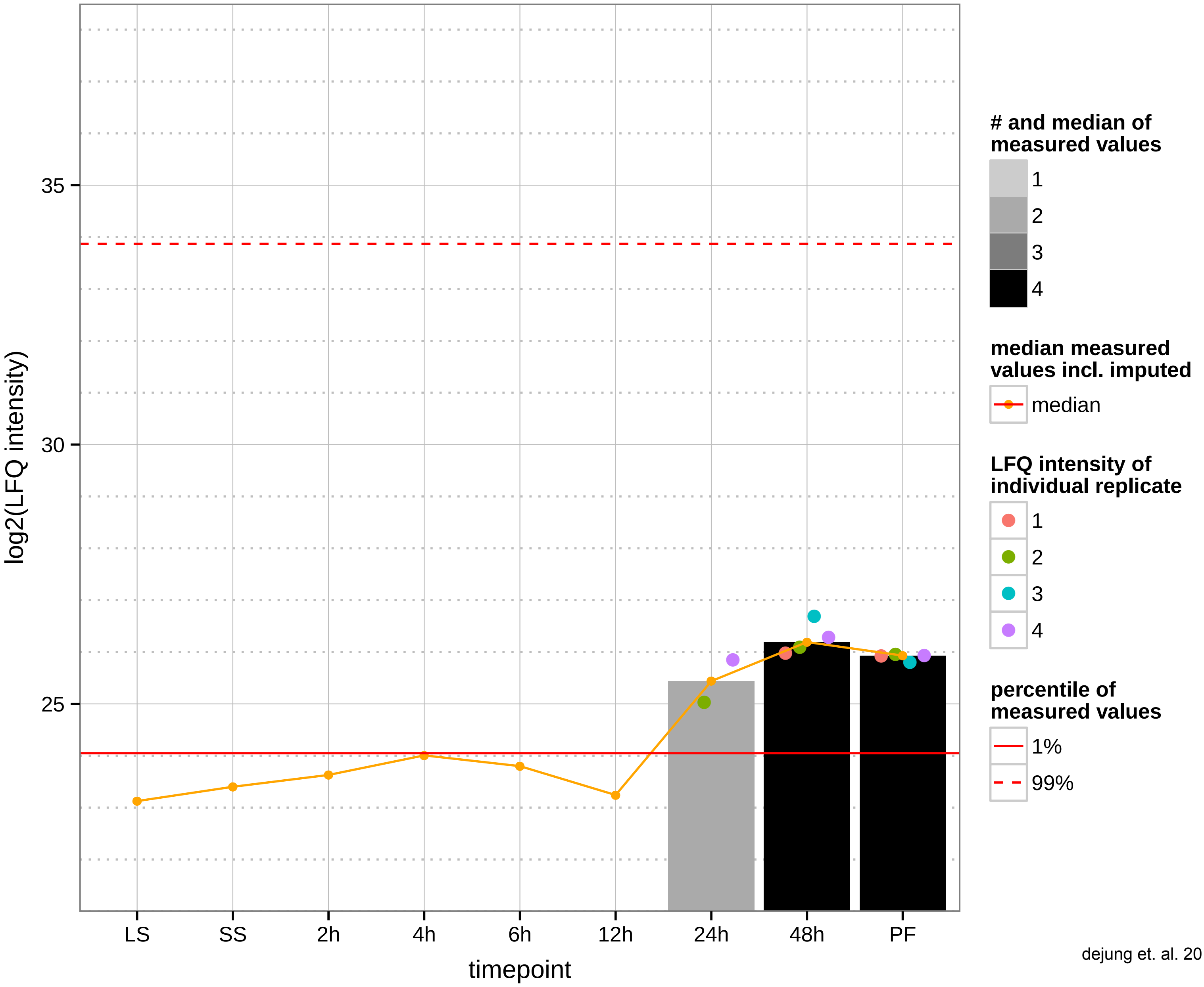

dejung et. al. 2015

60S acidic ribosomal protein, putative
Tb927.9.5690
AGOF: structural constituent of ribosome
AGOC: intracellular, ribosome
AGOP: translation, translational elongation
PGOF: structural constituent of ribosome
PGOC: intracellular, ribosome
PGOP: translational elongation

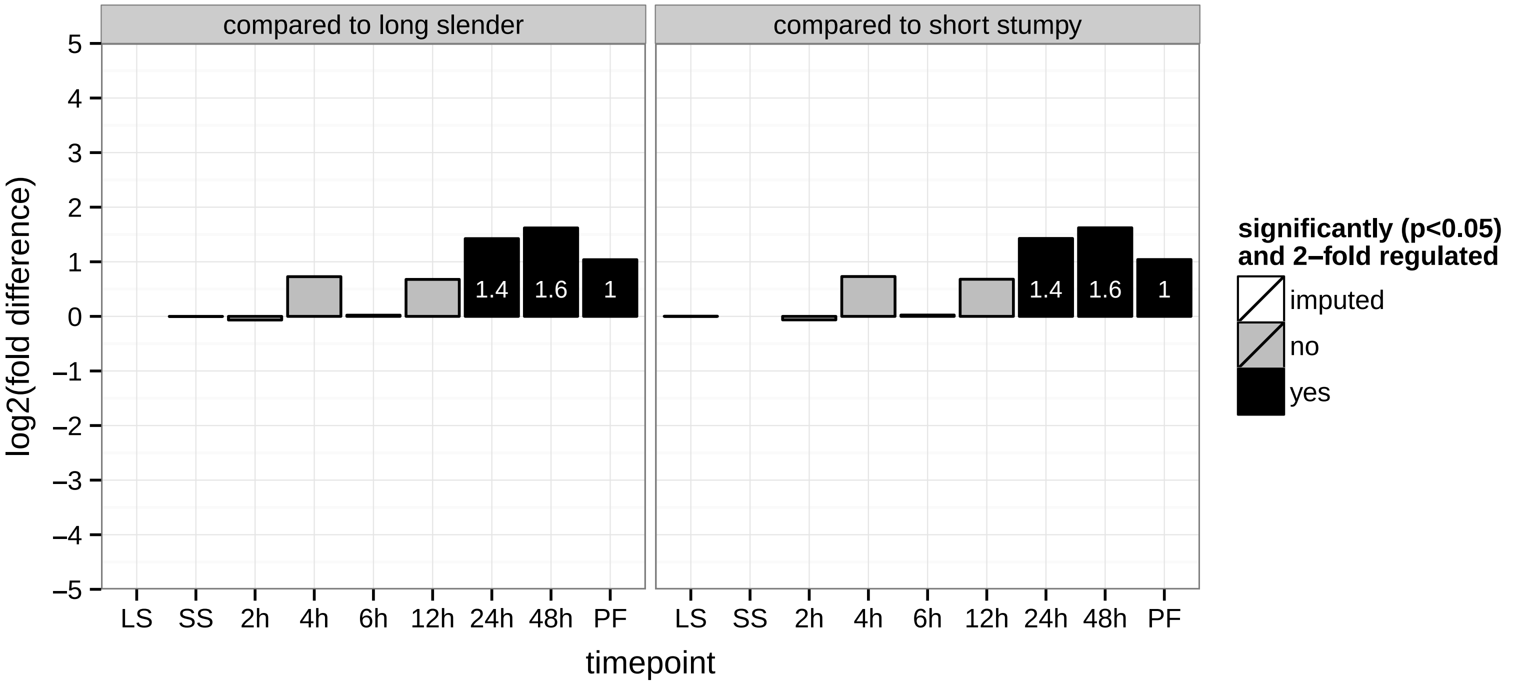

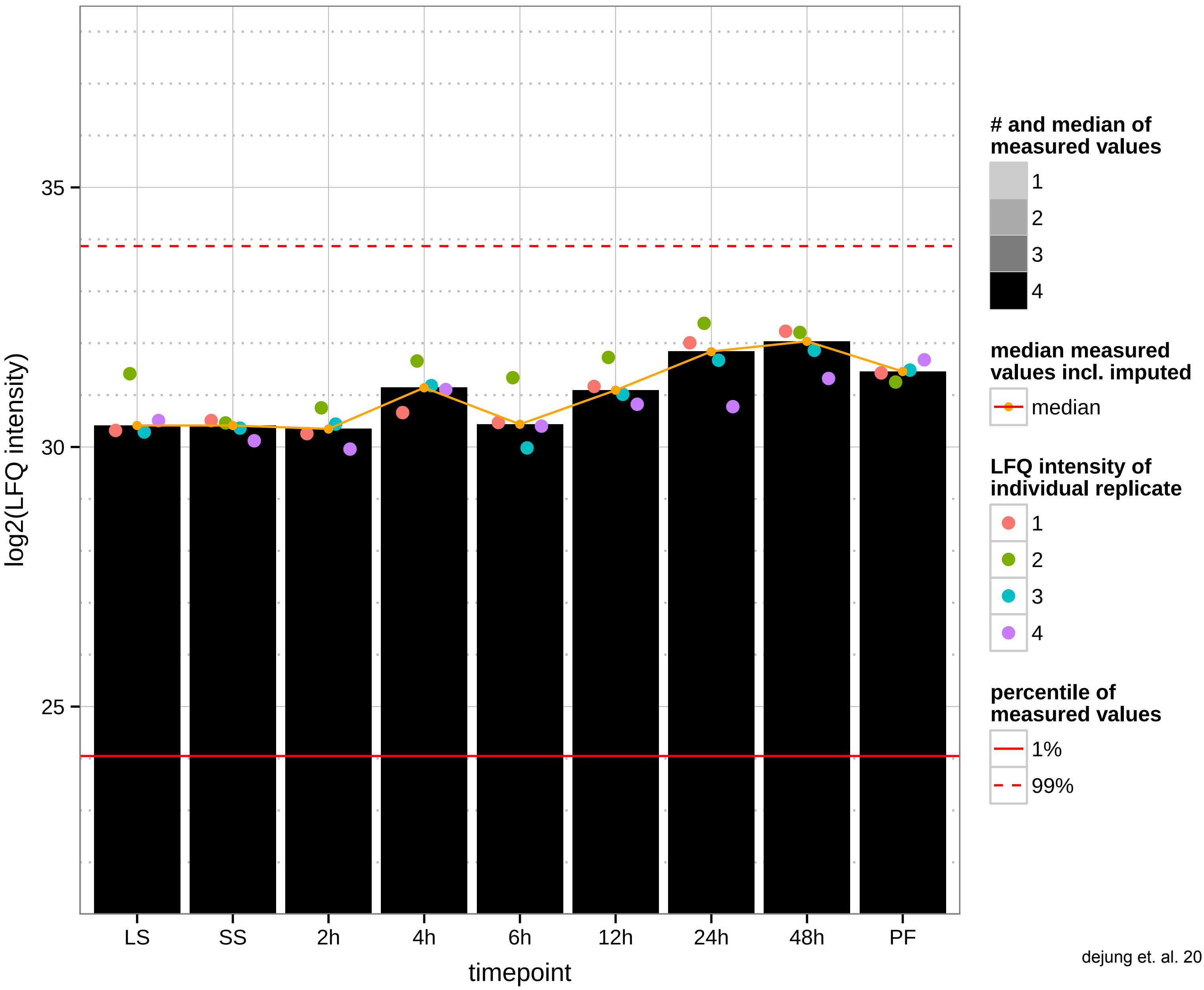

dejung et. al. 2015

succinate dehydrogenase, putative
Tb927.9.5960
AGOF: iron–sulfur cluster binding, oxidoreductase activity
AGOC: mitochondrion
AGOP: null
PGOF: iron–sulfur cluster binding
PGOC: null
PGOP: null

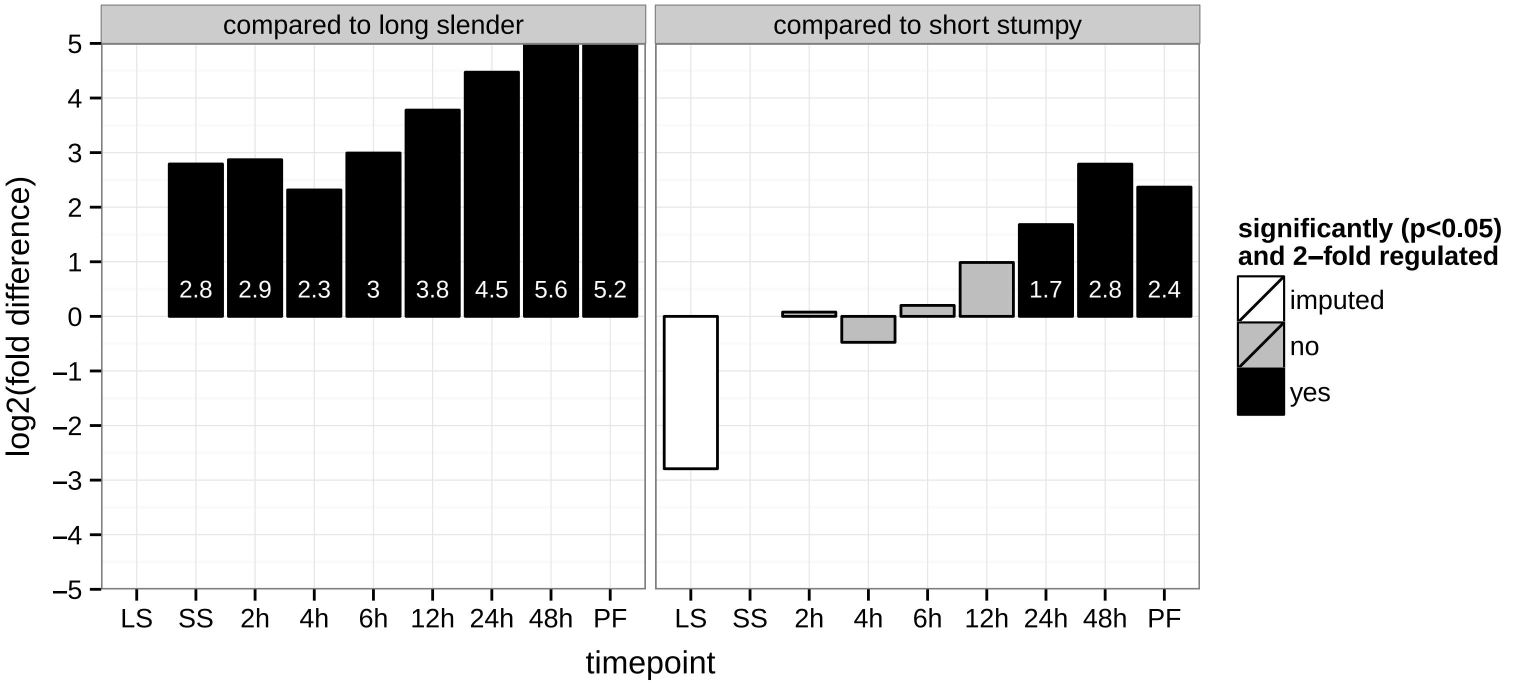

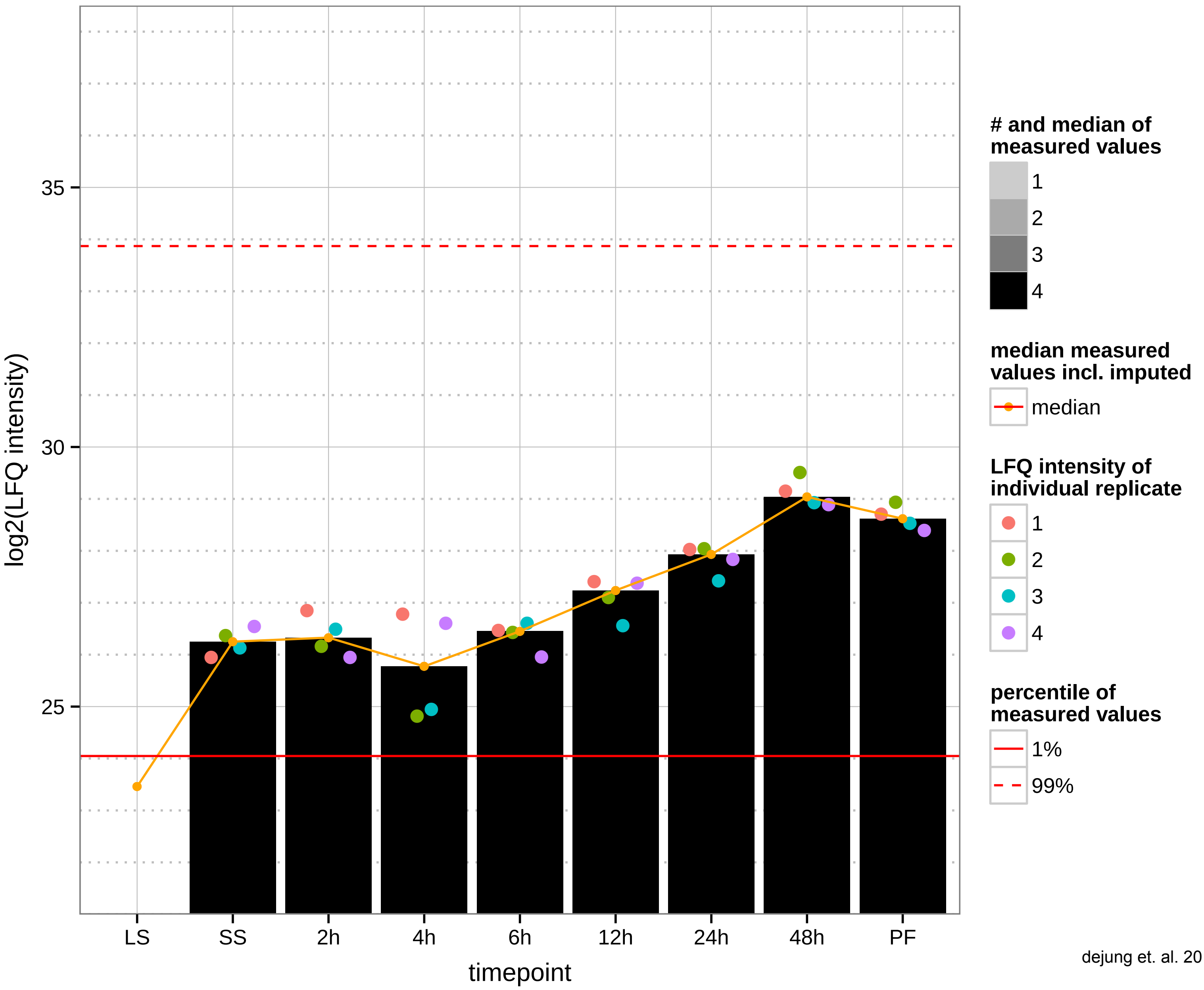

dejung et. al. 2015

hypothetical protein, conserved
Tb927.9.7830
AGOF: null
AGOC: mitochondrial membrane, mitochondrion
AGOP: null
PGOF: null
PGOC: null
PGOP: null

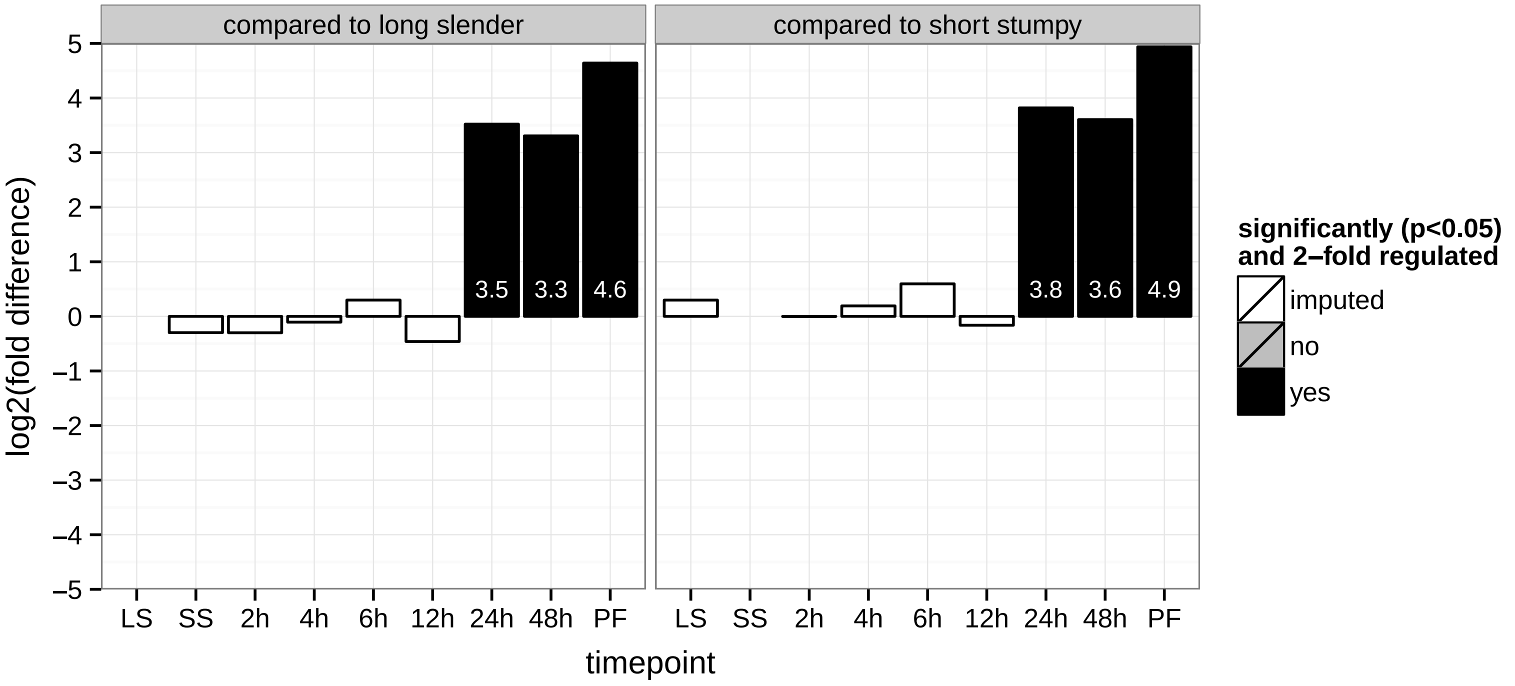

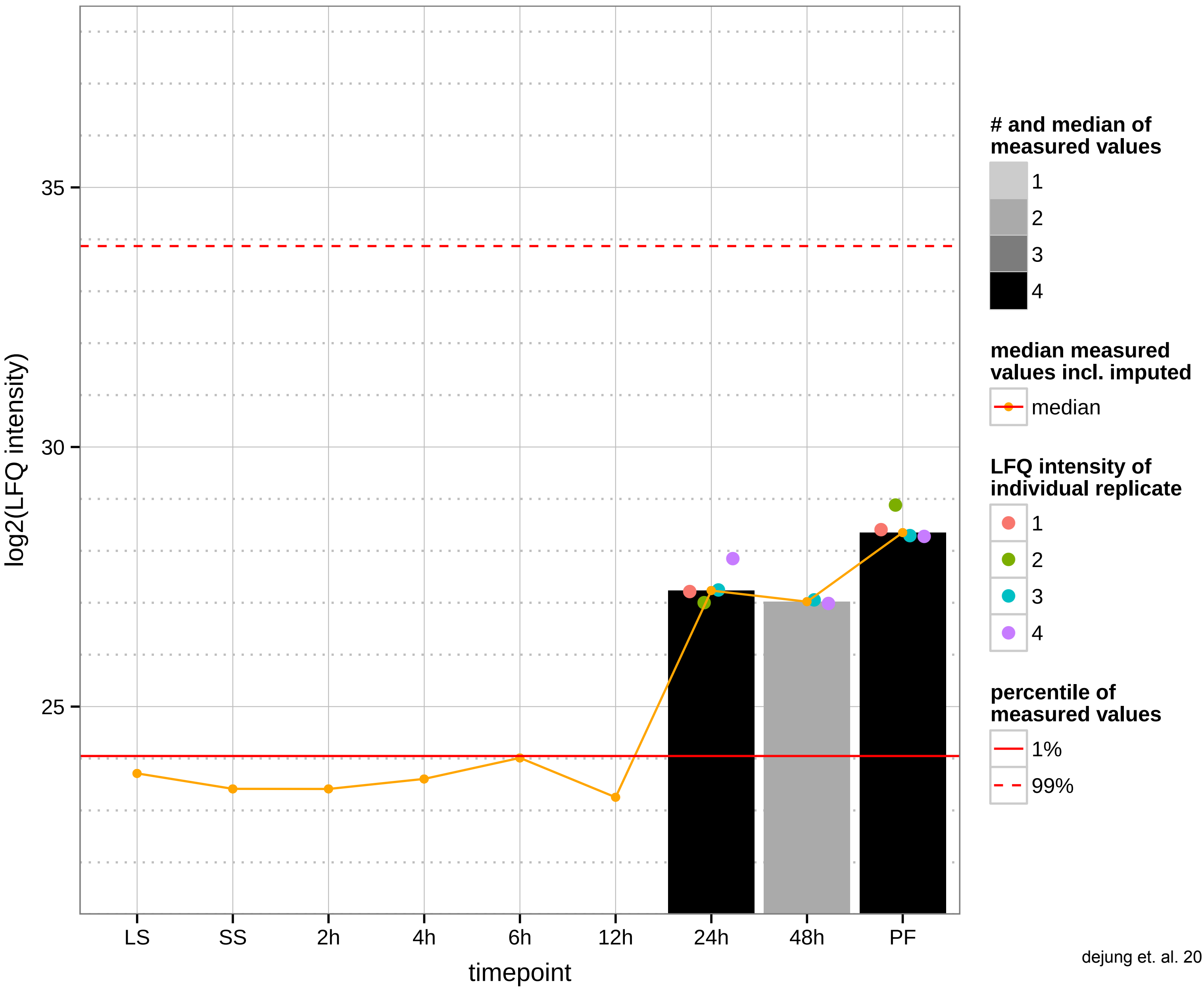

dejung et. al. 2015

nascent polypeptide associated complex subunit, putative
Tb927.9.8130;Tb927.9.8100
AGOF: transcription coactivator activity
AGOC: nascent polypeptide–associated complex
AGOP: 'de novo' cotranslational protein folding, translation
PGOF: null
PGOC: null
PGOP: null

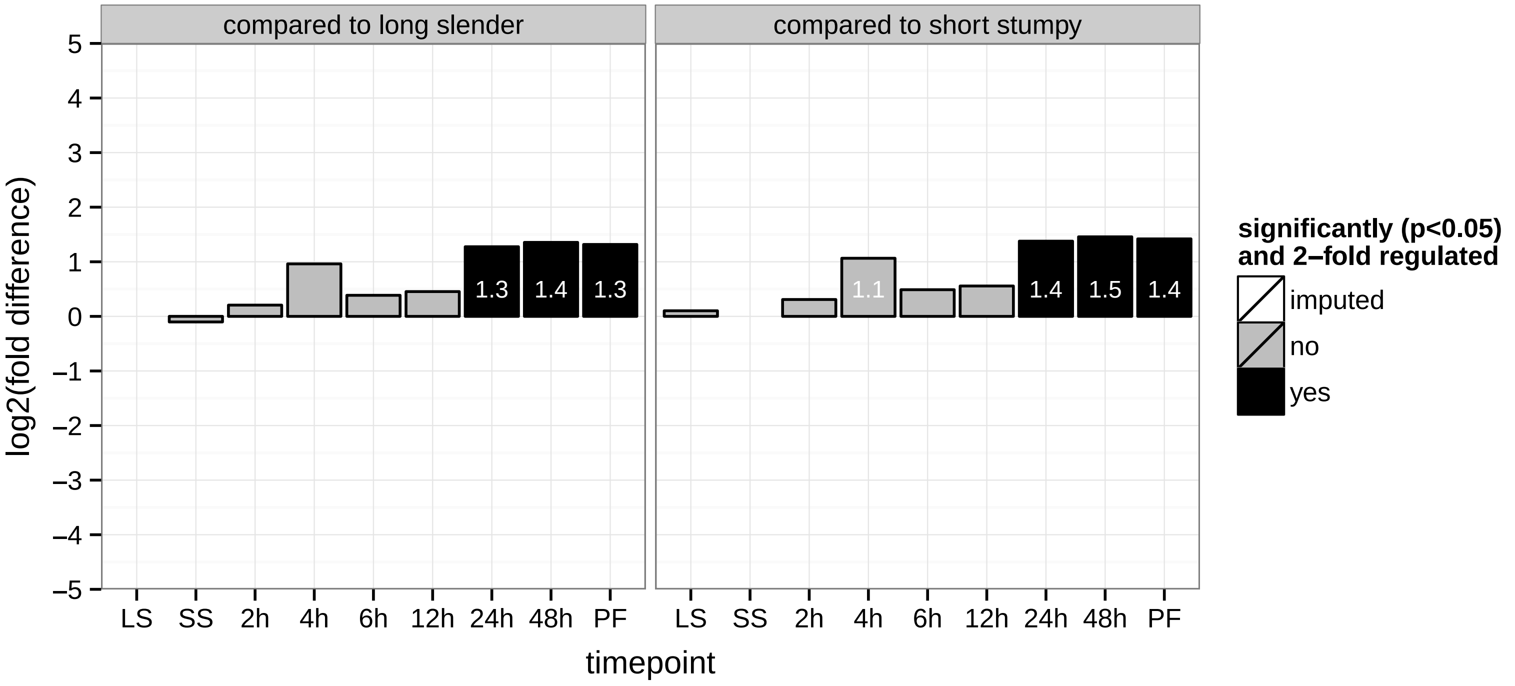

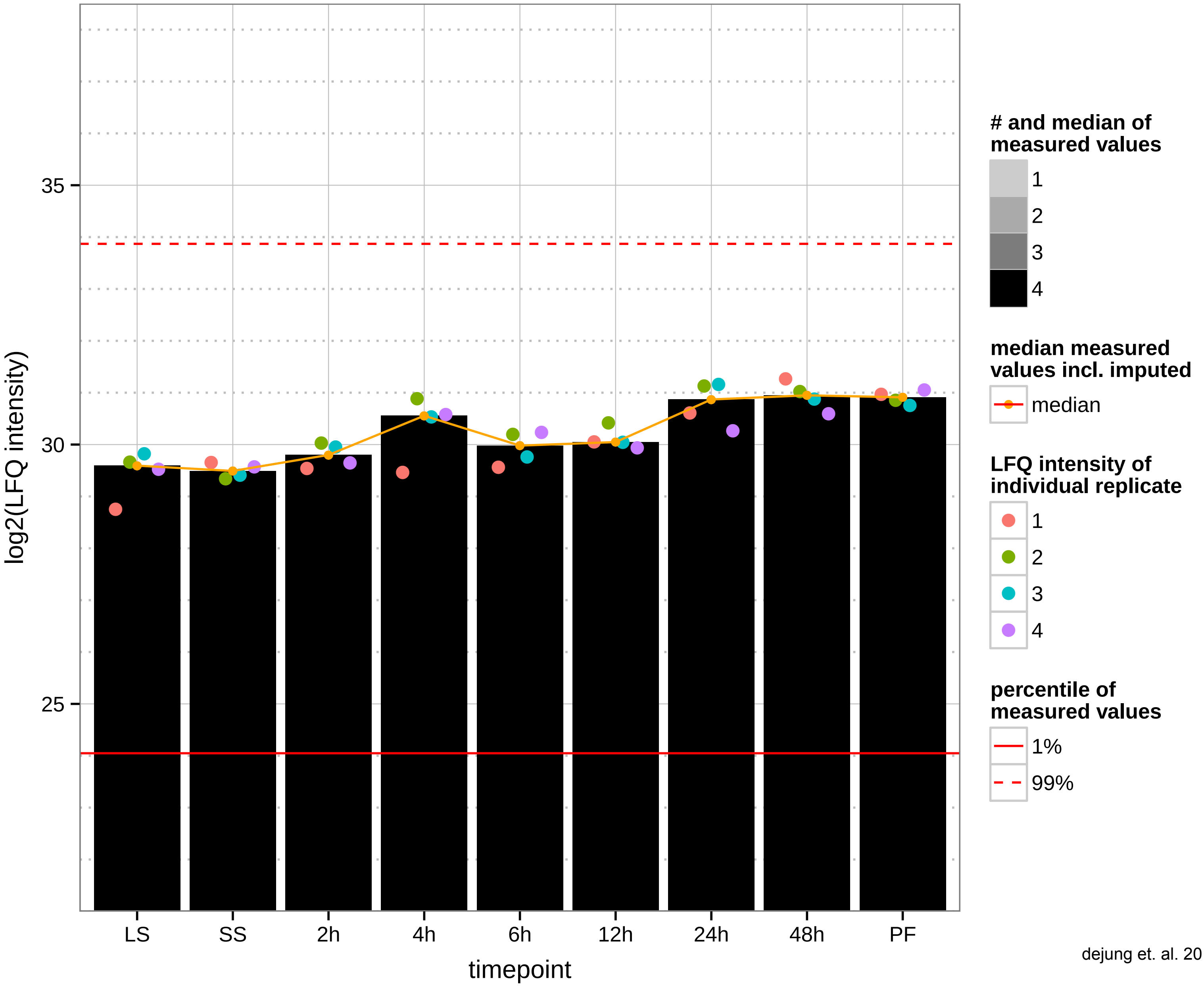

dejung et. al. 2015

ribosome−interacting GTPase 2, putative (RBG2)
Tb927.9.9020
AGOF: GTP binding
AGOC: cytoplasm, intracellular
AGOP: cytoplasmic translation
PGOF: GTP binding
PGOC: null
PGOP: null

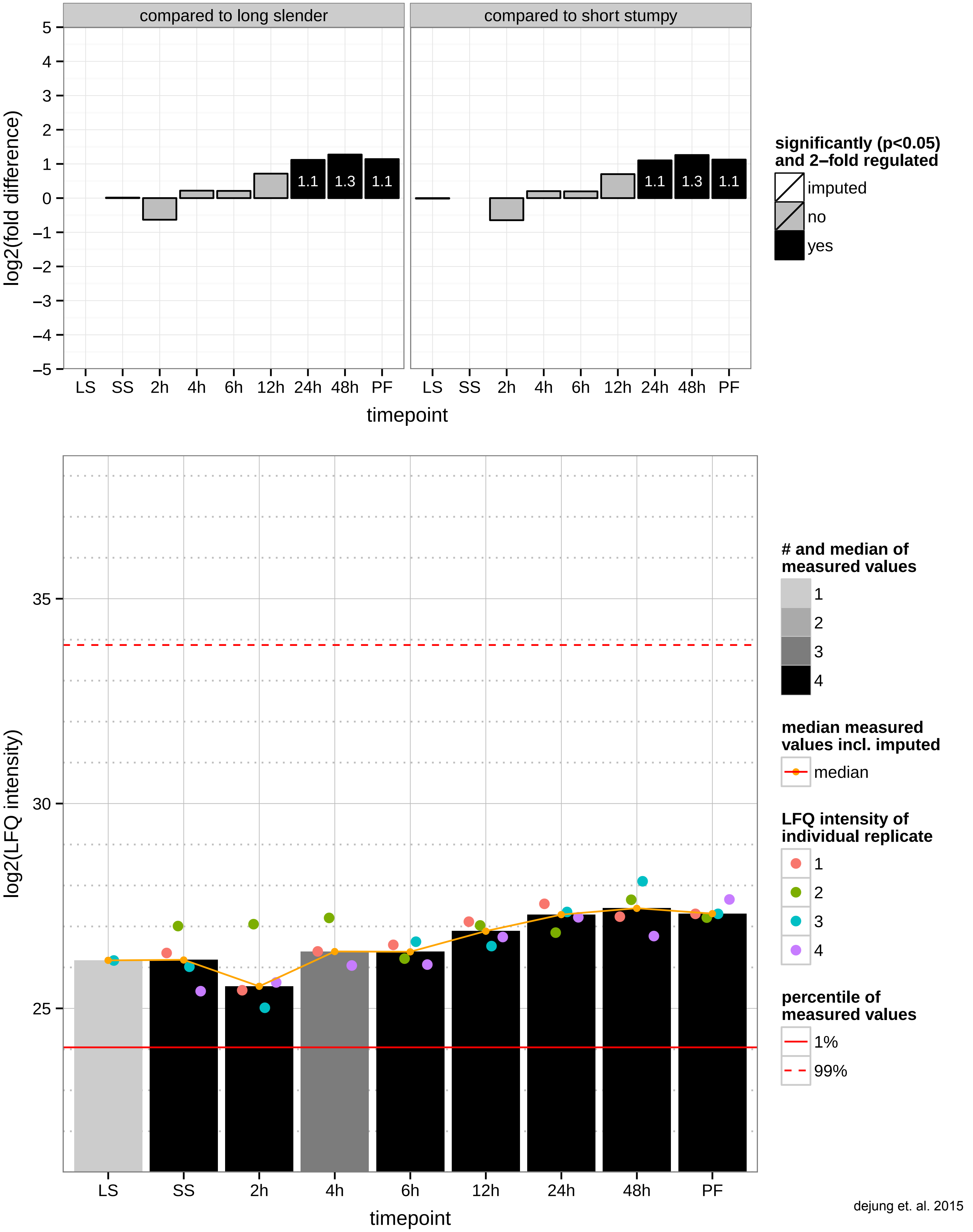

dejung et. al. 2015

zinc finger protein family member, putative (ZC3H28)
Tb927.9.9450
AGOF: RNA binding, zinc ion binding
AGOC: null
AGOP: null
PGOF: nucleic acid binding, zinc ion binding
PGOC: null
PGOP: null

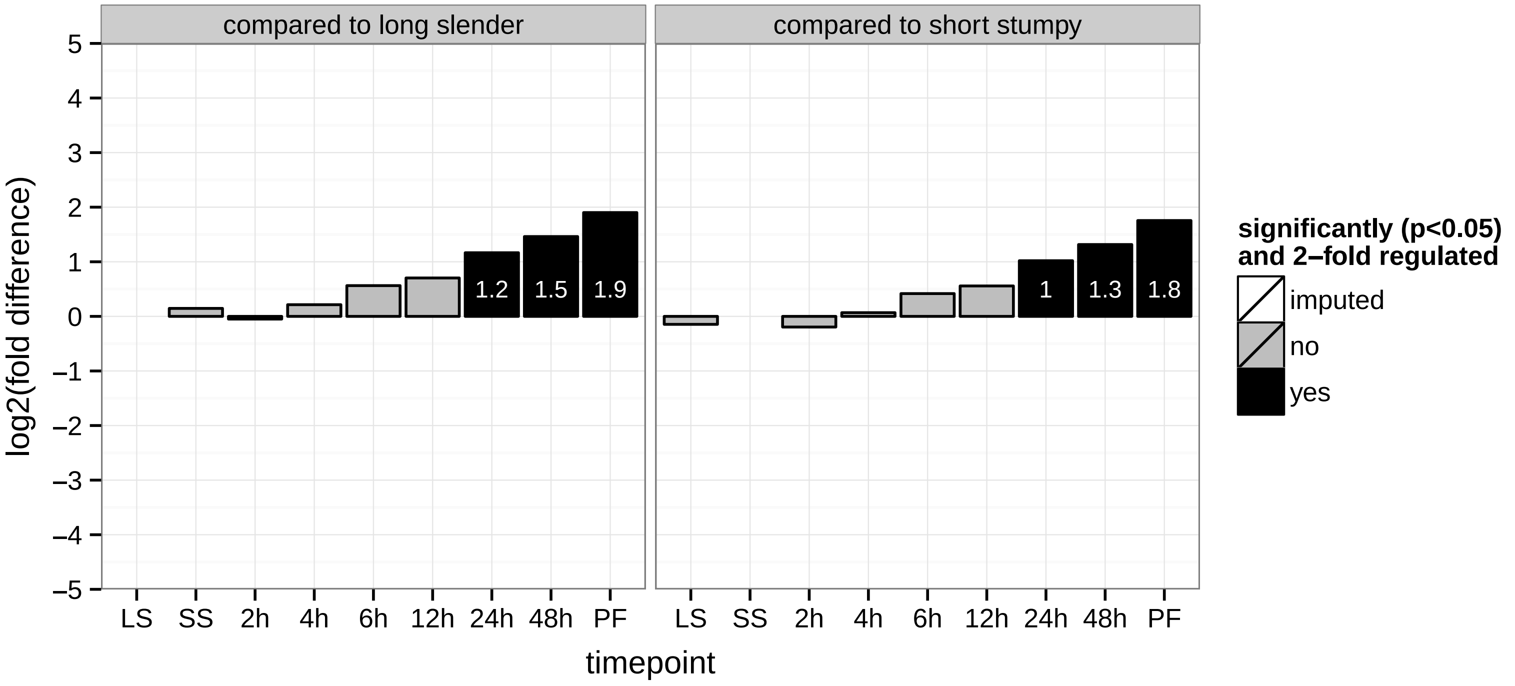

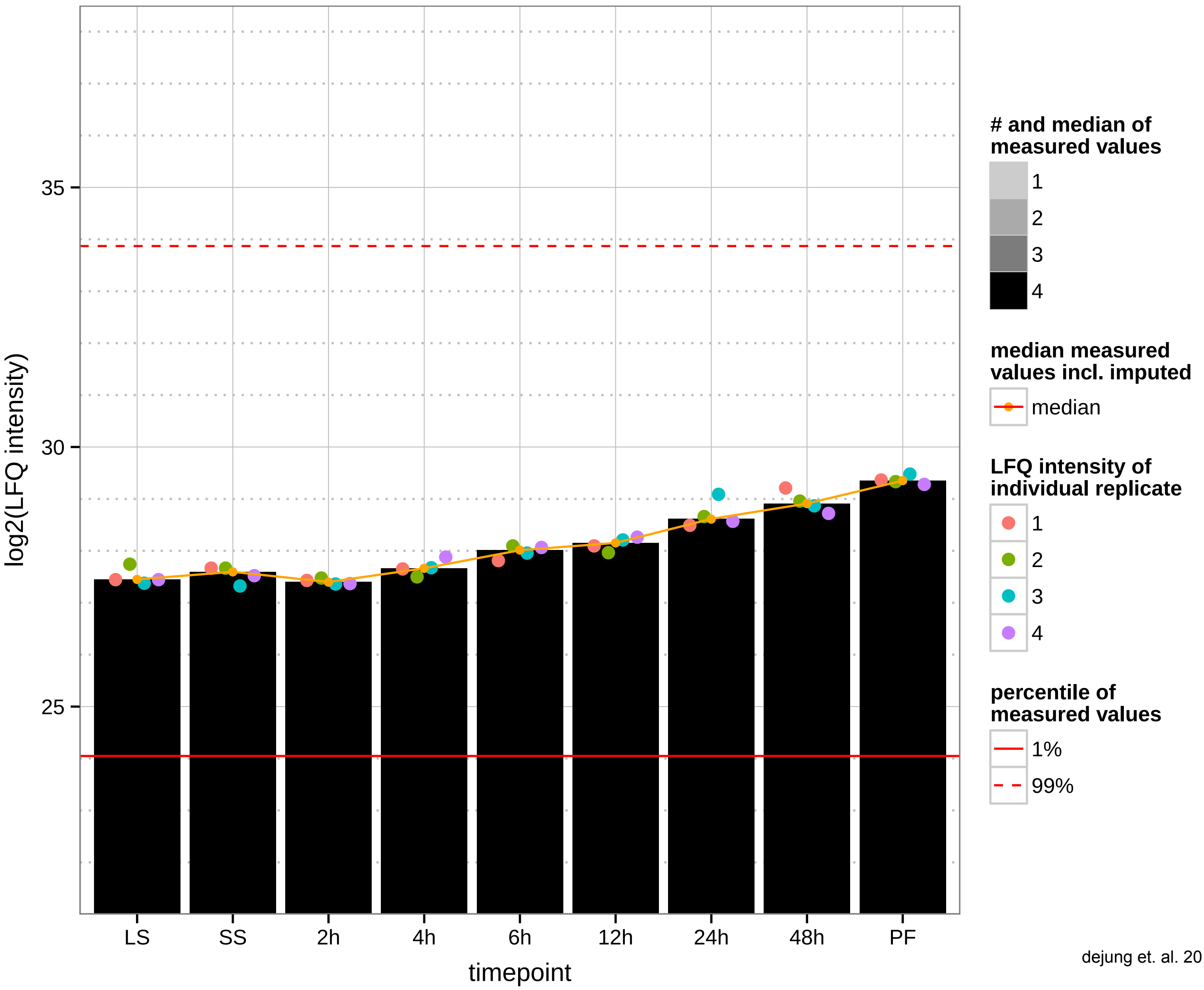

dejung et. al. 2015

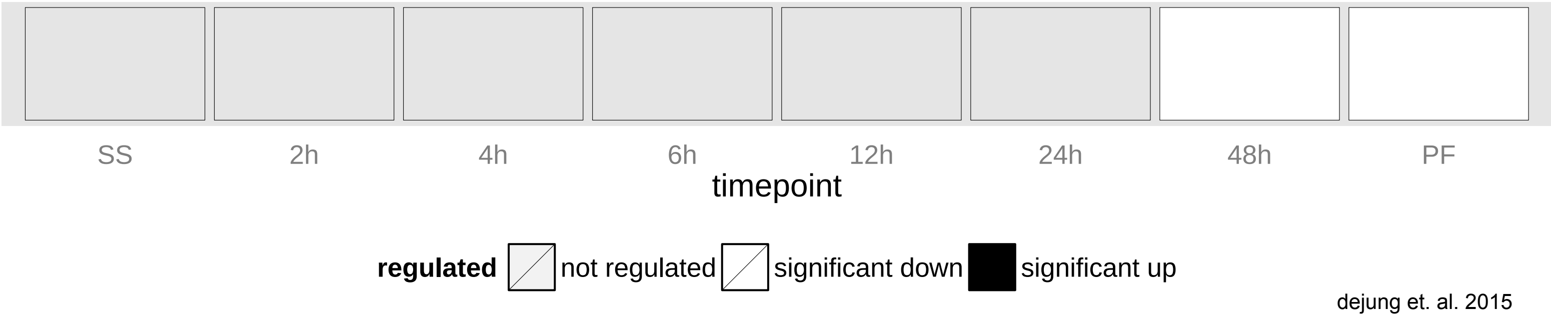
SS
2h
4h
6h
12h
24h
48h
PF
timepoint
regulated
not regulated
significant down
significant up
dejung et. al. 2015

hypothetical protein, BAC from homologous region on chr5, conserved
Tb927.5.4570;Tb05.5K5.210
AGOF: null
AGOC: null
AGOP: null
PGOF: null
PGOC: null
PGOP: null

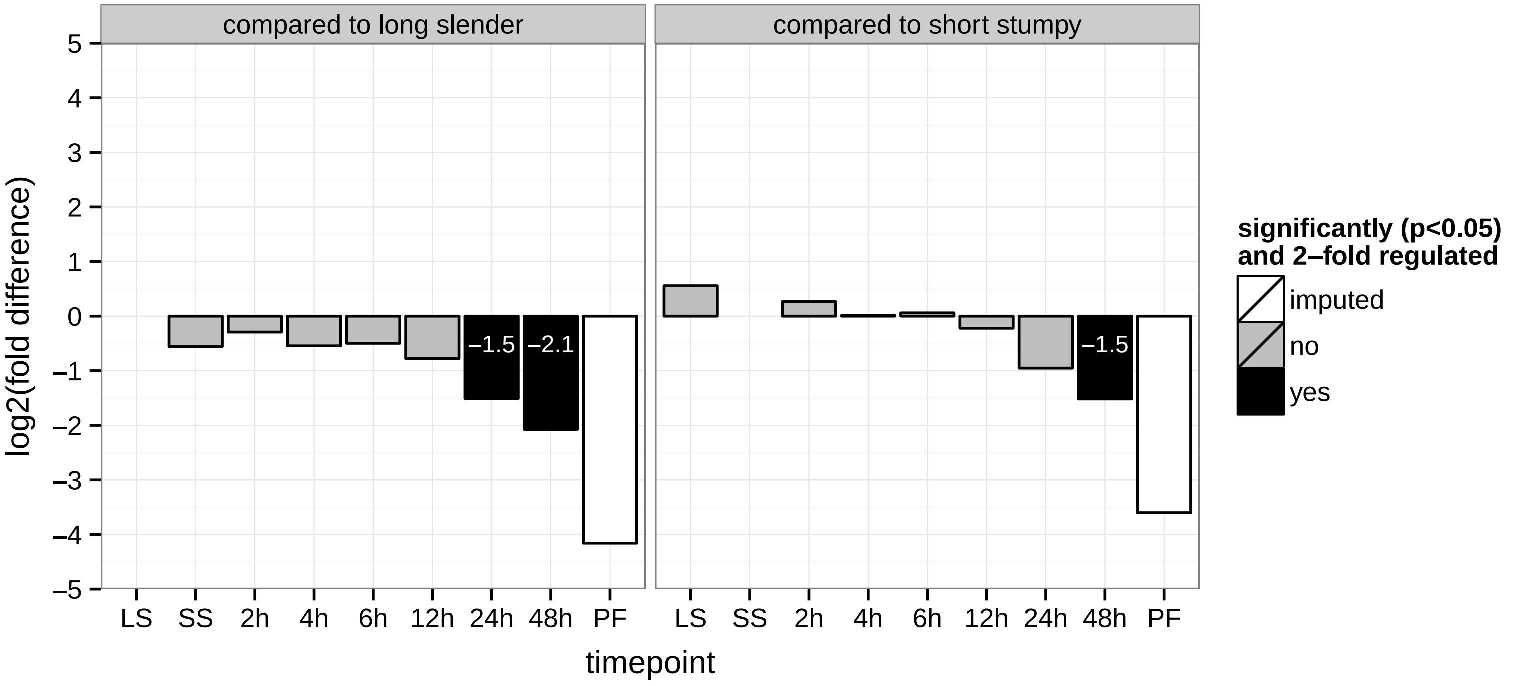

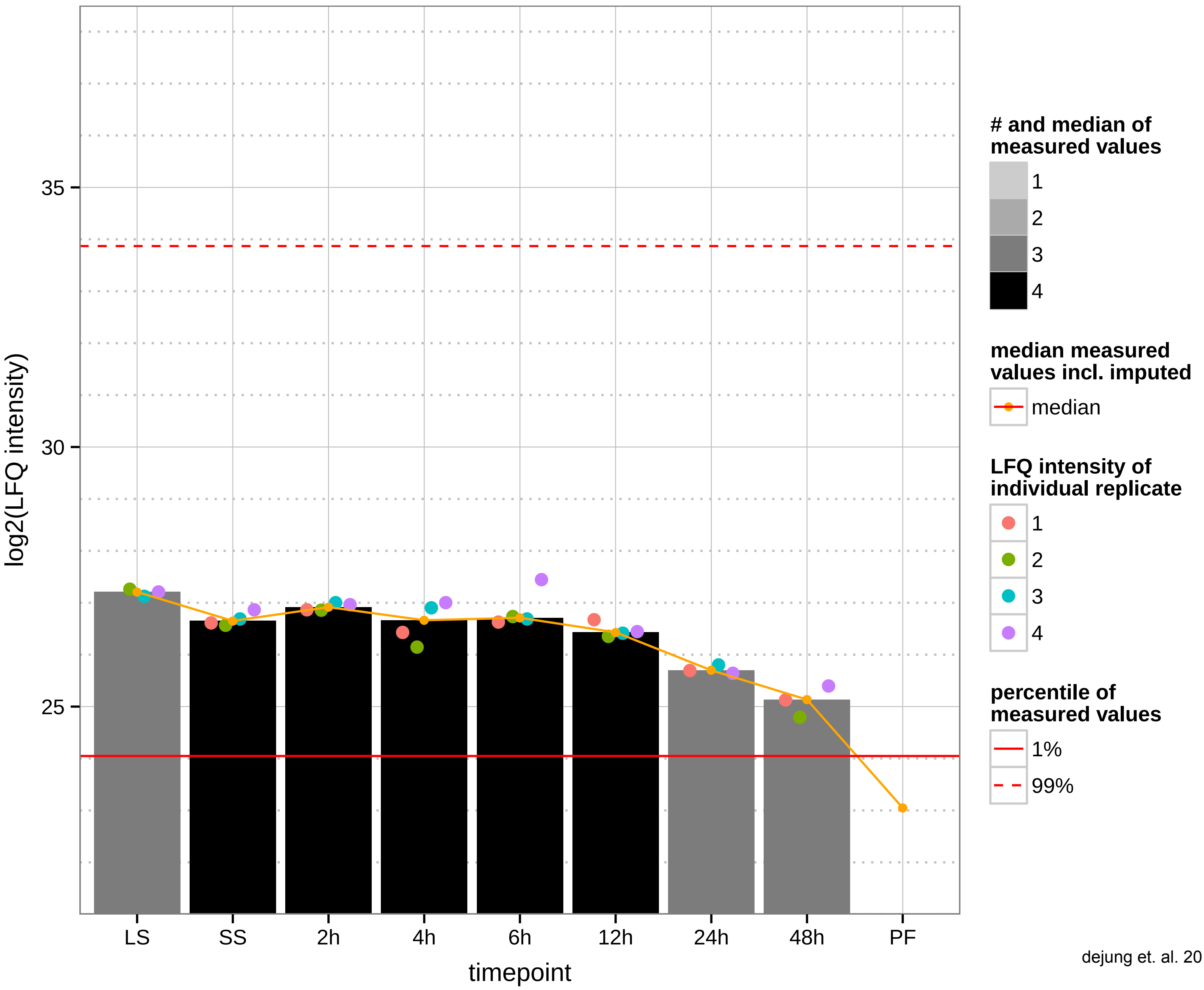

dejung et. al. 2015

hypothetical protein, chrIX additional, unordered contigs
Tb927.9.15870;Tb09.v4.0015
AGOF: null
AGOC: null
AGOP: null
PGOF: null
PGOC: null
PGOP: null

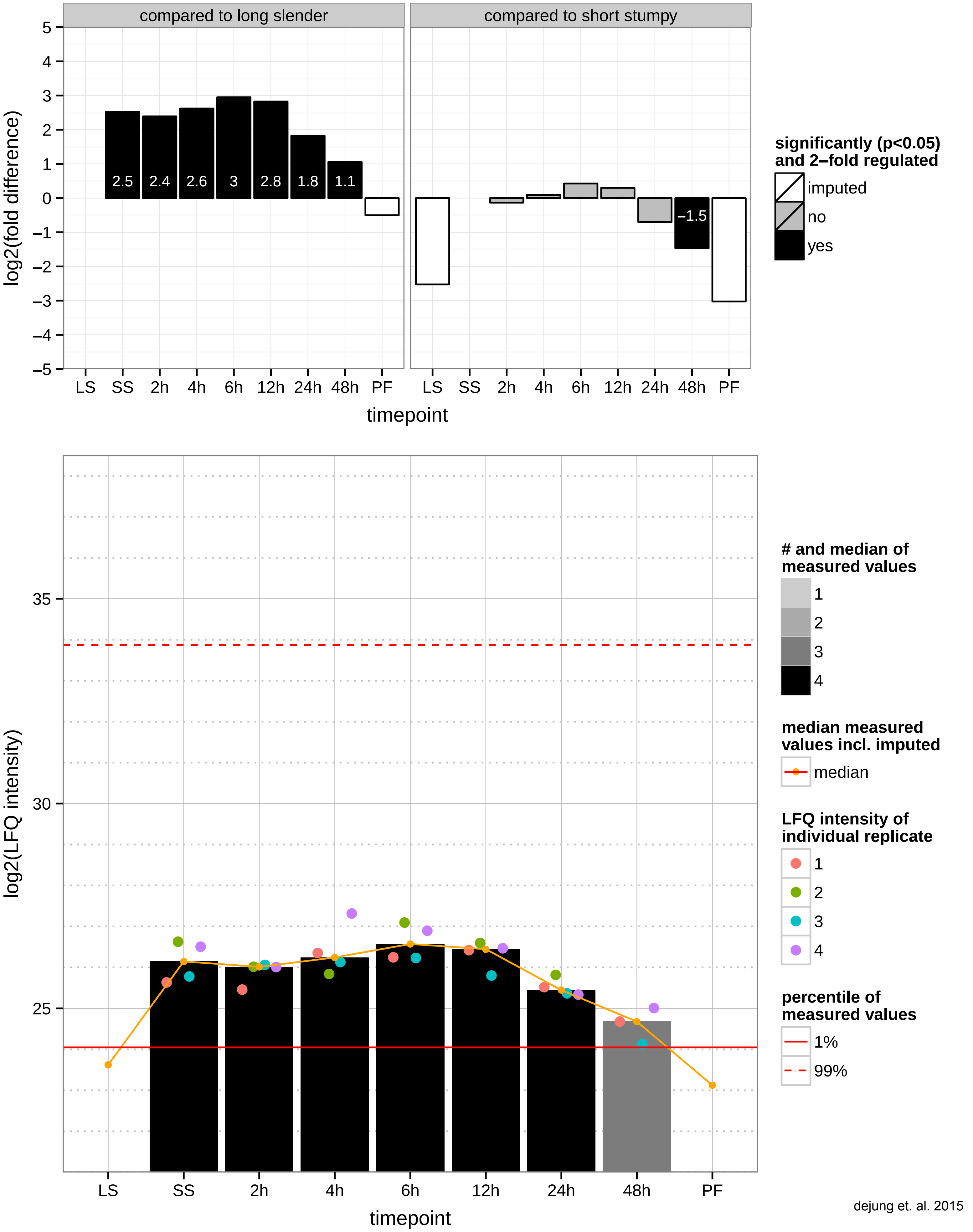

dejung et. al. 2015

hypothetical protein, conserved
Tb927.7.4500;Tb11.v5.0263
AGOF: null, phosphatidylinositol binding
AGOC: null
AGOP: null, cell communication
PGOF: phosphatidylinositol binding, protein binding
PGOC: null
PGOP: cell communication

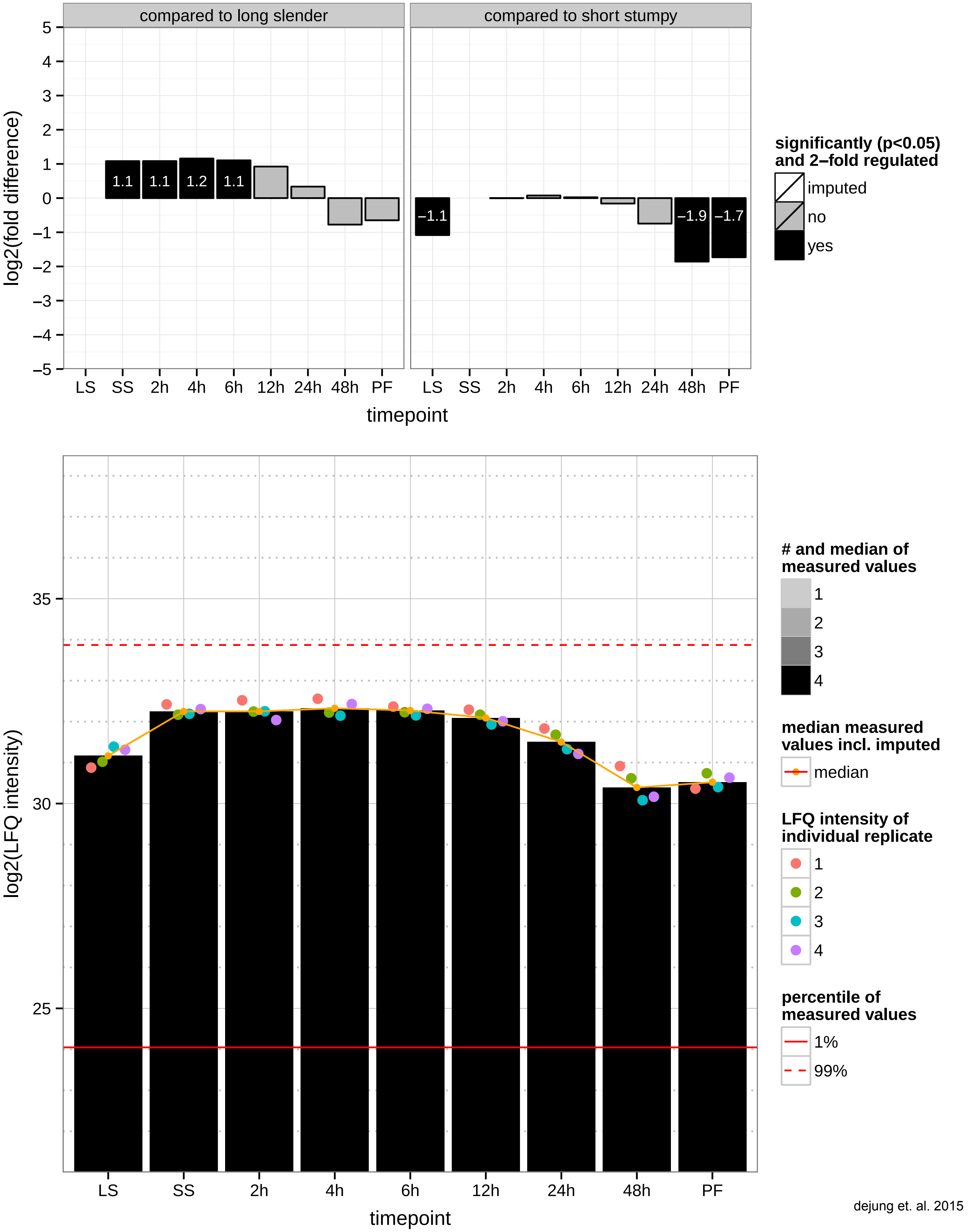

dejung et. al. 2015

adenylosuccinate synthetase, putative
Tb927.11.3650;Tb11.v5.0435
AGOF: null, GTP binding, adenylosuccinate synthase activity, magnesium ion binding
AGOC: null
AGOP: null, purine nucleotide biosynthetic process
PGOF: GTP binding, adenylosuccinate synthase activity, magnesium ion binding
PGOC: cytoplasm
PGOP: purine nucleotide biosynthetic process

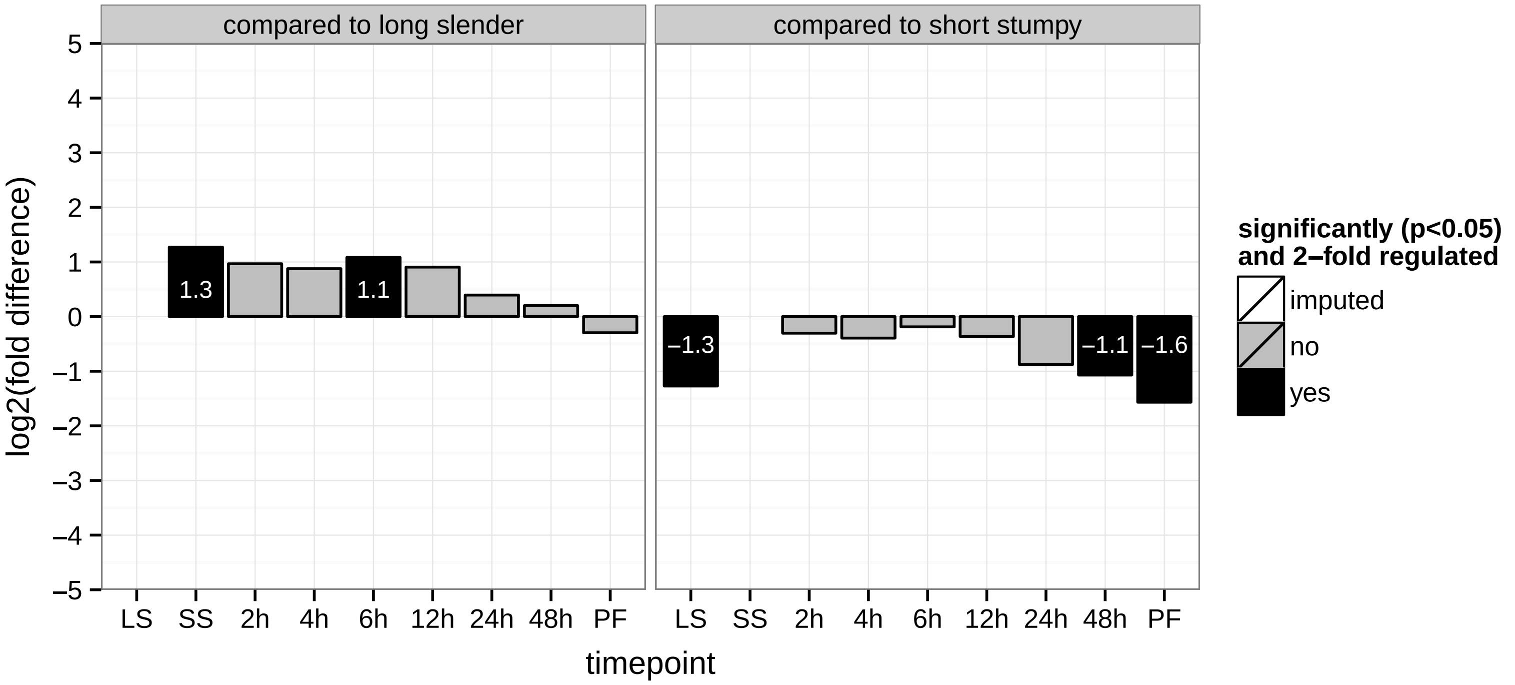

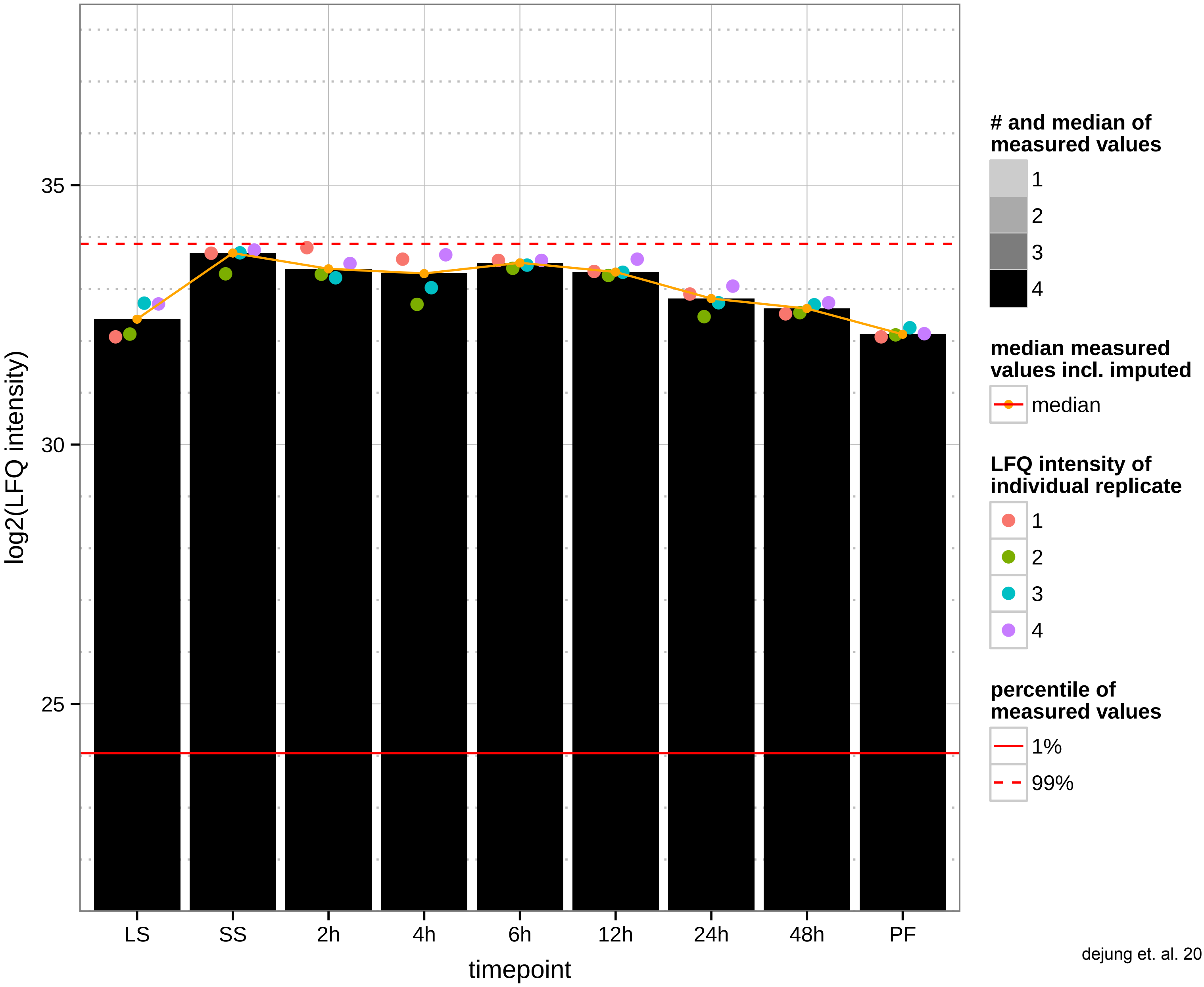

dejung et. al. 2015

vesicular–fusion protein nsf, putative, N–ethylmaleimide sensitive factor (NsF)
Tb927.1.1560;Tb11.v5.0464
AGOF: null, ATP binding, ATPase activity
AGOC: null
AGOP: null, exocytosis
PGOF: ATP binding, nucleoside–triphosphatase activity, nucleotide binding
PGOC: null
PGOP: null

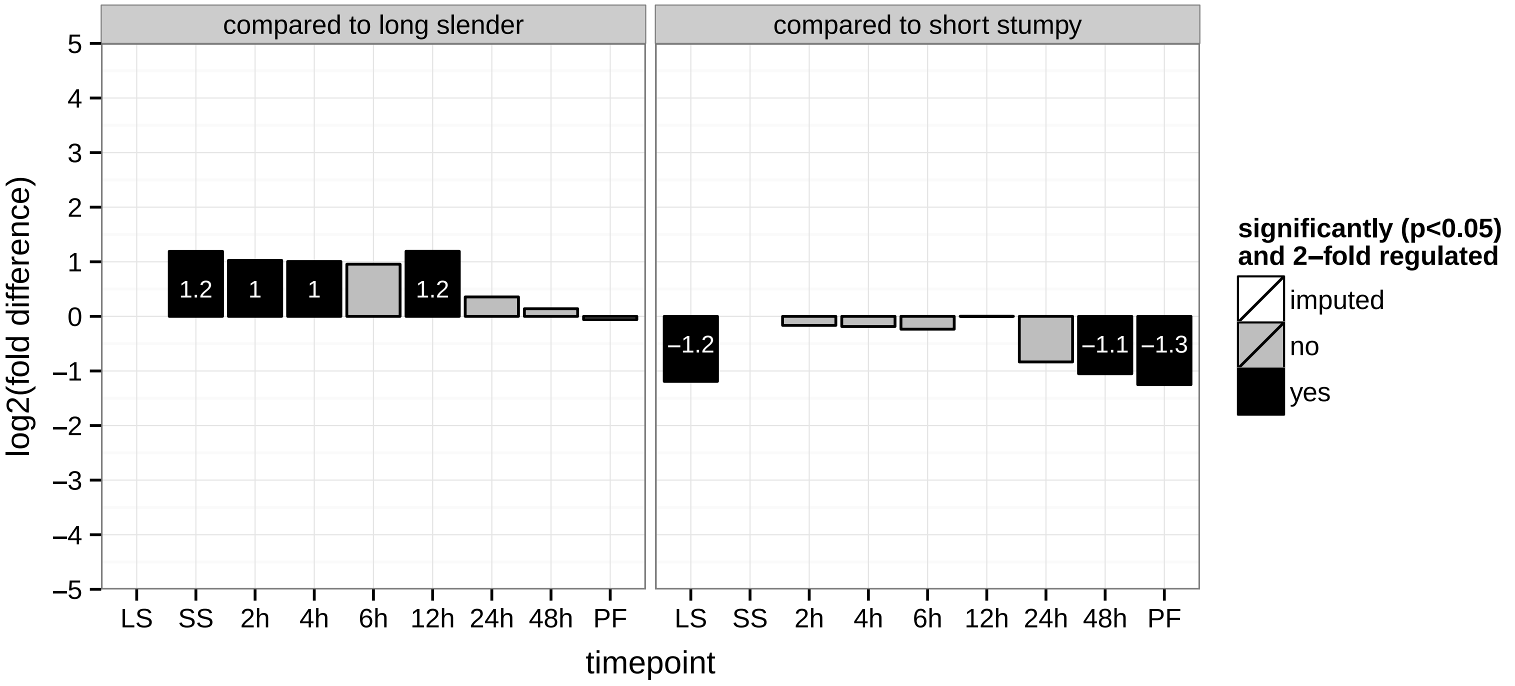

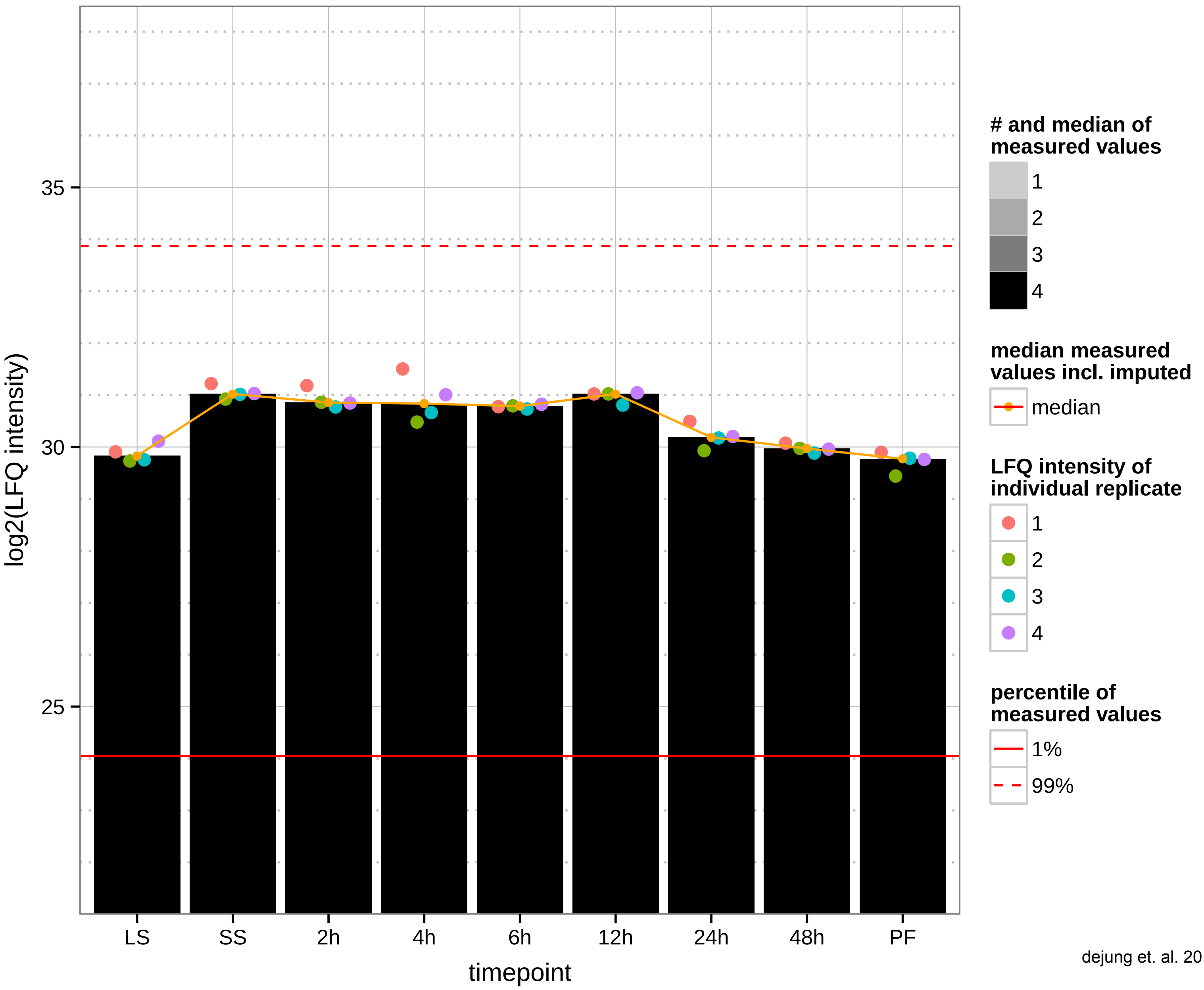

dejung et. al. 2015

fructose–bisphosphate aldolase, glycosomal, putative, glycosomal (ALD)
Tb927.10.5620;Tb11.v5.0531
AGOF: null, fructose–bisphosphate aldolase activity
AGOC: null, glycosome
AGOP: null, glycolysis
PGOF: fructose–bisphosphate aldolase activity
PGOC: null
PGOP: glycolysis

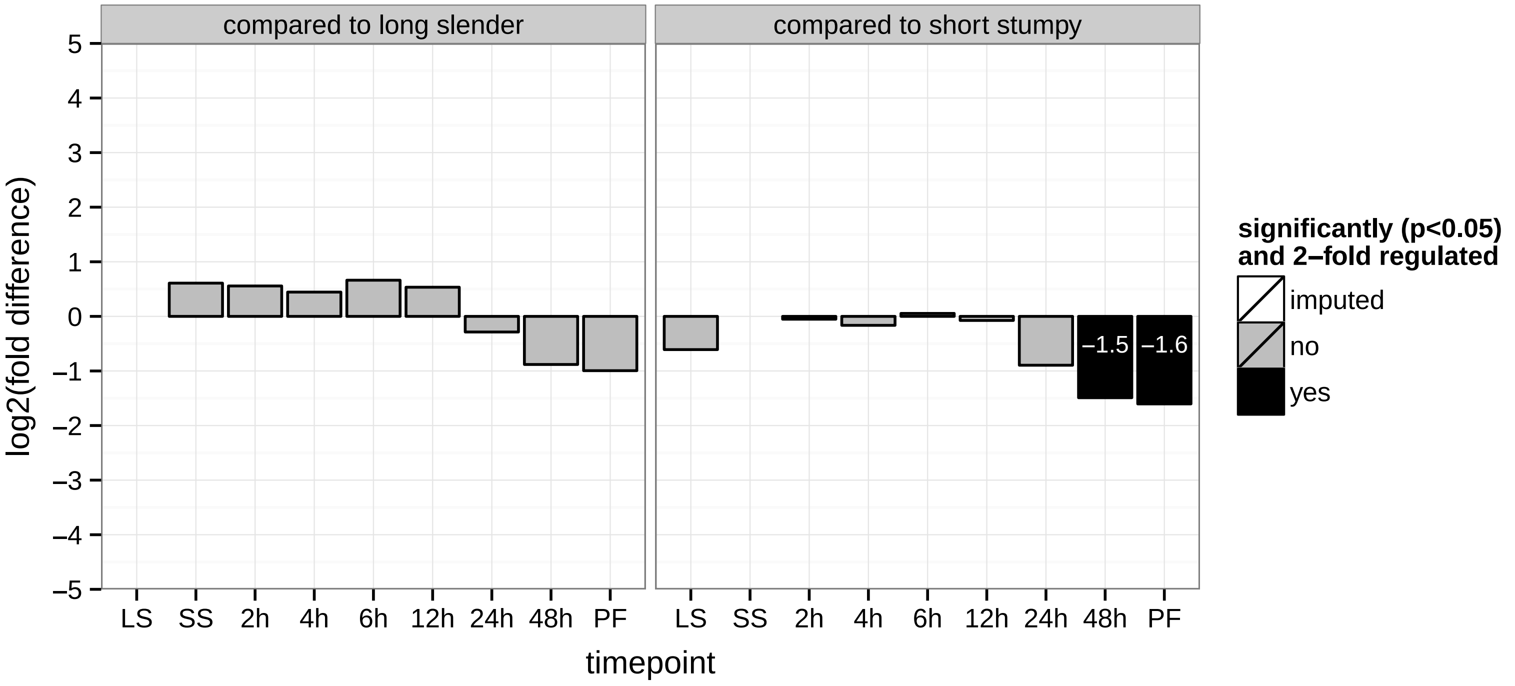

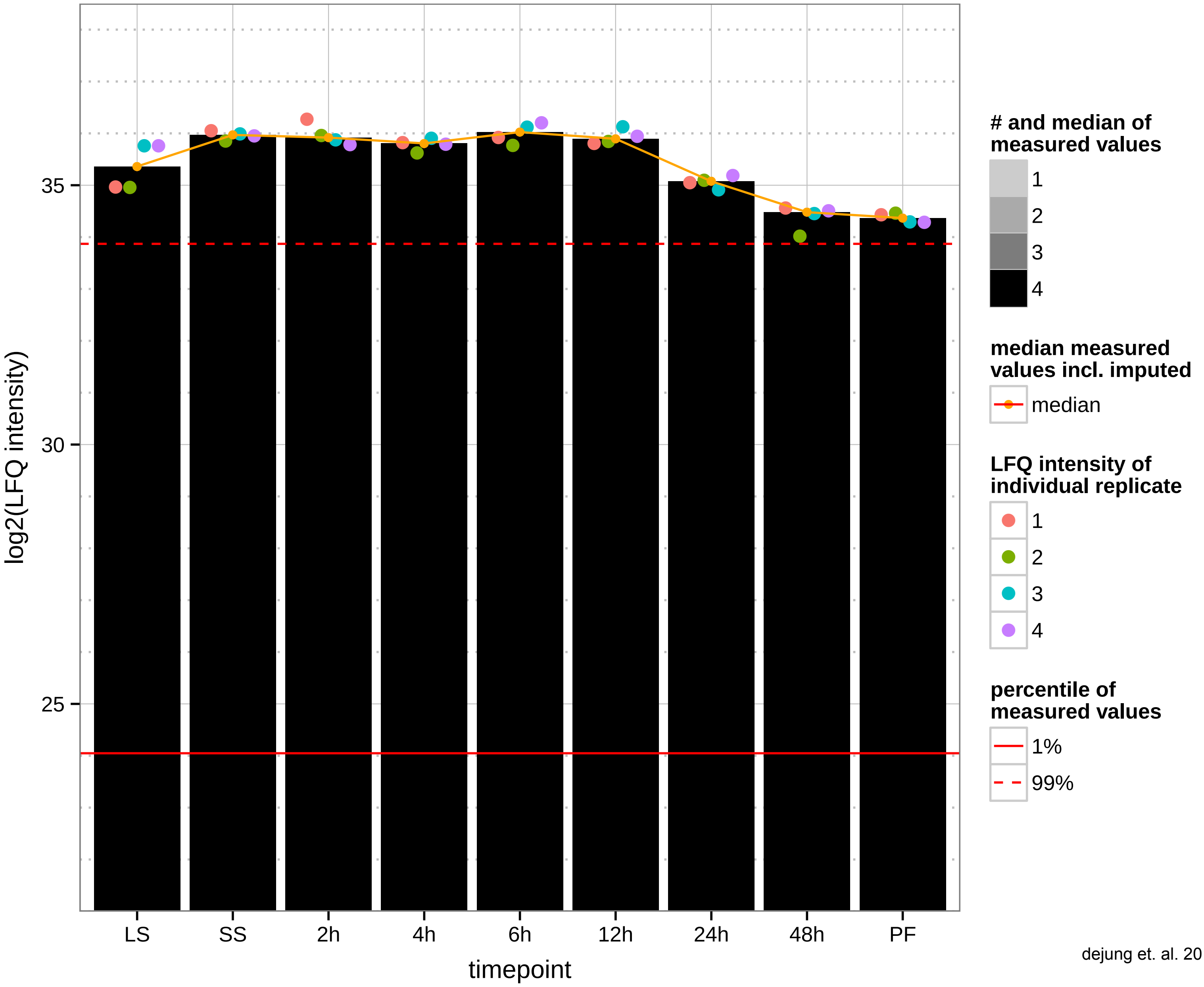

dejung et. al. 2015

protein disulfide isomerase, bloodstream– specific protein 2 precursor
Tb927.10.8230;Tb11.v5.0668
AGOF: null, protein disulfide isomerase activity
AGOC: null, cytoplasm
AGOP: null, cell redox homeostasis, protein folding
PGOF: isomerase activity
PGOC: null
PGOP: cell redox homeostasis

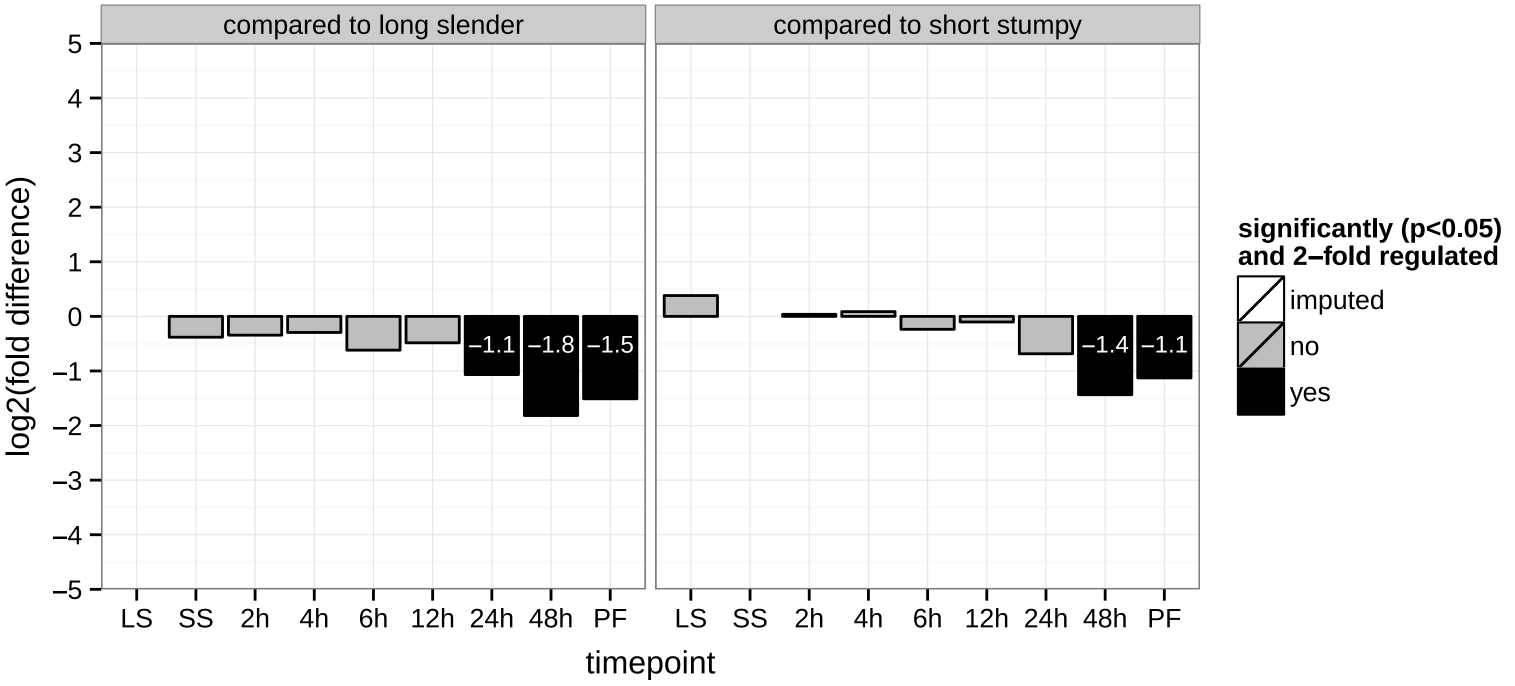

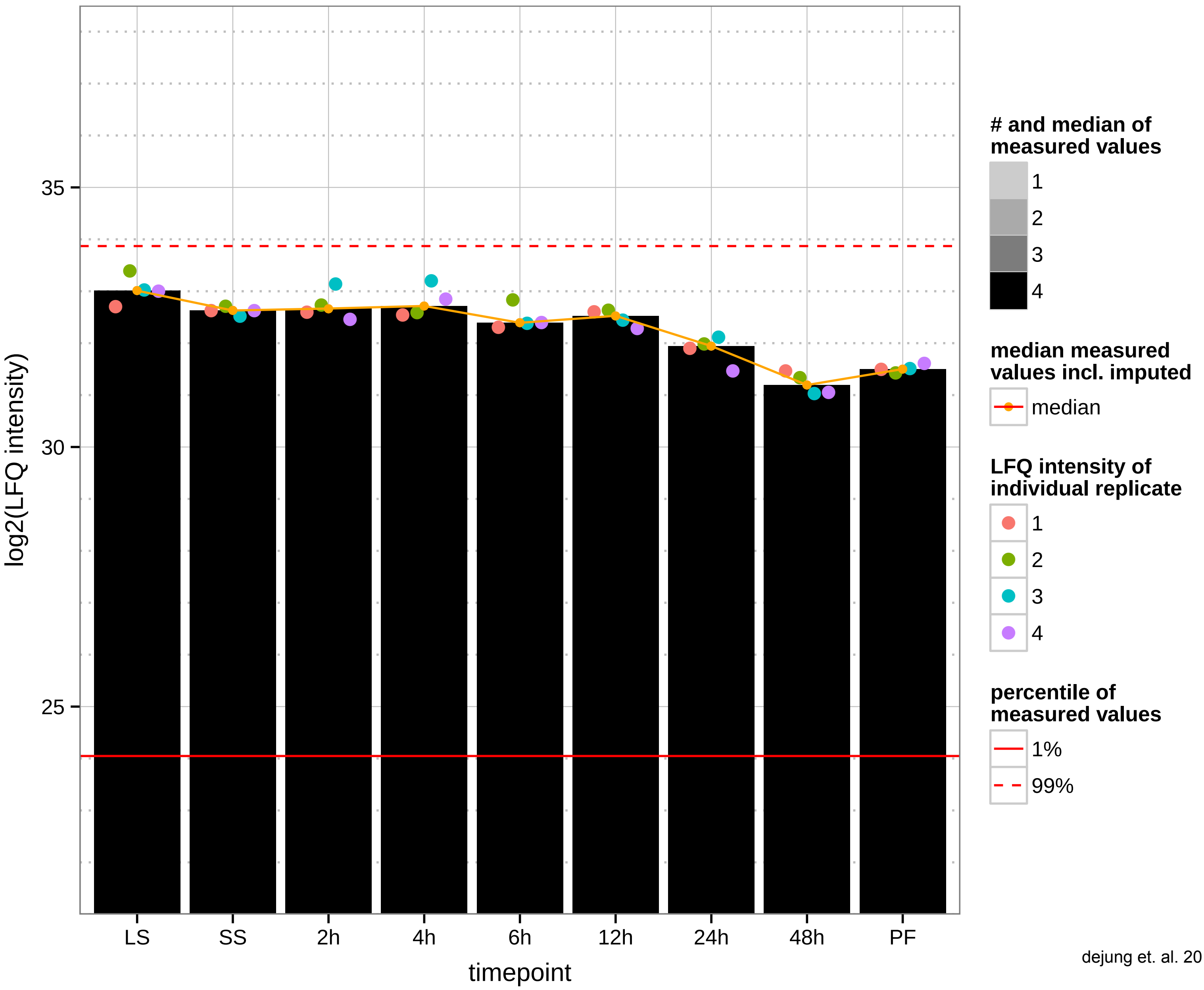

dejung et. al. 2015

hypothetical protein, conserved
Tb927.1.2970
AGOF: null
AGOC: null
AGOP: null
PGOF: null
PGOC: null
PGOP: null

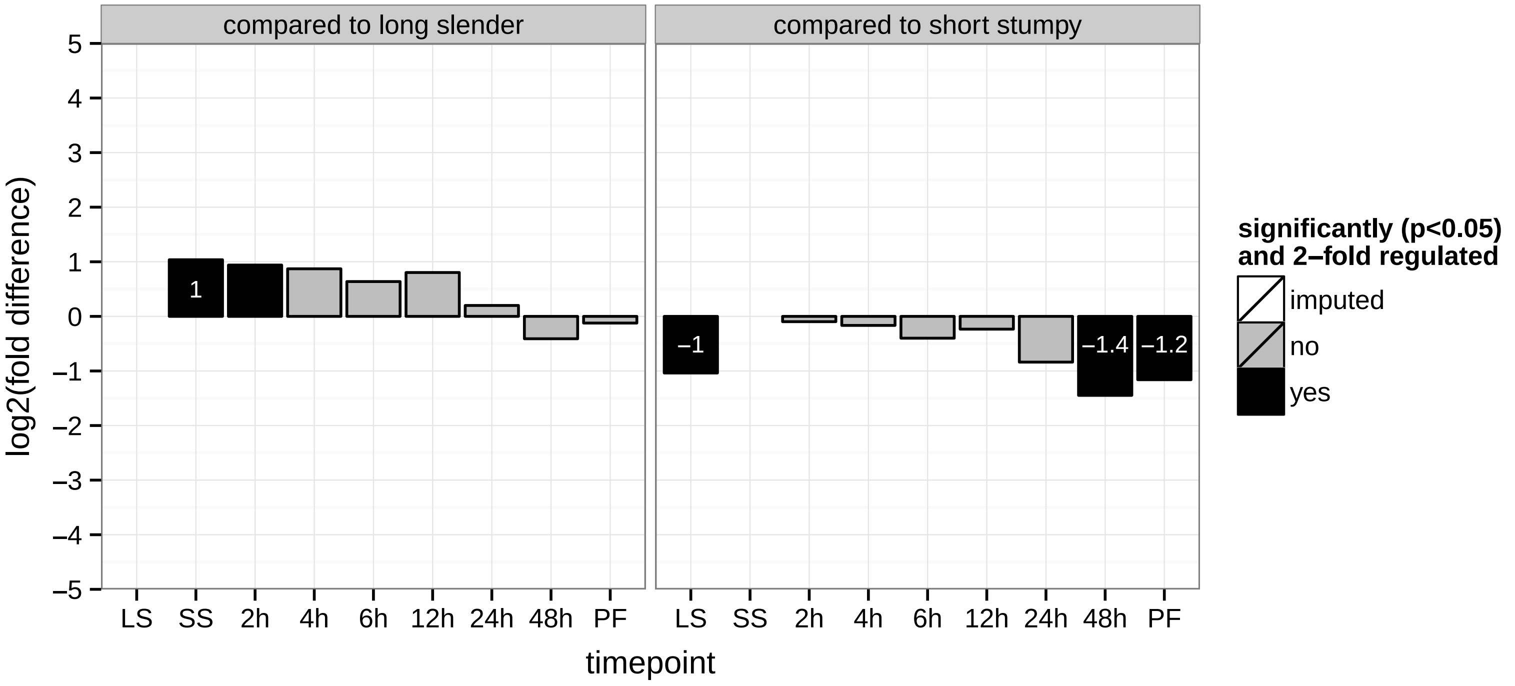

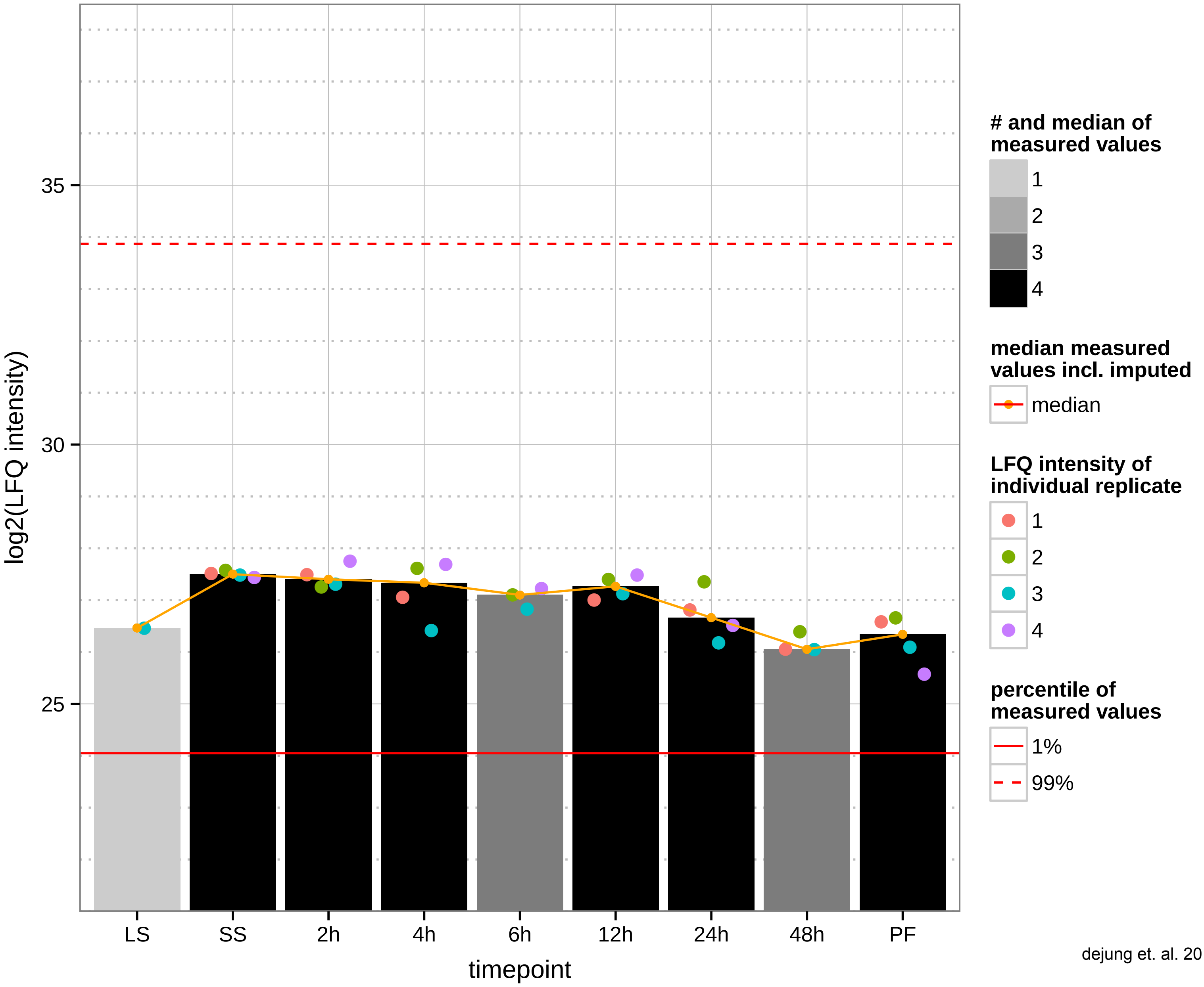

dejung et. al. 2015

acetyltransferase, putative
Tb927.1.4490
AGOF: N–acetyltransferase activity, palmitoyl–CoA oxidase activity
AGOC: null
AGOP: metabolic process
PGOF: N–acetyltransferase activity
PGOC: null
PGOP: null

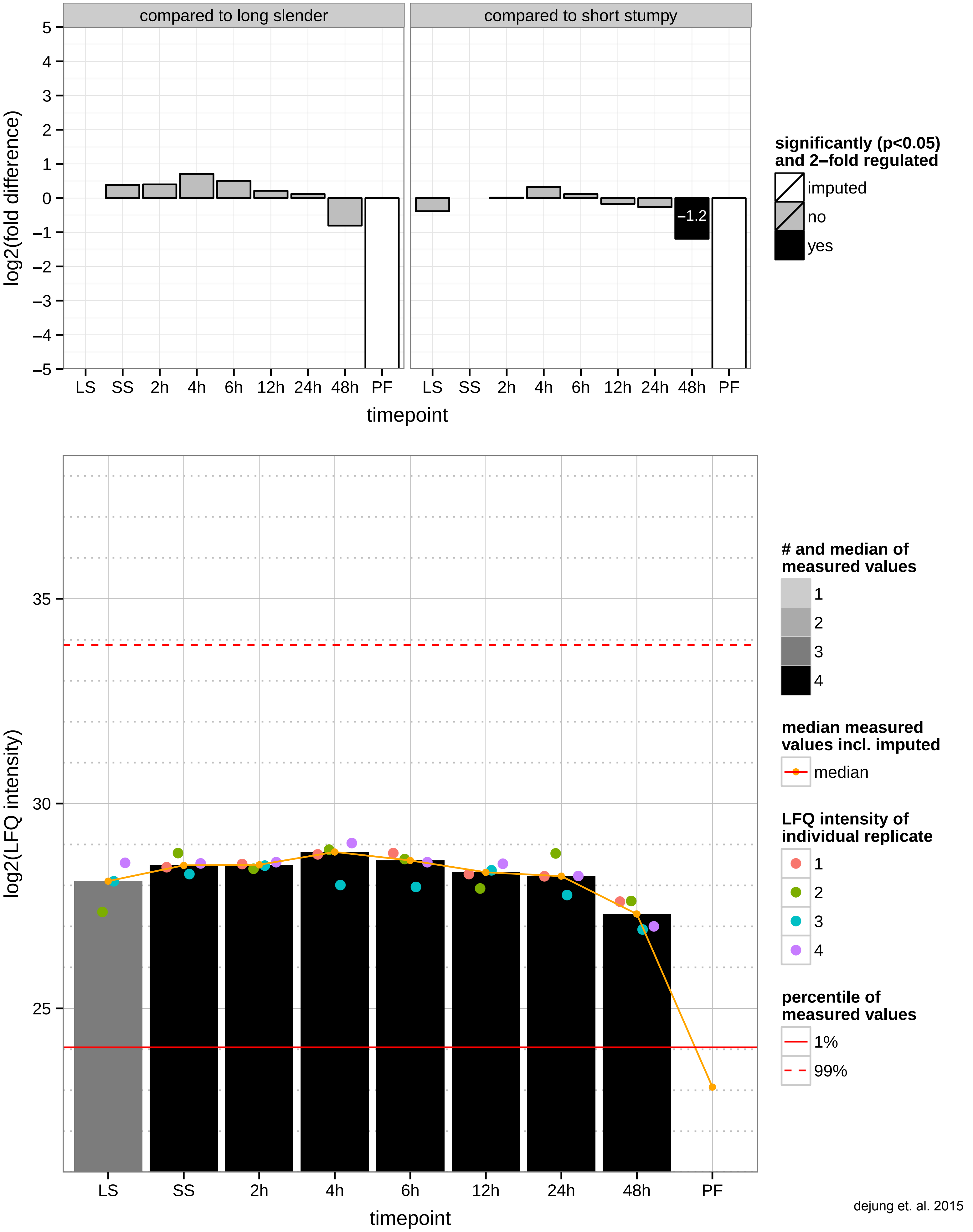

dejung et. al. 2015

serine carboxypeptidase III precursor, putative (CBP1), serine peptidase, Clan SC, Family S10
Tb927.10.1040;Tb11.v5.0638;Tb927.10.1050
AGOF: null, serine−type carboxypeptidase activity
AGOC: null
AGOP: null, proteolysis
PGOF: serine−type carboxypeptidase activity
PGOC: null
PGOP: proteolysis

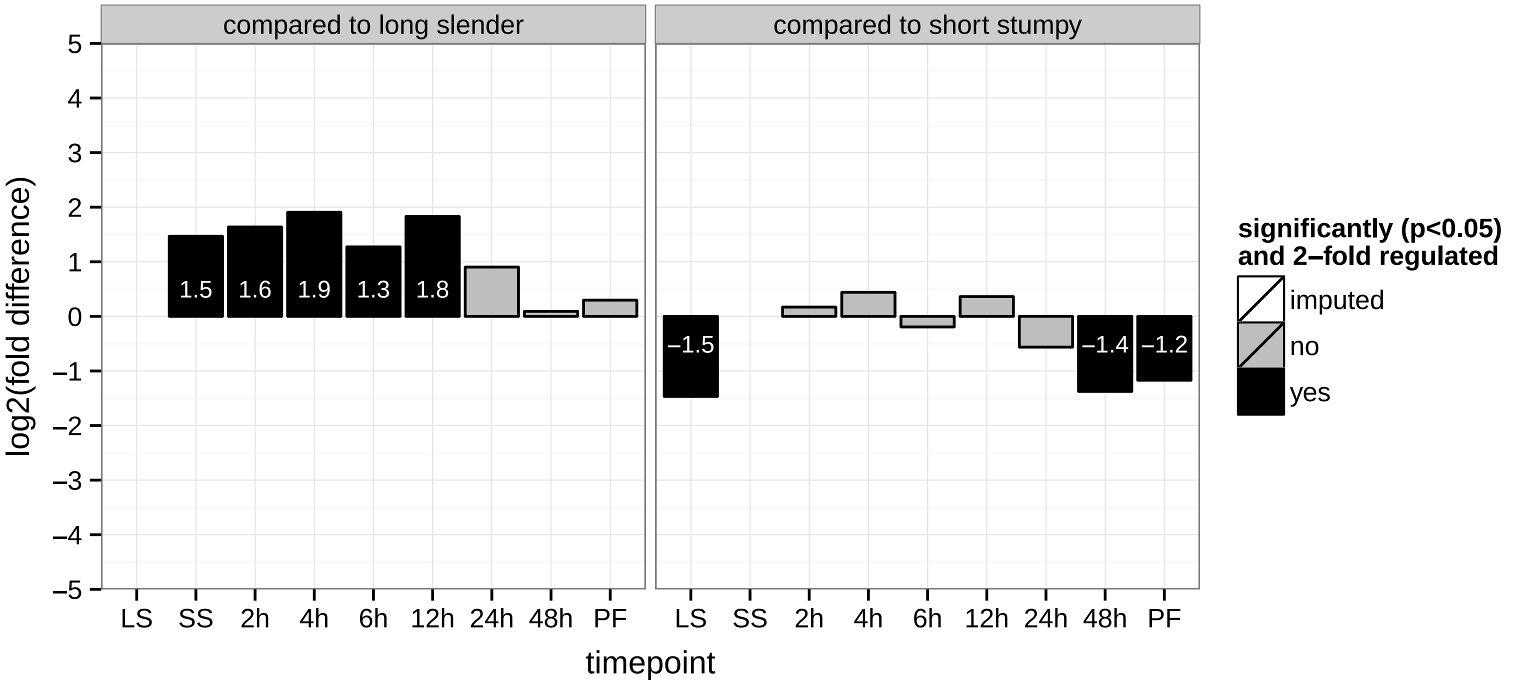

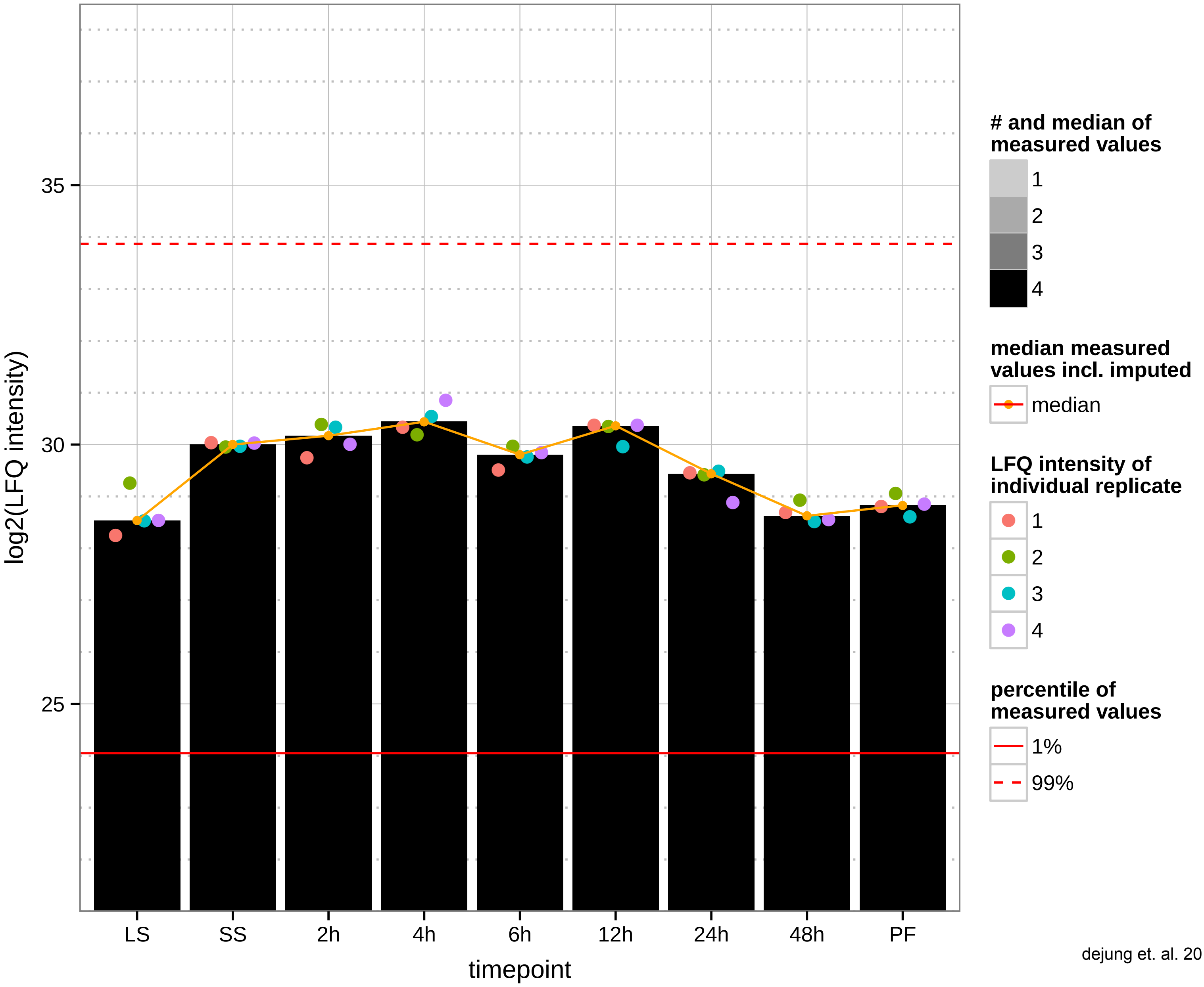

dejung et. al. 2015

hypothetical protein, conserved
Tb927.10.11140
AGOF: null
AGOC: null
AGOP: null
PGOF: null
PGOC: null
PGOP: null

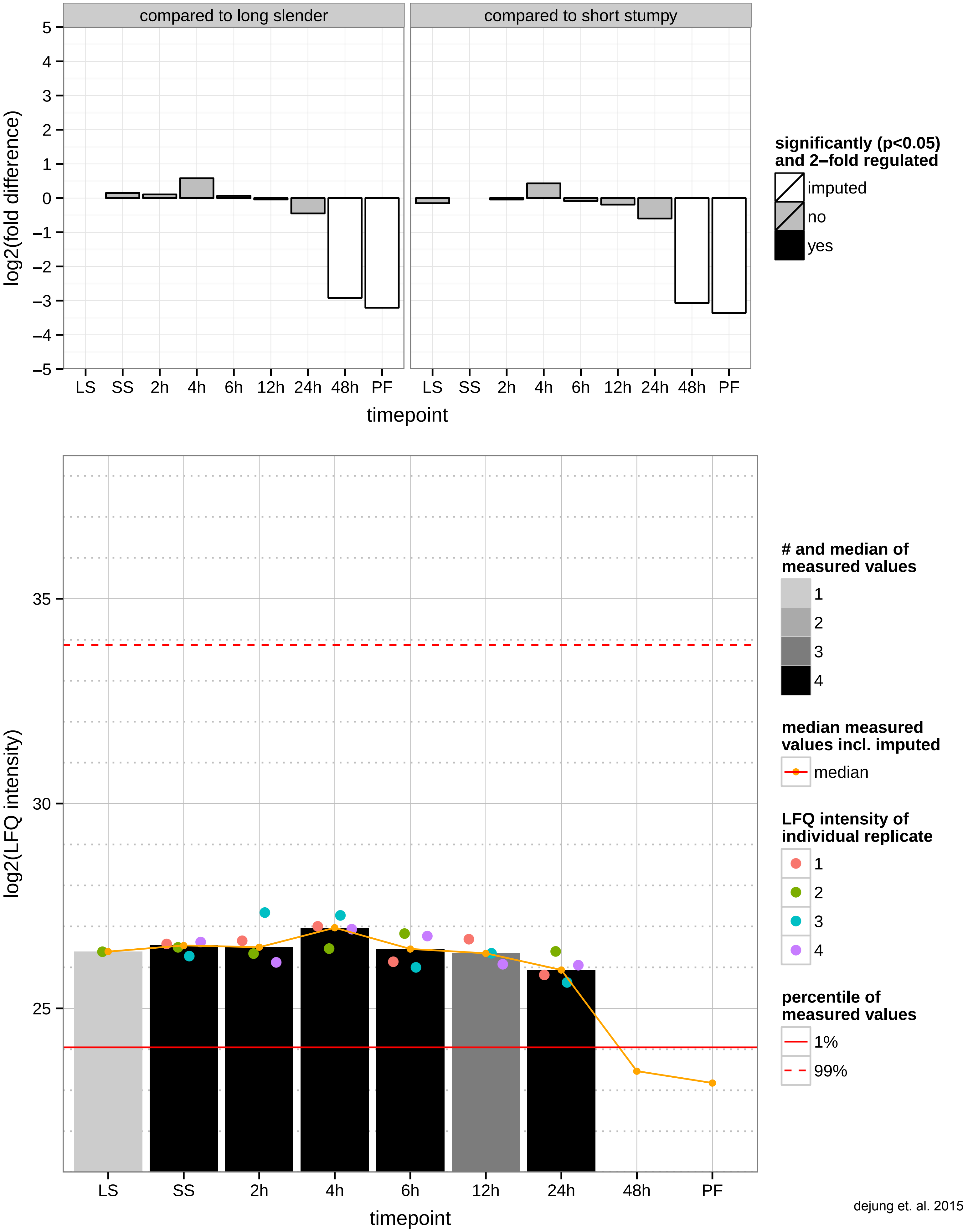

dejung et. al. 2015

chloride channel protein, putative
Tb927.10.11680
AGOF: hormone activity, voltage–gated chloride channel activity
AGOC: extracellular region, membrane
AGOP: chloride transport
PGOF: ion channel activity, protein binding, voltage–gated chloride channel activity
PGOC: membrane
PGOP: chloride transport, transmembrane transport

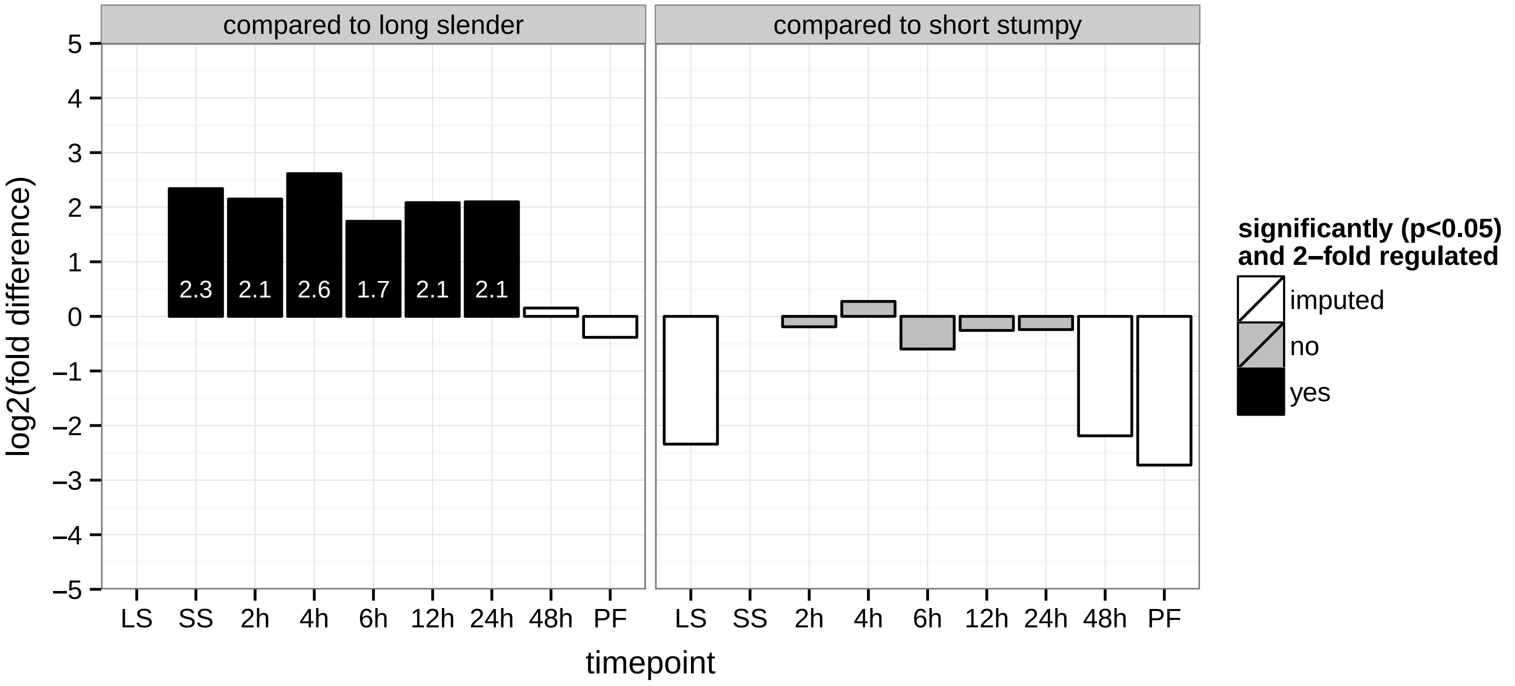

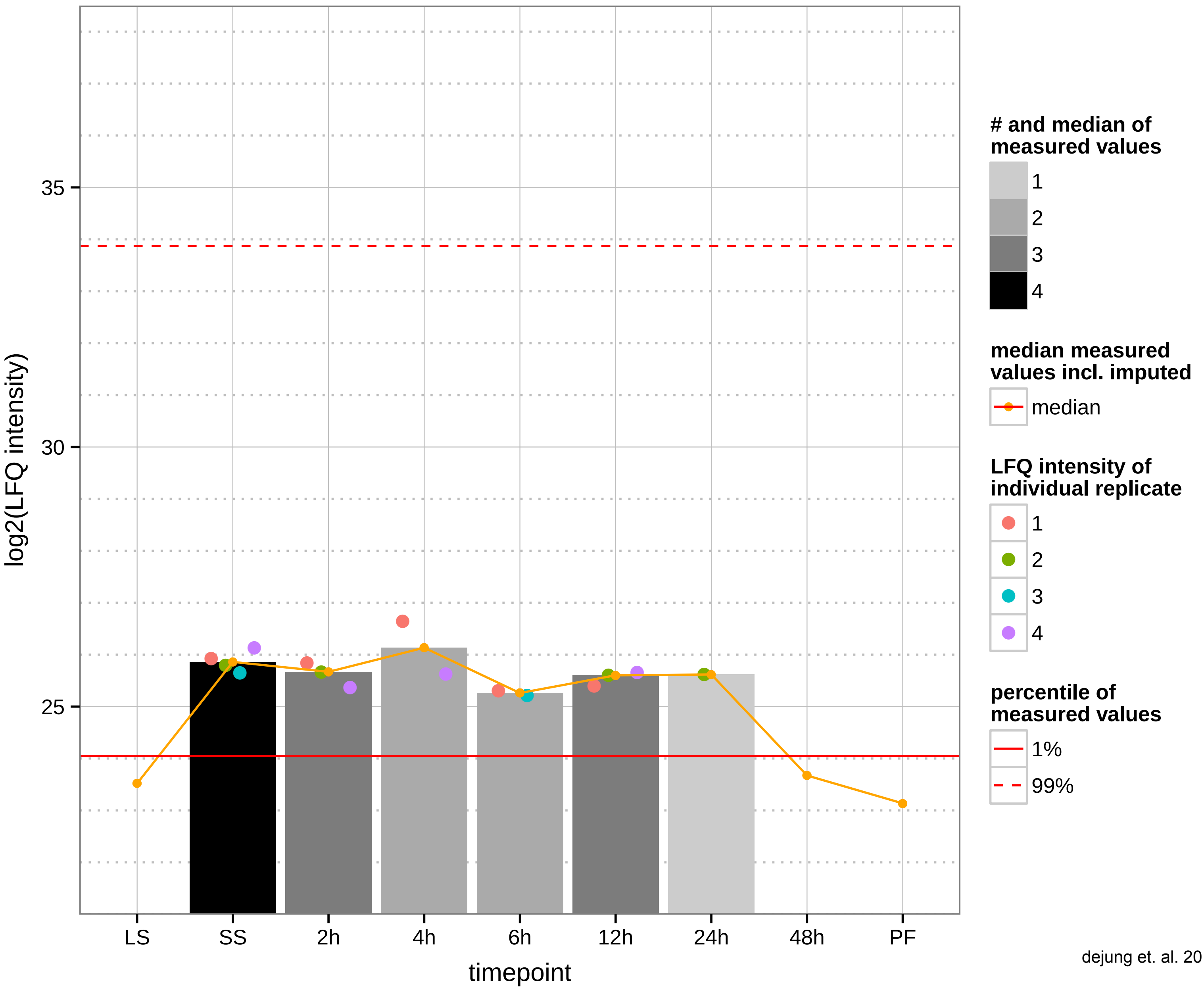

dejung et. al. 2015

hypothetical protein, conserved
Tb927.10.12010
AGOF: null
AGOC: null
AGOP: null
PGOF: null
PGOC: null
PGOP: null

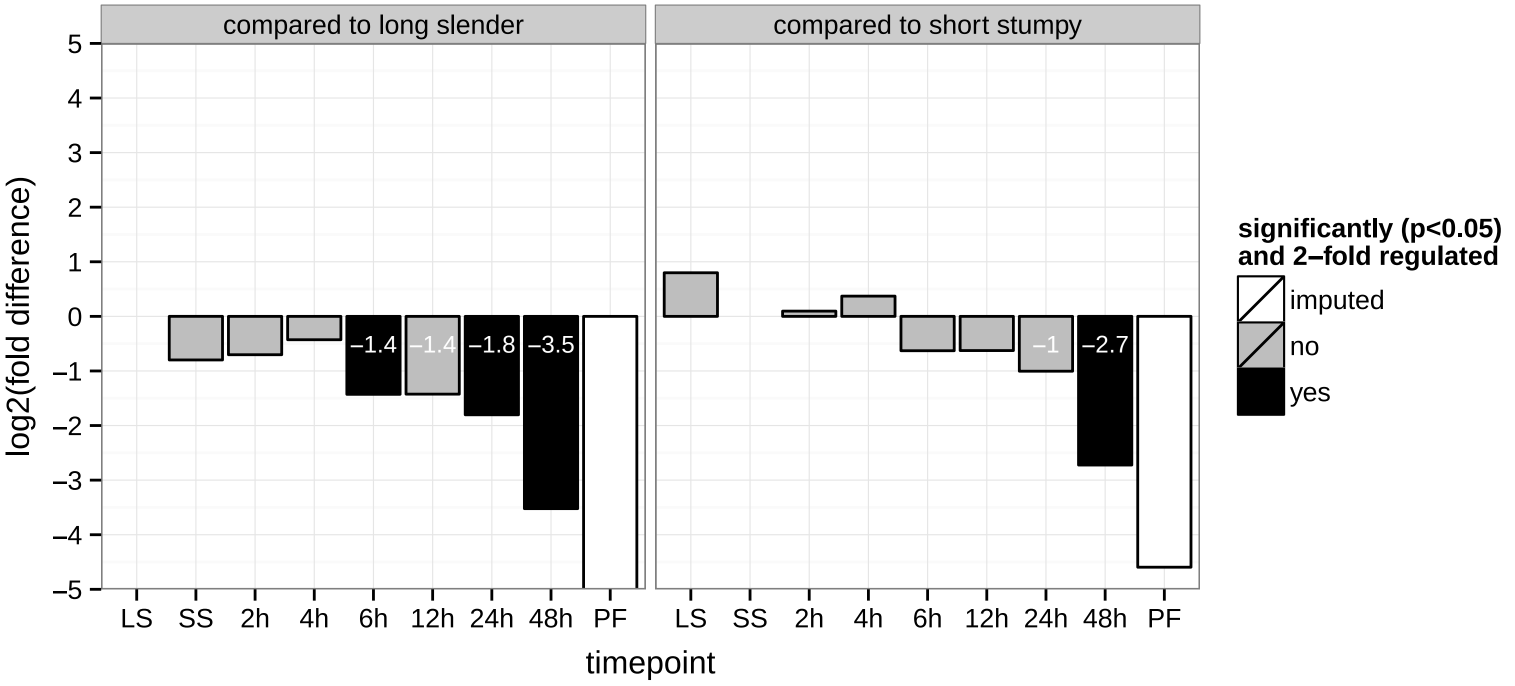

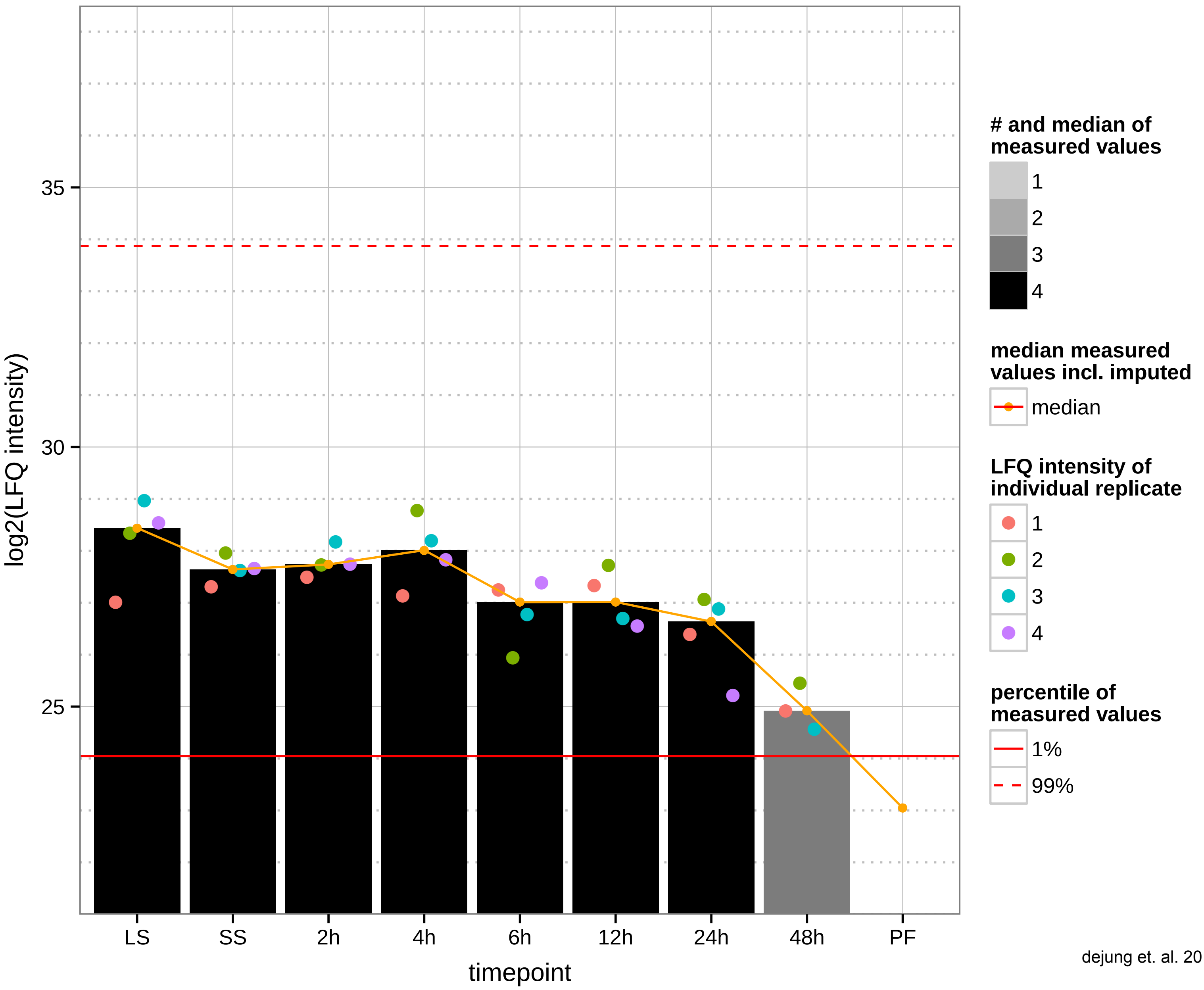

dejung et. al. 2015

hypothetical protein, conserved
Tb927.10.1230
AGOF: null
AGOC: null
AGOP: null
PGOF: null
PGOC: null
PGOP: null

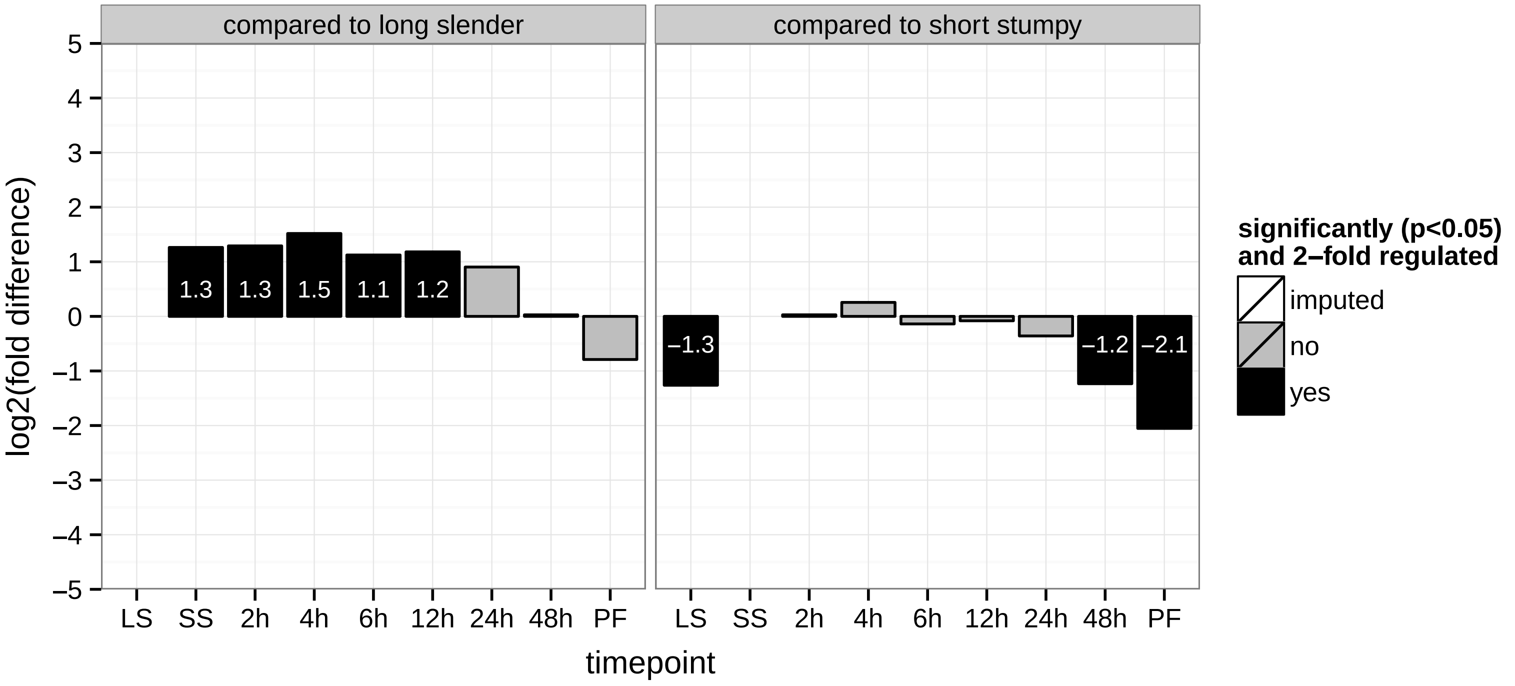

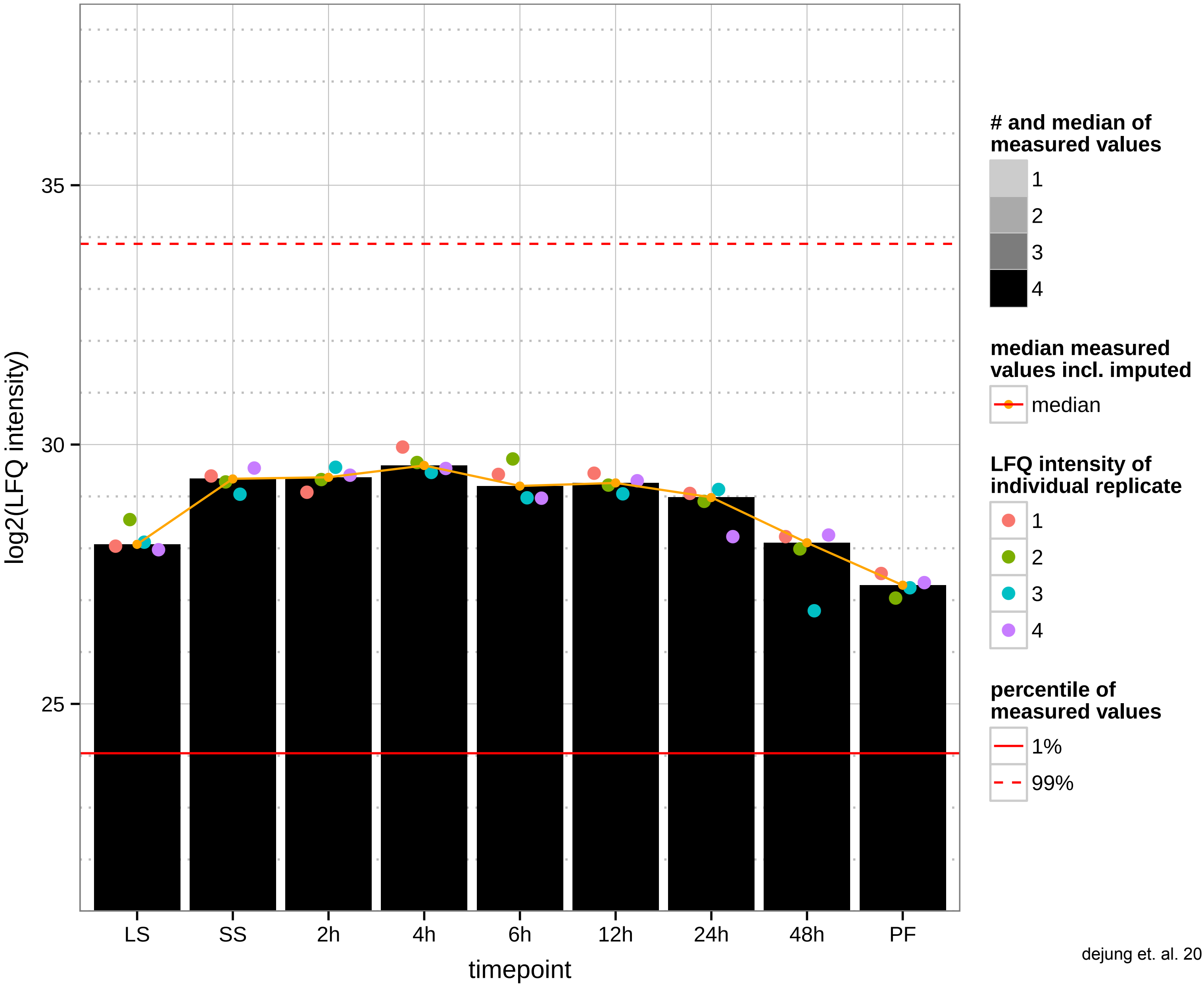

dejung et. al. 2015

cholinephosphate cytidylyltransferase A, putative
Tb927.10.12810
AGOF: cytidylyltransferase activity, nucleotidyltransferase activity
AGOC: cytosol
AGOP: biosynthetic process, phosphatidylcholine biosynthetic process
PGOF: catalytic activity, nucleotidyltransferase activity, phosphotransferase activity, for other substituted phosphate groups
PGOC: membrane
PGOP: biosynthetic process, phospholipid biosynthetic process

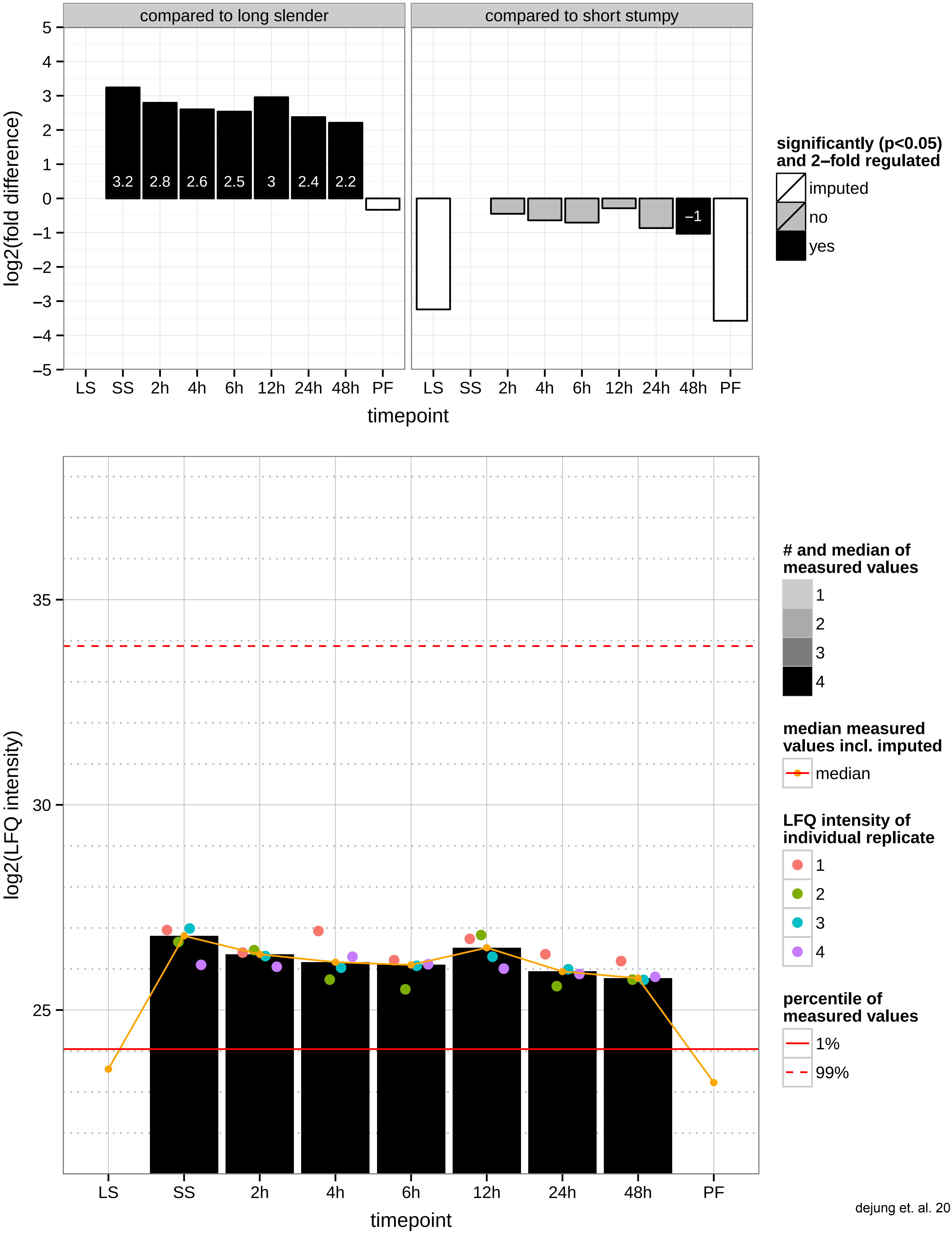

dejung et. al. 2015

predicted zinc finger protein
Tb927.10.12940
AGOF: zinc ion binding
AGOC: null
AGOP: null
PGOF: protein binding, zinc ion binding
PGOC: null
PGOP: null

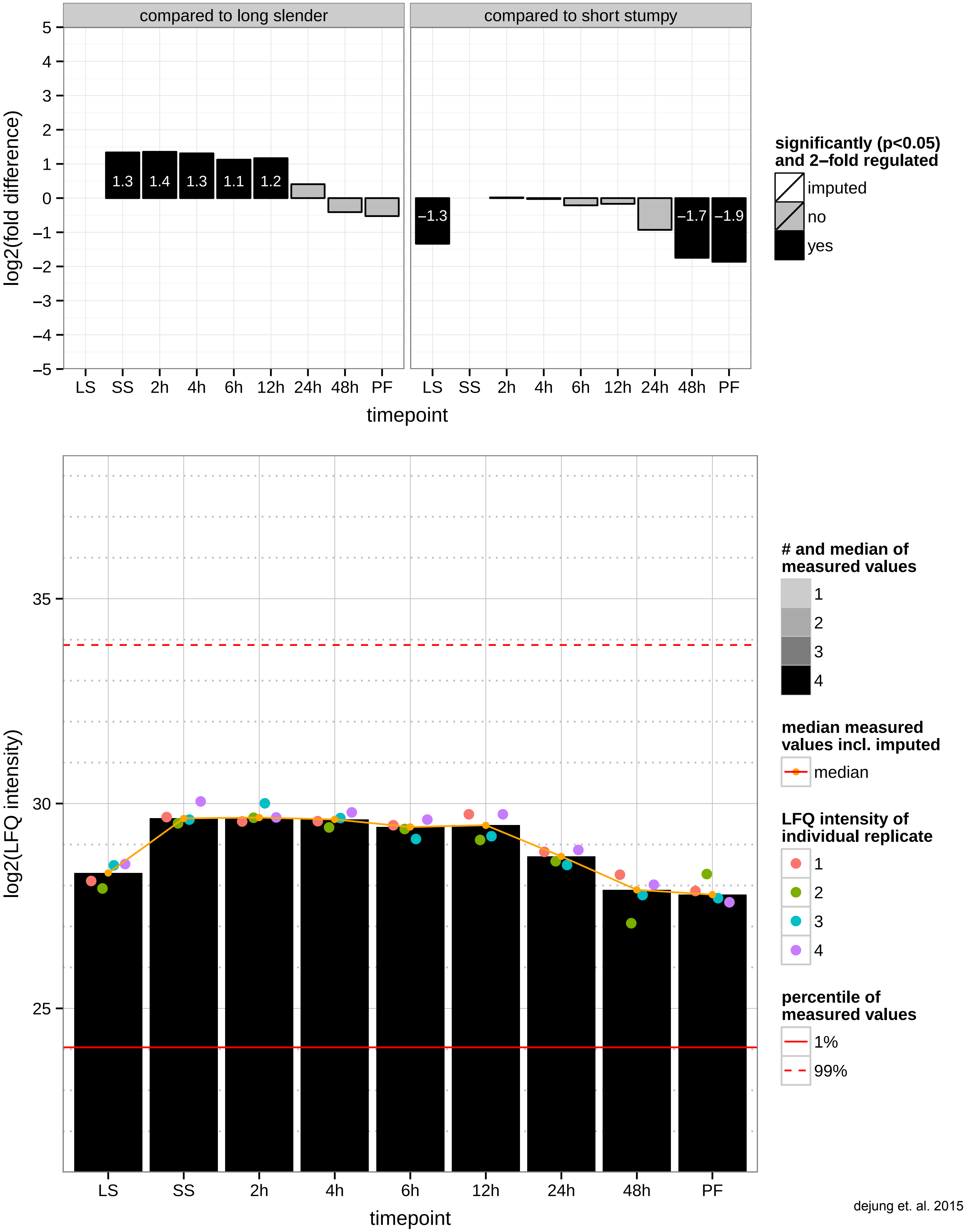

dejung et. al. 2015

Ras–related protein Rab5A (RAB5A)
Tb927.10.12960
AGOF: GTP binding, GTPase activity
AGOC: intracellular
AGOP: intracellular protein transport, nucleocytoplasmic transport, small GTPase mediated signal transduction
PGOF: GTP binding, GTPase activity
PGOC: intracellular, membrane
PGOP: GTP catabolic process, intracellular protein transport, nucleocytoplasmic transport, protein transport, signal transducti

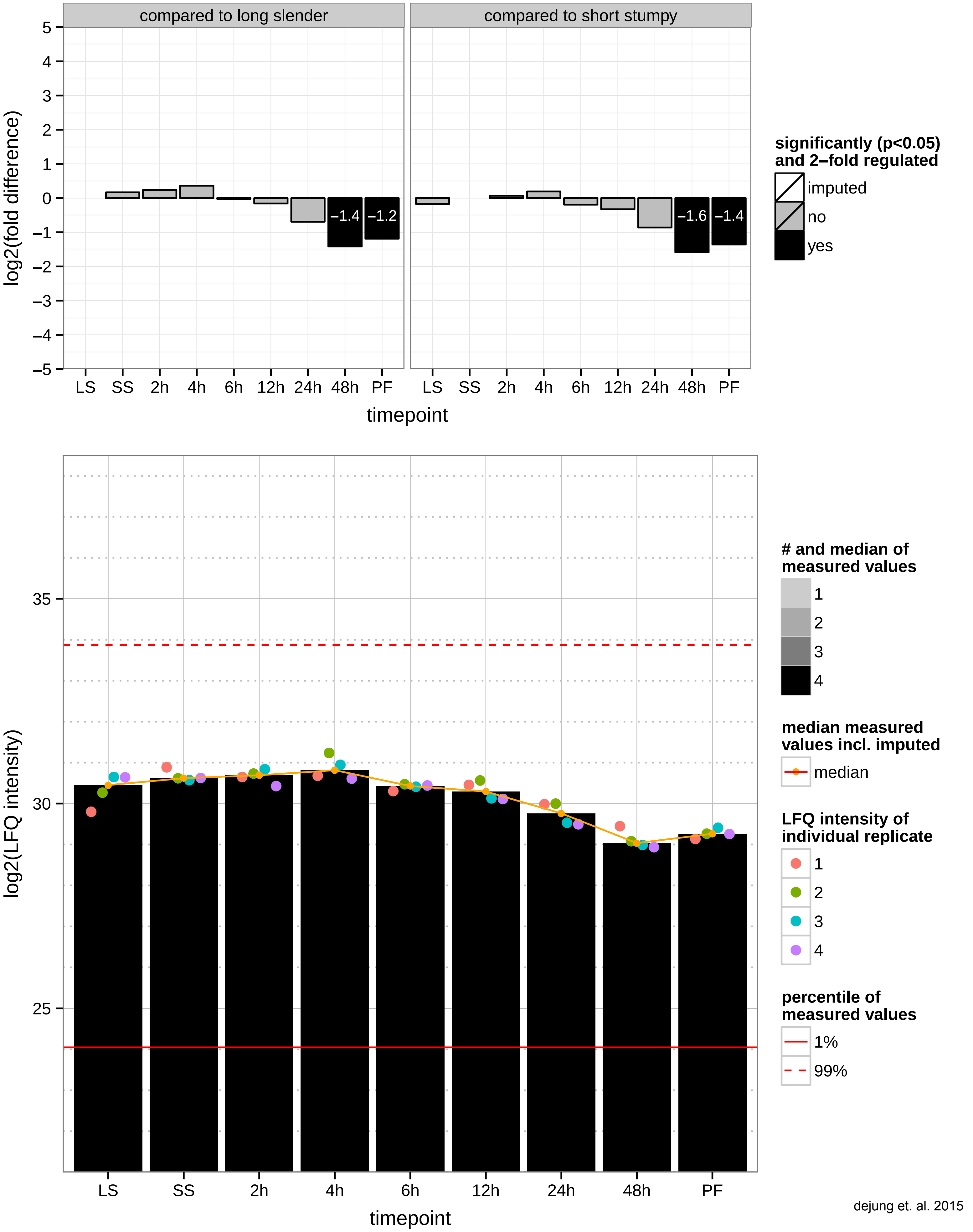

dejung et. al. 2015

RNA–binding protein, putative (RBP12)
Tb927.10.13540
AGOF: RNA binding
AGOC: null
AGOP: RNA processing
PGOF: nucleic acid binding
PGOC: null
PGOP: null

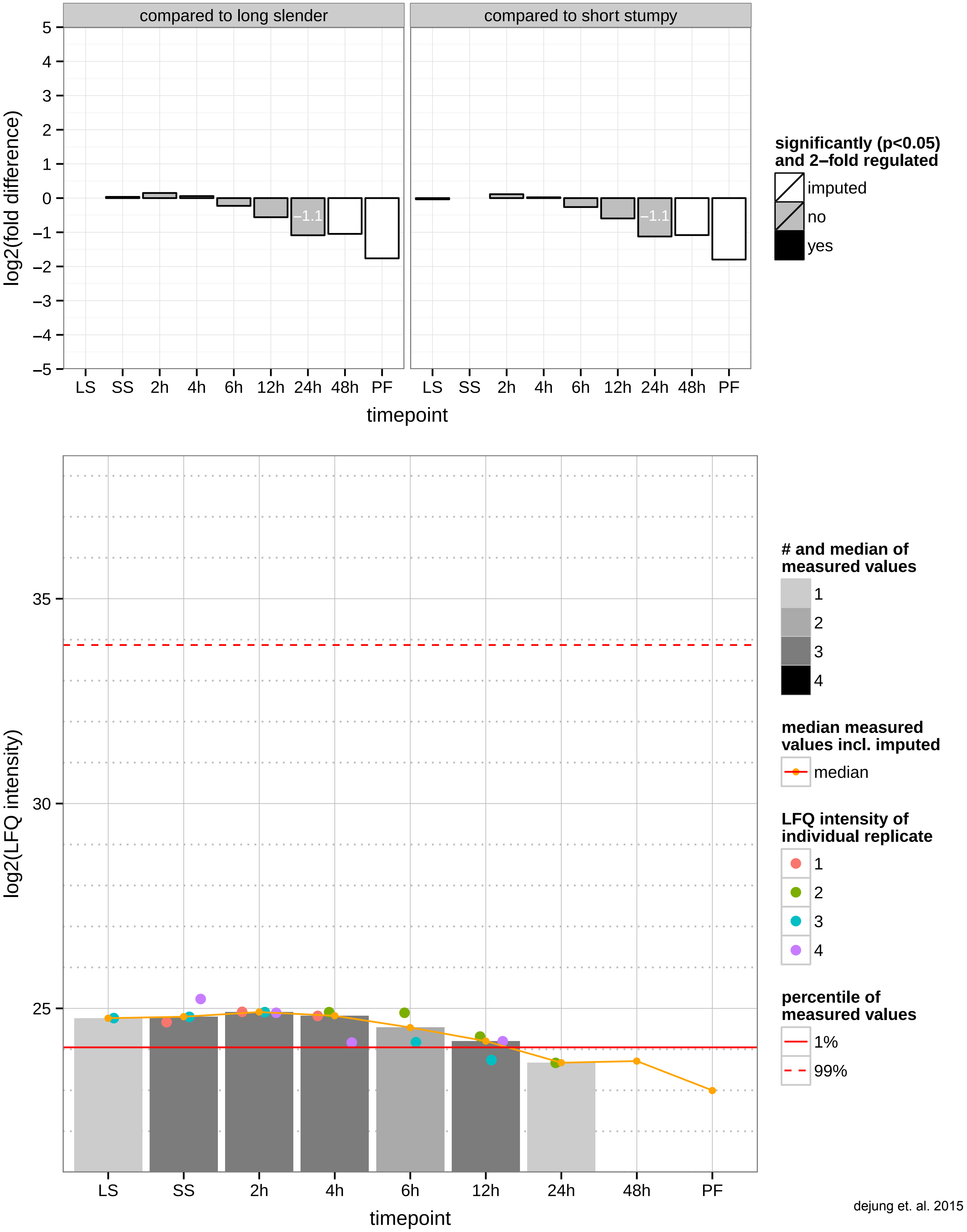

dejung et. al. 2015

hypothetical protein, conserved
Tb927.10.1430
AGOF: null
AGOC: null
AGOP: null
PGOF: null
PGOC: null
PGOP: null

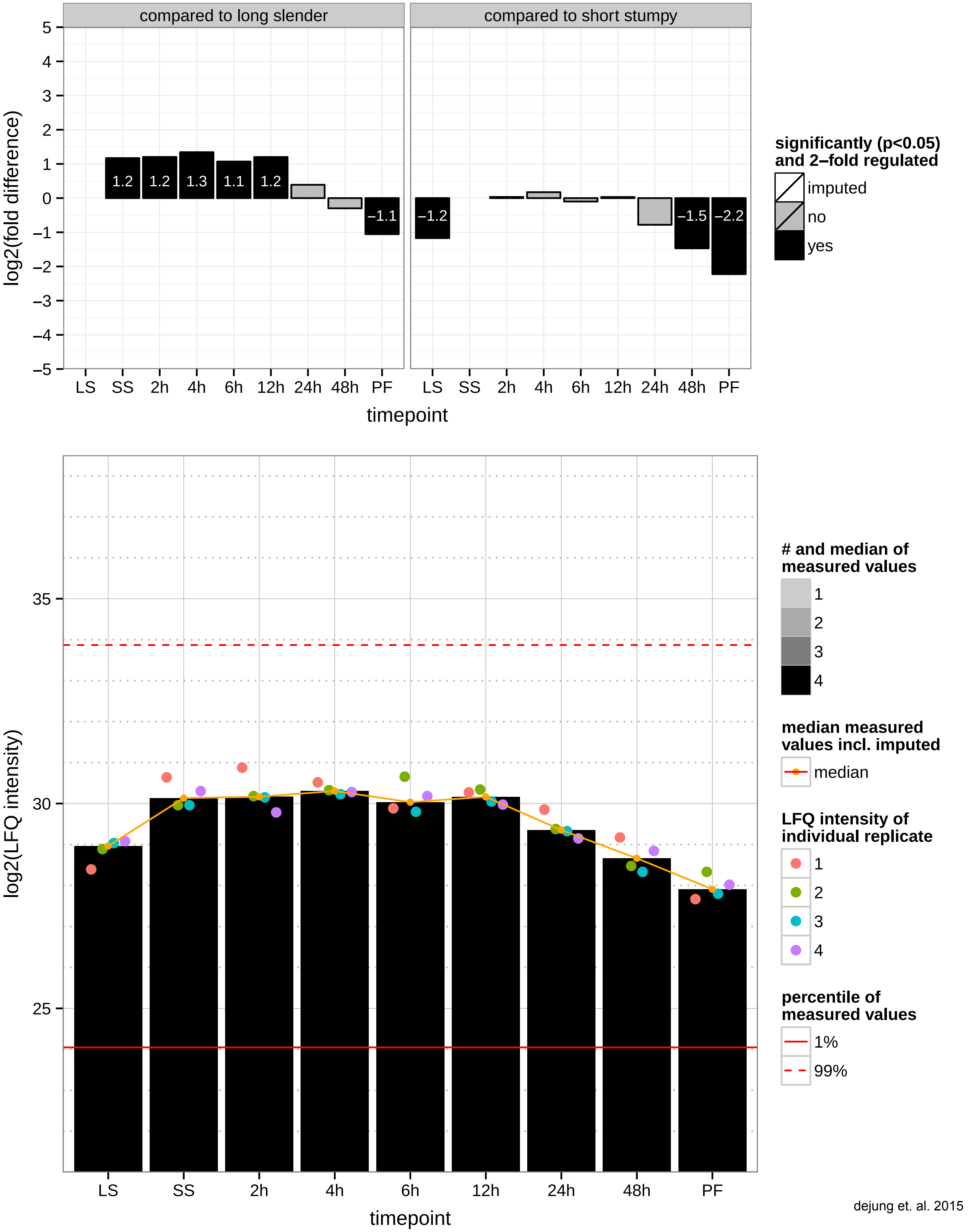

dejung et. al. 2015

syntaxin, putative
Tb927.10.14690
AGOF: transporter activity
AGOC: membrane
AGOP: vesicle−mediated transport
PGOF: protein binding
PGOC: null
PGOP: null

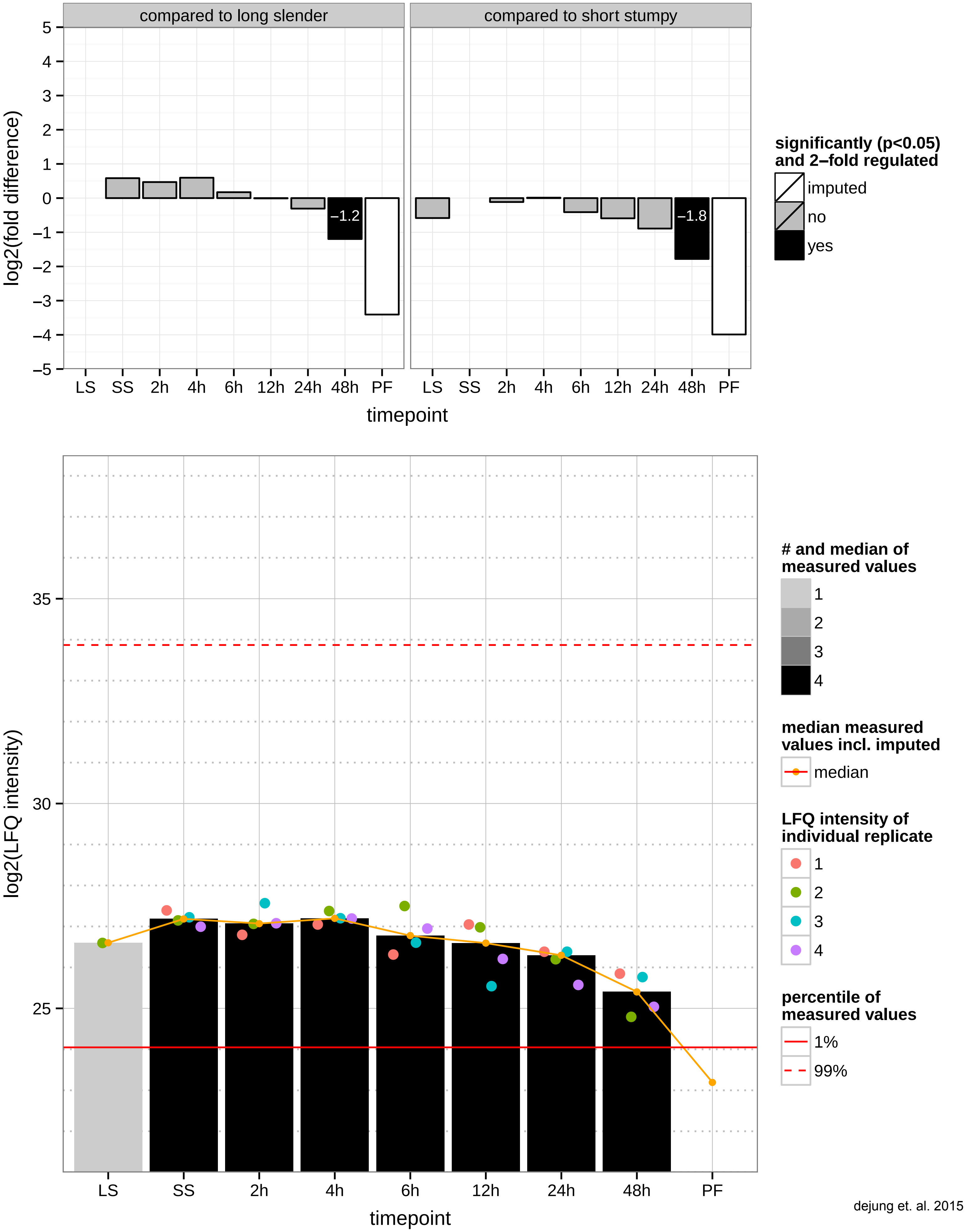

dejung et. al. 2015

ras–related protein Rab21, putative (RAB21)
Tb927.10.1520
AGOF: GTP binding, GTPase activity
AGOC: intracellular
AGOP: intracellular protein transport, nucleocytoplasmic transport, small GTPase mediated signal transduction
PGOF: GTP binding, GTPase activity
PGOC: intracellular, membrane
PGOP: GTP catabolic process, intracellular protein transport, nucleocytoplasmic transport, protein transport, signal transducti

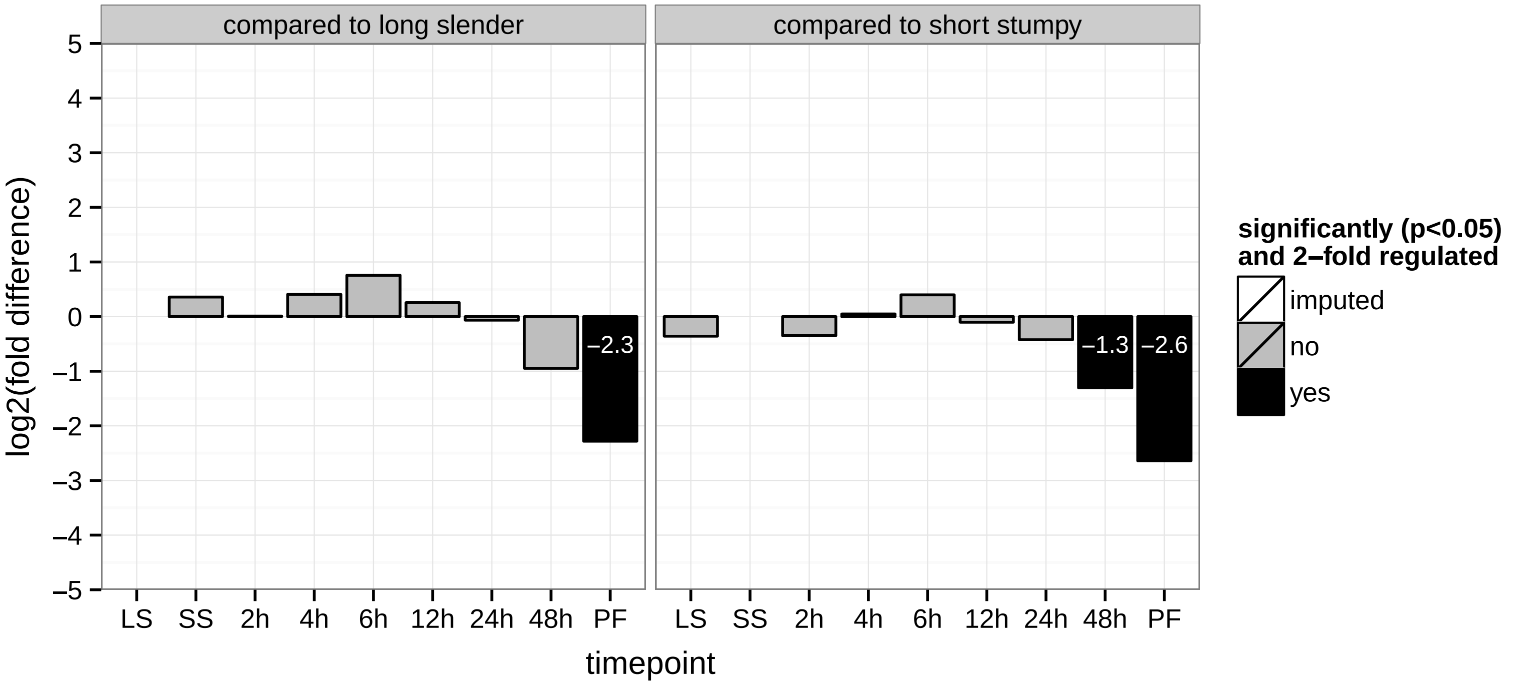

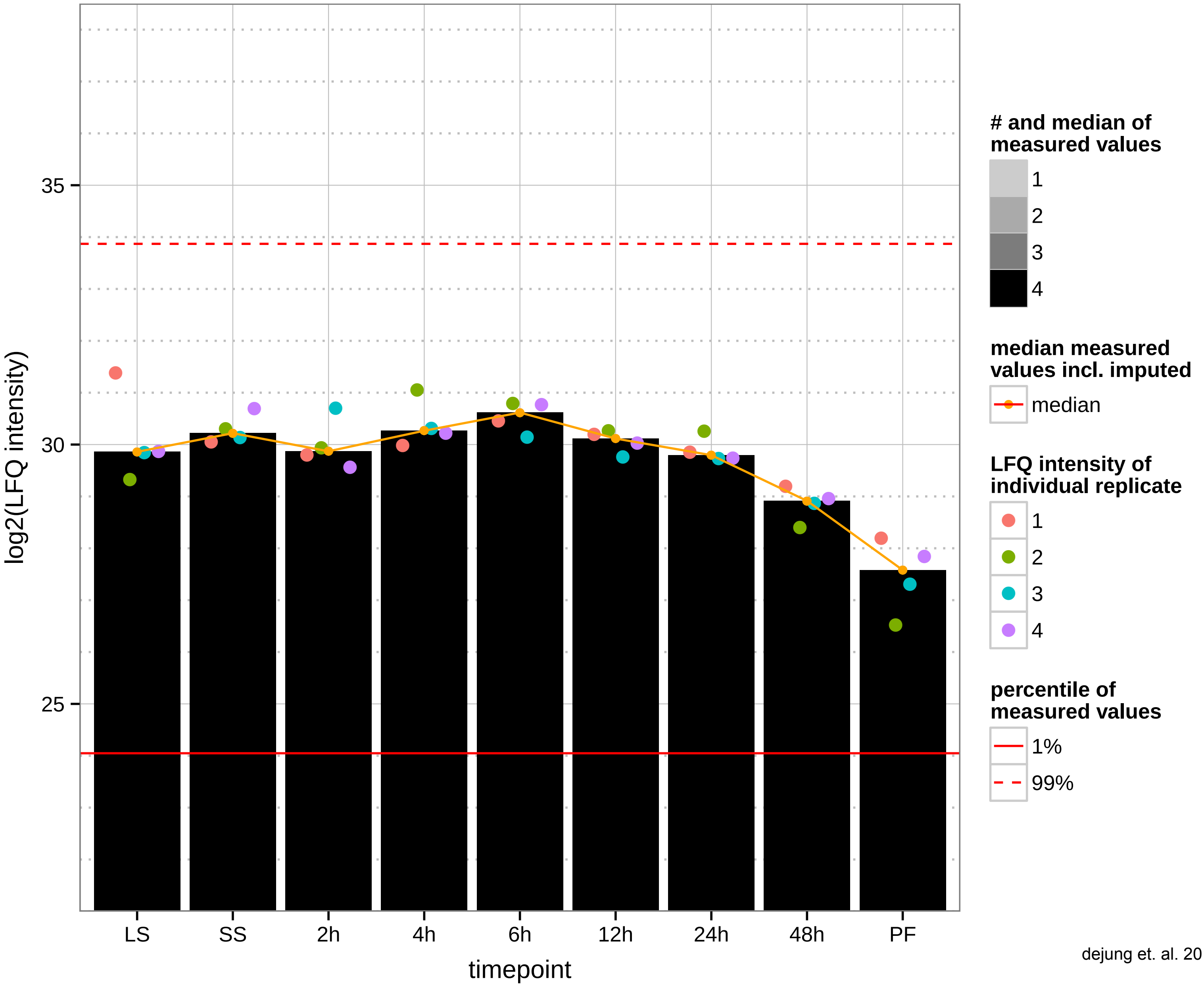

dejung et. al. 2015

hypothetical protein, conserved
Tb927.10.15200
AGOF: null
AGOC: null
AGOP: null
PGOF: null
PGOC: null
PGOP: null

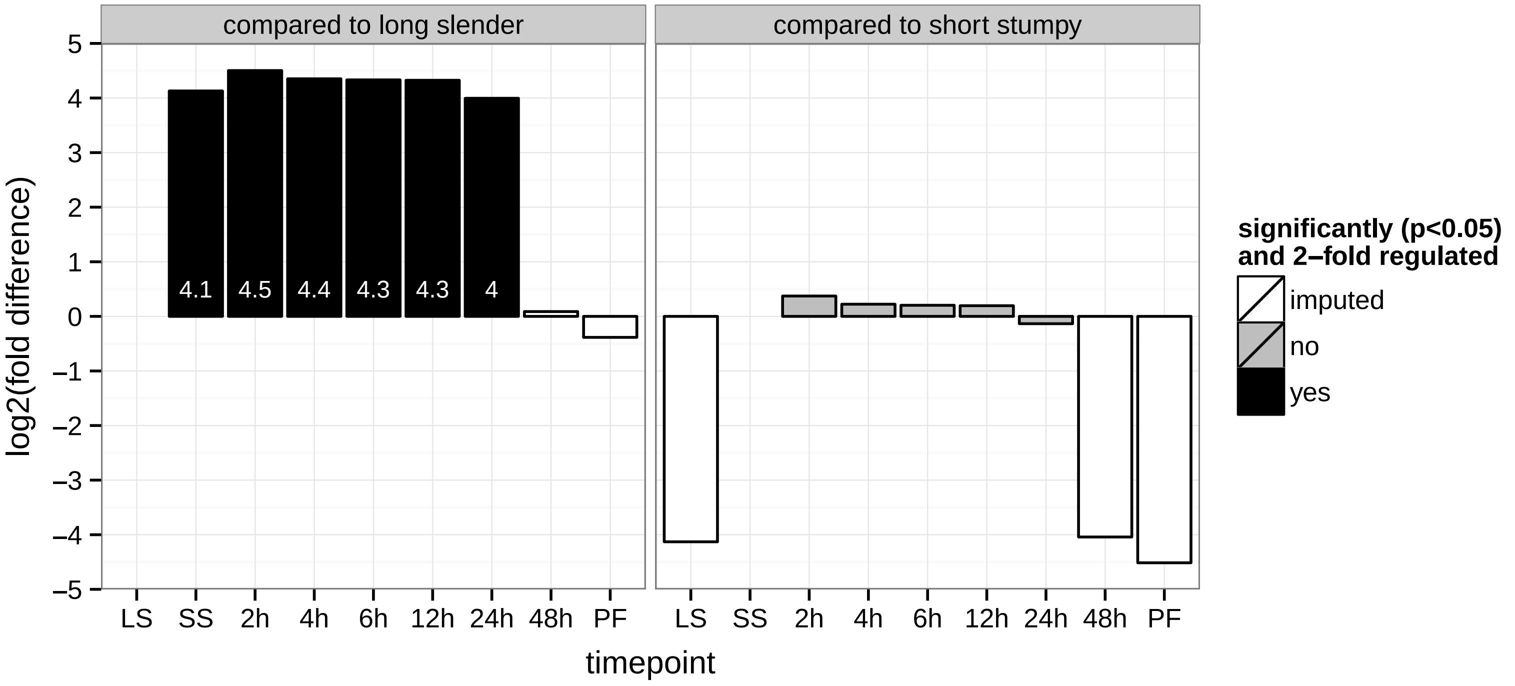

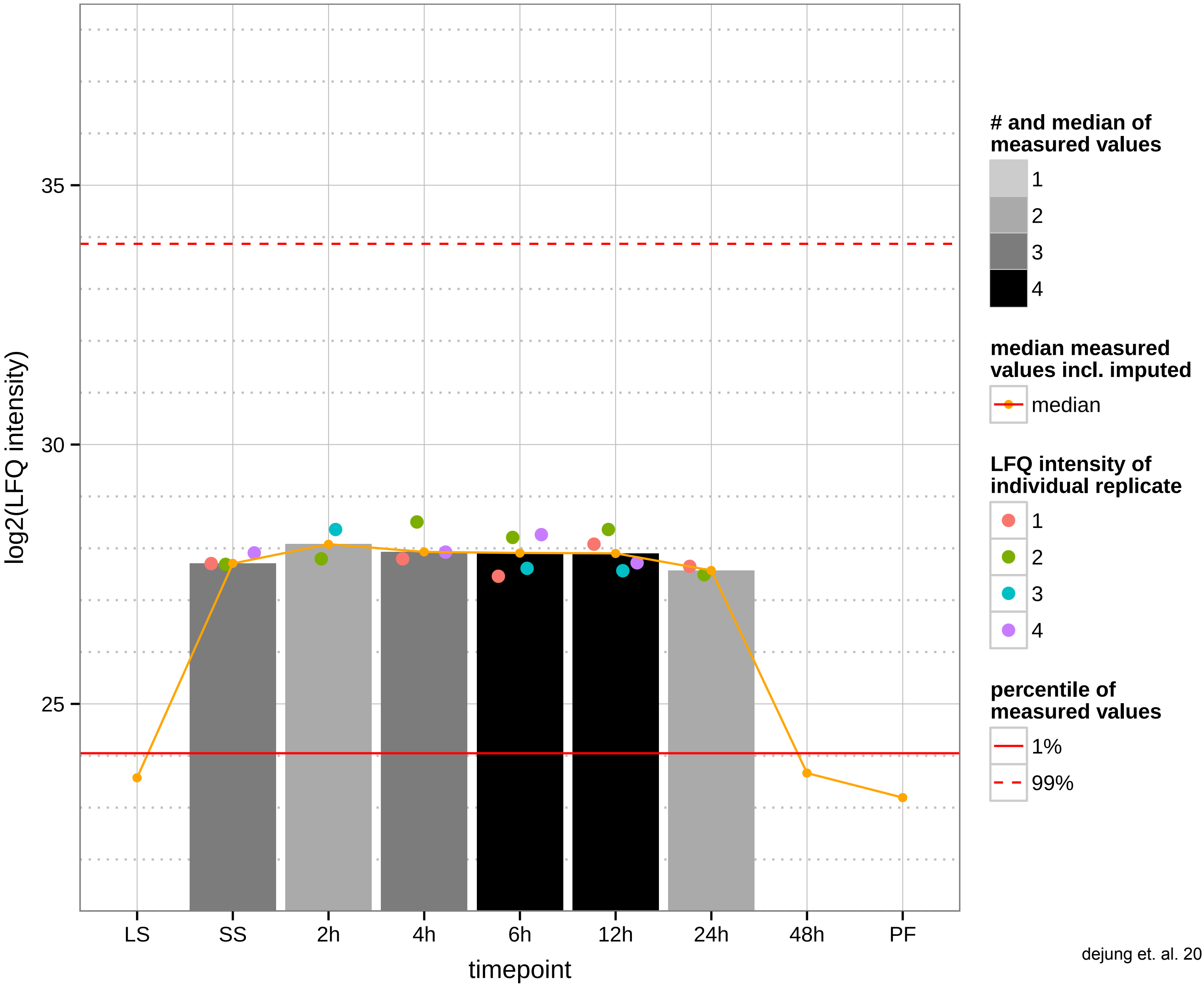

dejung et. al. 2015

fatty acyl CoA synthetase, putative, Long–chain–fatty–acid––CoA ligase 5 (EC 6.2.1.3) (Long–chain acyl–CoA synthetase 5)
Tb927.10.3260
AGOF: null, catalytic activity, long–chain fatty acid–CoA ligase activity
AGOC: null
AGOP: null, fatty acid elongation
PGOF: catalytic activity
PGOC: null
PGOP: metabolic process

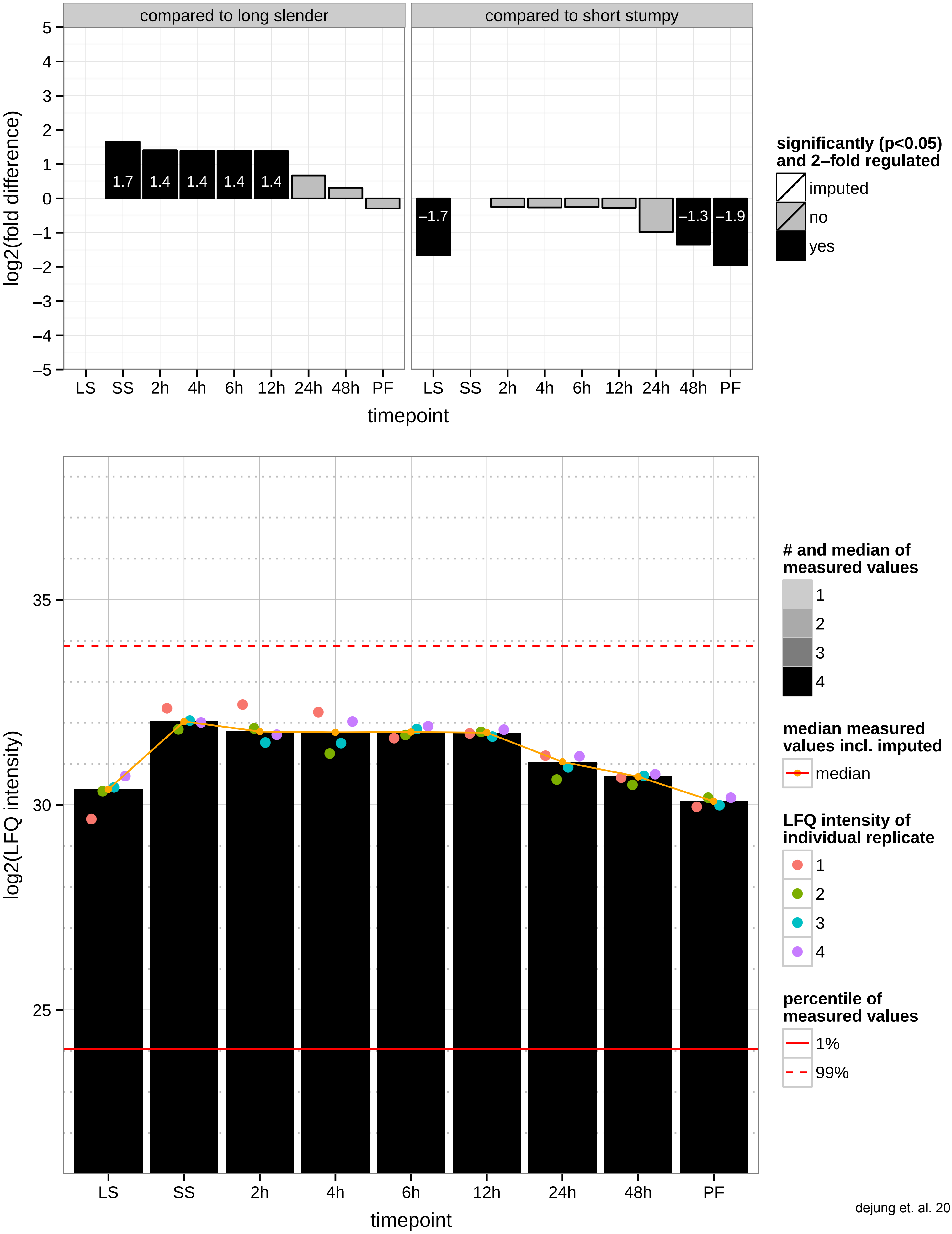

dejung et. al. 2015

aspartate aminotransferase
Tb927.10.3660
AGOF: L–aspartate:2–oxoglutarate aminotransferase activity, pyridoxal phosphate binding
AGOC: cytoplasm
AGOP: L–methionine salvage from methylthioadenosine, biosynthetic process
PGOF: pyridoxal phosphate binding, transferase activity
PGOC: null
PGOP: biosynthetic process

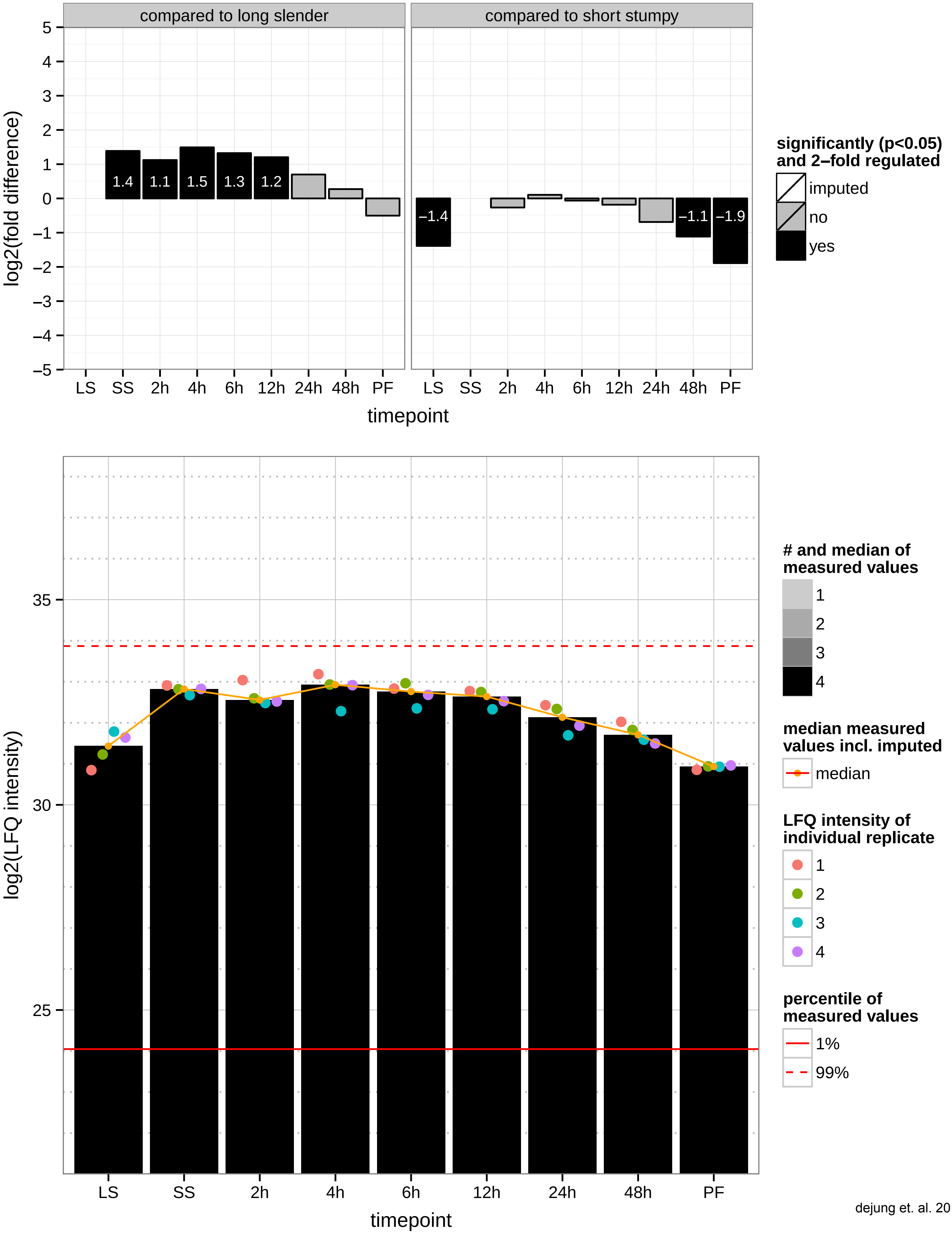

dejung et. al. 2015

hypothetical protein, conserved
Tb927.10.3670
AGOF: null
AGOC: null
AGOP: null
PGOF: null
PGOC: null
PGOP: null

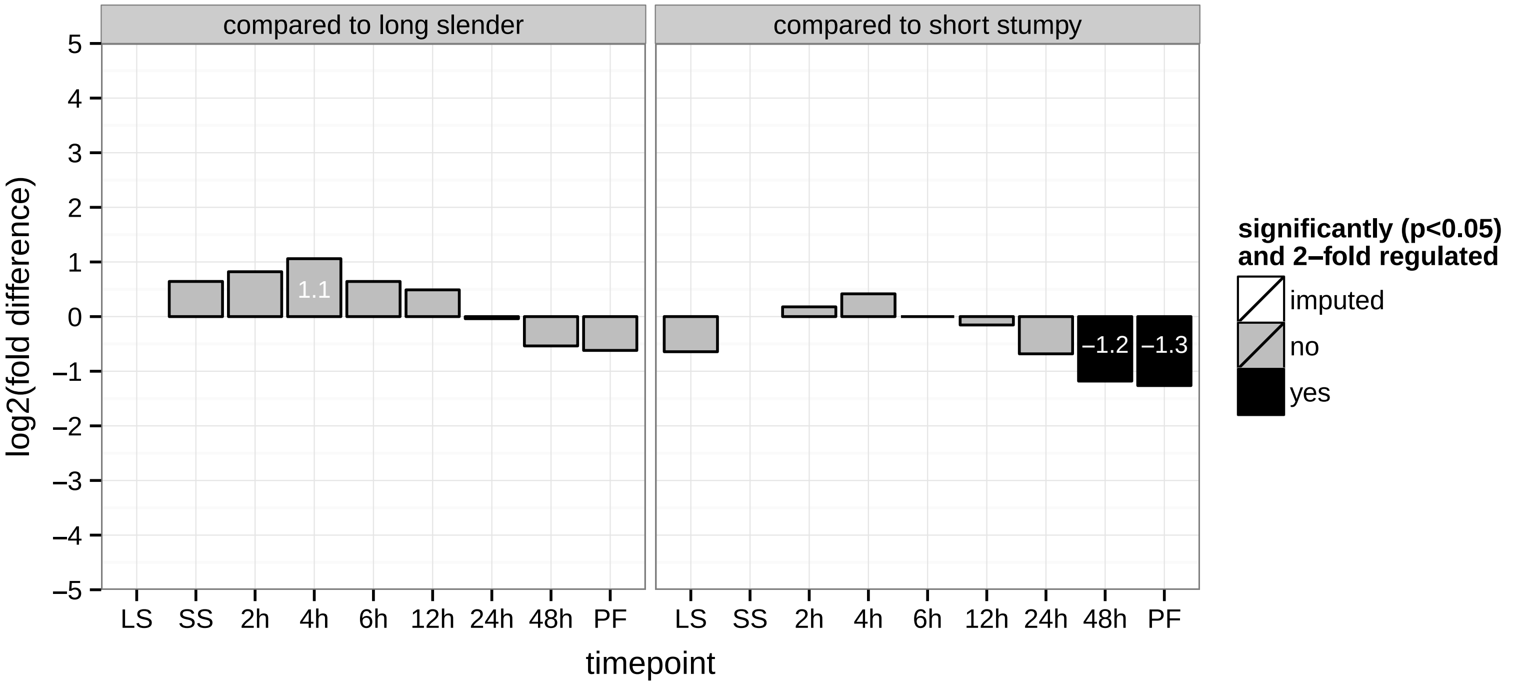

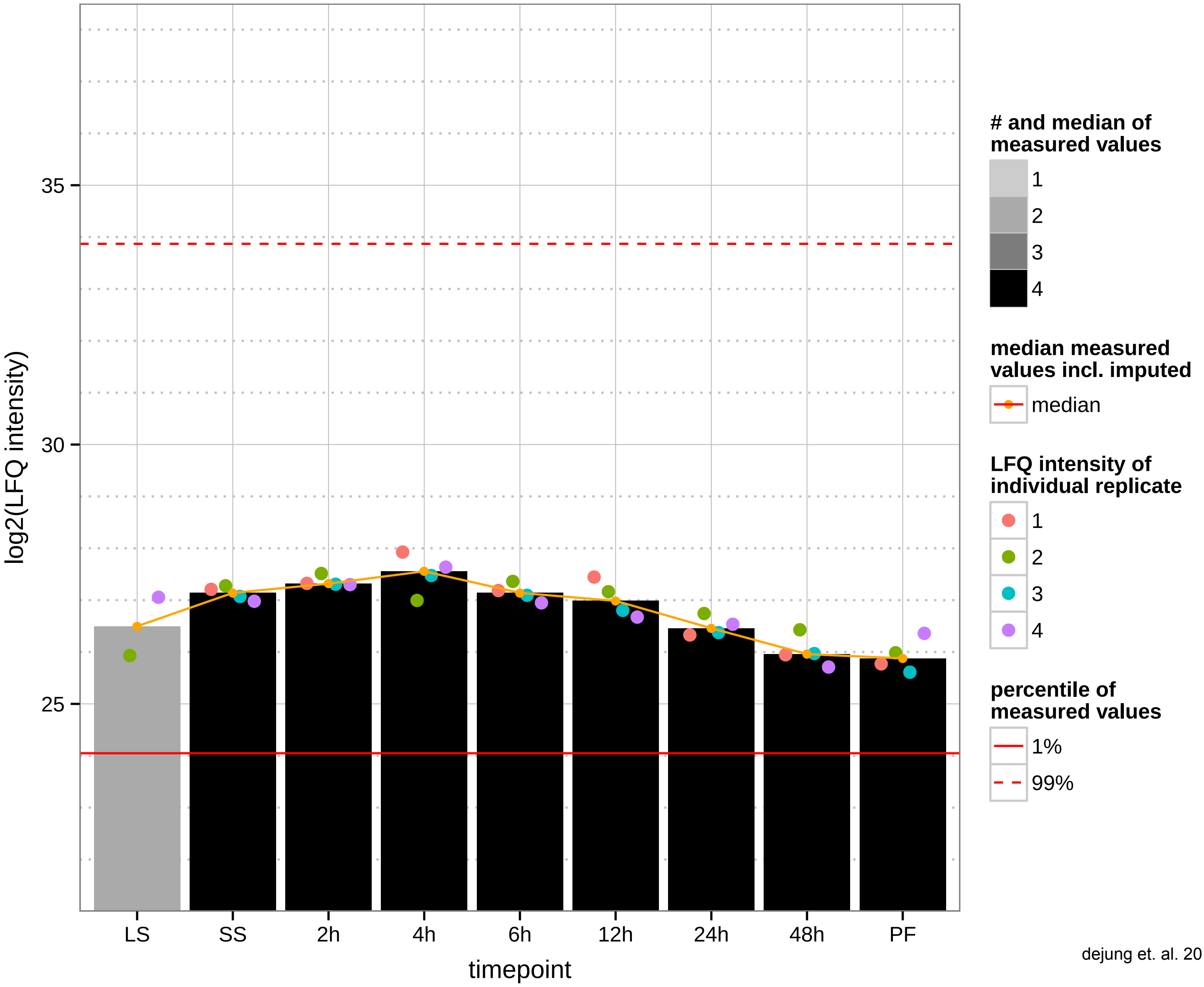

dejung et. al. 2015

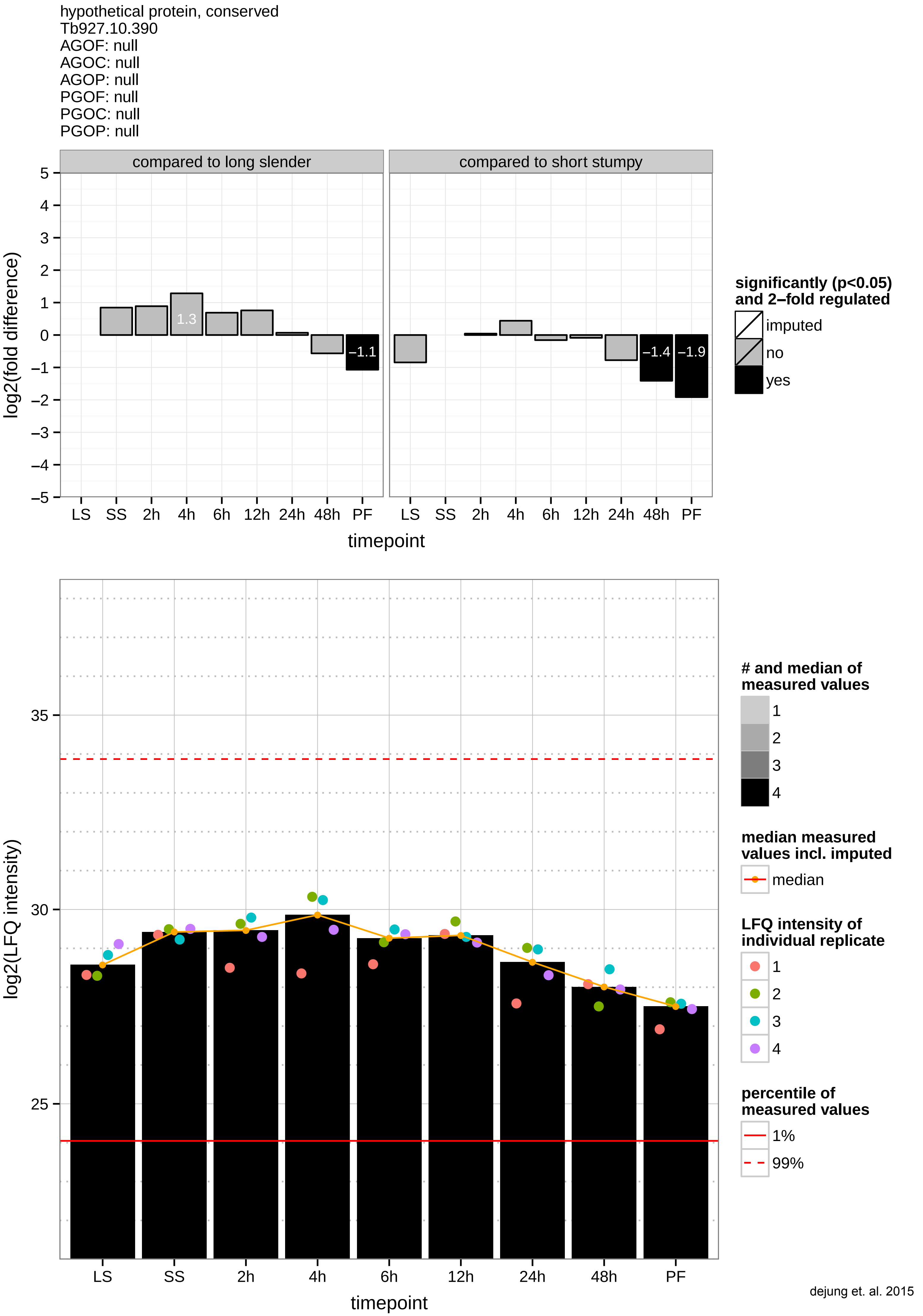

hypothetical protein, conserved
Tb927.10.390
AGOF: null
AGOC: null
AGOP: null
PGOF: null
PGOC: null
PGOP: null
compared to long slender
compared to short stumpy
log2(fold difference)
1.3
−1.1
−1.4
−1.9
LS SS 2h 4h 6h 12h 24h 48h PF
timepoint
significantly (p<0.05) and 2−fold regulated
imputed
no
yes
log2(LFQ intensity)
# and median of measured values
median measured values incl. imputed
median
LFQ intensity of individual replicate
percentile of measured values
1%
99%
dejung et. al. 2015

phosphatidylinositol−4−phosphate 5−kinase, putative
Tb927.10.4770
AGOF: 1−phosphatidylinositol−4−phosphate 5−kinase activity
AGOC: null
AGOP: phosphatidylinositol metabolic process
PGOF: phosphatidylinositol phosphate kinase activity
PGOC: null
PGOP: phosphatidylinositol metabolic process

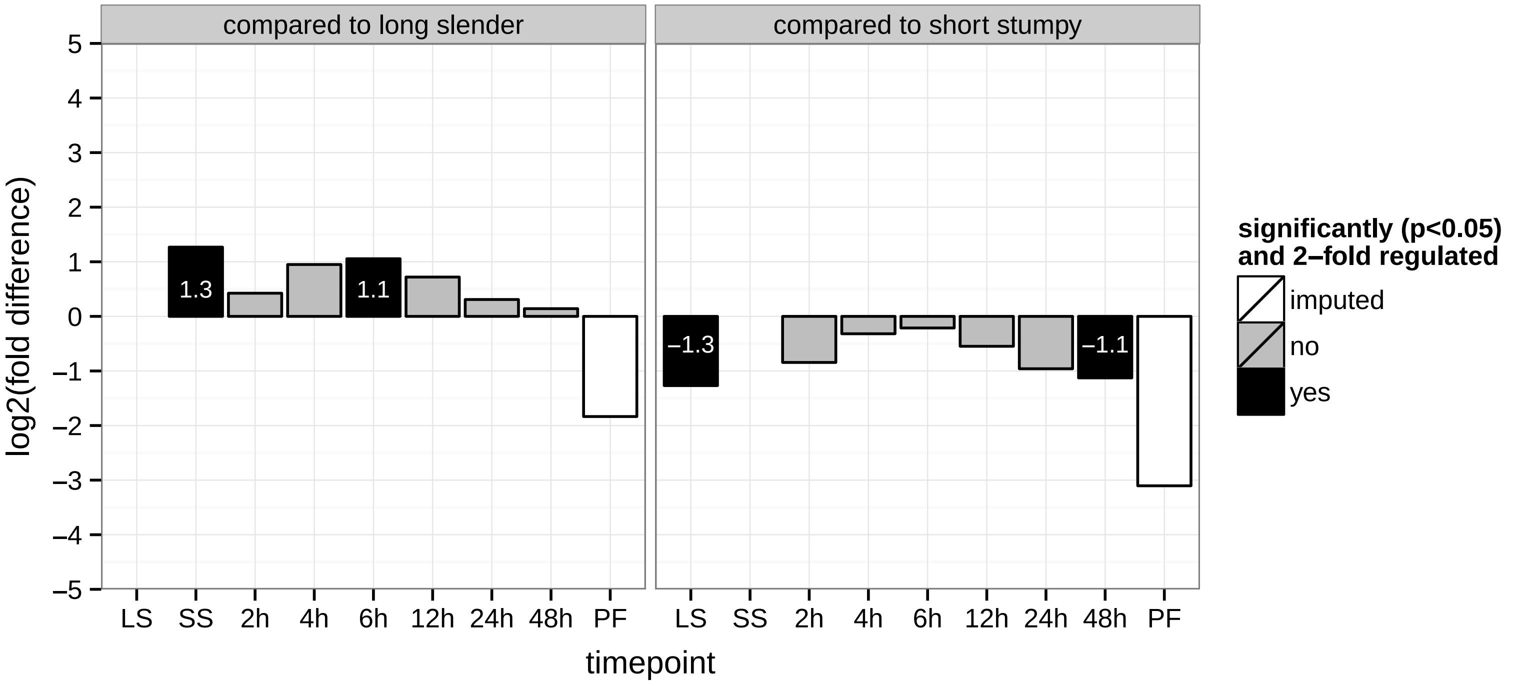

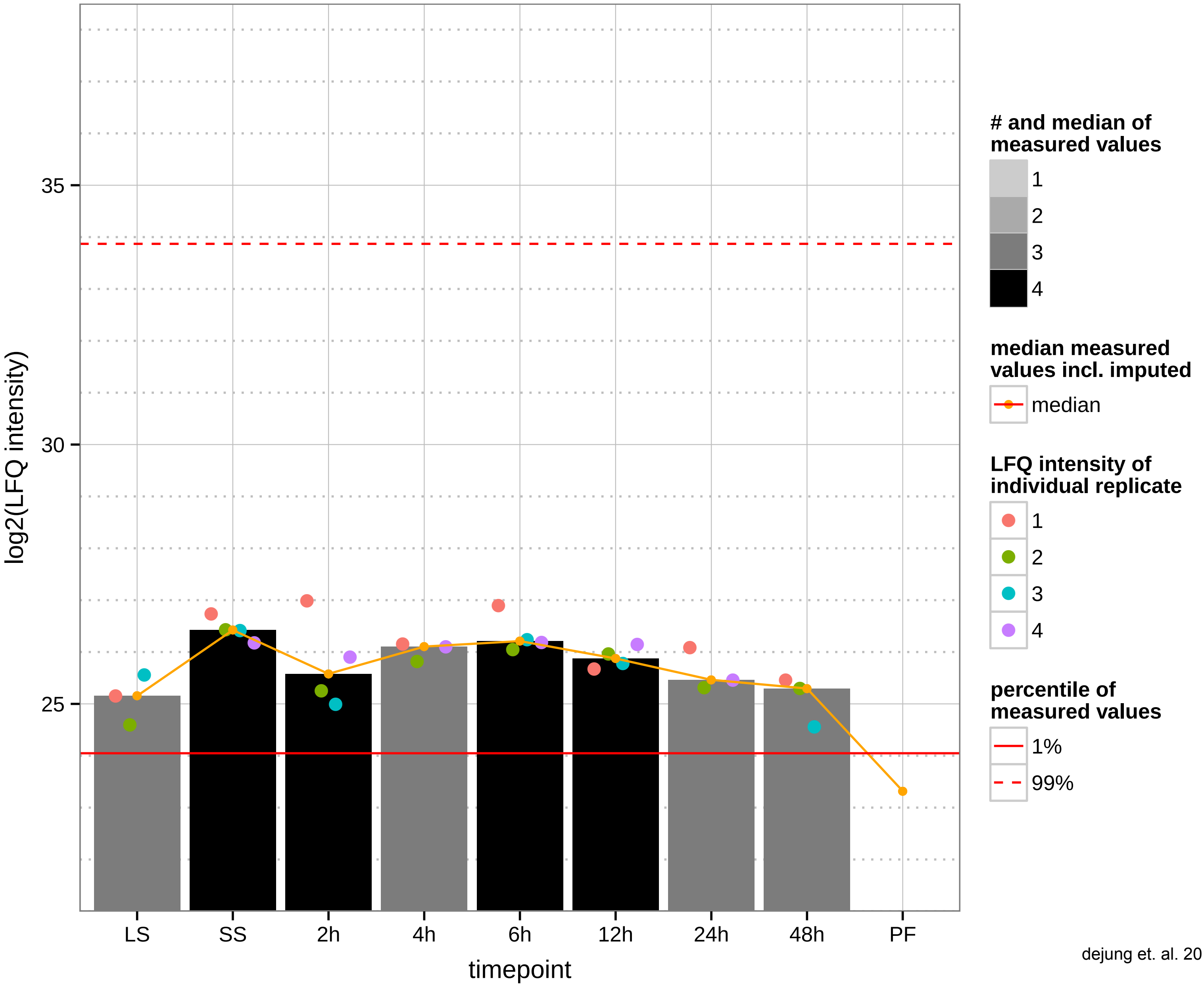

dejung et. al. 2015

mitogen–activated protein kinase 2, putative (MPK2)
Tb927.10.5140
AGOF: ATP binding, protein kinase activity, protein serine/threonine kinase activity, protein tyrosine kinase activity
AGOC: null
AGOP: cytokinesis after mitosis, protein phosphorylation
PGOF: ATP binding, protein kinase activity, transferase activity, transferring phosphorus–containing groups
PGOC: null
PGOP: protein phosphorylation

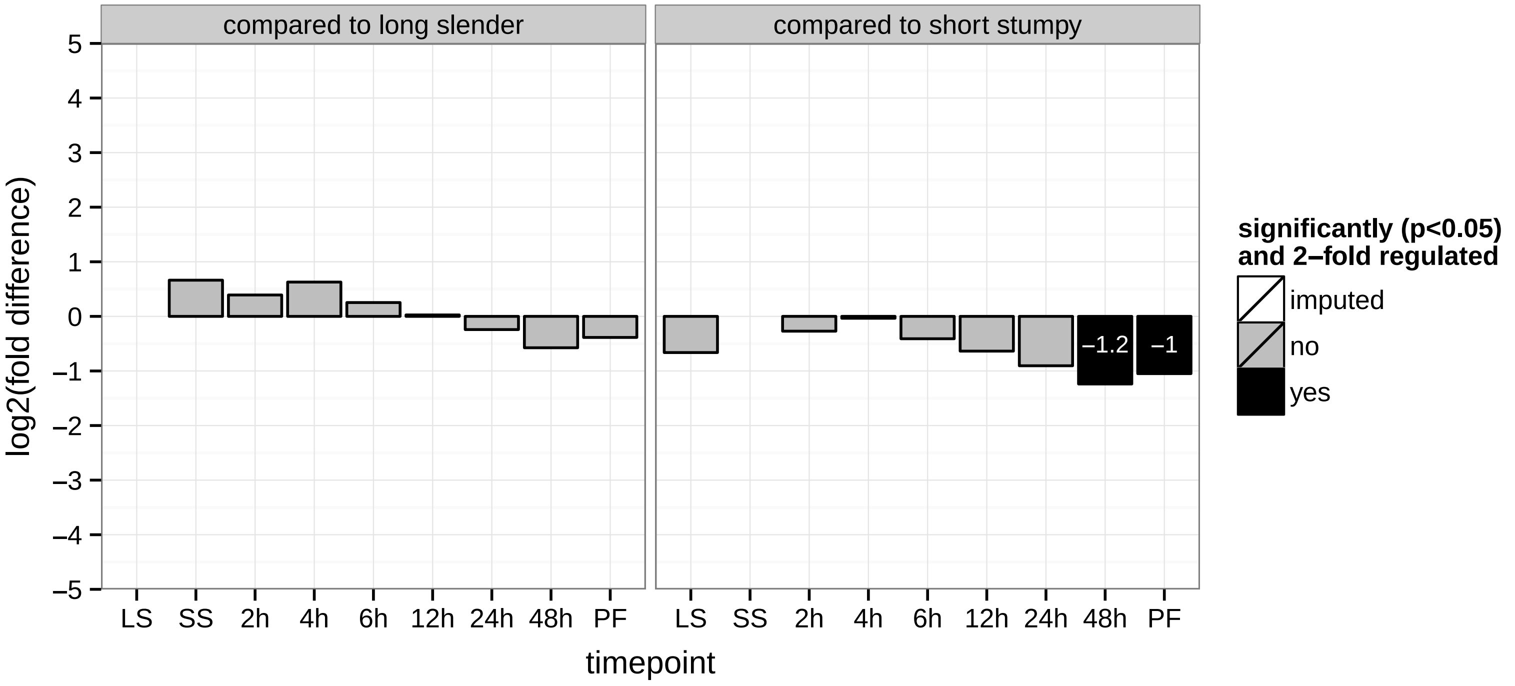

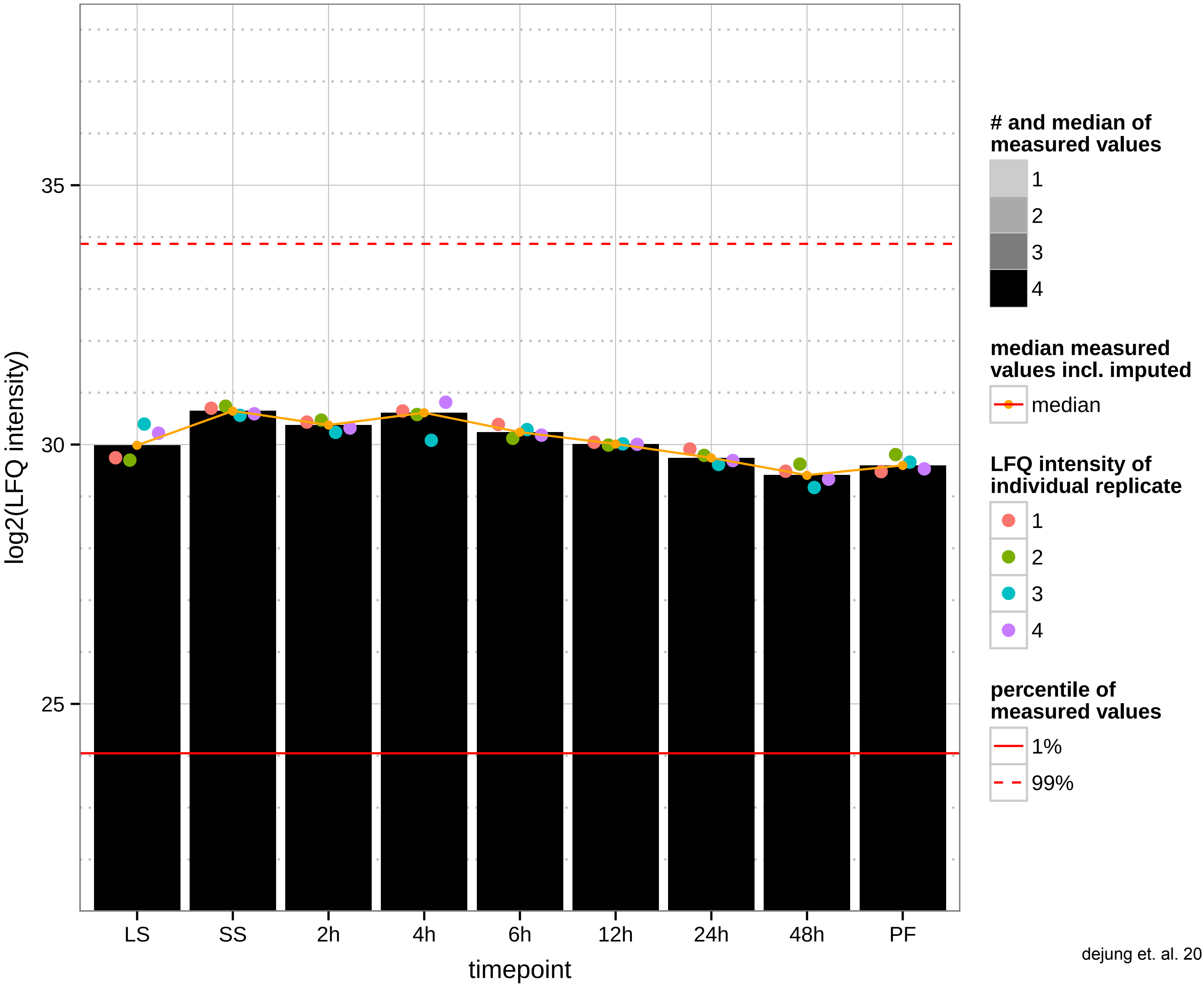

dejung et. al. 2015

hypothetical protein, conserved
Tb927.10.5810
AGOF: null
AGOC: null
AGOP: null
PGOF: null
PGOC: null
PGOP: null

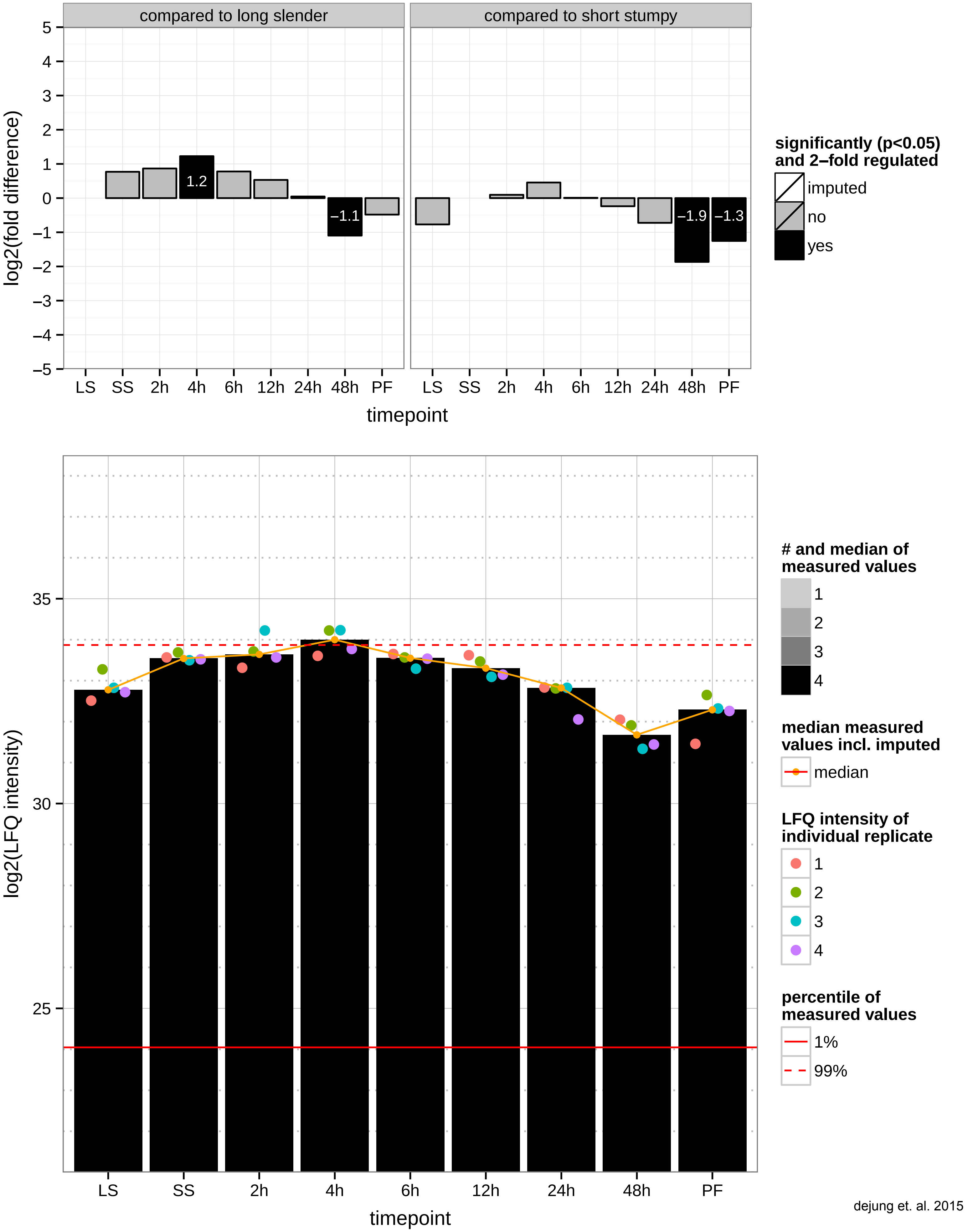

dejung et. al. 2015

clathrin heavy chain, putative, clathrin heavy chain (CHC)
Tb927.10.6050;Tb11.v5.0198
AGOF: null, structural molecule activity
AGOC: null, clathrin coat of coated pit, clathrin coat of trans−Golgi network vesicle
AGOP: null, intracellular protein transport, vesicle−mediated transport
PGOF: binding, structural molecule activity
PGOC: null, clathrin coat of coated pit, clathrin coat of trans−Golgi network vesicle
PGOP: intracellular protein transport, vesicle−mediated transport

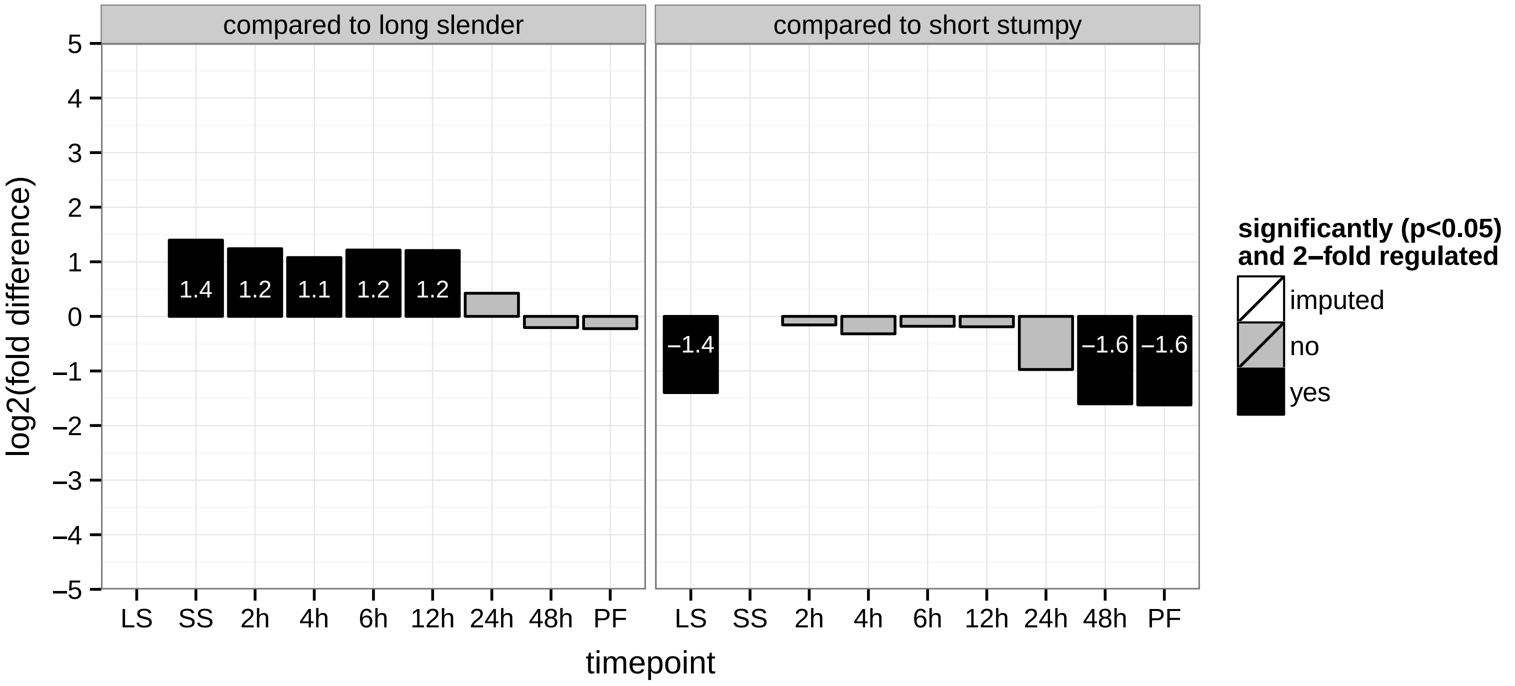

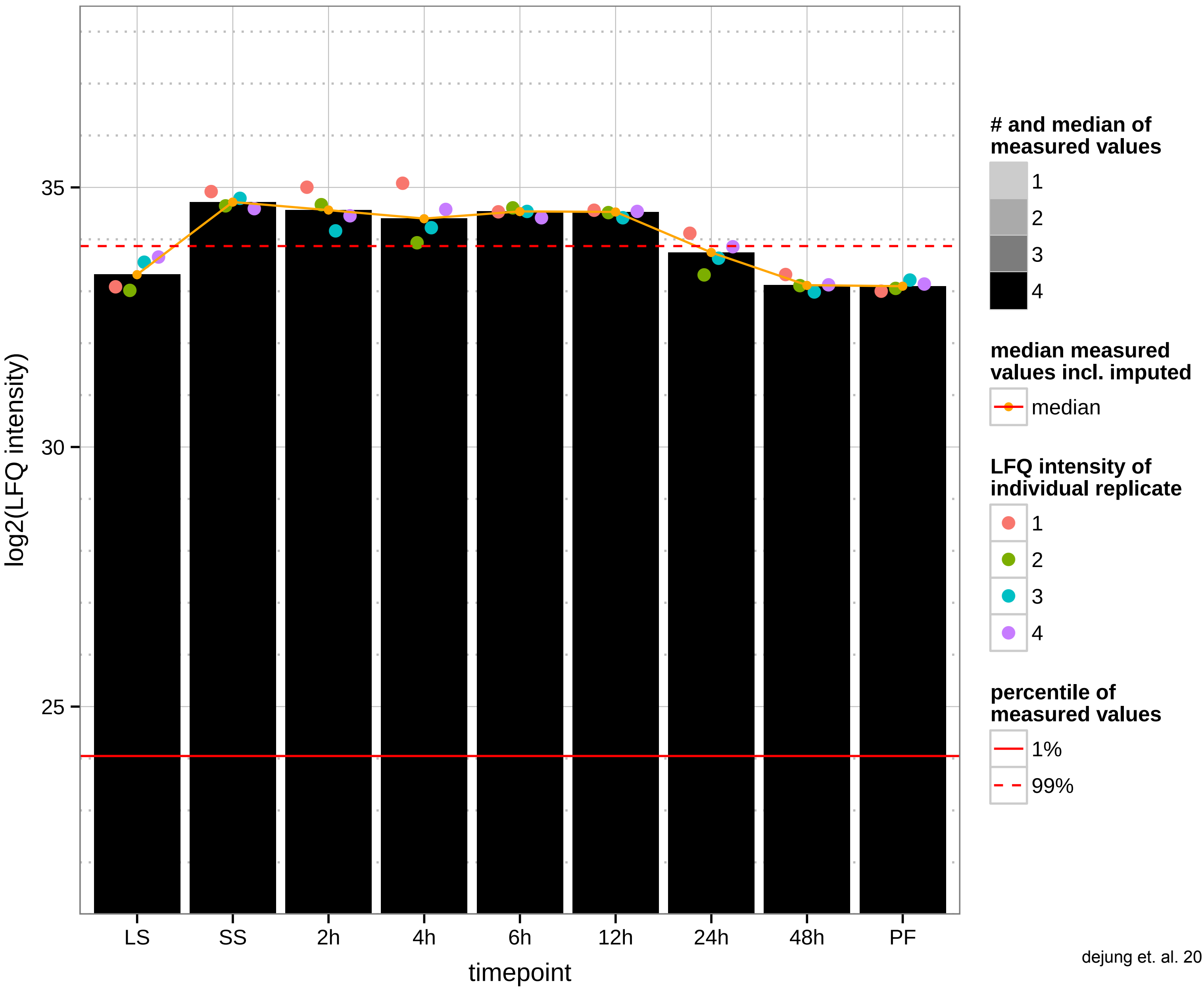

dejung et. al. 2015

alternative oxidase (AOX)
Tb927.10.7090
AGOF: alternative oxidase activity, ferric iron binding, oxidoreductase activity
AGOC: mitochondrial envelope, mitochondrial inner membrane, mitochondrion
AGOP: oxidation–reduction process, respiratory gaseous exchange
PGOF: null
PGOC: mitochondrial envelope
PGOP: oxidation–reduction process, respiratory gaseous exchange

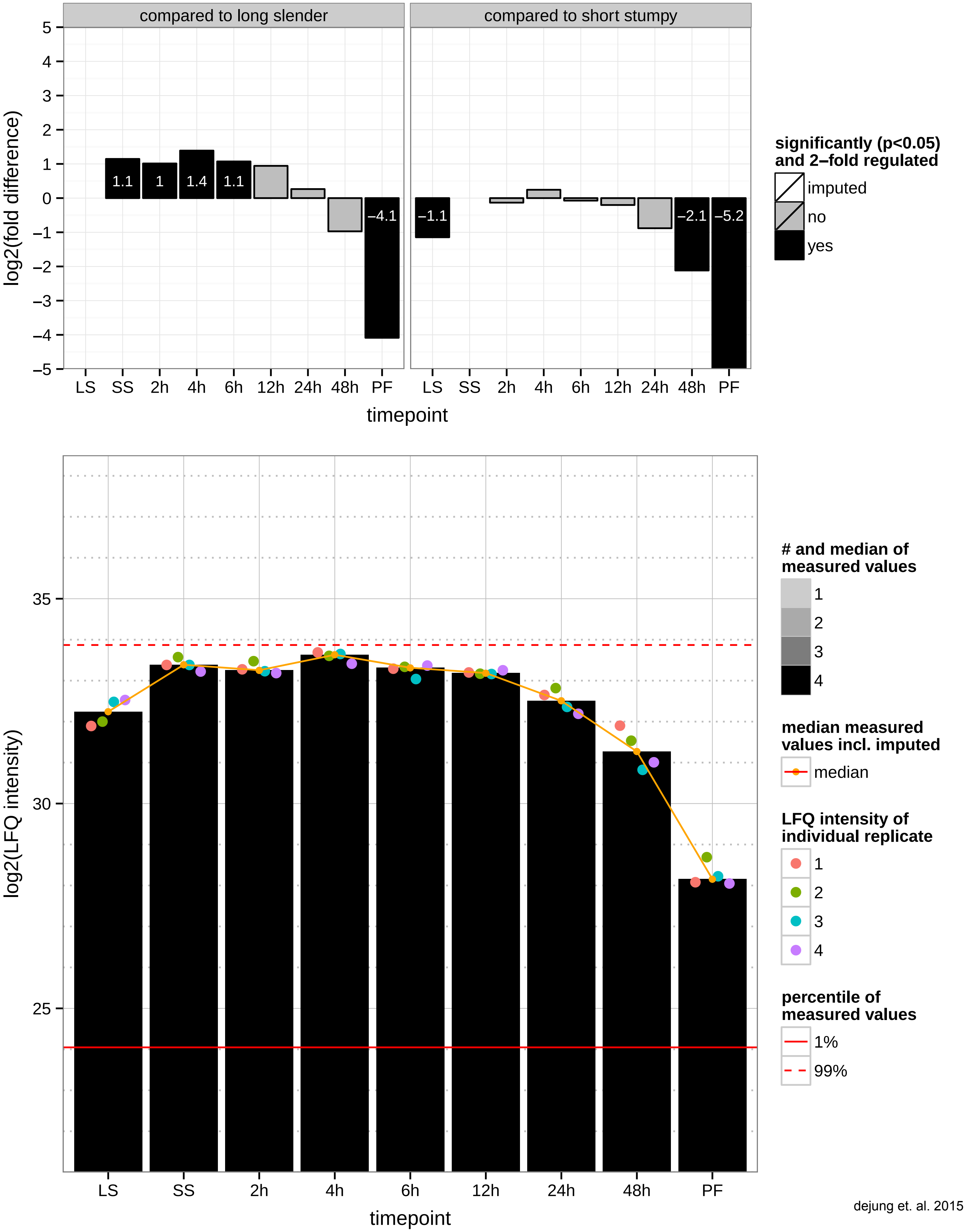

dejung et. al. 2015

inositol–3–phosphate synthase, putative
Tb927.10.7110
AGOF: inositol–3–phosphate synthase activity
AGOC: null
AGOP: inositol biosynthetic process, metabolic process, phospholipid biosynthetic process
PGOF: inositol–3–phosphate synthase activity
PGOC: null
PGOP: inositol biosynthetic process, phospholipid biosynthetic process

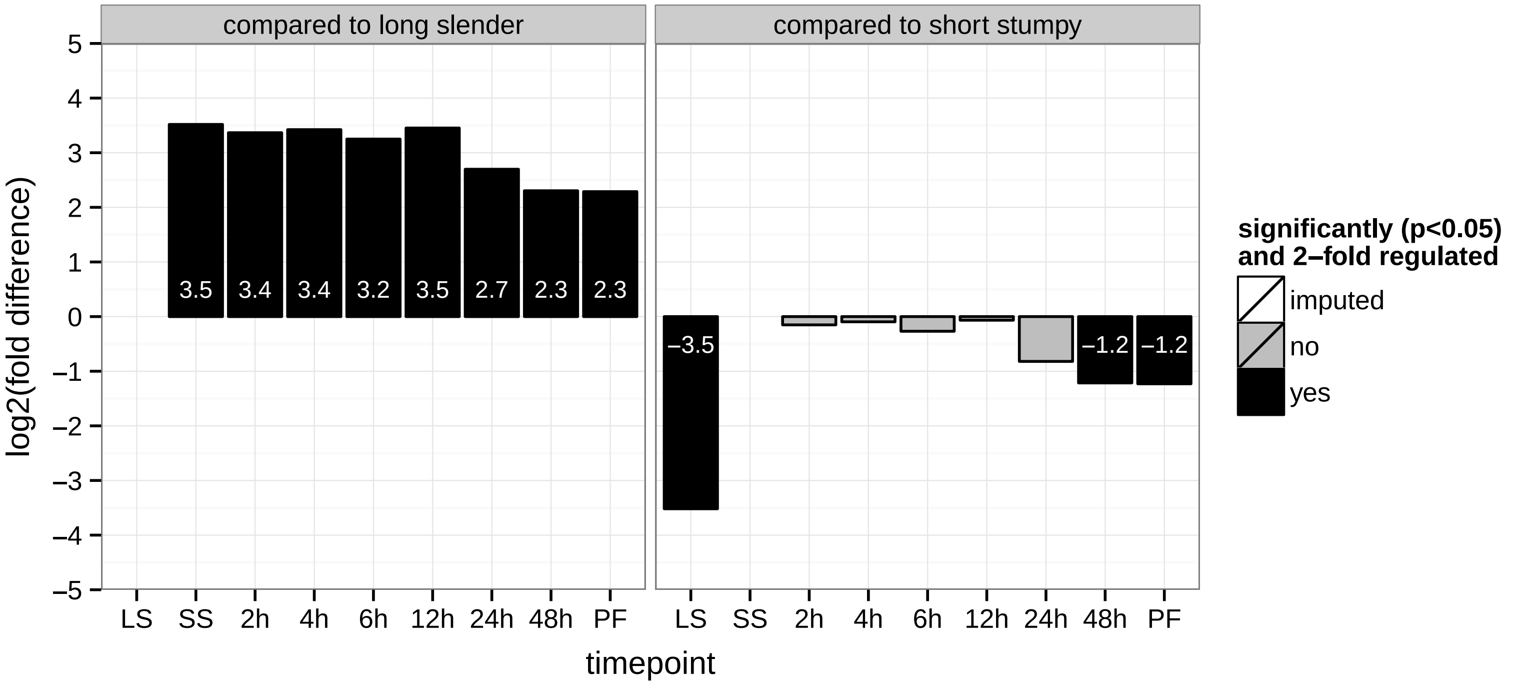

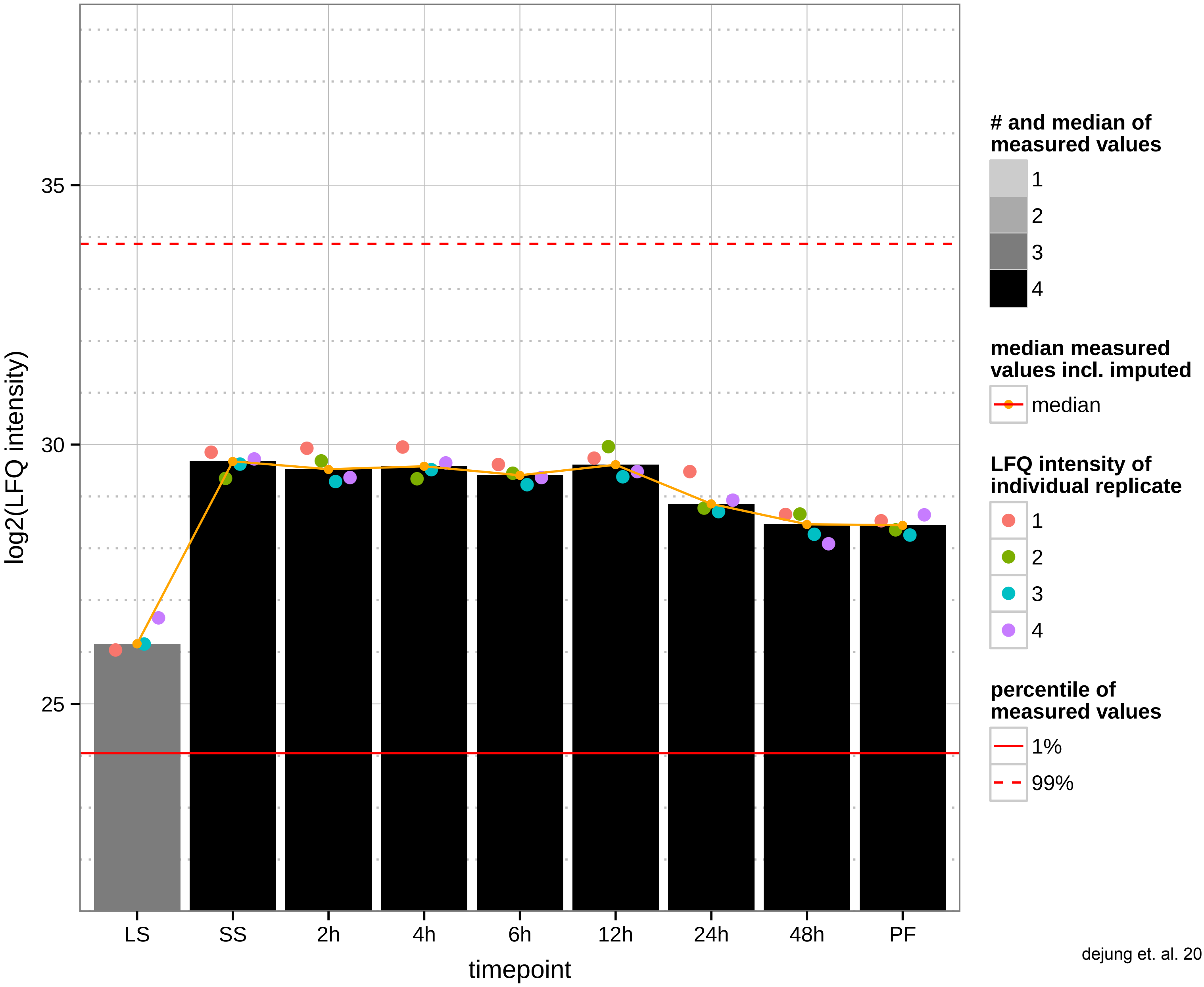

dejung et. al. 2015

hypothetical protein, conserved
Tb927.10.7820
AGOF: null
AGOC: null
AGOP: null
PGOF: null
PGOC: null
PGOP: null

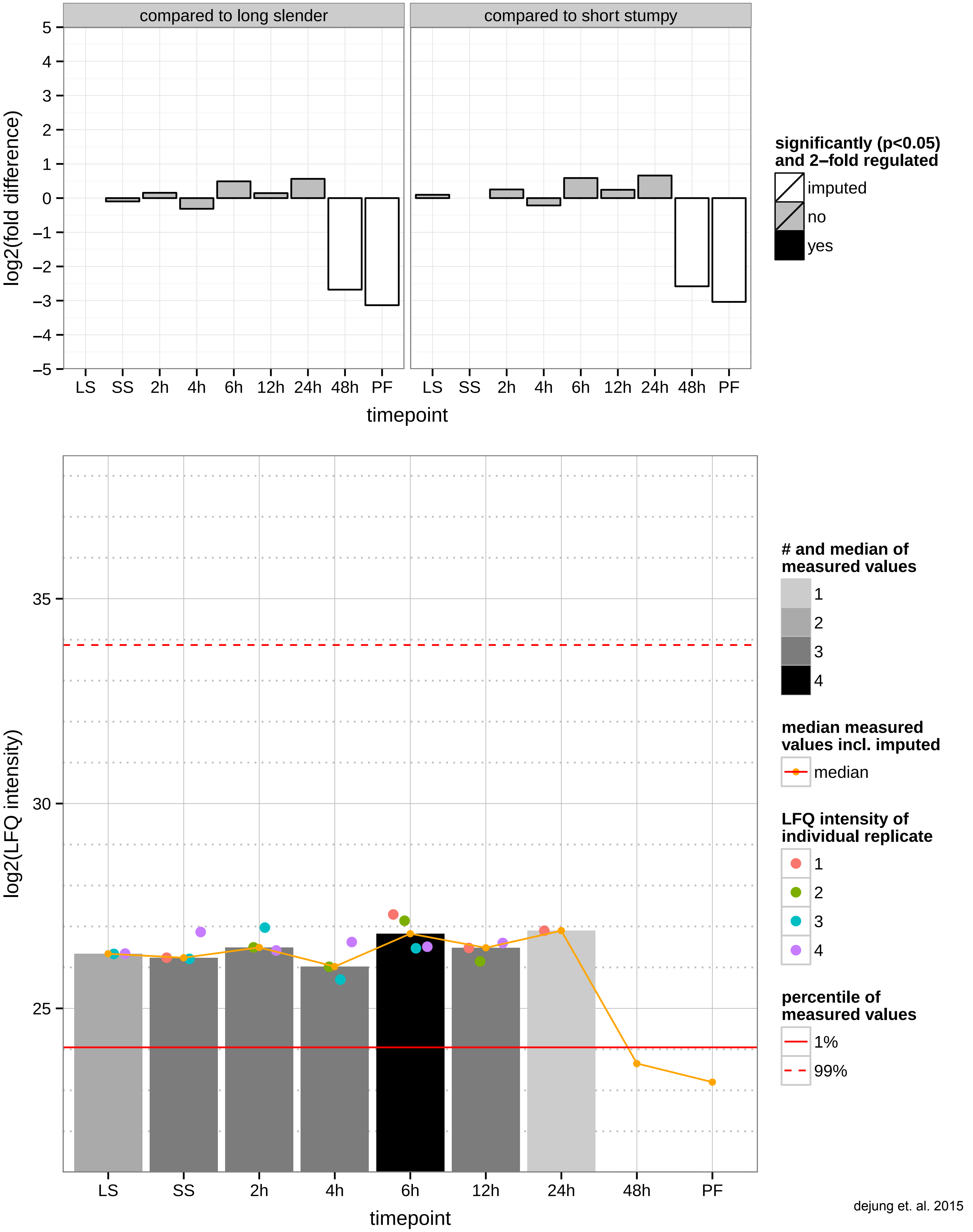

dejung et. al. 2015

DNA–(apurinic or apyrimidinic site) lyase, putative
Tb927.11.10640
AGOF: endonuclease activity
AGOC: null
AGOP: DNA repair
PGOF: nuclease activity
PGOC: null
PGOP: DNA repair

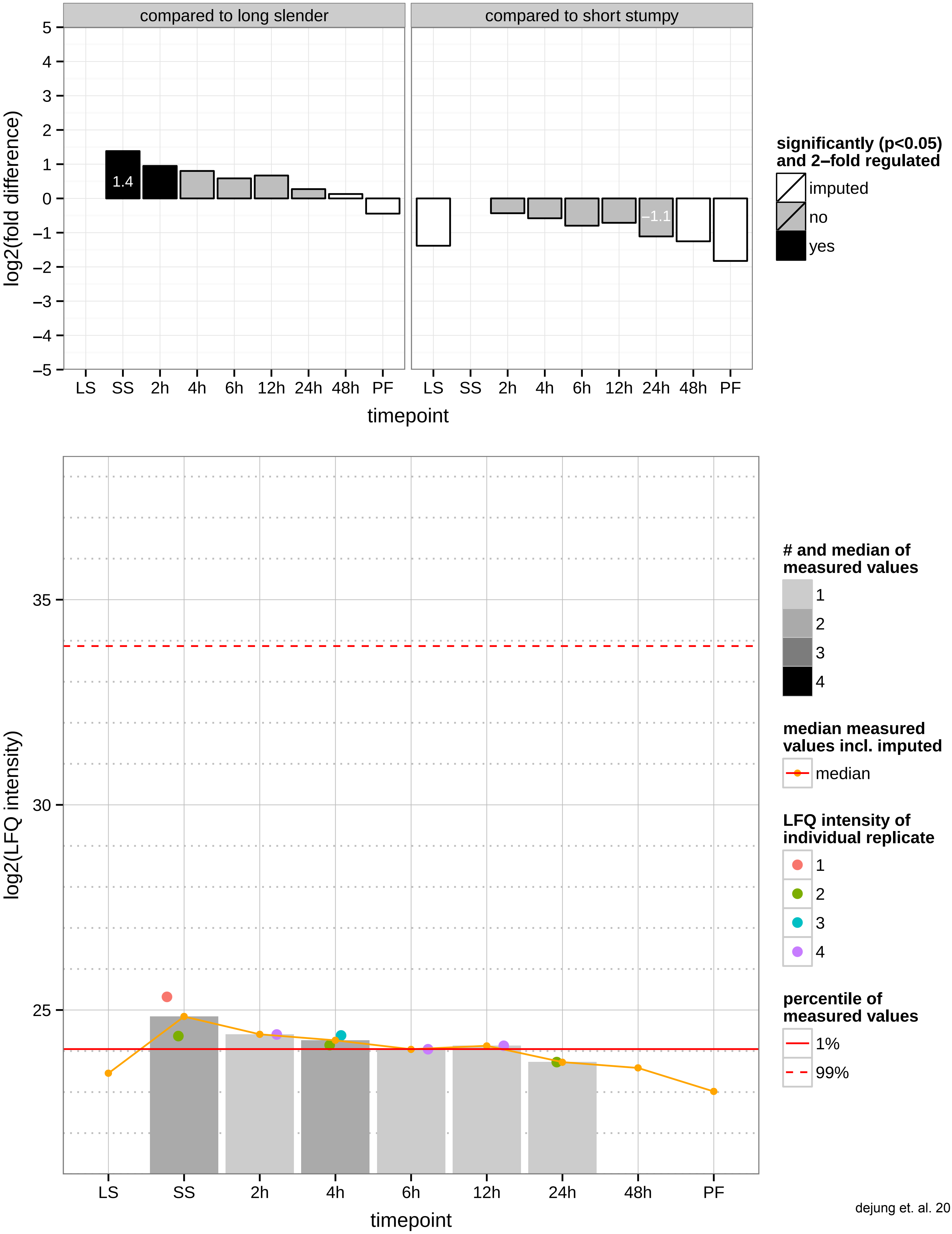

dejung et. al. 2015

trans−sialidase, putative
Tb927.11.11410
AGOF: exo−alpha−sialidase activity
AGOC: null
AGOP: pathogenesis
PGOF: null
PGOC: null
PGOP: null

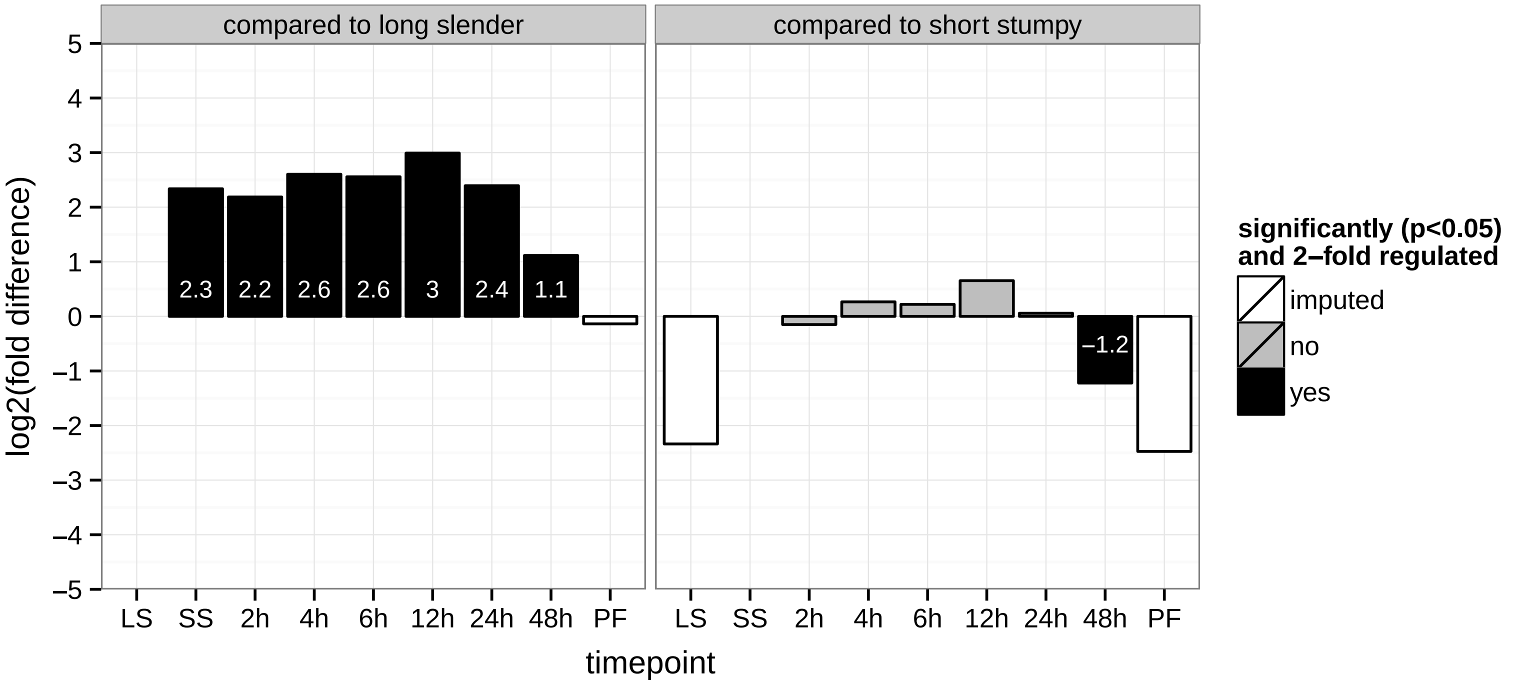

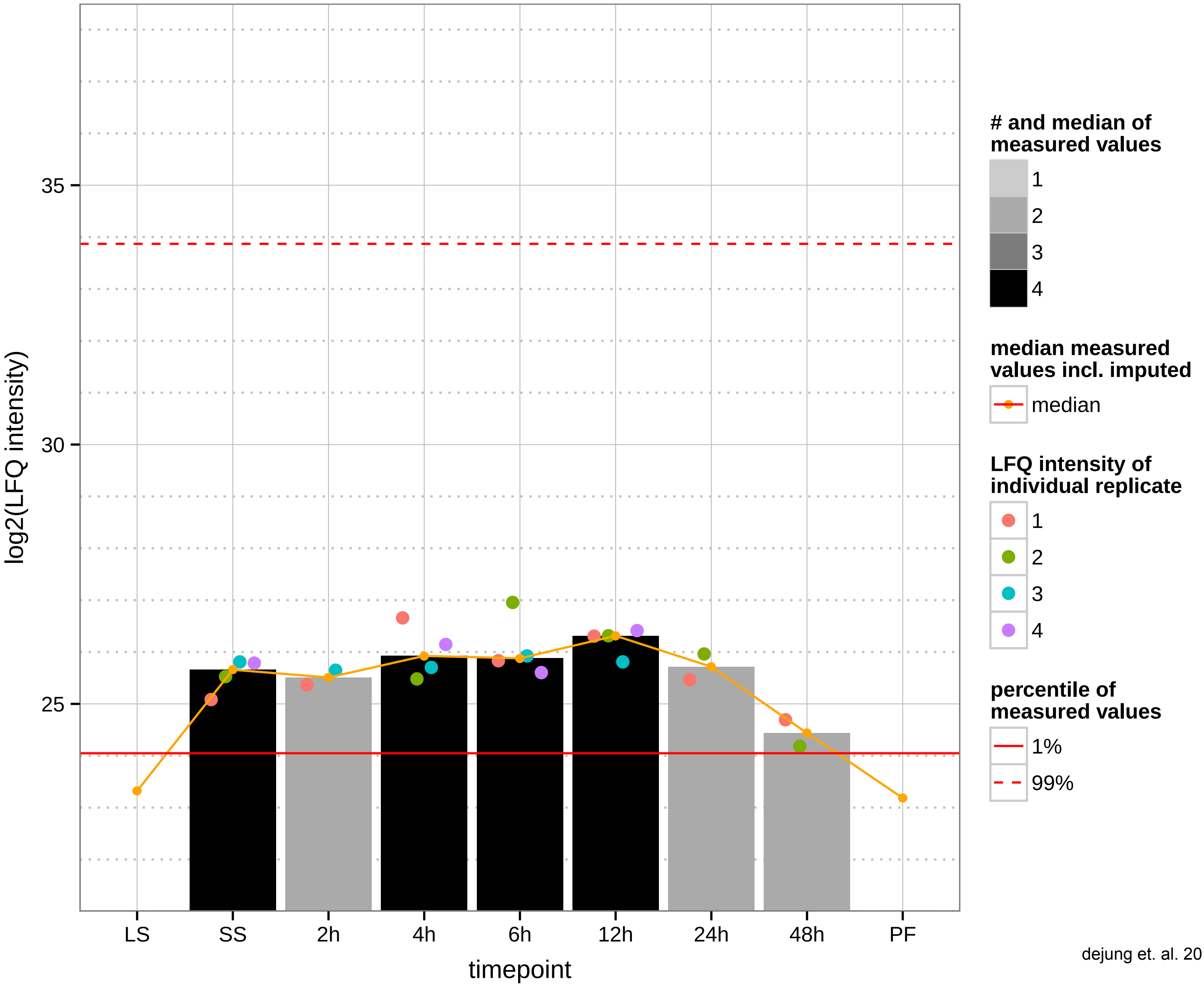

dejung et. al. 2015

Trichohyalin, putative
Tb927.11.11480
AGOF: null
AGOC: null
AGOP: modulation of development of symbiont involved in interaction with host, quorum sensing involved in interaction with ho
PGOF: null
PGOC: null
PGOP: null

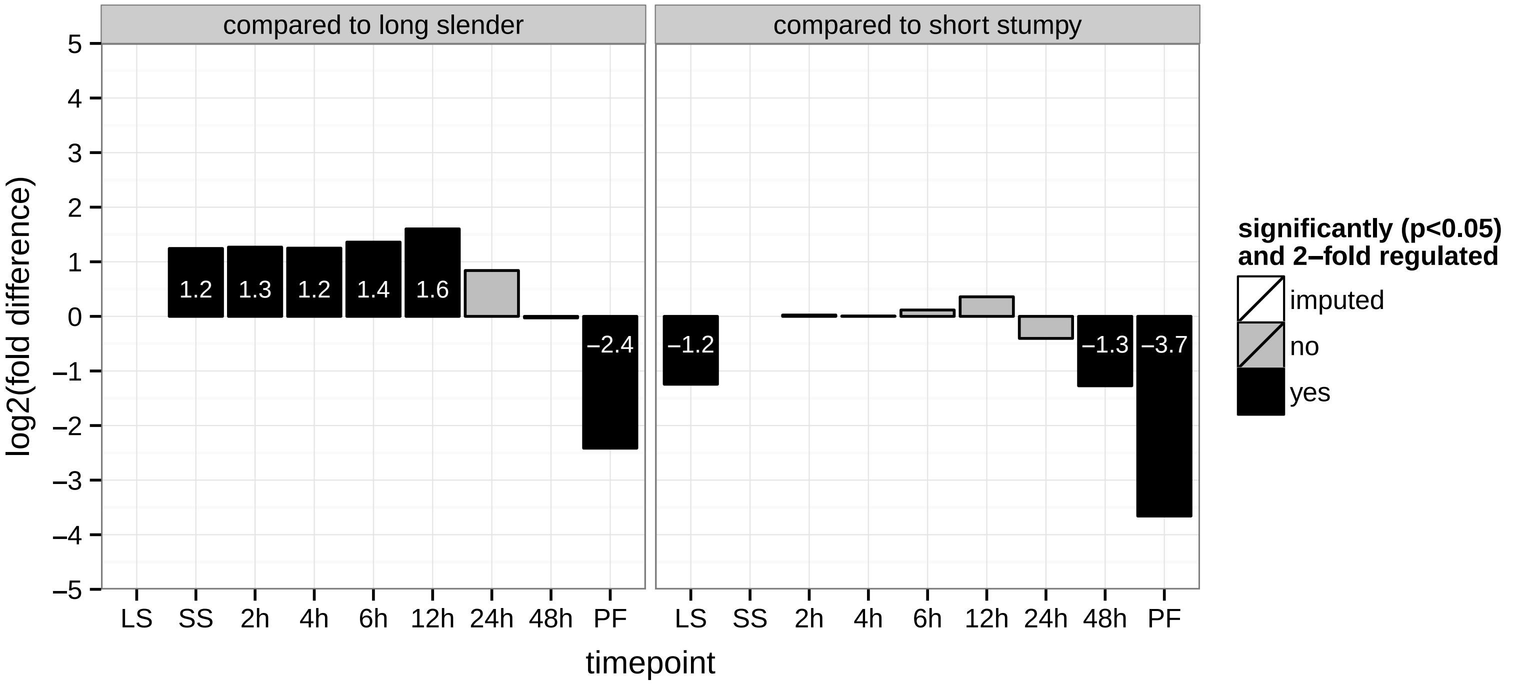

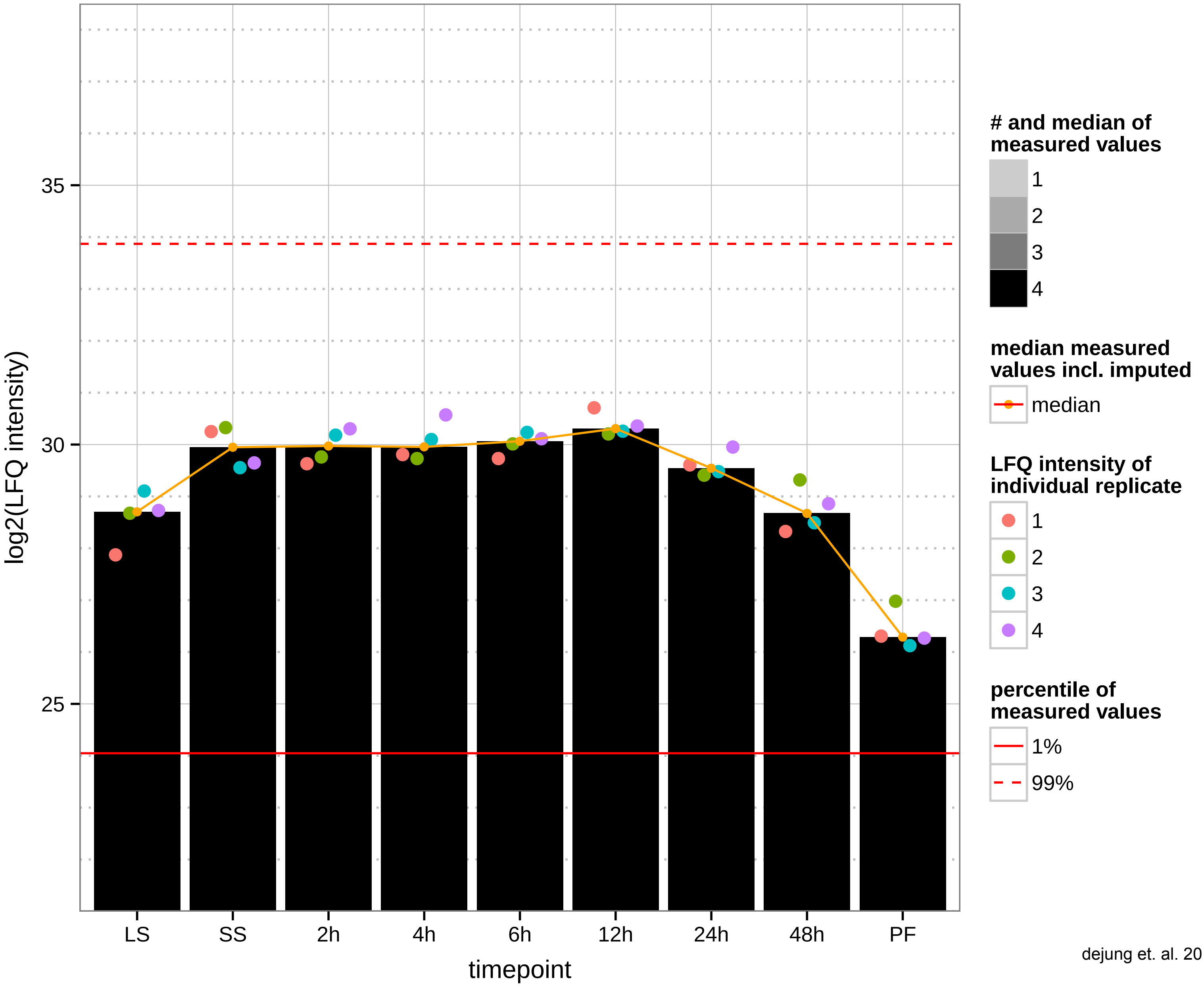

dejung et. al. 2015

microtubule–associated protein, corset–associated protein 15 (CAP15)
Tb927.11.11980
AGOF: null
AGOC: null
AGOP: null
PGOF: null
PGOC: null
PGOP: null

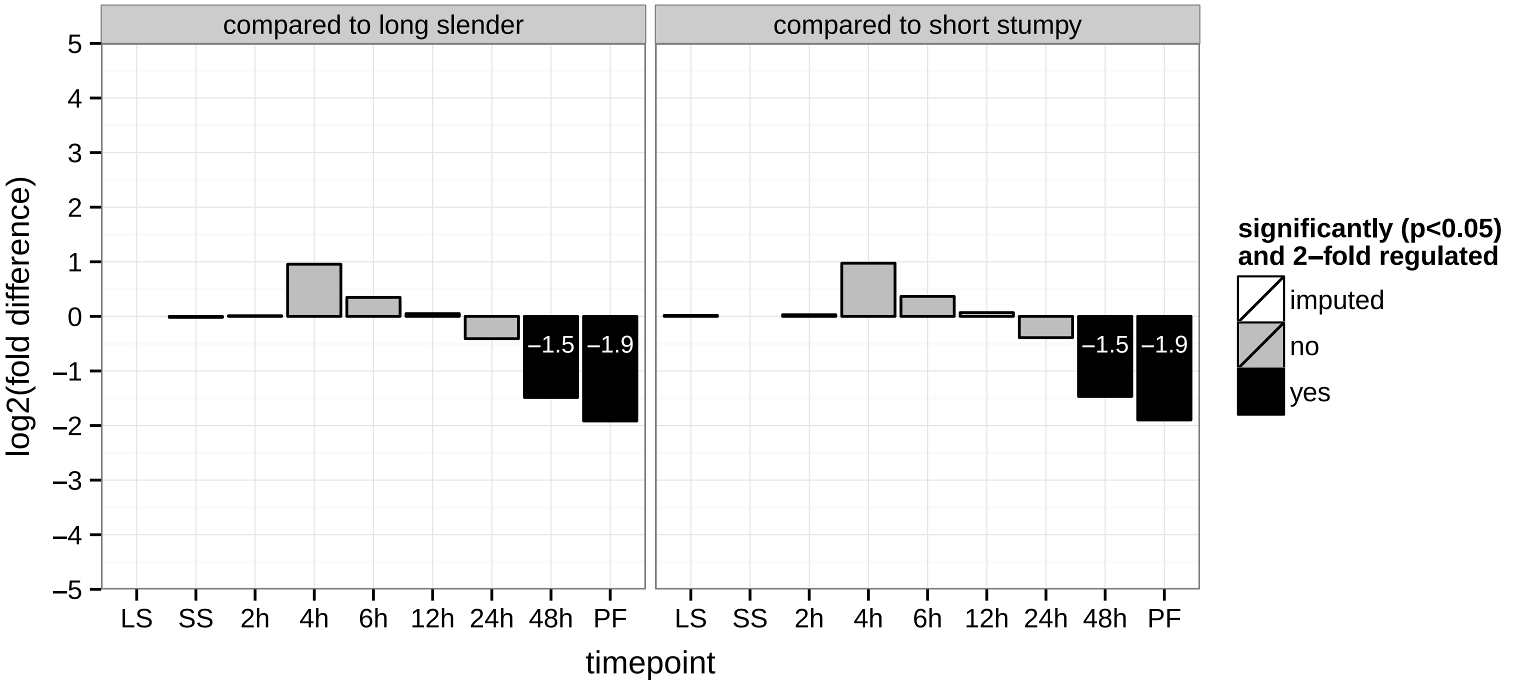

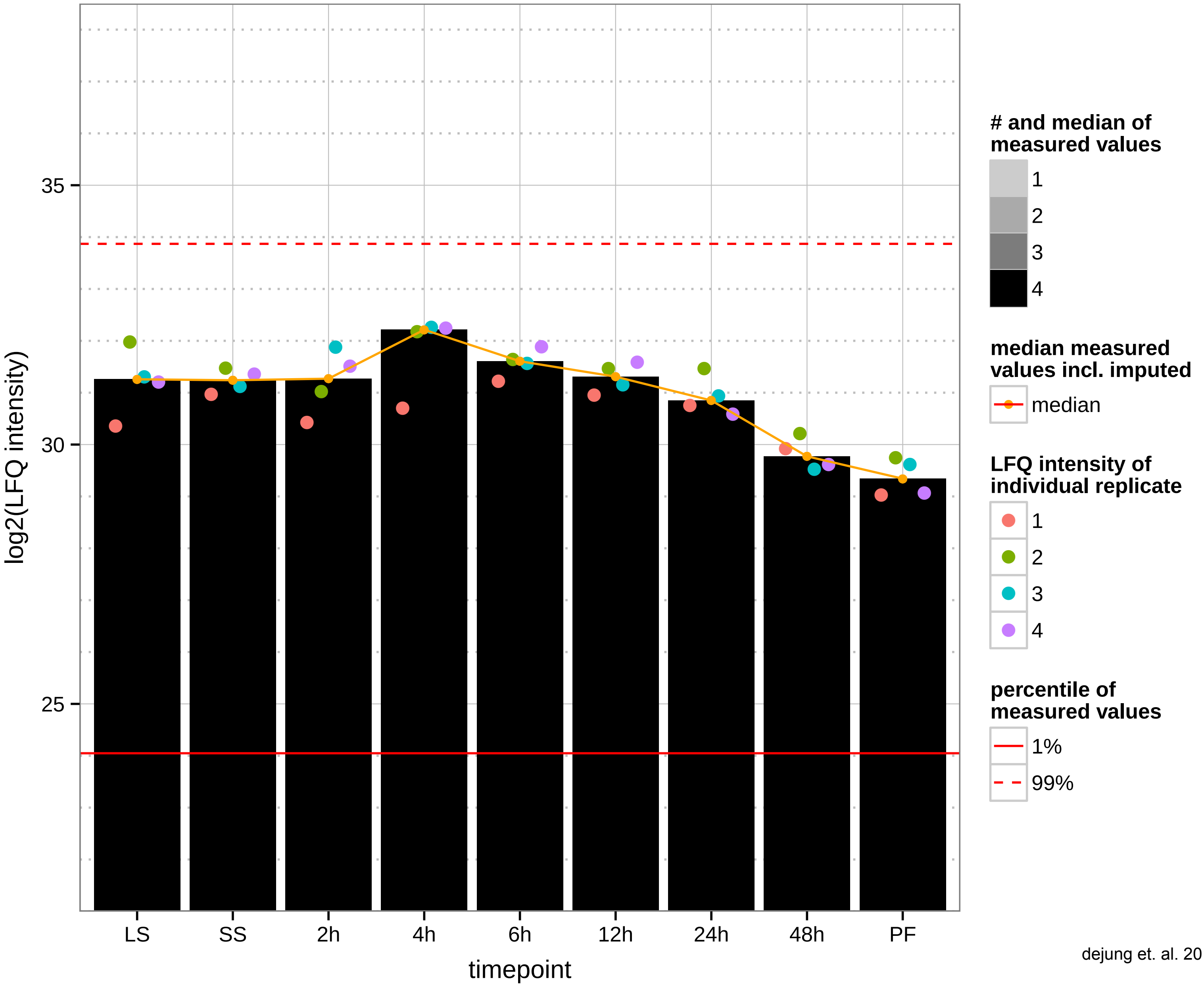

dejung et. al. 2015

hypothetical protein, conserved
Tb927.11.12060
AGOF: null
AGOC: null
AGOP: null
PGOF: null
PGOC: integral to peroxisomal membrane
PGOP: peroxisome fission

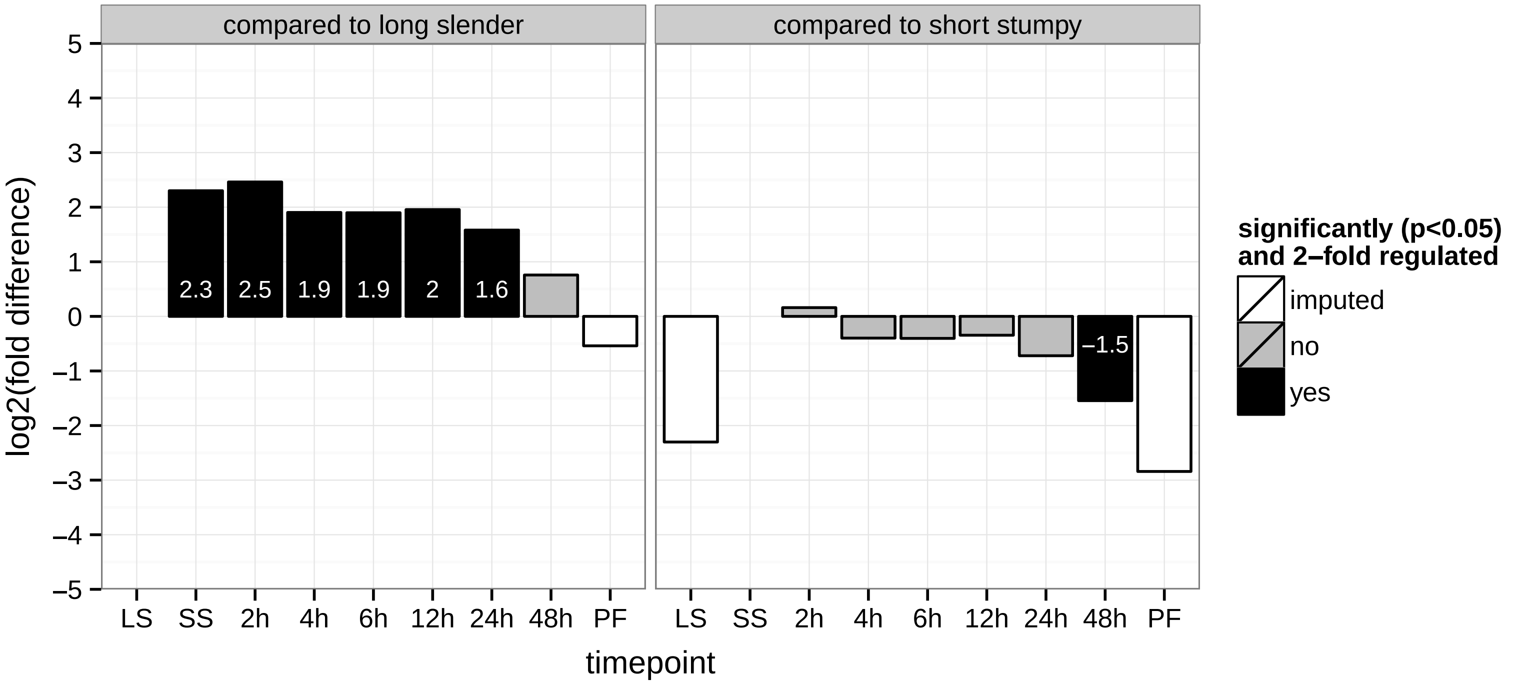

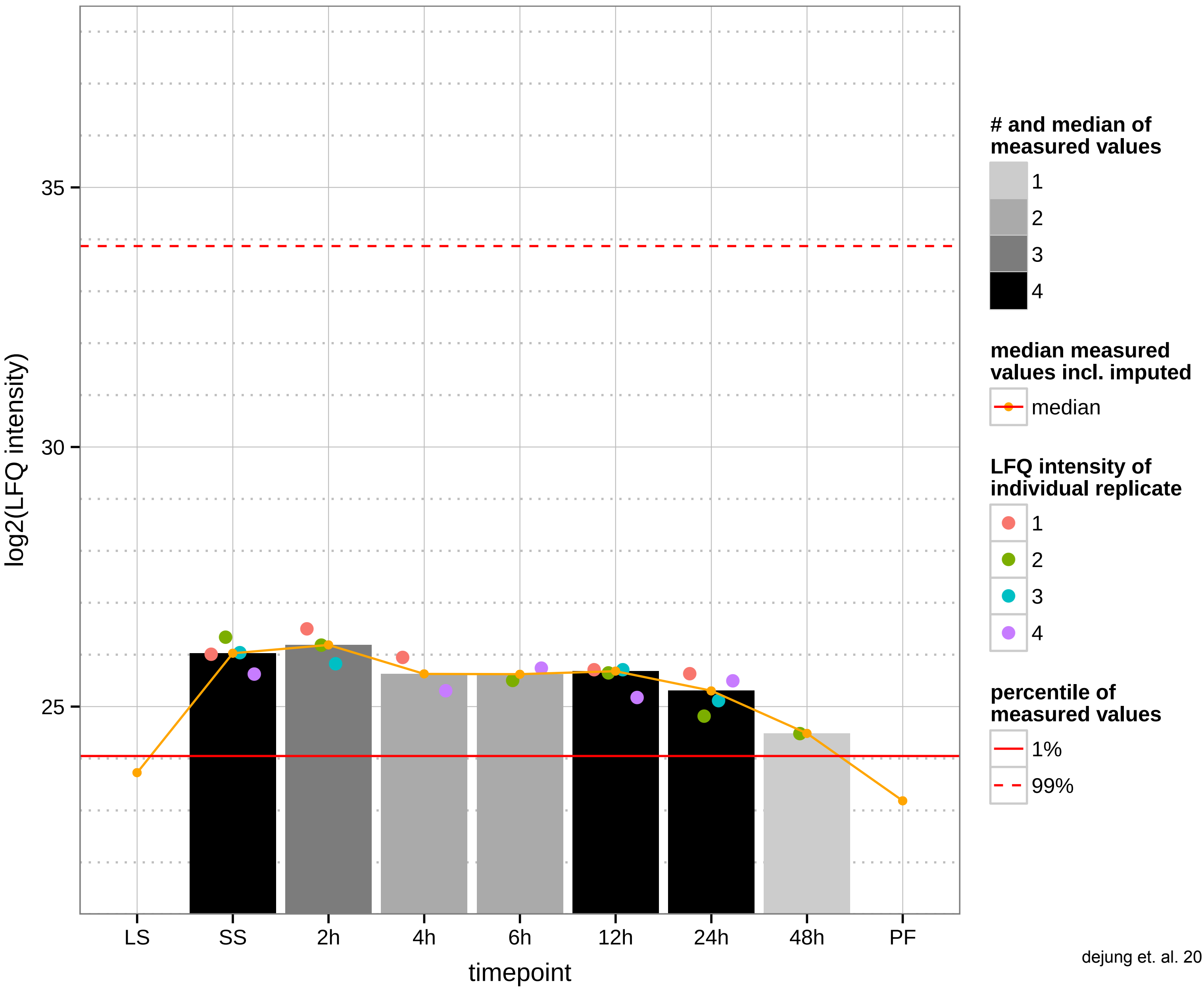

dejung et. al. 2015

hypothetical protein, conserved
Tb927.11.12870
AGOF: null
AGOC: null
AGOP: null
PGOF: null
PGOC: null
PGOP: null

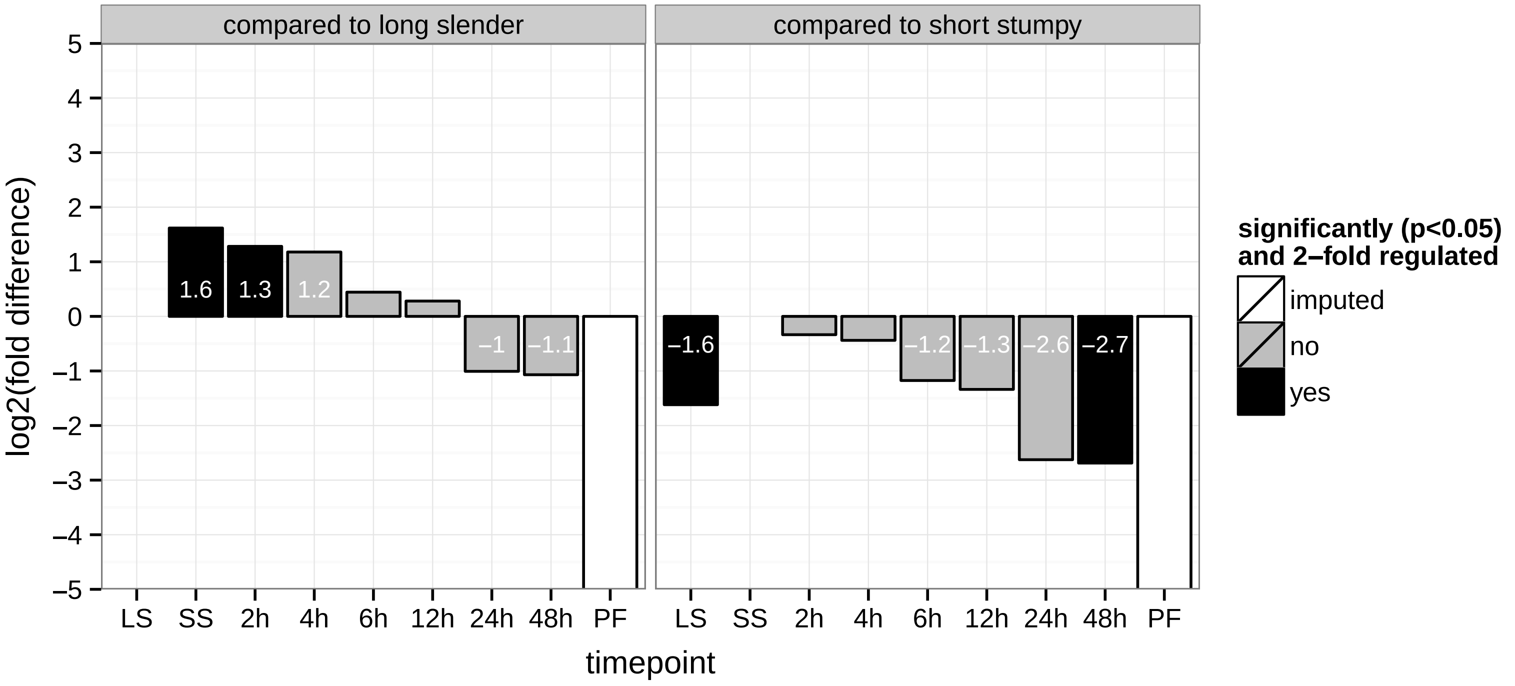

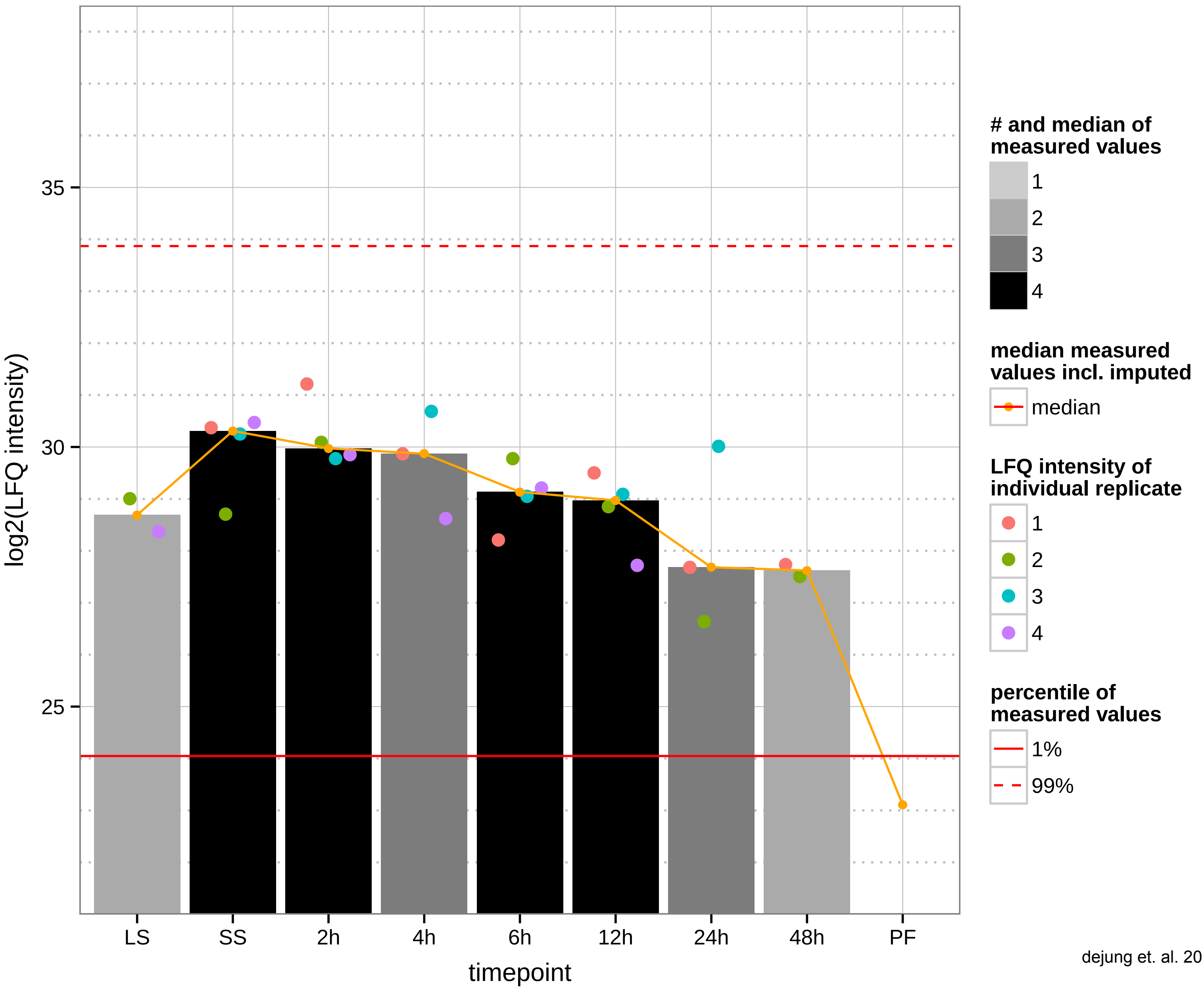

dejung et. al. 2015

kinesin–II 85 Kd subunit, putative
Tb927.11.13920
AGOF: ATP binding, microtubule motor activity, motor activity
AGOC: kinesin complex, microtubule associated complex
AGOP: microtubule–based movement
PGOF: ATP binding, microtubule motor activity
PGOC: null
PGOP: microtubule–based movement

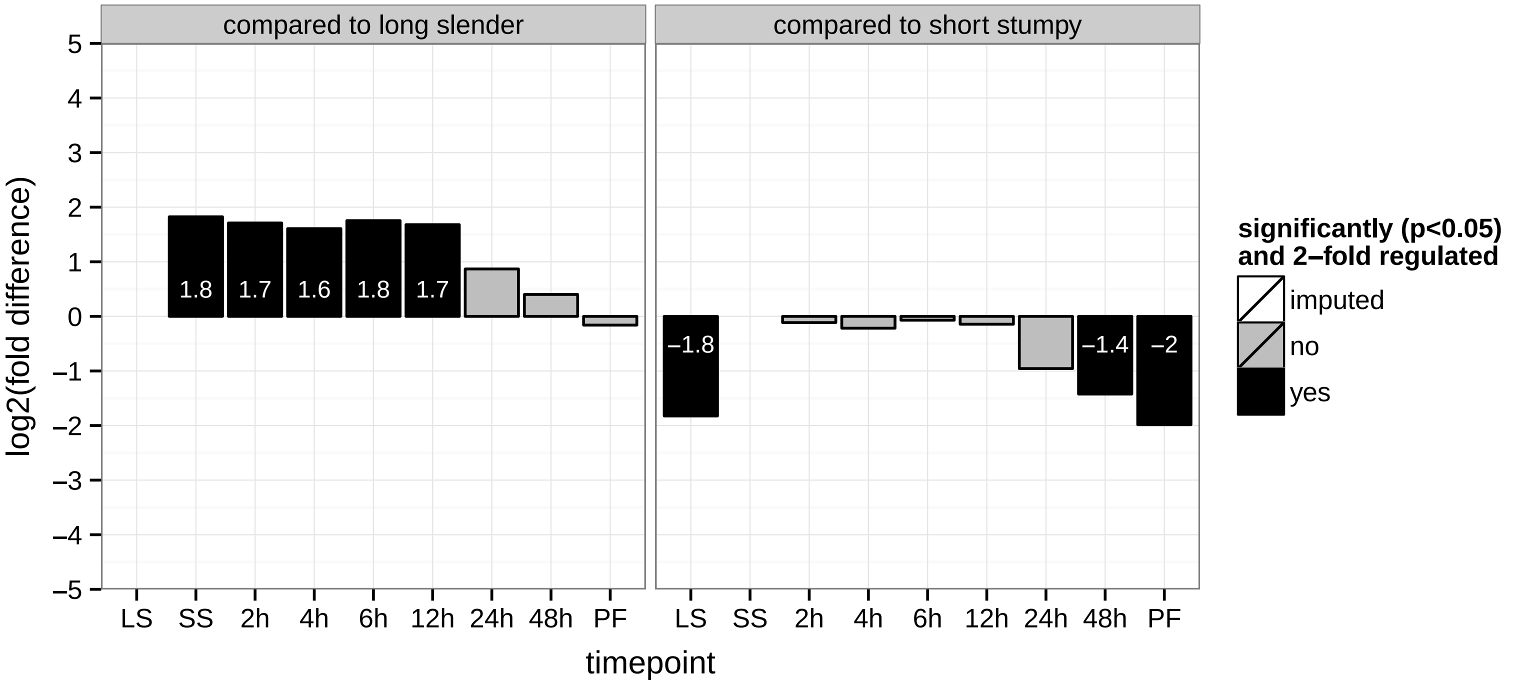

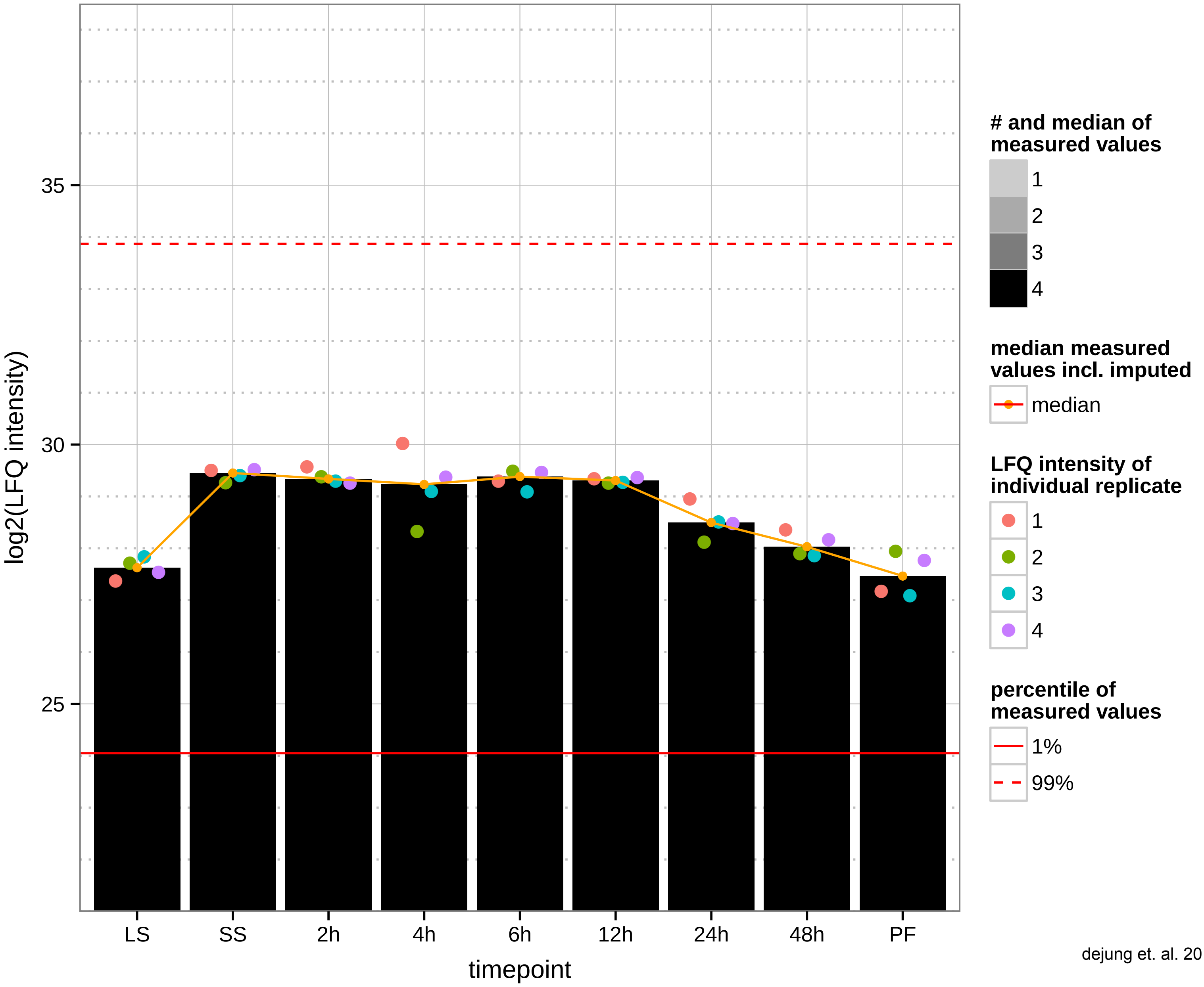

dejung et. al. 2015

hypothetical protein, conserved
Tb927.11.2320
AGOF: null
AGOC: null
AGOP: null
PGOF: null
PGOC: null
PGOP: null

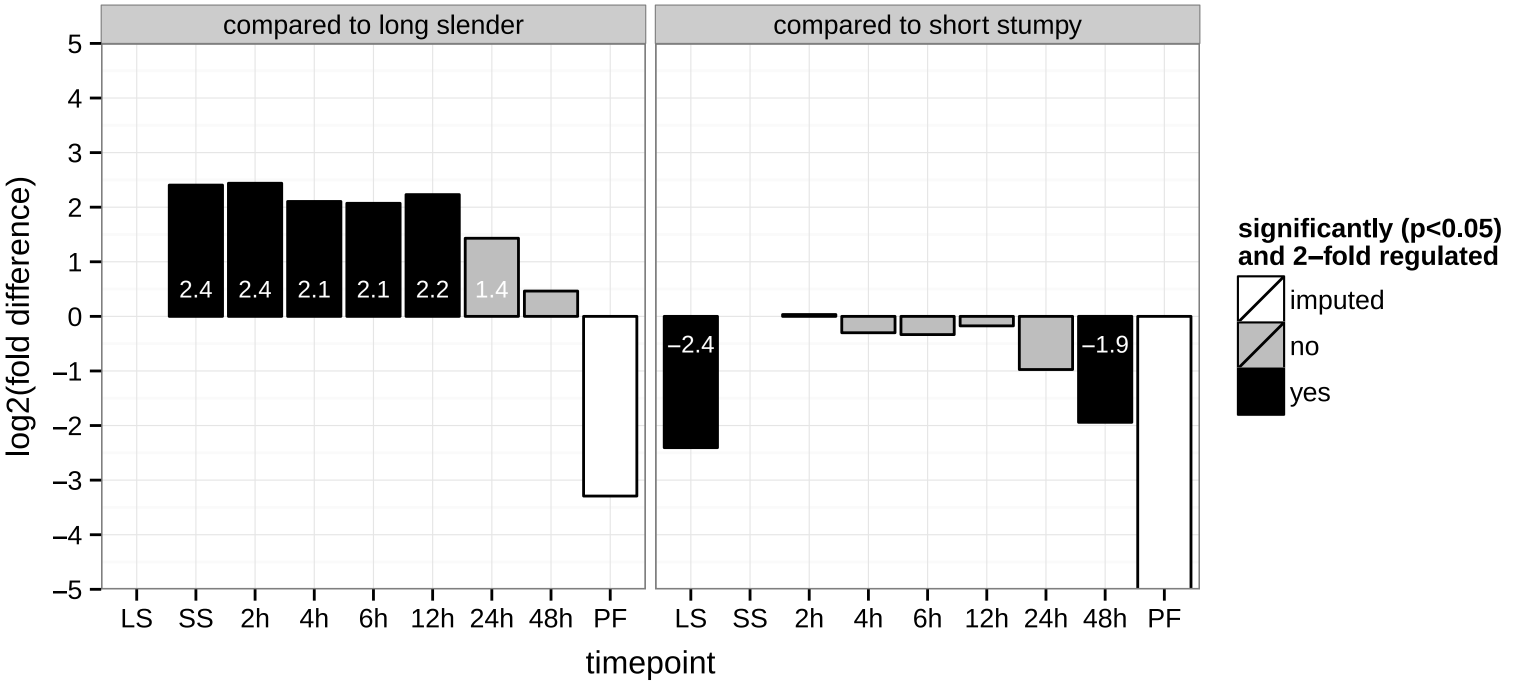

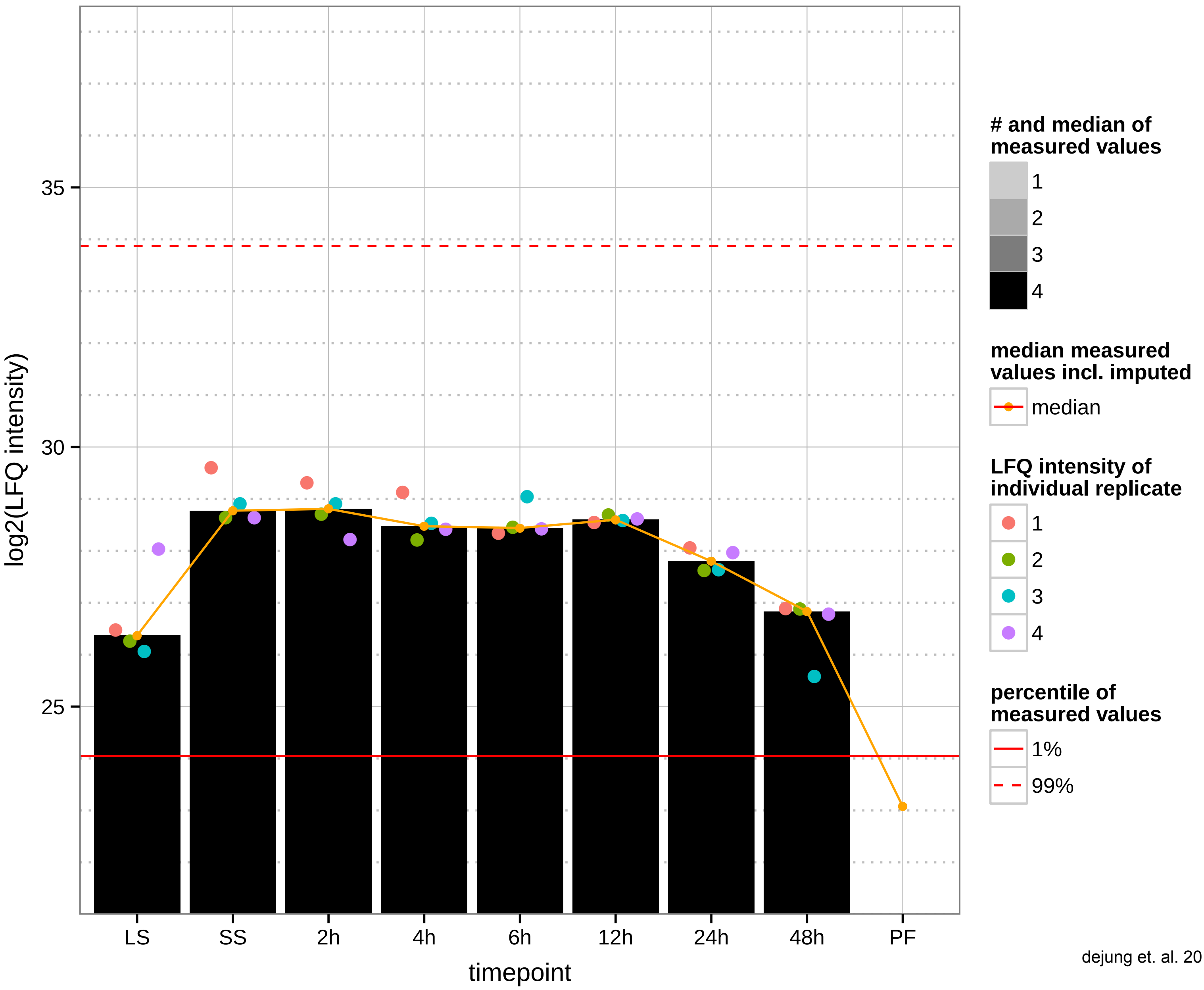

dejung et. al. 2015

hypothetical protein, conserved
Tb927.11.2400
AGOF: null
AGOC: null
AGOP: null
PGOF: null
PGOC: null
PGOP: null

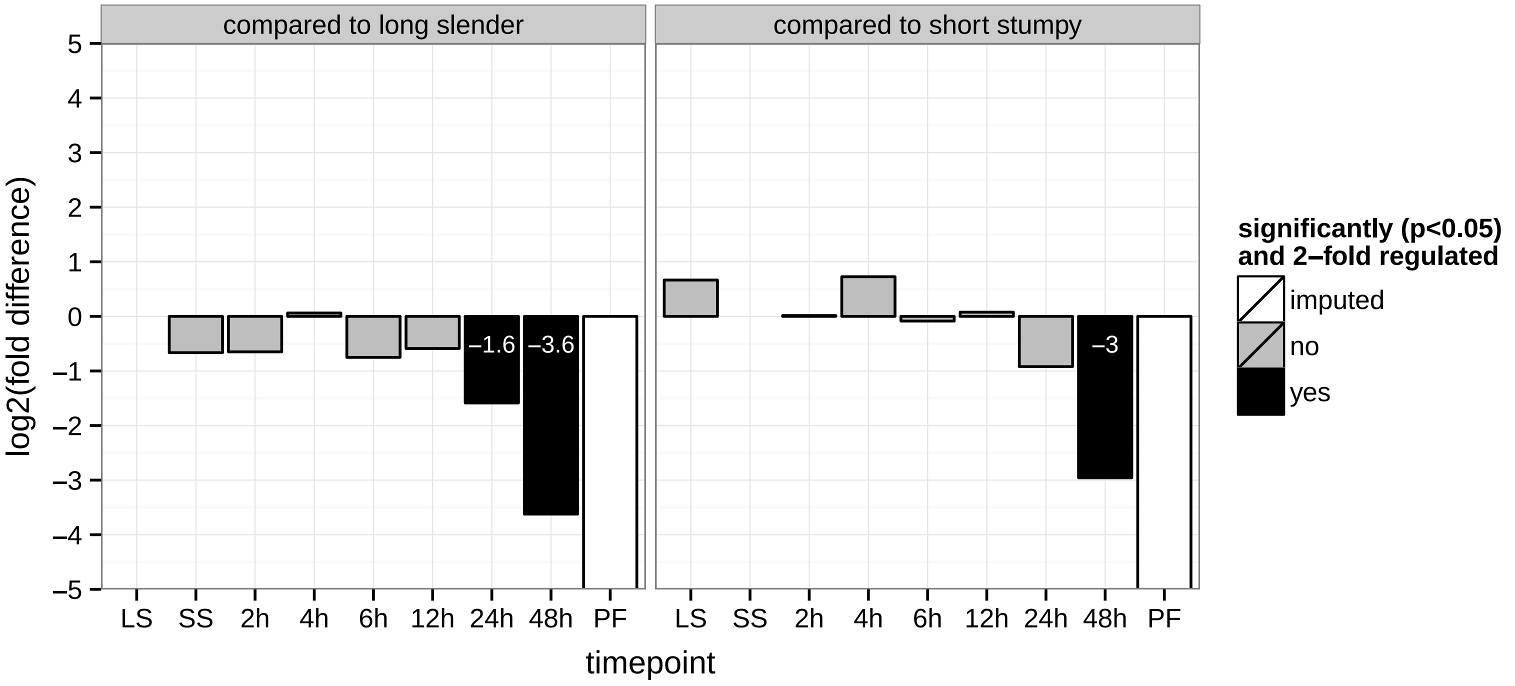

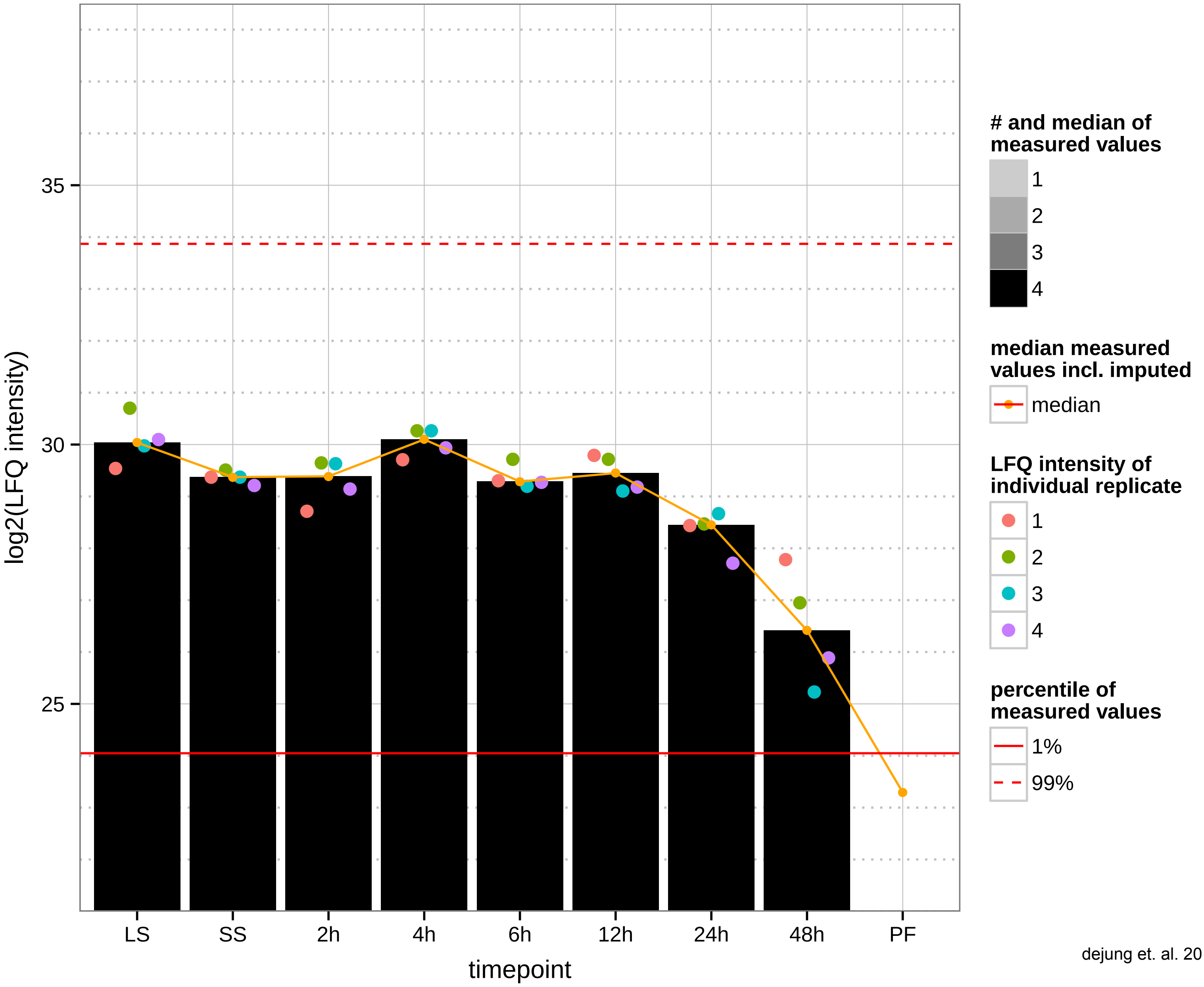

dejung et. al. 2015

Cytoplasmic dynein 2 heavy chain (DYNC2H1), putative, Cytoplasmic dynein 2 heavy chain (DYNC2H2), putative (DHC1b)
Tb927.11.2430
AGOF: ATP binding, ATPase activity, microtubule motor activity
AGOC: dynein complex
AGOP: chromosome segregation, microtubule–based movement, minus–end–directed vesicle transport along microtubule, m
PGOF: ATP binding, ATPase activity, microtubule motor activity
PGOC: dynein complex
PGOP: microtubule–based movement

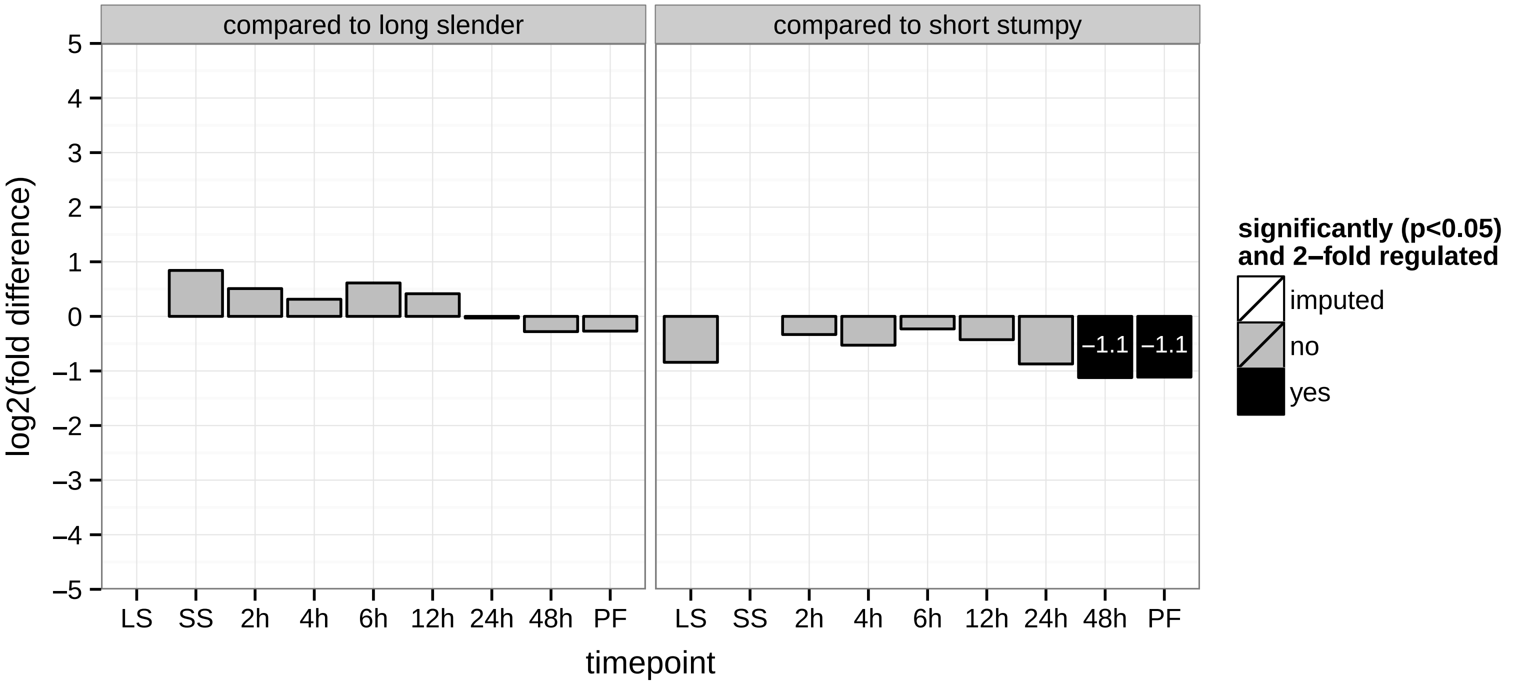

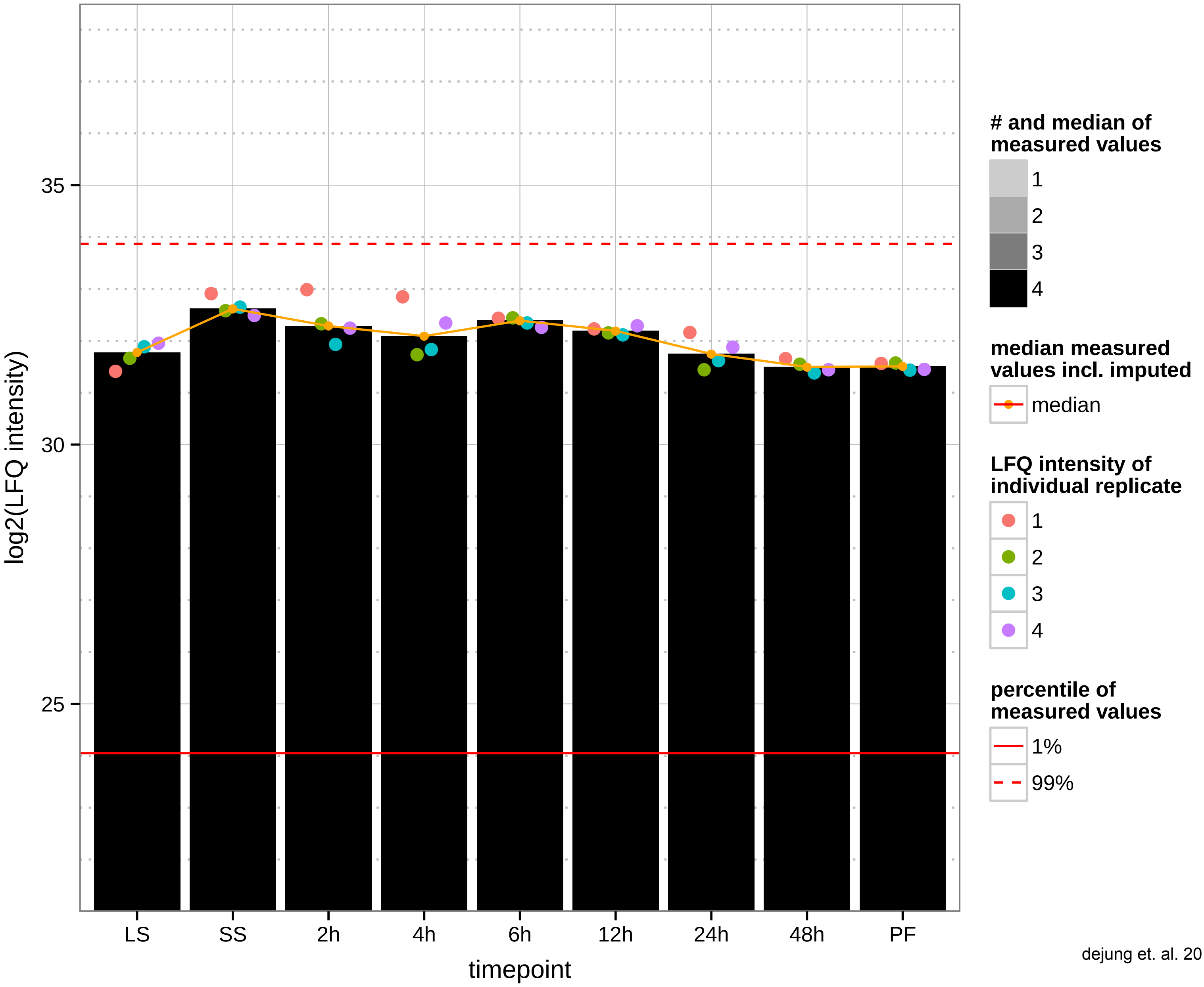

dejung et. al. 2015

UDP−galactose 4−epimerase (galE)
Tb927.11.2730
AGOF: UDP−glucose 4−epimerase activity, coenzyme binding
AGOC: null
AGOP: galactose metabolic process
PGOF: UDP−glucose 4−epimerase activity, catalytic activity, coenzyme binding
PGOC: null
PGOP: cellular metabolic process, galactose metabolic process

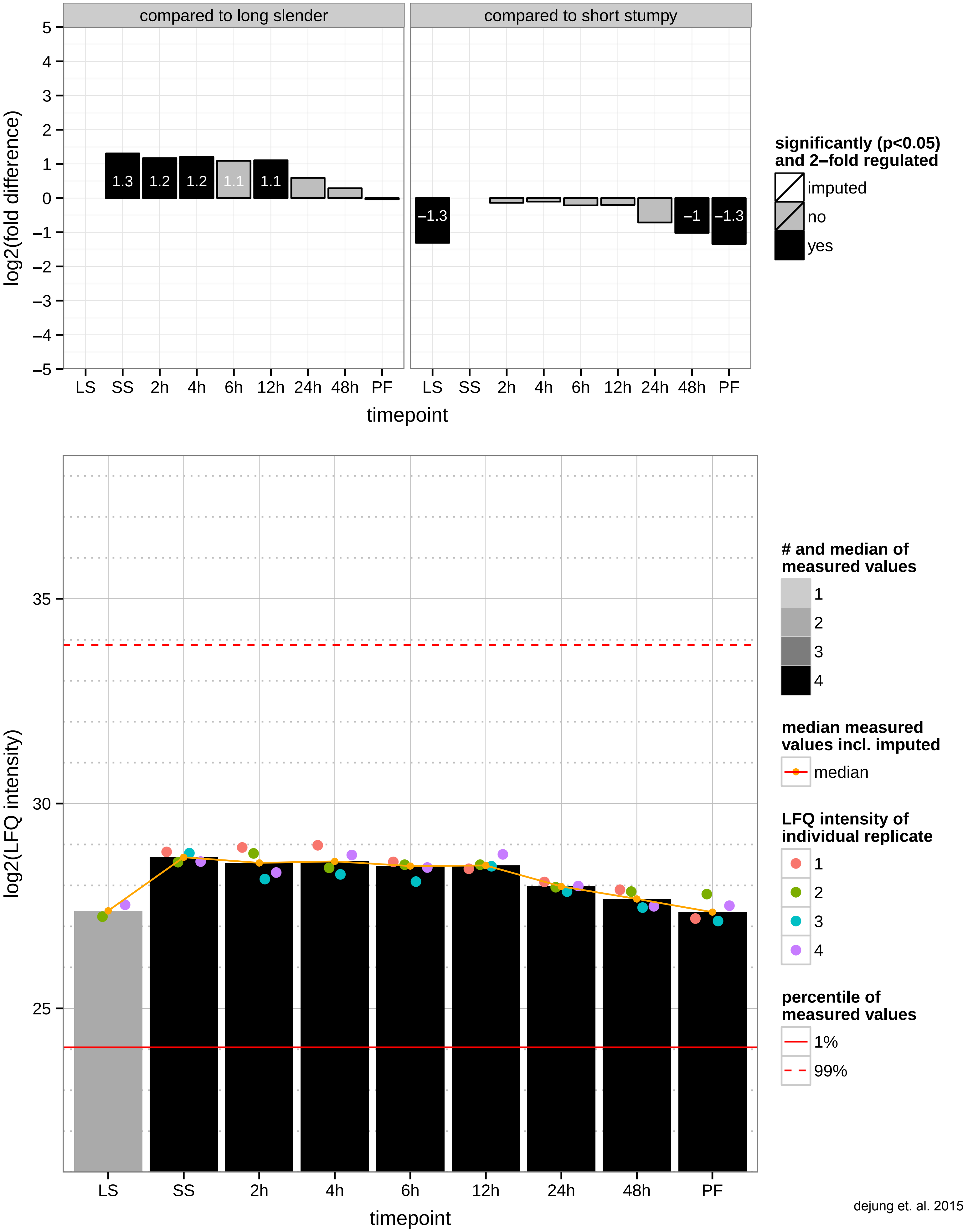

dejung et. al. 2015

ubiquitin–conjugating enzyme, putative, ubiquitin–conjugating enzyme–like protein
Tb927.11.3310
AGOF: ubiquitin–protein ligase activity
AGOC: null
AGOP: cellular protein modification process, post–translational protein modification, regulation of protein metabolic process
PGOF: acid–amino acid ligase activity
PGOC: null
PGOP: null

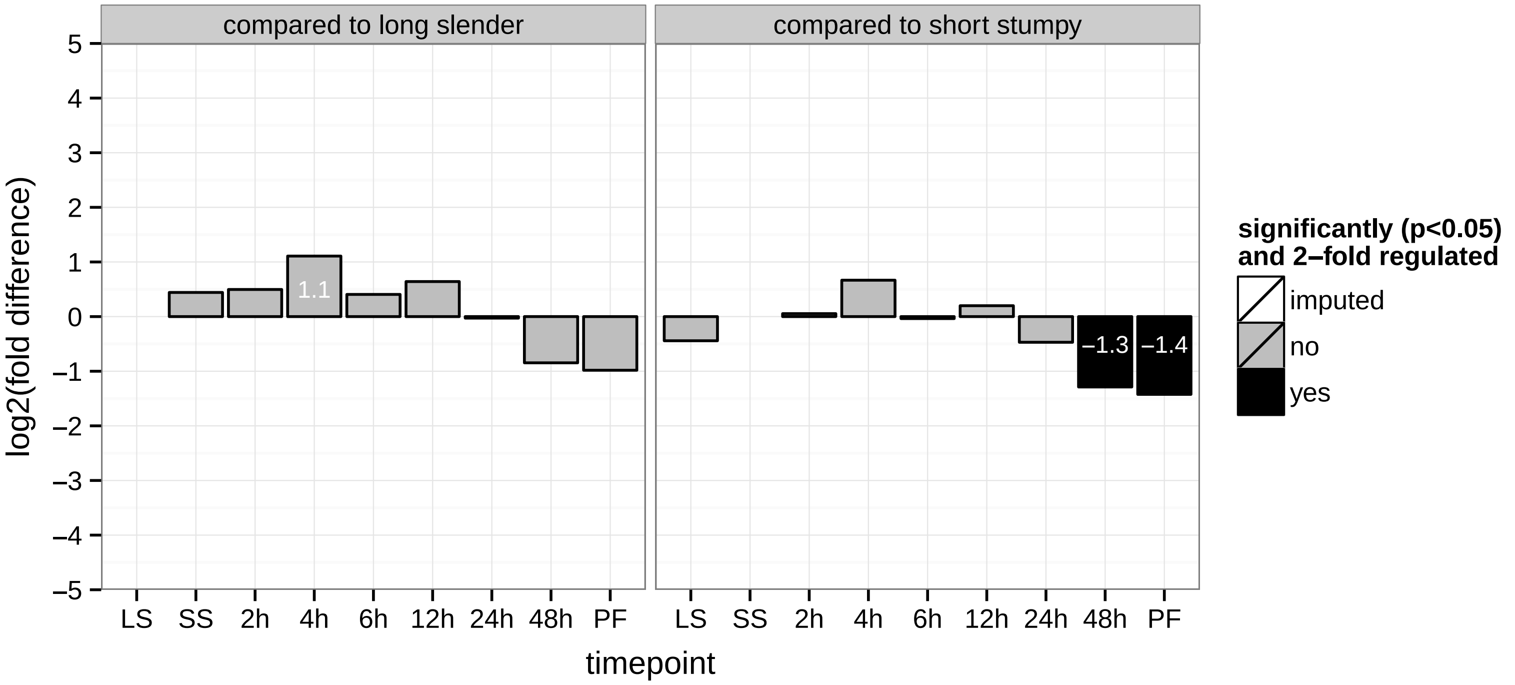

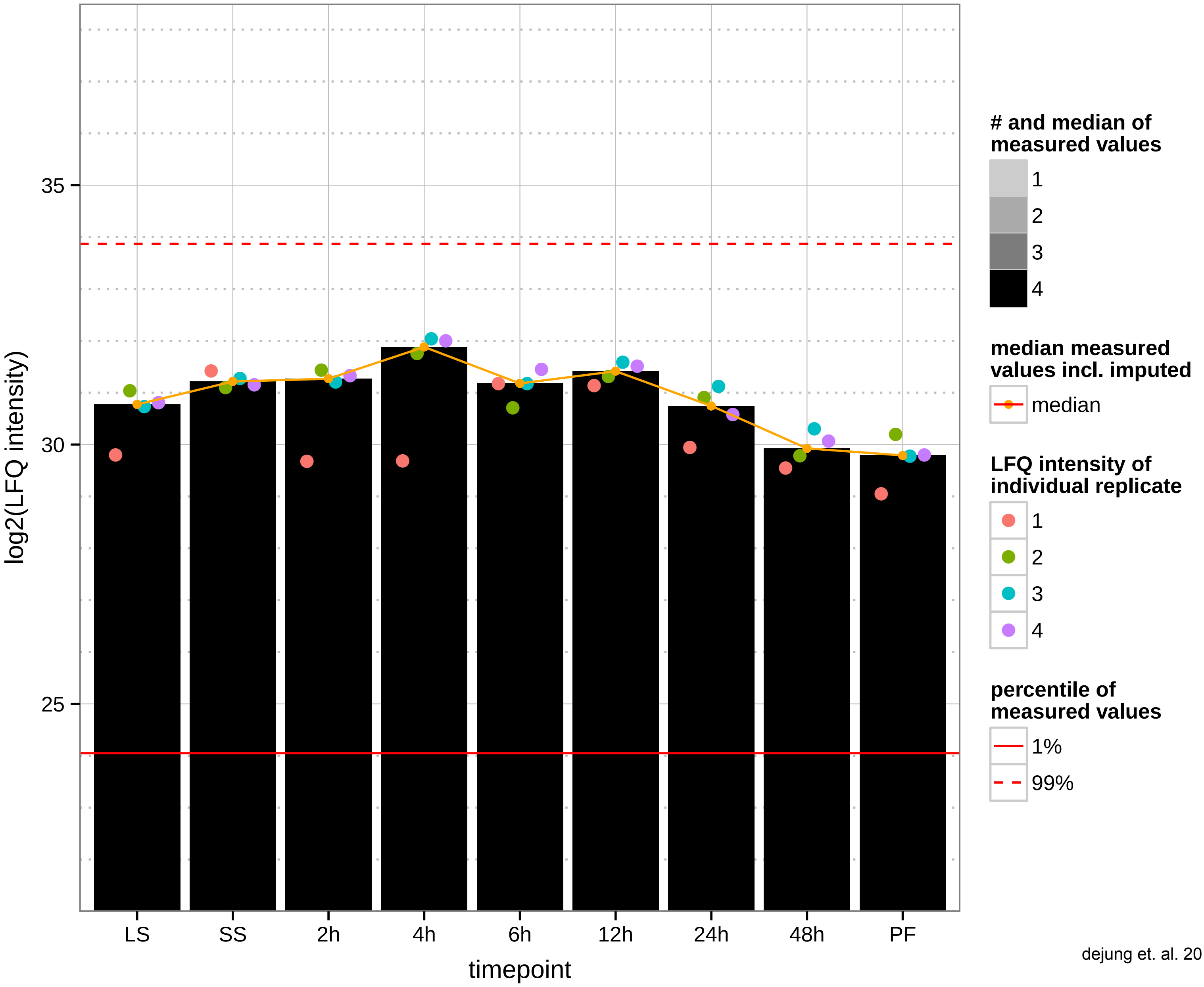

dejung et. al. 2015

kinetoplastid–specific dual specificity phosphatase, putative
Tb927.11.4170
AGOF: protein tyrosine/serine/threonine phosphatase activity
AGOC: null
AGOP: protein dephosphorylation
PGOF: phosphatase activity, protein tyrosine/serine/threonine phosphatase activity
PGOC: null
PGOP: dephosphorylation, protein dephosphorylation

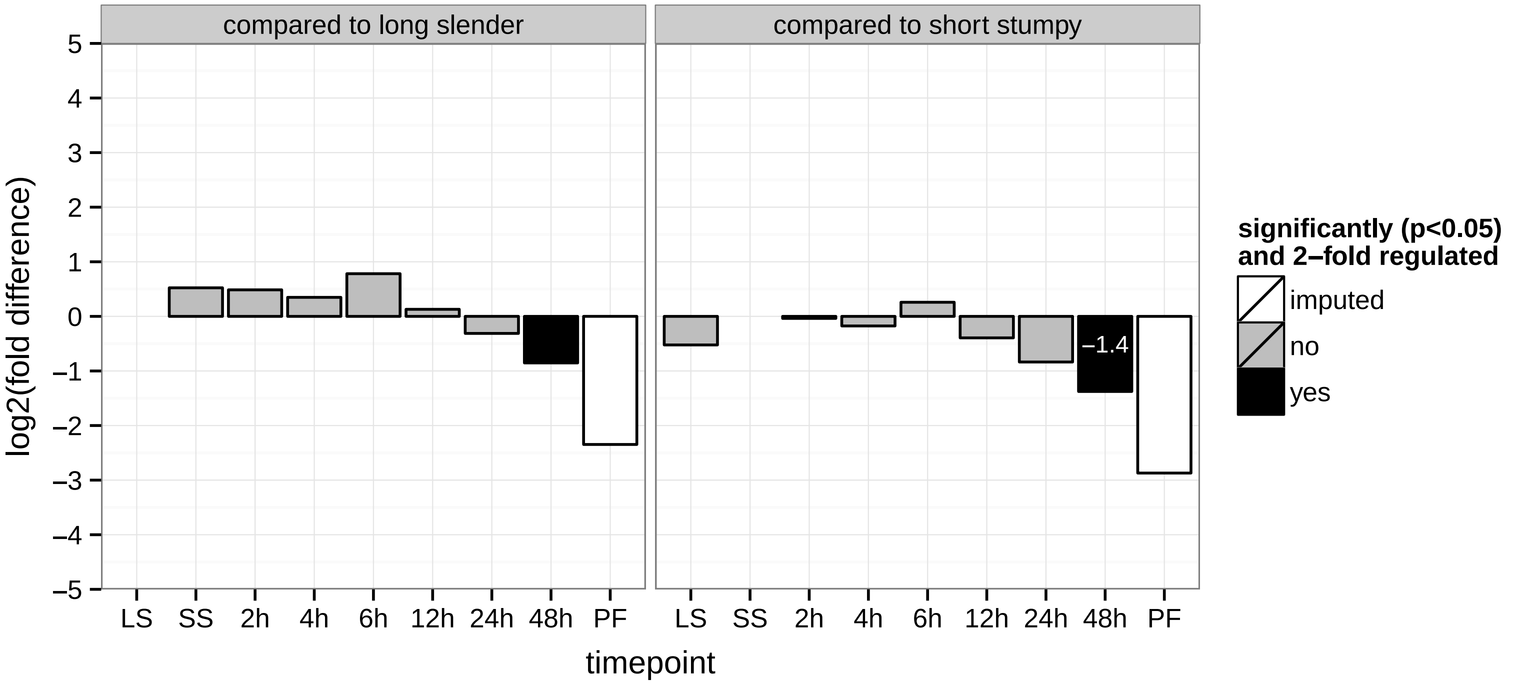

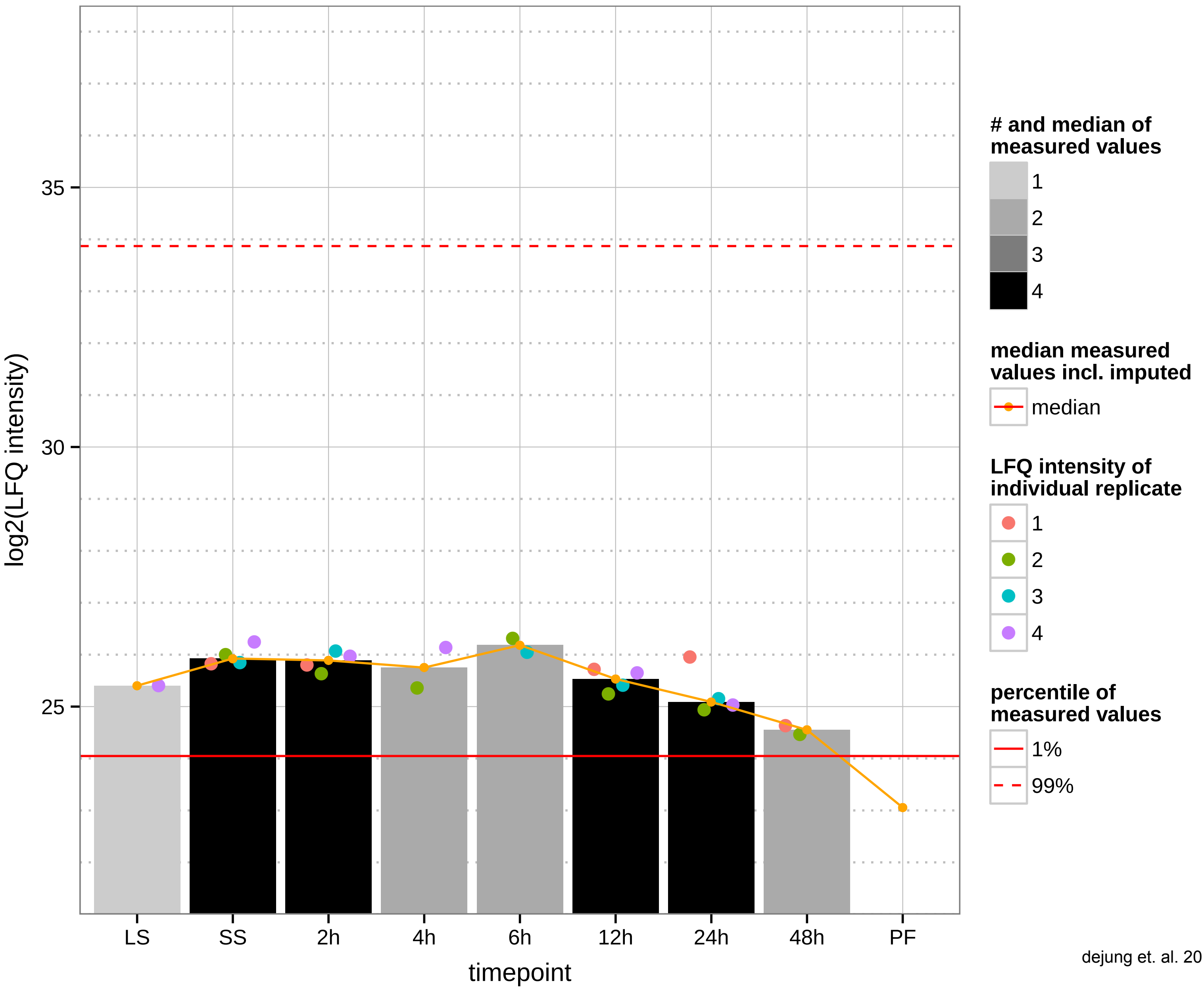

dejung et. al. 2015

Ras−related protein Rab5B (RAB5B)
Tb927.11.4570
AGOF: GTP binding, GTPase activity
AGOC: intracellular
AGOP: intracellular protein transport, nucleocytoplasmic transport, small GTPase mediated signal transduction
PGOF: GTP binding, GTPase activity
PGOC: intracellular, membrane
PGOP: GTP catabolic process, intracellular protein transport, nucleocytoplasmic transport, protein transport, signal transducti

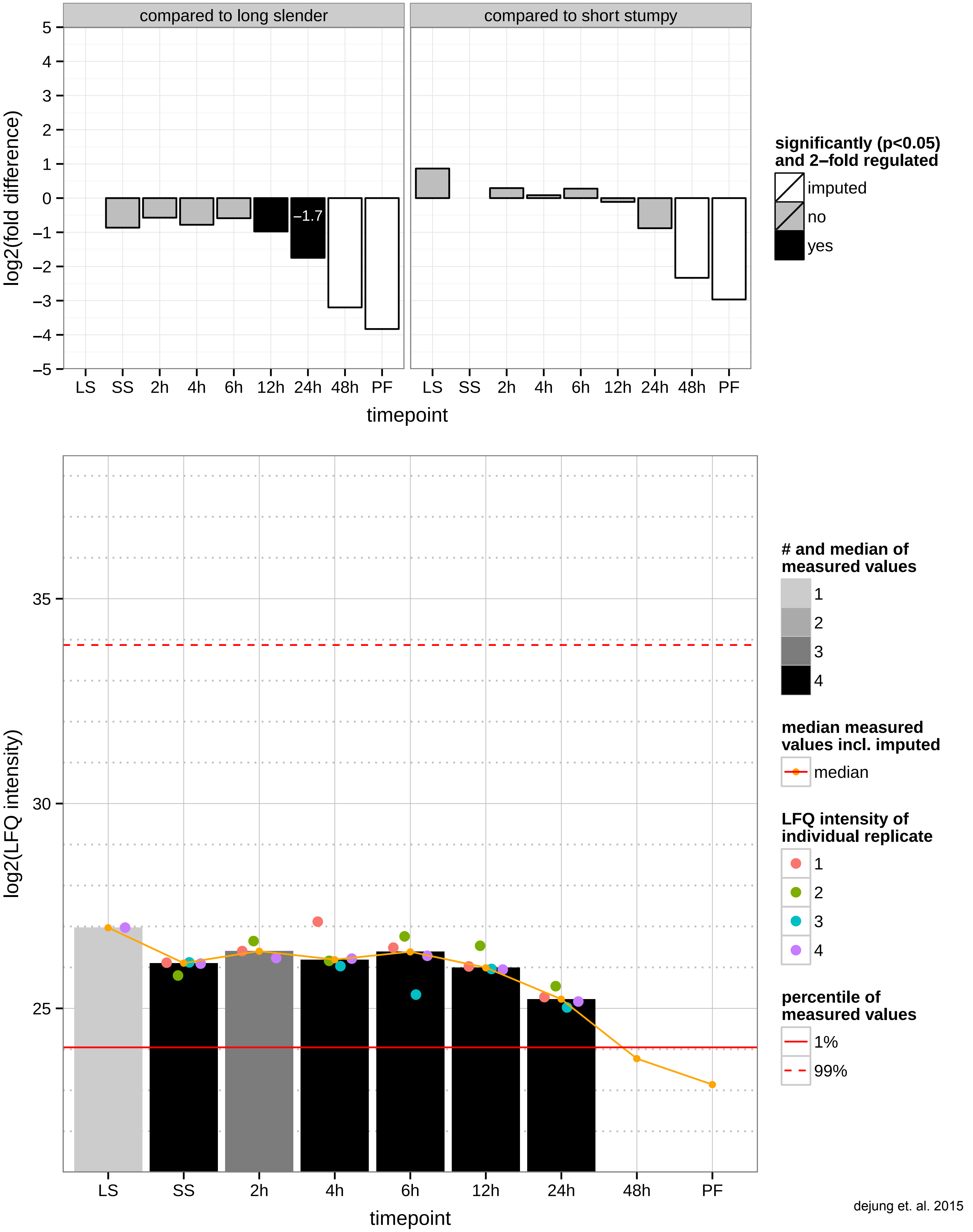

dejung et. al. 2015

hypothetical protein, conserved
Tb927.11.4860
AGOF: zinc ion binding
AGOC: null
AGOP: null
PGOF: protein binding, zinc ion binding
PGOC: null
PGOP: null

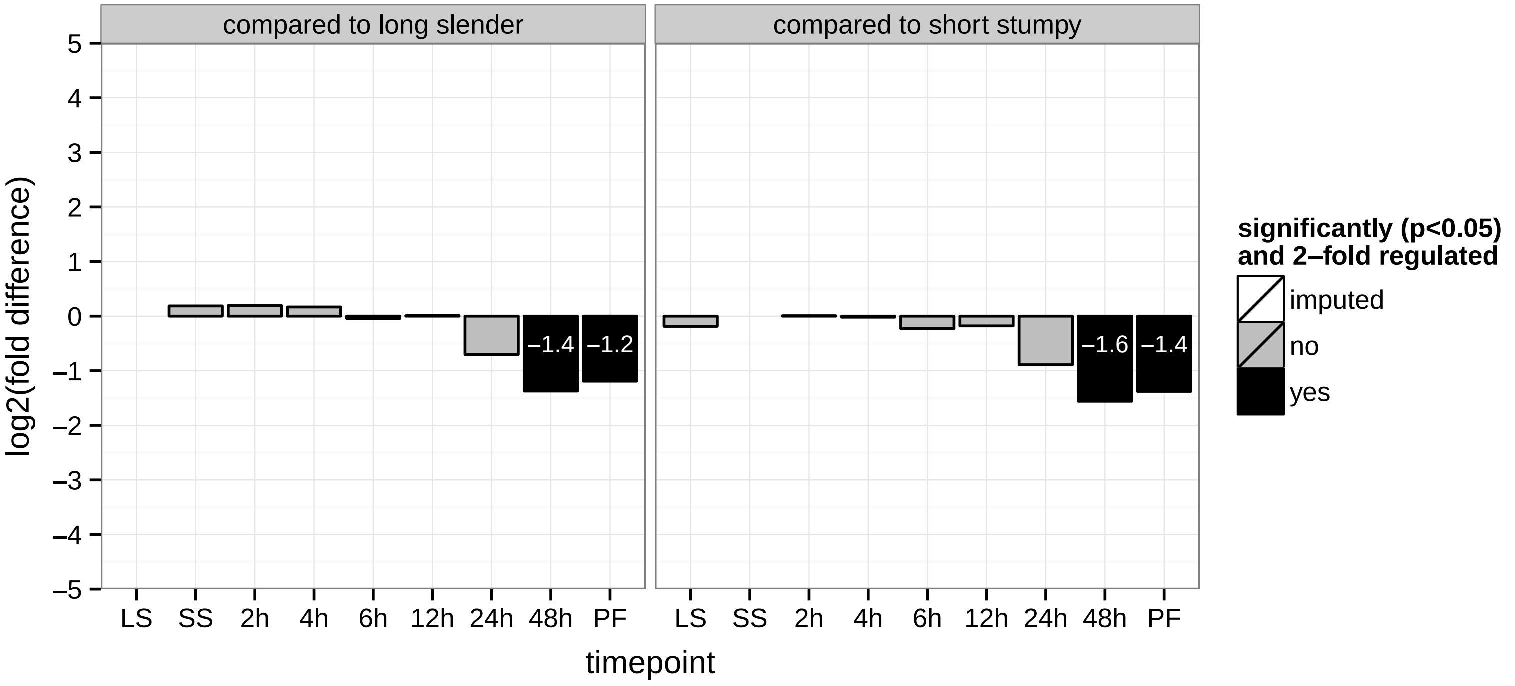

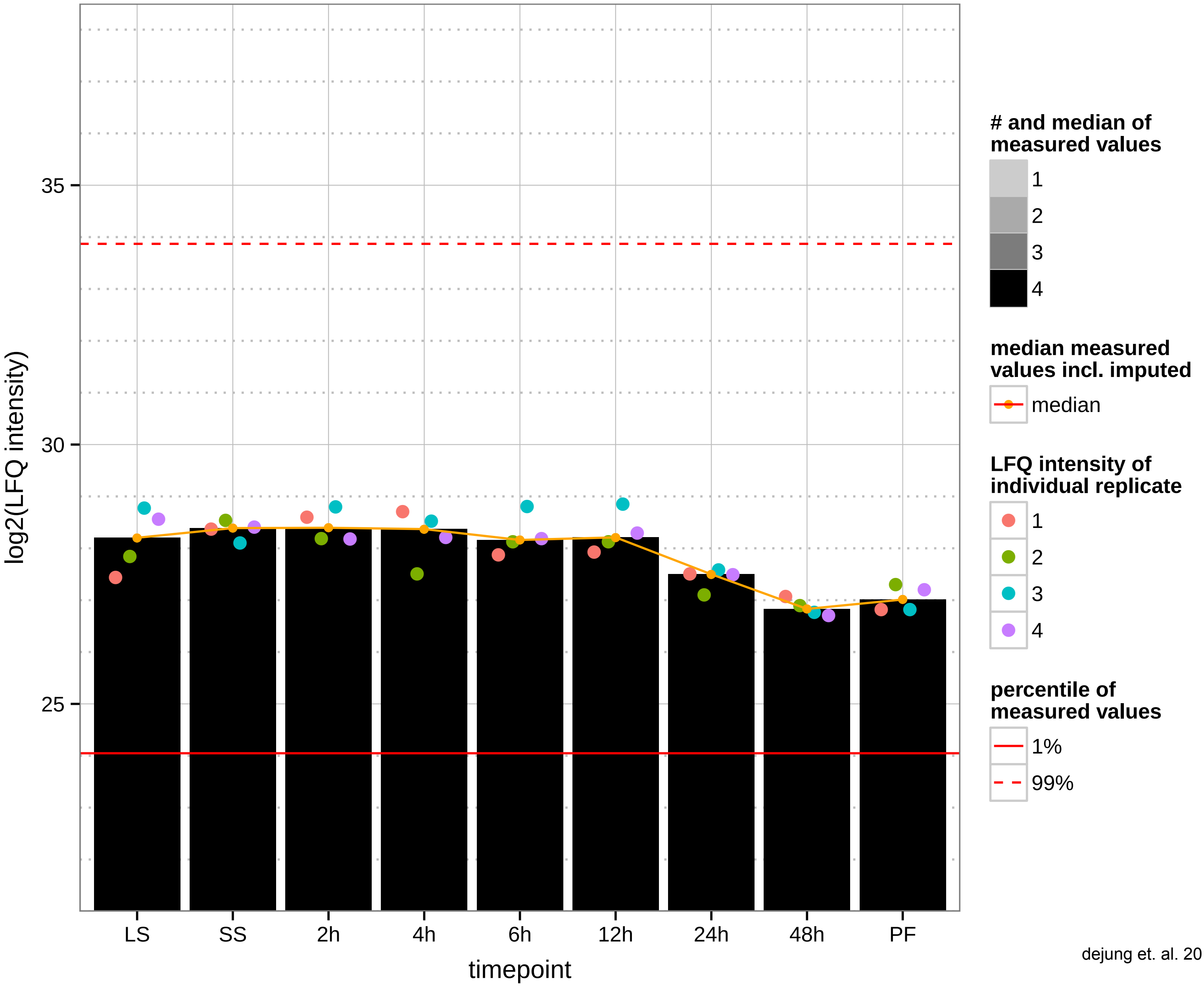

dejung et. al. 2015

hypothetical protein, conserved
Tb927.11.6390
AGOF: null
AGOC: null
AGOP: null
PGOF: null
PGOC: null
PGOP: null

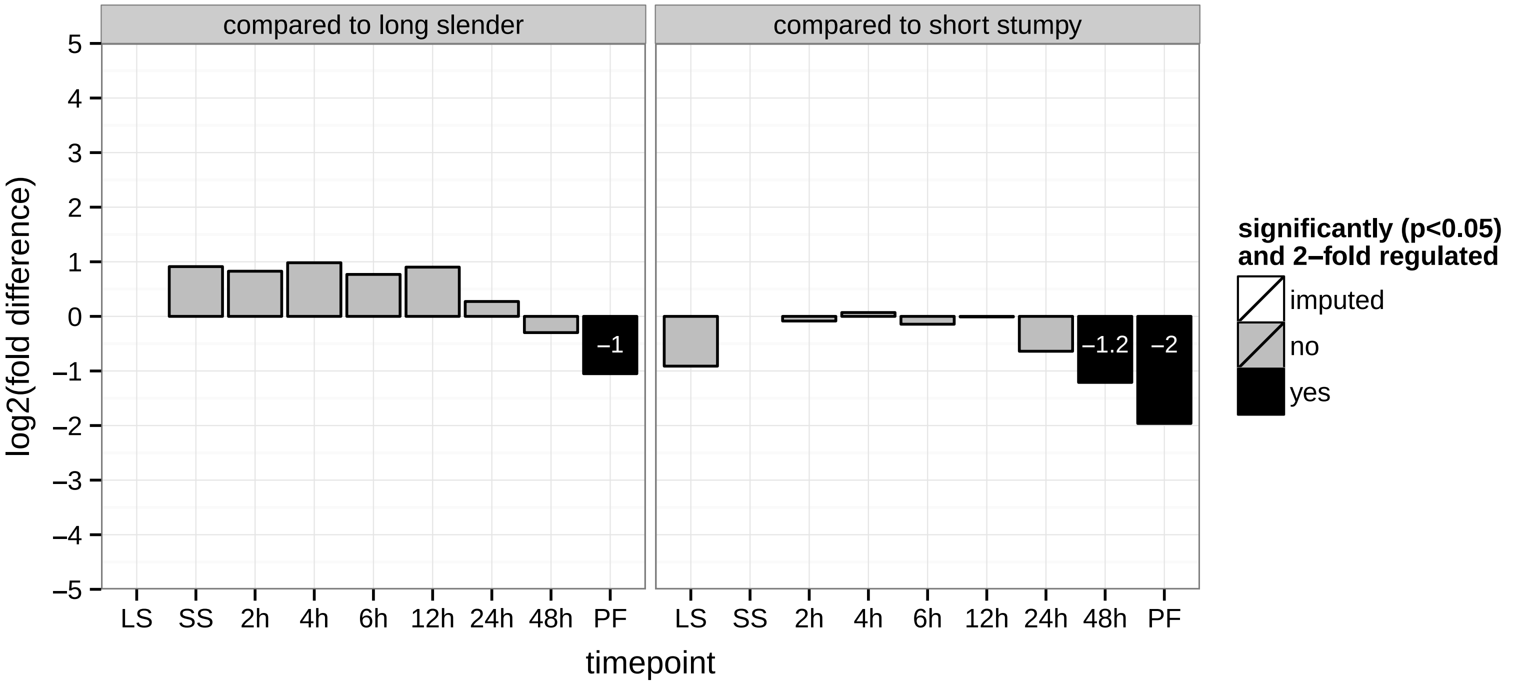

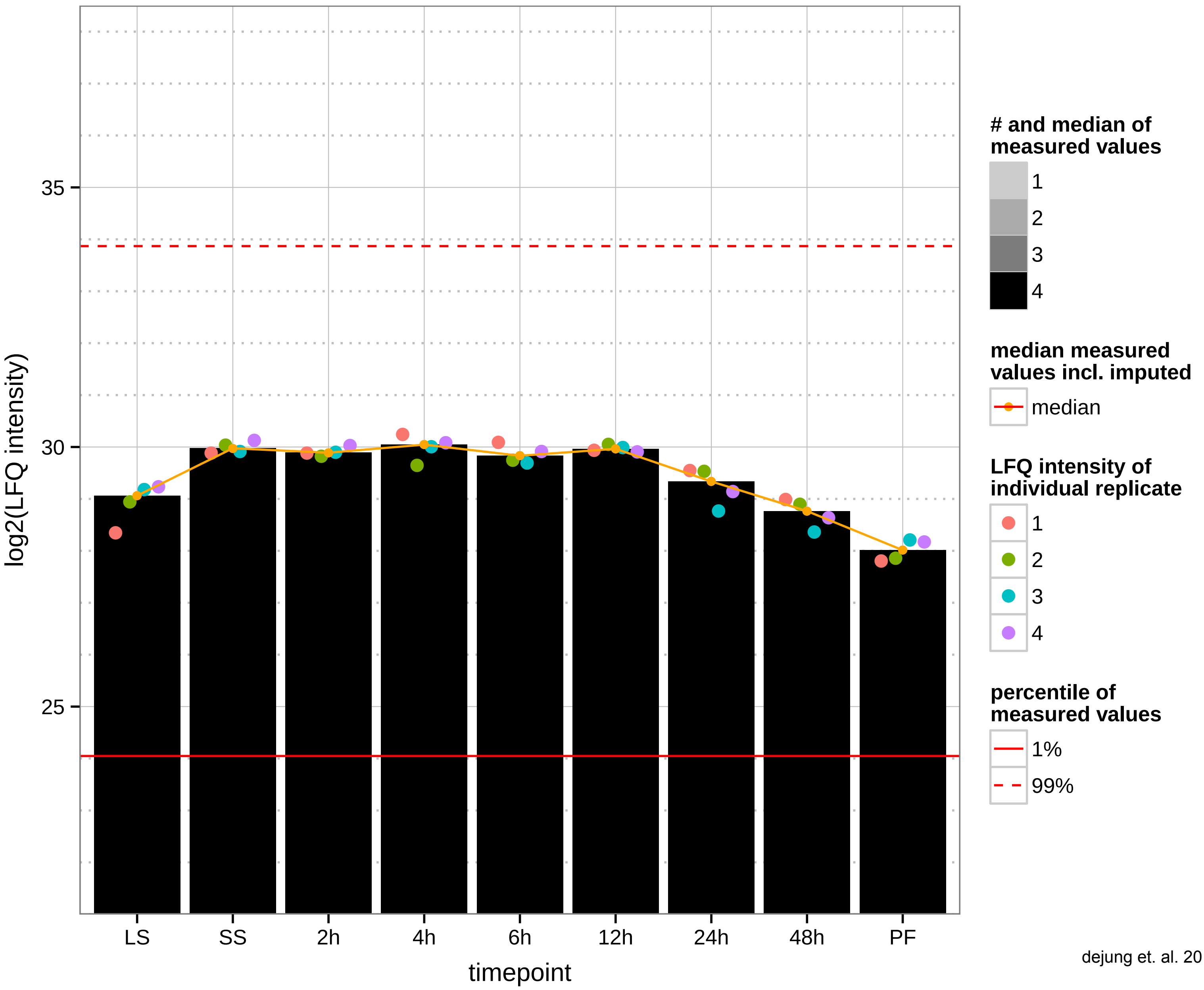

dejung et. al. 2015

hypothetical protein, conserved
Tb927.11.7570
AGOF: ATP binding, catalytic activity
AGOC: null
AGOP: null
PGOF: ATP binding, metal ion binding
PGOC: null
PGOP: null

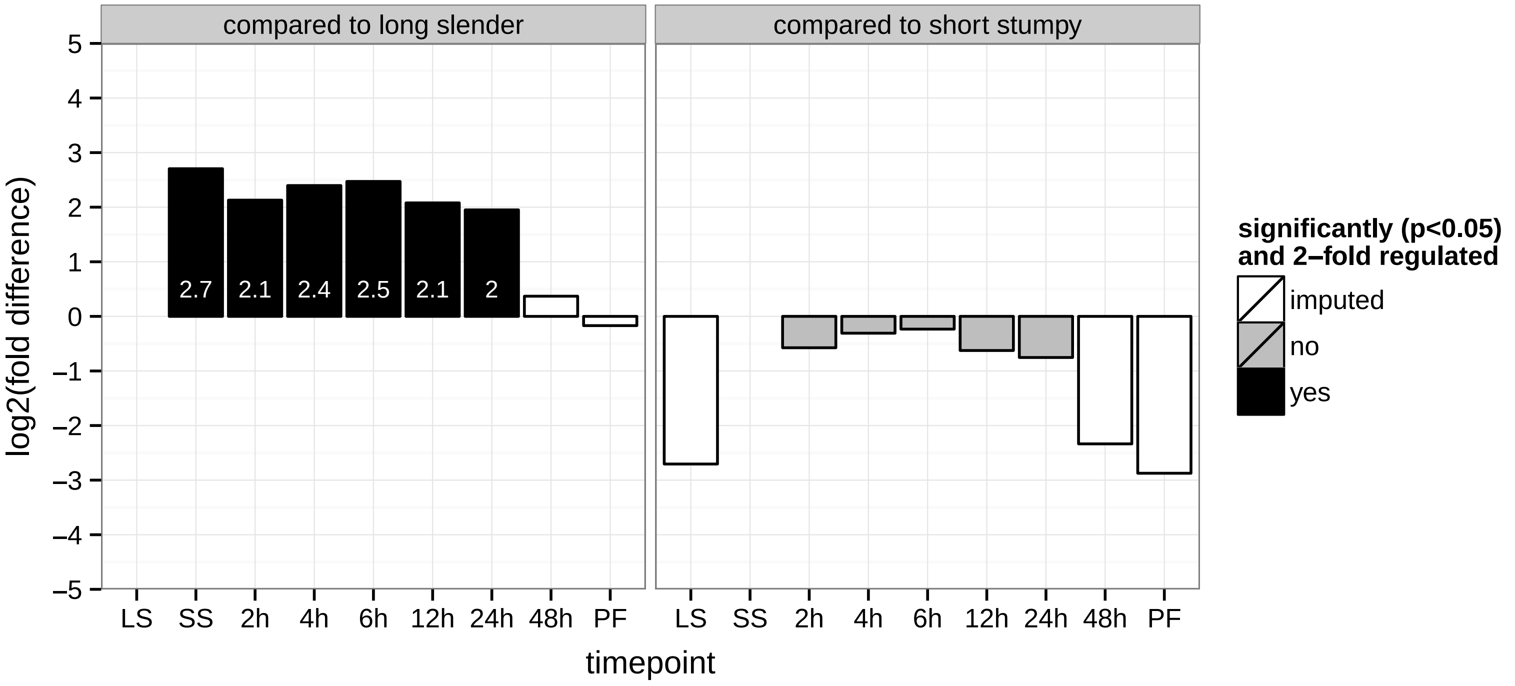

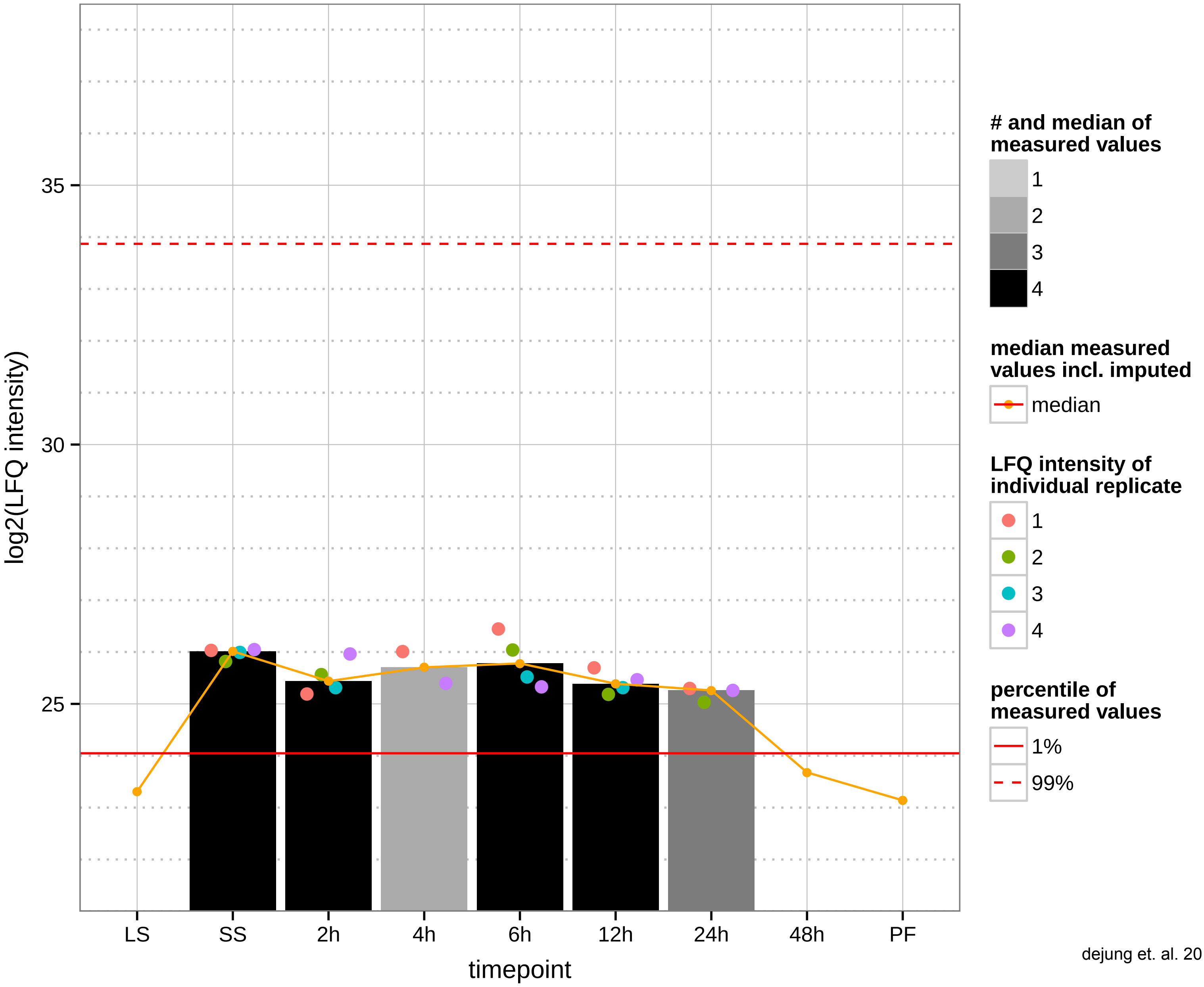

dejung et. al. 2015

haloacid dehalogenase–like hydrolase, putative
Tb927.11.8440
AGOF: hydrolase activity
AGOC: null
AGOP: metabolic process
PGOF: catalytic activity, hydrolase activity
PGOC: null
PGOP: metabolic process

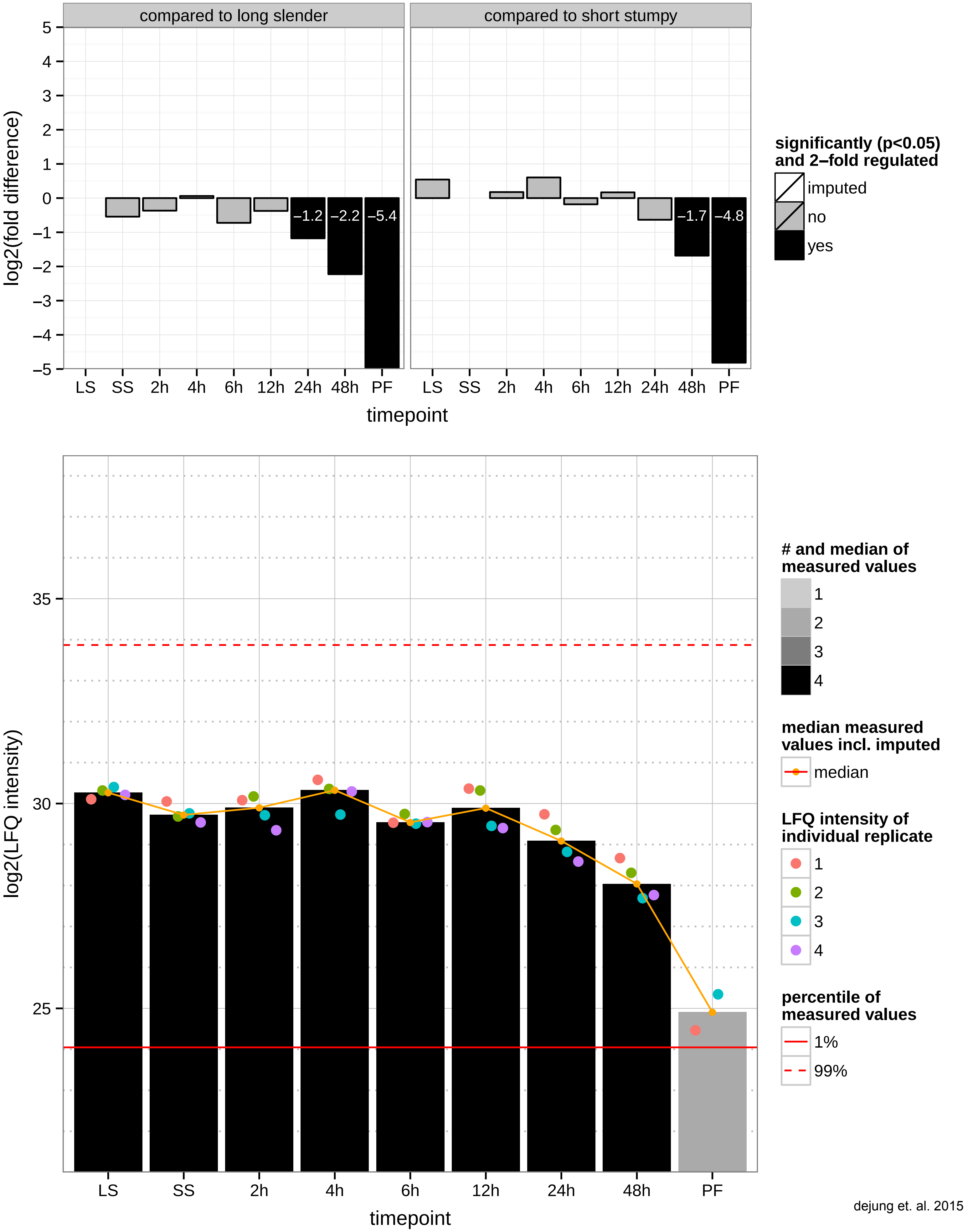

dejung et. al. 2015

target SNARE, putative
Tb927.11.8790
AGOF: SNAP receptor activity
AGOC: integral to membrane, membrane
AGOP: membrane fusion, vesicle–mediated transport
PGOF: protein binding
PGOC: membrane
PGOP: vesicle–mediated transport

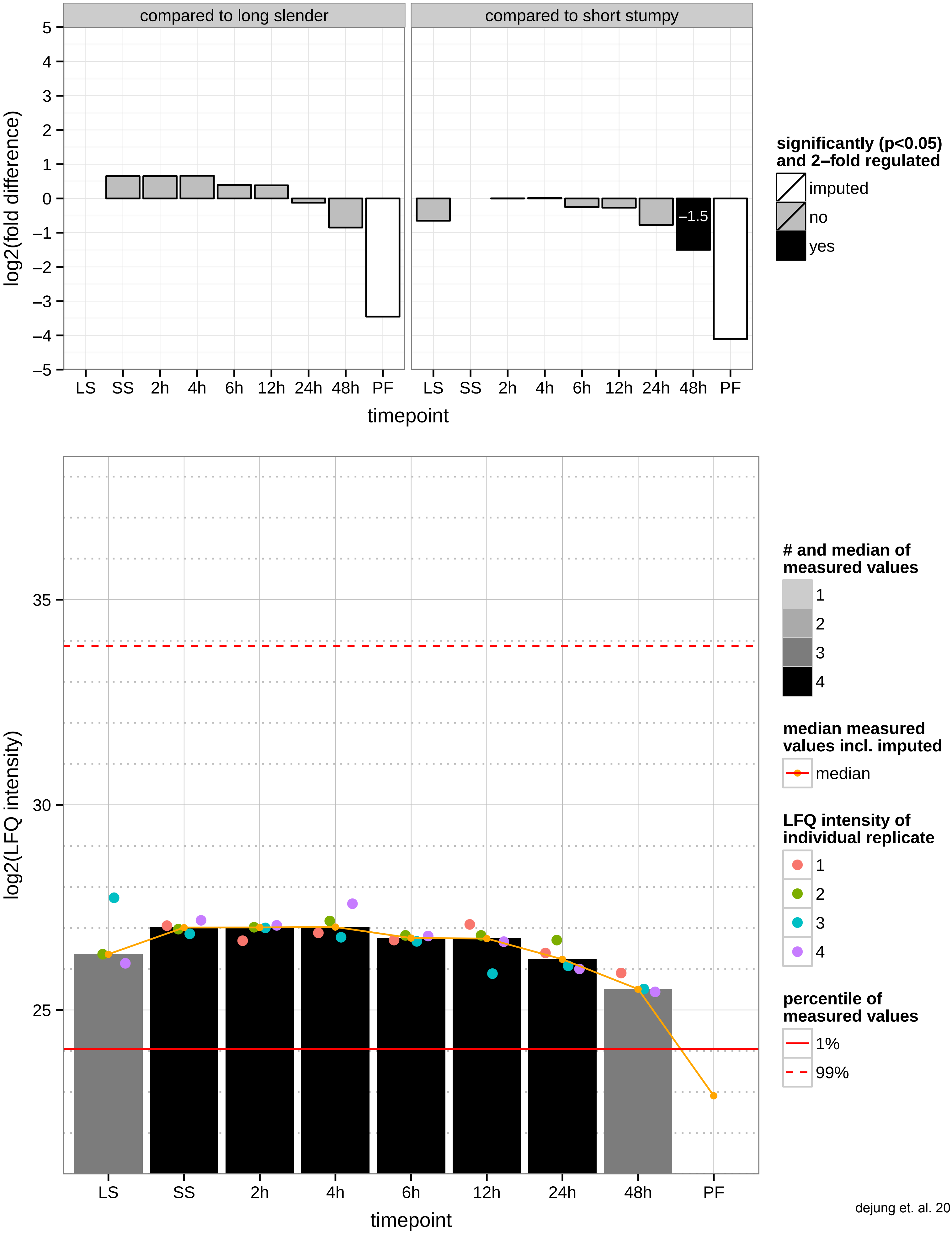

dejung et. al. 2015

ATP synthase, putative
Tb927.11.9420
AGOF: proton–transporting ATPase activity, rotational mechanism
AGOC: proton–transporting two–sector ATPase complex, catalytic domain
AGOP: ATP synthesis coupled proton transport
PGOF: proton–transporting ATPase activity, rotational mechanism
PGOC: proton–transporting two–sector ATPase complex, catalytic domain
PGOP: ATP hydrolysis coupled proton transport

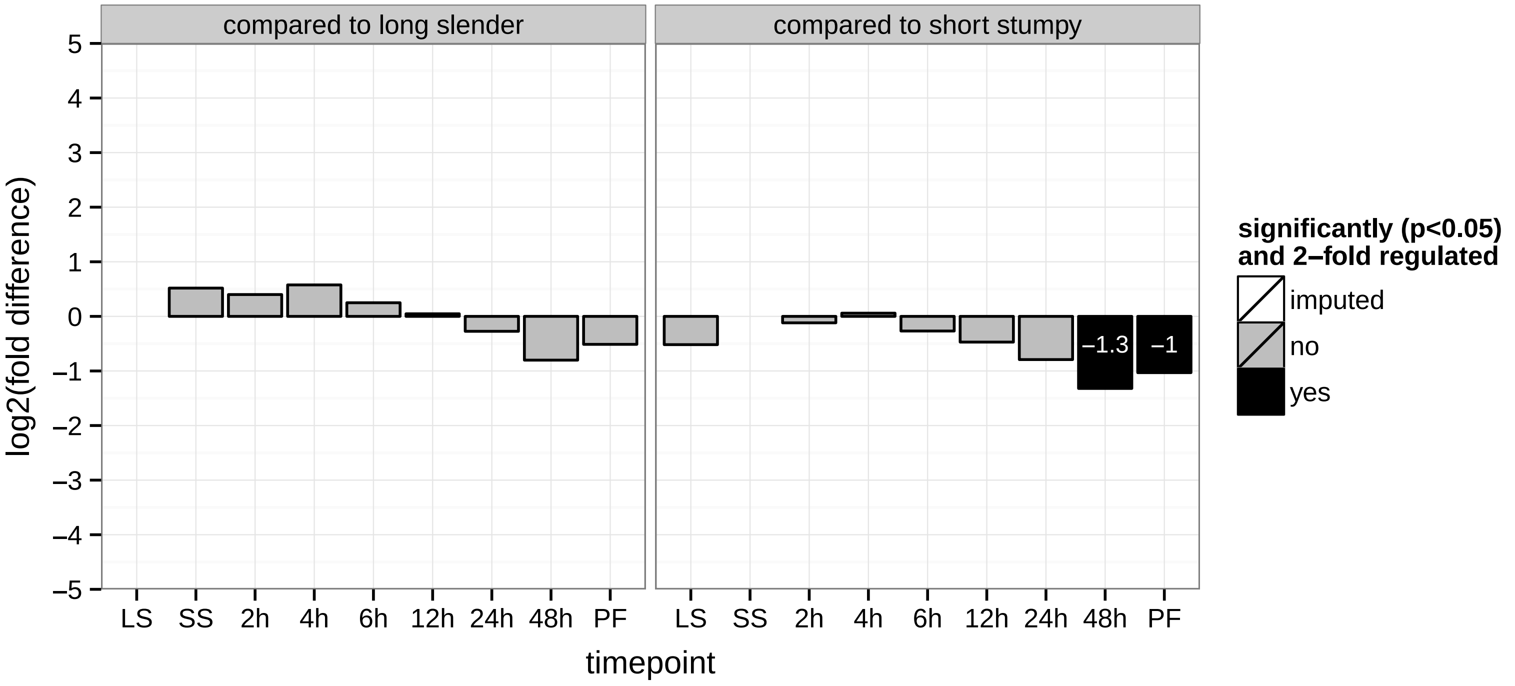

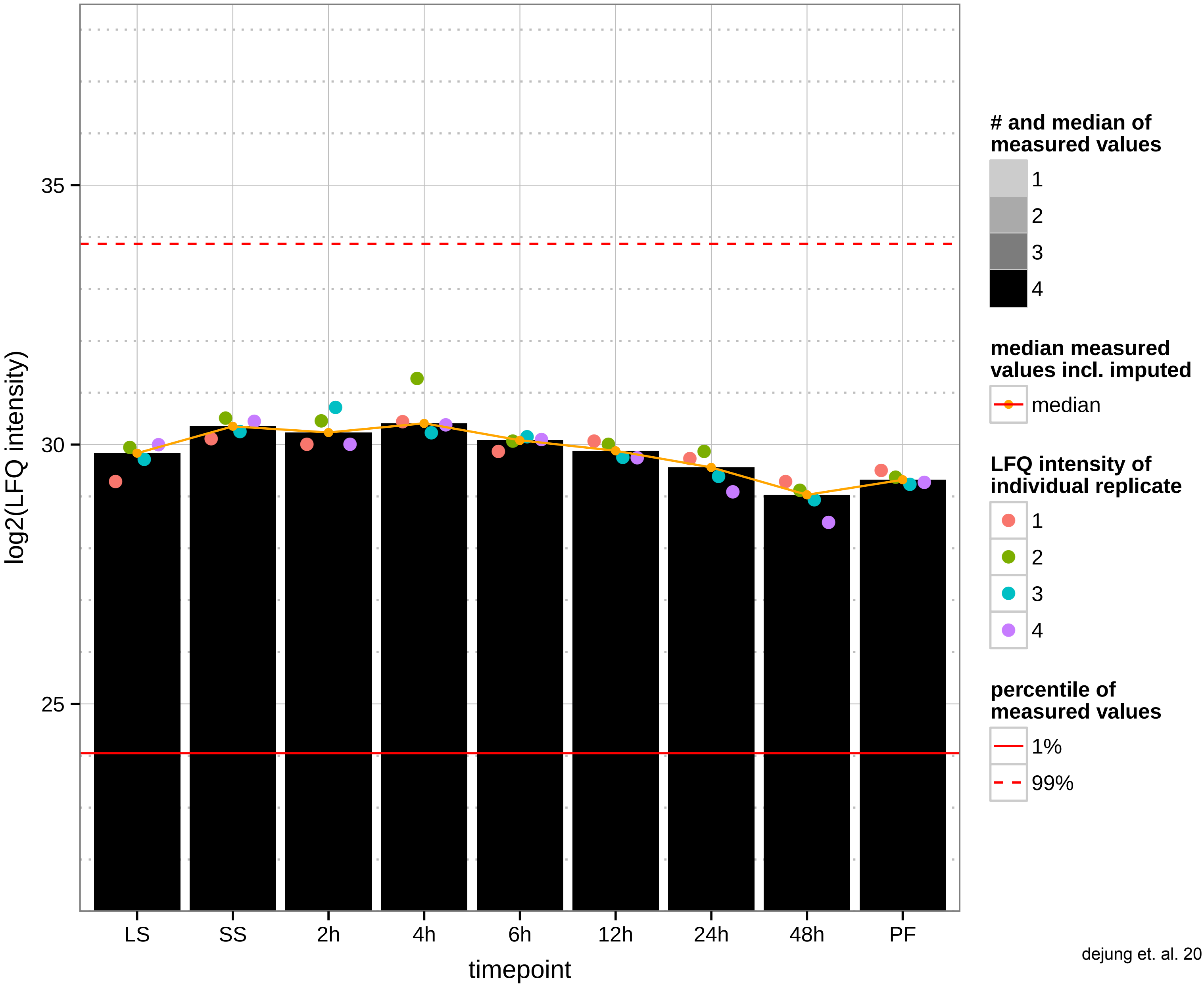

dejung et. al. 2015

histone H4 variant
Tb927.2.2670
AGOF: DNA binding
AGOC: nucleosome, nucleus
AGOP: chromosome organization, nucleosome assembly
PGOF: DNA binding
PGOC: nucleosome, nucleus
PGOP: nucleosome assembly

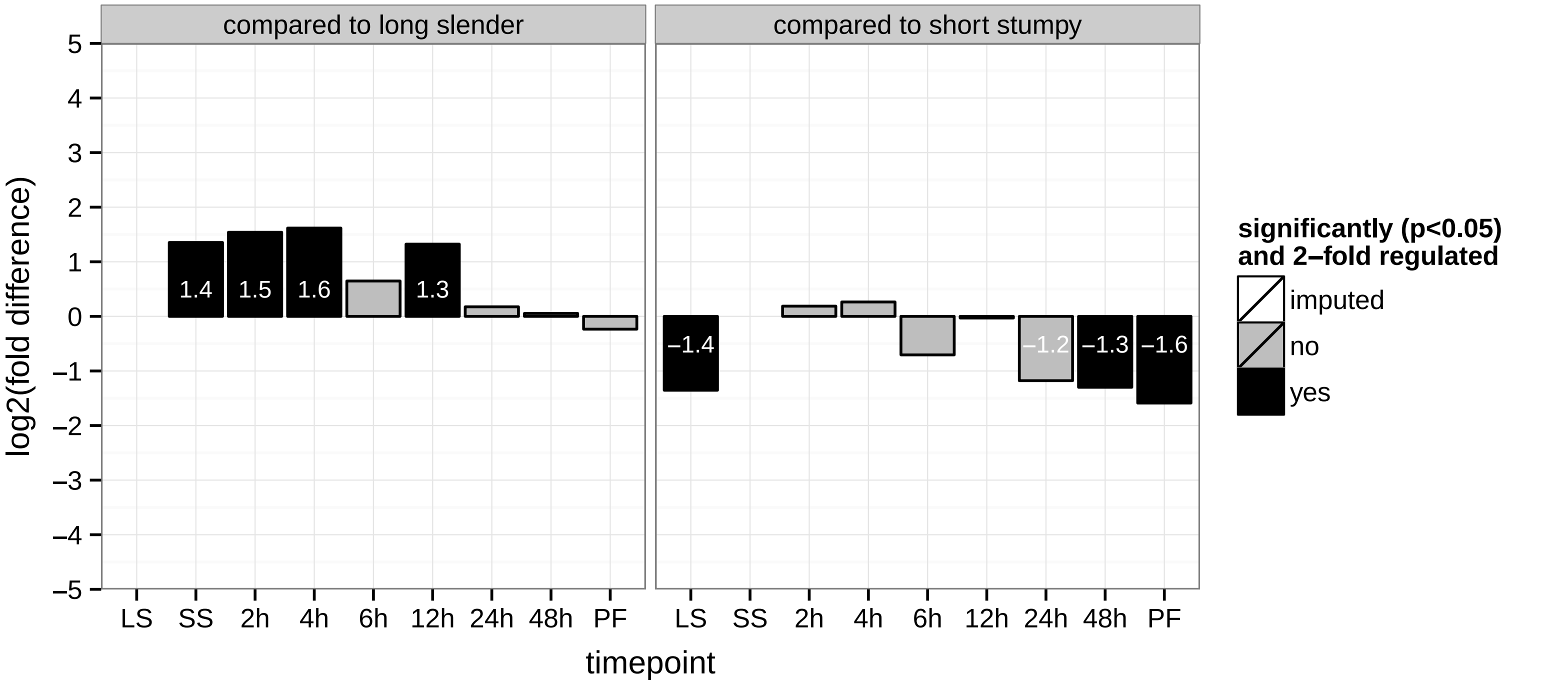

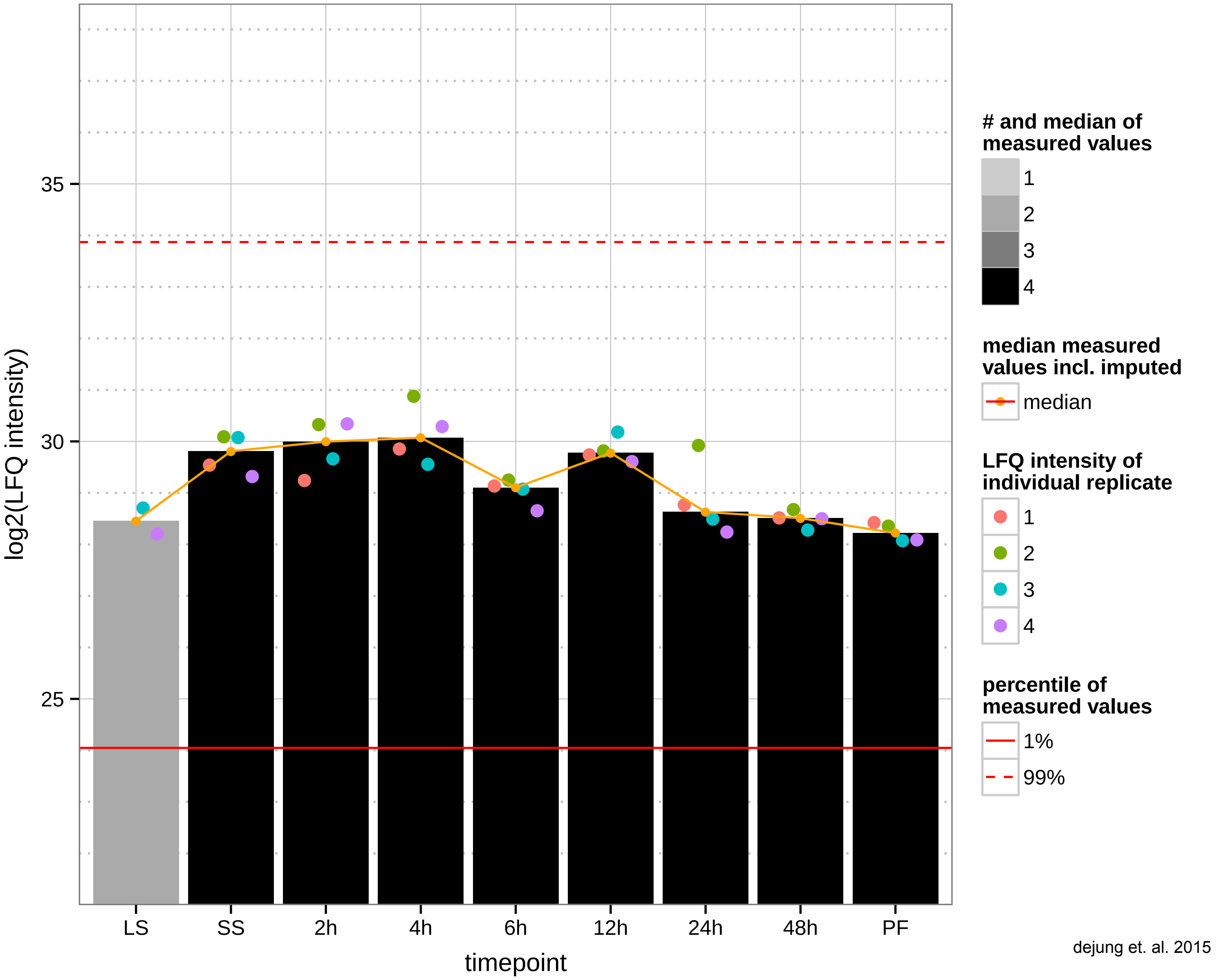

dejung et. al. 2015

hypothetical protein, conserved
Tb927.2.2770
AGOF: null
AGOC: null
AGOP: null
PGOF: null
PGOC: null
PGOP: null

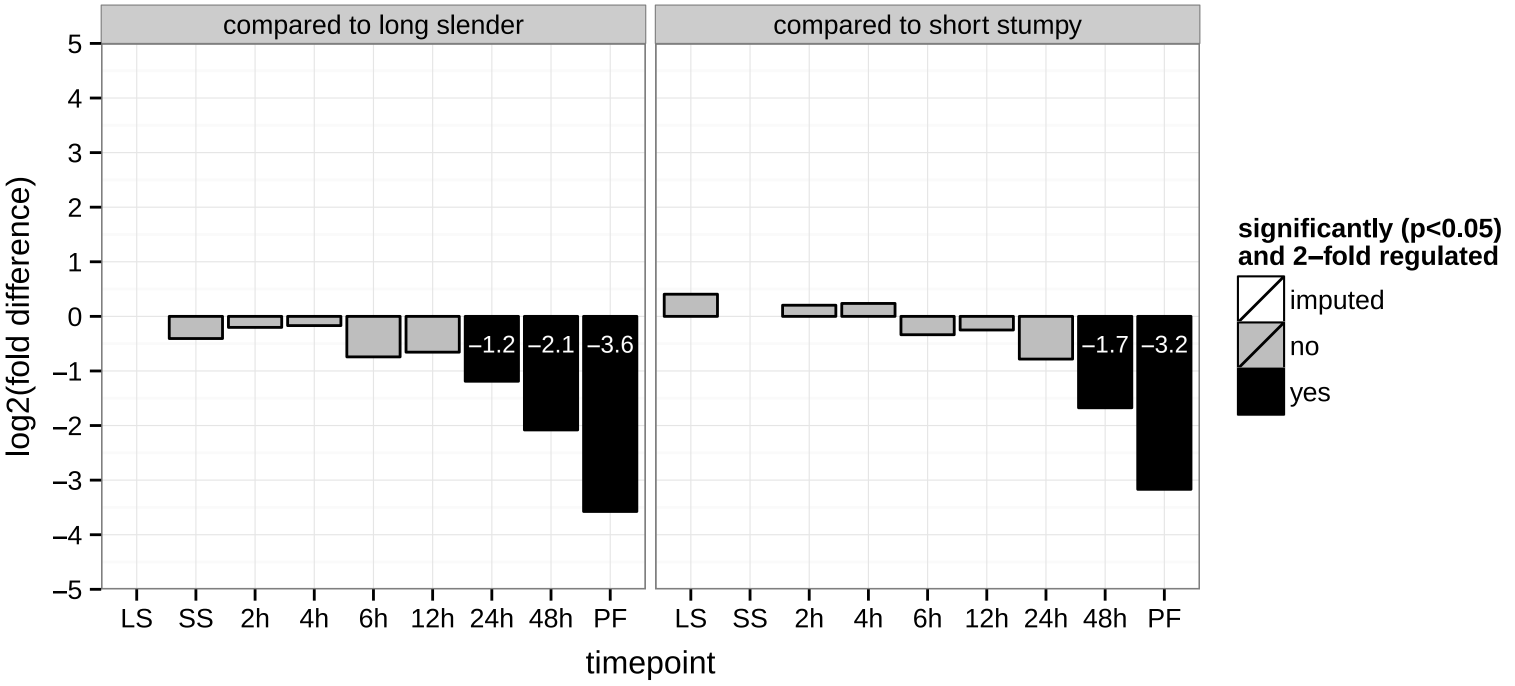

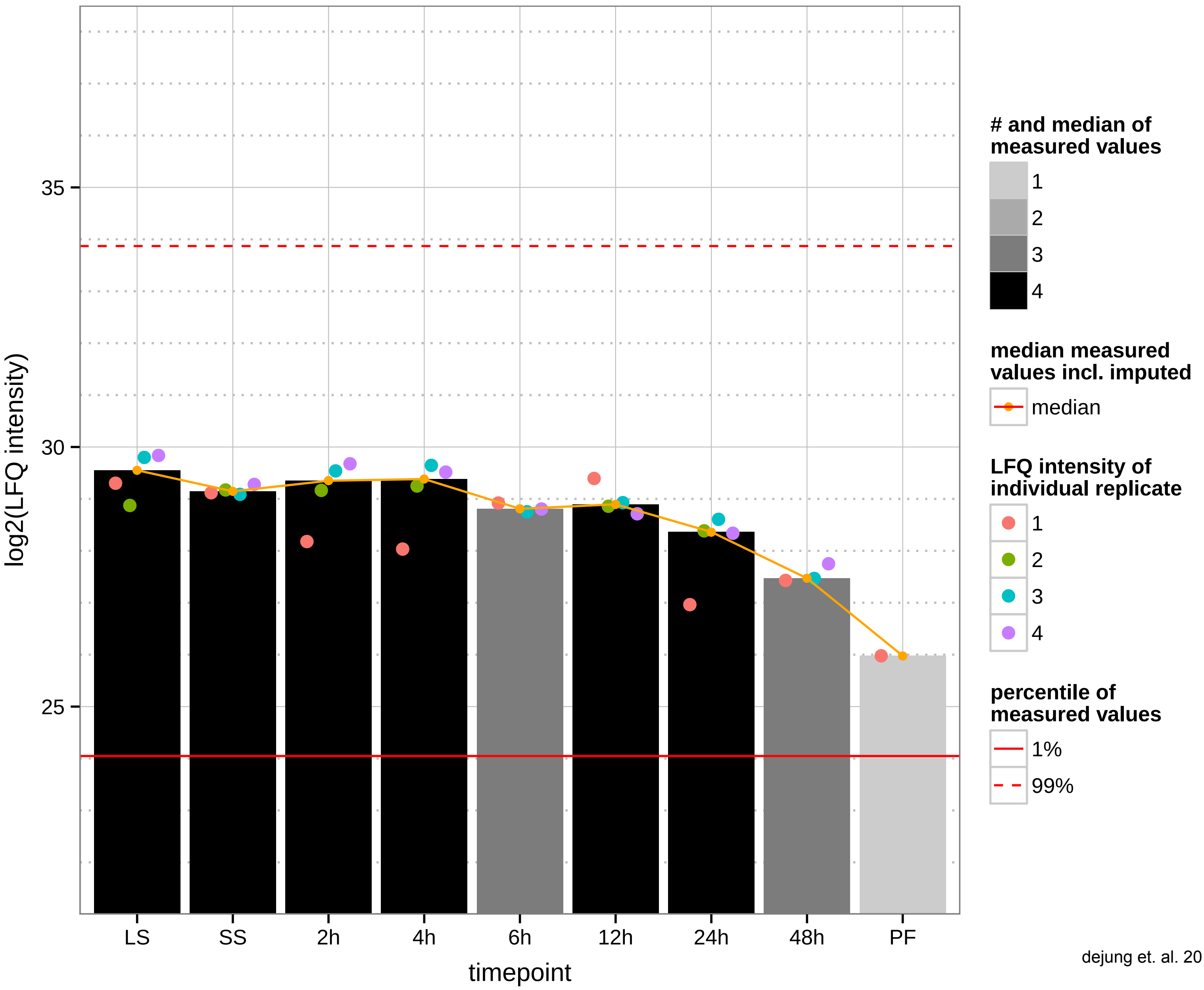

dejung et. al. 2015

GTP–binding elongation factor Tu family, putative
Tb927.2.3620
AGOF: GTP binding, GTPase activity, translation elongation factor activity
AGOC: null
AGOP: translational elongation
PGOF: GTP binding, GTPase activity
PGOC: null
PGOP: null

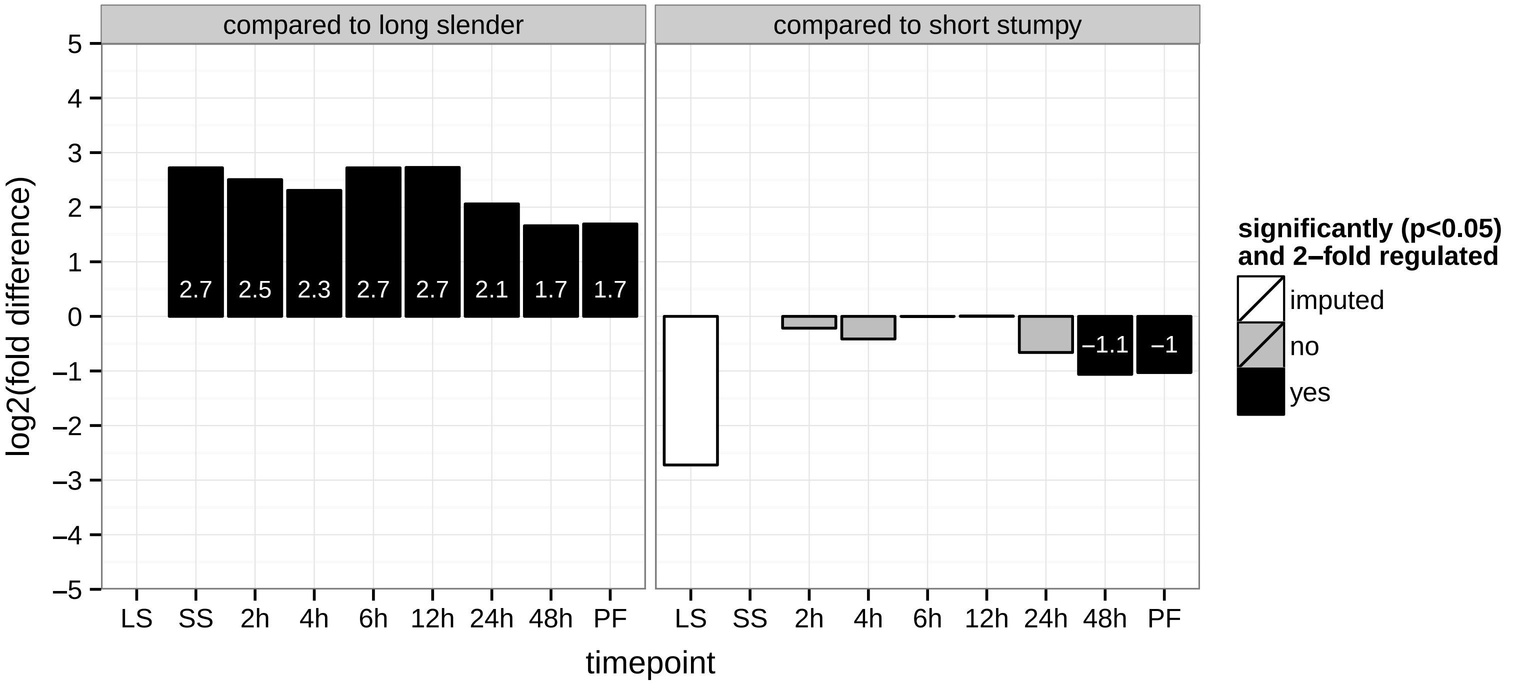

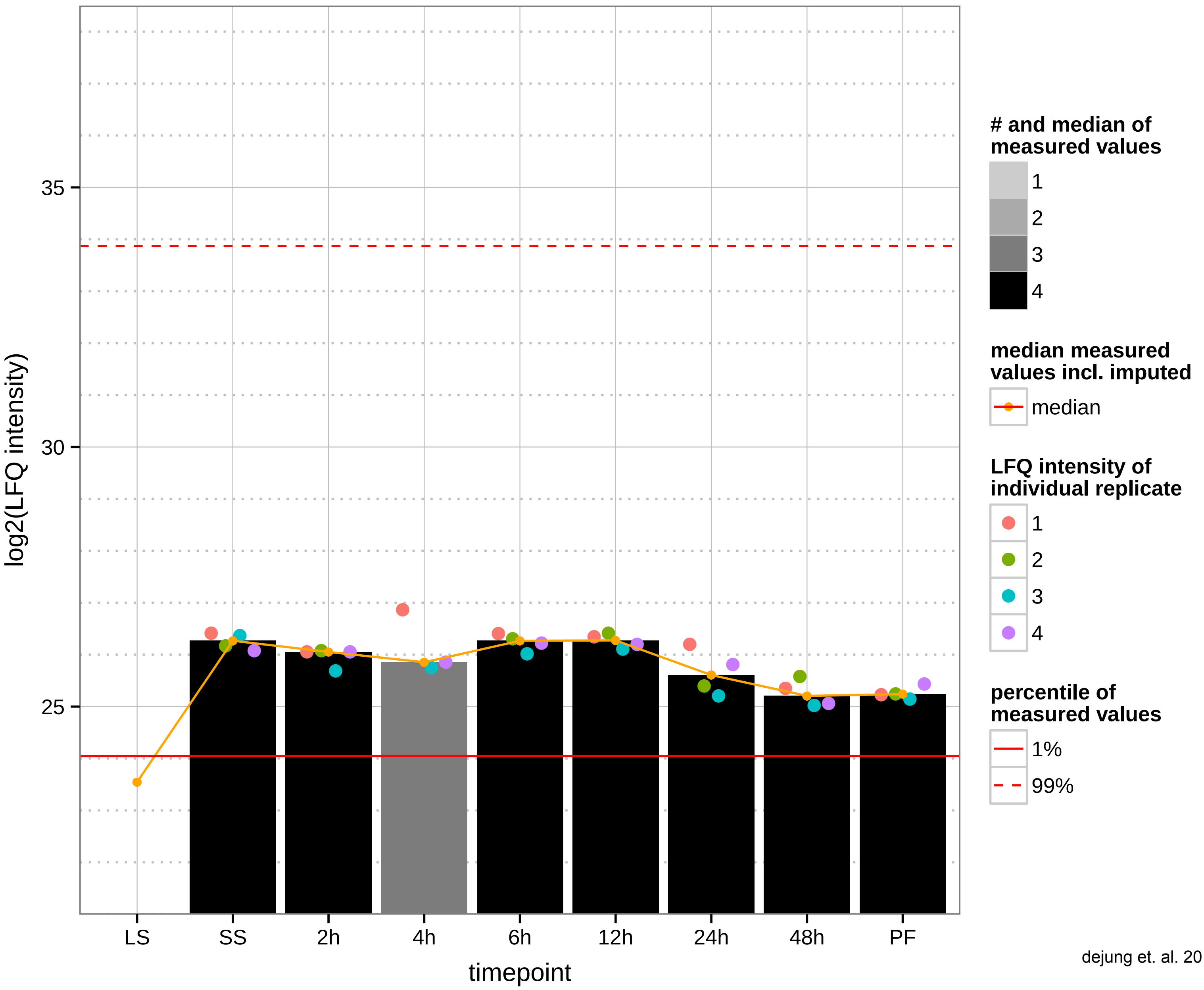

dejung et. al. 2015

heterogeneous nuclear ribonucleoprotein H/F, putative (HNRNPH)
Tb927.2.3880
AGOF: mRNA binding
AGOC: nucleus
AGOP: RNA processing
PGOF: nucleic acid binding
PGOC: null
PGOP: null

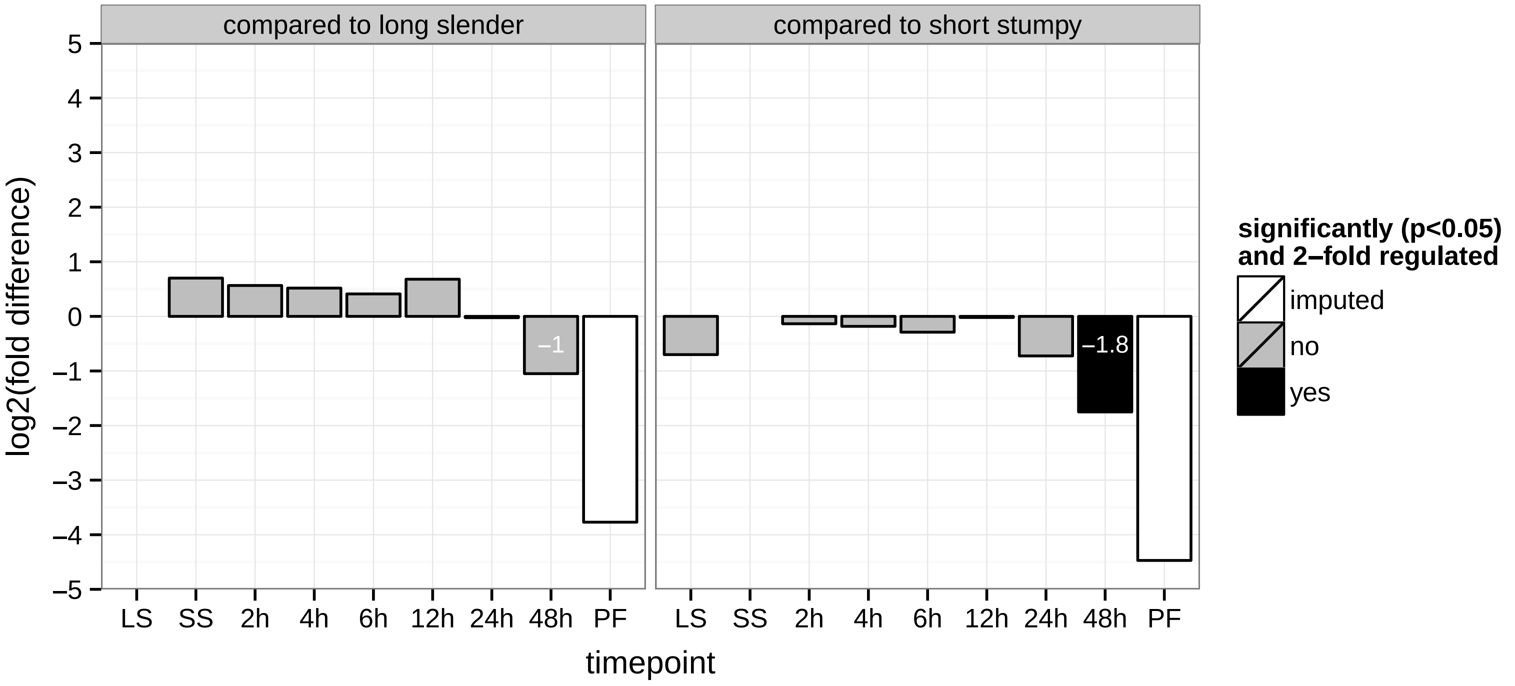

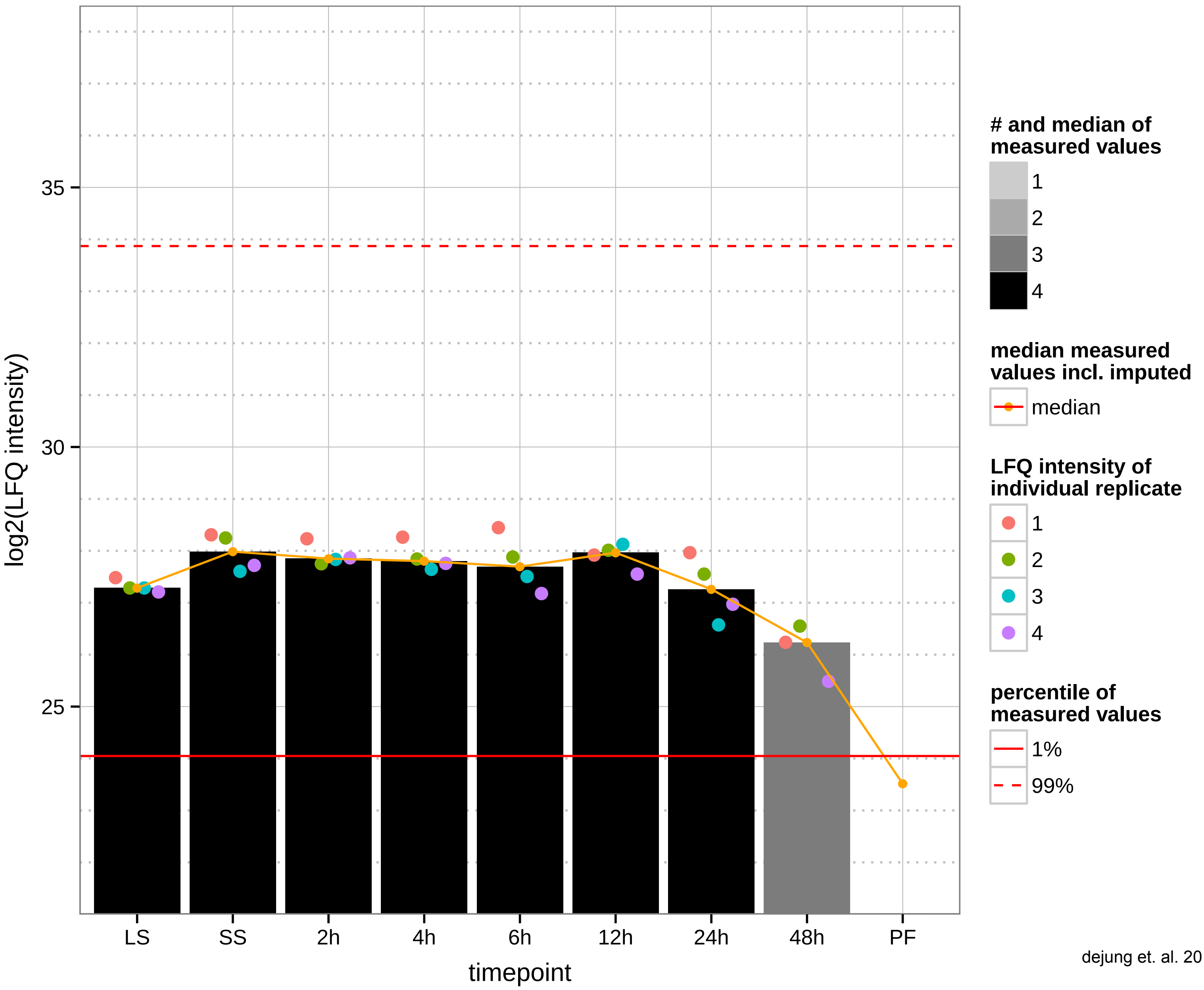

dejung et. al. 2015

trypanothione synthetase, putative, trypanothione synthetase (TRYS)
Tb927.2.4370;Tb11.v5.0653
AGOF: null, trypanothione−disulfide reductase activity
AGOC: null, cytosol
AGOP: null, trypanothione biosynthetic process
PGOF: null
PGOC: null
PGOP: null

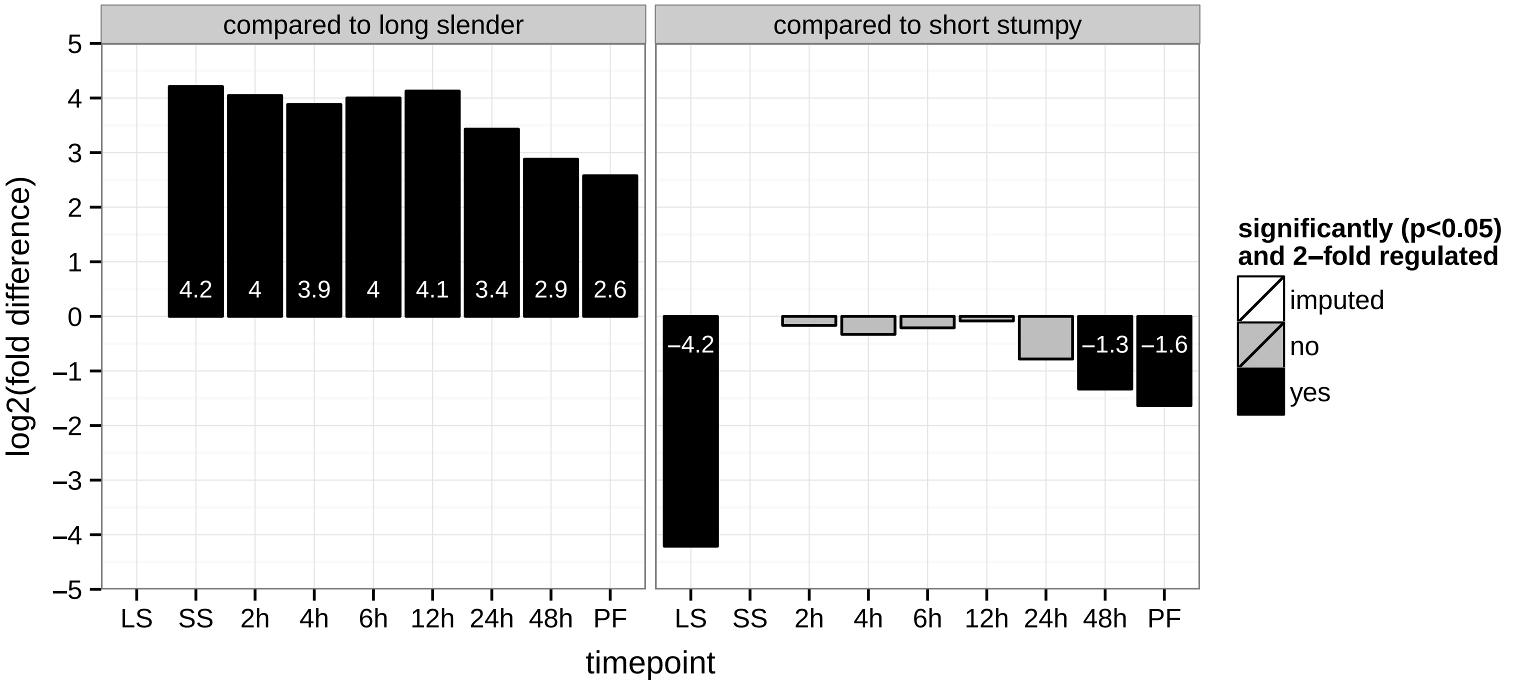

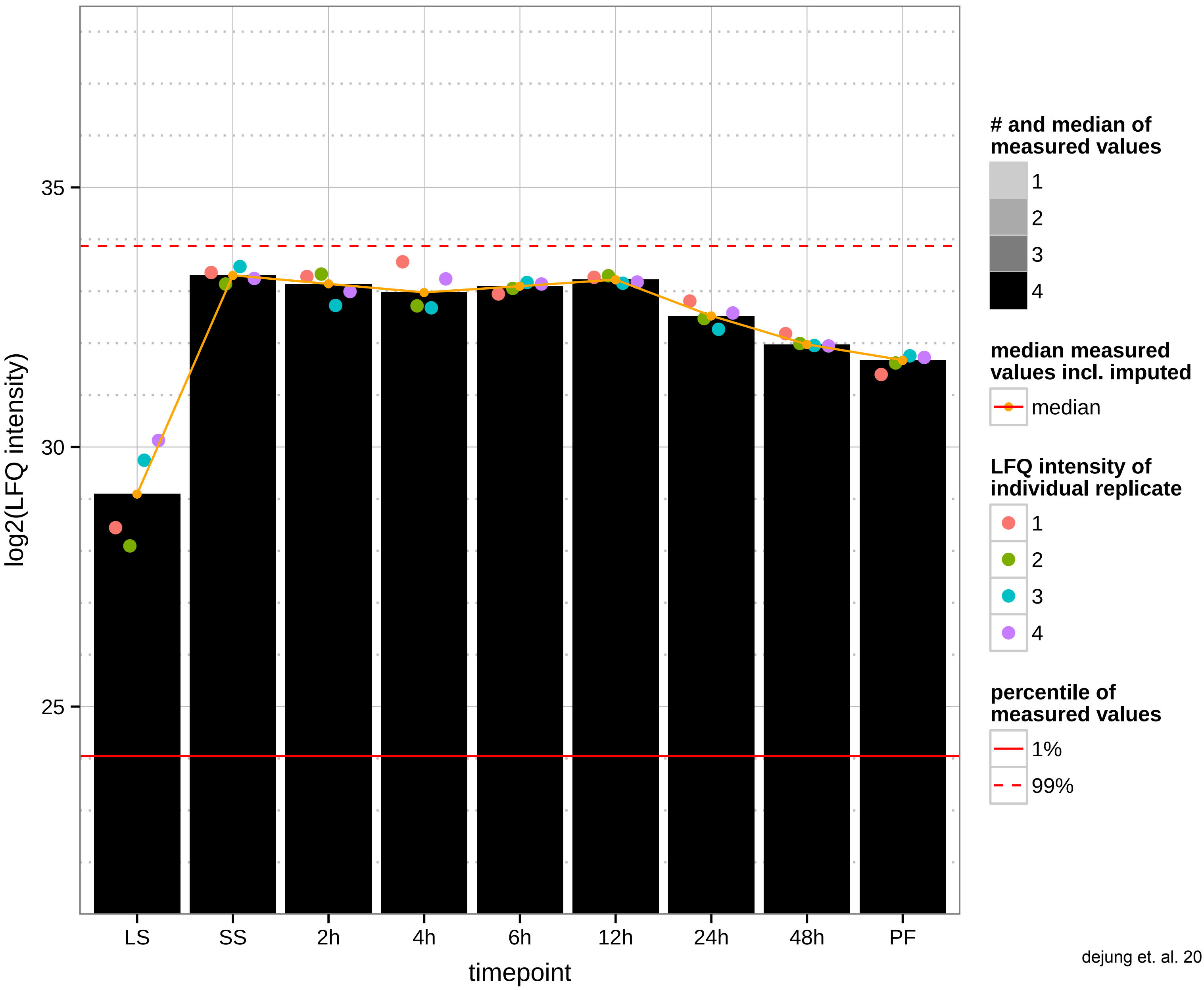

dejung et. al. 2015

sedoheptulose−1, 7−bisphosphatase (SBPase)
Tb927.2.5800
AGOF: molecular_function, phosphoric ester hydrolase activity
AGOC: glycosome
AGOP: carbohydrate metabolic process
PGOF: phosphoric ester hydrolase activity
PGOC: null
PGOP: carbohydrate metabolic process

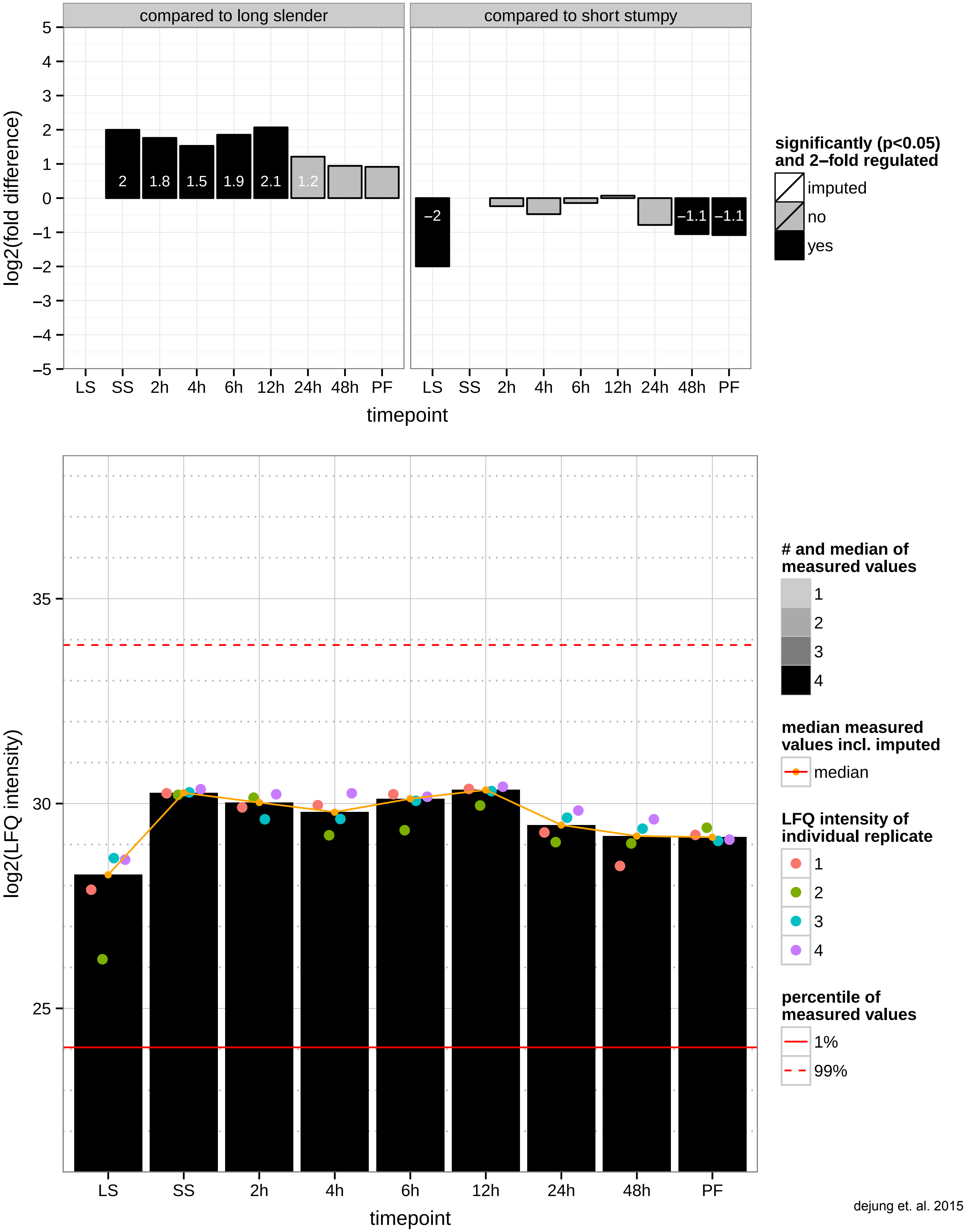

dejung et. al. 2015

hypothetical protein, conserved
Tb927.3.1390
AGOF: null
AGOC: null
AGOP: null
PGOF: null
PGOC: null
PGOP: null

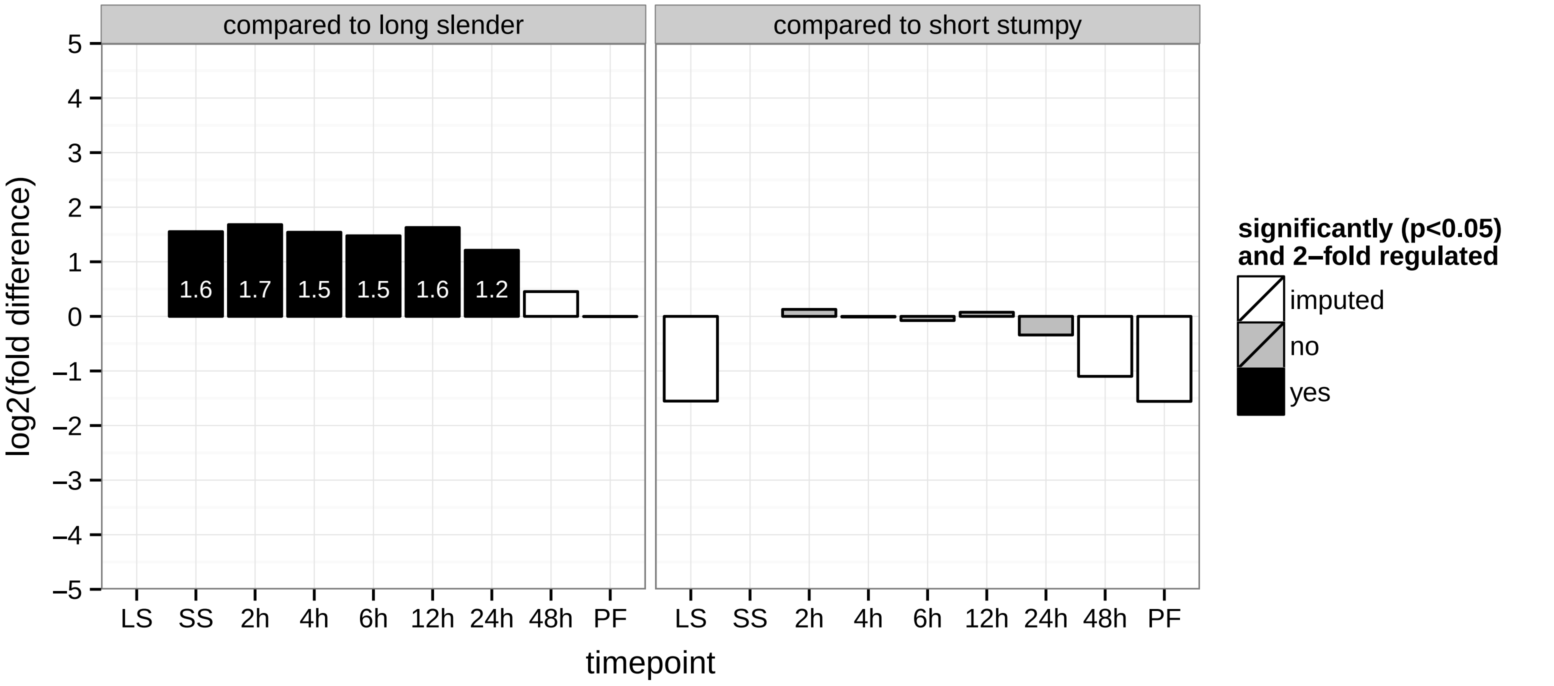

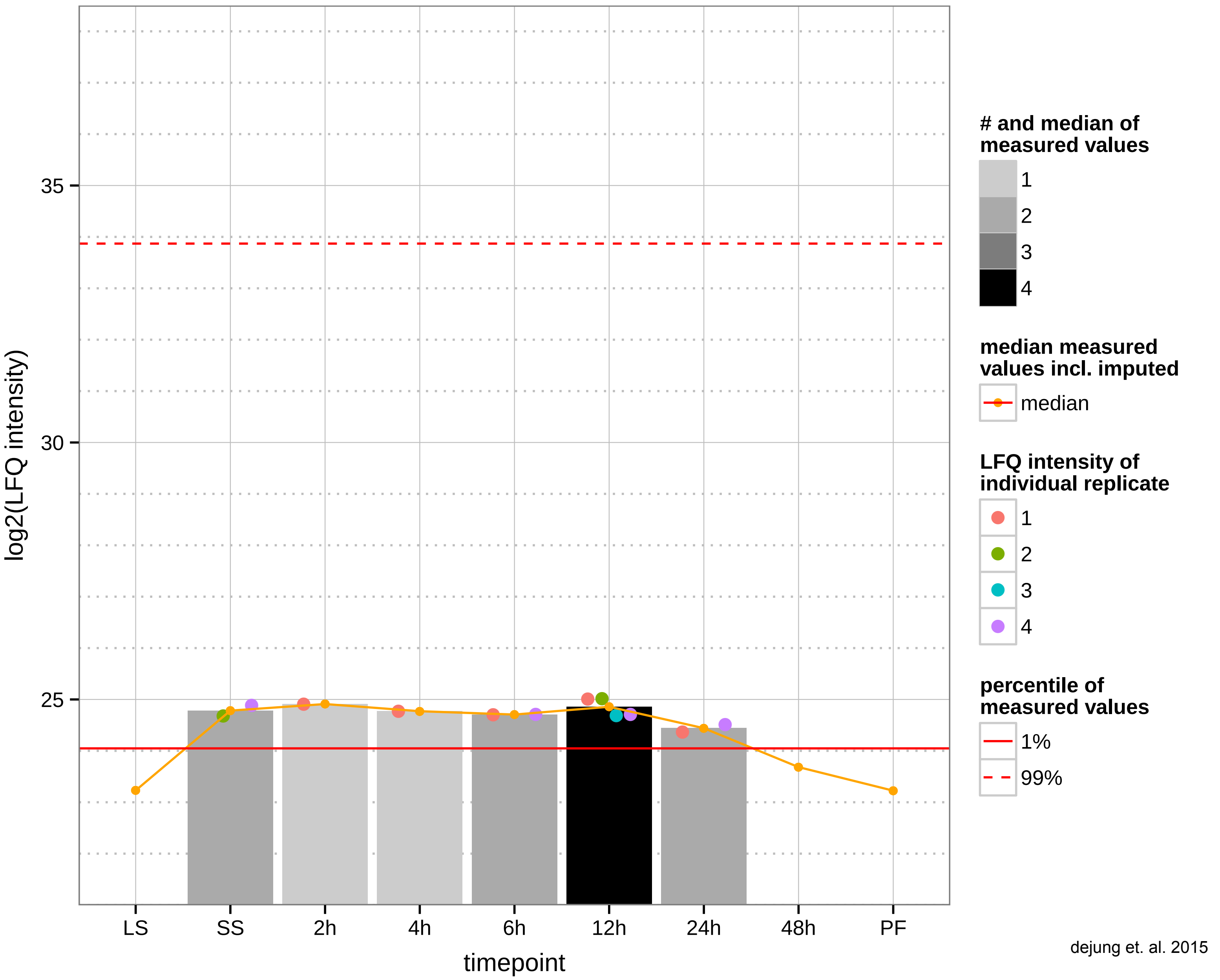

dejung et. al. 2015

dynamin, putative, vacuolar sortin protein 1
Tb927.3.4720
AGOF: GTP binding, GTPase activity
AGOC: null
AGOP: null
PGOF: GTP binding, GTPase activity
PGOC: null
PGOP: null

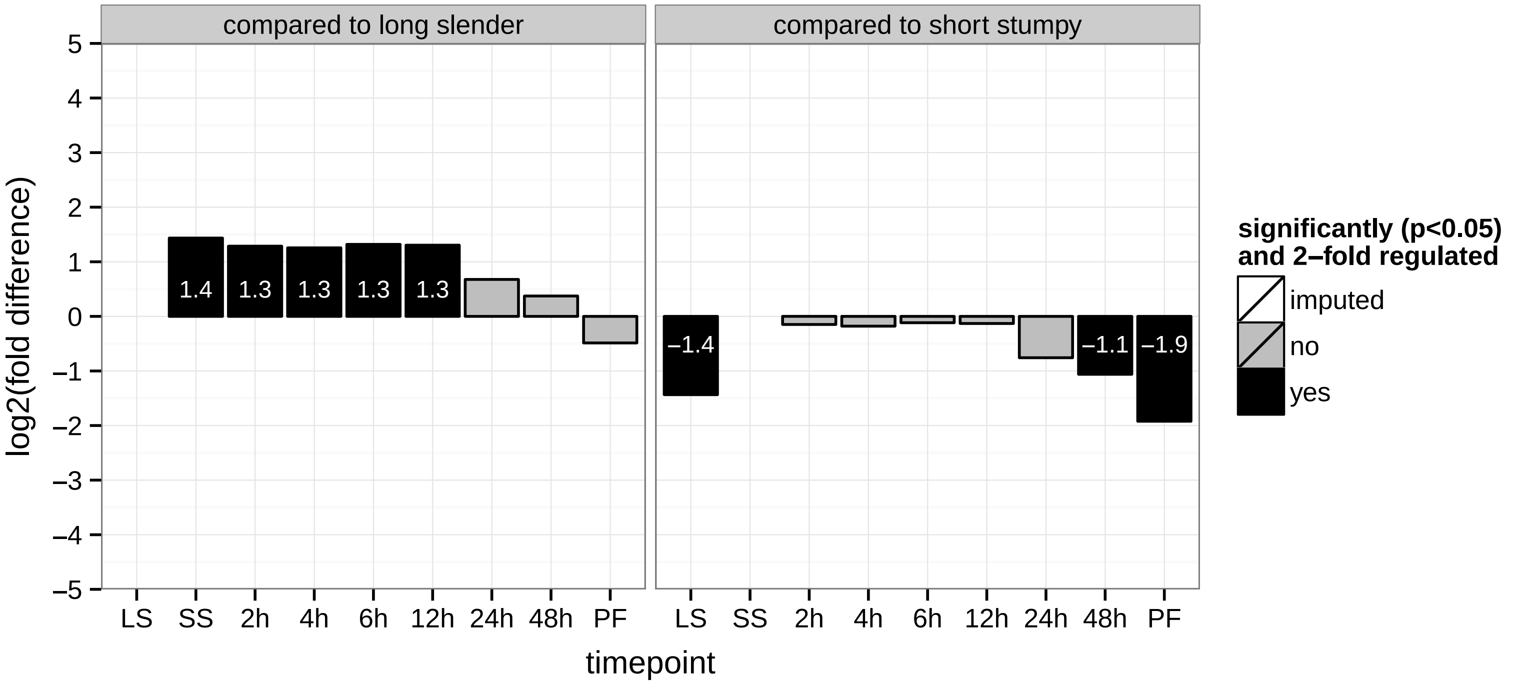

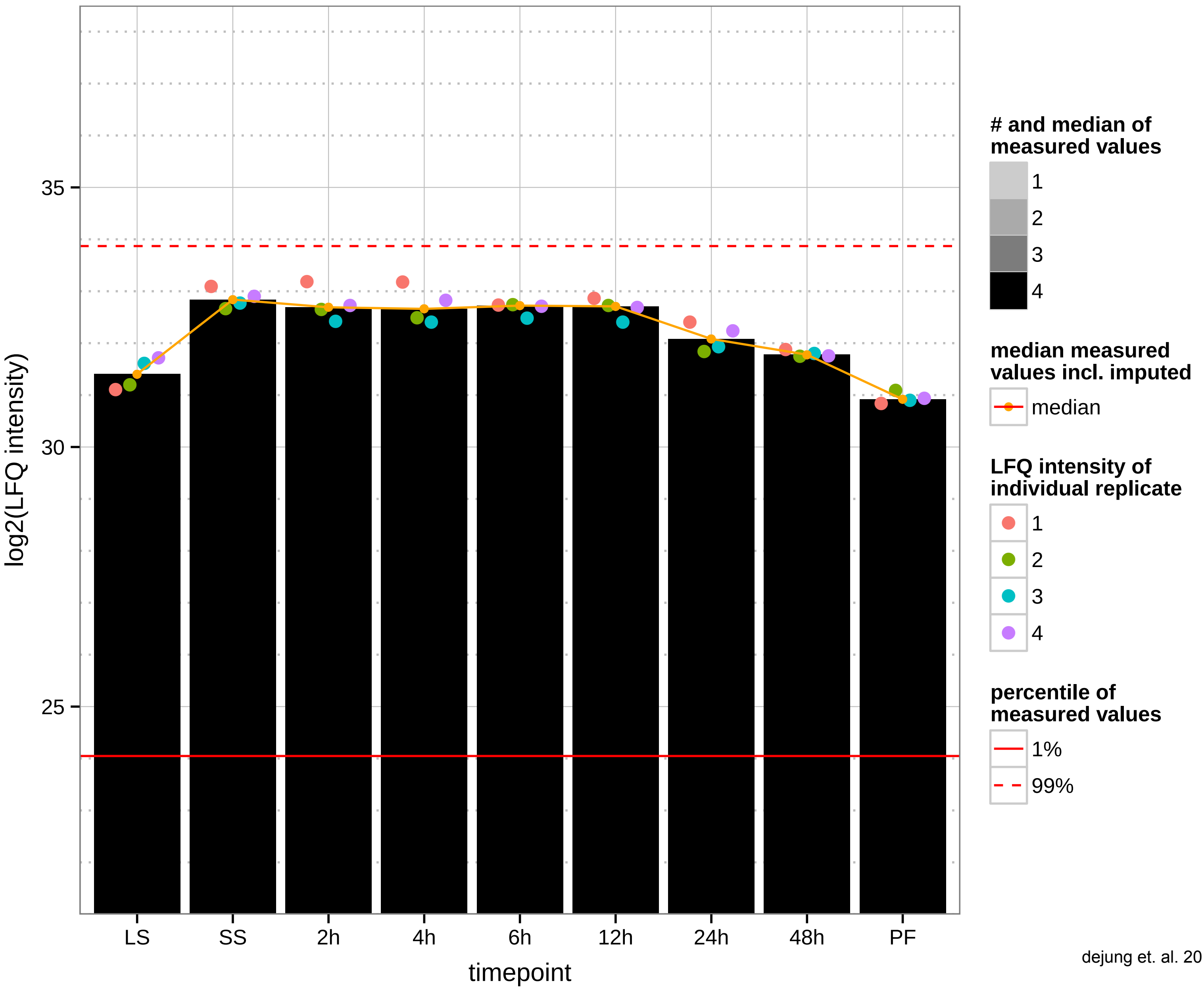

dejung et. al. 2015

flagellar transport protein, putative (PIFTB2)
Tb927.3.5490
AGOF: null
AGOC: null
AGOP: null
PGOF: protein binding
PGOC: null
PGOP: null

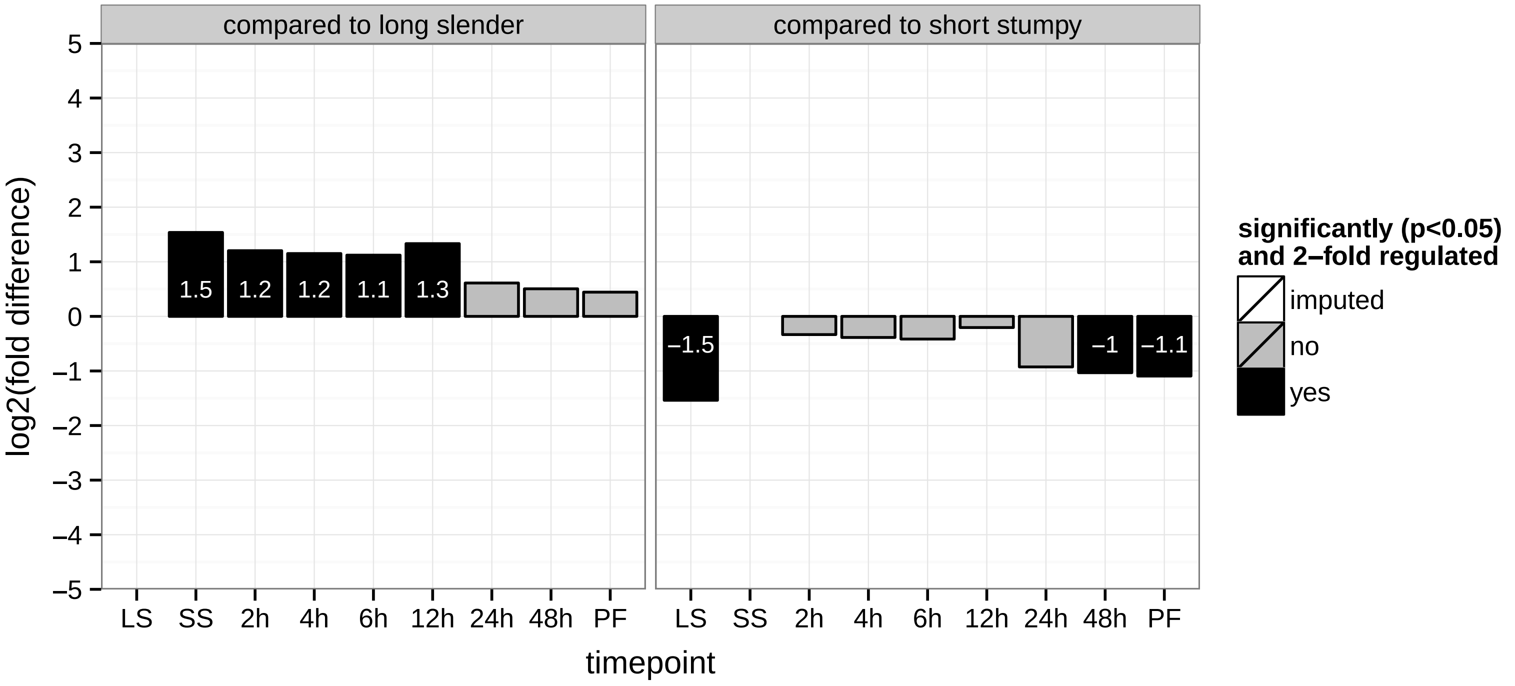

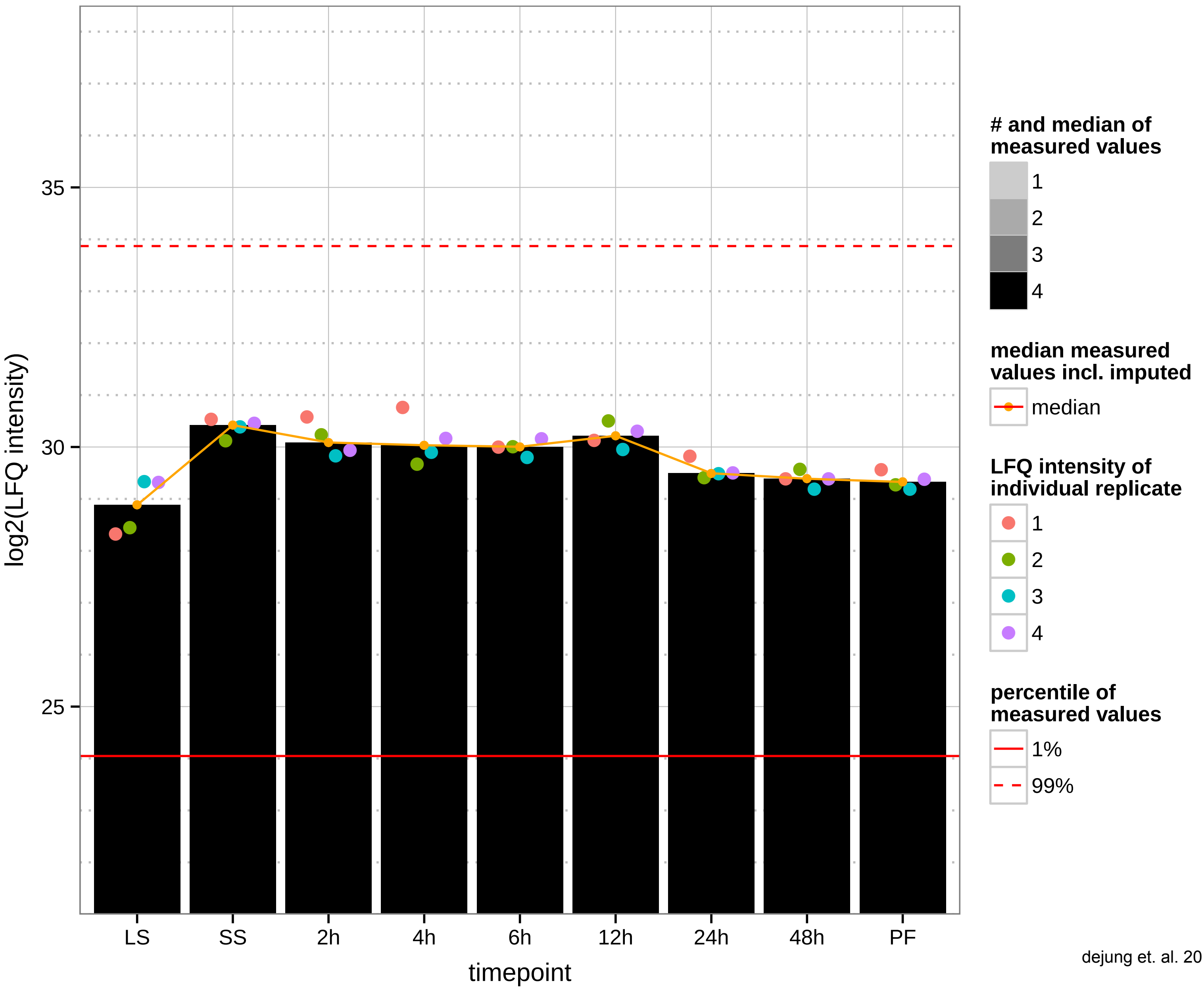

dejung et. al. 2015

ALBA–Domain Protein (ALBA4)
Tb927.4.2030
AGOF: nucleic acid binding
AGOC: cytoplasm, cytoplasmic stress granule
AGOP: null
PGOF: nucleic acid binding
PGOC: null
PGOP: null

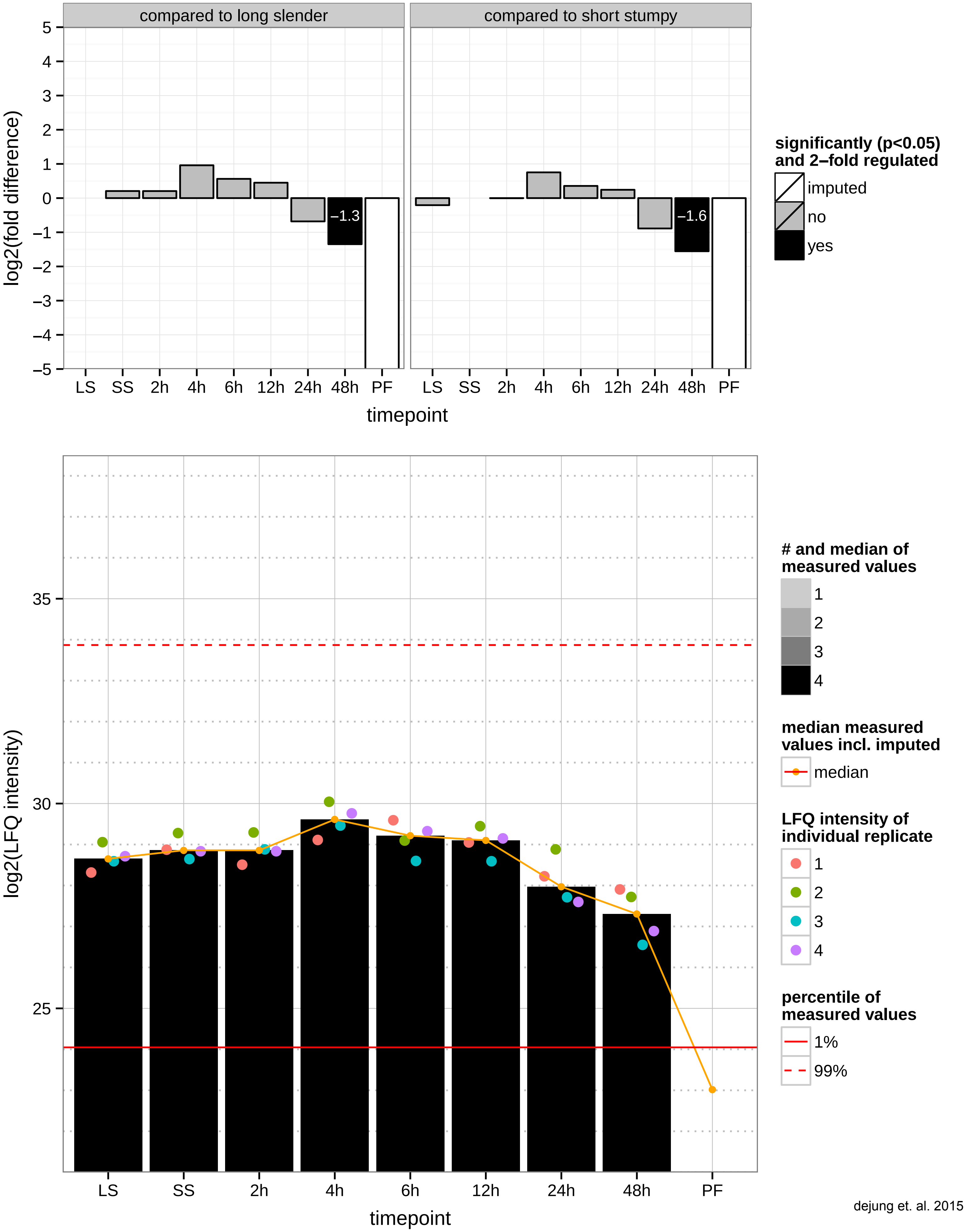

dejung et. al. 2015

hypothetical protein, conserved
Tb927.4.2170
AGOF: null
AGOC: null
AGOP: null
PGOF: null
PGOC: null
PGOP: null

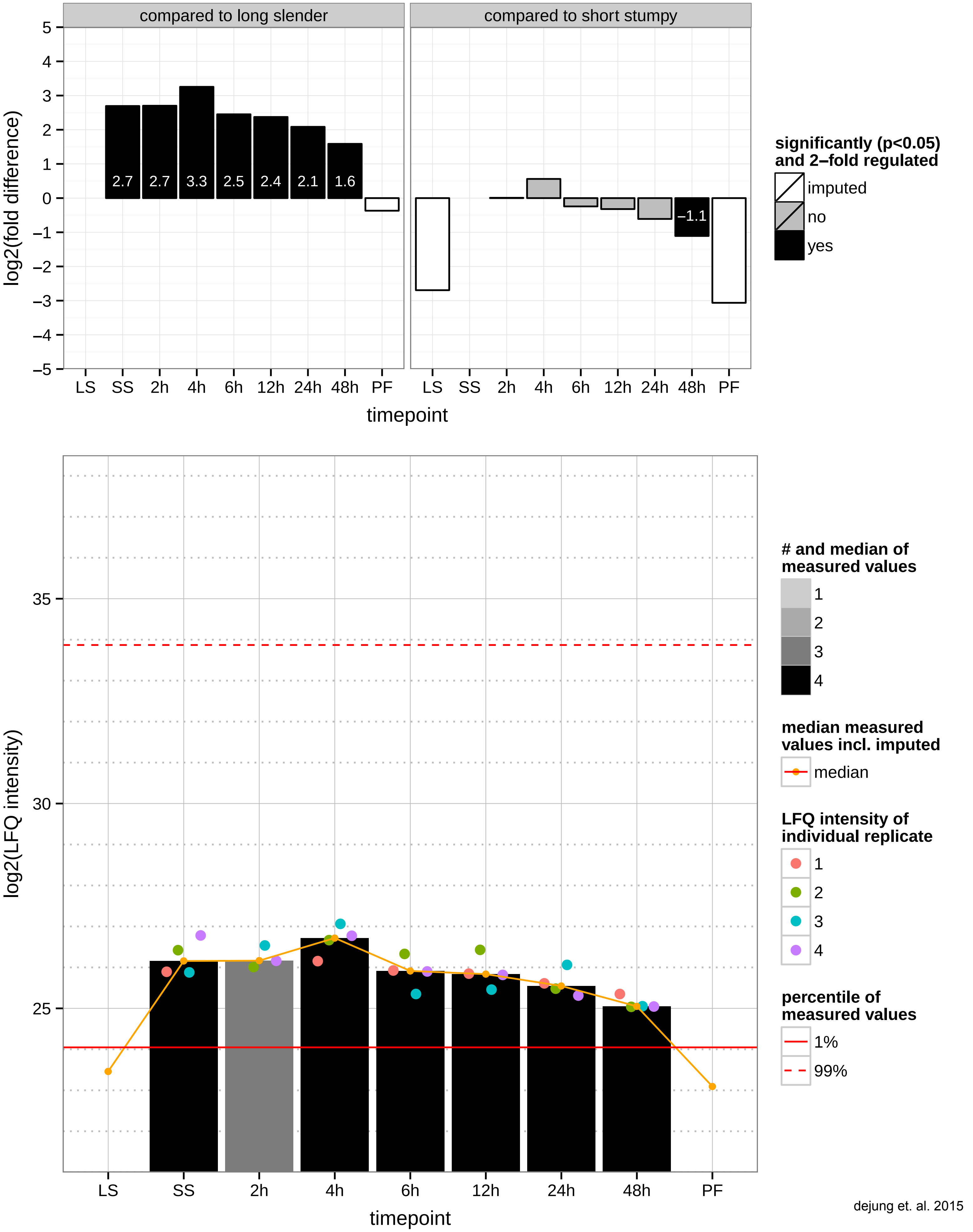

dejung et. al. 2015

hypothetical protein, conserved
Tb927.4.2640
AGOF: null
AGOC: null
AGOP: null
PGOF: protein binding
PGOC: null
PGOP: null

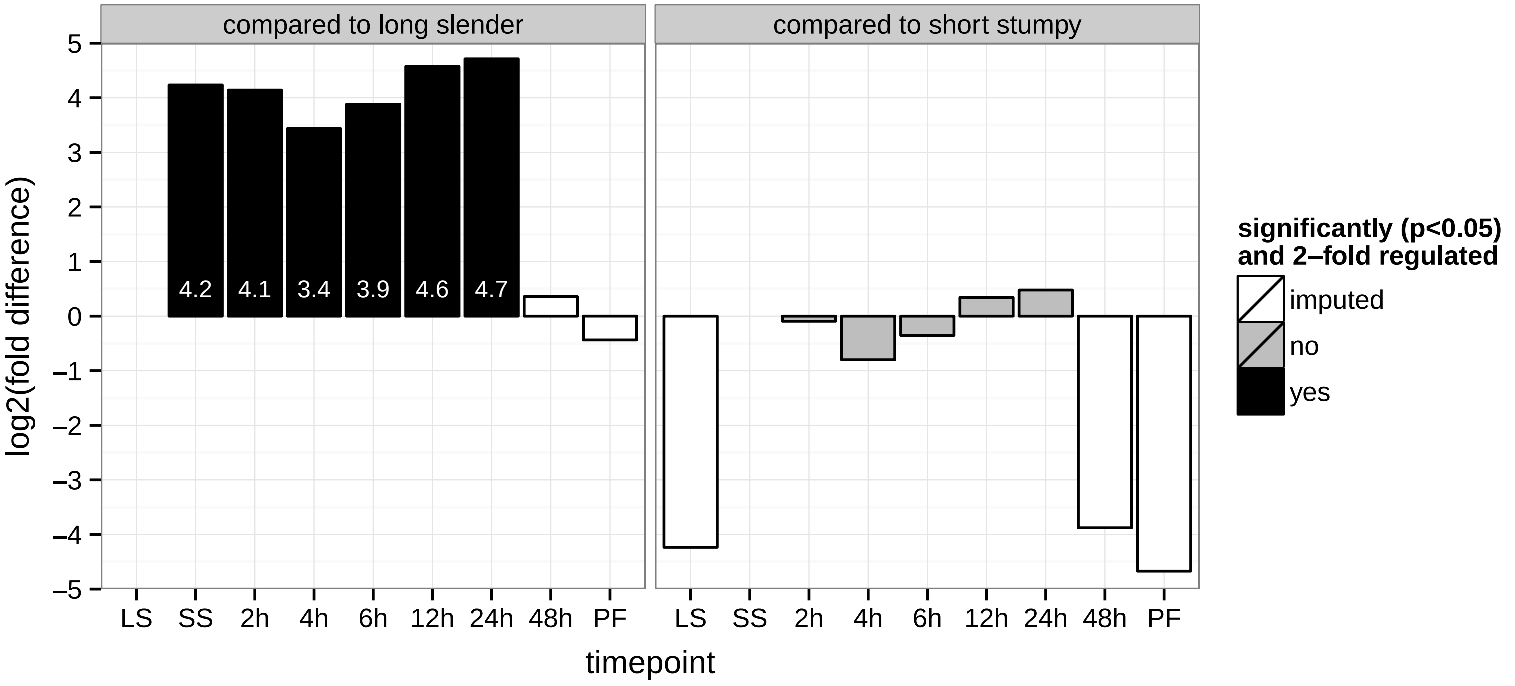

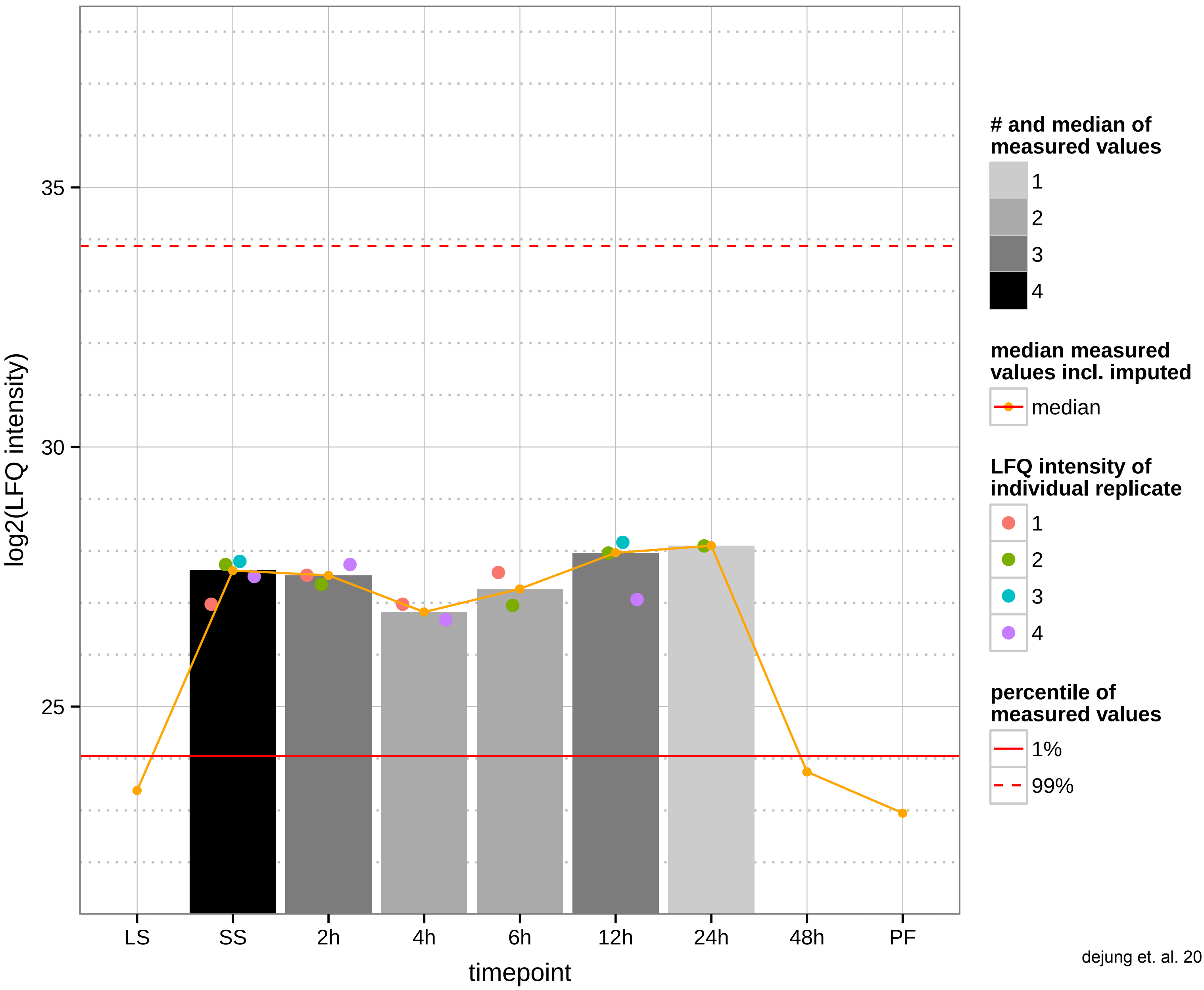

dejung et. al. 2015

hypothetical protein, conserved
Tb927.4.2760
AGOF: null
AGOC: null
AGOP: null
PGOF: null
PGOC: null
PGOP: null

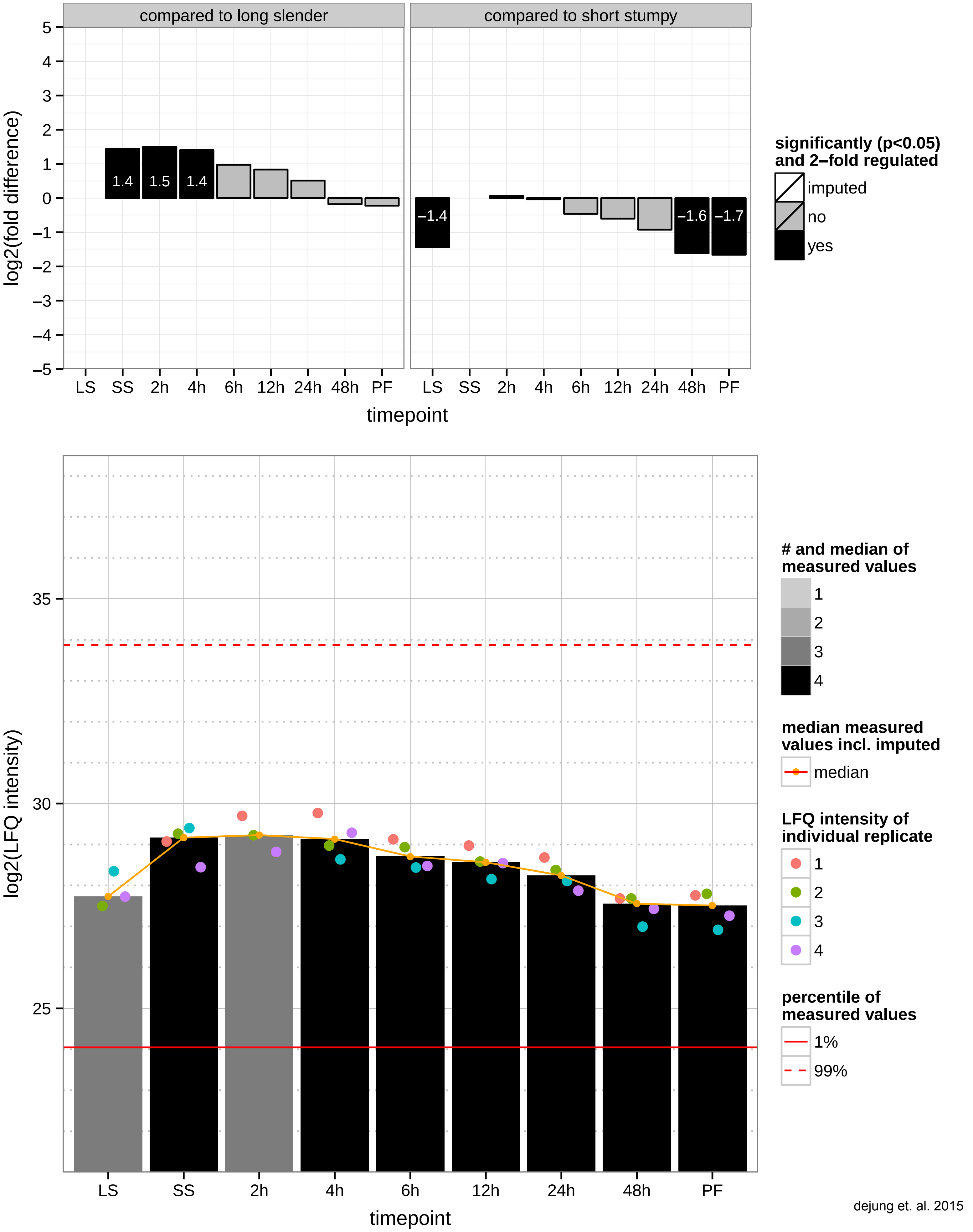

dejung et. al. 2015

hypothetical protein, conserved
Tb927.4.3400
AGOF: null
AGOC: null
AGOP: null
PGOF: binding
PGOC: null
PGOP: null

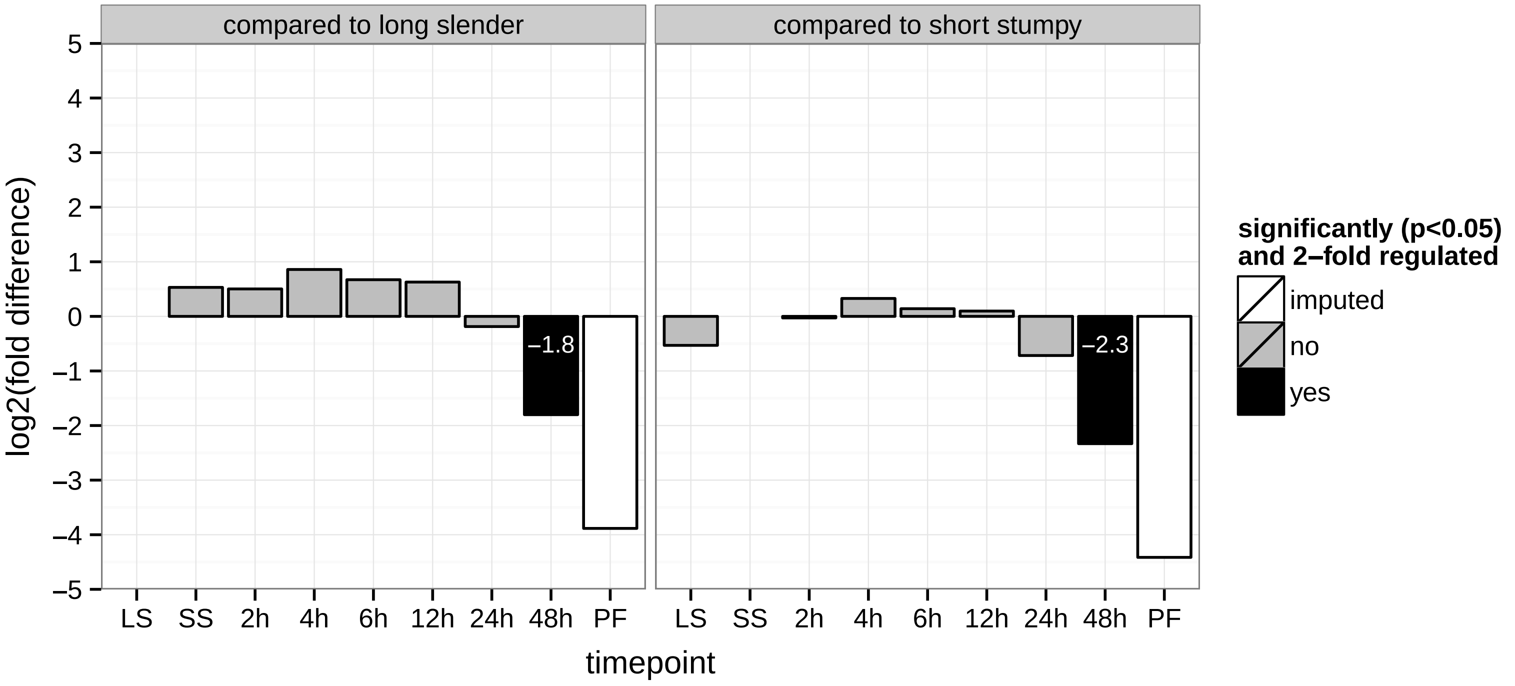

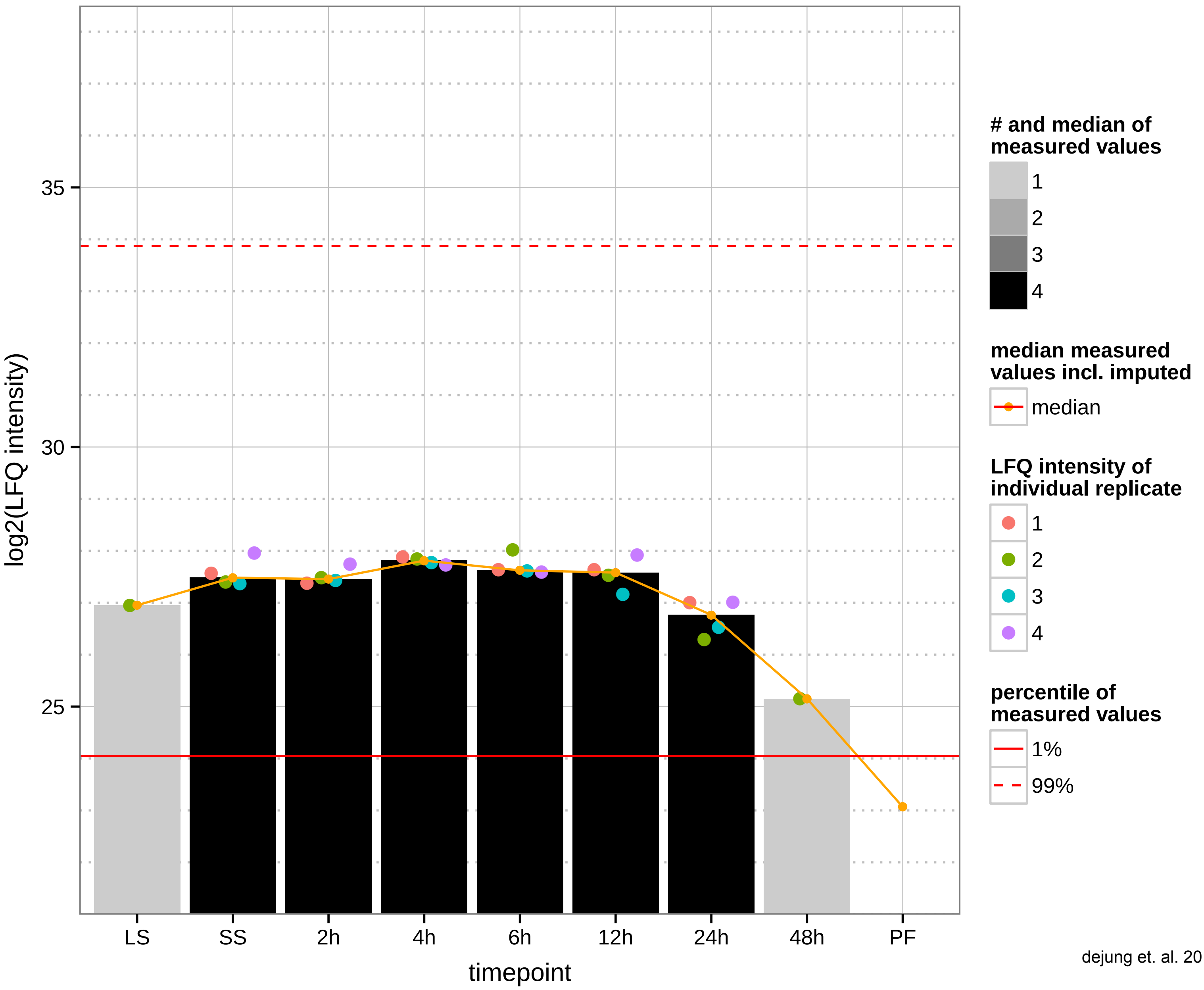

dejung et. al. 2015

hypothetical protein, conserved
Tb927.4.3970
AGOF: null
AGOC: null
AGOP: null
PGOF: null
PGOC: null
PGOP: null

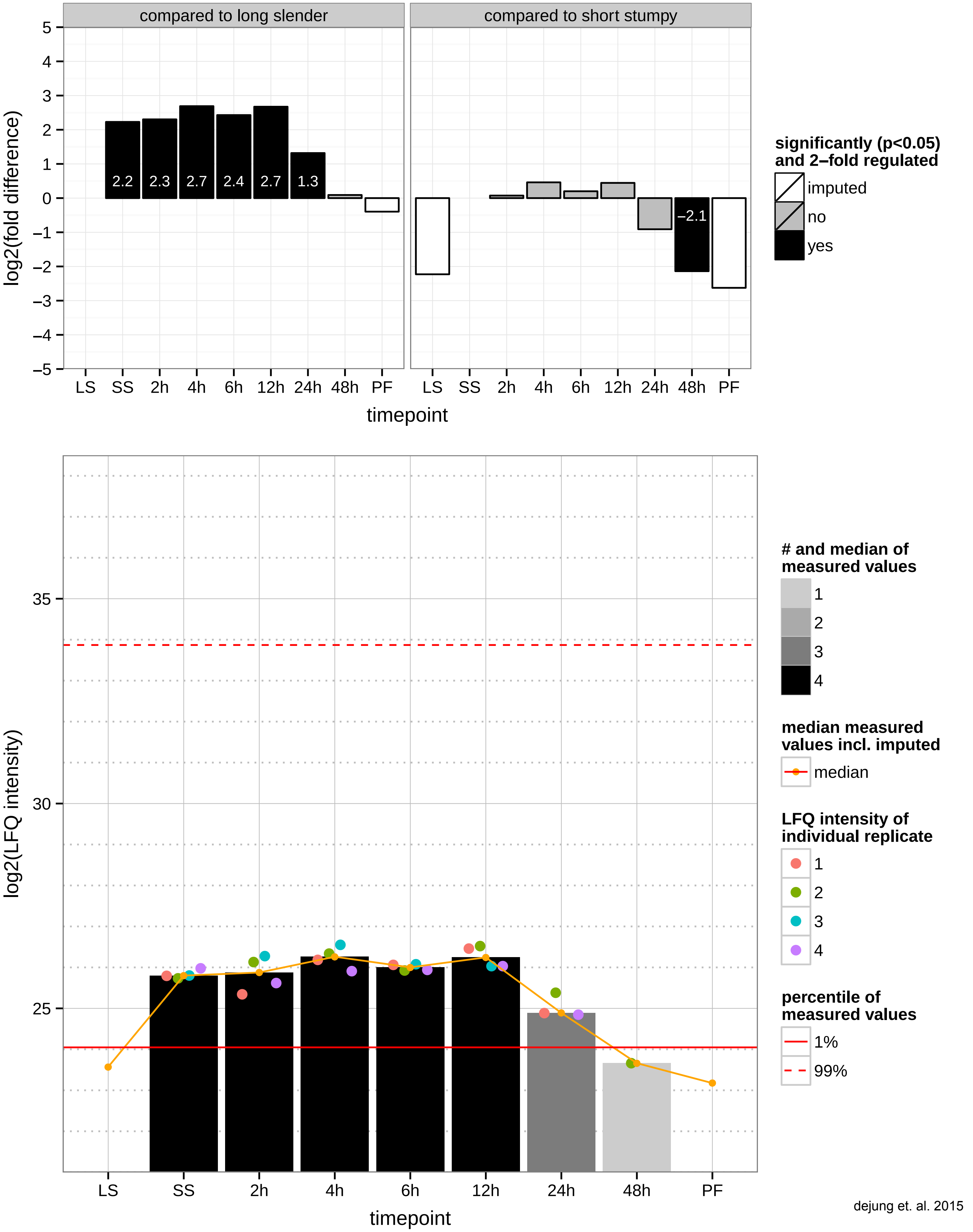

dejung et. al. 2015

hypothetical protein, conserved
Tb927.4.610
AGOF: phosphatidylinositol binding
AGOC: null
AGOP: cell communication
PGOF: phosphatidylinositol binding, protein binding
PGOC: null
PGOP: cell communication

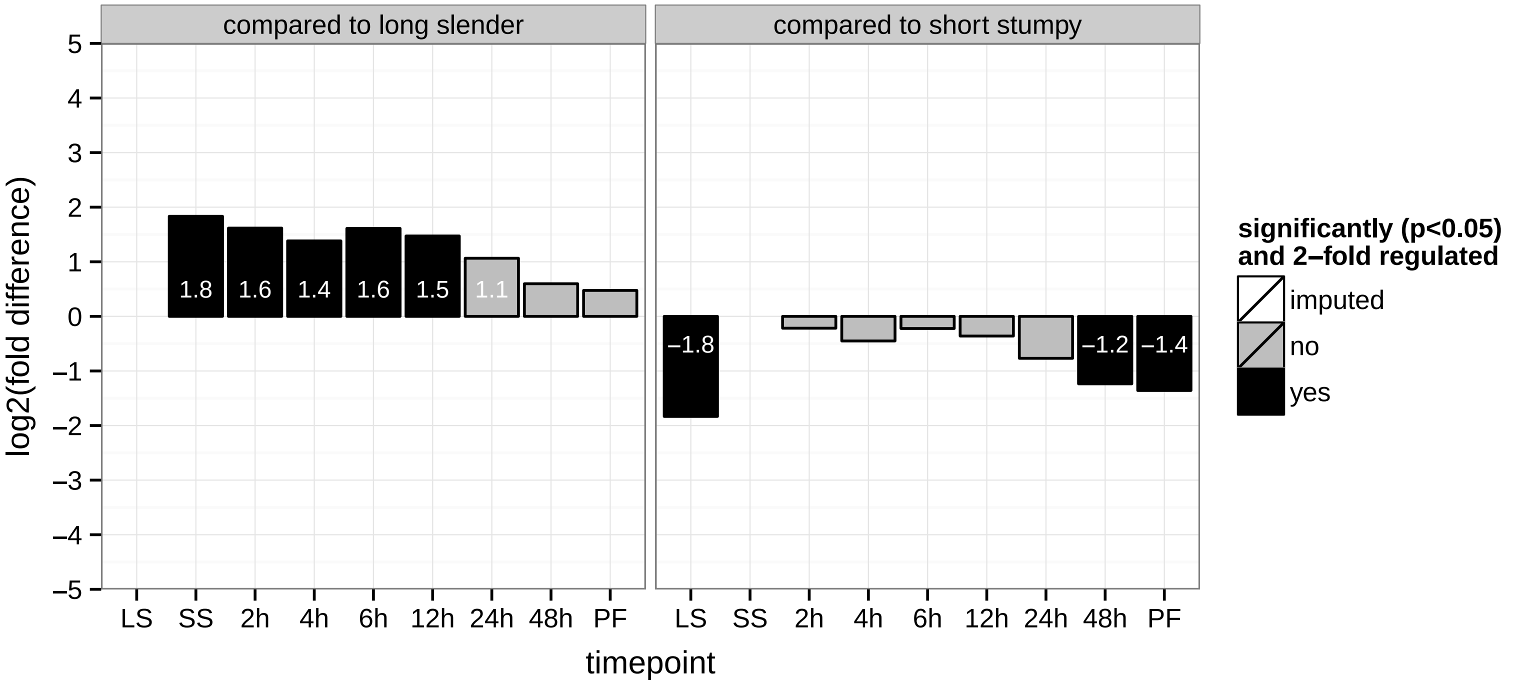

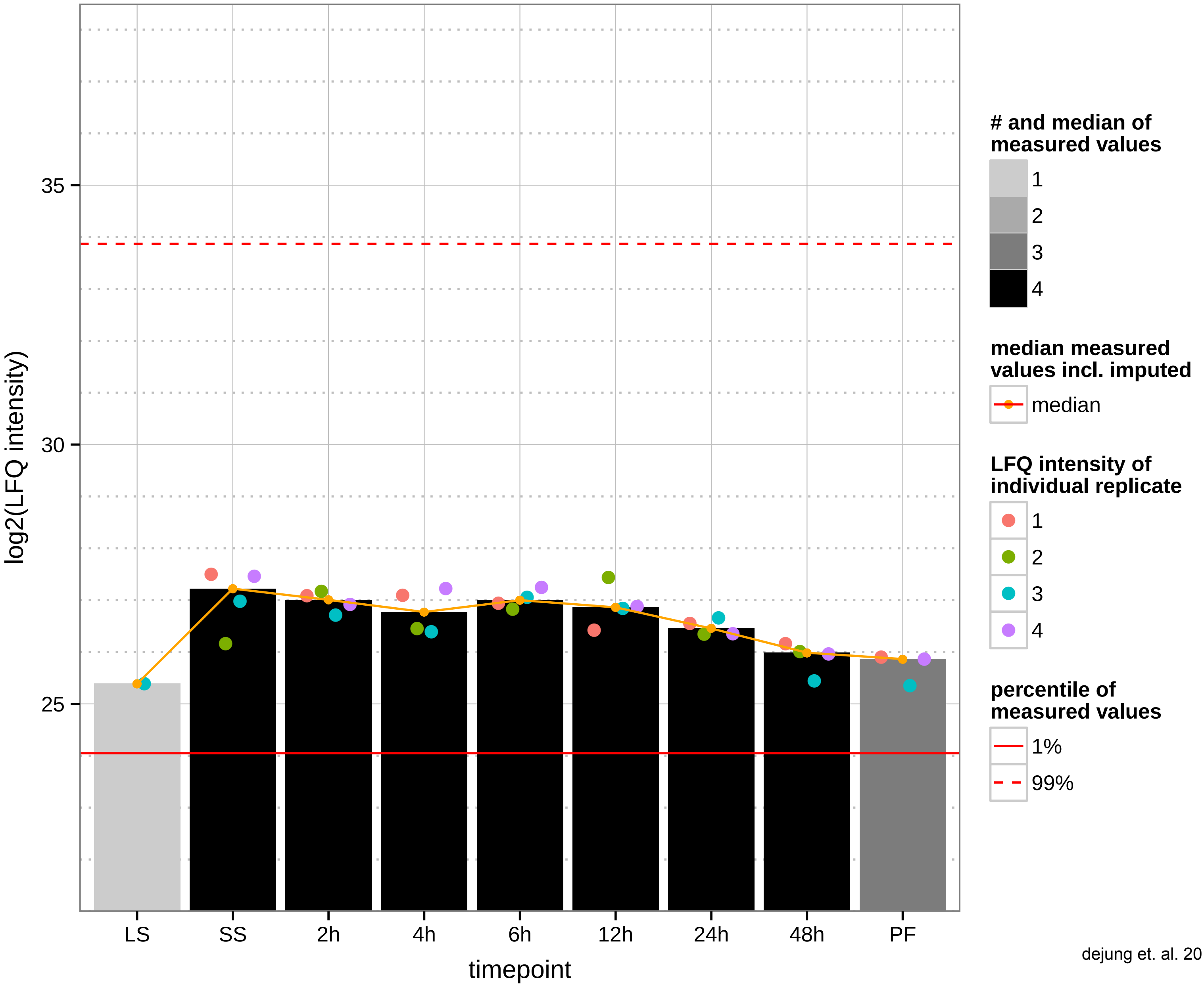

dejung et. al. 2015

hypothetical protein, conserved
Tb927.5.1160
AGOF: null
AGOC: null
AGOP: null
PGOF: null
PGOC: null
PGOP: null

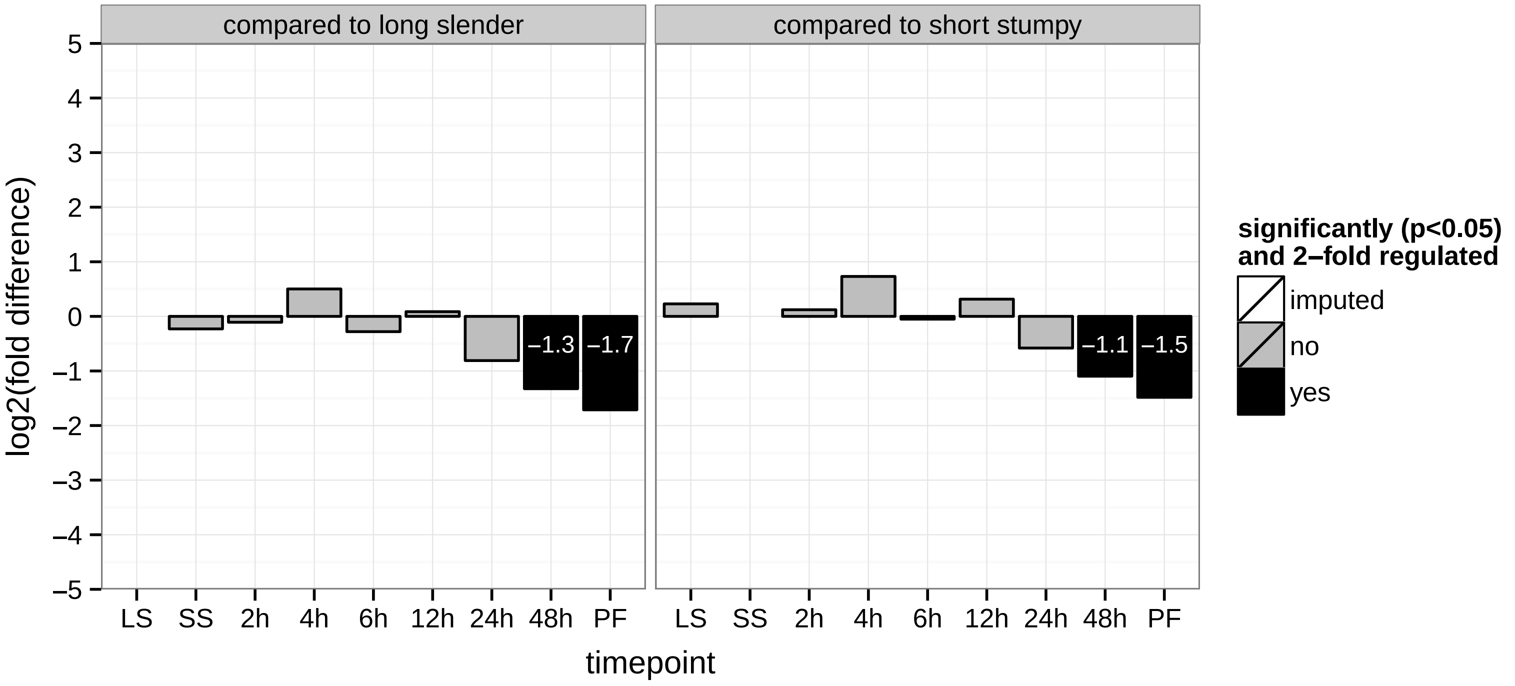

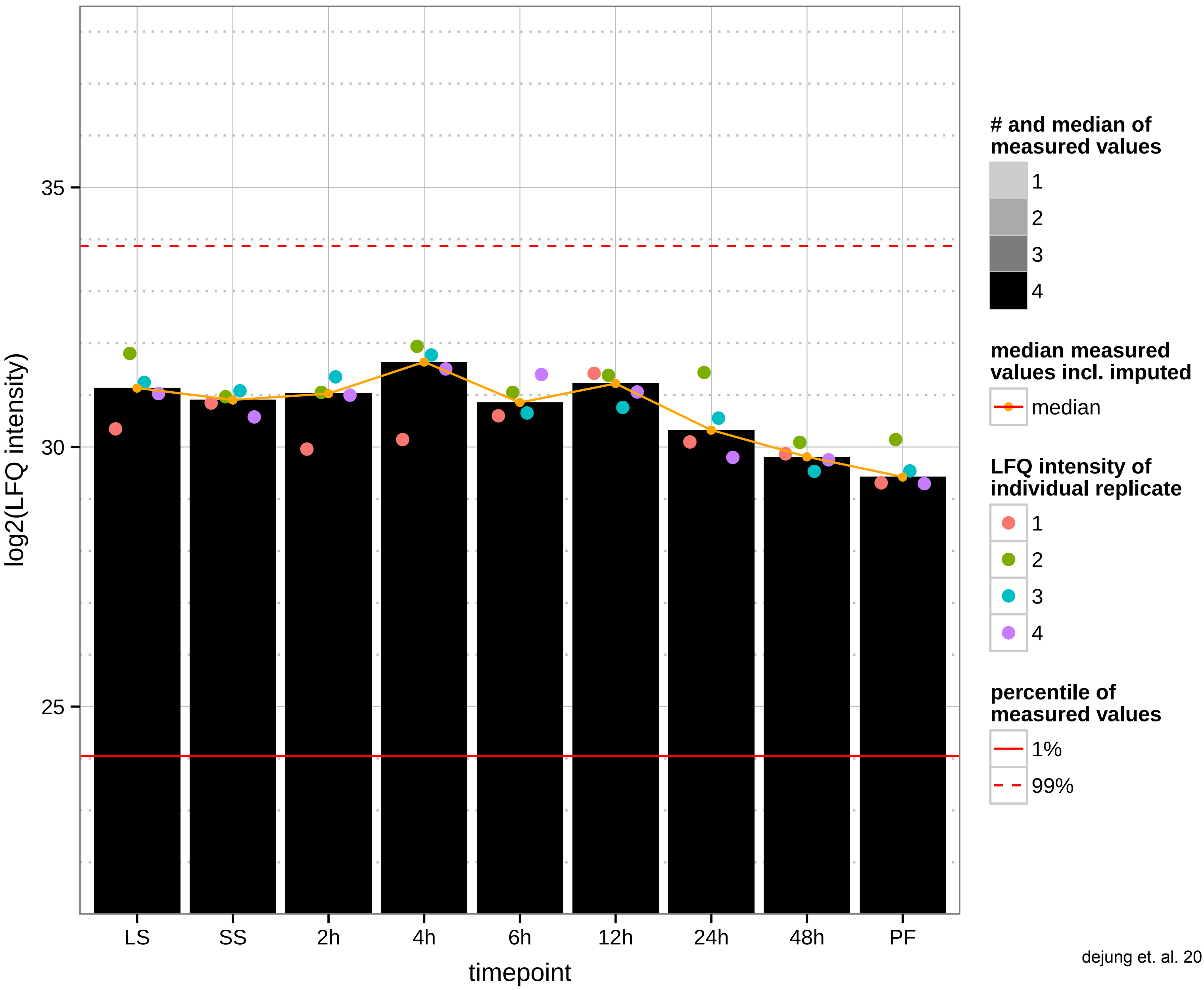

dejung et. al. 2015

hypothetical protein, conserved
Tb927.5.1260
AGOF: transporter activity
AGOC: integral to membrane
AGOP: transport
PGOF: sulfate transmembrane transporter activity
PGOC: integral to membrane
PGOP: sulfate transport

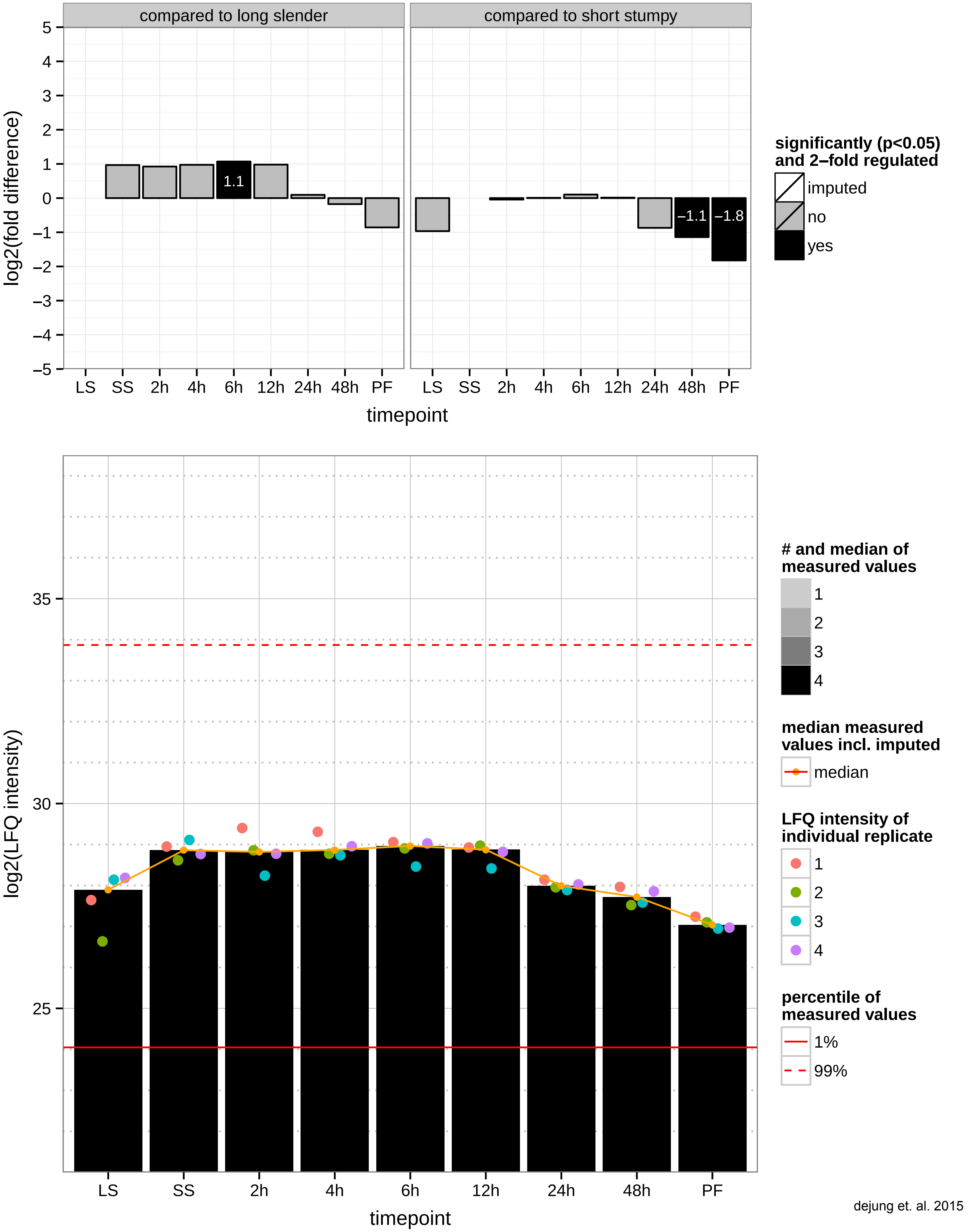

dejung et. al. 2015

inositol–1(or 4)–monophosphatase 1, putative (IMPase 1)
Tb927.5.2690
AGOF: null
AGOC: null
AGOP: null
PGOF: null
PGOC: null
PGOP: phosphatidylinositol phosphorylation

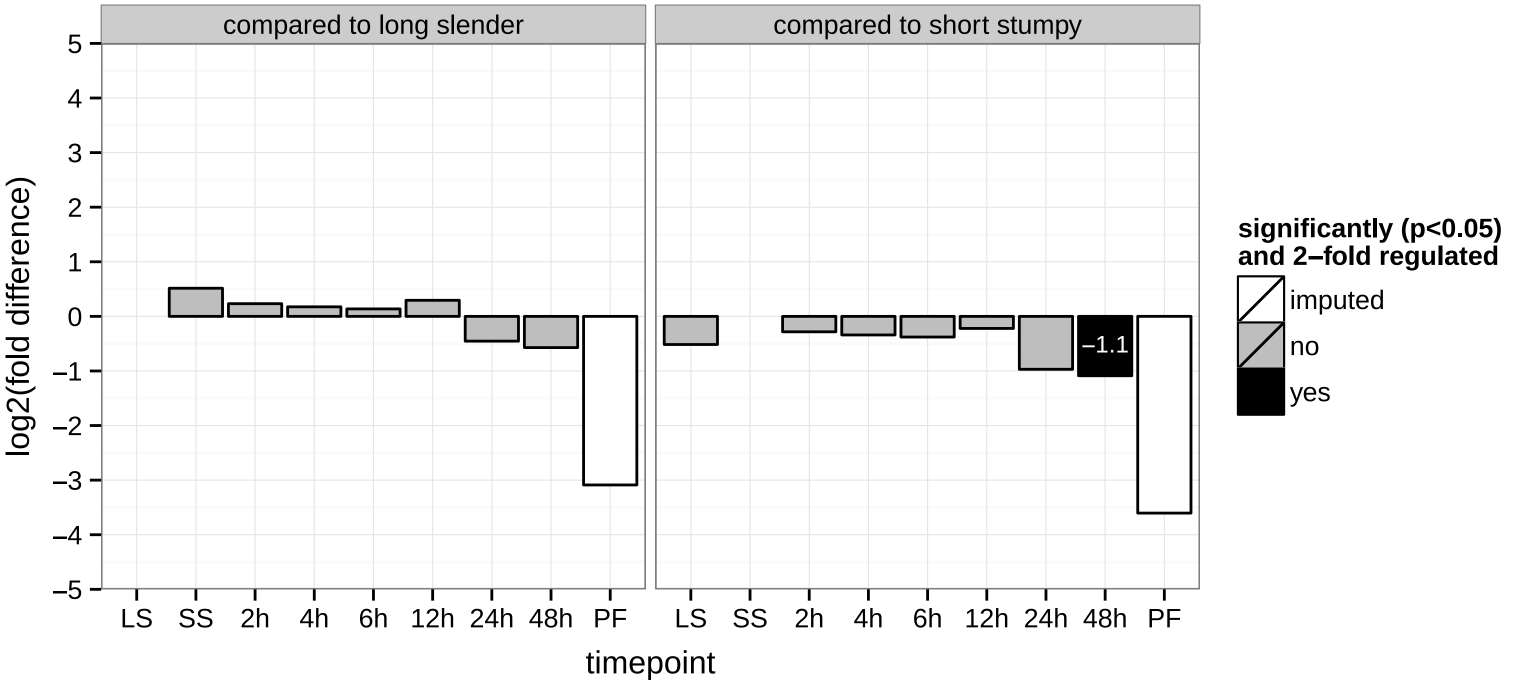

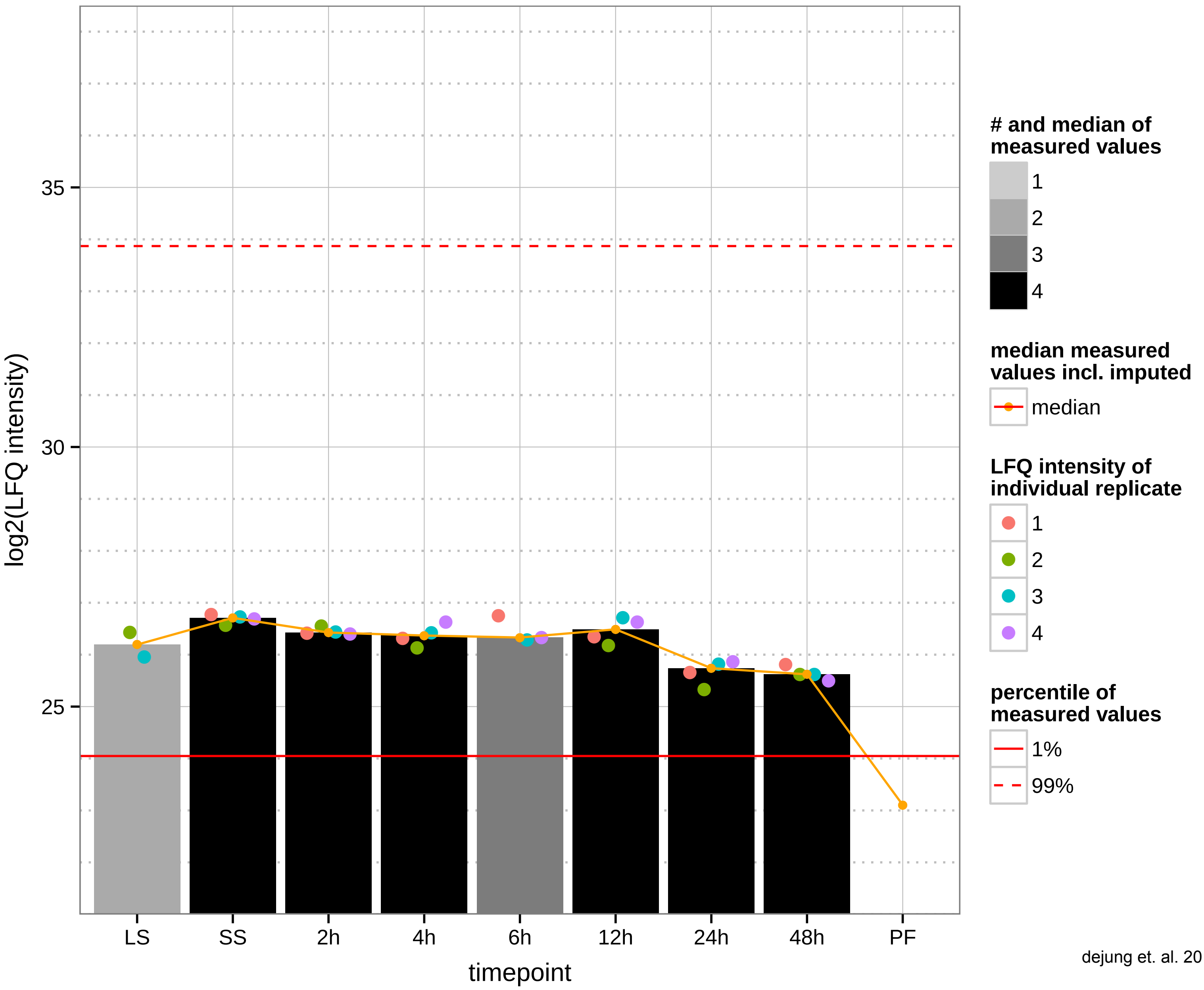

dejung et. al. 2015

vesicle–associated membrane protein, putative, synaptobrevin, putative (VAMP)
Tb927.5.3560
AGOF: null
AGOC: integral to membrane
AGOP: vesicle–mediated transport
PGOF: null
PGOC: integral to membrane
PGOP: transport, vesicle–mediated transport

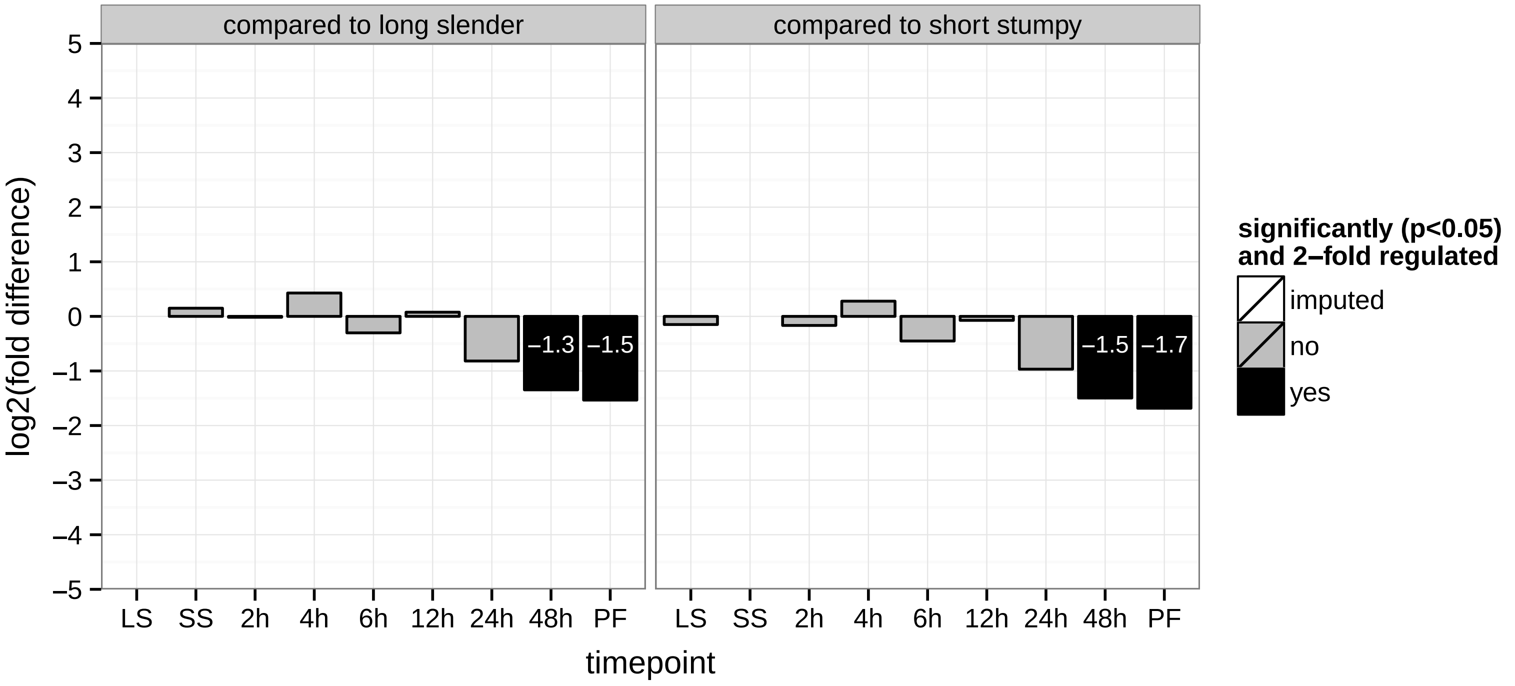

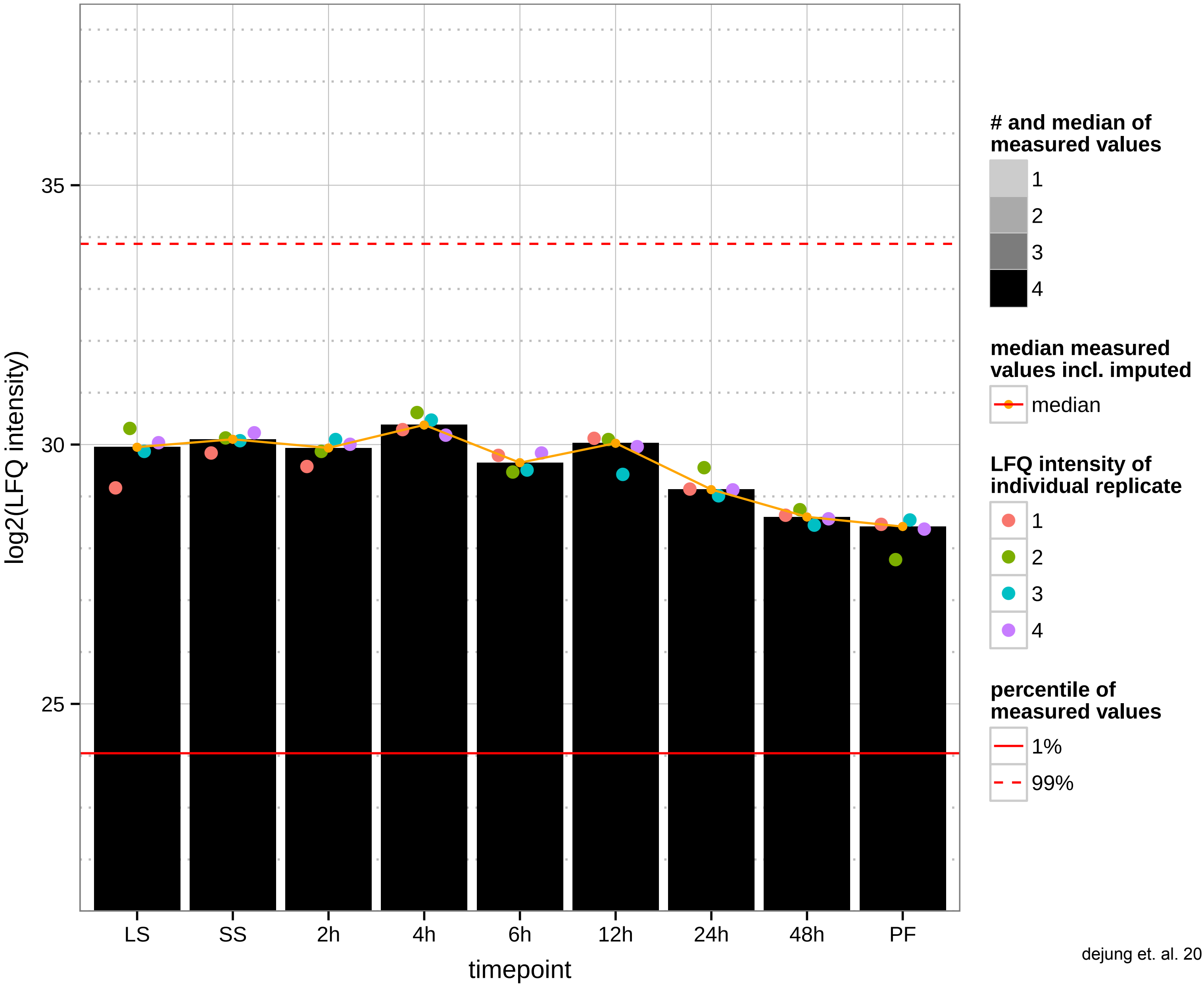

dejung et. al. 2015

cysteine peptidase, Clan CA, family C1, Cathepsin L–like (CP), Cathepsin L–like (Rhodesain)
Tb927.6.980;Tb927.6.970;Tb927.6.960;Tb927.6.1020;Tb927.6.1010
AGOF: cysteine–type peptidase activity
AGOC: null
AGOP: proteolysis
PGOF: cysteine–type endopeptidase activity, cysteine–type peptidase activity, null
PGOC: null
PGOP: proteolysis, null

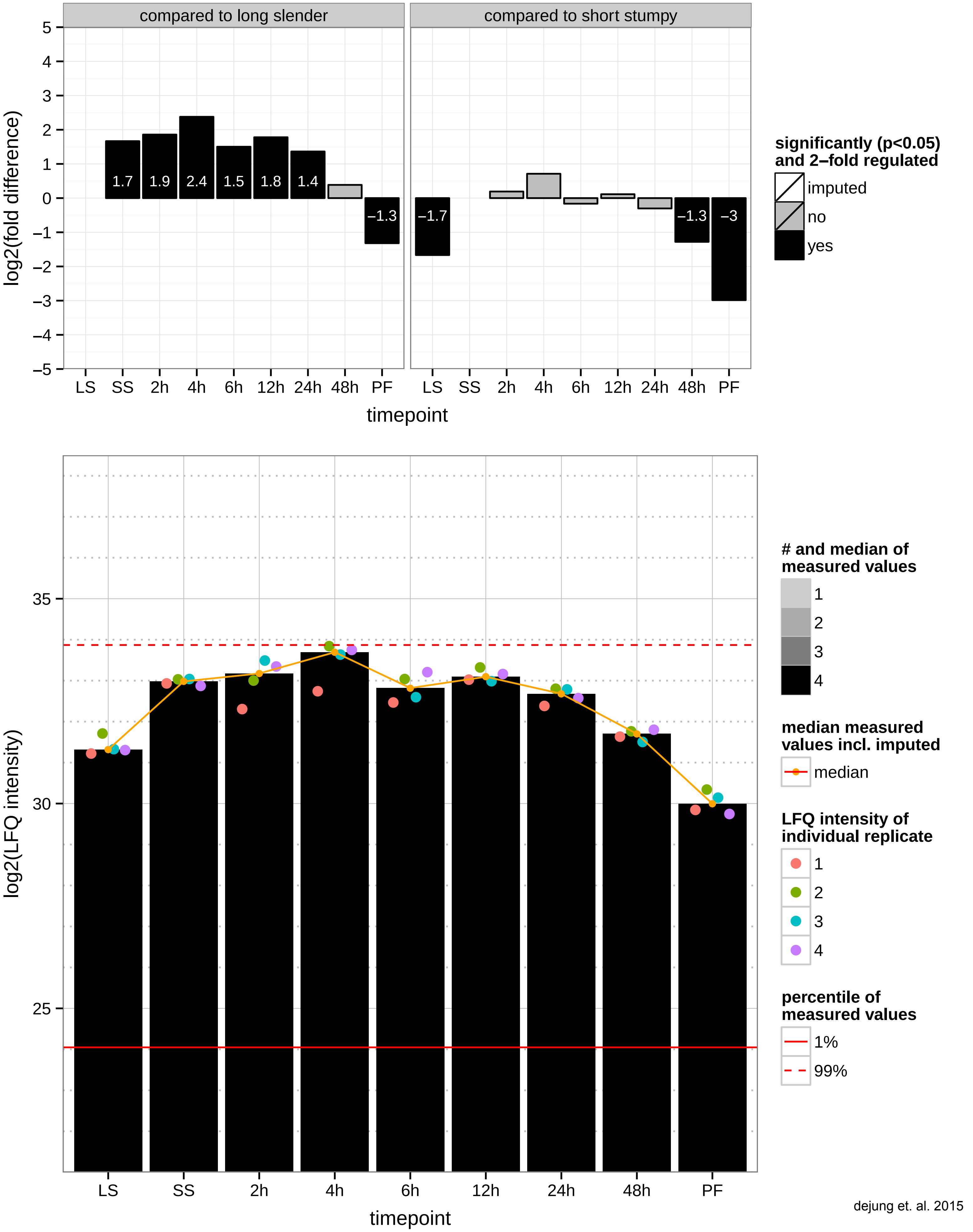

dejung et. al. 2015

cyclin 3, mitotic cyclin, putative (CYC3)
Tb927.6.1460
AGOF: molecular_function
AGOC: null
AGOP: G1/S transition of mitotic cell cycle
PGOF: null
PGOC: null
PGOP: null

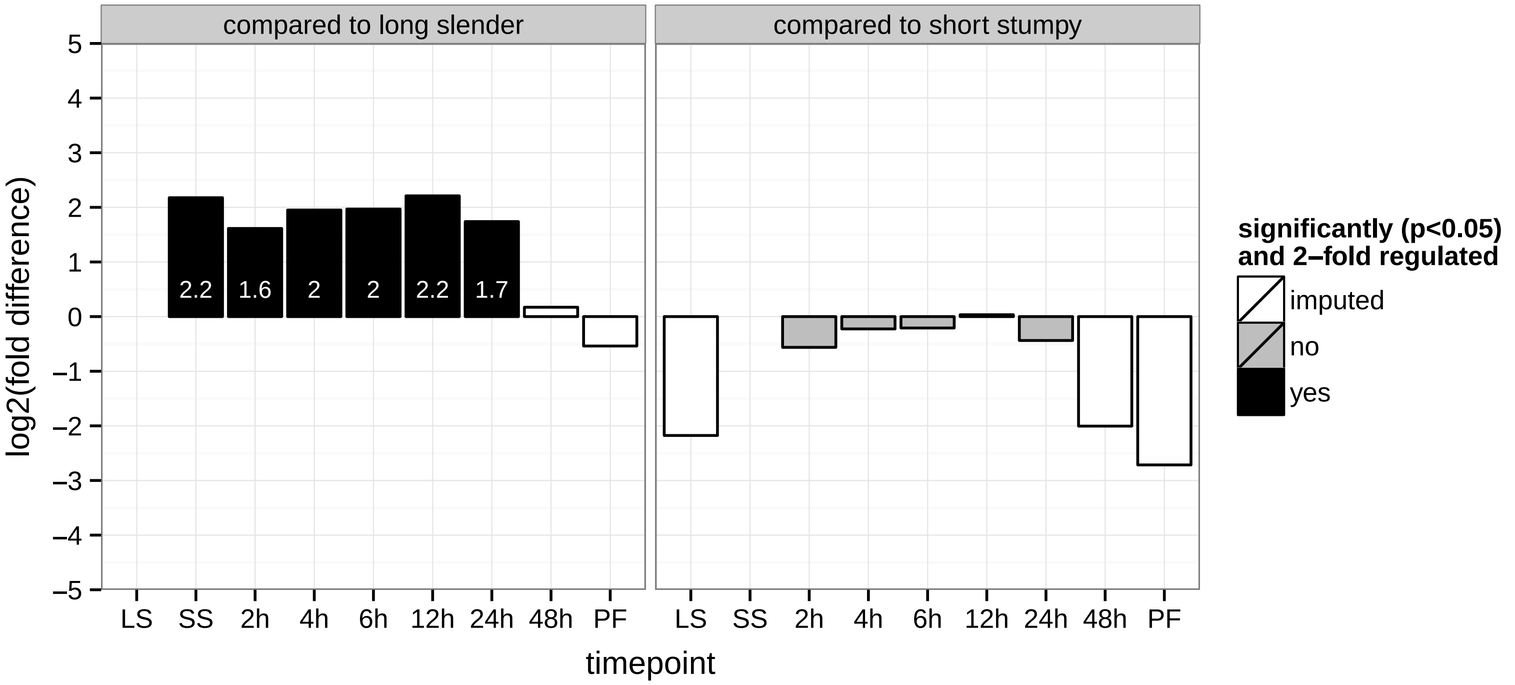

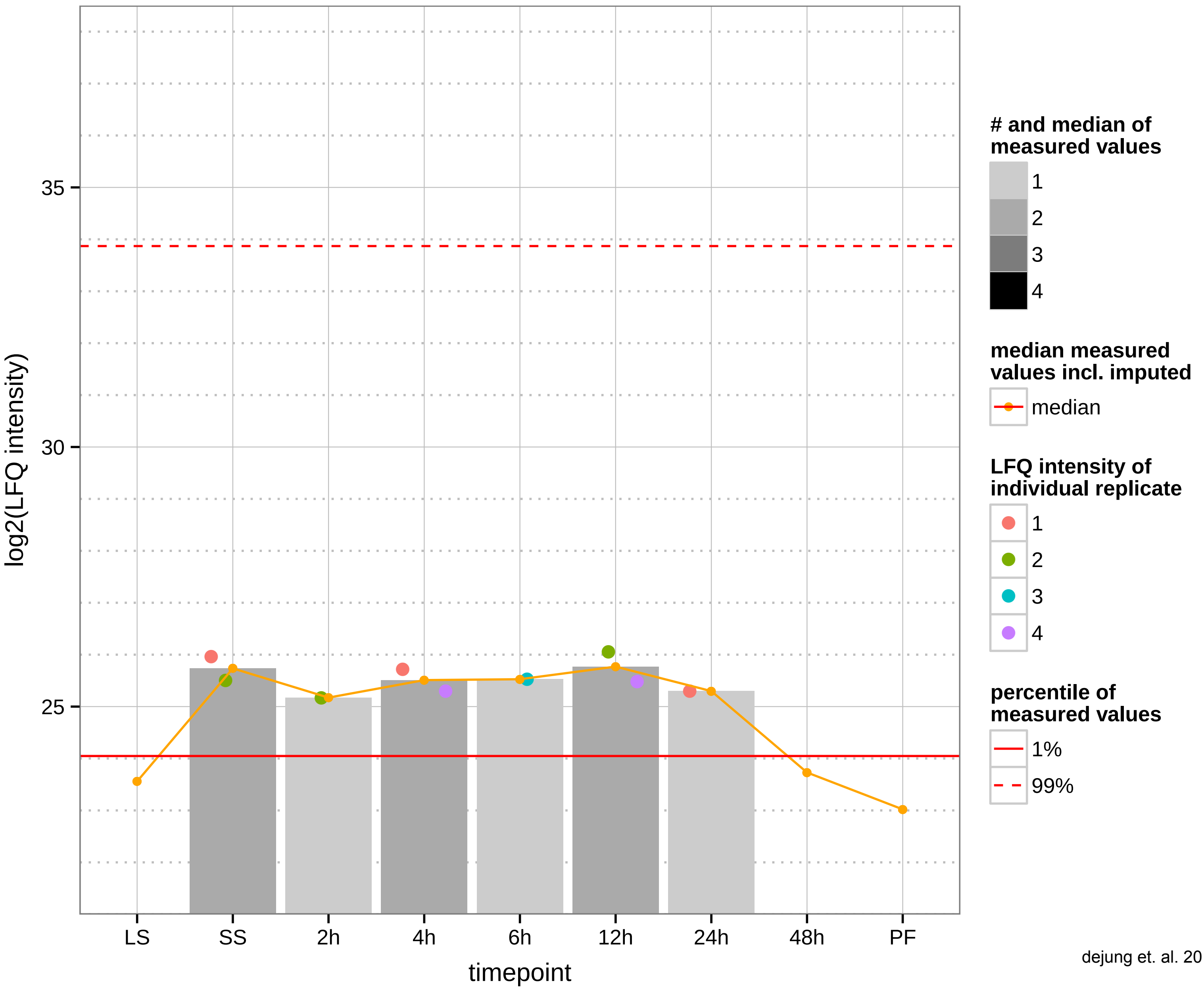

dejung et. al. 2015

hypothetical protein, conserved
Tb927.6.1530
AGOF: null
AGOC: null
AGOP: null
PGOF: null
PGOC: null
PGOP: null

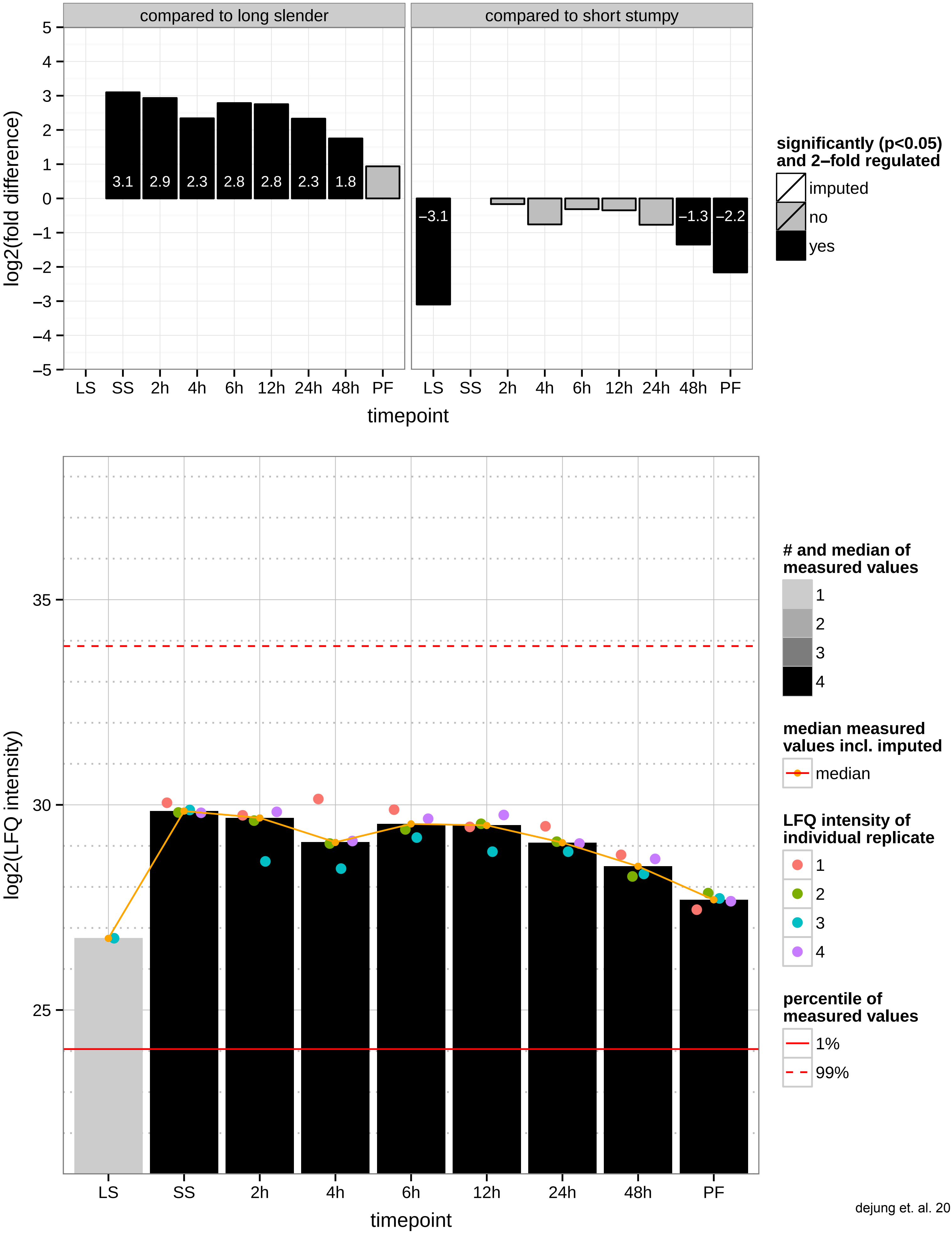

dejung et. al. 2015

hypothetical protein, conserved
Tb927.6.3240
AGOF: null
AGOC: null
AGOP: null
PGOF: null
PGOC: null
PGOP: null

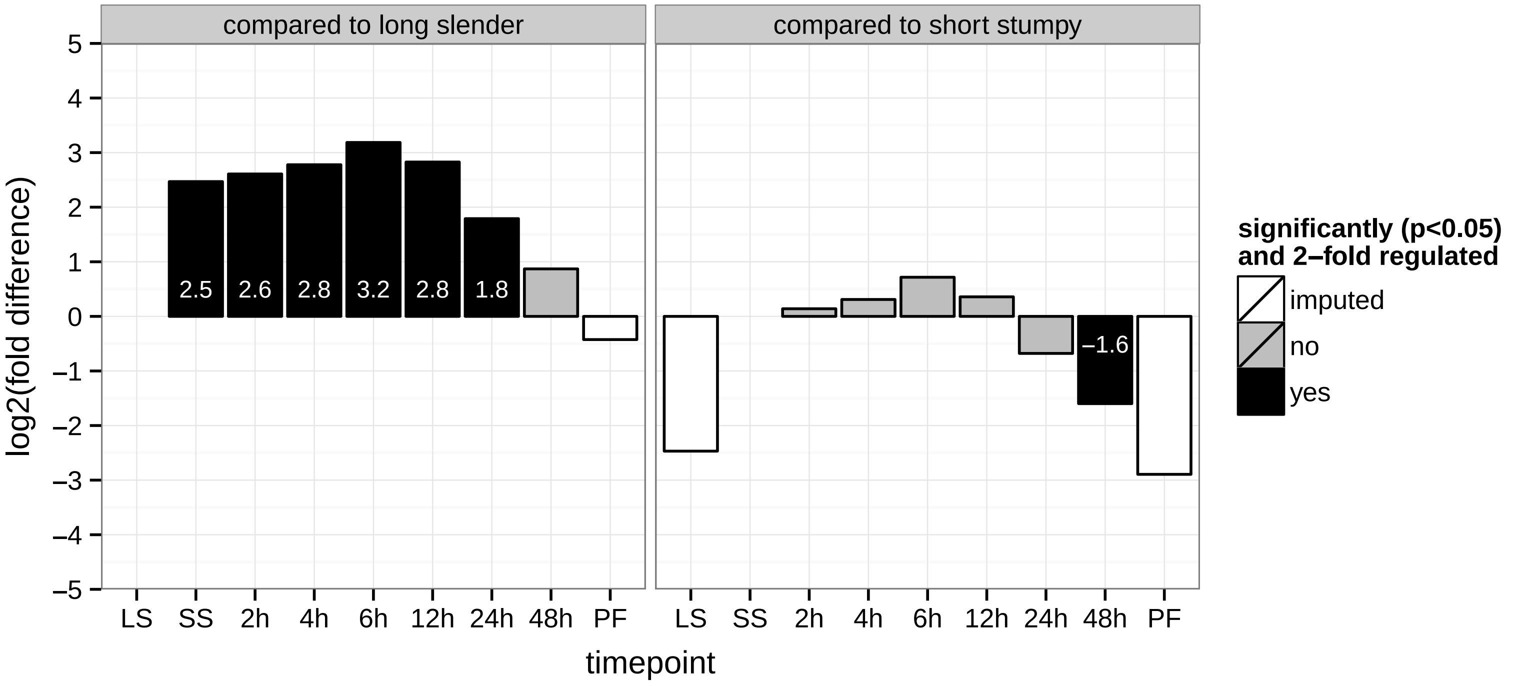

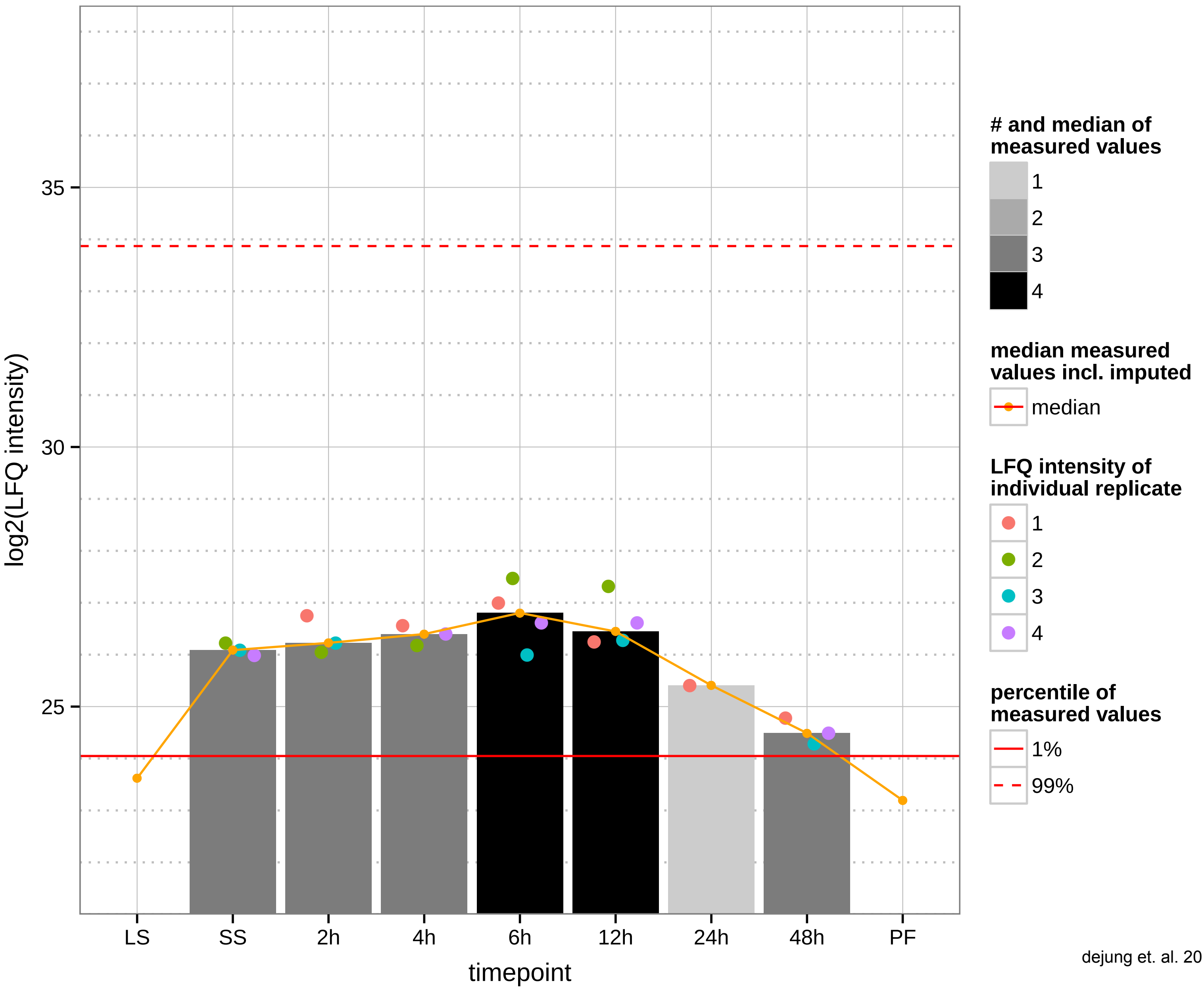

dejung et. al. 2015

hypothetical protein, conserved
Tb927.6.3720
AGOF: null
AGOC: null
AGOP: null
PGOF: protein binding
PGOC: null
PGOP: null

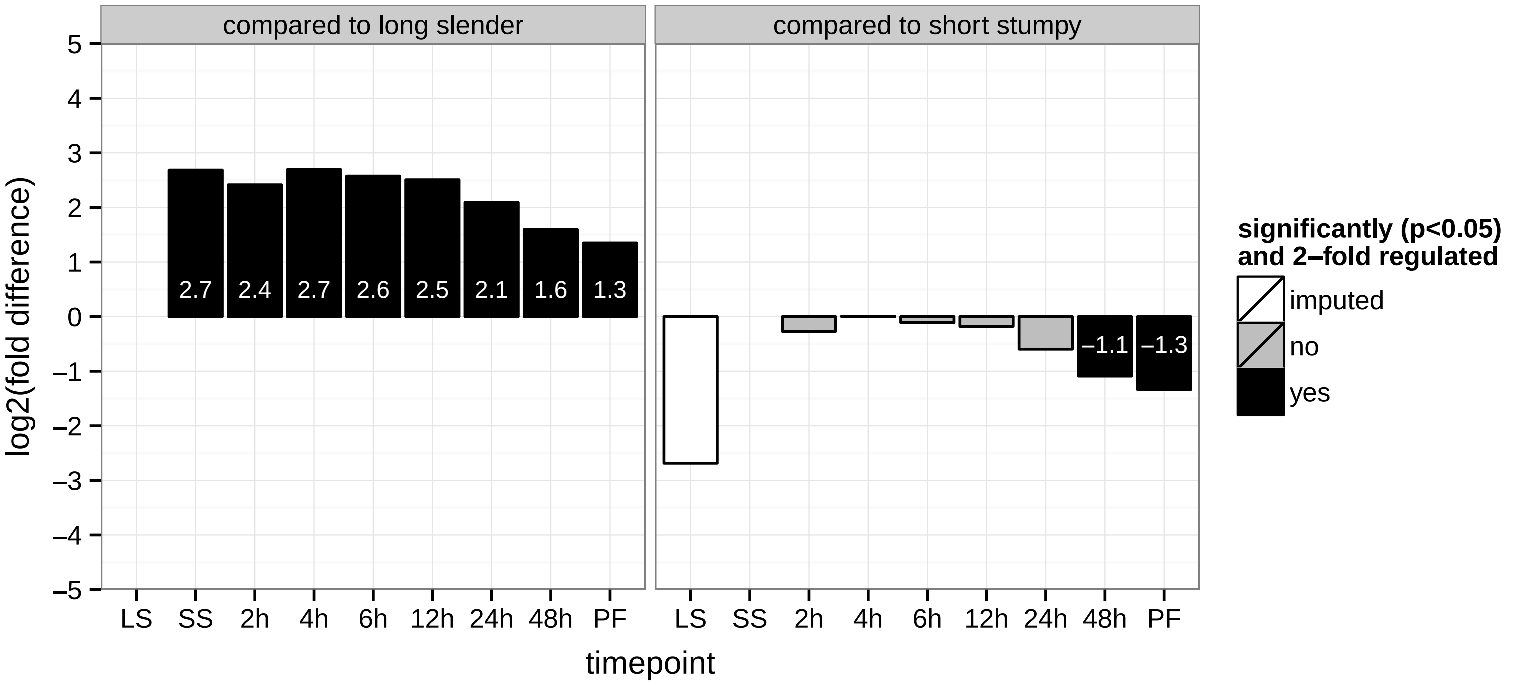

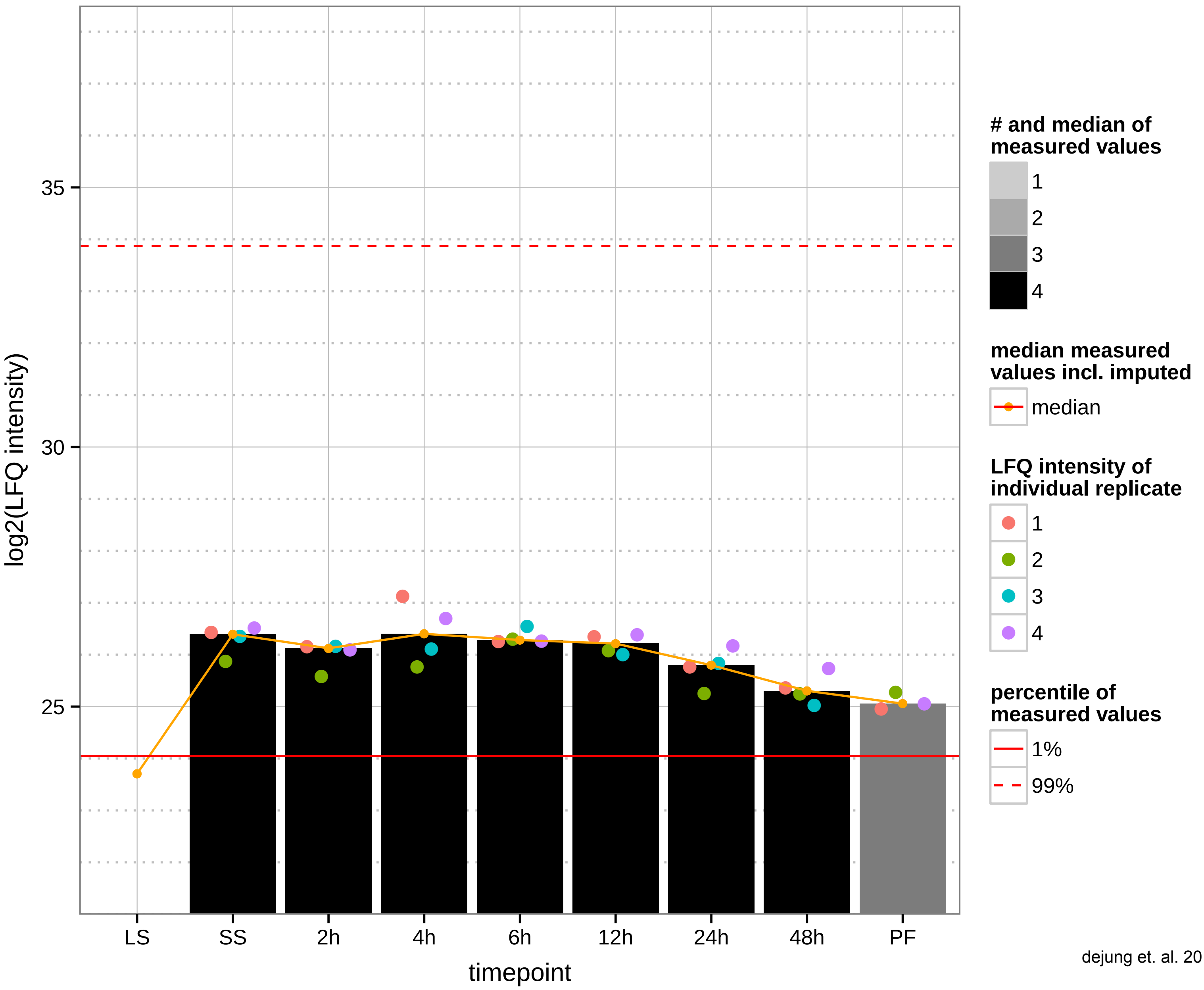

dejung et. al. 2015

hypothetical protein, conserved
Tb927.6.4550
AGOF: null
AGOC: null
AGOP: null
PGOF: null
PGOC: null
PGOP: null

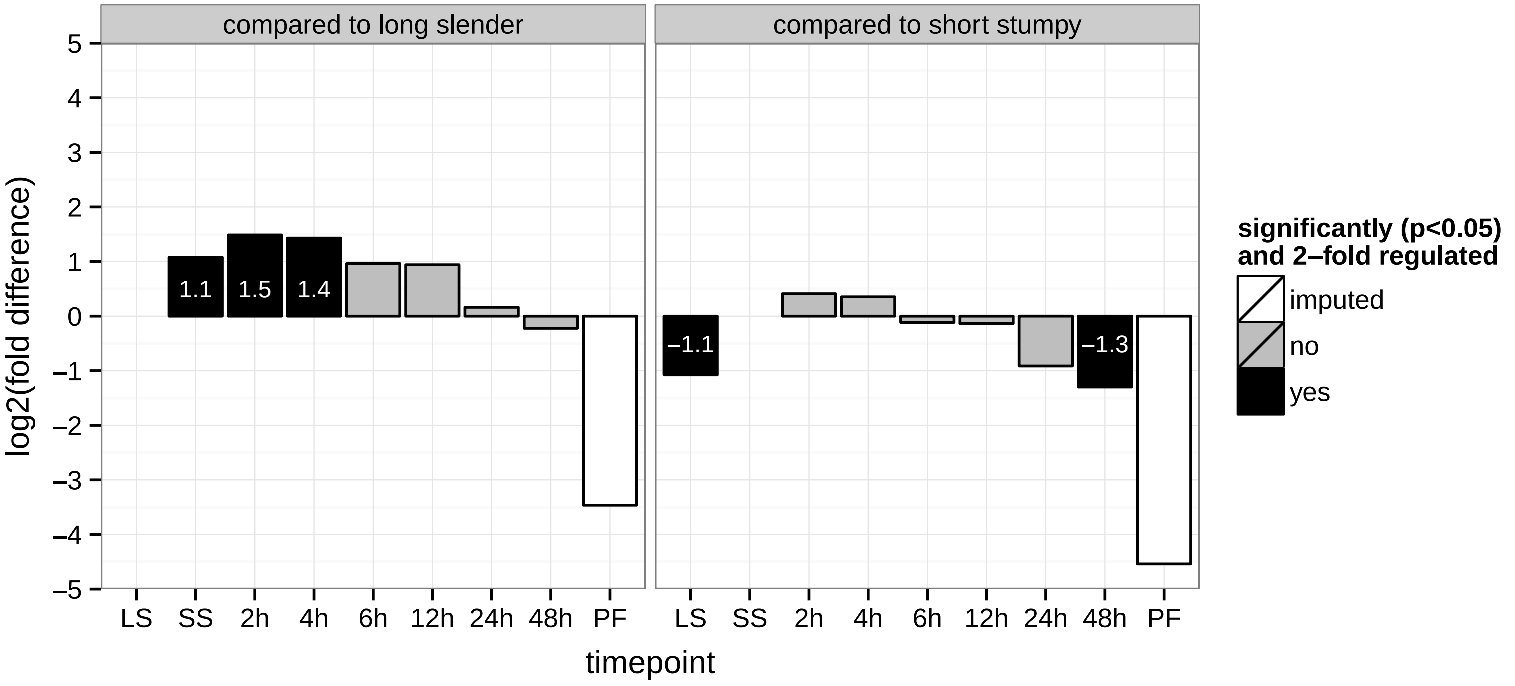

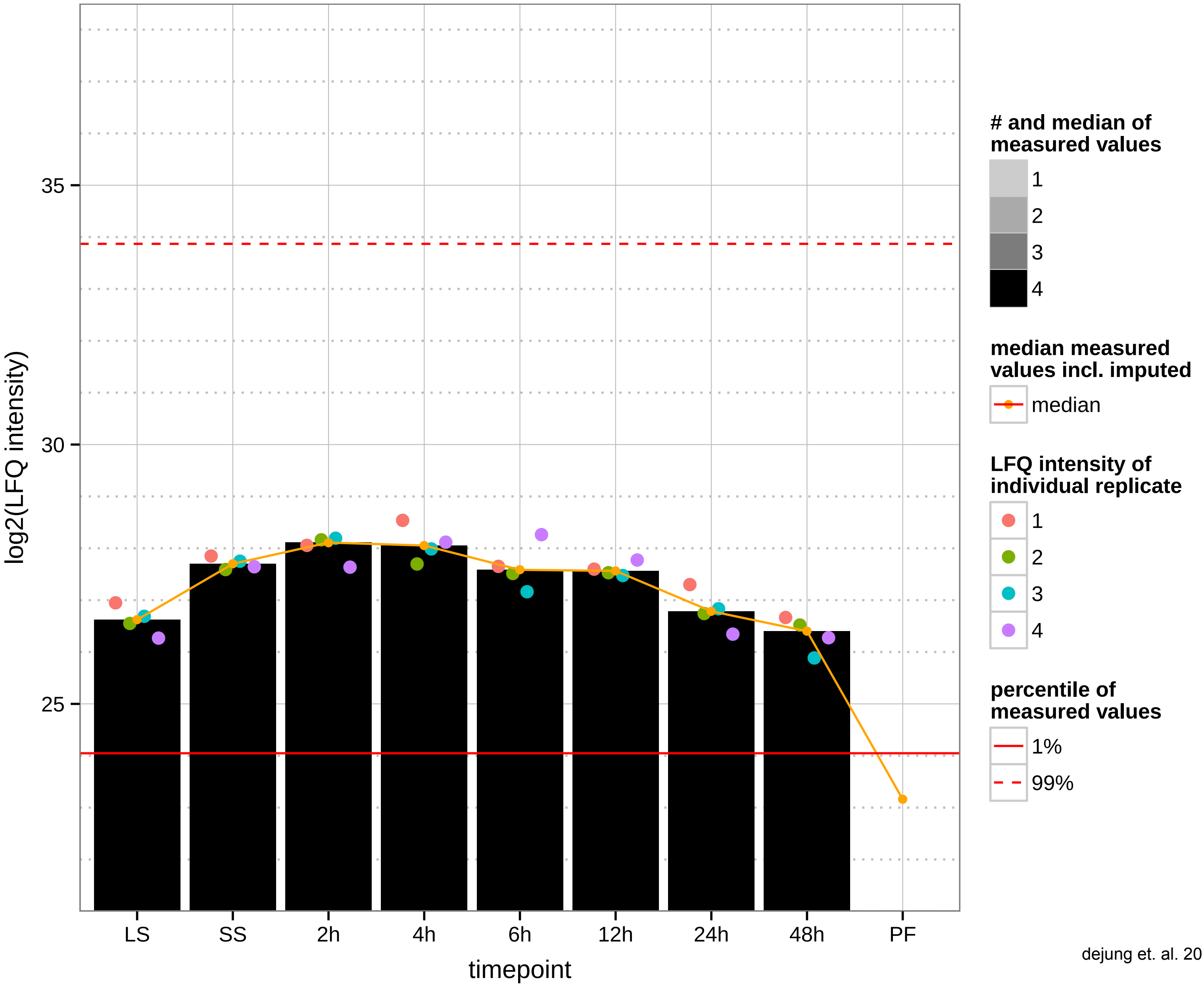

dejung et. al. 2015

C–1–tetrahydrofolate synthase, cytoplasmic, putative
Tb927.7.1600
AGOF: catalytic activity
AGOC: null
AGOP: folic acid–containing compound biosynthetic process
PGOF: catalytic activity, methylenetetrahydrofolate dehydrogenase (NADP+) activity
PGOC: null
PGOP: folic acid–containing compound biosynthetic process, oxidation–reduction process

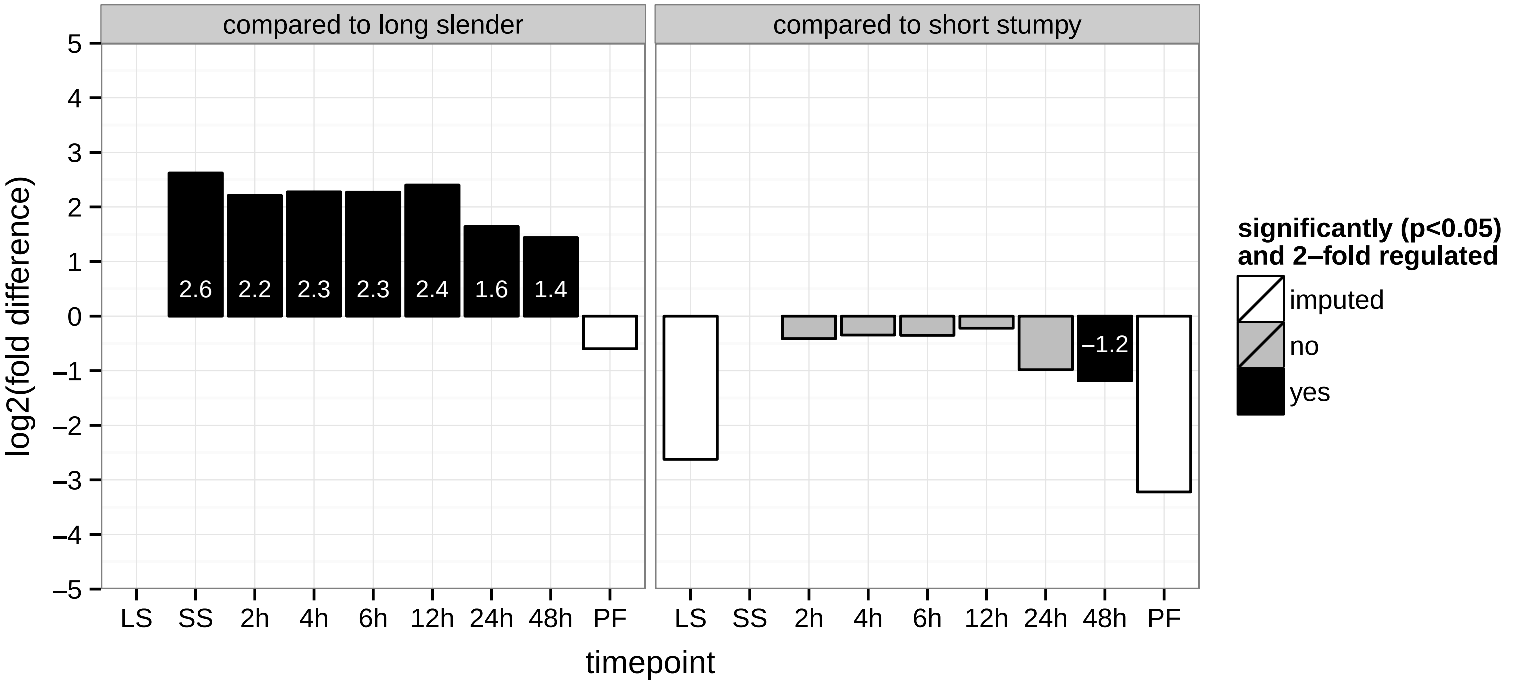

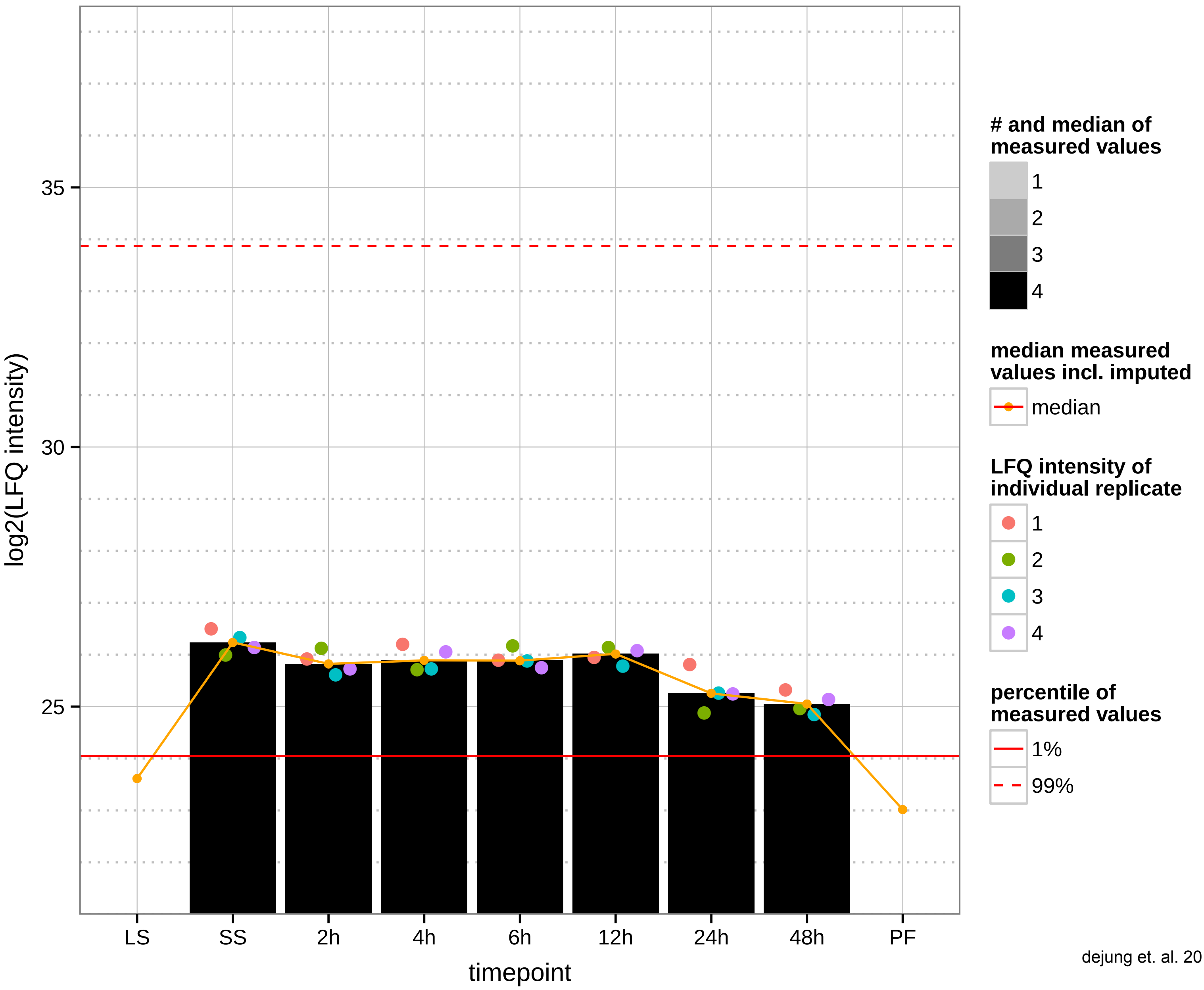

dejung et. al. 2015

hypothetical protein, conserved
Tb927.7.2650
AGOF: null
AGOC: null
AGOP: null
PGOF: null
PGOC: null
PGOP: null

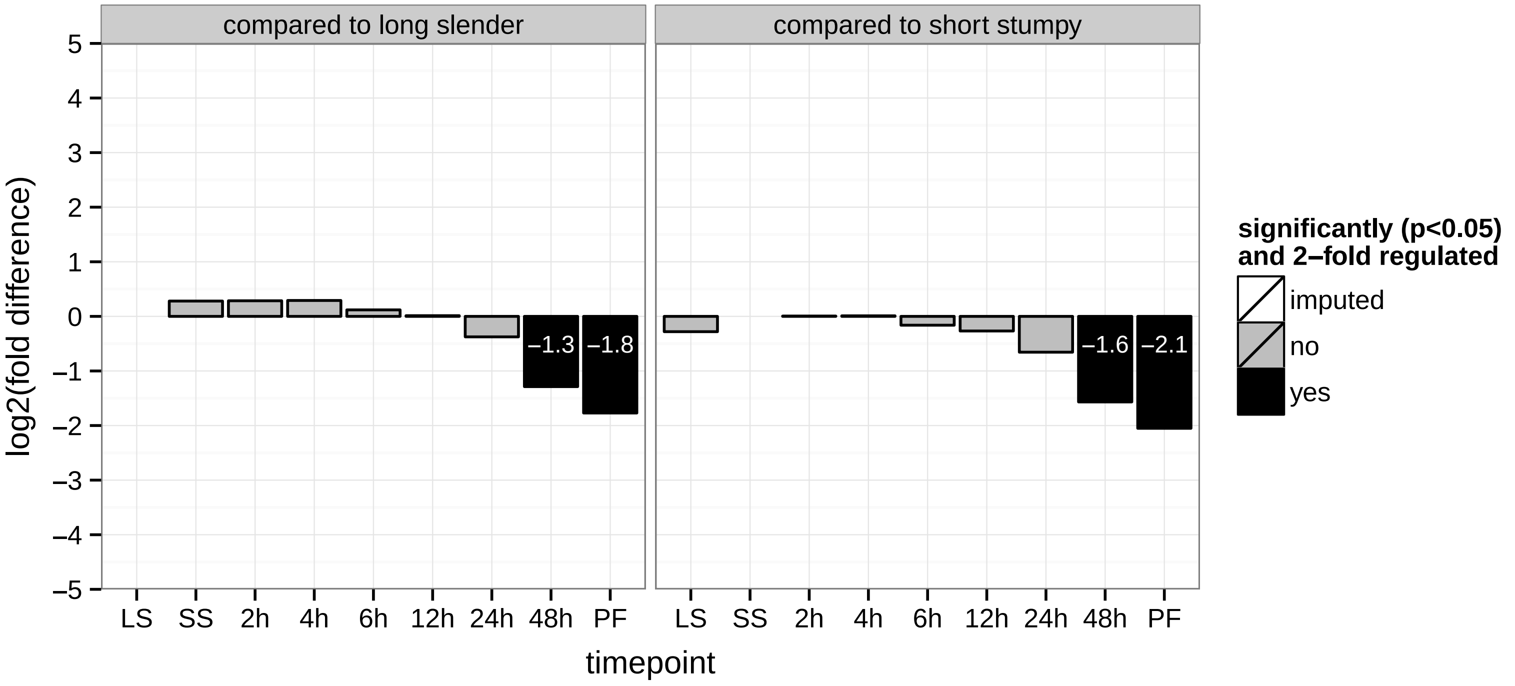

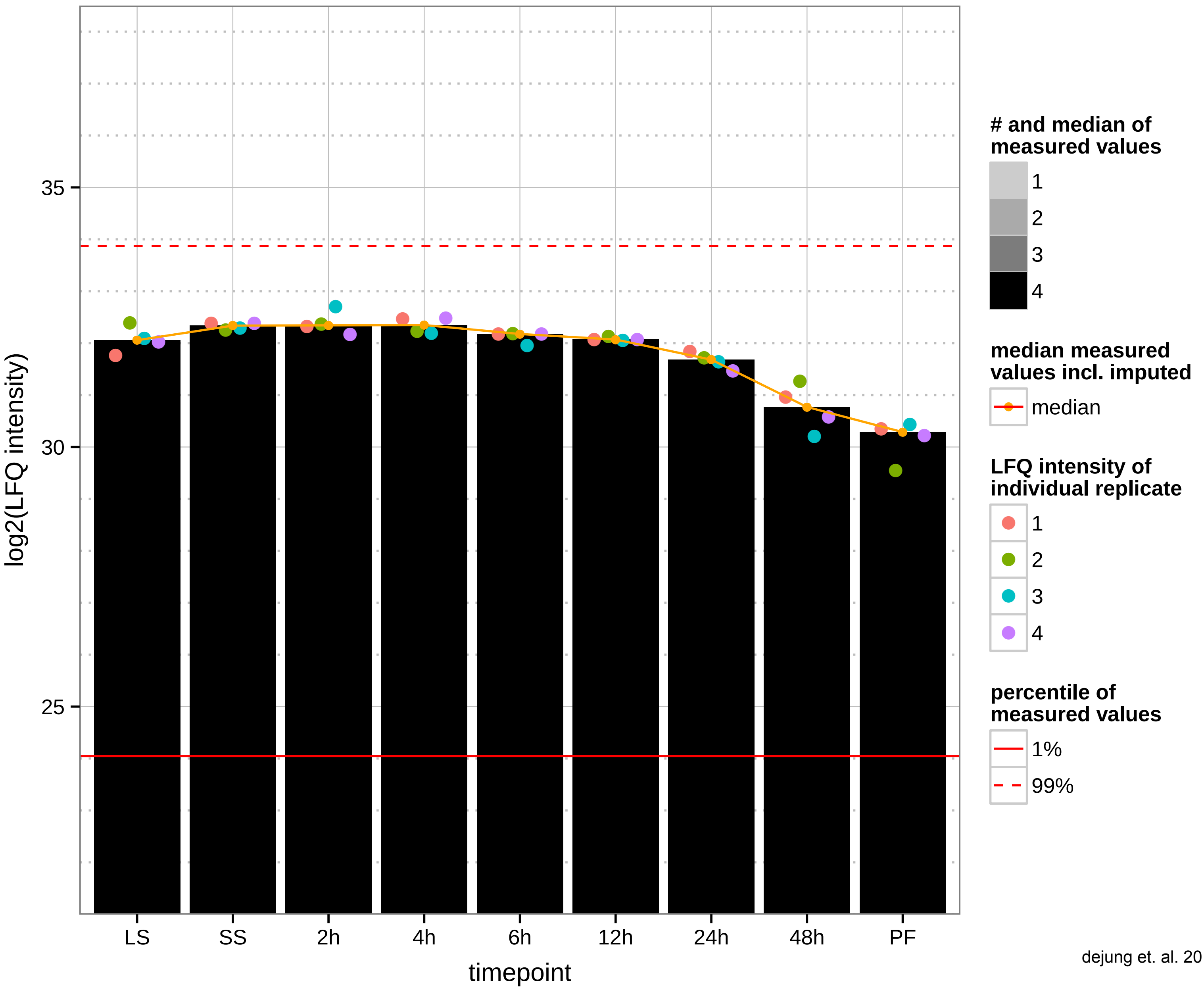

dejung et. al. 2015

hypothetical protein, conserved
Tb927.7.510;Tb11.v5.0890
AGOF: null
AGOC: null
AGOP: null
PGOF: null
PGOC: null
PGOP: null

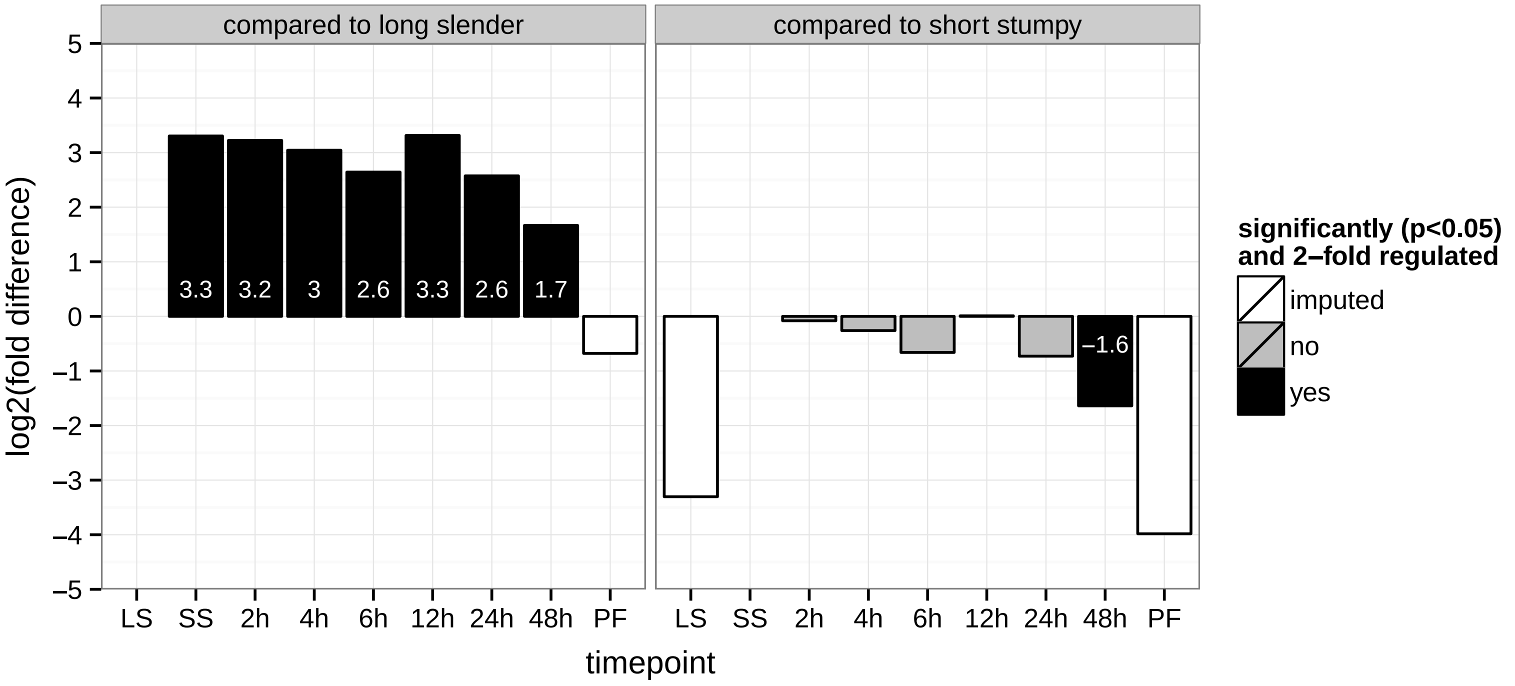

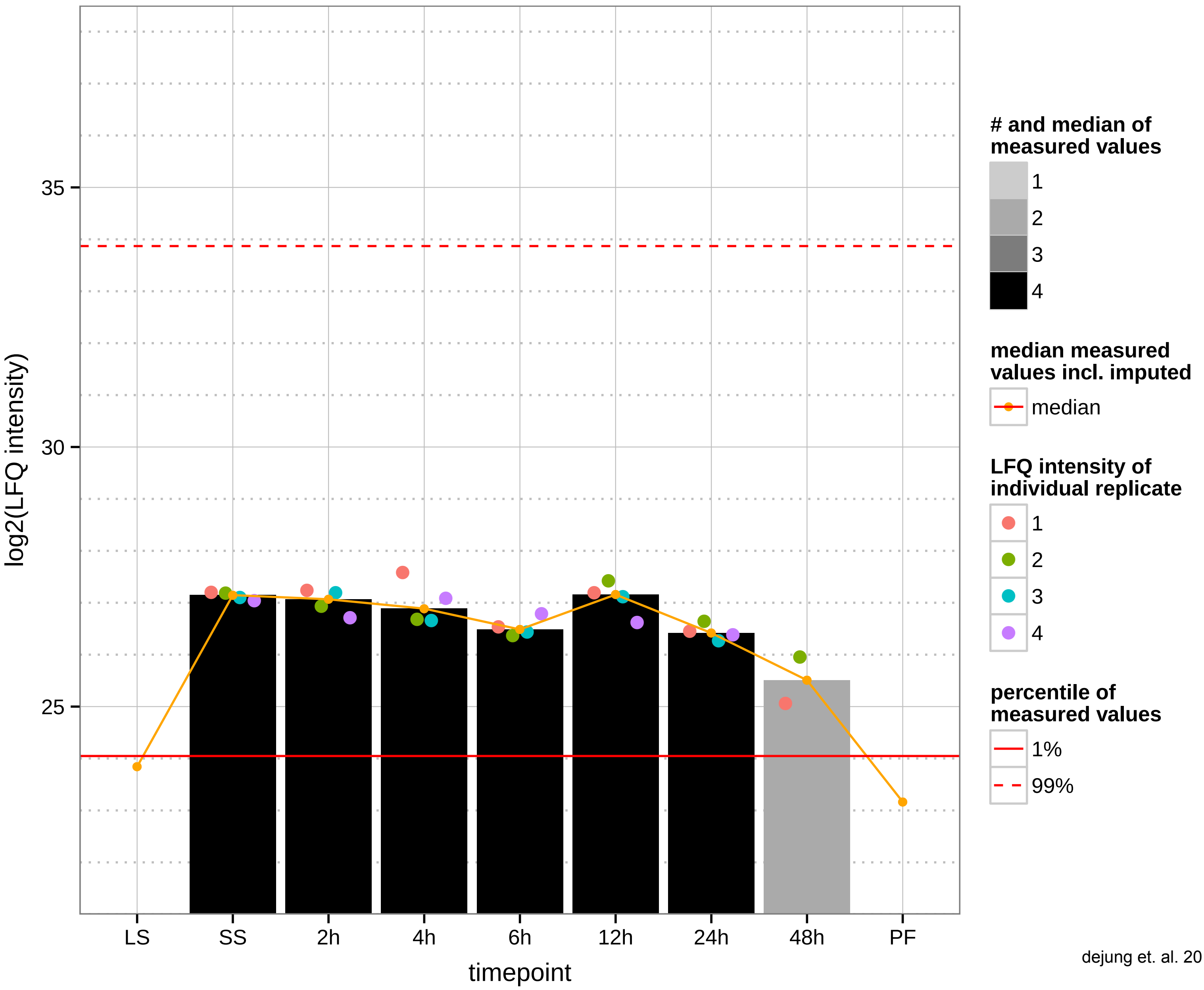

dejung et. al. 2015

hypothetical protein, conserved
Tb927.7.530
AGOF: zinc ion binding
AGOC: null
AGOP: null
PGOF: metal ion binding
PGOC: null
PGOP: null

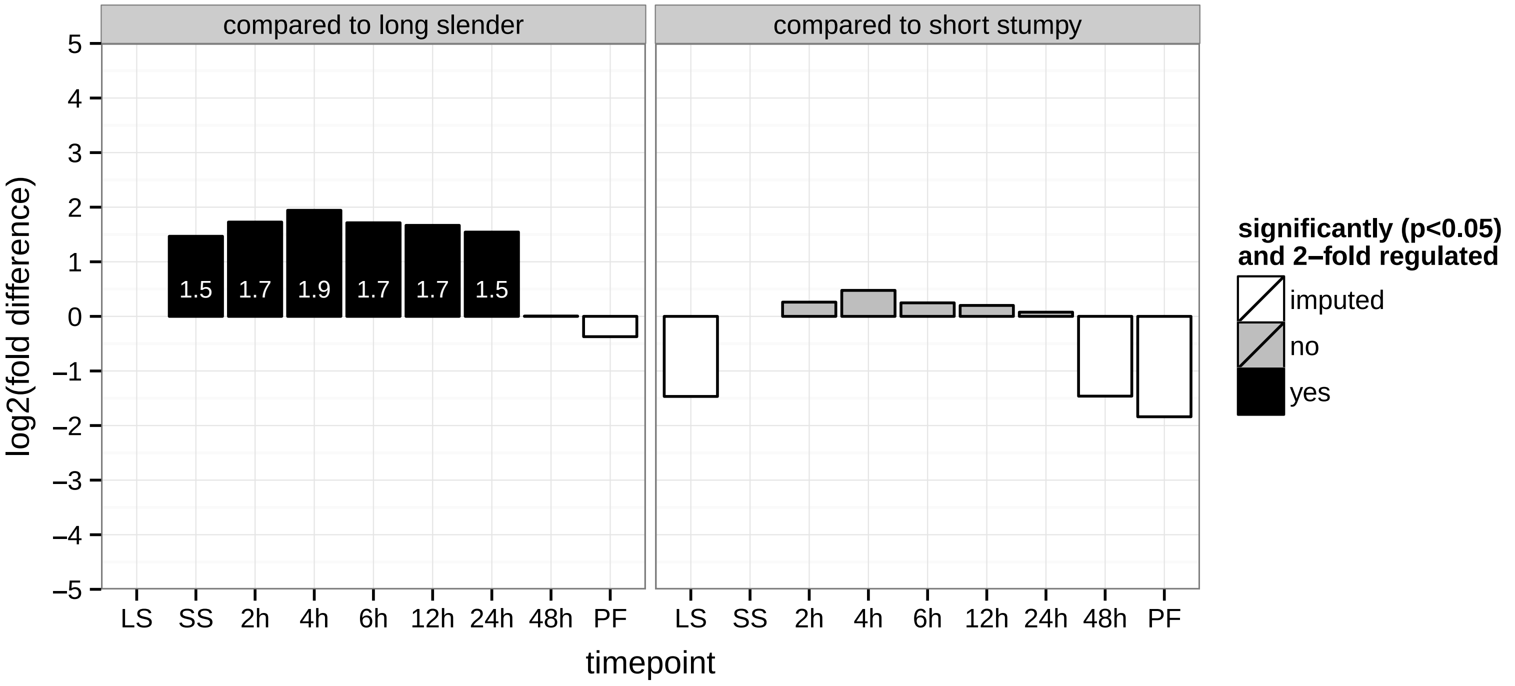

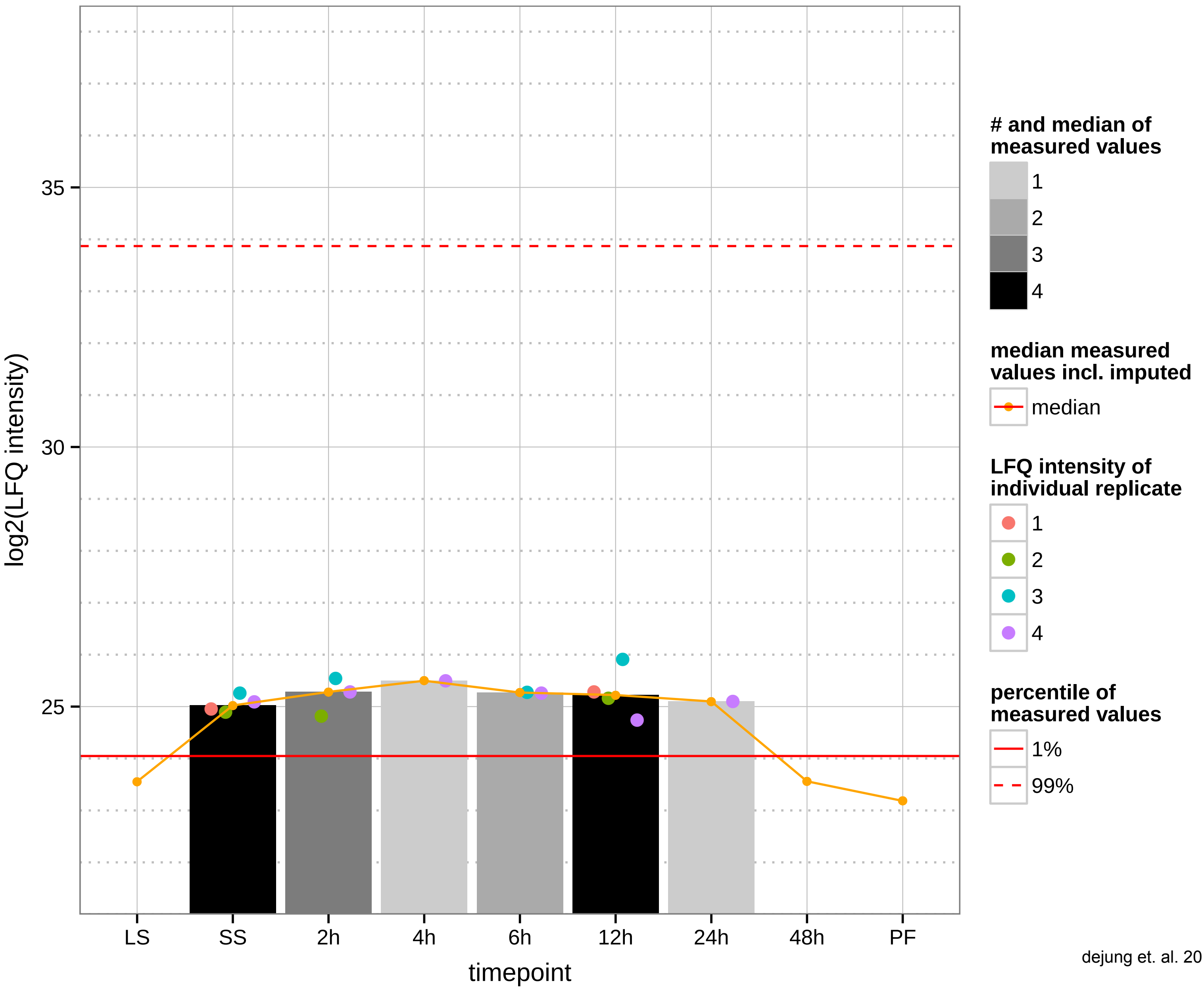

dejung et. al. 2015

hypothetical protein, conserved
Tb927.8.2080
AGOF: null
AGOC: null
AGOP: null
PGOF: ATP binding, RNA ligase (ATP) activity
PGOC: null
PGOP: tRNA splicing, via endonucleolytic cleavage and ligation

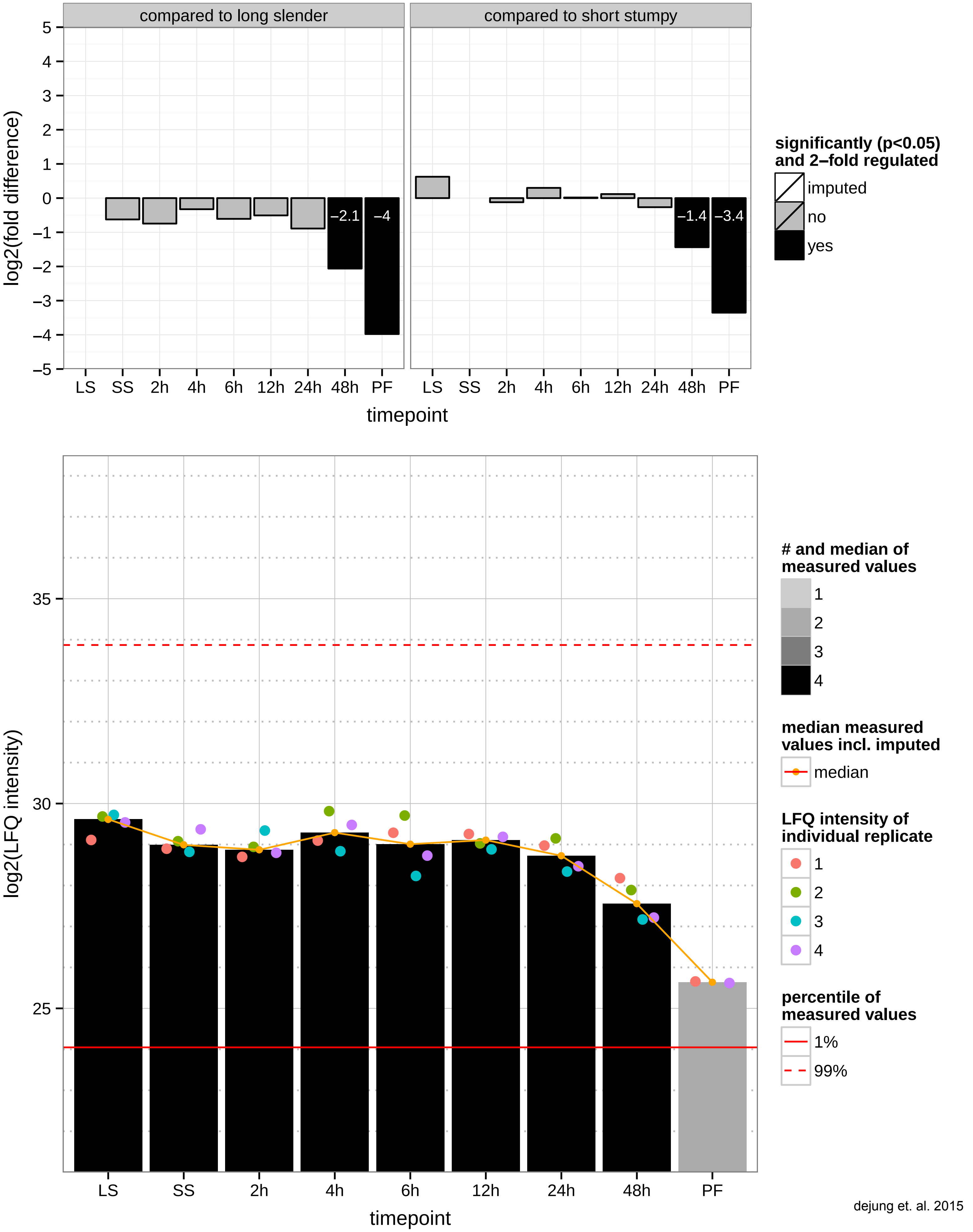

hypothetical protein, conserved
Tb927.8.2250
AGOF: null
AGOC: null
AGOP: null
PGOF: ATP binding, RNA ligase (ATP) activity
PGOC: null
PGOP: tRNA splicing, via endonucleolytic cleavage and ligation

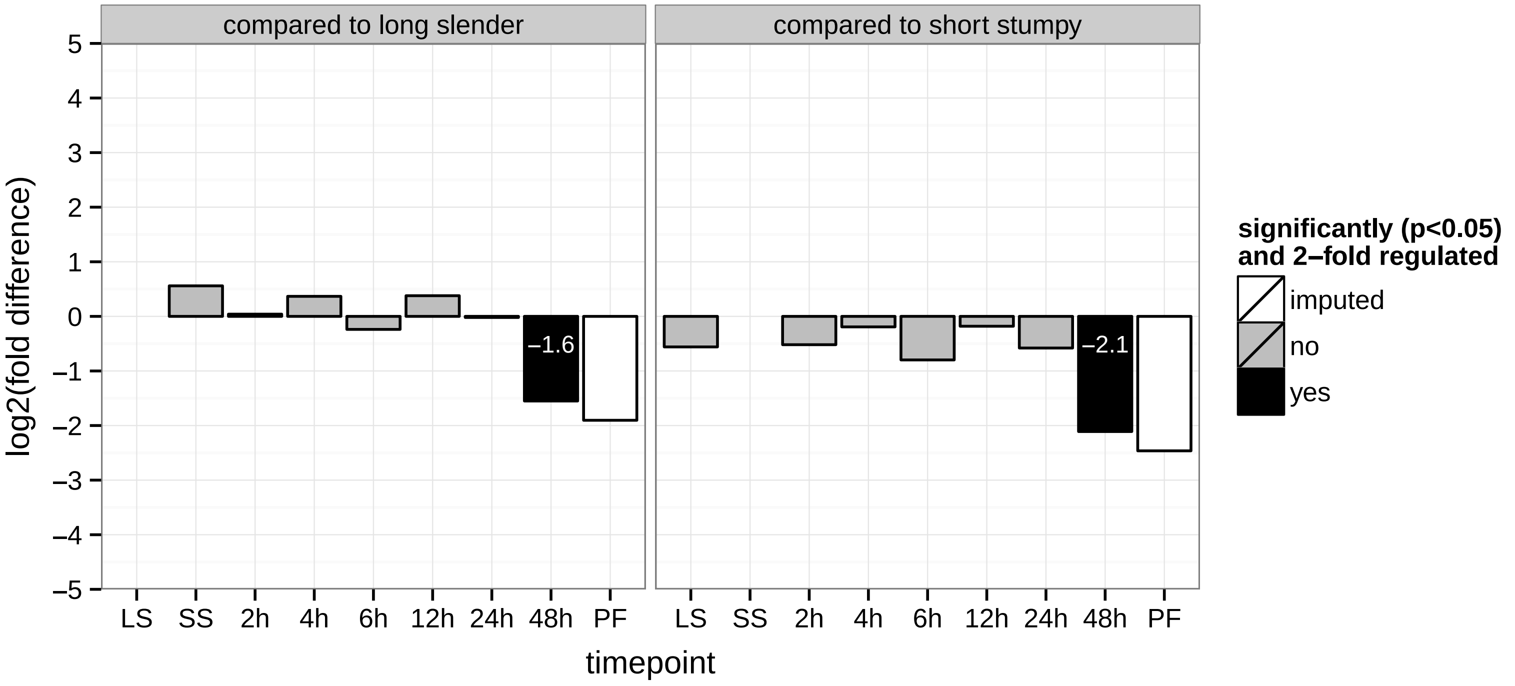

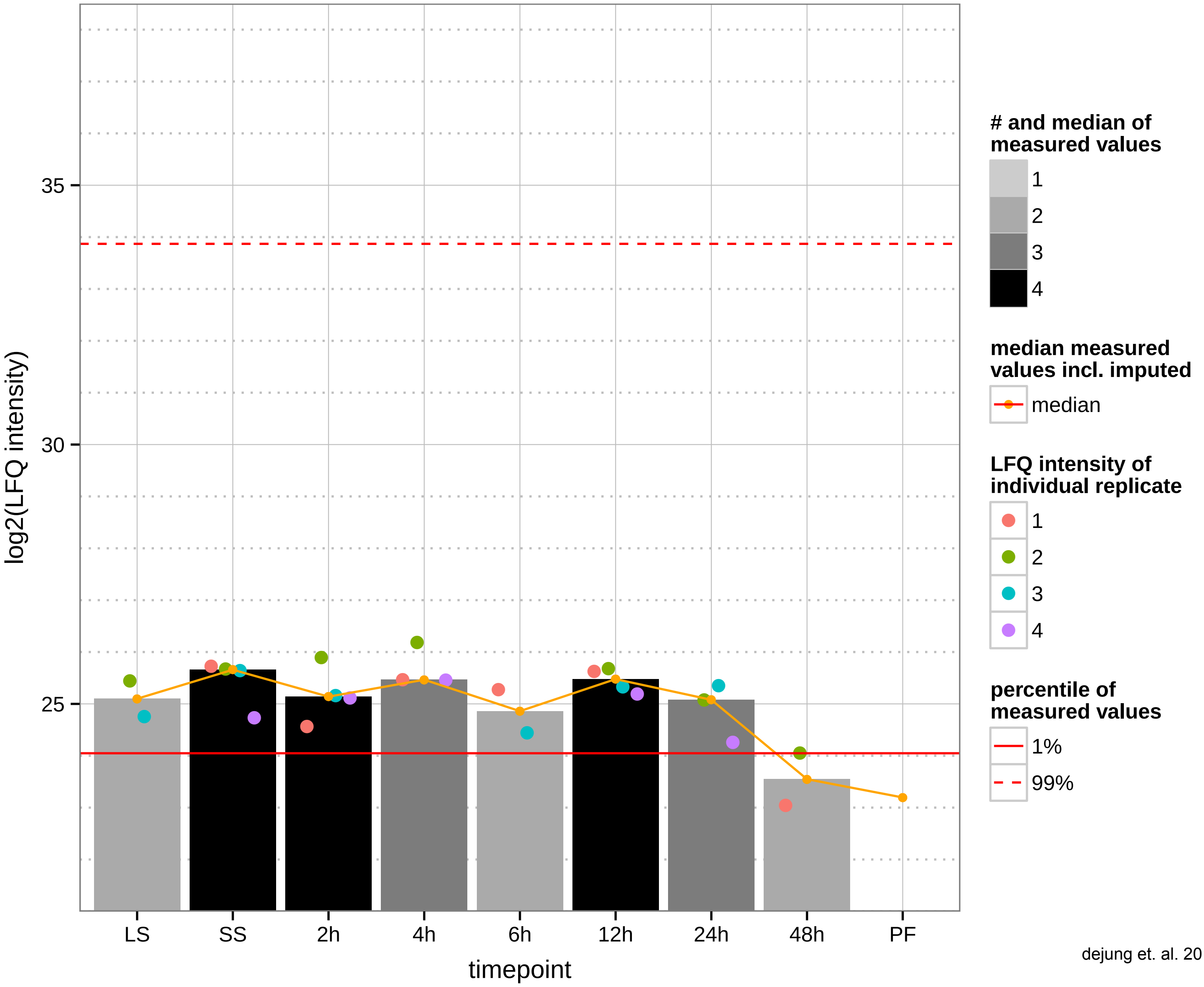

dejung et. al. 2015

hypothetical protein, conserved
Tb927.8.3520
AGOF: null
AGOC: null
AGOP: null
PGOF: null
PGOC: null
PGOP: null

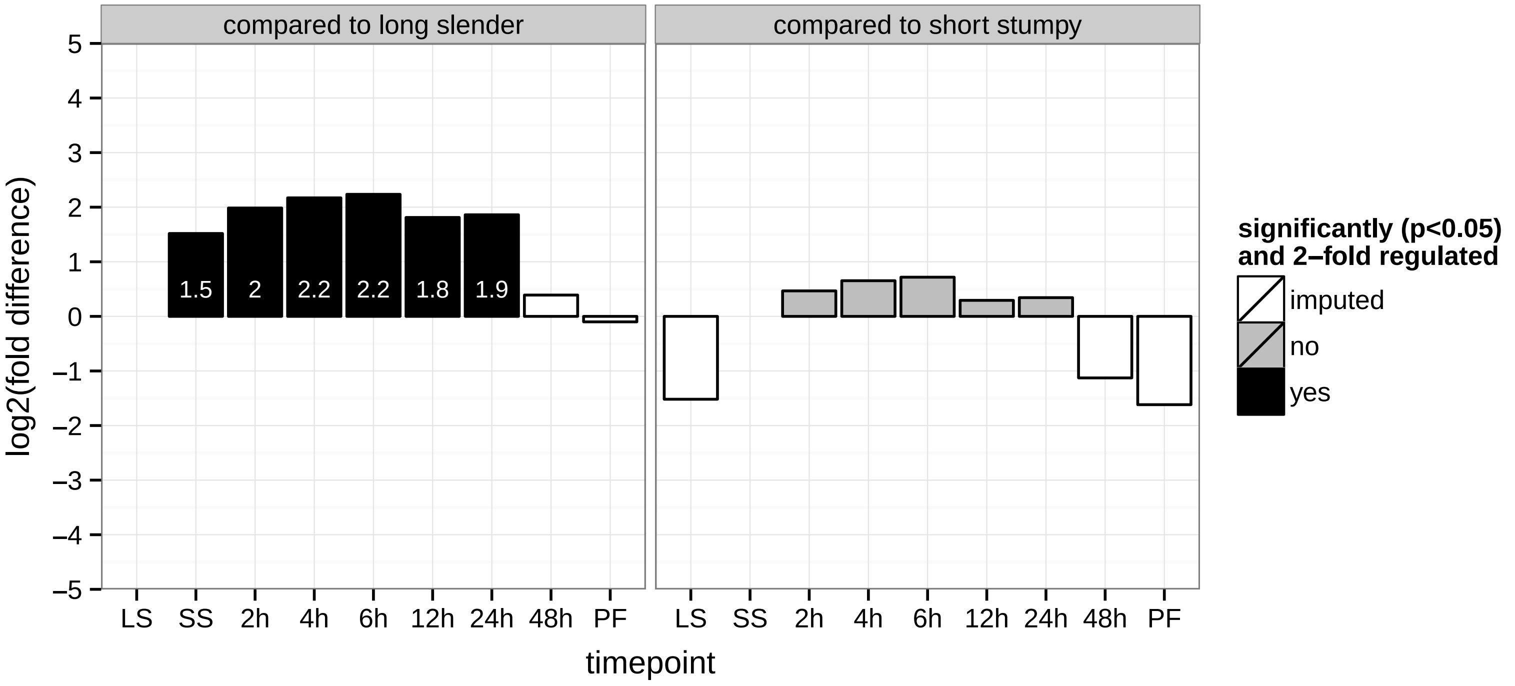

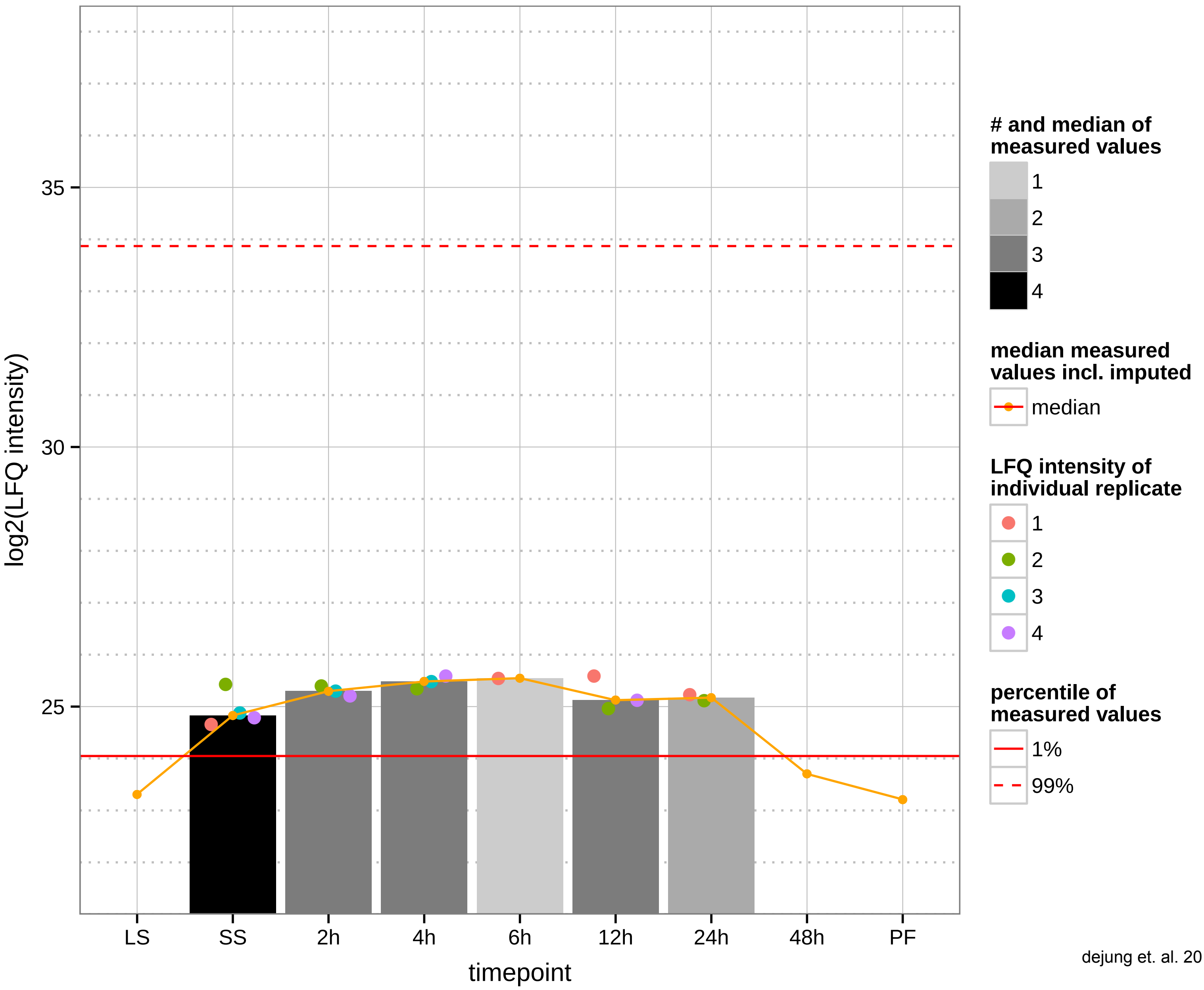

dejung et. al. 2015

flagellum–adhesion glycoprotein, putative
Tb927.8.4060
AGOF: null
AGOC: flagellar pocket, integral to membrane, motile cilium
AGOP: null
PGOF: null
PGOC: null
PGOP: null

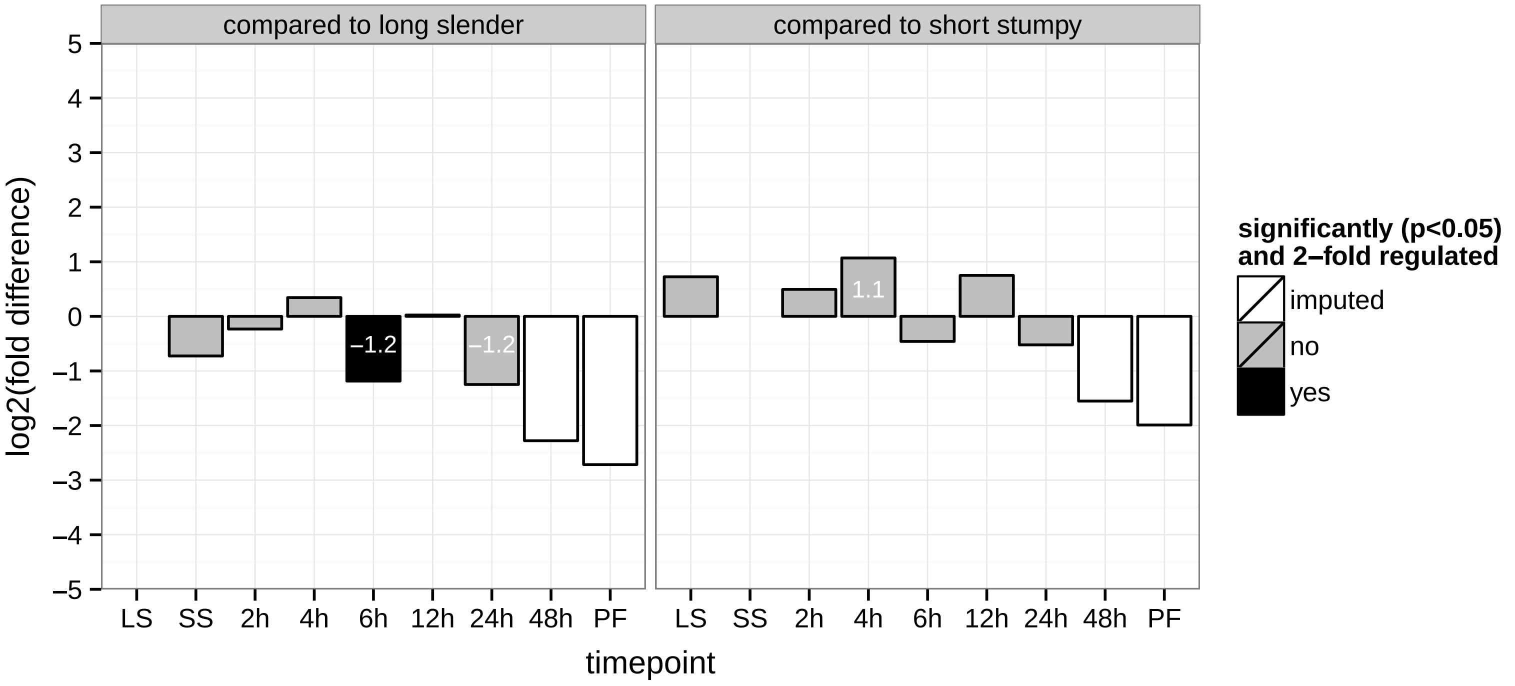

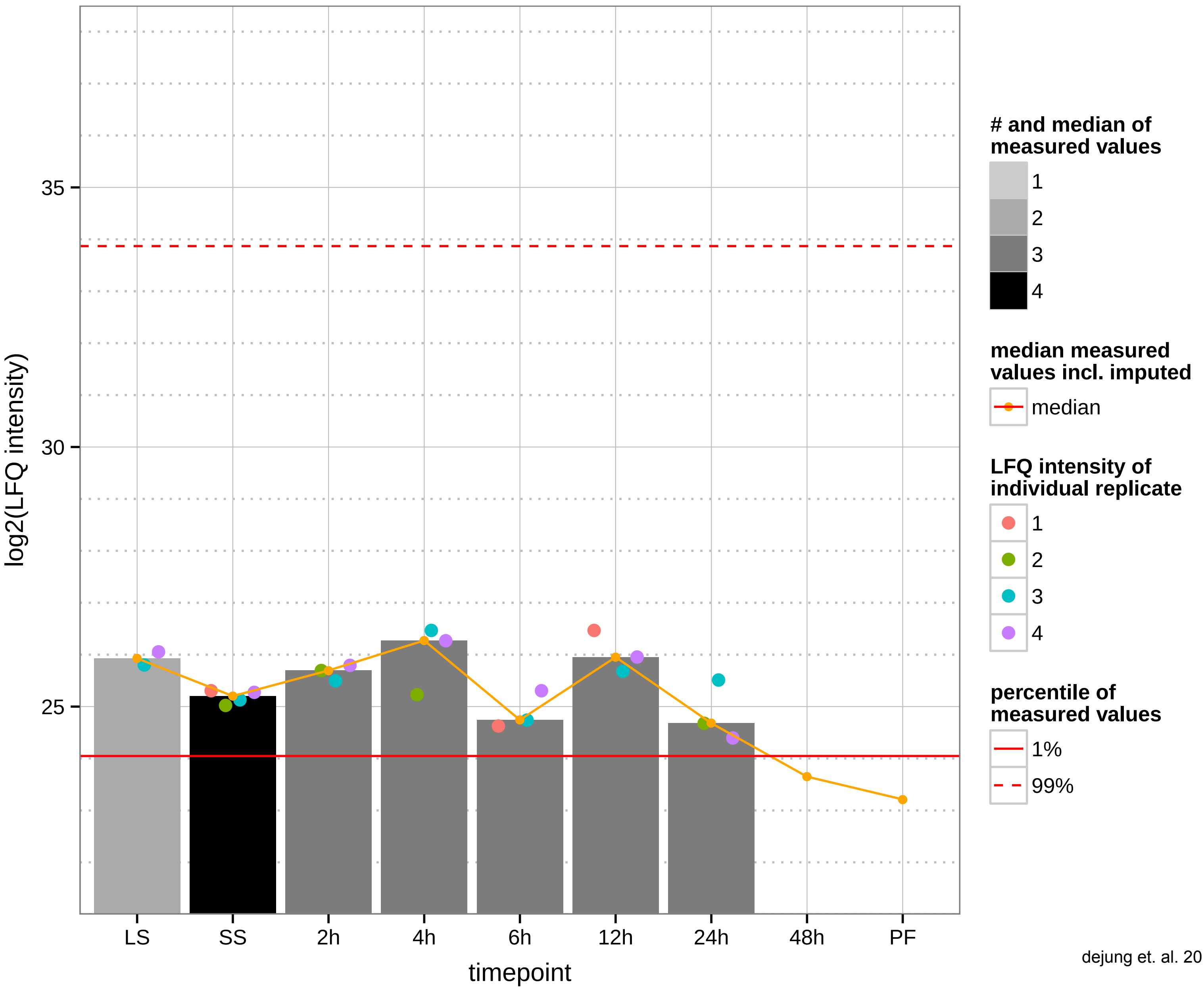

dejung et. al. 2015

small GTP–binding protein Rab11, Rab11A GTPase (RAB11)
Tb927.8.4330
AGOF: GTP binding, GTPase activity
AGOC: contractile vacuole, endomembrane system, endosome, intracellular
AGOP: endosomal transport, intracellular protein transport, nucleocytoplasmic transport, small GTPase mediated signal trans
PGOF: GTP binding, GTPase activity
PGOC: intracellular, membrane
PGOP: GTP catabolic process, intracellular protein transport, nucleocytoplasmic transport, protein transport, signal transducti

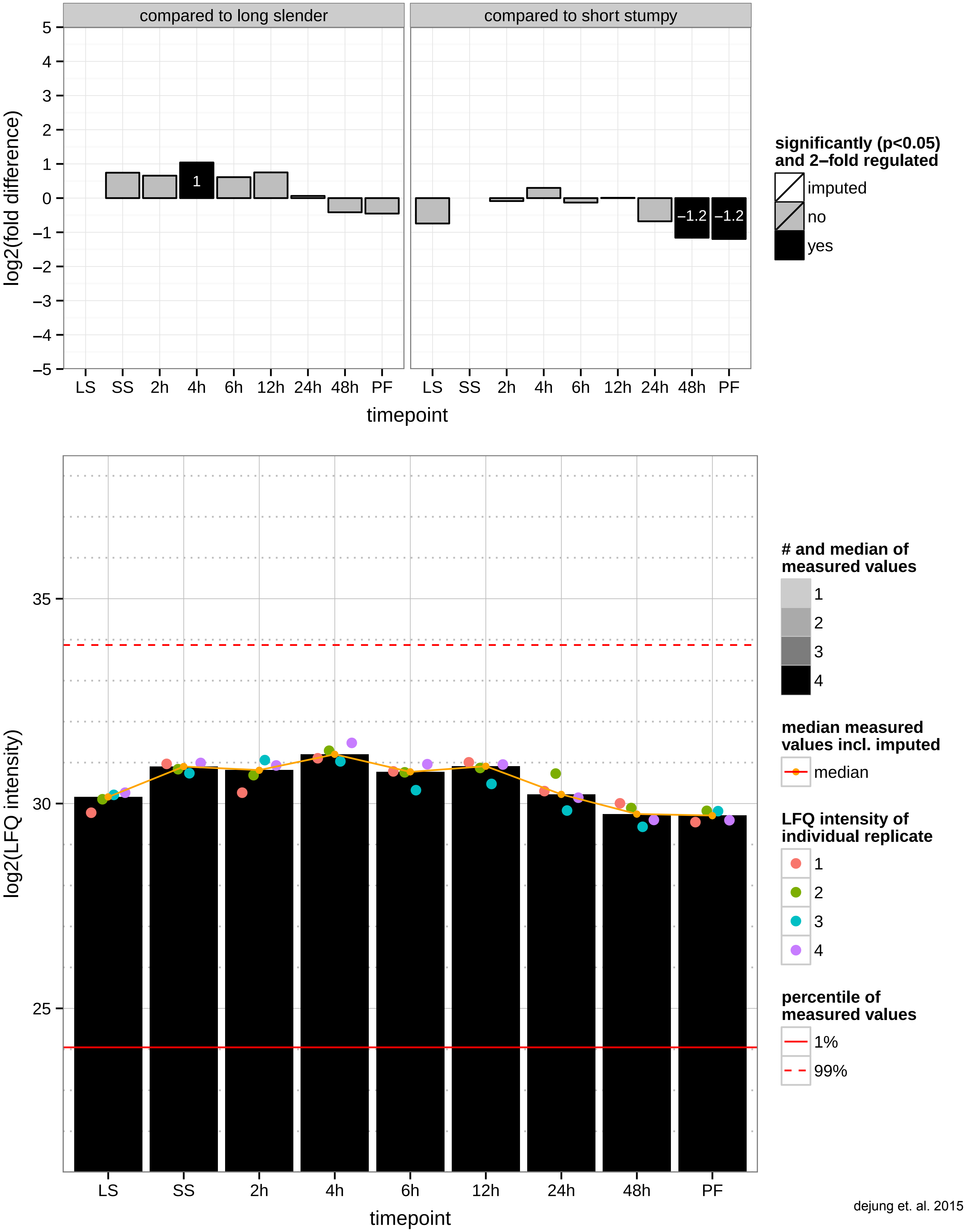

dejung et. al. 2015

hypothetical protein, conserved
Tb927.8.5240
AGOF: null
AGOC: membrane
AGOP: null
PGOF: null
PGOC: membrane
PGOP: null

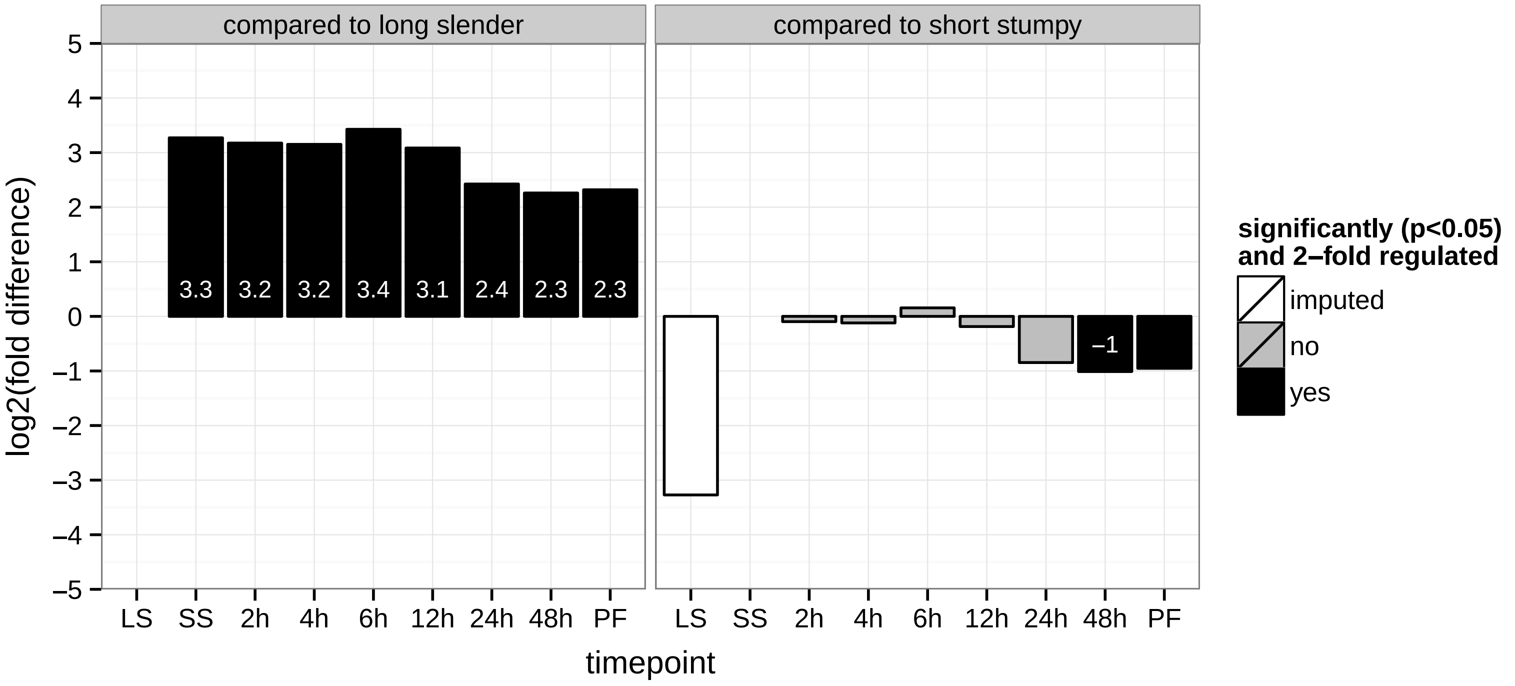

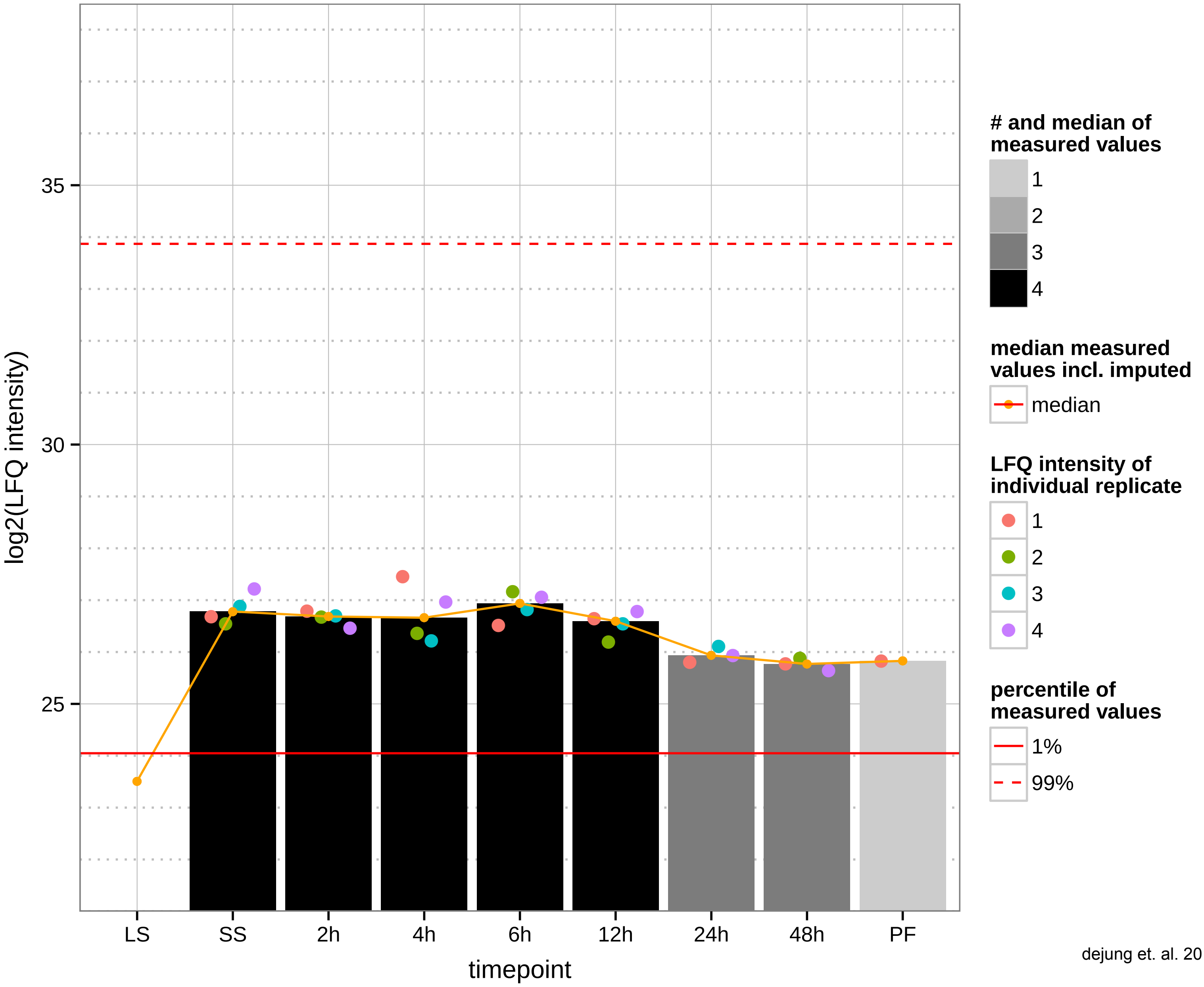

dejung et. al. 2015

hypothetical protein, conserved
Tb927.8.6620
AGOF: null
AGOC: integral to membrane, mitochondrion
AGOP: null
PGOF: protein binding
PGOC: null
PGOP: null

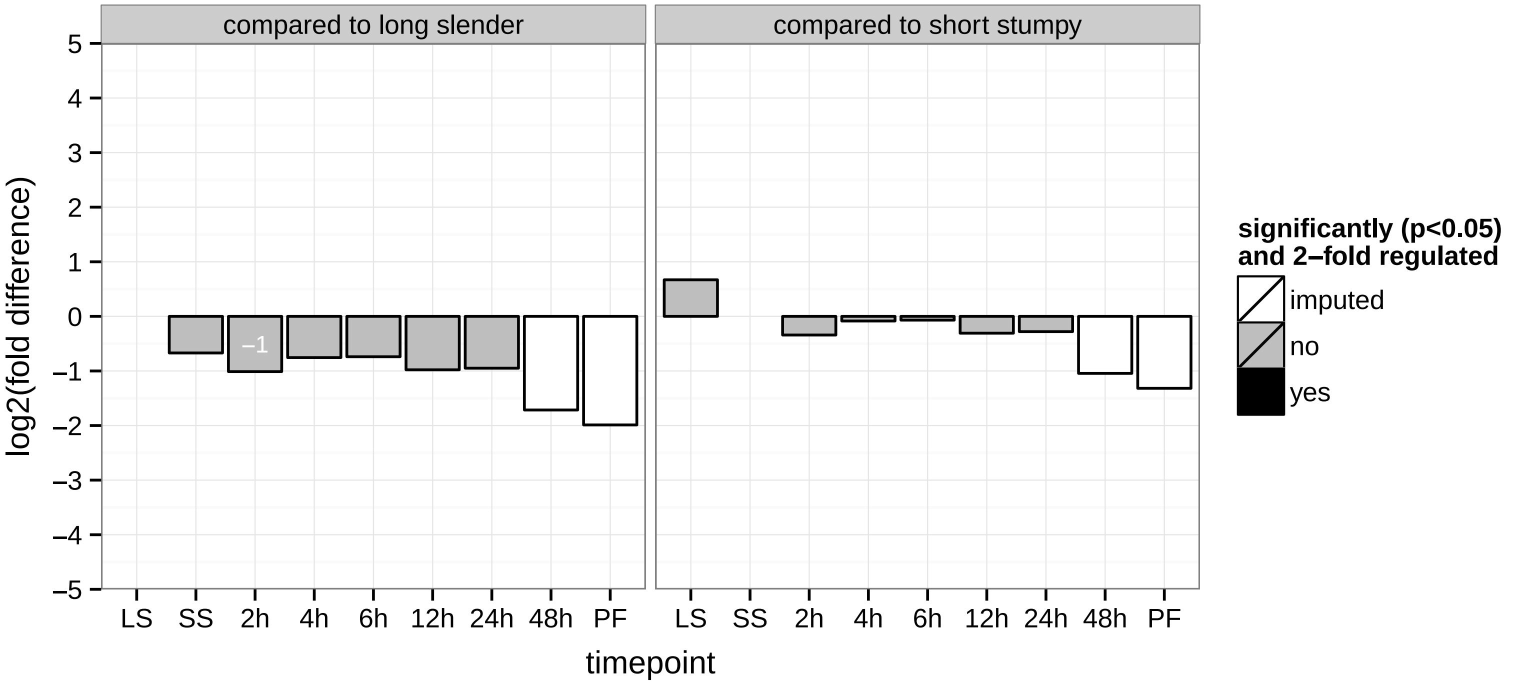

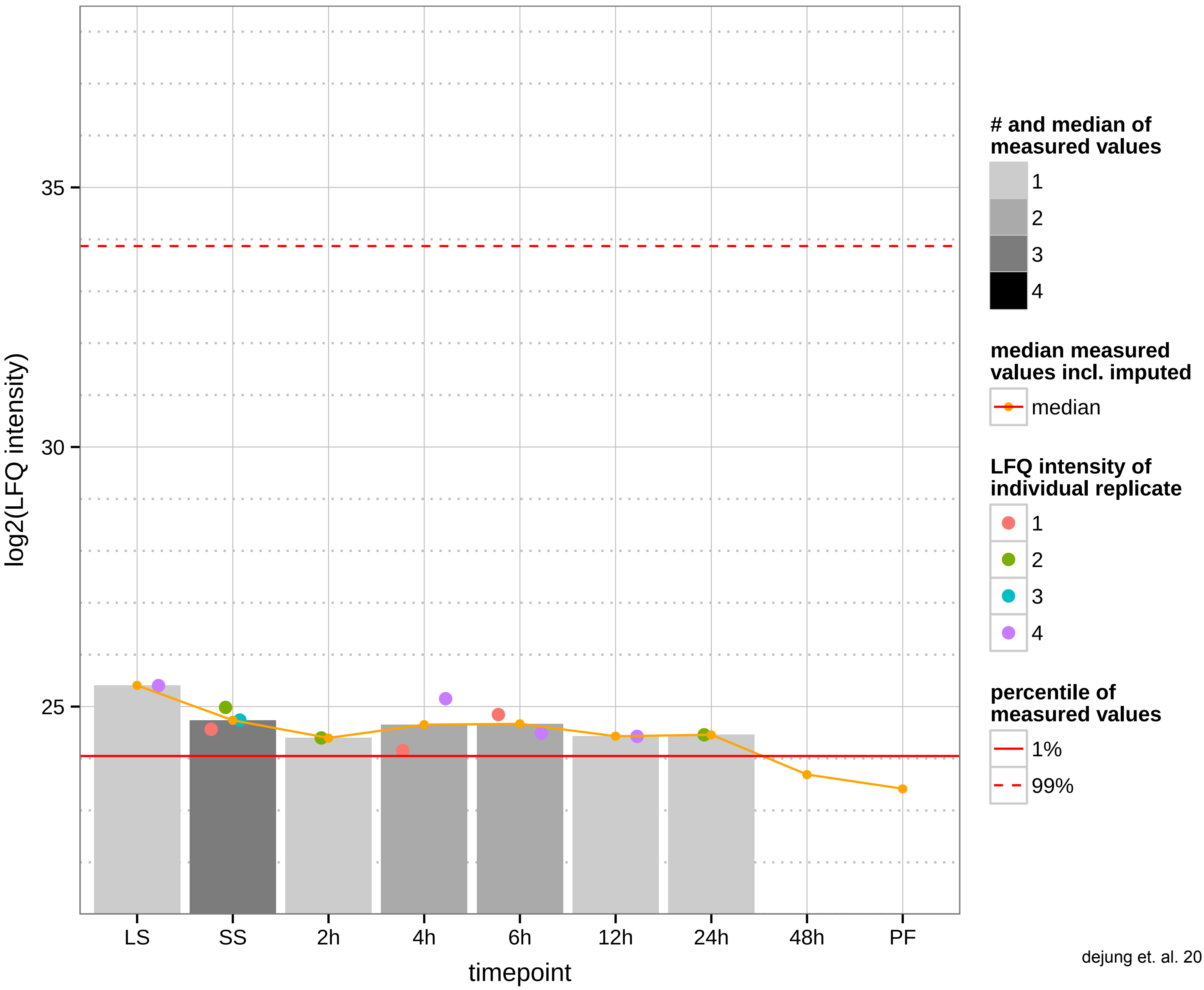

dejung et. al. 2015

hypothetical protein, conserved
Tb927.8.7720
AGOF: null
AGOC: integral to membrane
AGOP: null
PGOF: null
PGOC: null
PGOP: null

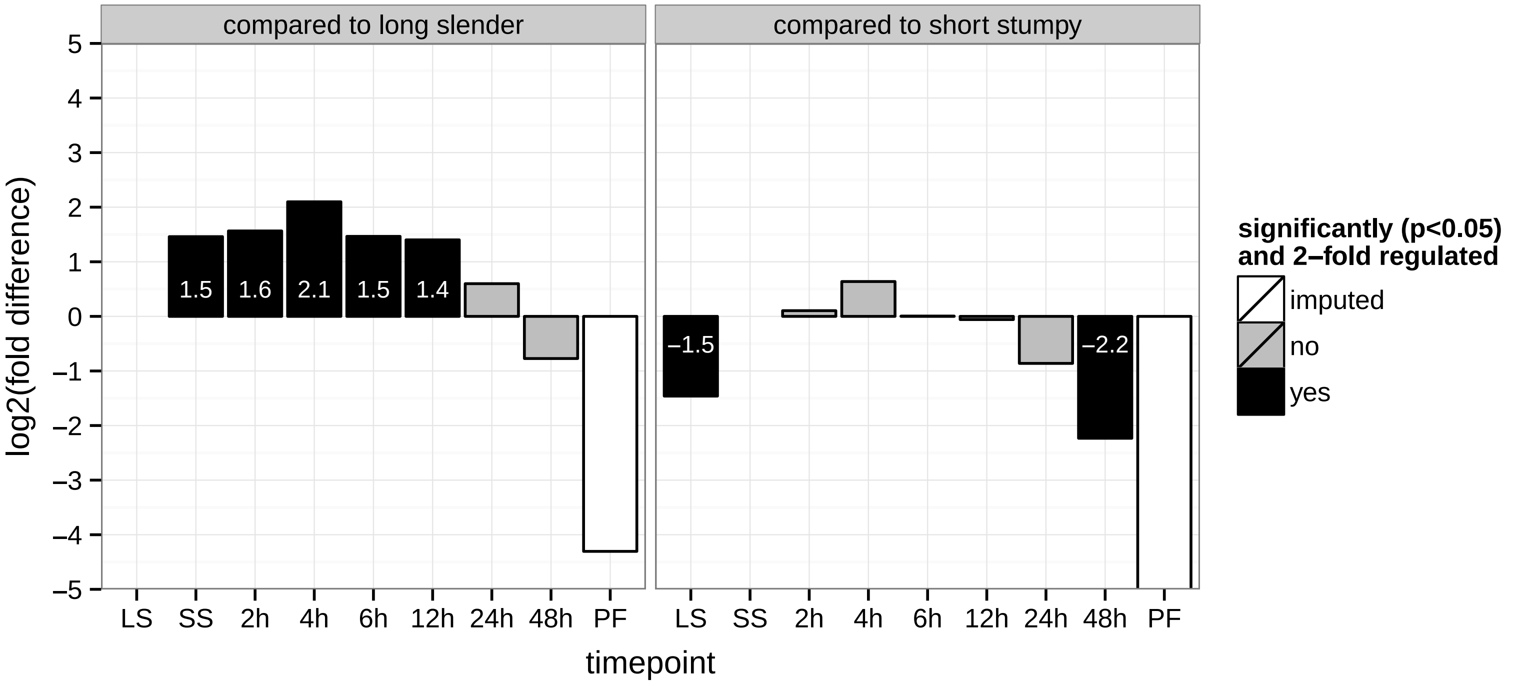

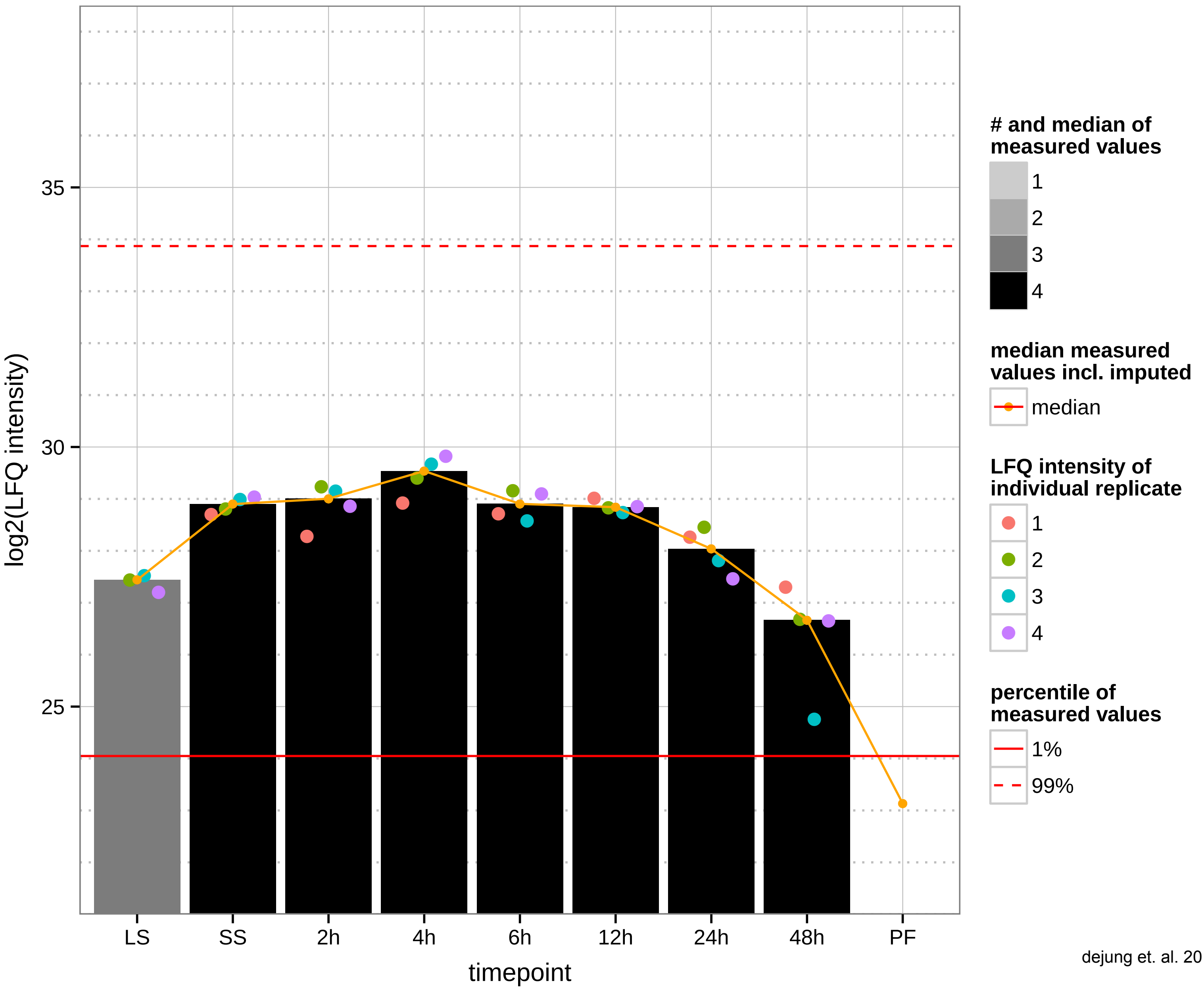

dejung et. al. 2015

hypothetical protein, conserved
Tb927.8.8000
AGOF: null
AGOC: null
AGOP: null
PGOF: null
PGOC: null
PGOP: null

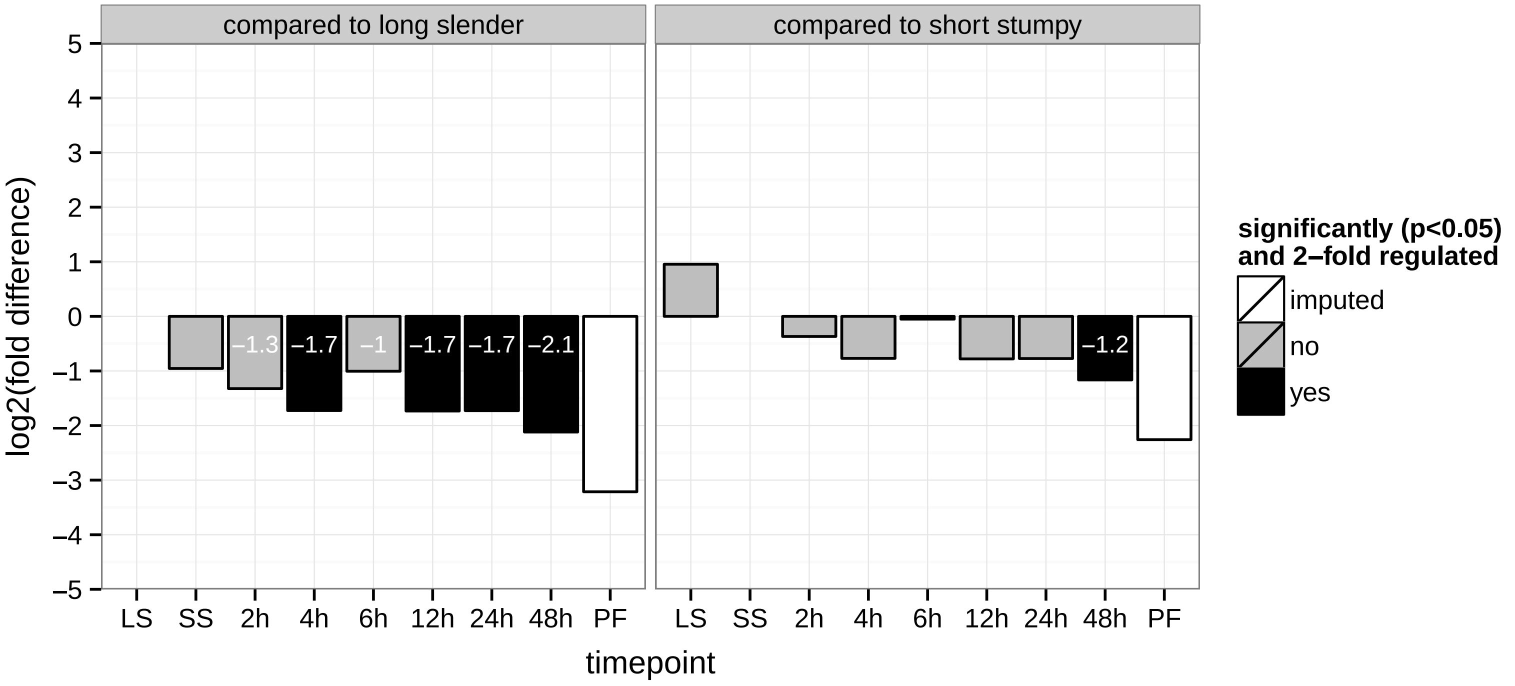

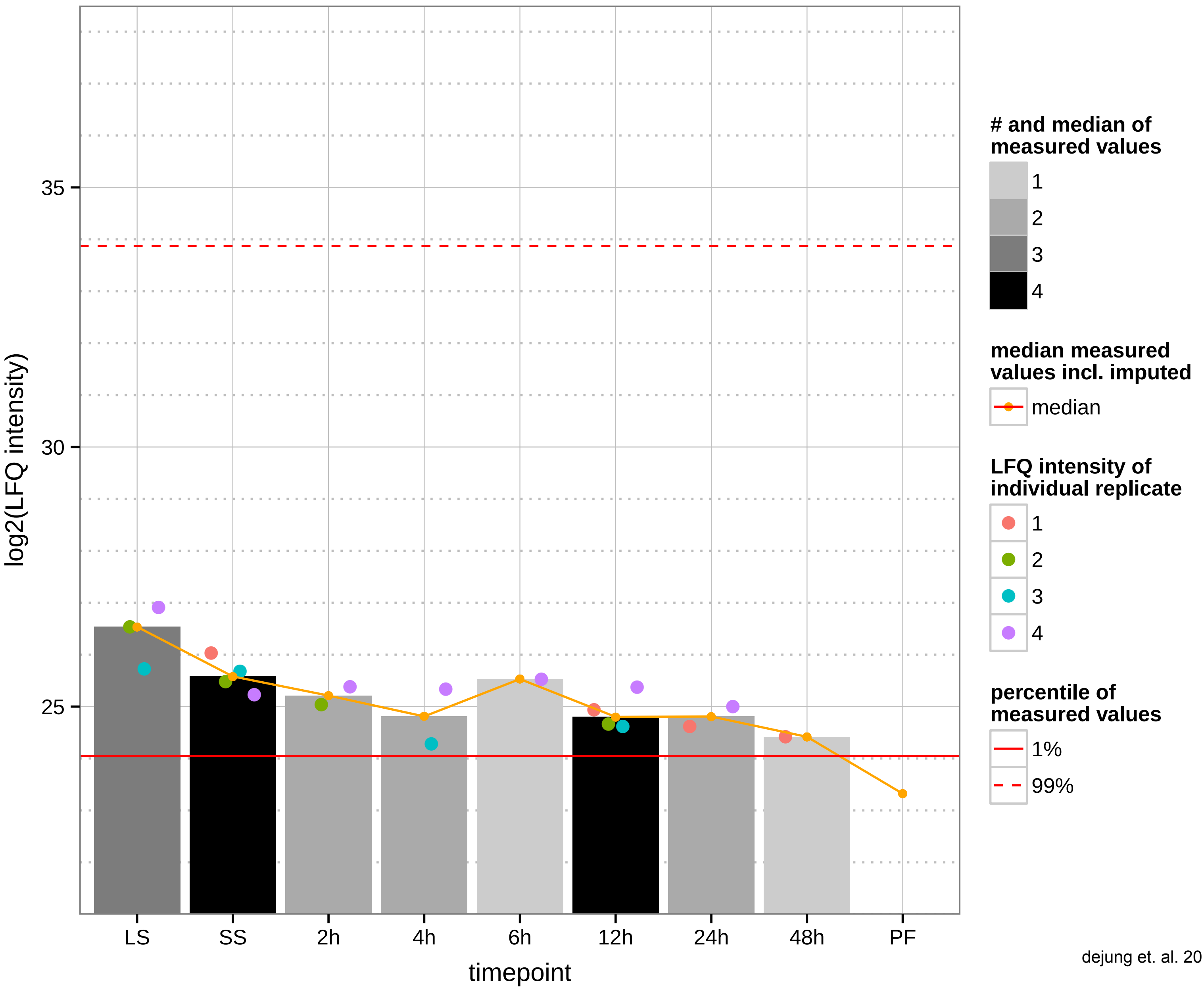

dejung et. al. 2015

hypothetical protein, conserved
Tb927.8.8030
AGOF: null
AGOC: integral to membrane
AGOP: transport
PGOF: null
PGOC: integral to membrane
PGOP: transport

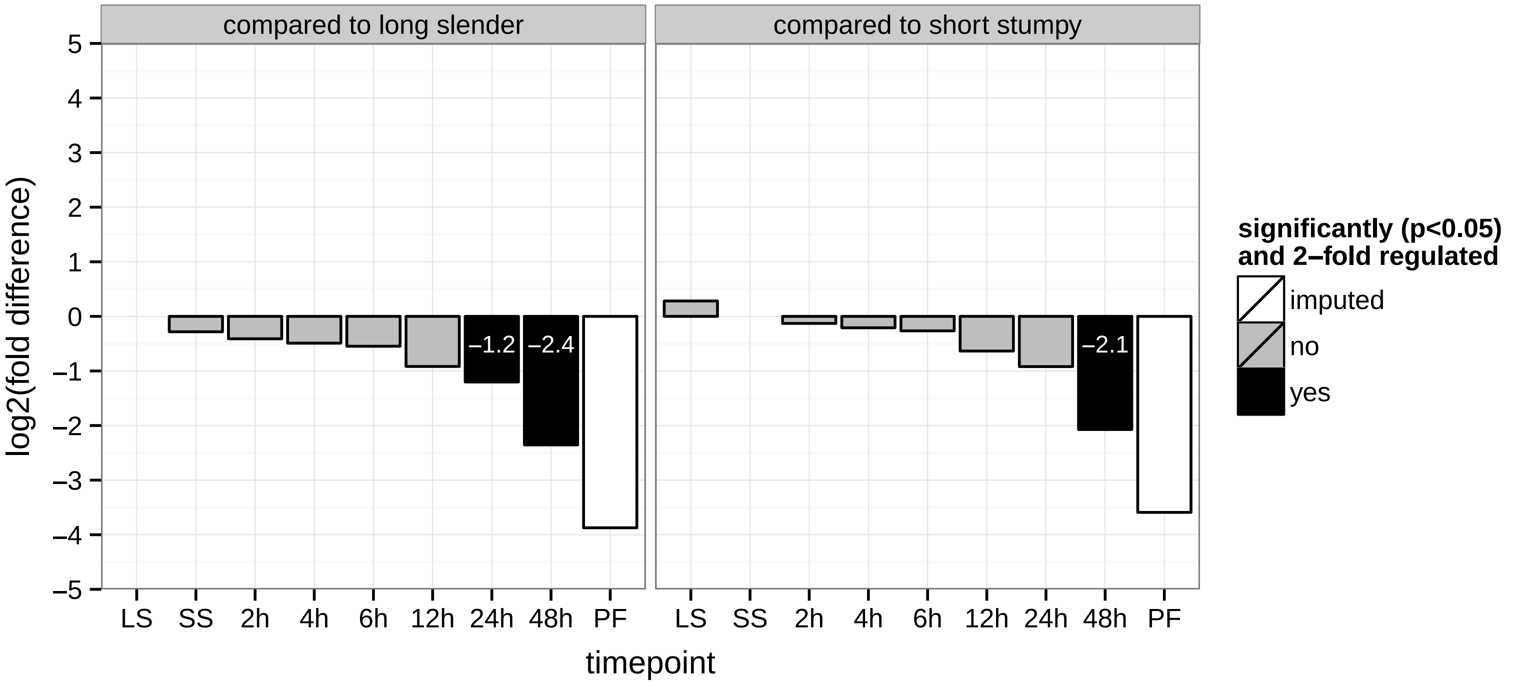

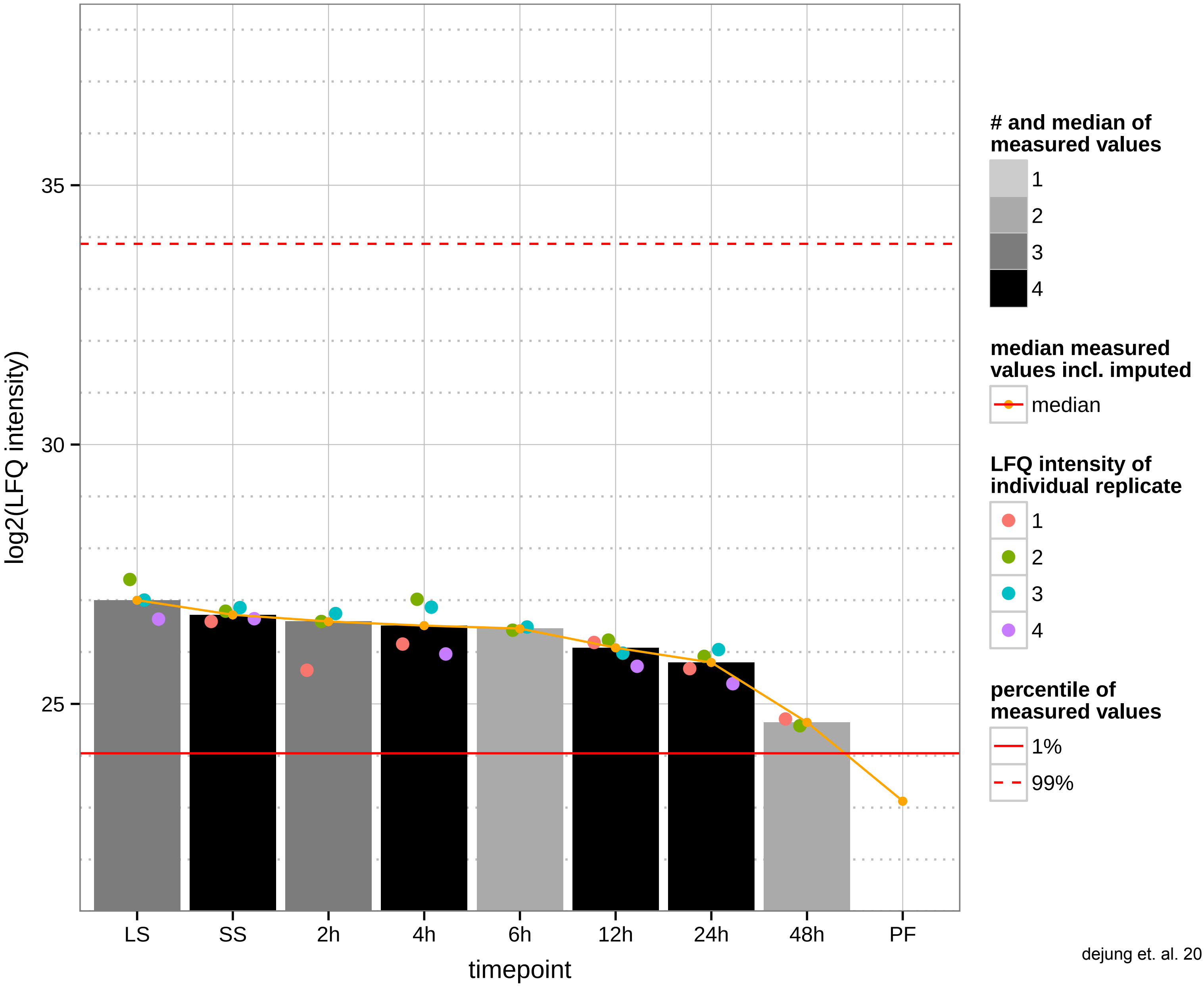

dejung et. al. 2015

small GTPase, putative, GTP–binding protein, putative (RAB7)
Tb927.9.11000
AGOF: GTP binding, GTPase activity
AGOC: intracellular
AGOP: protein transport, small GTPase mediated signal transduction
PGOF: GTP binding, GTPase activity
PGOC: membrane
PGOP: GTP catabolic process, protein transport, signal transduction, small GTPase mediated signal transduction

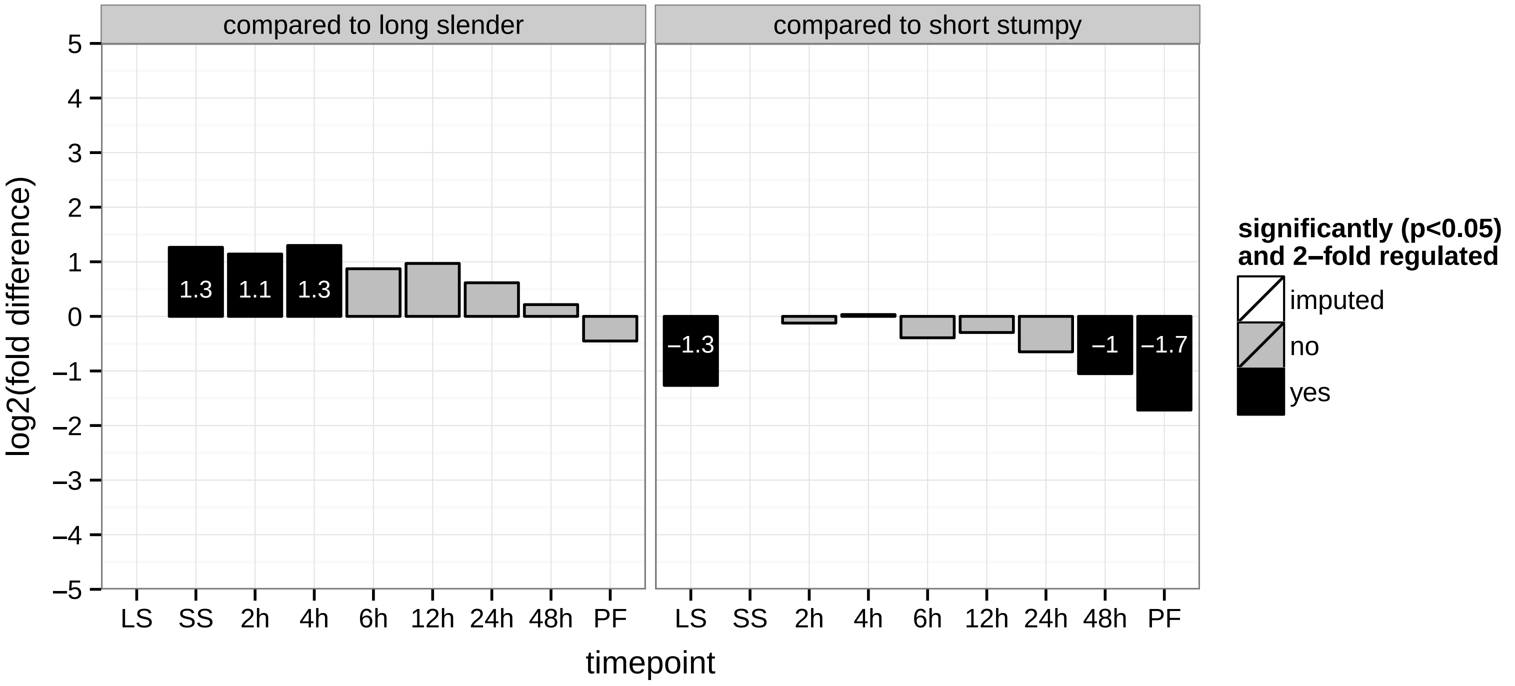

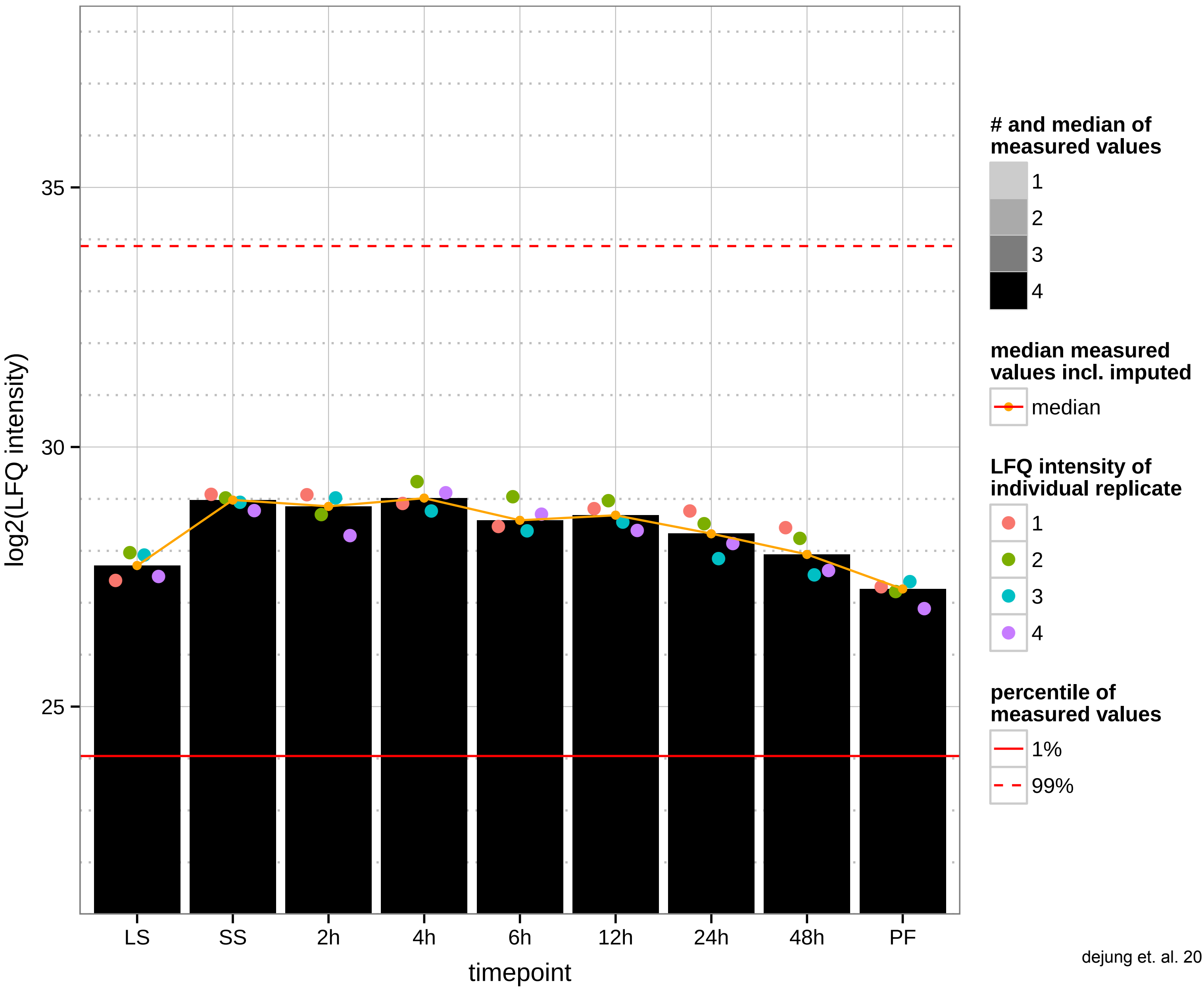

dejung et. al. 2015

cAMP–dependent protein kinase catalytic subunit 2 (PKAC2)
Tb927.9.11030
AGOF: ATP binding, protein serine/threonine kinase activity, protein tyrosine kinase activity
AGOC: null
AGOP: null
PGOF: ATP binding, protein kinase activity, protein serine/threonine kinase activity, transferase activity, transferring phosphoru
PGOC: null
PGOP: protein phosphorylation

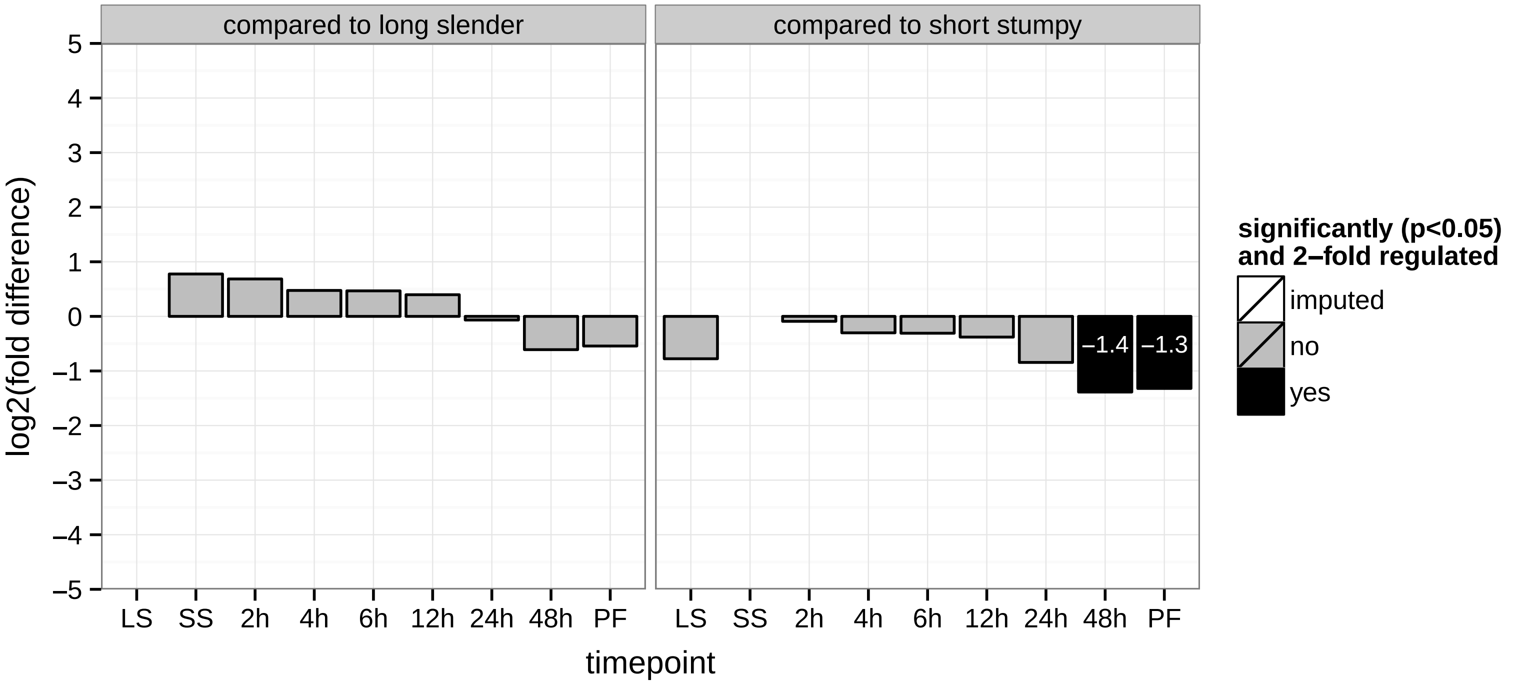

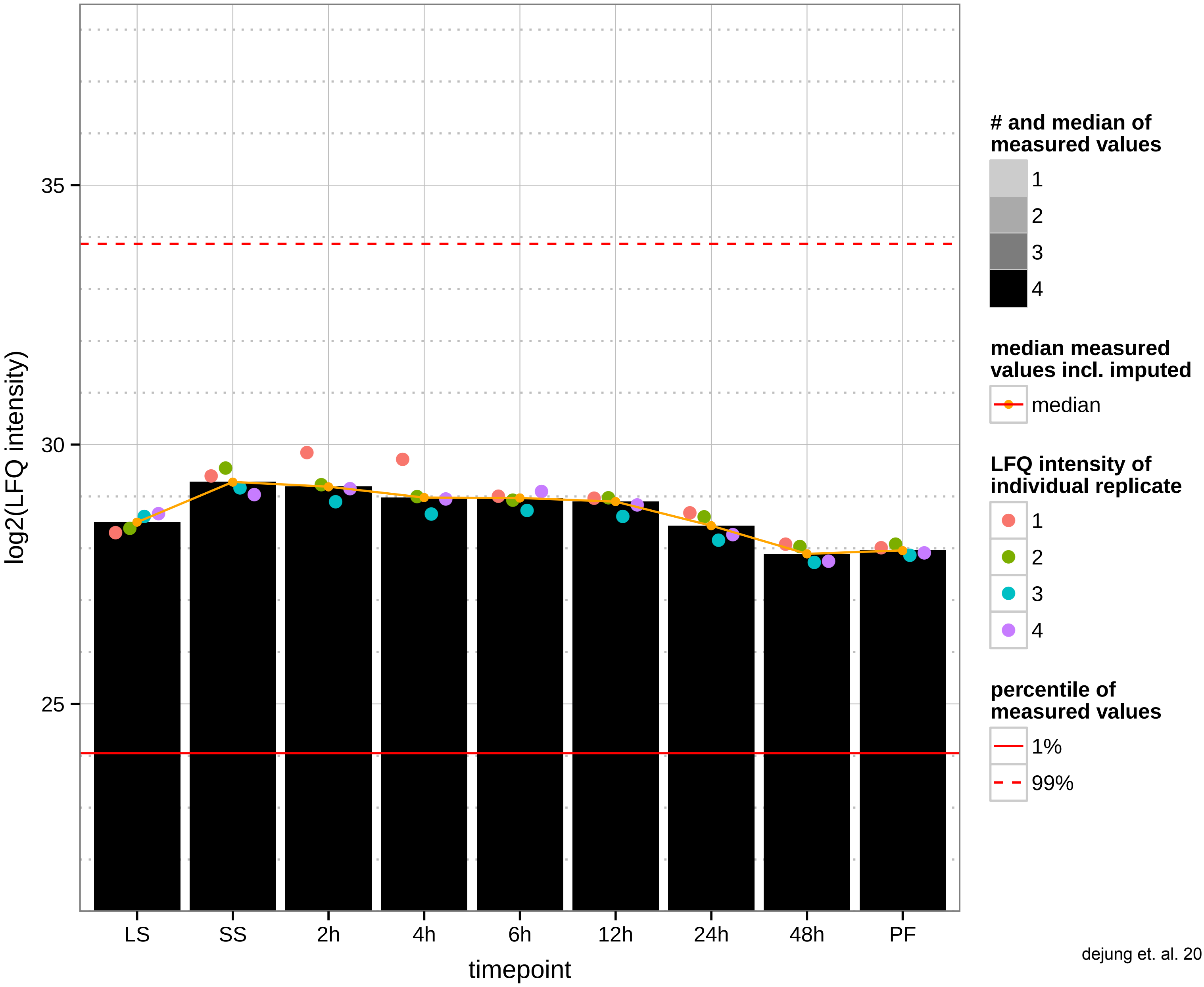

dejung et. al. 2015

cAMP–dependent protein kinase catalytic subunit 1 (PKAC1)
Tb927.9.11100
AGOF: ATP binding, protein serine/threonine kinase activity, protein tyrosine kinase activity
AGOC: null
AGOP: protein phosphorylation
PGOF: ATP binding, protein kinase activity, protein serine/threonine kinase activity, transferase activity, transferring phosphoru
PGOC: null
PGOP: protein phosphorylation

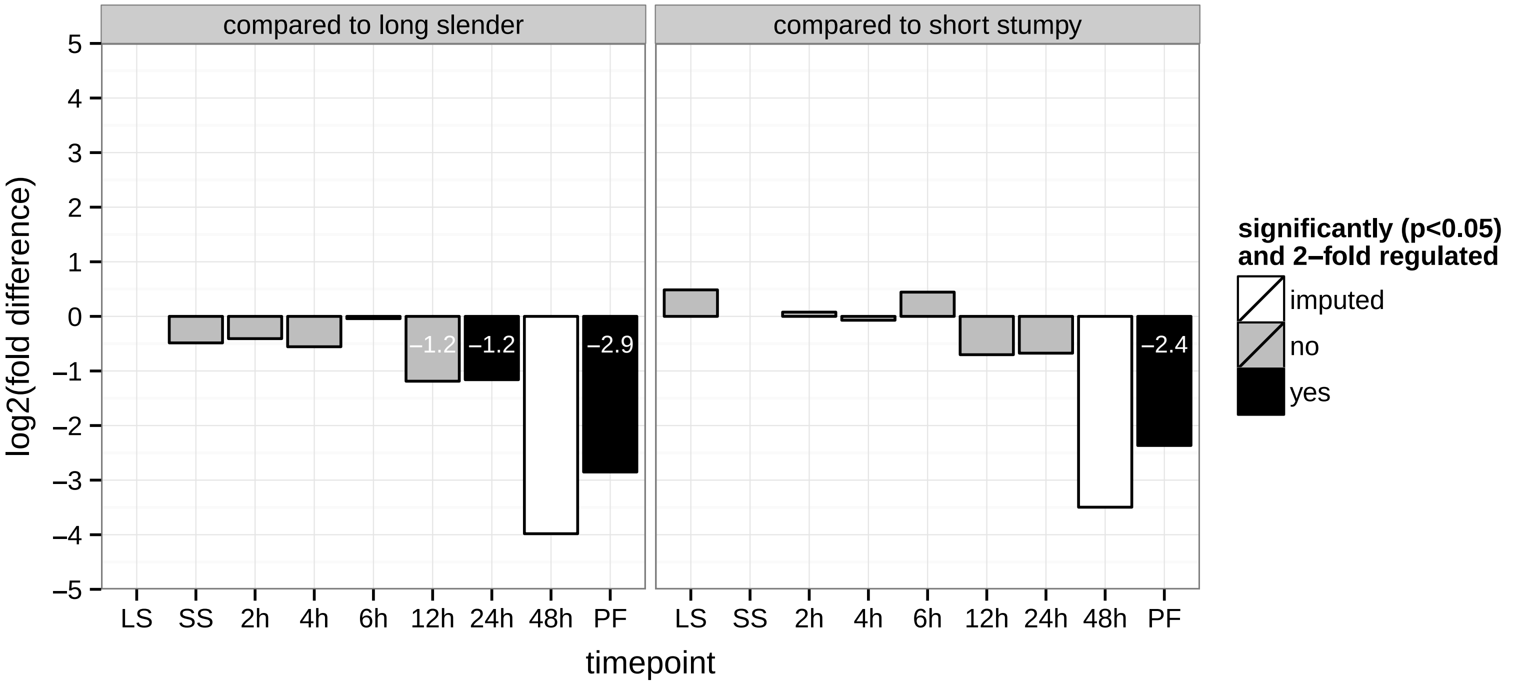

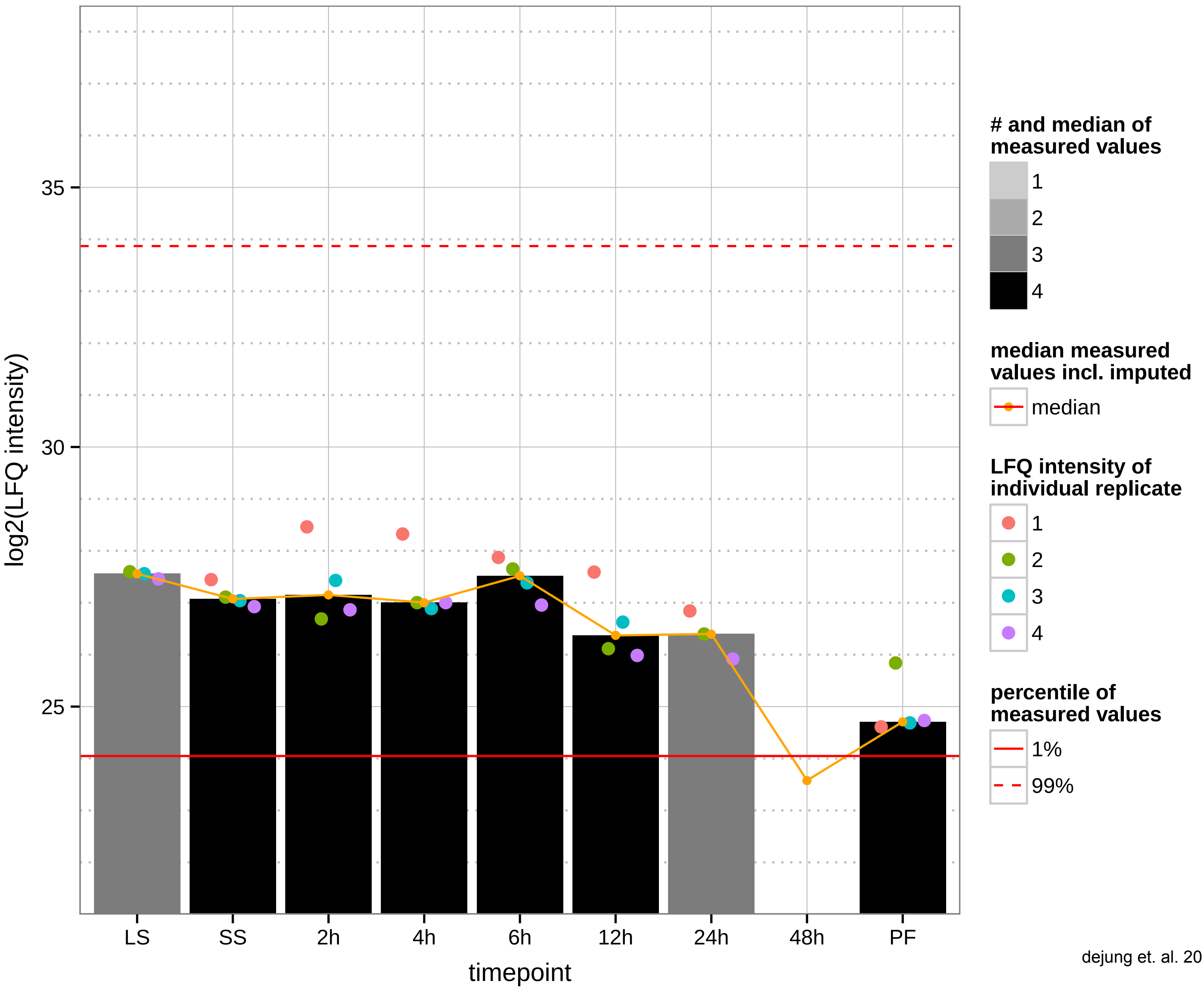

dejung et. al. 2015

ubiquitin–activating enzyme E1, putative, putative (UBA2)
Tb927.9.12650
AGOF: null, ATP binding, small protein activating enzyme activity, ubiquitin–protein ligase activity
AGOC: null, mitochondrion
AGOP: null, cellular protein modification process
PGOF: catalytic activity, ATP binding, catalytic activity, small protein activating enzyme activity
PGOC: null
PGOP: null, cellular protein modification process

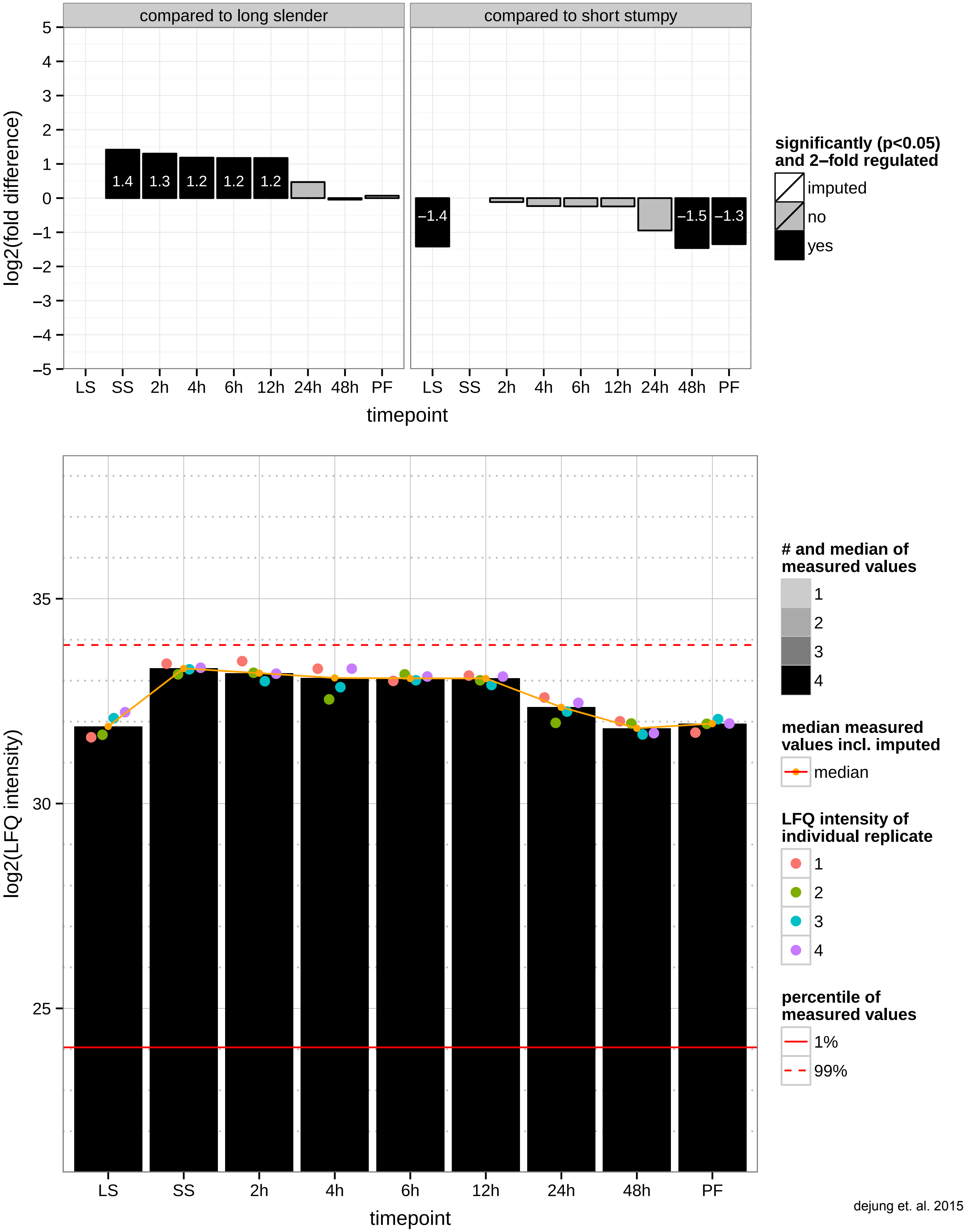

dejung et. al. 2015

predicted heat shock factor binding protein
Tb927.9.13070
AGOF: null
AGOC: null
AGOP: null
PGOF: null
PGOC: null
PGOP: null

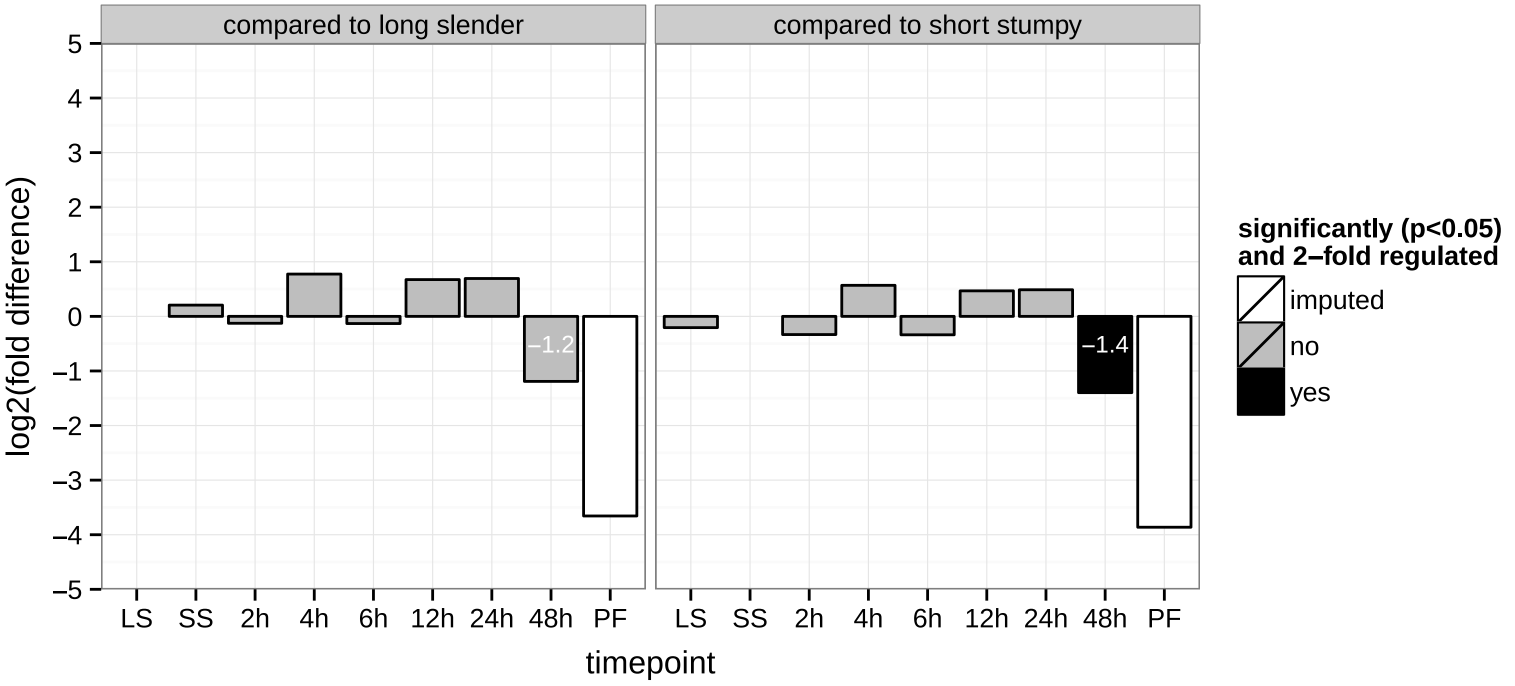

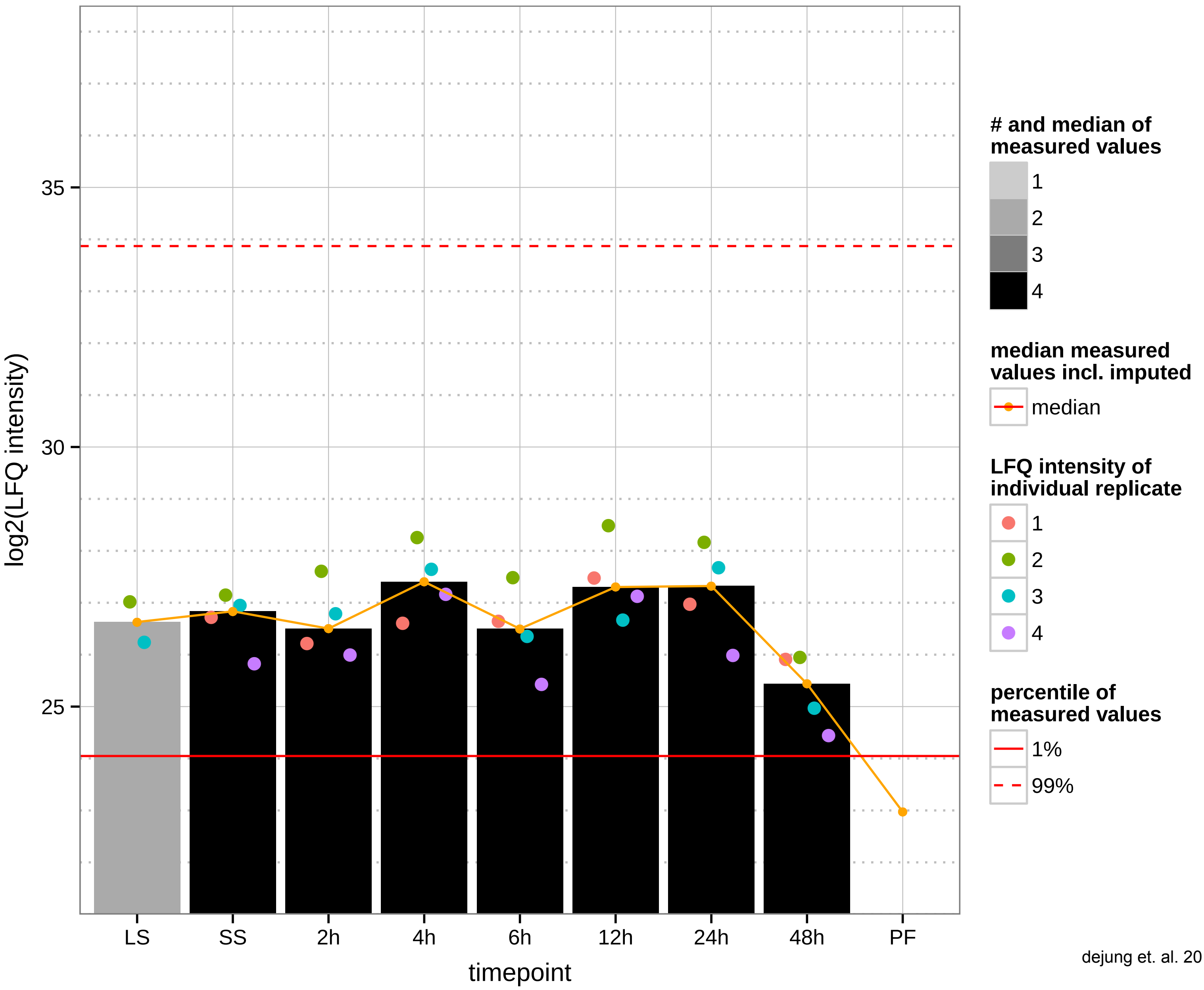

dejung et. al. 2015

phosphoinositide–binding protein, putative
Tb927.9.13380
AGOF: oxidoreductase activity, phosphatidylinositol binding, transition metal ion binding
AGOC: null
AGOP: cell communication, intracellular signal transduction
PGOF: phosphatidylinositol binding, protein binding
PGOC: null
PGOP: cell communication

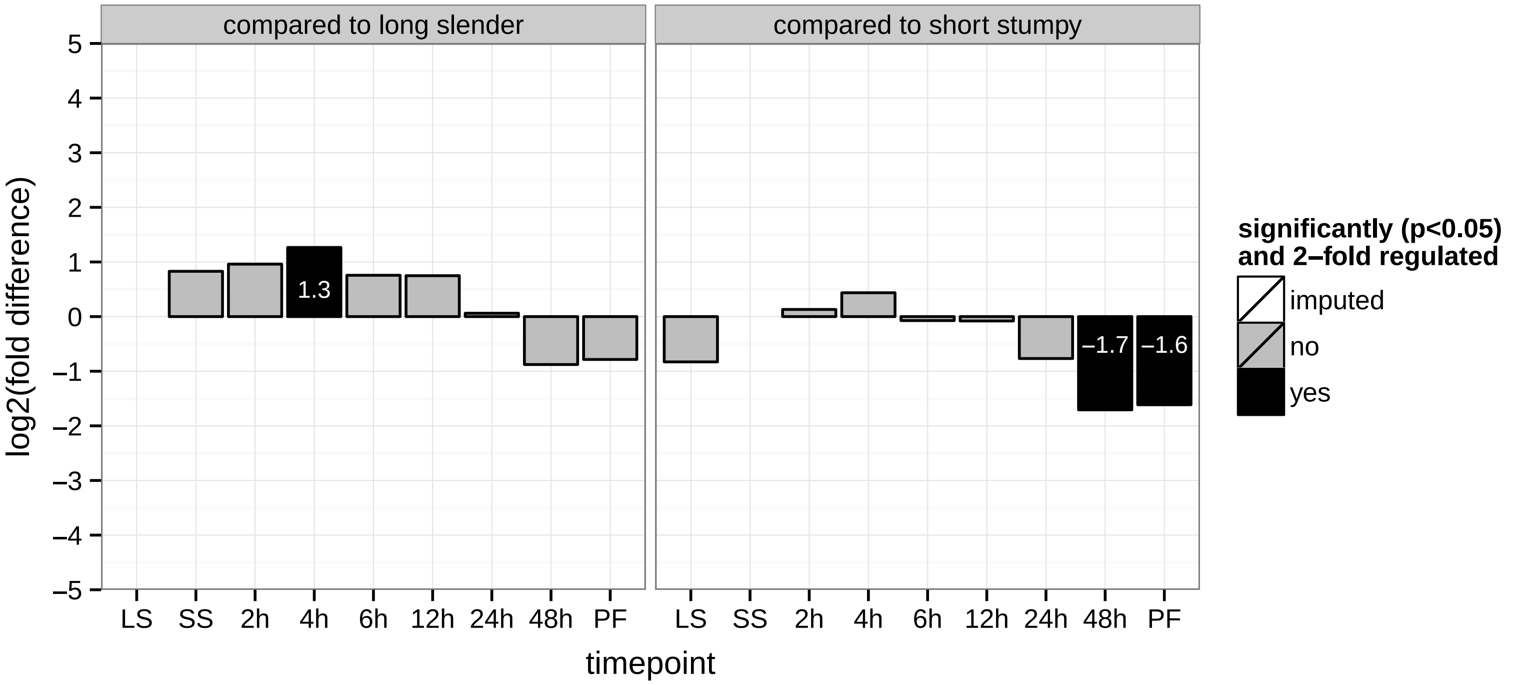

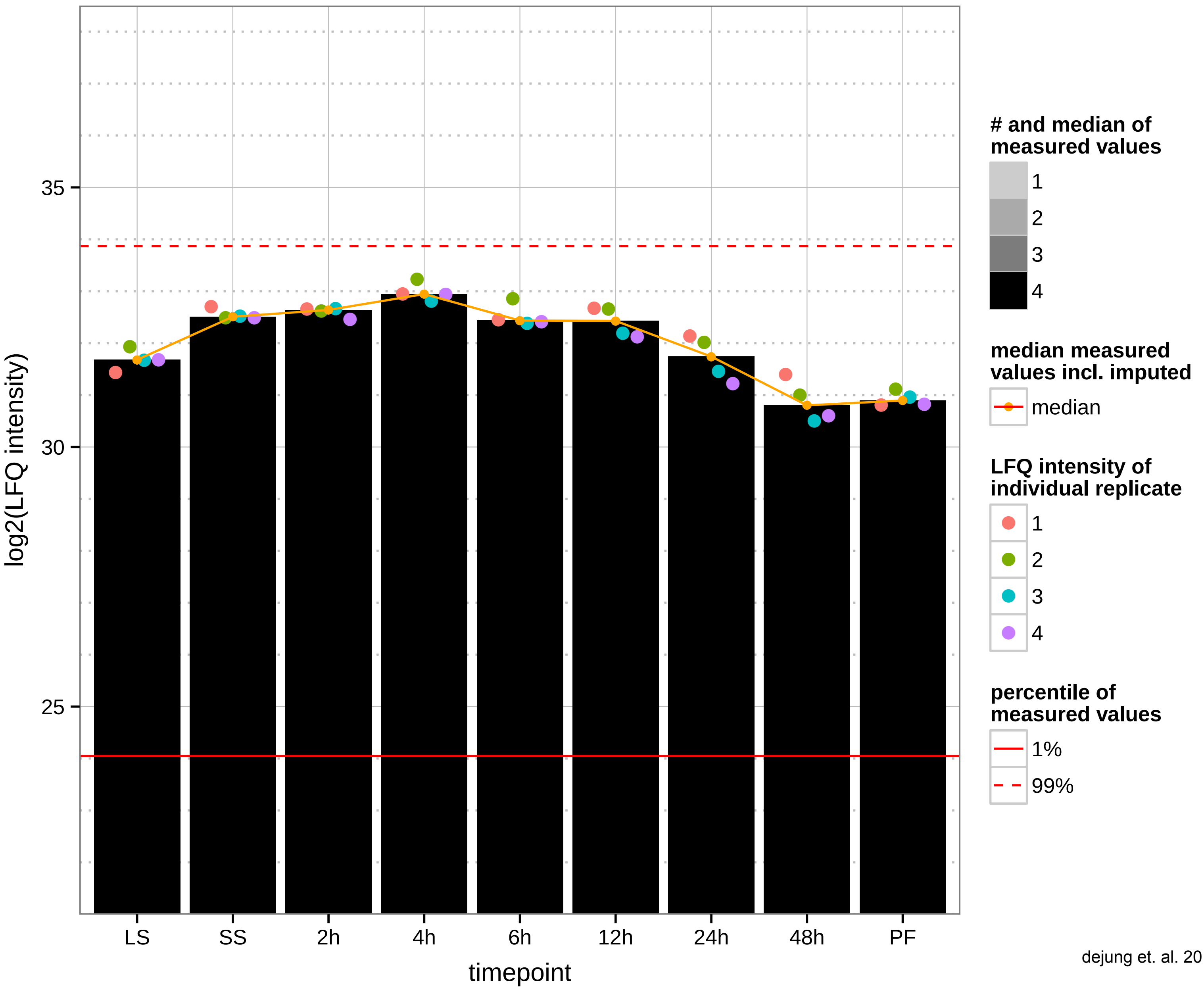

dejung et. al. 2015

hypothetical protein, conserved
Tb927.9.14100
AGOF: null
AGOC: null
AGOP: null
PGOF: null
PGOC: null
PGOP: null

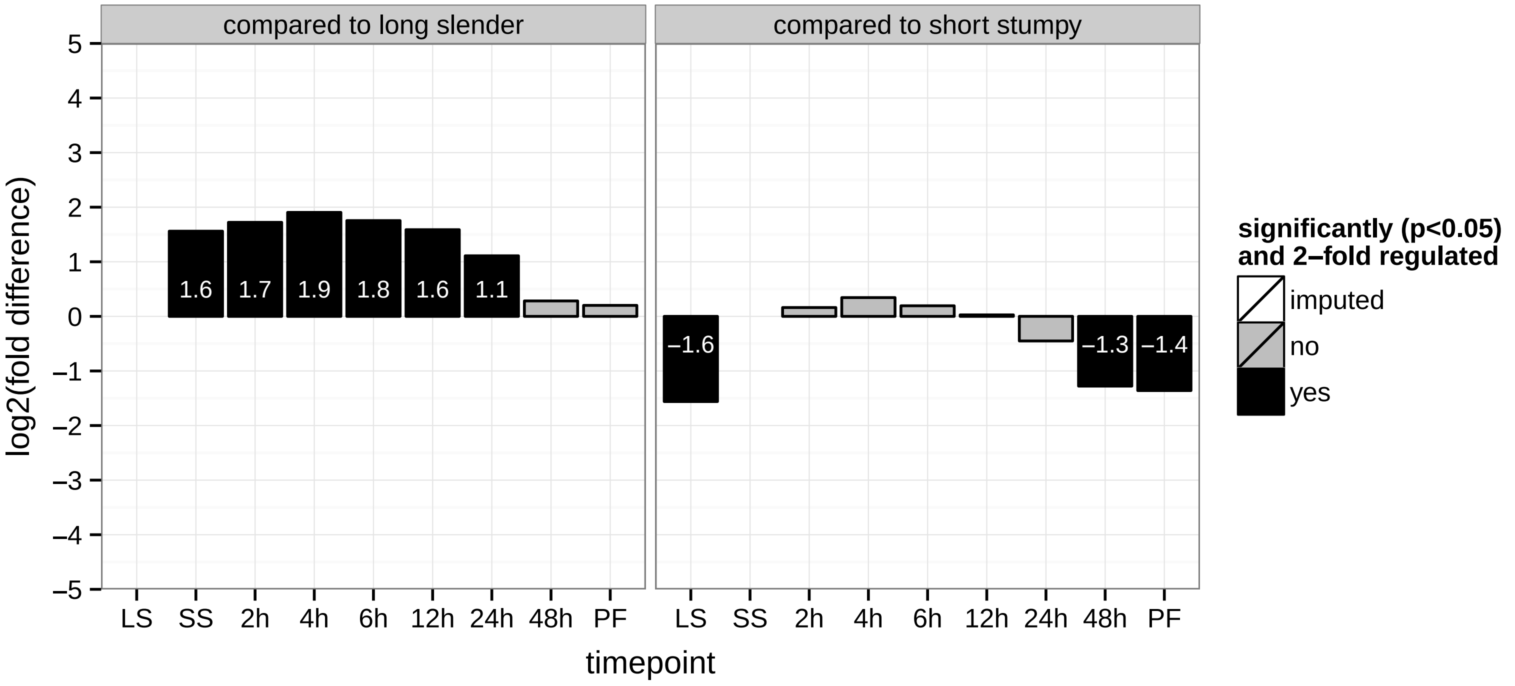

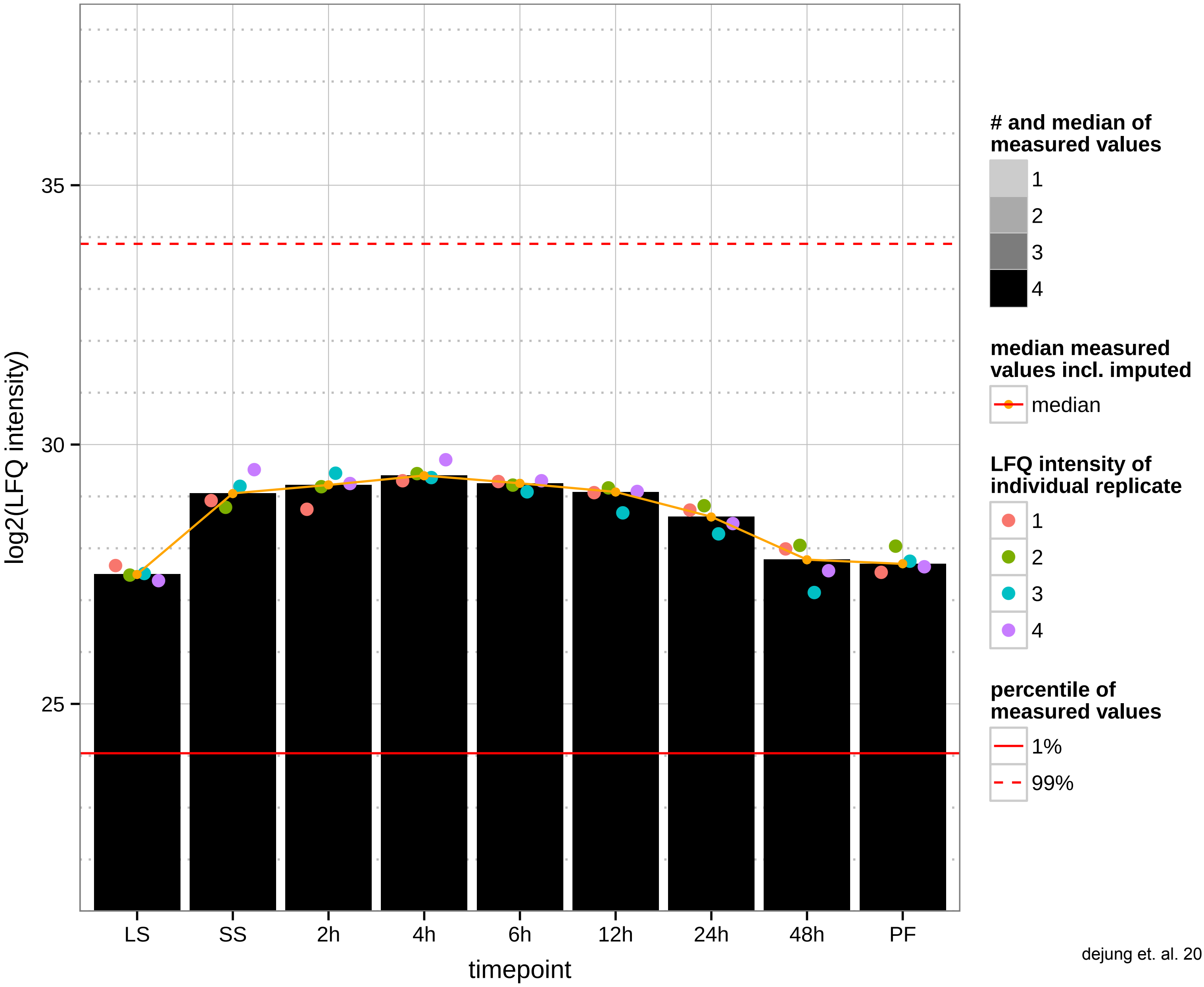

dejung et. al. 2015

Lys–63–specific deubiquitinase BRCC36, putative, JAB–MPN domain protein
Tb927.9.1680
AGOF: metallopeptidase activity, polyubiquitin binding, ubiquitin thiolesterase activity
AGOC: BRCA1–A complex, BRISC complex, nuclear ubiquitin ligase complex, nucleus
AGOP: G2/M transition DNA damage checkpoint, double–strand break repair, histone H2A K63–linked deubiquitination, prote
PGOF: protein binding
PGOC: null
PGOP: null

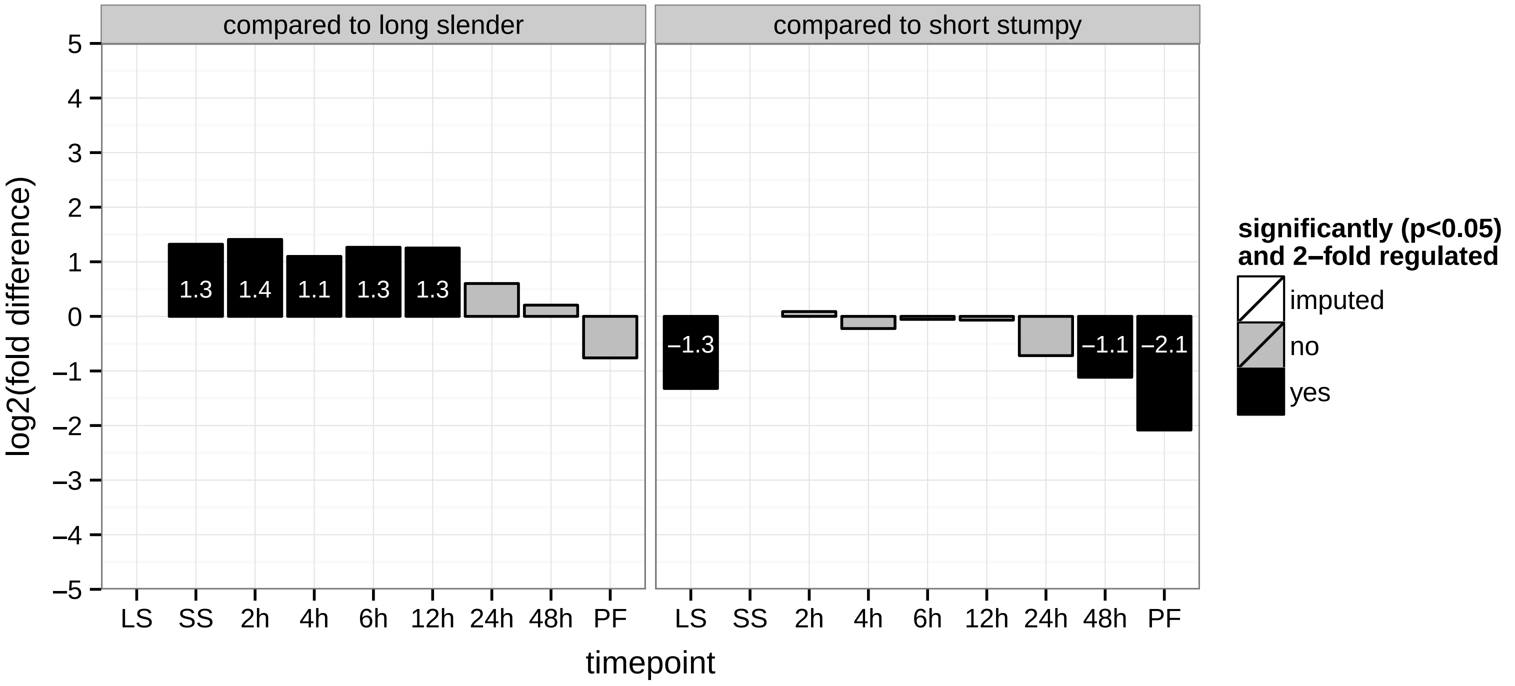

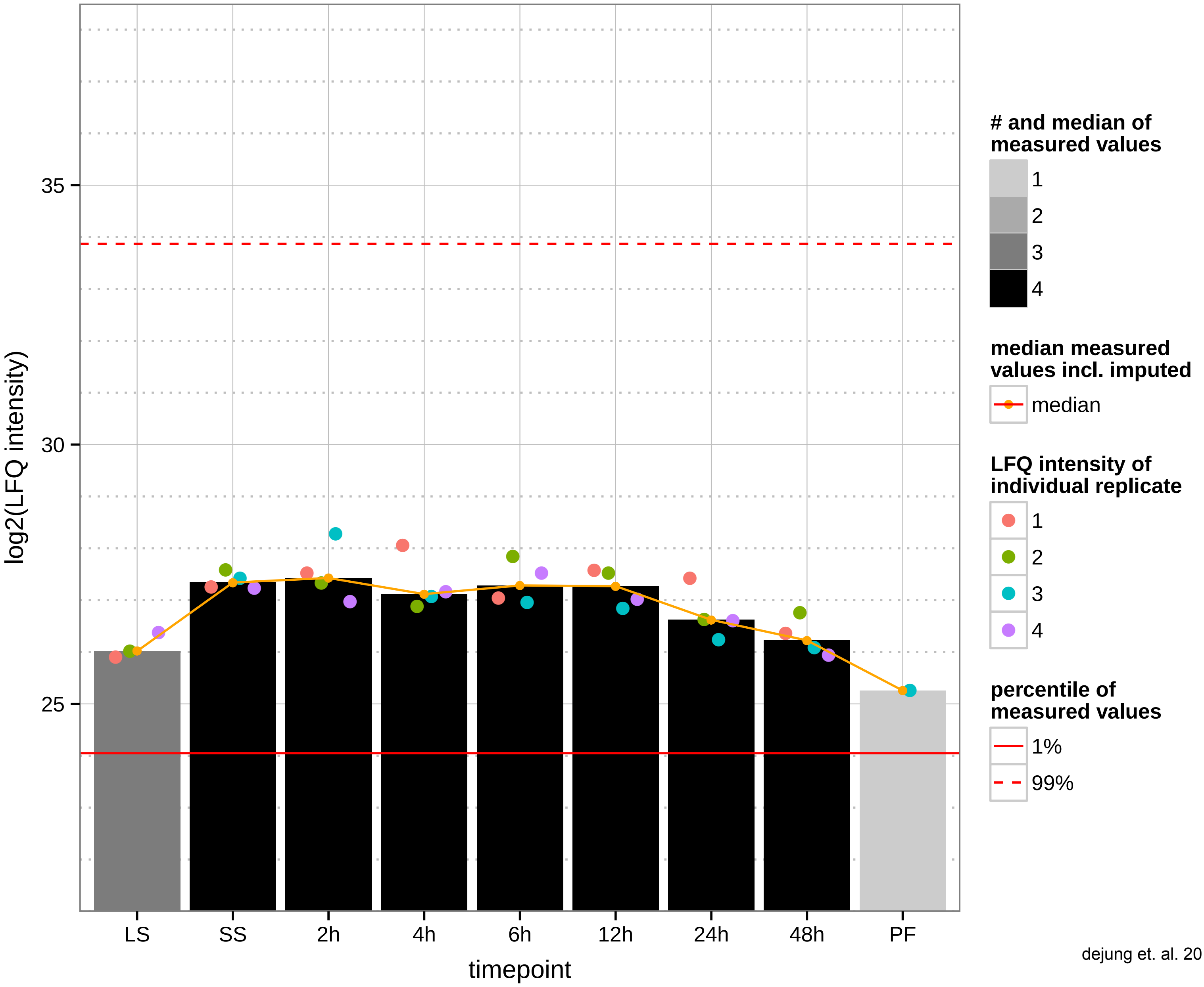

dejung et. al. 2015

GTPase activating protein, putative
Tb927.9.4370
AGOF: Rab GTPase activator activity, protein kinase activity
AGOC: intracellular
AGOP: regulation of Rab GTPase activity
PGOF: Rab GTPase activator activity
PGOC: intracellular
PGOP: regulation of Rab GTPase activity

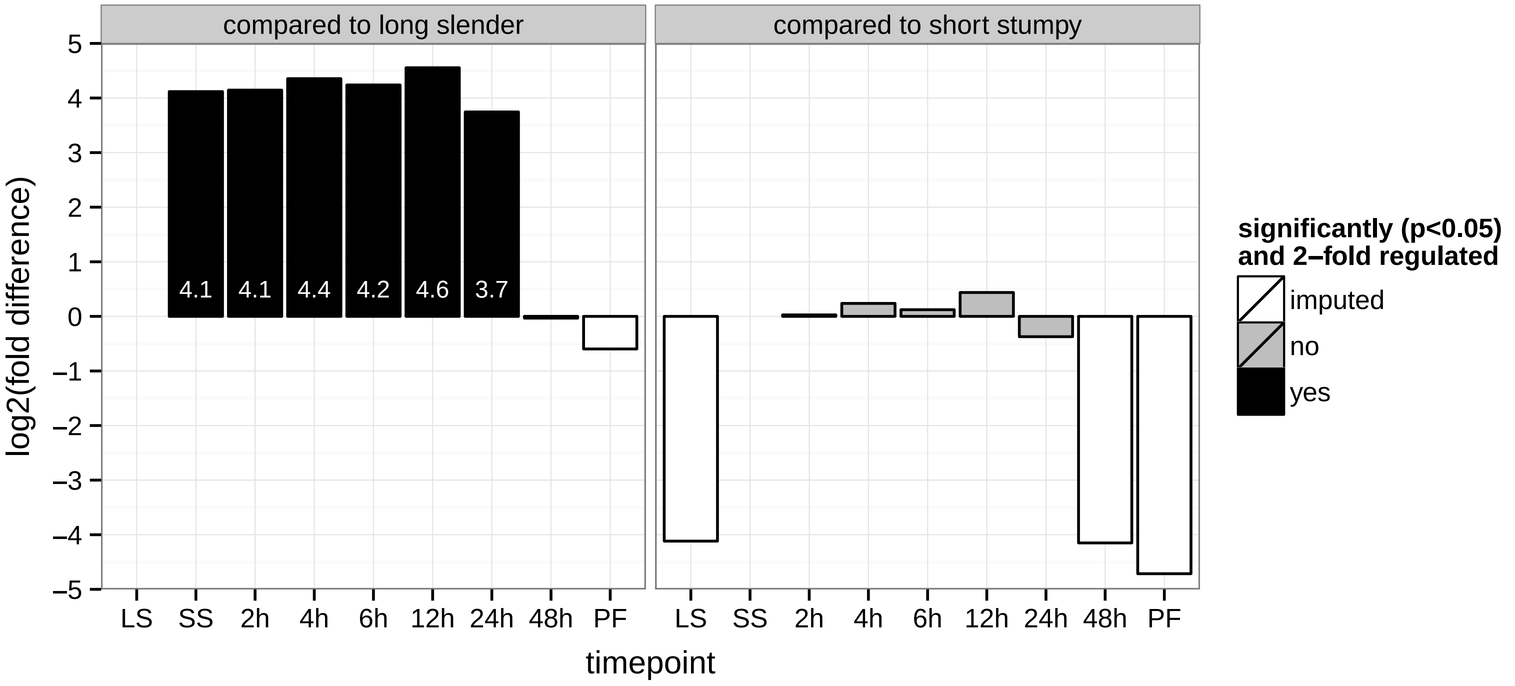

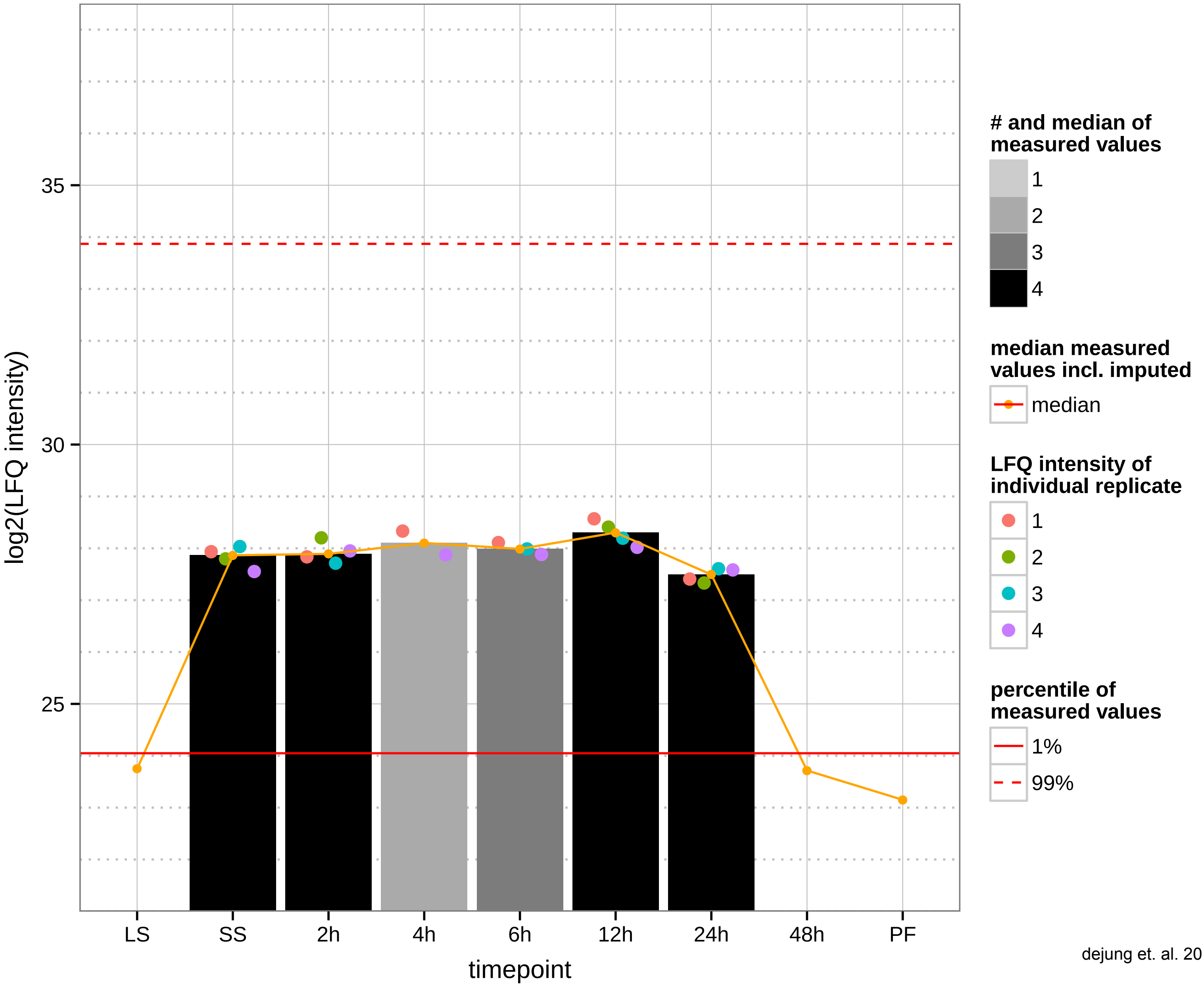

dejung et. al. 2015

inositol/phosphatidylinositol phosphatase, putative
Tb927.9.5680
AGOF: null
AGOC: intracellular
AGOP: signal transduction
PGOF: null
PGOC: intracellular
PGOP: phosphatidylinositol phosphorylation, signal transduction

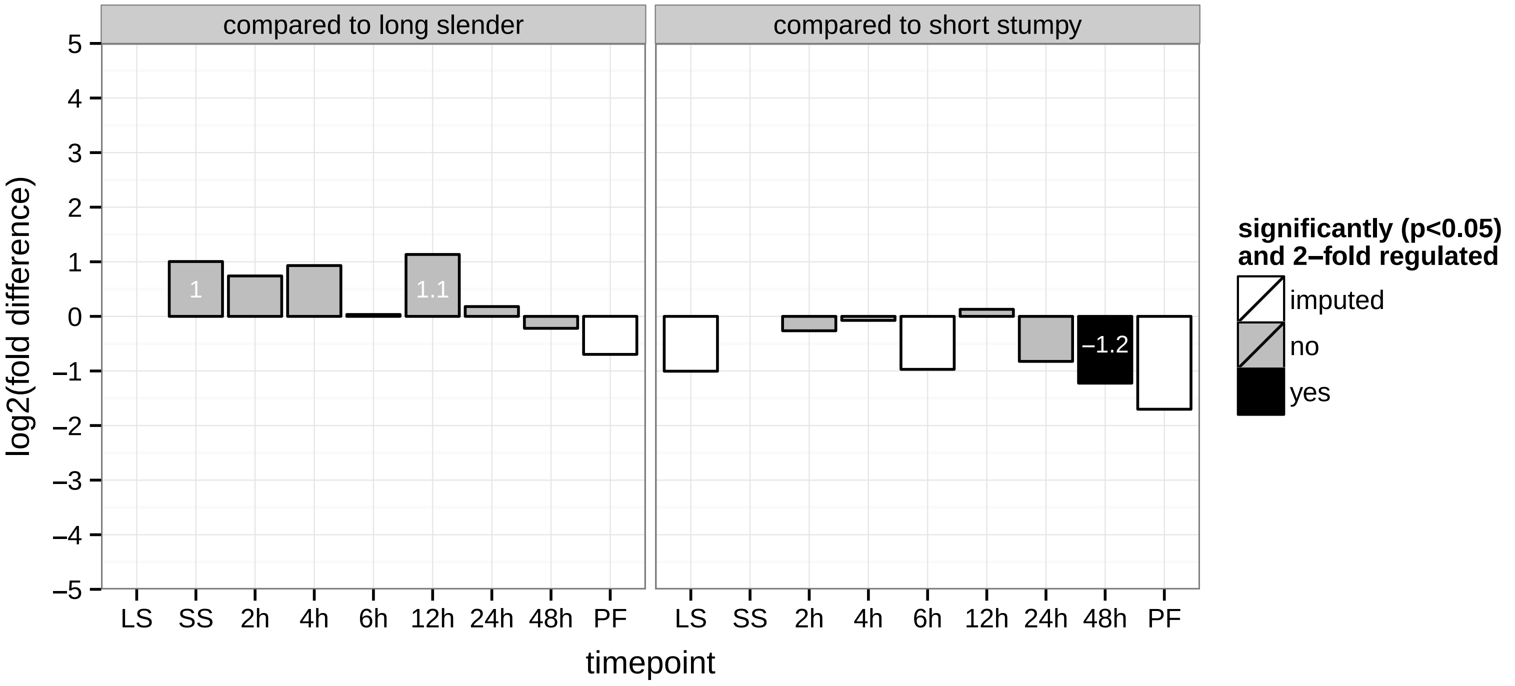

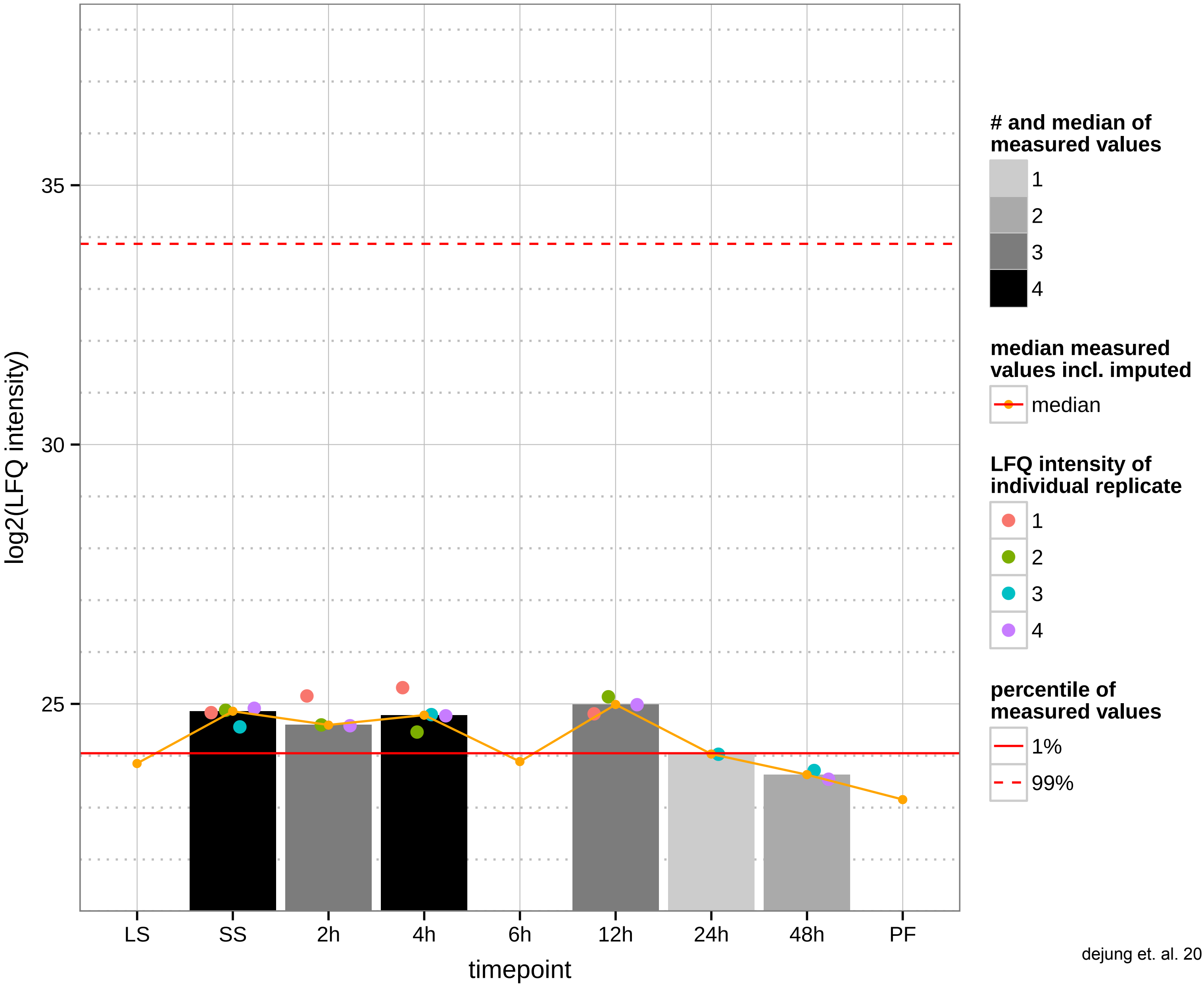

dejung et. al. 2015

hypothetical protein
Tb927.9.6530
AGOF: null
AGOC: null
AGOP: null
PGOF: null
PGOC: null
PGOP: null

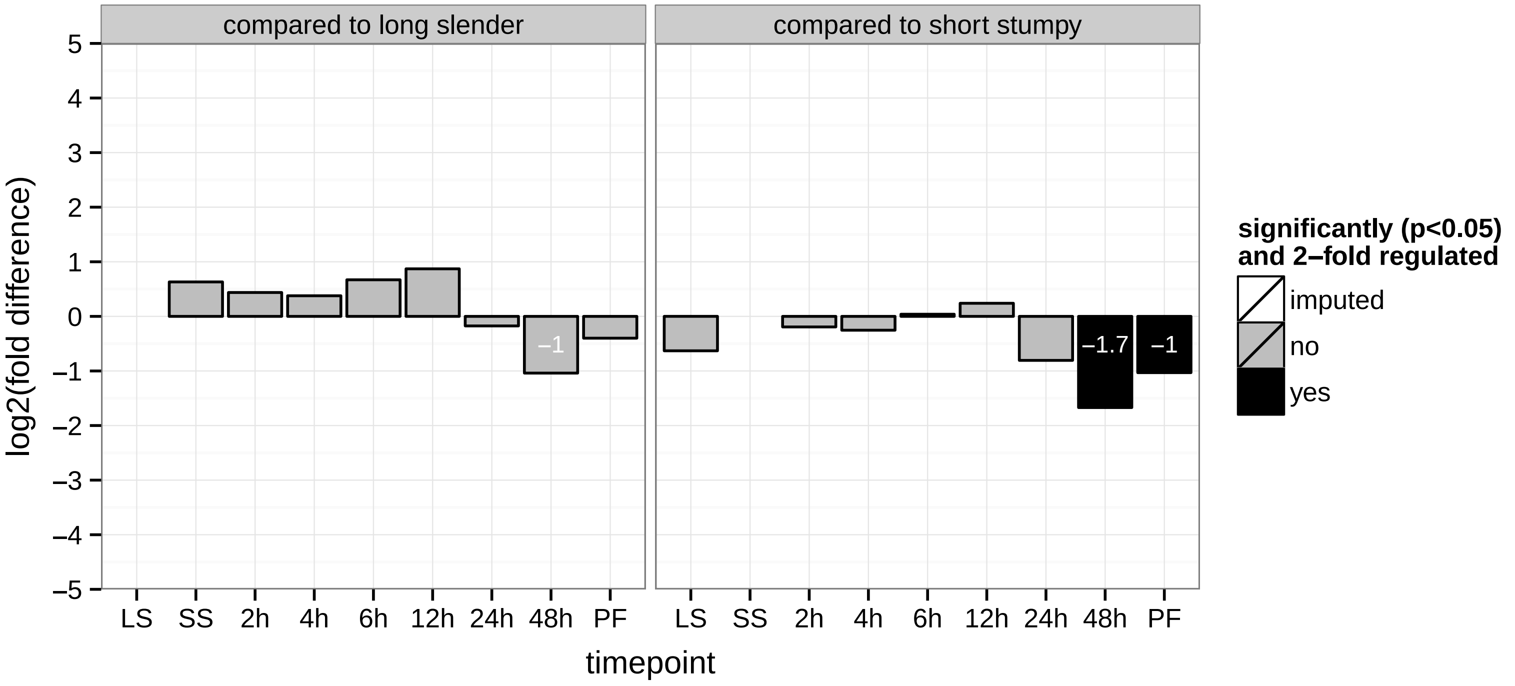

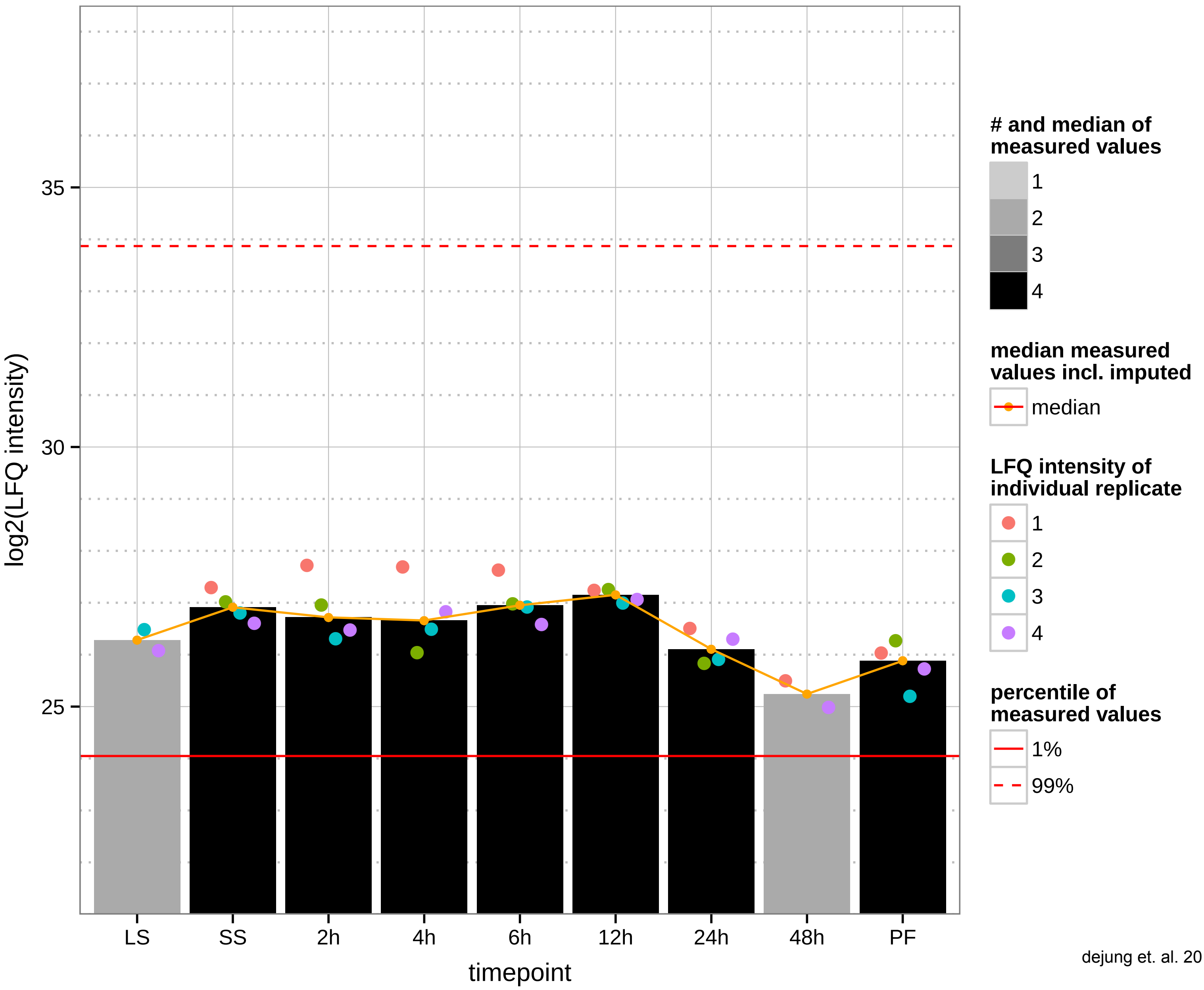

dejung et. al. 2015

ADP–ribosylation factor–like protein, putative (ARL1B)
Tb927.9.7230
AGOF: GTP binding
AGOC: intracellular
AGOP: intracellular protein transport, small GTPase mediated signal transduction
PGOF: GTP binding
PGOC: intracellular
PGOP: intracellular protein transport, protein transport, small GTPase mediated signal transduction

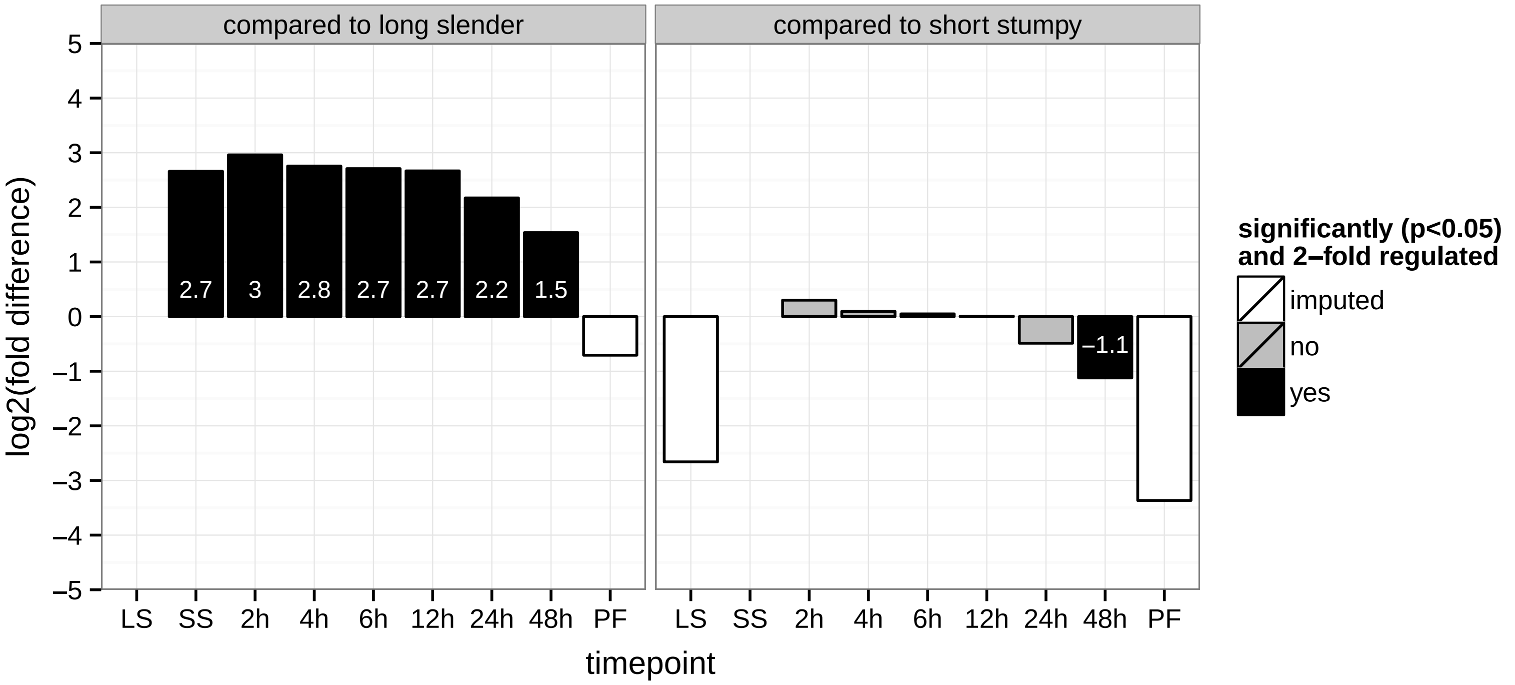

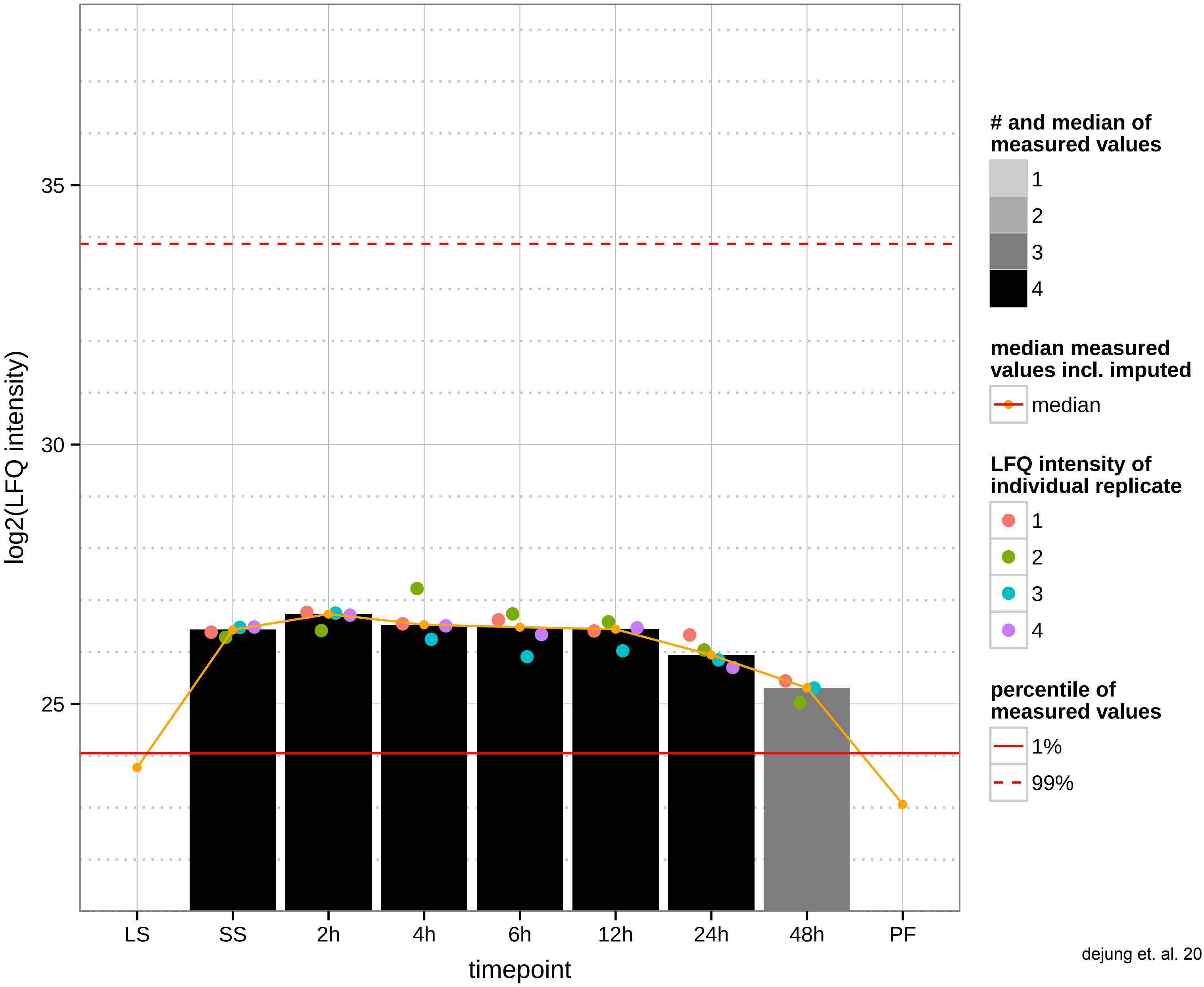

dejung et. al. 2015

hypothetical protein, conserved
Tb927.9.7520
AGOF: null
AGOC: null
AGOP: null
PGOF: protein binding
PGOC: null
PGOP: null

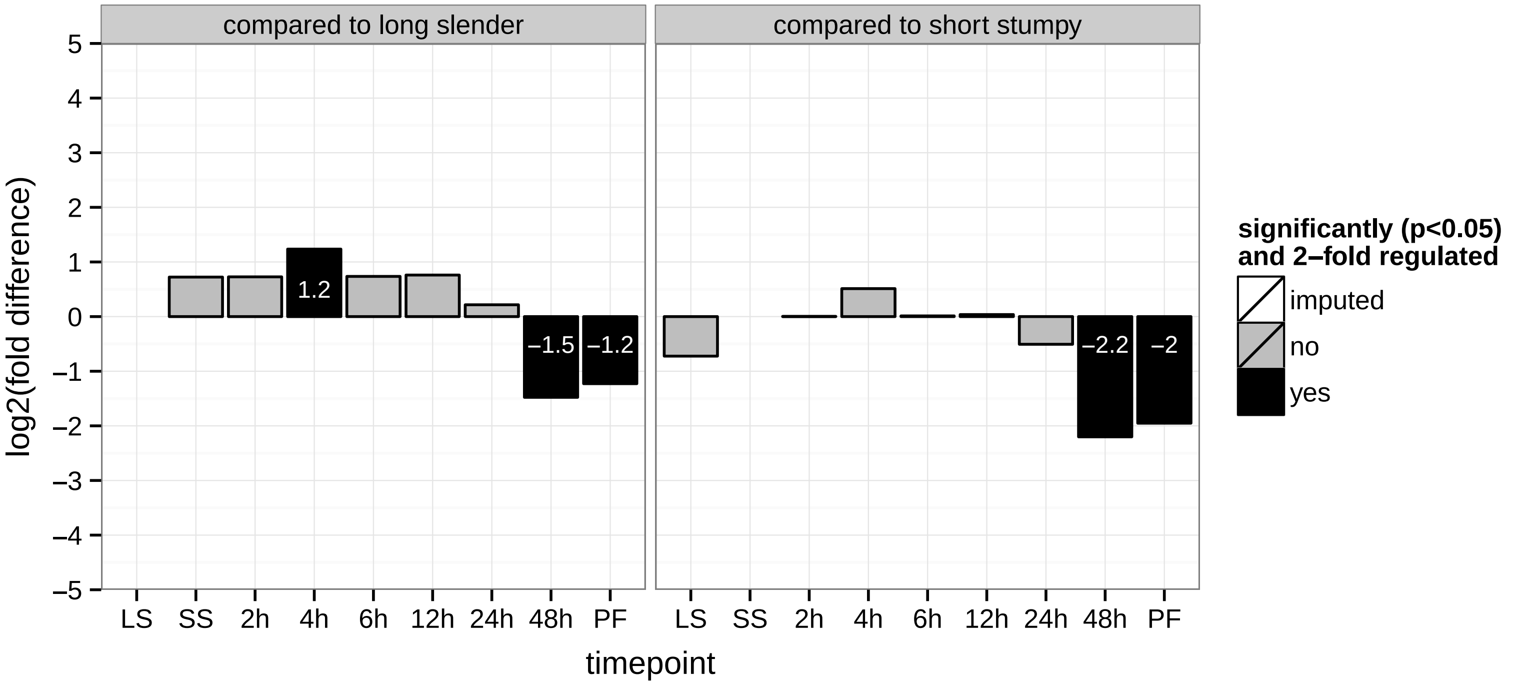

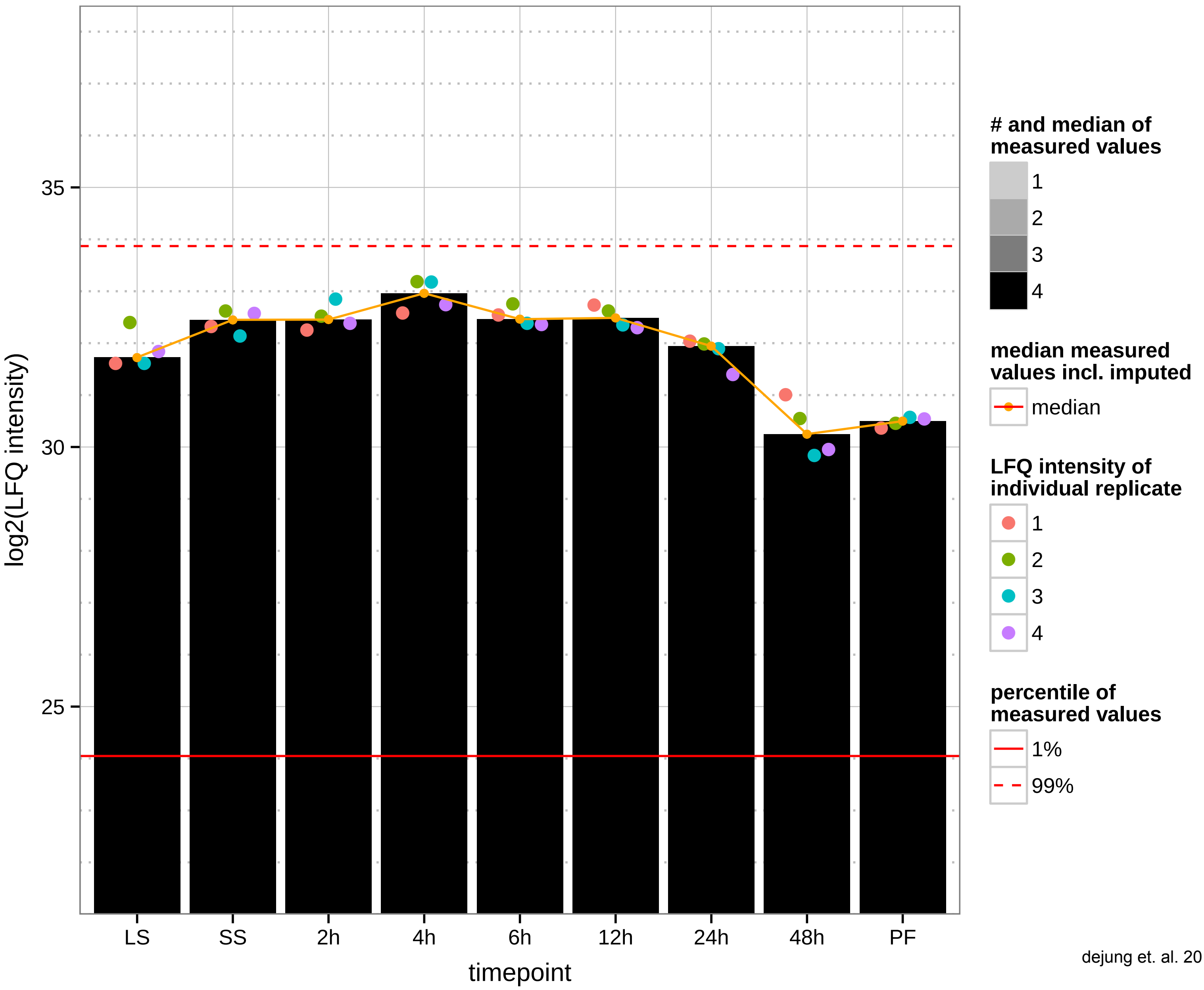

dejung et. al. 2015

ubiquitin−conjugating enzyme E2, putative, ubiquitin carrier protein, ubiquitin−protein ligase
Tb927.9.8000
AGOF: ubiquitin−protein ligase activity
AGOC: null
AGOP: cellular protein modification process, post−translational protein modification, regulation of protein metabolic process
PGOF: acid−amino acid ligase activity
PGOC: null
PGOP: null

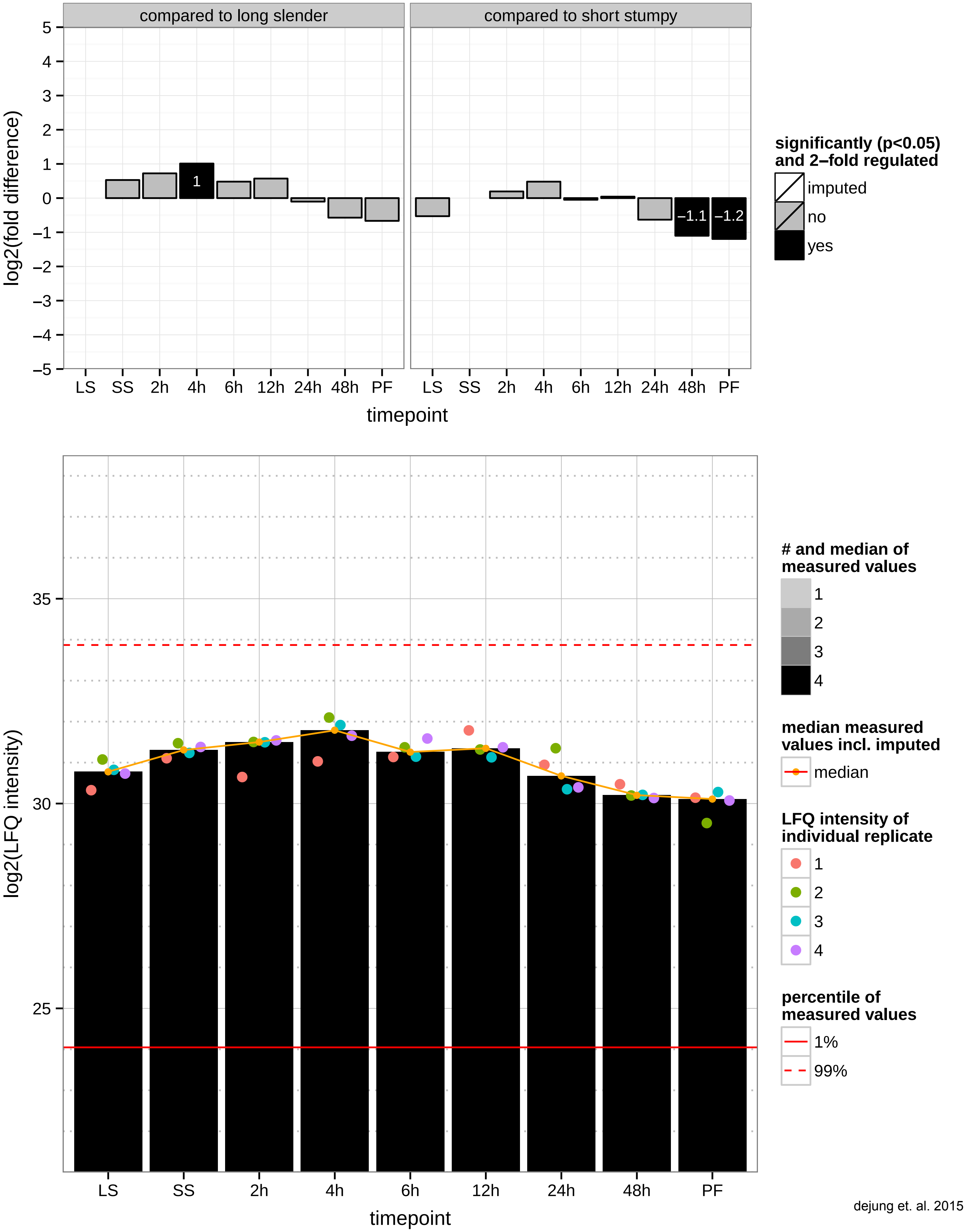

dejung et. al. 2015

hypothetical protein, conserved
Tb927.9.8060
AGOF: null
AGOC: null
AGOP: null
PGOF: null
PGOC: null
PGOP: null

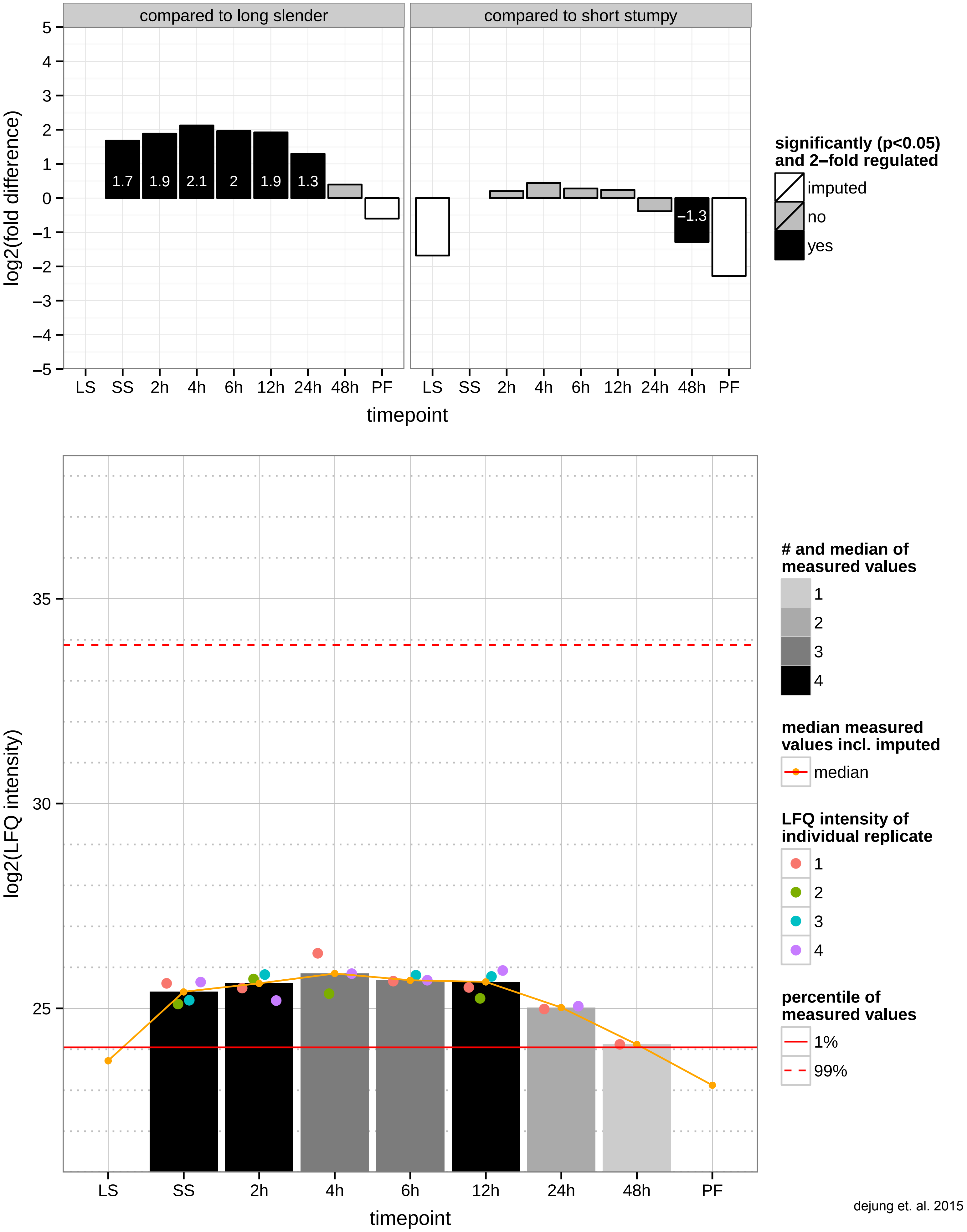

dejung et. al. 2015

fructose−1, 6−bisphosphatase (FBPase)
Tb927.9.8720
AGOF: fructose 1, 6−bisphosphate 1−phosphatase activity
AGOC: null
AGOP: carbohydrate metabolic process
PGOF: phosphoric ester hydrolase activity
PGOC: null
PGOP: carbohydrate metabolic process

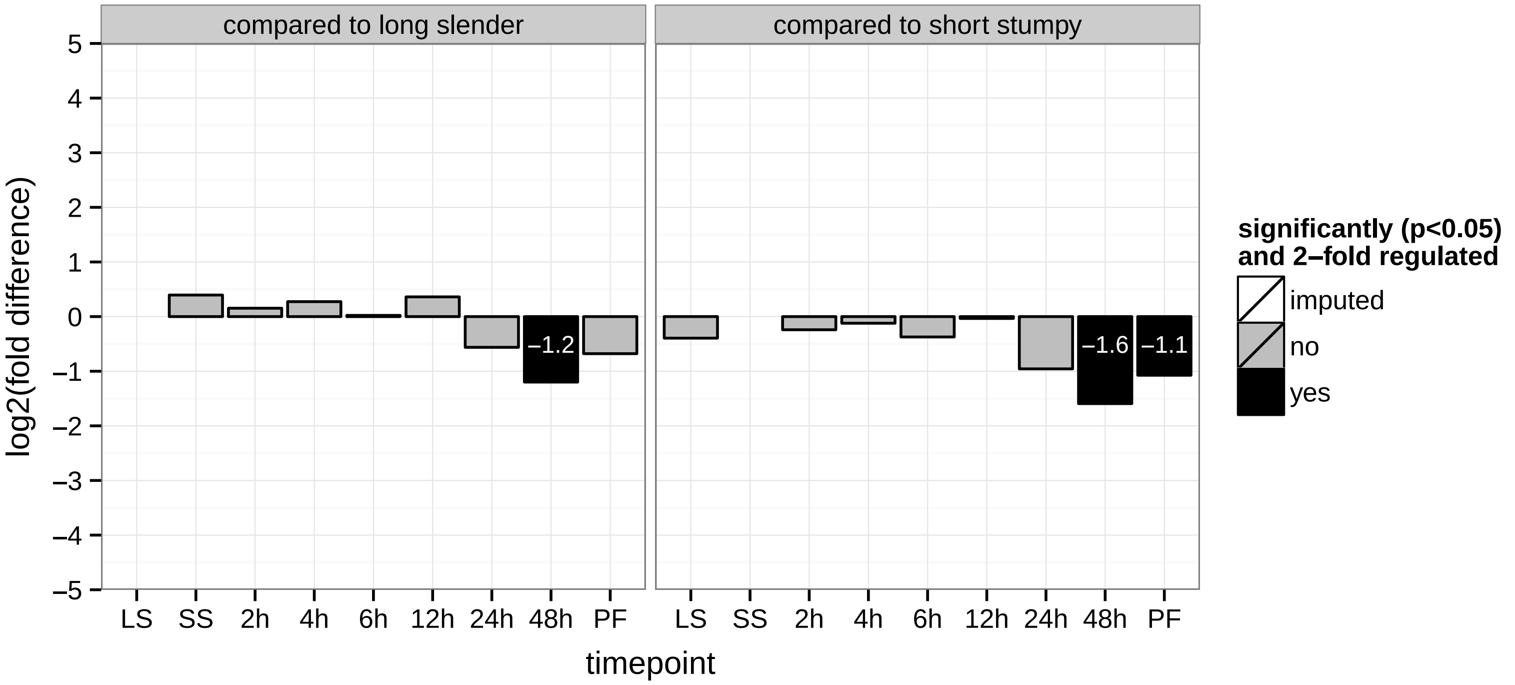

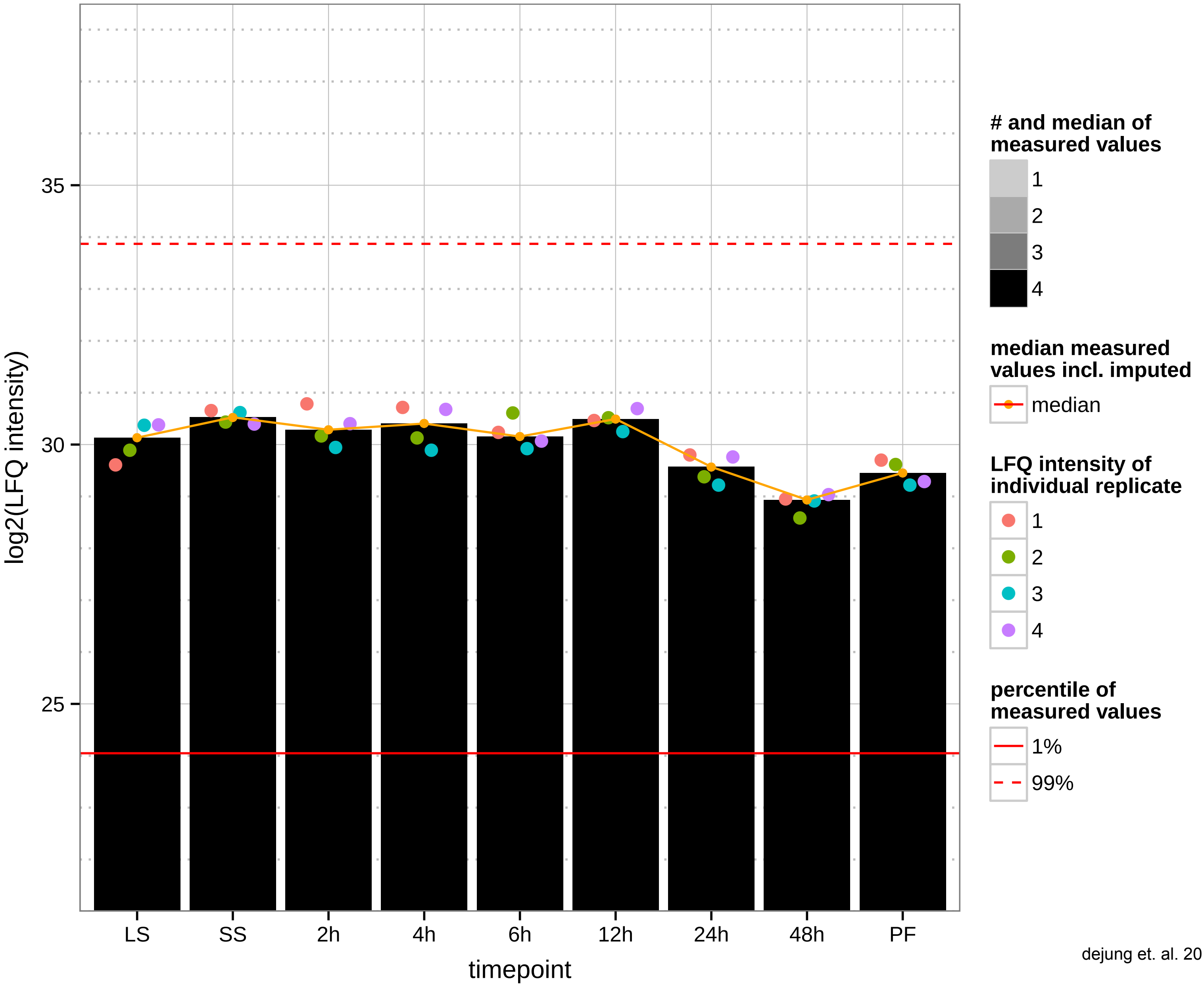

dejung et. al. 2015

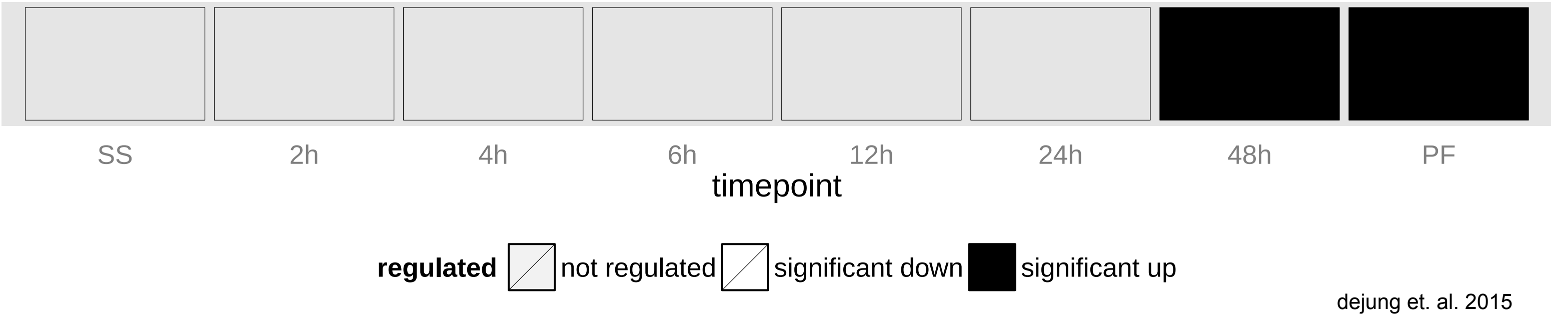
SS
2h
4h
6h
12h
24h
48h
PF
timepoint
regulated
not regulated
significant down
significant up
dejung et. al. 2015

serine/threonine protein phosphatase, putative, BAC from homologous region on chr5, kinetoplastid−specific phospho−protein
Tb927.5.4380;Tb05.5K5.30
AGOF: hydrolase activity, protein serine/threonine phosphatase activity
AGOC: null
AGOP: null, protein dephosphorylation
PGOF: hydrolase activity
PGOC: null
PGOP: null

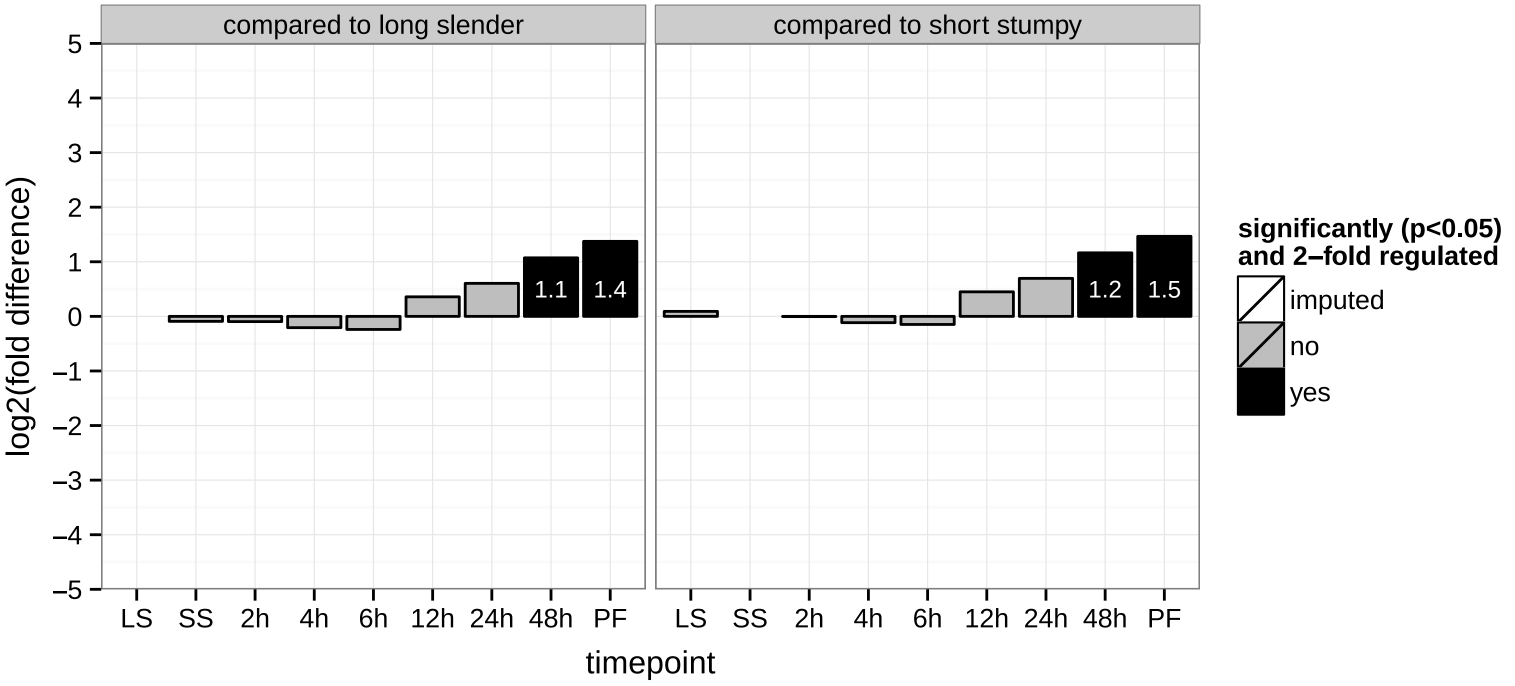

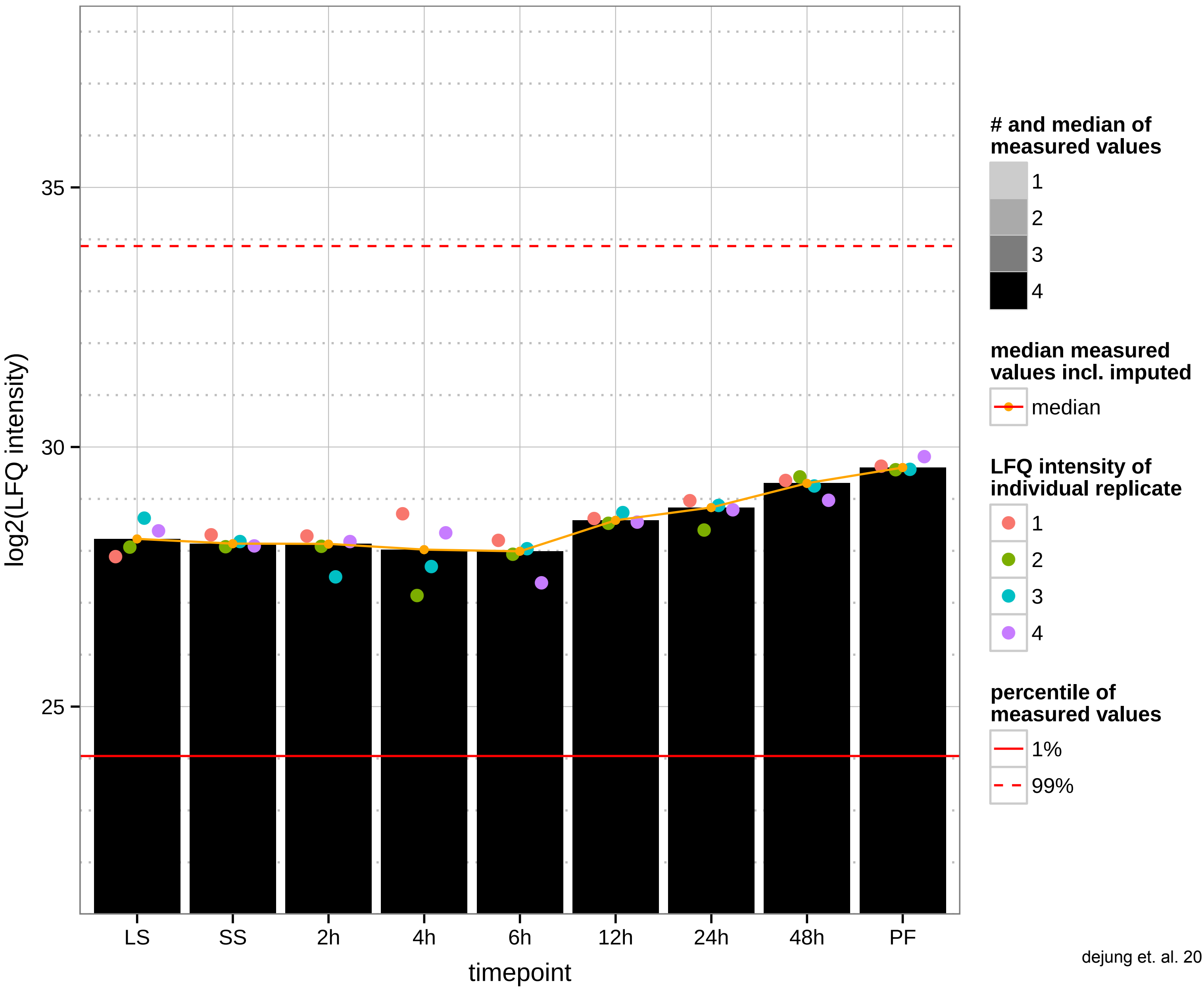

dejung et. al. 2015

60S ribosomal protein L21e, putative, chrXI additional, unordered contigs, ribosomal protein L21E (60S)
Tb927.4.1100;Tb927.11.680;Tb11.0880
AGOF: structural constituent of ribosome
AGOC: intracellular, ribosome
AGOP: translation
PGOF: structural constituent of ribosome
PGOC: intracellular, ribosome
PGOP: translation

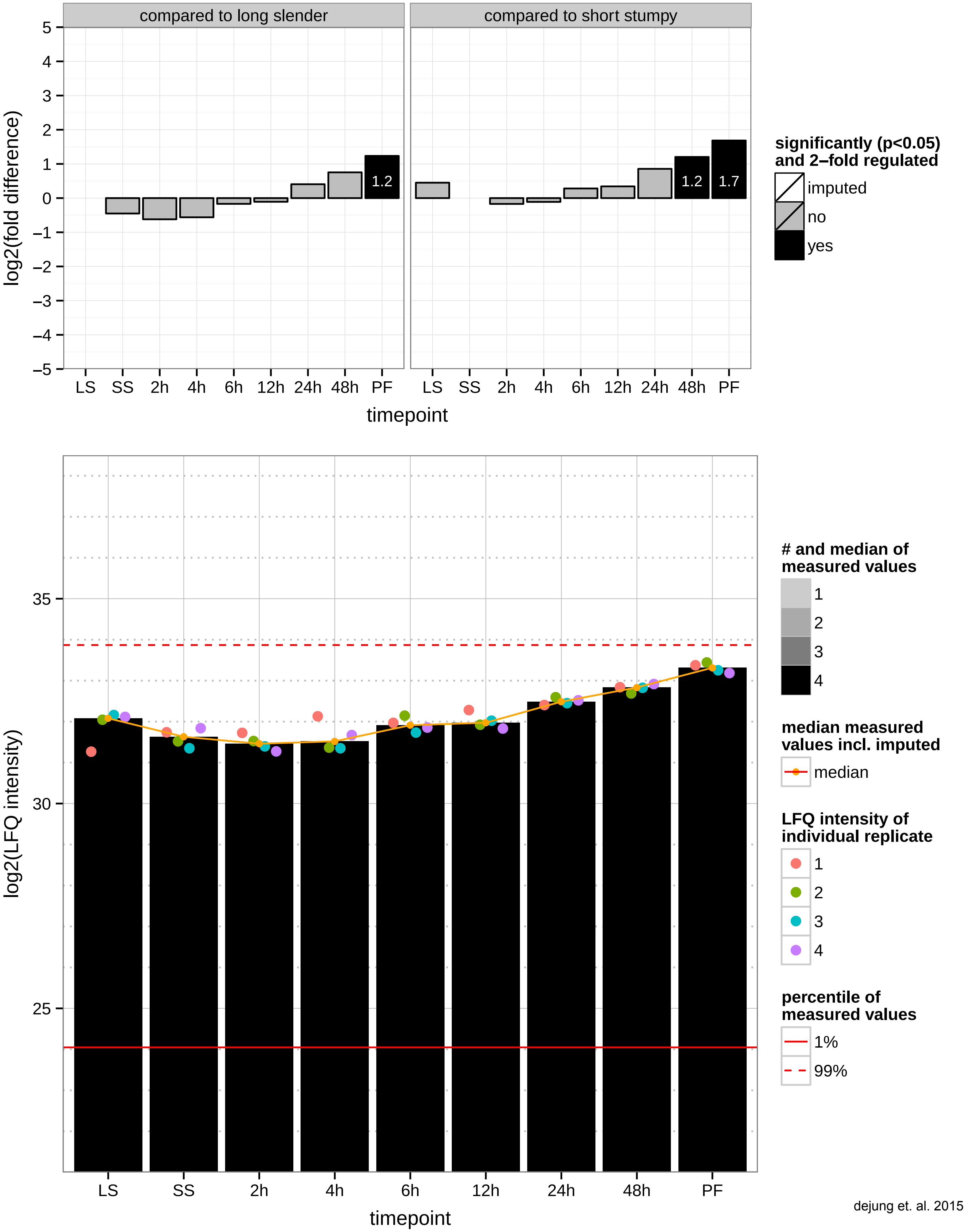

dejung et. al. 2015

hypothetical protein, chrXI additional, unordered contigs, methionine aminopeptidase, putative, metallo– peptidase, Clan MG,
Tb927.10.15270;Tb11.v5.0998;Tb11.1240
AGOF: aminopeptidase activity, metalloexopeptidase activity, null
AGOC: null
AGOP: cellular process, proteolysis, null
PGOF: aminopeptidase activity, metalloexopeptidase activity, zinc ion binding
PGOC: null
PGOP: cellular process, proteolysis

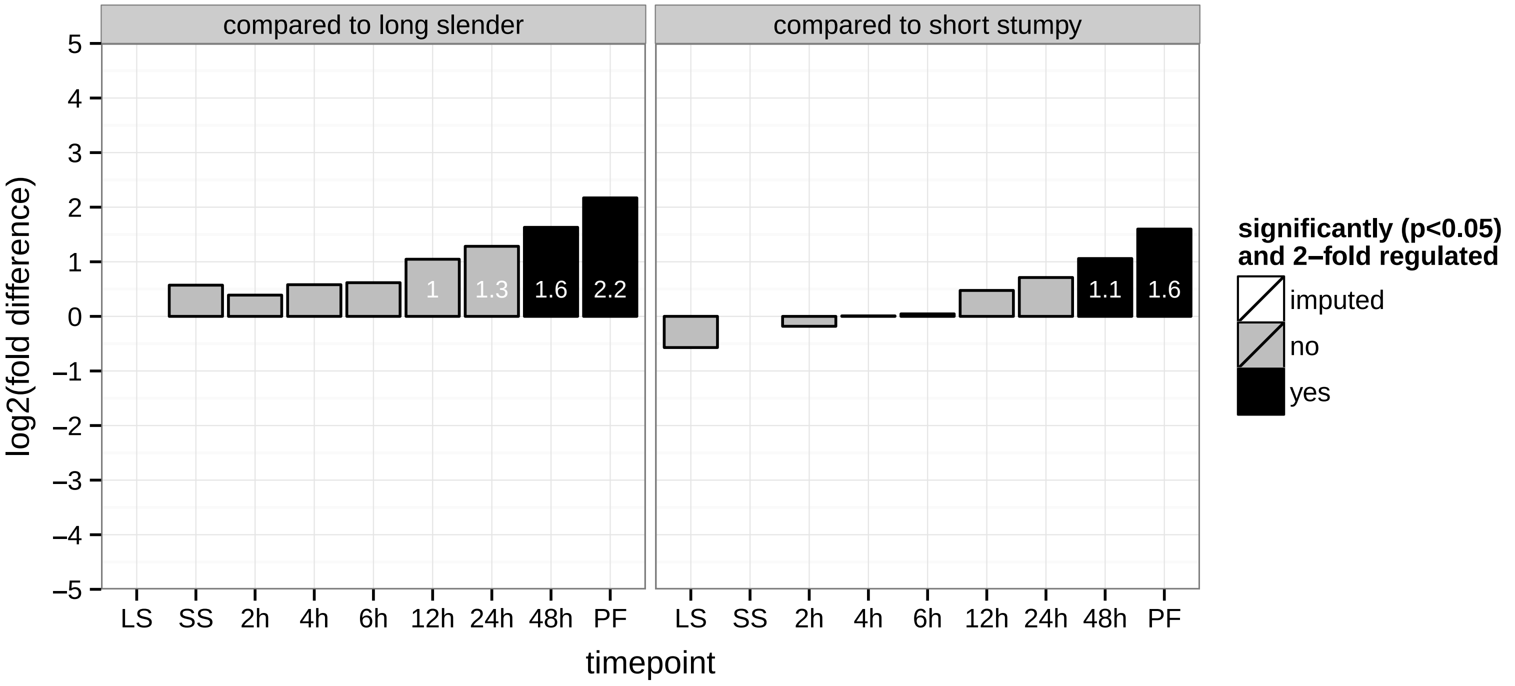

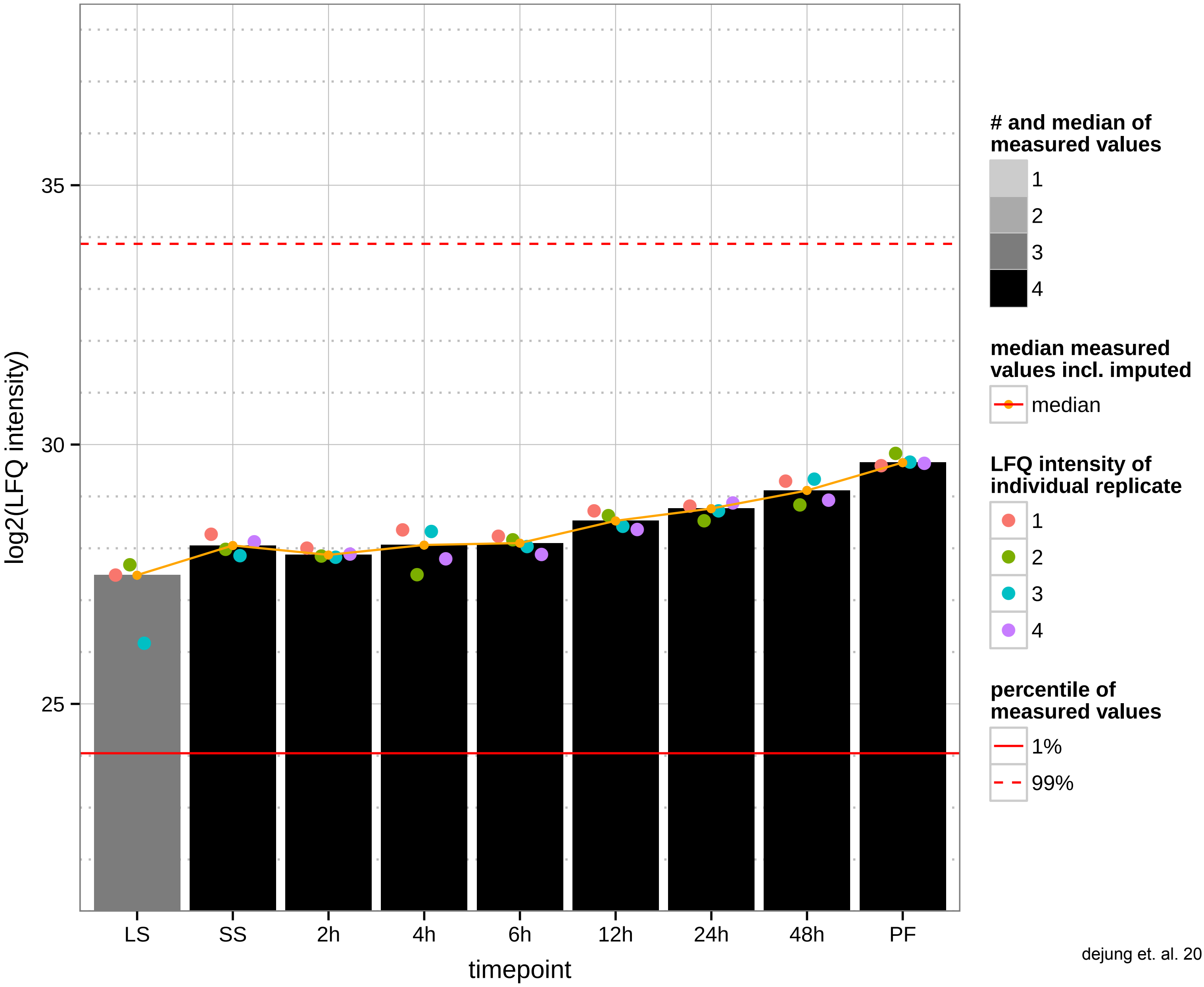

dejung et. al. 2015

hypothetical protein, conserved
Tb927.7.4890;Tb11.v5.0170
AGOF: null
AGOC: null
AGOP: null
PGOF: null
PGOC: null
PGOP: null

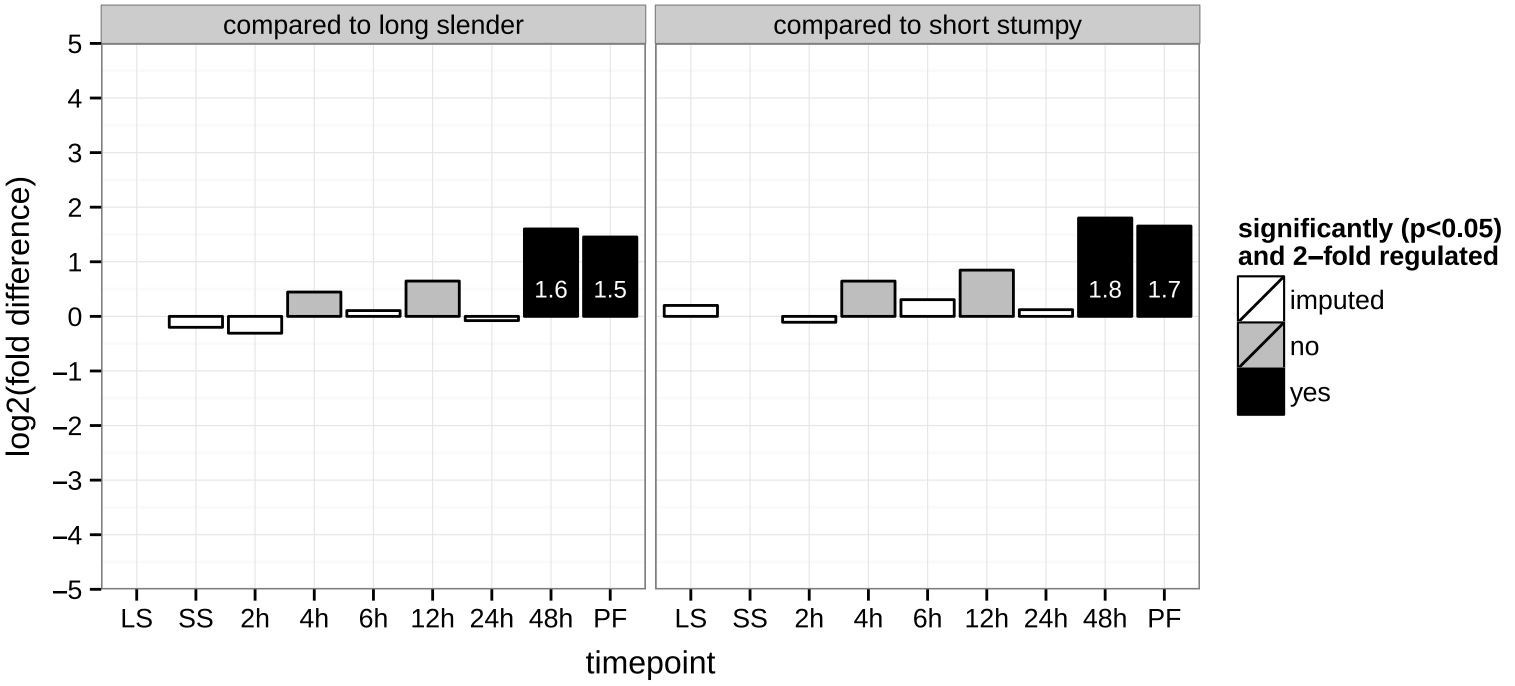

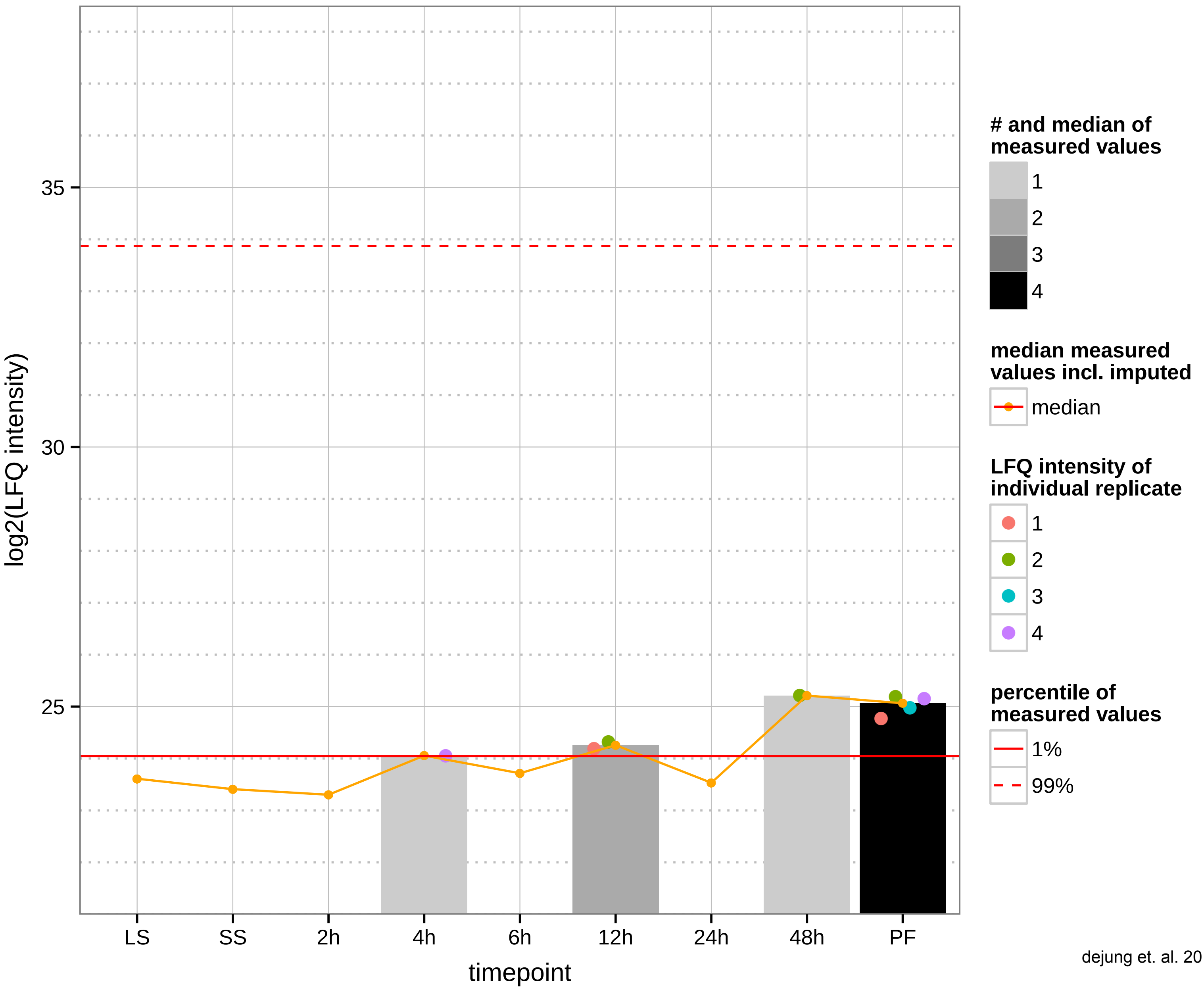

dejung et. al. 2015

pantothenate kinase subunit, putative
Tb927.11.7290;Tb11.v5.0254
AGOF: null, ATP binding, pantothenate kinase activity
AGOC: null
AGOP: null, coenzyme A biosynthetic process
PGOF: ATP binding, catalytic activity, pantothenate kinase activity
PGOC: null
PGOP: coenzyme A biosynthetic process, metabolic process

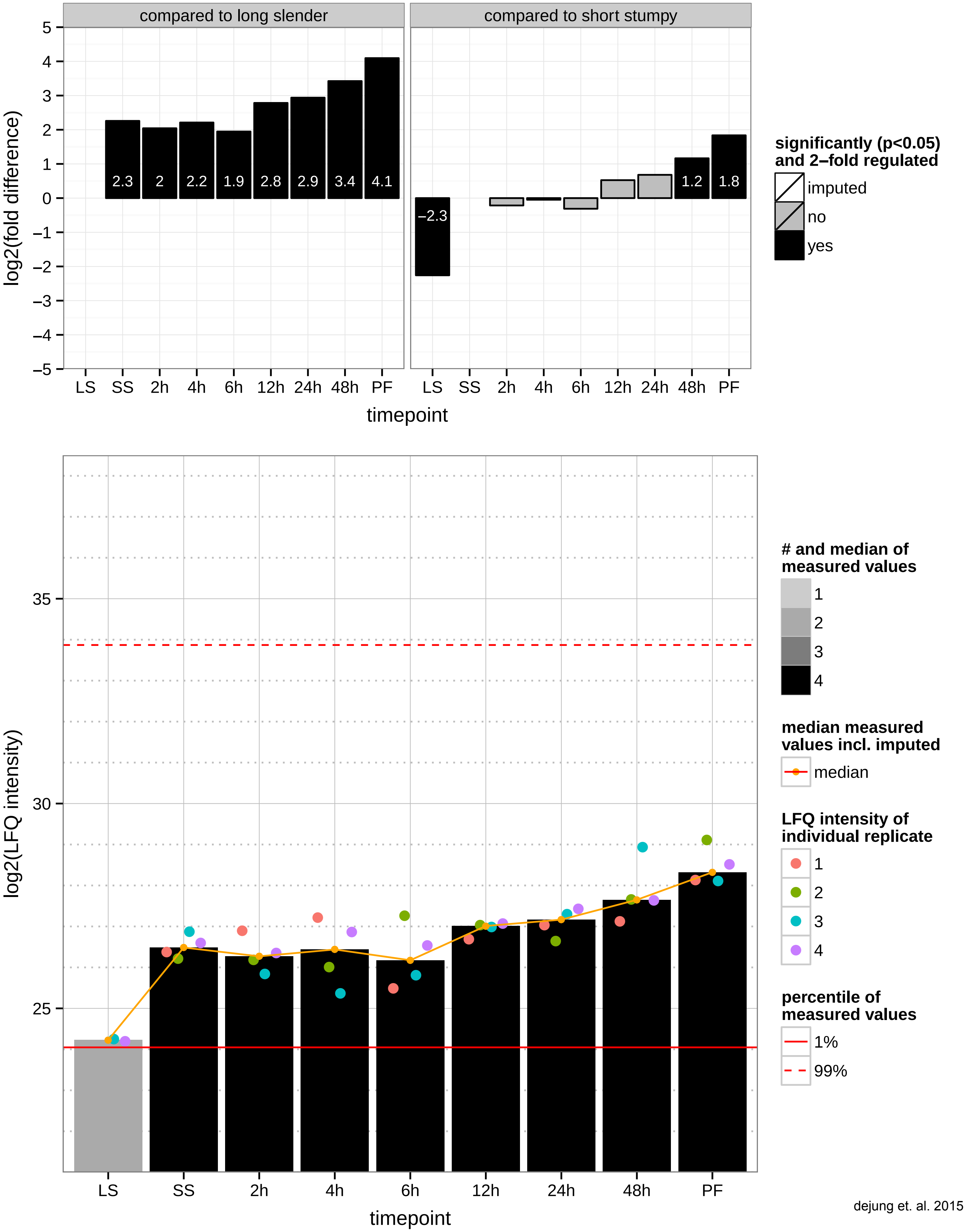

dejung et. al. 2015

hypothetical protein, conserved
Tb927.1.3270
AGOF: null
AGOC: null
AGOP: null
PGOF: null
PGOC: null
PGOP: null

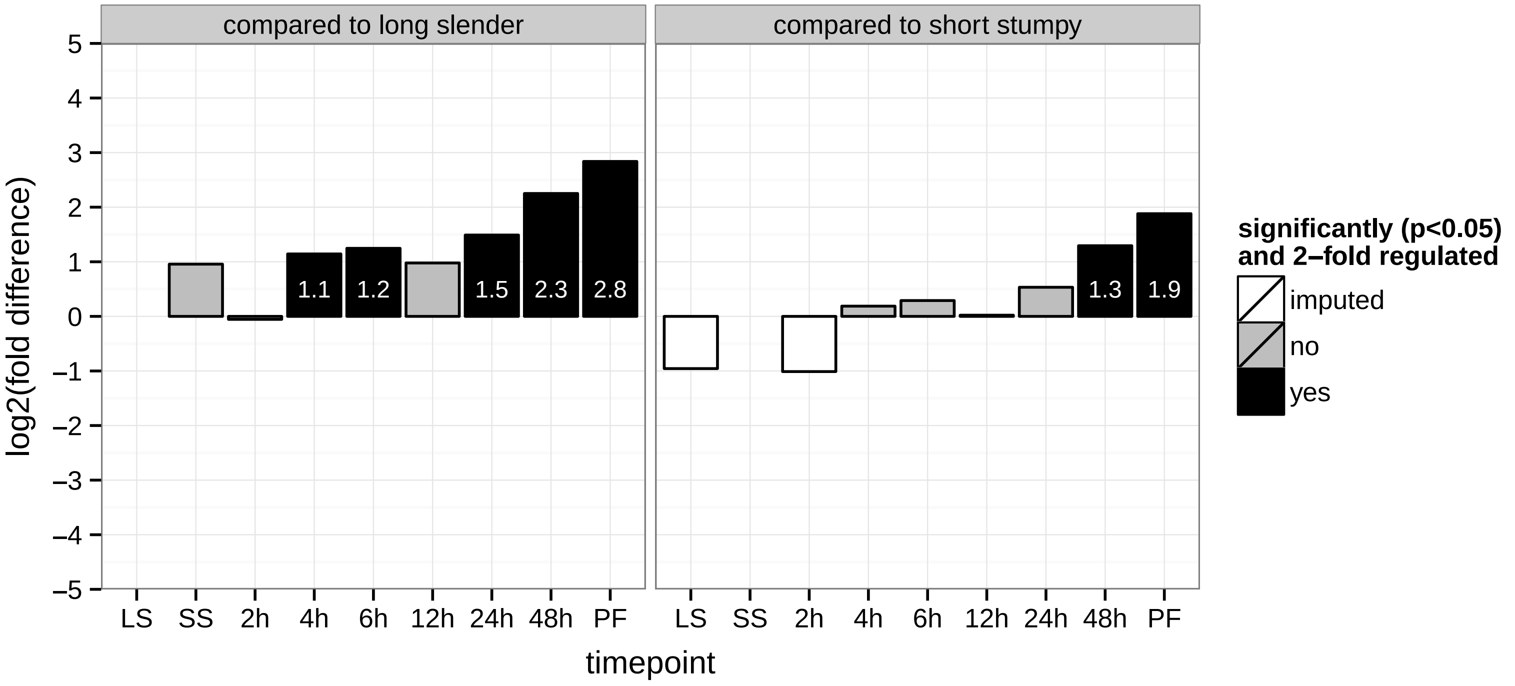

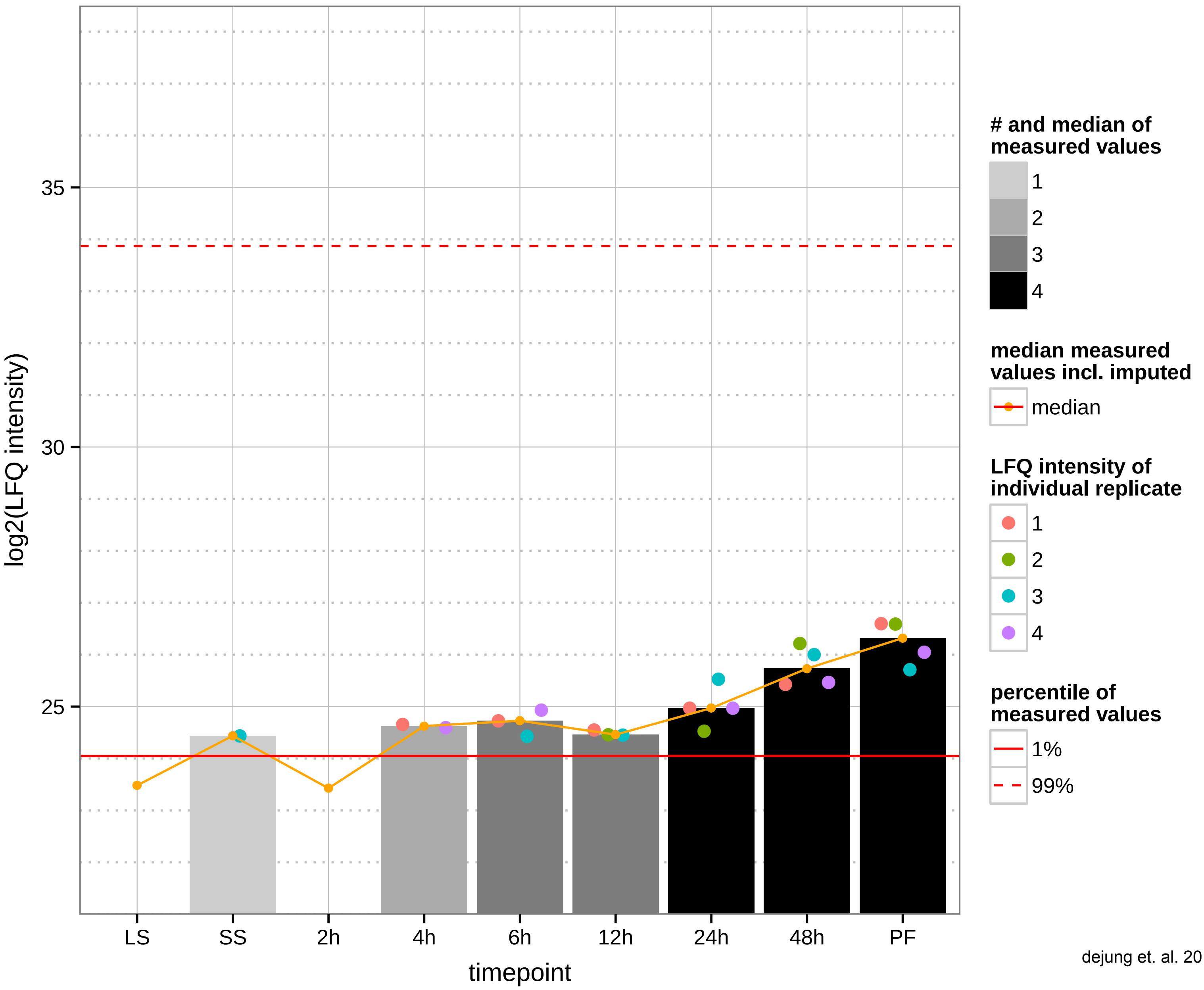

dejung et. al. 2015

protein tyrosine phosphatase, putative
Tb927.10.10610
AGOF: protein tyrosine phosphatase activity
AGOC: membrane, mitochondrial inner membrane
AGOP: null
PGOF: null
PGOC: null
PGOP: null

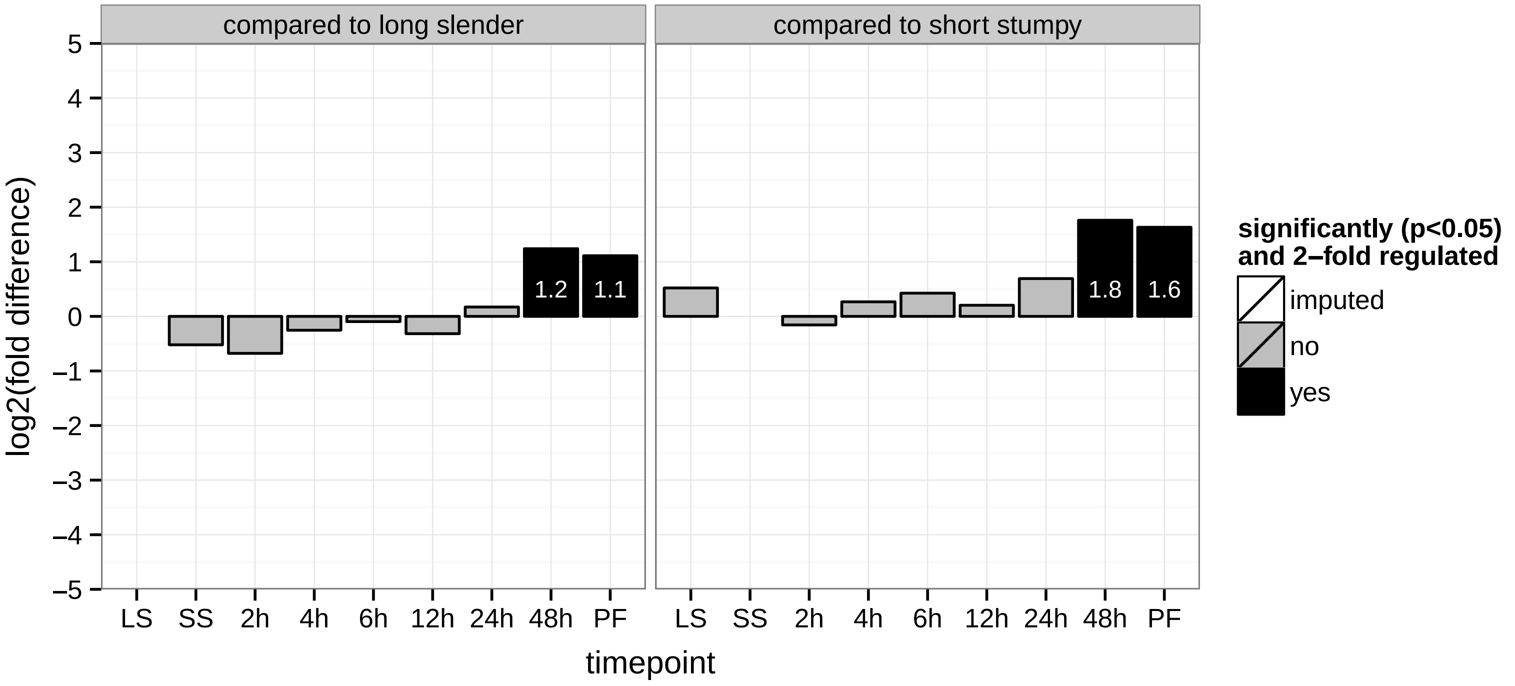

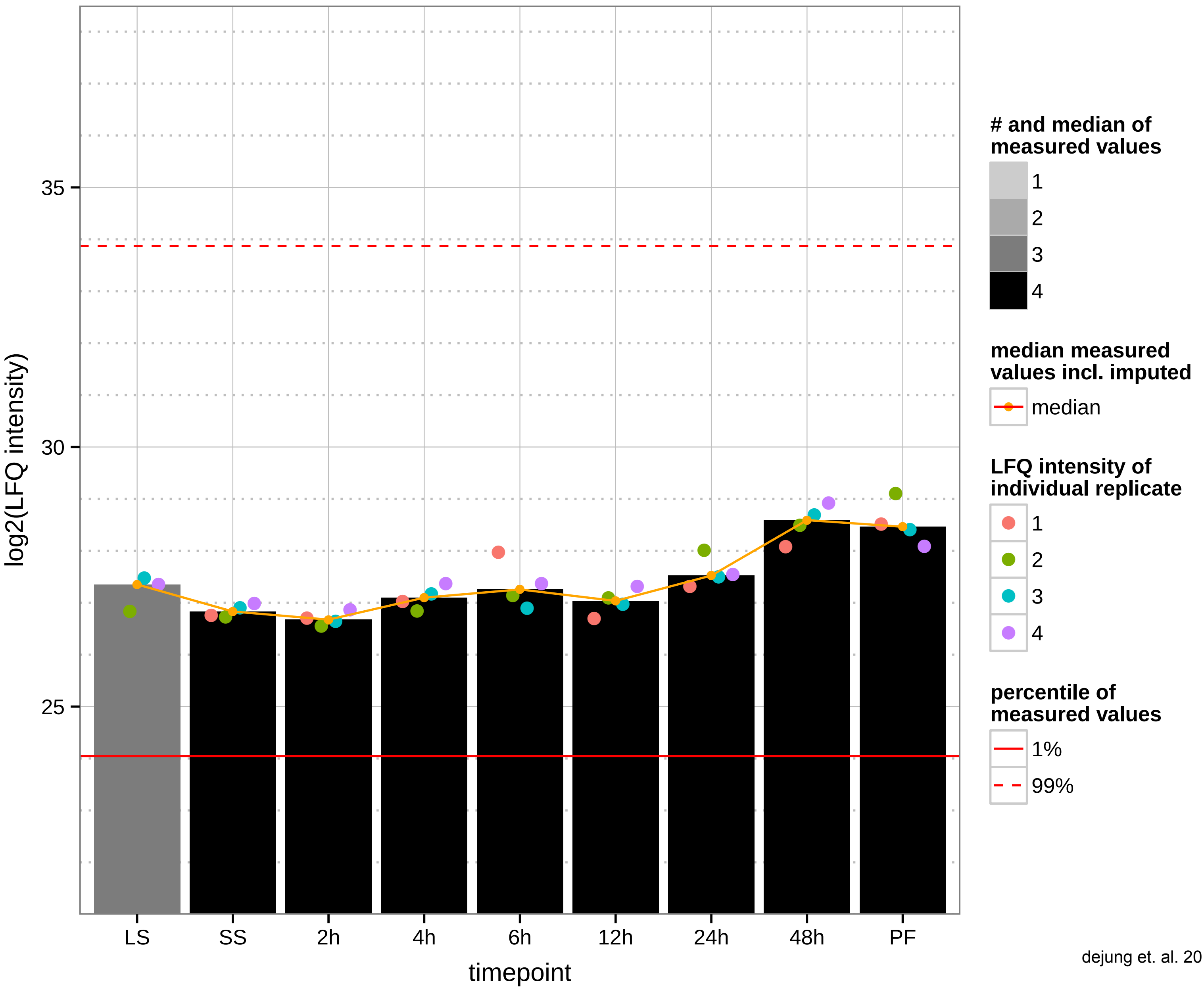

dejung et. al. 2015

ATP–binding cassette sub–family F member 1, putative, ATPase, putative (ABCF1)
Tb927.10.10880
AGOF: ATP binding, ATPase activity, coupled to transmembrane movement of substances
AGOC: ATP–binding cassette (ABC) transporter complex, integral to membrane, internal side of plasma membrane
AGOP: transport
PGOF: ATP binding, ATPase activity, nucleoside–triphosphatase activity, nucleotide binding
PGOC: null
PGOP: null

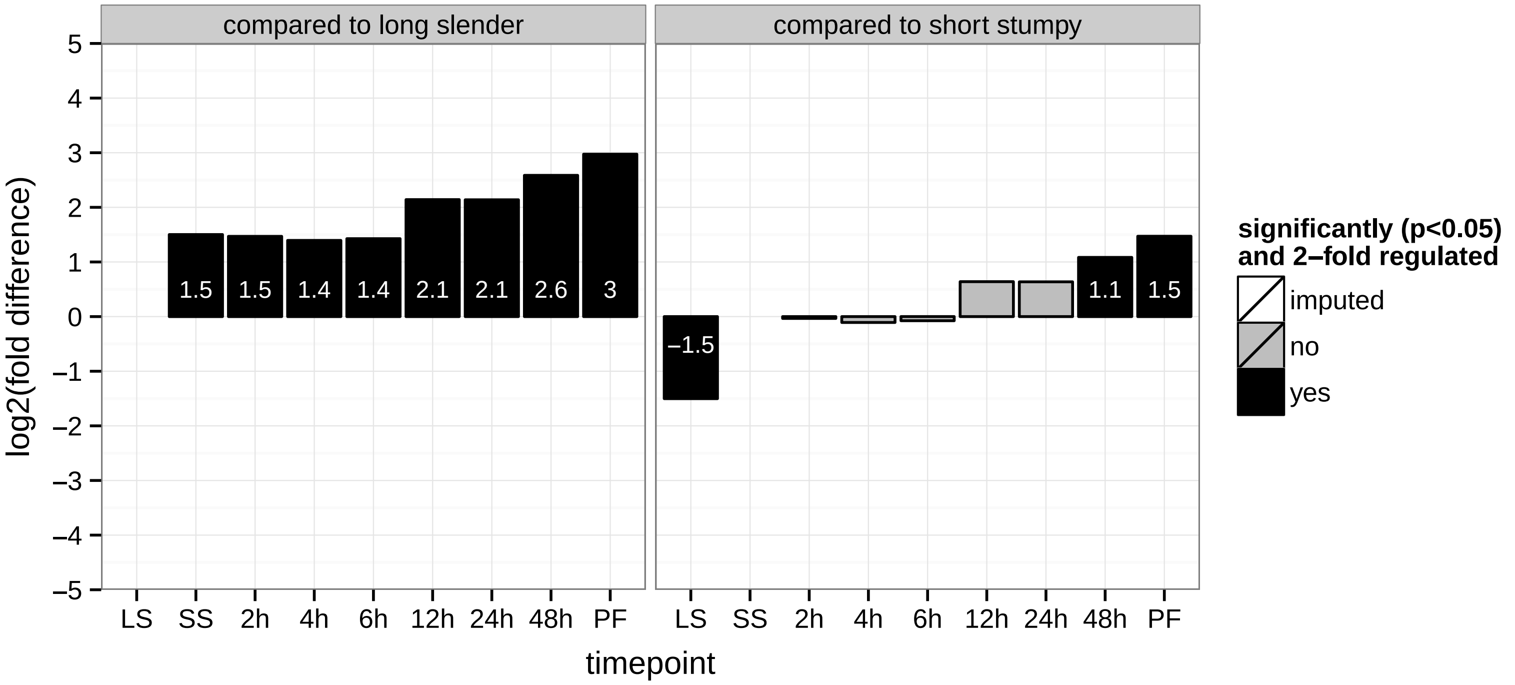

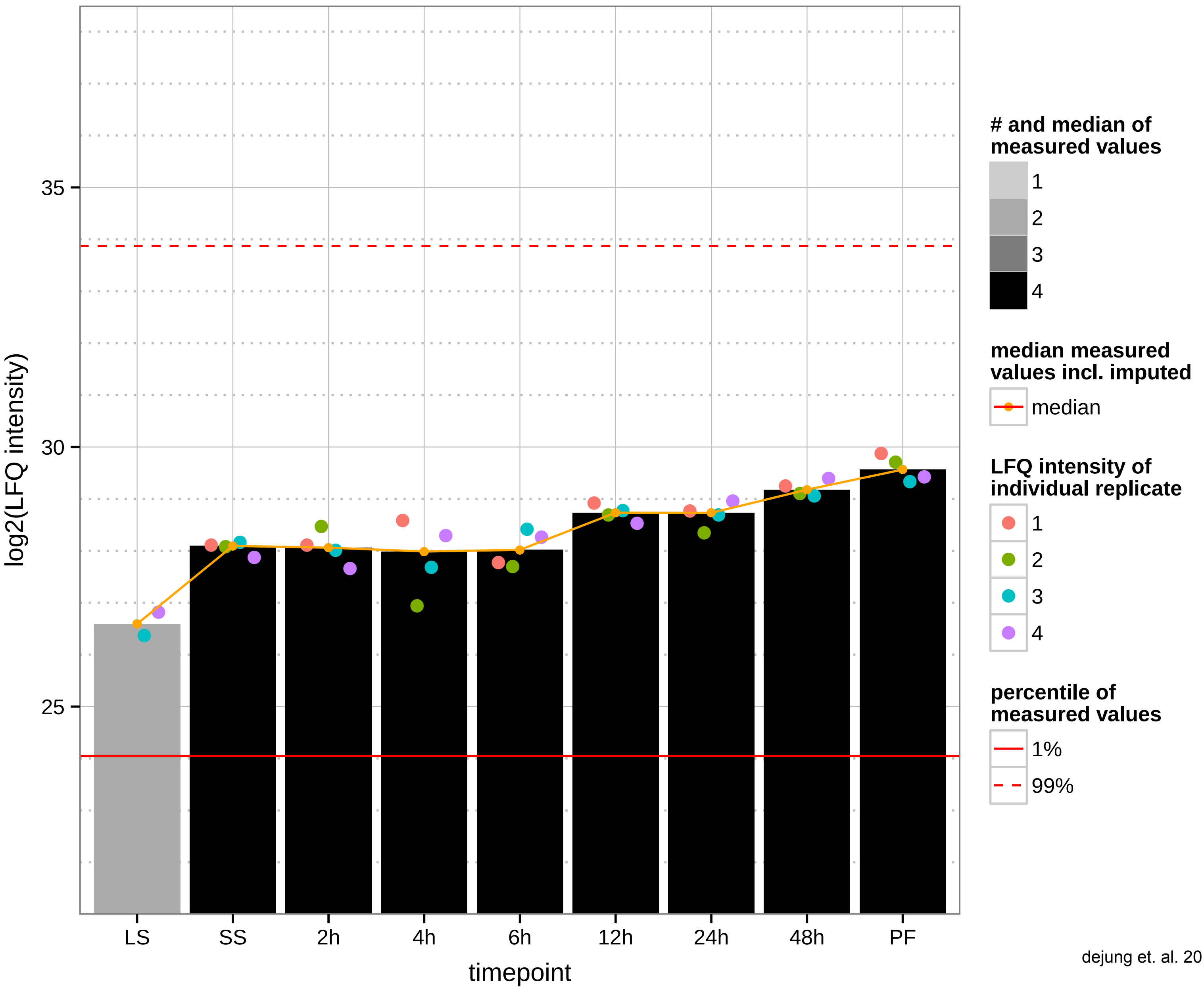

dejung et. al. 2015

60S ribosomal protein L6, putative
Tb927.10.11390
AGOF: structural constituent of ribosome
AGOC: intracellular, organellar large ribosomal subunit, ribosome
AGOP: translation
PGOF: structural constituent of ribosome
PGOC: intracellular, ribosome
PGOP: translation

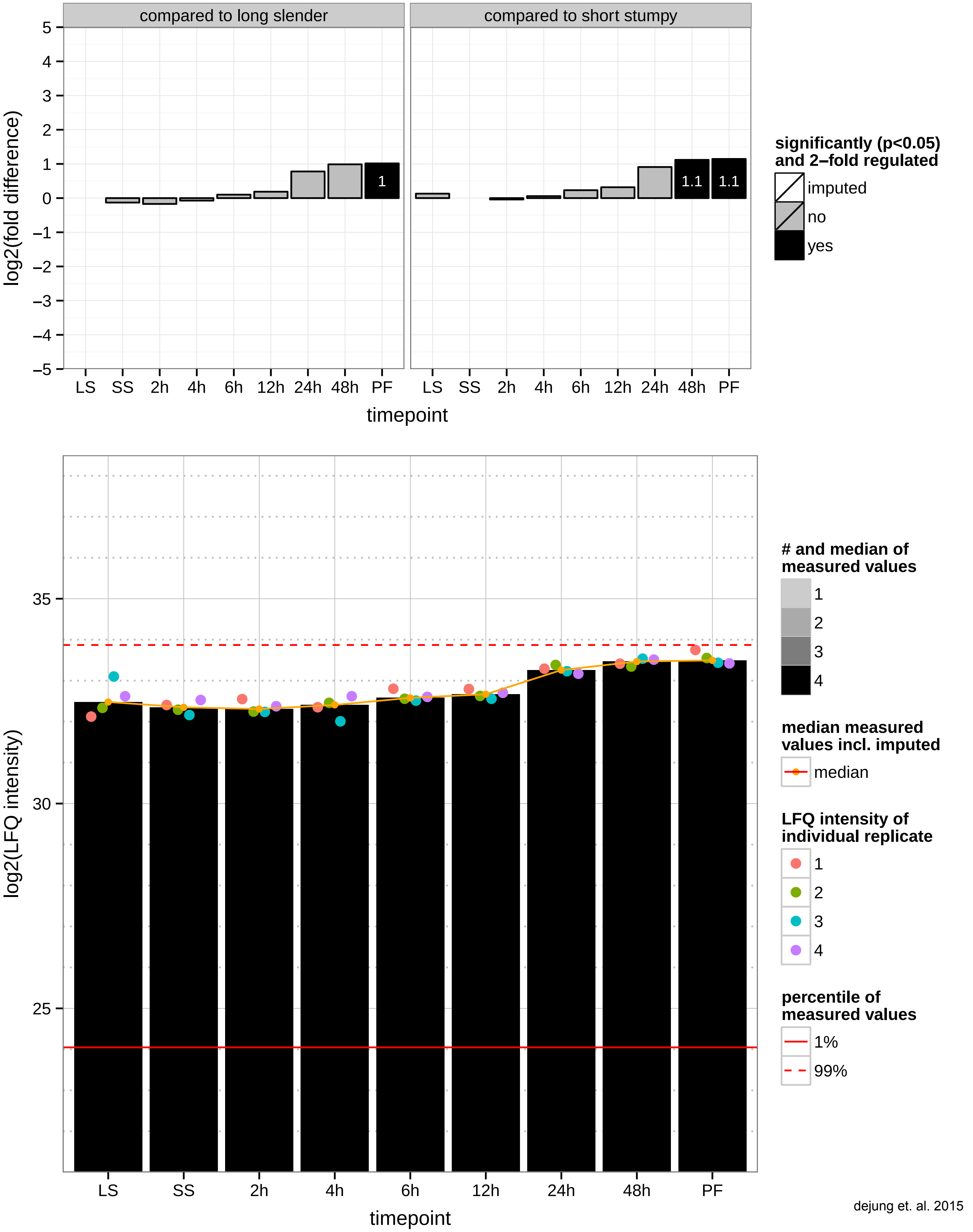

dejung et. al. 2015

3–ketoacyl–CoA reductase, putative
Tb927.10.12240
AGOF: oxidoreductase activity
AGOC: endoplasmic reticulum
AGOP: metabolic process
PGOF: oxidoreductase activity
PGOC: null
PGOP: metabolic process

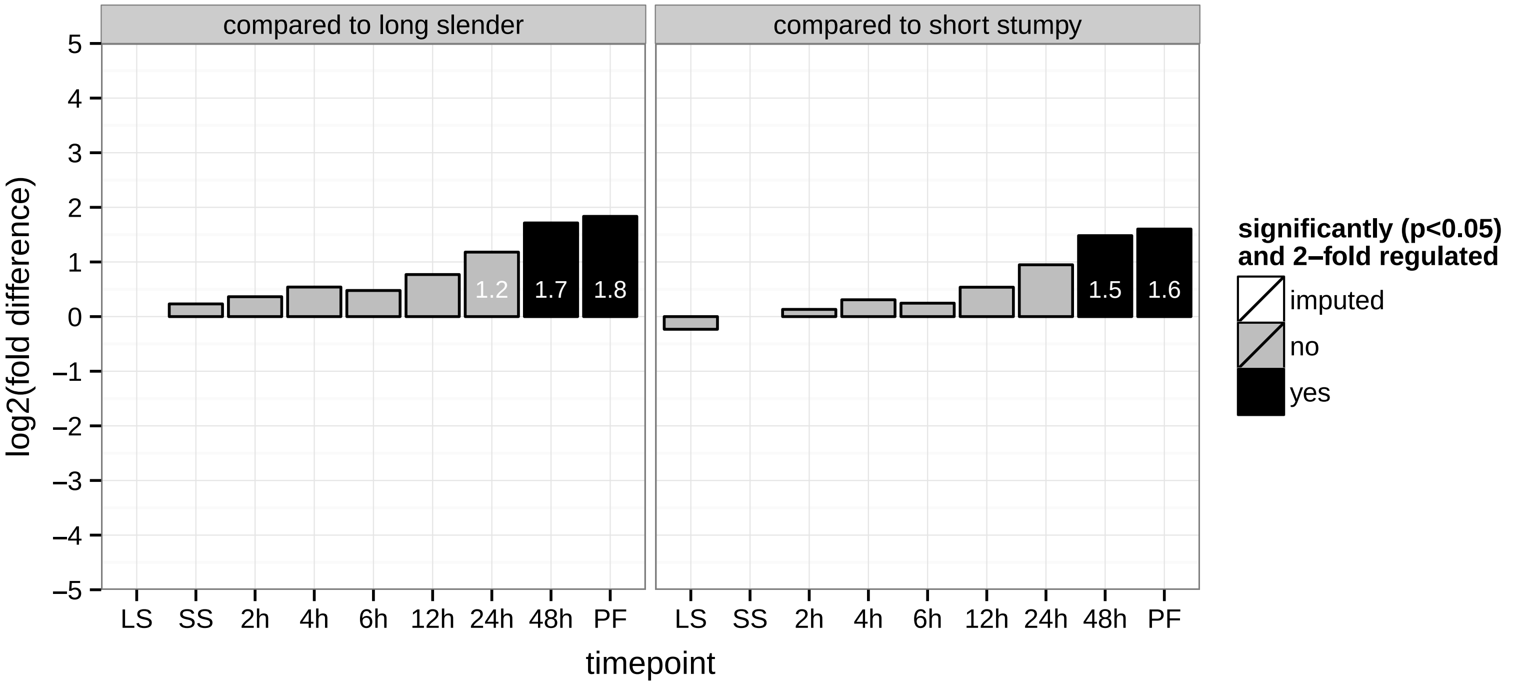

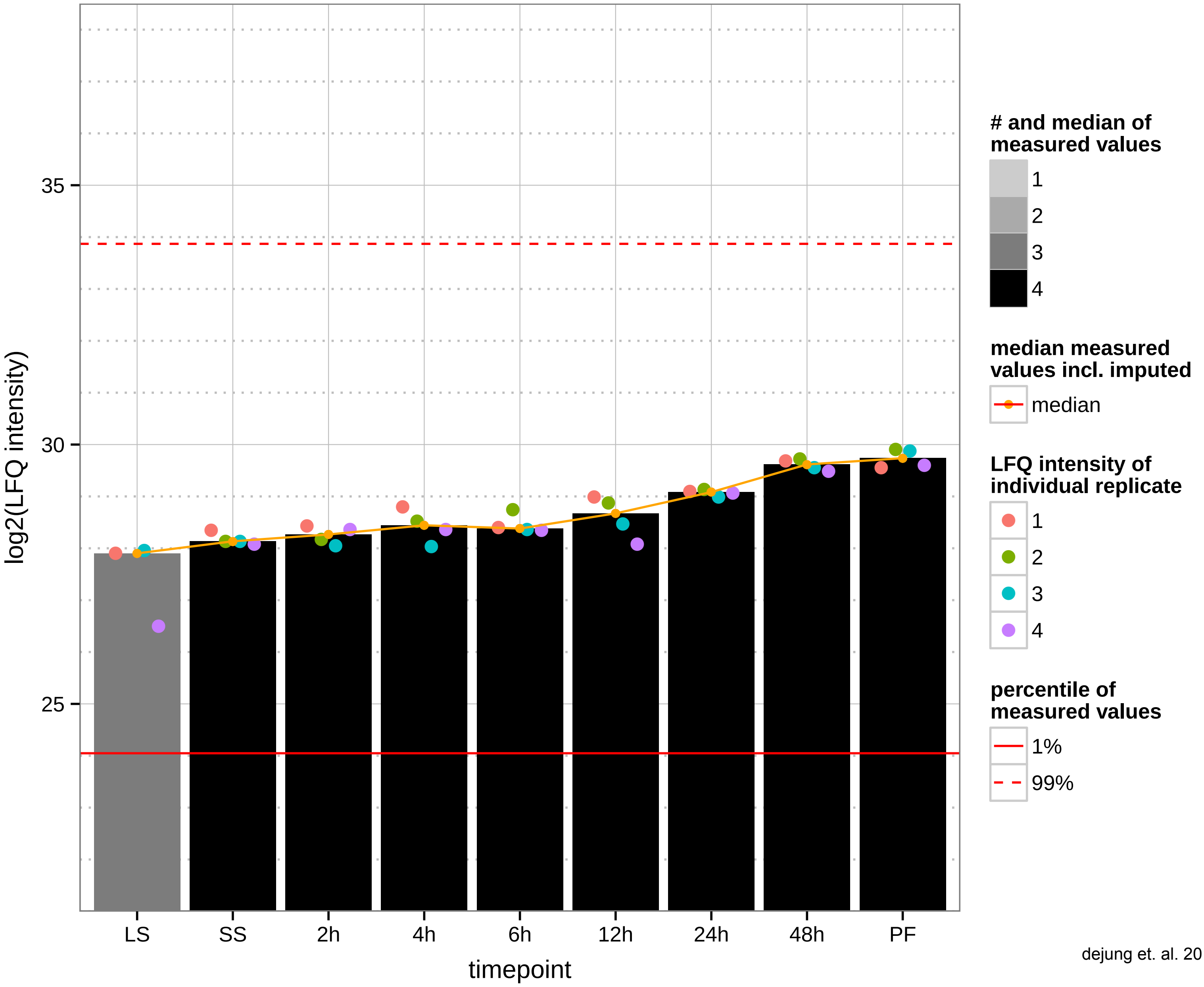

dejung et. al. 2015

cytosolic nonspecific dipeptidase, putative, peptidase (M20/M25/M40 family)
Tb927.10.12260
AGOF: hydrolase activity, metallopeptidase activity, protein dimerization activity
AGOC: null
AGOP: proteolysis
PGOF: hydrolase activity
PGOC: null
PGOP: metabolic process

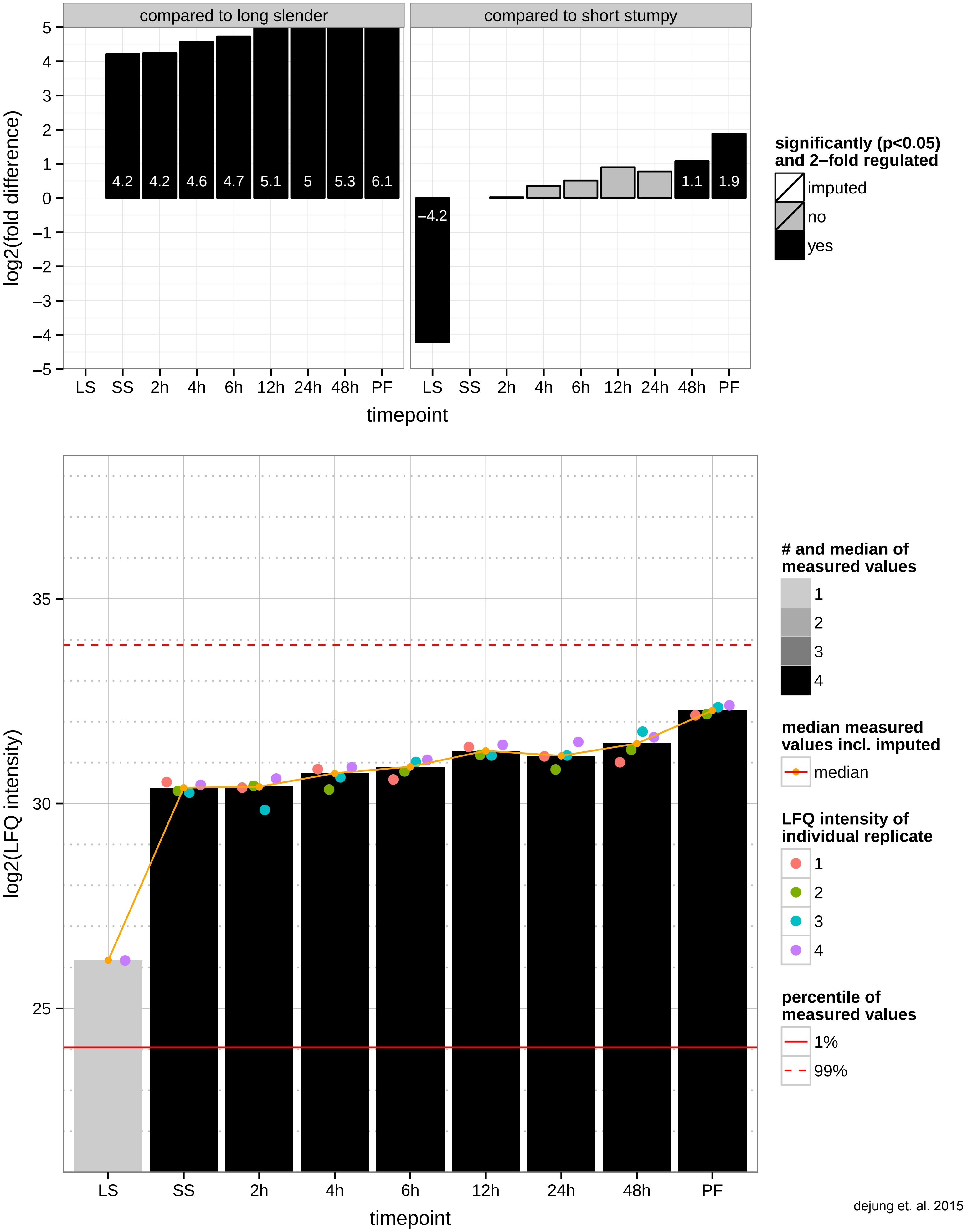

dejung et. al. 2015

pumilio/PUF RNA binding protein 2 (PUF2)
Tb927.10.12660
AGOF: RNA binding
AGOC: cytoplasm
AGOP: mRNA metabolic process
PGOF: RNA binding, binding
PGOC: null
PGOP: null

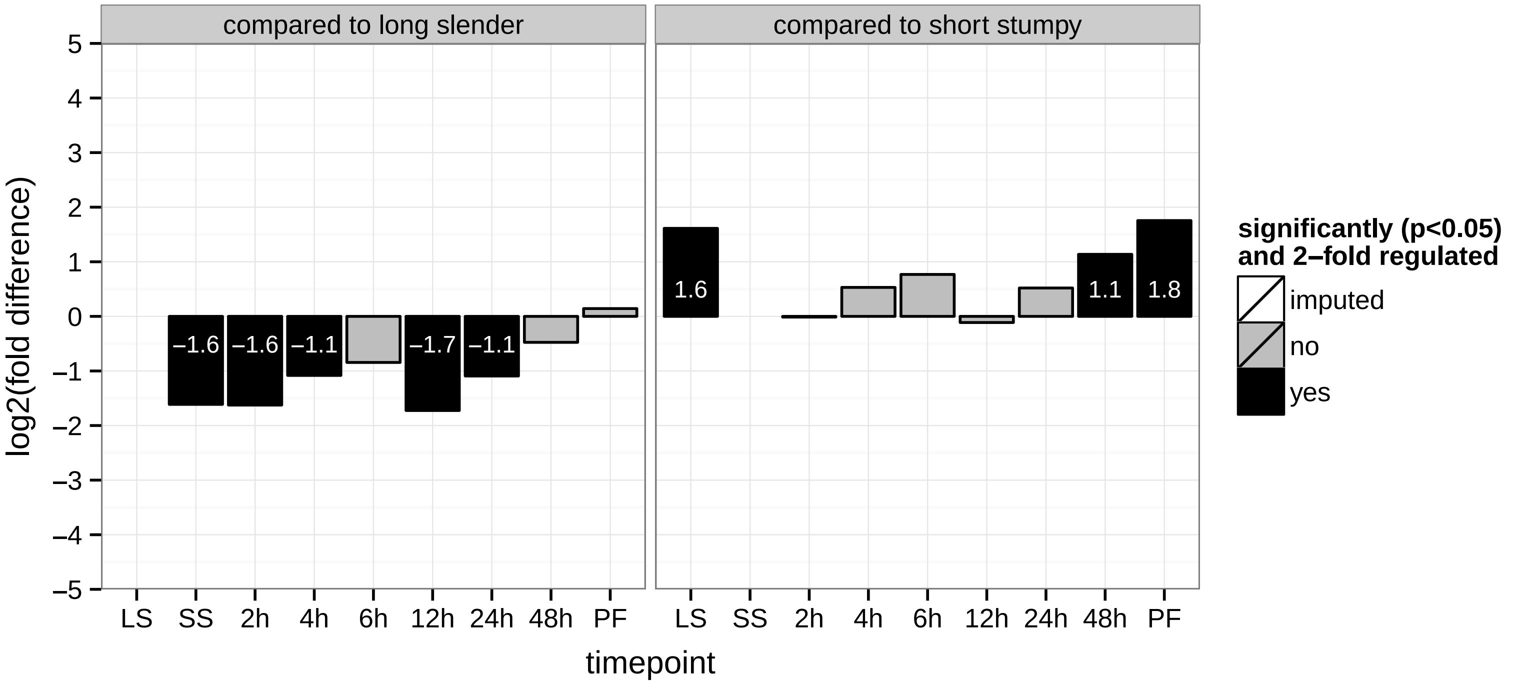

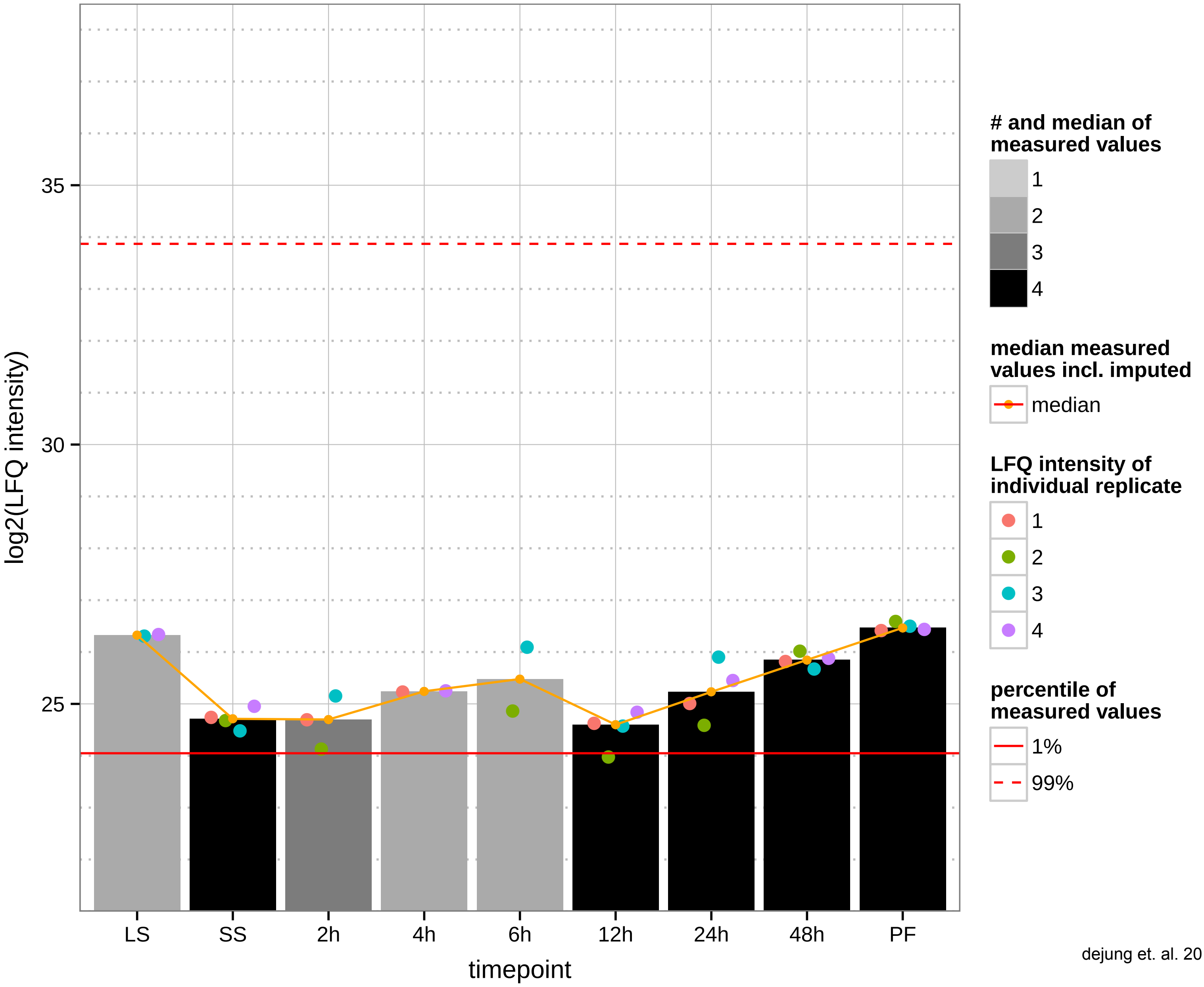

dejung et. al. 2015

tubulin–specific chaperone, putative
Tb927.10.13950
AGOF: null
AGOC: null
AGOP: null
PGOF: null
PGOC: null
PGOP: null

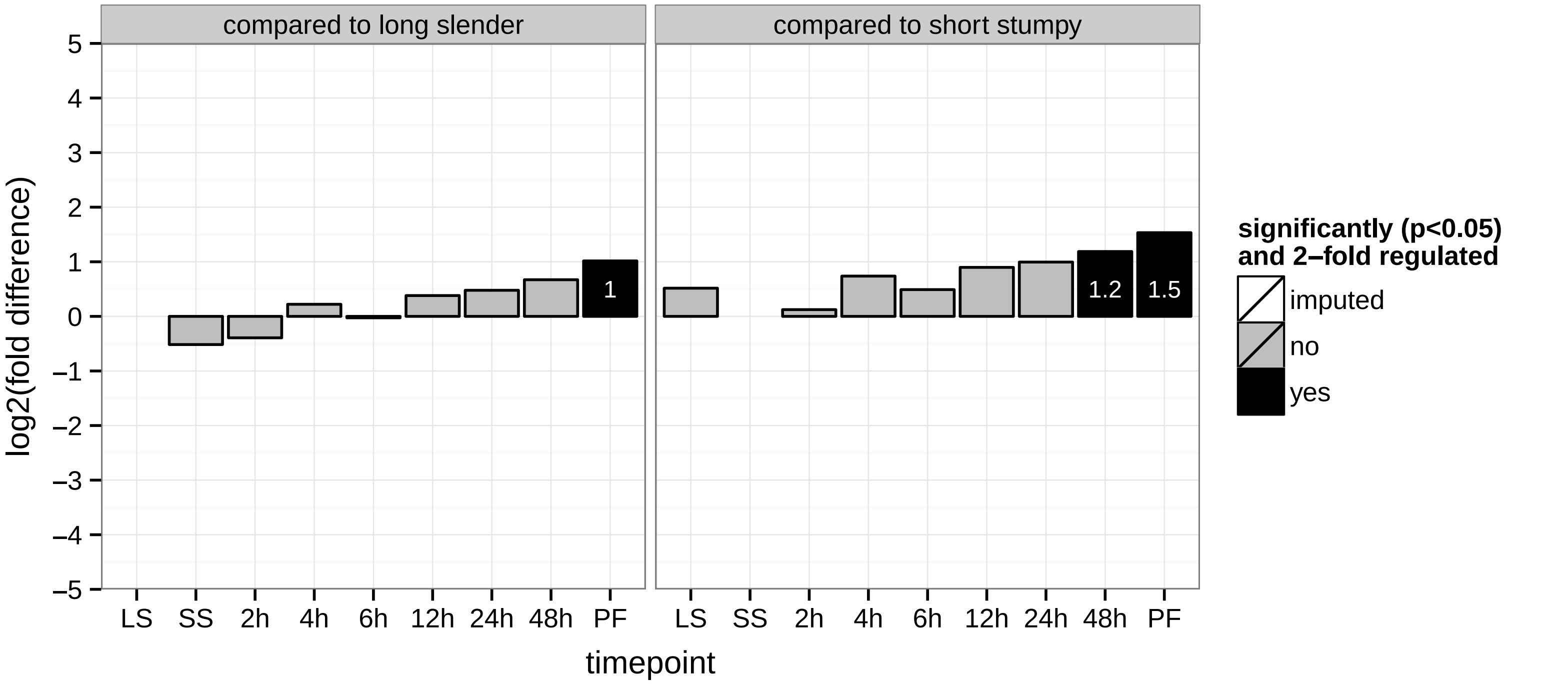

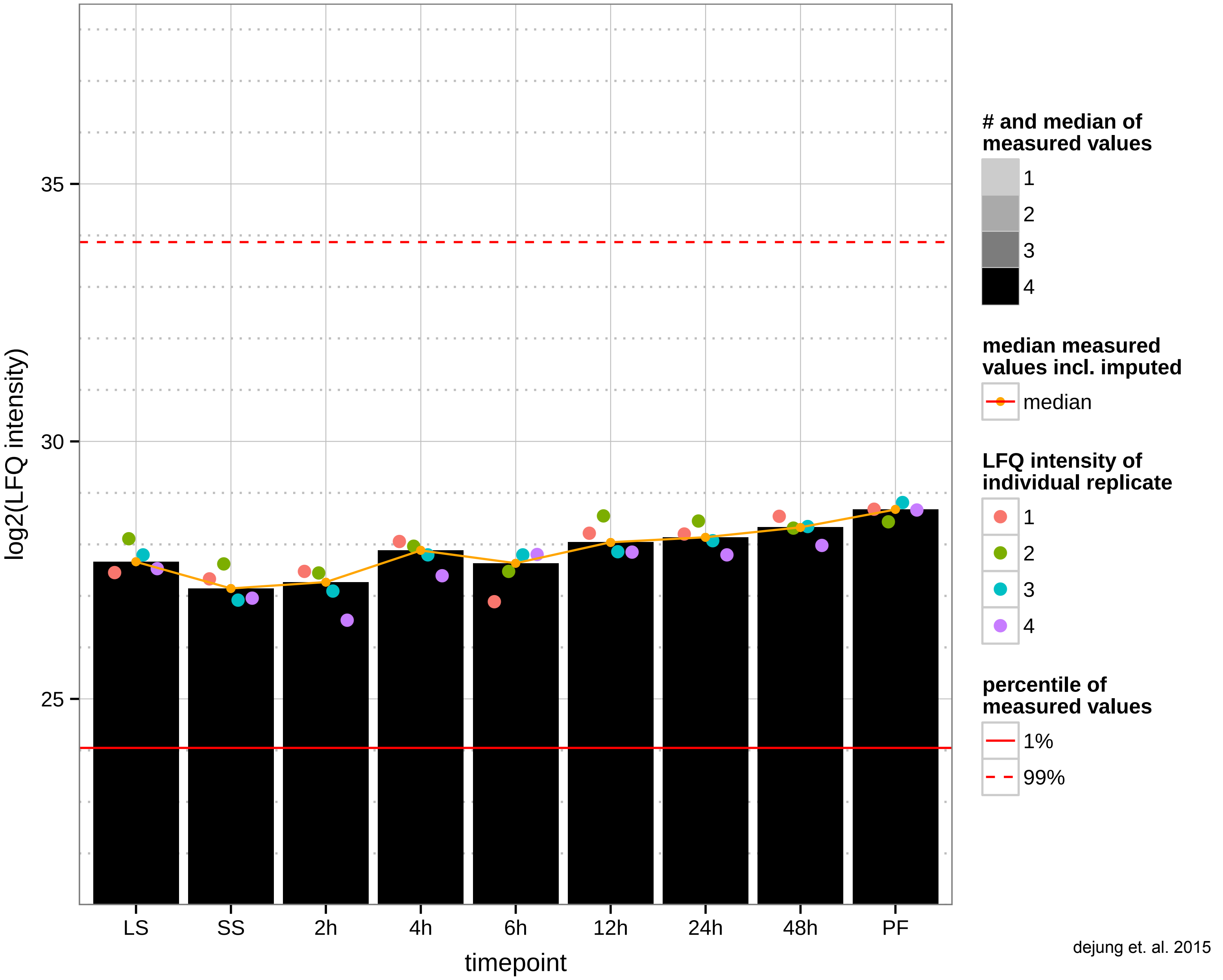

dejung et. al. 2015

60S ribosomal protein L17, putative
Tb927.11.4820;Tb927.10.14580
AGOF: structural constituent of ribosome
AGOC: intracellular, large ribosomal subunit, ribosome
AGOP: translation
PGOF: structural constituent of ribosome
PGOC: intracellular, large ribosomal subunit, ribosome
PGOP: translation

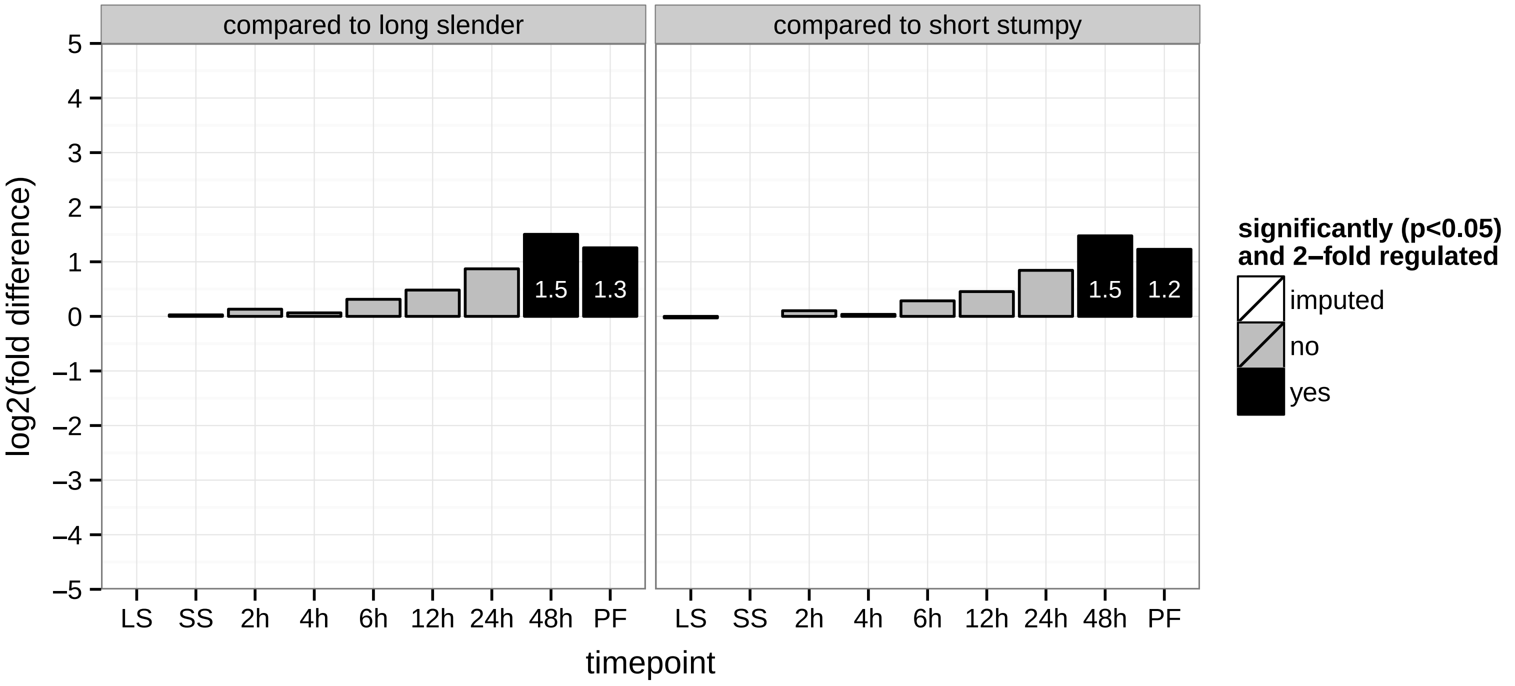

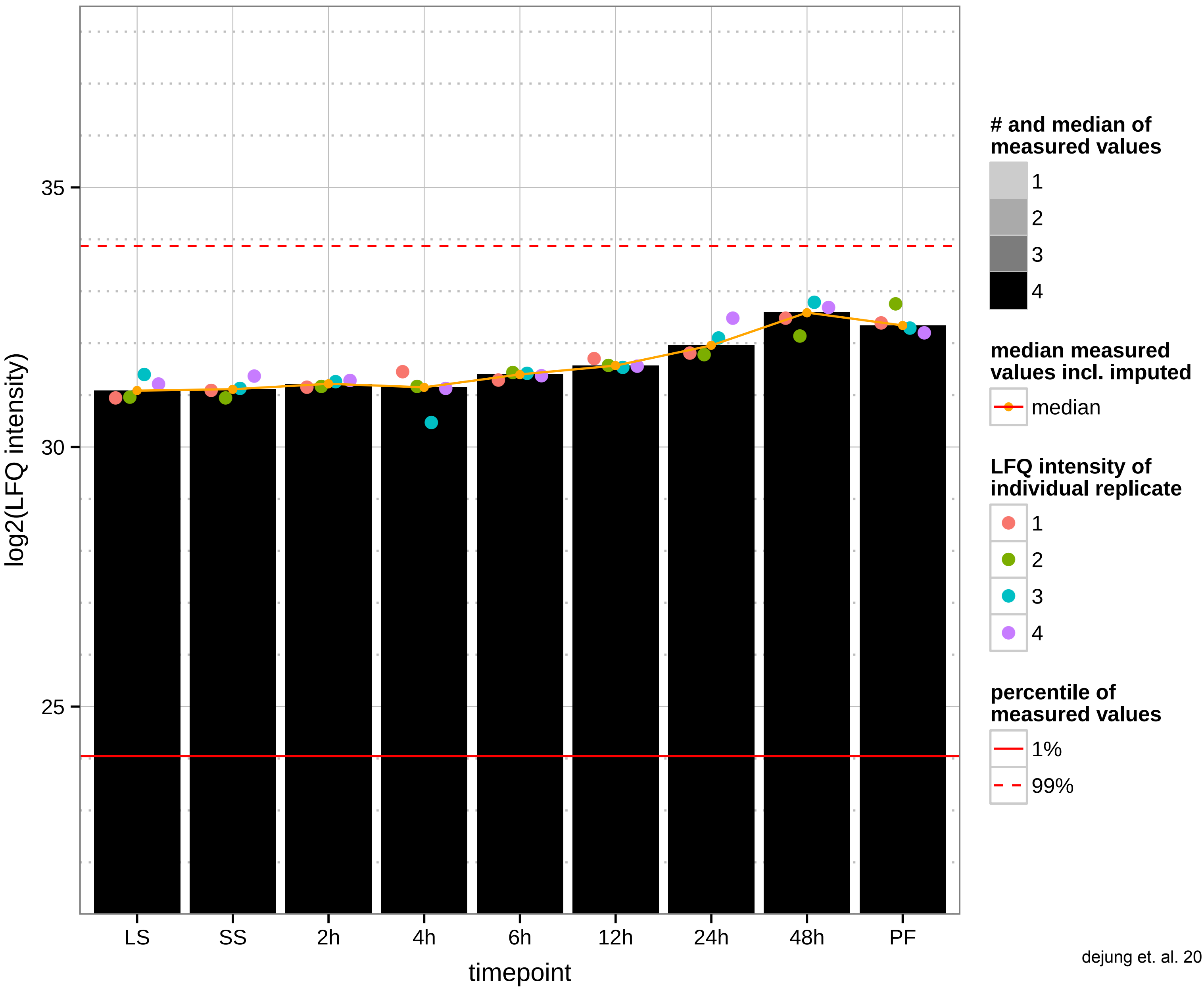

dejung et. al. 2015

hypothetical protein, conserved
Tb927.10.15220
AGOF: null
AGOC: endoplasmic reticulum
AGOP: null
PGOF: null
PGOC: null
PGOP: null

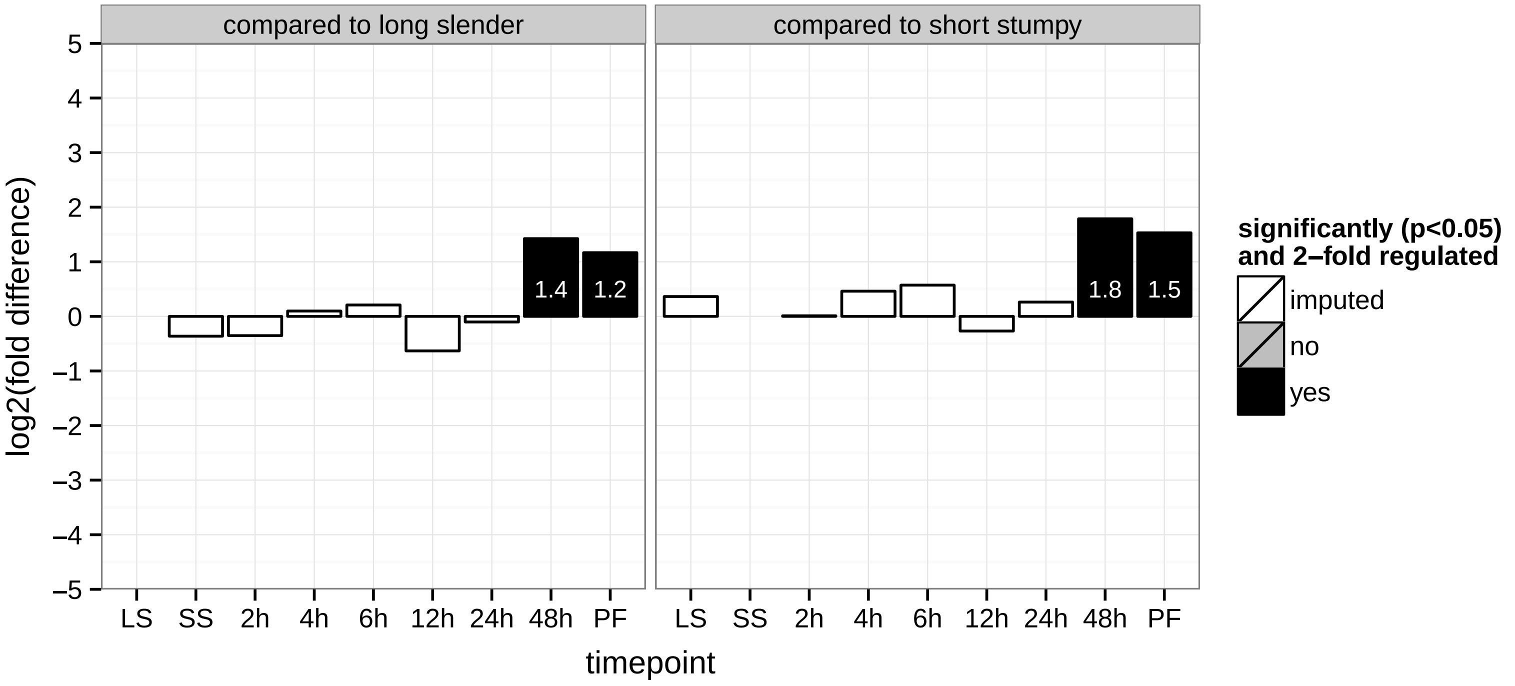

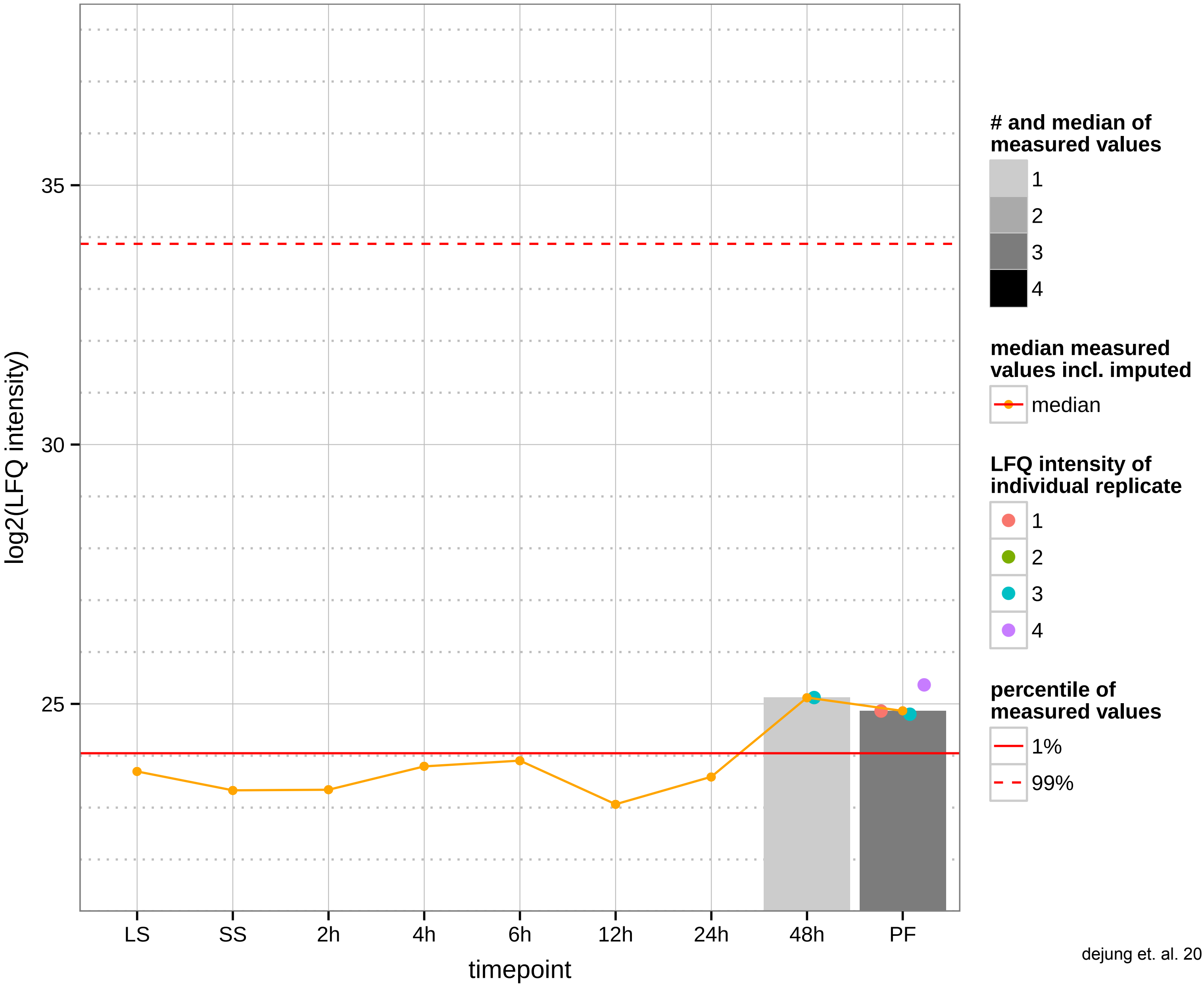

dejung et. al. 2015

ABC transporter, putative, ABC transport system ATP−binding protein
Tb927.10.15530;Tb11.v5.0748
AGOF: null, ATP binding, ATPase activity, uncoupled
AGOC: null, integral to membrane, internal side of plasma membrane
AGOP: null, regulation of ribosome biogenesis, ribosomal small subunit export from nucleus, transport
PGOF: ATP binding, ATPase activity, nucleoside−triphosphatase activity, nucleotide binding
PGOC: null
PGOP: null

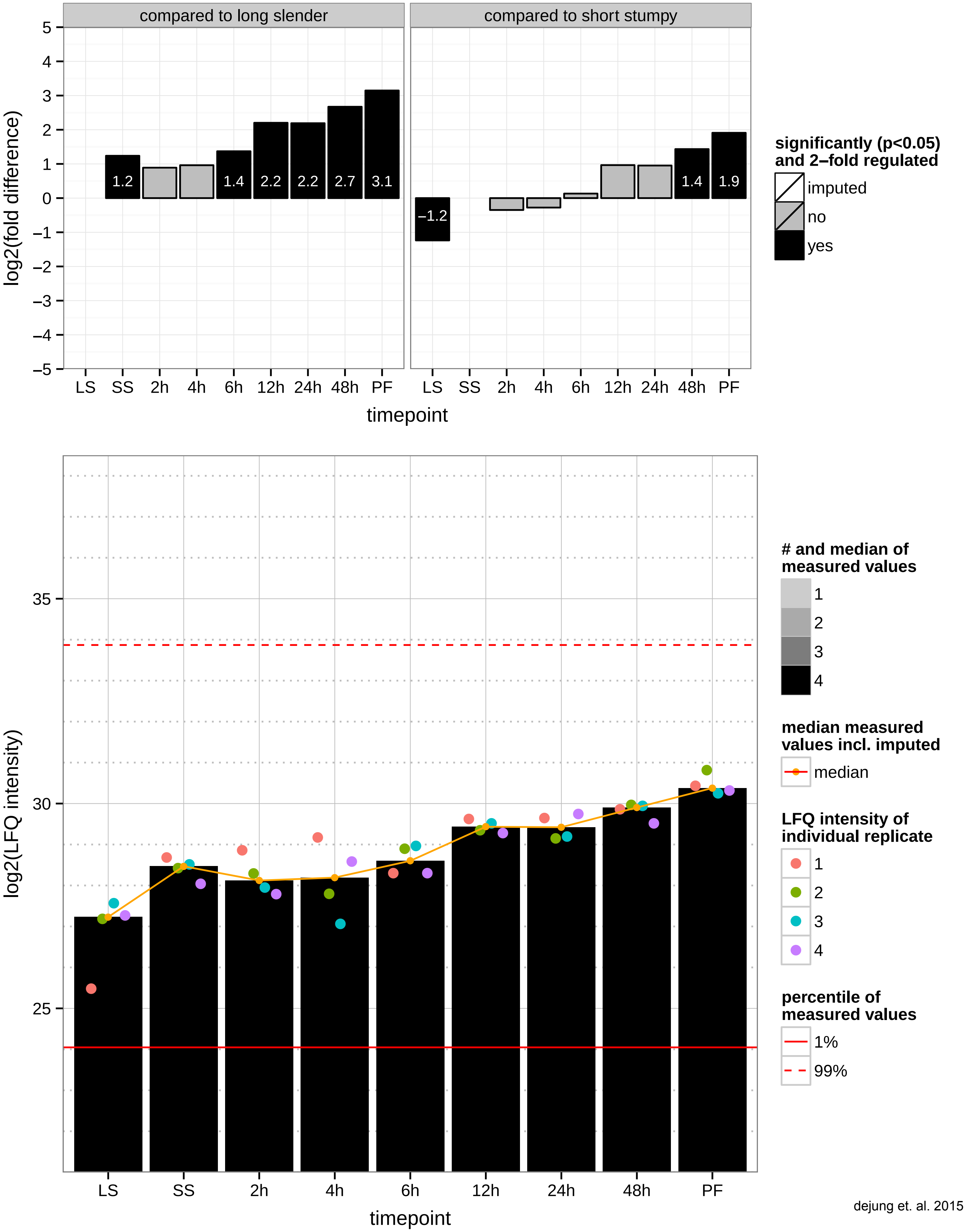

dejung et. al. 2015

glucose−6−phosphate 1−dehydrogenase (G6PD)
Tb927.10.2490
AGOF: glucose−6−phosphate dehydrogenase activity
AGOC: cytoplasm, glycosome
AGOP: cell proliferation, oxidation−reduction process, pentose−phosphate shunt, oxidative branch, response to drug, respons
PGOF: NADP binding, glucose−6−phosphate dehydrogenase activity
PGOC: null
PGOP: glucose metabolic process, oxidation−reduction process

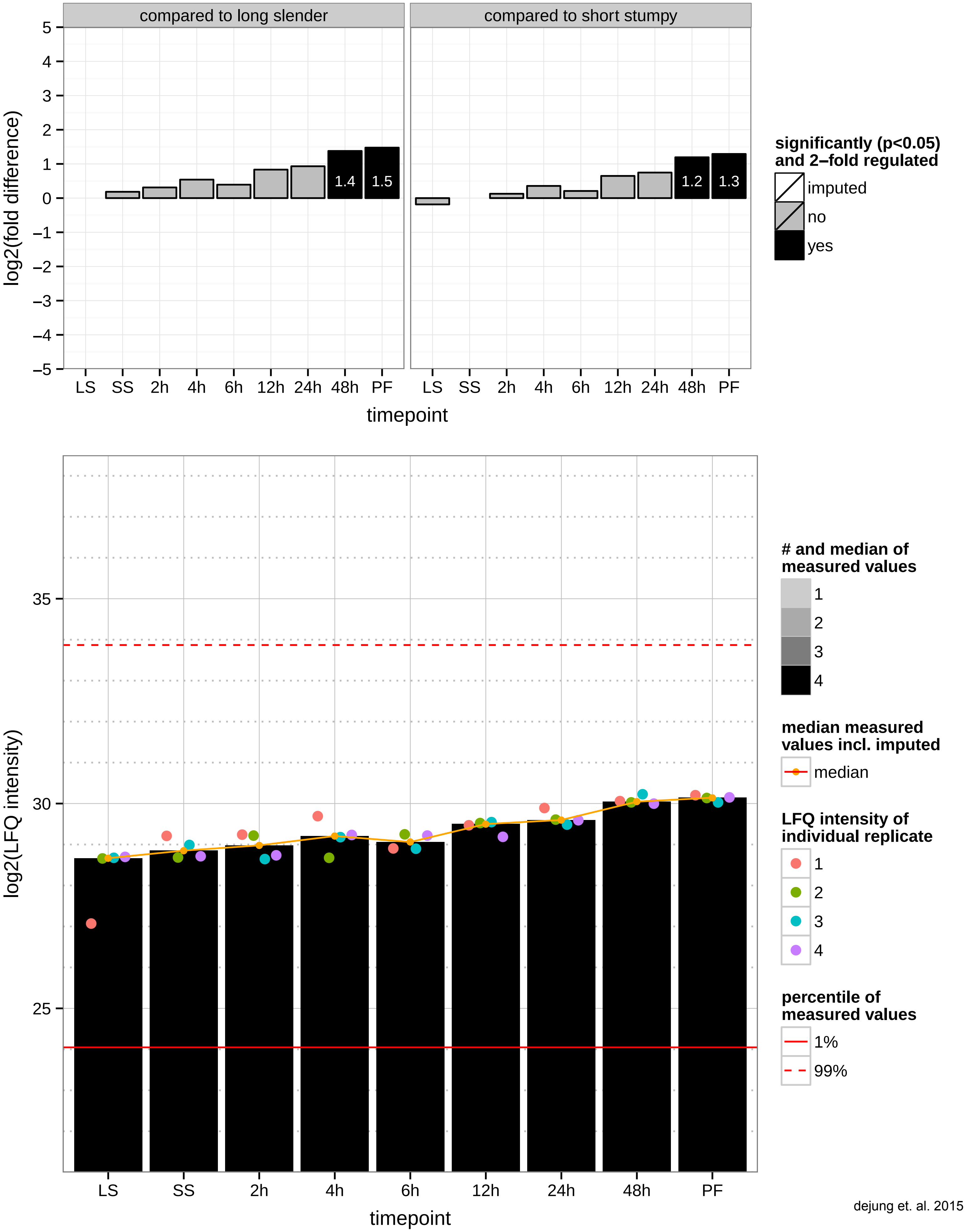

DNA primase large subunit, putative (pril)
Tb927.10.3110
AGOF: DNA primase activity
AGOC: alpha DNA polymerase:primase complex
AGOP: DNA replication, synthesis of RNA primer
PGOF: DNA primase activity, nucleotidyltransferase activity
PGOC: null
PGOP: DNA replication, synthesis of RNA primer

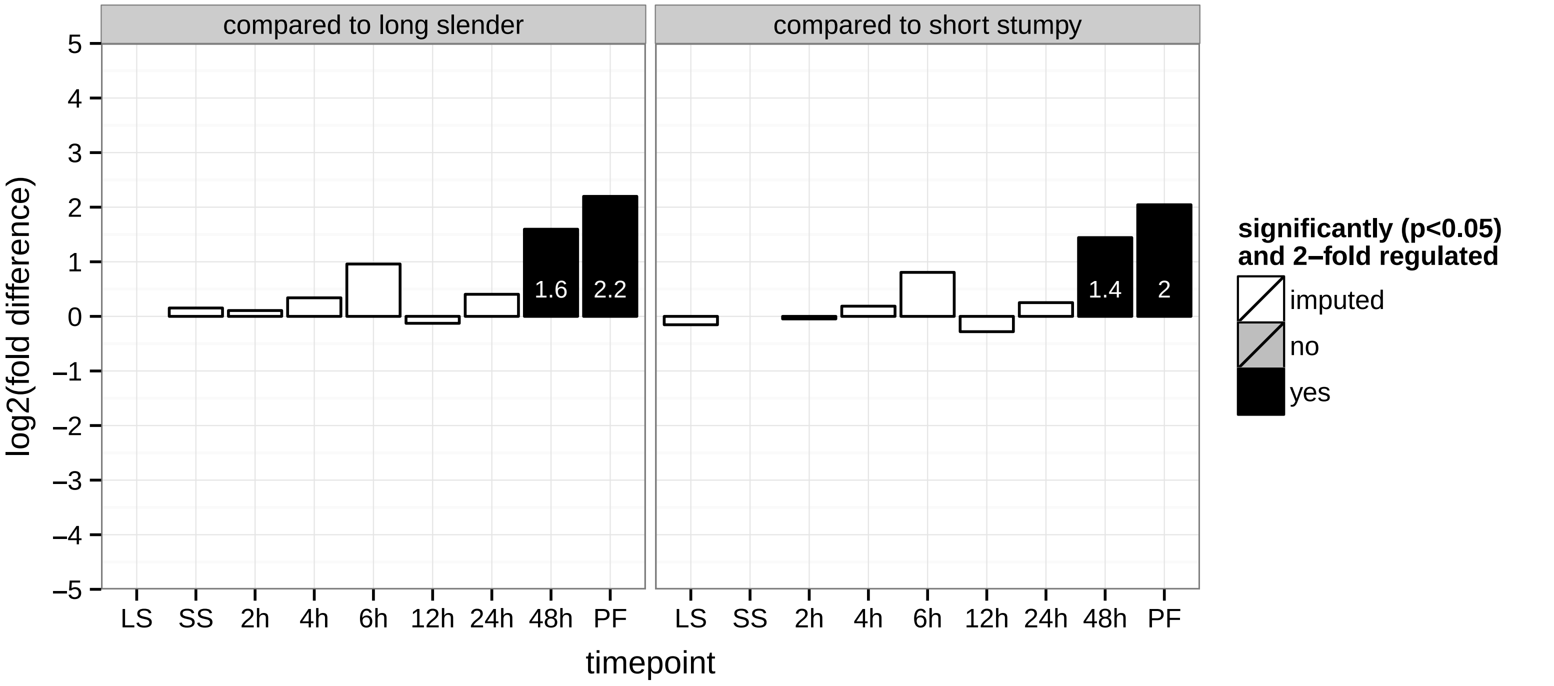

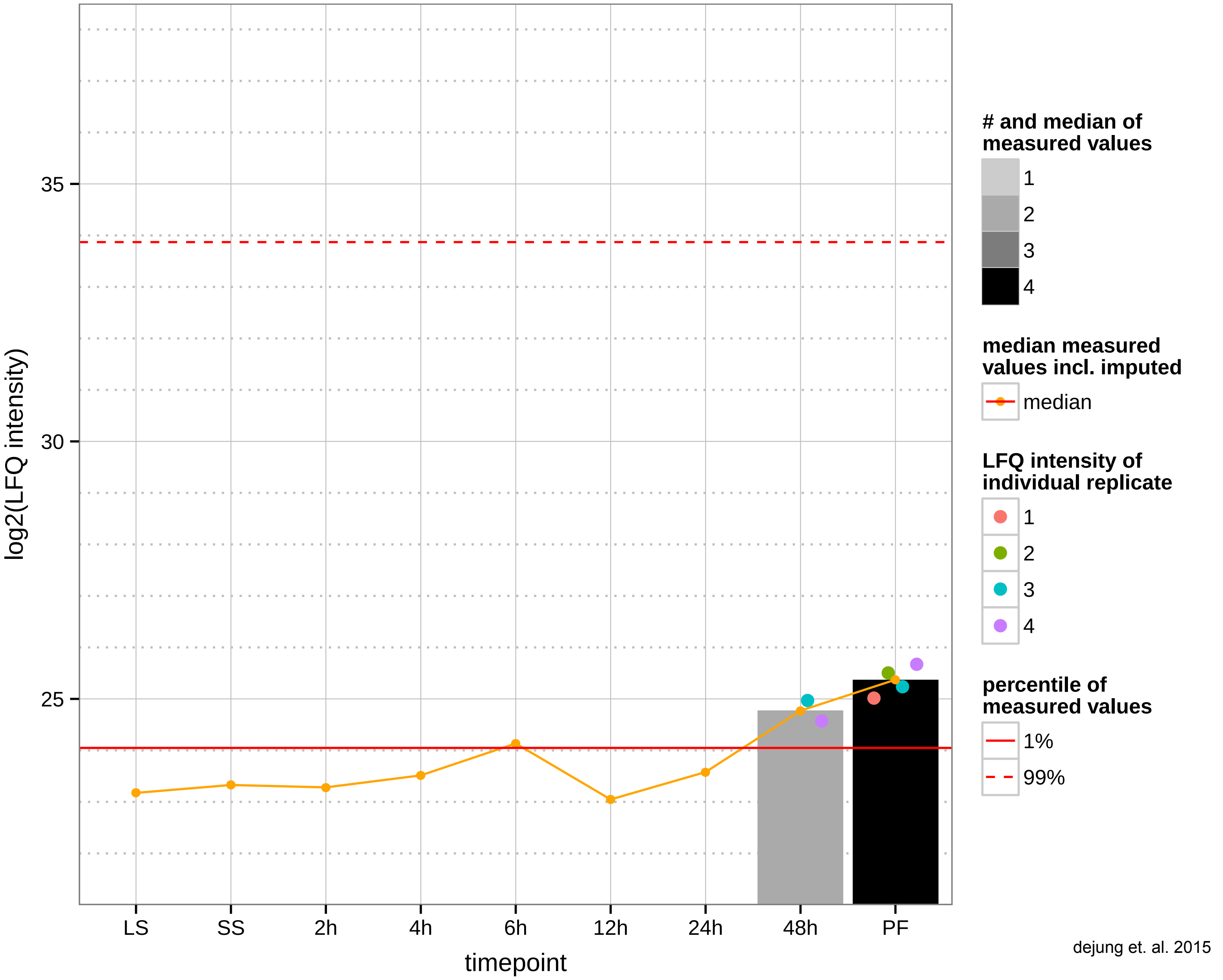

dejung et. al. 2015

ABC transporter, putative
Tb927.10.3170;Tb11.v5.0791
AGOF: null, ATP binding, ATPase activity, coupled to transmembrane movement of substances
AGOC: null, integral to membrane, internal side of plasma membrane
AGOP: null, transport
PGOF: ATP binding, ATPase activity, nucleoside−triphosphatase activity, nucleotide binding
PGOC: null
PGOP: null

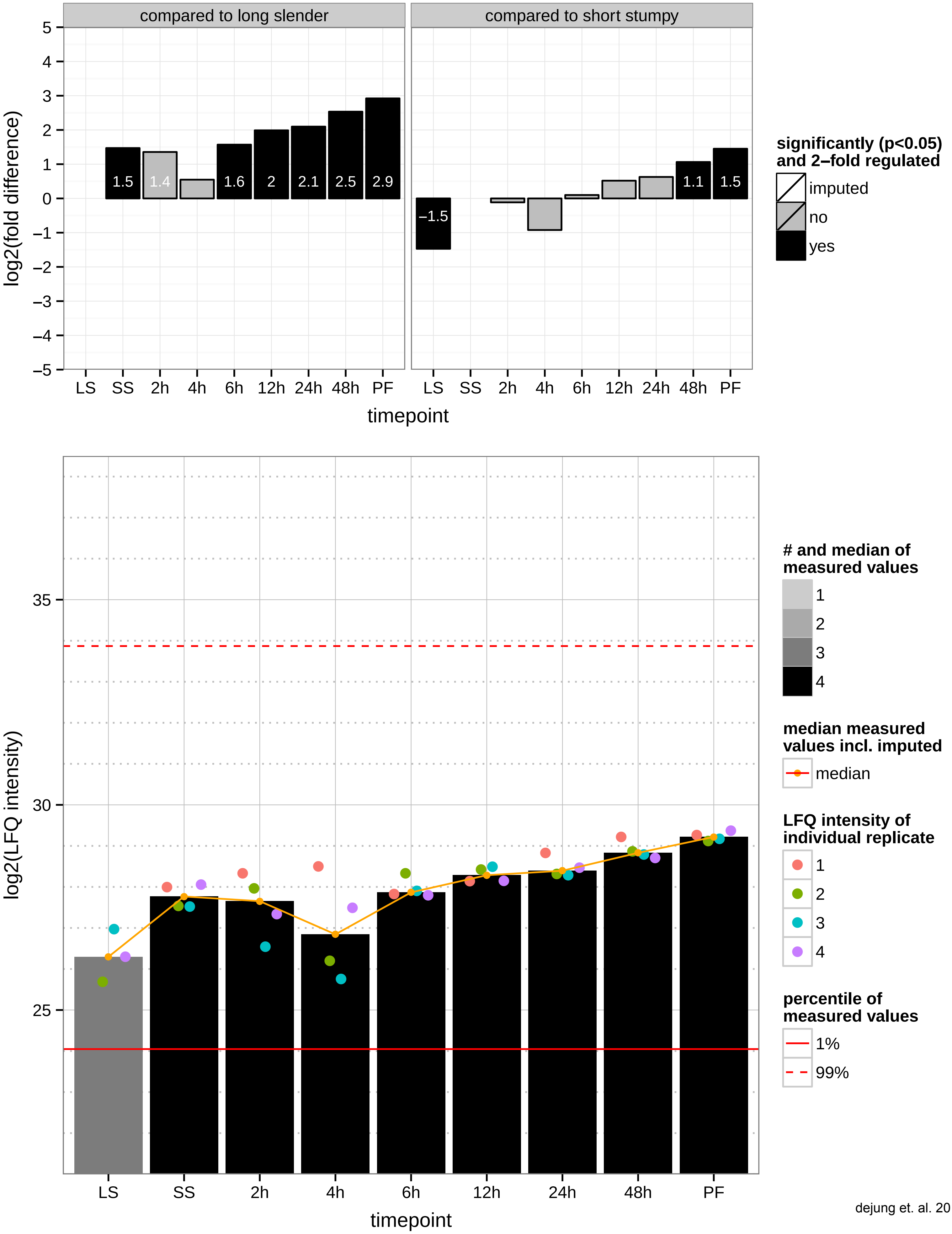

dejung et. al. 2015

40S ribosomal protein S10, putative
Tb927.10.5370;Tb927.10.5360
AGOF: RNA binding
AGOC: cytoplasm
AGOP: translation
PGOF: null
PGOC: null
PGOP: null

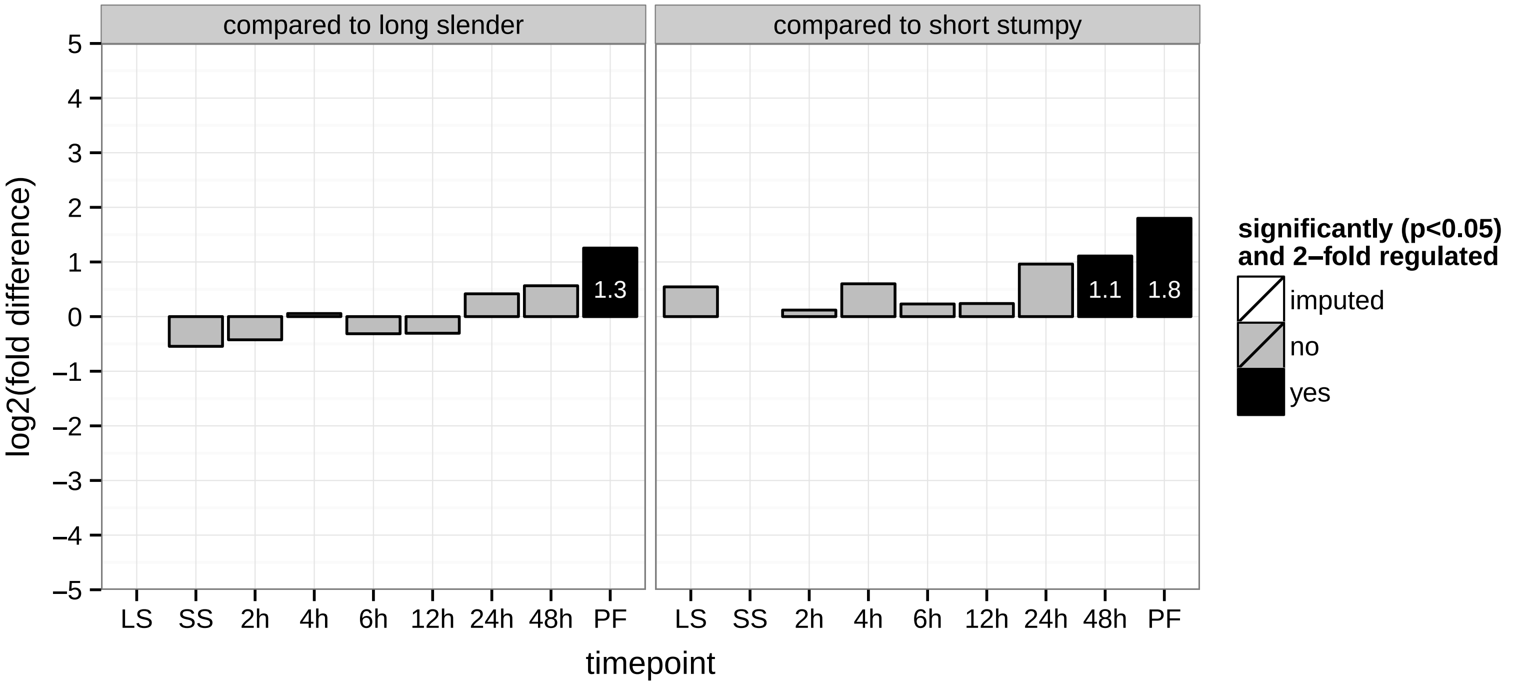

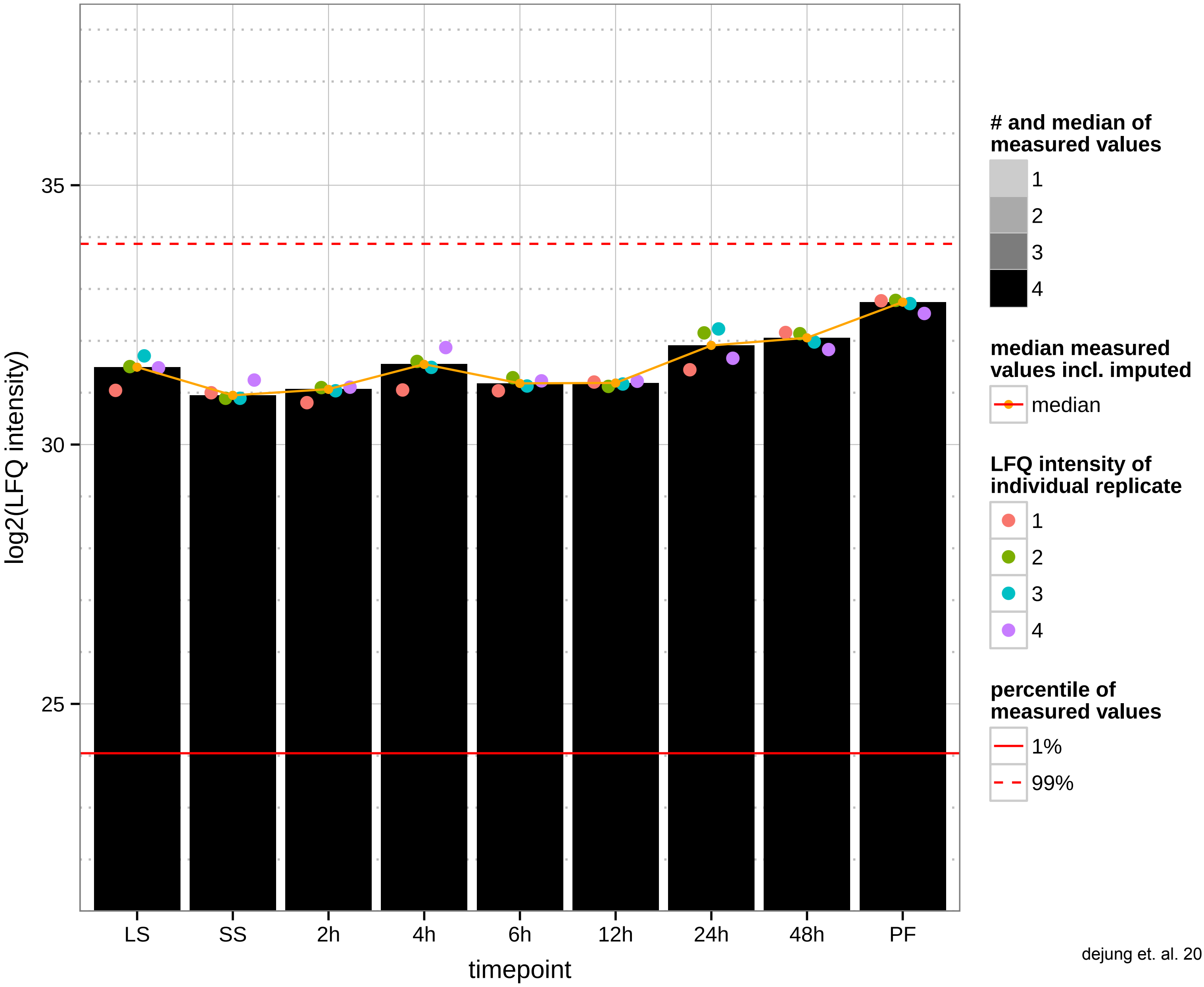

dejung et. al. 2015

60S ribosomal protein L24, putative
Tb927.10.5480;Tb927.10.5460
AGOF: structural constituent of ribosome
AGOC: intracellular, ribosome
AGOP: translation
PGOF: null
PGOC: null
PGOP: null

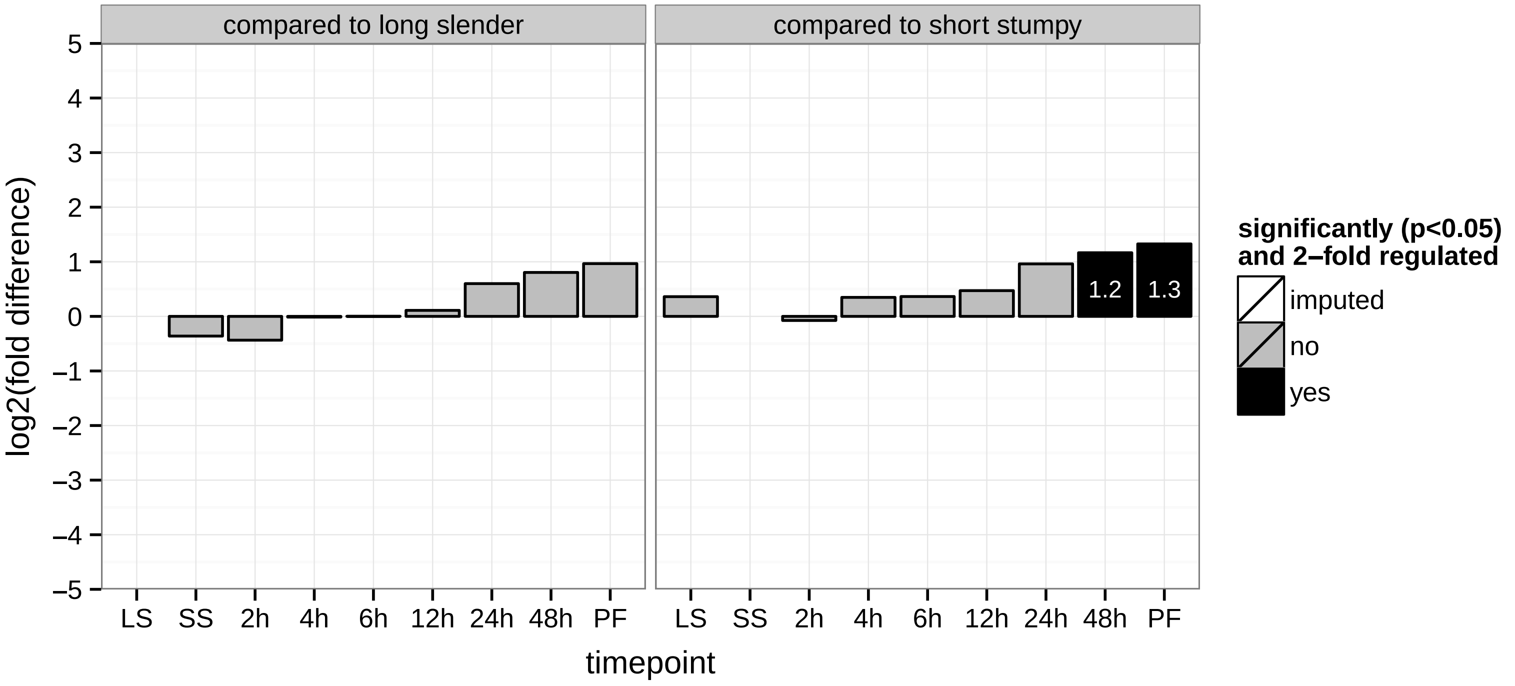

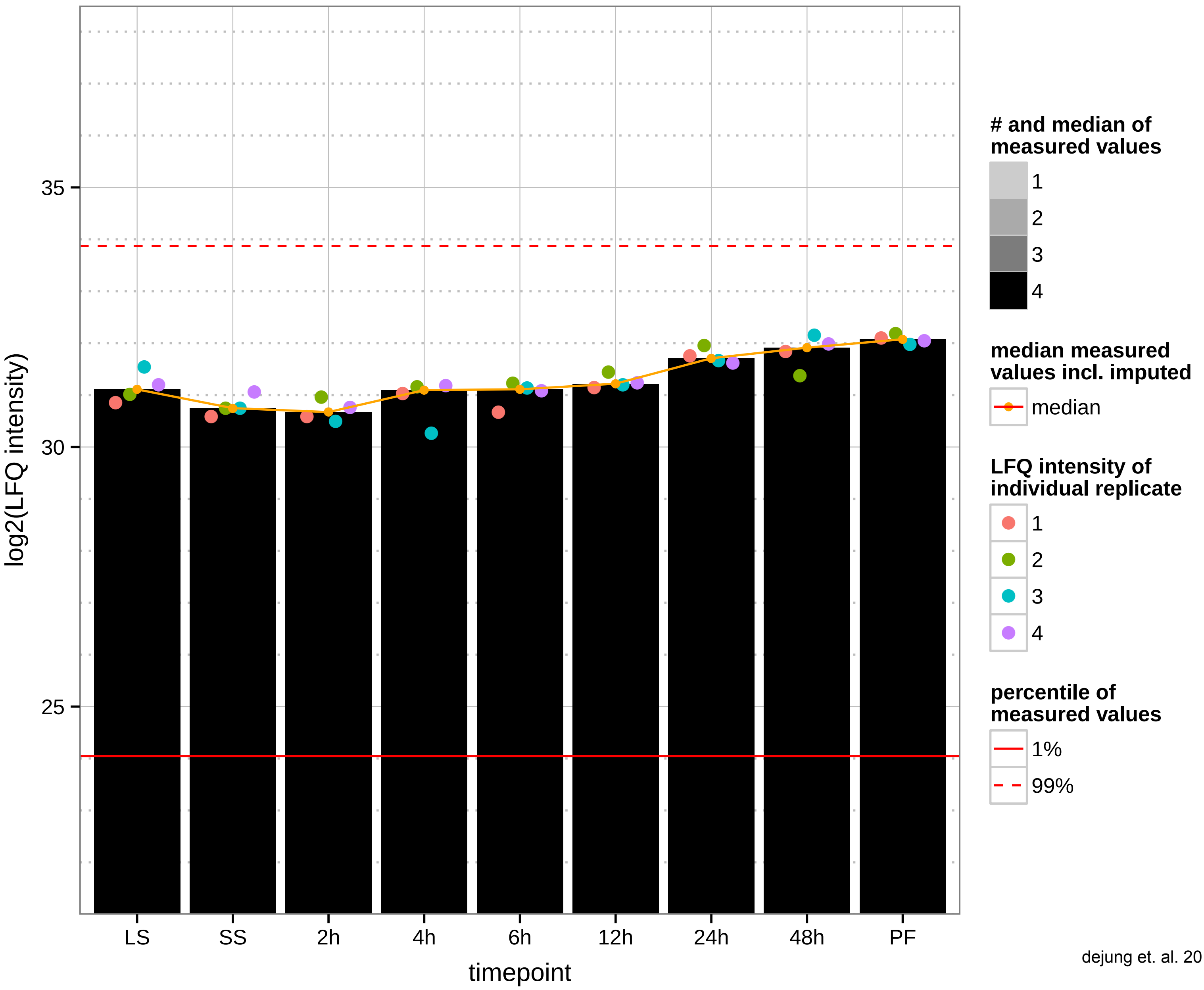

dejung et. al. 2015

structural maintenance of chromosome 4, putative, dual specificity protein phosphatase, putative (SMC4)
Tb927.10.740
AGOF: null, ATP binding
AGOC: null, chromosome, condensin complex, nucleus
AGOP: null, chromosome organization, chromosome segregation, mitosis
PGOF: null, ATP binding, protein binding
PGOC: null, chromosome
PGOP: null, chromosome organization, DNA recombination, DNA repair, chromosome condensation, chromosome organizati

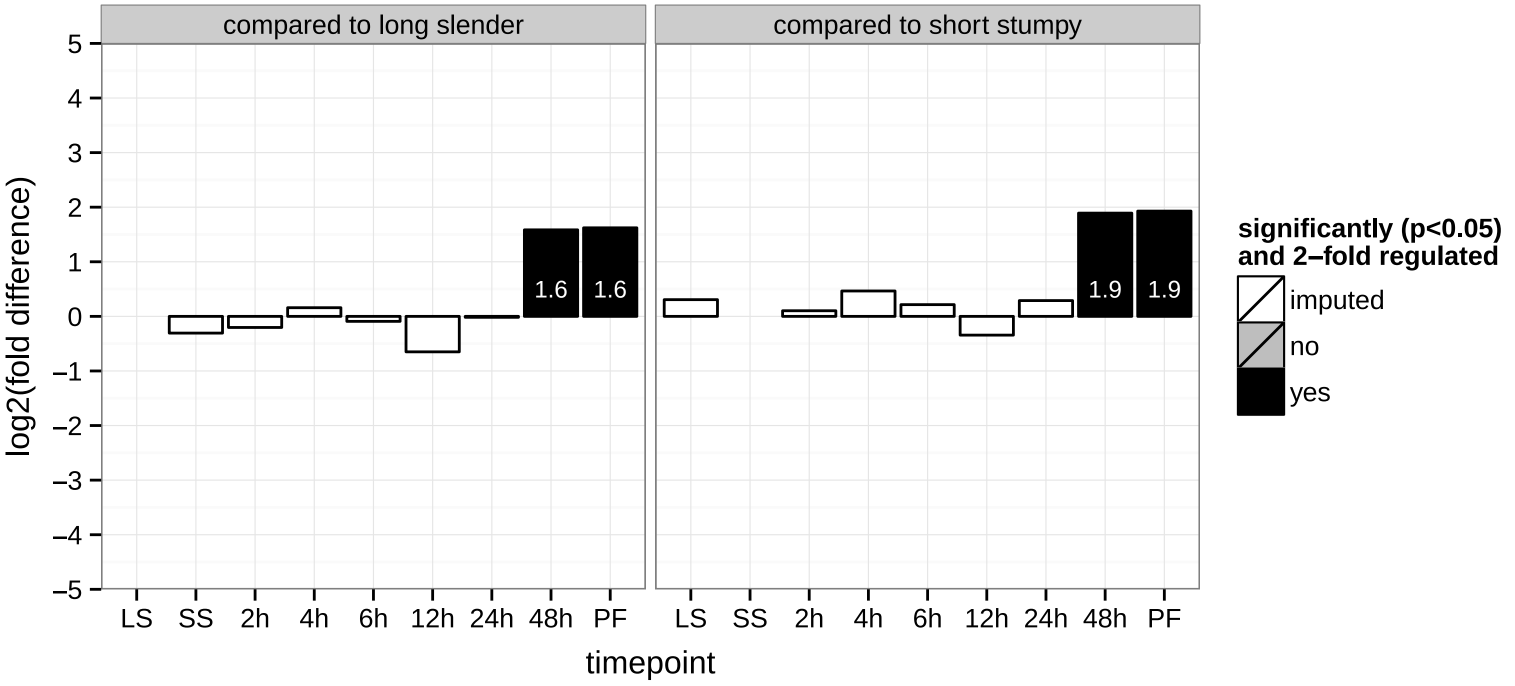

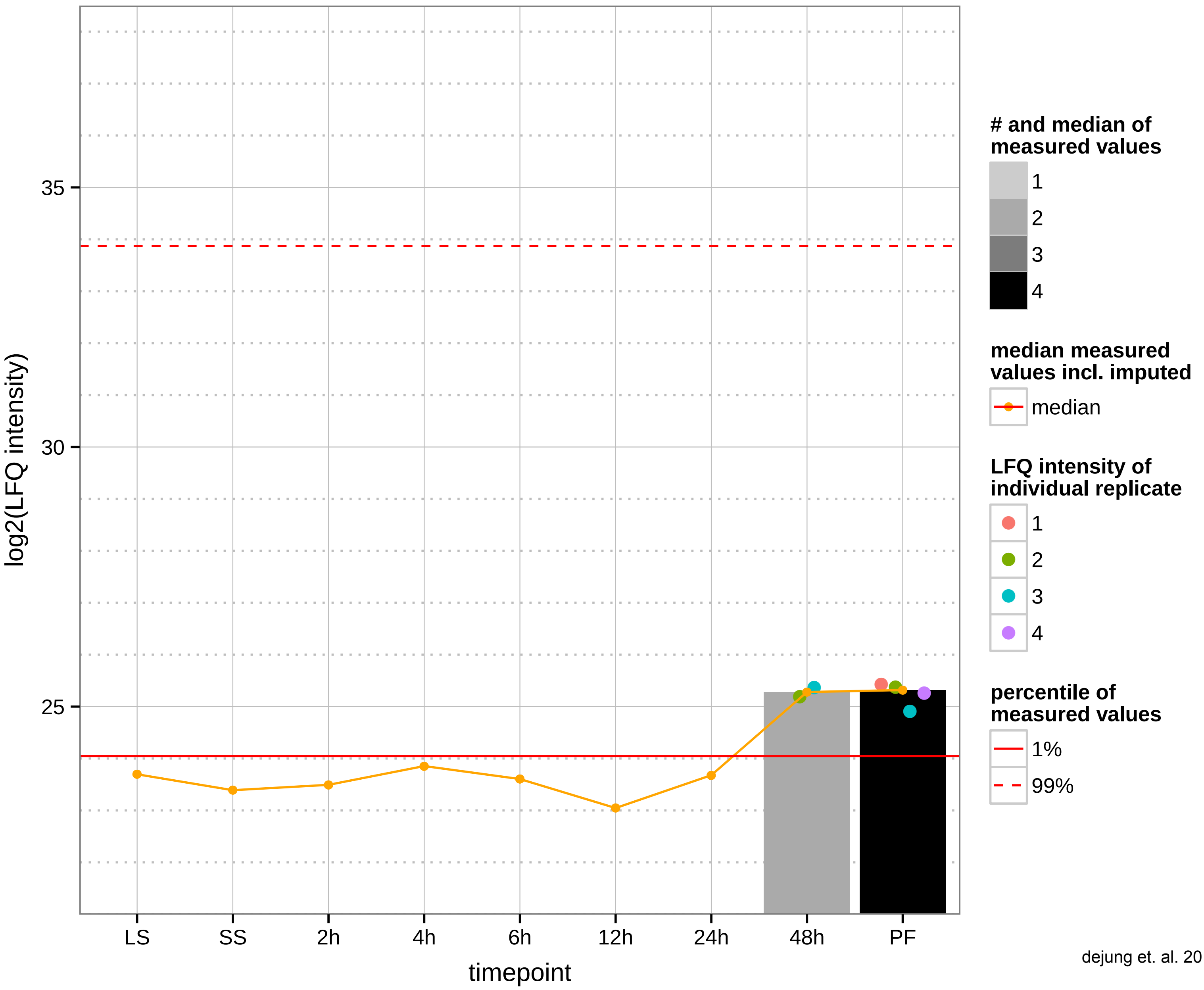

dejung et. al. 2015

hypothetical protein, conserved
Tb927.10.770
AGOF: null
AGOC: mitochondrion
AGOP: null
PGOF: translation release factor activity
PGOC: null
PGOP: translational termination

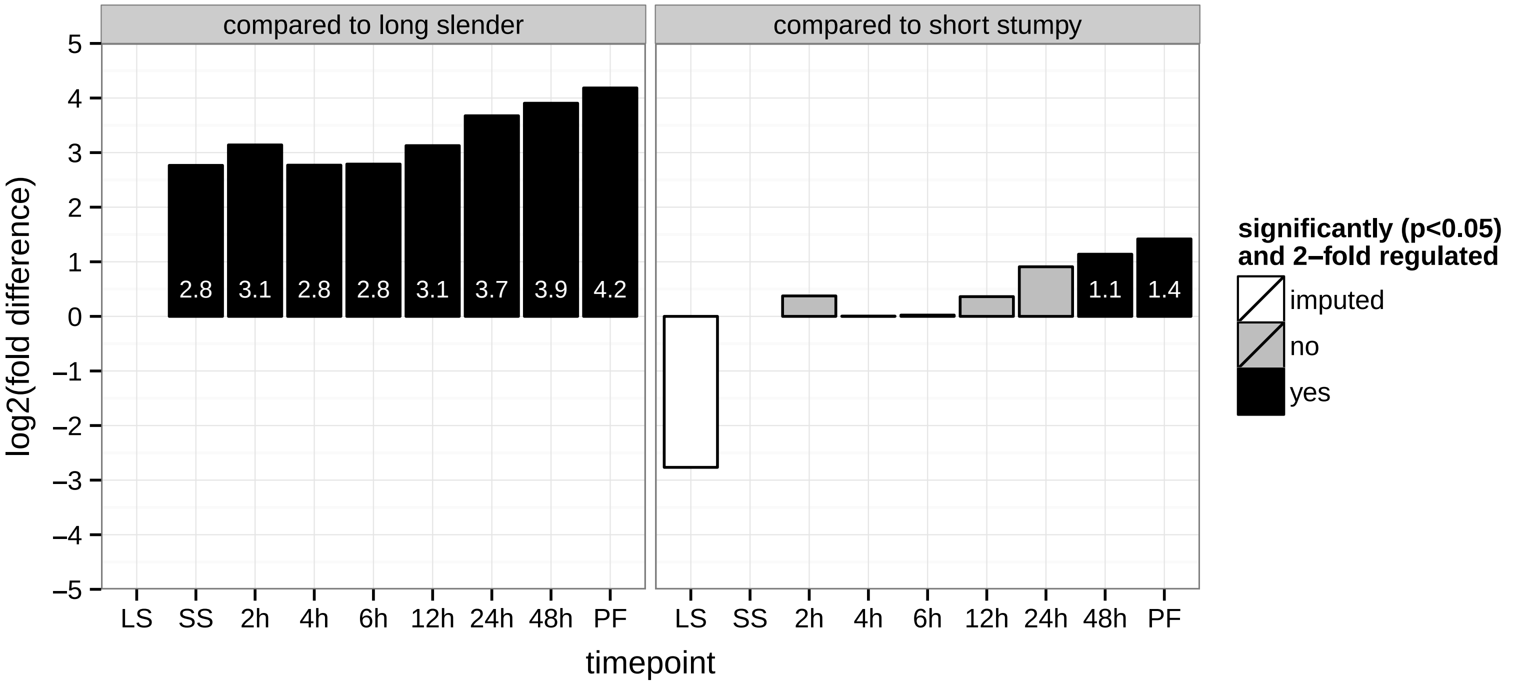

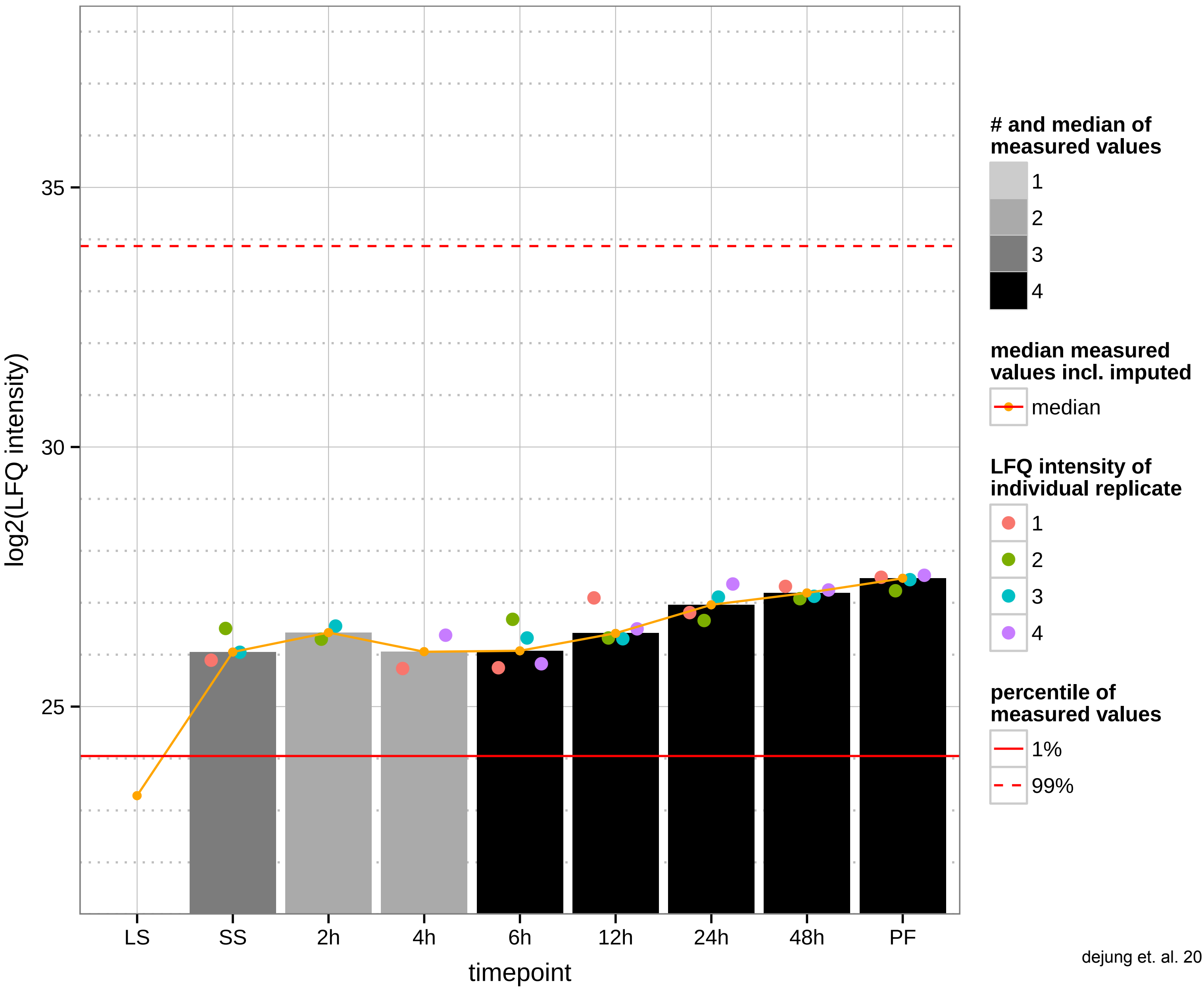

dejung et. al. 2015

ABC transporter, putative
Tb927.10.7700
AGOF: ATP binding, ATPase activity, coupled to transmembrane movement of substances
AGOC: integral to membrane, internal side of plasma membrane, membrane
AGOP: transport
PGOF: ATP binding, ATPase activity, nucleoside−triphosphatase activity, nucleotide binding
PGOC: membrane
PGOP: null

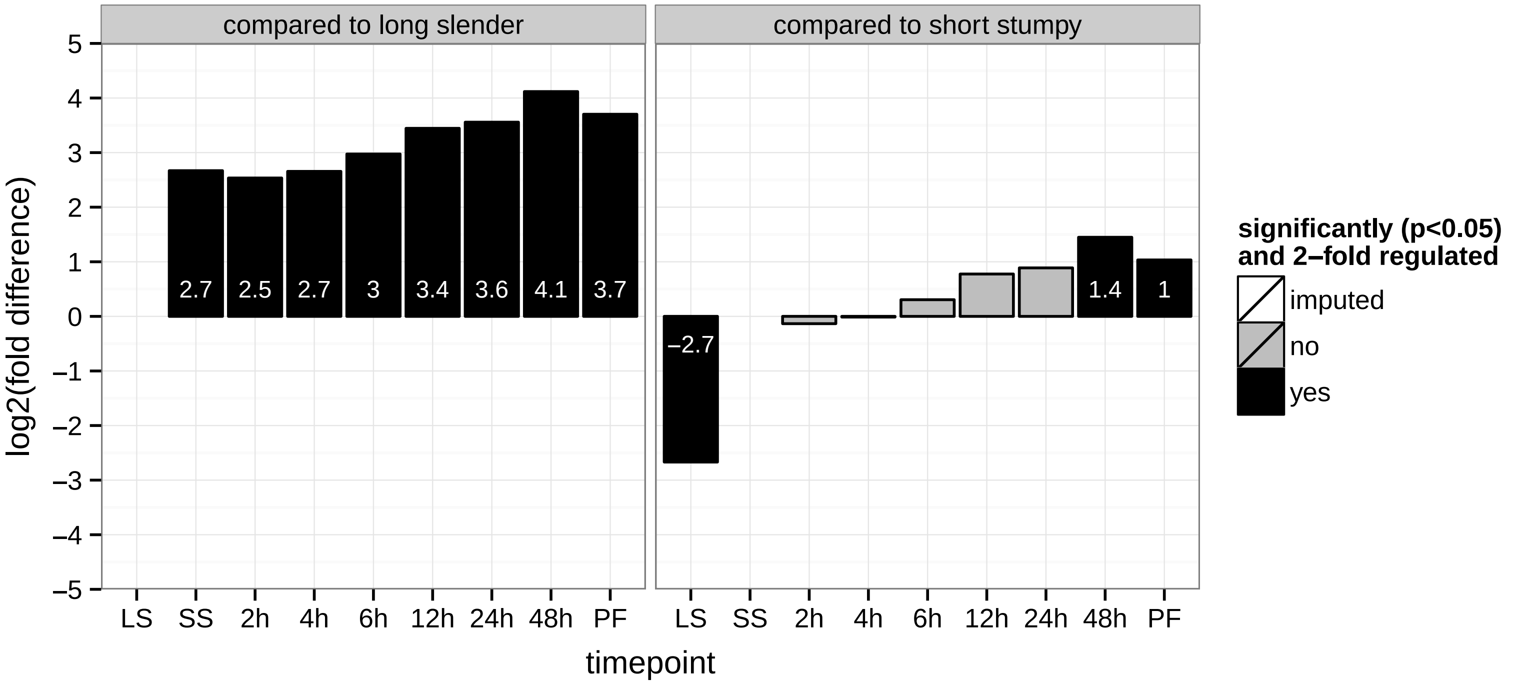

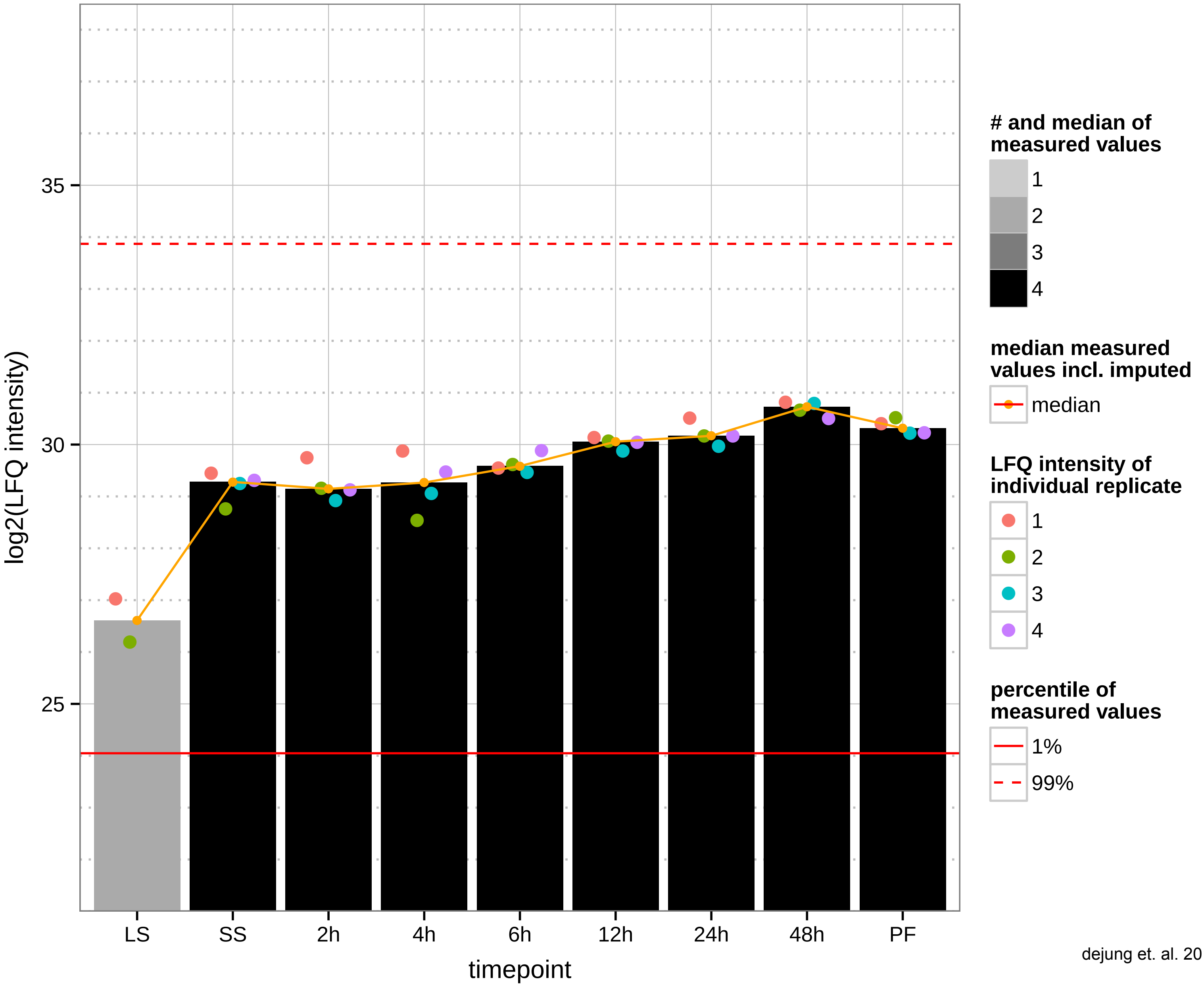

dejung et. al. 2015

hypothetical protein, conserved
Tb927.10.8630
AGOF: null
AGOC: mitochondrion
AGOP: null
PGOF: null
PGOC: null
PGOP: null

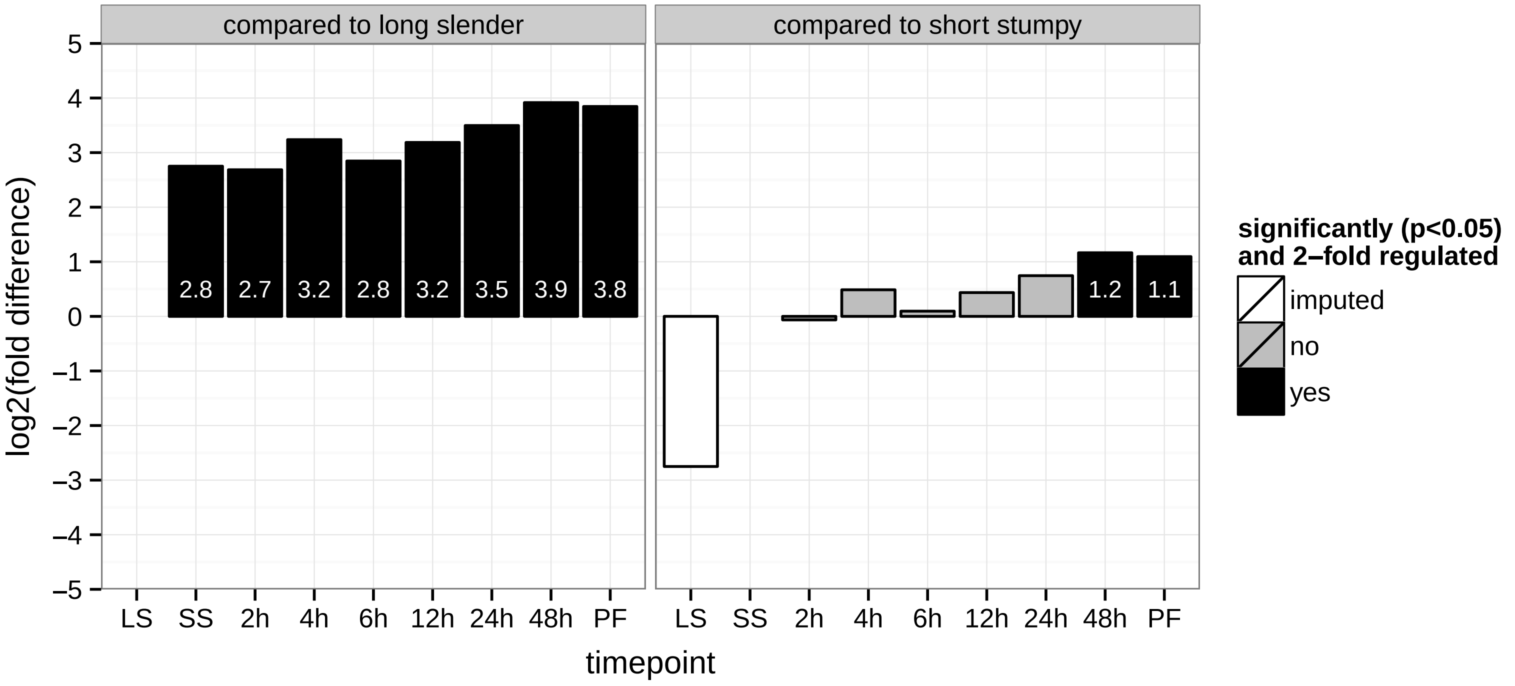

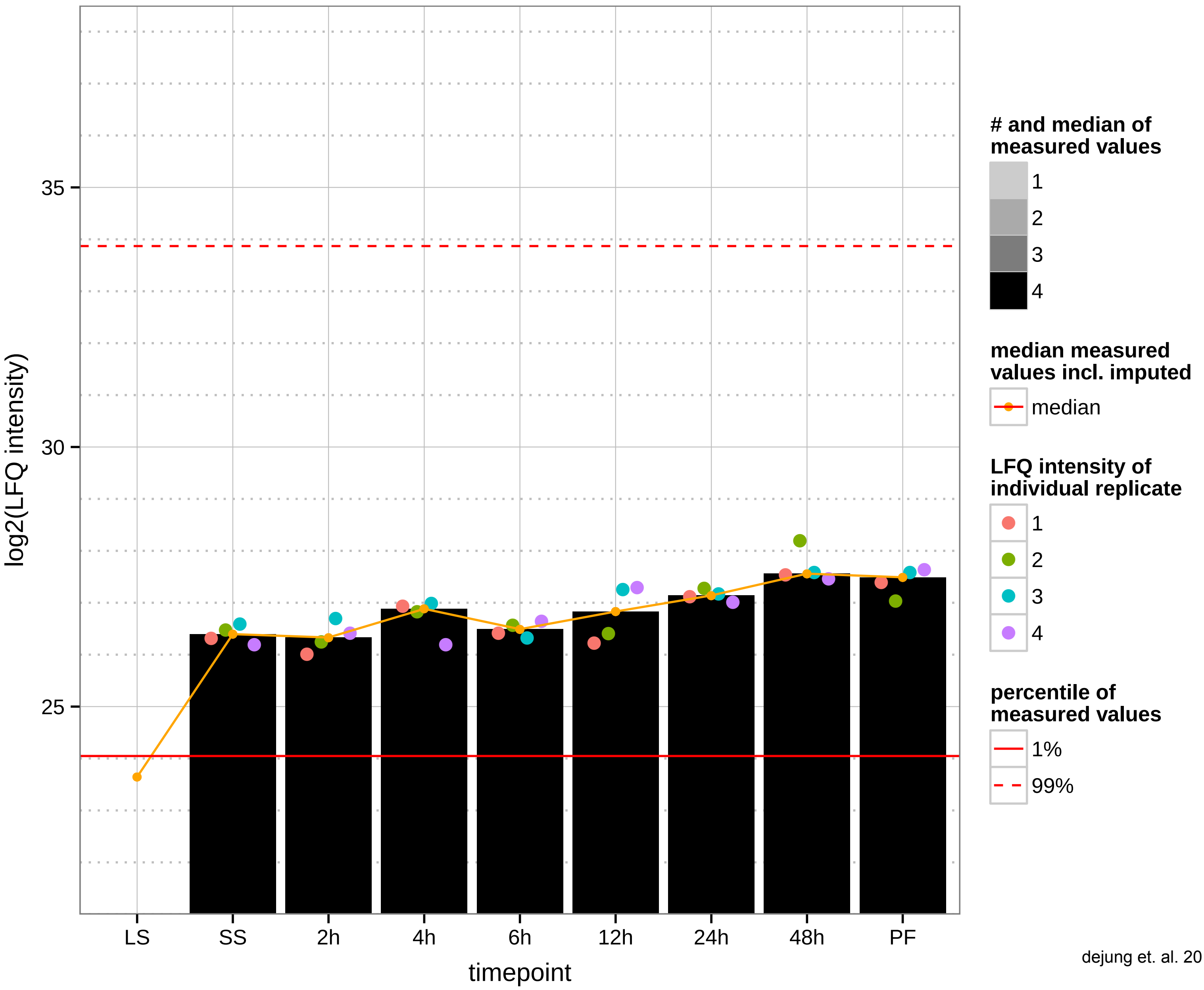

dejung et. al. 2015

hypothetical protein, conserved
Tb927.10.8730
AGOF: ATP binding, protein kinase activity
AGOC: mitochondrial inner membrane, mitochondrion
AGOP: protein phosphorylation
PGOF: ATP binding, protein kinase activity, transferase activity, transferring phosphorus–containing groups
PGOC: null
PGOP: protein phosphorylation

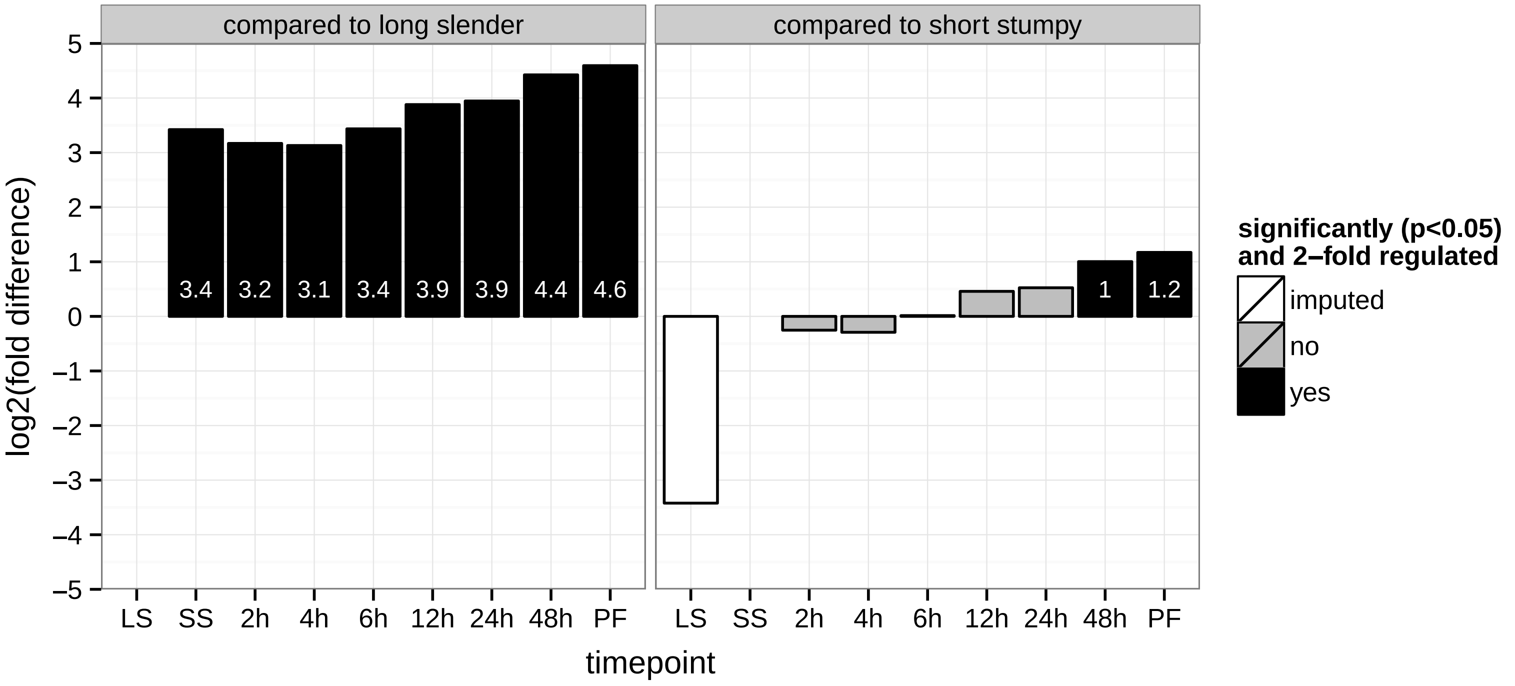

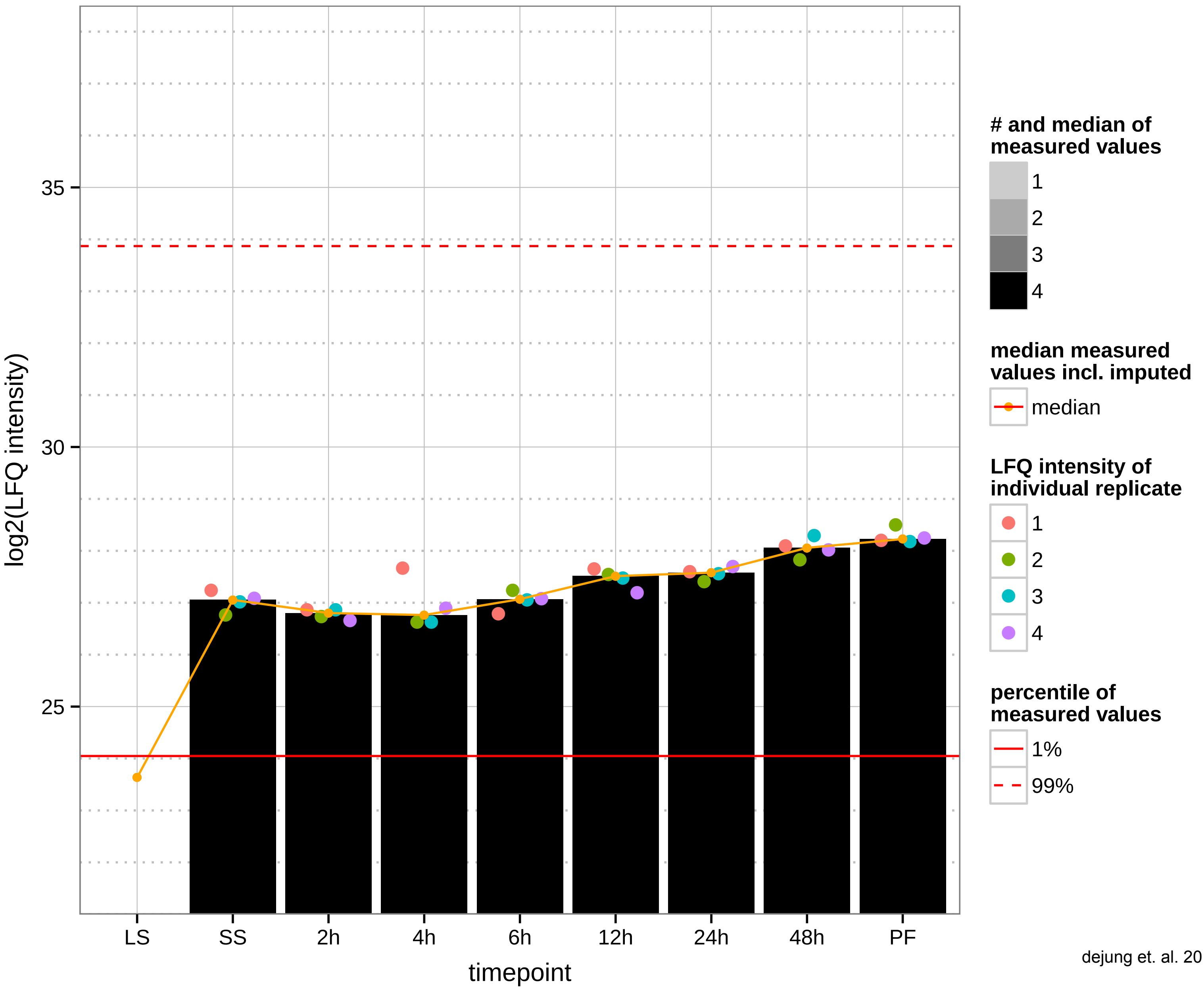

dejung et. al. 2015

hypothetical protein, conserved
Tb927.11.10320
AGOF: endodeoxyribonuclease activity, producing 5'–phosphomonoesters
AGOC: null
AGOP: null
PGOF: endodeoxyribonuclease activity, producing 5'–phosphomonoesters
PGOC: null
PGOP: null

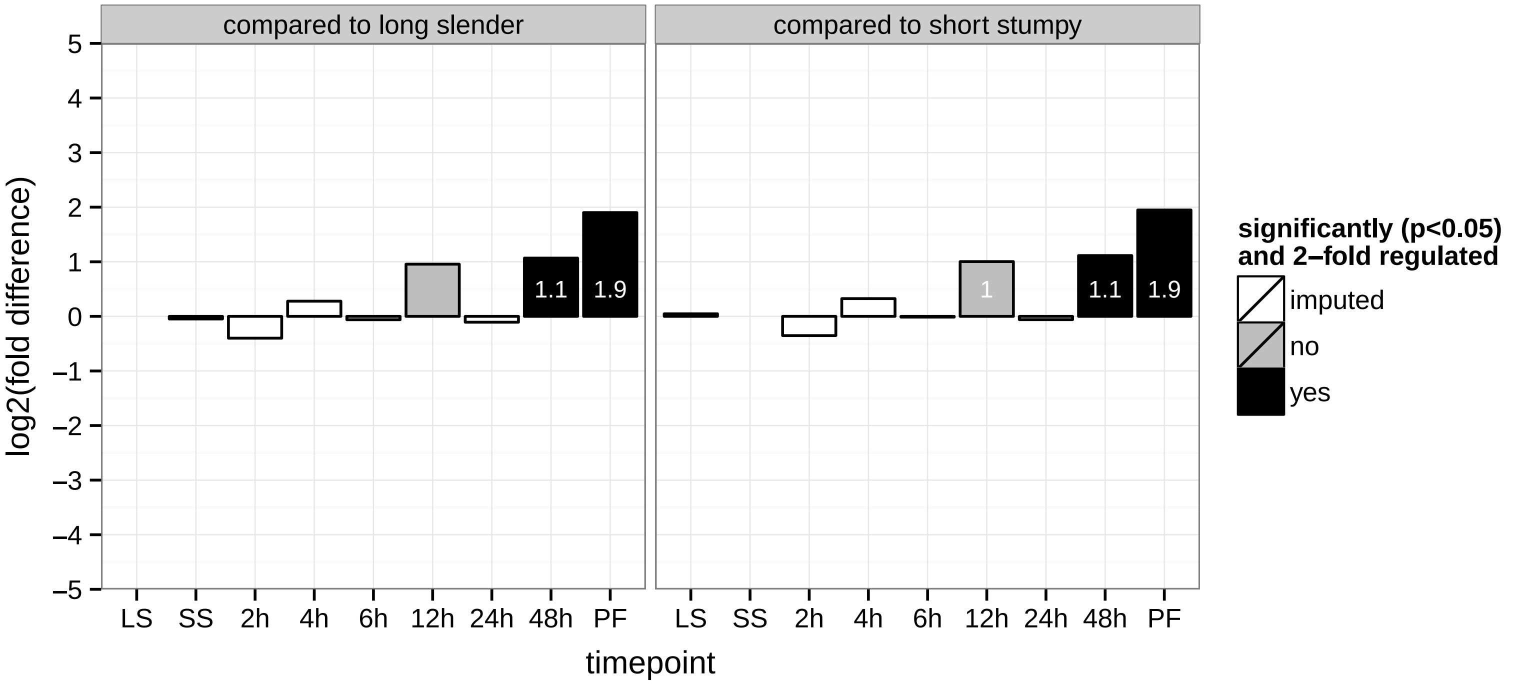

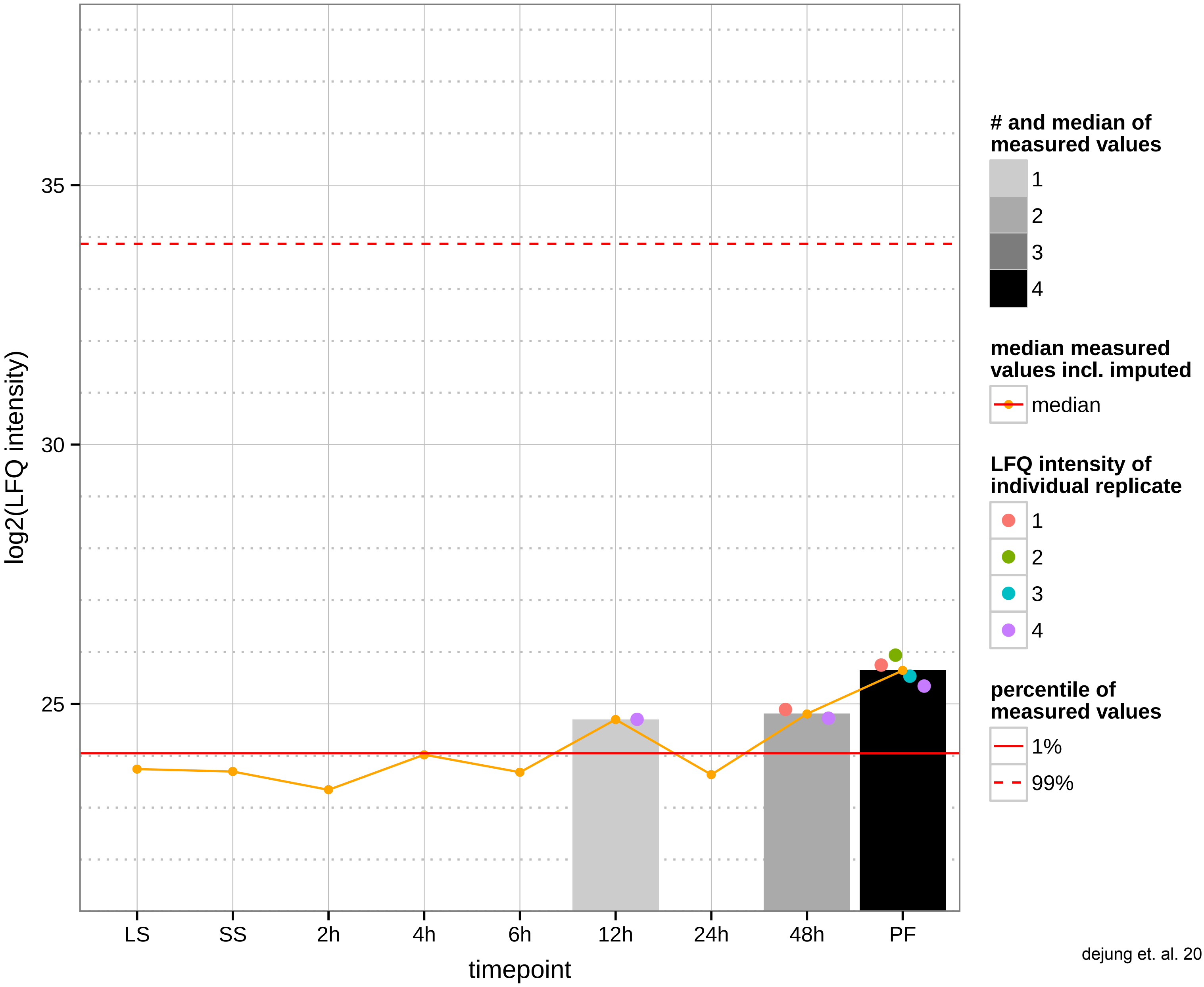

dejung et. al. 2015

hypothetical protein, conserved
Tb927.11.11460
AGOF: null
AGOC: mitochondrion
AGOP: null
PGOF: null
PGOC: null
PGOP: null

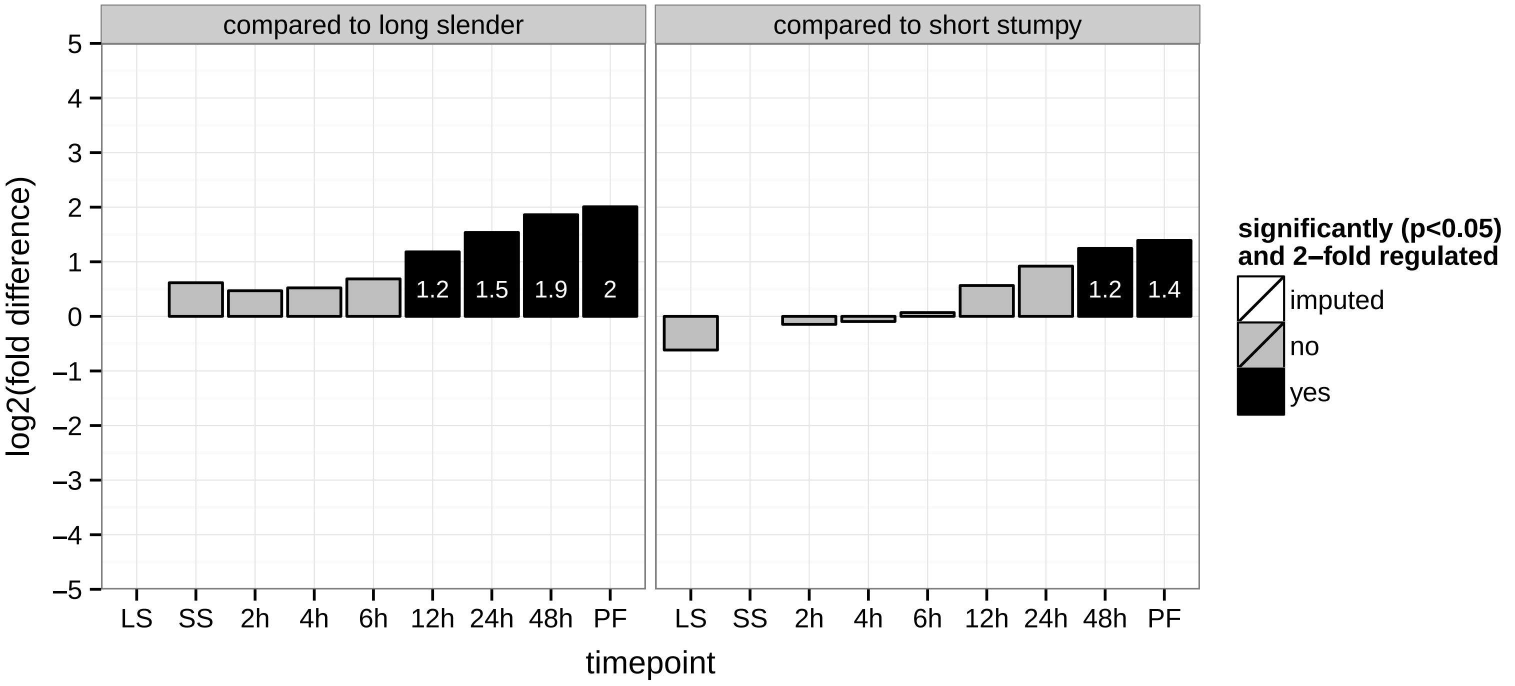

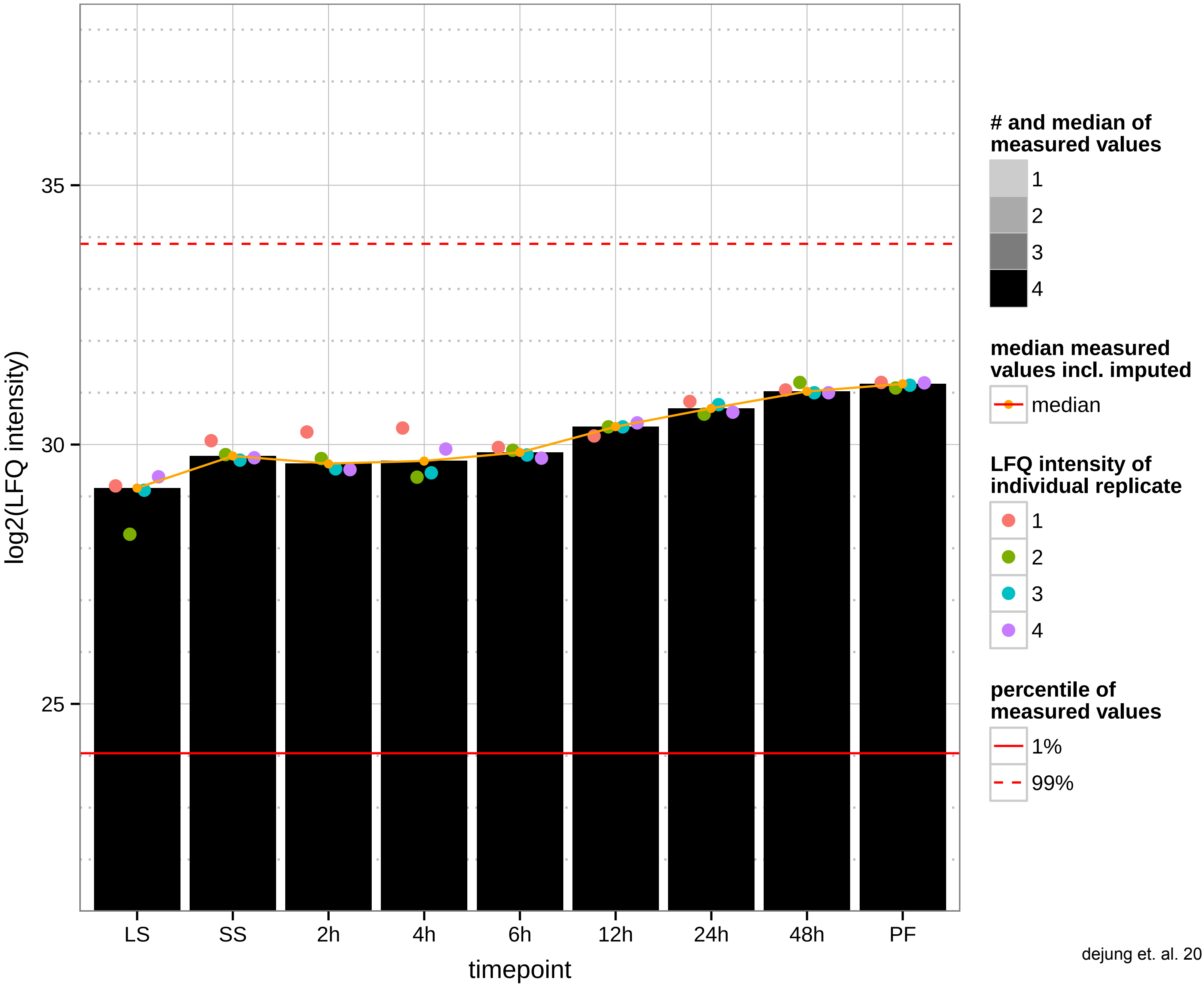

dejung et. al. 2015

glutaredoxin, putative
Tb927.11.1370
AGOF: disulfide oxidoreductase activity, electron carrier activity, protein disulfide isomerase activity, protein disulfide oxidoredu
AGOC: mitochondrion
AGOP: cell redox homeostasis, oxidation–reduction process
PGOF: electron carrier activity, protein disulfide oxidoreductase activity
PGOC: null
PGOP: cell redox homeostasis

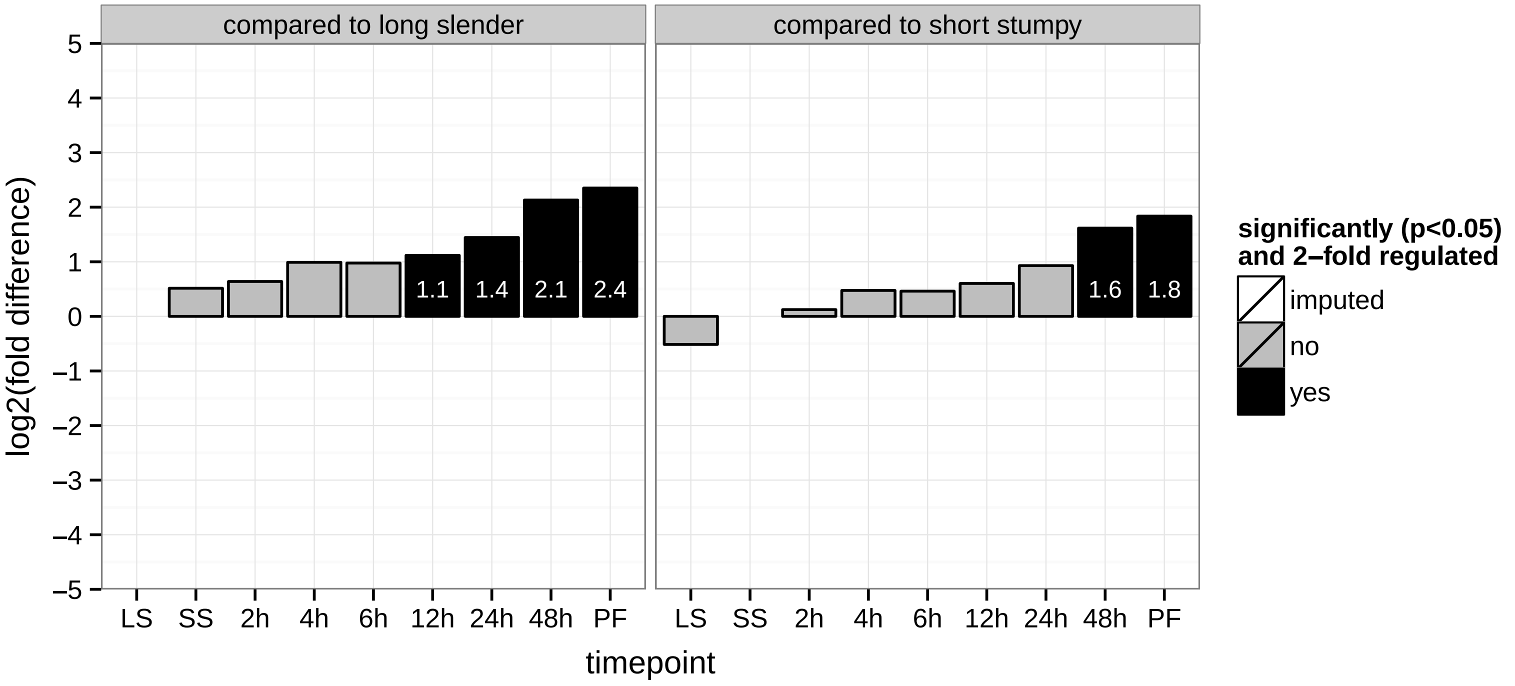

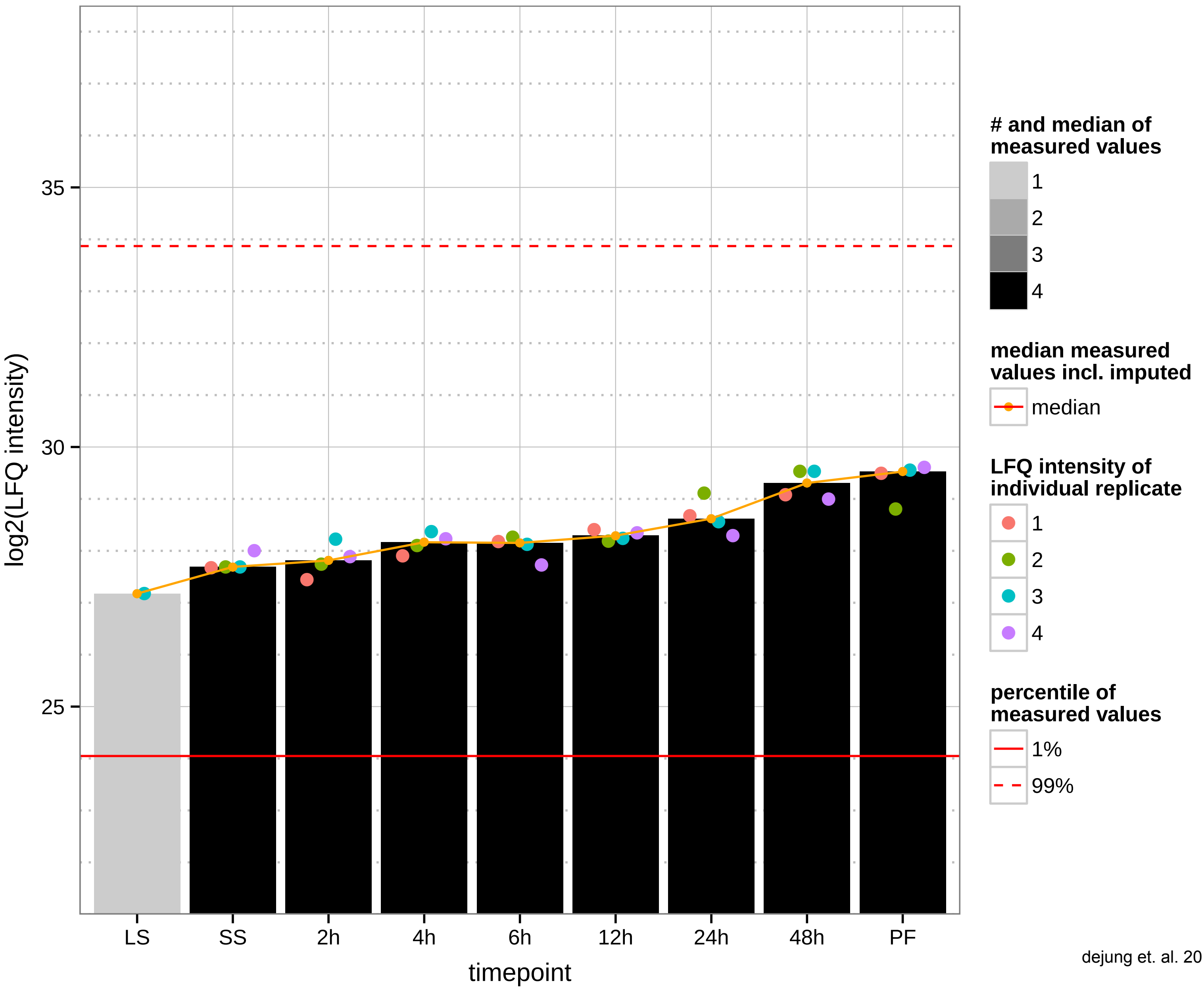

dejung et. al. 2015

hypothetical protein, conserved
Tb927.11.14170
AGOF: null
AGOC: null
AGOP: null
PGOF: null
PGOC: null
PGOP: null

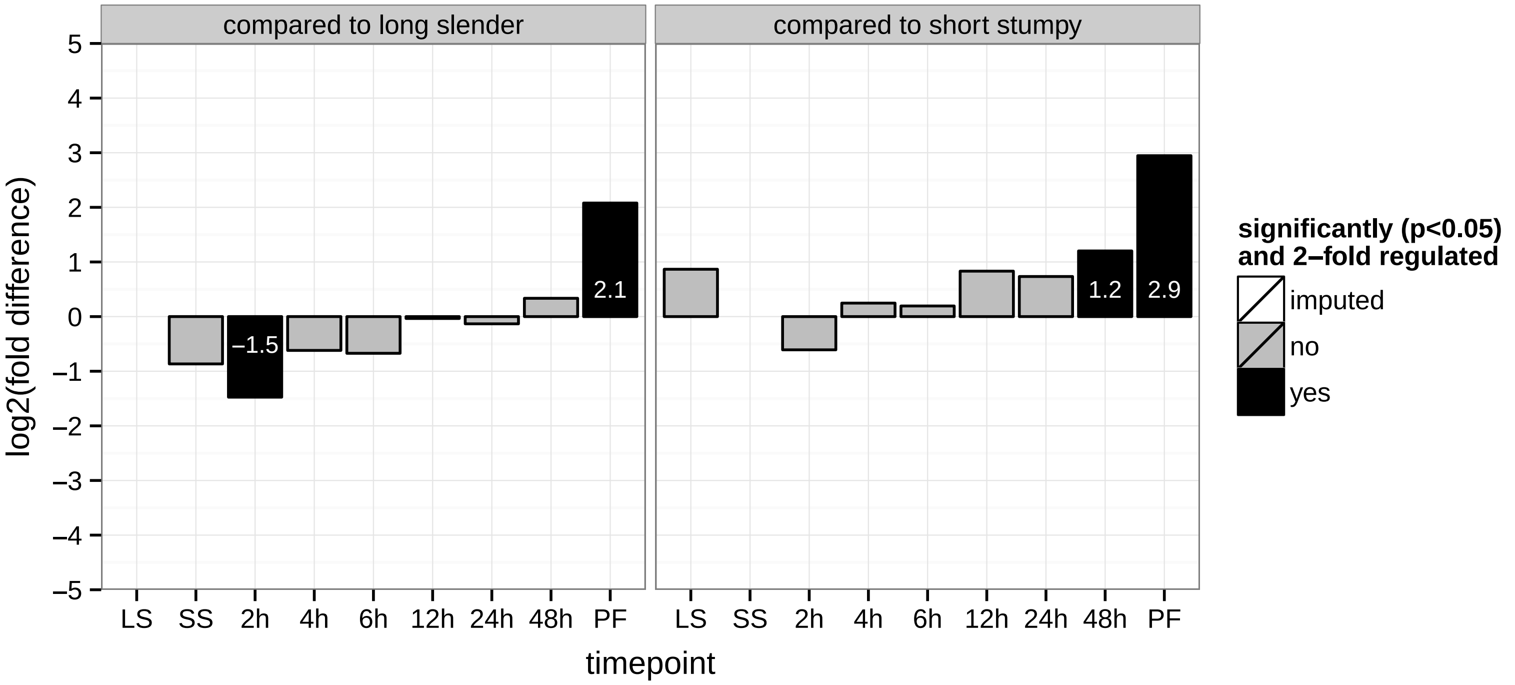

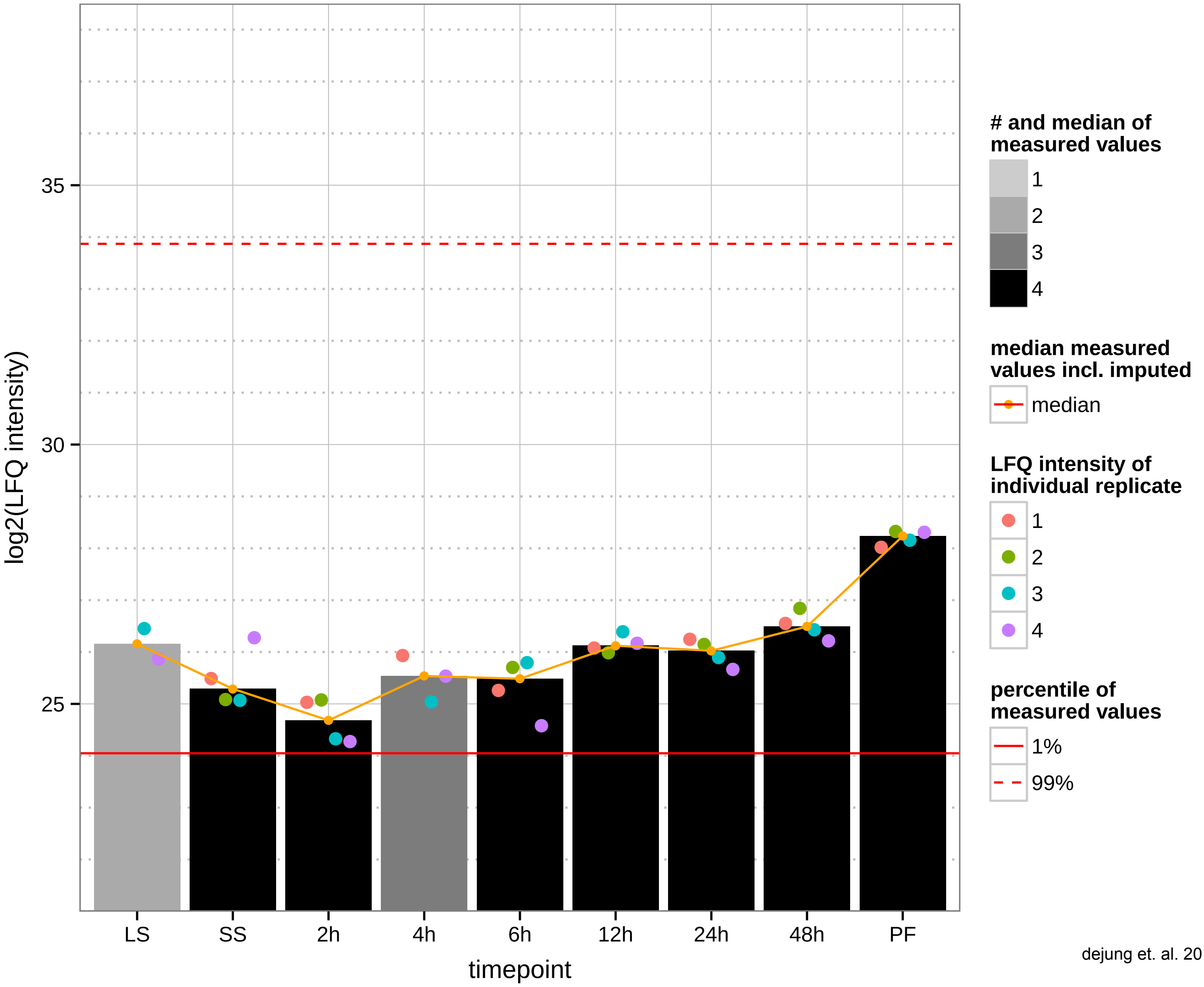

dejung et. al. 2015

ubiquitin–conjugating enzyme E2, putative, ubiquitin carrier protein, ubiquitin–protein ligase
Tb927.11.14200
AGOF: ubiquitin–protein ligase activity
AGOC: null
AGOP: cellular protein modification process, post–translational protein modification, regulation of protein metabolic process
PGOF: acid–amino acid ligase activity
PGOC: null
PGOP: null

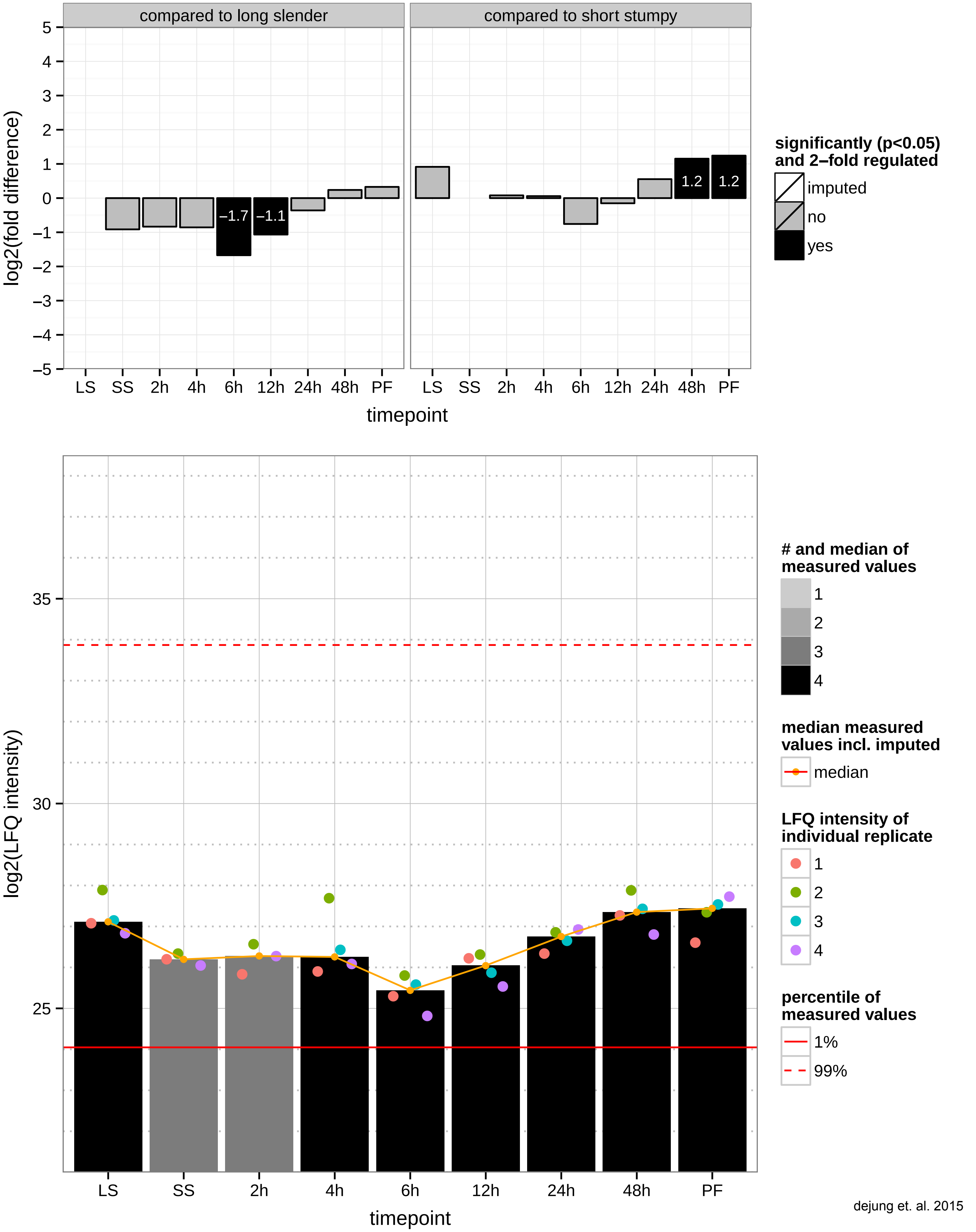

dejung et. al. 2015

hypothetical protein, conserved
Tb927.11.14980
AGOF: null
AGOC: mitochondrion
AGOP: null
PGOF: null
PGOC: null
PGOP: null

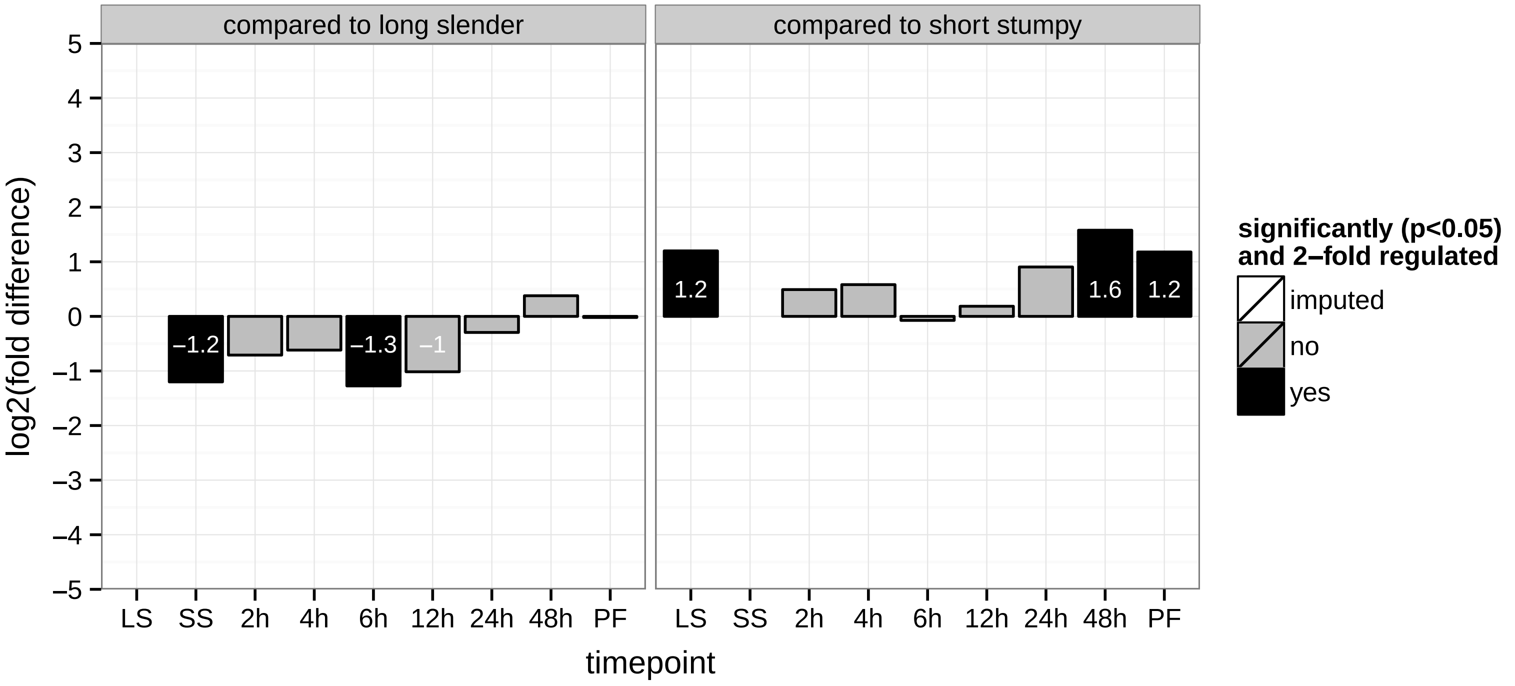

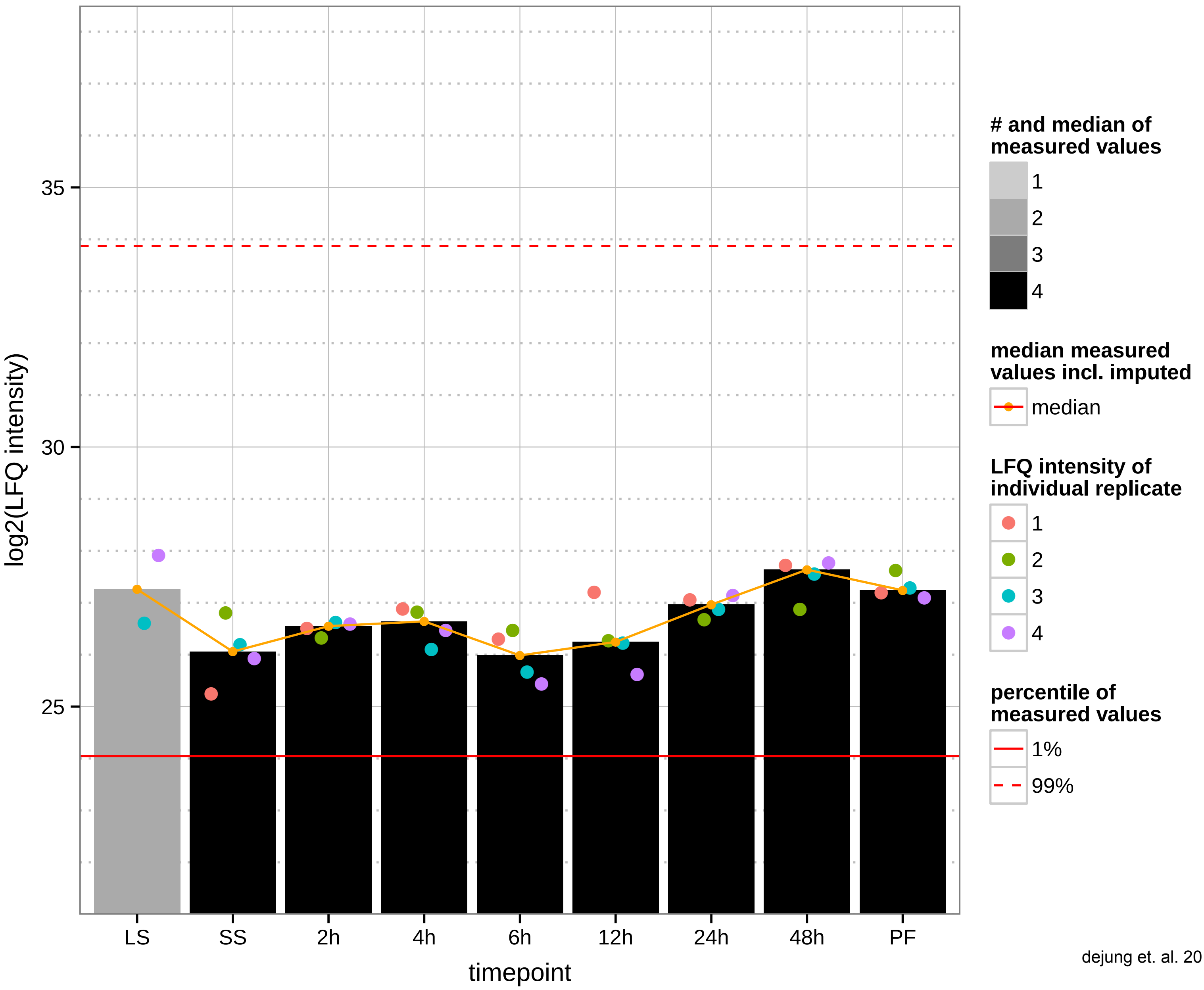

dejung et. al. 2015

hypothetical protein, conserved
Tb927.11.2120
AGOF: null
AGOC: null
AGOP: null
PGOF: binding
PGOC: null
PGOP: null

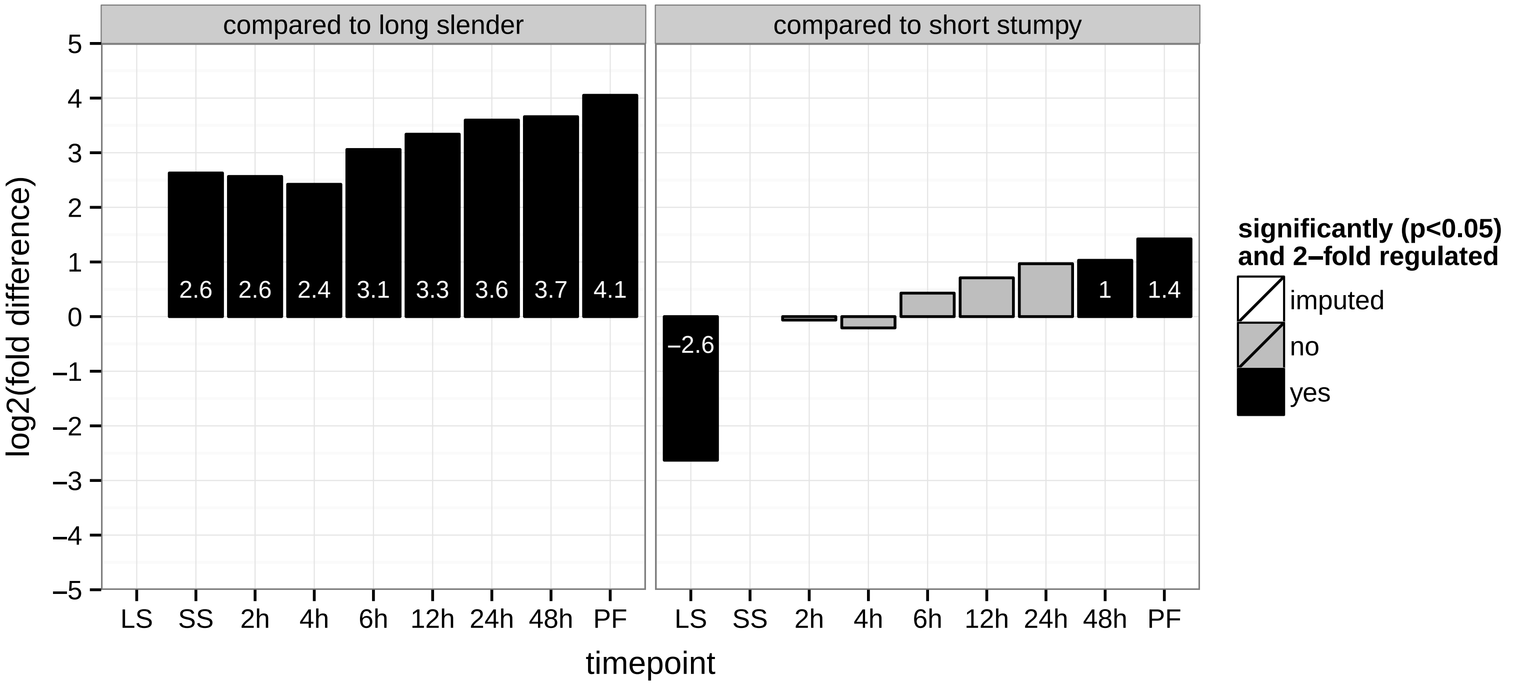

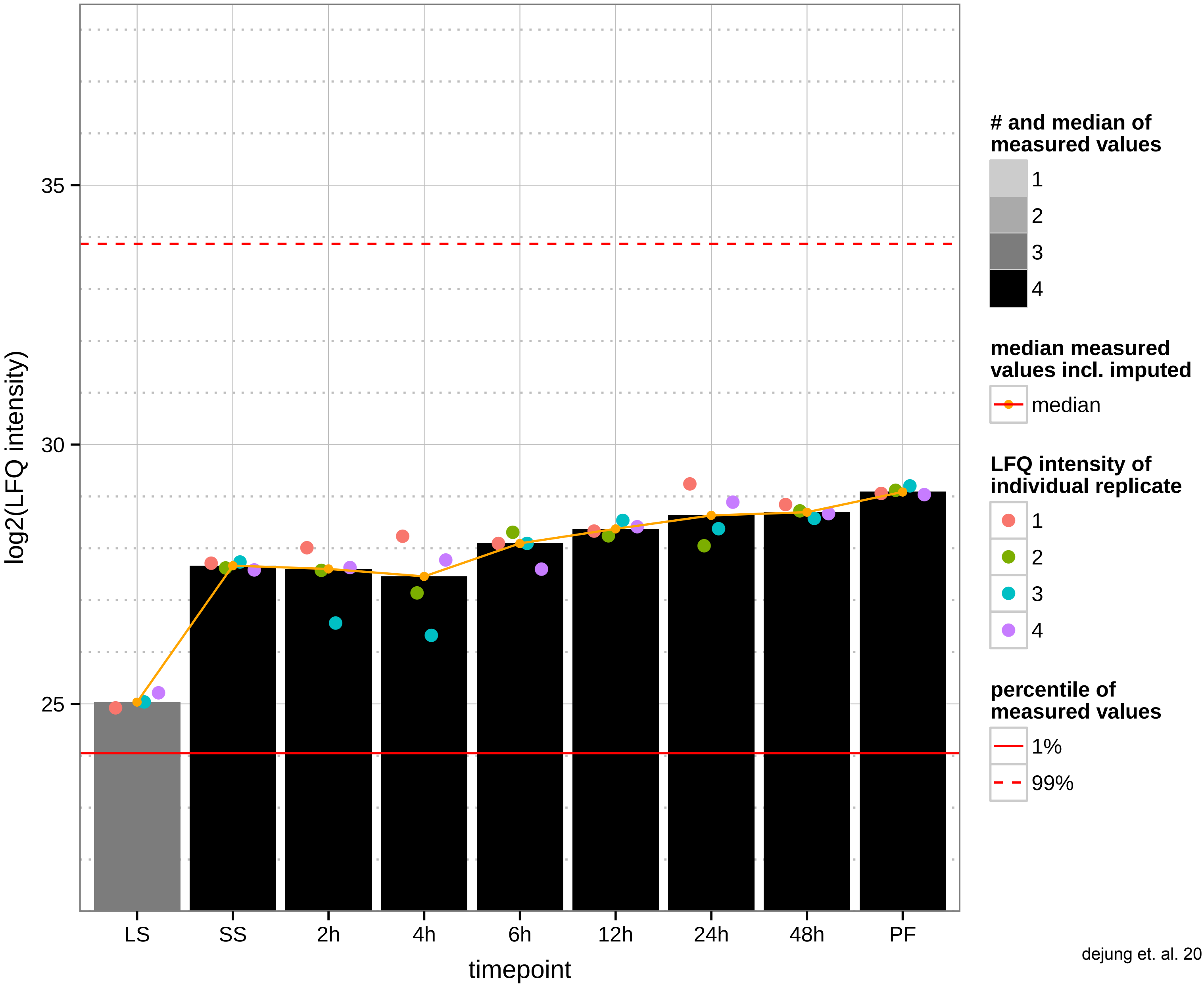

dejung et. al. 2015

hypothetical protein, conserved
Tb927.11.2840
AGOF: null
AGOC: null
AGOP: null
PGOF: phosphatase activity
PGOC: null
PGOP: null

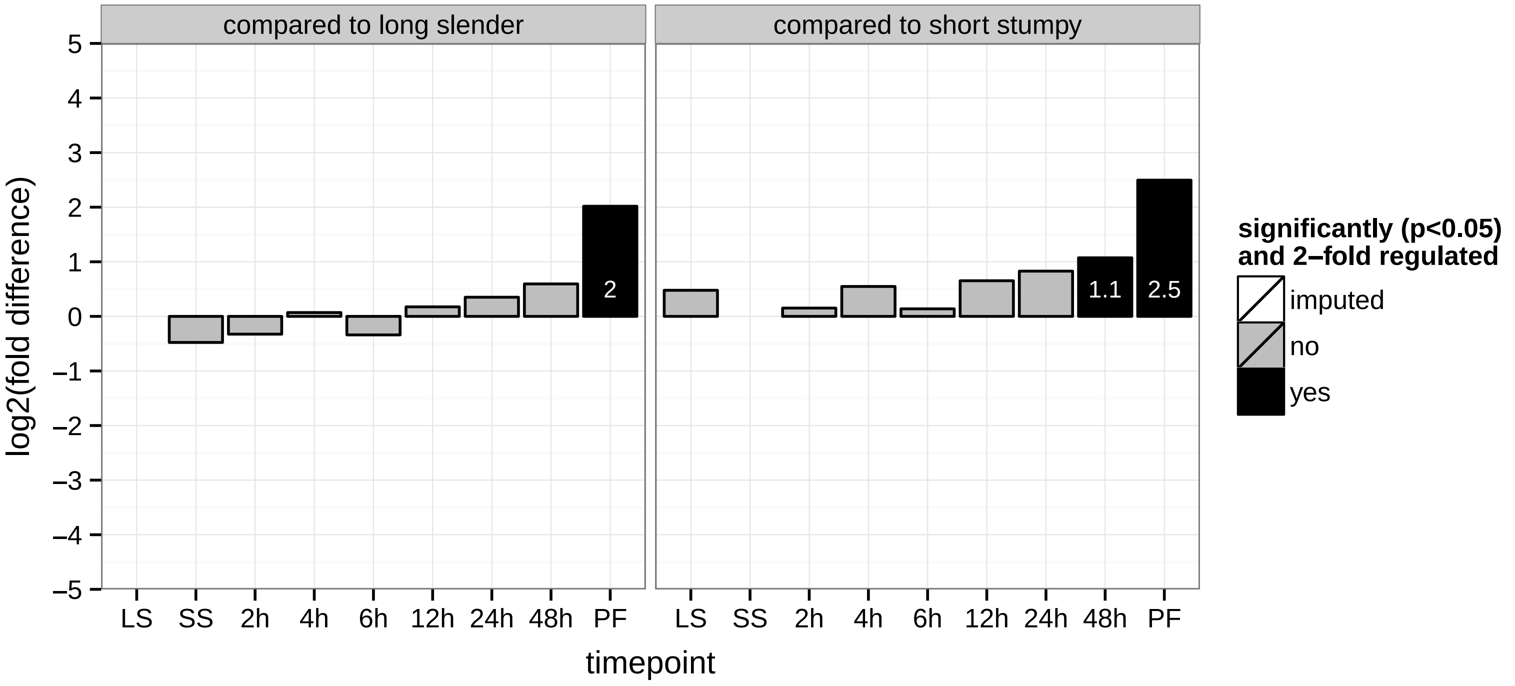

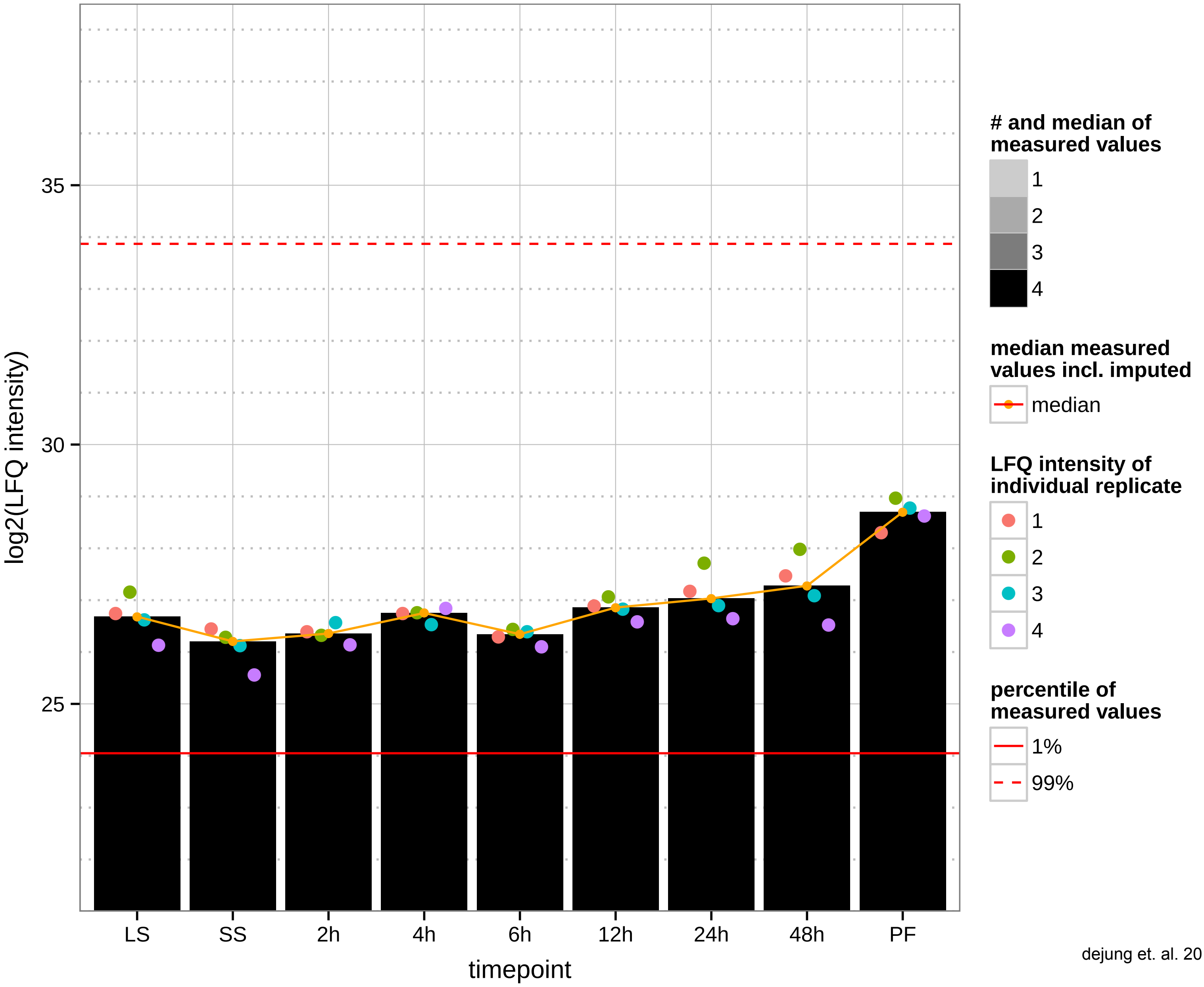

dejung et. al. 2015

Ribosome assembly protein rrb1, putative
Tb927.11.3070
AGOF: null
AGOC: nucleolus
AGOP: ribosome biogenesis
PGOF: protein binding
PGOC: null
PGOP: null

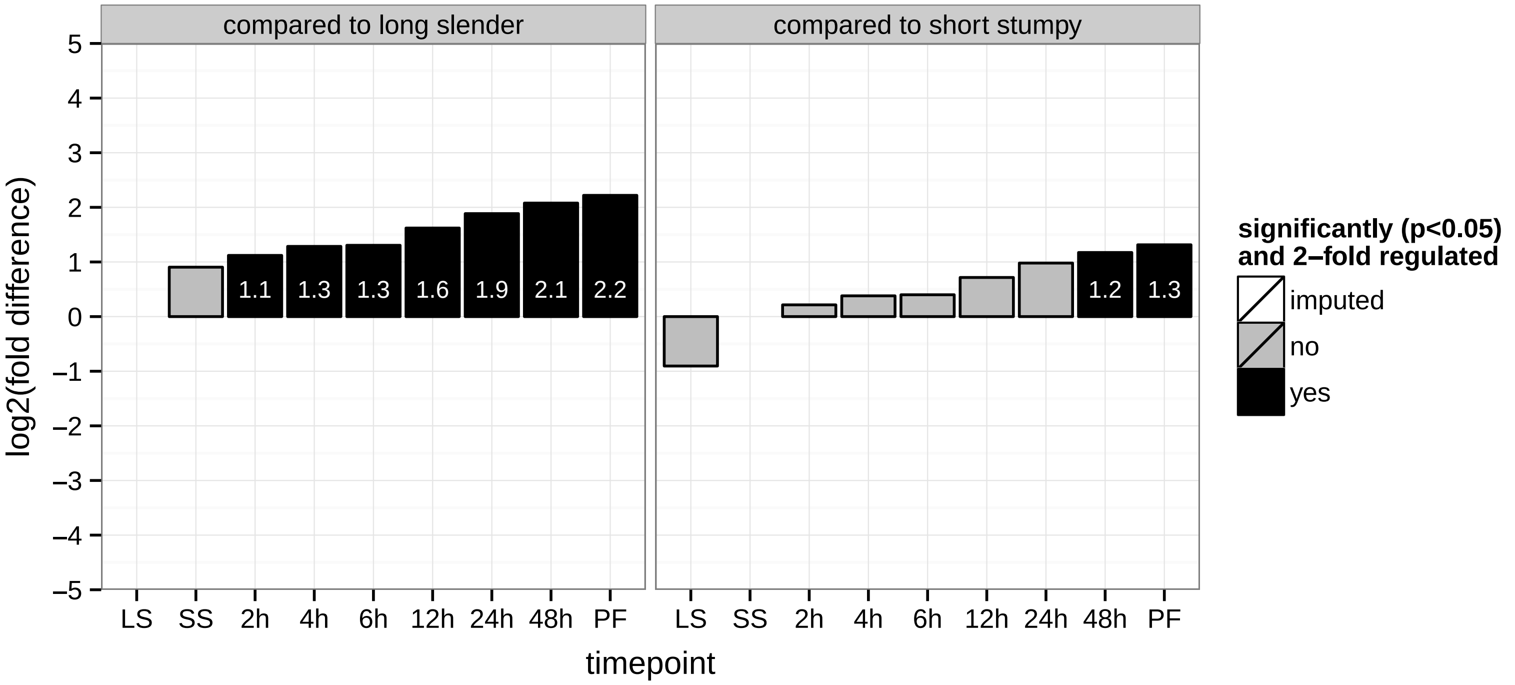

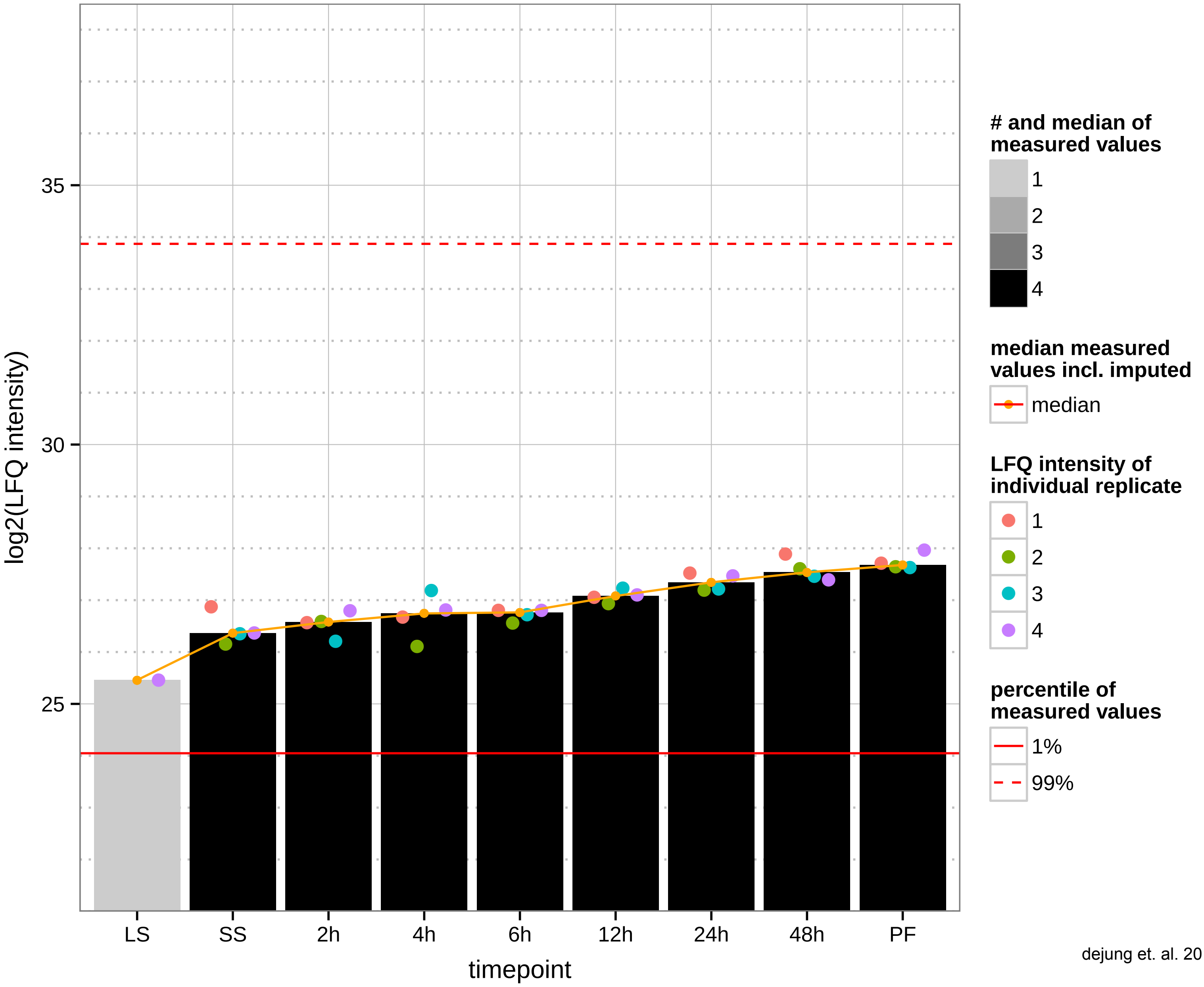

dejung et. al. 2015

hypothetical protein, conserved
Tb927.11.4420
AGOF: null
AGOC: null
AGOP: null
PGOF: null
PGOC: null
PGOP: null

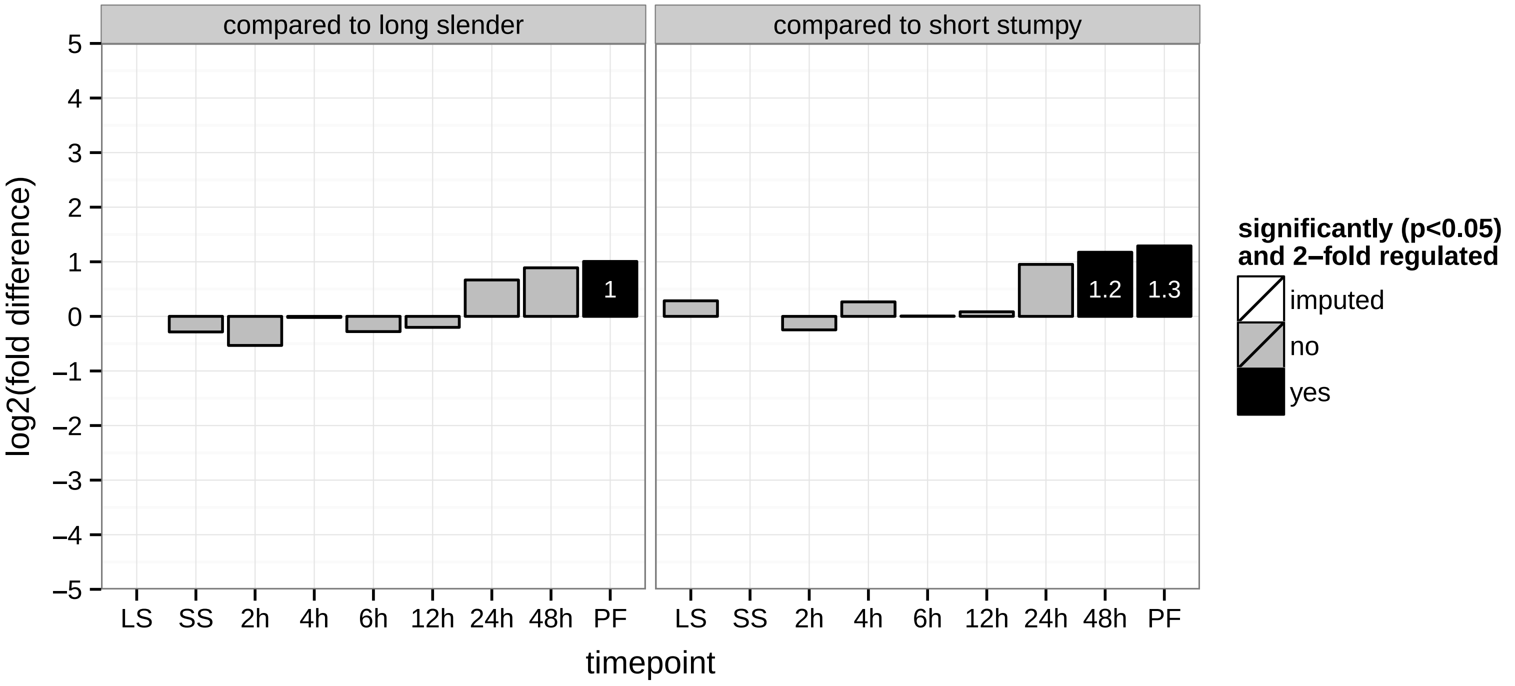

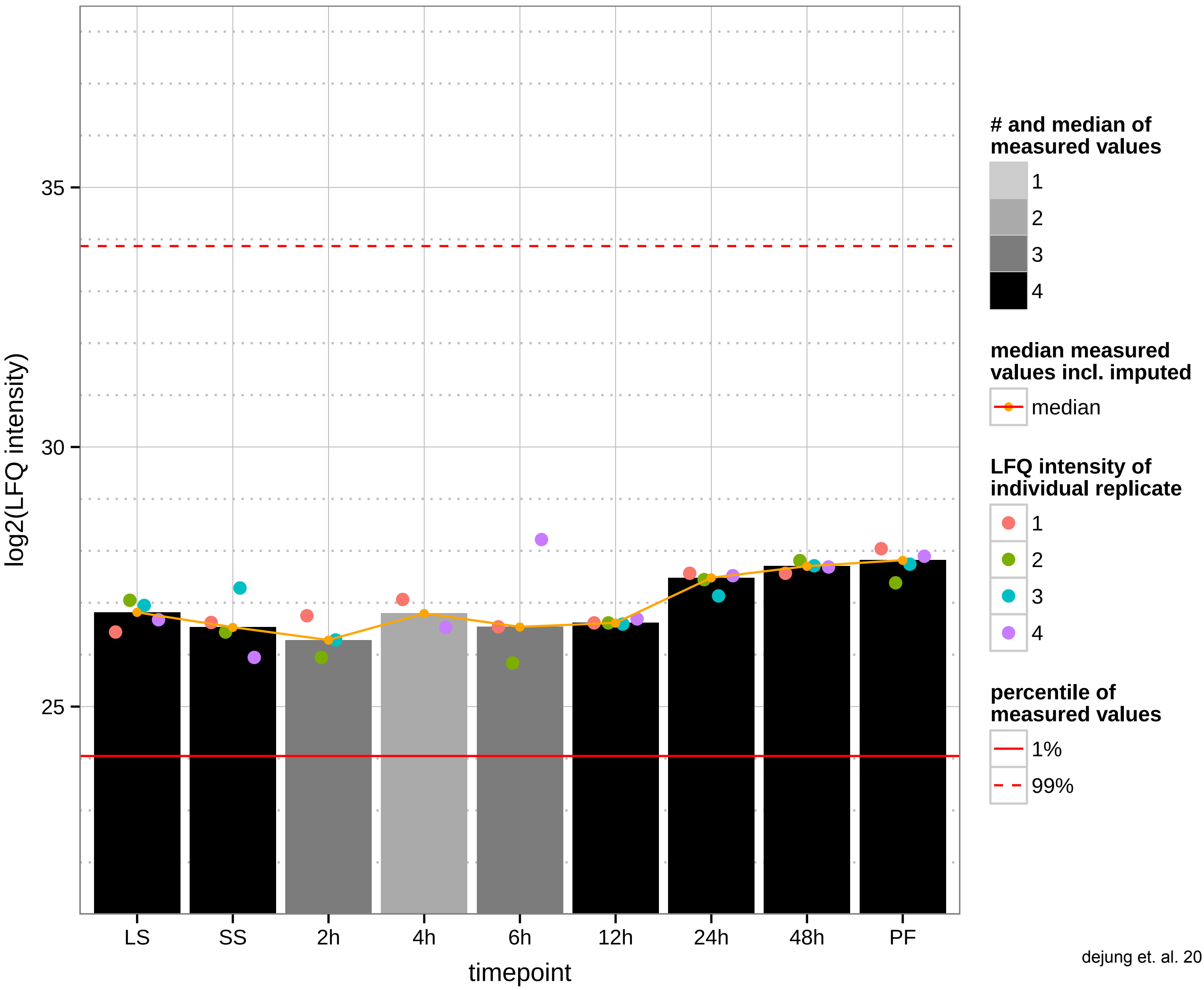

dejung et. al. 2015

pyruvate dehydrogenase (lipoamide) kinase, putative
Tb927.11.4780
AGOF: ATP binding, pyruvate dehydrogenase (acetyl−transferring) kinase activity
AGOC: mitochondrion
AGOP: null
PGOF: ATP binding
PGOC: null
PGOP: null

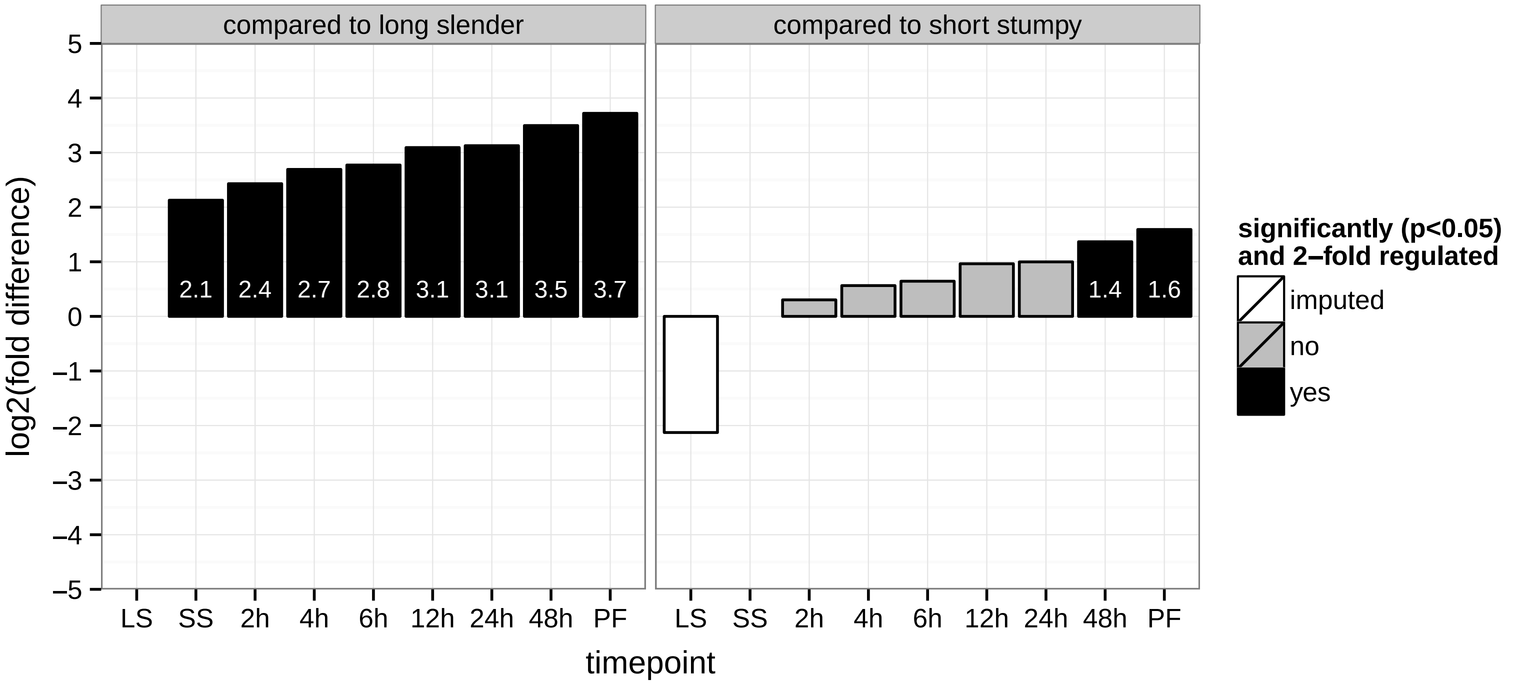

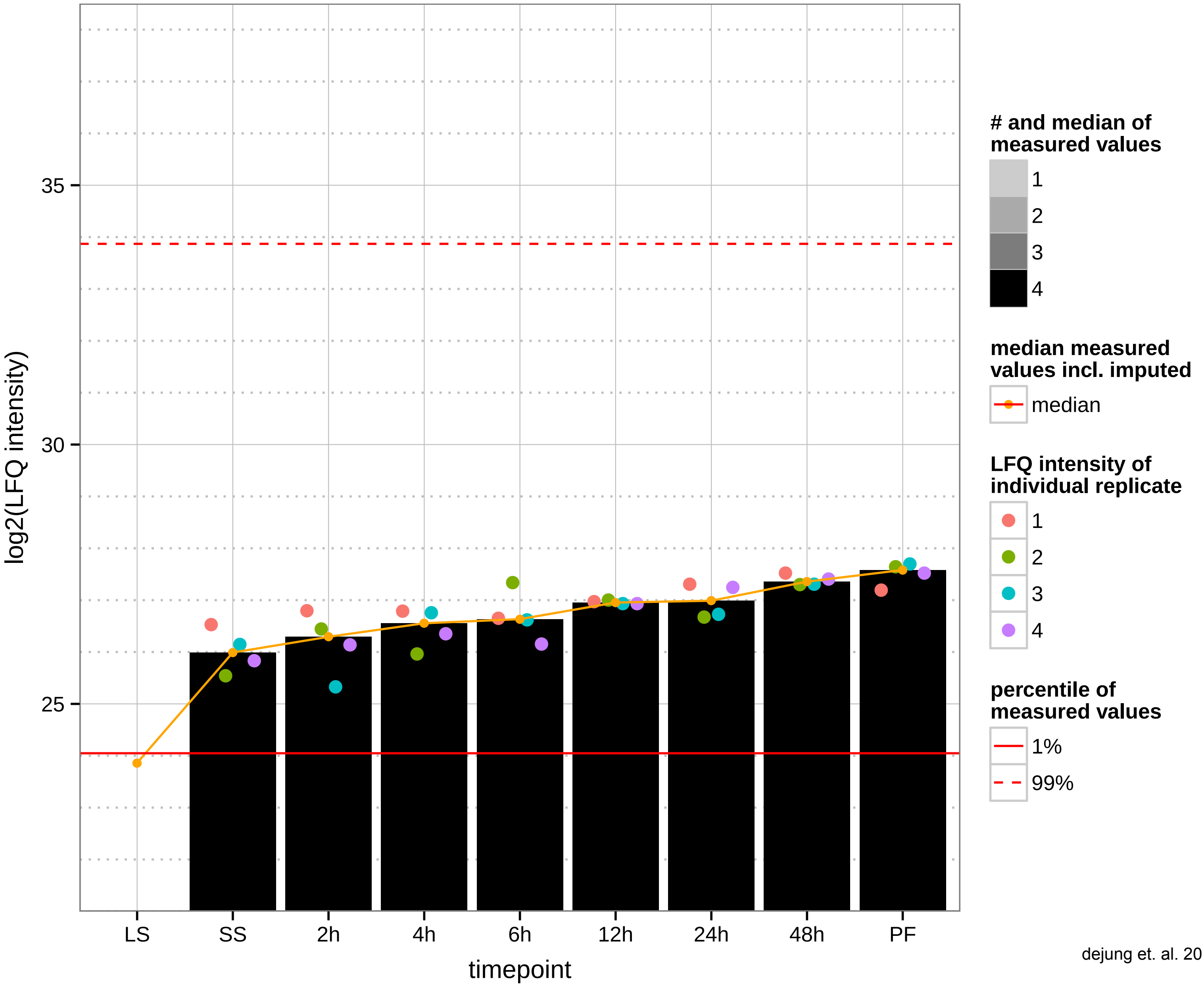

dejung et. al. 2015

chaperone protein DNAj, putative
Tb927.11.5220
AGOF: heat shock protein binding
AGOC: null
AGOP: protein folding
PGOF: heat shock protein binding
PGOC: null
PGOP: null

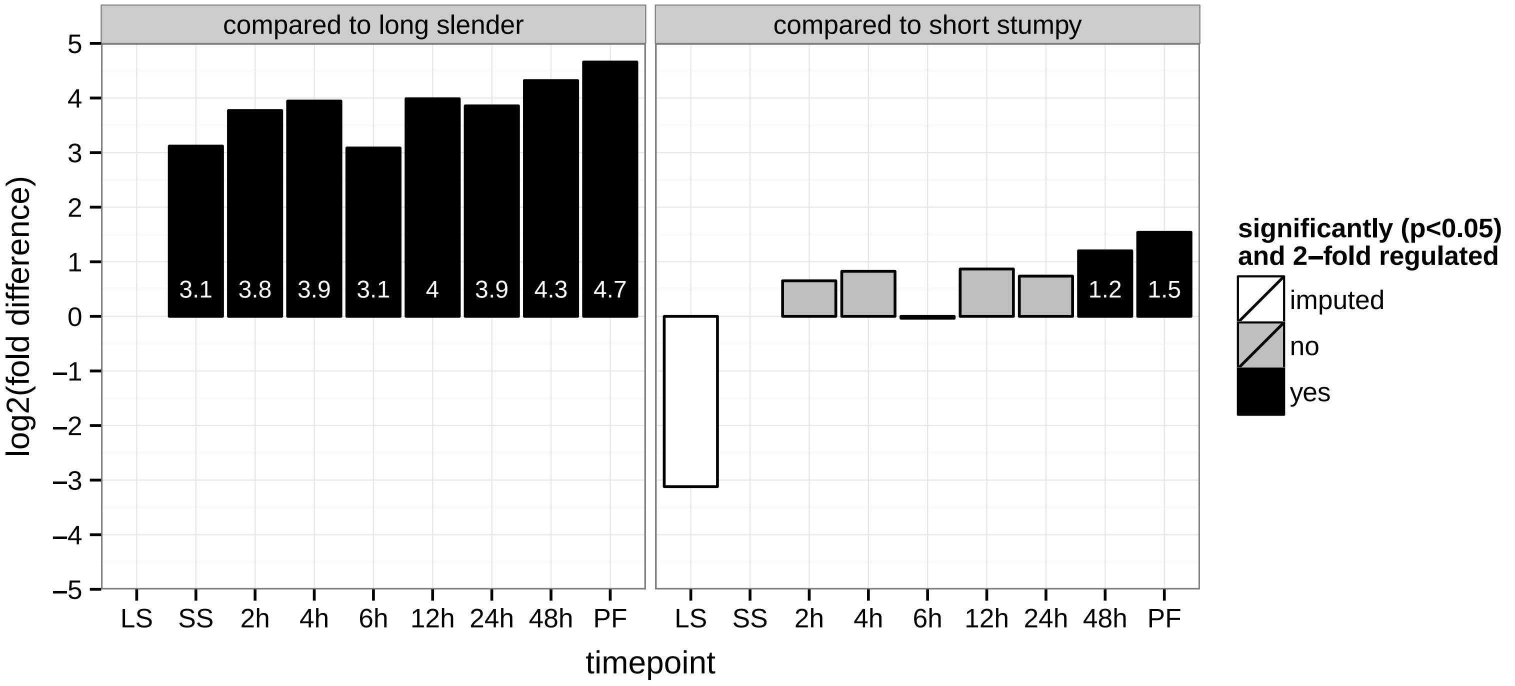

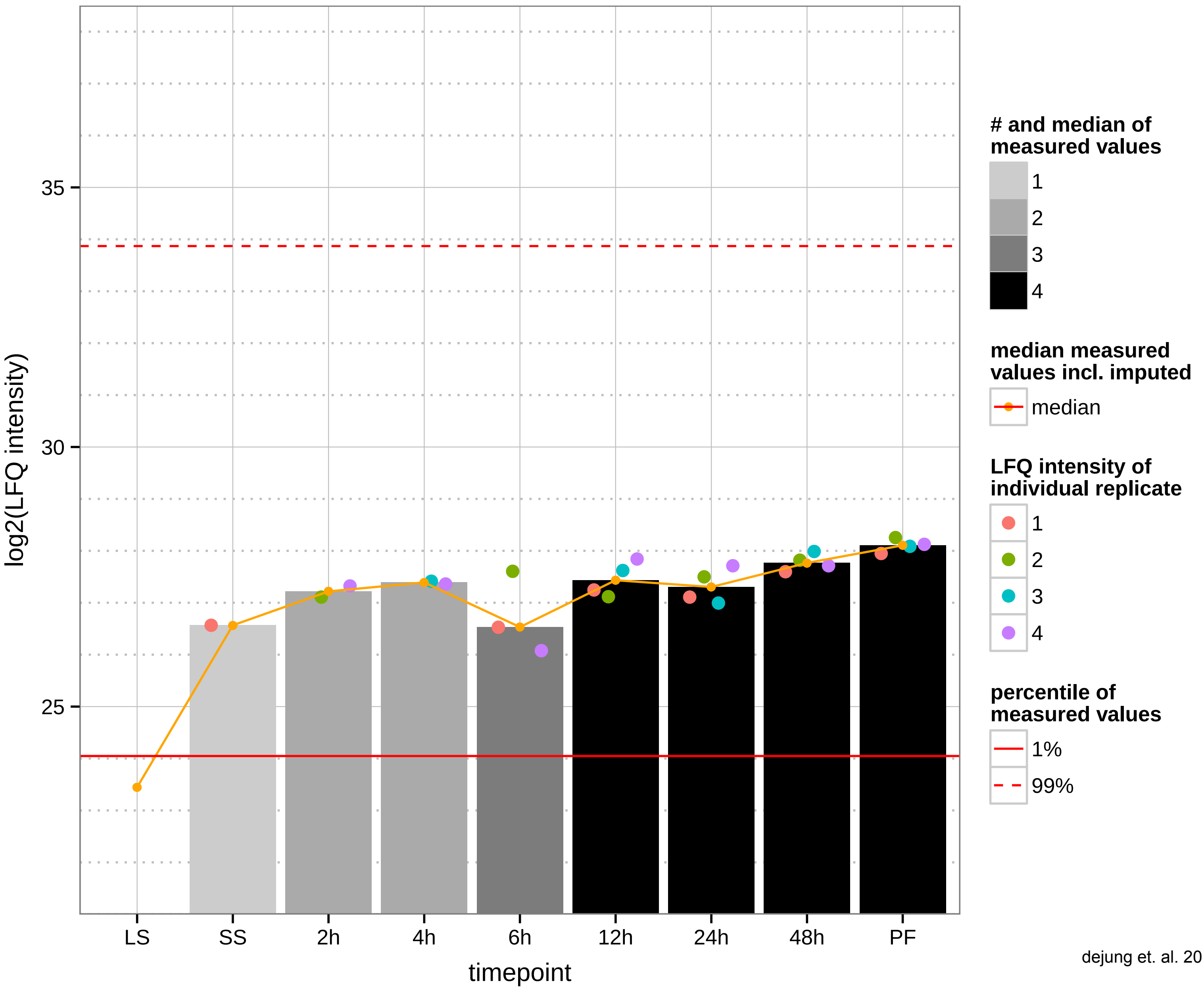

dejung et. al. 2015

hypothetical protein, conserved
Tb927.11.6000
AGOF: structural constituent of ribosome
AGOC: intracellular, mitochondrion, ribosome
AGOP: translation
PGOF: structural constituent of ribosome
PGOC: ribosome
PGOP: translation

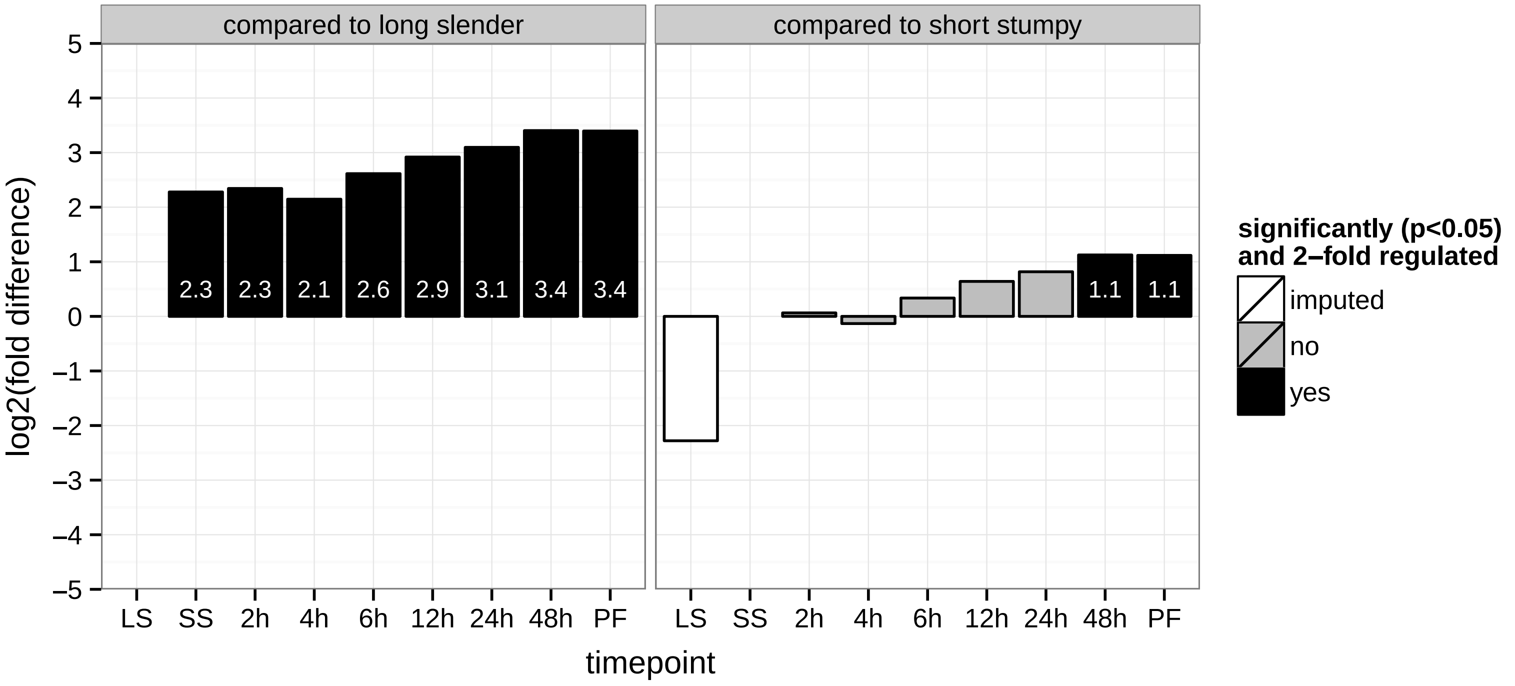

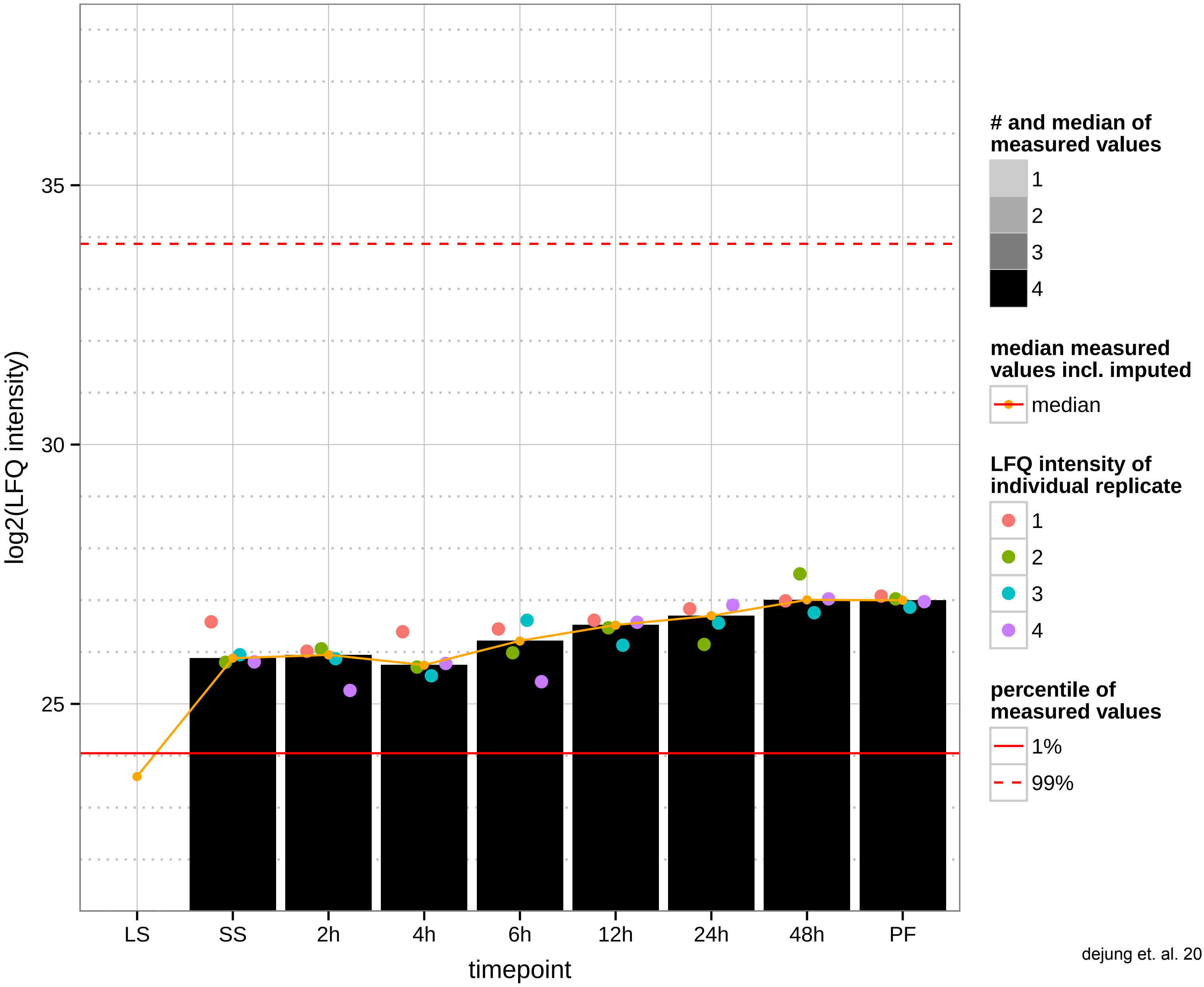

dejung et. al. 2015

40S ribosomal protein S5, putative
Tb927.11.6300
AGOF: structural constituent of ribosome
AGOC: intracellular, ribosome, small ribosomal subunit
AGOP: translation
PGOF: structural constituent of ribosome
PGOC: intracellular, ribosome, small ribosomal subunit
PGOP: translation

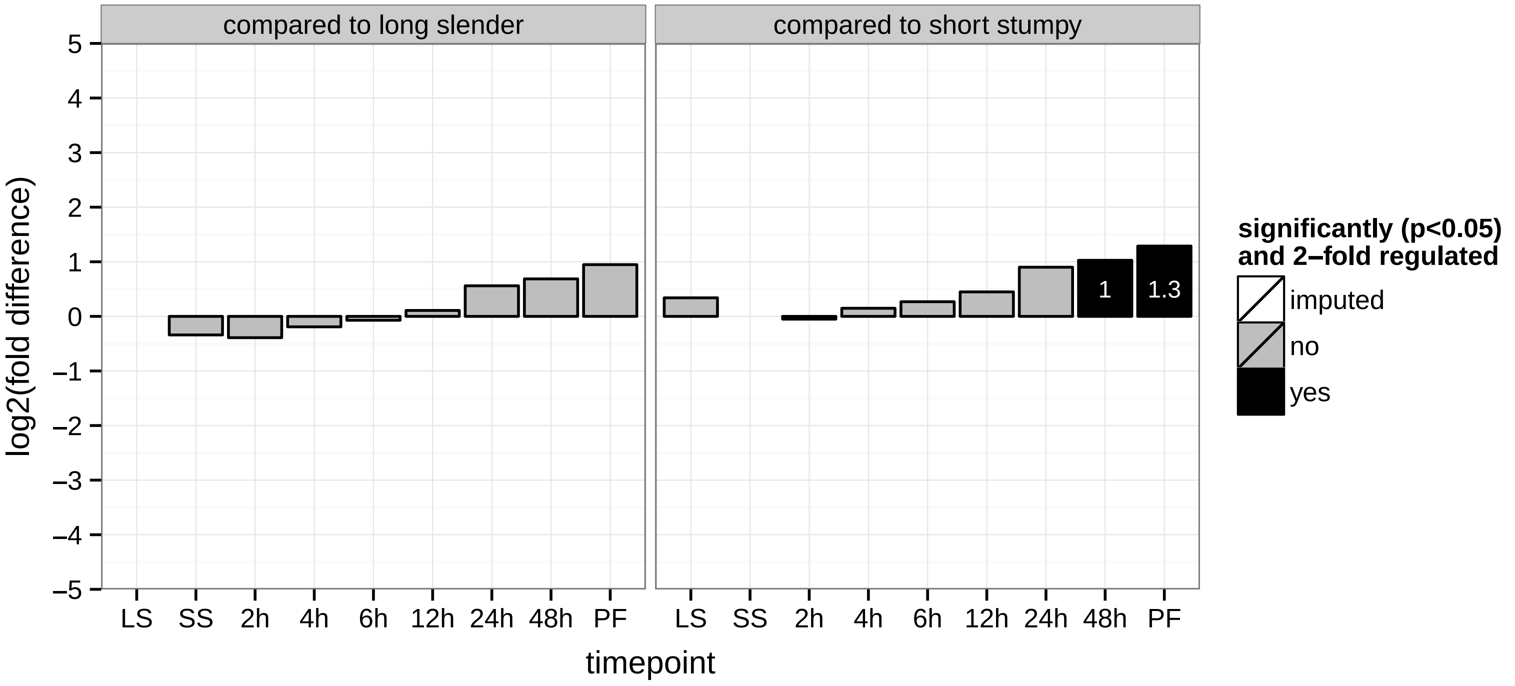

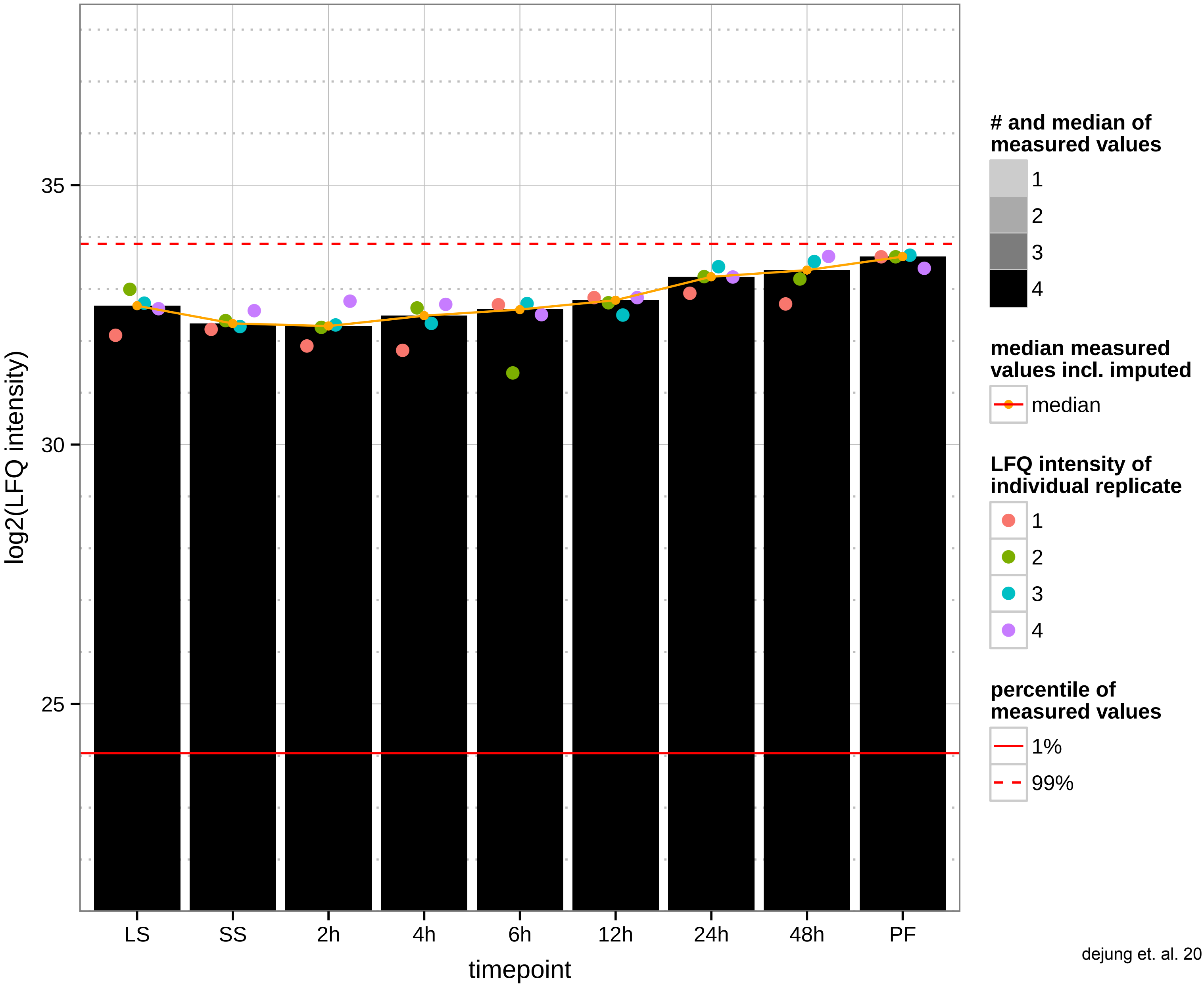

dejung et. al. 2015

hypothetical protein, conserved
Tb927.11.6420
AGOF: null
AGOC: null
AGOP: null
PGOF: binding
PGOC: null
PGOP: null

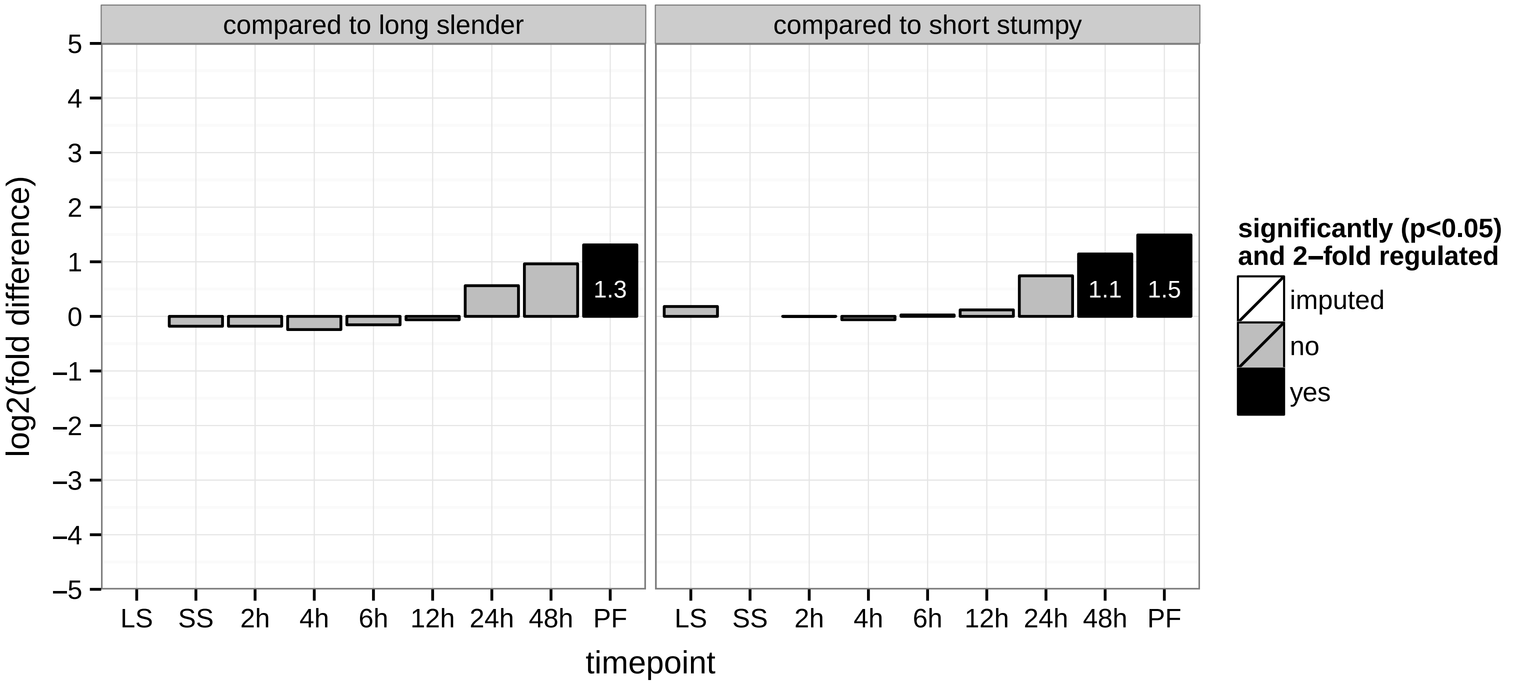

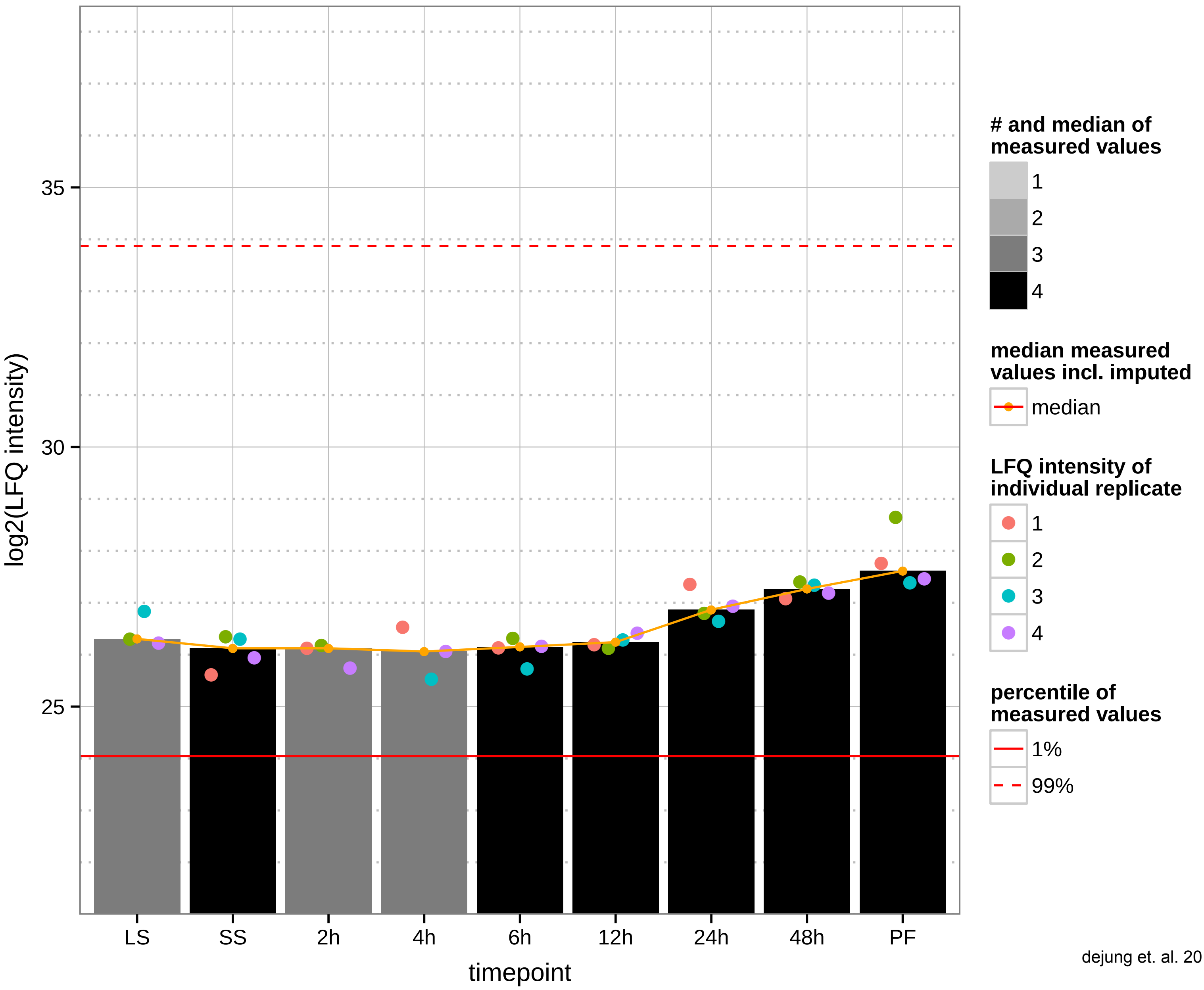

dejung et. al. 2015

40S ribosomal protein S21, putative
Tb927.11.6510;Tb927.11.6500
AGOF: structural constituent of ribosome
AGOC: intracellular, ribosome
AGOP: translation
PGOF: structural constituent of ribosome
PGOC: intracellular, ribosome
PGOP: translation

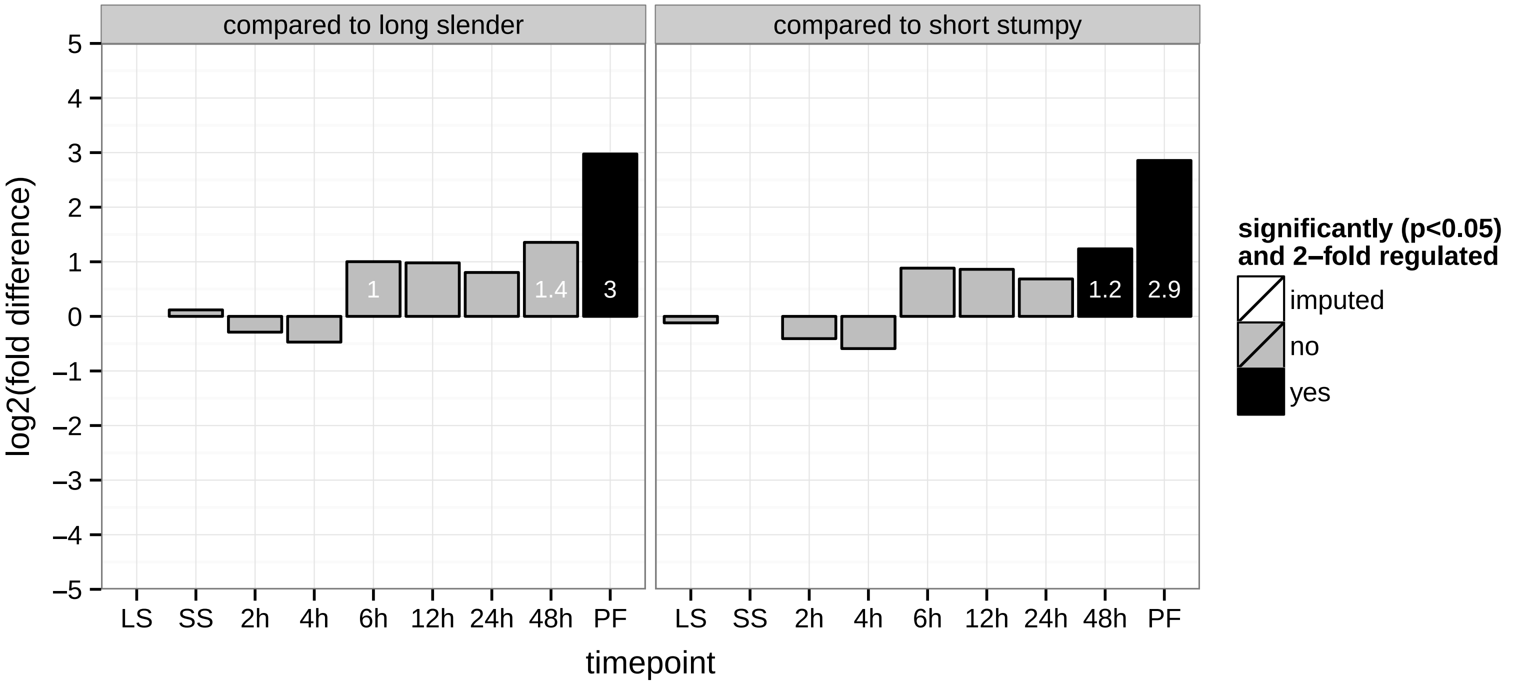

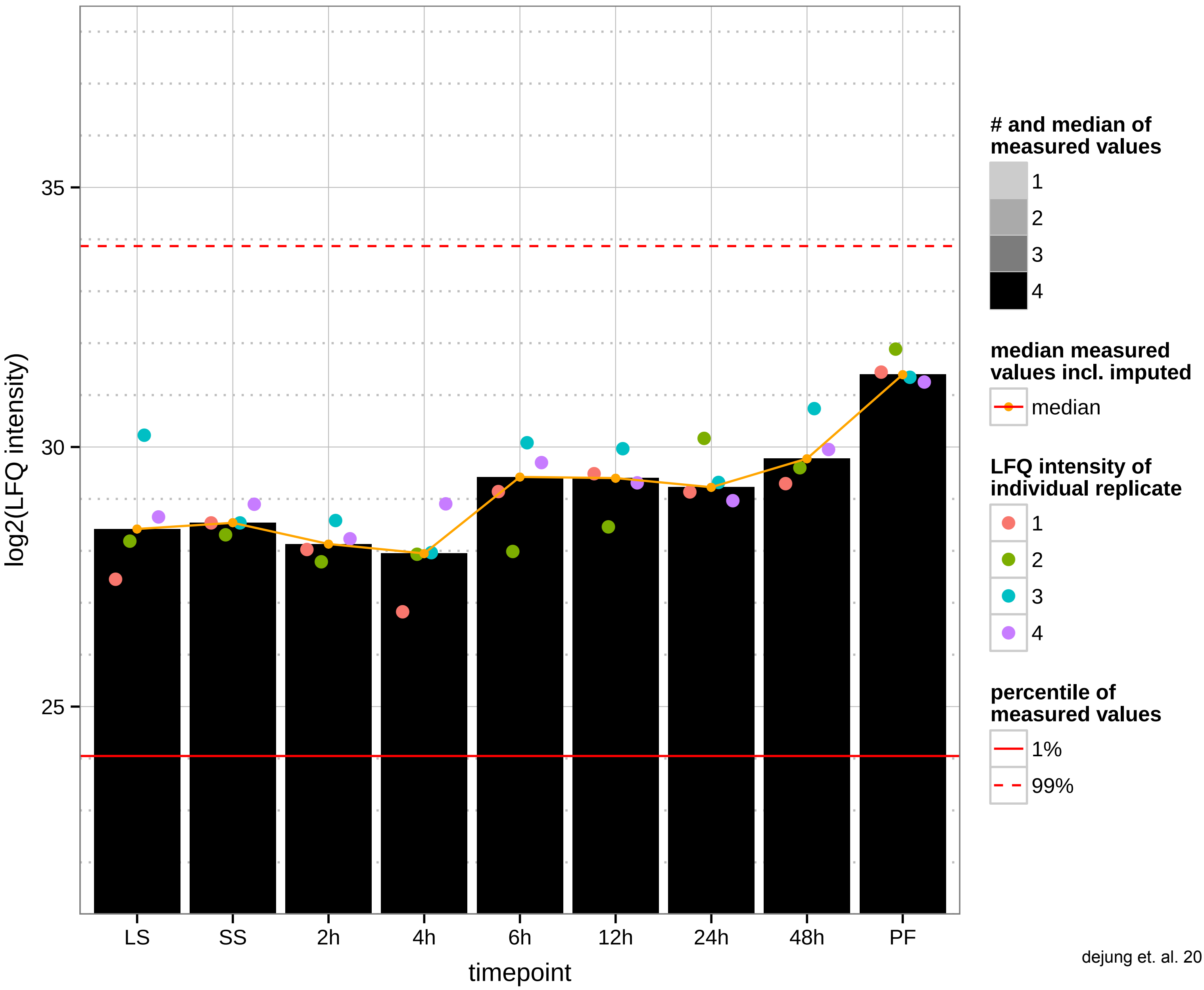

dejung et. al. 2015

tubulin−tyrsoine ligase−like protein, putative, putative (TTL)
Tb927.11.6810
AGOF: null, tubulin−tyrosine ligase activity
AGOC: null
AGOP: null, cellular protein modification process
PGOF: null, tubulin−tyrosine ligase activity
PGOC: null
PGOP: null, cellular protein modification process

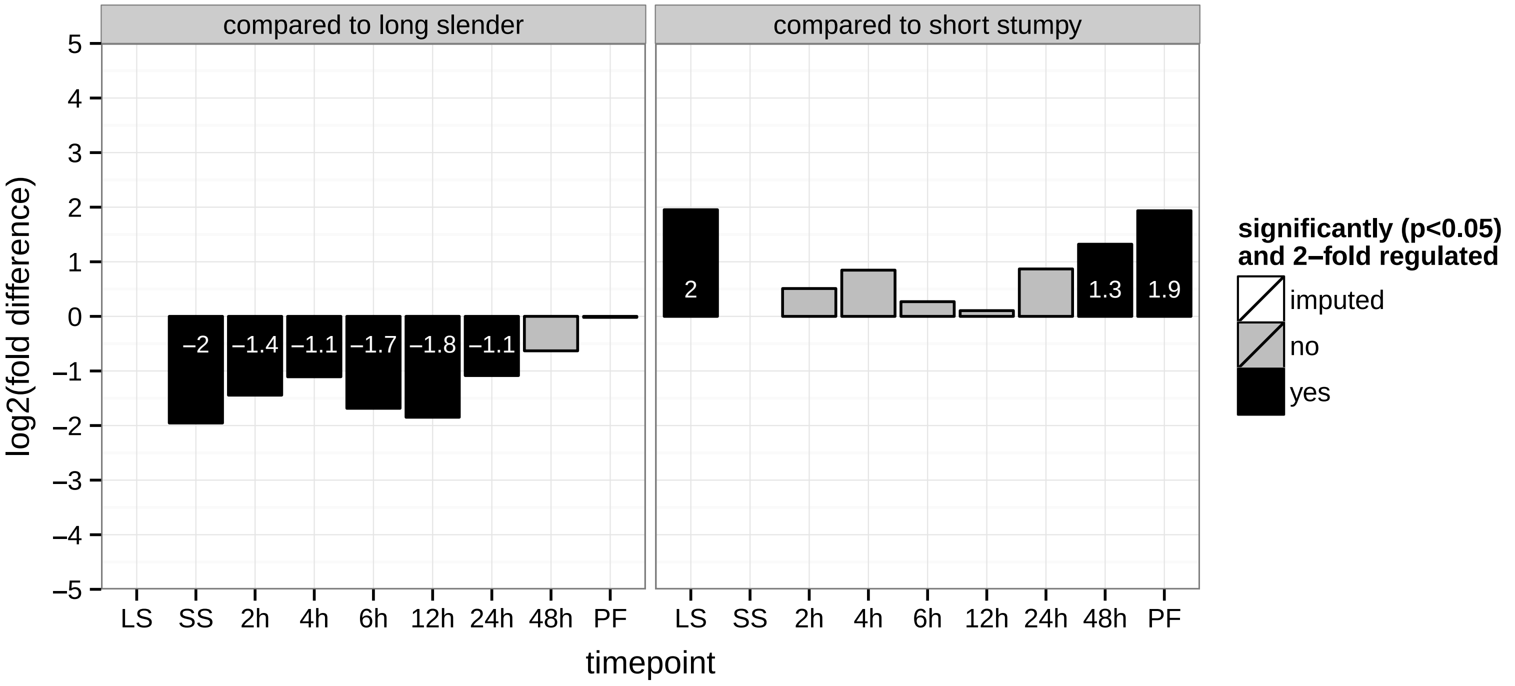

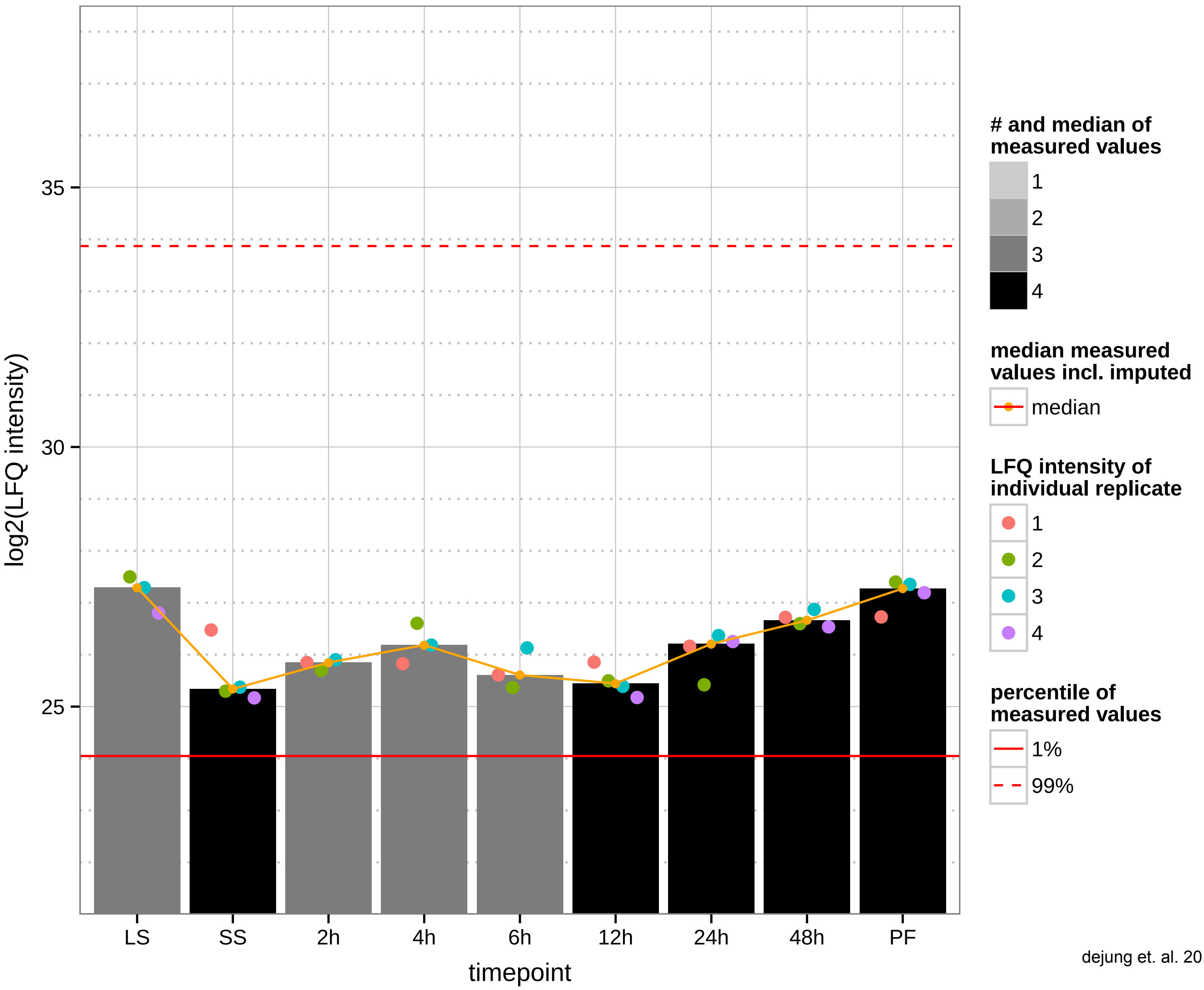

dejung et. al. 2015

ribosomal protein S26, putative
Tb927.11.8200
AGOF: structural constituent of ribosome
AGOC: intracellular, ribosome
AGOP: translation
PGOF: structural constituent of ribosome
PGOC: intracellular, ribosome
PGOP: translation

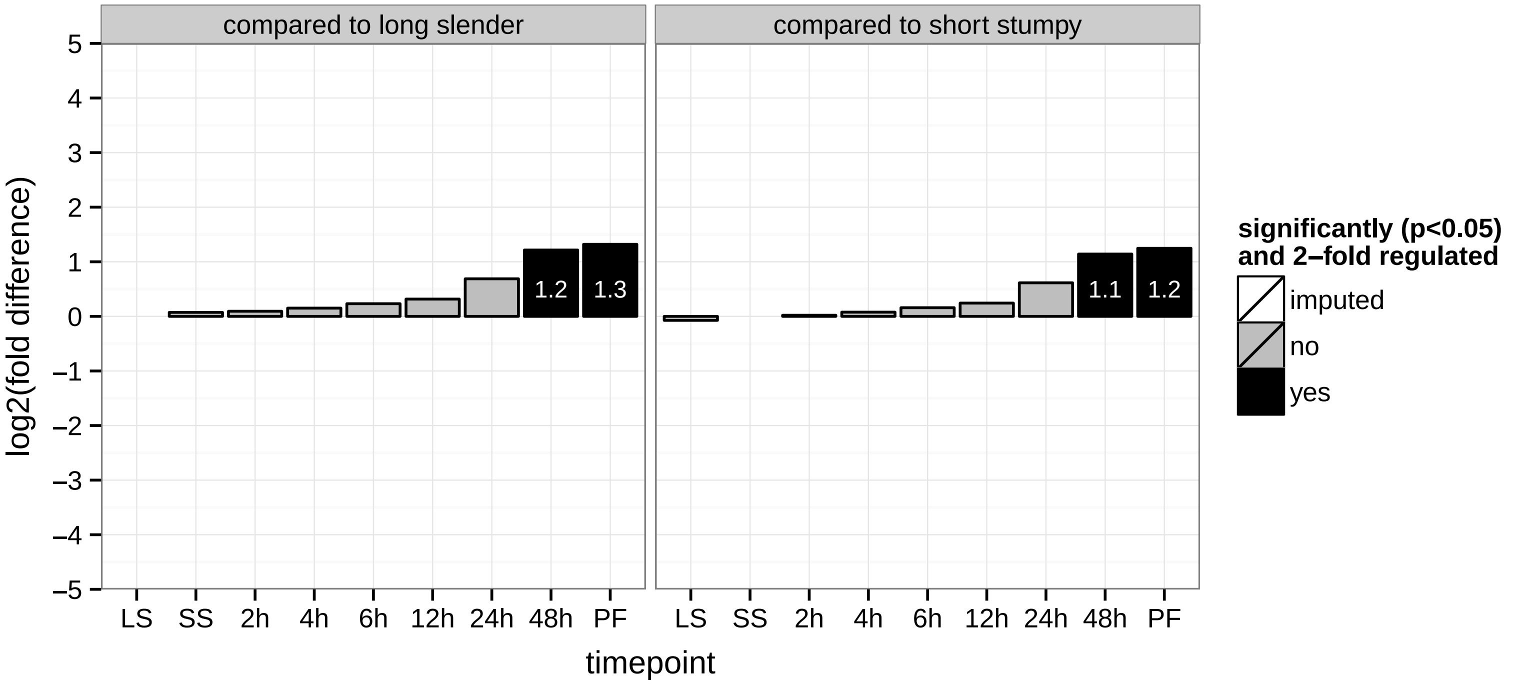

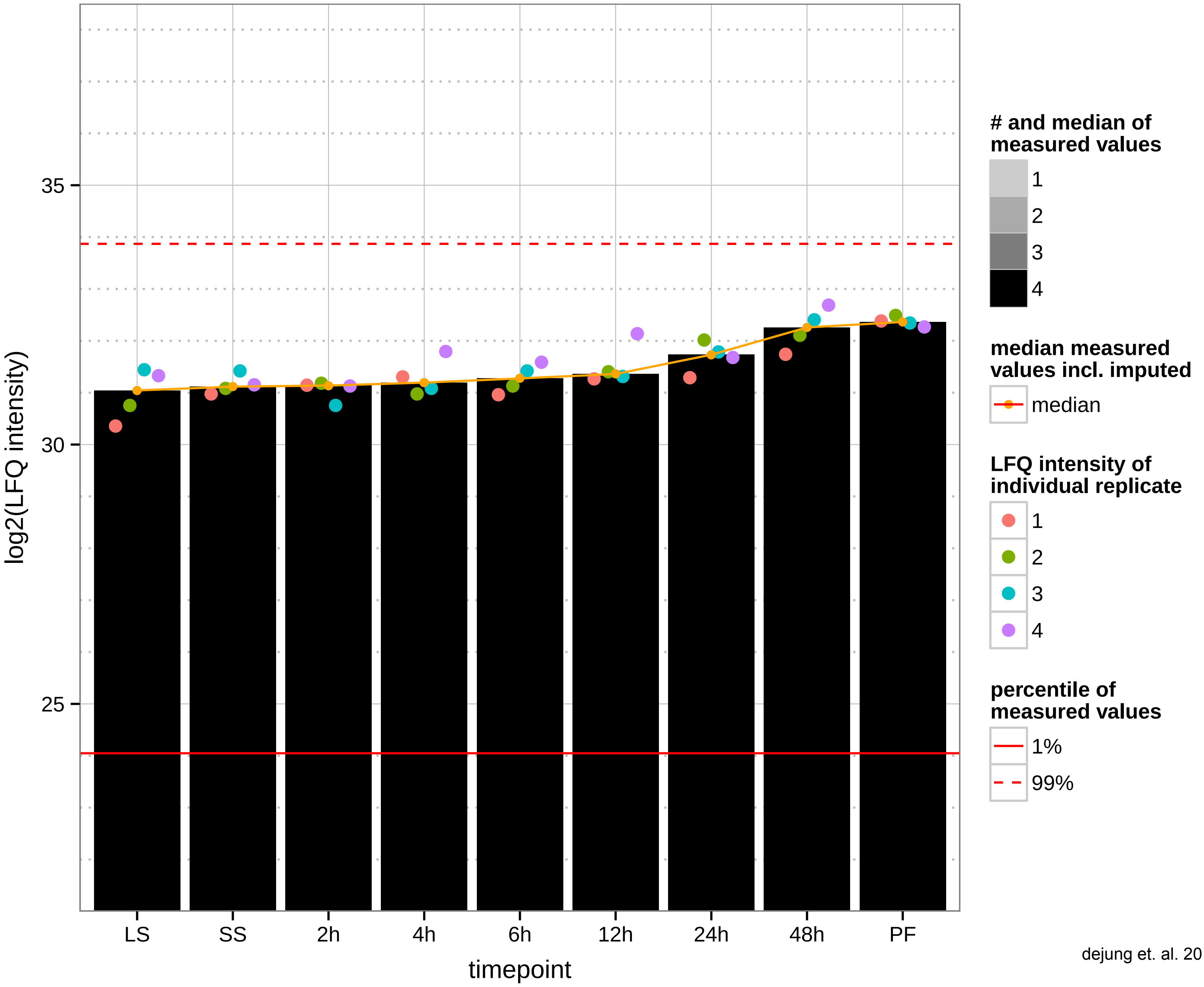

dejung et. al. 2015

hypothetical protein, conserved
Tb927.11.870
AGOF: null
AGOC: mitochondrion
AGOP: null
PGOF: structural constituent of ribosome
PGOC: intracellular, ribosome
PGOP: translation

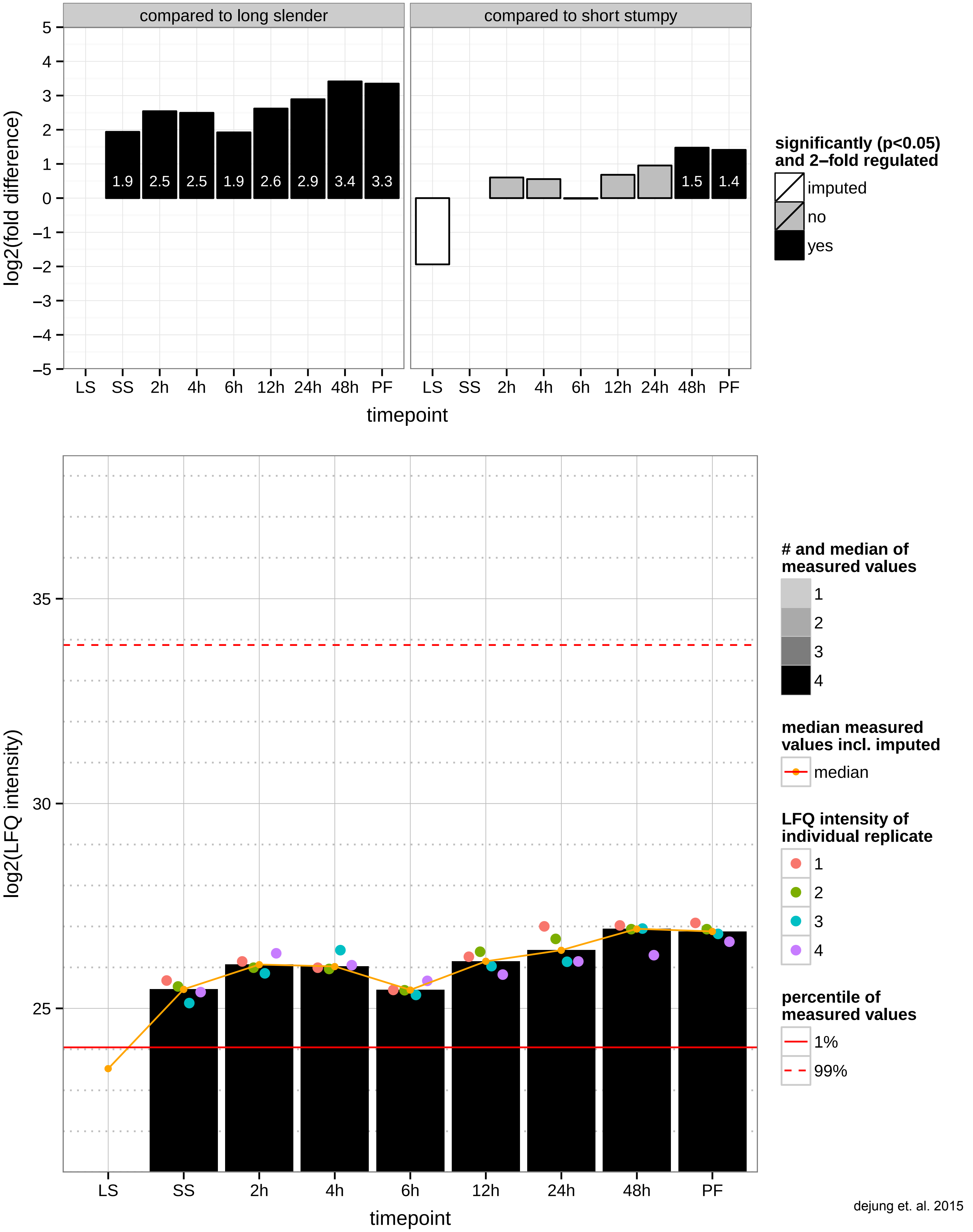

dejung et. al. 2015

hypothetical protein, conserved
Tb927.11.8880
AGOF: null
AGOC: null
AGOP: null
PGOF: null
PGOC: null
PGOP: null

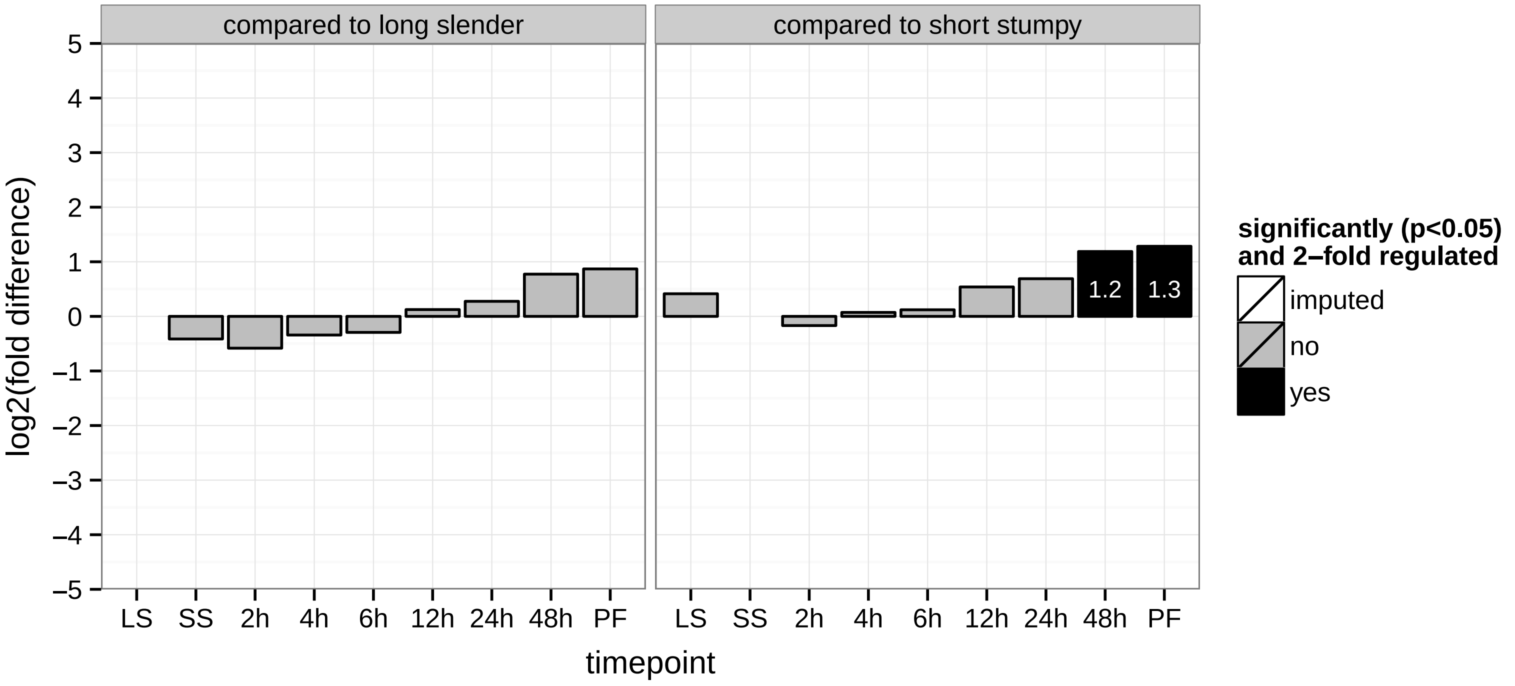

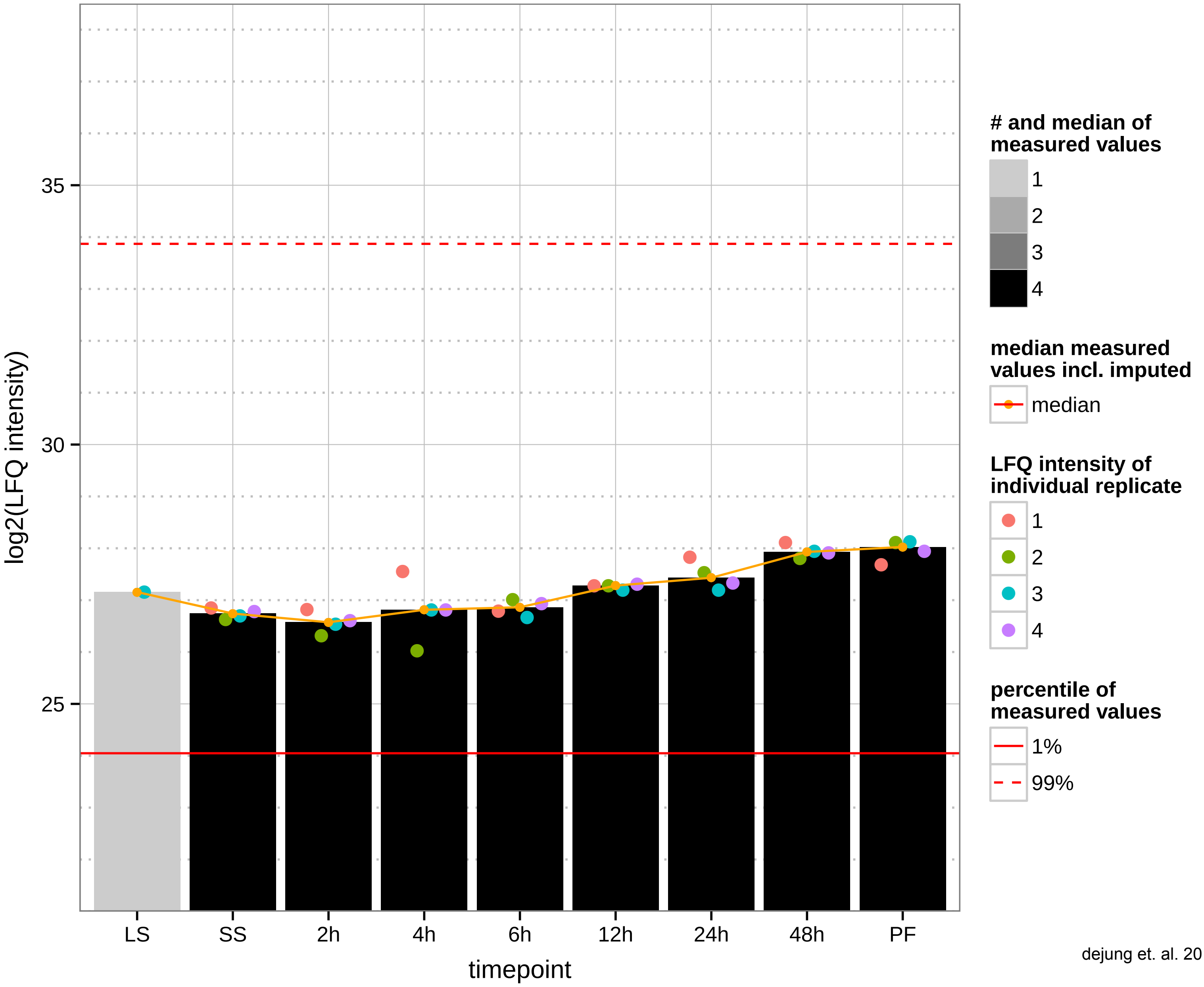

dejung et. al. 2015

glycine synthase, putative (GCVT1)
Tb927.11.9670
AGOF: aminomethyltransferase activity
AGOC: cytoplasm, mitochondrion
AGOP: glycine decarboxylation via glycine cleavage system
PGOF: aminomethyltransferase activity
PGOC: cytoplasm
PGOP: glycine catabolic process

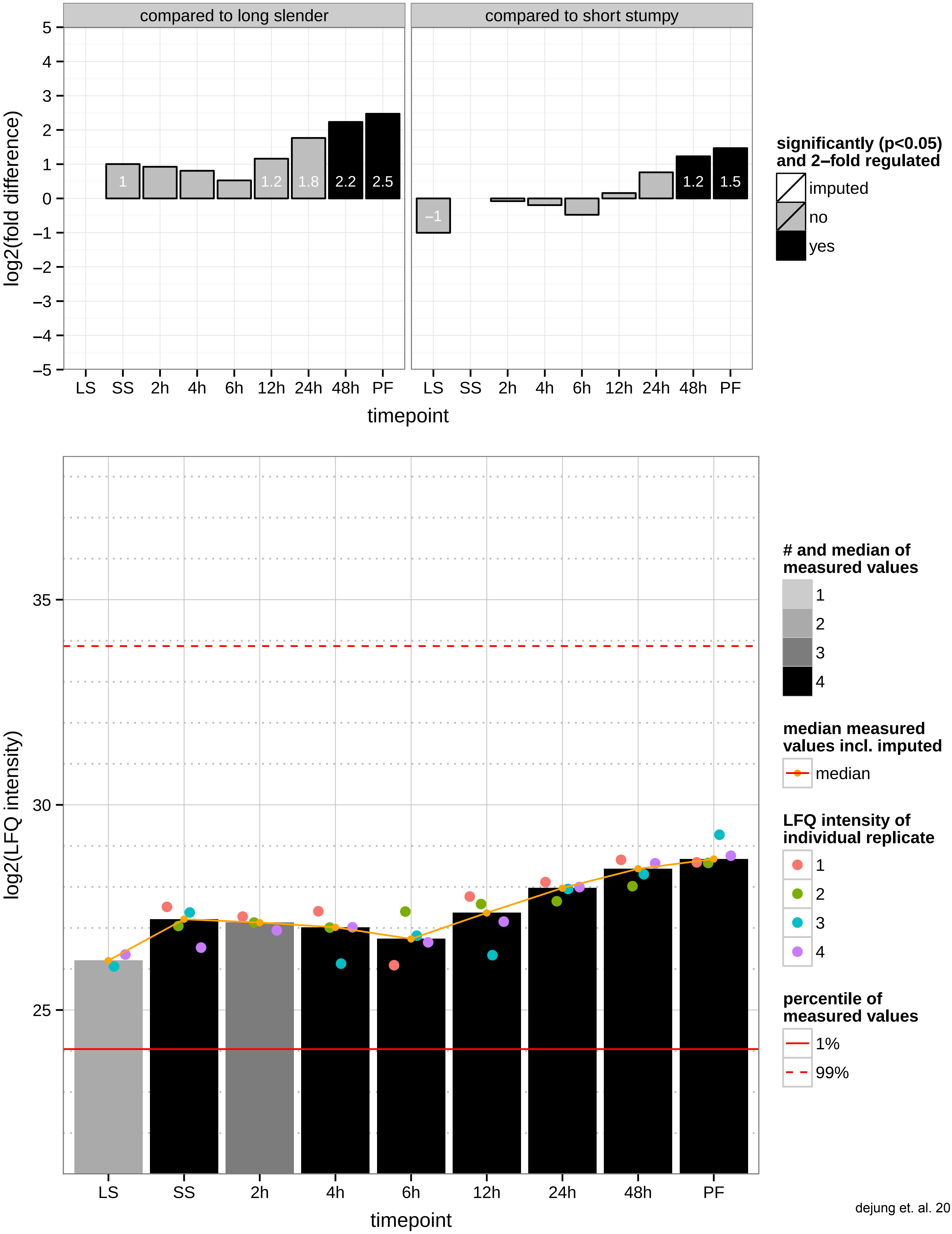

dejung et. al. 2015

hypothetical protein, conserved
Tb927.11.9820
AGOF: null
AGOC: null
AGOP: null
PGOF: null
PGOC: null
PGOP: null

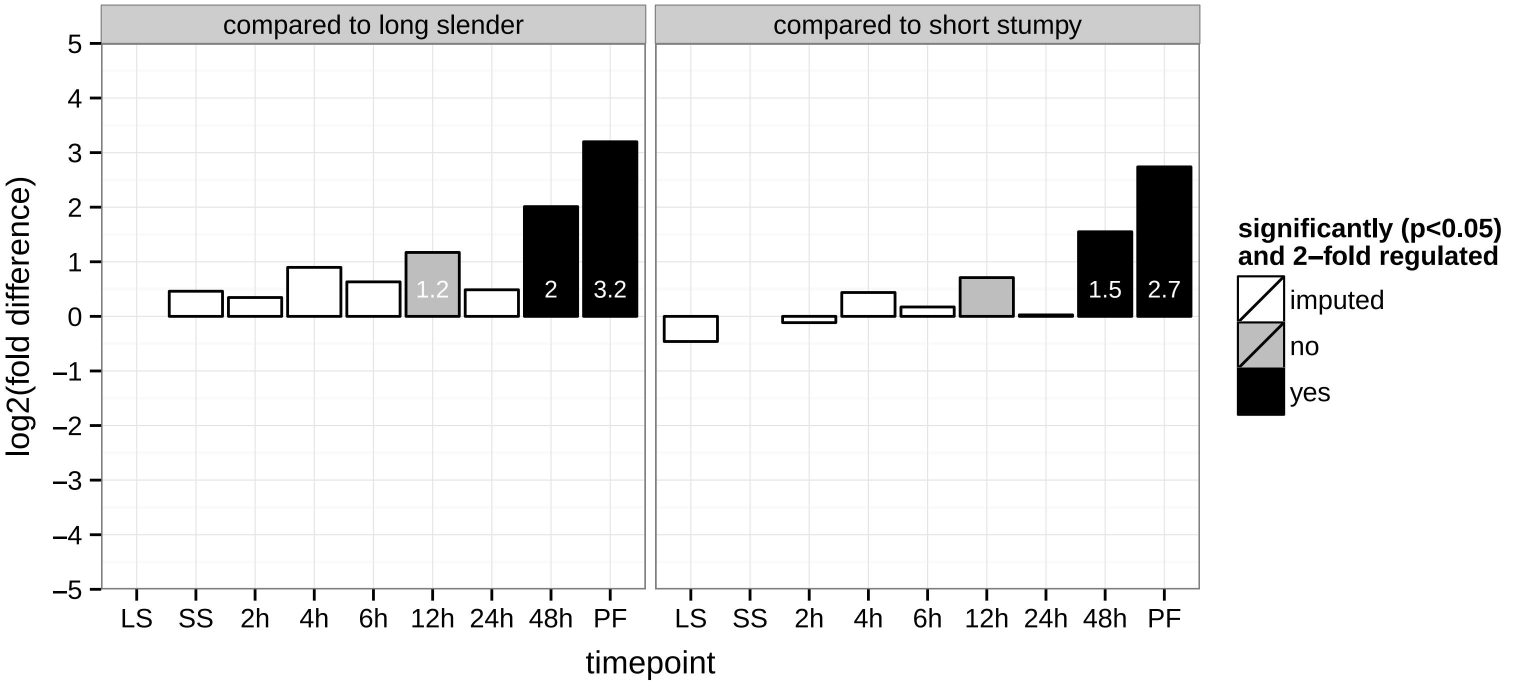

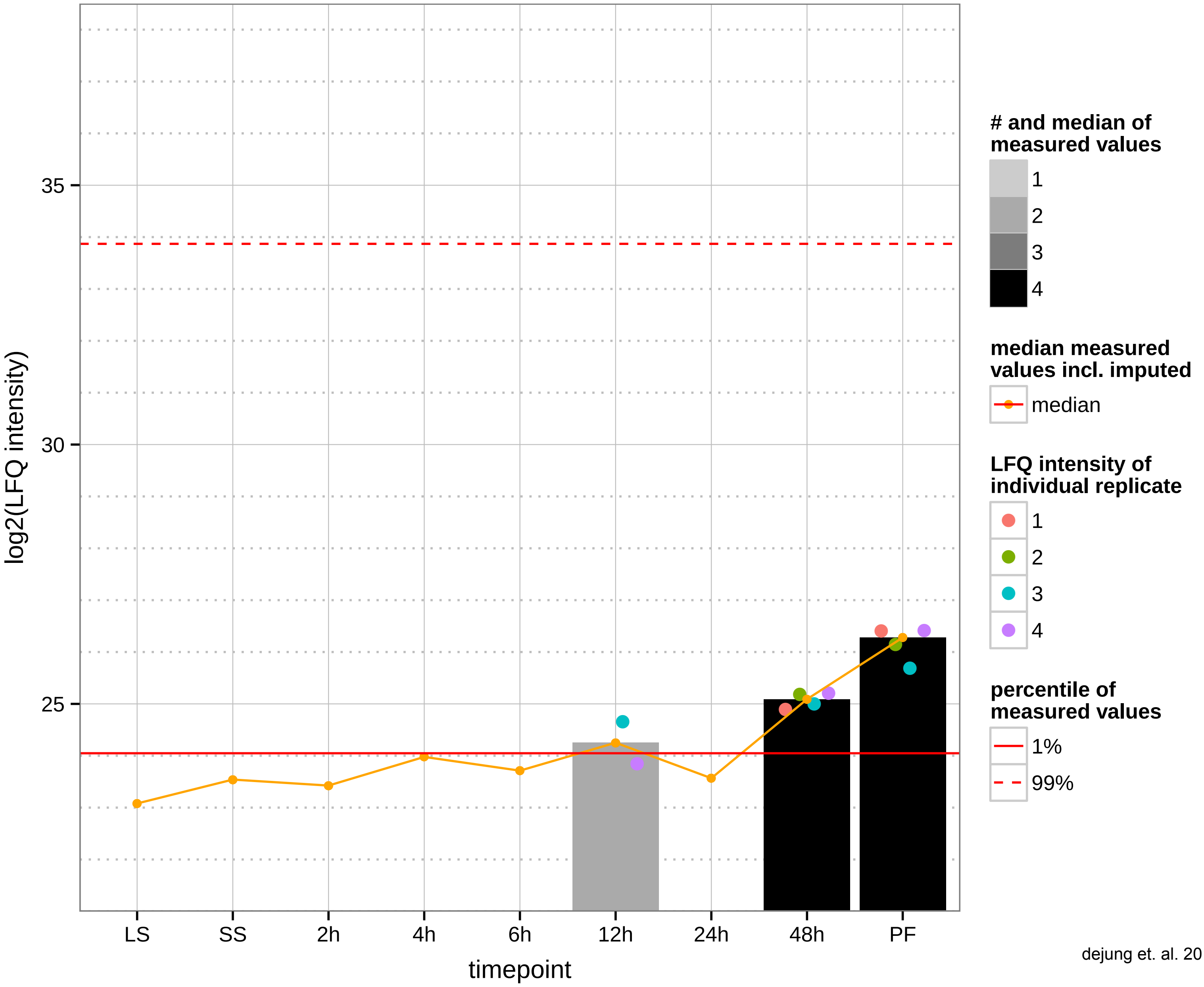

dejung et. al. 2015

voltage−dependent anion−selective channel (VDAC), voltage−dependent anion−selective channel
Tb927.2.2520;Tb927.2.2510
AGOF: porin activity, voltage−gated anion channel activity, null
AGOC: eukaryotic translation initiation factor 4F complex, mitochondrial outer membrane, pore complex, integral to mitochond
AGOP: ion transport, mitochondrial transport
PGOF: null
PGOC: null
PGOP: null

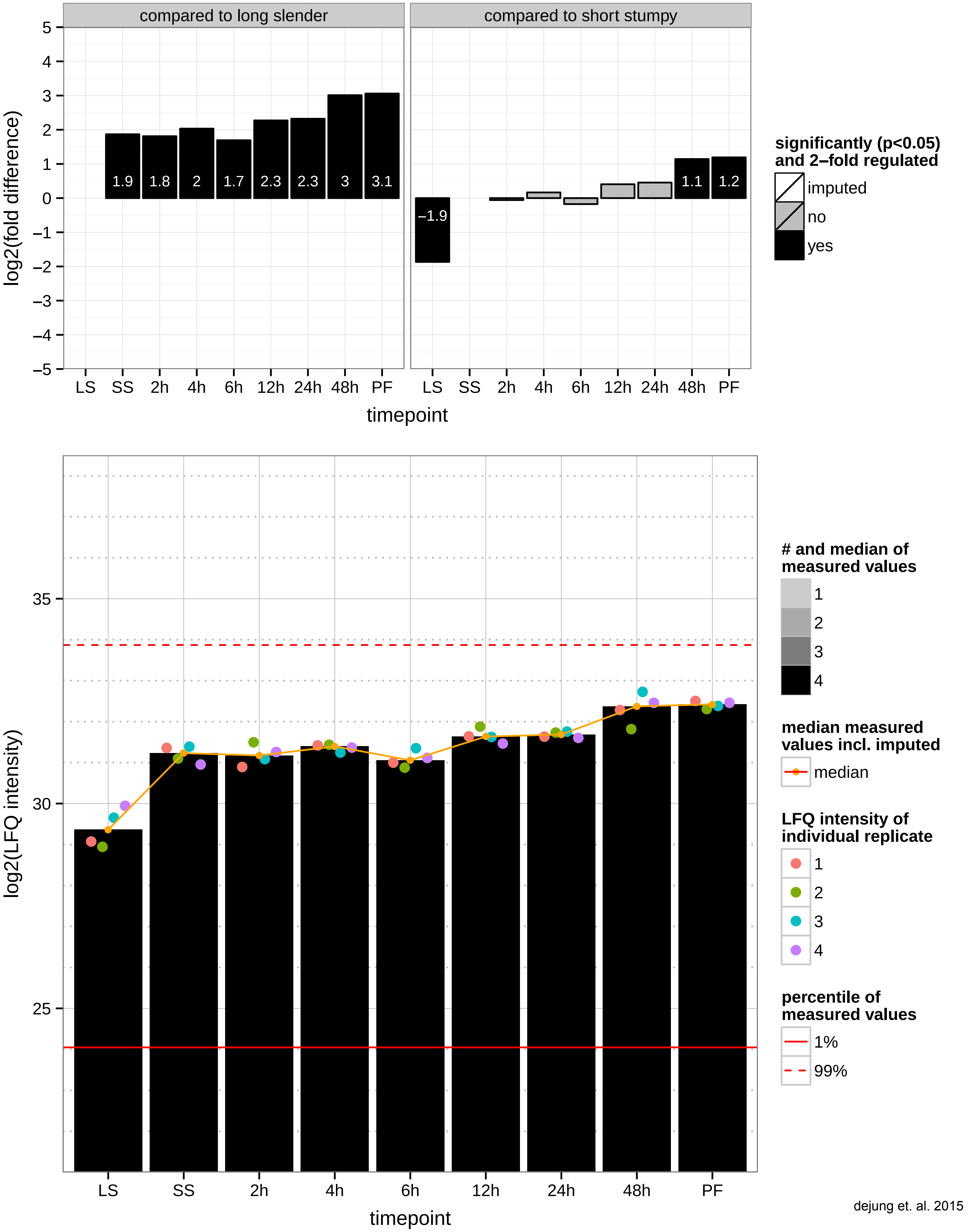

dejung et. al. 2015

enoyl–CoA hydratase/Enoyl–CoA isomerase/3–hydroxyacyl–CoA dehydrogenase, putative
Tb927.2.4130
AGOF: isomerase activity, oxidoreductase activity
AGOC: mitochondrion
AGOP: fatty acid metabolic process, metabolic process
PGOF: 3–hydroxyacyl–CoA dehydrogenase activity, catalytic activity, oxidoreductase activity
PGOC: null
PGOP: fatty acid metabolic process, metabolic process, oxidation–reduction process

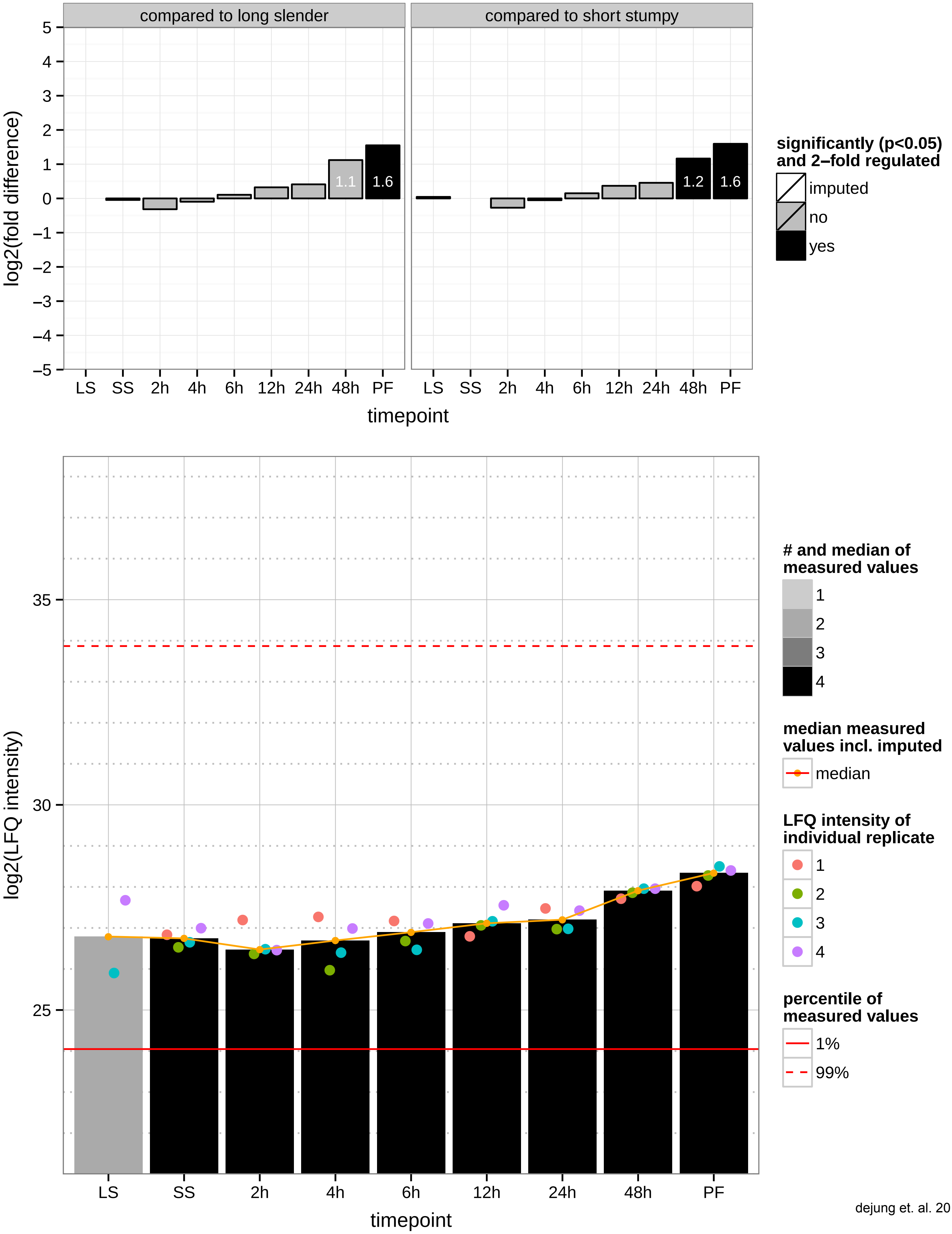

dejung et. al. 2015

ATP−dependent DEAD/H RNA helicase, putative
Tb927.3.2600
AGOF: ATP binding, ATP−dependent helicase activity, helicase activity, nucleic acid binding
AGOC: null
AGOP: null
PGOF: ATP binding, ATP−dependent helicase activity, helicase activity, nucleic acid binding
PGOC: null
PGOP: null

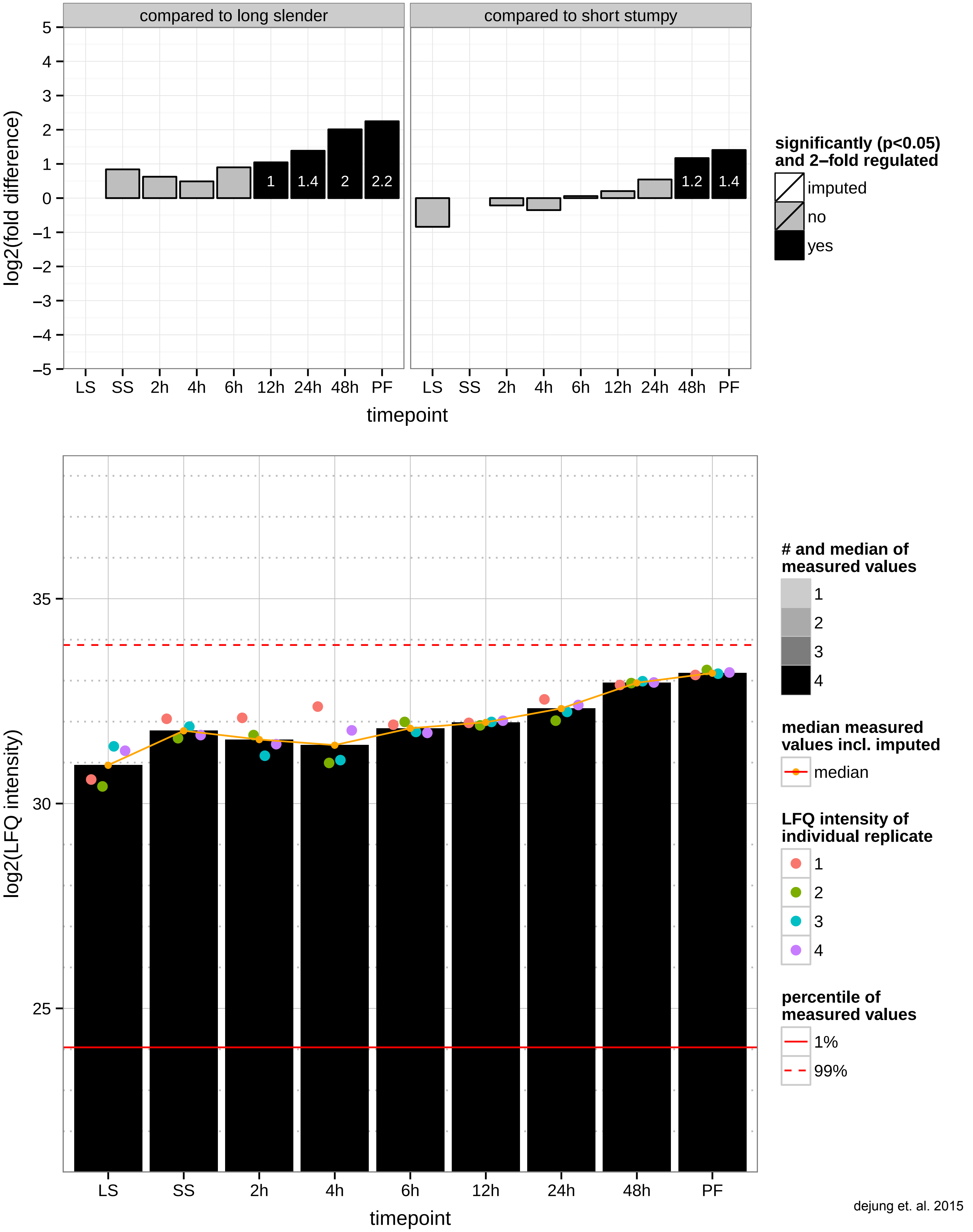

dejung et. al. 2015

hypothetical protein, conserved
Tb927.3.3130
AGOF: null
AGOC: null
AGOP: null
PGOF: protein binding
PGOC: null
PGOP: null

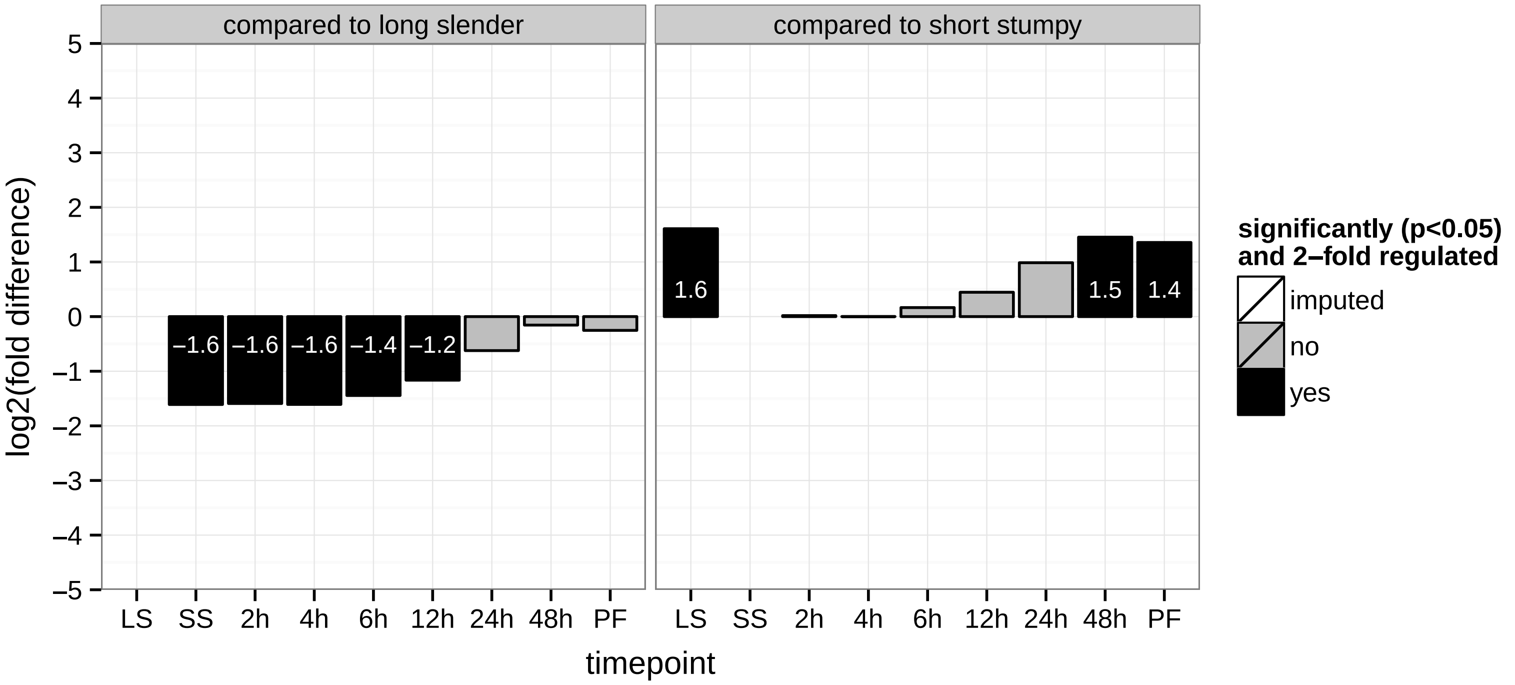

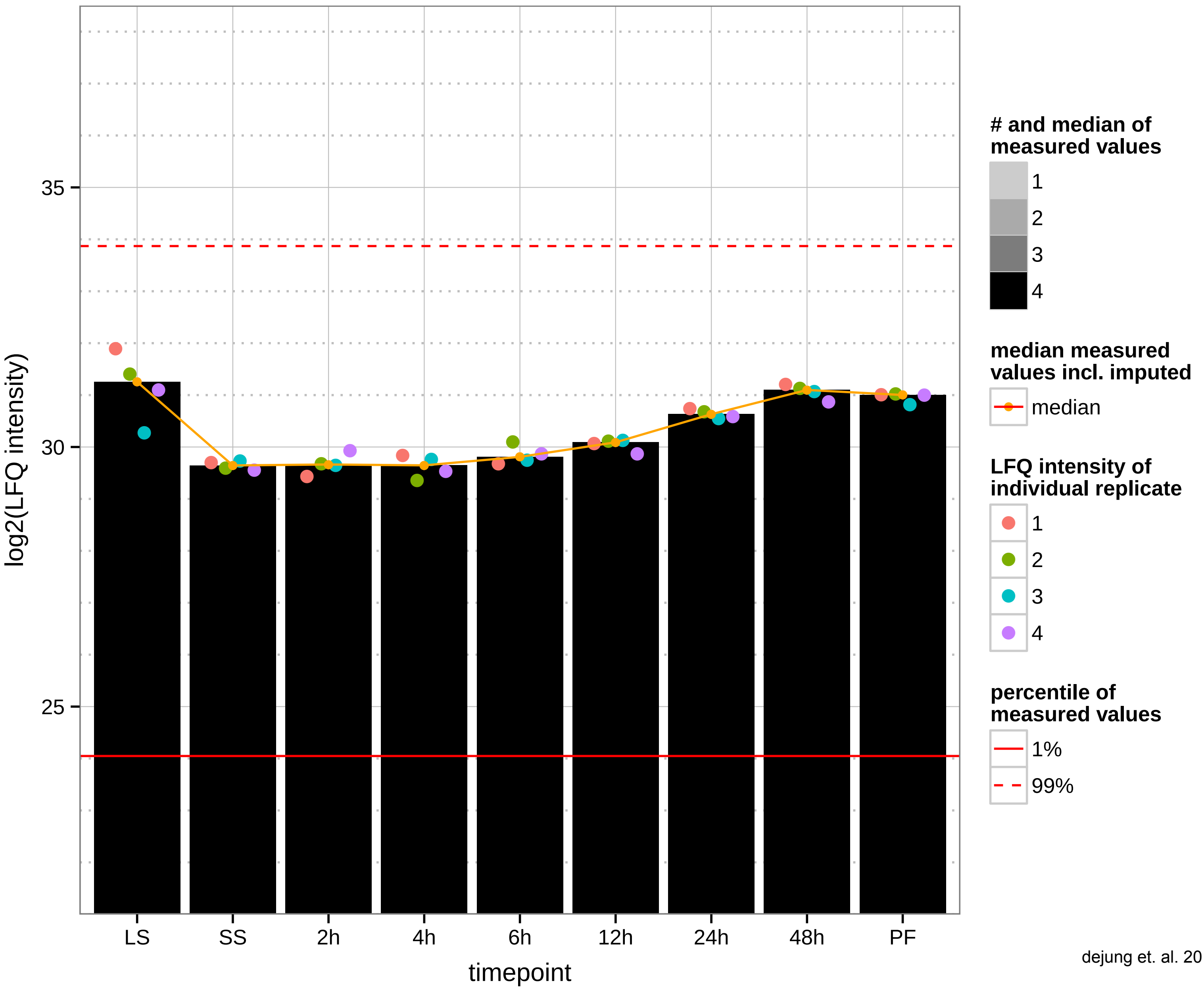

dejung et. al. 2015

hypothetical protein, conserved (pseudogene), conserved, frameshift
Tb927.3.4980
AGOF: null
AGOC: null
AGOP: null
PGOF: null
PGOC: null
PGOP: null

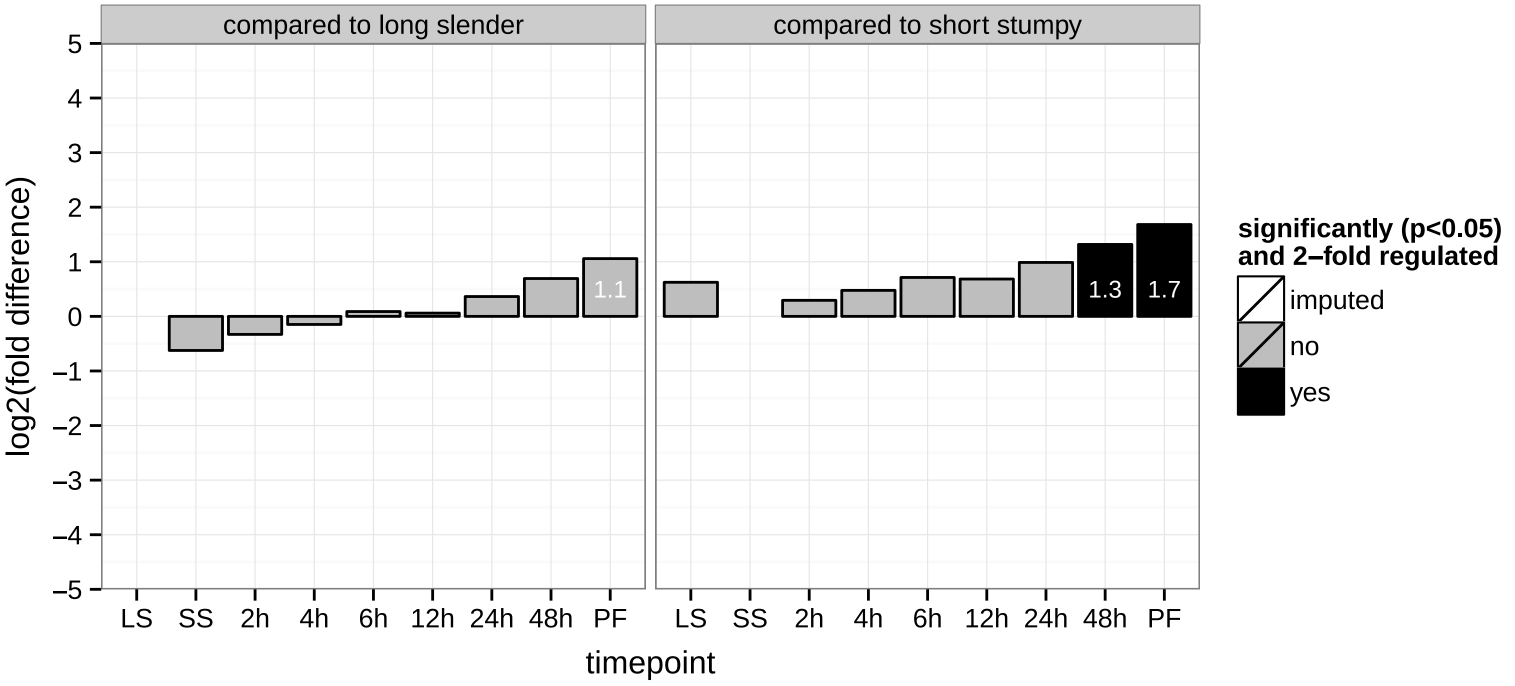

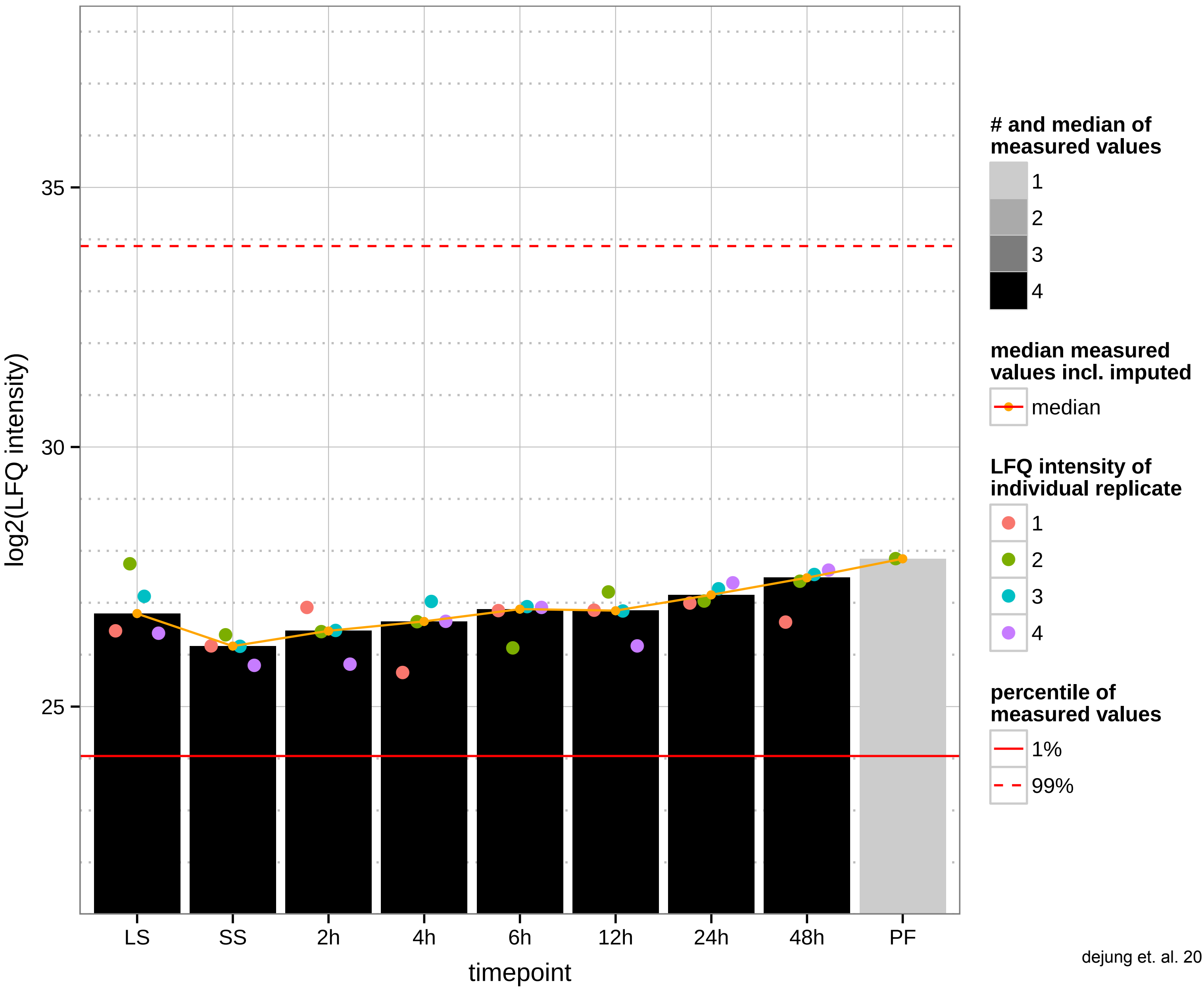

dejung et. al. 2015

hypothetical protein, conserved
Tb927.3.5130
AGOF: exonuclease activity, nucleic acid binding
AGOC: intracellular
AGOP: null
PGOF: exonuclease activity, nucleic acid binding
PGOC: intracellular
PGOP: null

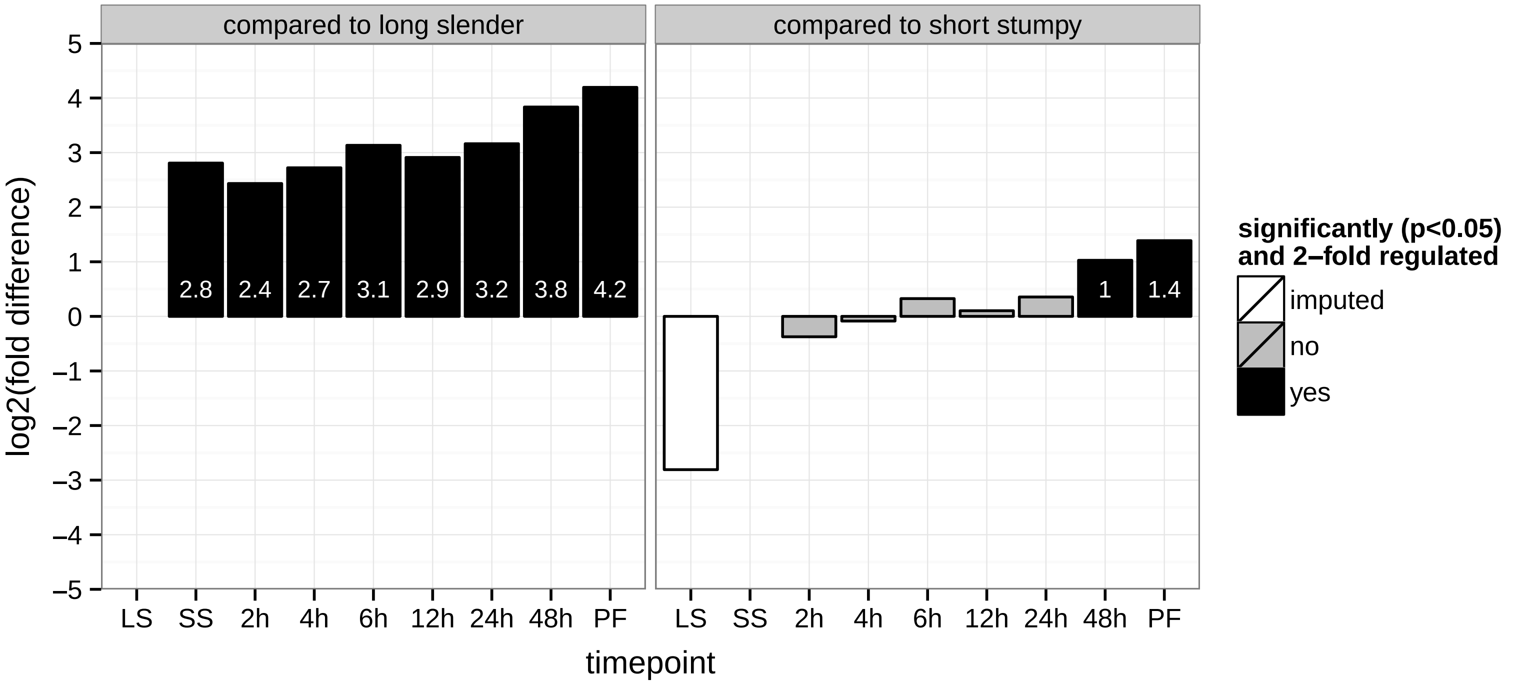

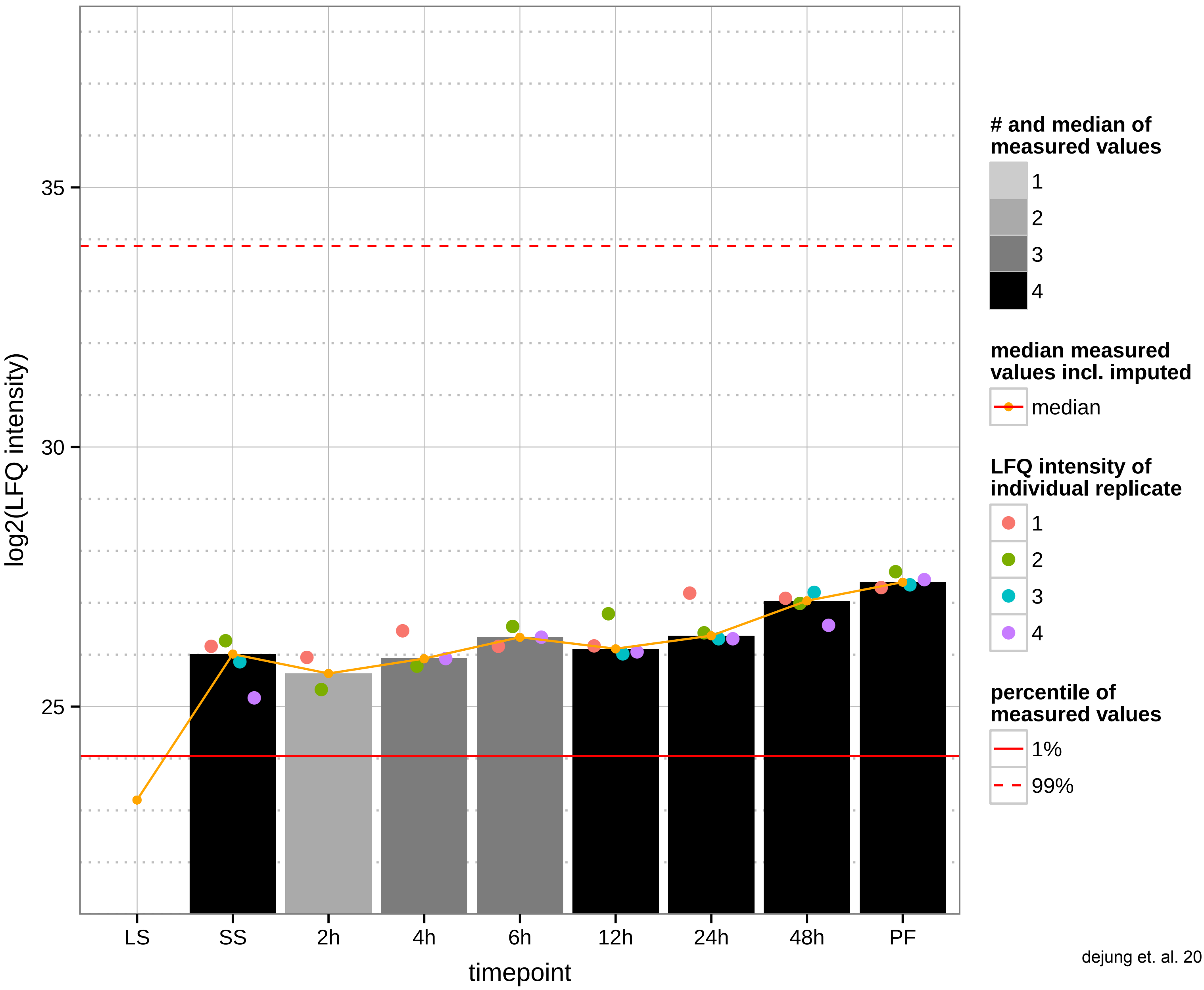

dejung et. al. 2015

hypothetical protein, conserved
Tb927.4.1360
AGOF: carbohydrate binding, isomerase activity
AGOC: null
AGOP: carbohydrate metabolic process
PGOF: carbohydrate binding, catalytic activity, isomerase activity
PGOC: null
PGOP: carbohydrate metabolic process

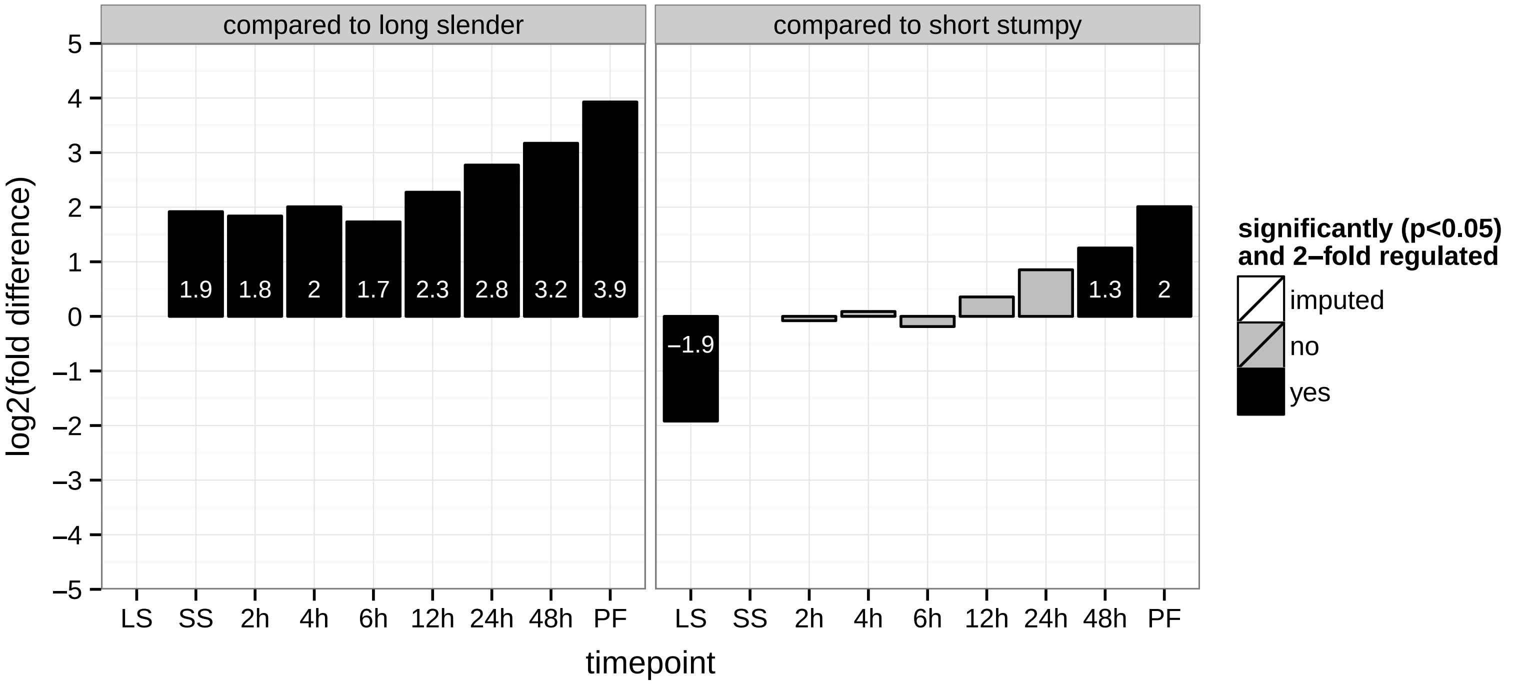

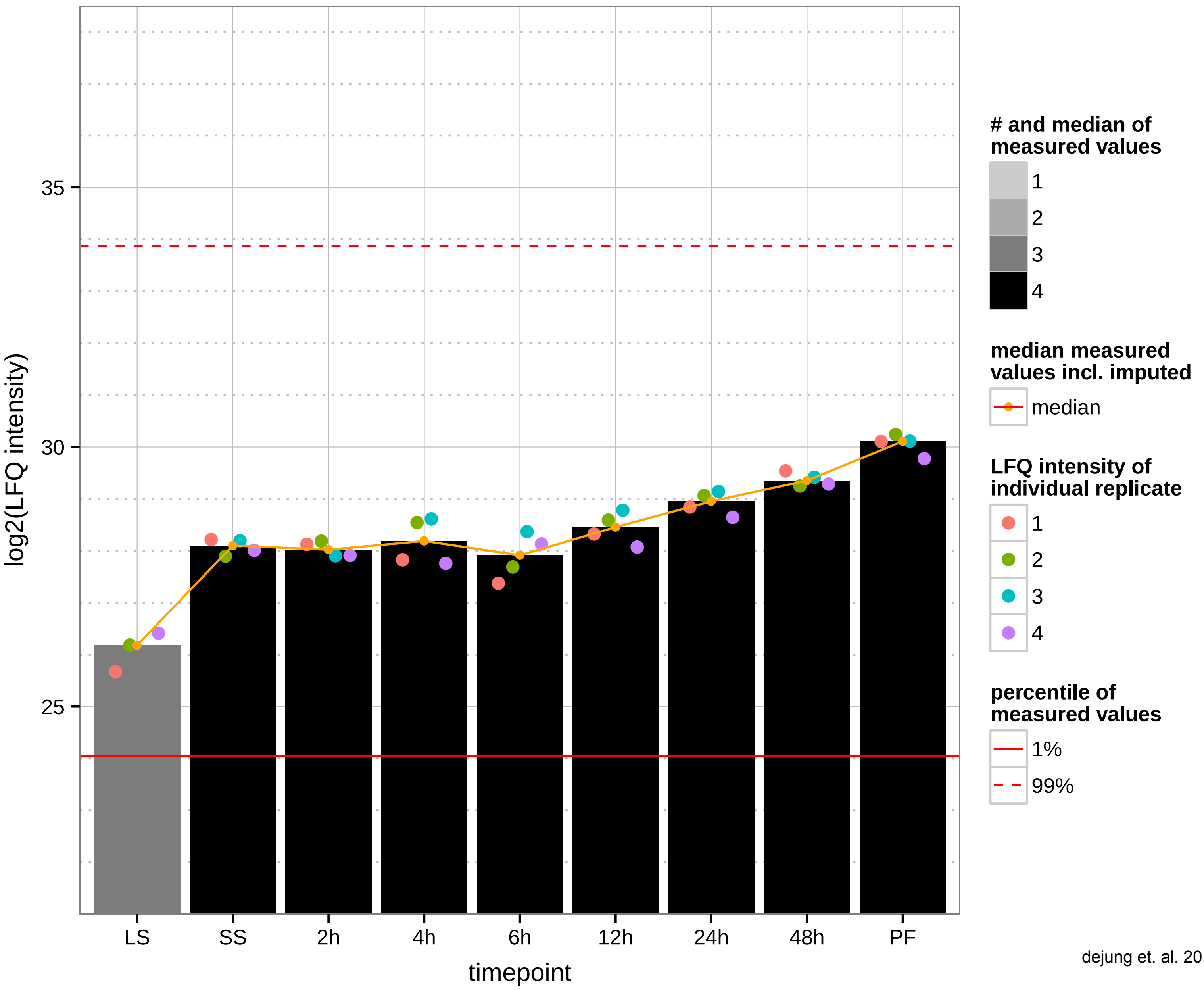

dejung et. al. 2015

60S ribosomal protein L35a, putative
Tb927.4.2180
AGOF: structural constituent of ribosome
AGOC: intracellular, ribosome
AGOP: translation
PGOF: structural constituent of ribosome
PGOC: intracellular, ribosome
PGOP: translation

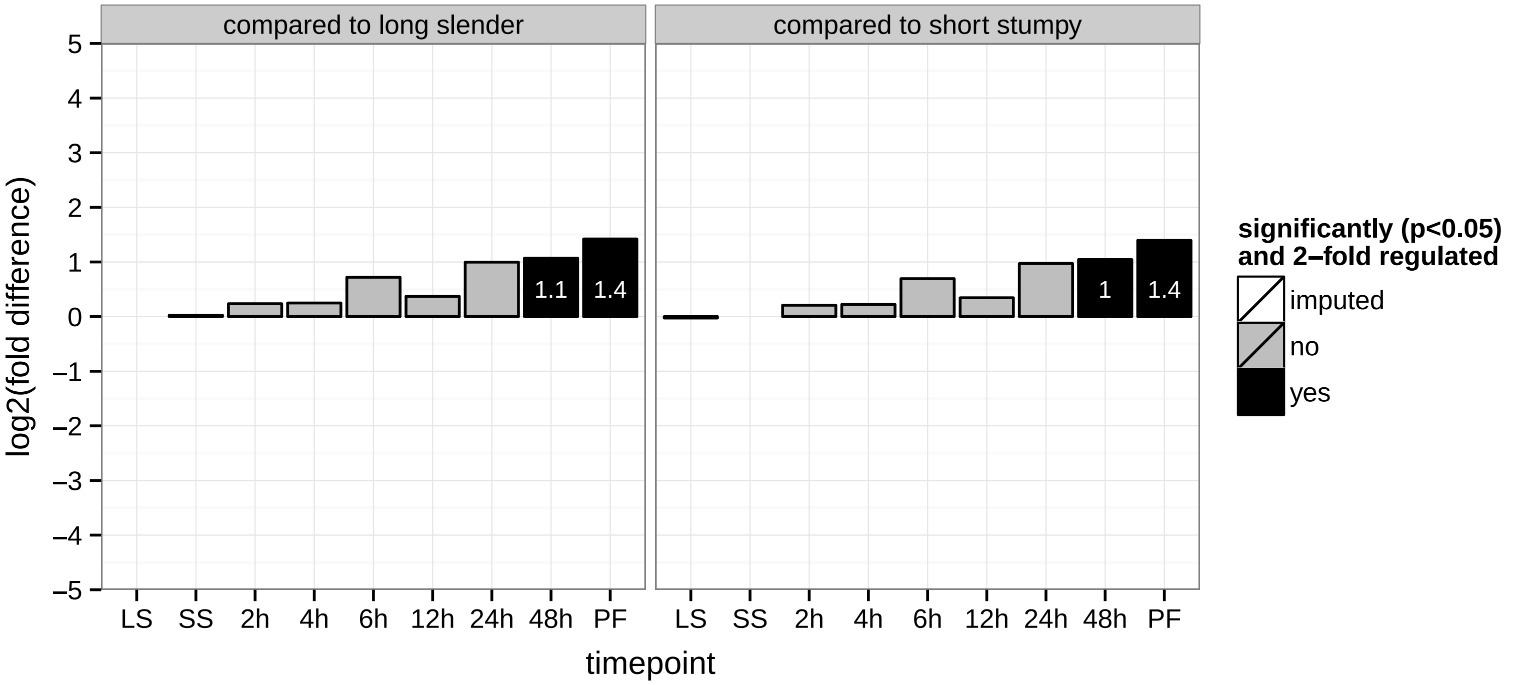

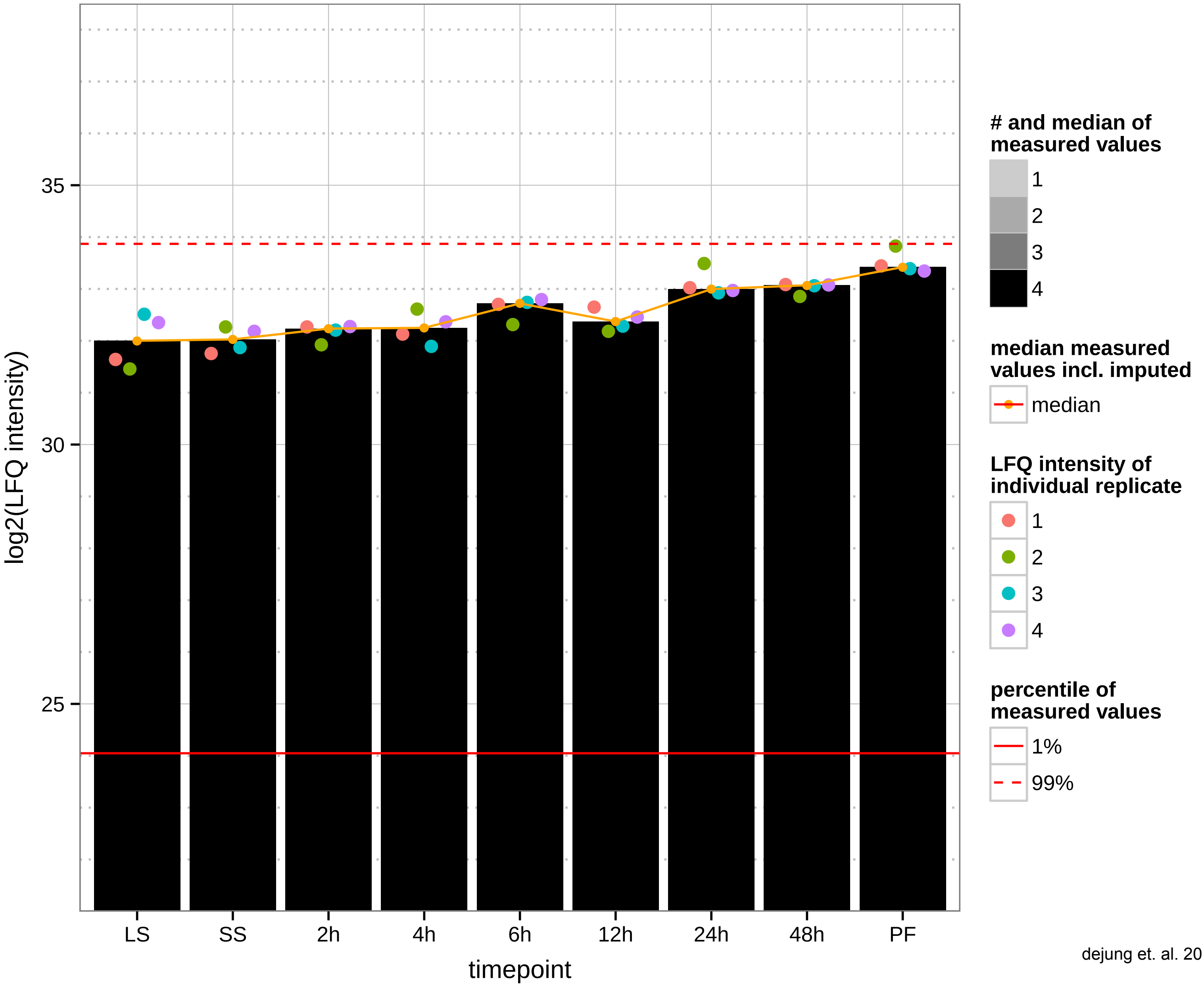

dejung et. al. 2015

ATP−dependent RNA helicase, putative
Tb927.4.3890
AGOF: ATP binding, helicase activity, nucleic acid binding
AGOC: null
AGOP: null
PGOF: ATP binding, ATP−dependent helicase activity, helicase activity, nucleic acid binding
PGOC: null
PGOP: null

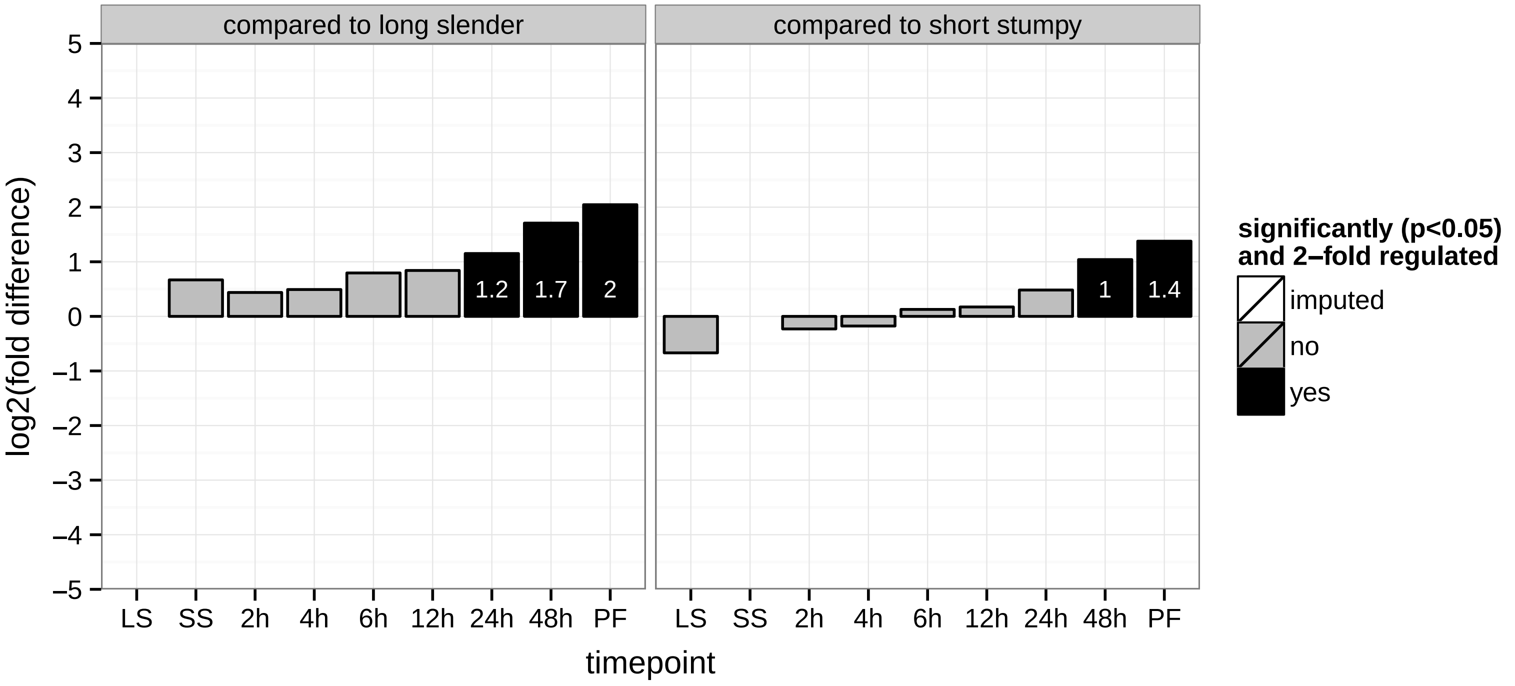

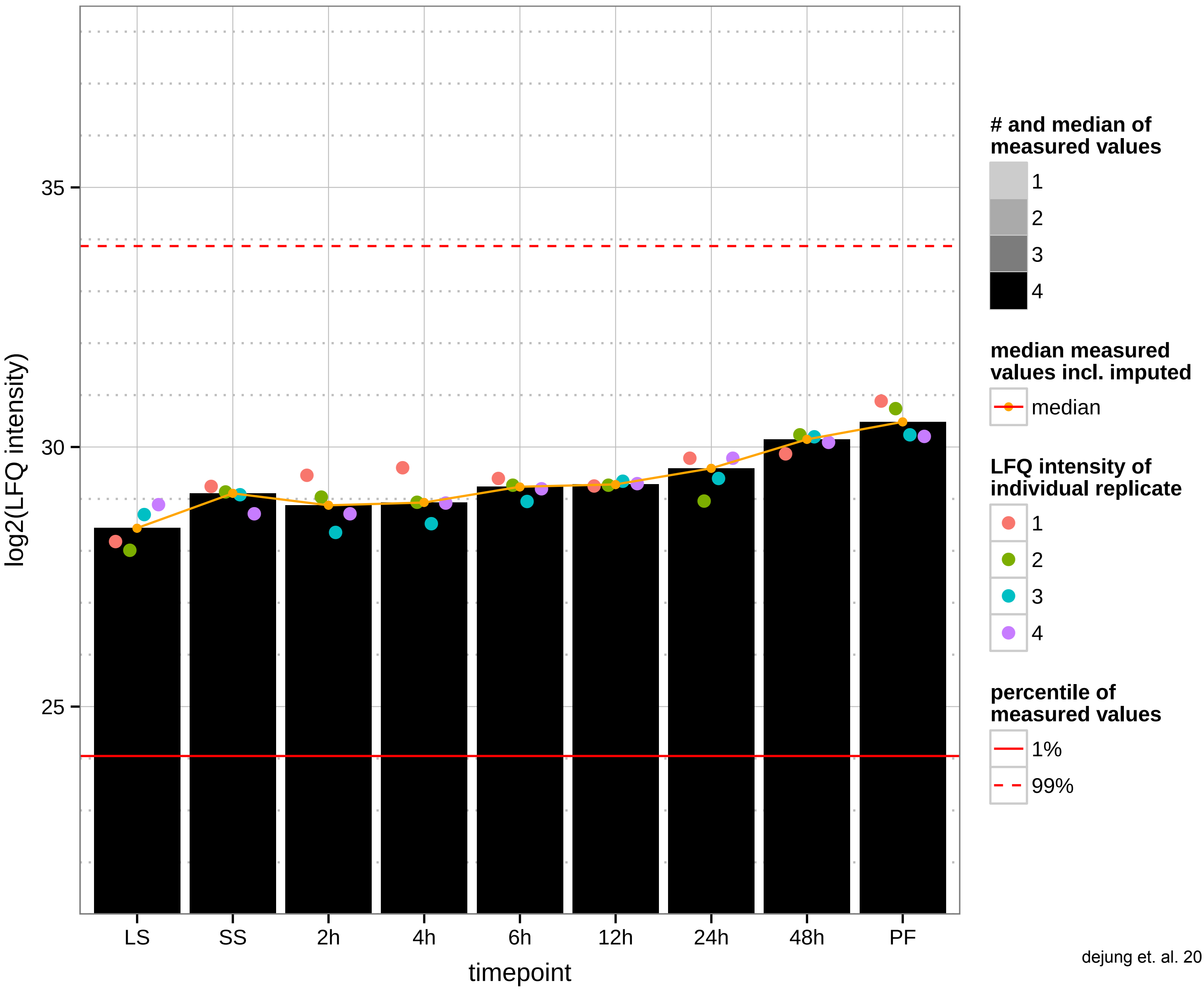

dejung et. al. 2015

hypothetical protein, conserved
Tb927.4.4040
AGOF: null
AGOC: null
AGOP: null
PGOF: null
PGOC: null
PGOP: null

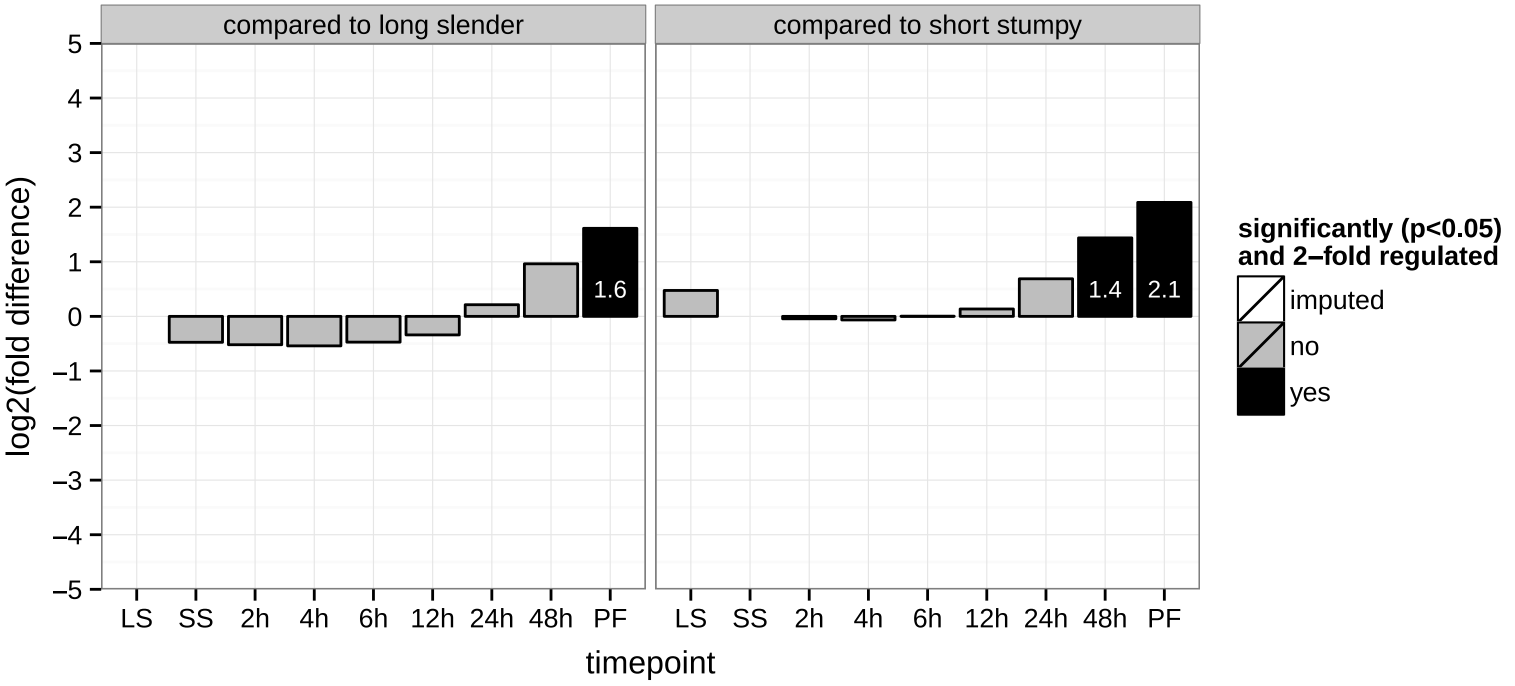

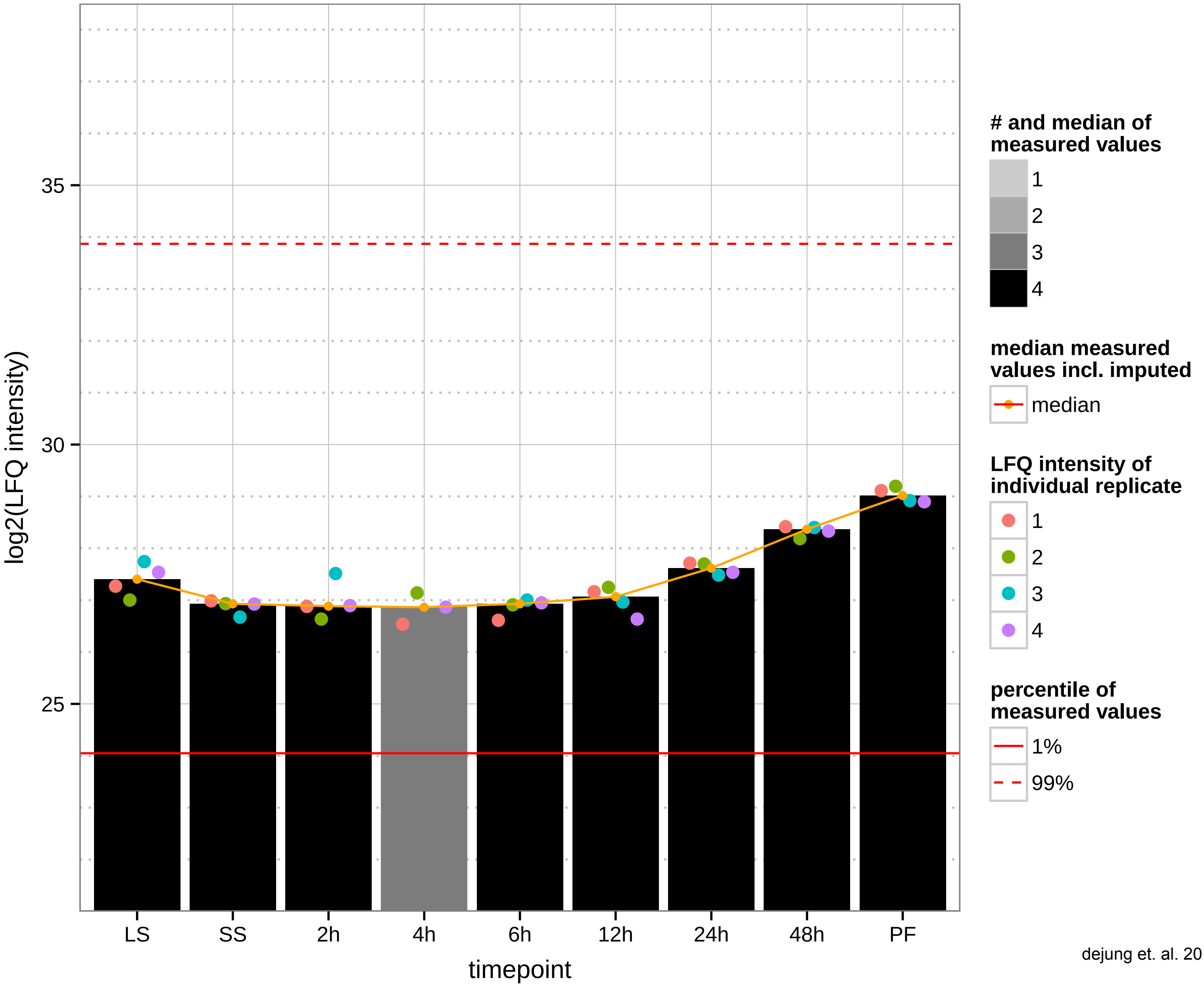

dejung et. al. 2015

ATP−dependent zinc metallopeptidase, putative, metallo−peptidase, Clan MA(E) Family M41
Tb927.4.4210
AGOF: ATP binding, metalloendopeptidase activity, nucleoside−triphosphatase activity, zinc ion binding
AGOC: integral to membrane, mitochondrial inner membrane
AGOP: proteolysis
PGOF: ATP binding, metalloendopeptidase activity, nucleoside−triphosphatase activity, nucleotide binding
PGOC: null
PGOP: proteolysis

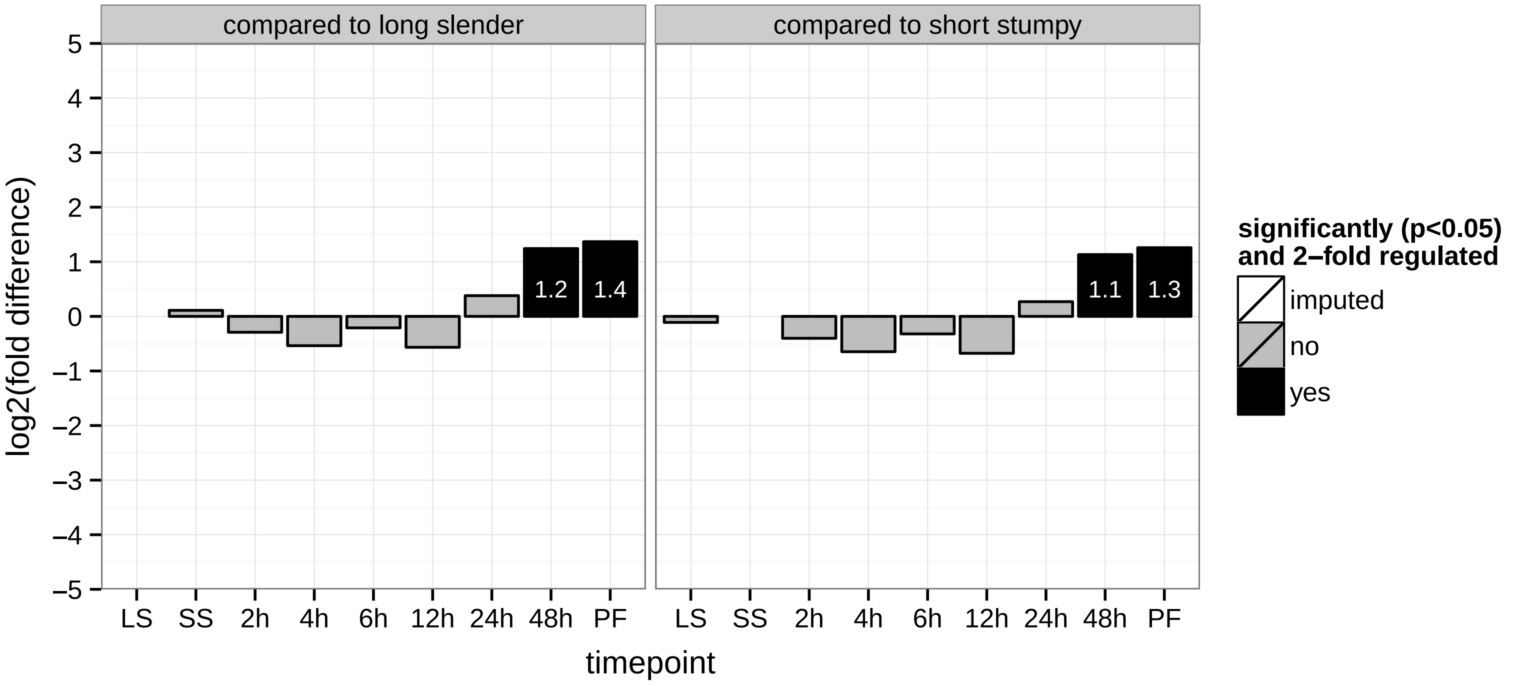

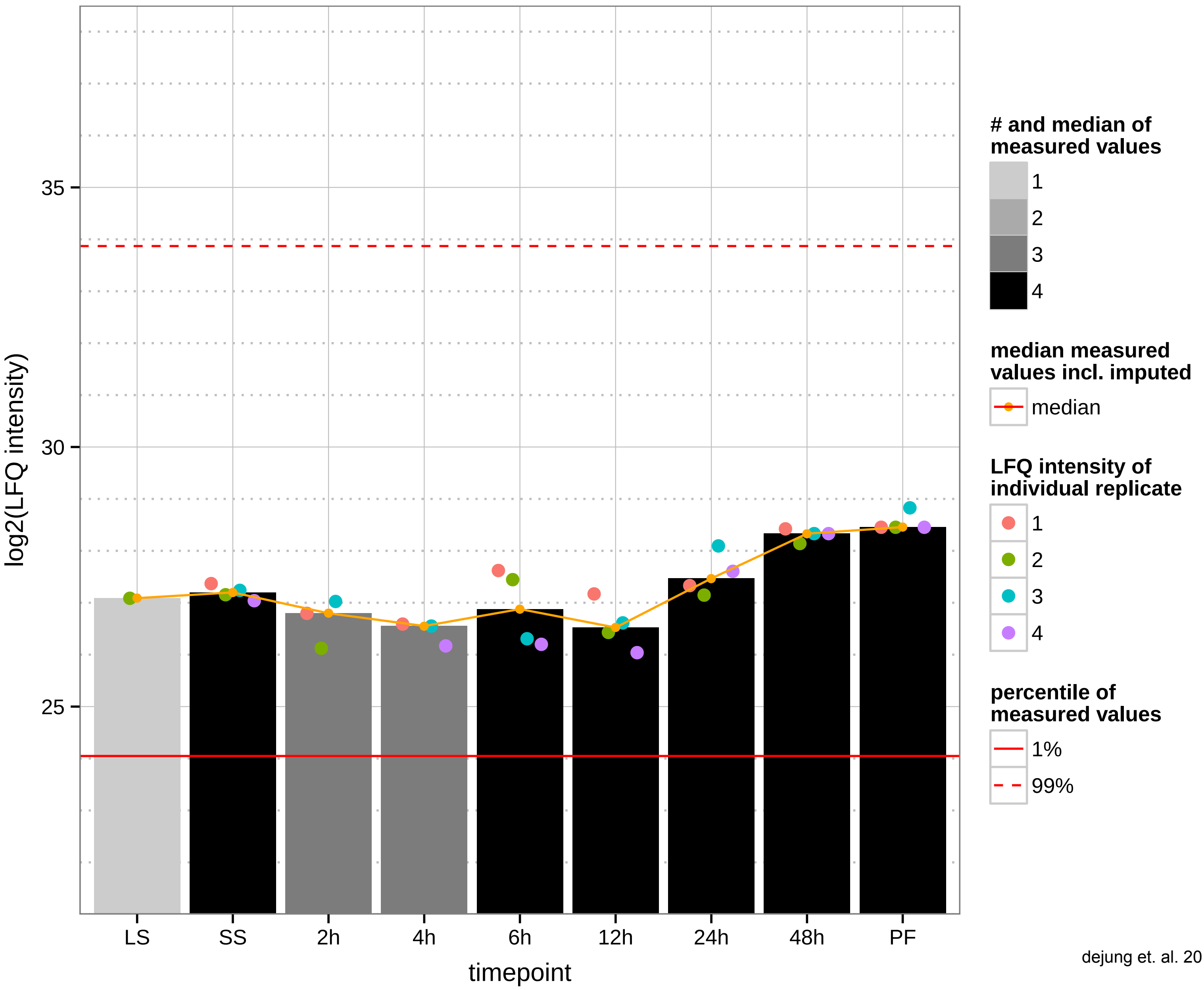

dejung et. al. 2015

short–chain dehydrogenase, putative
Tb927.5.1210
AGOF: oxidoreductase activity
AGOC: mitochondrial inner membrane
AGOP: metabolic process
PGOF: oxidoreductase activity
PGOC: null
PGOP: metabolic process

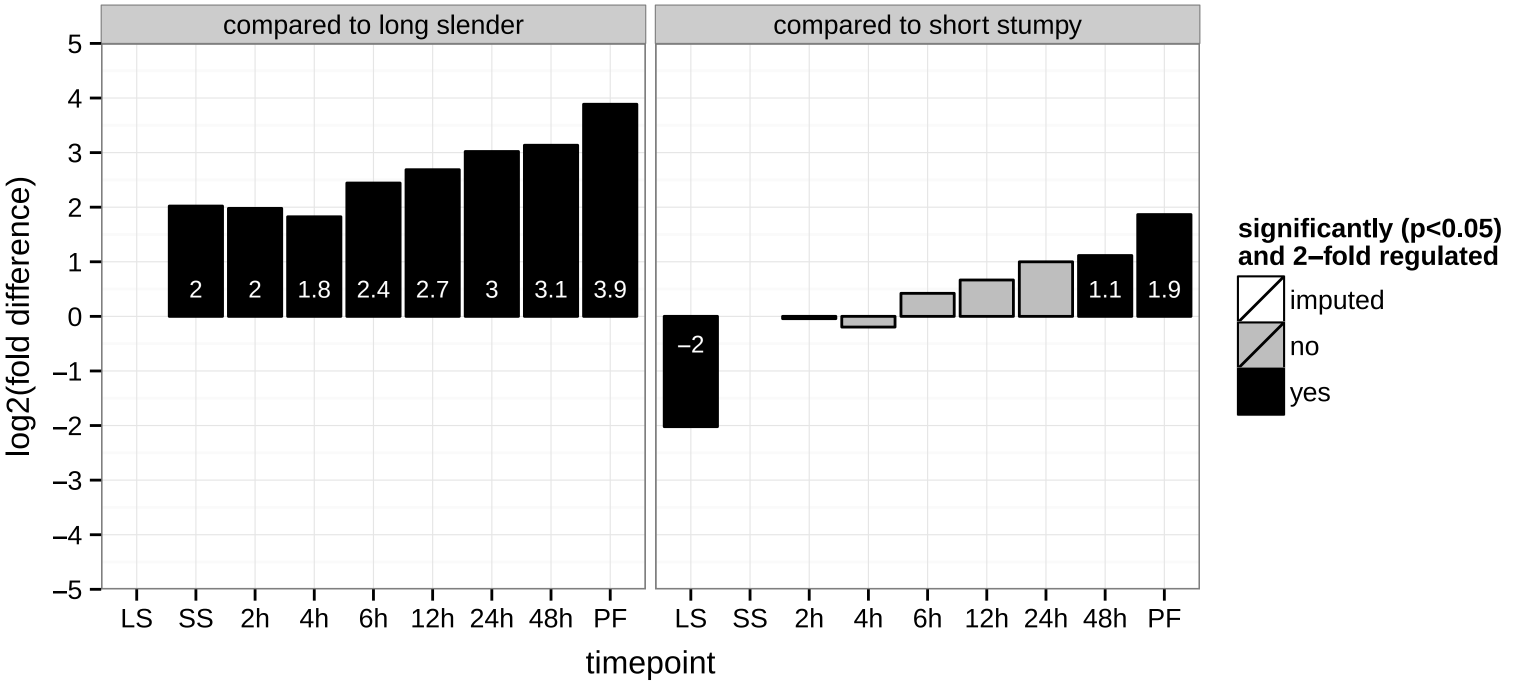

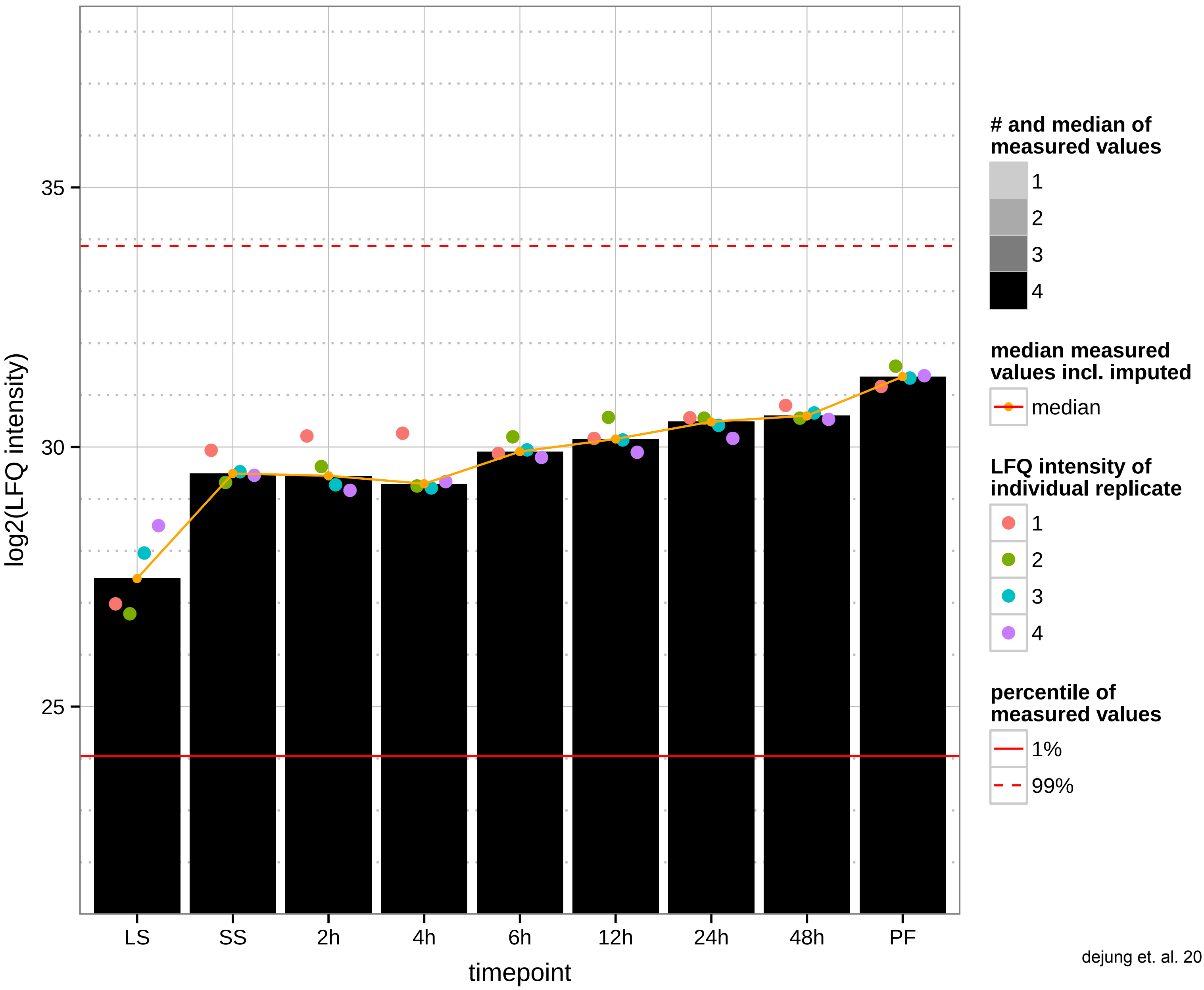

dejung et. al. 2015

hypothetical protein, conserved
Tb927.5.1790
AGOF: null
AGOC: mitochondrion
AGOP: null
PGOF: null
PGOC: null
PGOP: null

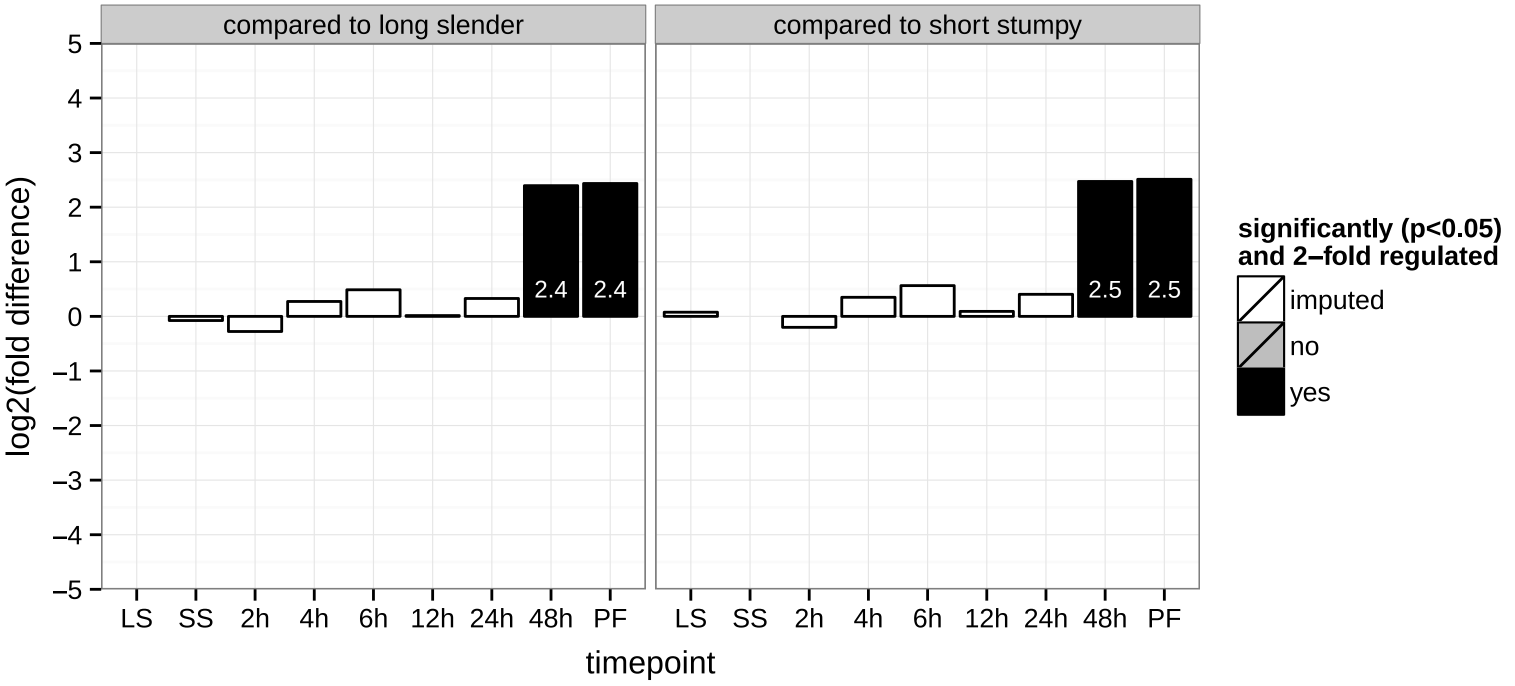

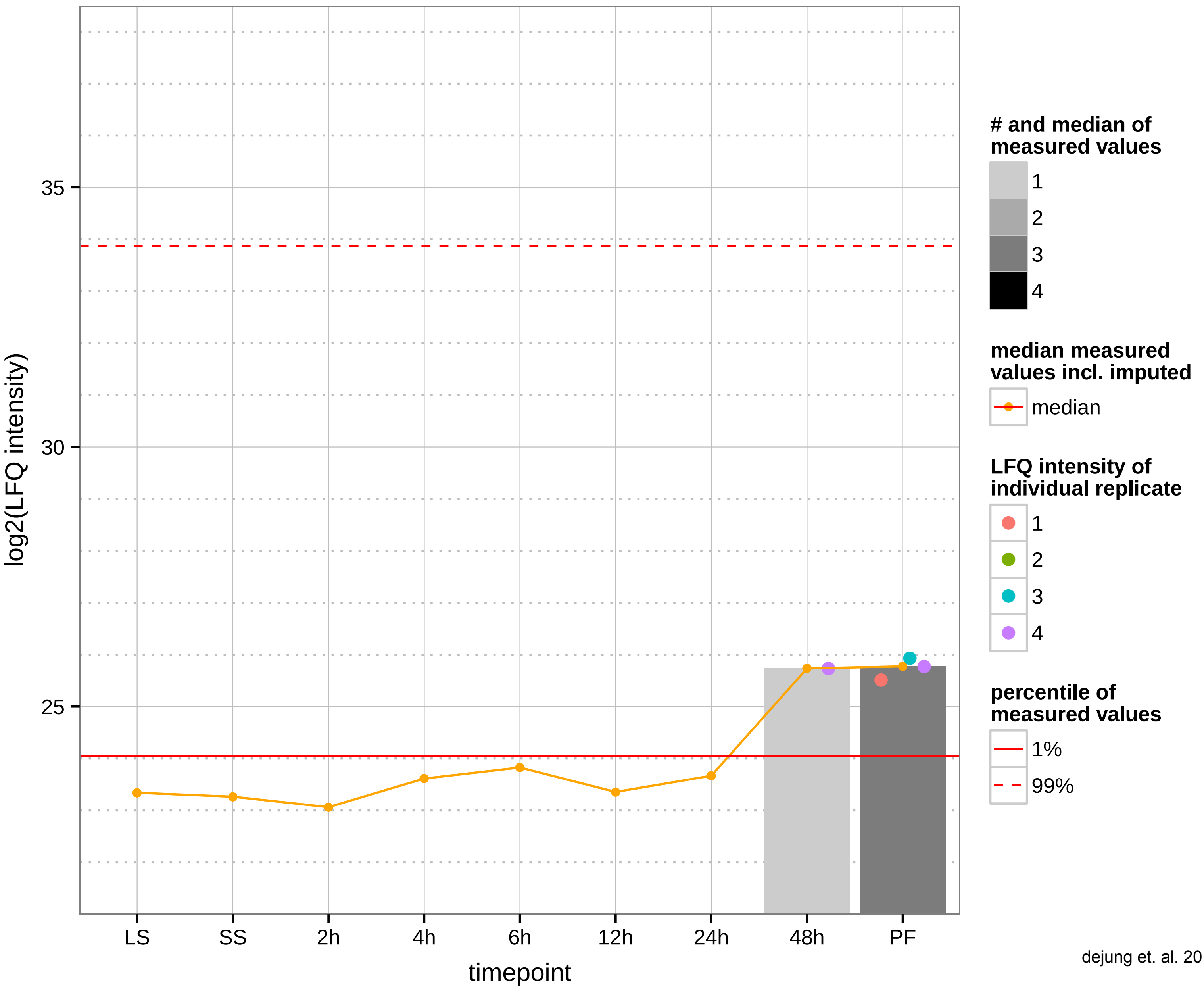

dejung et. al. 2015

regulator of nonsense transcripts UPF1 (upf1)
Tb927.5.2140
AGOF: ATP binding, DNA binding, helicase activity, nucleoside−triphosphatase activity, zinc ion binding
AGOC: cytoplasm
AGOP: nuclear−transcribed mRNA catabolic process, nonsense−mediated decay
PGOF: ATP binding, DNA binding, helicase activity, nucleoside−triphosphatase activity, nucleotide binding, zinc ion binding
PGOC: cytoplasm
PGOP: nuclear−transcribed mRNA catabolic process, nonsense−mediated decay

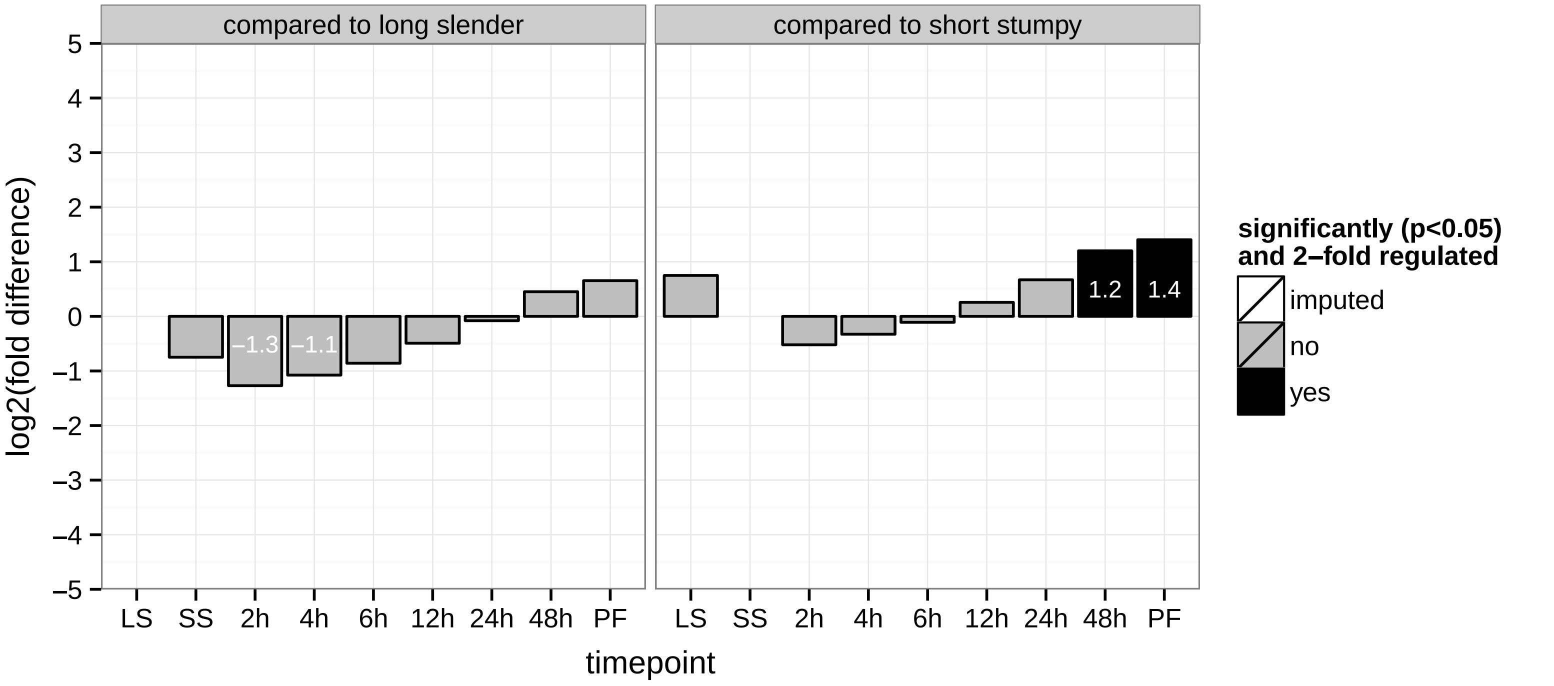

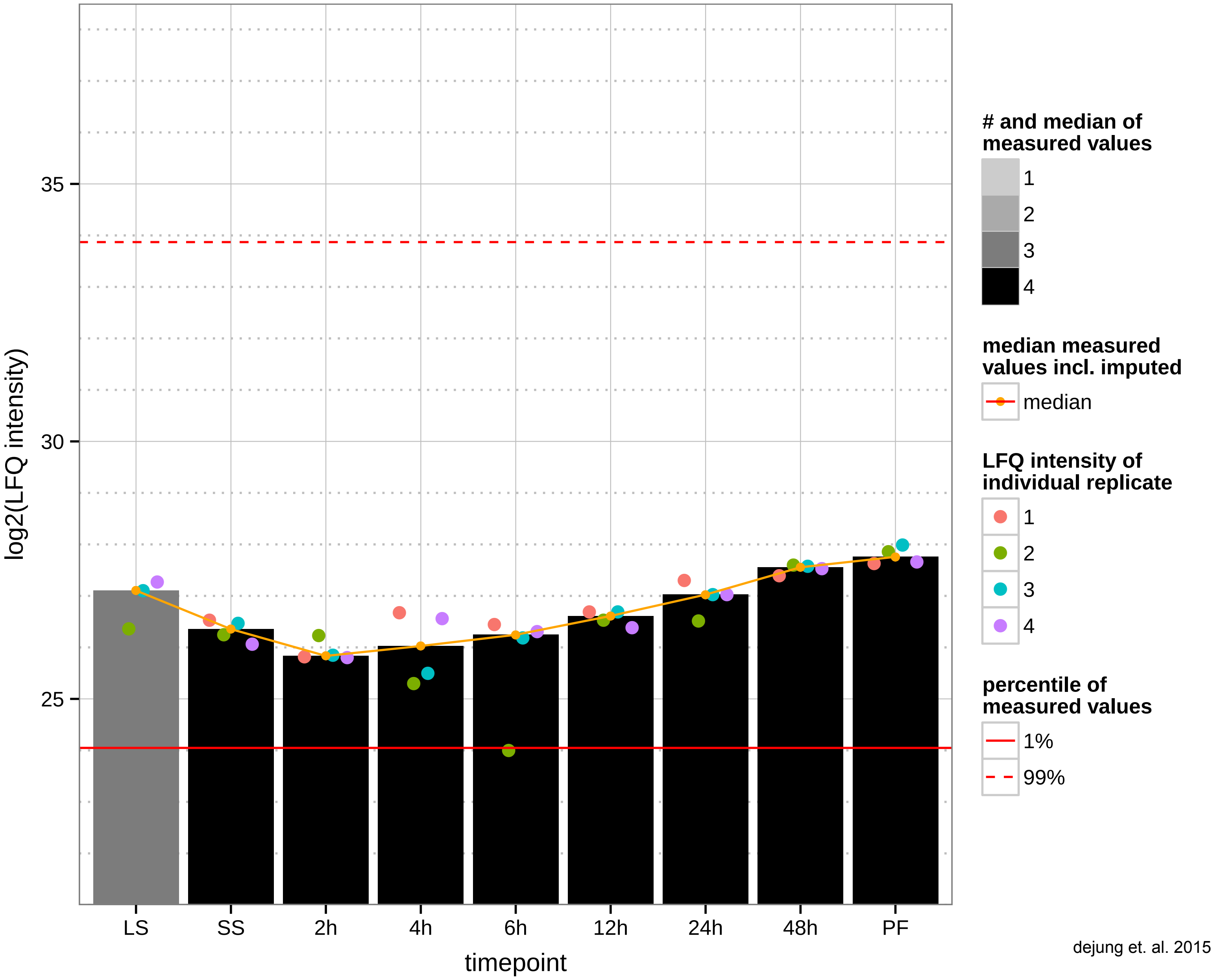

dejung et. al. 2015

hypothetical protein, conserved
Tb927.5.2320
AGOF: null
AGOC: null
AGOP: null
PGOF: null
PGOC: null
PGOP: null

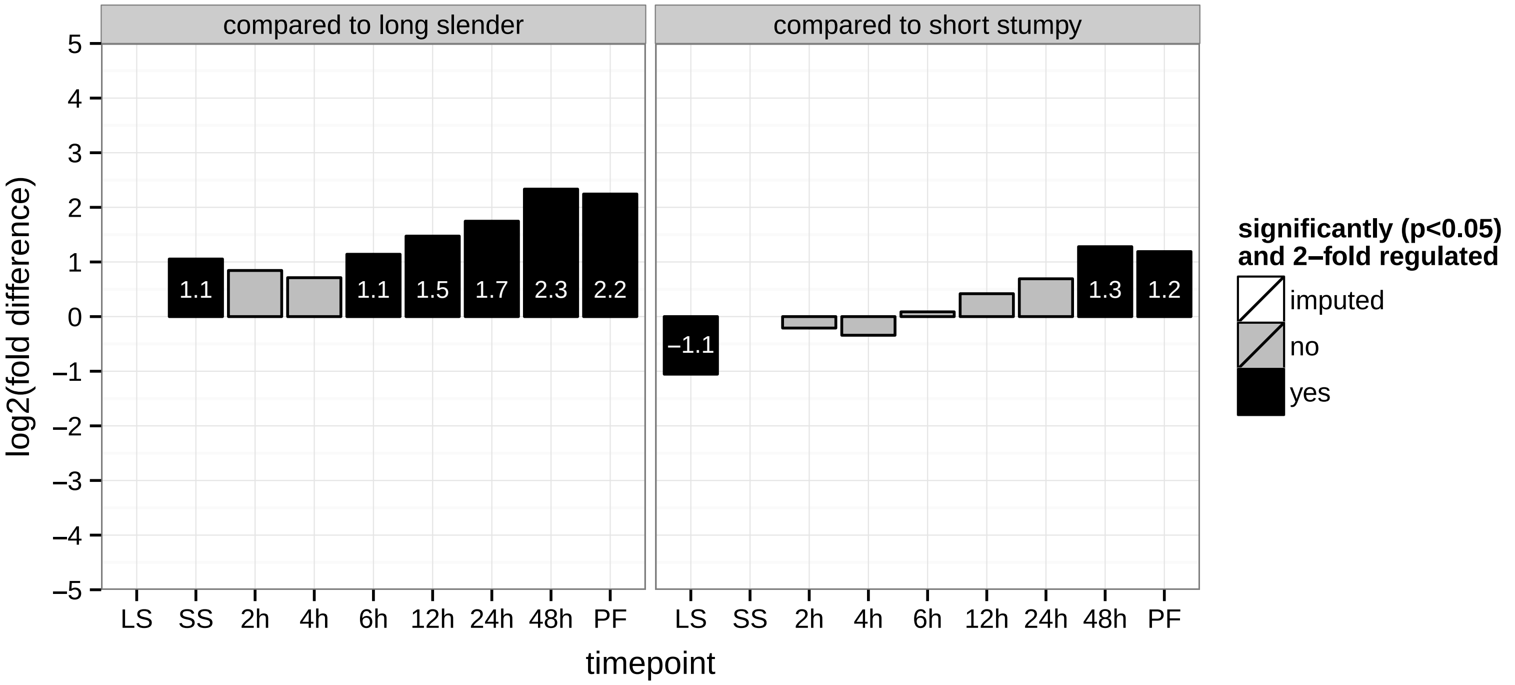

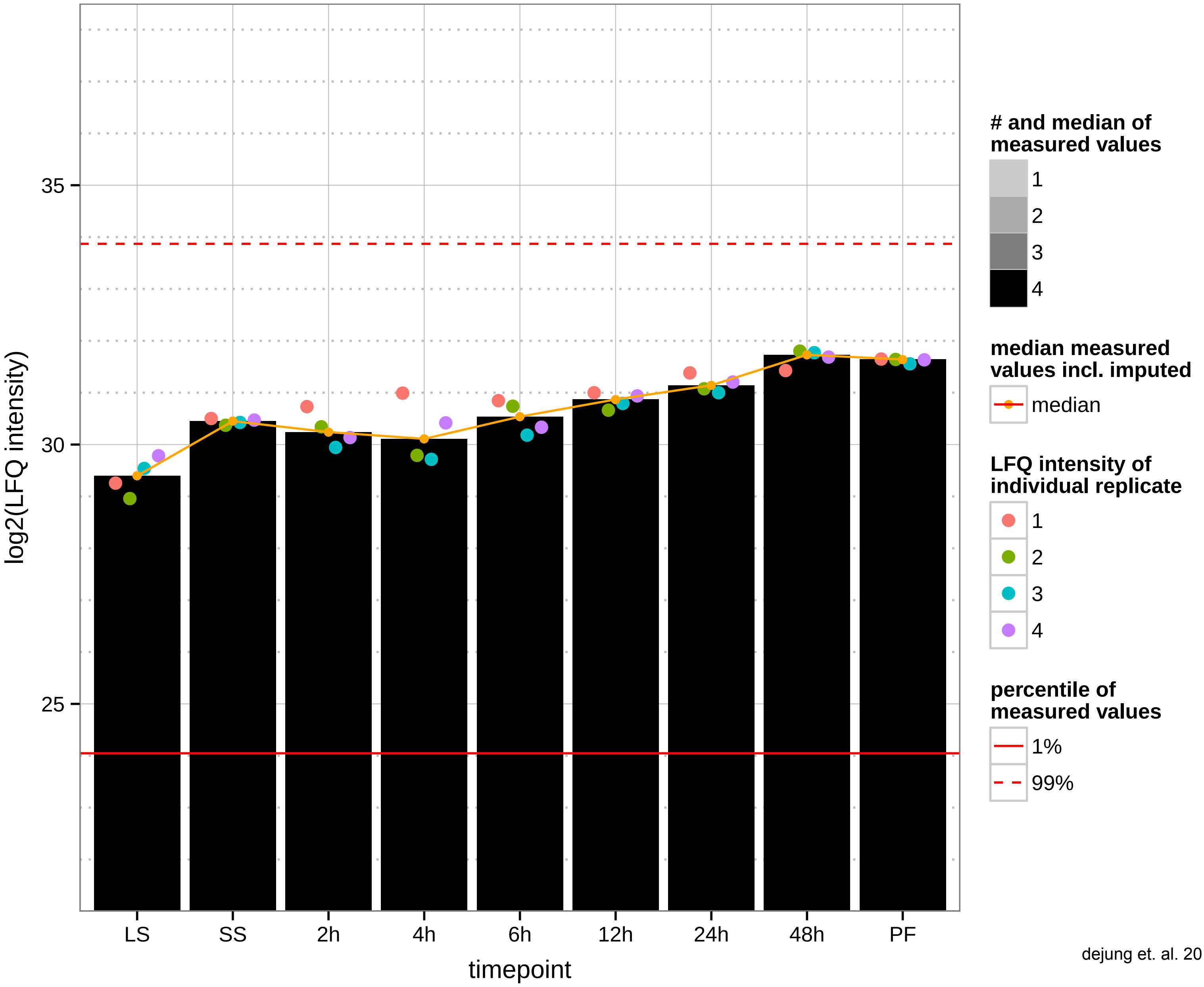

dejung et. al. 2015

mitochondrial DNA polymerase beta
Tb927.5.2780
AGOF: DNA binding, DNA–directed DNA polymerase activity, catalytic activity
AGOC: mitochondrion
AGOP: DNA repair, DNA replication
PGOF: DNA binding, DNA–directed DNA polymerase activity, catalytic activity, nucleotidyltransferase activity
PGOC: null
PGOP: null

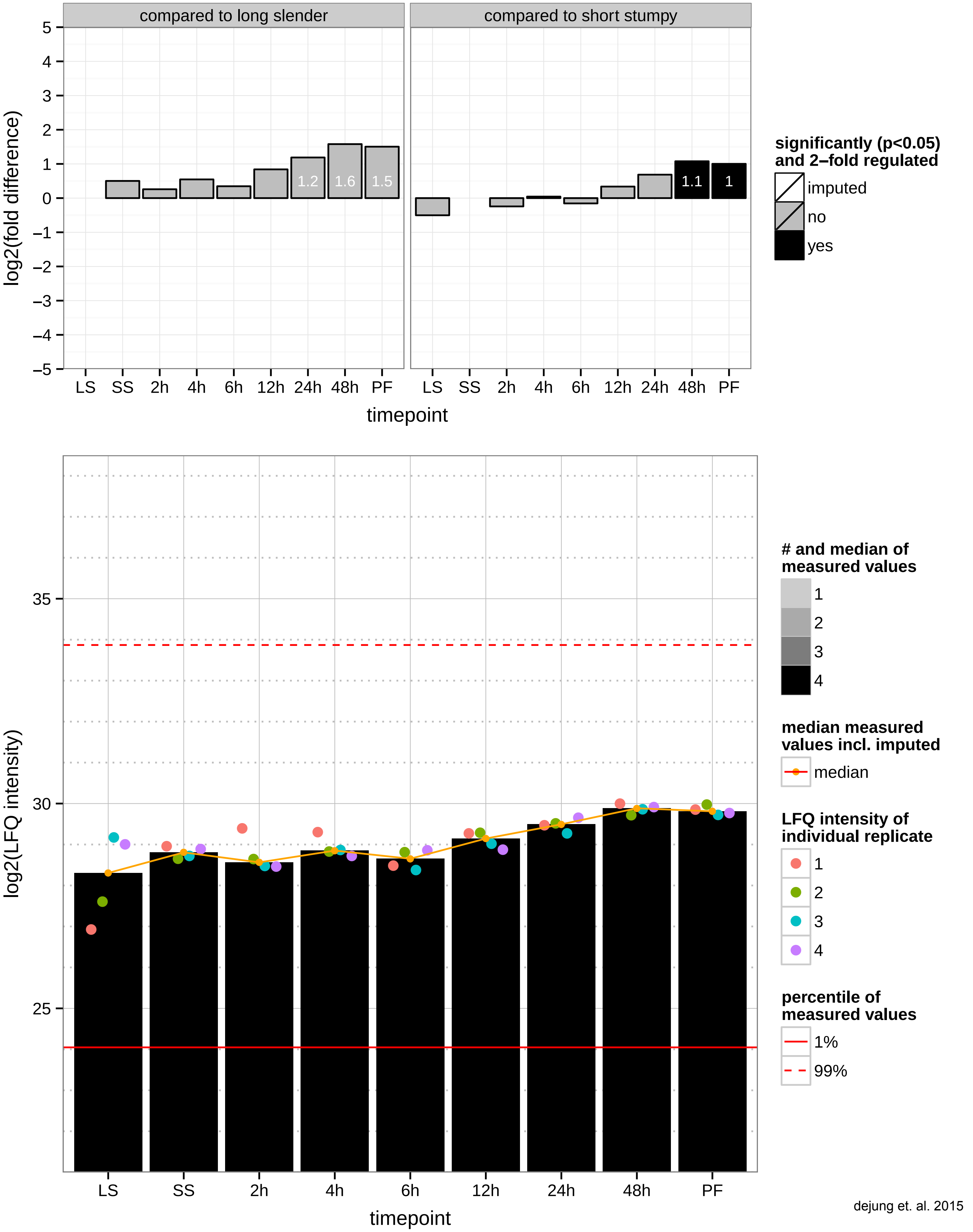

dejung et. al. 2015

mitochondrial DNA polymerase beta–PAK (Pol beta–PAK)
Tb927.5.2790
AGOF: DNA binding, DNA–directed DNA polymerase activity, catalytic activity
AGOC: kinetoplast
AGOP: DNA replication
PGOF: DNA binding, DNA–directed DNA polymerase activity, catalytic activity, nucleotidyltransferase activity
PGOC: null
PGOP: null

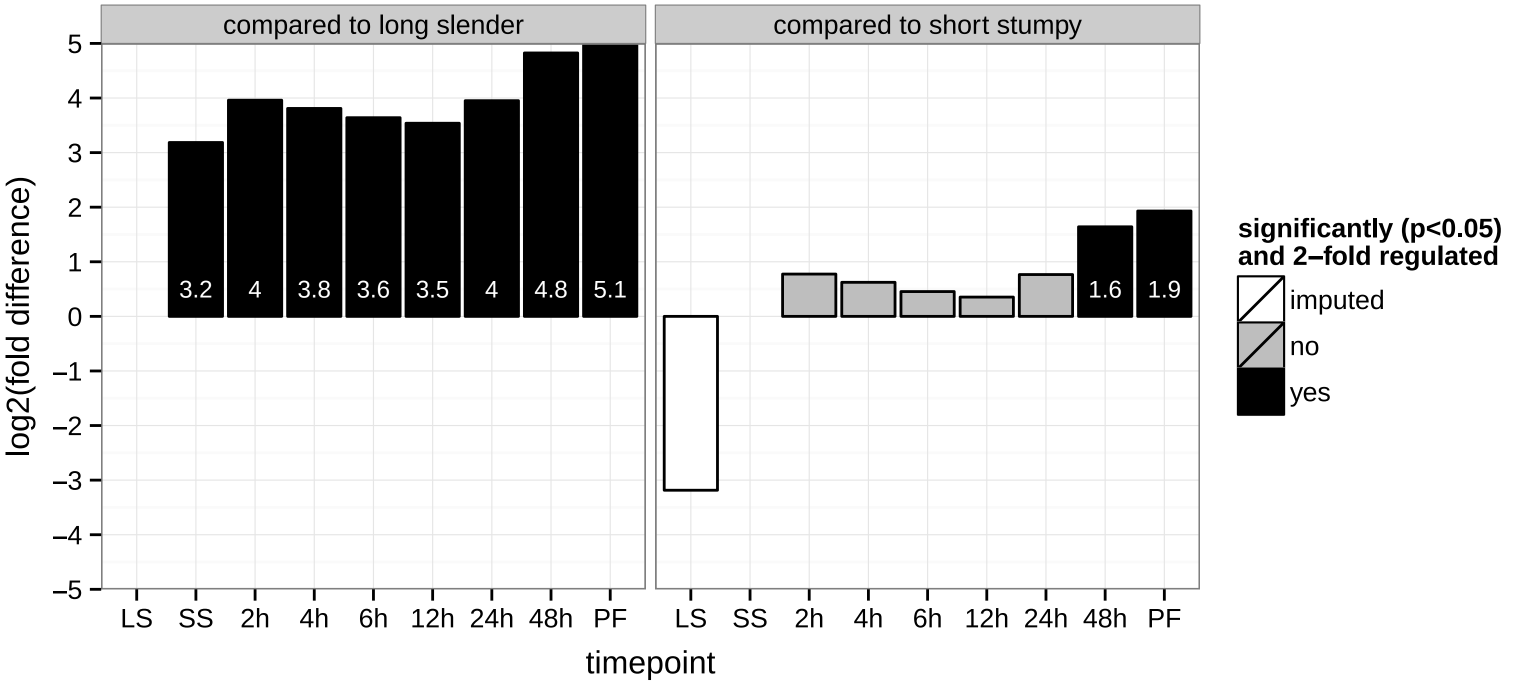

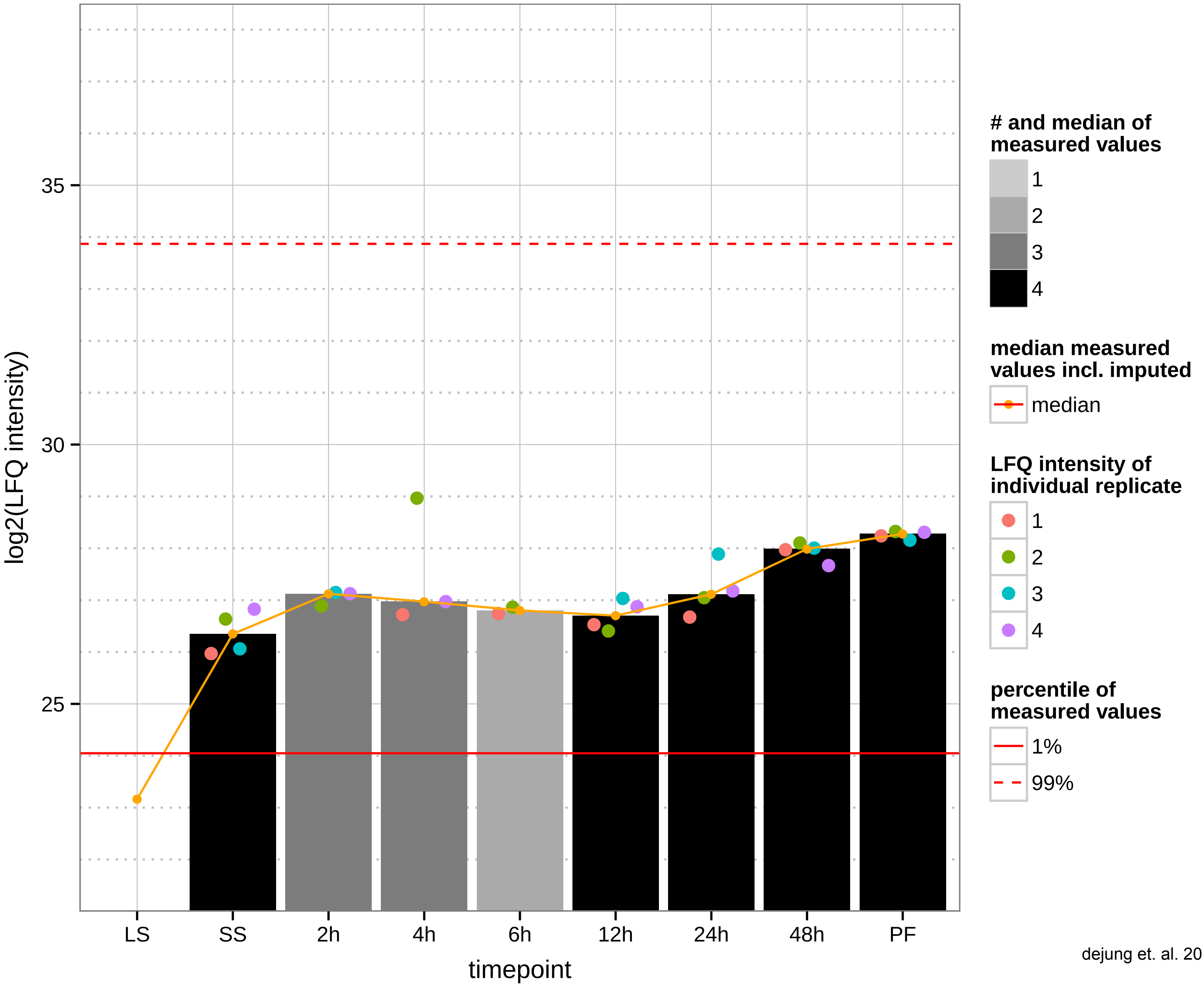

dejung et. al. 2015

hypothetical protein, conserved
Tb927.5.3680
AGOF: null
AGOC: null
AGOP: null
PGOF: phosphatidylinositol N–acetylglucosaminyltransferase activity
PGOC: null
PGOP: null

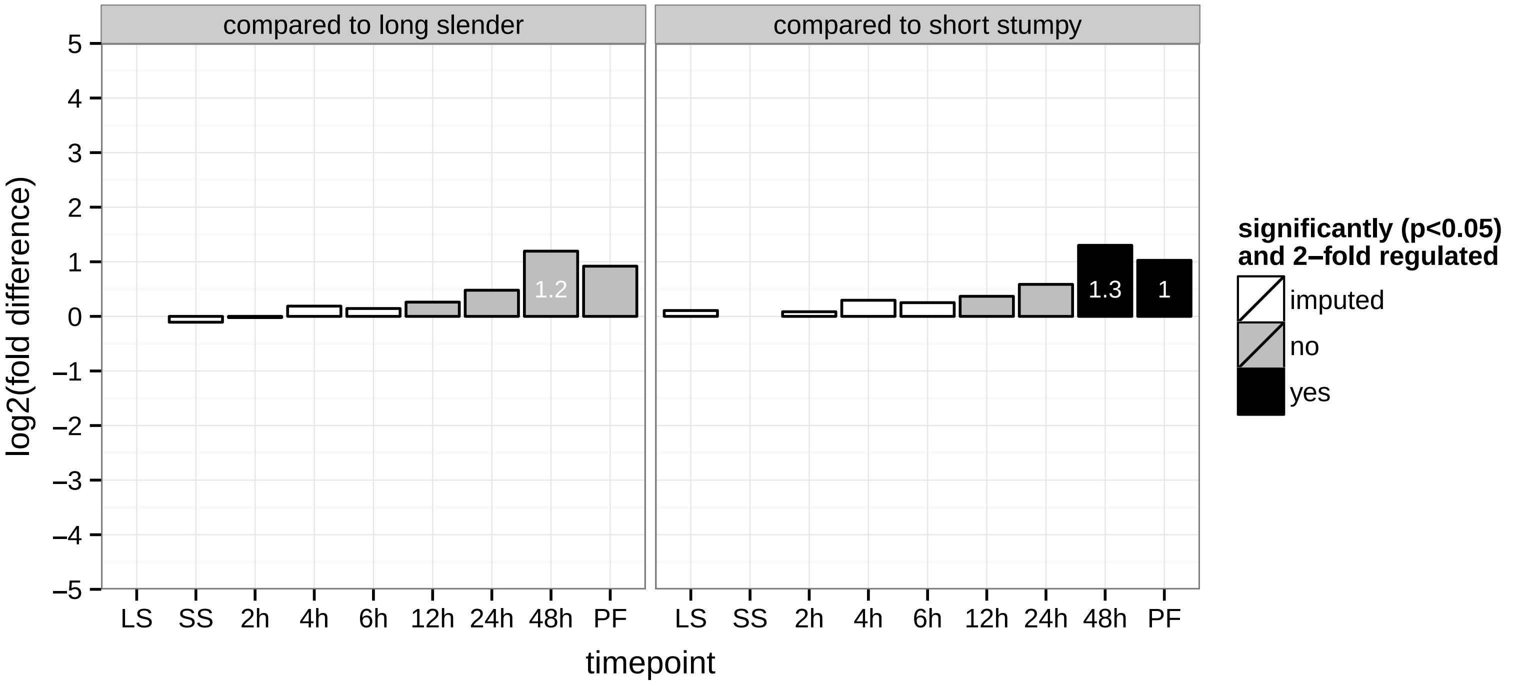

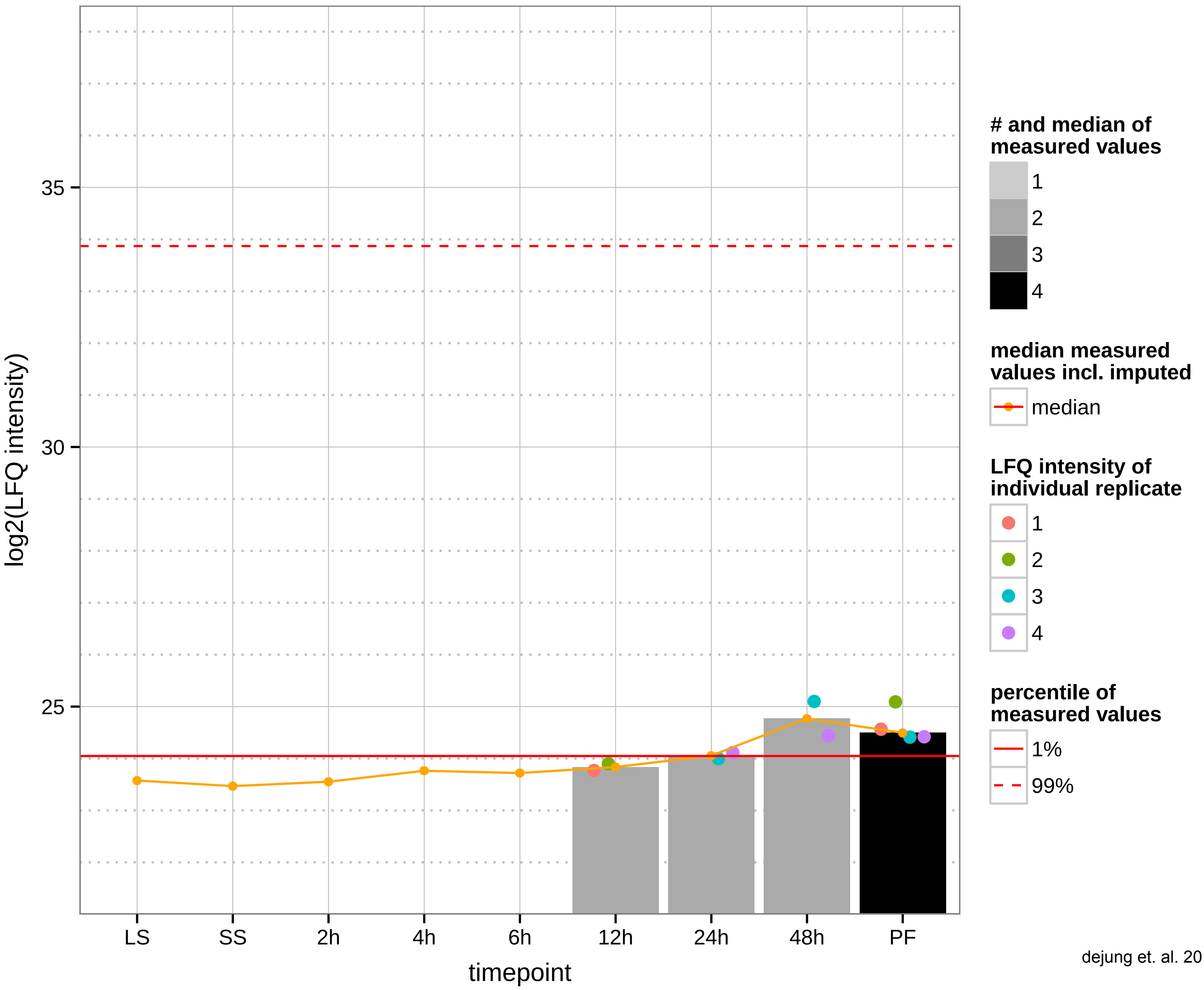

dejung et. al. 2015

monothiol glutaredoxin, putative
Tb927.5.950
AGOF: electron carrier activity, glutathione−disulfide reductase activity, protein disulfide oxidoreductase activity
AGOC: null
AGOP: cell redox homeostasis, oxidation−reduction process
PGOF: electron carrier activity, protein disulfide oxidoreductase activity
PGOC: null
PGOP: cell redox homeostasis

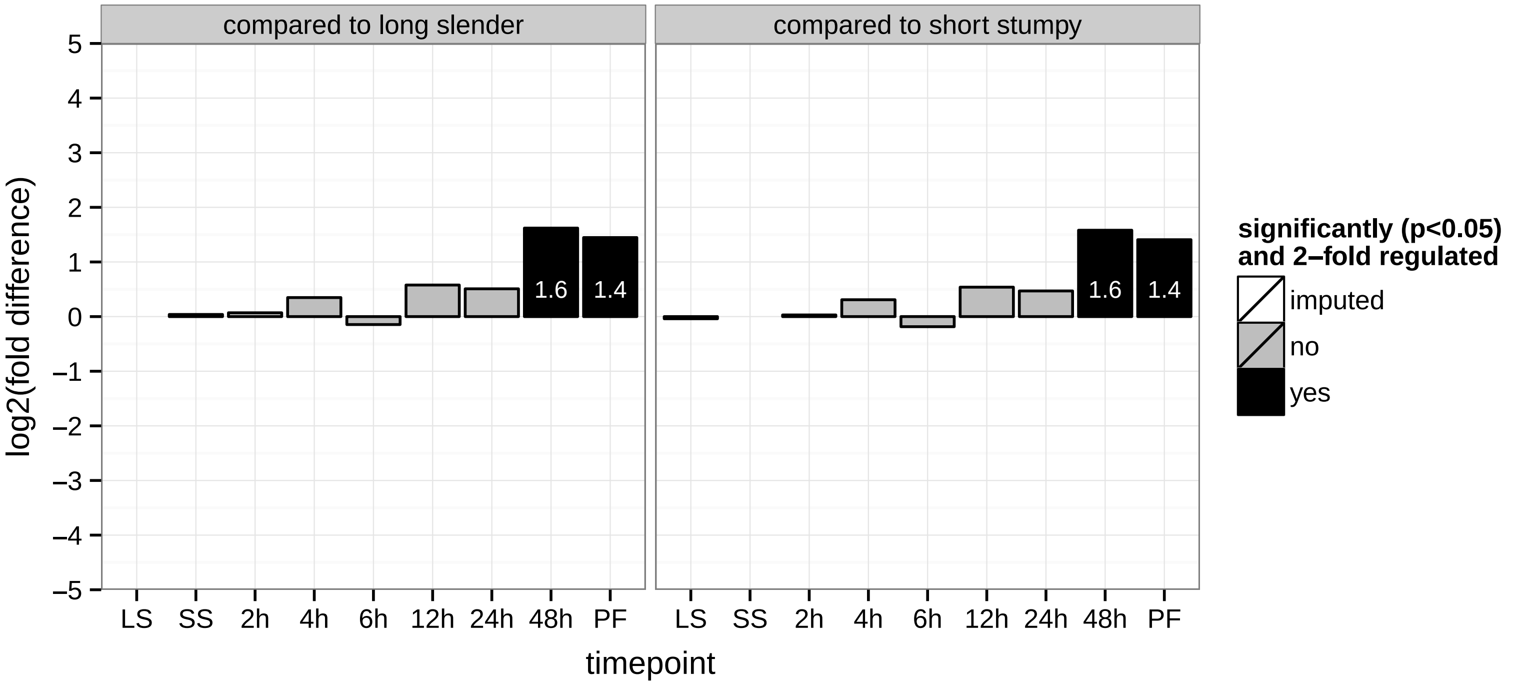

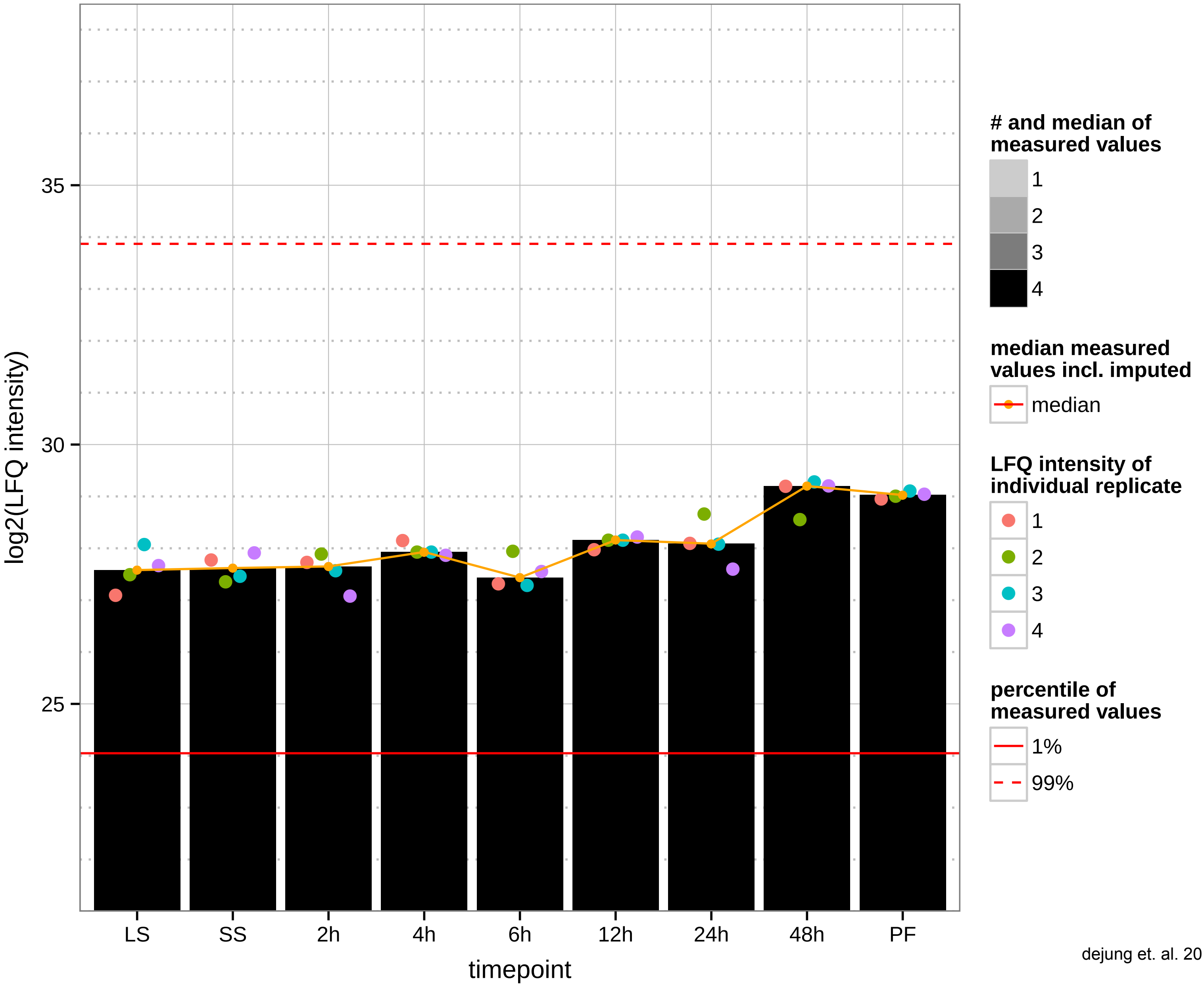

dejung et. al. 2015

hypothetical protein, conserved
Tb927.6.1430
AGOF: null
AGOC: null
AGOP: null
PGOF: protein binding
PGOC: null
PGOP: null

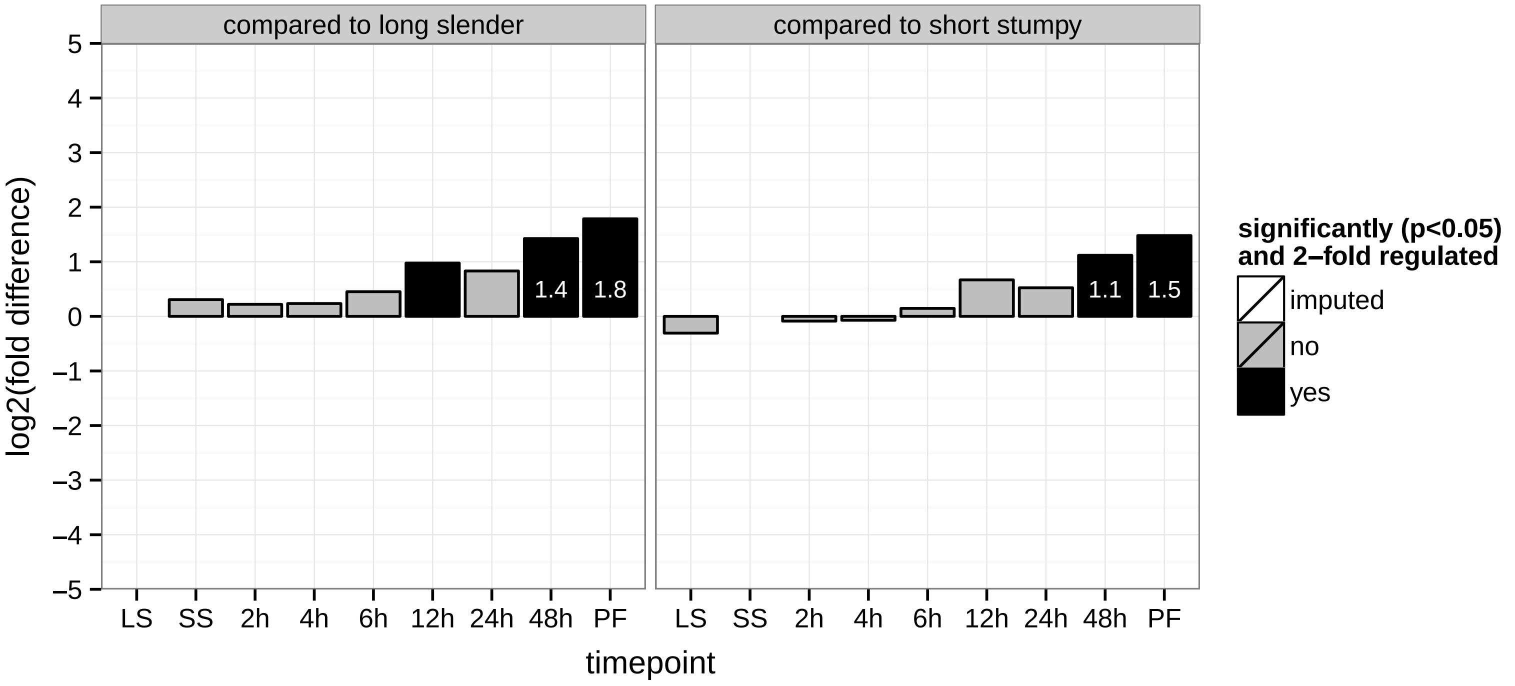

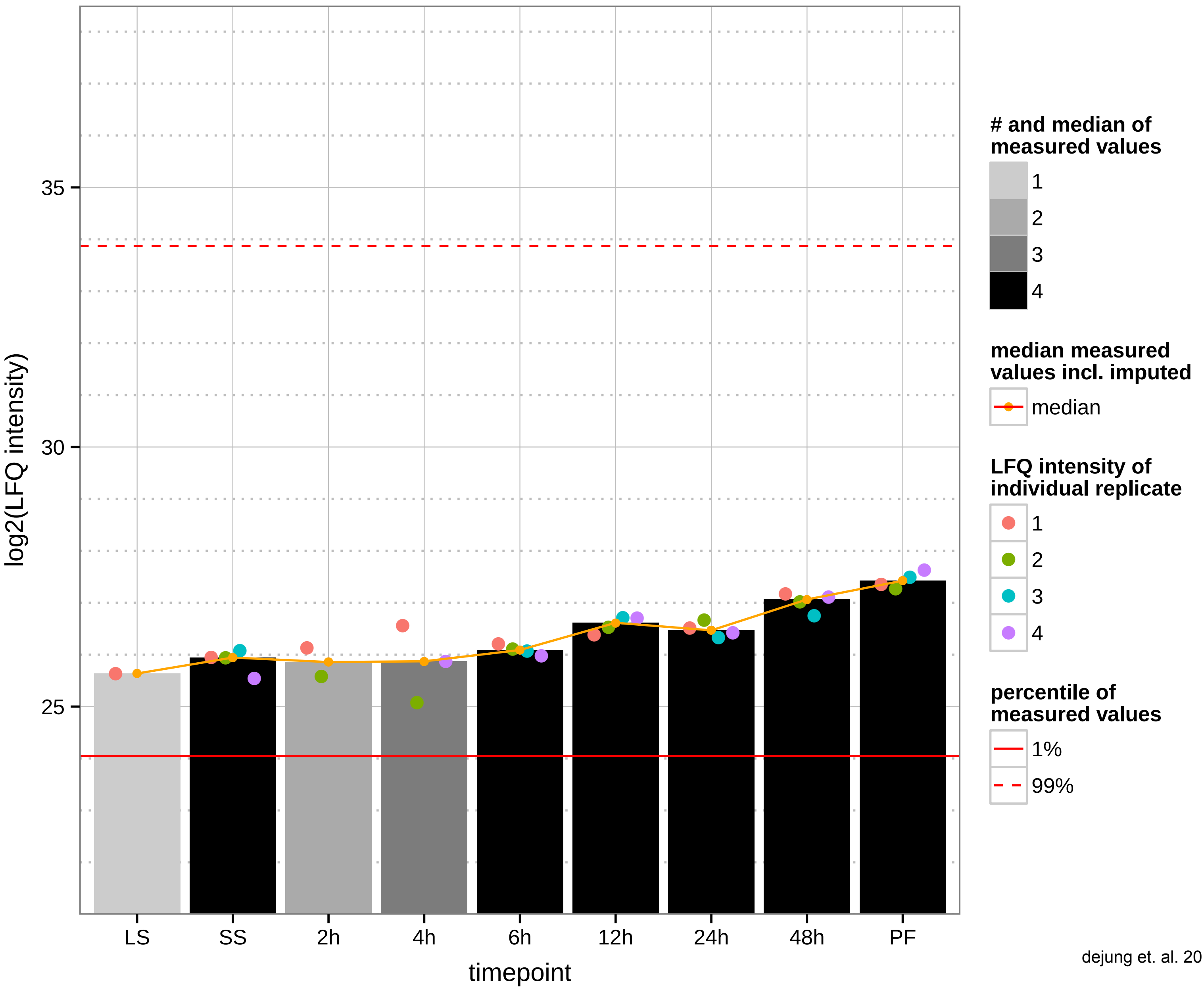

dejung et. al. 2015

hypothetical protein, conserved
Tb927.6.1440
AGOF: null
AGOC: mitochondrion
AGOP: null
PGOF: null
PGOC: null
PGOP: null

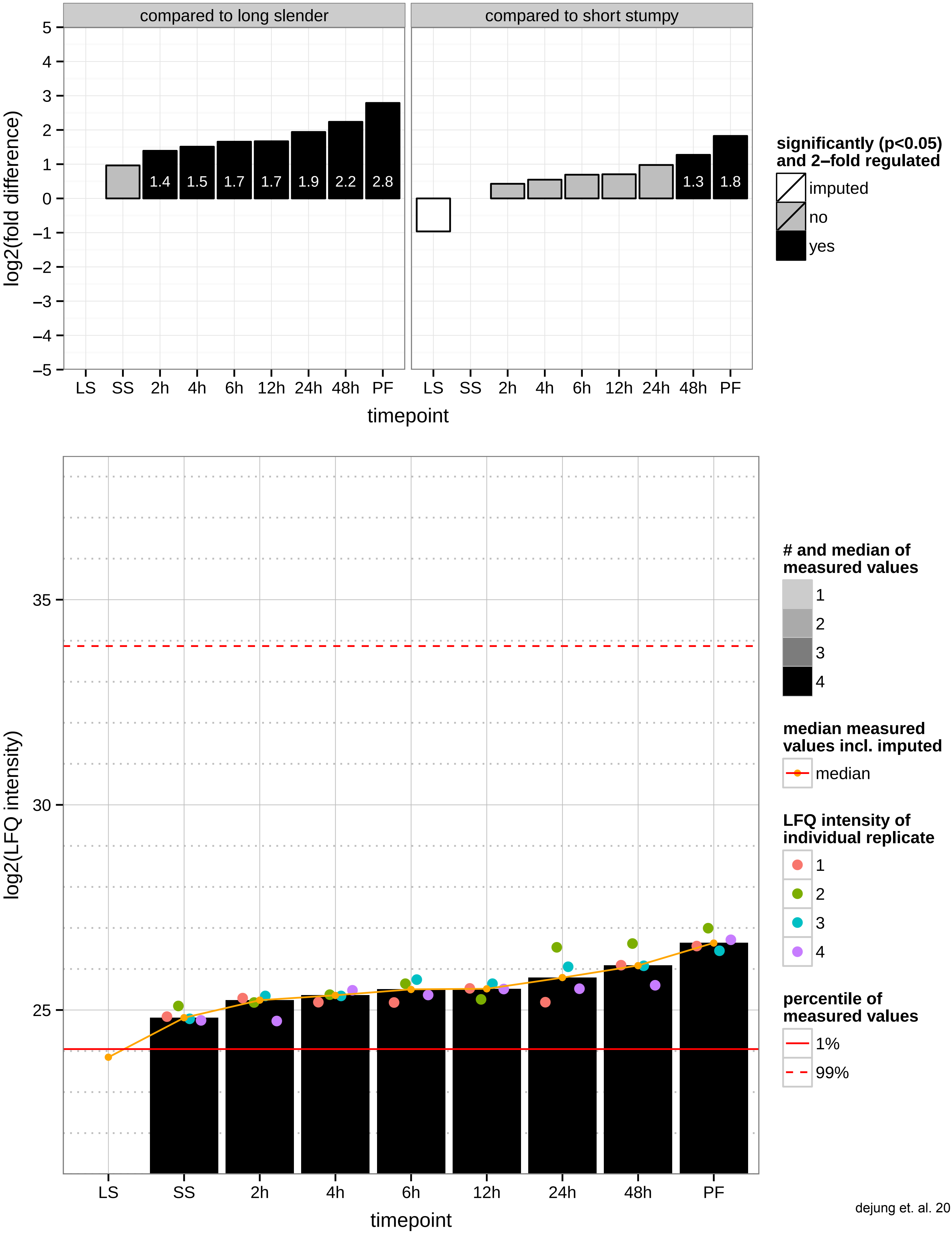

dejung et. al. 2015

hypothetical protein, conserved
Tb927.6.2010
AGOF: catalytic activity
AGOC: mitochondrion
AGOP: metabolic process
PGOF: catalytic activity
PGOC: null
PGOP: metabolic process

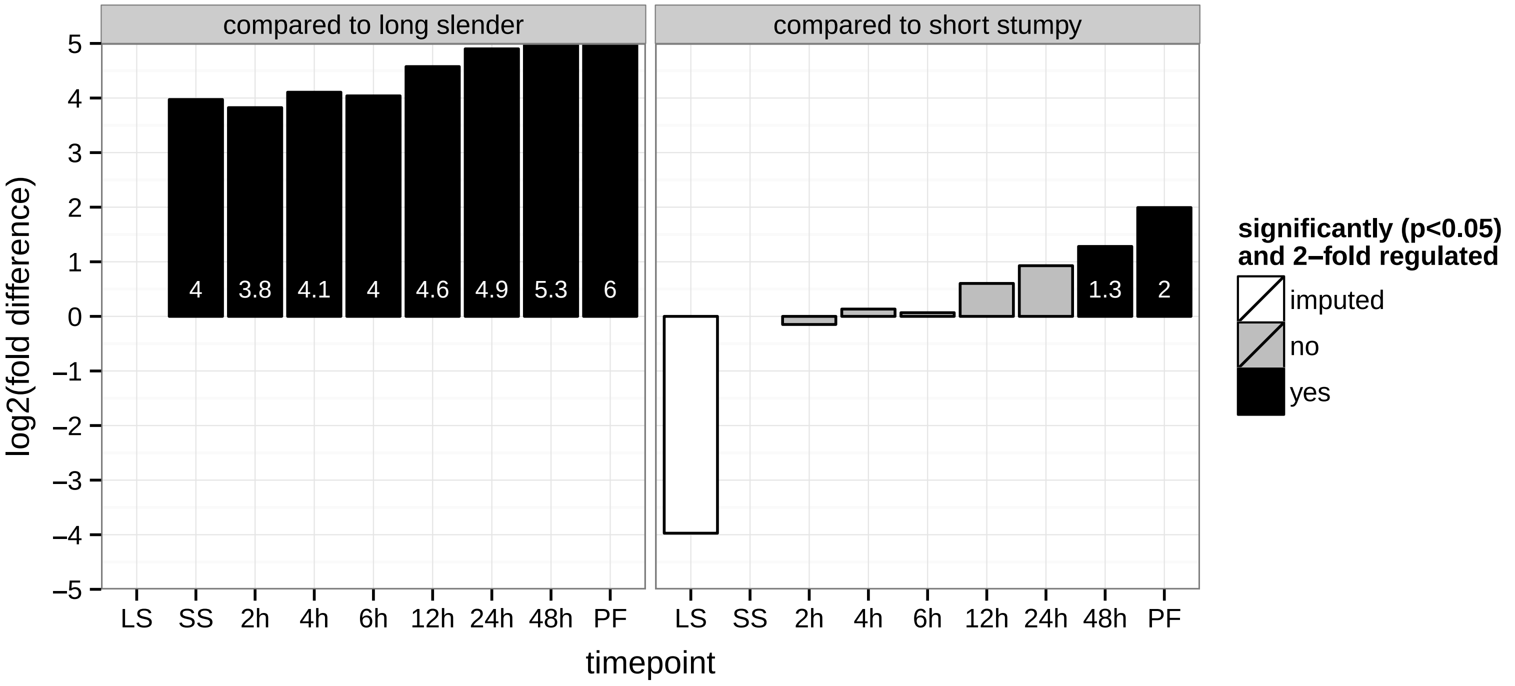

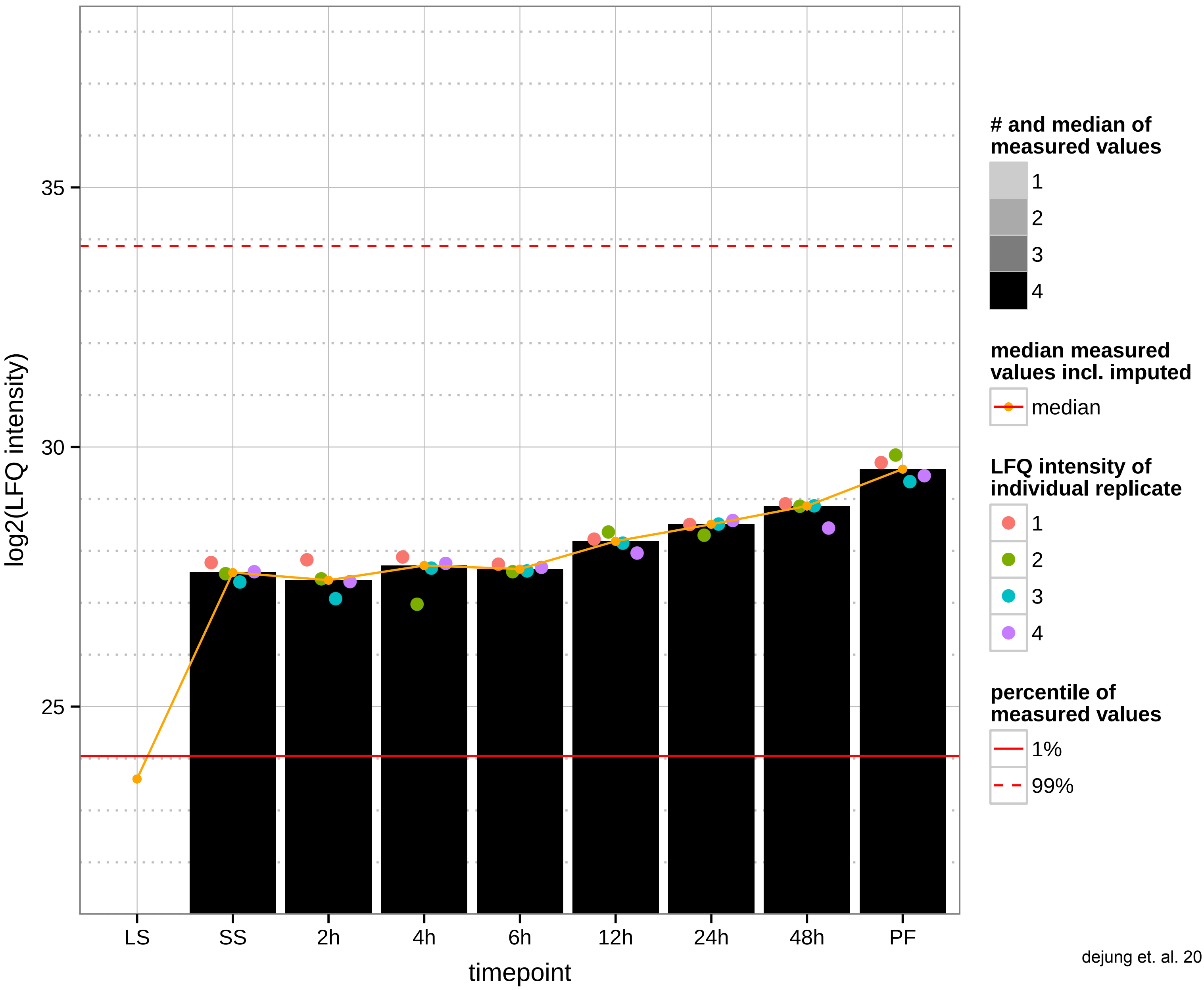

dejung et. al. 2015

hypothetical protein, conserved
Tb927.6.3220;Tb11.v5.0720
AGOF: null
AGOC: null
AGOP: null
PGOF: null
PGOC: null
PGOP: null

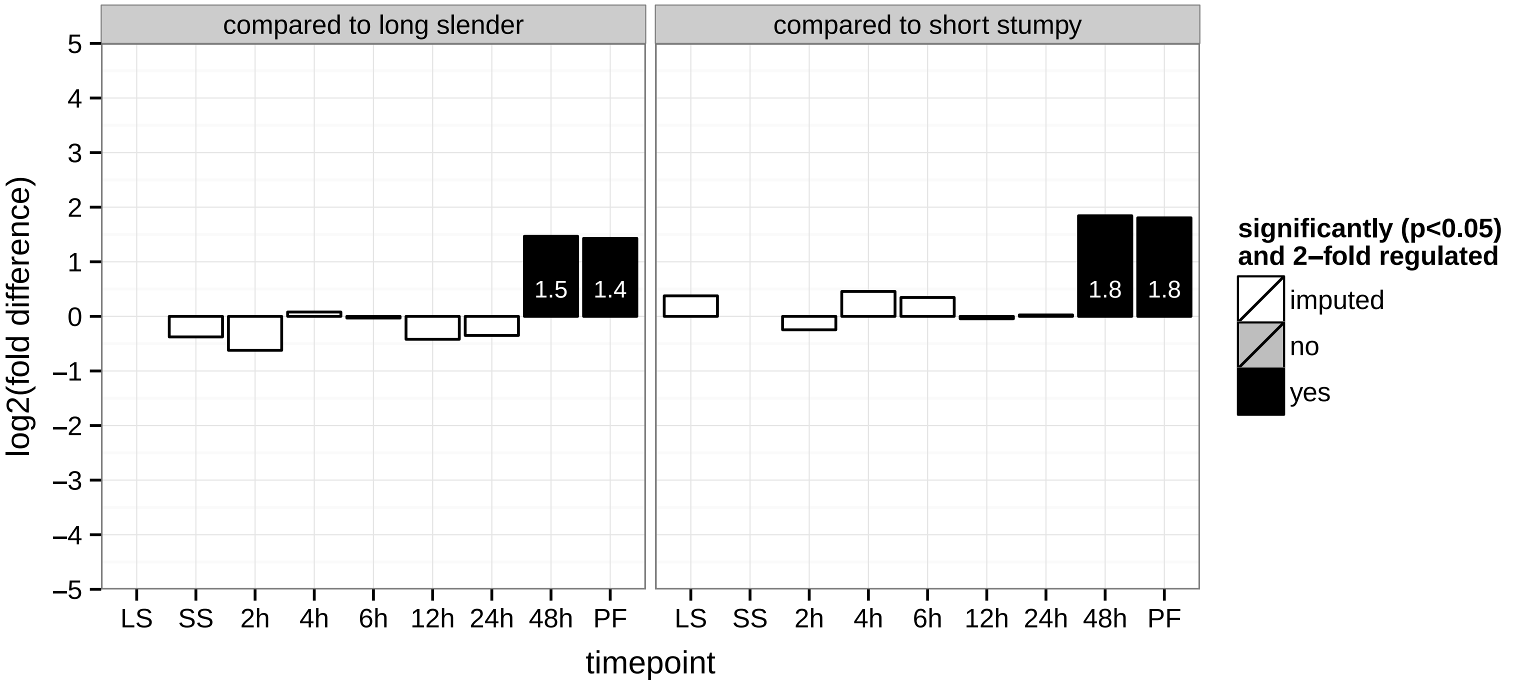

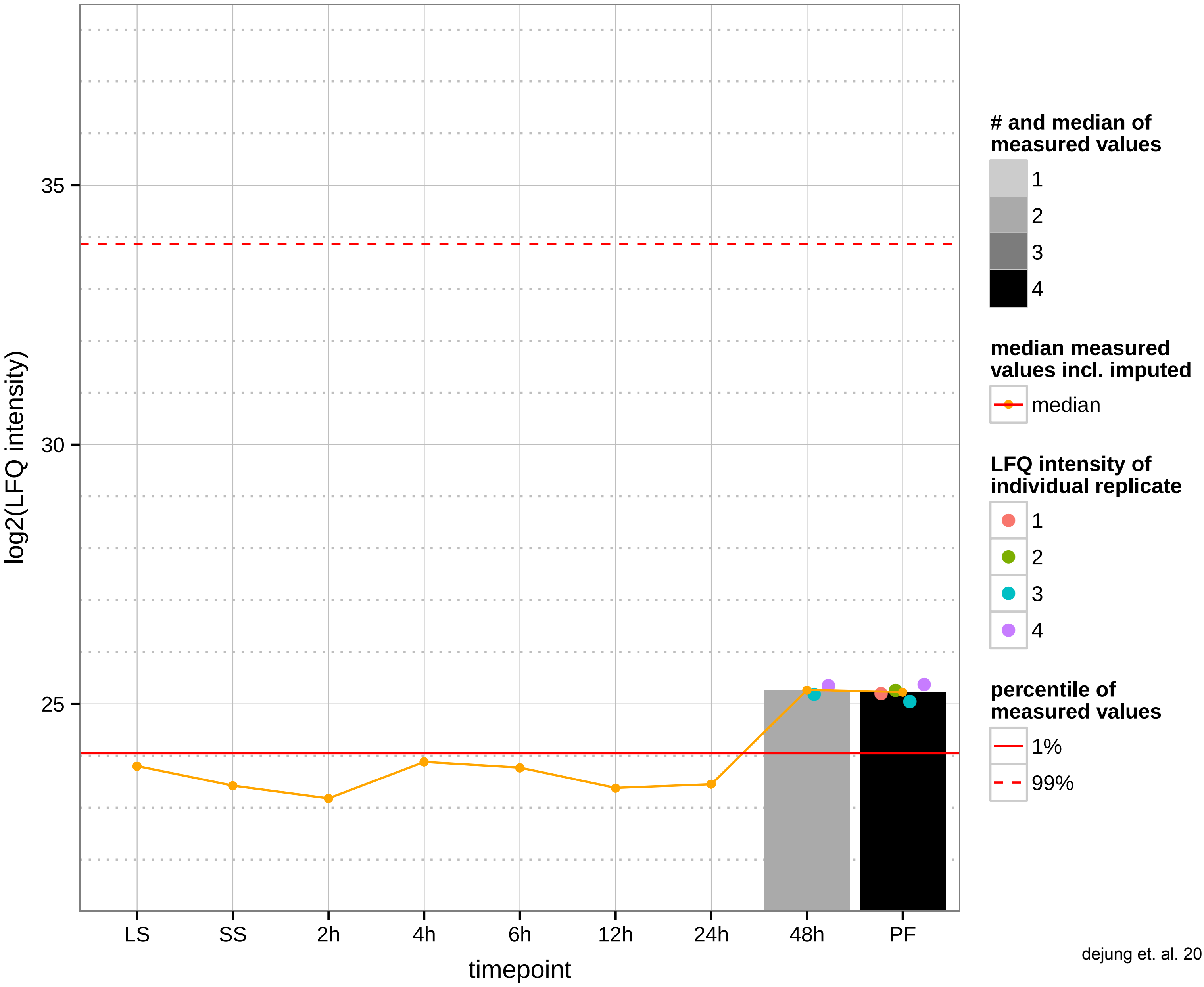

dejung et. al. 2015

hypothetical protein, conserved
Tb927.6.3930
AGOF: null
AGOC: mitochondrion
AGOP: null
PGOF: null
PGOC: null
PGOP: null

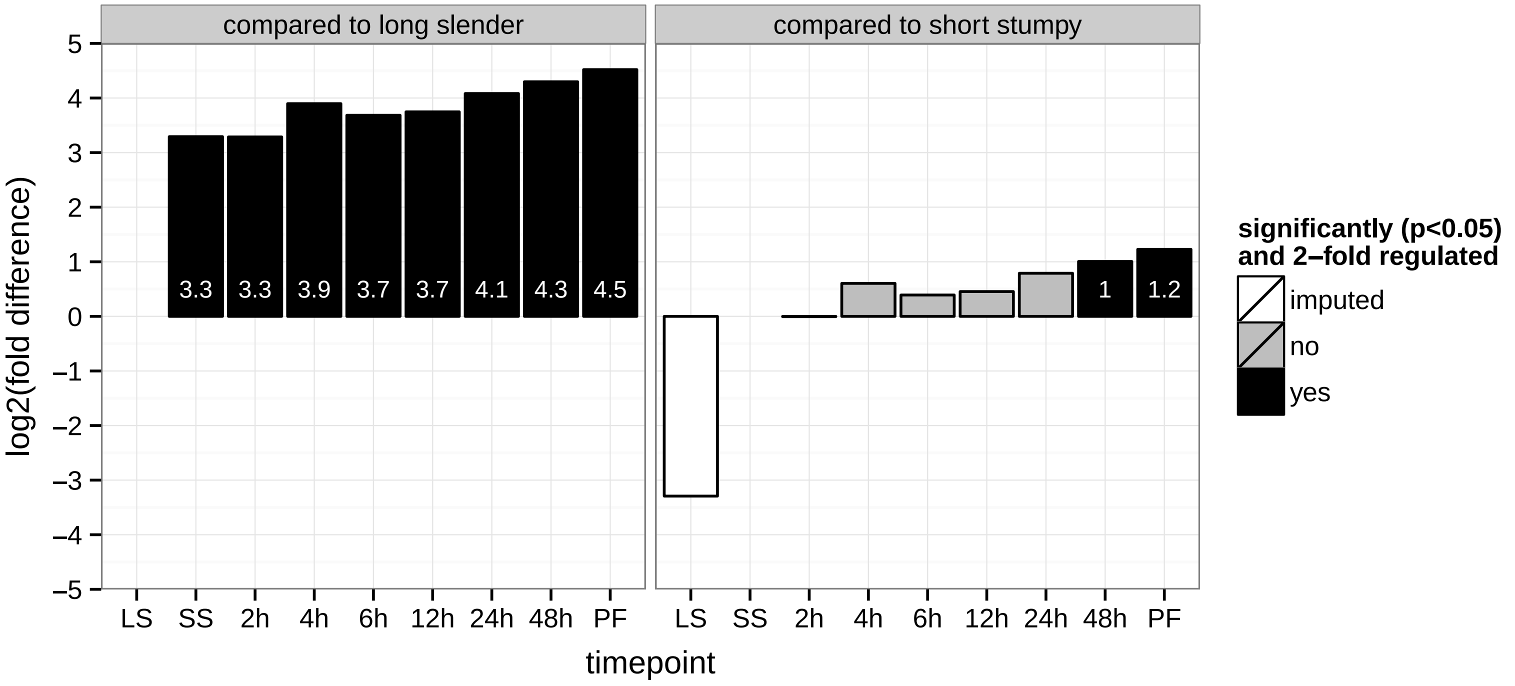

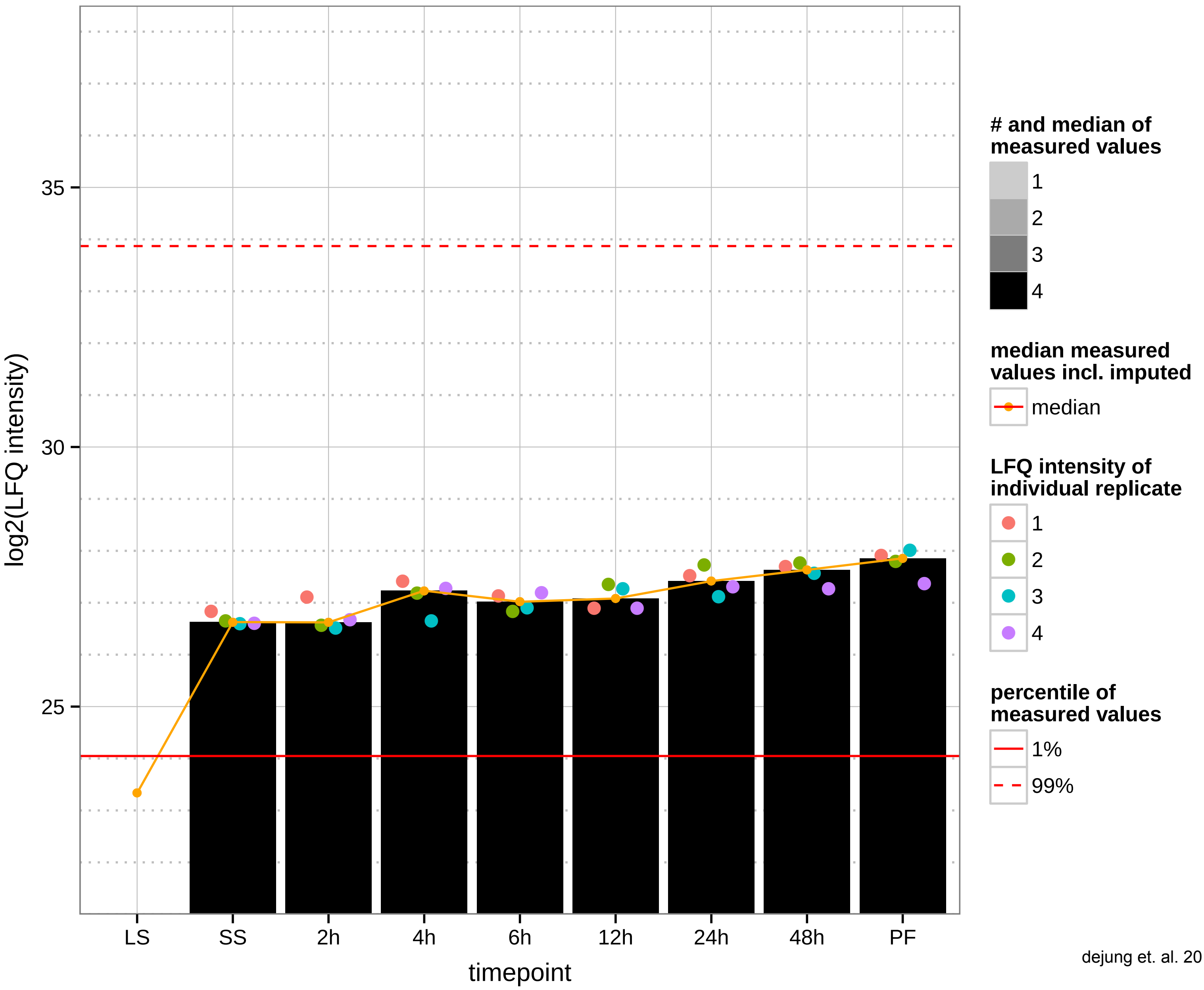

dejung et. al. 2015

hypothetical protein, conserved
Tb927.6.4200
AGOF: null
AGOC: mitochondrion
AGOP: null
PGOF: null
PGOC: null
PGOP: null

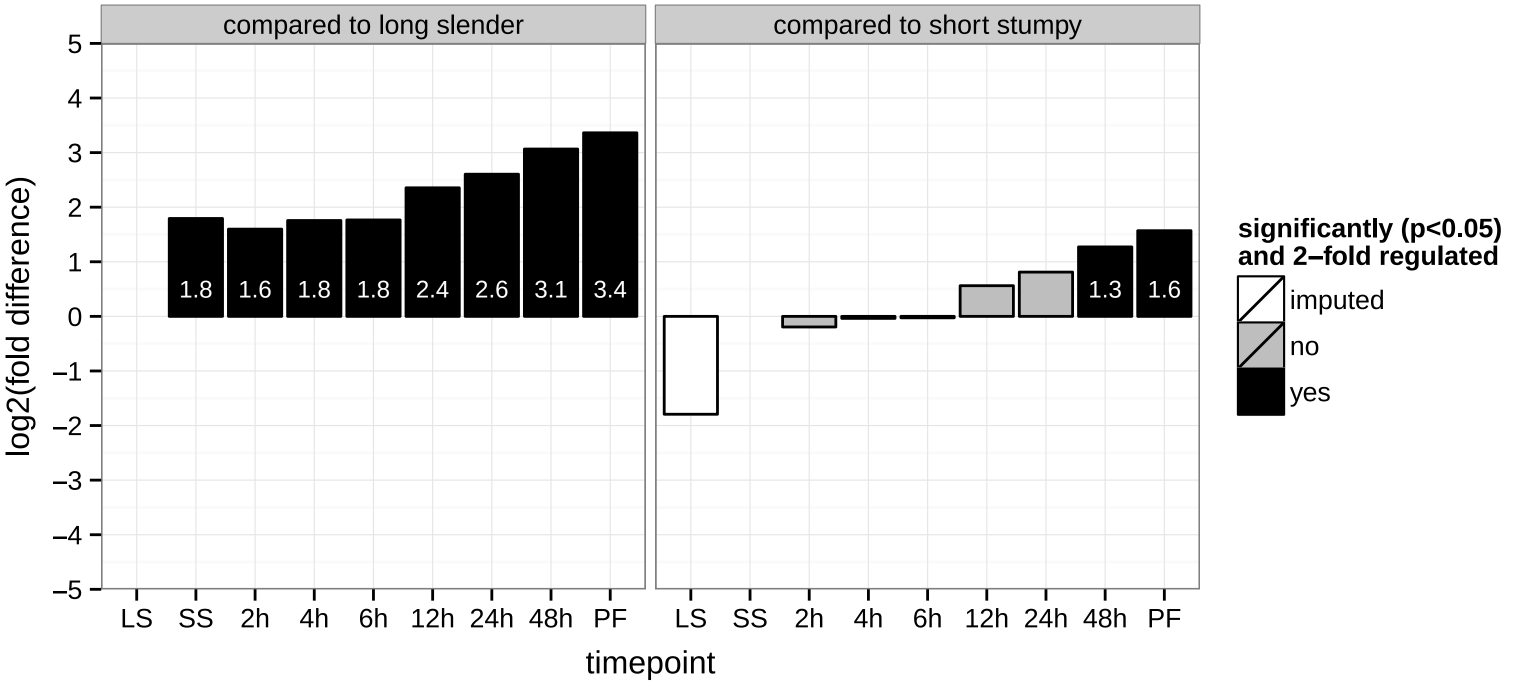

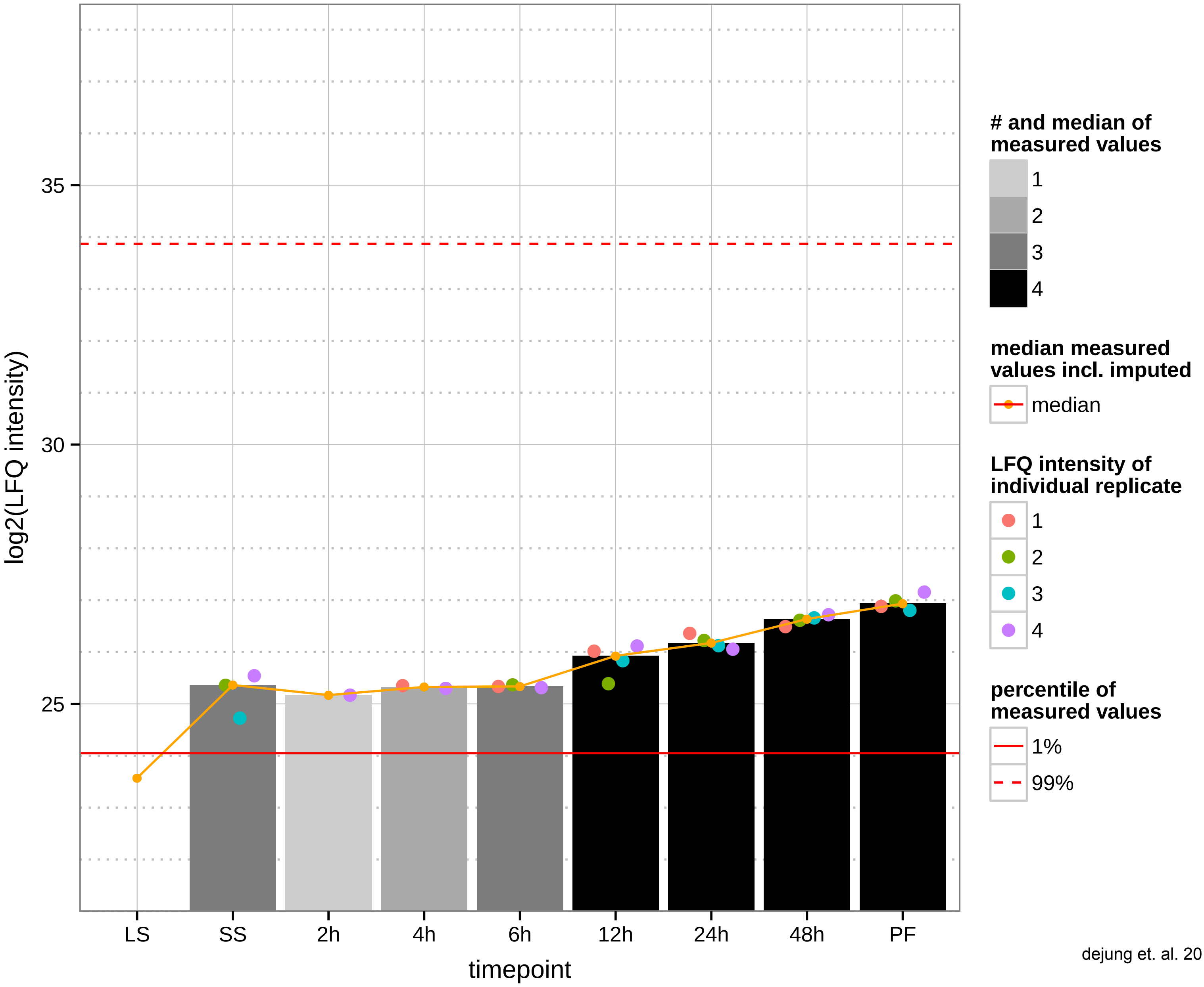

dejung et. al. 2015

valyl–tRNA synthetase, putative (ValRS)
Tb927.6.4480
AGOF: ATP binding, valine–tRNA ligase activity
AGOC: cytoplasm
AGOP: translation, valyl–tRNA aminoacylation
PGOF: ATP binding, aminoacyl–tRNA editing activity, aminoacyl–tRNA ligase activity, nucleotide binding, valine–tRNA ligase a
PGOC: cytoplasm
PGOP: tRNA aminoacylation for protein translation, valyl–tRNA aminoacylation

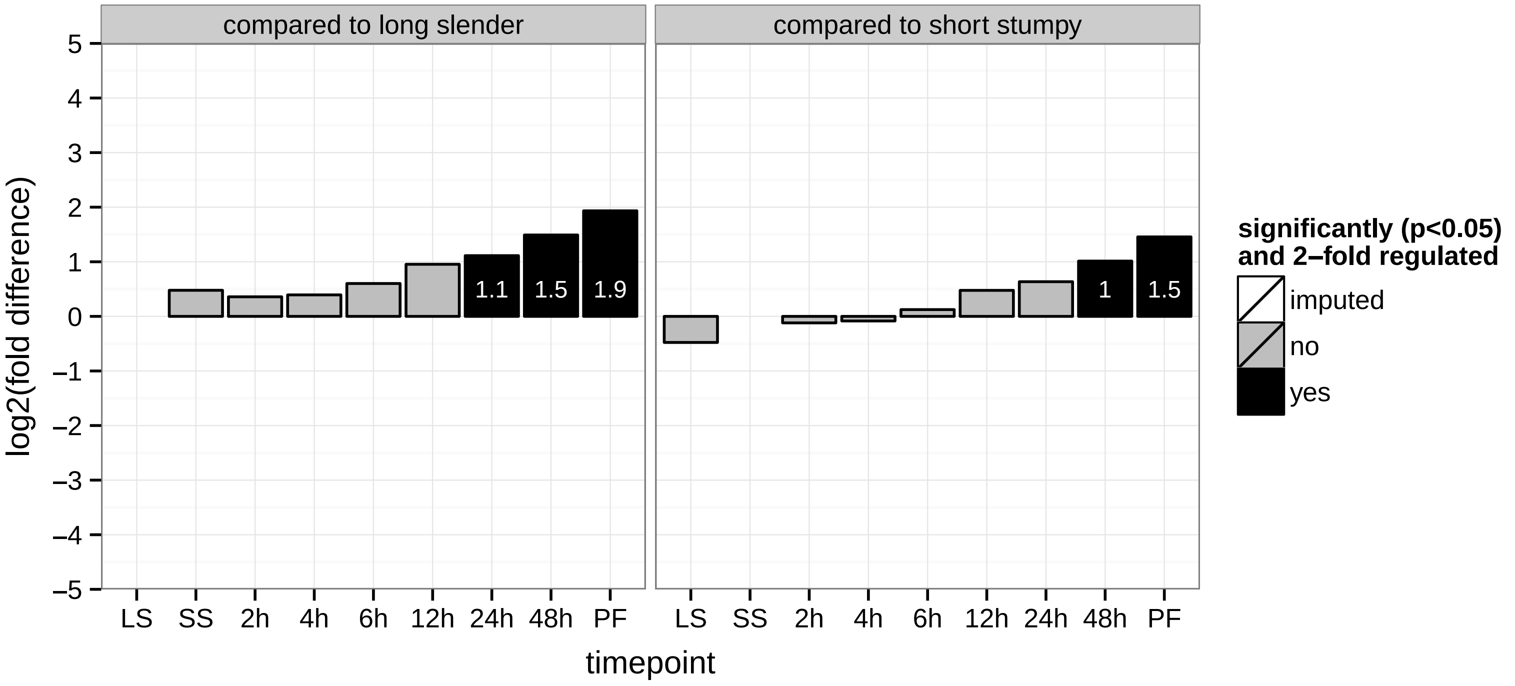

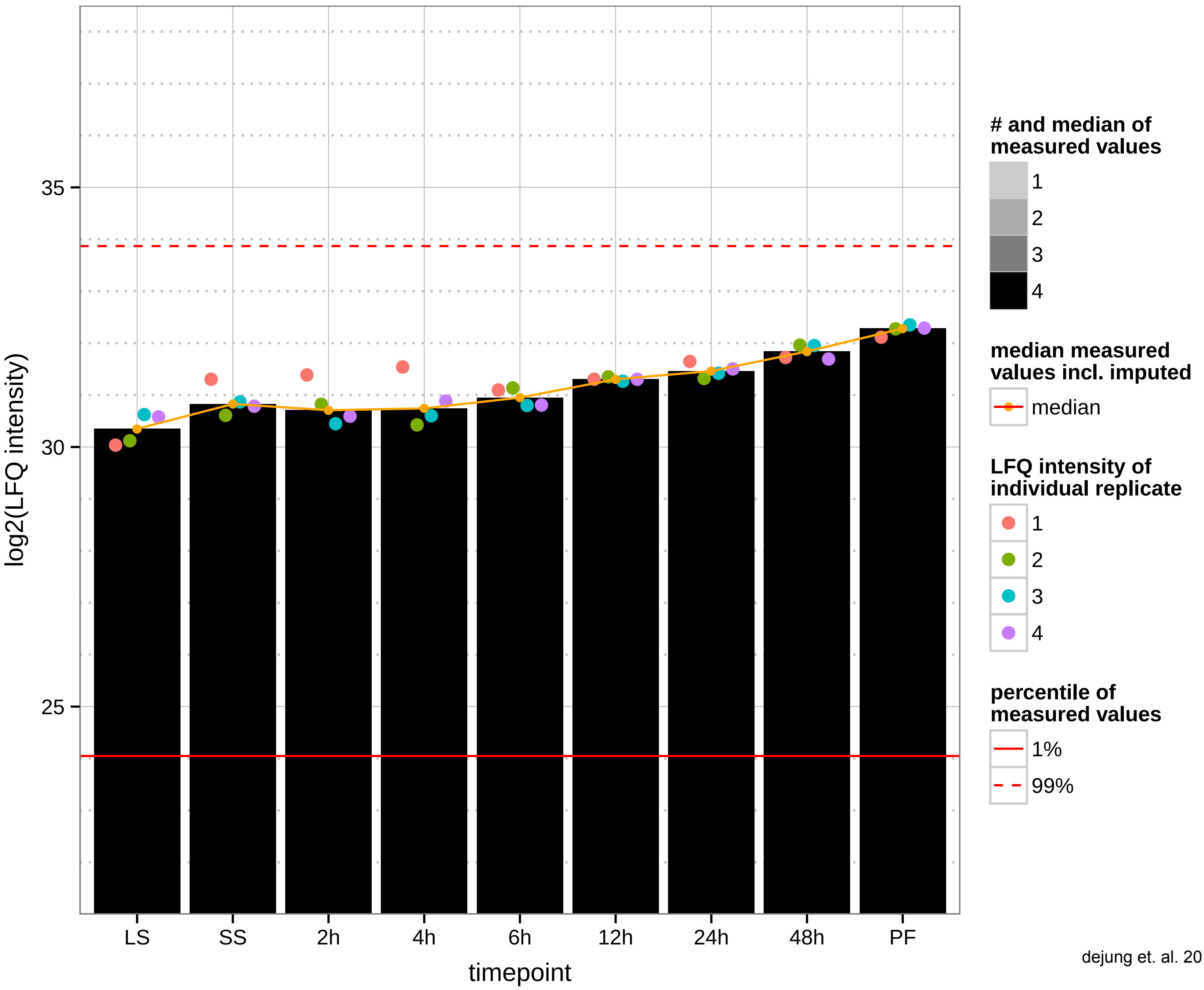

dejung et. al. 2015

hypothetical protein, conserved
Tb927.6.4750
AGOF: null
AGOC: null
AGOP: null
PGOF: protein binding
PGOC: null
PGOP: null

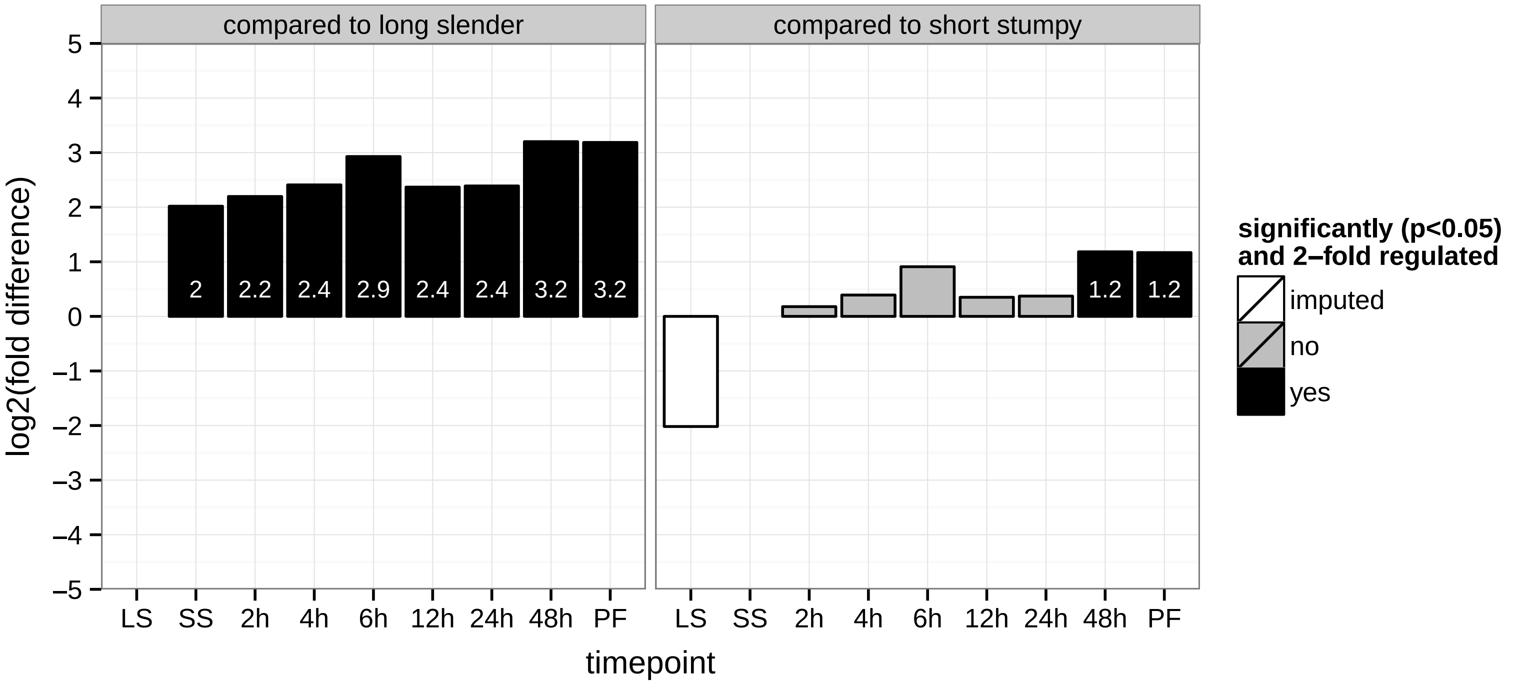

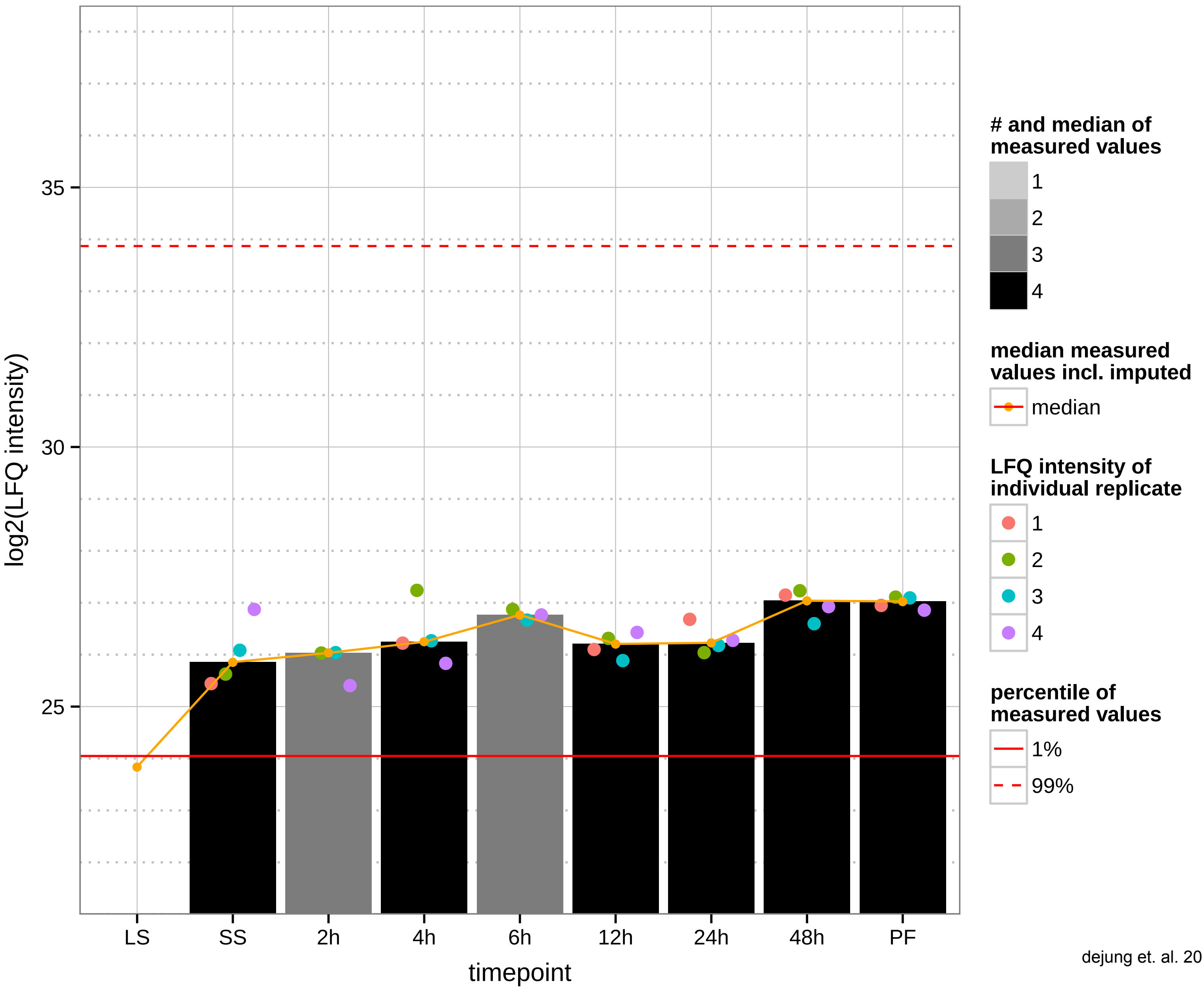

dejung et. al. 2015

N–acetyltransferase, putative
Tb927.7.2360
AGOF: N–acetyltransferase activity
AGOC: null
AGOP: metabolic process
PGOF: N–acetyltransferase activity
PGOC: null
PGOP: null

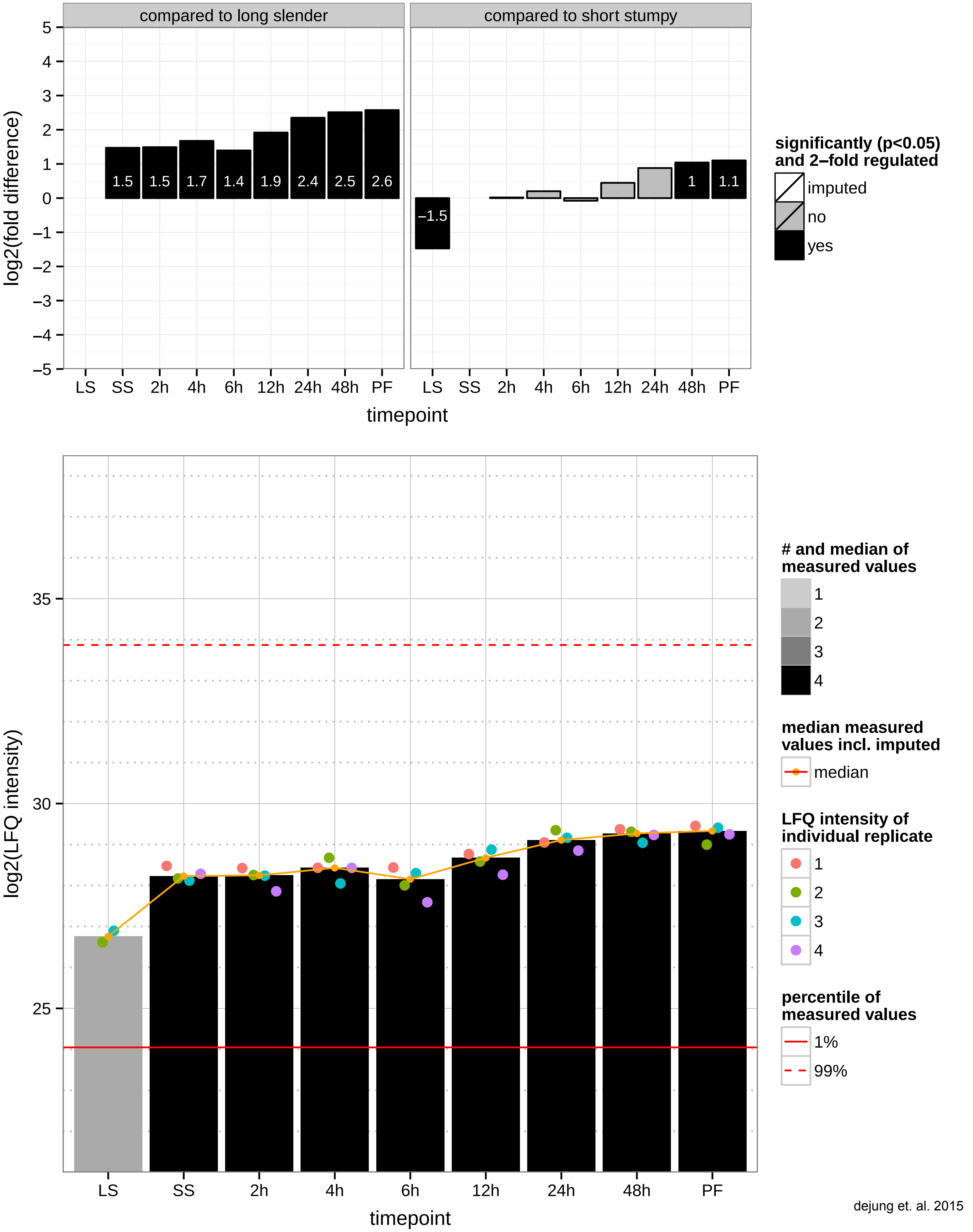

dejung et. al. 2015

hypothetical protein, conserved
Tb927.7.2410
AGOF: GTP binding
AGOC: intracellular, mitochondrion
AGOP: null
PGOF: GTP binding, GTPase activity
PGOC: null
PGOP: null

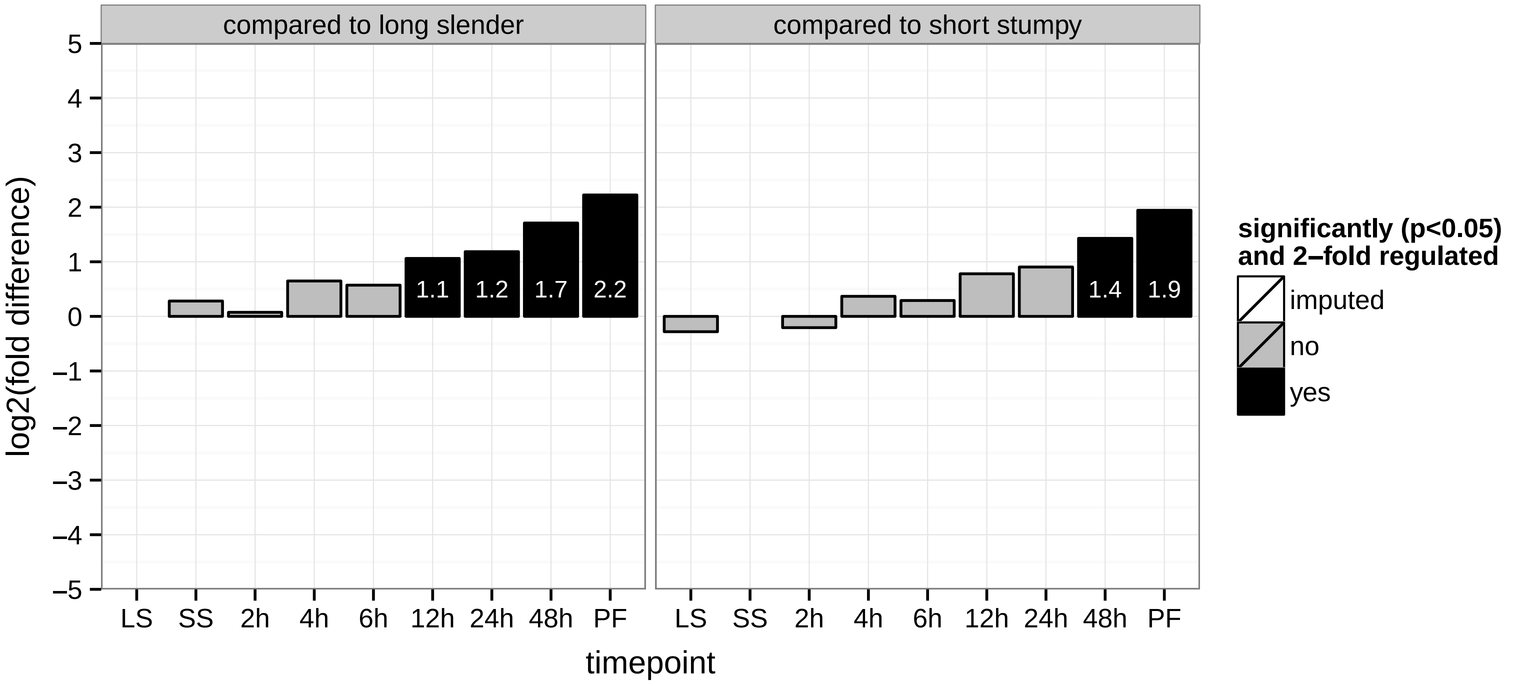

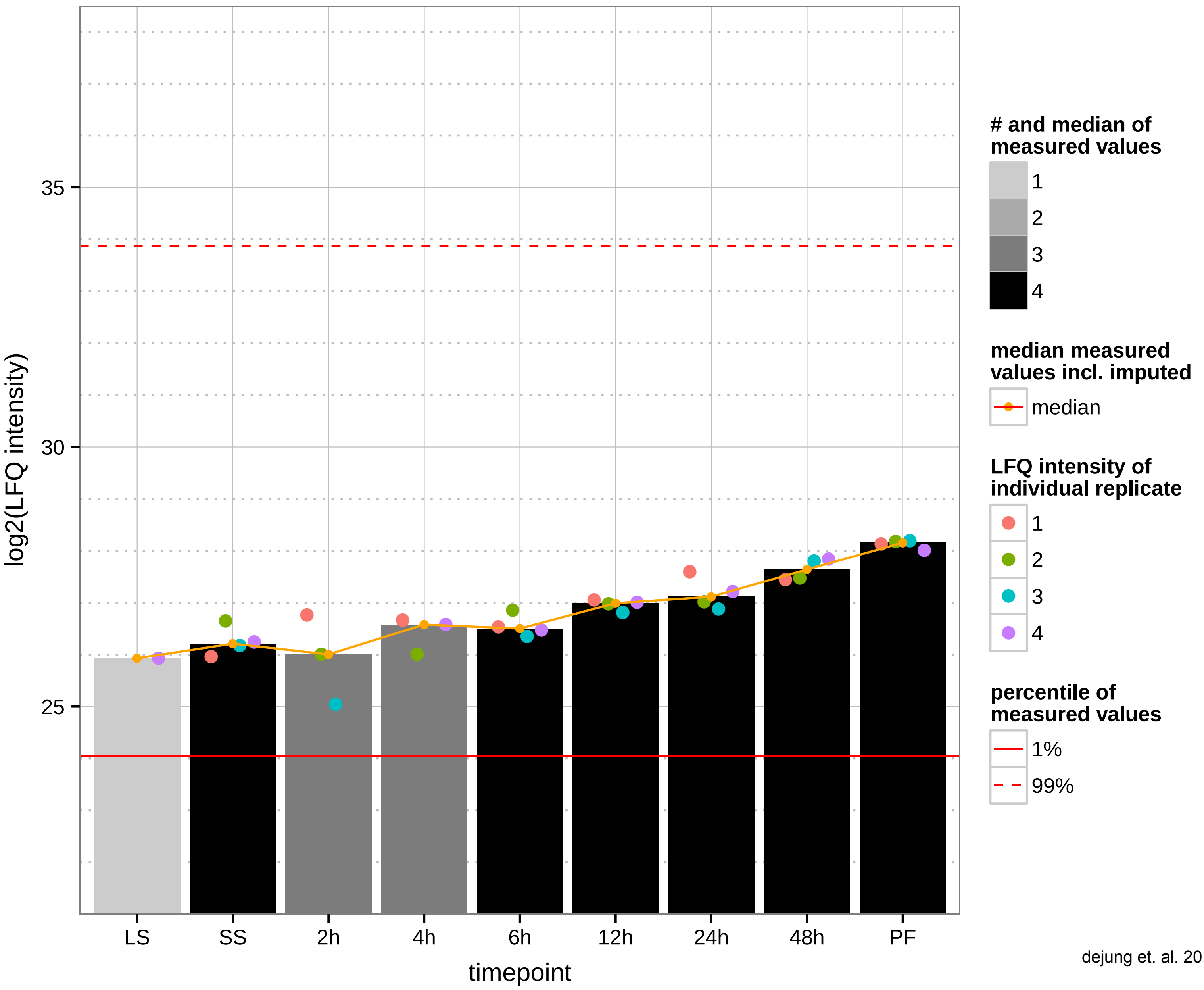

dejung et. al. 2015

hypothetical protein, conserved
Tb927.7.3030;Tb927.7.2990
AGOF: null
AGOC: mitochondrion, null
AGOP: null
PGOF: null
PGOC: null
PGOP: null

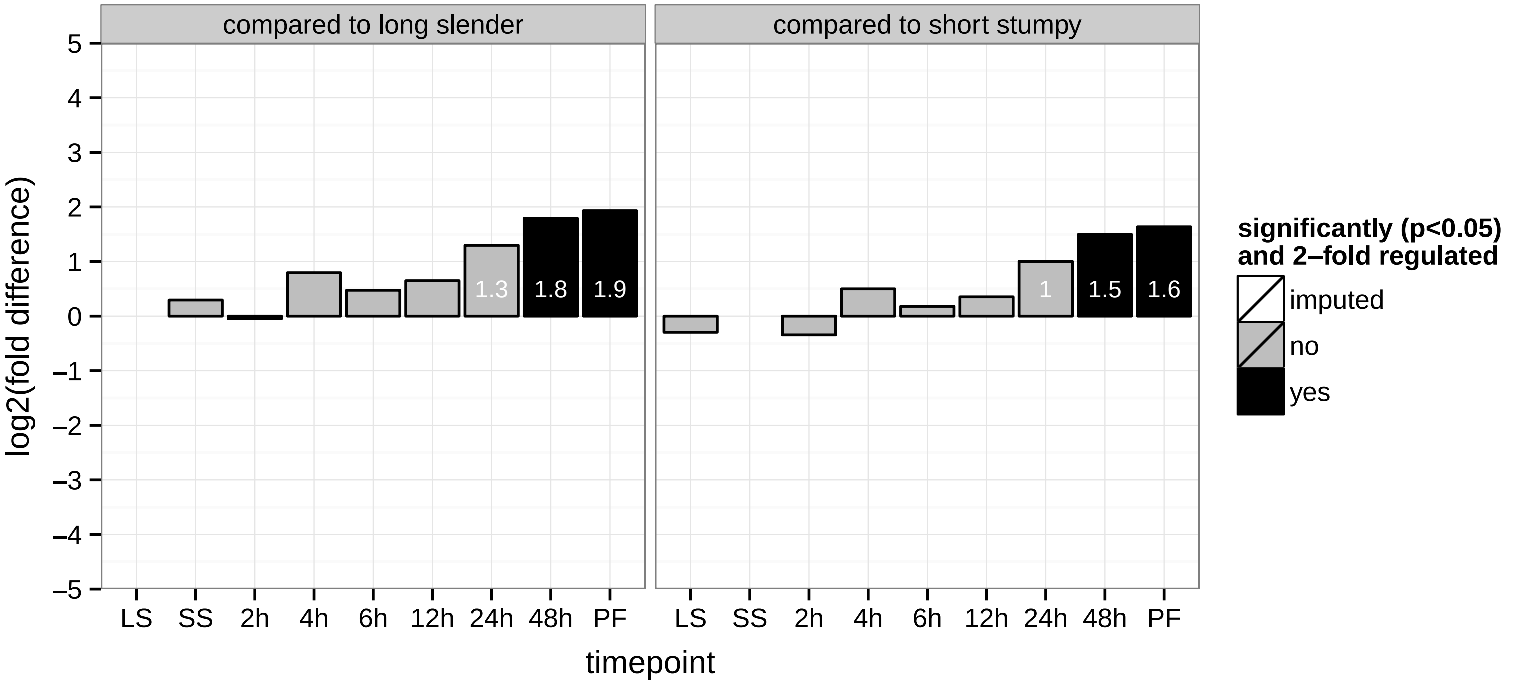

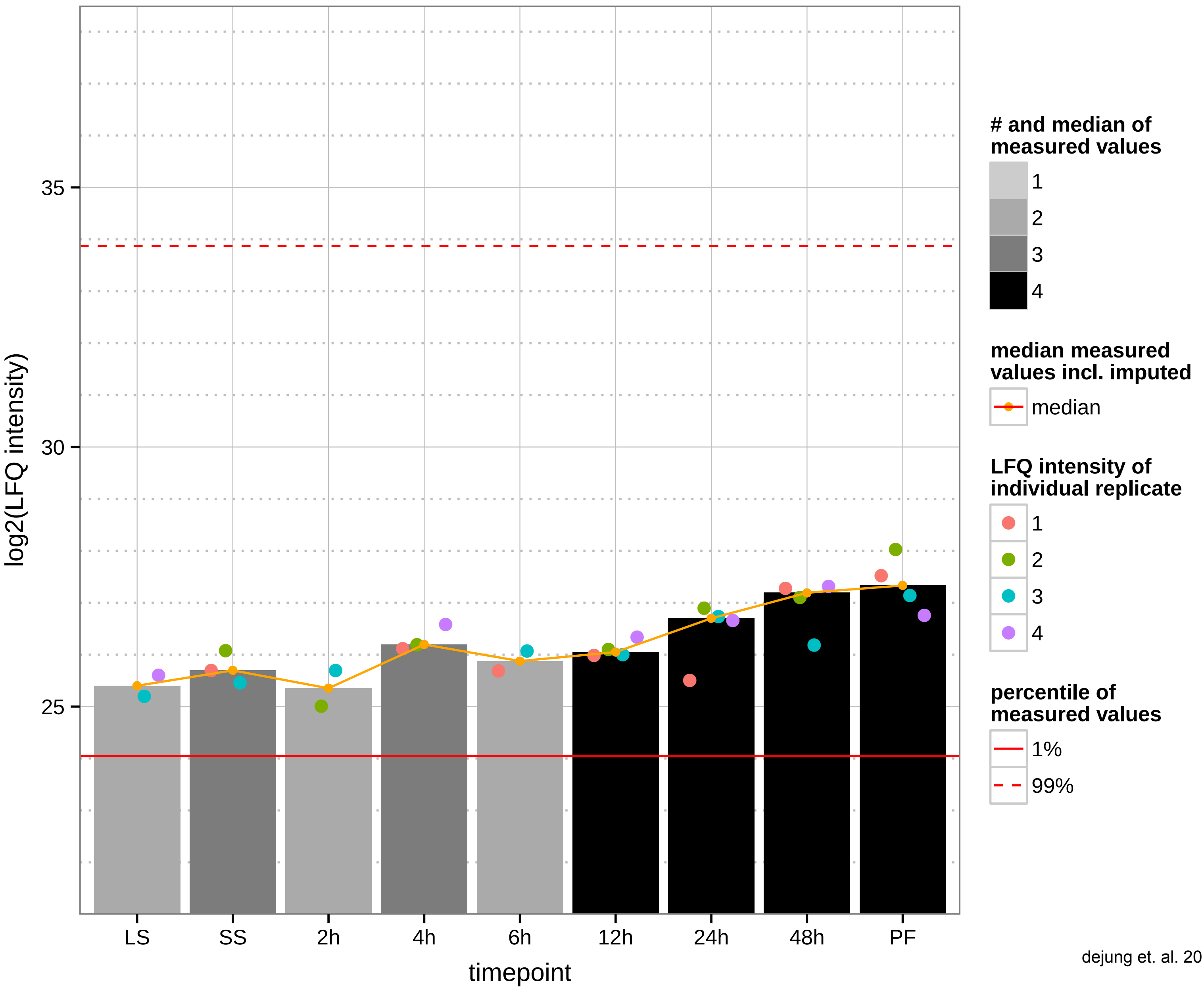

dejung et. al. 2015

hypothetical protein, conserved
Tb927.7.4880
AGOF: null
AGOC: null
AGOP: null
PGOF: null
PGOC: null
PGOP: null

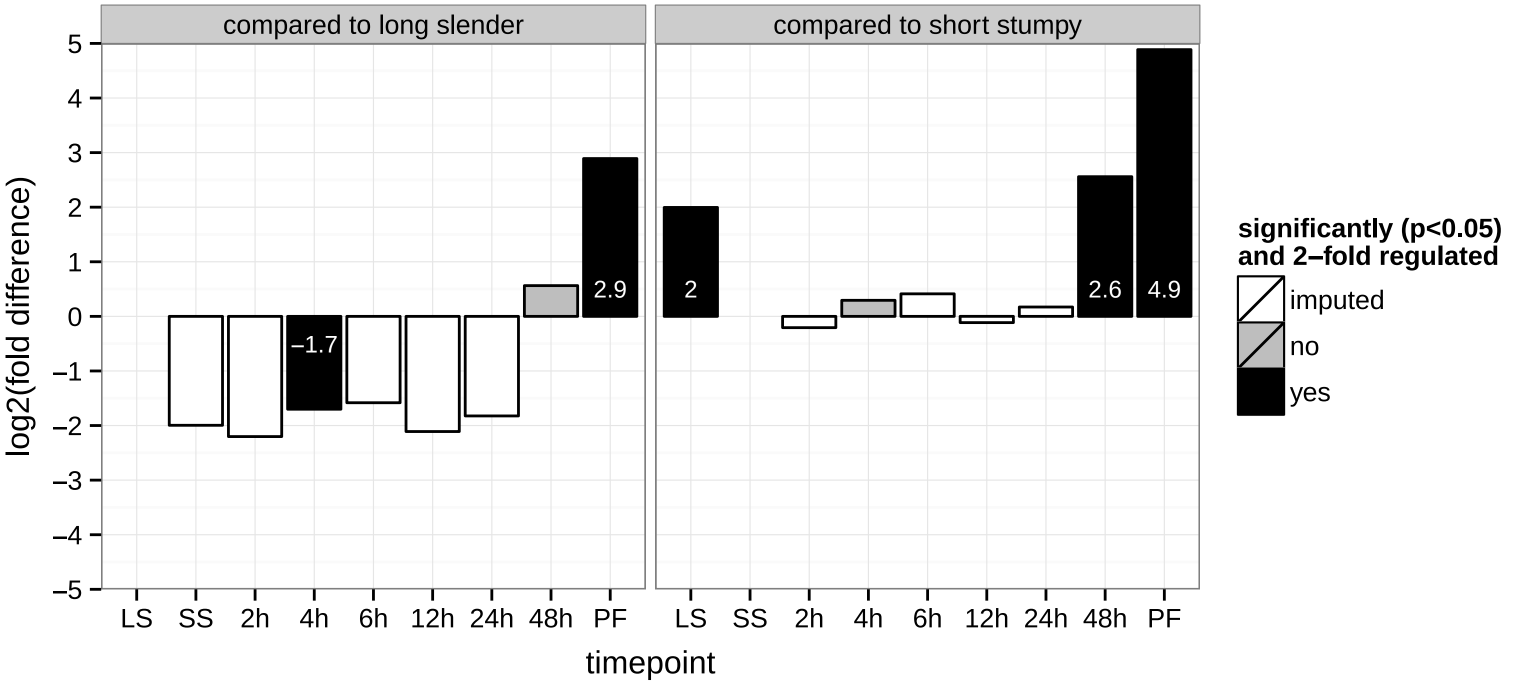

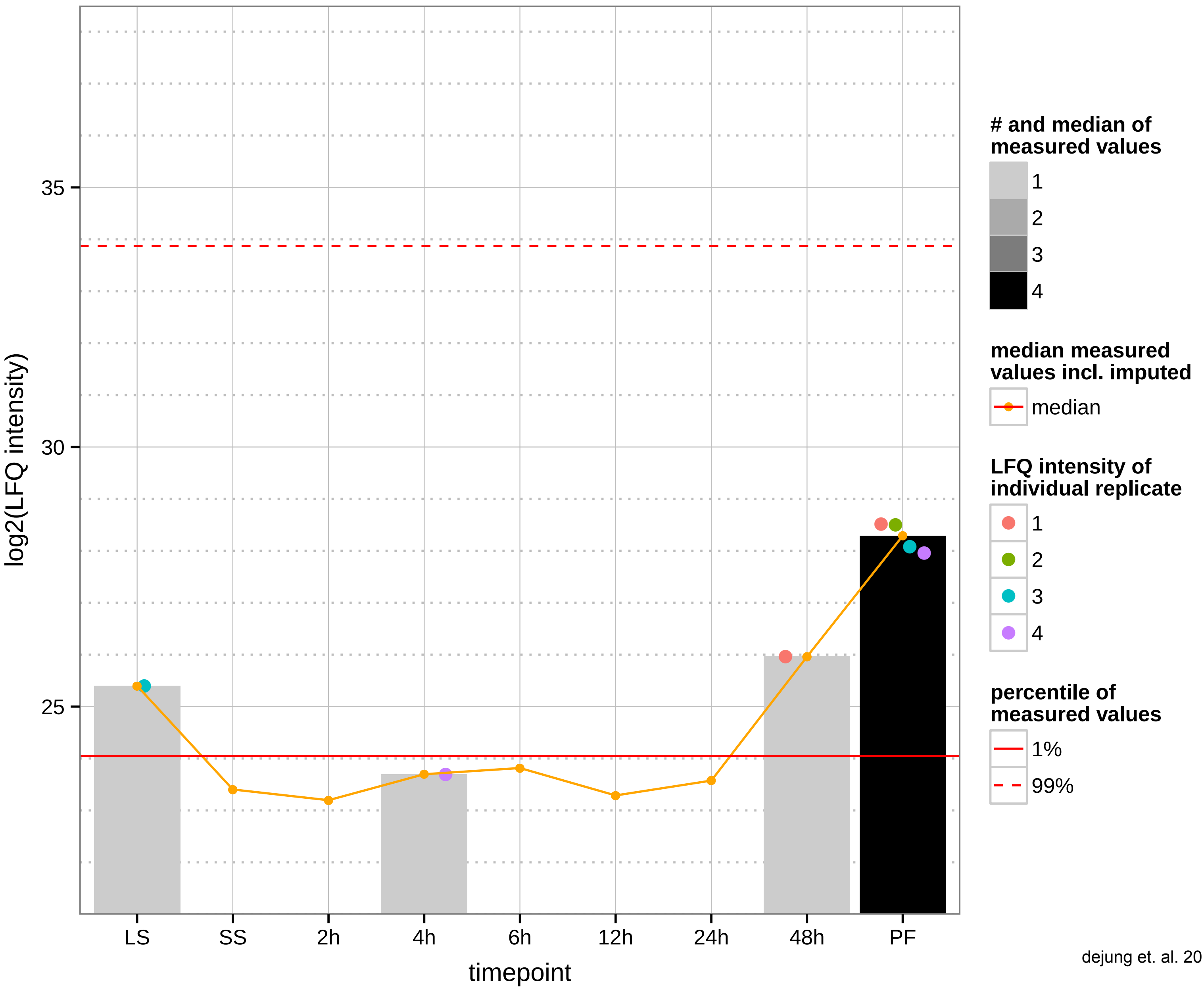

dejung et. al. 2015

60S ribosomal protein L23a, 60S ribosomal protein L25, putative
Tb927.7.5180;Tb927.7.5170
AGOF: RNA binding, structural constituent of ribosome
AGOC: intracellular, ribosome
AGOP: ribosome biogenesis, translation
PGOF: structural constituent of ribosome, null
PGOC: intracellular, ribosome, null
PGOP: translation, null

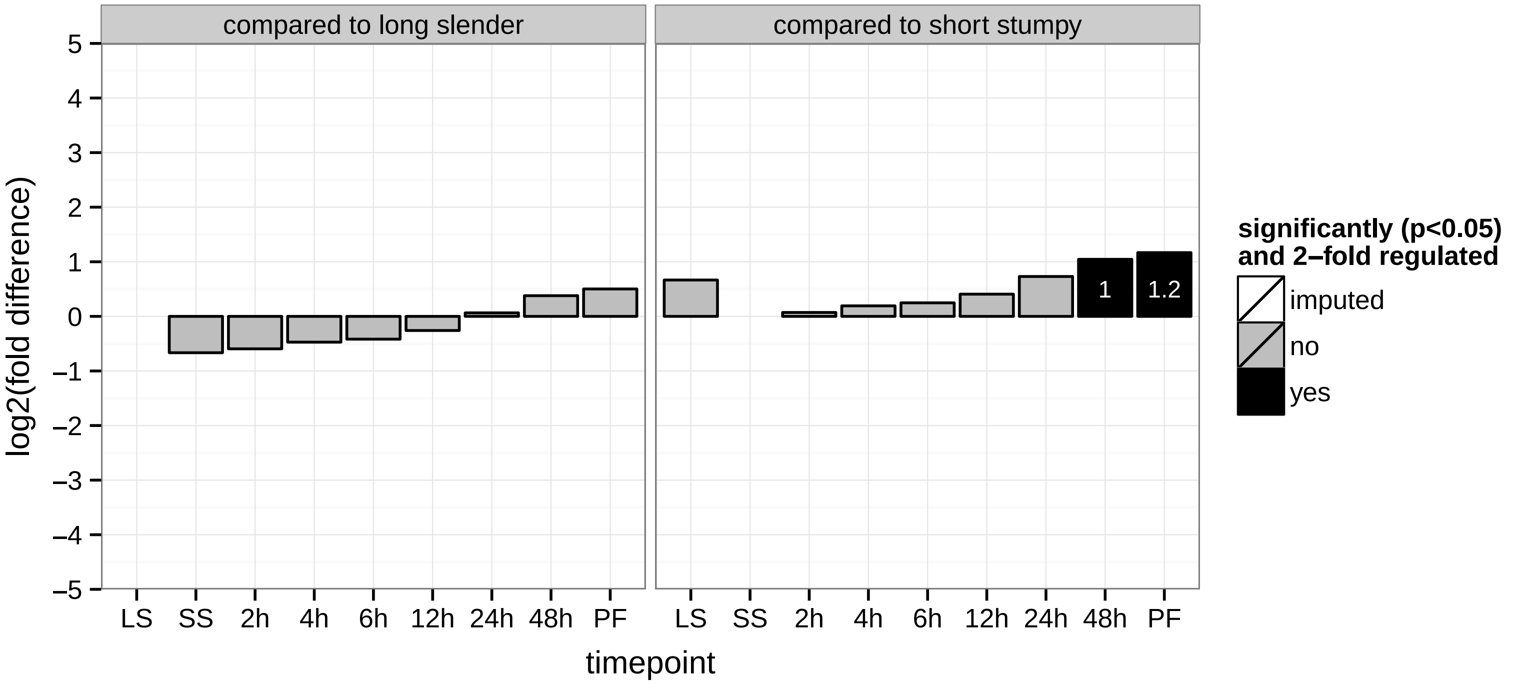

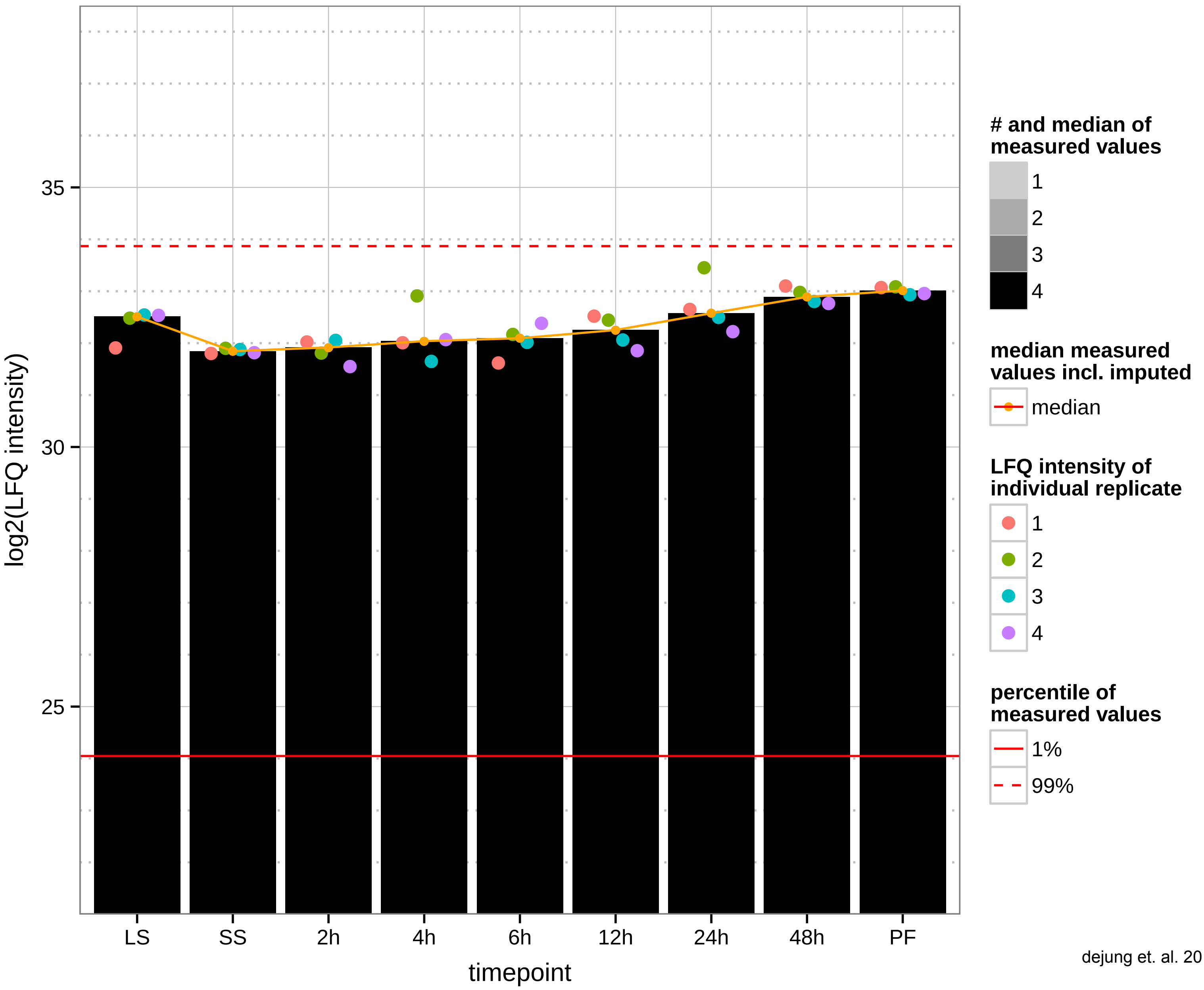

dejung et. al. 2015

hypothetical protein, conserved
Tb927.7.5210
AGOF: phosphatase activity
AGOC: null
AGOP: null
PGOF: phosphatase activity
PGOC: null
PGOP: null

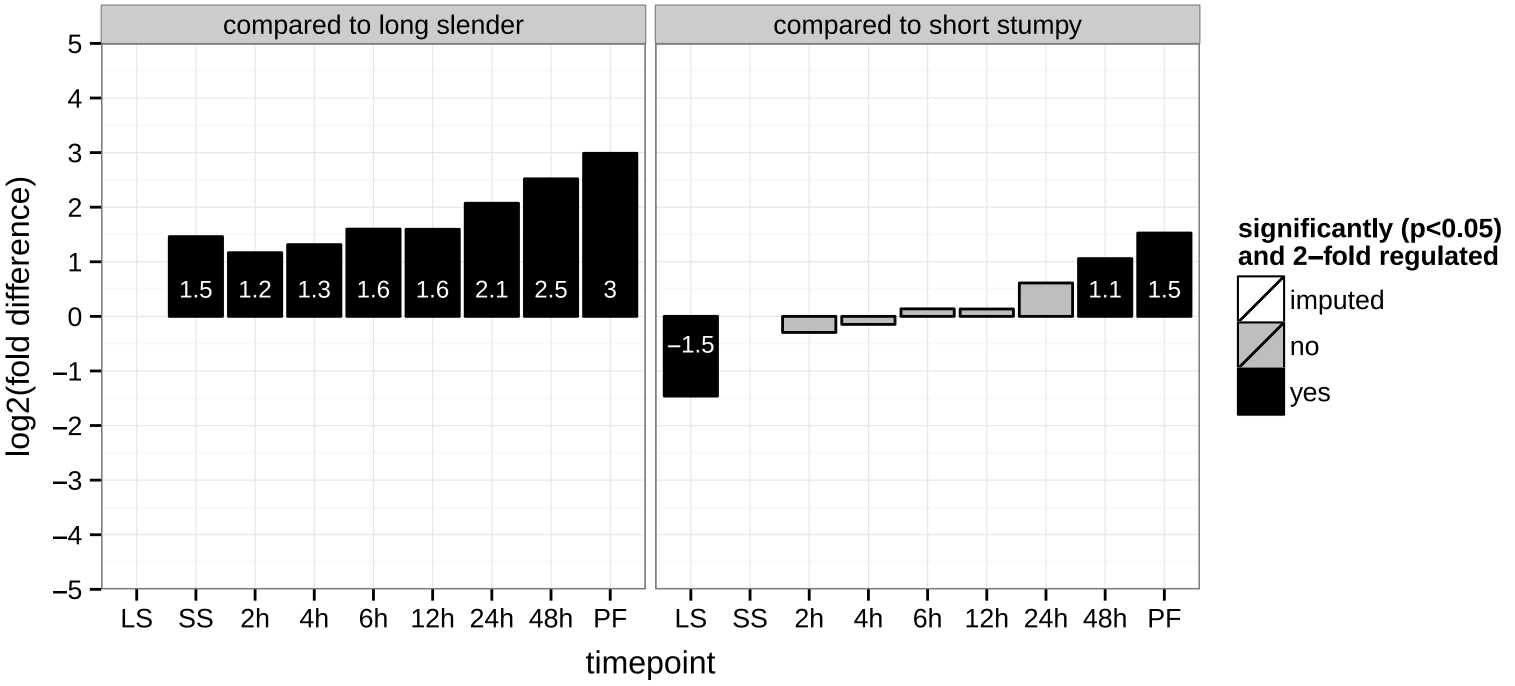

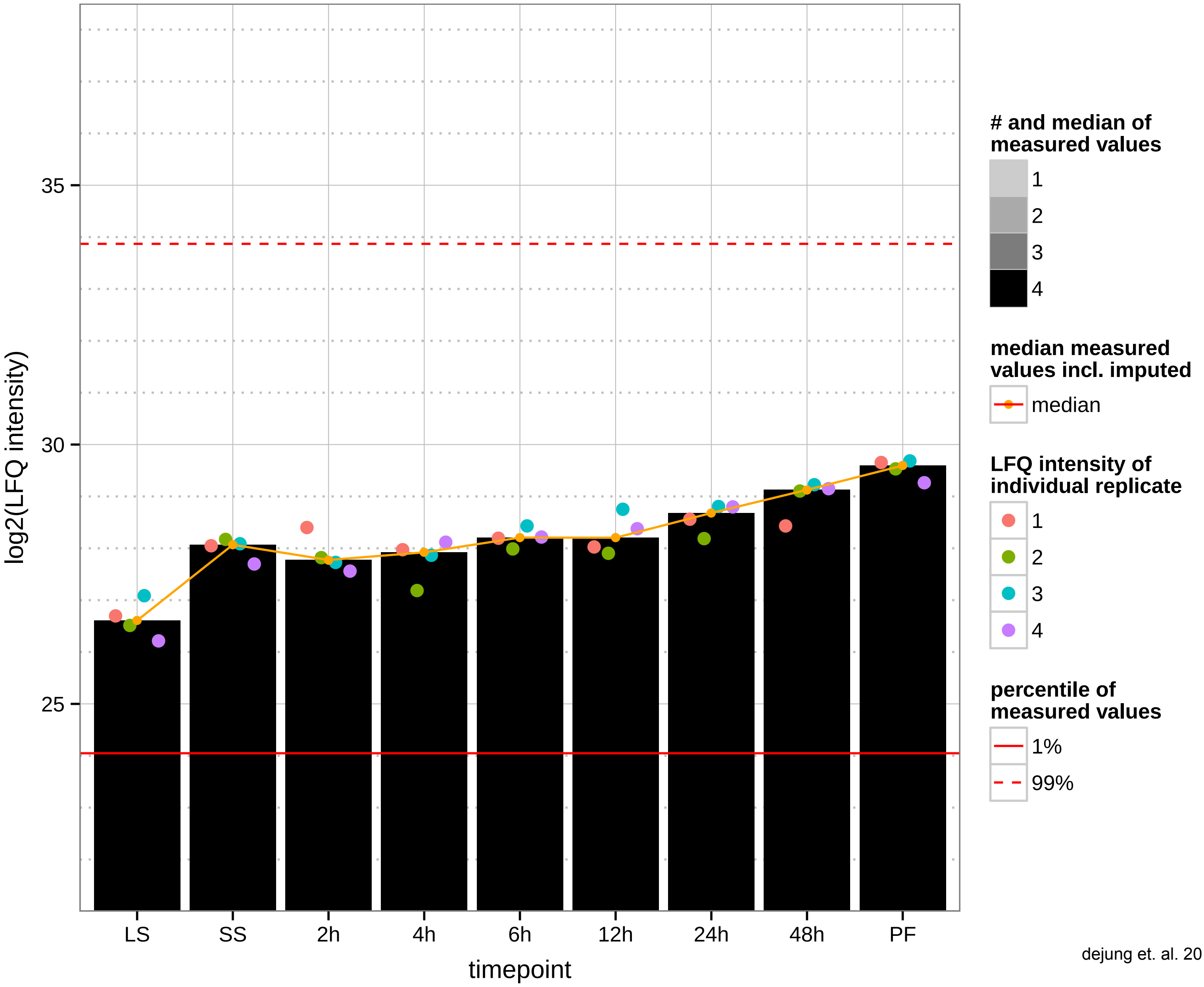

dejung et. al. 2015

dihydrofolate reductase–thymidylate synthase (DHFR–TS)
Tb927.7.5480
AGOF: dihydrofolate reductase activity, thymidylate synthase activity
AGOC: null
AGOP: dTMP biosynthetic process, glycine biosynthetic process, nucleotide biosynthetic process, one–carbon metabolic proc
PGOF: dihydrofolate reductase activity, thymidylate synthase activity
PGOC: null
PGOP: dTMP biosynthetic process, glycine biosynthetic process, nucleotide biosynthetic process, one–carbon metabolic proc

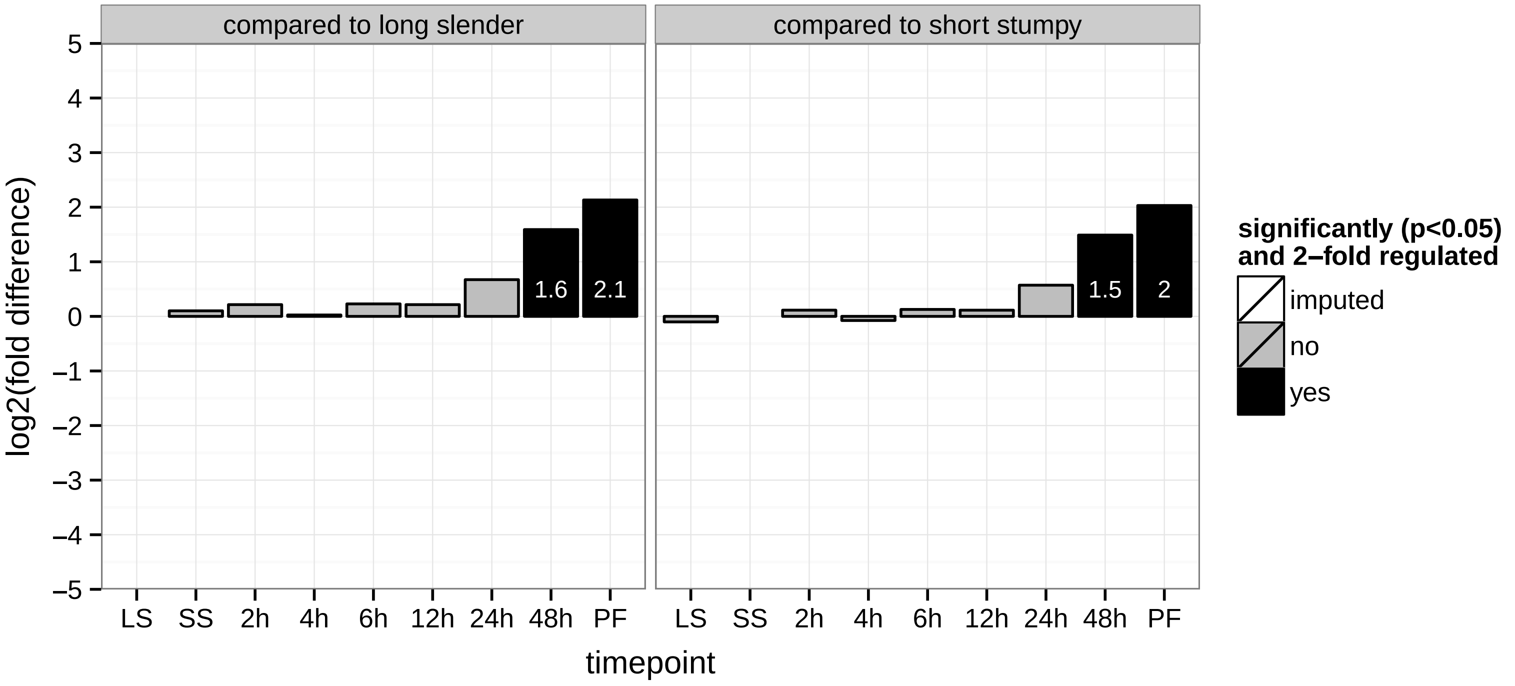

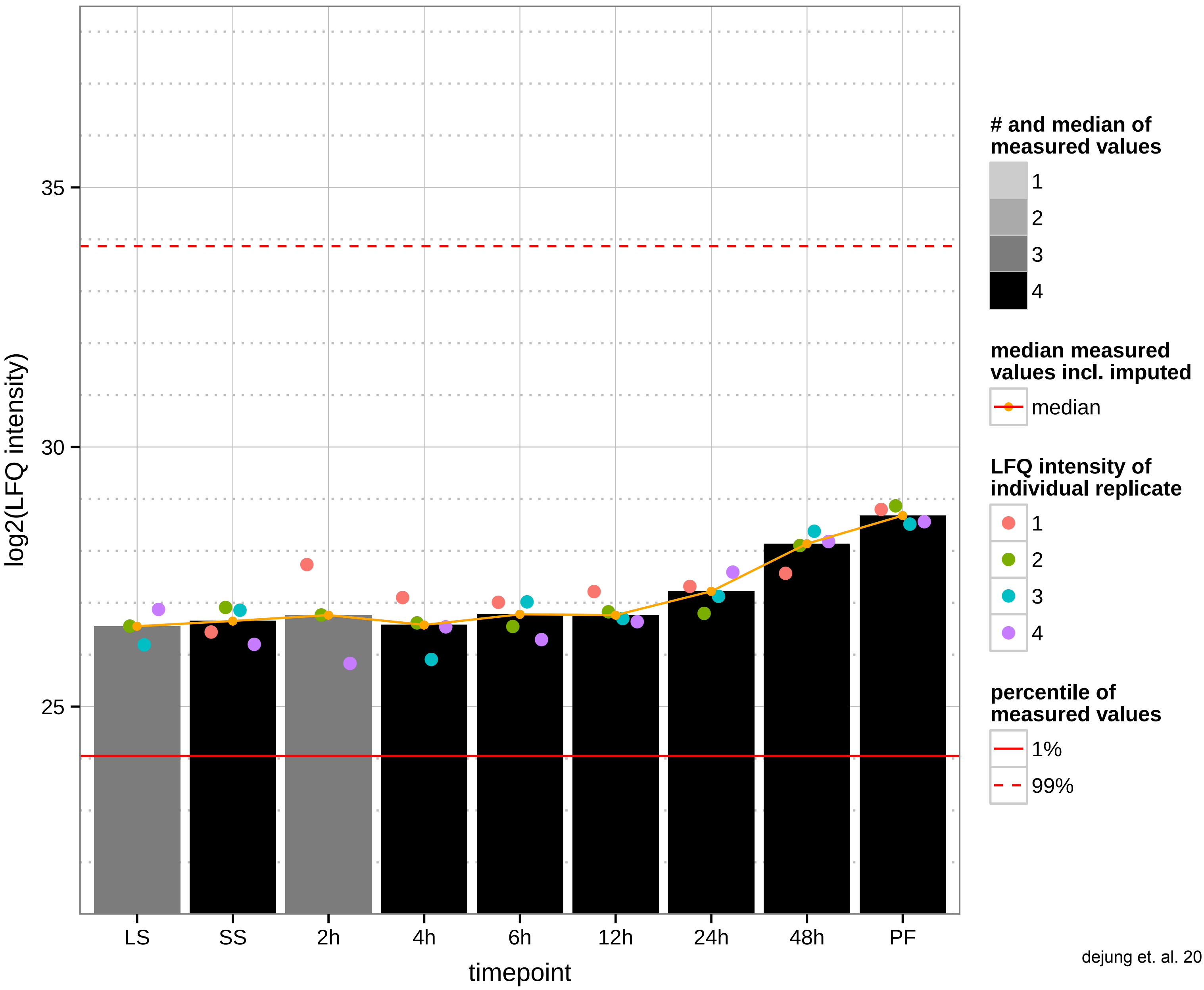

dejung et. al. 2015

hypothetical protein, conserved
Tb927.7.5670
AGOF: null
AGOC: null
AGOP: null
PGOF: oxidoreductase activity, oxidoreductase activity, acting on paired donors, with incorporation or reduction of molecular o
PGOC: null
PGOP: oxidation–reduction process

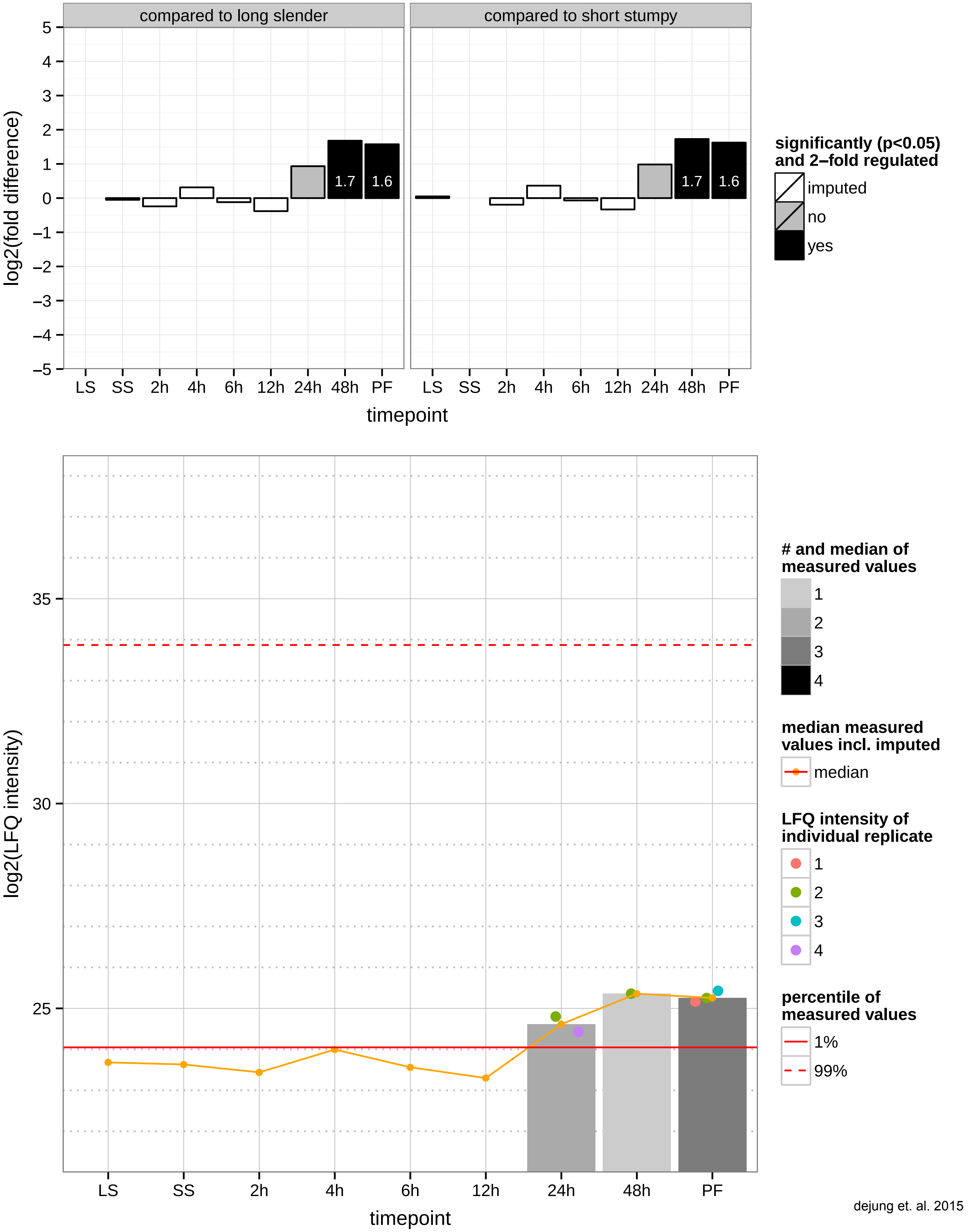

dejung et. al. 2015

hypothetical protein, conserved
Tb927.7.6620
AGOF: null
AGOC: null
AGOP: null
PGOF: null
PGOC: null
PGOP: null

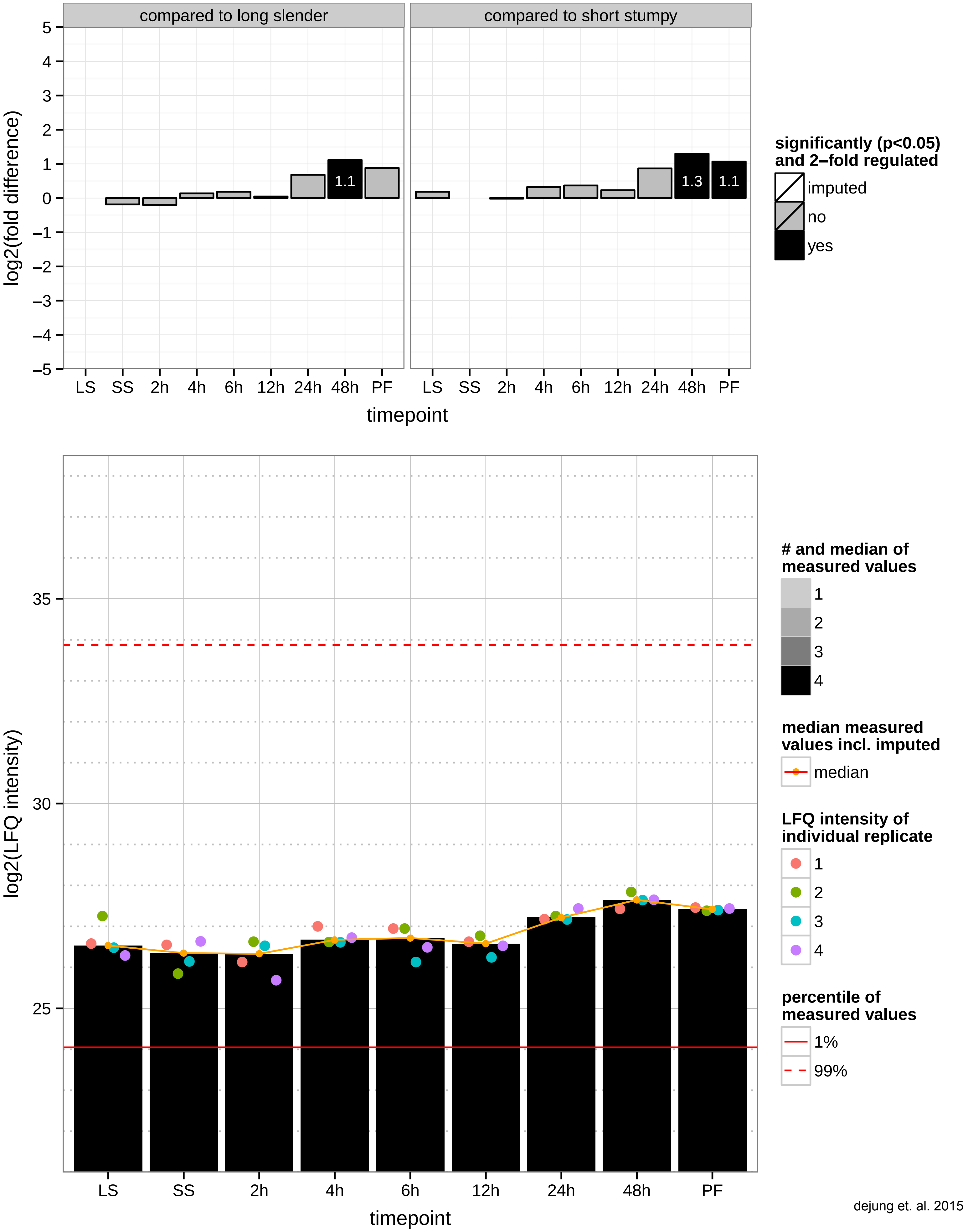

dejung et. al. 2015

hypothetical protein, conserved
Tb927.7.7050
AGOF: null
AGOC: null
AGOP: null
PGOF: null
PGOC: null
PGOP: null

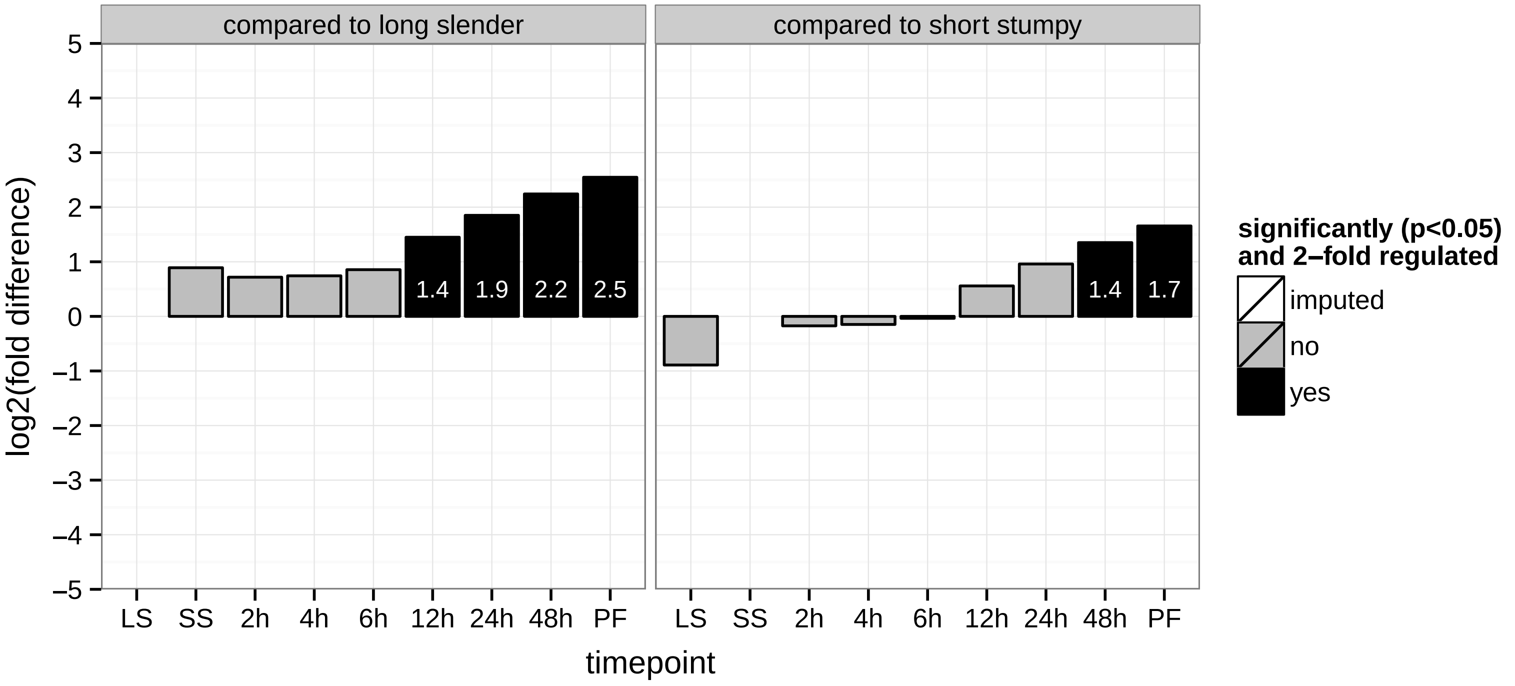

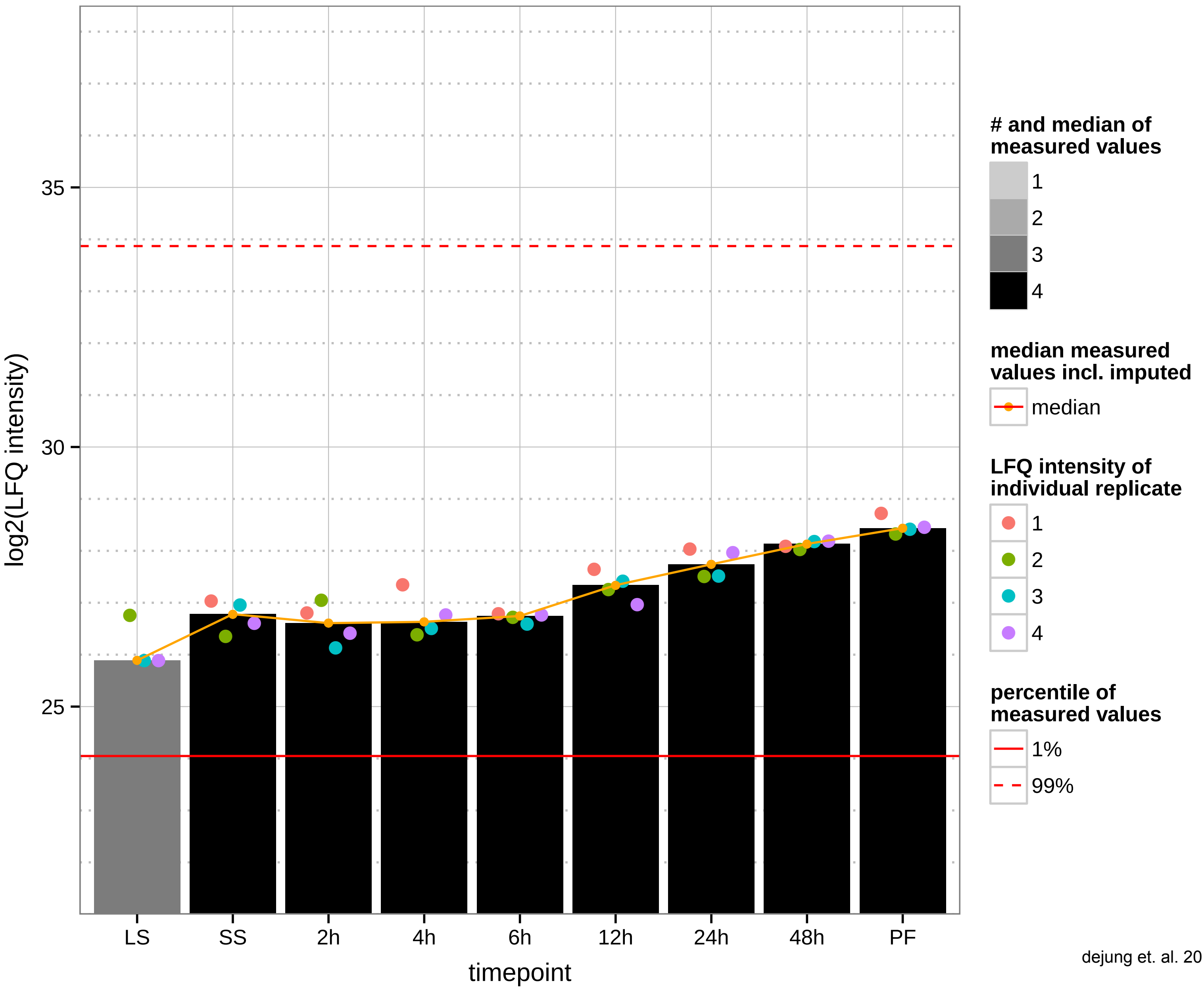

dejung et. al. 2015

60S ribosomal protein L7a, putative
Tb927.8.1340;Tb927.8.1330
AGOF: structural constituent of ribosome
AGOC: ribosome
AGOP: translation
PGOF: null
PGOC: null
PGOP: null

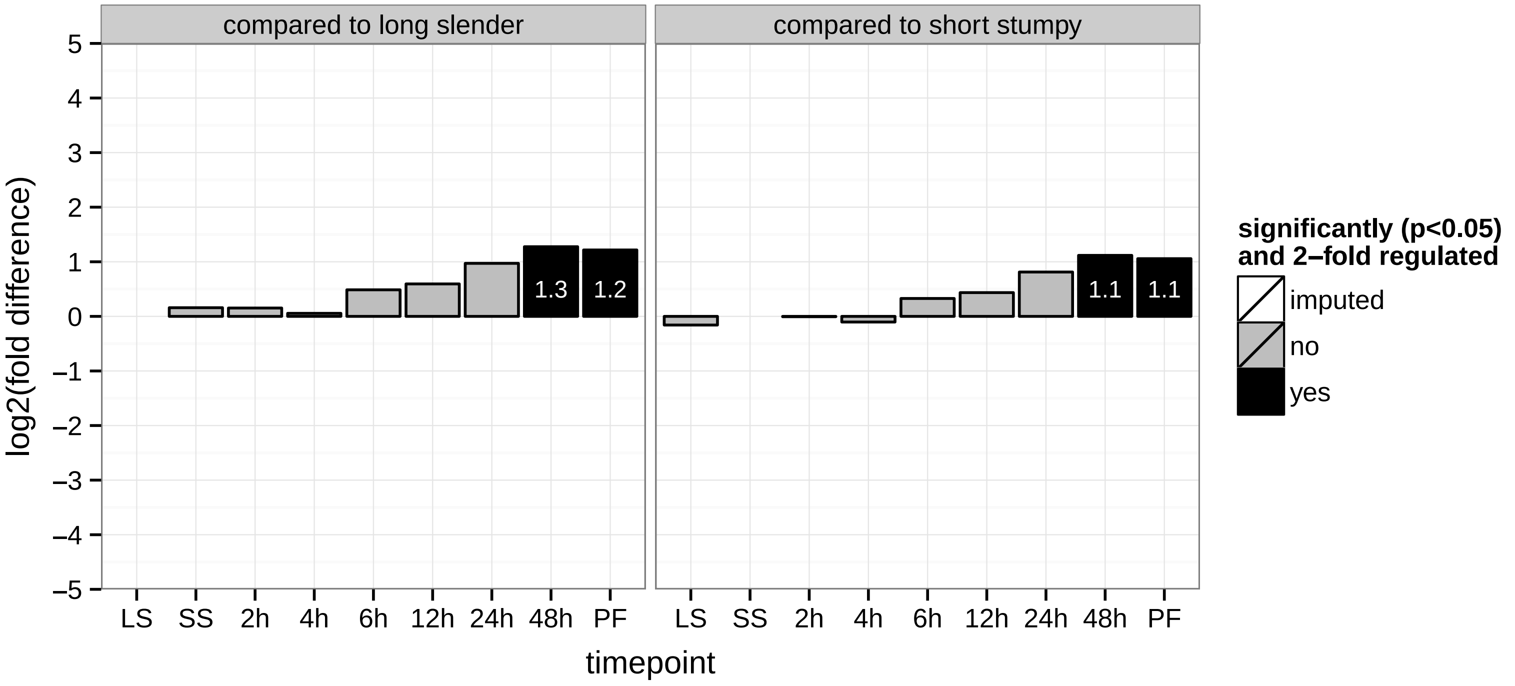

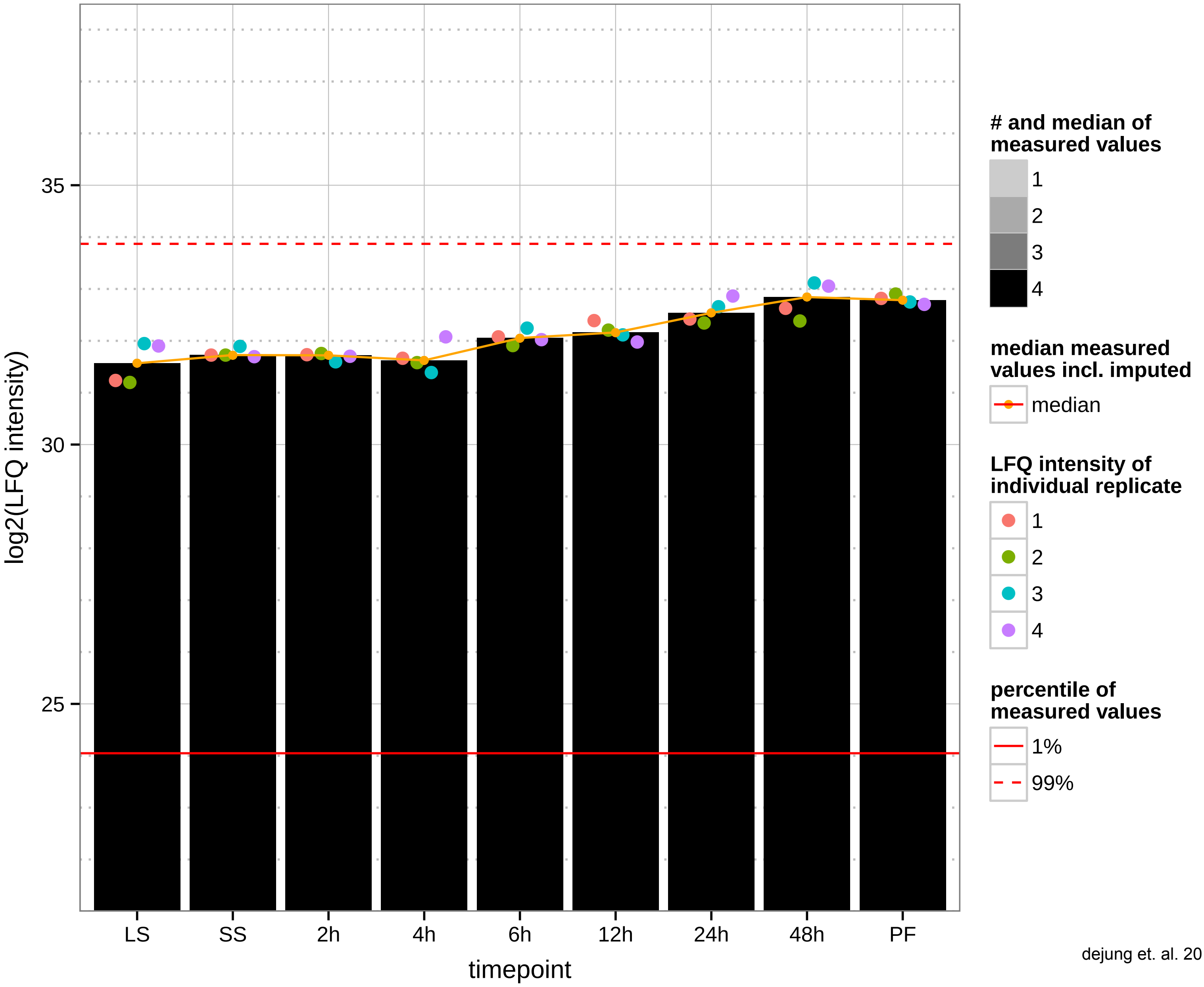

dejung et. al. 2015

ATP−dependent DEAD/H RNA helicase, putative (DBP2.1)
Tb927.8.1510
AGOF: ATP binding, ATP−dependent helicase activity, helicase activity, nucleic acid binding
AGOC: null
AGOP: nucleobase−containing compound metabolic process
PGOF: ATP binding, ATP−dependent helicase activity, helicase activity, nucleic acid binding
PGOC: null
PGOP: null

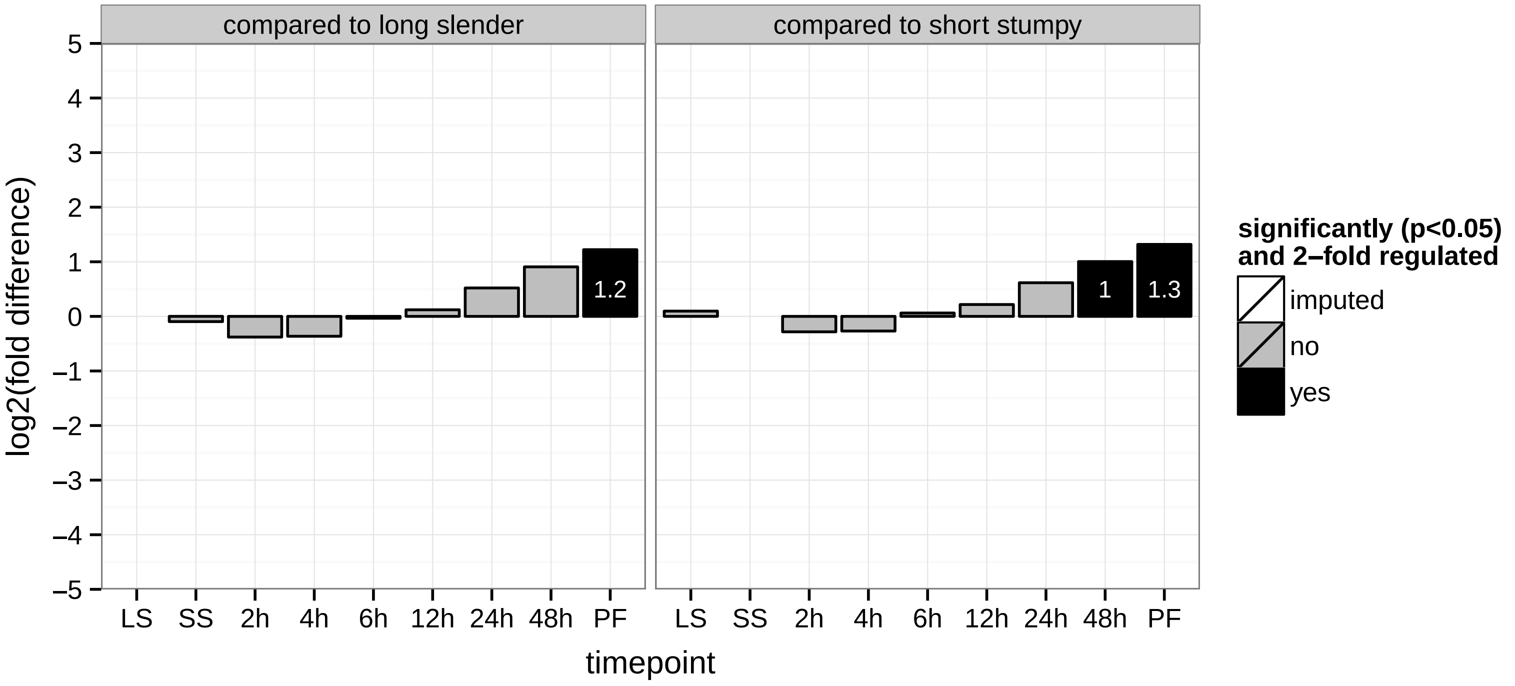

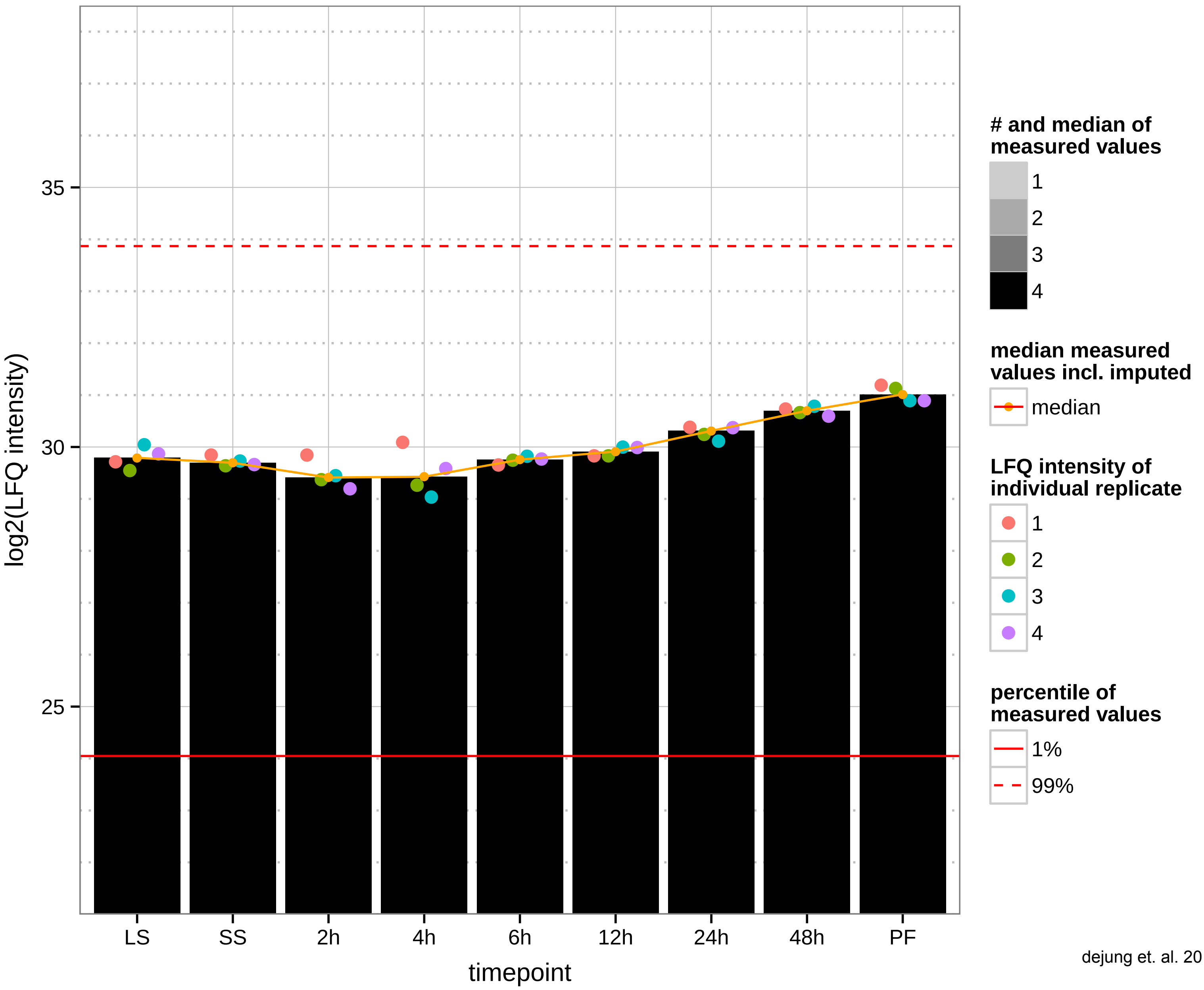

dejung et. al. 2015

hypothetical protein, conserved
Tb927.8.1570
AGOF: null
AGOC: null
AGOP: null
PGOF: null
PGOC: null
PGOP: null

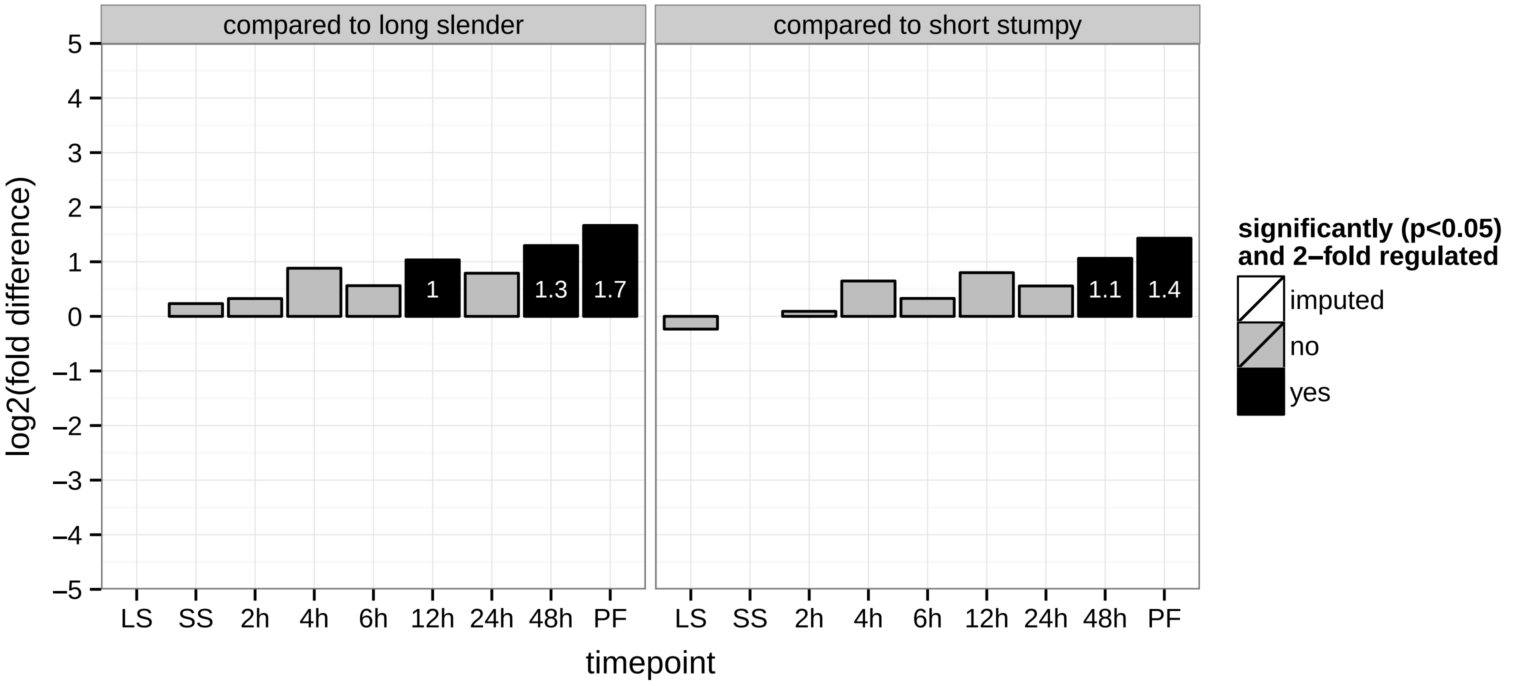

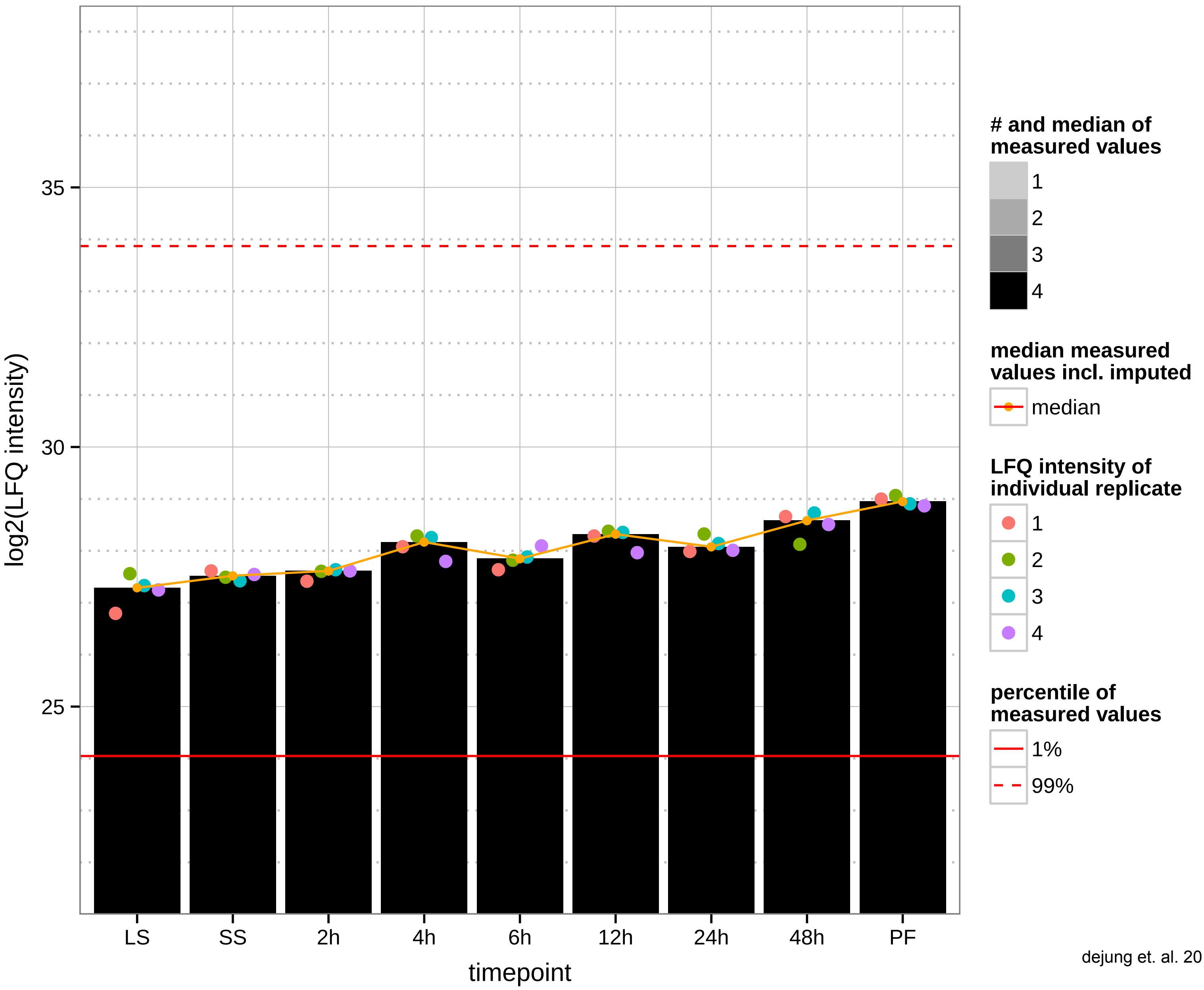

dejung et. al. 2015

electron transfer protein, putative
Tb927.8.3380
AGOF: electron carrier activity, iron–sulfur cluster binding, oxidoreductase activity
AGOC: null
AGOP: oxidation–reduction process, tricarboxylic acid cycle
PGOF: electron carrier activity, iron–sulfur cluster binding, oxidoreductase activity
PGOC: null
PGOP: oxidation–reduction process, tricarboxylic acid cycle

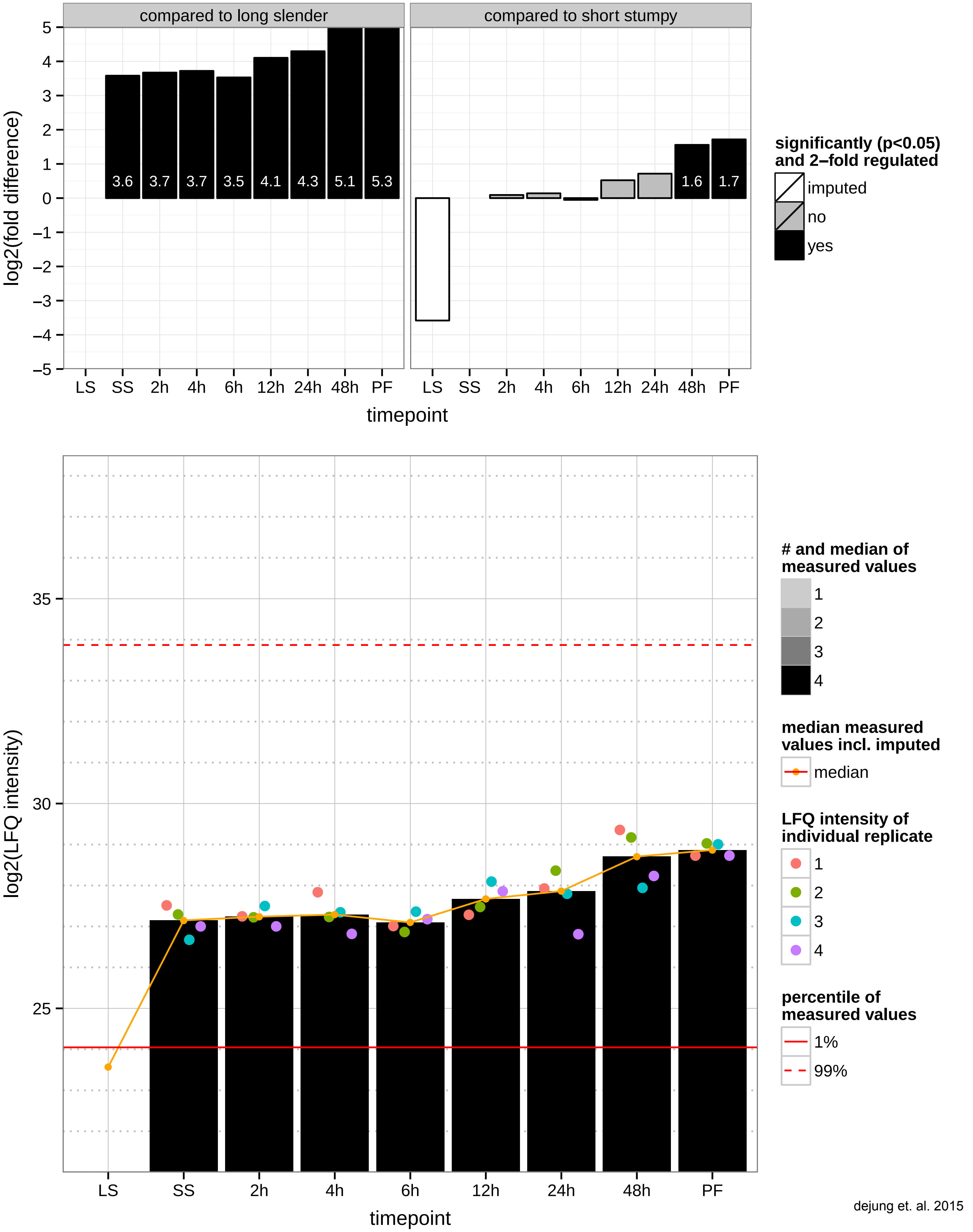

dejung et. al. 2015

hypothetical protein, conserved
Tb927.8.4290
AGOF: null
AGOC: null
AGOP: null
PGOF: null
PGOC: null
PGOP: null

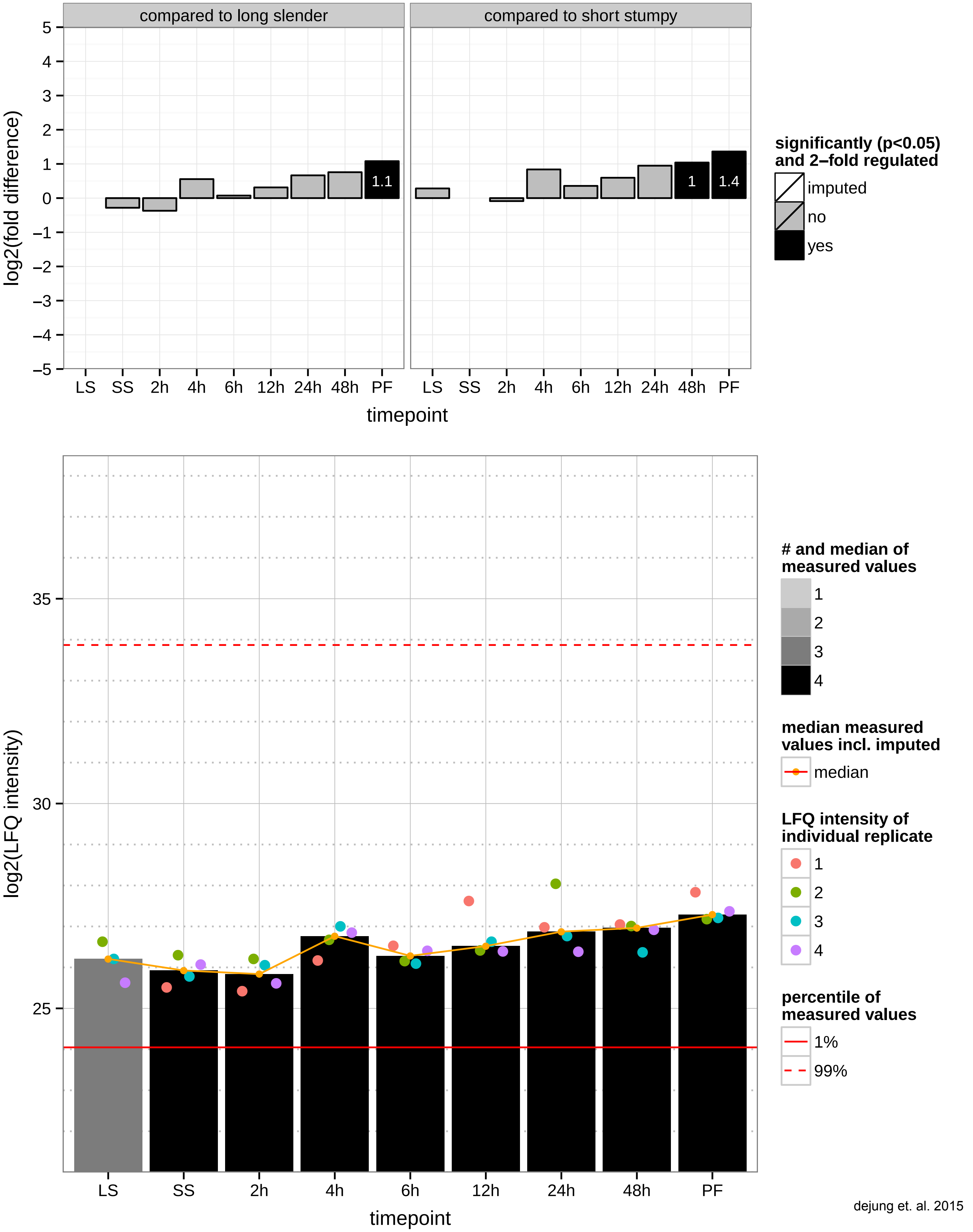

dejung et. al. 2015

RNA–binding protein, putative (RBP11)
Tb927.8.4450
AGOF: RNA binding
AGOC: null
AGOP: RNA processing
PGOF: nucleic acid binding
PGOC: null
PGOP: null

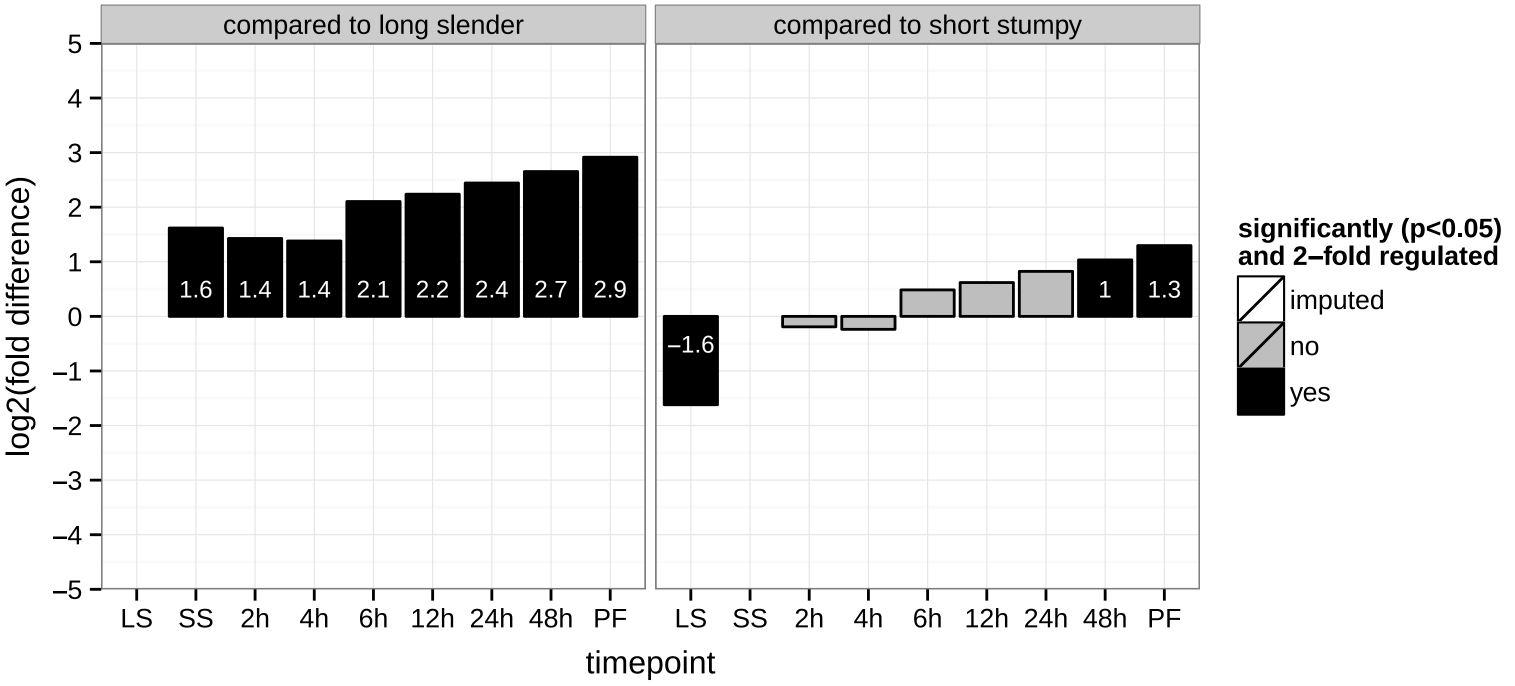

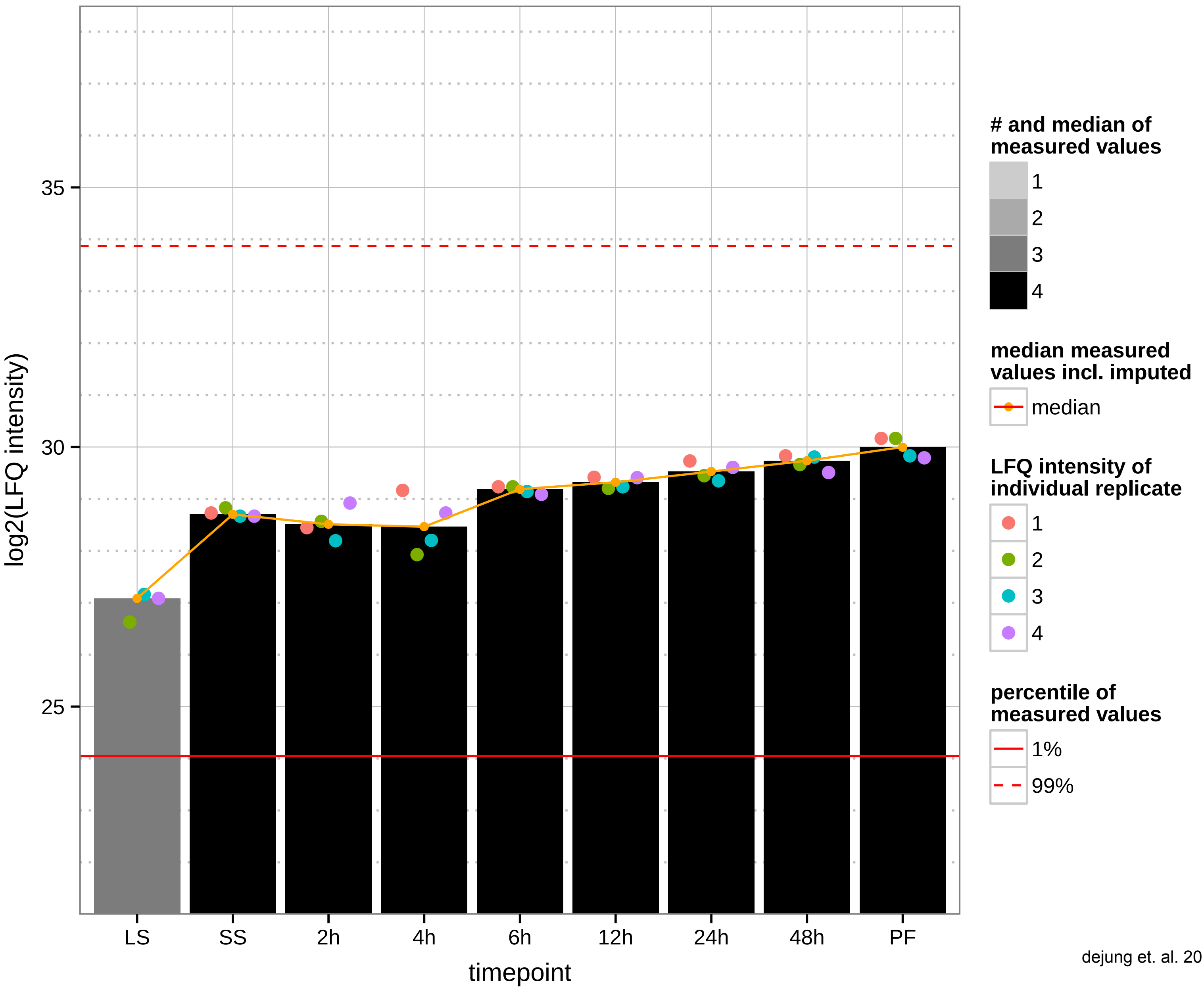

dejung et. al. 2015

phosphatase of regenerating liver–type phosphatase, putative
Tb927.8.5780
AGOF: phosphoprotein phosphatase activity
AGOC: null
AGOP: protein dephosphorylation
PGOF: phosphatase activity, protein tyrosine/serine/threonine phosphatase activity
PGOC: null
PGOP: dephosphorylation, protein dephosphorylation

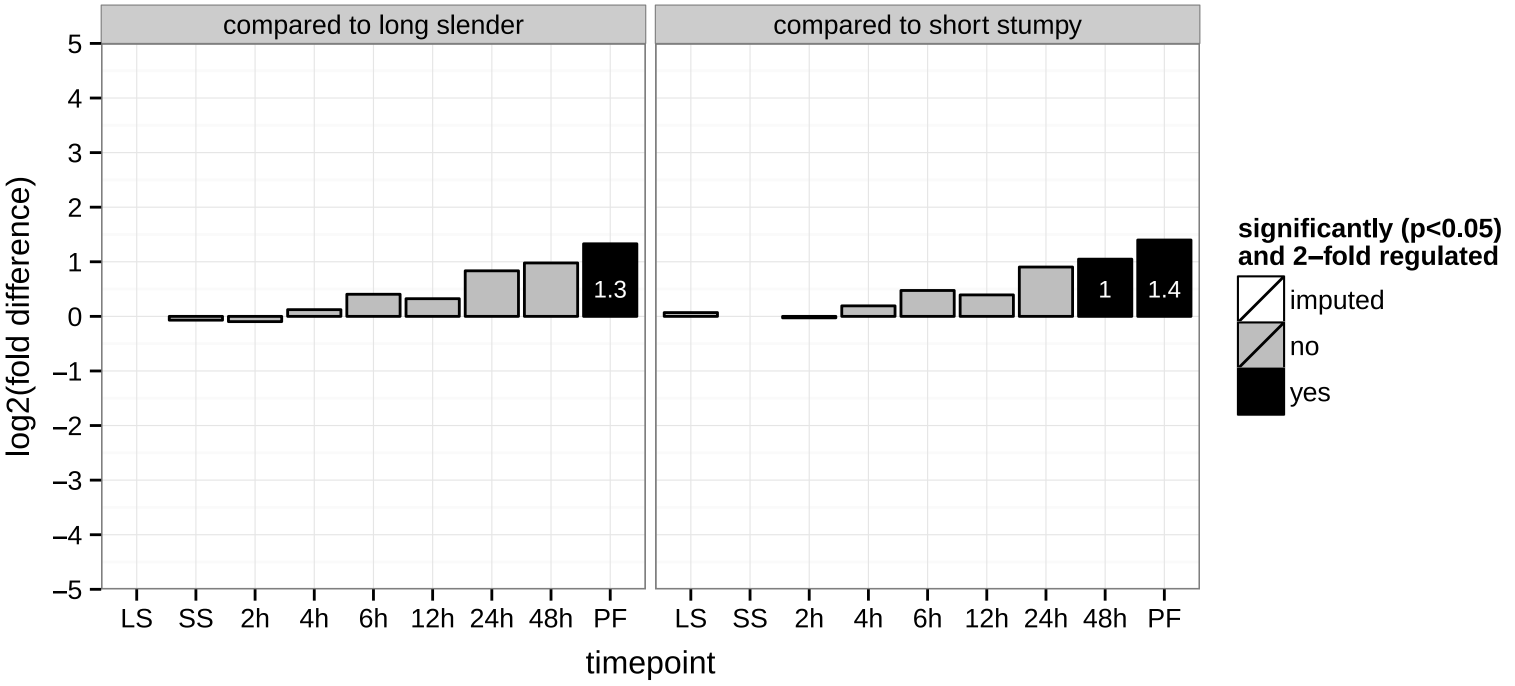

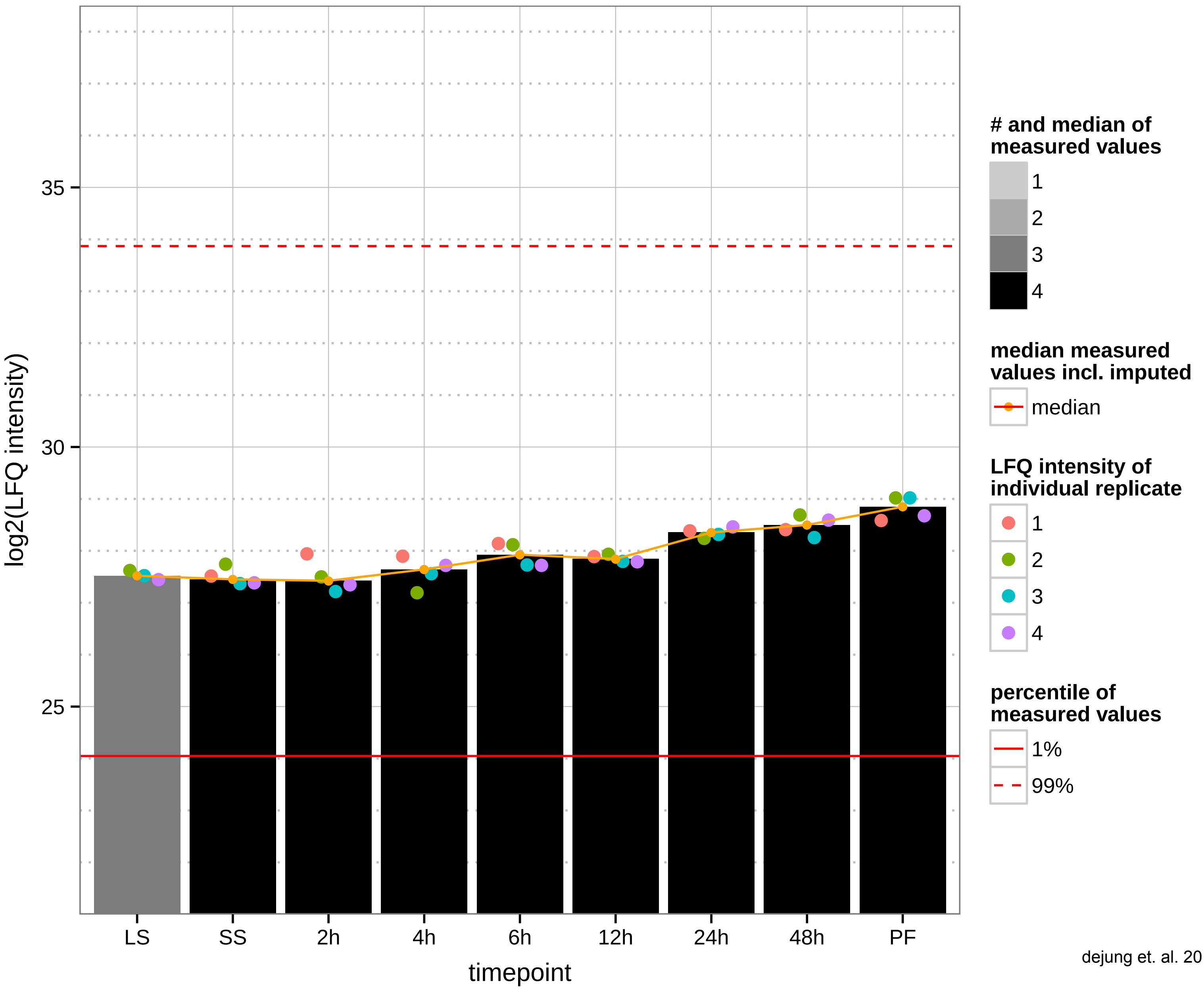

dejung et. al. 2015

3–hydroxy–3–methylglutaryl–CoA synthase, putative (HMGS)
Tb927.8.6110
AGOF: hydroxymethylglutaryl–CoA synthase activity
AGOC: null
AGOP: ergosterol biosynthetic process
PGOF: catalytic activity, hydroxymethylglutaryl–CoA synthase activity
PGOC: null
PGOP: isoprenoid biosynthetic process, metabolic process

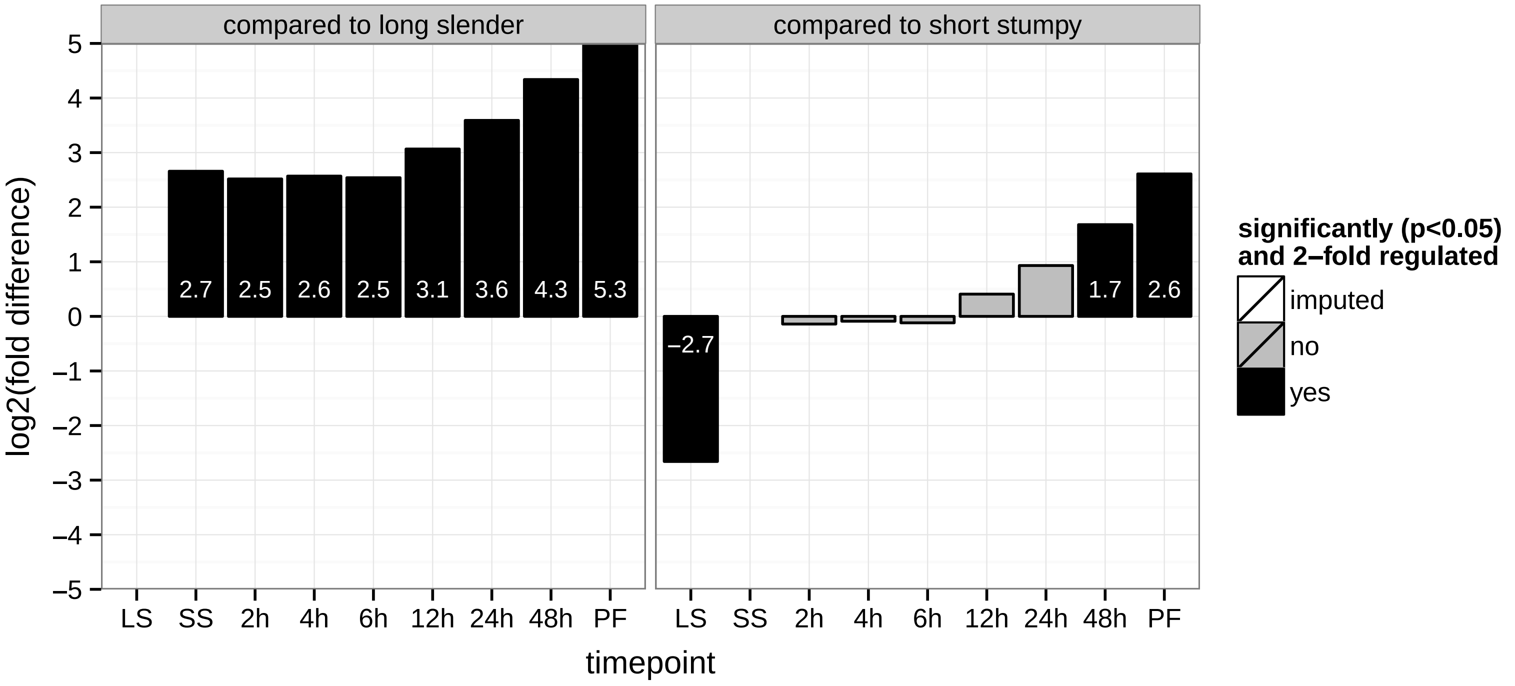

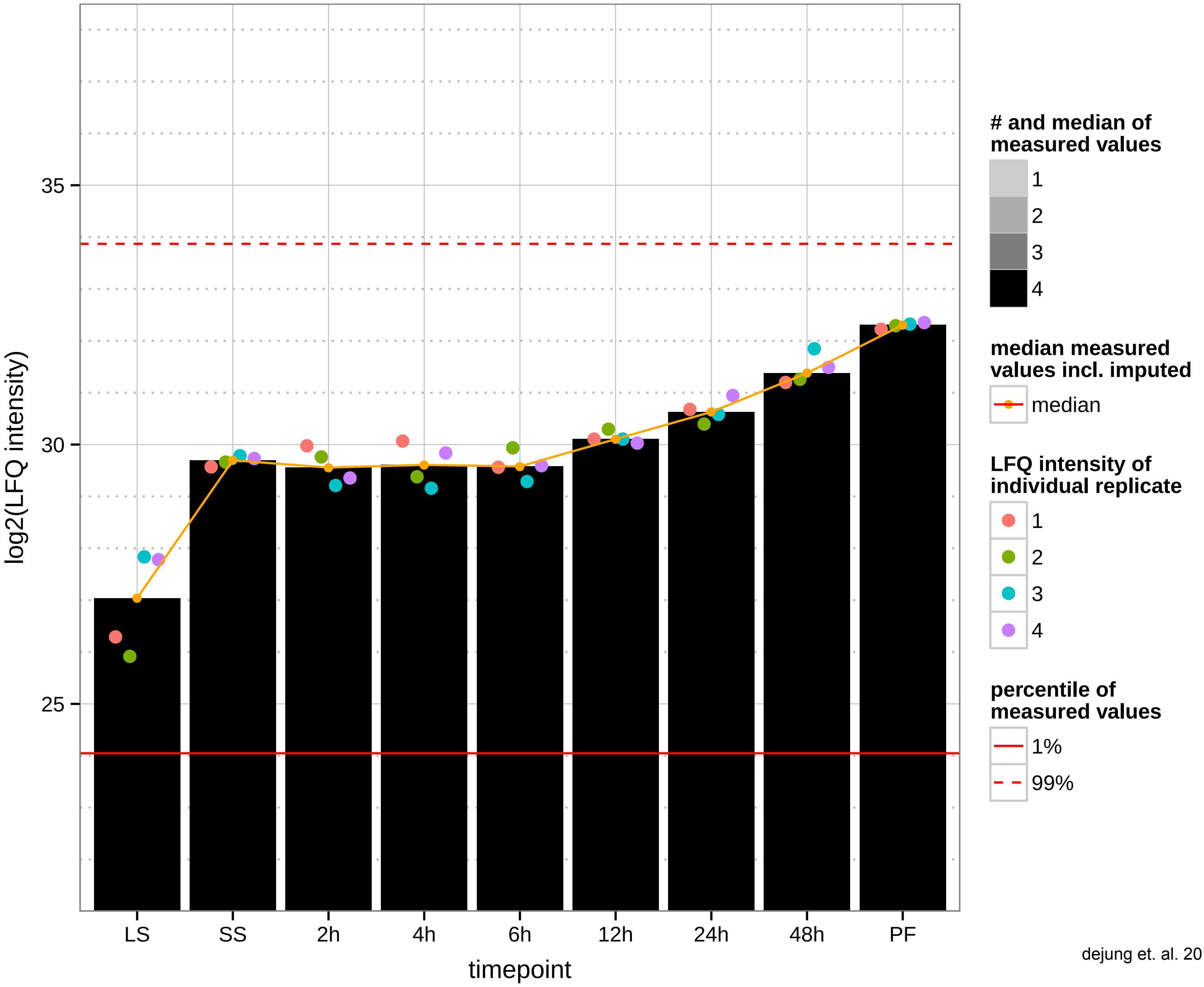

dejung et. al. 2015

hypothetical protein, conserved
Tb927.8.6330
AGOF: null
AGOC: null
AGOP: null
PGOF: protein binding
PGOC: null
PGOP: null

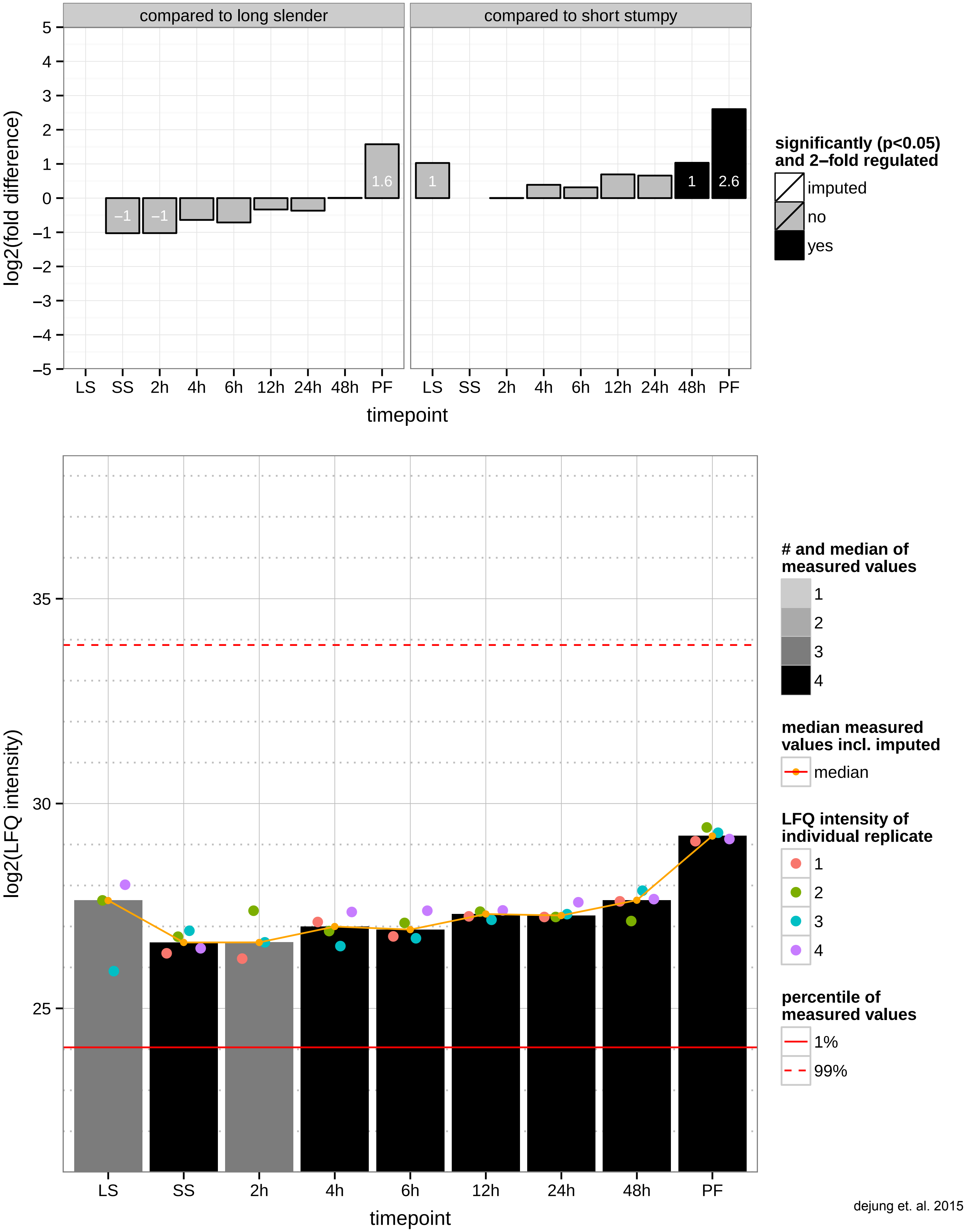

dejung et. al. 2015

3–methylcrotonyl–CoA carboxylase alpha subunit, putative
Tb927.8.6970
AGOF: ATP binding, biotin binding, methylcrotonoyl–CoA carboxylase activity
AGOC: mitochondrion
AGOP: pyrimidine nucleobase biosynthetic process, urea cycle
PGOF: ATP binding, biotin carboxylase activity, catalytic activity, ligase activity, metal ion binding
PGOC: null
PGOP: metabolic process

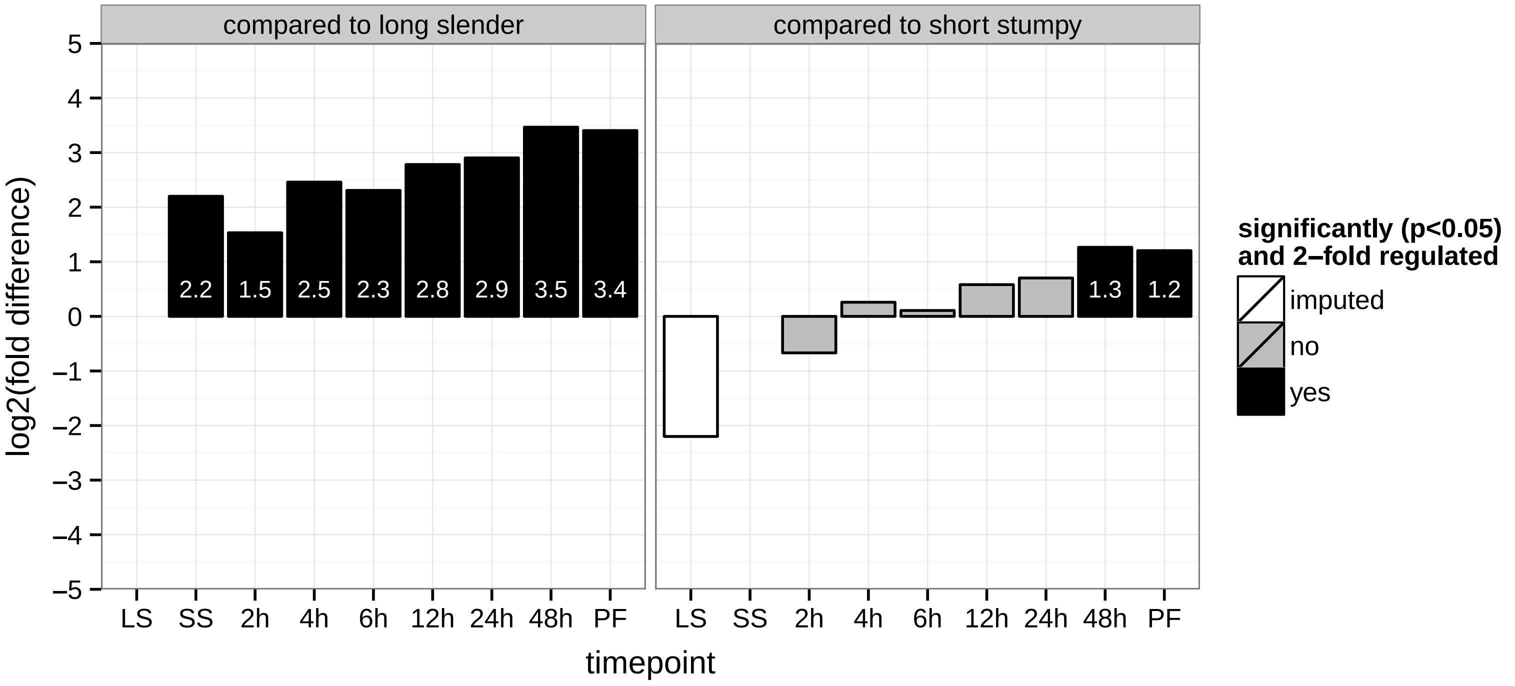

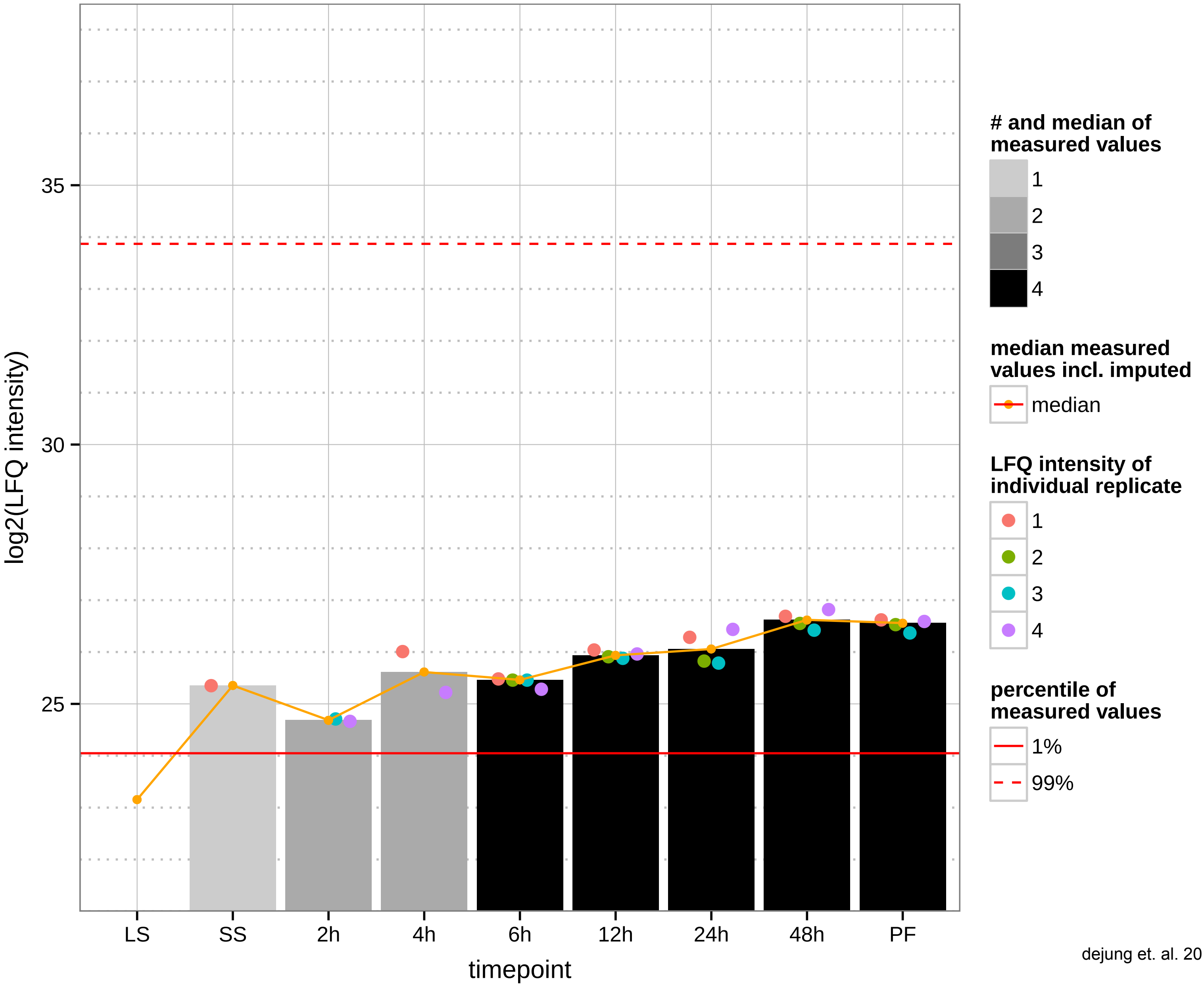

dejung et. al. 2015

hypothetical protein, conserved
Tb927.8.7040
AGOF: null
AGOC: mitochondrion
AGOP: null
PGOF: null
PGOC: null
PGOP: null

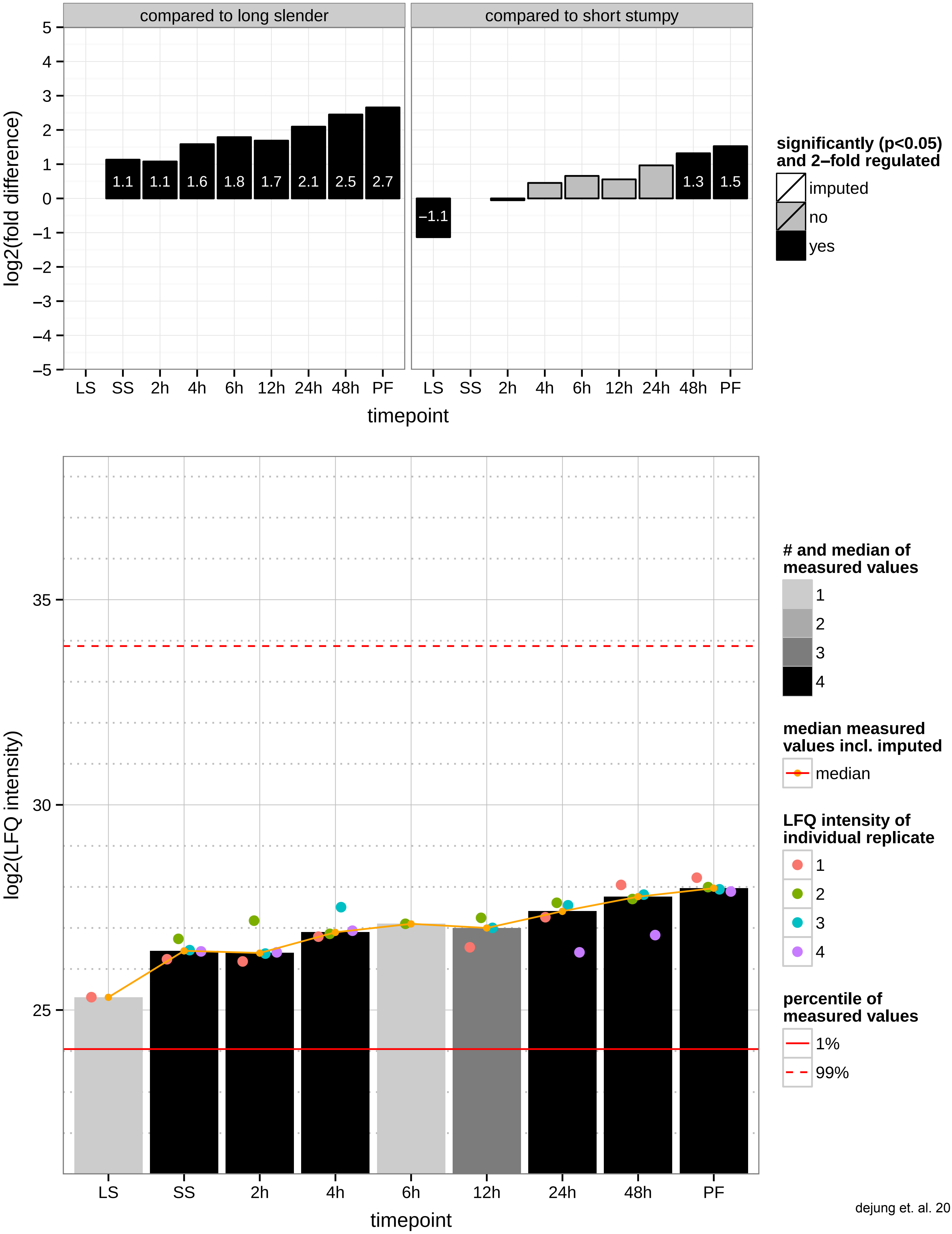

dejung et. al. 2015

Polyadenylate–binding protein 2 (Poly(A)–binding protein 2) (Poly(A)–binding protein II) (PABII) (Polyadenylate–binding nuclea
Tb927.9.10770
AGOF: RNA binding, poly(A) RNA binding
AGOC: null
AGOP: RNA processing
PGOF: RNA binding, nucleic acid binding
PGOC: null
PGOP: null

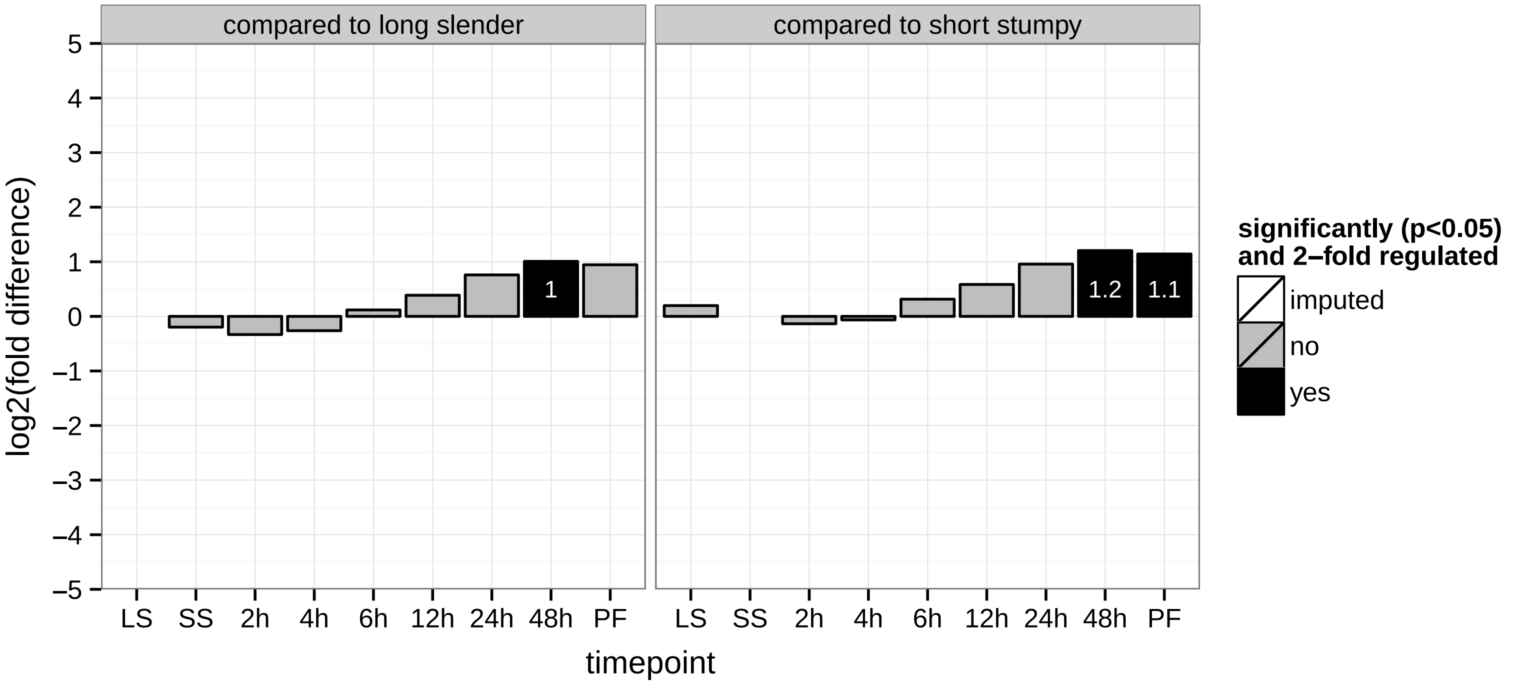

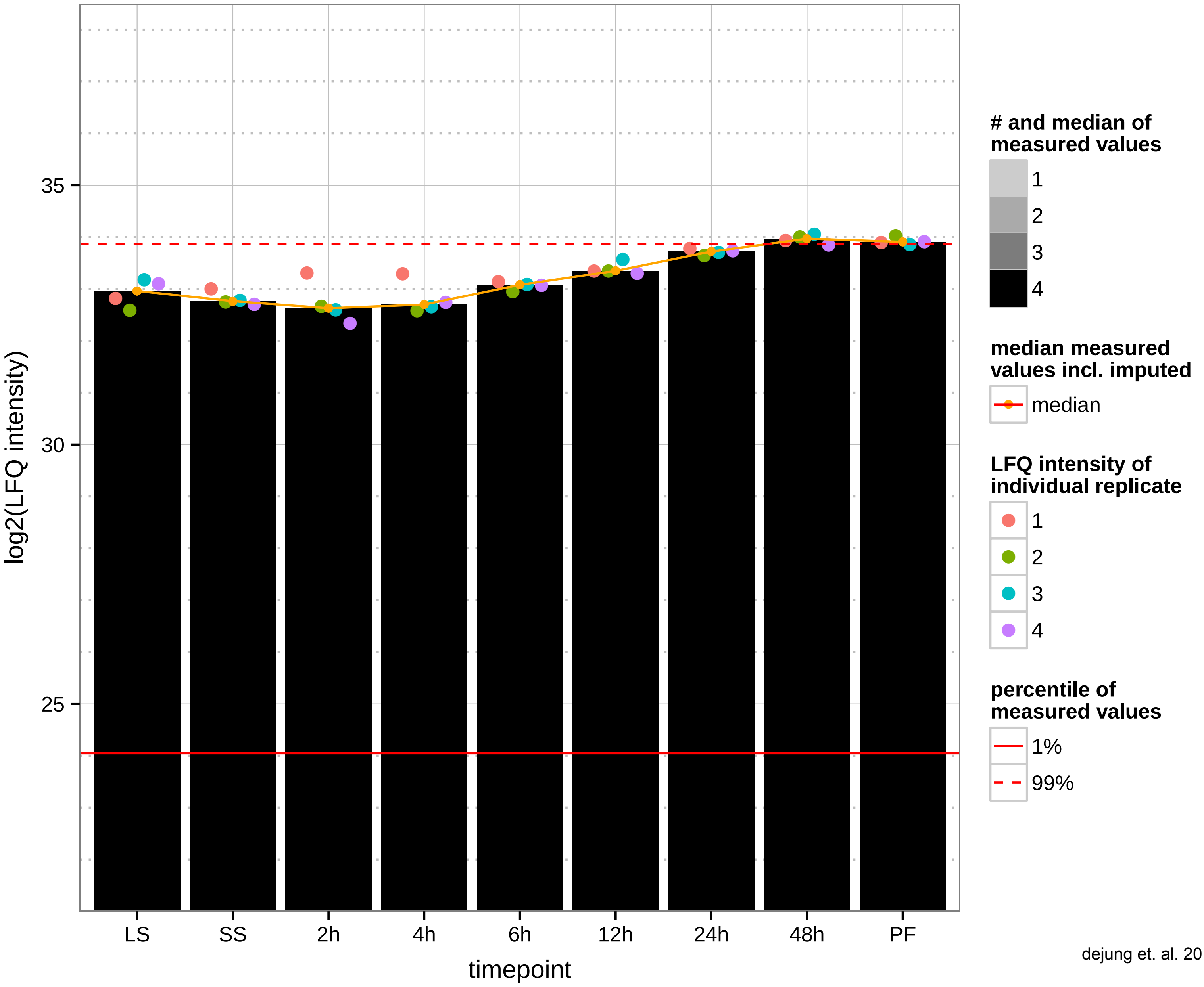

dejung et. al. 2015

kinetoplastid membrane protein KMP−11, unspecified product, kinetoplastid membrane protein KMP−11 (KMP−11)
Tb927.9.13920;Tb927.9.13880;Tb927.9.13820
AGOF: null
AGOC: mitochondrion, null
AGOP: defense response, positive regulation of cell proliferation, null
PGOF: null
PGOC: null
PGOP: defense response, positive regulation of cell proliferation, null

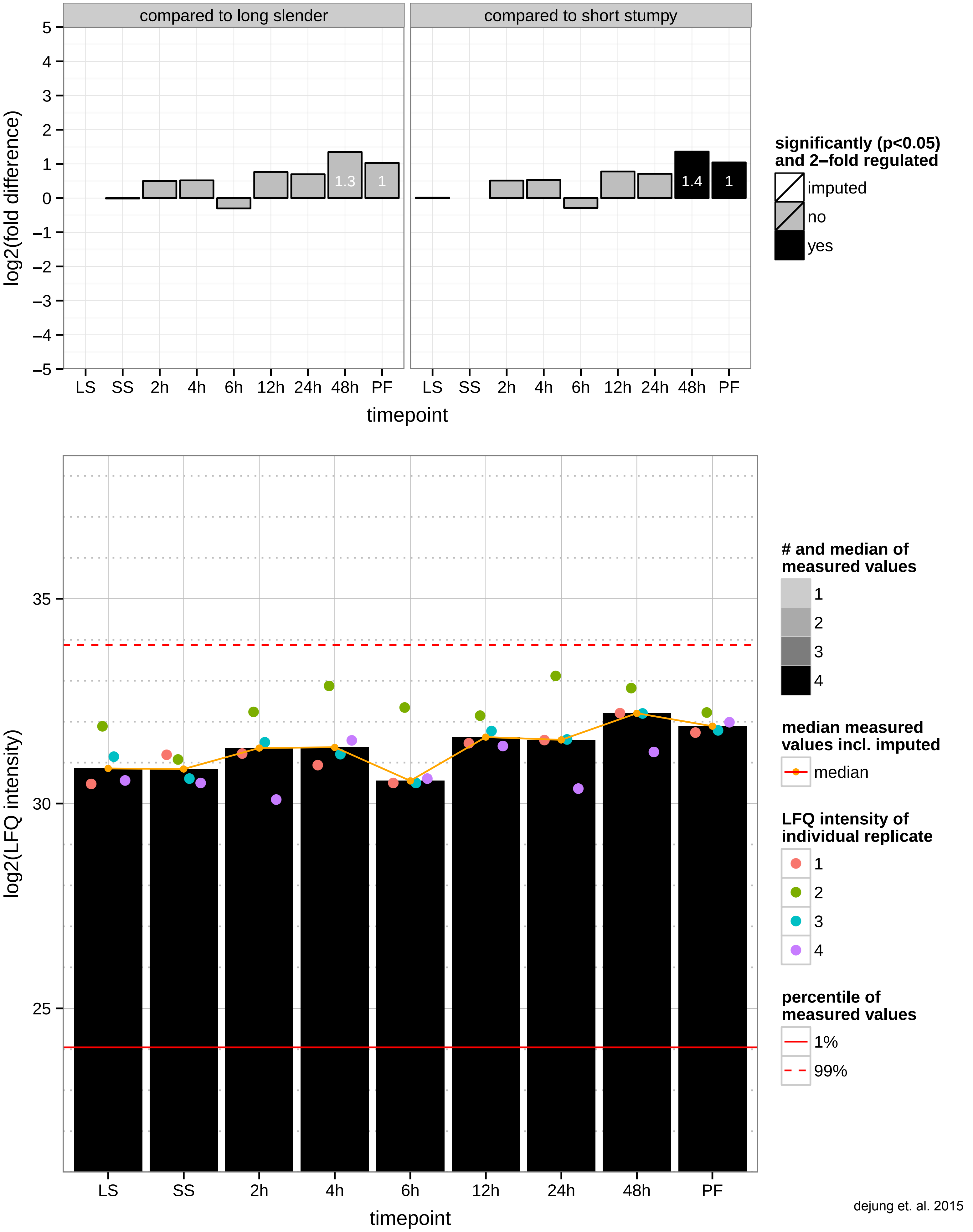

dejung et. al. 2015

hypothetical protein, conserved
Tb927.9.2900
AGOF: null
AGOC: null
AGOP: null
PGOF: binding
PGOC: null
PGOP: null

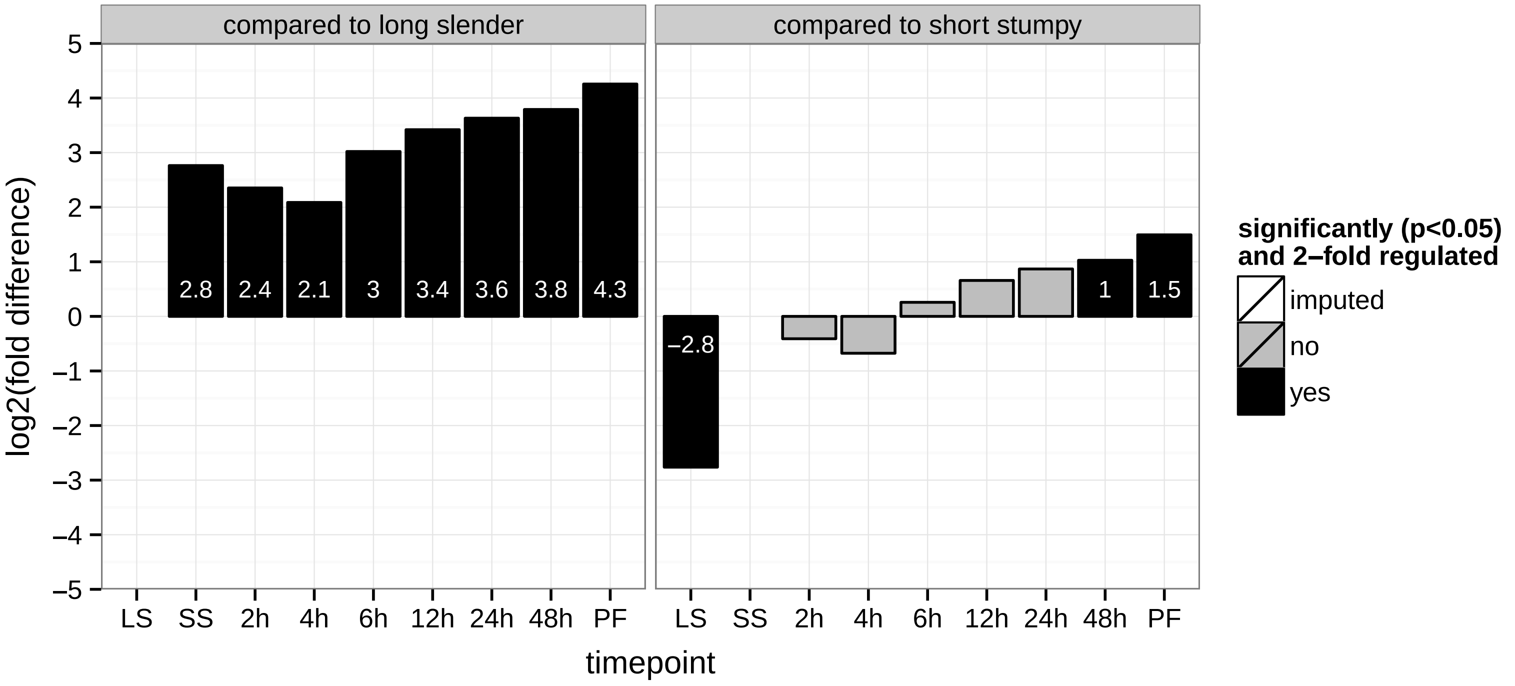

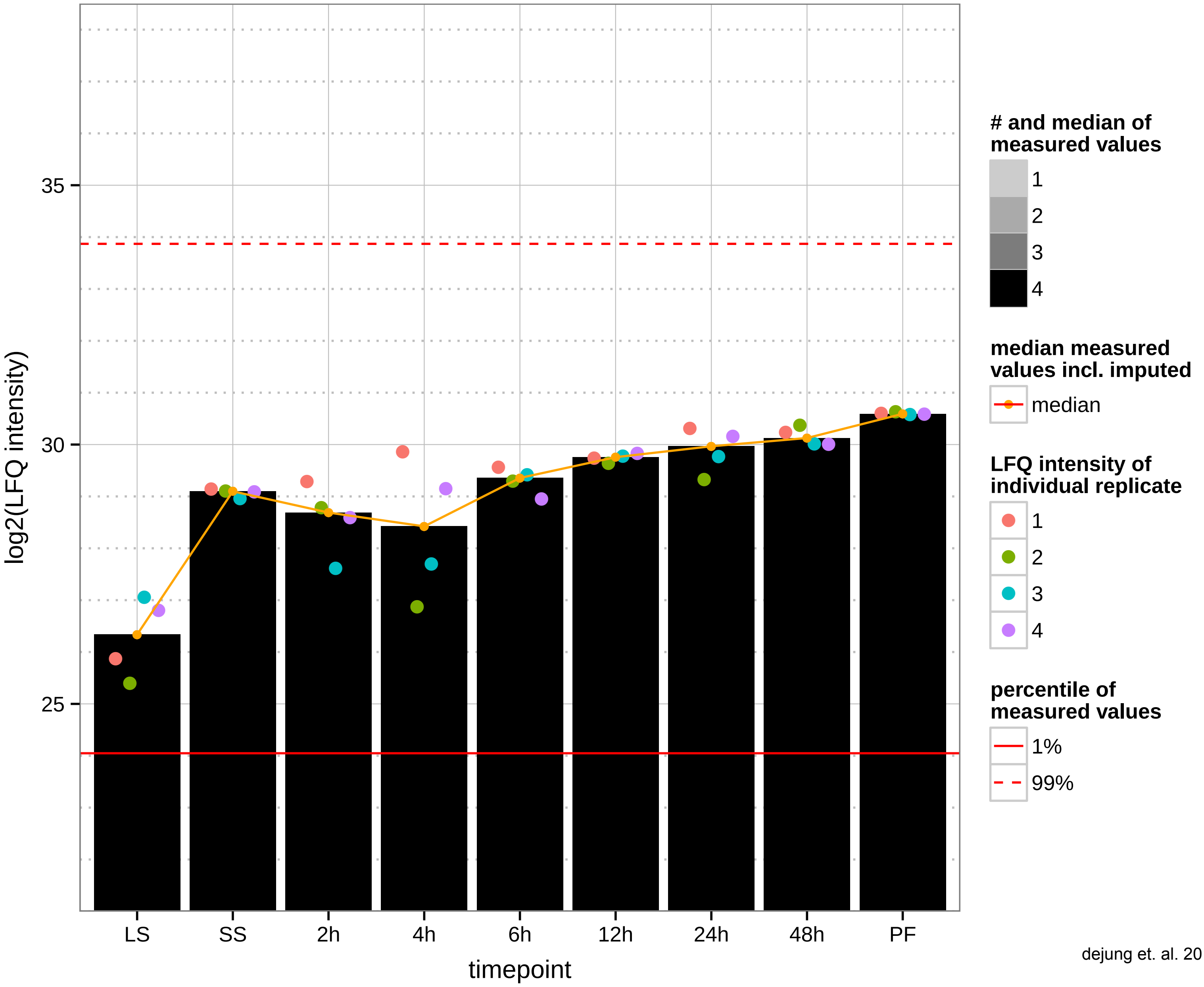

dejung et. al. 2015

kinesin (Kif–13–1)
Tb927.9.3650
AGOF: ATP binding, microtubule motor activity, motor activity
AGOC: microtubule associated complex, nucleus
AGOP: microtubule–based movement, mitotic spindle organization, regulation of chromosome segregation
PGOF: ATP binding, microtubule motor activity
PGOC: null
PGOP: microtubule–based movement

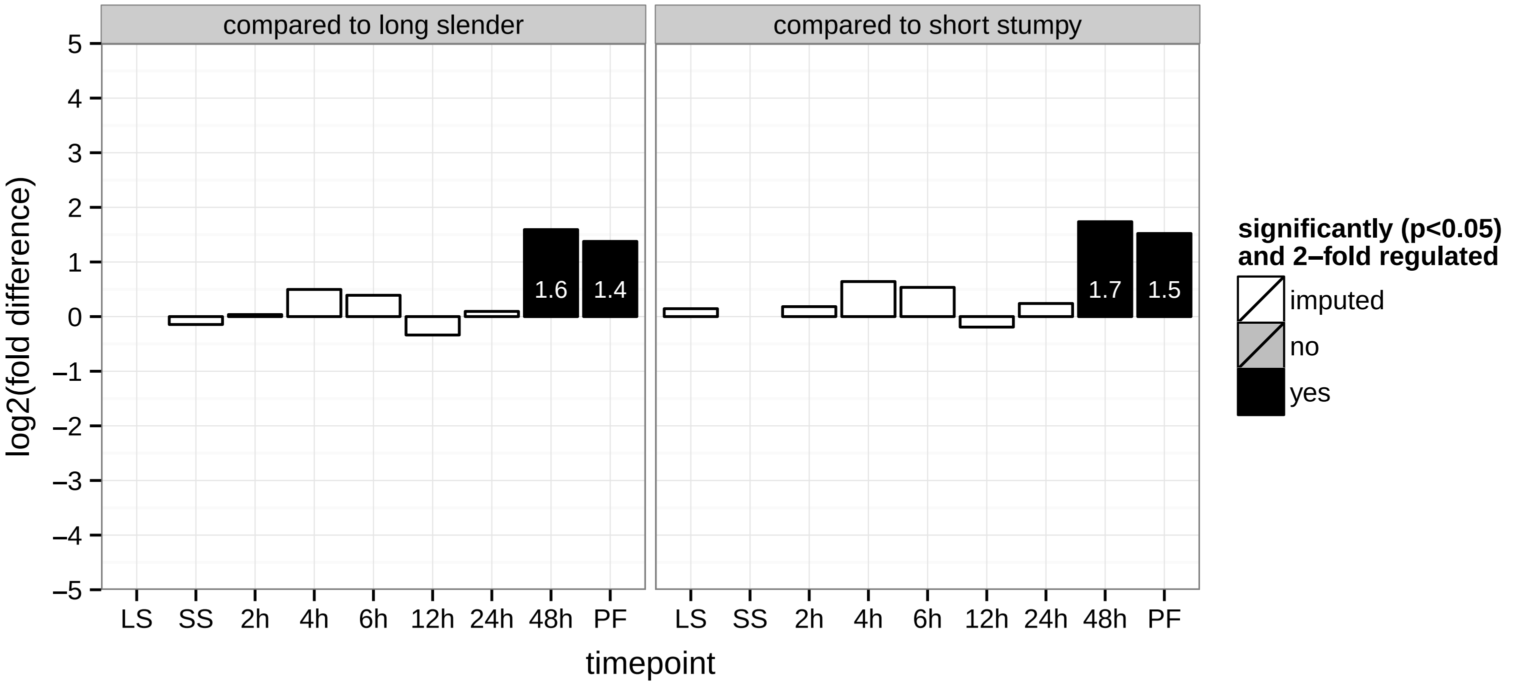

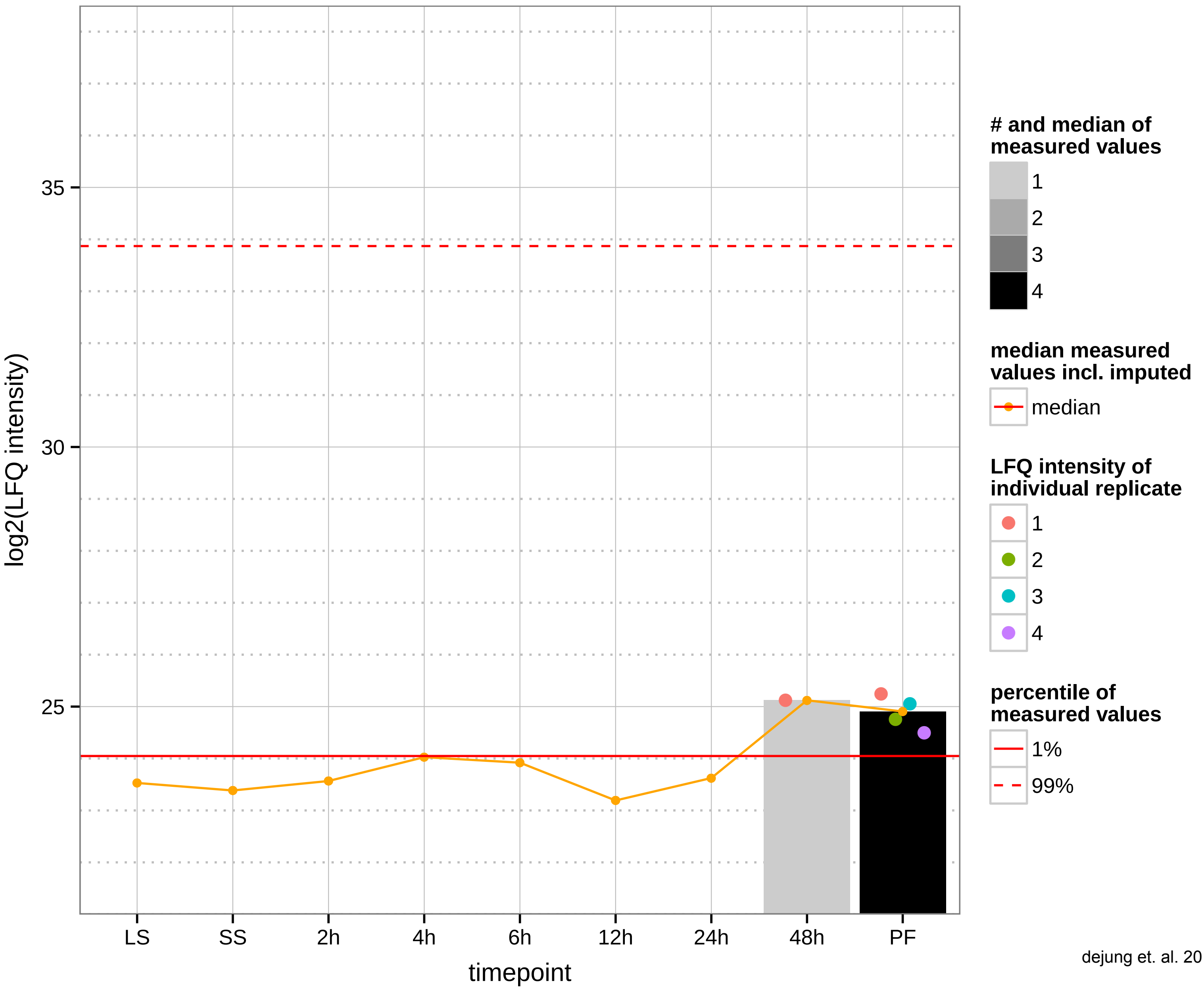

dejung et. al. 2015

condensin subunit 1, putative
Tb927.9.4900
AGOF: null
AGOC: condensin complex
AGOP: mitotic chromosome condensation
PGOF: binding
PGOC: null
PGOP: null

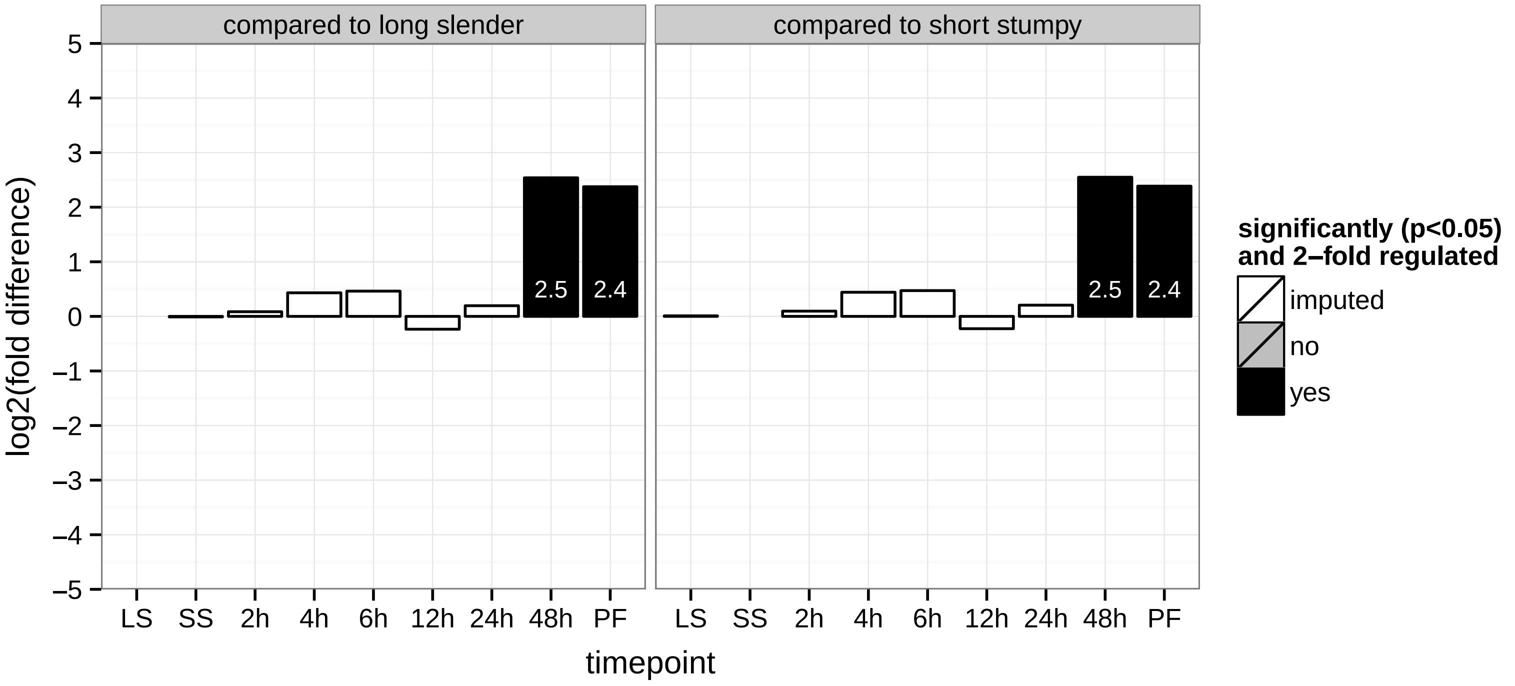

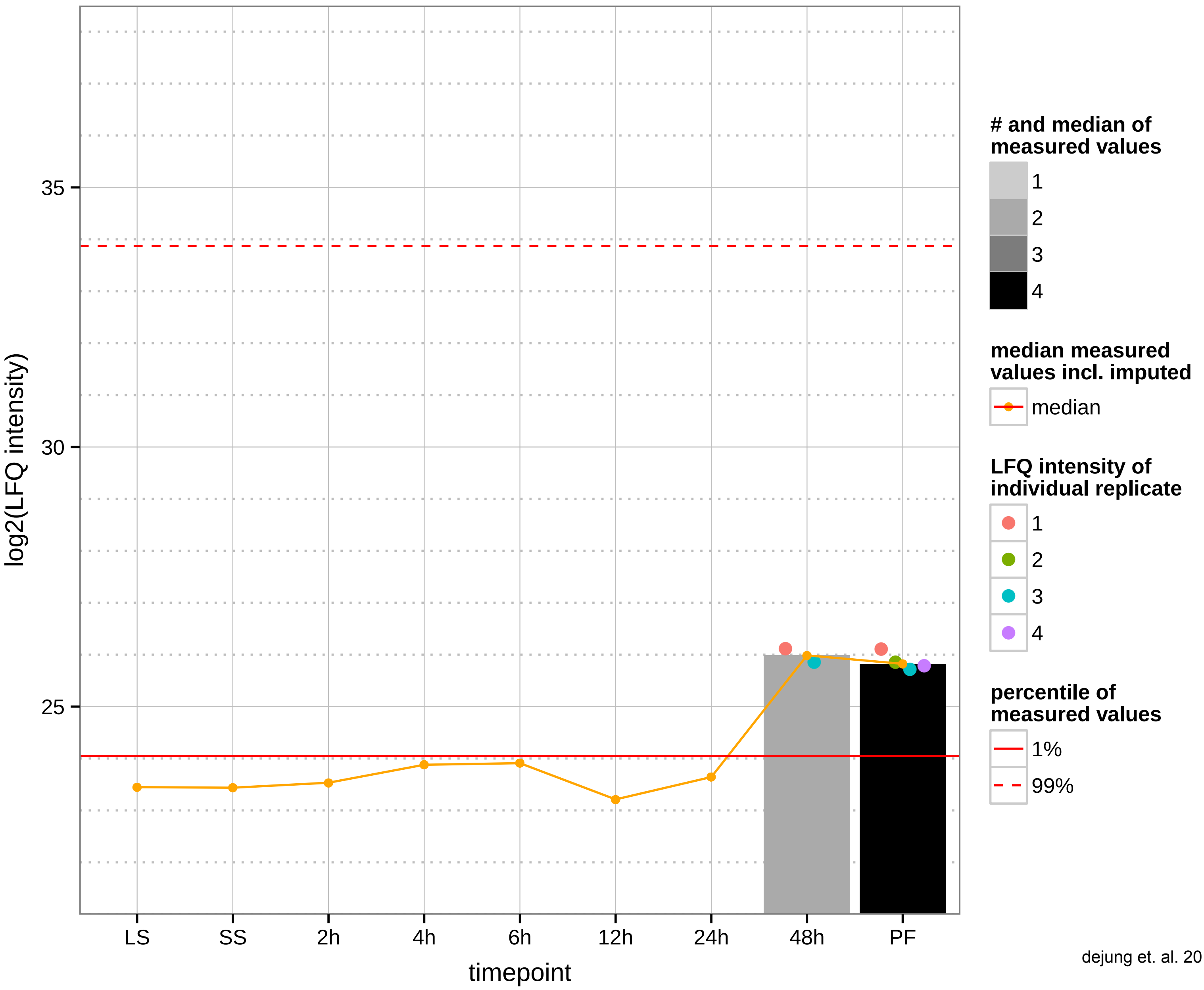

dejung et. al. 2015

glutamate dehydrogenase (GDH)
Tb927.9.5900
AGOF: glutamate dehydrogenase (NAD+) activity, oxidoreductase activity
AGOC: mitochondrion
AGOP: cellular amino acid metabolic process, glutamate catabolic process to 2–oxoglutarate, oxidation–reduction process
PGOF: glutamate dehydrogenase (NAD+) activity, oxidoreductase activity
PGOC: null
PGOP: cellular amino acid metabolic process, glutamate catabolic process to 2–oxoglutarate, oxidation–reduction process

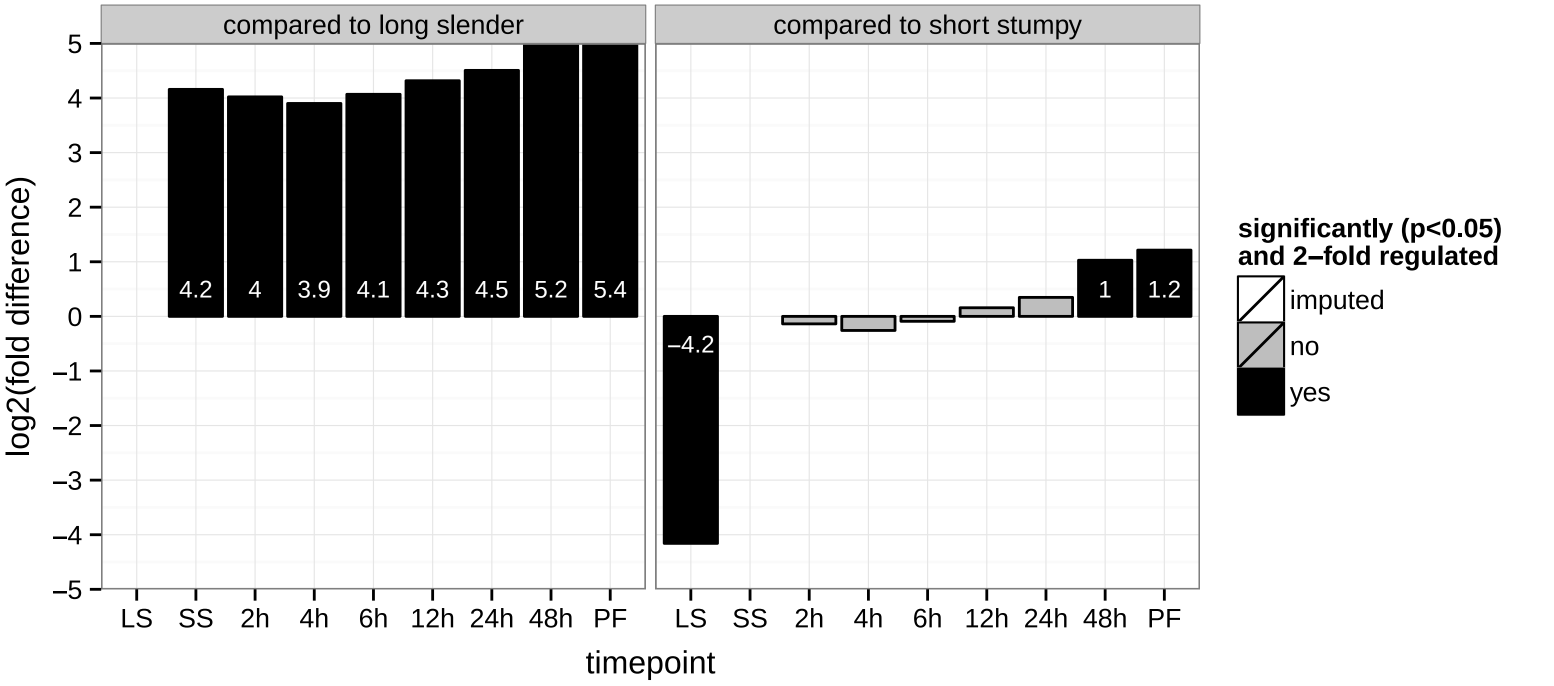

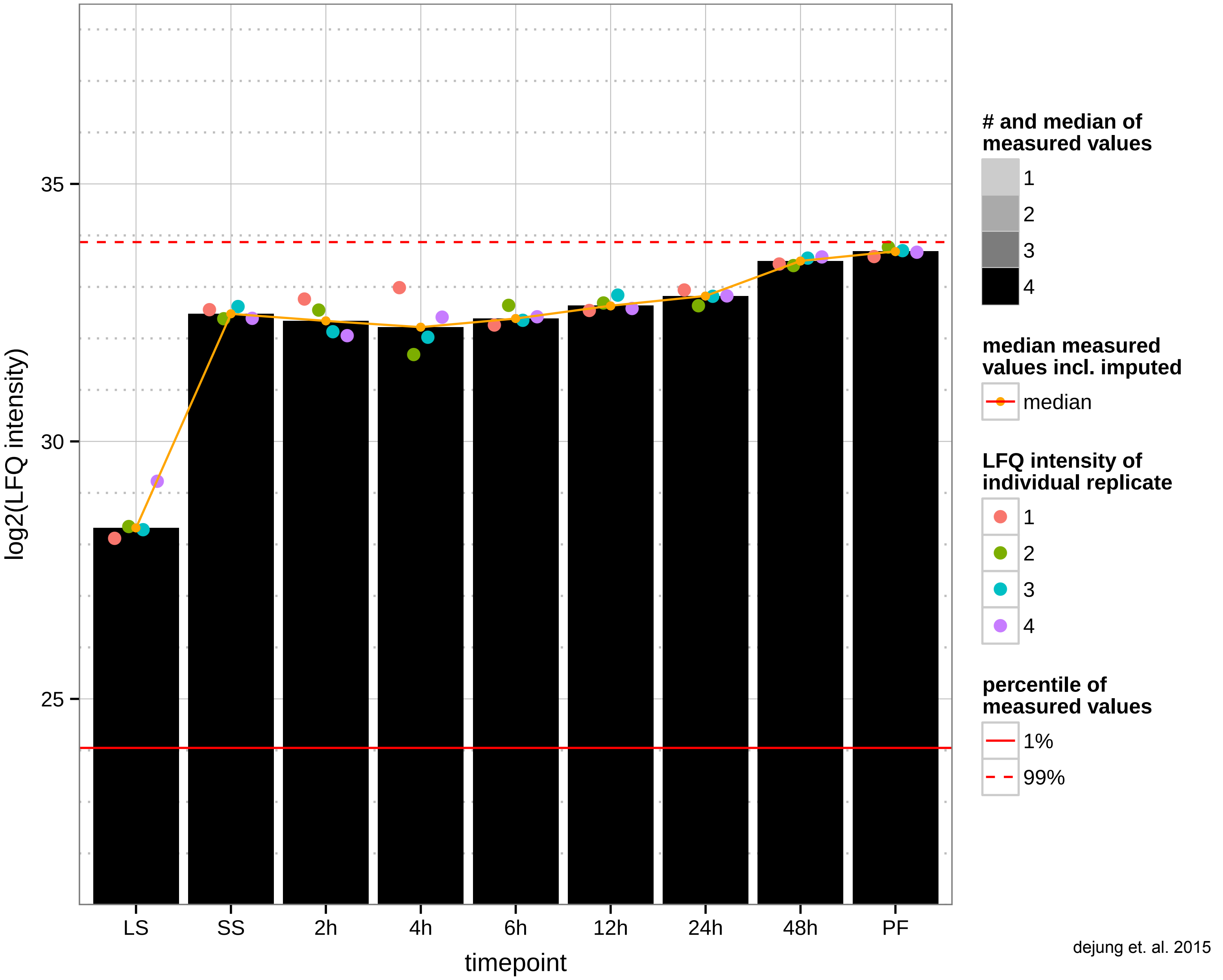

dejung et. al. 2015

ABC transporter, putative
Tb927.9.6310
AGOF: ATP binding, ATPase activity, coupled to transmembrane movement of substances
AGOC: integral to membrane, internal side of plasma membrane, membrane
AGOP: transport
PGOF: ATP binding, ATPase activity, nucleoside−triphosphatase activity, nucleotide binding
PGOC: membrane
PGOP: null

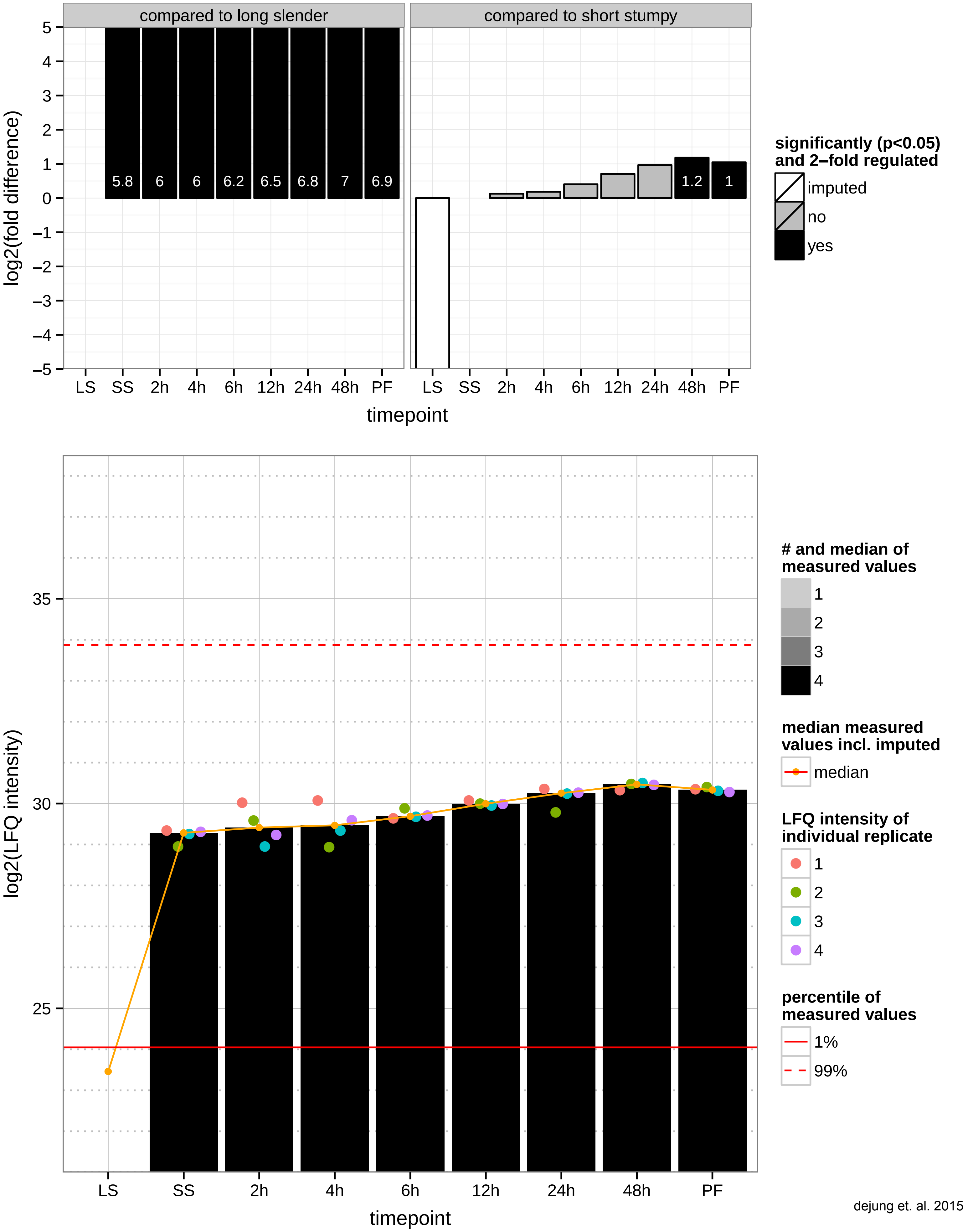

dejung et. al. 2015

oxidoreductase, putative
Tb927.9.7190
AGOF: alcohol dehydrogenase activity, zinc−dependent, oxidoreductase activity, zinc ion binding
AGOC: mitochondrion
AGOP: metabolic process
PGOF: oxidoreductase activity
PGOC: null
PGOP: oxidation−reduction process

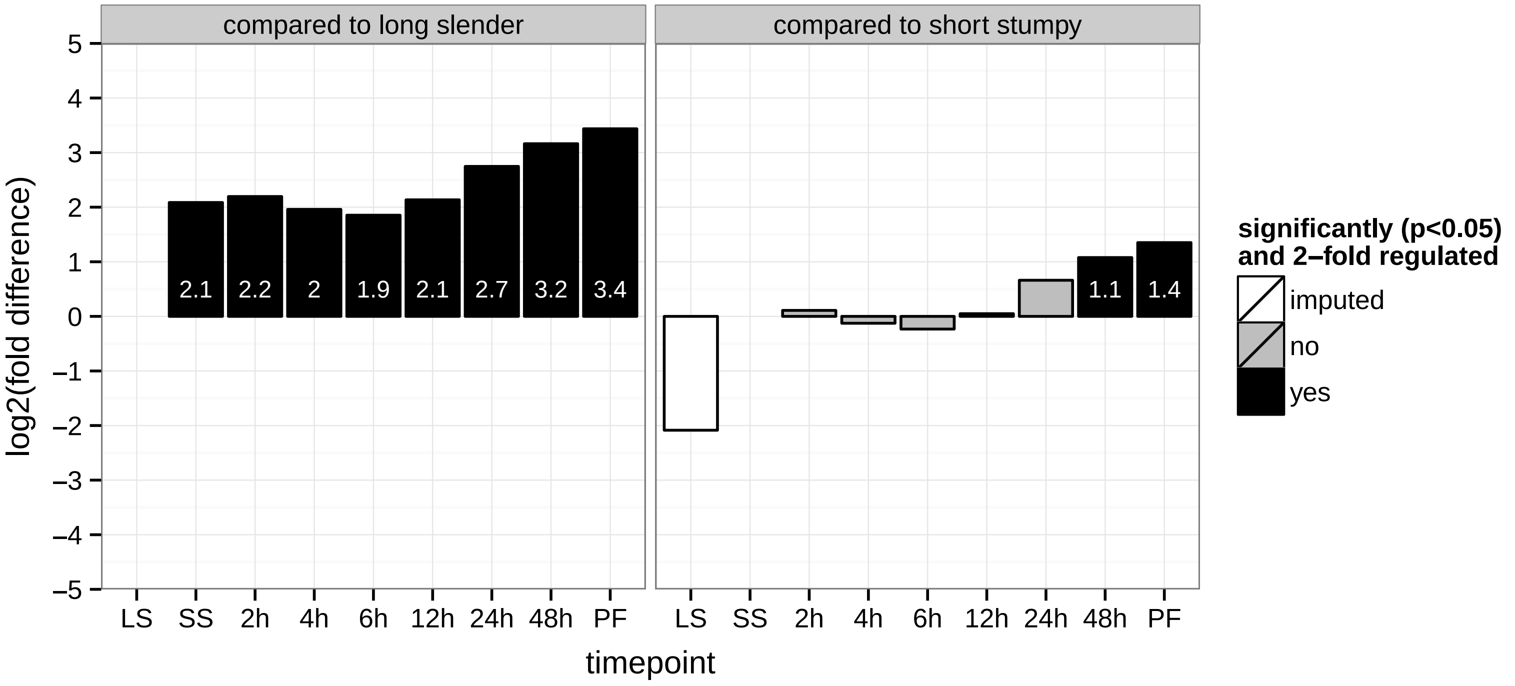

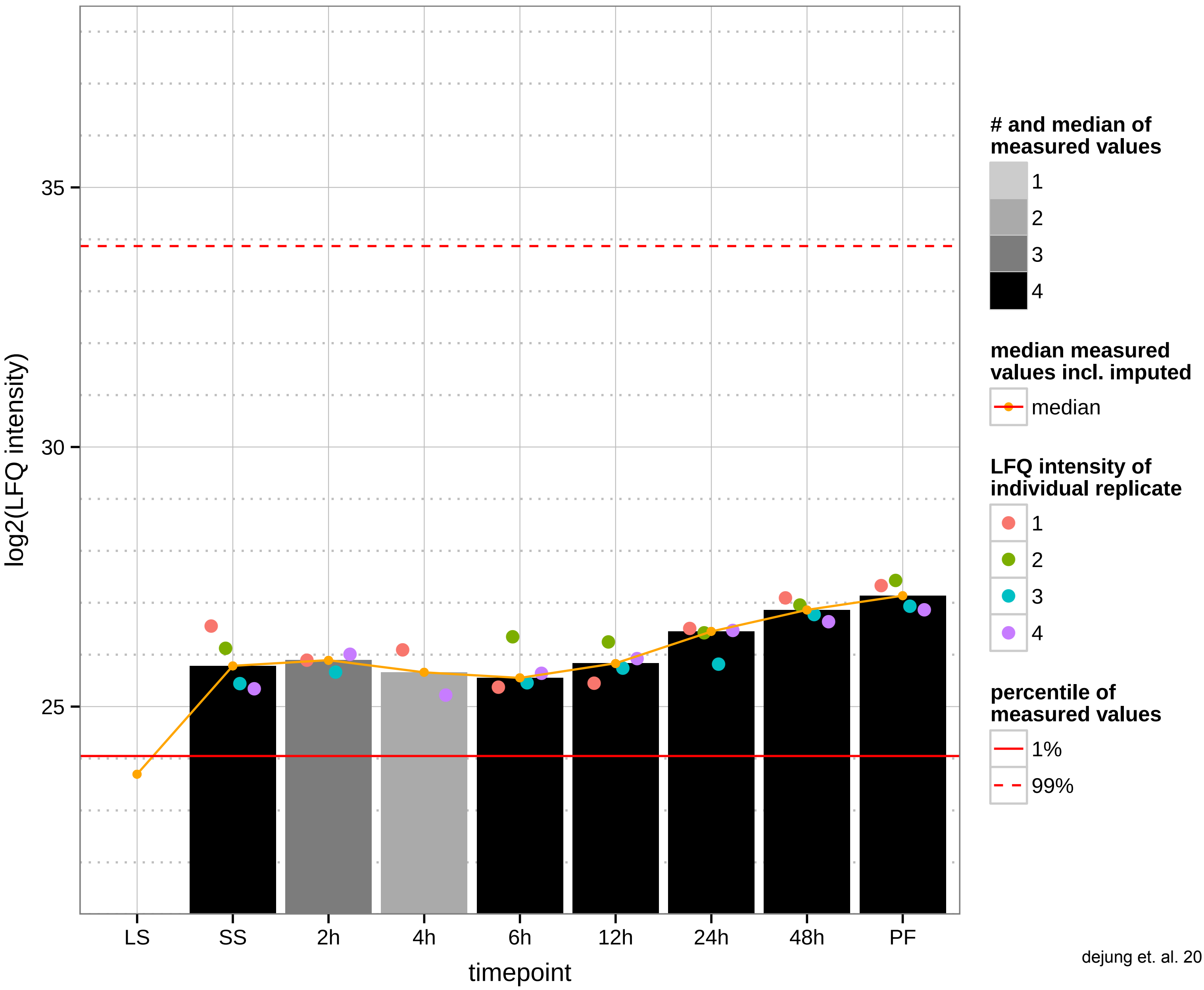

dejung et. al. 2015

60S ribosomal protein L10, putative, QM–like protein (QM), QM–like protein
Tb927.9.8420;Tb927.9.8070
AGOF: structural constituent of ribosome
AGOC: intracellular, ribosome
AGOP: translation
PGOF: structural constituent of ribosome
PGOC: intracellular, ribosome
PGOP: translation

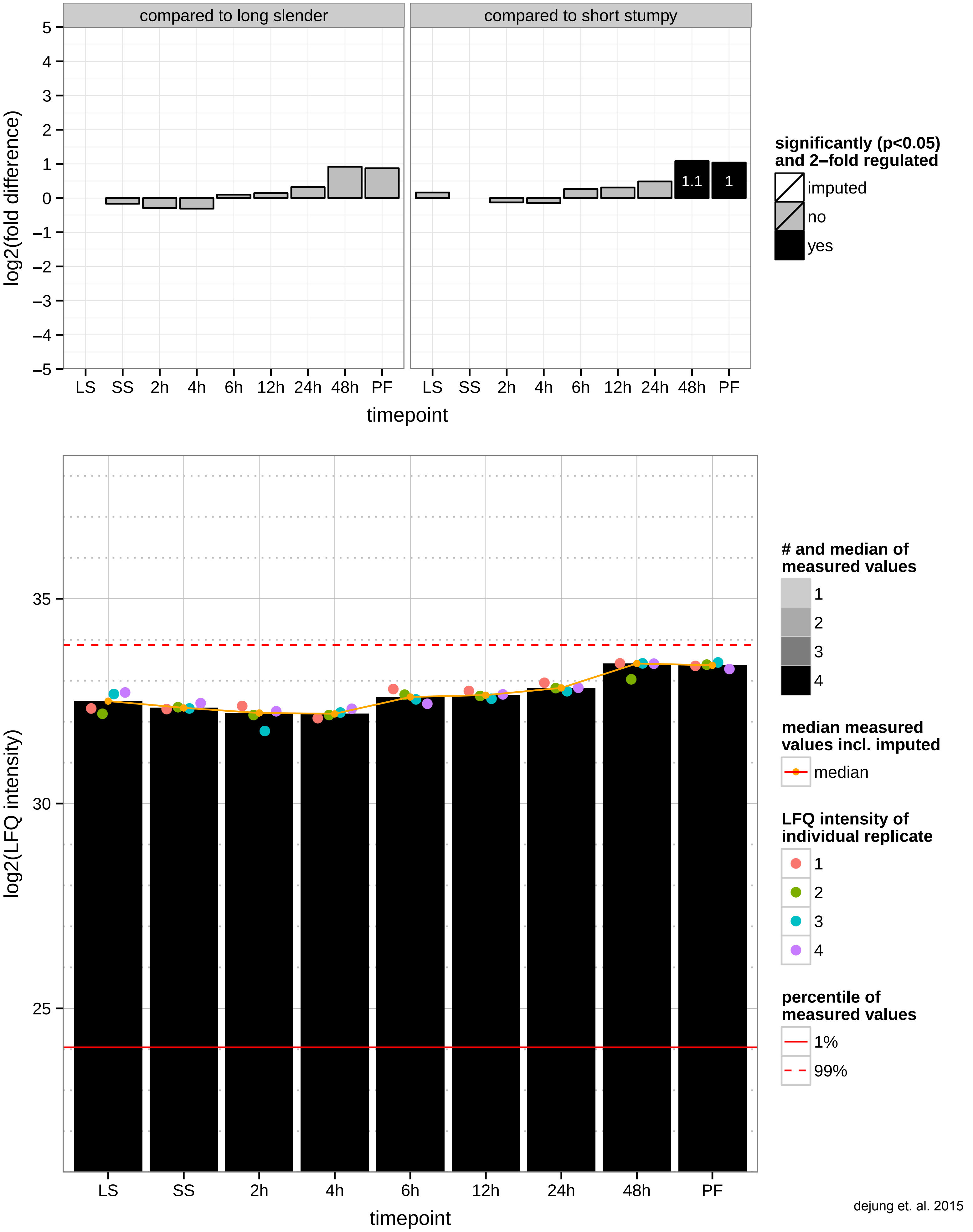

dejung et. al. 2015

adenylate kinase (ADKG)
Tb927.9.8450
AGOF: ATP binding, adenylate kinase activity, cytidylate kinase activity, uridylate kinase activity
AGOC: null
AGOP: nucleobase–containing compound metabolic process, nucleobase–containing small molecule interconversion
PGOF: ATP binding, nucleobase–containing compound kinase activity
PGOC: null
PGOP: nucleobase–containing compound metabolic process

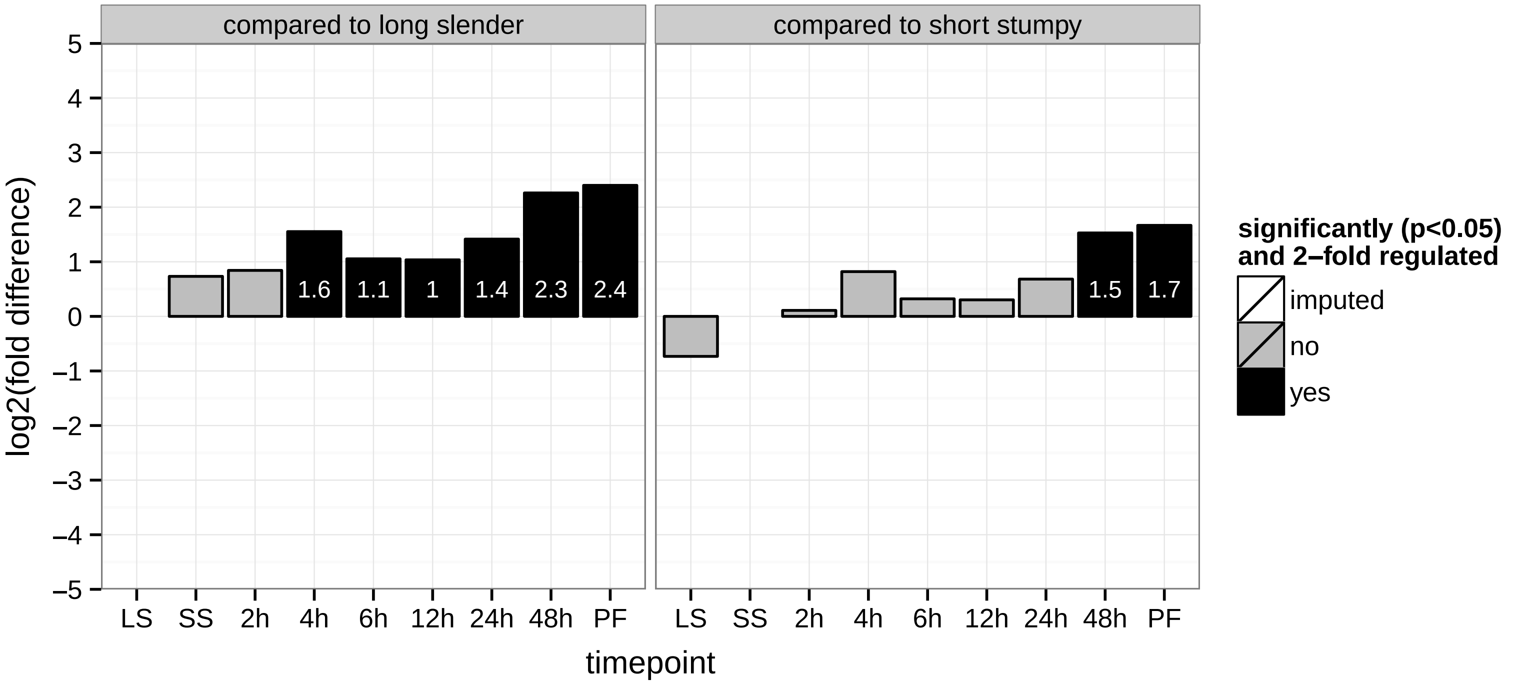

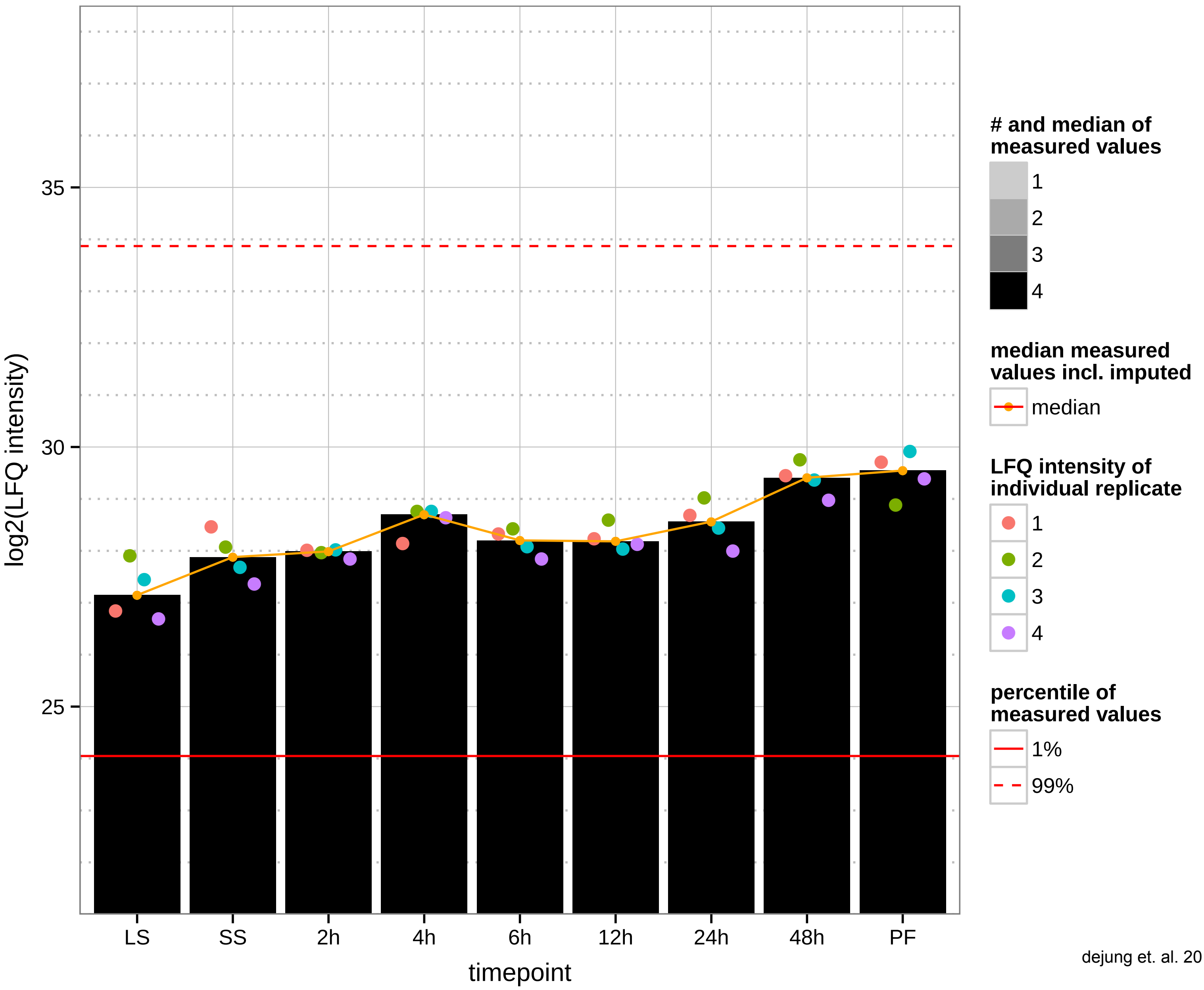

dejung et. al. 2015

hypothetical protein, conserved
Tb927.9.8520
AGOF: null
AGOC: null
AGOP: null
PGOF: null
PGOC: null
PGOP: null

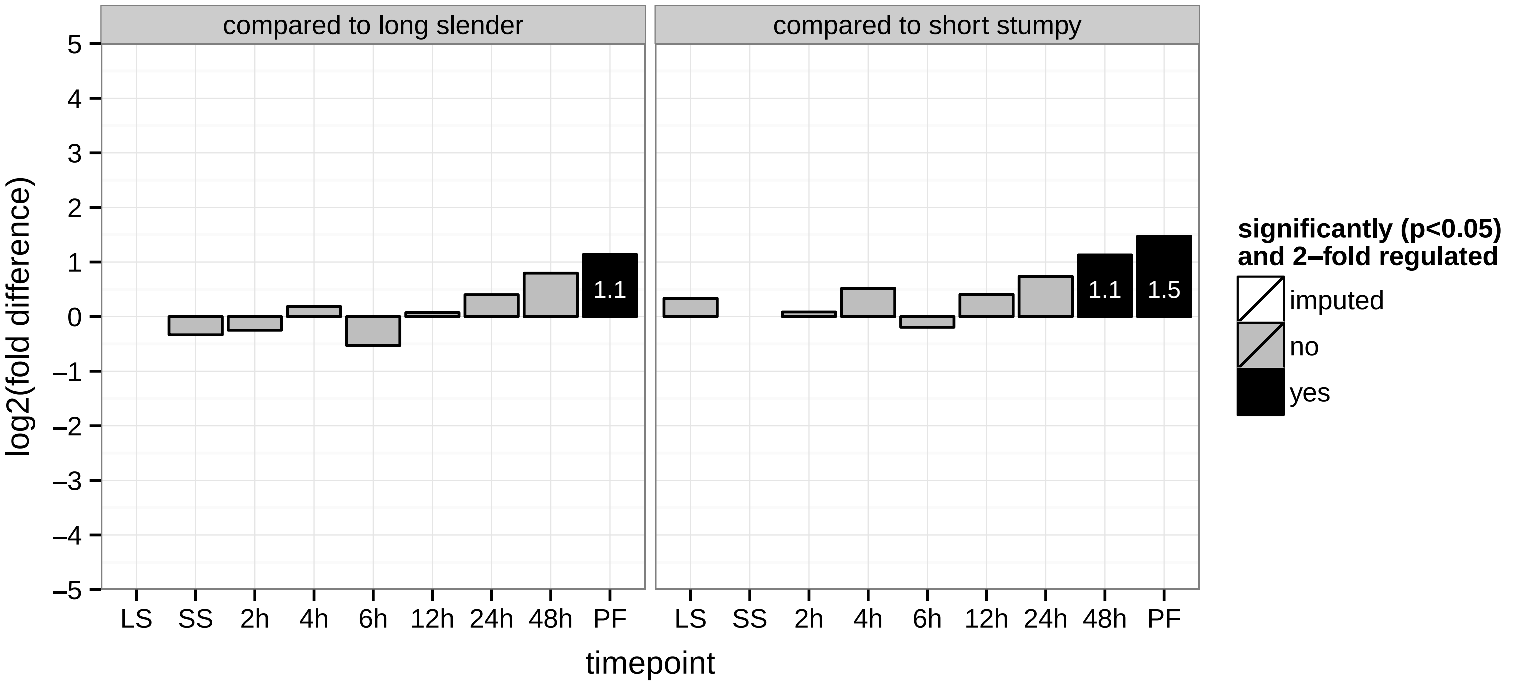

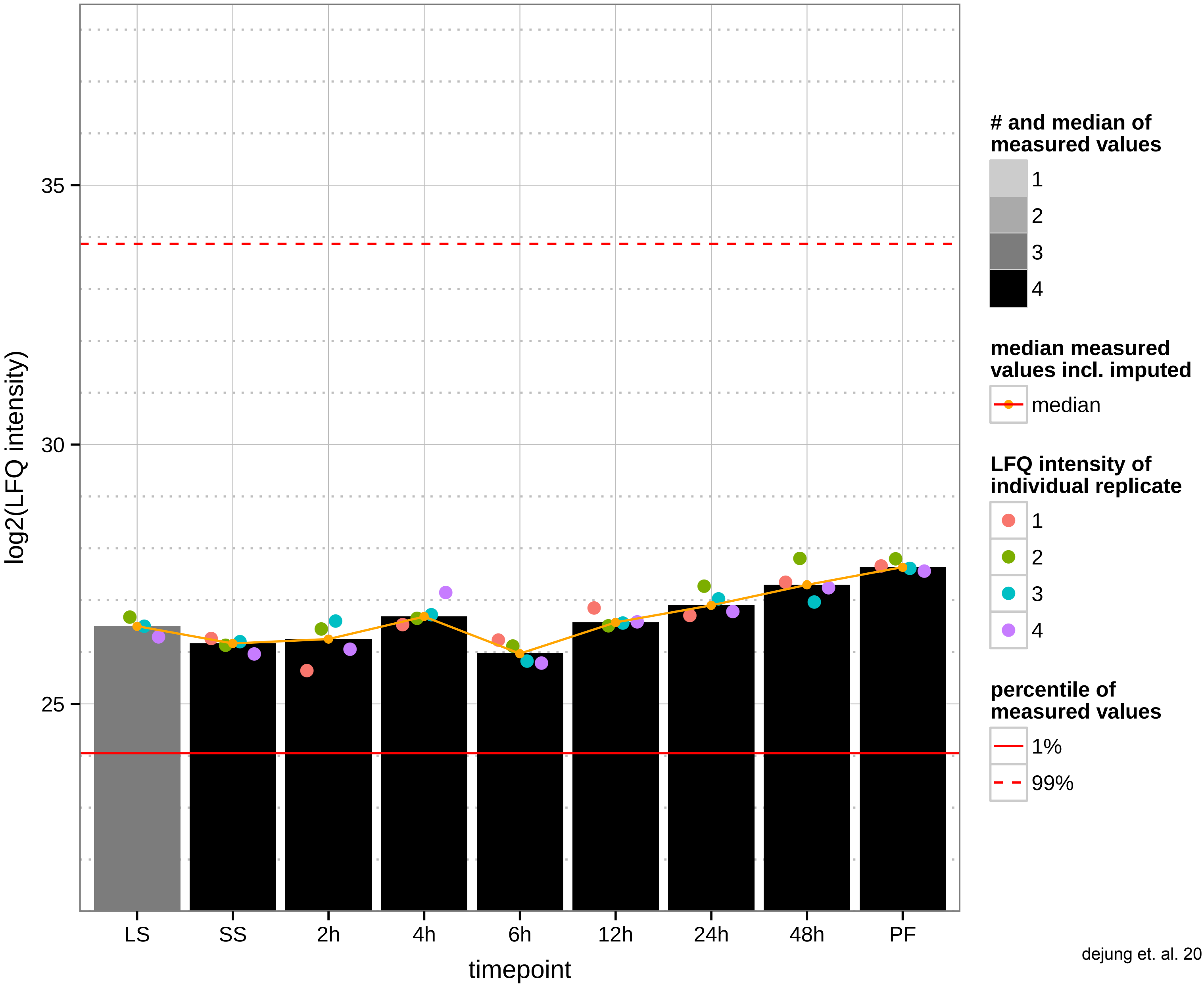

dejung et. al. 2015

isopentenyl–diphosphate delta–isomerase (type II) (idi1)
Tb927.9.9000
AGOF: FMN binding, oxidoreductase activity
AGOC: cytoplasm
AGOP: isoprenoid biosynthetic process
PGOF: FMN binding, isopentenyl–diphosphate delta–isomerase activity, oxidoreductase activity
PGOC: cytoplasm
PGOP: isoprenoid biosynthetic process

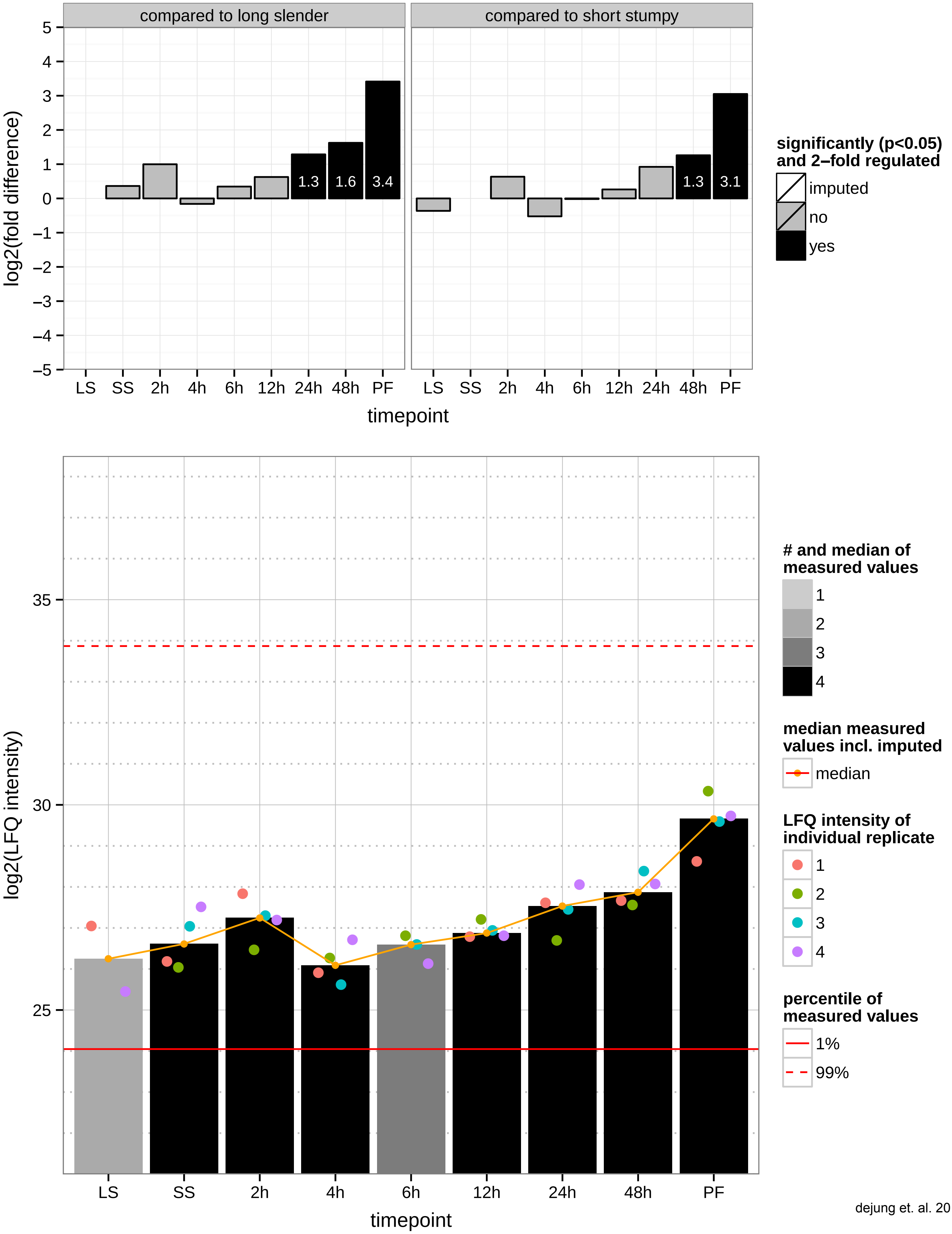

dejung et. al. 2015

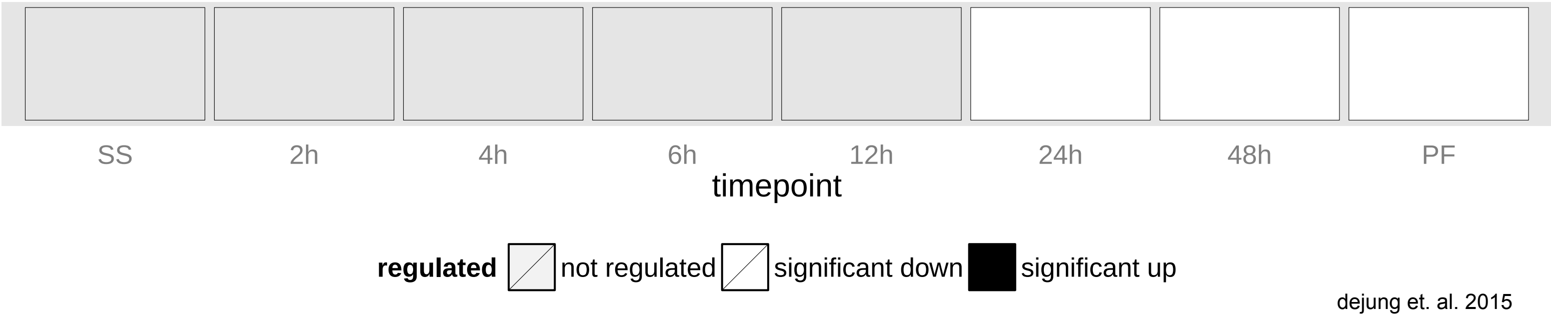
SS
2h
4h
6h
12h
24h
48h
PF
timepoint
regulated
not regulated
significant down
significant up
dejung et. al. 2015

expression site– associated gene (ESAG) protein, putative, expression site associated gene 5 (ESAG5) protein, chrIX additio
Tb927.9.15890;Tb11.v5.0463;Tb09.v4.0016
AGOF: lipid binding, null
AGOC: null, plasma membrane
AGOP: null
PGOF: lipid binding
PGOC: null
PGOP: null

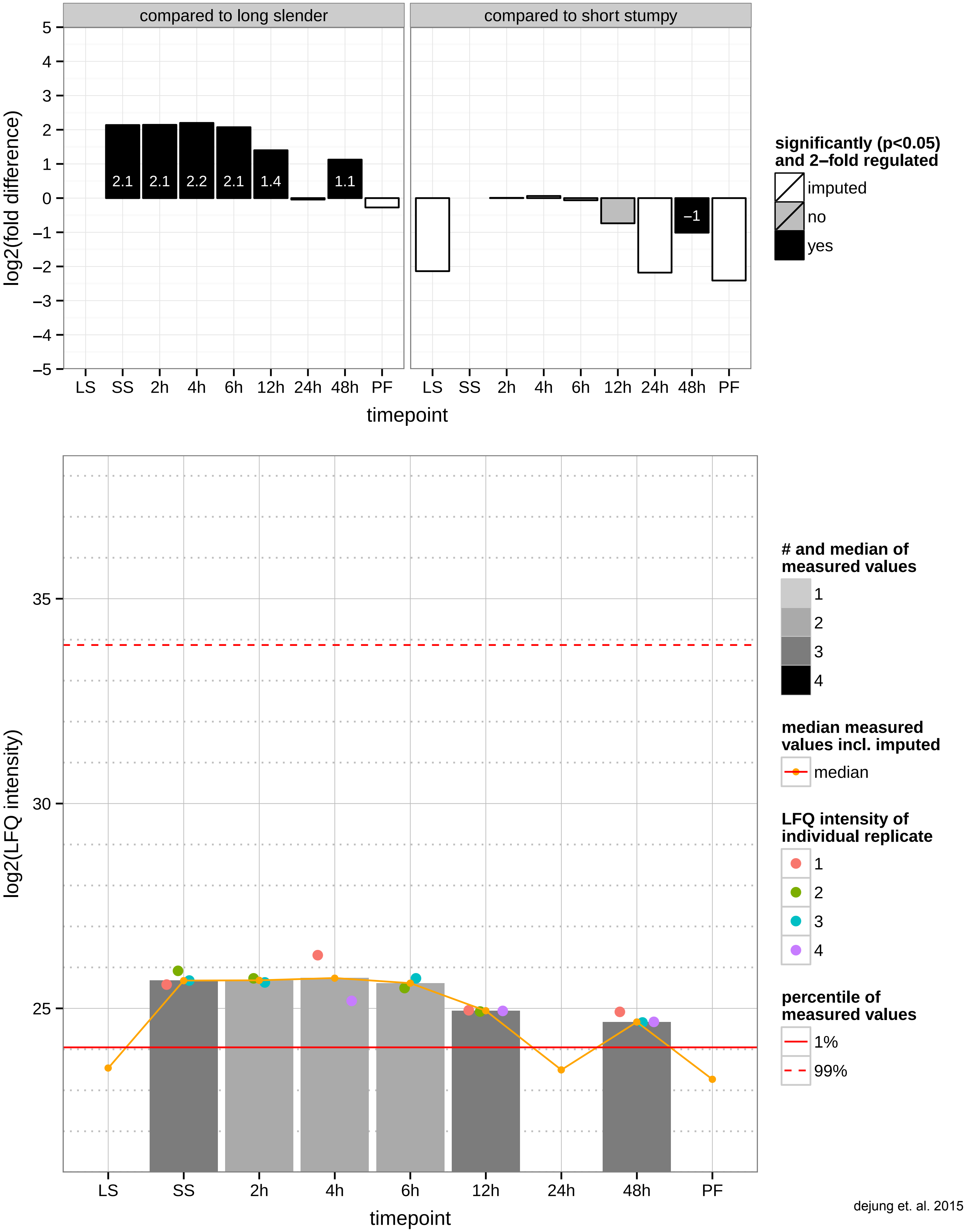

dejung et. al. 2015

cell–division control protein 2 homolog 6, putative, protein kinase, cdc2–related kinase, putative (CRK6)
Tb927.11.1180;Tb11.v5.0387
AGOF: null, ATP binding, protein serine/threonine kinase activity, protein tyrosine kinase activity
AGOC: null
AGOP: null, growth, protein phosphorylation
PGOF: ATP binding, protein kinase activity, transferase activity, transferring phosphorus–containing groups
PGOC: null
PGOP: protein phosphorylation

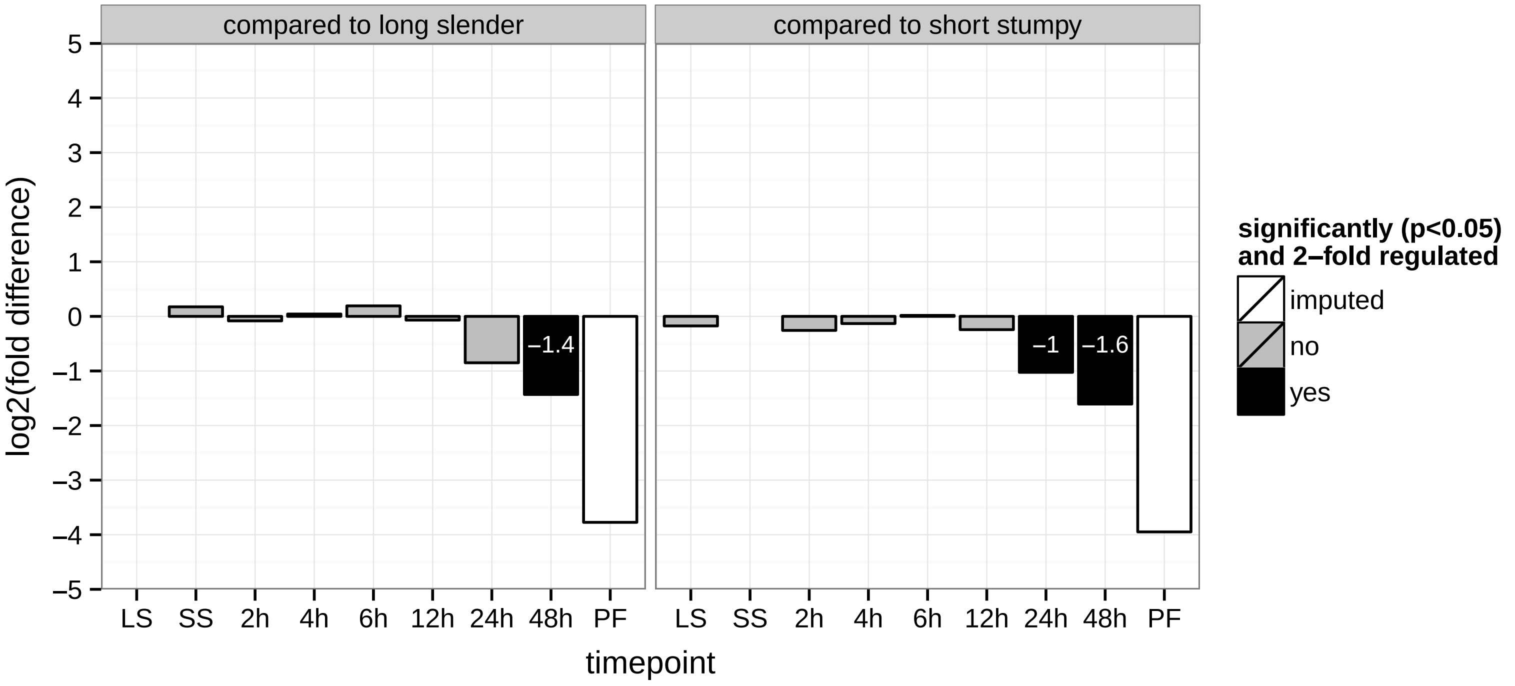

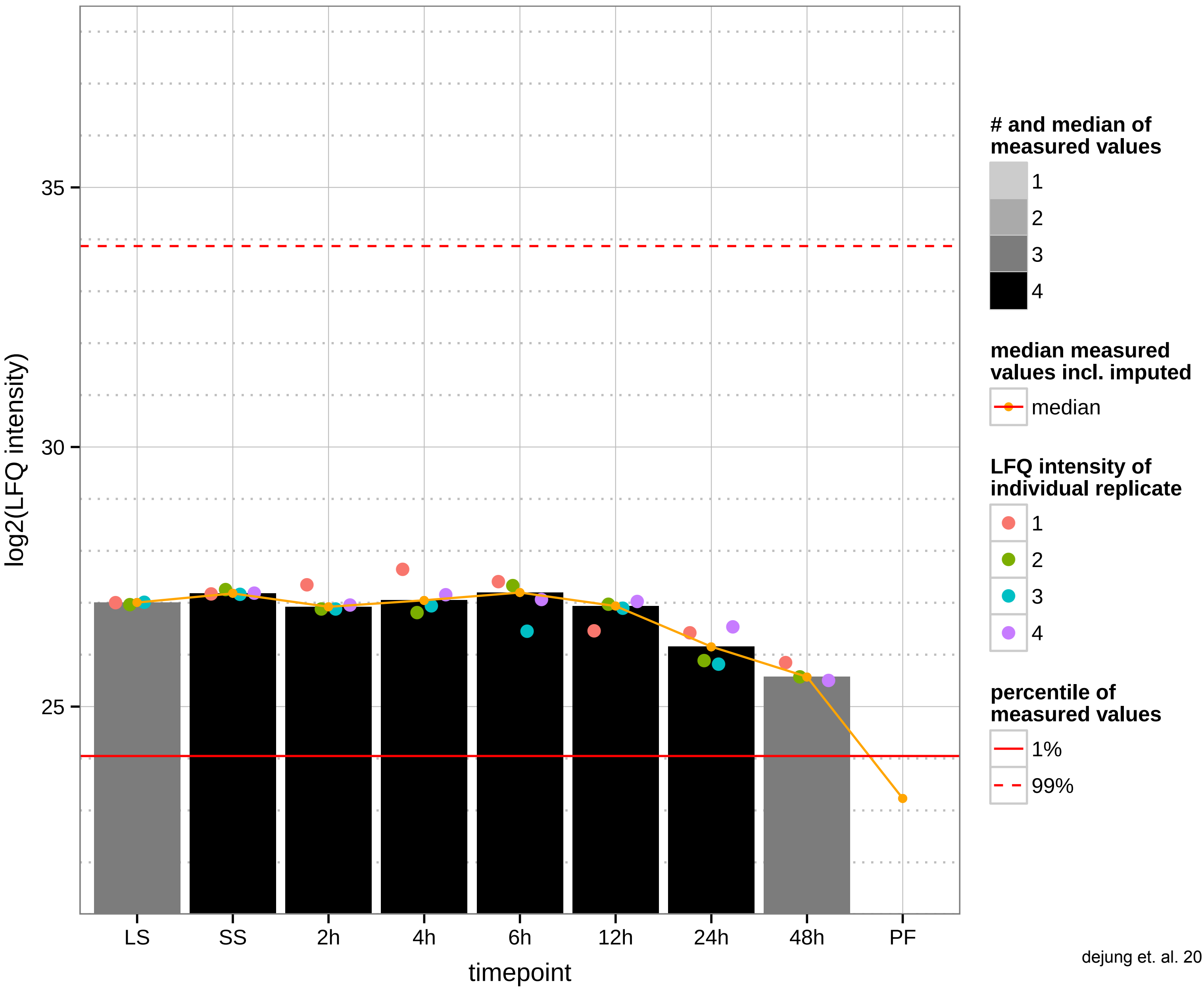

dejung et. al. 2015

glycerol uptake protein, putative
Tb927.10.15910;Tb11.v5.0406
AGOF: null
AGOC: null
AGOP: null
PGOF: null
PGOC: null
PGOP: null

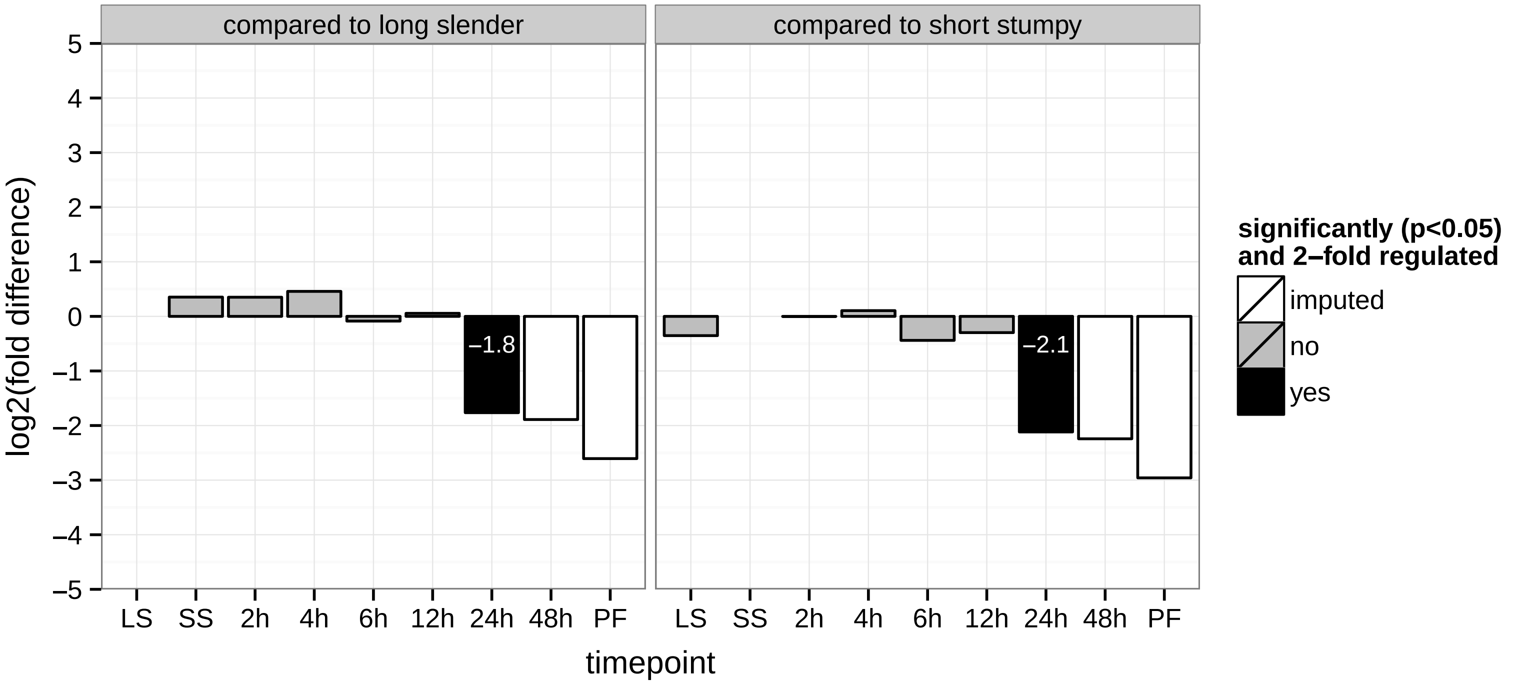

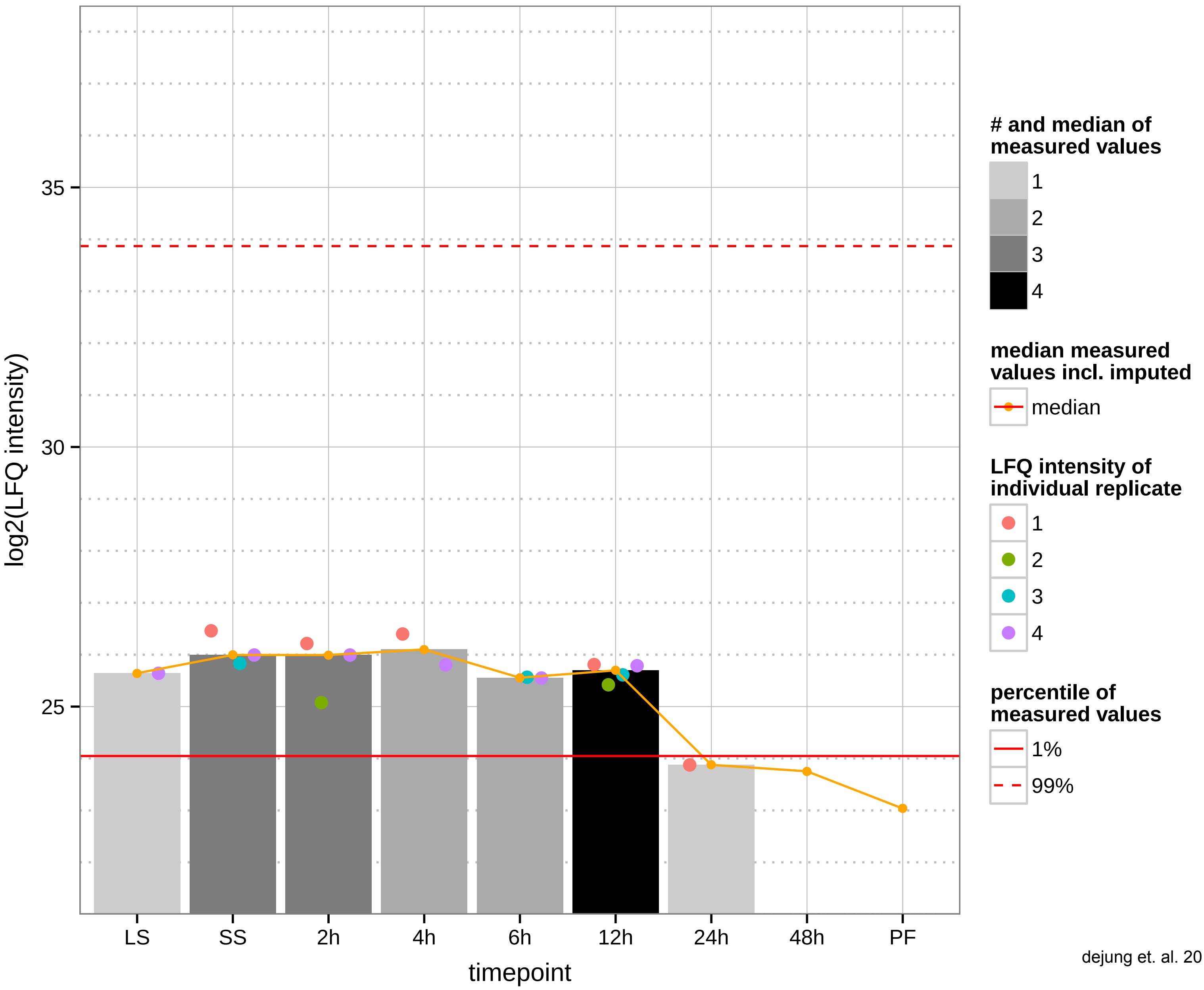

dejung et. al. 2015

protein kinase A regulatory subunit, putative, protein kinase A regulatory subunit (PKA−R)
Tb927.11.4610;Tb11.v5.0587
AGOF: null, cAMP−dependent protein kinase regulator activity
AGOC: null, cAMP−dependent protein kinase complex
AGOP: null, intracellular signal transduction, modulation of development of symbiont involved in interaction with host, protein p
PGOF: null
PGOC: null
PGOP: null

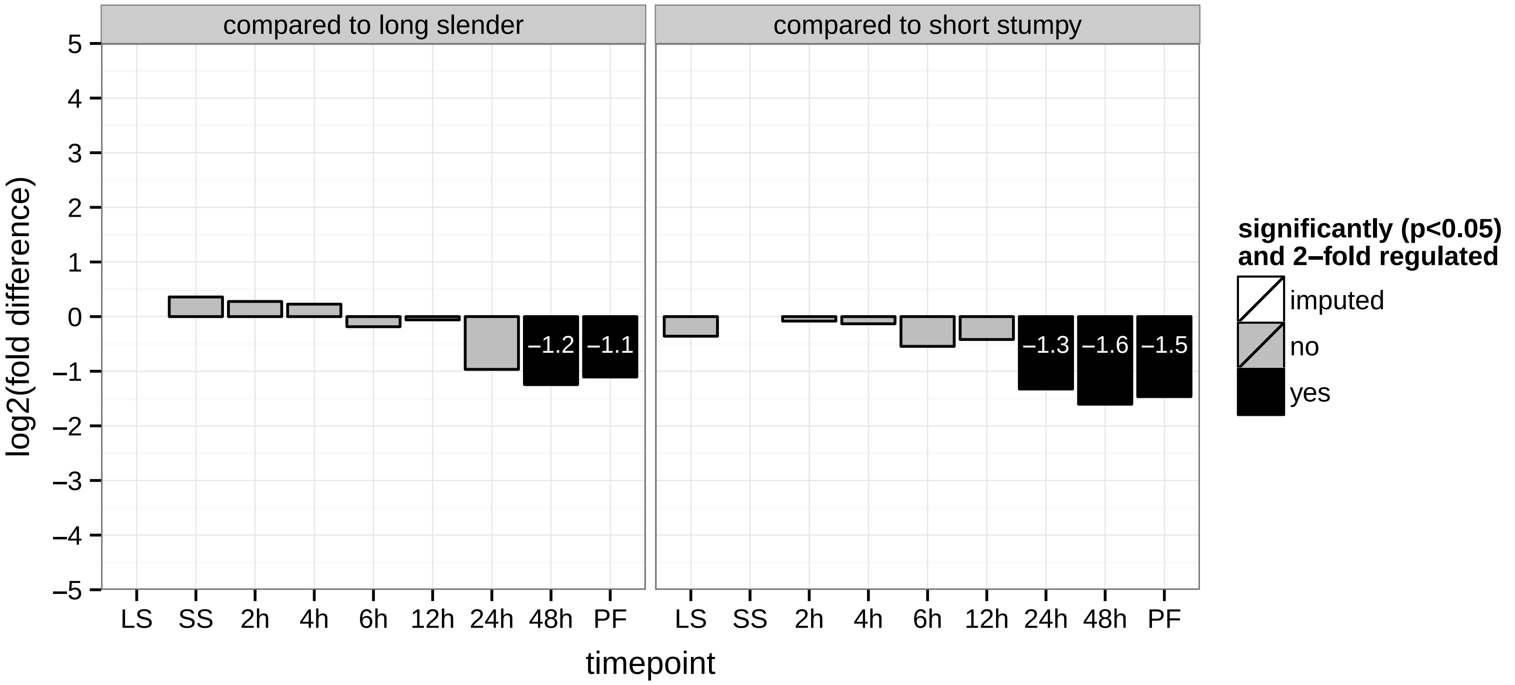

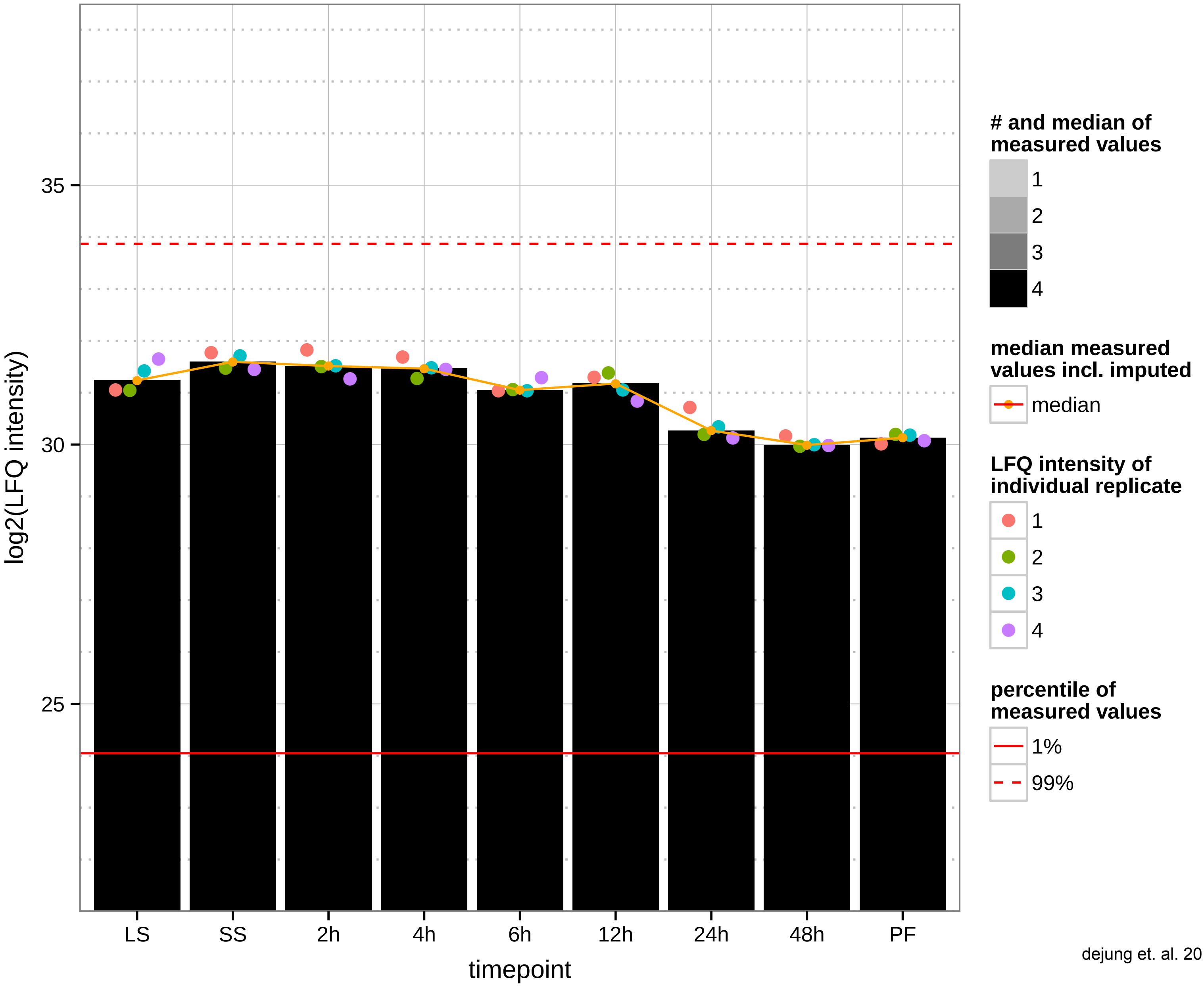

dejung et. al. 2015

kinesin, putative, C–terminal motor kinesin, putative (TBKIFC1)
Tb927.10.14890;Tb11.v5.0636
AGOF: null, ATP binding, microtubule motor activity, motor activity
AGOC: null, cytoplasm, cytoplasmic microtubule, kinesin complex, kinesin complex, microtubule associated complex
AGOP: null, intracellular protein transport, microtubule–based movement, microtubule–based movement
PGOF: ATP binding, microtubule motor activity
PGOC: null
PGOP: microtubule–based movement, intracellular protein transport, microtubule–based movement

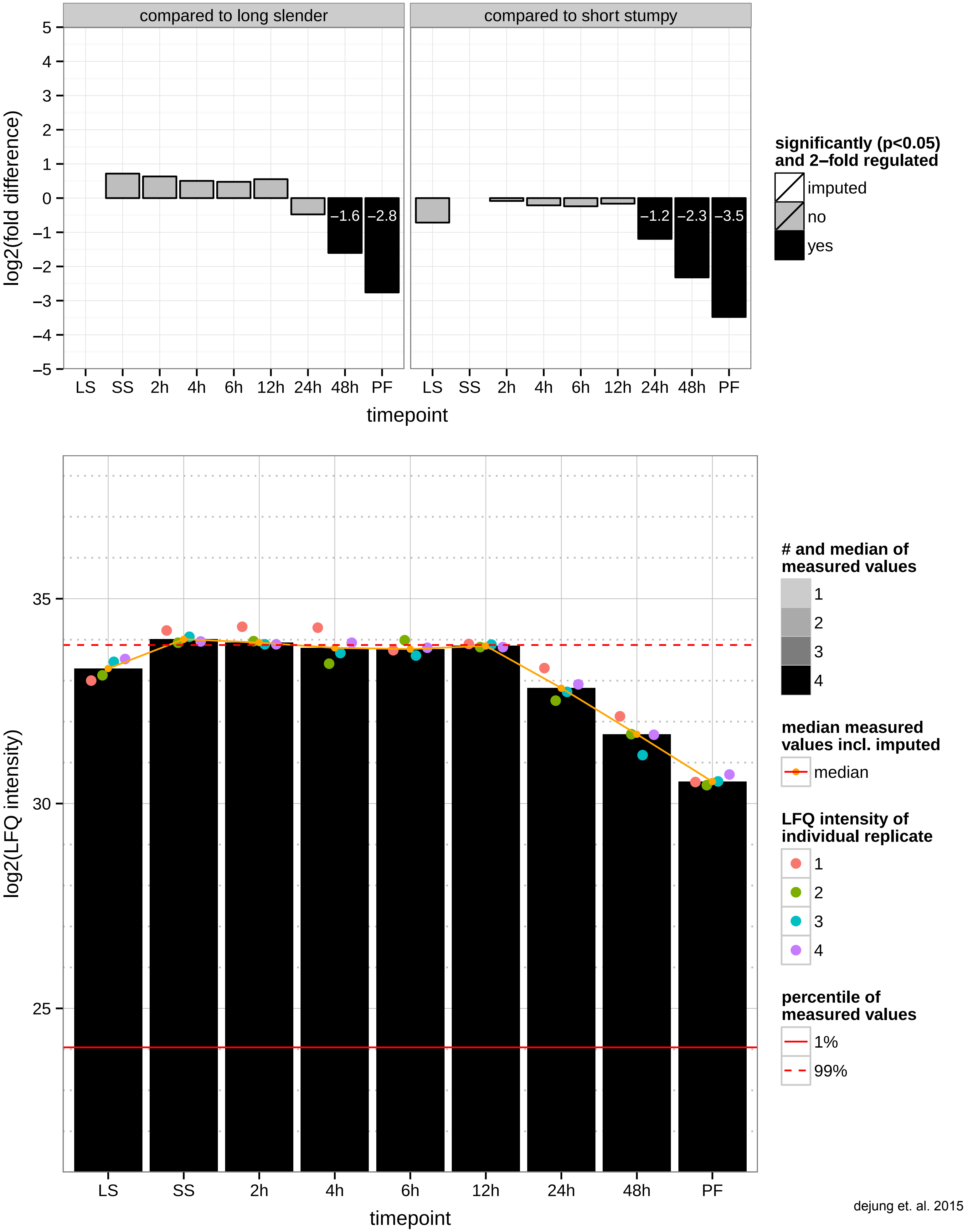

dejung et. al. 2015

expression site–associated gene (ESAG) protein, putative, expression site–associated gene 5 (ESAG5) protein
Tb11.v5.0826;Tb927.7.6860
AGOF: null, lipid binding
AGOC: null
AGOP: null
PGOF: lipid binding
PGOC: null
PGOP: null

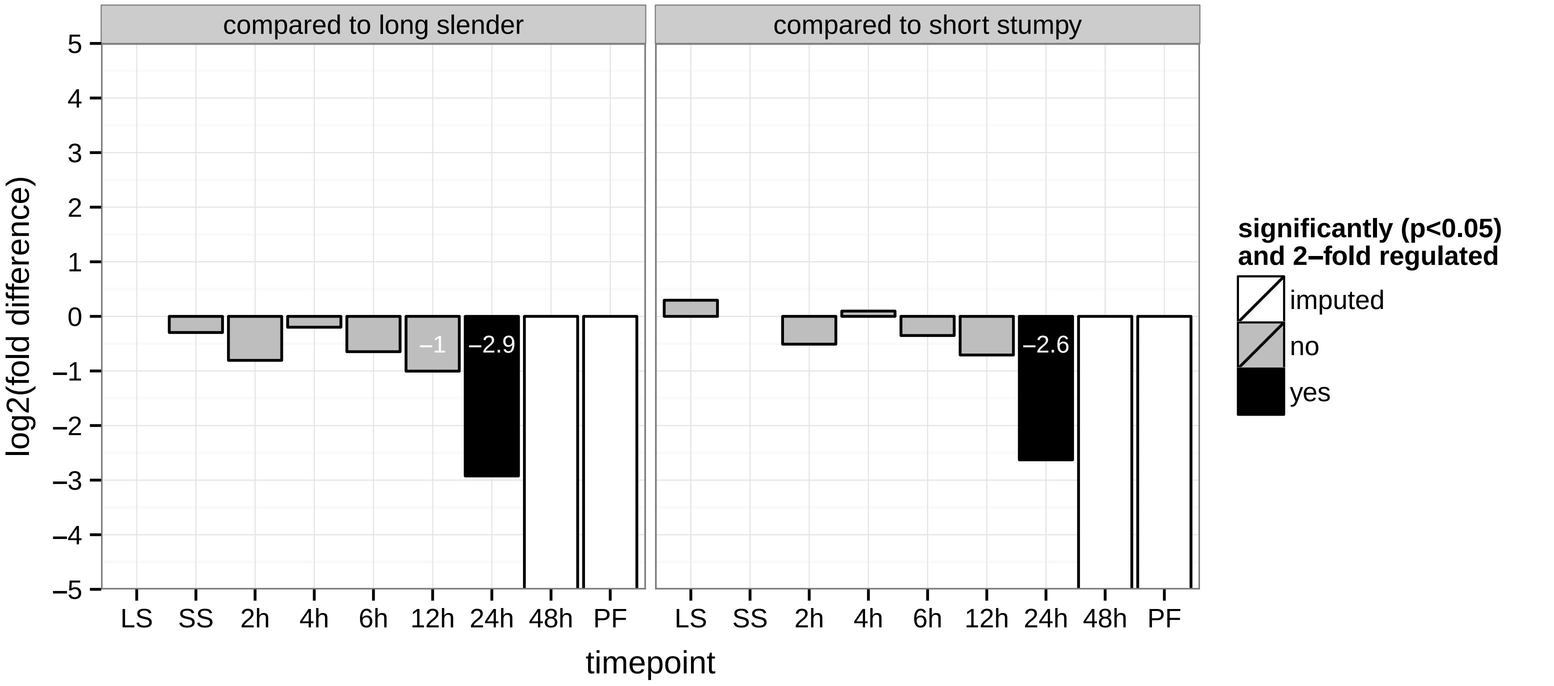

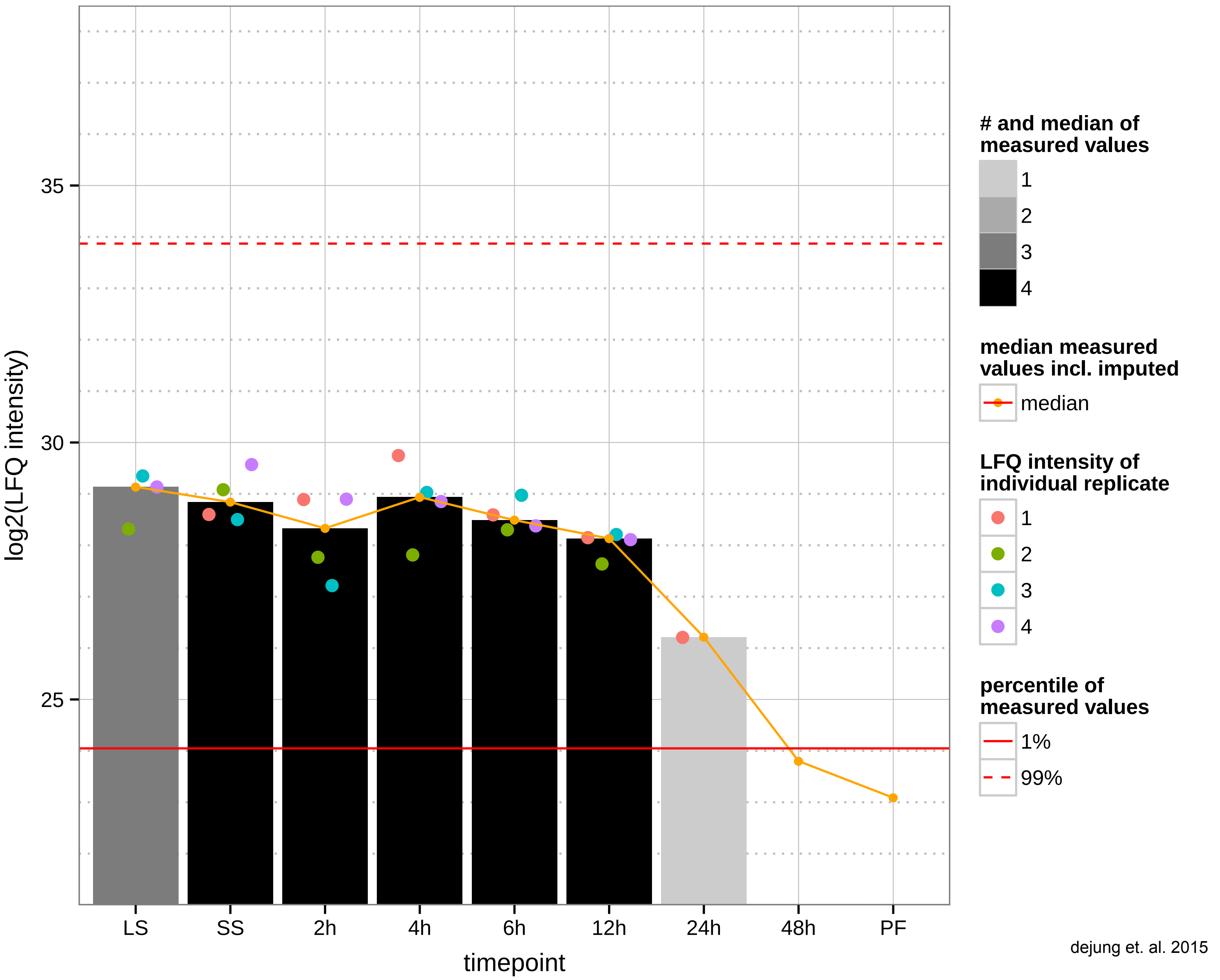

dejung et. al. 2015

aminoacylase, putative, N–acyl–L–amino acid amidohydrolase
Tb927.1.3000
AGOF: hydrolase activity, metallopeptidase activity, protein dimerization activity
AGOC: null
AGOP: proteolysis
PGOF: hydrolase activity
PGOC: null
PGOP: metabolic process

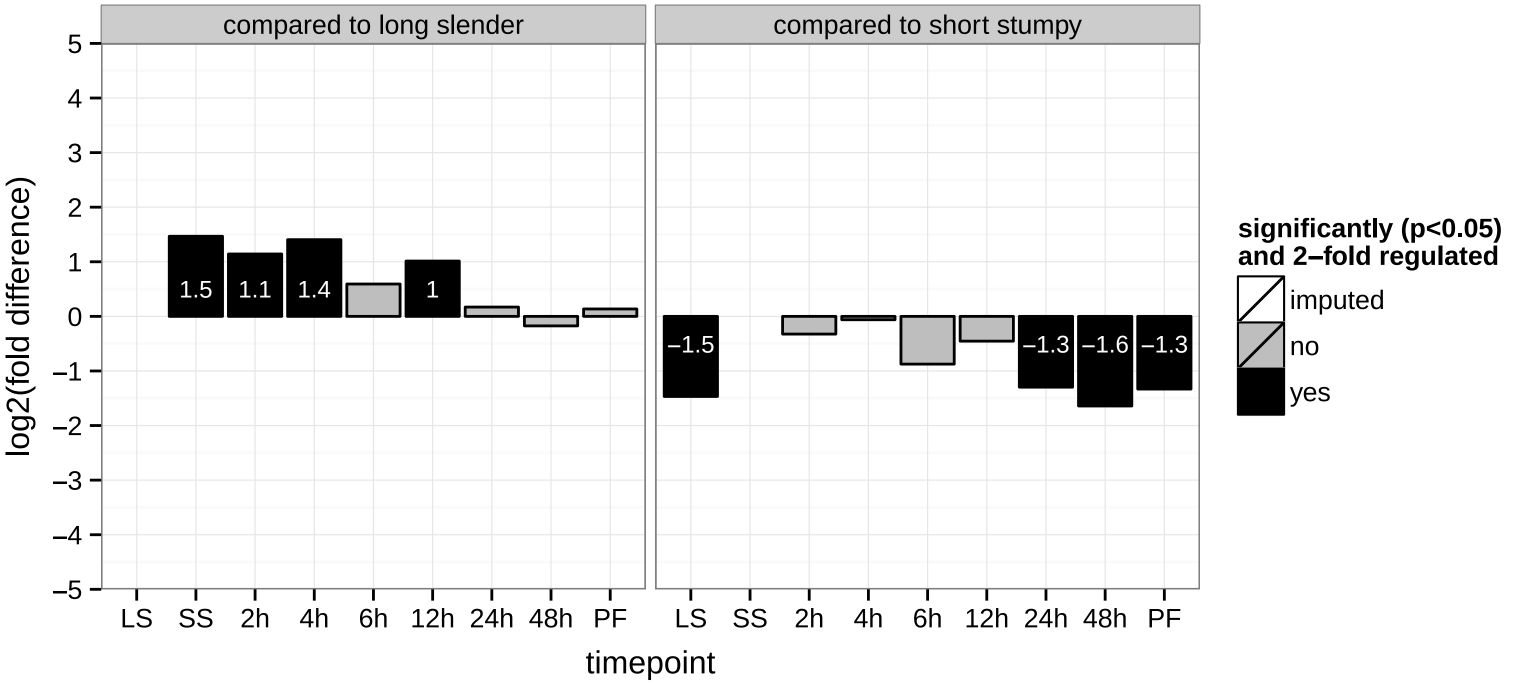

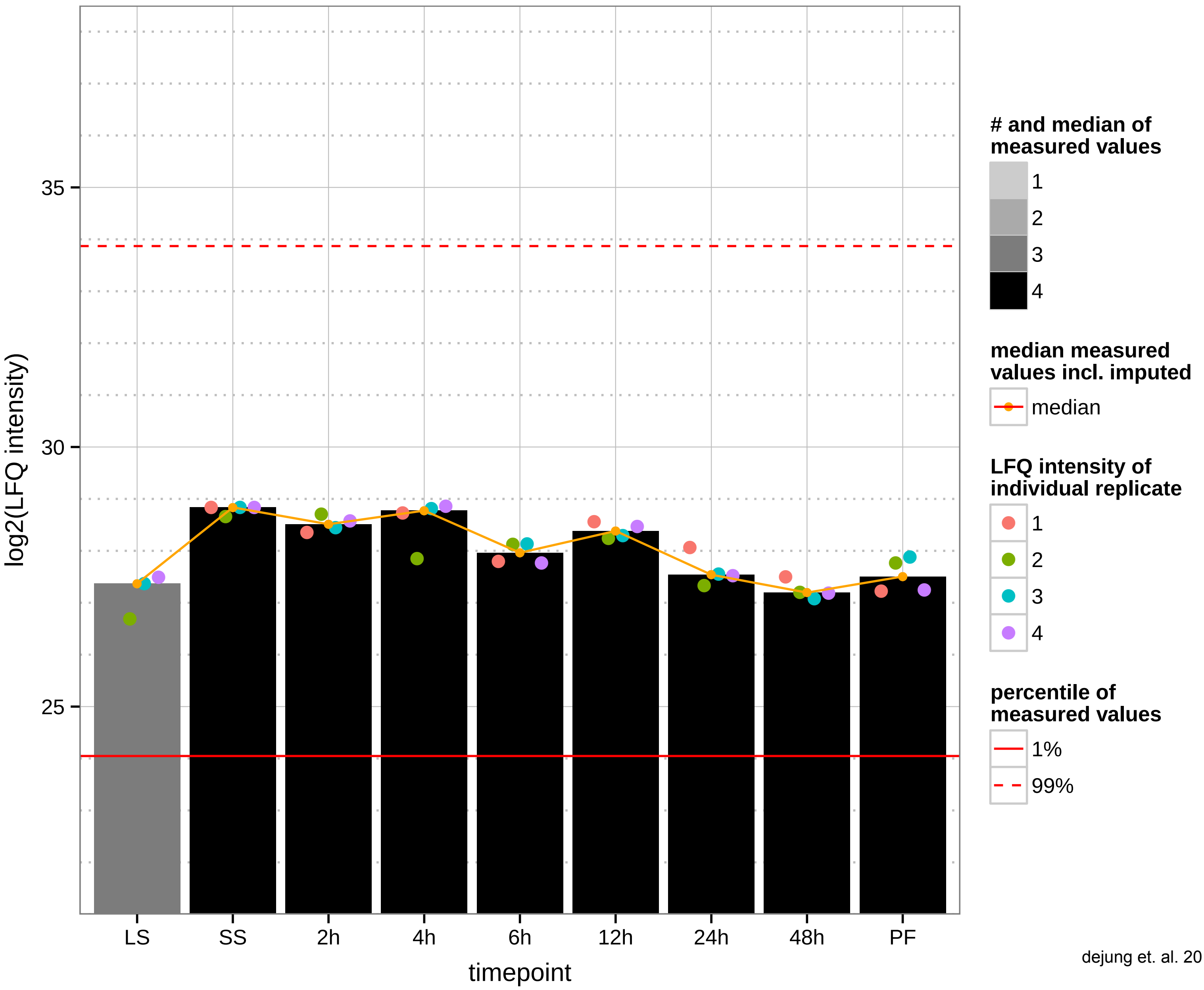

dejung et. al. 2015

hypothetical protein, conserved
Tb927.1.3260
AGOF: null
AGOC: null
AGOP: null
PGOF: binding
PGOC: null
PGOP: null

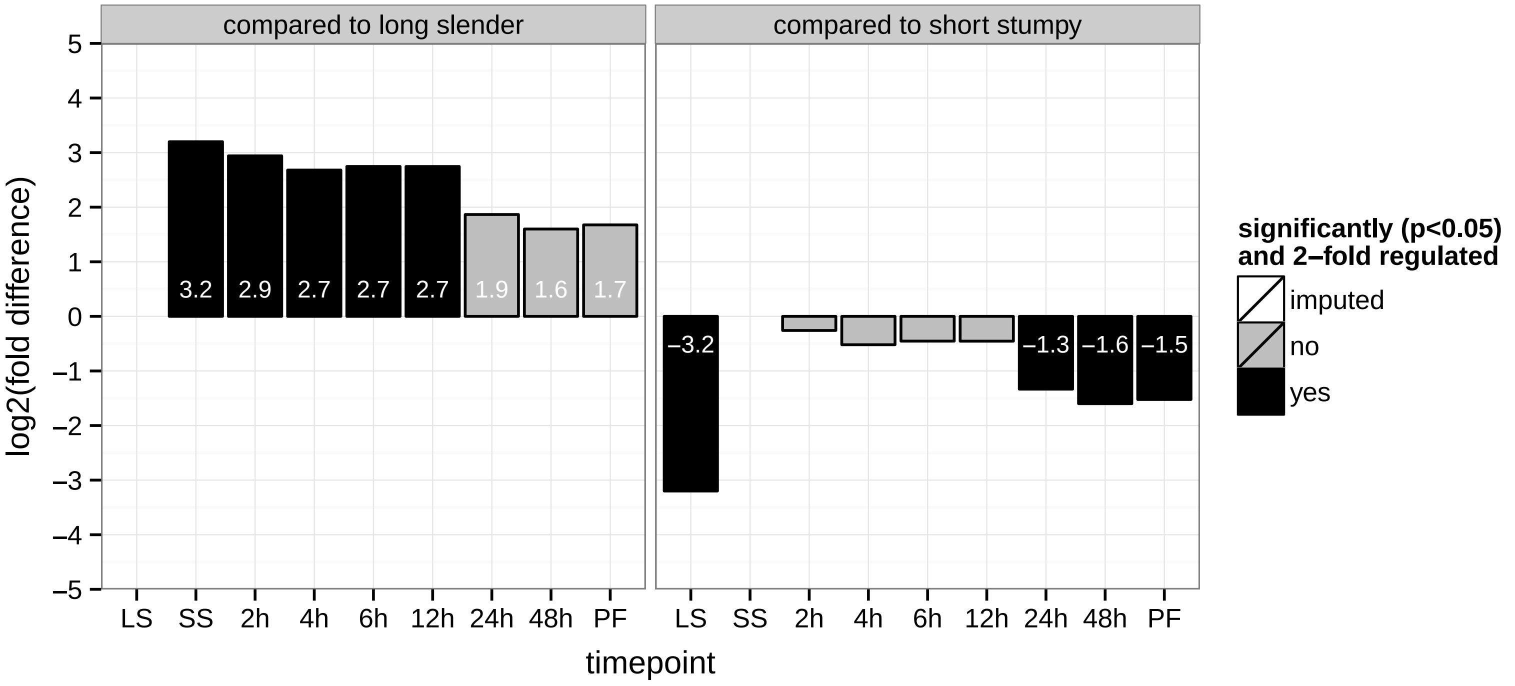

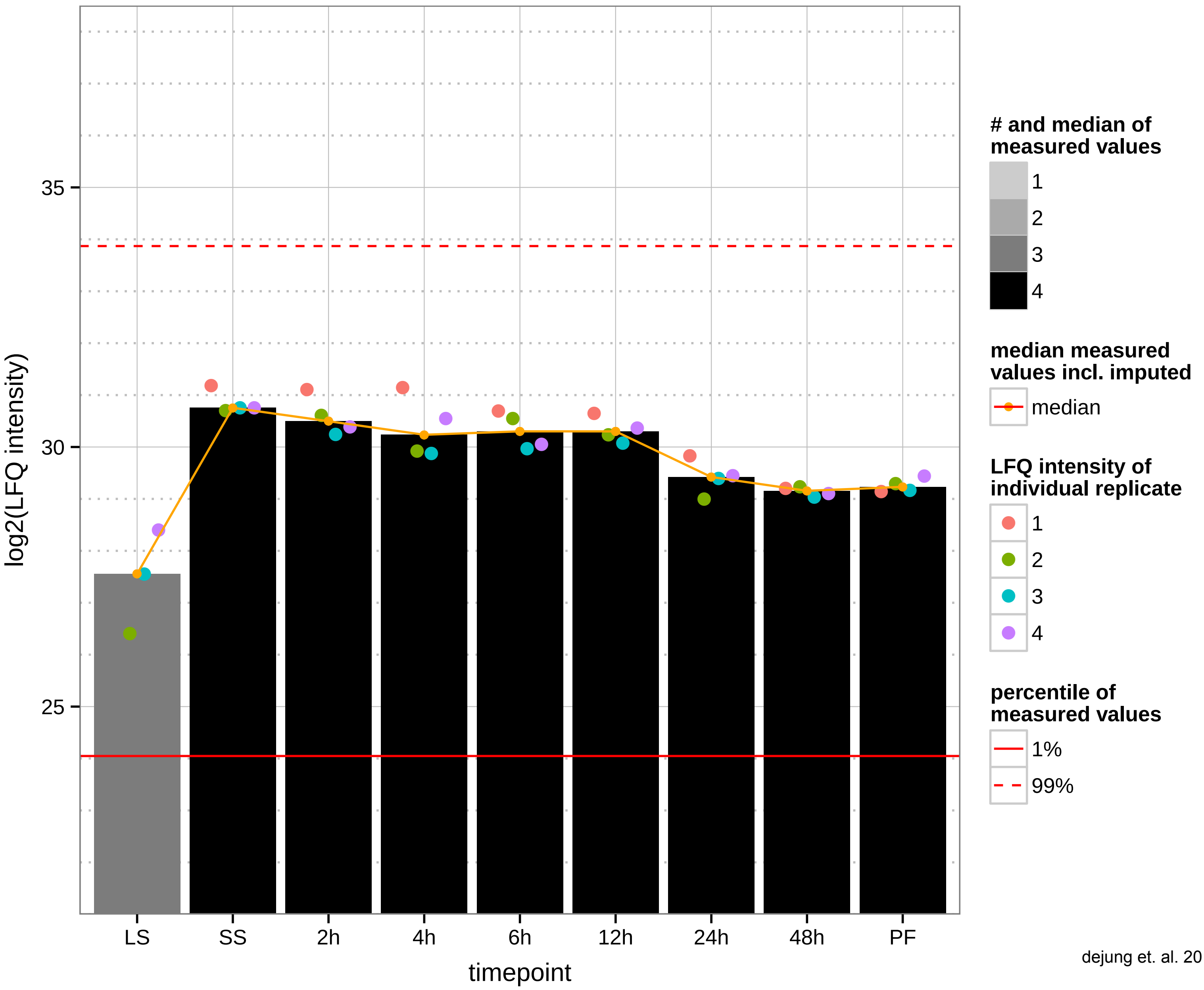

dejung et. al. 2015

glucose−6−phosphate isomerase, glycosomal (PGI)
Tb927.1.3830
AGOF: glucose−6−phosphate isomerase activity
AGOC: glycosome
AGOP: gluconeogenesis, glycolysis
PGOF: glucose−6−phosphate isomerase activity
PGOC: null
PGOP: gluconeogenesis, glycolysis

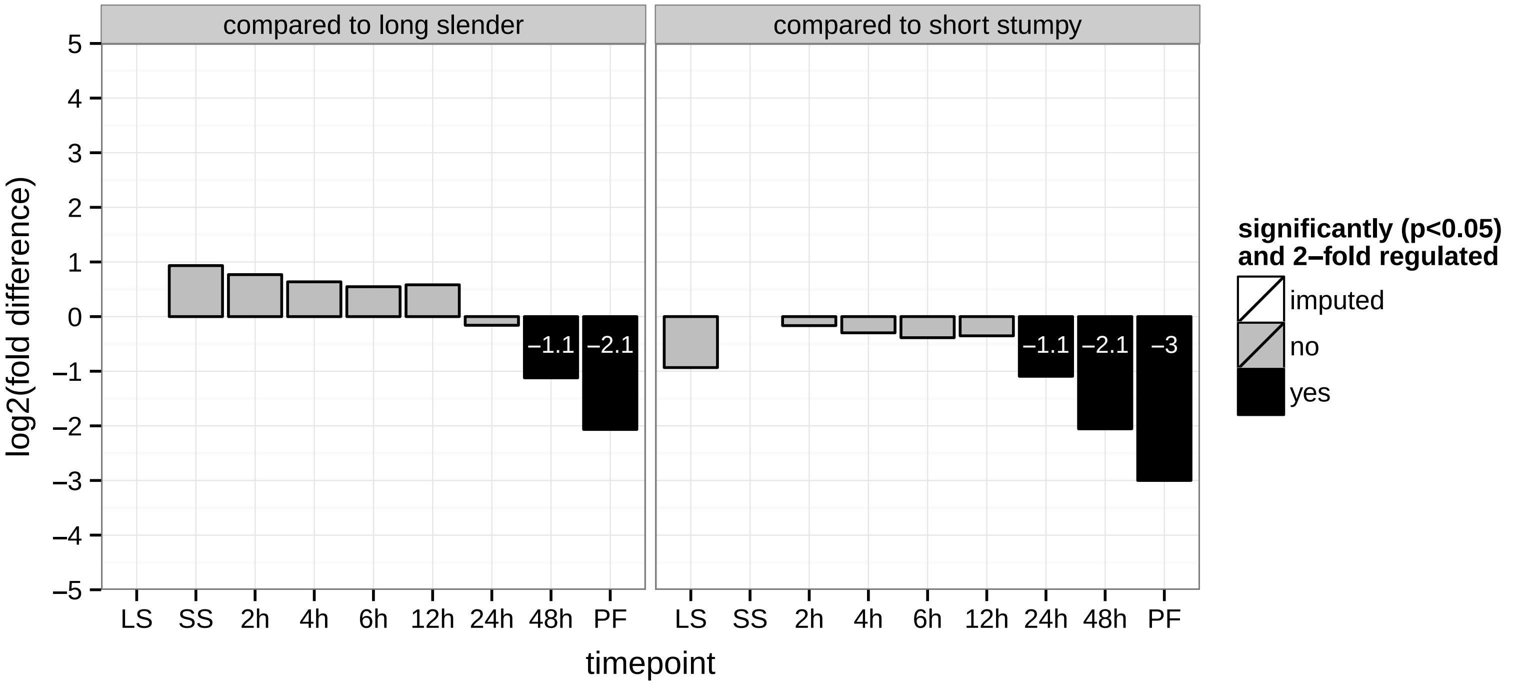

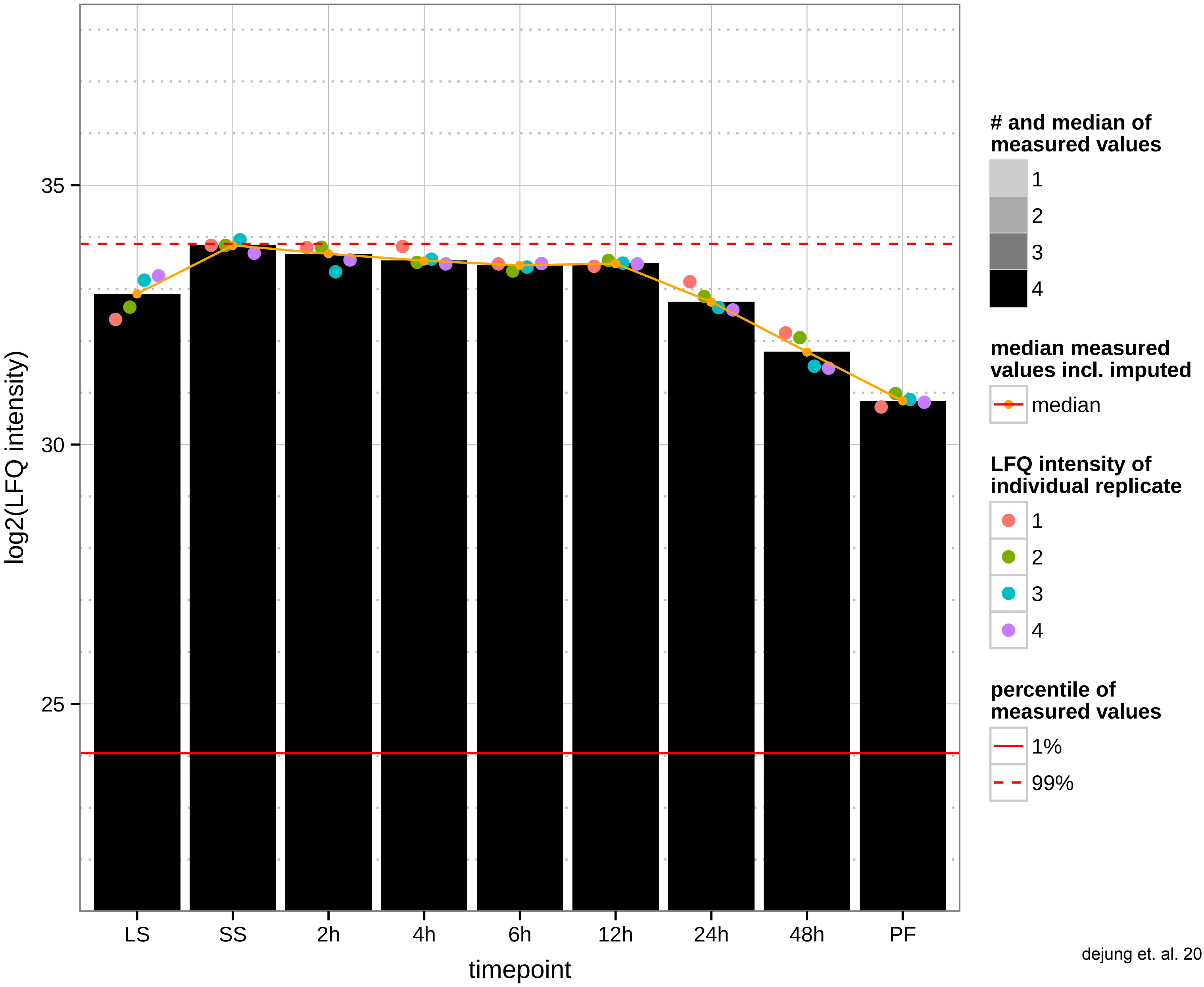

dejung et. al. 2015

RNA–binding protein, putative (RBP7A)
Tb927.10.12090
AGOF: RNA binding
AGOC: null
AGOP: RNA processing, modulation of development of symbiont involved in interaction with host, quorum sensing involved in
PGOF: nucleic acid binding
PGOC: null
PGOP: null

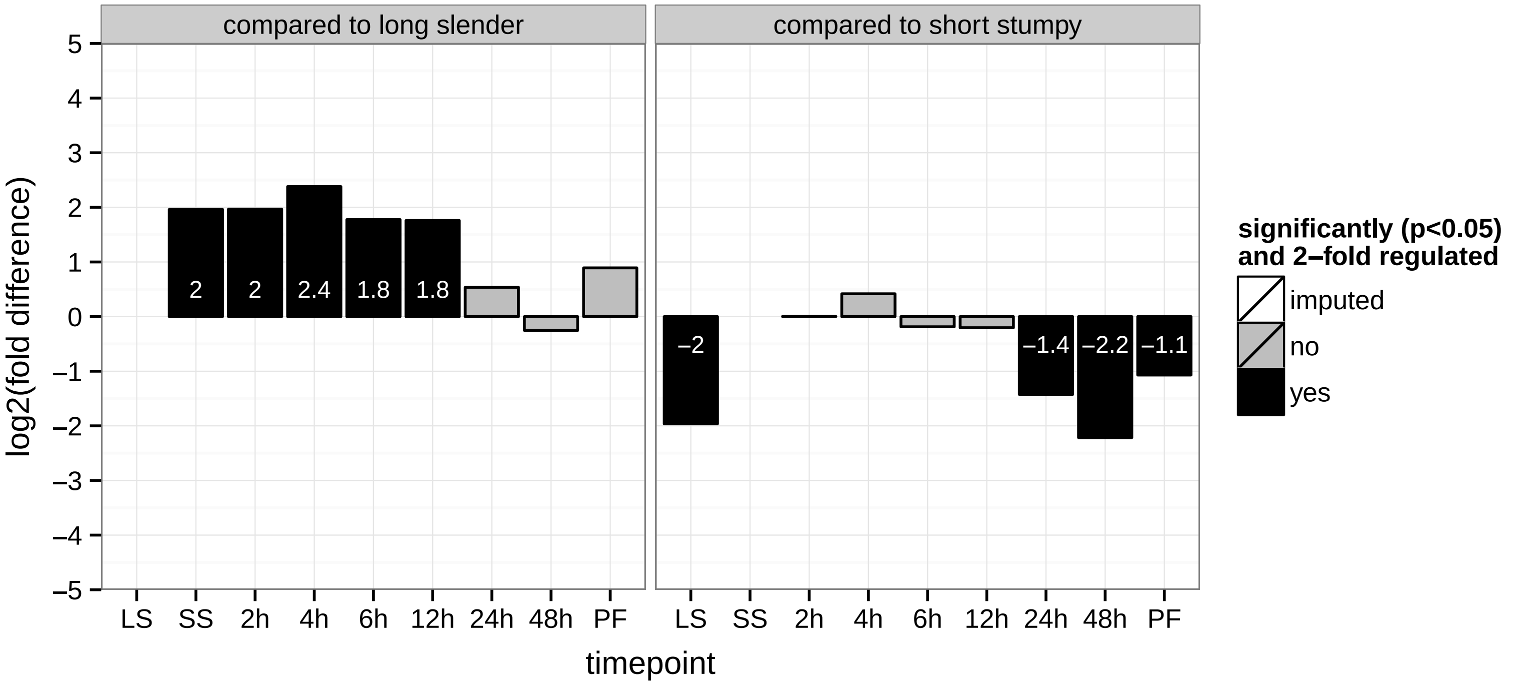

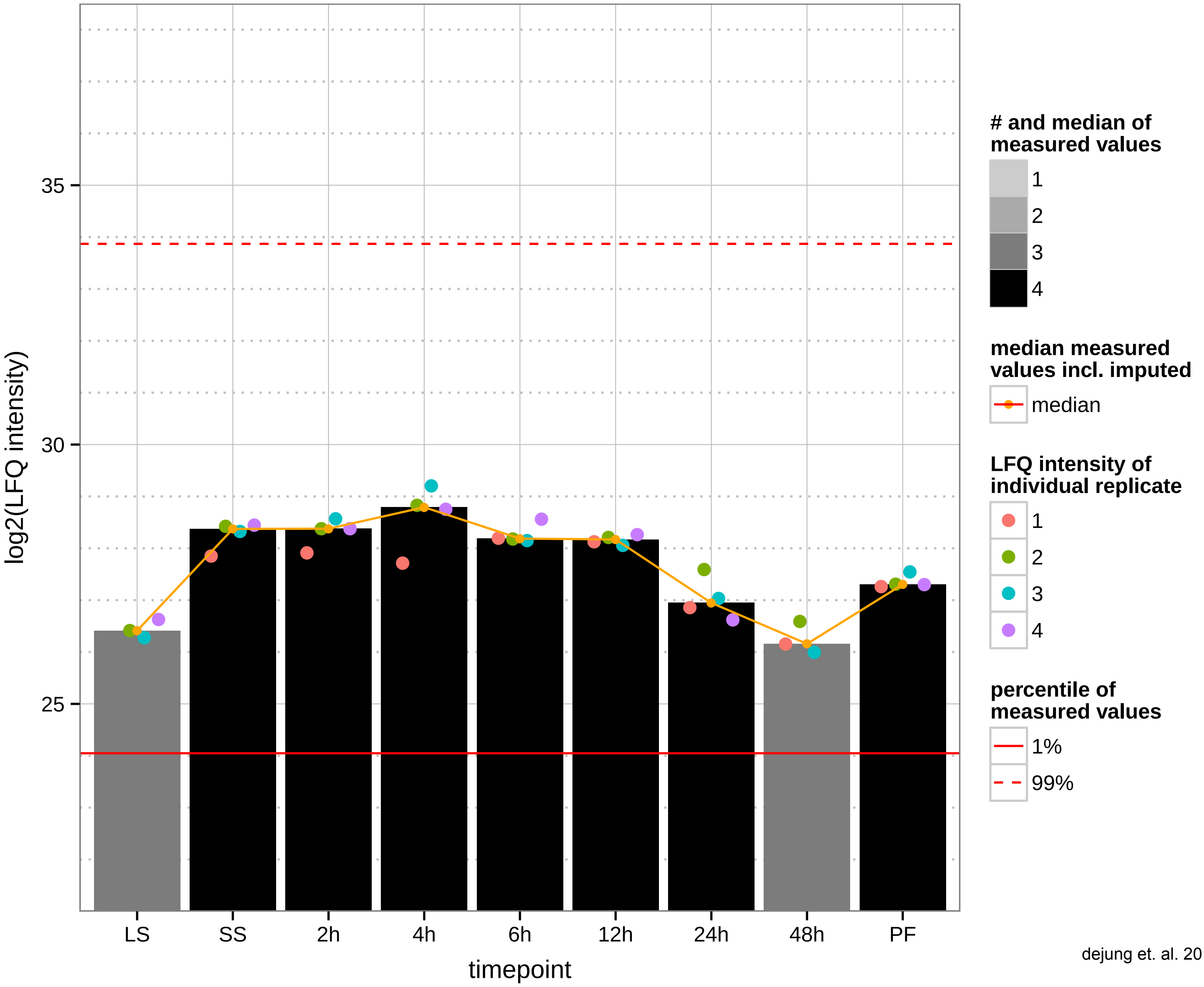

dejung et. al. 2015

hypothetical protein, conserved
Tb927.10.12590
AGOF: null
AGOC: null
AGOP: null
PGOF: protein binding
PGOC: null
PGOP: null

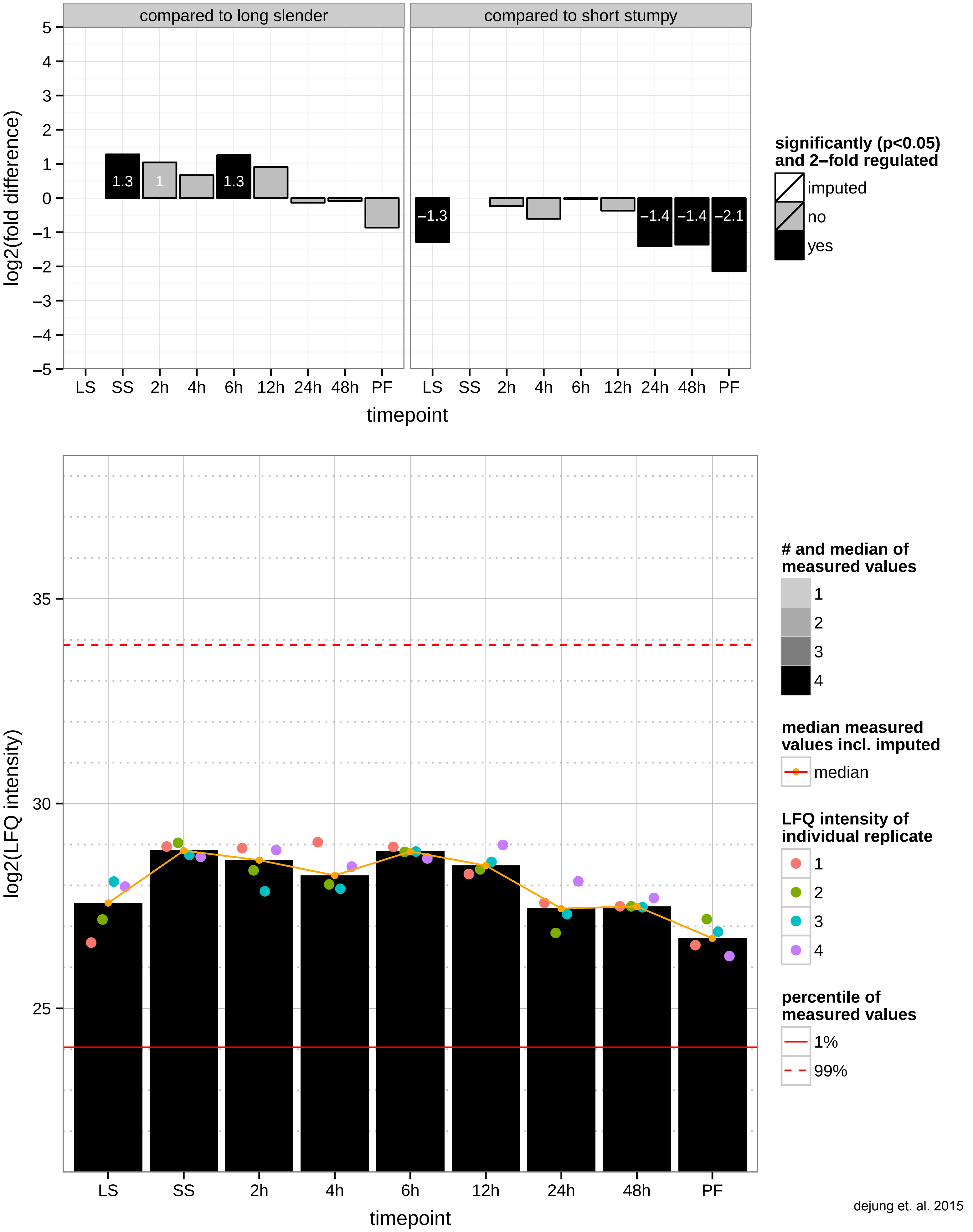

dejung et. al. 2015

hypothetical protein, conserved
Tb927.10.12820
AGOF: null
AGOC: null
AGOP: null
PGOF: null
PGOC: null
PGOP: null

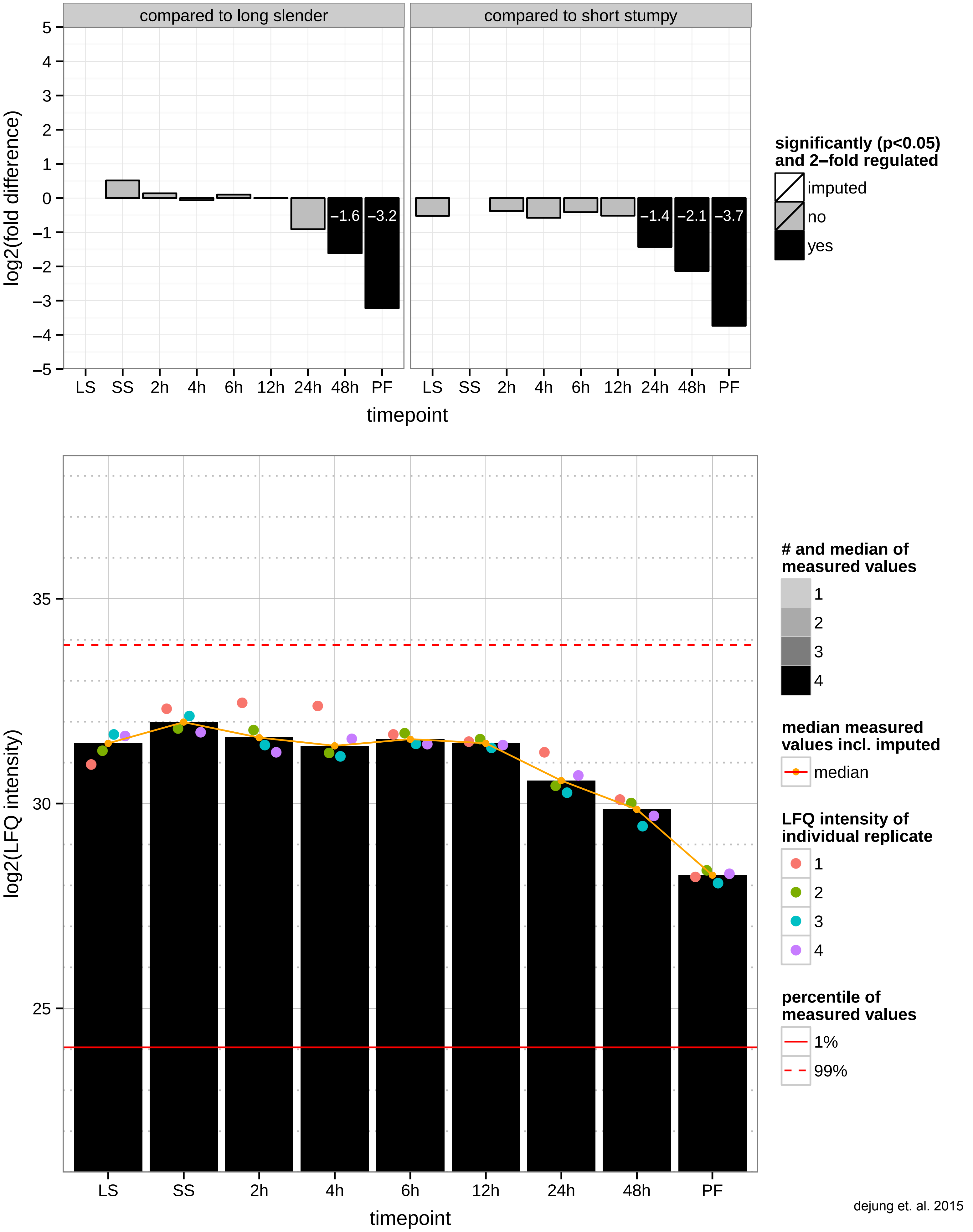

dejung et. al. 2015

hypothetical protein, conserved
Tb927.10.13790
AGOF: null
AGOC: null
AGOP: null
PGOF: null
PGOC: null
PGOP: null

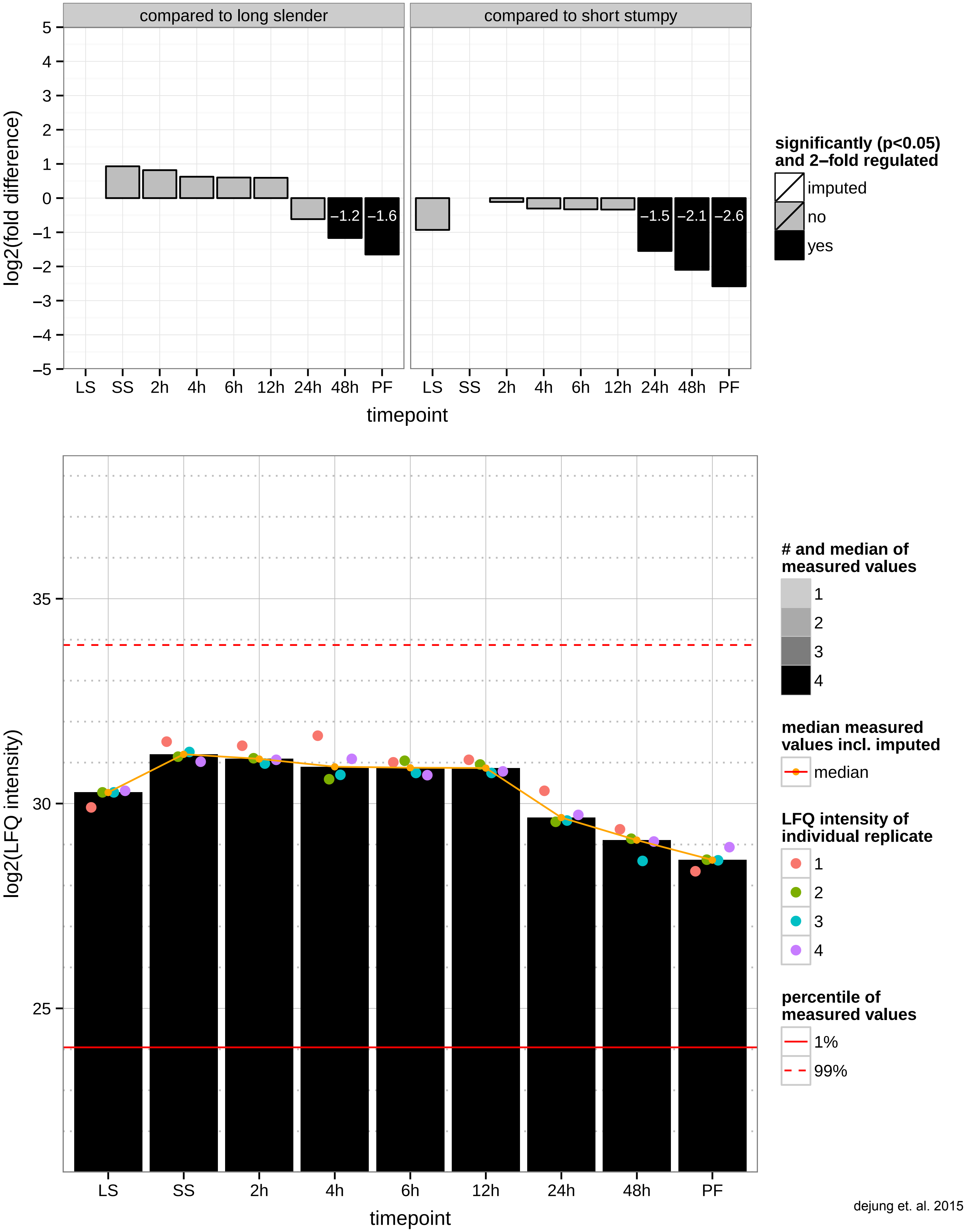

pyruvate kinase 1, putative, pyruvate kinase 1 (PYK1)
Tb927.10.14140;Tb11.v5.0605
AGOF: null, magnesium ion binding, potassium ion binding, pyruvate kinase activity
AGOC: null, cytoplasm
AGOP: null, glycolysis
PGOF: catalytic activity, magnesium ion binding, potassium ion binding, pyruvate kinase activity
PGOC: null
PGOP: glycolysis

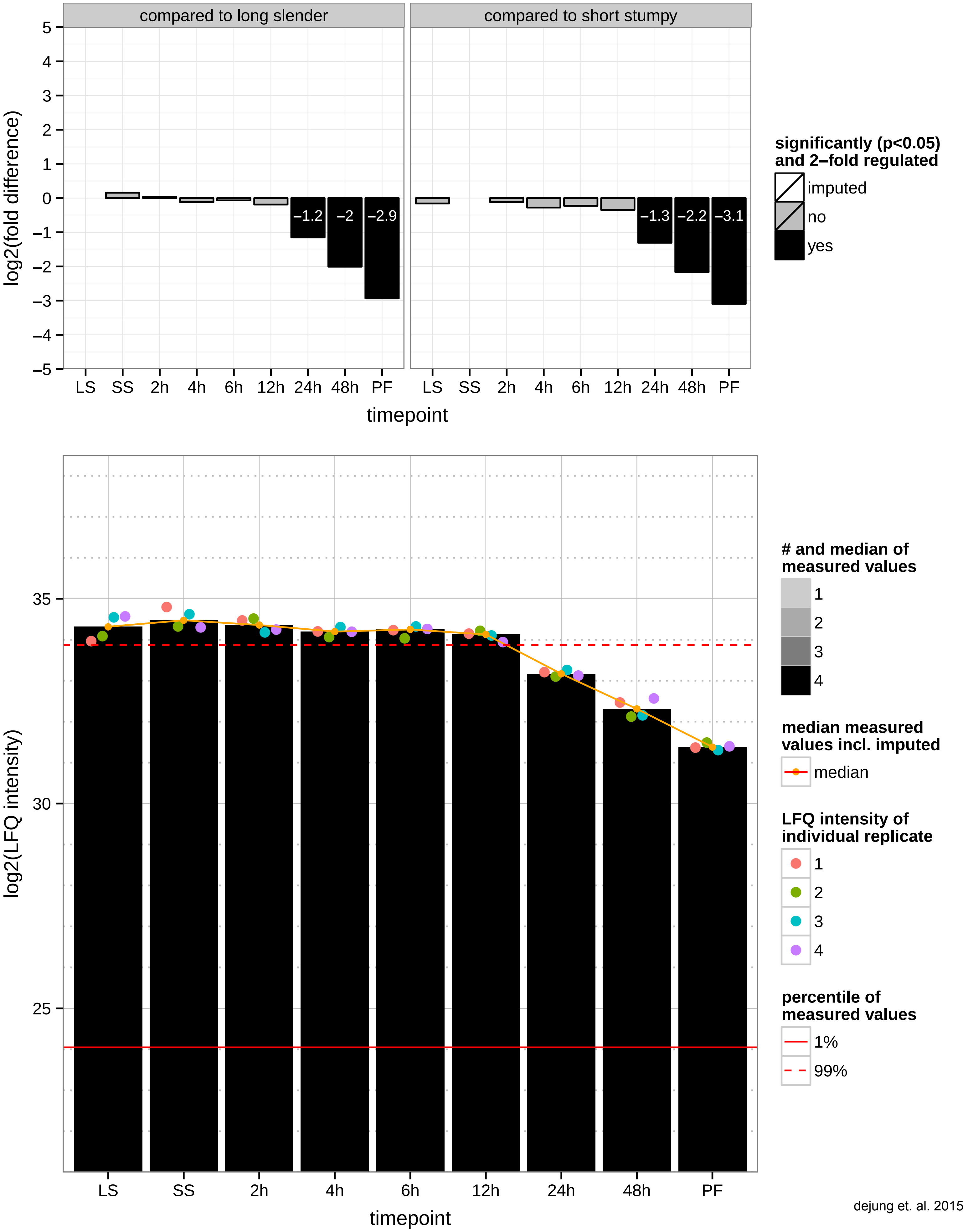

dejung et. al. 2015

Aquaglyceroporin 3 (AQP3)
Tb927.10.14160
AGOF: glycerol channel activity, water channel activity
AGOC: plasma membrane
AGOP: glycerol transport
PGOF: transporter activity
PGOC: integral to membrane, membrane
PGOP: transmembrane transport, transport, water transport

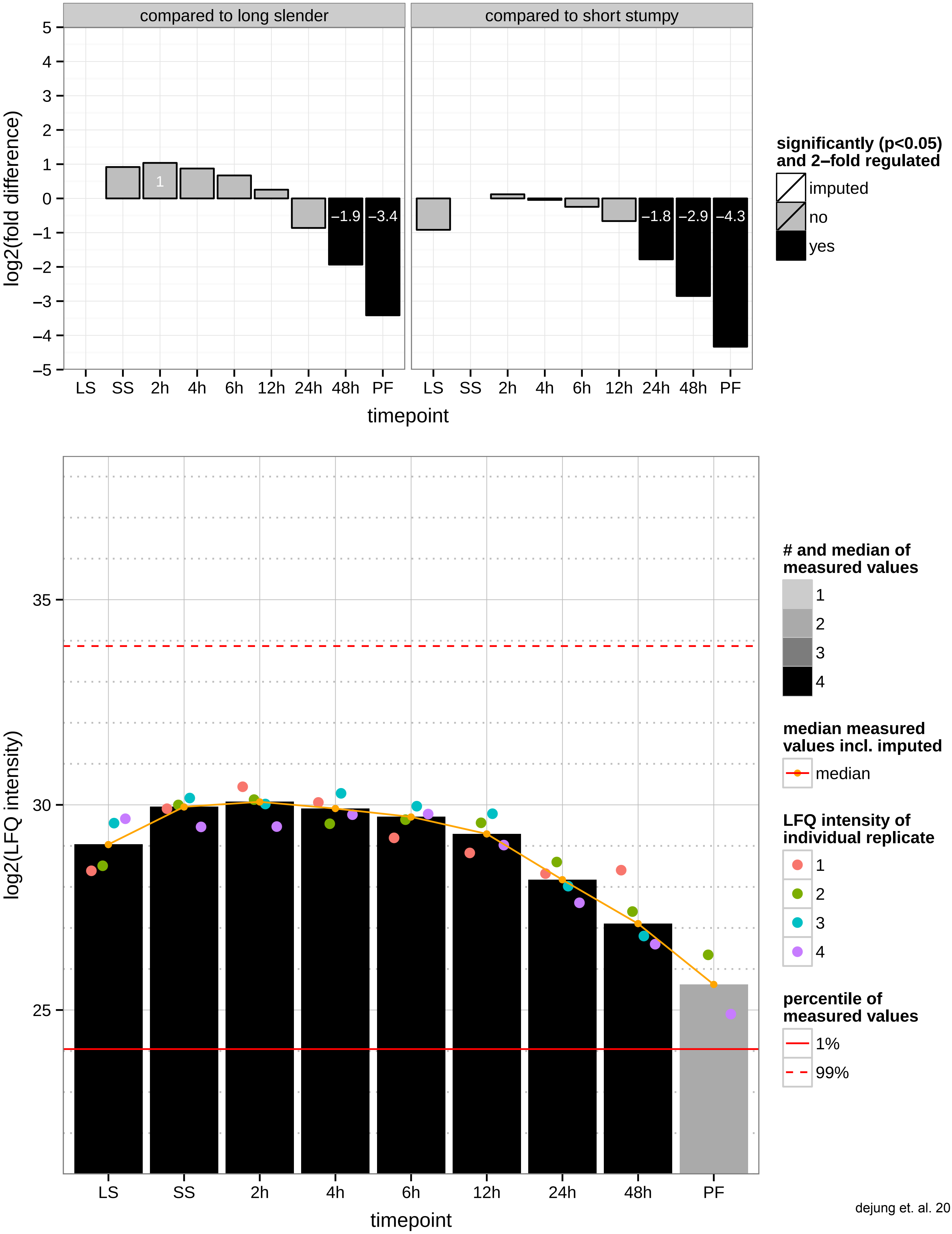

dejung et. al. 2015

hypothetical protein, conserved
Tb927.10.15440
AGOF: null
AGOC: null
AGOP: null
PGOF: null
PGOC: null
PGOP: null

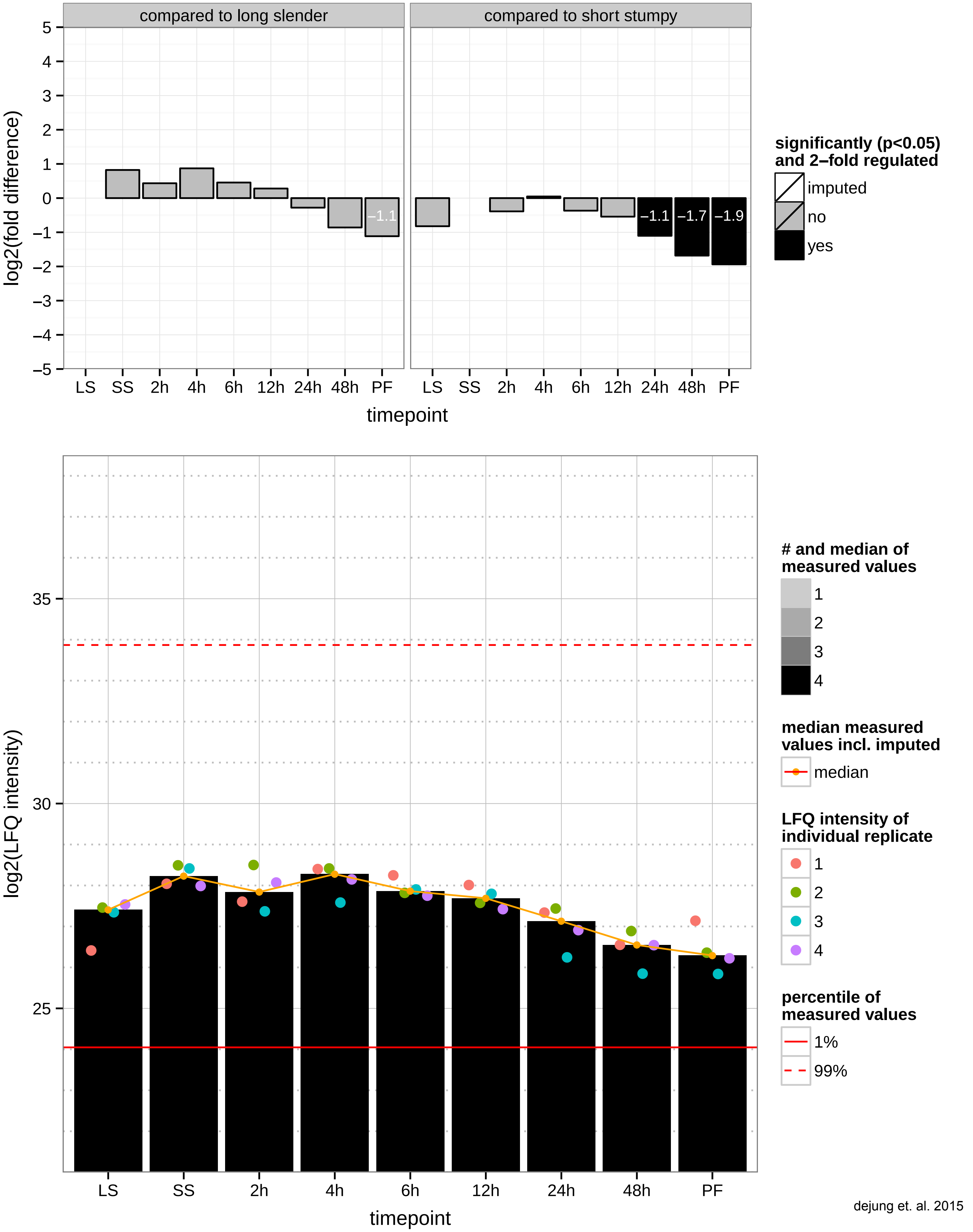

dejung et. al. 2015

Metacaspase−4 (MCA4)
Tb927.10.2440
AGOF: cysteine−type endopeptidase activity, peptidase activity
AGOC: nucleus
AGOP: aerobic respiration, proteolysis
PGOF: cysteine−type endopeptidase activity
PGOC: null
PGOP: proteolysis

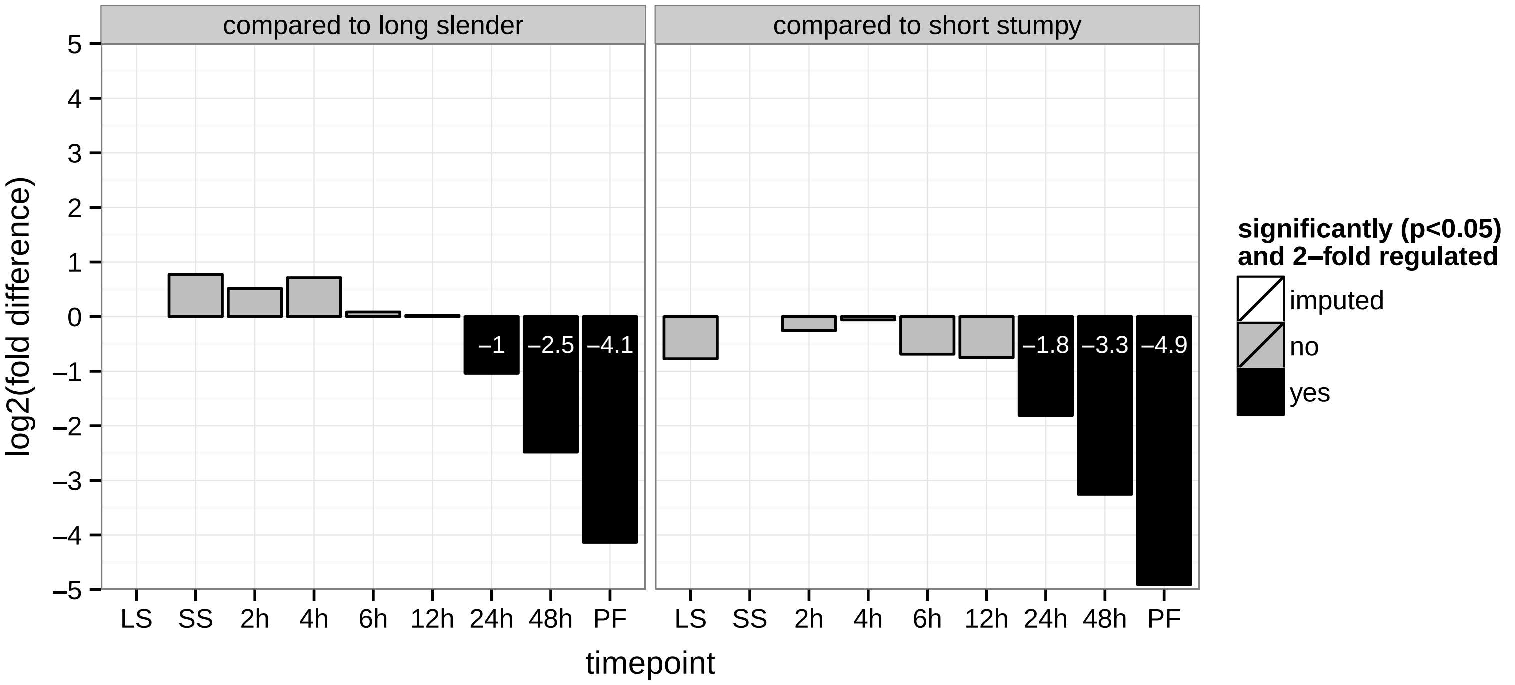

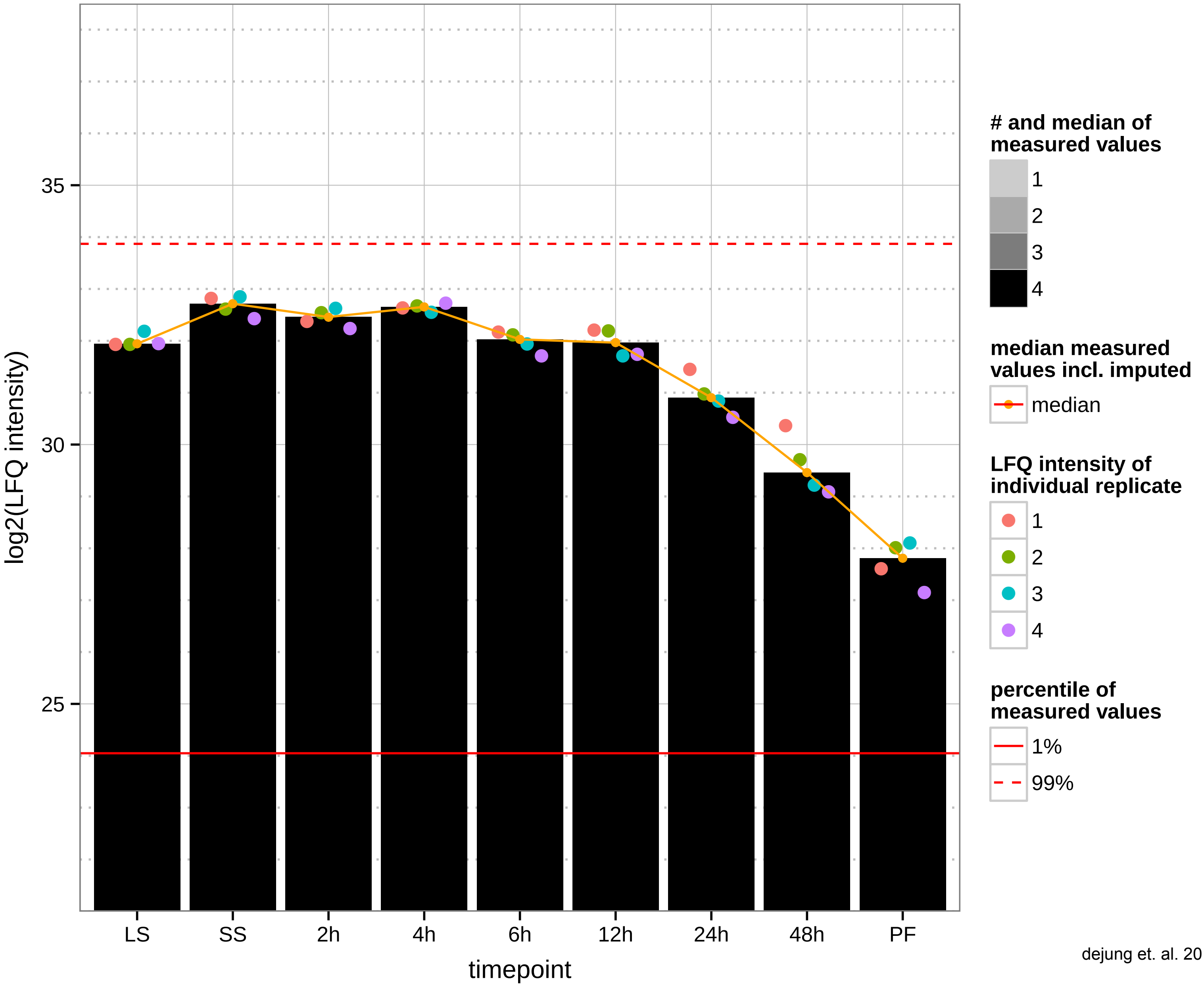

dejung et. al. 2015

lysosomal alpha–mannosidase precursor, putative
Tb927.10.2570;Tb11.v5.0498
AGOF: null, alpha–mannosidase activity, carbohydrate binding, zinc ion binding
AGOC: null, lysosome
AGOP: null, carbohydrate metabolic process, mannose metabolic process
PGOF: carbohydrate binding, catalytic activity, hydrolase activity, hydrolyzing O–glycosyl compounds, mannosidase activity, zi
PGOC: null
PGOP: carbohydrate metabolic process, mannose metabolic process

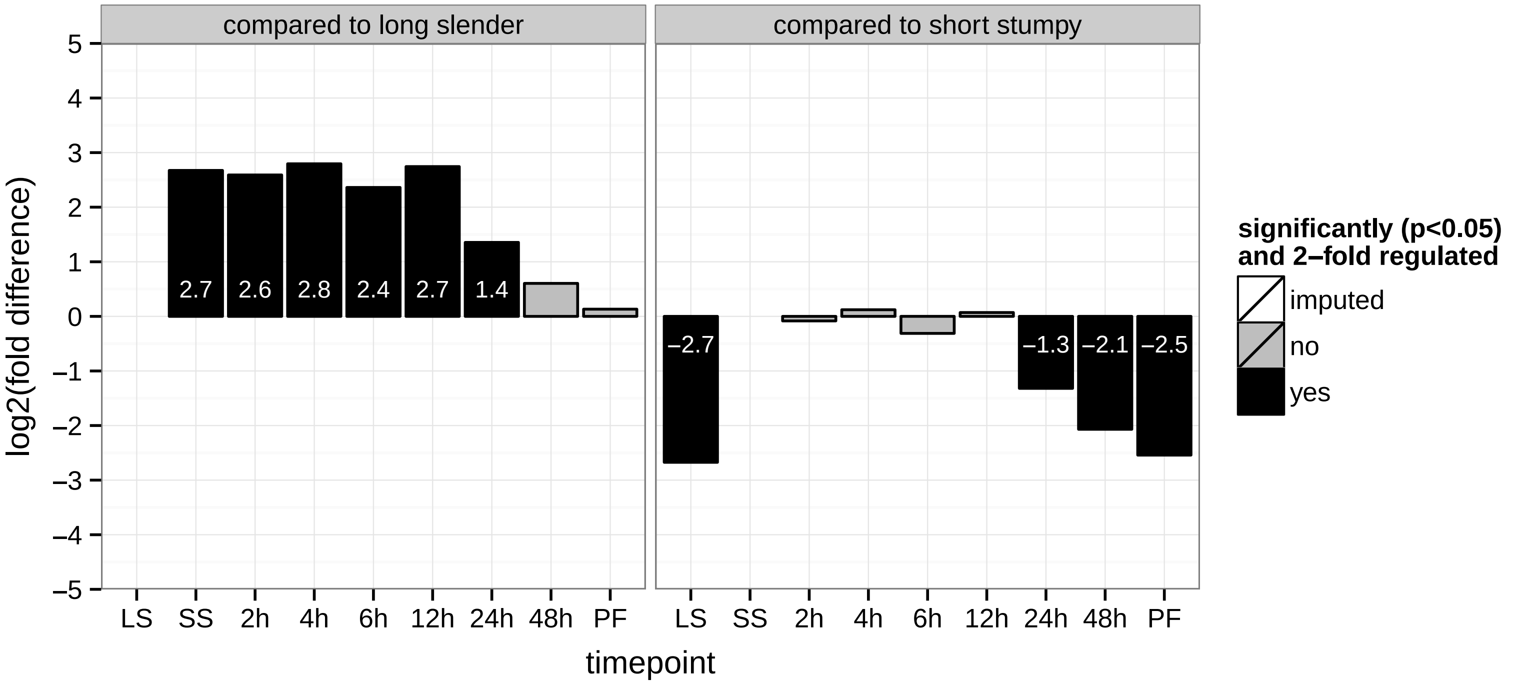

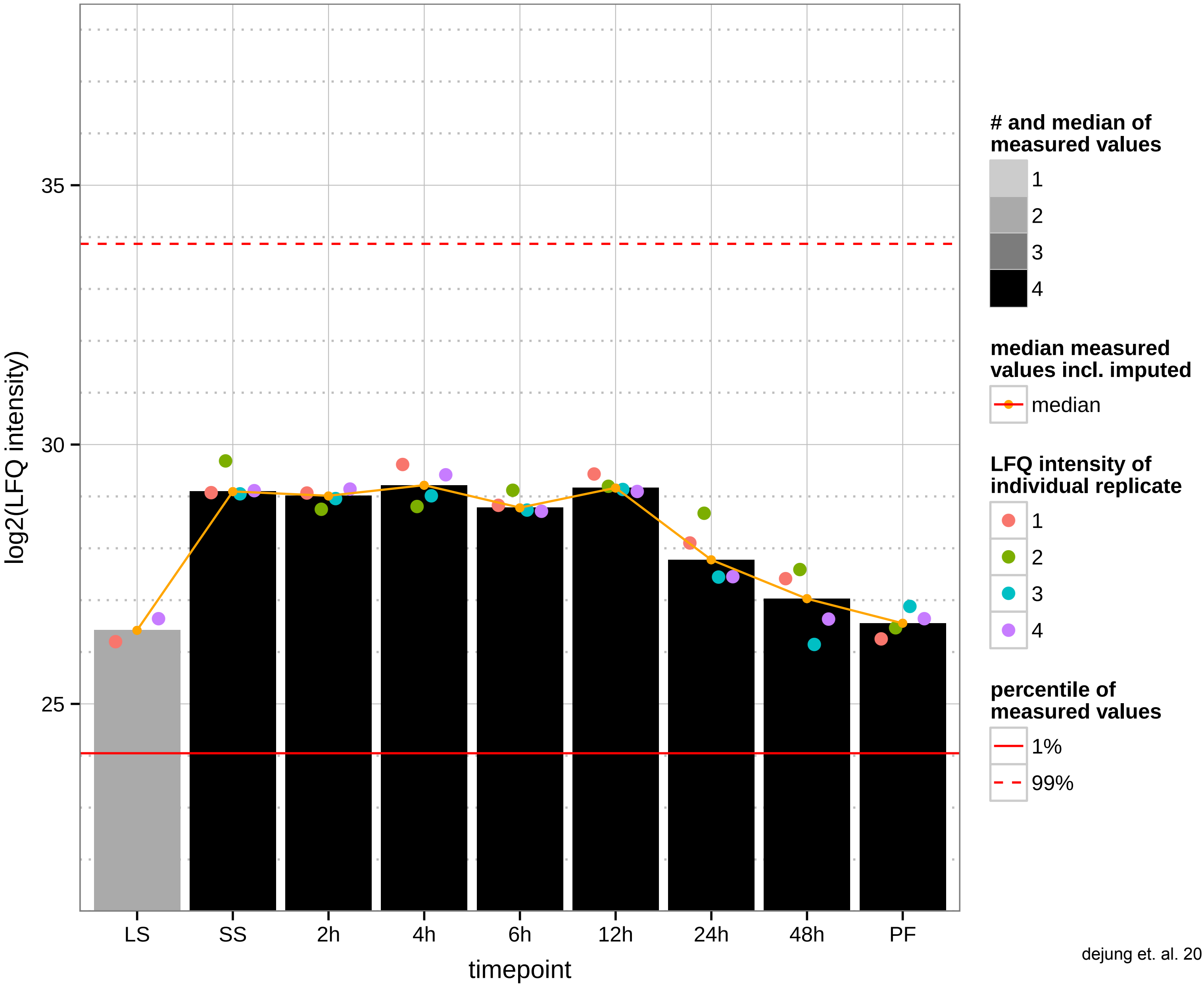

dejung et. al. 2015

TFIIF−stimulated CTD phosphatase, putative
Tb927.10.4180
AGOF: null
AGOC: null
AGOP: null
PGOF: protein binding
PGOC: null
PGOP: null

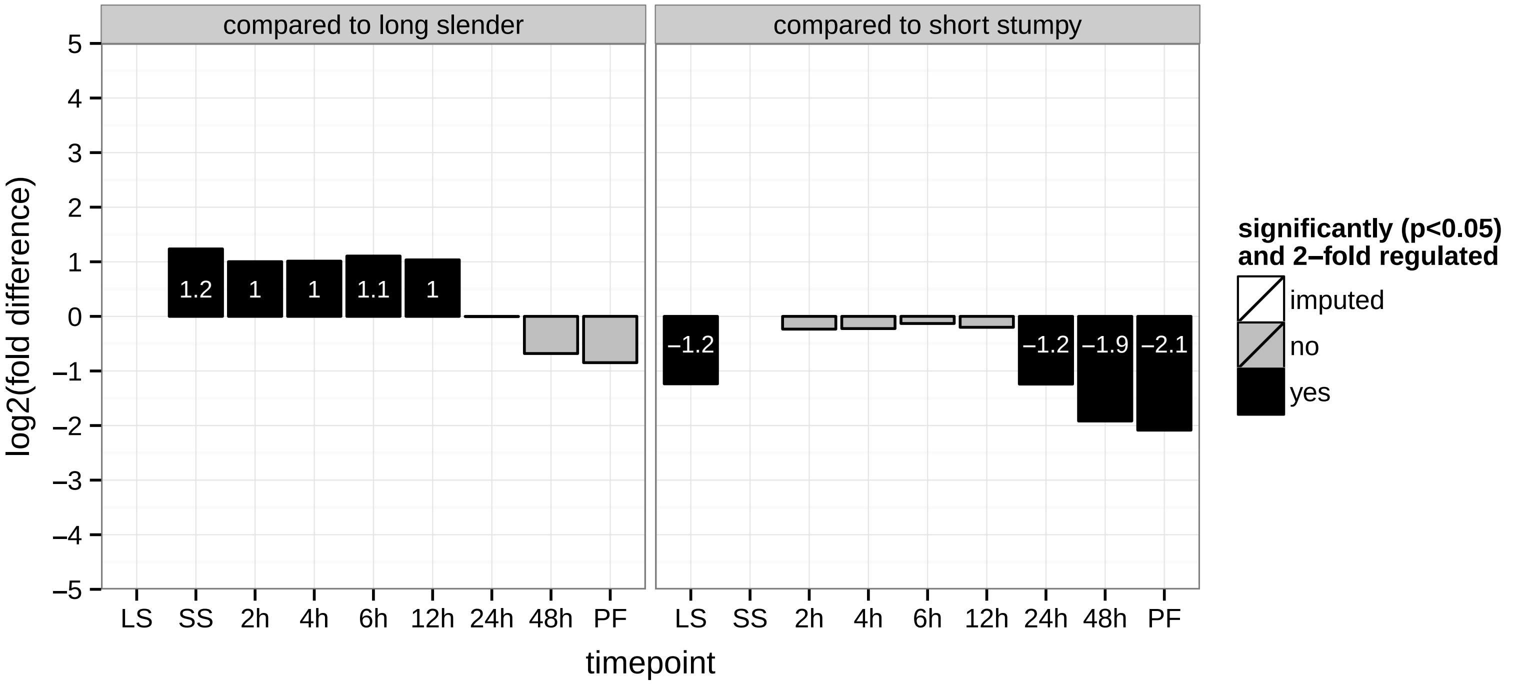

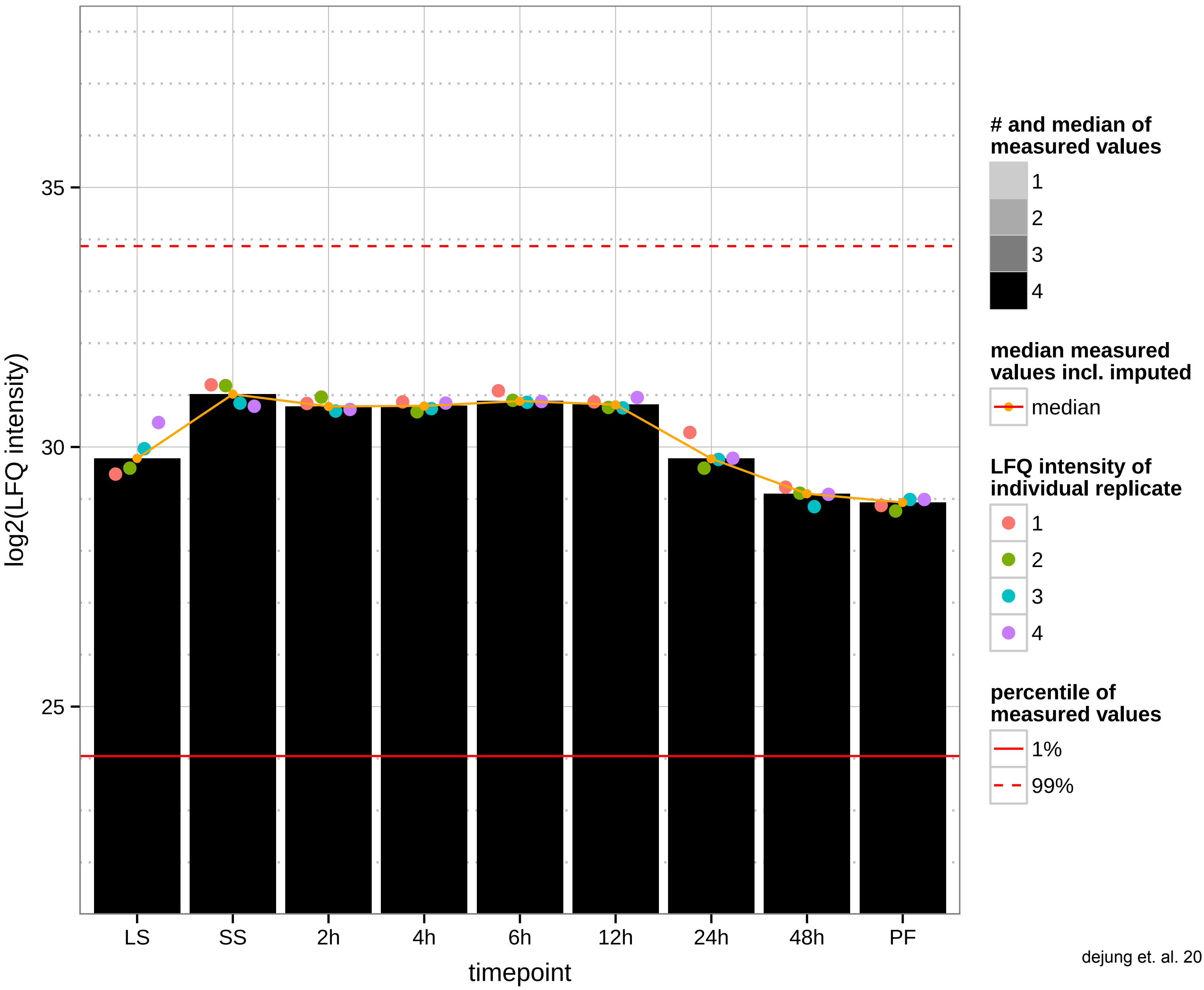

dejung et. al. 2015

hypothetical protein, conserved, zinc finger protein family member, putative (ZC3H32)
Tb927.10.5250;Tb11.v5.0232
AGOF: null, RNA binding, zinc ion binding
AGOC: null
AGOP: null
PGOF: nucleic acid binding, zinc ion binding
PGOC: null
PGOP: null

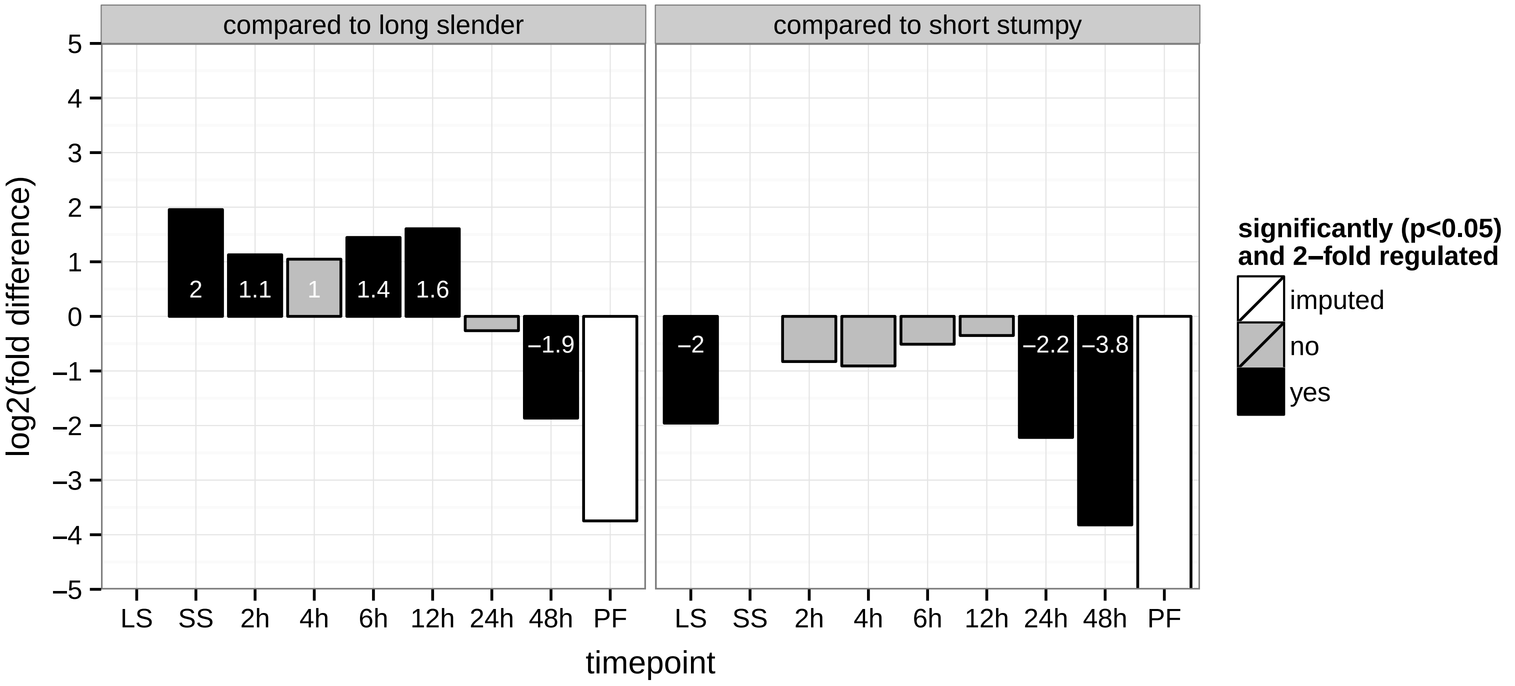

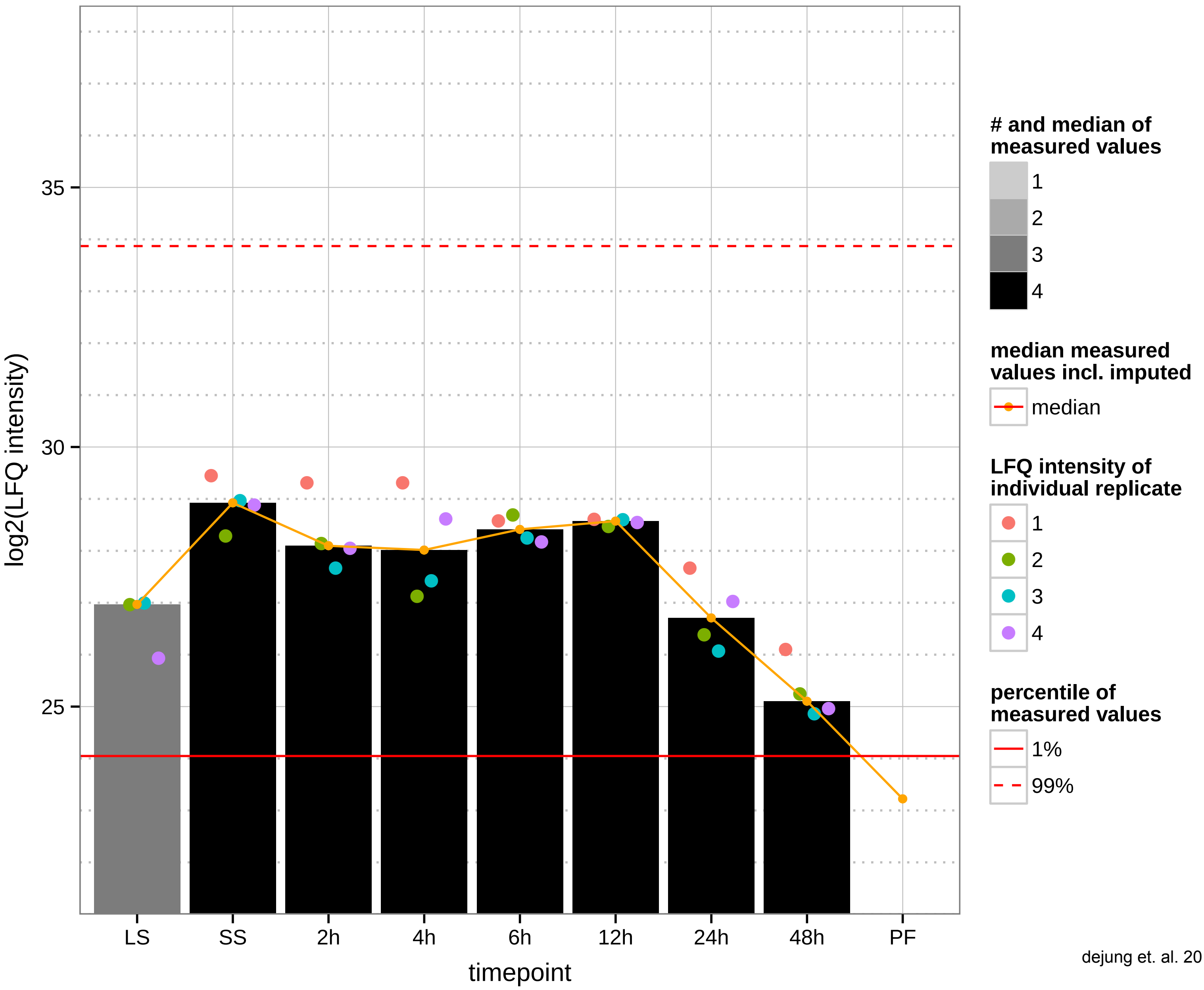

dejung et. al. 2015

hypothetical protein, conserved
Tb927.10.6750;Tb927.10.6760
AGOF: null
AGOC: null
AGOP: null
PGOF: null
PGOC: null
PGOP: null

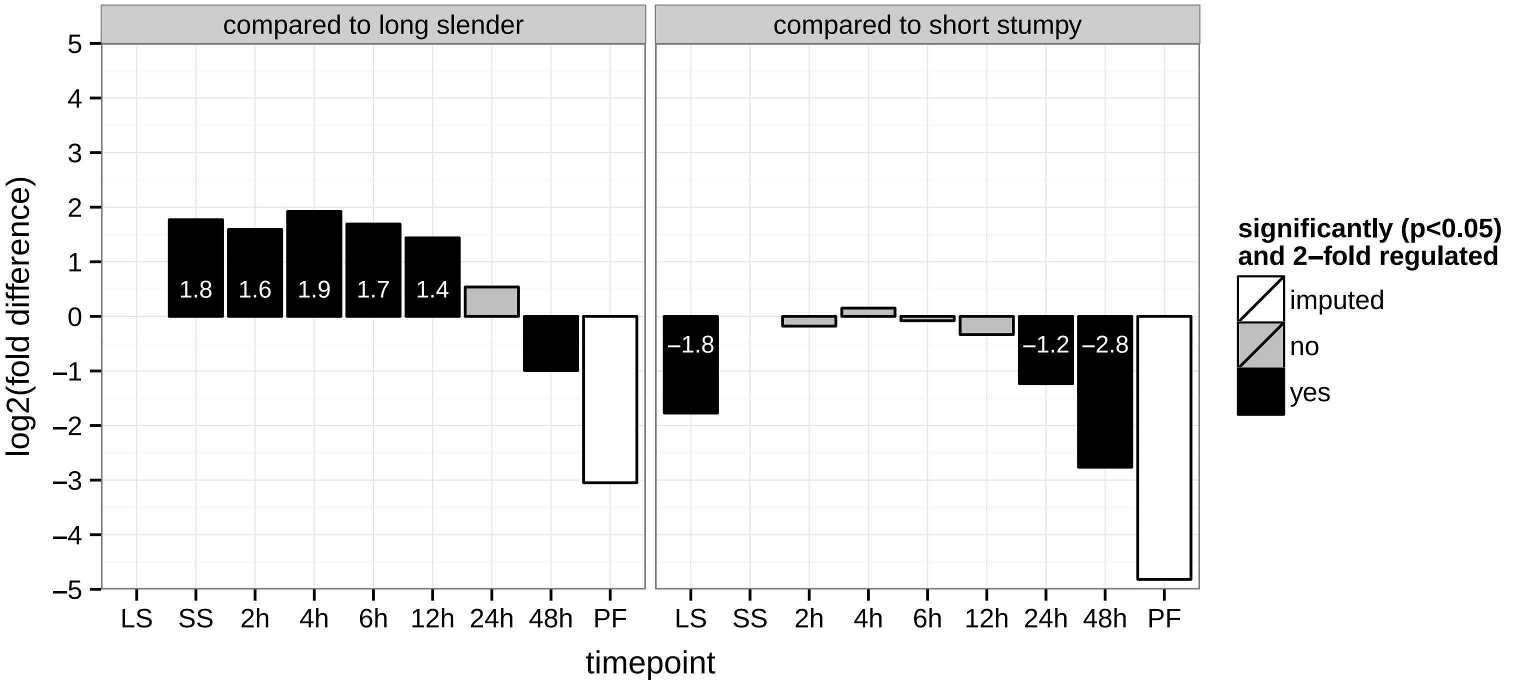

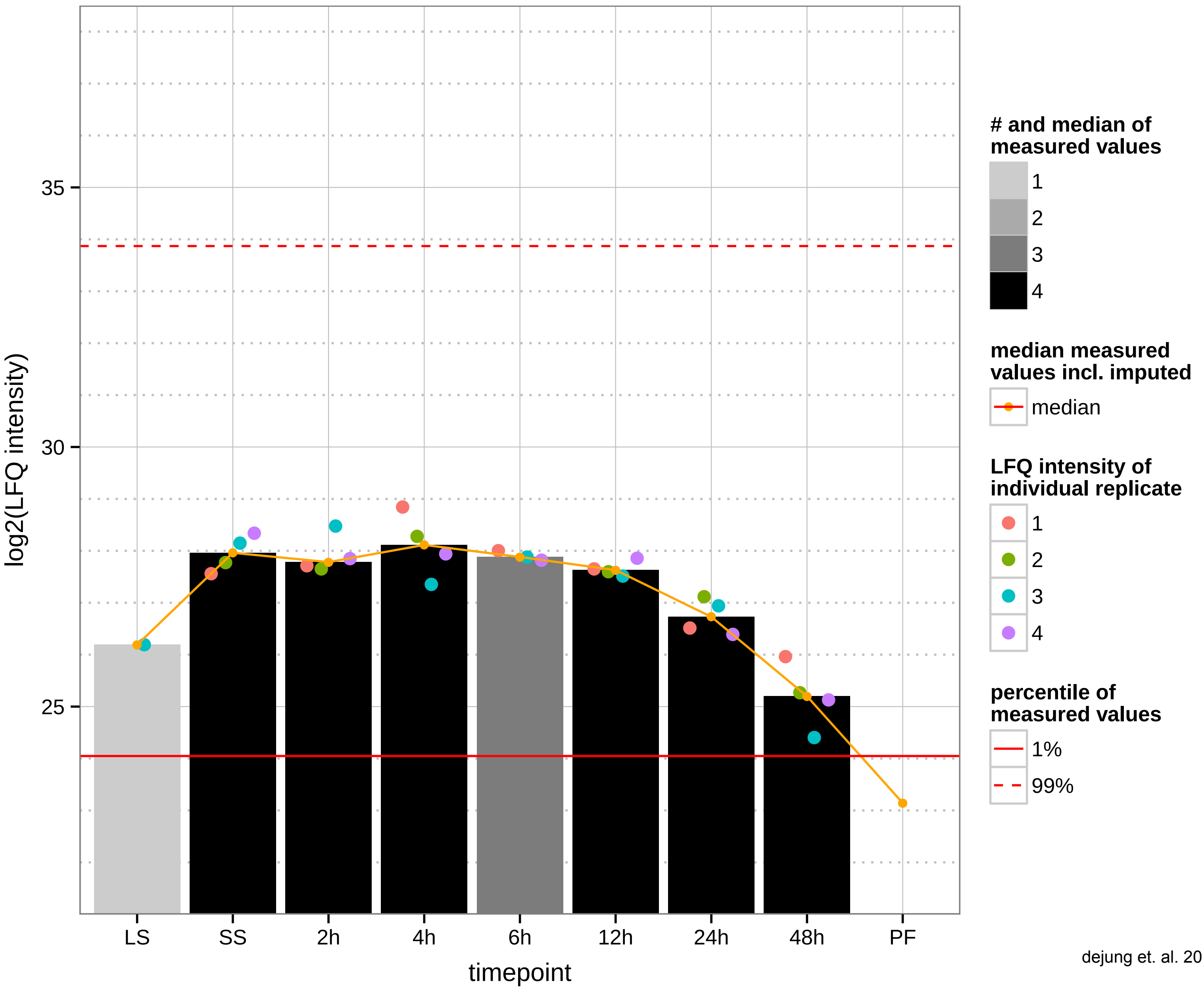

dejung et. al. 2015

hypothetical protein, conserved
Tb927.10.840;Tb11.v5.0753
AGOF: null
AGOC: null
AGOP: null
PGOF: null, protein binding
PGOC: null
PGOP: null

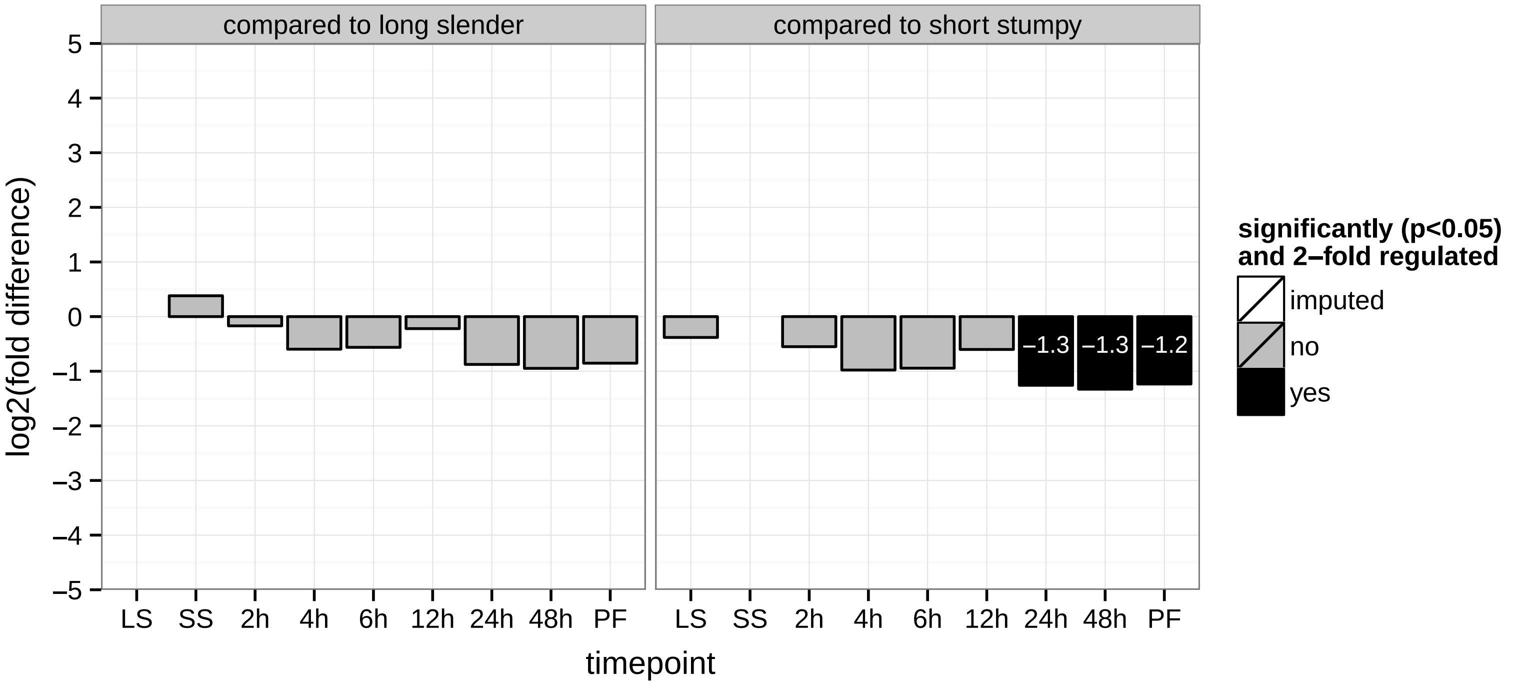

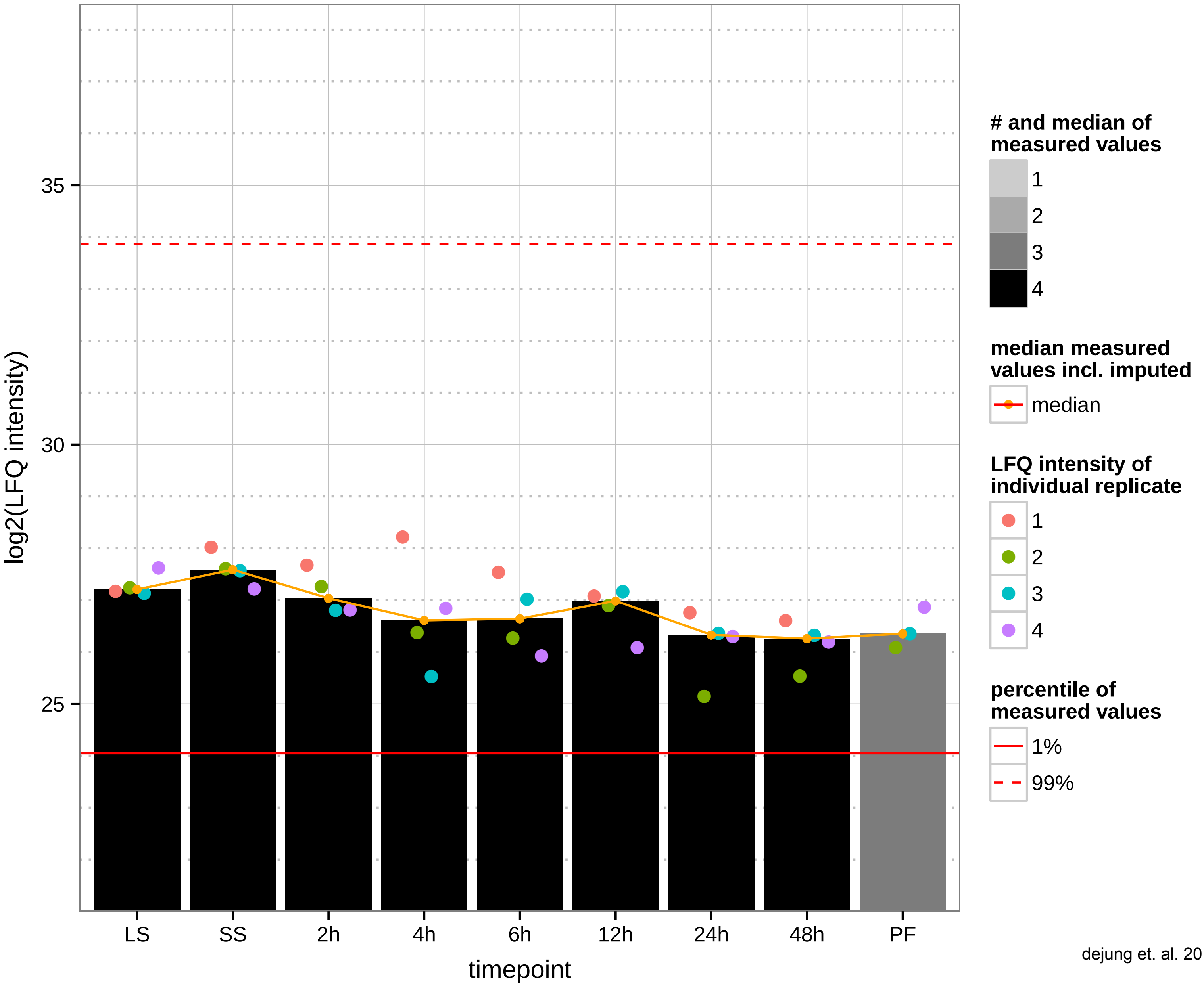

dejung et. al. 2015

hypothetical protein, conserved
Tb927.10.9360
AGOF: oxidoreductase activity
AGOC: null
AGOP: null
PGOF: oxidoreductase activity
PGOC: null
PGOP: oxidation−reduction process

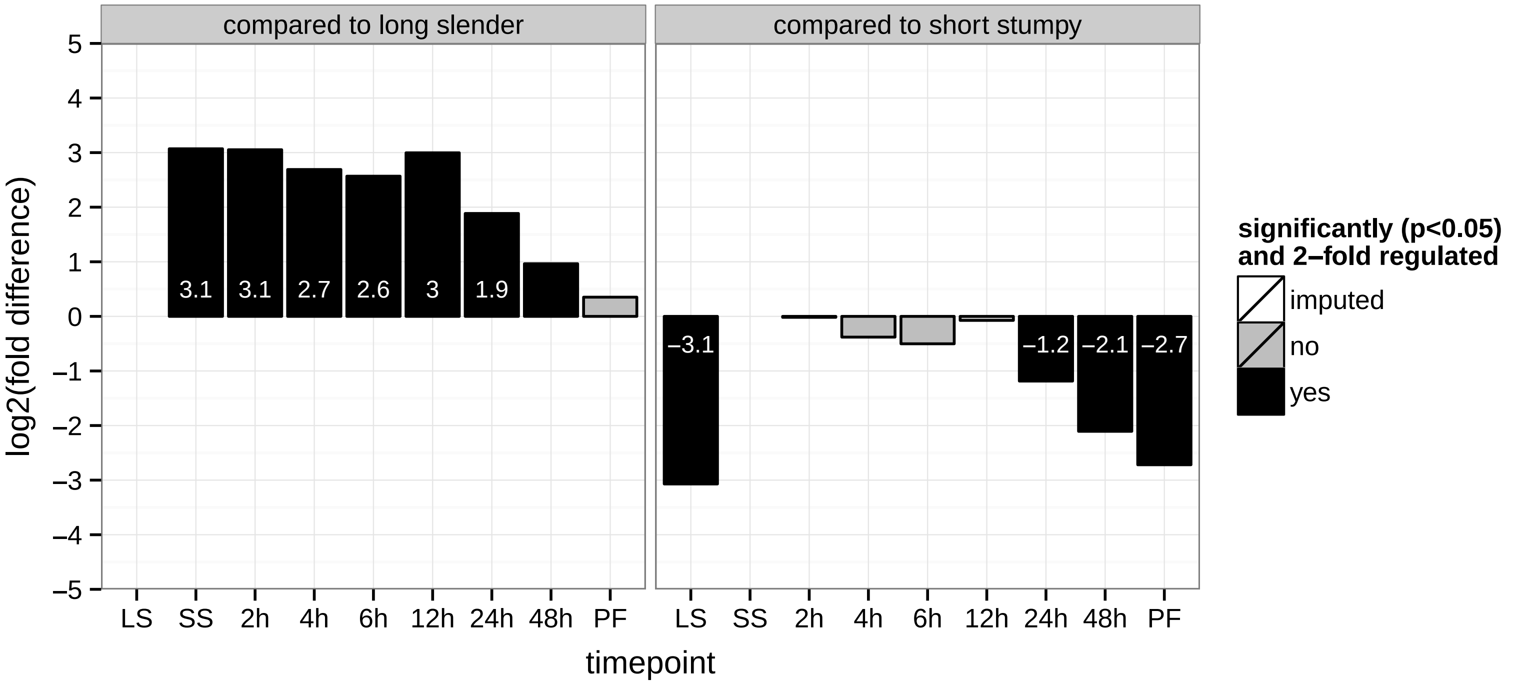

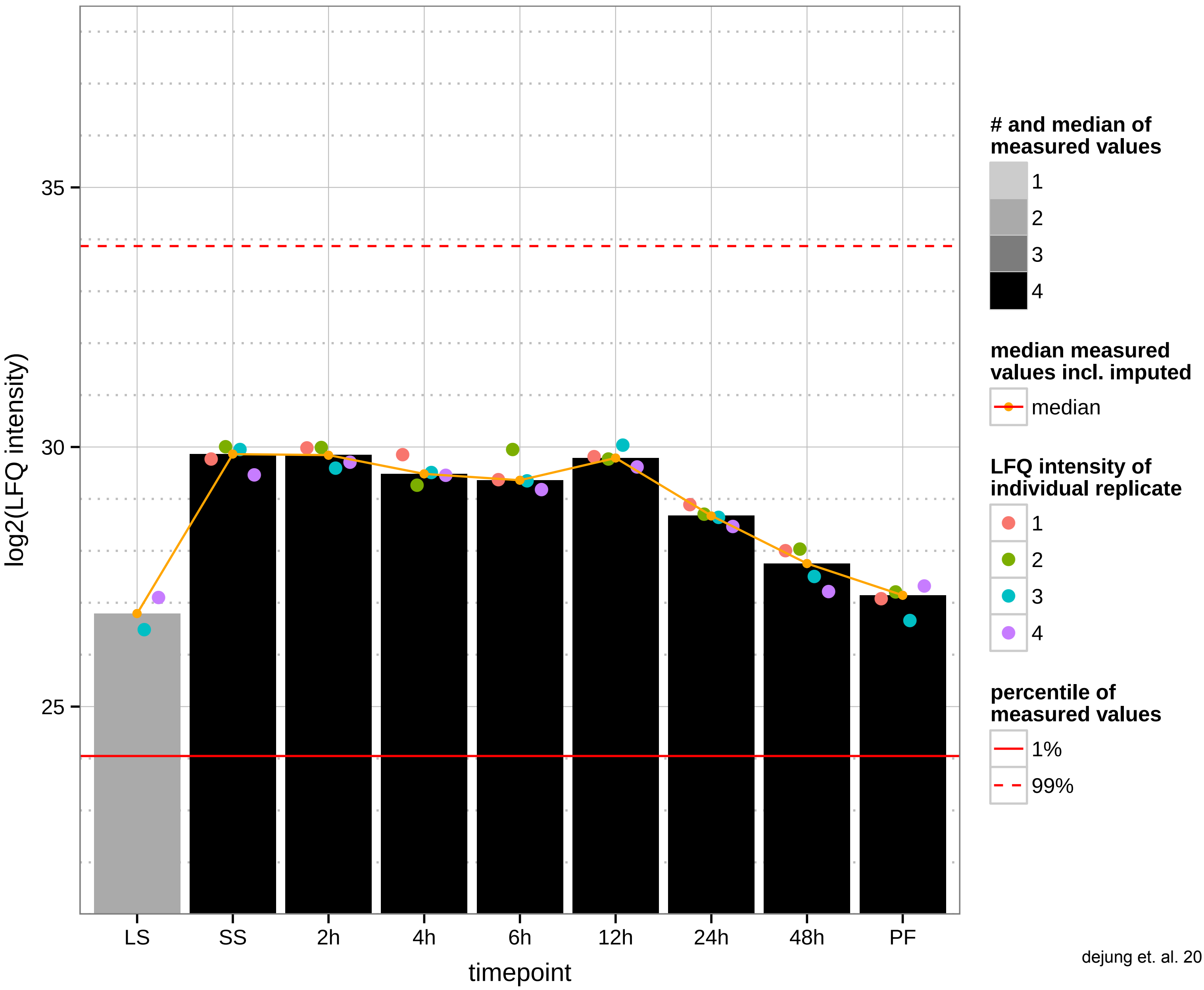

dejung et. al. 2015

hypothetical protein, conserved
Tb927.11.11710
AGOF: null
AGOC: null
AGOP: null
PGOF: null
PGOC: null
PGOP: null

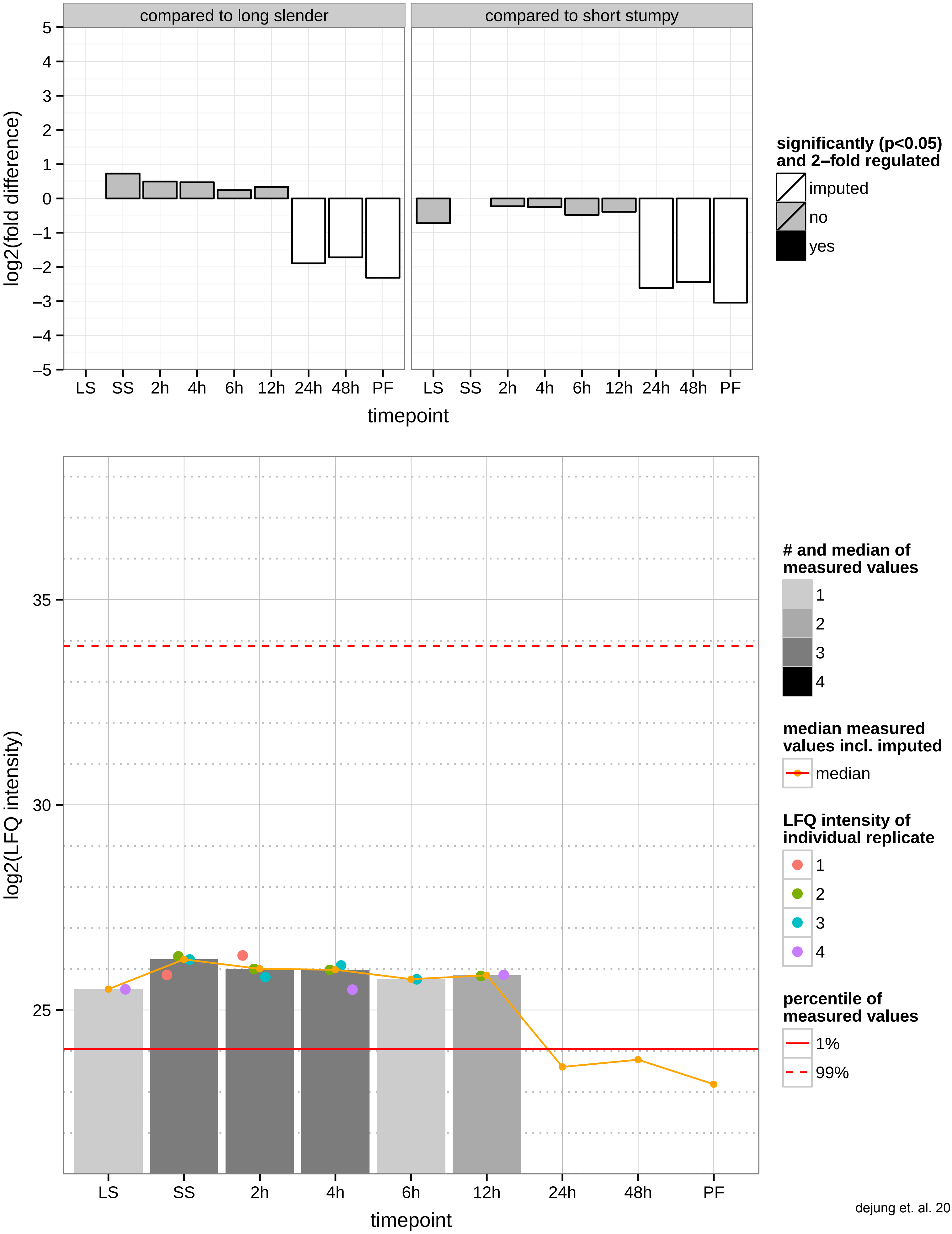

dejung et. al. 2015

hypothetical protein, conserved
Tb927.11.1230
AGOF: null
AGOC: null
AGOP: null
PGOF: null
PGOC: null
PGOP: null

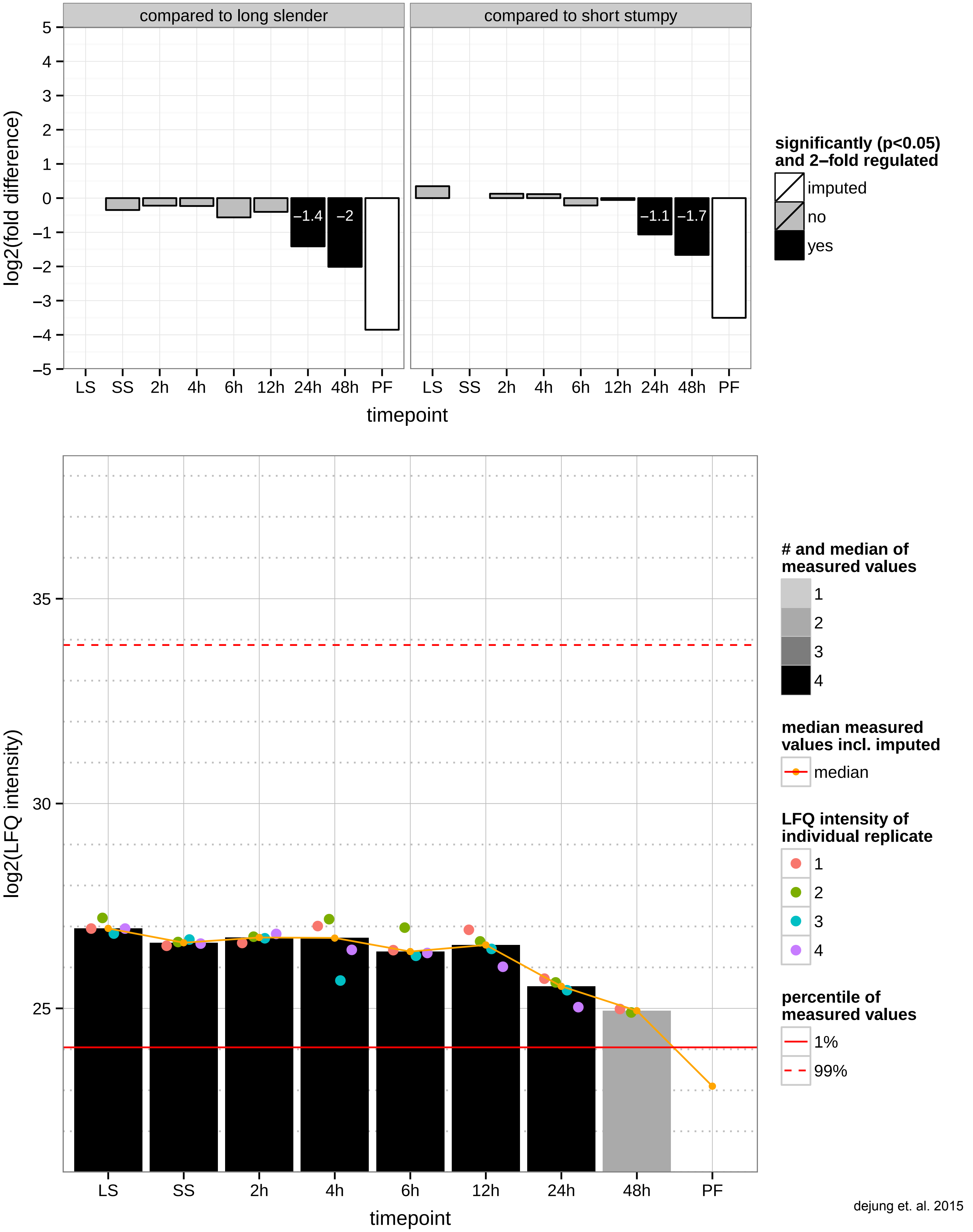

dejung et. al. 2015

membrane−bound acid phosphatase 1 precursor (MBAP1)
Tb927.11.13130
AGOF: null
AGOC: integral to membrane, lysosomal membrane
AGOP: null
PGOF: acid phosphatase activity
PGOC: null
PGOP: null

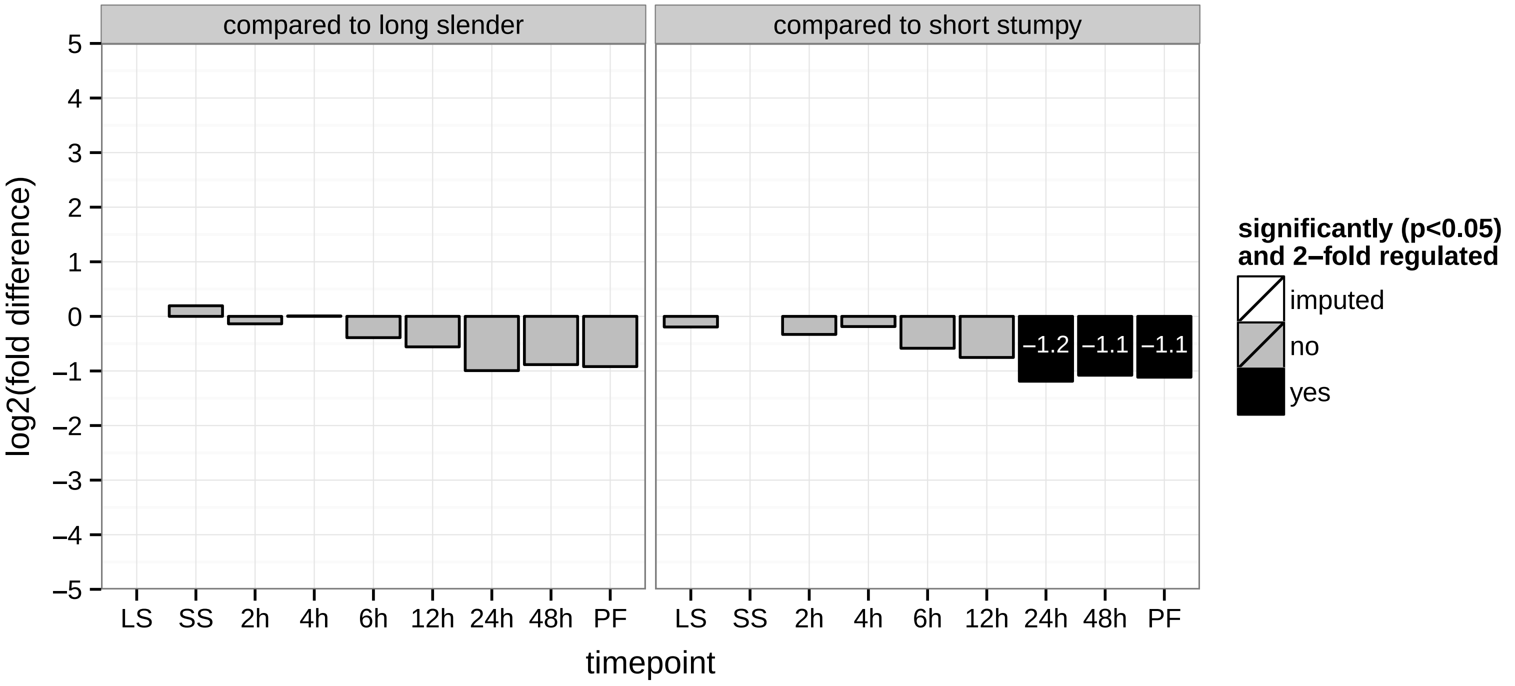

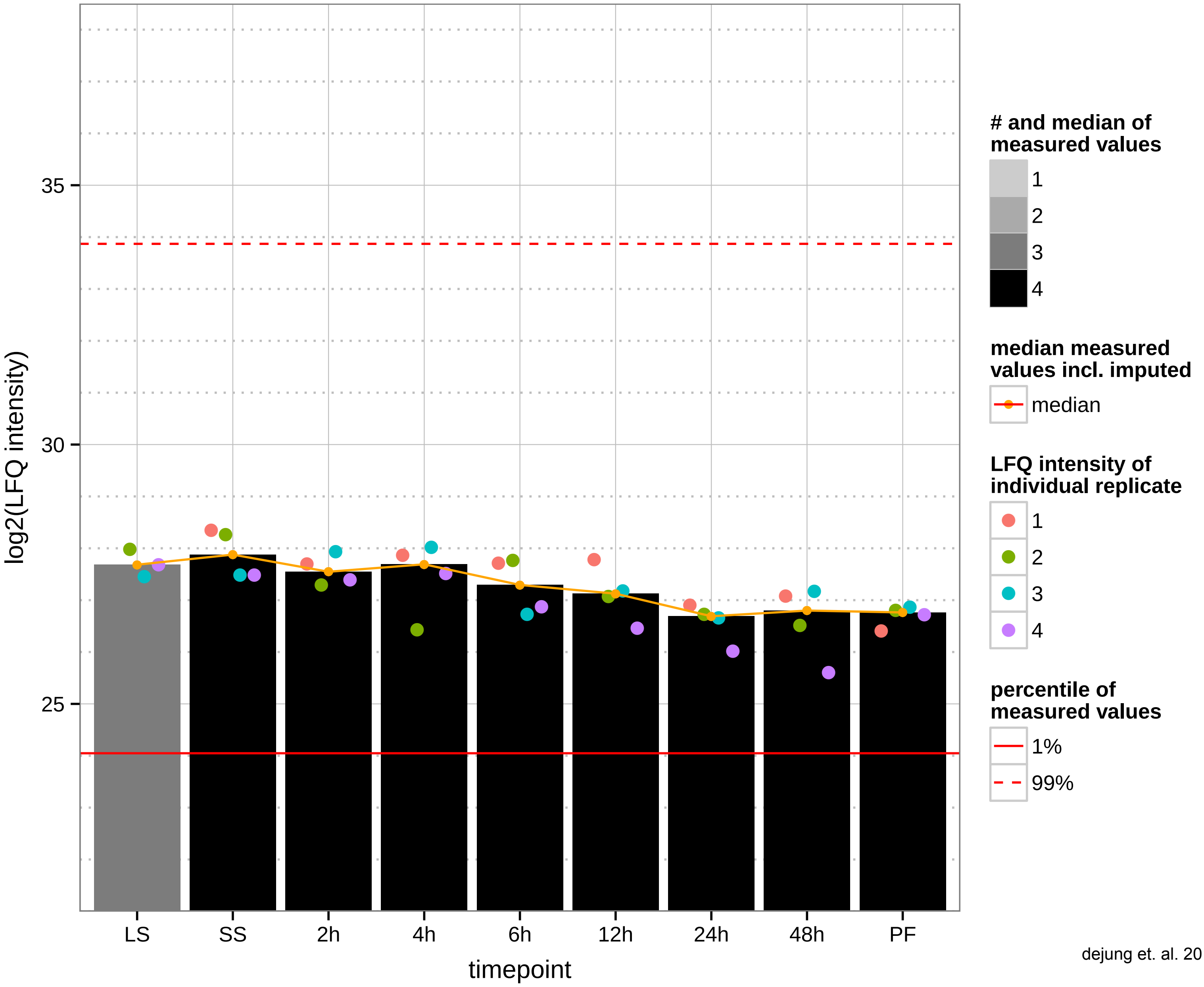

dejung et. al. 2015

hypothetical protein, conserved
Tb927.11.15870
AGOF: null
AGOC: null
AGOP: null
PGOF: null
PGOC: null
PGOP: null

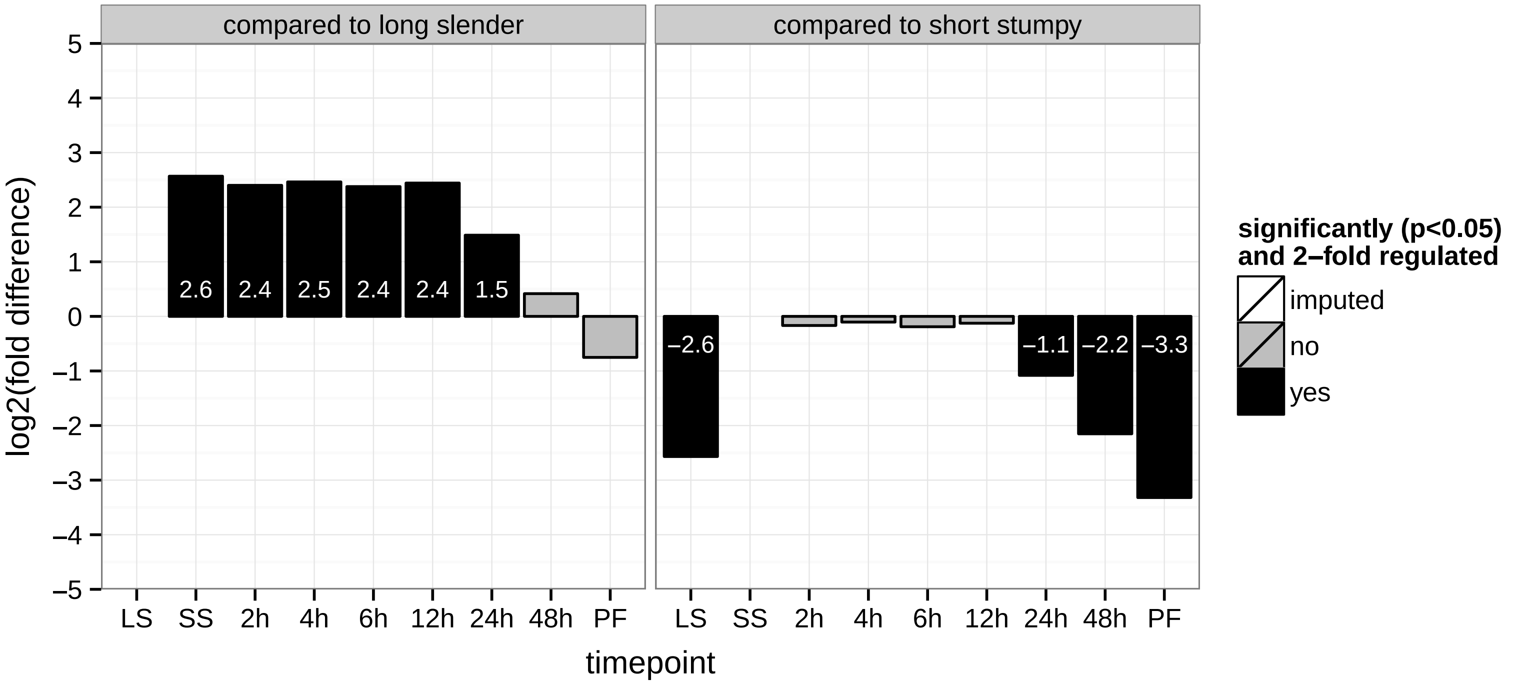

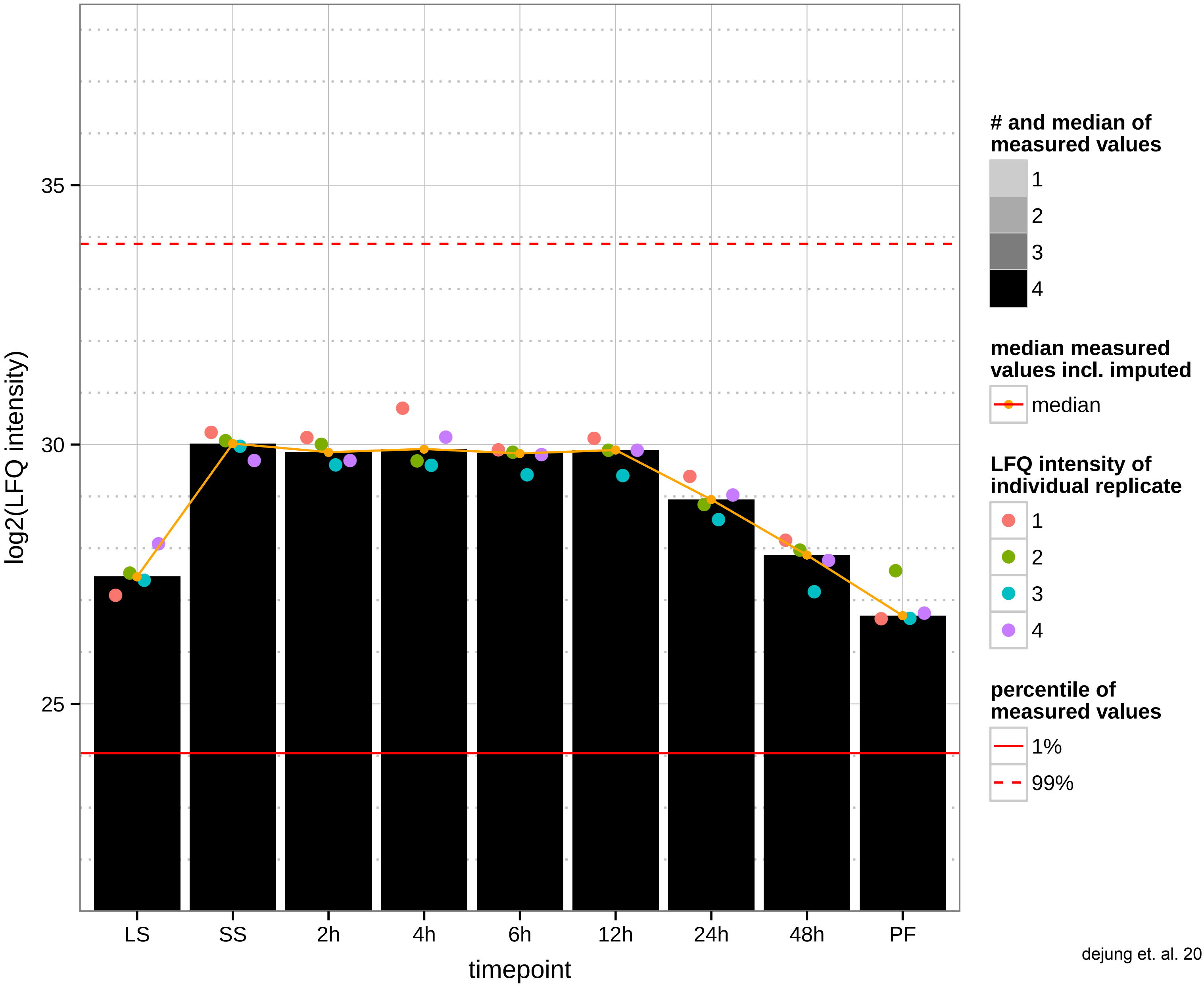

dejung et. al. 2015

diacylglycerol acyltransferase, putative
Tb927.11.2210
AGOF: diacylglycerol O−acyltransferase activity
AGOC: integral to membrane
AGOP: triglyceride biosynthetic process
PGOF: transferase activity, transferring acyl groups other than amino−acyl groups
PGOC: null
PGOP: null

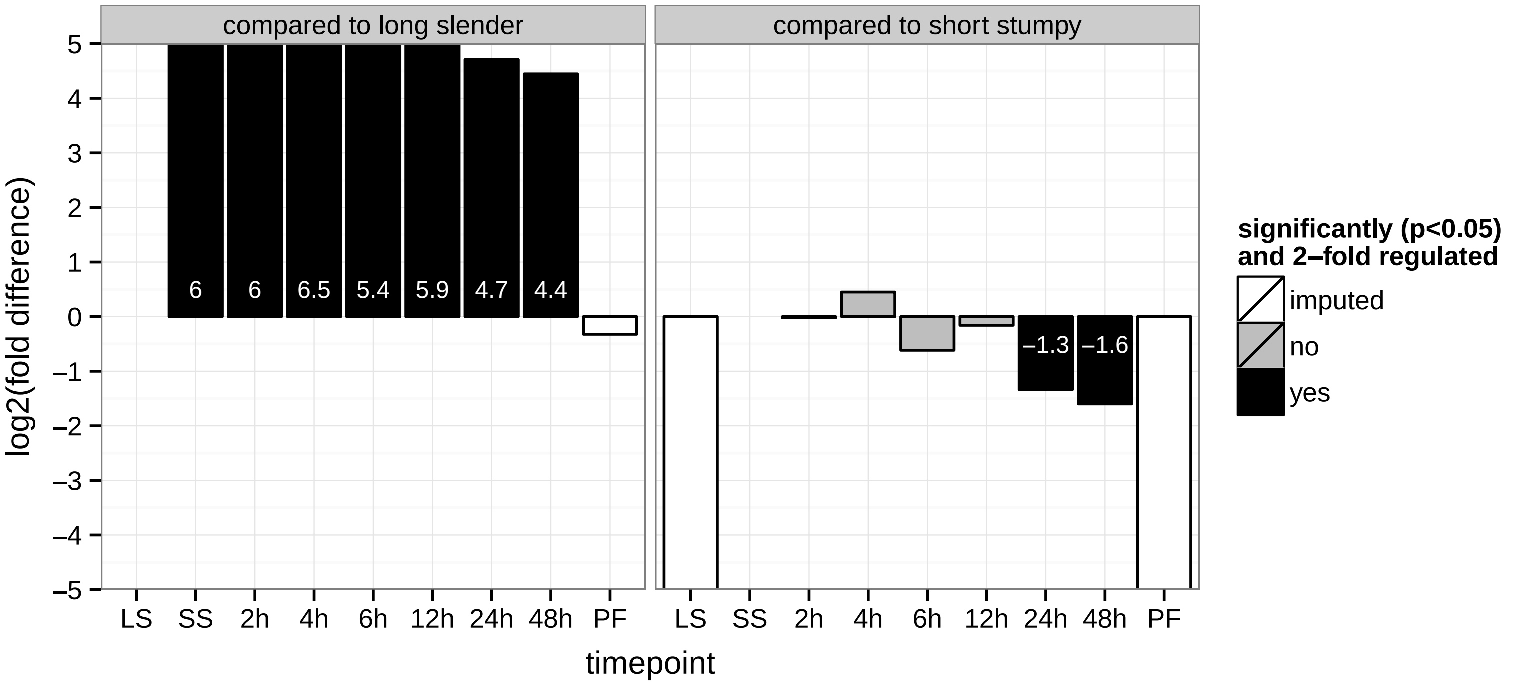

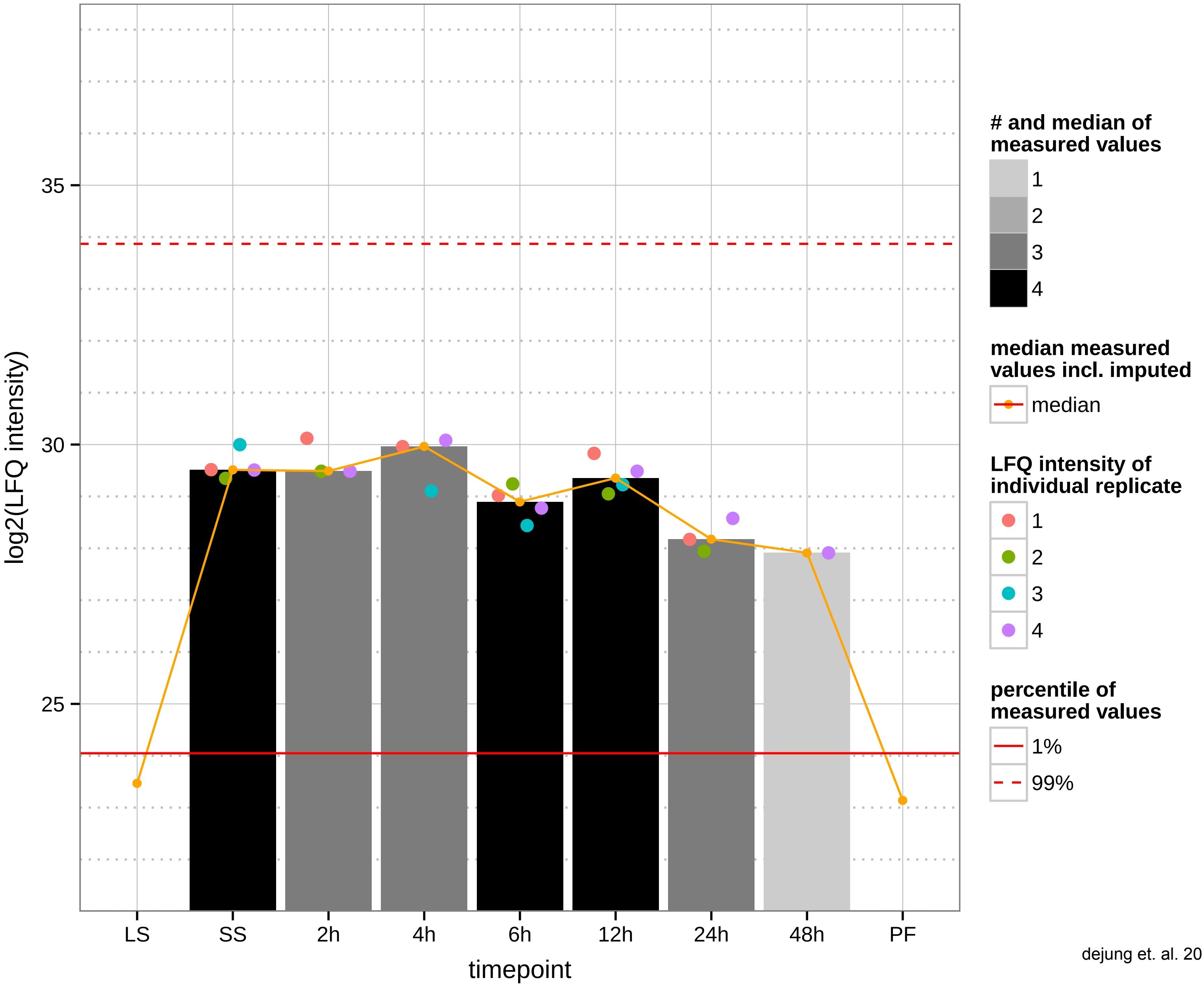

dejung et. al. 2015

Unc104–like kinesin, putative
Tb927.11.2490
AGOF: ATP binding, microtubule motor activity, motor activity
AGOC: kinesin complex, microtubule associated complex
AGOP: microtubule–based movement, vesicle transport along microtubule
PGOF: ATP binding, microtubule motor activity, protein binding
PGOC: null
PGOP: microtubule–based movement

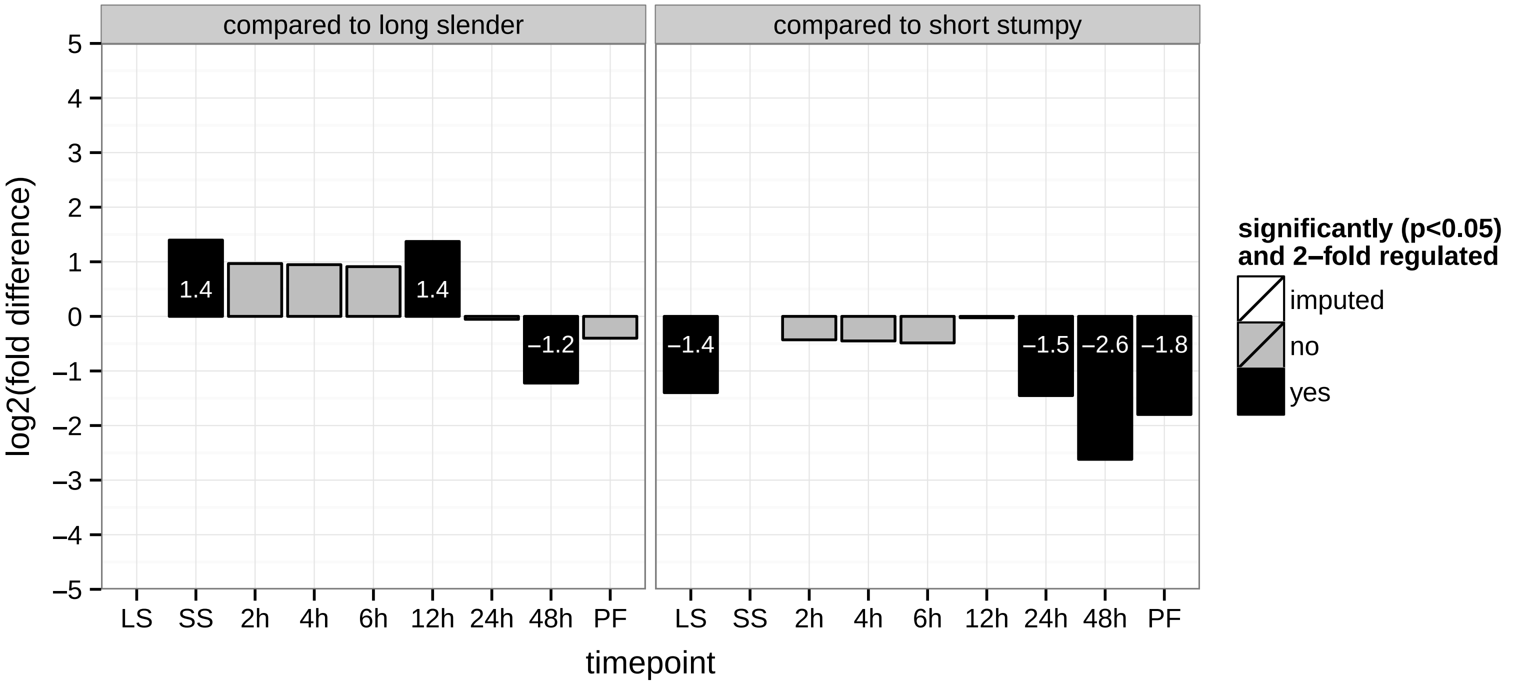

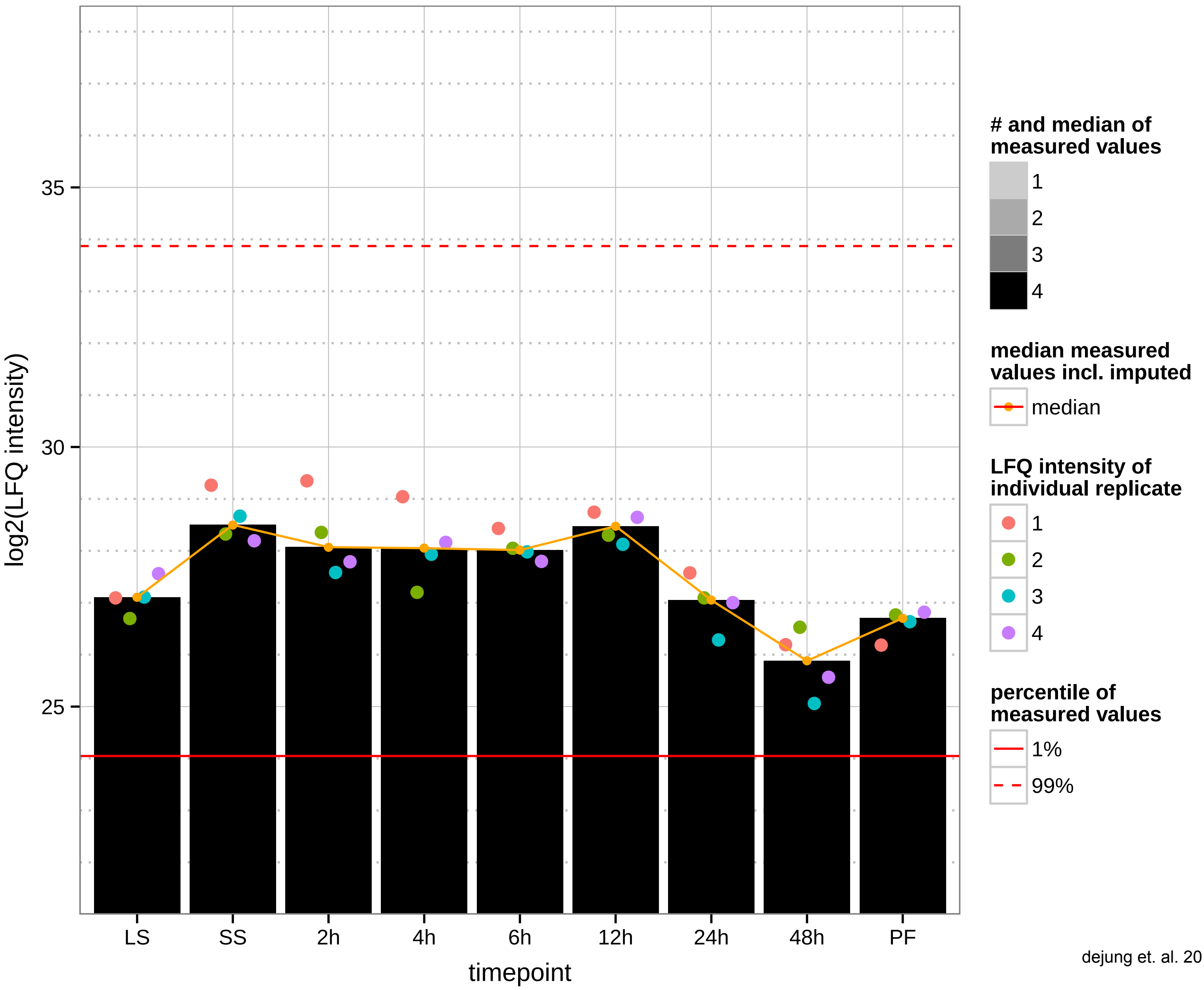

dejung et. al. 2015

hypothetical protein, conserved
Tb927.11.2860
AGOF: null
AGOC: null
AGOP: null
PGOF: protein binding
PGOC: null
PGOP: null

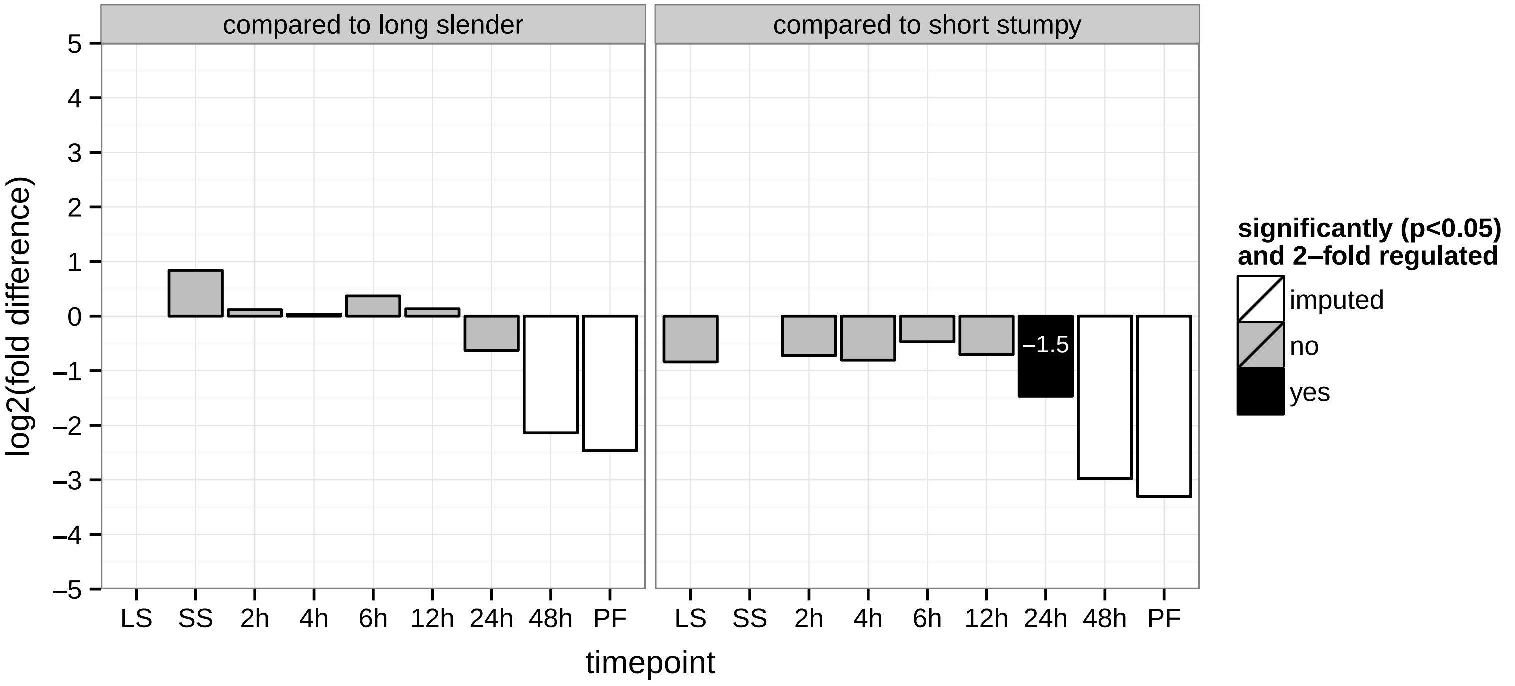

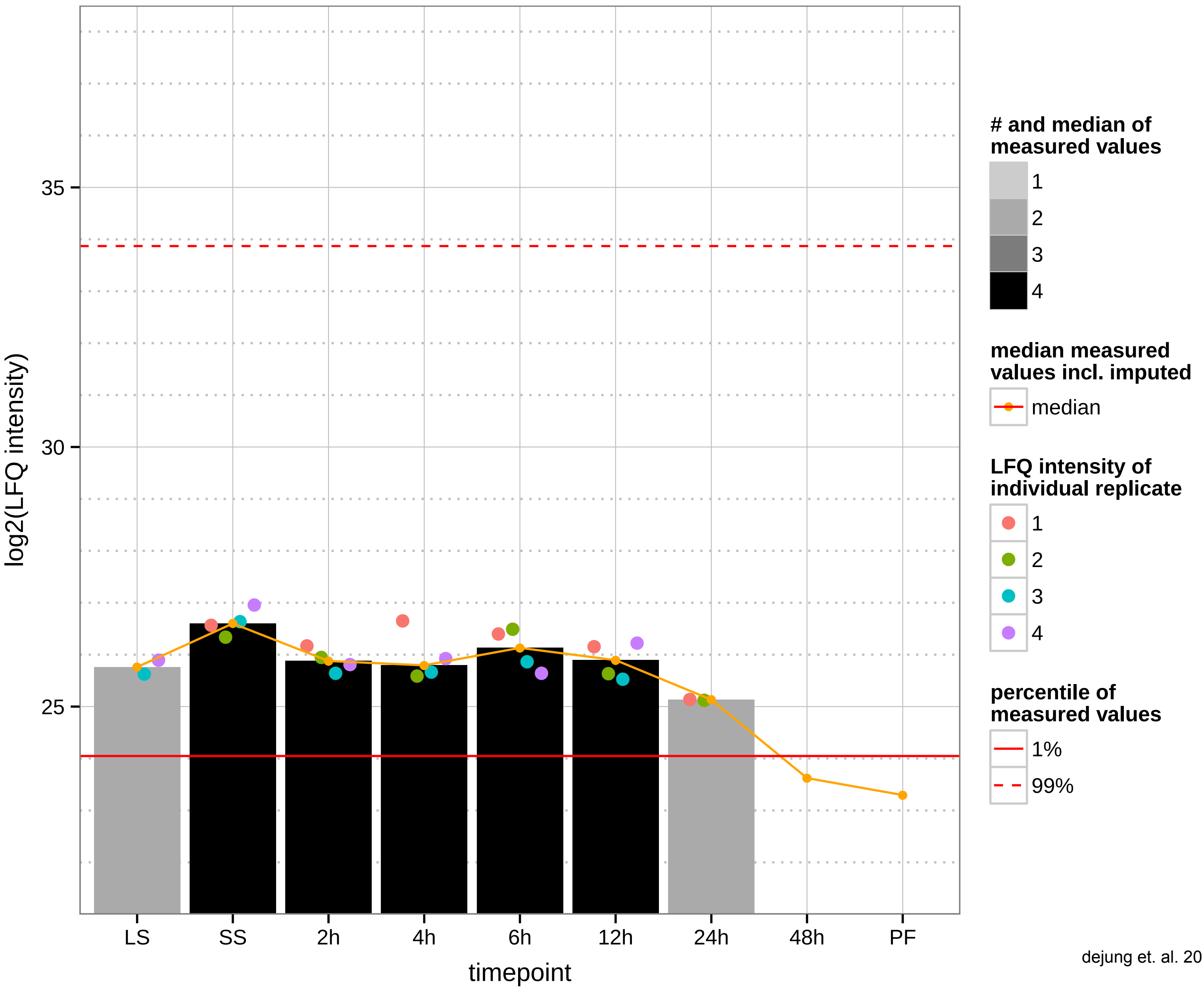

dejung et. al. 2015

phosphatase and tensin homologue, putative
Tb927.11.290
AGOF: protein tyrosine/serine/threonine phosphatase activity
AGOC: null
AGOP: modulation of development of symbiont involved in interaction with host, protein dephosphorylation, quorum sensing i
PGOF: phosphatase activity, protein tyrosine/serine/threonine phosphatase activity
PGOC: null
PGOP: dephosphorylation, protein dephosphorylation

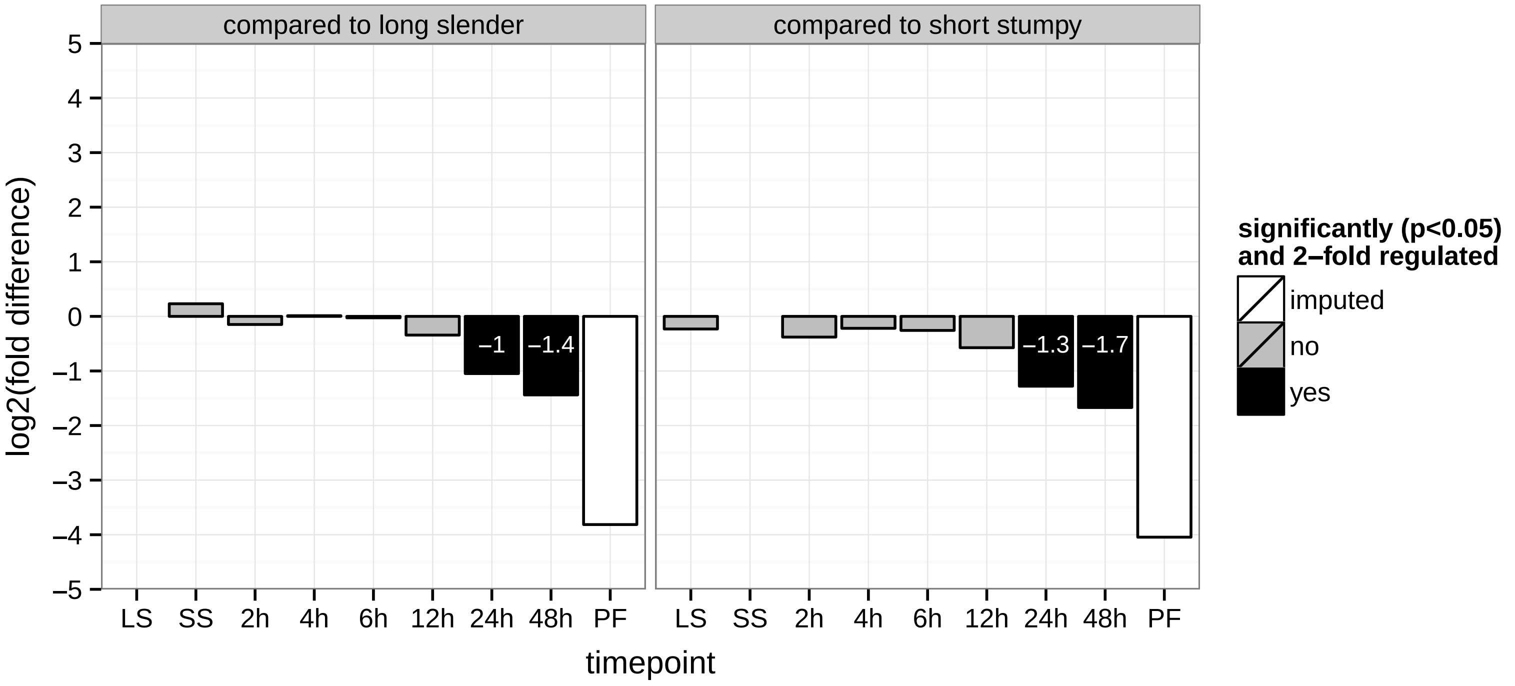

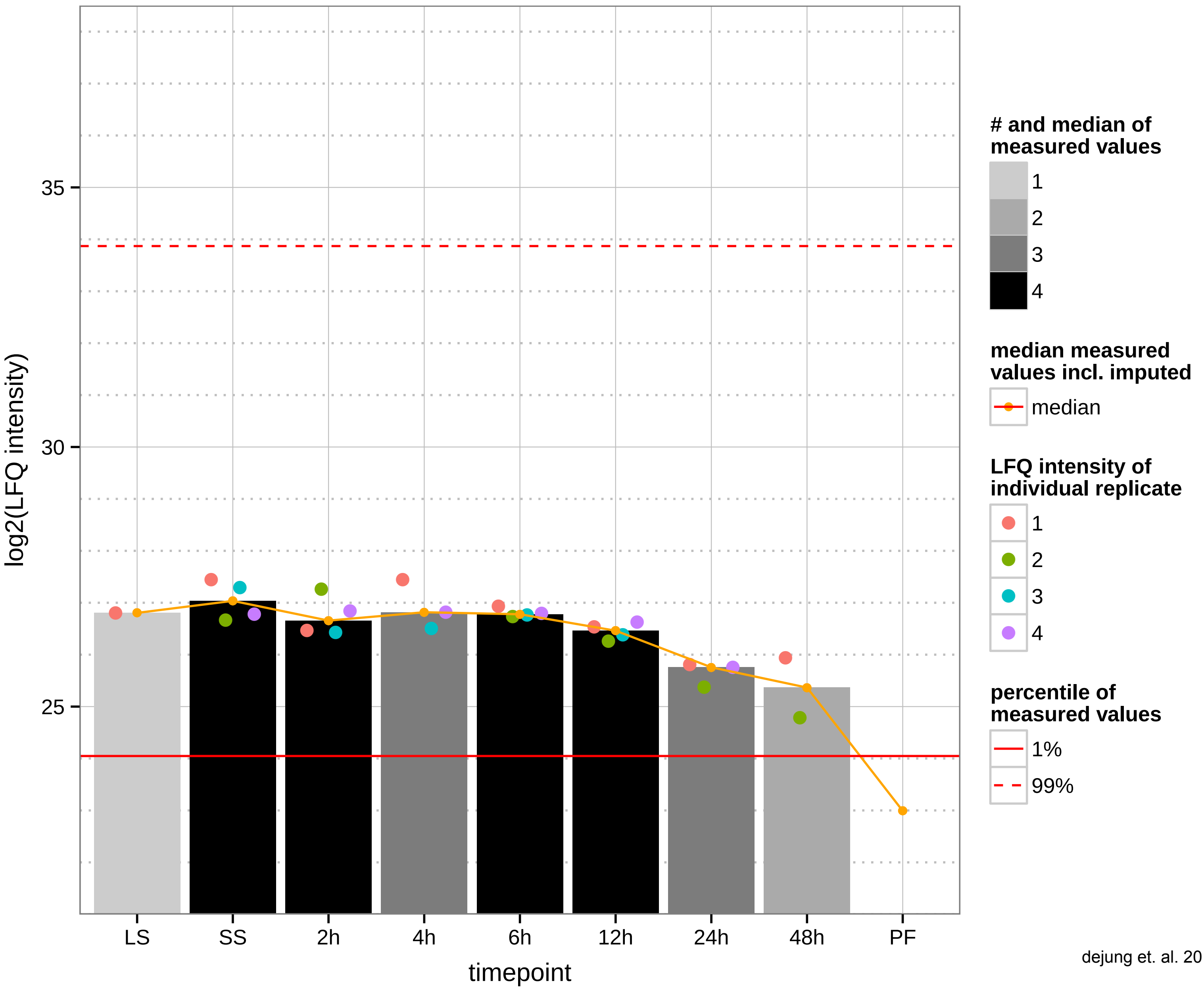

dejung et. al. 2015

metacaspase, cysteine peptidase, Clan CD, family C13 (mca1)
Tb927.11.3220
AGOF: cysteine−type endopeptidase activity
AGOC: mitochondrion
AGOP: mitochondrion organization, proteolysis
PGOF: cysteine−type endopeptidase activity
PGOC: null
PGOP: proteolysis

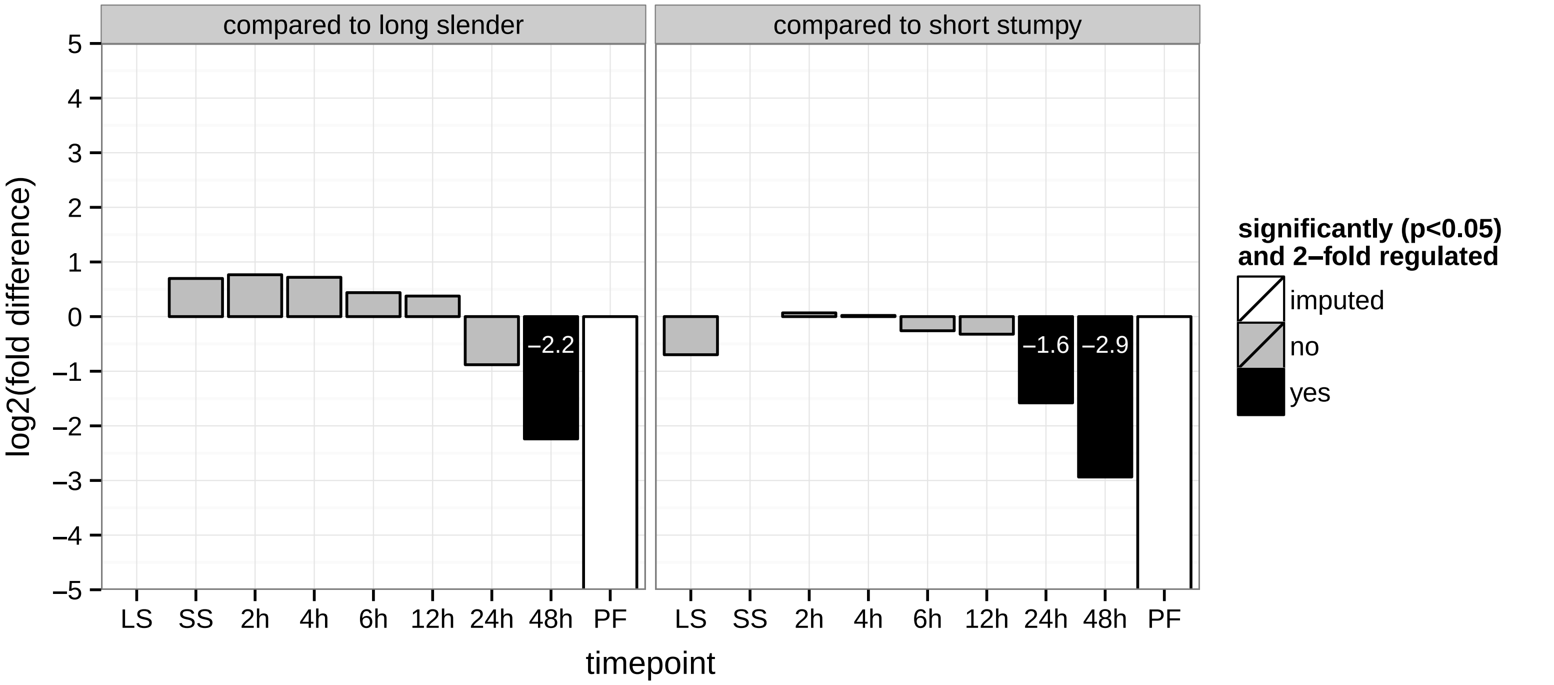

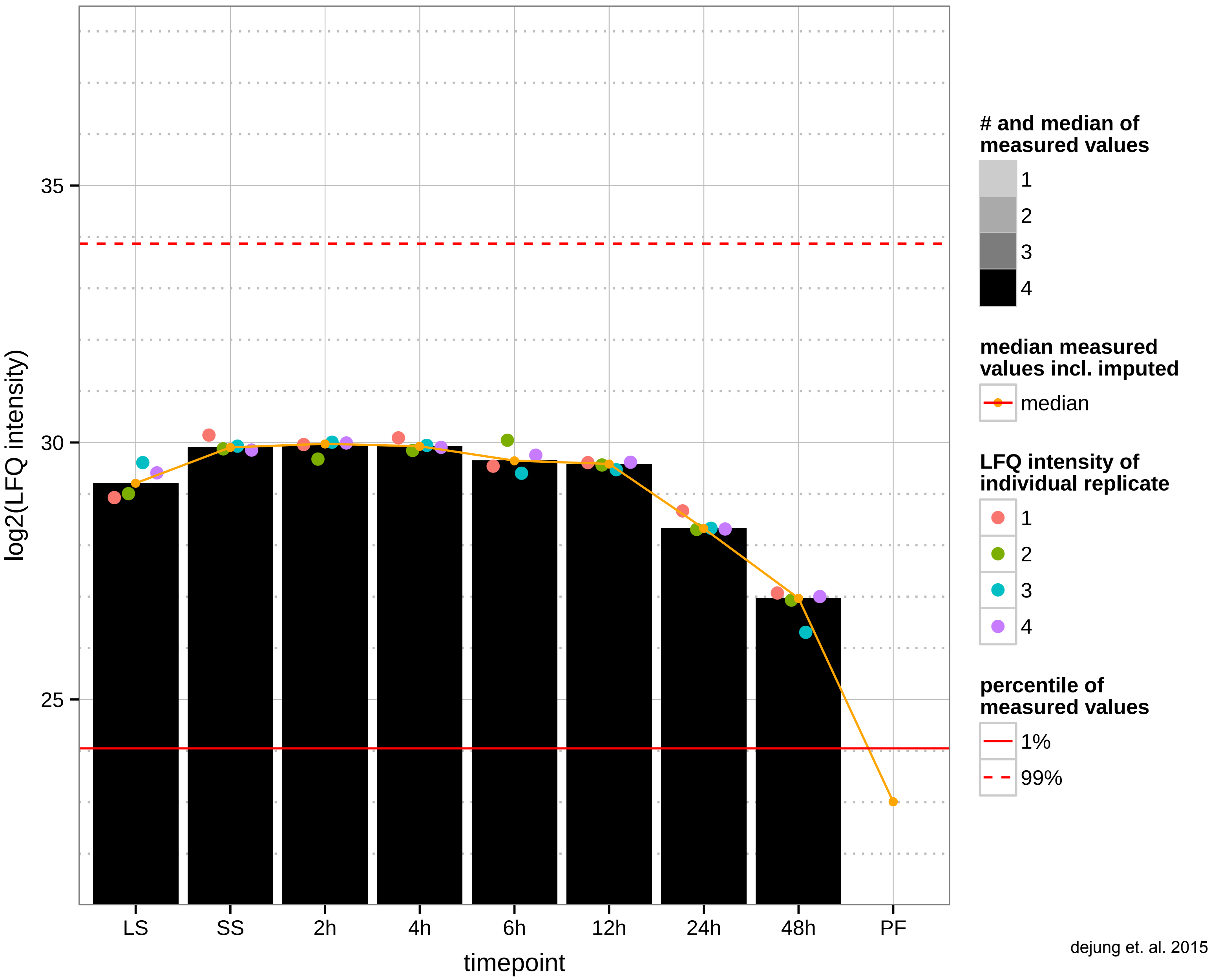

dejung et. al. 2015

AMP deaminase, putative
Tb927.11.3850
AGOF: deaminase activity
AGOC: null
AGOP: purine ribonucleoside monophosphate biosynthetic process
PGOF: deaminase activity
PGOC: null
PGOP: purine ribonucleoside monophosphate biosynthetic process

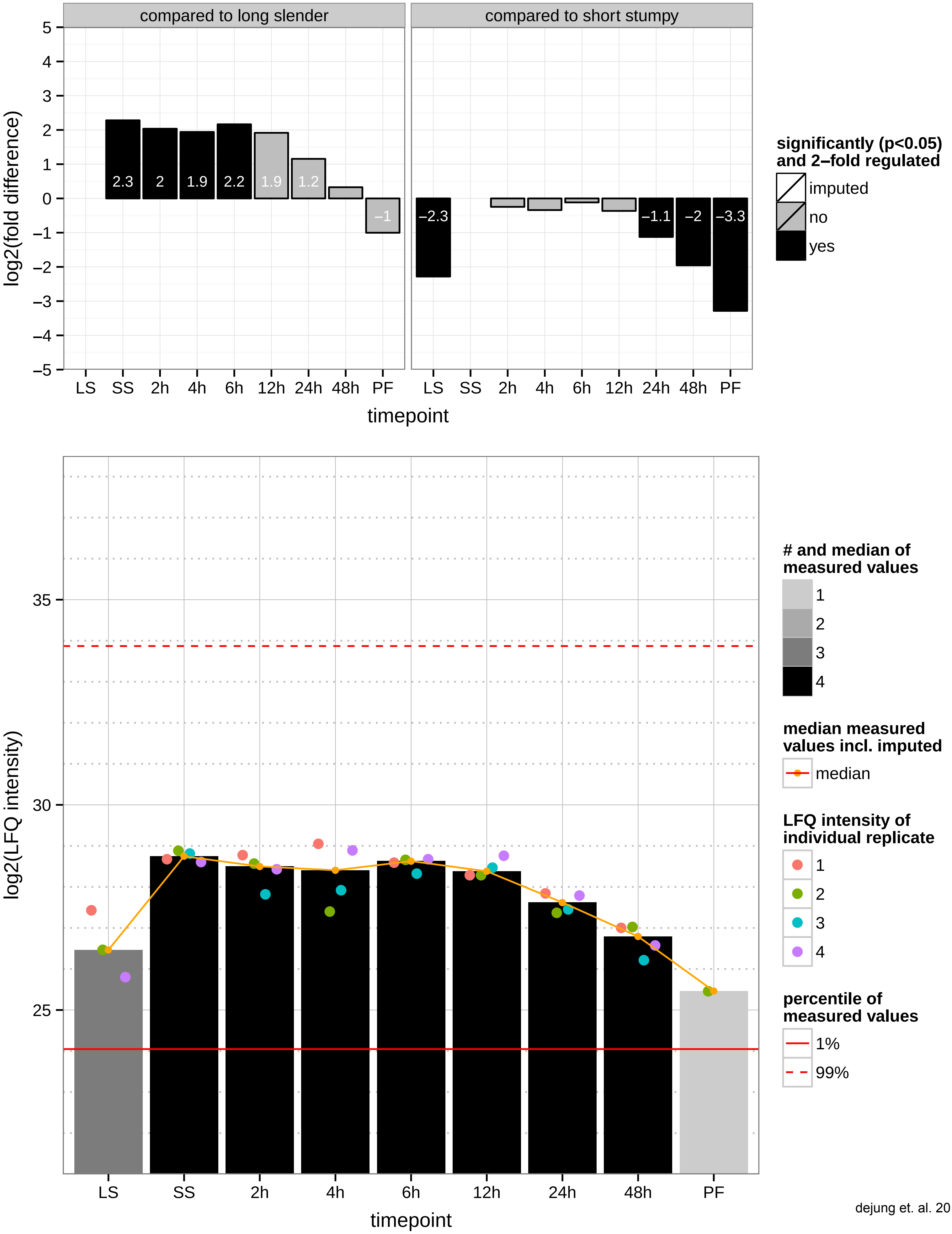

dejung et. al. 2015

clathrin coat assembly protein, putative
Tb927.11.4800
AGOF: clathrin binding, phospholipid binding
AGOC: clathrin coat of coated pit, contractile vacuole
AGOP: receptor–mediated endocytosis
PGOF: phospholipid binding
PGOC: null
PGOP: null

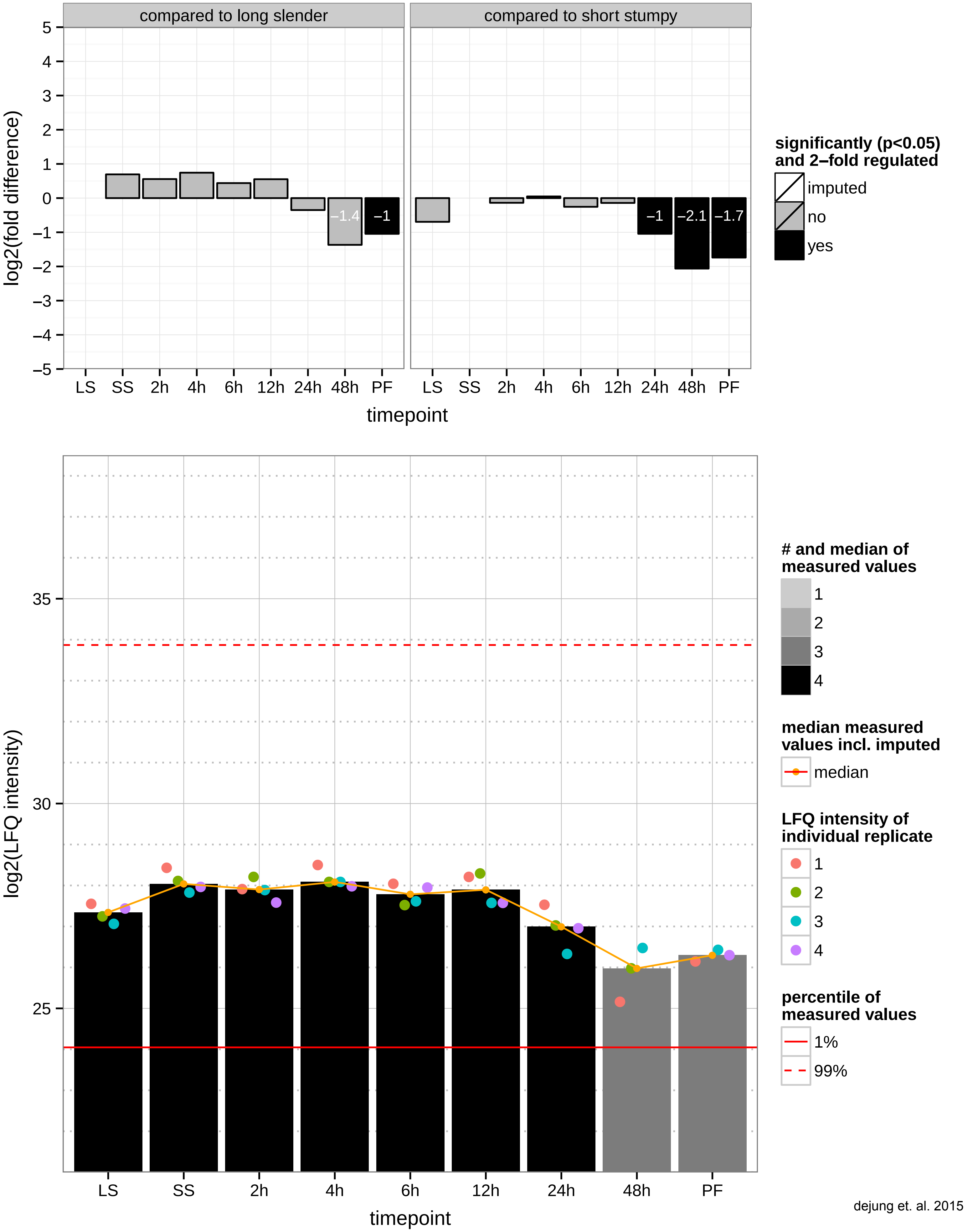

dejung et. al. 2015

hypothetical protein, conserved
Tb927.11.5370
AGOF: null
AGOC: null
AGOP: null
PGOF: null
PGOC: null
PGOP: null

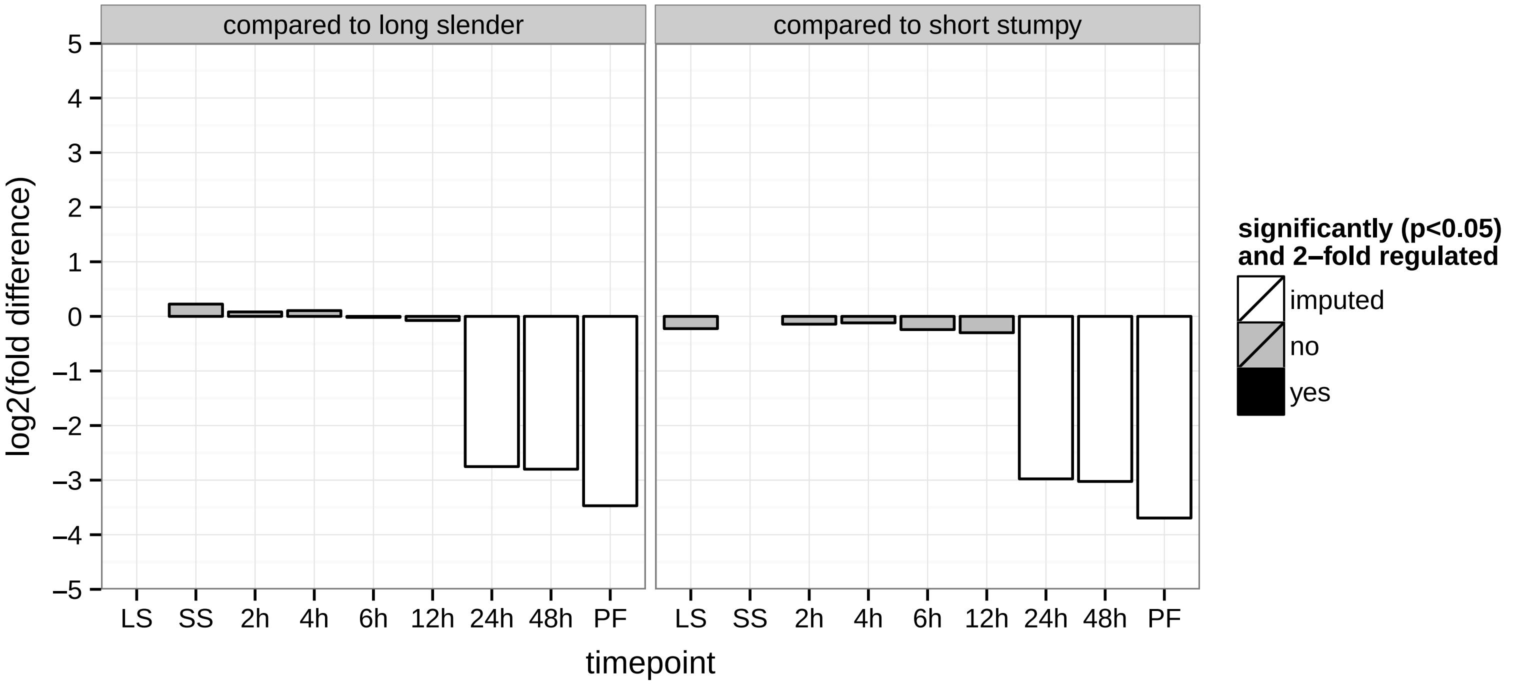

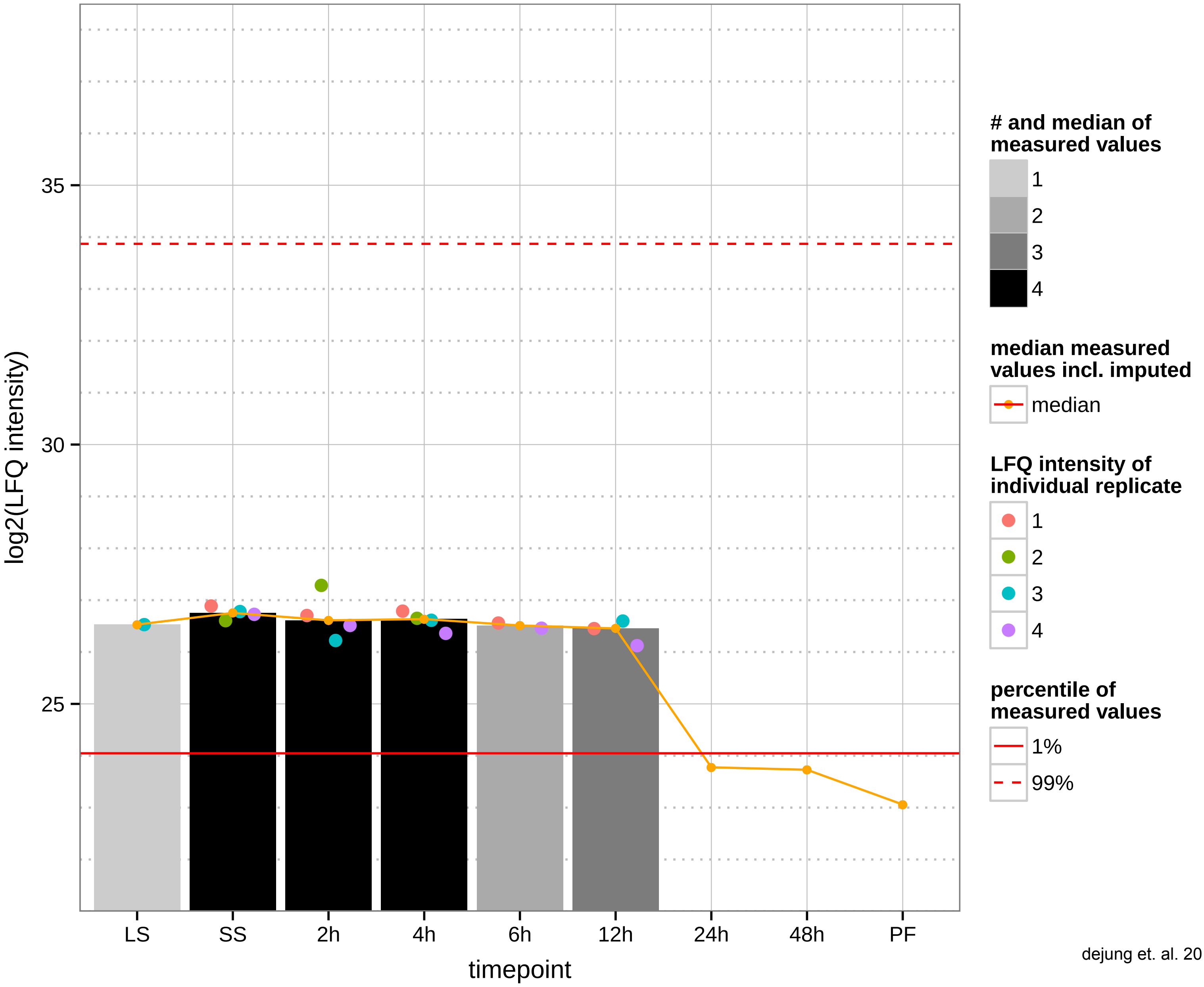

dejung et. al. 2015

protein kinase, putative
Tb927.11.5860
AGOF: ATP binding, protein serine/threonine kinase activity, protein tyrosine kinase activity
AGOC: null
AGOP: protein phosphorylation
PGOF: ATP binding, protein kinase activity, transferase activity, transferring phosphorus–containing groups
PGOC: null
PGOP: protein phosphorylation

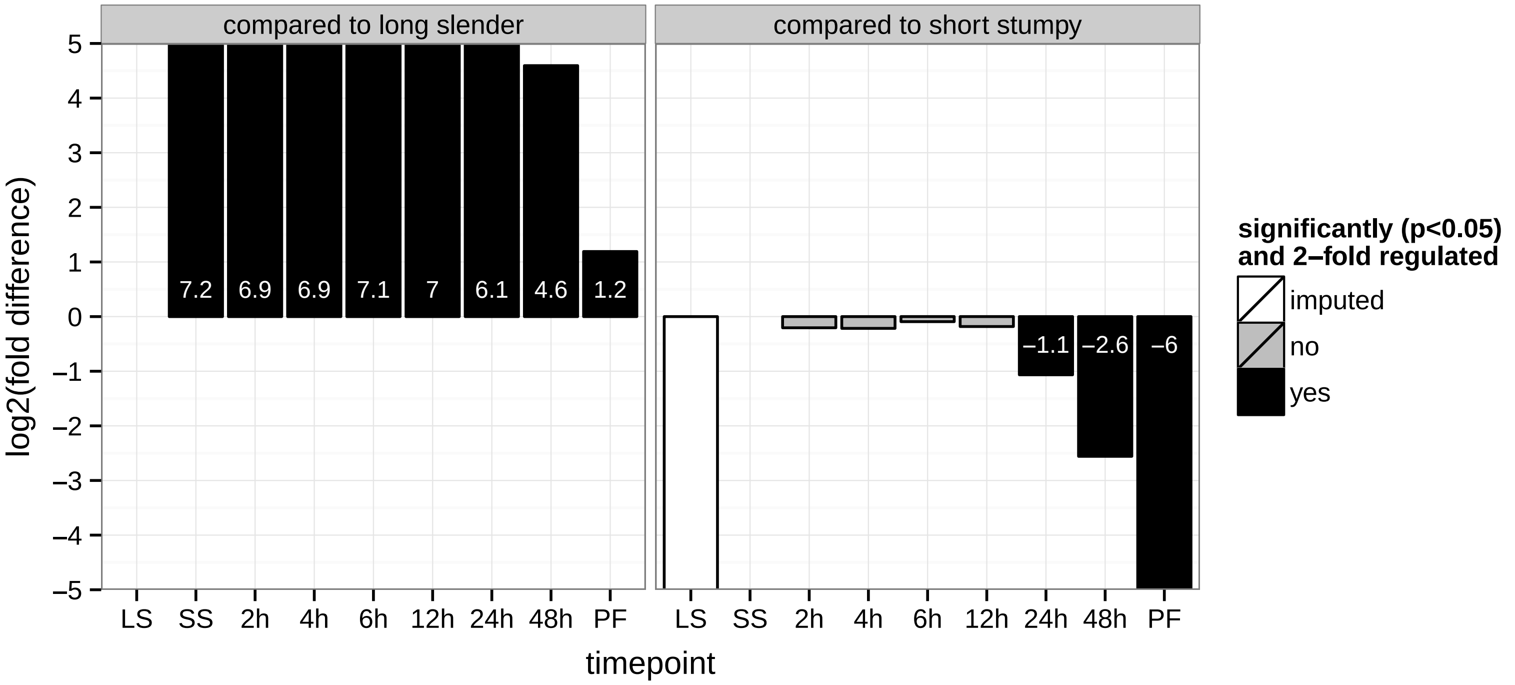

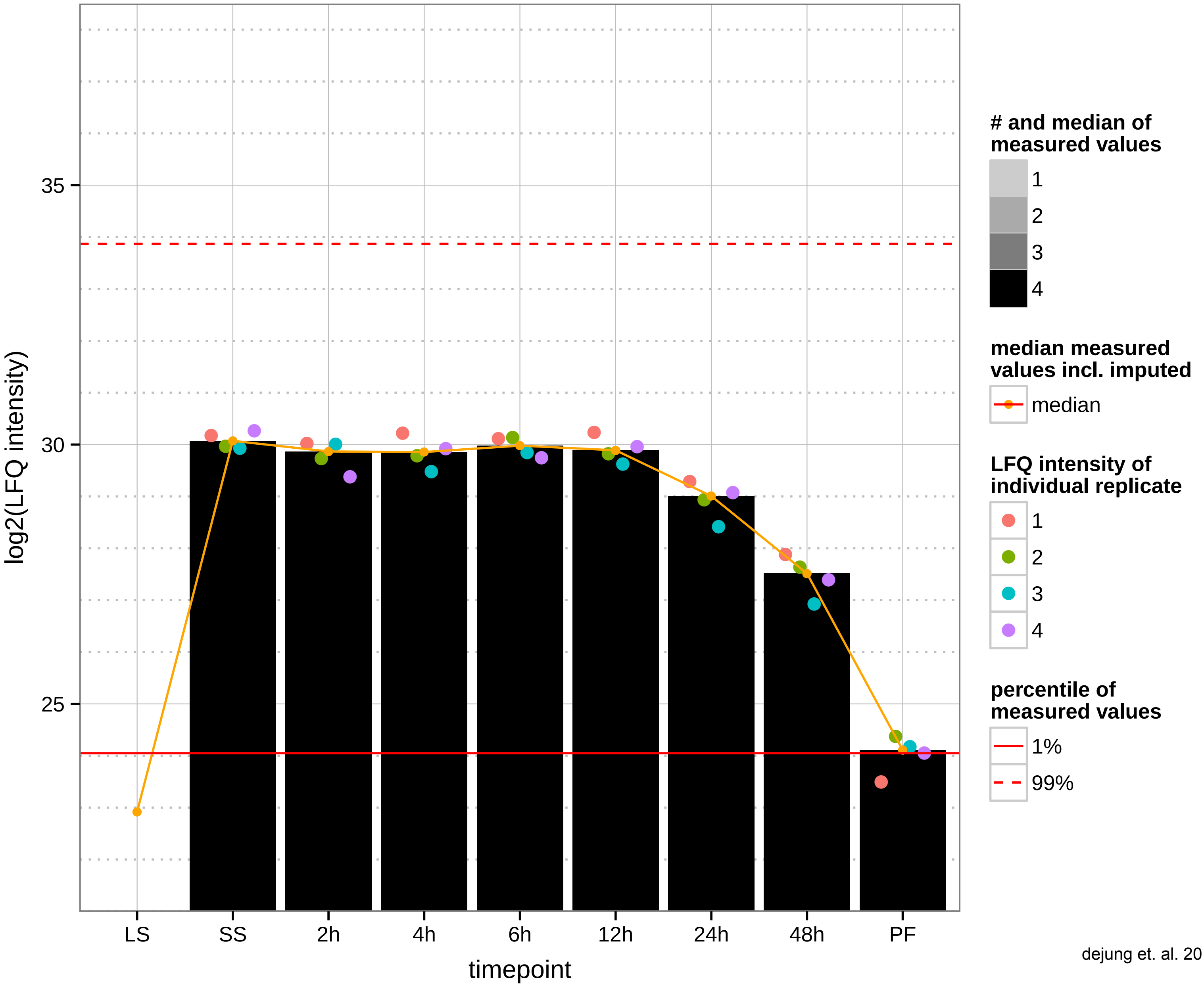

dejung et. al. 2015

hypothetical protein, conserved
Tb927.11.5910
AGOF: null
AGOC: null
AGOP: null
PGOF: null
PGOC: null
PGOP: null

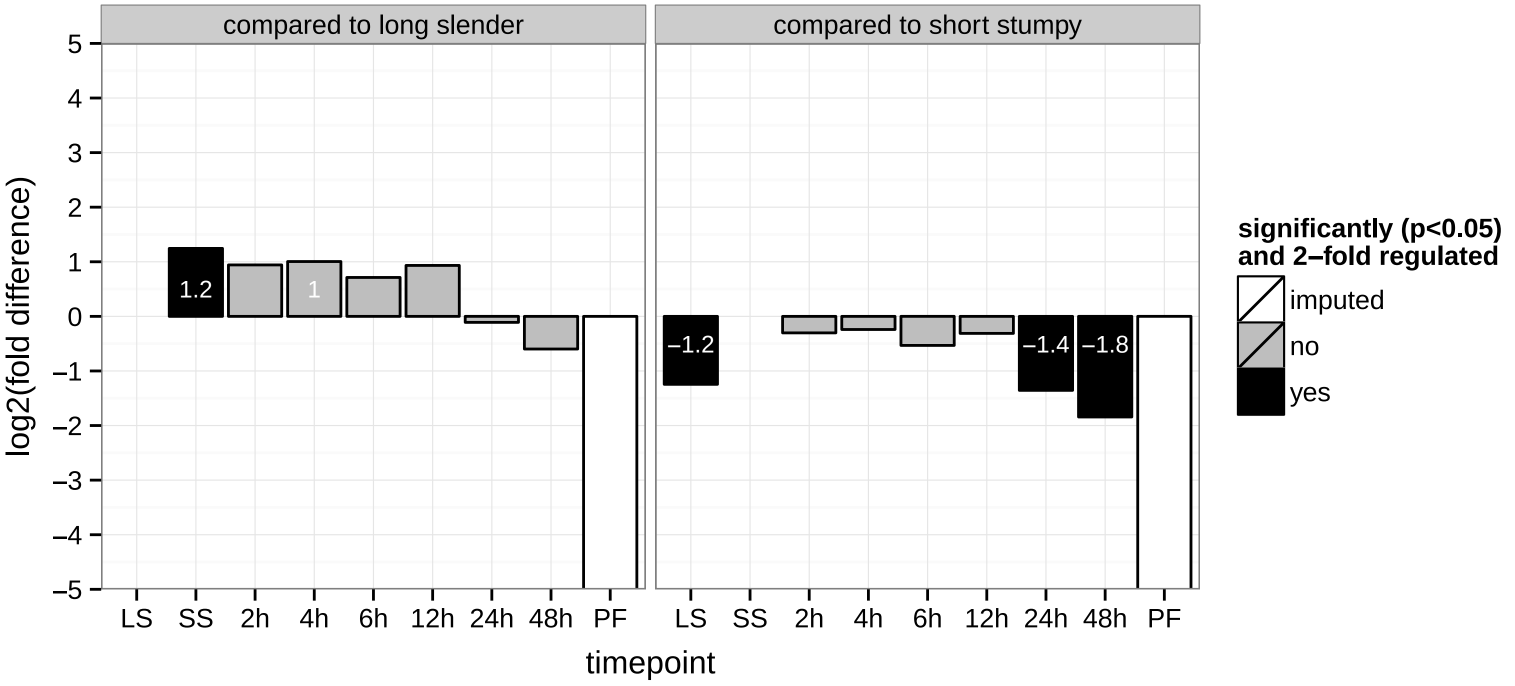

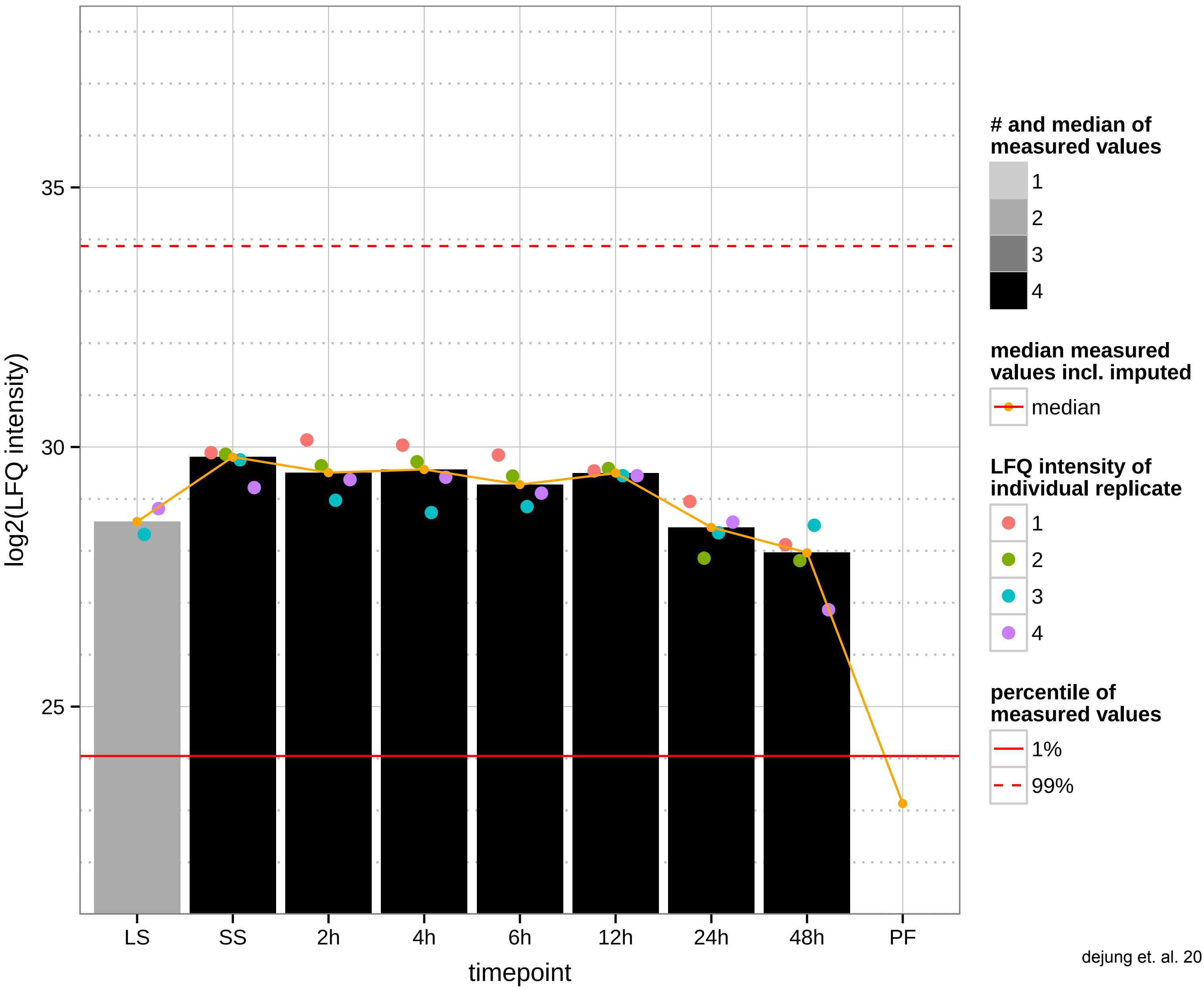

dejung et. al. 2015

acidocalcisomal pyrophosphatase
Tb927.11.7080
AGOF: calcium ion binding, inorganic diphosphatase activity, magnesium ion binding, pyrophosphatase activity, zinc ion bindi
AGOC: acidocalcisome, cytoplasm
AGOP: phosphate–containing compound metabolic process
PGOF: inorganic diphosphatase activity, magnesium ion binding
PGOC: cytoplasm
PGOP: phosphate–containing compound metabolic process

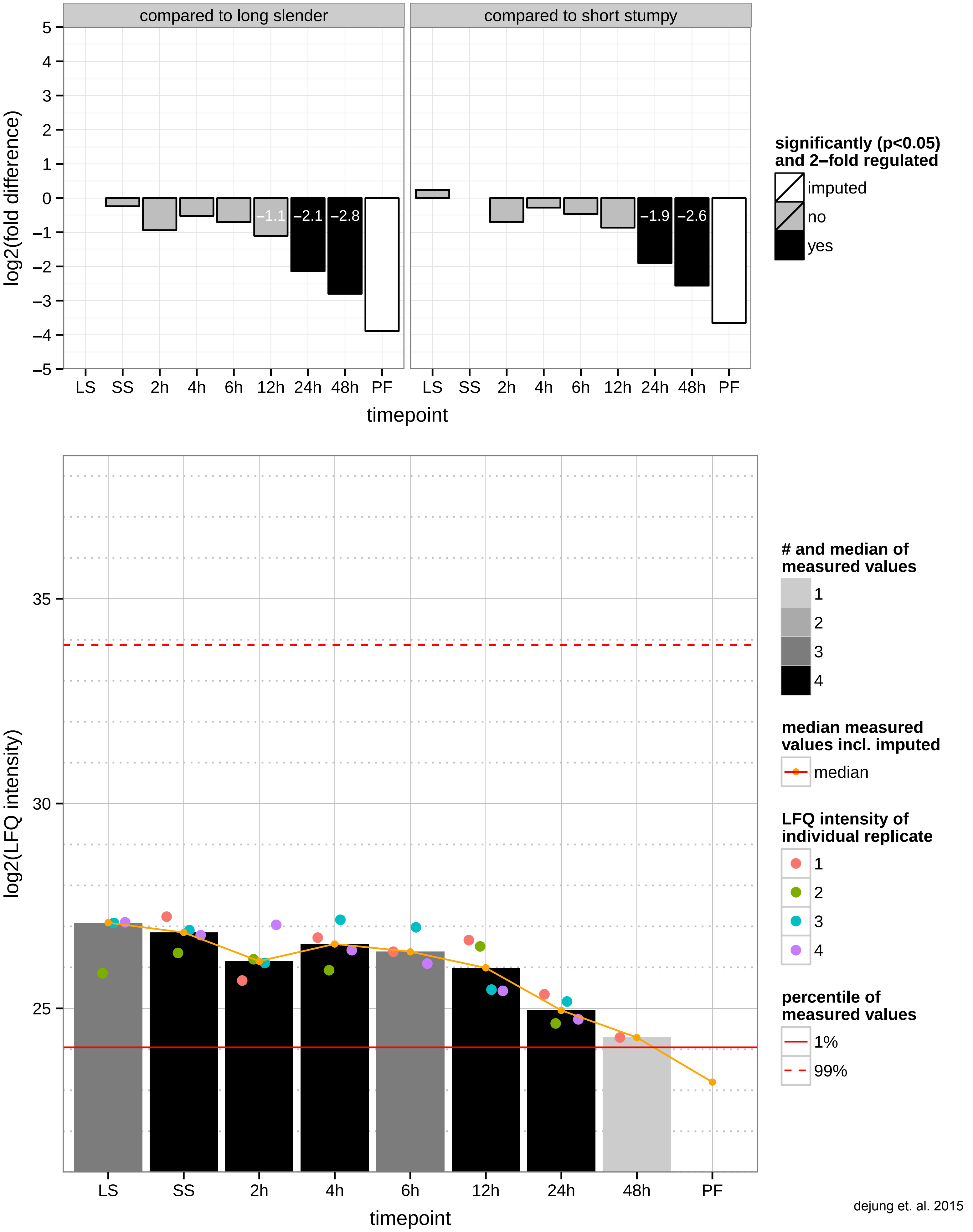

dejung et. al. 2015

hypothetical protein, conserved
Tb927.11.7120
AGOF: null
AGOC: exocyst
AGOP: vesicle docking involved in exocytosis
PGOF: null
PGOC: null
PGOP: null

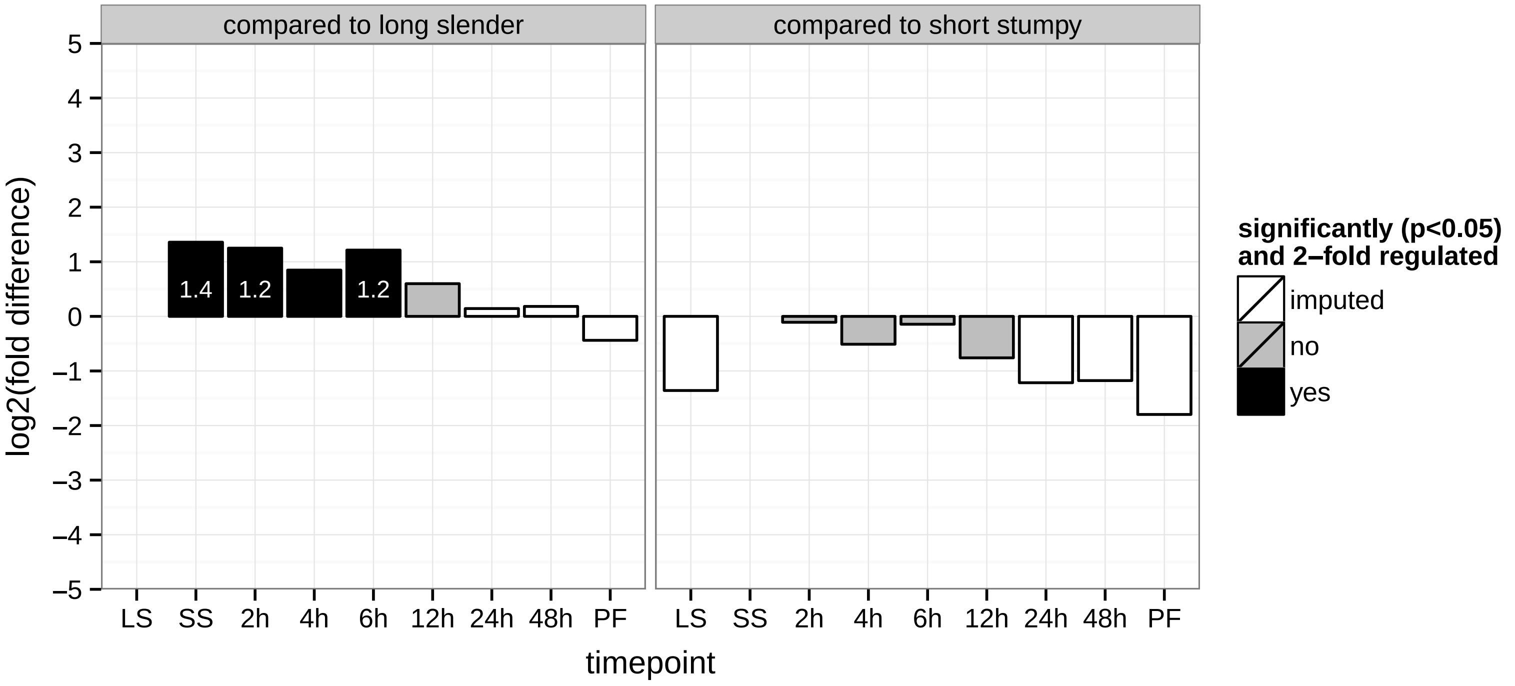

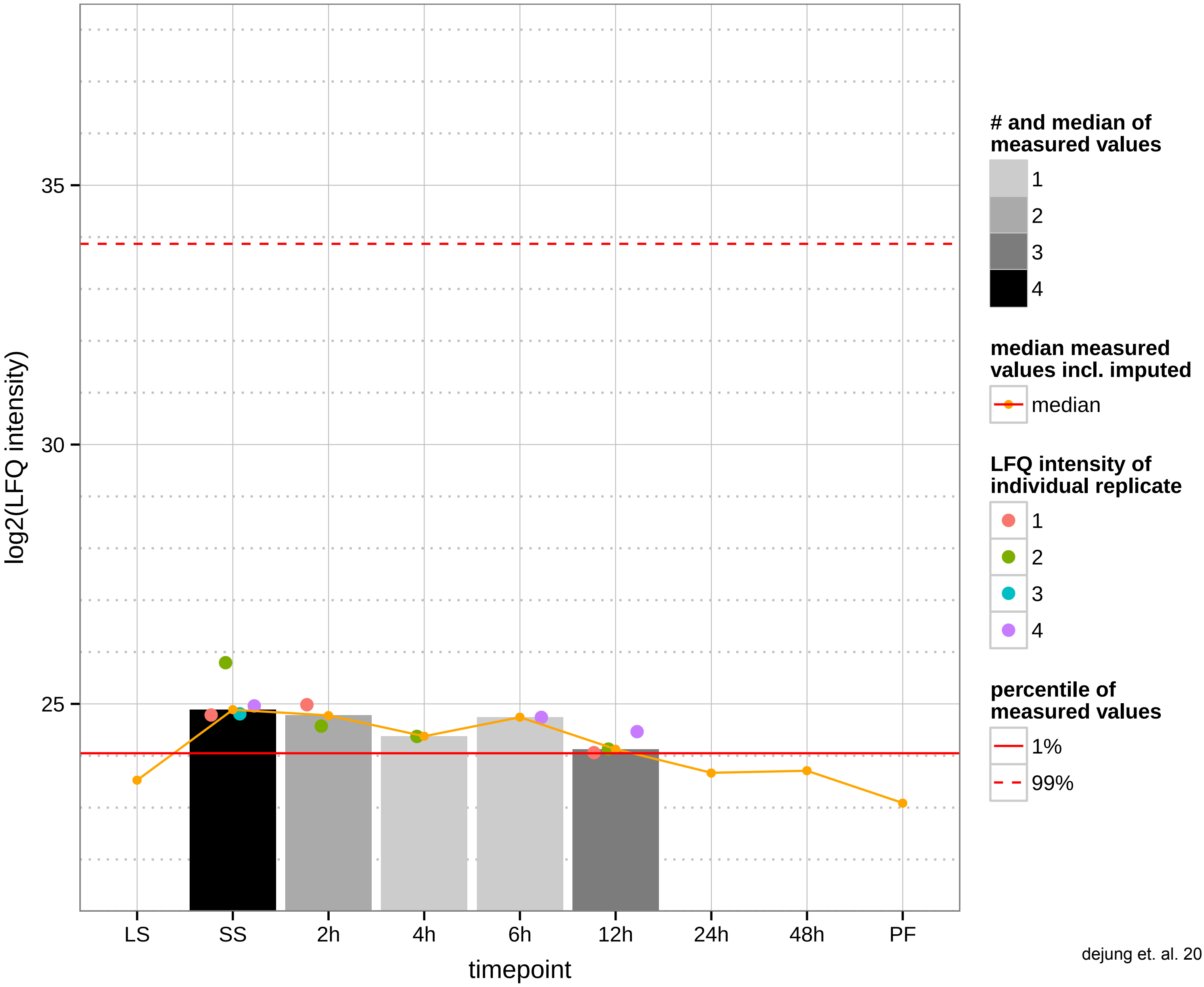

dejung et. al. 2015

palmitoyl acyltransferase 9, putative
Tb927.11.7360
AGOF: zinc ion binding
AGOC: integral to membrane
AGOP: endocytosis, protein palmitoylation, transport
PGOF: zinc ion binding
PGOC: null
PGOP: null

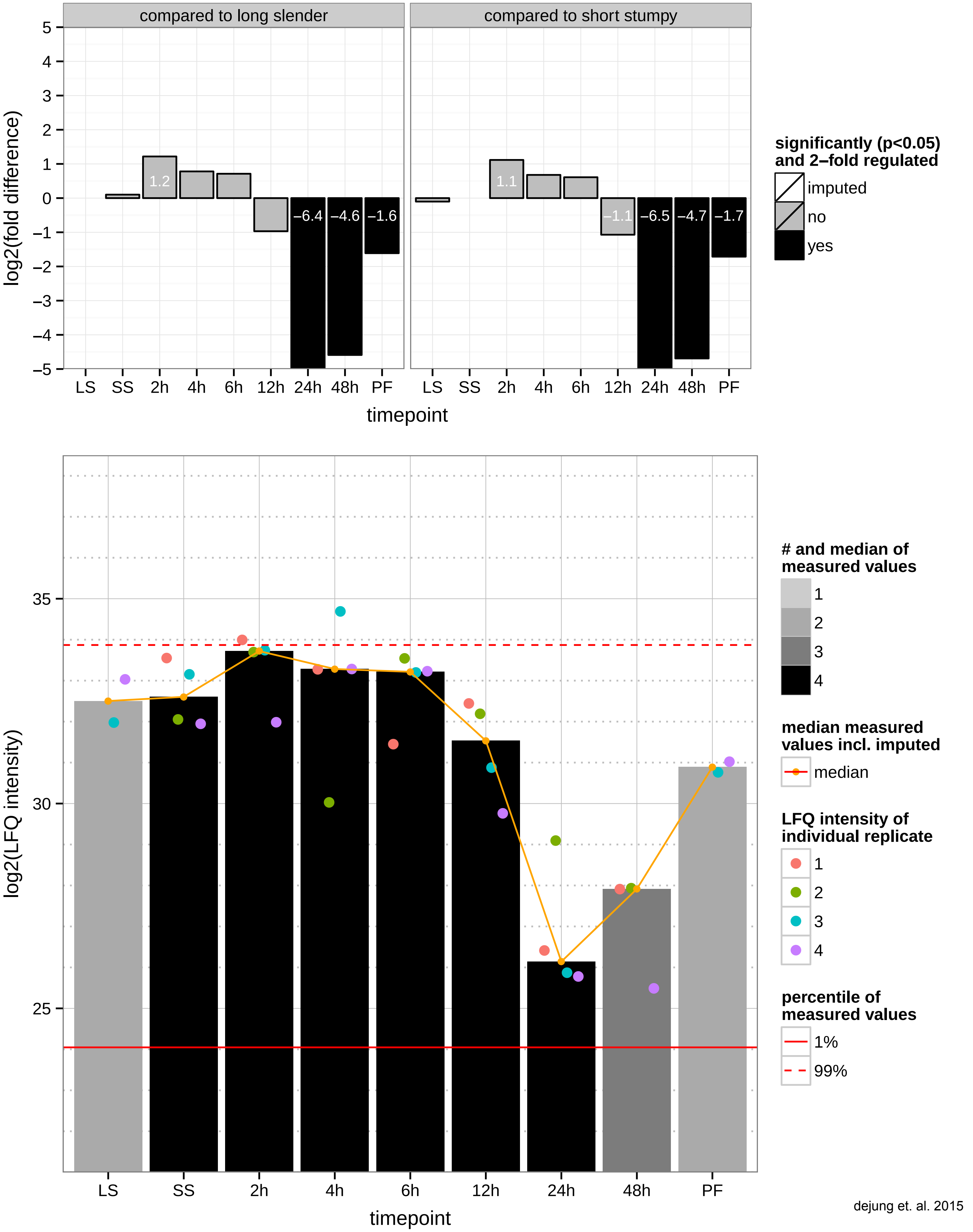

dejung et. al. 2015

MSP−A, putative, chrXI additional, unordered contigs, Gp63−3 surface protease homolog, Gp63−1 surface protease homolog
Tb927.11.7710;Tb11.0380
AGOF: metalloendopeptidase activity, zinc ion binding, null
AGOC: membrane, null
AGOP: cell adhesion, proteolysis, null
PGOF: metalloendopeptidase activity, zinc ion binding
PGOC: membrane
PGOP: cell adhesion, proteolysis

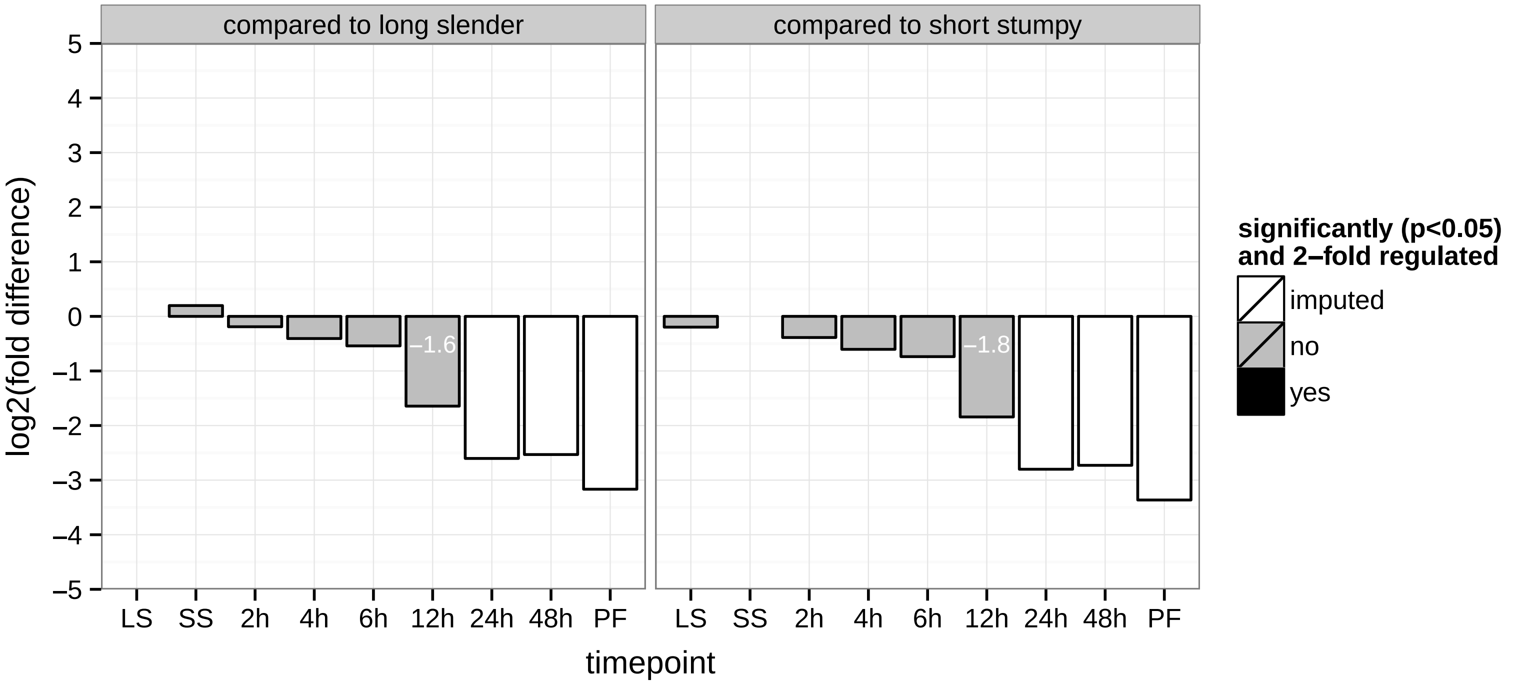

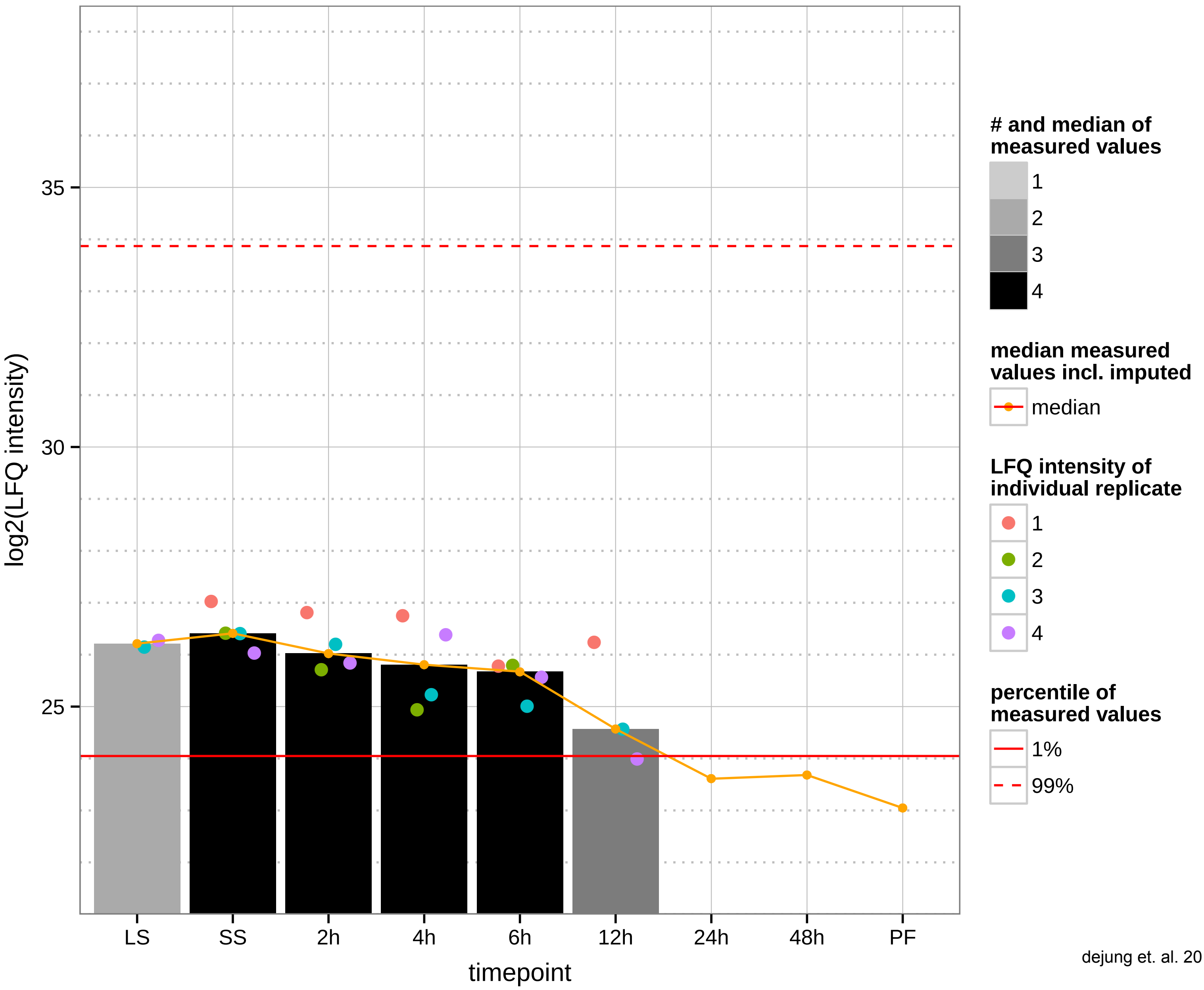

dejung et. al. 2015

hypothetical protein, conserved
Tb927.11.810
AGOF: null
AGOC: null
AGOP: null
PGOF: null
PGOC: null
PGOP: null

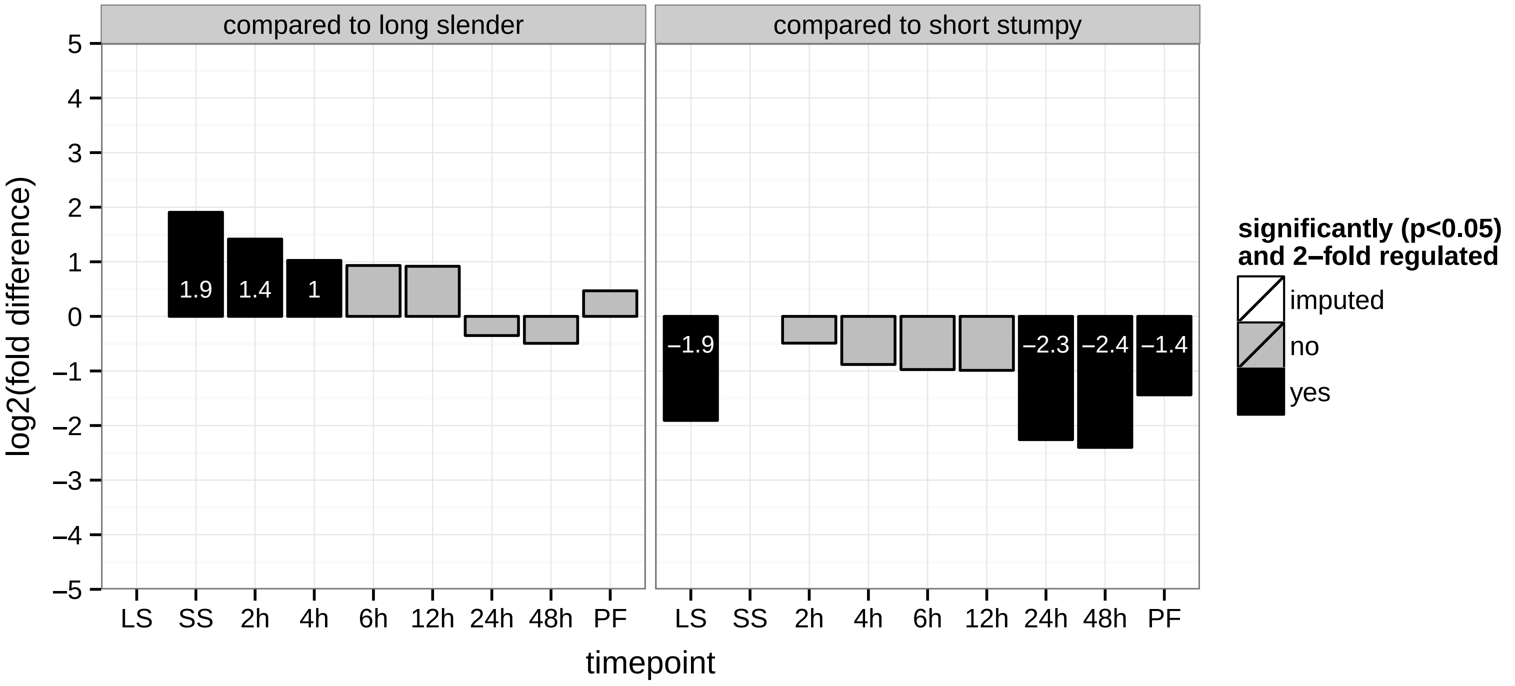

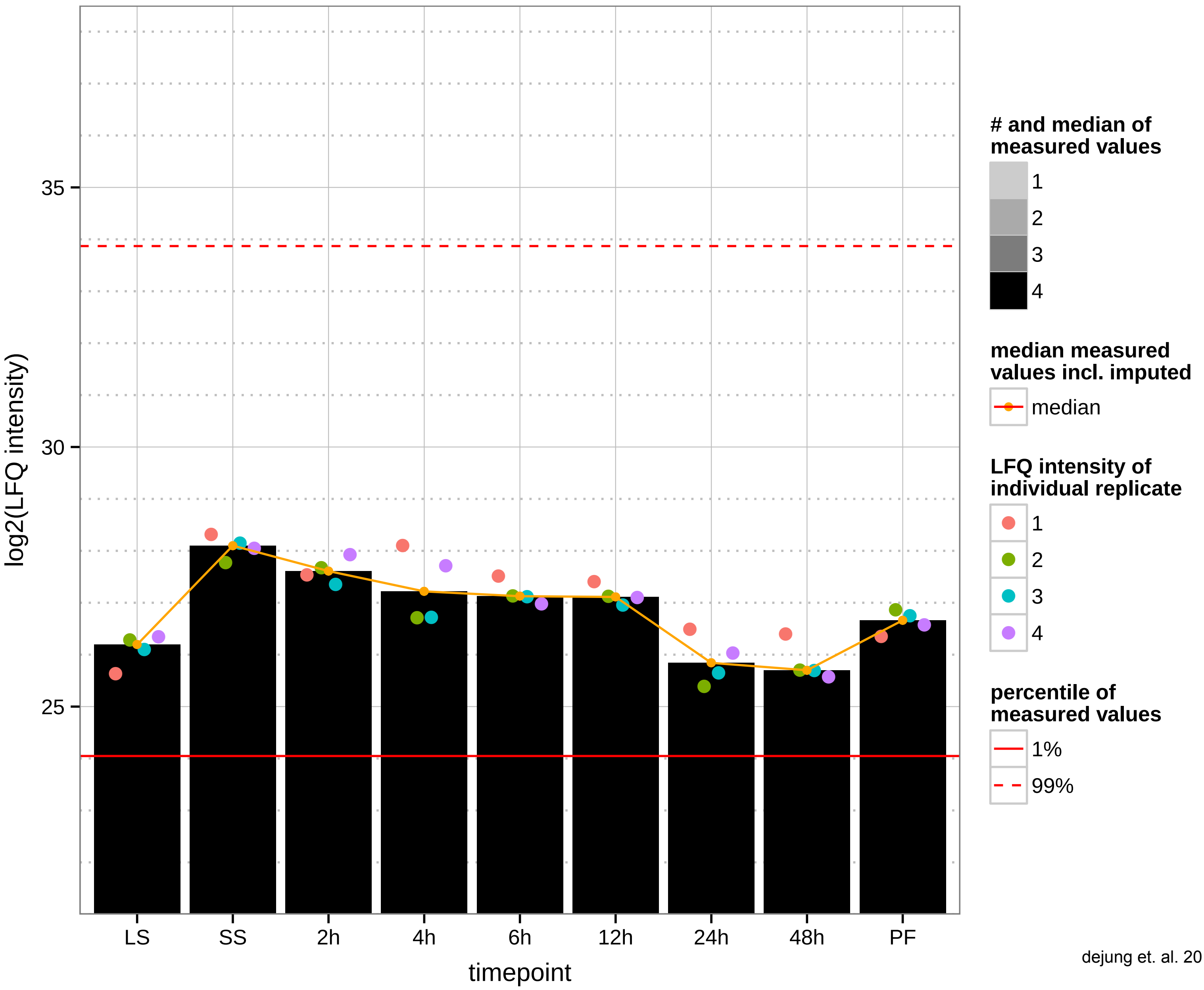

dejung et. al. 2015

phosphonopyruvate decarboxylase–like protein, putative
Tb927.11.9350
AGOF: carboxy–lyase activity, thiamine pyrophosphate binding
AGOC: null
AGOP: null
PGOF: catalytic activity, thiamine pyrophosphate binding
PGOC: null
PGOP: null

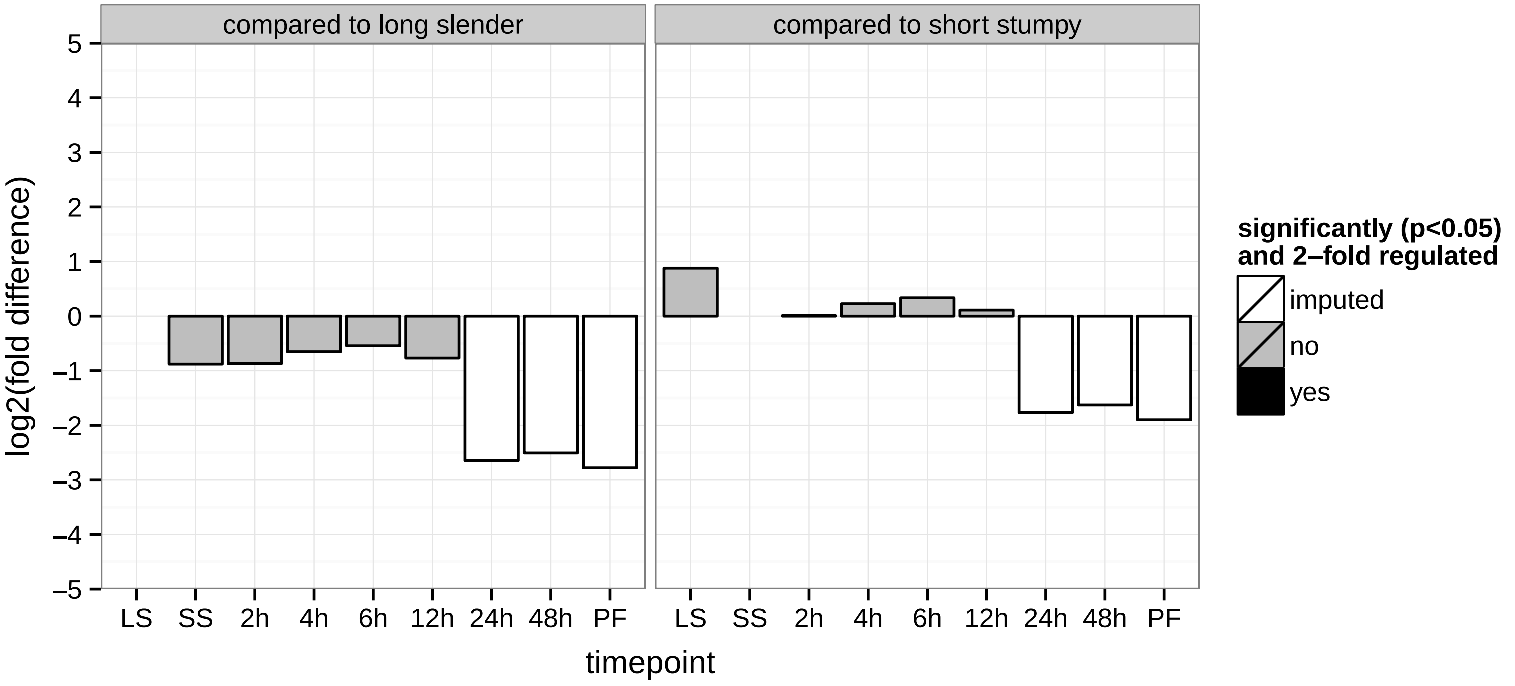

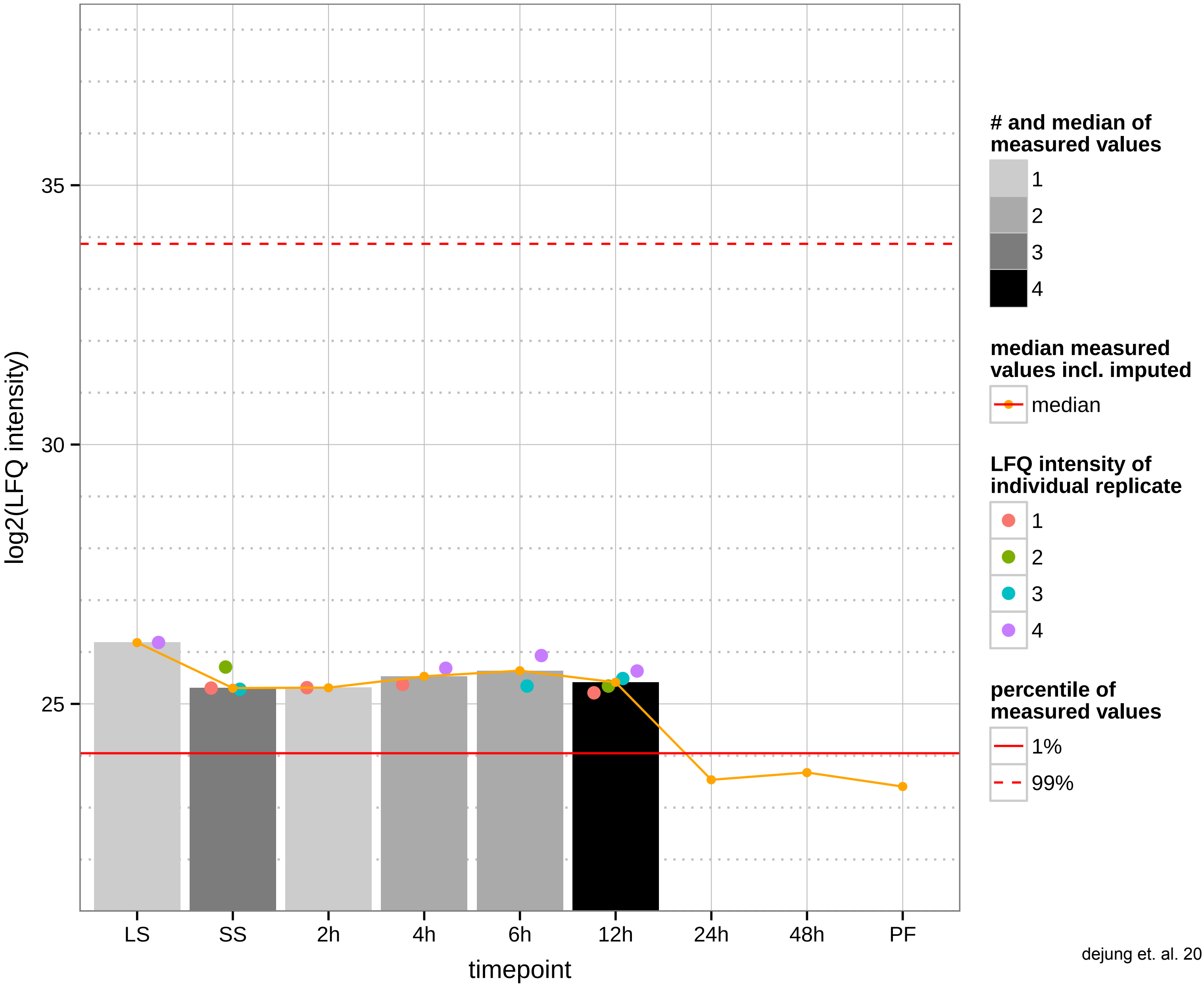

dejung et. al. 2015

ADP–ribosylation factor GTPase activating protein, putative
Tb927.11.9470
AGOF: ARF GTPase activator activity, DNA binding, zinc ion binding
AGOC: nucleus
AGOP: regulation of ARF GTPase activity
PGOF: ARF GTPase activator activity, zinc ion binding
PGOC: null
PGOP: regulation of ARF GTPase activity

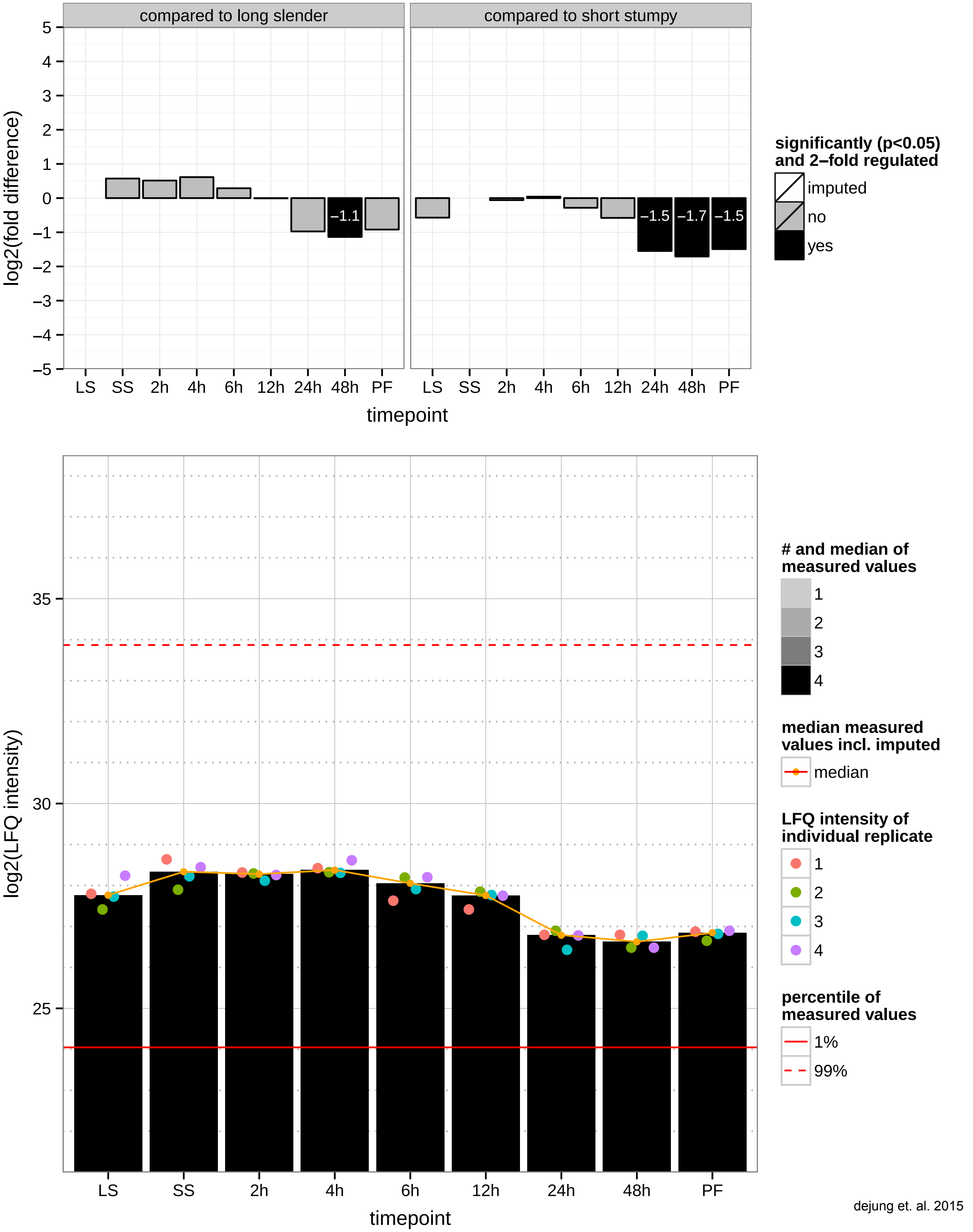

dejung et. al. 2015

protein kinase, putative, NIMA/Nek Serine/threonine–protein kinase family
Tb927.2.2120
AGOF: ATP binding, protein kinase activity, protein serine/threonine kinase activity, protein tyrosine kinase activity
AGOC: null
AGOP: protein phosphorylation
PGOF: ATP binding, protein kinase activity, transferase activity, transferring phosphorus–containing groups
PGOC: null
PGOP: protein phosphorylation

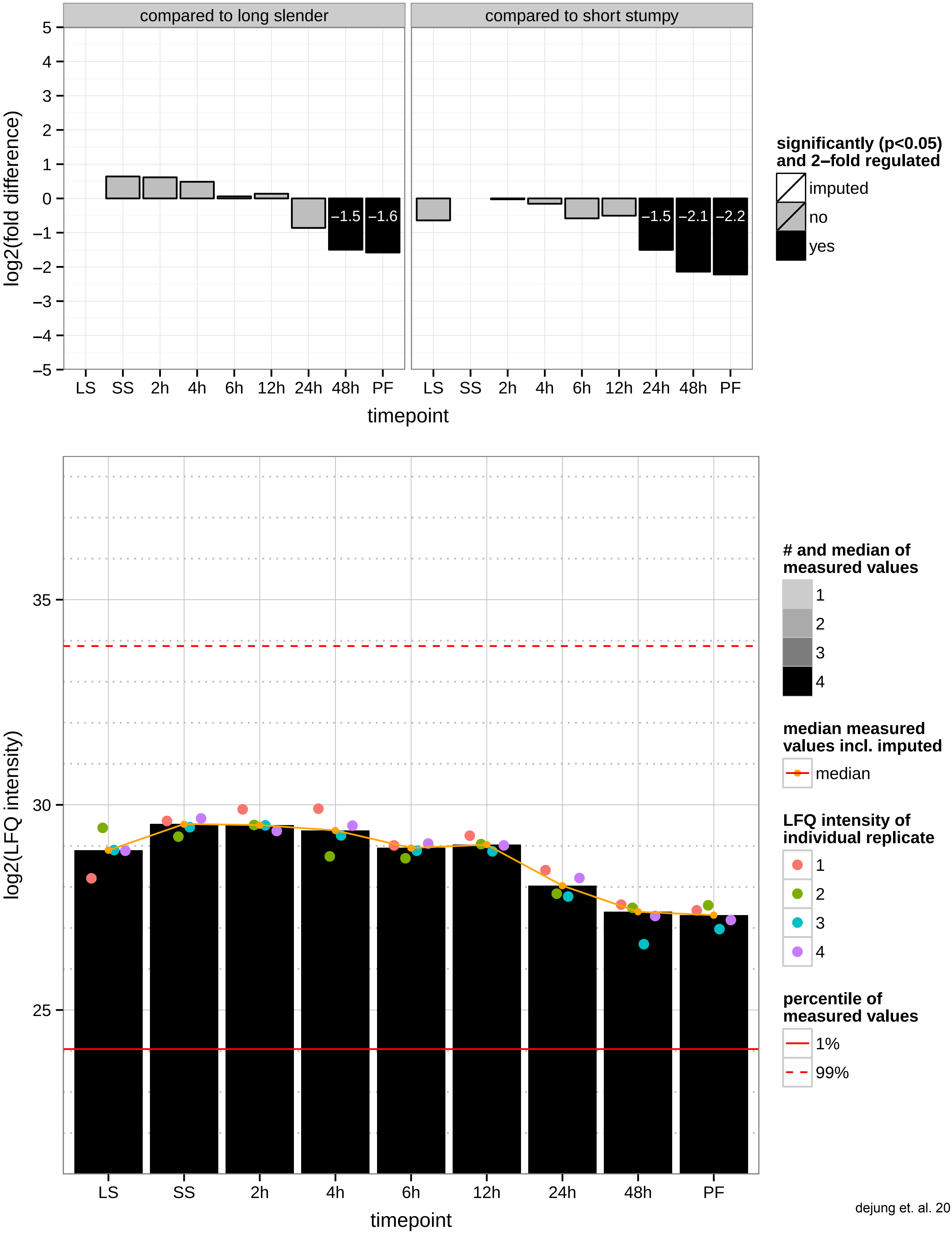

dejung et. al. 2015

hypothetical protein, conserved
Tb927.2.5010
AGOF: null
AGOC: null
AGOP: null
PGOF: phospholipid binding, protein binding
PGOC: cell cortex
PGOP: cortical protein anchoring

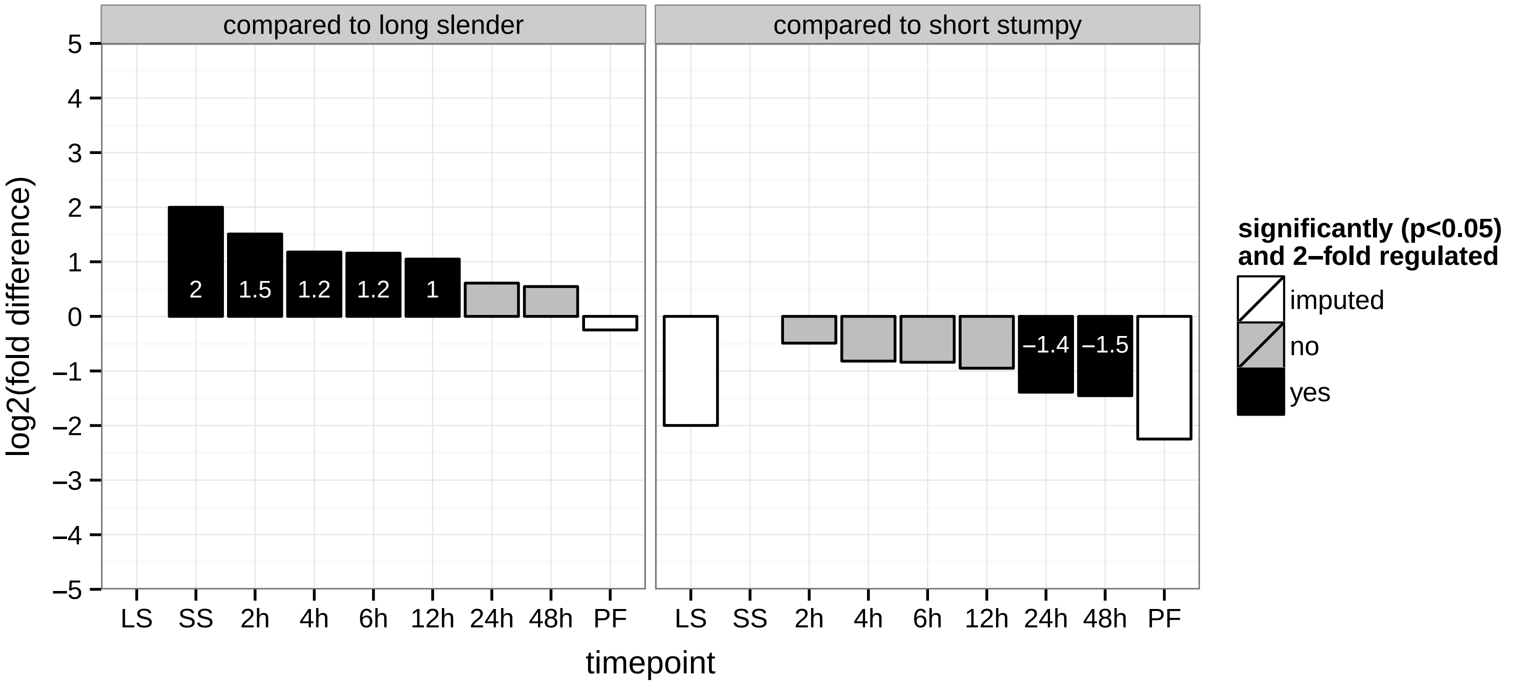

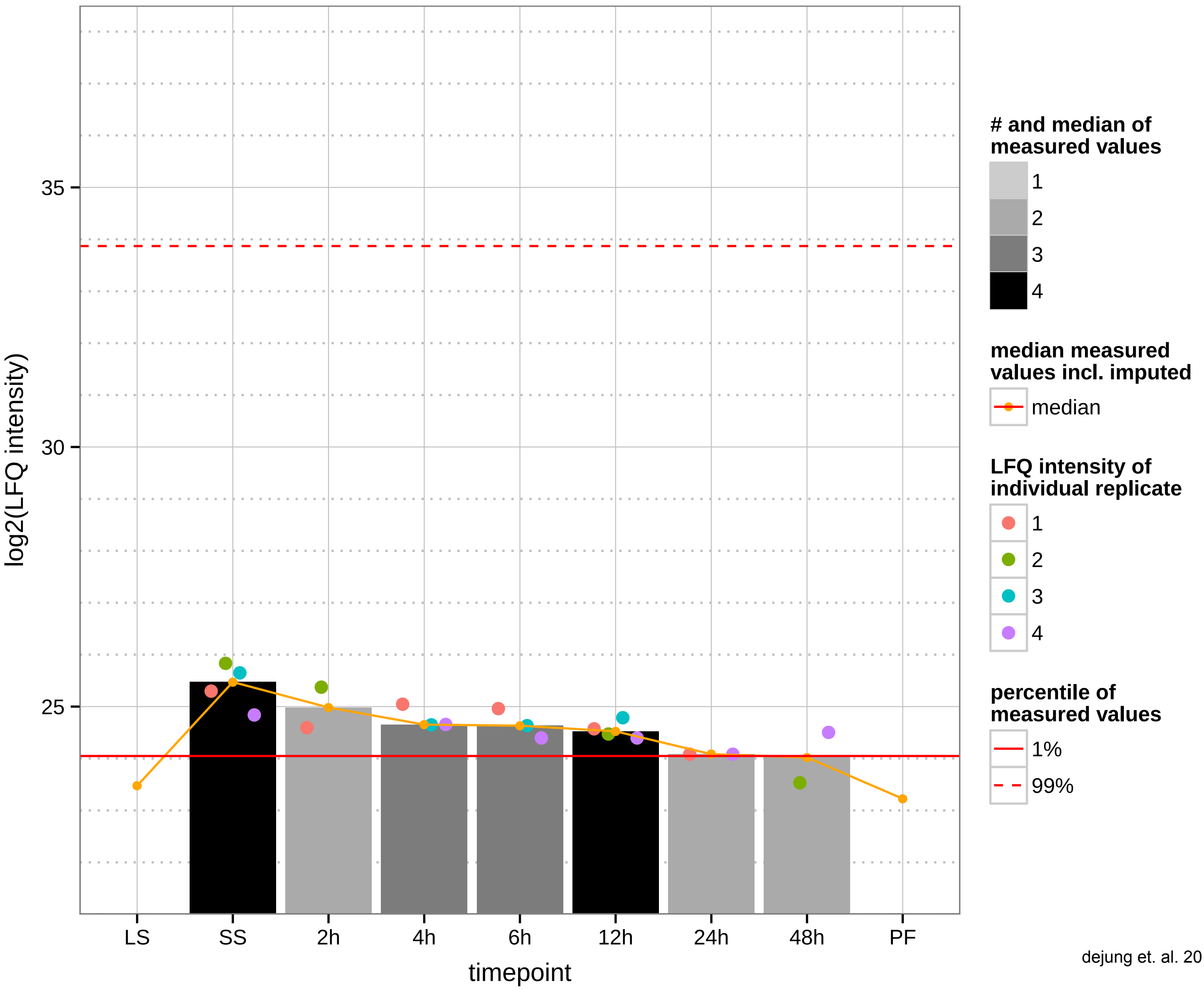

dejung et. al. 2015

ATP−dependent Clp protease subunit, heat shock protein 104 (HSP104), putative, atp−dependent chaperone (HSP104)
Tb927.2.5980
AGOF: ATP binding, ATPase activity
AGOC: null
AGOP: protein metabolic process
PGOF: ATP binding, nucleoside−triphosphatase activity, nucleotide binding
PGOC: null
PGOP: protein metabolic process

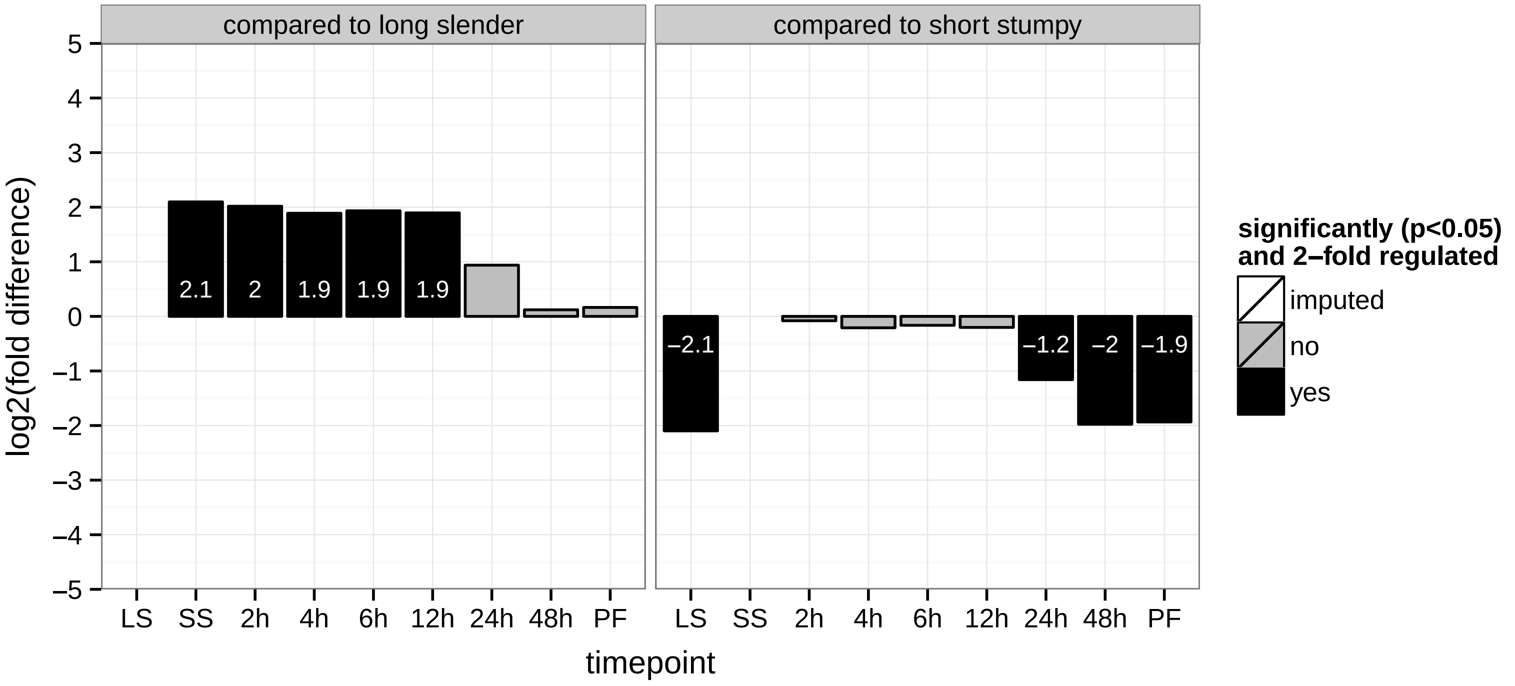

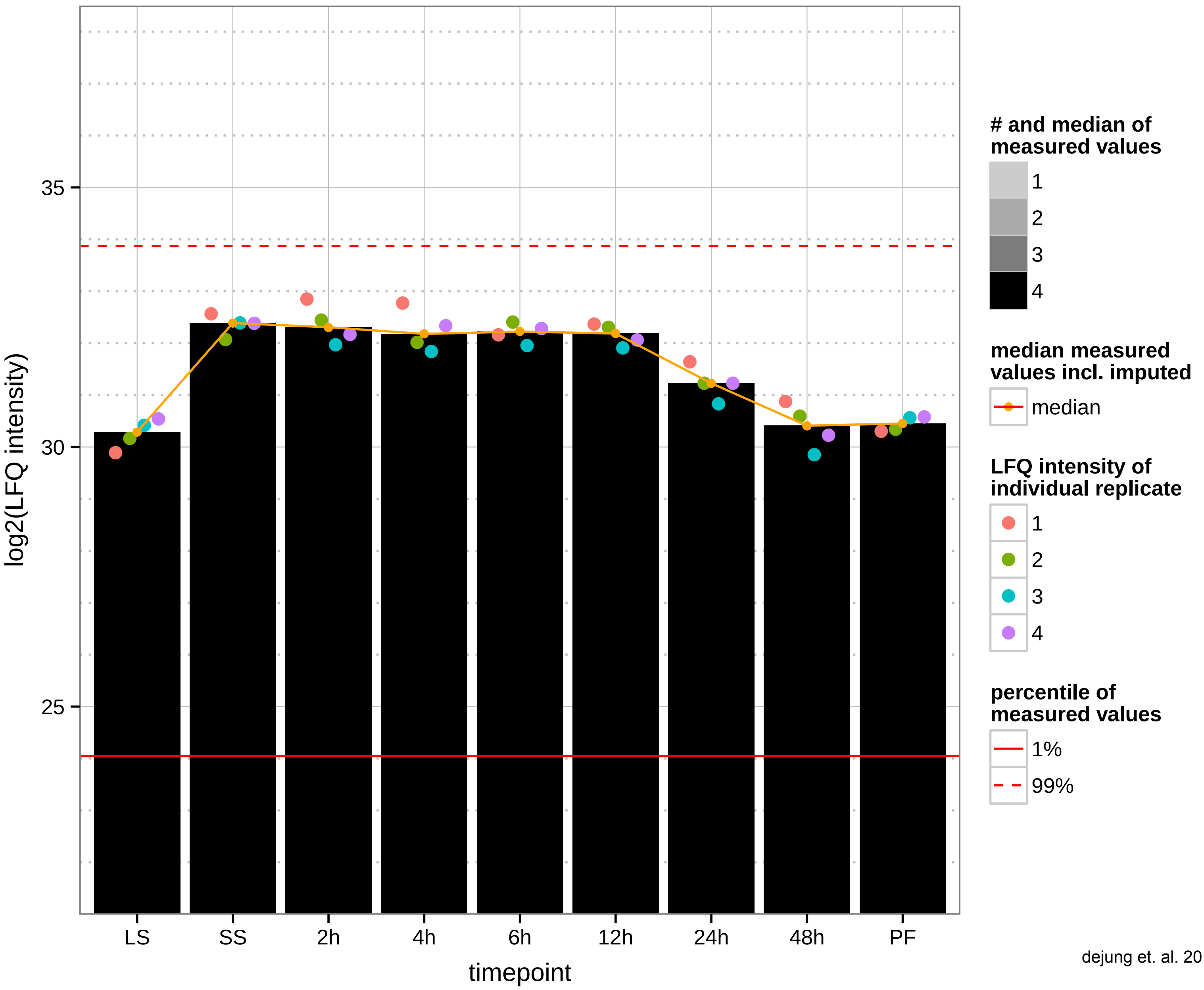

dejung et. al. 2015

glycosylphosphatidylinositol–specific phospholipase C, VSG lipase (GPI–PLC)
Tb927.2.6000
AGOF: glycosylphosphatidylinositol diacylglycerol–lyase activity, phospholipase C activity
AGOC: plasma membrane
AGOP: GPI anchor release, glycerophospholipid metabolic process, intracellular signal transduction, lipid metabolic process
PGOF: glycosylphosphatidylinositol diacylglycerol–lyase activity, phospholipase C activity, phosphoric diester hydrolase activit
PGOC: null
PGOP: glycerophospholipid metabolic process, intracellular signal transduction, lipid metabolic process

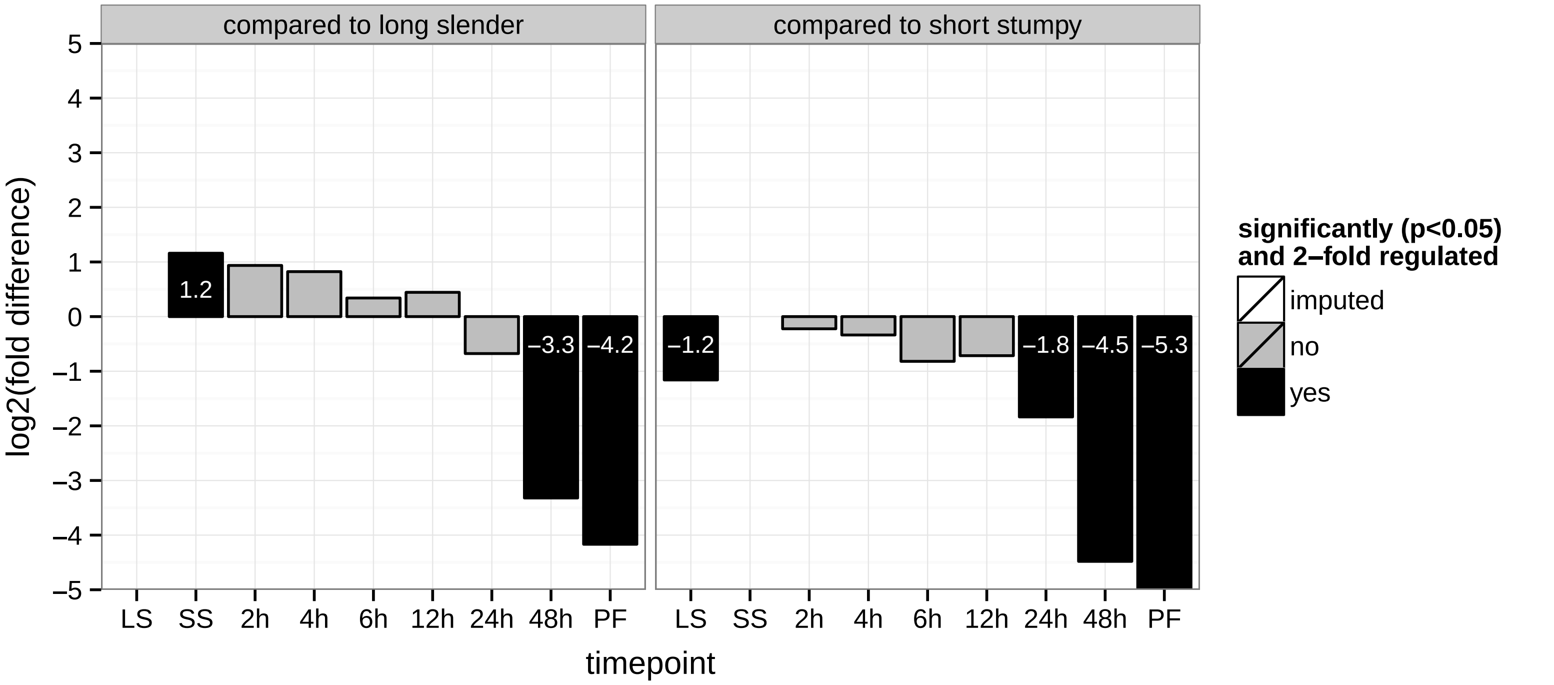

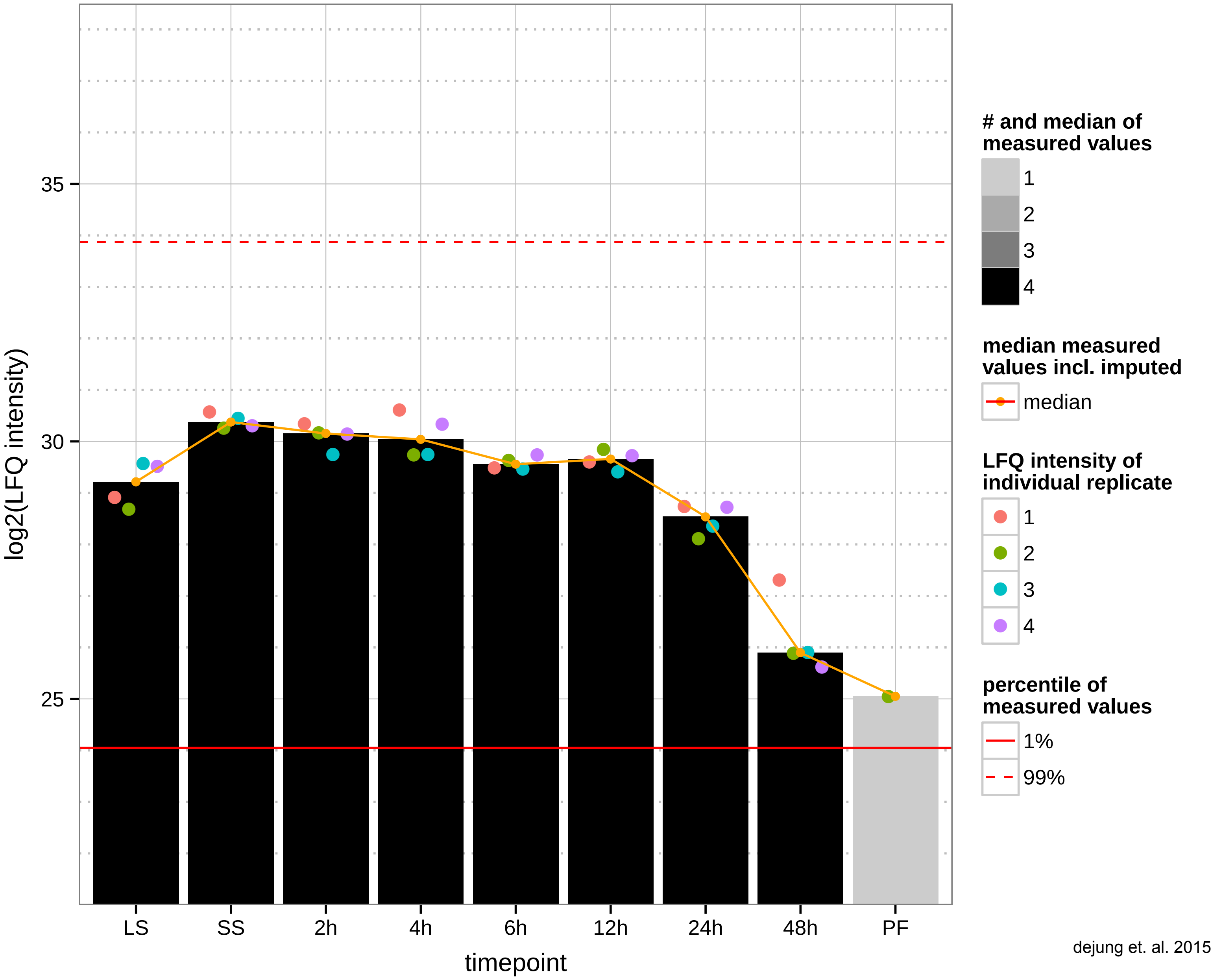

dejung et. al. 2015

ATP−dependent phosphofructokinase (PFK)
Tb927.3.3270
AGOF: 6−phosphofructokinase activity, ATP binding
AGOC: 6−phosphofructokinase complex, glycosome
AGOP: glycolysis
PGOF: 6−phosphofructokinase activity, ATP binding
PGOC: 6−phosphofructokinase complex
PGOP: glycolysis

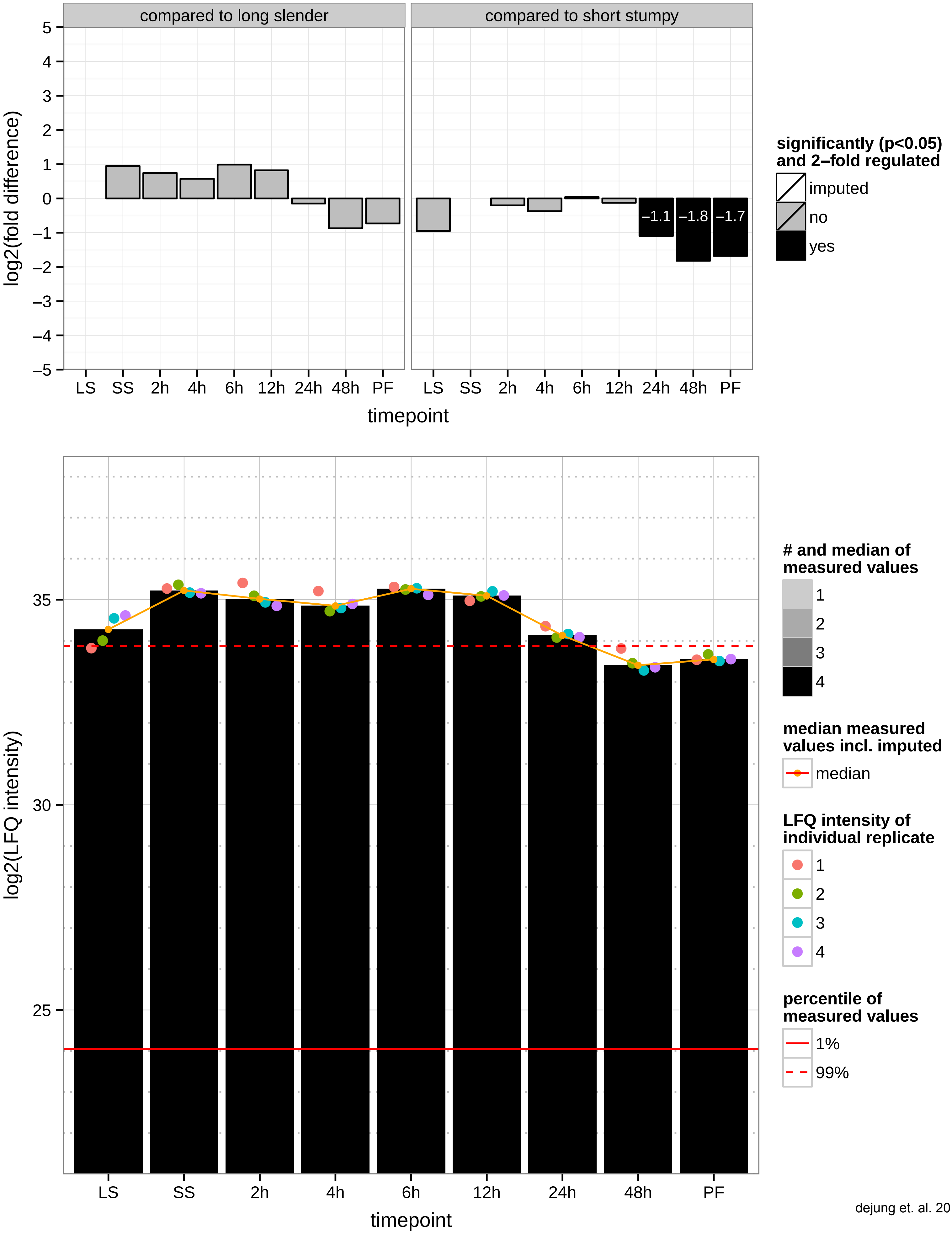

dejung et. al. 2015

hypothetical protein, conserved
Tb927.3.4660
AGOF: null
AGOC: null
AGOP: null
PGOF: protein binding
PGOC: null
PGOP: null

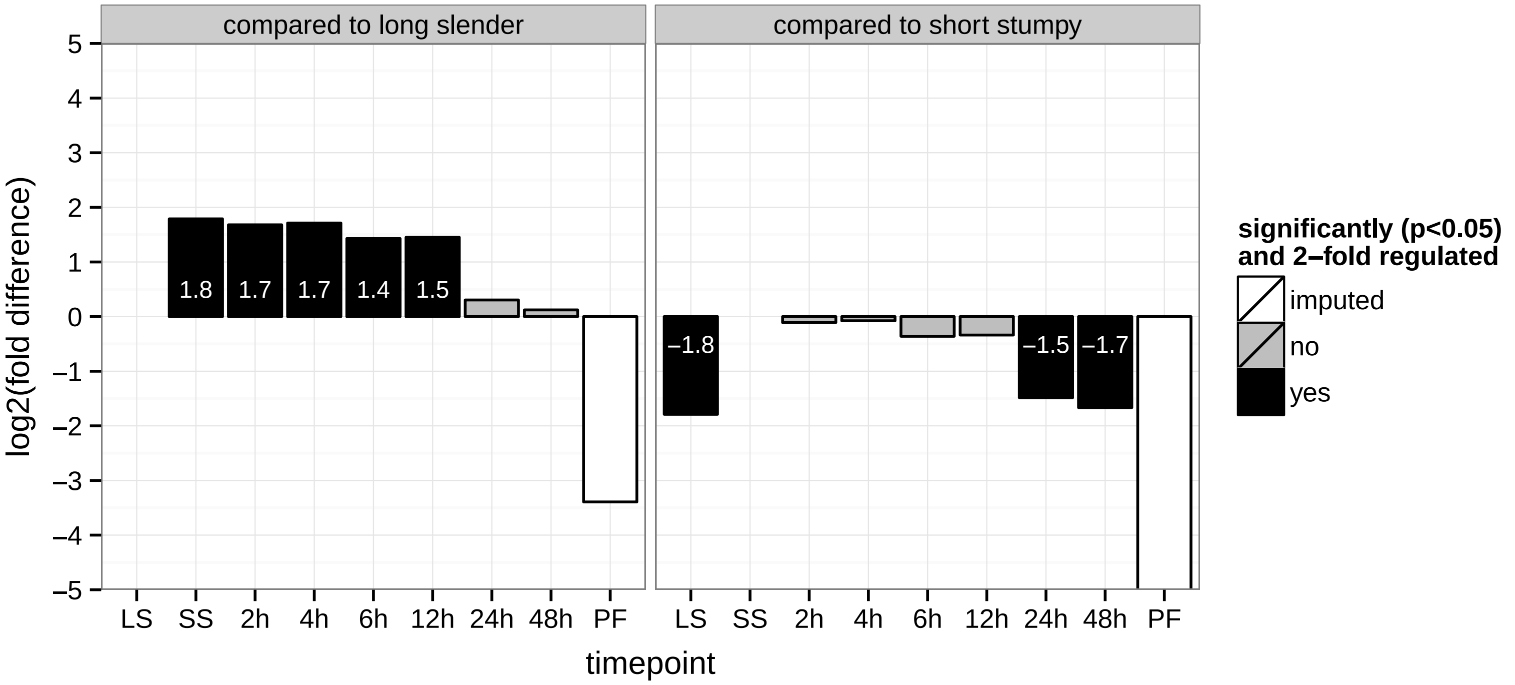

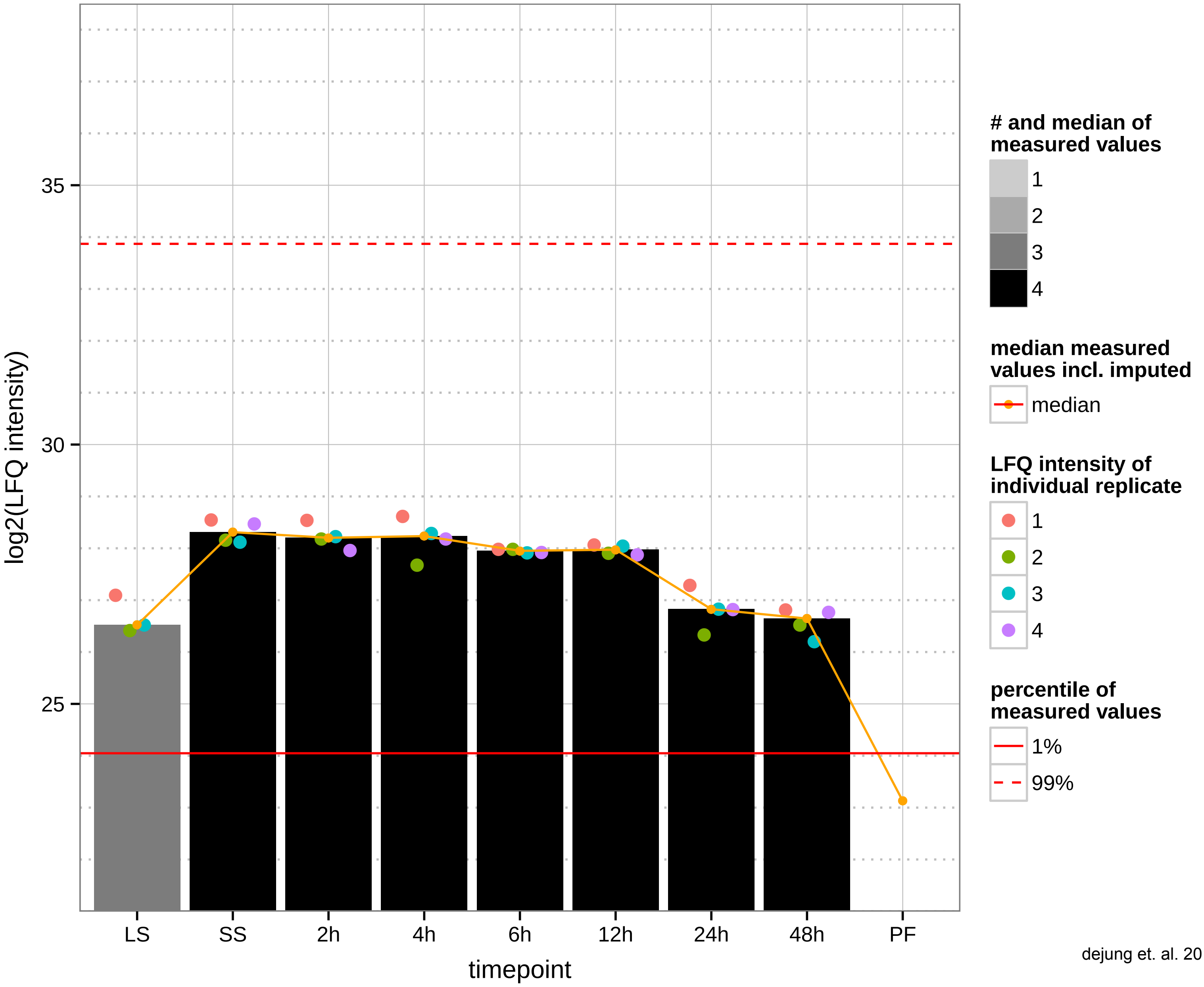

dejung et. al. 2015

hypothetical protein, conserved
Tb927.3.4710
AGOF: null
AGOC: null
AGOP: null
PGOF: null
PGOC: null
PGOP: null

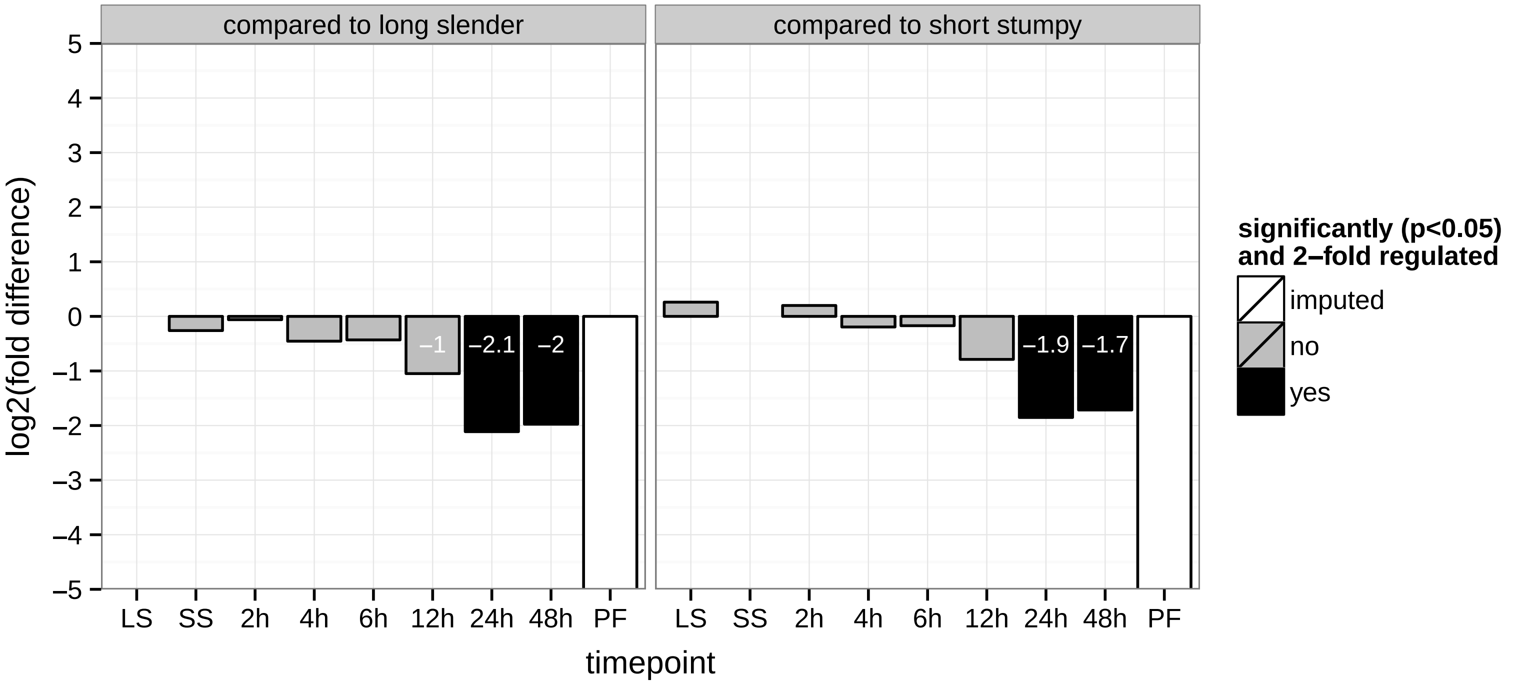

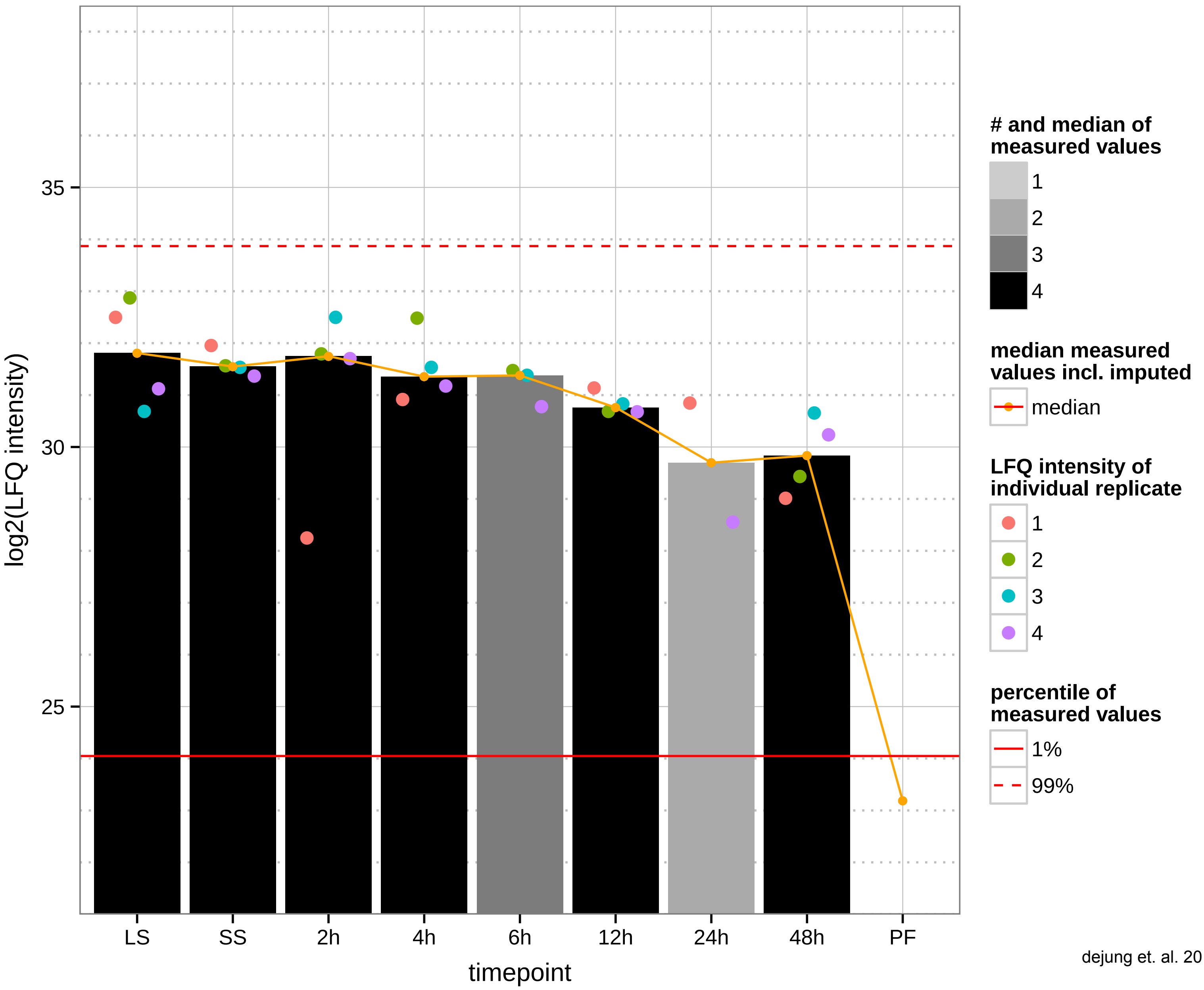

dejung et. al. 2015

tryparedoxin 2, putative (TXN2)
Tb927.3.5090
AGOF: antioxidant activity, oxidoreductase activity
AGOC: null
AGOP: cell redox homeostasis
PGOF: null
PGOC: null
PGOP: null

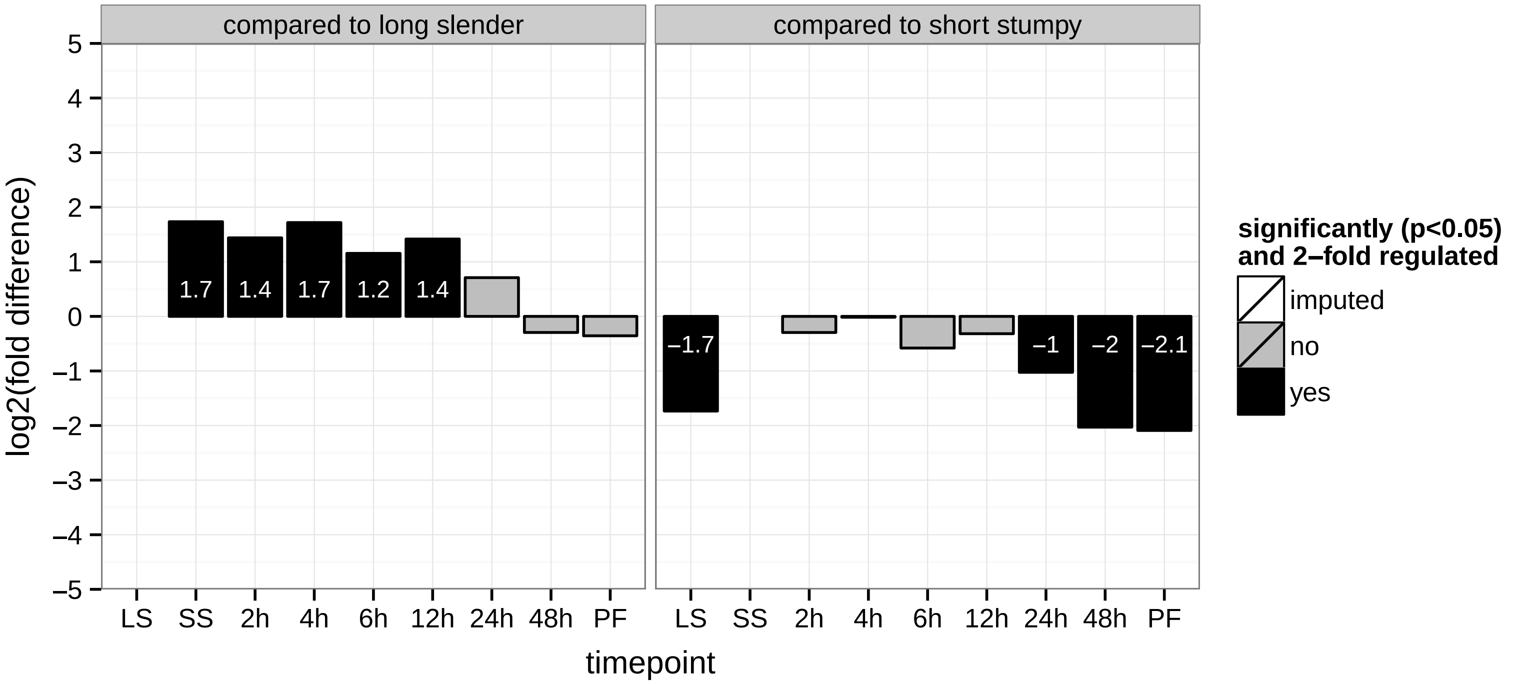

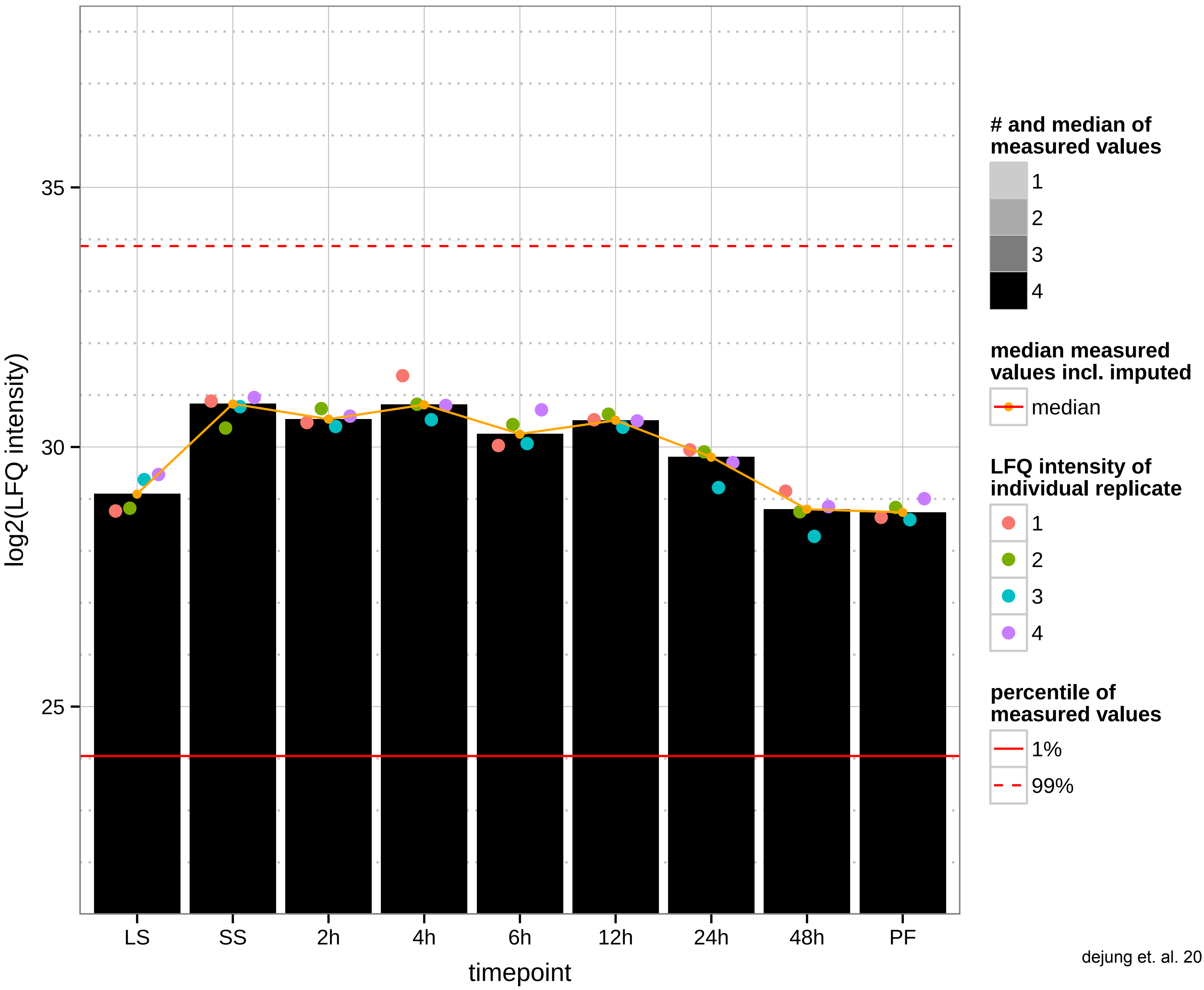

dejung et. al. 2015

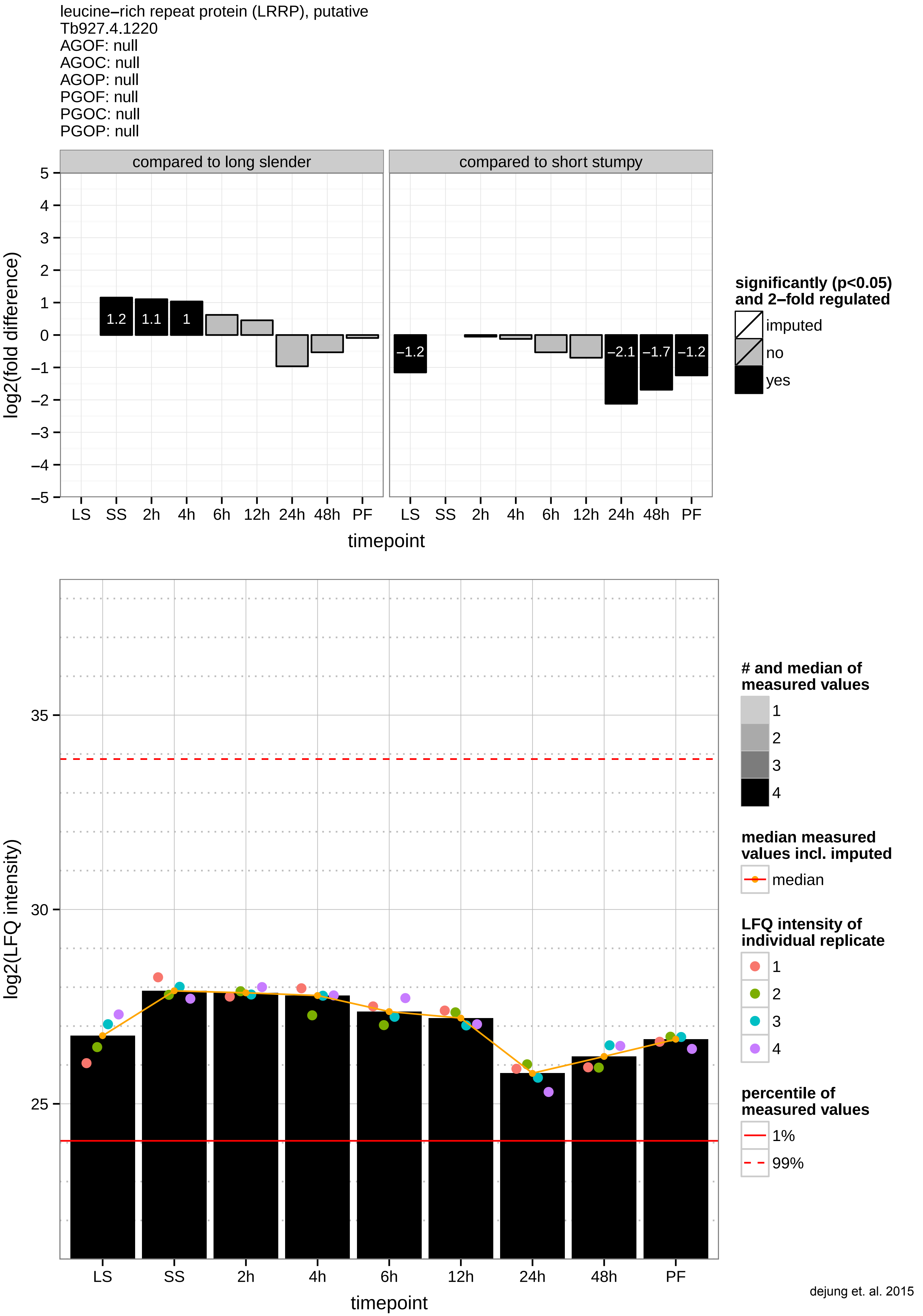

leucine−rich repeat protein (LRRP), putative
Tb927.4.1220
AGOF: null
AGOC: null
AGOP: null
PGOF: null
PGOC: null
PGOP: null
compared to long slender
compared to short stumpy
log2(fold difference)
1.2
1.1
1
−1.2
−2.1
−1.7
−1.2
LS
SS
2h
4h
6h
12h
24h
48h
PF
timepoint
significantly (p<0.05) and 2−fold regulated
imputed
no
yes
log2(LFQ intensity)
# and median of measured values
median measured values incl. imputed
median
LFQ intensity of individual replicate
percentile of measured values
1%
99%
dejung et. al. 2015

hypothetical protein, conserved
Tb927.4.2240
AGOF: catalytic activity
AGOC: null
AGOP: metabolic process
PGOF: catalytic activity
PGOC: null
PGOP: metabolic process

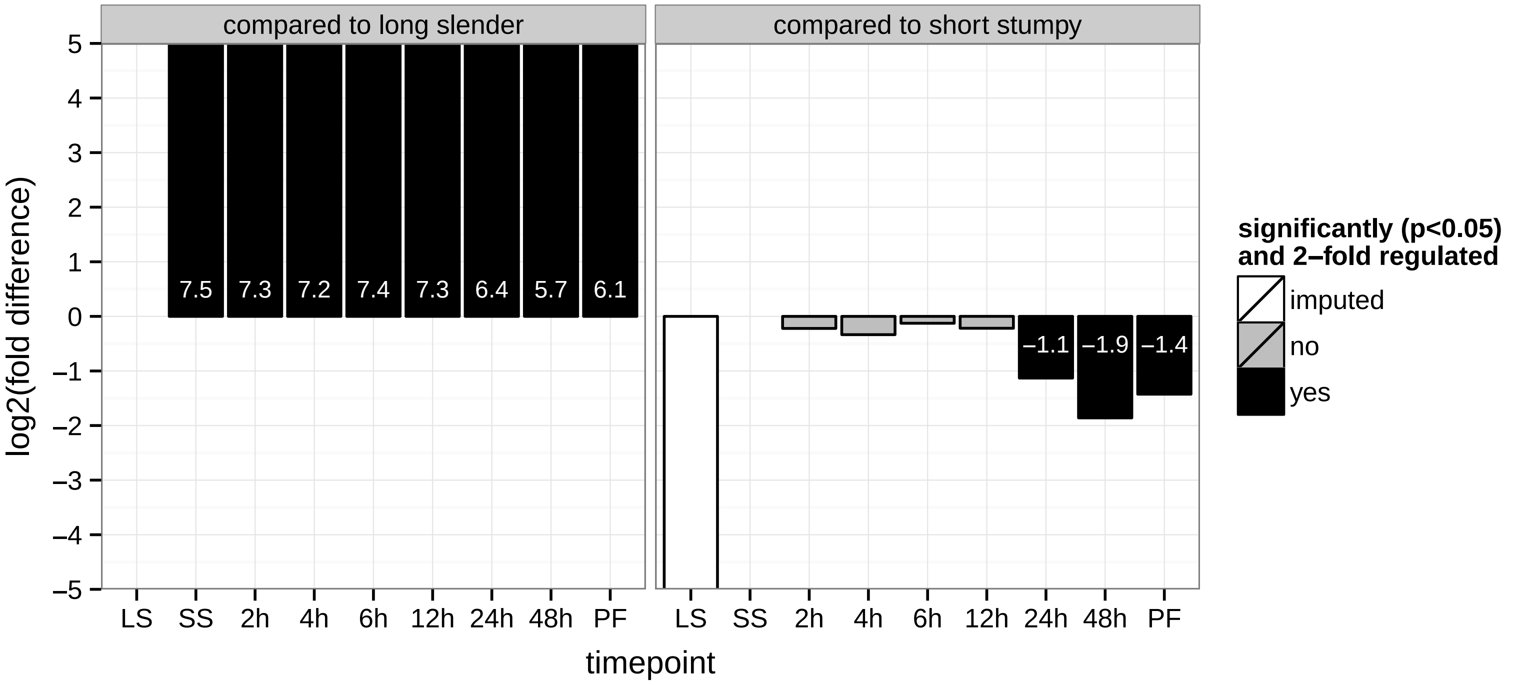

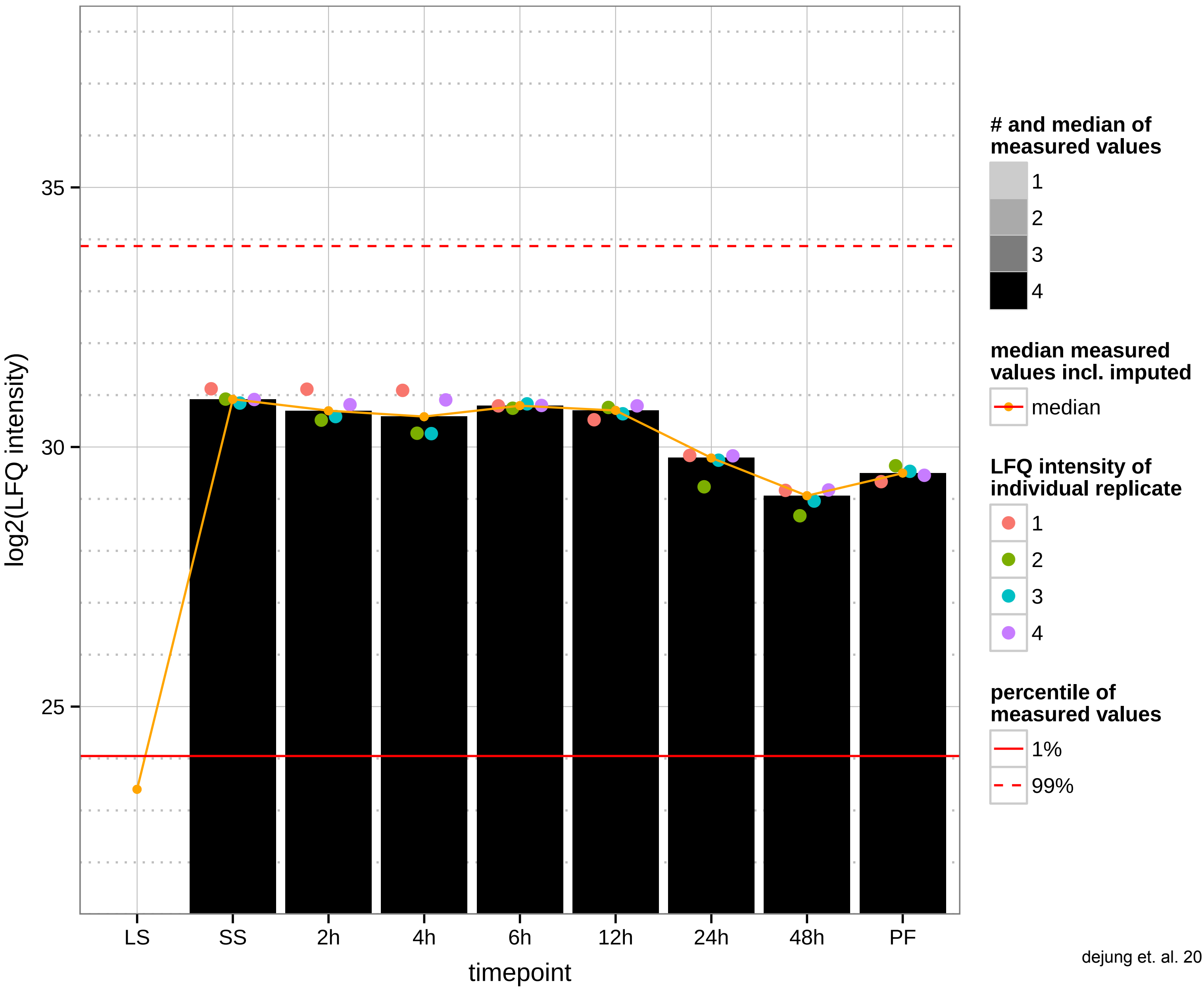

dejung et. al. 2015

hypothetical protein, conserved
Tb927.4.2370
AGOF: null
AGOC: null
AGOP: null
PGOF: null
PGOC: null
PGOP: null

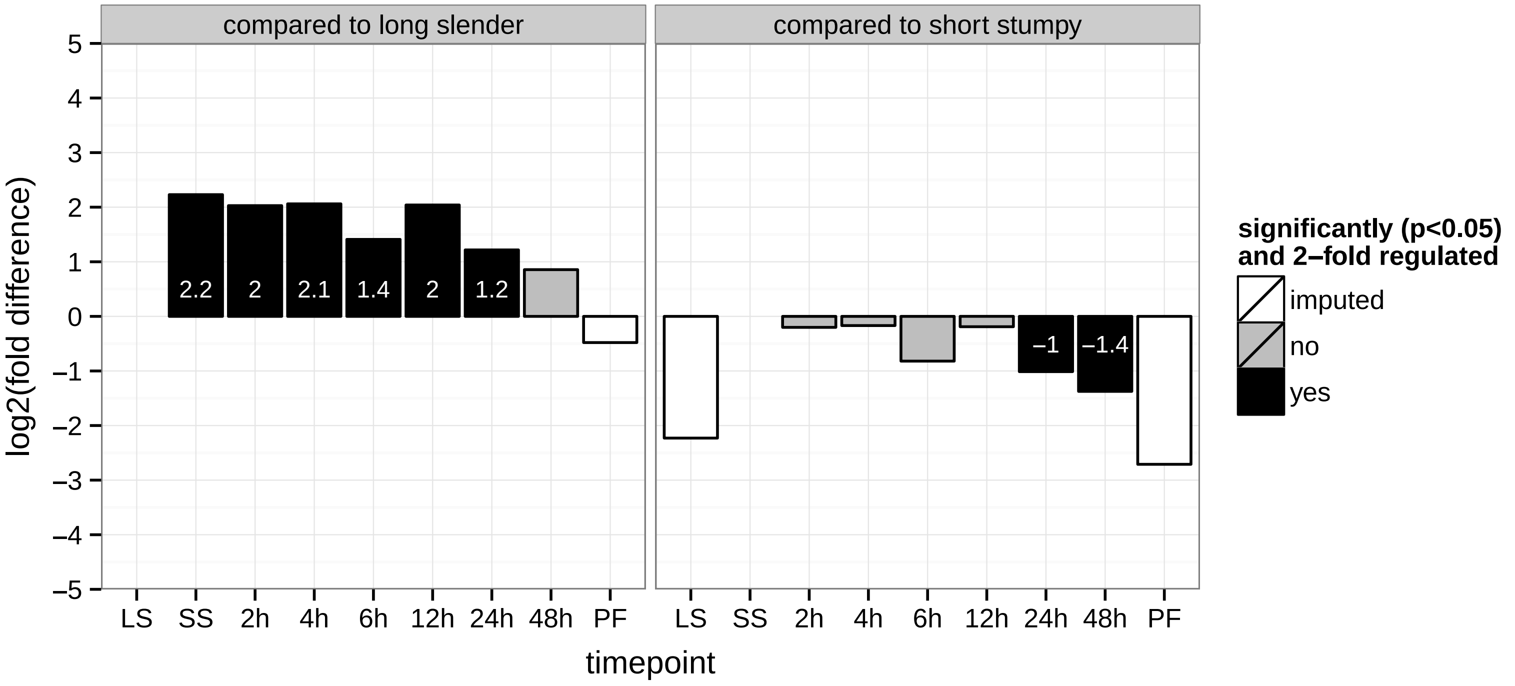

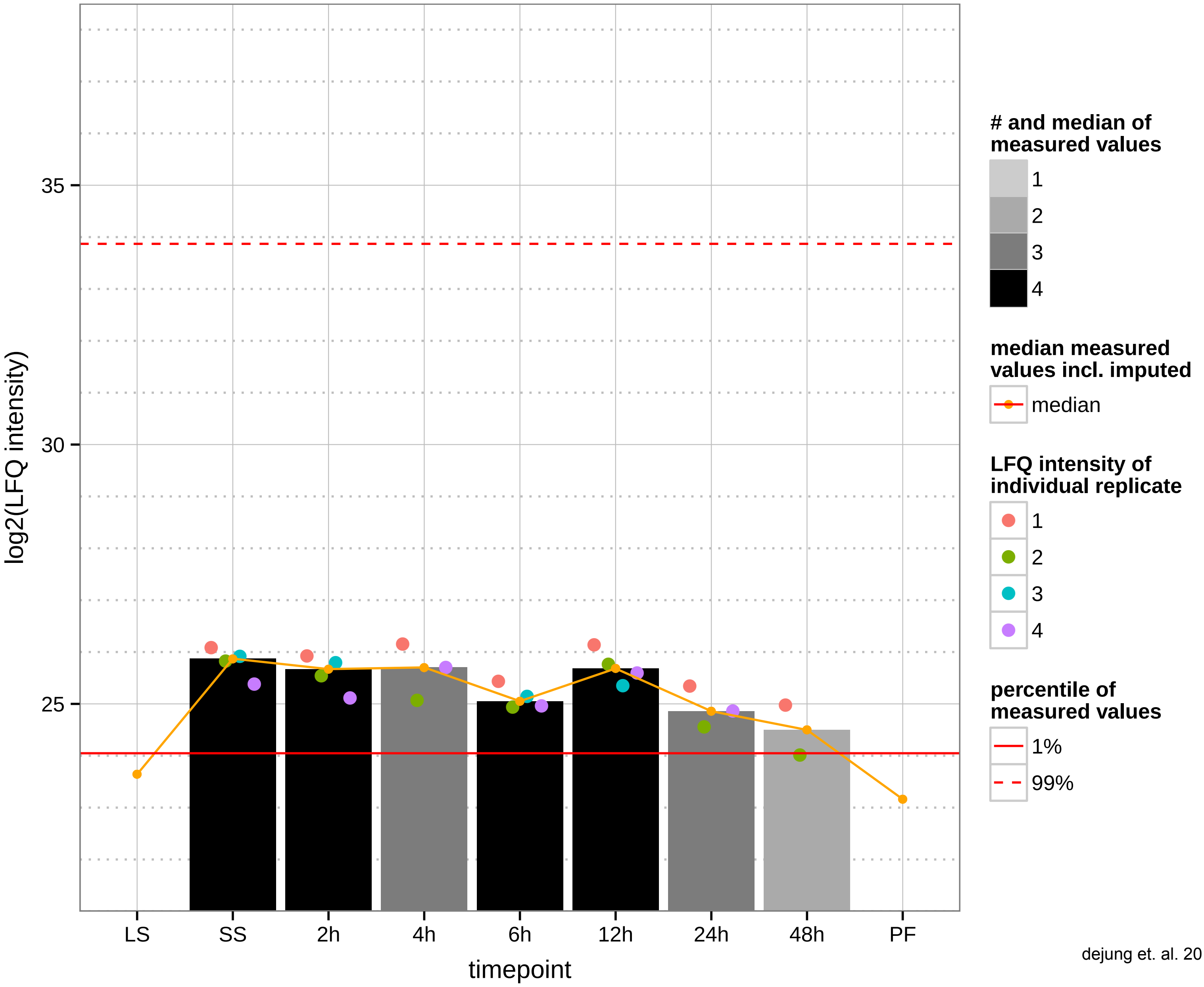

dejung et. al. 2015

sarcoplasmic reticulum glycoprotein, putative
Tb927.4.2380
AGOF: GTP binding, calcium ion binding
AGOC: intracellular, sarcoplasmic reticulum
AGOP: calcium ion transport
PGOF: GTP binding, GTPase activity
PGOC: null
PGOP: null

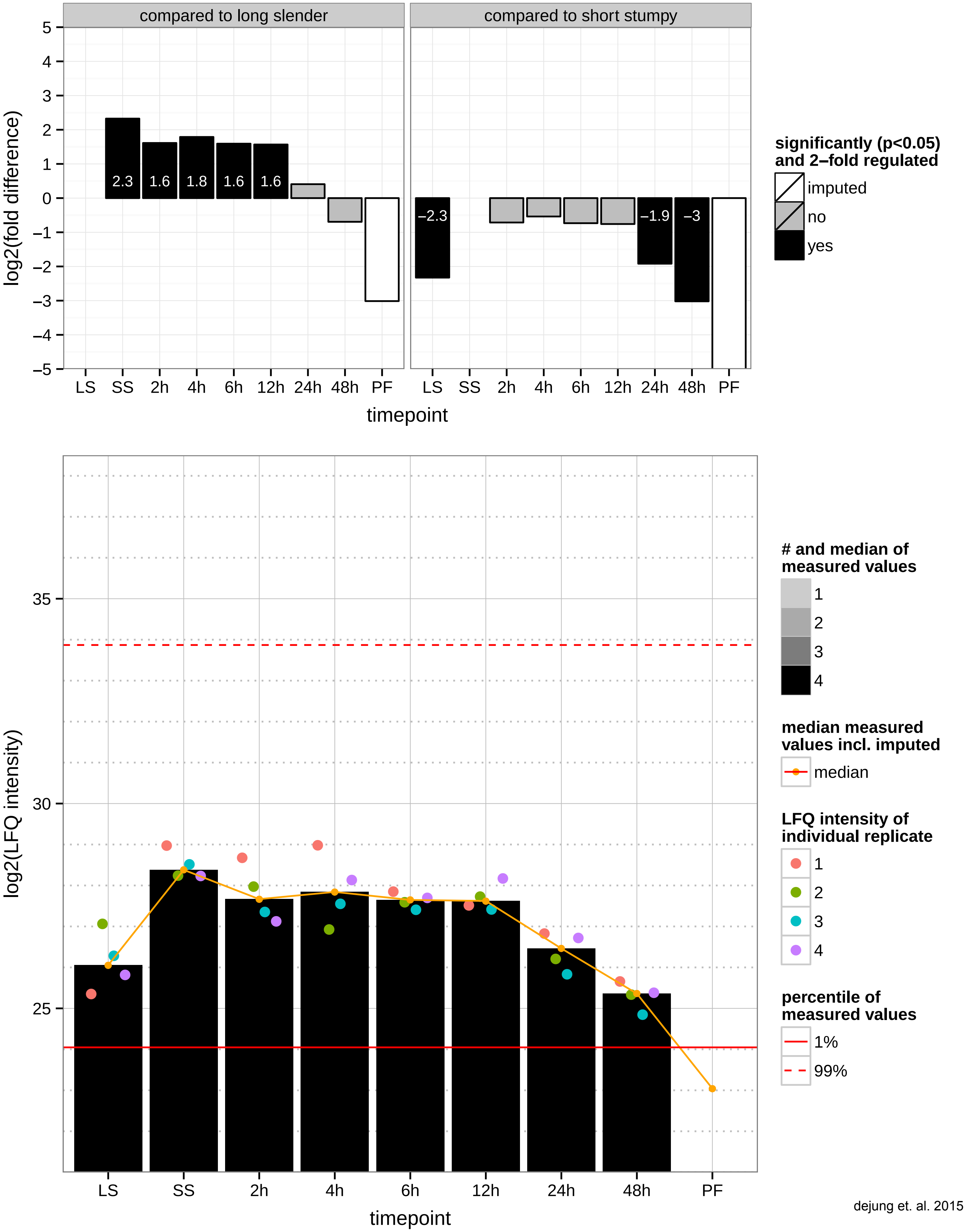

dejung et. al. 2015

myosin IB heavy chain, putative
Tb927.4.3380
AGOF: ATP binding, motor activity, zinc ion binding
AGOC: myosin complex
AGOP: null
PGOF: ATP binding, motor activity, protein binding
PGOC: myosin complex
PGOP: null

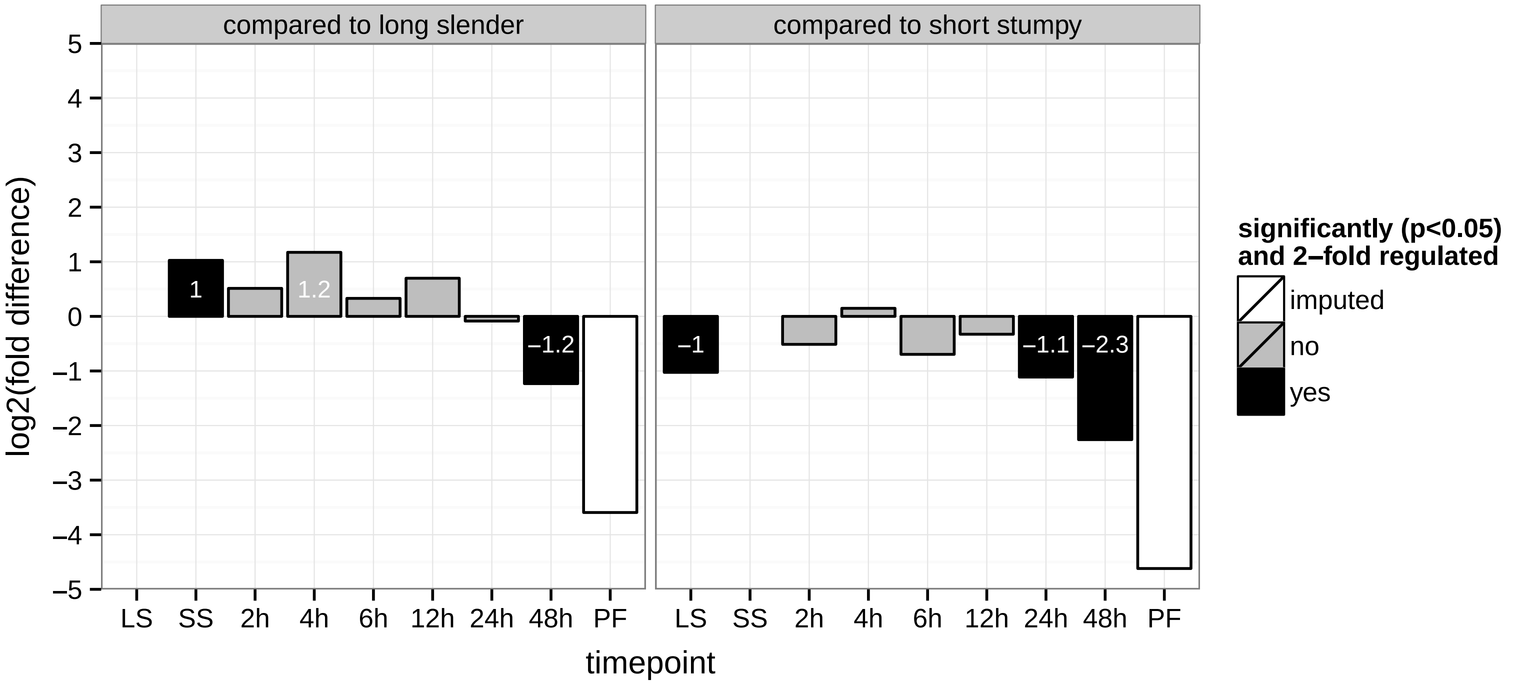

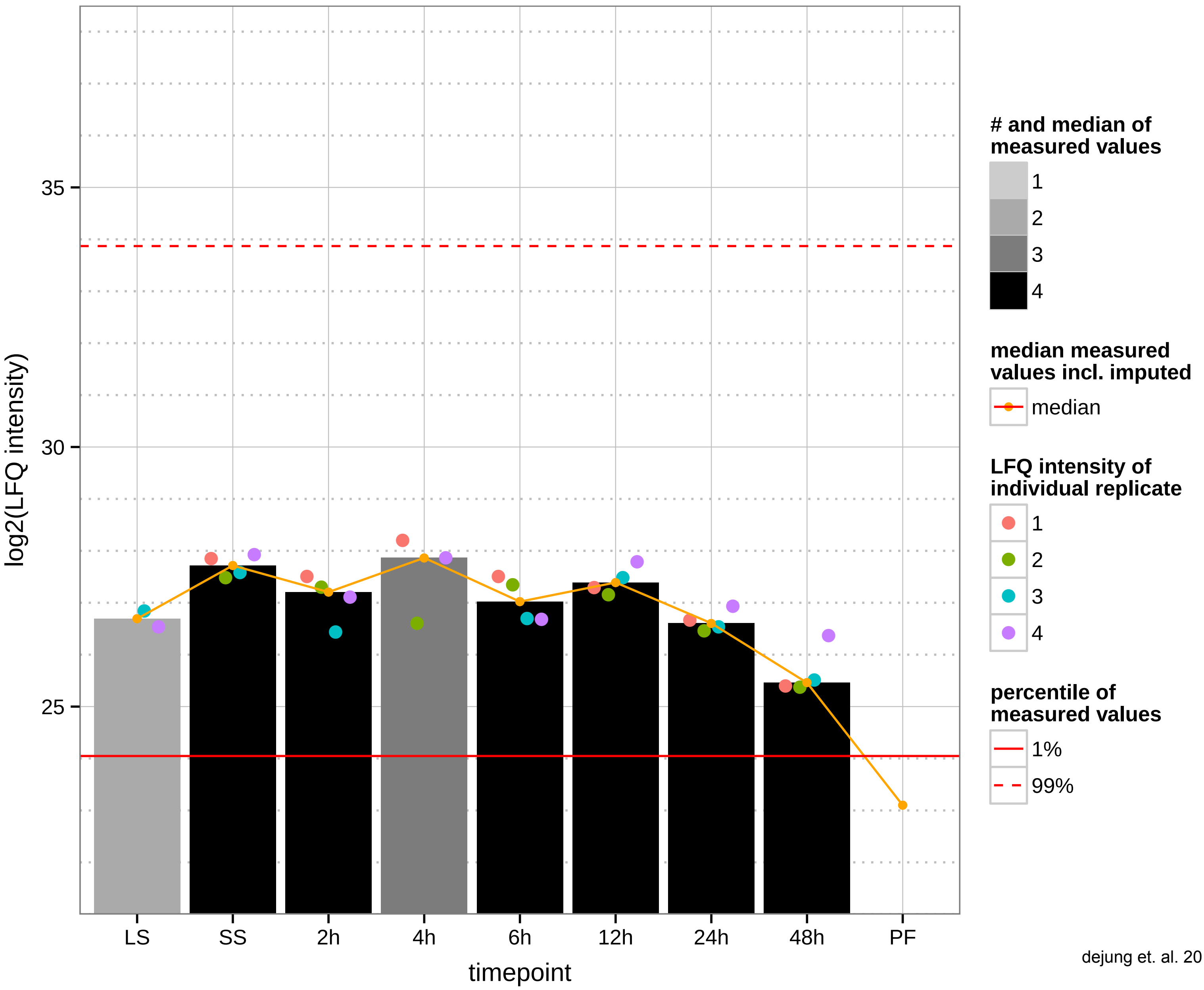

dejung et. al. 2015

hypothetical protein
Tb927.4.3480
AGOF: null
AGOC: null
AGOP: null
PGOF: null
PGOC: null
PGOP: null

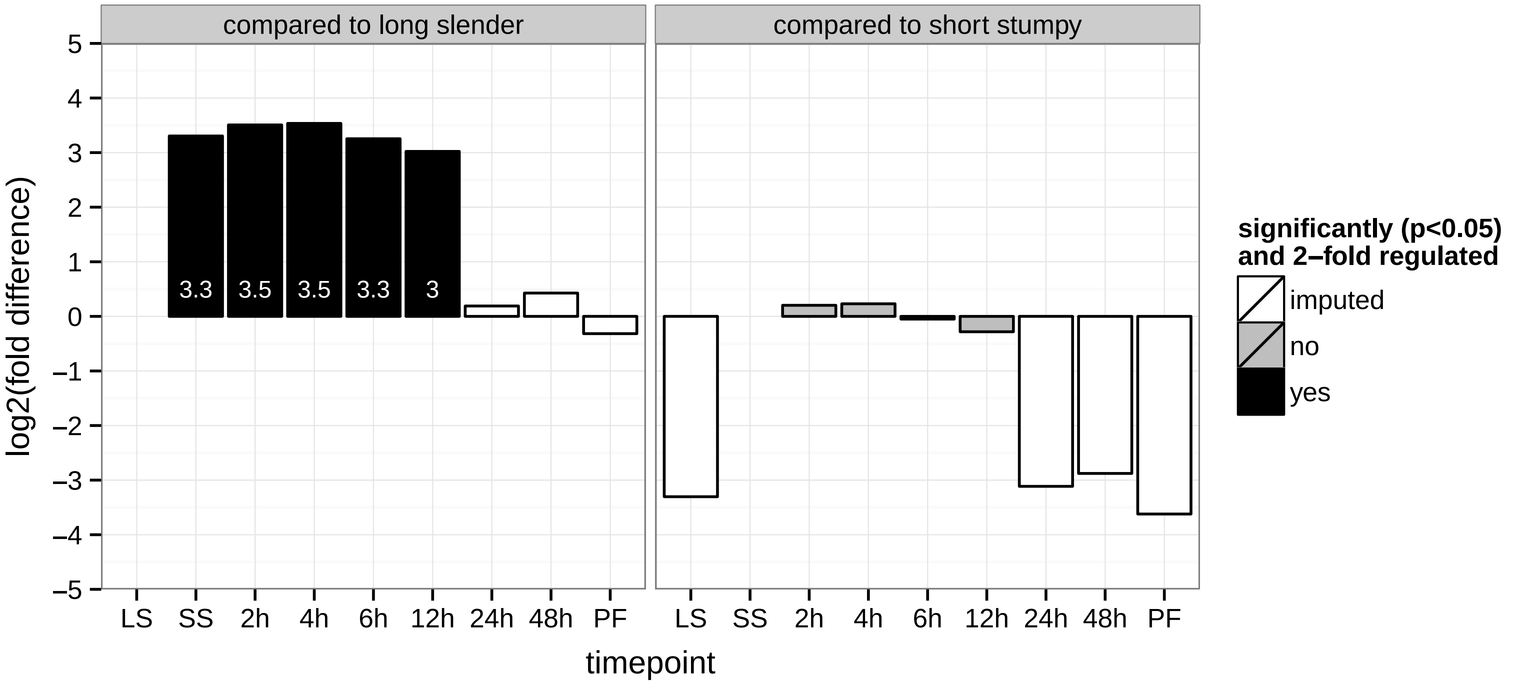

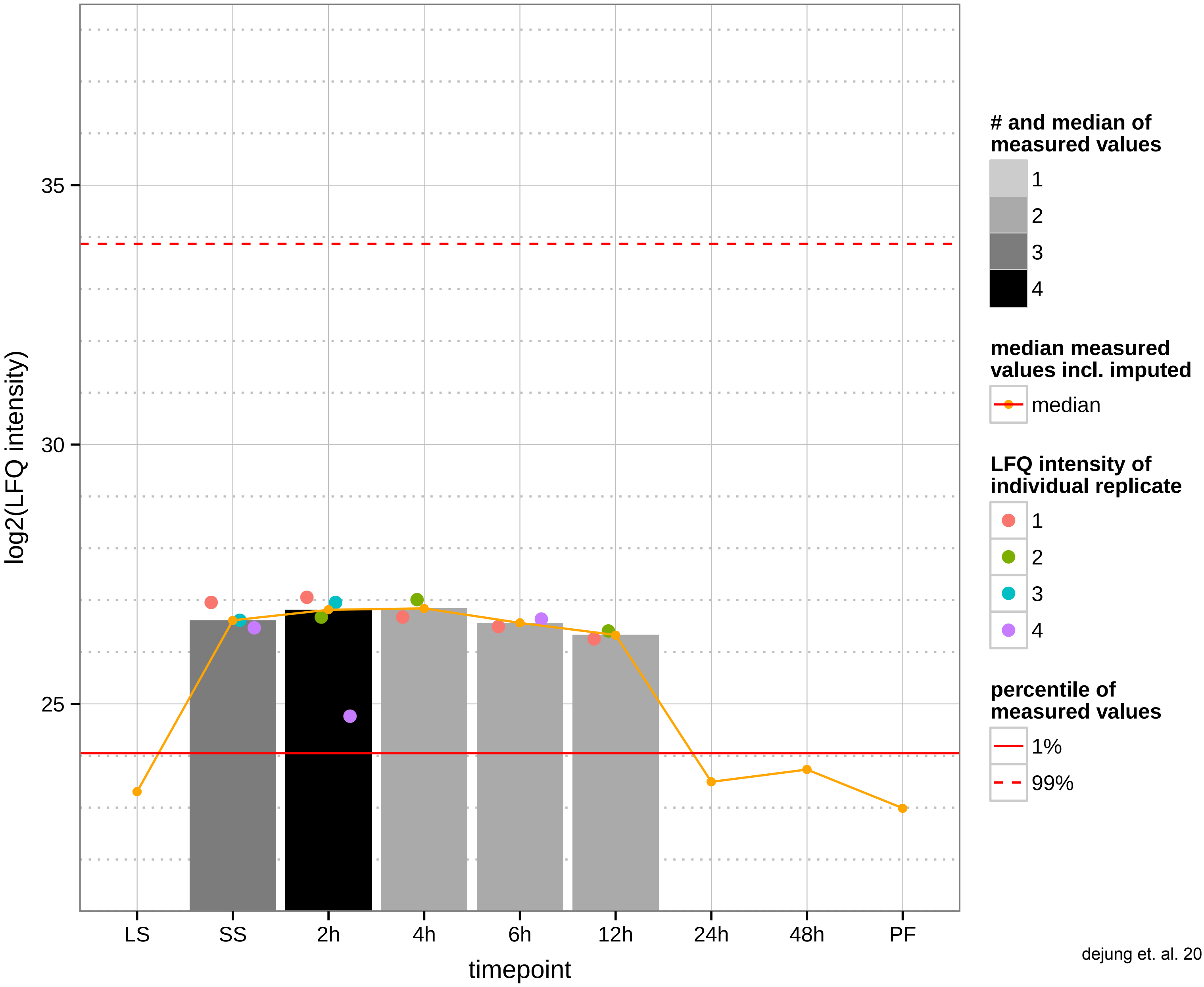

dejung et. al. 2015

Repressor of differentiation kinase 2 (RDK2), serine/threonine−protein kinase a, putative, protein kinase
Tb927.4.5310
AGOF: ATP binding, protein kinase activity, protein serine/threonine kinase activity, protein tyrosine kinase activity
AGOC: null
AGOP: modulation of development of symbiont involved in interaction with host, protein phosphorylation, protein phosphorylat
PGOF: ATP binding, phospholipid binding, protein binding, protein kinase activity, transferase activity, transferring phosphorus
PGOC: null
PGOP: protein phosphorylation

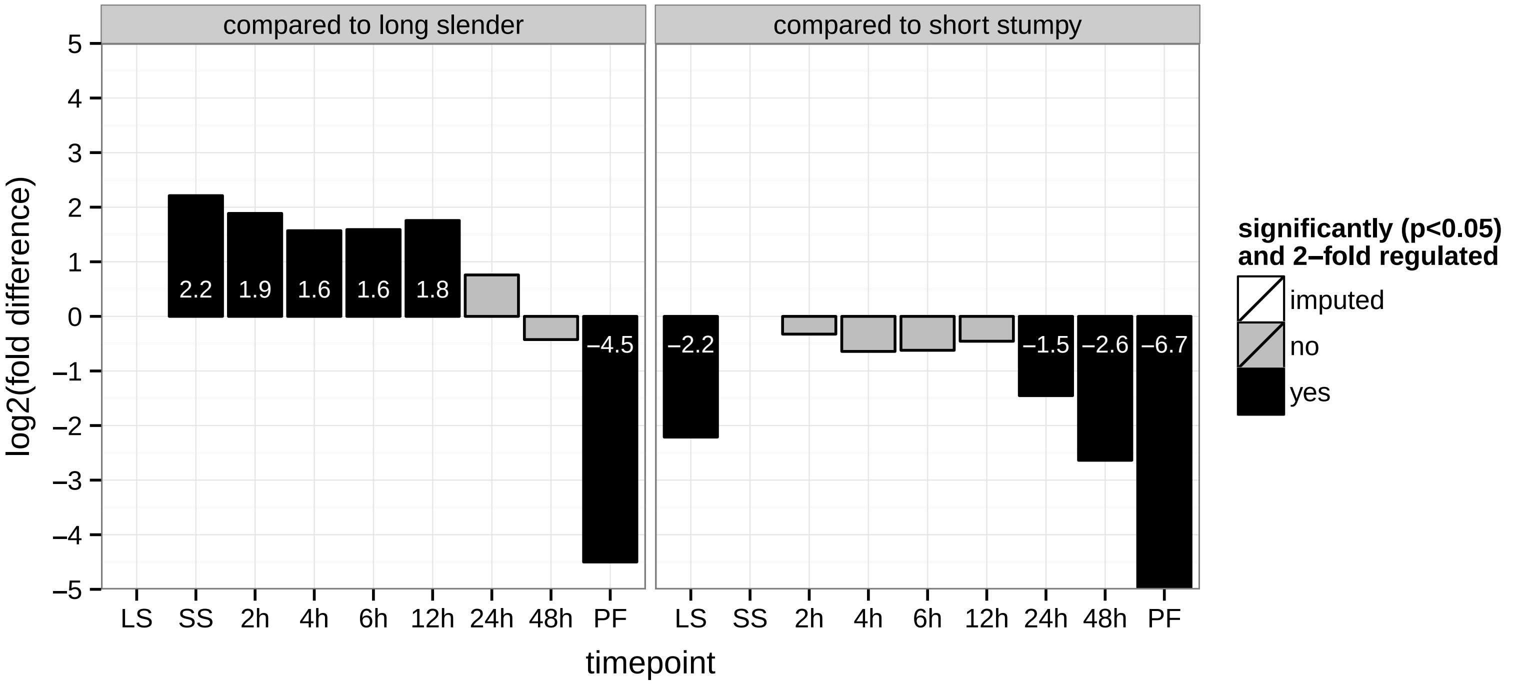

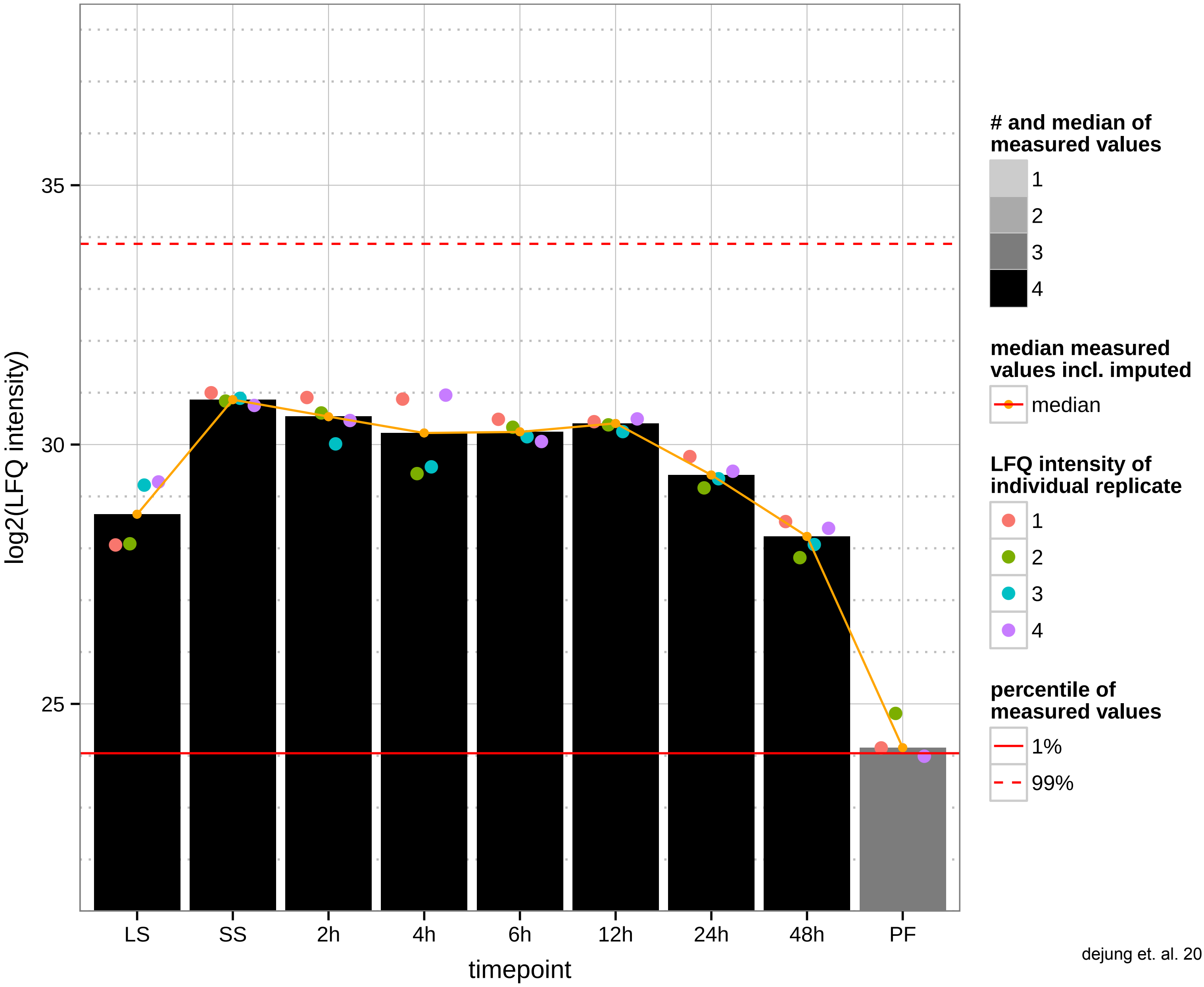

dejung et. al. 2015

Cytoplasmic dynein 2 heavy chain (DYNC2H1), putative
Tb927.4.560
AGOF: ATP binding, ATPase activity, microtubule motor activity
AGOC: dynein complex
AGOP: chromosome segregation, microtubule–based movement, minus–end–directed vesicle transport along microtubule, m
PGOF: ATP binding, ATPase activity, microtubule motor activity, nucleoside–triphosphatase activity, nucleotide binding
PGOC: dynein complex
PGOP: microtubule–based movement

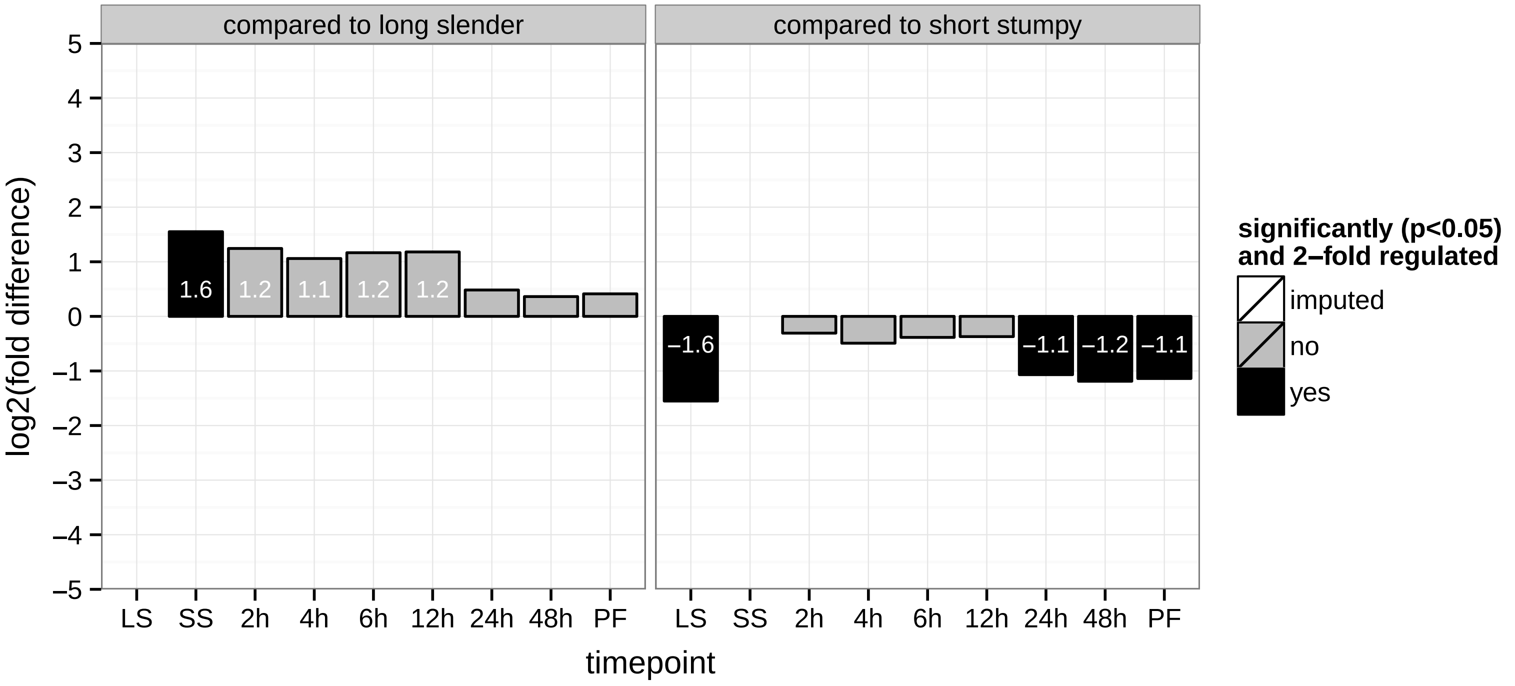

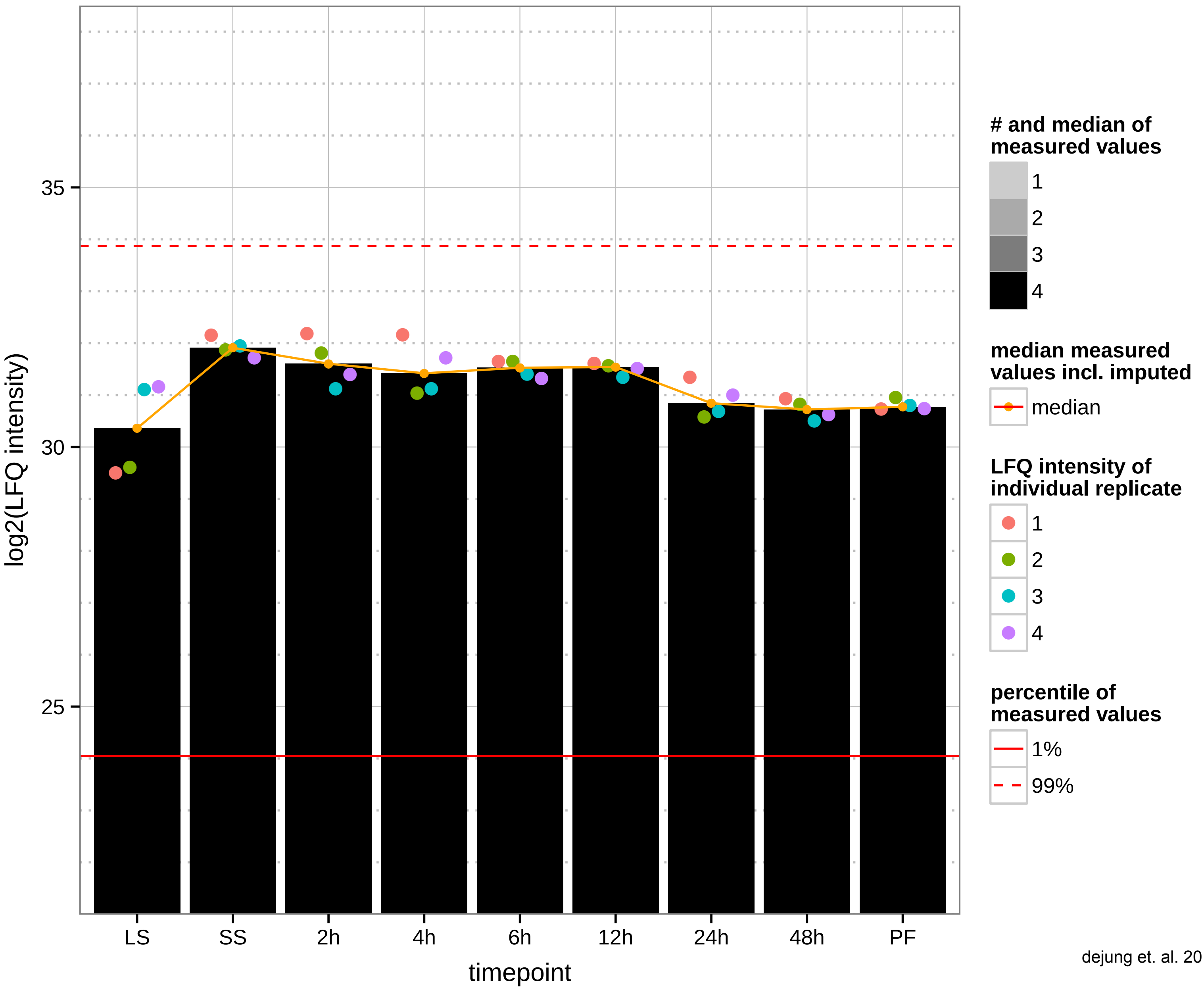

dejung et. al. 2015

gamma–adaptin 1, putative, AP–1 adapter complex gamma subunit
Tb927.4.760
AGOF: protein transporter activity
AGOC: Golgi apparatus, Golgi apparatus part, clathrin adaptor complex, membrane coat
AGOP: intracellular protein transport, vesicle–mediated transport
PGOF: binding, protein transporter activity
PGOC: Golgi apparatus, Golgi apparatus part, clathrin adaptor complex, membrane coat
PGOP: intracellular protein transport, protein transport, vesicle–mediated transport

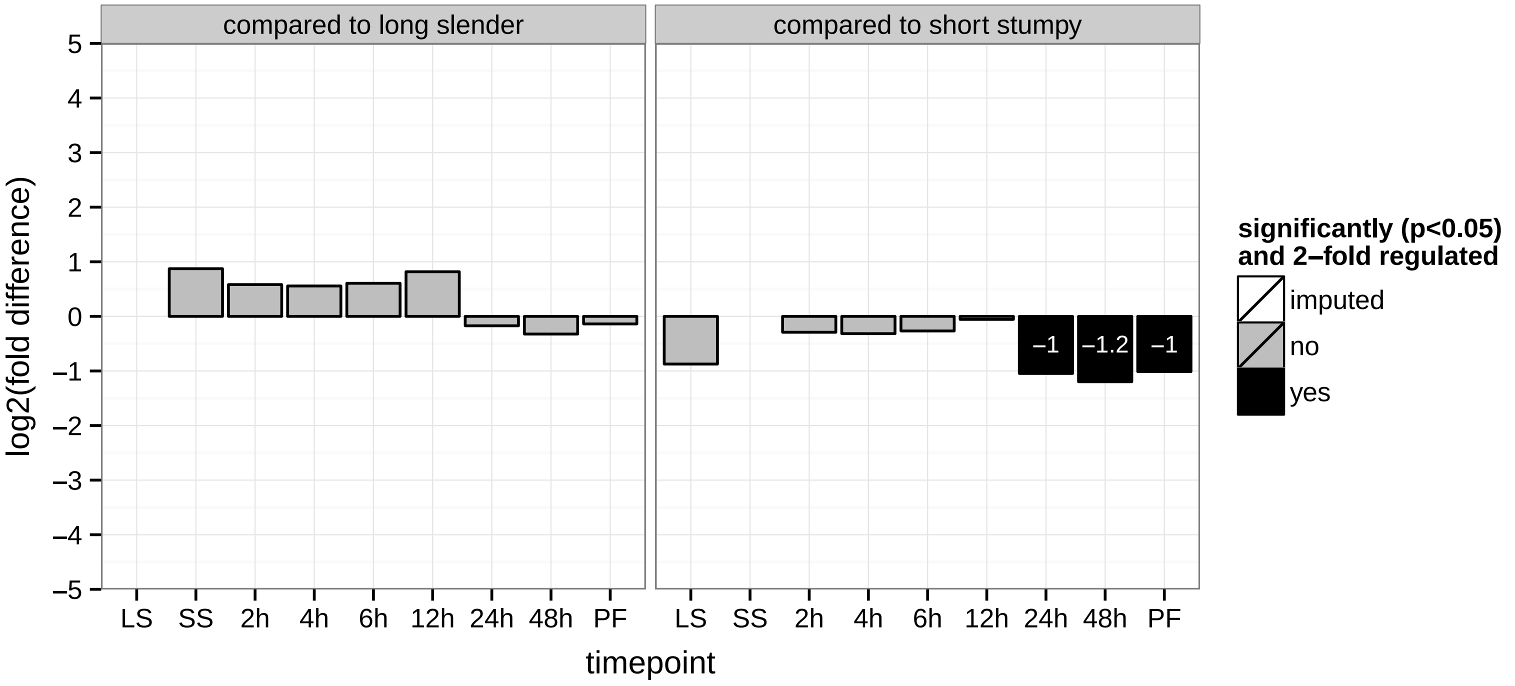

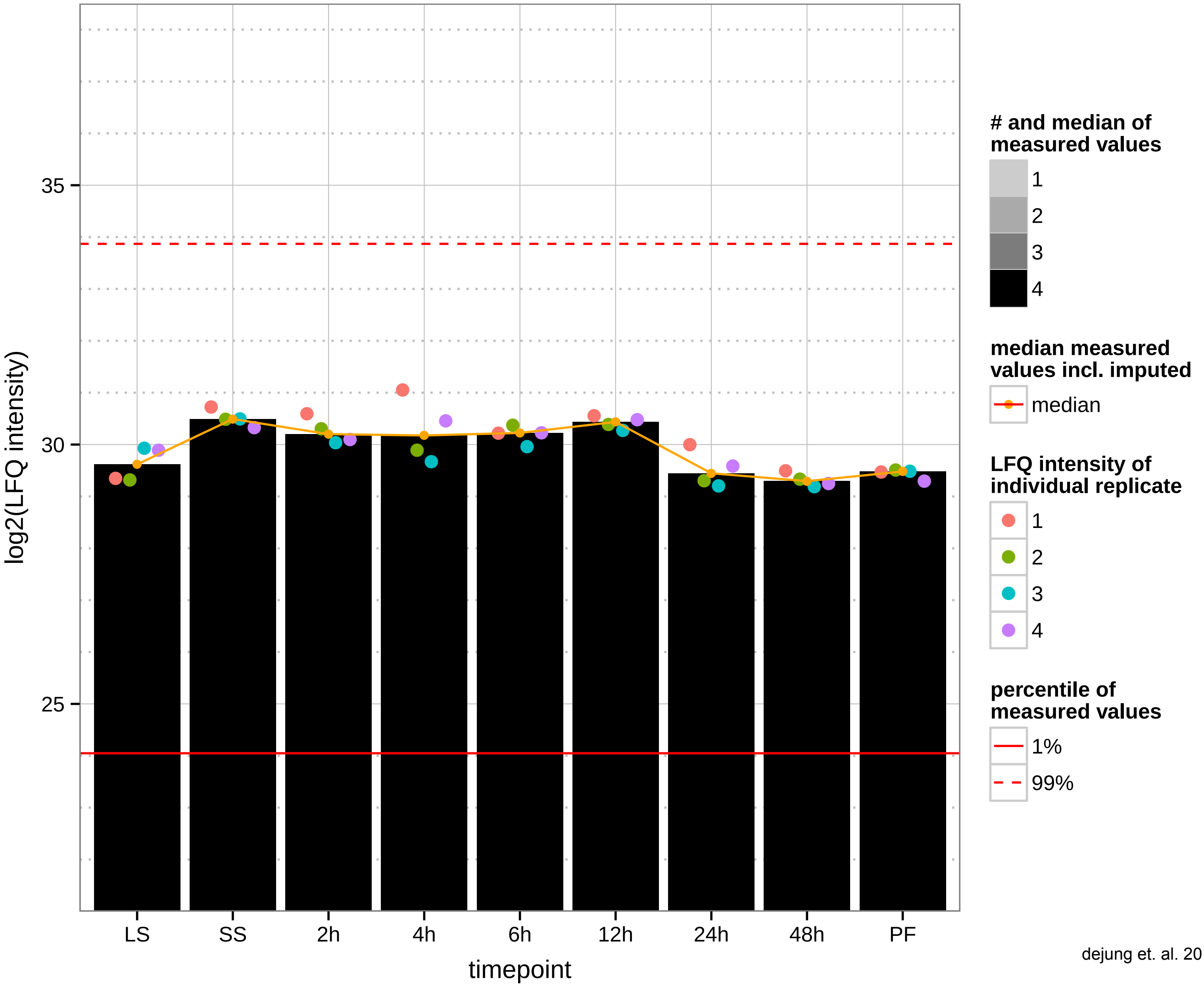

dejung et. al. 2015

expression site–associated gene (ESAG) protein, putative, expression site–associated gene 5 (ESAG5) protein
Tb927.4.810
AGOF: lipid binding
AGOC: null
AGOP: null
PGOF: lipid binding
PGOC: null
PGOP: null

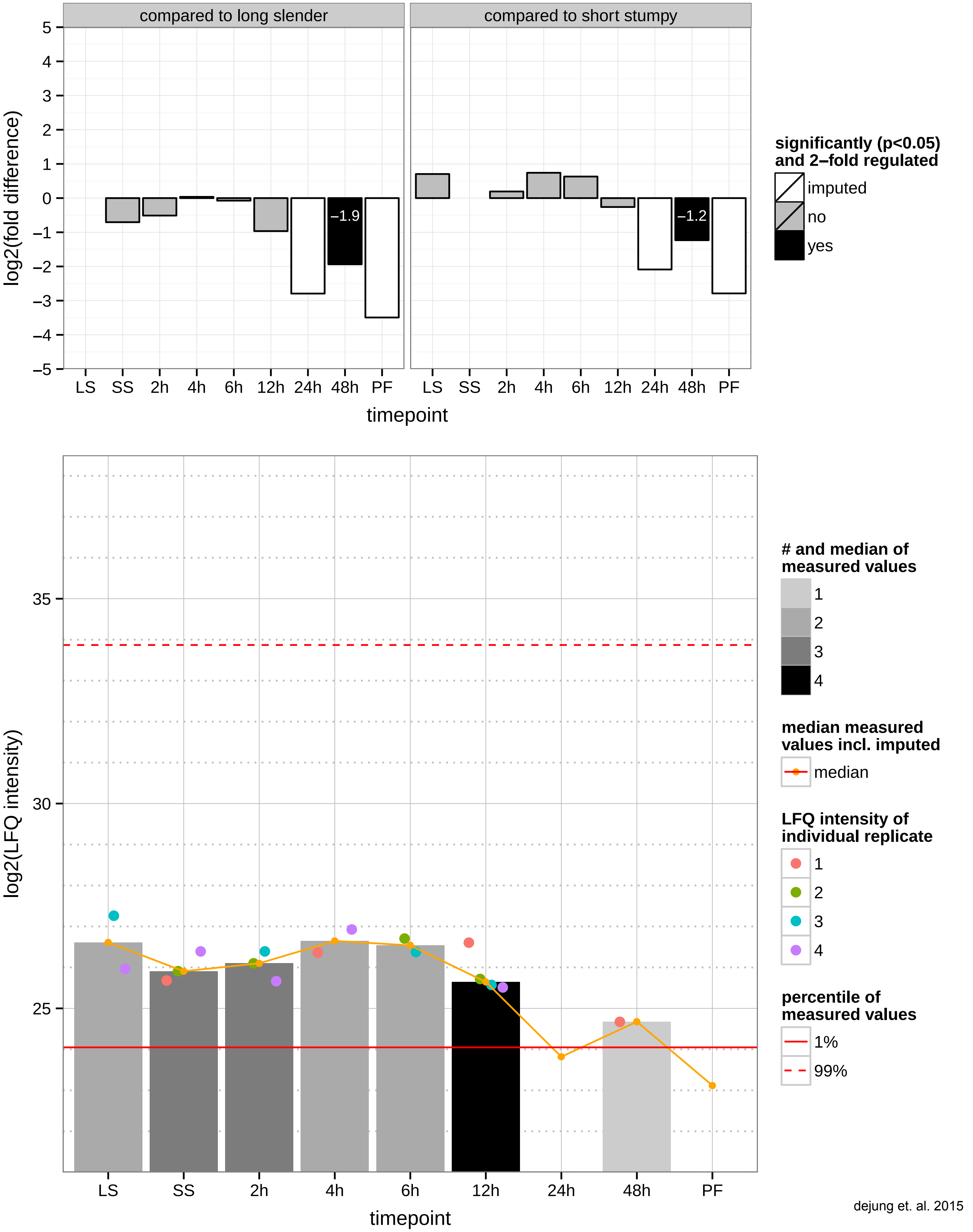

dejung et. al. 2015

64 kDa invariant surface glycoprotein (ISG64)
Tb927.5.1410
AGOF: null
AGOC: null
AGOP: null
PGOF: null
PGOC: null
PGOP: null

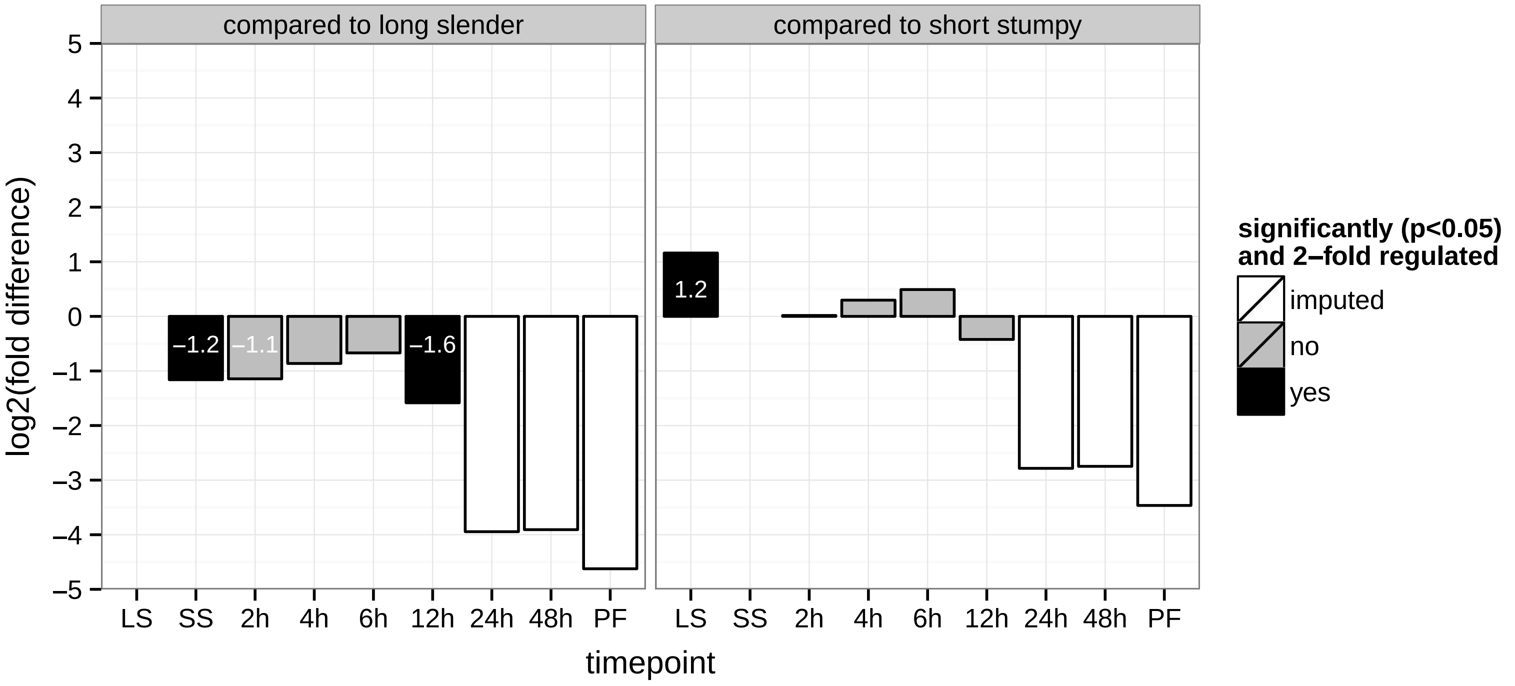

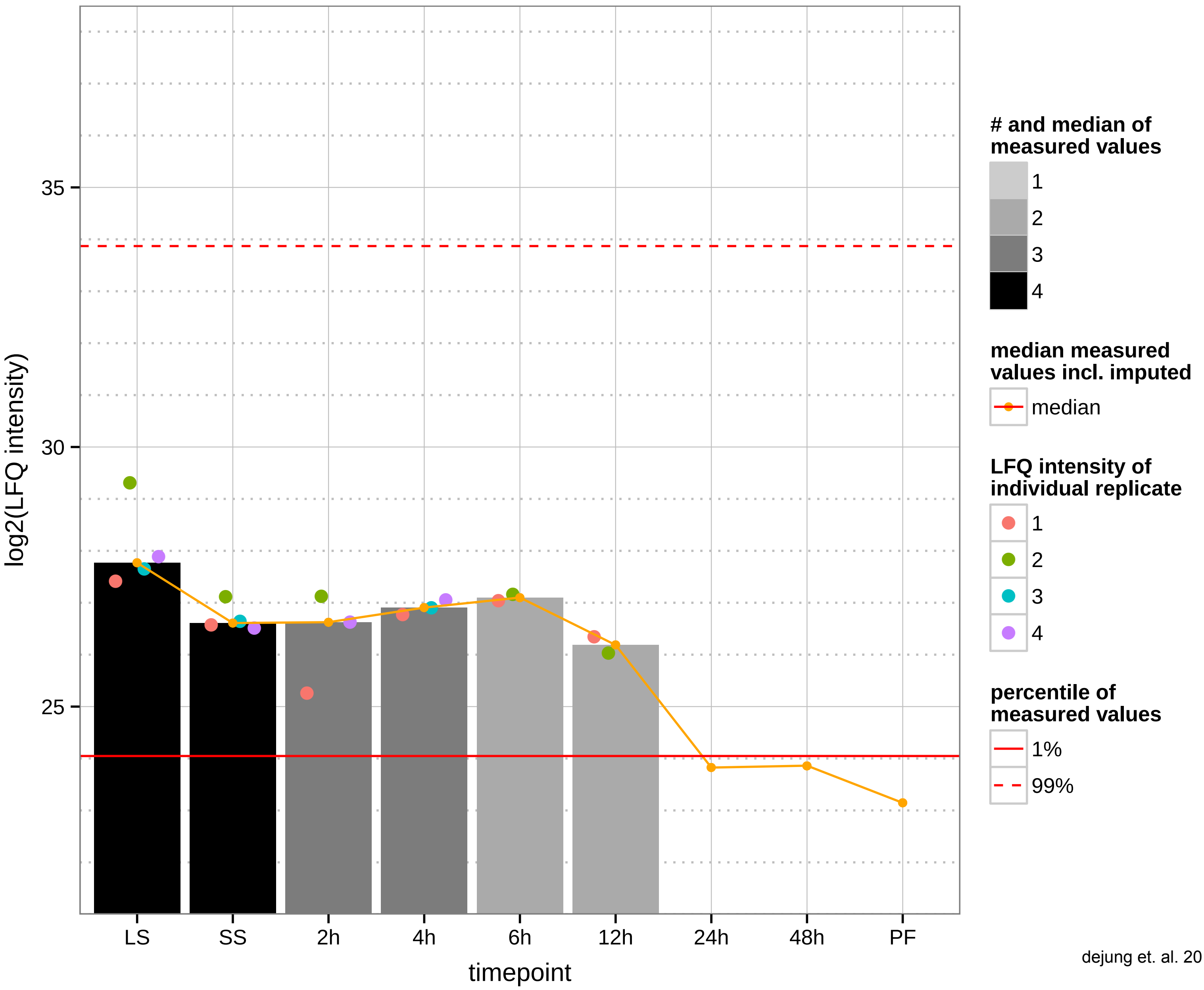

dejung et. al. 2015

kinesin, putative
Tb927.5.2090
AGOF: ATP binding, microtubule motor activity, motor activity
AGOC: microtubule associated complex, kinesin complex, microtubule associated complex
AGOP: microtubule–based movement
PGOF: ATP binding, microtubule motor activity
PGOC: null
PGOP: microtubule–based movement

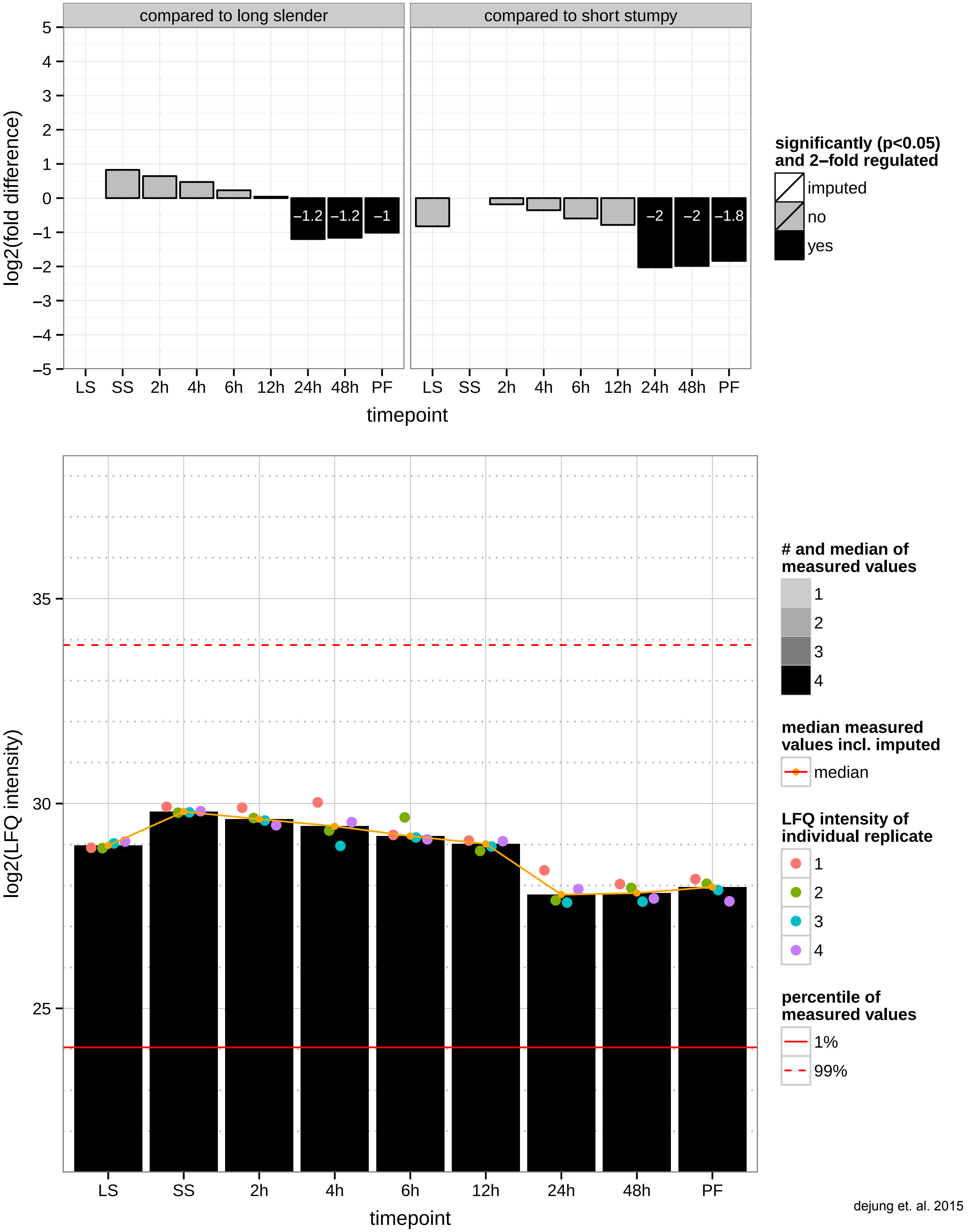

dejung et. al. 2015

hypothetical protein, conserved
Tb927.5.310;Tb11.v5.0208
AGOF: null
AGOC: null
AGOP: null
PGOF: null
PGOC: null
PGOP: null

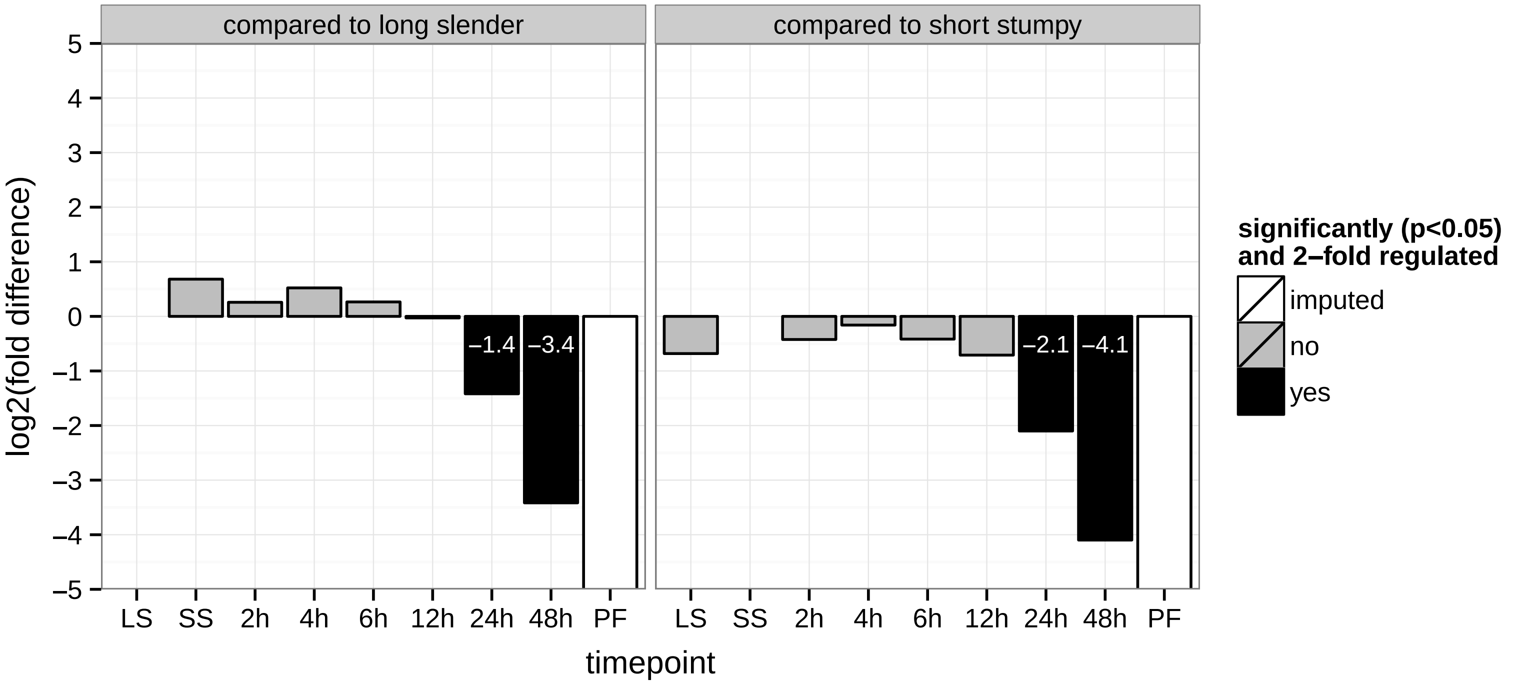

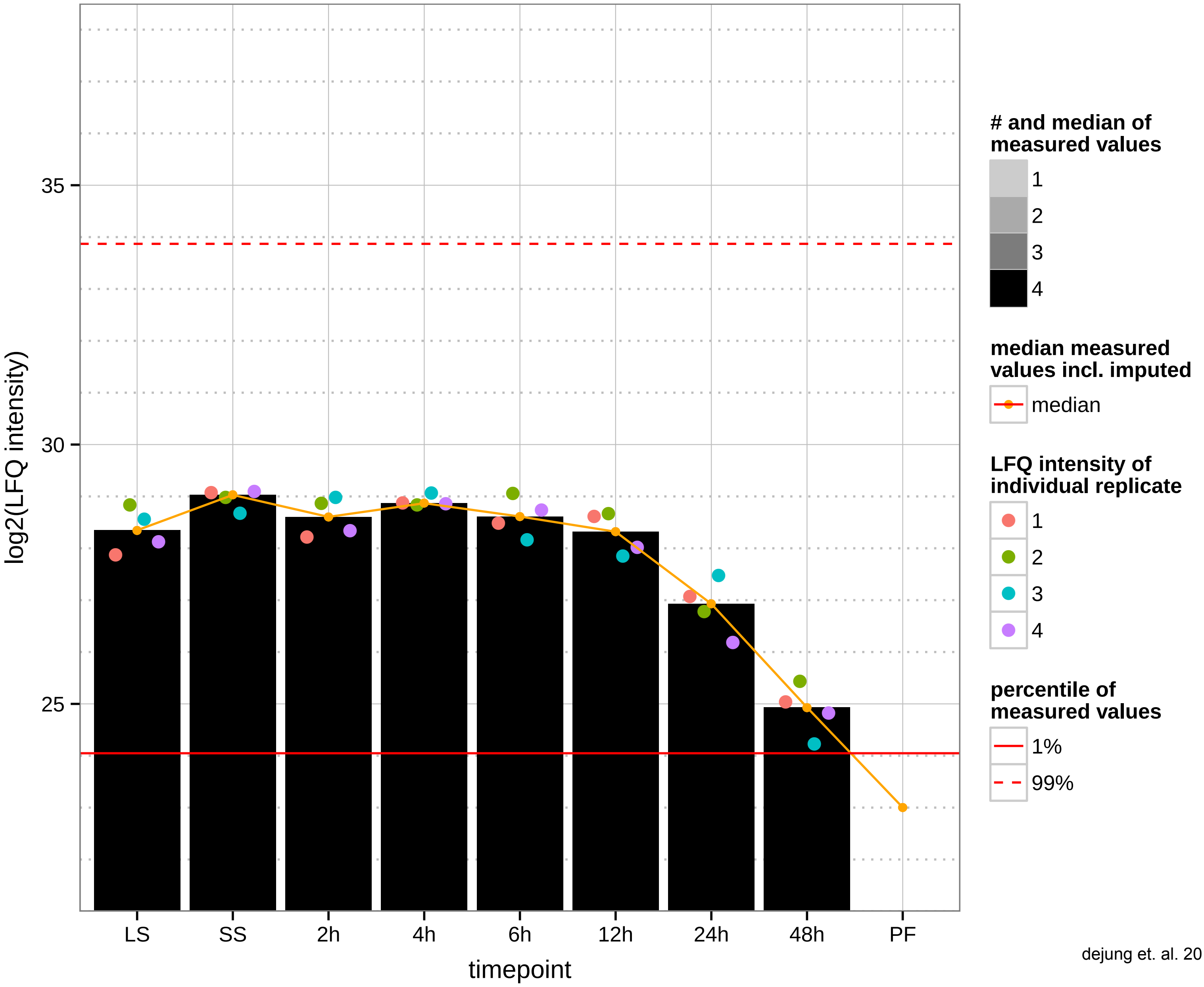

dejung et. al. 2015

hypothetical protein, conserved, acidic phosphatase, putative
Tb927.5.630
AGOF: null
AGOC: null
AGOP: null
PGOF: nucleic acid binding, null
PGOC: null
PGOP: null

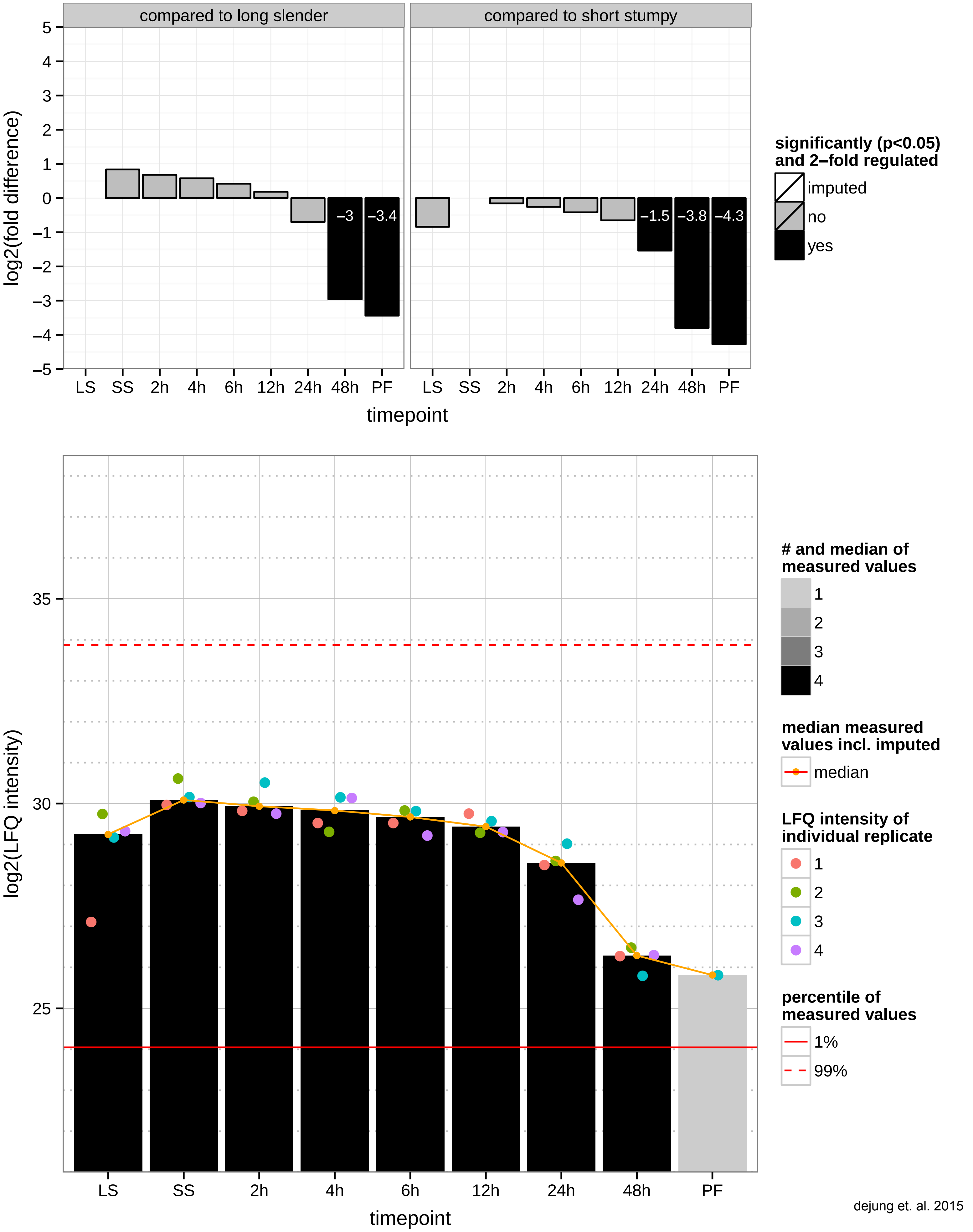

dejung et. al. 2015

hypothetical protein, conserved, oligosaccharyl transferase subunit, putative
Tb927.5.890
AGOF: oligosaccharyl transferase activity
AGOC: membrane
AGOP: protein glycosylation, protein mannosylation
PGOF: oligosaccharyl transferase activity
PGOC: membrane
PGOP: protein glycosylation

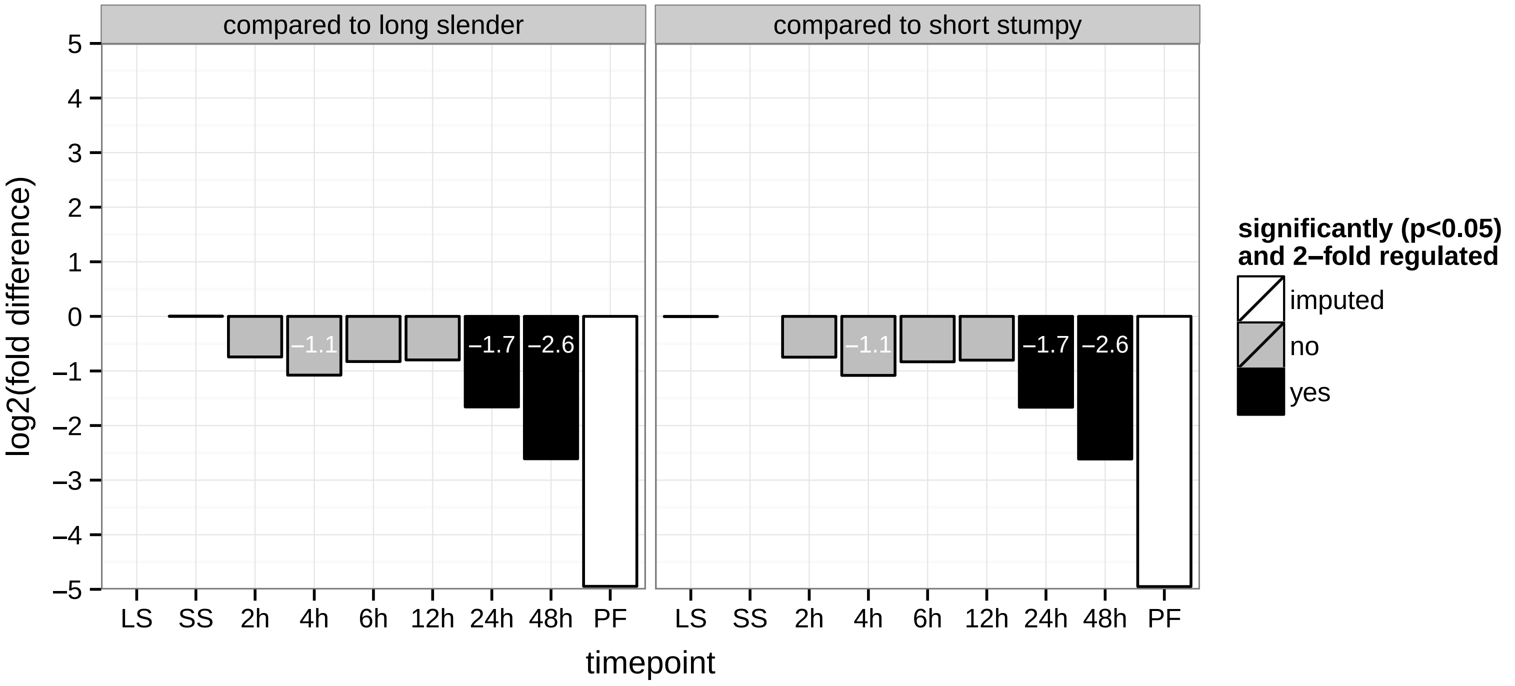

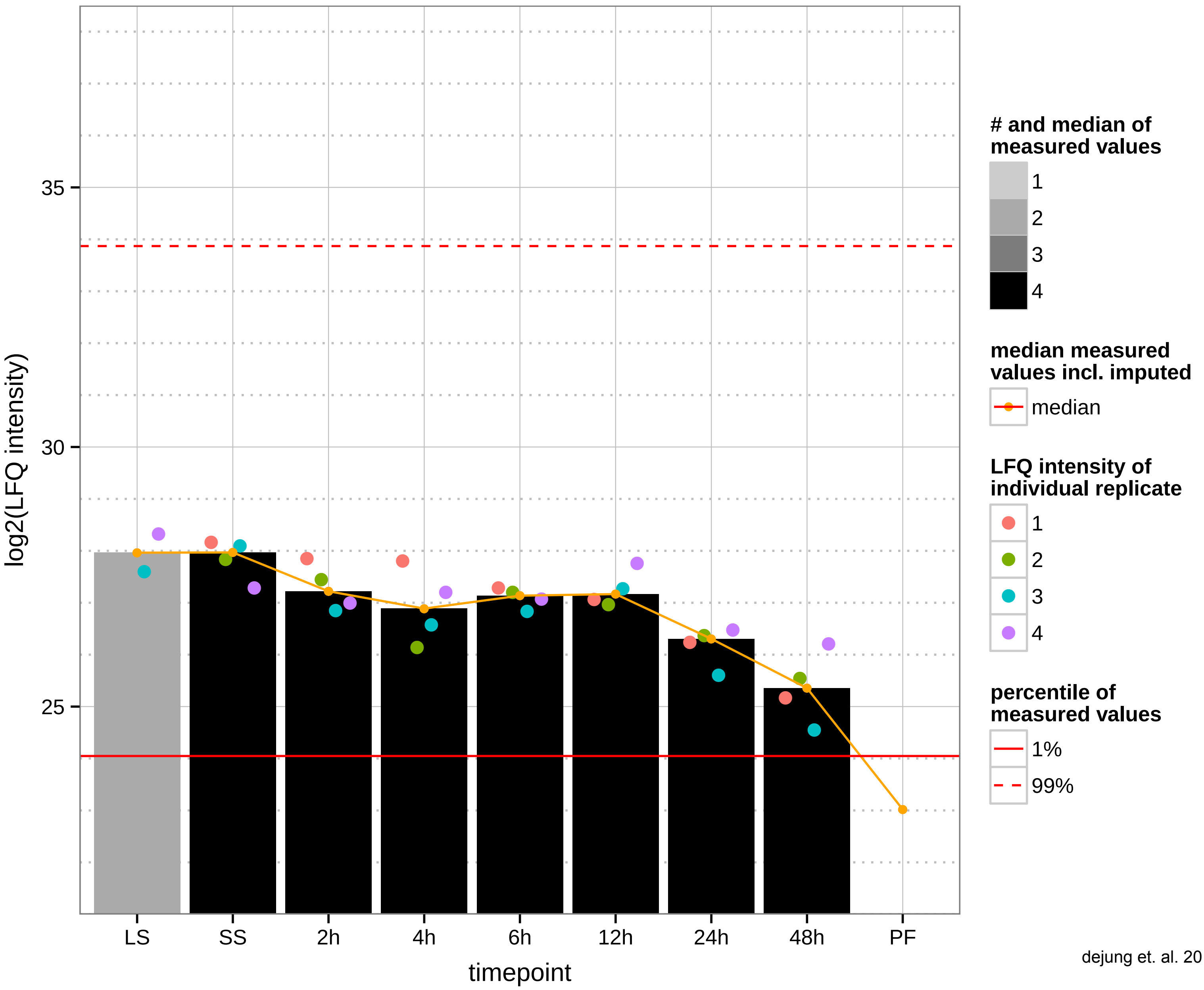

dejung et. al. 2015

hypothetical protein, conserved
Tb927.6.1730
AGOF: null
AGOC: null
AGOP: null
PGOF: null
PGOC: null
PGOP: null

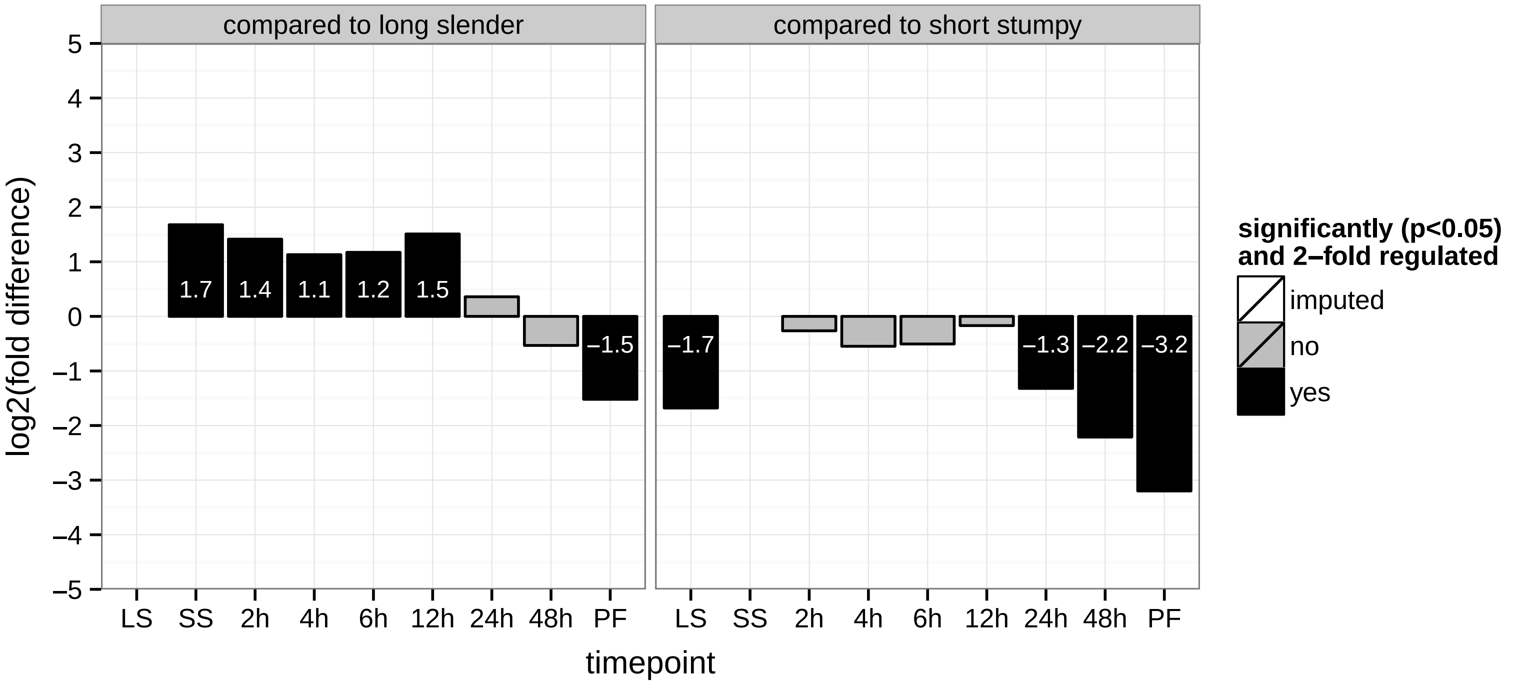

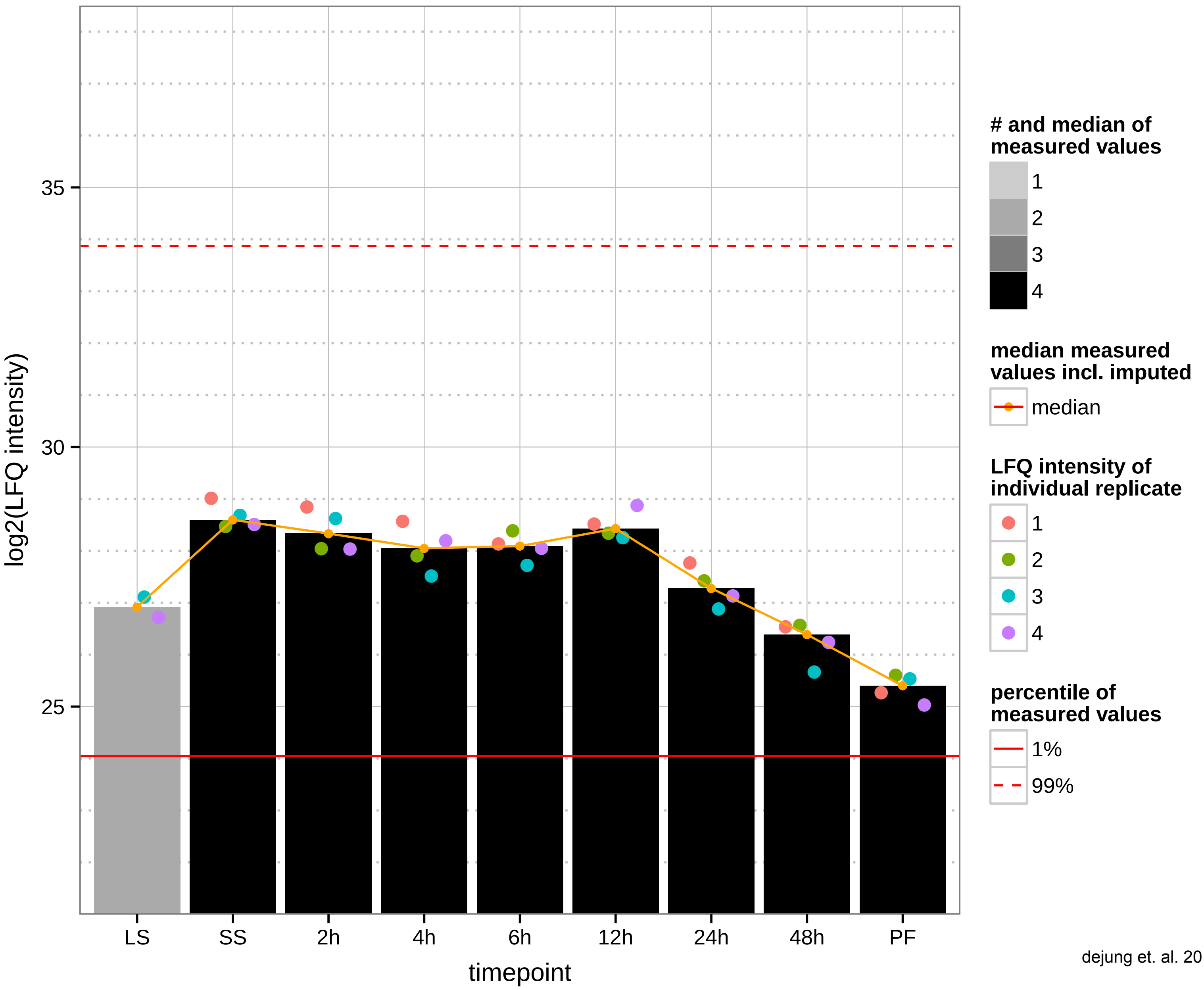

dejung et. al. 2015

rac serine−threonine kinase, putative, protein kinase
Tb927.6.2250
AGOF: ATP binding, protein serine/threonine kinase activity, protein tyrosine kinase activity
AGOC: null
AGOP: protein phosphorylation
PGOF: ATP binding, phospholipid binding, protein binding, protein kinase activity, protein serine/threonine kinase activity, tran
PGOC: null
PGOP: protein phosphorylation

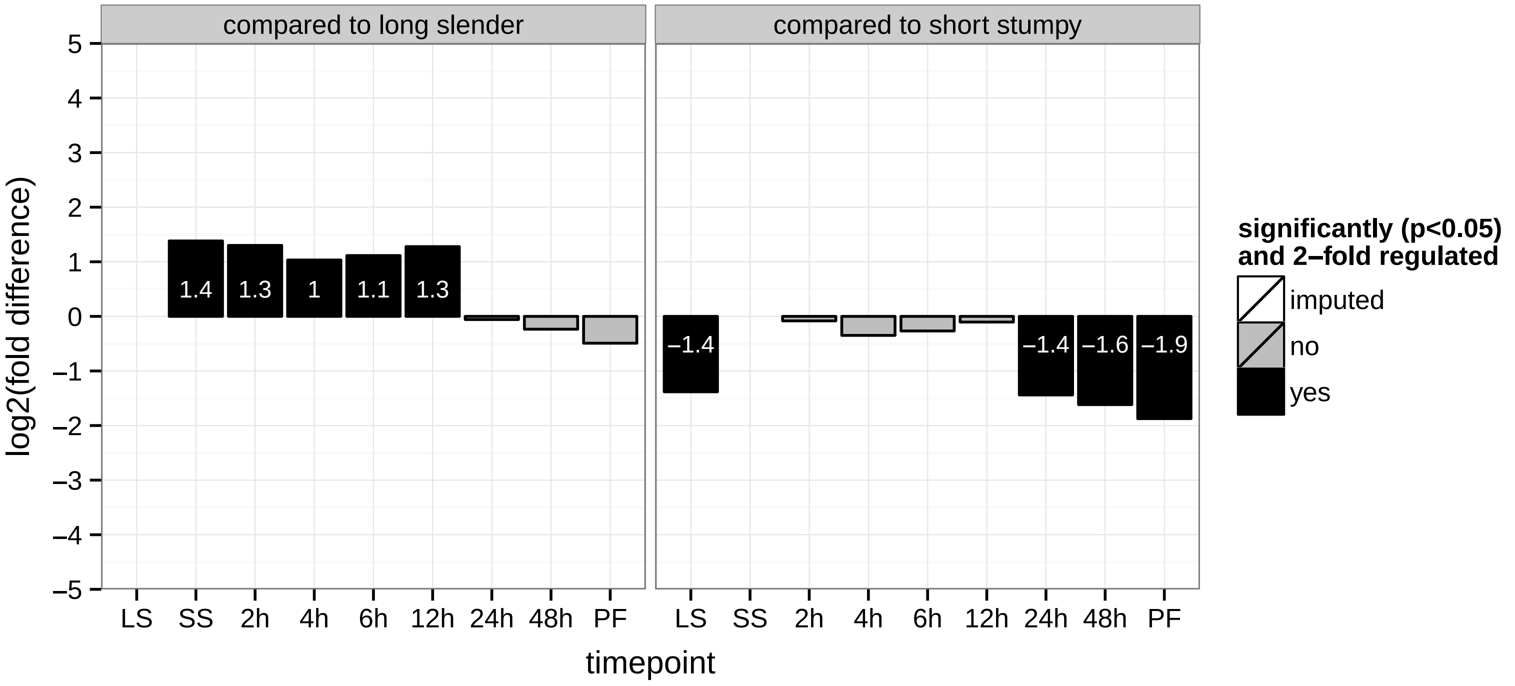

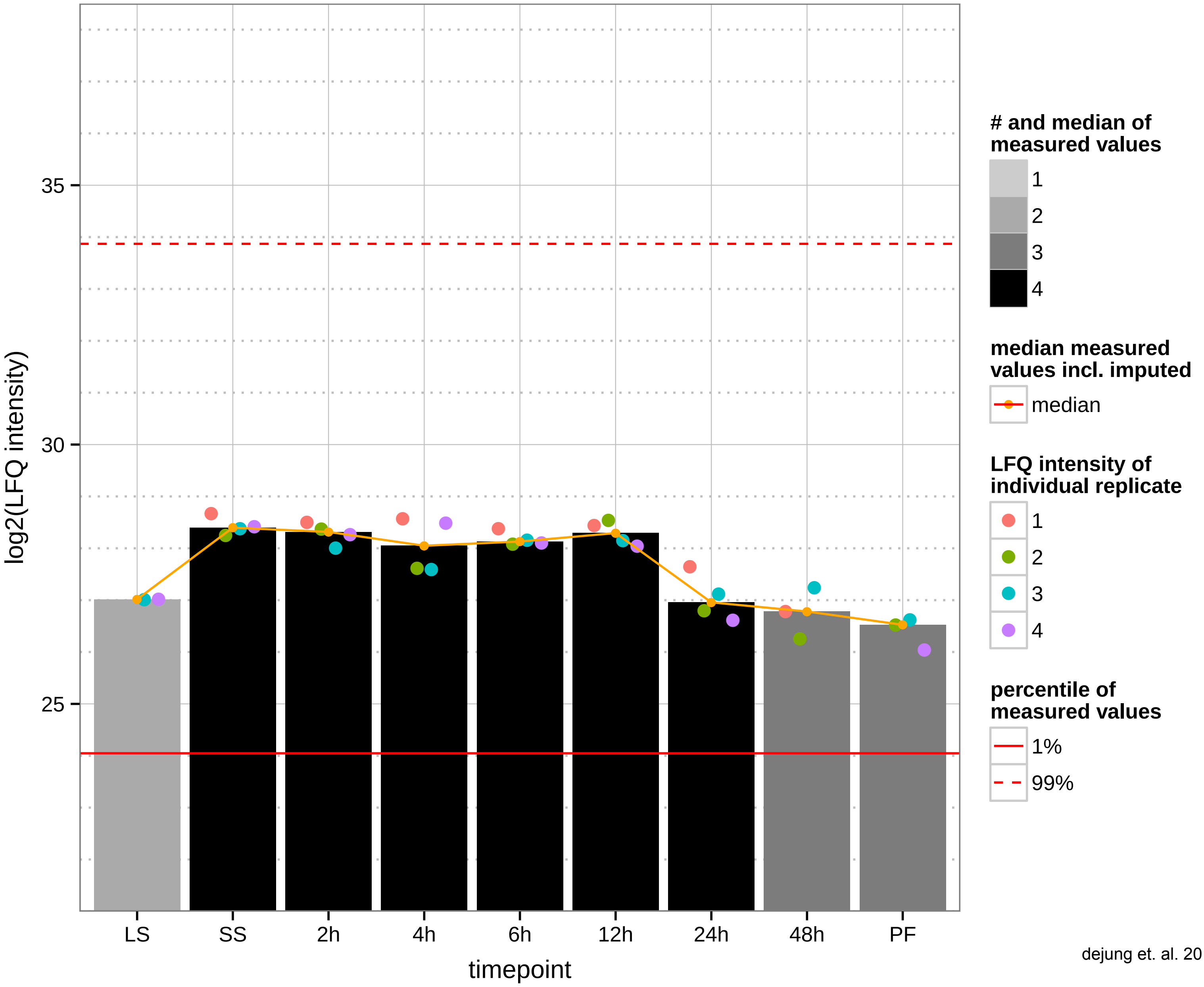

dejung et. al. 2015

hypothetical protein, conserved
Tb927.6.3640
AGOF: zinc ion binding
AGOC: intracellular
AGOP: null
PGOF: protein binding, zinc ion binding
PGOC: intracellular
PGOP: null

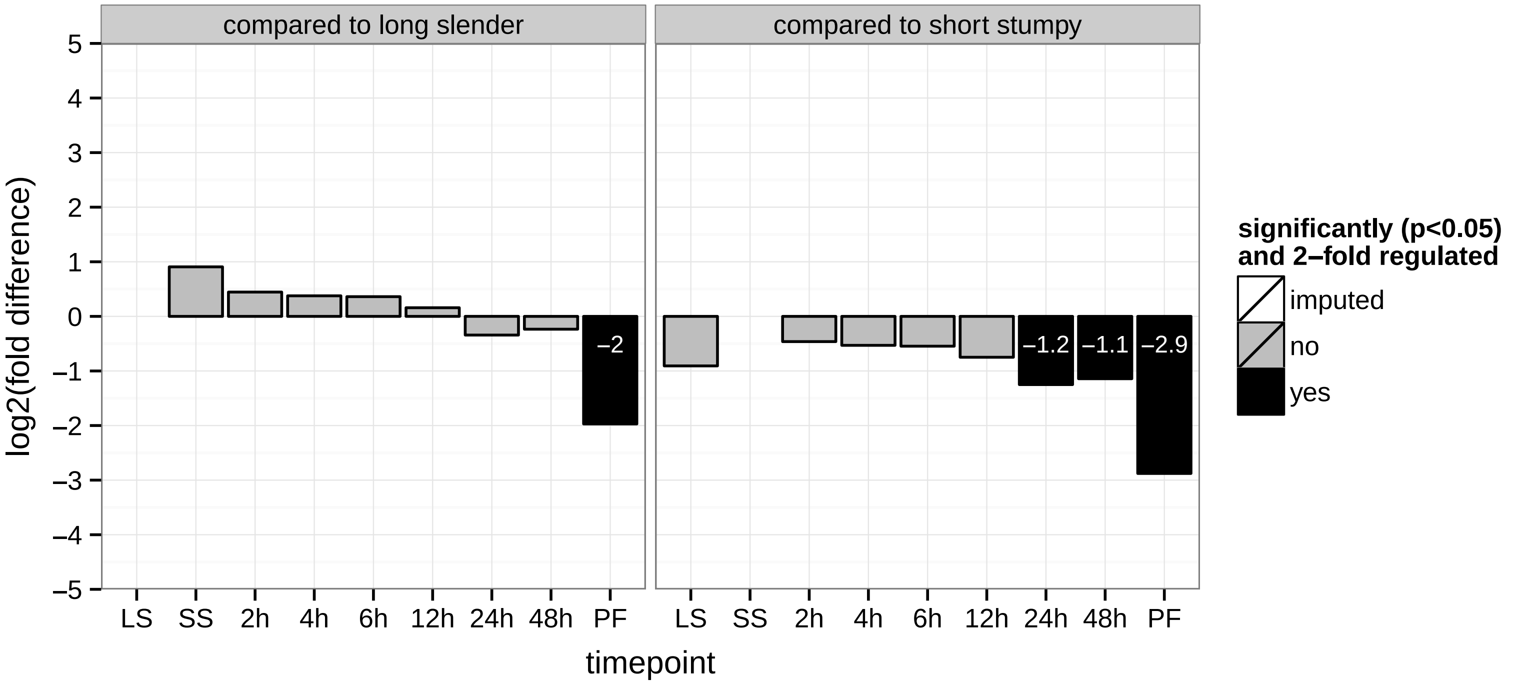

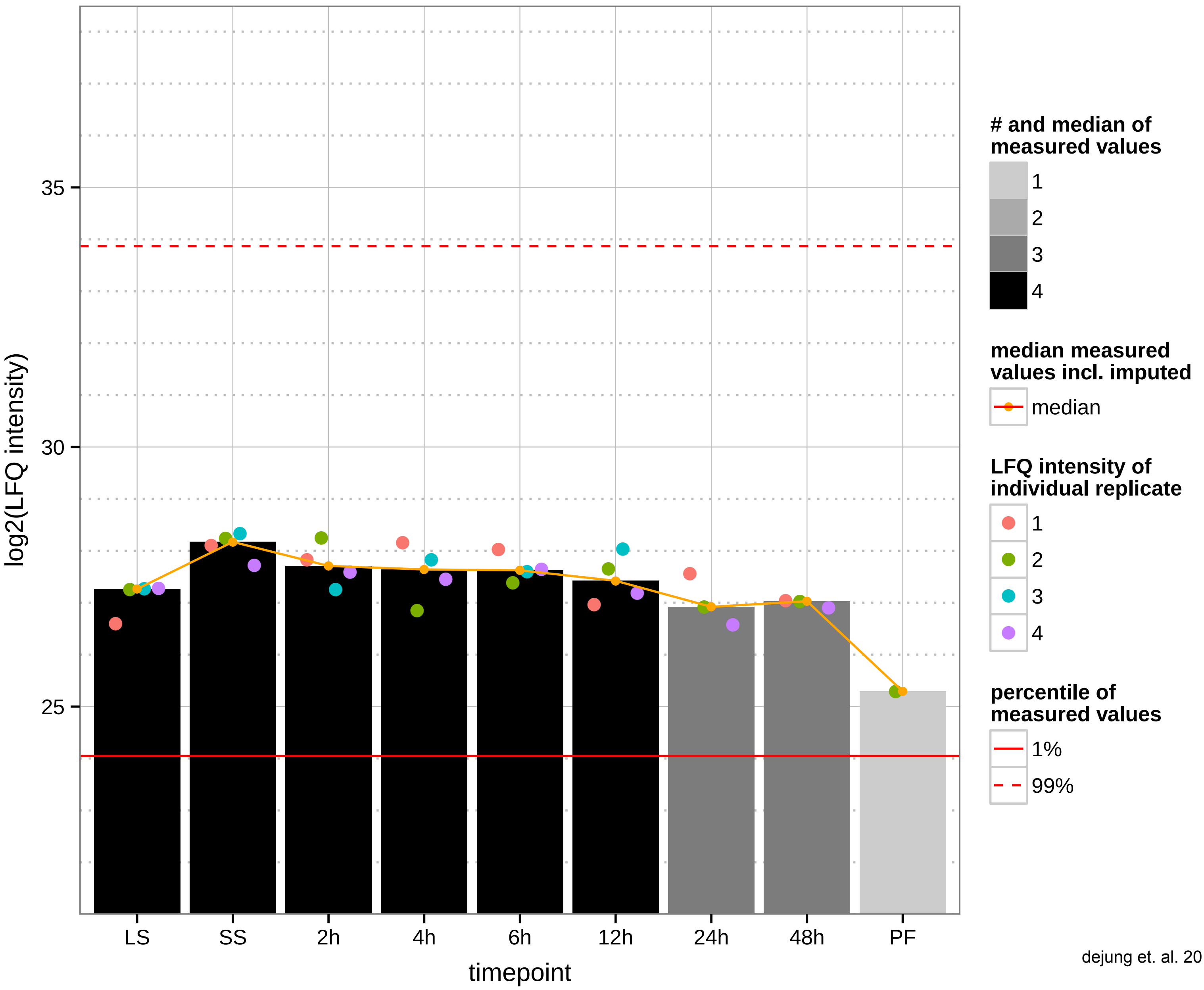

dejung et. al. 2015

kinesin, putative
Tb927.6.4390
AGOF: ATP binding, microtubule motor activity, motor activity
AGOC: microtubule associated complex
AGOP: microtubule–based movement
PGOF: ATP binding, microtubule motor activity
PGOC: null
PGOP: microtubule–based movement

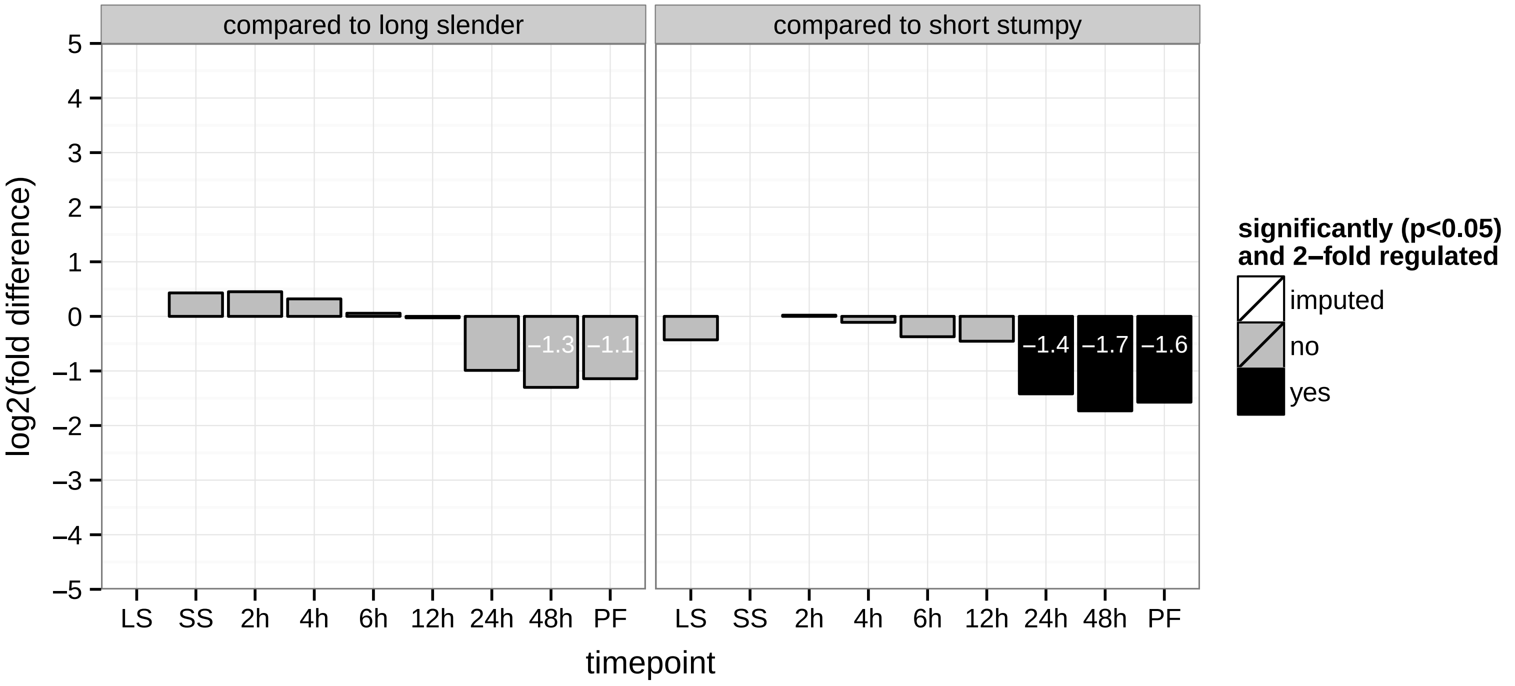

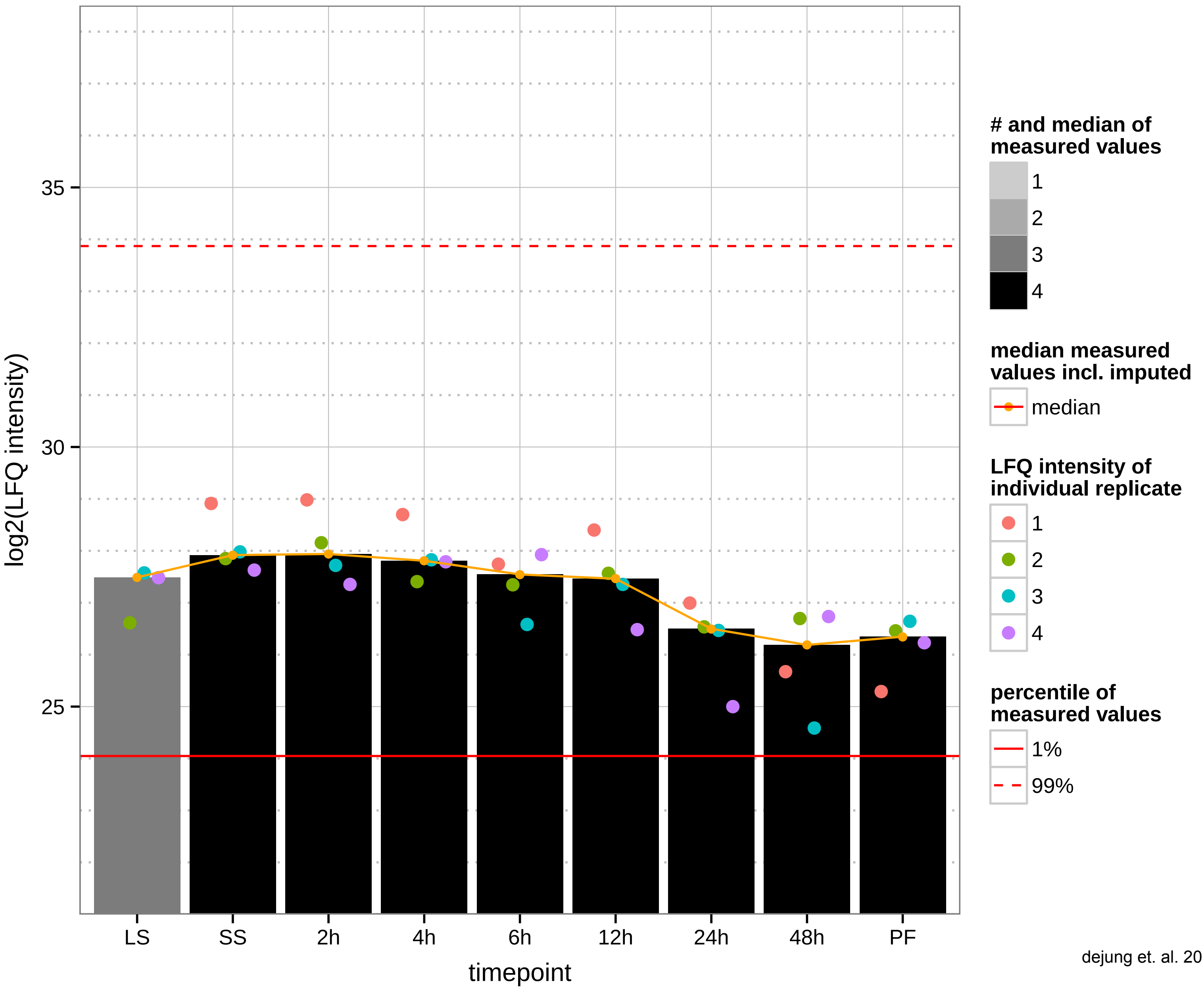

dejung et. al. 2015

metacaspase MCA3, cysteine peptidase, Clan CD, family C13, putative (TbMCA4), metacaspase MCA2 (MCA2)
Tb927.6.940;Tb927.6.930
AGOF: cysteine–type endopeptidase activity, peptidase activity, cysteine–type peptidase activity
AGOC: nucleus
AGOP: aerobic respiration, proteolysis, proteolysis
PGOF: cysteine–type endopeptidase activity
PGOC: null
PGOP: proteolysis

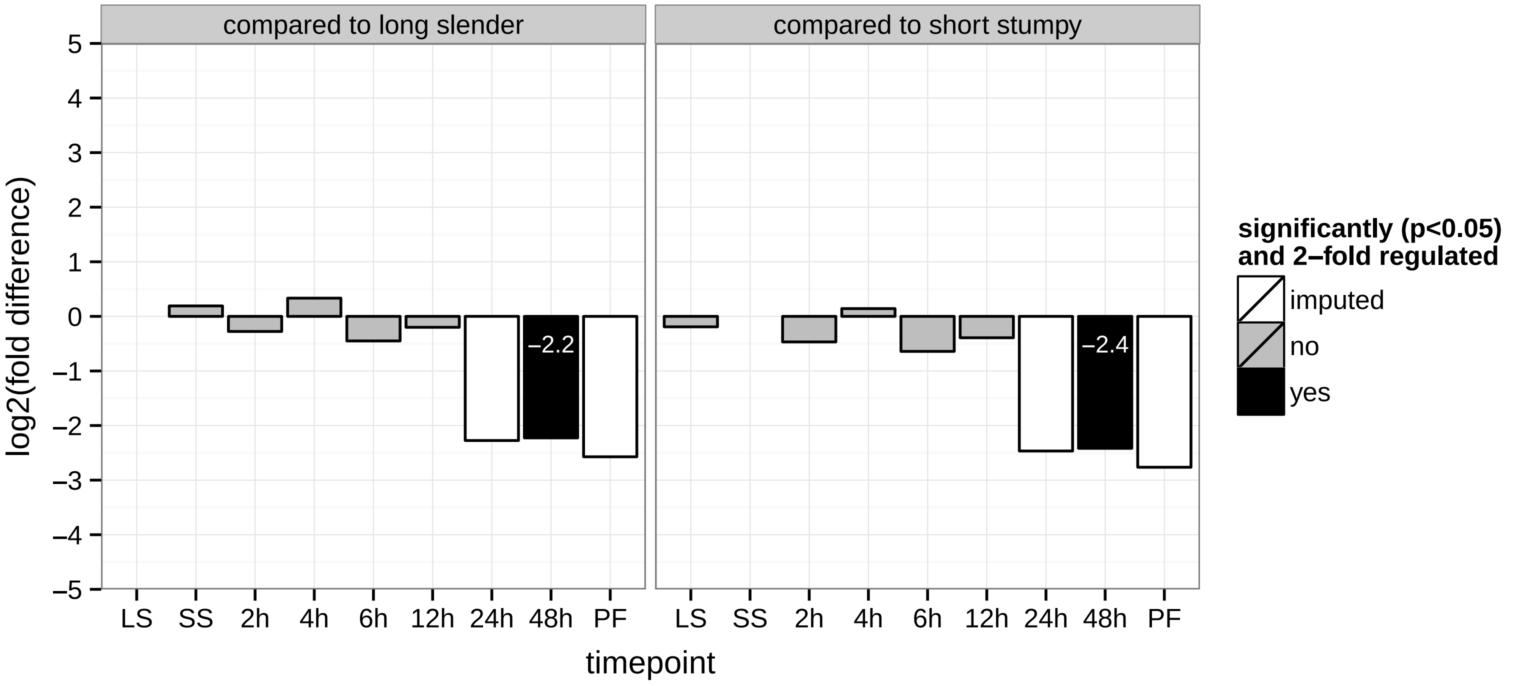

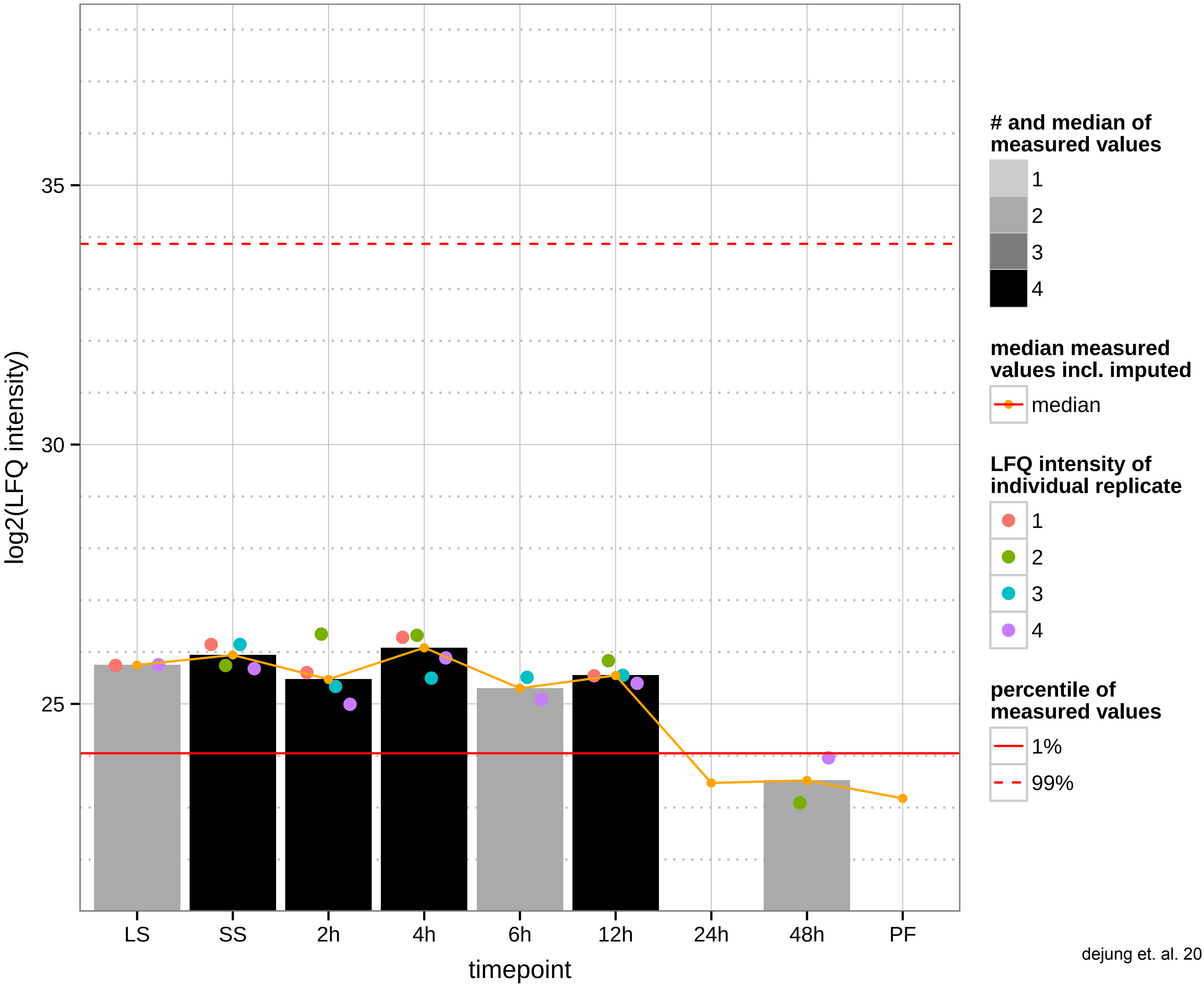

dejung et. al. 2015

synaptojanin (N−terminal domain), putative
Tb927.7.3490
AGOF: inositol−polyphosphate 5−phosphatase activity
AGOC: null
AGOP: endocytosis
PGOF: phosphoric ester hydrolase activity
PGOC: null
PGOP: null

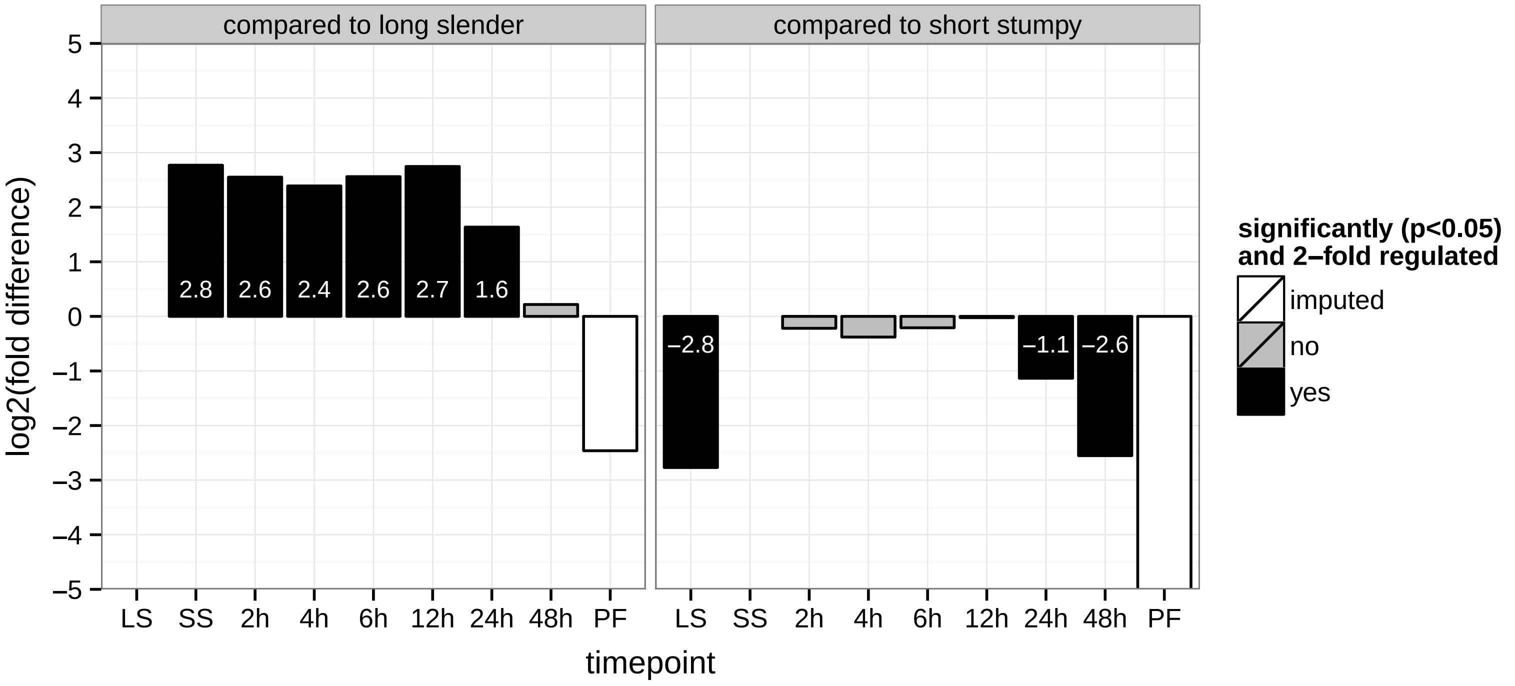

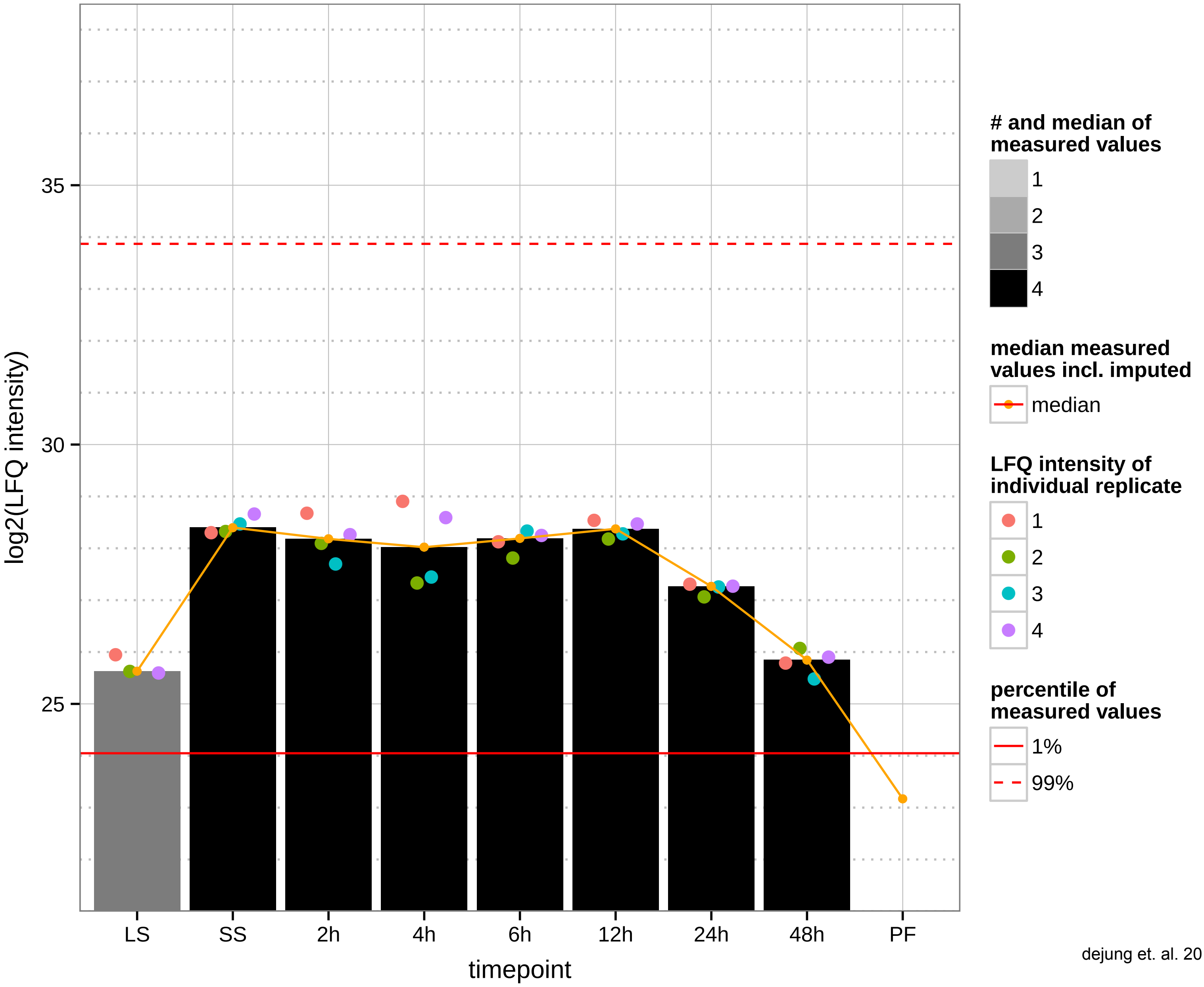

dejung et. al. 2015

Protein Associated with Differentiation (PAD1)
Tb927.7.5930
AGOF: null
AGOC: cell surface, plasma membrane
AGOP: citrate transport, regulation of developmental process, response to temperature stimulus
PGOF: null
PGOC: integral to membrane
PGOP: transmembrane transport

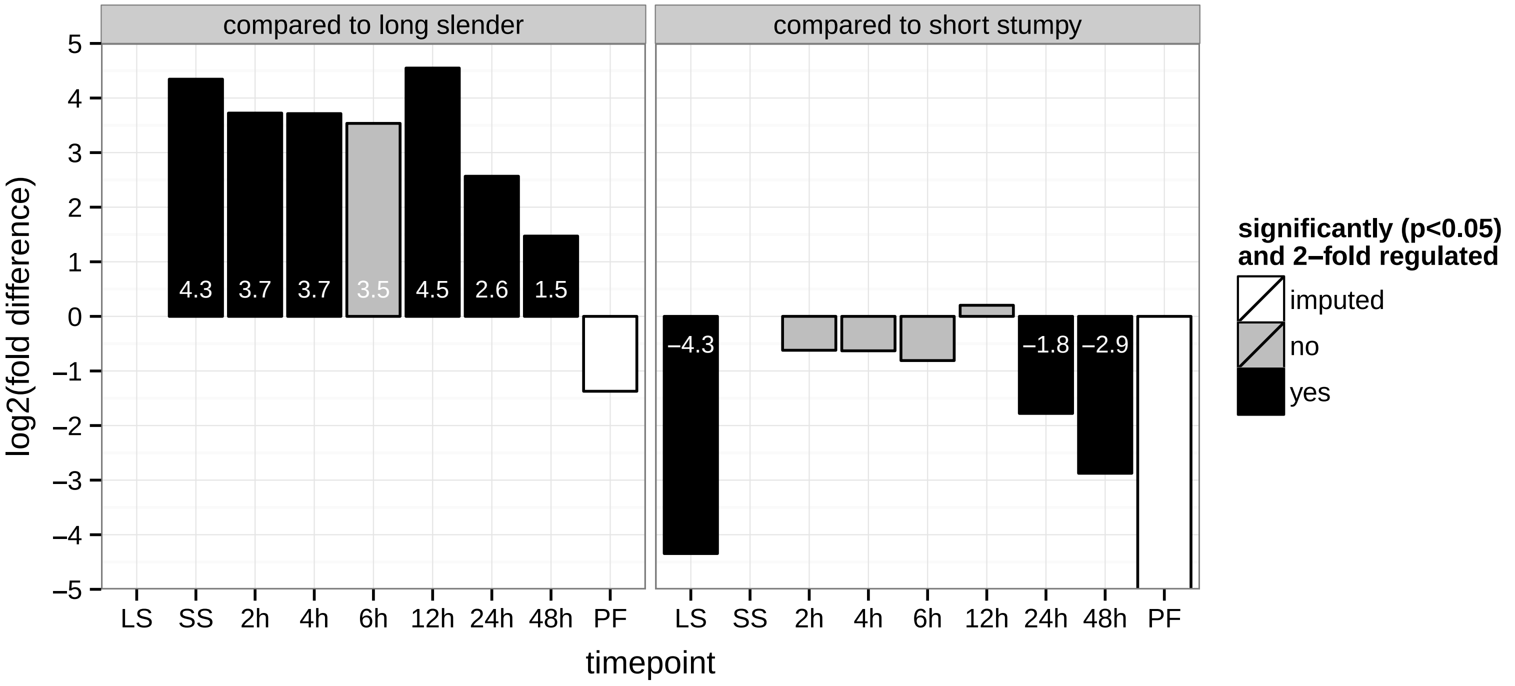

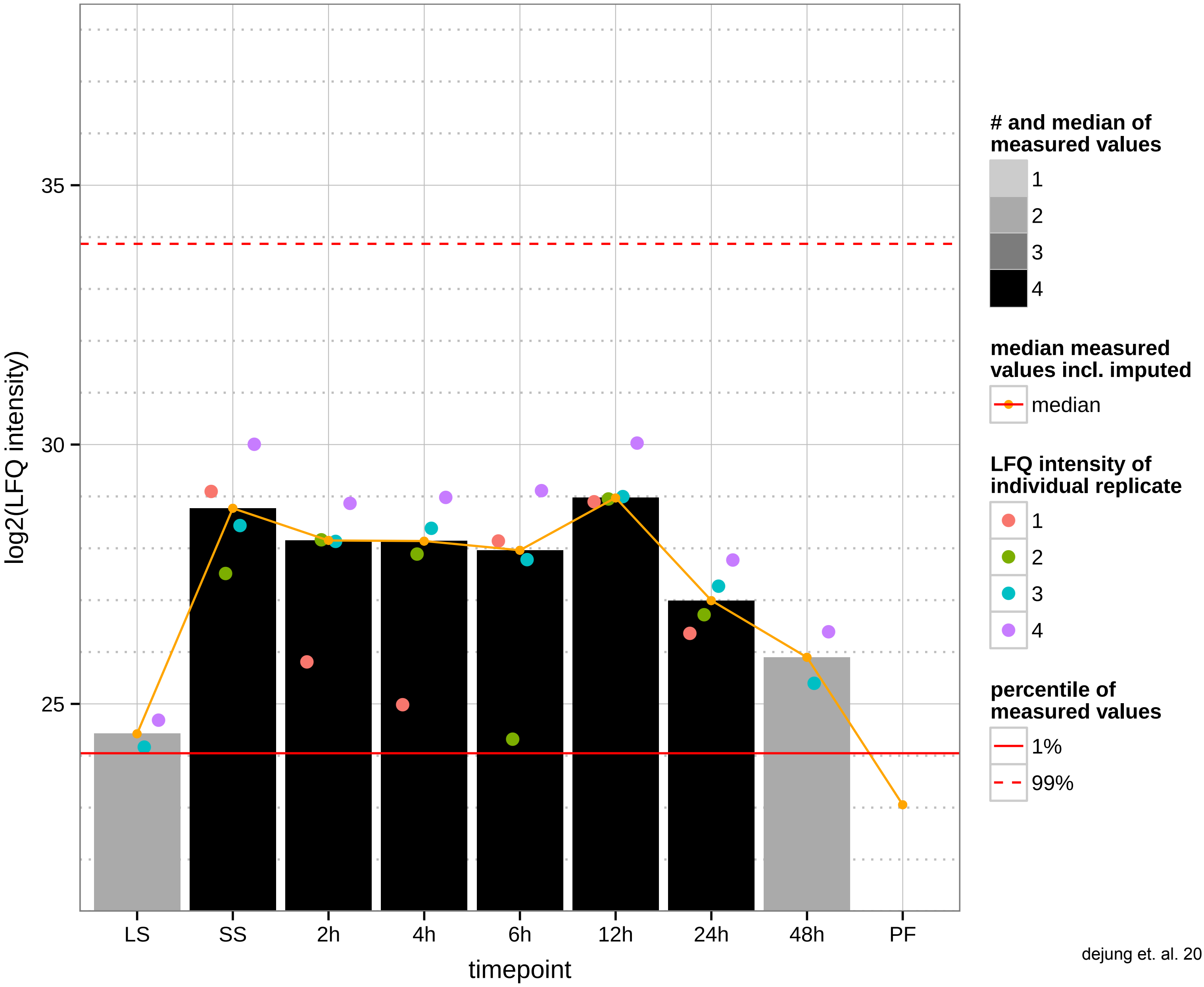

dejung et. al. 2015

vesicle transport v–SNARE protein, putative
Tb927.7.6440
AGOF: SNAP receptor activity
AGOC: SNARE complex
AGOP: intracellular protein transport
PGOF: null
PGOC: null
PGOP: null

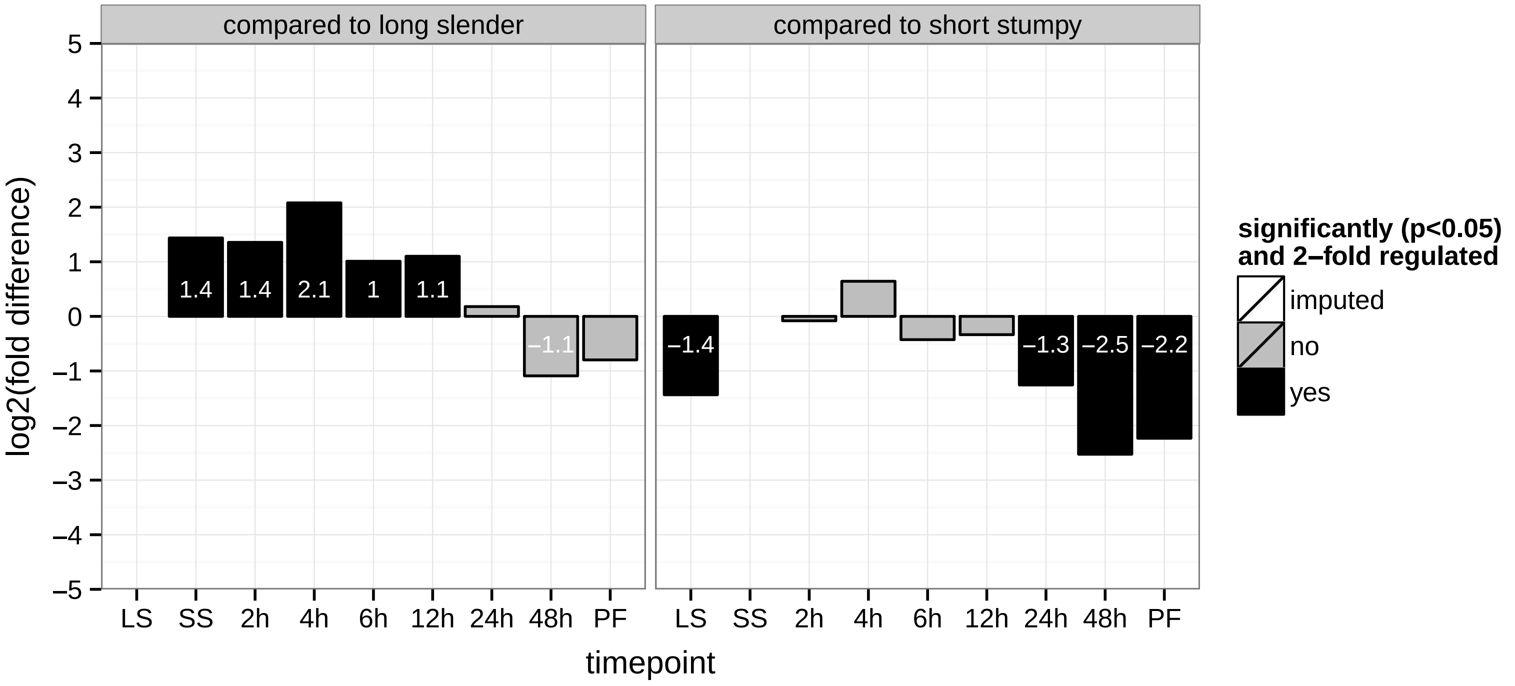

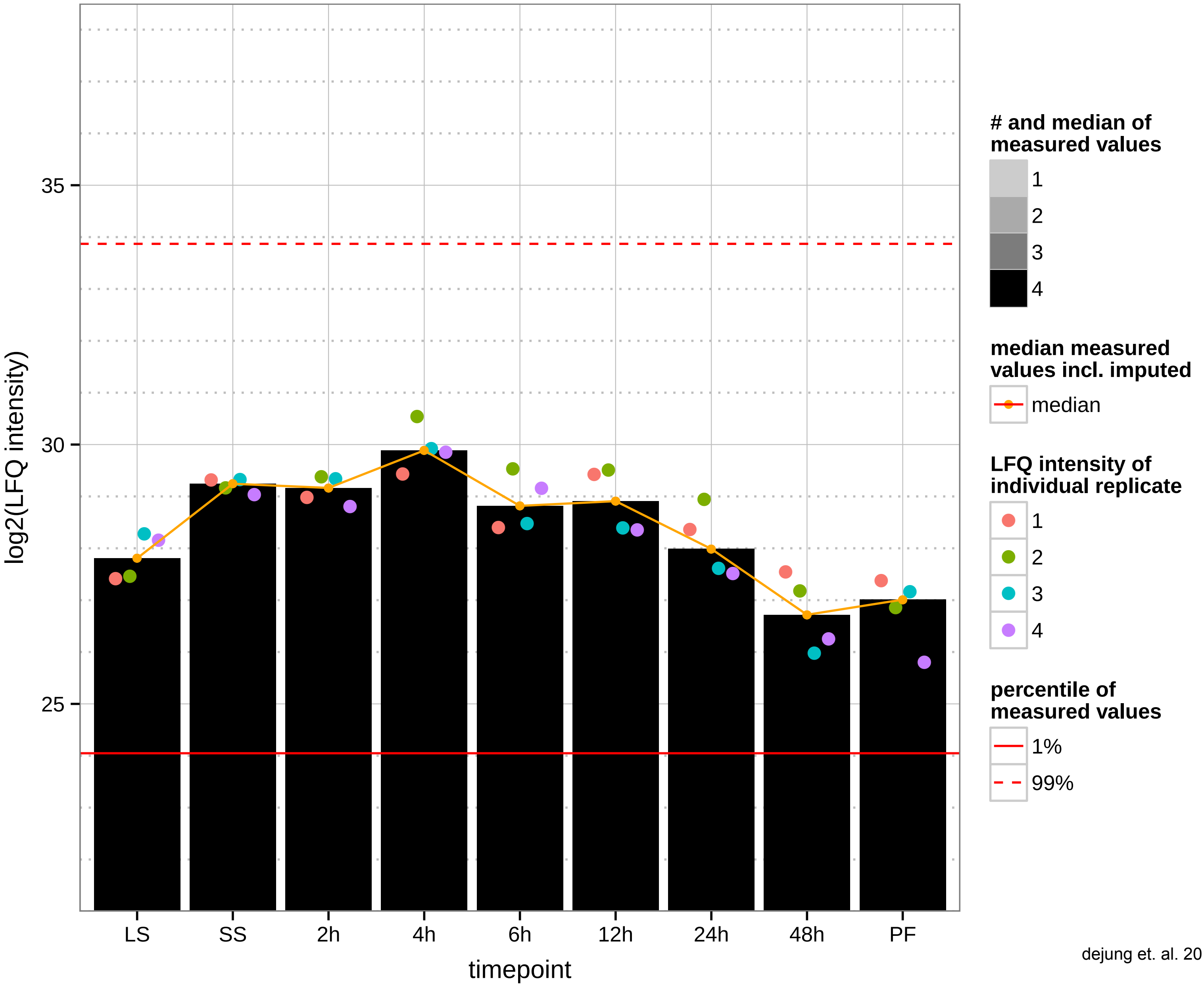

dejung et. al. 2015

receptor–type adenylate cyclase GRESAG 4, putative
Tb927.7.7530;Tb927.7.7520
AGOF: phosphorus–oxygen lyase activity, adenylate cyclase activity
AGOC: integral to membrane
AGOP: cyclic nucleotide biosynthetic process, intracellular signal transduction
PGOF: phosphorus–oxygen lyase activity
PGOC: null
PGOP: cyclic nucleotide biosynthetic process, intracellular signal transduction

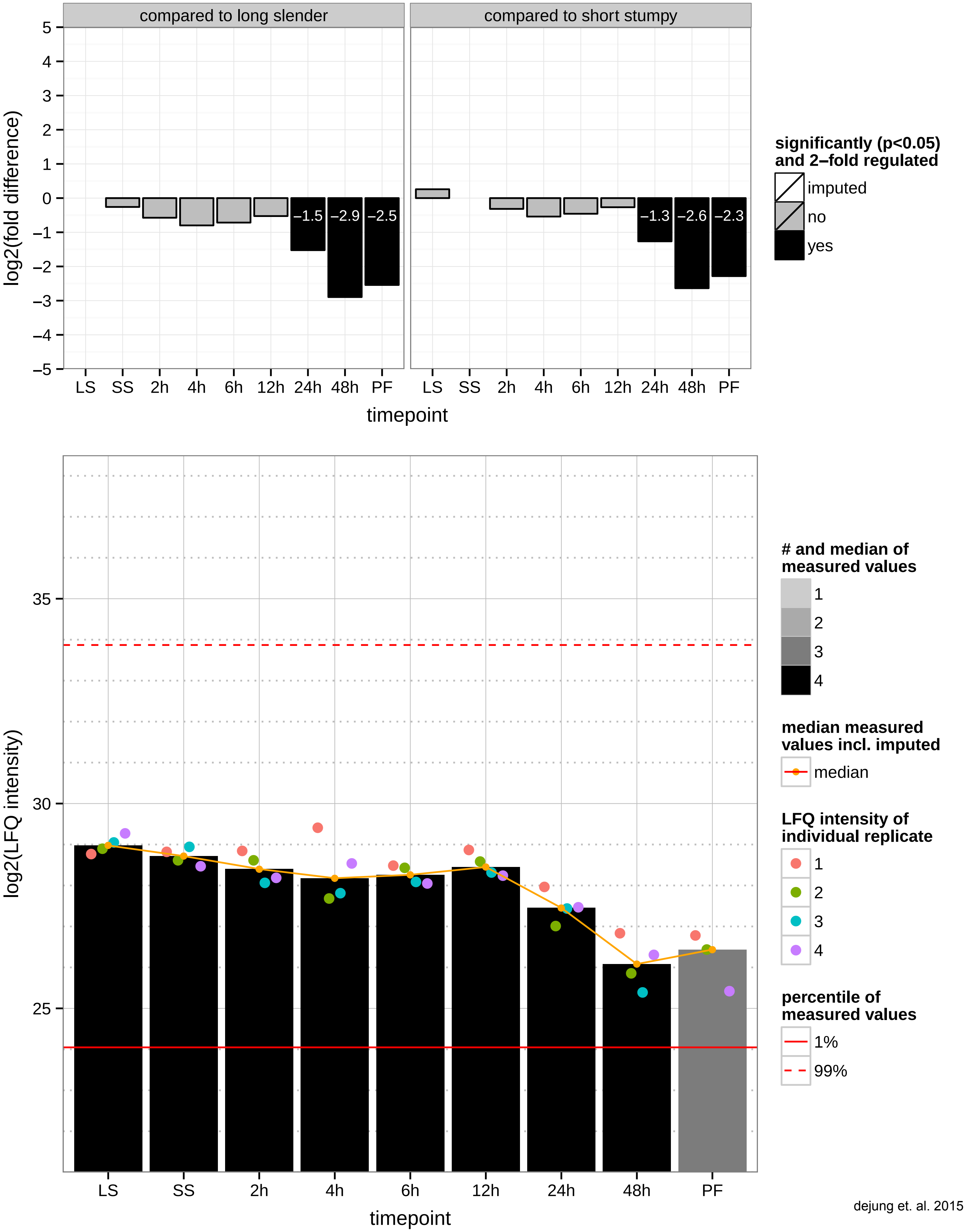

dejung et. al. 2015

hypothetical protein, conserved
Tb927.8.2070;Tb927.8.2270
AGOF: null
AGOC: null
AGOP: null
PGOF: null
PGOC: null
PGOP: null

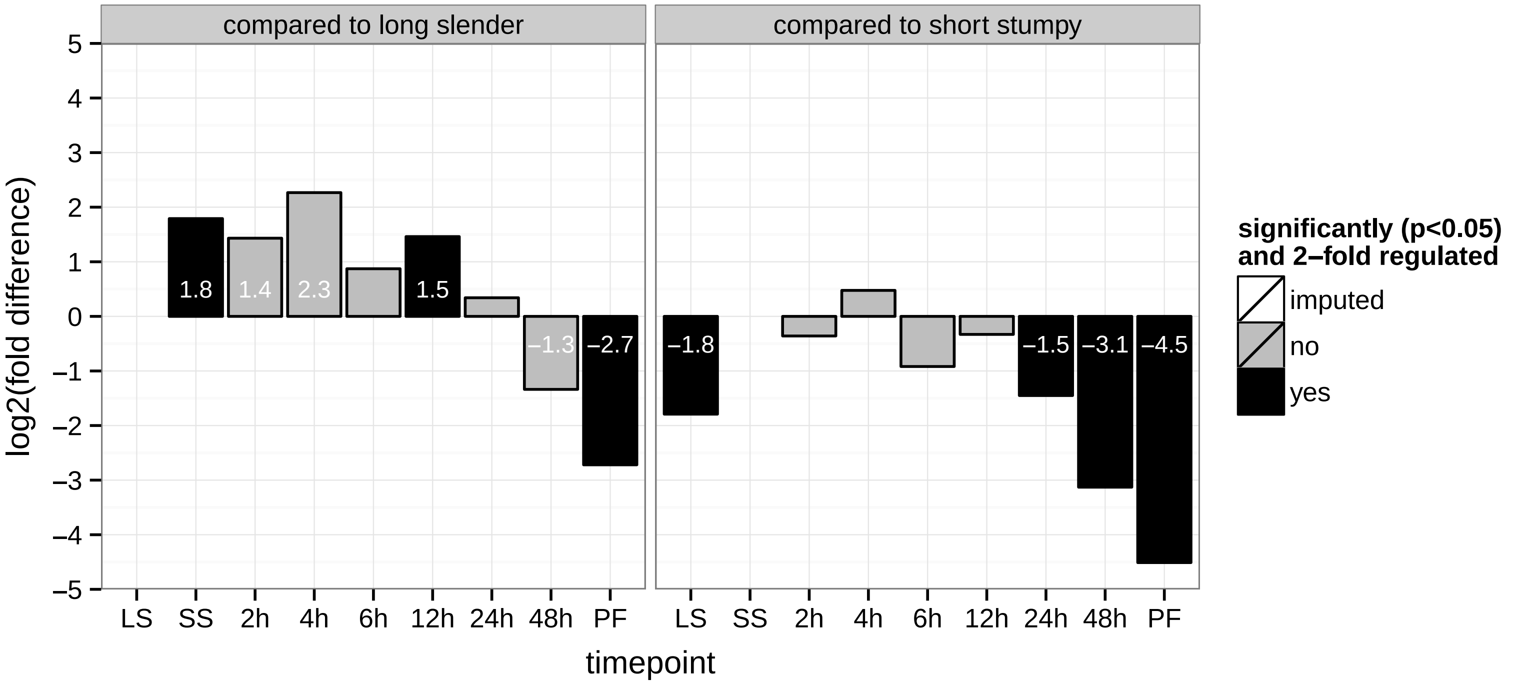

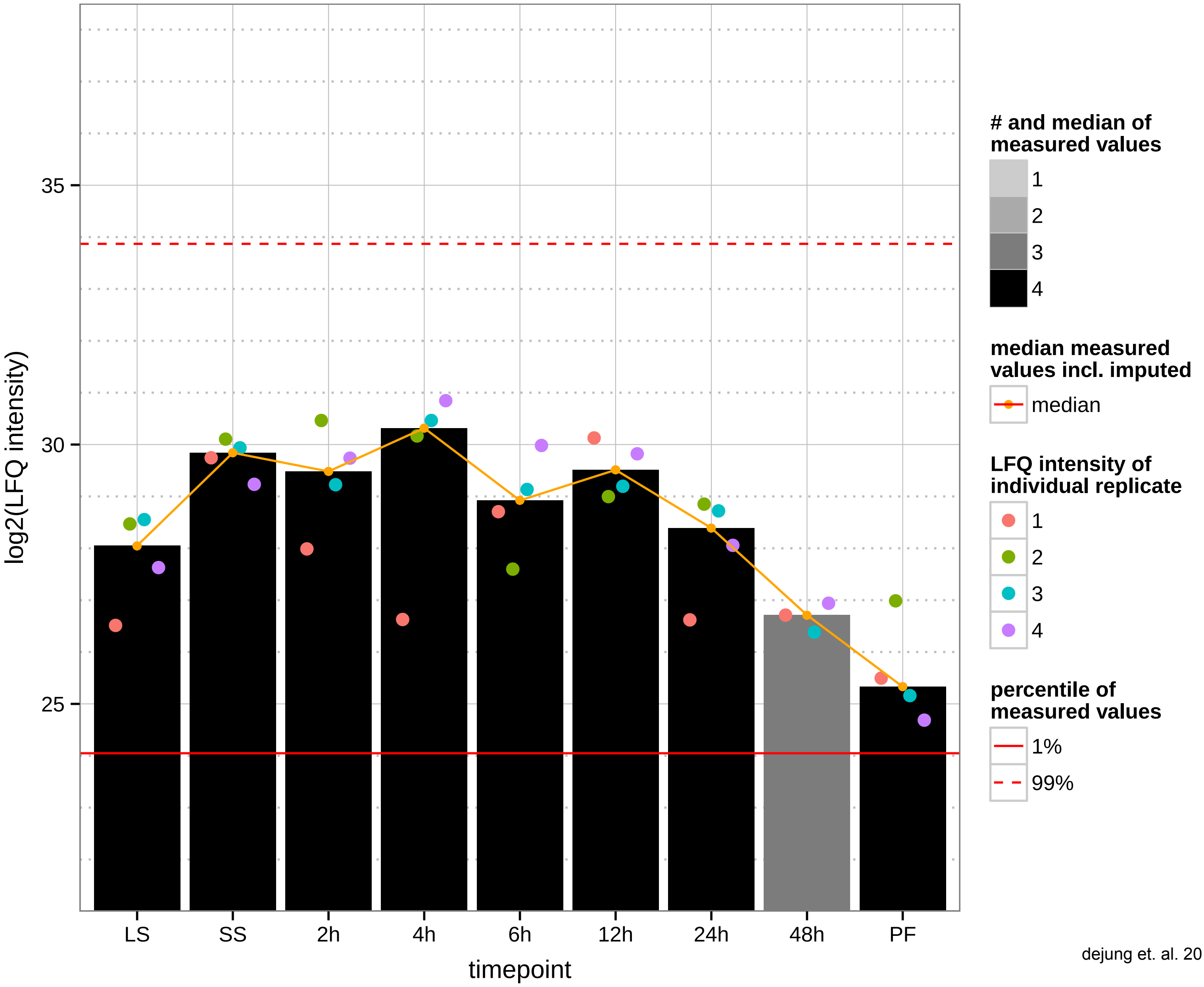

dejung et. al. 2015

hypothetical protein, conserved
Tb927.8.2280;Tb927.8.2260
AGOF: null
AGOC: null
AGOP: null
PGOF: null
PGOC: null
PGOP: null

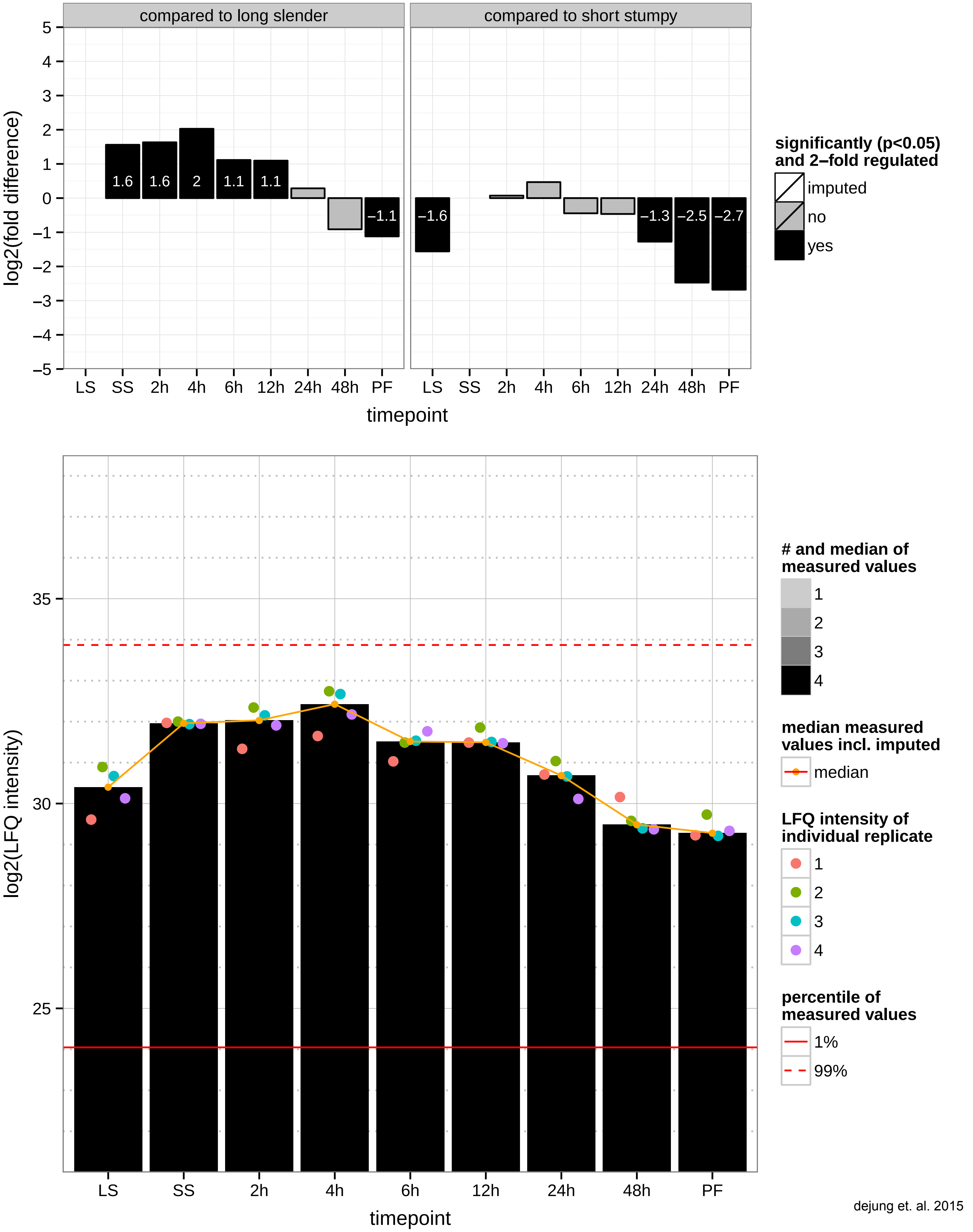

dejung et. al. 2015

hypothetical protein, conserved
Tb927.8.2720
AGOF: null
AGOC: null
AGOP: null
PGOF: null
PGOC: null
PGOP: null

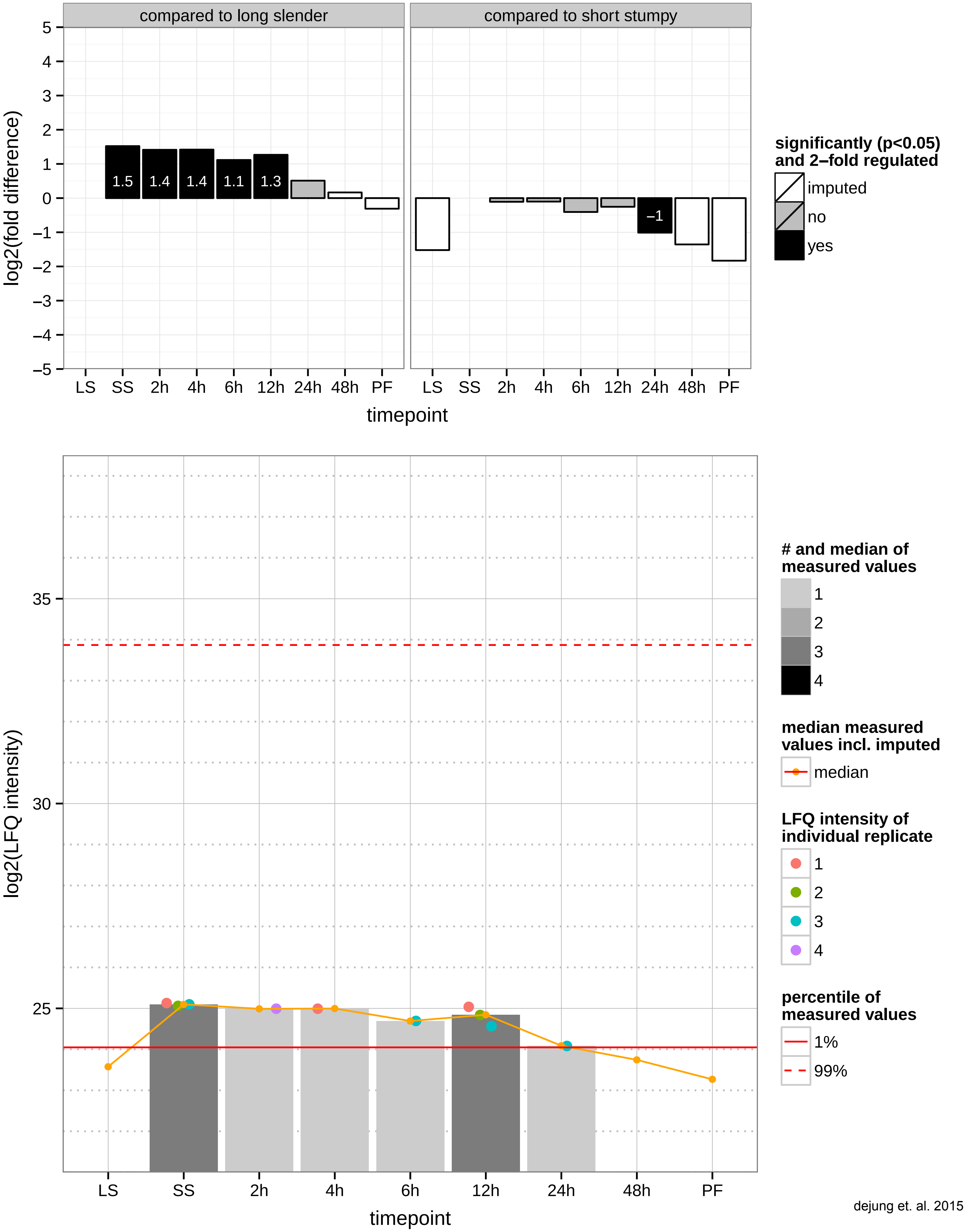

dejung et. al. 2015

hypothetical protein, conserved
Tb927.8.2860
AGOF: null
AGOC: null
AGOP: modulation of development of symbiont involved in interaction with host, quorum sensing involved in interaction with ho
PGOF: null
PGOC: null
PGOP: null

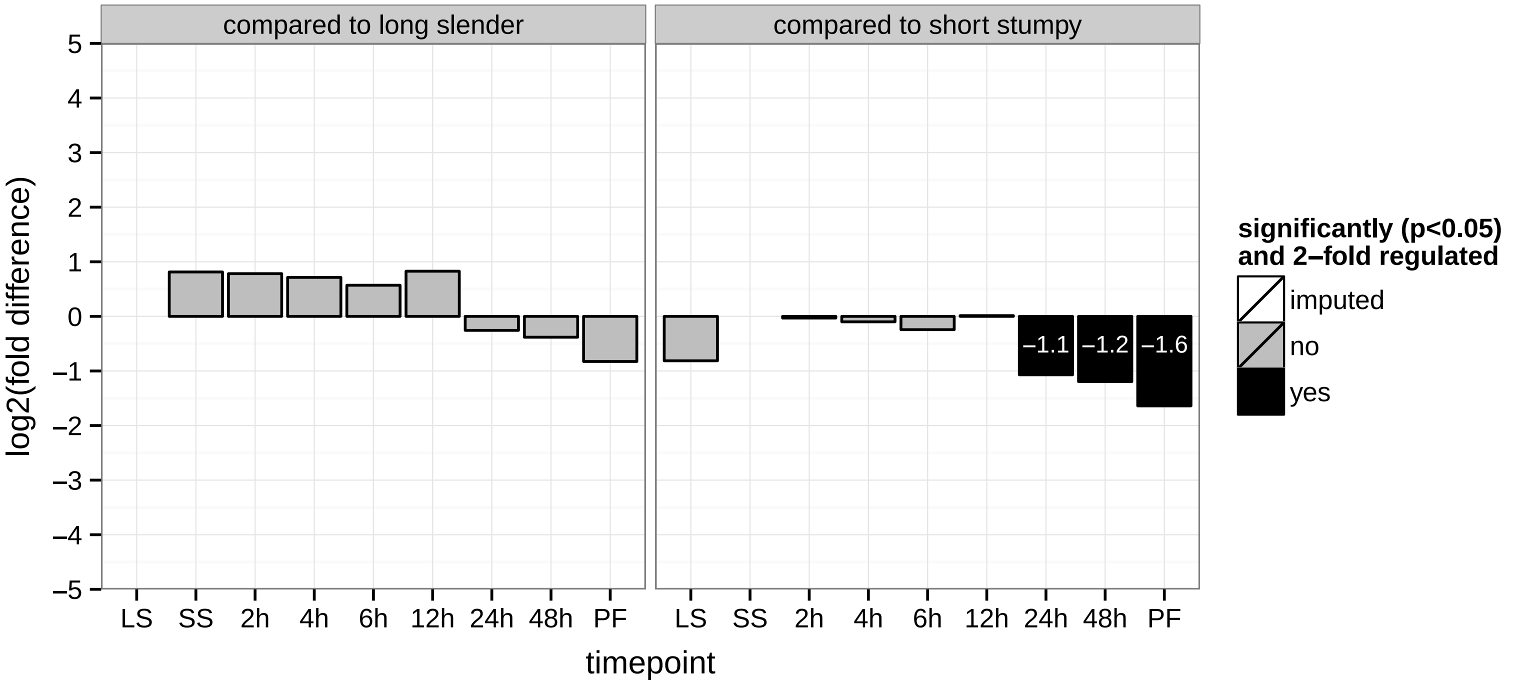

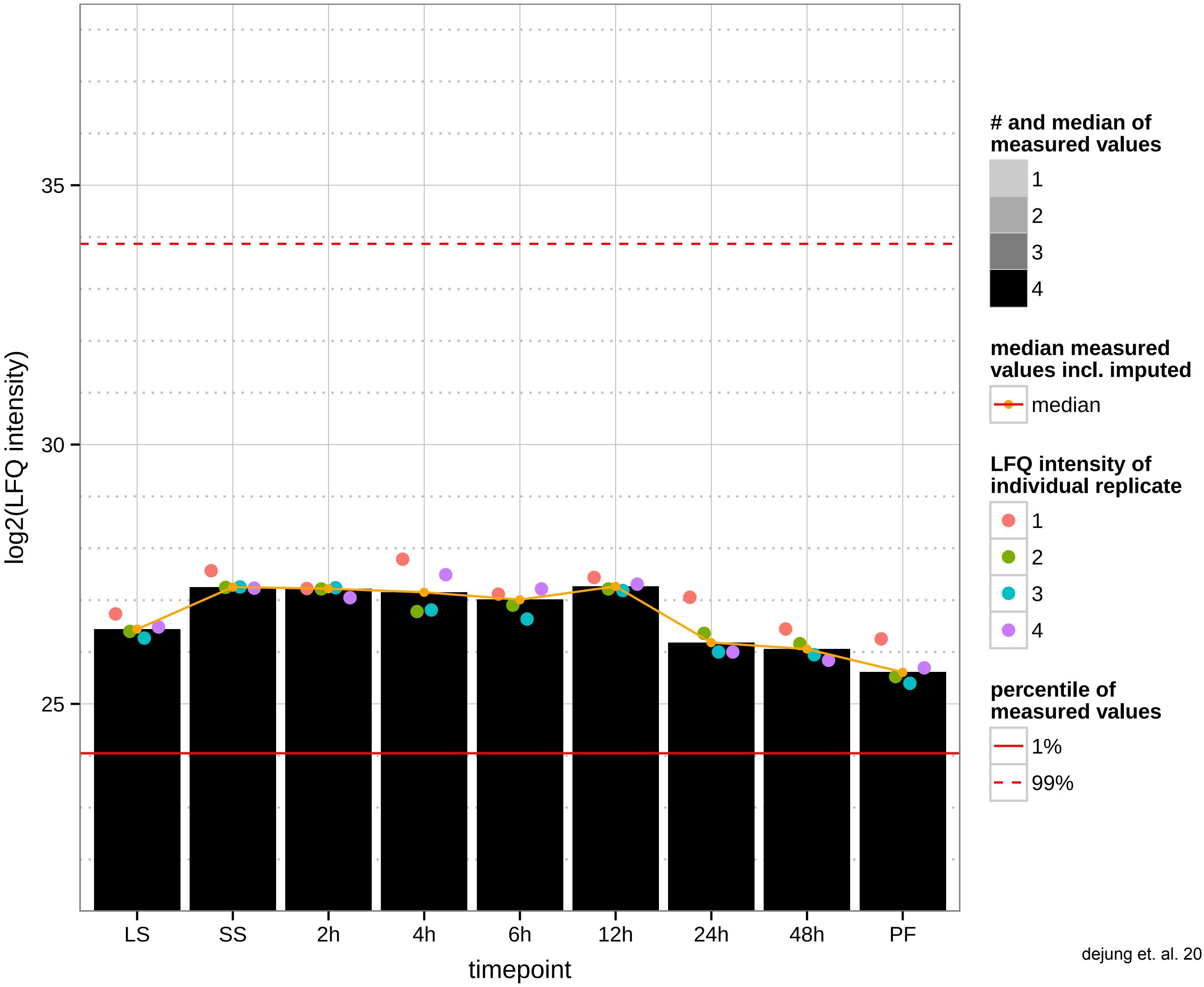

dejung et. al. 2015

mitogen–activated protein kinase 3, putative
Tb927.8.3550
AGOF: ATP binding, protein serine/threonine kinase activity, protein tyrosine kinase activity
AGOC: null
AGOP: null
PGOF: ATP binding, protein kinase activity, transferase activity, transferring phosphorus–containing groups
PGOC: null
PGOP: protein phosphorylation

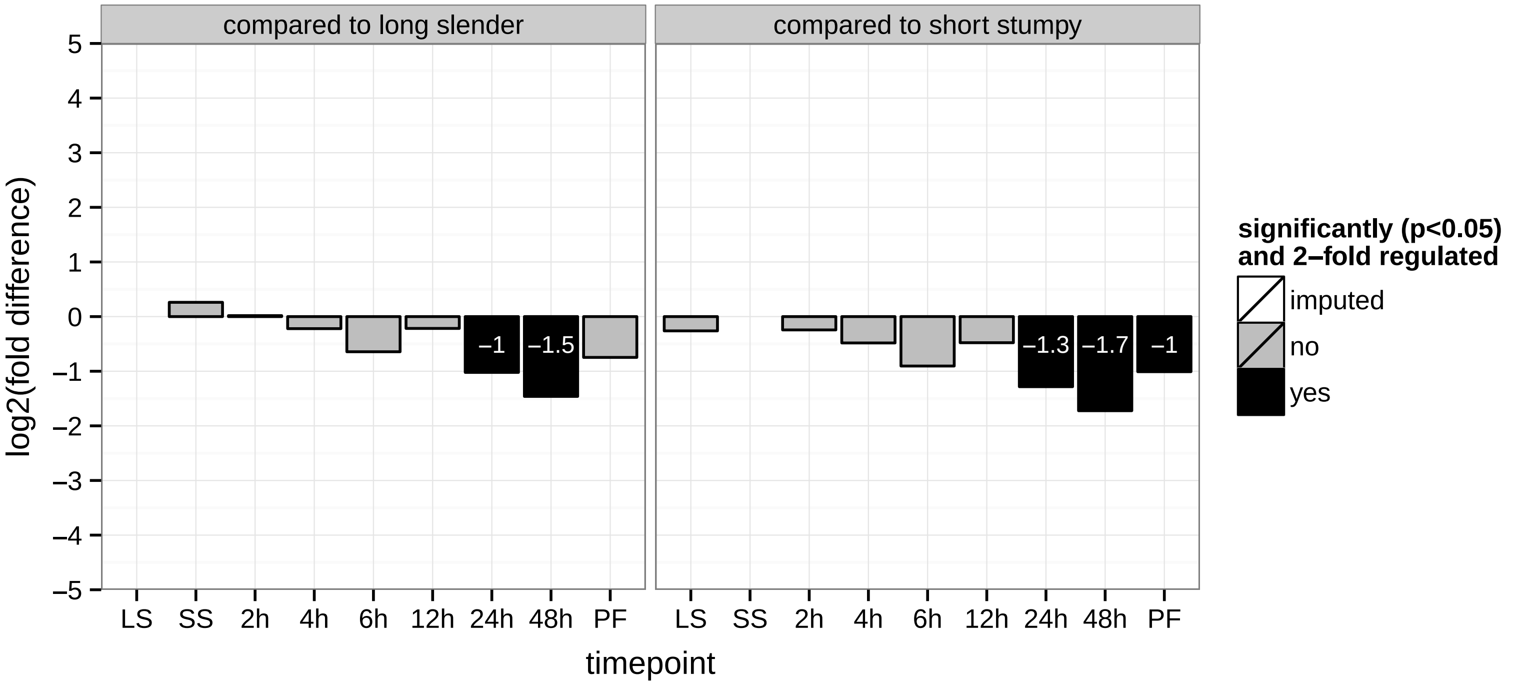

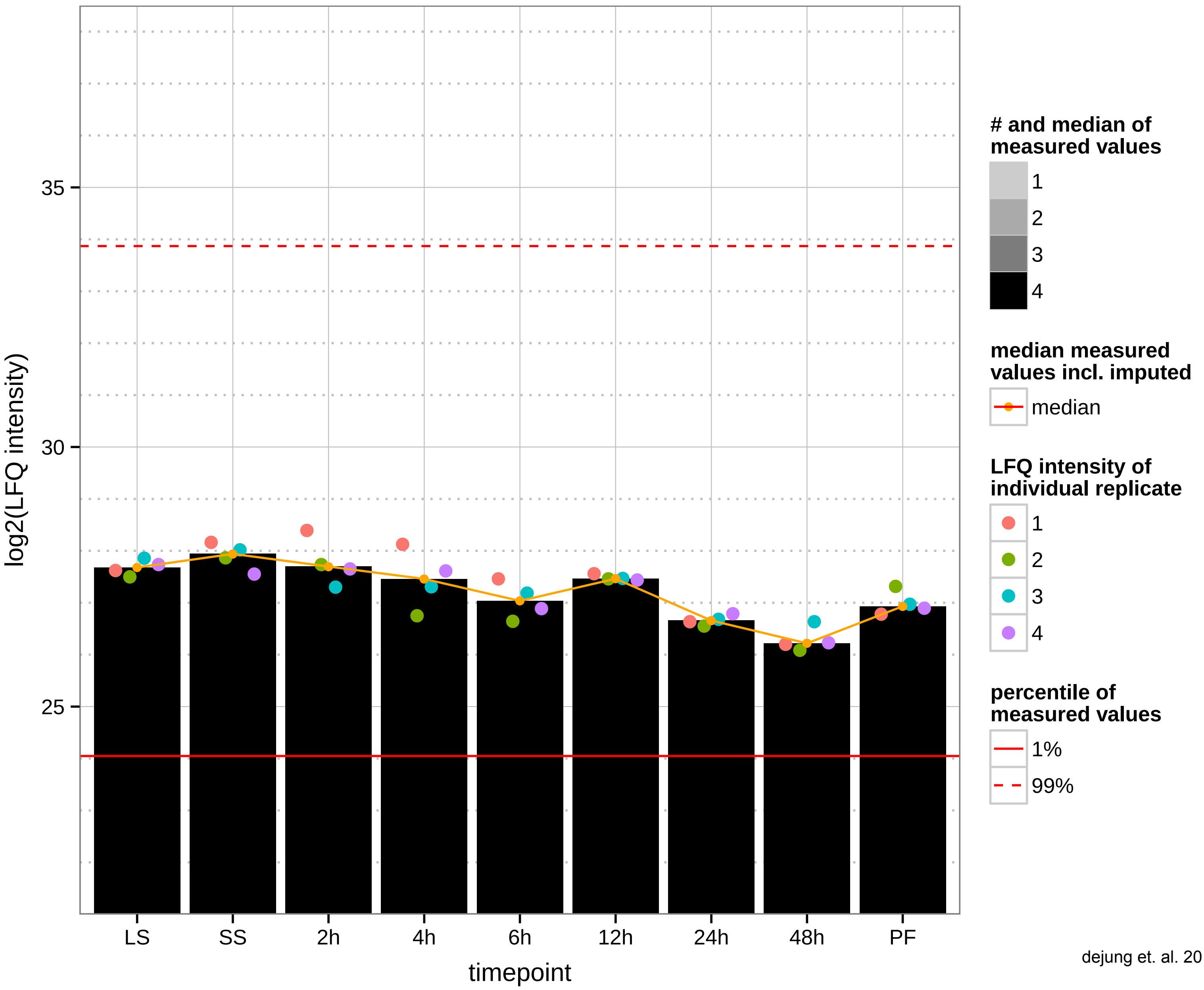

dejung et. al. 2015

mitogen–activated protein kinase, putative (MPK10)
Tb927.8.3770
AGOF: ATP binding, protein kinase activity, protein serine/threonine kinase activity, protein tyrosine kinase activity
AGOC: null
AGOP: null
PGOF: ATP binding, protein kinase activity, transferase activity, transferring phosphorus–containing groups
PGOC: null
PGOP: protein phosphorylation

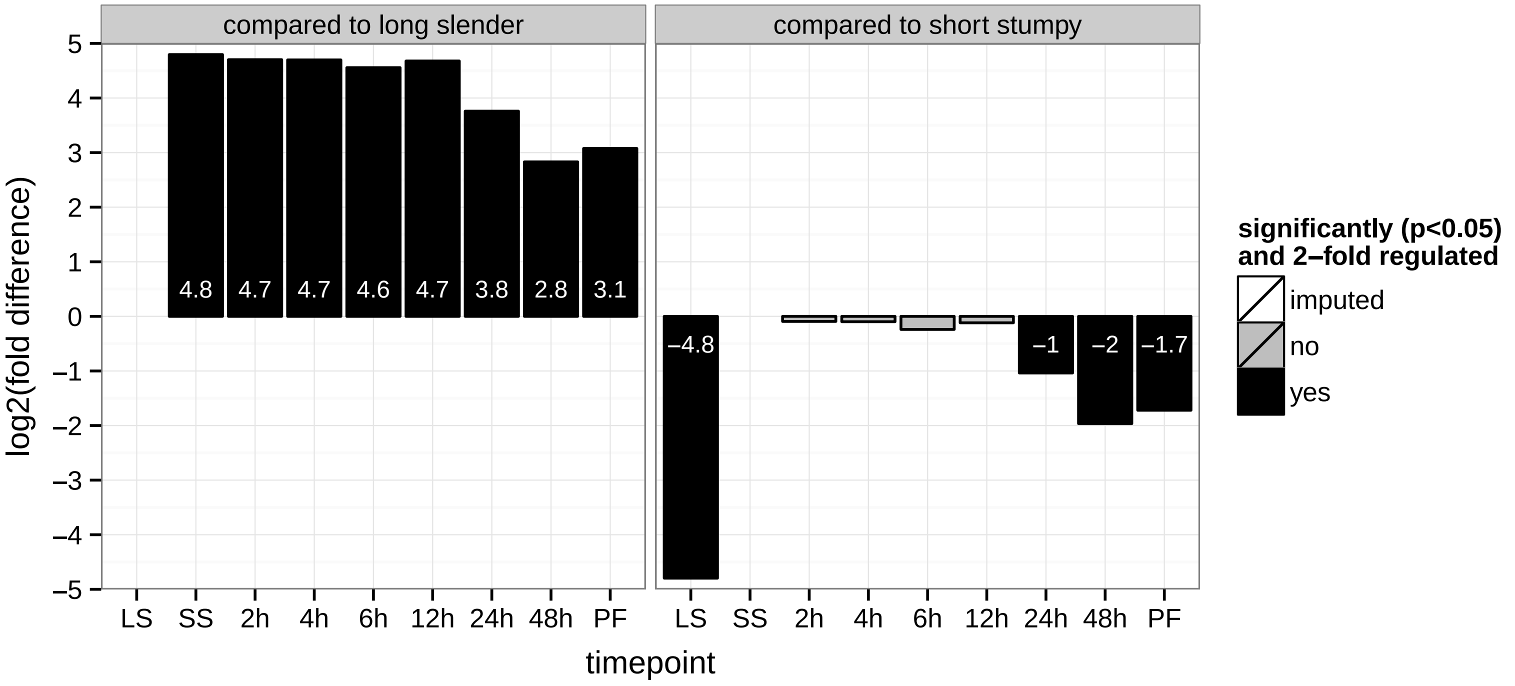

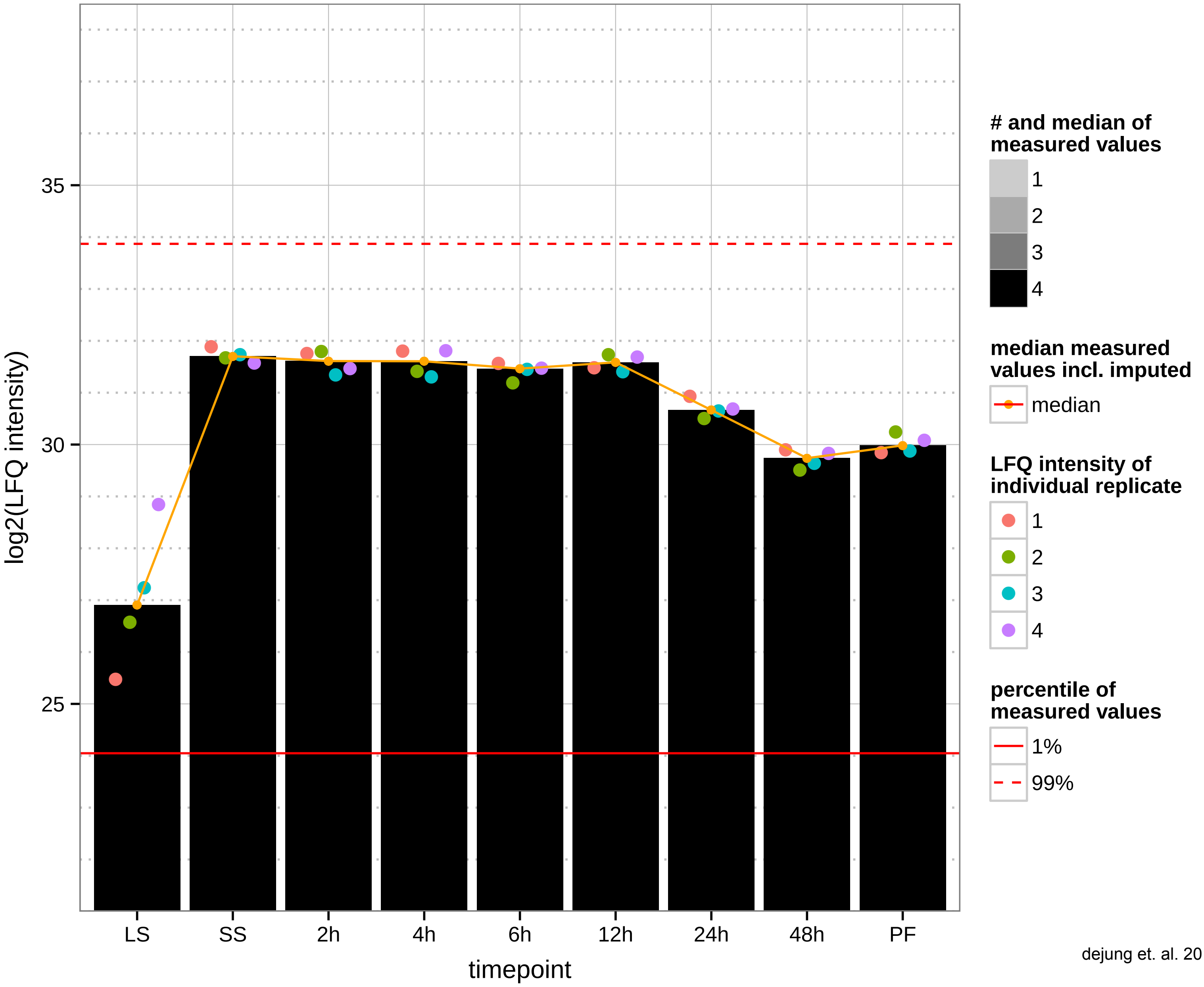

dejung et. al. 2015

endoplasmic reticulum oxidoreductin, putative, pol–associated gene 1
Tb927.8.4890
AGOF: disulfide oxidoreductase activity, electron carrier activity, flavin adenine dinucleotide binding, oxidoreductase activity, ac
AGOC: endoplasmic reticulum membrane, integral to membrane
AGOP: oxidation–reduction process, protein thiol–disulfide exchange
PGOF: flavin adenine dinucleotide binding, oxidoreductase activity, acting on a sulfur group of donors, disulfide as acceptor
PGOC: endoplasmic reticulum membrane
PGOP: oxidation–reduction process, protein thiol–disulfide exchange

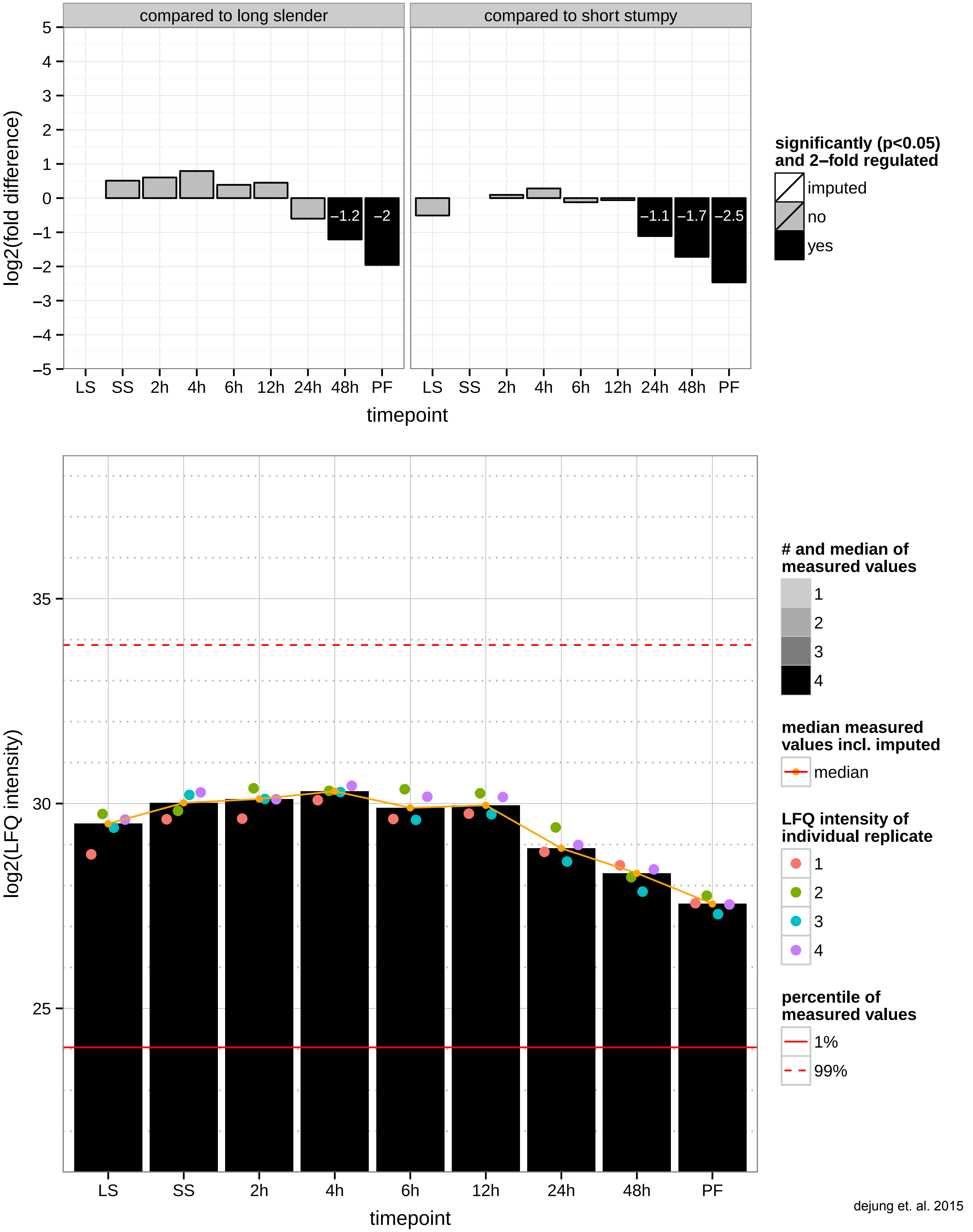

dejung et. al. 2015

hypothetical protein, conserved
Tb927.8.5050
AGOF: null
AGOC: null
AGOP: null
PGOF: null
PGOC: null
PGOP: null

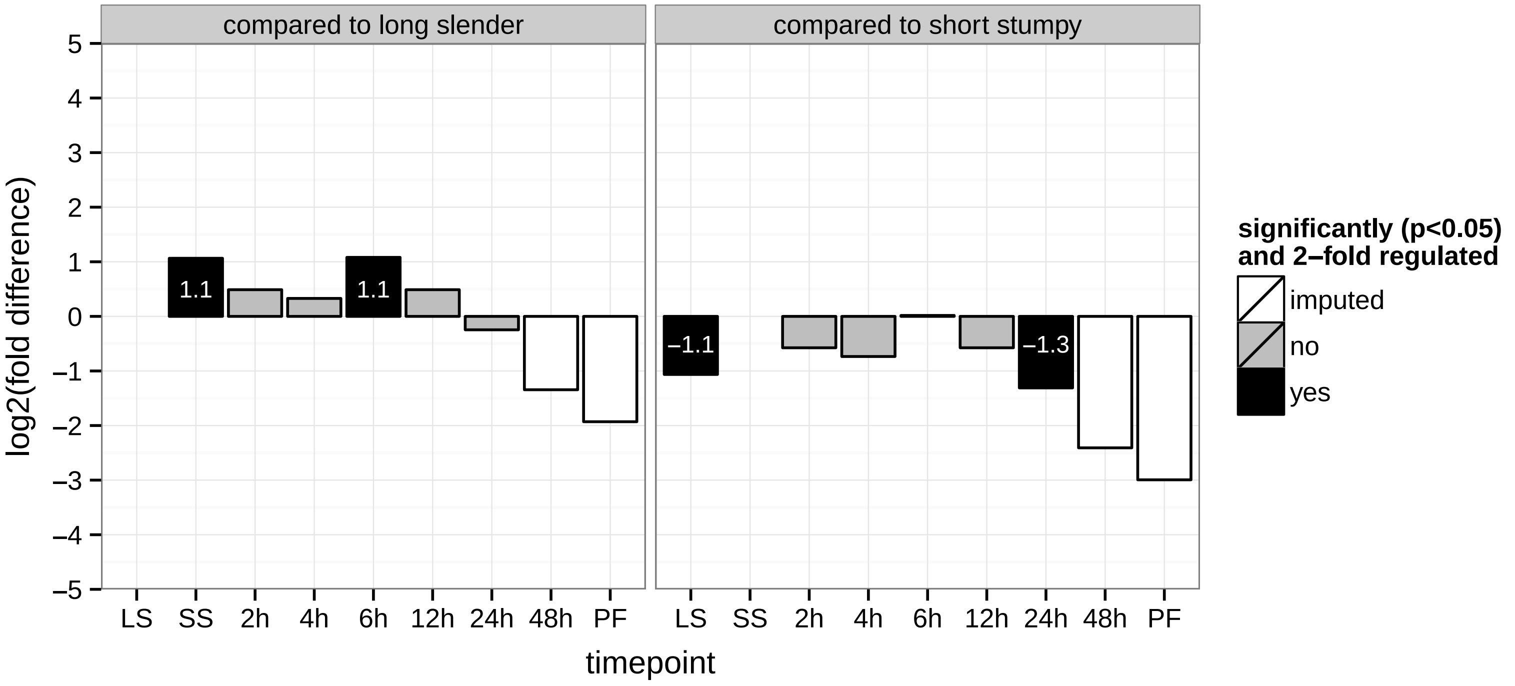

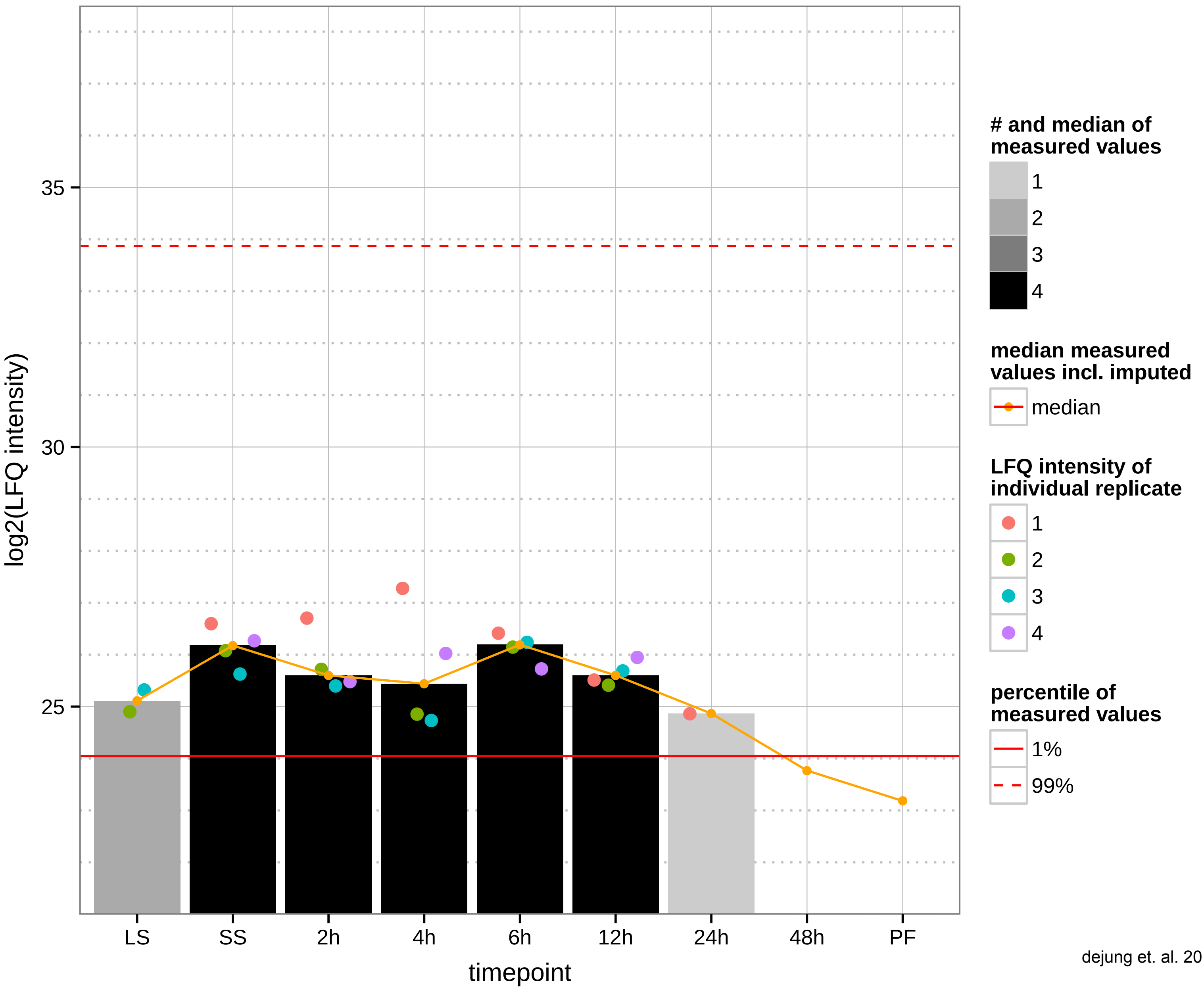

dejung et. al. 2015

hypothetical protein, conserved
Tb927.8.5080
AGOF: carbon−nitrogen ligase activity, with glutamine as amido−N−donor
AGOC: null
AGOP: null
PGOF: carbon−nitrogen ligase activity, with glutamine as amido−N−donor
PGOC: null
PGOP: null

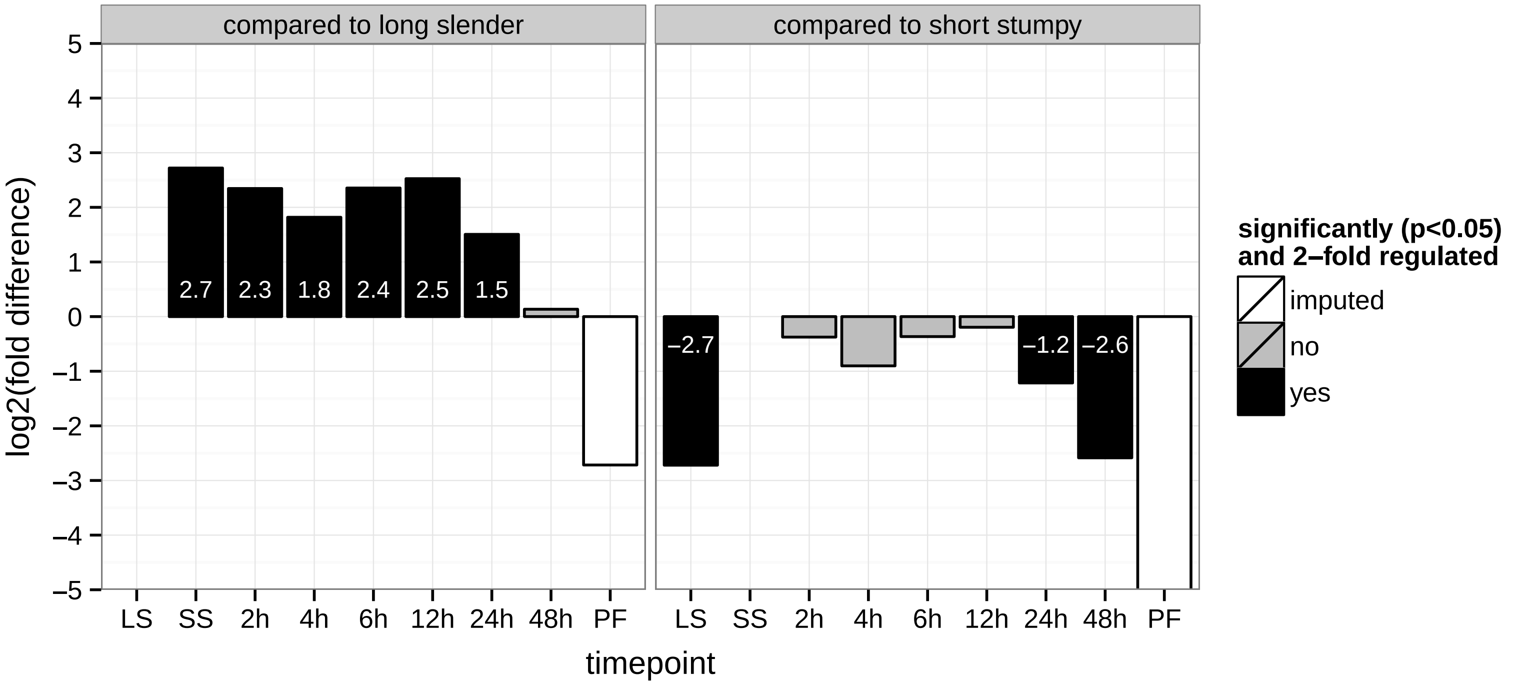

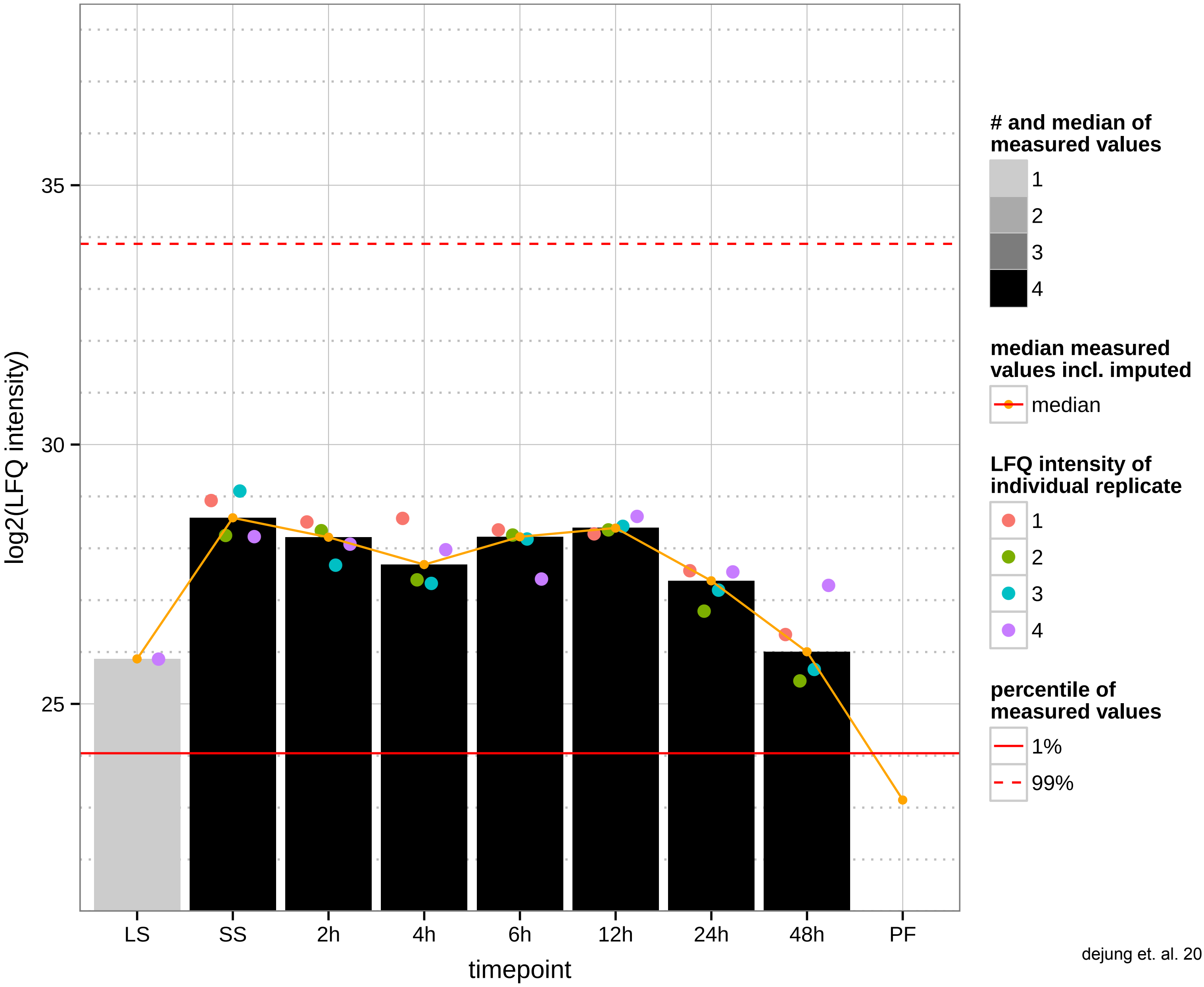

dejung et. al. 2015

serine/threonine–protein kinase NrkA (NRKB)
Tb927.8.6930
AGOF: ATP binding, protein serine/threonine kinase activity, protein tyrosine kinase activity
AGOC: null
AGOP: protein phosphorylation
PGOF: ATP binding, phospholipid binding, protein binding, protein kinase activity, transferase activity, transferring phosphorus
PGOC: null
PGOP: protein phosphorylation

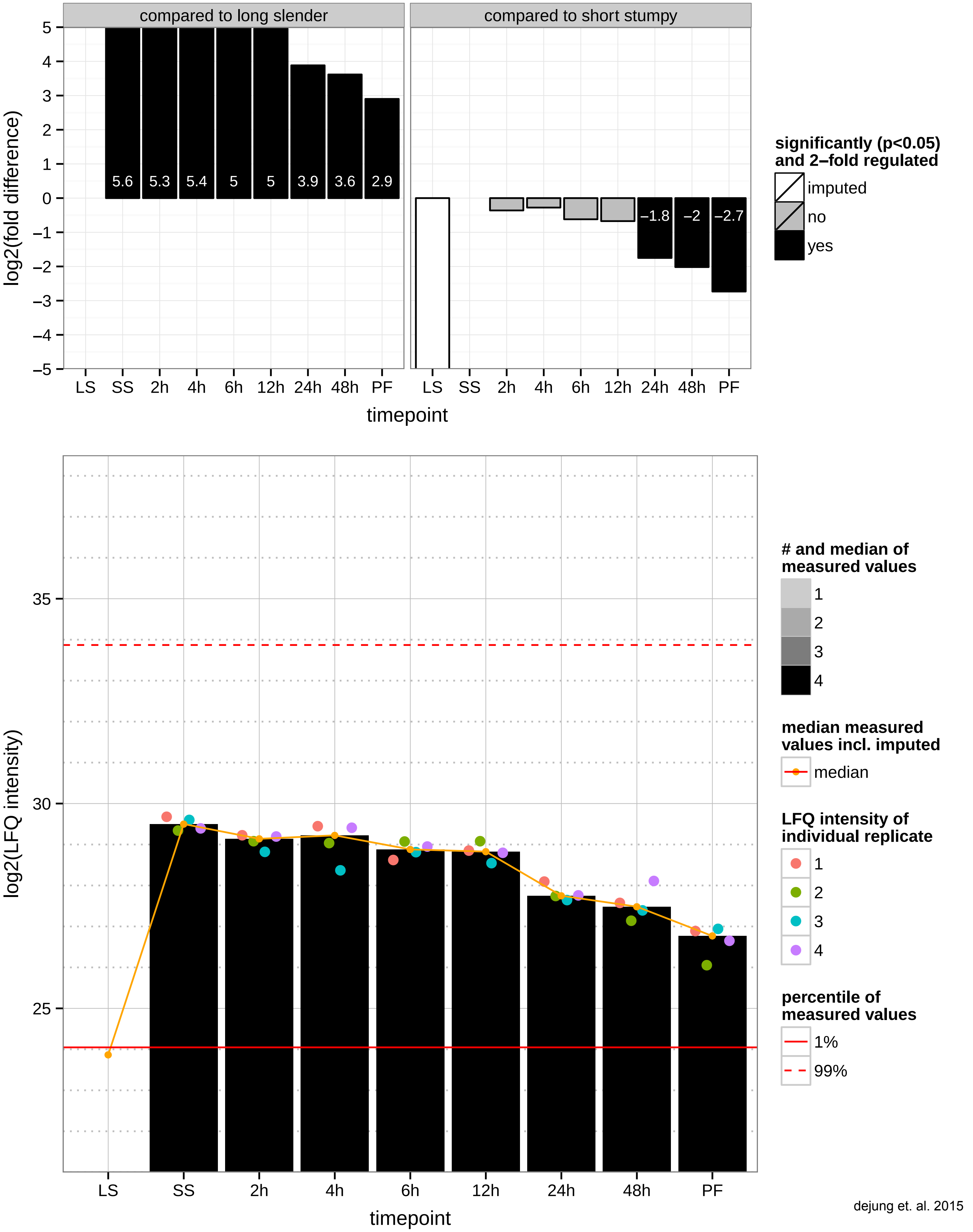

dejung et. al. 2015

hypothetical protein
Tb927.8.7970
AGOF: null
AGOC: null
AGOP: null
PGOF: null
PGOC: null
PGOP: null

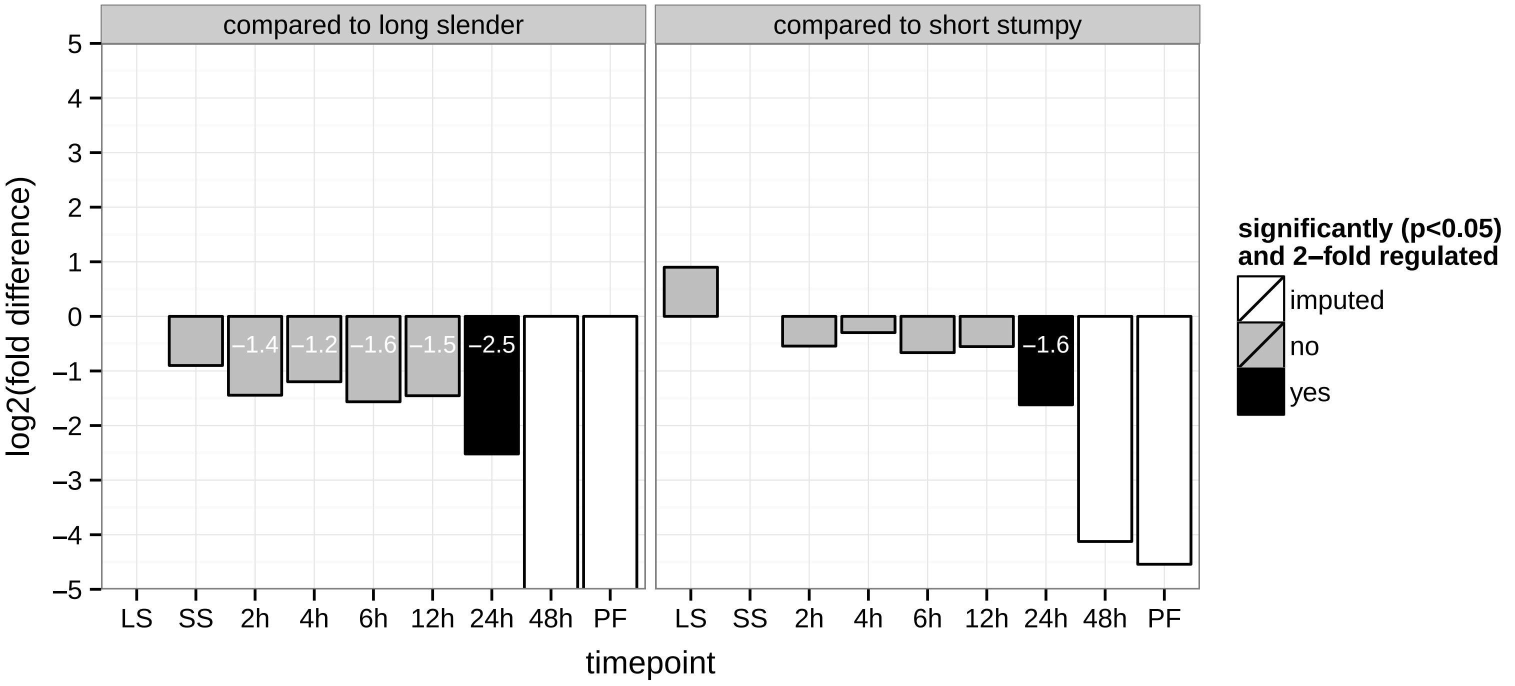

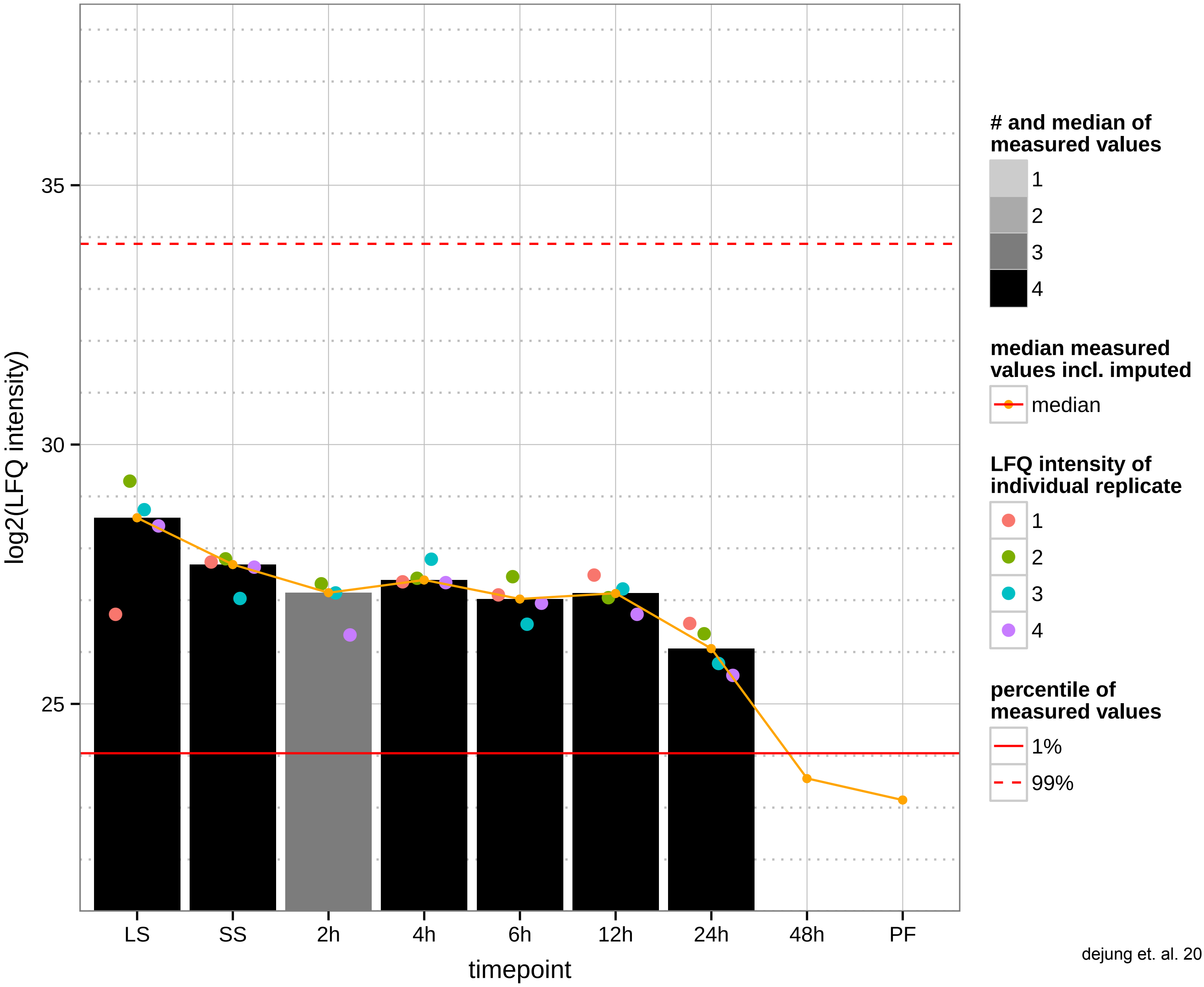

dejung et. al. 2015

small GTP–binding rab protein, putative
Tb927.8.8140
AGOF: GTP binding
AGOC: intracellular
AGOP: protein transport, small GTPase mediated signal transduction
PGOF: GTP binding, GTPase activity
PGOC: intracellular, membrane
PGOP: GTP catabolic process, protein transport, signal transduction, small GTPase mediated signal transduction

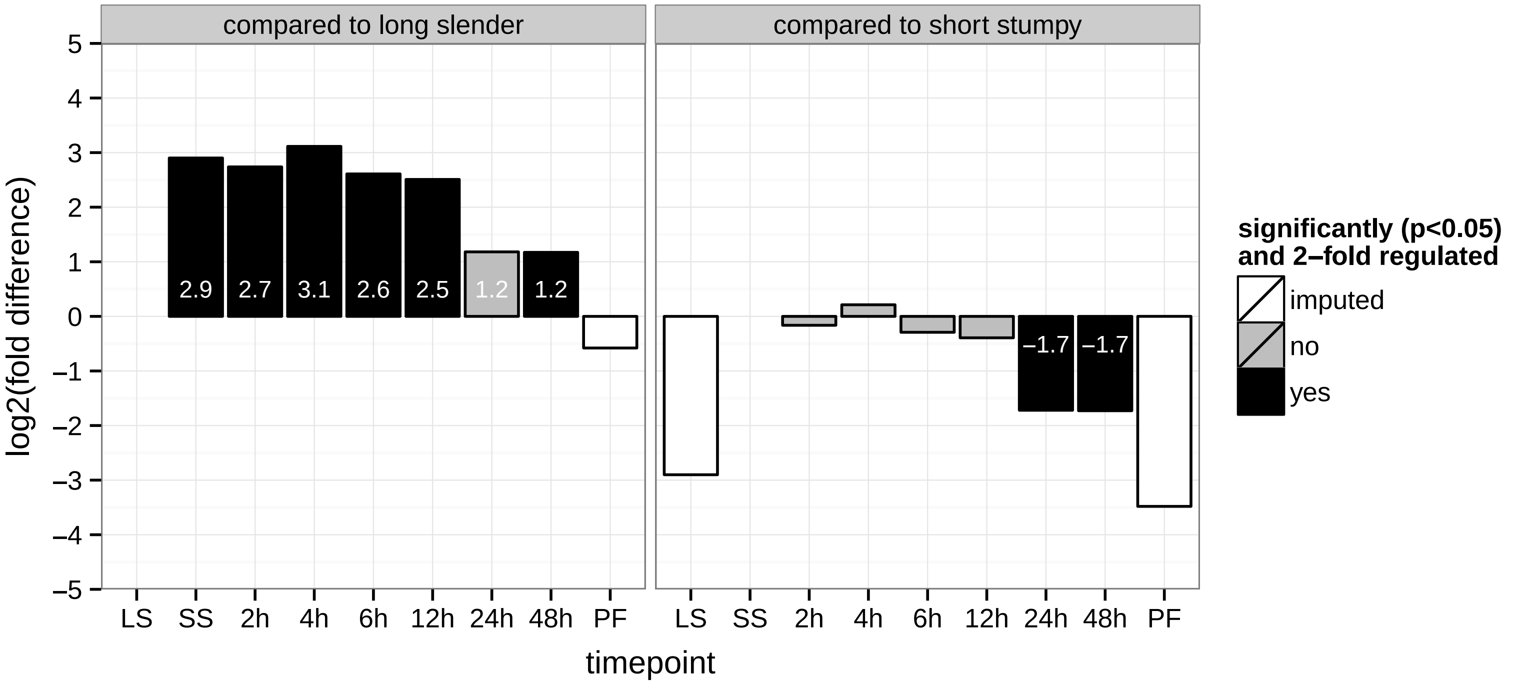

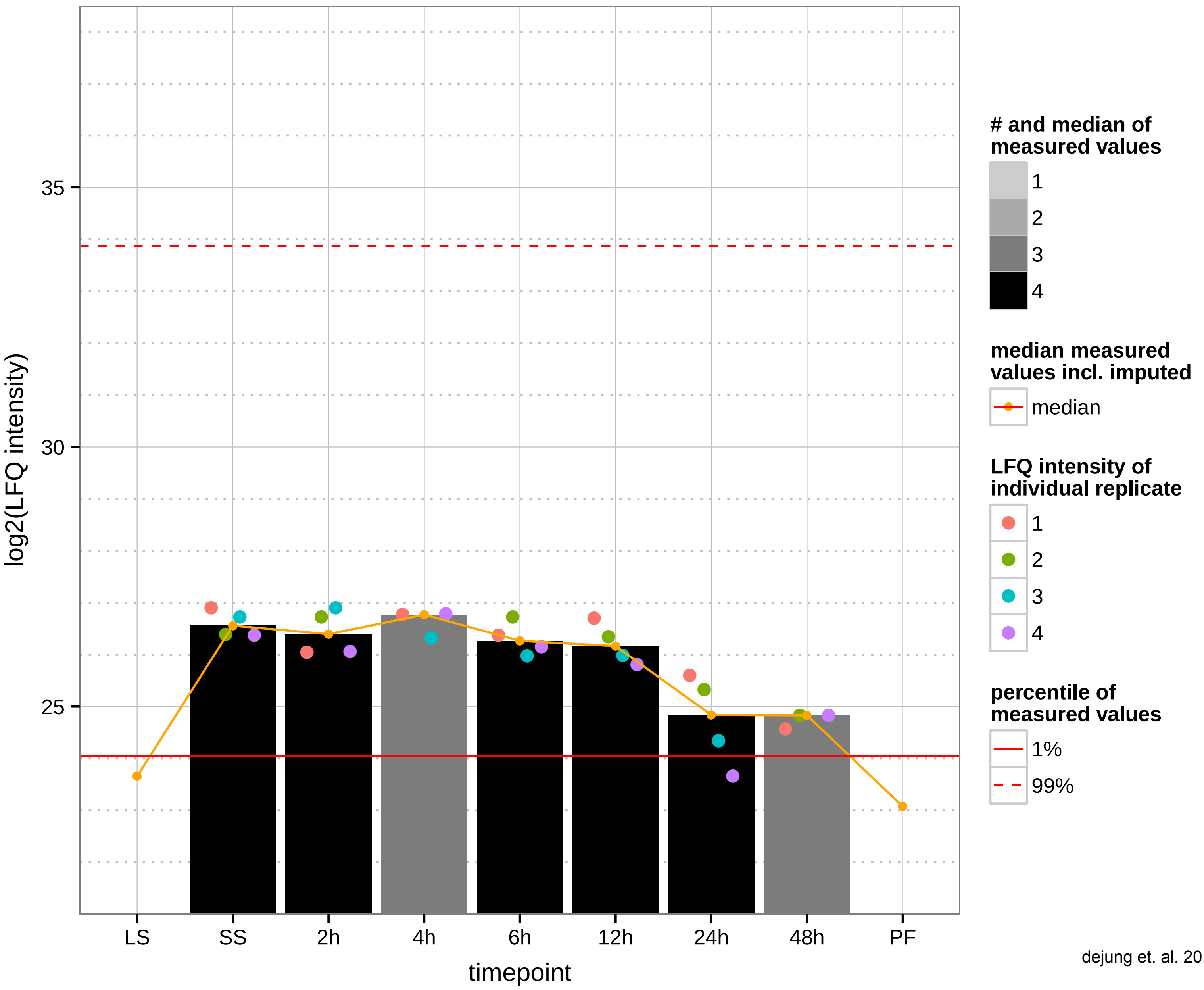

dejung et. al. 2015

hypothetical protein, conserved
Tb927.8.8150
AGOF: null
AGOC: null
AGOP: null
PGOF: protein binding
PGOC: null
PGOP: null

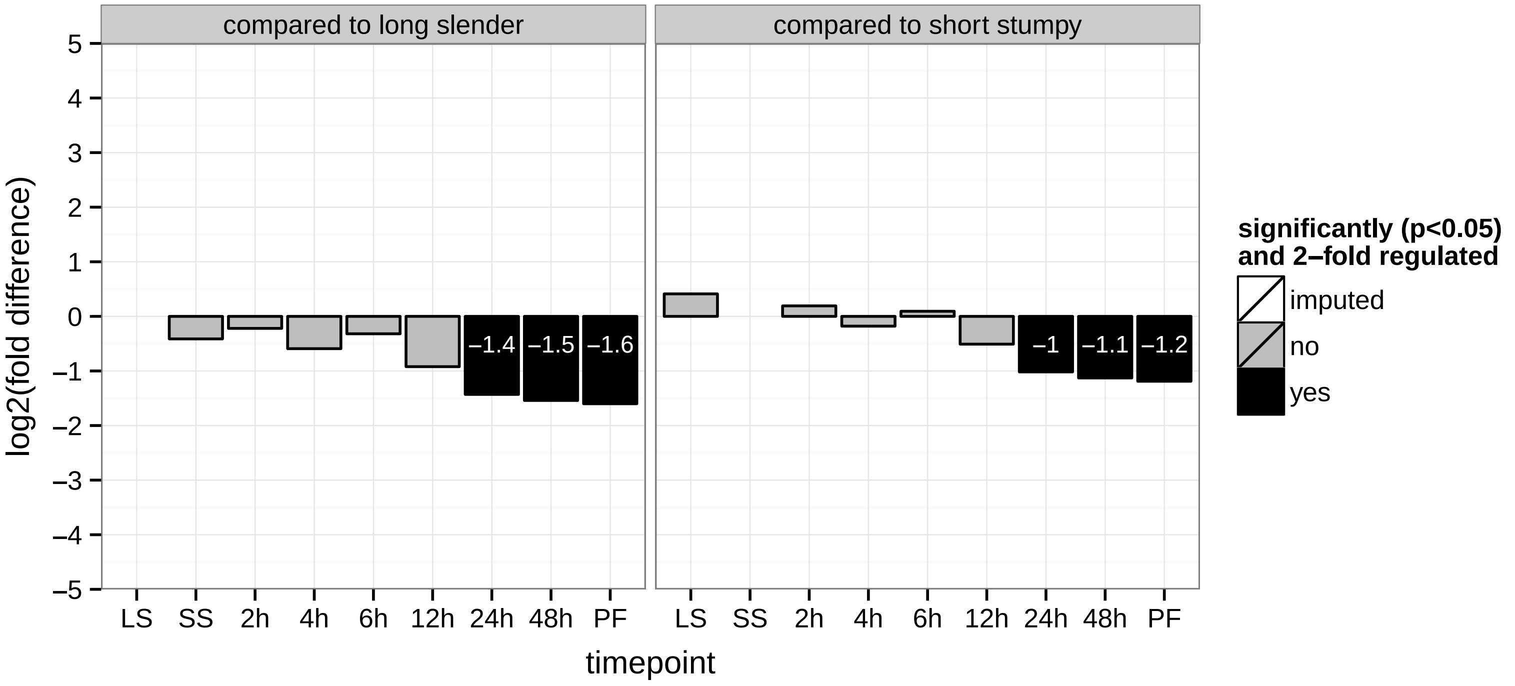

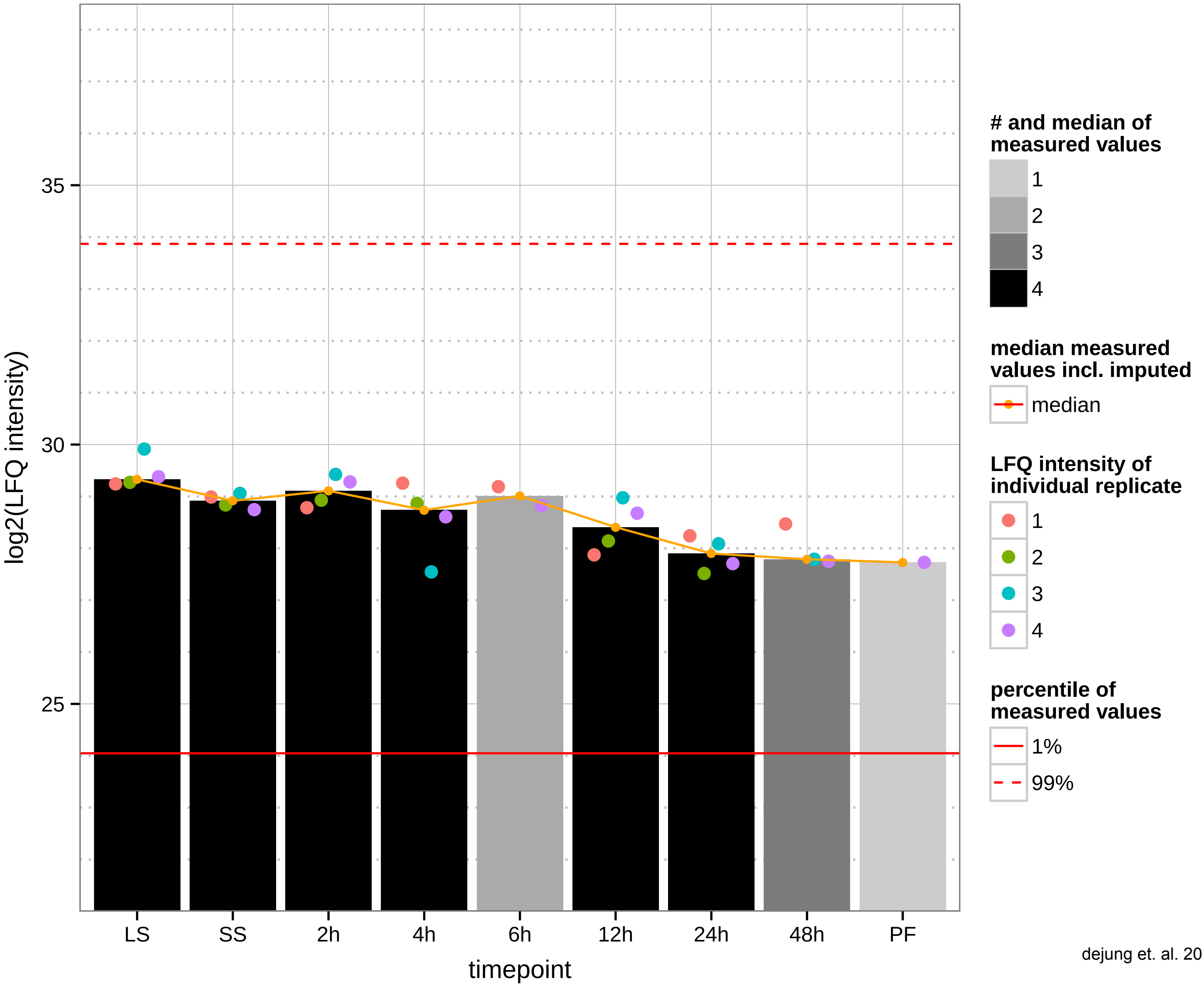

dejung et. al. 2015

amino acid transporter, putative
Tb927.8.8230
AGOF: amine transmembrane transporter activity
AGOC: integral to membrane
AGOP: amino acid transport
PGOF: null
PGOC: null
PGOP: null

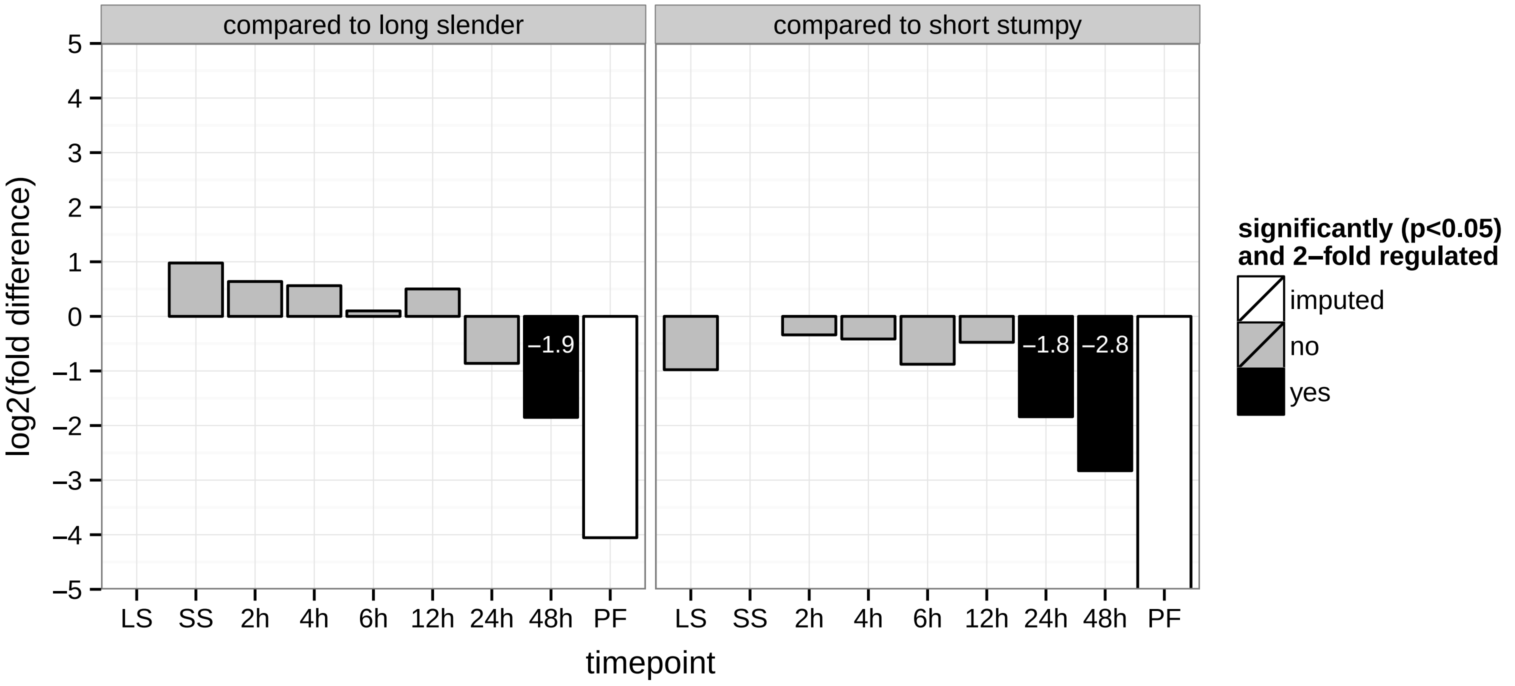

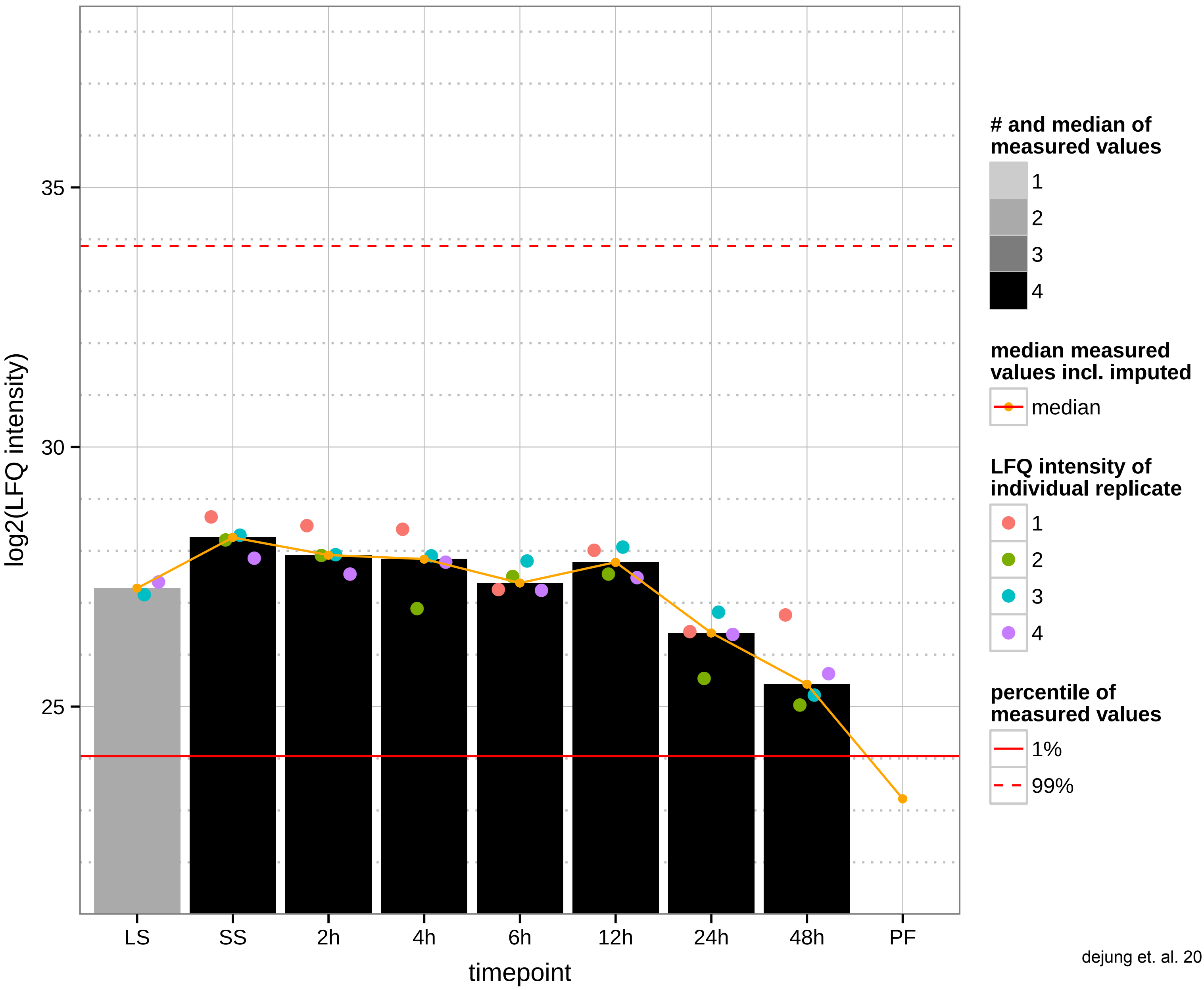

dejung et. al. 2015

hypothetical protein, conserved
Tb927.8.8280
AGOF: null
AGOC: null
AGOP: null
PGOF: null
PGOC: null
PGOP: null

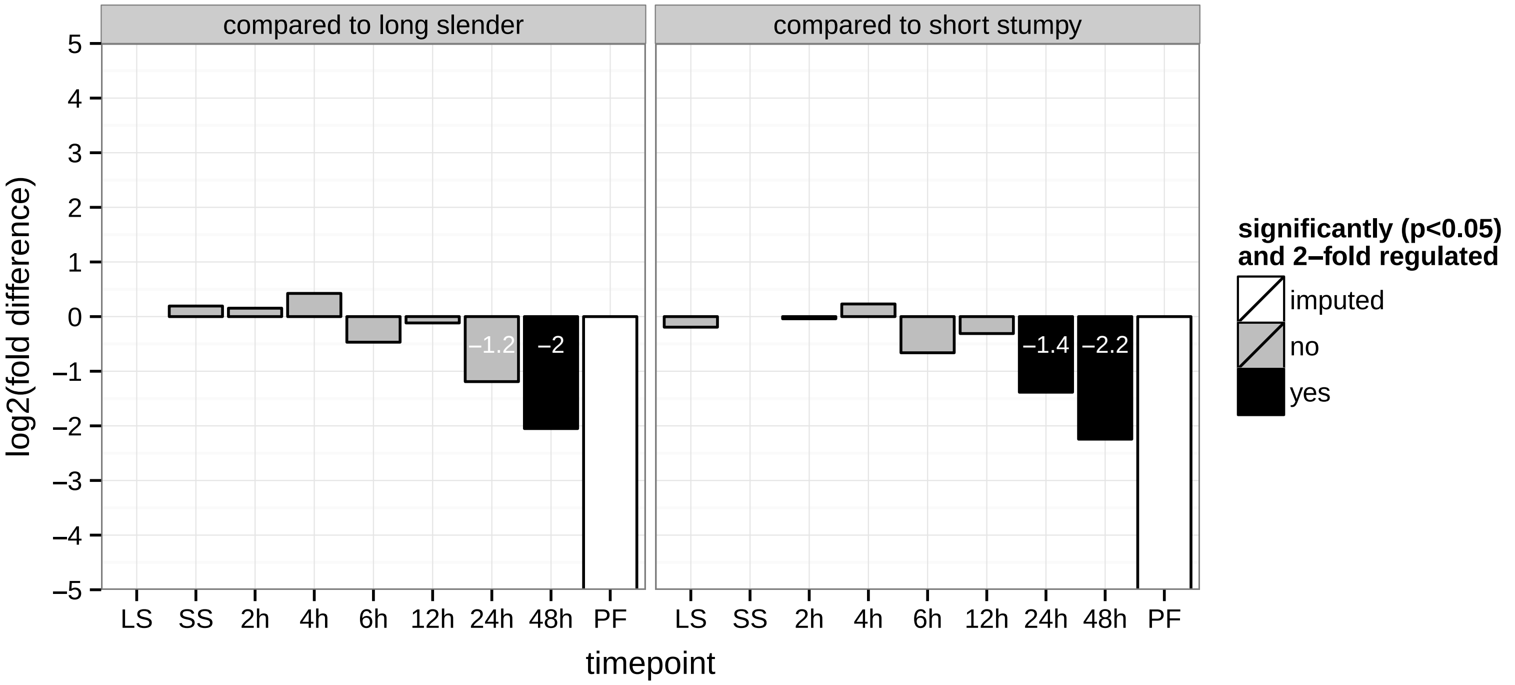

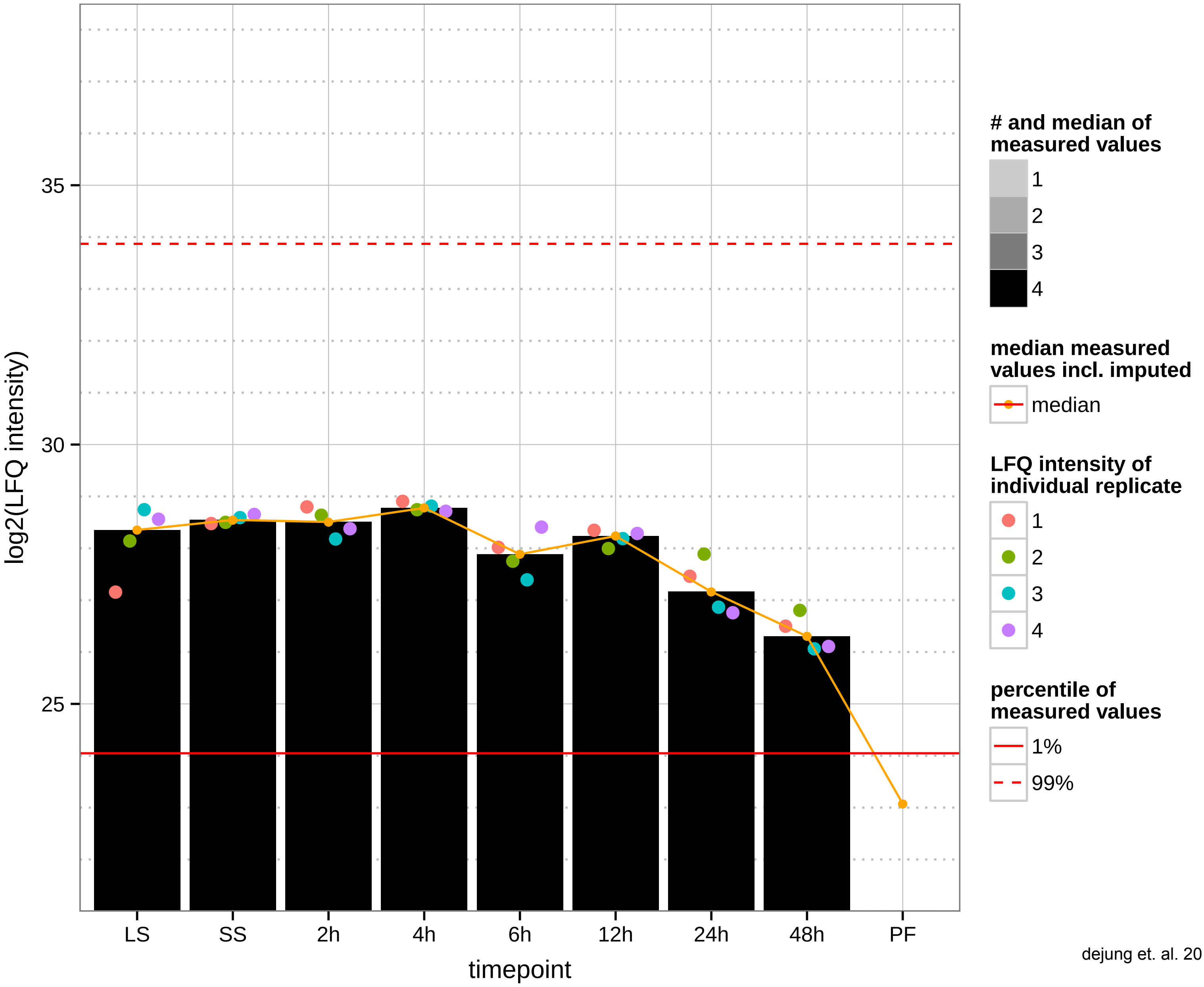

dejung et. al. 2015

glycerol kinase, glycosomal (glk1)
Tb927.9.12630;Tb927.9.12590;Tb927.9.12570;Tb927.9.12550;Tb927.9.12610
AGOF: ATP binding, glycerol kinase activity
AGOC: glycosome
AGOP: carbohydrate metabolic process, glycerol–3–phosphate metabolic process
PGOF: glycerol kinase activity, phosphotransferase activity, alcohol group as acceptor
PGOC: null
PGOP: carbohydrate metabolic process, glycerol–3–phosphate metabolic process

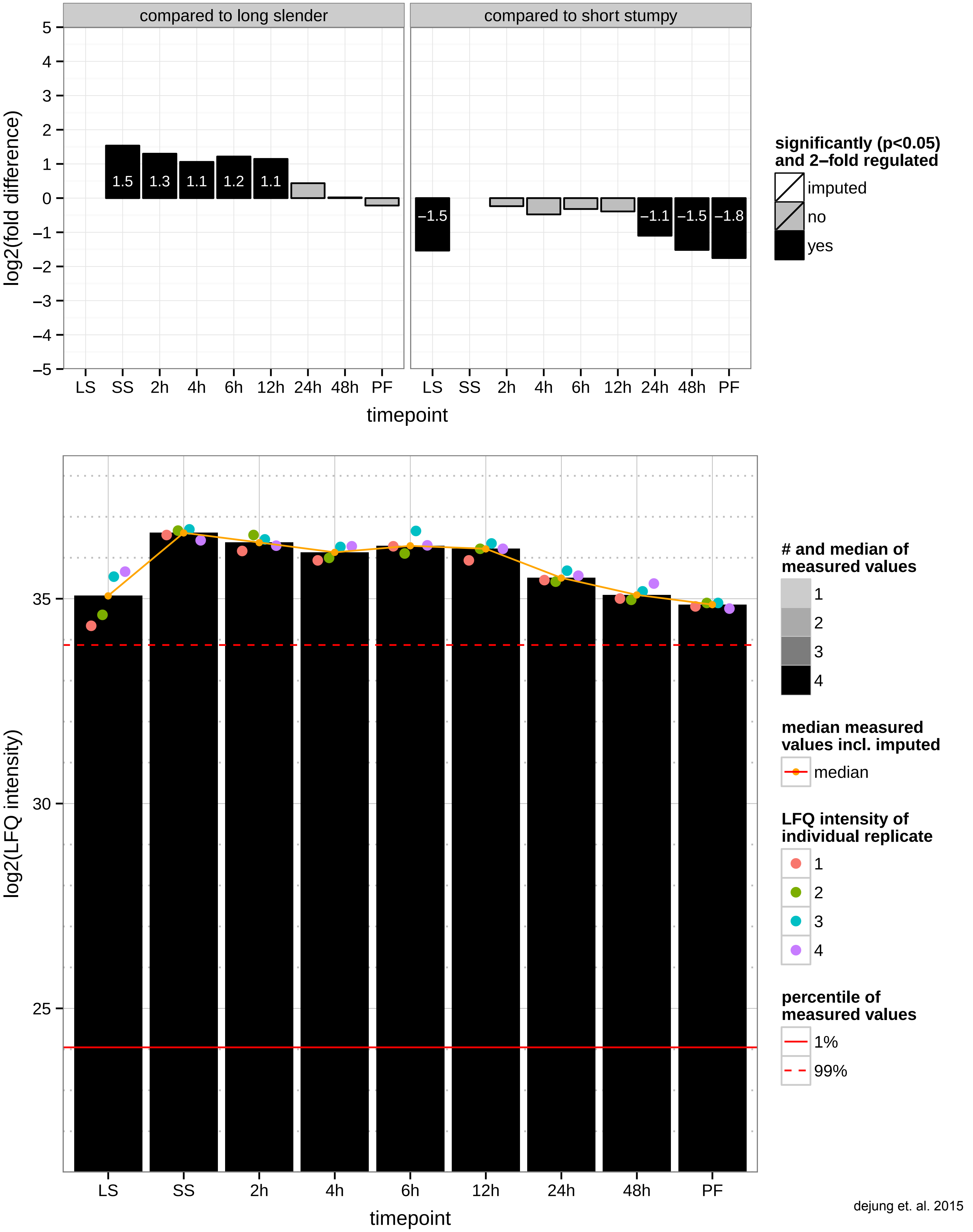

dejung et. al. 2015

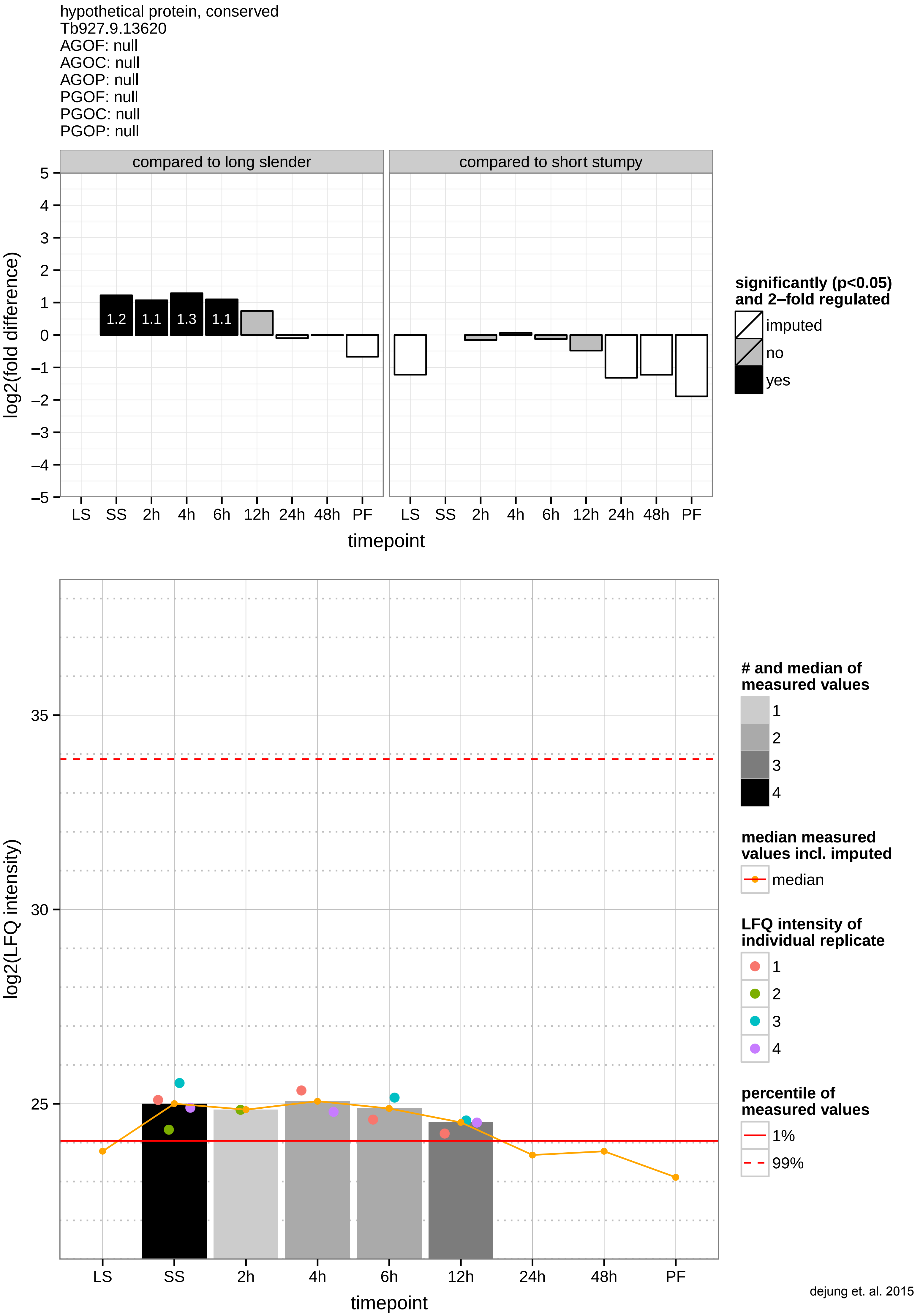
hypothetical protein, conserved
Tb927.9.13620
AGOF: null
AGOC: null
AGOP: null
PGOF: null
PGOC: null
PGOP: null
compared to long slender
compared to short stumpy
log2(fold difference)
1.2
1.1
1.3
1.1
LS
SS
2h
4h
6h
12h
24h
48h
PF
timepoint
significantly (p<0.05) and 2–fold regulated
imputed
no
yes
log2(LFQ intensity)
# and median of measured values
1
2
3
4
median measured values incl. imputed
median
LFQ intensity of individual replicate
1
2
3
4
percentile of measured values
1%
99%
dejung et. al. 2015

ubiquitin carboxyl–terminal hydrolase, putative, cysteine peptidase, Clan CA, family C19
Tb927.9.14470
AGOF: cysteine–type endopeptidase activity, ubiquitin thiolesterase activity
AGOC: null
AGOP: ubiquitin–dependent protein catabolic process
PGOF: ubiquitin thiolesterase activity
PGOC: null
PGOP: ubiquitin–dependent protein catabolic process

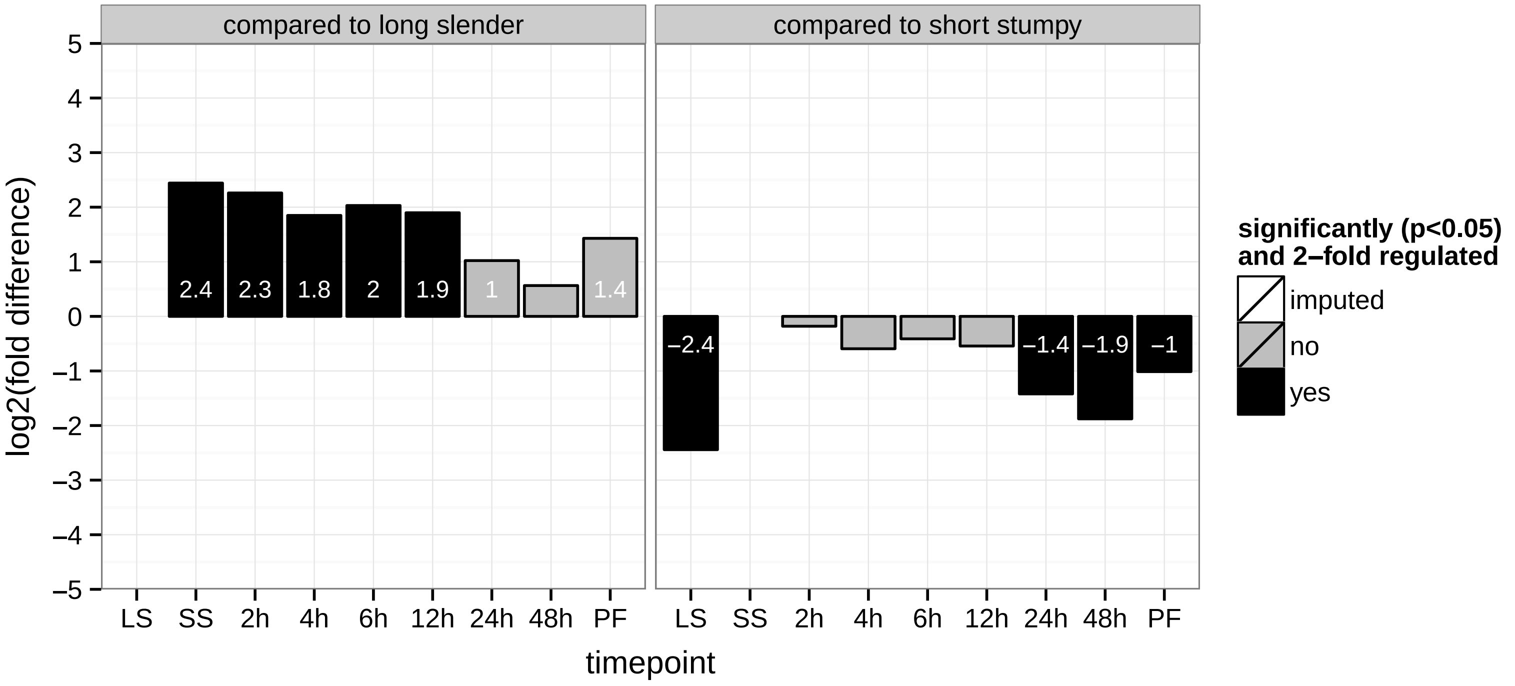

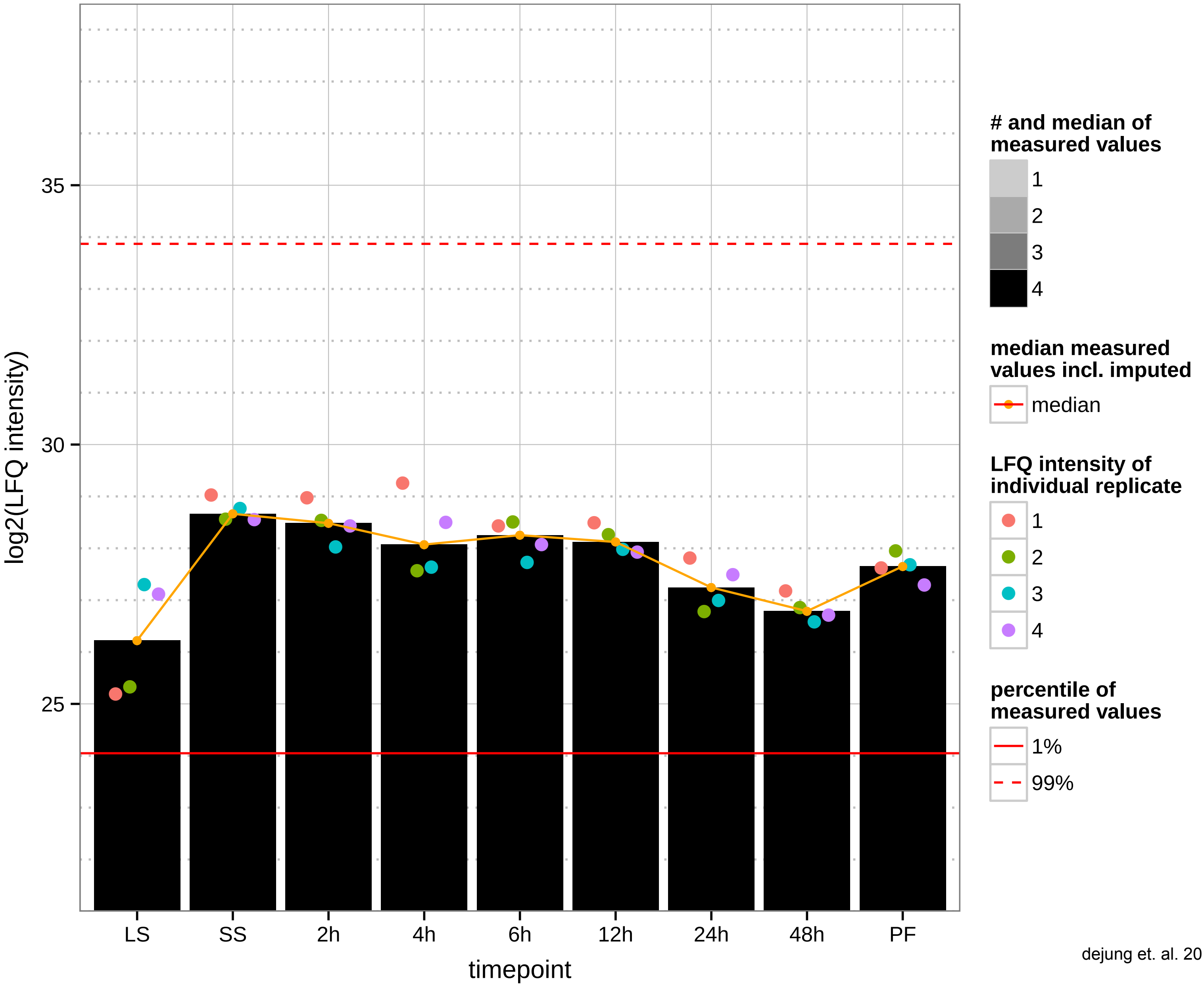

dejung et. al. 2015

hypothetical protein, conserved
Tb927.9.1600
AGOF: null
AGOC: null
AGOP: null
PGOF: null
PGOC: null
PGOP: null

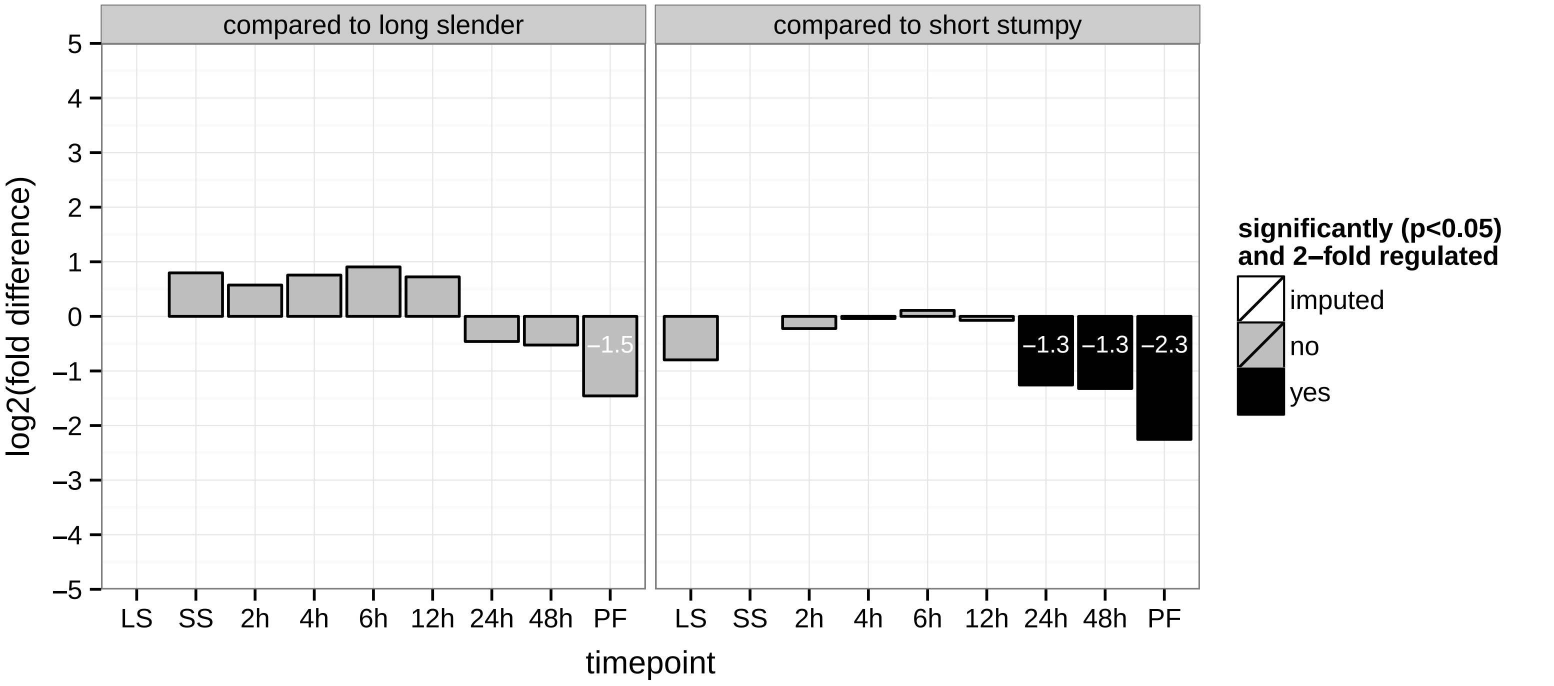

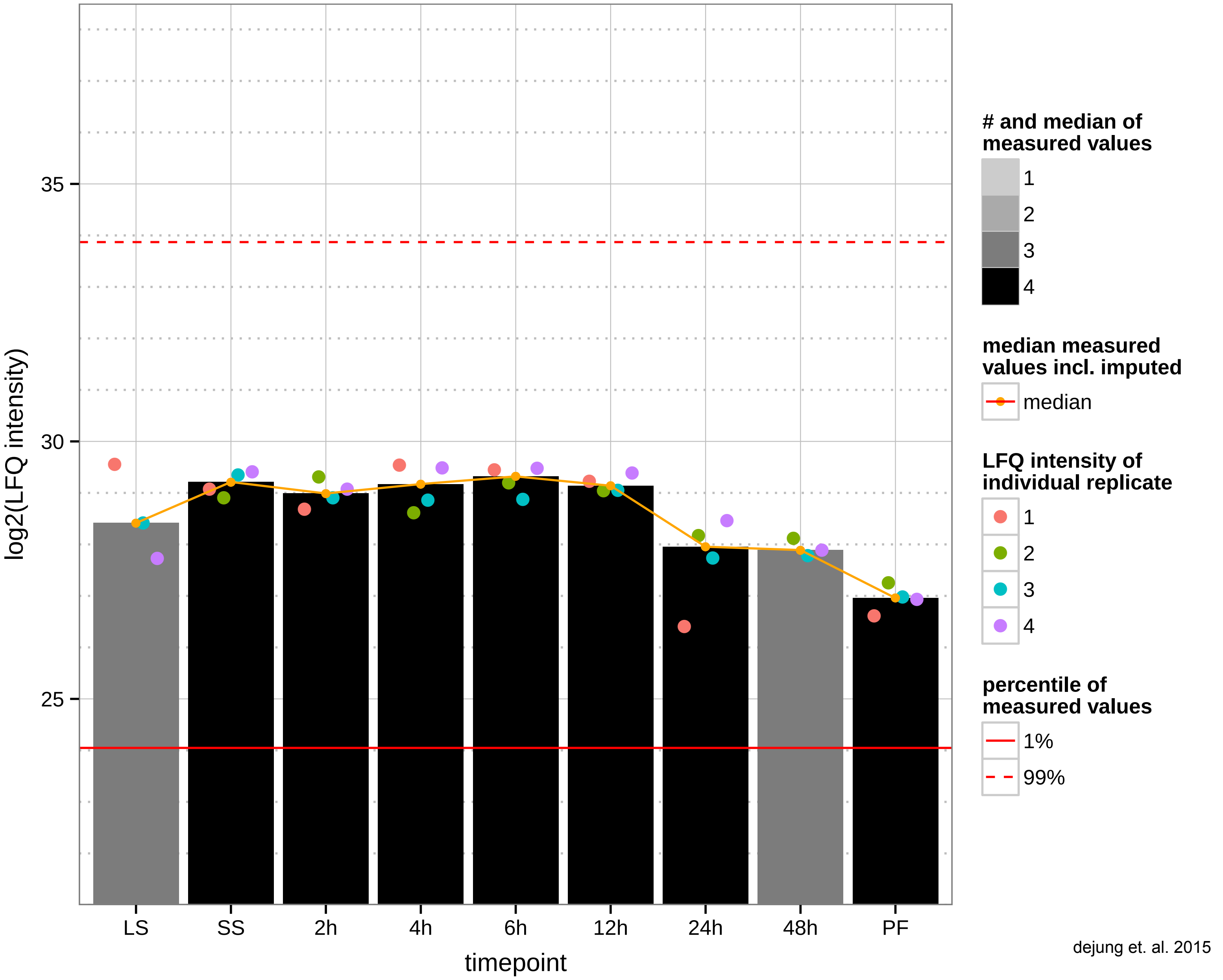

dejung et. al. 2015

syntaxin binding protein 1, putative
Tb927.9.1970
AGOF: SNARE binding, protein transporter activity
AGOC: null
AGOP: vesicle docking involved in exocytosis, vesicle–mediated transport
PGOF: null
PGOC: null
PGOP: vesicle docking involved in exocytosis, vesicle–mediated transport

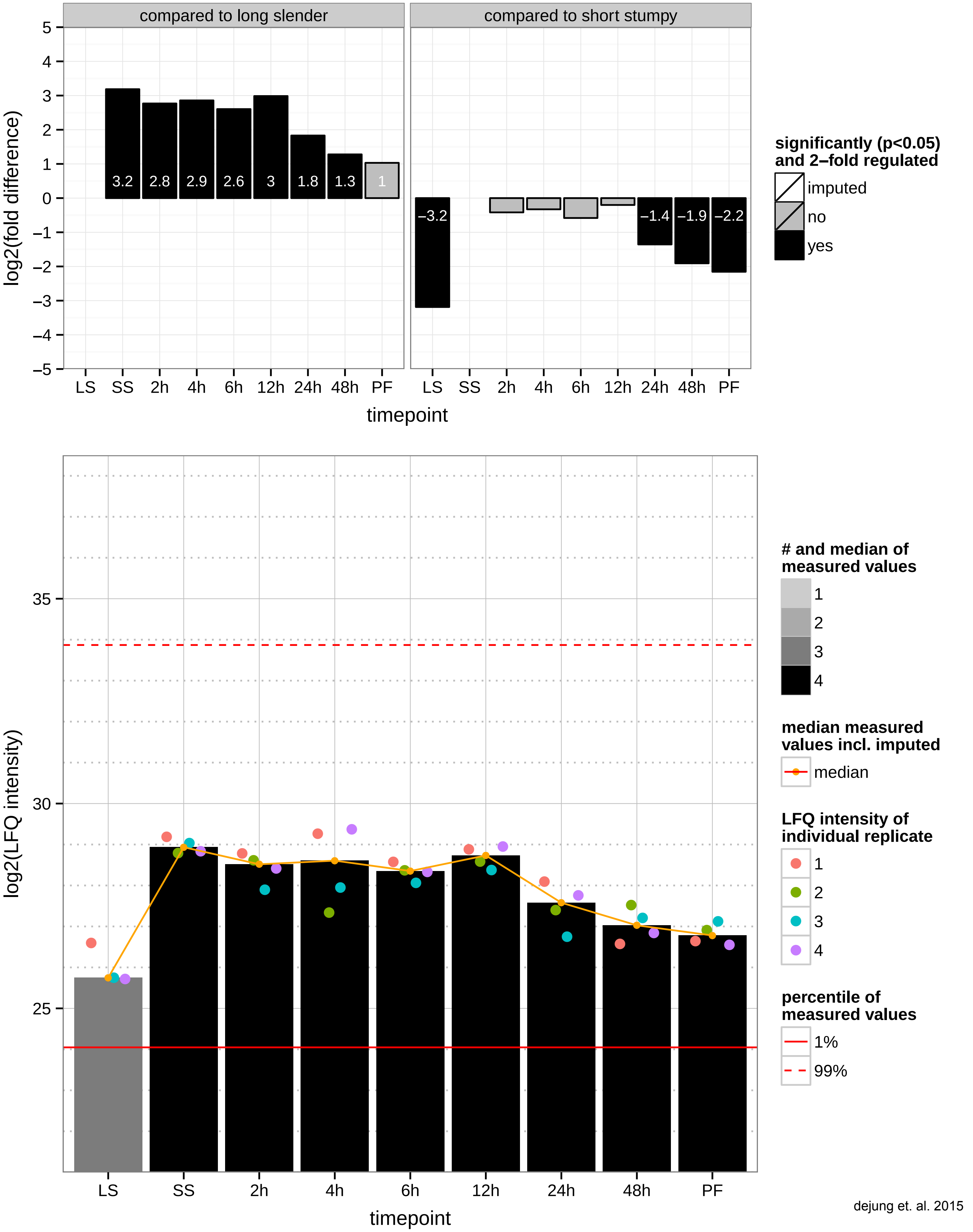

dejung et. al. 2015

hypothetical protein, conserved
Tb927.9.2290
AGOF: null
AGOC: null
AGOP: null
PGOF: null
PGOC: null
PGOP: null

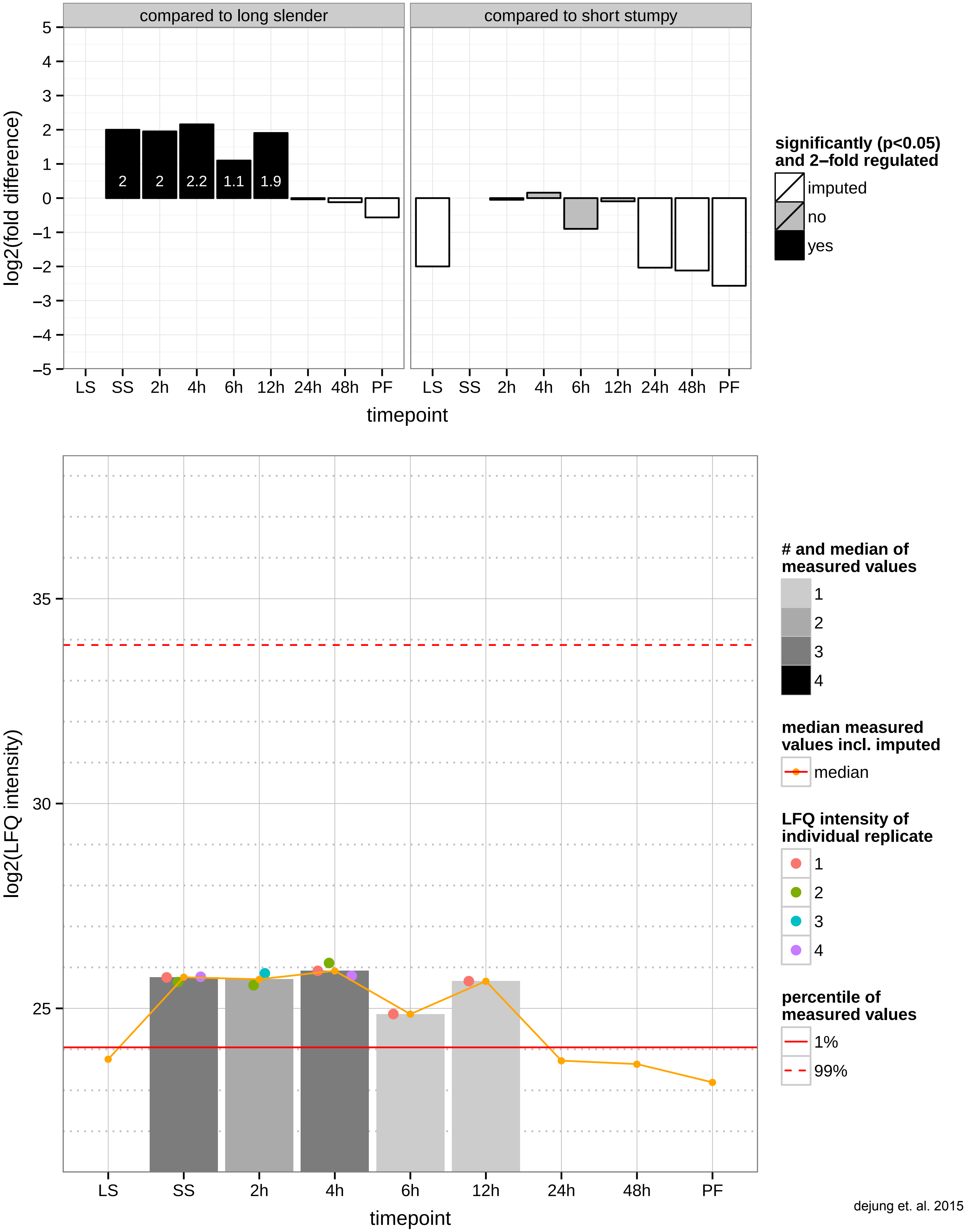

syntaxin, putative
Tb927.9.3820
AGOF: transporter activity
AGOC: membrane
AGOP: vesicle−mediated transport
PGOF: protein binding
PGOC: null
PGOP: null

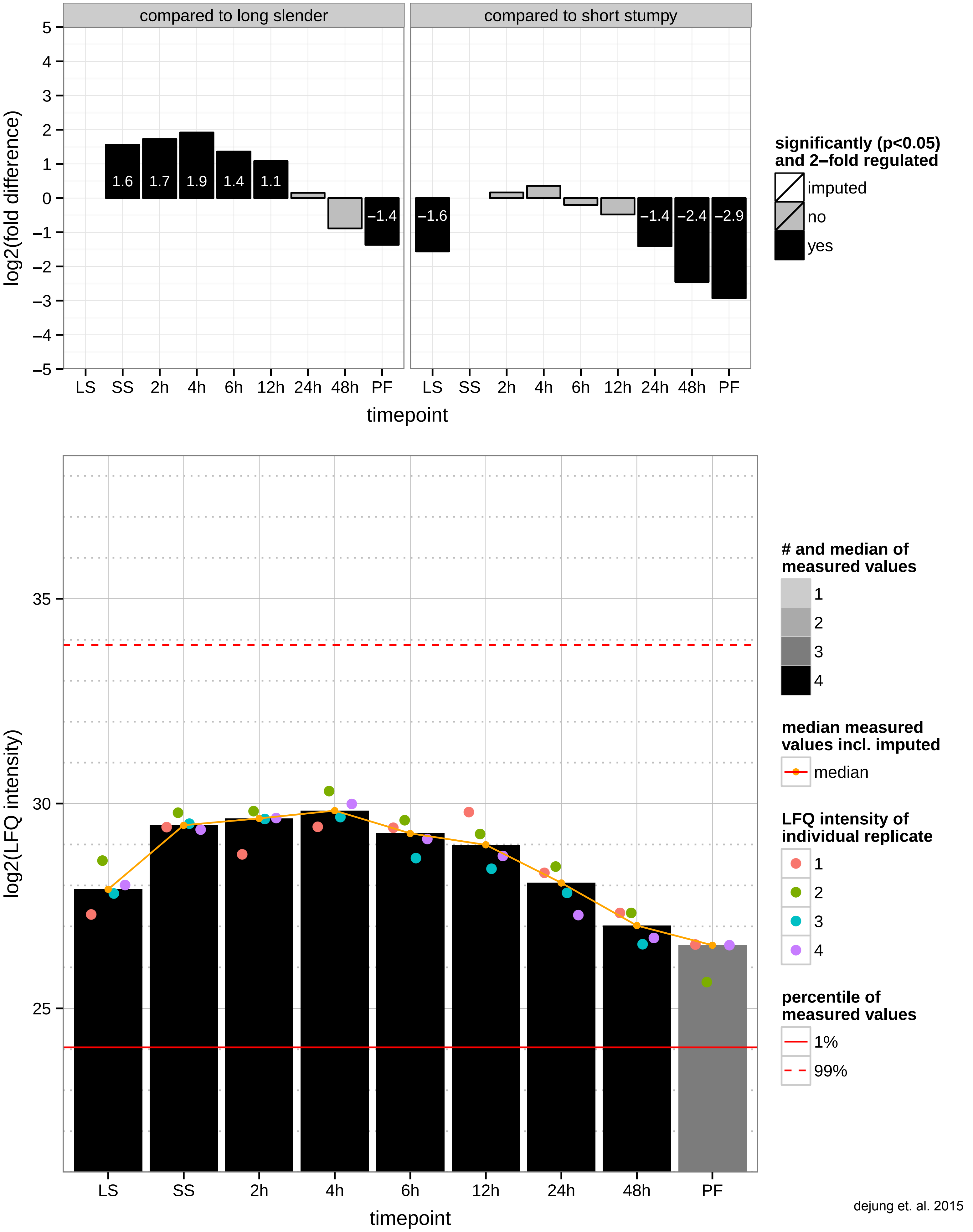

dejung et. al. 2015

hypothetical protein, conserved
Tb927.9.7760
AGOF: null
AGOC: null
AGOP: null
PGOF: null
PGOC: null
PGOP: null

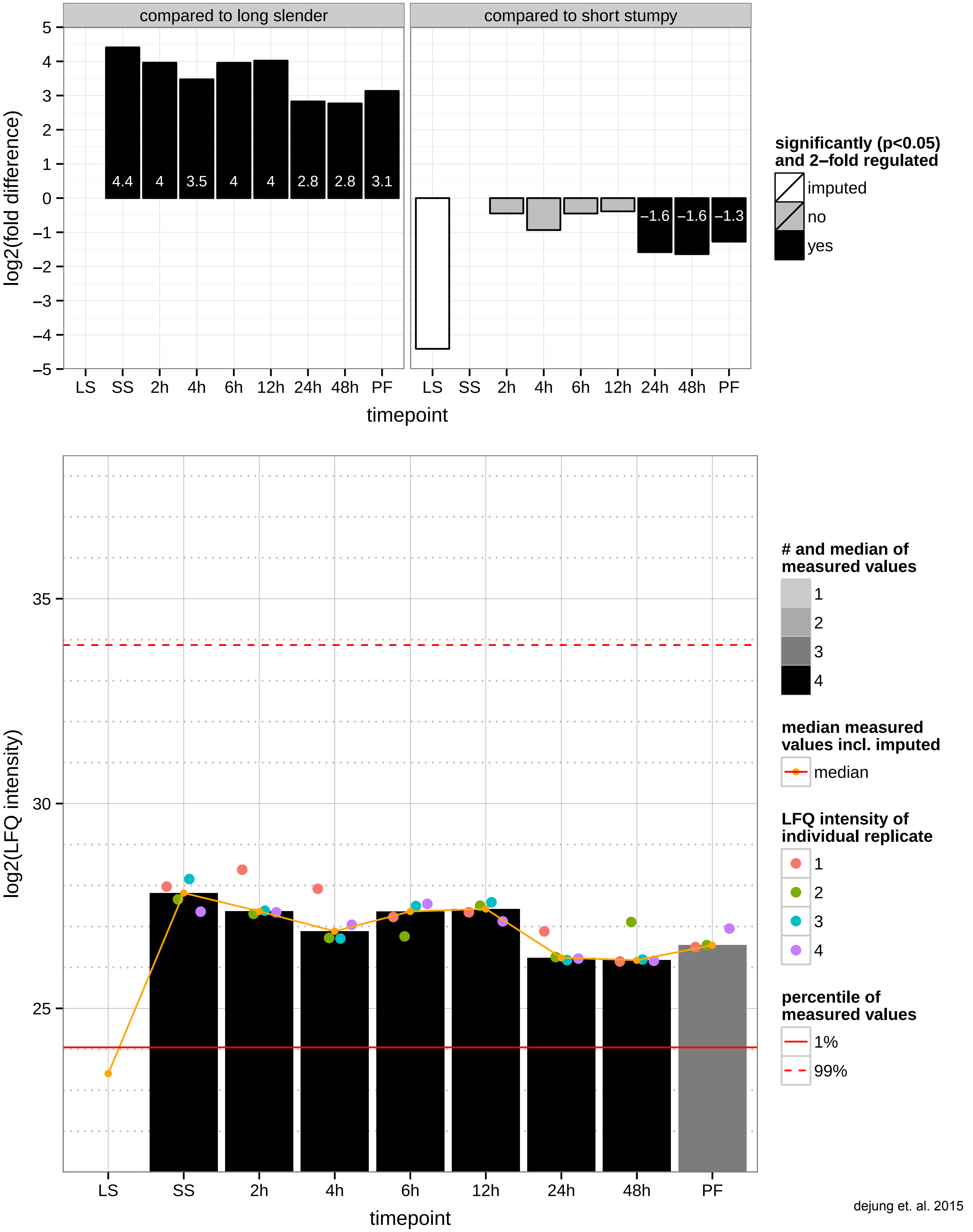

dejung et. al. 2015

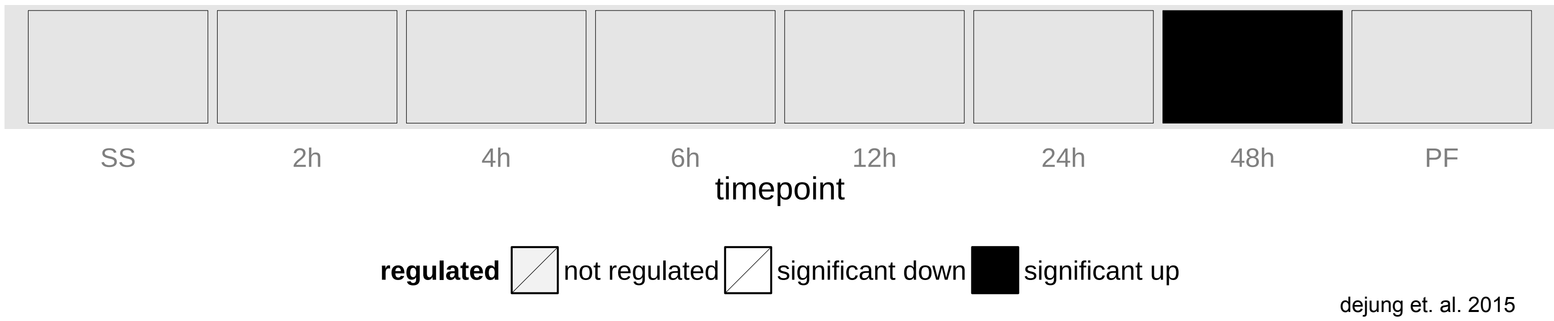
SS
2h
4h
6h
12h
24h
48h
PF
timepoint
regulated
not regulated
significant down
significant up
dejung et. al. 2015

hypothetical protein, conserved
Tb11.02.5430b;Tb11.02.5430
AGOF: null
AGOC: null
AGOP: null
PGOF: null
PGOC: null
PGOP: null

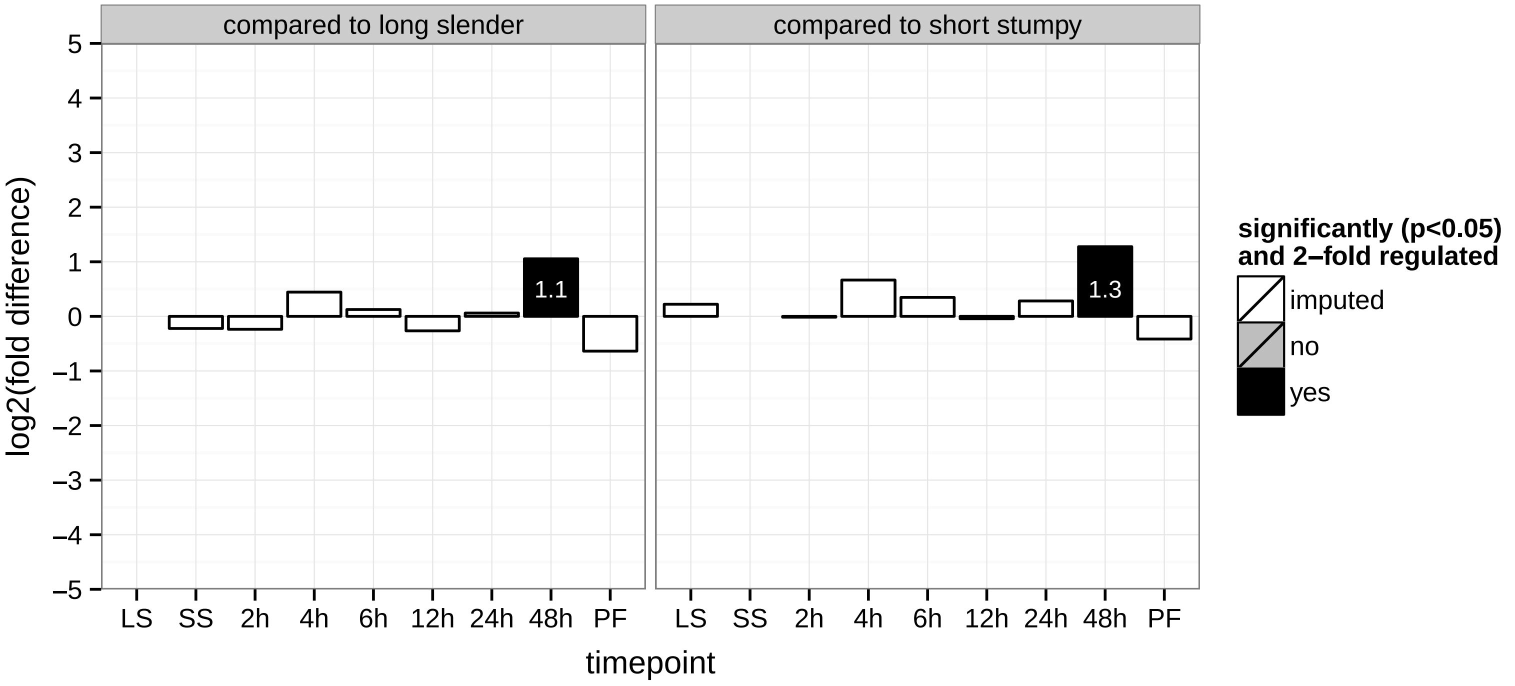

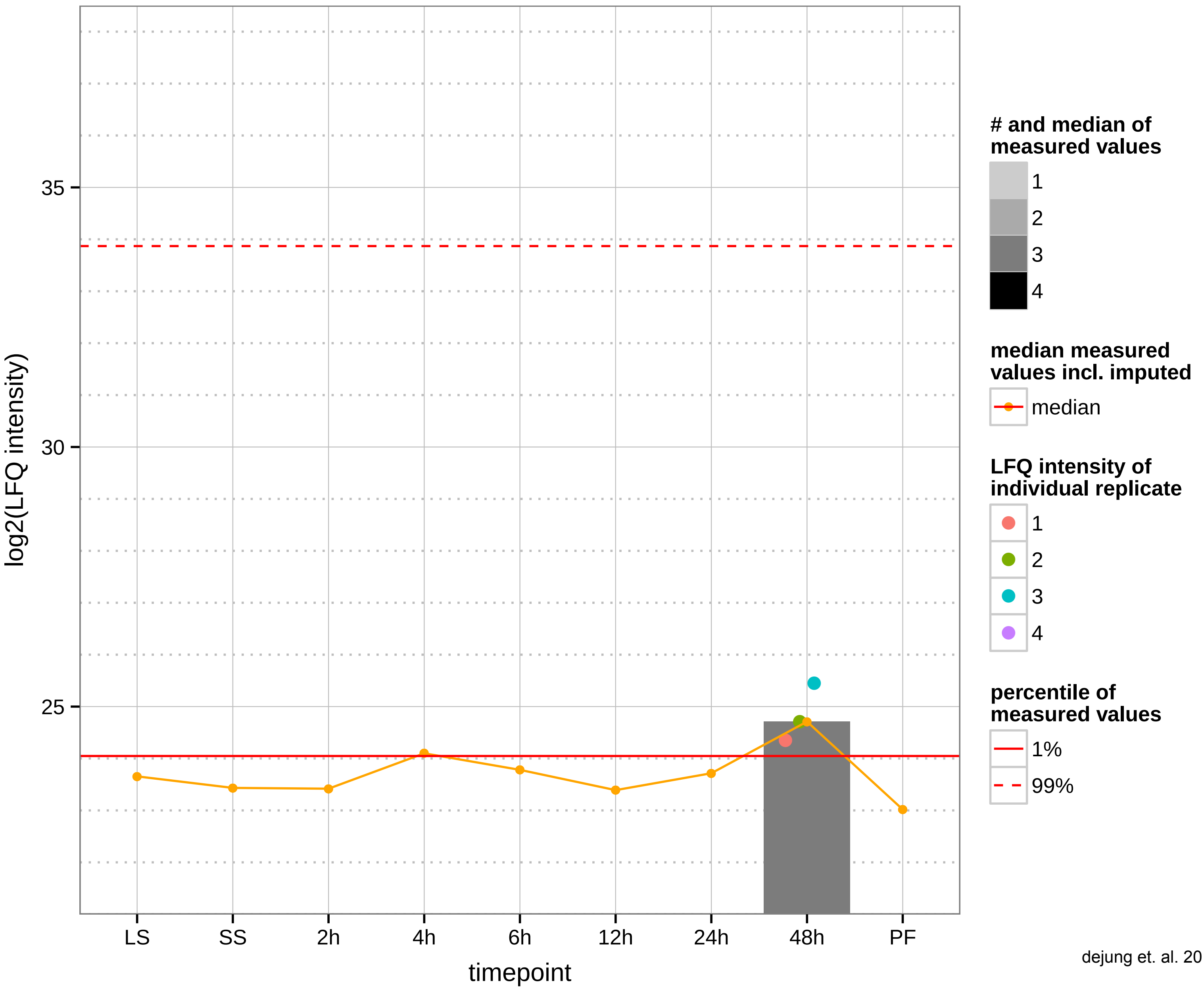

dejung et. al. 2015

60S ribosomal protein L19, putative
Tb927.7.5020;Tb927.7.5000;Tb11.v5.0182;Tb11.v5.0181
AGOF: null, structural constituent of ribosome
AGOC: null, intracellular, ribosome
AGOP: null, ribosome biogenesis, translation
PGOF: null, structural constituent of ribosome
PGOC: null, intracellular, ribosome
PGOP: null, translation

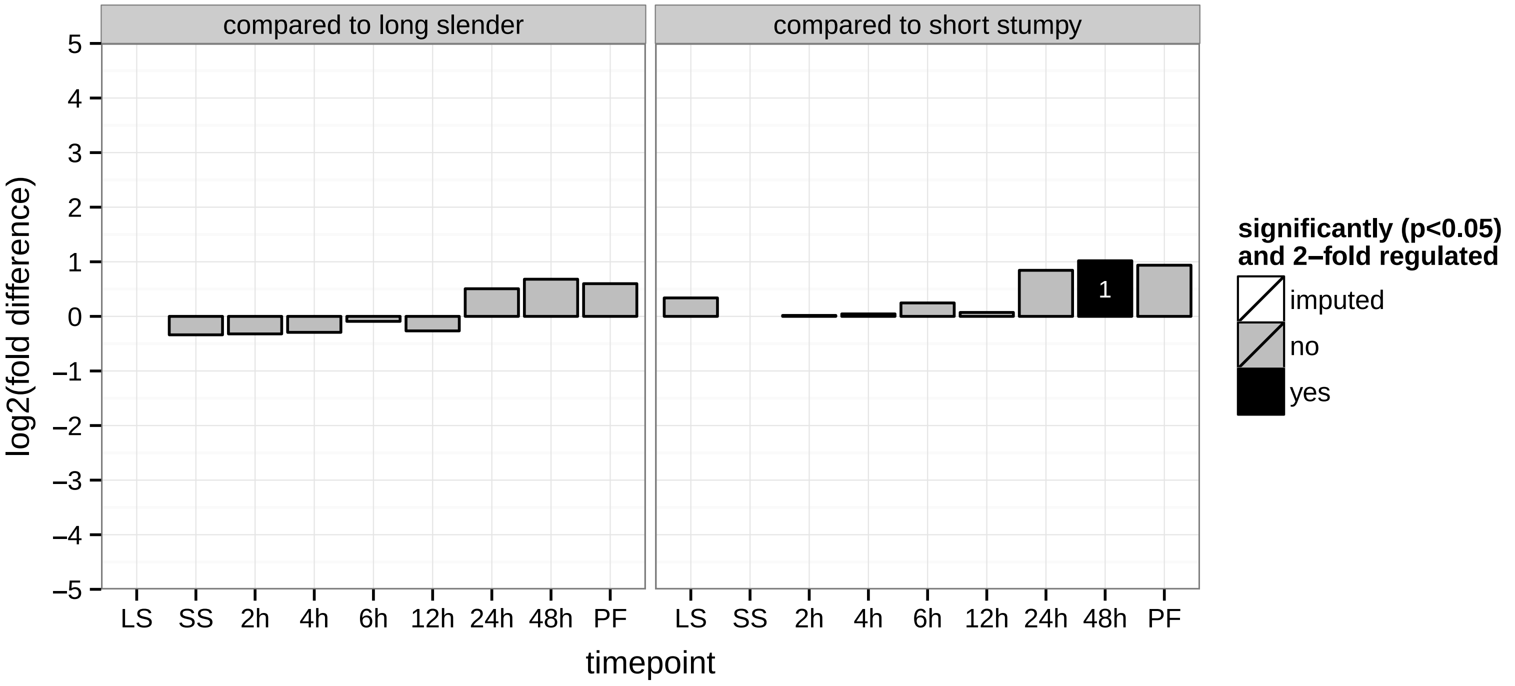

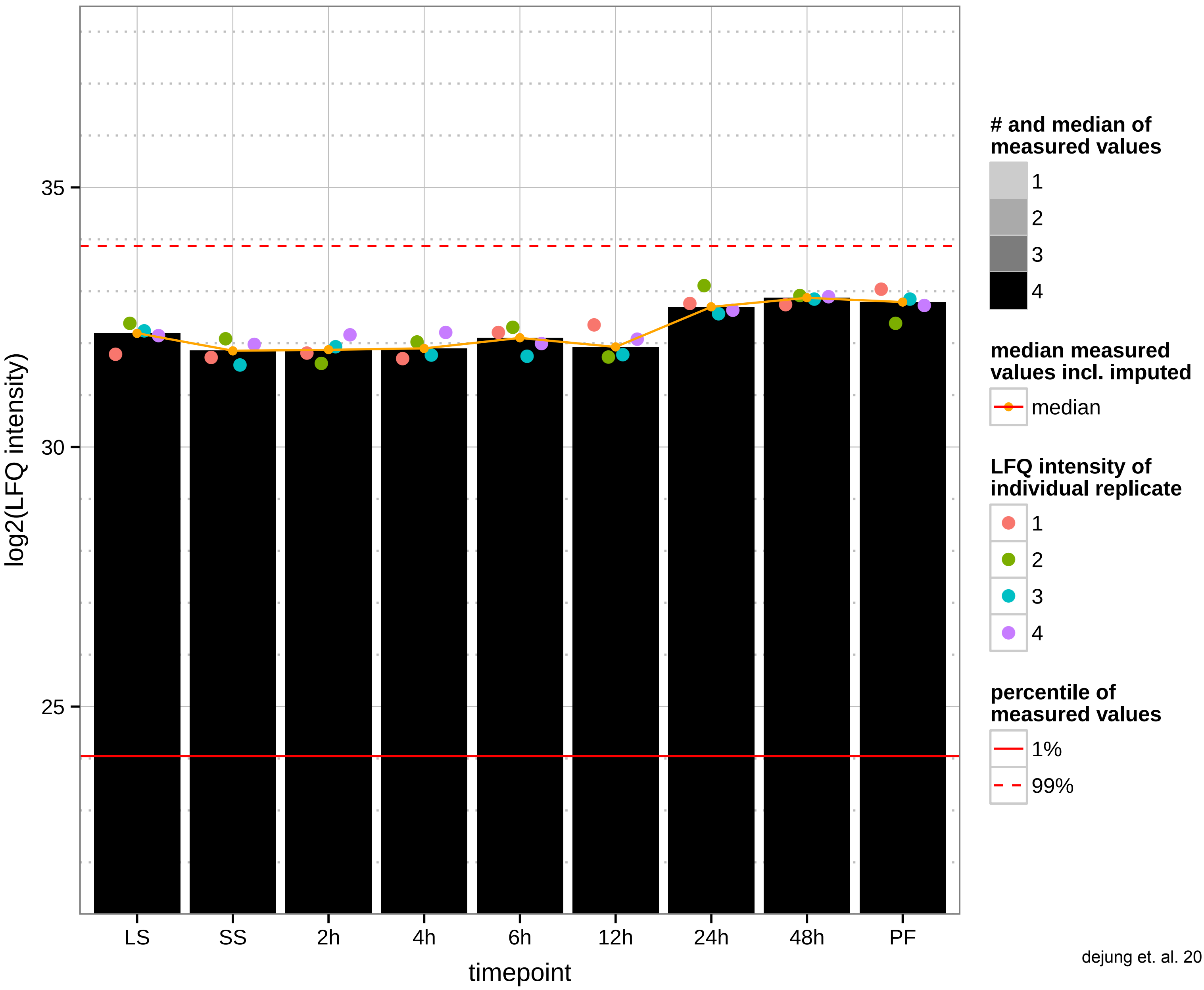

dejung et. al. 2015

40S ribosomal protein S18, putative, variant surface glycoprotein (VSG), putative (RPS18)
Tb927.10.5340;Tb927.10.5330;Tb11.v5.1060;Tb11.v5.0243
AGOF: null, RNA binding, structural constituent of ribosome
AGOC: null, intracellular, organellar small ribosomal subunit, ribosome
AGOP: null, translation
PGOF: null, RNA binding, nucleic acid binding, structural constituent of ribosome
PGOC: null, intracellular, ribosome
PGOP: null, translation

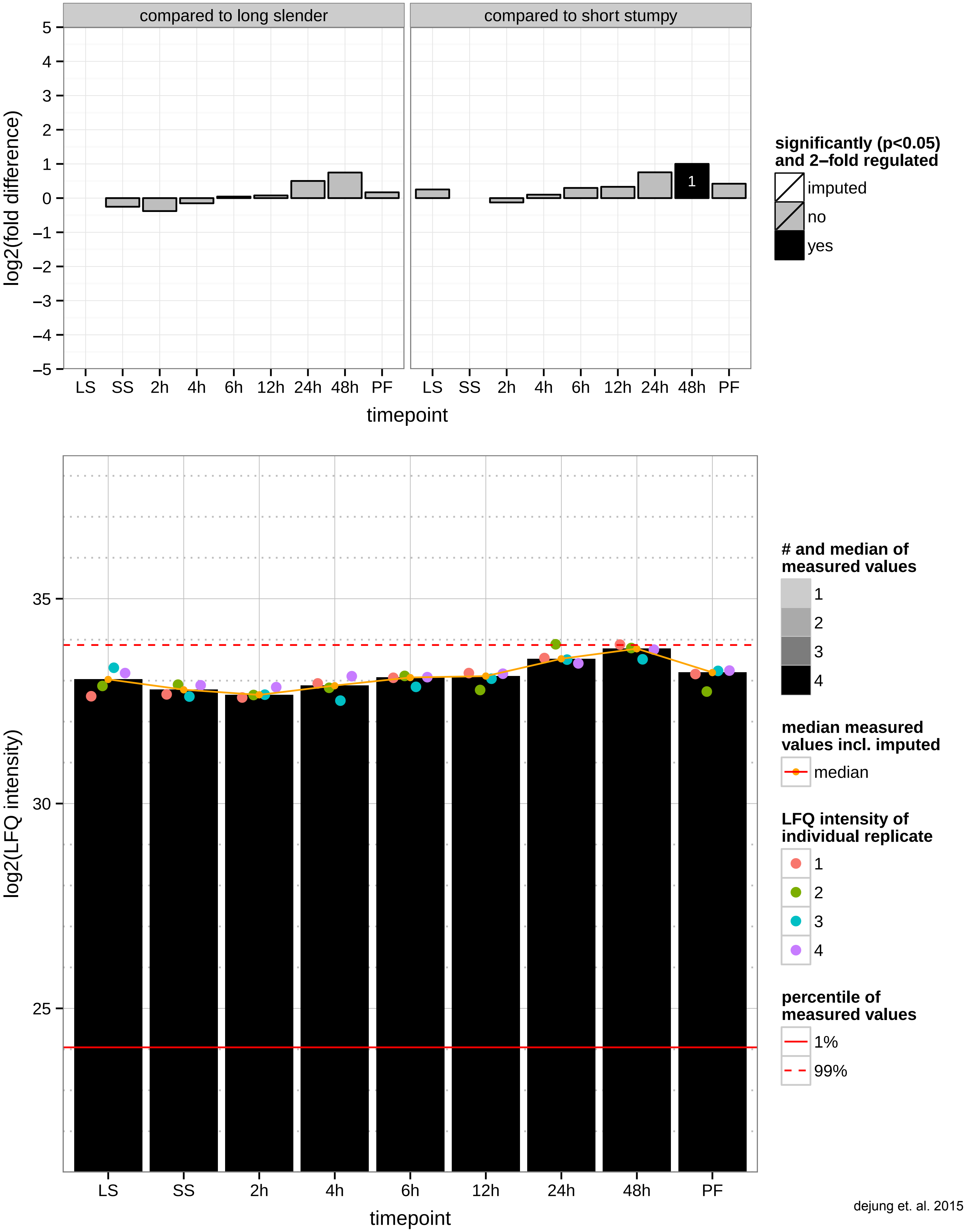

dejung et. al. 2015

periodic tryptophan protein 2, putative, Periodic tryptophan protein 2 homolog
Tb927.10.13270;Tb11.v5.0563
AGOF: null, snoRNA binding
AGOC: null, Pwp2p−containing subcomplex of 90S preribosome, small−subunit processome
AGOP: null, cytokinesis, completion of separation
PGOF: protein binding
PGOC: null
PGOP: null

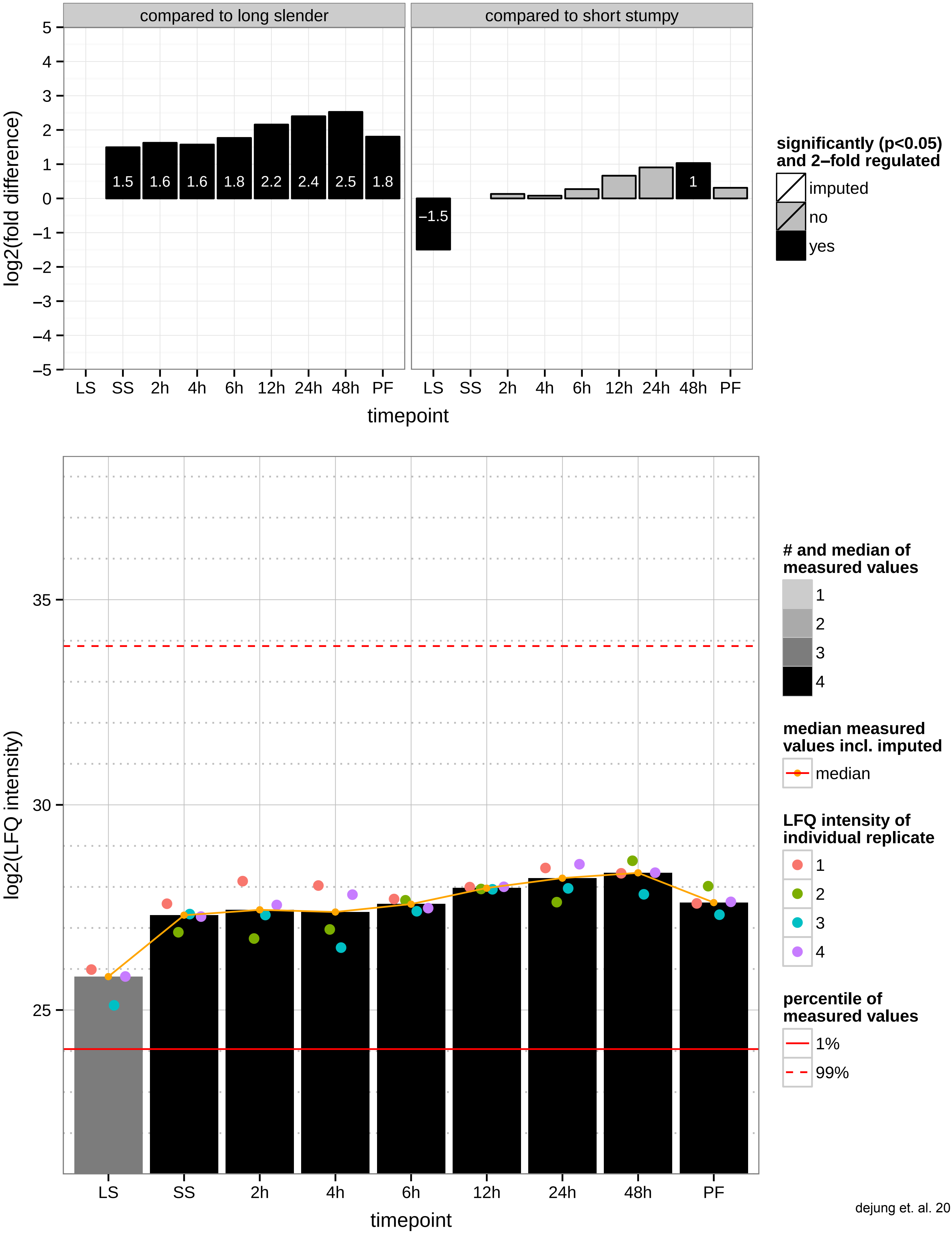

dejung et. al. 2015

hypothetical protein, conserved, NADH−ubiquinone oxidoreductase complex I subunit, putative, NDUFA9 subunit
Tb927.9.15380;Tb11.v5.0686
AGOF: null, catalytic activity
AGOC: null, mitochondrion, respiratory chain complex I
AGOP: null, metabolic process
PGOF: null
PGOC: null
PGOP: null

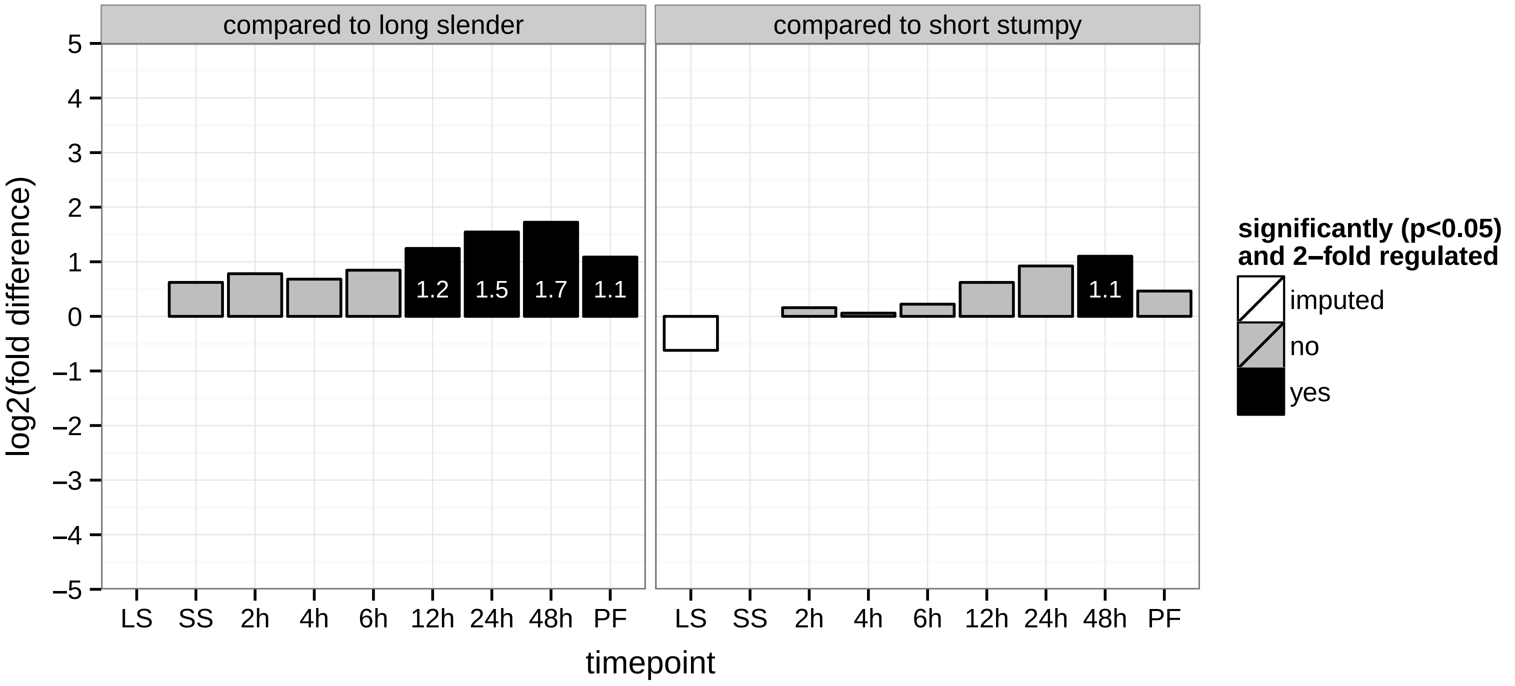

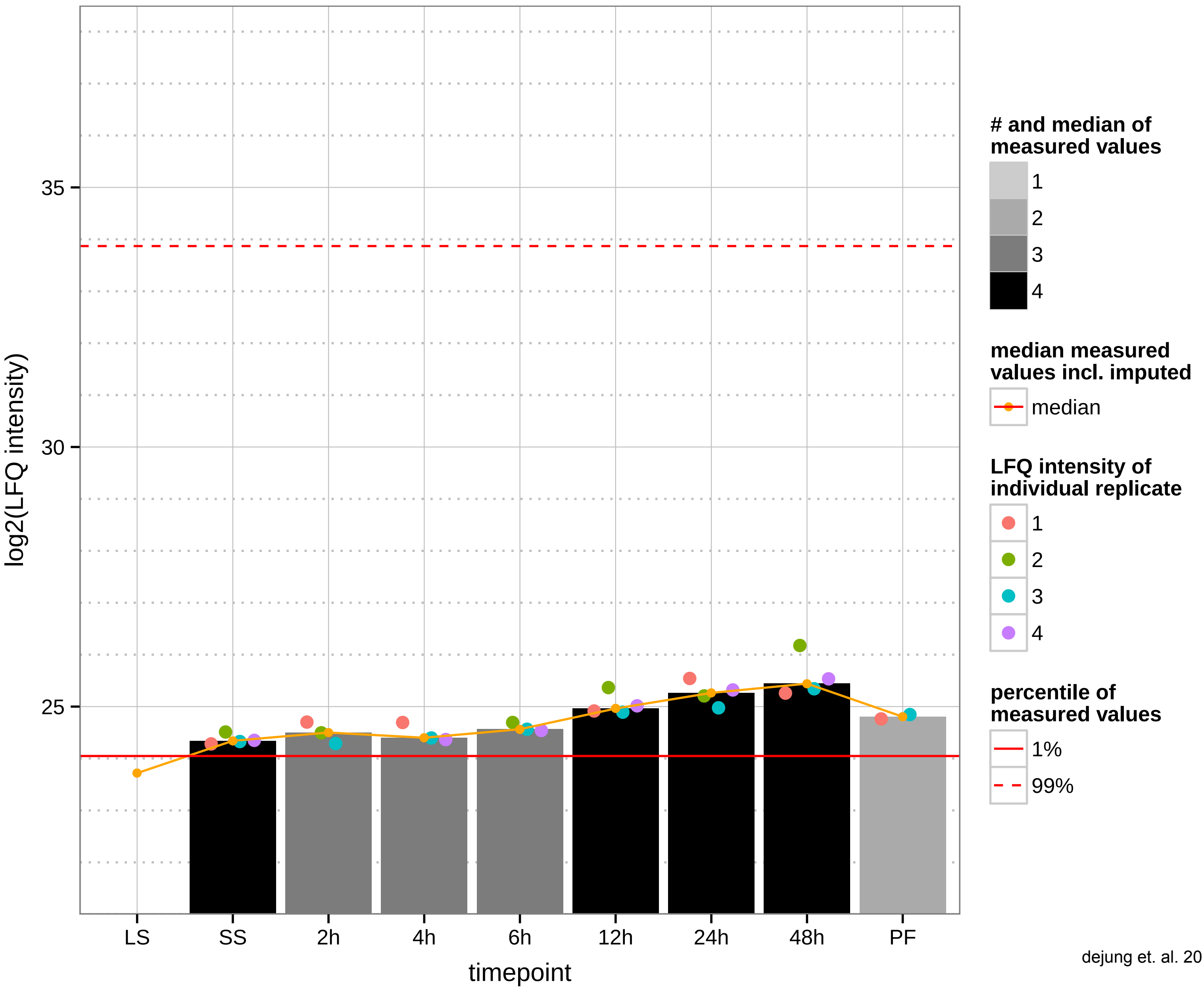

dejung et. al. 2015

retrotransposon hot spot (RHS) protein, putative
Tb11.v5.0713
AGOF: null
AGOC: null
AGOP: null
PGOF: null
PGOC: null
PGOP: null

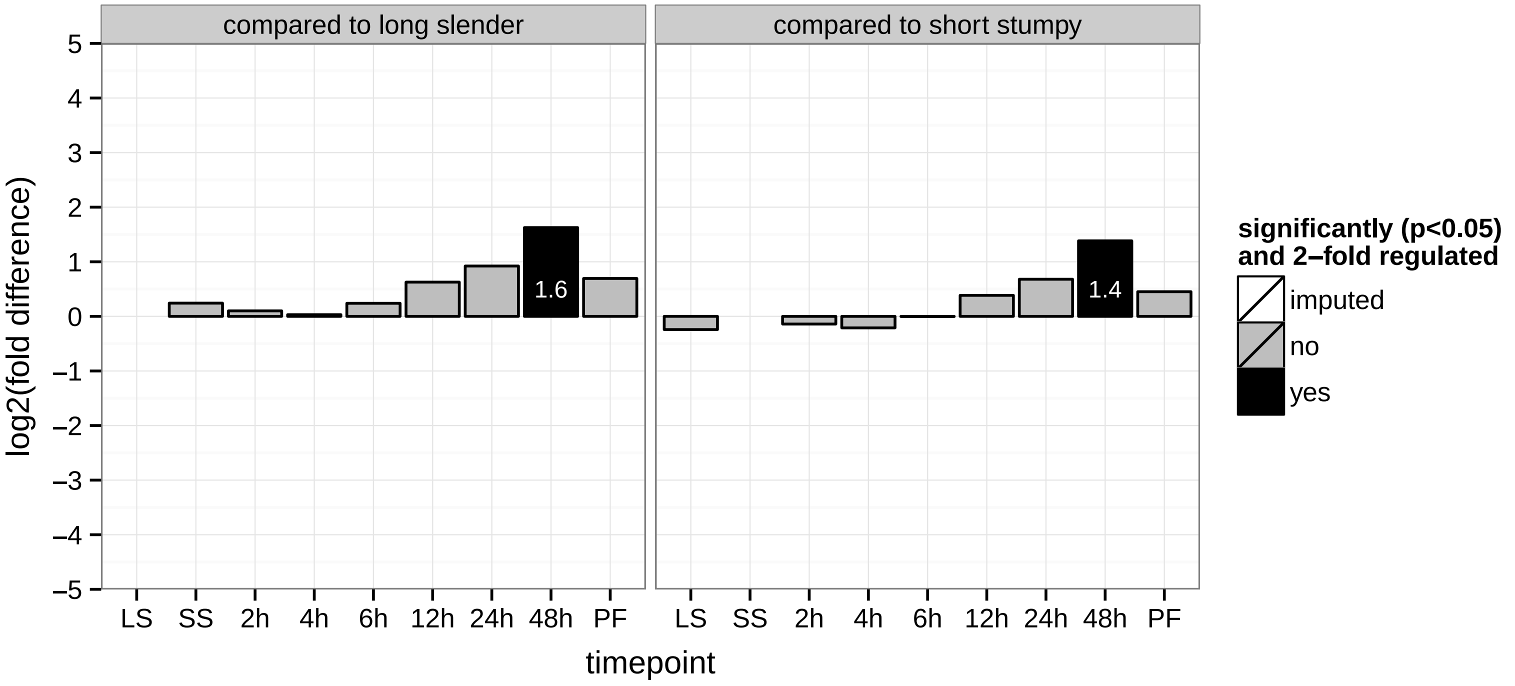

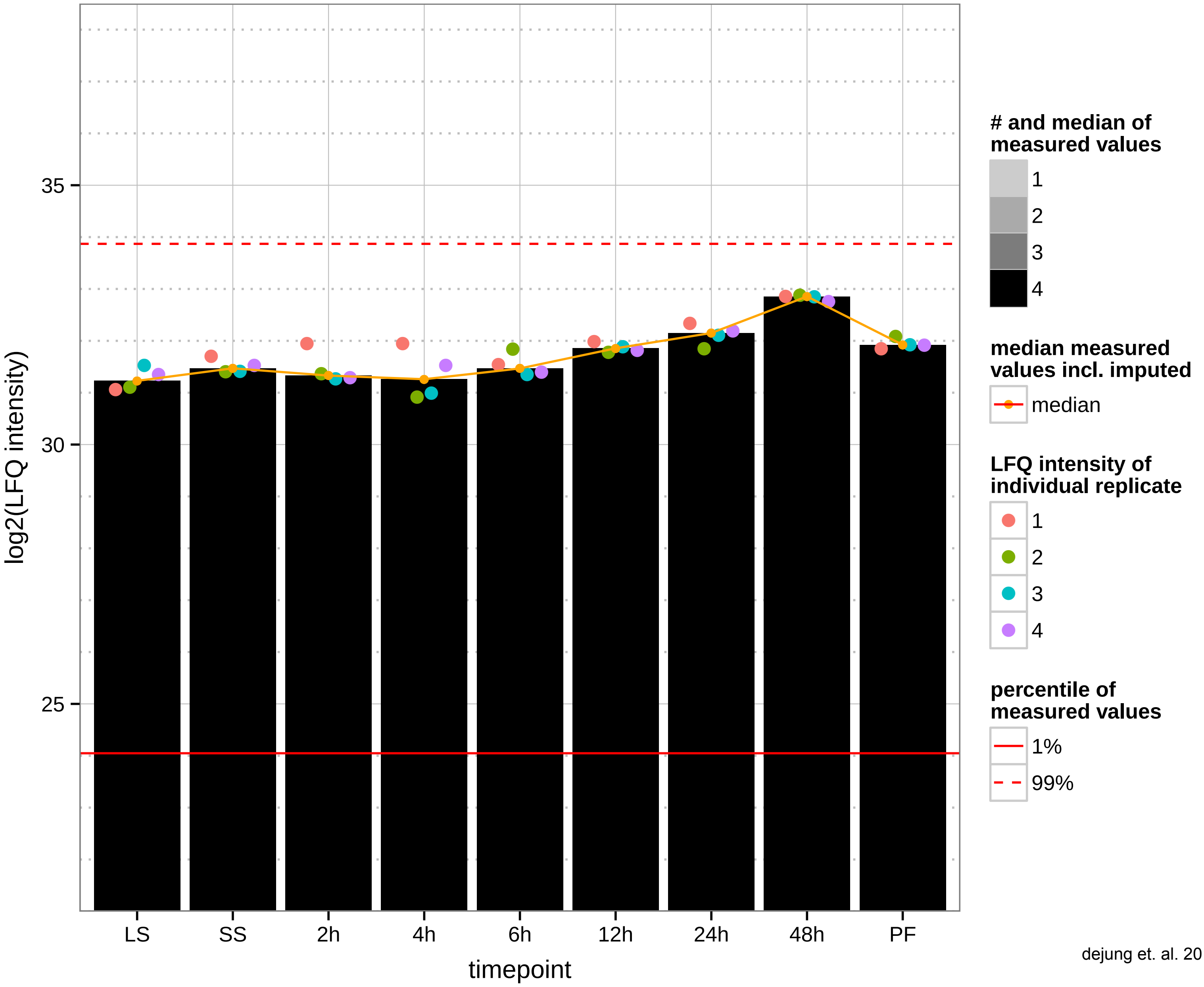

dejung et. al. 2015

hypothetical protein, conserved, ATP dependent DEAD–box helicase (RH)
Tb11.v5.0842;Tb927.4.2720
AGOF: null, ATP binding, helicase activity, nucleic acid binding, ribosome binding
AGOC: null, mitochondrial ribosome, mitochondrion
AGOP: null
PGOF: ATP binding, ATP–dependent helicase activity, helicase activity, nucleic acid binding
PGOC: null
PGOP: null

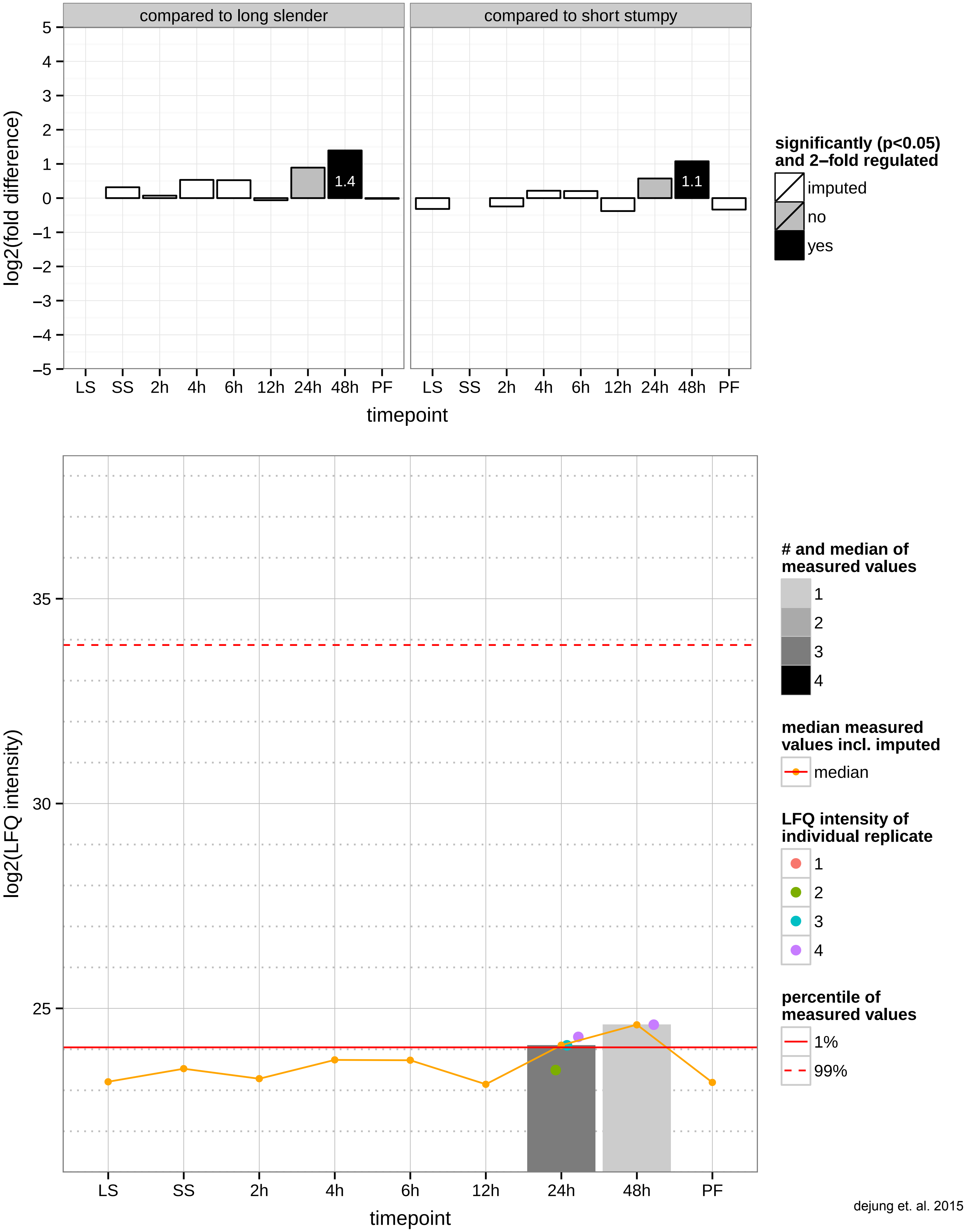

dejung et. al. 2015

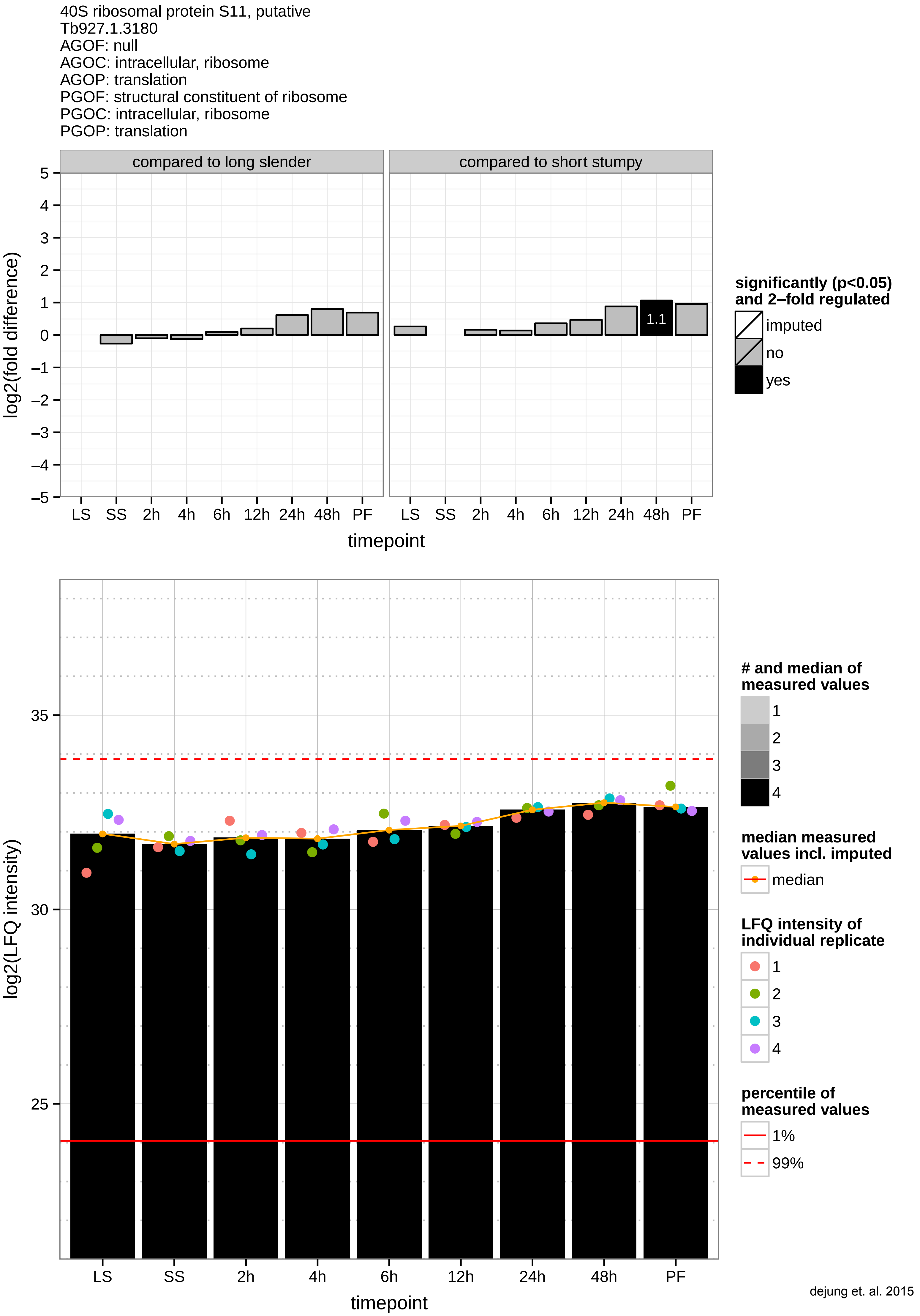
40S ribosomal protein S11, putative
Tb927.1.3180
AGOF: null
AGOC: intracellular, ribosome
AGOP: translation
PGOF: structural constituent of ribosome
PGOC: intracellular, ribosome
PGOP: translation
compared to long slender
compared to short stumpy
log2(fold difference)
1.1
timepoint
LS
SS
2h
4h
6h
12h
24h
48h
PF
significantly (p<0.05) and 2–fold regulated
imputed
no
yes
log2(LFQ intensity)
# and median of measured values
median measured values incl. imputed
median
LFQ intensity of individual replicate
percentile of measured values
1%
99%
dejung et. al. 2015

hypothetical protein, conserved
Tb927.1.3910
AGOF: null
AGOC: null
AGOP: null
PGOF: protein binding
PGOC: null
PGOP: null

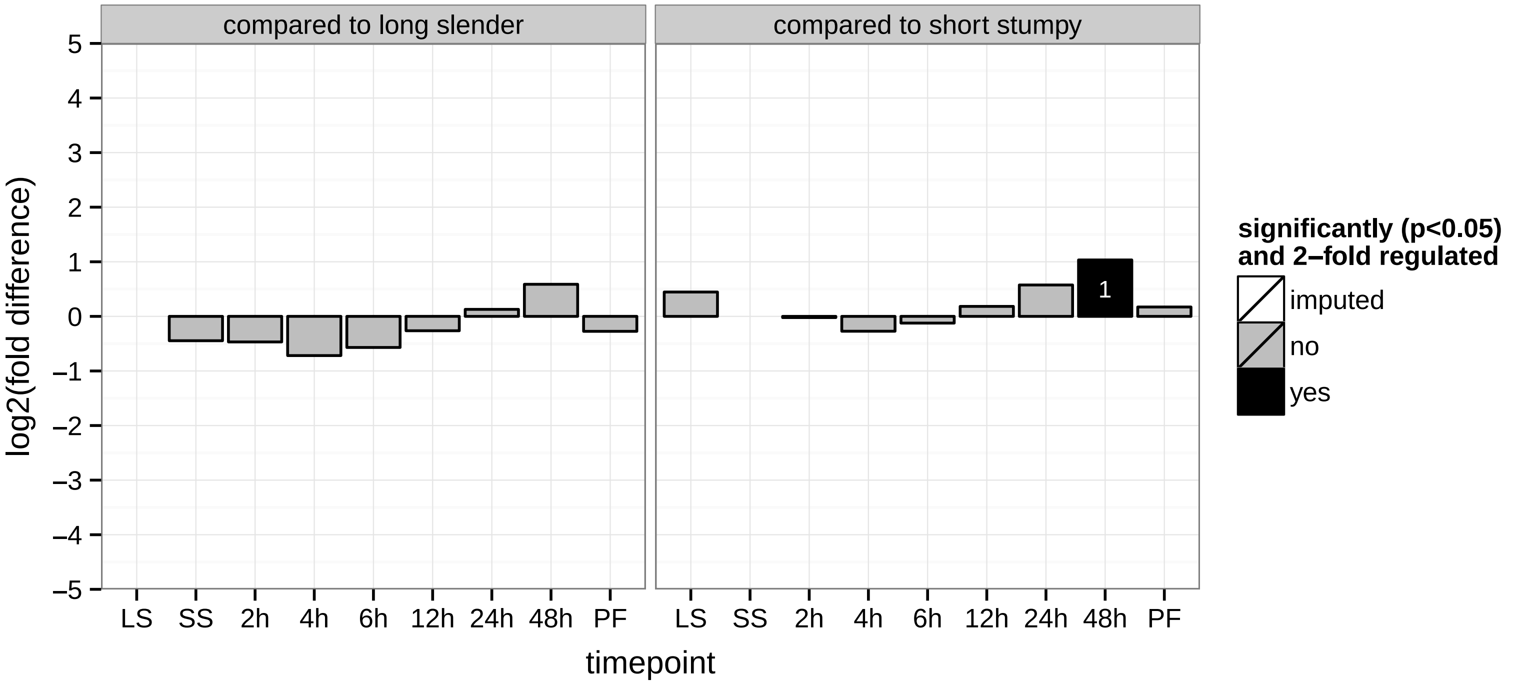

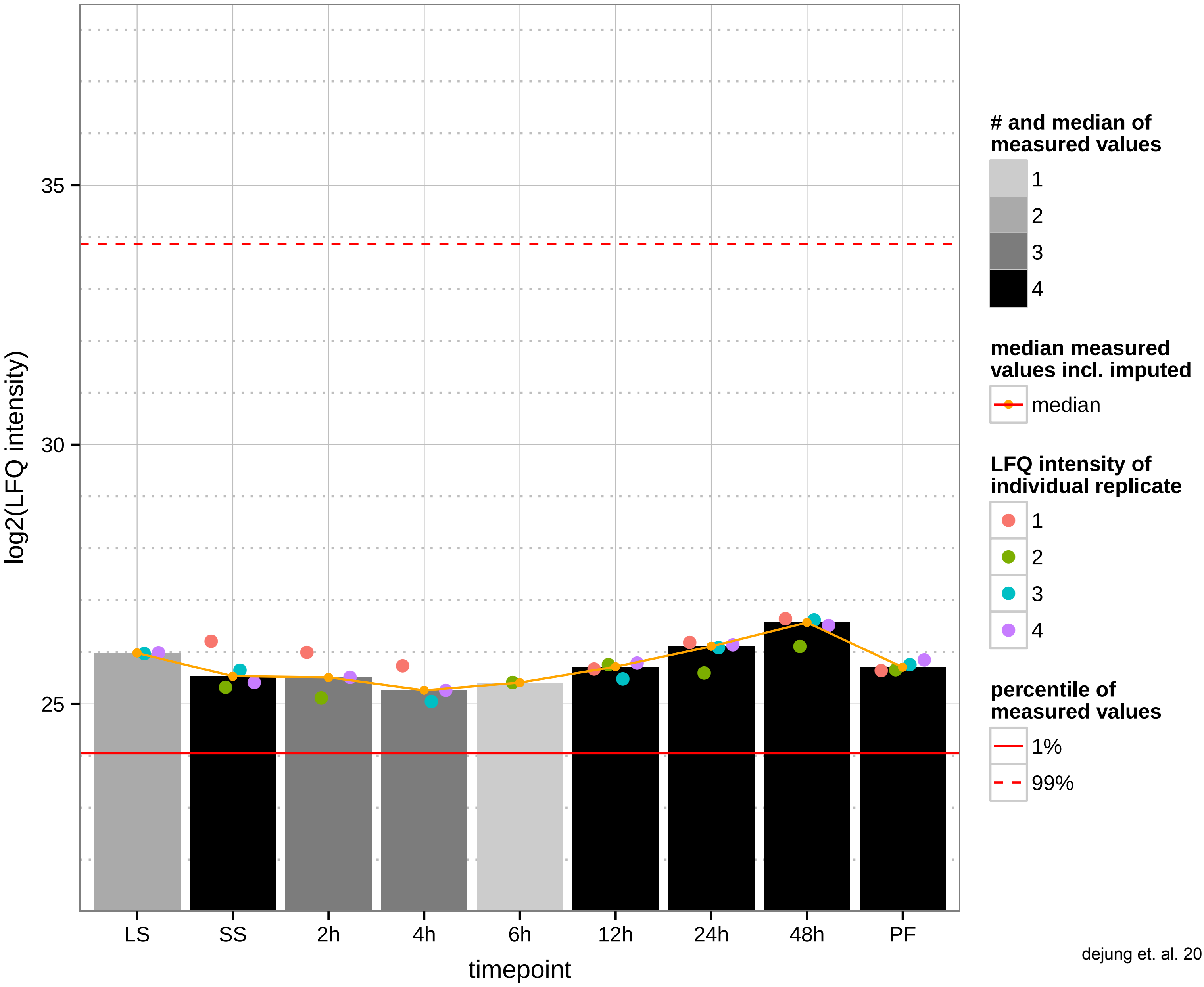

dejung et. al. 2015

ABC transporter, putative
Tb927.1.4420
AGOF: ATP binding, ATPase activity, coupled to transmembrane movement of substances
AGOC: ATP−binding cassette (ABC) transporter complex
AGOP: transport
PGOF: ATP binding, ATPase activity, nucleoside−triphosphatase activity, nucleotide binding
PGOC: null
PGOP: null

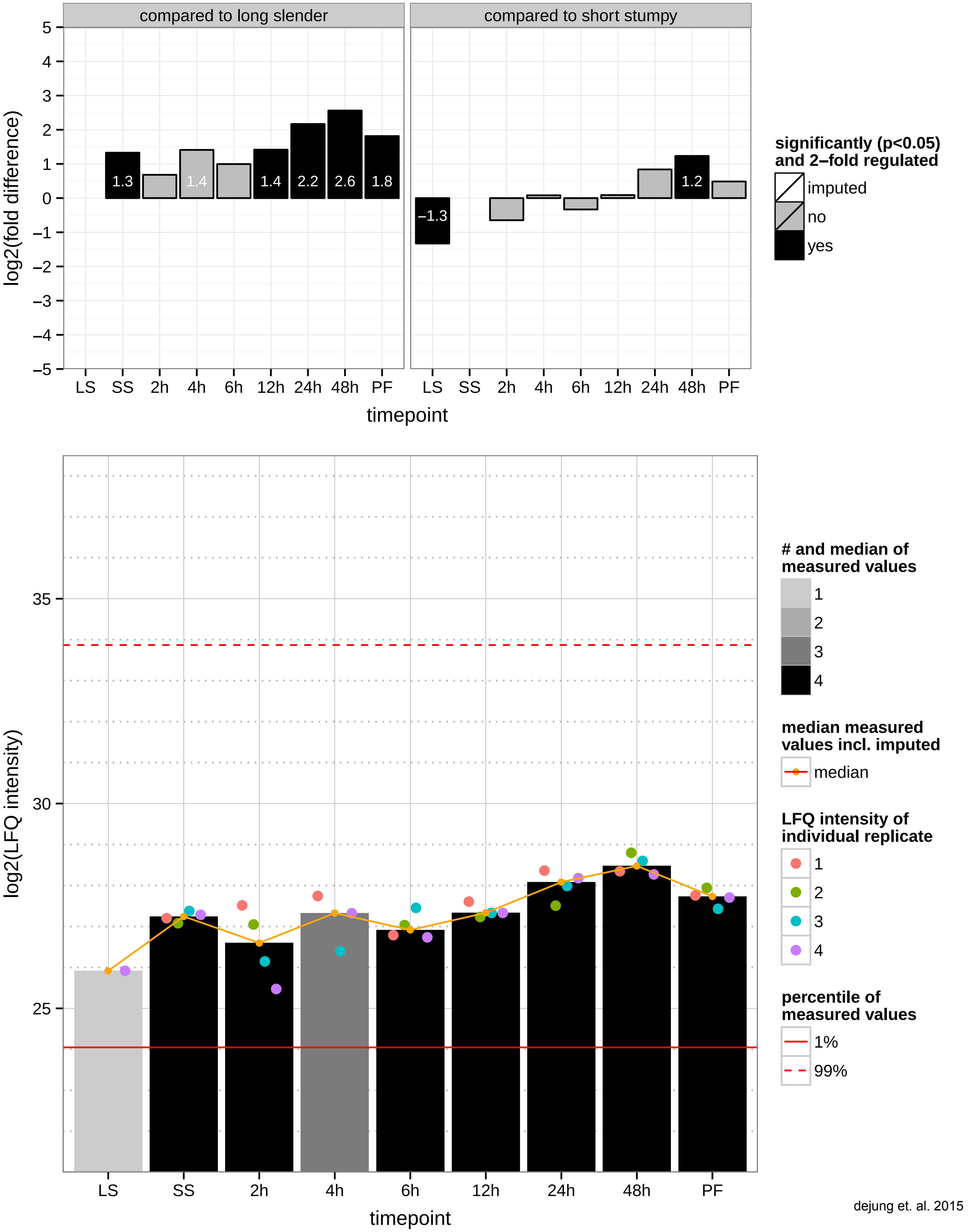

dejung et. al. 2015

hypothetical protein, conserved
Tb927.1.4480
AGOF: null
AGOC: null
AGOP: null
PGOF: protein binding
PGOC: null
PGOP: null

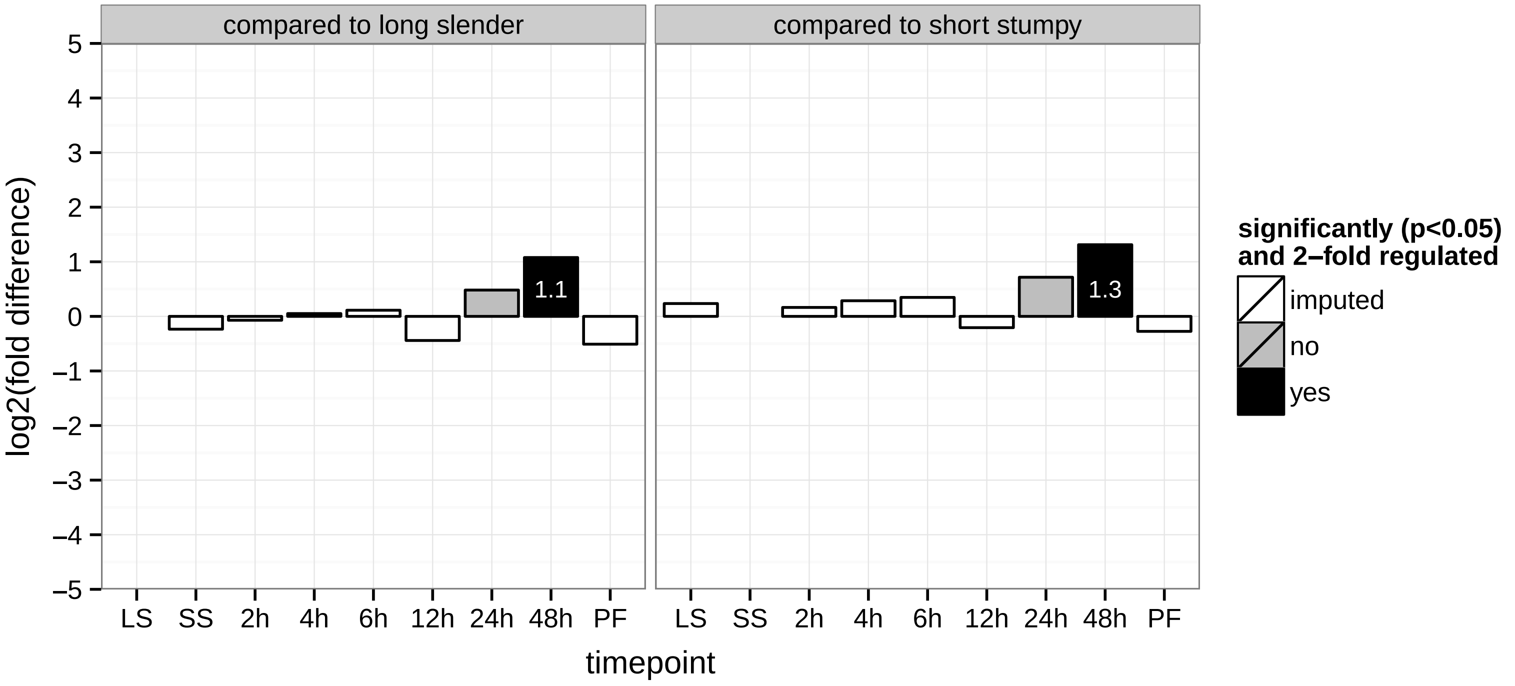

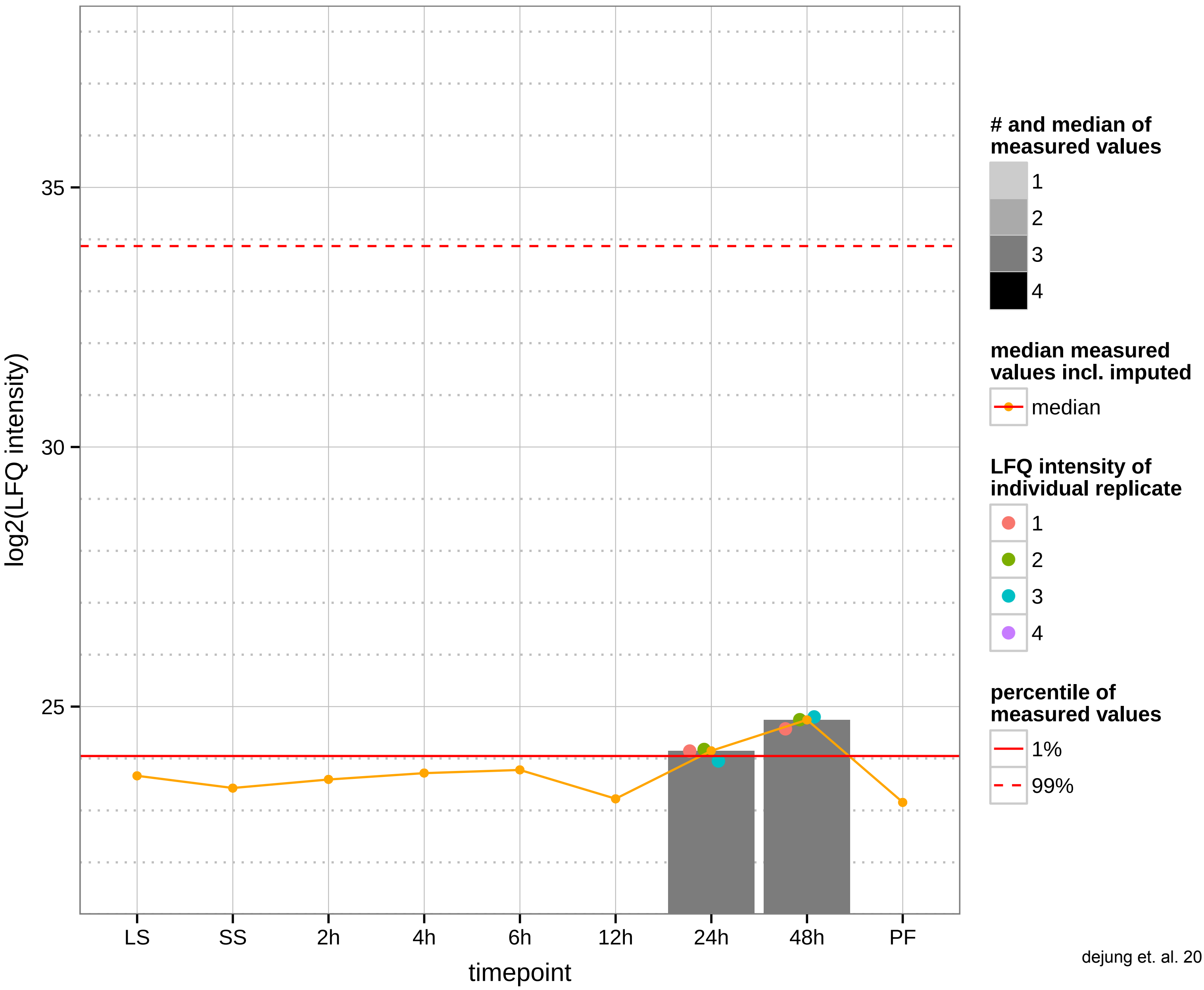

dejung et. al. 2015

arginine N–methyltransferase (PRMT1)
Tb927.1.4690
AGOF: arginine N–methyltransferase activity, protein–arginine N–methyltransferase activity
AGOC: null
AGOP: peptidyl–arginine methylation, to asymmetrical–dimethyl arginine, protein methylation
PGOF: null
PGOC: null
PGOP: null

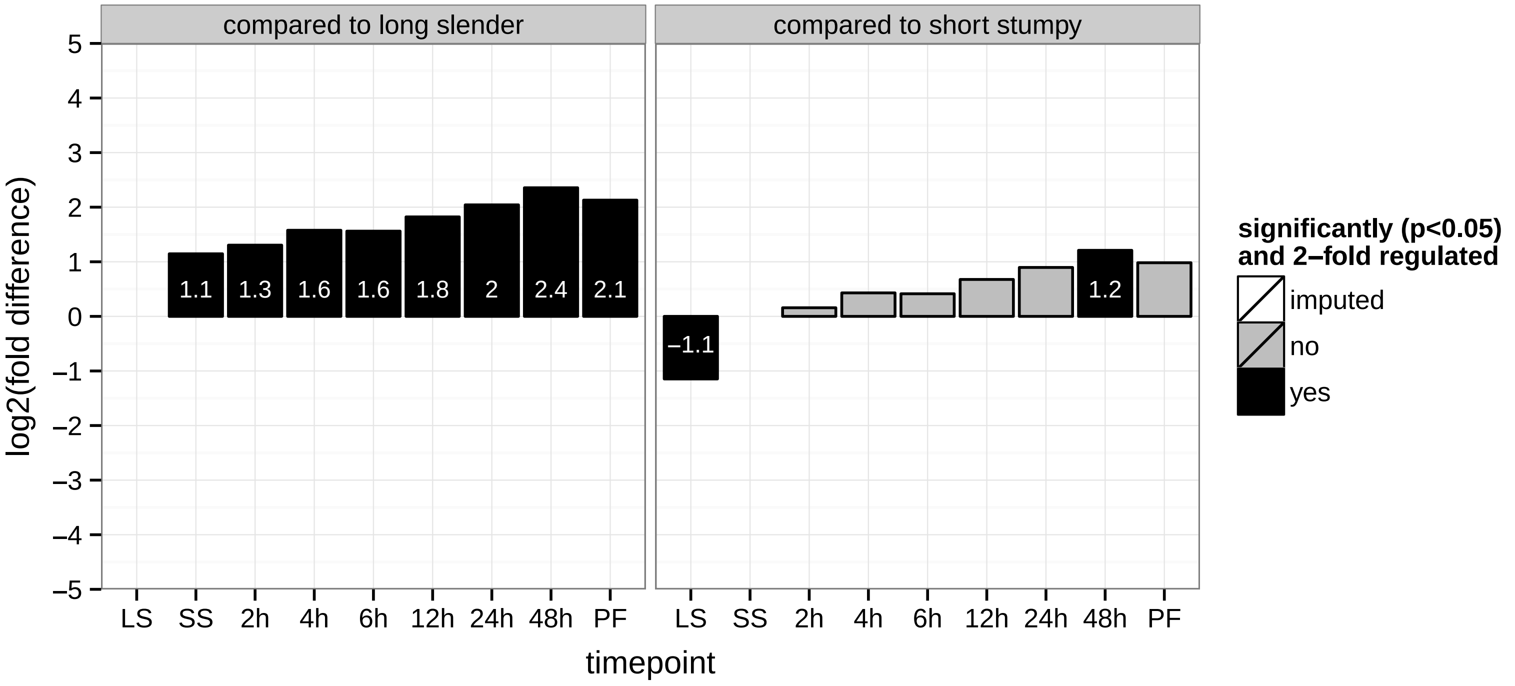

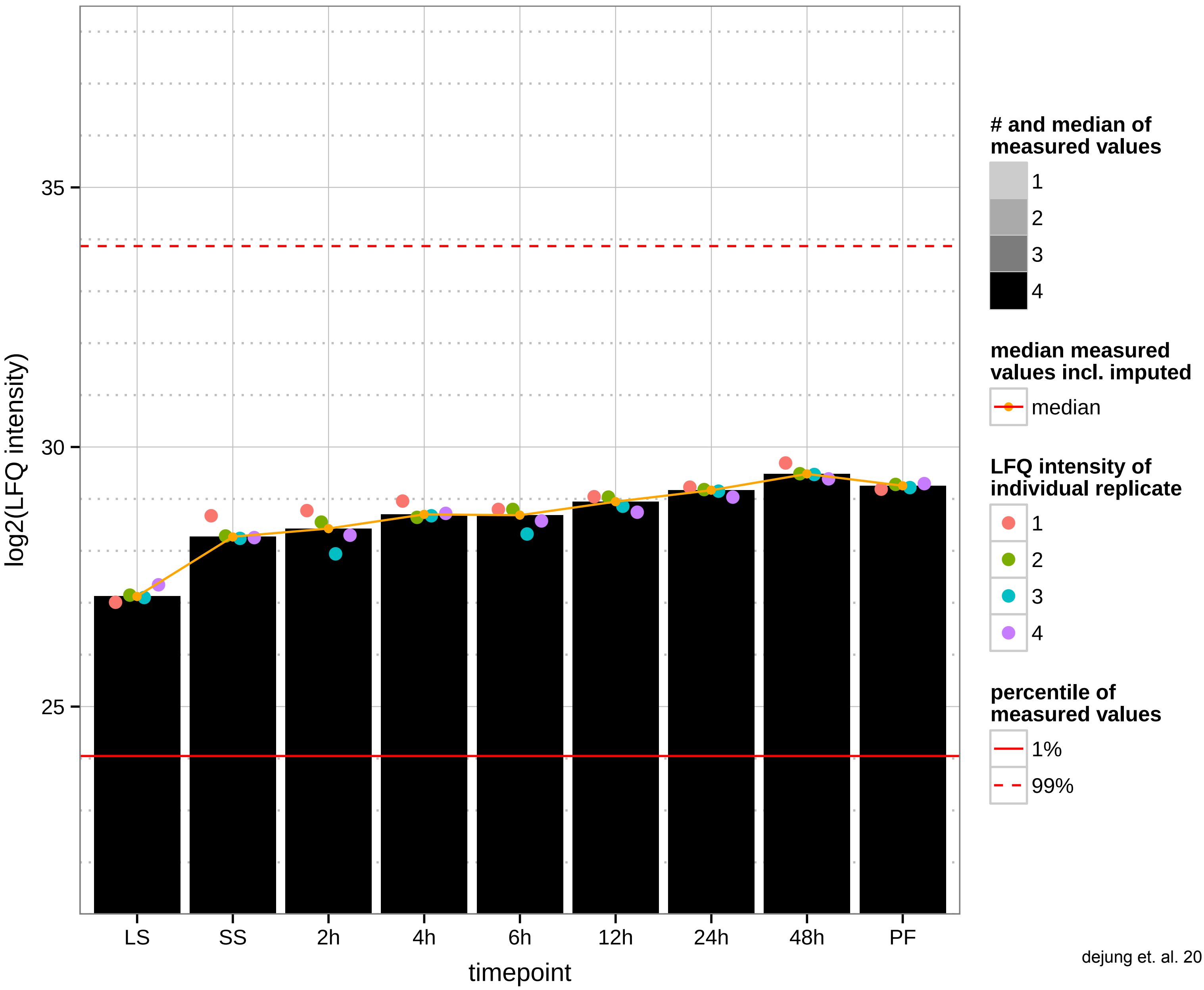

dejung et. al. 2015

hypothetical protein, conserved
Tb927.1.5000
AGOF: null
AGOC: mitochondrion
AGOP: null
PGOF: null
PGOC: null
PGOP: null

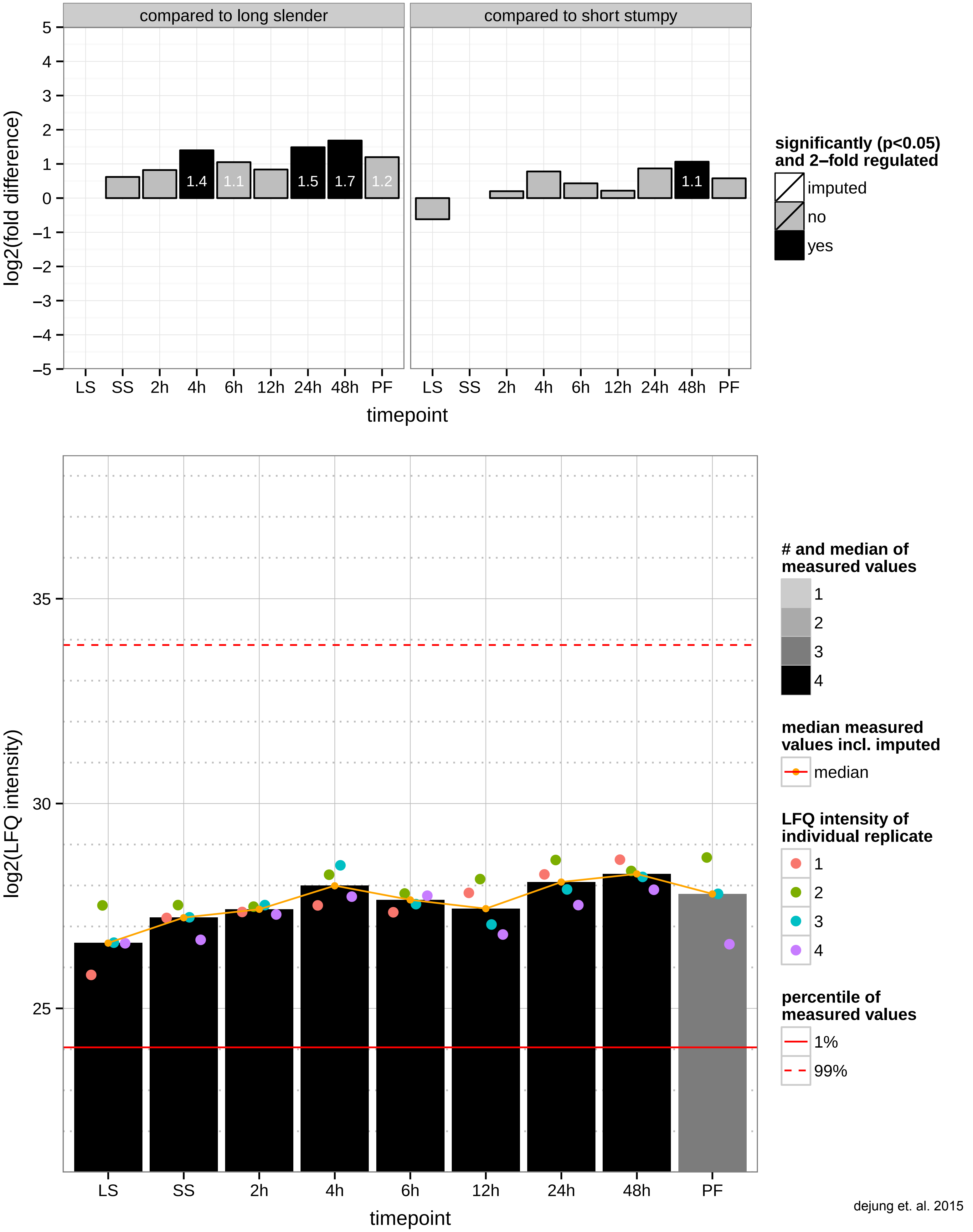

dejung et. al. 2015

phosphate–repressible phosphate permease, putative
Tb927.1.600
AGOF: inorganic phosphate transmembrane transporter activity, phosphate ion transmembrane transporter activity
AGOC: membrane
AGOP: null
PGOF: inorganic phosphate transmembrane transporter activity
PGOC: membrane
PGOP: phosphate ion transport

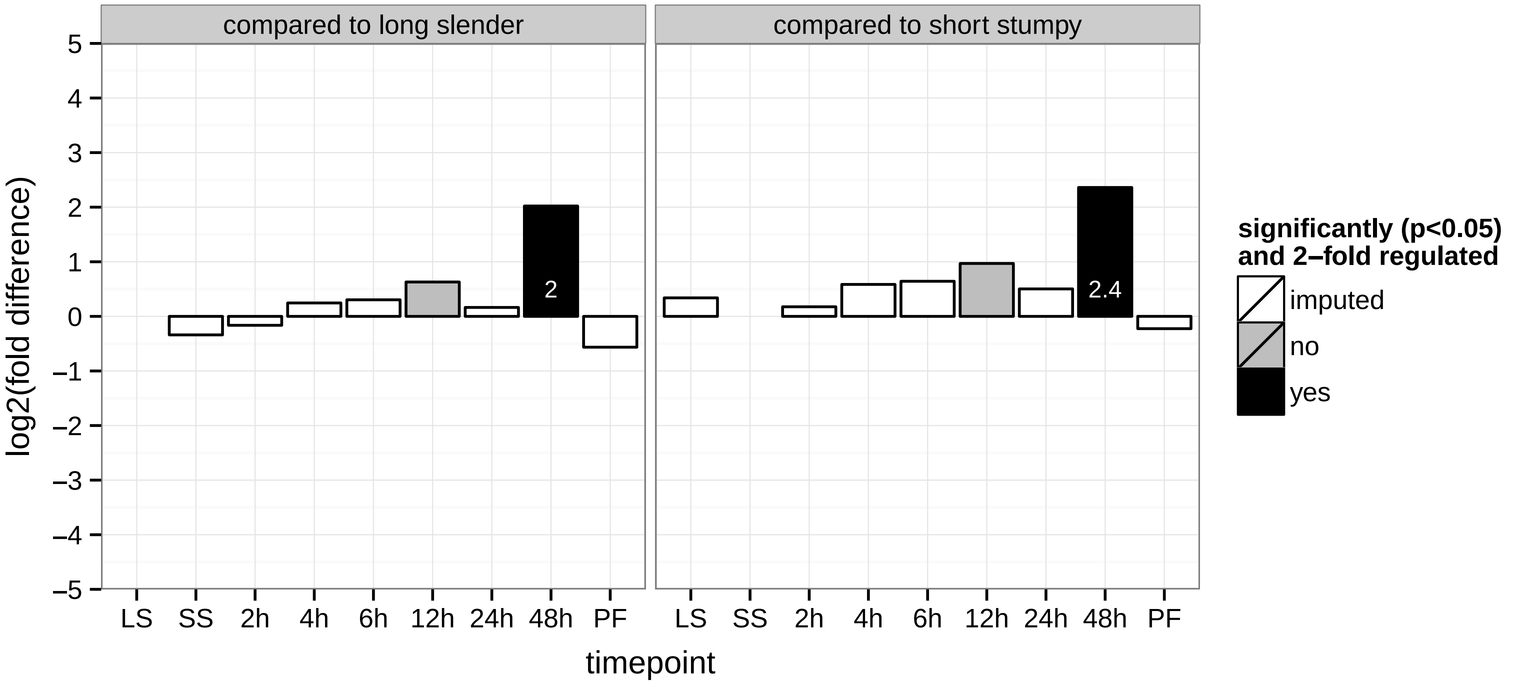

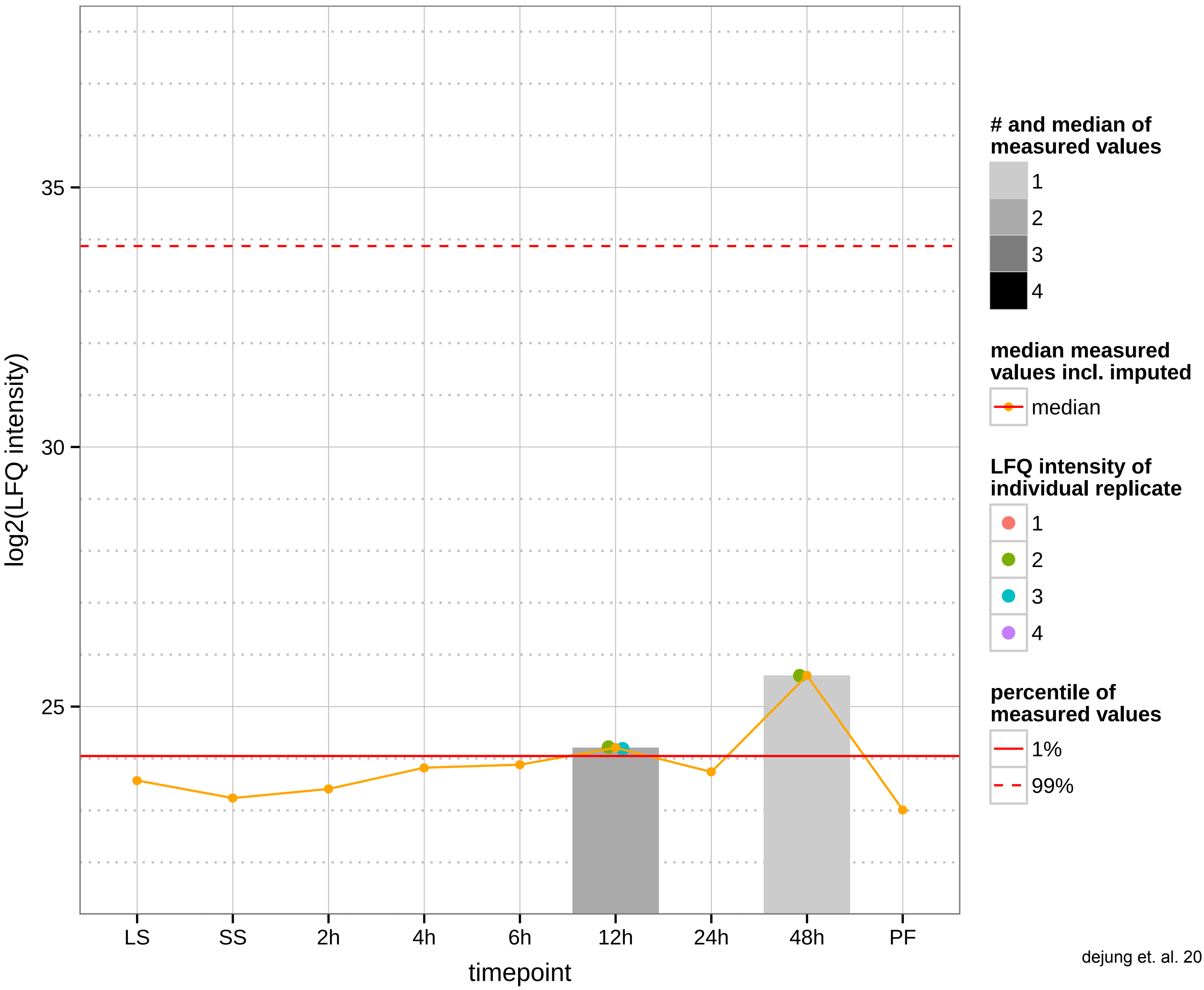

dejung et. al. 2015

Metal−binding domain−containing protein
Tb927.1.750
AGOF: null
AGOC: null
AGOP: null
PGOF: null
PGOC: null
PGOP: null

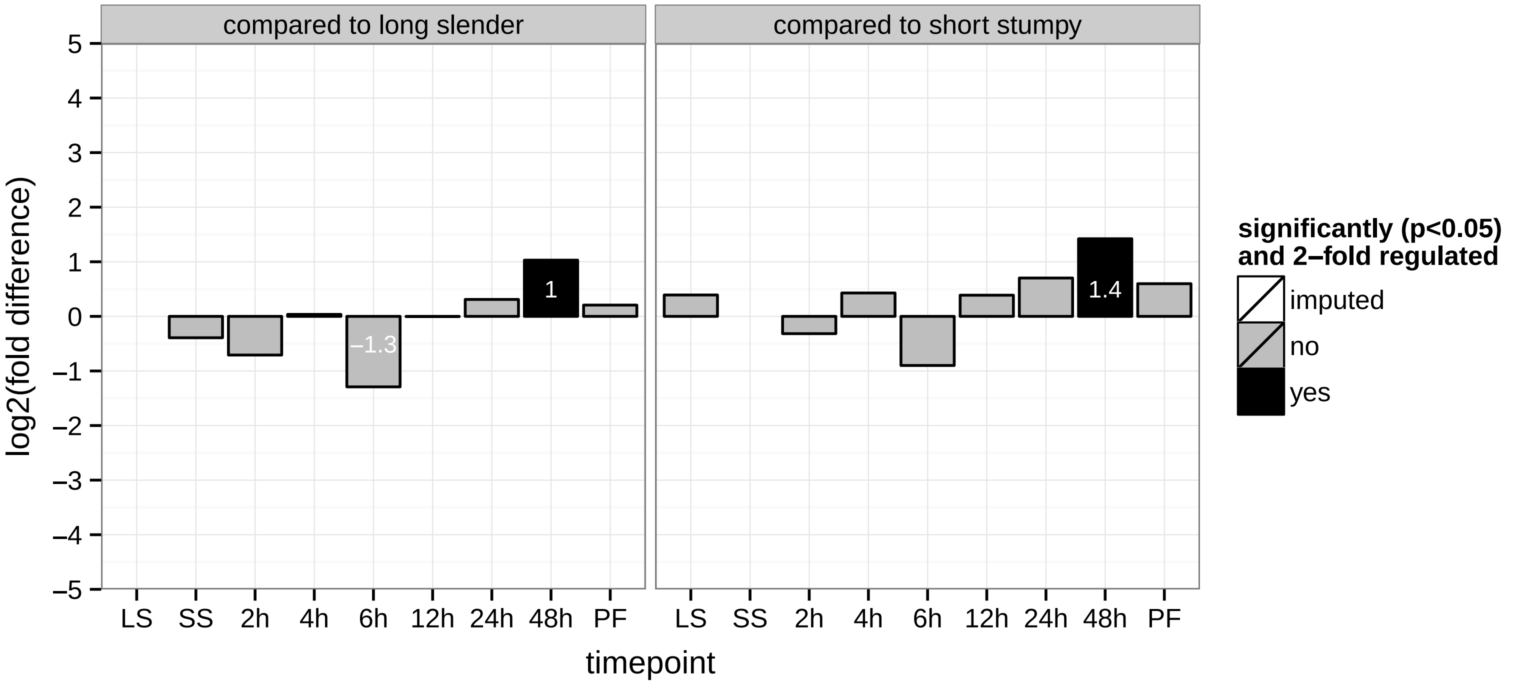

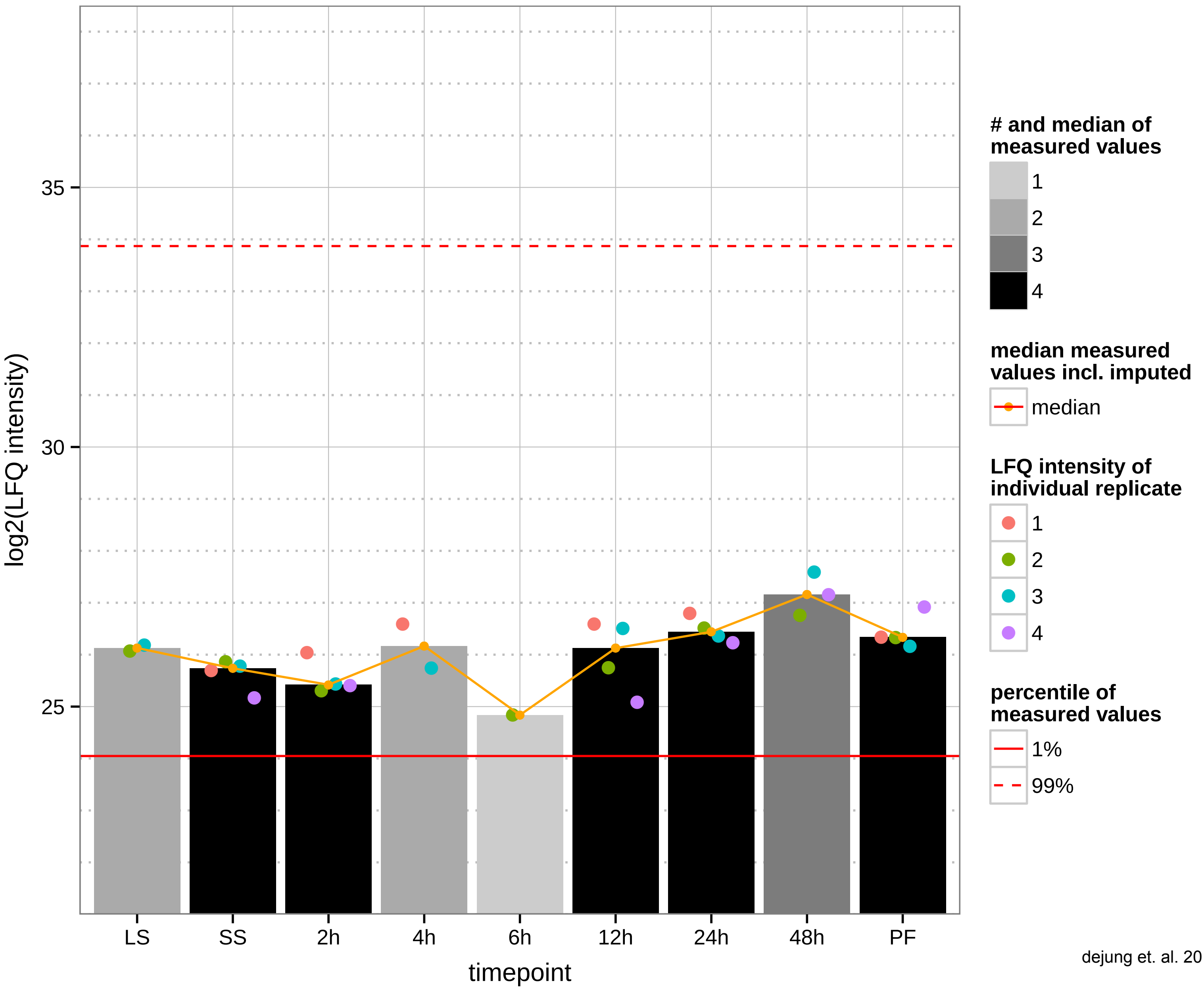

dejung et. al. 2015

T–complex protein 1, delta subunit, putative (TCP–1–delta)
Tb927.10.1060
AGOF: ATP binding, unfolded protein binding
AGOC: chaperonin–containing T–complex
AGOP: protein folding, regulation of cell cycle
PGOF: ATP binding, unfolded protein binding
PGOC: null
PGOP: cellular protein metabolic process, protein folding

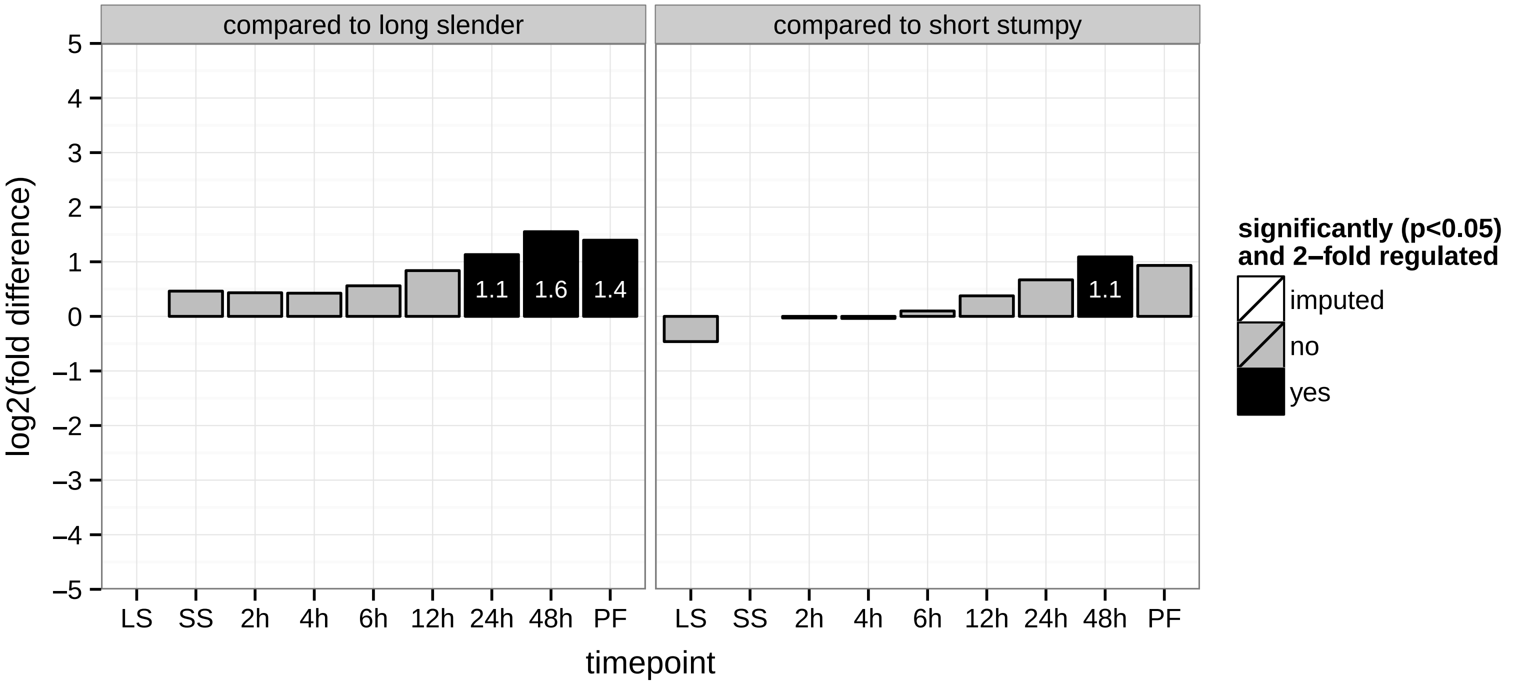

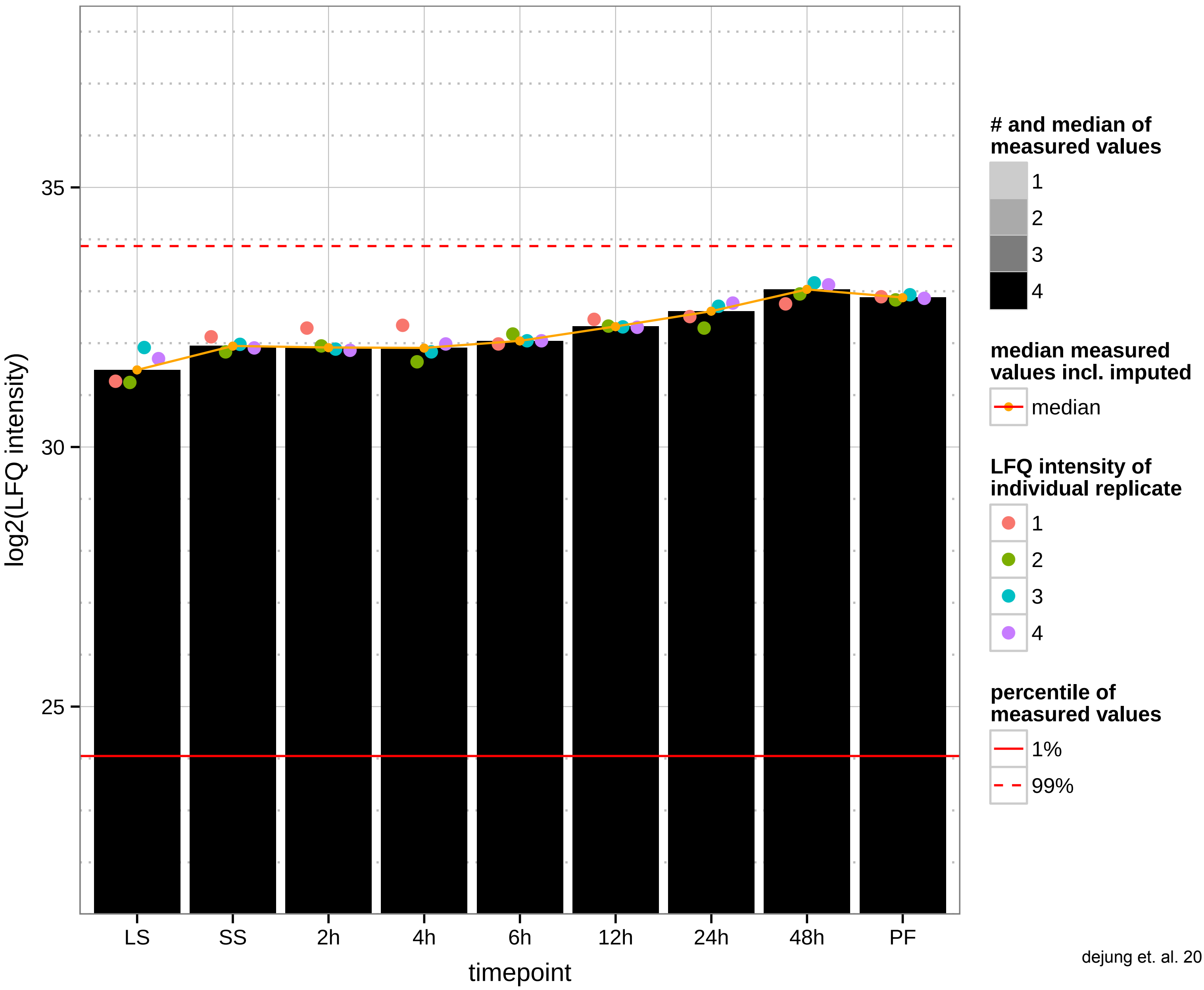

dejung et. al. 2015

hypothetical protein, conserved
Tb927.10.11050
AGOF: null
AGOC: mitochondrion
AGOP: null
PGOF: null
PGOC: null
PGOP: null

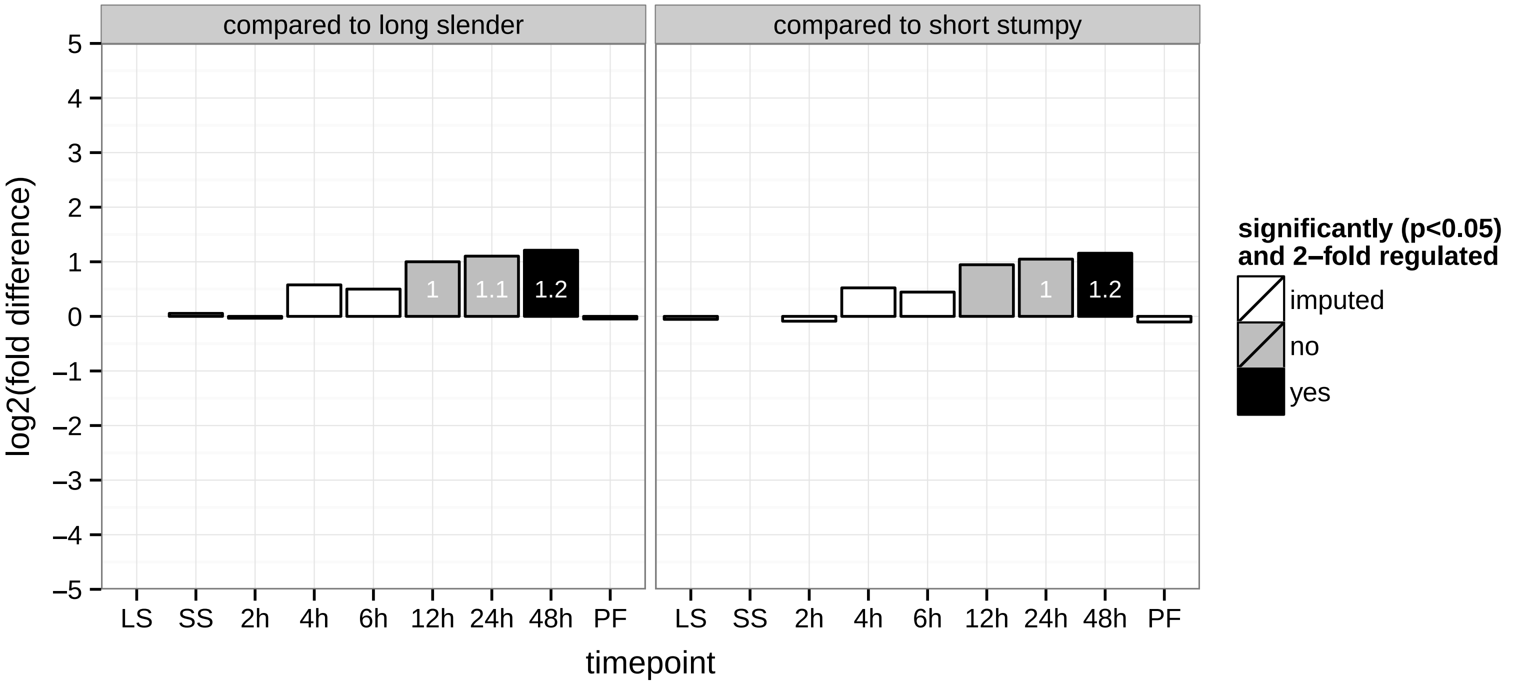

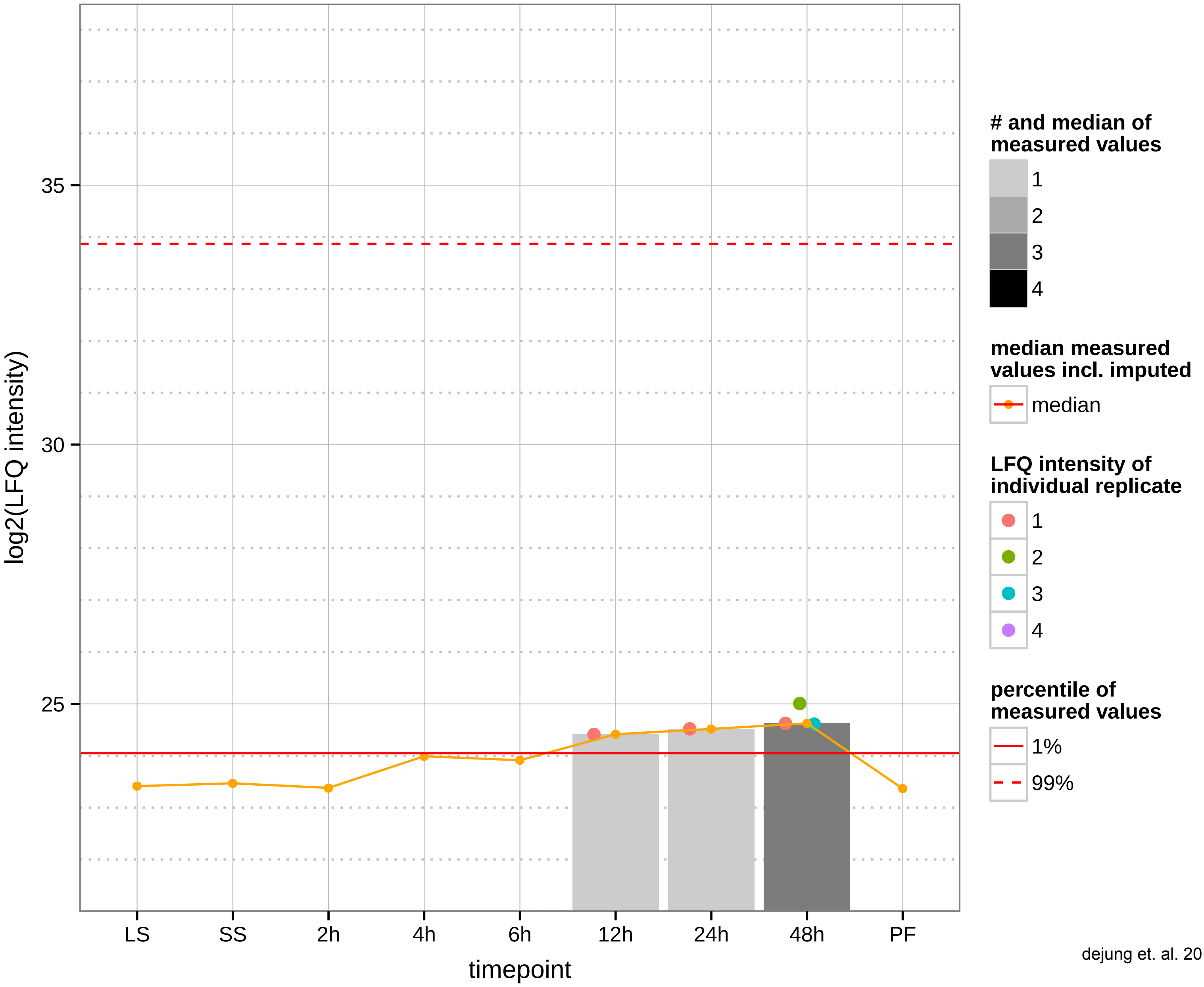

dejung et. al. 2015

hypothetical protein, conserved
Tb927.10.11120
AGOF: null
AGOC: null
AGOP: null
PGOF: null
PGOC: null
PGOP: null

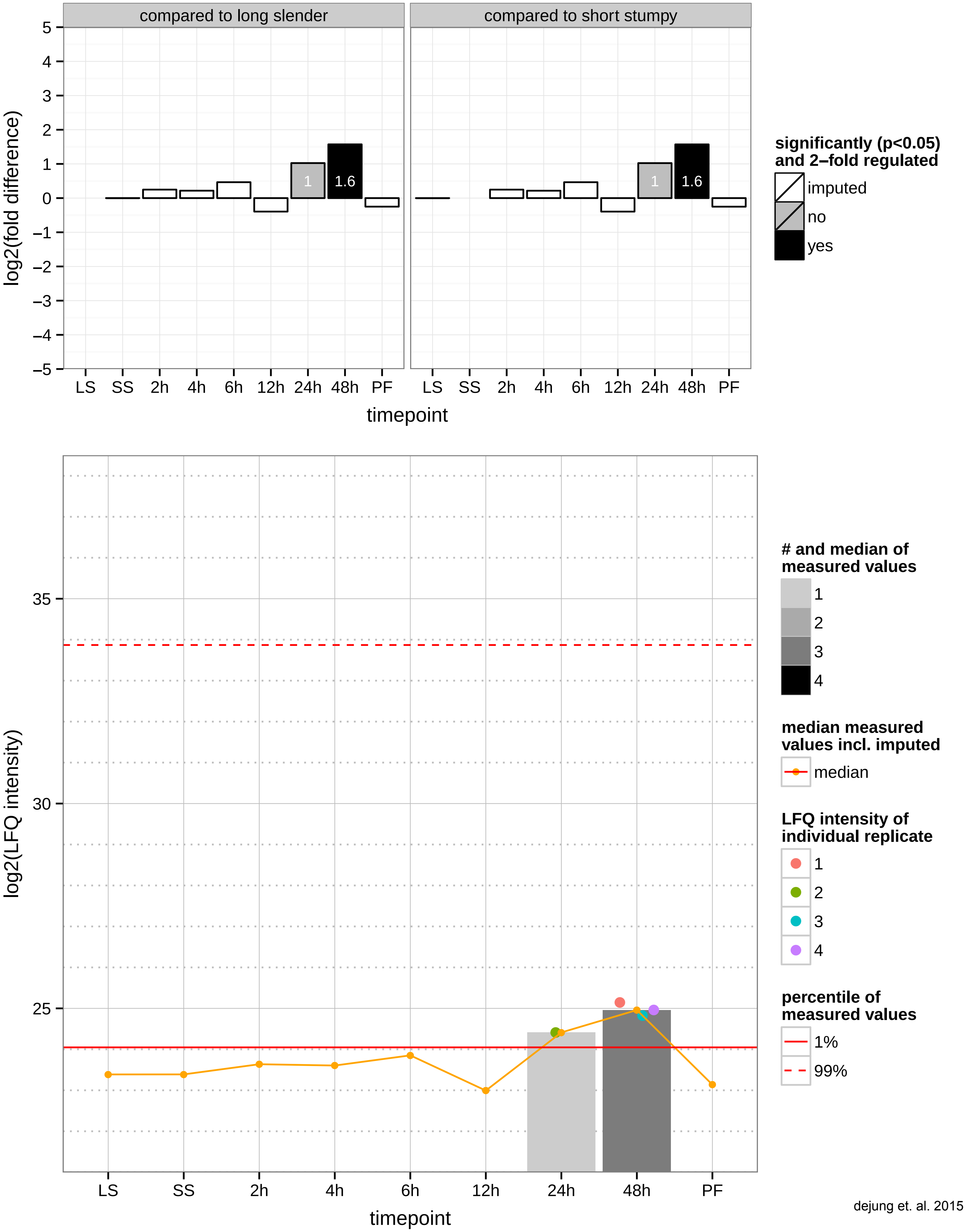

dejung et. al. 2015

hypothetical protein, conserved
Tb927.10.14130
AGOF: 7S RNA binding
AGOC: signal recognition particle
AGOP: SRP−dependent cotranslational protein targeting to membrane
PGOF: 7S RNA binding
PGOC: signal recognition particle
PGOP: SRP−dependent cotranslational protein targeting to membrane

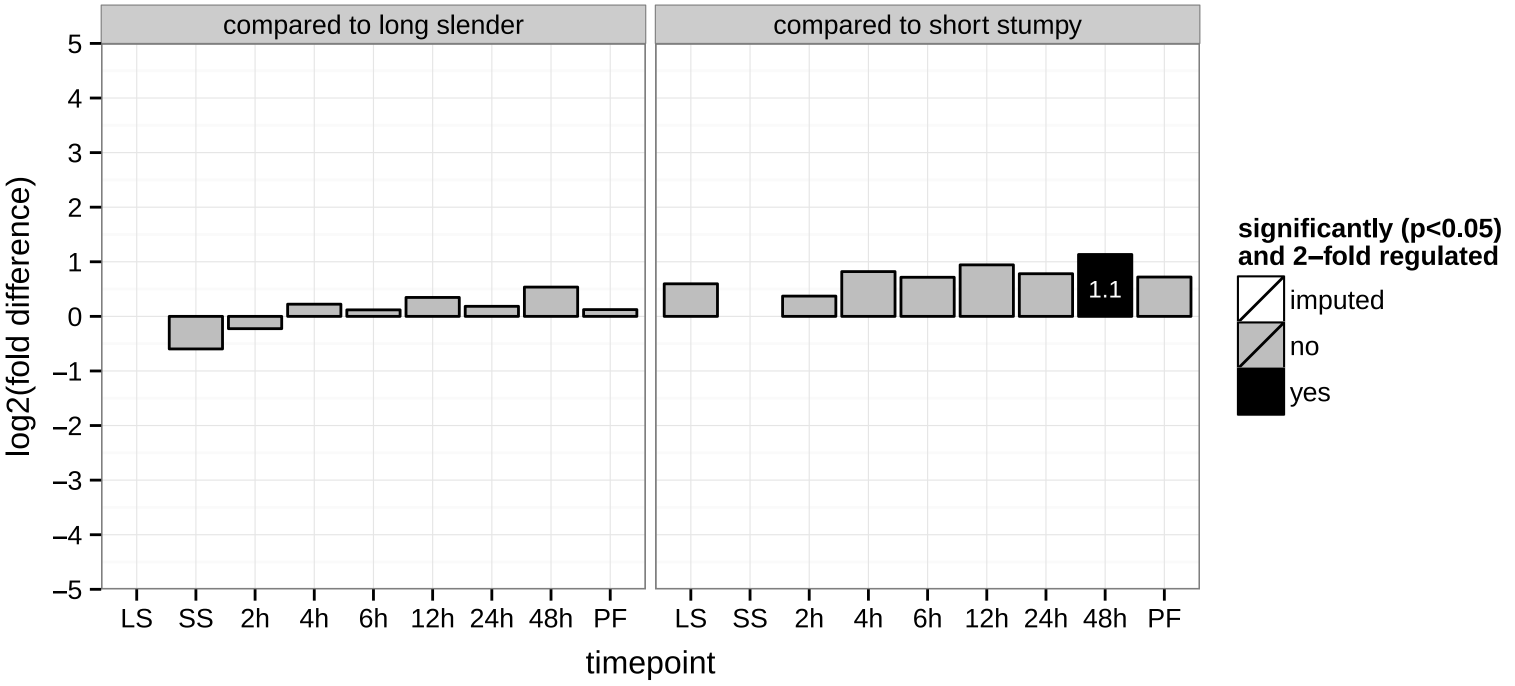

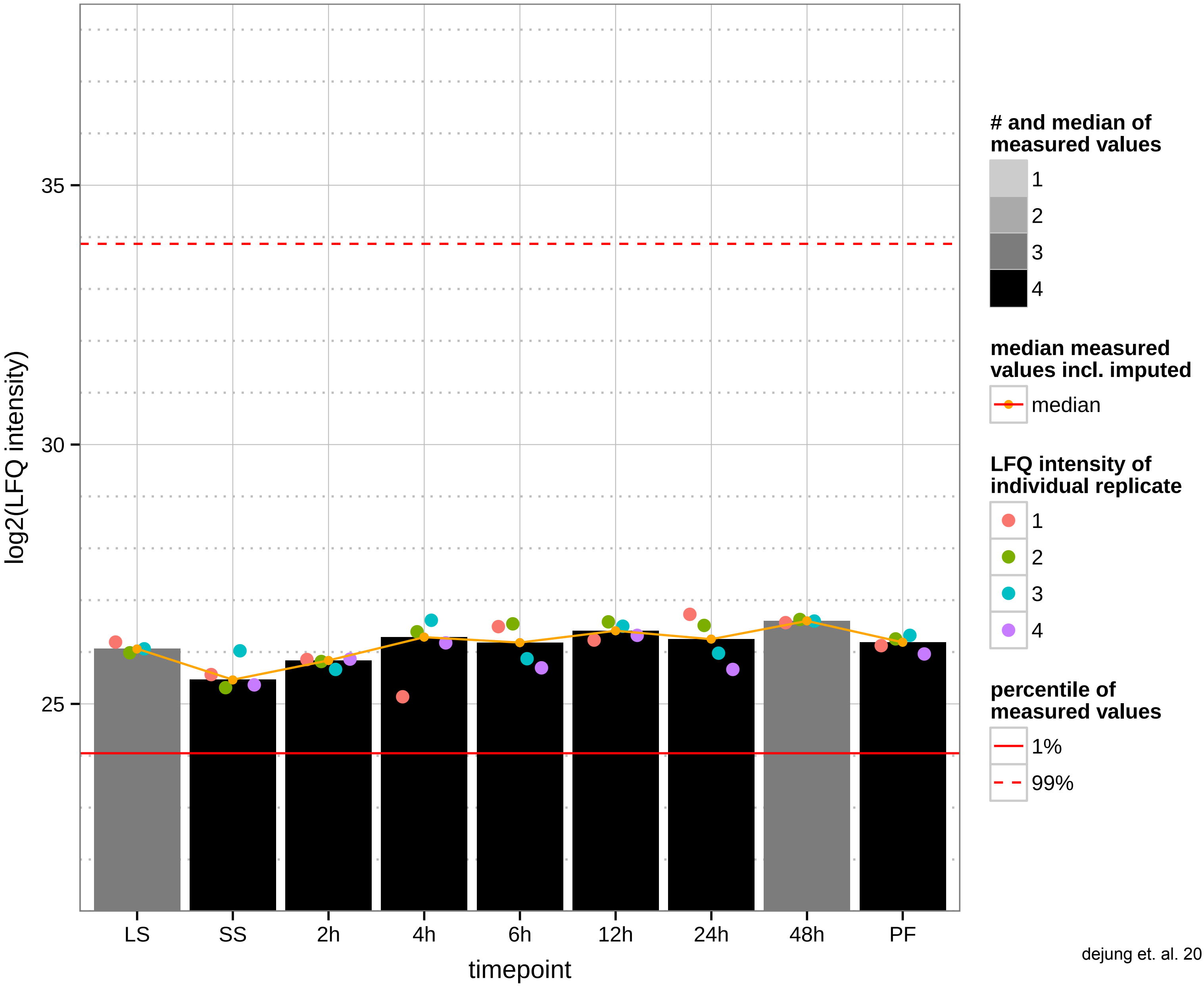

dejung et. al. 2015

NA, ATP−dependent DEAD/H RNA helicase, putative
Tb927.10.14550
AGOF: NA, ATP binding, ATP−dependent helicase activity, helicase activity, nucleic acid binding
AGOC: NA, null
AGOP: NA, nucleobase−containing compound metabolic process
PGOF: NA, ATP binding, ATP−dependent helicase activity, helicase activity, nucleic acid binding
PGOC: NA, null
PGOP: NA, null

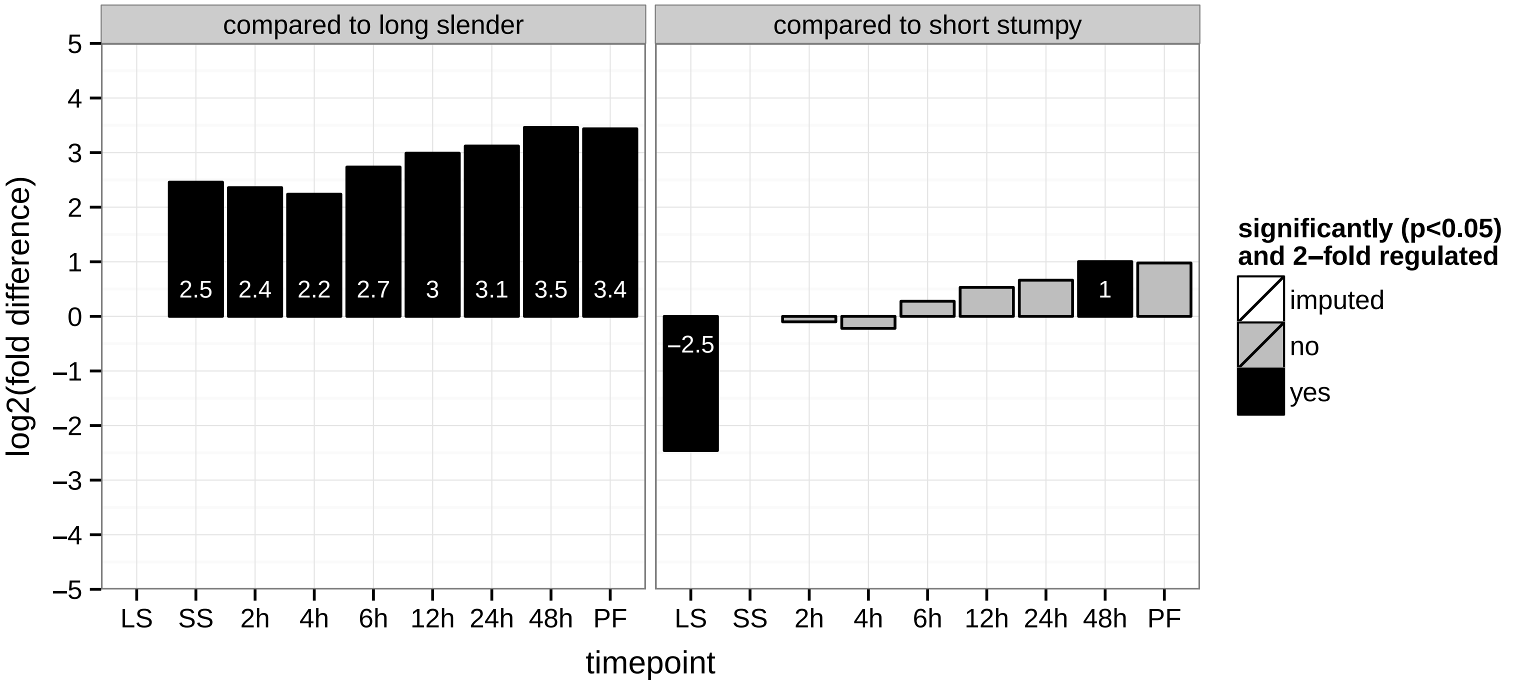

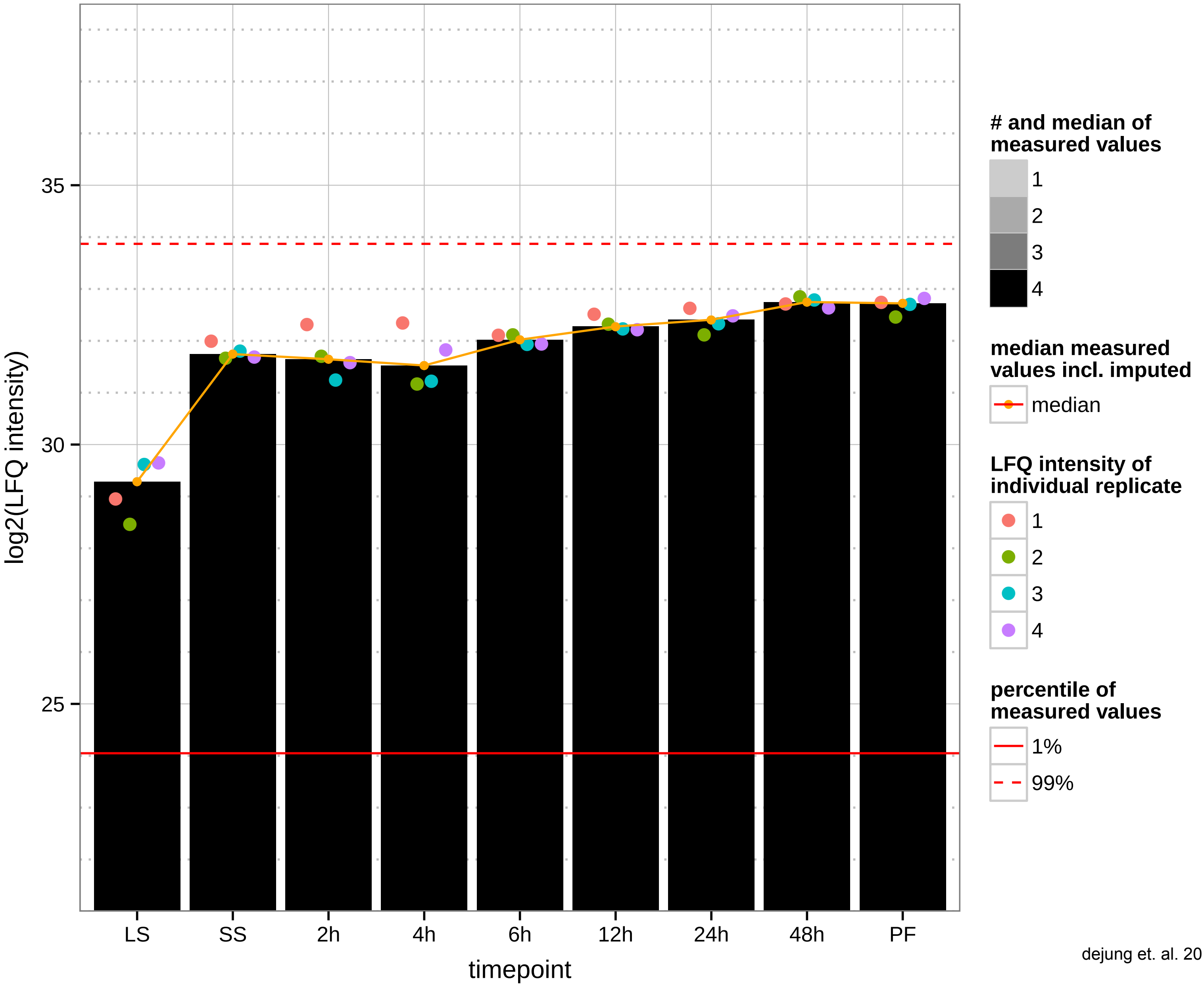

dejung et. al. 2015

hypothetical protein, conserved
Tb927.10.14560;Tb11.v5.0477
AGOF: null
AGOC: null
AGOP: null
PGOF: protein binding
PGOC: null
PGOP: null

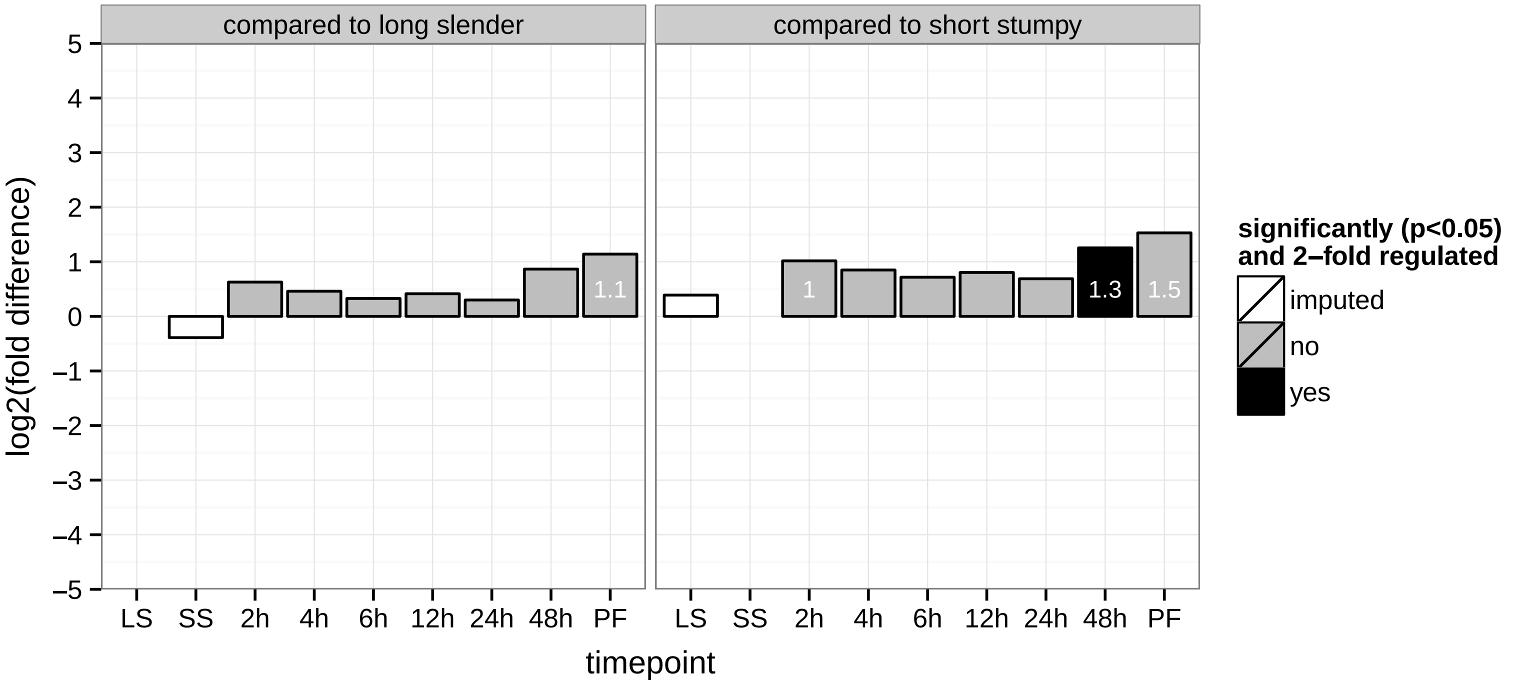

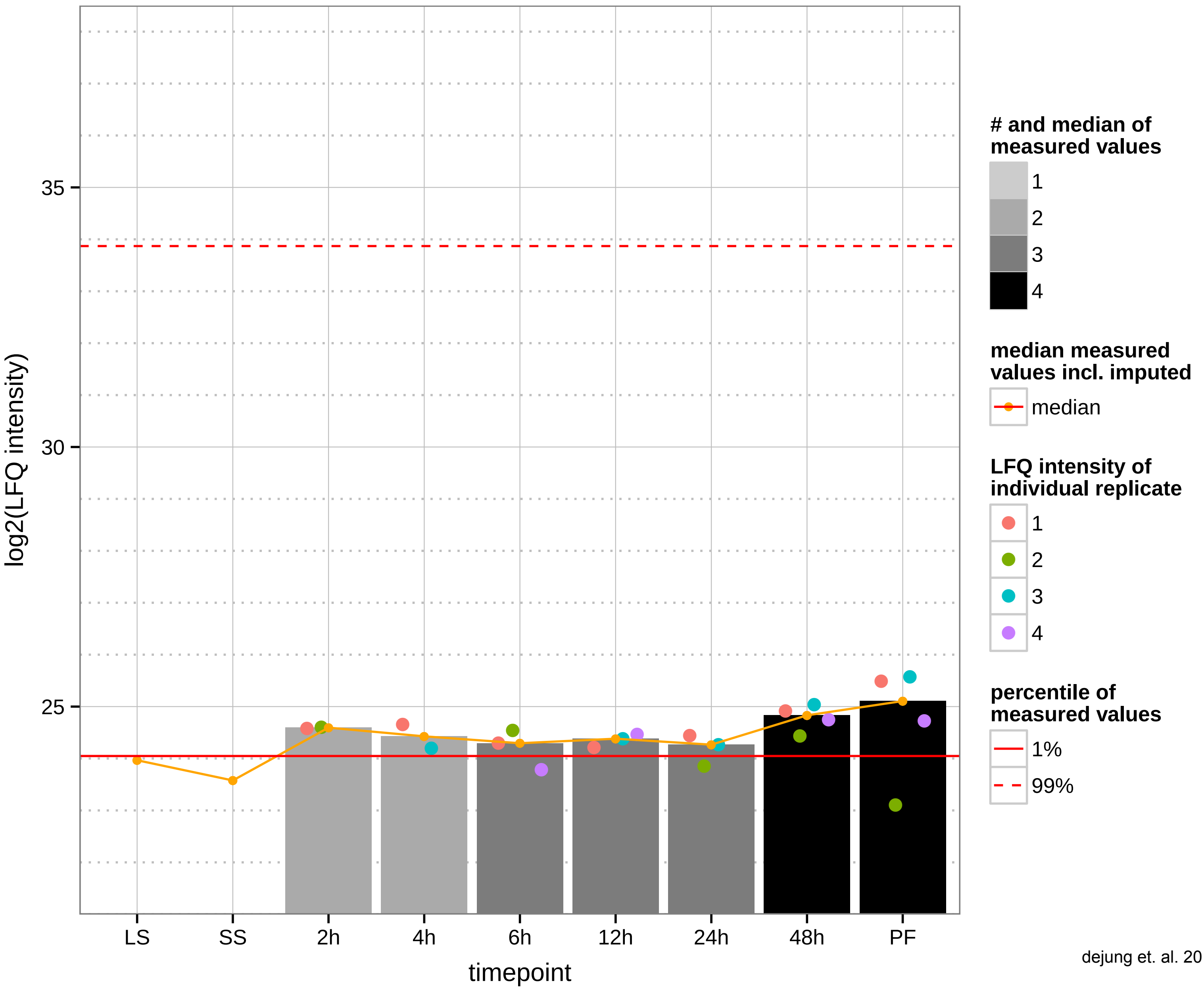

dejung et. al. 2015

metallo–peptidase, Clan MG, Family M24, aminopeptidase, putative
Tb927.10.14790
AGOF: null
AGOC: null
AGOP: cellular process
PGOF: null
PGOC: null
PGOP: cellular process

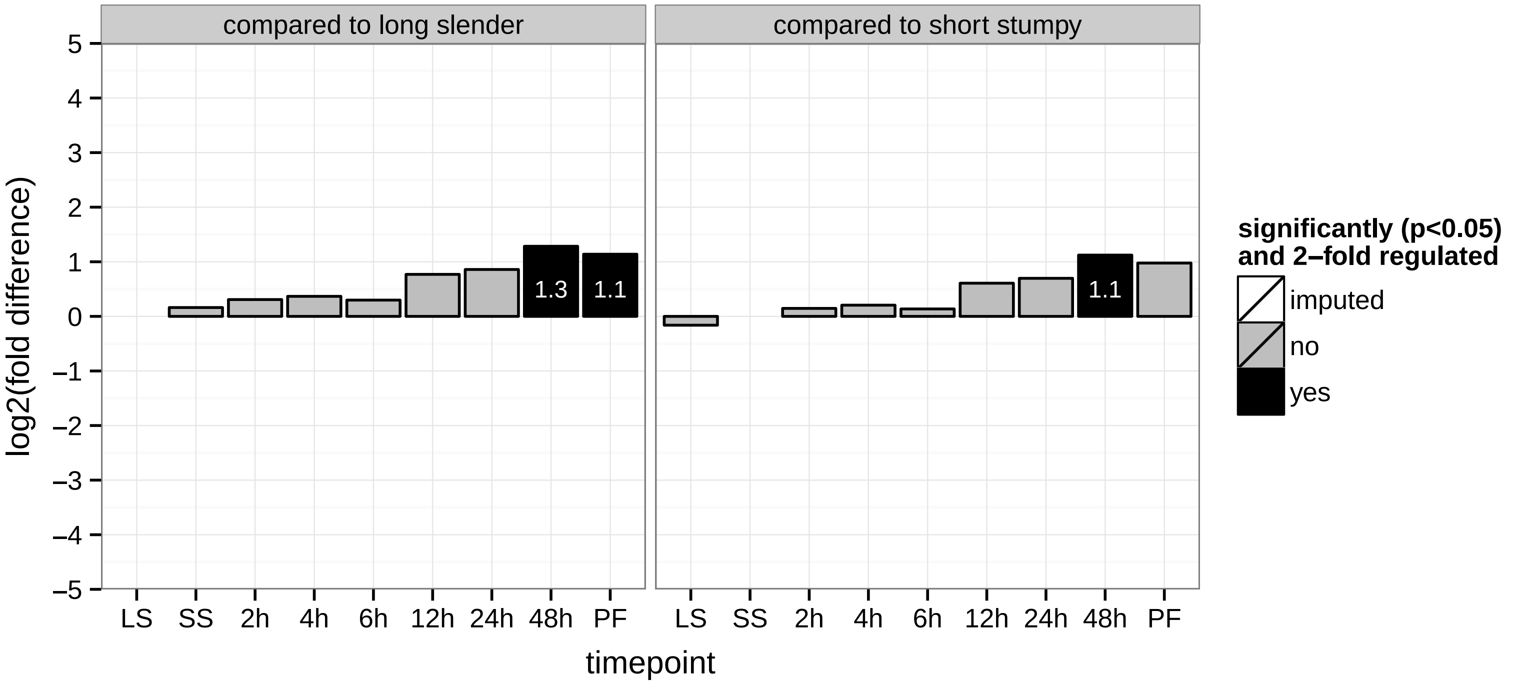

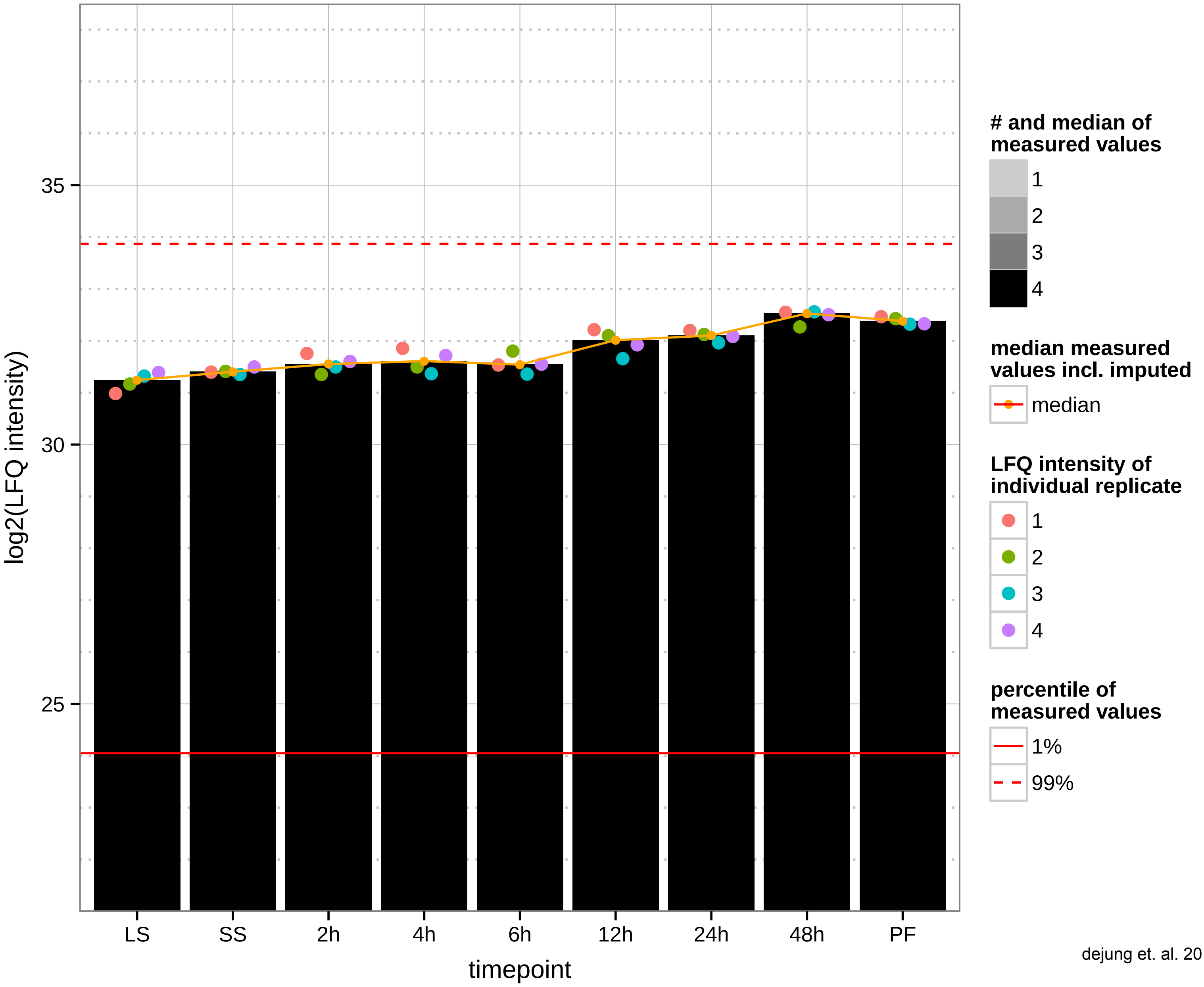

dejung et. al. 2015

hypothetical protein, conserved
Tb927.10.1730
AGOF: null
AGOC: null
AGOP: null
PGOF: null
PGOC: null
PGOP: null

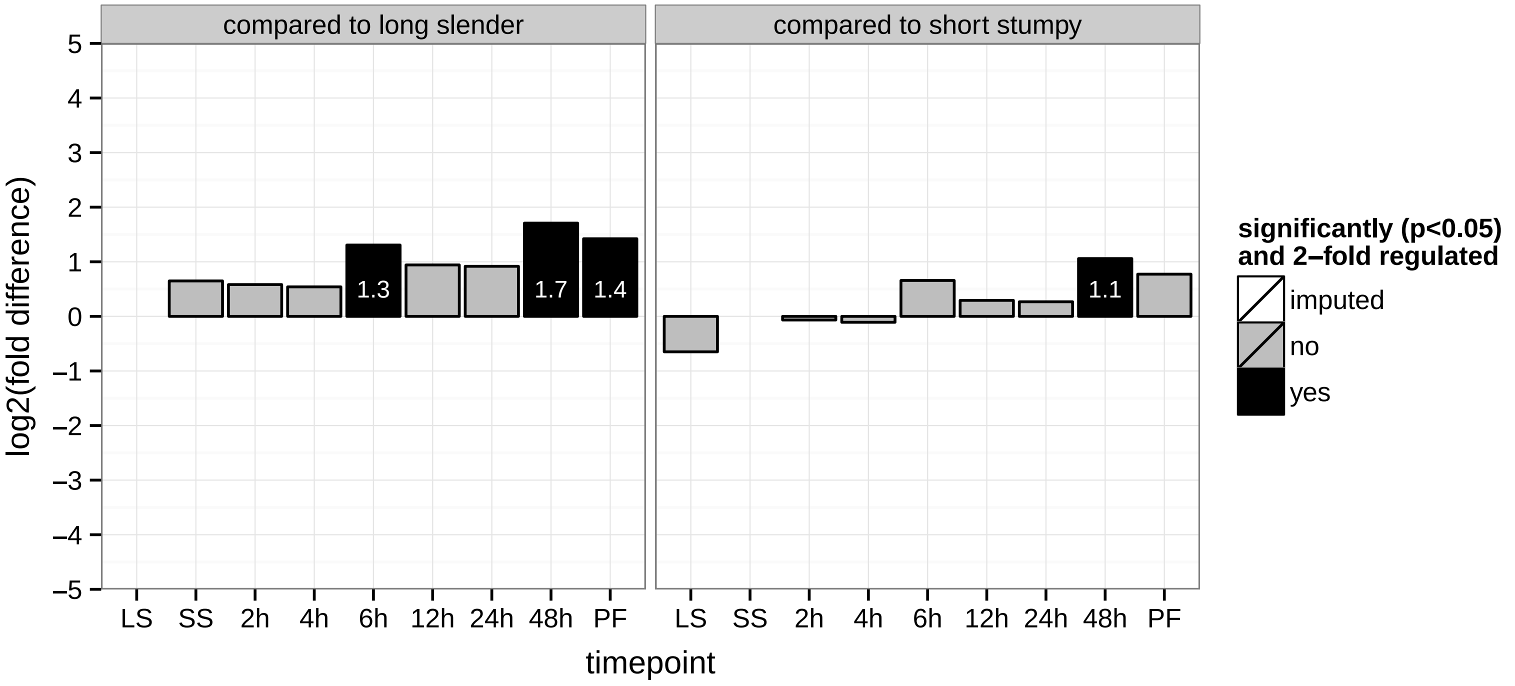

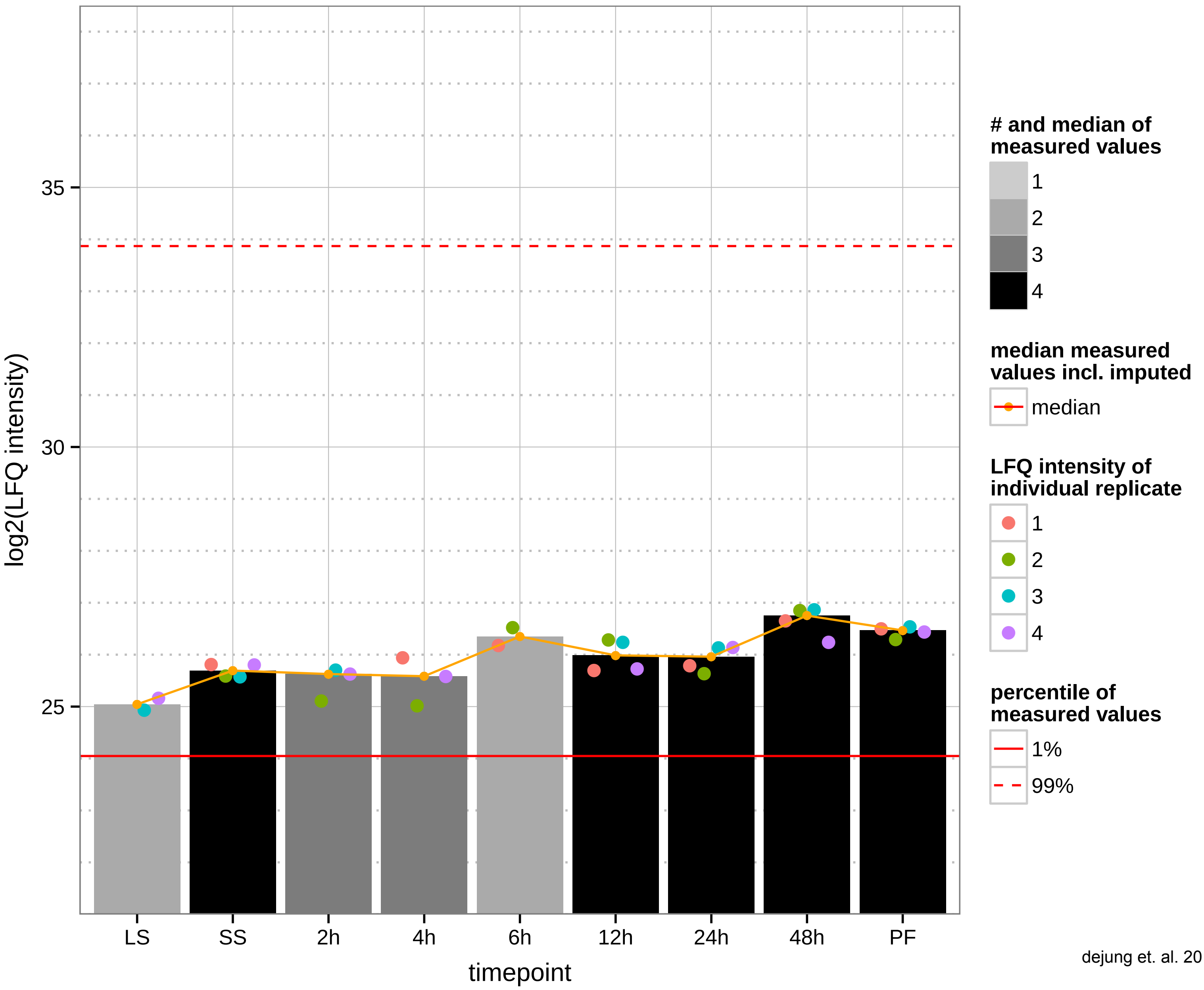

dejung et. al. 2015

40S ribosomal protein S9, putative
Tb927.8.1110;Tb927.10.5610
AGOF: RNA binding, rRNA binding, structural constituent of ribosome, rRNA binding
AGOC: intracellular, ribosome, small ribosomal subunit
AGOP: translation
PGOF: RNA binding, rRNA binding, structural constituent of ribosome
PGOC: intracellular, small ribosomal subunit
PGOP: translation

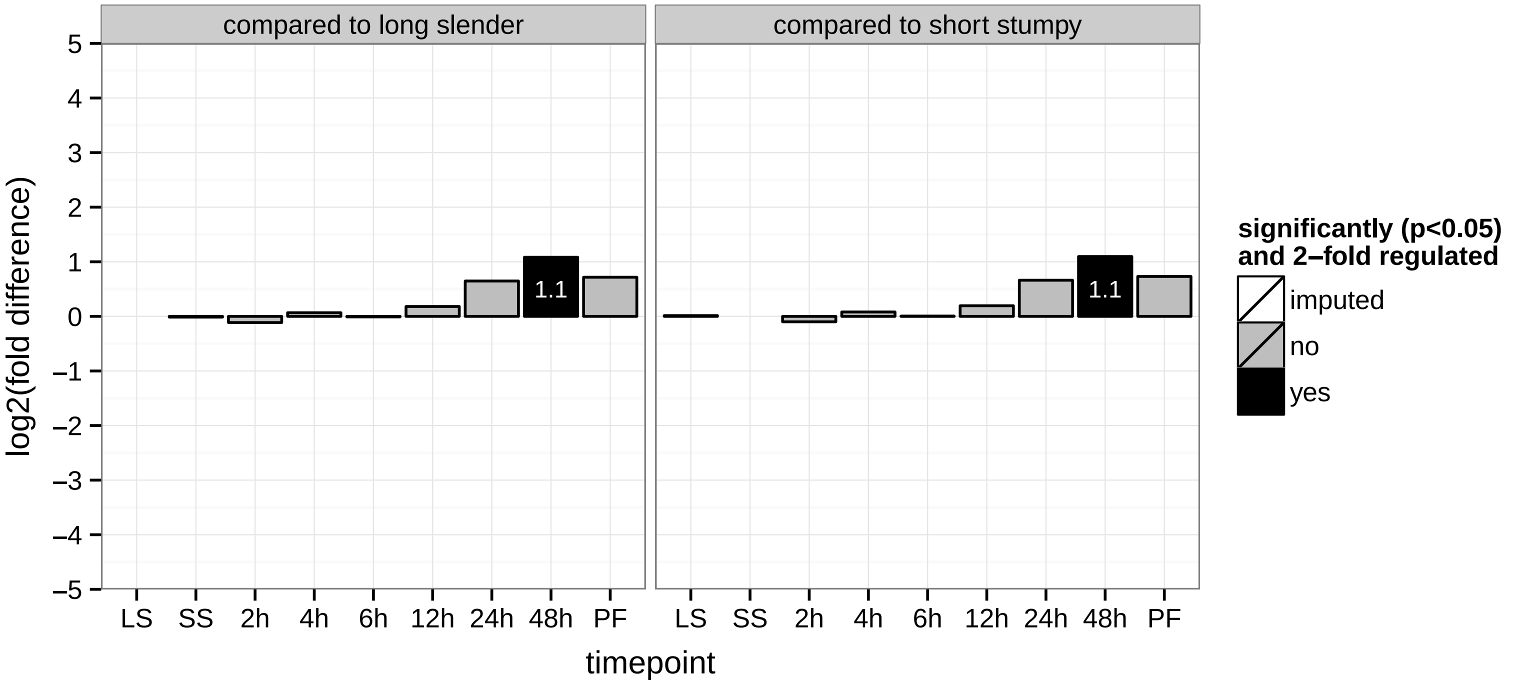

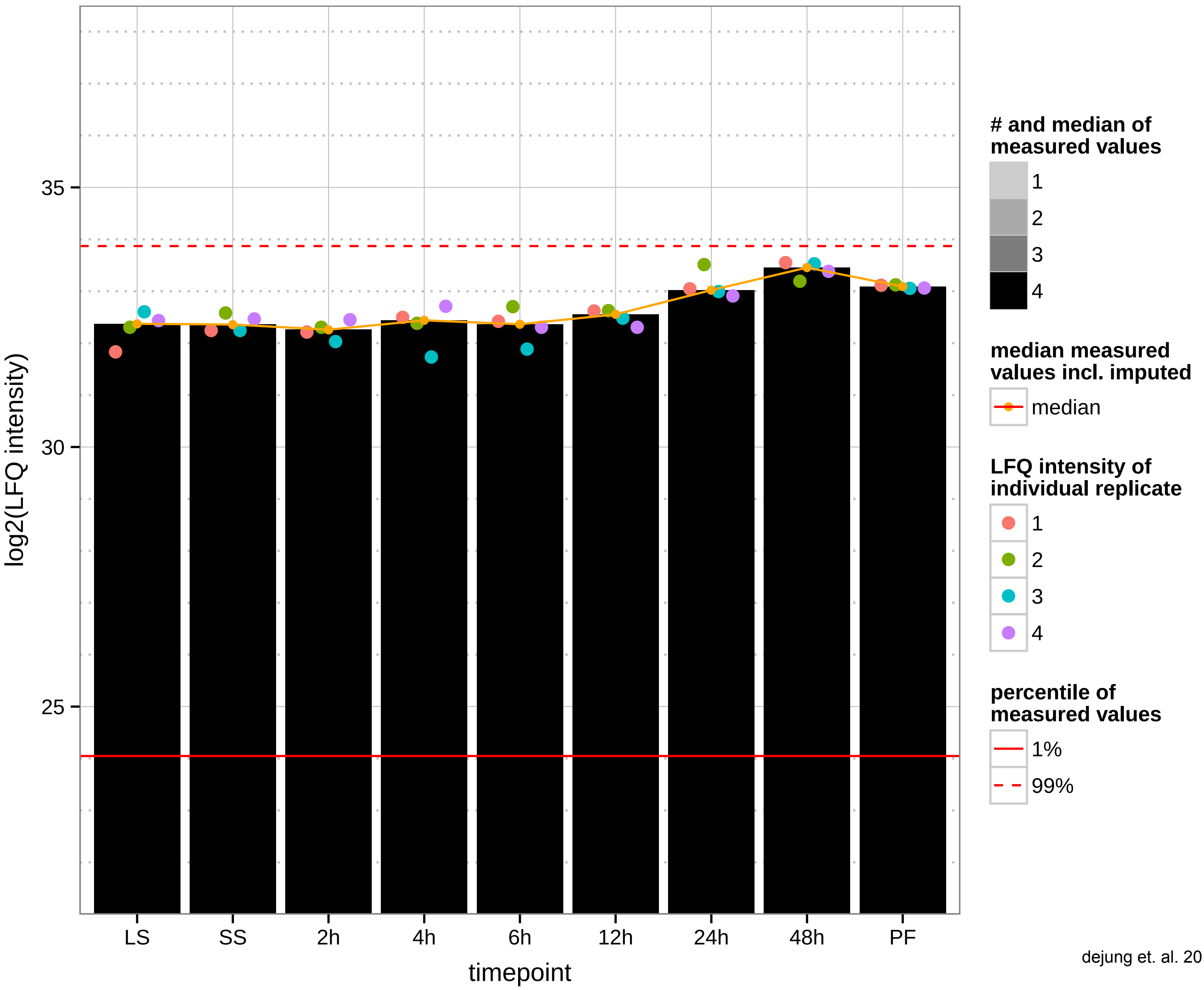

dejung et. al. 2015

Proteophosphoglycan, putative
Tb927.10.5880
AGOF: null
AGOC: null
AGOP: null
PGOF: null
PGOC: null
PGOP: null

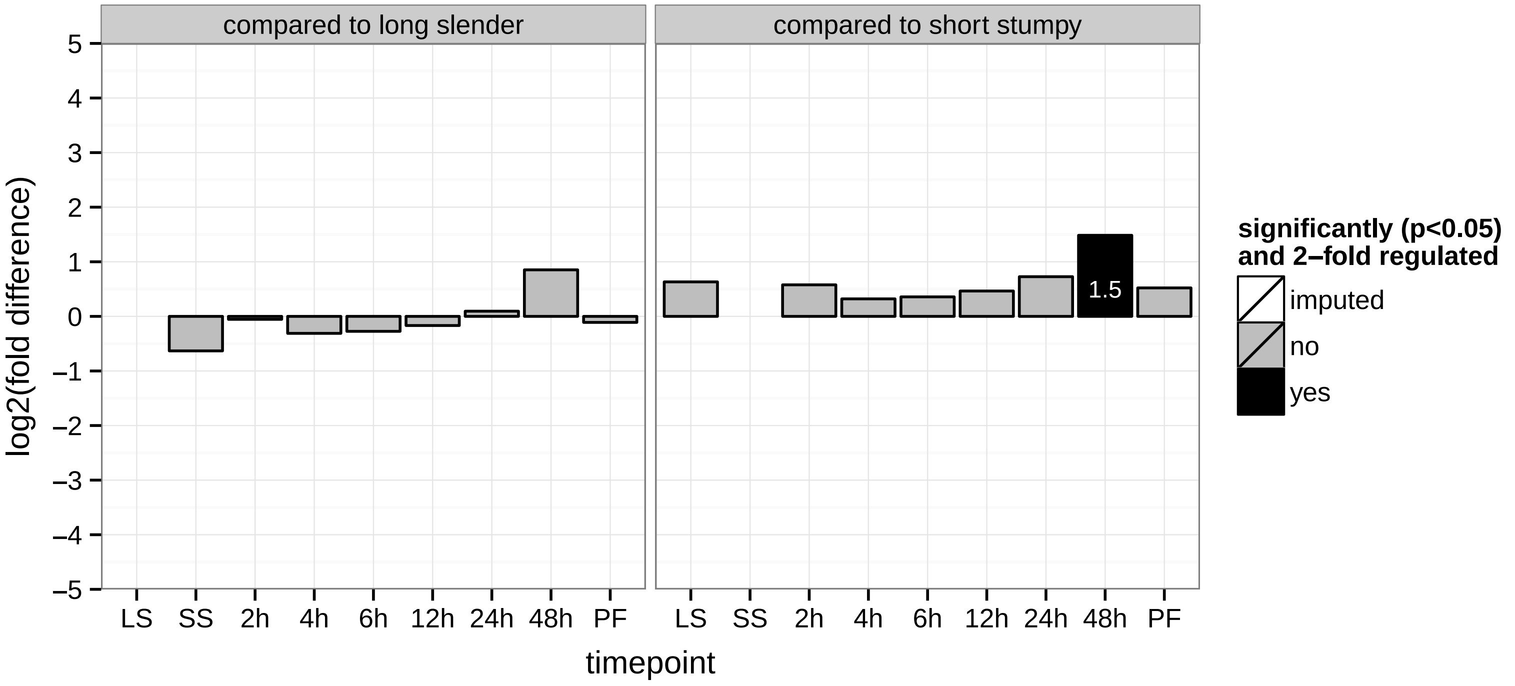

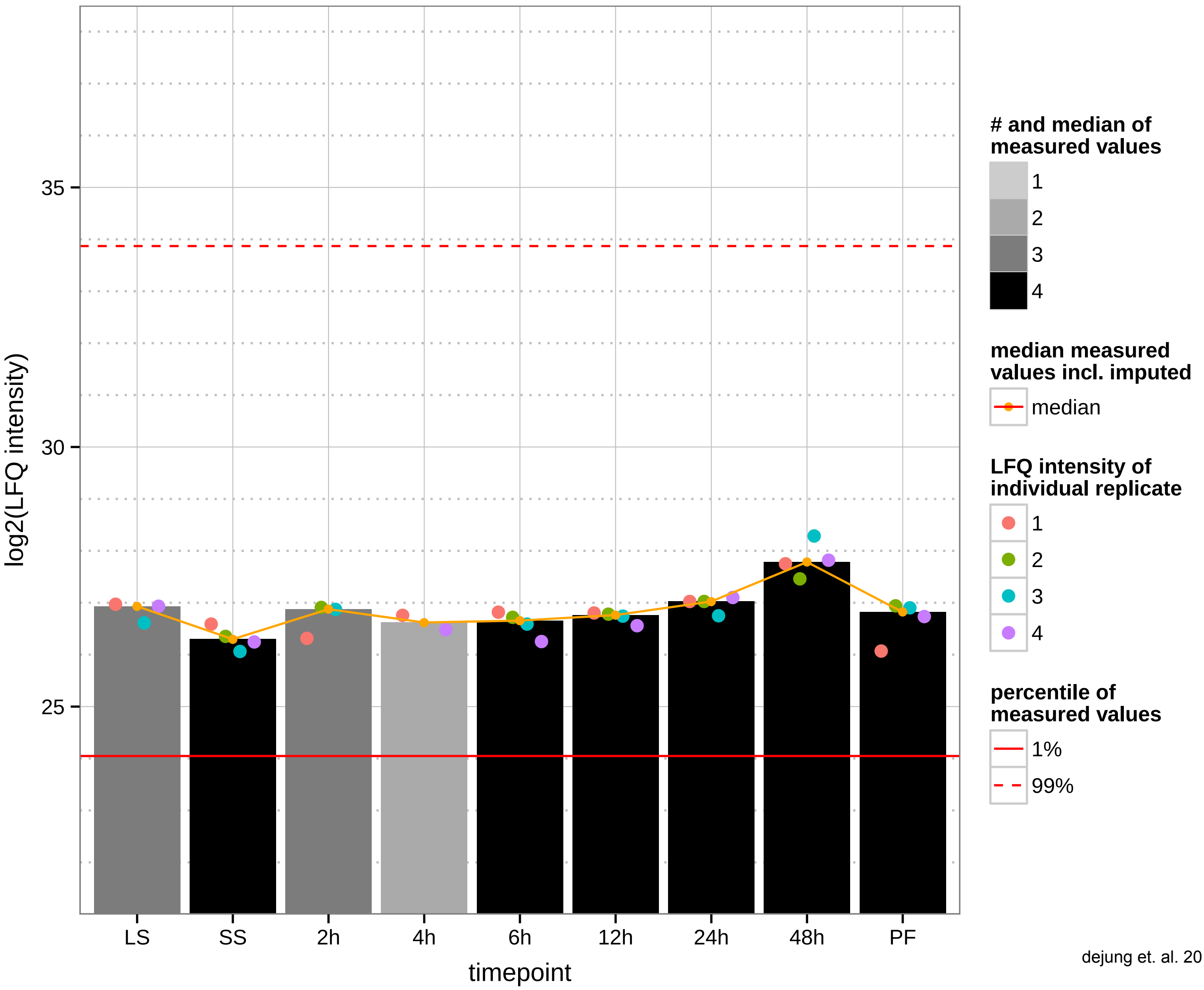

dejung et. al. 2015

T–complex protein 1, theta subunit, putative, CCT–theta, putative (TCP–1–theta)
Tb927.10.8190
AGOF: ATP binding, unfolded protein binding
AGOC: chaperonin–containing T–complex
AGOP: protein folding, regulation of cell cycle
PGOF: ATP binding, unfolded protein binding
PGOC: null
PGOP: cellular protein metabolic process, protein folding

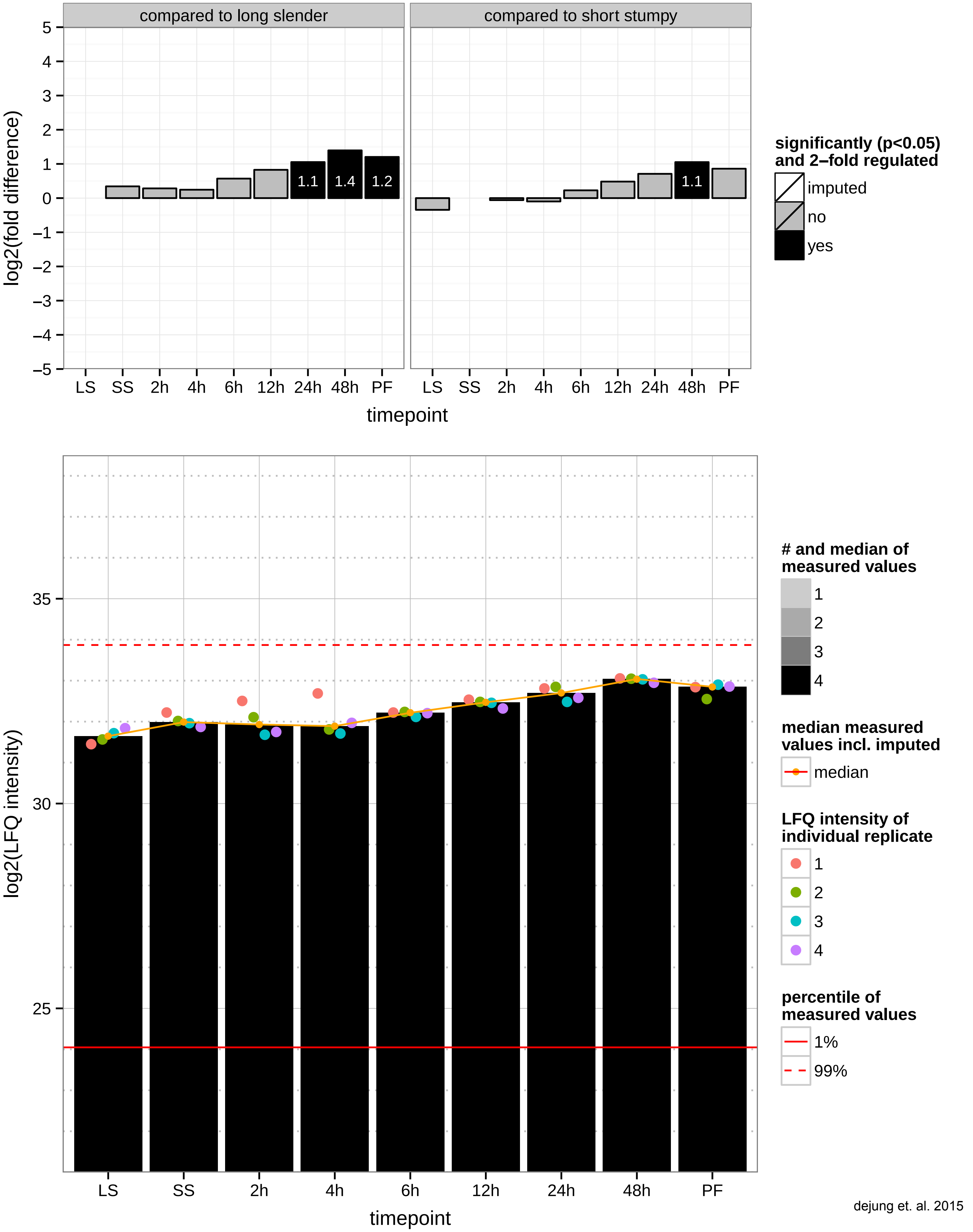

dejung et. al. 2015

hypothetical protein, conserved
Tb927.10.820
AGOF: null
AGOC: null
AGOP: null
PGOF: null
PGOC: null
PGOP: null

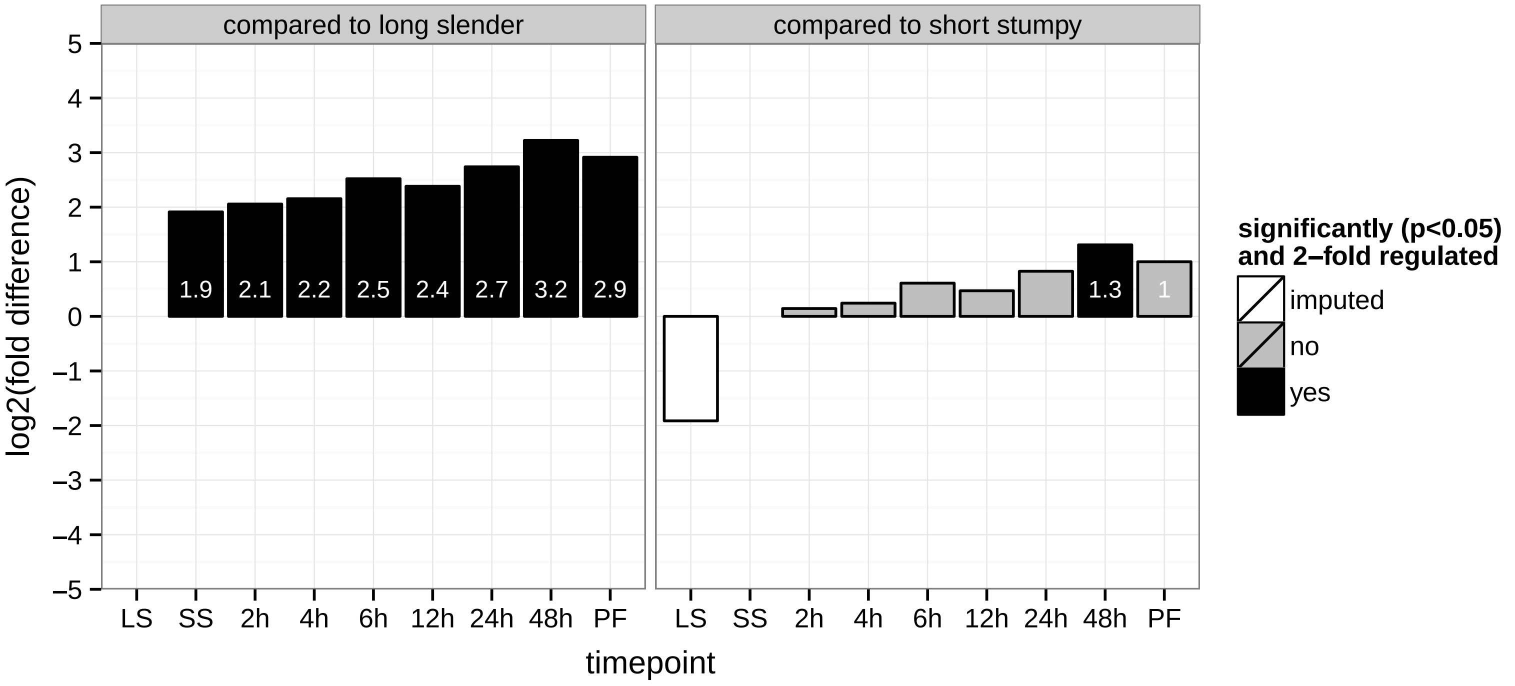

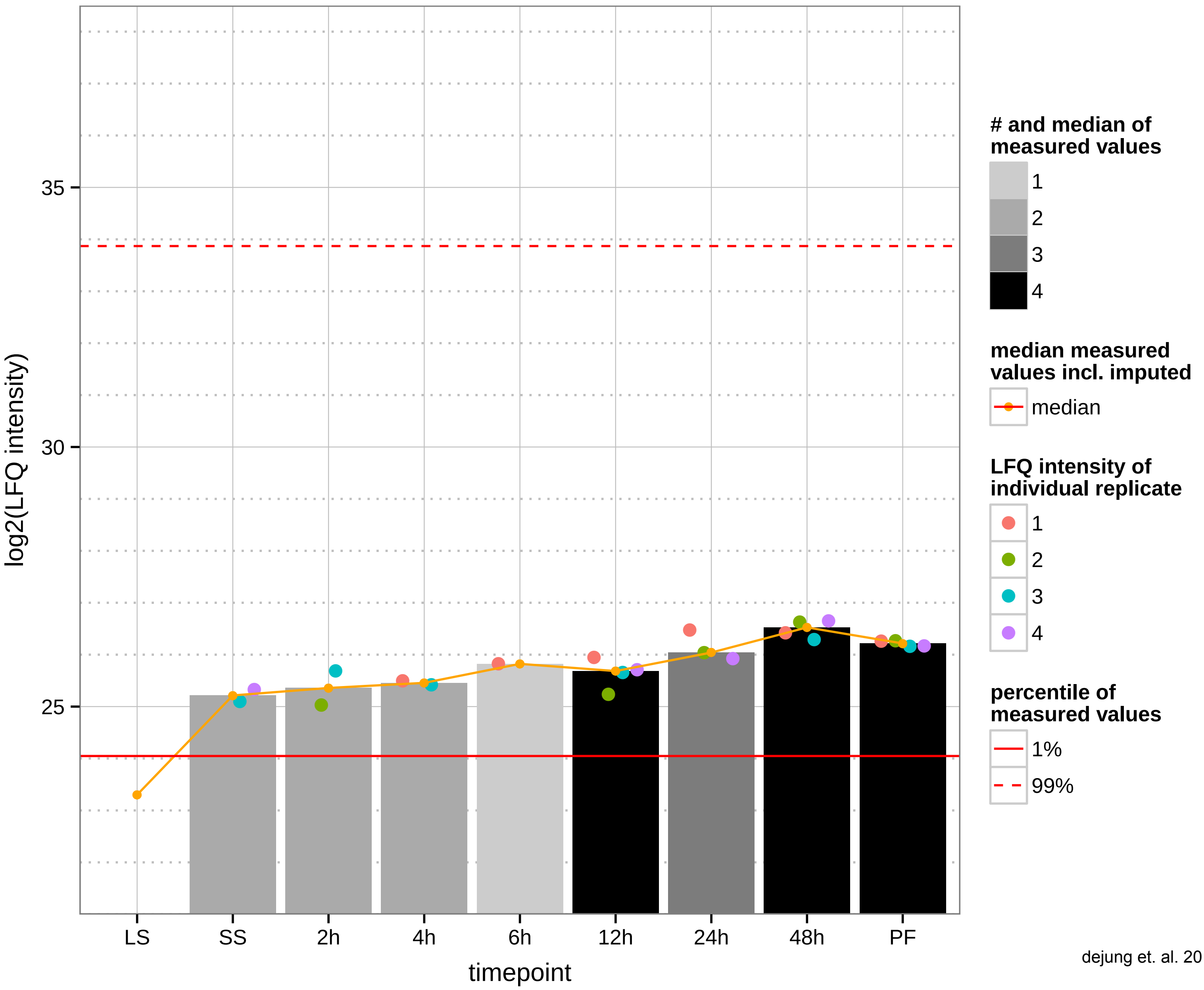

dejung et. al. 2015

40S ribosomal protein S12, putative
Tb927.10.8430
AGOF: structural constituent of ribosome
AGOC: intracellular, ribosome
AGOP: translation
PGOF: null
PGOC: null
PGOP: null

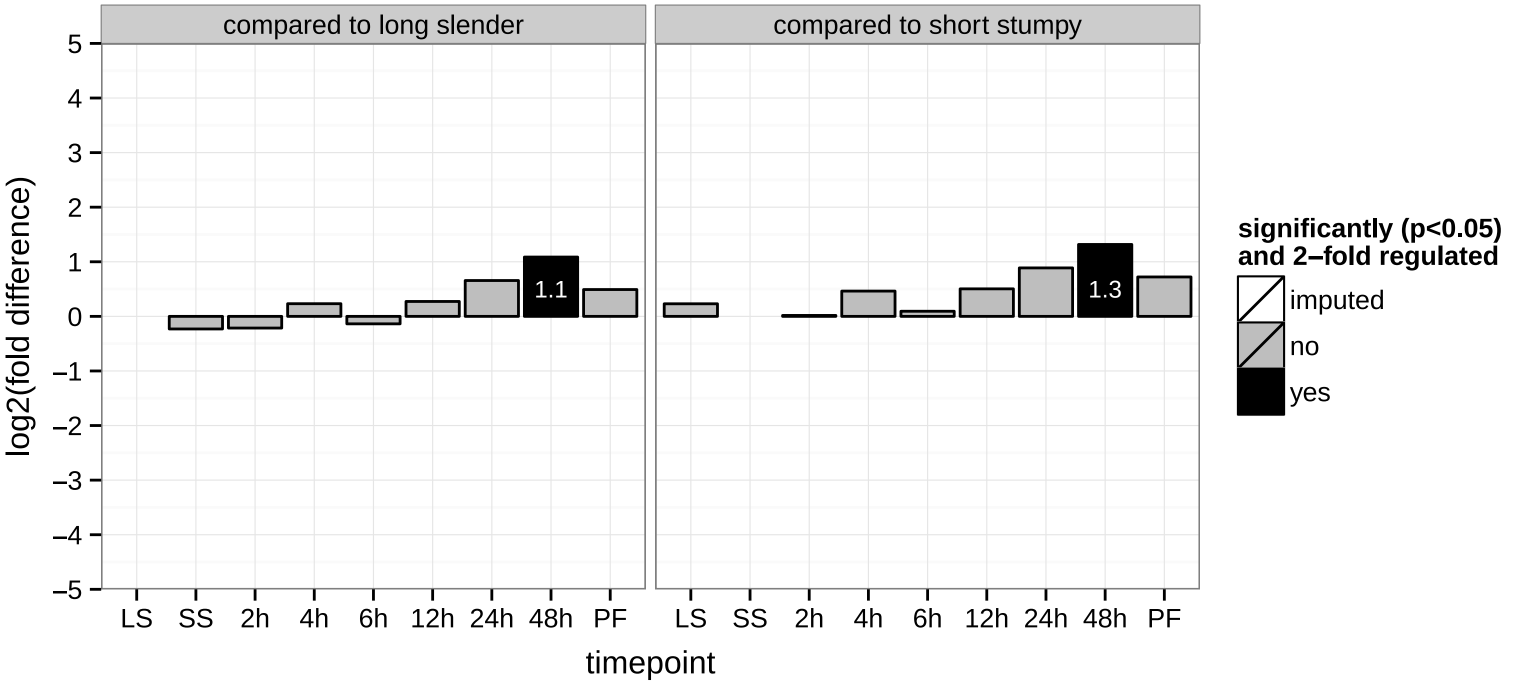

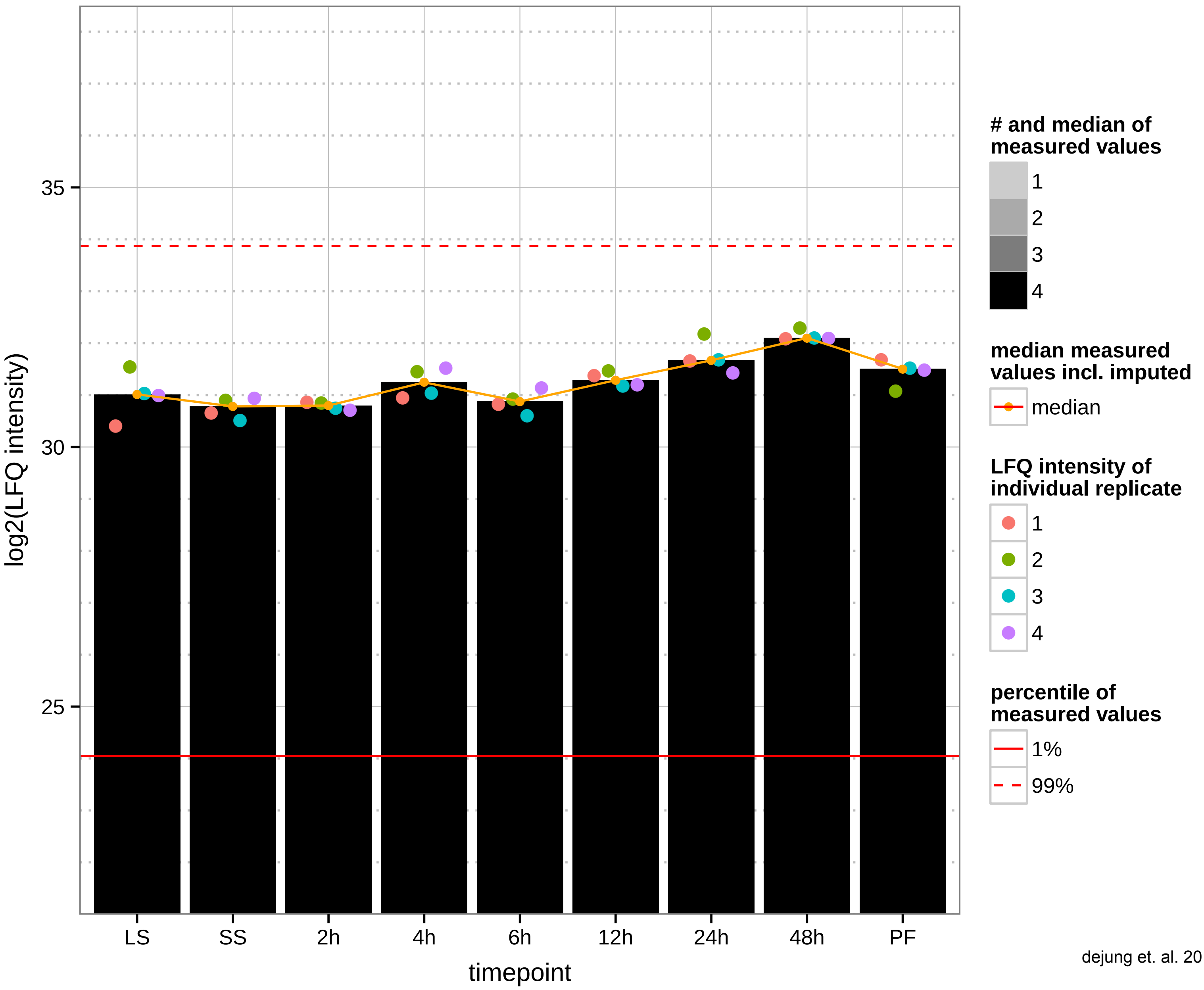

dejung et. al. 2015

hypothetical protein, conserved
Tb927.10.9200
AGOF: null
AGOC: null
AGOP: null
PGOF: null
PGOC: null
PGOP: null

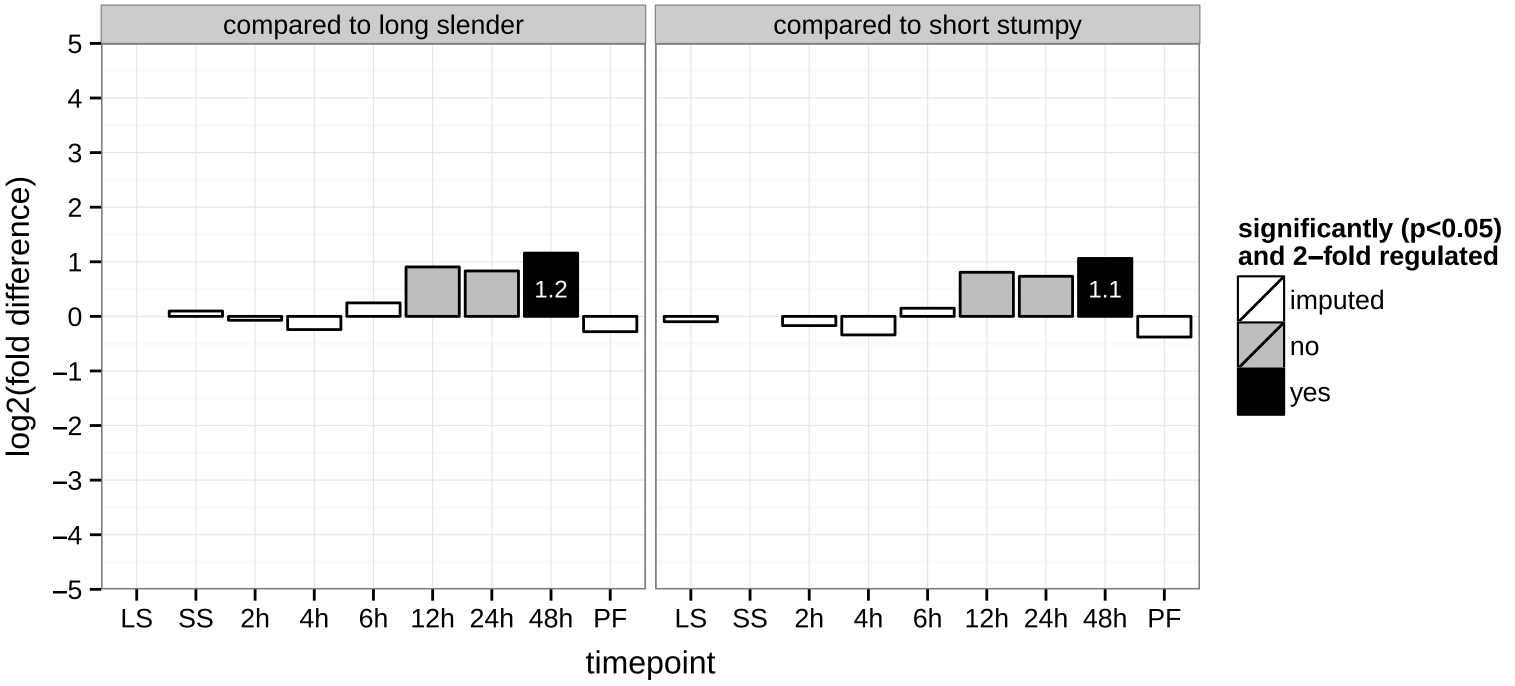

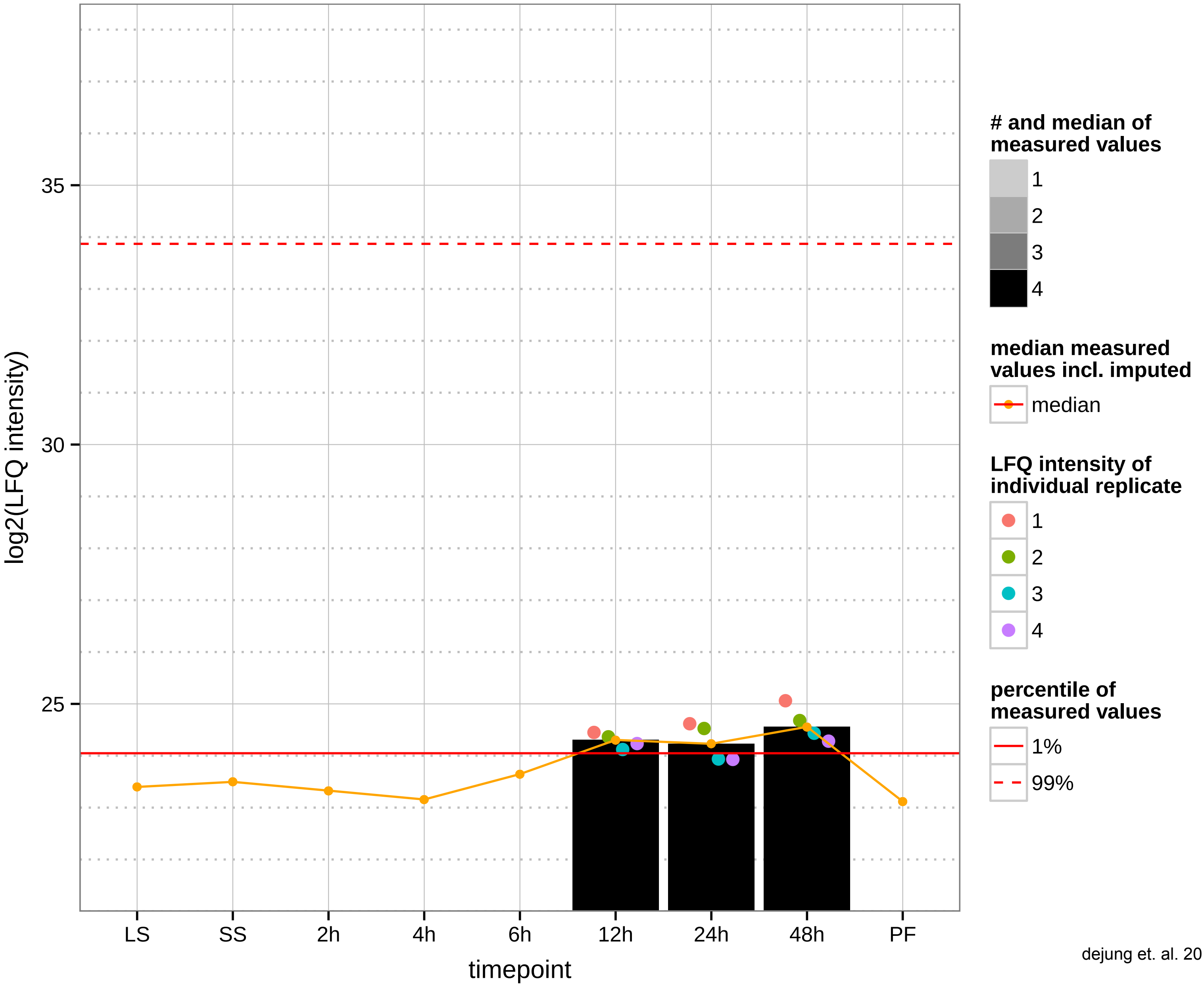

dejung et. al. 2015

RNA–editing–associated protein 1, RNA–binding protein, oligo (U) binding protein, RNA editing complex protein (REAP–1)
Tb927.10.9720
AGOF: null
AGOC: mitochondrial mRNA editing complex
AGOP: RNA modification
PGOF: null
PGOC: null
PGOP: null

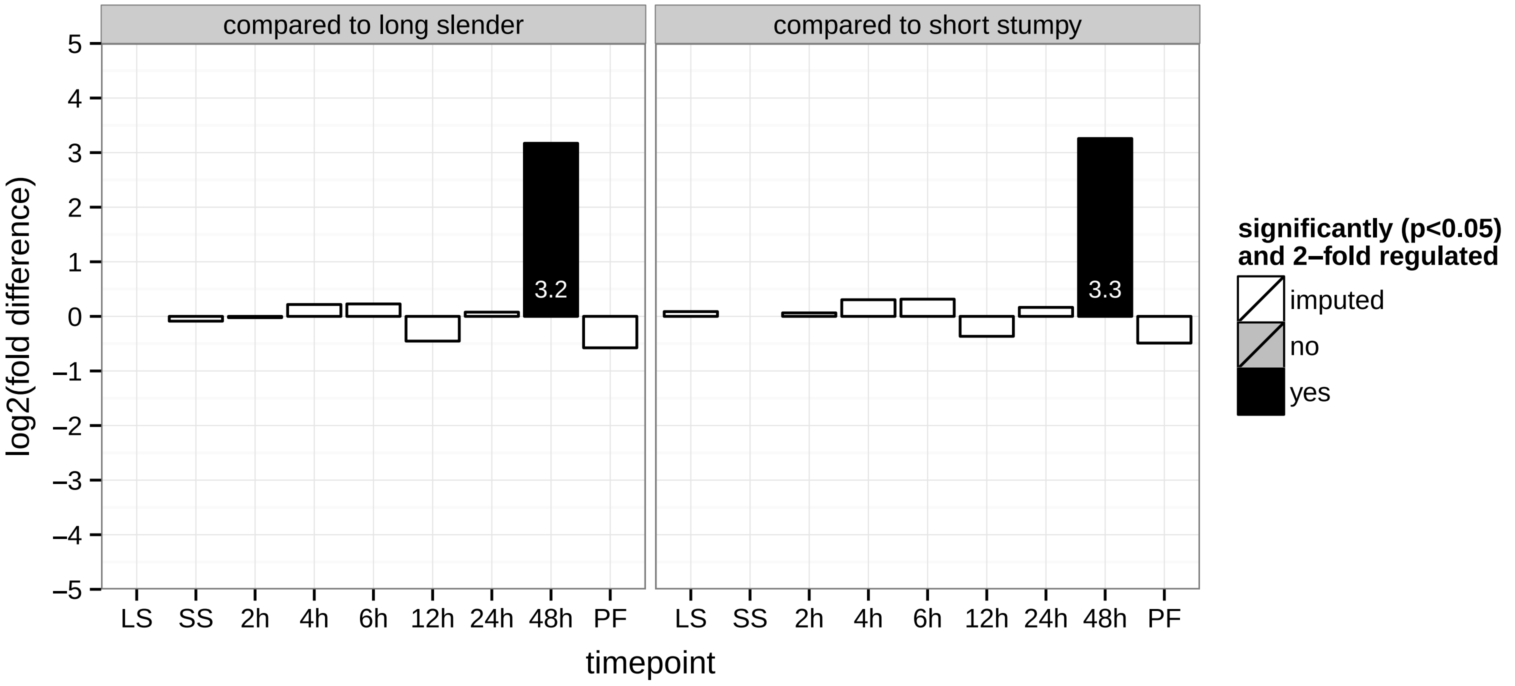

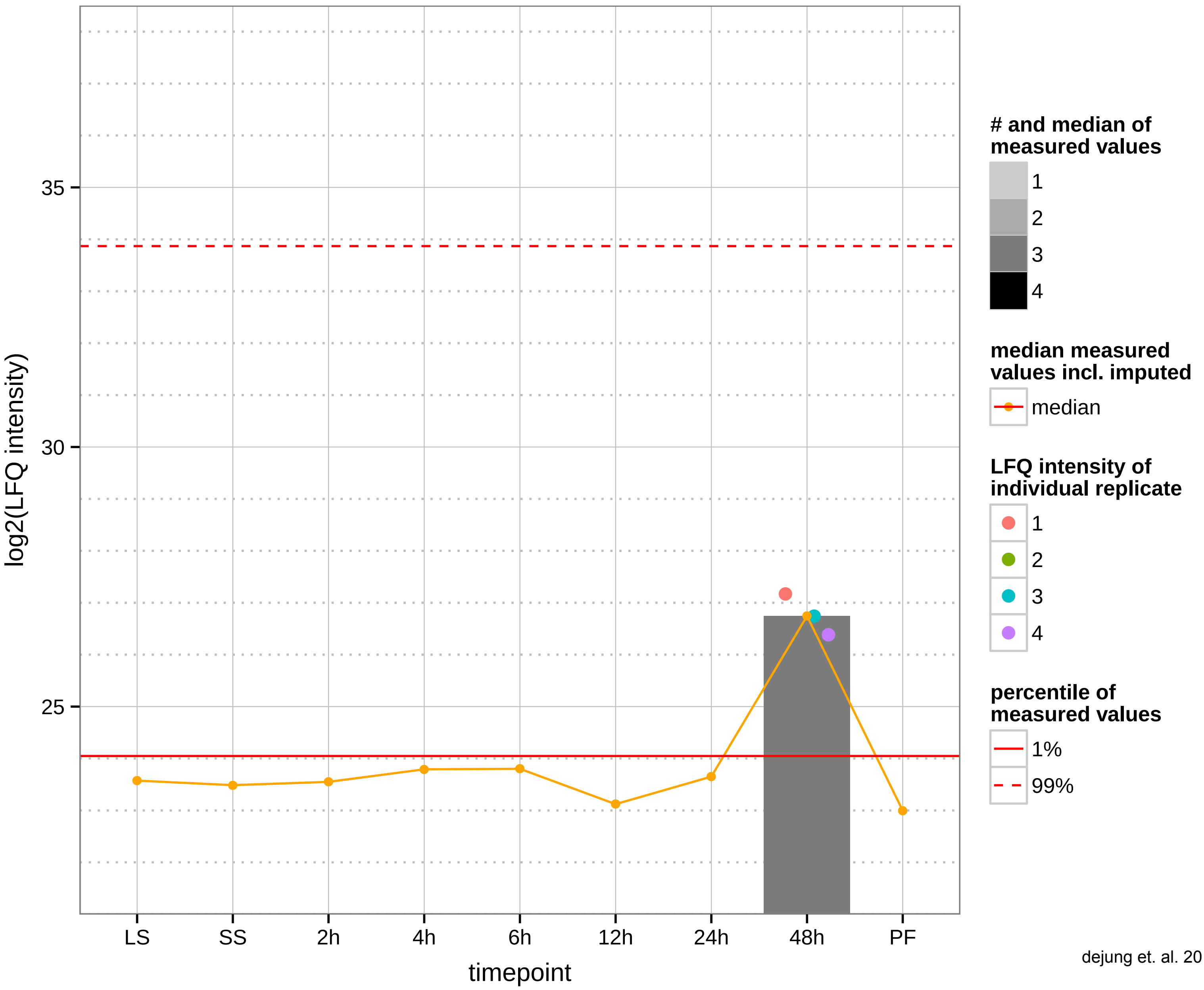

dejung et. al. 2015

hypothetical protein, conserved
Tb927.11.10220
AGOF: null
AGOC: null
AGOP: null
PGOF: null
PGOC: null
PGOP: null

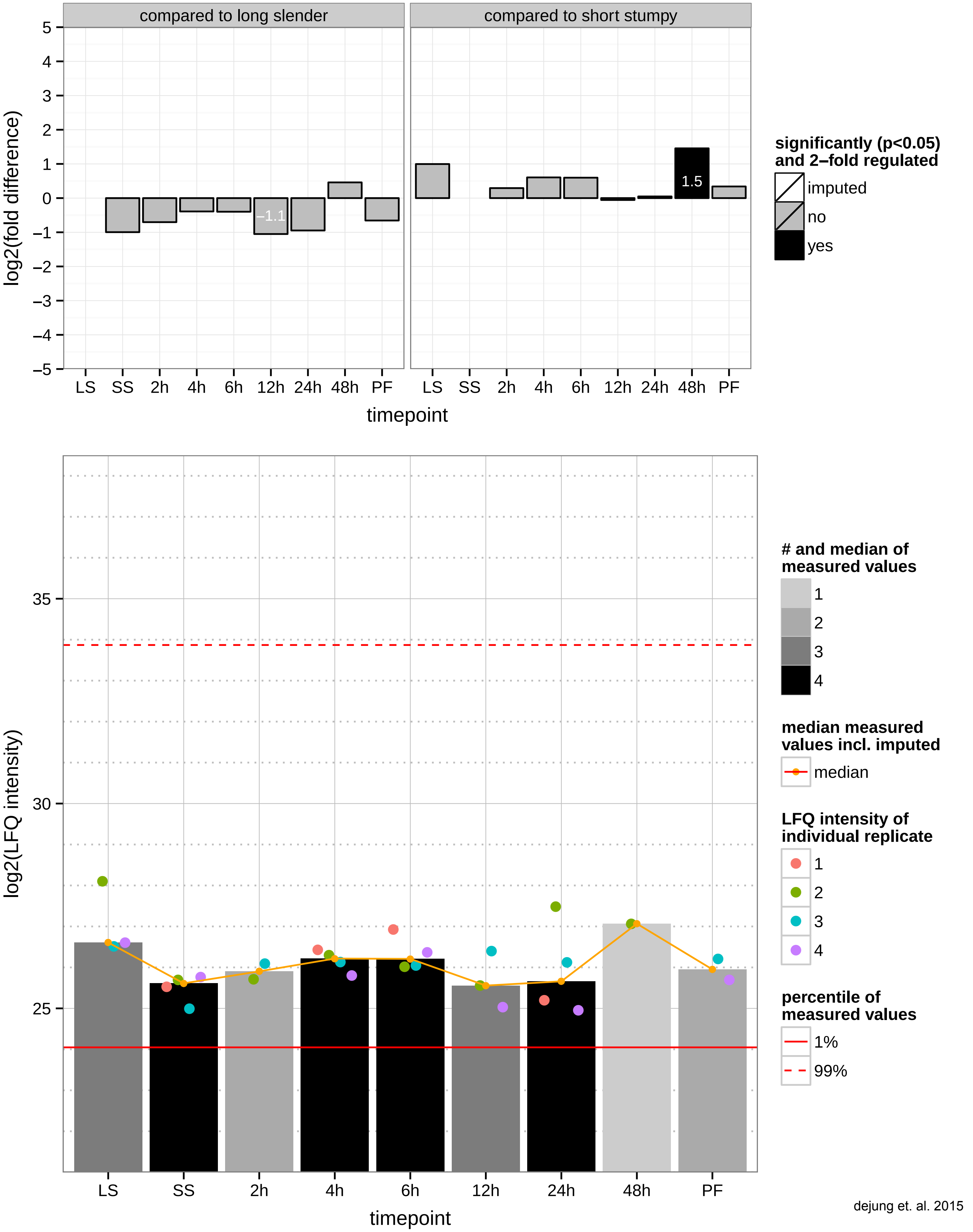

dejung et. al. 2015

hypothetical protein, conserved
Tb927.11.10300
AGOF: null
AGOC: null
AGOP: null
PGOF: protein binding
PGOC: null
PGOP: null

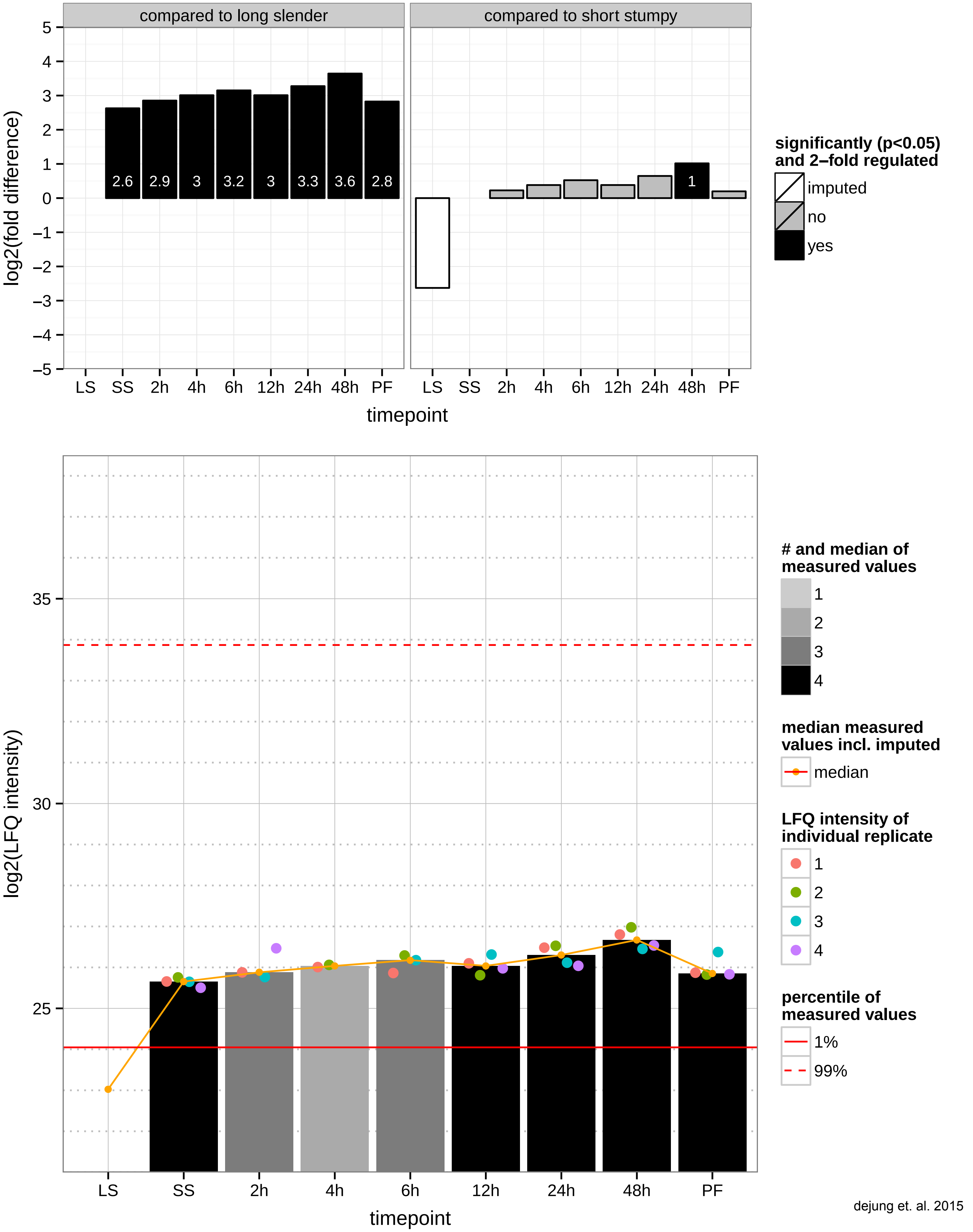

dejung et. al. 2015

heat shock protein HslVU, ATPase subunit HslU, putative, ATP−dependent hsl protease ATP−binding subunit hslU
Tb927.11.12230
AGOF: ATP binding, ATP−dependent peptidase activity, ATPase activity, peptidase activity, acting on L−amino acid peptides
AGOC: HslUV protease complex
AGOP: null
PGOF: ATP binding, ATPase activity, nucleoside−triphosphatase activity, nucleotide binding, peptidase activity, acting on L−an
PGOC: HslUV protease complex, cytoplasm
PGOP: null

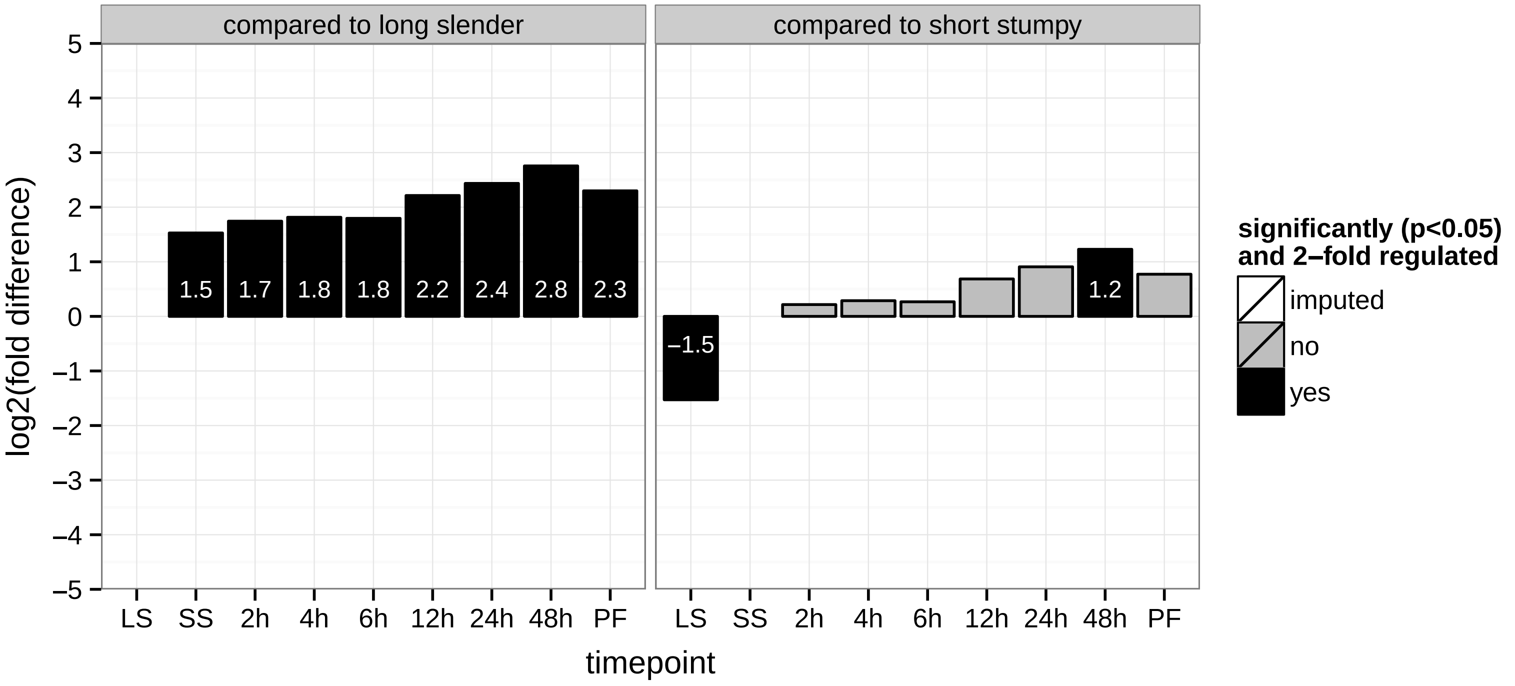

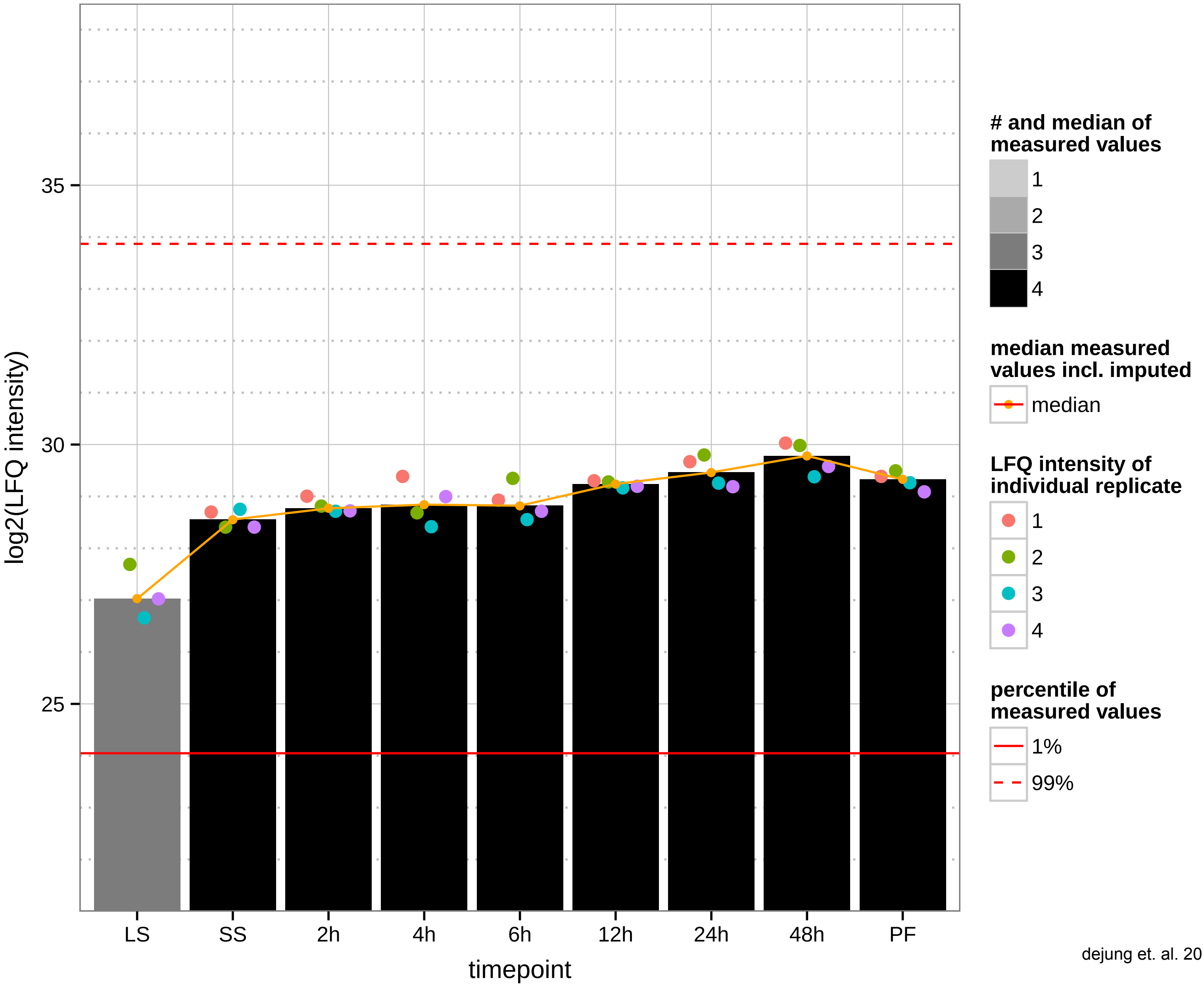

dejung et. al. 2015

hypothetical protein, conserved
Tb927.11.13340
AGOF: null
AGOC: null
AGOP: null
PGOF: null
PGOC: null
PGOP: null

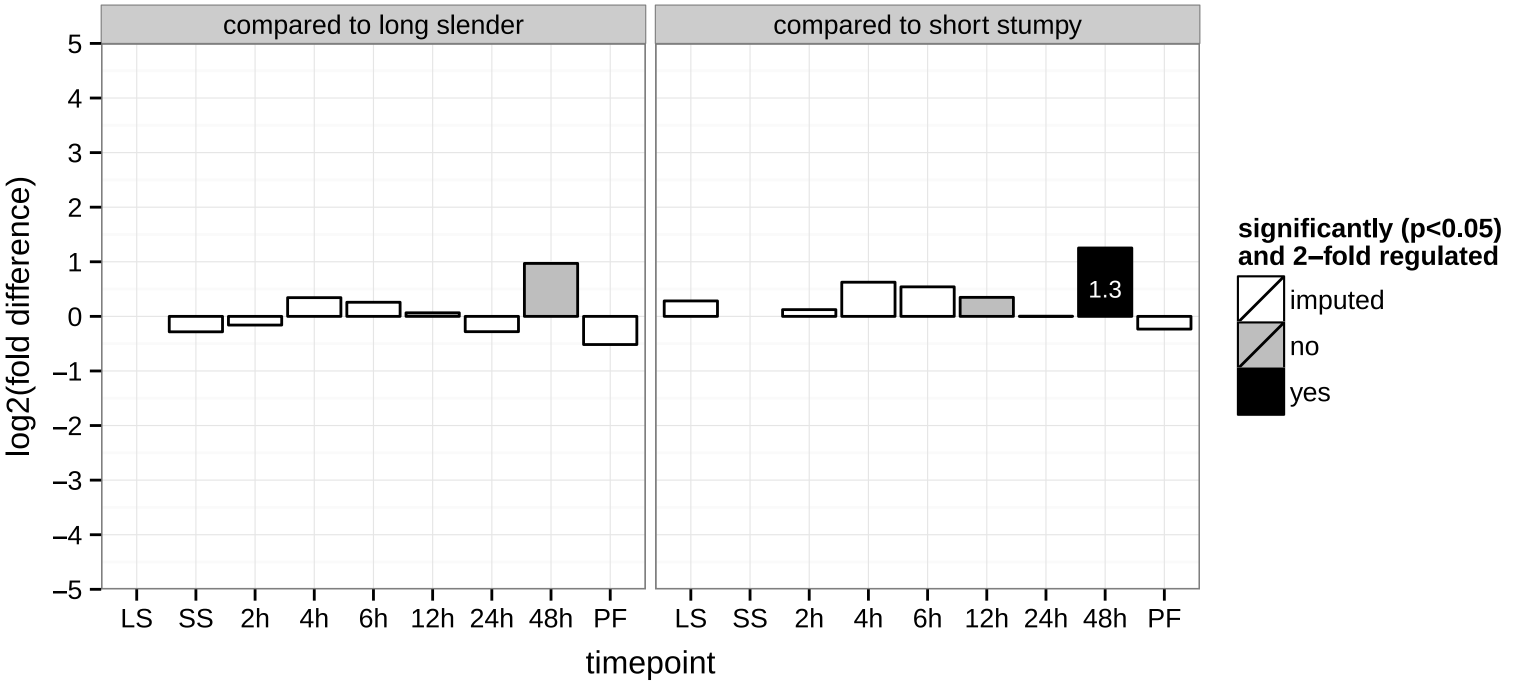

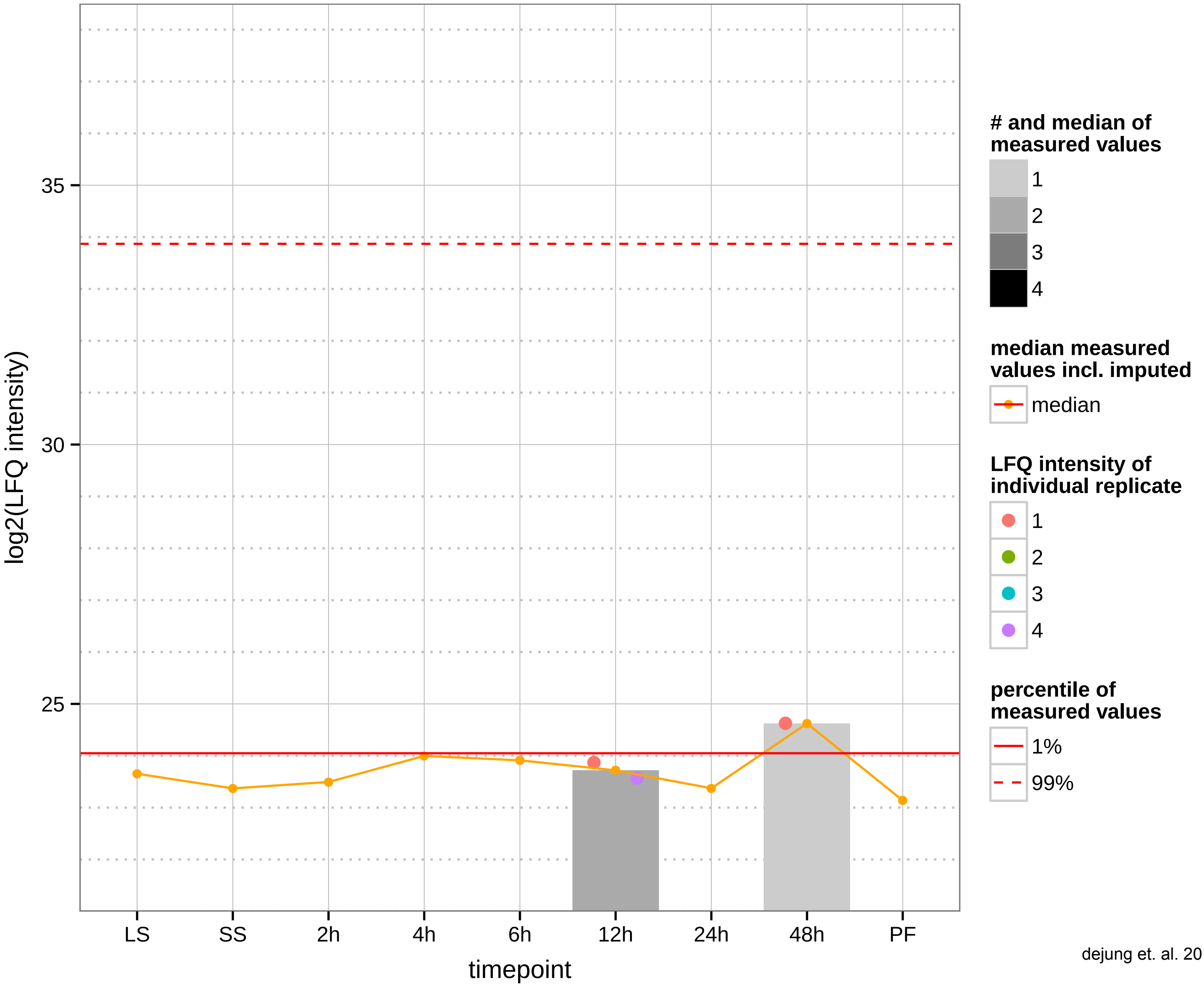

dejung et. al. 2015

phosphatidylinositol 3–related kinase, putative (ATR)
Tb927.11.14680
AGOF: 1–phosphatidylinositol–3–kinase activity, phosphotransferase activity, alcohol group as acceptor
AGOC: null
AGOP: cytokinesis checkpoint, growth
PGOF: phosphotransferase activity, alcohol group as acceptor, protein binding, transferase activity, transferring phosphorus–c
PGOC: null
PGOP: null

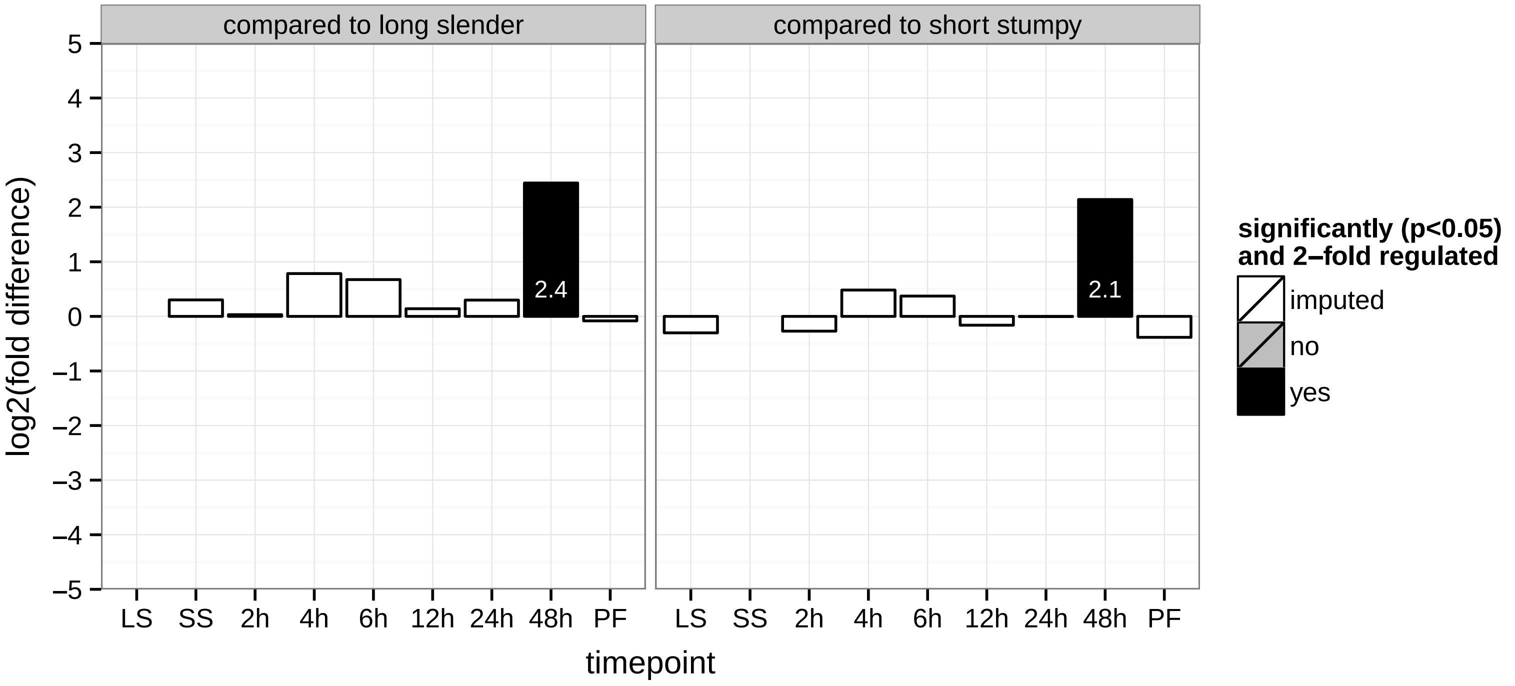

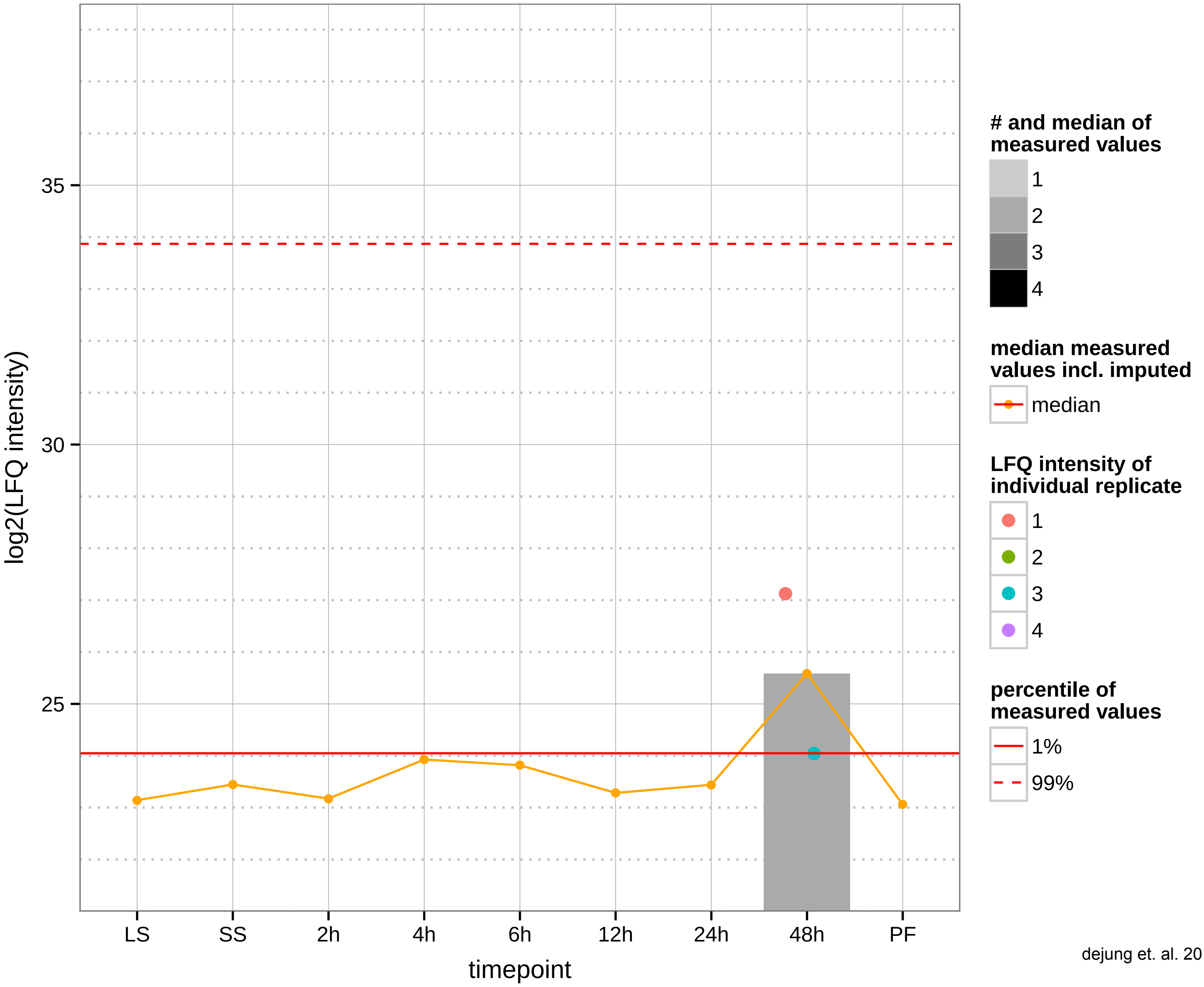

dejung et. al. 2015

metalloprotease, putative, cell division protein FtsH homologue
Tb927.11.14730
AGOF: ATP binding, ATPase activity, metalloendopeptidase activity, zinc ion binding
AGOC: integral to membrane, mitochondrial inner membrane
AGOP: proteolysis
PGOF: ATP binding, metalloendopeptidase activity, nucleoside−triphosphatase activity, nucleotide binding
PGOC: null
PGOP: proteolysis

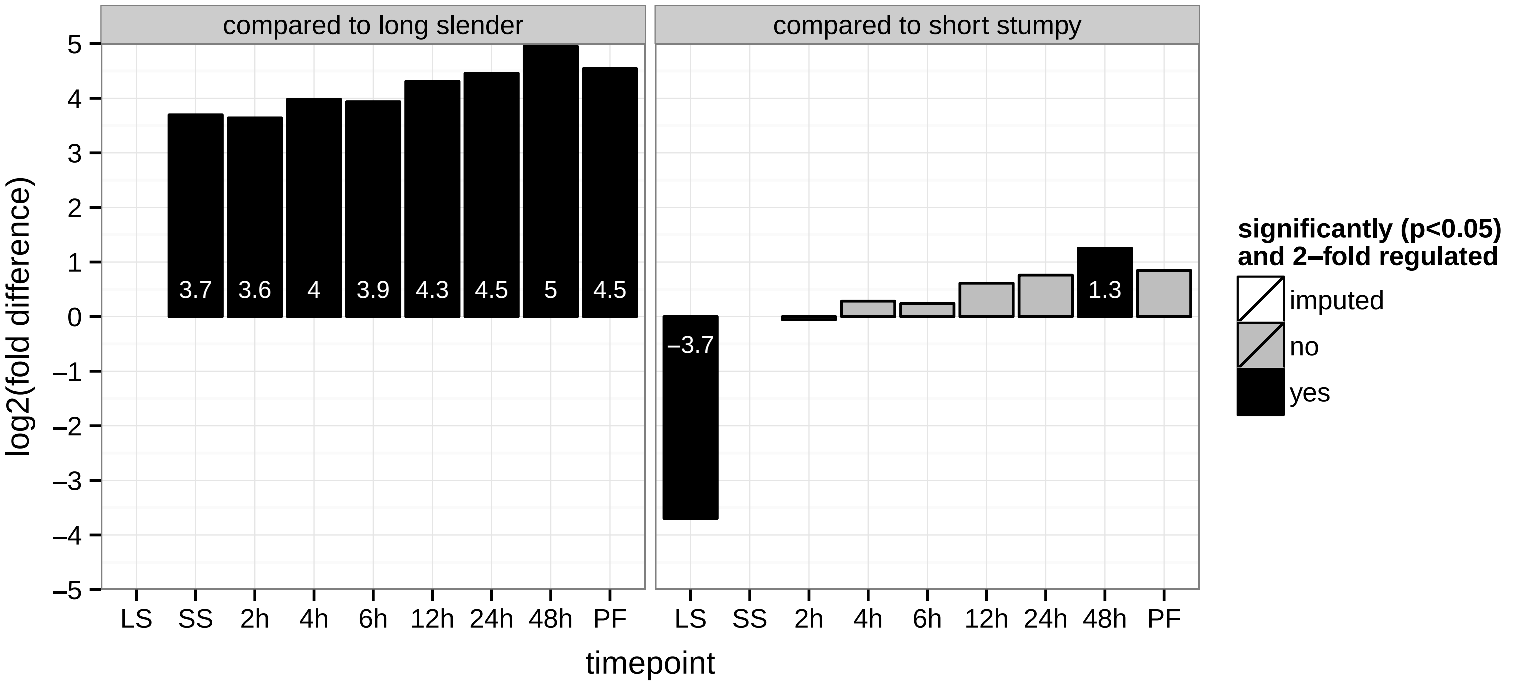

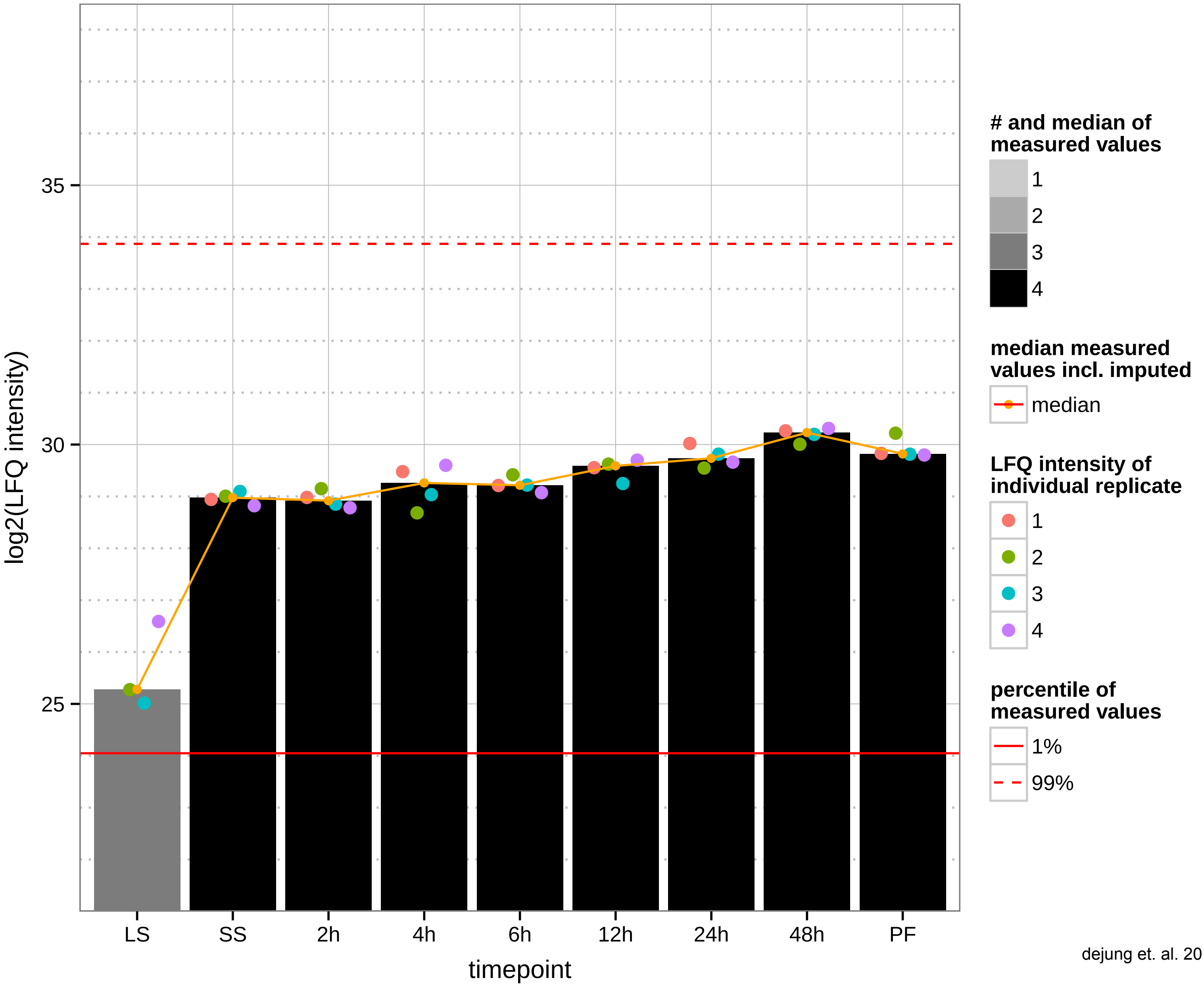

dejung et. al. 2015

iron superoxide dismutase
Tb927.11.15910
AGOF: metal ion binding, superoxide dismutase activity
AGOC: null
AGOP: oxidation–reduction process, superoxide metabolic process
PGOF: metal ion binding, superoxide dismutase activity
PGOC: null
PGOP: oxidation–reduction process, superoxide metabolic process

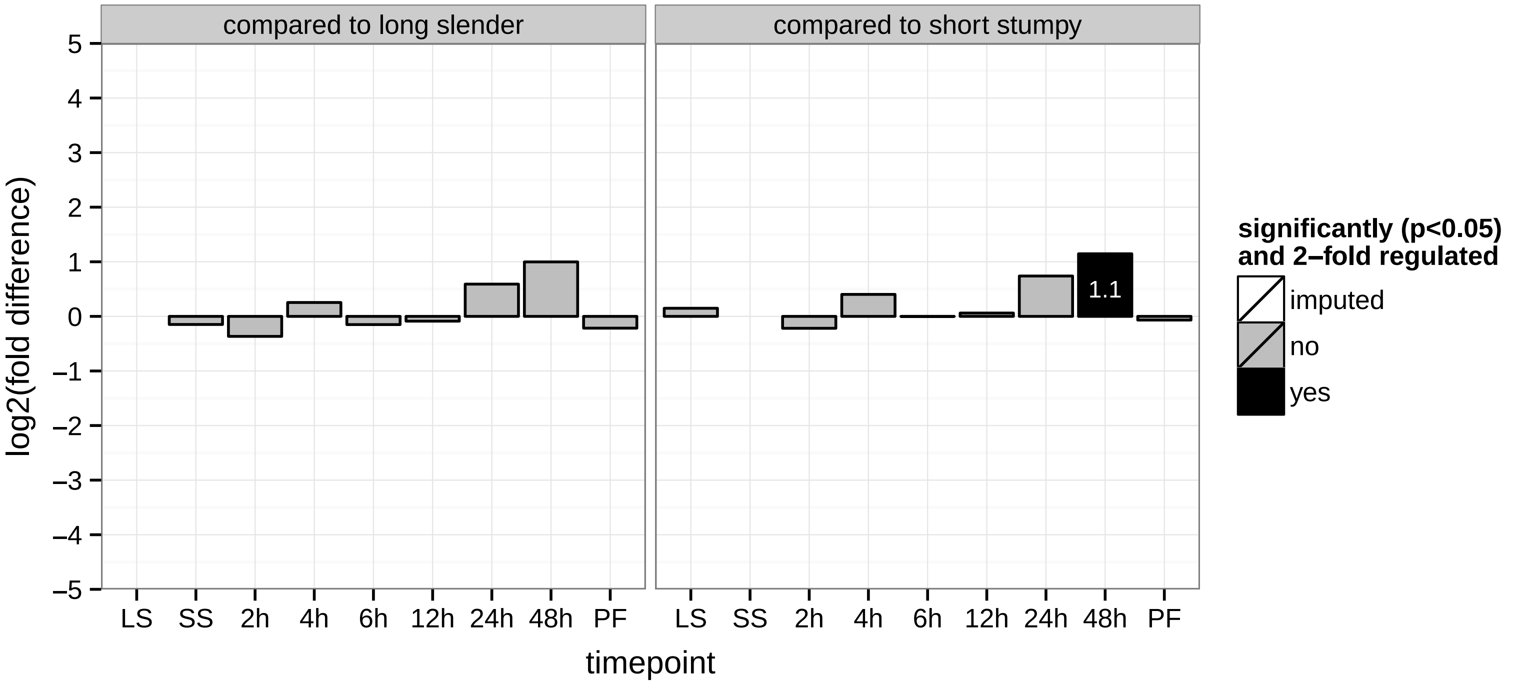

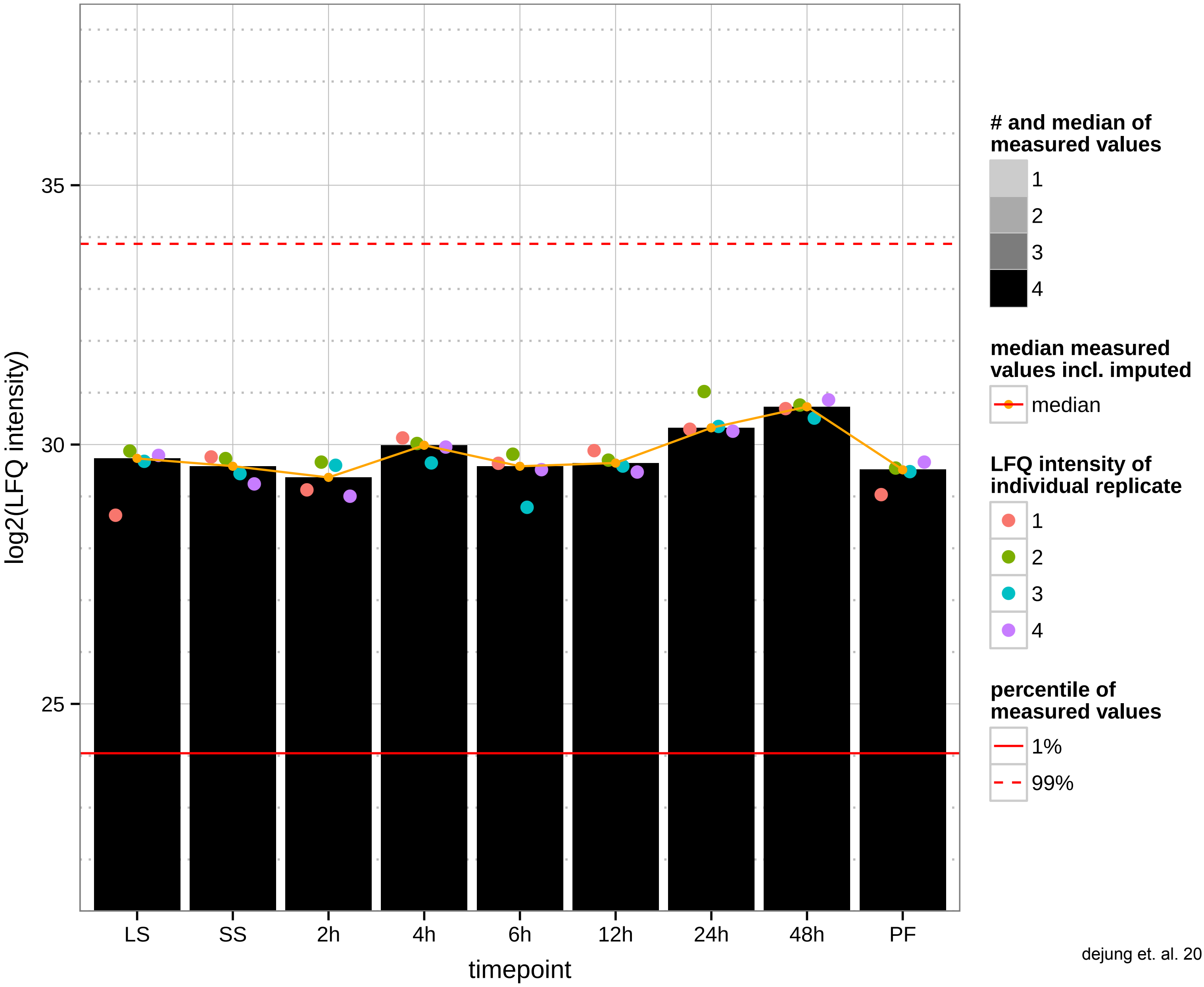

dejung et. al. 2015

hypothetical protein, conserved
Tb927.11.1950
AGOF: RNA binding, ribonuclease III activity
AGOC: mitochondrion
AGOP: RNA processing
PGOF: RNA binding, ribonuclease III activity
PGOC: null
PGOP: RNA processing

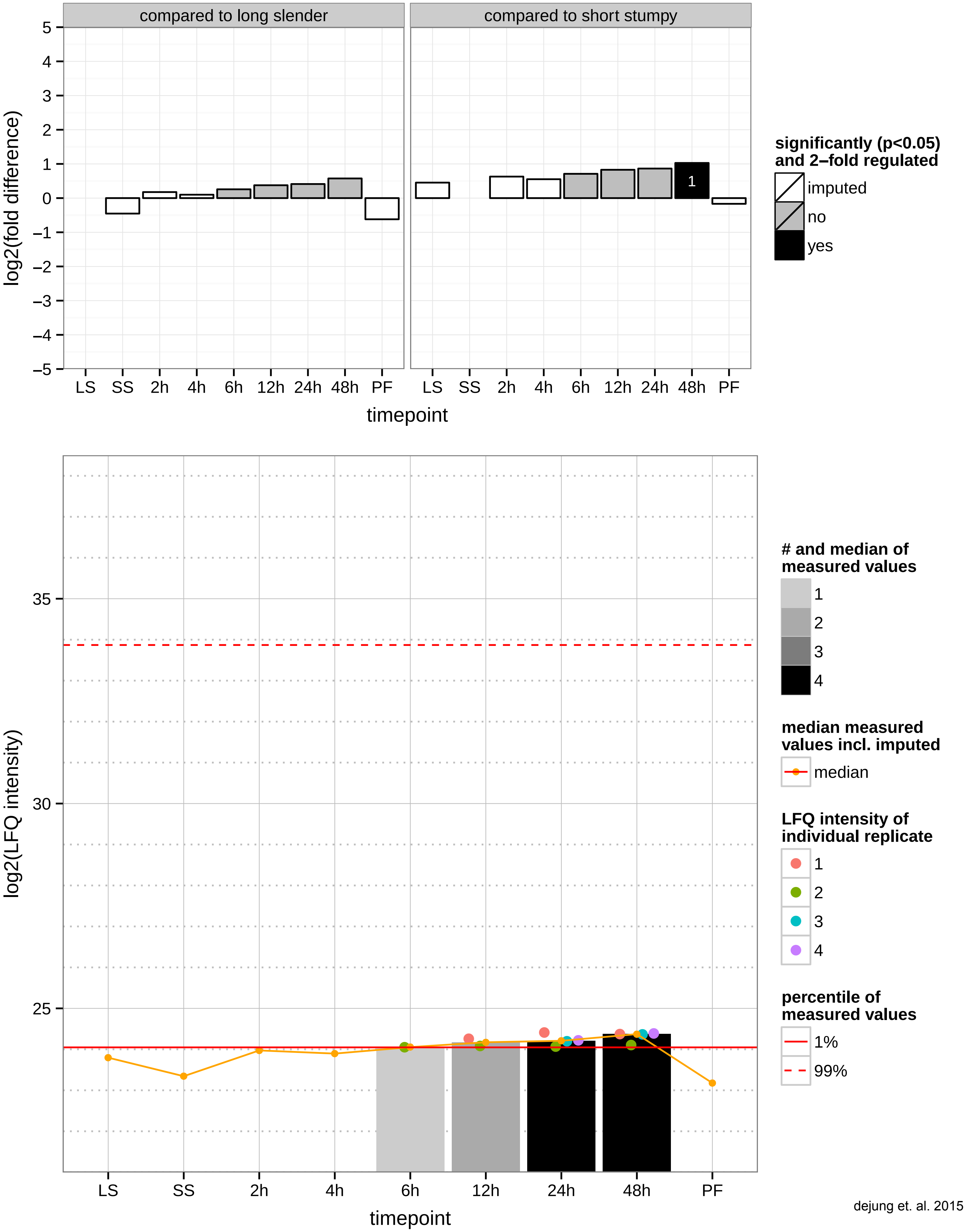

dejung et. al. 2015

arginyl–tRNA synthetase, putative
Tb927.11.1990
AGOF: ATP binding, arginine–tRNA ligase activity
AGOC: cytoplasm
AGOP: arginyl–tRNA aminoacylation, translation
PGOF: ATP binding, aminoacyl–tRNA ligase activity, arginine–tRNA ligase activity, nucleotide binding
PGOC: cytoplasm
PGOP: arginyl–tRNA aminoacylation, tRNA aminoacylation for protein translation

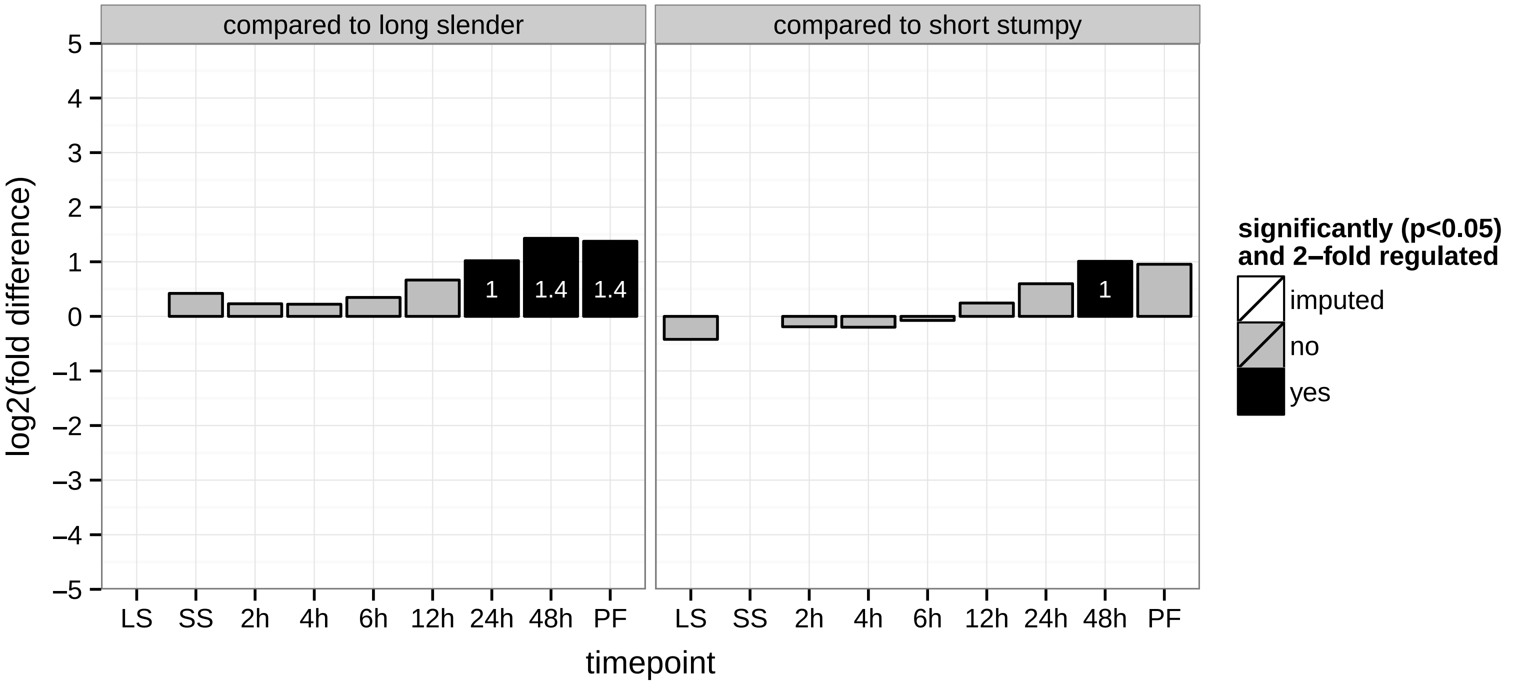

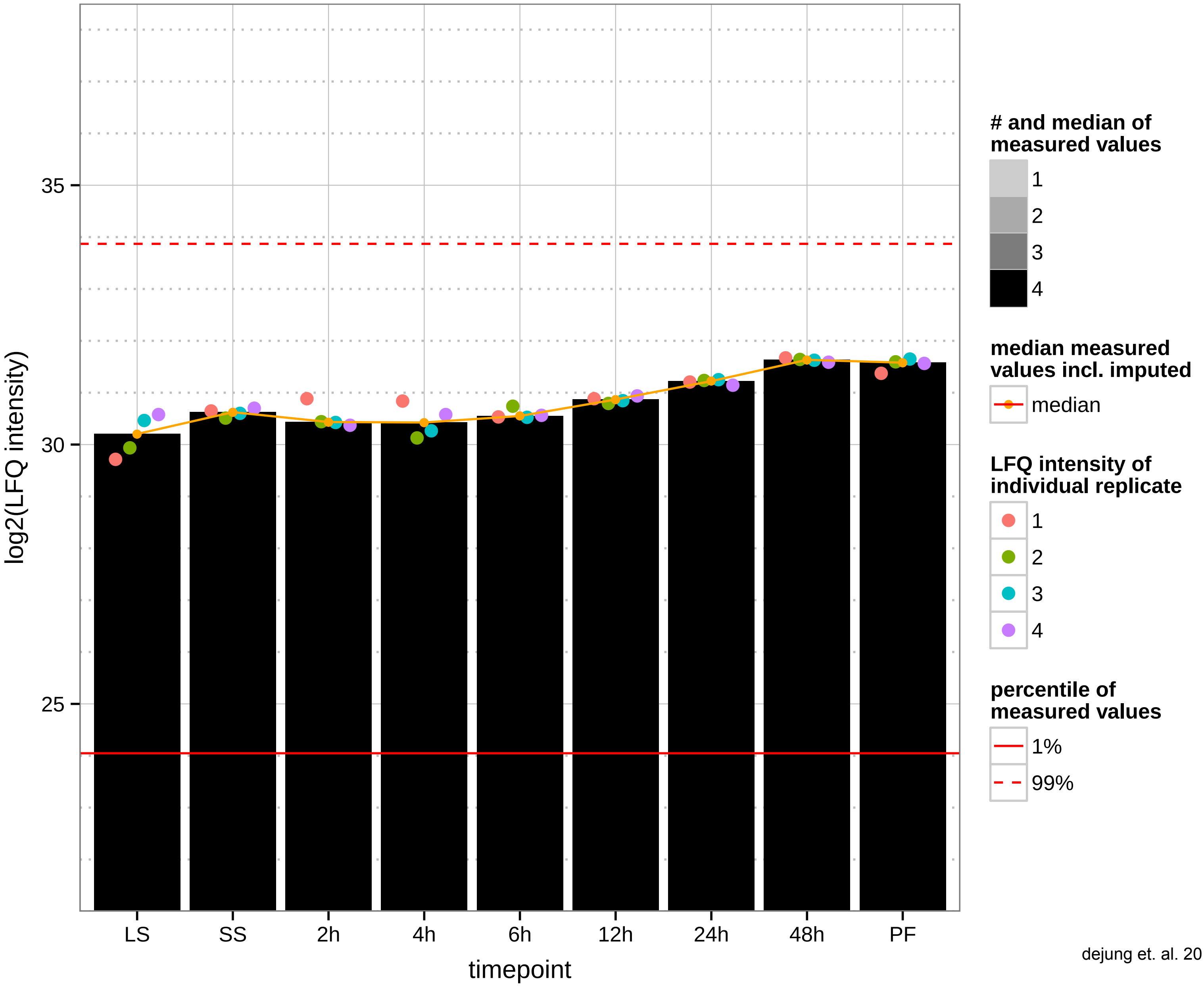

dejung et. al. 2015

hypothetical protein, conserved
Tb927.11.4330
AGOF: null
AGOC: mitochondrion
AGOP: null
PGOF: null
PGOC: null
PGOP: null

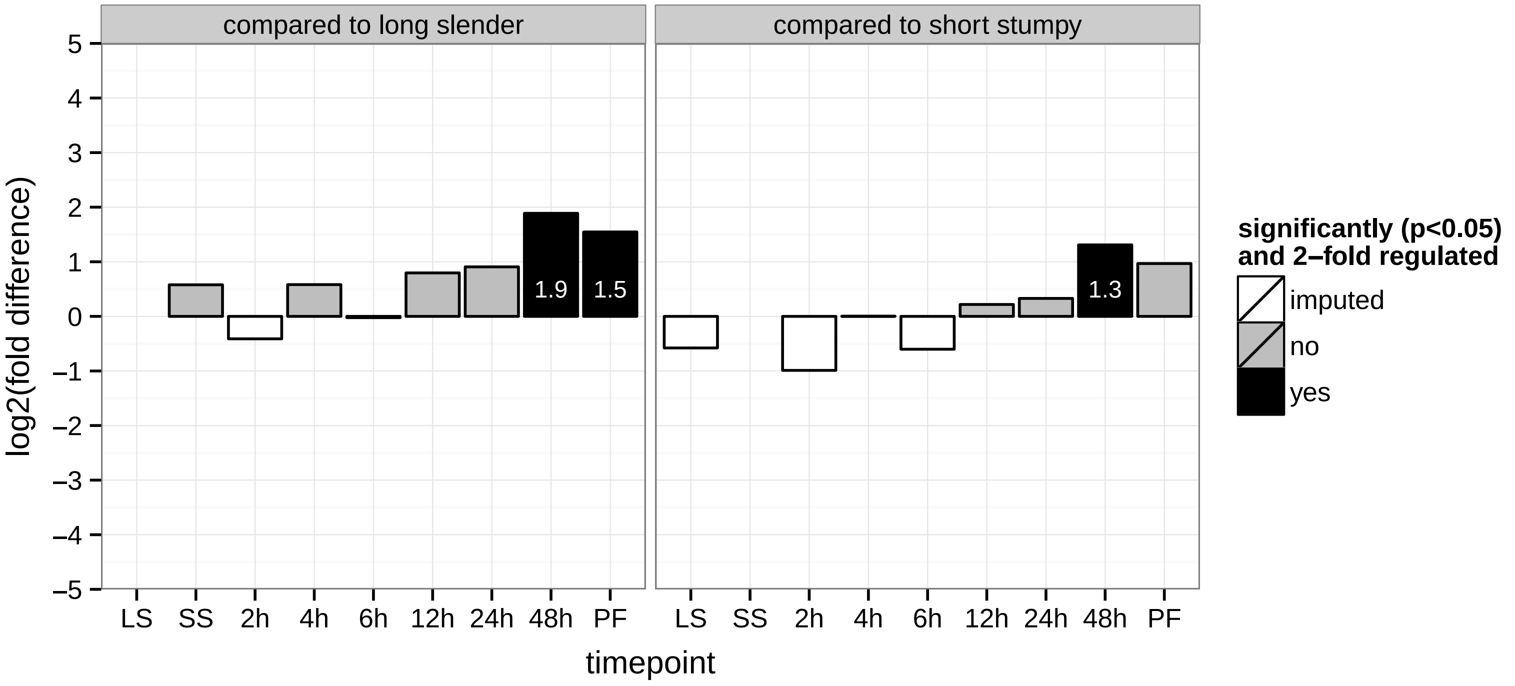

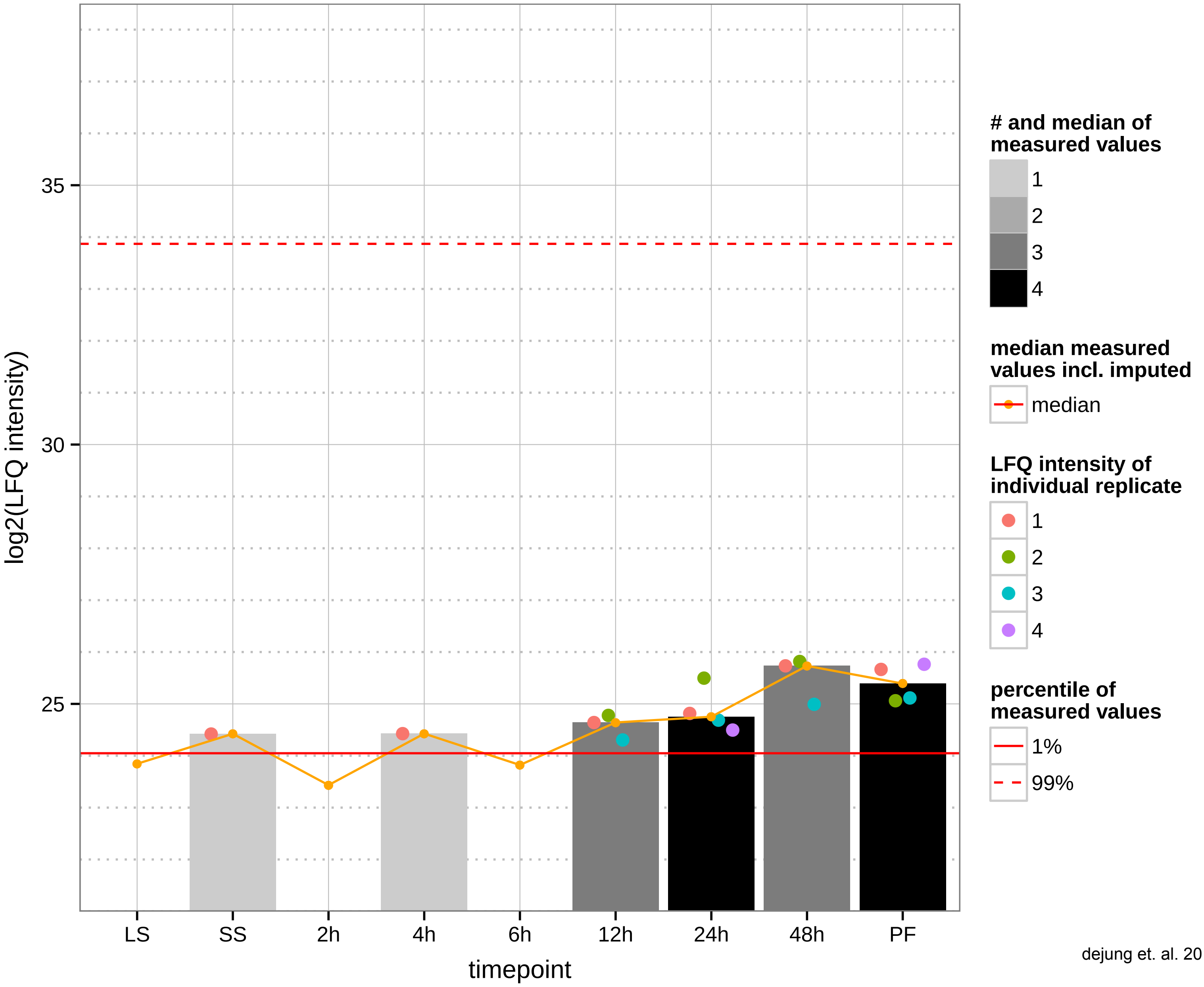

dejung et. al. 2015

aspartate aminotransferase, mitochondrial
Tb927.11.5090
AGOF: L−alanine:2−oxoglutarate aminotransferase activity, pyridoxal phosphate binding, transaminase activity
AGOC: mitochondrion
AGOP: L−methionine salvage from methylthioadenosine, biosynthetic process
PGOF: pyridoxal phosphate binding, transferase activity
PGOC: null
PGOP: biosynthetic process

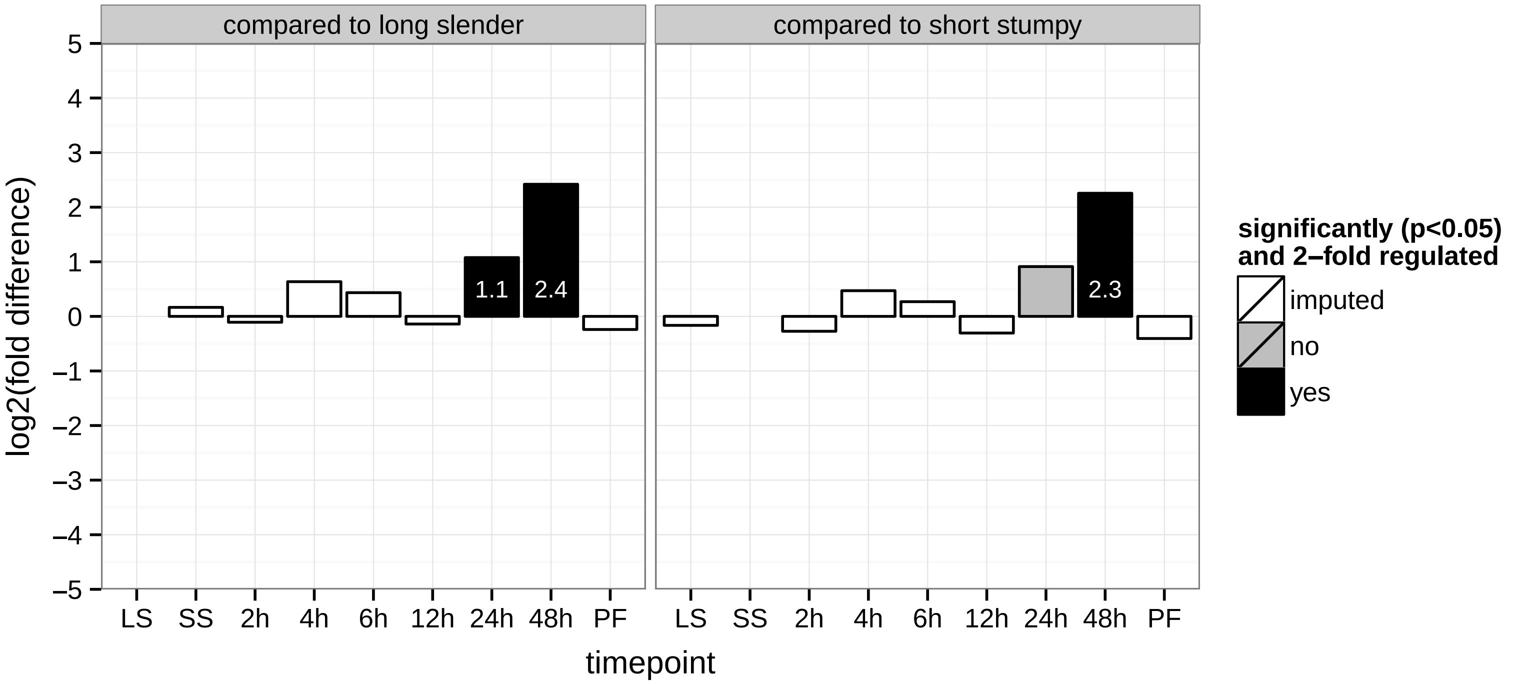

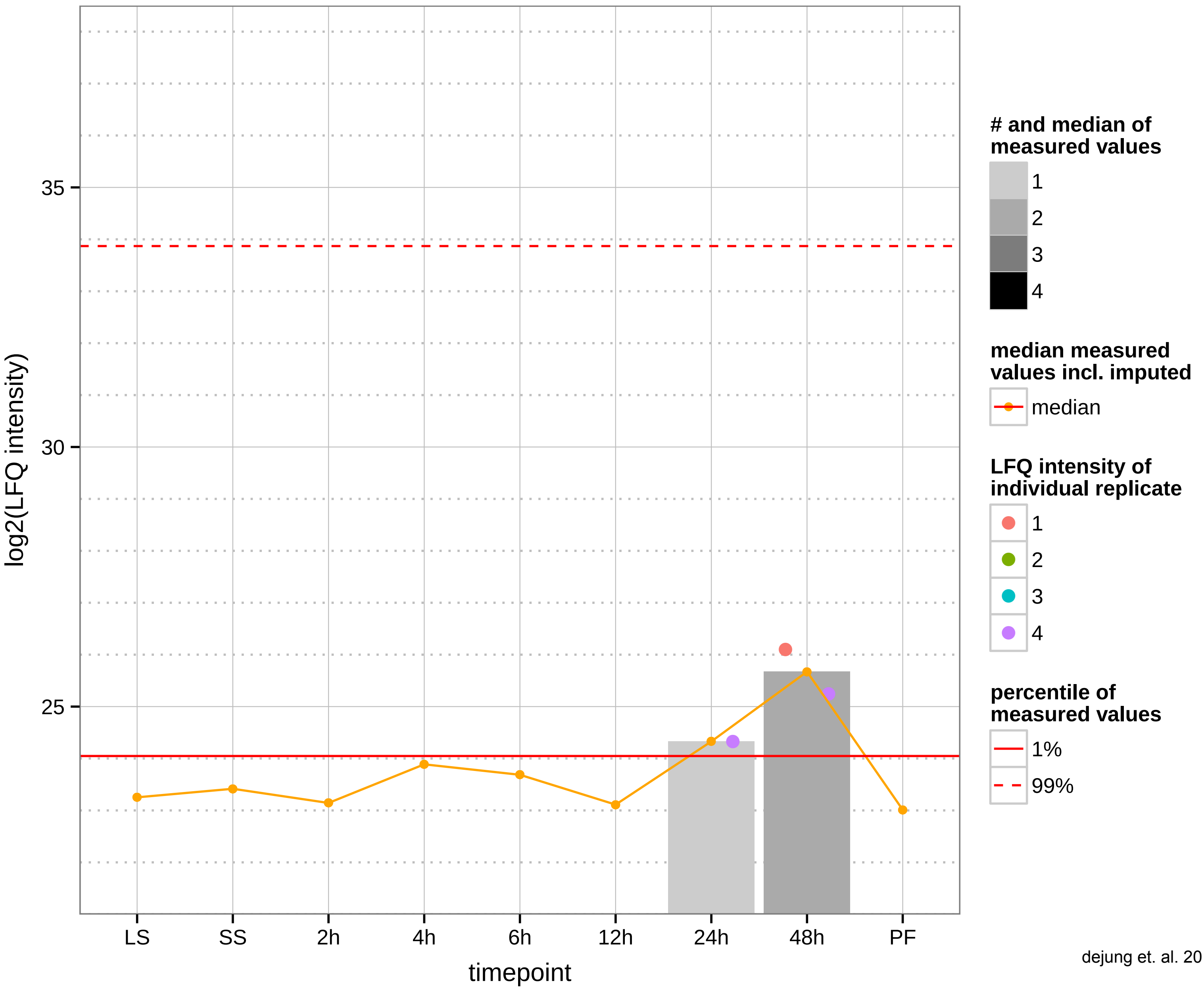

dejung et. al. 2015

malic enzyme
Tb927.11.5440
AGOF: NAD binding, malic enzyme activity, oxidoreductase activity, acting on the CH–OH group of donors, NAD or NADP as a
AGOC: cytosol
AGOP: malate metabolic process
PGOF: NAD binding, malate dehydrogenase (oxaloacetate–decarboxylating) activity, malic enzyme activity, oxidoreductase ac
PGOC: null
PGOP: malate metabolic process, oxidation–reduction process

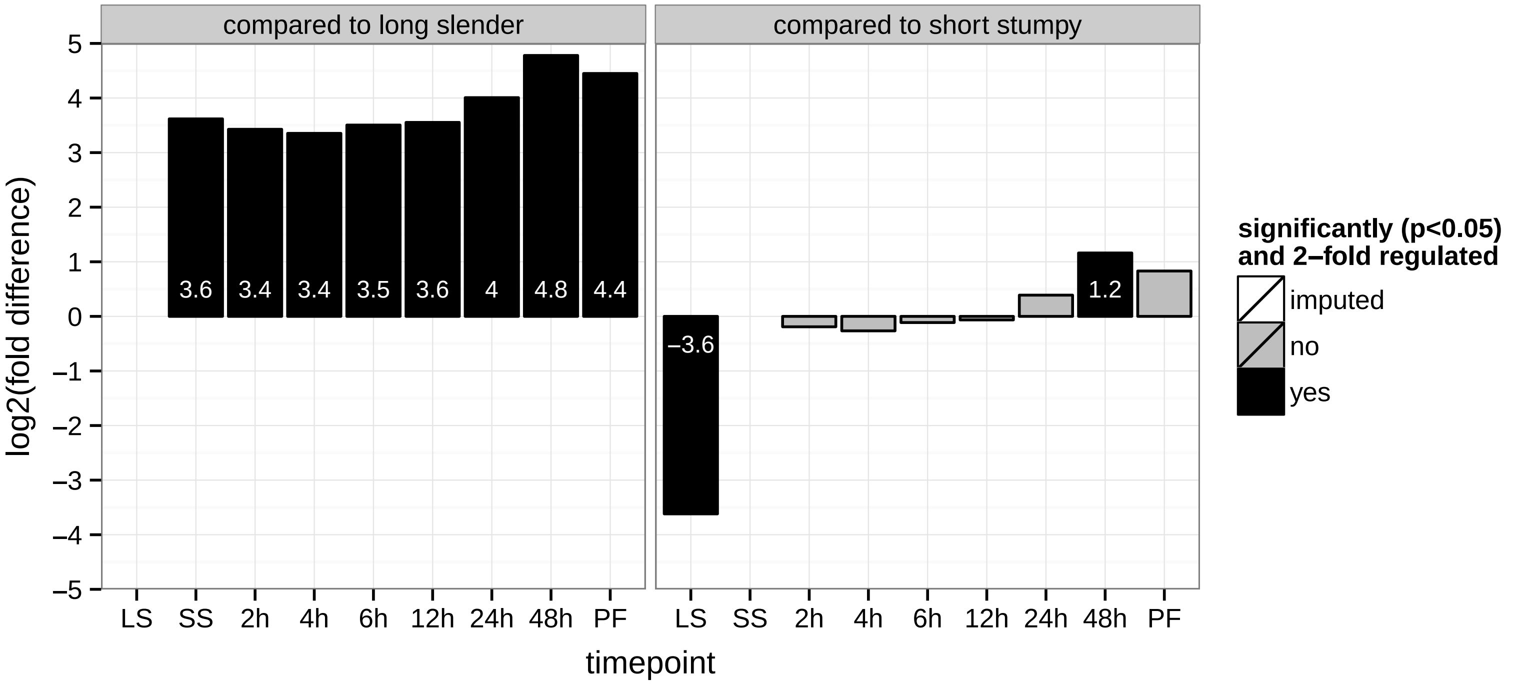

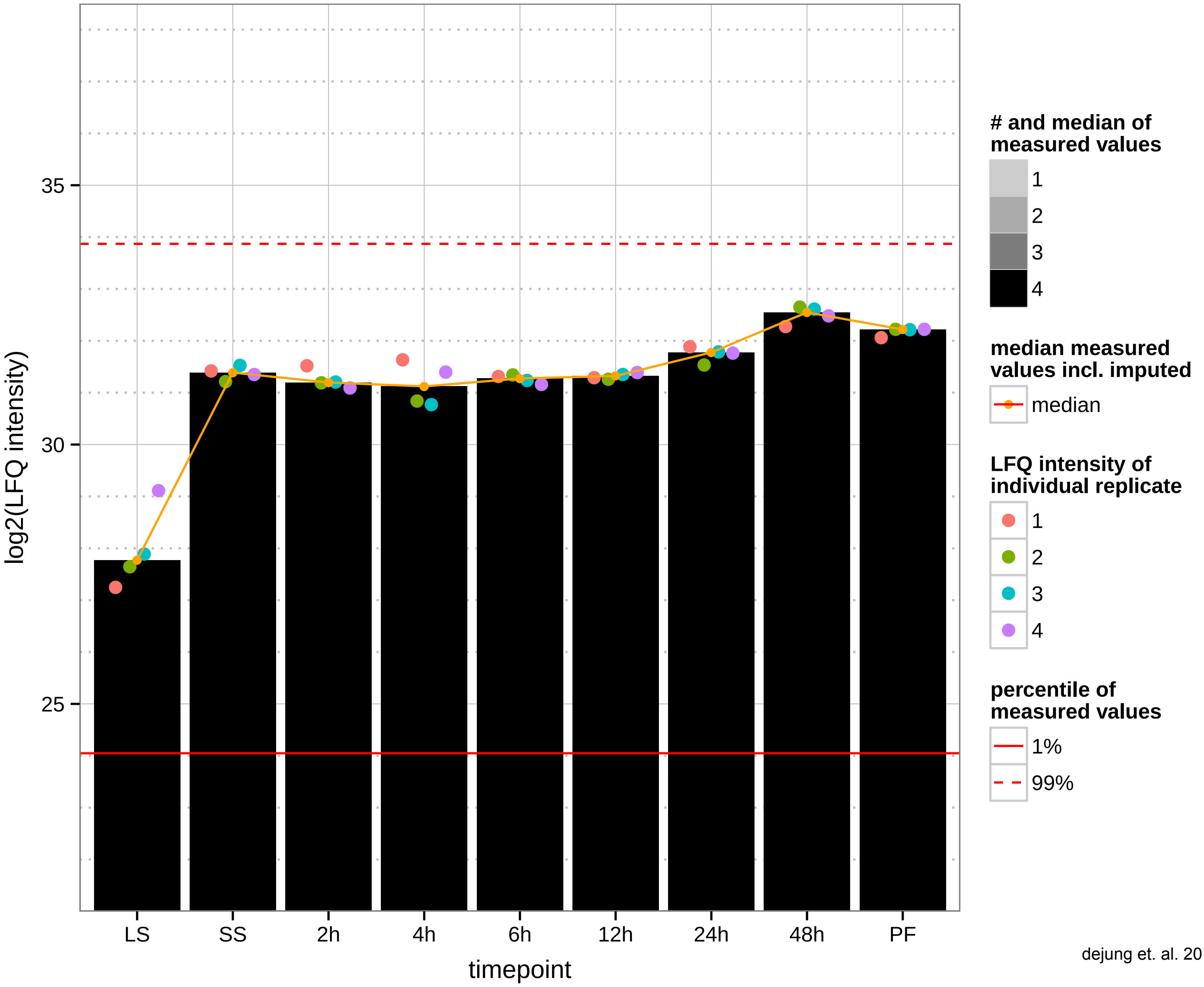

dejung et. al. 2015

hypothetical protein, conserved
Tb927.11.6040
AGOF: null
AGOC: integral to membrane
AGOP: null
PGOF: null
PGOC: null
PGOP: null

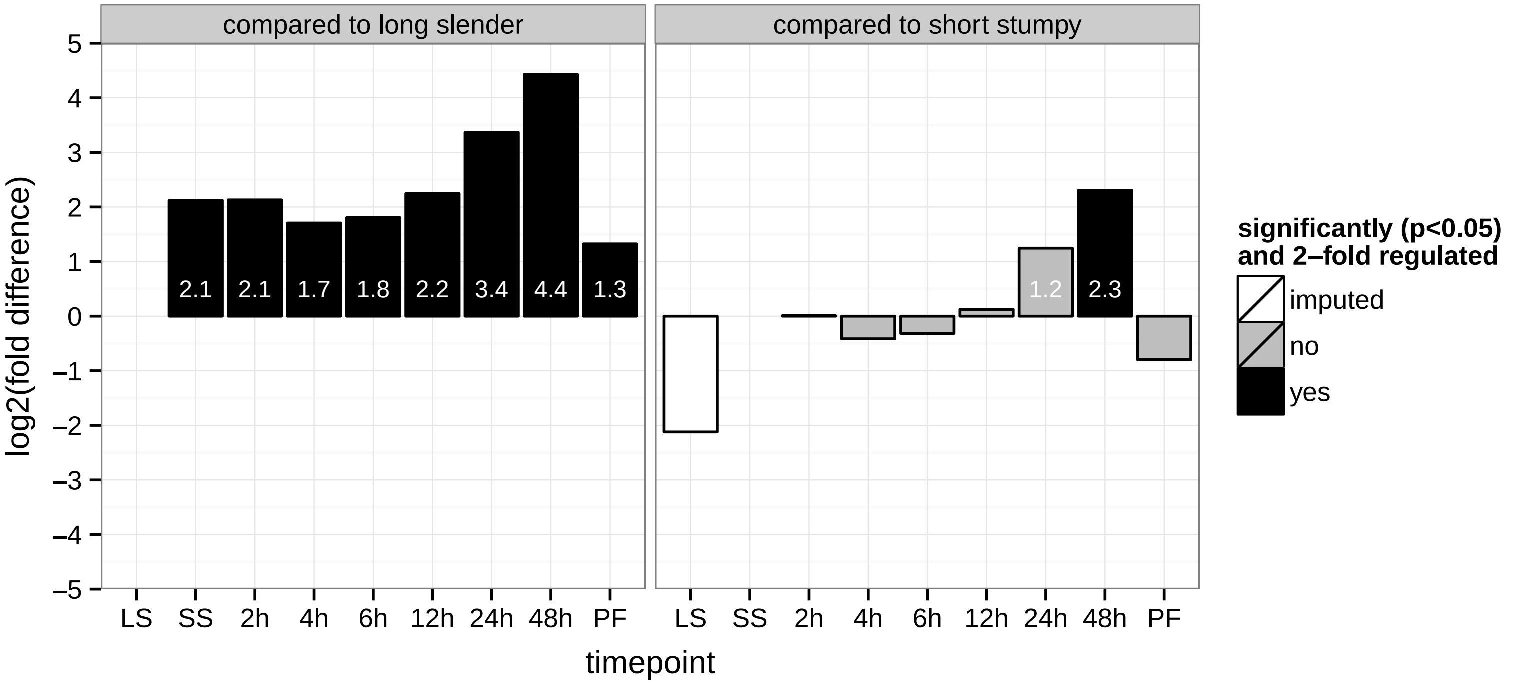

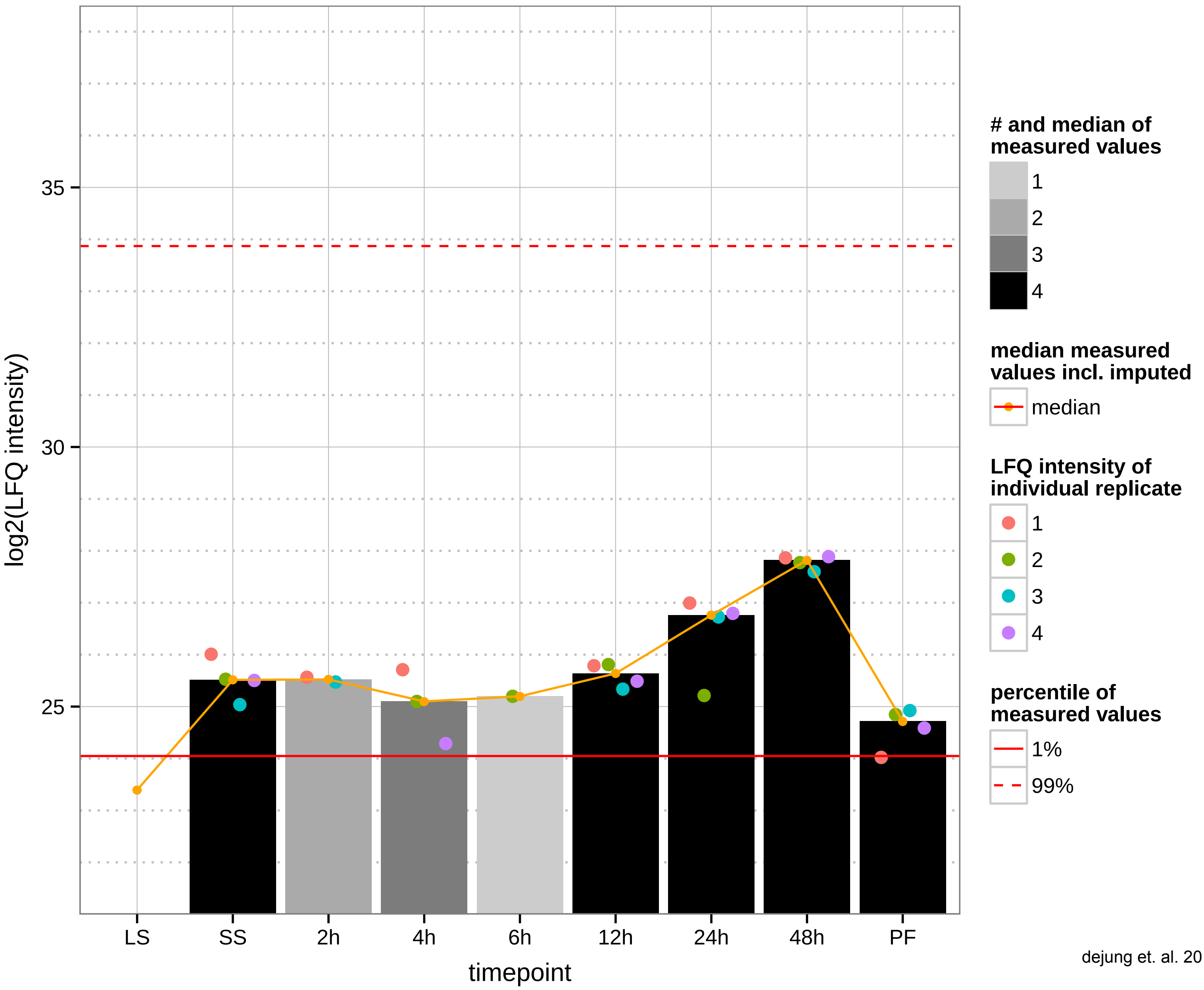

dejung et. al. 2015

hypothetical protein, conserved
Tb927.11.6570
AGOF: calcium ion binding
AGOC: integral to membrane
AGOP: null
PGOF: null
PGOC: null
PGOP: null

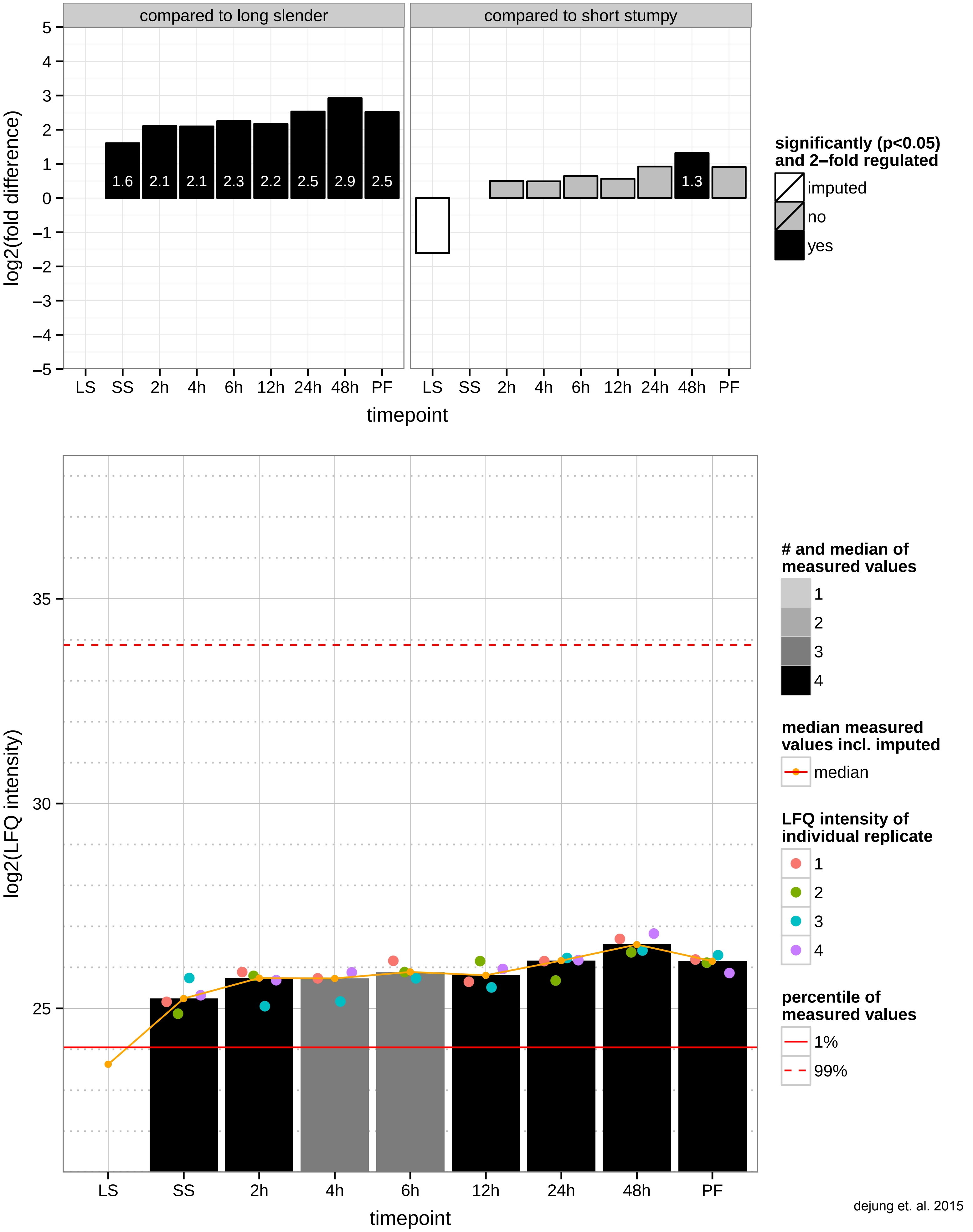

dejung et. al. 2015

replication factor A, 51kDa subunit, putative
Tb927.11.9130
AGOF: DNA binding
AGOC: nucleus
AGOP: DNA replication
PGOF: DNA binding, nucleic acid binding
PGOC: nucleus
PGOP: DNA replication

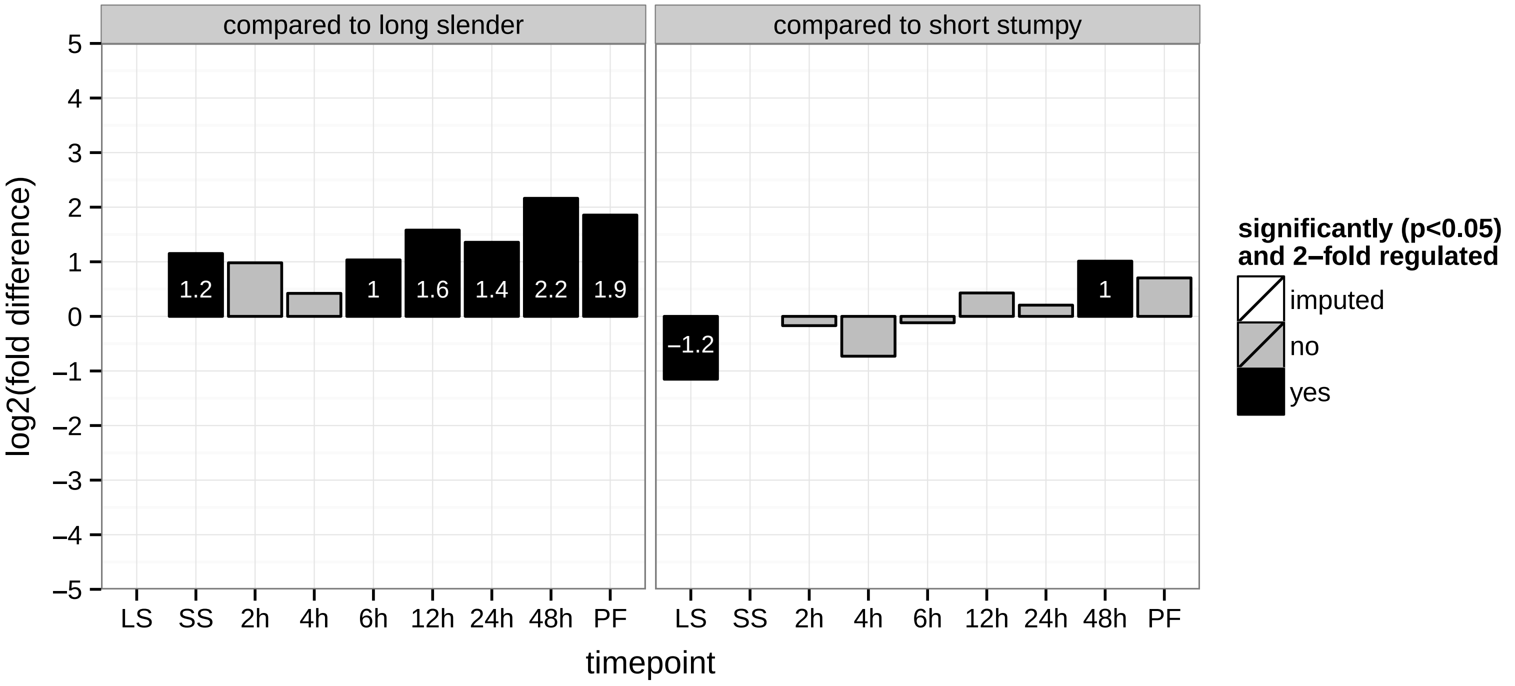

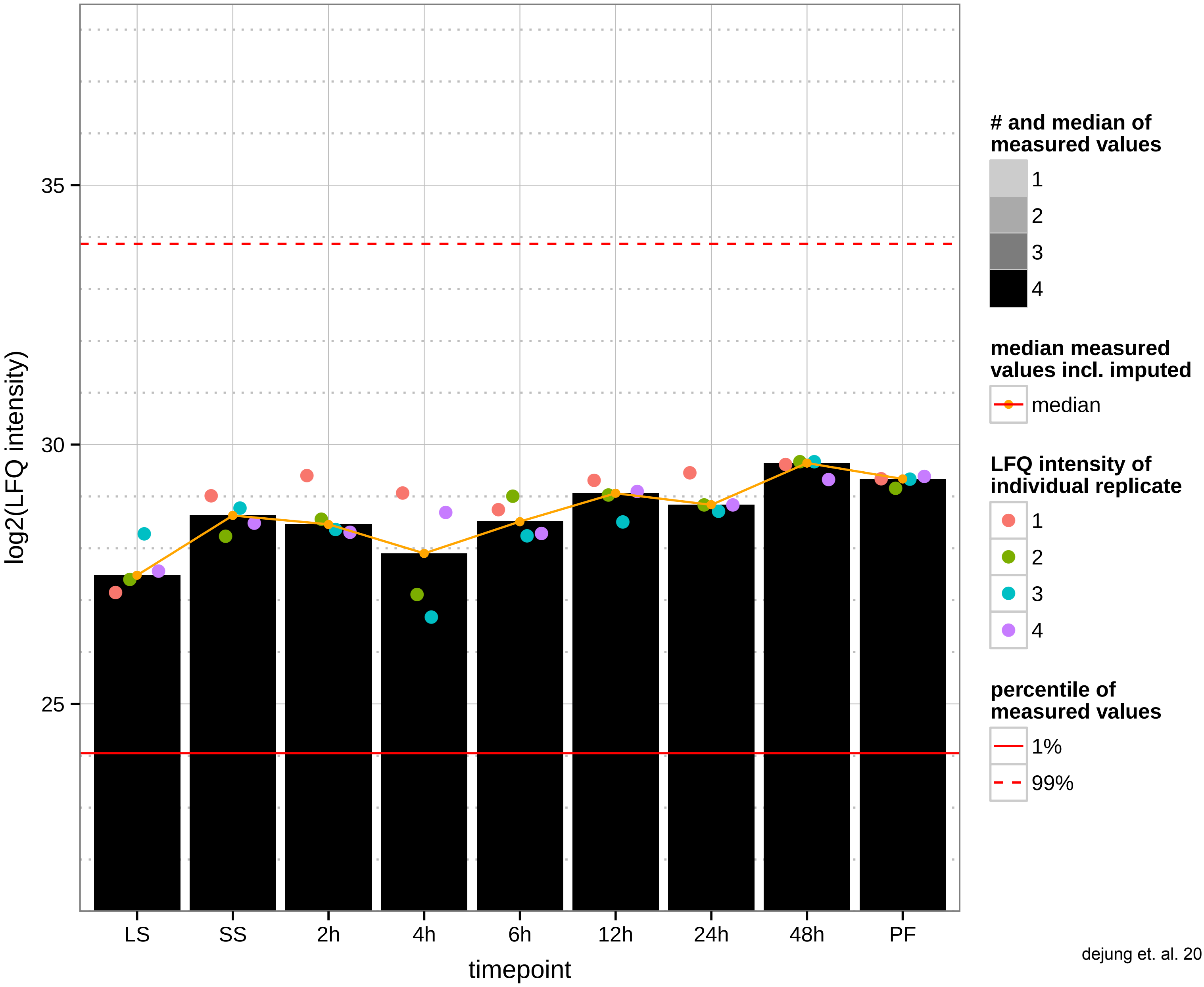

dejung et. al. 2015

hypothetical protein, conserved
Tb927.11.9210
AGOF: null
AGOC: null
AGOP: null
PGOF: null
PGOC: null
PGOP: null

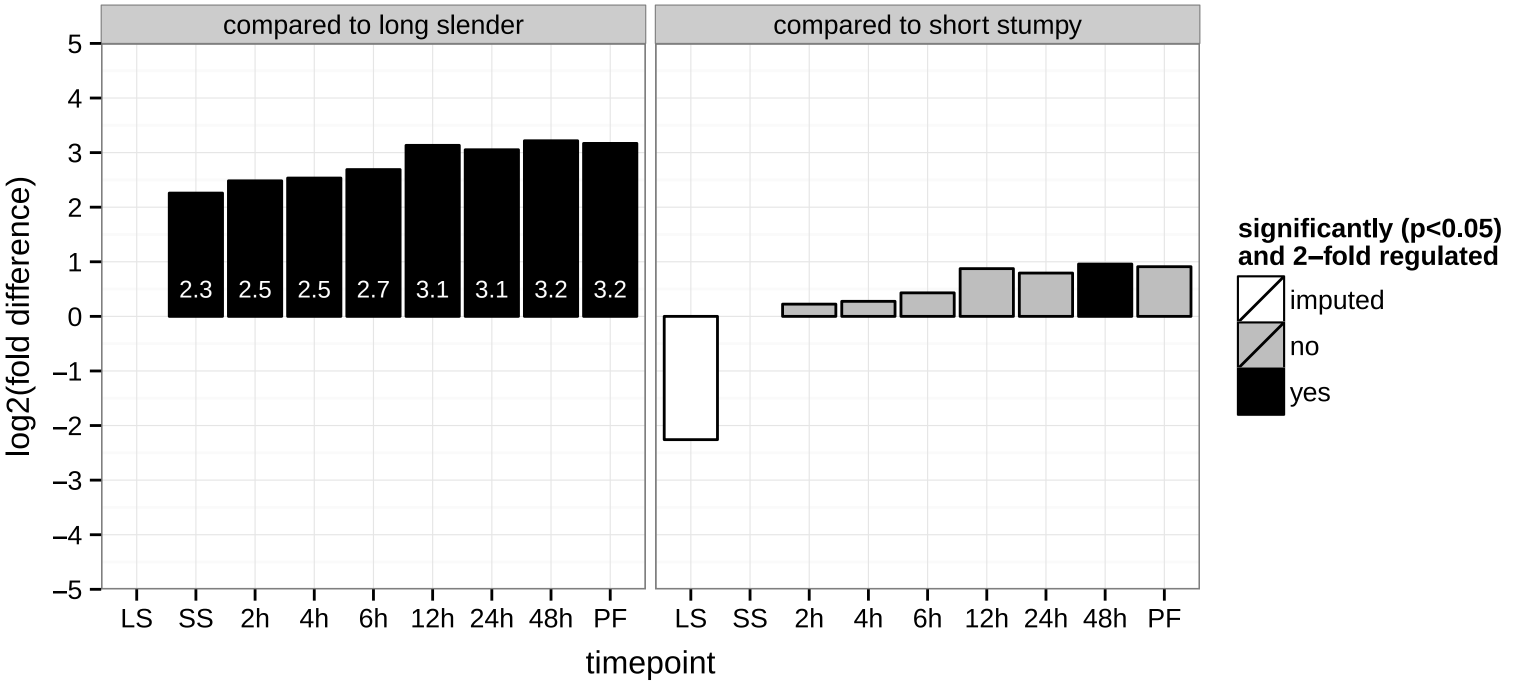

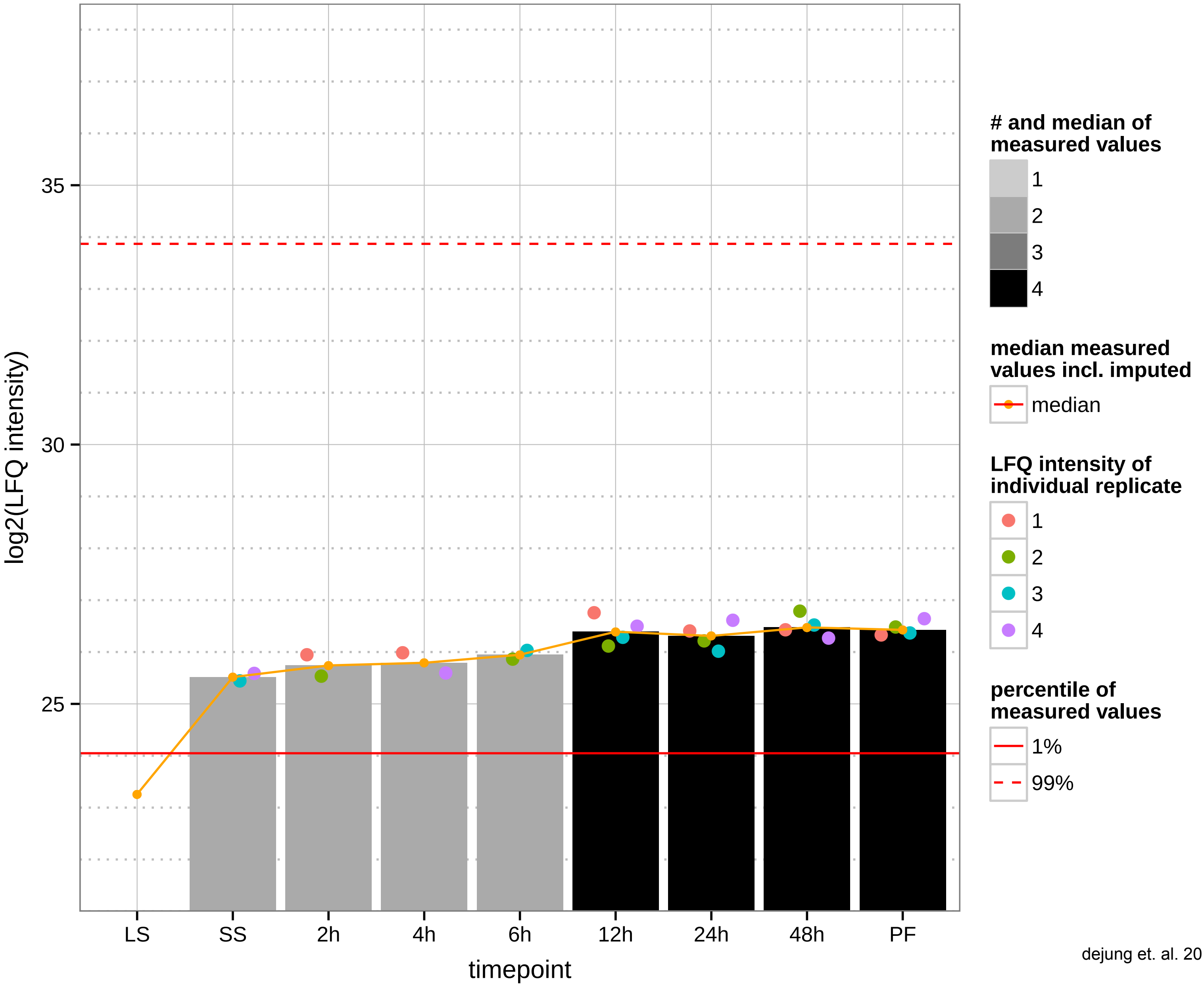

dejung et. al. 2015

hypothetical protein, conserved
Tb927.11.9230
AGOF: null
AGOC: null
AGOP: null
PGOF: null
PGOC: null
PGOP: null

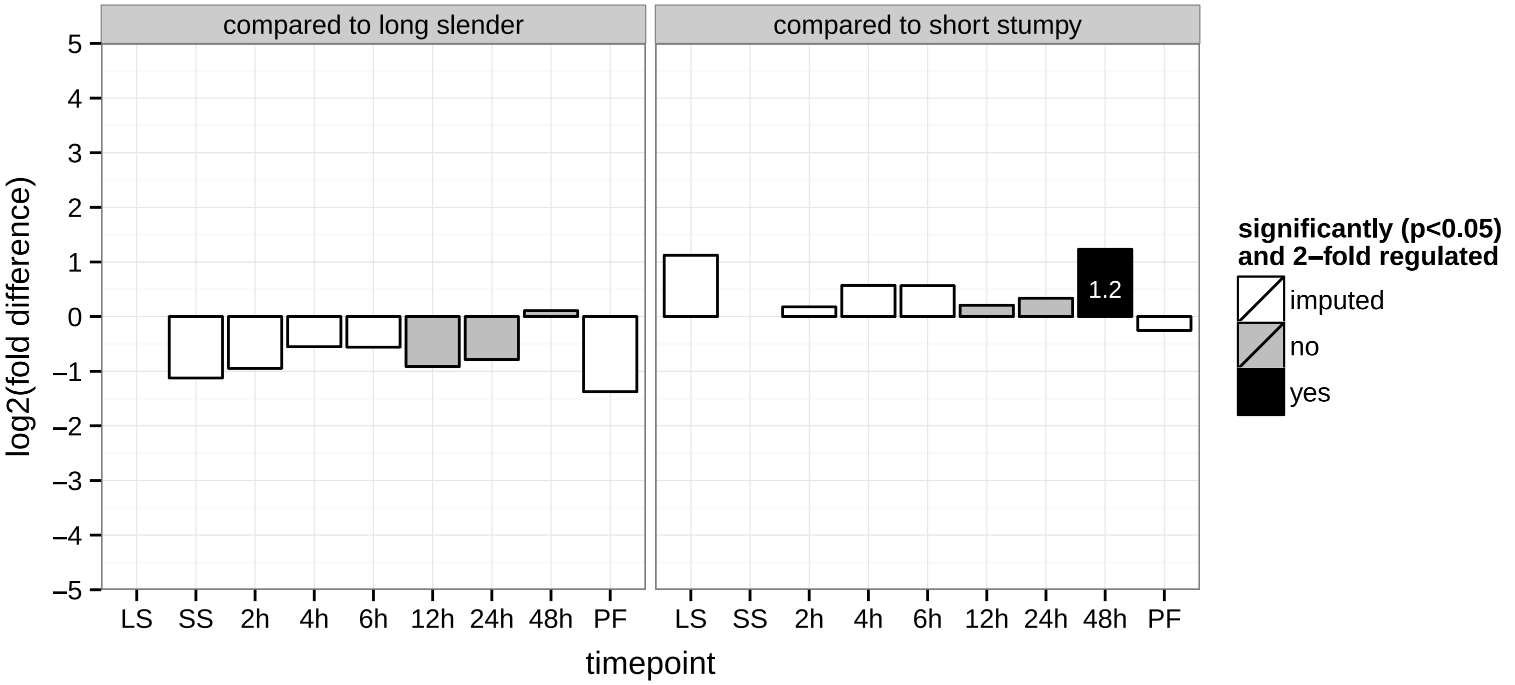

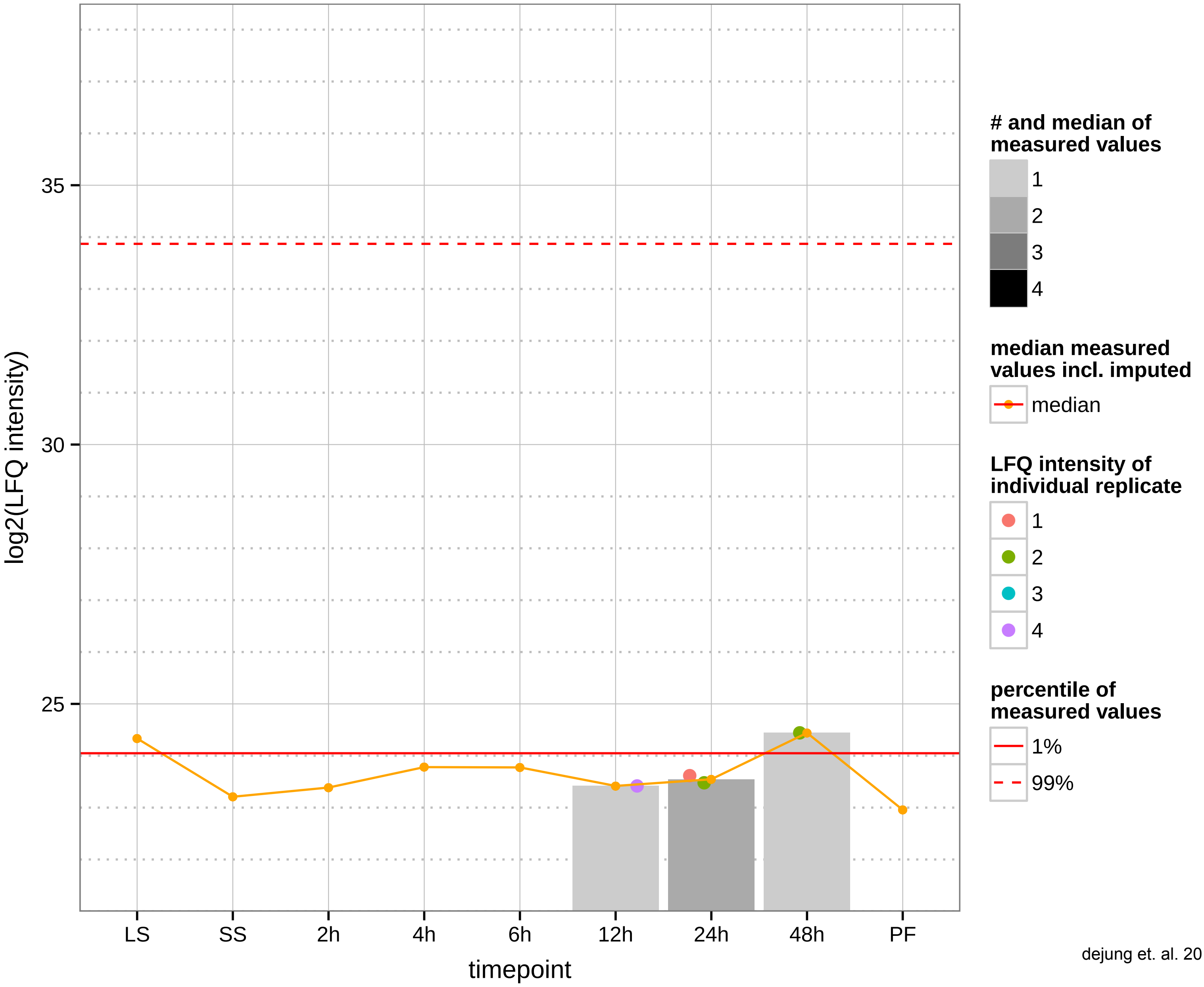

dejung et. al. 2015

calmodulin, putative
Tb927.11.9790
AGOF: calcium ion binding
AGOC: null
AGOP: null
PGOF: calcium ion binding
PGOC: null
PGOP: null

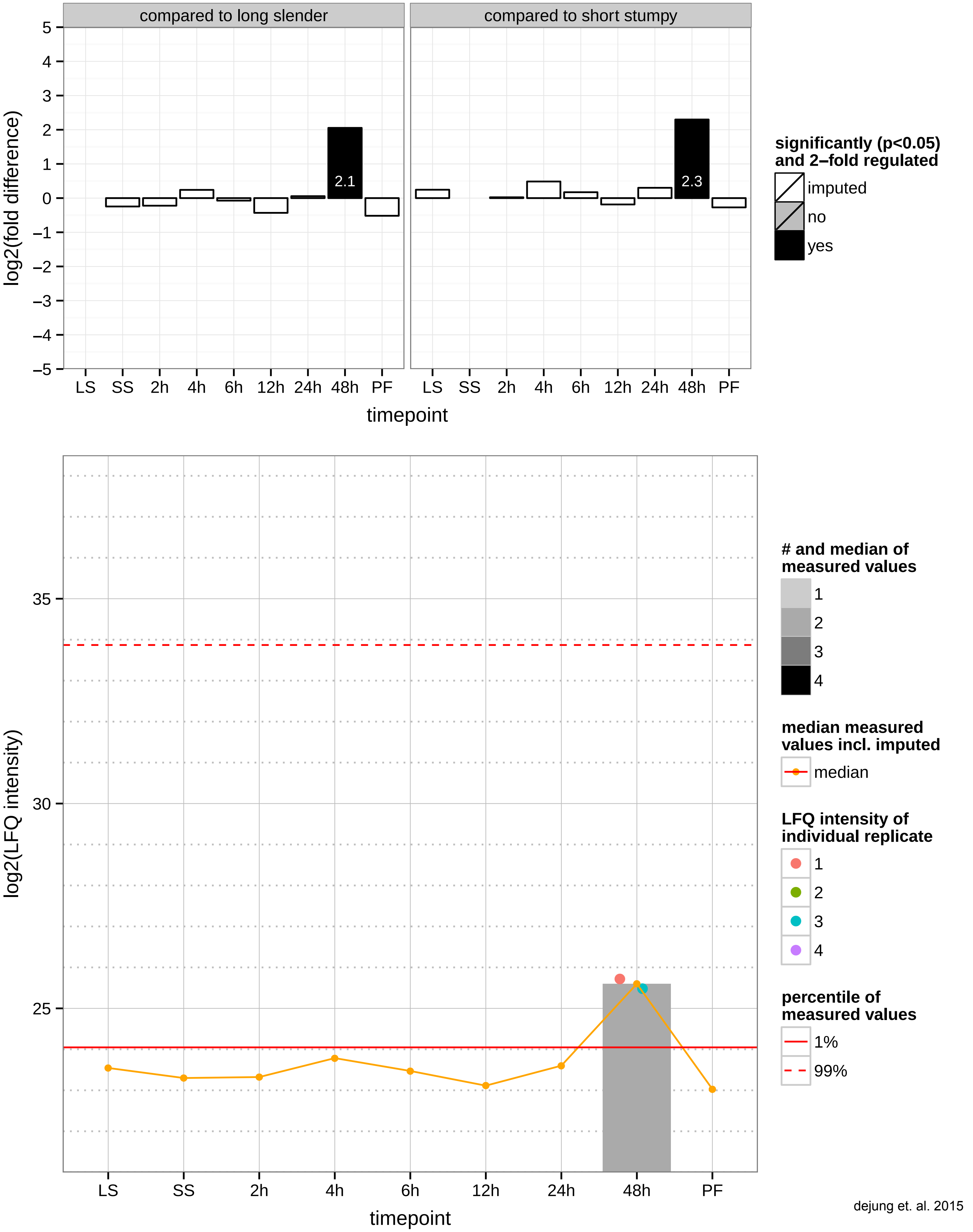

dejung et. al. 2015

protein kinase, putative
Tb927.2.1820
AGOF: ATP binding, calcium ion binding, protein kinase activity, protein serine/threonine kinase activity, protein tyrosine kinase
AGOC: null
AGOP: protein phosphorylation
PGOF: ATP binding, calcium ion binding, protein kinase activity, transferase activity, transferring phosphorus–containing group
PGOC: null
PGOP: protein phosphorylation

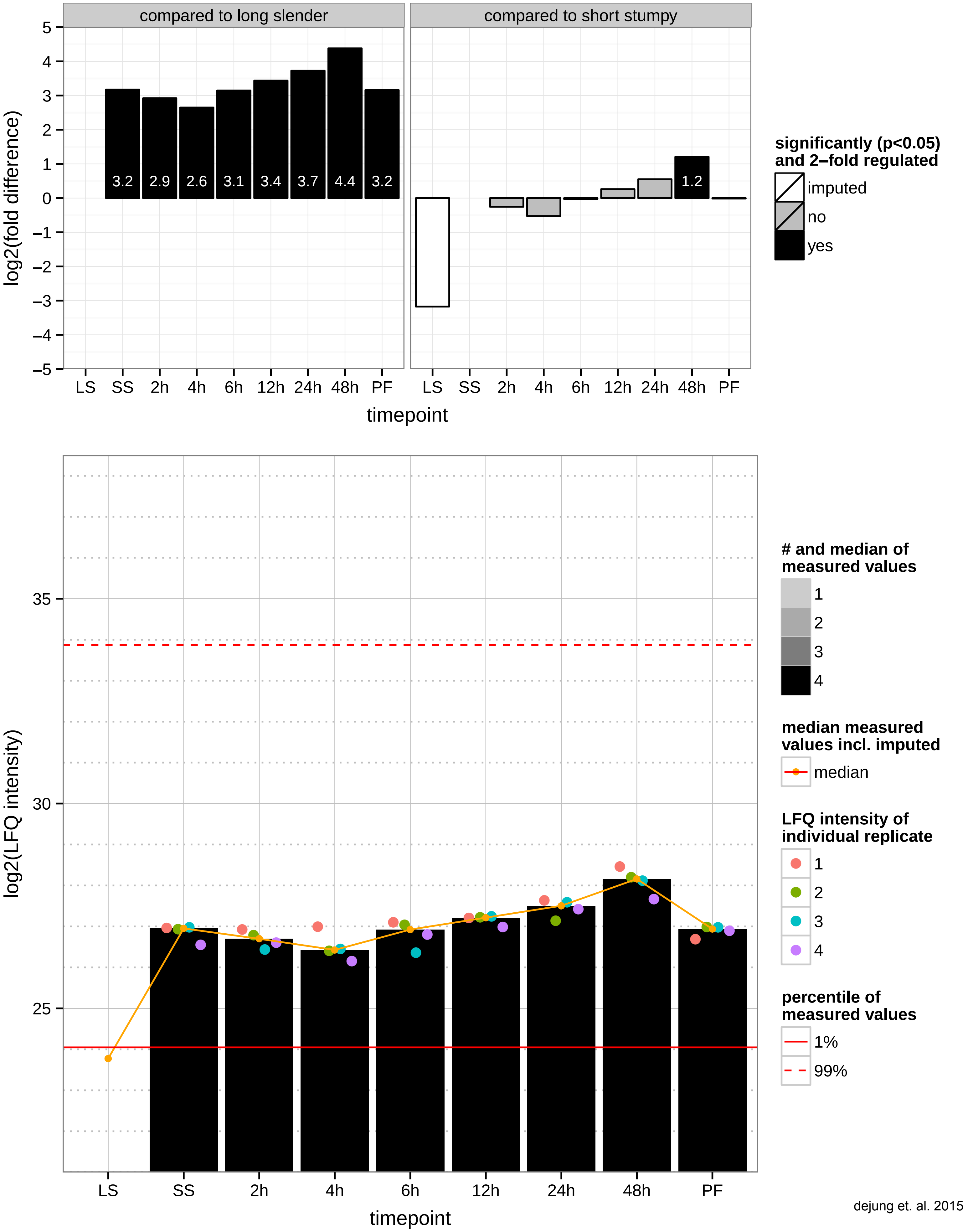

dejung et. al. 2015

retrotransposon hot spot protein 5 (RHS5), putative, retrotransposon hot spot protein (RHS, pseudogene), degenerate
Tb927.2.240
AGOF: null
AGOC: nucleus, null
AGOP: null
PGOF: null
PGOC: null
PGOP: null

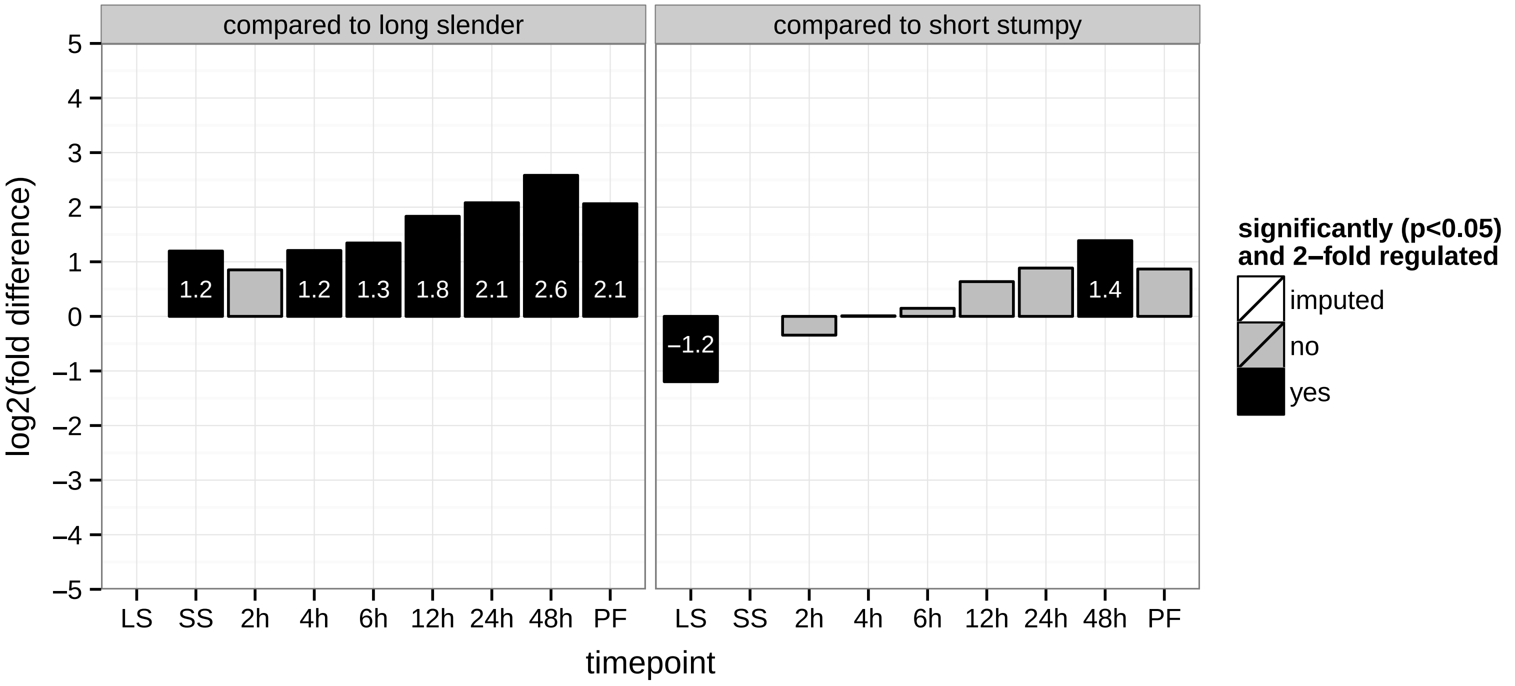

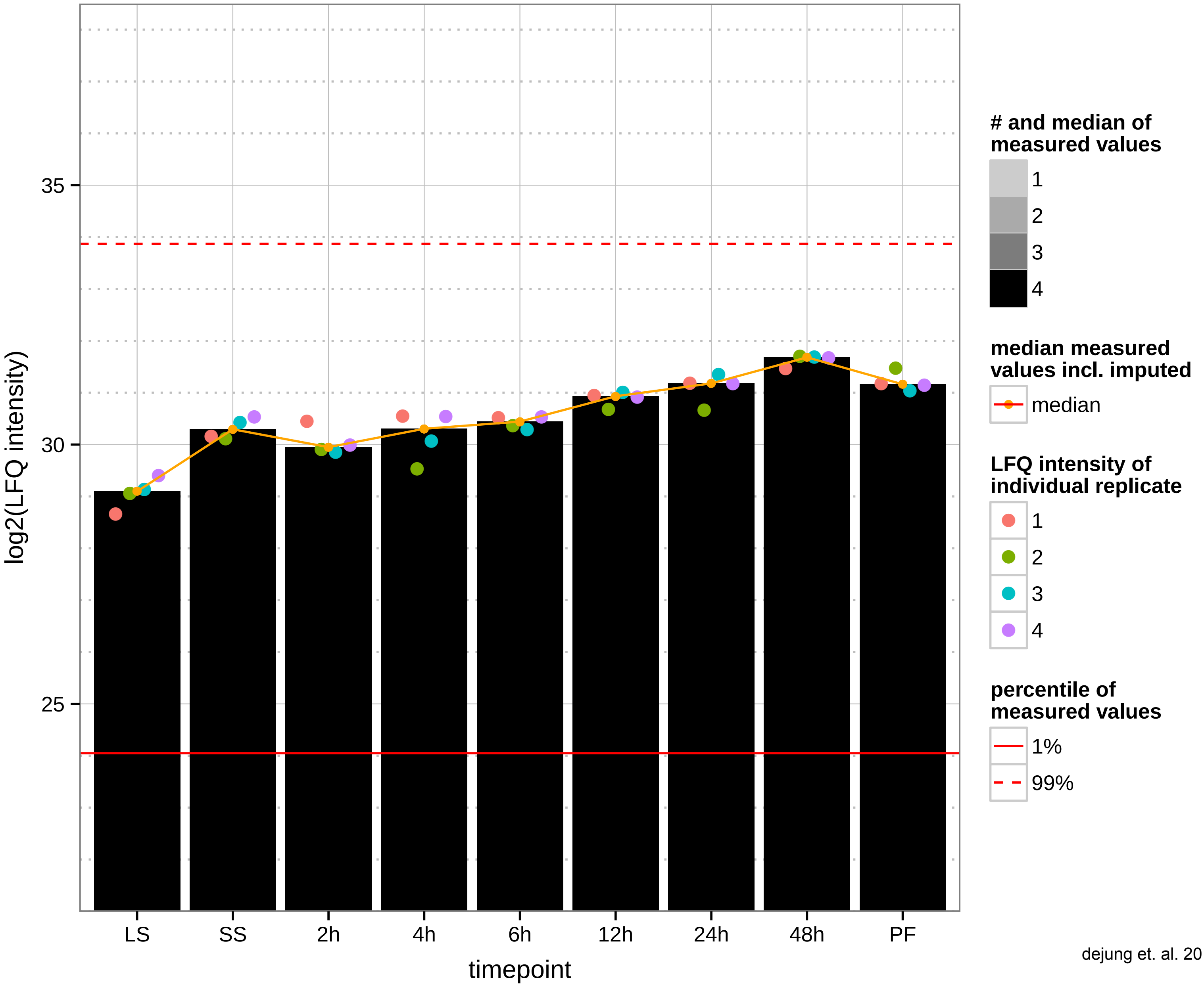

dejung et. al. 2015

RNA–editing complex protein, KREPA1, RNA–editing complex protein (KREPA1)
Tb927.2.2470
AGOF: alpha–catenin binding, zinc ion binding
AGOC: intracellular, mitochondrial mRNA editing complex, mitochondrion
AGOP: RNA nucleotide deletion, RNA nucleotide insertion
PGOF: zinc ion binding
PGOC: intracellular
PGOP: null

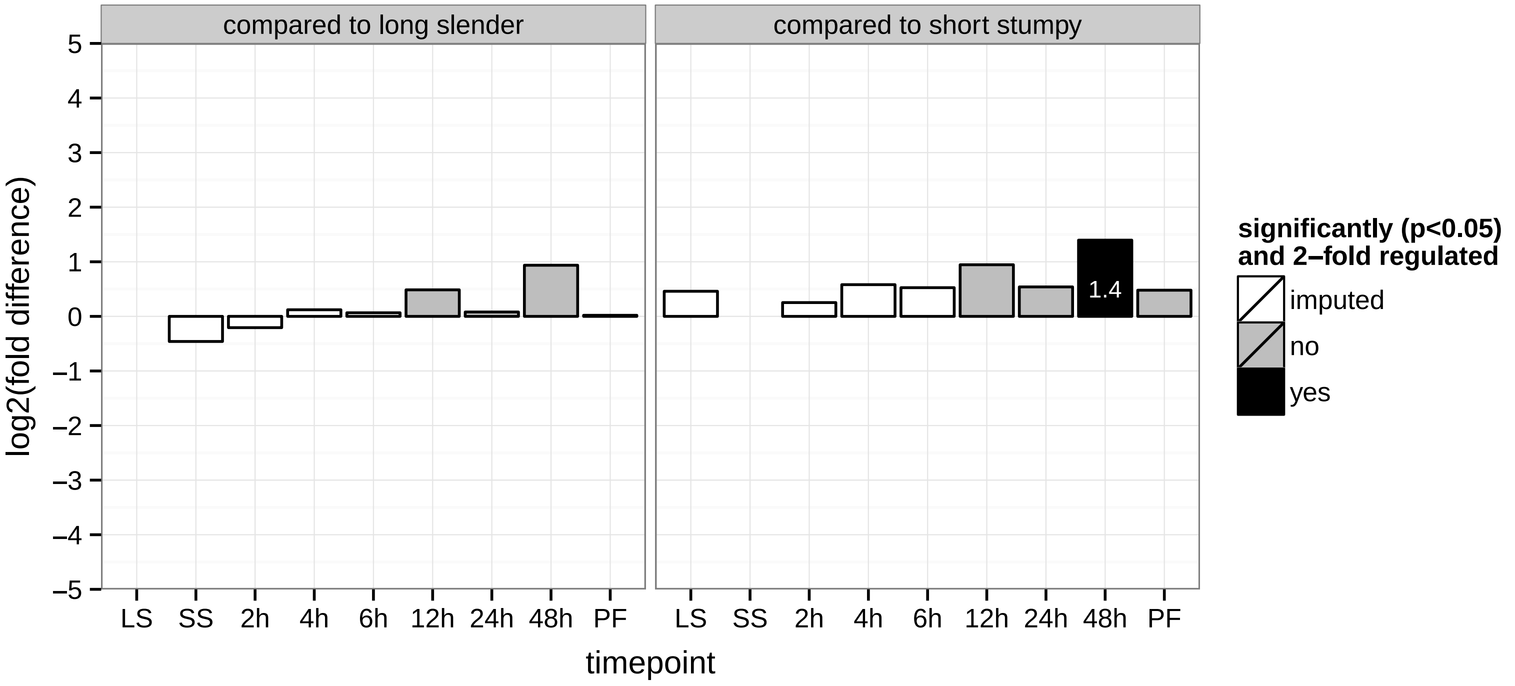

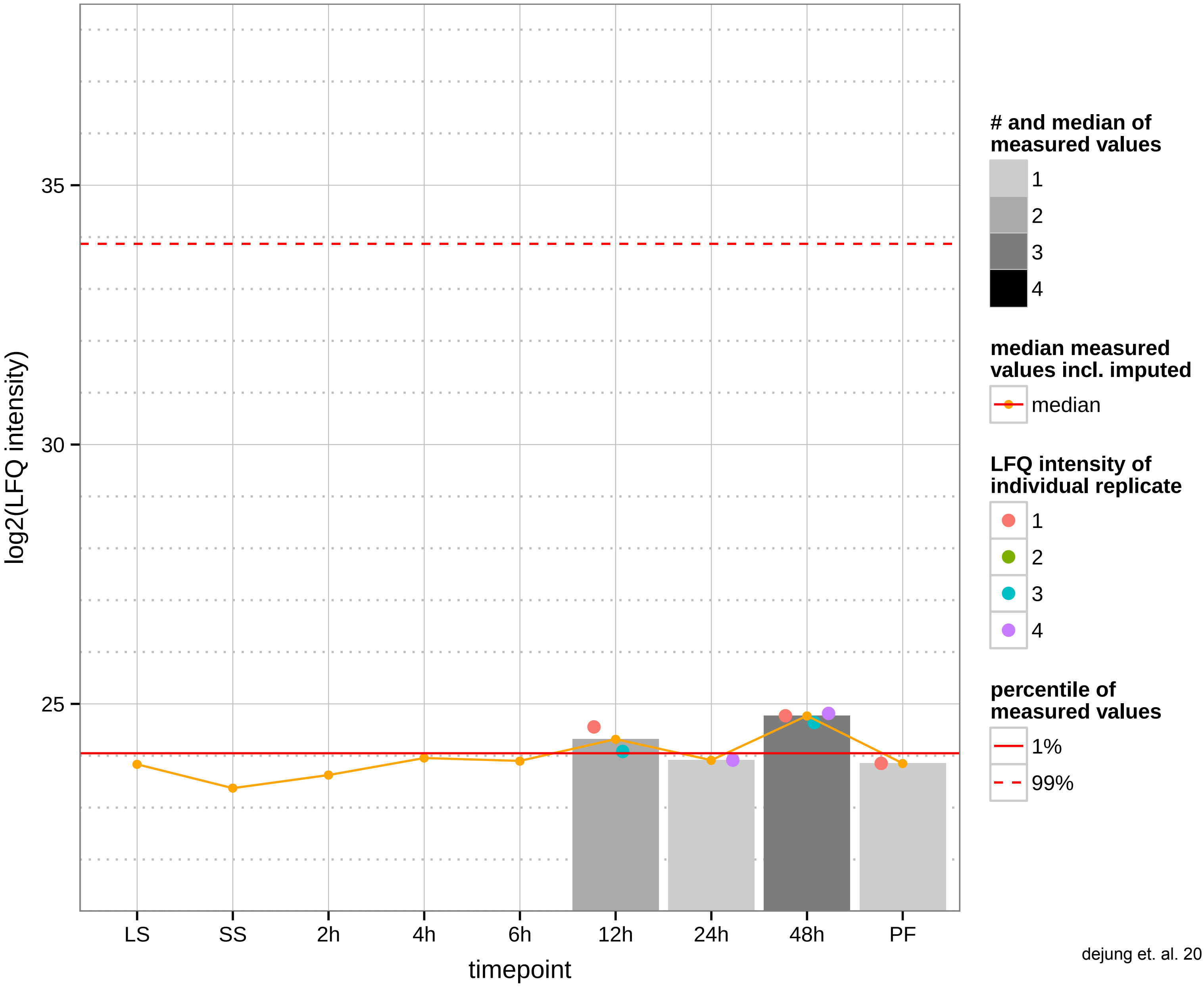

dejung et. al. 2015

NUP−1 protein, putative
Tb927.2.4230
AGOF: null
AGOC: null, nuclear lamina
AGOP: null
PGOF: null
PGOC: null
PGOP: null

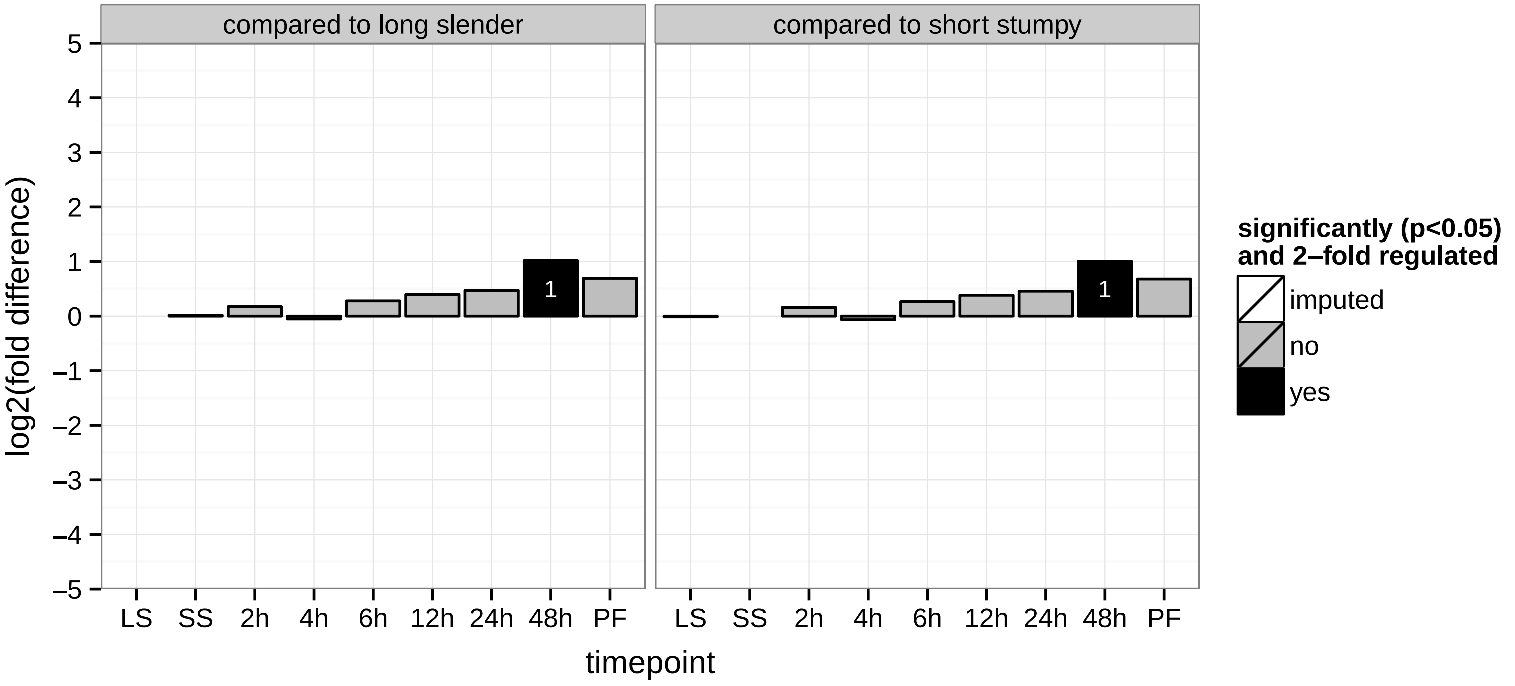

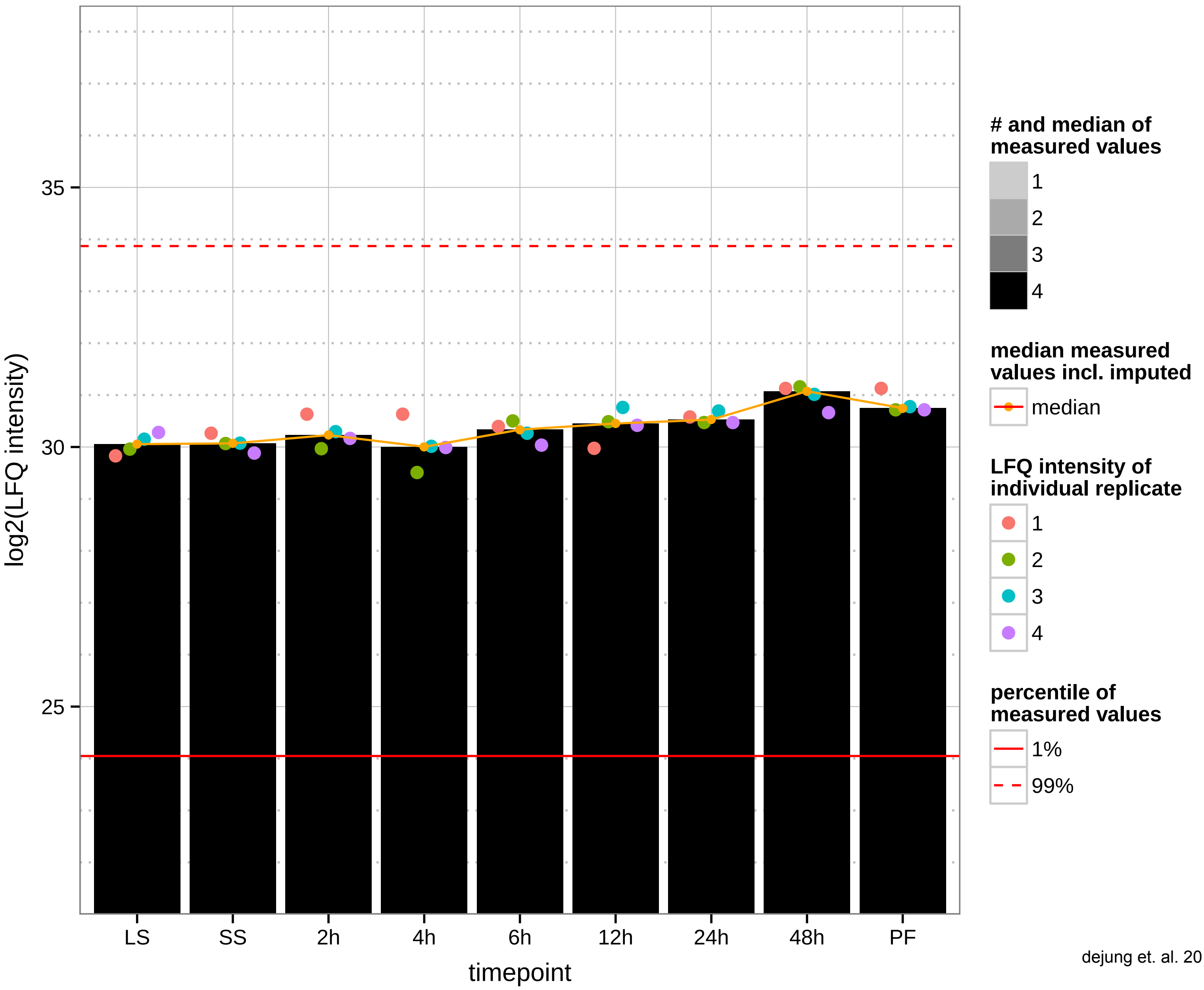

dejung et. al. 2015

FtsJ cell division protein, putative
Tb927.2.4550
AGOF: methyltransferase activity, nucleic acid binding
AGOC: nucleus
AGOP: rRNA methylation
PGOF: methyltransferase activity, nucleic acid binding
PGOC: nucleus
PGOP: methylation, rRNA processing

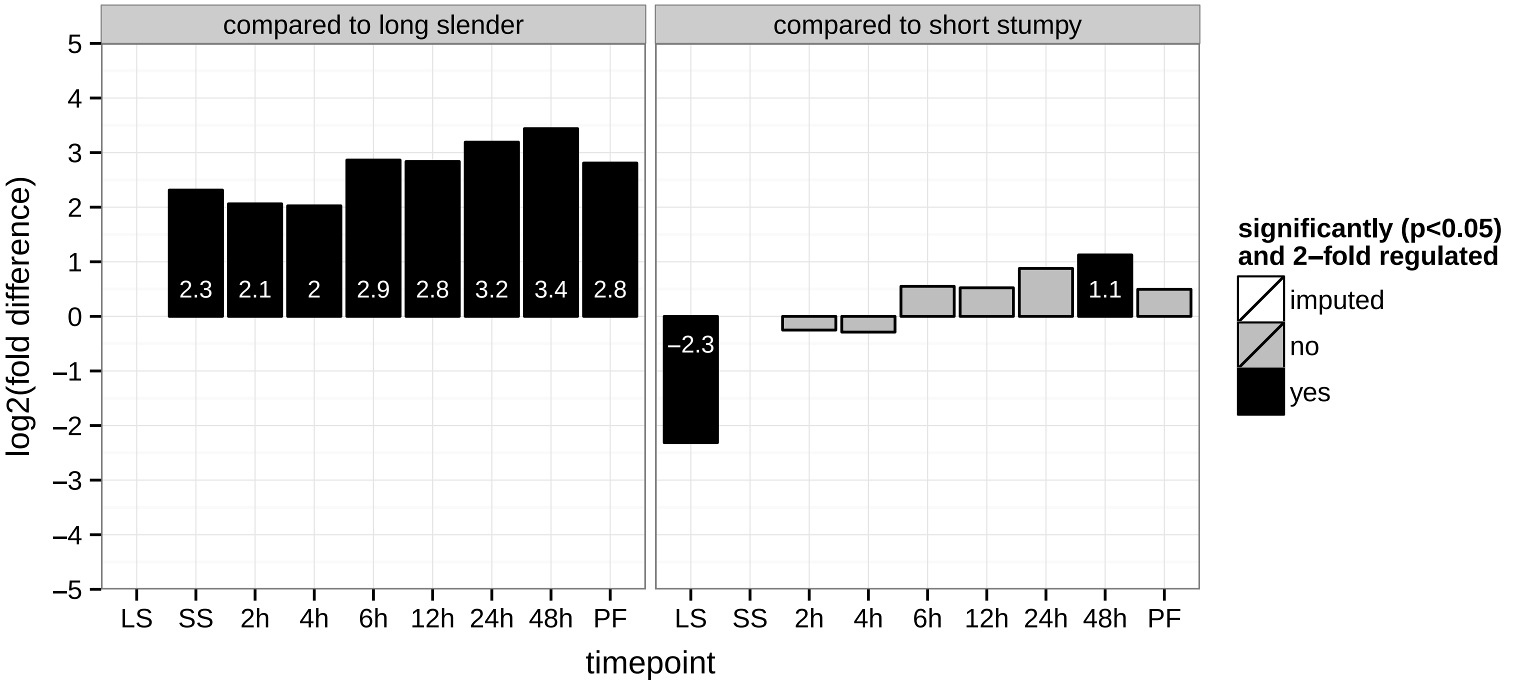

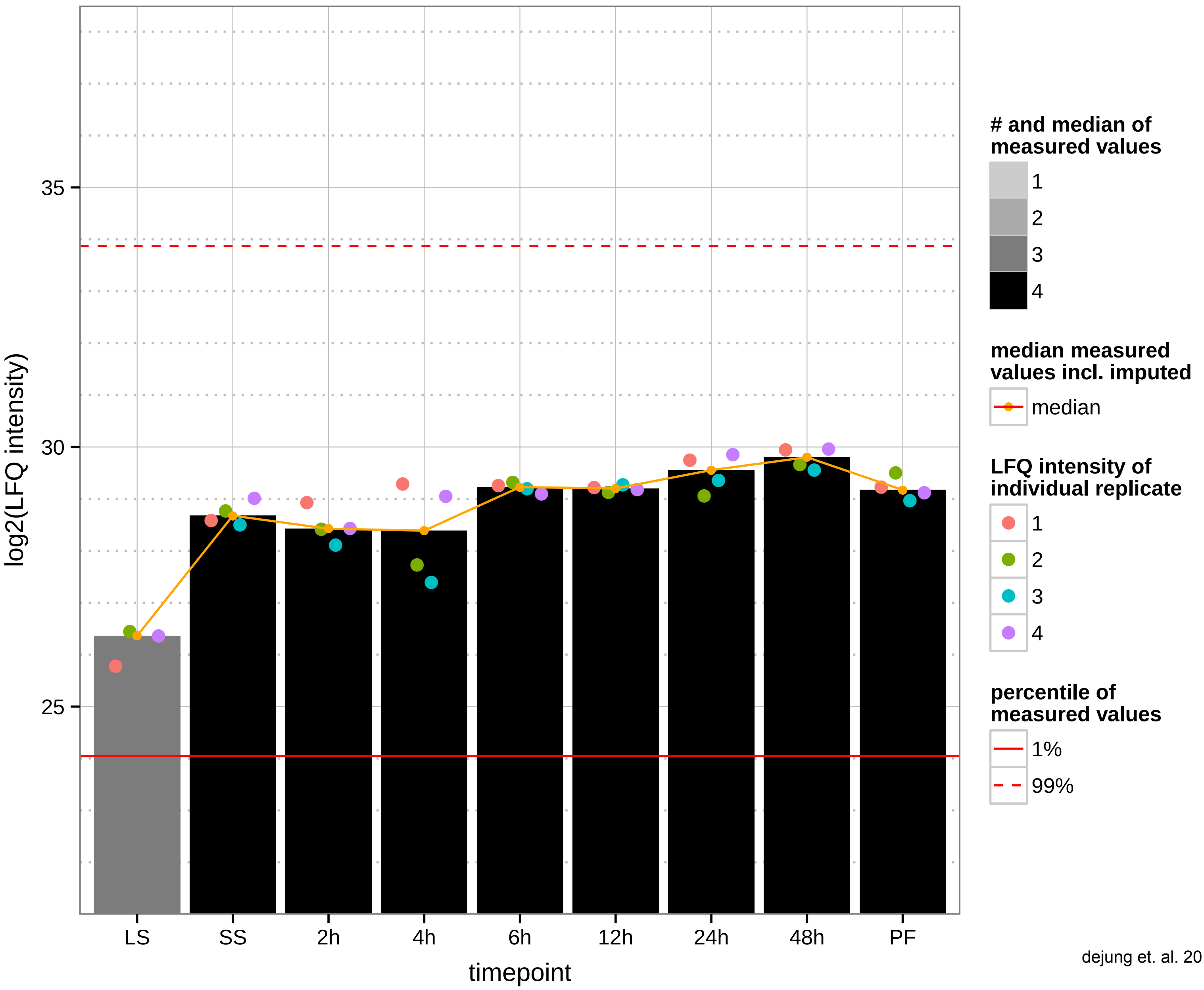

dejung et. al. 2015

U6 snRNA–associated Sm–like protein LSm8p (TbLSm8)
Tb927.3.1780
AGOF: U6 snRNA 3'–end binding
AGOC: U4/U6 x U5 tri–snRNP complex, nucleus
AGOP: assembly of spliceosomal tri–snRNP, nuclear mRNA splicing, via spliceosome
PGOF: null
PGOC: null
PGOP: null

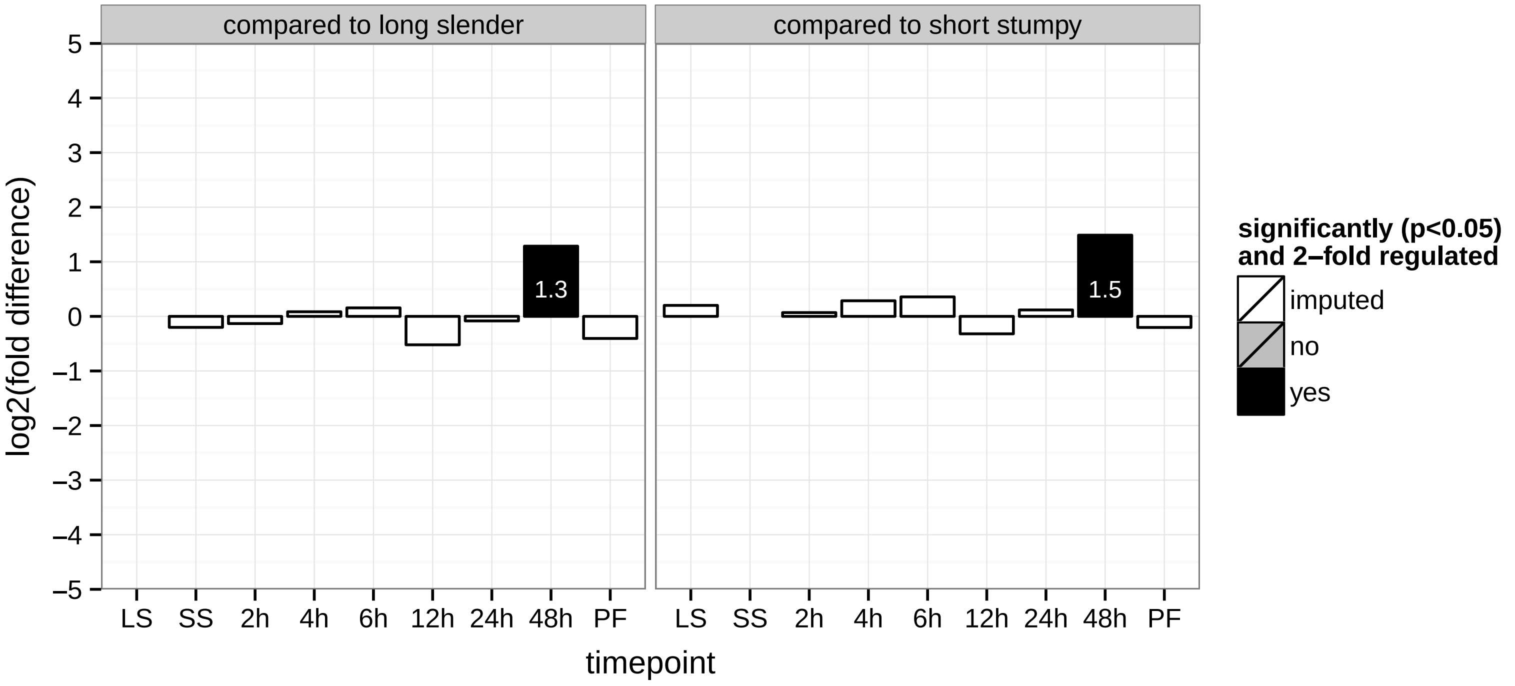

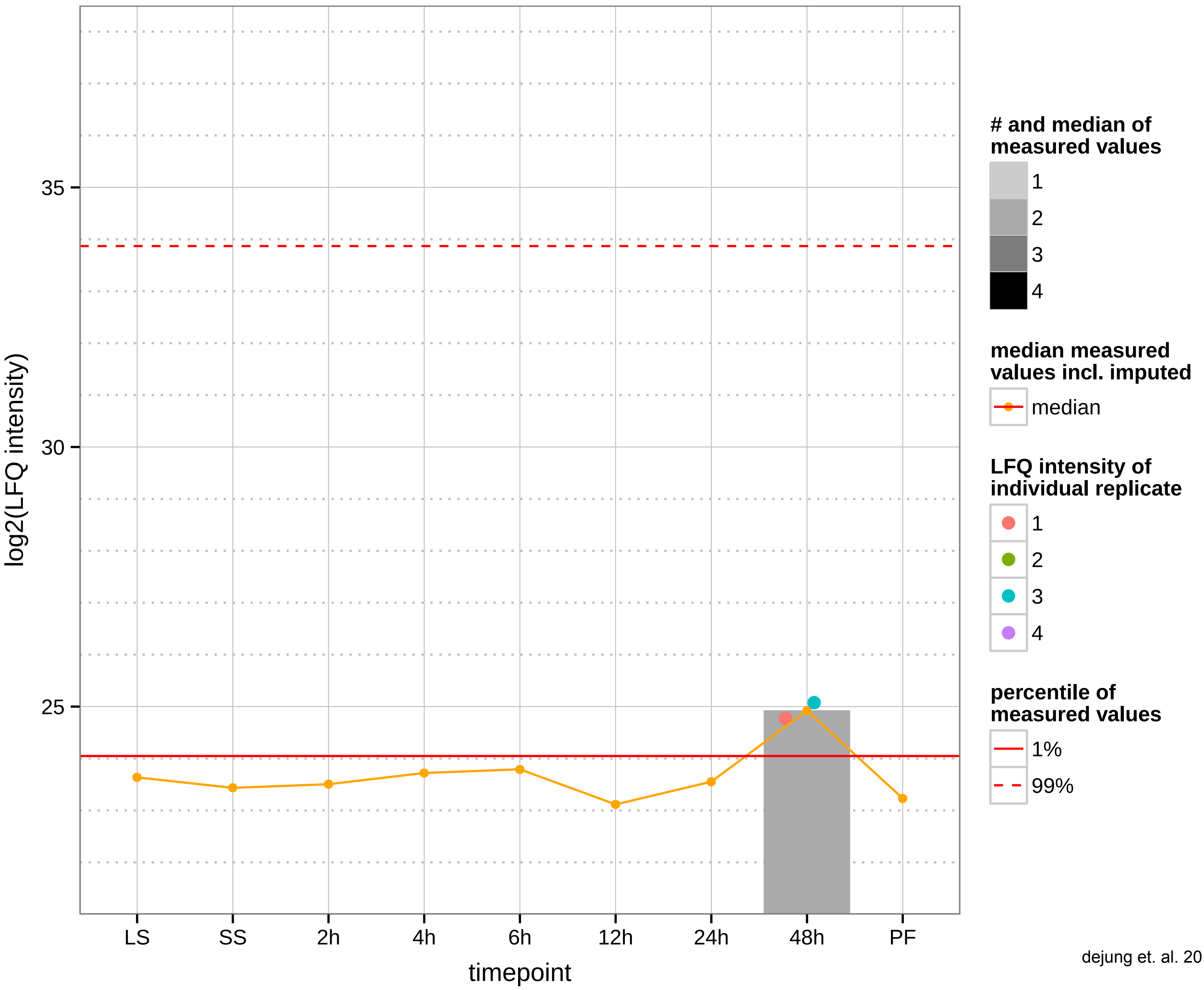

dejung et. al. 2015

hypothetical protein, conserved
Tb927.3.2100
AGOF: null
AGOC: null
AGOP: null
PGOF: null
PGOC: null
PGOP: null

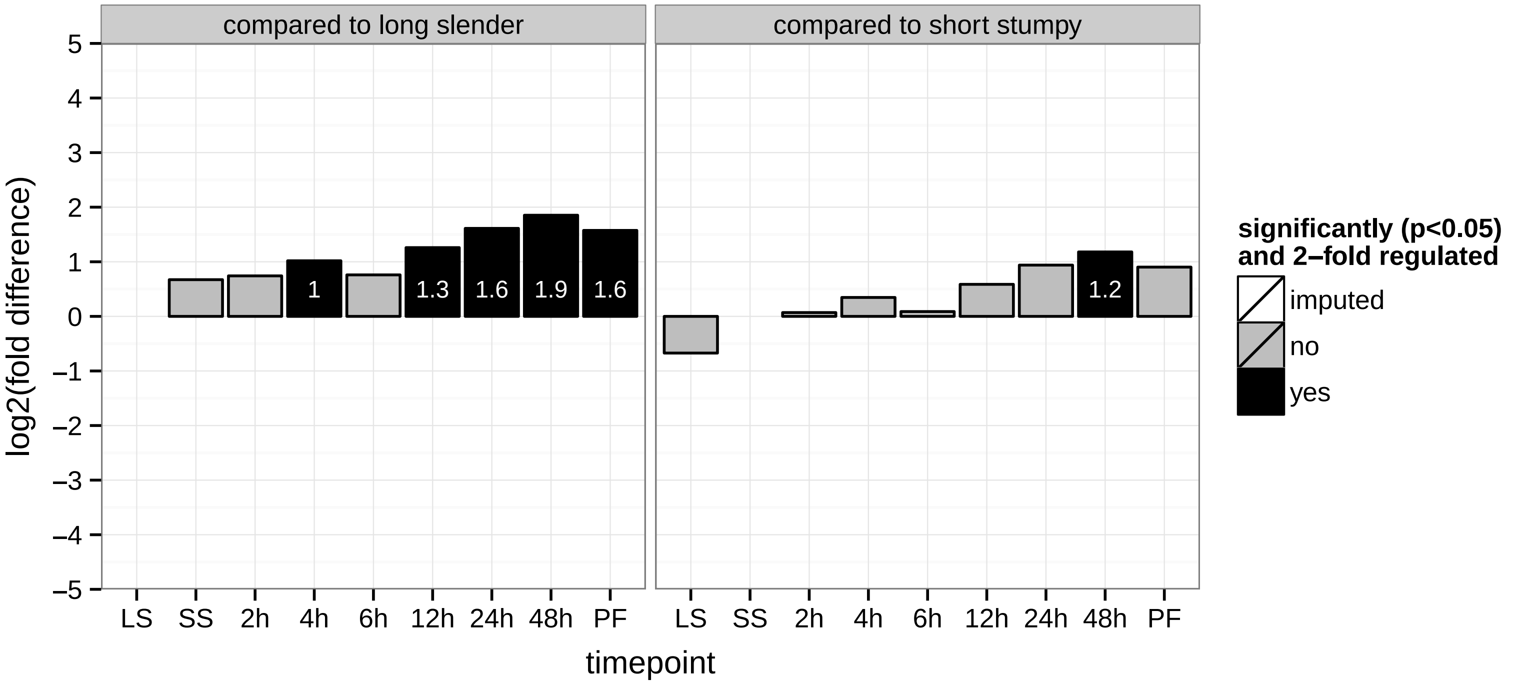

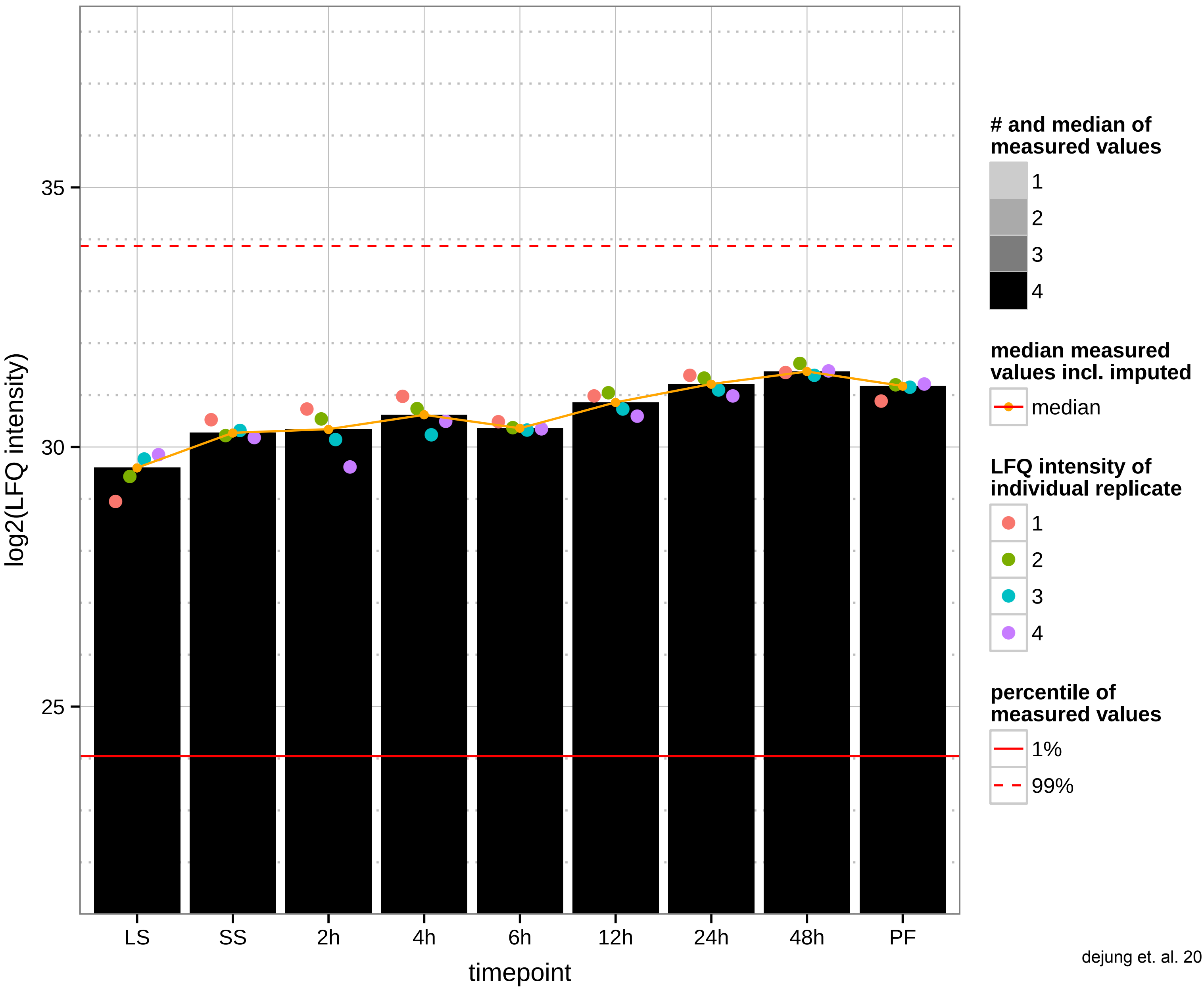

dejung et. al. 2015

hypothetical protein, conserved
Tb927.3.2660
AGOF: endodeoxyribonuclease activity, producing 5'−phosphomonoesters
AGOC: null
AGOP: null
PGOF: endodeoxyribonuclease activity, producing 5'−phosphomonoesters
PGOC: null
PGOP: null

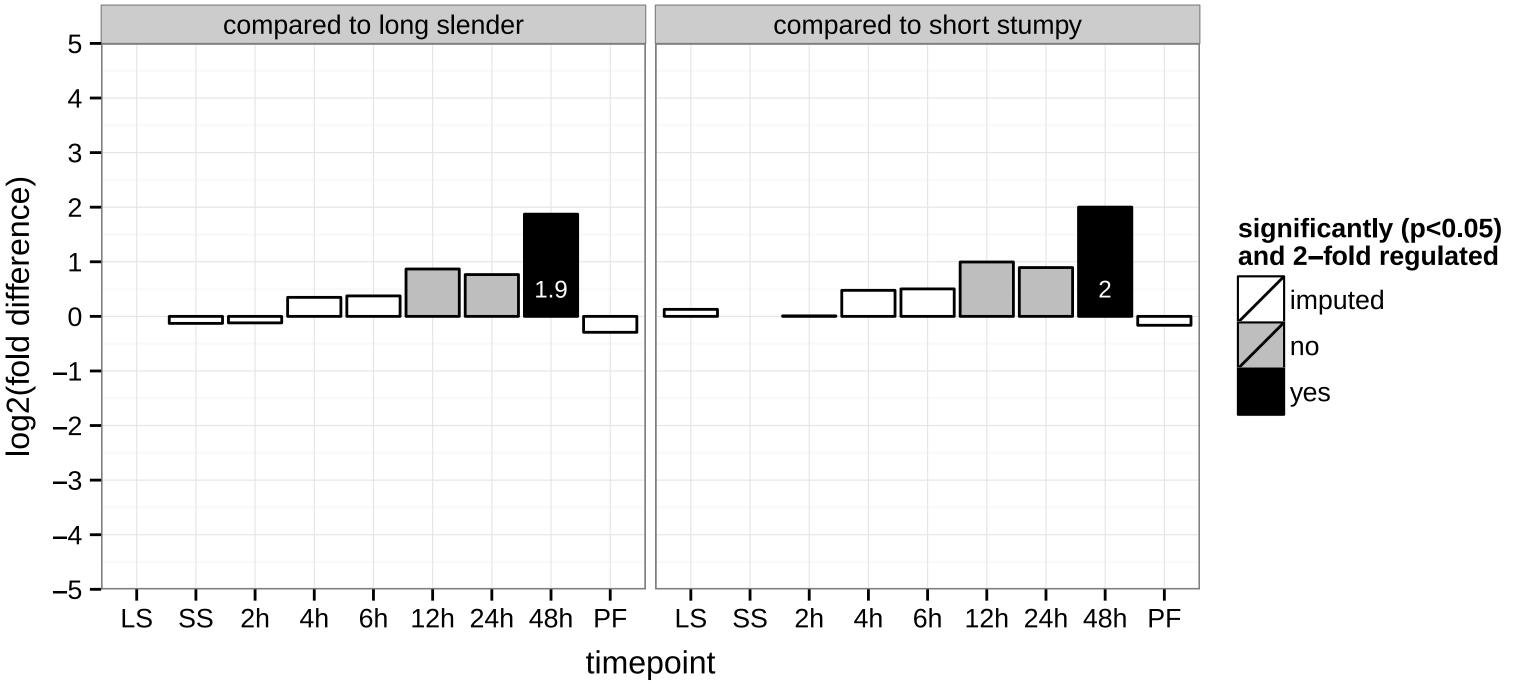

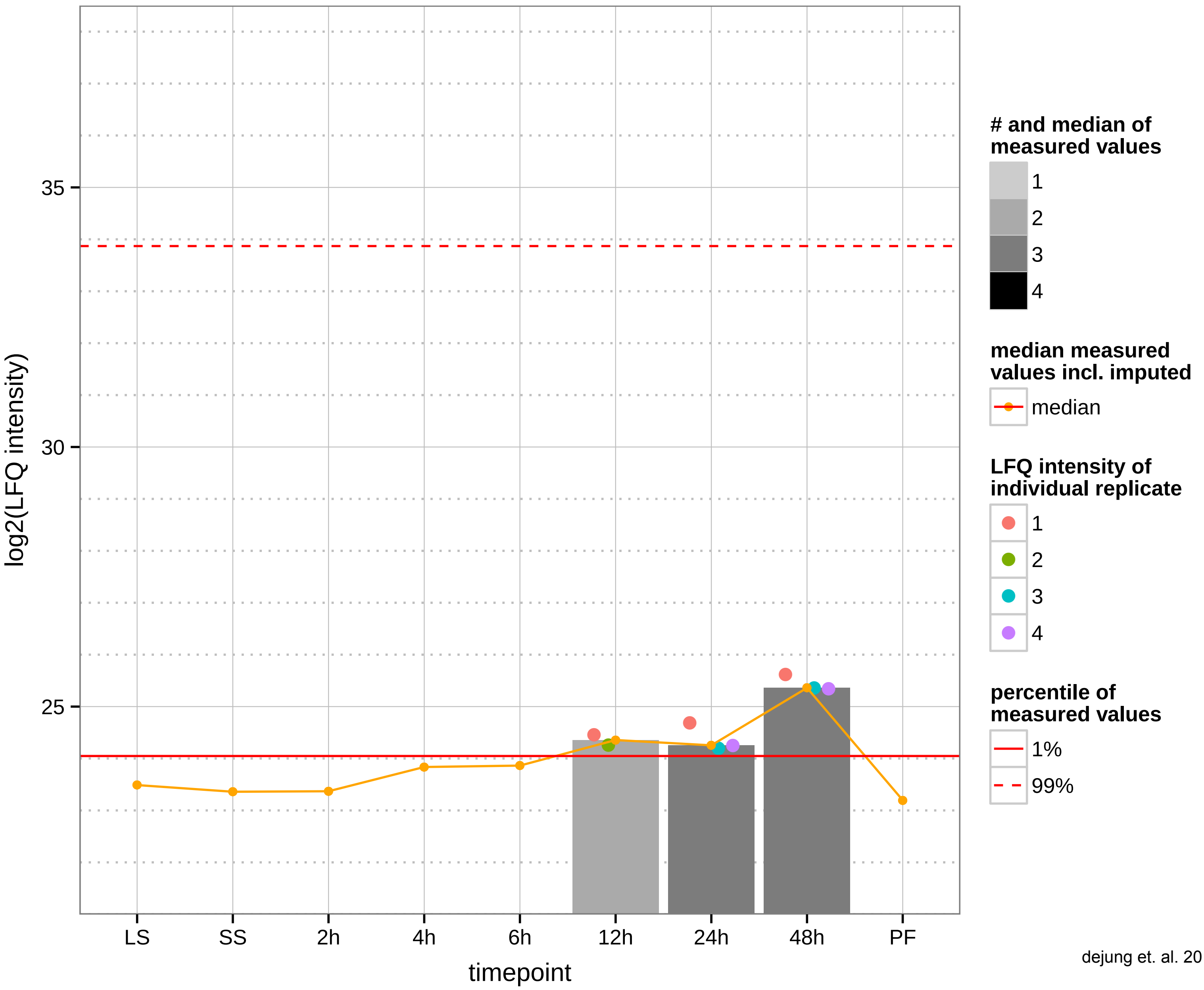

dejung et. al. 2015

hypothetical protein, conserved
Tb927.3.3150;Tb11.v5.0796
AGOF: null
AGOC: null
AGOP: null
PGOF: null
PGOC: null
PGOP: null

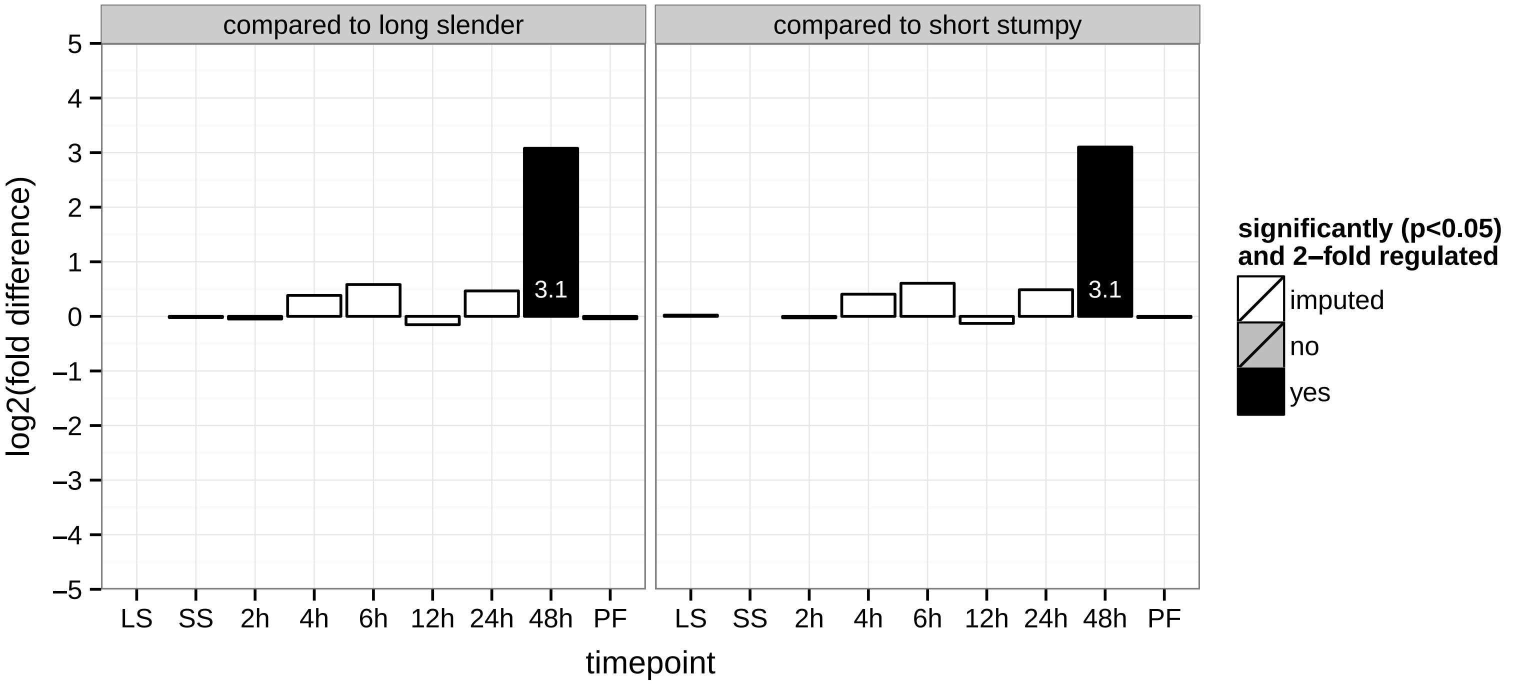

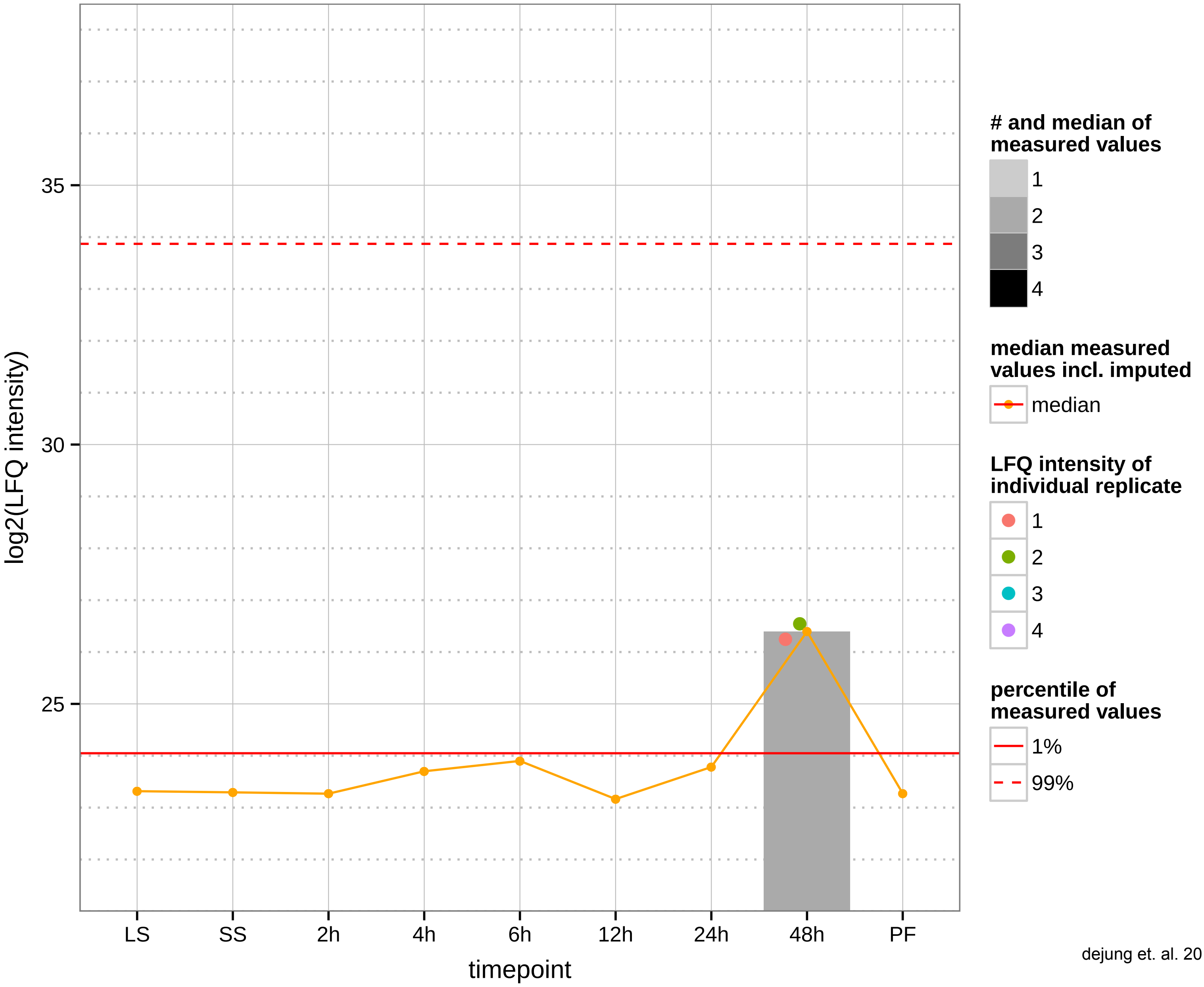

dejung et. al. 2015

60S ribosomal protein L13, putative
Tb927.3.3320;Tb927.3.3310
AGOF: structural constituent of ribosome
AGOC: intracellular, ribosome
AGOP: null
PGOF: structural constituent of ribosome, null
PGOC: intracellular, ribosome, null
PGOP: translation, null

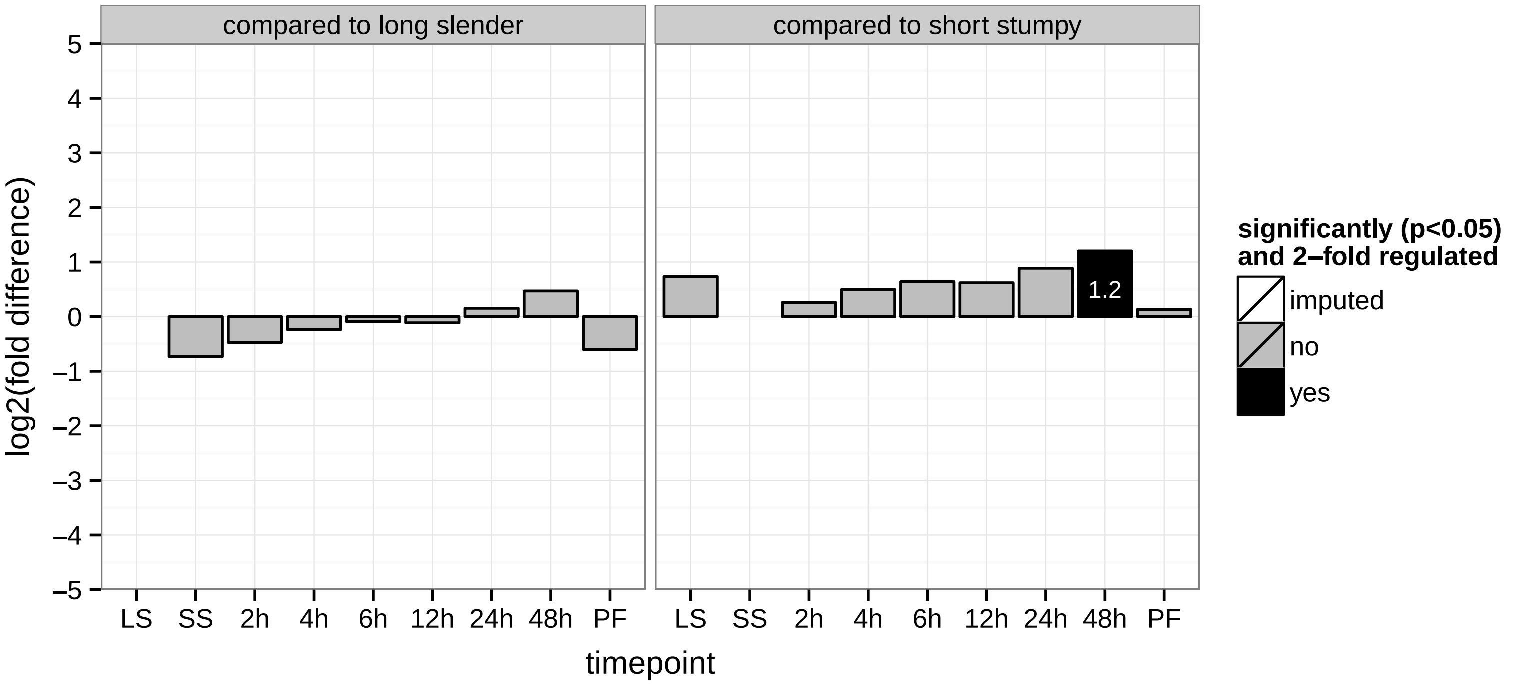

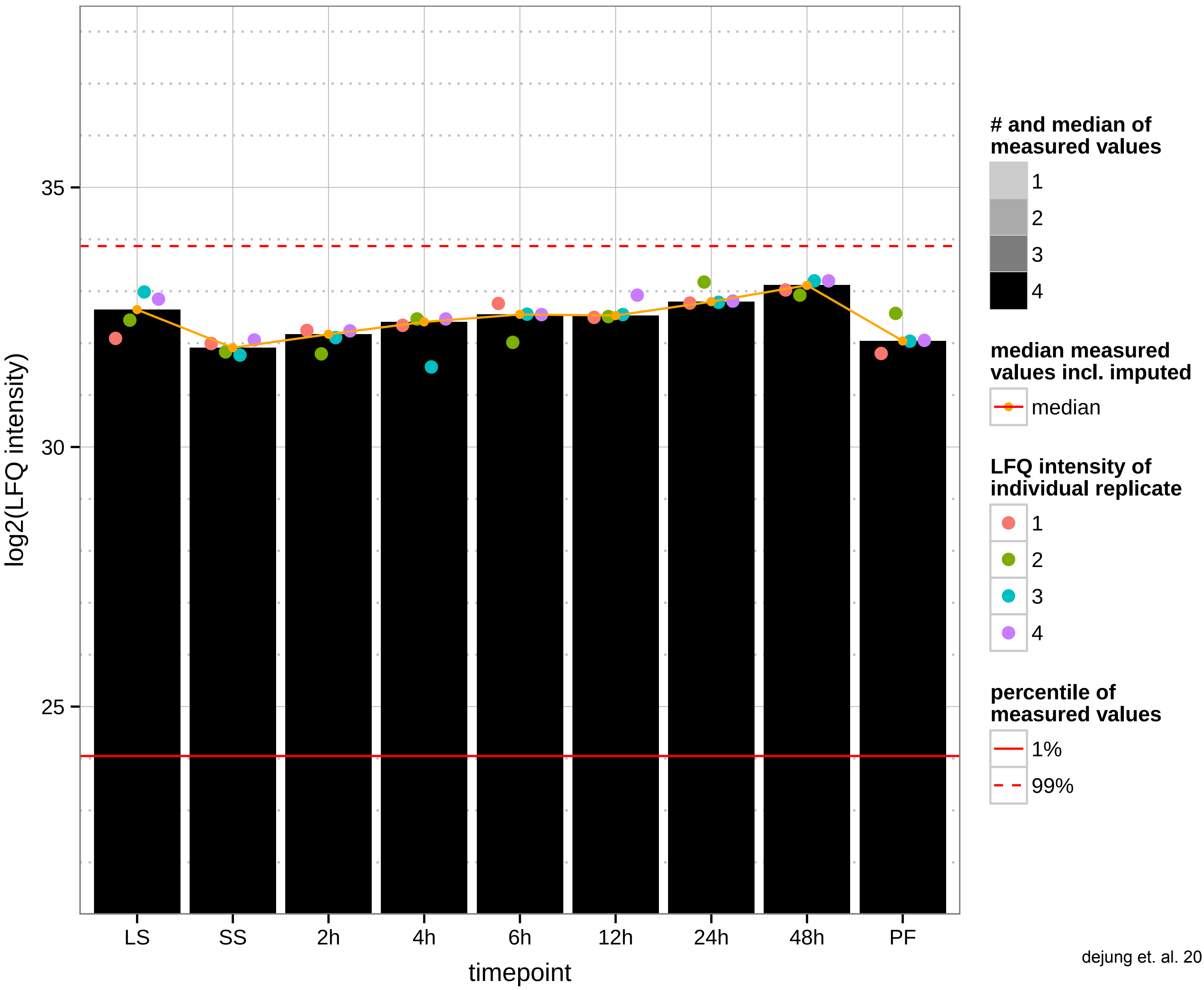

dejung et. al. 2015

RNA–binding protein, putative (TRRM3)
Tb927.3.3670
AGOF: RNA binding
AGOC: nucleus
AGOP: RNA processing
PGOF: nucleic acid binding
PGOC: null
PGOP: null

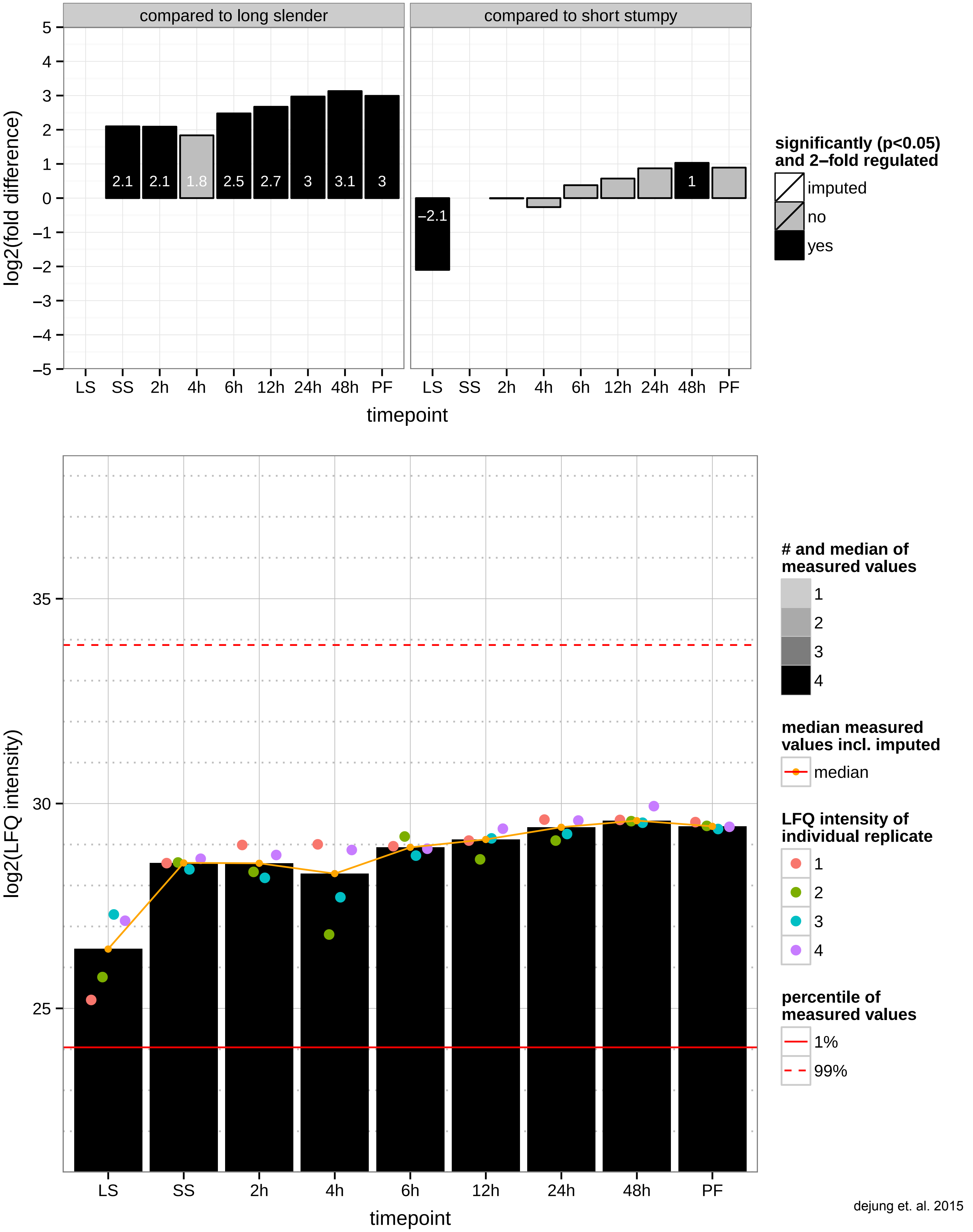

dejung et. al. 2015

hypothetical protein, conserved
Tb927.3.3740
AGOF: zinc ion binding
AGOC: intracellular
AGOP: null
PGOF: zinc ion binding
PGOC: intracellular
PGOP: null

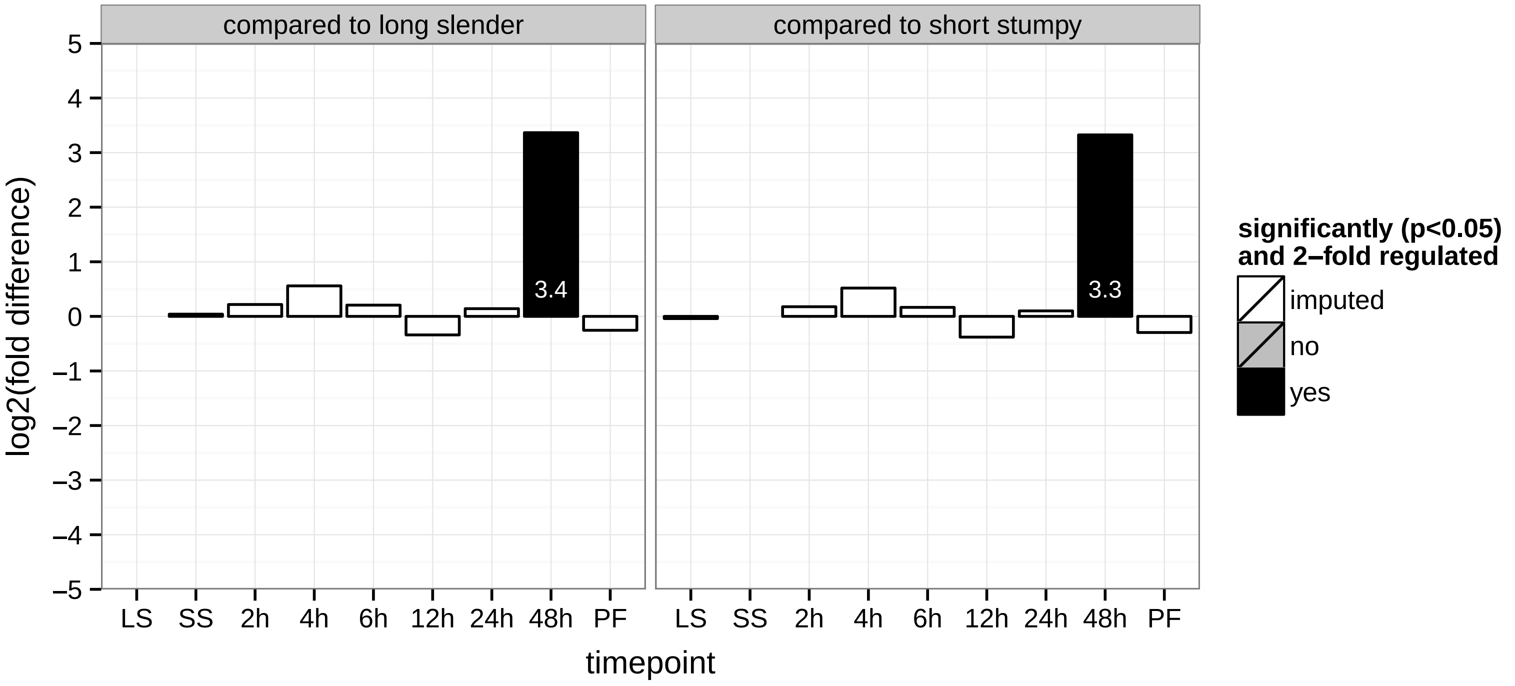

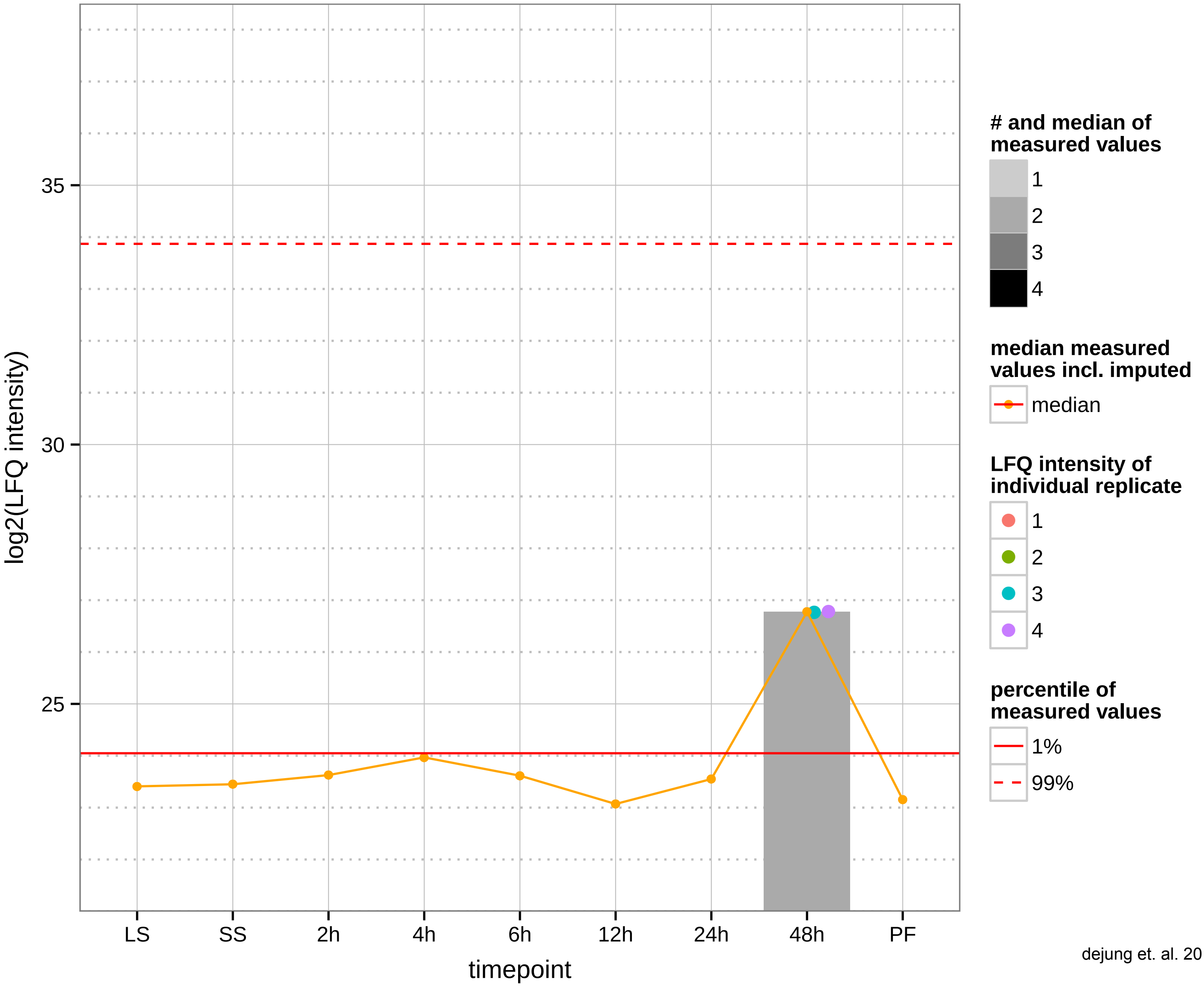

dejung et. al. 2015

hypothetical protein, conserved
Tb927.3.4620
AGOF: nucleic acid binding, zinc ion binding
AGOC: null
AGOP: null
PGOF: nucleic acid binding, zinc ion binding
PGOC: null
PGOP: null

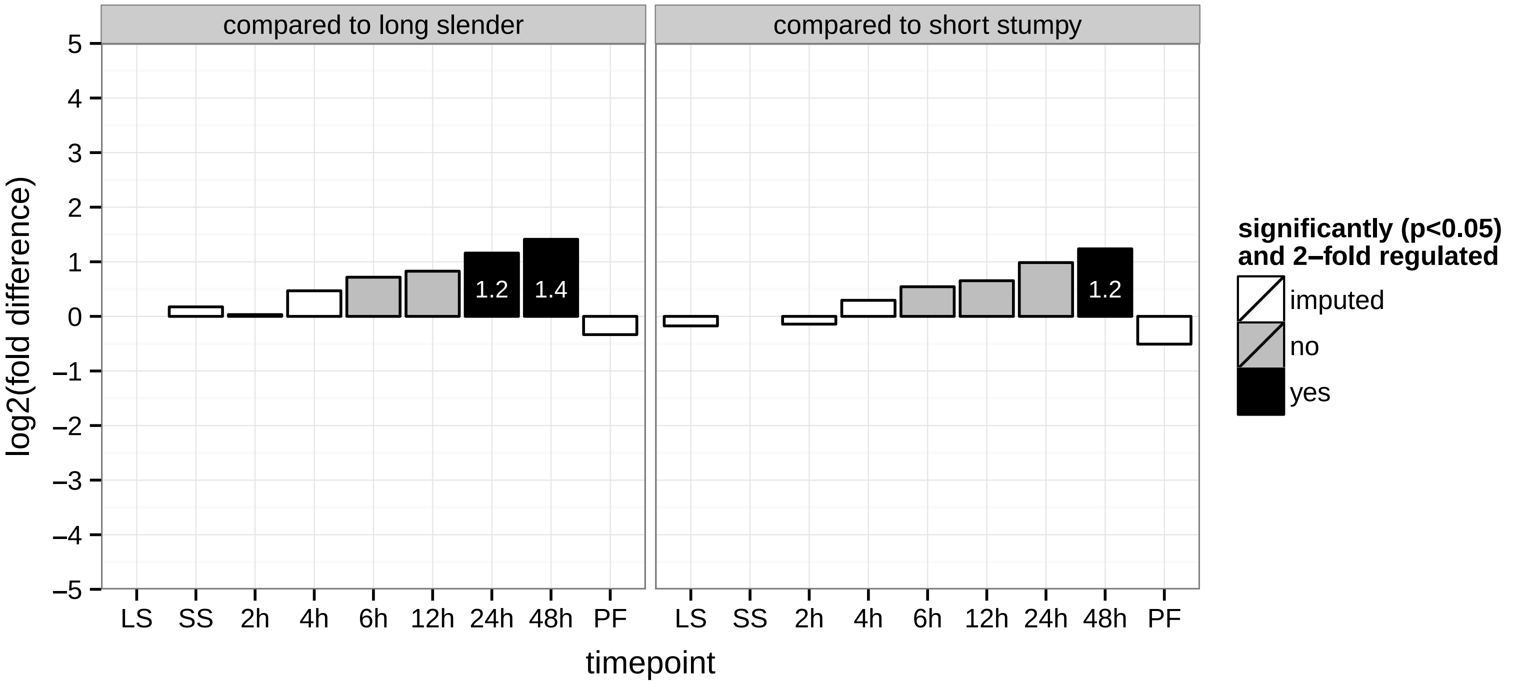

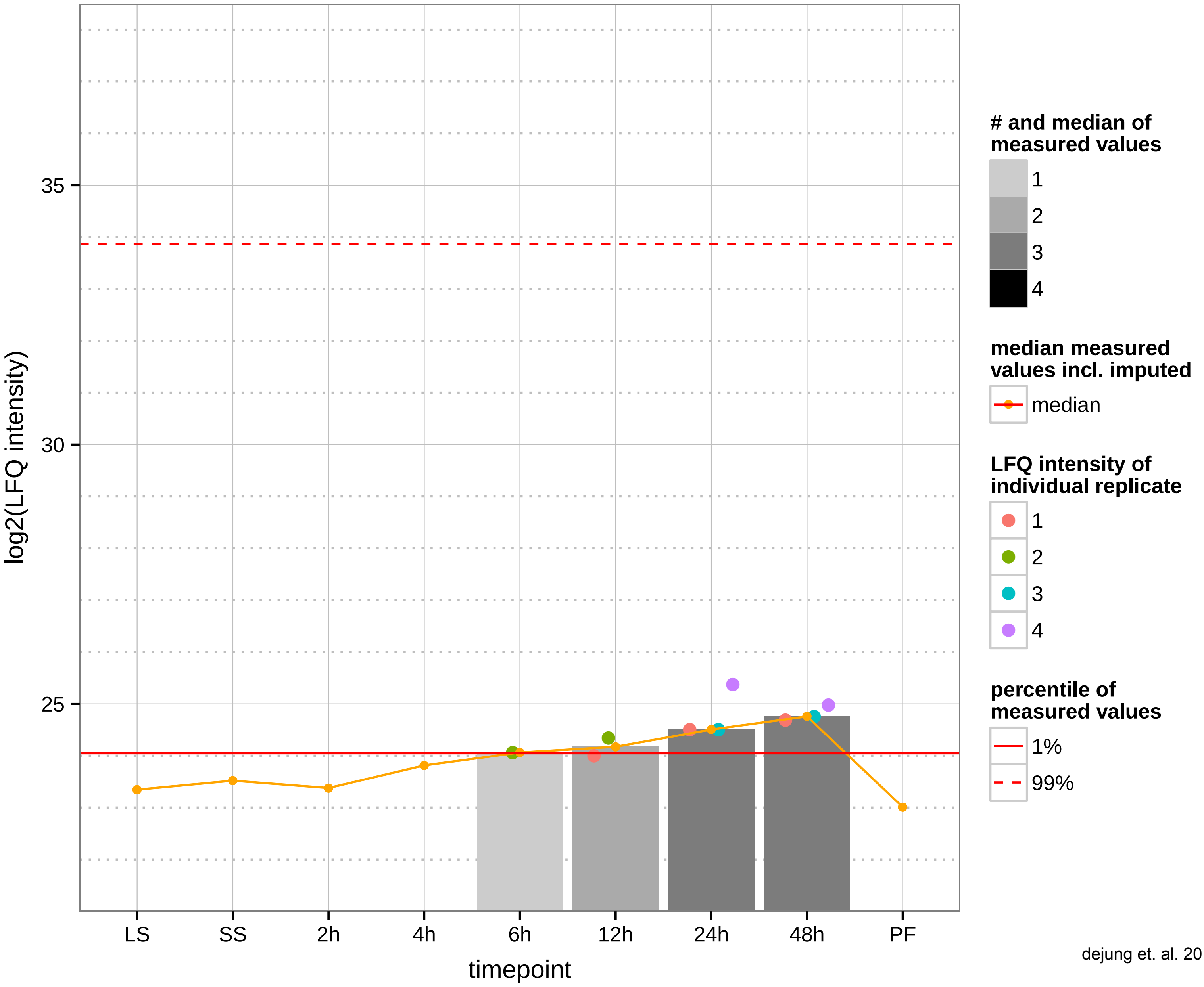

dejung et. al. 2015

paraflagellar rod protein, I2 antigen
Tb927.3.5310
AGOF: null
AGOC: null
AGOP: null
PGOF: null
PGOC: null
PGOP: null

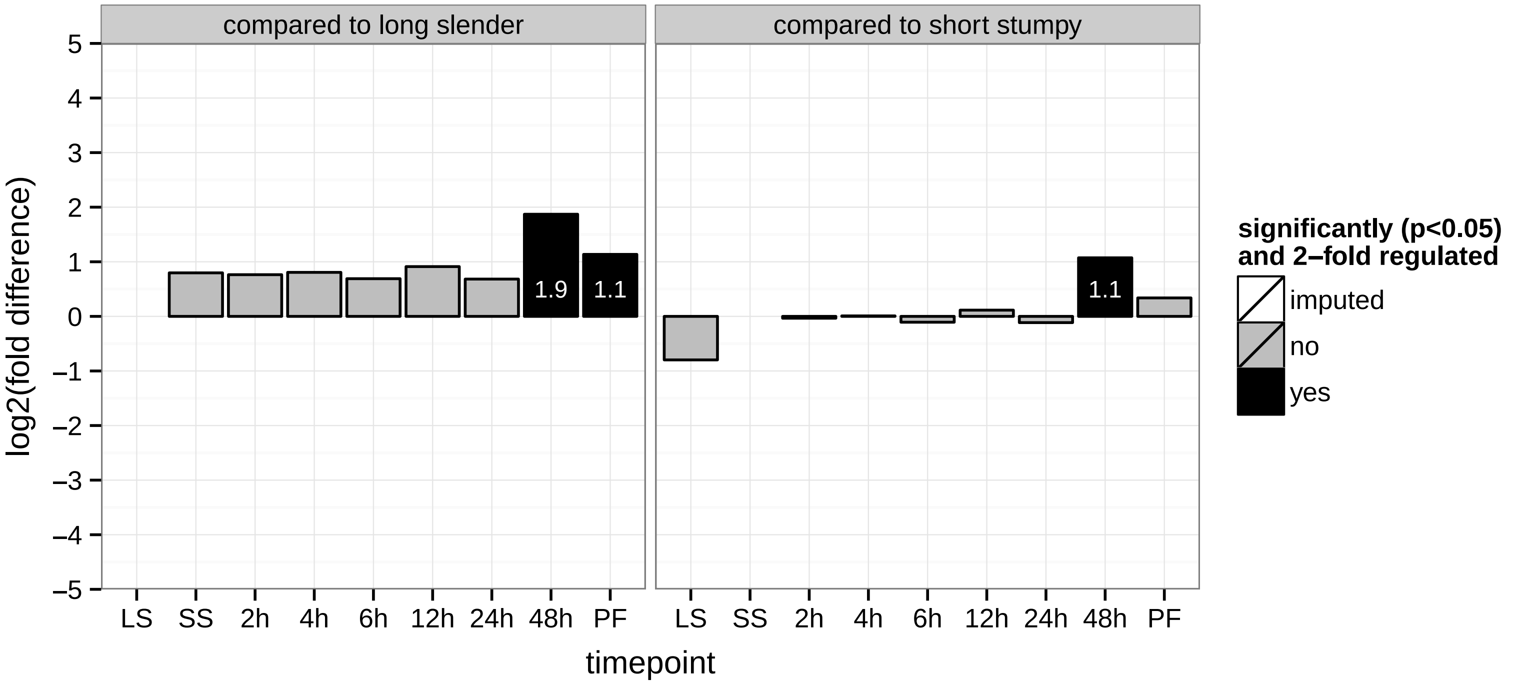

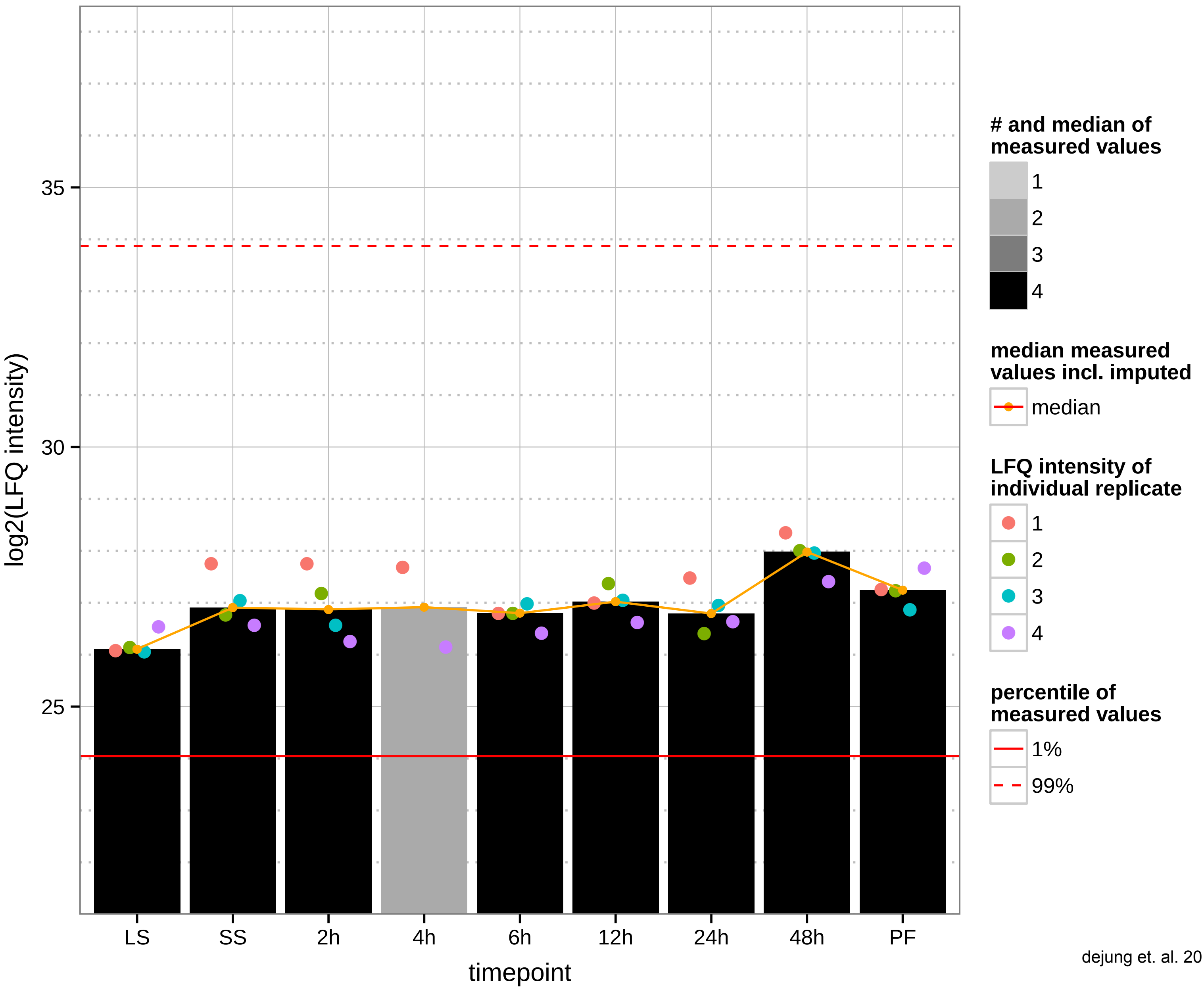

dejung et. al. 2015

hypothetical protein, conserved
Tb927.3.5400
AGOF: ATP binding, protein serine/threonine kinase activity
AGOC: null
AGOP: growth
PGOF: ATP binding, catalytic activity, protein serine/threonine kinase activity, transferase activity, transferring phosphorus–con
PGOC: null
PGOP: null

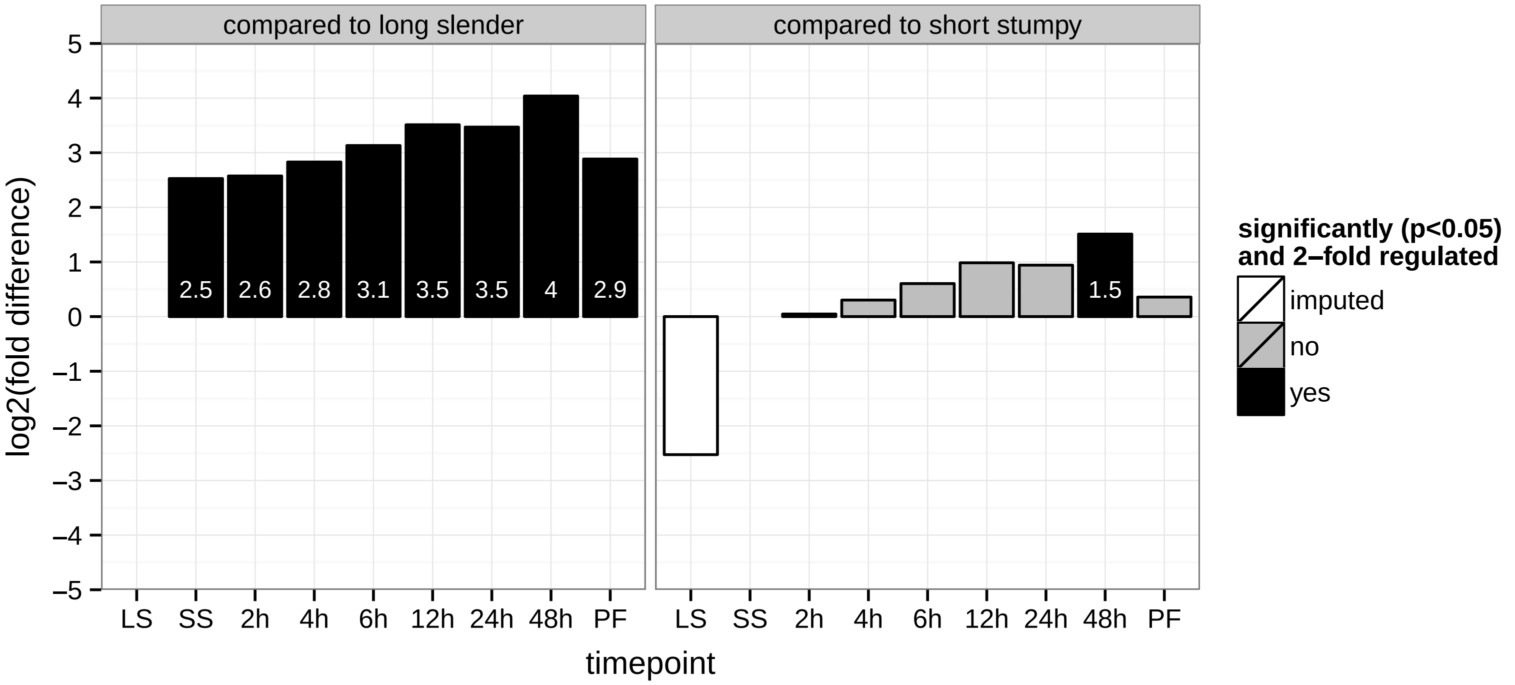

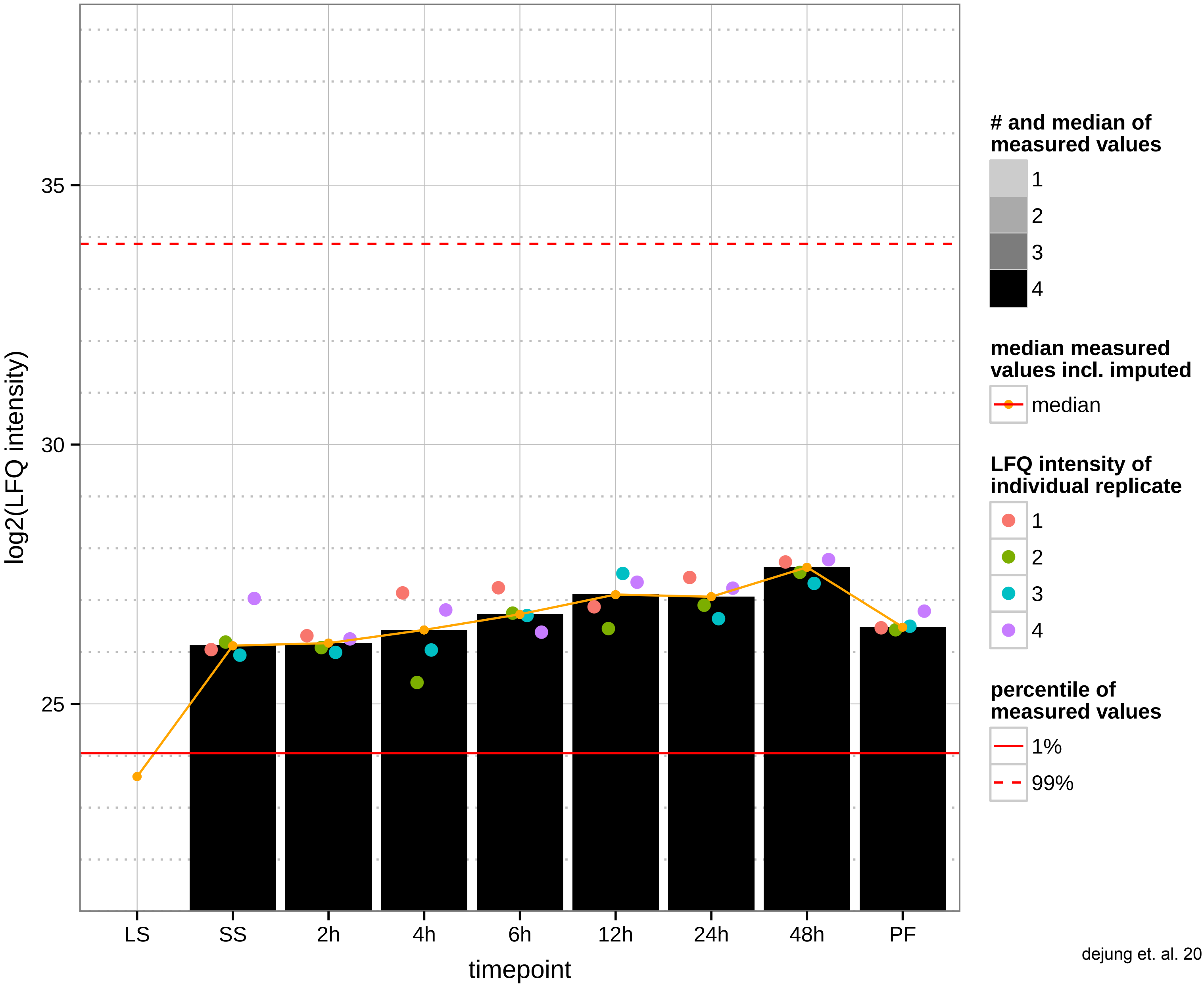

dejung et. al. 2015

hypothetical protein, conserved
Tb927.4.1520
AGOF: null
AGOC: null
AGOP: viral protein processing, viral reproduction
PGOF: null
PGOC: null
PGOP: viral protein processing, viral reproduction

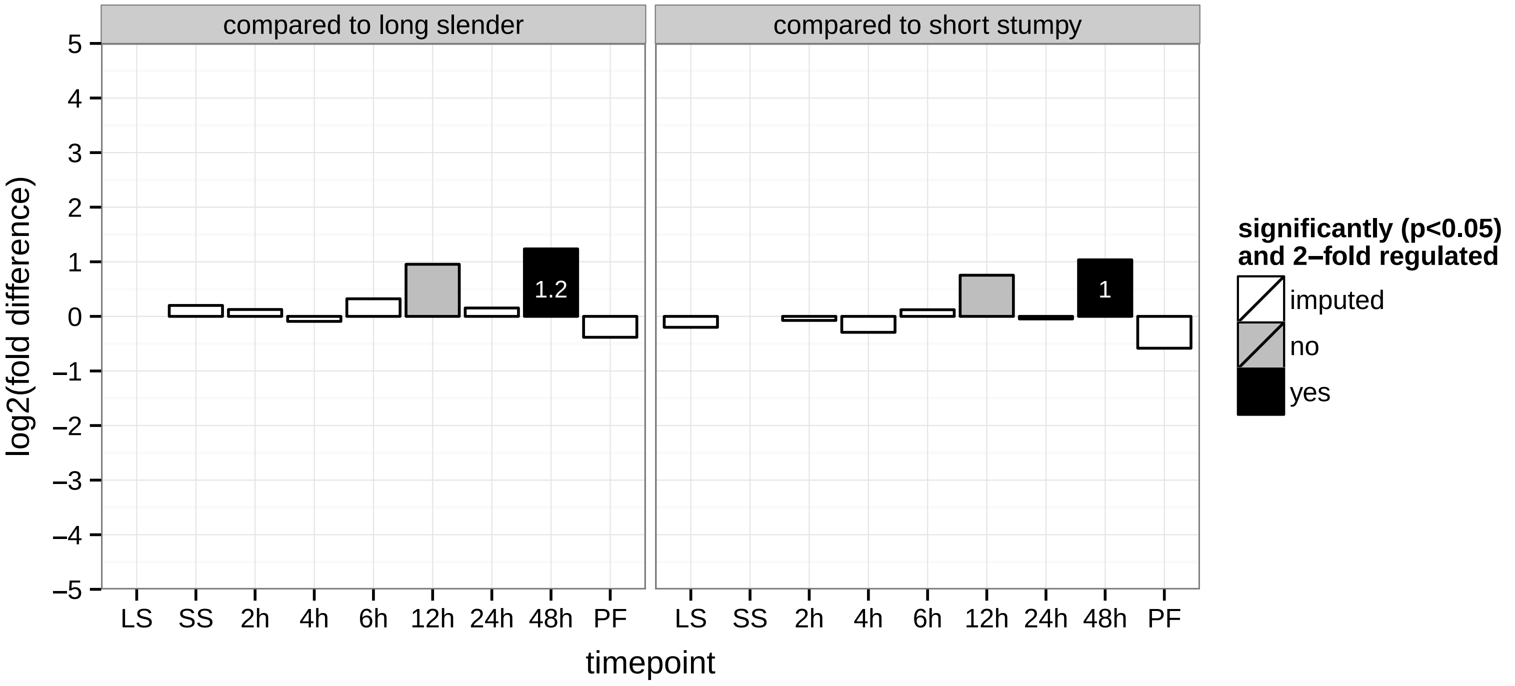

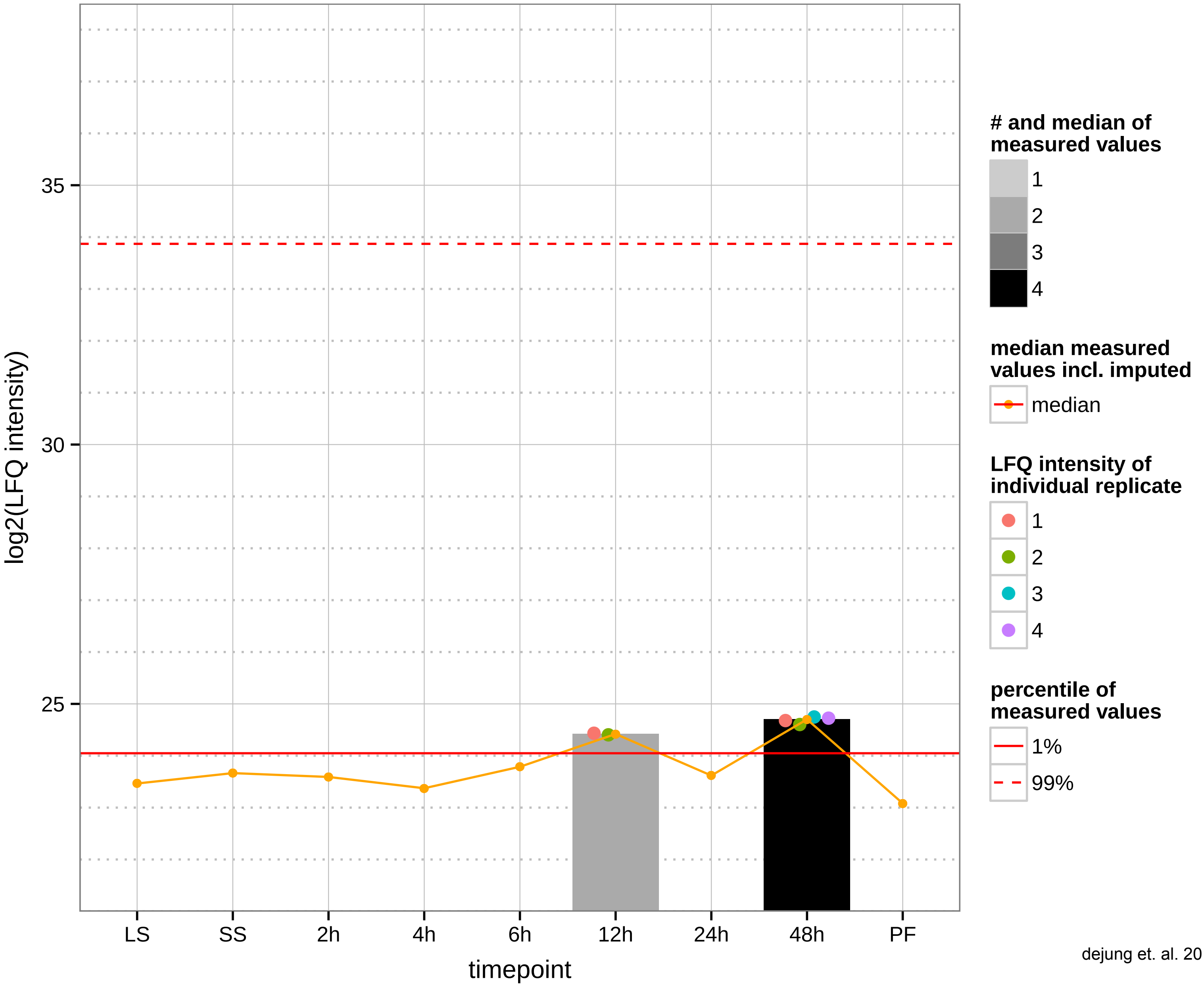

dejung et. al. 2015

hypothetical protein, conserved
Tb927.4.720
AGOF: null
AGOC: mitochondrion
AGOP: null
PGOF: null
PGOC: null
PGOP: null

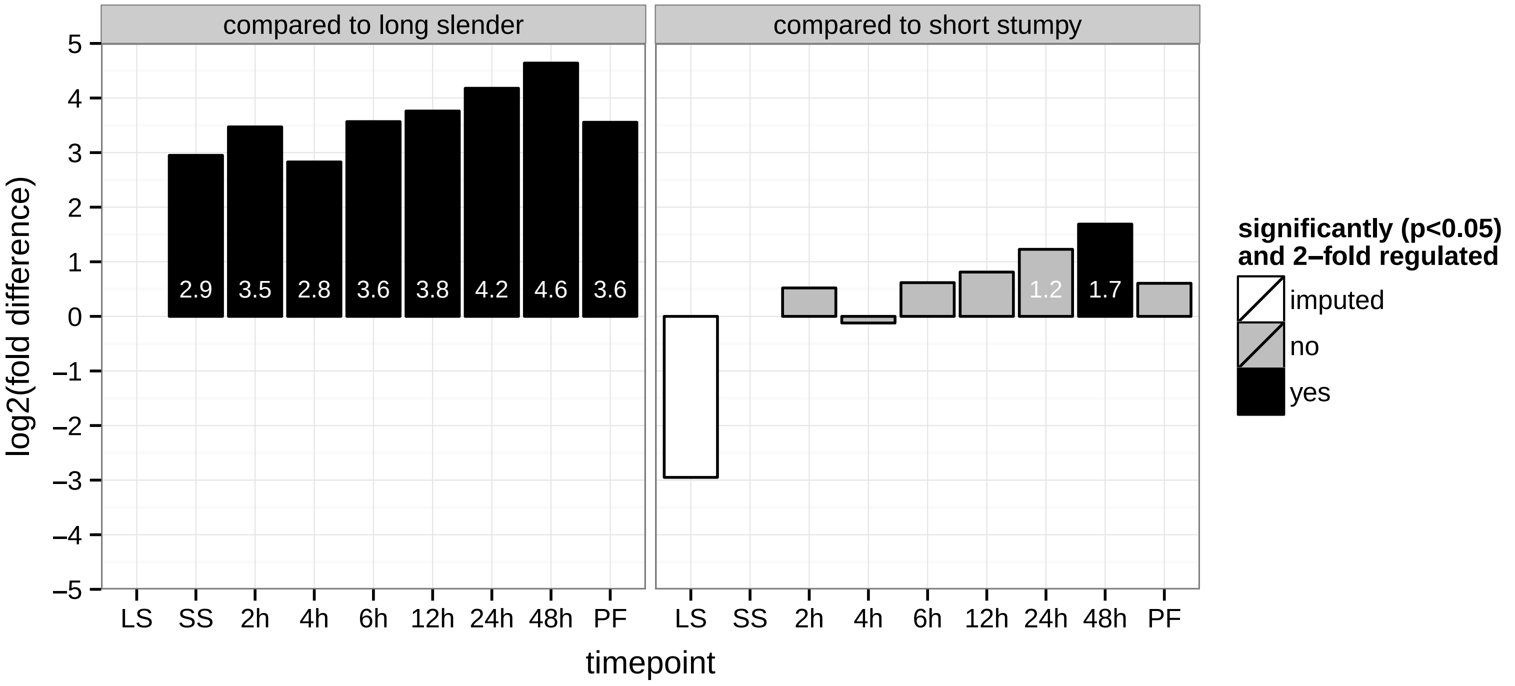

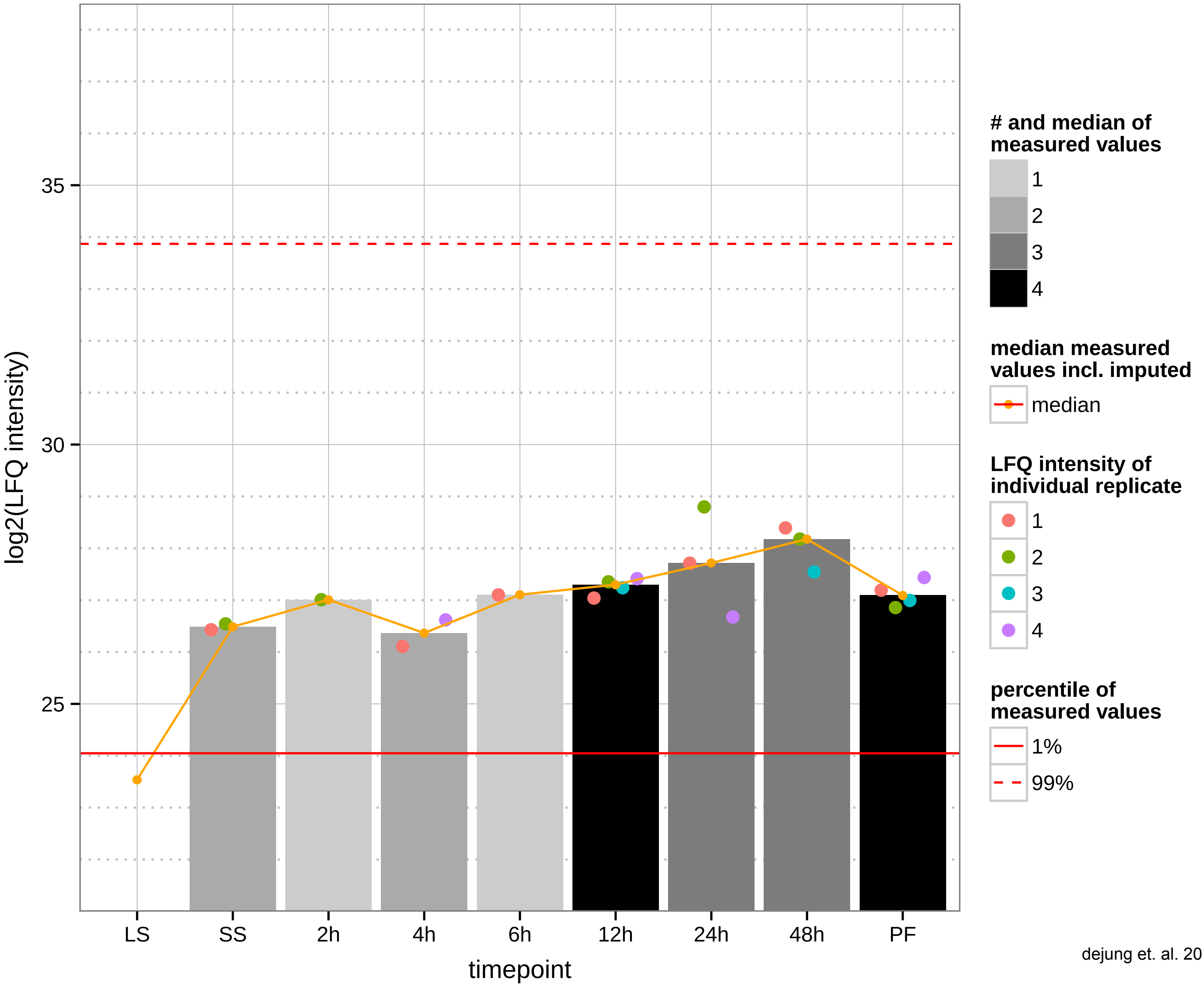

dejung et. al. 2015

ubiquitin–protein ligase, putative (upl3)
Tb927.4.770
AGOF: acid–amino acid ligase activity
AGOC: intracellular
AGOP: cellular protein modification process
PGOF: acid–amino acid ligase activity
PGOC: intracellular
PGOP: cellular protein modification process

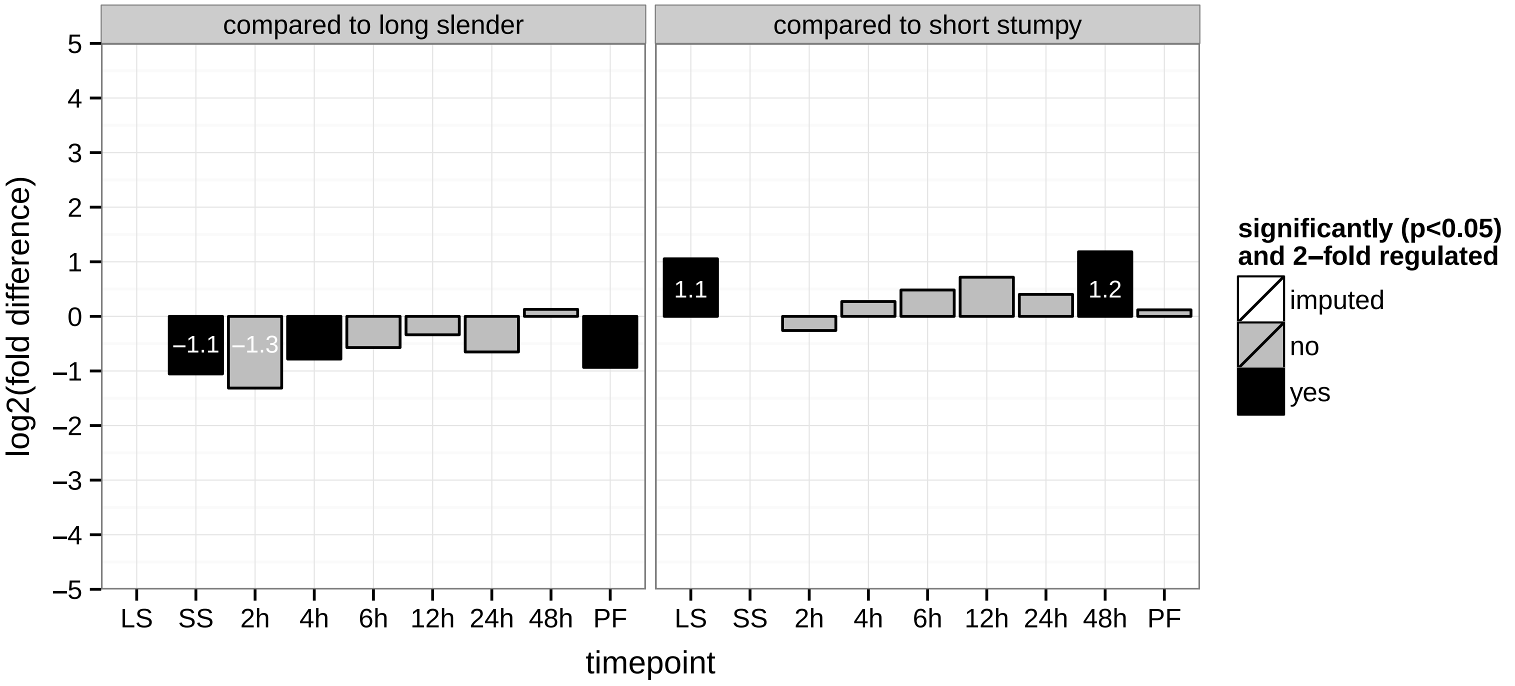

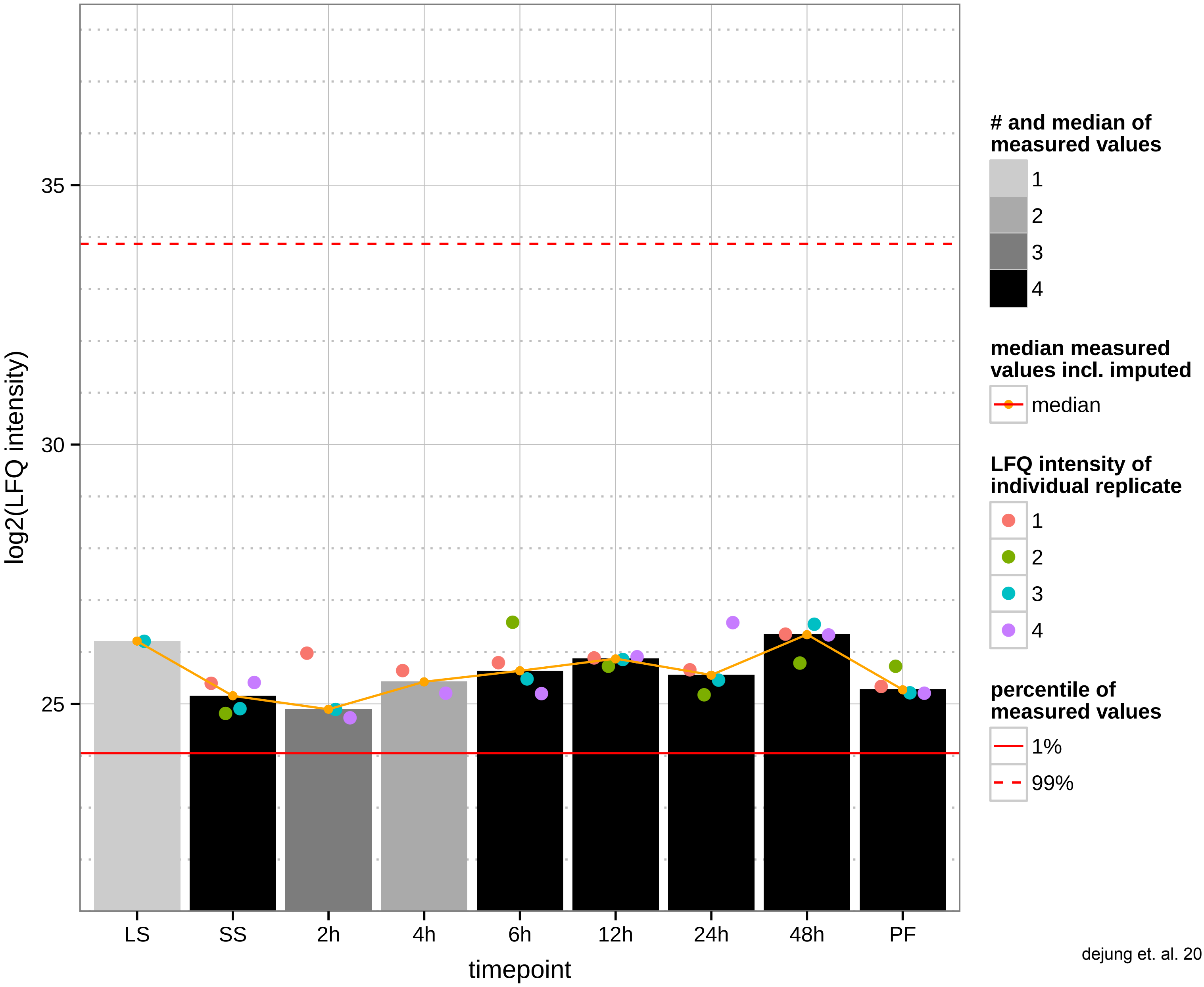

dejung et. al. 2015

ADG1, pseudogene
Tb927.5.3390
AGOF: null
AGOC: null
AGOP: null
PGOF: null
PGOC: null
PGOP: null

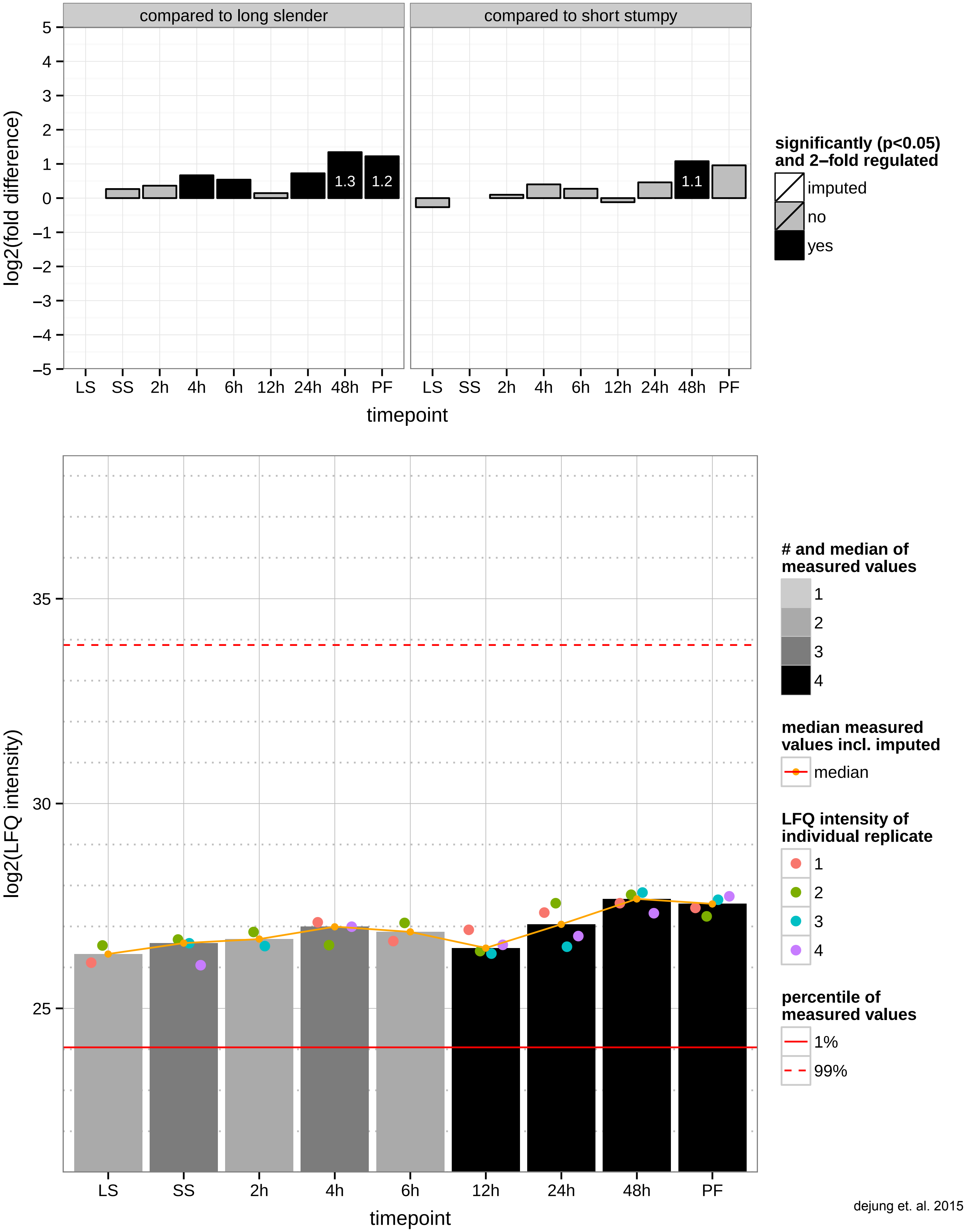

dejung et. al. 2015

hypothetical protein, conserved
Tb927.5.770
AGOF: null
AGOC: null
AGOP: null
PGOF: null
PGOC: null
PGOP: null

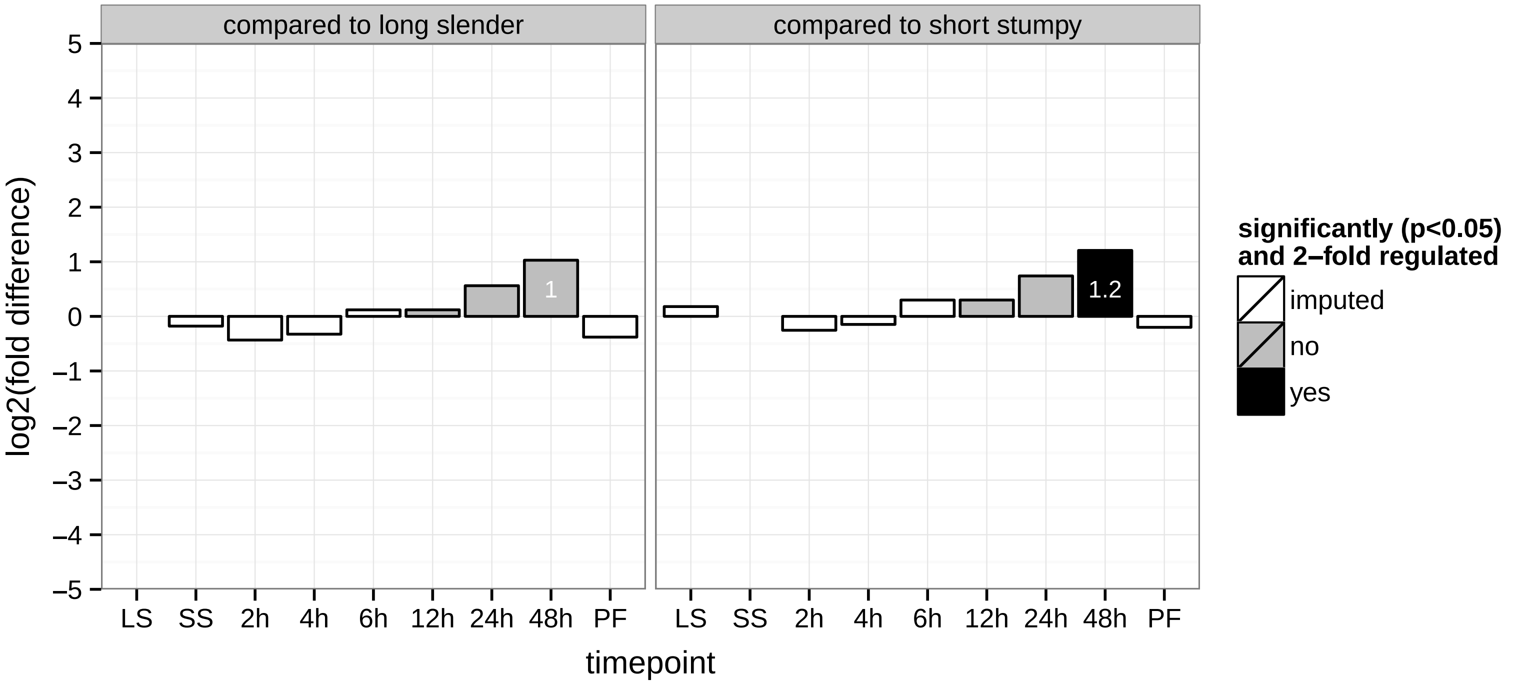

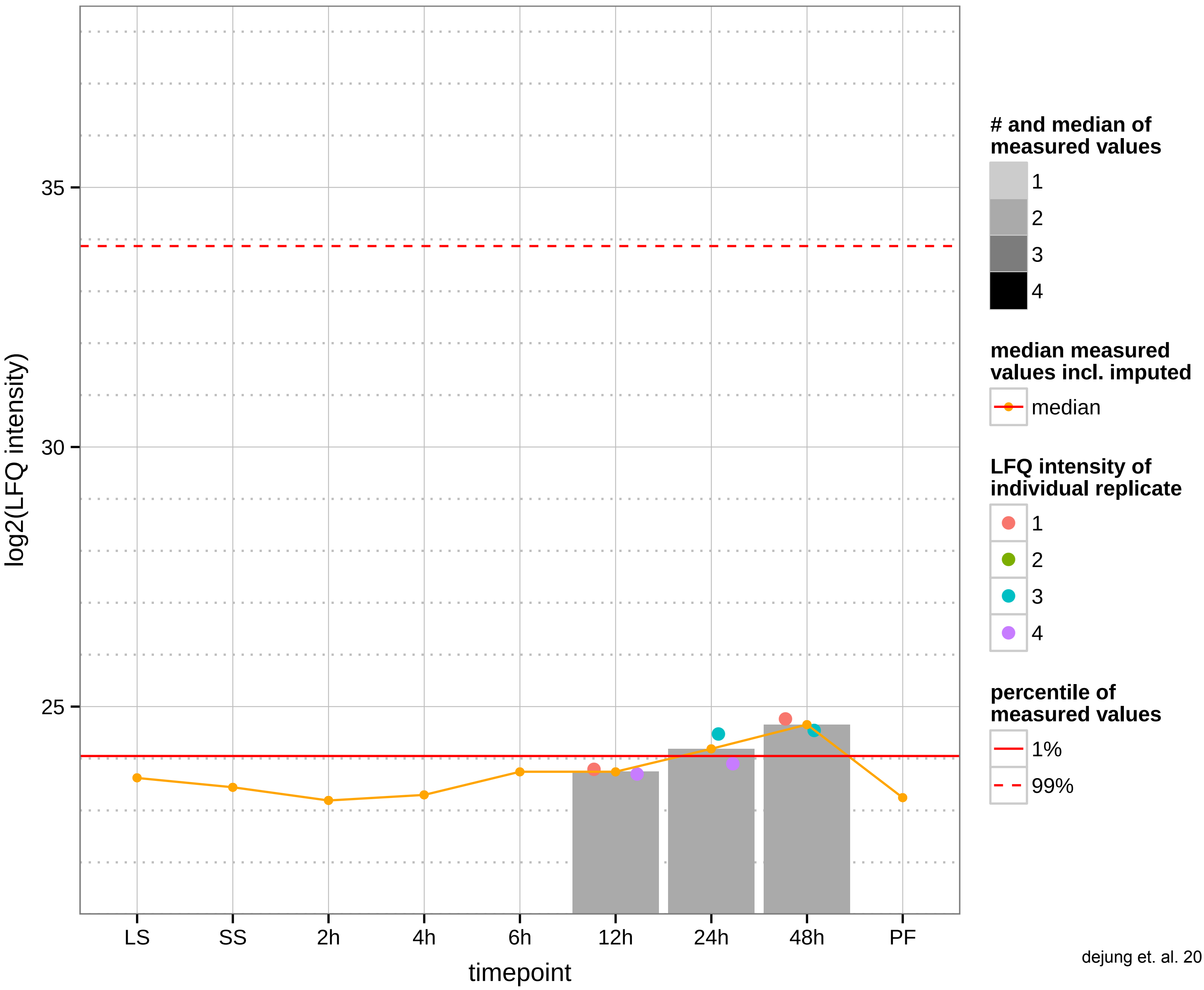

dejung et. al. 2015

ubiquitin carboxyl–terminal hydrolase, putative, cysteine peptidase, Clan CA, family C19
Tb927.6.1110
AGOF: ubiquitin thiolesterase activity
AGOC: null
AGOP: ubiquitin–dependent protein catabolic process
PGOF: ubiquitin thiolesterase activity
PGOC: null
PGOP: ubiquitin–dependent protein catabolic process

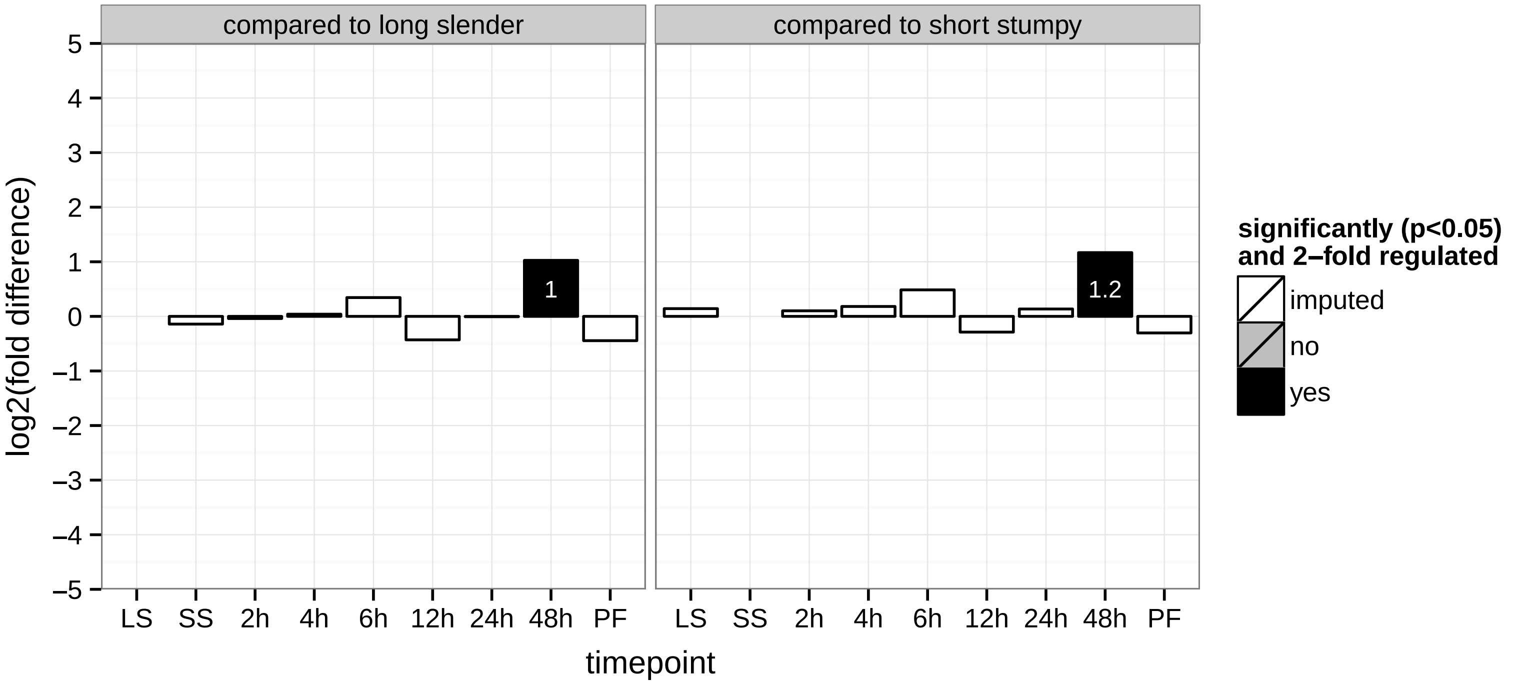

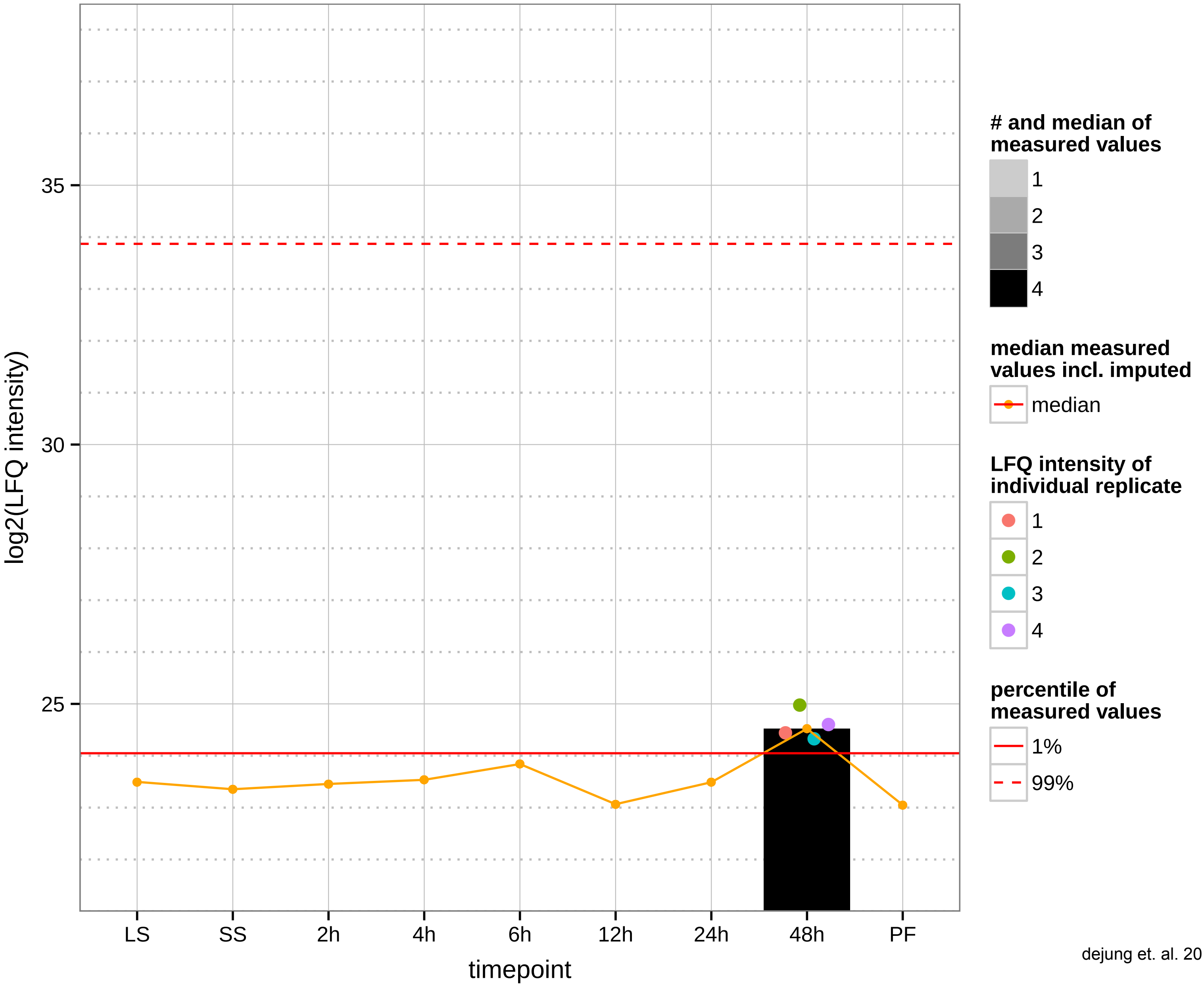

dejung et. al. 2015

cell division cycle protein 16 homolog, putative
Tb927.6.2150
AGOF: null
AGOC: null
AGOP: null
PGOF: protein binding
PGOC: null
PGOP: null

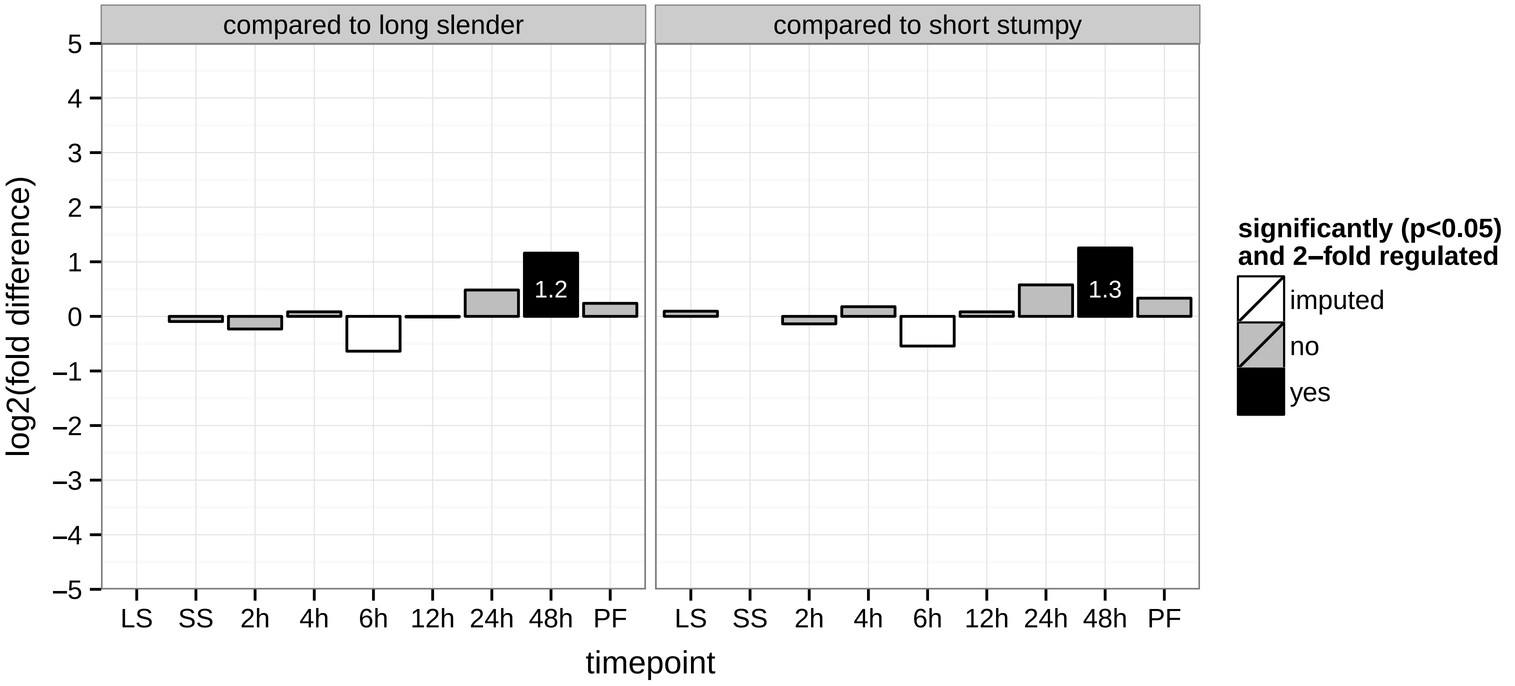

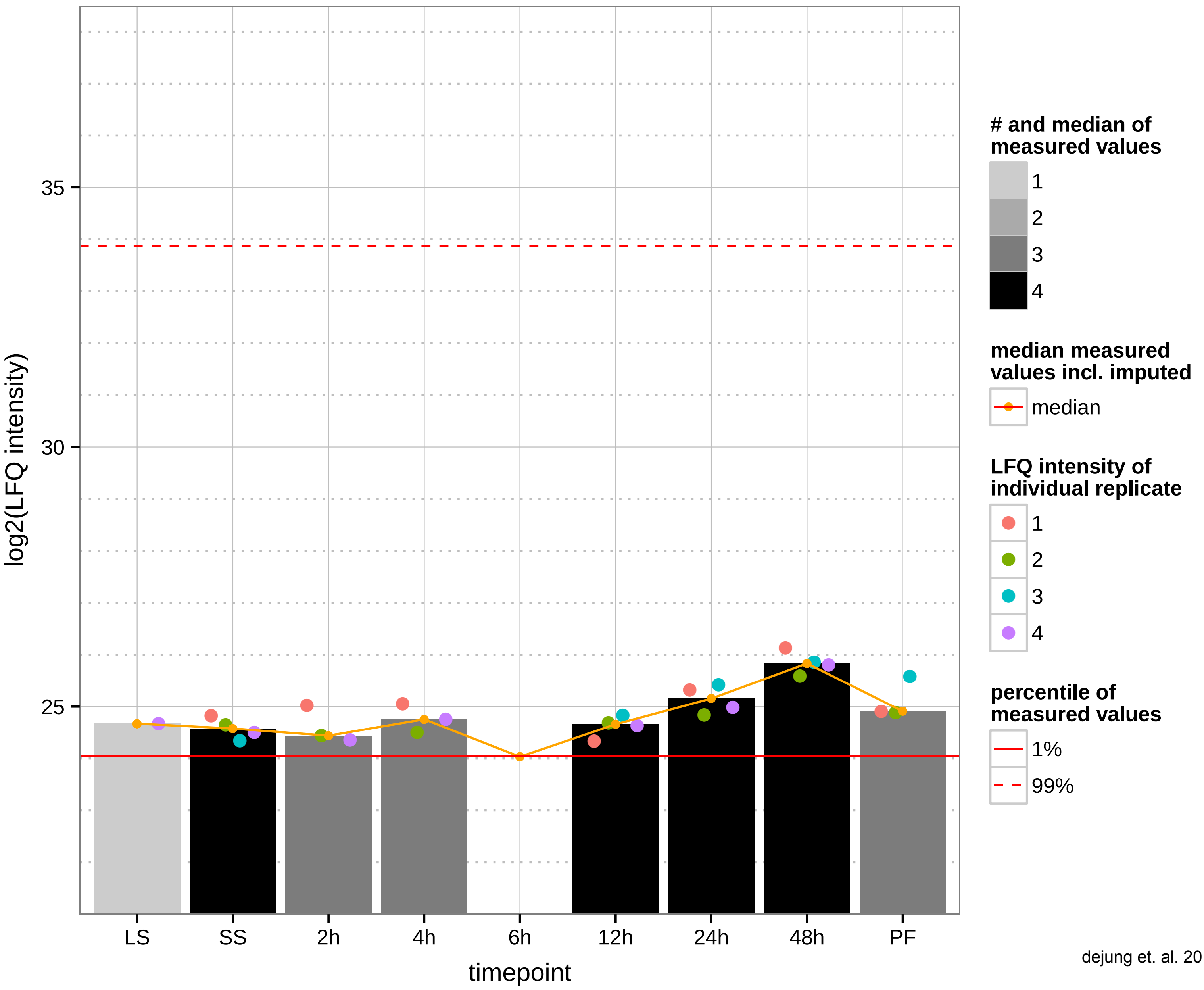

dejung et. al. 2015

hypothetical protein, conserved
Tb927.6.2710
AGOF: zinc ion binding
AGOC: integral to membrane, intracellular
AGOP: null
PGOF: protein binding, zinc ion binding
PGOC: intracellular
PGOP: null

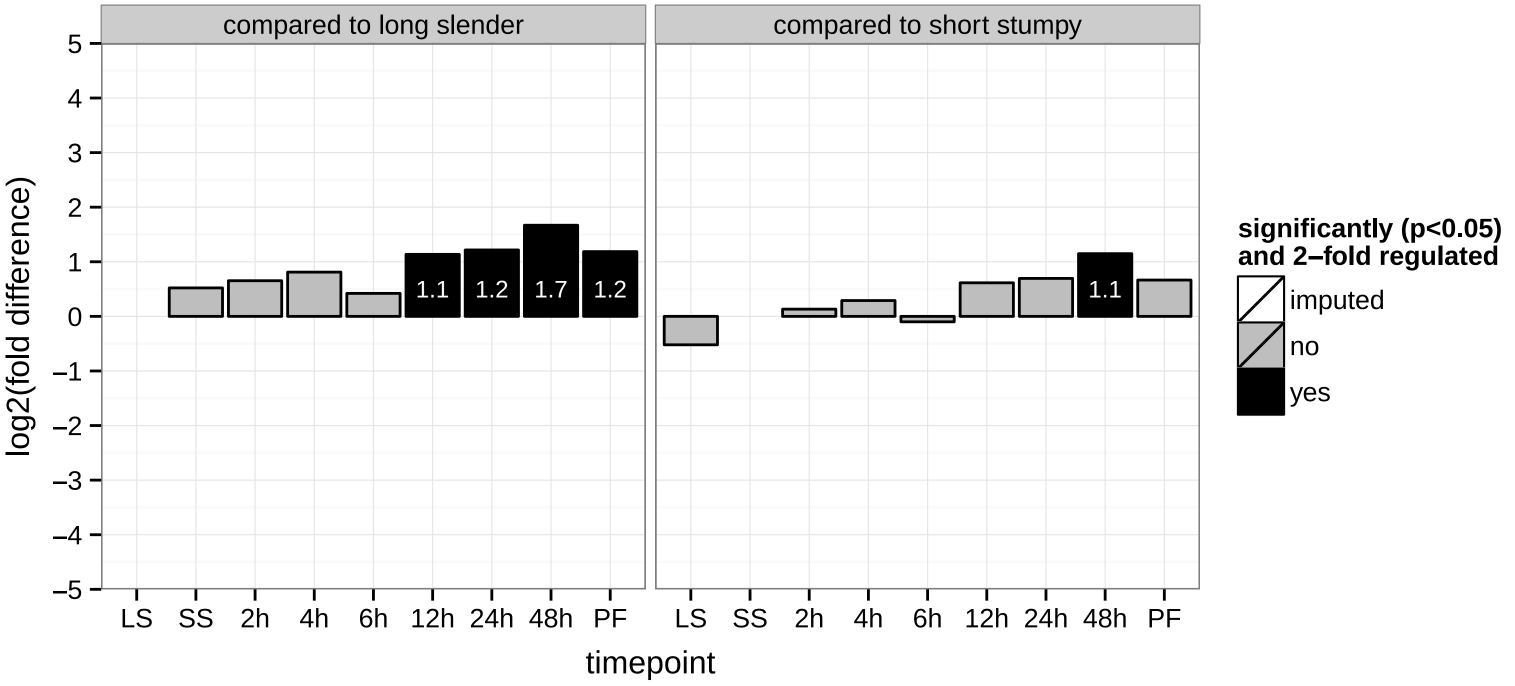

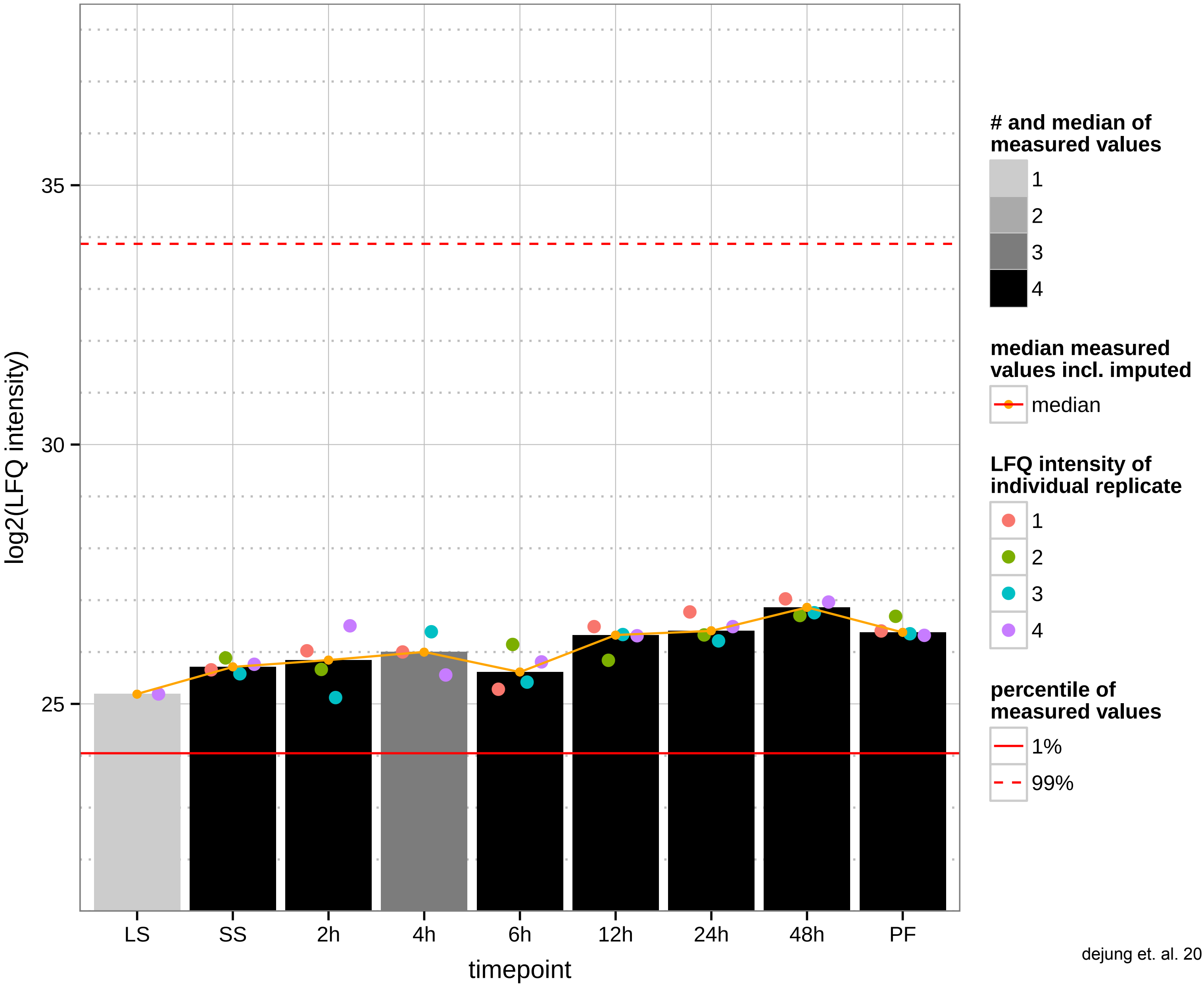

dejung et. al. 2015

serine/arginine–rich protein specific kinase SRPK, putative, protein kinase
Tb927.6.4970
AGOF: ATP binding, protein serine/threonine kinase activity, protein tyrosine kinase activity
AGOC: null
AGOP: growth, protein phosphorylation
PGOF: ATP binding, protein kinase activity, transferase activity, transferring phosphorus–containing groups
PGOC: null
PGOP: protein phosphorylation

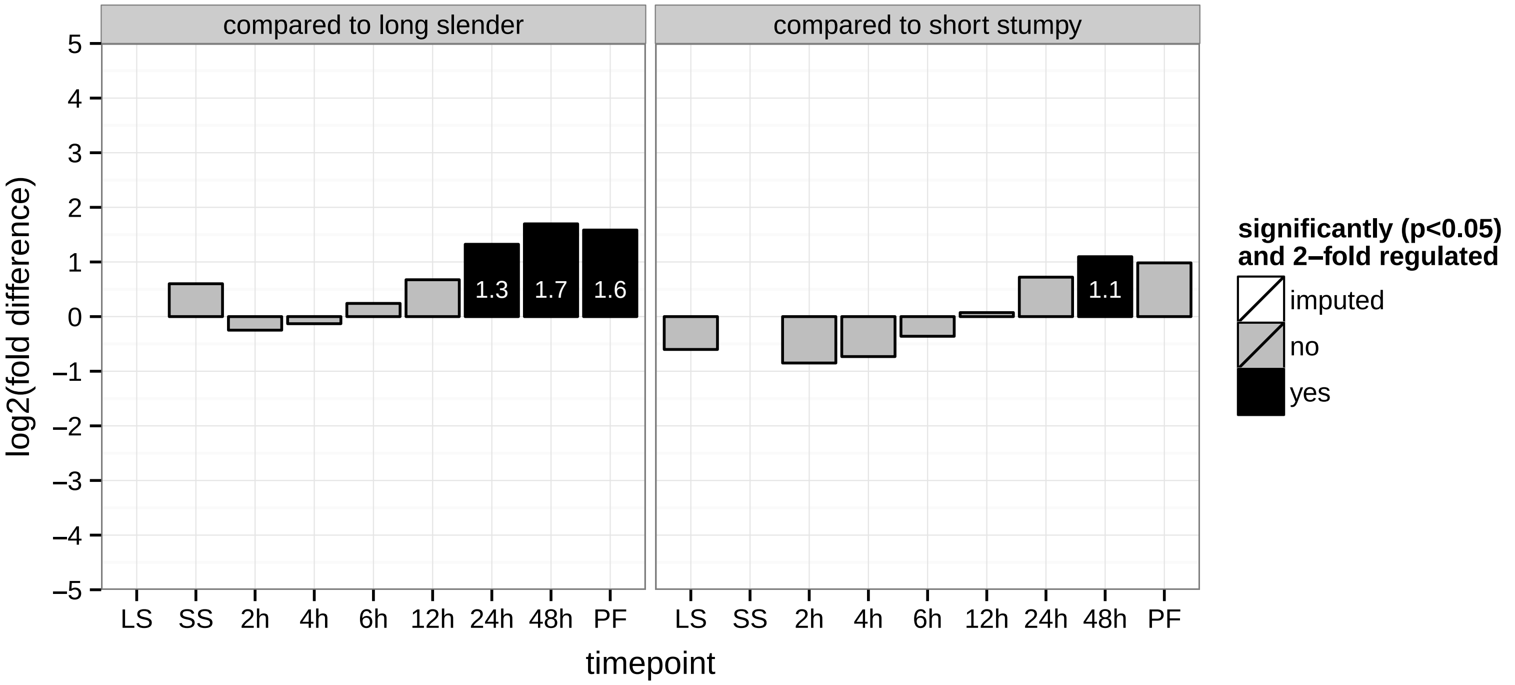

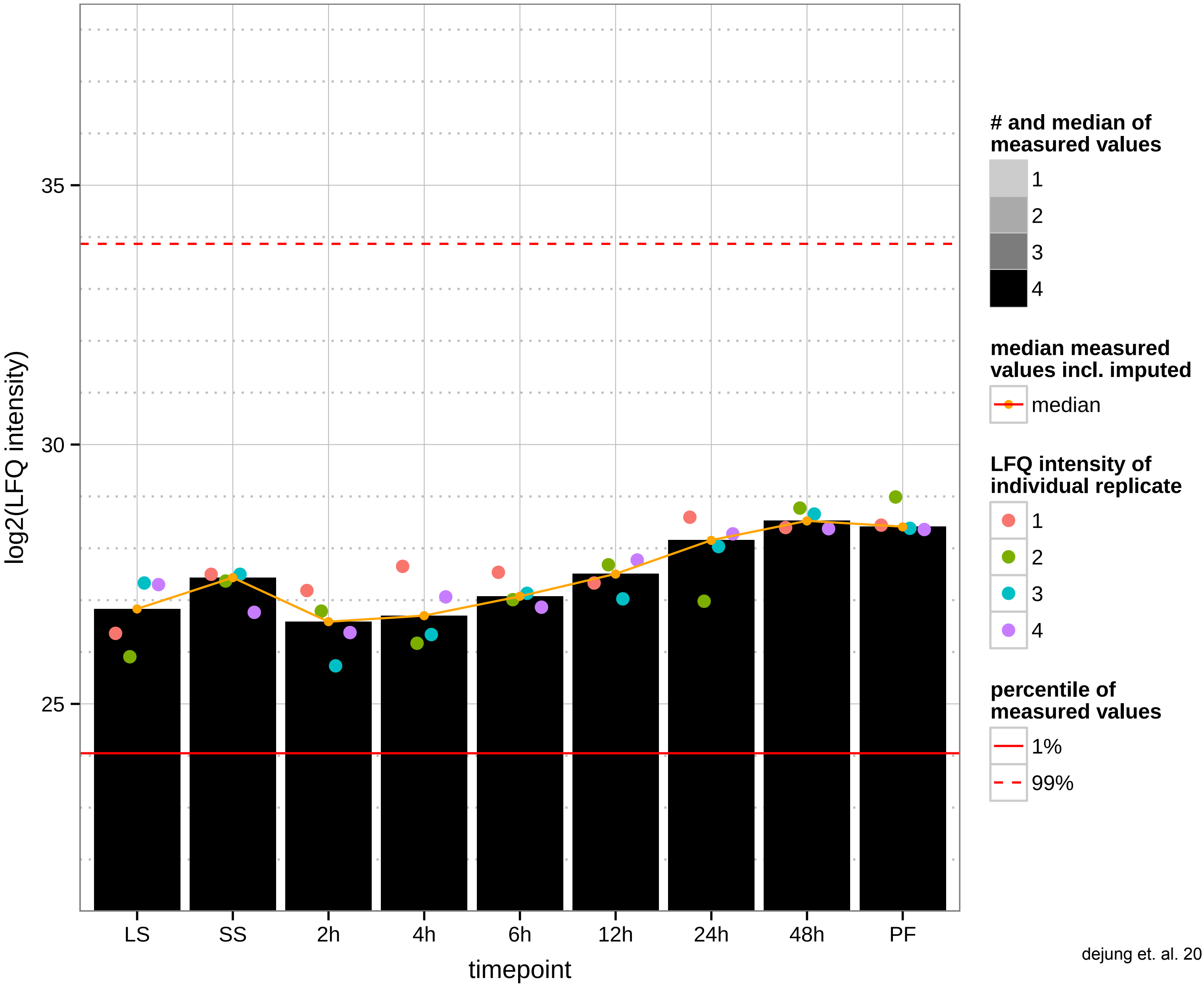

dejung et. al. 2015

hypothetical protein, conserved
Tb927.7.2390
AGOF: null
AGOC: mitochondrion
AGOP: null
PGOF: null
PGOC: null
PGOP: null

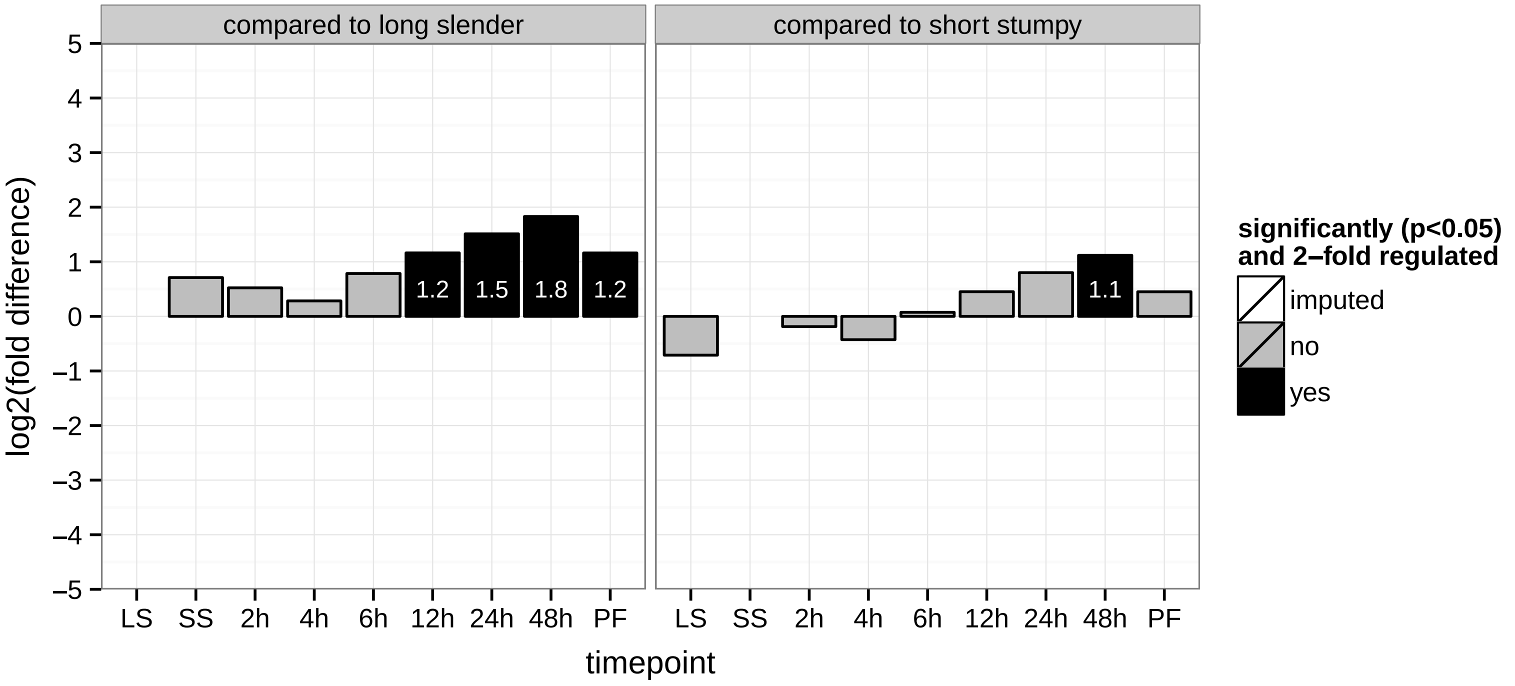

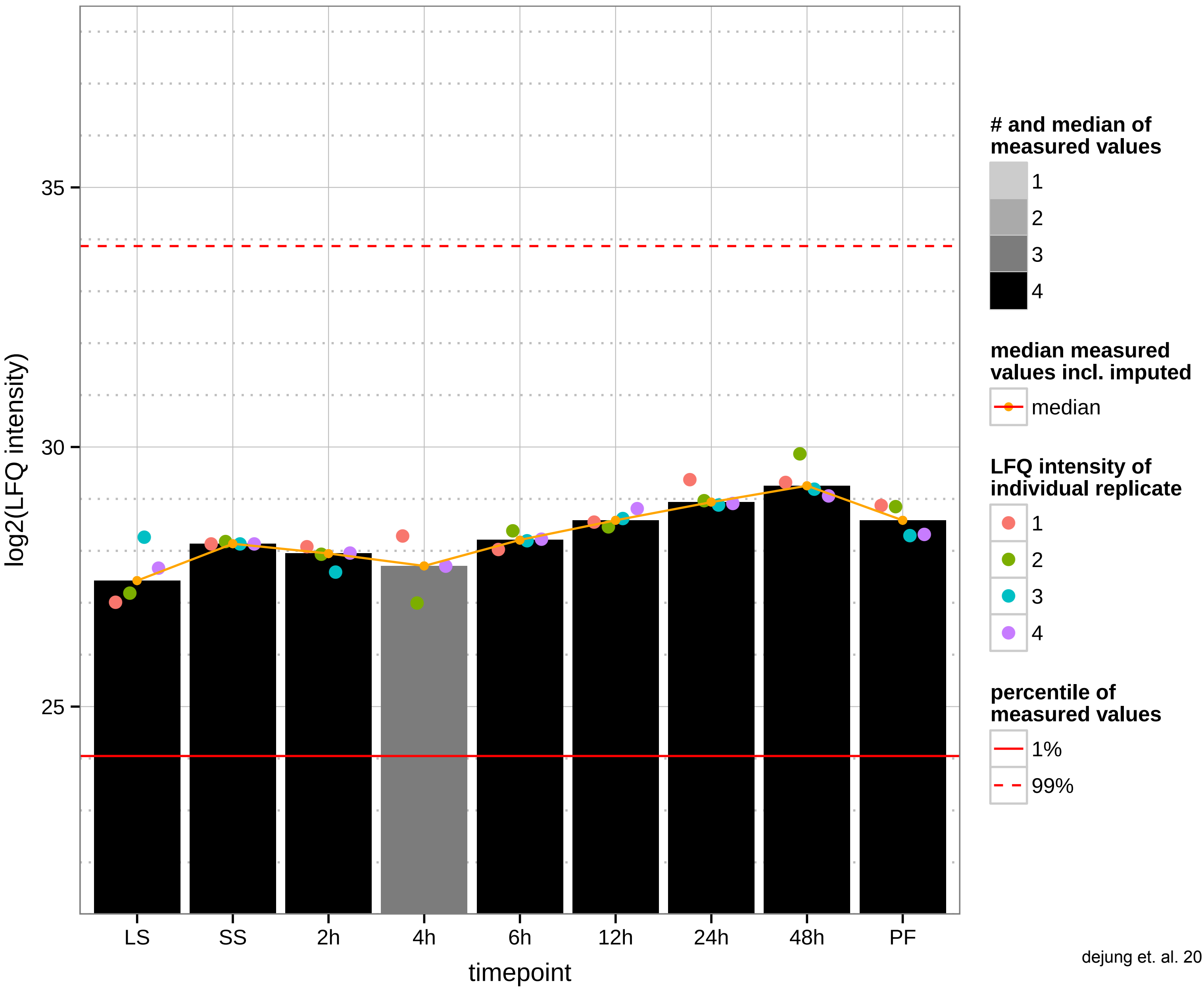

dejung et. al. 2015

peptidyl–prolyl cis–trans isomerase, putative, parvulin (PAR45)
Tb927.7.2480
AGOF: peptidyl–prolyl cis–trans isomerase activity
AGOC: nucleus
AGOP: null
PGOF: isomerase activity, protein binding
PGOC: null
PGOP: null

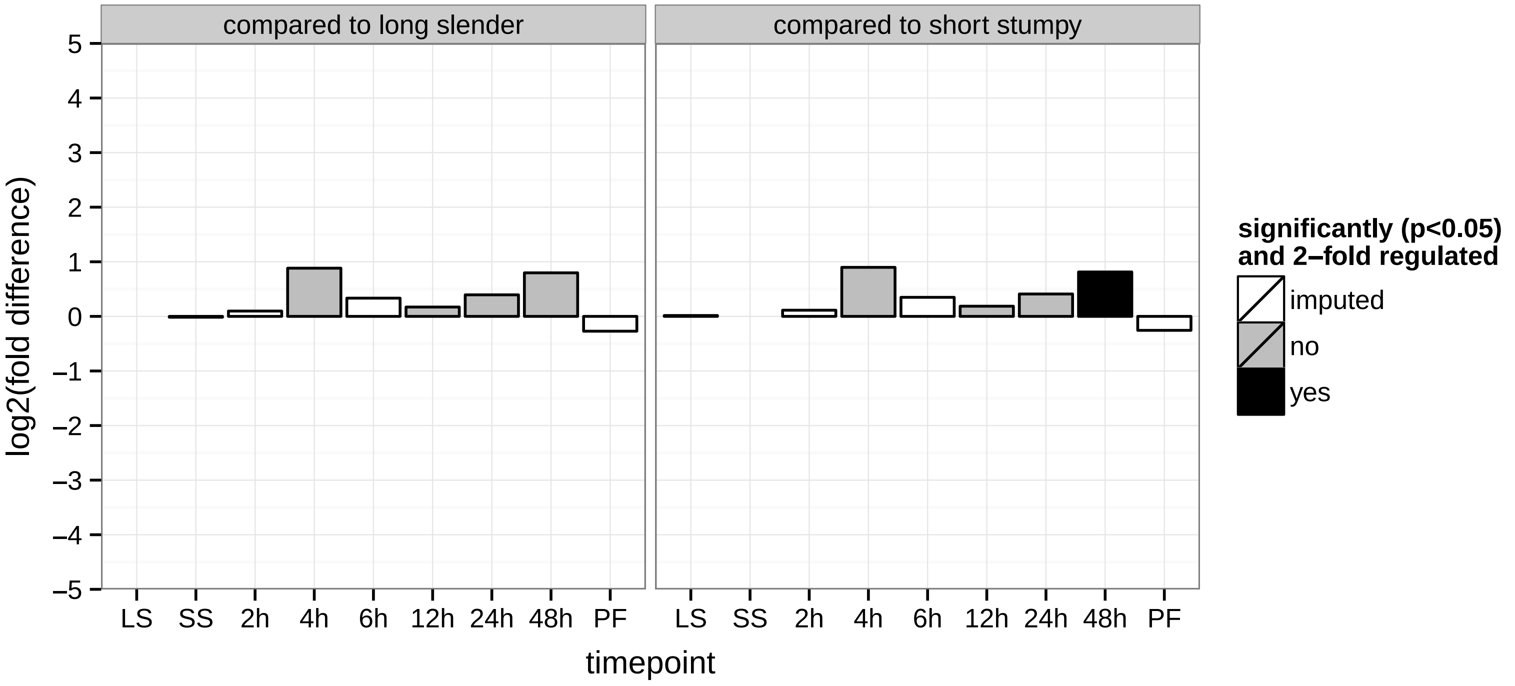

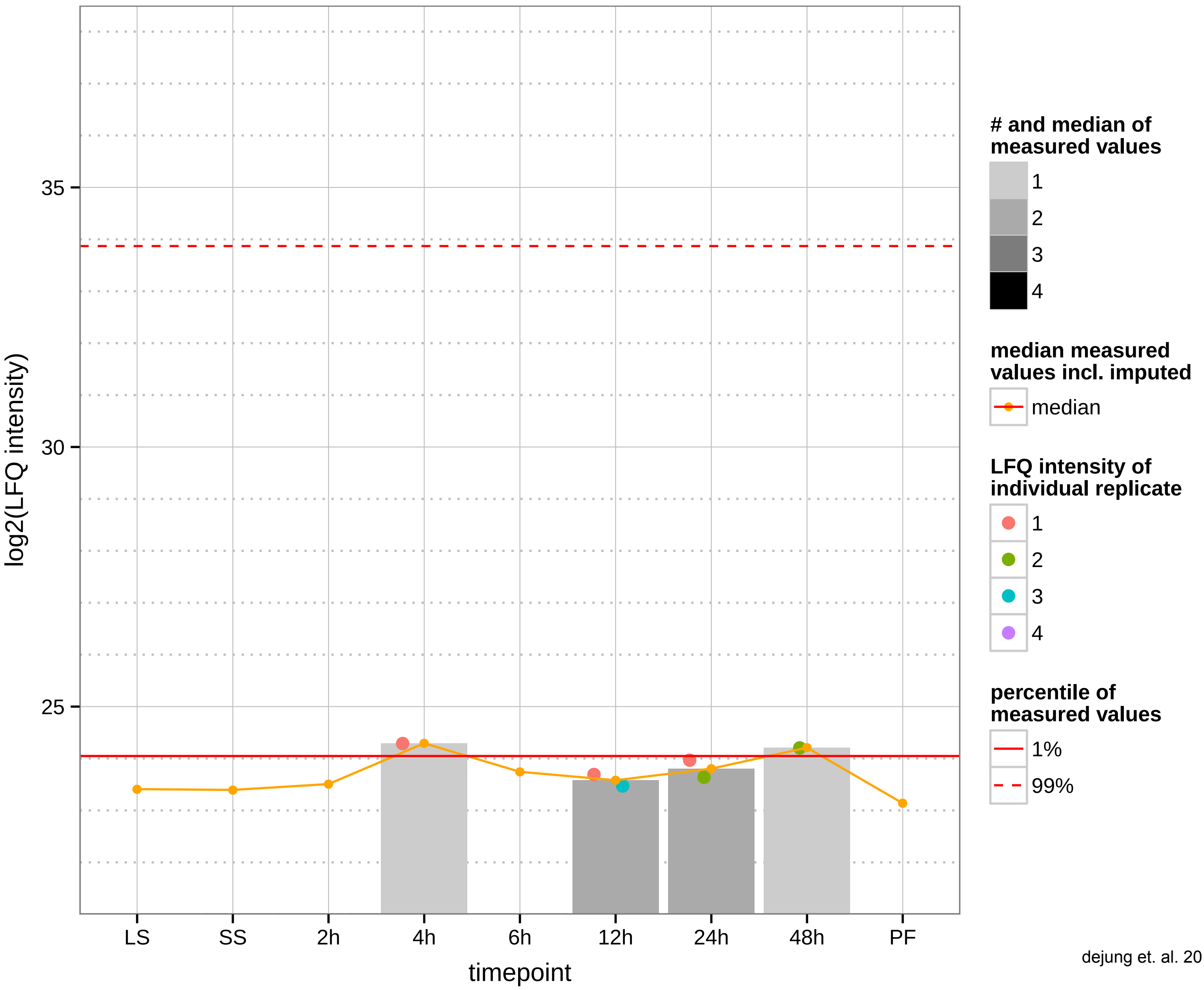

dejung et. al. 2015

hypothetical protein, conserved
Tb927.7.3040
AGOF: null
AGOC: null
AGOP: null
PGOF: protein binding
PGOC: null
PGOP: null

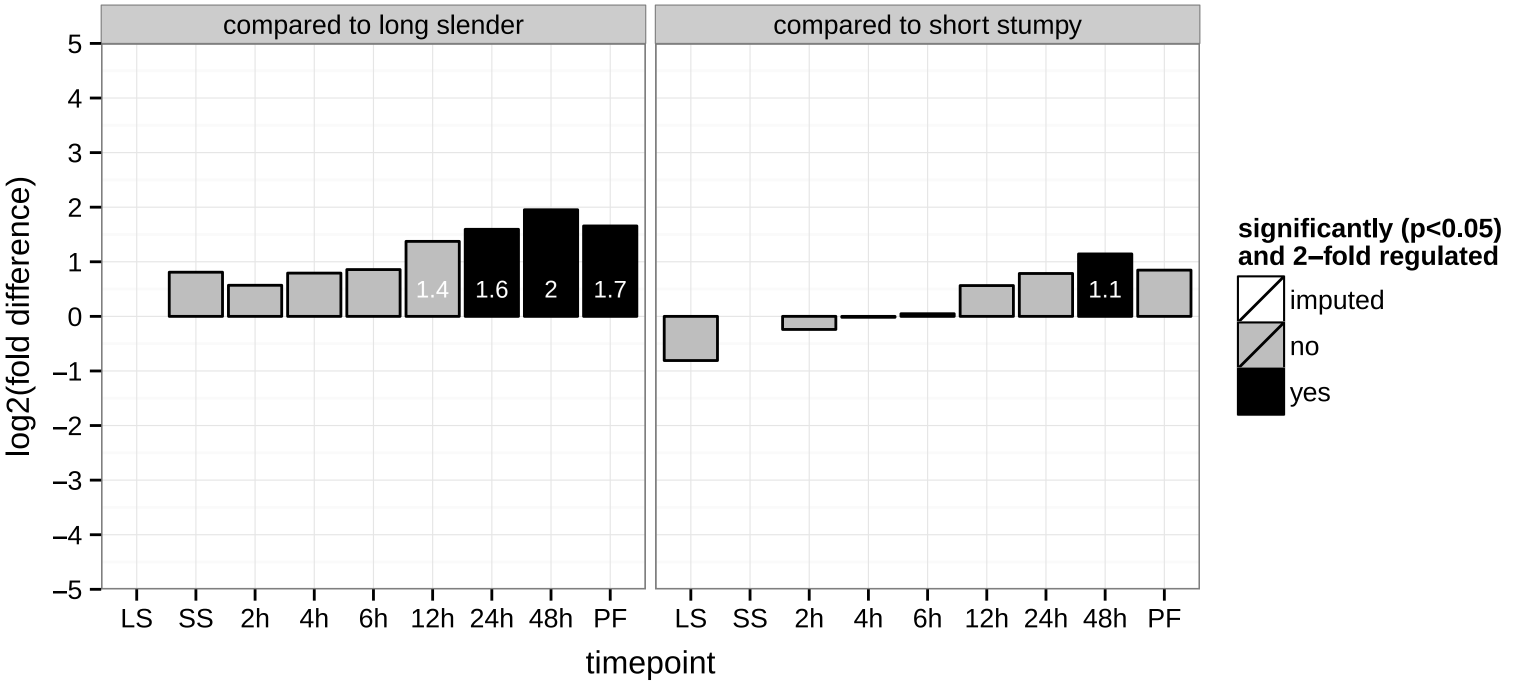

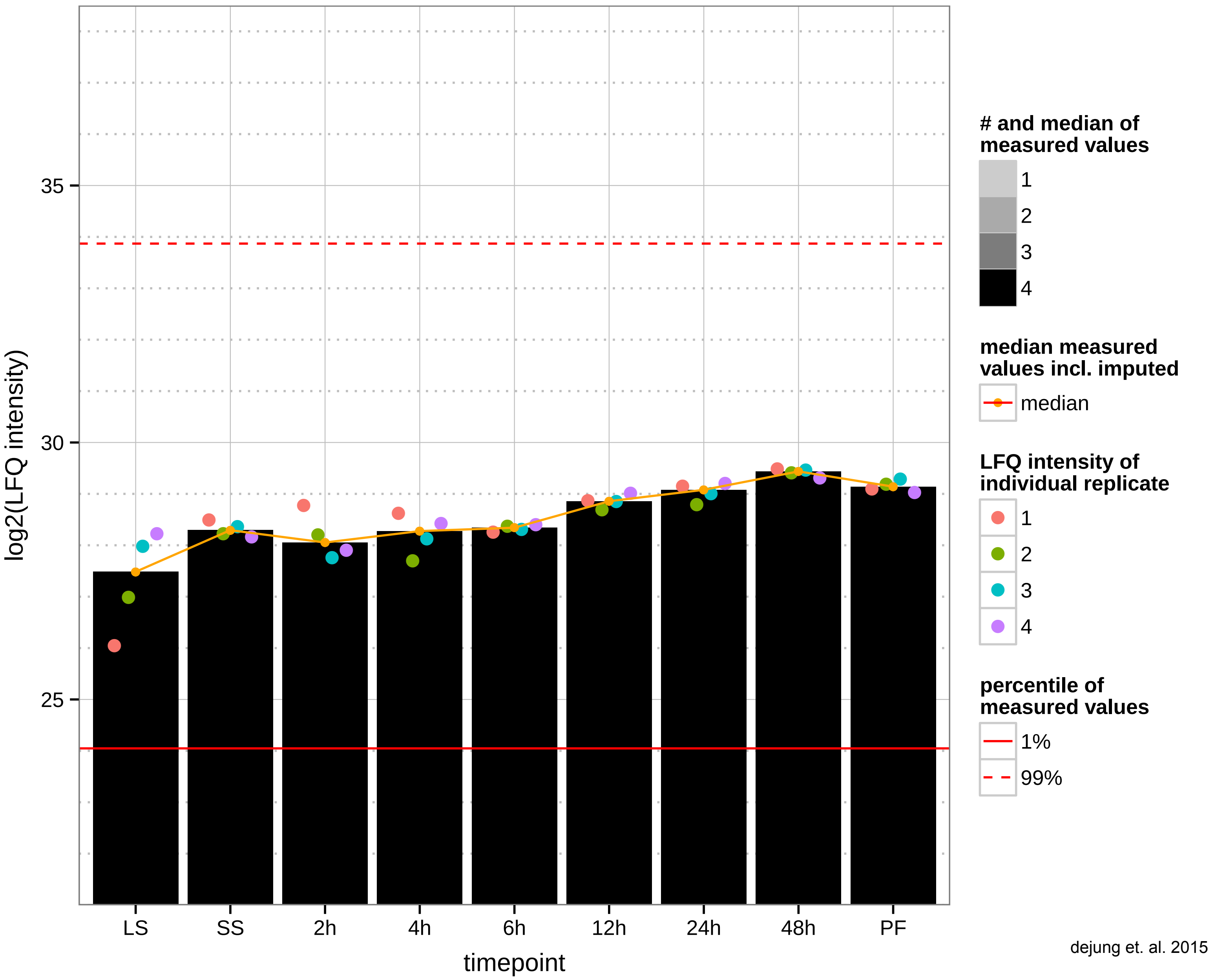

dejung et. al. 2015

farnesyl pyrophosphate synthase (FPPS)
Tb927.7.3360
AGOF: dimethylallyltranstransferase activity, geranyltranstransferase activity
AGOC: cytosol, endoplasmic reticulum
AGOP: cholesterol biosynthetic process, farnesyl diphosphate biosynthetic process
PGOF: null
PGOC: null
PGOP: isoprenoid biosynthetic process

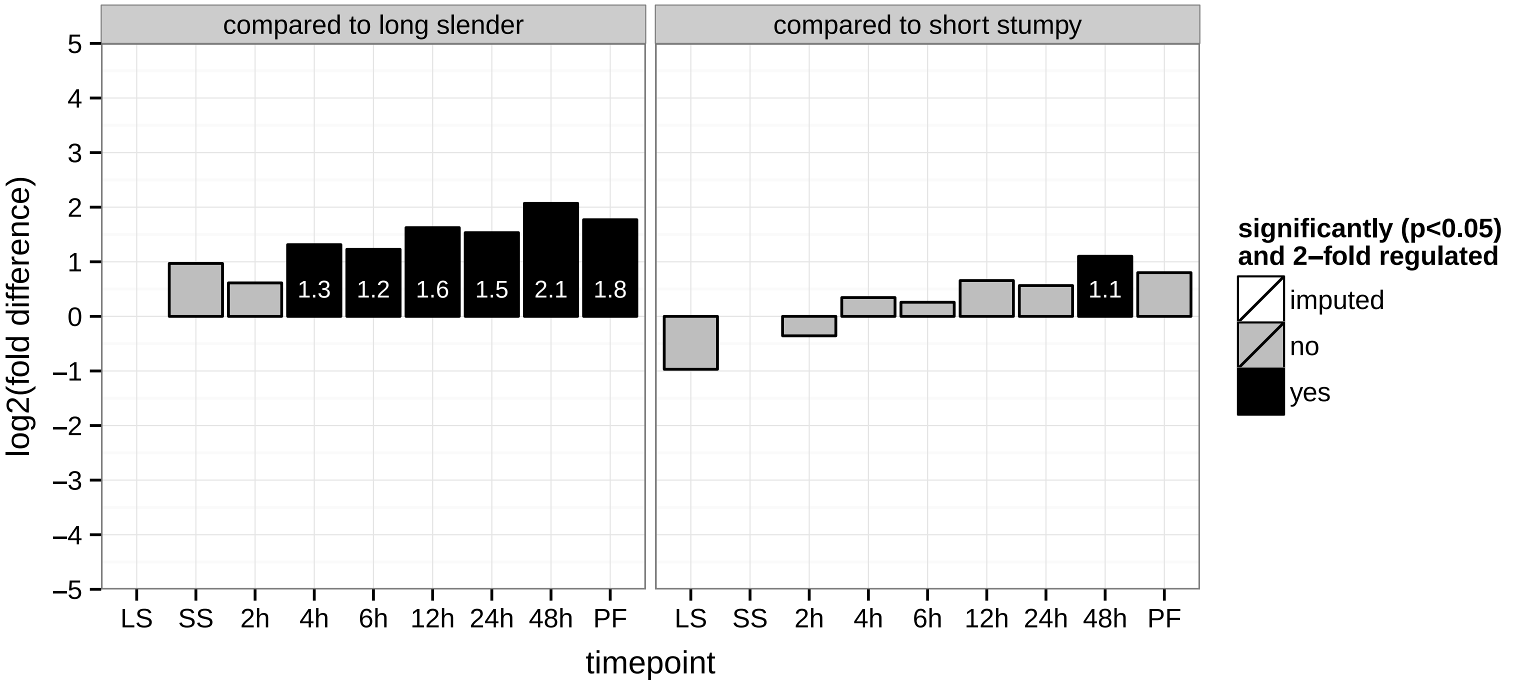

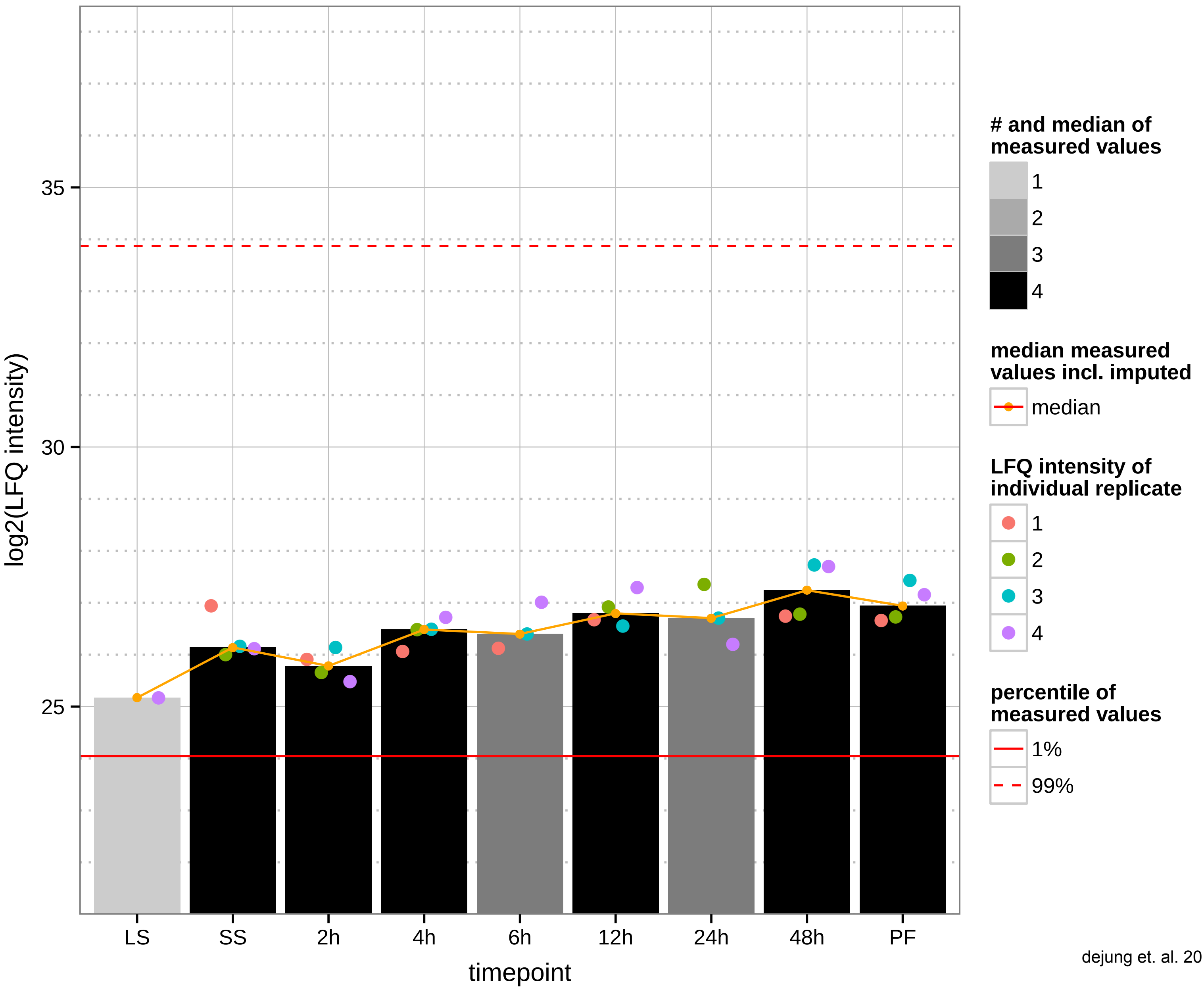

dejung et. al. 2015

calpain–like cysteine peptidase, putative, cysteine peptidase, Clan CA, family C2
Tb927.7.4060
AGOF: null
AGOC: null
AGOP: null
PGOF: null
PGOC: null
PGOP: null

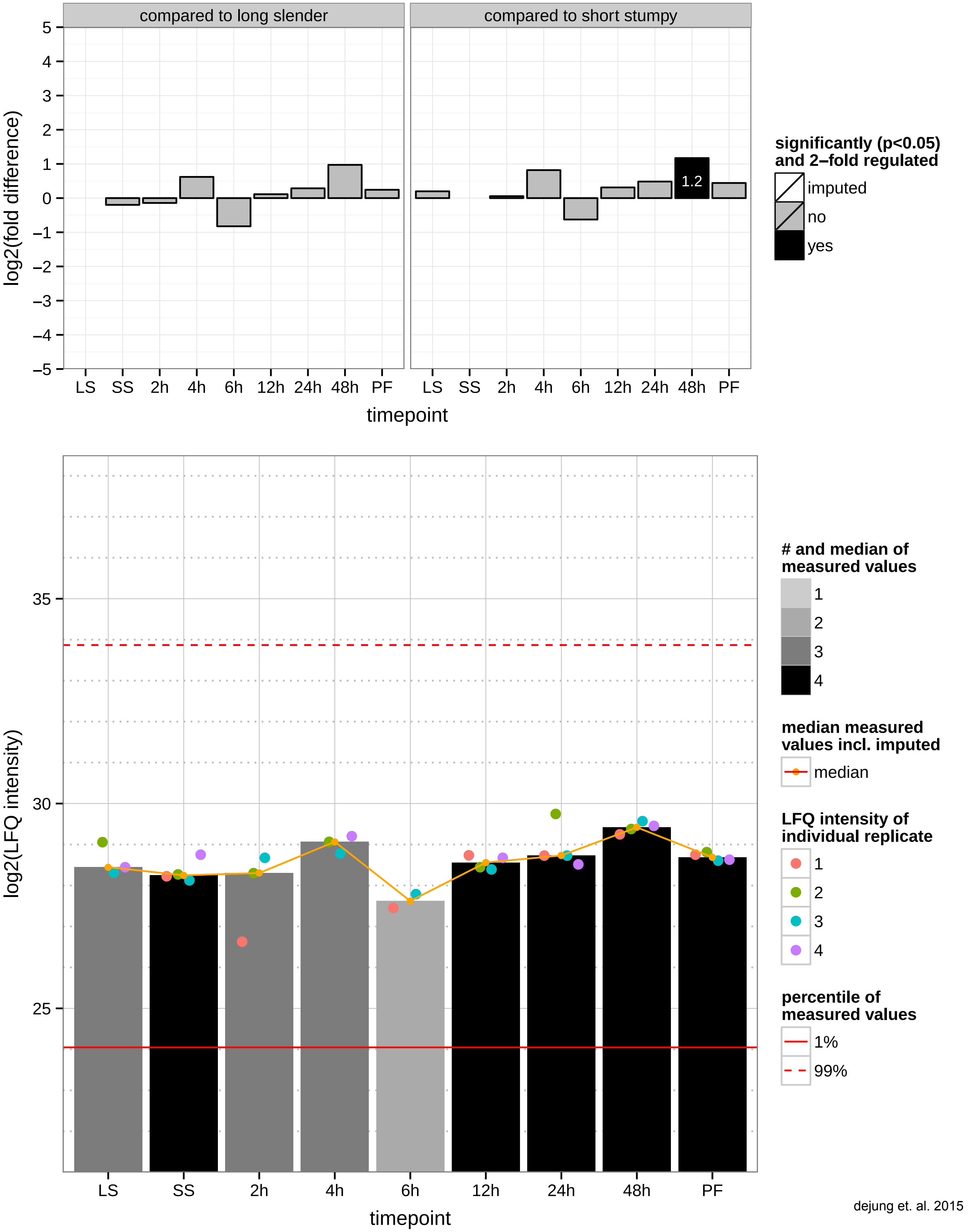

dejung et. al. 2015

leucine–rich repeat protein (LRRP), putative
Tb927.7.5600
AGOF: null
AGOC: null
AGOP: null
PGOF: null
PGOC: null
PGOP: null

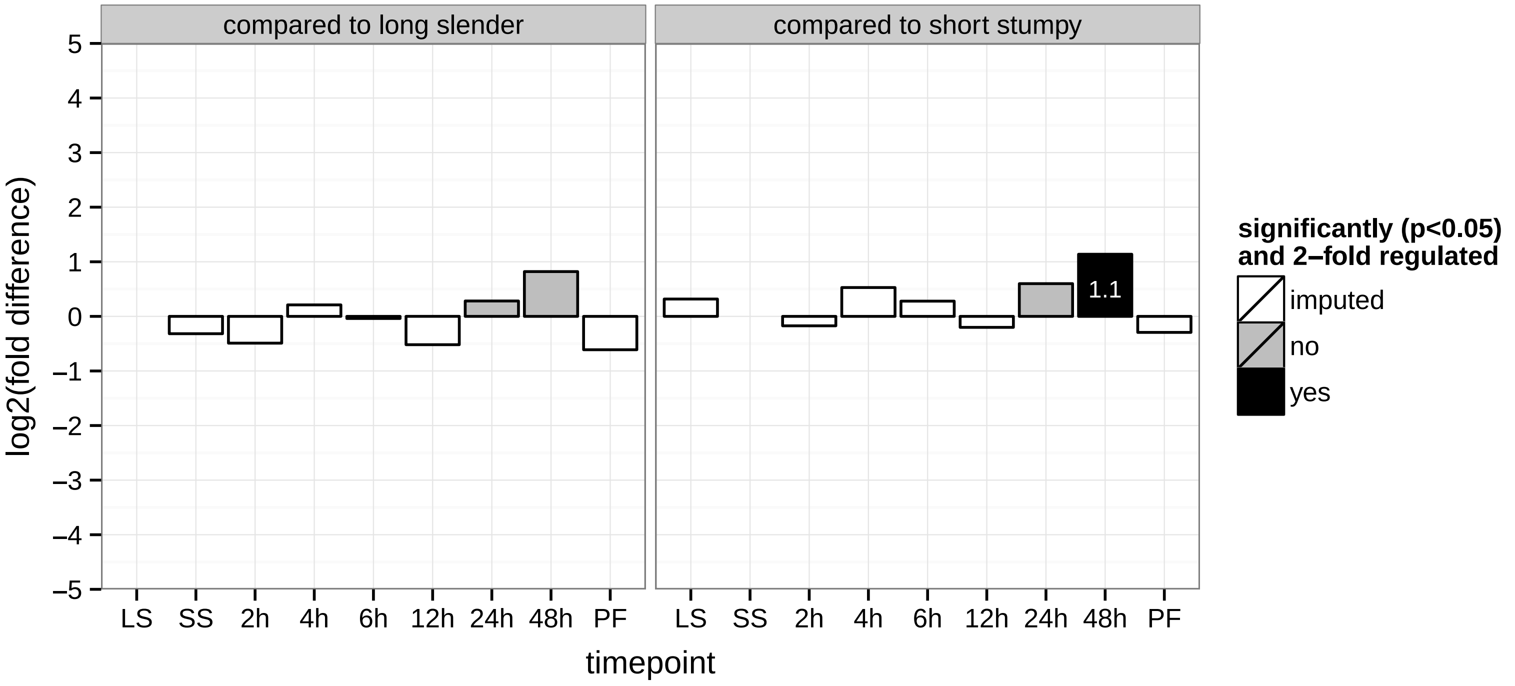

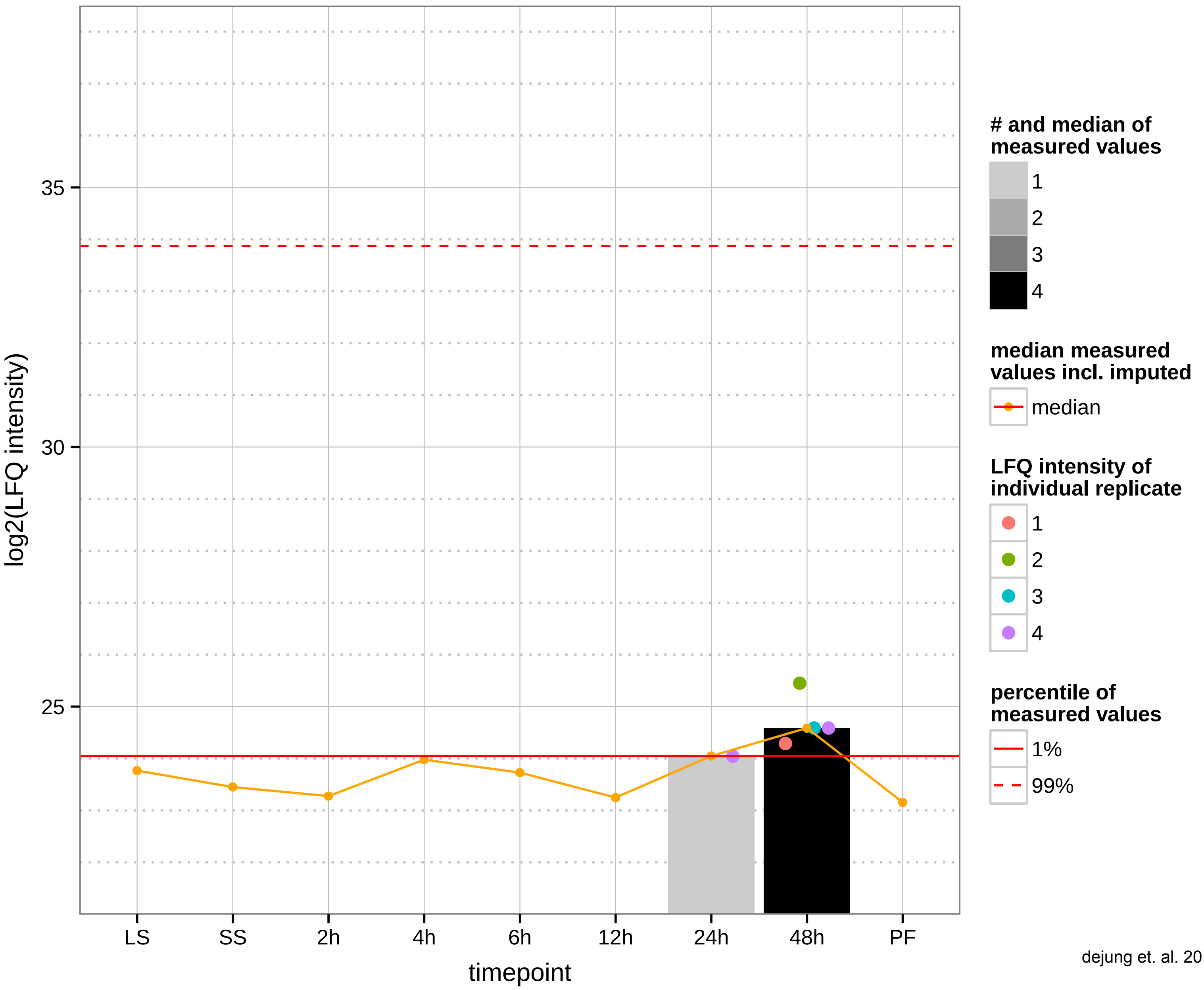

dejung et. al. 2015

hypothetical protein, conserved
Tb927.7.6770
AGOF: fatty–acyl–CoA binding
AGOC: null
AGOP: null
PGOF: fatty–acyl–CoA binding
PGOC: null
PGOP: null

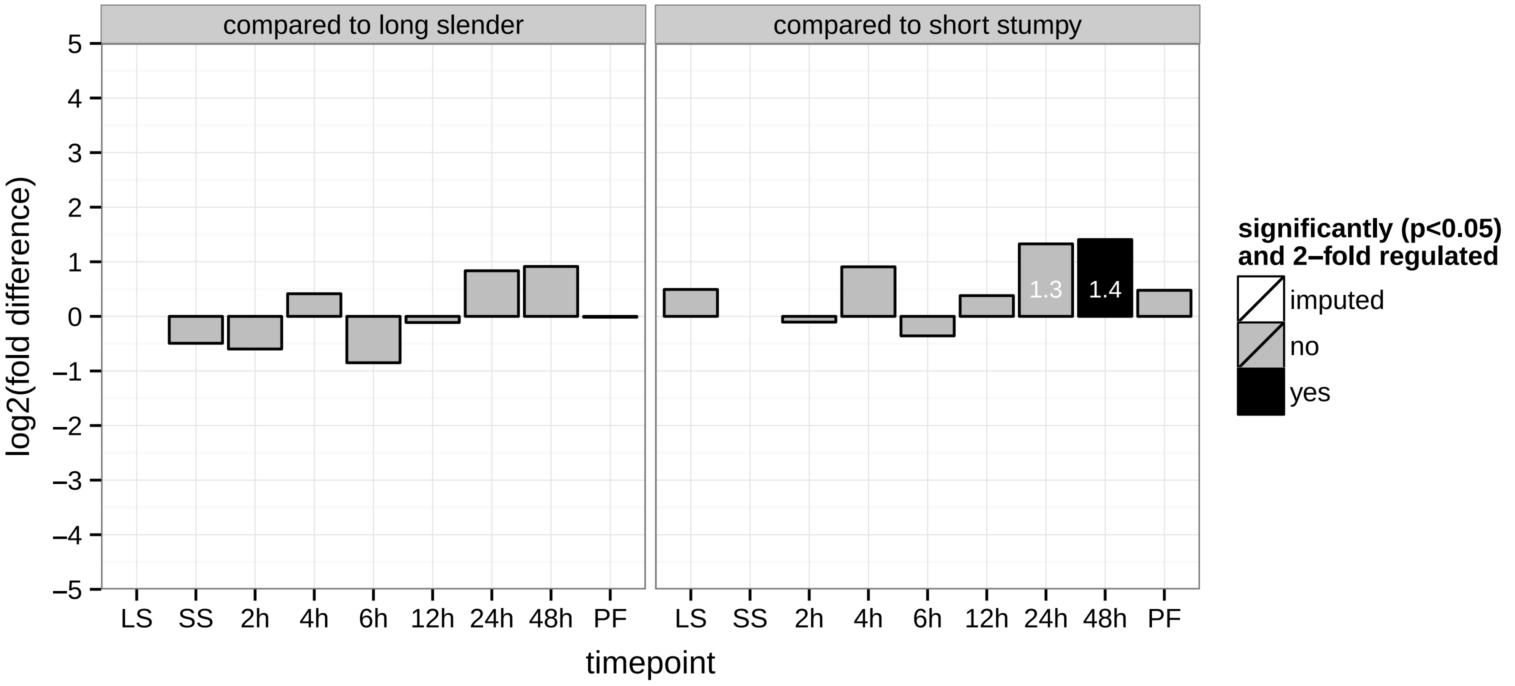

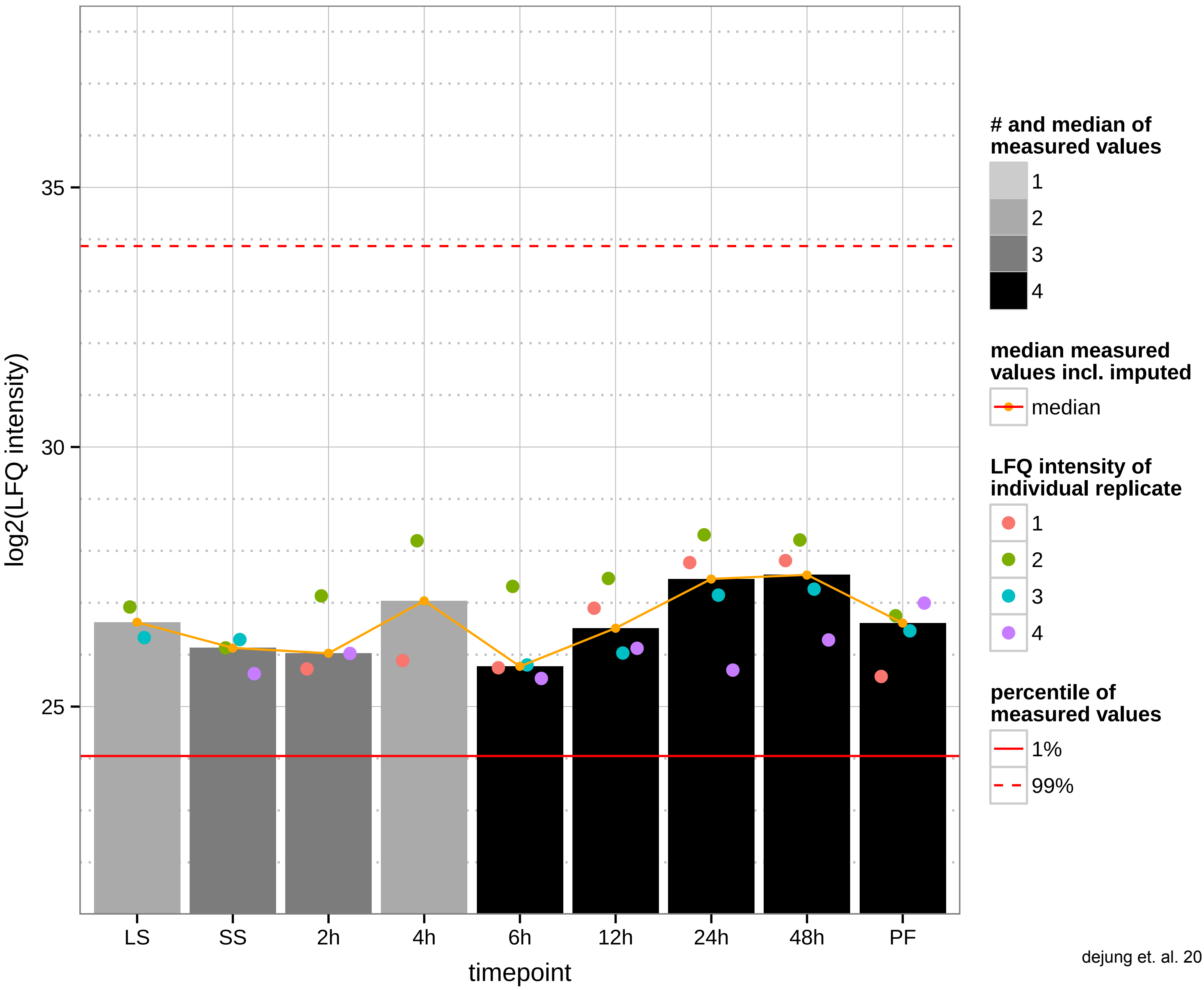

dejung et. al. 2015

hypothetical protein, conserved
Tb927.7.7210
AGOF: null
AGOC: null
AGOP: null
PGOF: null
PGOC: null
PGOP: null

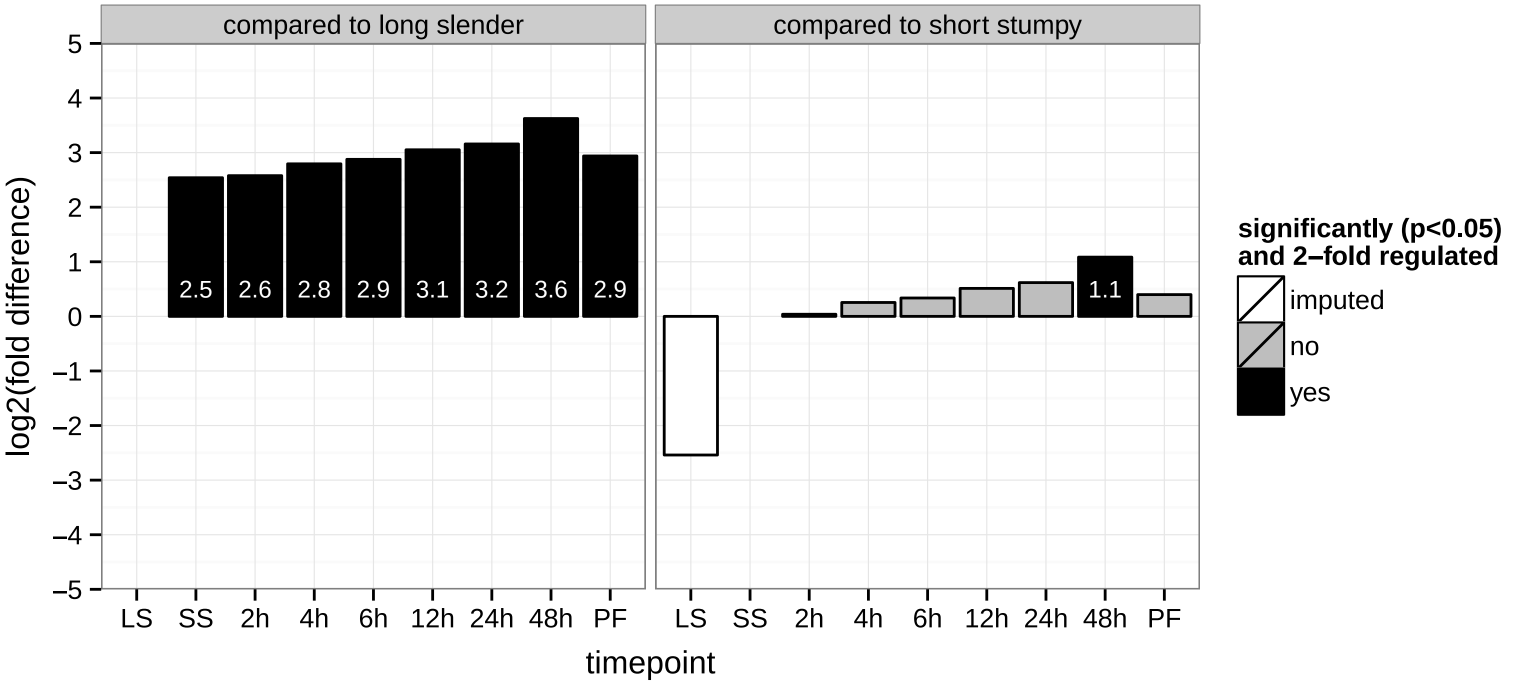

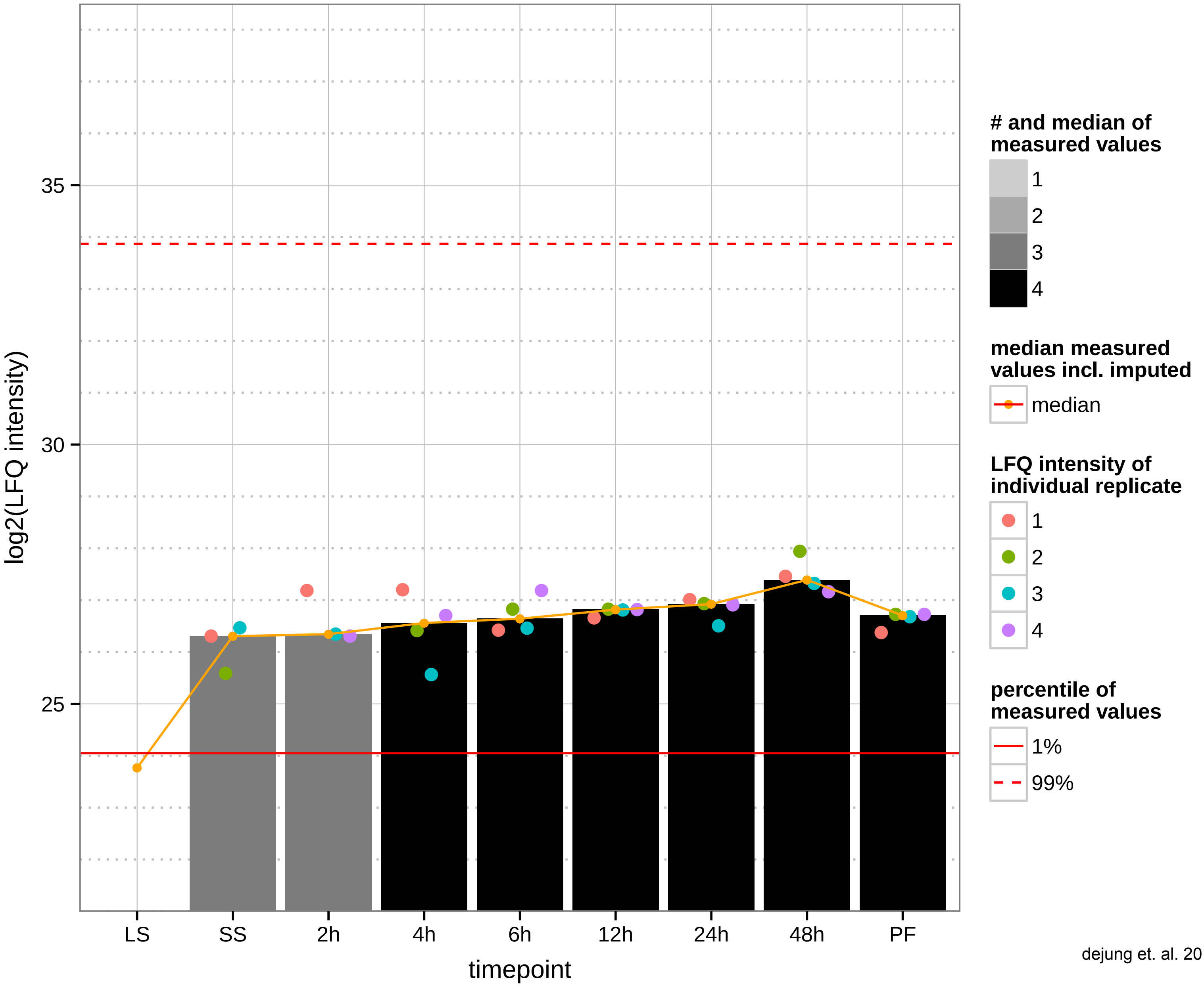

dejung et. al. 2015

mitochondrial RNA binding protein (TbRBP38)
Tb927.8.2740
AGOF: RNA binding
AGOC: mitochondrion
AGOP: mRNA stabilization
PGOF: null
PGOC: null
PGOP: null

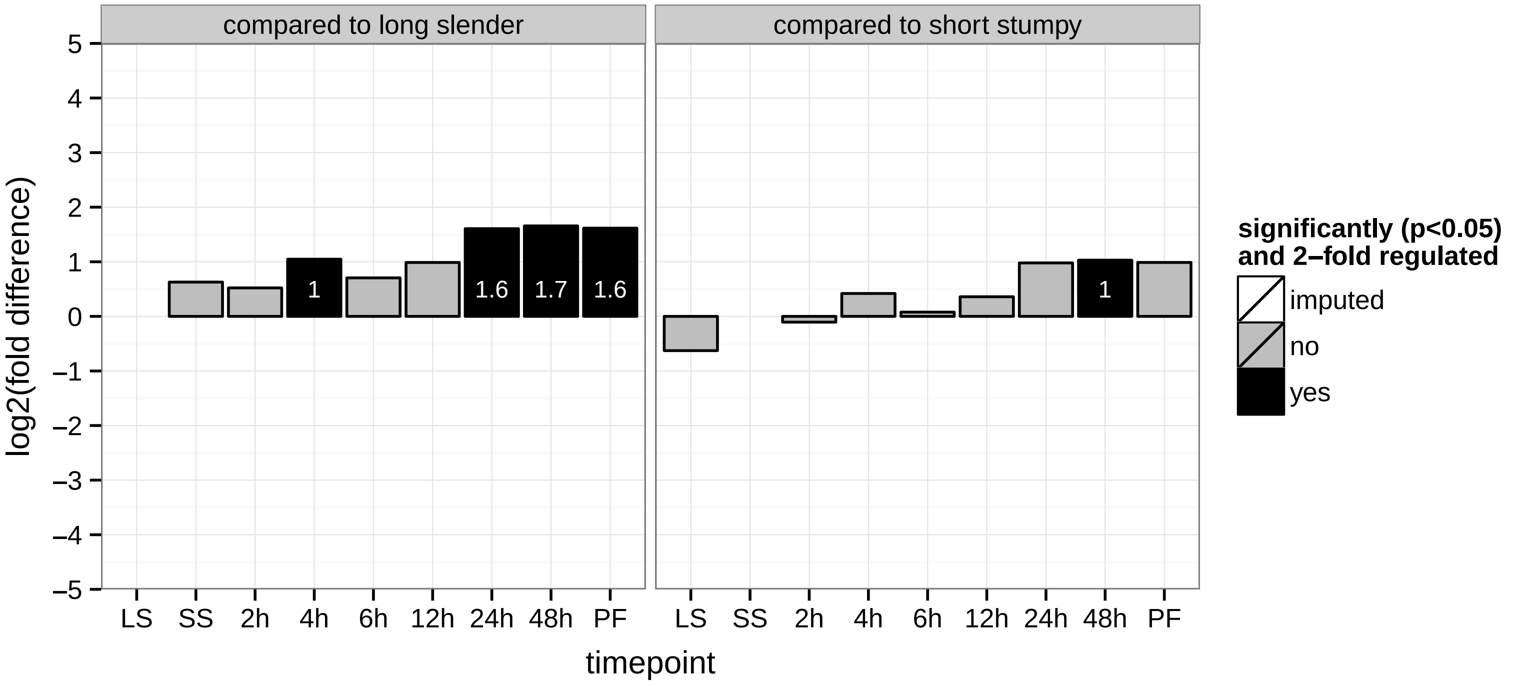

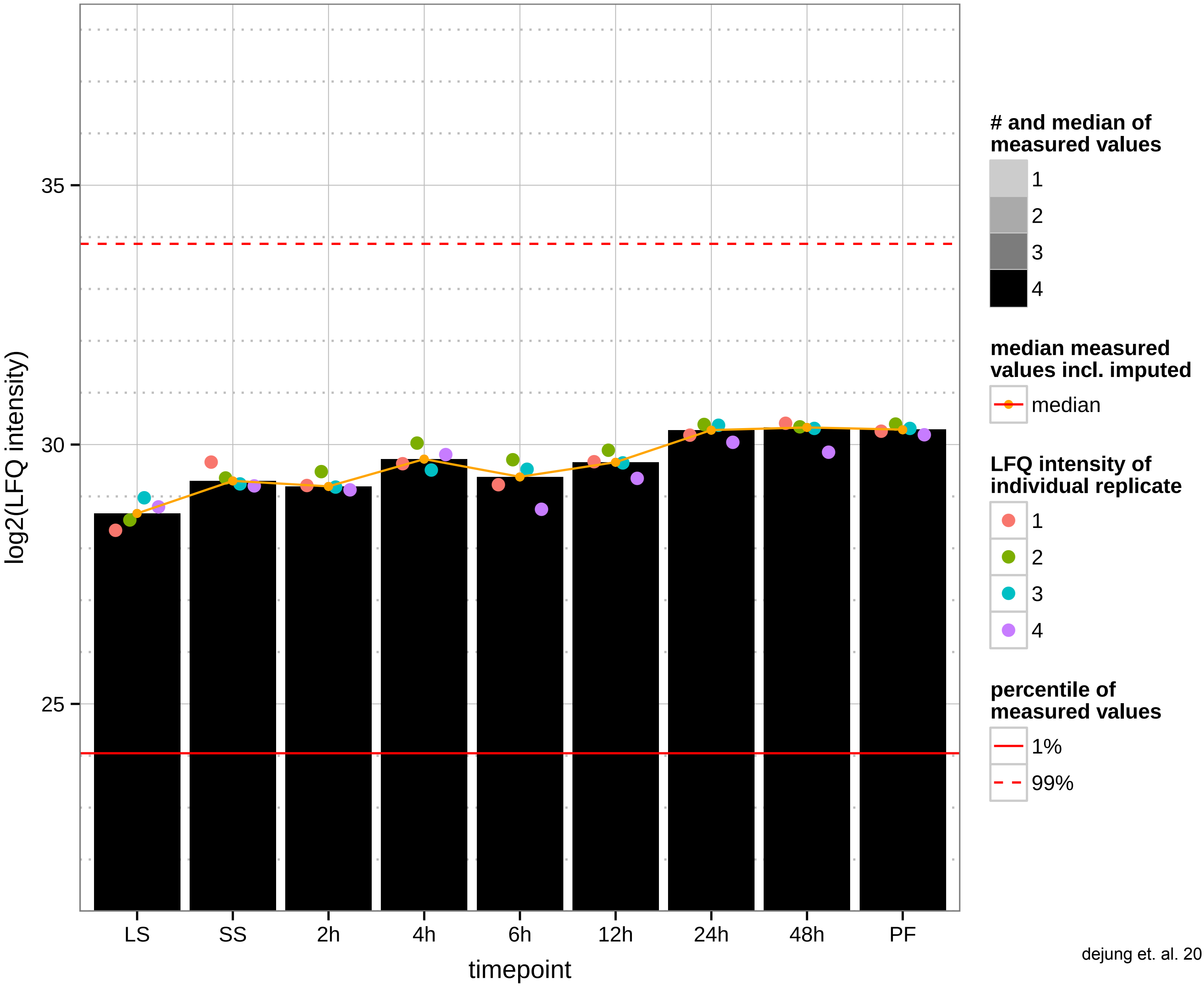

dejung et. al. 2015

hypothetical protein, conserved
Tb927.8.3260
AGOF: null
AGOC: null
AGOP: null
PGOF: null
PGOC: null
PGOP: null

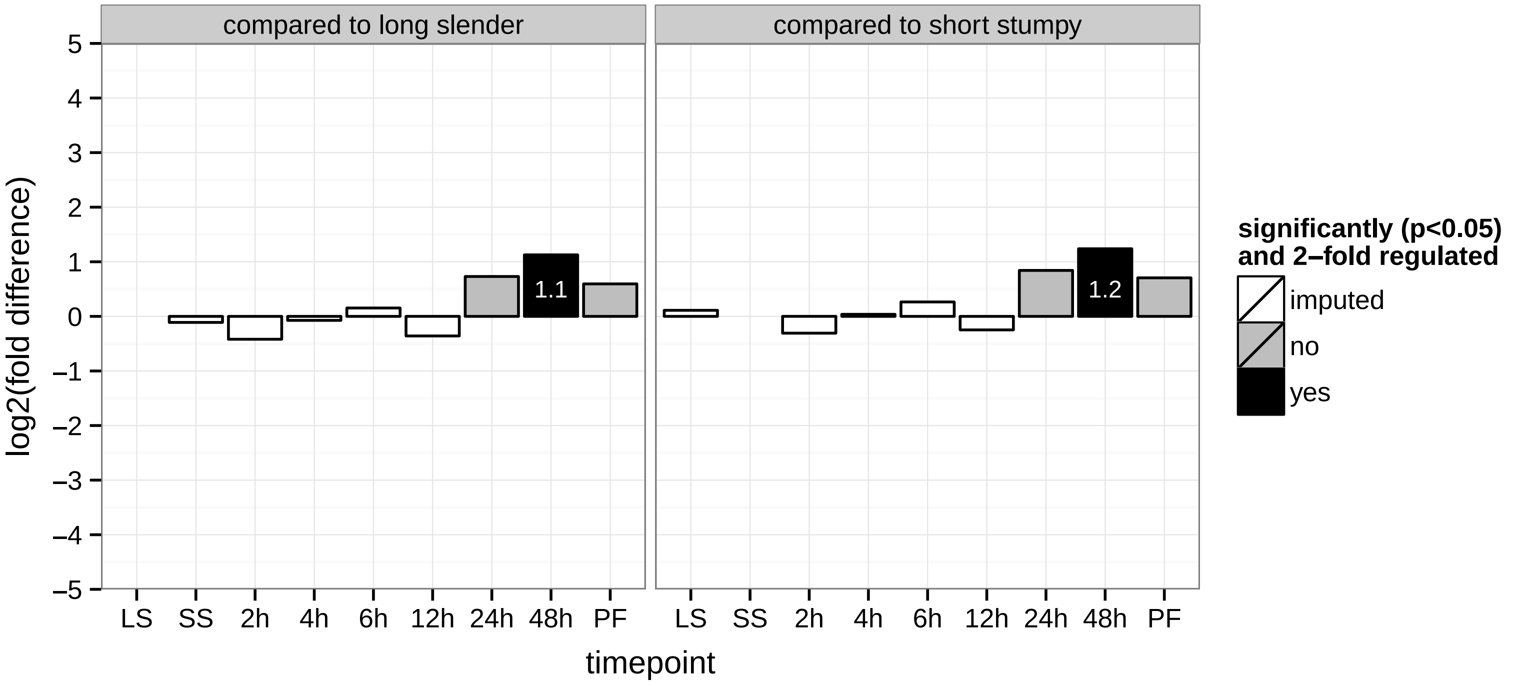

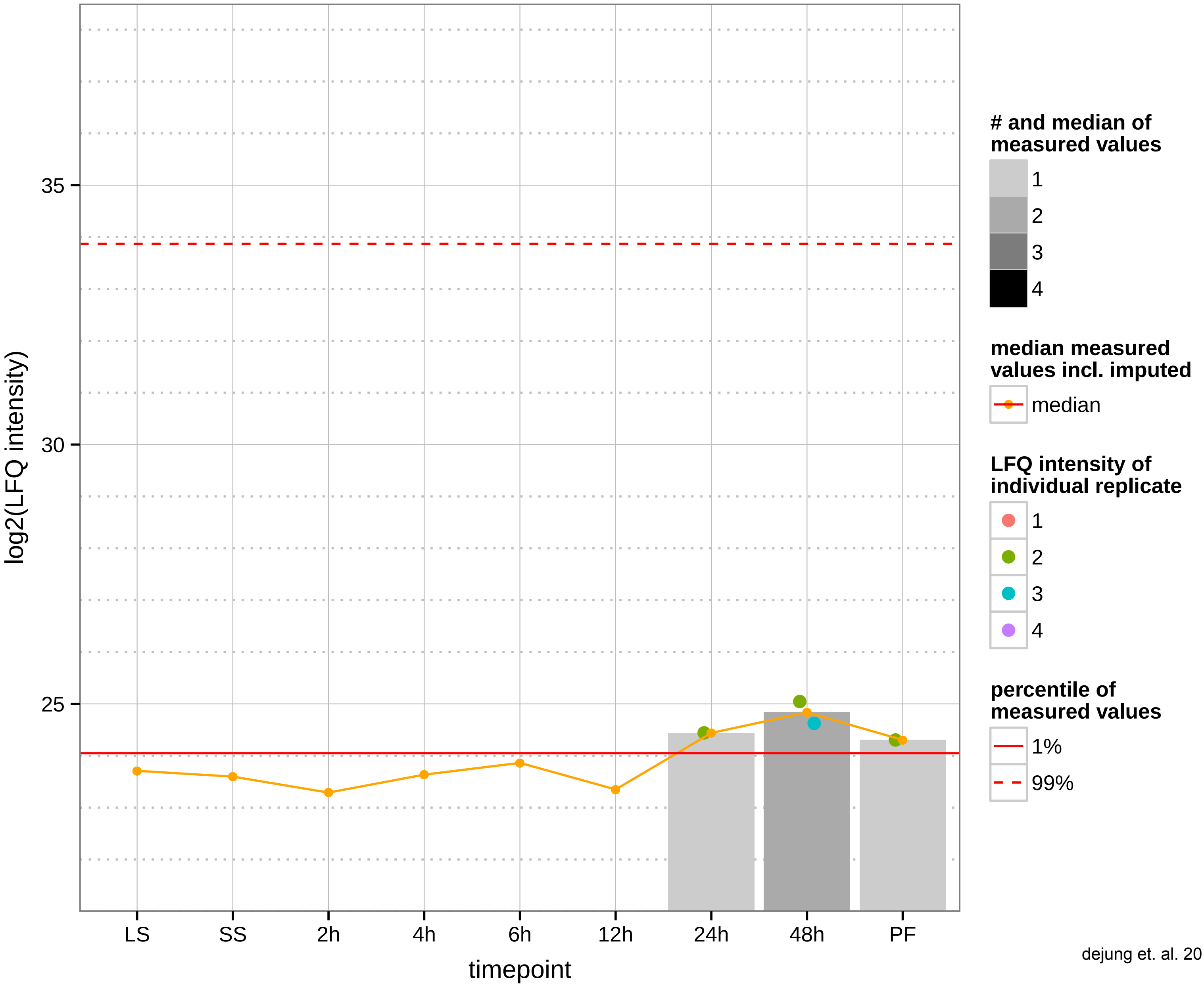

dejung et. al. 2015

hypothetical protein, conserved
Tb927.8.5610
AGOF: null
AGOC: null
AGOP: null
PGOF: null
PGOC: null
PGOP: null

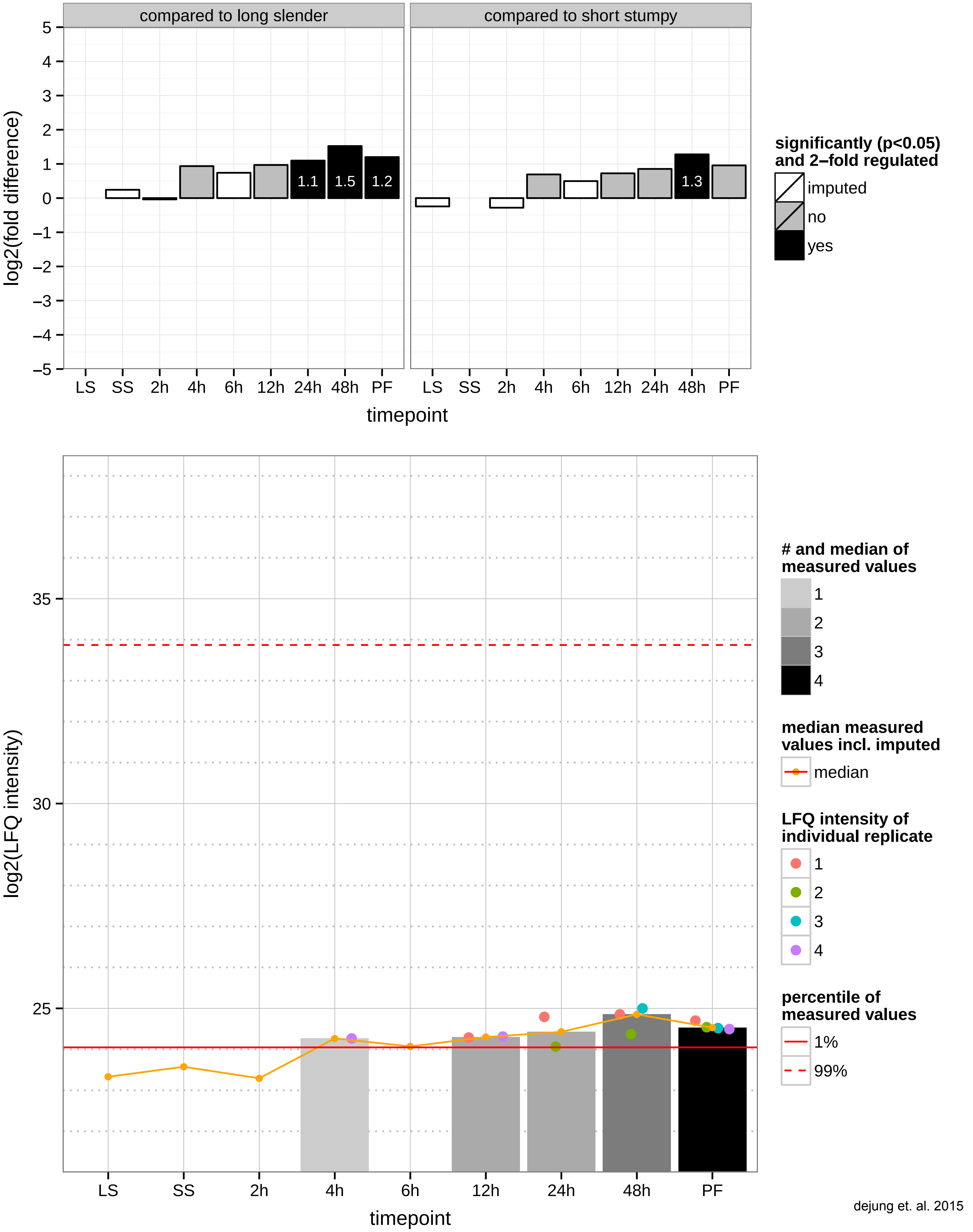

dejung et. al. 2015

hypothetical protein, conserved
Tb927.8.6280
AGOF: peptidyl−prolyl cis−trans isomerase activity
AGOC: null
AGOP: null
PGOF: peptidyl−prolyl cis−trans isomerase activity
PGOC: null
PGOP: protein folding

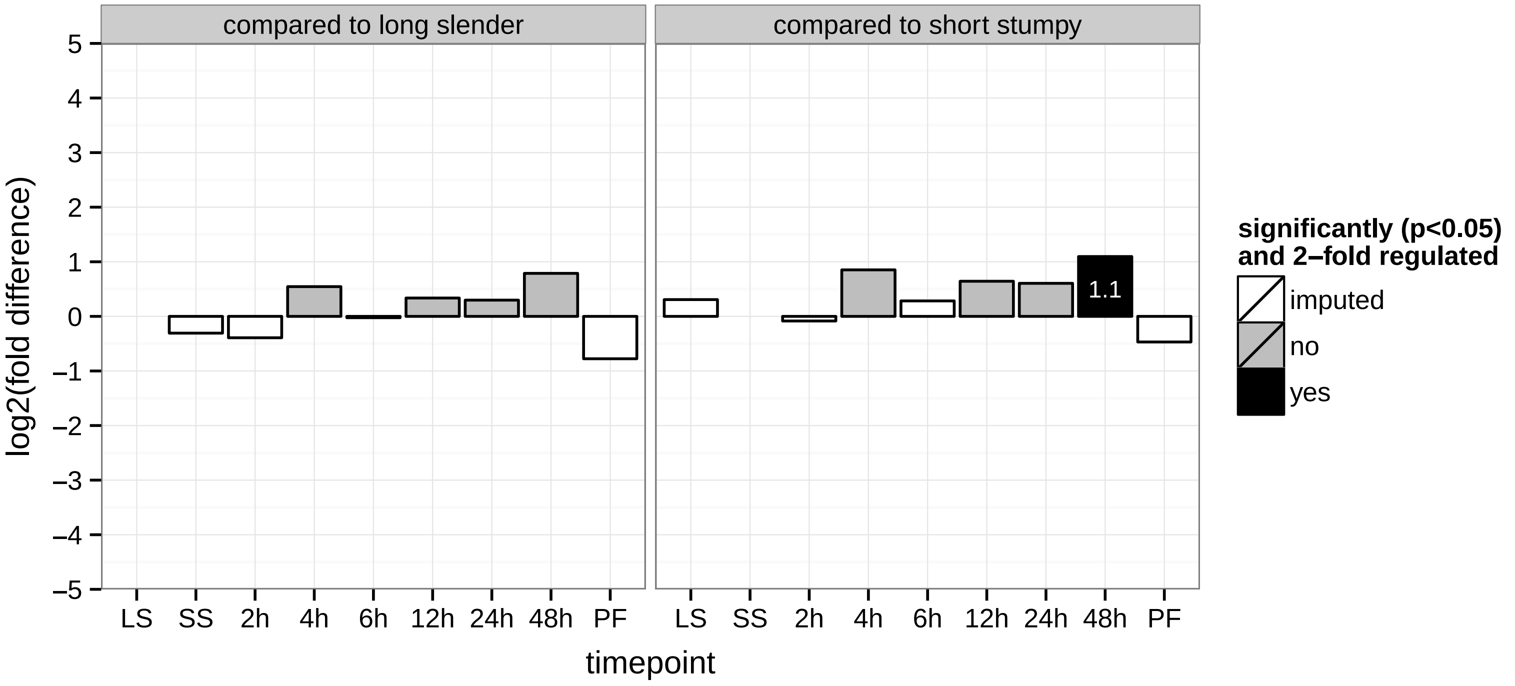

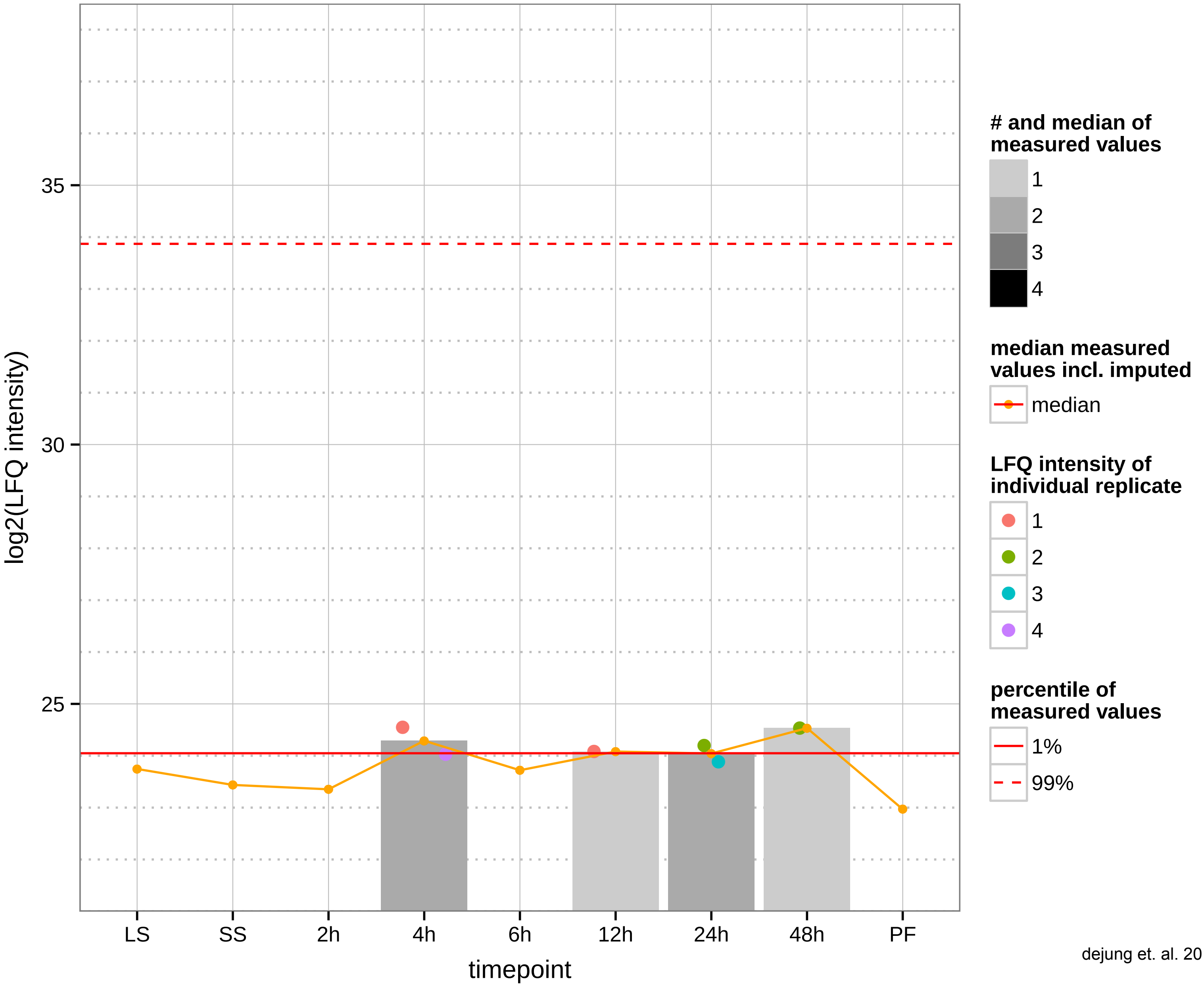

dejung et. al. 2015

mitochondrial RNA binding complex 1 subunit (MRB8180)
Tb927.8.8180
AGOF: RNA binding
AGOC: mitochondrion
AGOP: cytidine to uridine editing, mRNA stabilization
PGOF: null
PGOC: null
PGOP: null

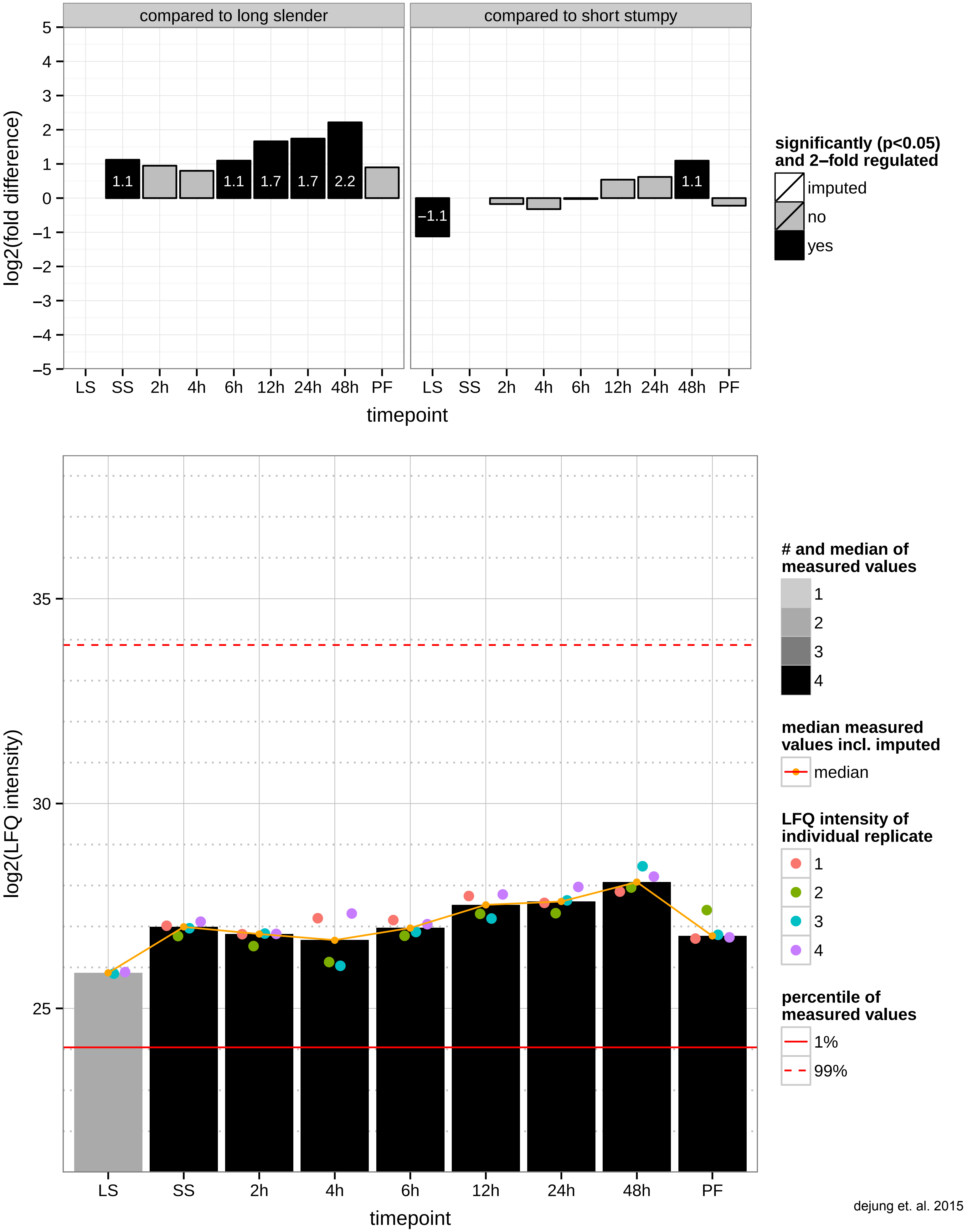

dejung et. al. 2015

mitochondrial carrier protein, mitochondrial carrier protein (MCP11)
Tb927.9.10310
AGOF: null
AGOC: mitochondrial inner membrane, mitochondrial part
AGOP: transport
PGOF: null
PGOC: null
PGOP: null

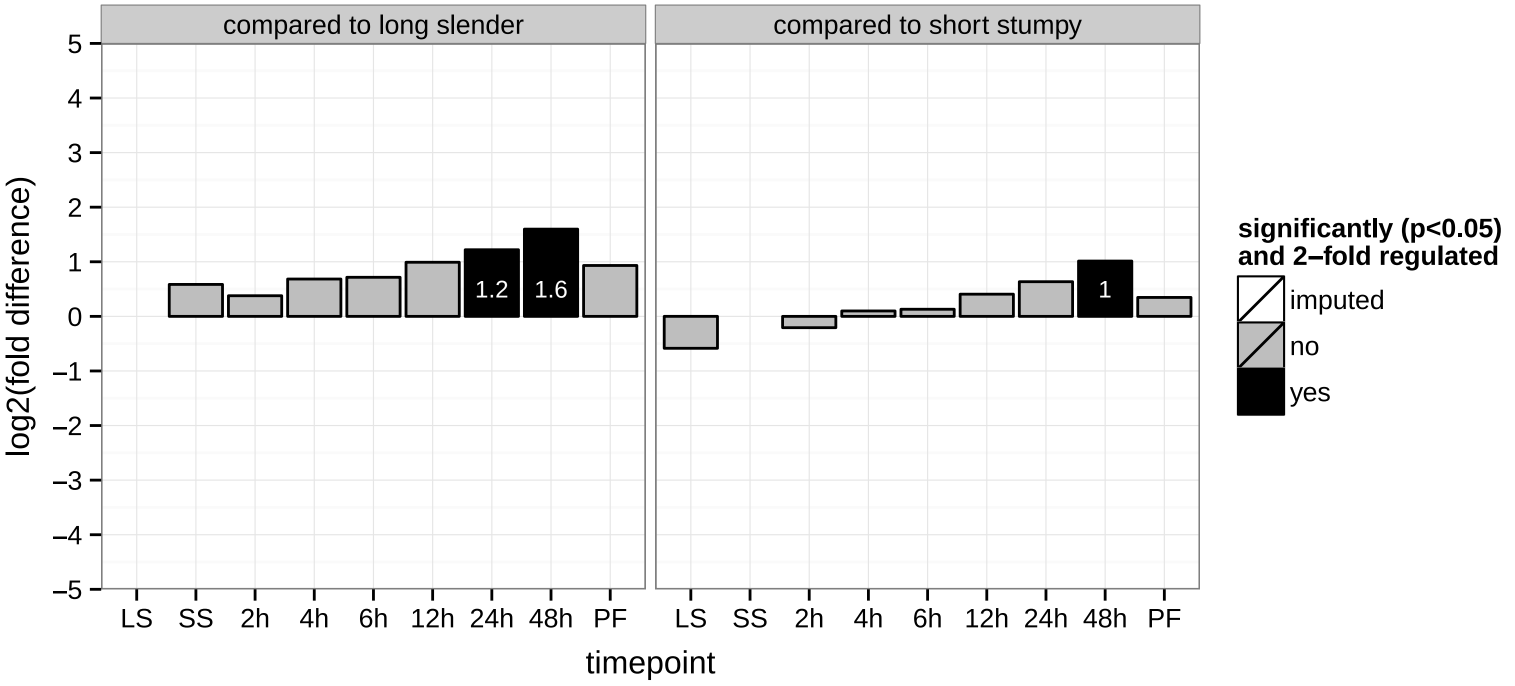

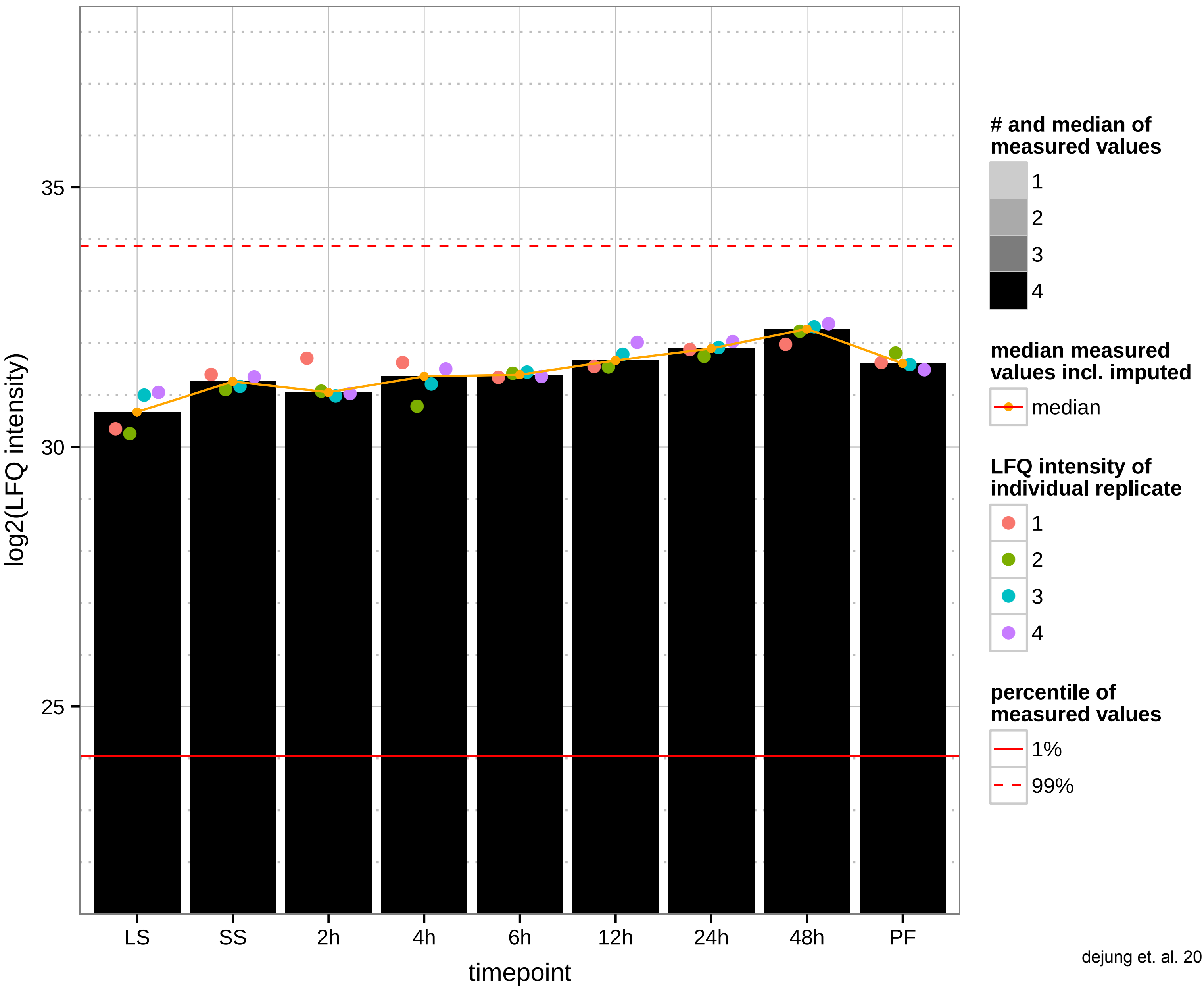

dejung et. al. 2015

elongation factor 1–alpha, putative, hsp70 subfamily B suppressor 1
Tb927.9.10710
AGOF: GTP binding, GTPase activity, translation elongation factor activity
AGOC: eukaryotic translation elongation factor 1 complex
AGOP: translational elongation
PGOF: GTP binding, GTPase activity
PGOC: null
PGOP: null

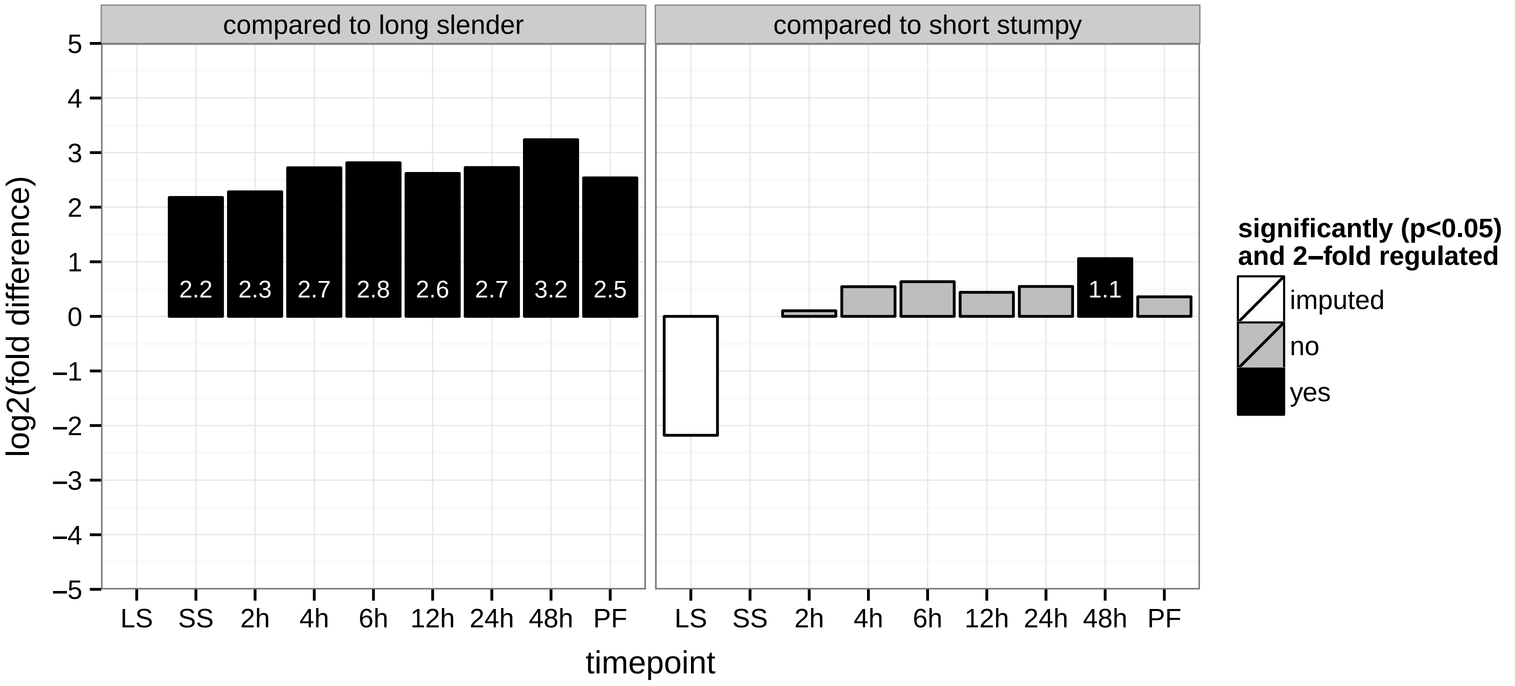

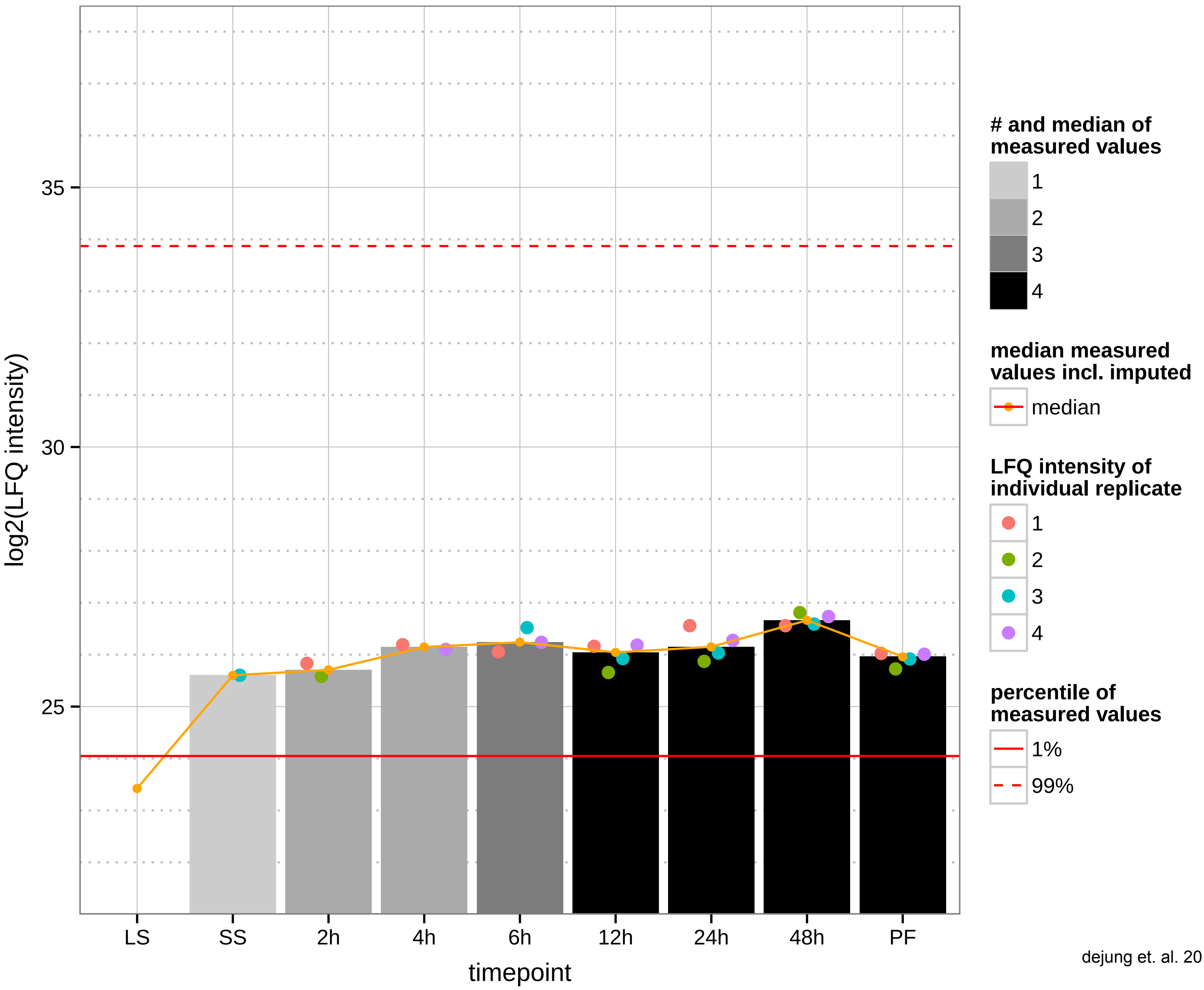

dejung et. al. 2015

hypothetical protein
Tb927.9.10790
AGOF: null
AGOC: null
AGOP: null
PGOF: null
PGOC: null
PGOP: null

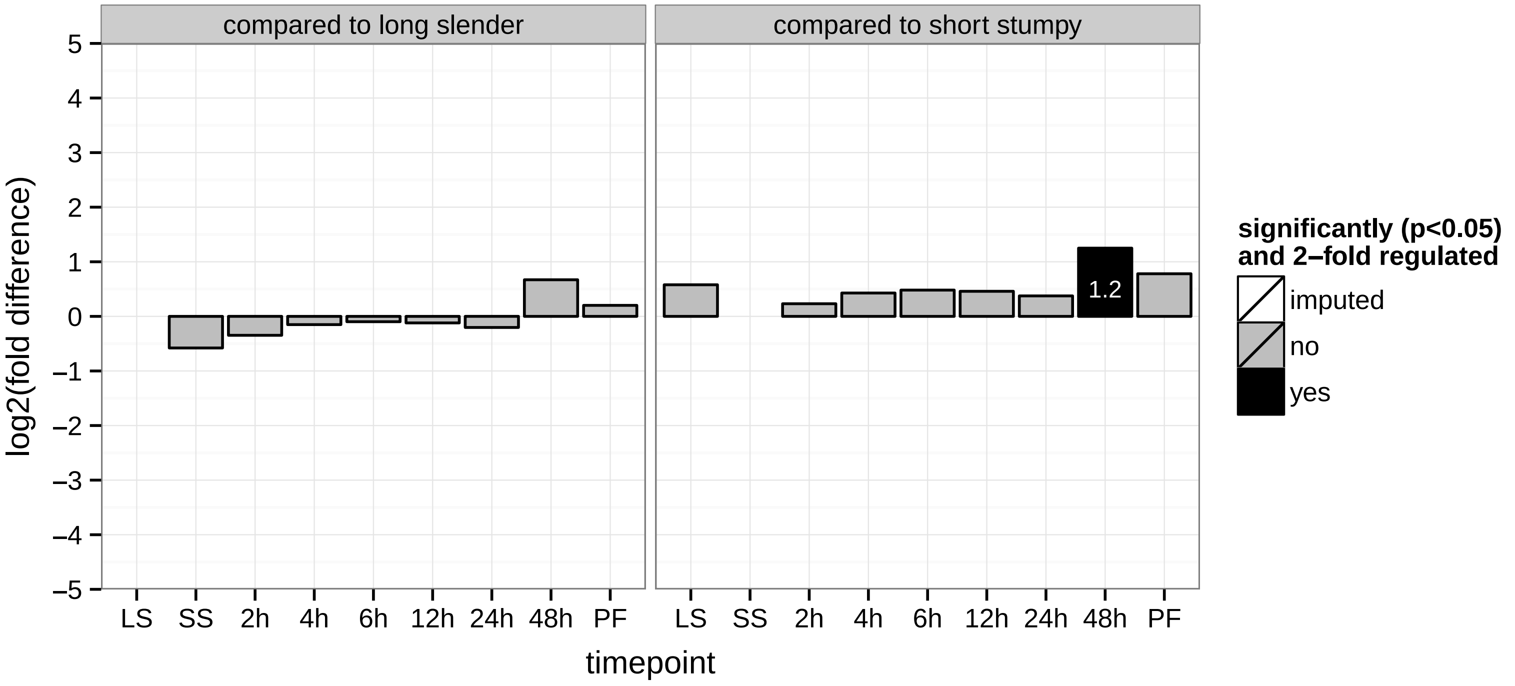

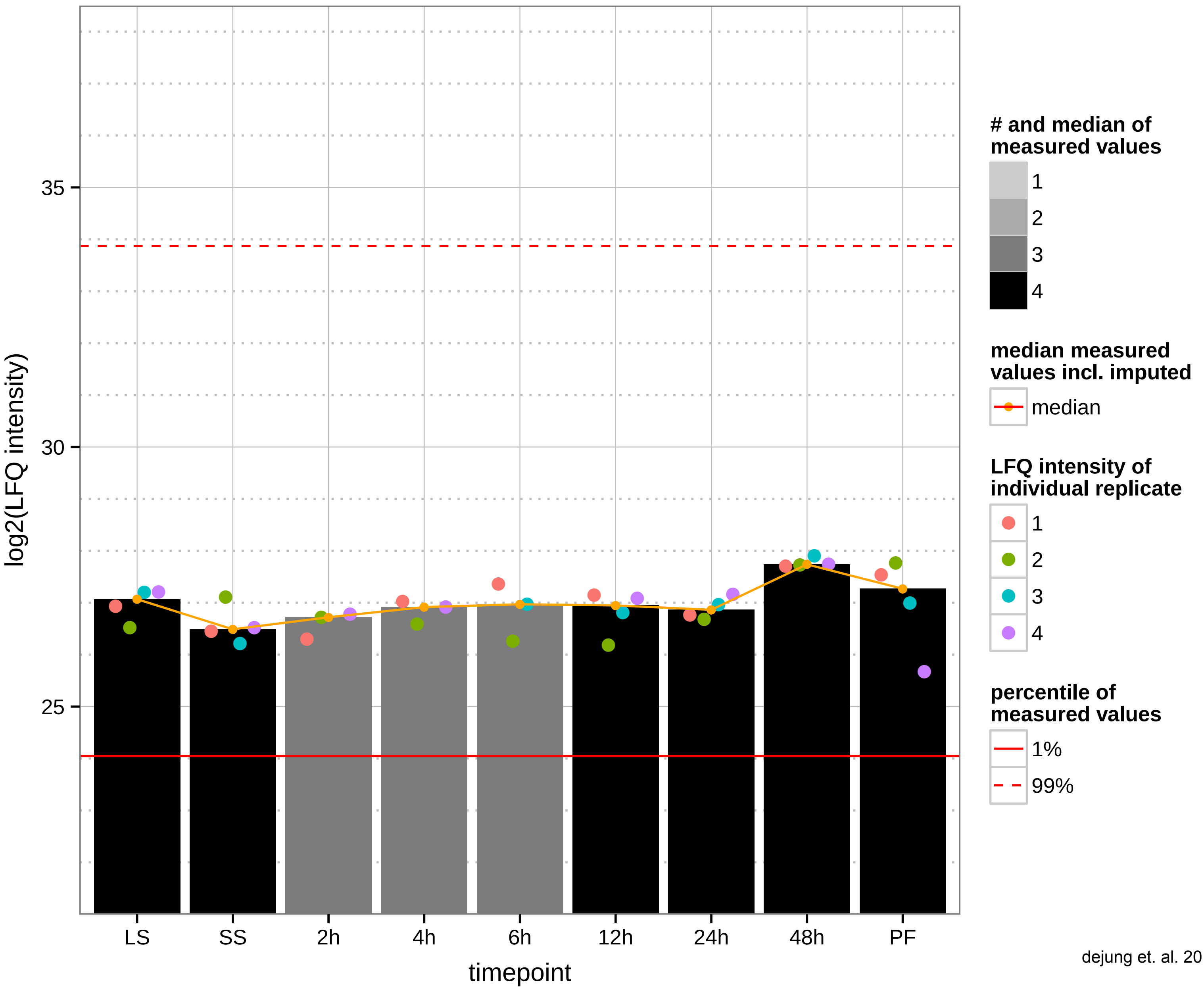

dejung et. al. 2015

hypothetical protein, conserved
Tb927.9.11540
AGOF: null
AGOC: null
AGOP: null
PGOF: null
PGOC: null
PGOP: null

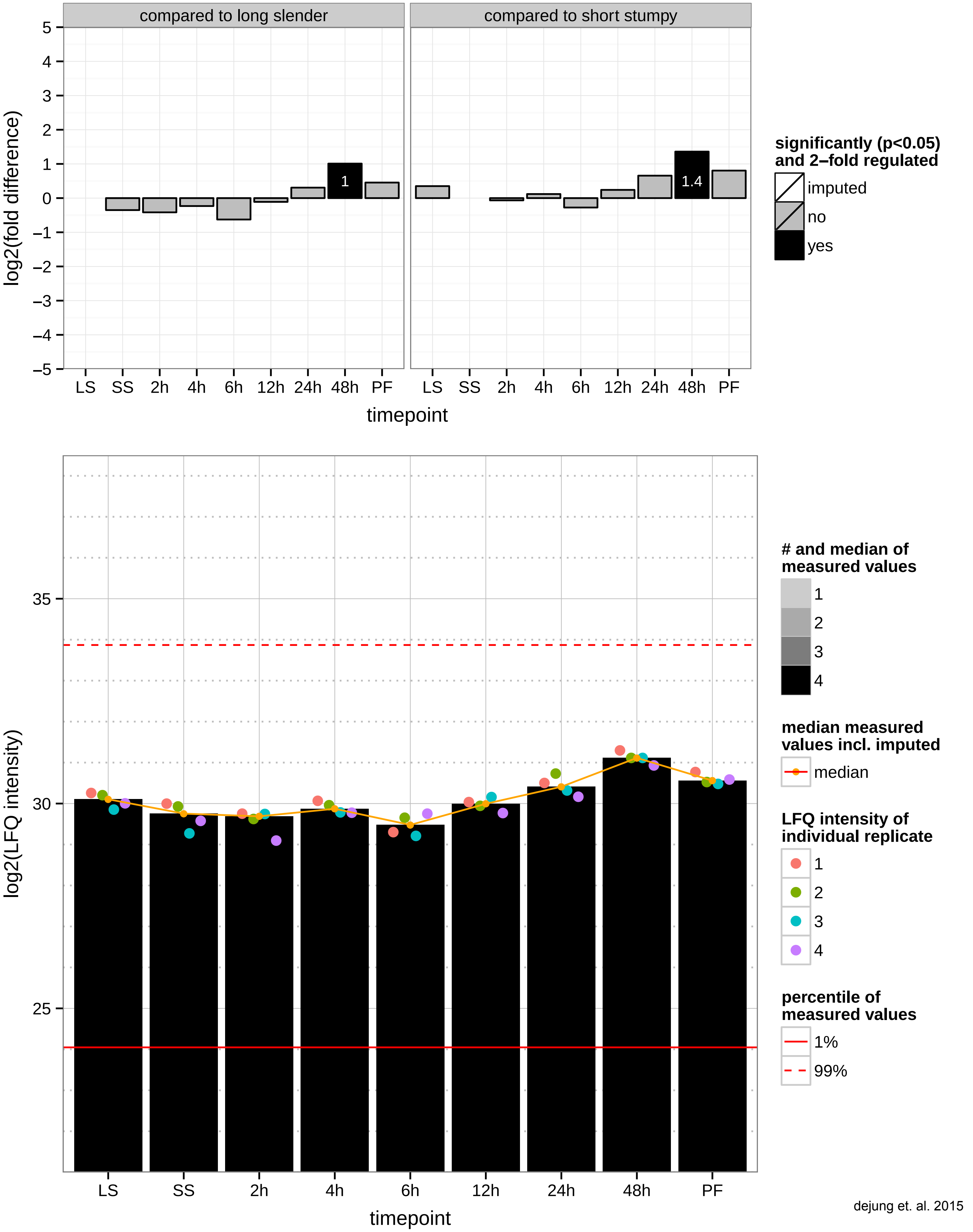

dejung et. al. 2015

leucine–rich repeat protein (LRRP), putative
Tb927.9.12170
AGOF: null
AGOC: null
AGOP: null
PGOF: protein binding
PGOC: null
PGOP: null

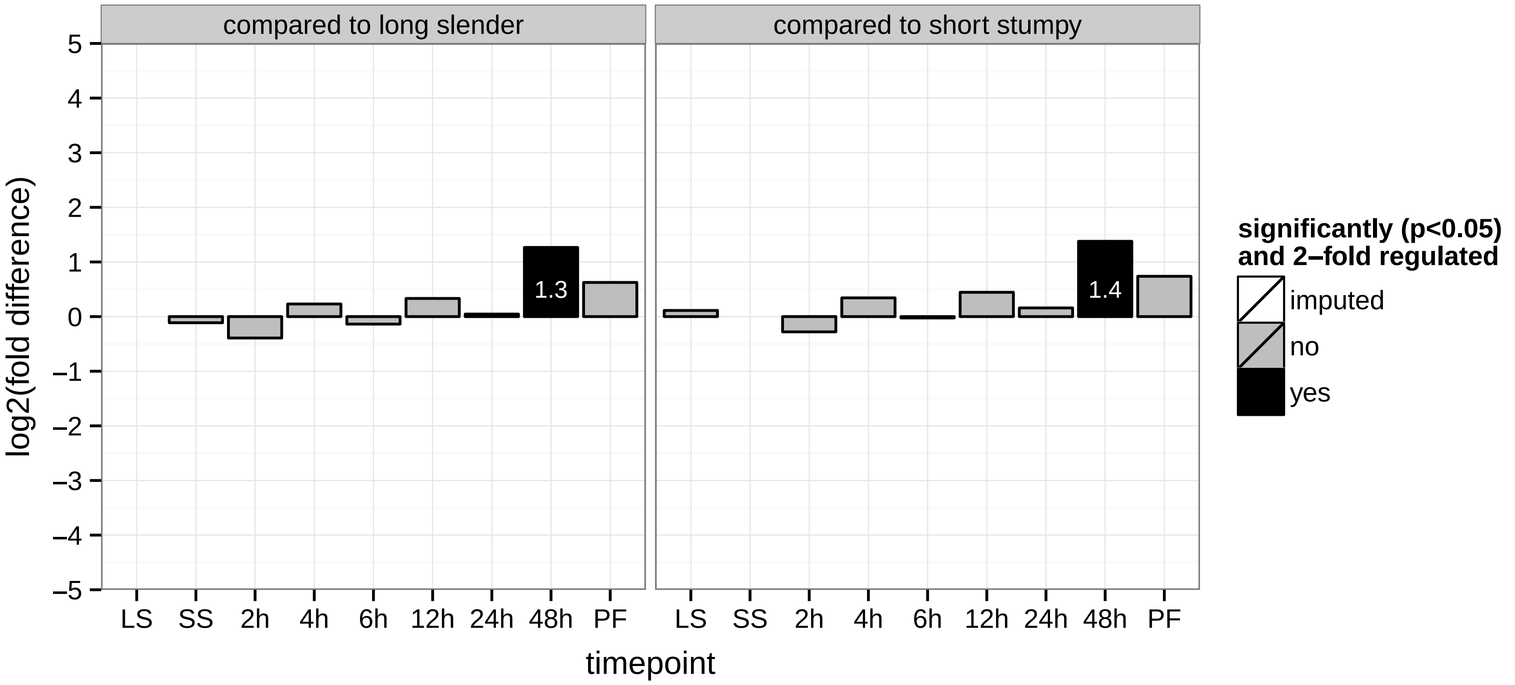

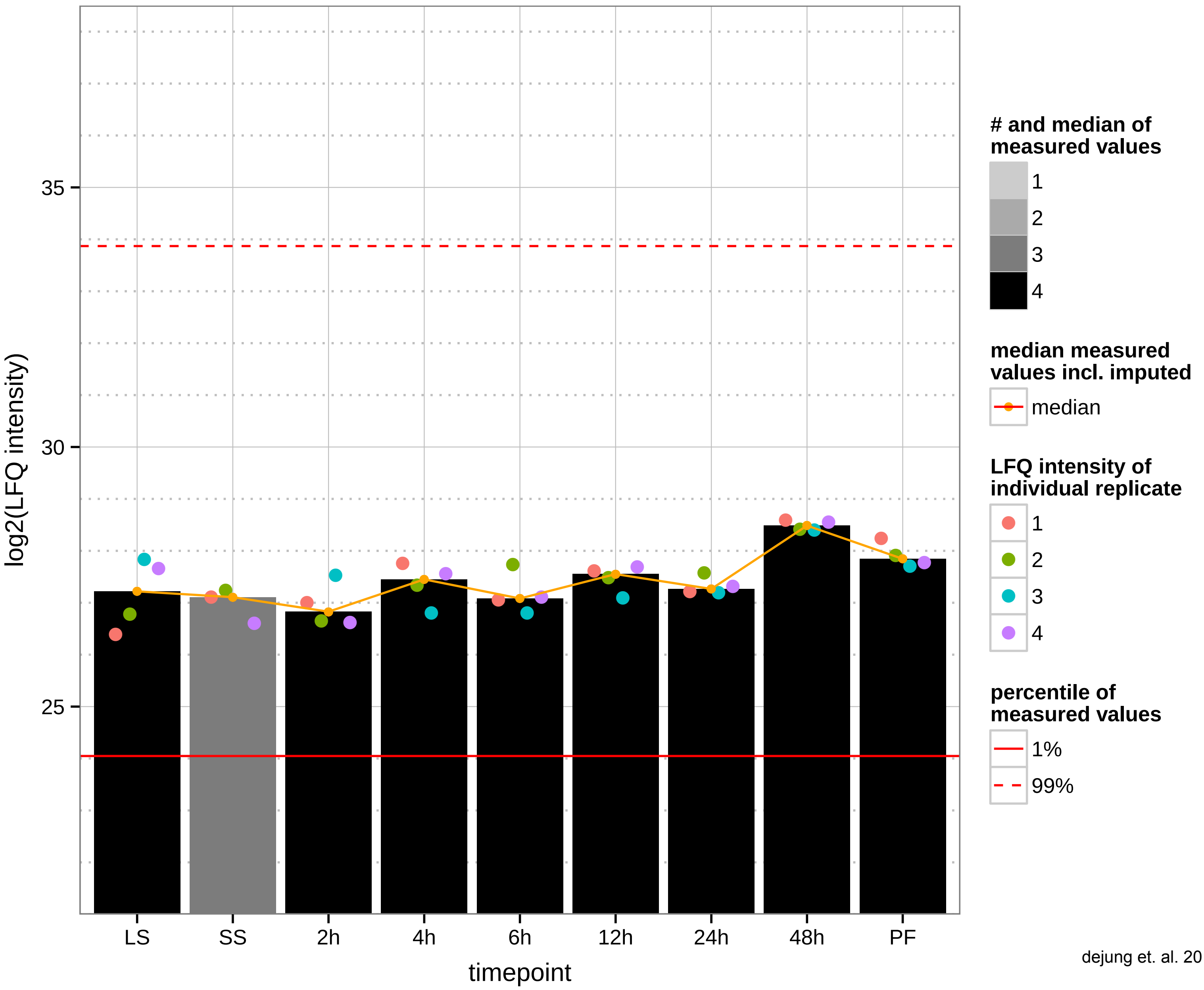

dejung et. al. 2015

ATP−dependent DEAD/H RNA helicase, putative (DED1)
Tb927.9.12510
AGOF: ATP binding, ATP−dependent helicase activity, helicase activity, nucleic acid binding
AGOC: null
AGOP: nucleobase−containing compound metabolic process
PGOF: ATP binding, ATP−dependent helicase activity, helicase activity, nucleic acid binding
PGOC: null
PGOP: null

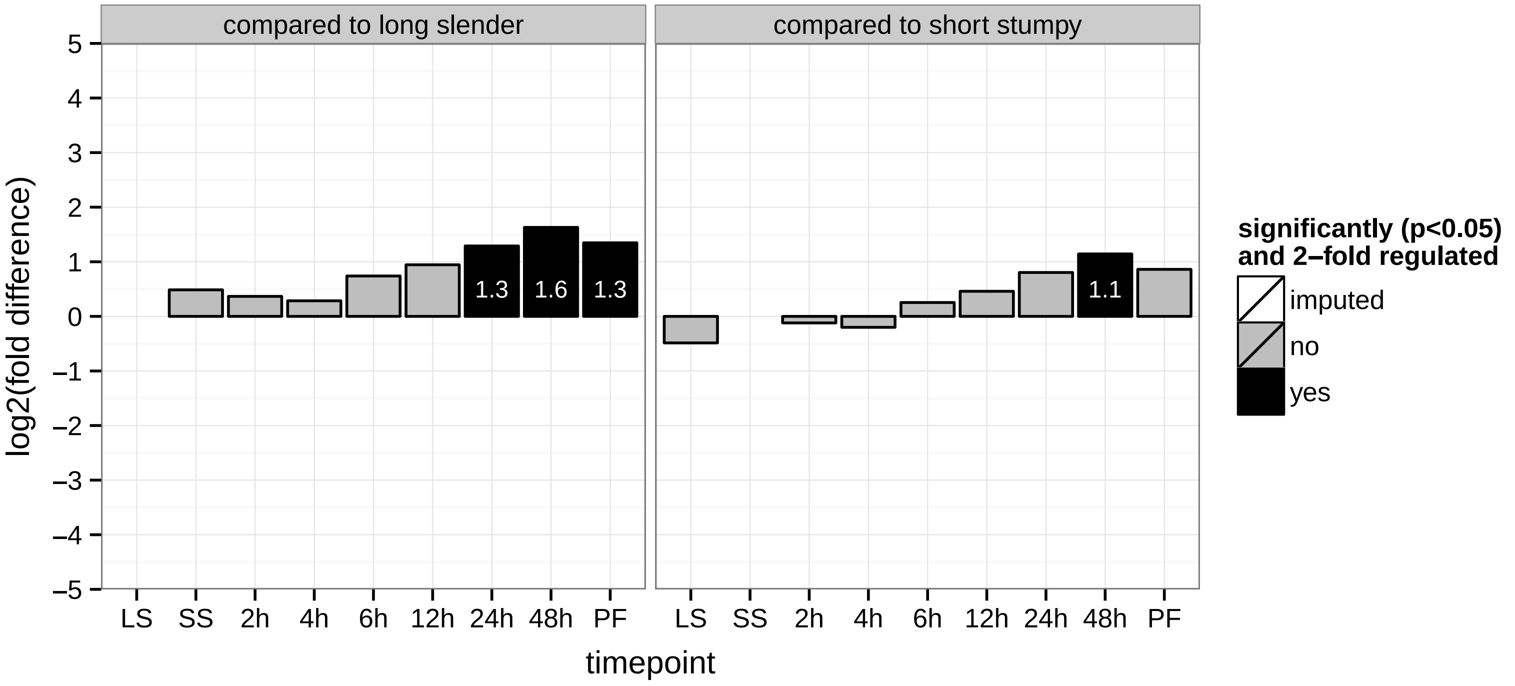

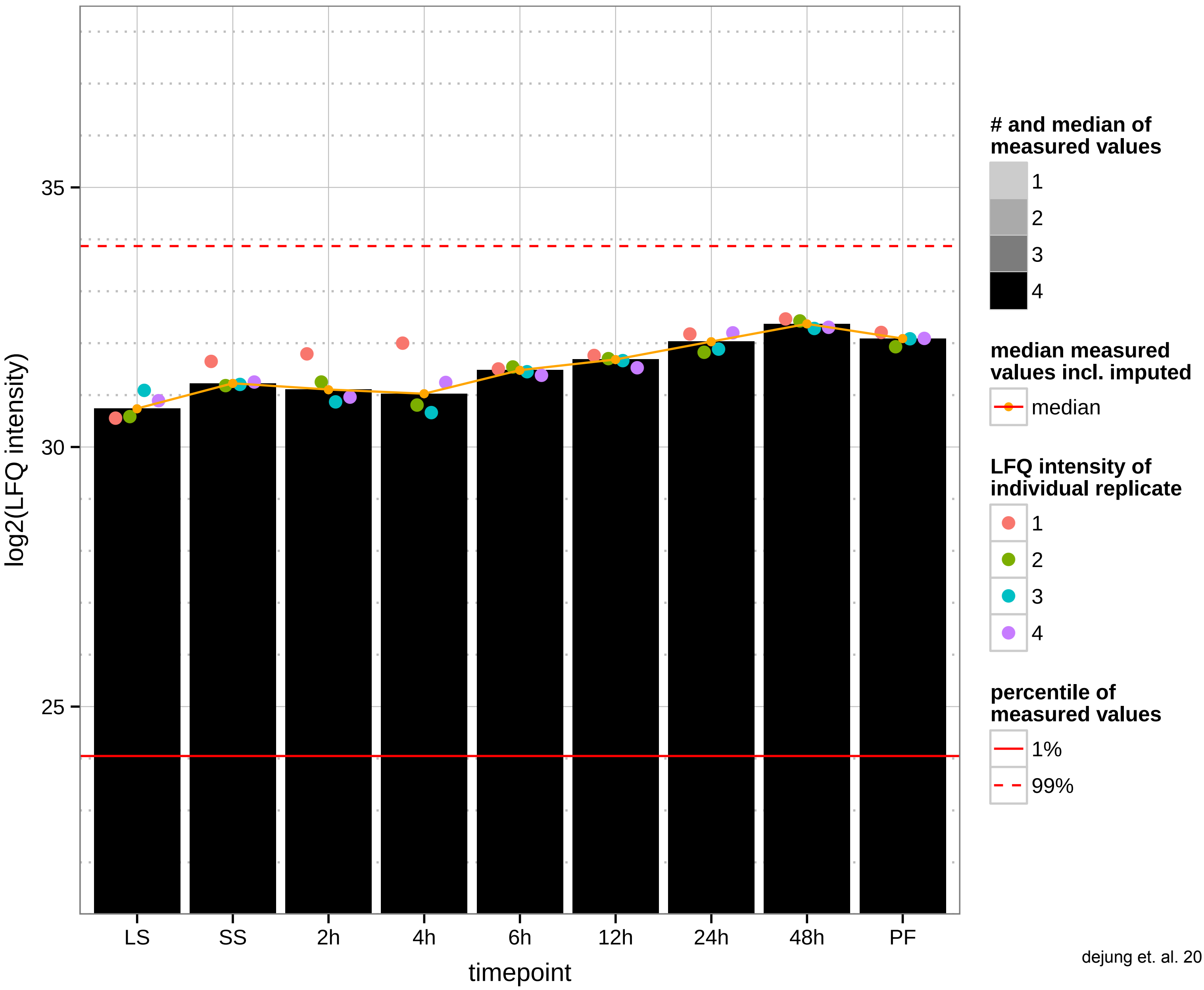

dejung et. al. 2015

hypothetical protein, conserved
Tb927.9.13520
AGOF: null
AGOC: null
AGOP: null
PGOF: binding
PGOC: null
PGOP: null

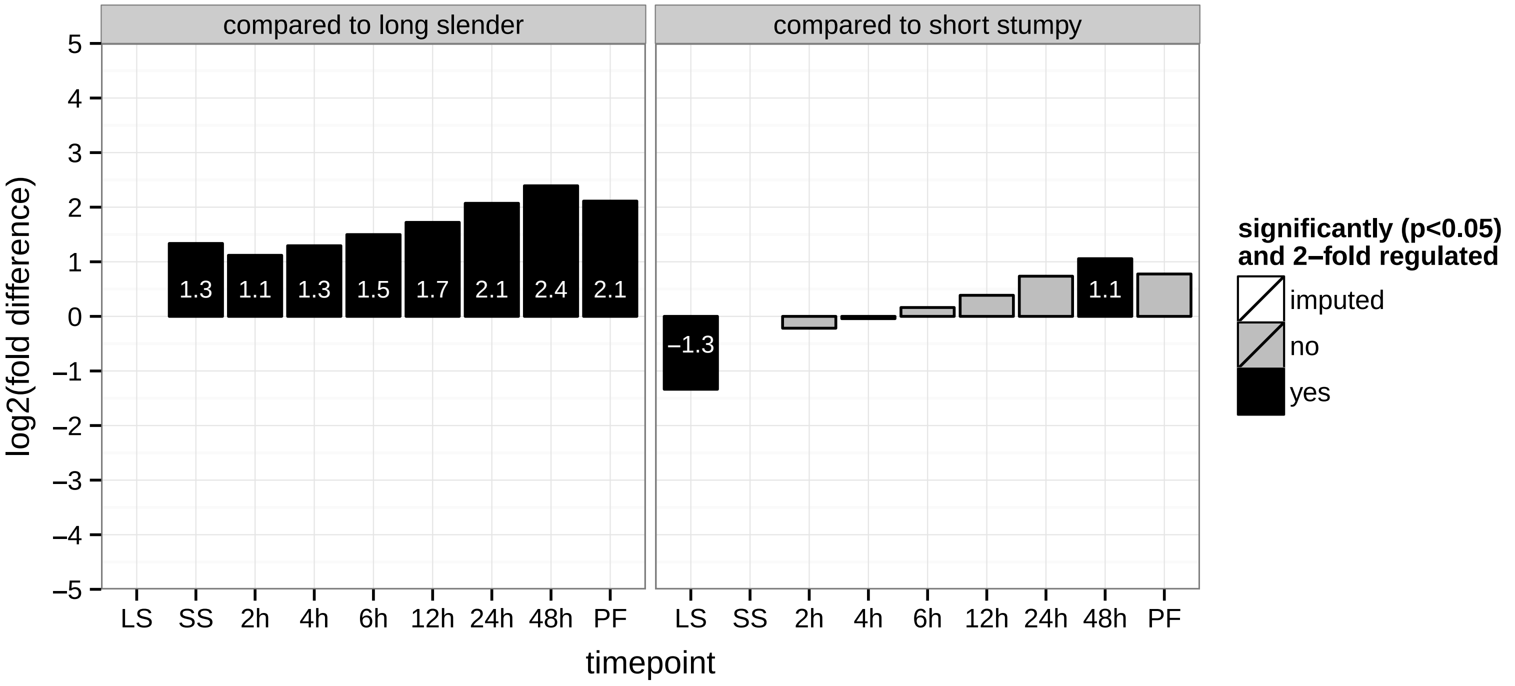

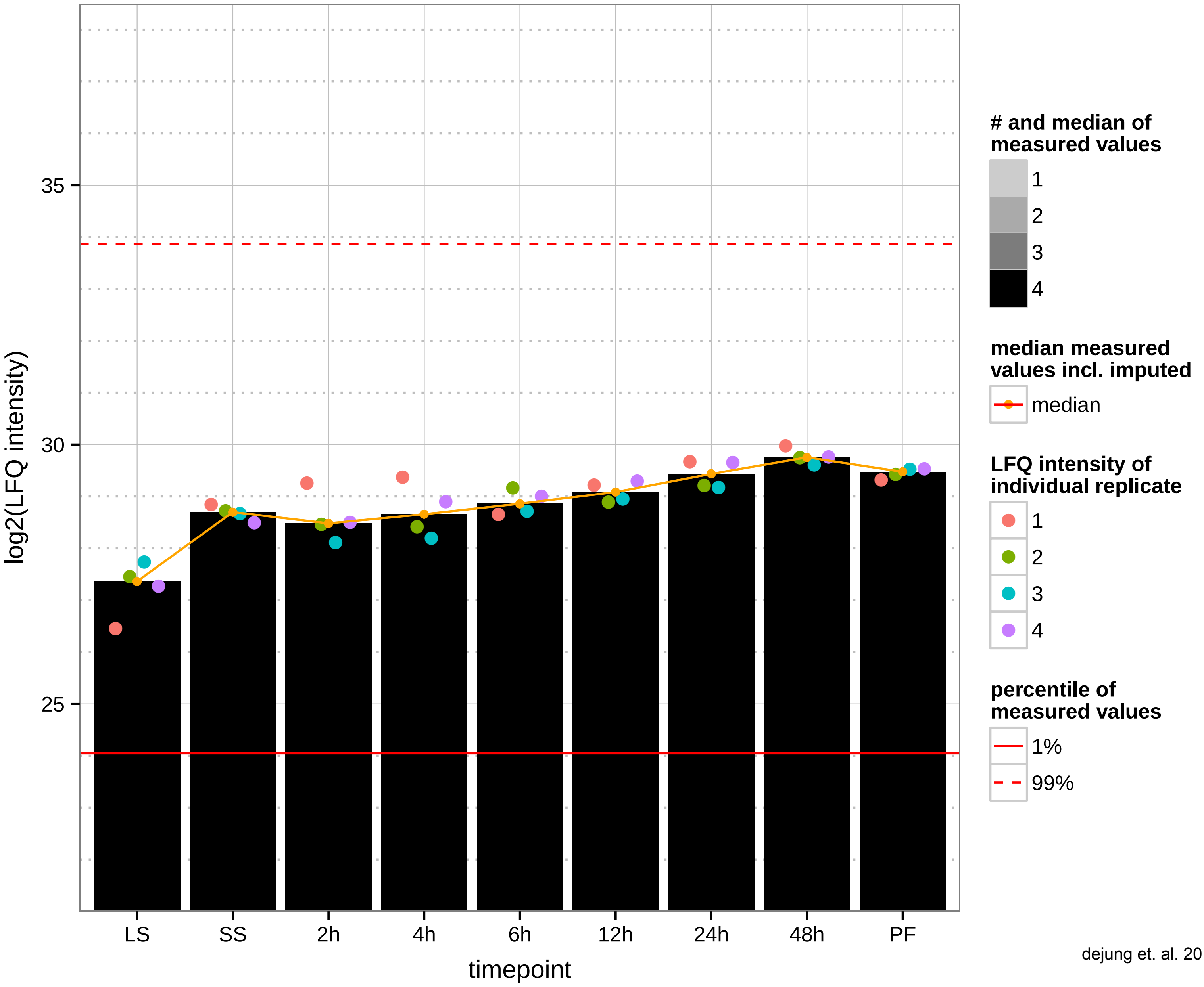

dejung et. al. 2015

ribosomal protein L15, putative
Tb927.9.15190
AGOF: structural constituent of ribosome
AGOC: intracellular, ribosome
AGOP: translation
PGOF: structural constituent of ribosome
PGOC: intracellular, ribosome
PGOP: translation

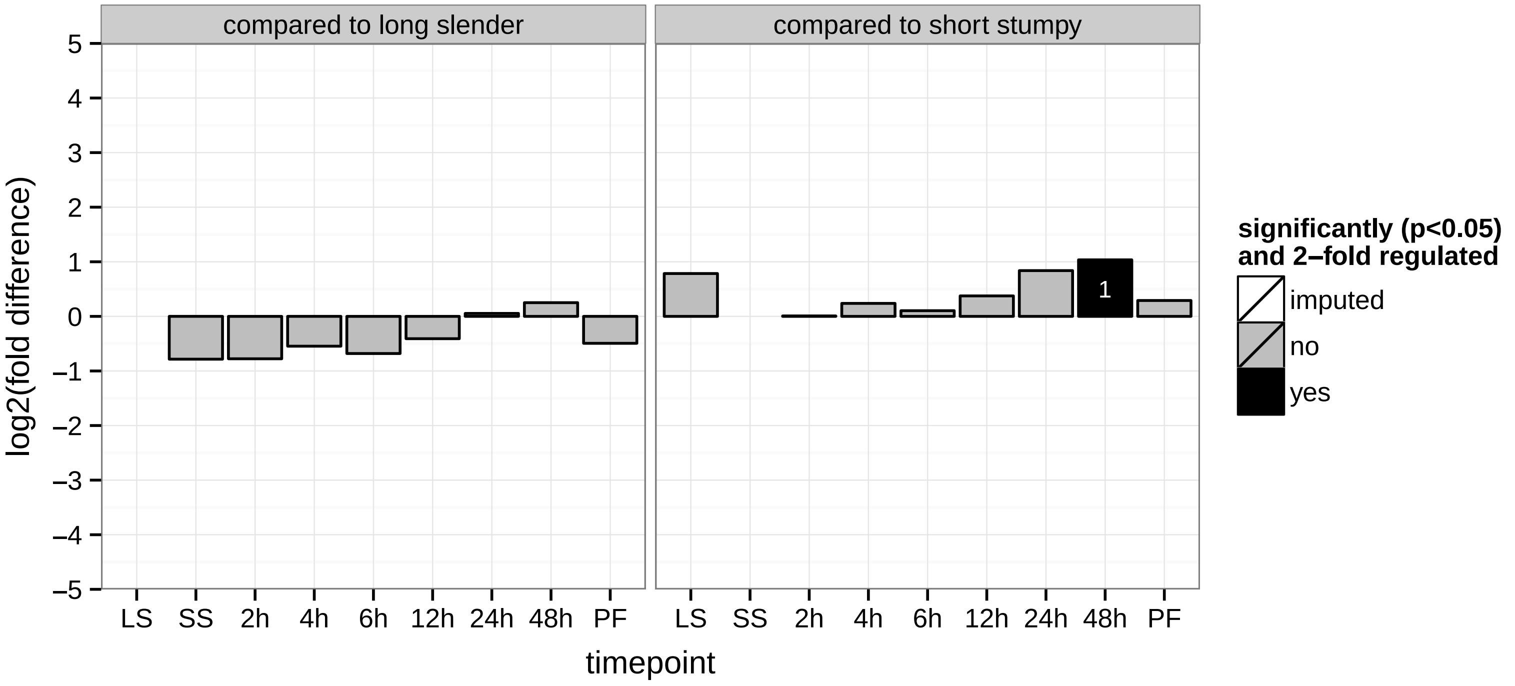

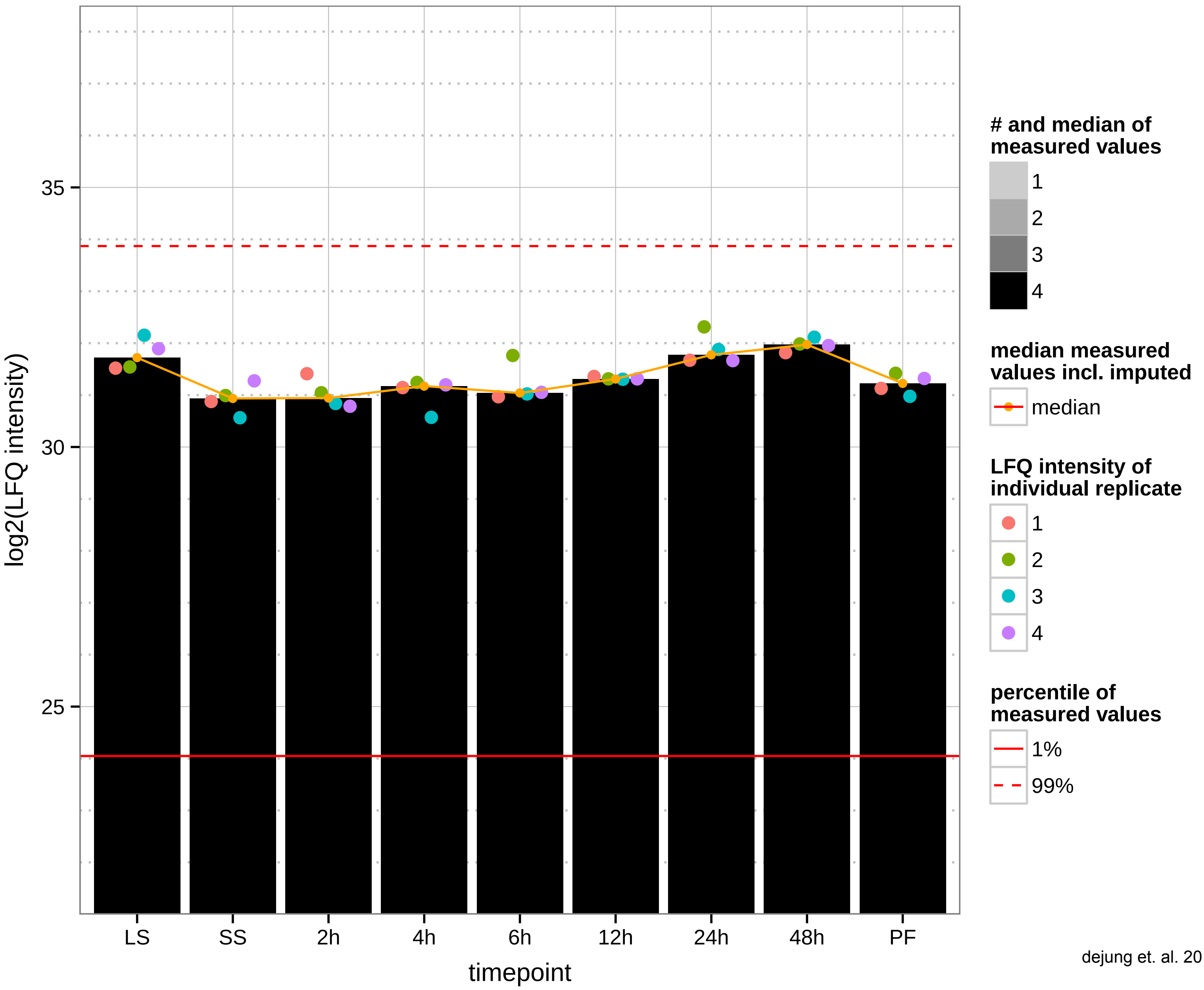

dejung et. al. 2015

hypothetical protein, conserved
Tb927.9.1590
AGOF: null
AGOC: null
AGOP: null
PGOF: null
PGOC: null
PGOP: null

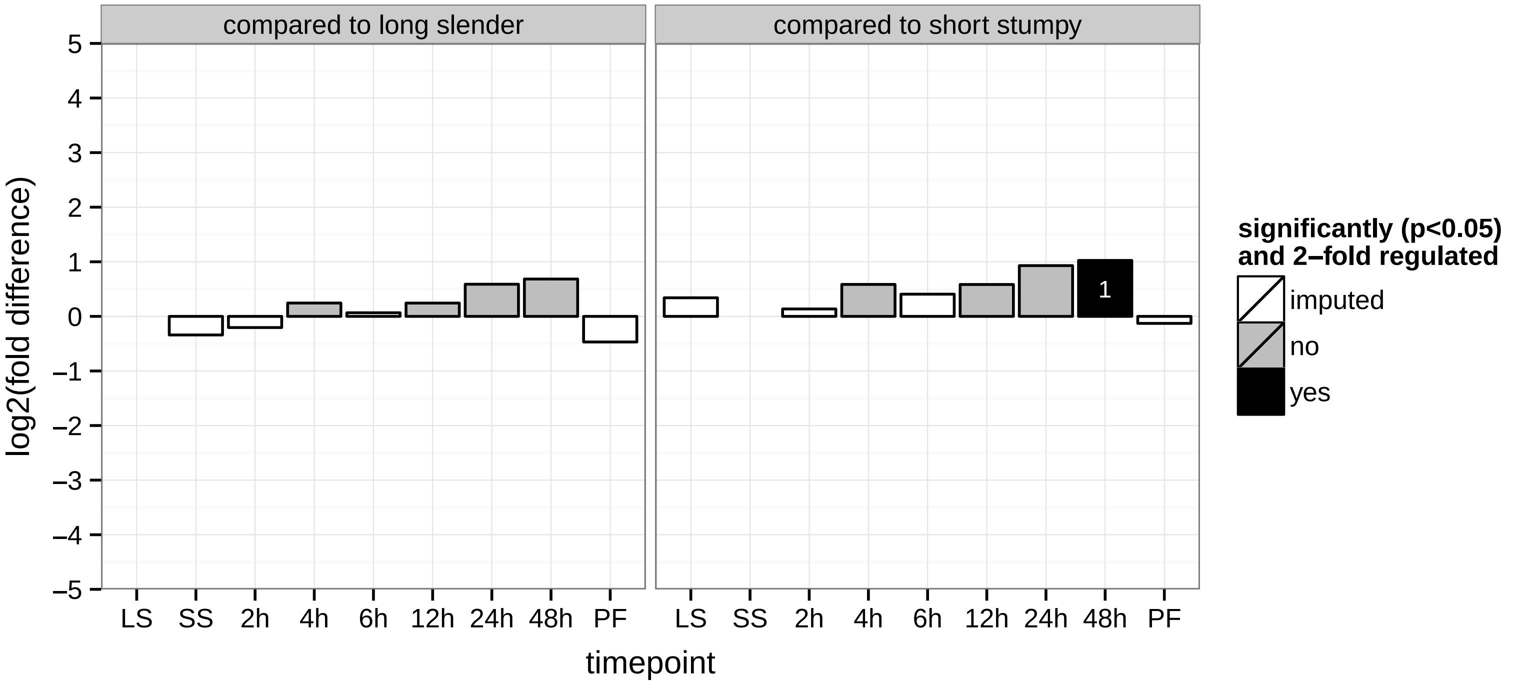

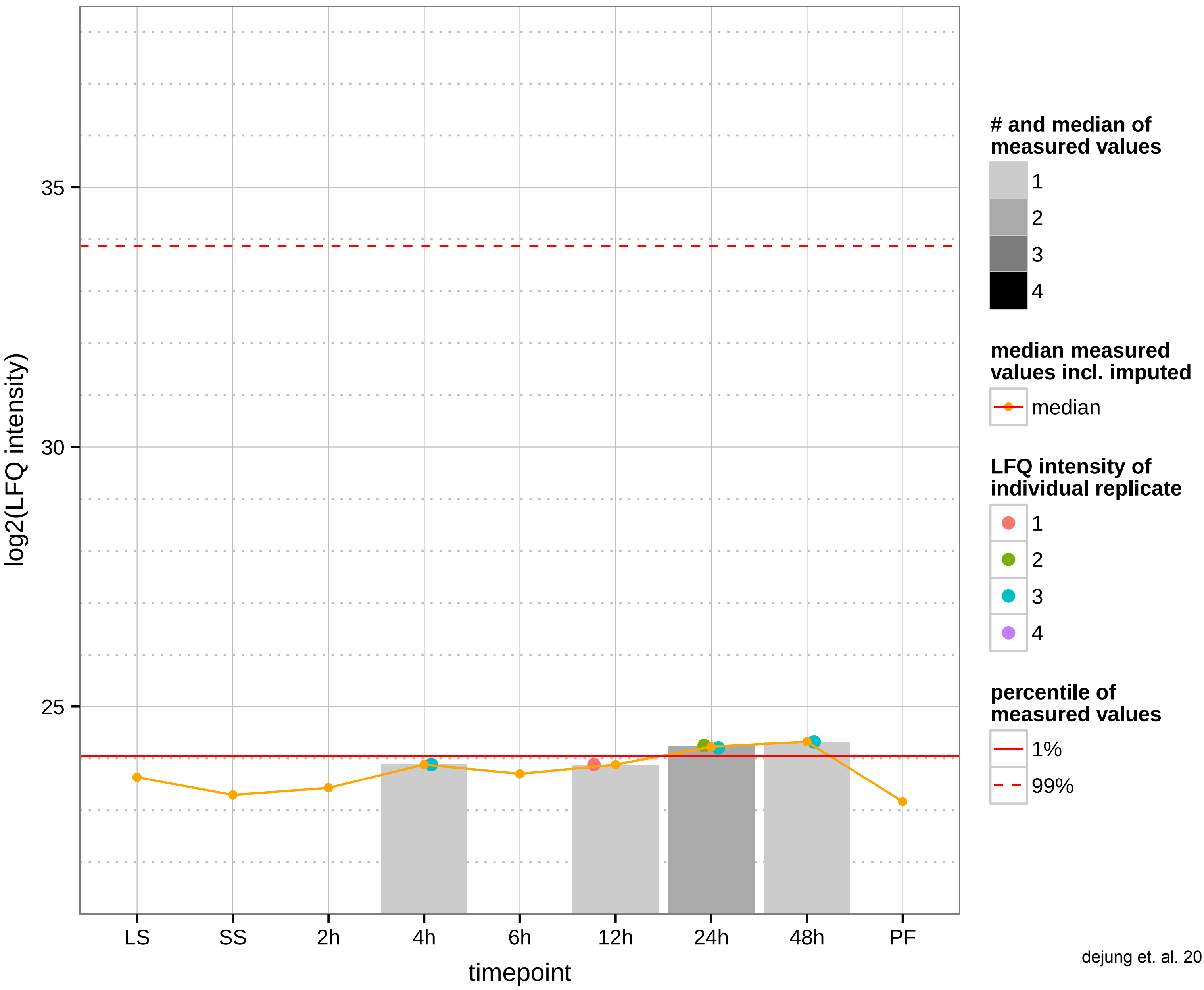

dejung et. al. 2015

hypothetical protein, conserved
Tb927.9.2620
AGOF: null
AGOC: mitochondrion
AGOP: null
PGOF: null
PGOC: null
PGOP: null

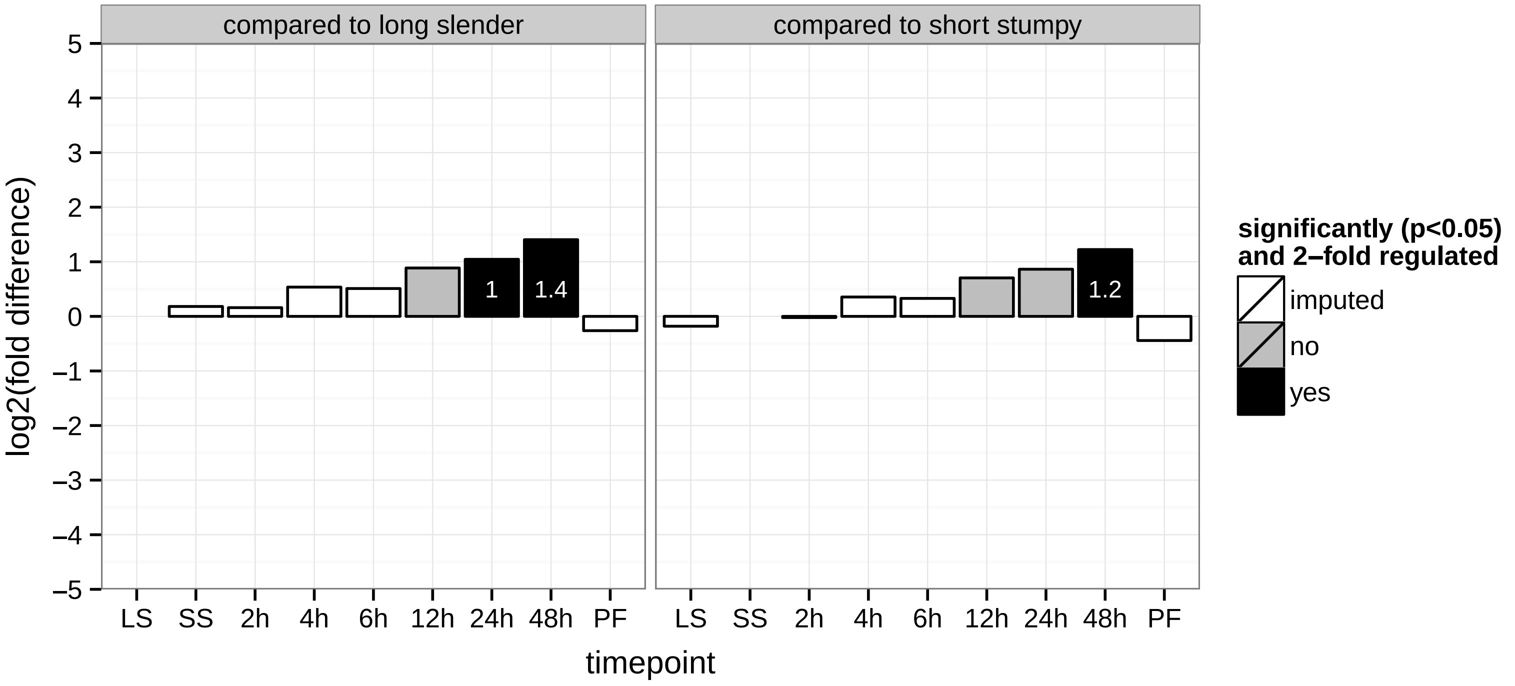

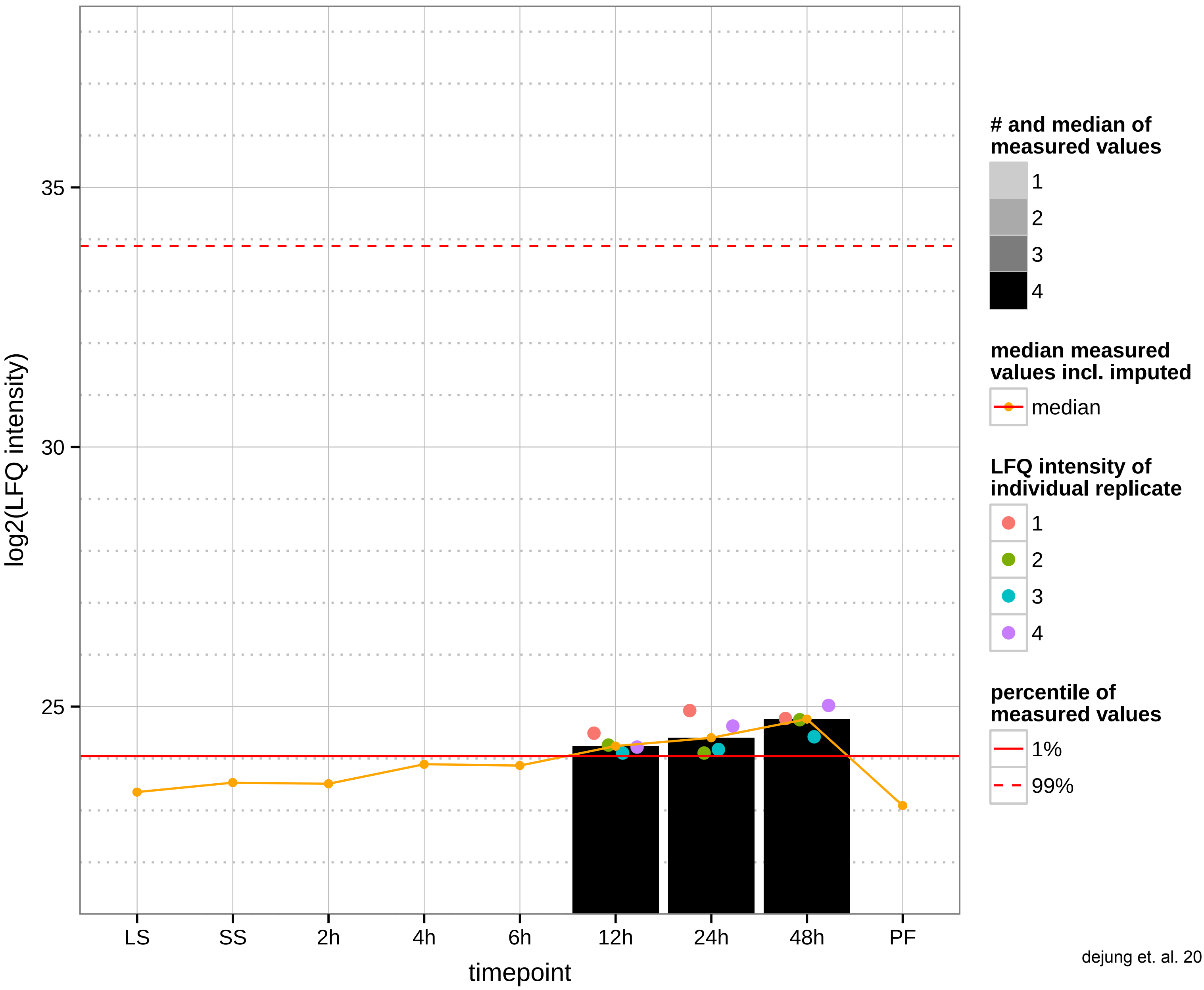

dejung et. al. 2015

cAMP–specific phosphodiesterase (PDEB1)
Tb927.9.5040
AGOF: 3', 5'–cyclic–nucleotide phosphodiesterase activity
AGOC: cytoplasm
AGOP: cell proliferation, signal transduction
PGOF: 3', 5'–cyclic–nucleotide phosphodiesterase activity, catalytic activity, protein binding
PGOC: null
PGOP: signal transduction

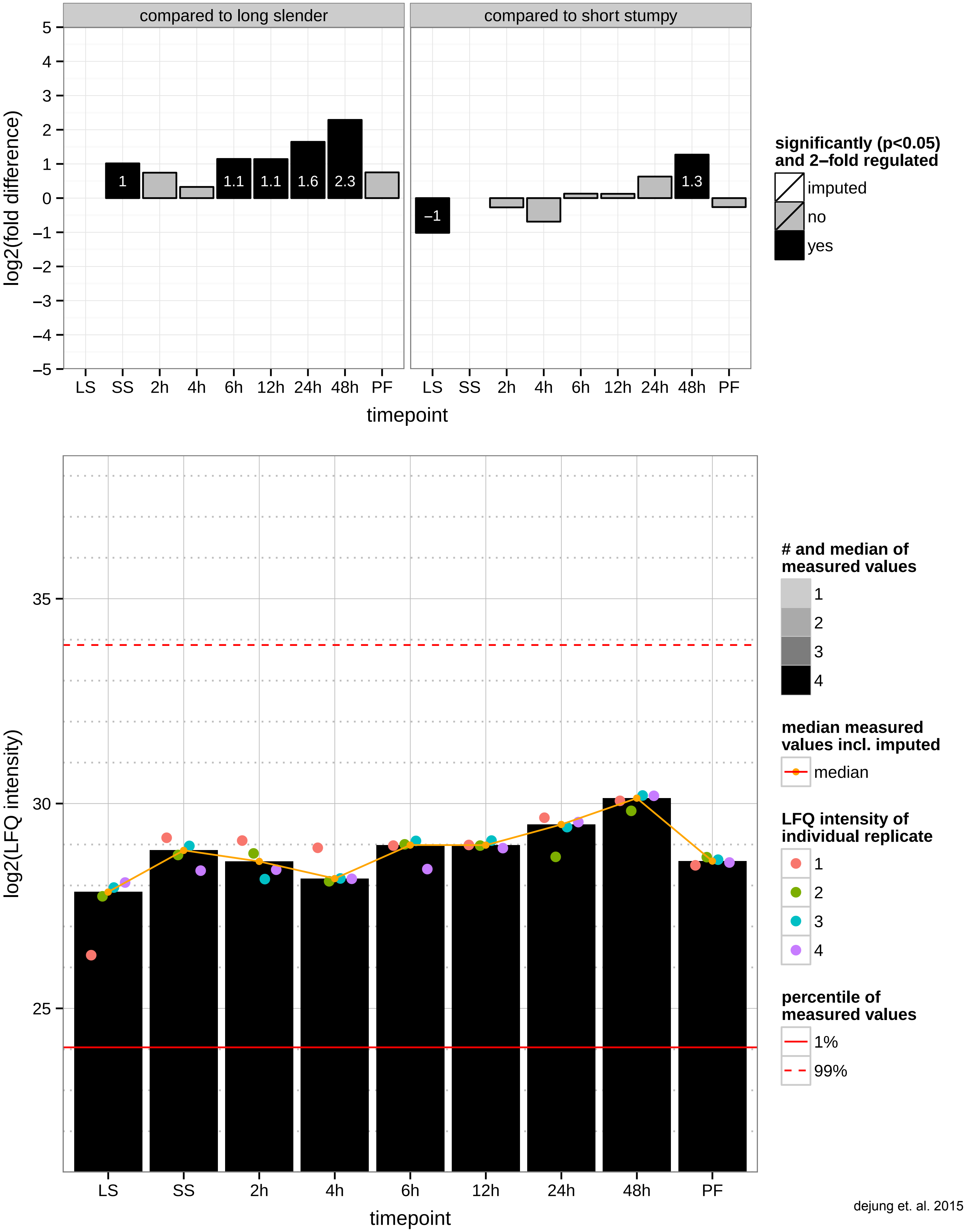

dejung et. al. 2015

adenylosuccinate lyase, putative (ADSL)
Tb927.9.7550
AGOF: N6−(1, 2−dicarboxyethyl)AMP AMP−lyase (fumarate−forming) activity
AGOC: null
AGOP: IMP biosynthetic process, purine nucleobase metabolic process, purine ribonucleotide biosynthetic process
PGOF: N6−(1, 2−dicarboxyethyl)AMP AMP−lyase (fumarate−forming) activity, catalytic activity
PGOC: null
PGOP: IMP biosynthetic process, purine ribonucleotide biosynthetic process

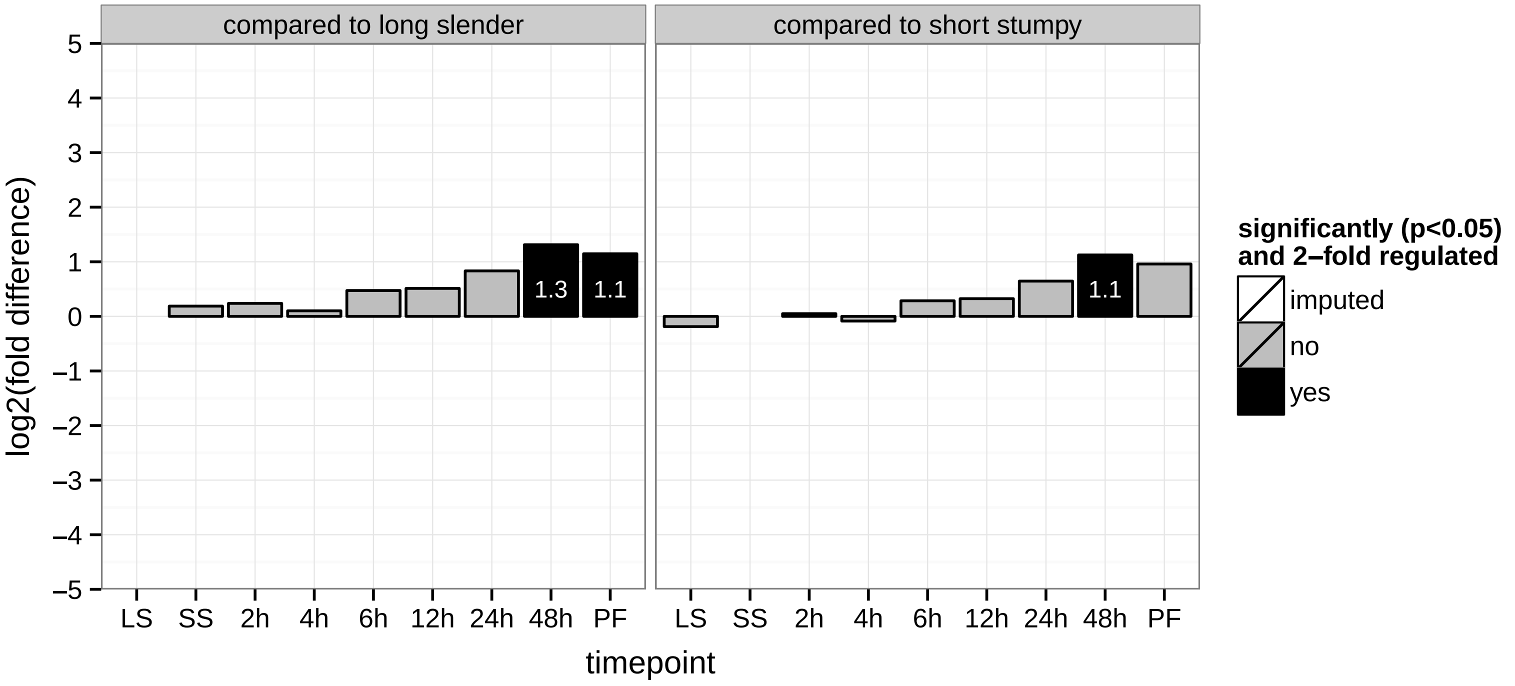

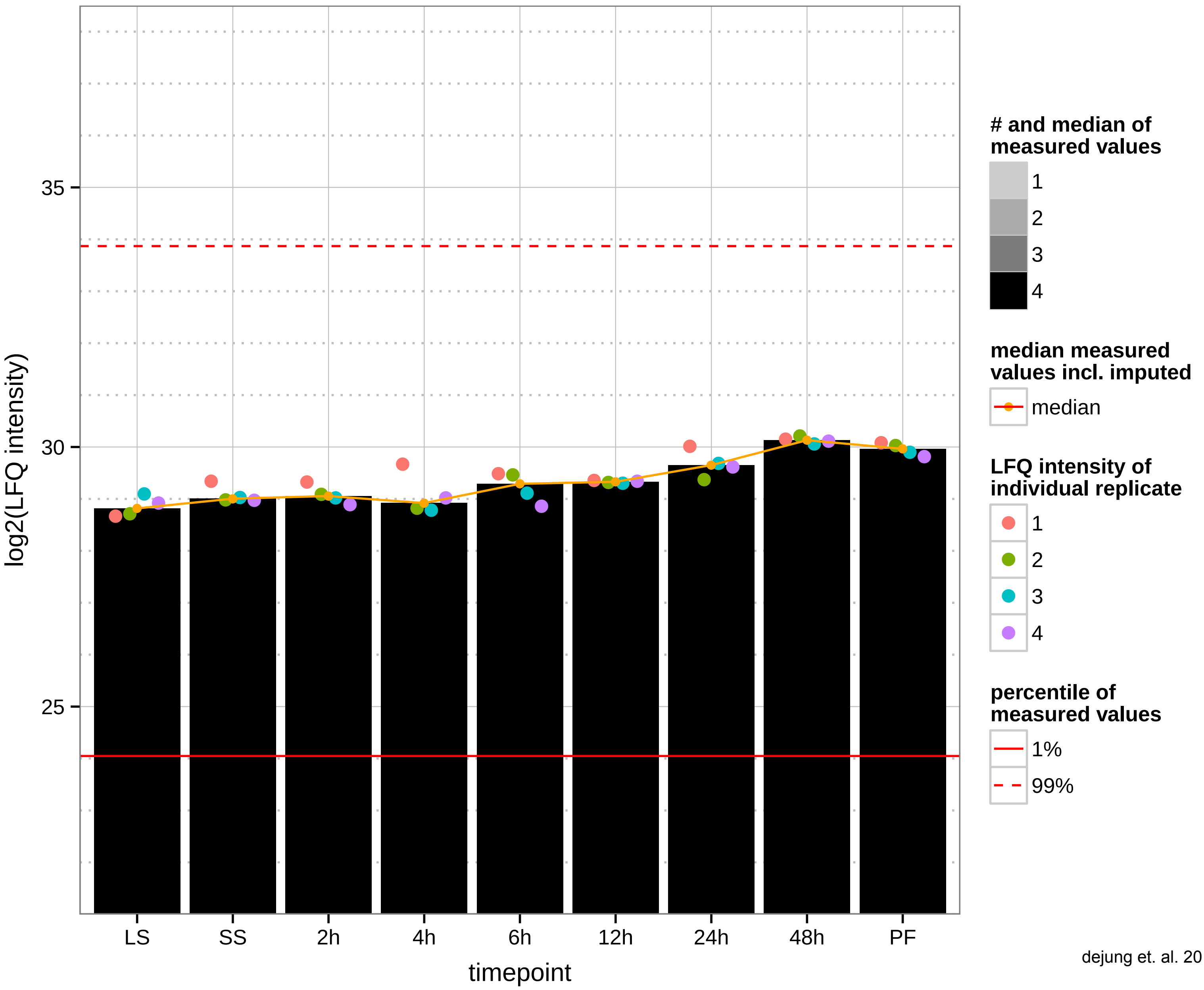

dejung et. al. 2015

hypothetical protein, conserved
Tb927.9.9660
AGOF: null
AGOC: mitochondrion
AGOP: null
PGOF: null
PGOC: null
PGOP: null

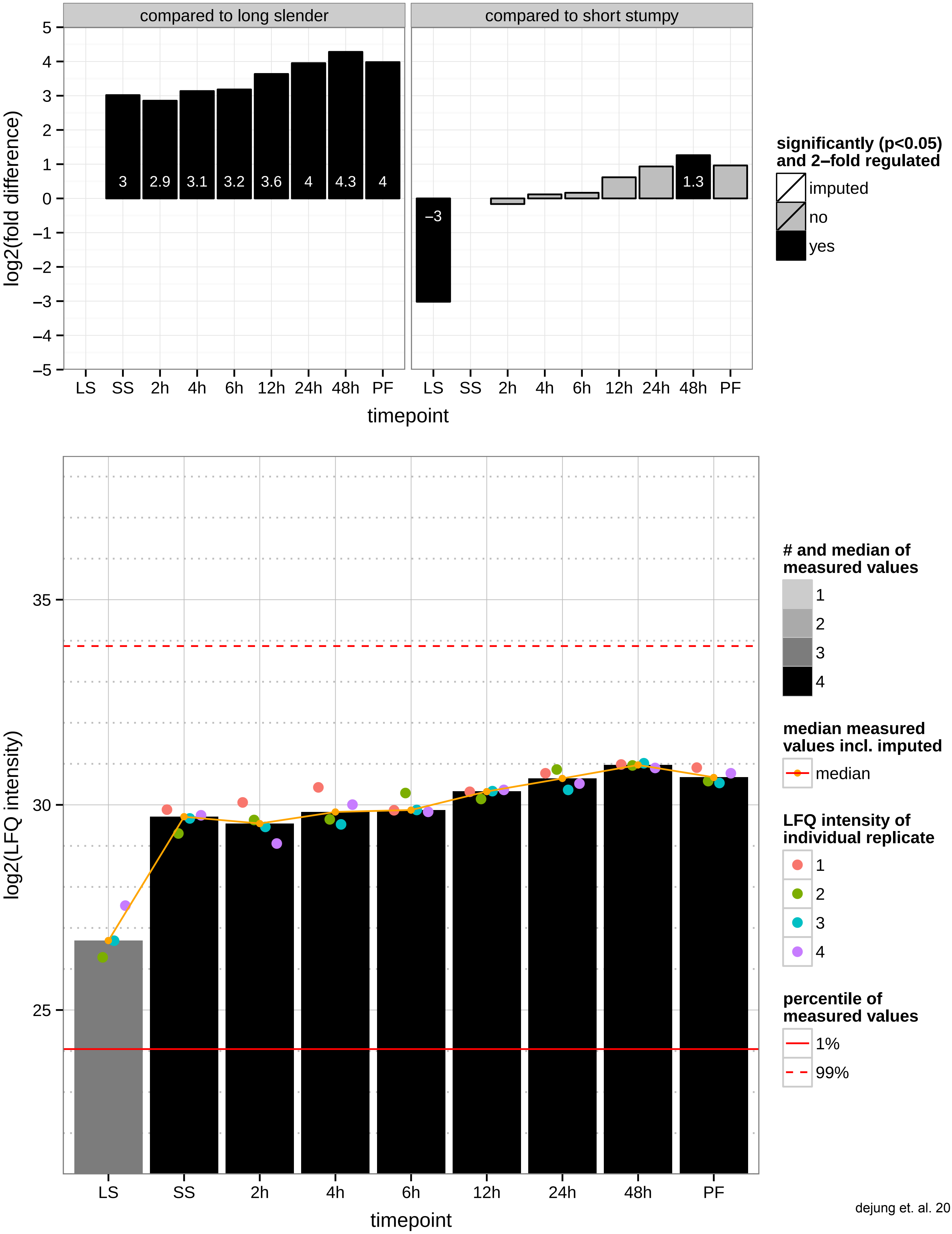

dejung et. al. 2015

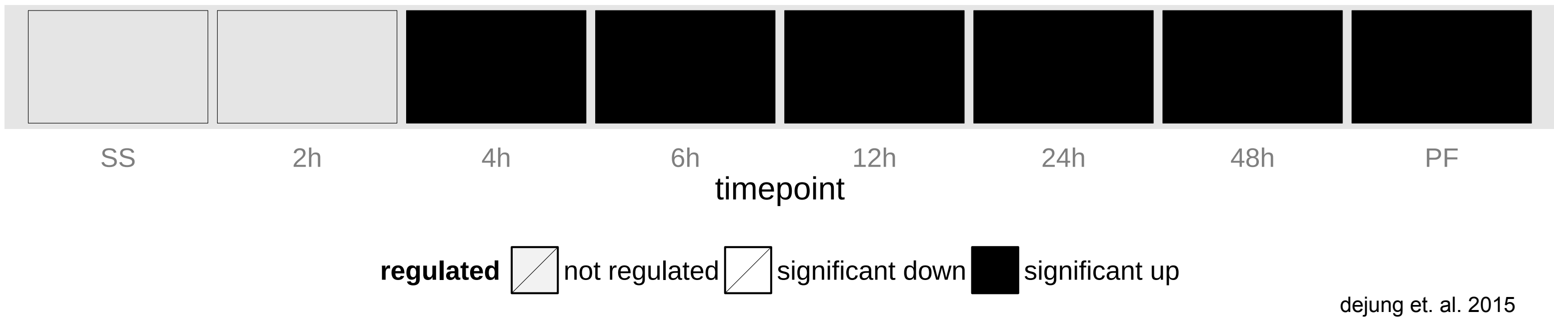
SS
2h
4h
6h
12h
24h
48h
PF
timepoint
regulated
not regulated
significant down
significant up
dejung et. al. 2015

hypothetical protein, conserved, trans–sialidase, putative
Tb927.5.440;Tb11.v5.0223
AGOF: null, exo–alpha–sialidase activity
AGOC: null
AGOP: null, pathogenesis
PGOF: null
PGOC: null
PGOP: null

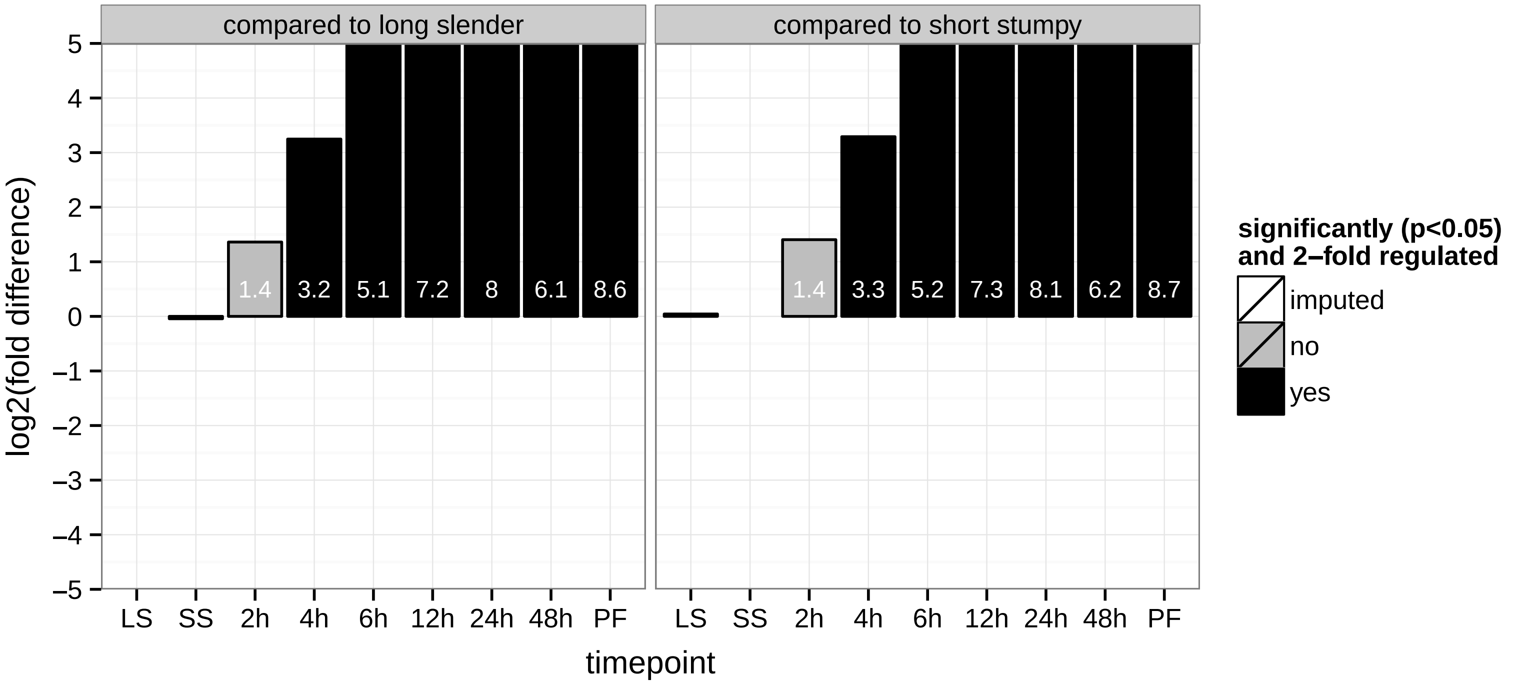

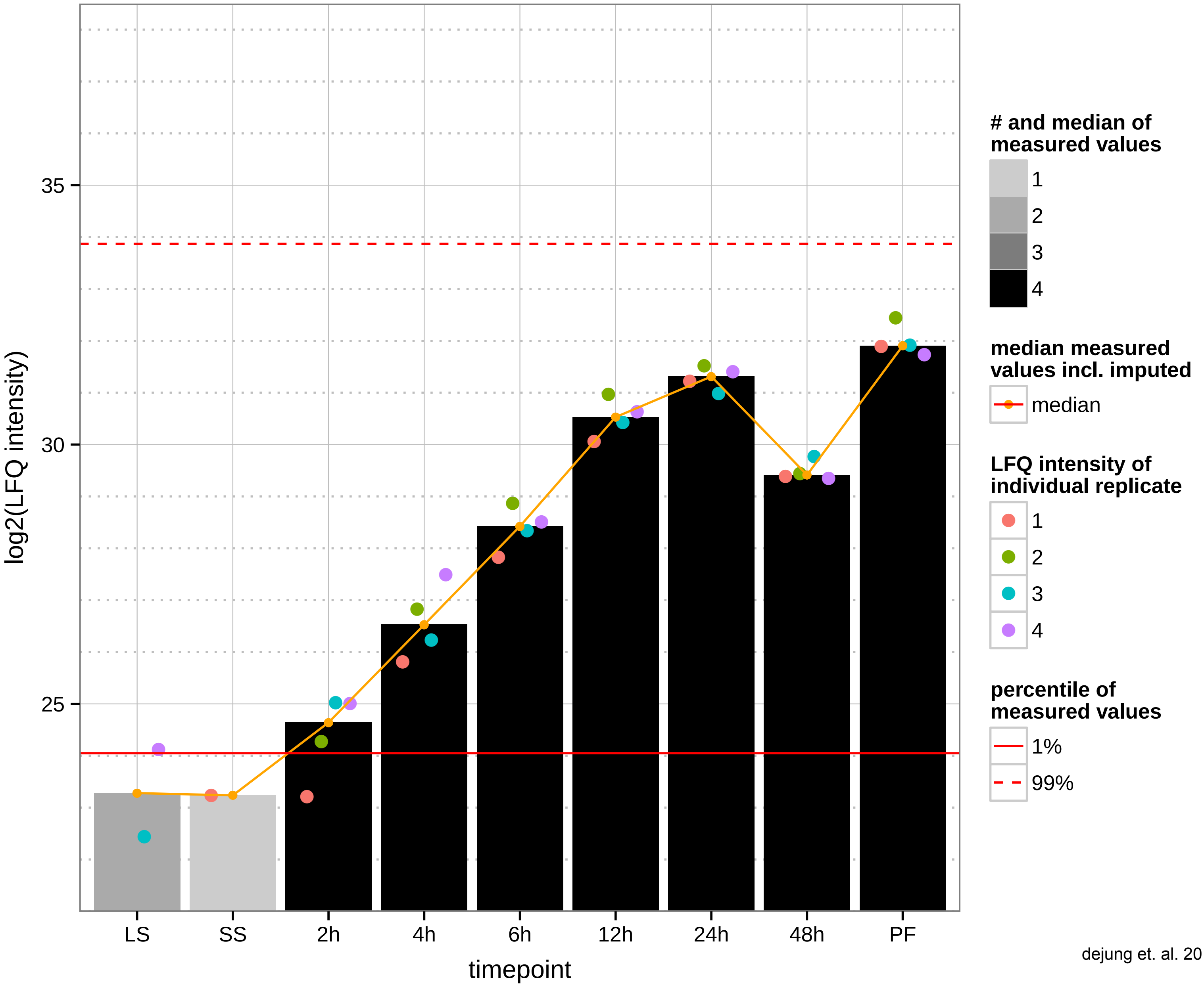

dejung et. al. 2015

hypothetical protein, conserved, mitochondrial RNA binding complex 1 subunit (PPR5)
Tb927.10.380;Tb11.v5.0637
AGOF: null, RNA binding
AGOC: null, mitochondrion
AGOP: null, RNA polyadenylation
PGOF: null
PGOC: null
PGOP: null

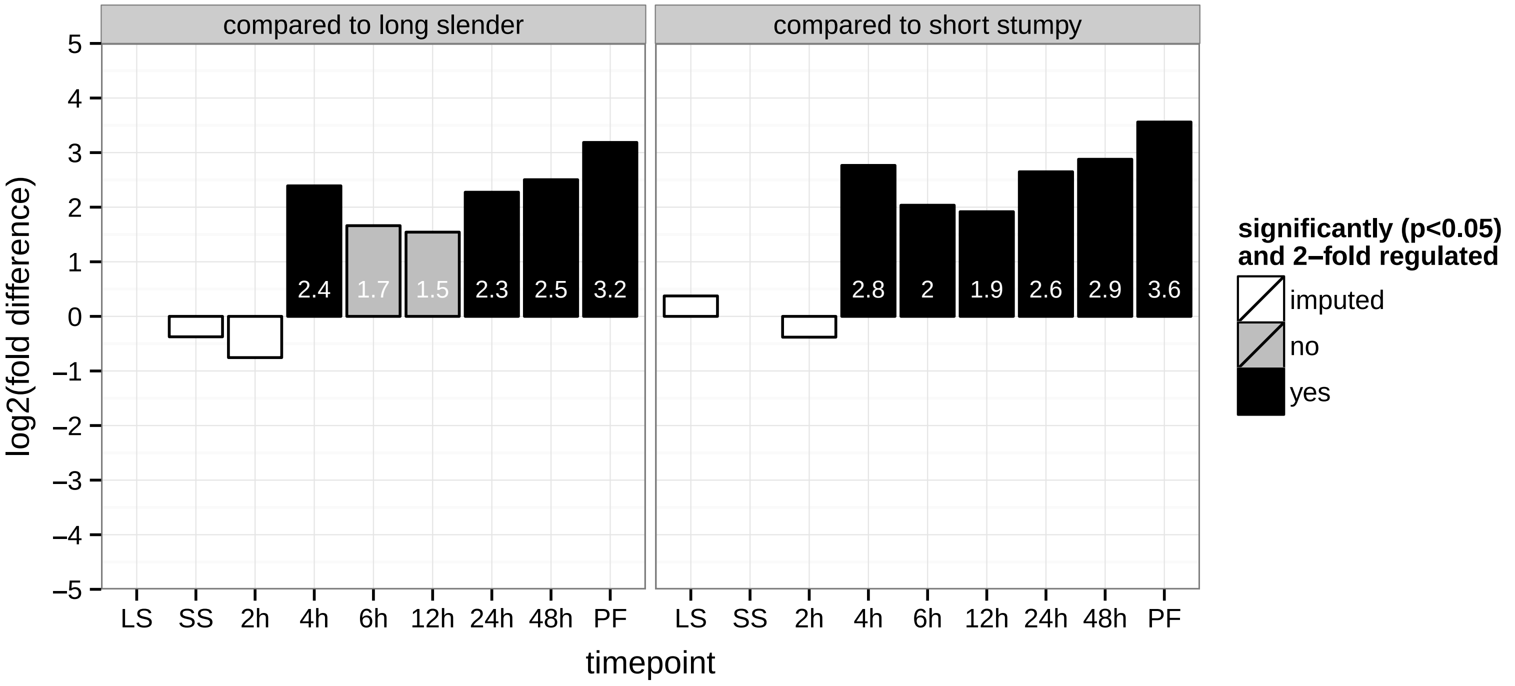

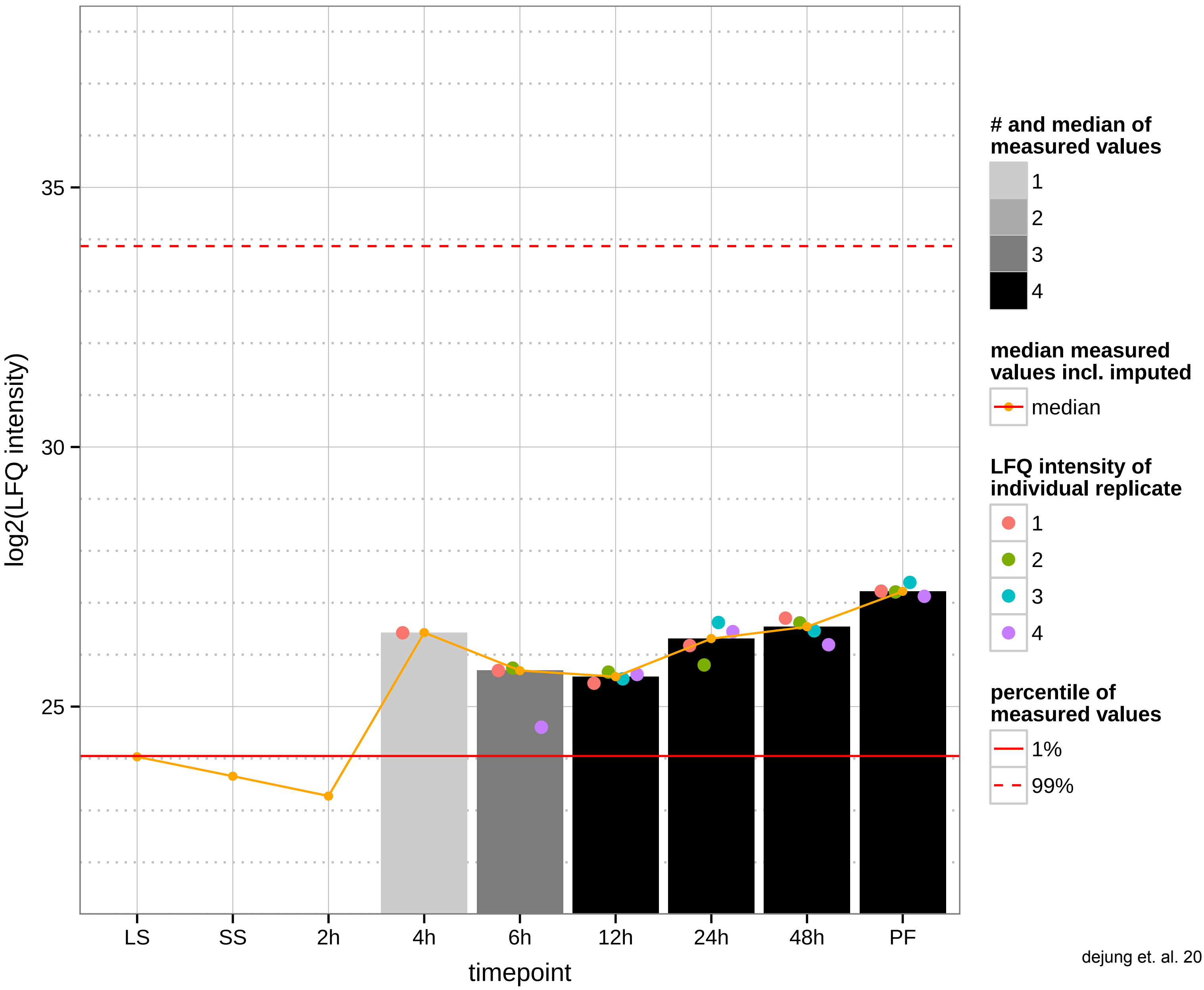

dejung et. al. 2015

hypothetical protein, conserved
Tb11.v5.0775;Tb927.10.7560
AGOF: null, methyltransferase activity
AGOC: null
AGOP: null, metabolic process
PGOF: methyltransferase activity
PGOC: null
PGOP: metabolic process

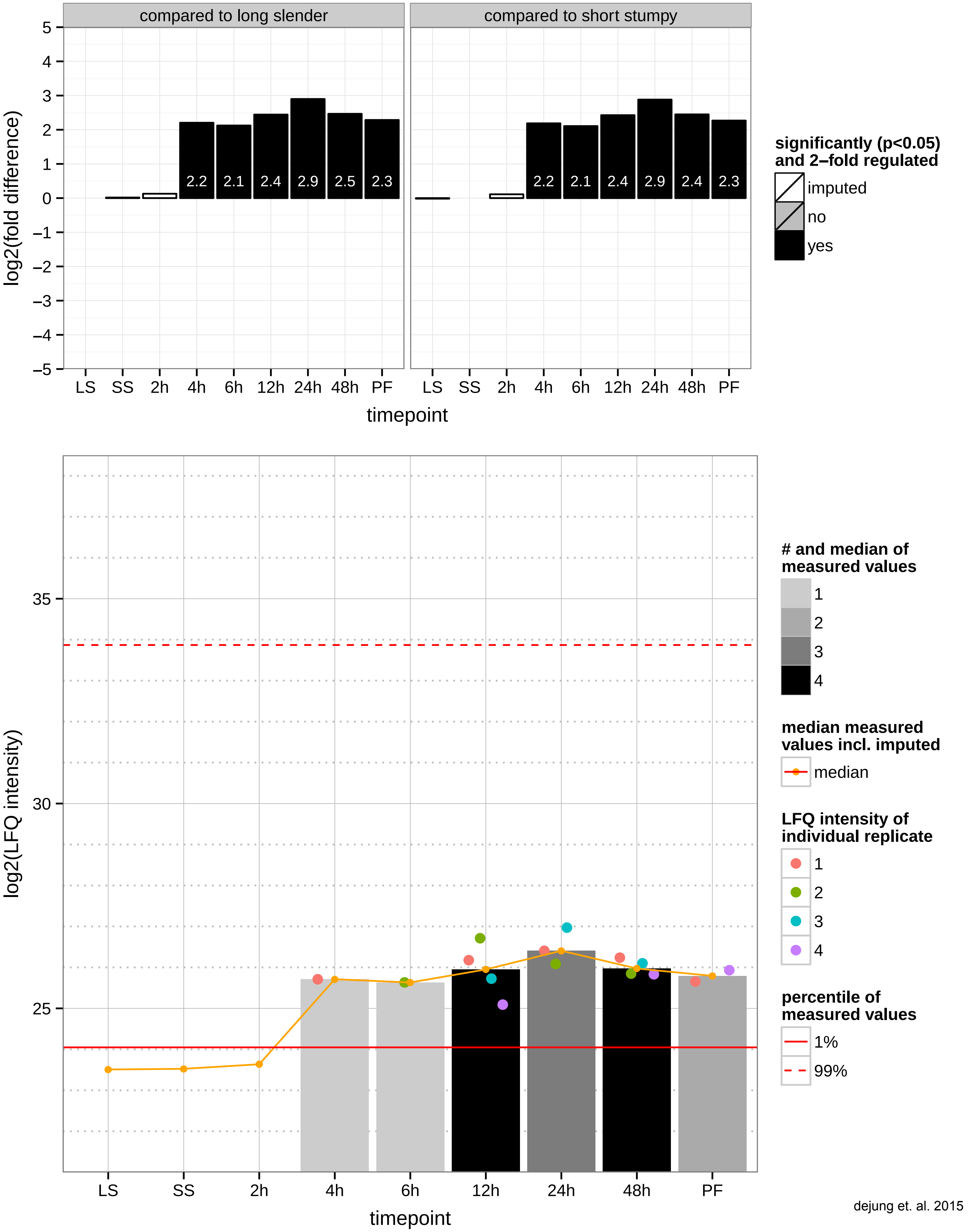

dejung et. al. 2015

hypothetical protein, conserved
Tb927.1.2210
AGOF: null
AGOC: null
AGOP: null
PGOF: null
PGOC: nucleus
PGOP: nucleosome assembly

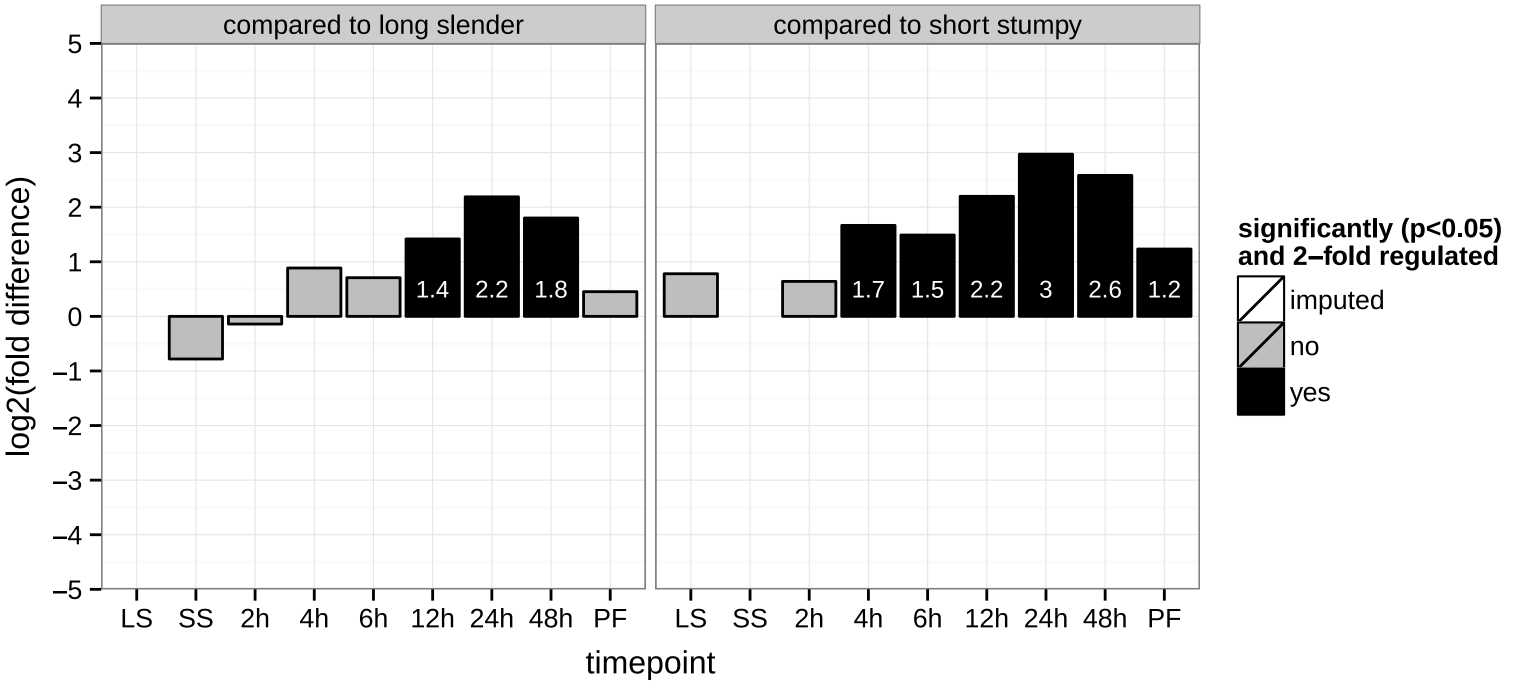

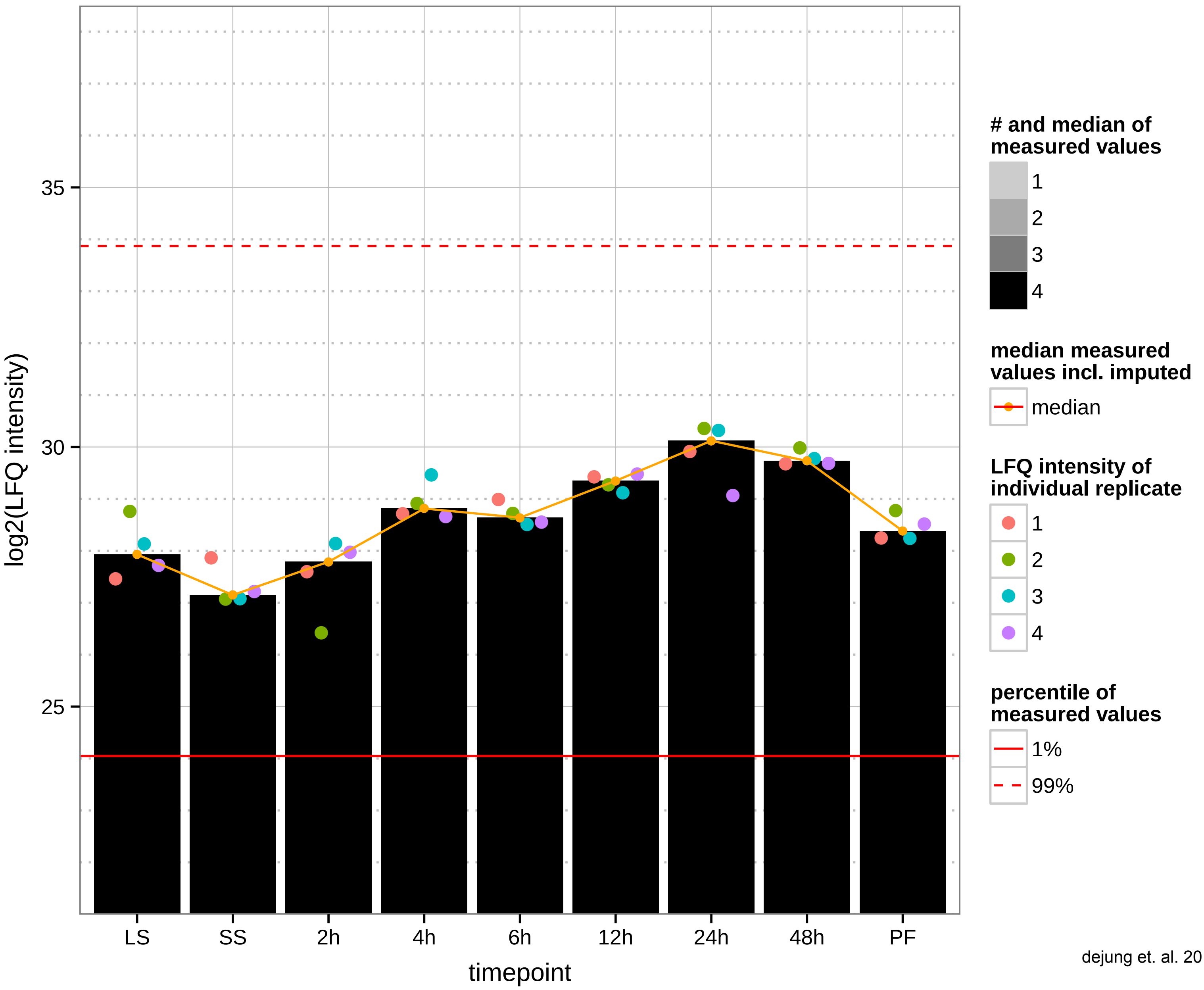

dejung et. al. 2015

hypothetical protein, conserved
Tb927.1.3070
AGOF: null
AGOC: null
AGOP: null
PGOF: null
PGOC: null
PGOP: null

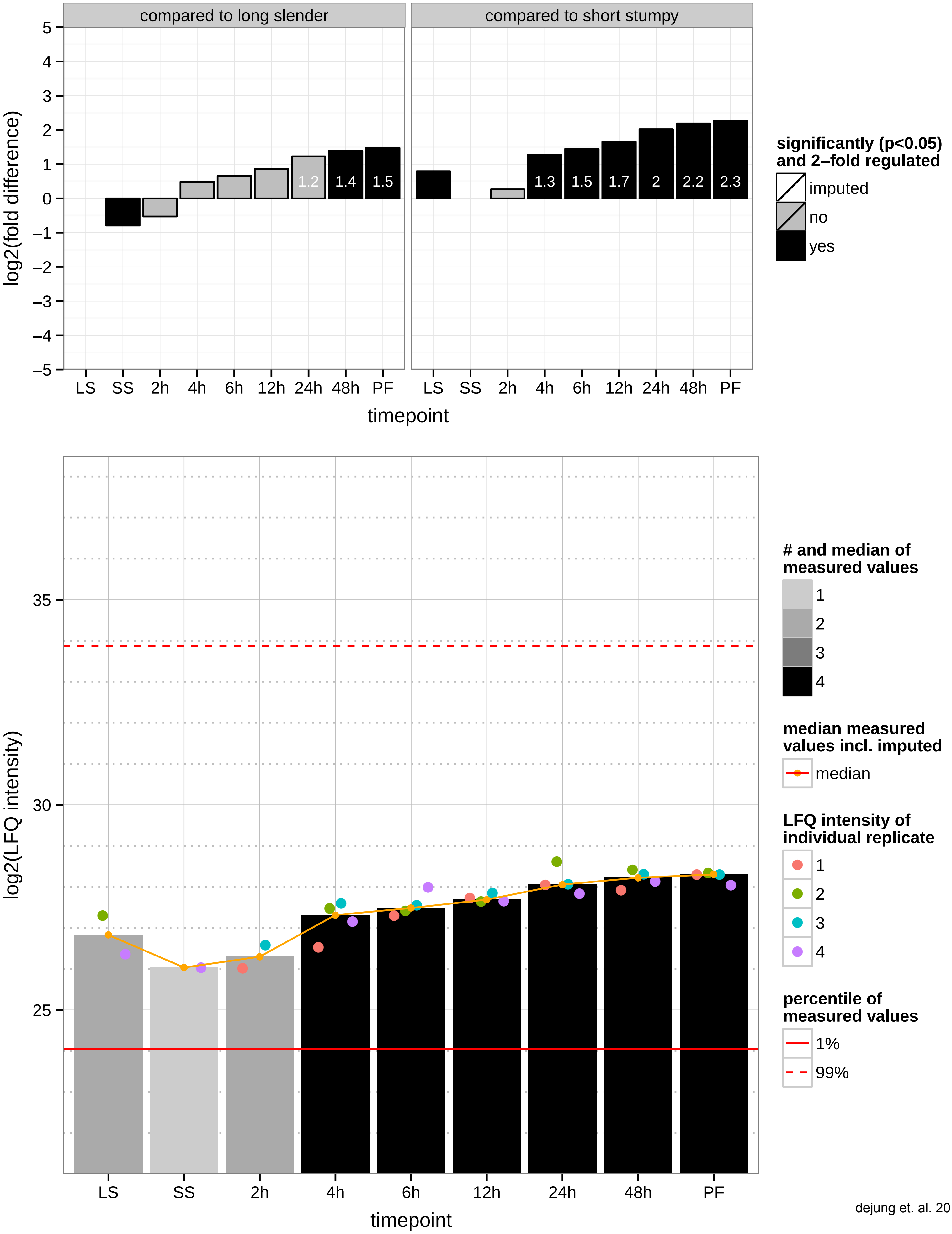

dejung et. al. 2015

cytochrome oxidase subunit IV (COXIV)
Tb927.1.4100
AGOF: cytochrome−c oxidase activity
AGOC: mitochondrial respiratory chain complex IV, mitochondrion
AGOP: oxidation−reduction process
PGOF: null
PGOC: null
PGOP: null

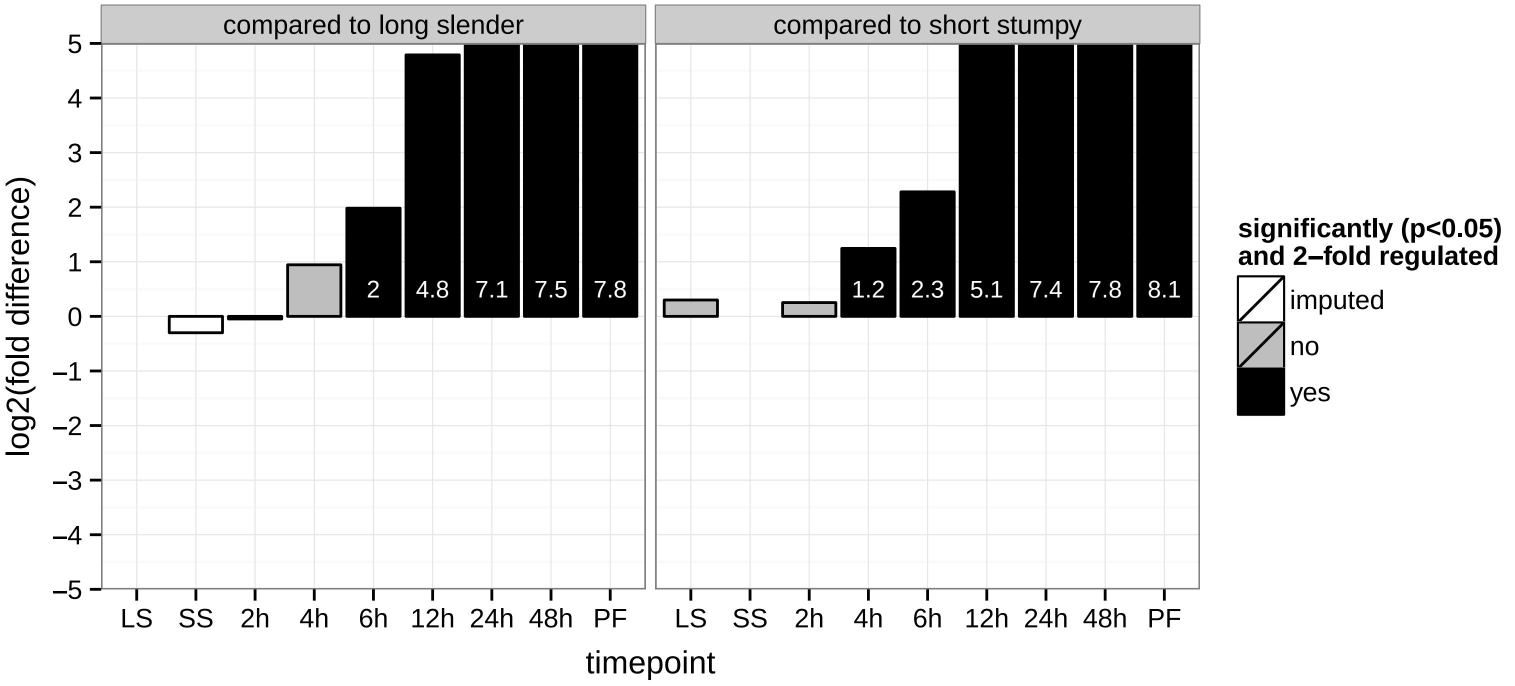

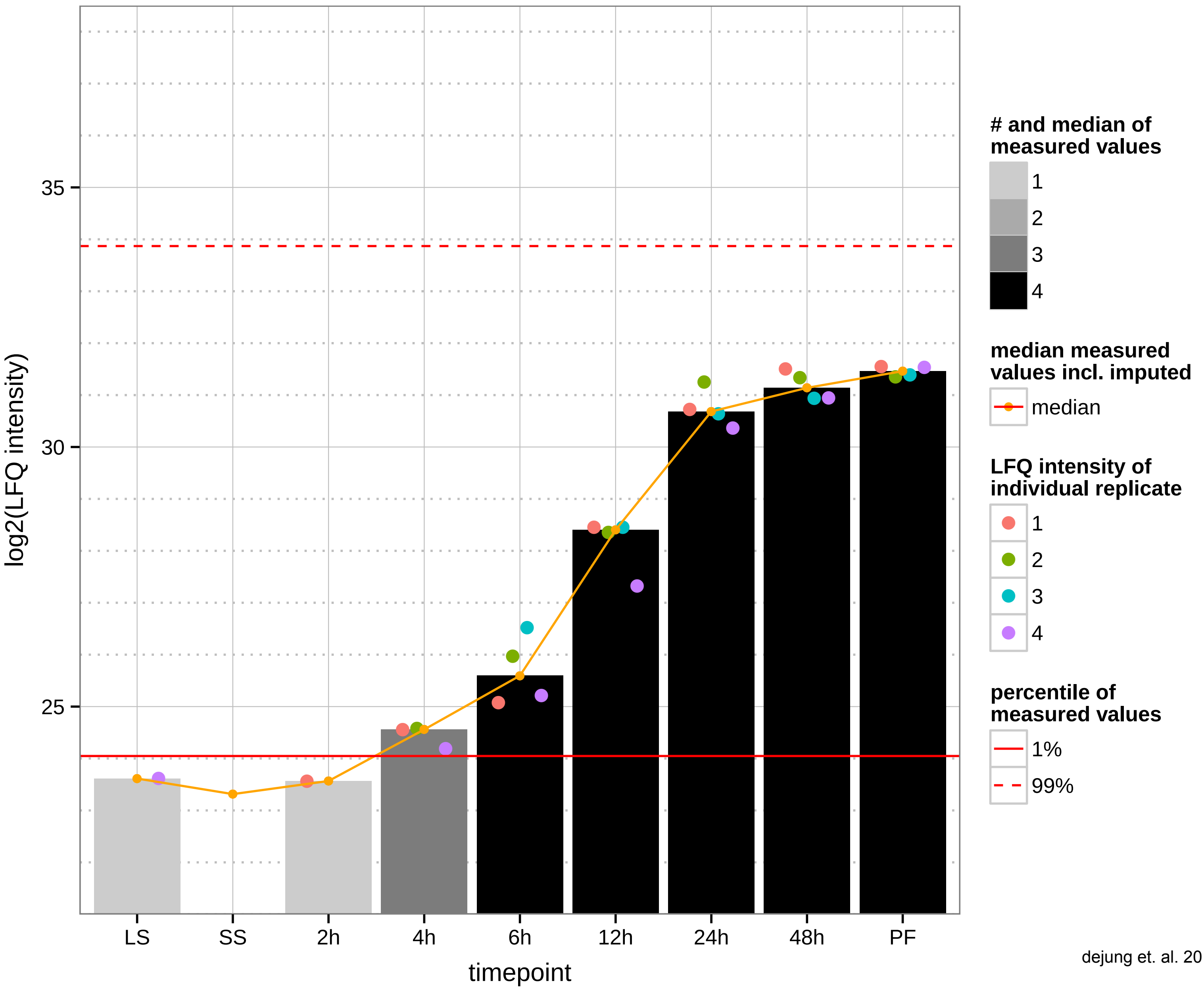

dejung et. al. 2015

hypothetical protein, conserved
Tb927.1.560
AGOF: null
AGOC: null
AGOP: null
PGOF: protein binding
PGOC: null
PGOP: null

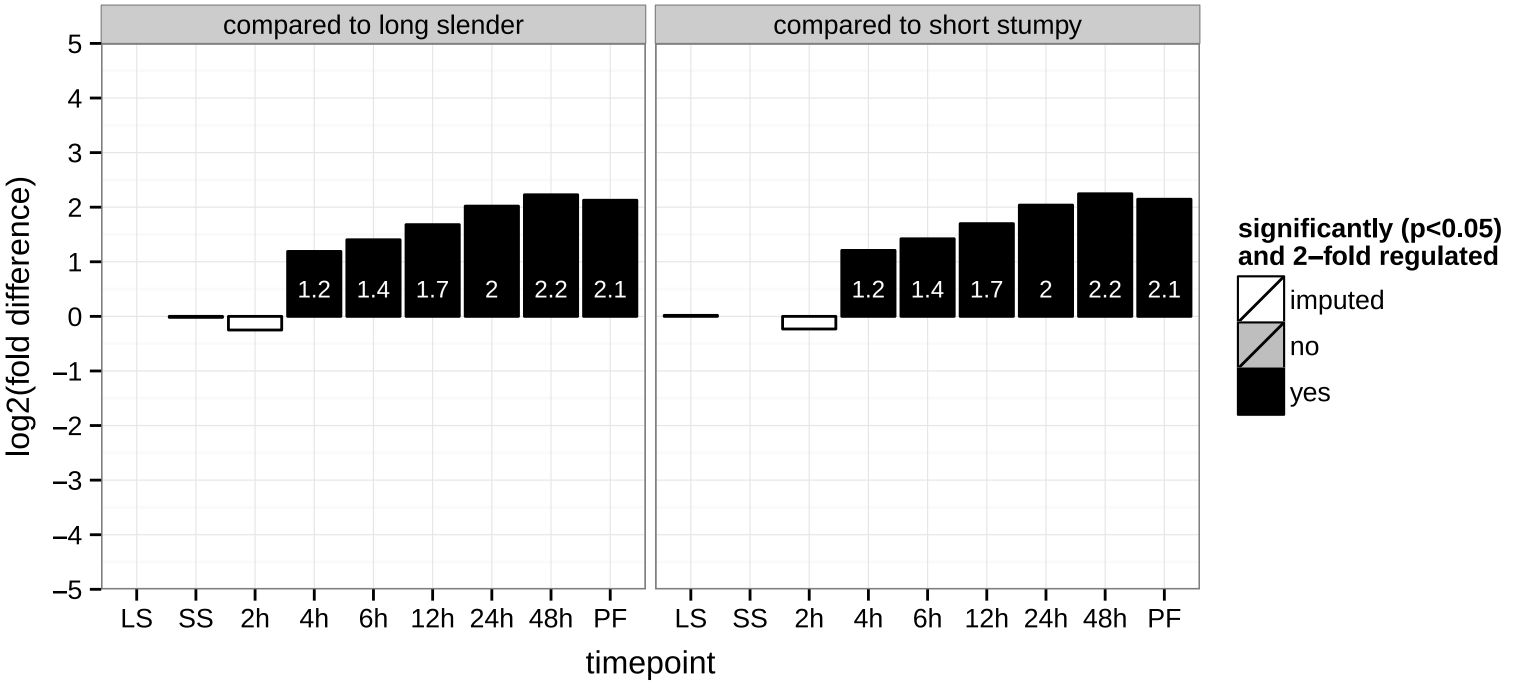

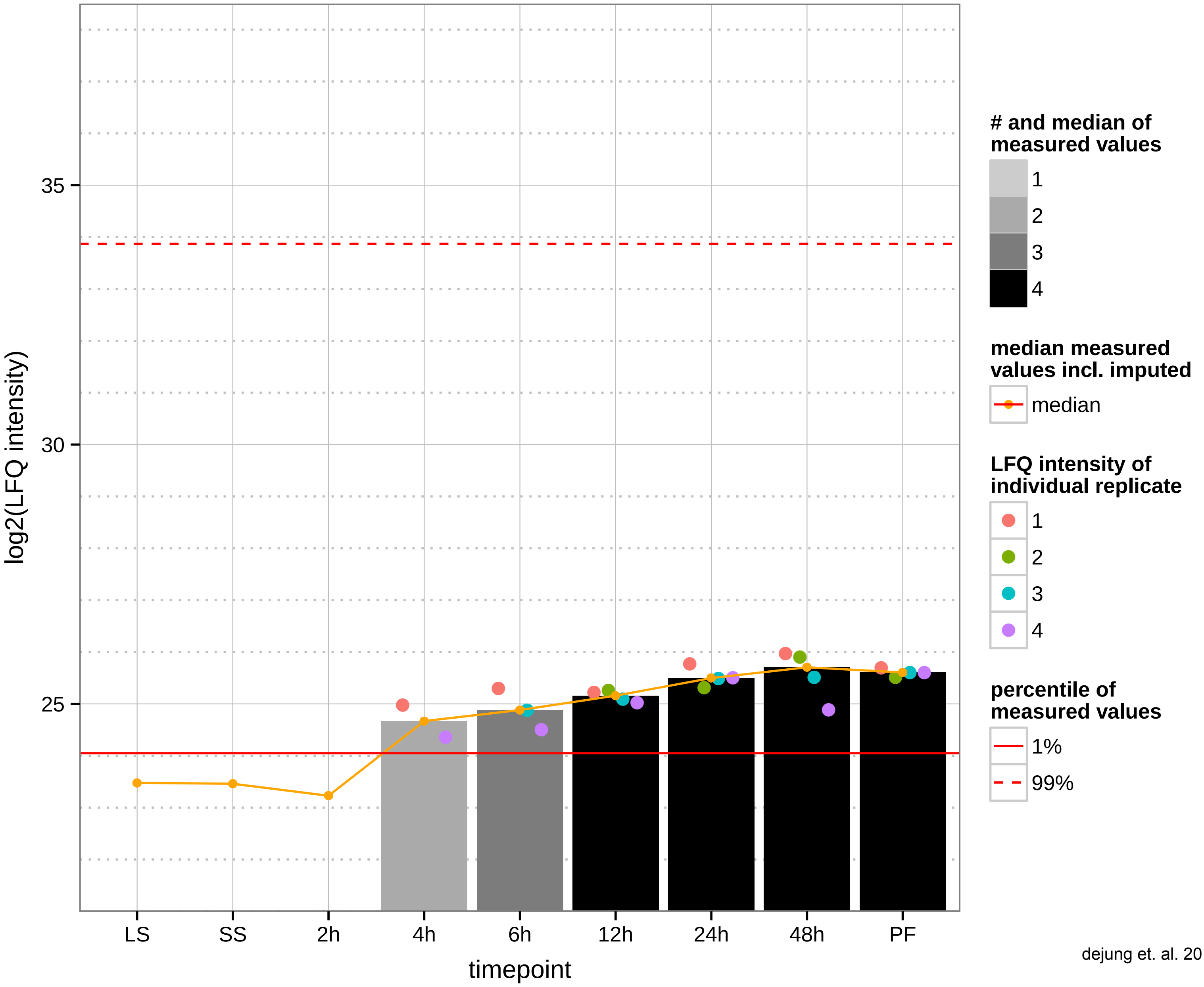

dejung et. al. 2015

splicing factor Prp31
Tb927.10.10700
AGOF: RNA binding
AGOC: nucleus
AGOP: RNA splicing
PGOF: null
PGOC: null
PGOP: null

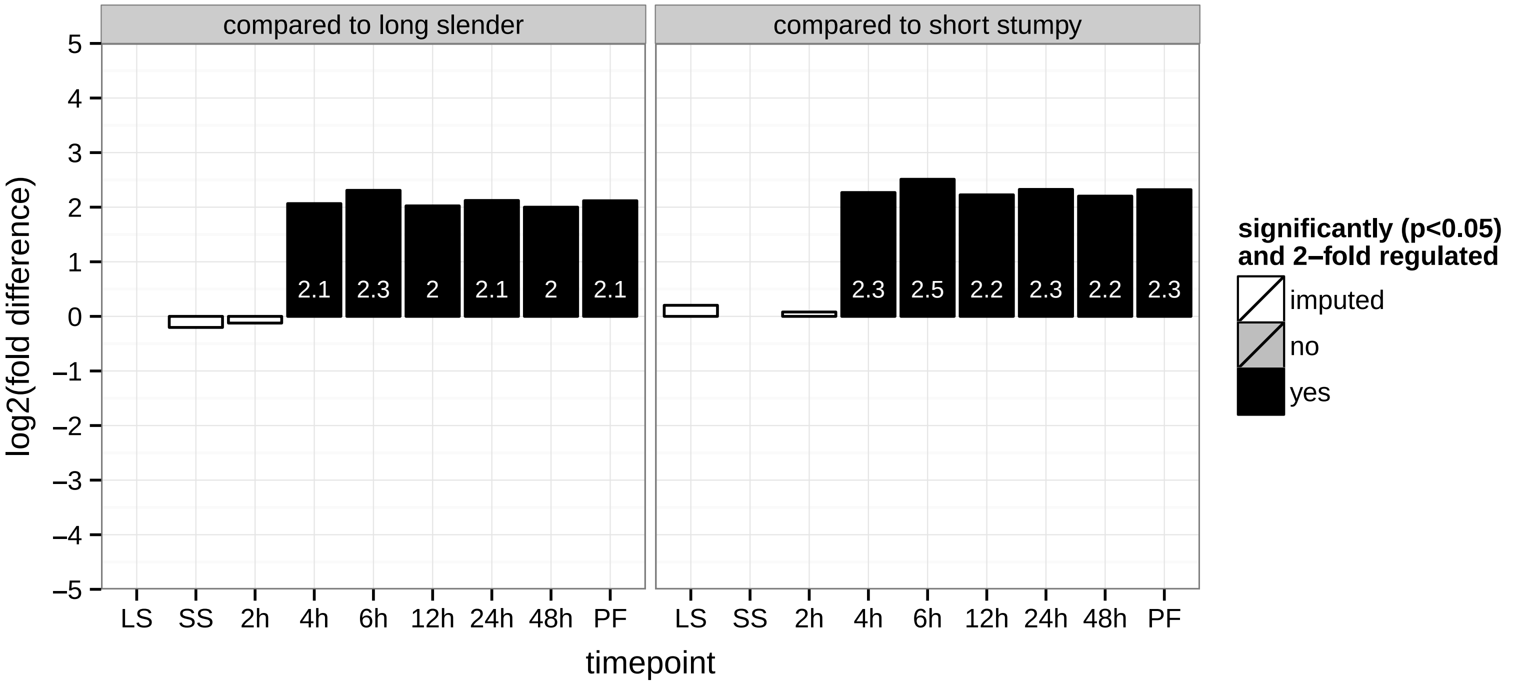

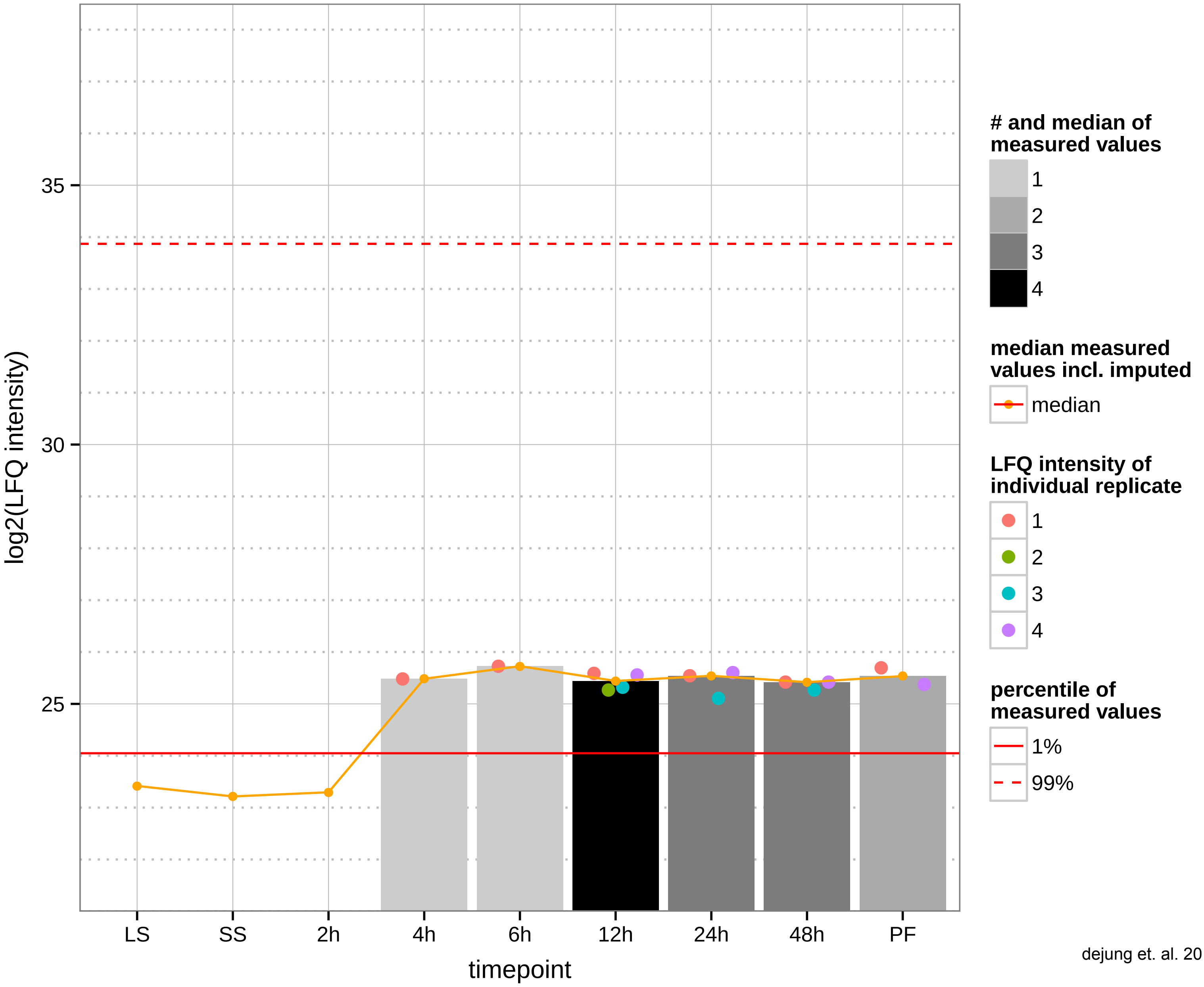

dejung et. al. 2015

Ubiquitin–like modifier–activating enzyme ATG7, putative, Autophagy–related protein 7 (ATG7)
Tb927.10.11180
AGOF: catalytic activity
AGOC: null
AGOP: autophagy, protein targeting to vacuole
PGOF: catalytic activity
PGOC: null
PGOP: null

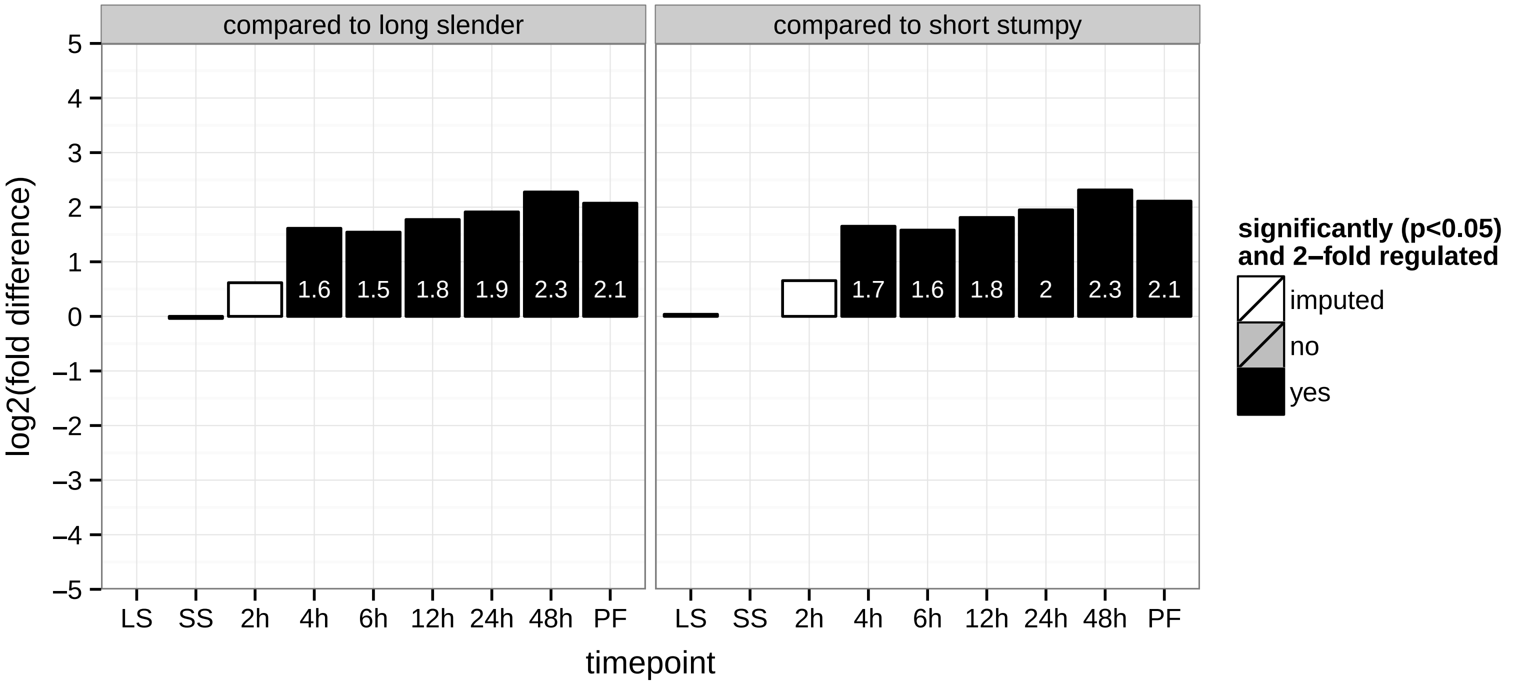

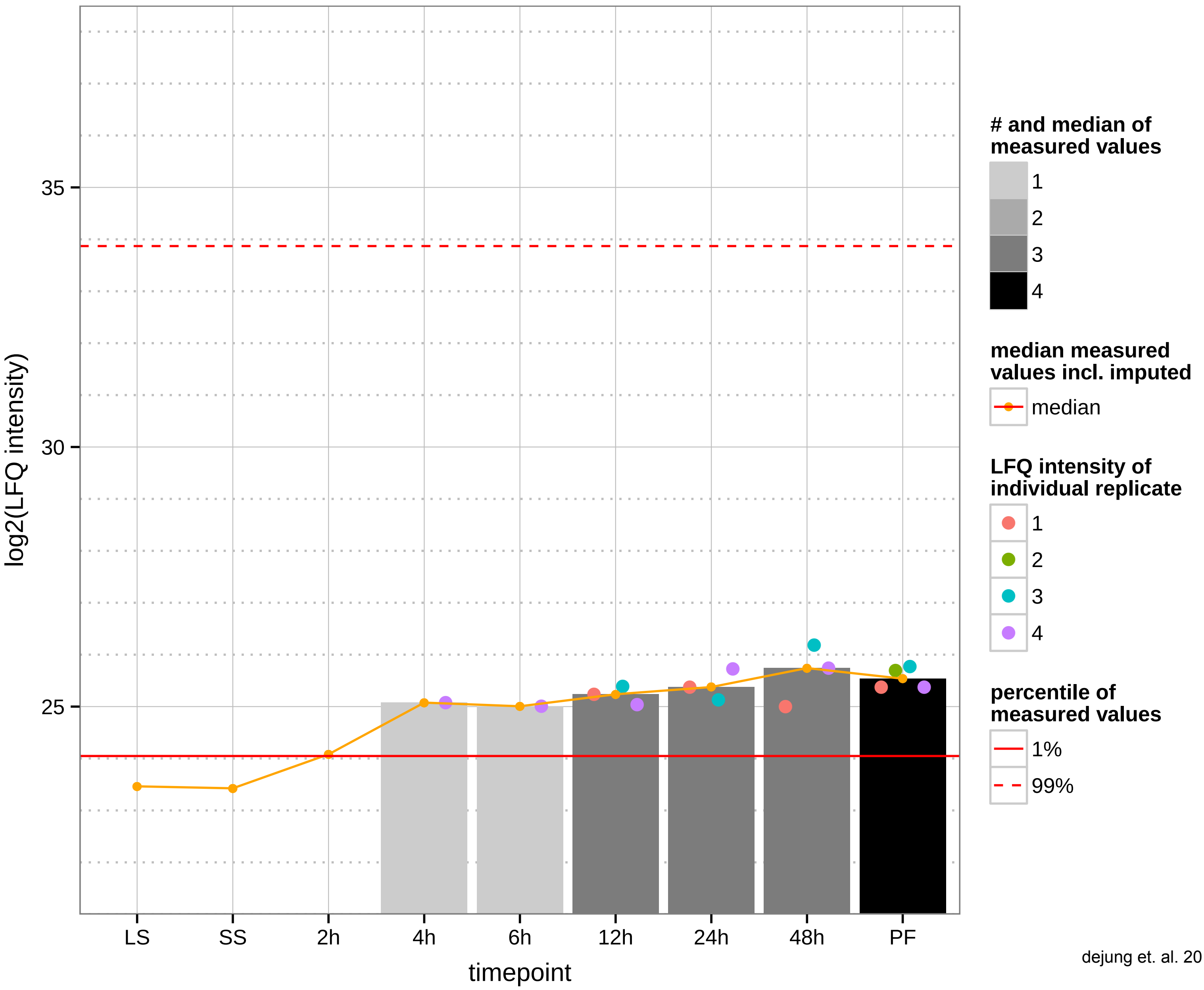

dejung et. al. 2015

hypothetical protein, conserved
Tb927.10.11370
AGOF: null
AGOC: mitochondrion
AGOP: null
PGOF: null
PGOC: null
PGOP: null

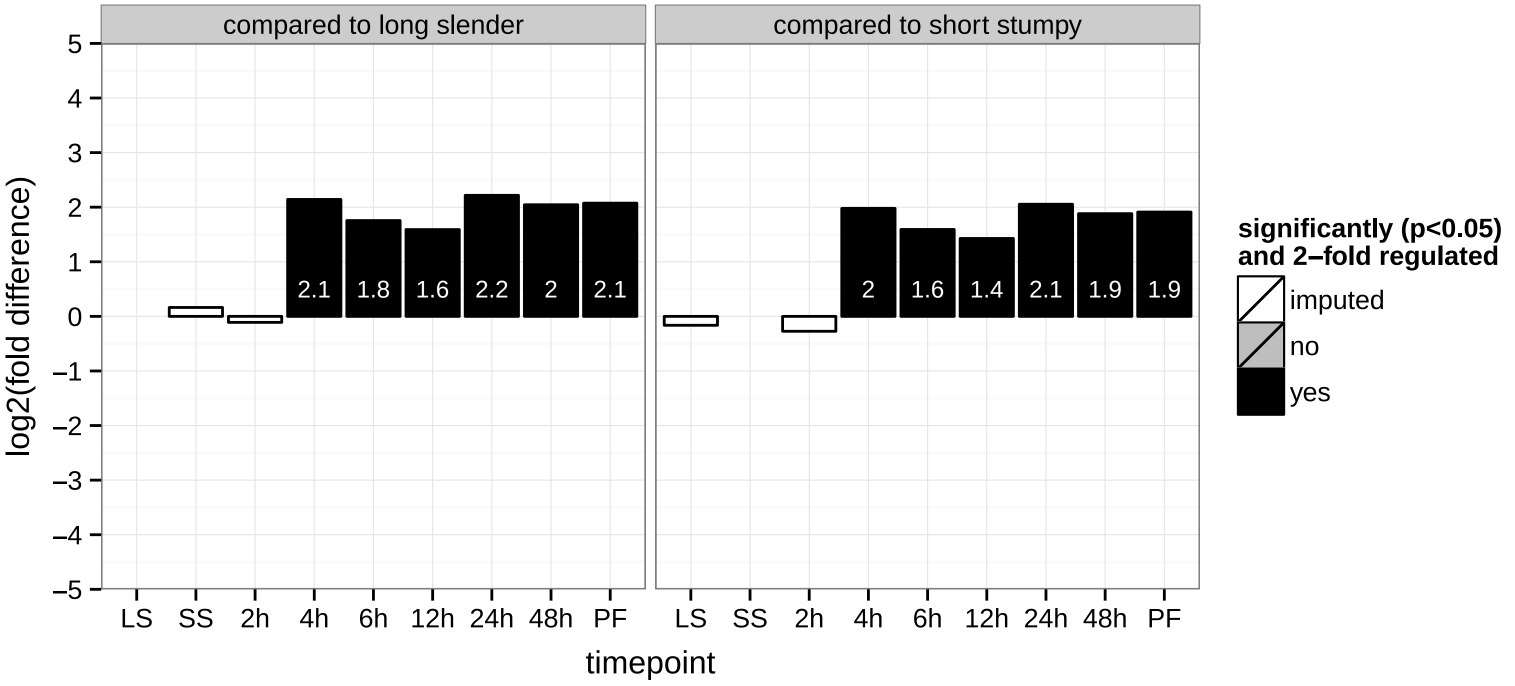

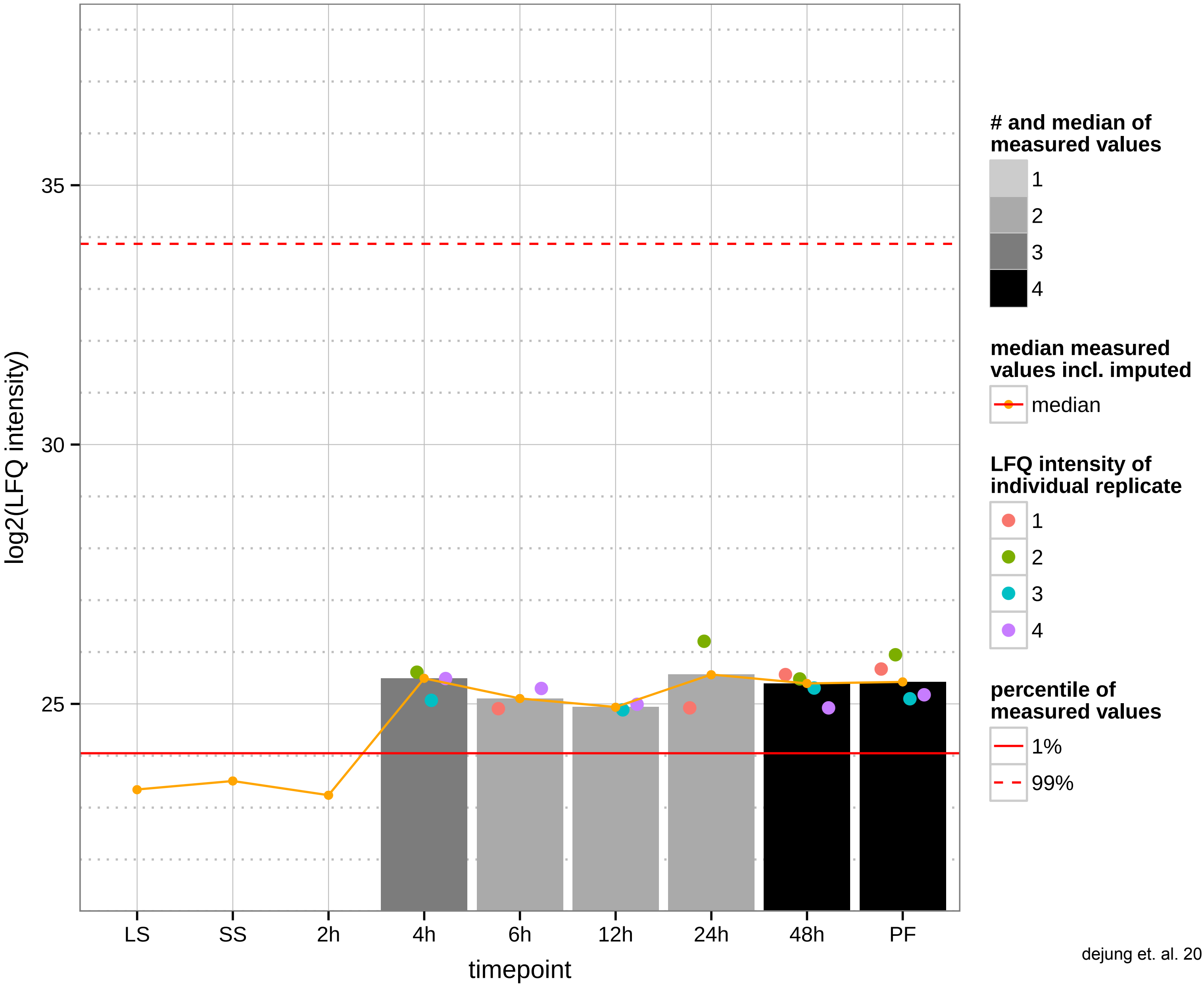

dejung et. al. 2015

hypothetical protein, conserved
Tb927.10.1310
AGOF: null
AGOC: null
AGOP: null
PGOF: binding
PGOC: null
PGOP: null

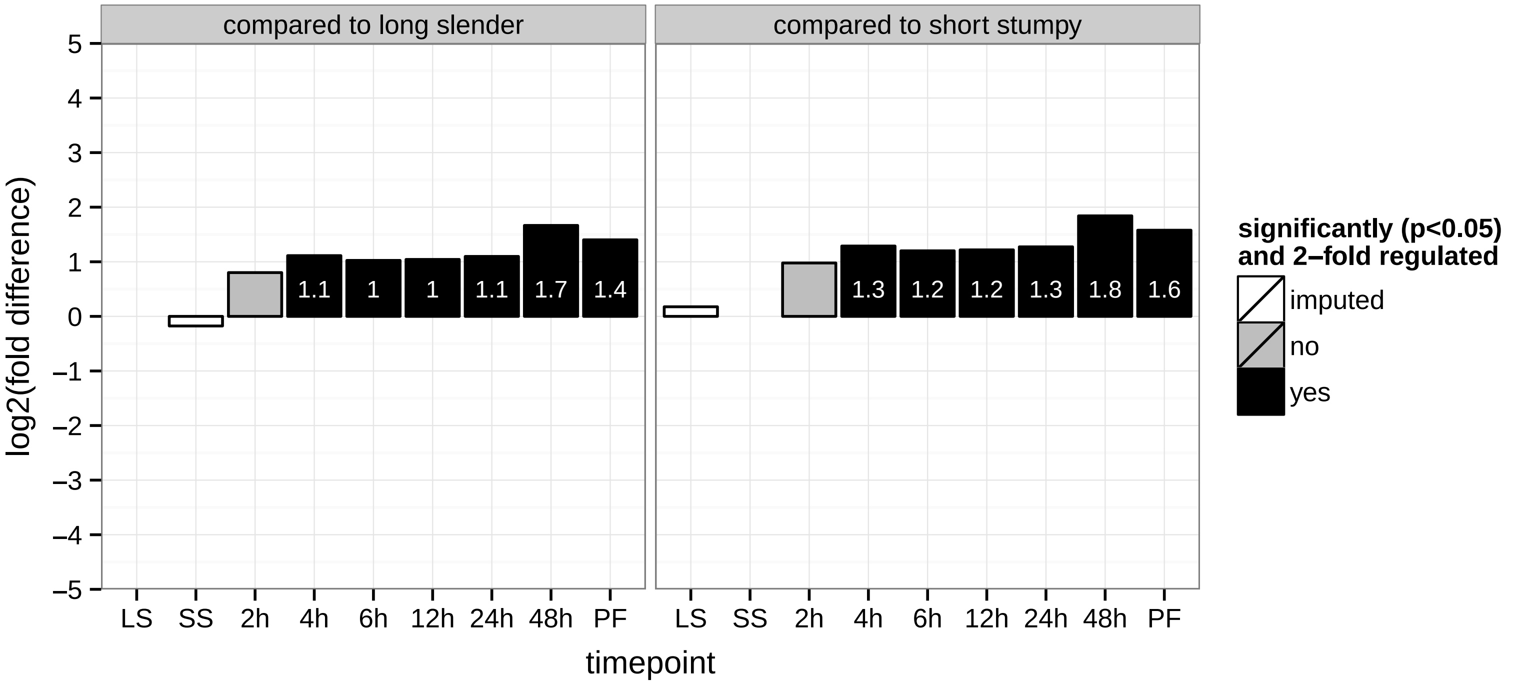

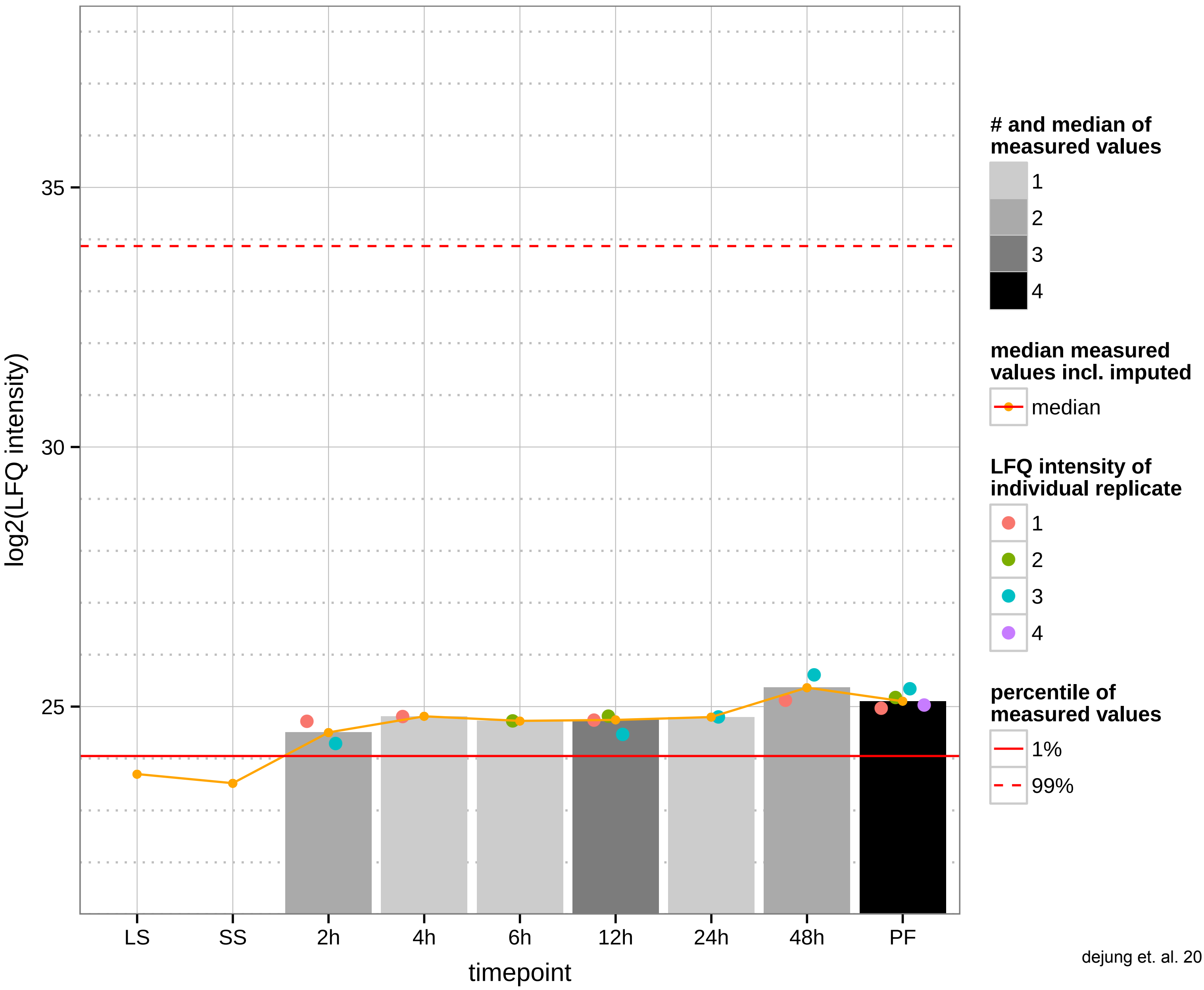

dejung et. al. 2015

hypothetical protein, conserved
Tb927.10.14330
AGOF: null
AGOC: small−subunit processome
AGOP: rRNA processing
PGOF: null
PGOC: small−subunit processome
PGOP: rRNA processing

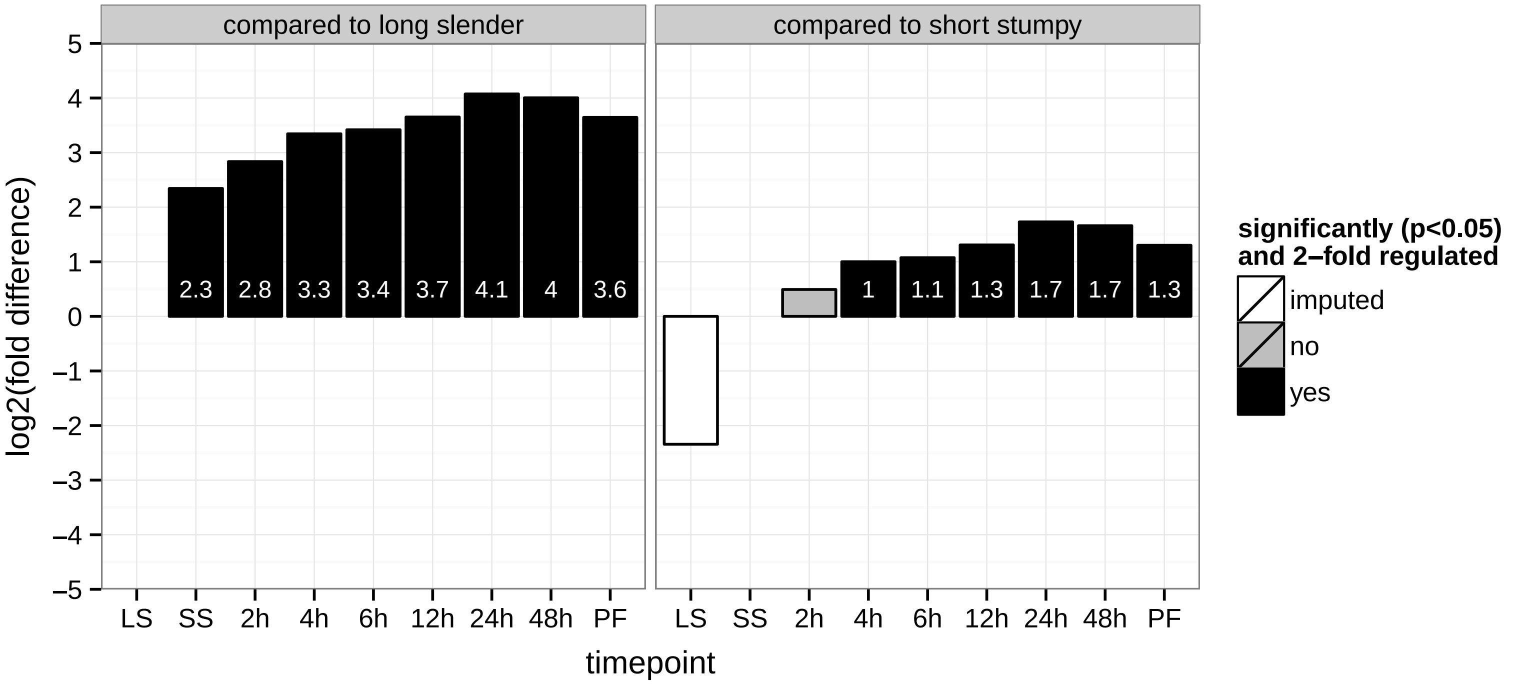

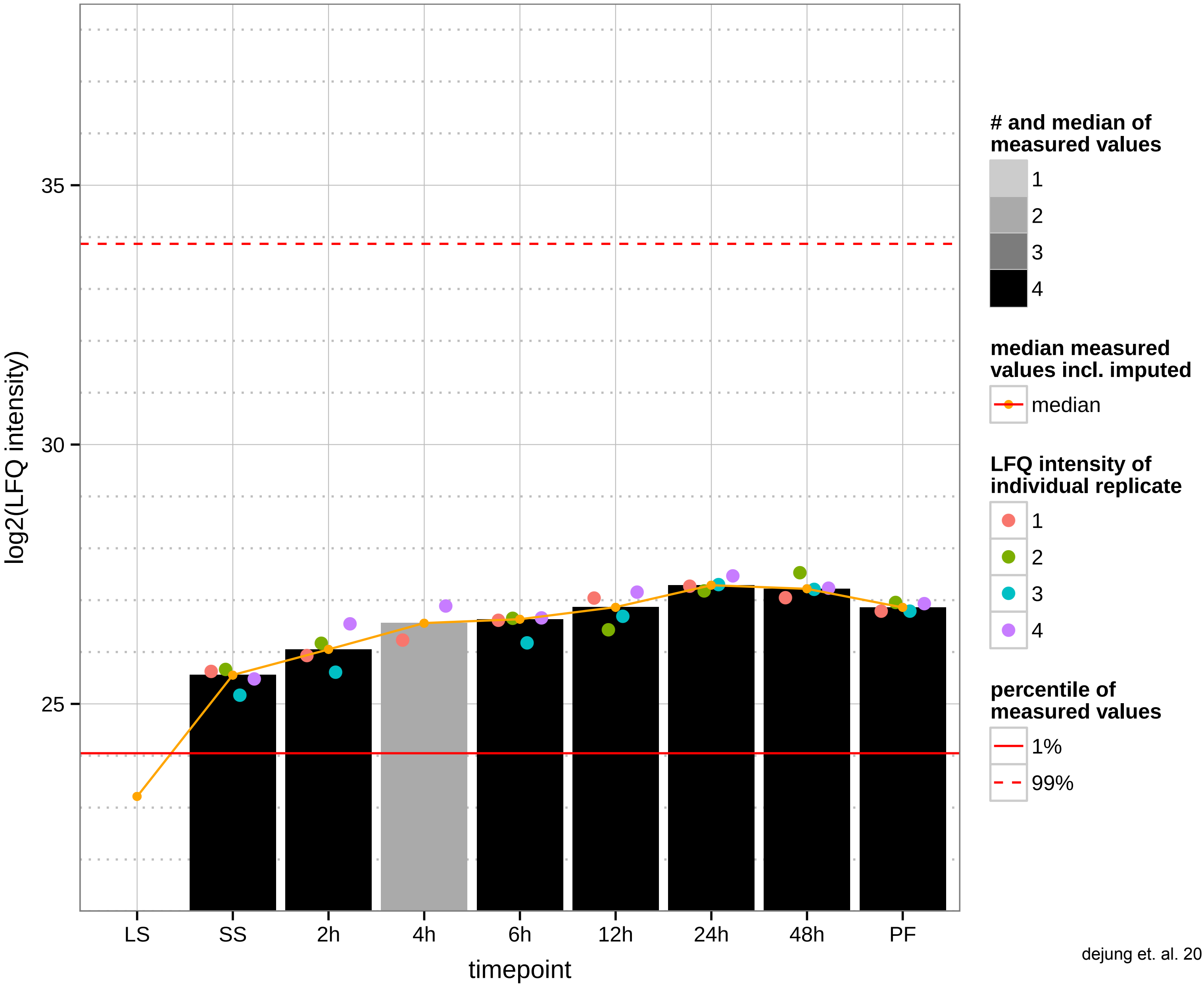

dejung et. al. 2015

chaperone protein DNAj, putative, variant surface glycoprotein (VSG)
Tb927.10.14730;Tb11.v5.1062
AGOF: null, heat shock protein binding, unfolded protein binding
AGOC: null
AGOP: null, protein folding
PGOF: heat shock protein binding, null
PGOC: null
PGOP: null

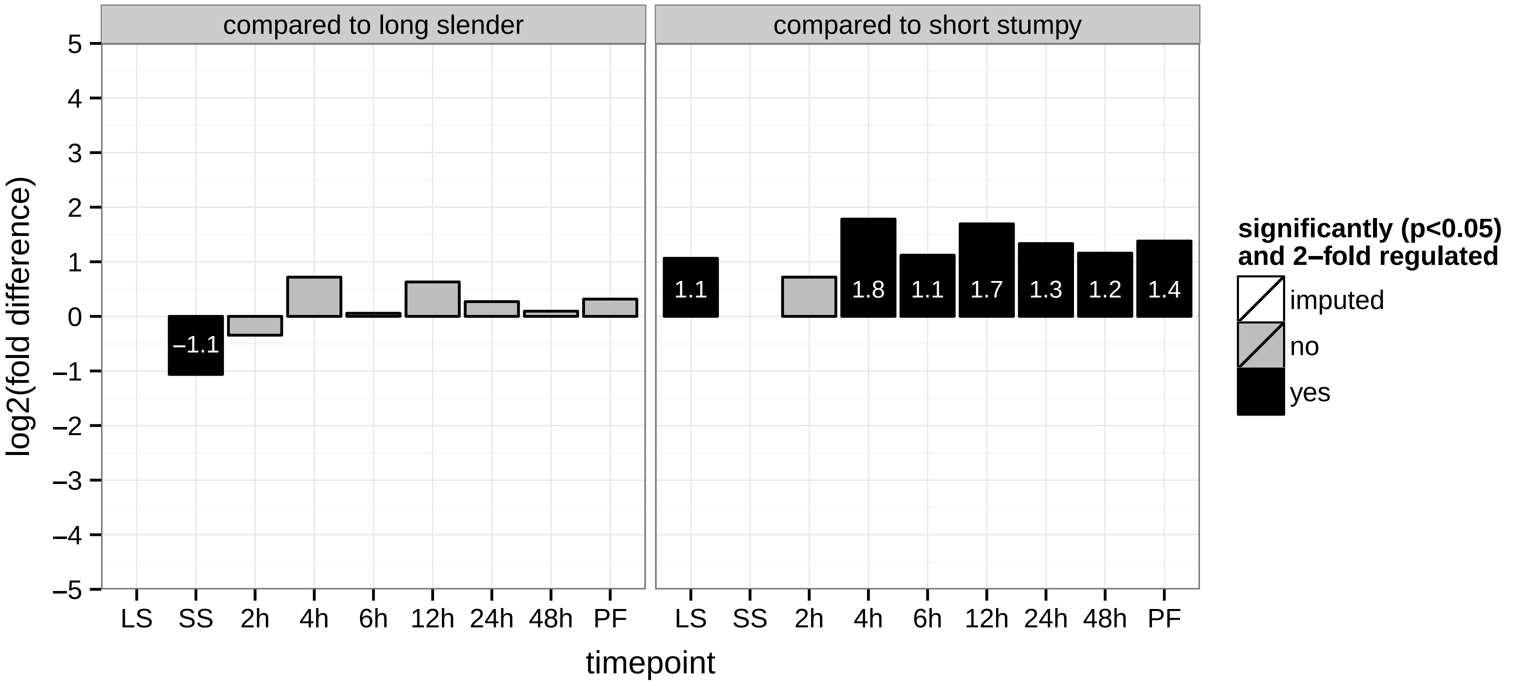

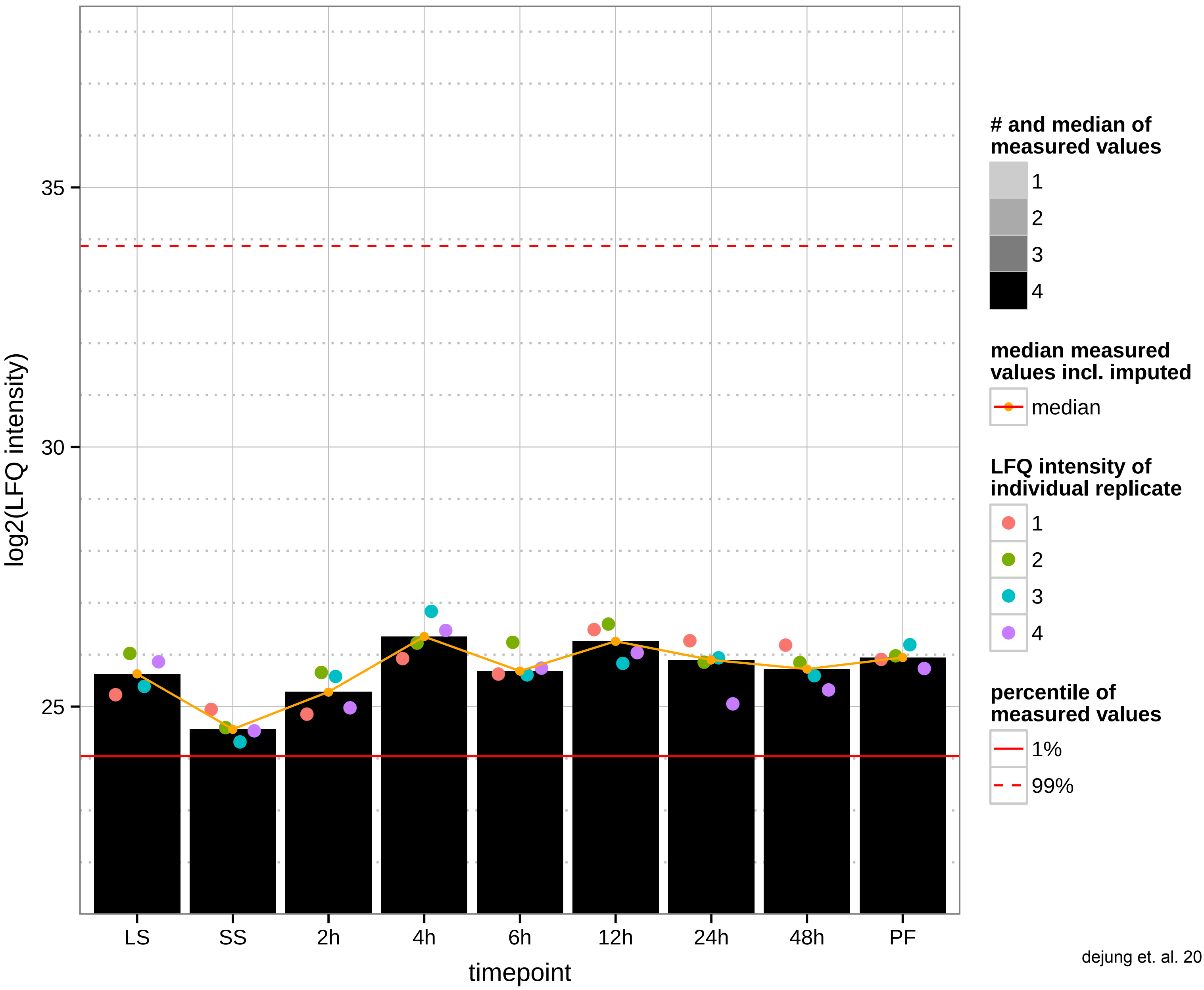

dejung et. al. 2015

dual specificity protein phosphatase, putative, phosphoserine/threonine/tyrosine−binding protein
Tb927.10.1620;Tb11.v5.0643
AGOF: null, protein tyrosine/serine/threonine phosphatase activity
AGOC: null
AGOP: null, protein dephosphorylation
PGOF: phosphatase activity, protein tyrosine/serine/threonine phosphatase activity
PGOC: null
PGOP: dephosphorylation, protein dephosphorylation

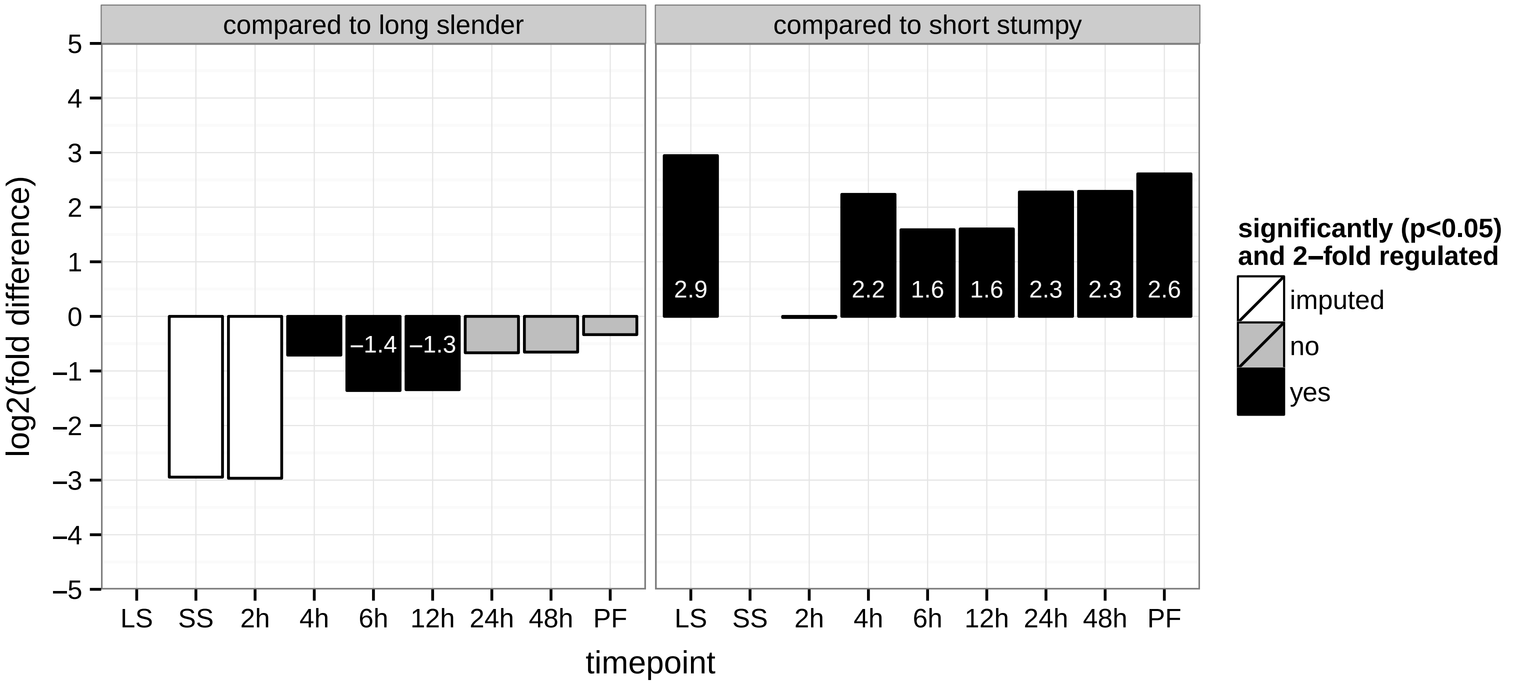

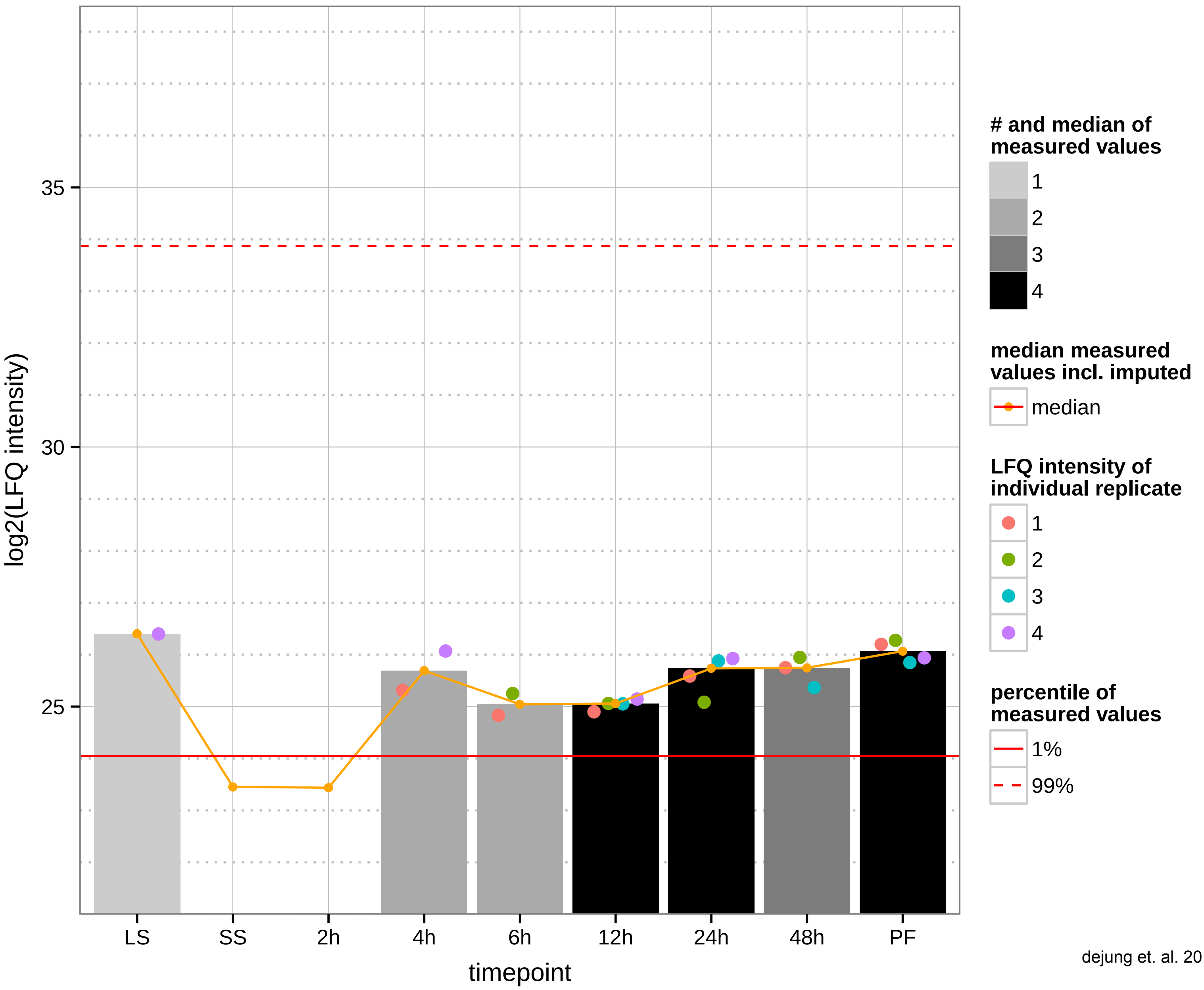

dejung et. al. 2015

malate dehydrogenase–related
Tb927.10.2550
AGOF: L–malate dehydrogenase activity
AGOC: mitochondrion
AGOP: glycolysis, malate metabolic process, oxidation–reduction process
PGOF: catalytic activity, oxidoreductase activity, acting on the CH–OH group of donors, NAD or NADP as acceptor
PGOC: null
PGOP: carbohydrate metabolic process, cellular carbohydrate metabolic process, oxidation–reduction process

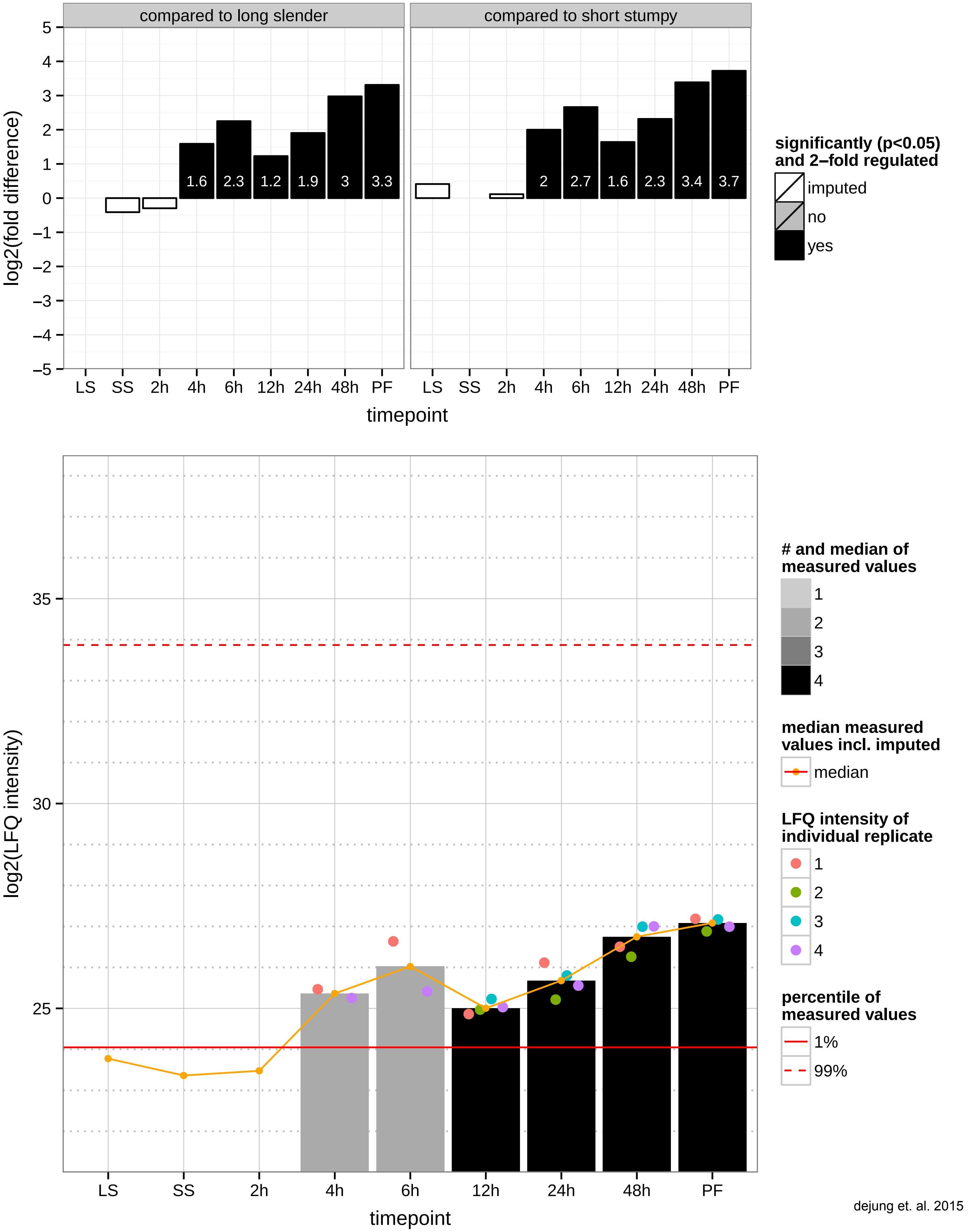

dejung et. al. 2015

NDUFA5/B13 subunit, putative
Tb927.10.4130
AGOF: null
AGOC: mitochondrion
AGOP: null
PGOF: null
PGOC: null
PGOP: null

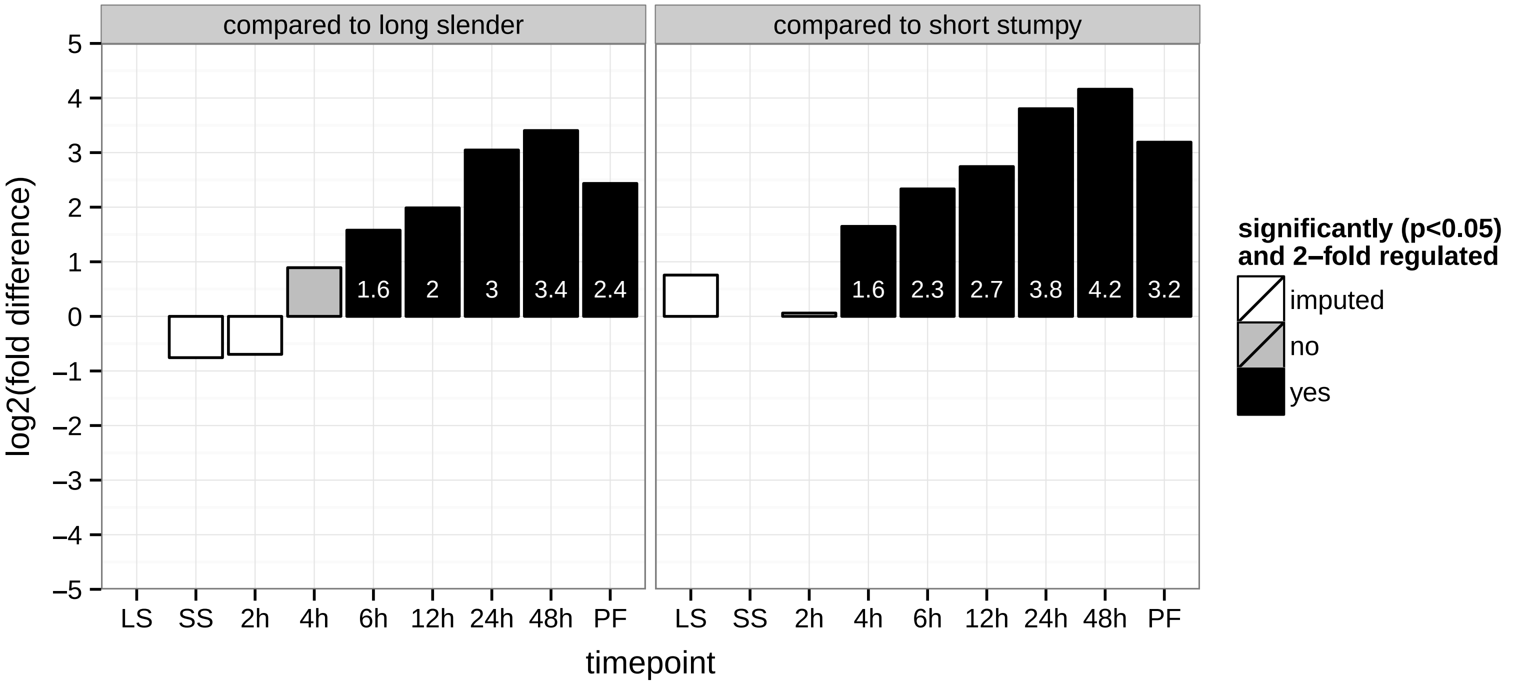

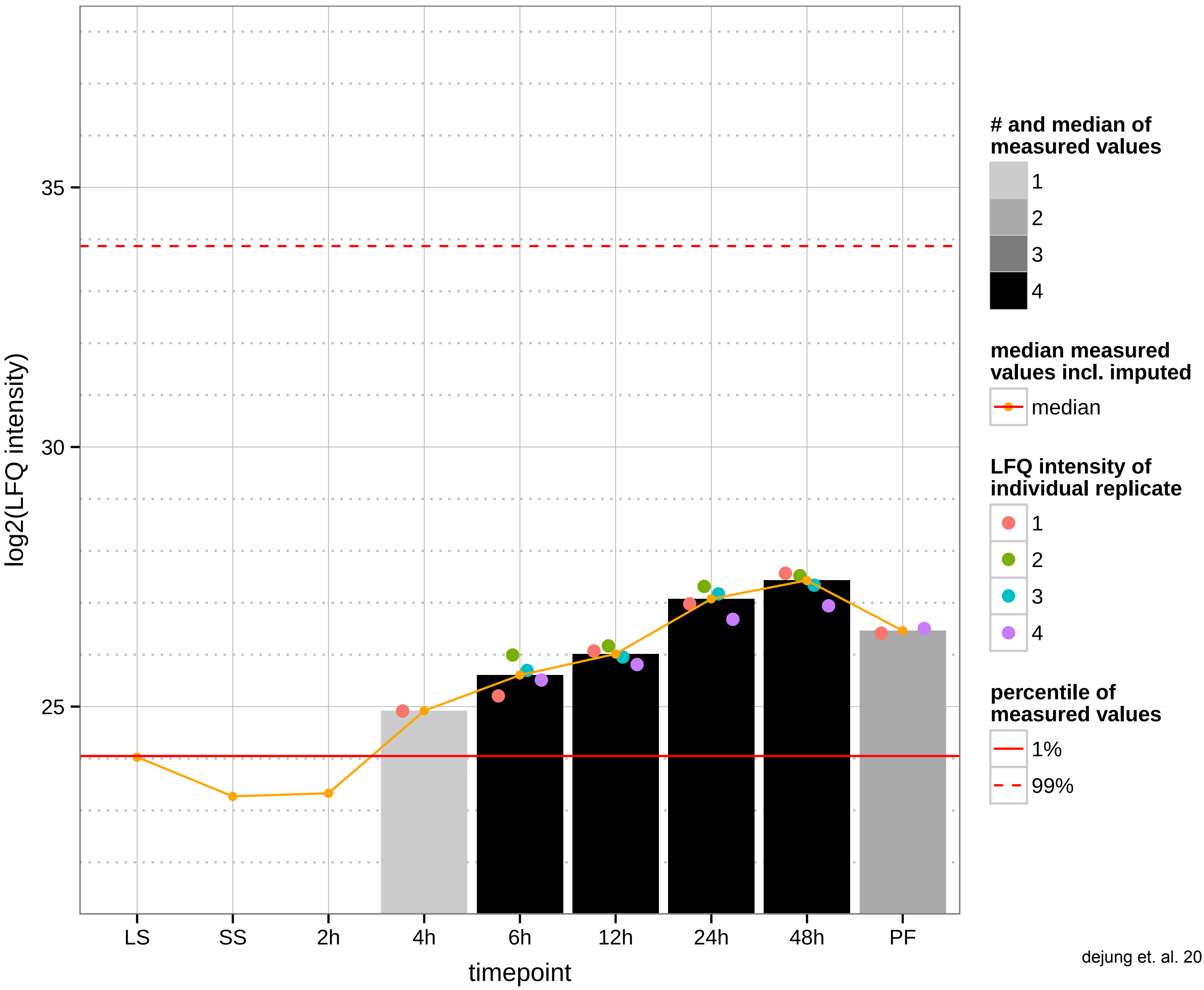

dejung et. al. 2015

protein phosphatase 2C, putative
Tb927.10.5590
AGOF: catalytic activity, phosphatase activity
AGOC: mitochondrion
AGOP: dephosphorylation
PGOF: catalytic activity
PGOC: null
PGOP: null

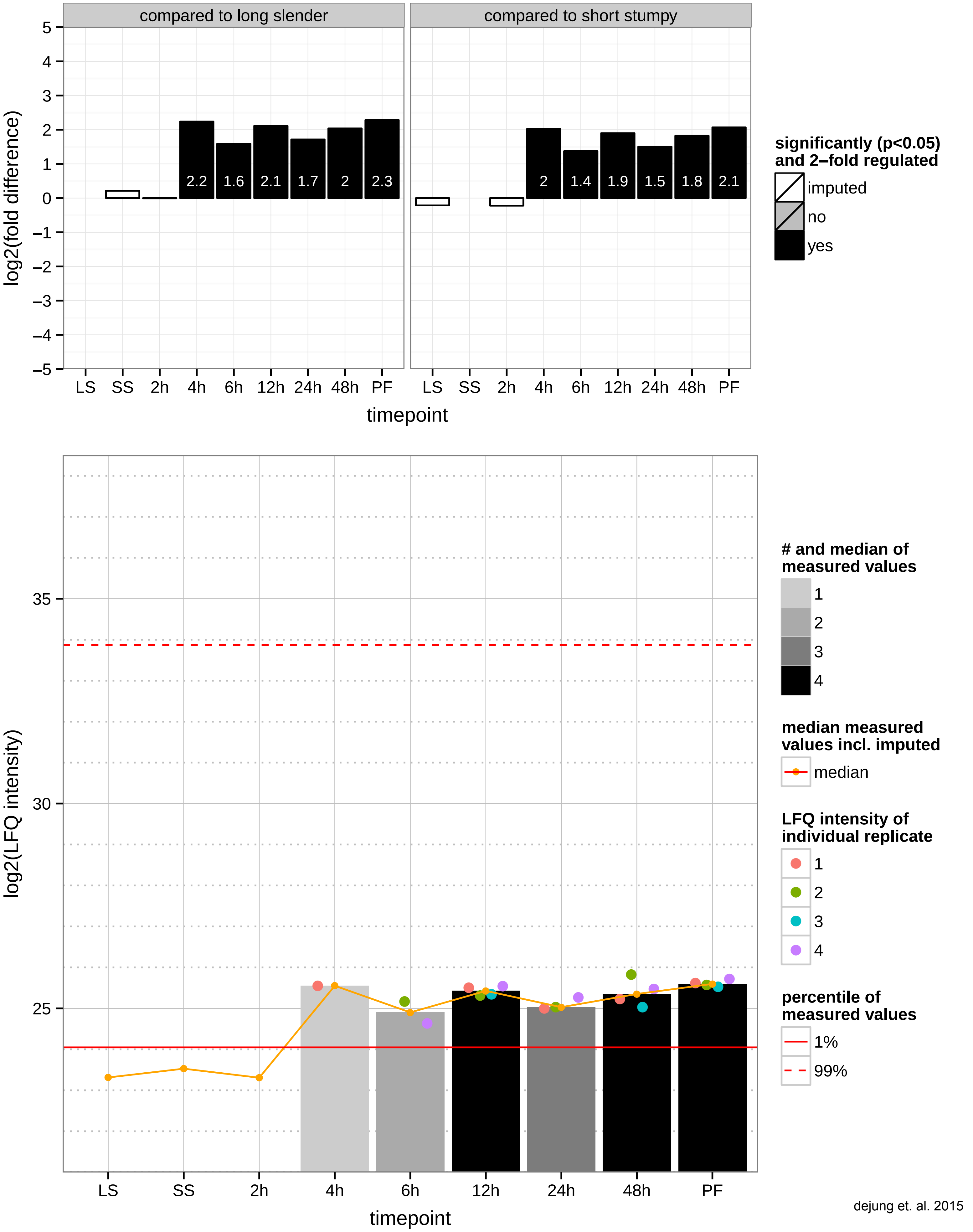

dejung et. al. 2015

mitochondrial RNA binding complex 1 subunit, mitochondrial edited mRNA stability factor 1 subunit, kinteoplast poly(A) polym
Tb927.10.6850
AGOF: RNA binding, structural constituent of ribosome
AGOC: intracellular, mitochondrion, ribosome
AGOP: RNA polyadenylation, mRNA stabilization, translation
PGOF: structural constituent of ribosome
PGOC: intracellular, ribosome
PGOP: translation

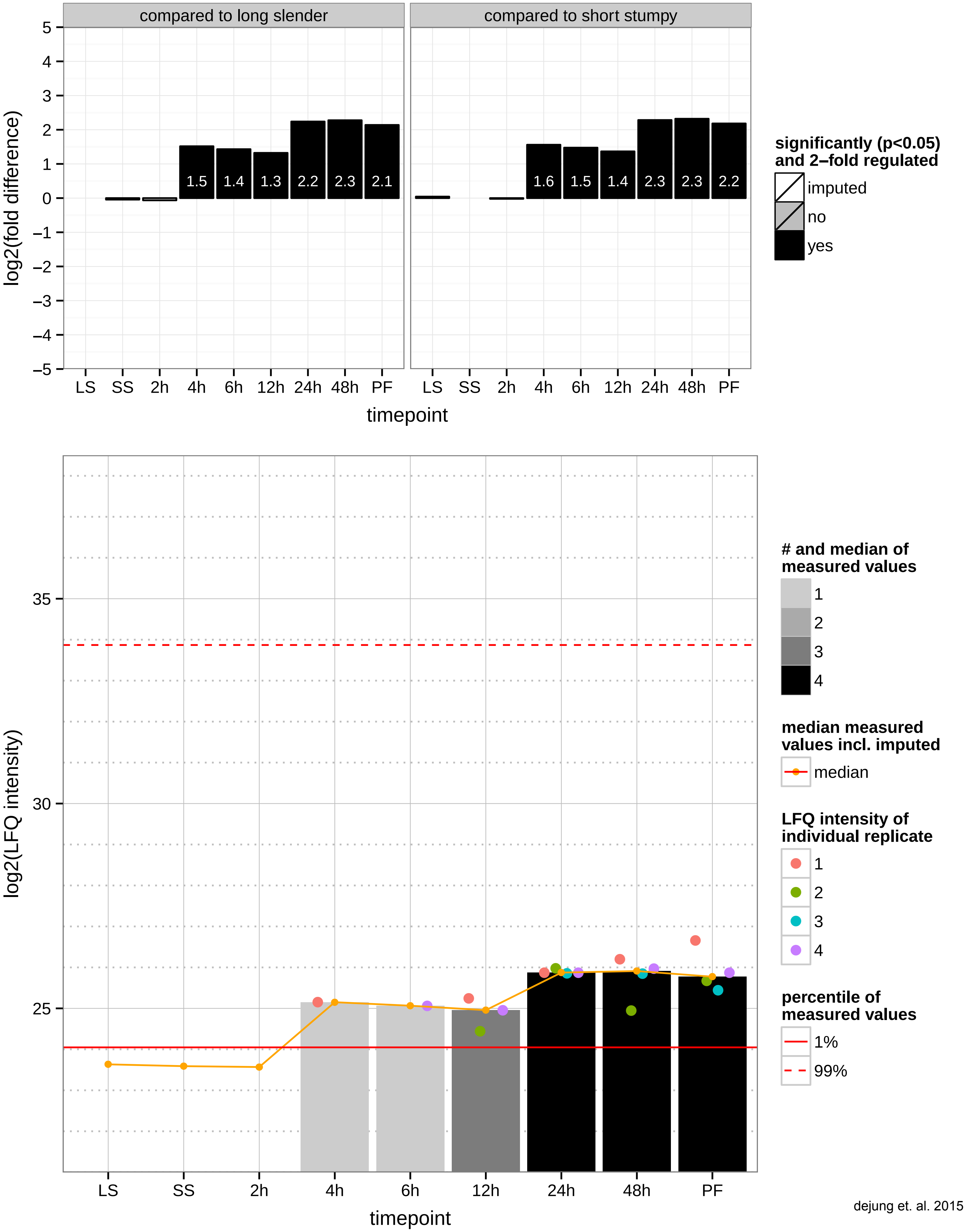

dejung et. al. 2015

mitochondrial ATP−dependent zinc metallopeptidase, putative, metallo−peptidase, Clan MA(E) Family M41
Tb927.10.7620
AGOF: ATP binding, metalloendopeptidase activity, nucleoside−triphosphatase activity, unfolded protein binding
AGOC: membrane, mitochondrion
AGOP: cell cycle, protein catabolic process, proteolysis
PGOF: ATP binding, metalloendopeptidase activity, nucleoside−triphosphatase activity, nucleotide binding
PGOC: membrane
PGOP: protein catabolic process, proteolysis

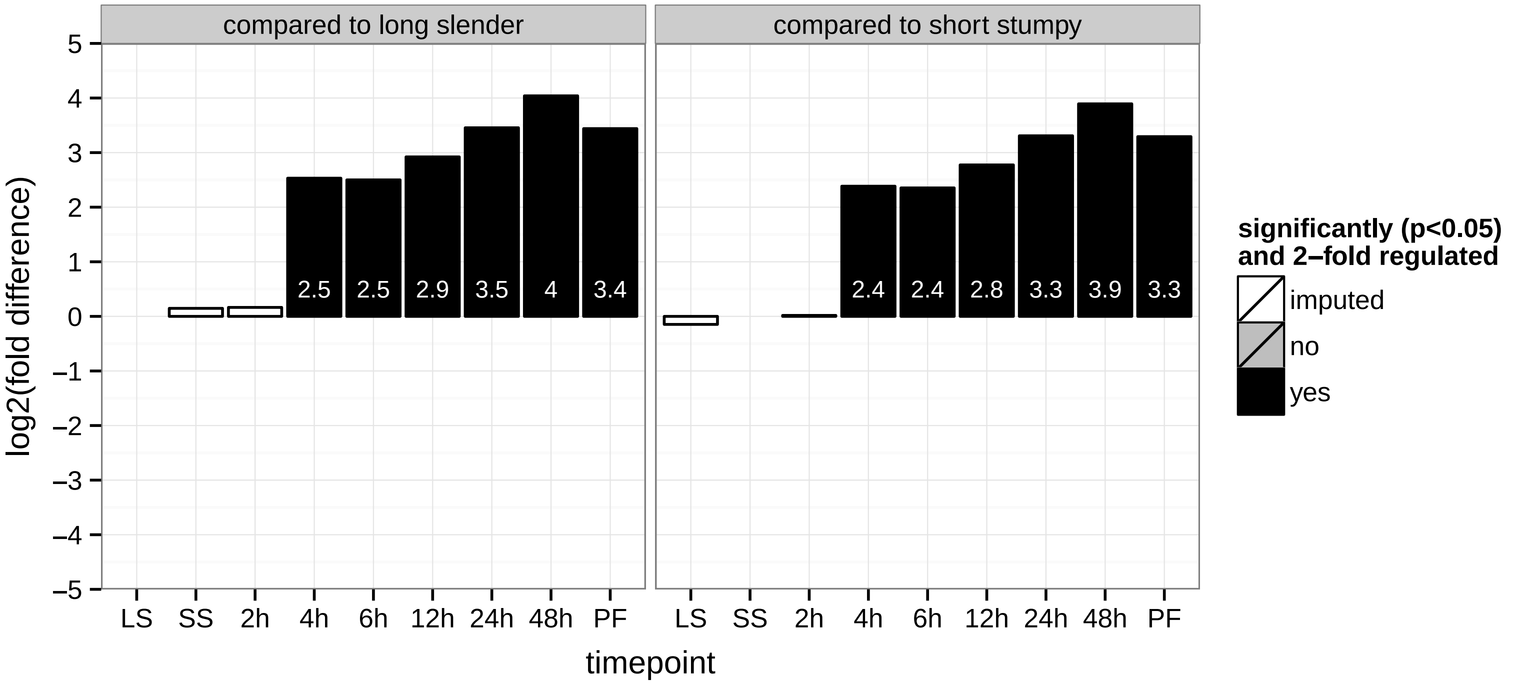

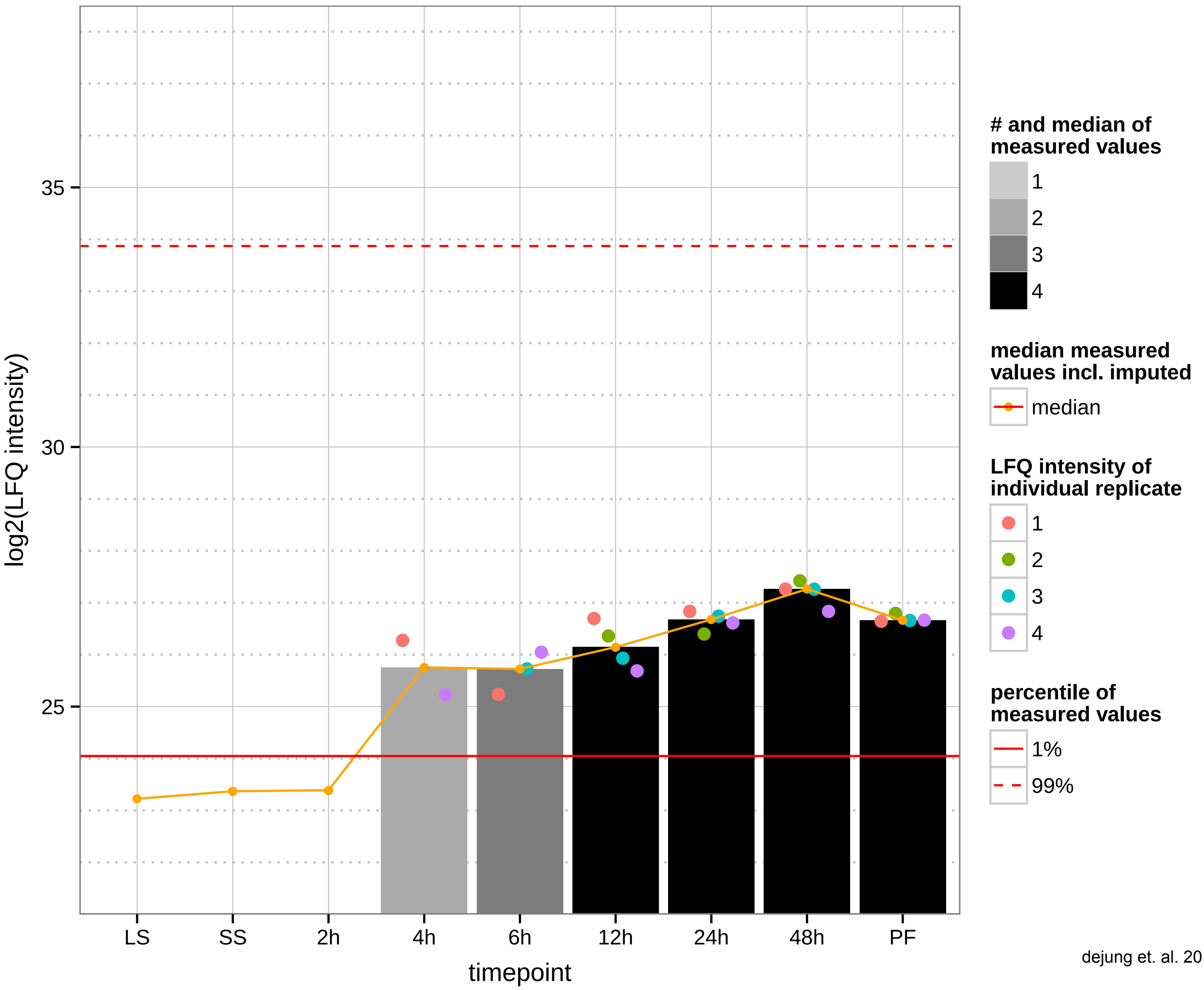

dejung et. al. 2015

40S ribosomal protein S8, putative
Tb927.10.7710
AGOF: structural constituent of ribosome
AGOC: intracellular, ribosome
AGOP: translation
PGOF: null
PGOC: null
PGOP: null

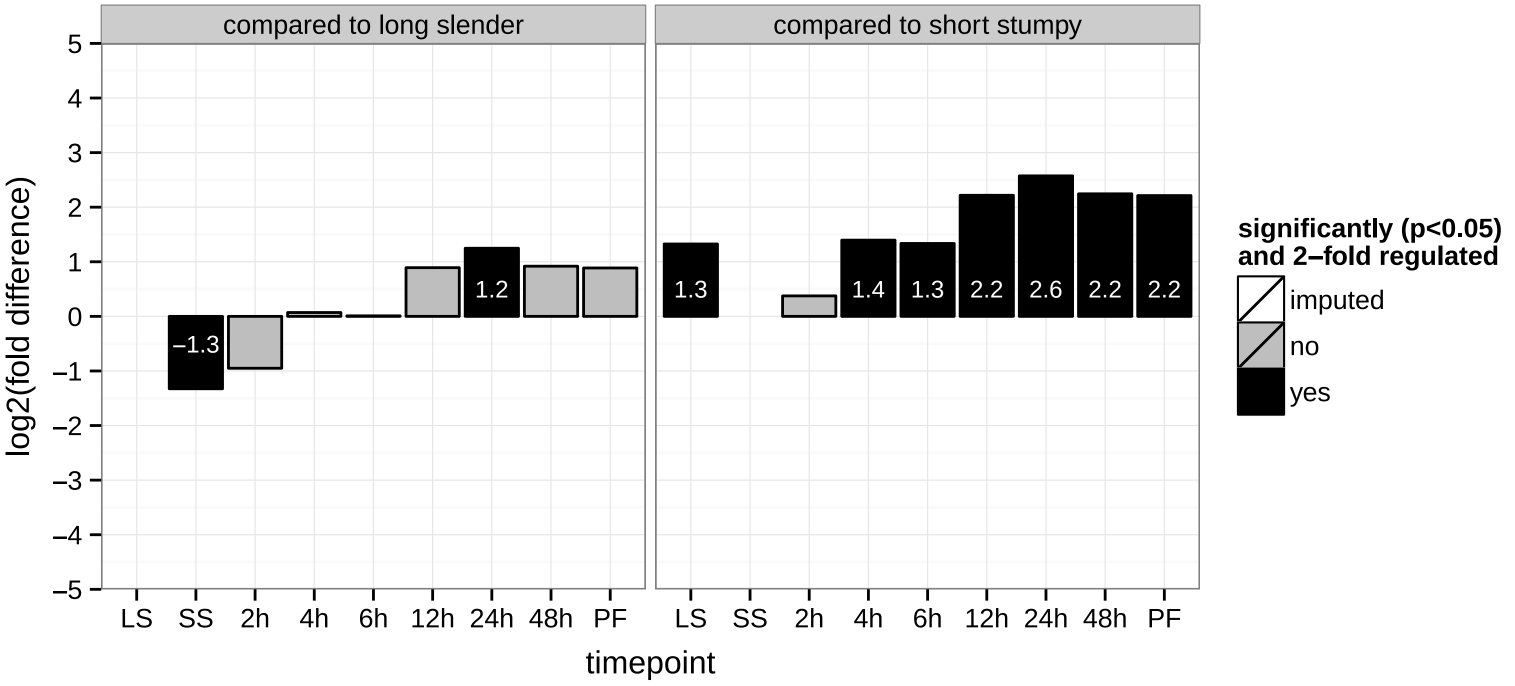

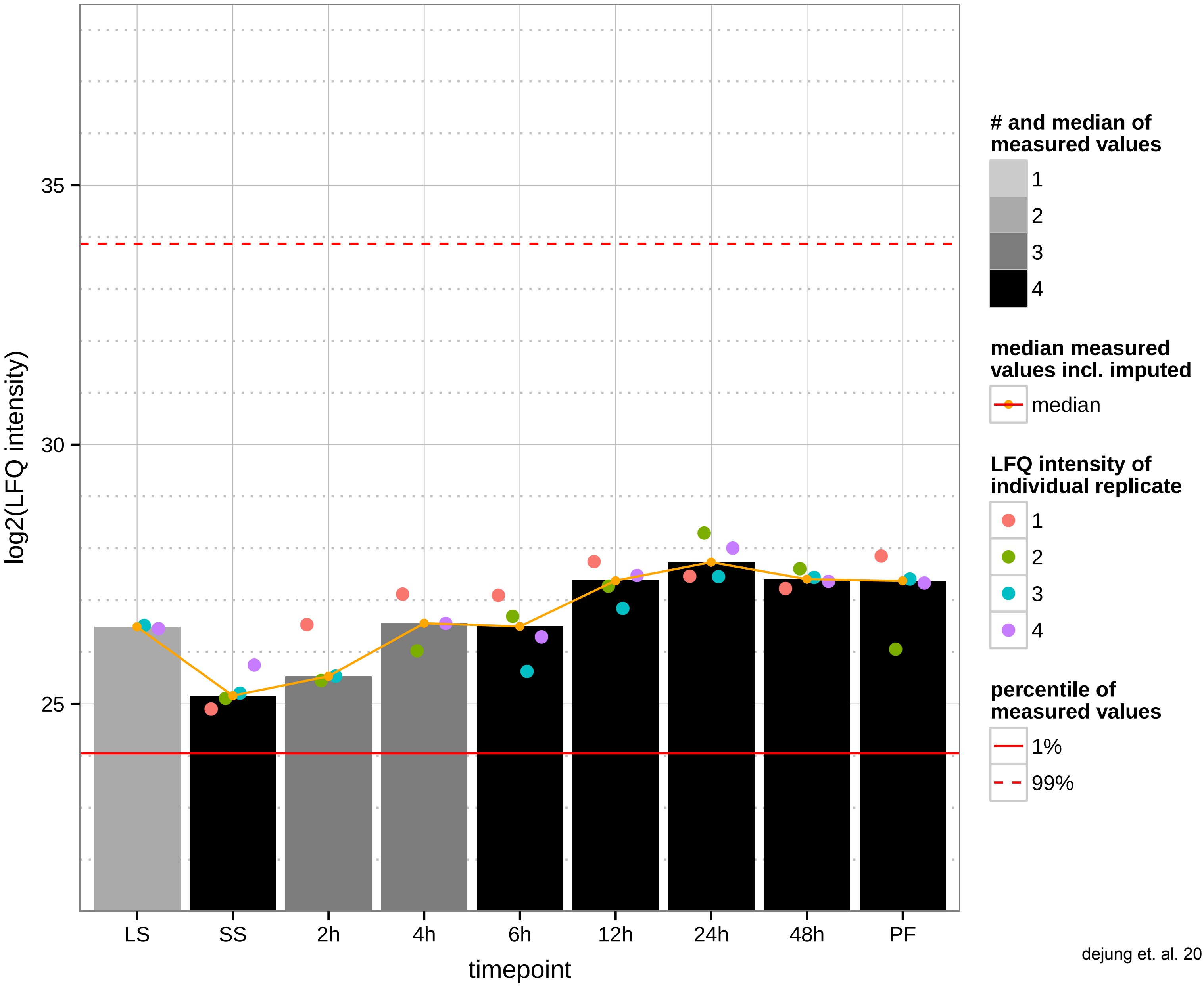

dejung et. al. 2015

hypothetical protein, conserved
Tb927.11.13710
AGOF: null
AGOC: mitochondrion
AGOP: null
PGOF: null
PGOC: null
PGOP: null

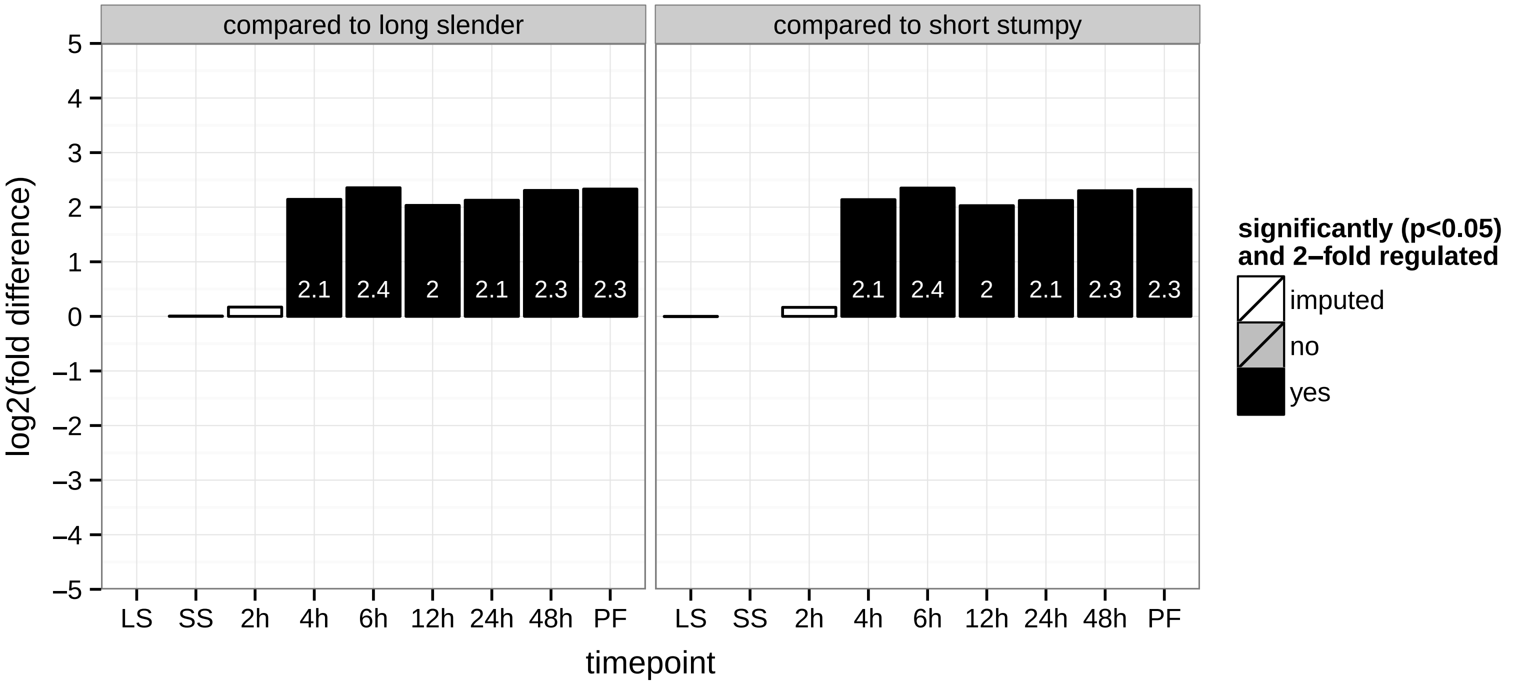

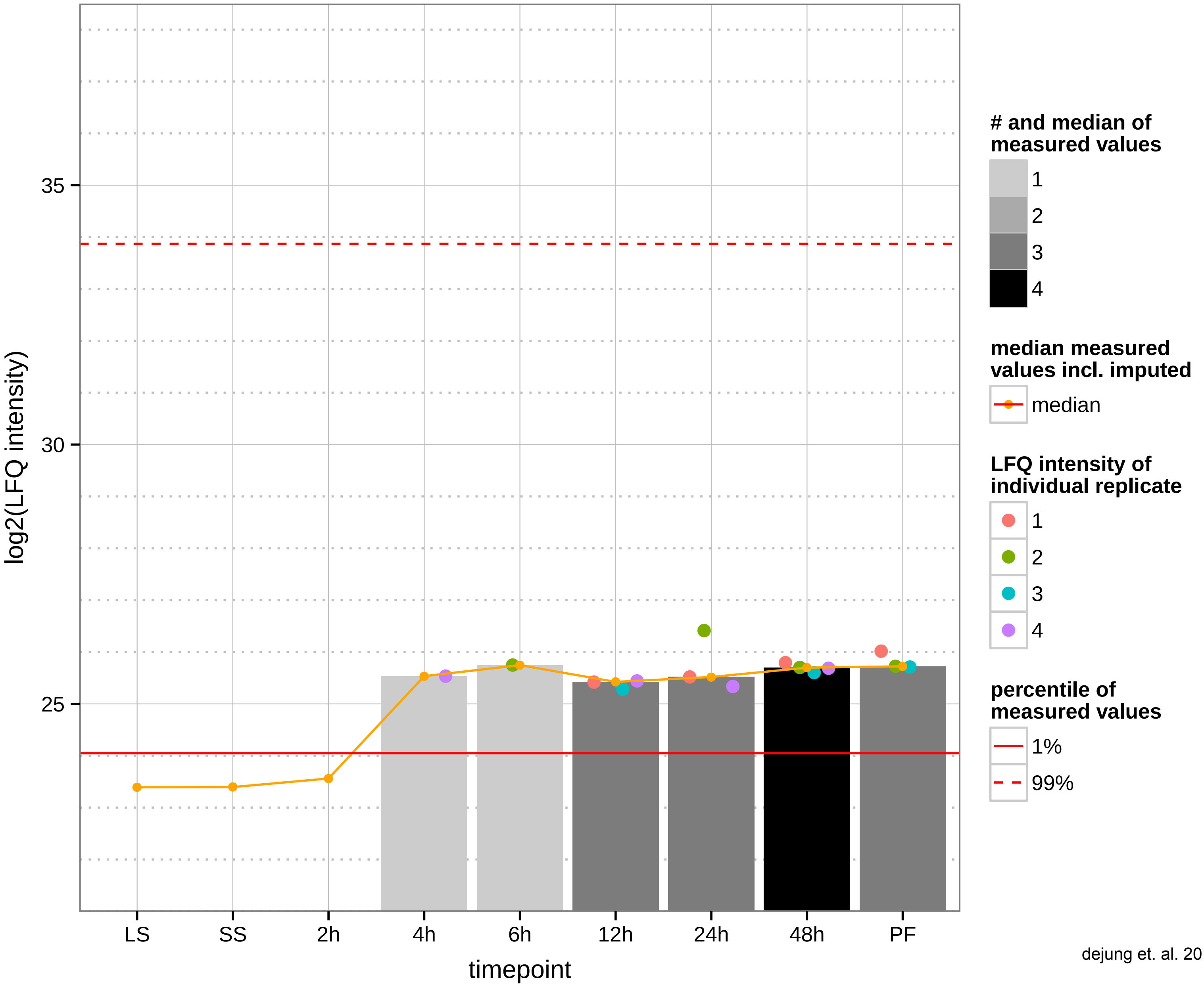

dejung et. al. 2015

hypothetical protein, conserved
Tb927.11.14390
AGOF: null
AGOC: null
AGOP: null
PGOF: null
PGOC: null
PGOP: null

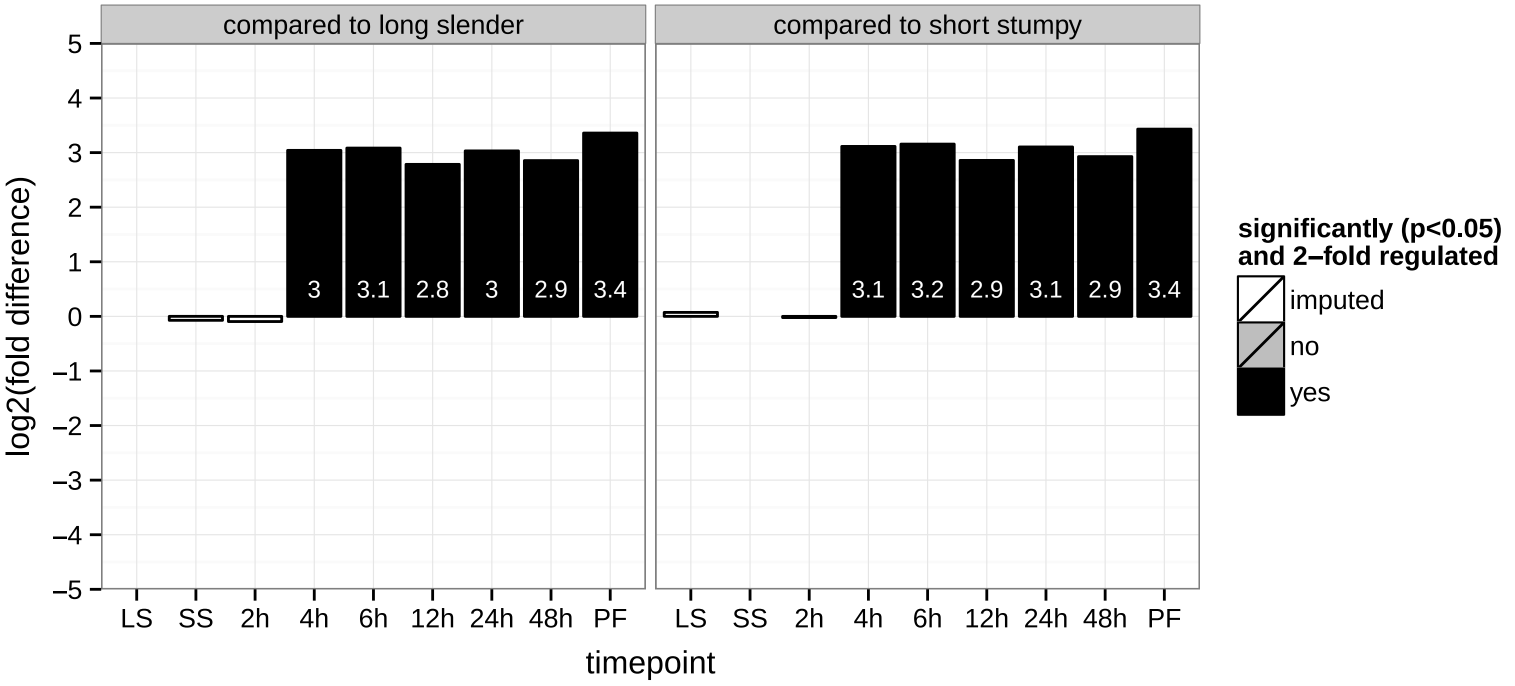

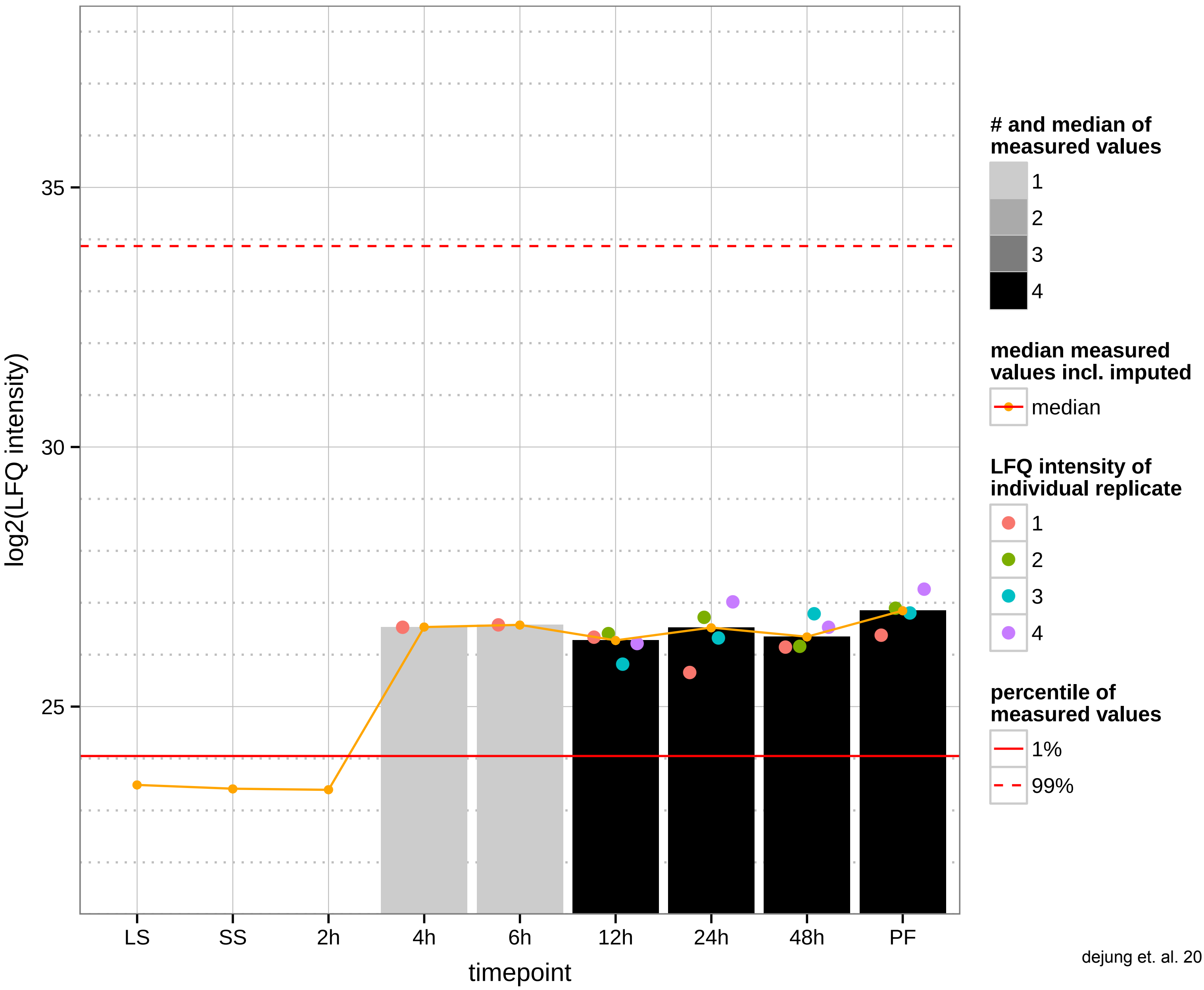

dejung et. al. 2015

hypothetical protein, conserved
Tb927.11.15590
AGOF: catalytic activity
AGOC: null
AGOP: metabolic process
PGOF: null
PGOC: null
PGOP: null

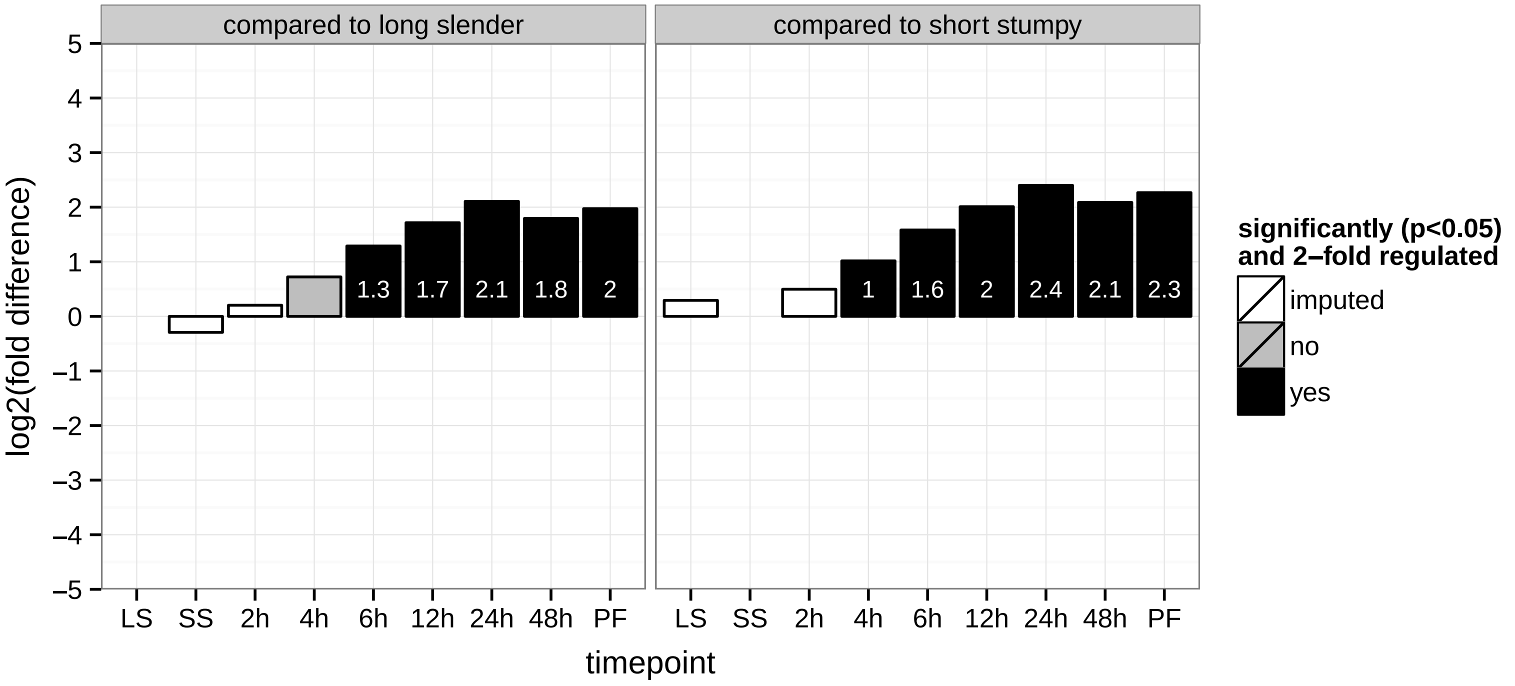

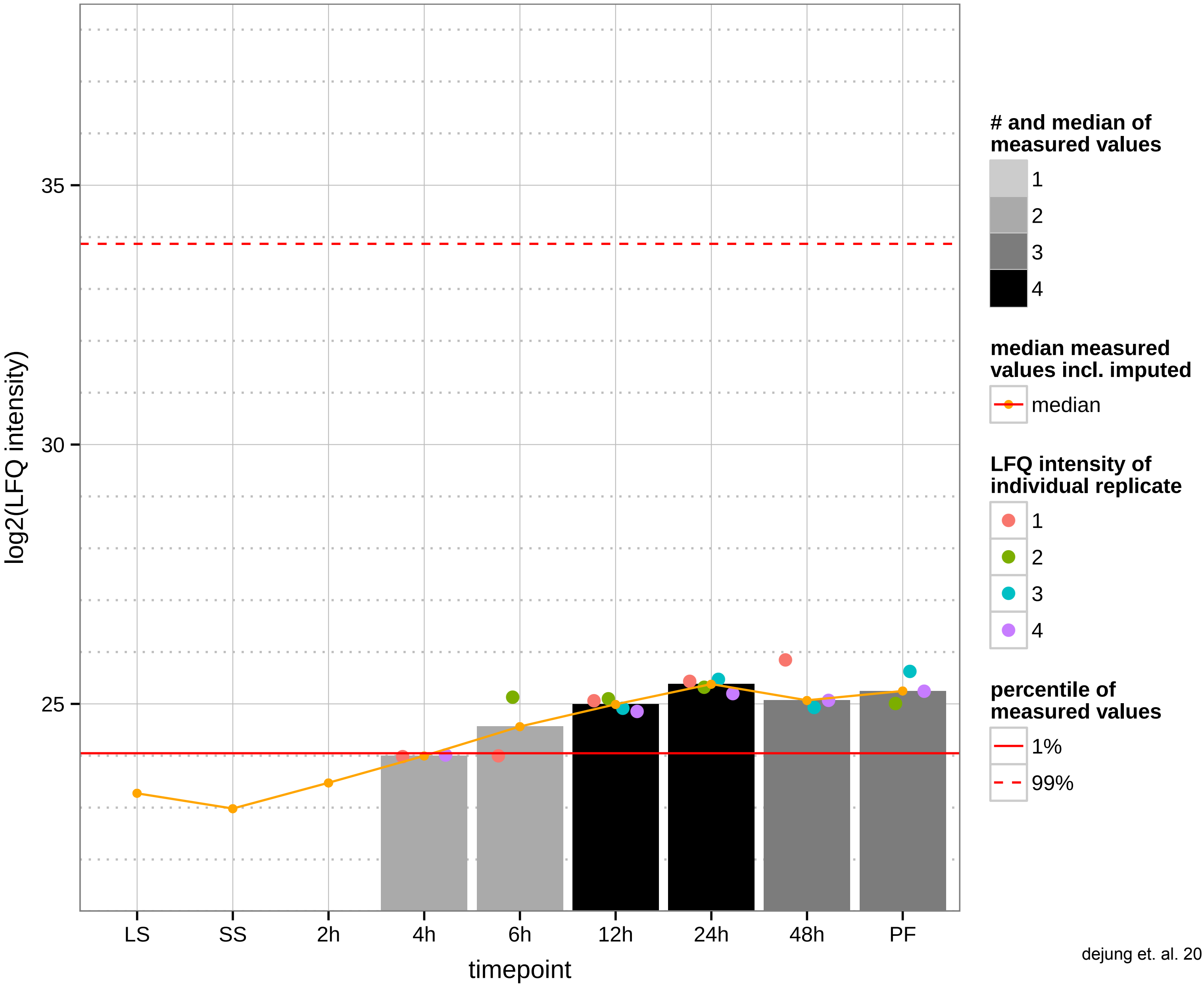

dejung et. al. 2015

hypothetical protein, conserved
Tb927.11.16750
AGOF: null
AGOC: mitochondrion
AGOP: null
PGOF: null
PGOC: null
PGOP: null

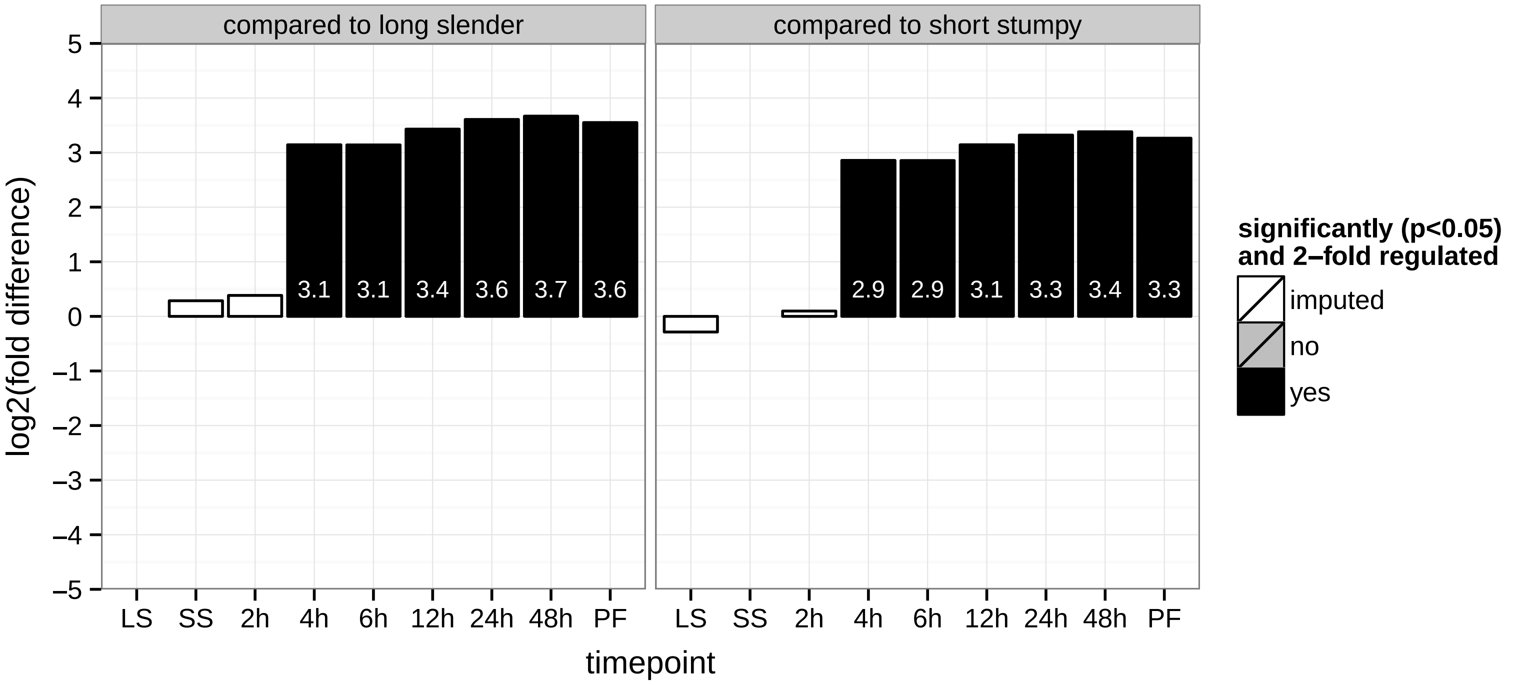

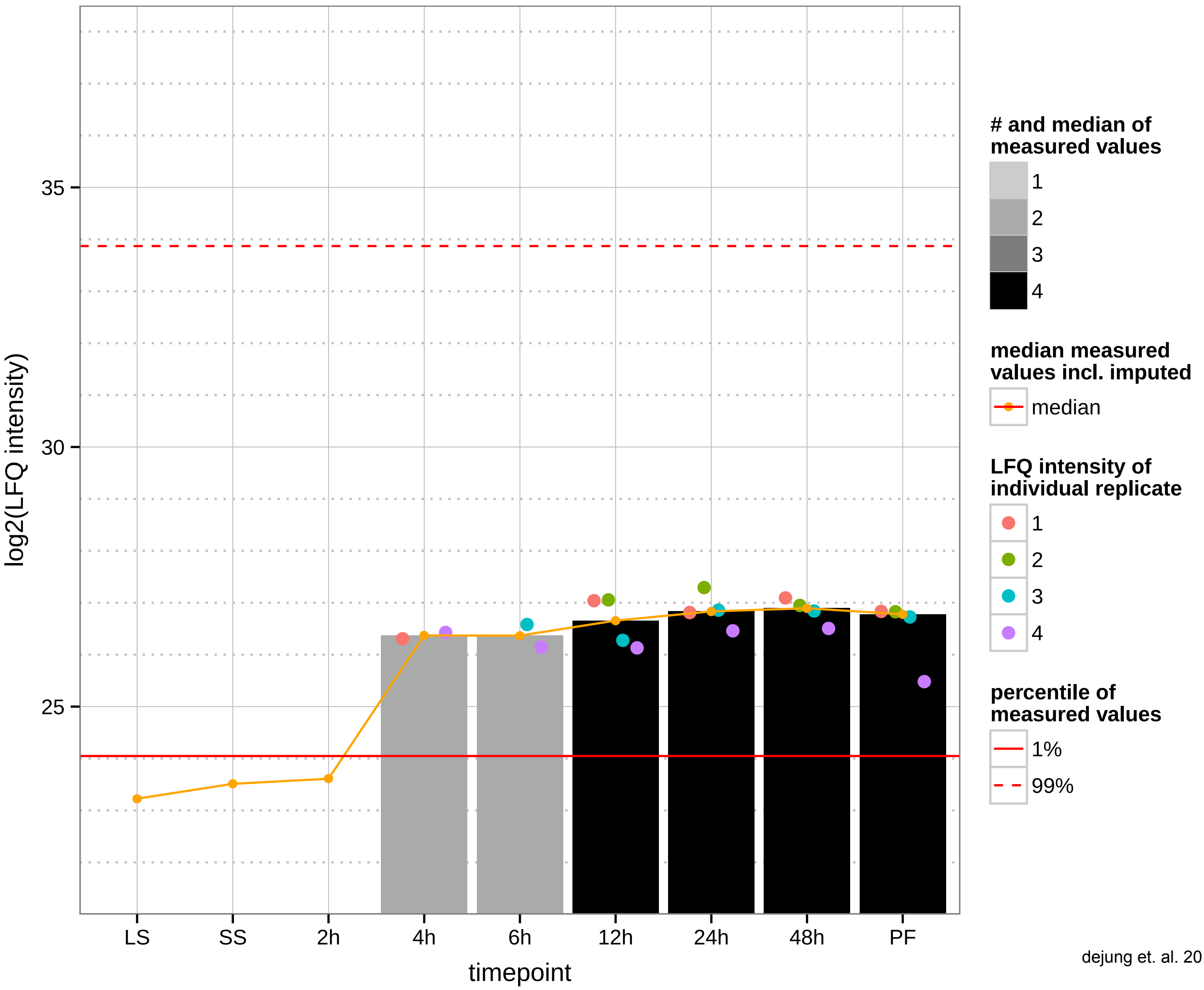

dejung et. al. 2015

hypothetical protein, conserved
Tb927.11.1720
AGOF: null
AGOC: null
AGOP: null
PGOF: null
PGOC: null
PGOP: null

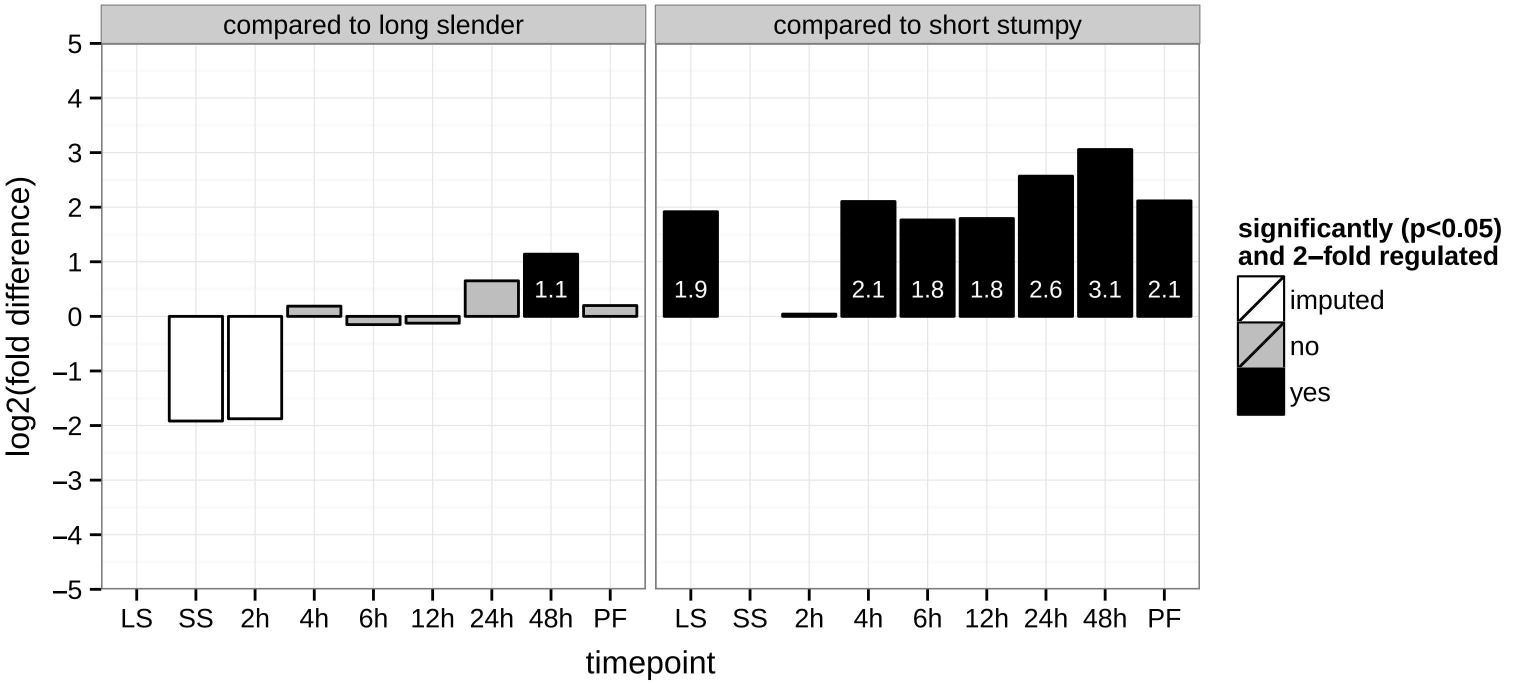

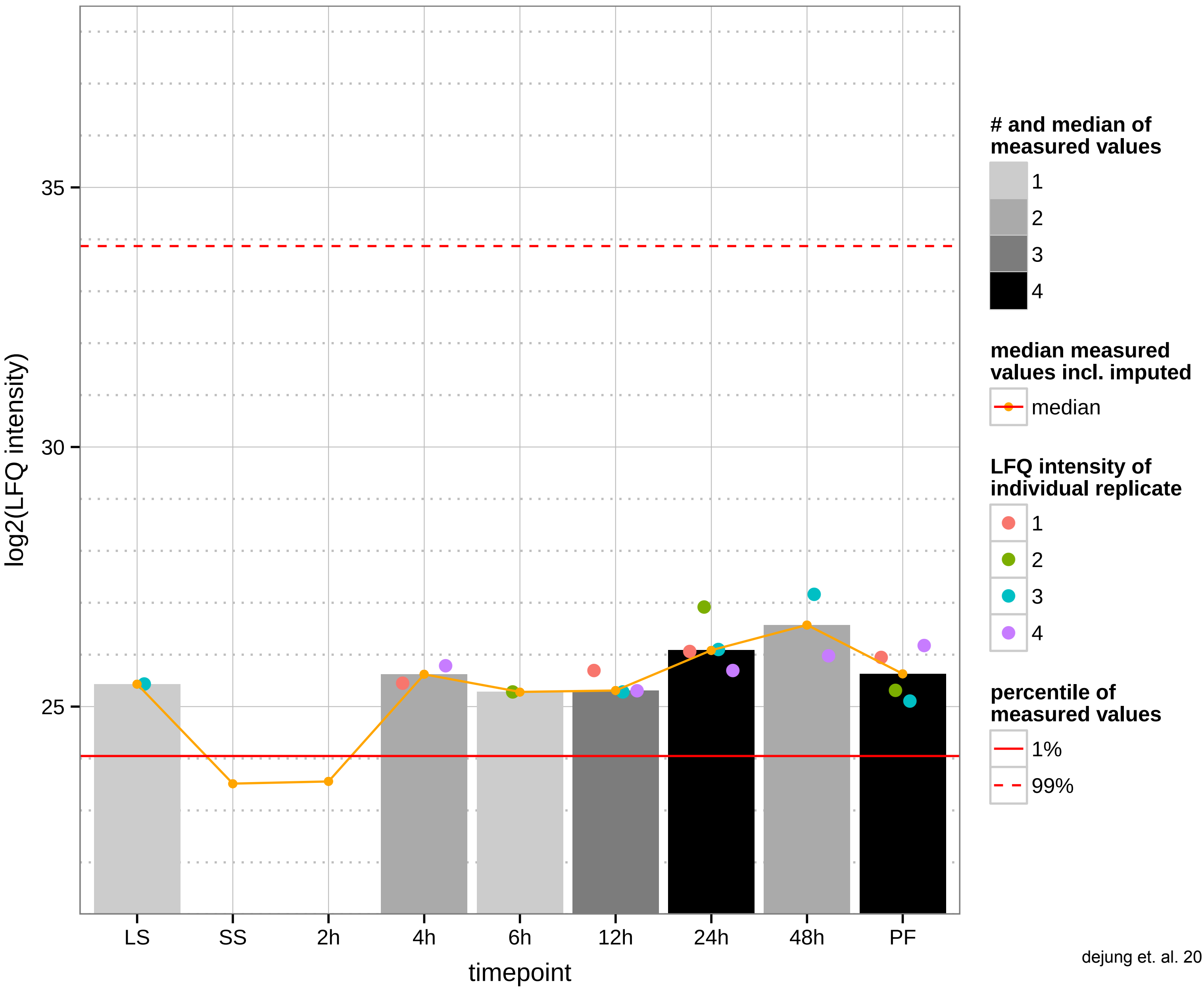

dejung et. al. 2015

hypothetical protein, conserved
Tb927.11.2420
AGOF: tubulin–tyrosine ligase activity
AGOC: null
AGOP: cellular protein modification process
PGOF: tubulin–tyrosine ligase activity
PGOC: null
PGOP: cellular protein modification process

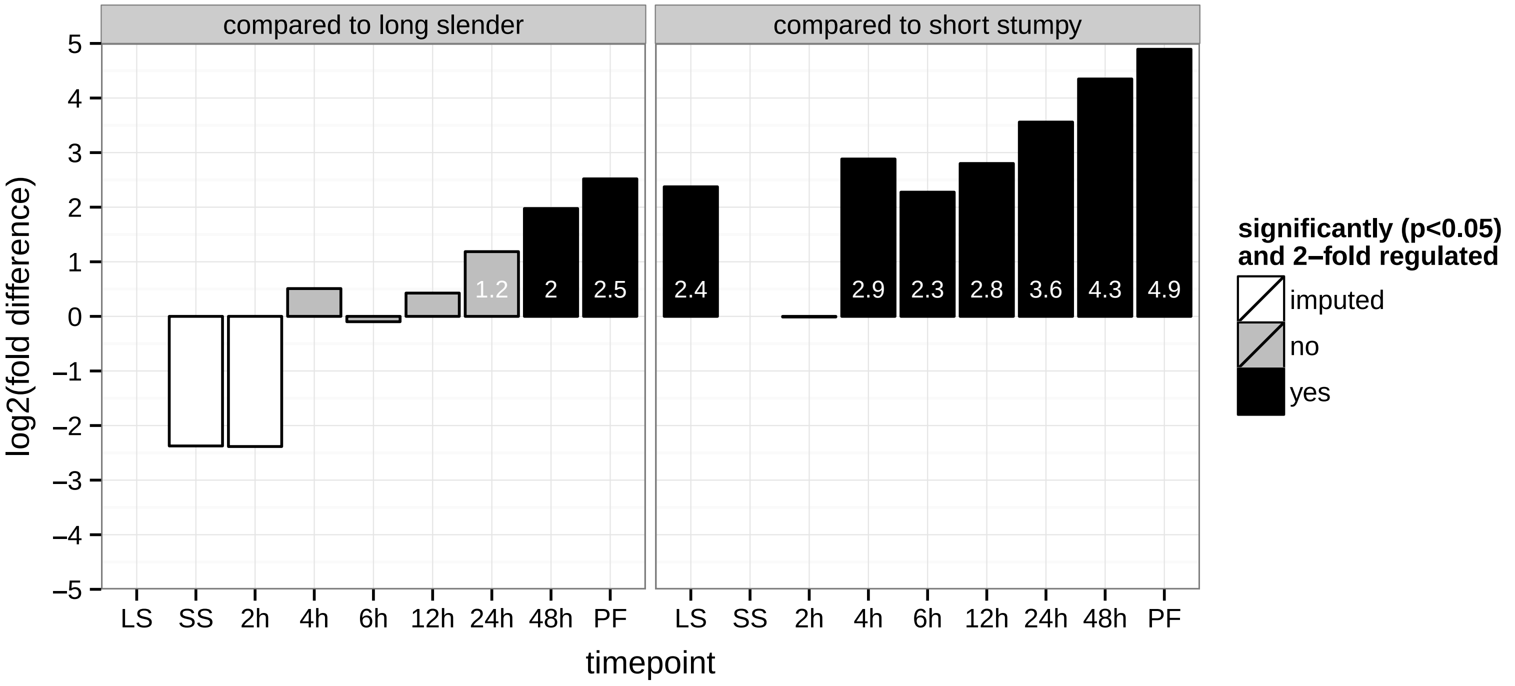

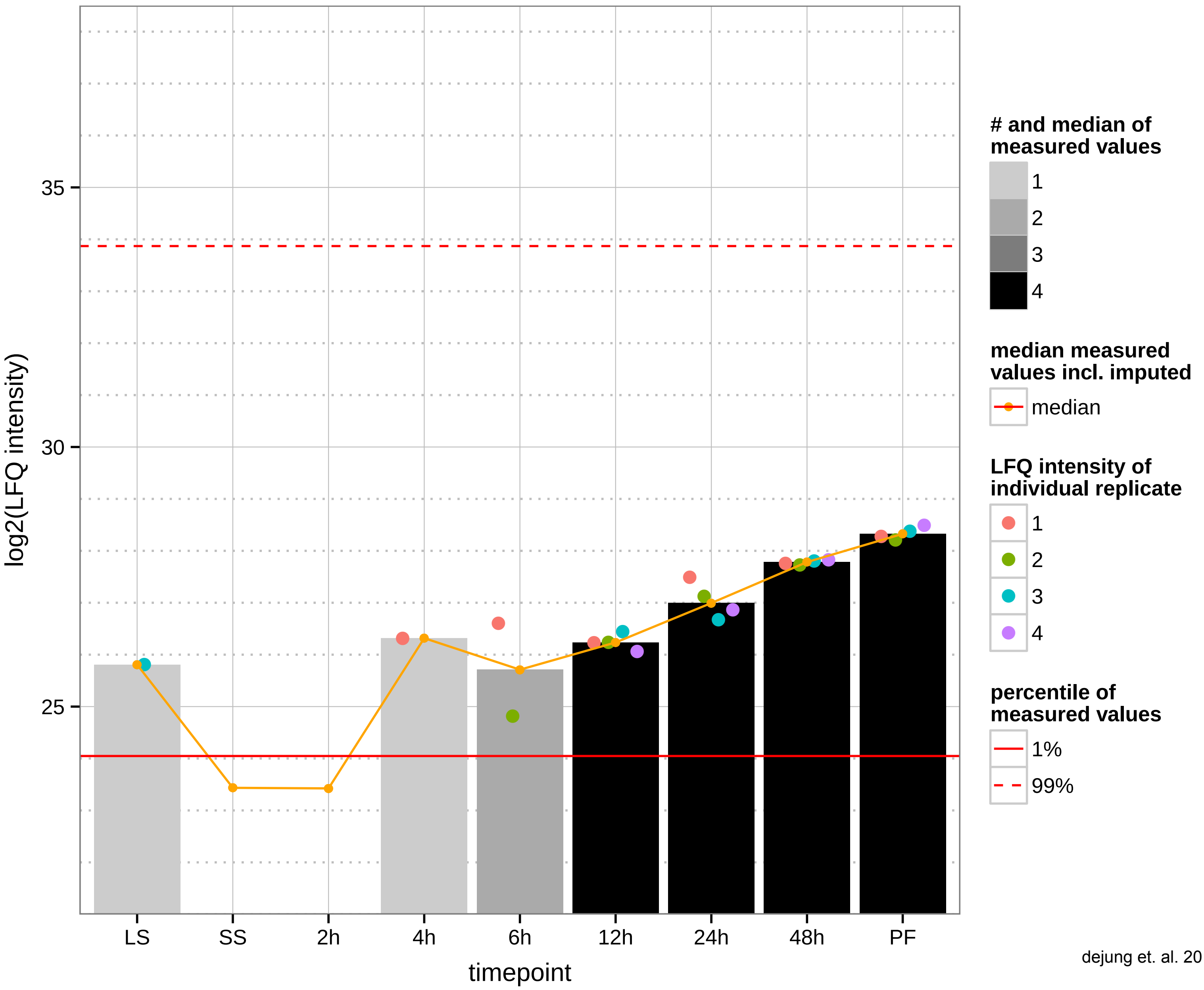

dejung et. al. 2015

hypothetical protein, conserved
Tb927.11.3100
AGOF: null
AGOC: null
AGOP: null
PGOF: protein binding
PGOC: null
PGOP: null

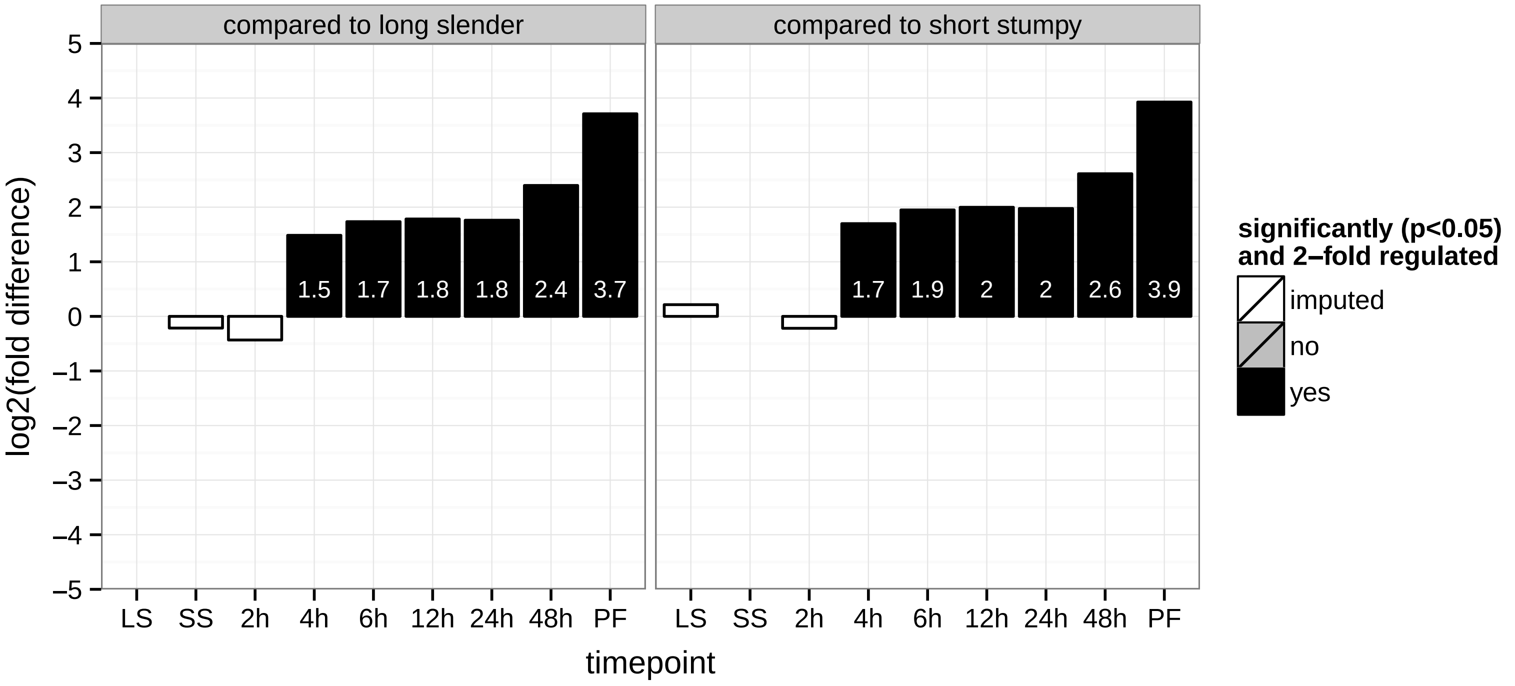

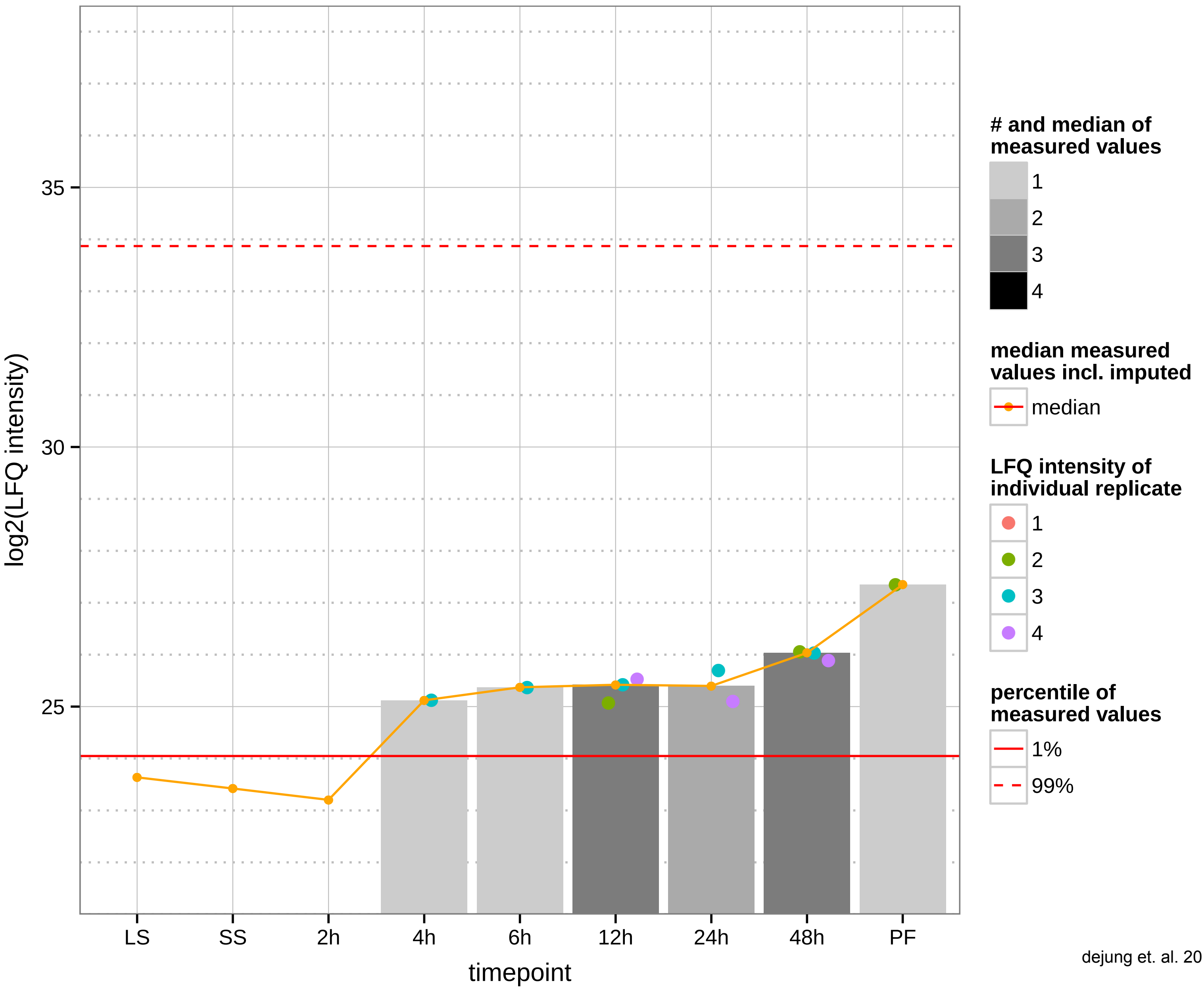

dejung et. al. 2015

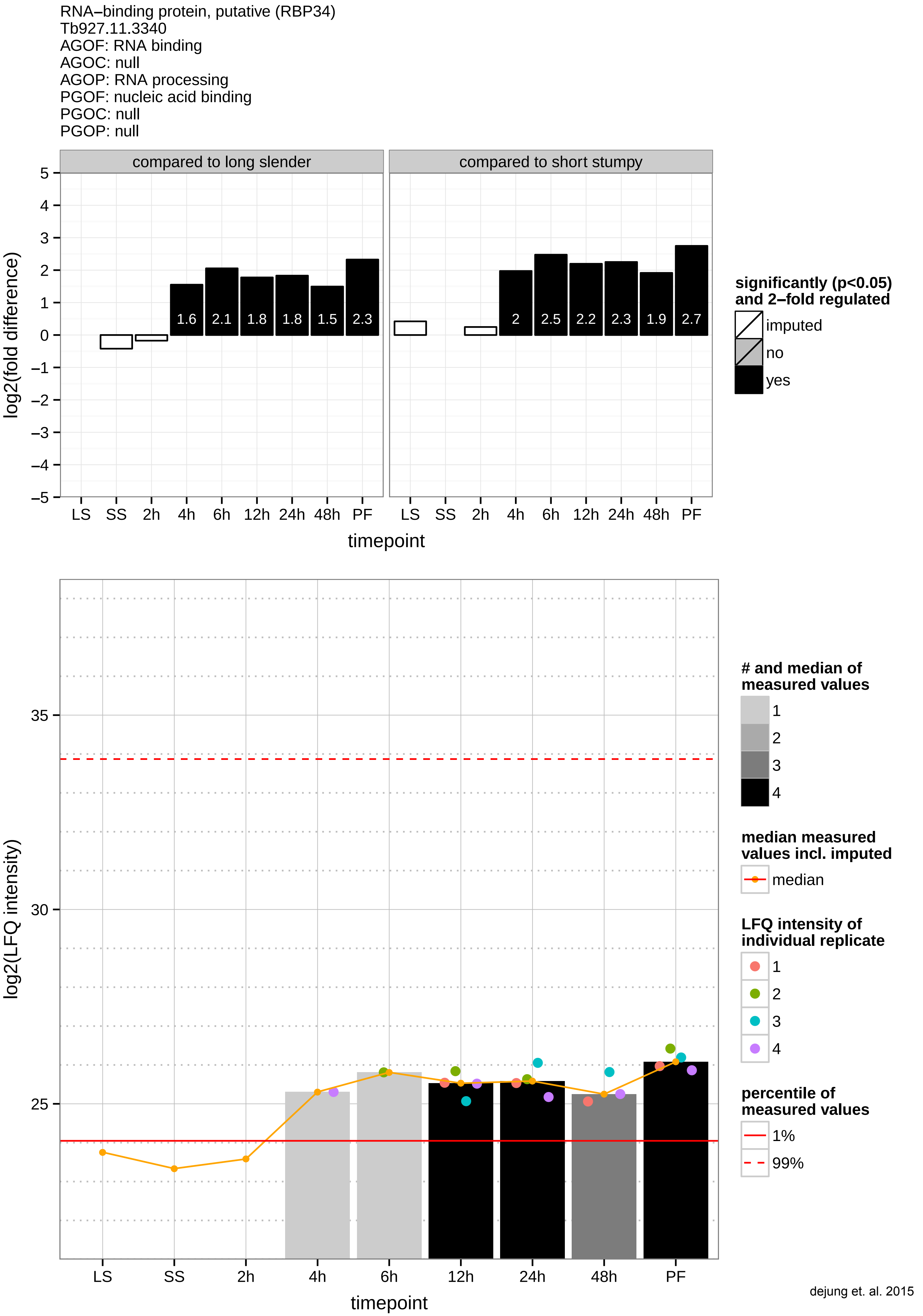
RNA–binding protein, putative (RBP34)
Tb927.11.3340
AGOF: RNA binding
AGOC: null
AGOP: RNA processing
PGOF: nucleic acid binding
PGOC: null
PGOP: null
compared to long slender
compared to short stumpy
log2(fold difference)
1.6
2.1
1.8
1.8
1.5
2.3
2
2.5
2.2
2.3
1.9
2.7
LS
SS
2h
4h
6h
12h
24h
48h
PF
timepoint
significantly (p<0.05) and 2–fold regulated
imputed
no
yes
log2(LFQ intensity)
# and median of measured values
1
2
3
4
median measured values incl. imputed
median
LFQ intensity of individual replicate
percentile of measured values
1%
99%
dejung et. al. 2015

hypothetical protein, conserved
Tb927.11.3640
AGOF: null
AGOC: mitochondrion
AGOP: null
PGOF: null
PGOC: null
PGOP: null

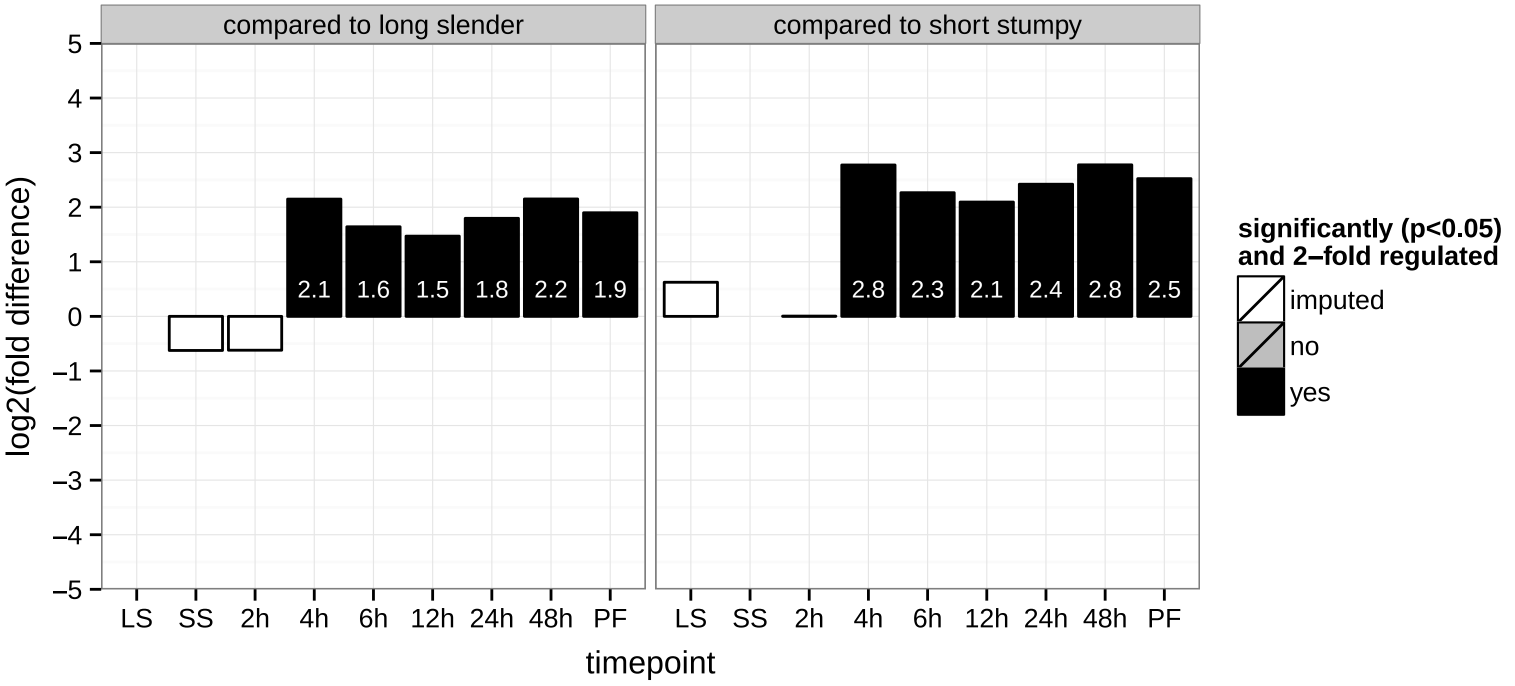

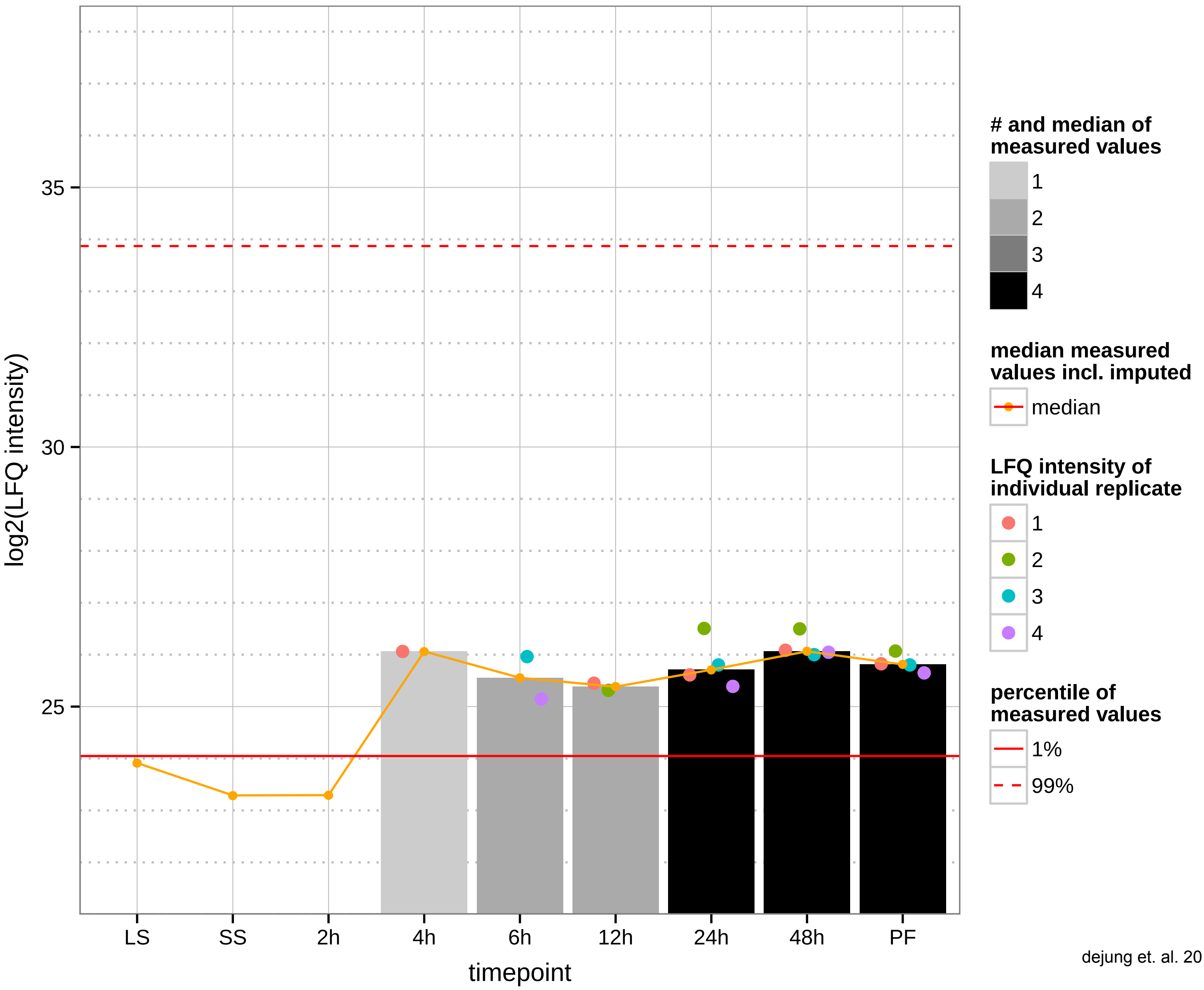

dejung et. al. 2015

mitochondrial processing peptidase alpha subunit, putative, metallo–peptidase, Clan ME, Family M16
Tb927.11.3980
AGOF: catalytic activity, metalloendopeptidase activity, zinc ion binding
AGOC: mitochondrion
AGOP: proteolysis
PGOF: catalytic activity, metal ion binding, metalloendopeptidase activity, zinc ion binding
PGOC: null
PGOP: proteolysis

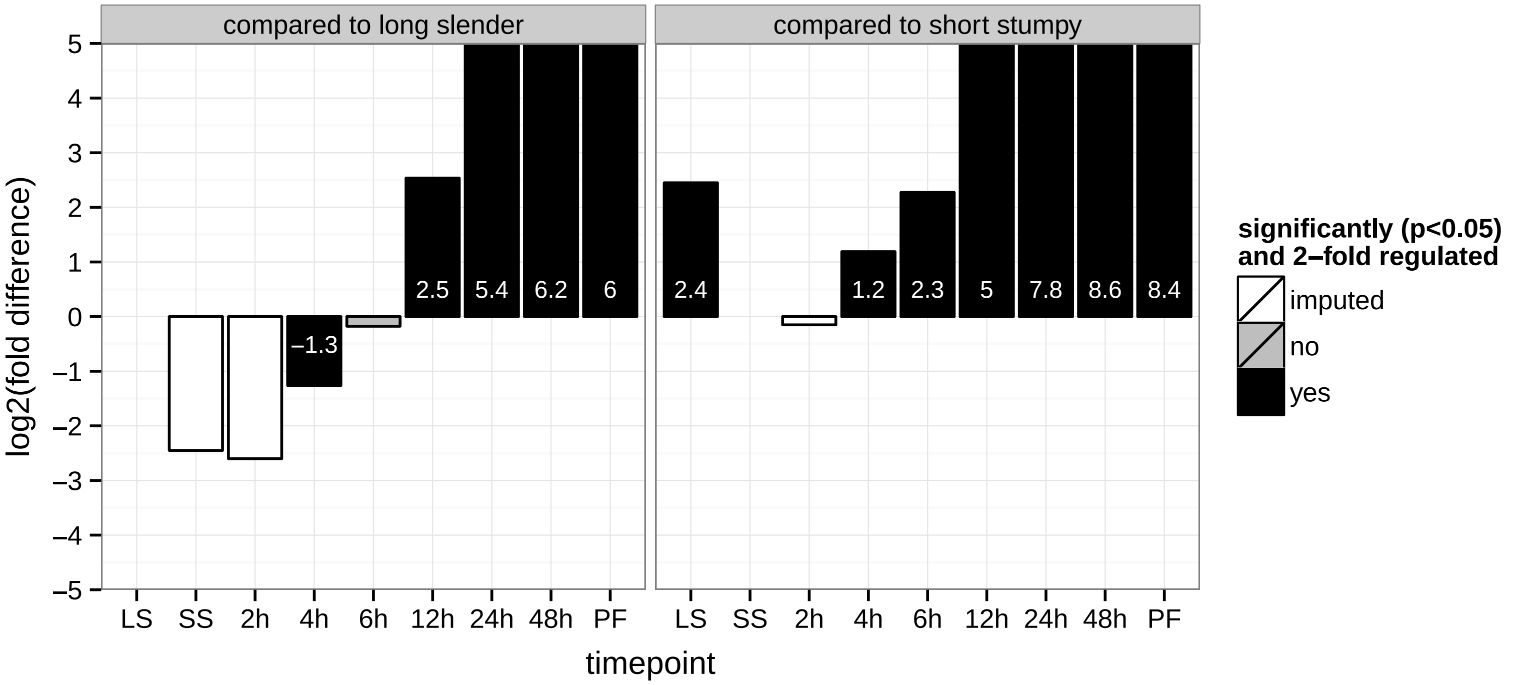

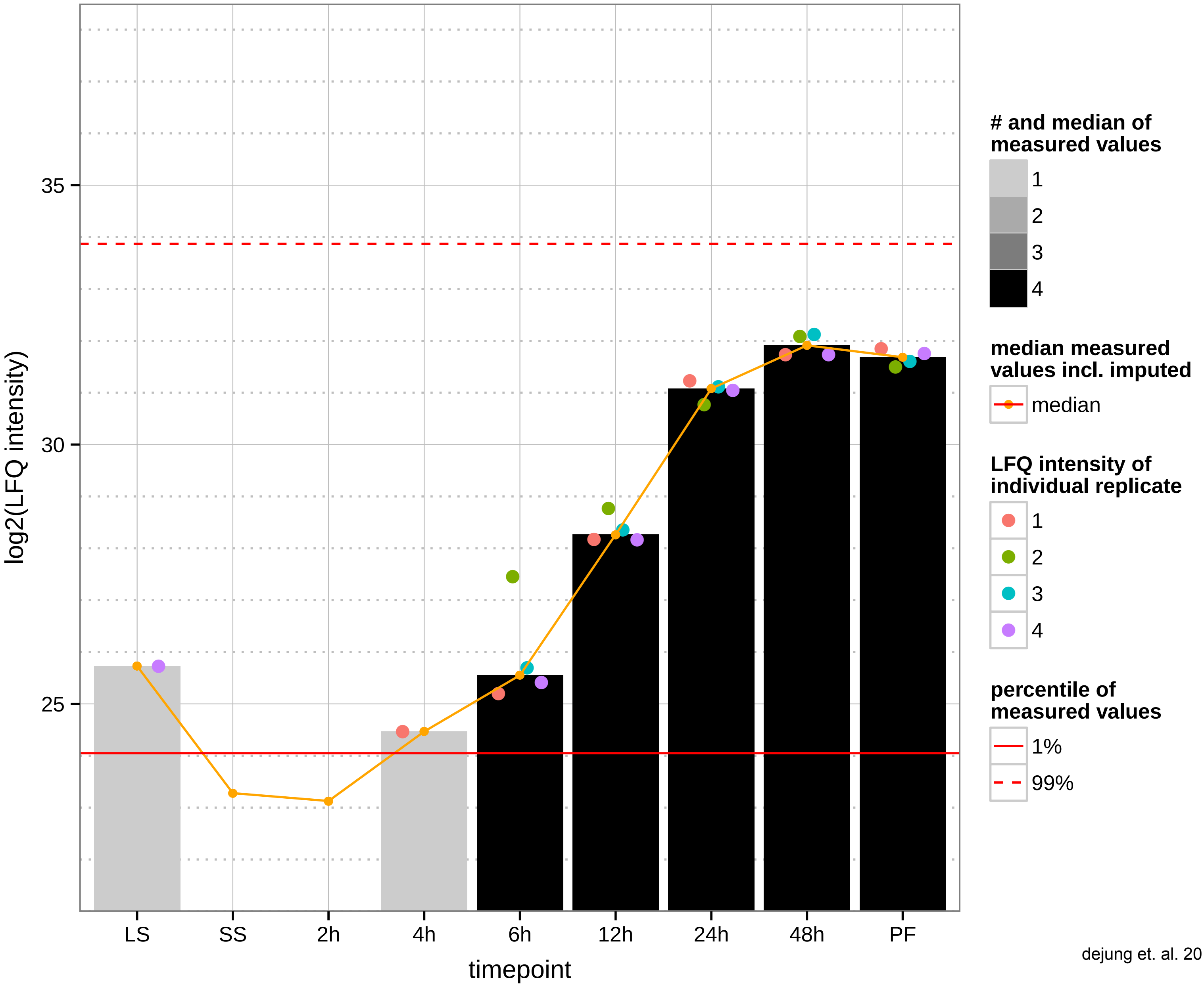

dejung et. al. 2015

ATP−dependent RNA helicase, putative
Tb927.11.4380
AGOF: ATP binding, ATP−dependent helicase activity, helicase activity, nucleic acid binding
AGOC: null
AGOP: null
PGOF: ATP binding, ATP−dependent helicase activity, helicase activity, nucleic acid binding, nucleoside−triphosphatase activi
PGOC: null
PGOP: null

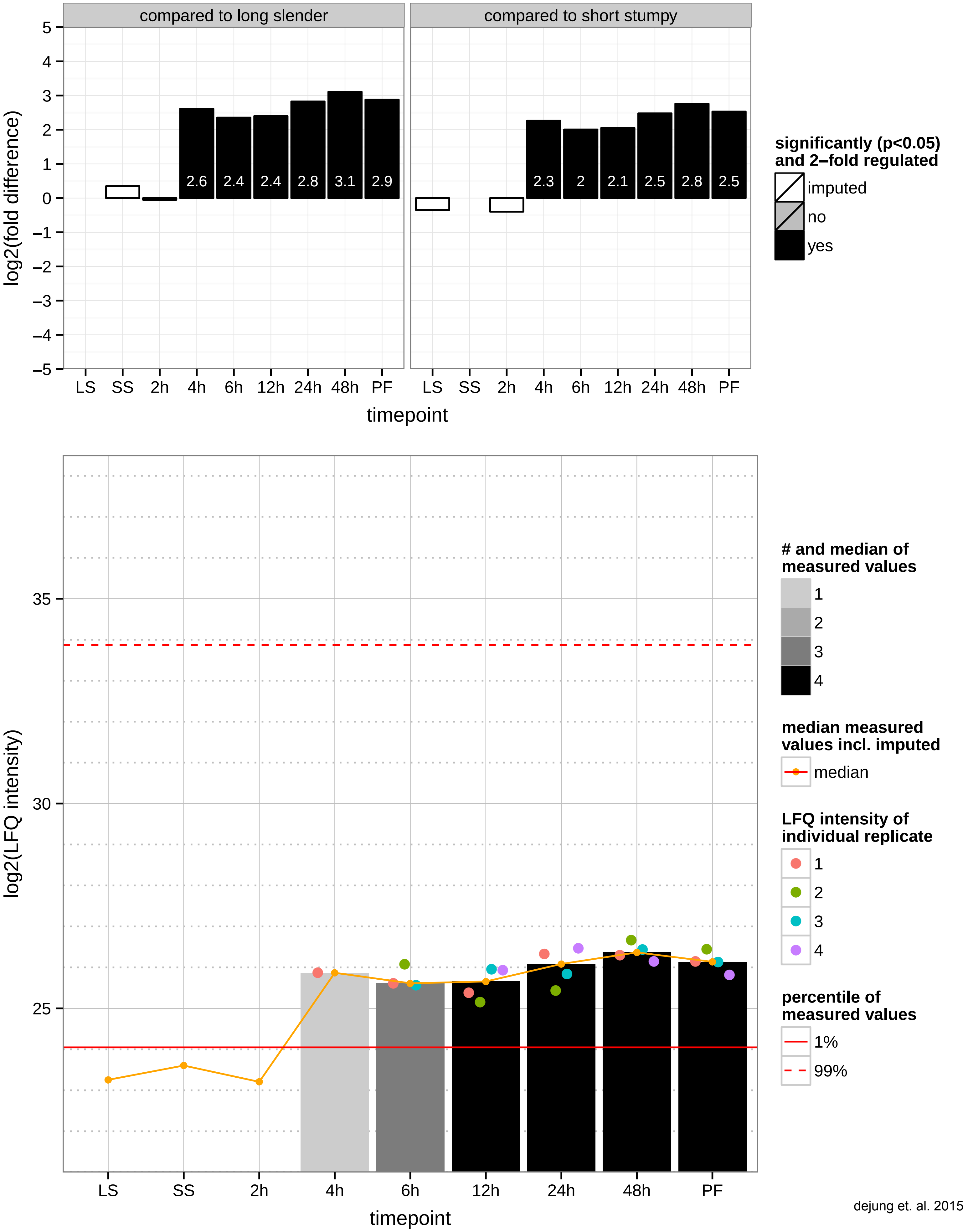

dejung et. al. 2015

hypothetical protein, conserved
Tb927.11.4650
AGOF: null
AGOC: mitochondrion
AGOP: null
PGOF: null
PGOC: null
PGOP: null

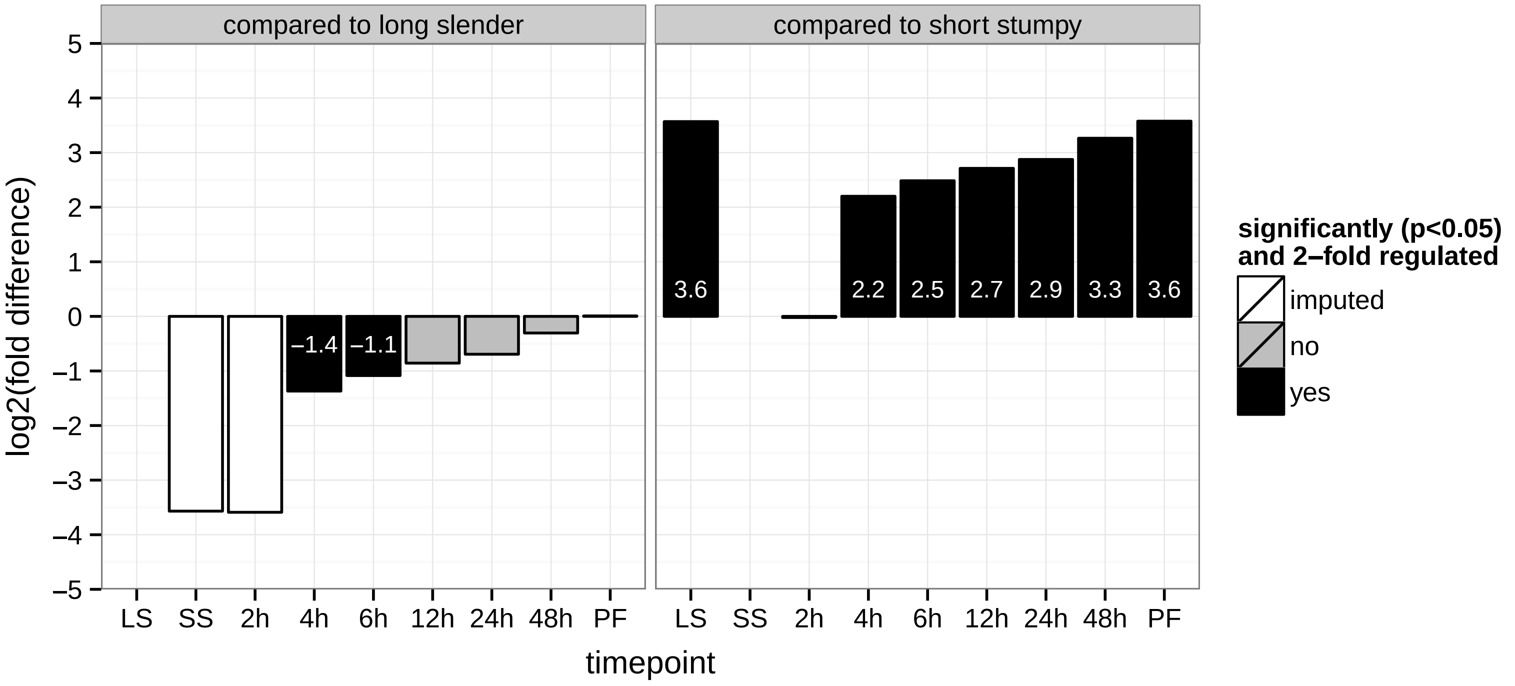

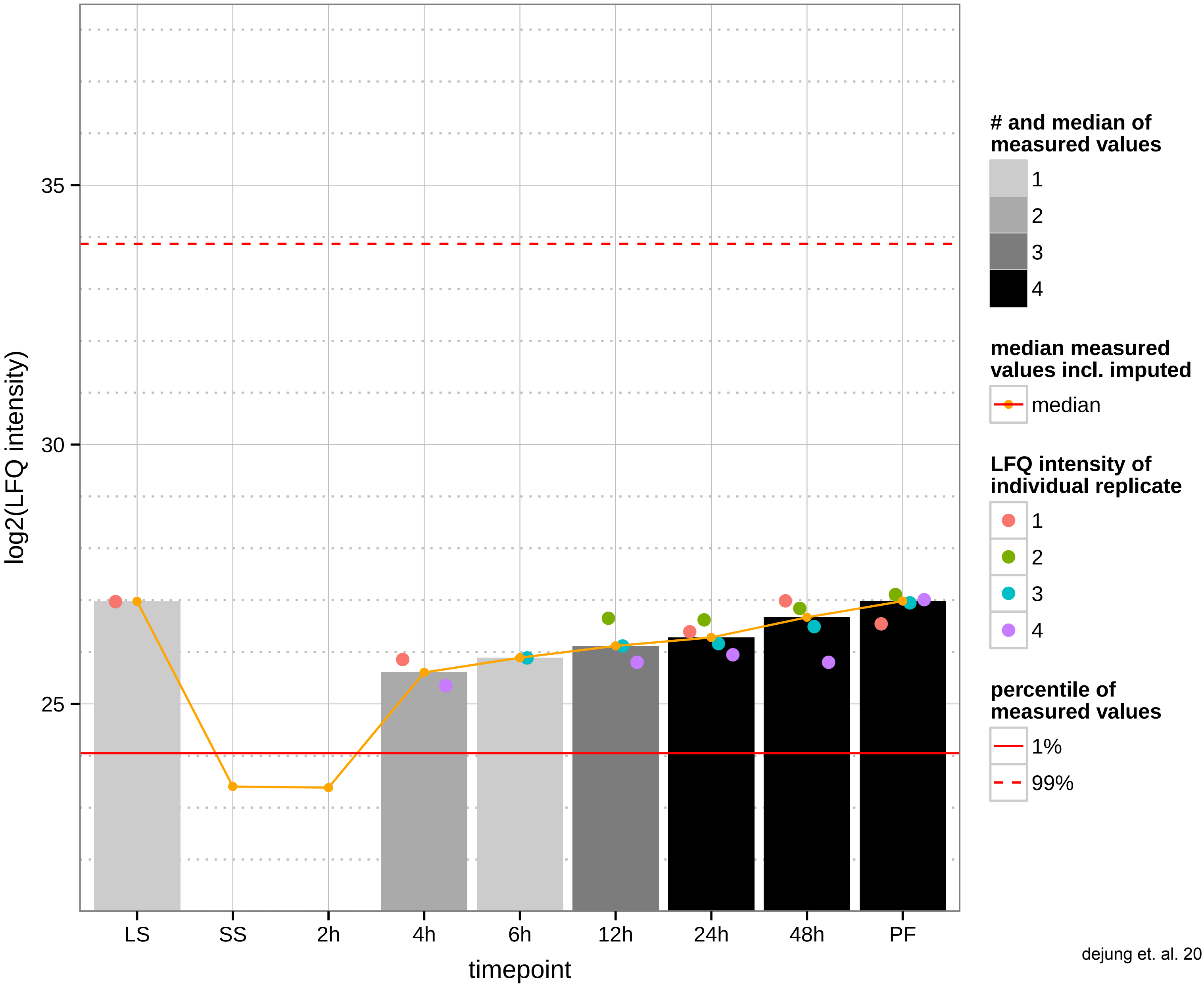

dejung et. al. 2015

hypothetical protein, conserved
Tb927.11.5880
AGOF: null
AGOC: mitochondrion
AGOP: null
PGOF: null
PGOC: null
PGOP: null

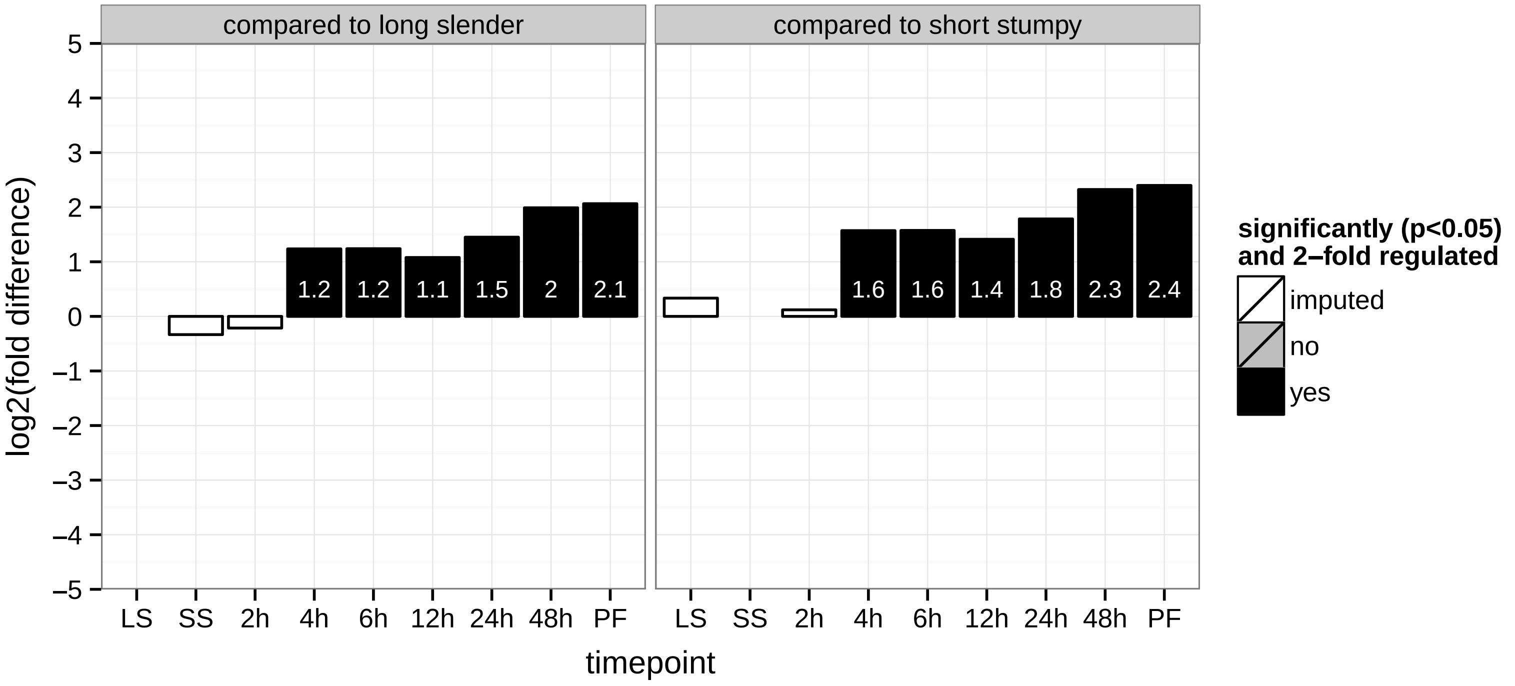

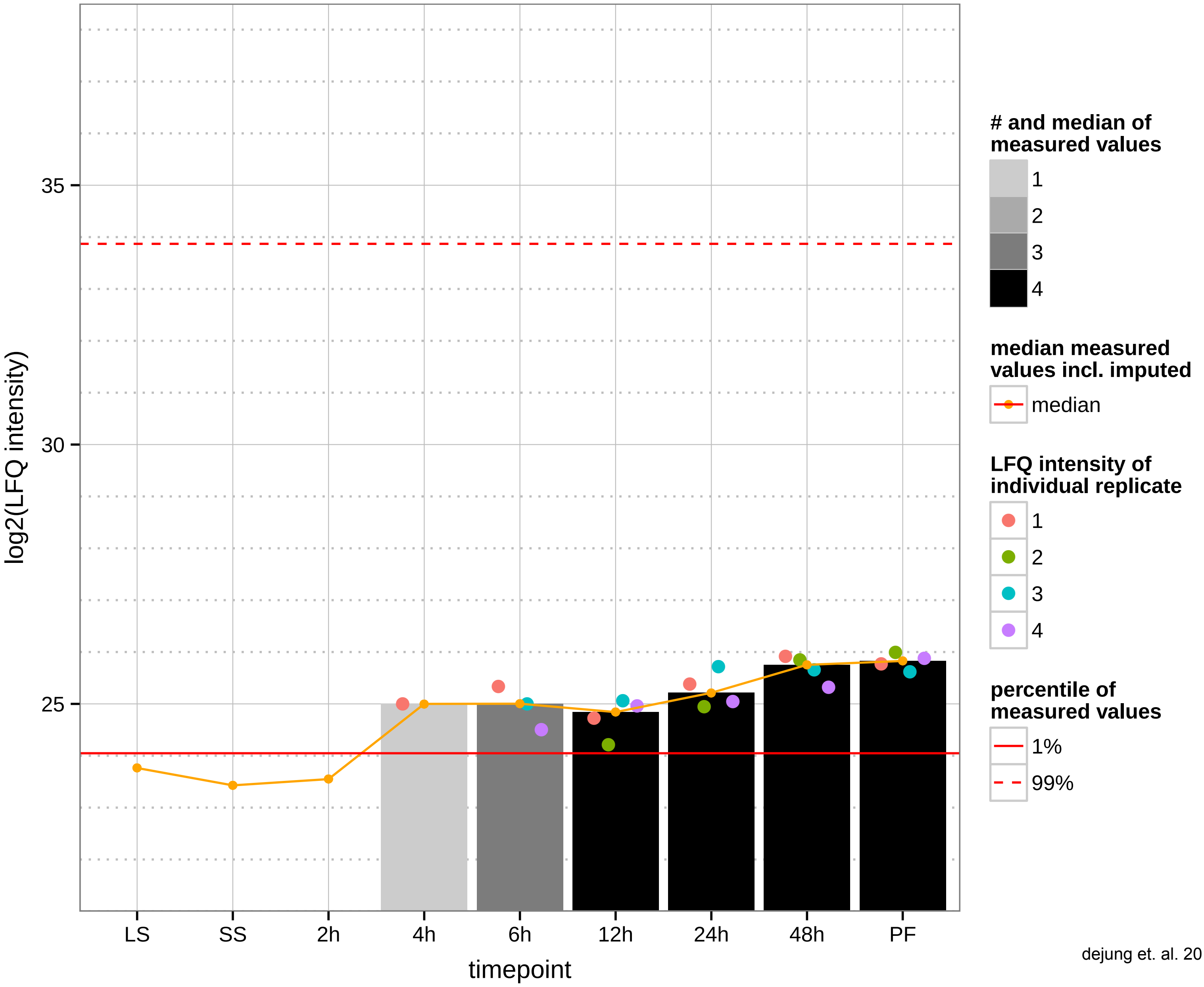

dejung et. al. 2015

hypothetical protein, conserved
Tb927.11.600
AGOF: null
AGOC: mitochondrion
AGOP: null
PGOF: null
PGOC: null
PGOP: null

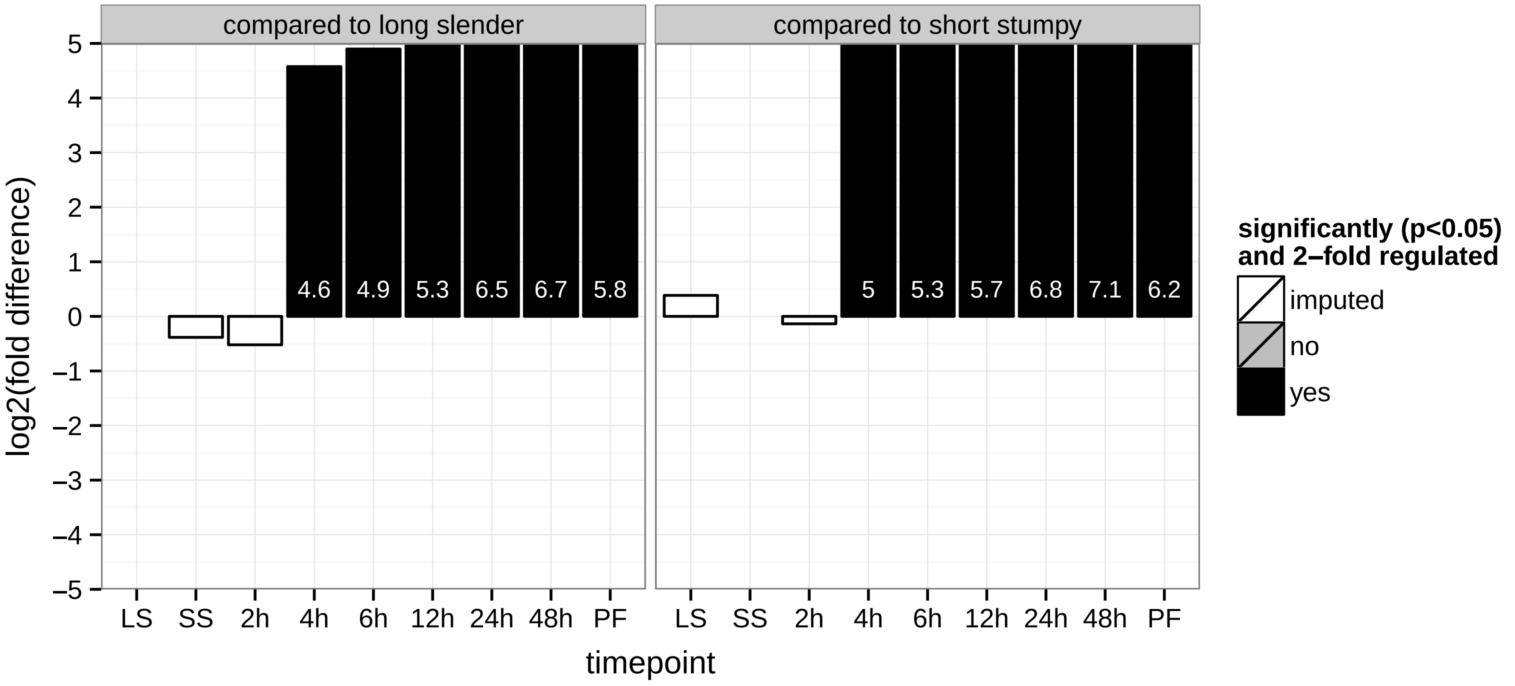

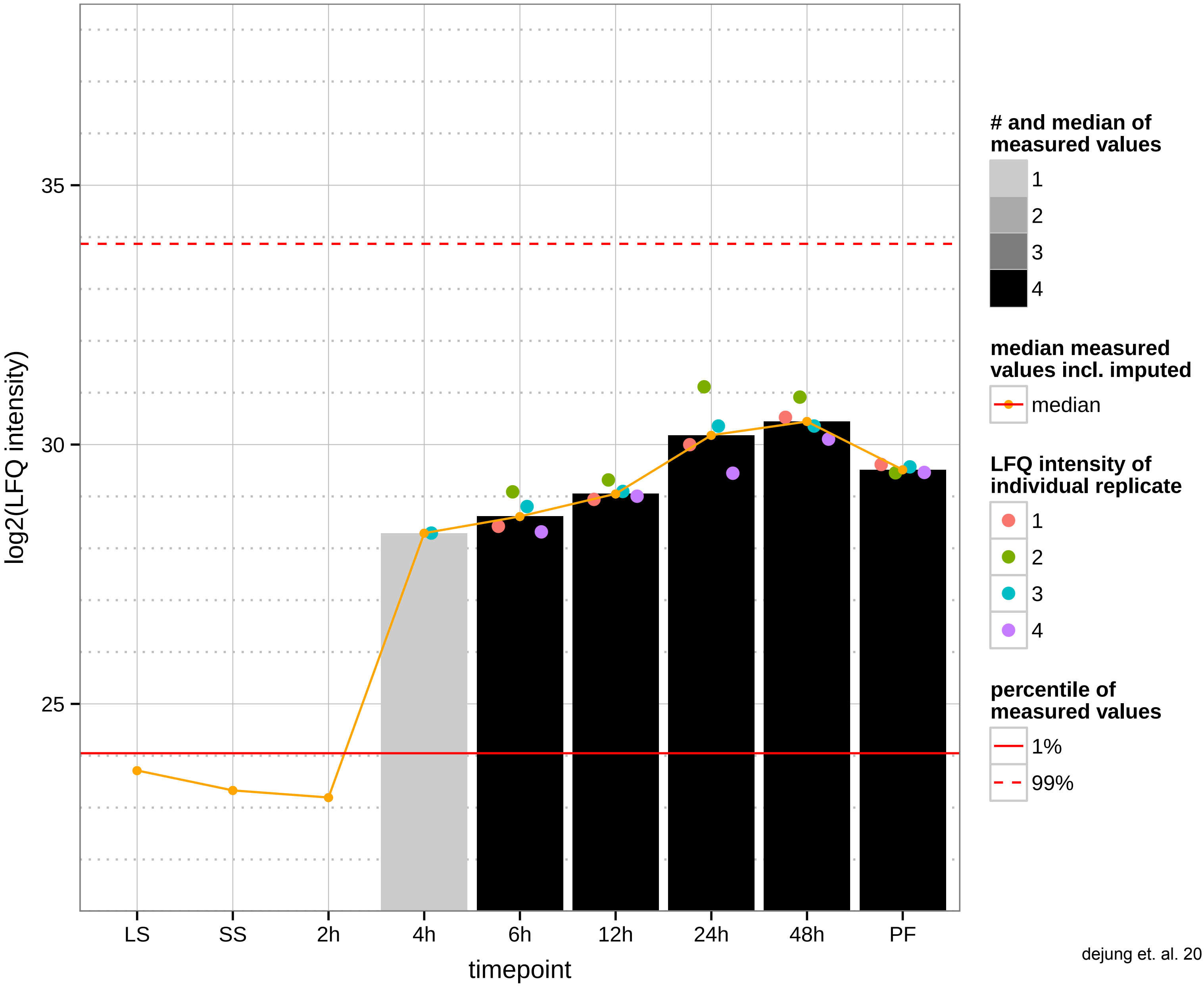

dejung et. al. 2015

hypothetical protein, conserved
Tb927.11.6430
AGOF: null
AGOC: null
AGOP: null
PGOF: null
PGOC: null
PGOP: null

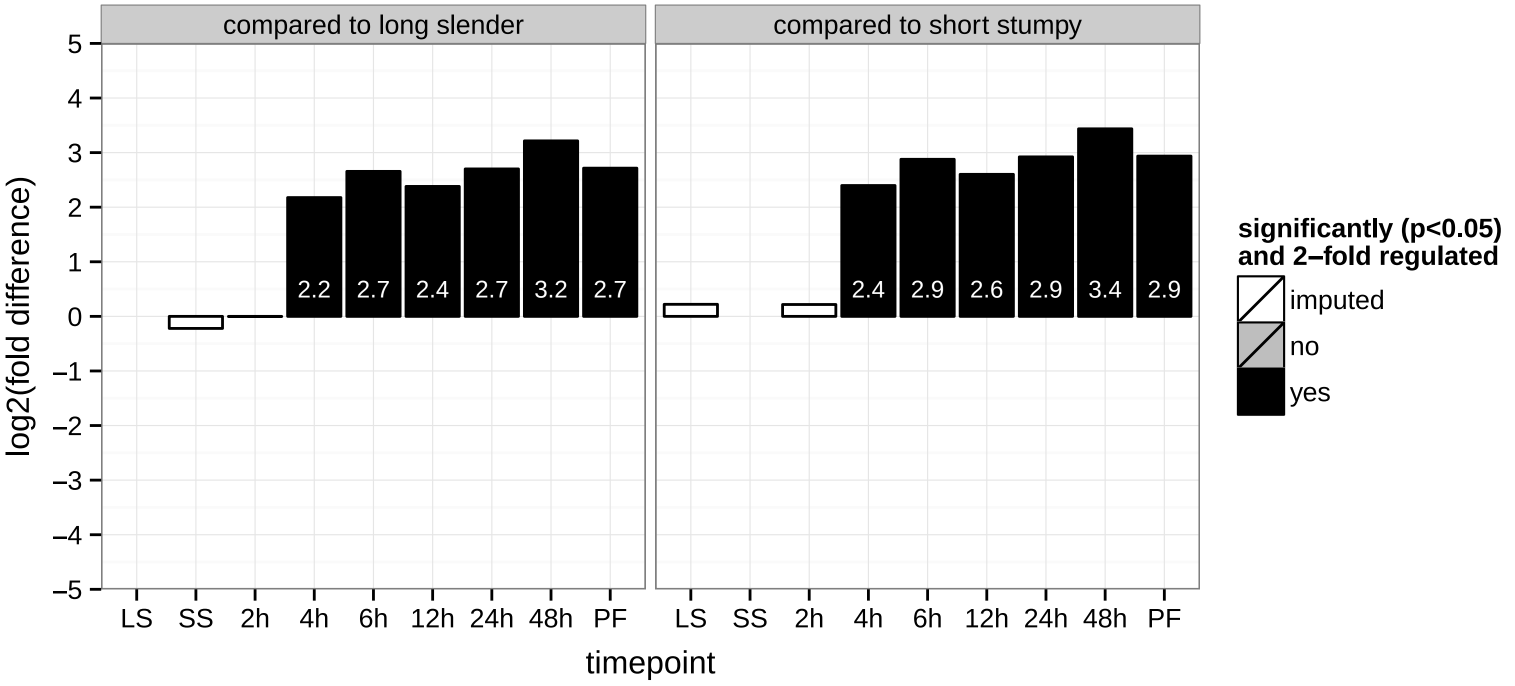

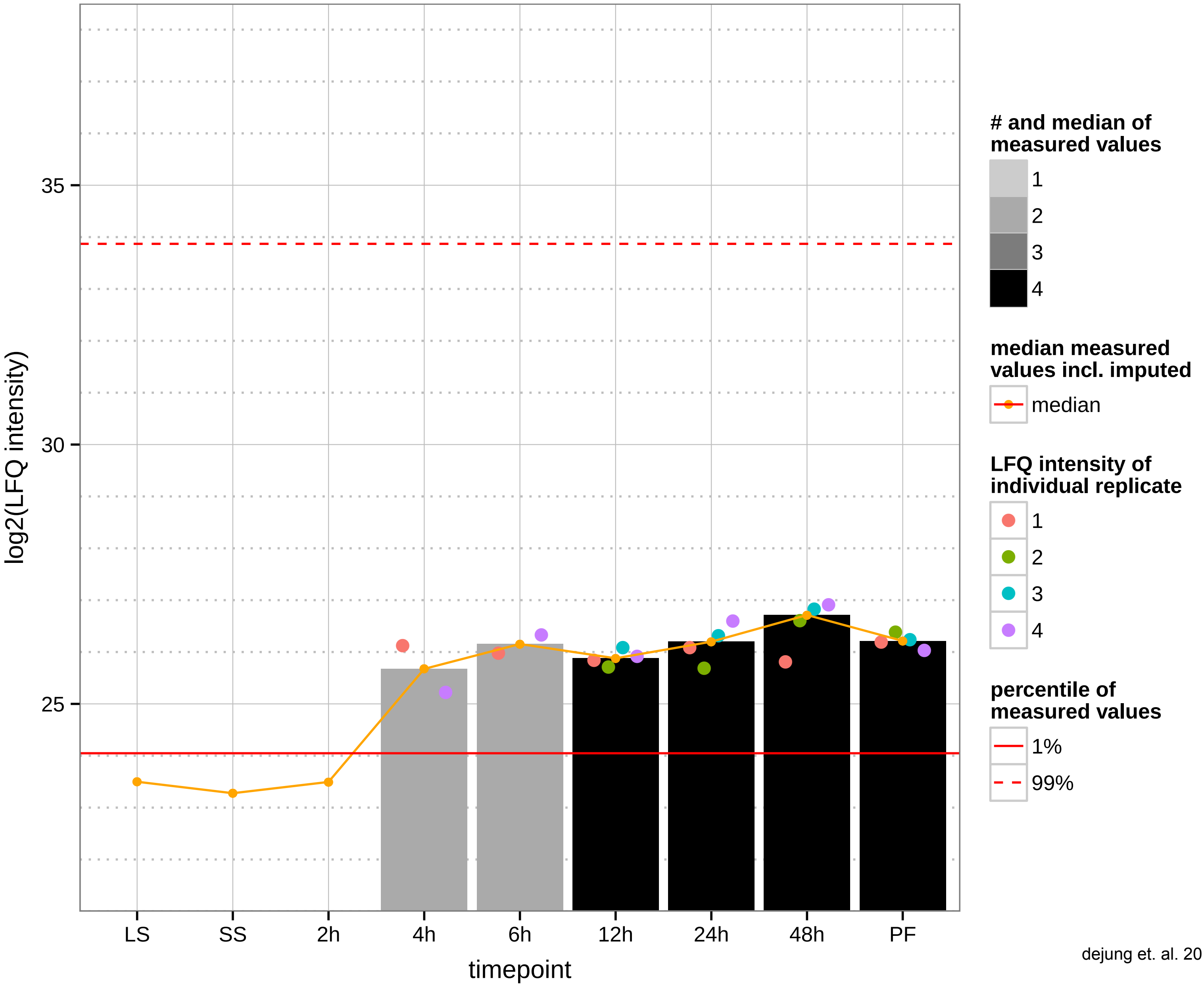

dejung et. al. 2015

hypothetical protein, conserved
Tb927.11.8180
AGOF: null
AGOC: null
AGOP: null
PGOF: null
PGOC: null
PGOP: null

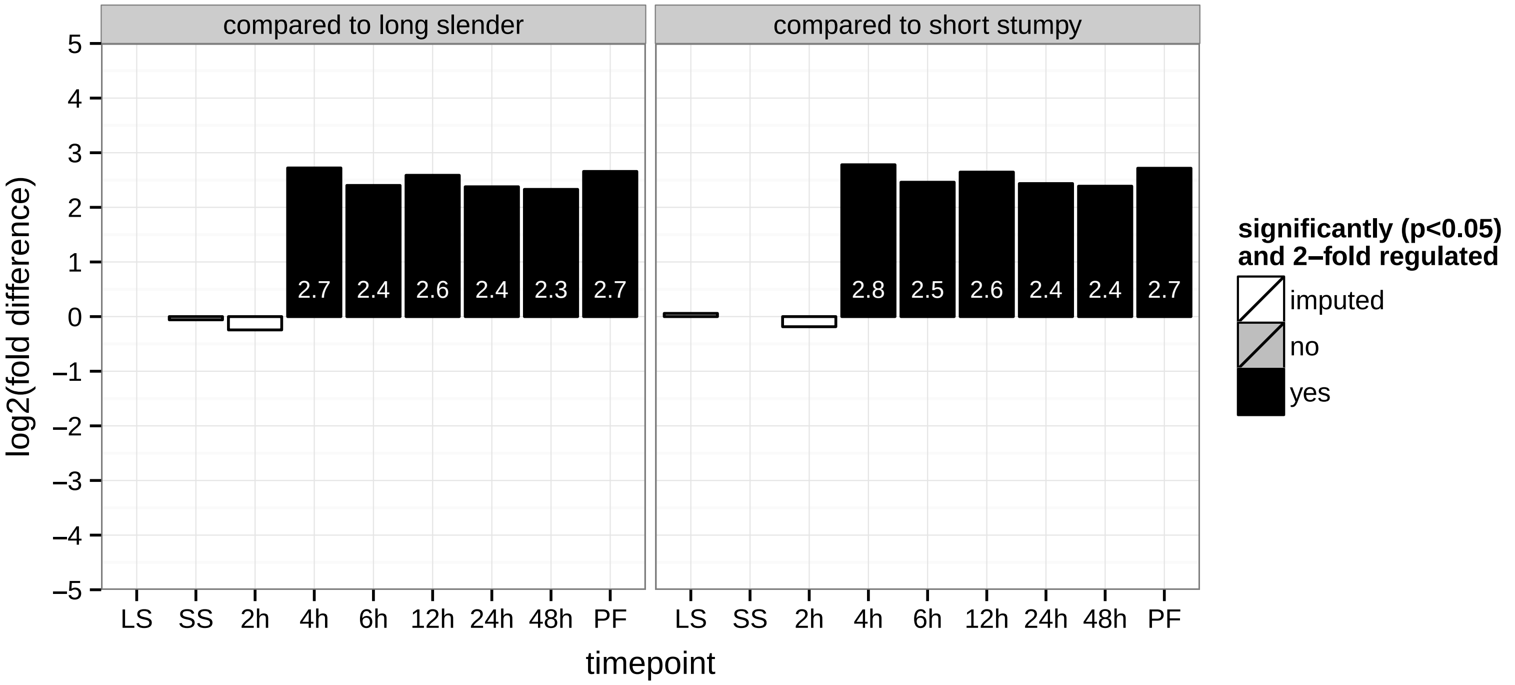

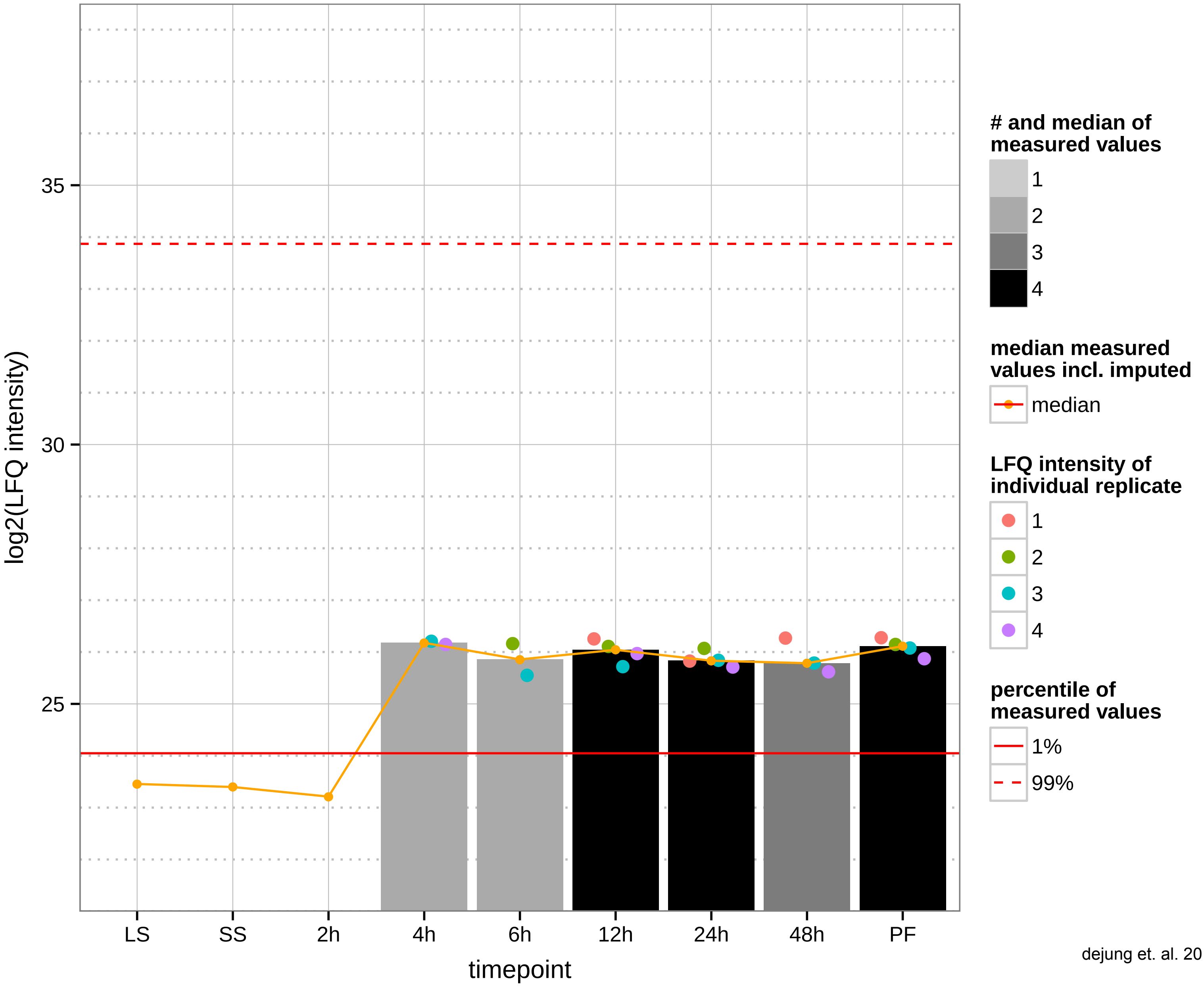

dejung et. al. 2015

nucleic acid binding protein, putative
Tb927.11.9510
AGOF: nucleic acid binding, zinc ion binding
AGOC: null
AGOP: null
PGOF: nucleic acid binding, zinc ion binding
PGOC: null
PGOP: null

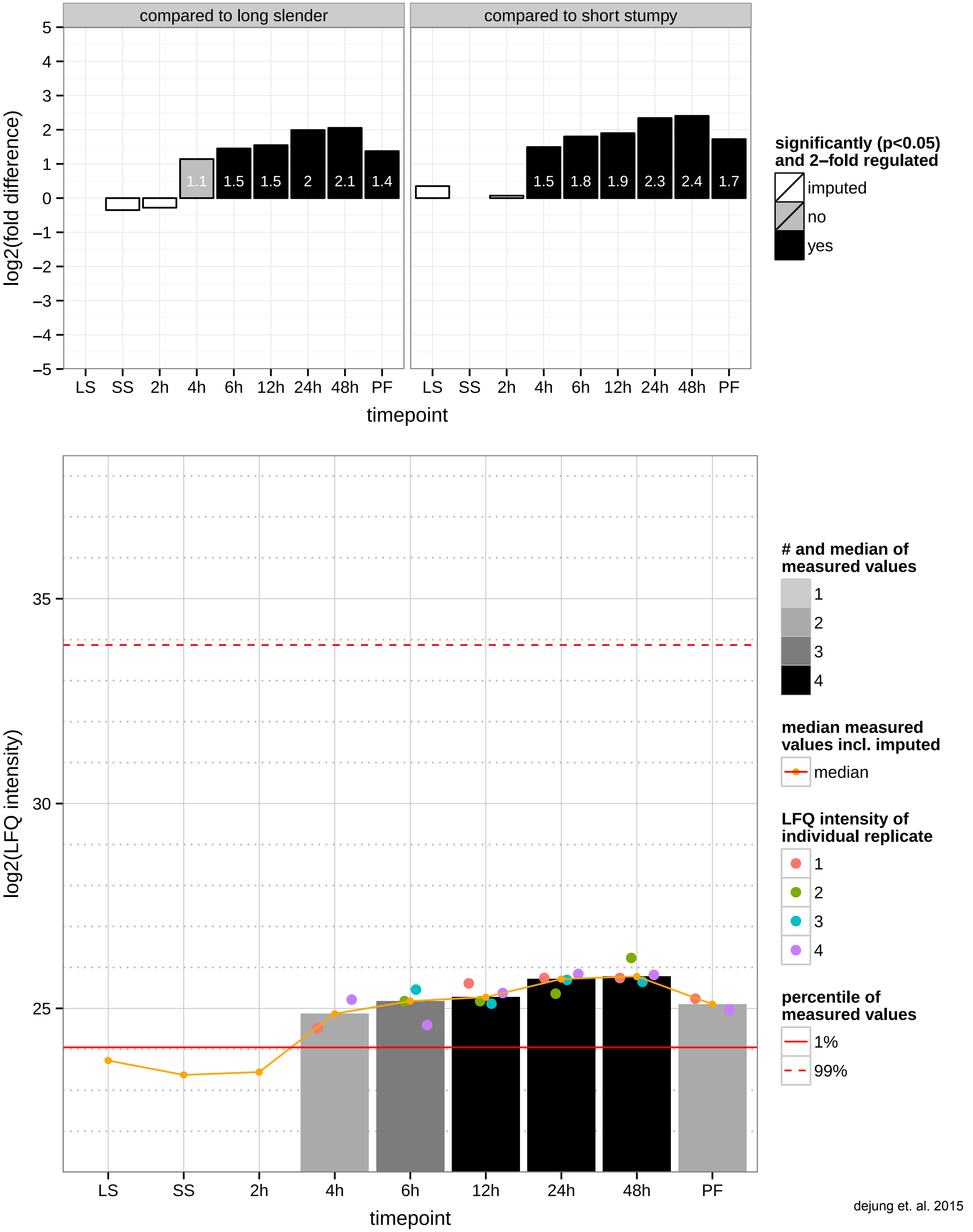

dejung et. al. 2015

retrotransposon hot spot protein (RHS, pseudogene), putative, retrotransposon hot spot protein 3 (RHS3), interrupted
Tb927.2.1140
AGOF: null
AGOC: null
AGOP: null
PGOF: null
PGOC: null
PGOP: null

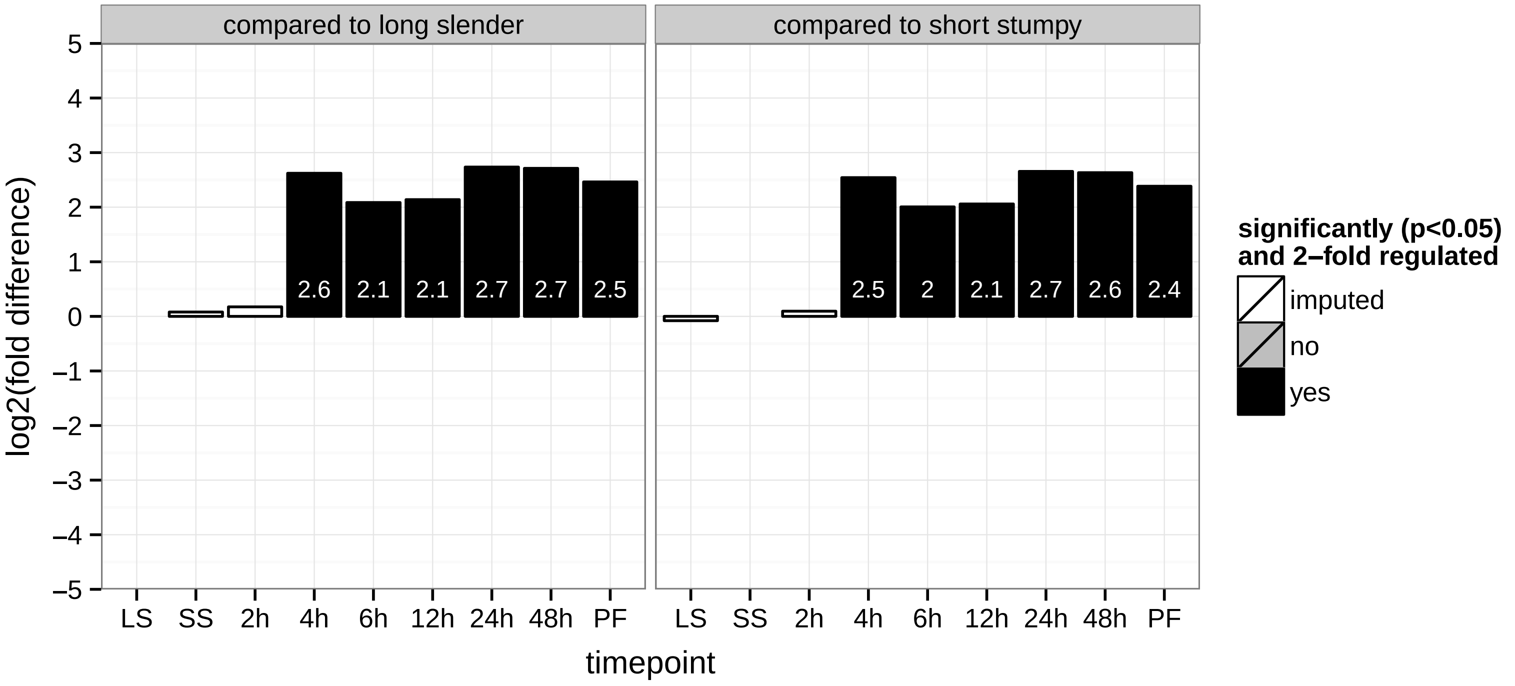

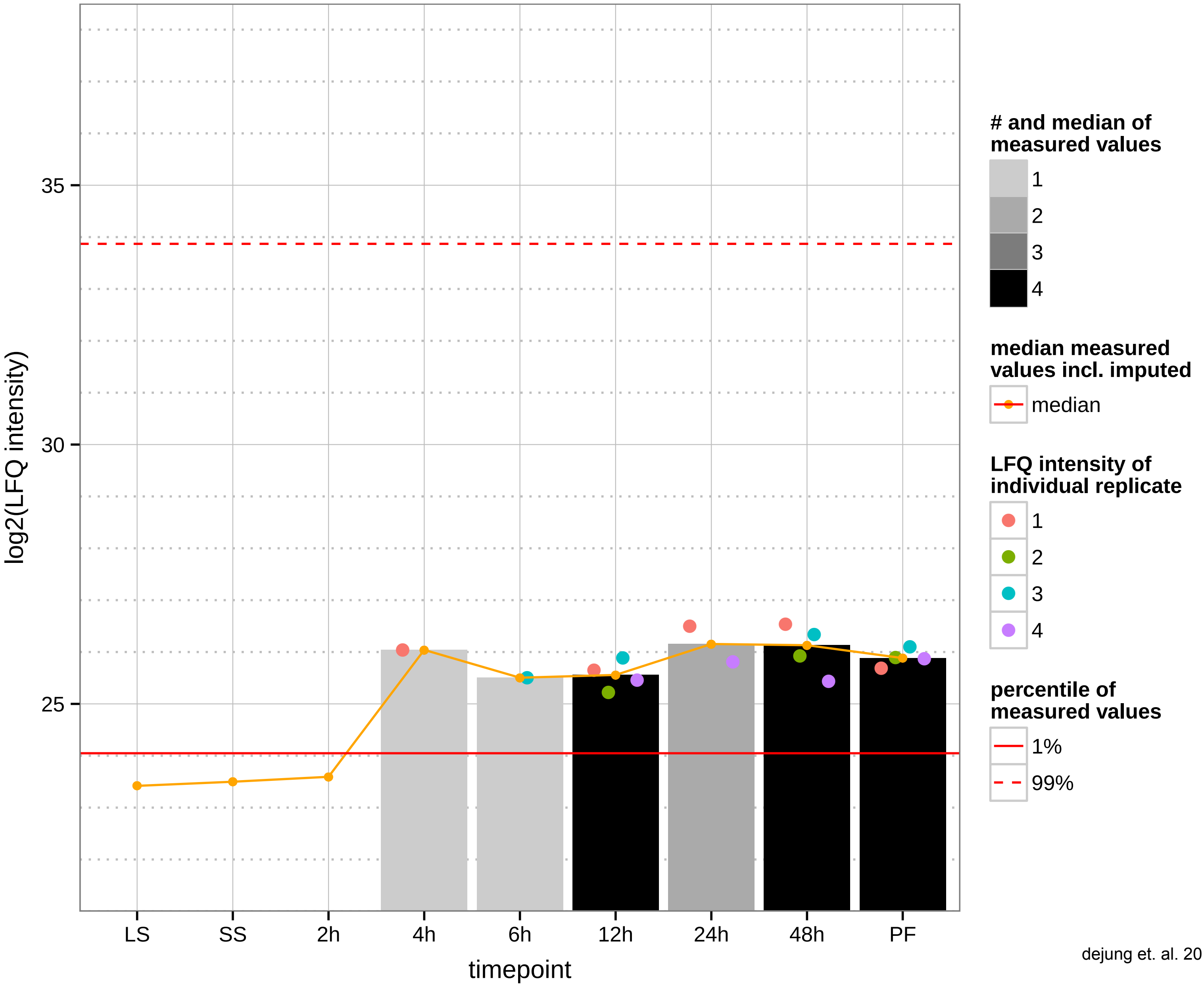

dejung et. al. 2015

hypothetical protein, conserved
Tb927.3.4160
AGOF: damaged DNA binding
AGOC: null
AGOP: nucleotide–excision repair, proteasomal ubiquitin–dependent protein catabolic process
PGOF: damaged DNA binding, protein binding
PGOC: null
PGOP: nucleotide–excision repair, proteasomal ubiquitin–dependent protein catabolic process

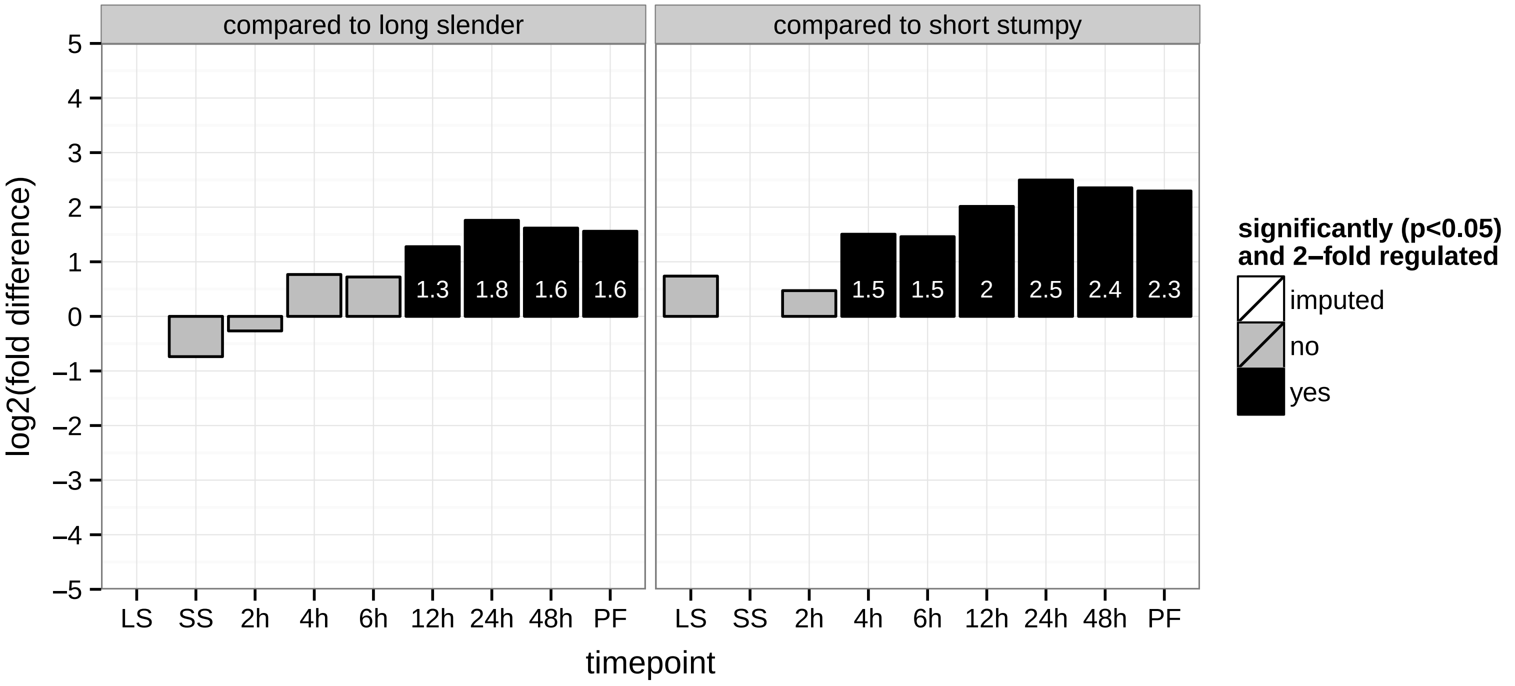

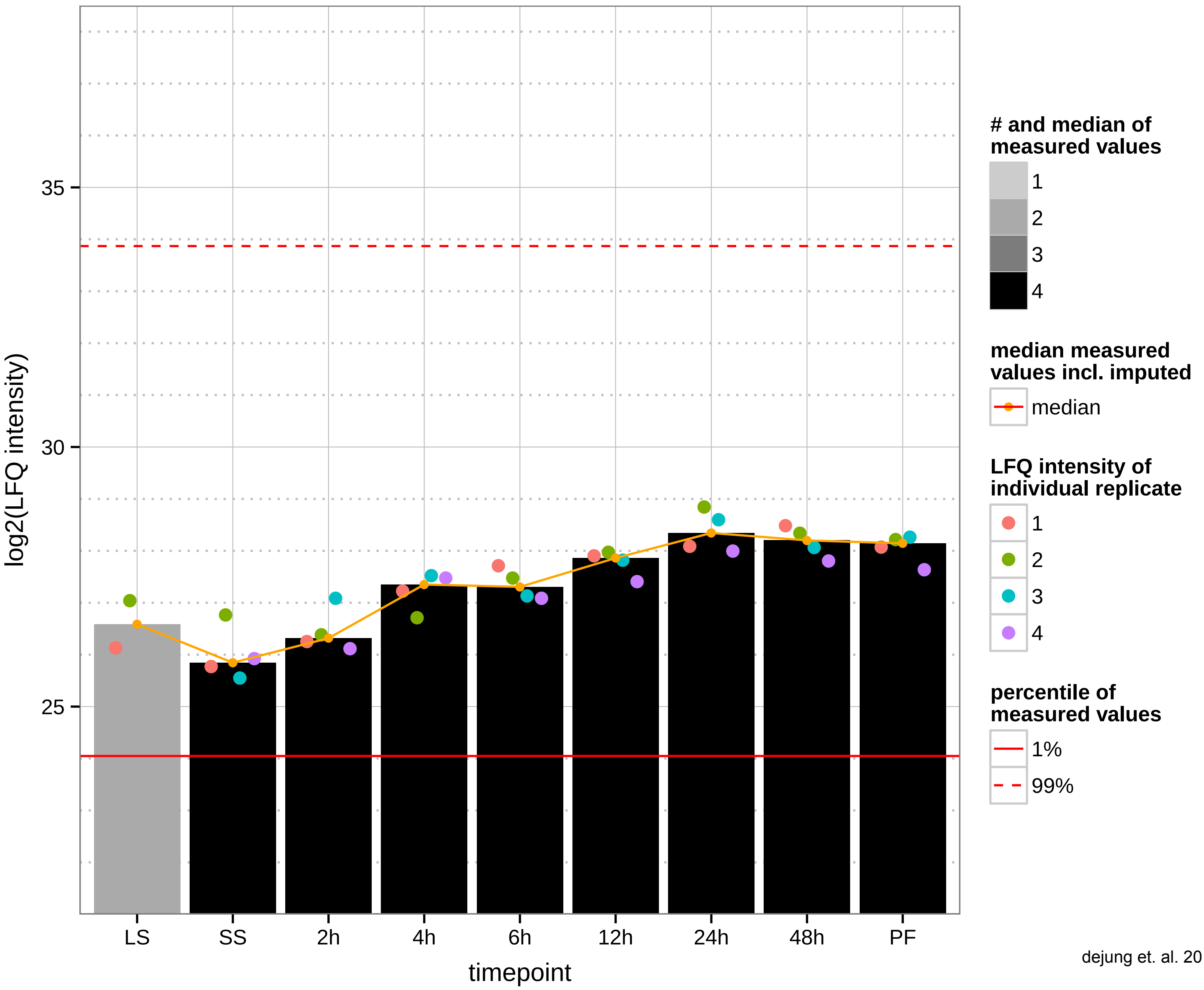

dejung et. al. 2015

uracil phosphoribosyltransferase, putative
Tb927.4.3320
AGOF: null
AGOC: null
AGOP: nucleoside metabolic process
PGOF: null
PGOC: null
PGOP: nucleoside metabolic process

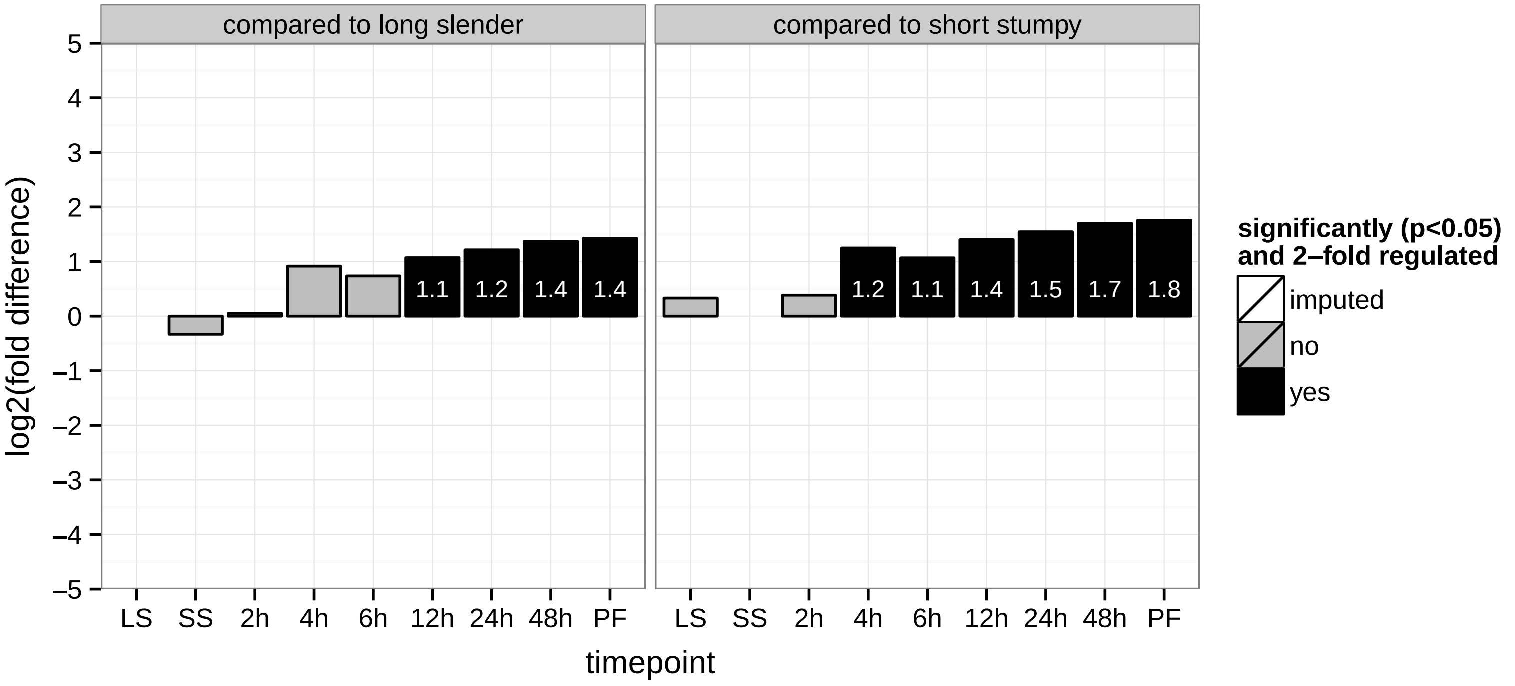

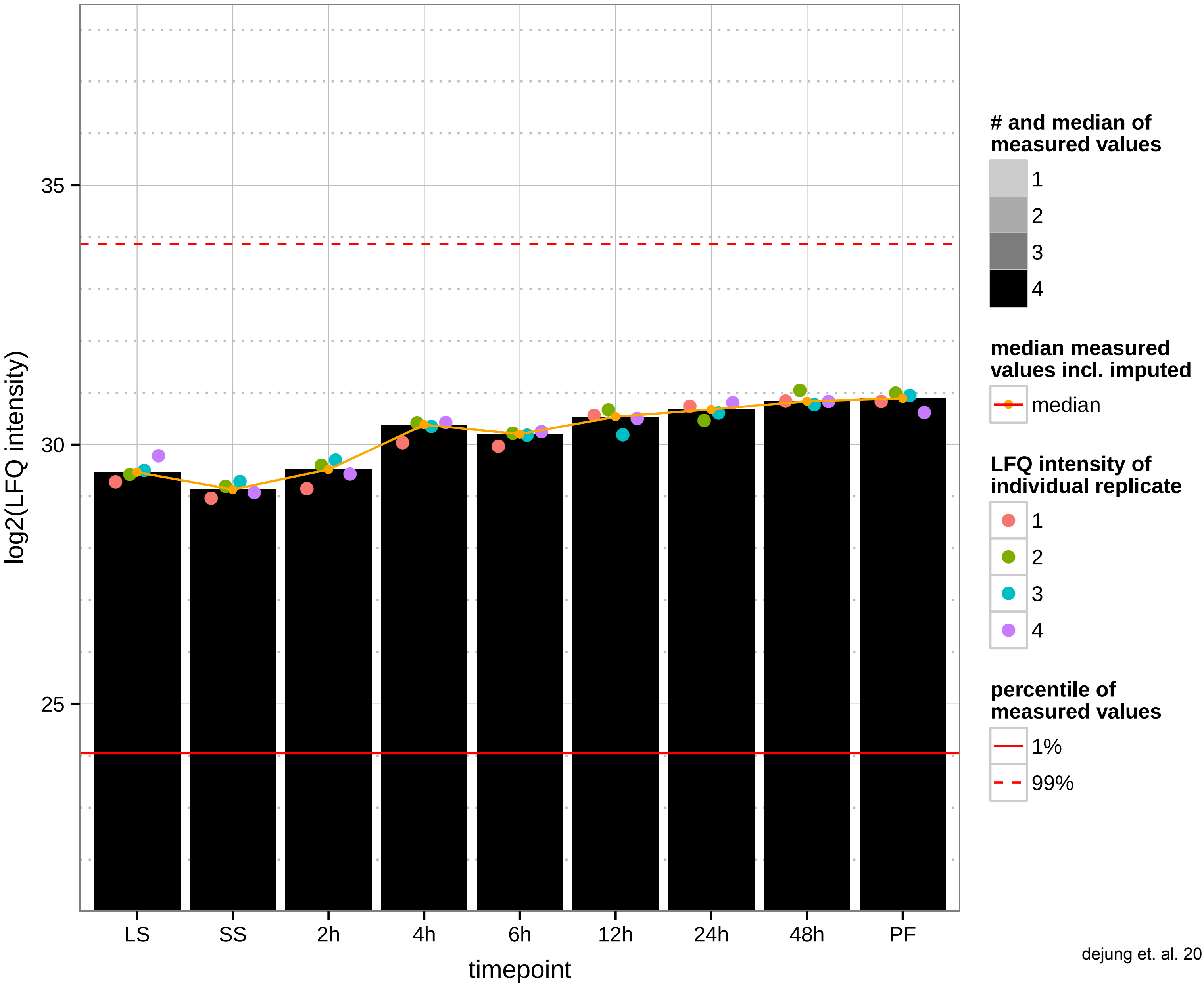

dejung et. al. 2015

hypothetical protein, conserved
Tb927.4.3500
AGOF: null
AGOC: null
AGOP: null
PGOF: null
PGOC: null
PGOP: null

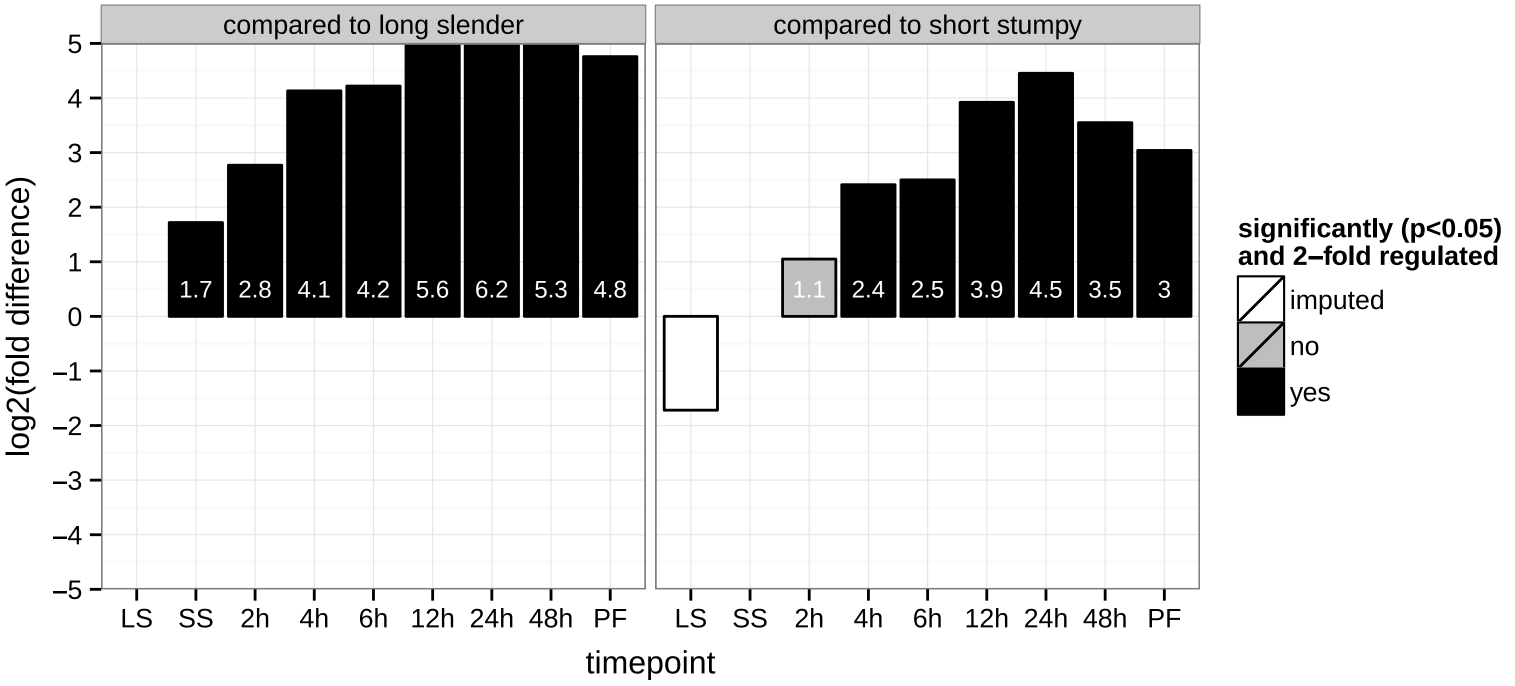

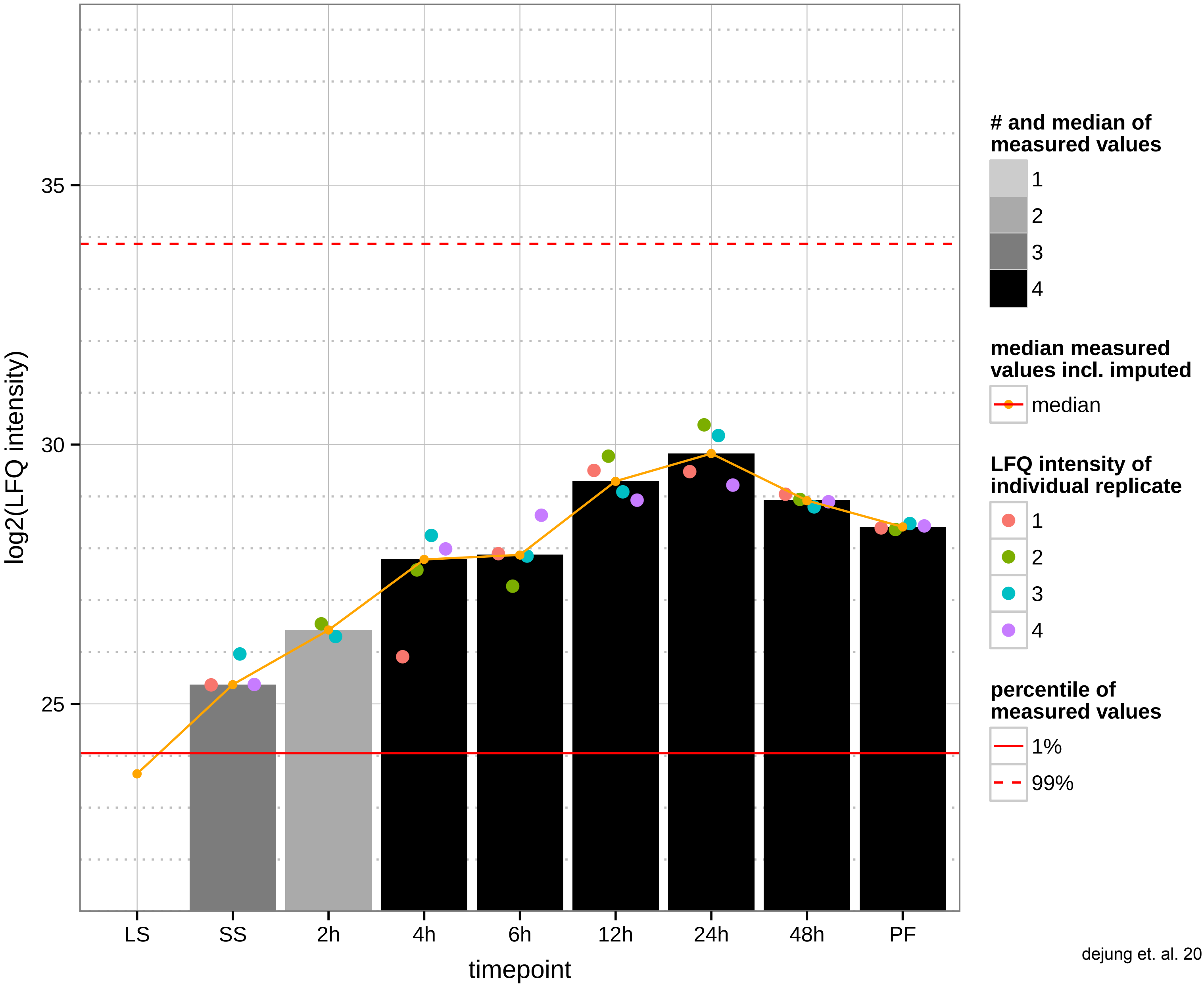

dejung et. al. 2015

hypothetical protein, conserved
Tb927.4.3900
AGOF: zinc ion binding
AGOC: intracellular
AGOP: null
PGOF: zinc ion binding
PGOC: intracellular
PGOP: null

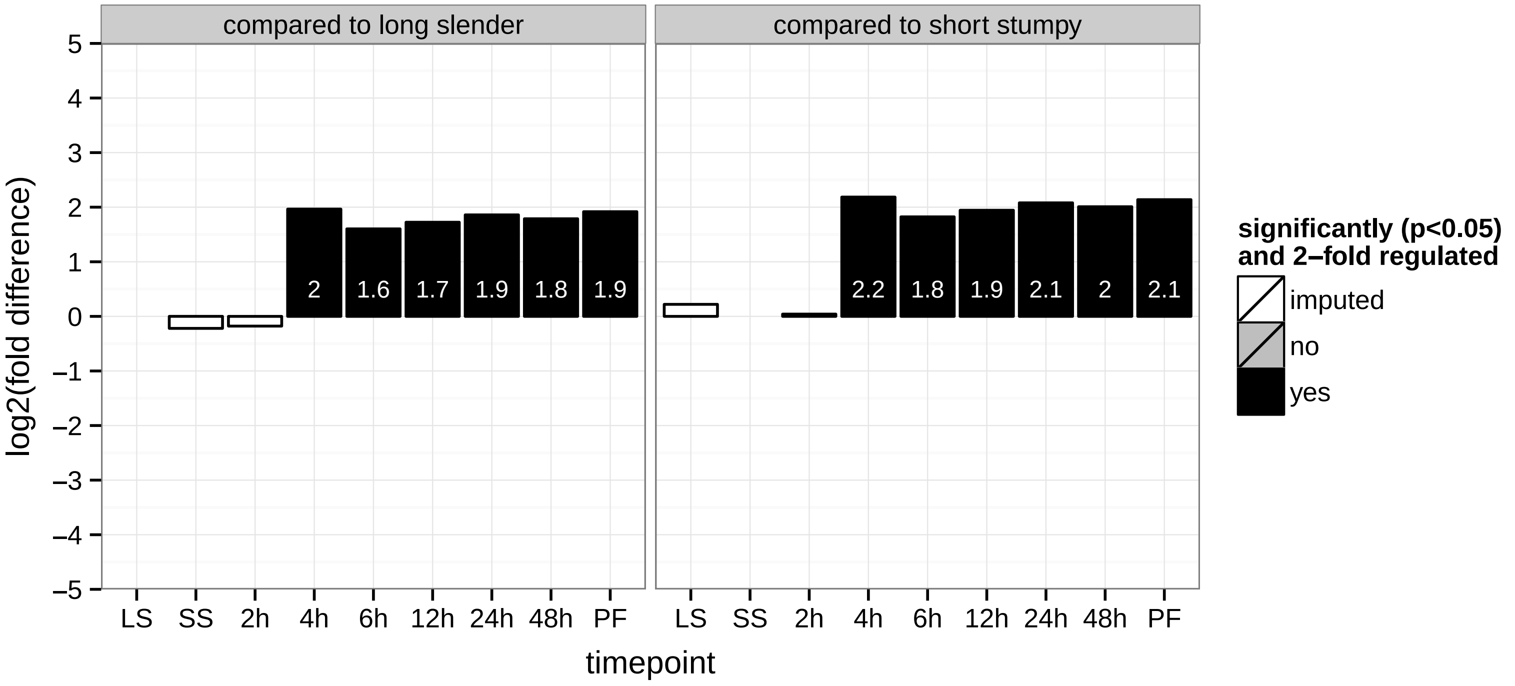

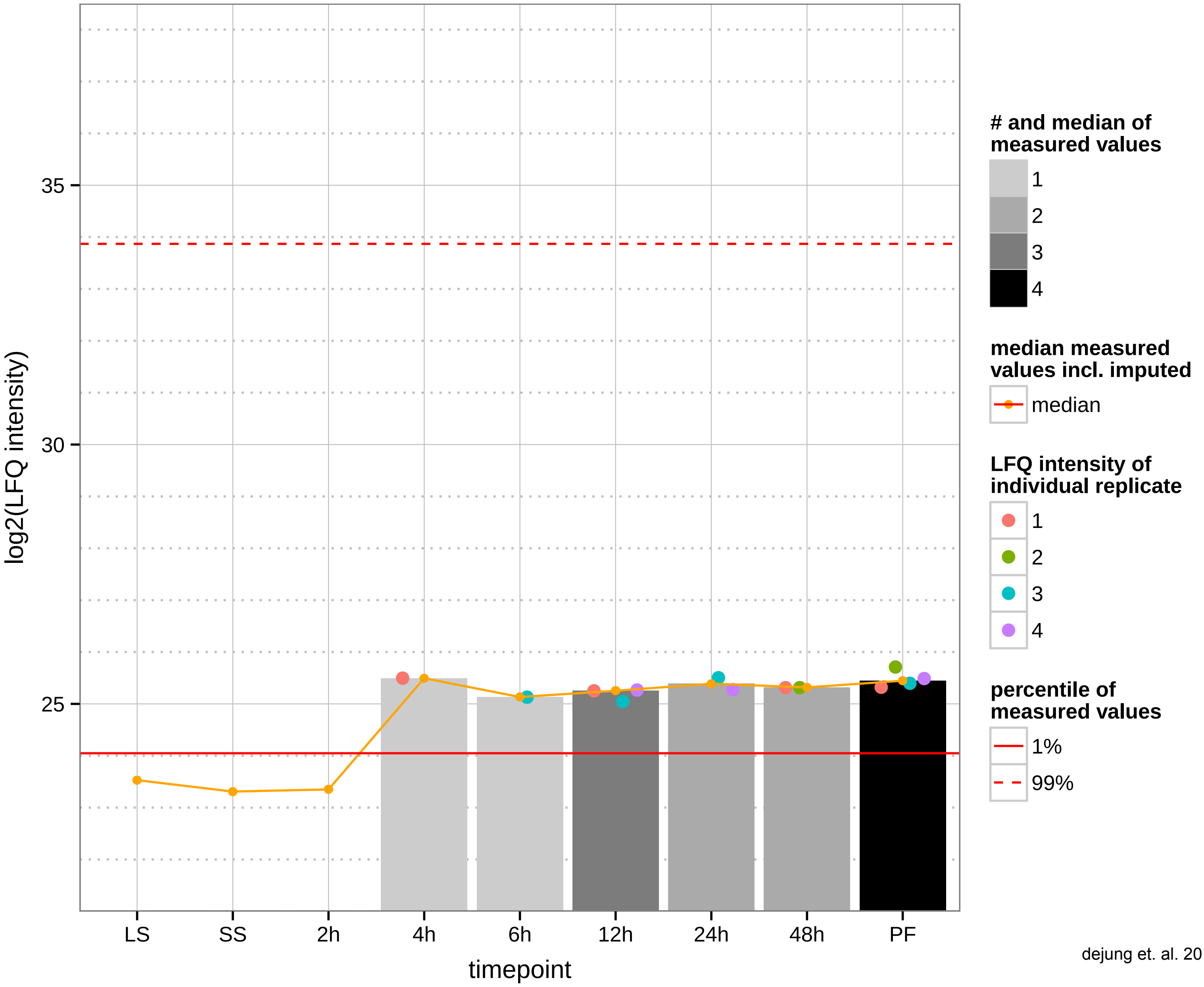

dejung et. al. 2015

cytoskeleton–associated protein CAP5.5, putative, cysteine peptidase, Clan CA, family C2, putative (CAP5.5)
Tb927.4.3950
AGOF: calcium–dependent cysteine–type endopeptidase activity
AGOC: intracellular
AGOP: proteolysis
PGOF: calcium–dependent cysteine–type endopeptidase activity
PGOC: intracellular
PGOP: proteolysis

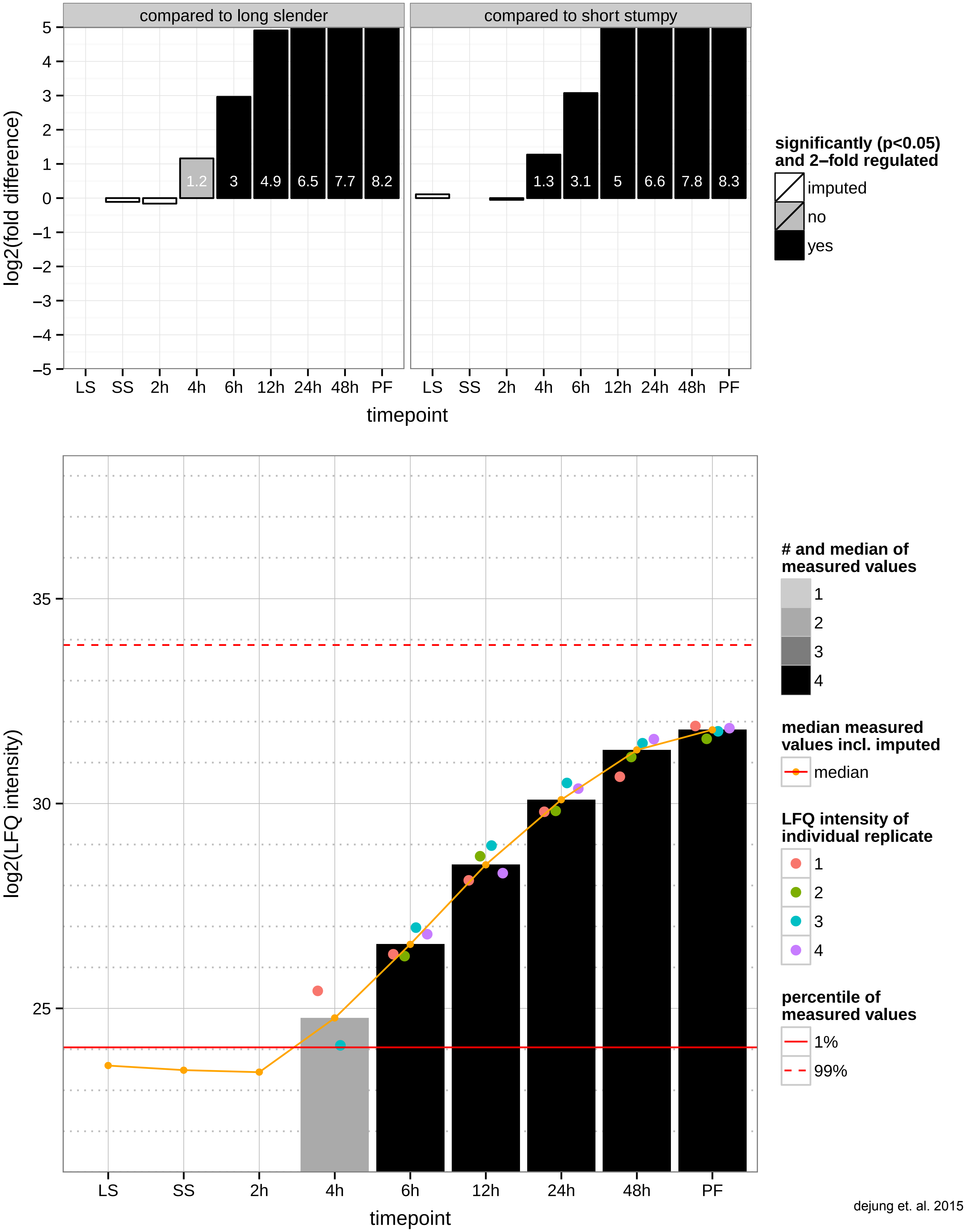

dejung et. al. 2015

mevalonate kinase, putative
Tb927.4.4070
AGOF: ATP binding, mevalonate kinase activity
AGOC: cytoplasm
AGOP: isoprenoid biosynthetic process, phosphorylation
PGOF: ATP binding, mevalonate kinase activity
PGOC: cytoplasm
PGOP: isoprenoid biosynthetic process

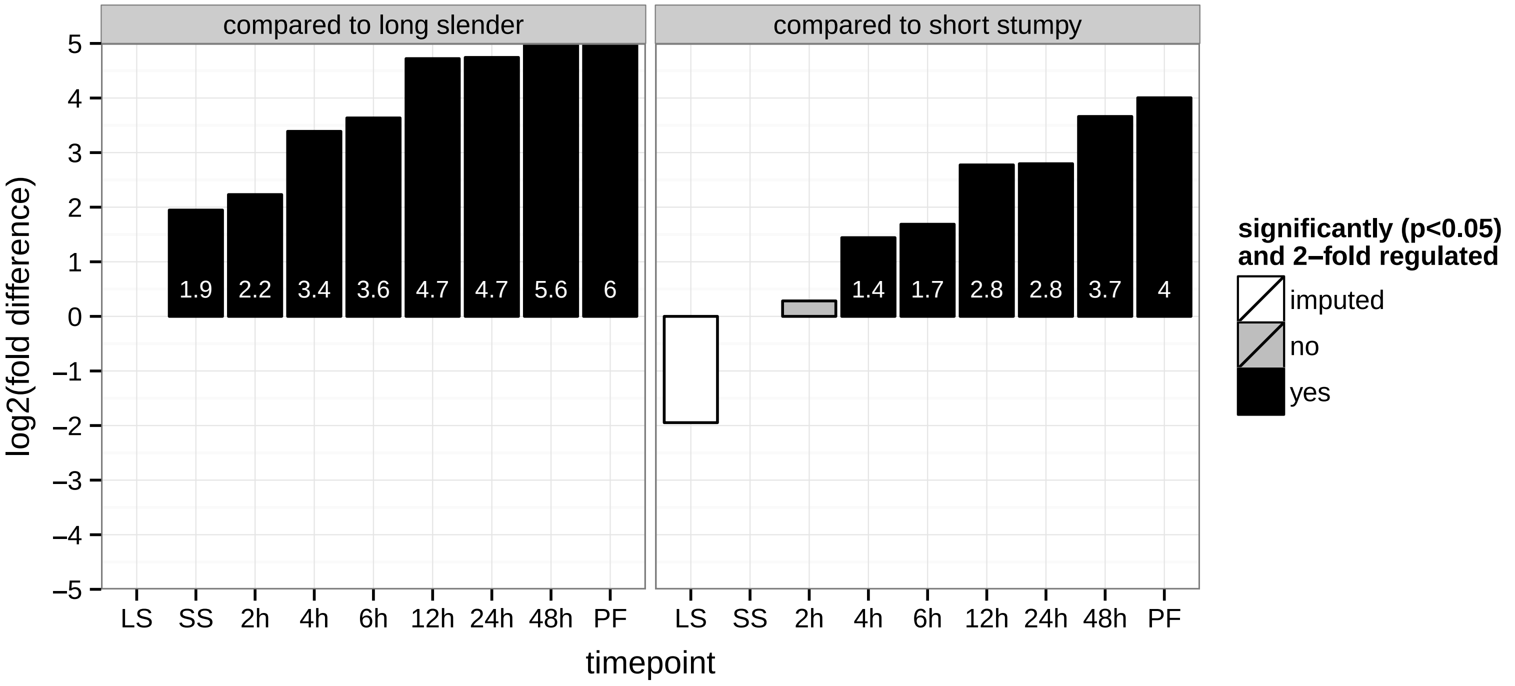

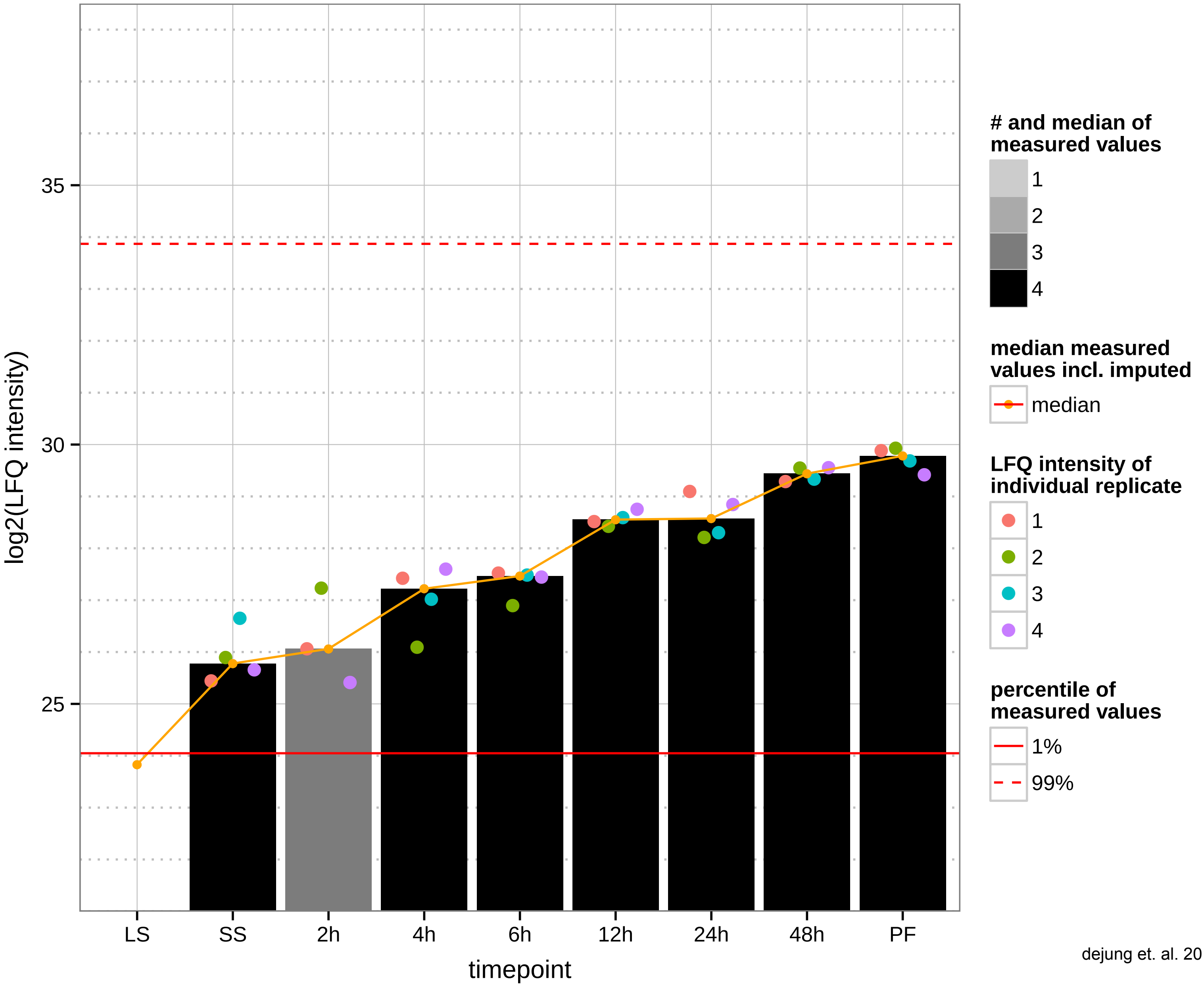

dejung et. al. 2015

hypothetical protein, conserved
Tb927.6.1250
AGOF: null
AGOC: null, mitochondrion
AGOP: null
PGOF: null
PGOC: null
PGOP: null

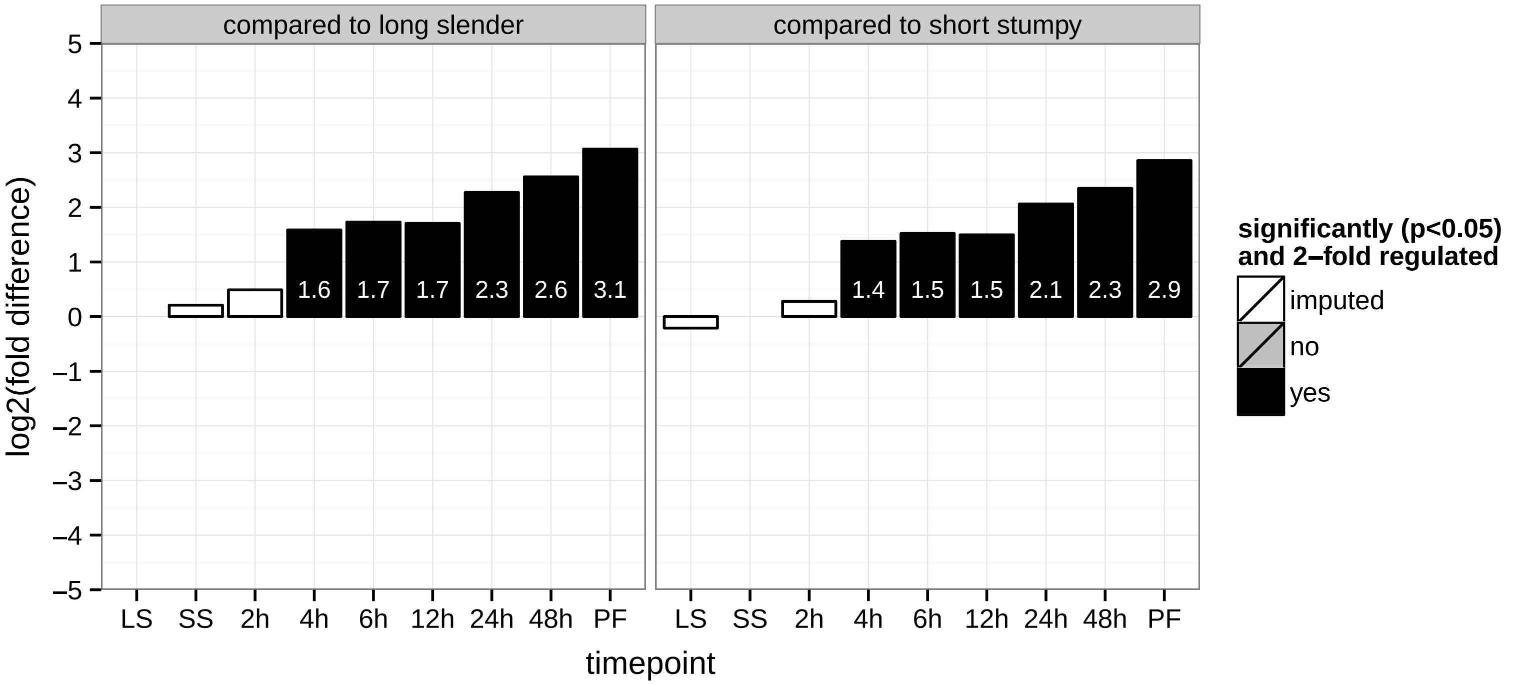

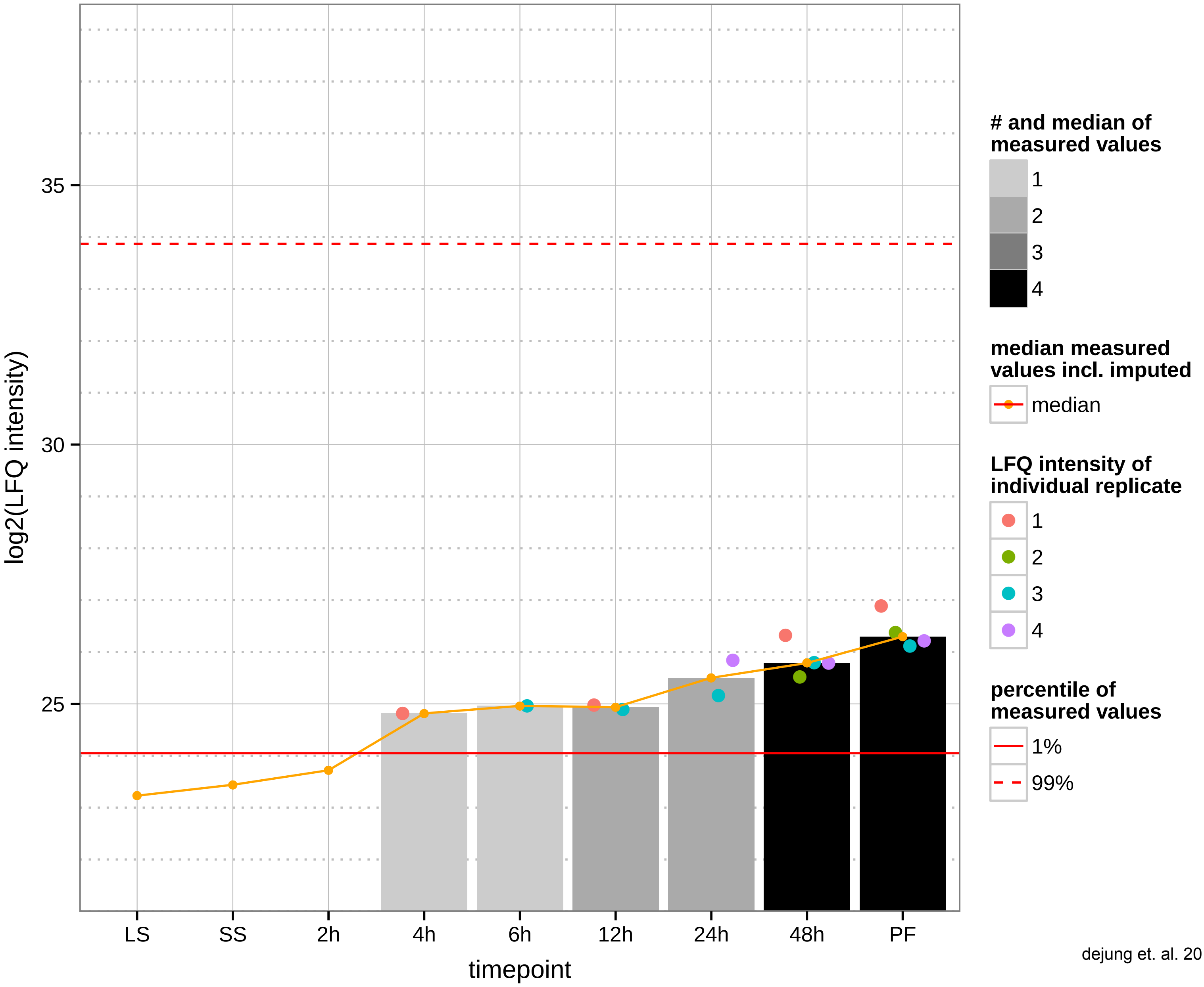

dejung et. al. 2015

hypothetical protein, conserved
Tb927.6.2380
AGOF: null
AGOC: mitochondrion
AGOP: null
PGOF: null
PGOC: null
PGOP: null

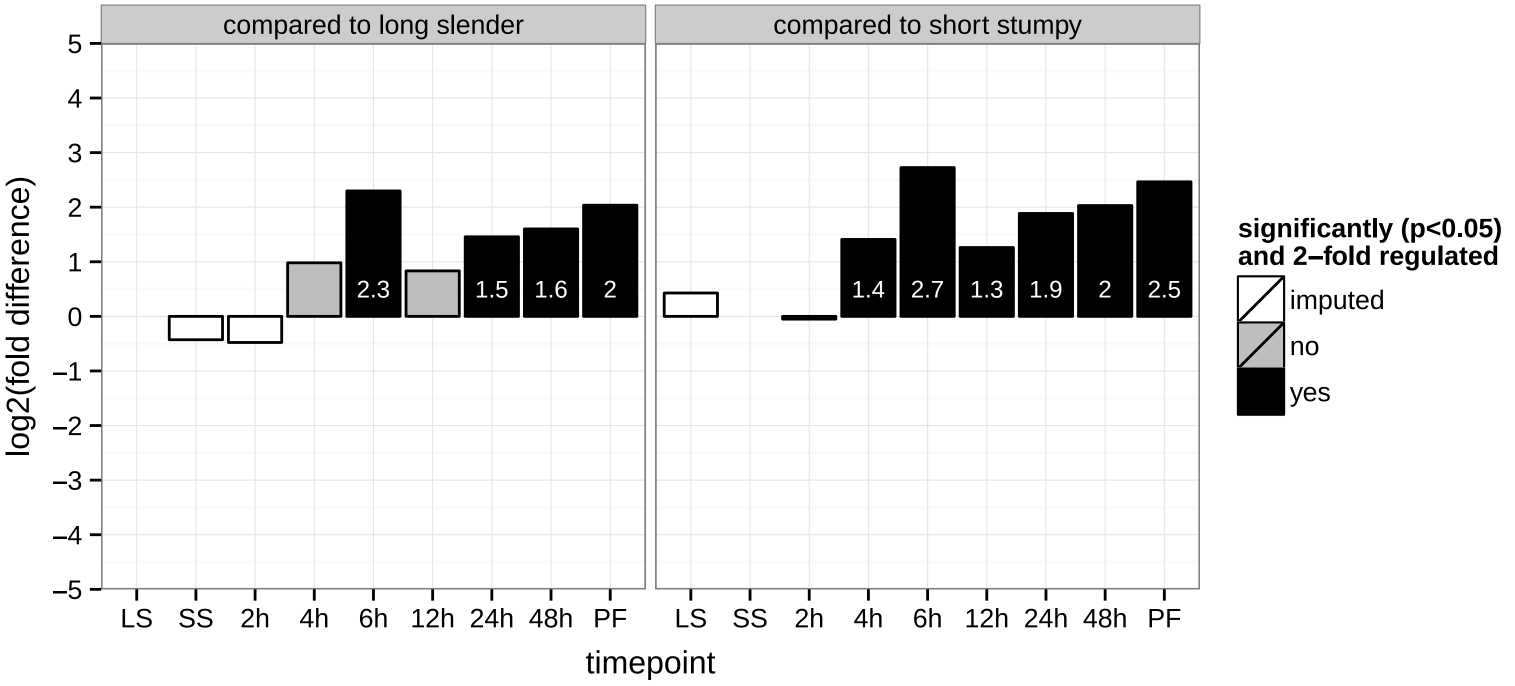

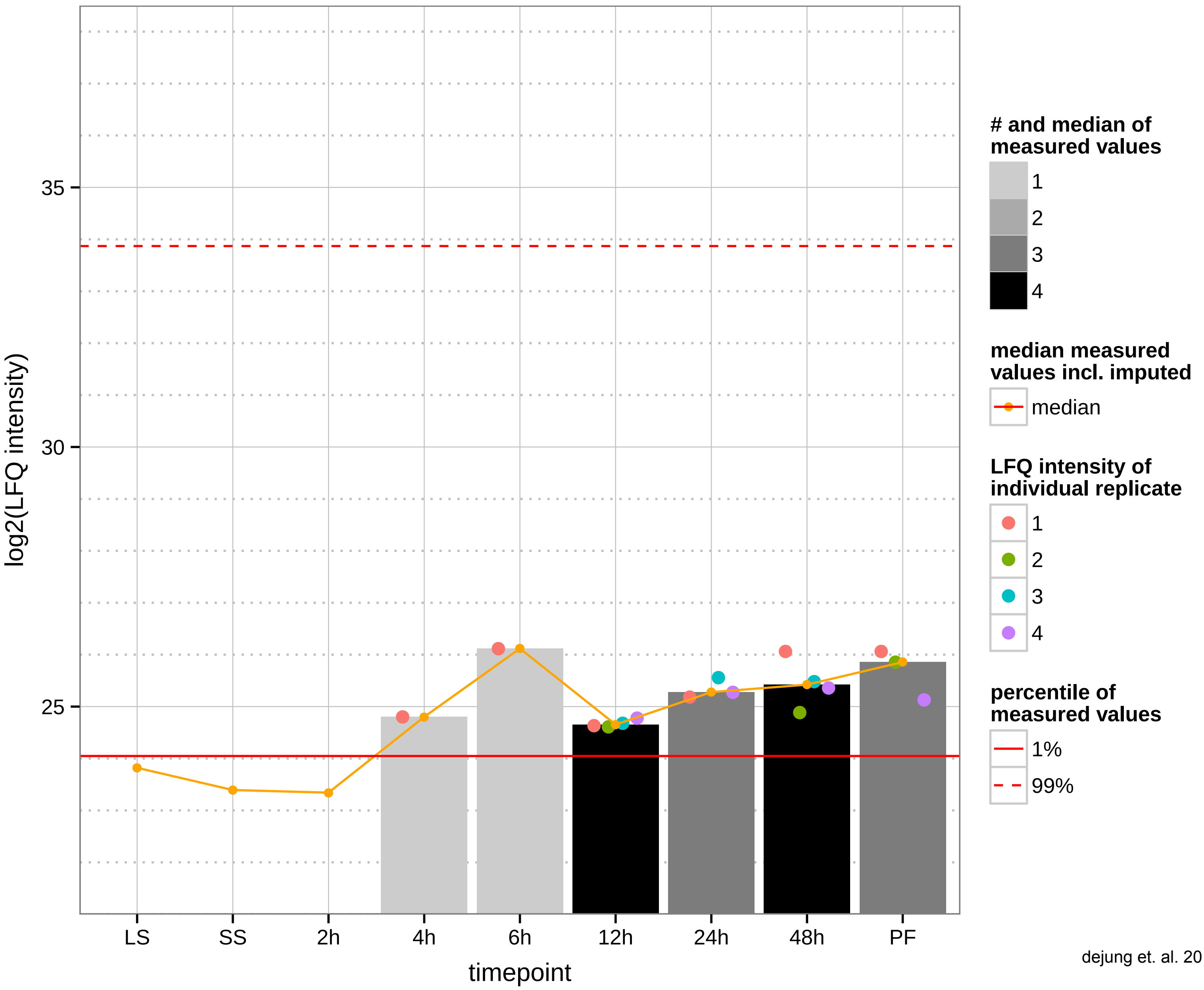

dejung et. al. 2015

hypothetical protein, conserved
Tb927.7.2980;Tb927.7.3020
AGOF: oxidoreductase activity
AGOC: null
AGOP: null
PGOF: oxidoreductase activity
PGOC: null
PGOP: null

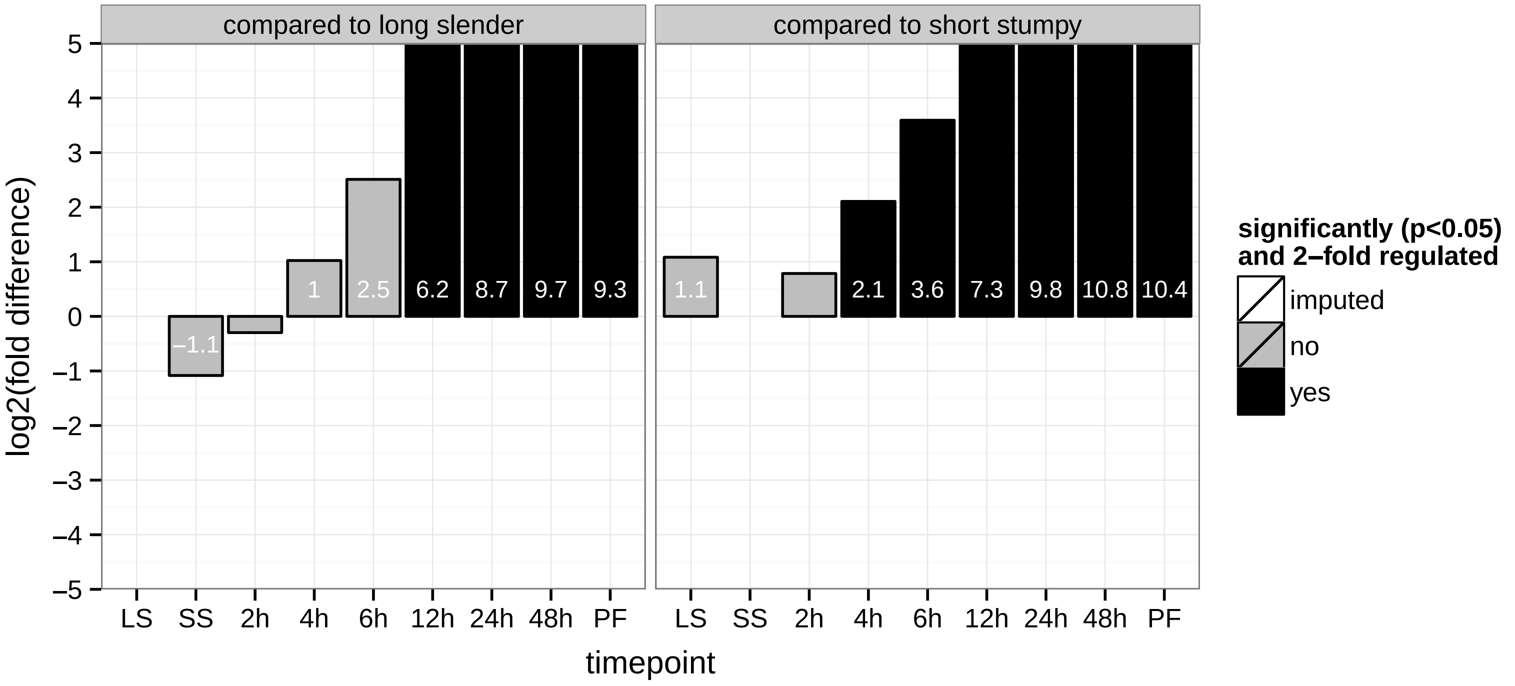

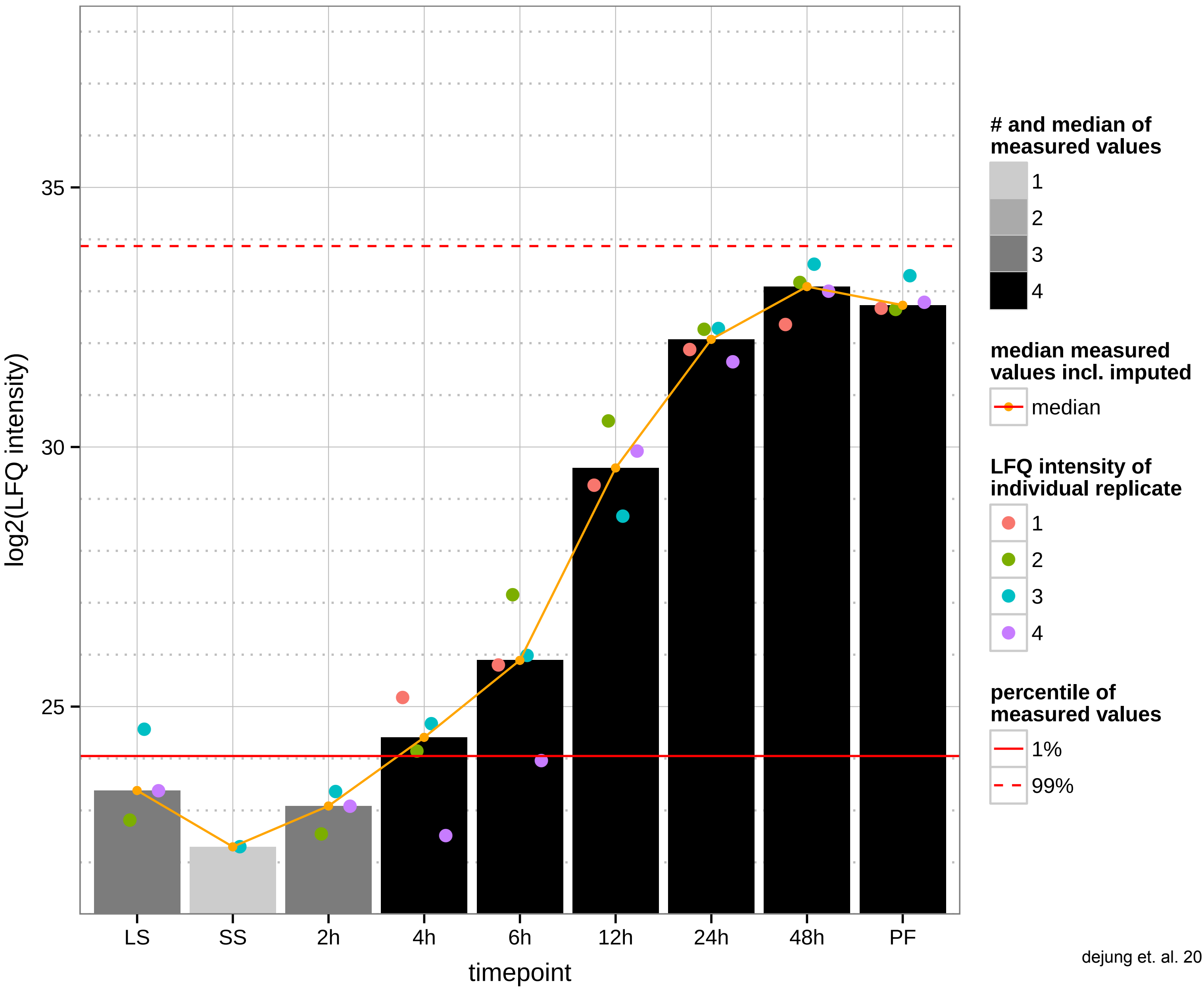

dejung et. al. 2015

hypothetical protein, conserved
Tb927.7.3810
AGOF: DNA binding
AGOC: mitochondrion
AGOP: regulation of transcription, DNA–dependent
PGOF: DNA binding
PGOC: null
PGOP: regulation of transcription, DNA–dependent

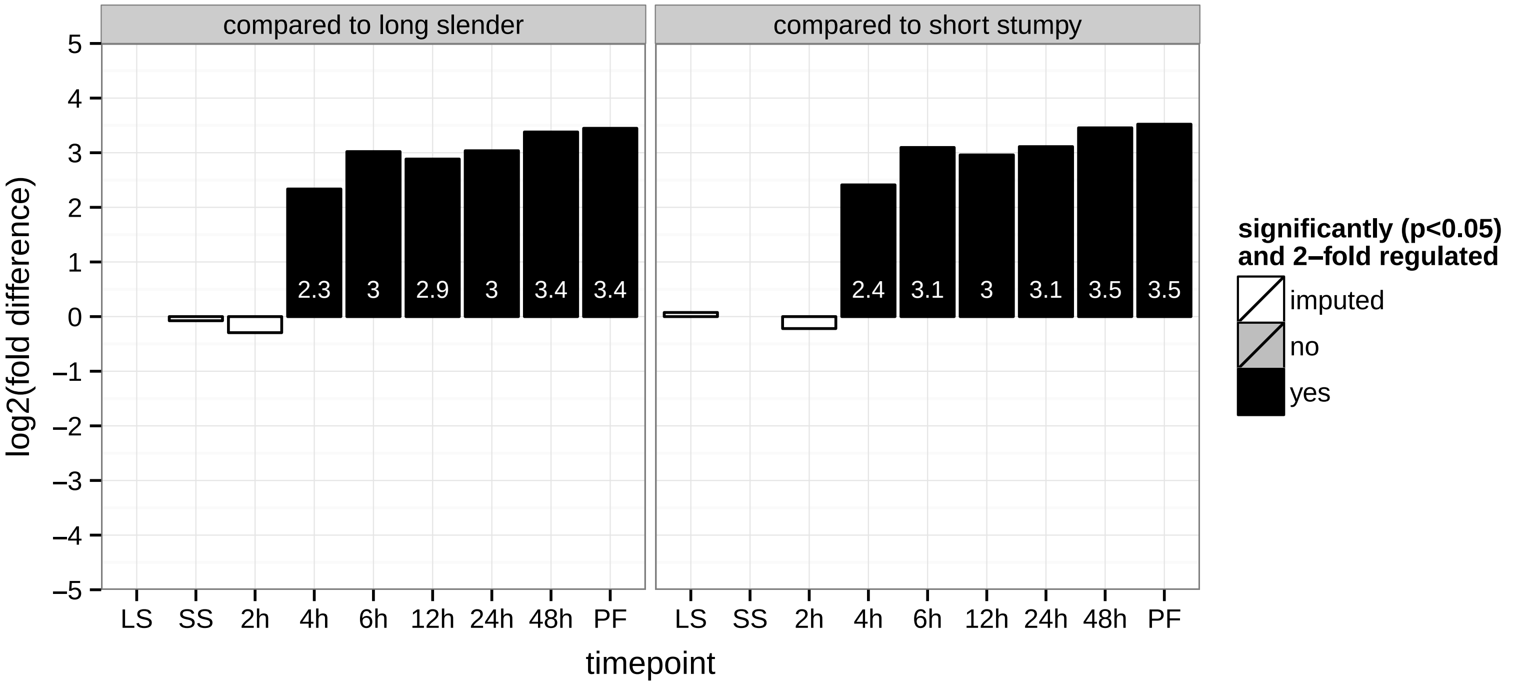

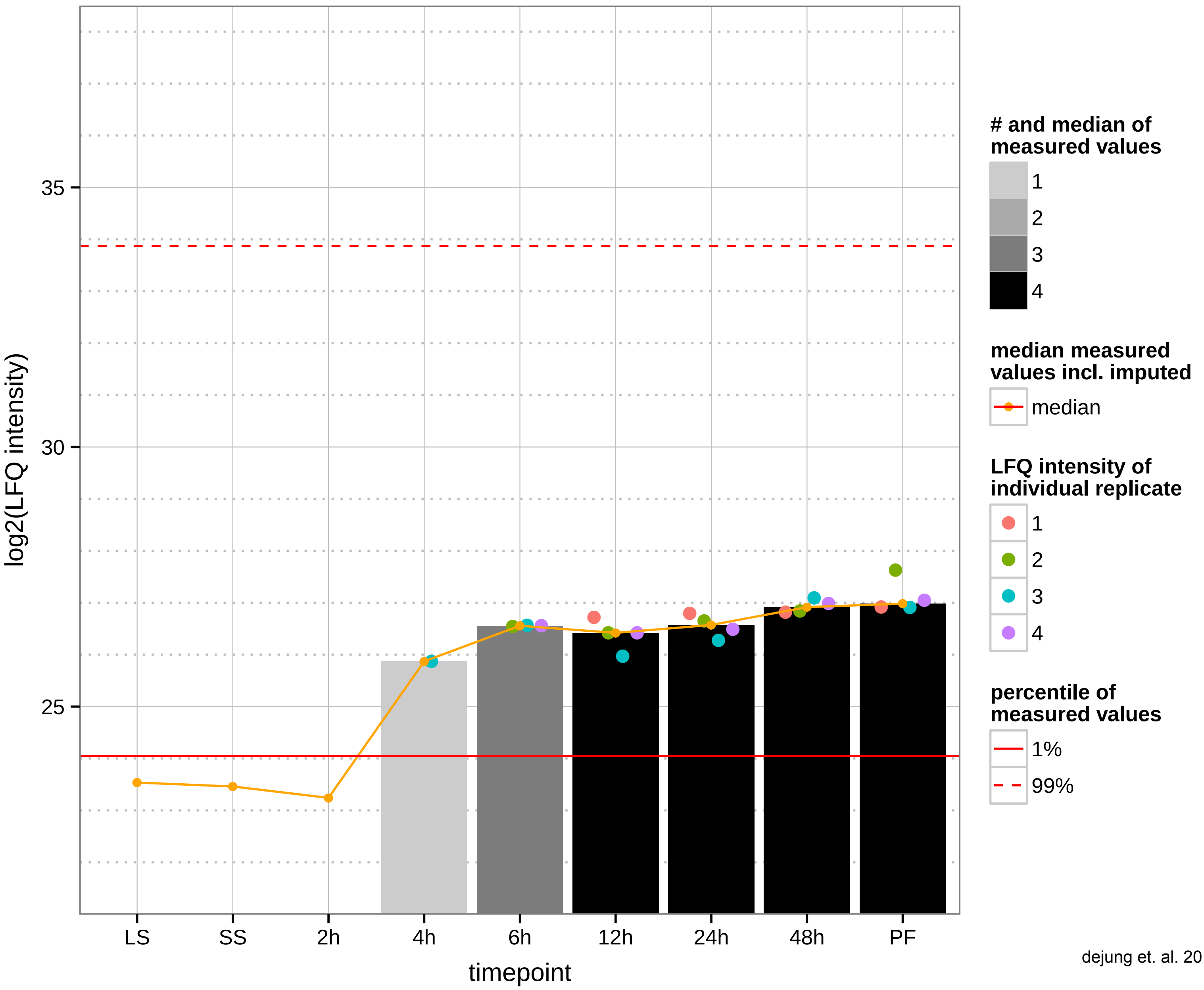

dejung et. al. 2015

hypothetical protein, conserved
Tb927.7.4270
AGOF: null
AGOC: null
AGOP: null
PGOF: null
PGOC: null
PGOP: null

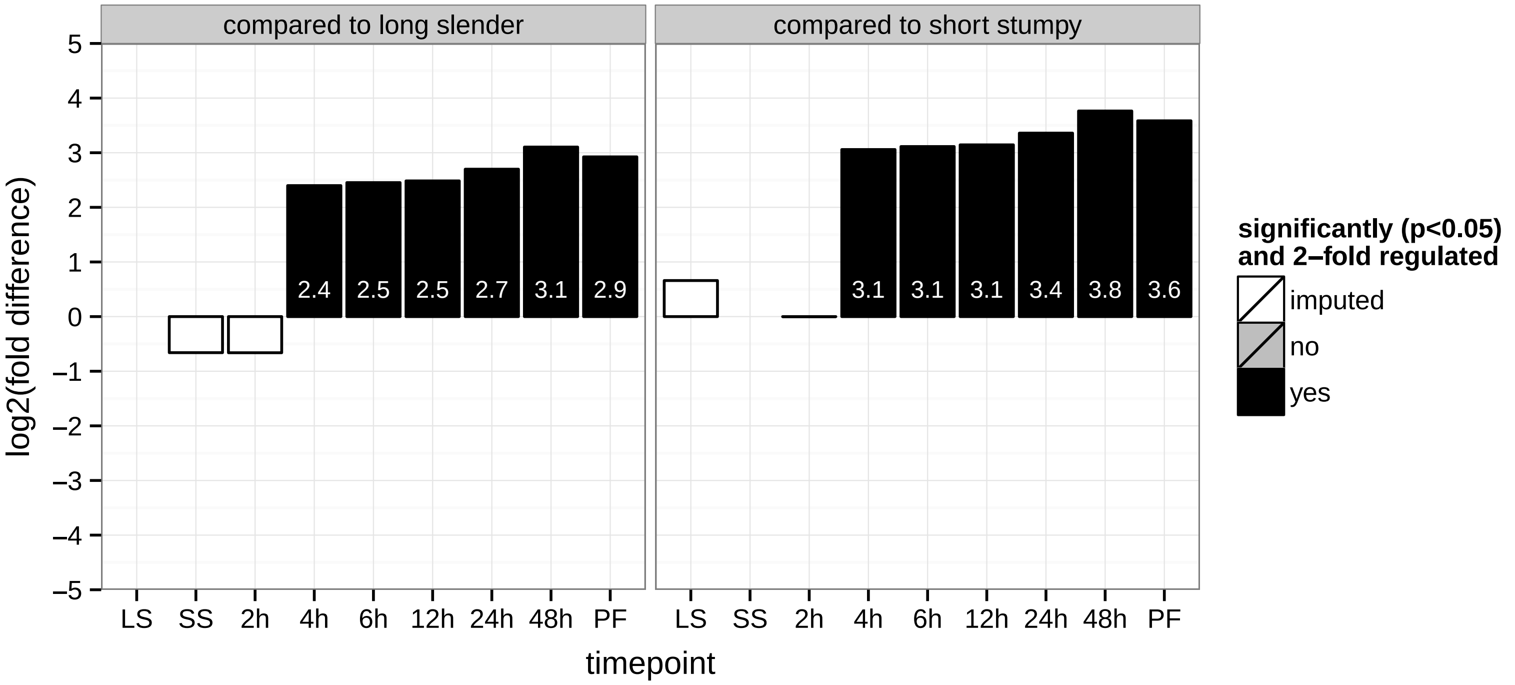

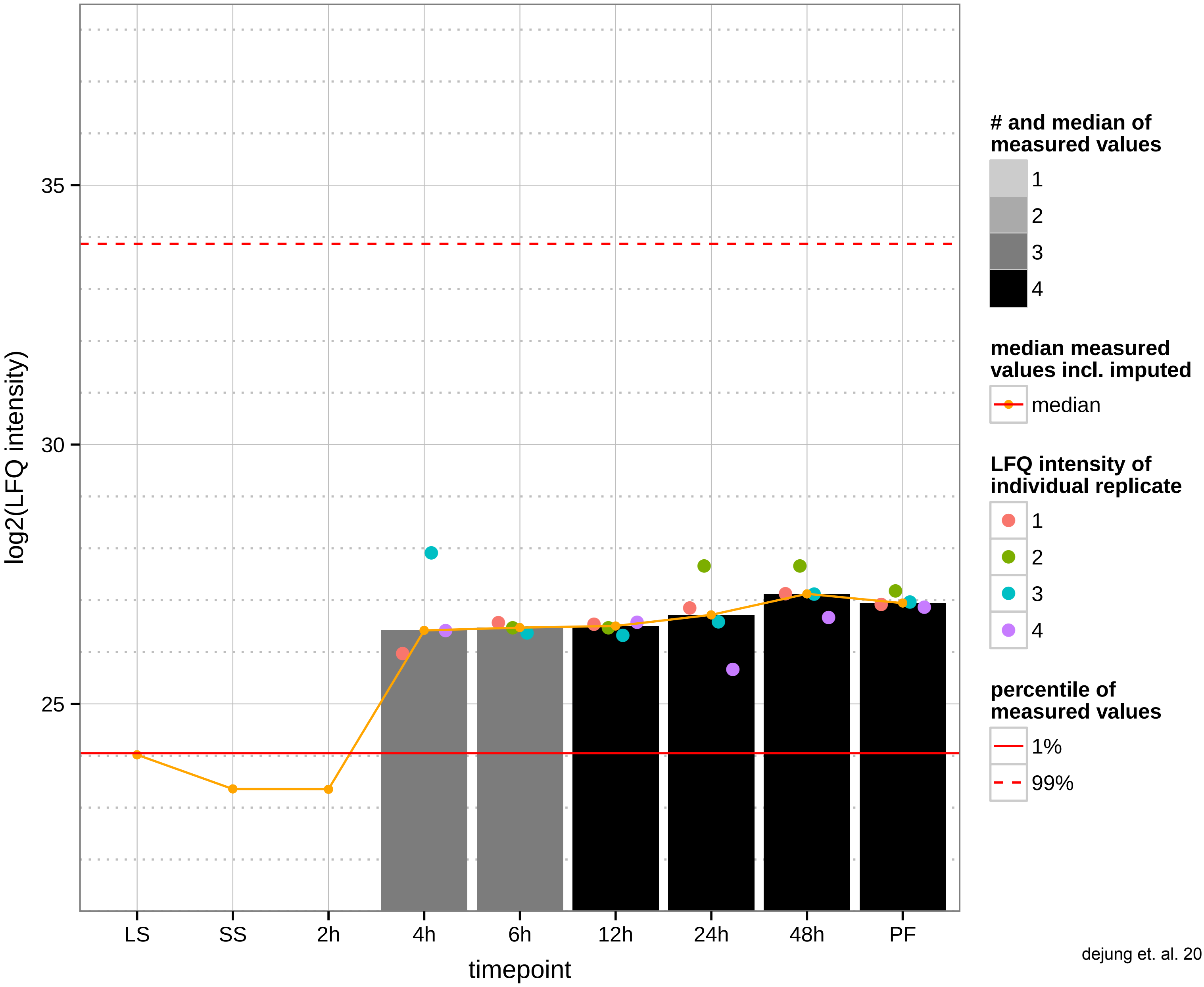

dejung et. al. 2015

hypothetical protein, conserved
Tb927.7.6820
AGOF: null
AGOC: null
AGOP: null
PGOF: null
PGOC: null
PGOP: null

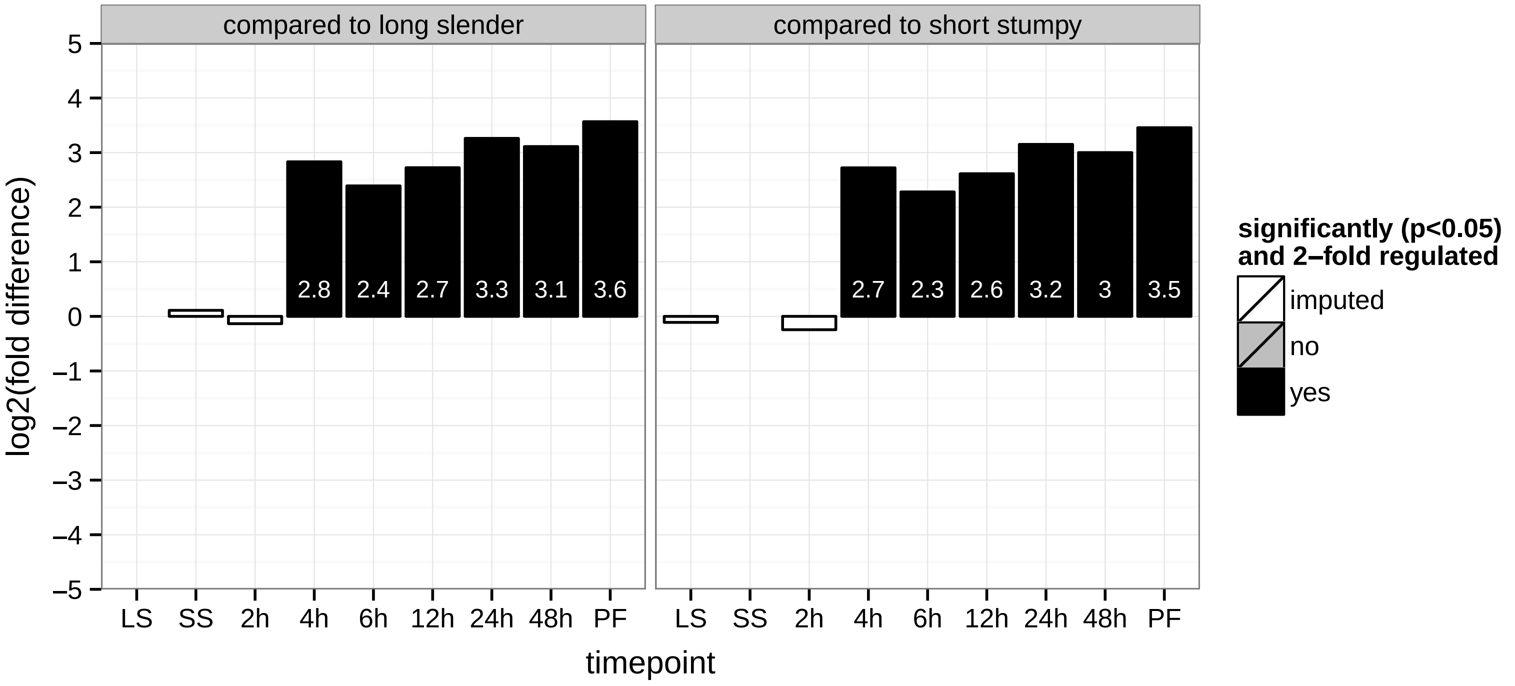

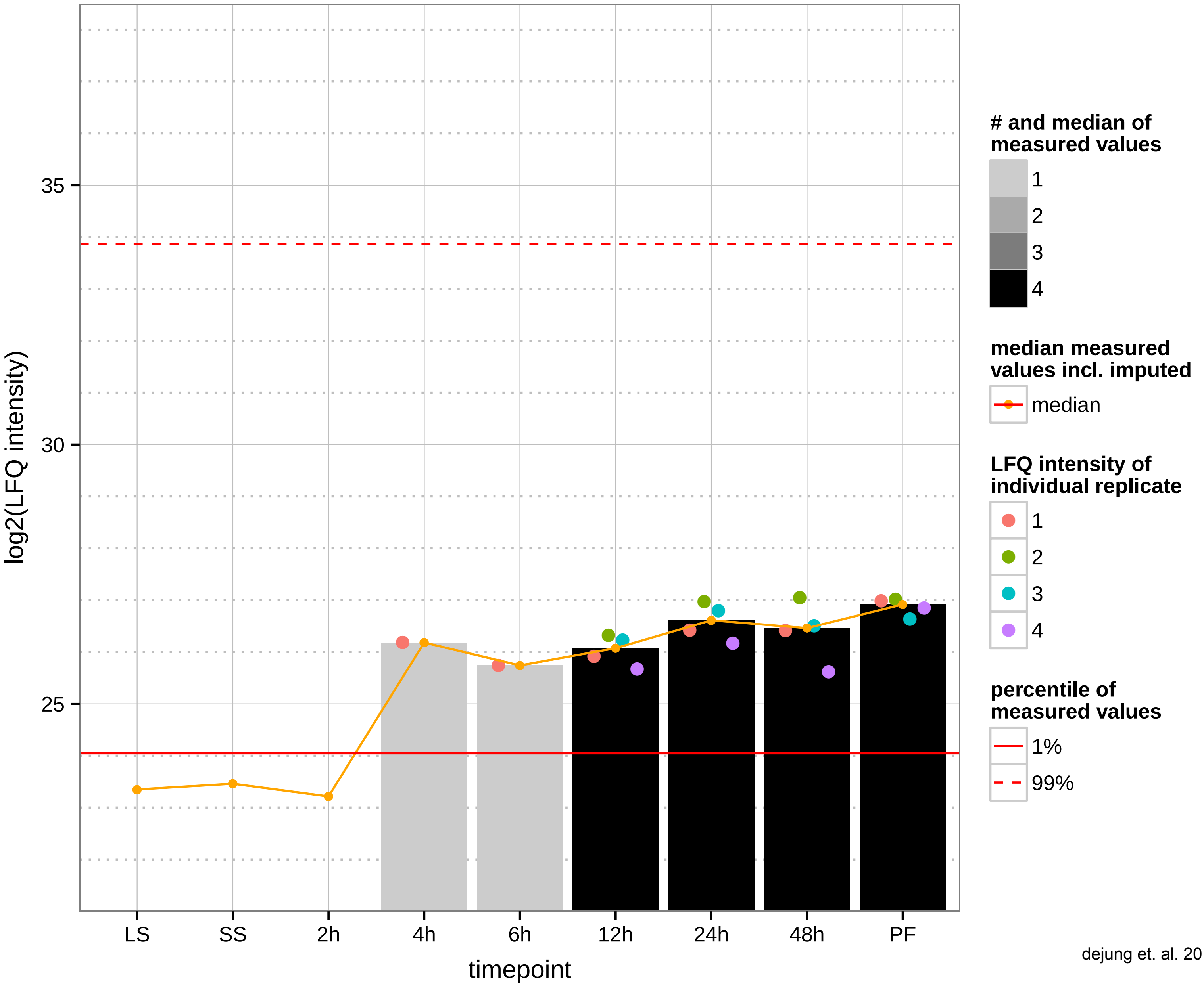

dejung et. al. 2015

receptor–type adenylate cyclase GRESAG 4, putative, adenylyl cyclase, ATP pyrophosphate–lyase, BAC from homologous re
Tb927.7.7470
AGOF: phosphorus–oxygen lyase activity, null, adenylate cyclase activity
AGOC: null, integral to membrane
AGOP: cyclic nucleotide biosynthetic process, intracellular signal transduction, null
PGOF: phosphorus–oxygen lyase activity
PGOC: null
PGOP: cyclic nucleotide biosynthetic process, intracellular signal transduction

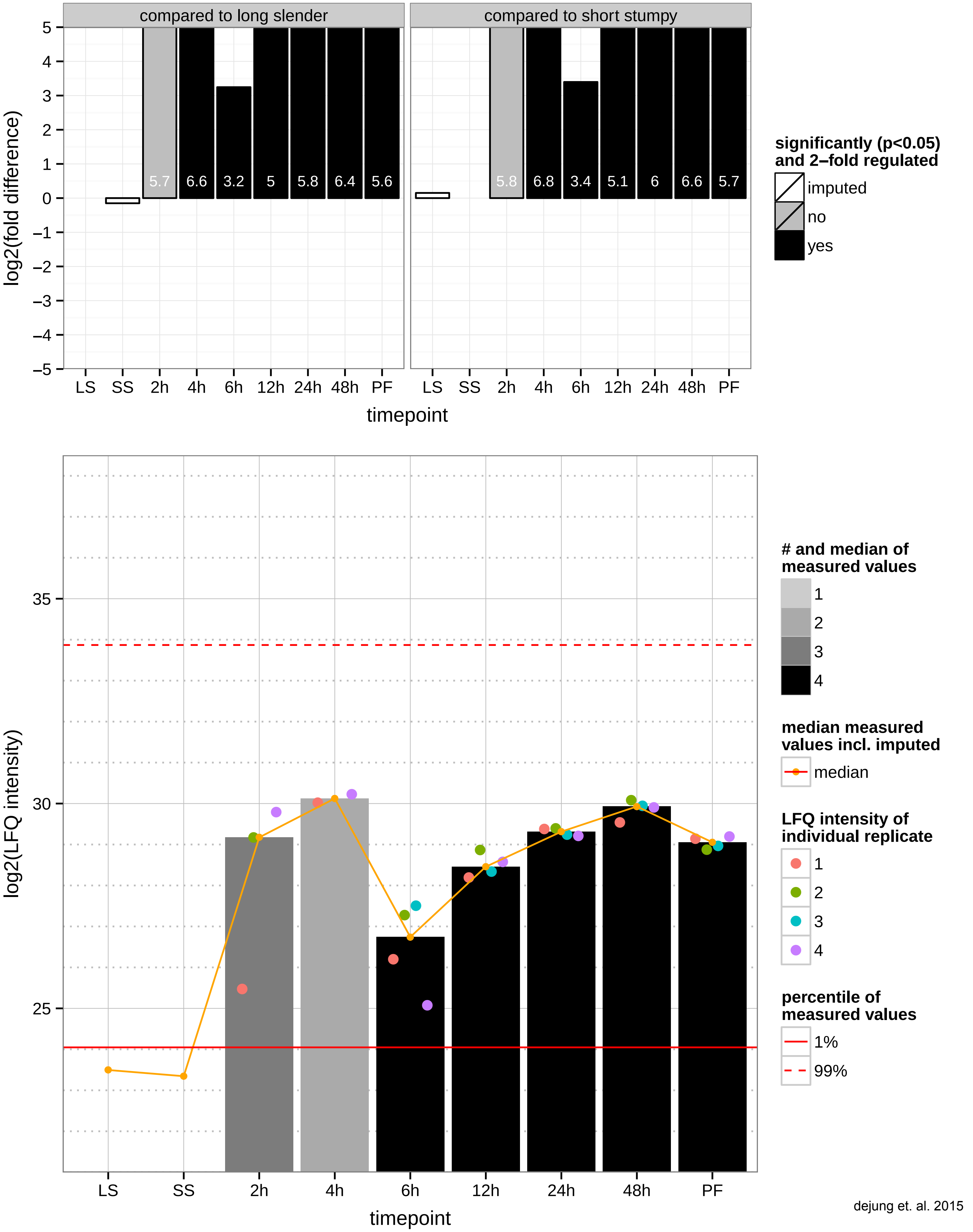

dejung et. al. 2015

phosphatidylglycerolphosphate synthase, mitochondrial (pgps)
Tb927.8.1720
AGOF: CDP−diacylglycerol−glycerol−3−phosphate 3−phosphatidyltransferase activity
AGOC: membrane, mitochondrion
AGOP: cardiolipin biosynthetic process, glycerophospholipid biosynthetic process, inner mitochondrial membrane organizatio
PGOF: catalytic activity
PGOC: null
PGOP: metabolic process

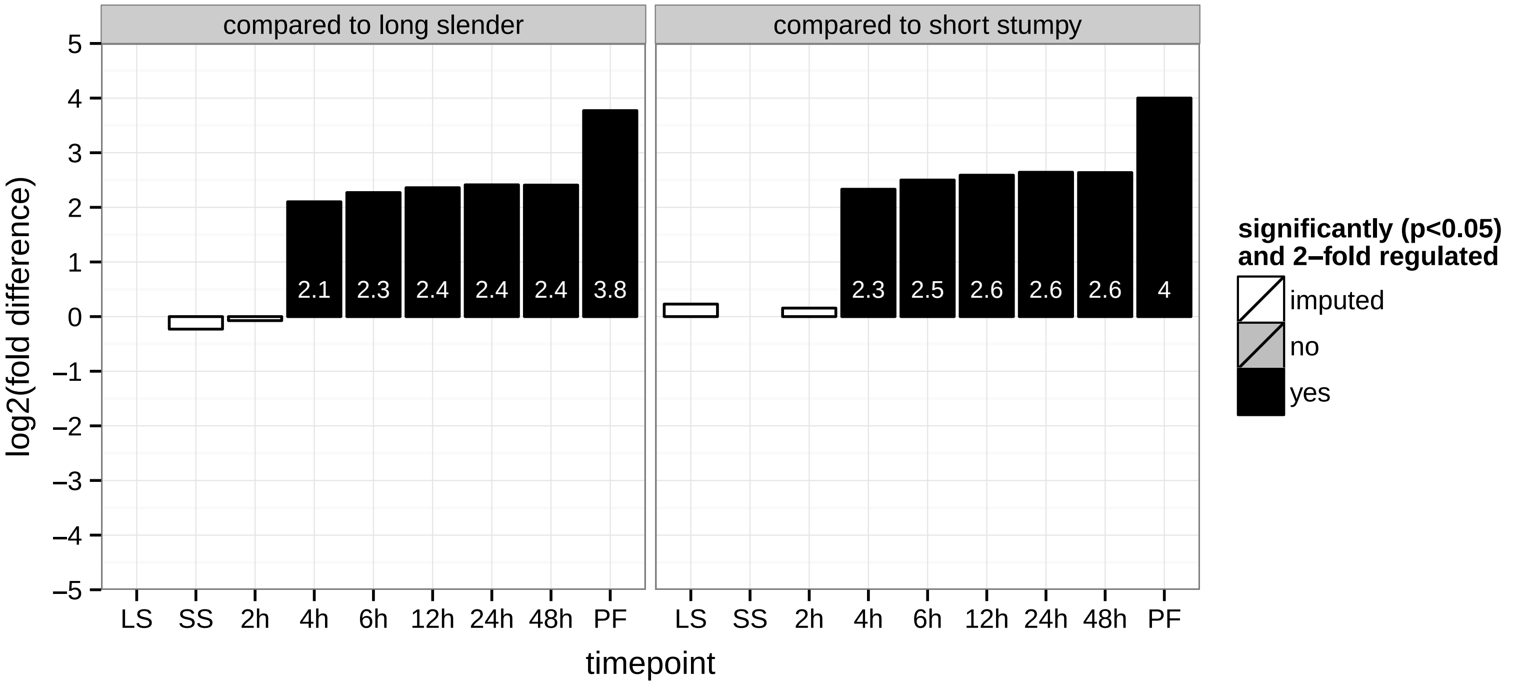

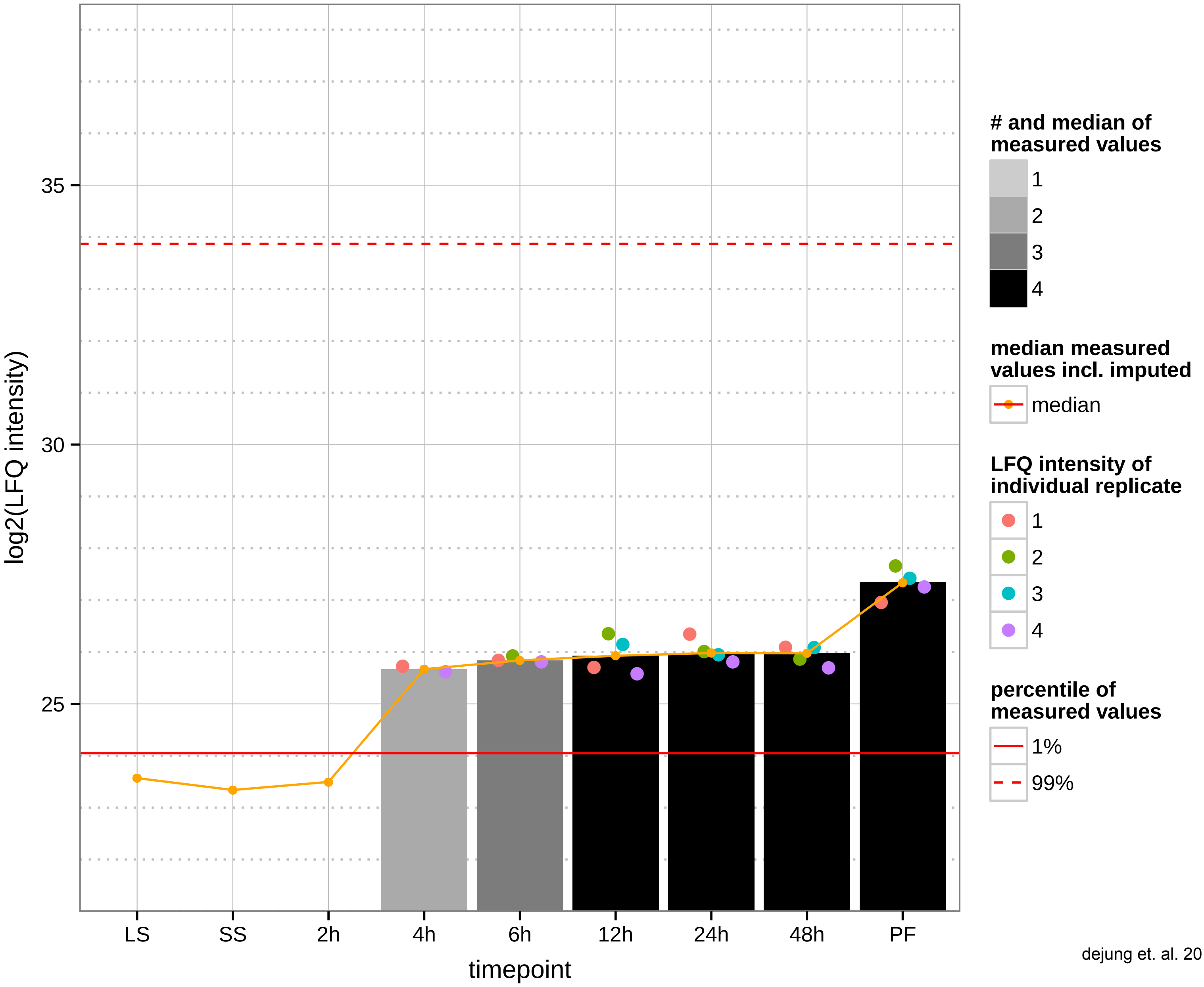

dejung et. al. 2015

hypothetical protein, conserved
Tb927.8.2330
AGOF: null
AGOC: null
AGOP: null
PGOF: null
PGOC: null
PGOP: null

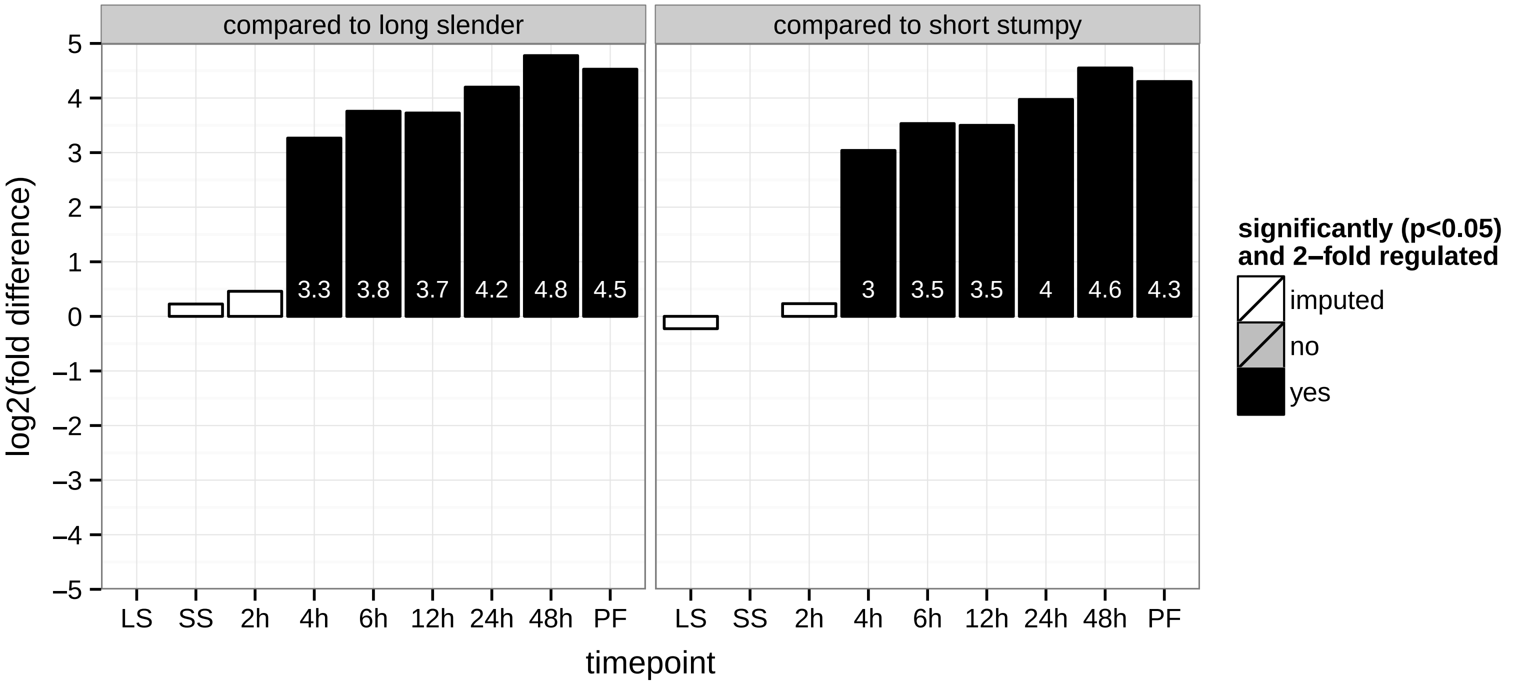

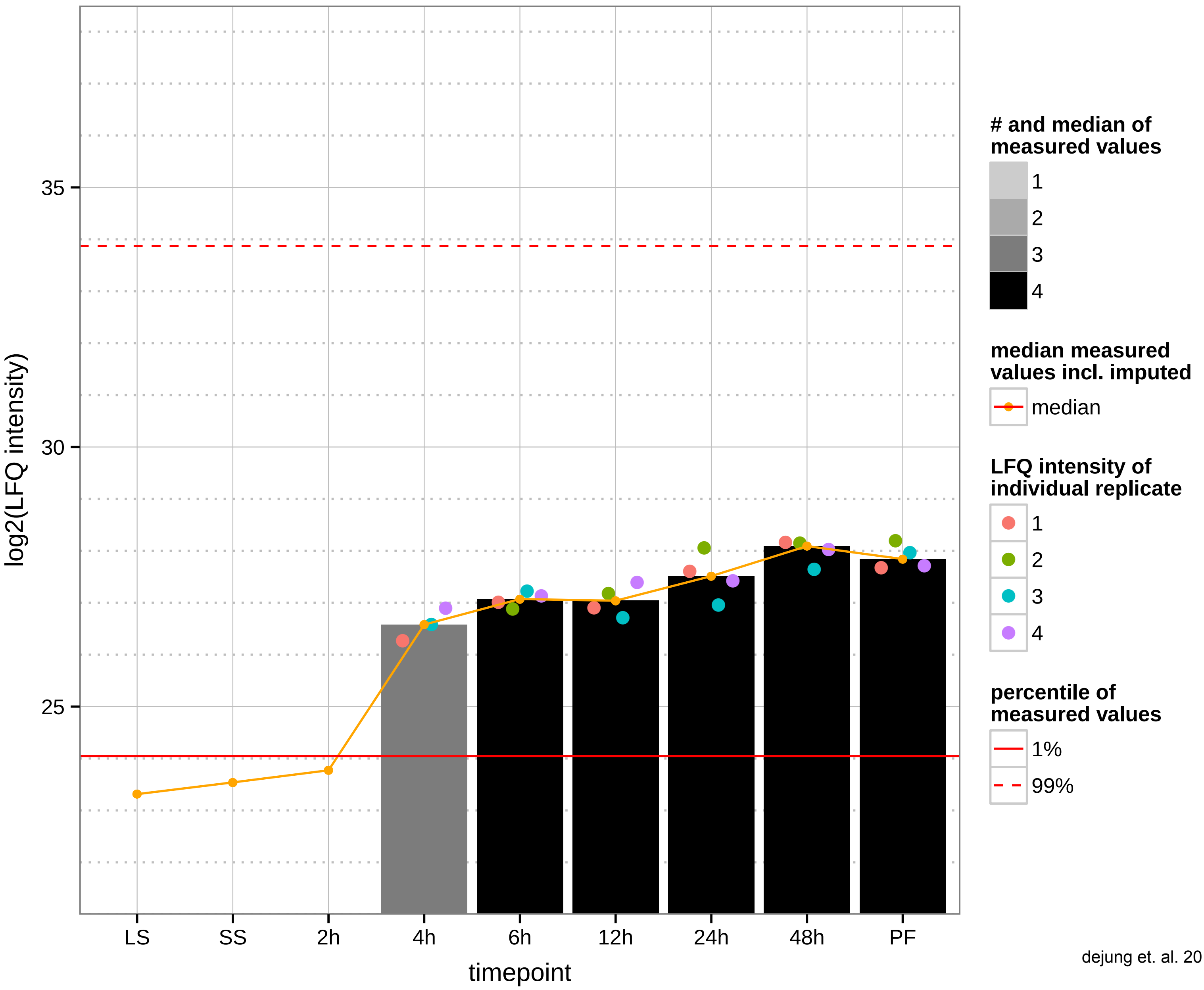

dejung et. al. 2015

flagellum–adhesion glycoprotein (fla1)
Tb927.8.4010
AGOF: null
AGOC: cilium, flagellar pocket, integral to membrane
AGOP: null
PGOF: null
PGOC: null
PGOP: null

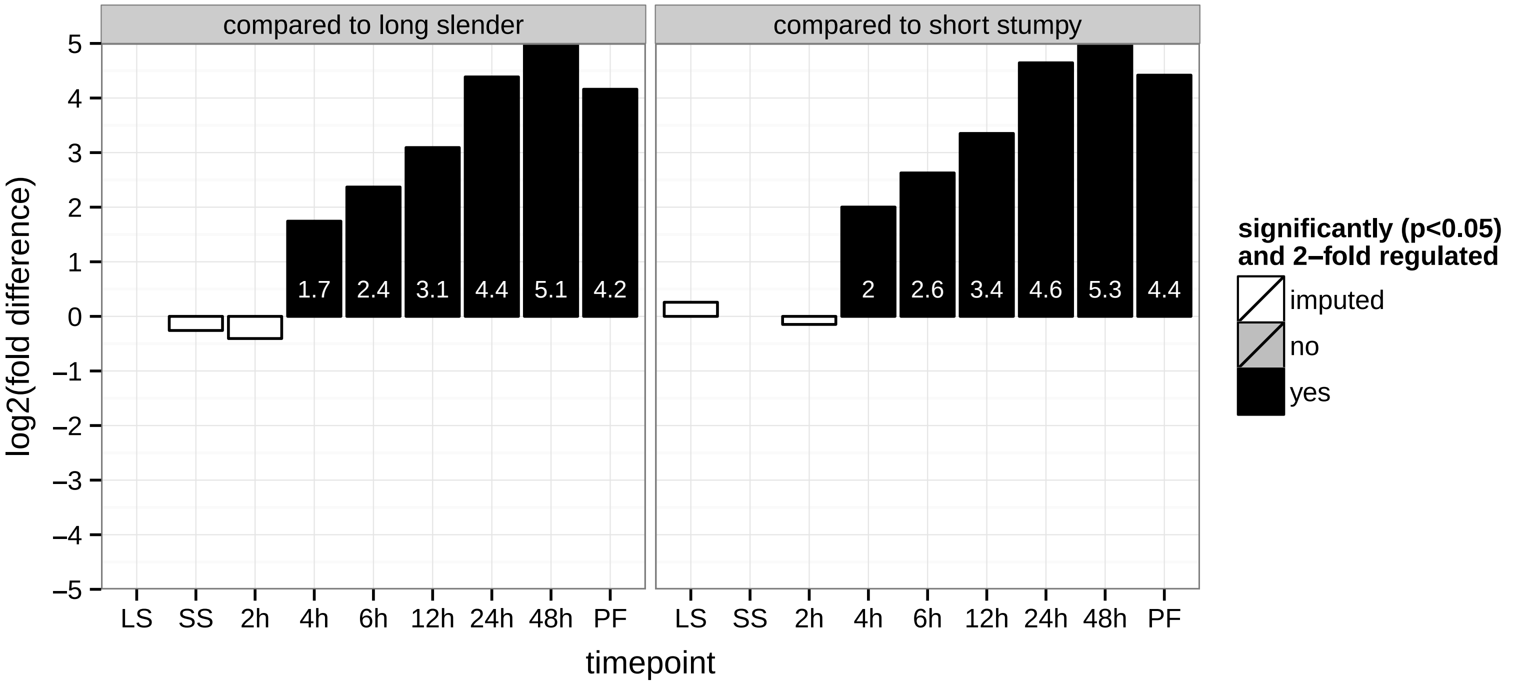

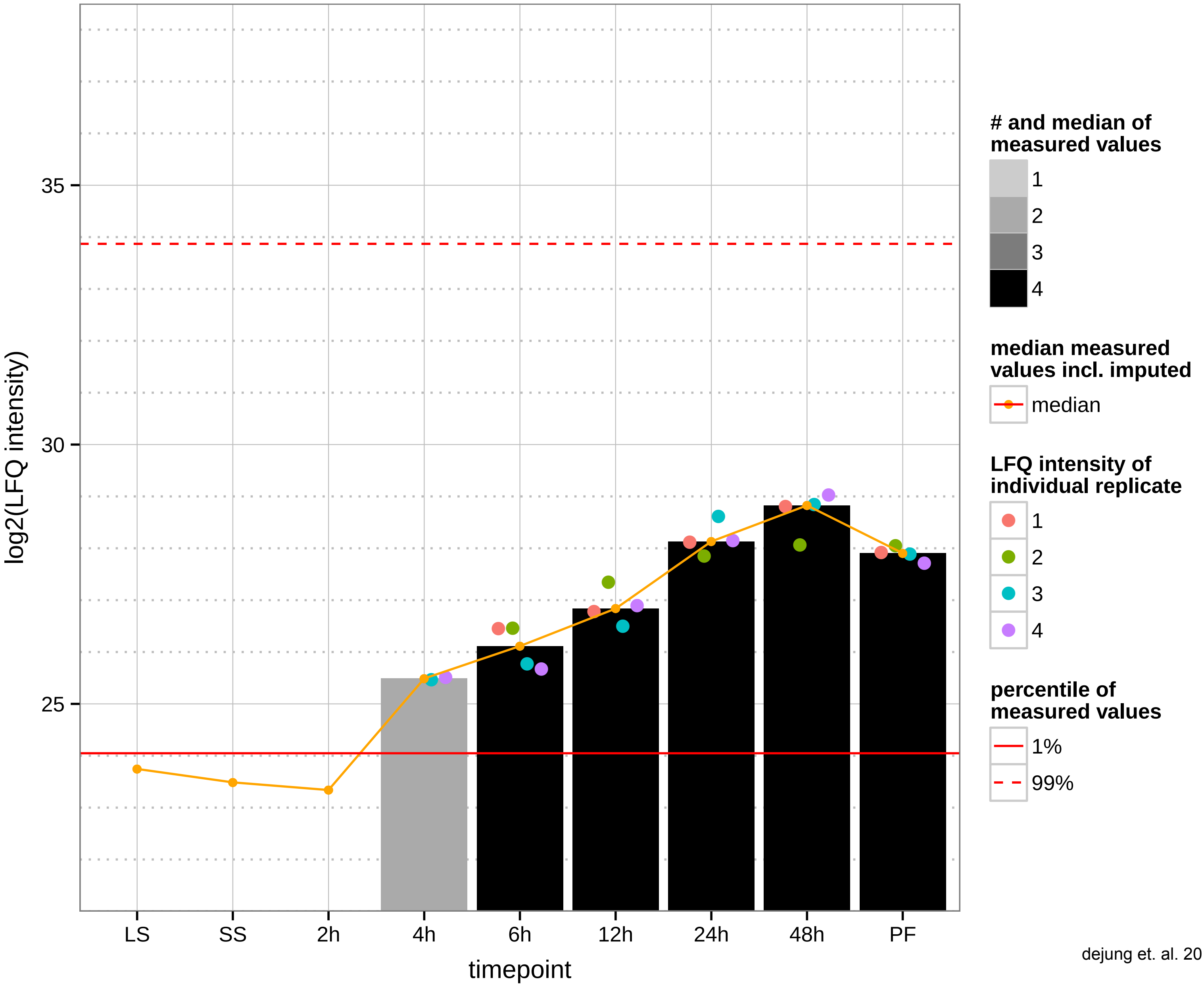

dejung et. al. 2015

hypothetical protein, conserved
Tb927.8.4100;Tb927.8.4050
AGOF: null
AGOC: integral to membrane
AGOP: null
PGOF: null
PGOC: null
PGOP: null

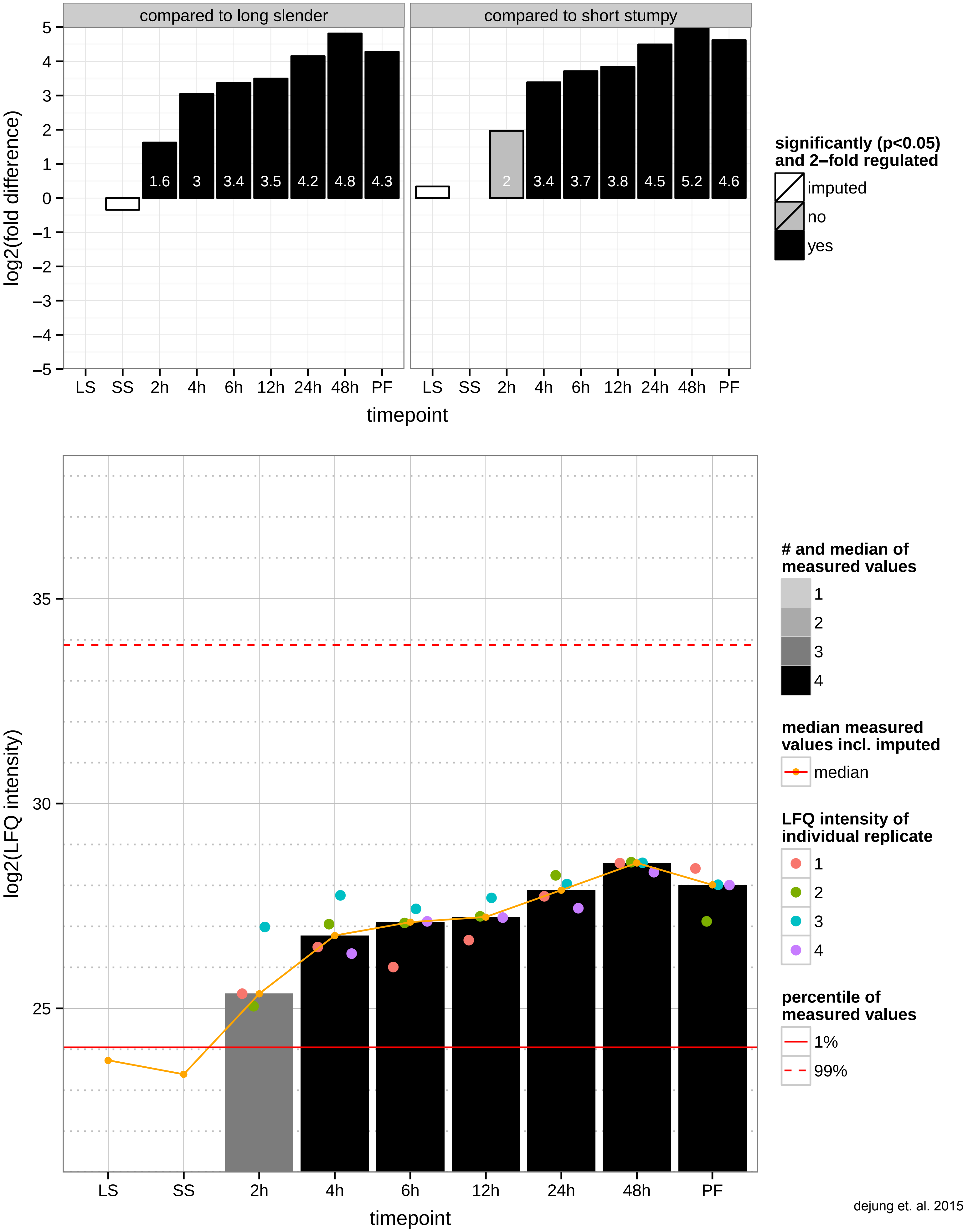

dejung et. al. 2015

hypothetical protein, conserved
Tb927.8.4150
AGOF: null
AGOC: mitochondrion
AGOP: null
PGOF: null
PGOC: null
PGOP: null

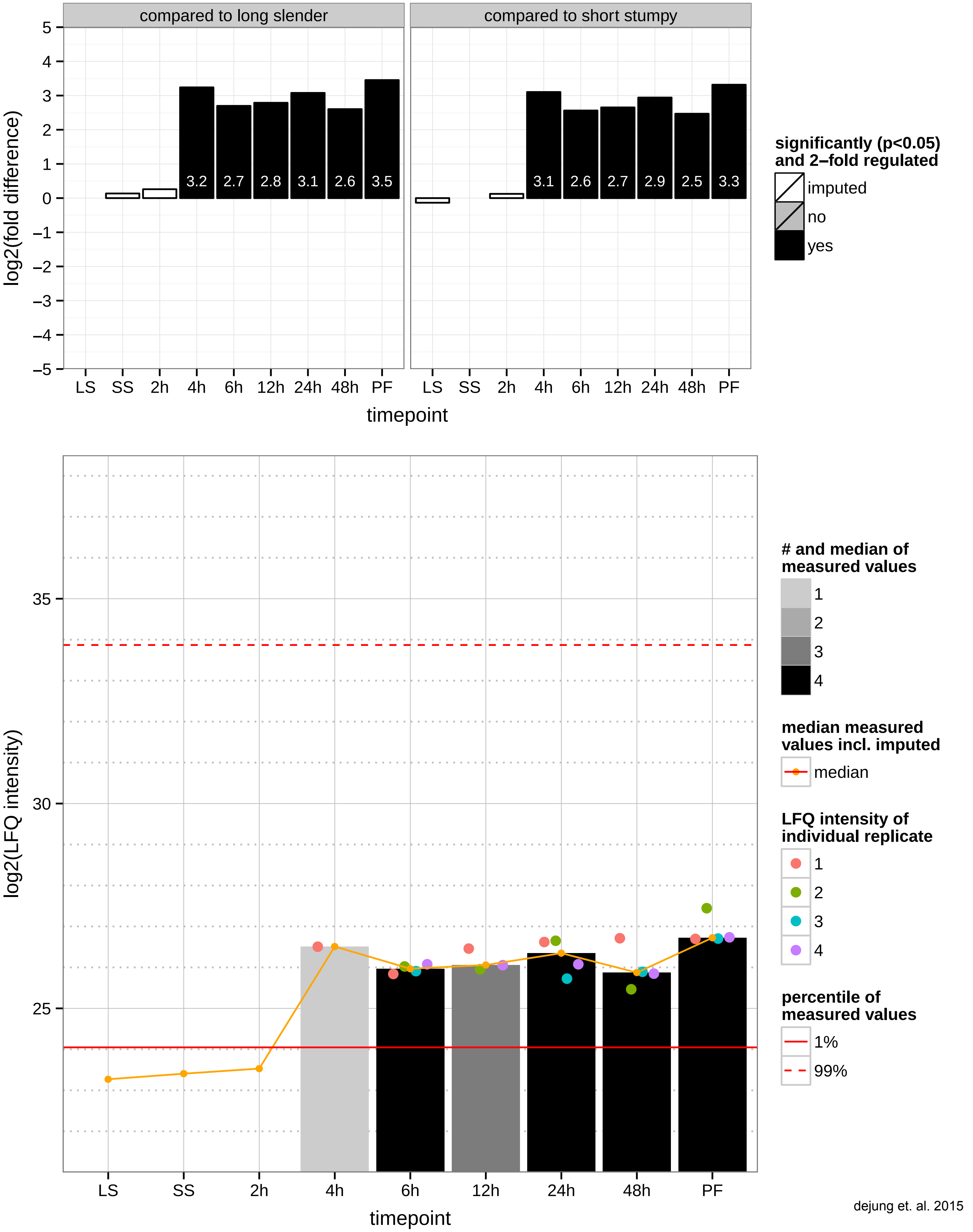

dejung et. al. 2015

hypothetical protein, conserved
Tb927.8.4190
AGOF: null
AGOC: null
AGOP: null
PGOF: null
PGOC: null
PGOP: null

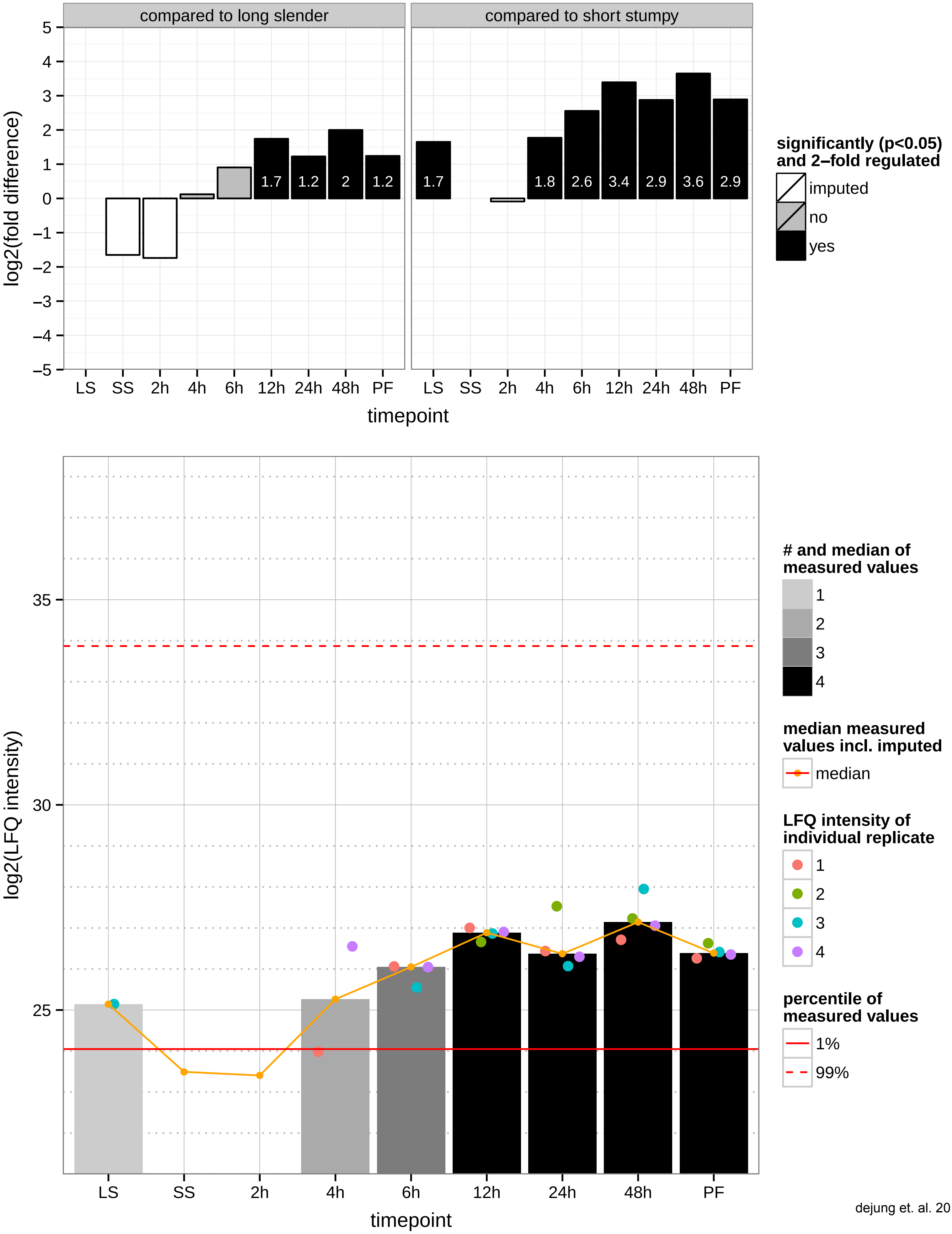

dejung et. al. 2015

cytochrome c
Tb927.8.5120
AGOF: electron carrier activity, heme binding, iron ion binding
AGOC: mitochondrion
AGOP: aerobic respiration
PGOF: electron carrier activity, heme binding, iron ion binding
PGOC: null
PGOP: null

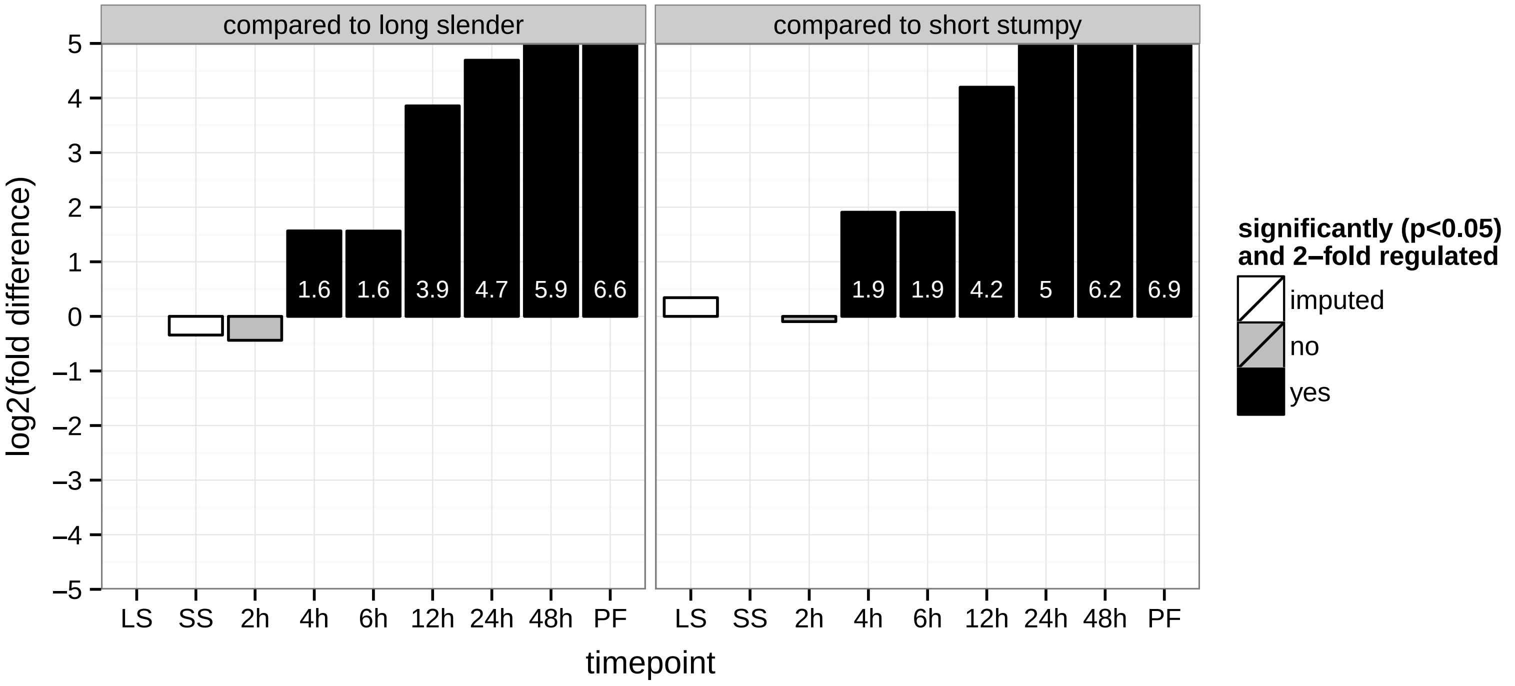

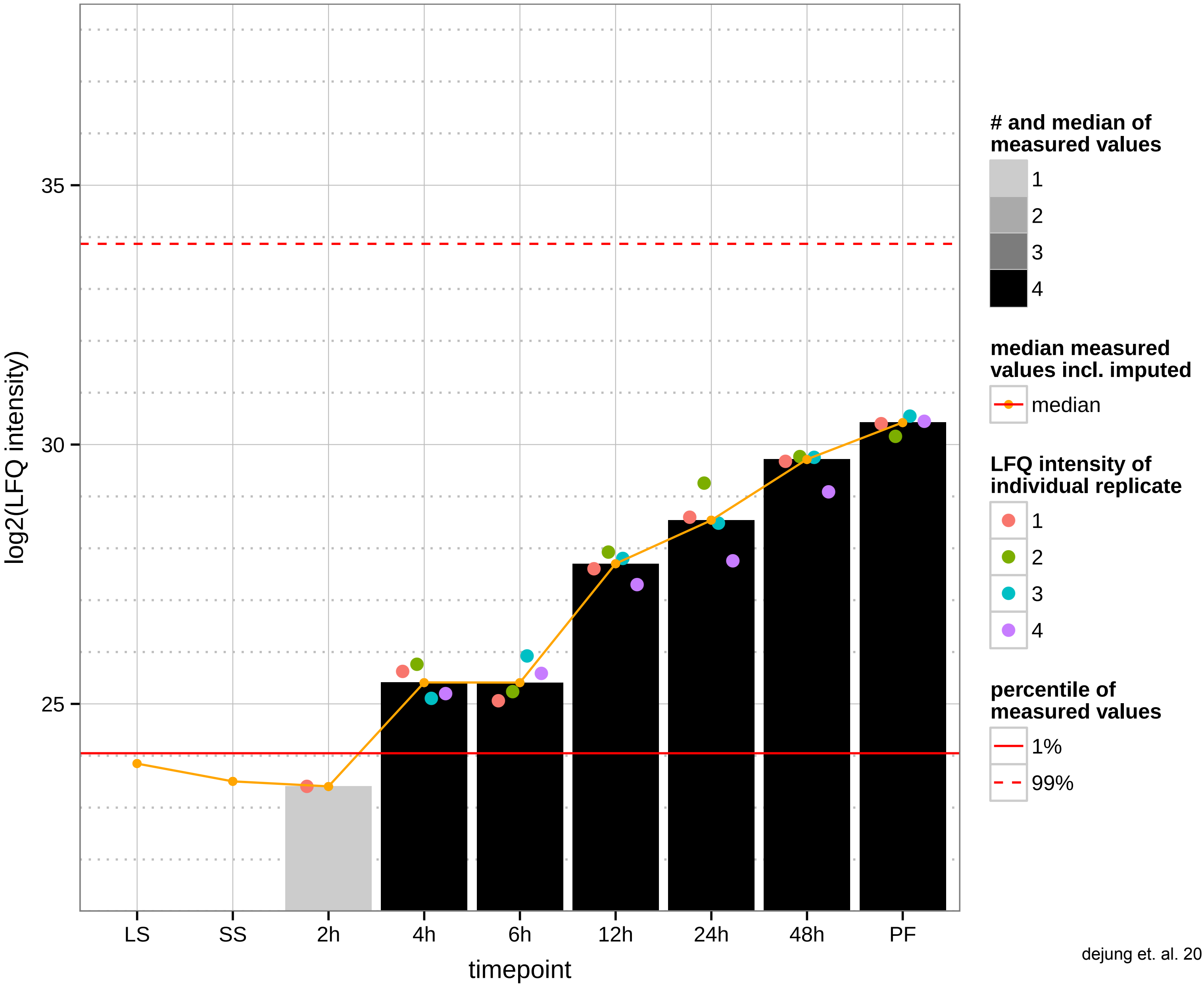

dejung et. al. 2015

hypothetical protein, conserved
Tb927.8.5560
AGOF: null
AGOC: mitochondrion
AGOP: null
PGOF: null
PGOC: null
PGOP: null

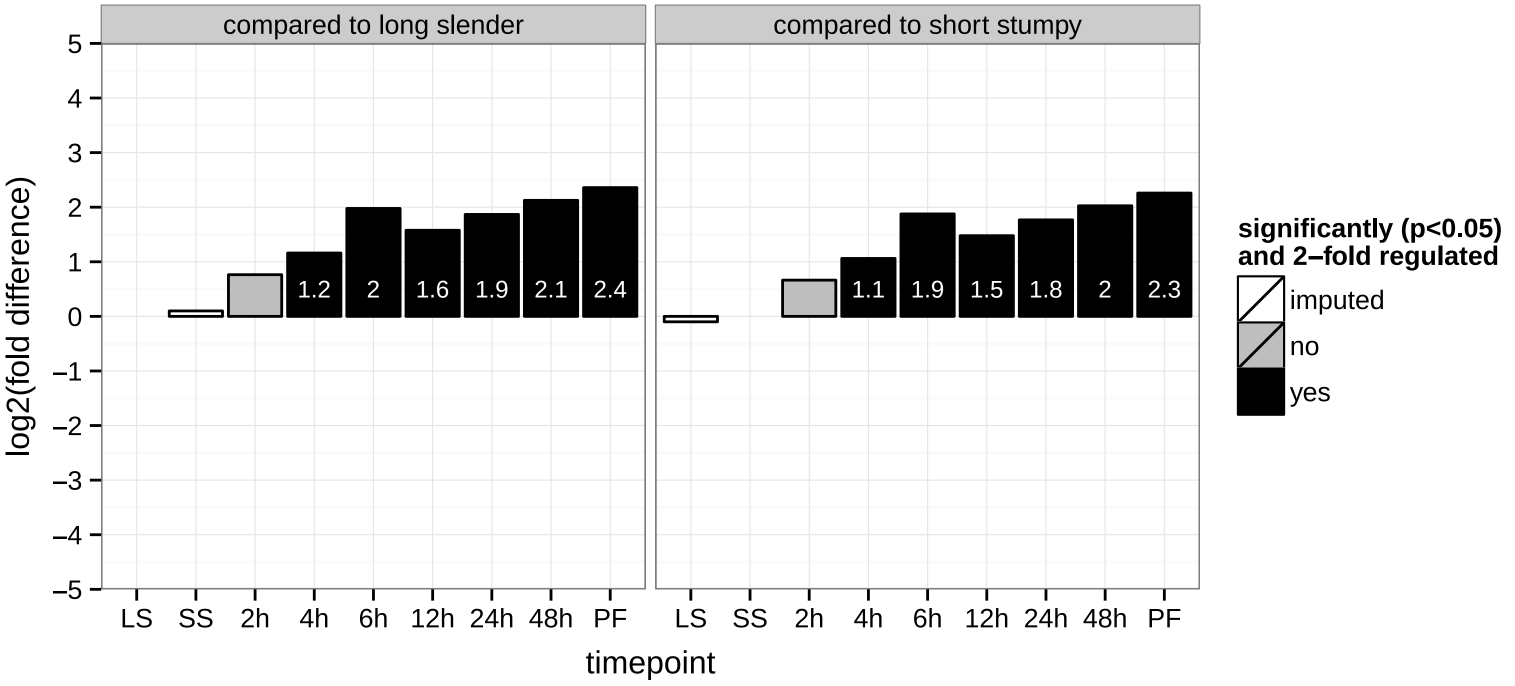

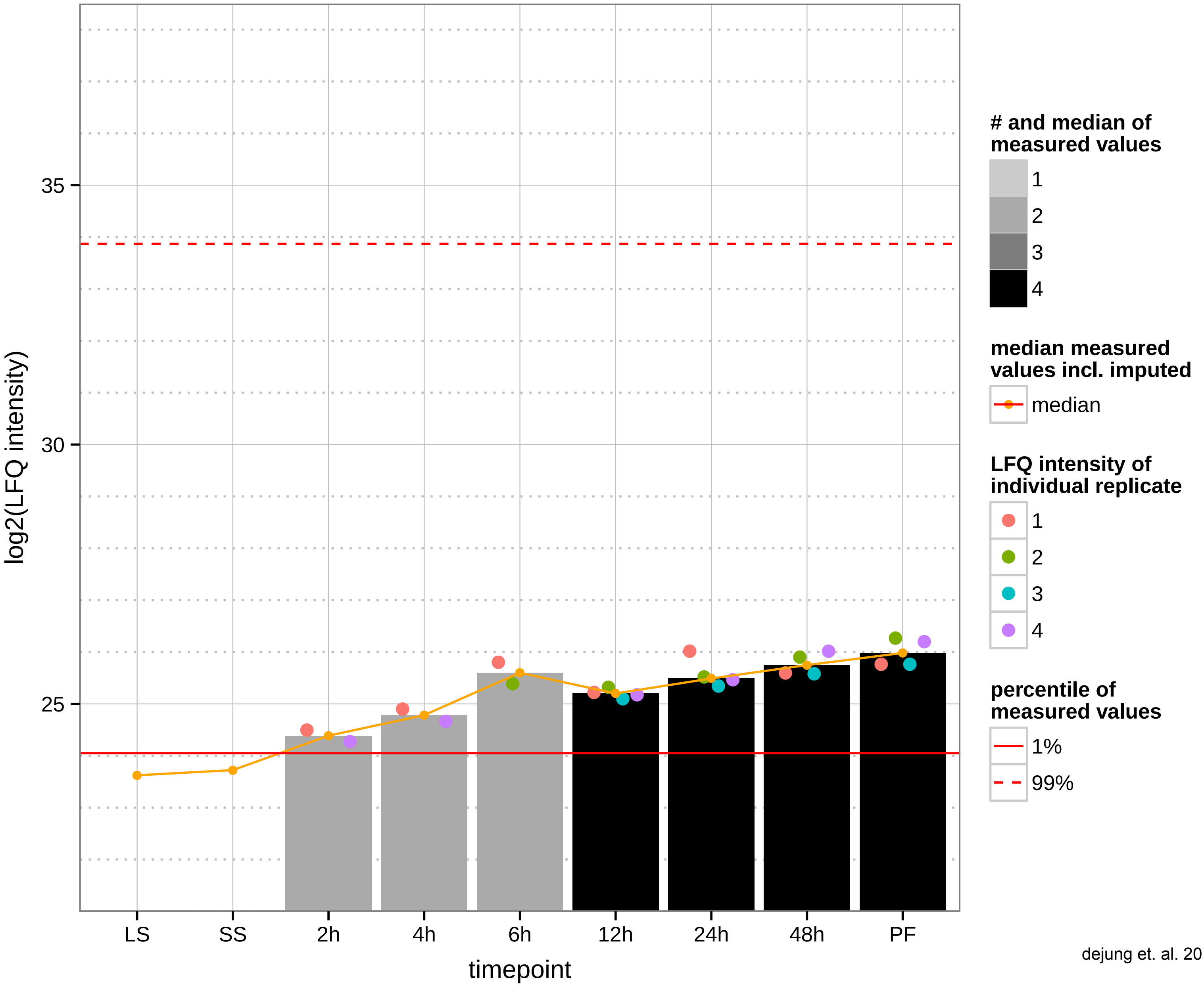

dejung et. al. 2015

proteasome 26S non–ATPase subunit 9, putative
Tb927.8.5740
AGOF: endopeptidase activity
AGOC: proteasome regulatory particle
AGOP: proteasomal ubiquitin–dependent protein catabolic process
PGOF: protein binding
PGOC: null
PGOP: null

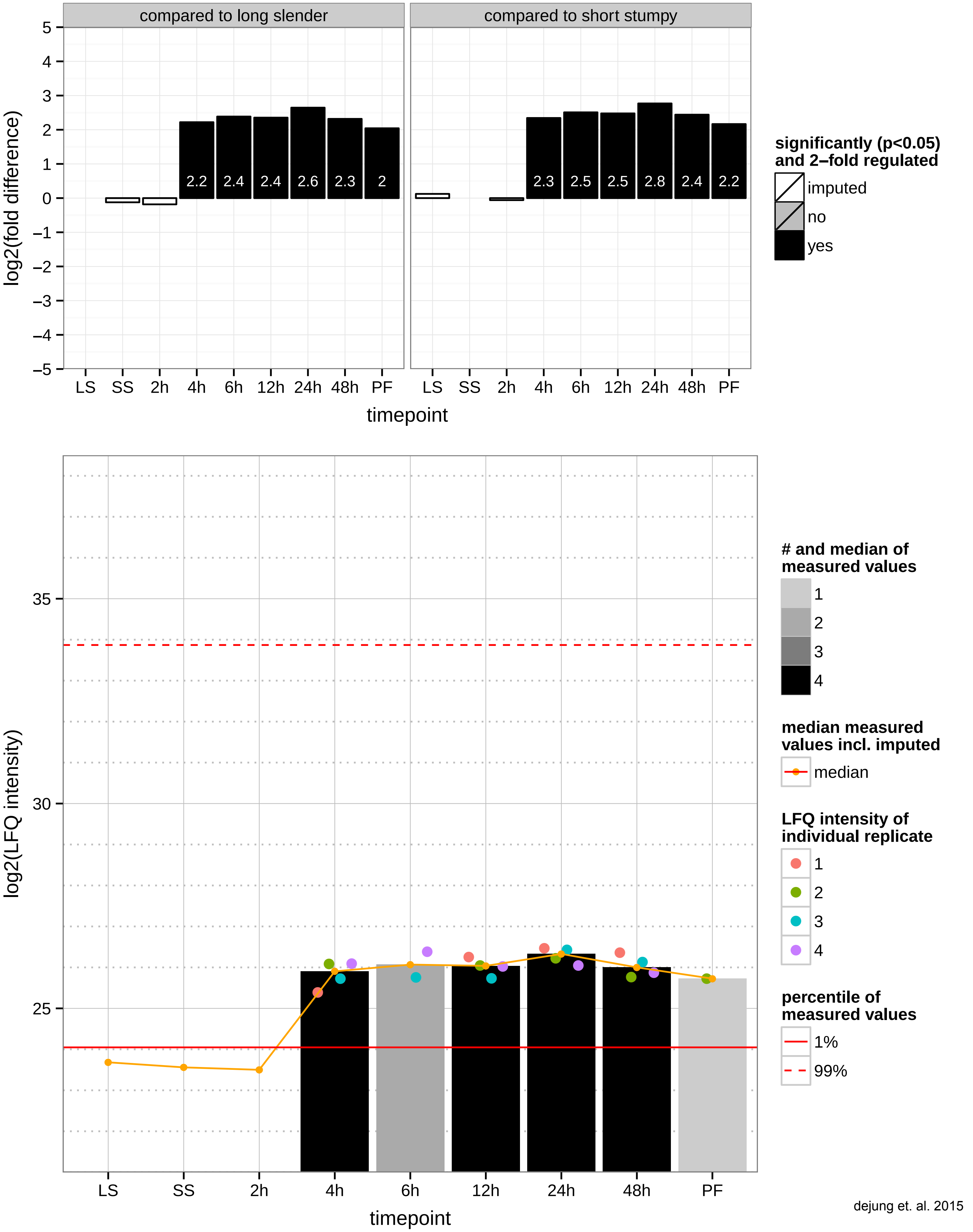

dejung et. al. 2015

hypothetical protein, conserved
Tb927.8.6640
AGOF: catalytic activity
AGOC: mitochondrion
AGOP: metabolic process
PGOF: oxidoreductase activity, acting on the aldehyde or oxo group of donors, NAD or NADP as acceptor
PGOC: null
PGOP: oxidation–reduction process

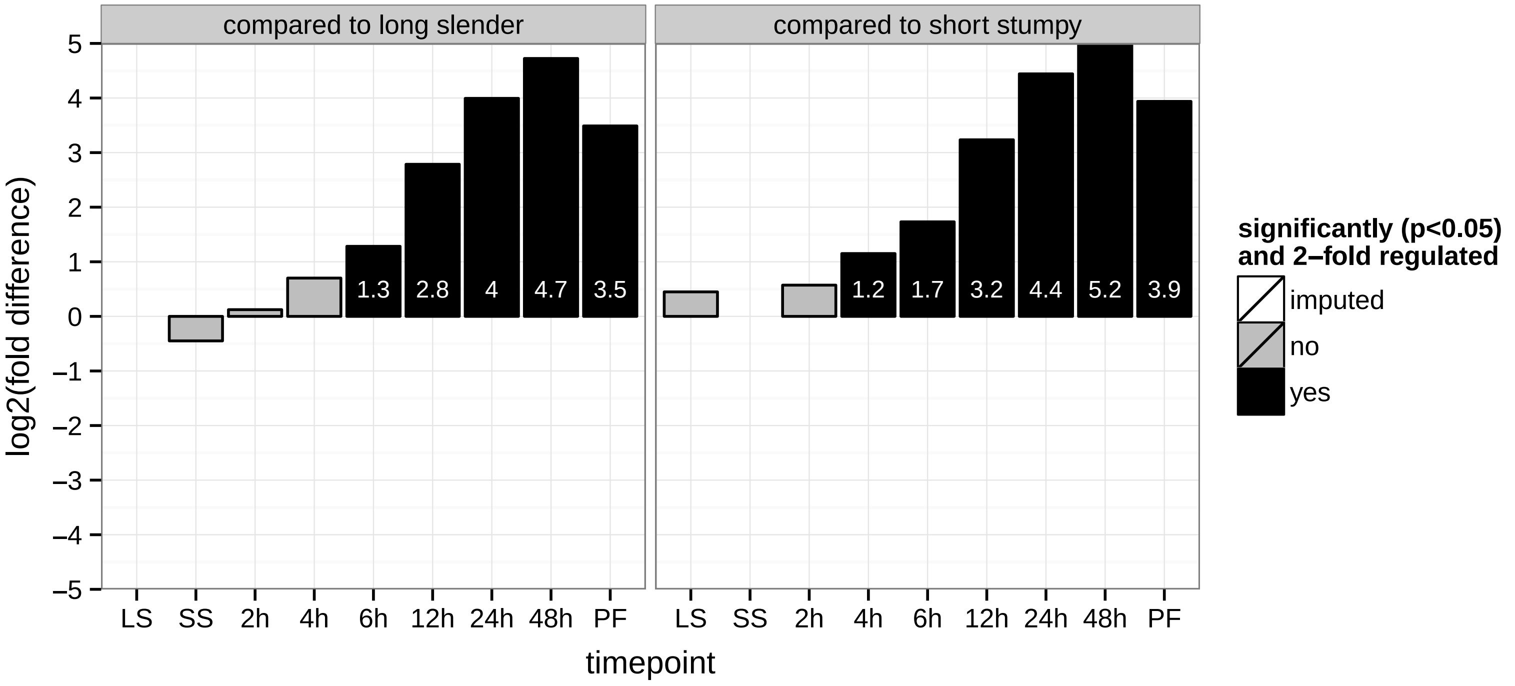

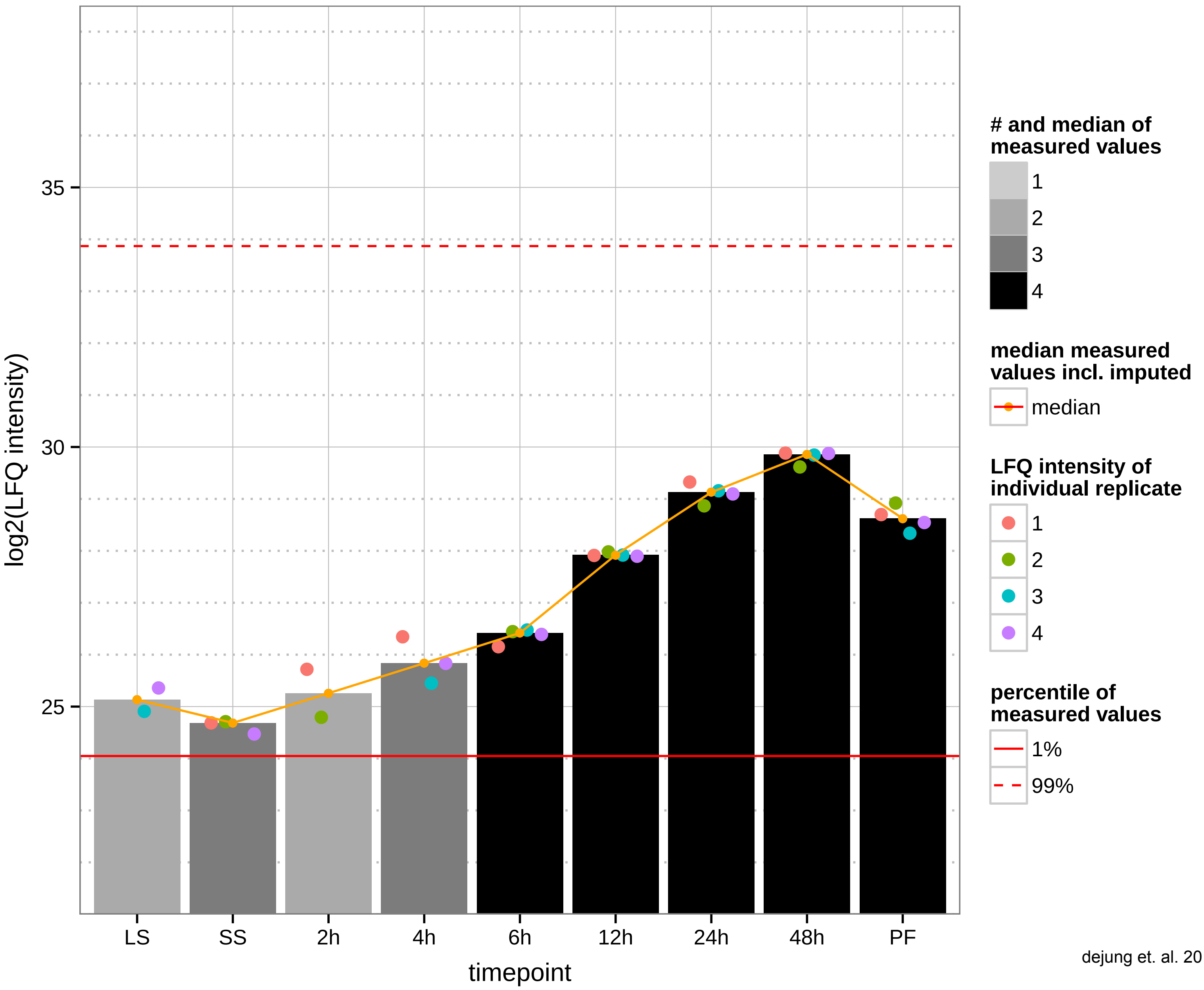

dejung et. al. 2015

hypothetical protein, conserved
Tb927.8.6860
AGOF: null
AGOC: null
AGOP: null
PGOF: null
PGOC: null
PGOP: null

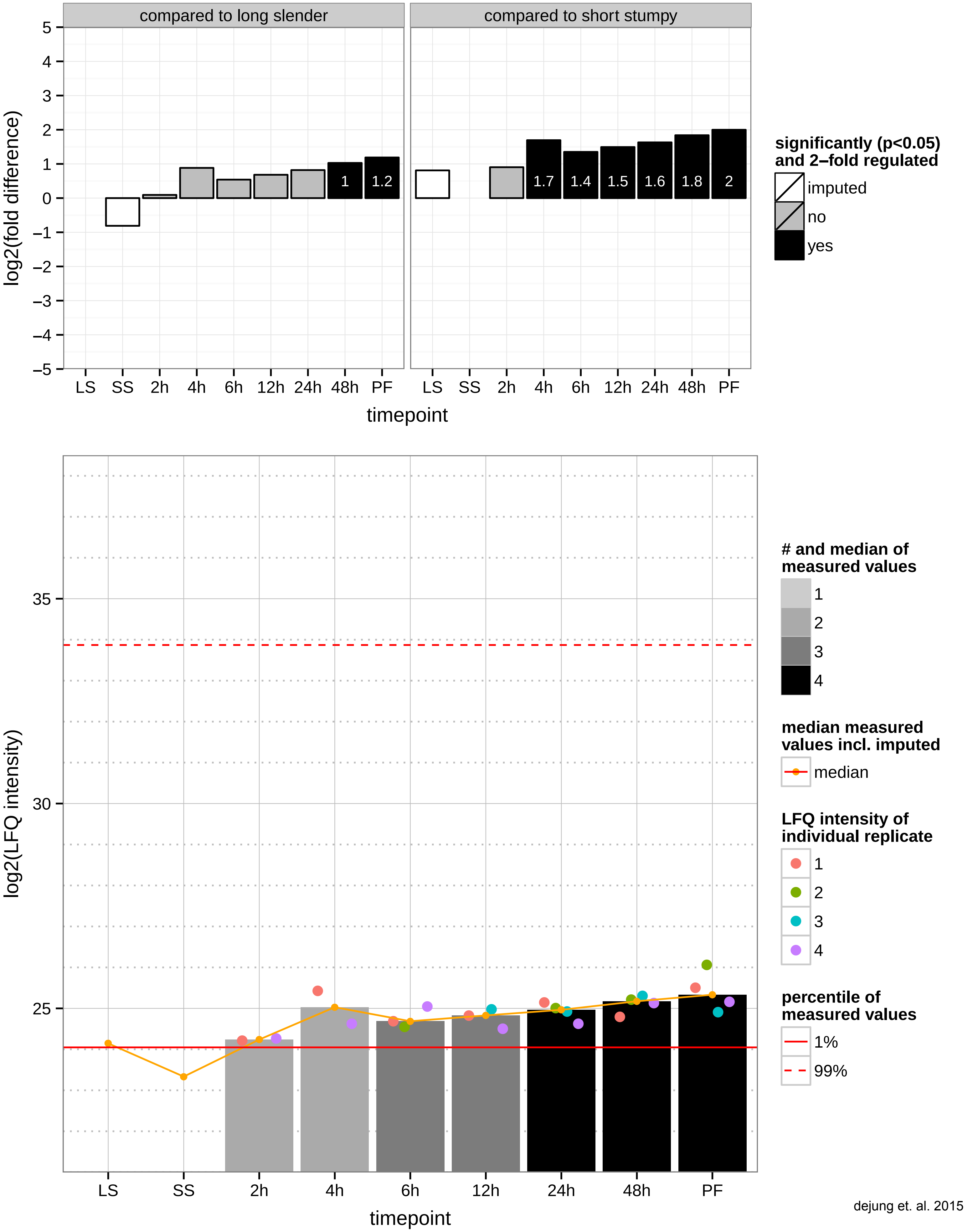

dejung et. al. 2015

squalene synthase, putative
Tb927.8.7120
AGOF: farnesyl−diphosphate farnesyltransferase activity, oxidoreductase activity
AGOC: endoplasmic reticulum membrane, integral to membrane, mitochondrial outer membrane
AGOP: cholesterol biosynthetic process, ergosterol biosynthetic process, isoprenoid biosynthetic process, oxidation−reductior
PGOF: transferase activity
PGOC: null
PGOP: biosynthetic process

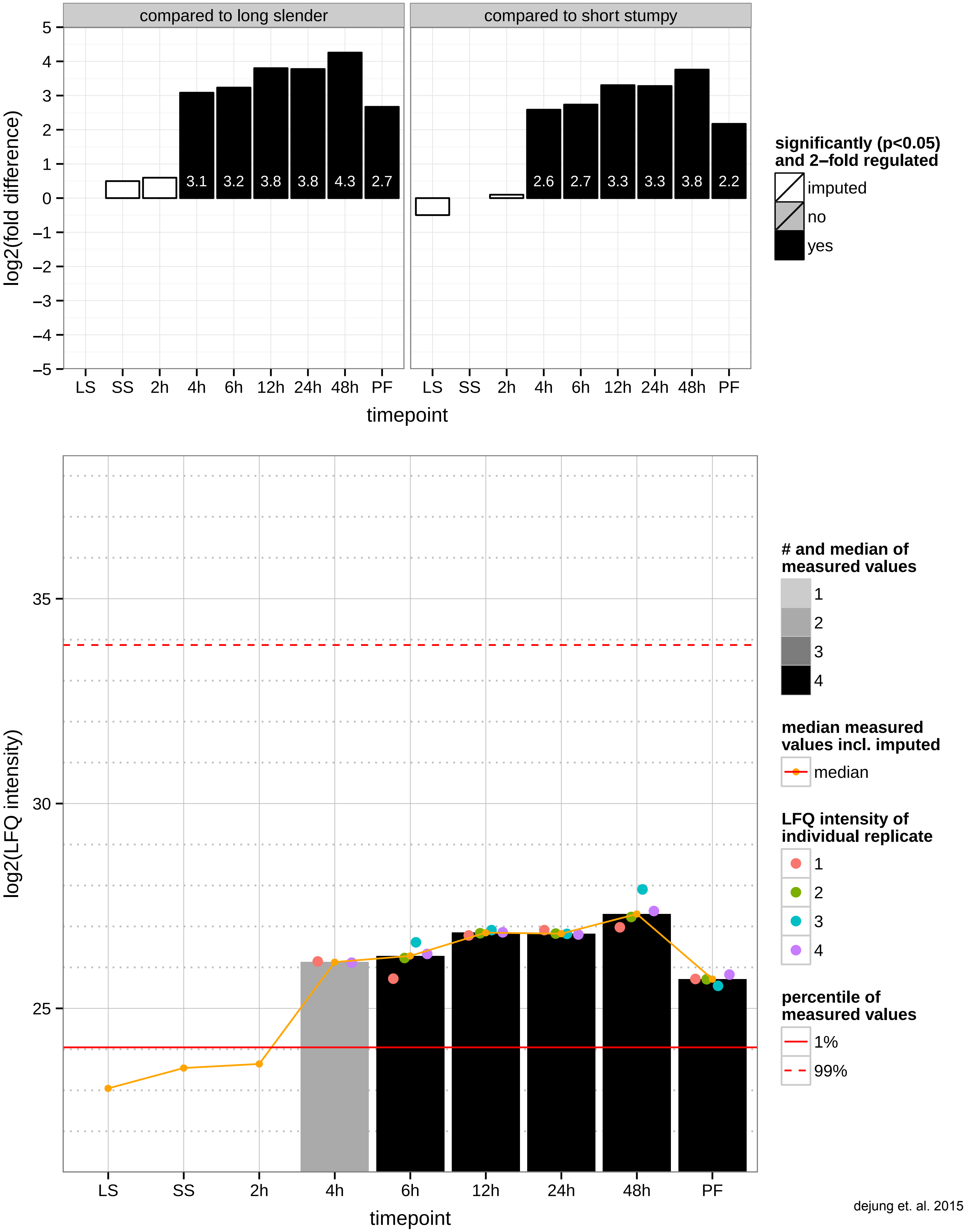

dejung et. al. 2015

zinc finger protein, putative, predicted zinc finger protein
Tb927.9.11870
AGOF: zinc ion binding
AGOC: null
AGOP: null
PGOF: metal ion binding
PGOC: null
PGOP: null

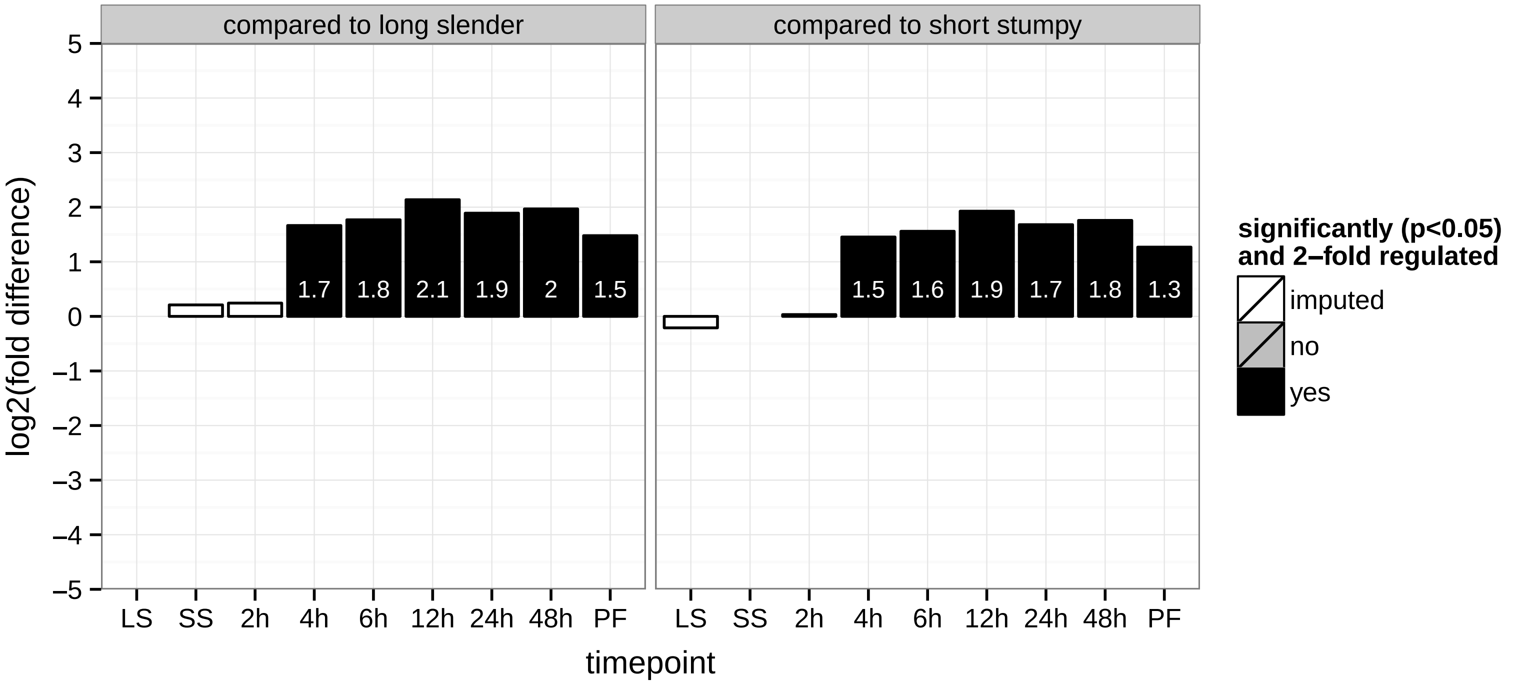

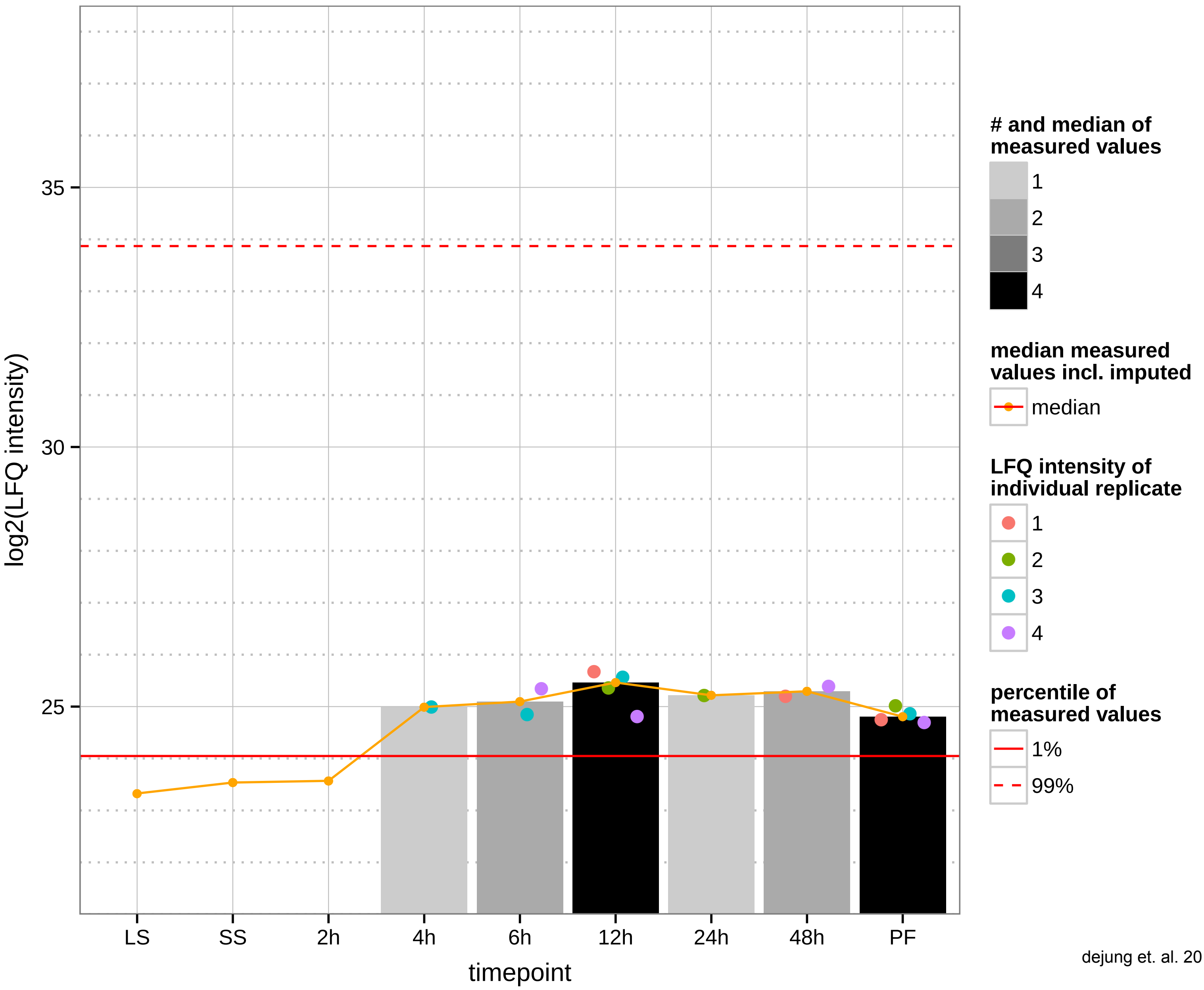

dejung et. al. 2015

hypothetical protein, conserved
Tb927.9.11910
AGOF: heme binding, peroxidase activity
AGOC: mitochondrion
AGOP: oxidation−reduction process, response to oxidative stress
PGOF: heme binding, peroxidase activity
PGOC: null
PGOP: oxidation−reduction process, response to oxidative stress

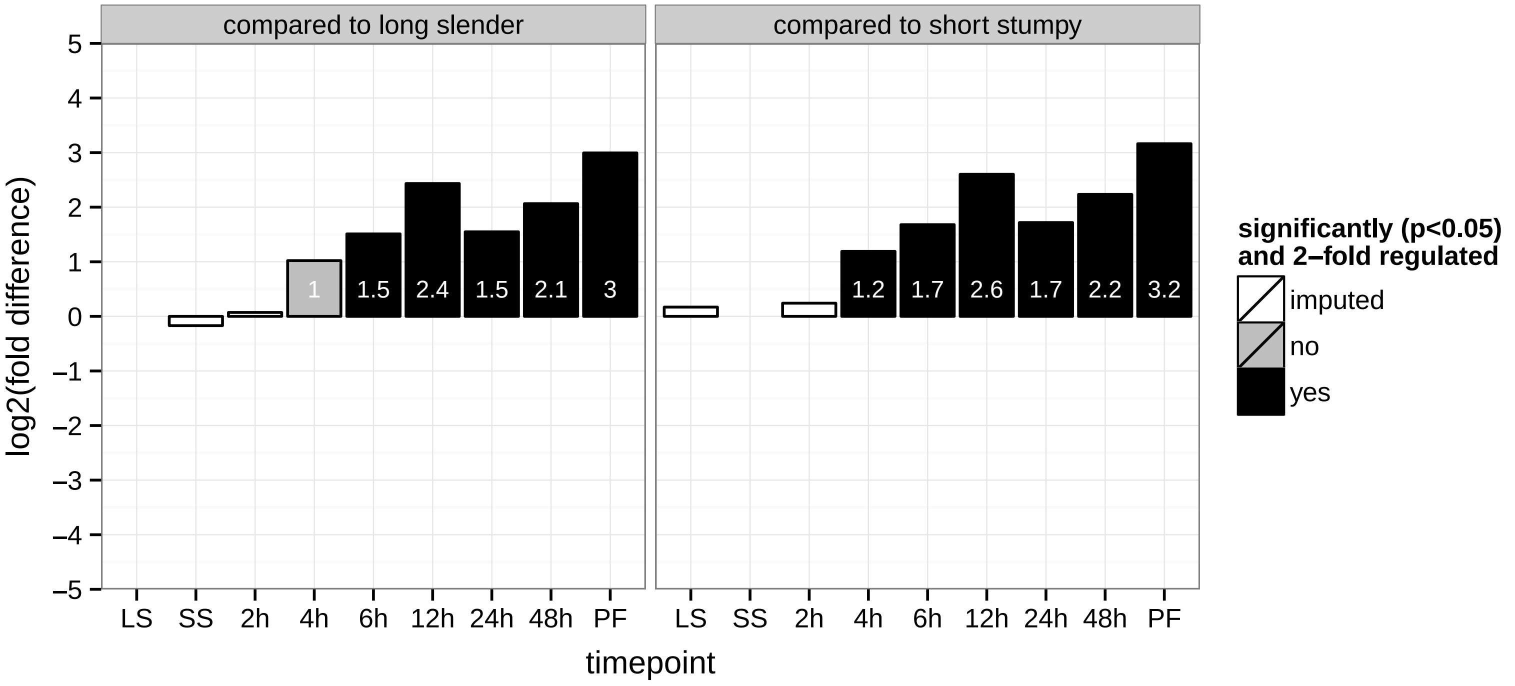

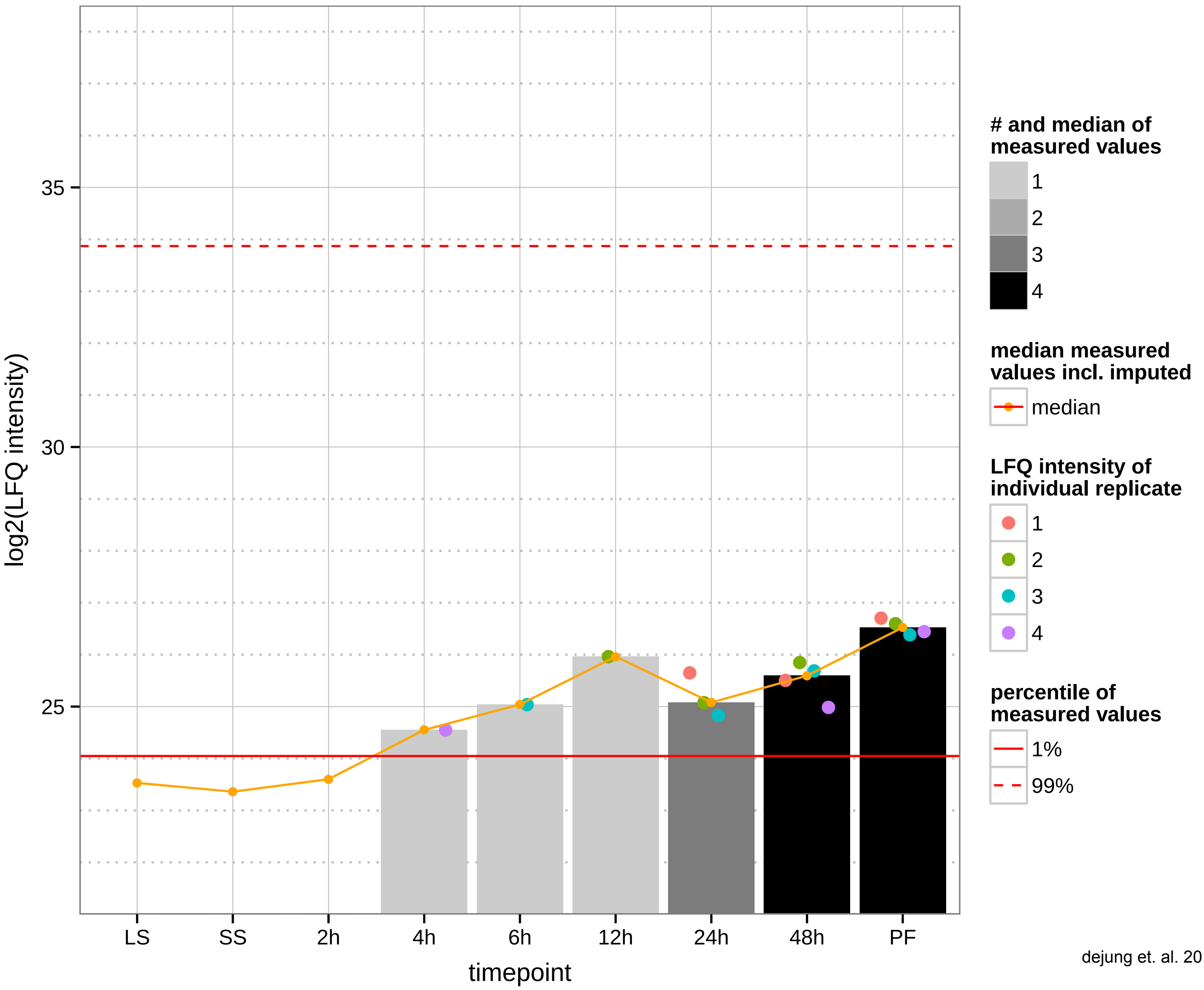

dejung et. al. 2015

hypothetical protein, conserved
Tb927.9.1710
AGOF: null
AGOC: null
AGOP: null
PGOF: null
PGOC: null
PGOP: null

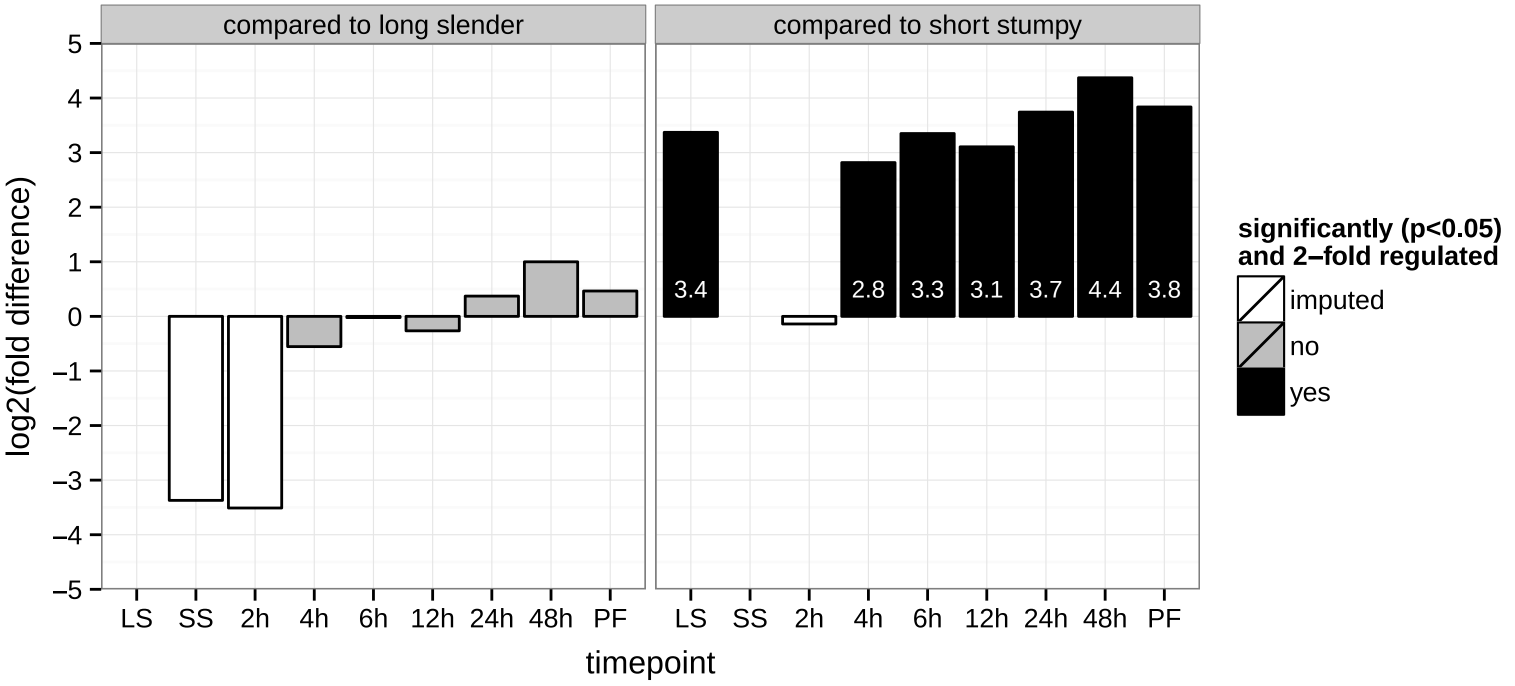

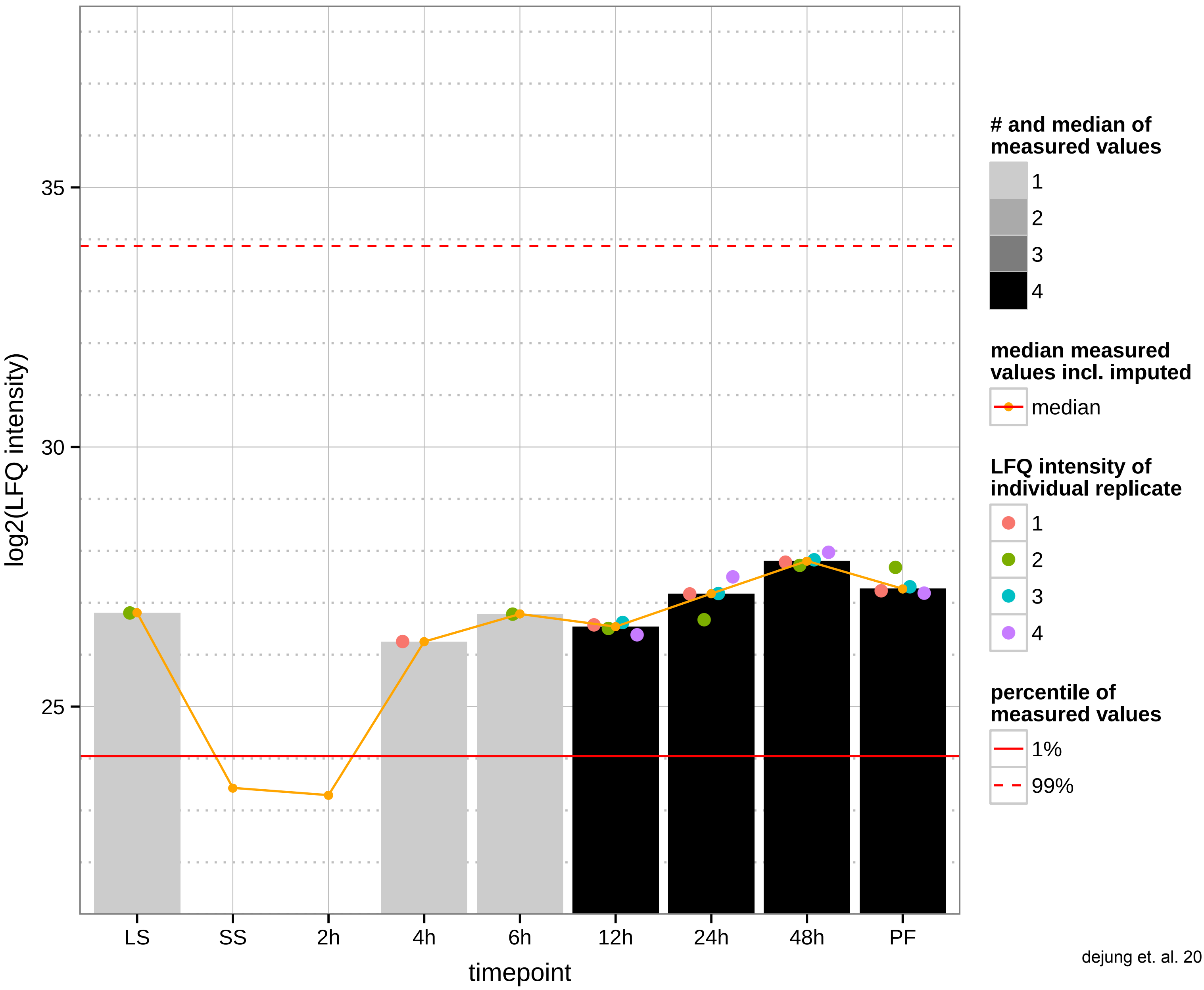

dejung et. al. 2015

hypothetical protein, conserved
Tb927.9.2390
AGOF: null
AGOC: null
AGOP: null
PGOF: null
PGOC: null
PGOP: null

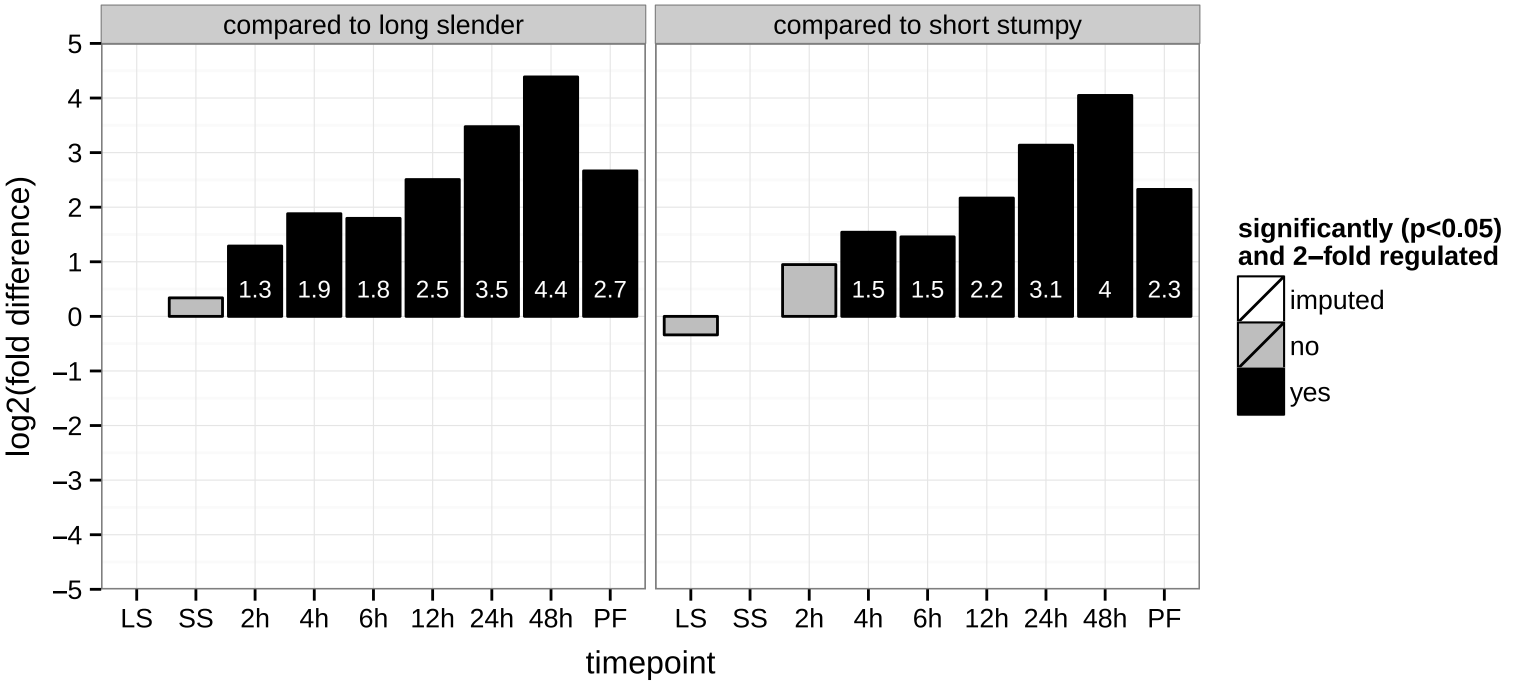

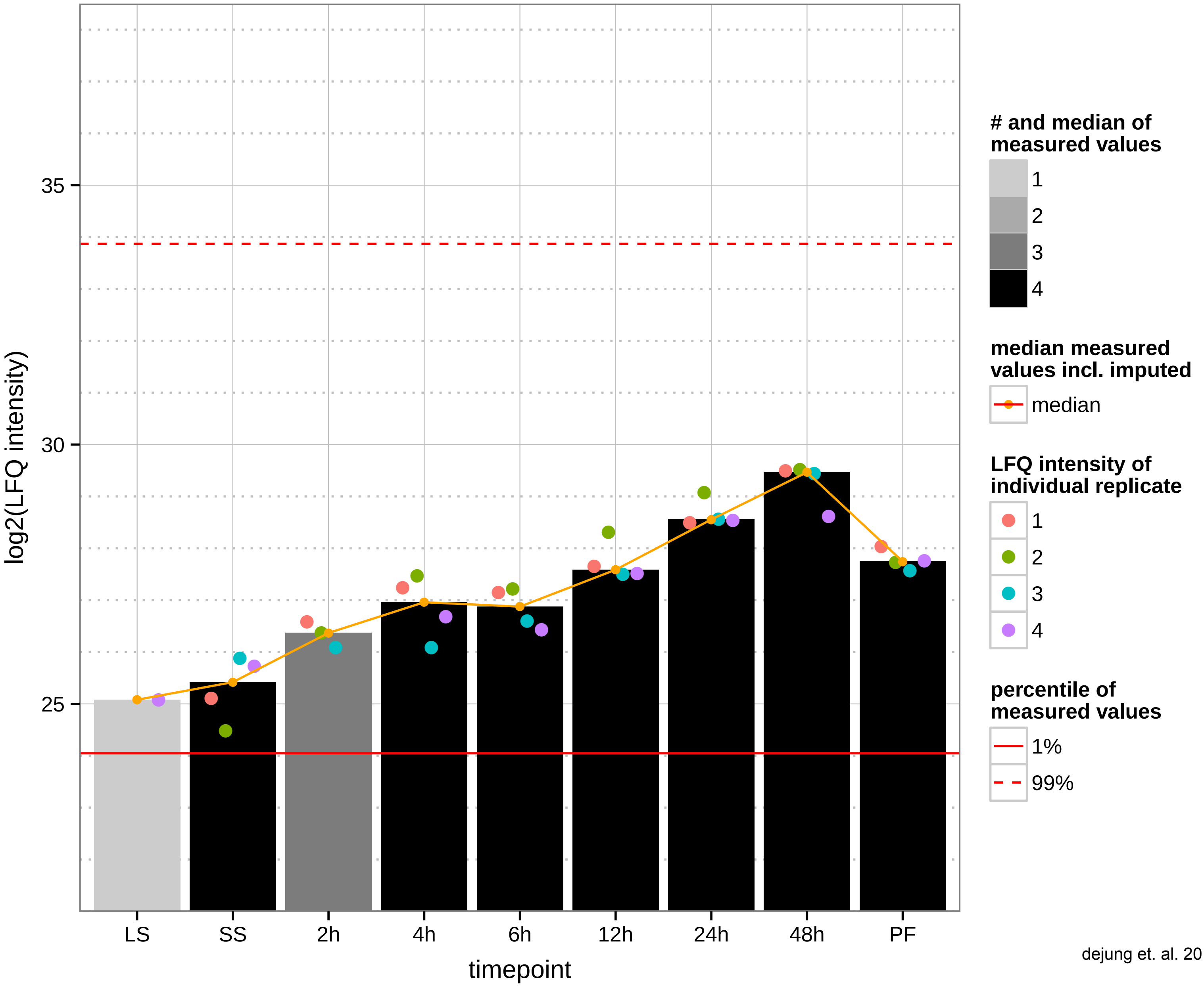

dejung et. al. 2015

cytochrome oxidase subunit V (COXV)
Tb927.9.3170
AGOF: null
AGOC: mitochondrion
AGOP: null
PGOF: null
PGOC: null
PGOP: null

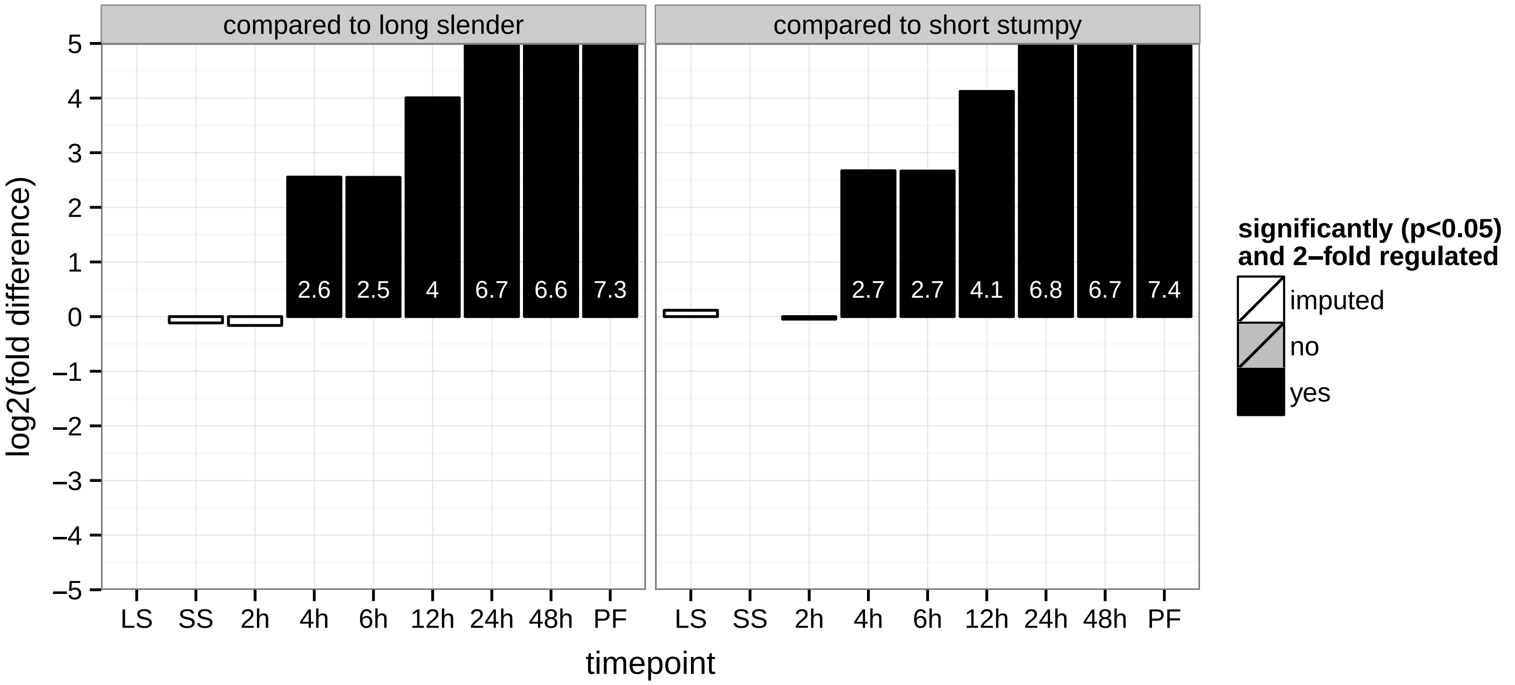

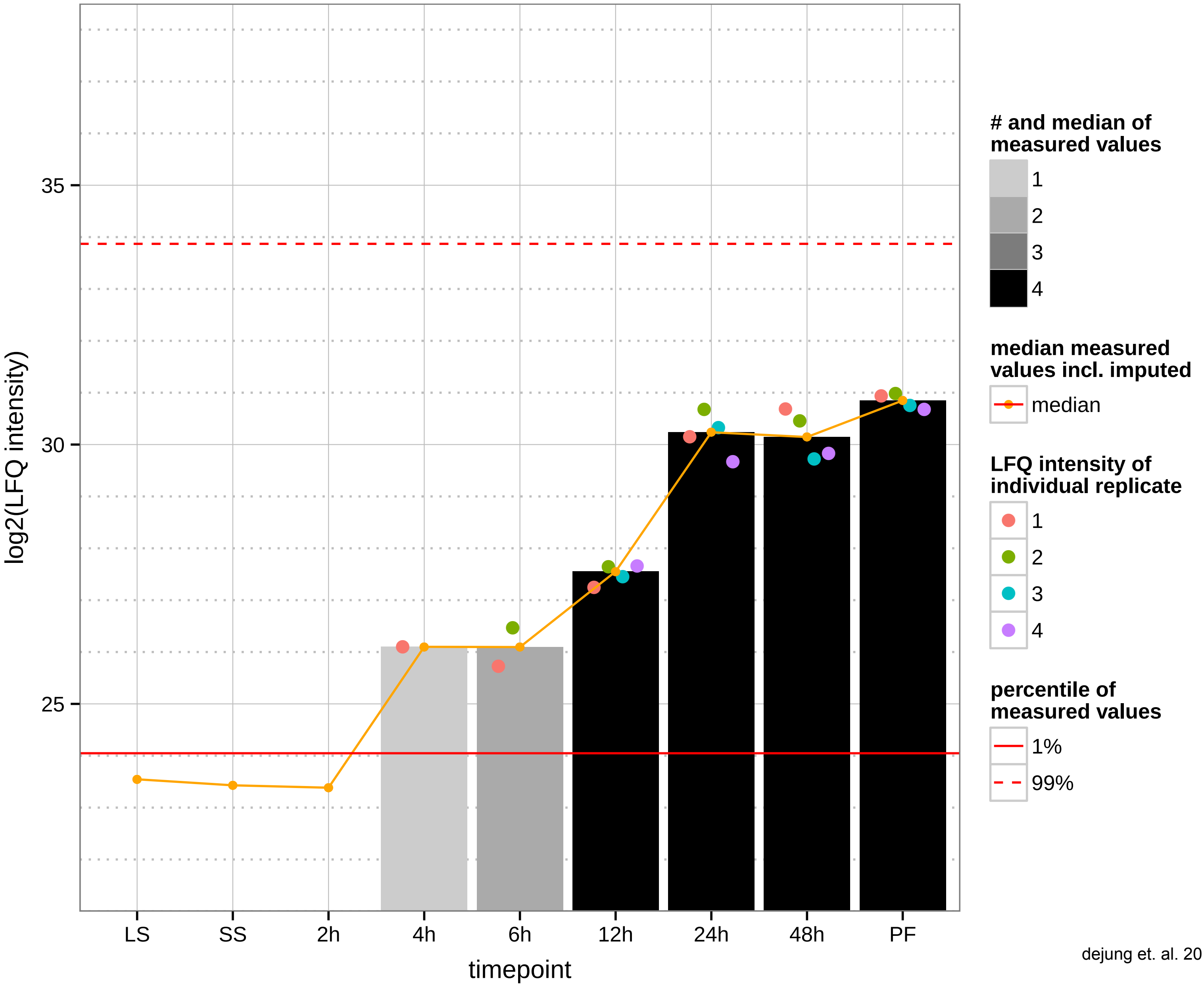

dejung et. al. 2015

2Fe–2S iron–sulfur cluster binding domain containing protein, putative
Tb927.9.6060
AGOF: thiol oxidase activity
AGOC: mitochondrial intermembrane space, mitochondrion
AGOP: oxidation–reduction process, protein import into mitochondrial intermembrane space
PGOF: thiol oxidase activity
PGOC: null
PGOP: oxidation–reduction process

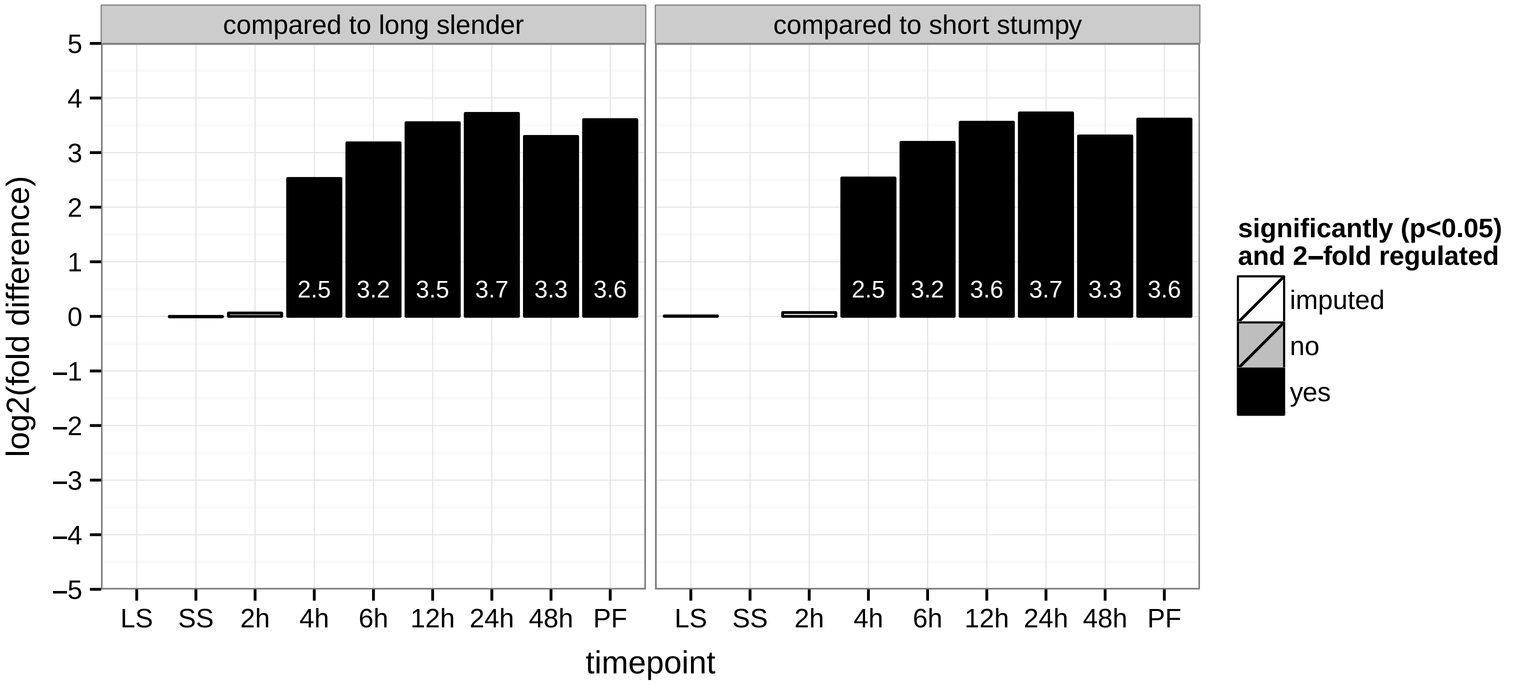

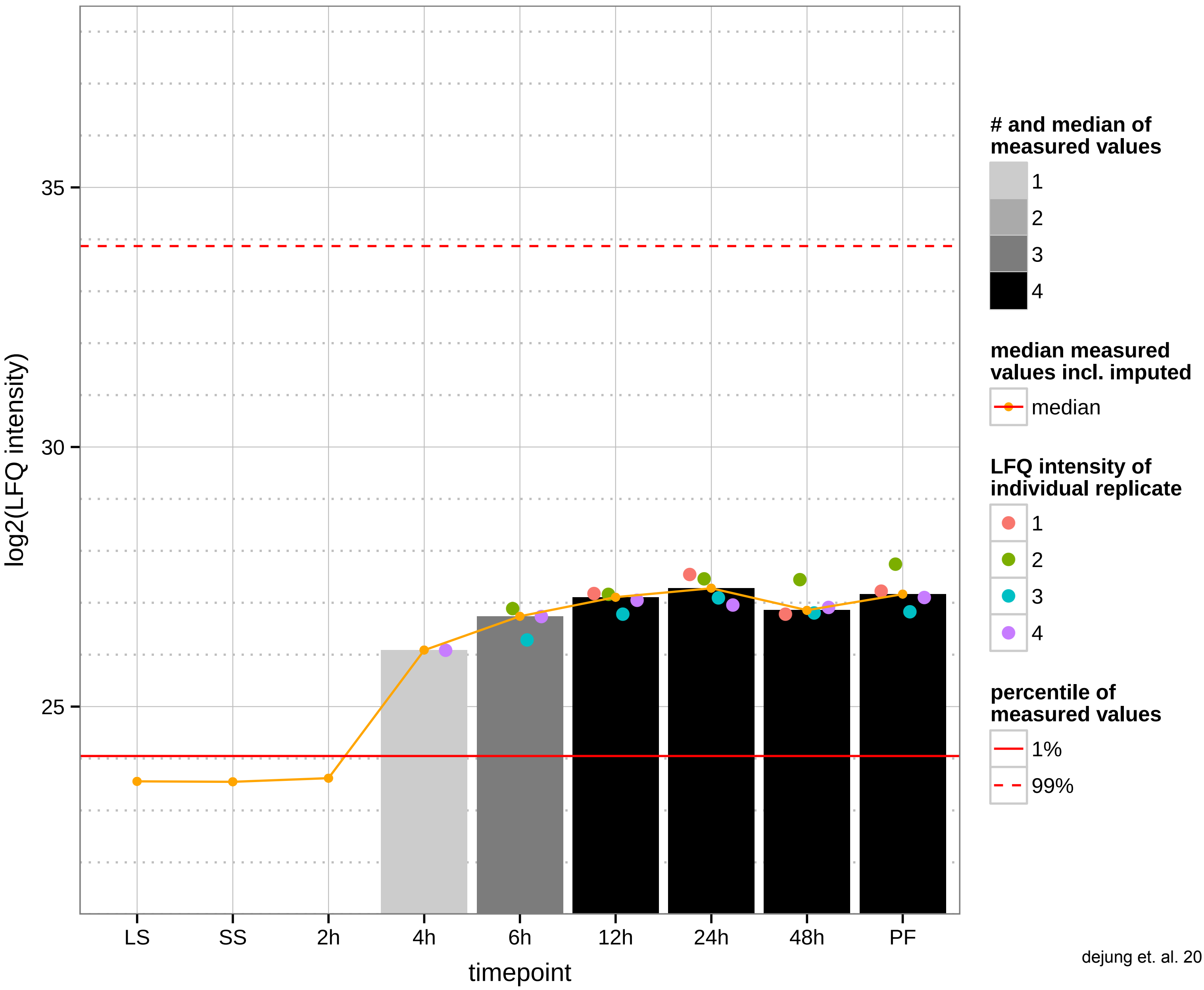

dejung et. al. 2015

hypothetical protein, conserved
Tb927.9.6620
AGOF: null
AGOC: mitochondrion
AGOP: null
PGOF: null
PGOC: null
PGOP: null

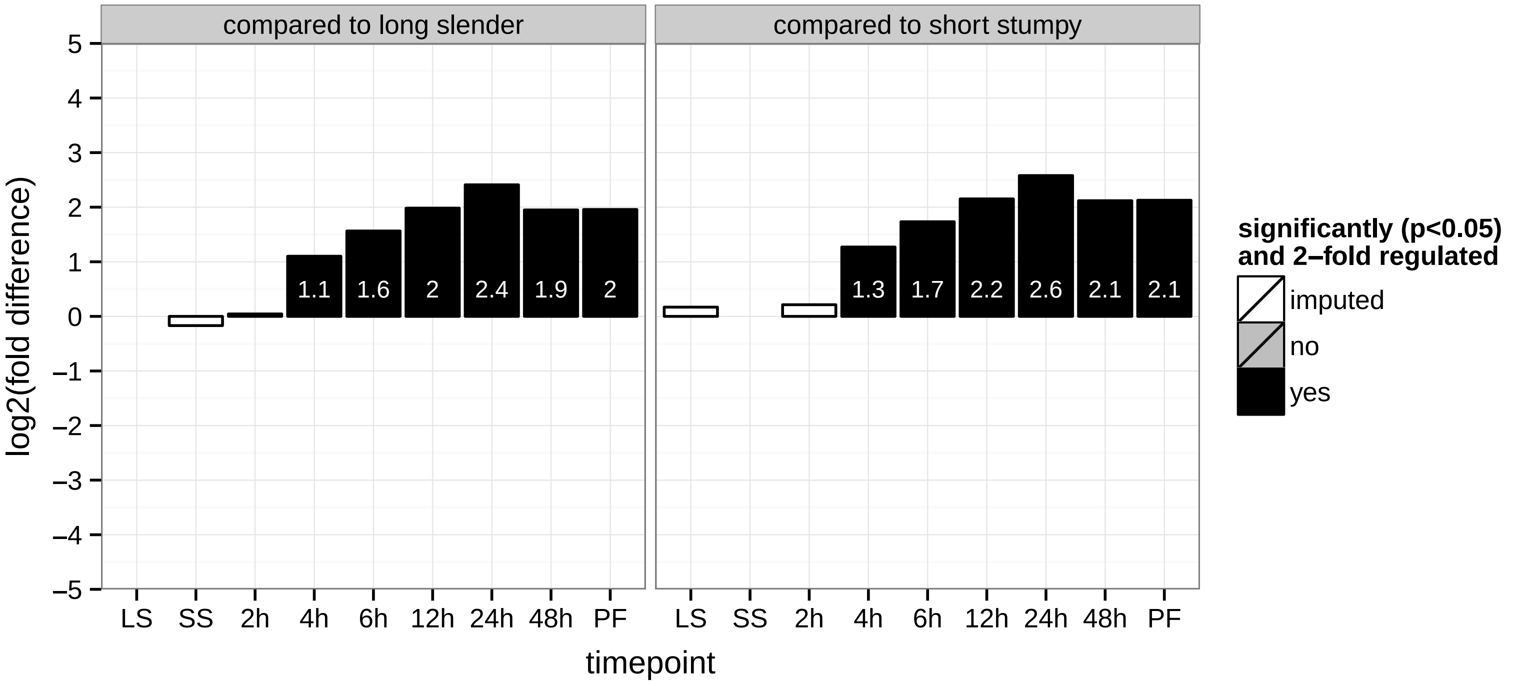

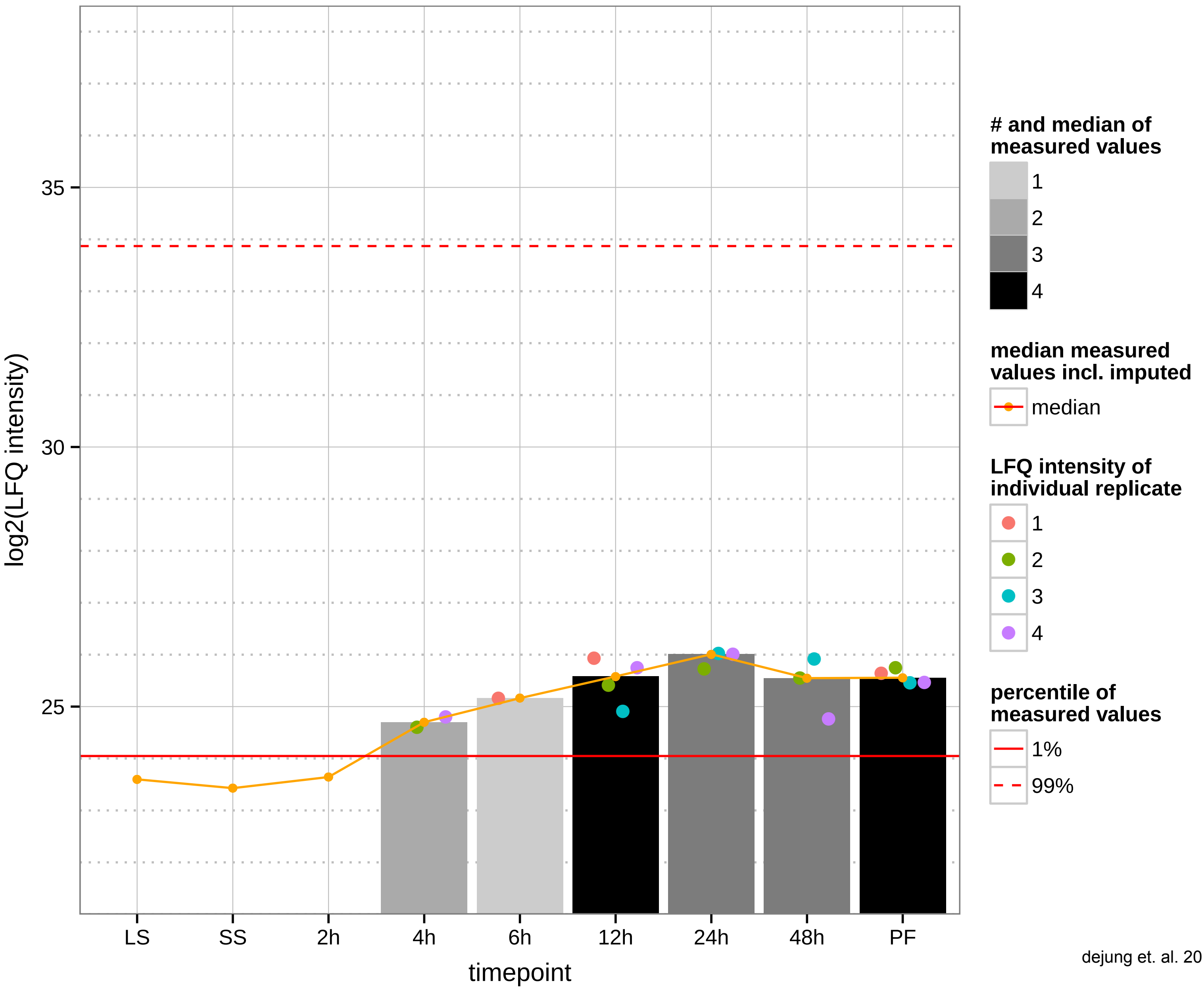

dejung et. al. 2015

hypothetical protein, conserved
Tb927.9.7170
AGOF: structural constituent of ribosome
AGOC: mitochondrial ribosome, mitochondrion
AGOP: translation
PGOF: structural constituent of ribosome
PGOC: mitochondrial ribosome
PGOP: translation

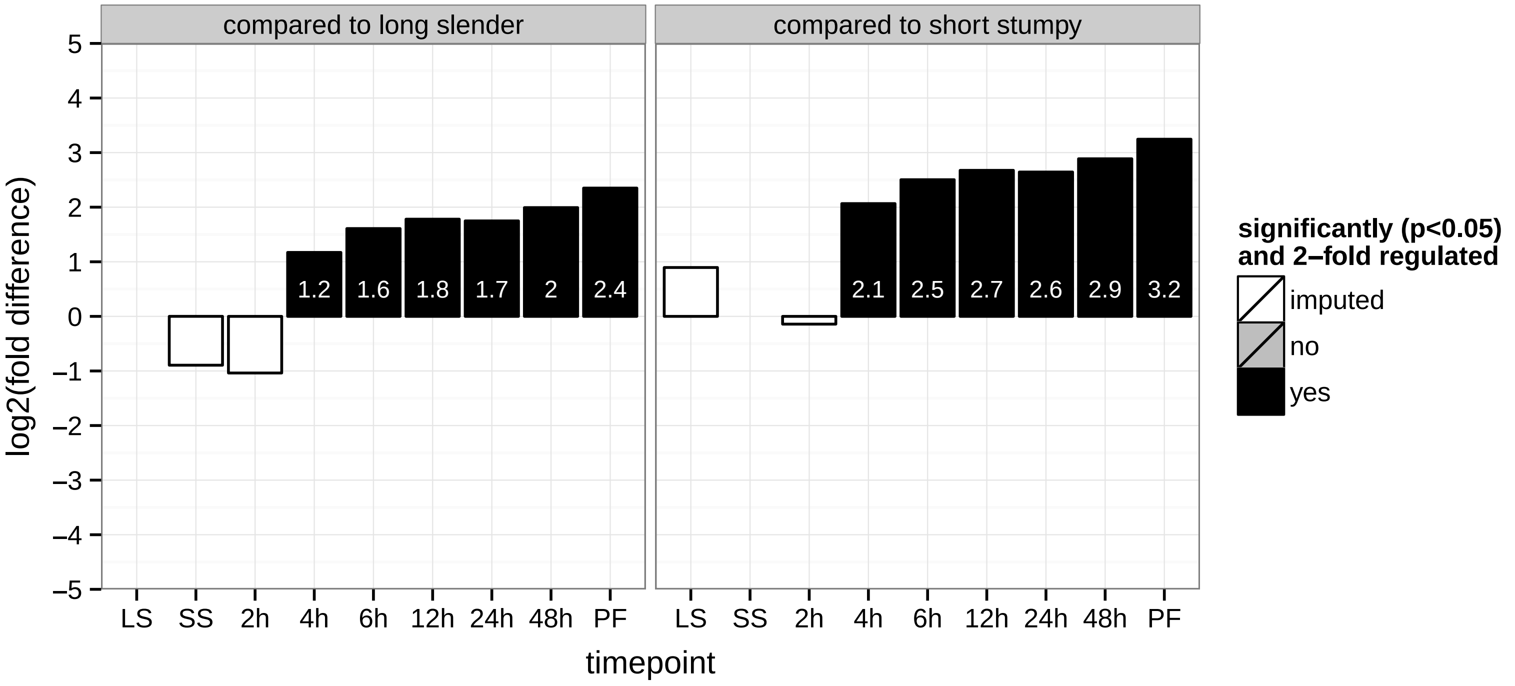

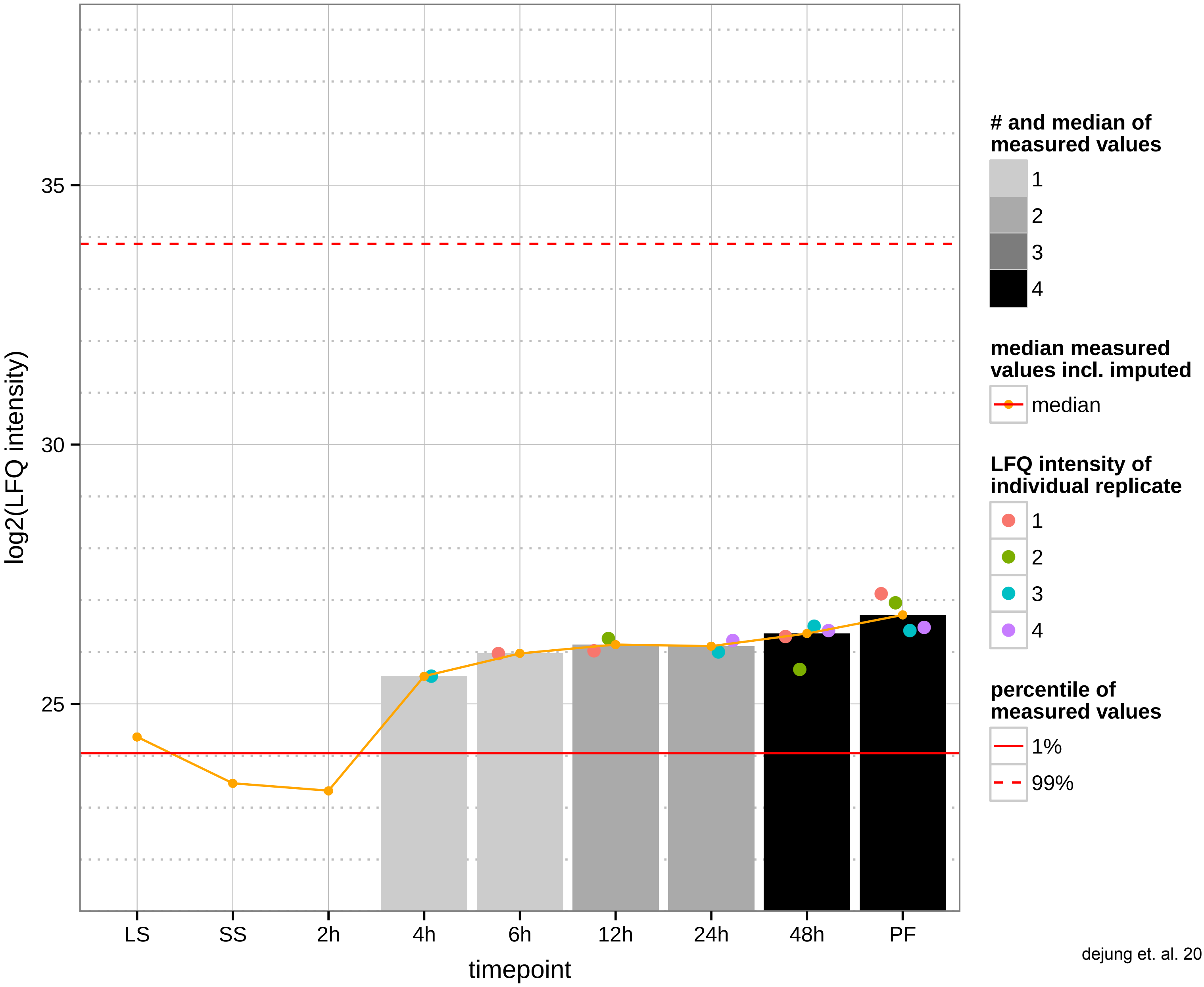

dejung et. al. 2015

hypothetical protein, conserved
Tb927.9.8820
AGOF: null
AGOC: null
AGOP: null
PGOF: null
PGOC: null
PGOP: null

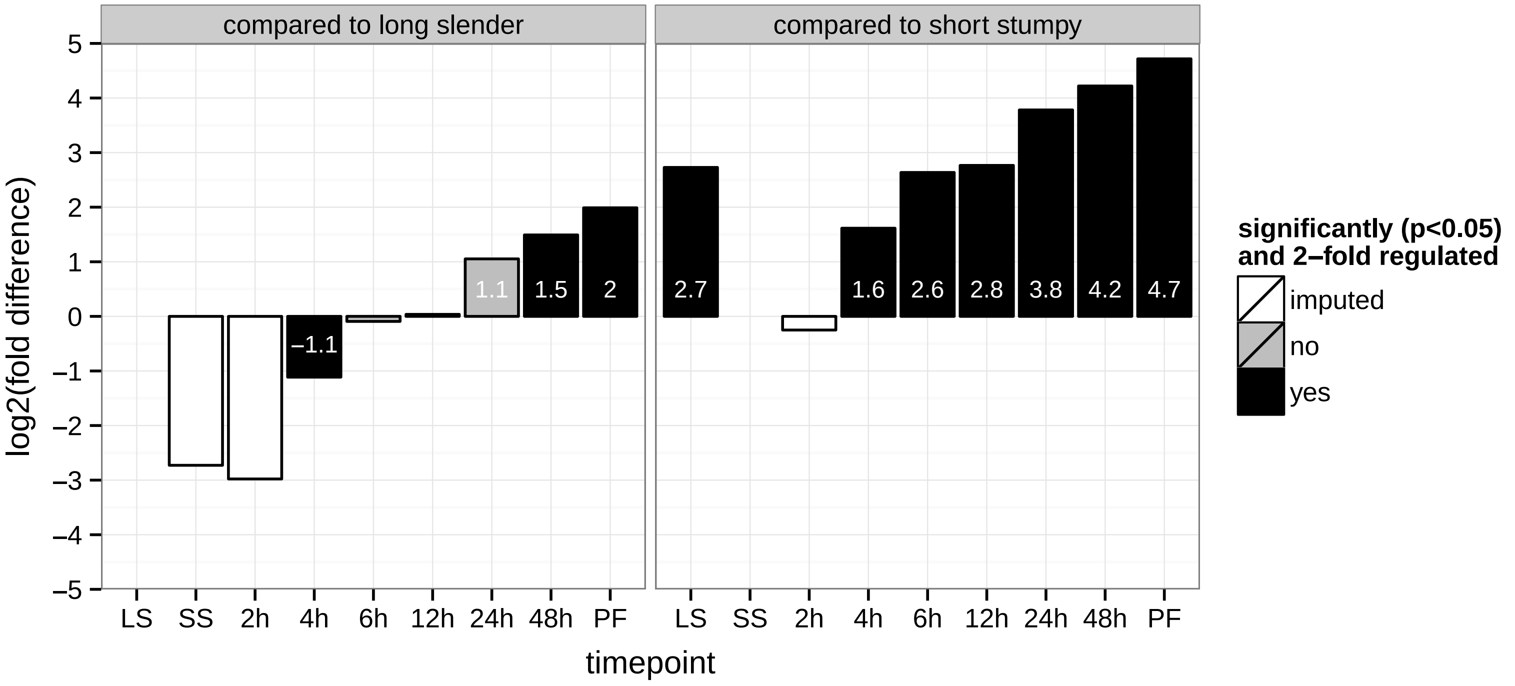

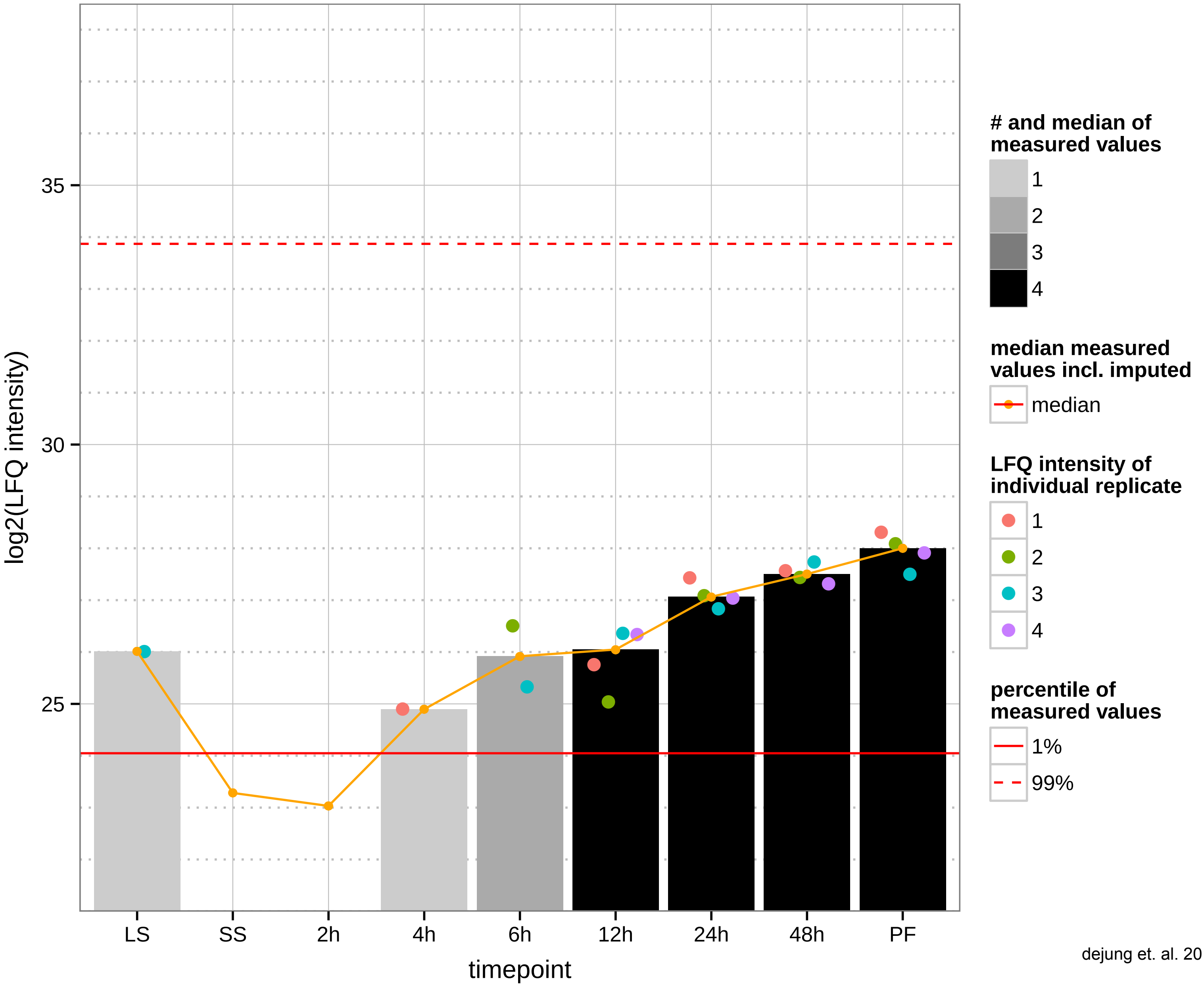

dejung et. al. 2015

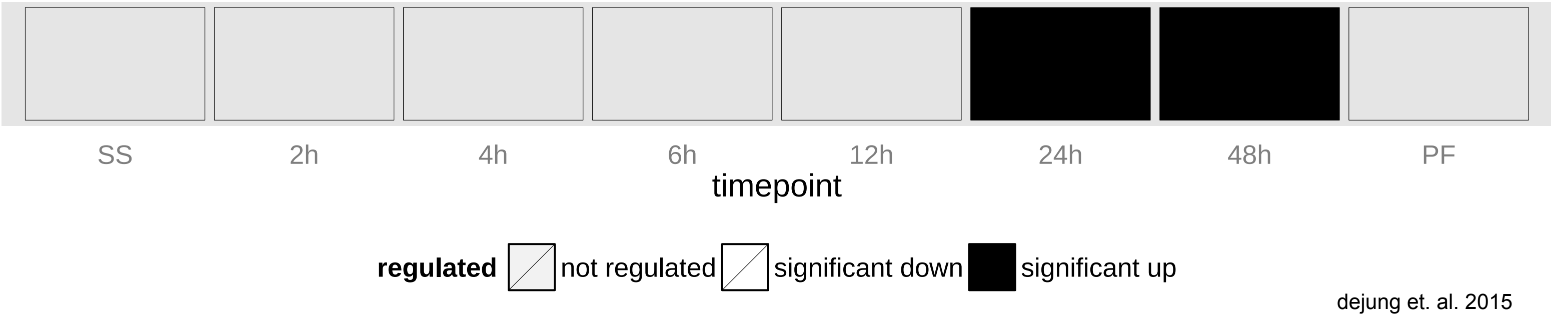
SS
2h
4h
6h
12h
24h
48h
PF
timepoint
regulated
not regulated
significant down
significant up
dejung et. al. 2015

hypothetical protein, conserved, chrX additional, unordered contigs
Tb10.v4.0041;Tb927.10.4510
AGOF: null
AGOC: null
AGOP: null
PGOF: null
PGOC: null
PGOP: null

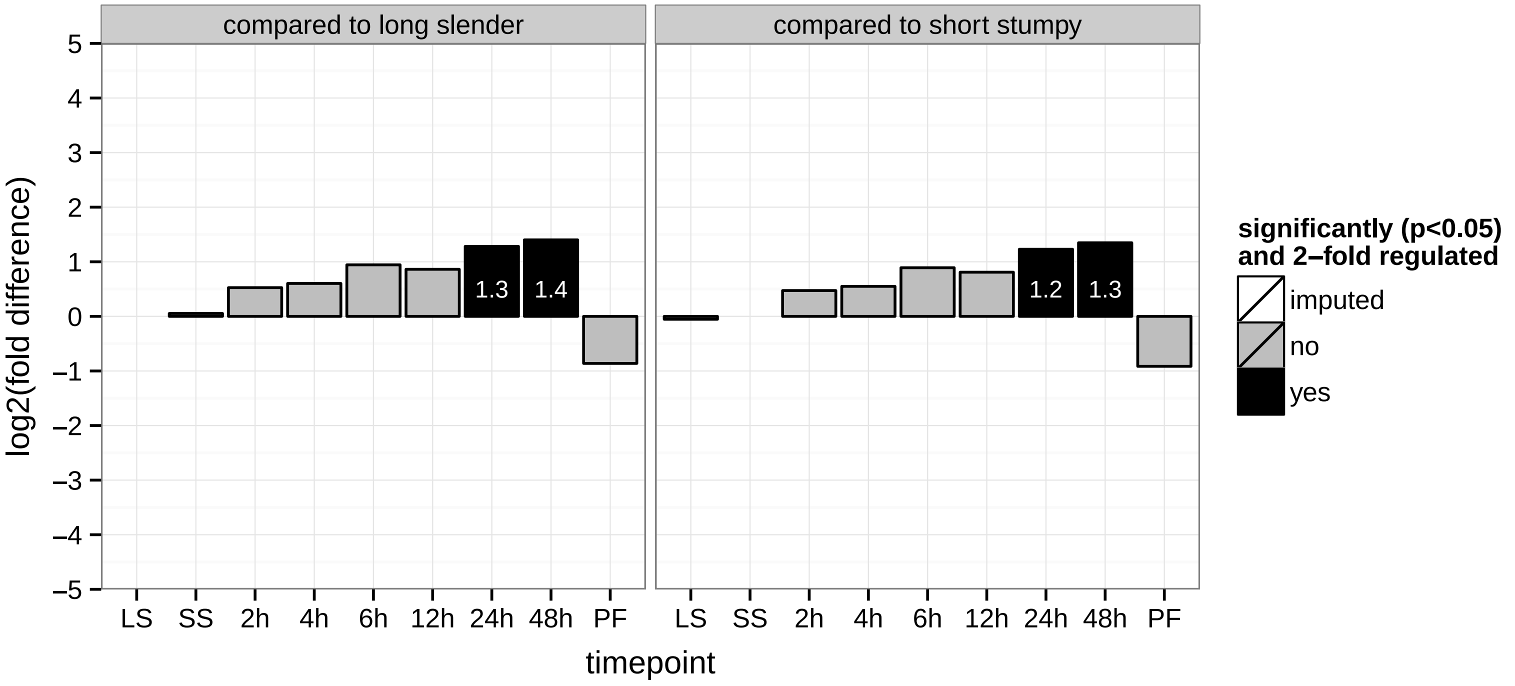

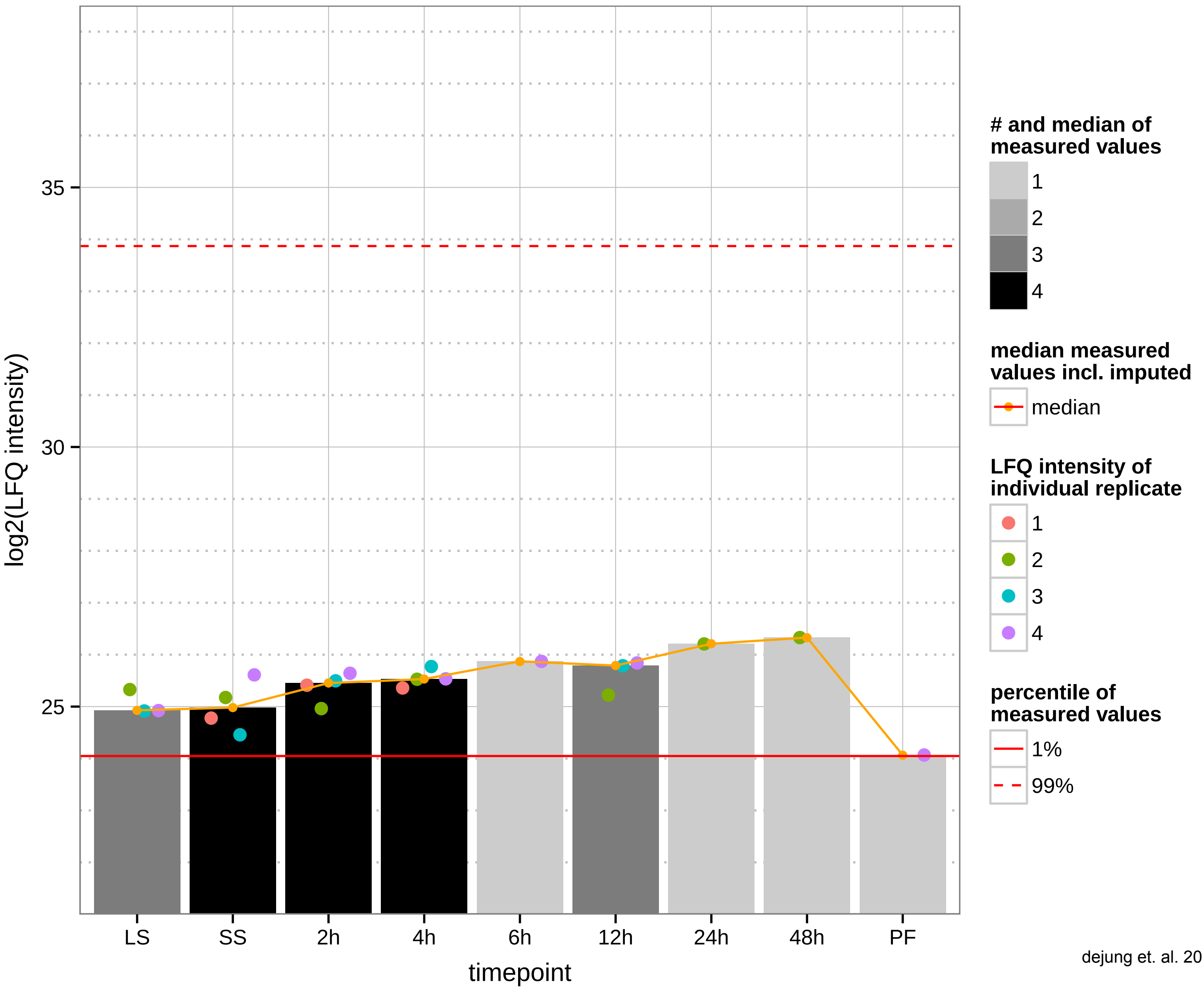

dejung et. al. 2015

prohibitin, putative, chrX additional, unordered contigs, prohibitin 2, putative (PHB2)
Tb927.10.4310;Tb10.v4.0045
AGOF: null
AGOC: mitochondrial inner membrane, membrane, mitochondrion
AGOP: null, proteolysis, regulation of mitochondrial translation, replicative cell aging
PGOF: null
PGOC: null
PGOP: null

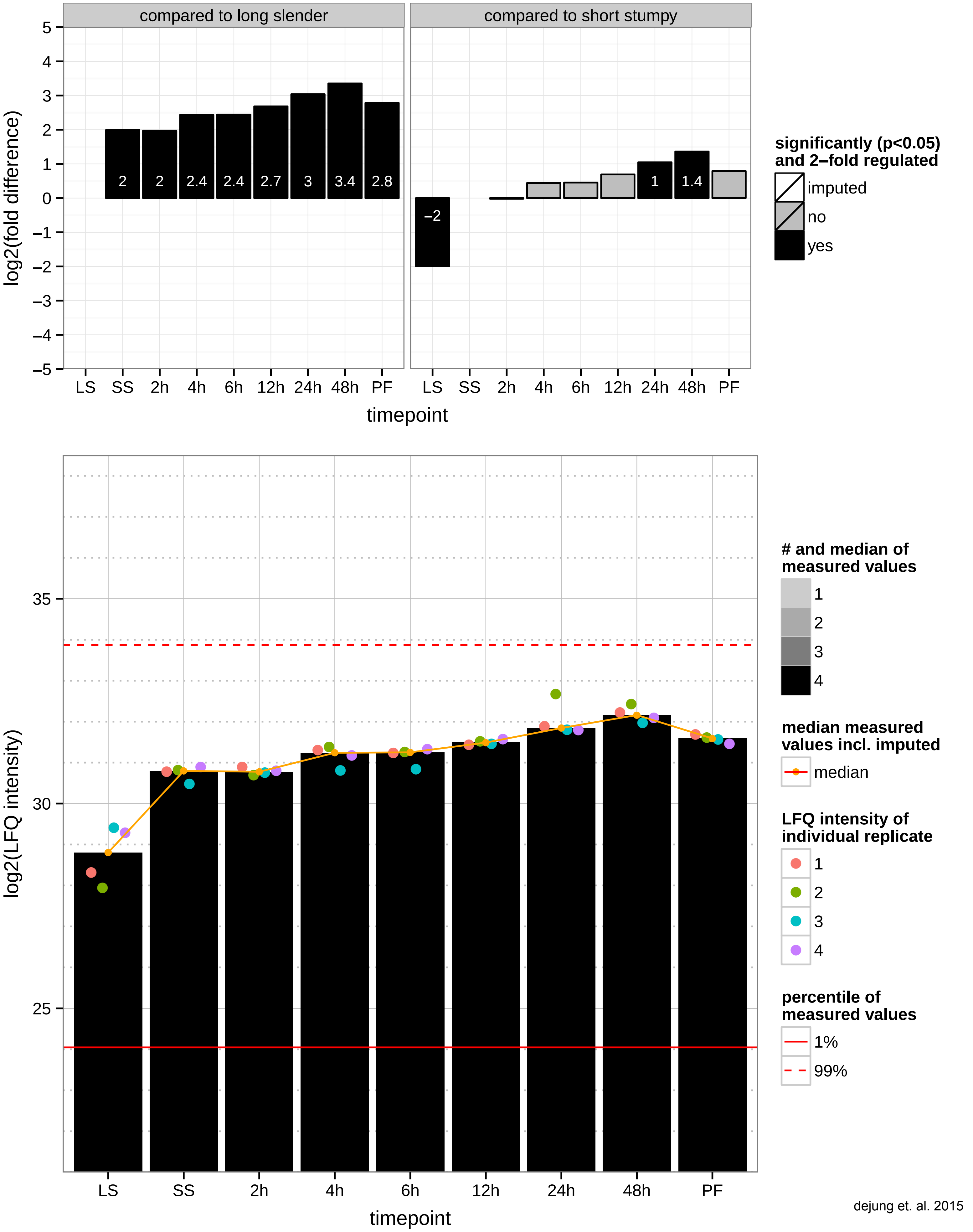

dejung et. al. 2015

hypothetical protein, conserved
Tb11.02.5070b;Tb927.11.7212
AGOF: transferase activity
AGOC: null
AGOP: null
PGOF: transferase activity
PGOC: null
PGOP: null

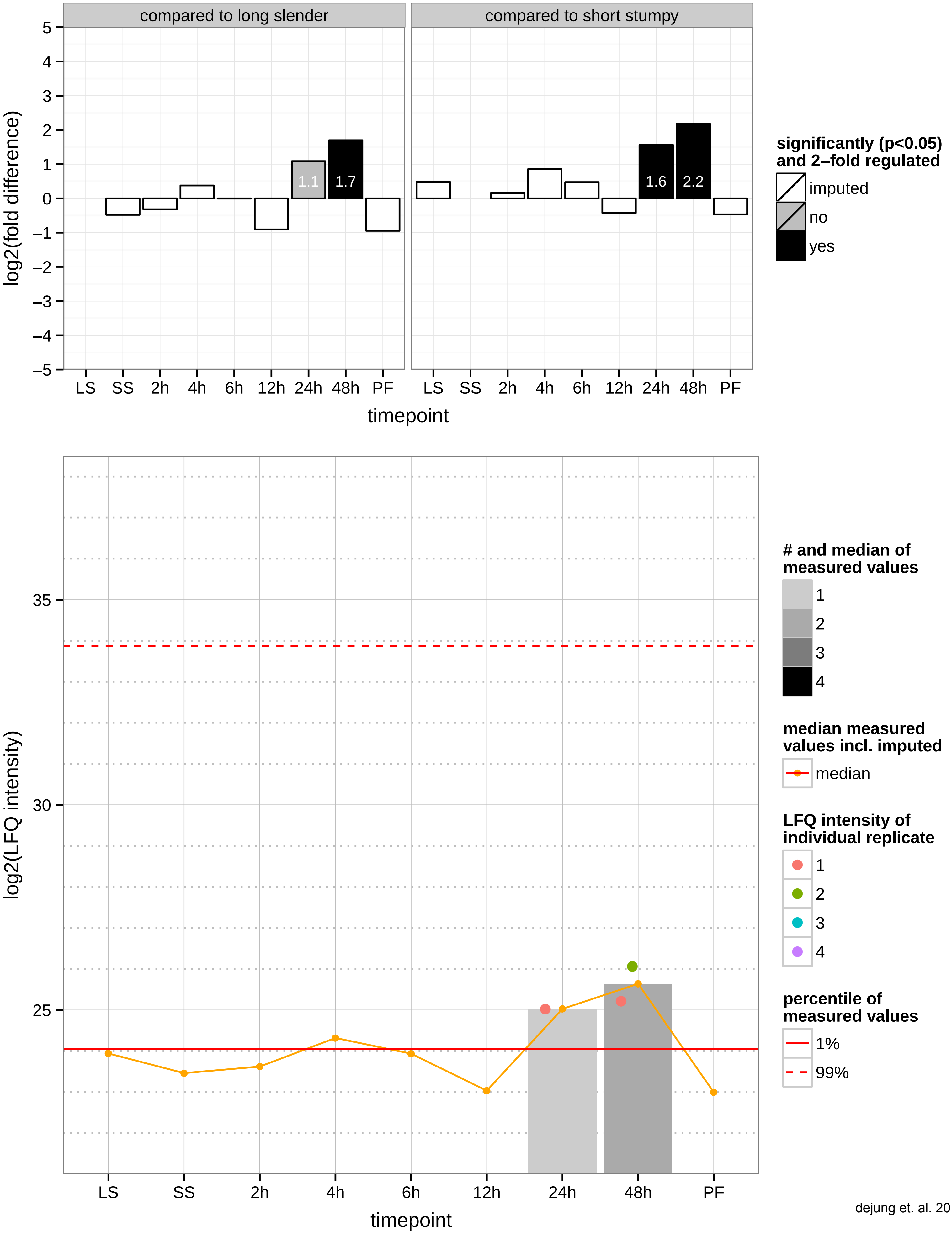

dejung et. al. 2015

retrotransposon hot spot (RHS) protein, putative, retrotransposon hotspot (RHS) protein 1
Tb927.5.295b;Tb11.v5.0214
AGOF: null
AGOC: null
AGOP: null
PGOF: null
PGOC: null
PGOP: null

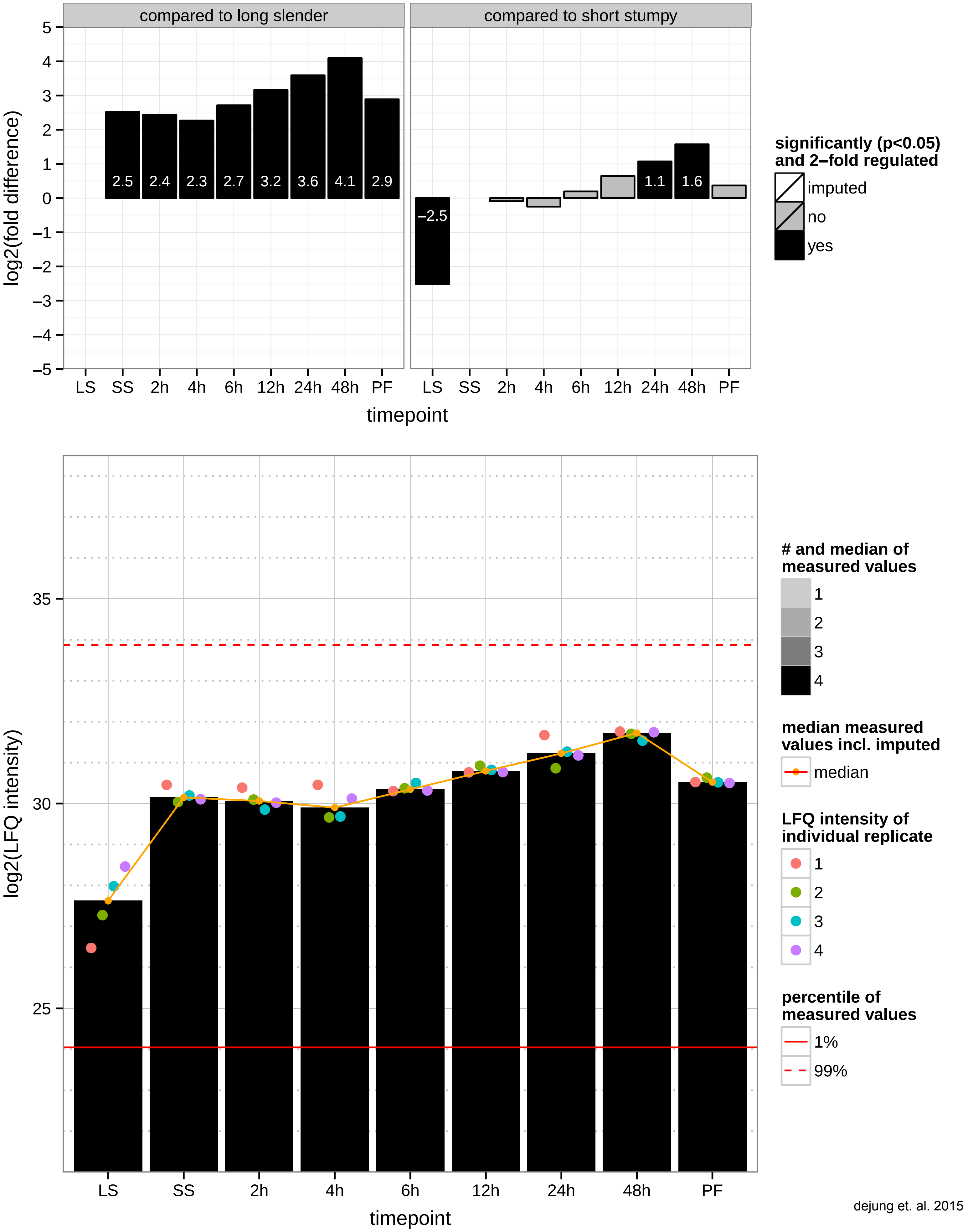

dejung et. al. 2015

hypothetical protein, conserved
Tb927.8.2470;Tb11.v5.0799
AGOF: null
AGOC: null, mitochondrion
AGOP: null
PGOF: null
PGOC: null
PGOP: null

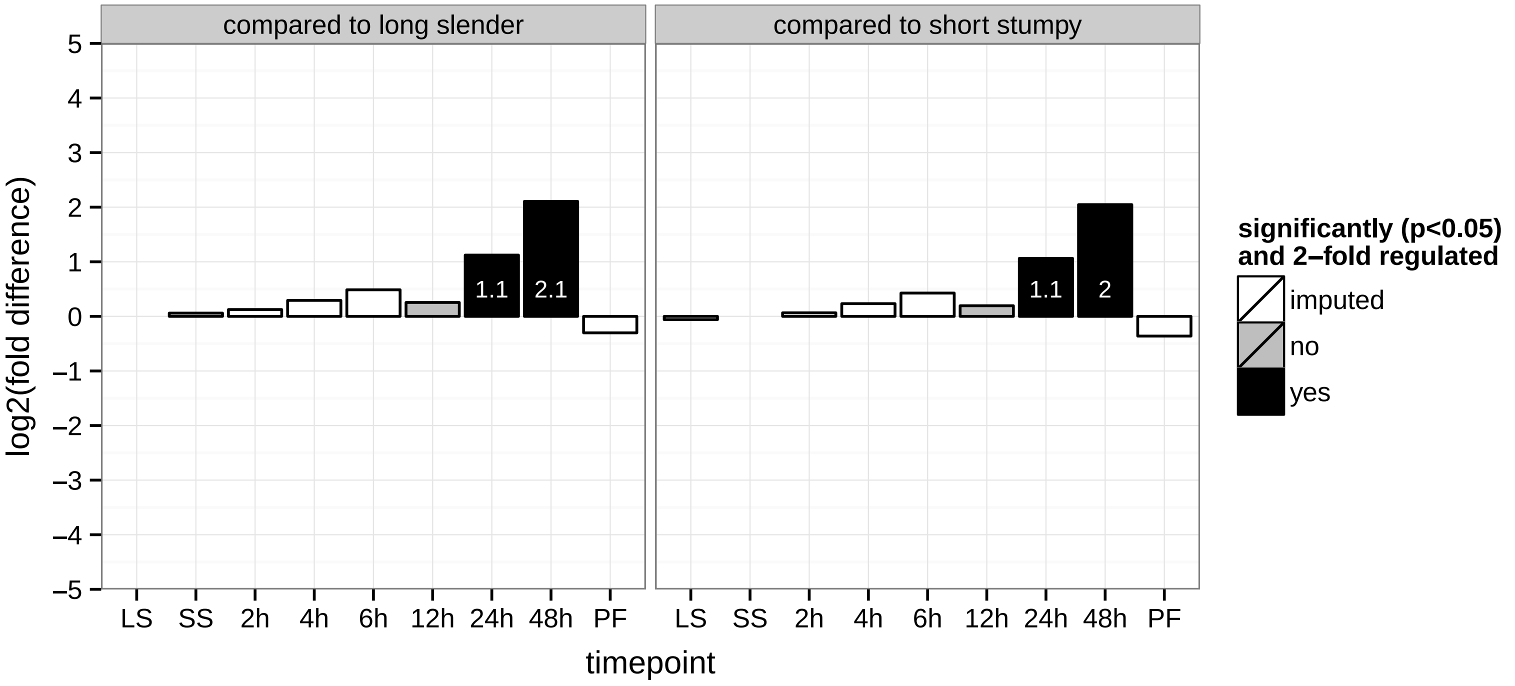

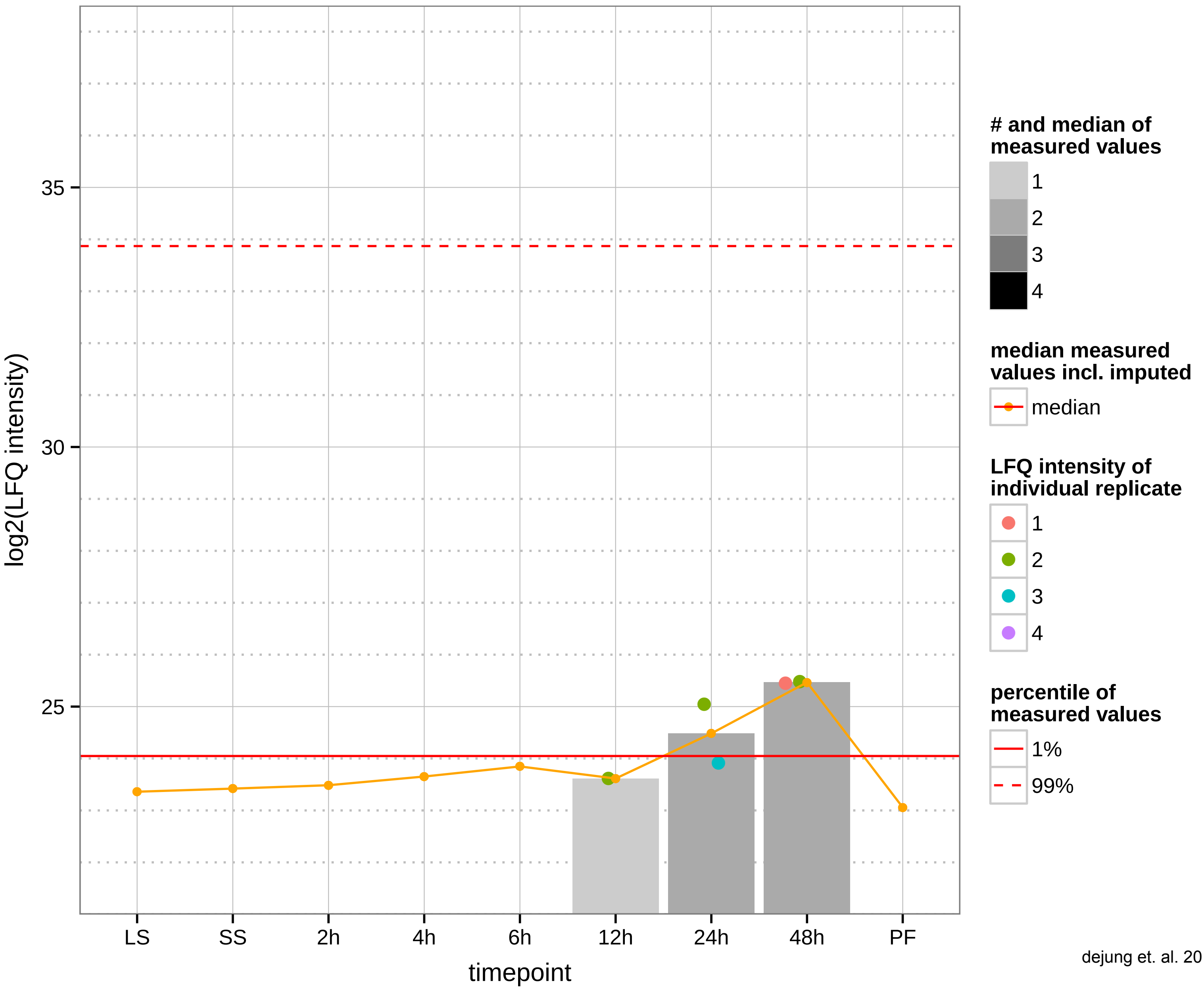

dejung et. al. 2015

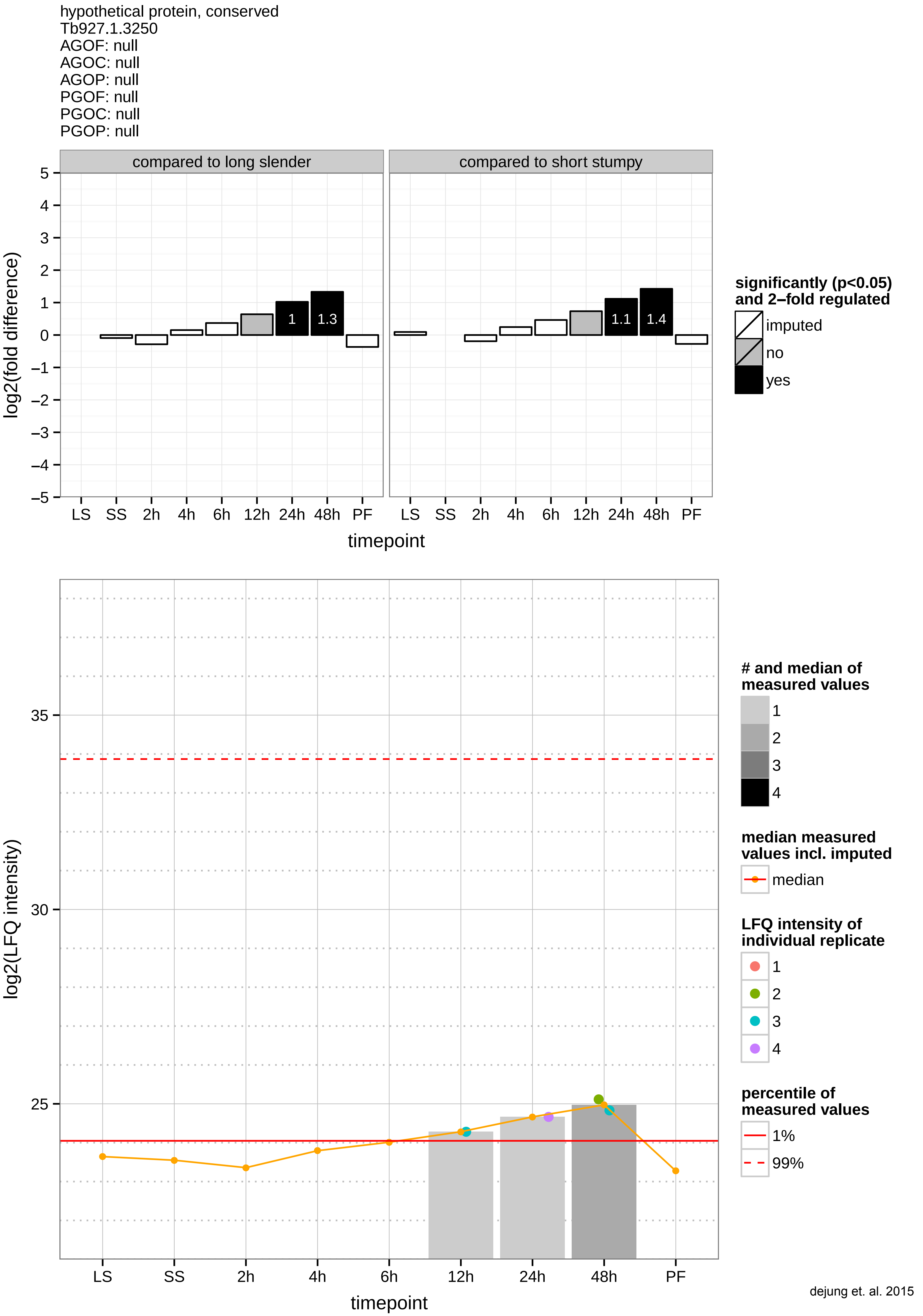

dejung et. al. 2015

minichromosome maintenance (MCM) complex subunit, putative, DNA replication licensing factor MCM8
Tb927.10.10410;Tb11.v5.0535
AGOF: null, ATP binding, DNA binding
AGOC: null
AGOP: null, DNA replication, DNA–dependent DNA replication initiation
PGOF: ATP binding, DNA binding
PGOC: null
PGOP: DNA replication

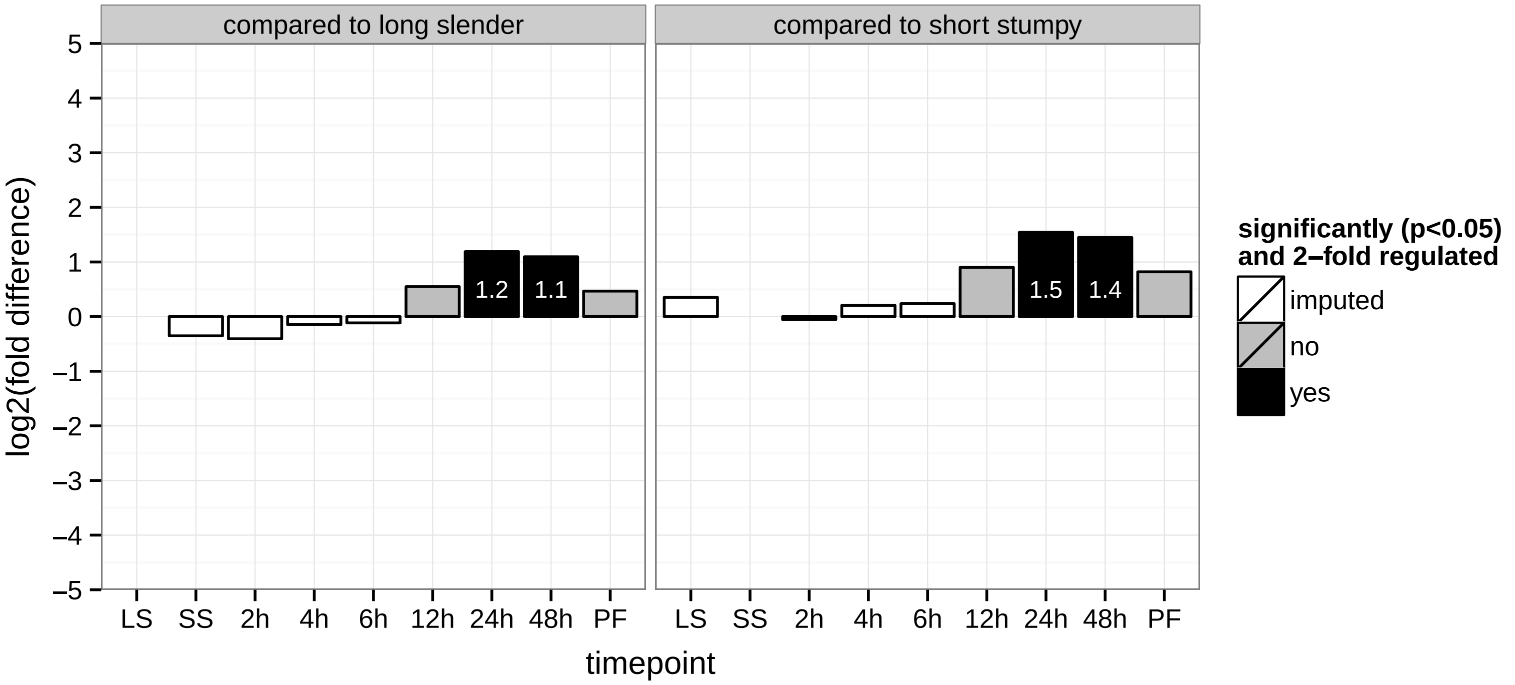

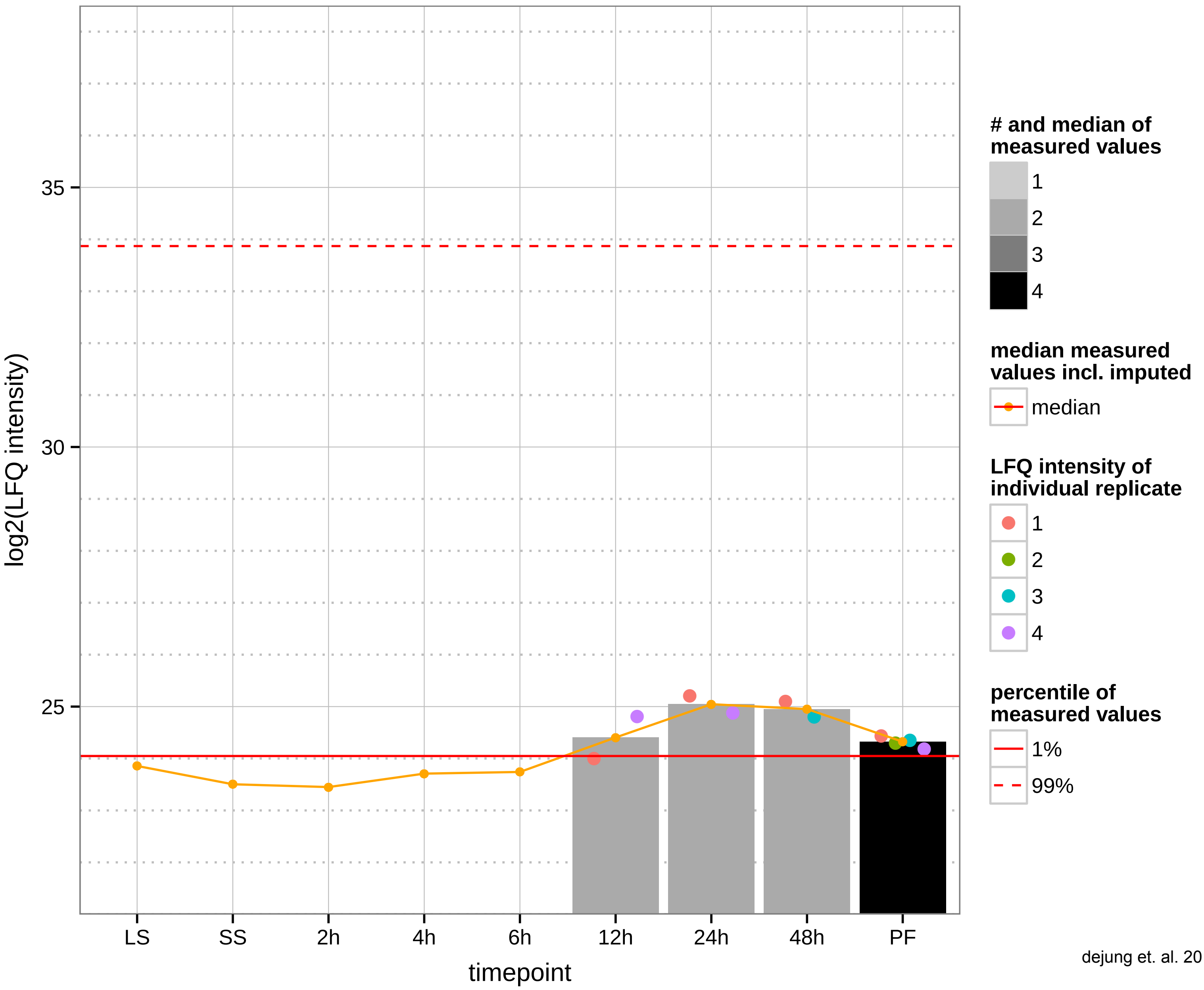

dejung et. al. 2015

cAMP phosphodiesterase A, putative, 3', 5'−cyclic nucleotide phosphodiesterase, phosphodiesterase, putative (PDEA)
Tb927.10.13000
AGOF: 3', 5'−cyclic−AMP phosphodiesterase activity, 3', 5'−cyclic−nucleotide phosphodiesterase activity
AGOC: cytosol
AGOP: negative regulation of reactive oxygen species metabolic process, signal transduction
PGOF: 3', 5'−cyclic−nucleotide phosphodiesterase activity, catalytic activity
PGOC: null
PGOP: signal transduction

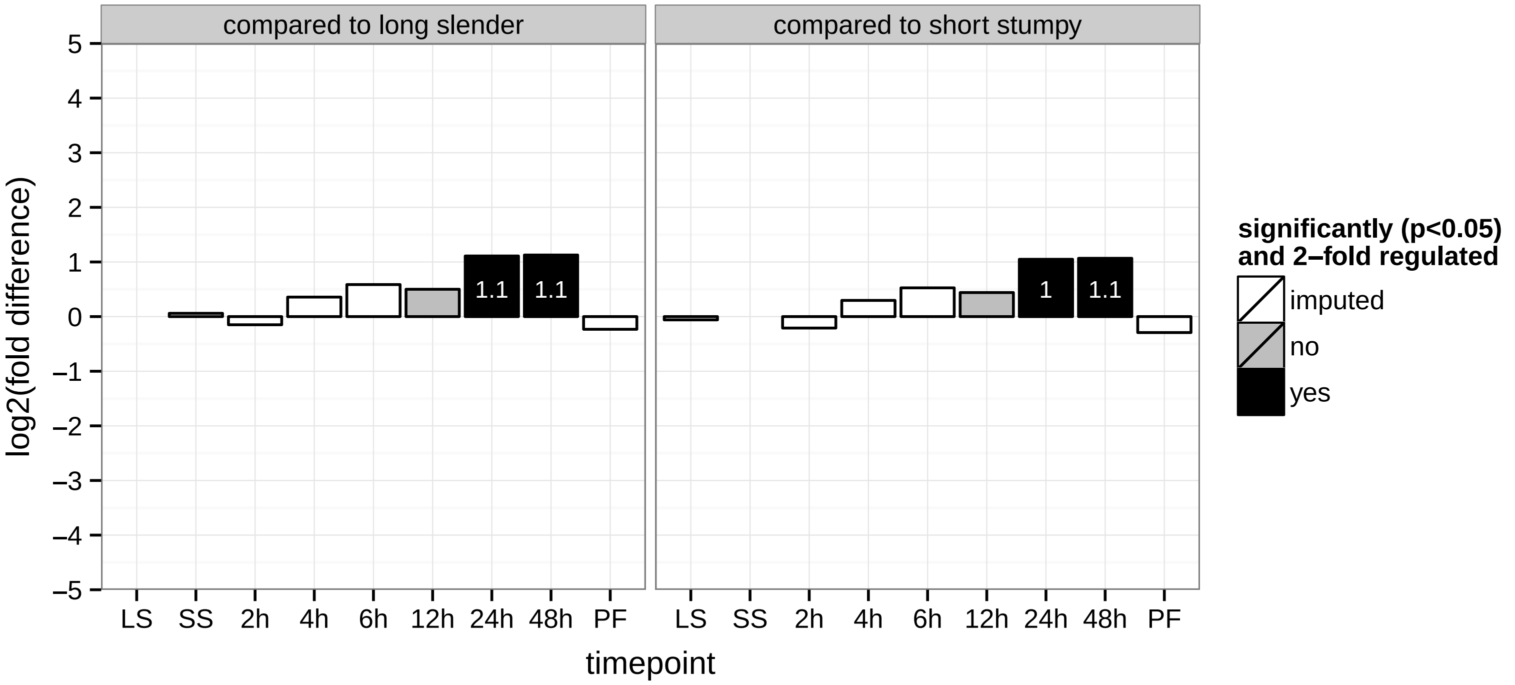

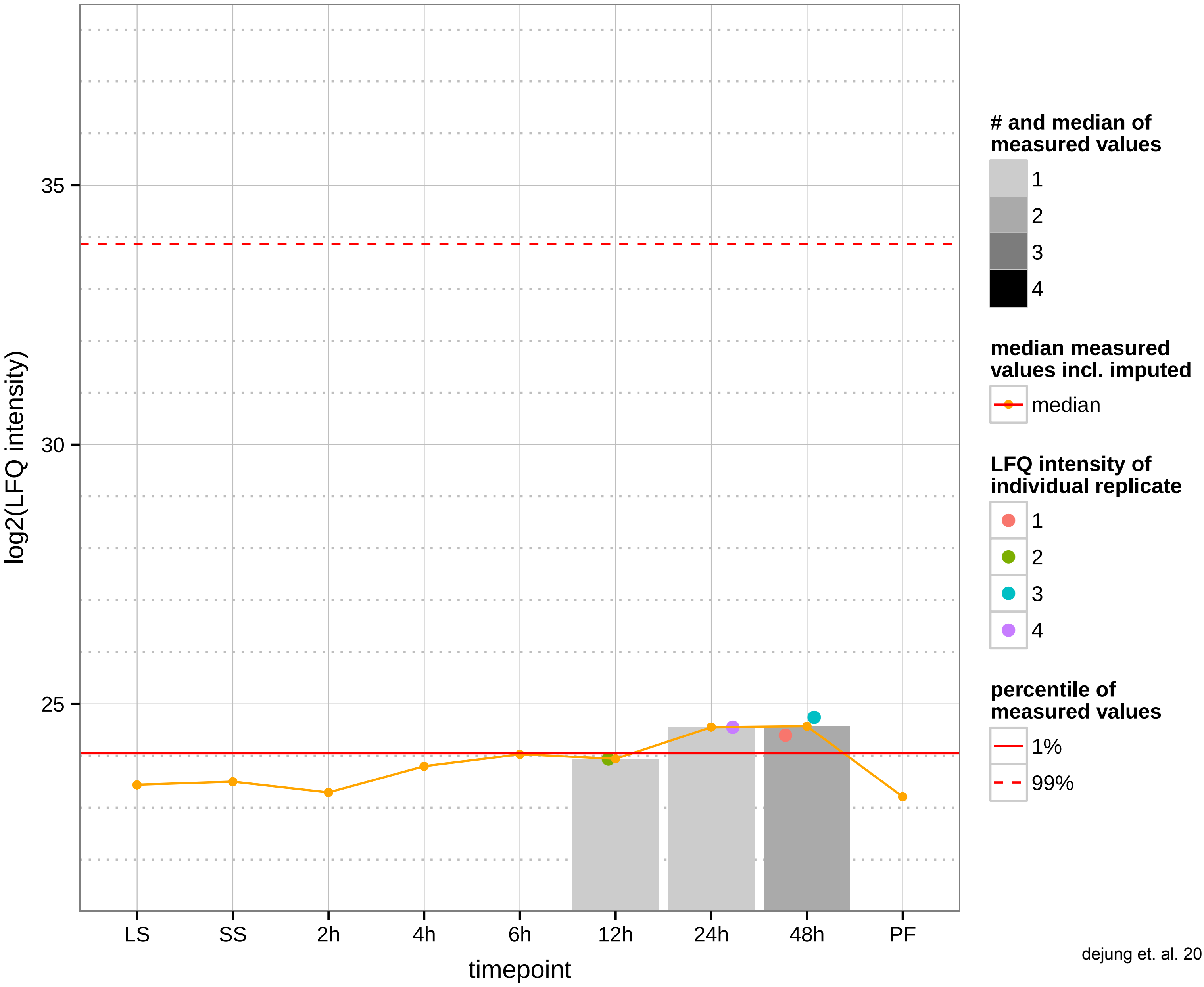

dejung et. al. 2015

30S ribosomal protein S8, putative
Tb927.10.13300
AGOF: structural constituent of ribosome
AGOC: intracellular, mitochondrion, organellar small ribosomal subunit, ribosome
AGOP: translation
PGOF: structural constituent of ribosome
PGOC: intracellular, ribosome
PGOP: translation

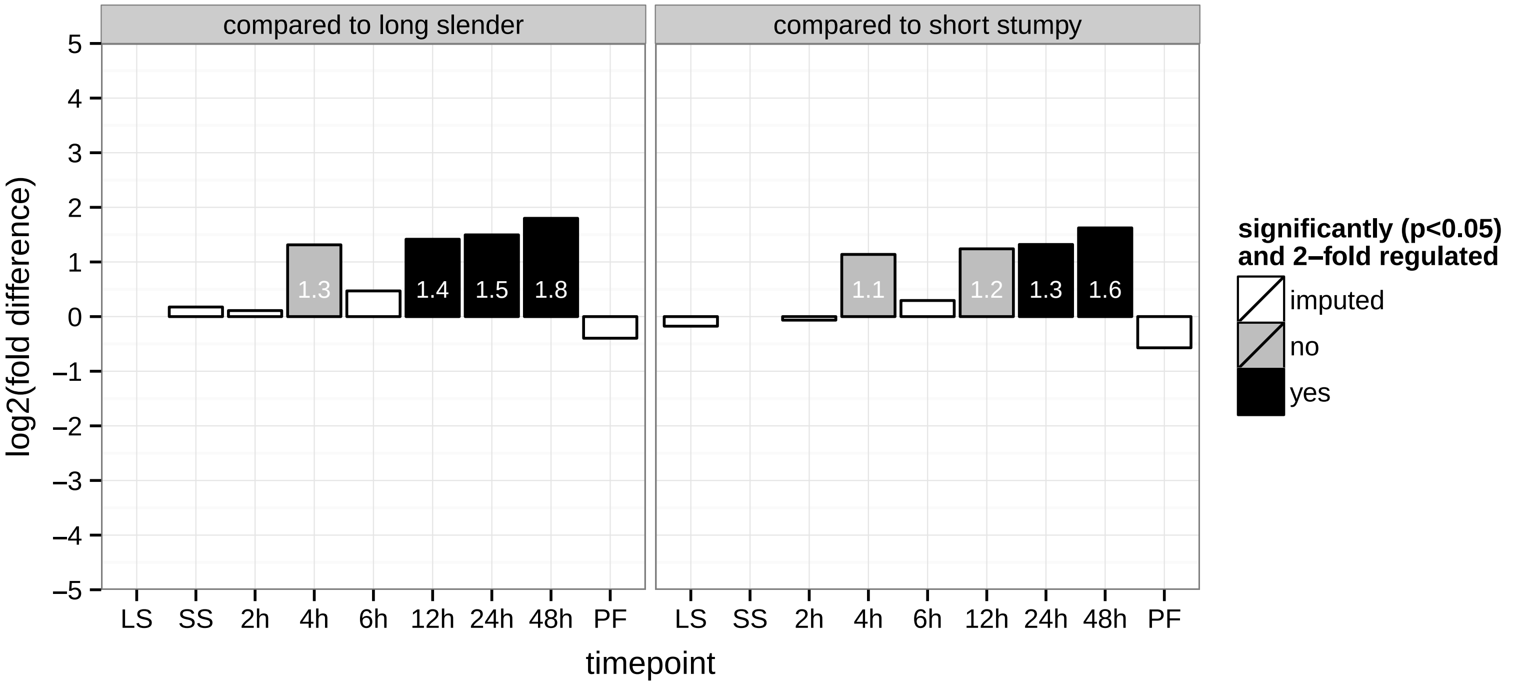

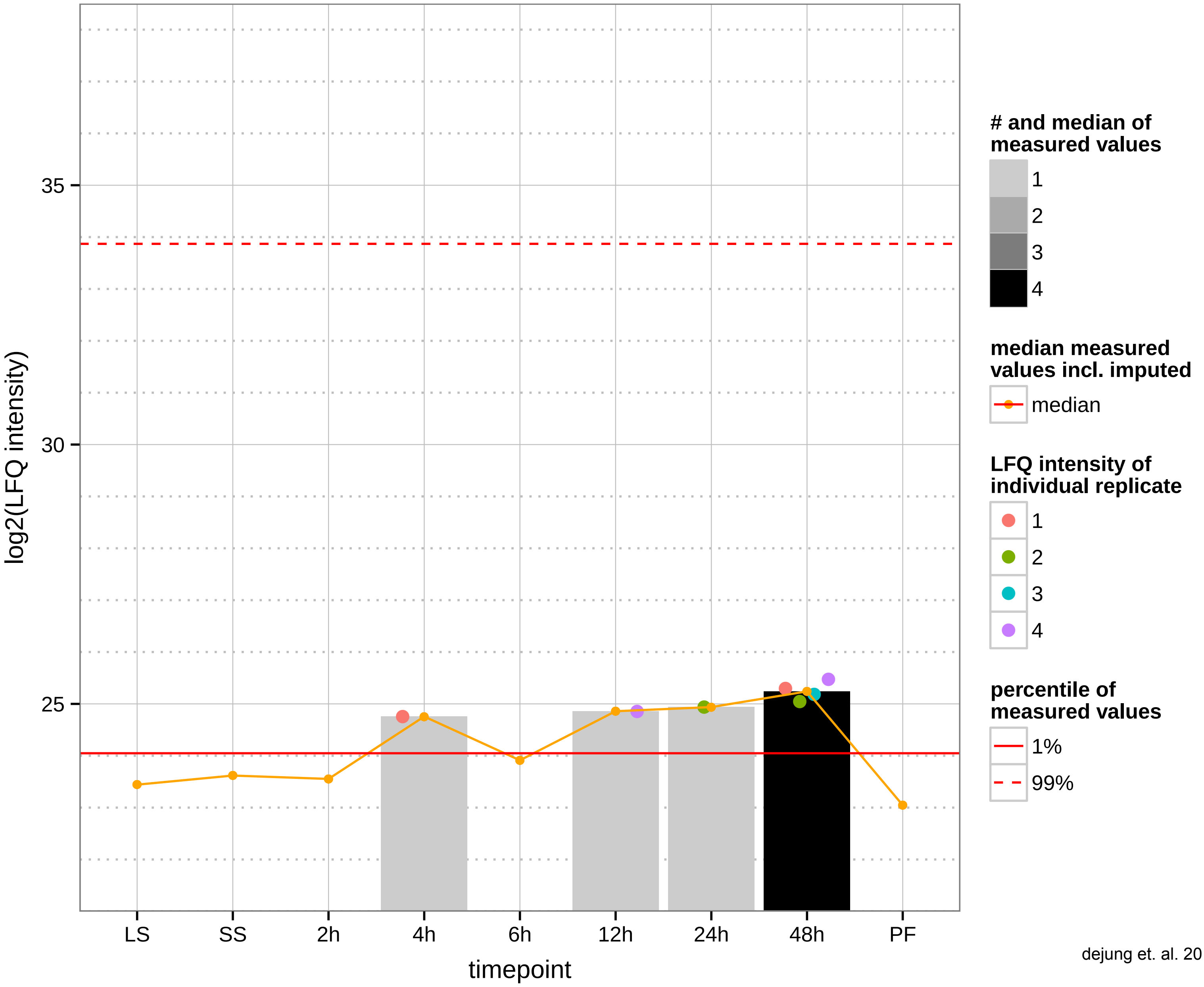

dejung et. al. 2015

hypothetical protein, conserved
Tb927.10.14700
AGOF: null
AGOC: null
AGOP: null
PGOF: null
PGOC: null
PGOP: null

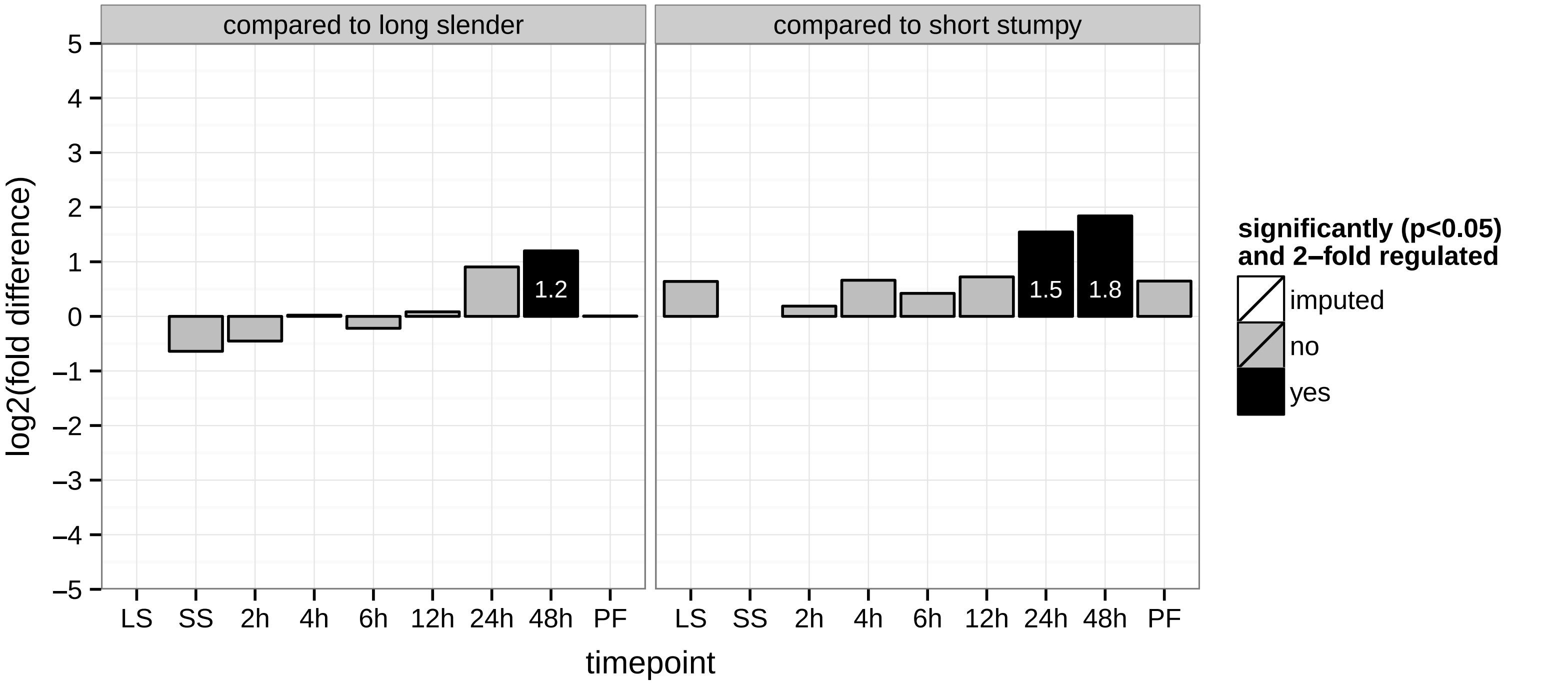

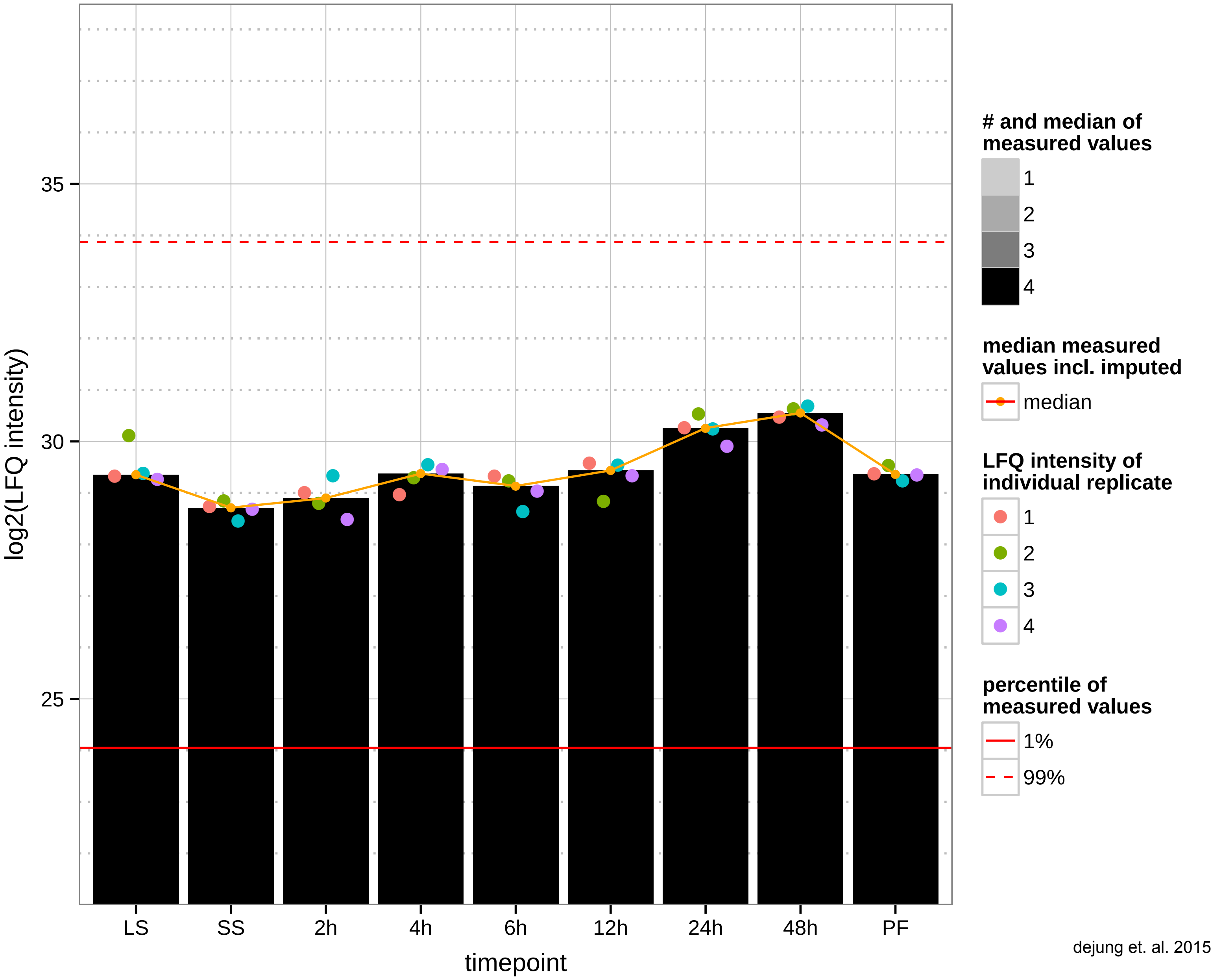

dejung et. al. 2015

Mitochondrial ADP/ATP carrier protein 5c (MCP5c), Mitochondrial ADP/ATP carrier protein 5b, putative (MCP5b), Mitochondri
Tb927.10.14840;Tb927.10.14830;Tb927.10.14820
AGOF: transporter activity
AGOC: mitochondrial inner membrane, mitochondrion
AGOP: transport
PGOF: null
PGOC: null
PGOP: null

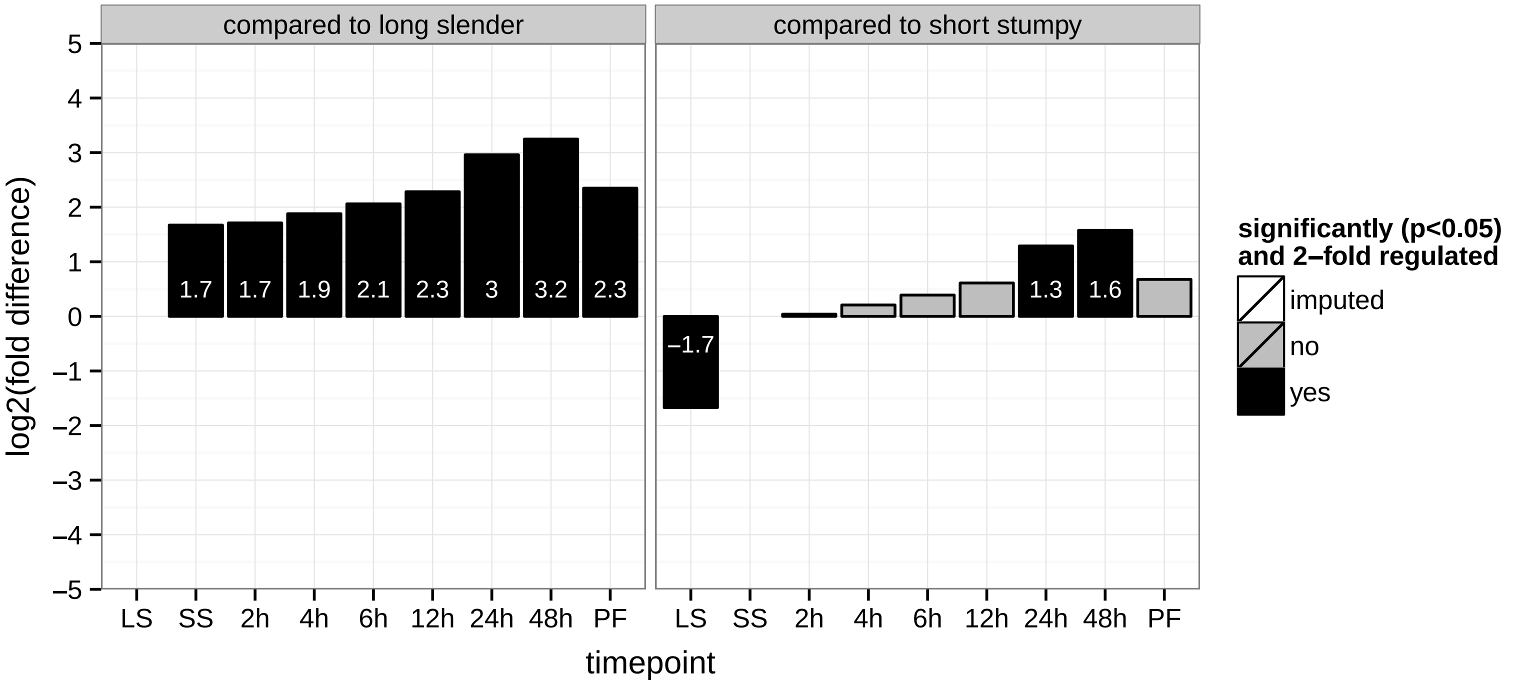

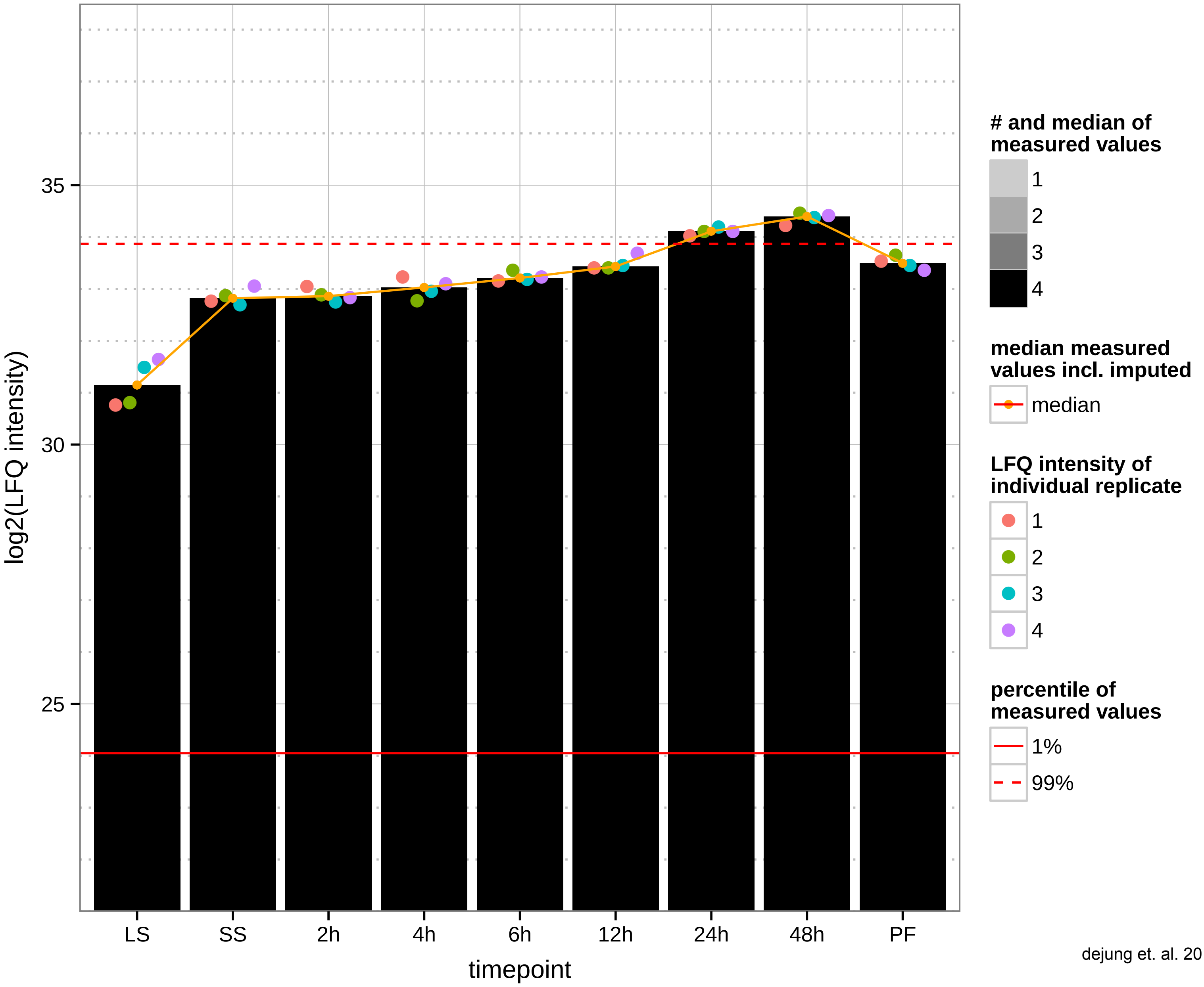

dejung et. al. 2015

hypothetical protein, conserved
Tb927.10.2620
AGOF: null
AGOC: null
AGOP: null
PGOF: null
PGOC: null
PGOP: null

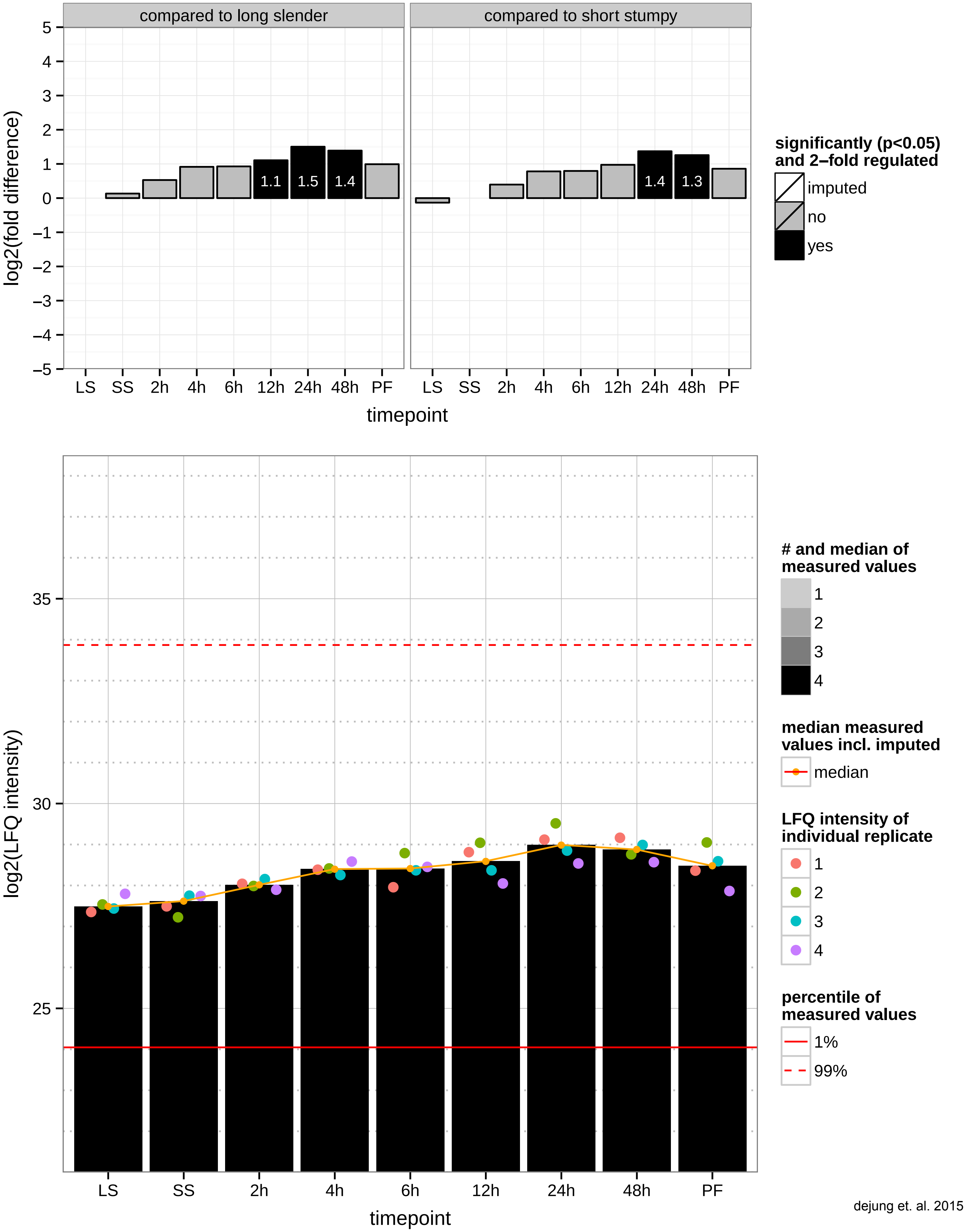

dejung et. al. 2015

hypothetical protein, conserved
Tb927.10.9330
AGOF: null
AGOC: null
AGOP: null
PGOF: null
PGOC: null
PGOP: null

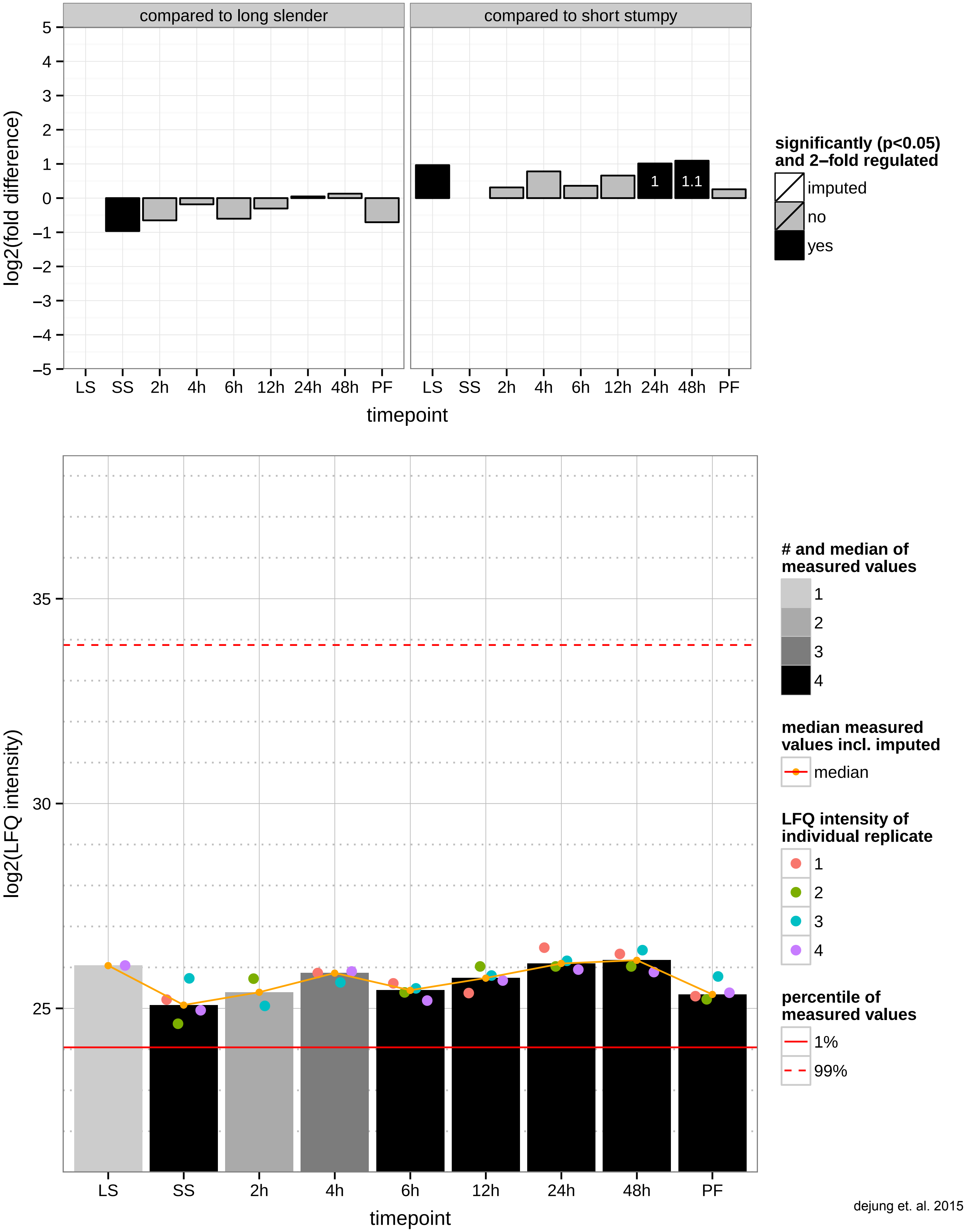

dejung et. al. 2015

hypothetical protein, conserved
Tb927.11.10260
AGOF: null
AGOC: null
AGOP: null
PGOF: null
PGOC: null
PGOP: null

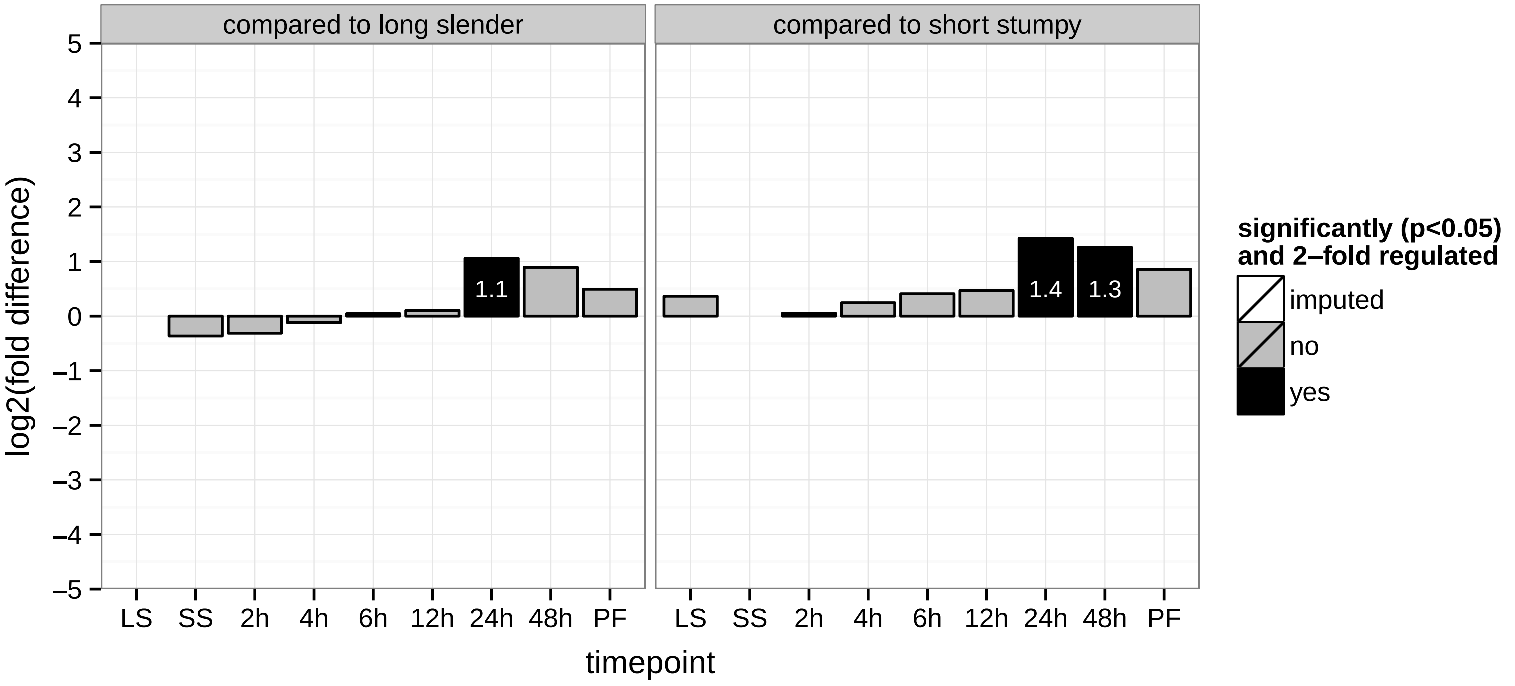

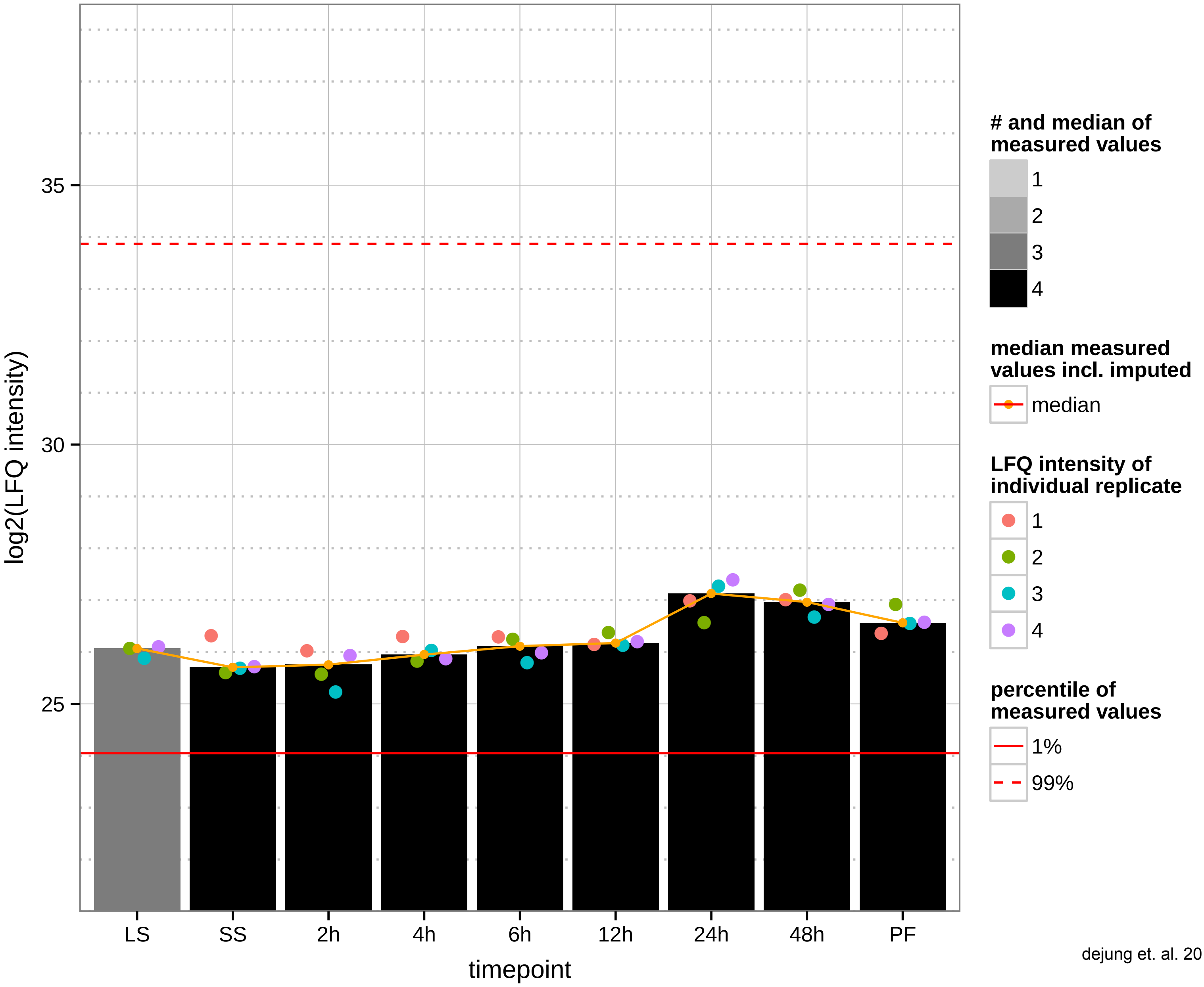

dejung et. al. 2015

hypothetical protein, conserved
Tb927.11.10960
AGOF: null
AGOC: mitochondrion
AGOP: null
PGOF: oxidoreductase activity, oxidoreductase activity, acting on paired donors, with incorporation or reduction of molecular o
PGOC: null
PGOP: oxidation−reduction process

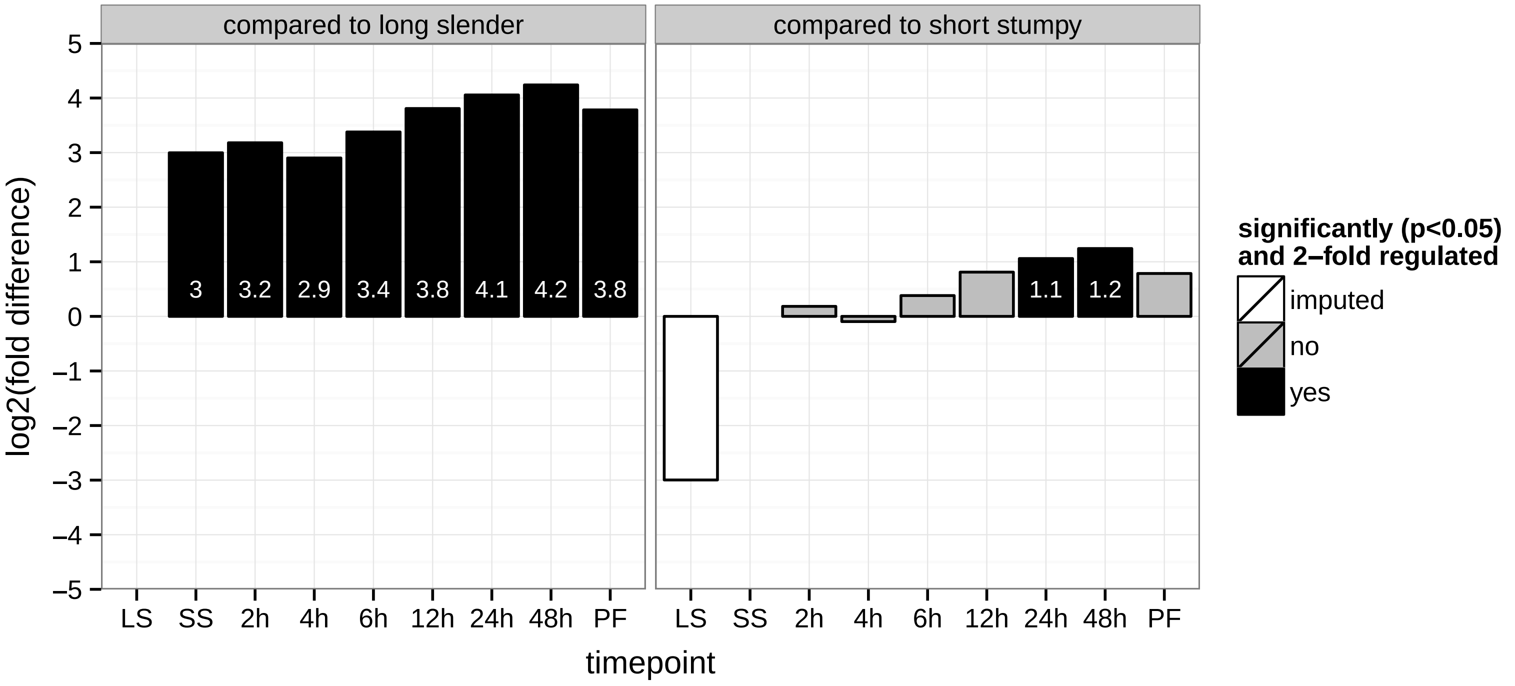

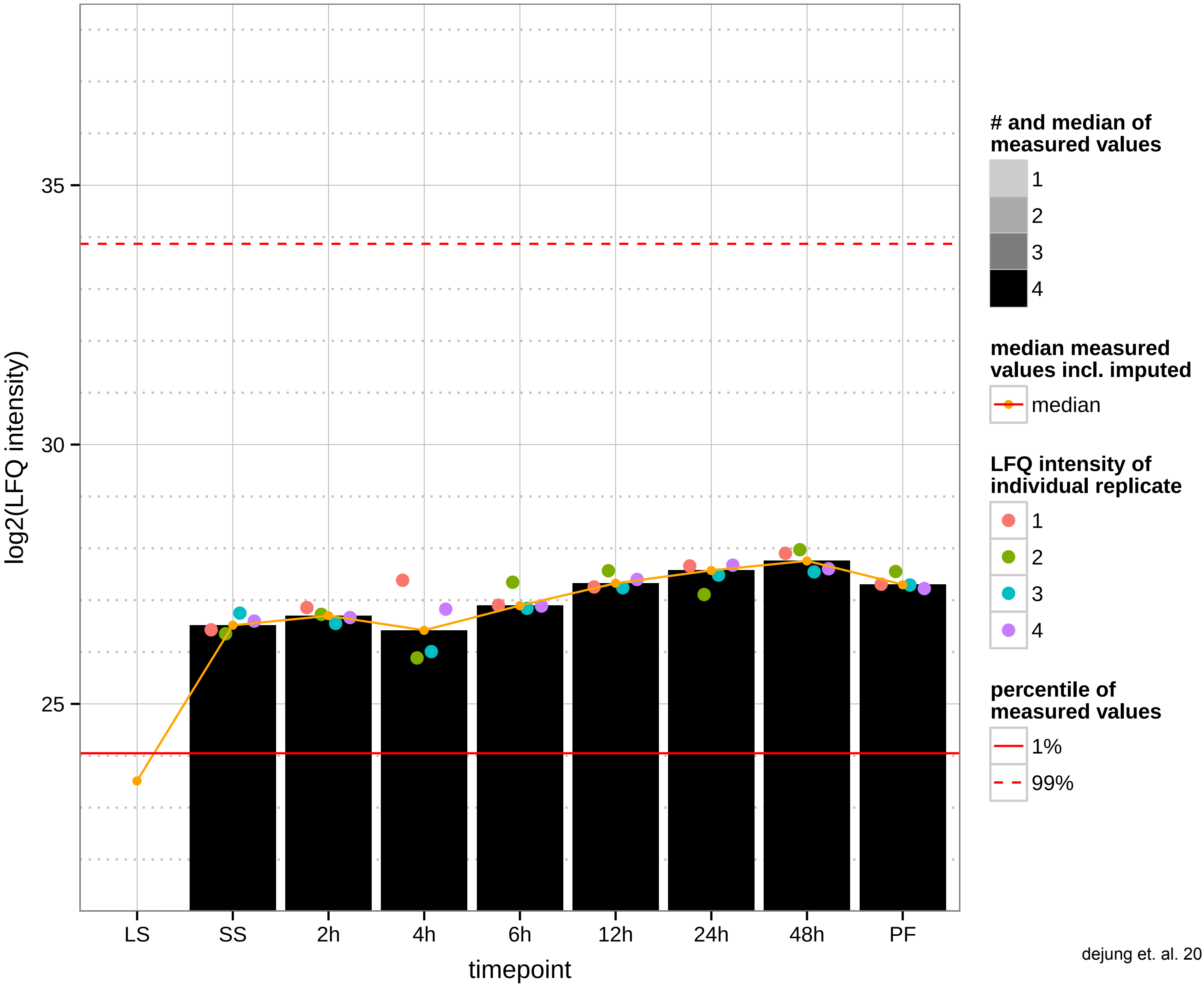

dejung et. al. 2015

hypothetical protein, conserved
Tb927.11.11760
AGOF: null
AGOC: null
AGOP: null
PGOF: null
PGOC: null
PGOP: null

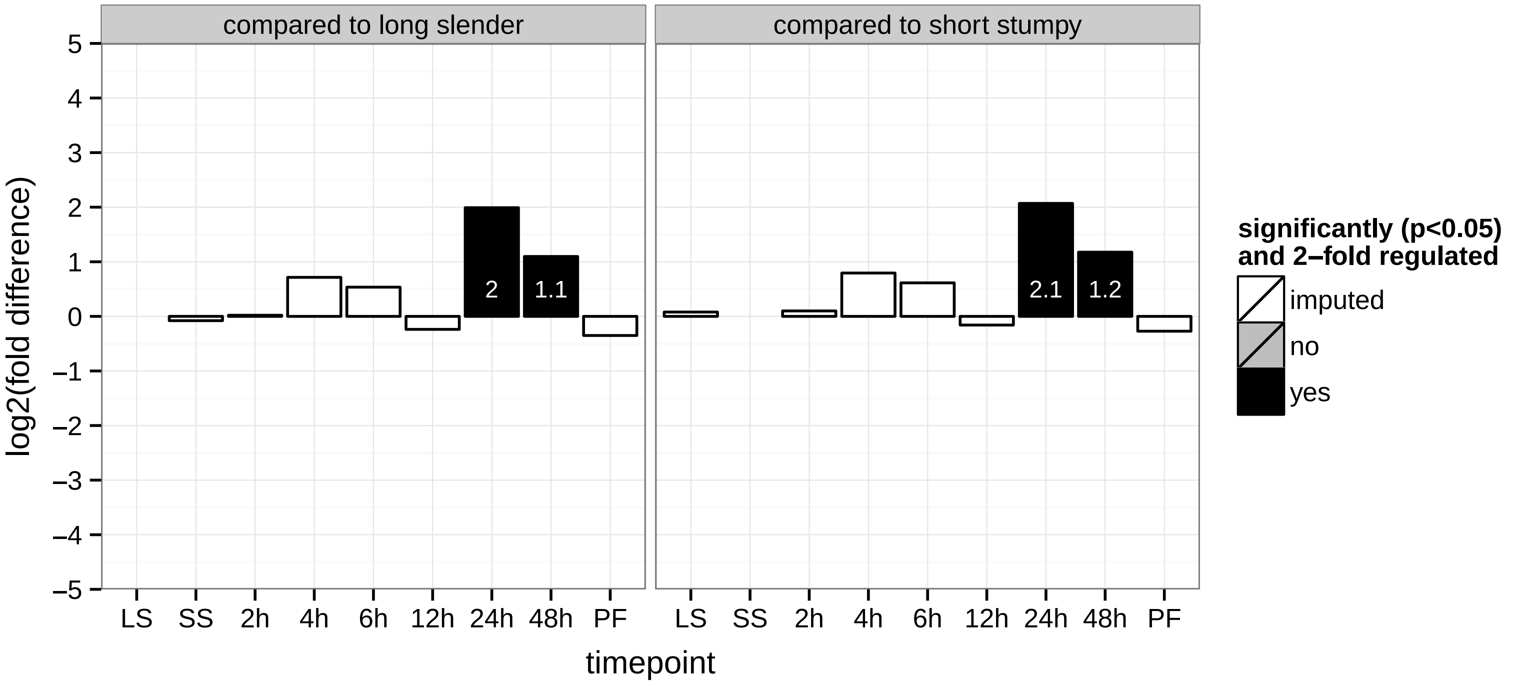

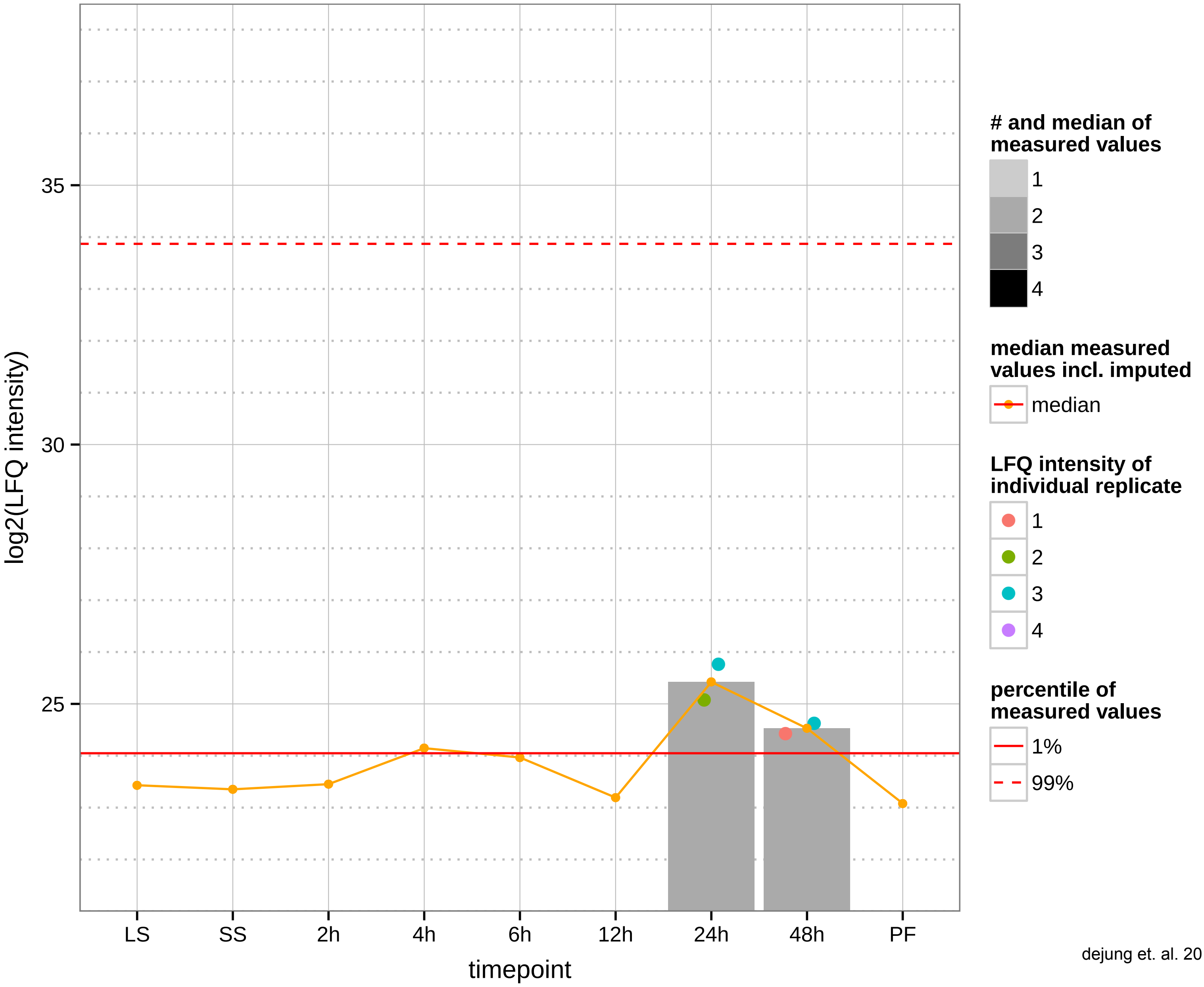

dejung et. al. 2015

cytochrome oxidase assembly protein, putative
Tb927.11.11950
AGOF: null
AGOC: mitochondrial membrane, mitochondrion
AGOP: protein complex assembly
PGOF: oxidoreductase activity, acting on the CH–CH group of donors
PGOC: membrane
PGOP: heme a biosynthetic process, oxidation–reduction process

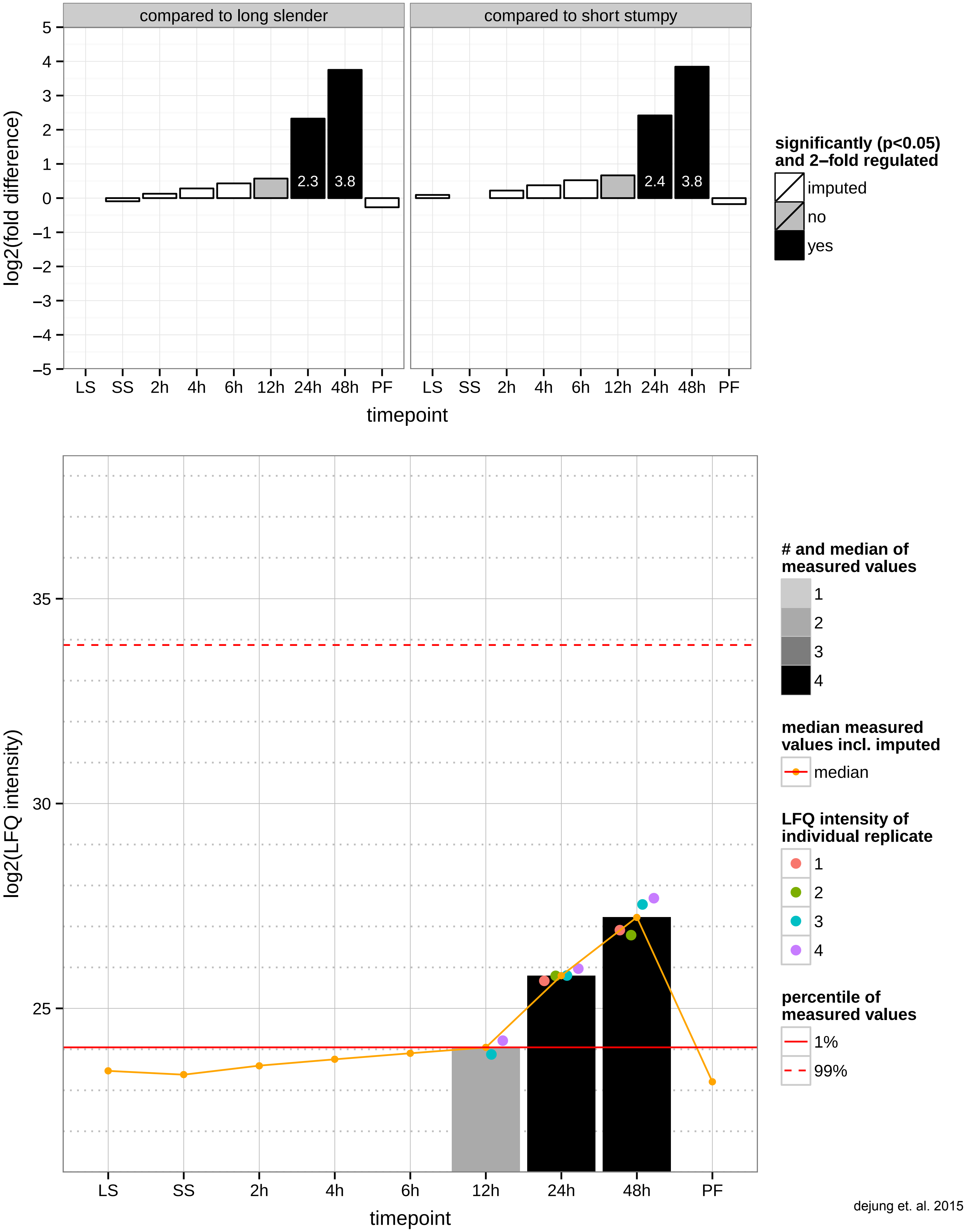

dejung et. al. 2015

hypothetical protein, conserved
Tb927.11.1290
AGOF: null
AGOC: mitochondrion
AGOP: null
PGOF: null
PGOC: null
PGOP: null

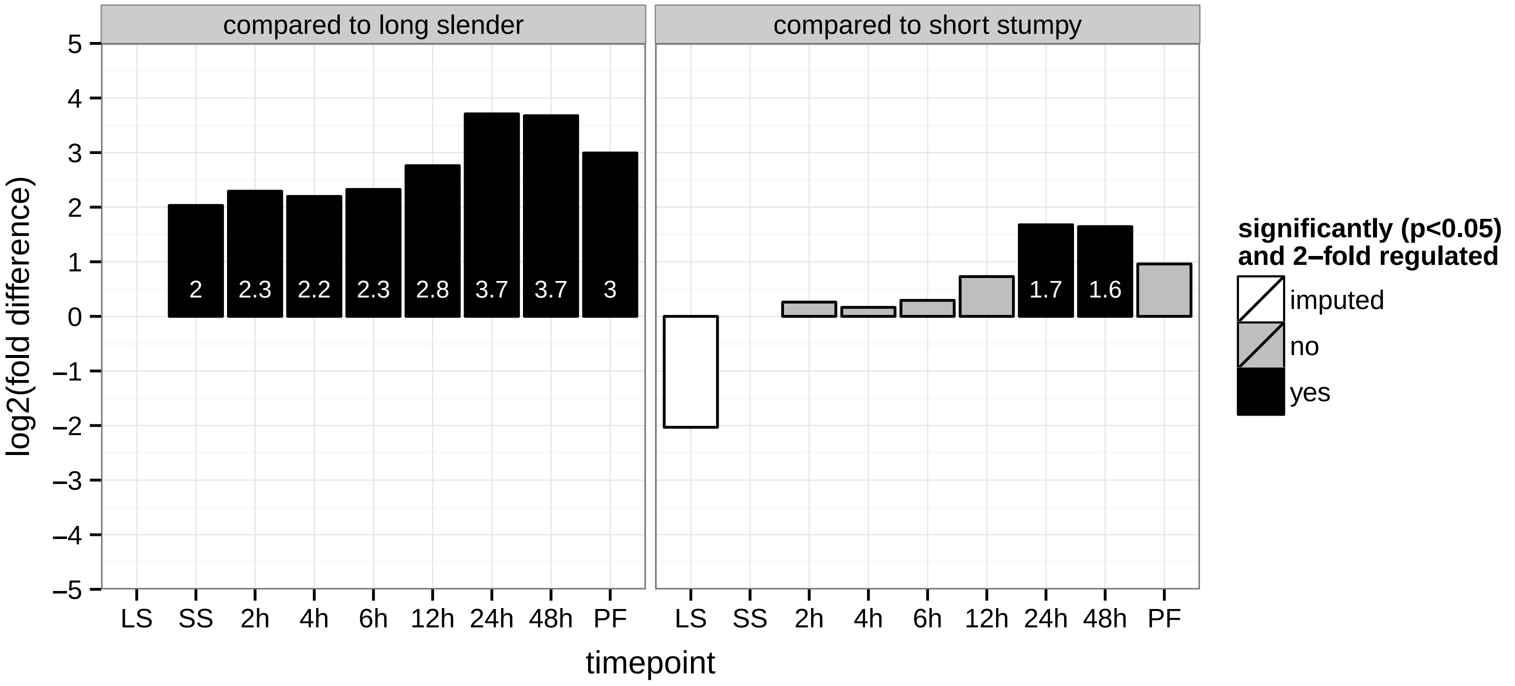

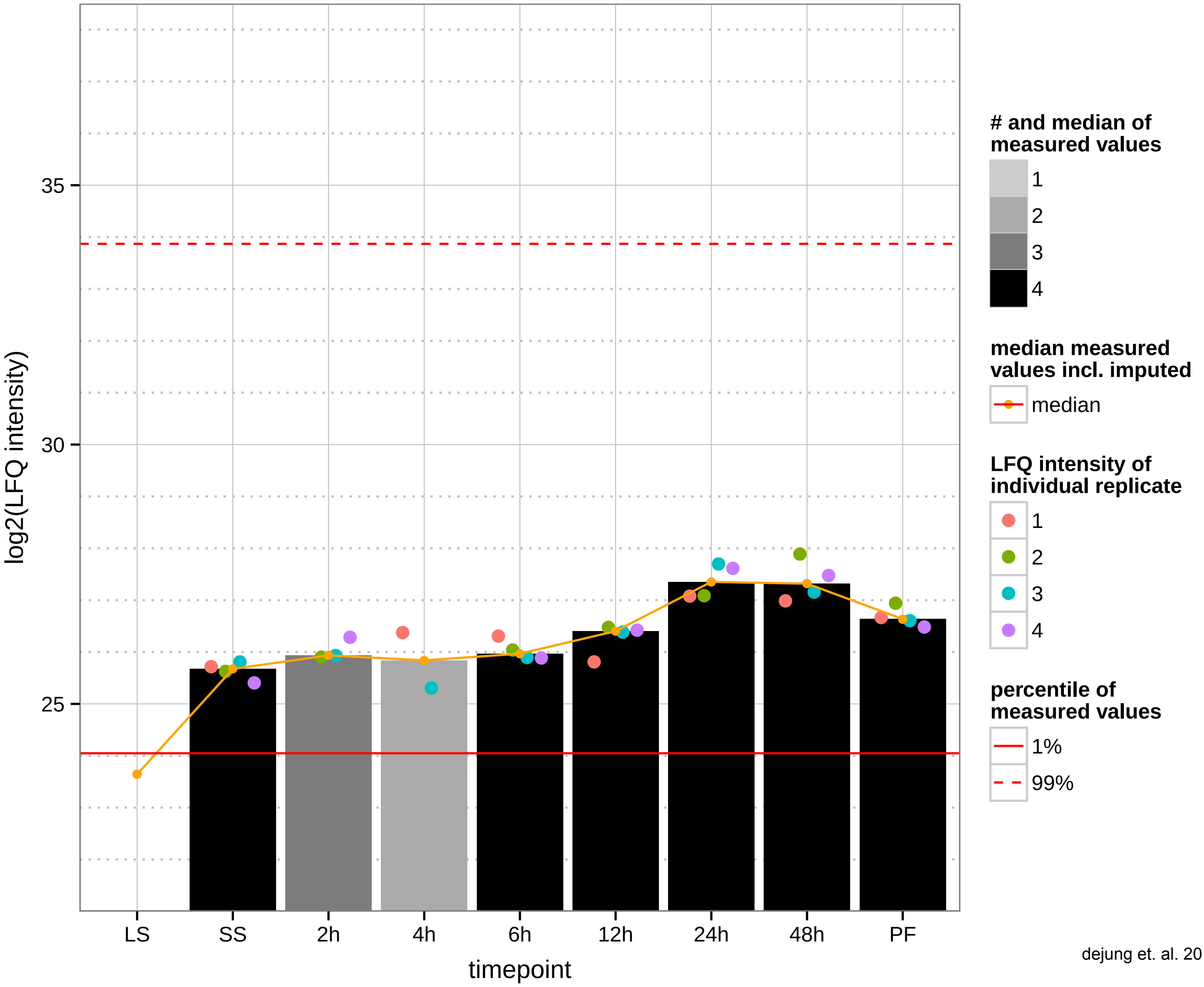

dejung et. al. 2015

hypothetical protein, conserved
Tb927.11.1550
AGOF: null
AGOC: null
AGOP: null
PGOF: exonuclease activity, nucleic acid binding
PGOC: intracellular
PGOP: null

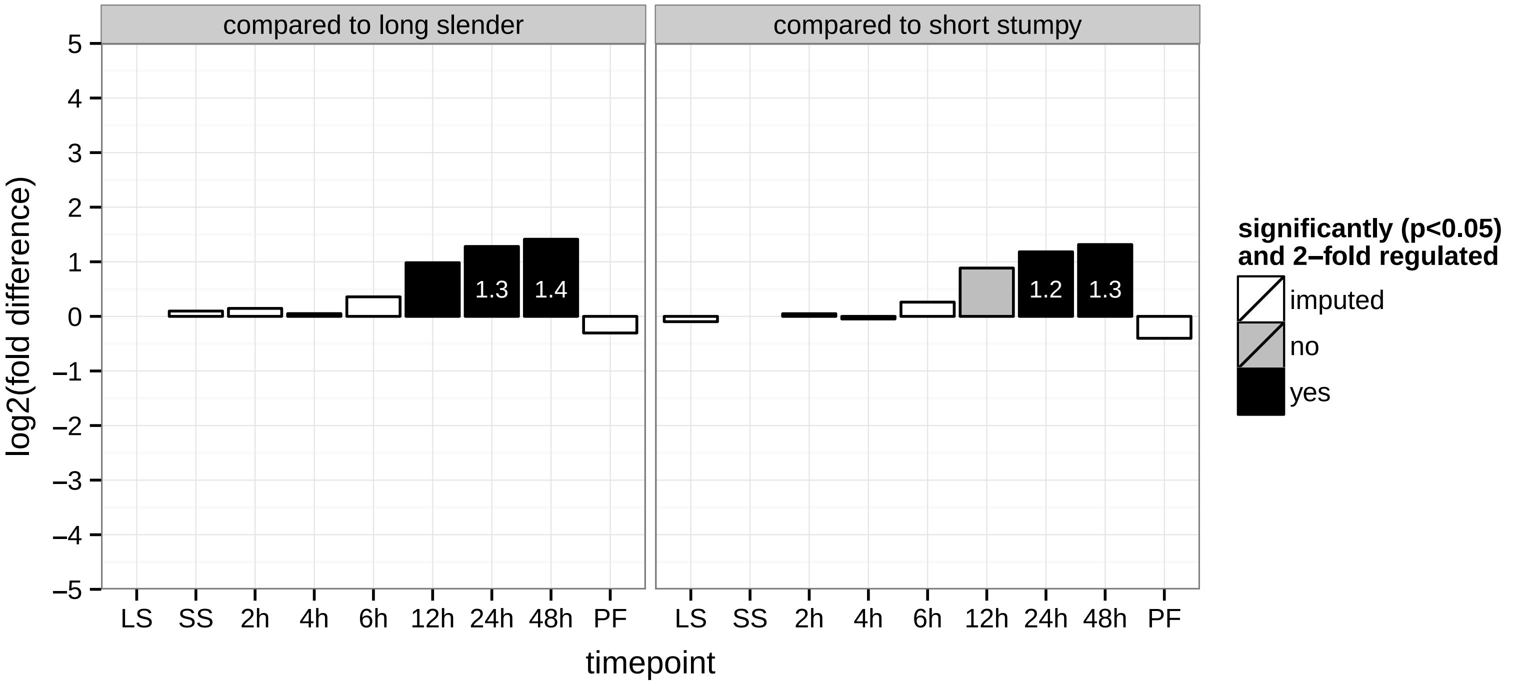

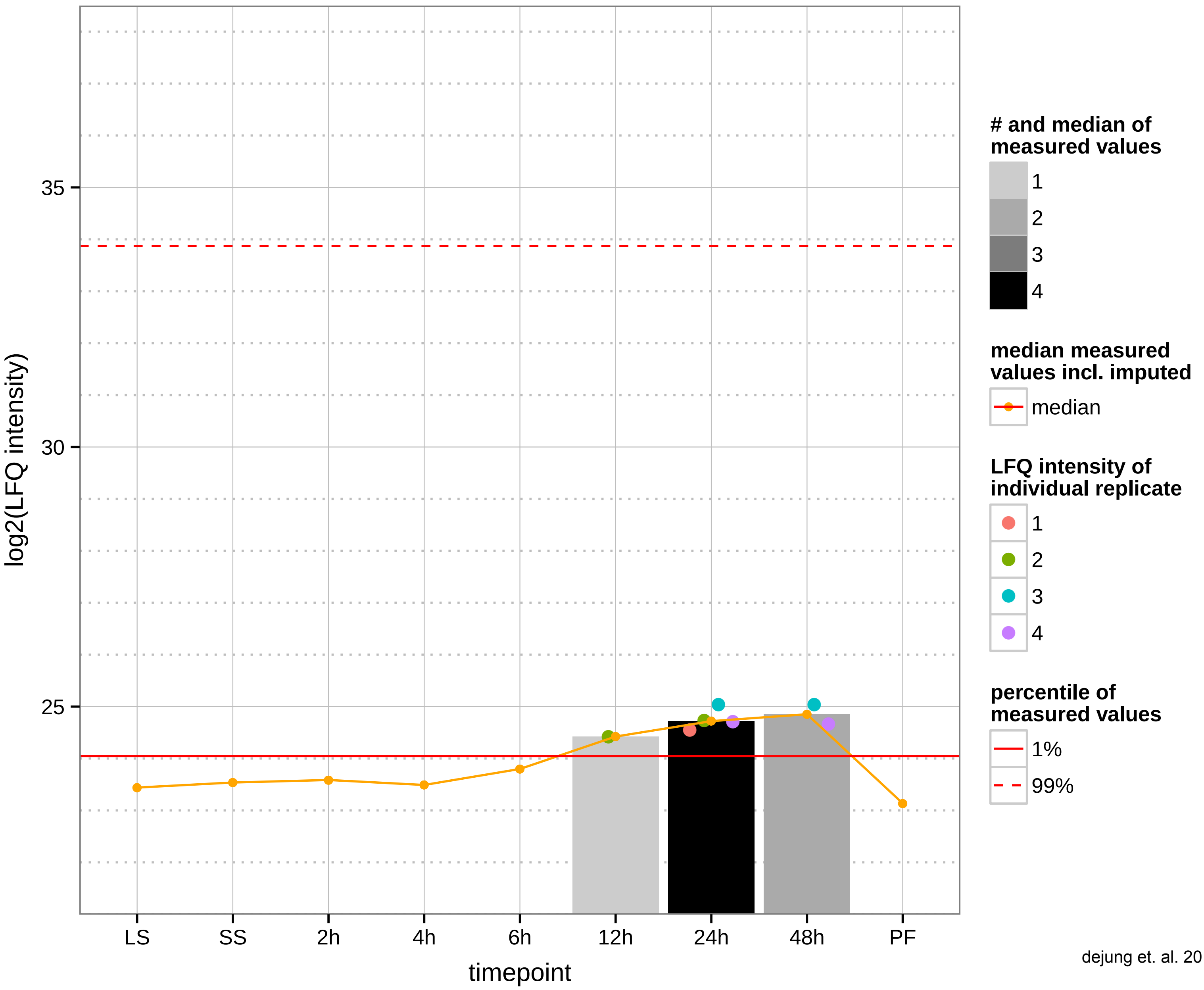

dejung et. al. 2015

cyclin dependent kinases regulatory subunit, putative (CKS1)
Tb927.11.16390
AGOF: cyclin–dependent protein kinase activity, cyclin–dependent protein kinase regulator activity
AGOC: protein kinase CK2 complex
AGOP: cell cycle
PGOF: cyclin–dependent protein kinase regulator activity
PGOC: null
PGOP: cell cycle

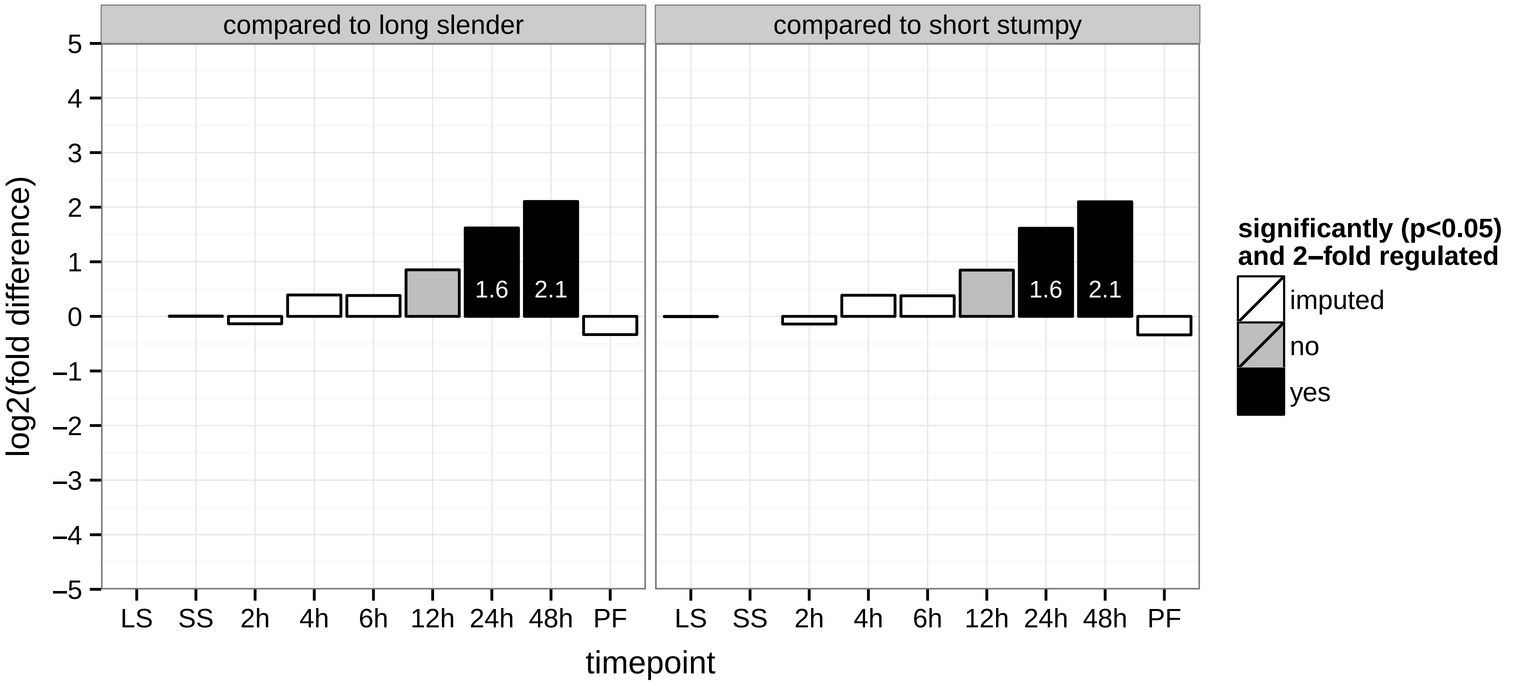

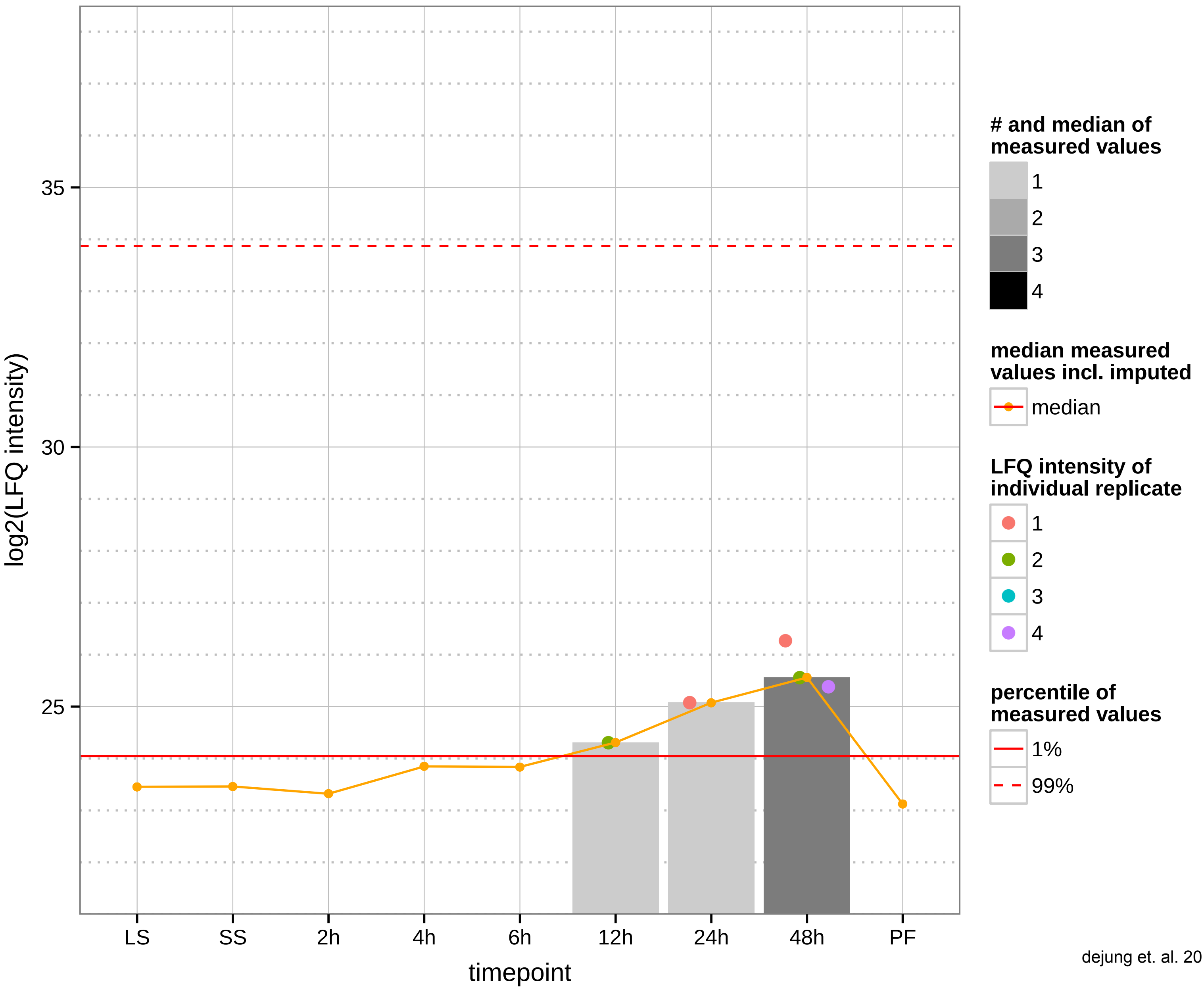

dejung et. al. 2015

hypothetical protein, conserved
Tb927.11.480
AGOF: GTP binding, RNA binding
AGOC: intracellular, mitochondrion
AGOP: null
PGOF: GTP binding
PGOC: null
PGOP: null

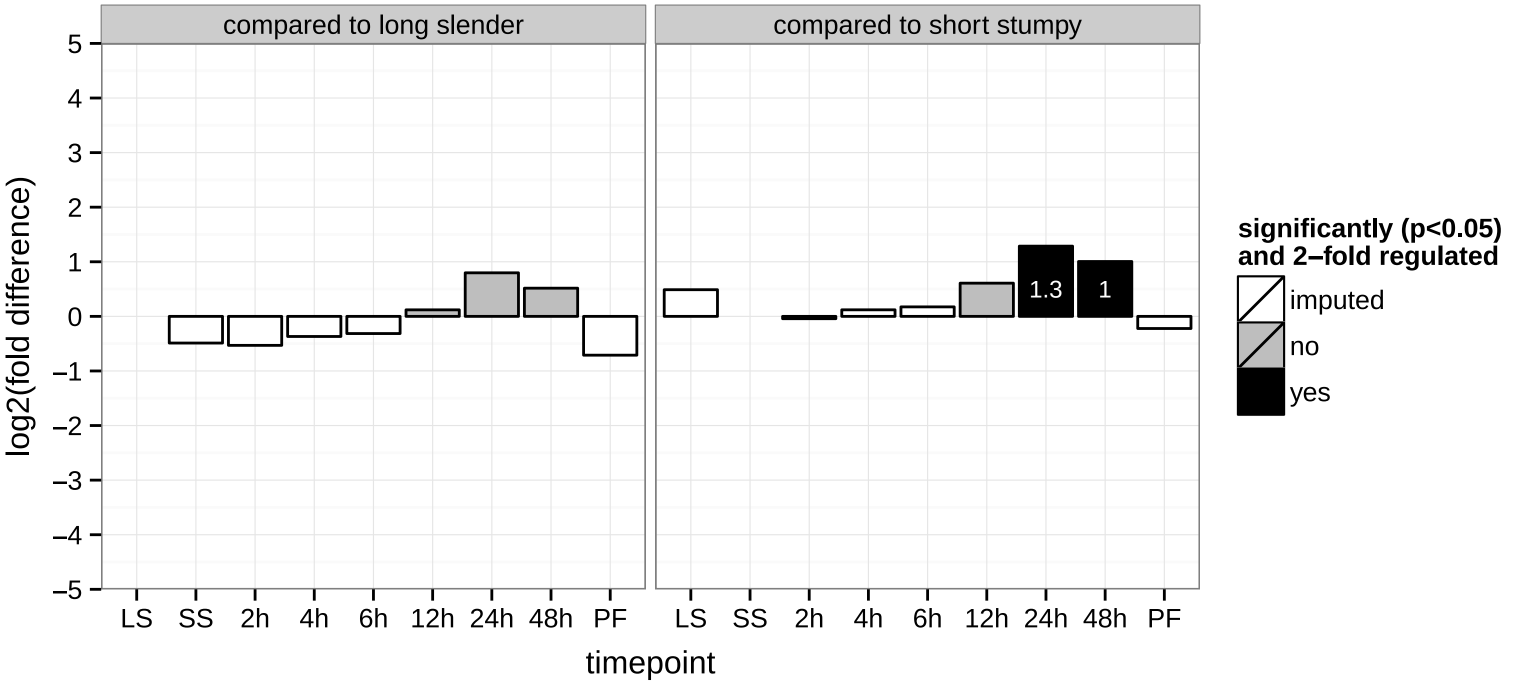

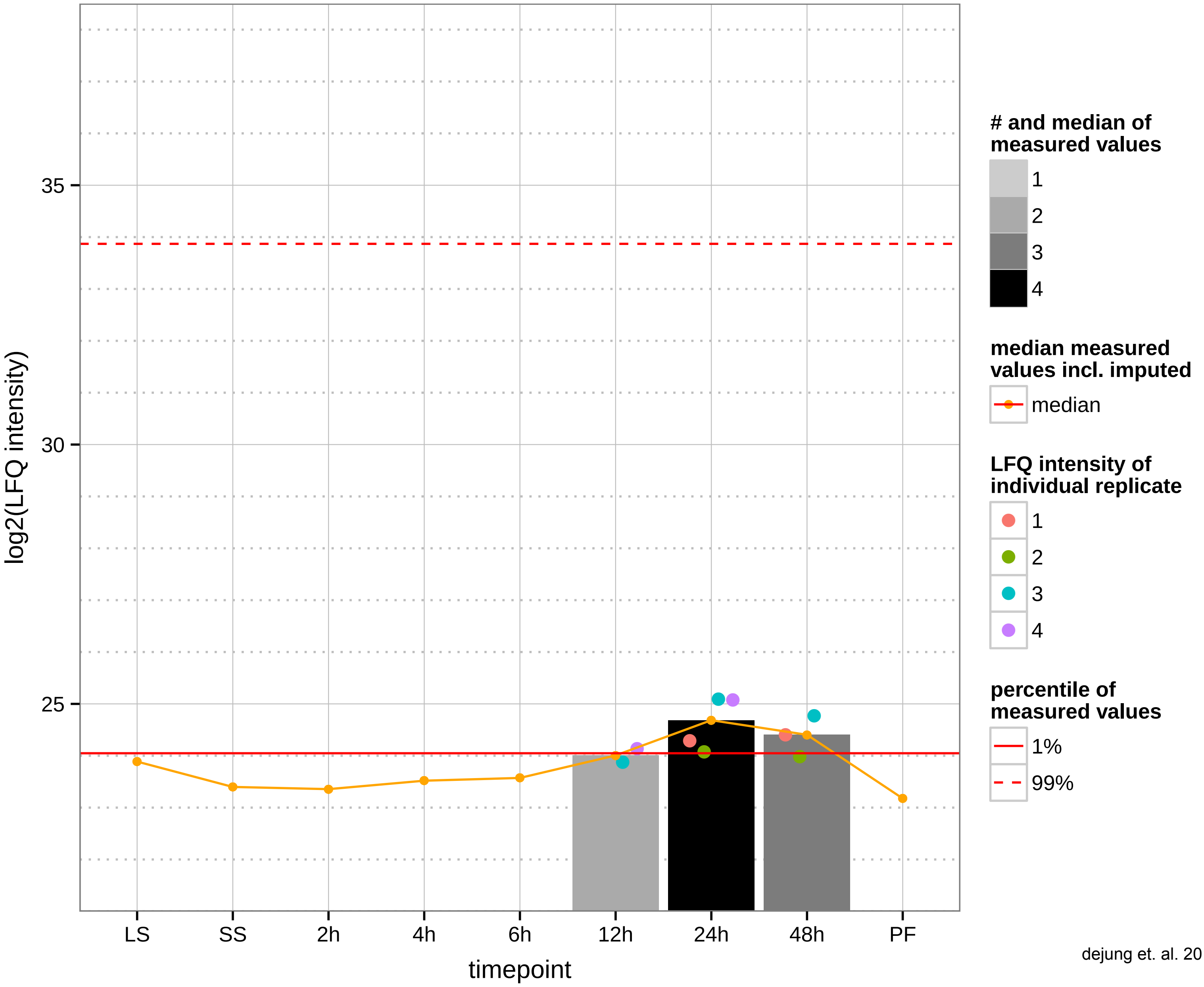

dejung et. al. 2015

hypothetical protein, conserved
Tb927.11.4840
AGOF: null
AGOC: null
AGOP: null
PGOF: null
PGOC: null
PGOP: null

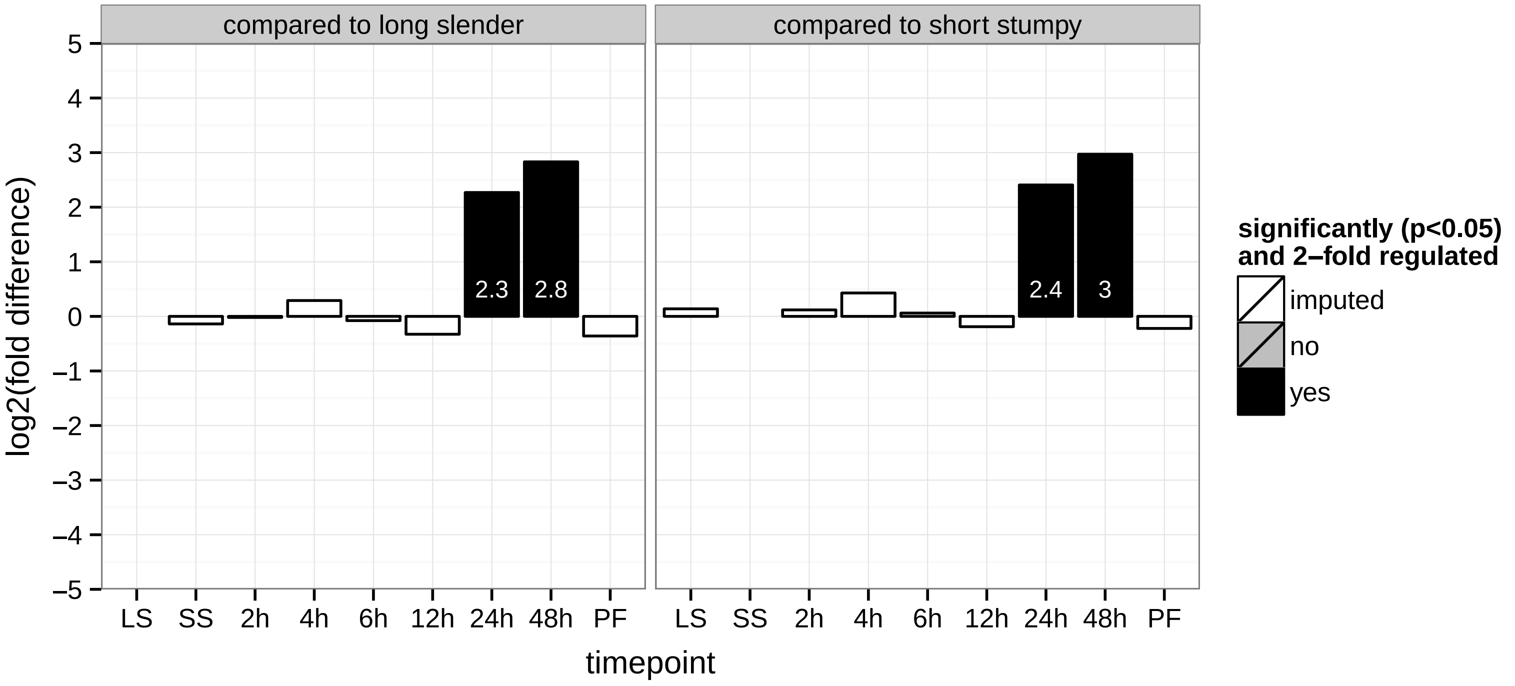

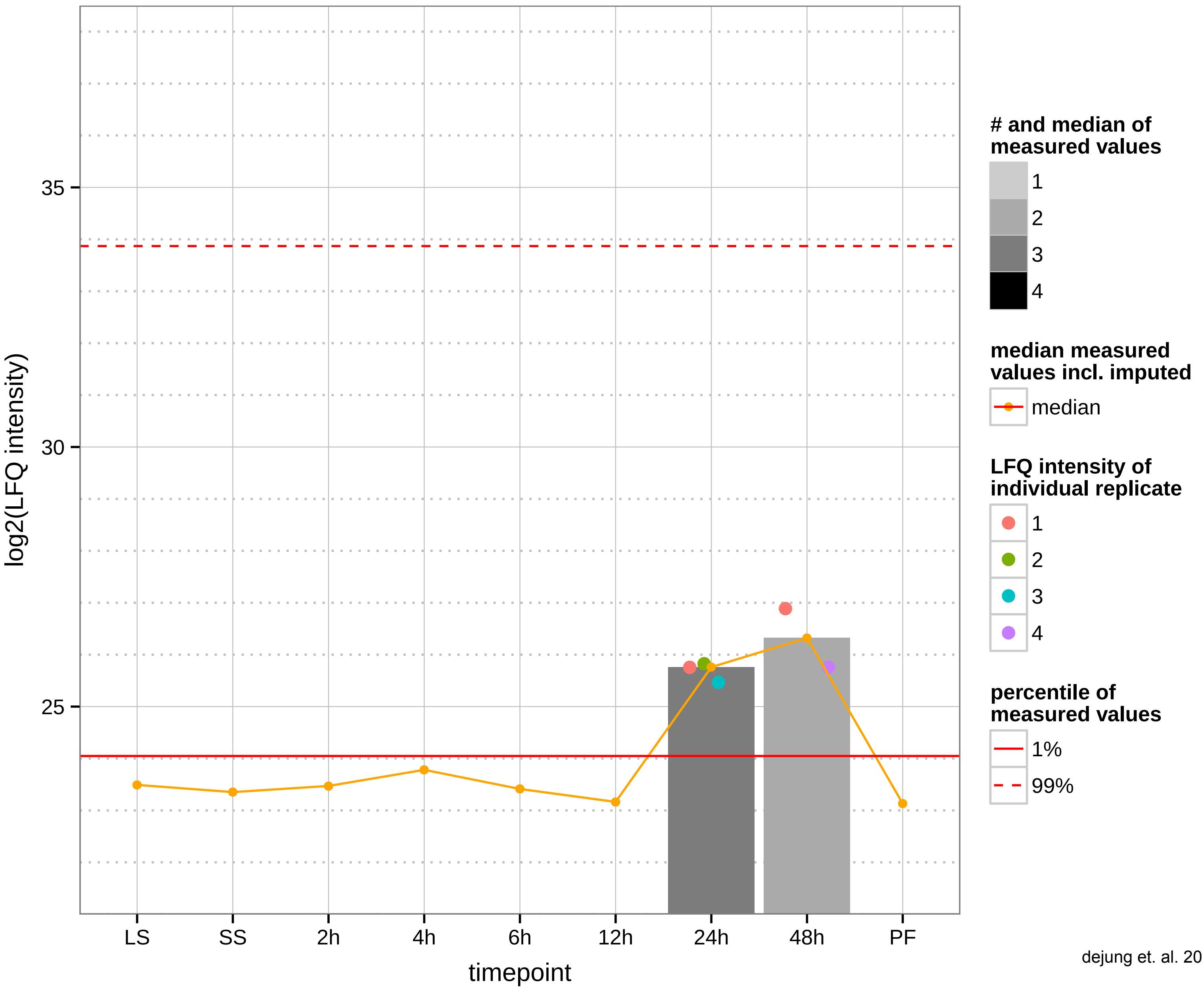

dejung et. al. 2015

hypothetical protein, conserved
Tb927.11.7340
AGOF: null
AGOC: null
AGOP: metabolic process
PGOF: null
PGOC: null
PGOP: null

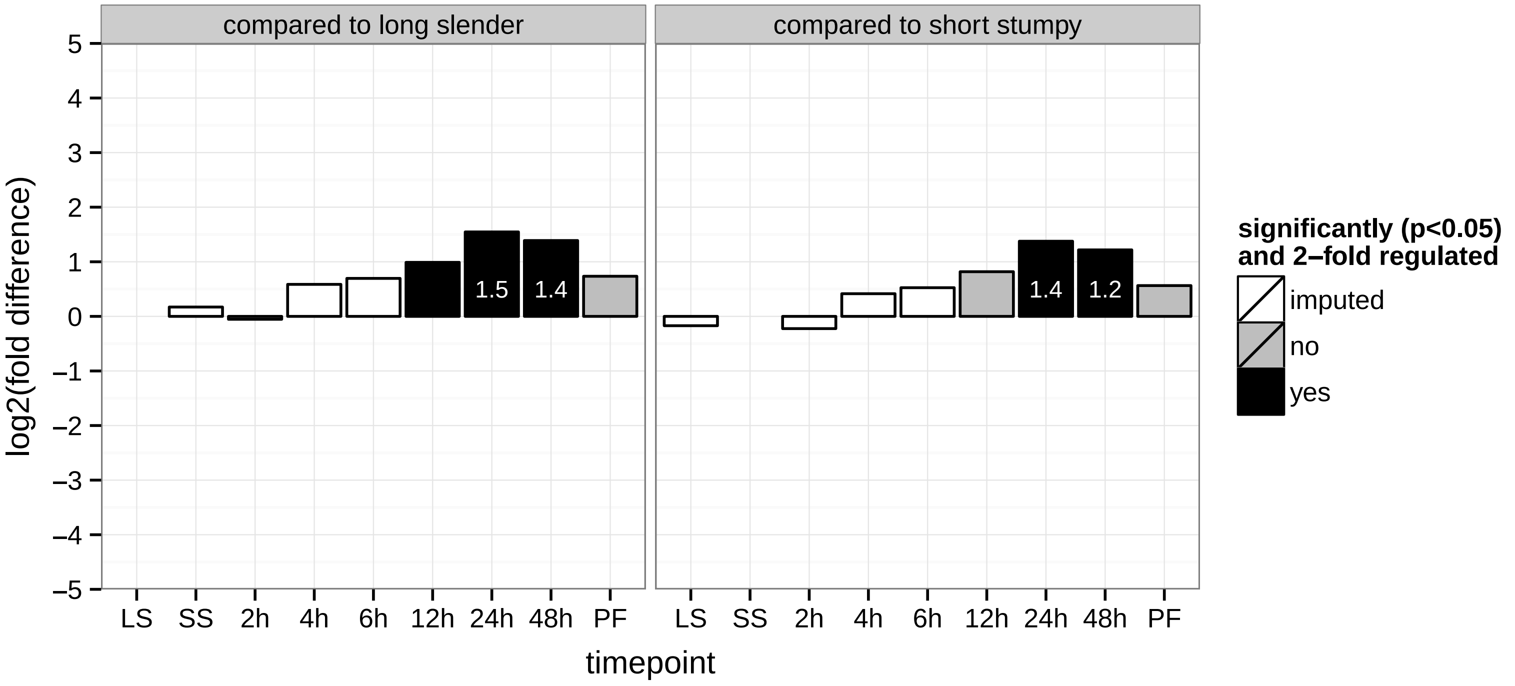

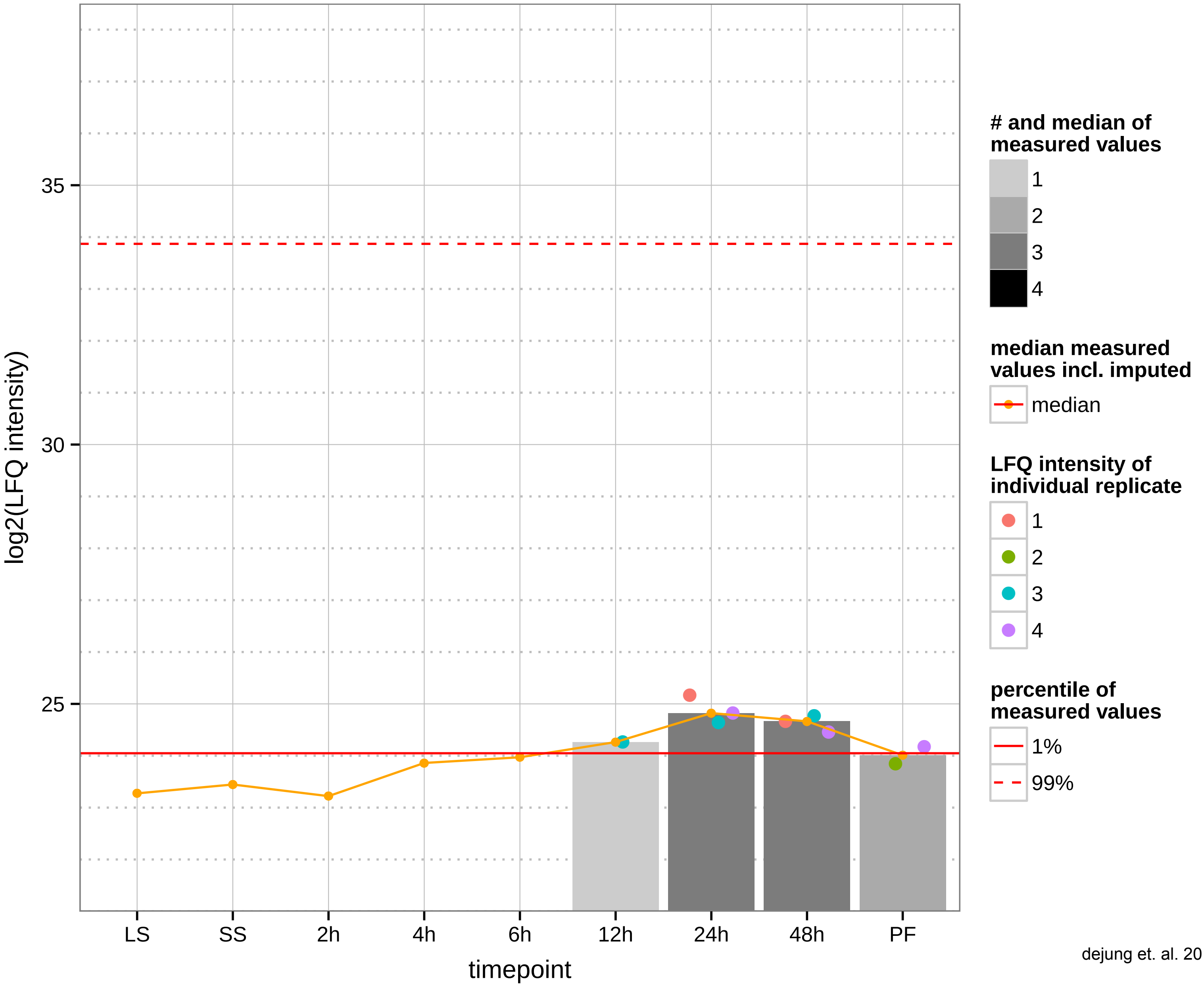

dejung et. al. 2015

mitochondrial RNA binding protein 16 (RBP16)
Tb927.11.7900
AGOF: DNA binding, RNA binding
AGOC: mitochondrion
AGOP: RNA modification, regulation of transcription, DNA–dependent
PGOF: DNA binding, nucleic acid binding
PGOC: null
PGOP: regulation of transcription, DNA–dependent

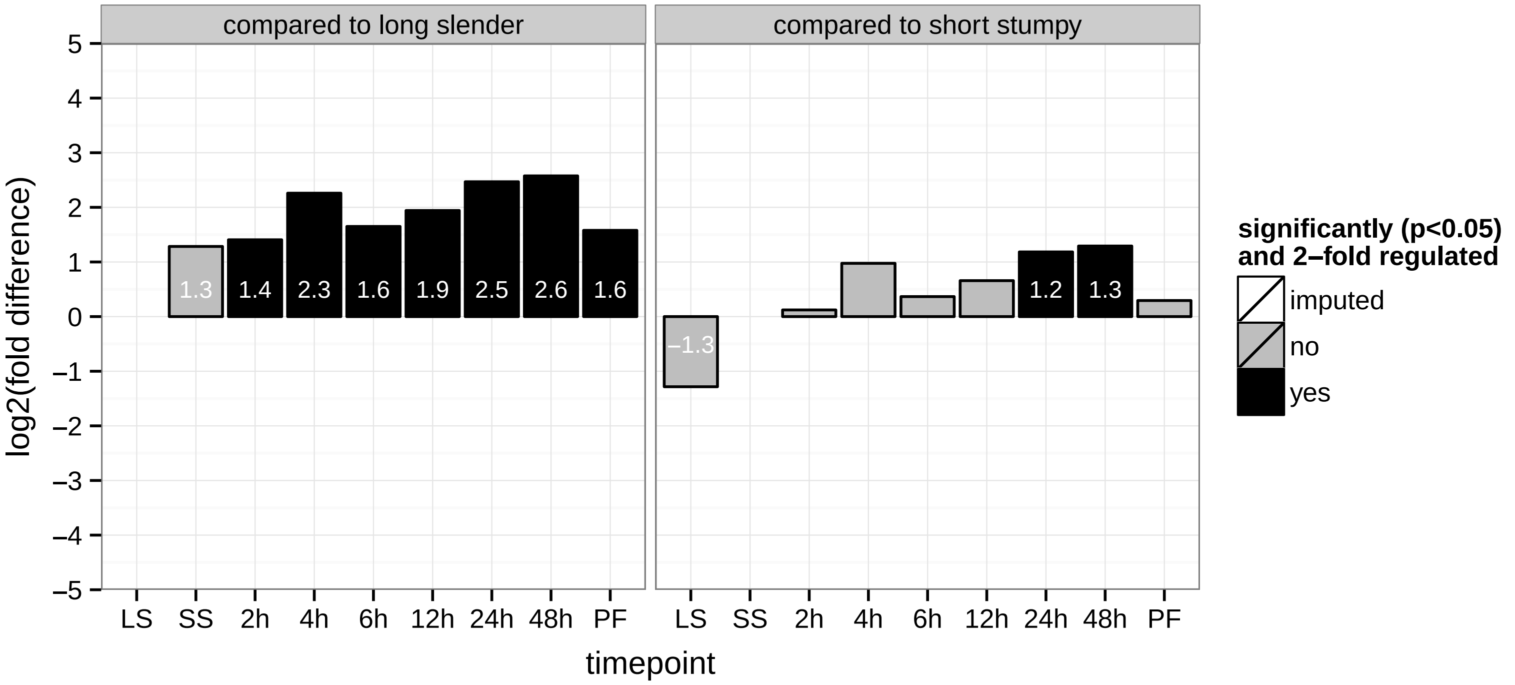

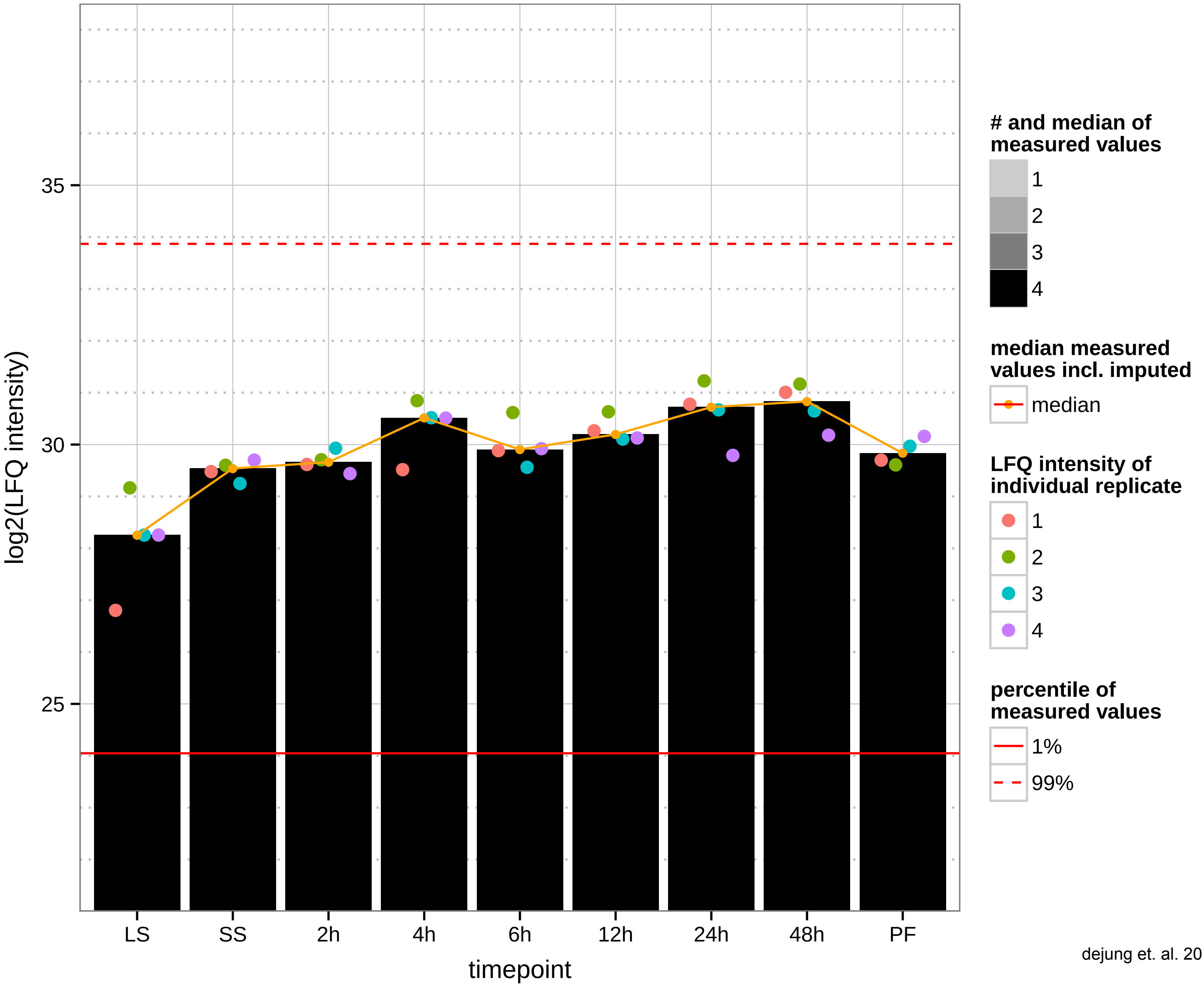

dejung et. al. 2015

palmitoyl acyltransferase 4, putative
Tb927.11.9070
AGOF: transferase activity, transferring acyl groups, zinc ion binding
AGOC: integral to membrane
AGOP: endocytosis, protein palmitoylation, transport
PGOF: zinc ion binding
PGOC: null
PGOP: null

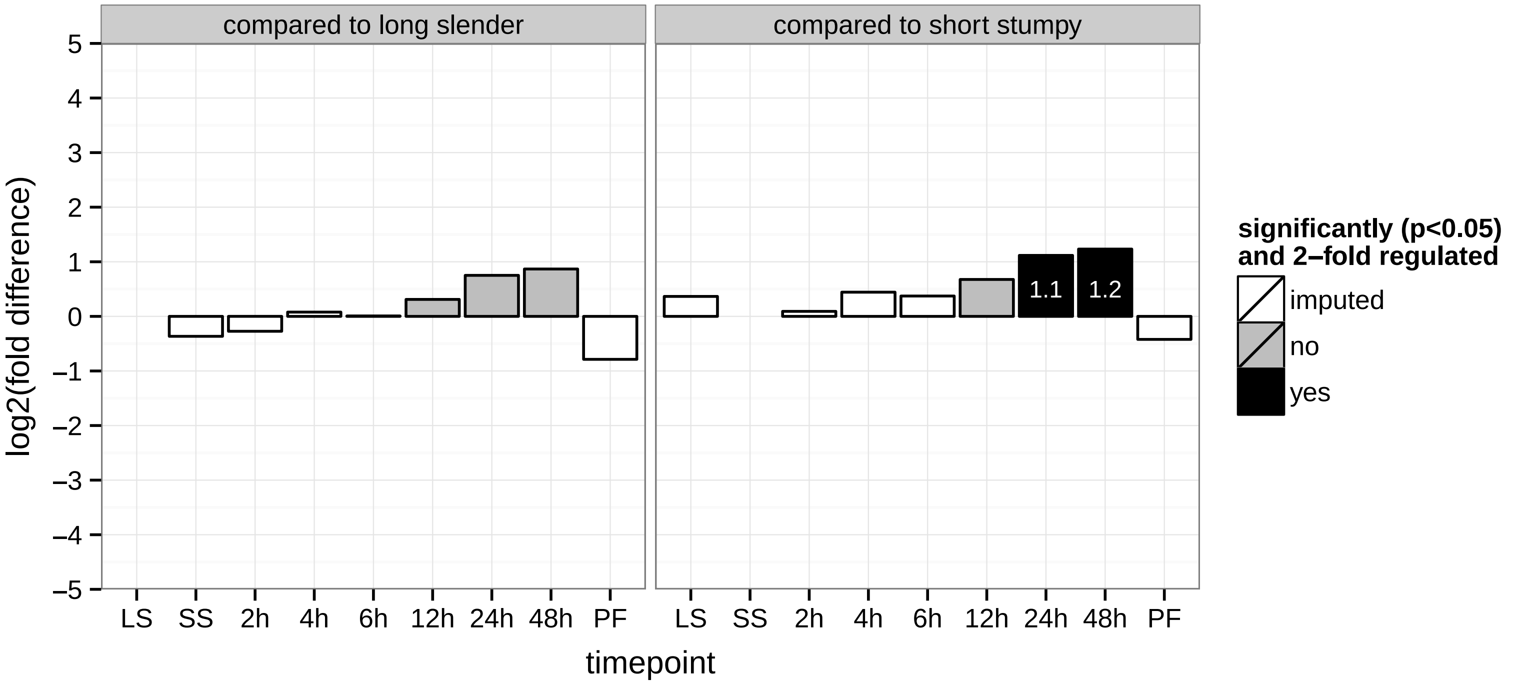

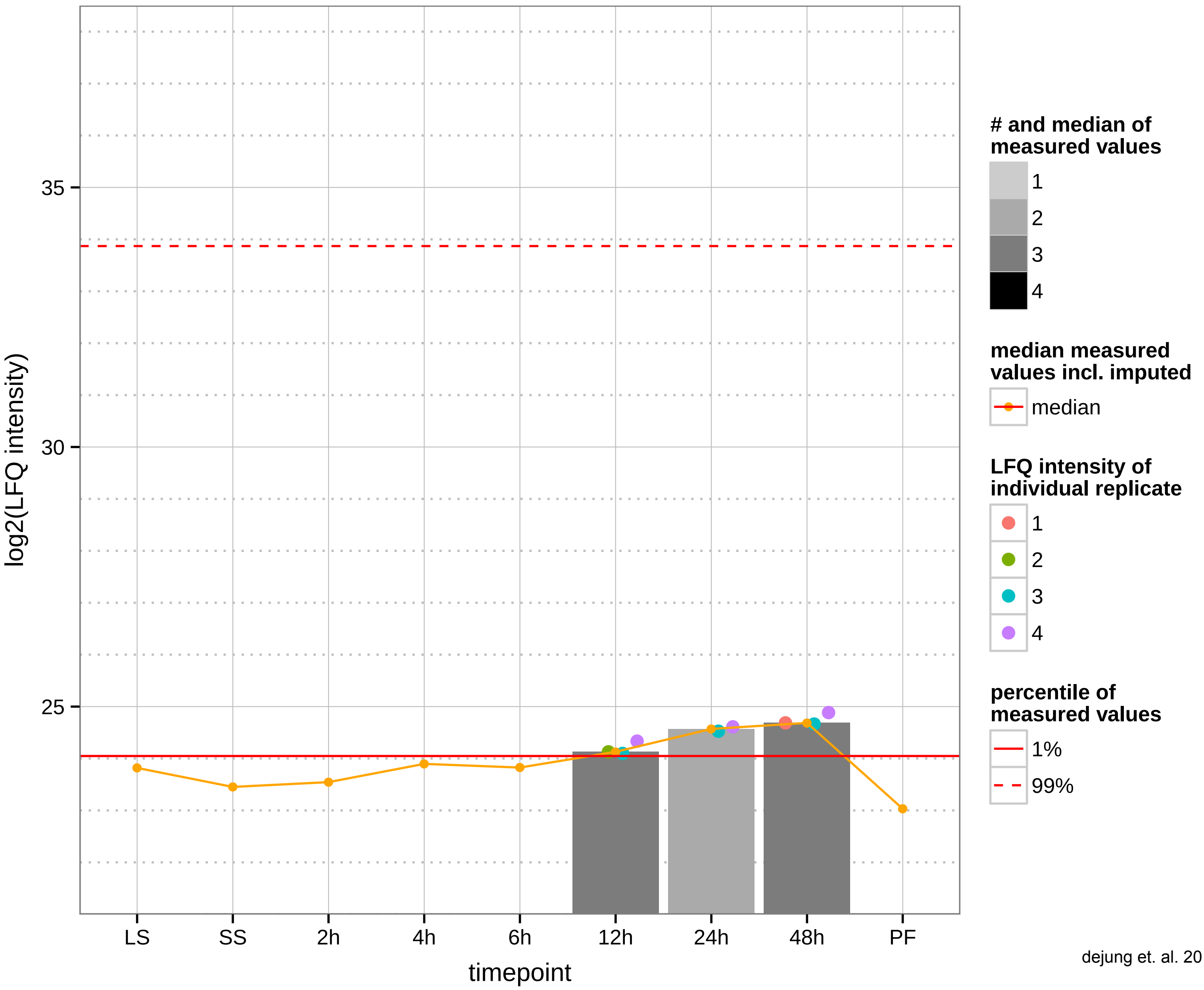

dejung et. al. 2015

hypothetical protein, conserved
Tb927.11.910
AGOF: null
AGOC: null
AGOP: null
PGOF: binding
PGOC: null
PGOP: null

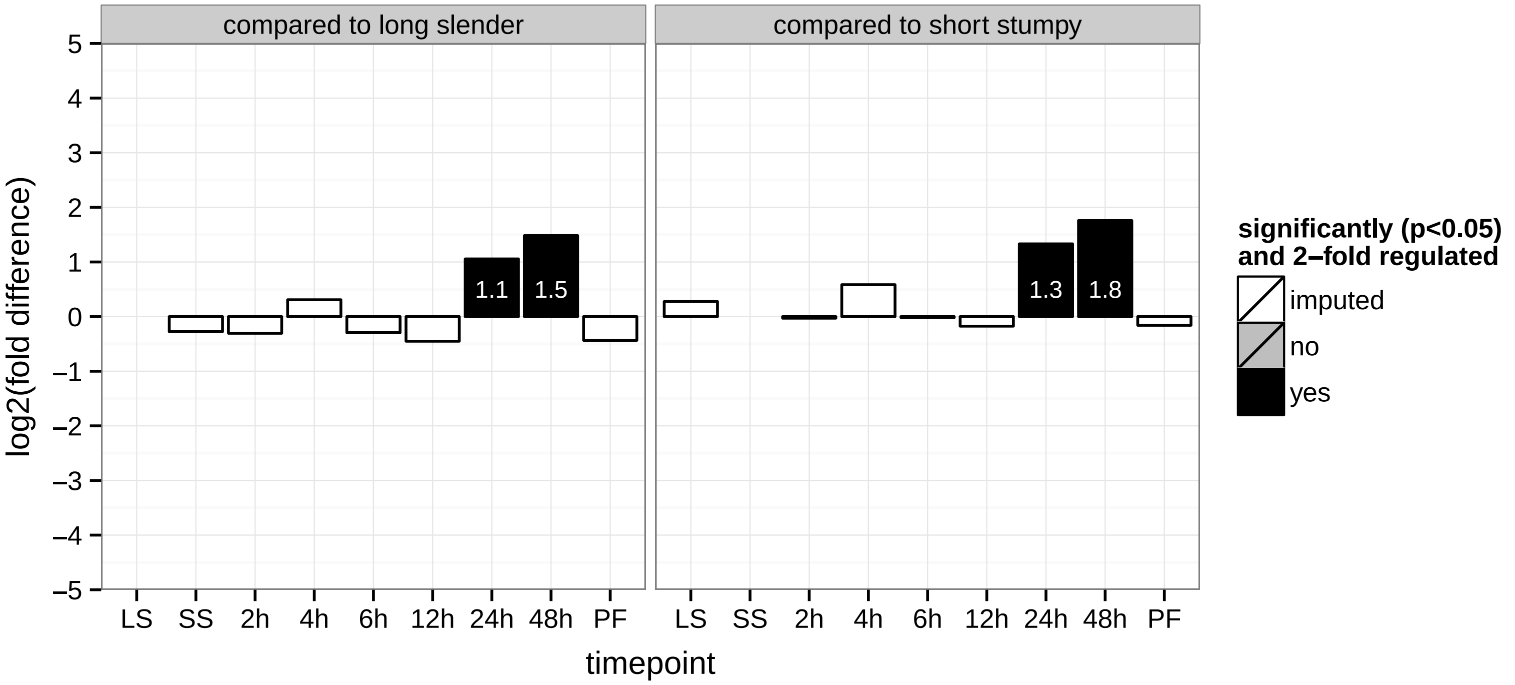

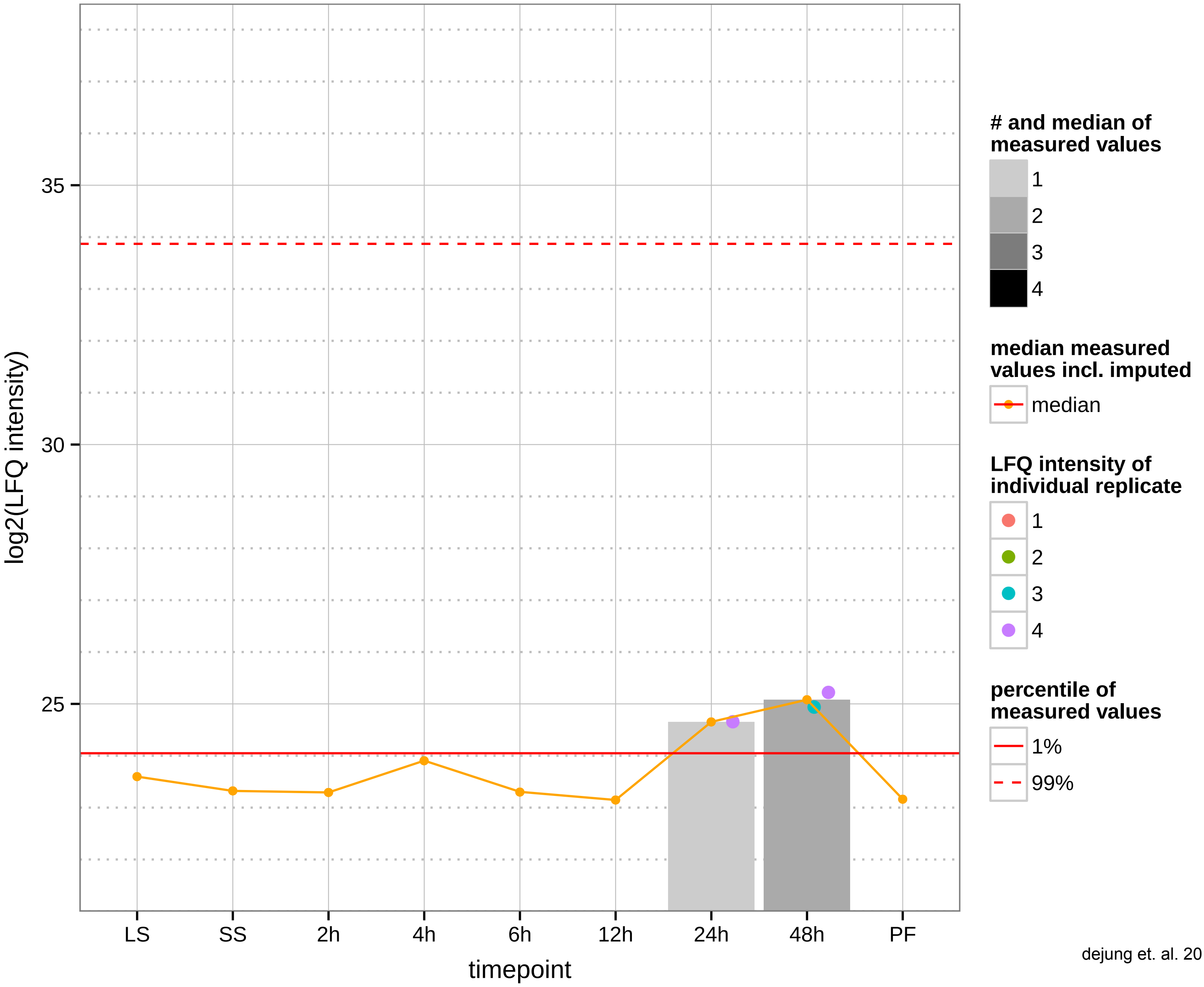

dejung et. al. 2015

small GTP–binding protein RAB6, putative
Tb927.2.2130
AGOF: GTP binding, GTPase activity
AGOC: intracellular, plasma membrane
AGOP: intracellular protein transport, mitosis, nucleocytoplasmic transport, small GTPase mediated signal transduction, synap
PGOF: GTP binding, GTPase activity
PGOC: intracellular, membrane
PGOP: GTP catabolic process, intracellular protein transport, nucleocytoplasmic transport, protein transport, signal transducti

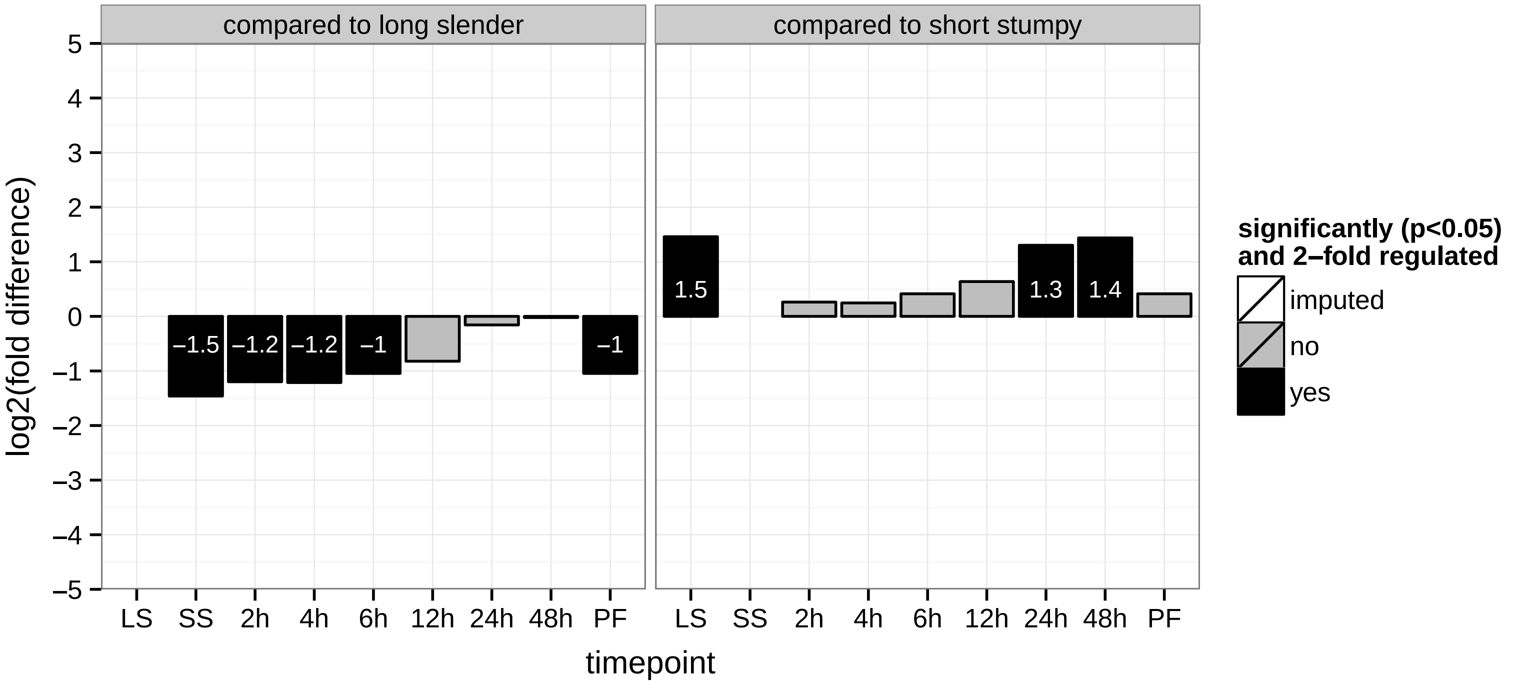

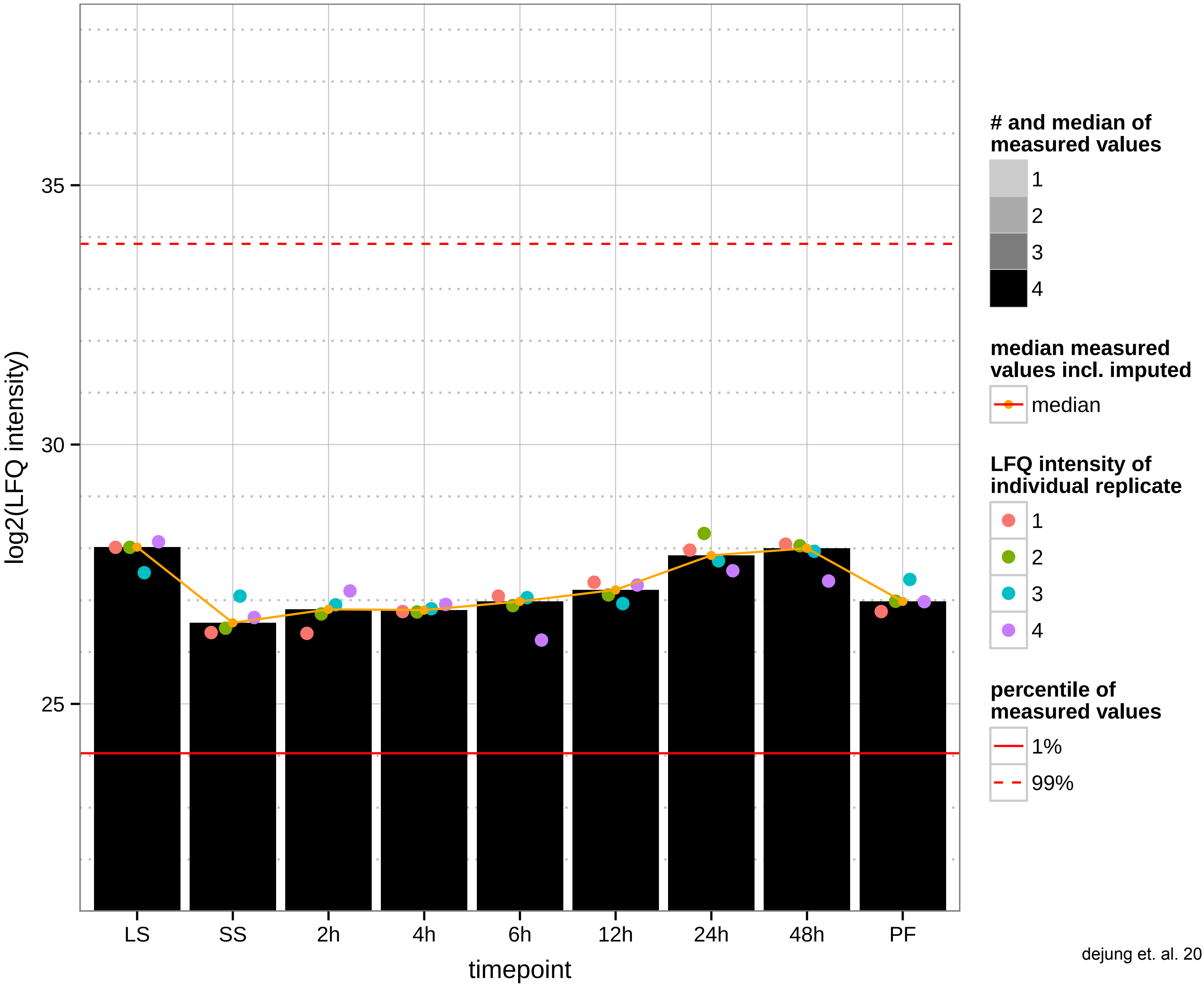

dejung et. al. 2015

cdc2−related kinase, putative (CRK9)
Tb927.2.4510
AGOF: ATP binding, protein kinase activity, protein serine/threonine kinase activity, protein tyrosine kinase activity
AGOC: null
AGOP: null
PGOF: ATP binding, protein kinase activity, transferase activity, transferring phosphorus−containing groups
PGOC: null
PGOP: protein phosphorylation

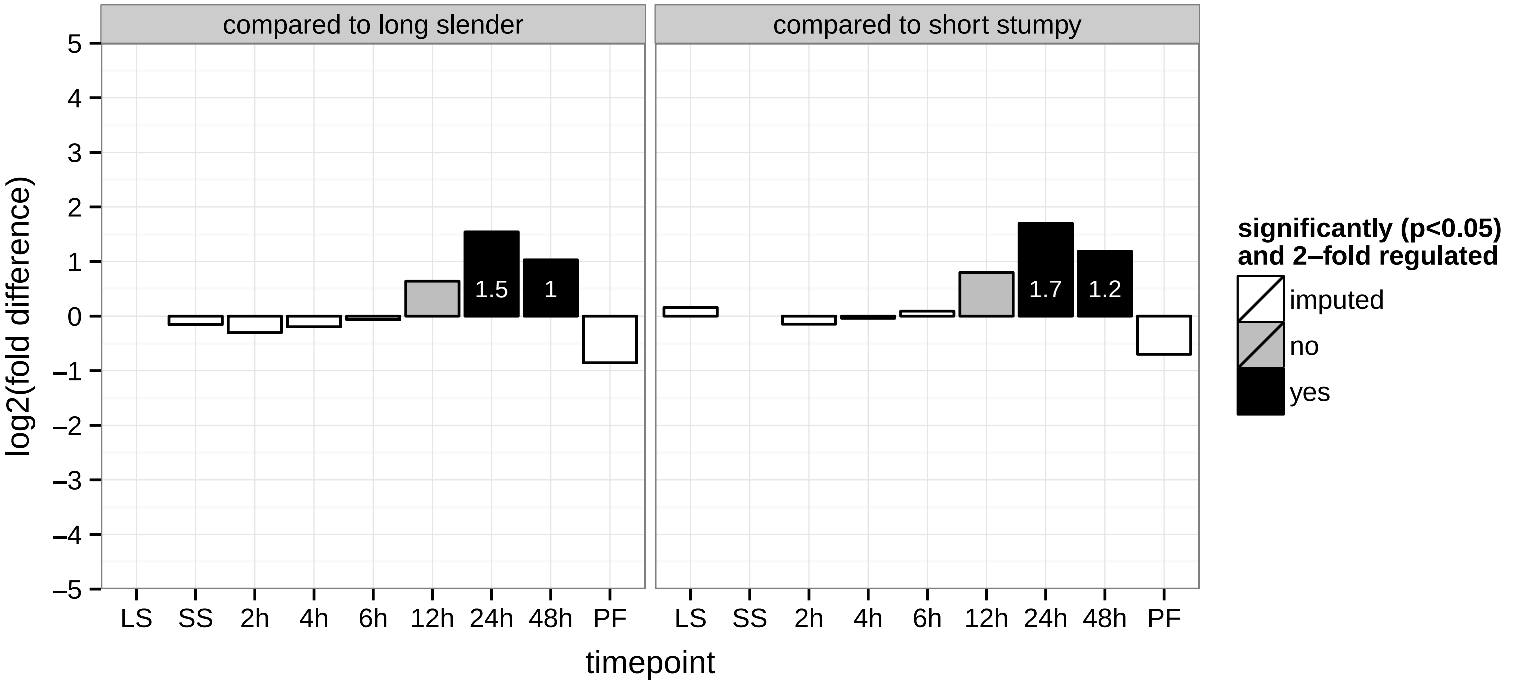

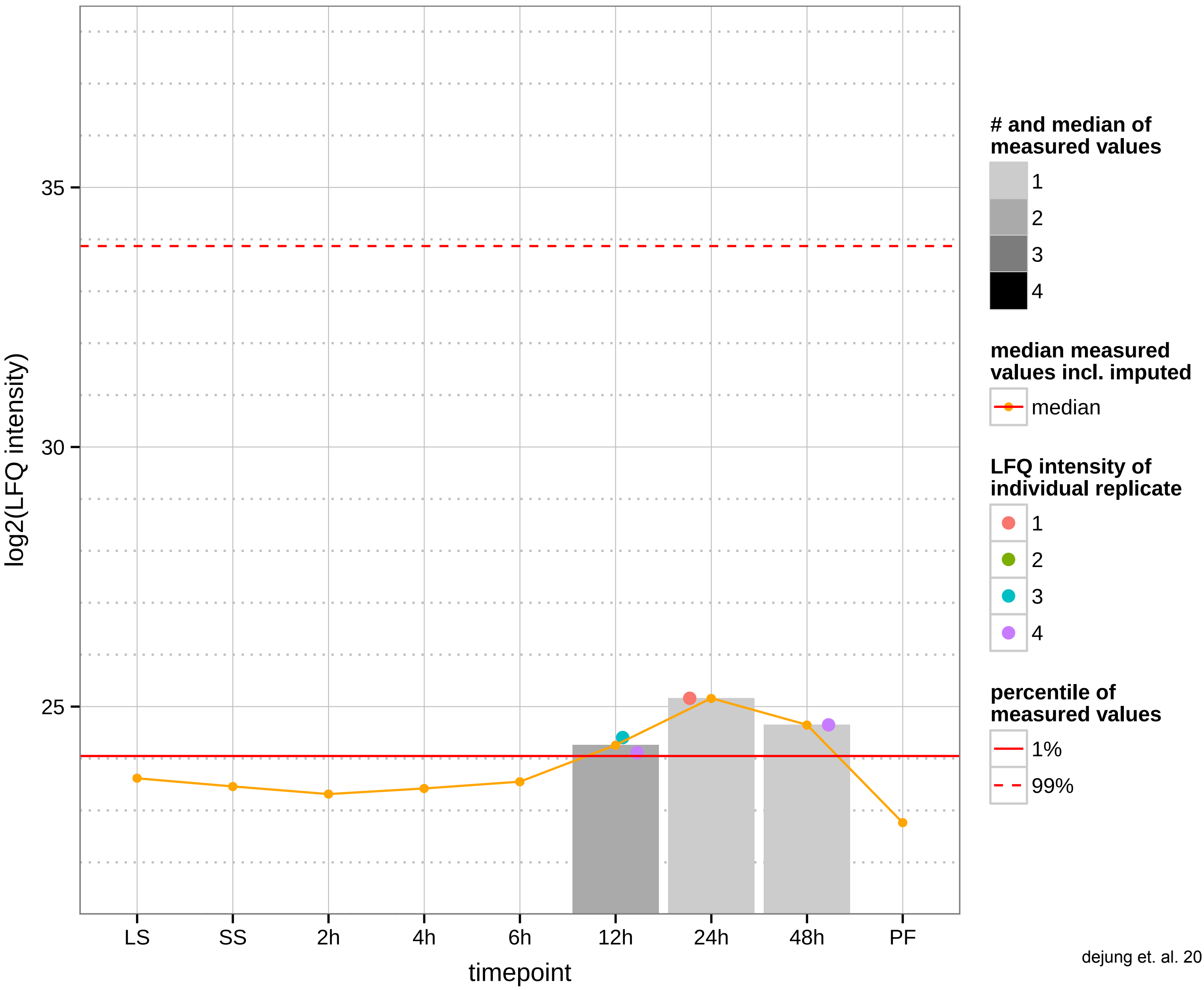

dejung et. al. 2015

hypothetical protein, conserved
Tb927.3.3030
AGOF: null
AGOC: null
AGOP: null
PGOF: null
PGOC: null
PGOP: null

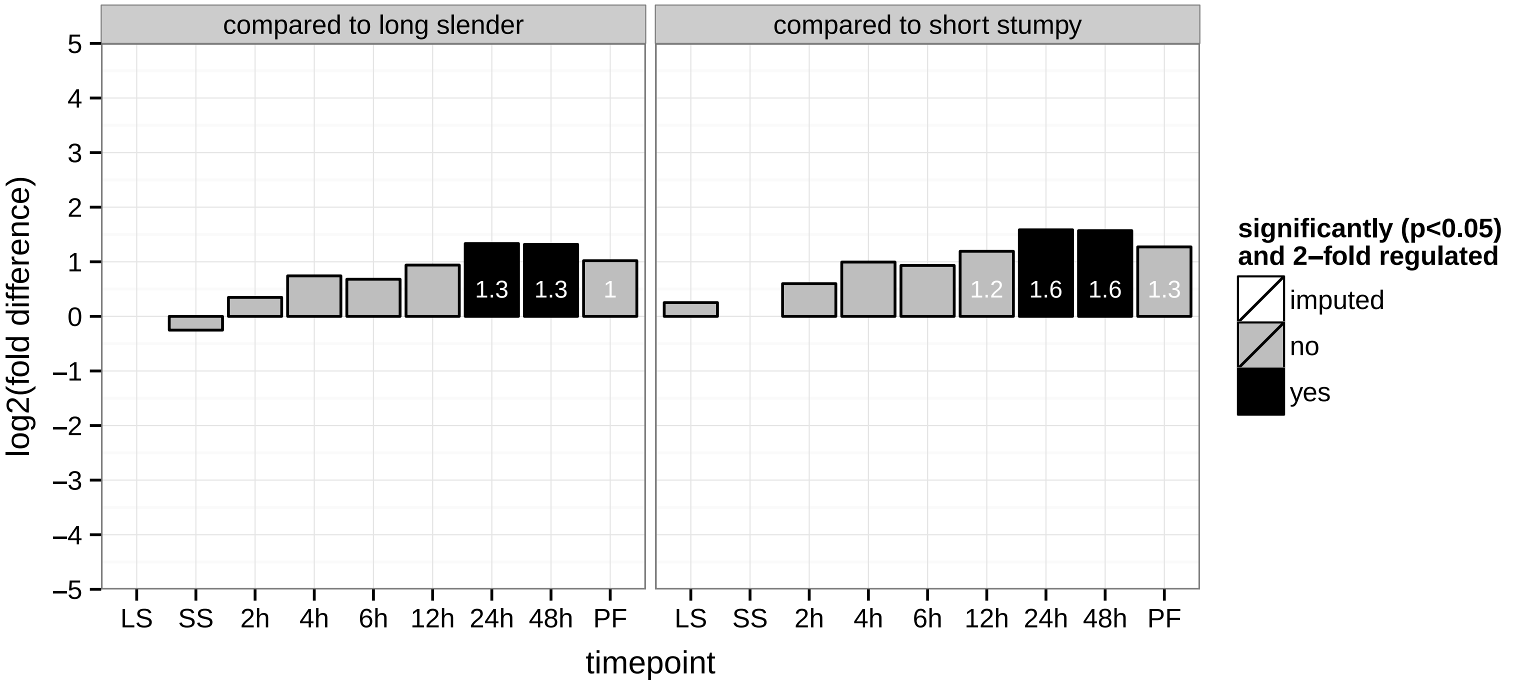

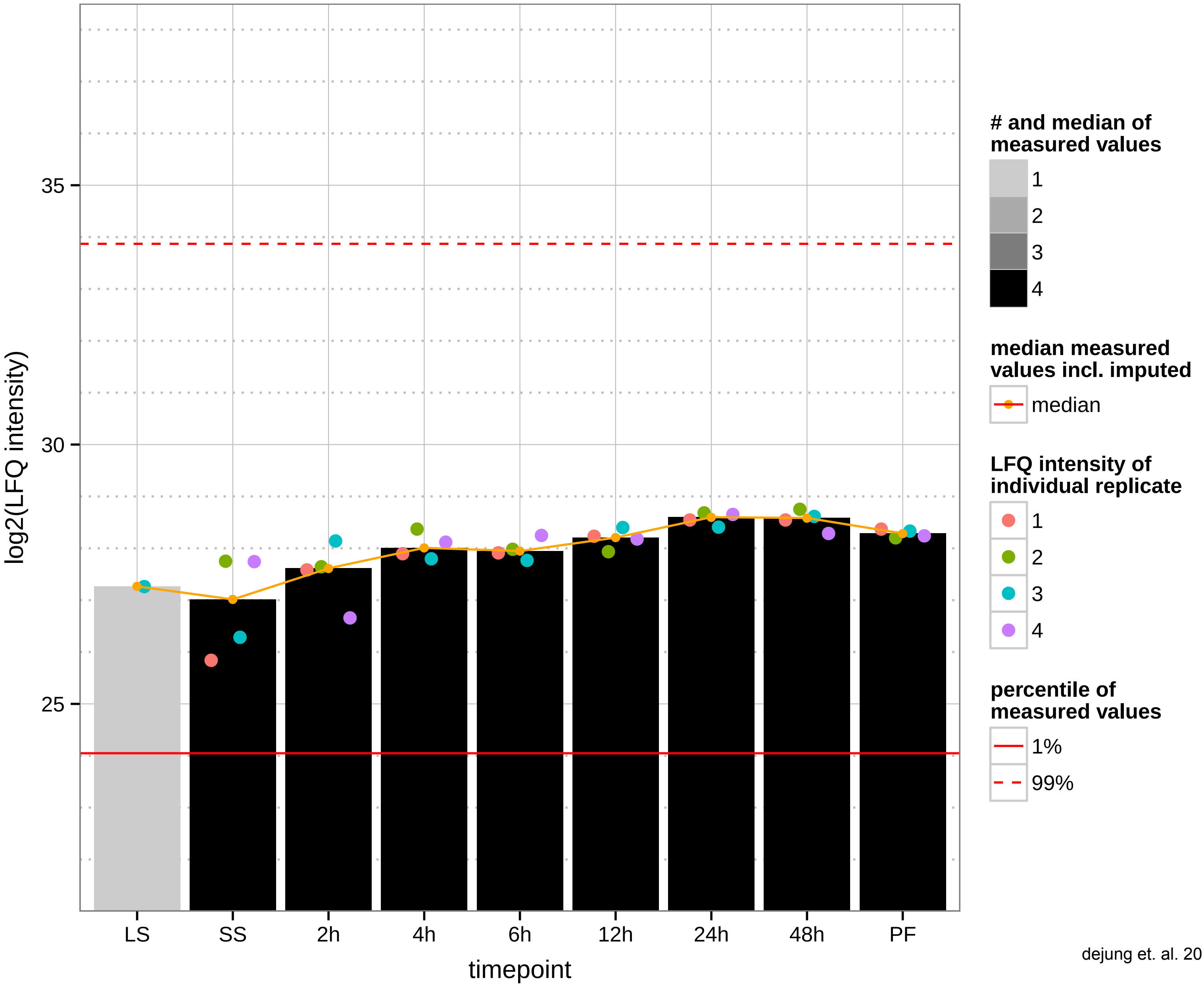

dejung et. al. 2015

Cell growth−regulating nucleolar protein, putative
Tb927.3.3970
AGOF: null
AGOC: null
AGOP: null
PGOF: null
PGOC: null
PGOP: null

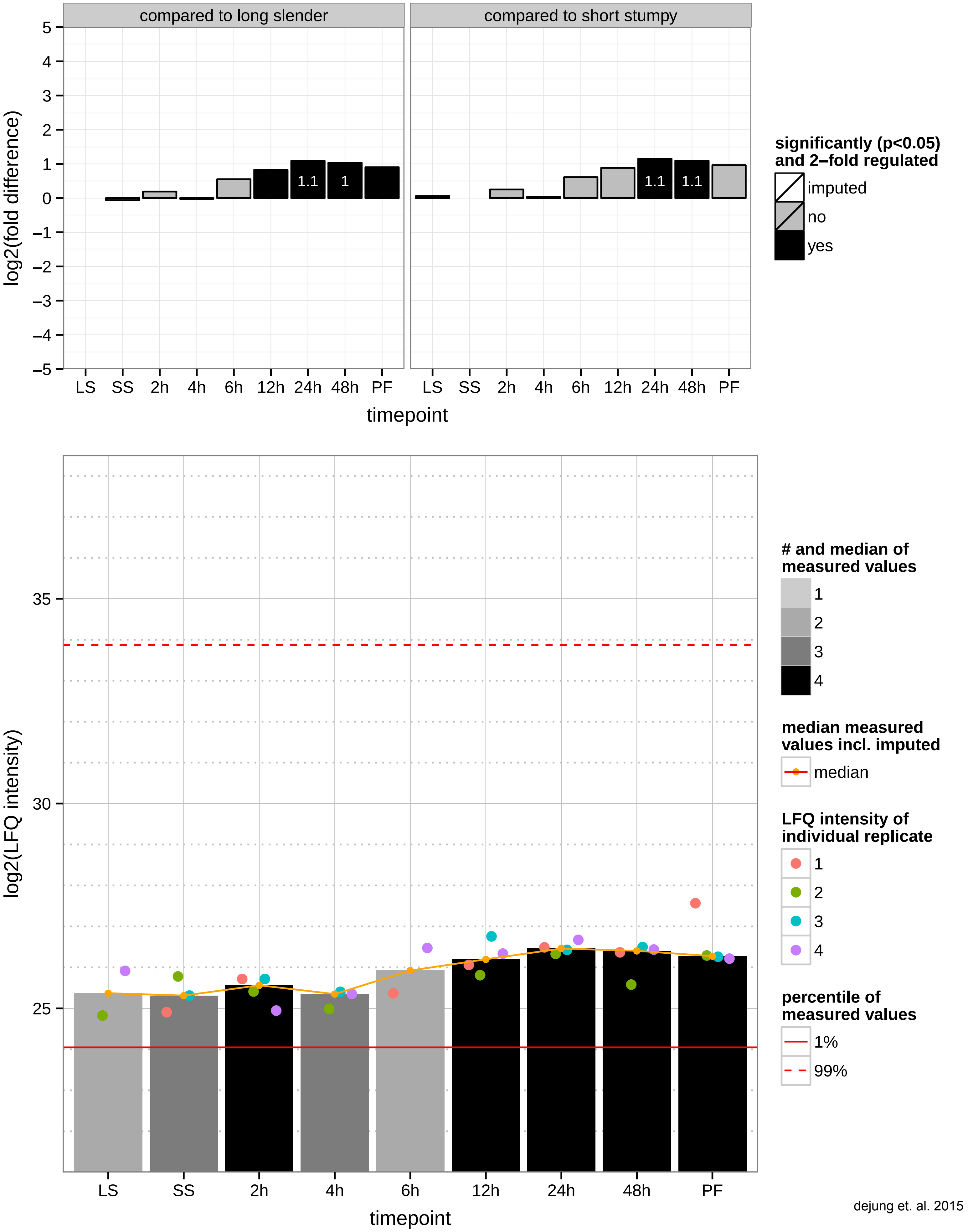

dejung et. al. 2015

hypothetical protein, conserved
Tb927.3.4250
AGOF: null
AGOC: null
AGOP: null
PGOF: null
PGOC: null
PGOP: null

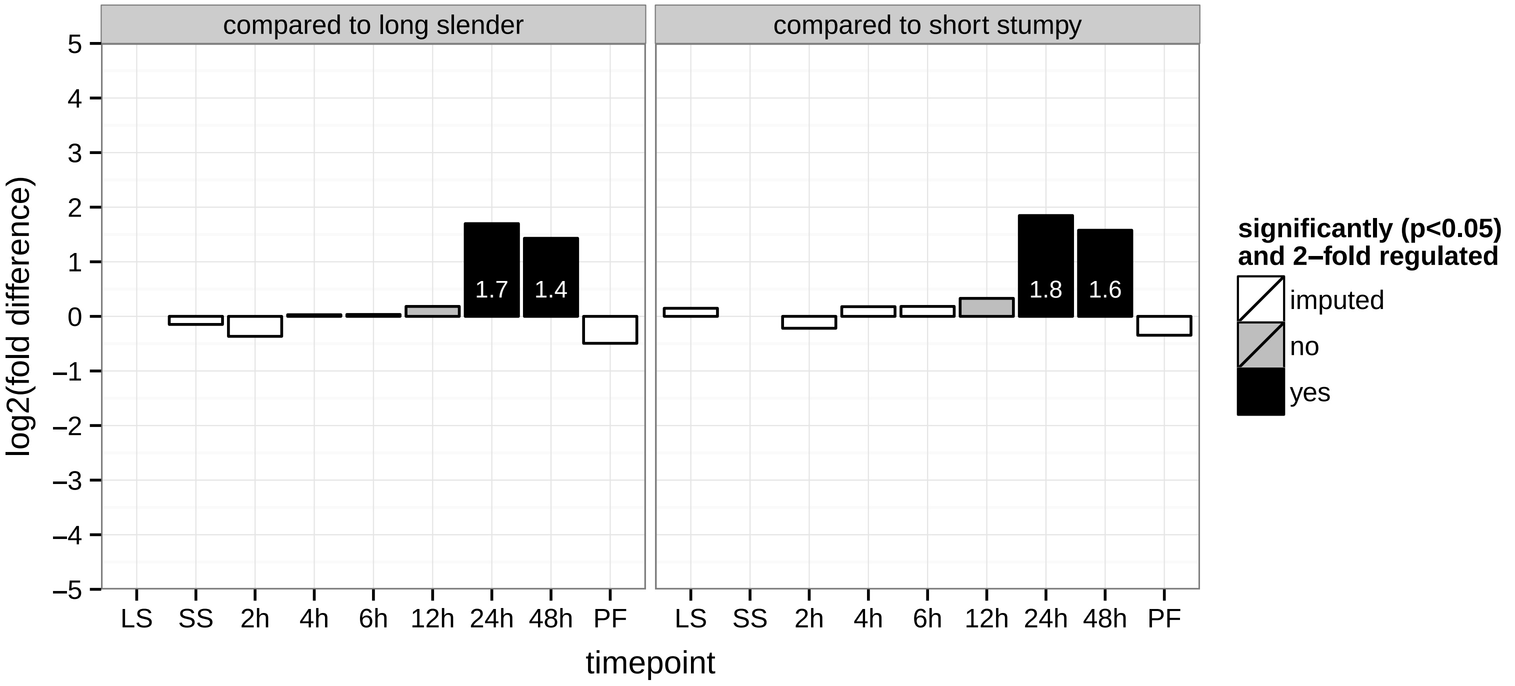

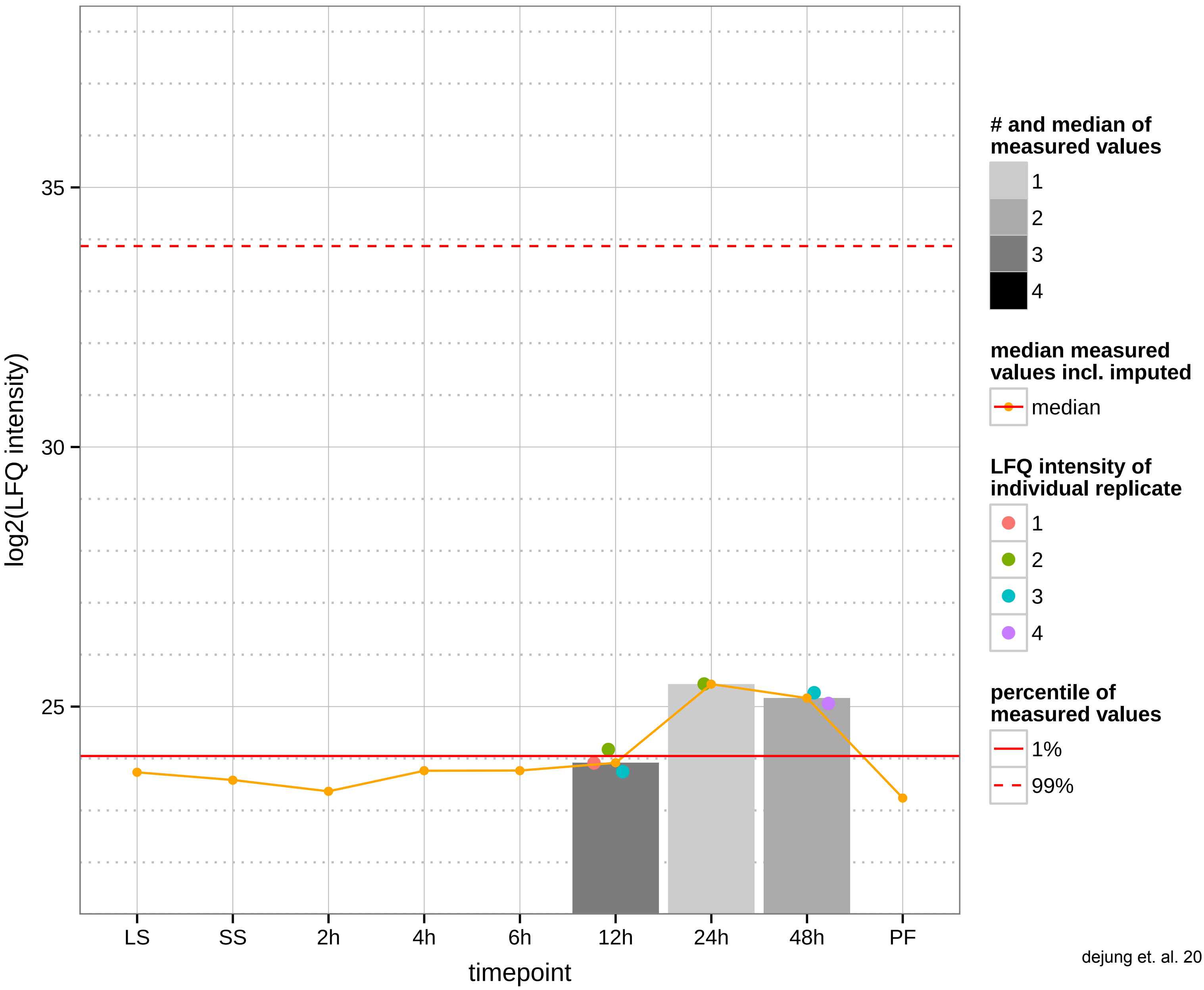

dejung et. al. 2015

hypothetical protein, conserved
Tb927.3.4780;Tb927.3.4740
AGOF: catalytic activity
AGOC: mitochondrion
AGOP: metabolic process
PGOF: NAD binding, oxidoreductase activity, acting on the CH–OH group of donors, NAD or NADP as acceptor
PGOC: cytoplasm
PGOP: glycerol–3–phosphate catabolic process, oxidation–reduction process

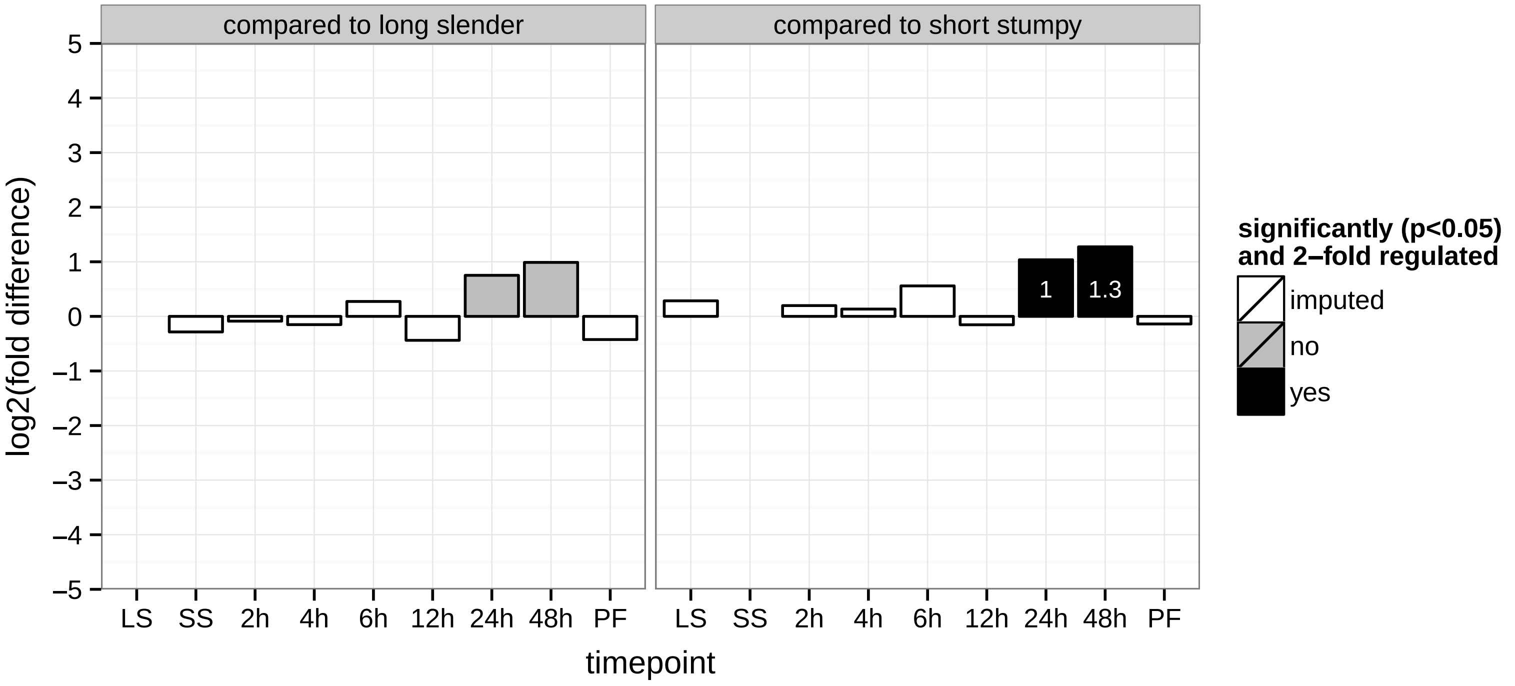

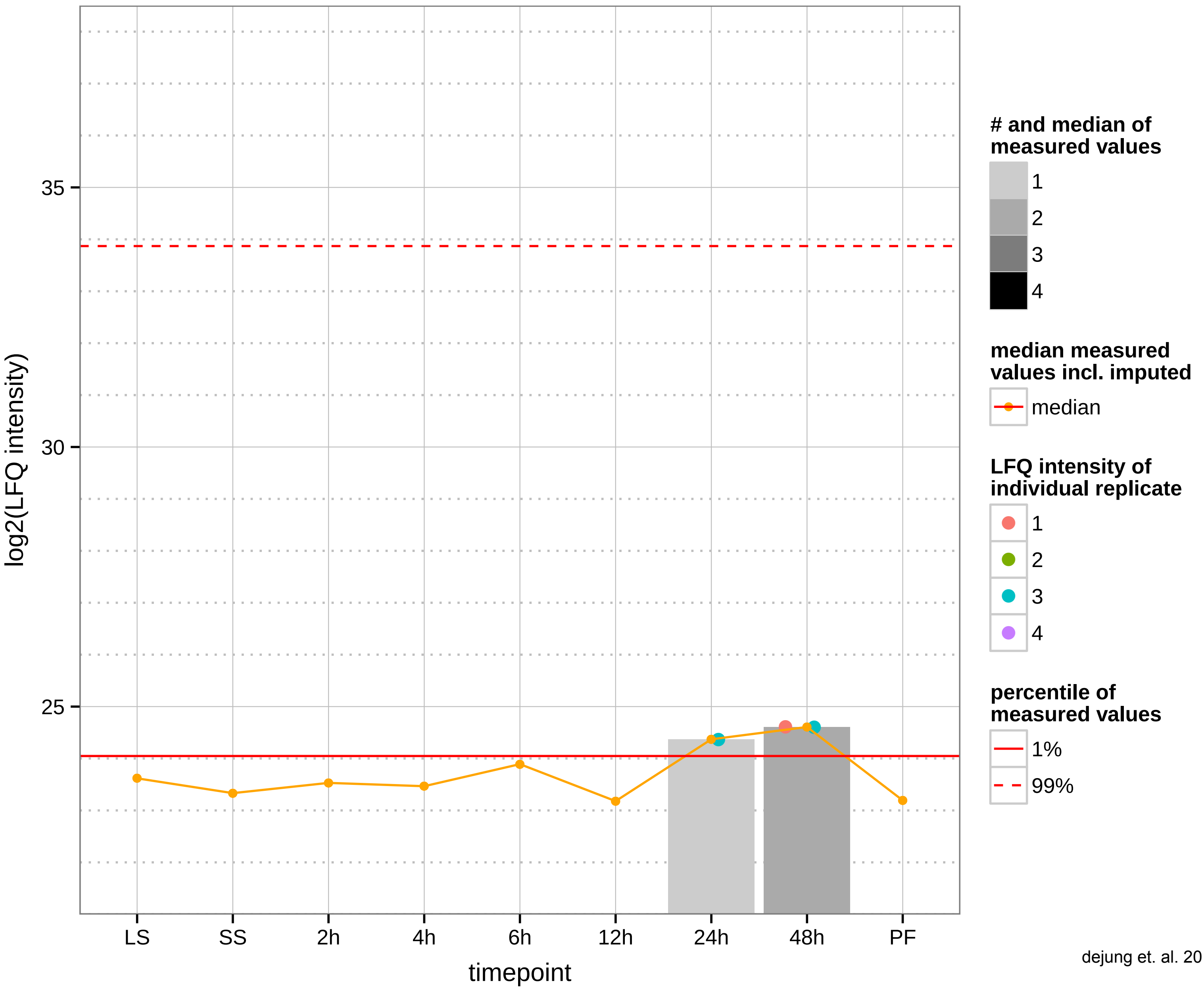

dejung et. al. 2015

enoyl–CoA hydratase, mitochondrial precursor, putative
Tb927.3.4850
AGOF: catalytic activity
AGOC: null
AGOP: metabolic process
PGOF: catalytic activity
PGOC: null
PGOP: metabolic process

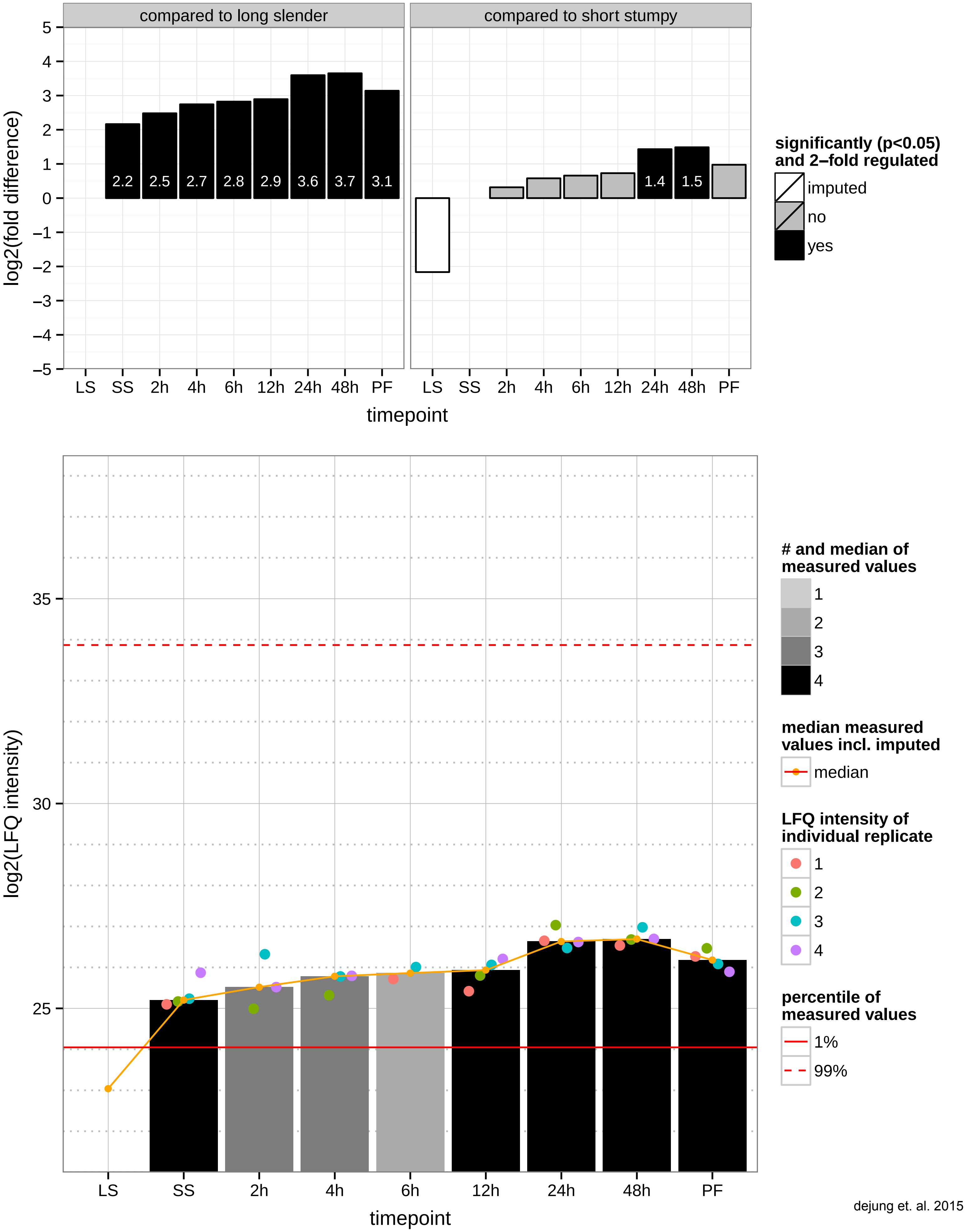

dejung et. al. 2015

hypothetical protein, conserved
Tb927.3.4880
AGOF: null
AGOC: nucleus
AGOP: nucleosome assembly
PGOF: null
PGOC: nucleus
PGOP: nucleosome assembly

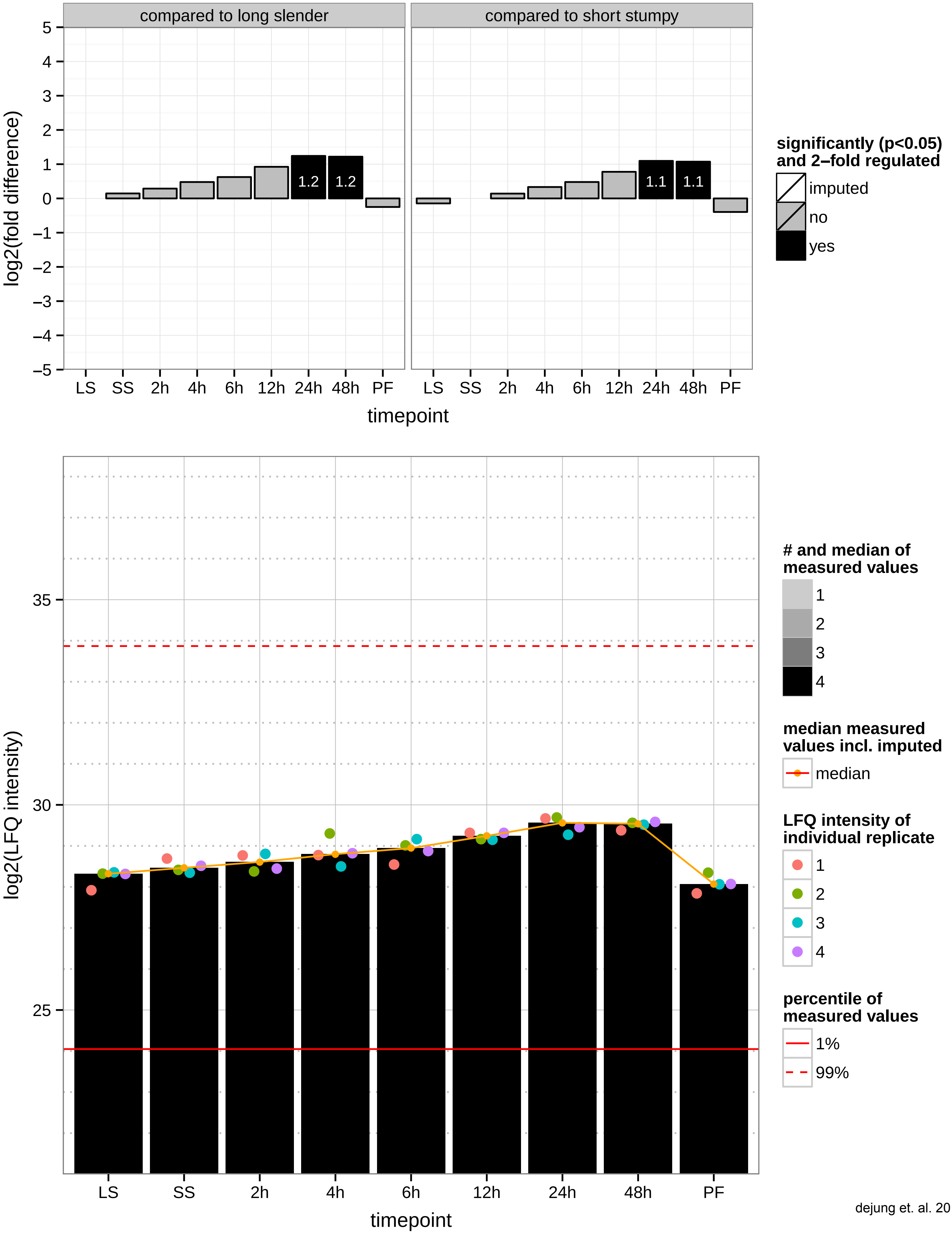

dejung et. al. 2015

acyl carrier protein, mitochondrial precursor, putative (ACP)
Tb927.3.860
AGOF: ACP phosphopantetheine attachment site binding involved in fatty acid biosynthetic process, cofactor binding
AGOC: mitochondrion
AGOP: fatty acid biosynthetic process
PGOF: null
PGOC: null
PGOP: fatty acid biosynthetic process

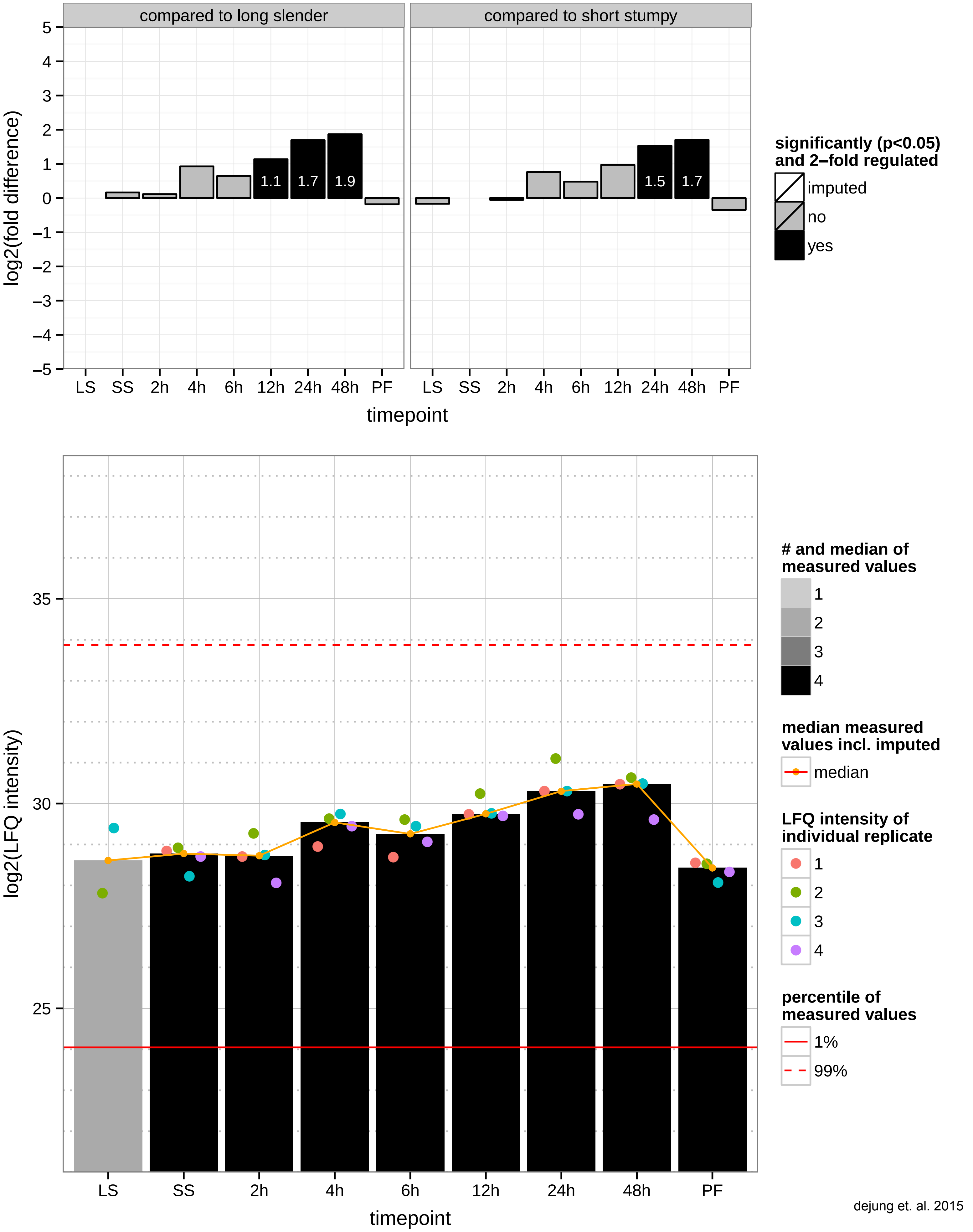

dejung et. al. 2015

hypothetical protein, conserved
Tb927.3.940
AGOF: null
AGOC: mitochondrion
AGOP: null
PGOF: null
PGOC: null
PGOP: null

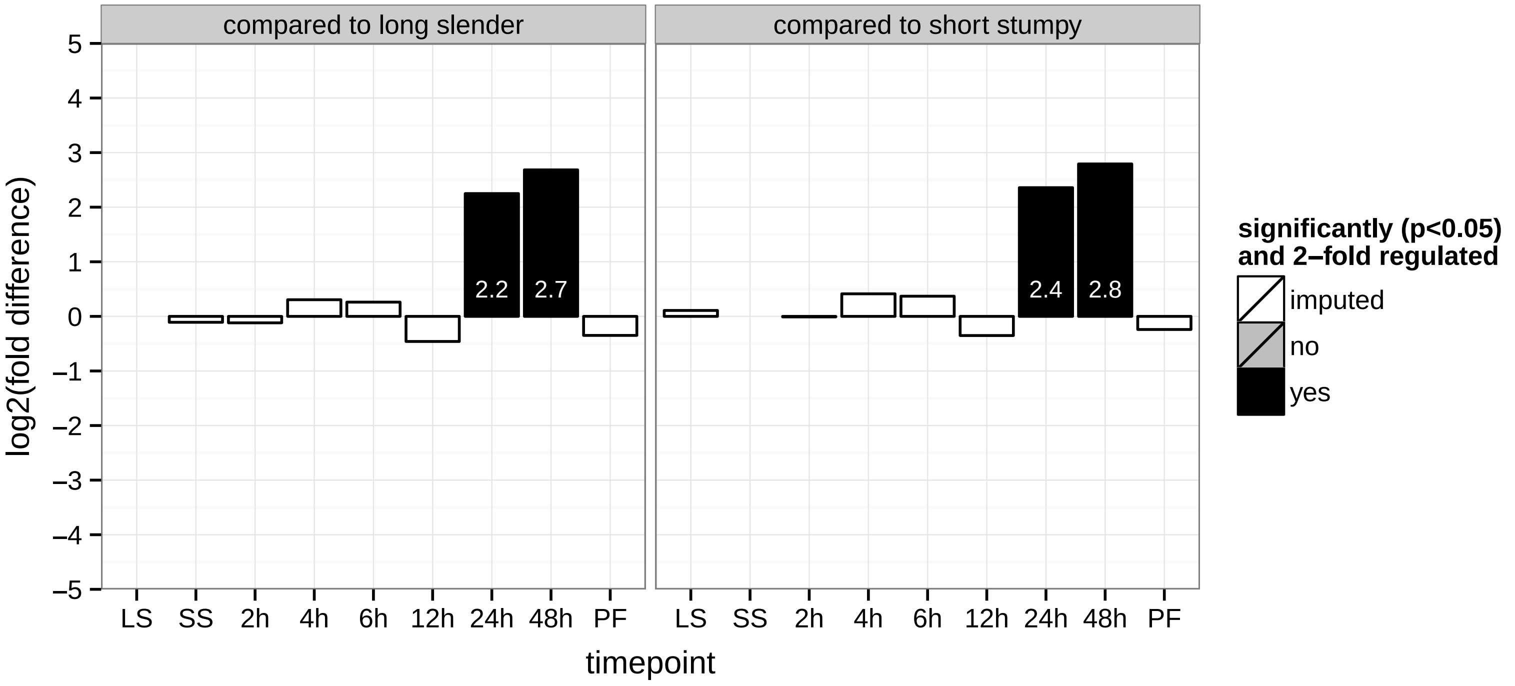

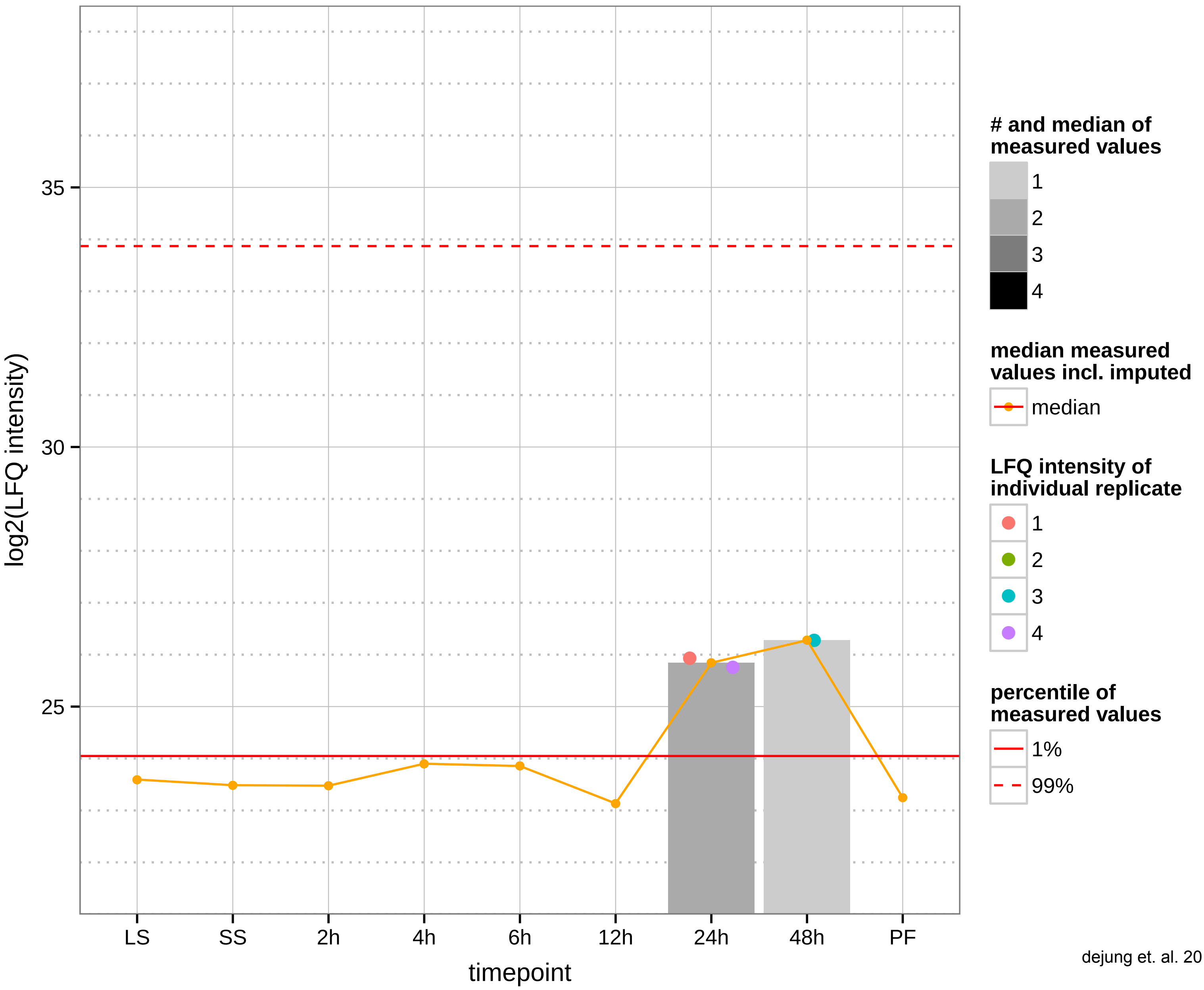

dejung et. al. 2015

hypothetical protein, conserved
Tb927.4.2320
AGOF: null
AGOC: null
AGOP: null
PGOF: null
PGOC: null
PGOP: null

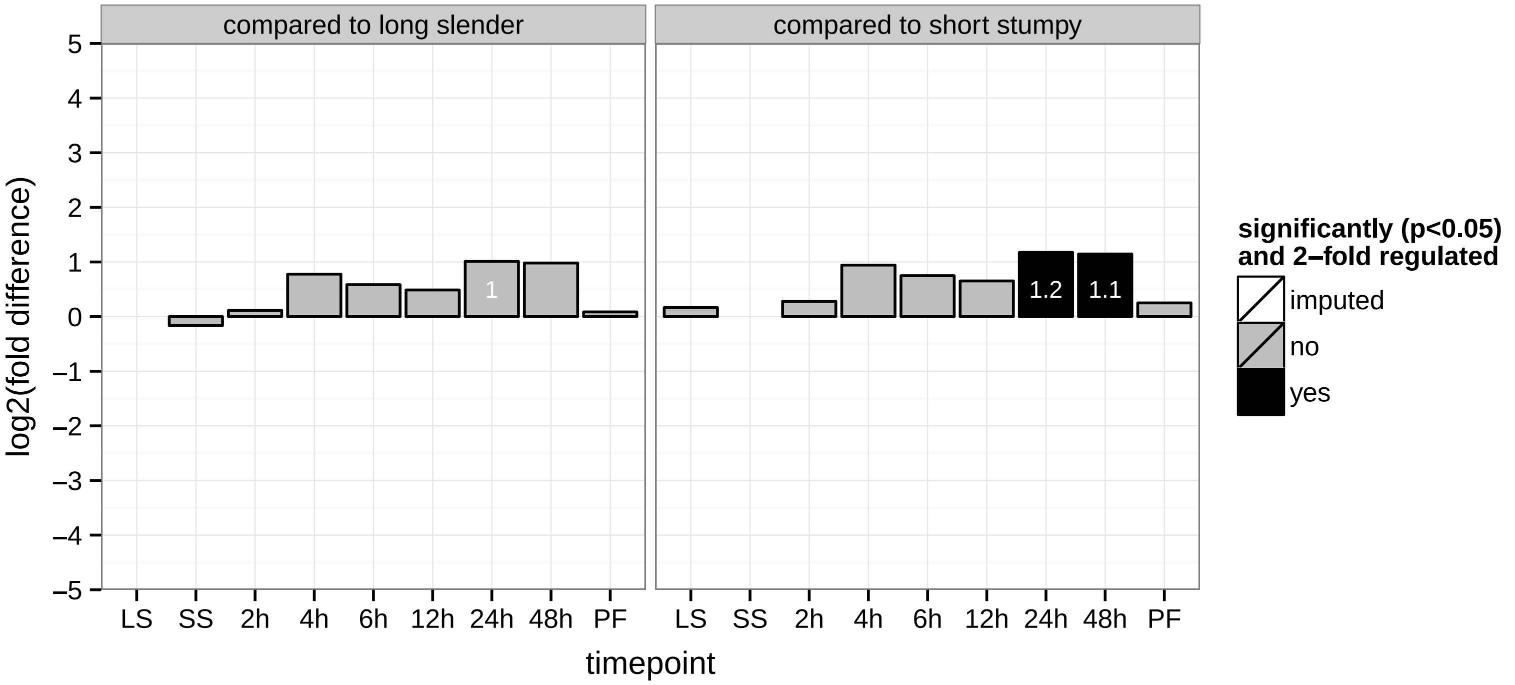

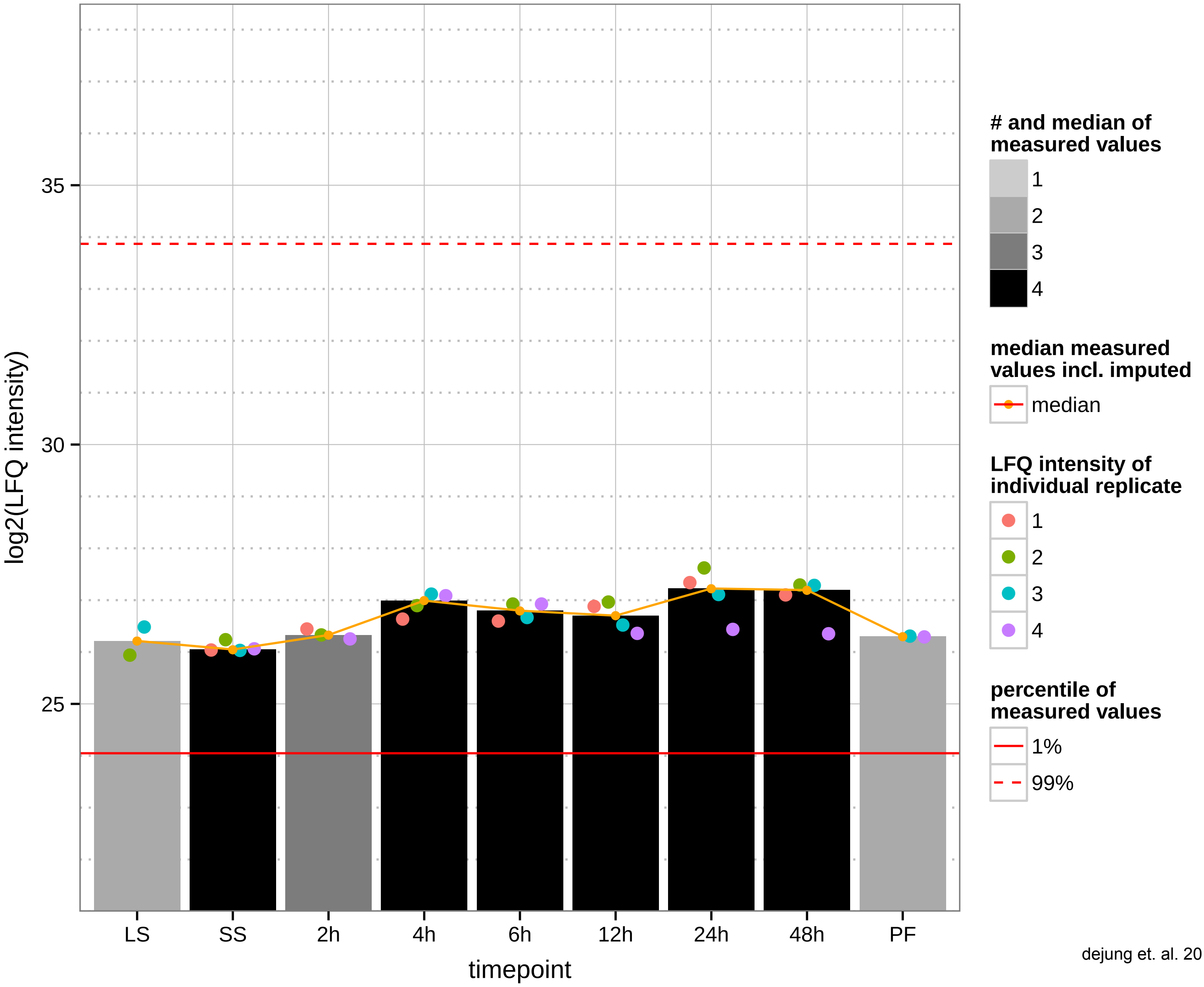

dejung et. al. 2015

hypothetical protein, conserved
Tb927.4.2400
AGOF: null
AGOC: null
AGOP: null
PGOF: null
PGOC: null
PGOP: null

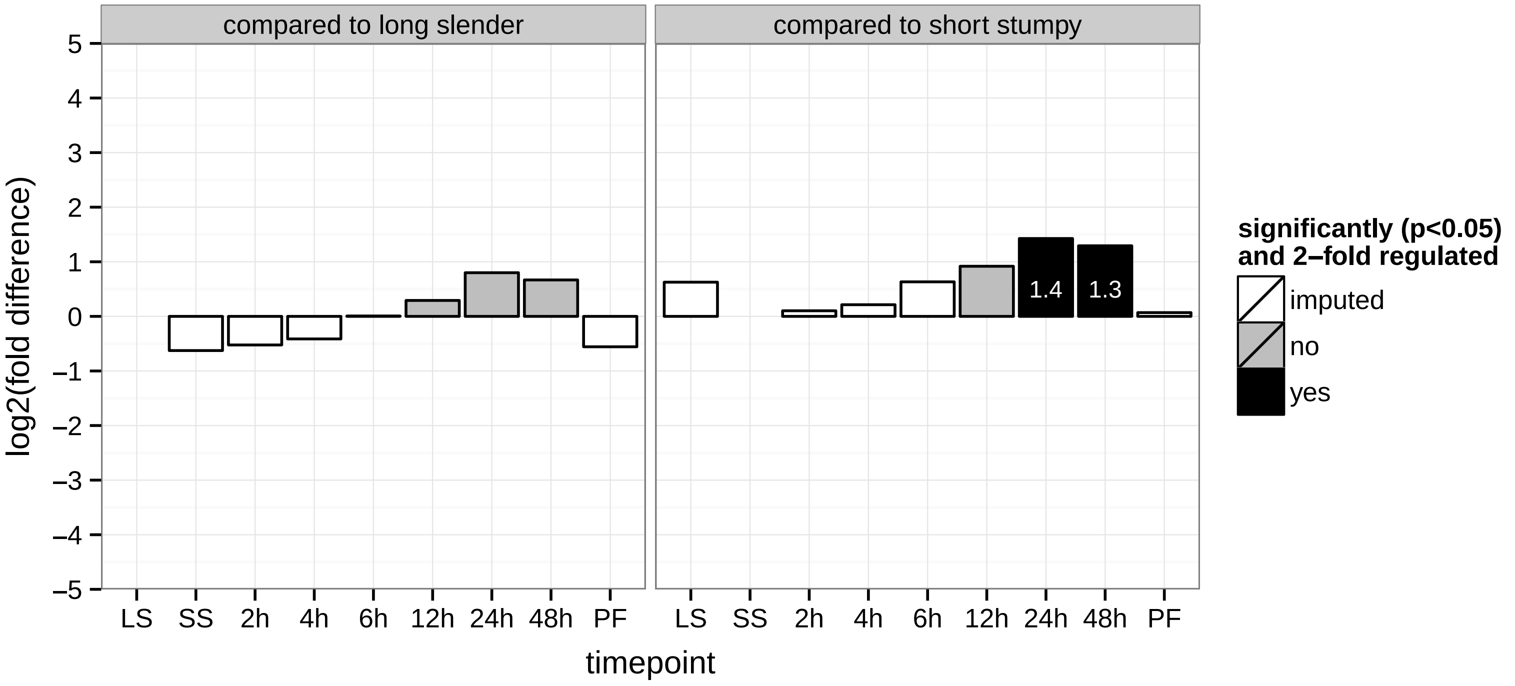

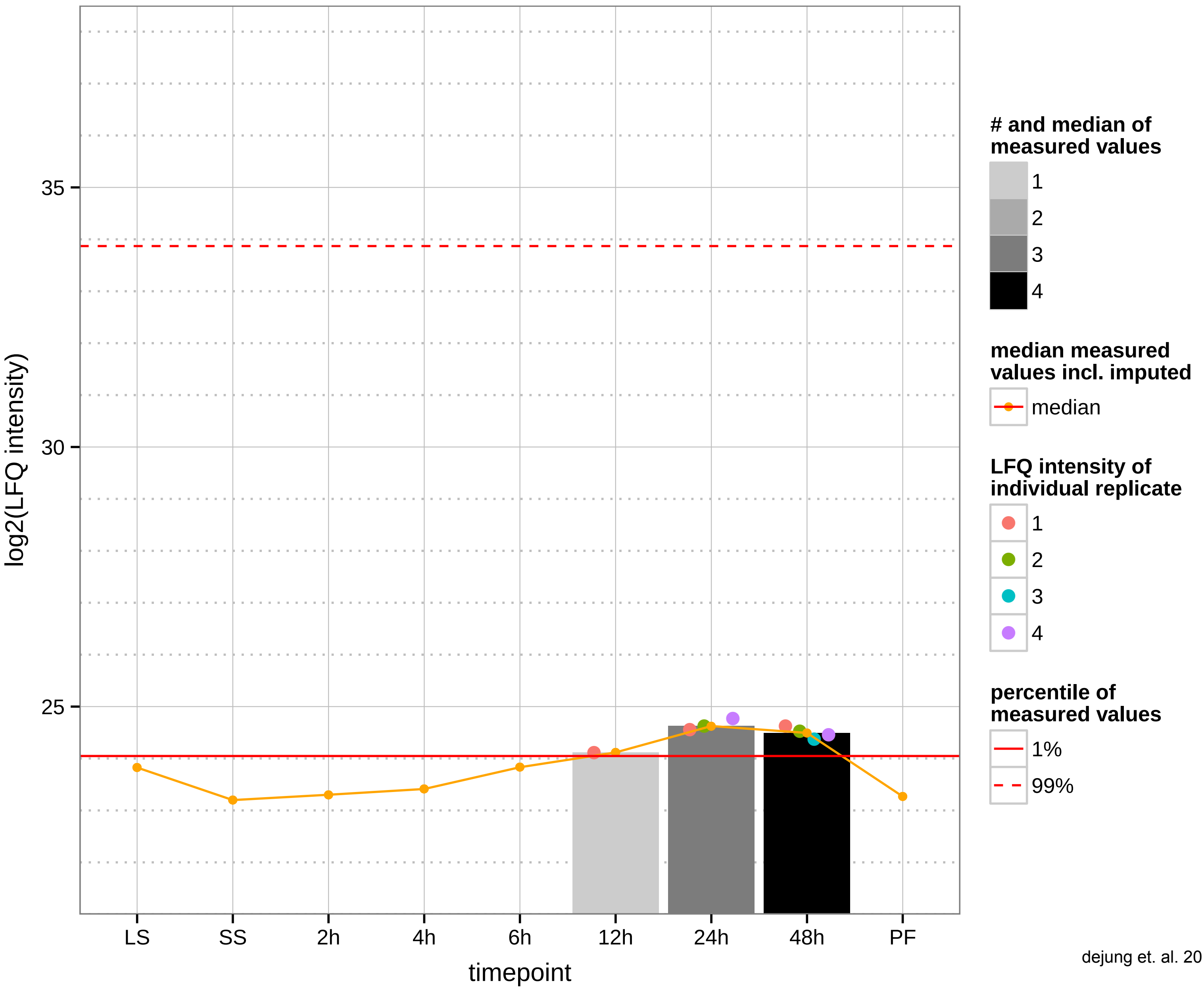

dejung et. al. 2015

hypothetical protein, conserved
Tb927.4.2740
AGOF: null
AGOC: null
AGOP: null
PGOF: null
PGOC: null
PGOP: null

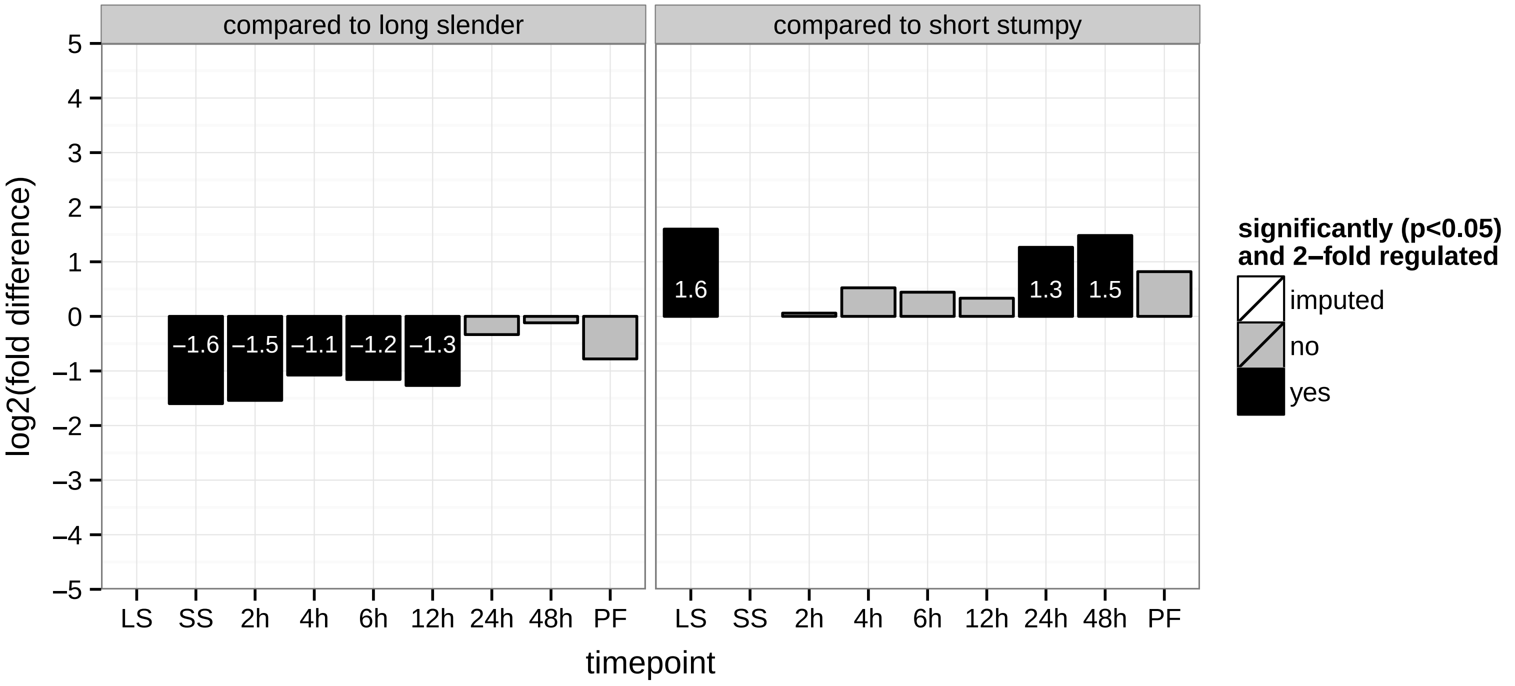

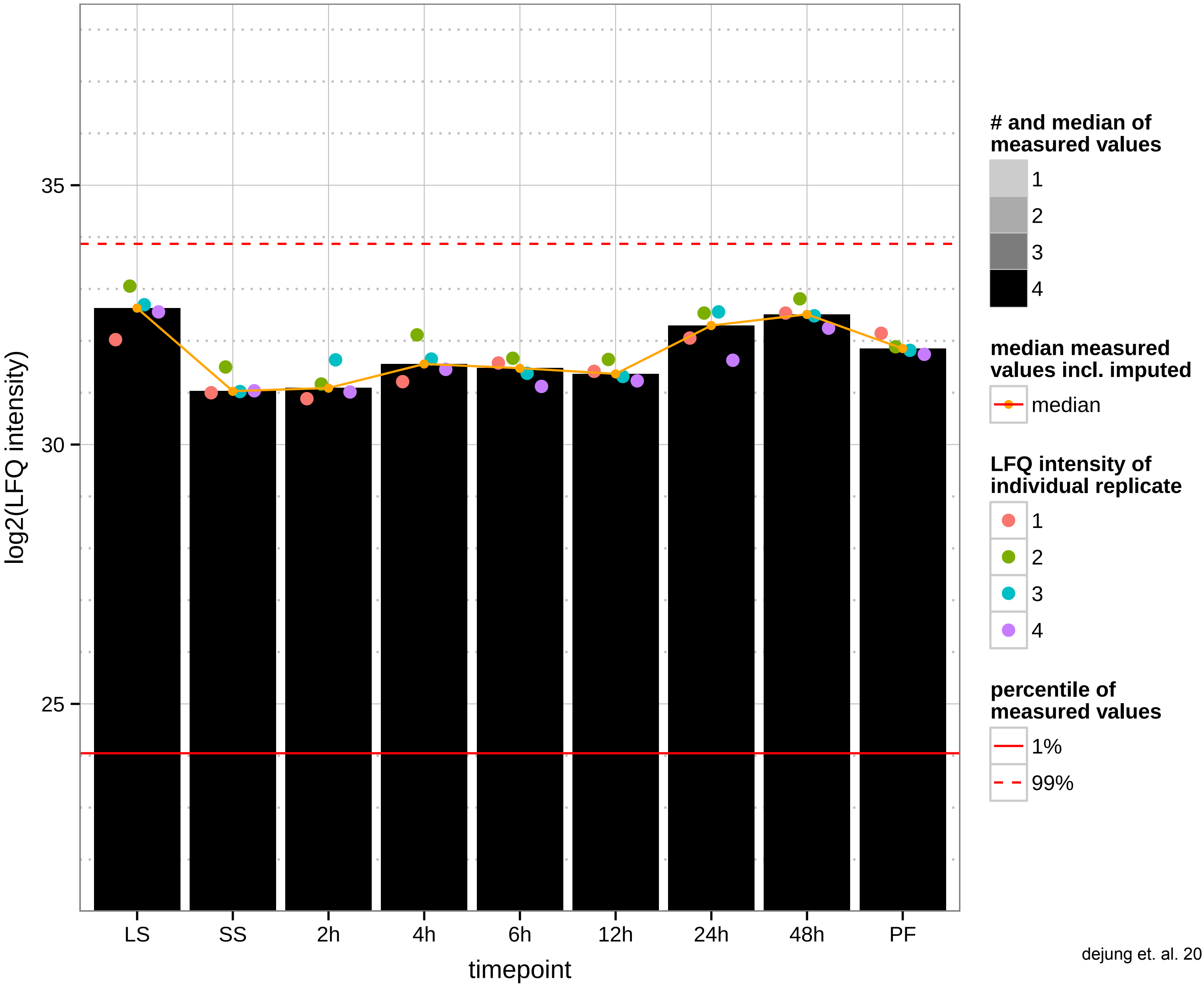

dejung et. al. 2015

ubiquinol–cytochrome c reductase, putative
Tb927.4.4990;Tb927.8.7430
AGOF: null, ubiquinol–cytochrome–c reductase activity
AGOC: null, mitochondrial respiratory chain complex III
AGOP: null, mitochondrial electron transport, ubiquinol to cytochrome c
PGOF: null
PGOC: null
PGOP: null

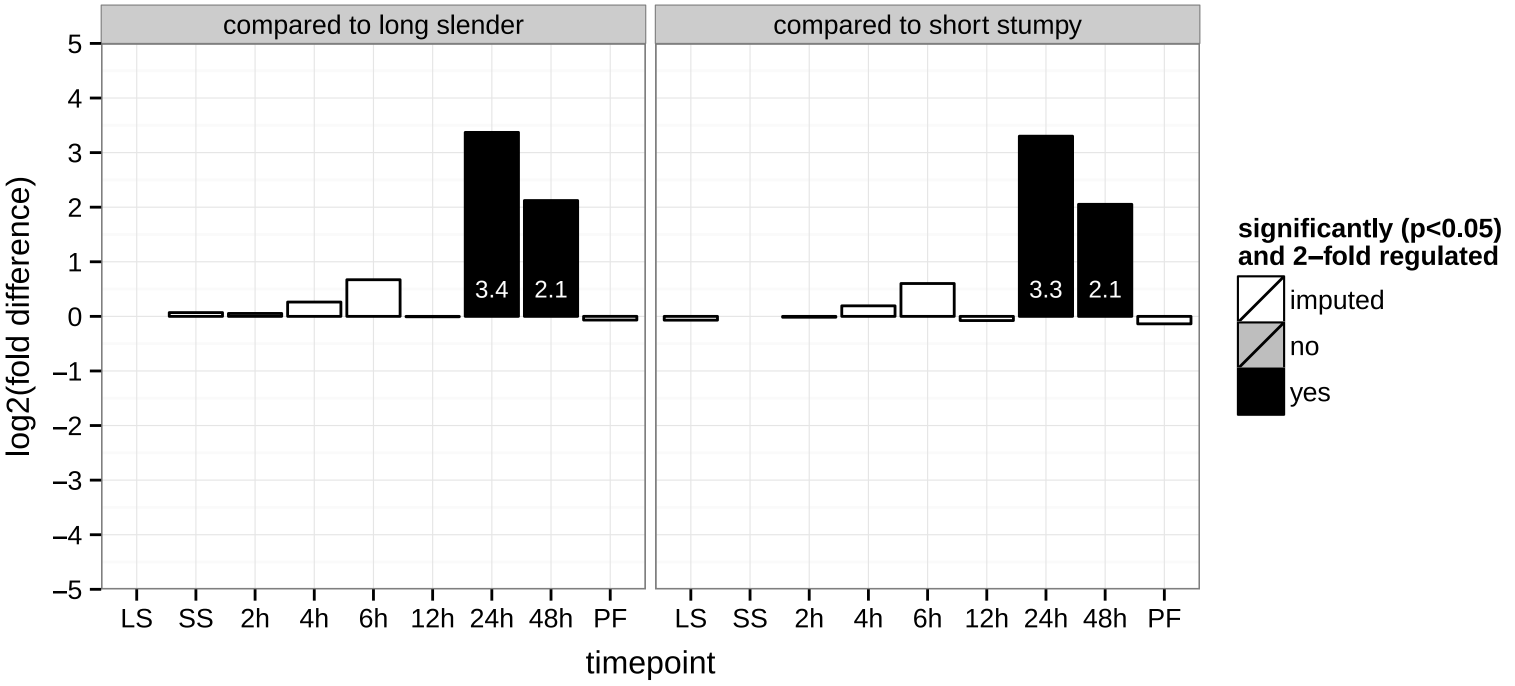

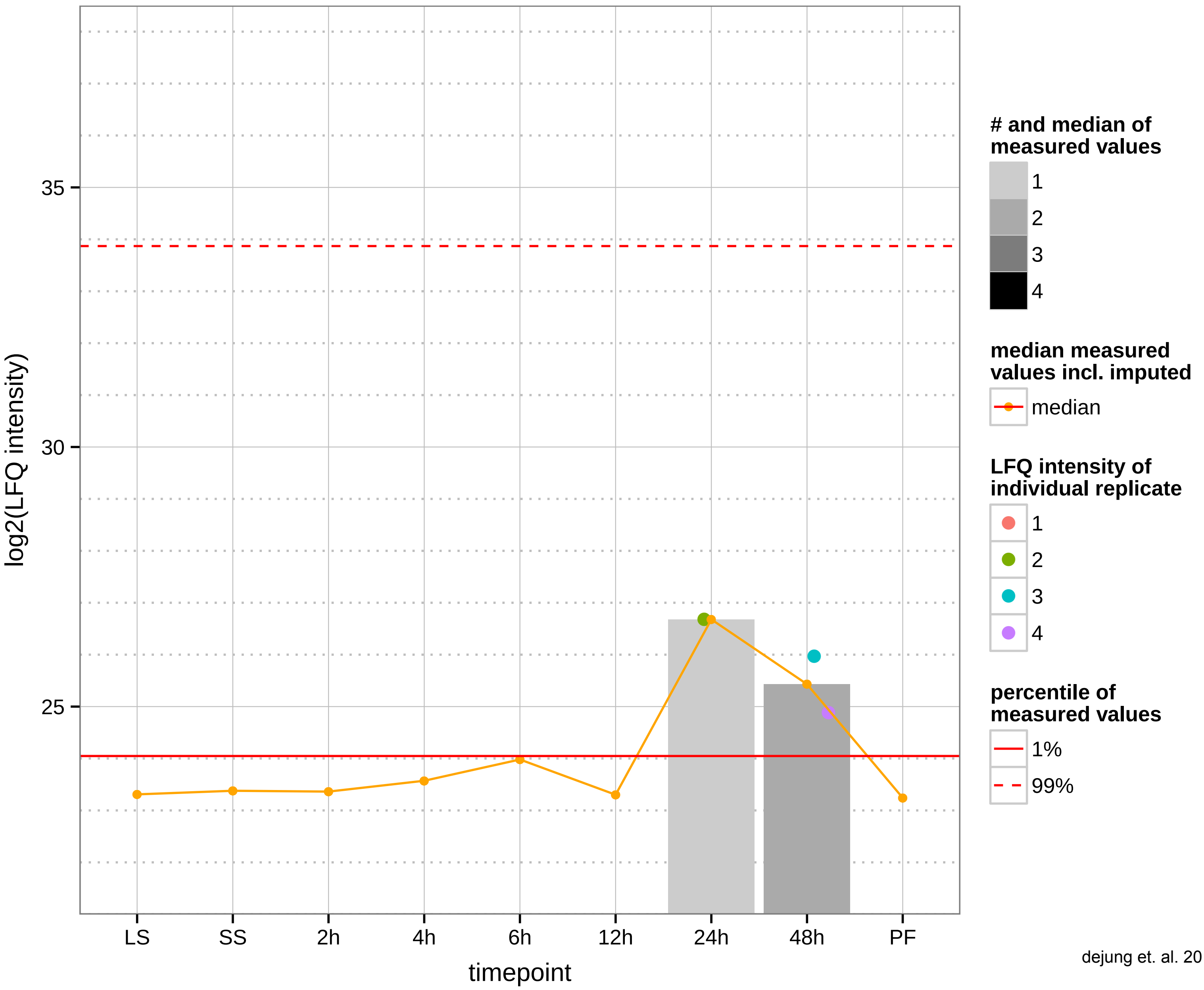

dejung et. al. 2015

hypothetical protein, conserved
Tb927.4.510
AGOF: null
AGOC: null
AGOP: null
PGOF: null
PGOC: null
PGOP: null

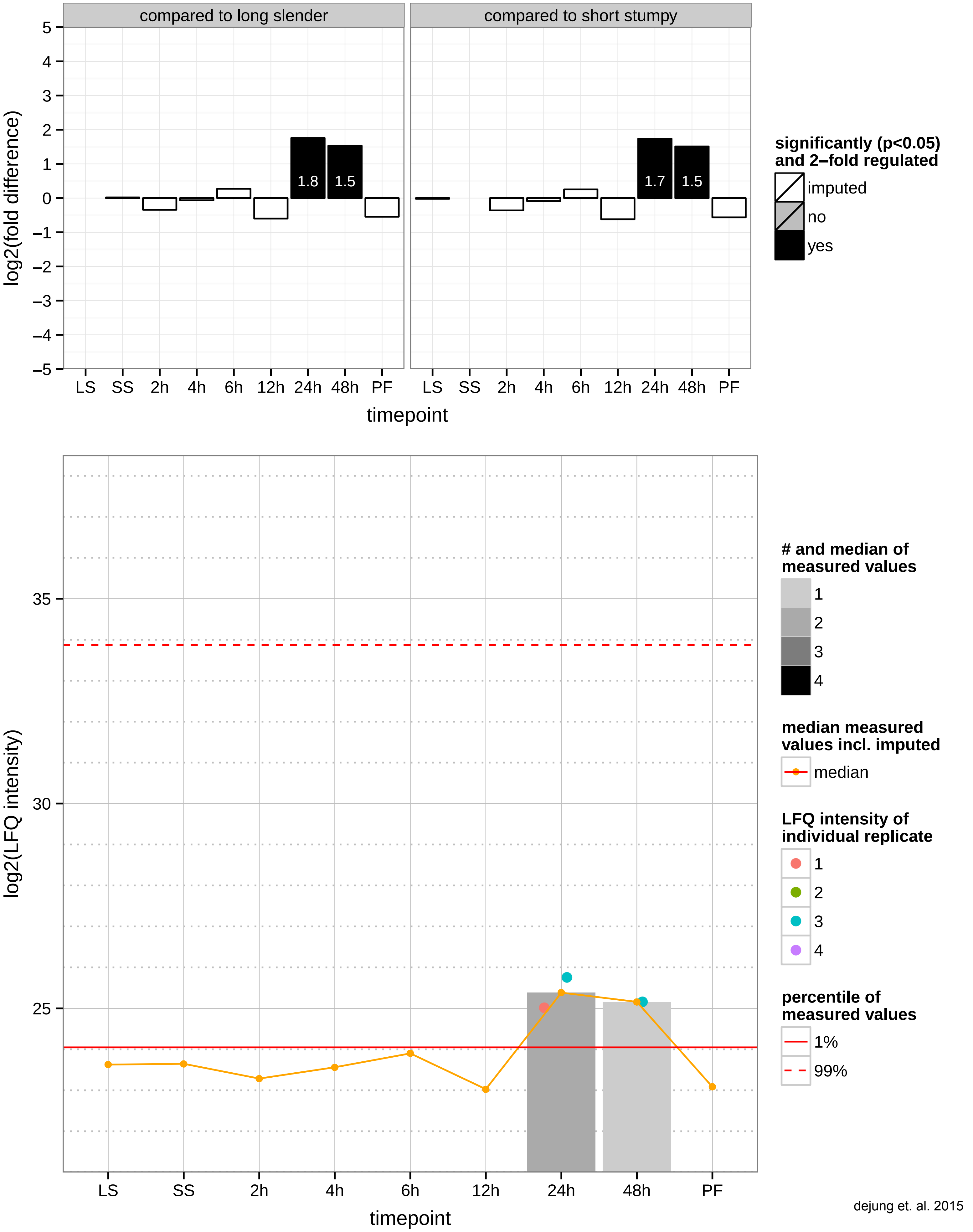

dejung et. al. 2015

hypothetical protein, conserved
Tb927.5.2580
AGOF: null
AGOC: mitochondrion
AGOP: null
PGOF: null
PGOC: null
PGOP: null

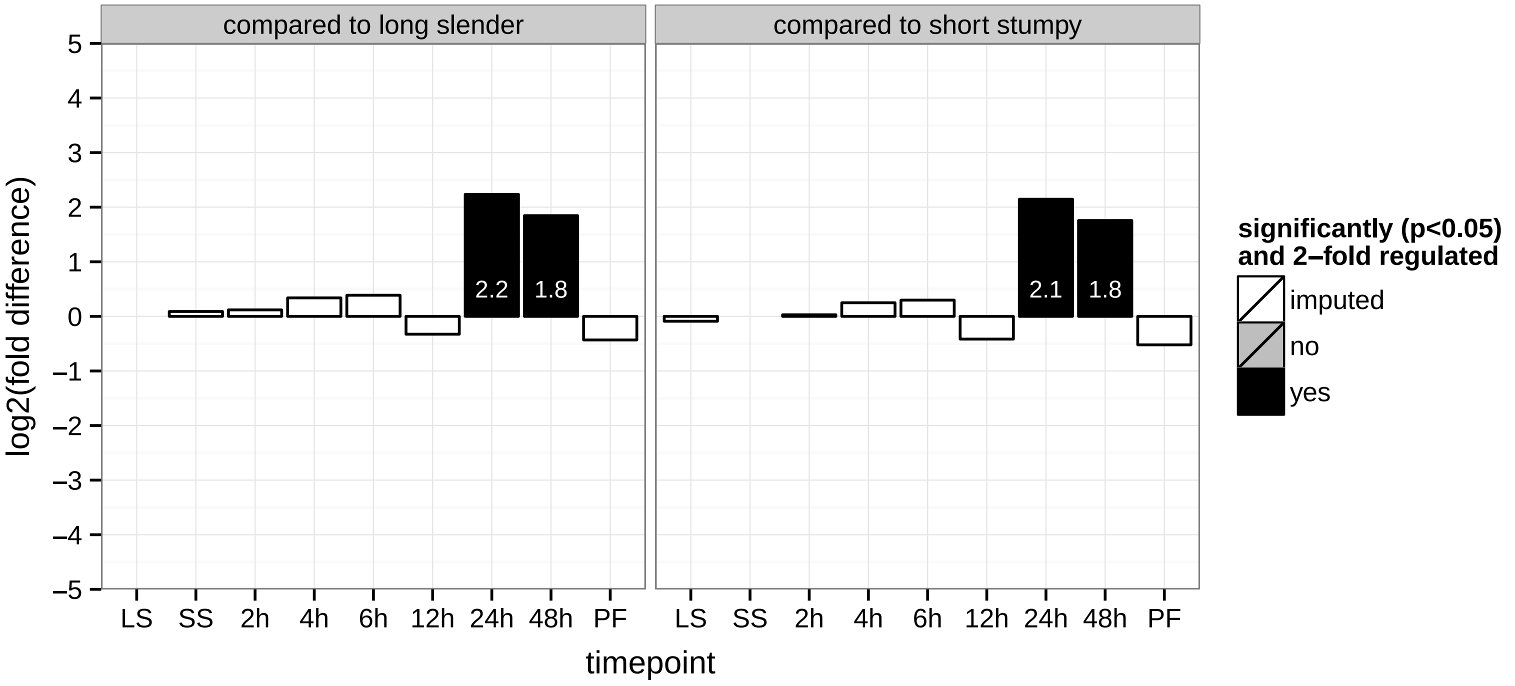

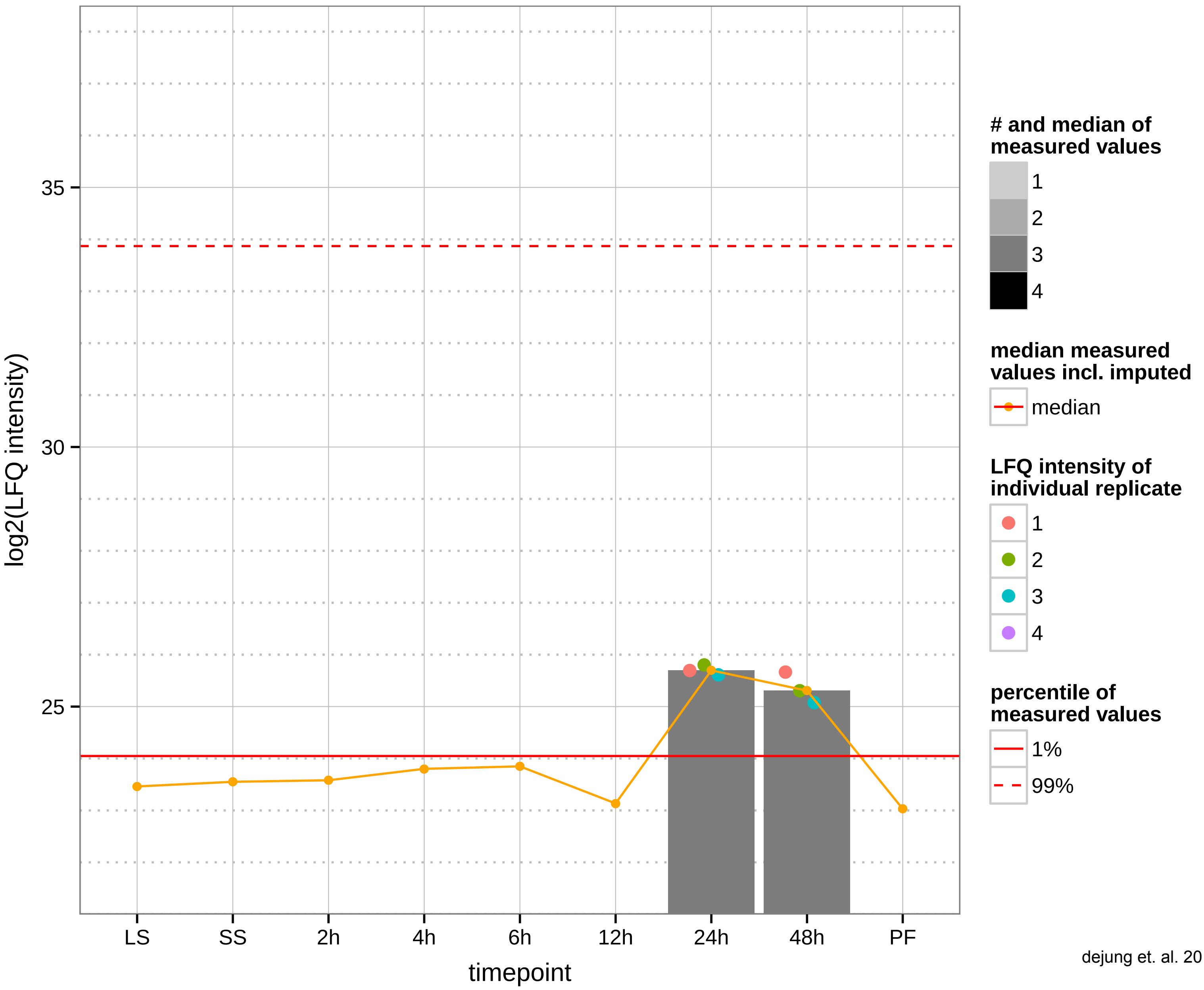

dejung et. al. 2015

MIX protein
Tb927.5.3040
AGOF: null
AGOC: cytochrome complex, mitochondrial inner membrane, mitochondrion
AGOP: electron transport chain, mitochondrial respiratory chain complex IV assembly
PGOF: null
PGOC: null
PGOP: null

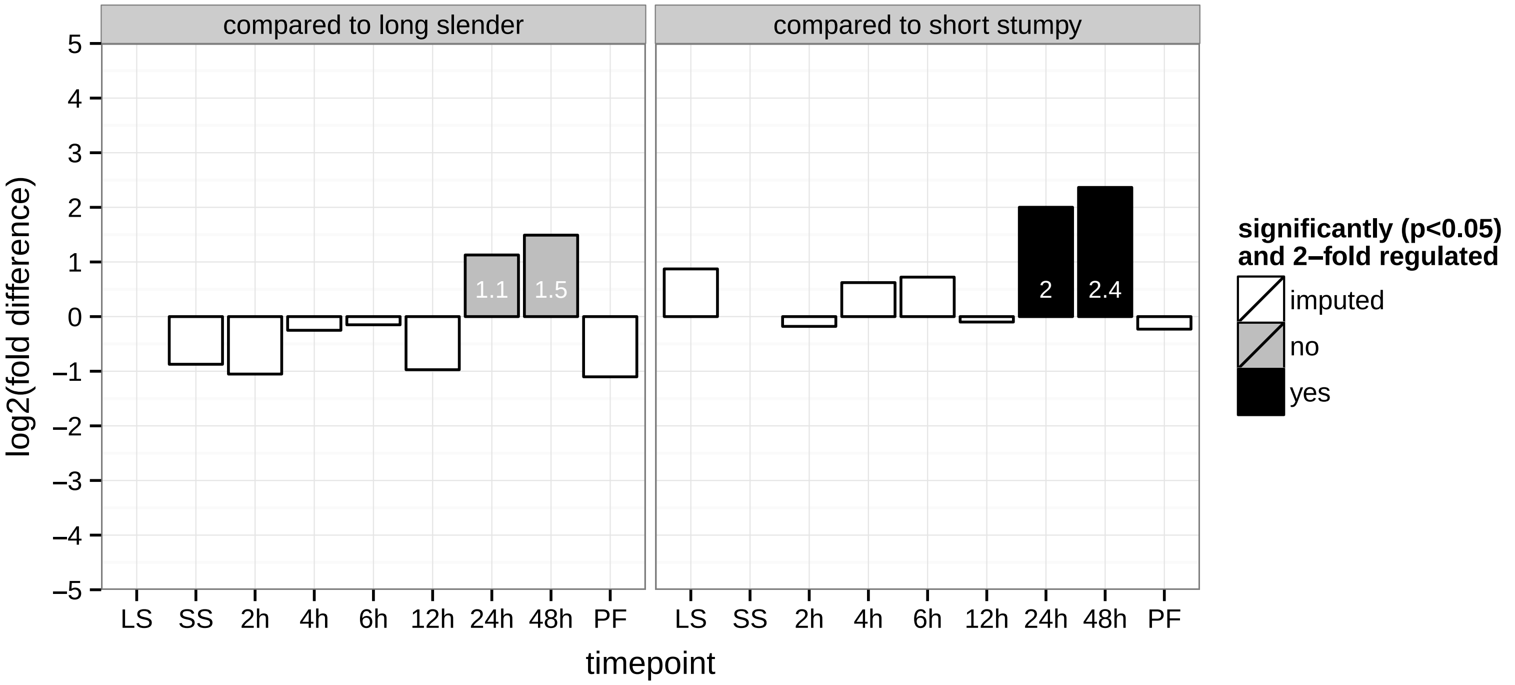

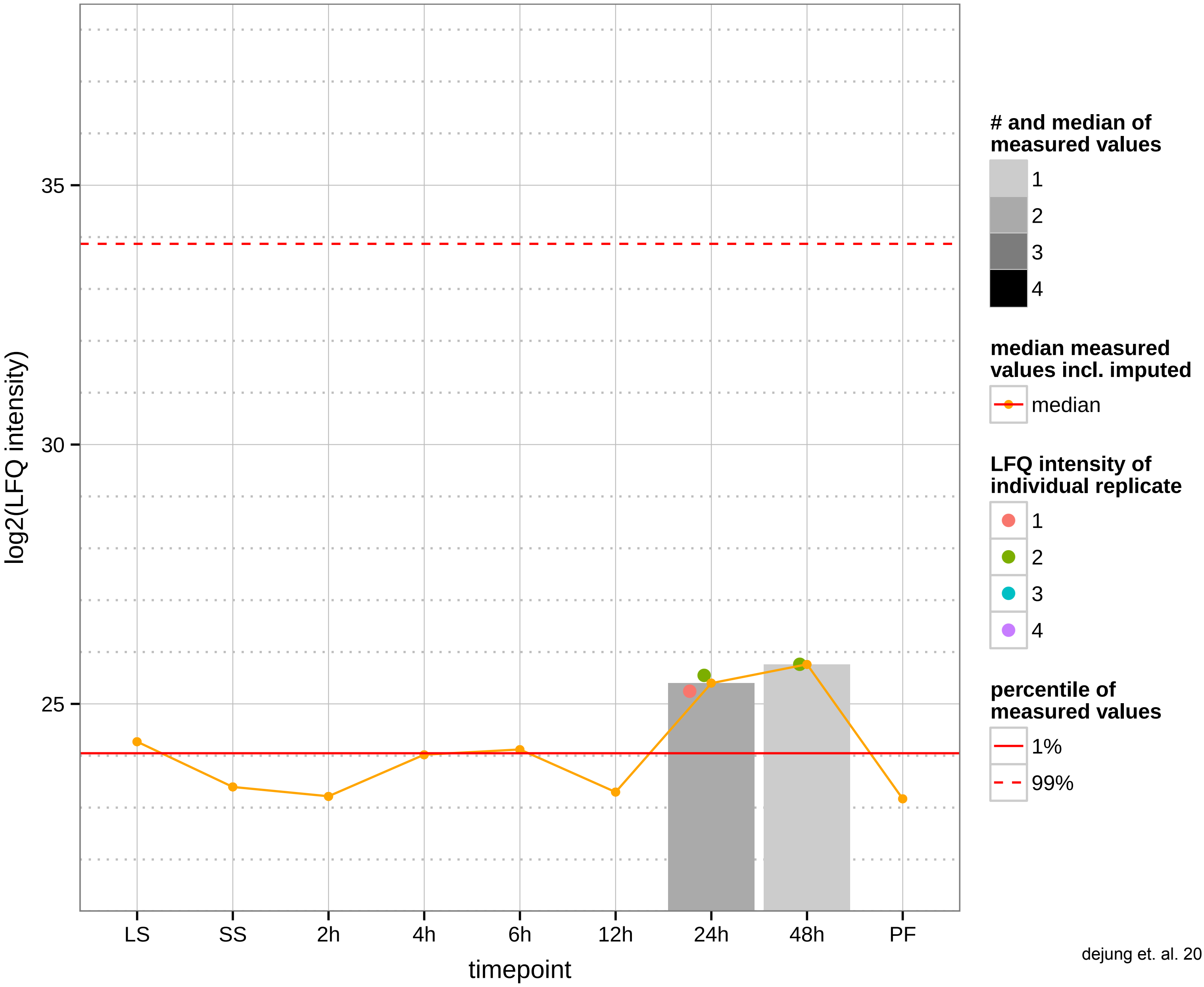

dejung et. al. 2015

U6 snRNA–associated Sm–like protein LSm7p (TbLSm7)
Tb927.5.4030
AGOF: null
AGOC: U6 snRNP, nucleolus, nucleus
AGOP: maturation of SSU–rRNA, nuclear mRNA splicing, via spliceosome, nuclear–transcribed mRNA catabolic process
PGOF: null
PGOC: null
PGOP: null

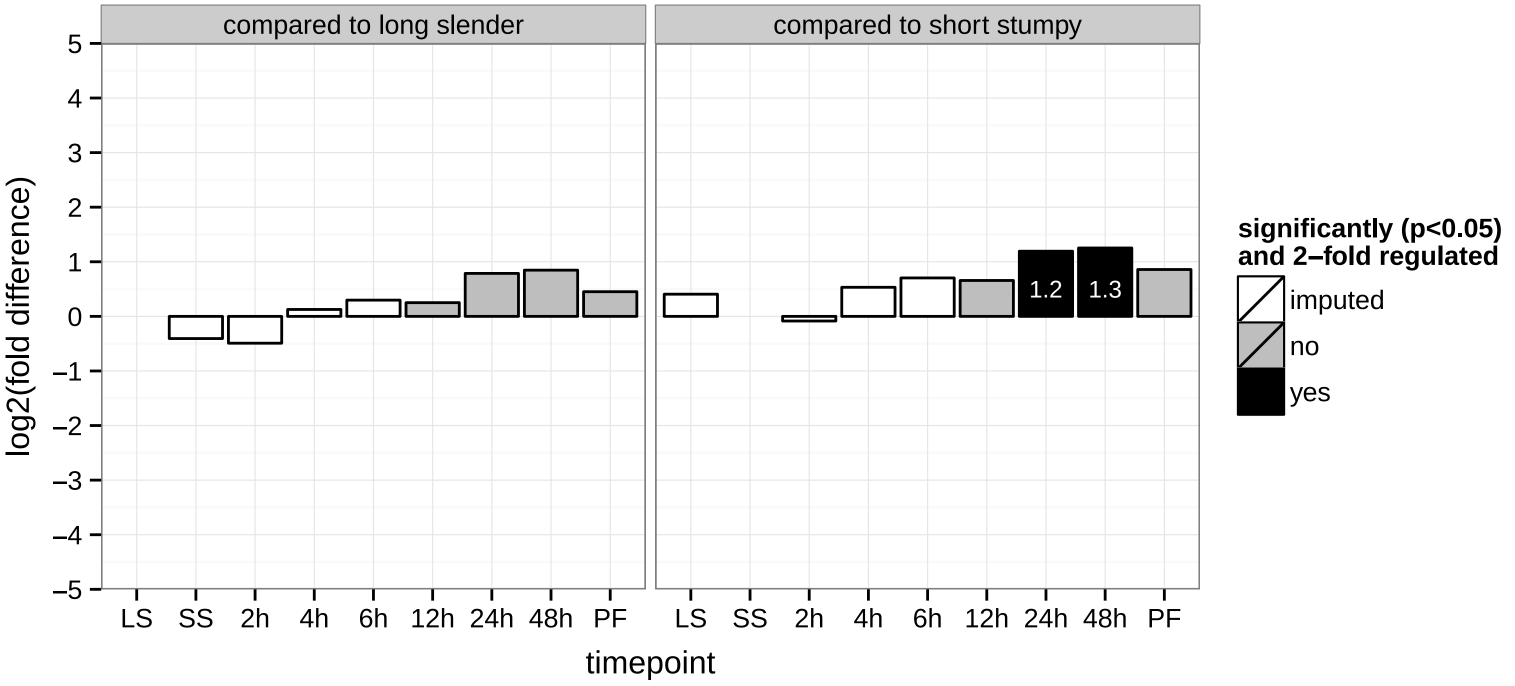

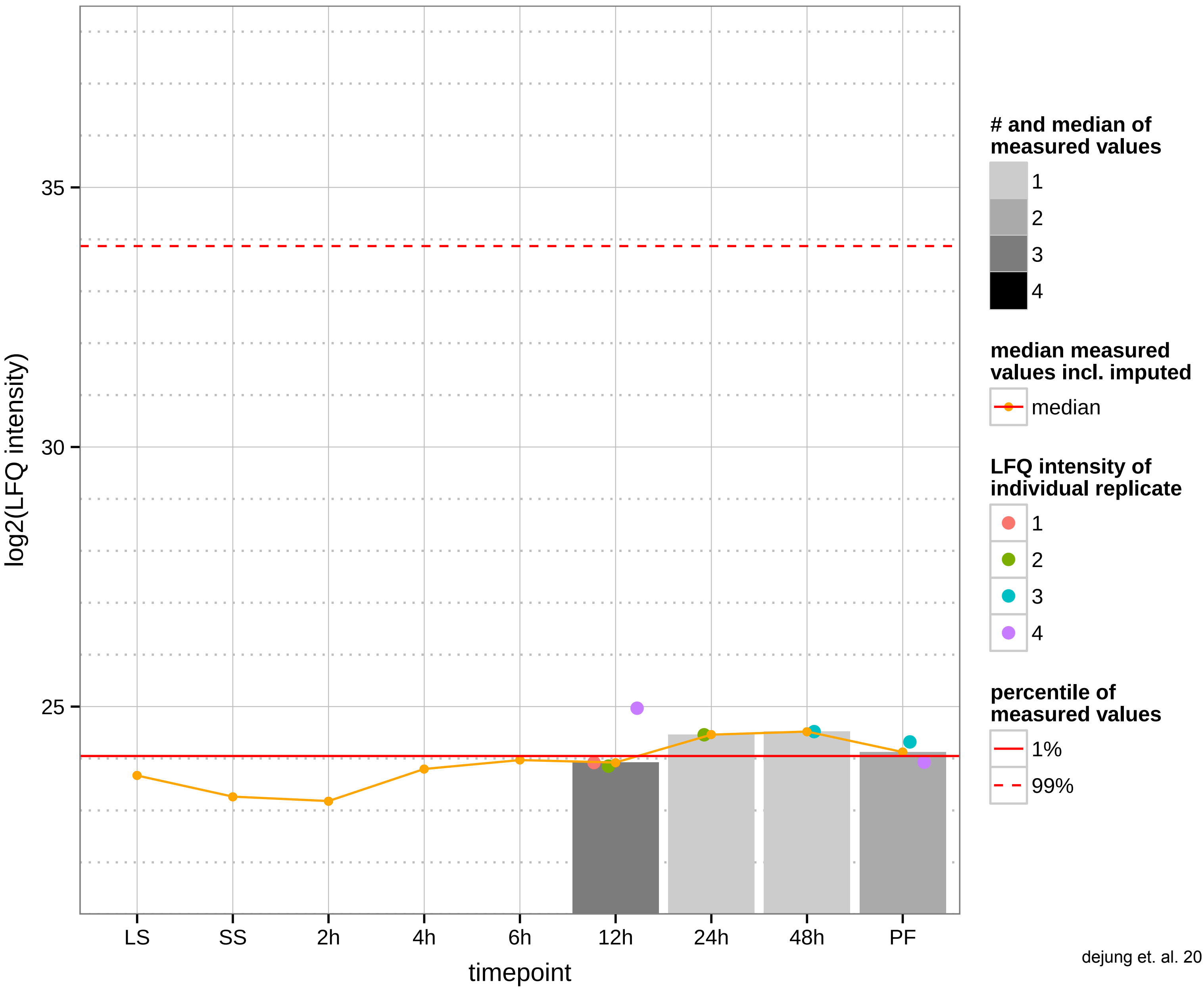

dejung et. al. 2015

protein phosphatase 1, regulatory subunit, putative
Tb927.5.590
AGOF: null
AGOC: null
AGOP: null
PGOF: protein binding
PGOC: null
PGOP: null

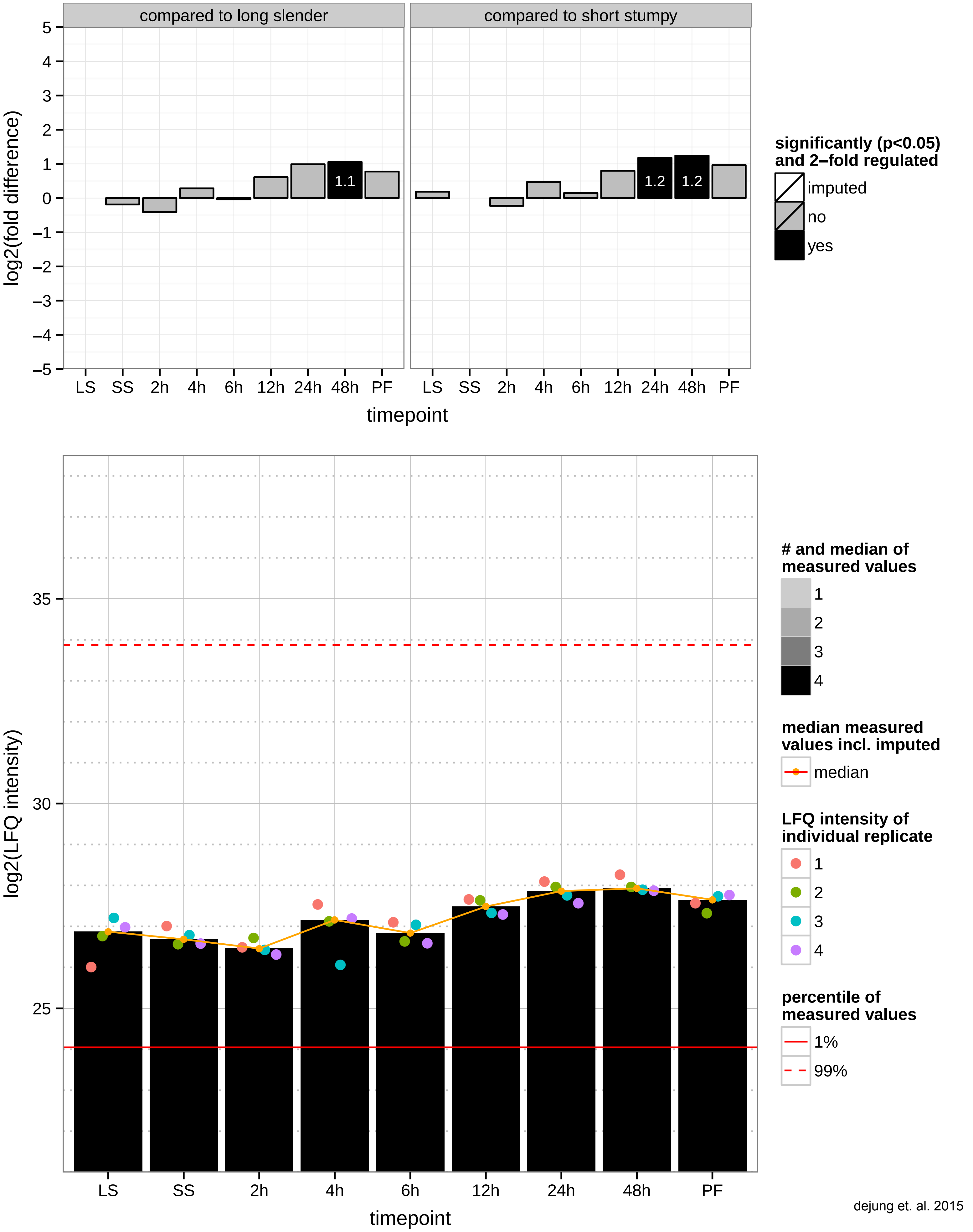

dejung et. al. 2015

hypothetical protein, conserved
Tb927.5.980
AGOF: oxidoreductase activity
AGOC: null
AGOP: null
PGOF: oxidoreductase activity, oxidoreductase activity, acting on paired donors, with incorporation or reduction of molecular o
PGOC: null
PGOP: oxidation−reduction process

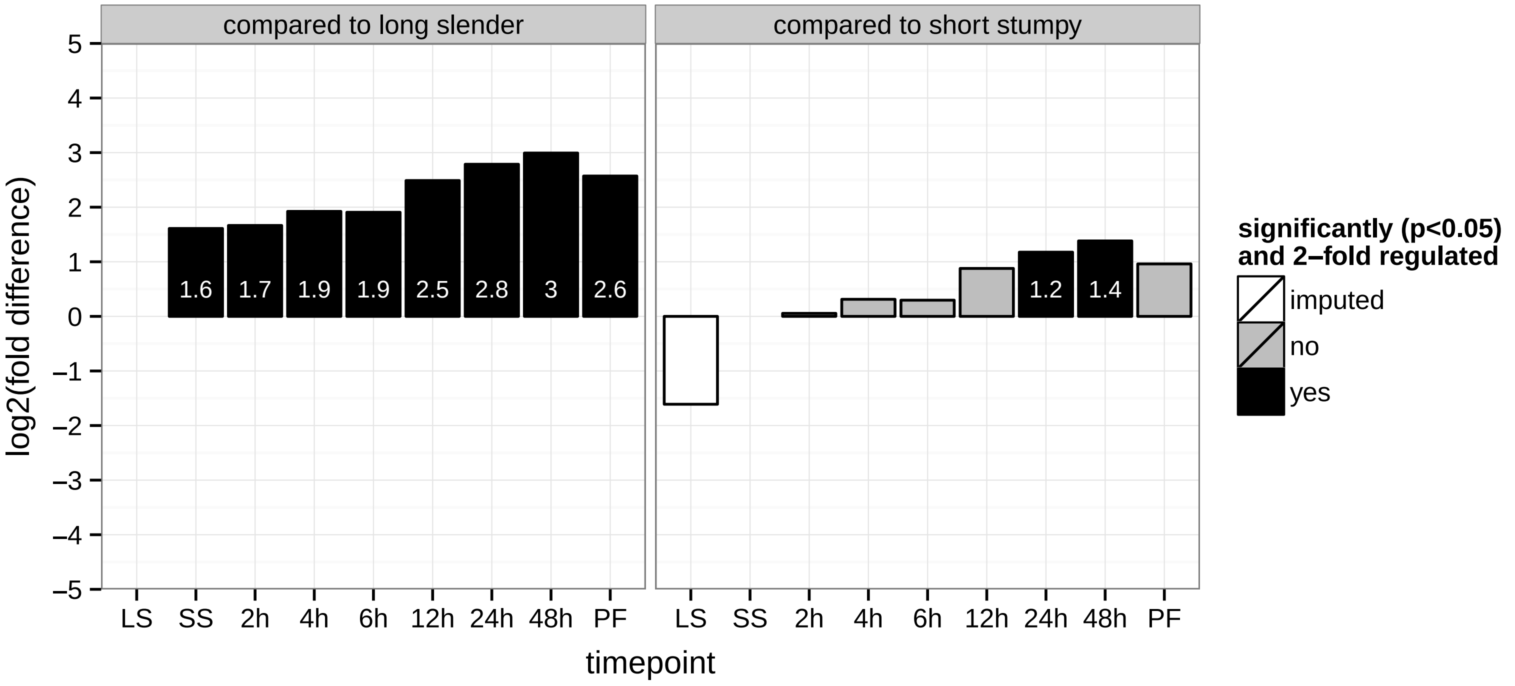

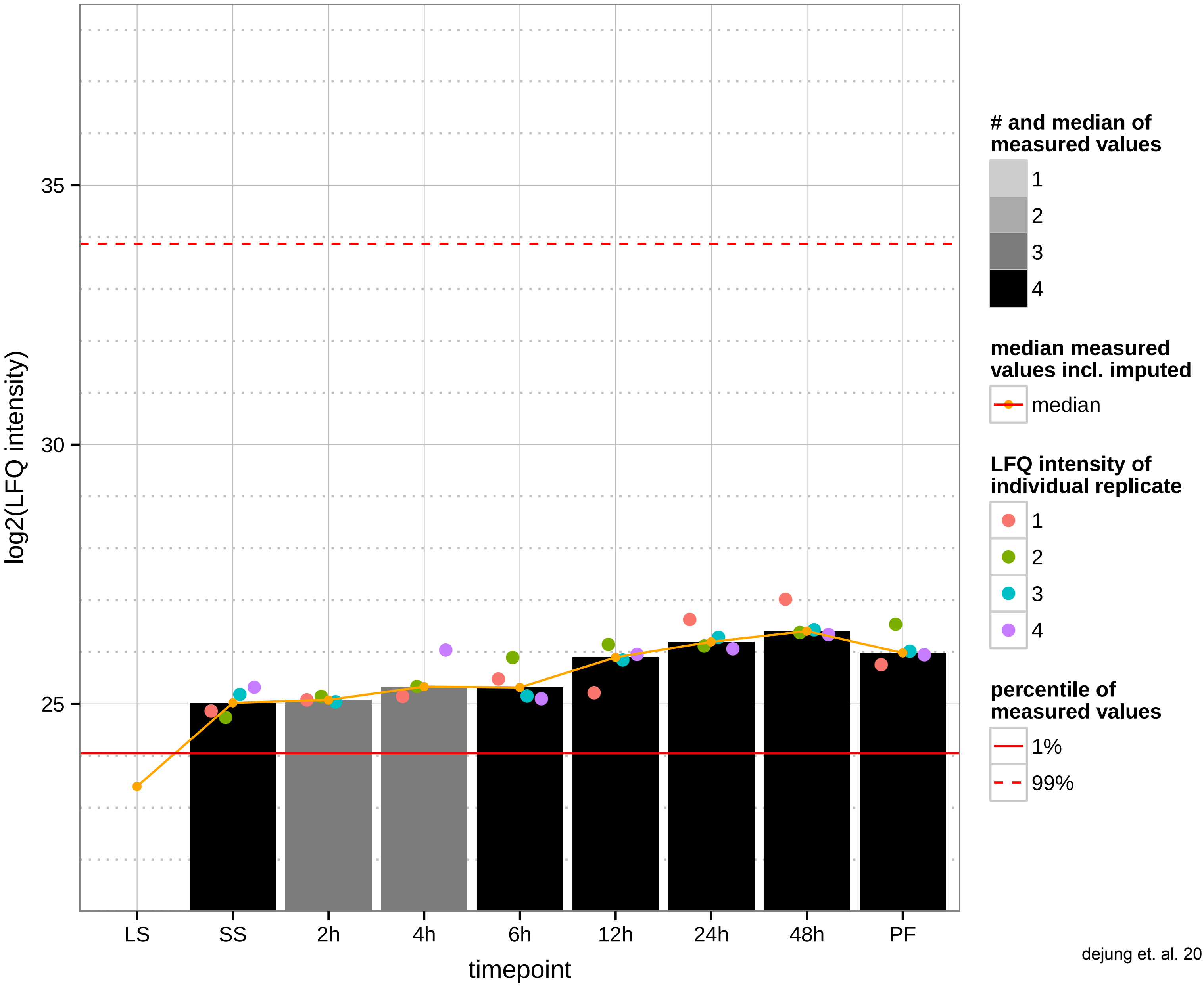

dejung et. al. 2015

hypothetical protein, conserved
Tb927.6.1910
AGOF: null
AGOC: null
AGOP: null
PGOF: null
PGOC: null
PGOP: null

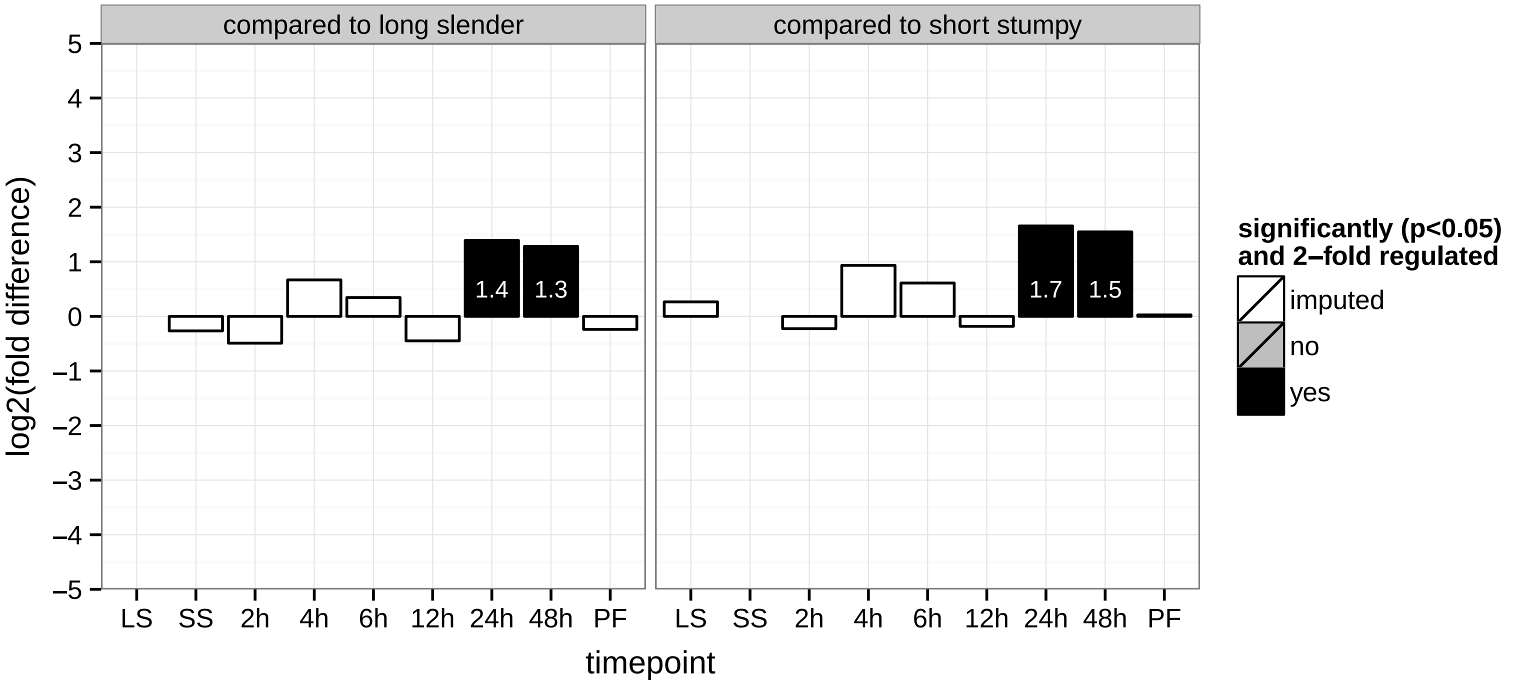

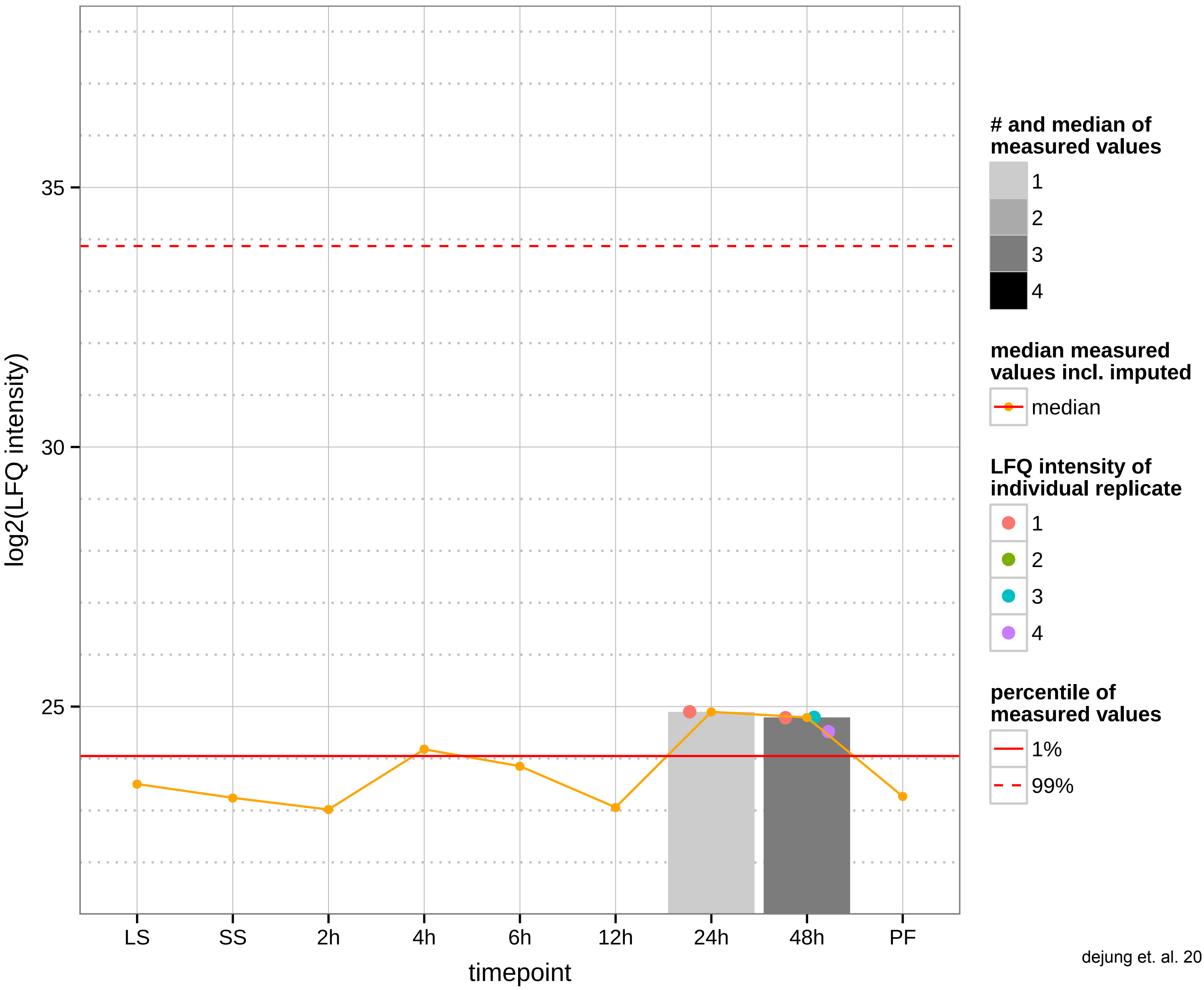

dejung et. al. 2015

hypothetical protein, conserved
Tb927.6.3530
AGOF: null
AGOC: membrane
AGOP: transport
PGOF: null
PGOC: null
PGOP: null

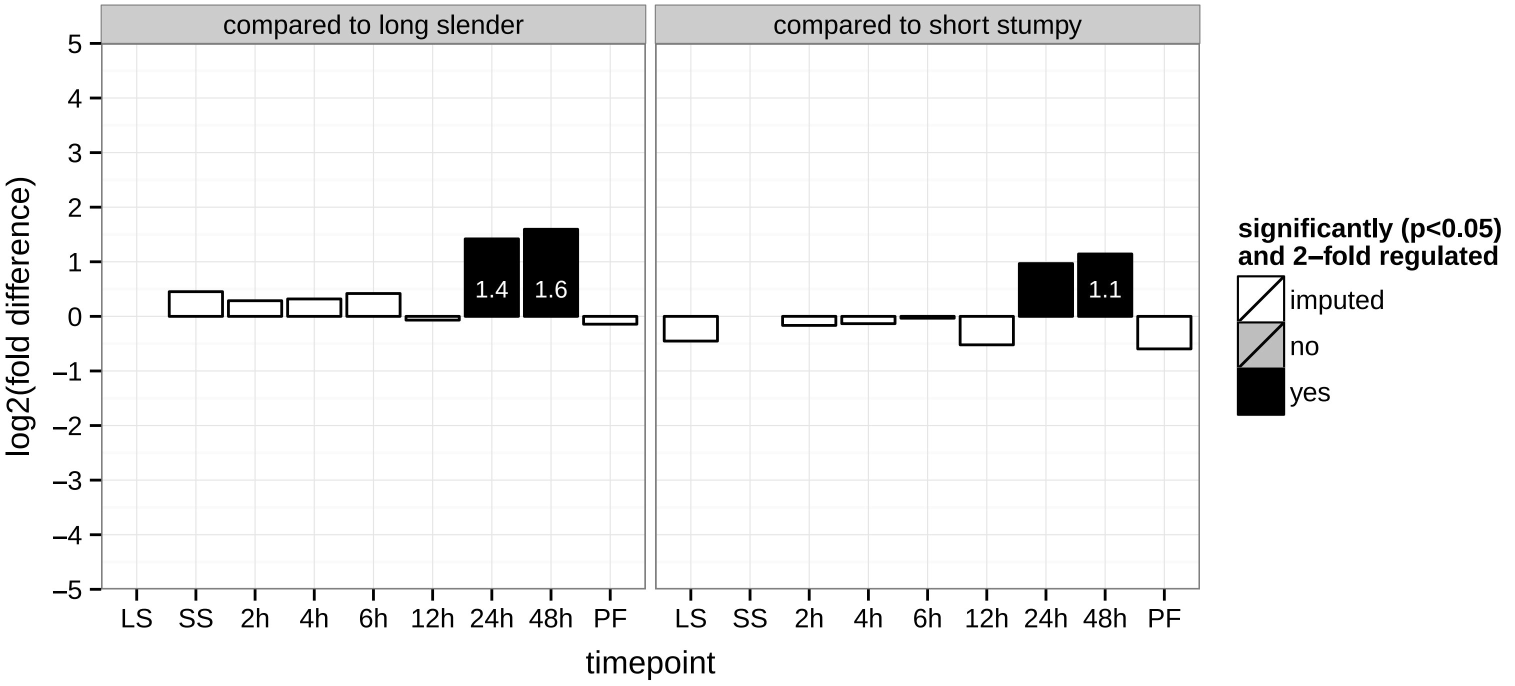

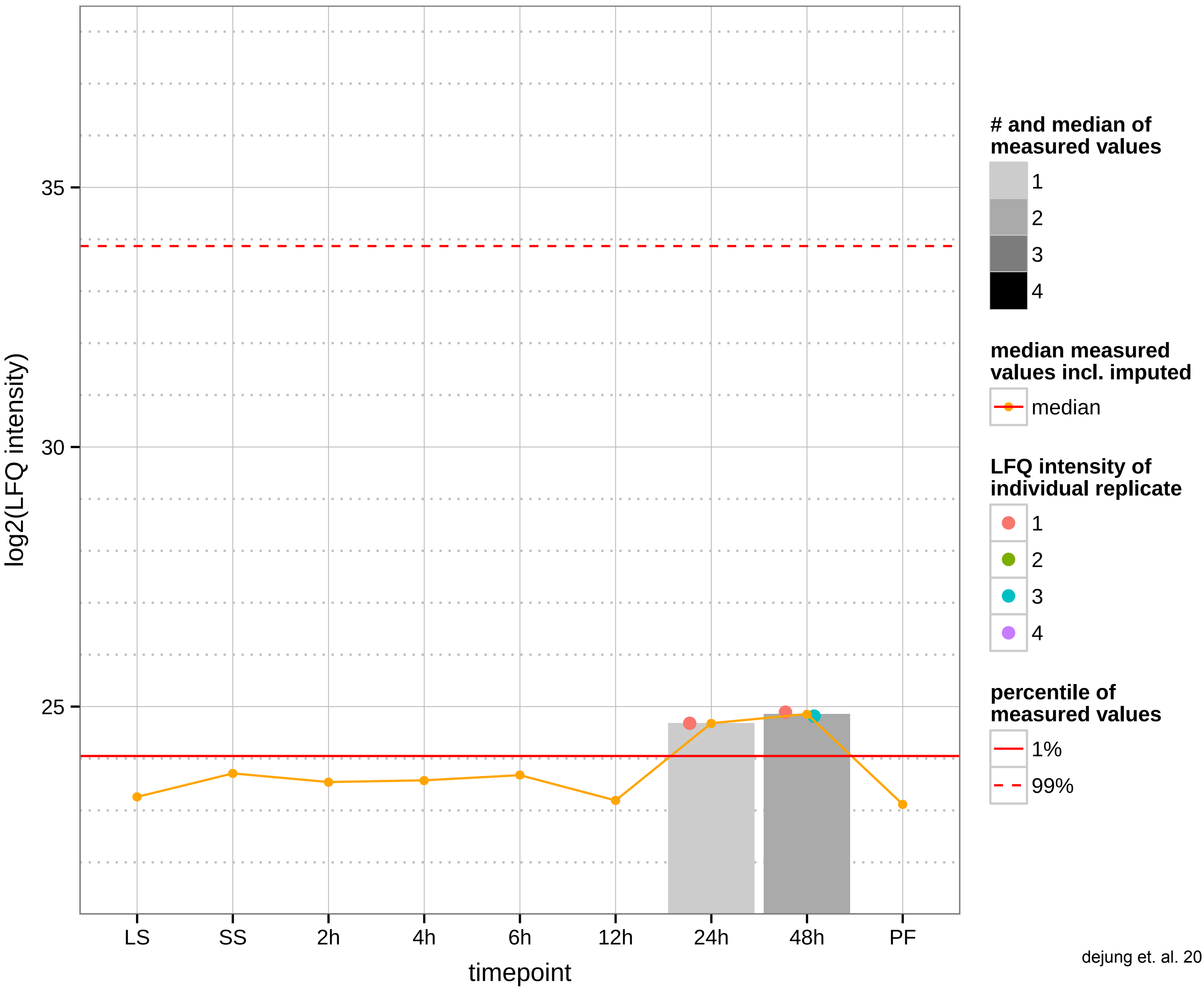

dejung et. al. 2015

tubulin–tyrosine ligase–like protein, conserved
Tb927.6.3570
AGOF: tubulin–tyrosine ligase activity
AGOC: null
AGOP: cellular protein modification process
PGOF: tubulin–tyrosine ligase activity
PGOC: null
PGOP: cellular protein modification process

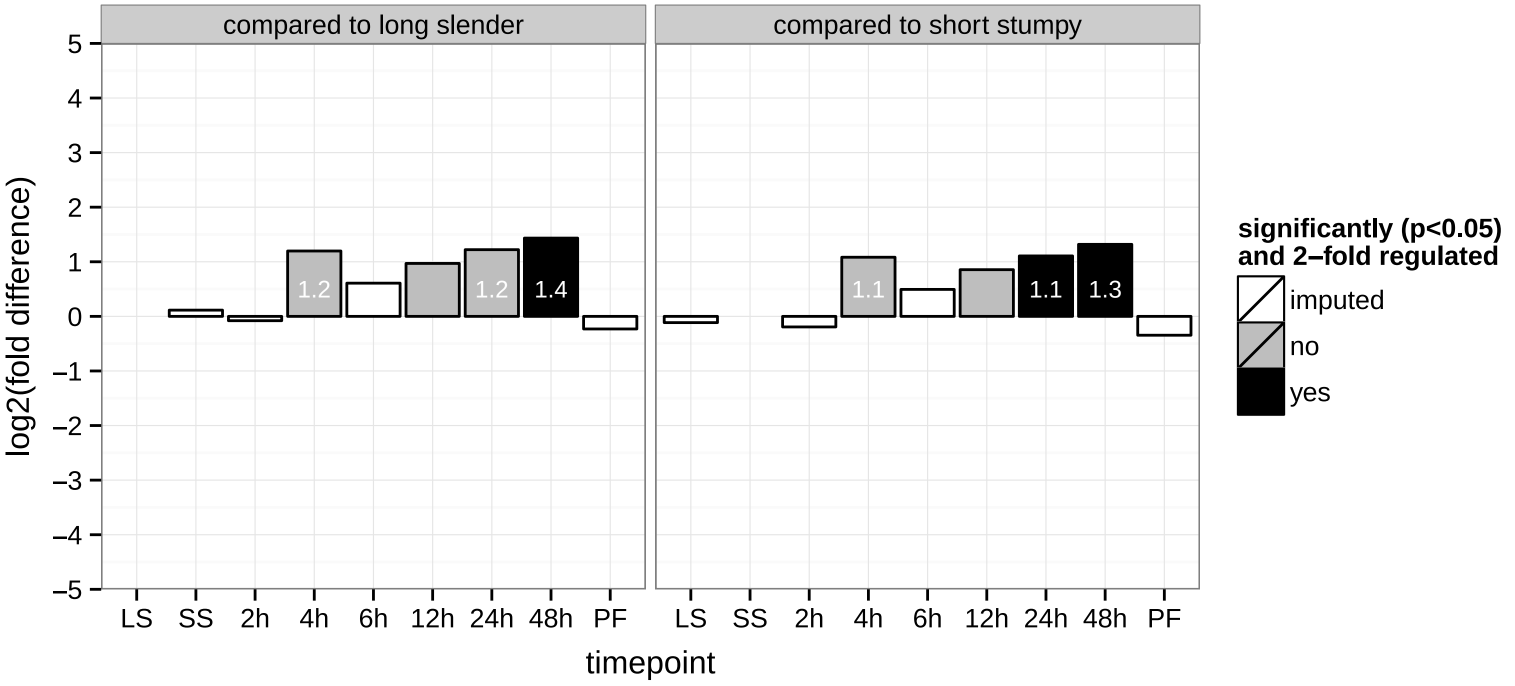

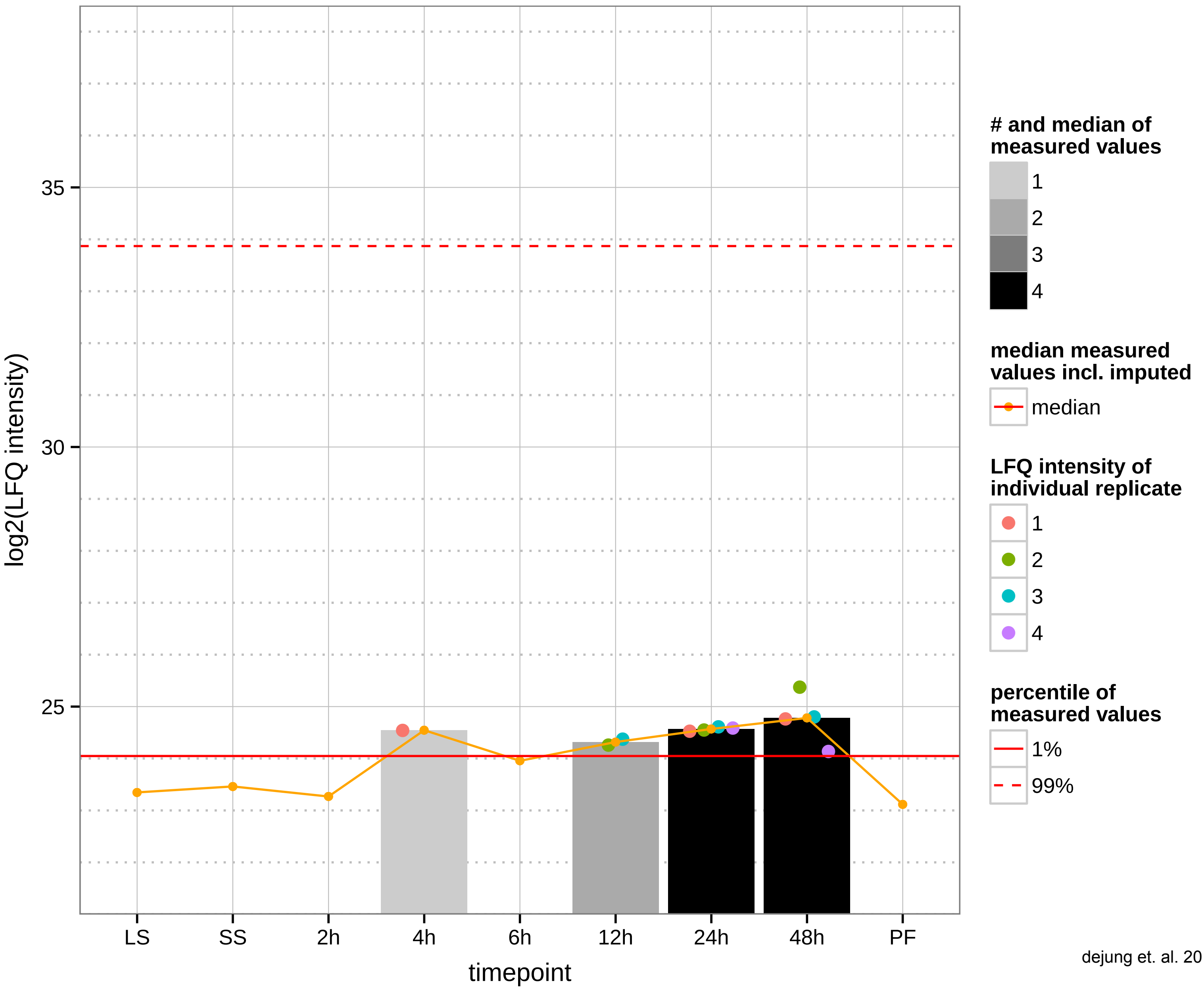

dejung et. al. 2015

hypothetical protein, conserved
Tb927.6.3600
AGOF: null
AGOC: mitochondrion
AGOP: null
PGOF: null
PGOC: null
PGOP: null

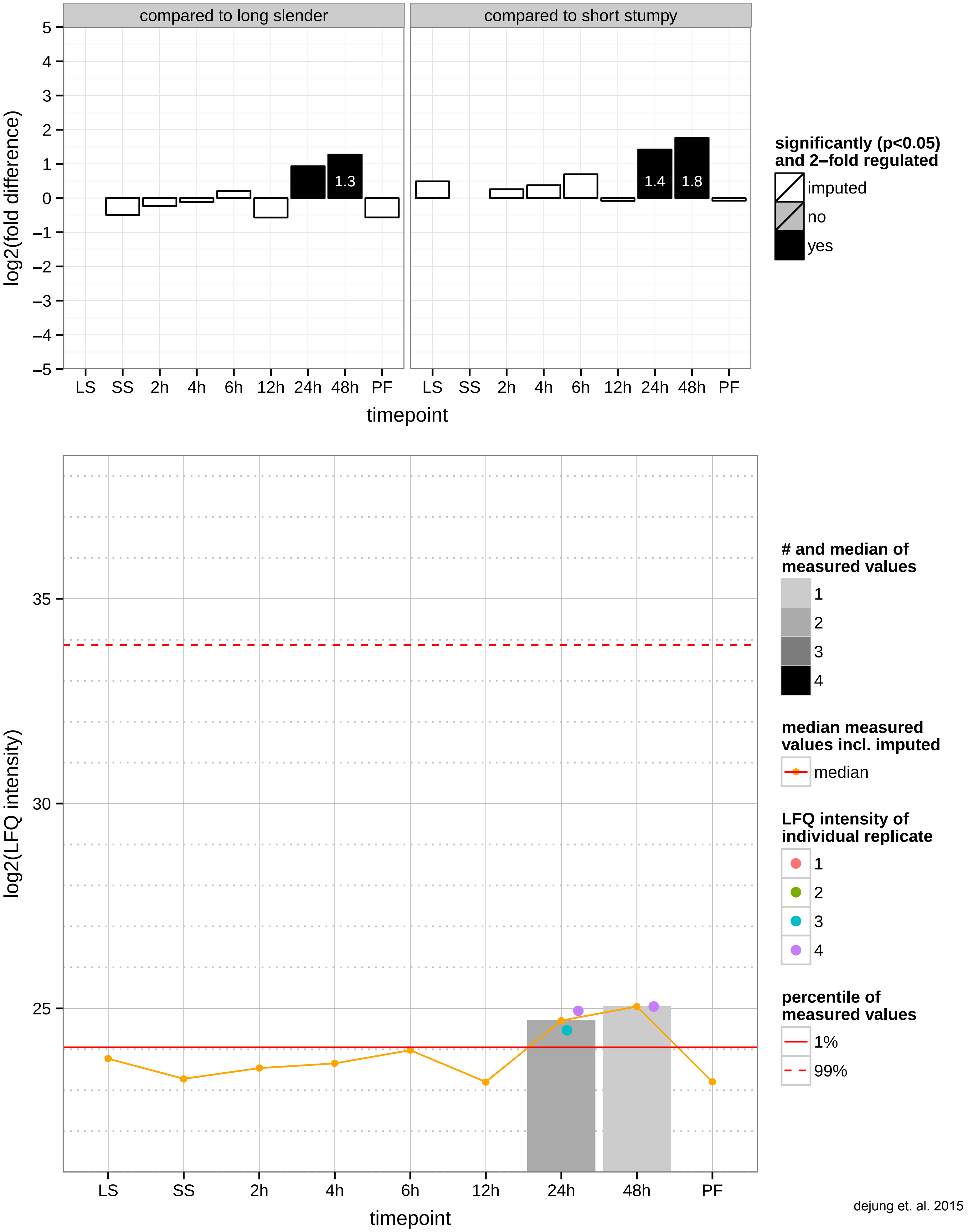

dejung et. al. 2015

S–adenosylmethionine synthetase, putative (METK1)
Tb927.6.4920;Tb927.6.4910;Tb927.6.4900;Tb927.6.4880;Tb927.6.4860;Tb927.6.4850;Tb927.6.4840;Tb927.6.4890;Tb927.6
AGOF: ATP binding, methionine adenosyltransferase activity
AGOC: null
AGOP: one–carbon metabolic process
PGOF: null, ATP binding, methionine adenosyltransferase activity
PGOC: null
PGOP: null, S–adenosylmethionine biosynthetic process

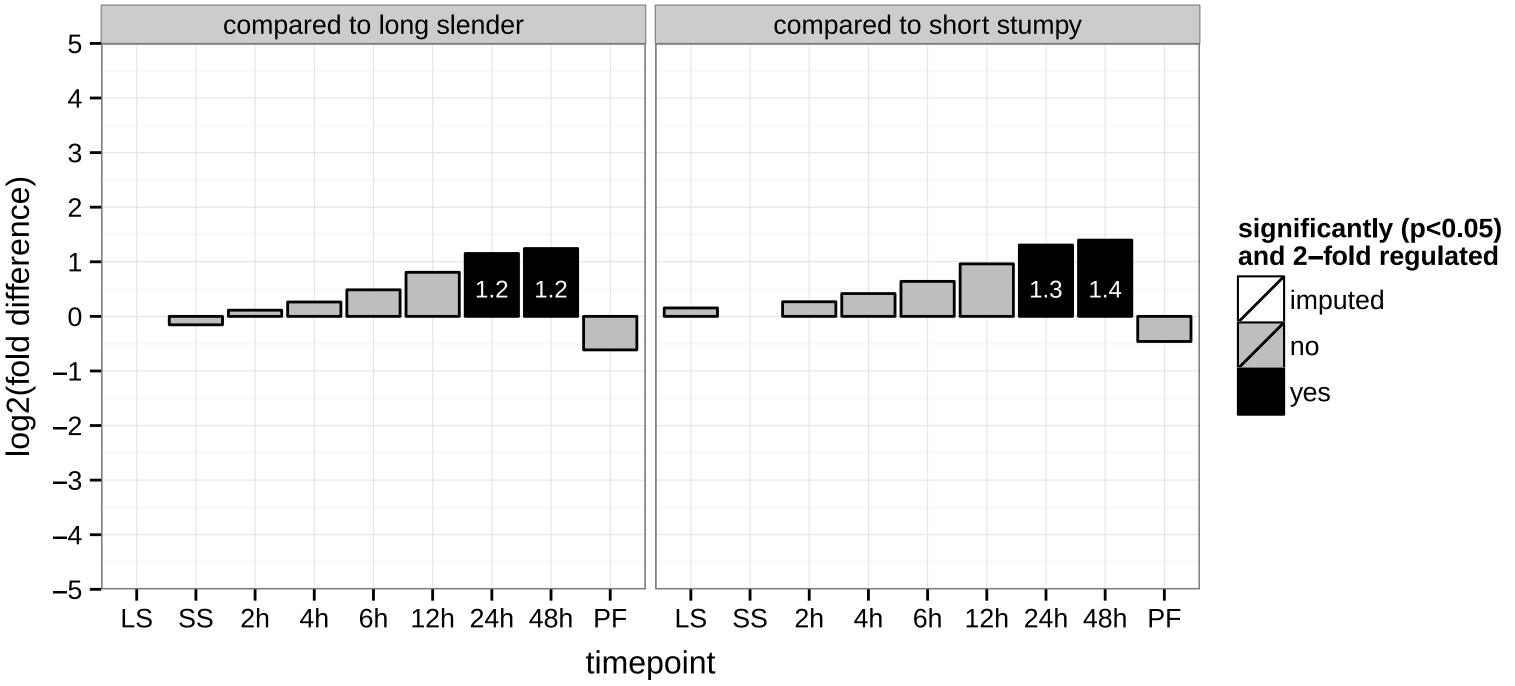

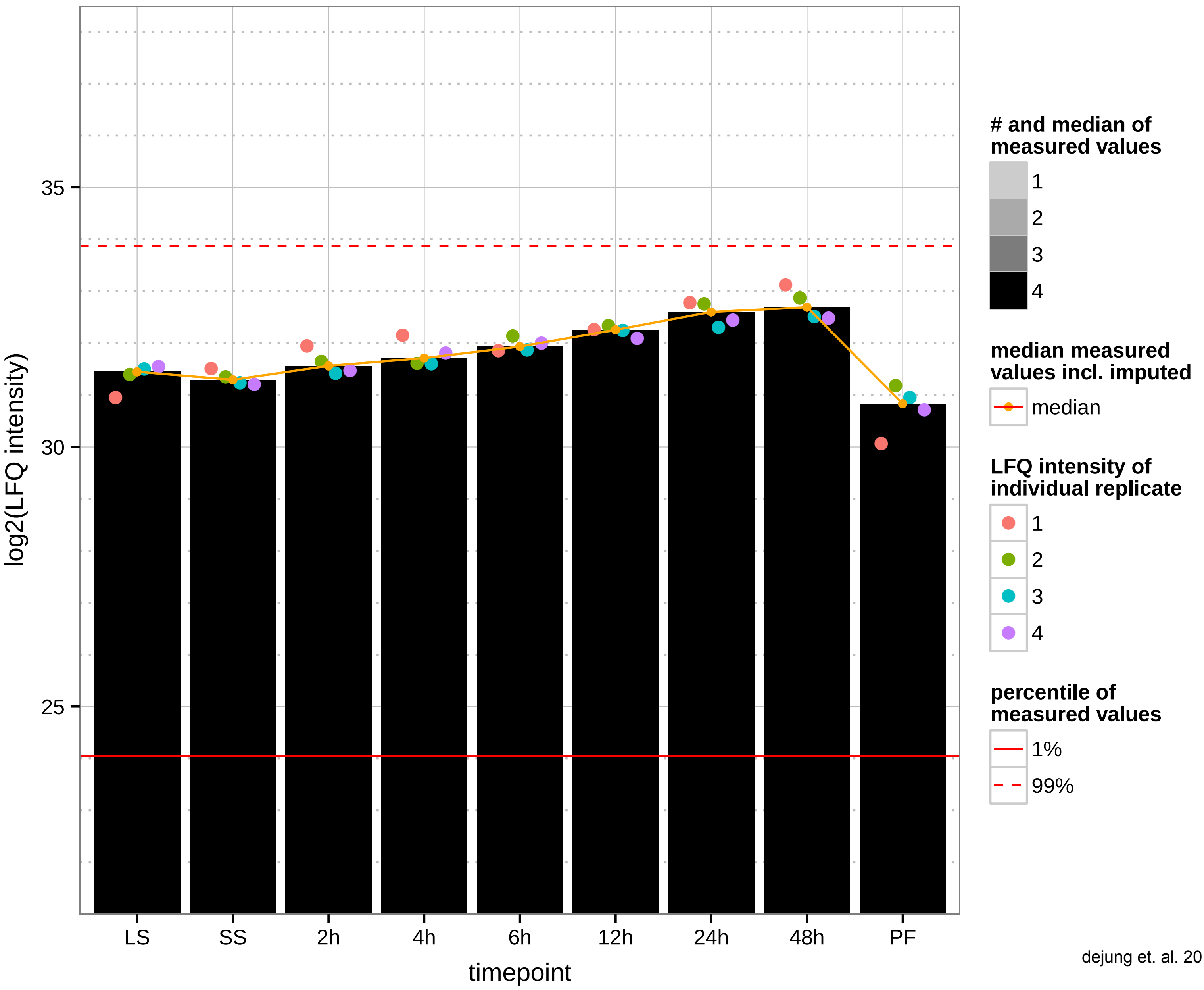

dejung et. al. 2015

Zinc finger CCCH domain–containing protein 47 (zc3h47)
Tb927.6.4960
AGOF: RNA binding, nucleic acid binding, zinc ion binding
AGOC: null
AGOP: null
PGOF: nucleic acid binding, zinc ion binding
PGOC: null
PGOP: null

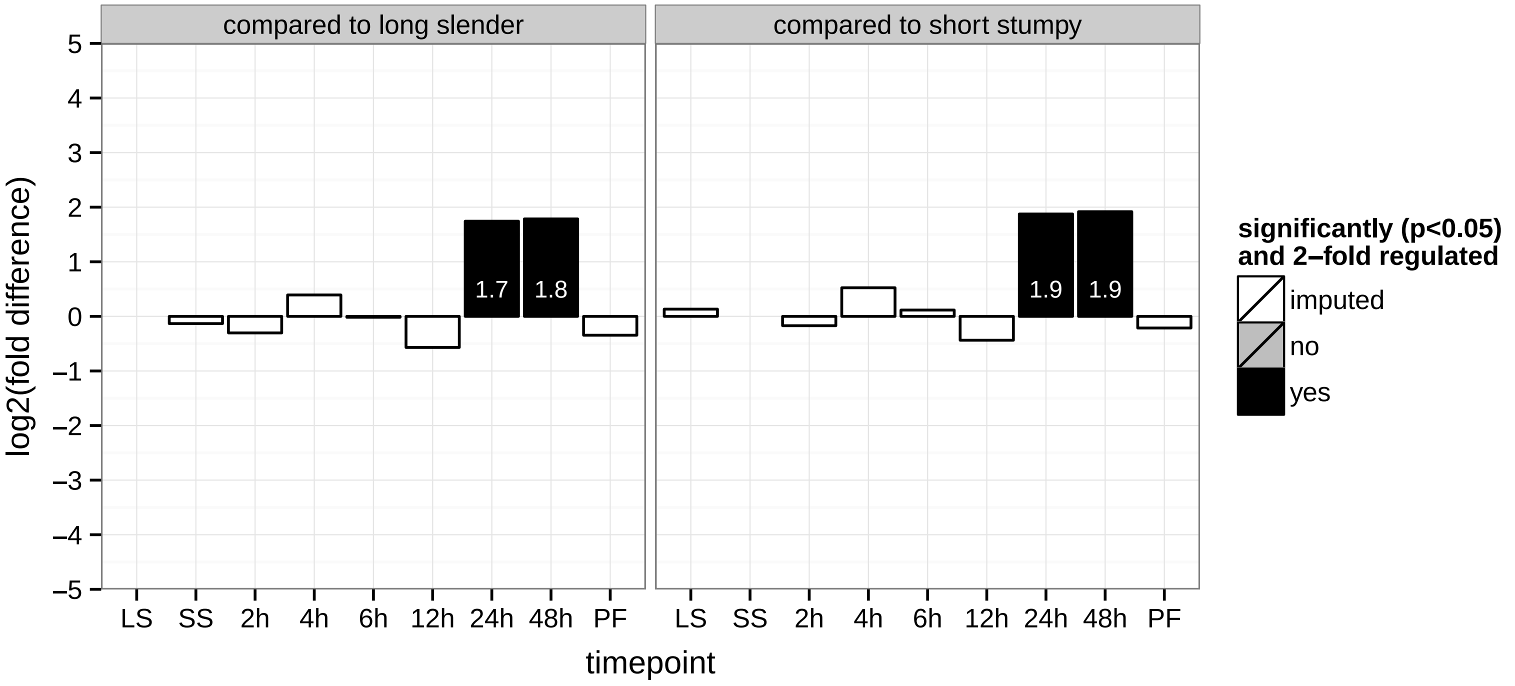

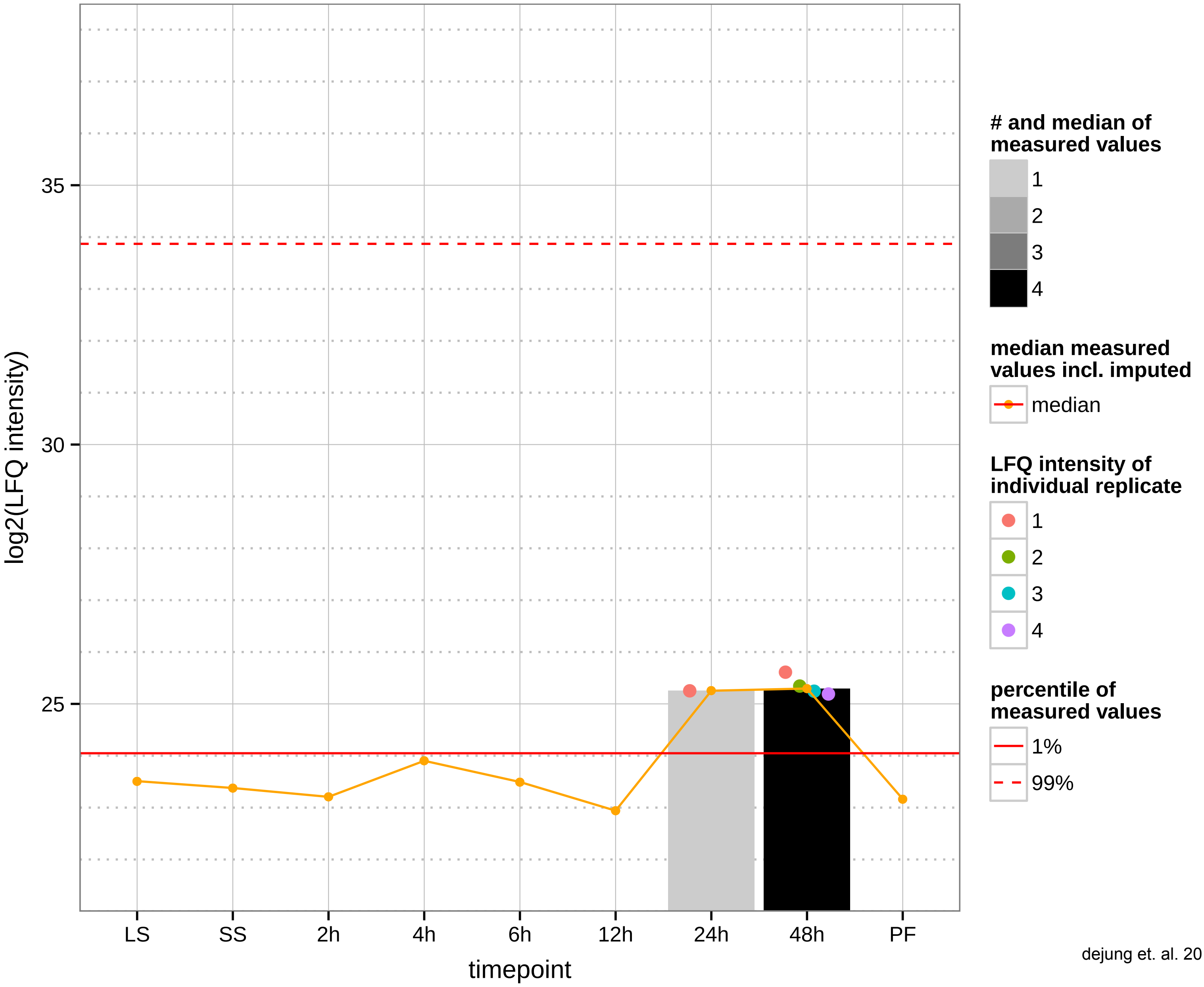

dejung et. al. 2015

40S ribosomal protein S33, putative
Tb927.7.240;Tb927.7.230
AGOF: structural constituent of ribosome
AGOC: intracellular, ribosome
AGOP: translation
PGOF: null, structural constituent of ribosome
PGOC: null, intracellular, ribosome
PGOP: null, translation

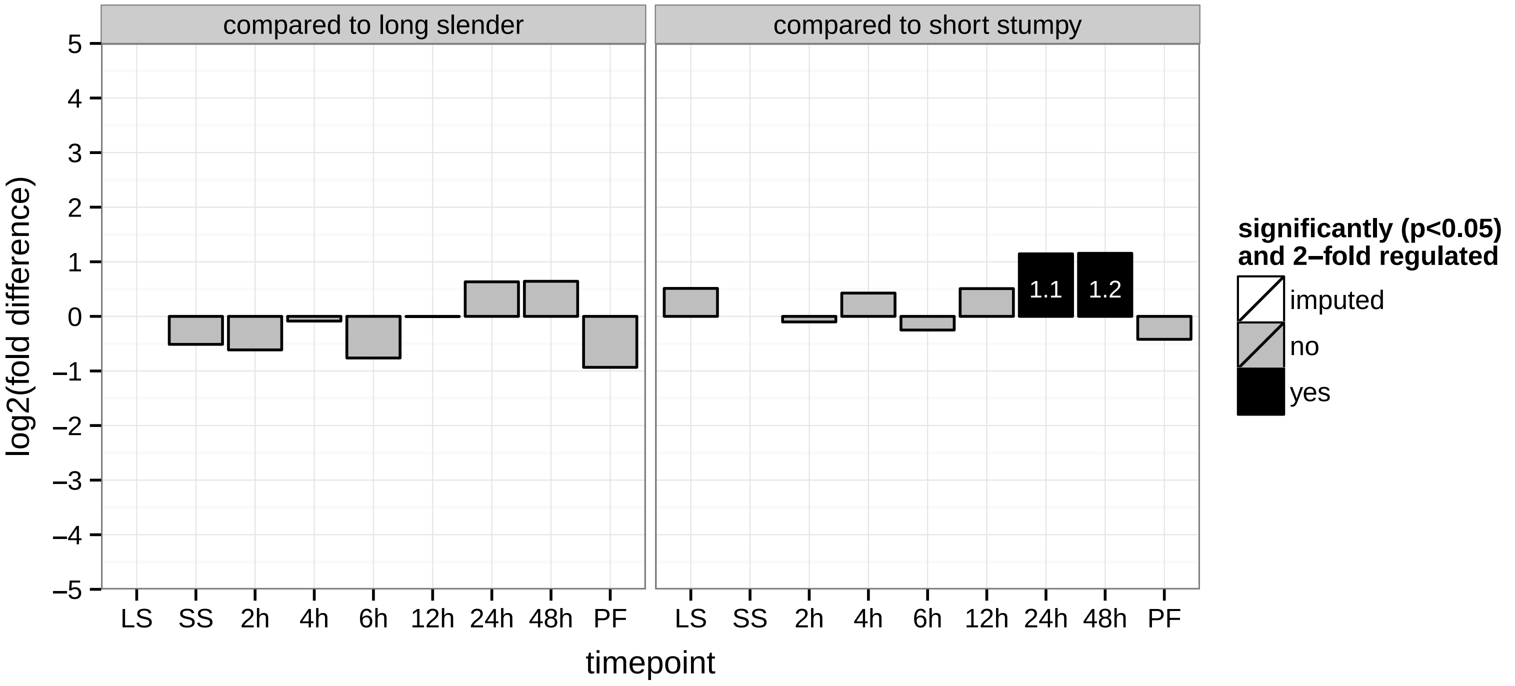

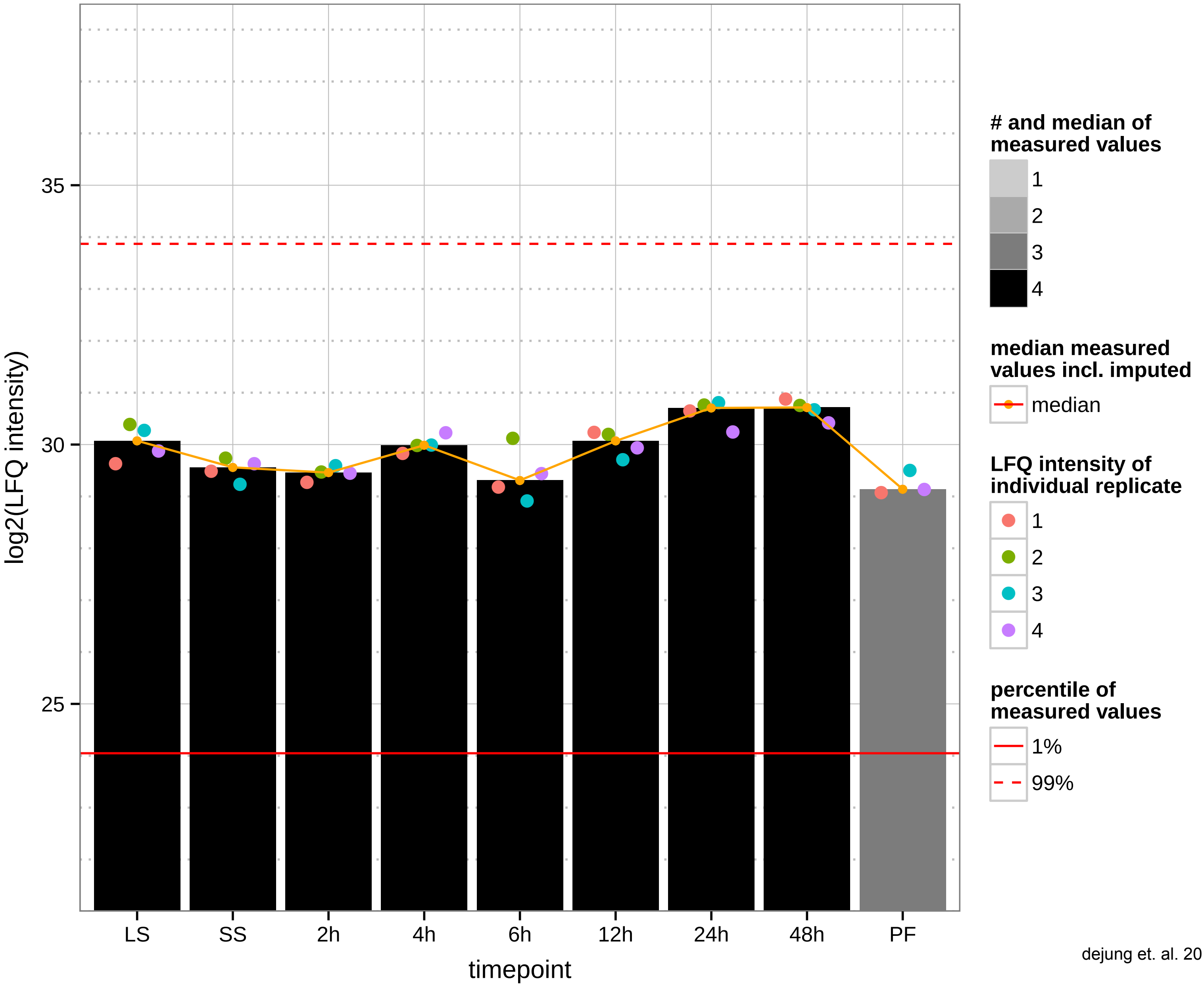

dejung et. al. 2015

hypothetical protein, conserved
Tb927.7.7130
AGOF: FMN binding, iron–sulfur cluster binding, oxidoreductase activity
AGOC: null
AGOP: null
PGOF: FMN binding, catalytic activity, iron–sulfur cluster binding, oxidoreductase activity
PGOC: null
PGOP: null

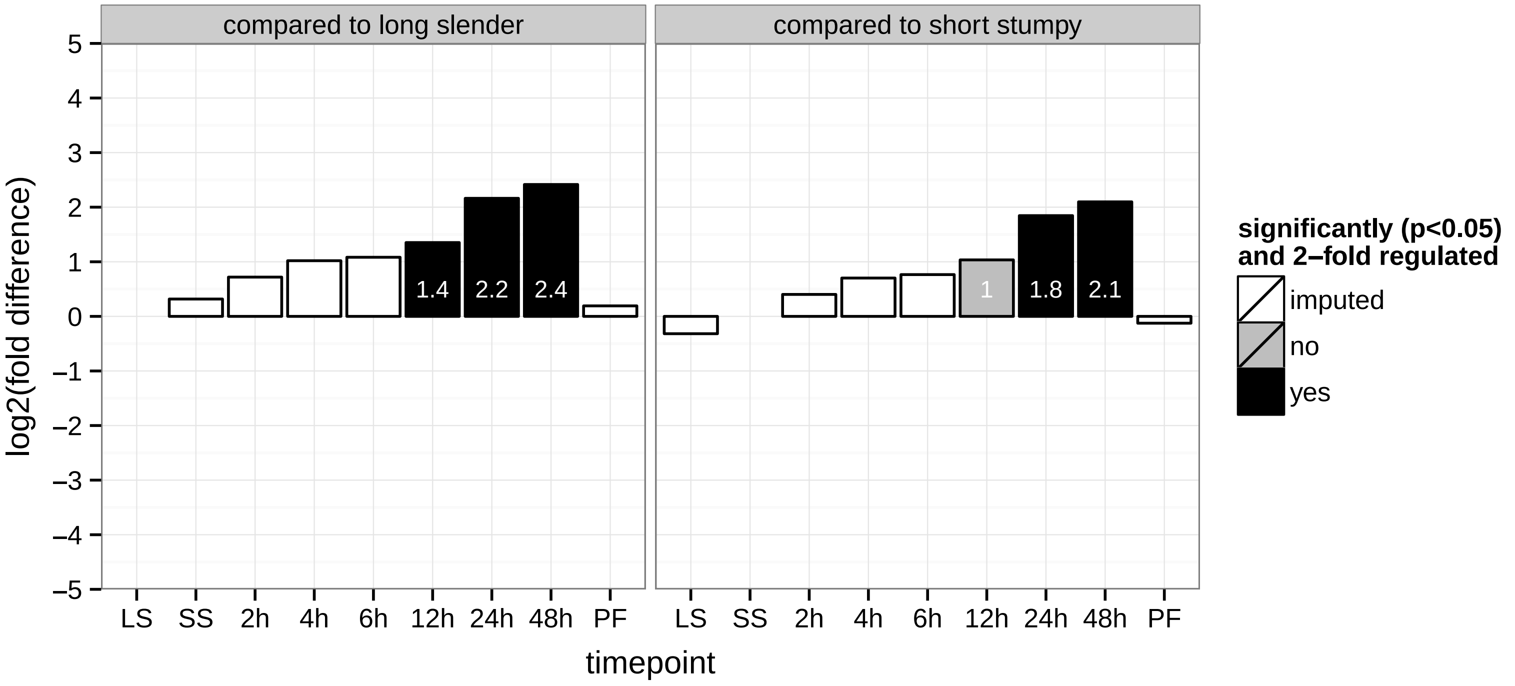

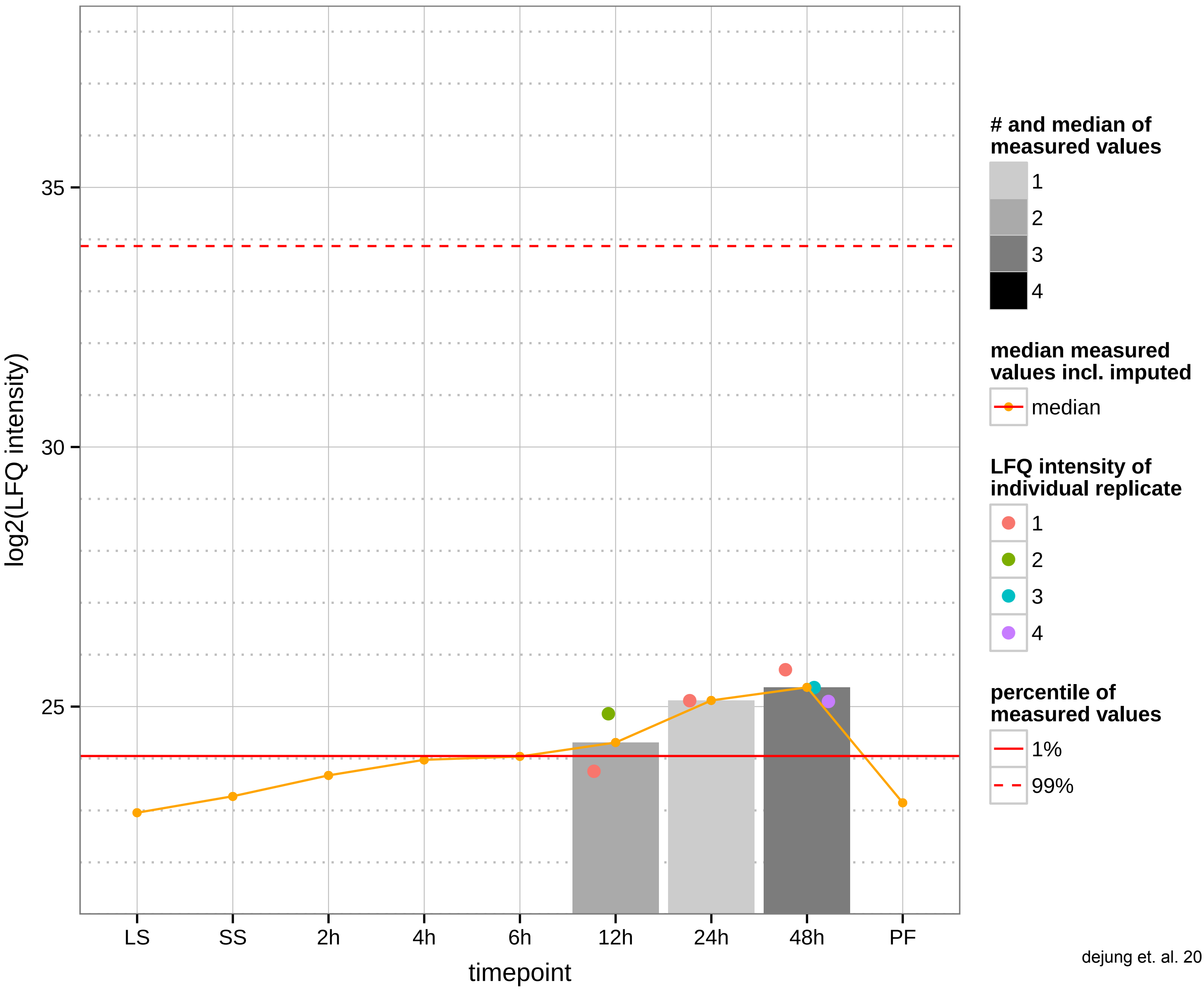

dejung et. al. 2015

thymine−7−hydroxylase, putative (TLP7)
Tb927.7.7500
AGOF: ferrous iron binding, oxidoreductase activity
AGOC: null
AGOP: metabolic process
PGOF: oxidoreductase activity, oxidoreductase activity, acting on paired donors, with incorporation or reduction of molecular o
PGOC: null
PGOP: oxidation−reduction process

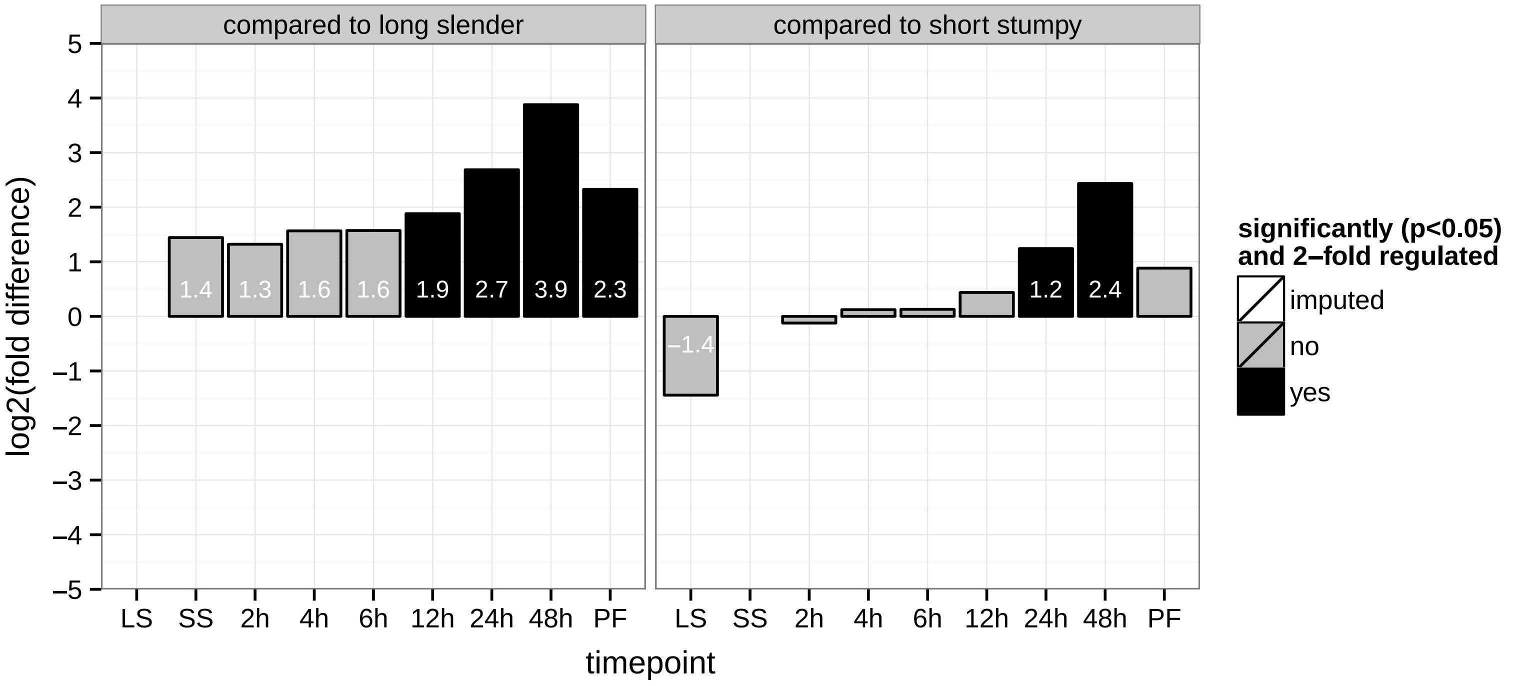

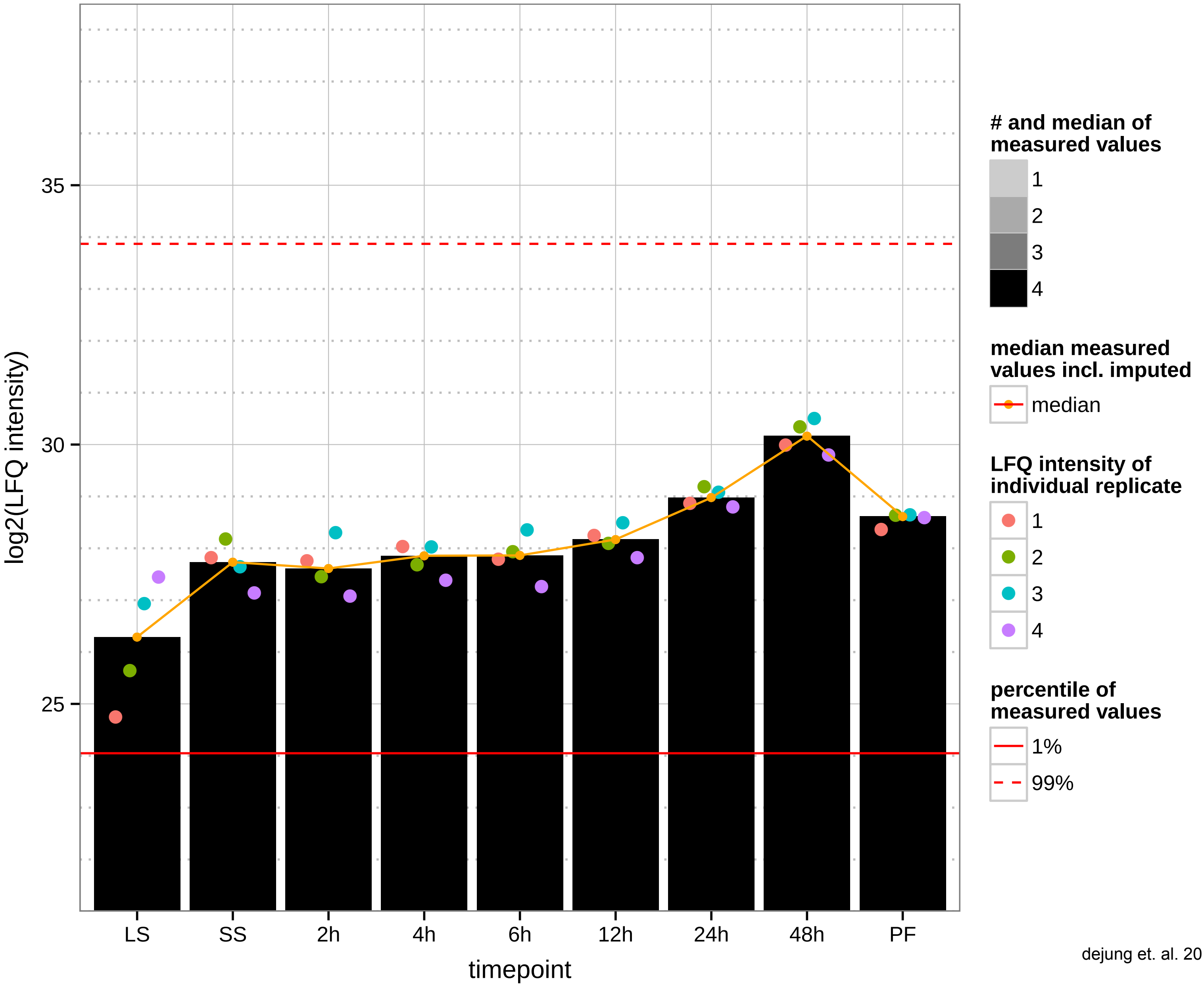

dejung et. al. 2015

conserved protein
Tb927.8.1270
AGOF: null
AGOC: null
AGOP: null
PGOF: null
PGOC: null
PGOP: null

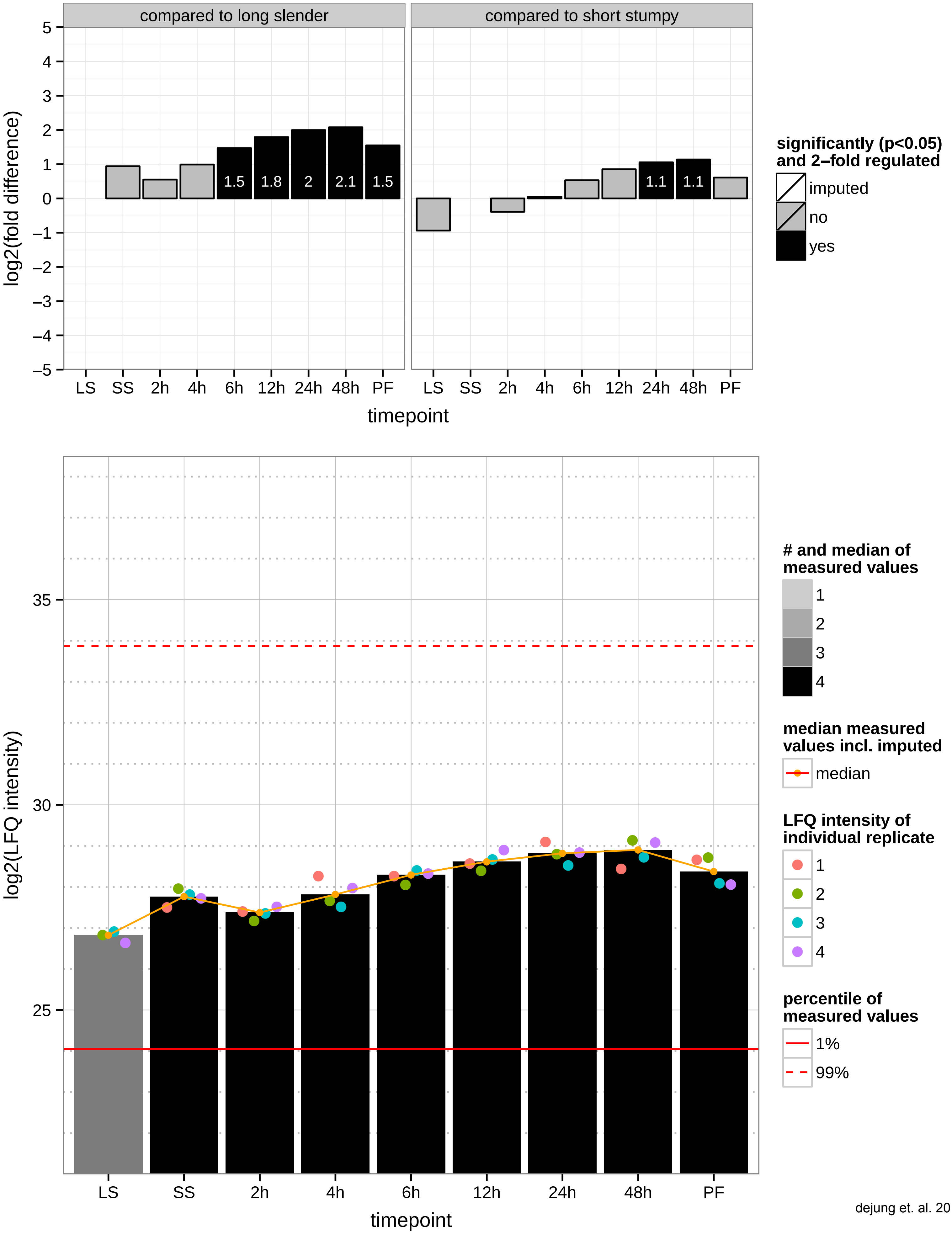

dejung et. al. 2015

hypothetical protein, conserved
Tb927.8.3050
AGOF: null
AGOC: null
AGOP: null
PGOF: null
PGOC: null
PGOP: null

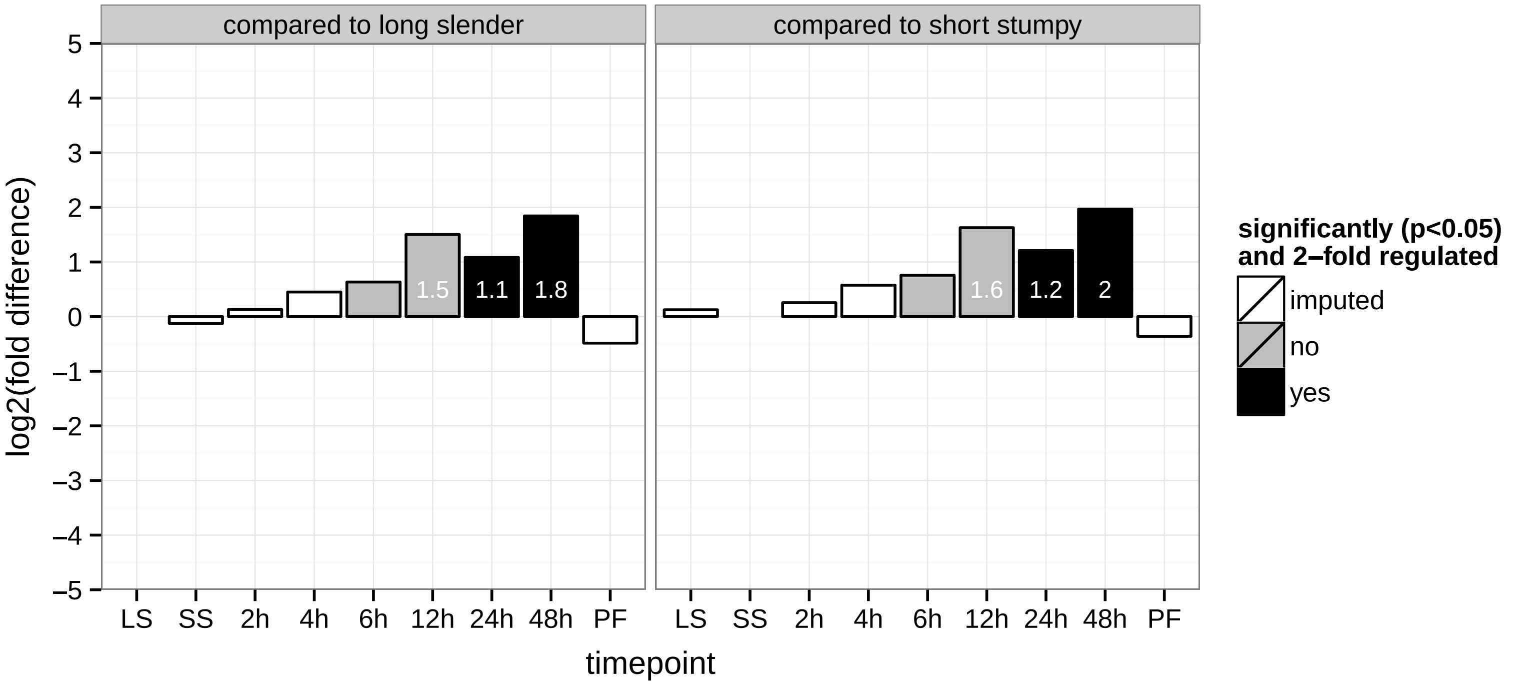

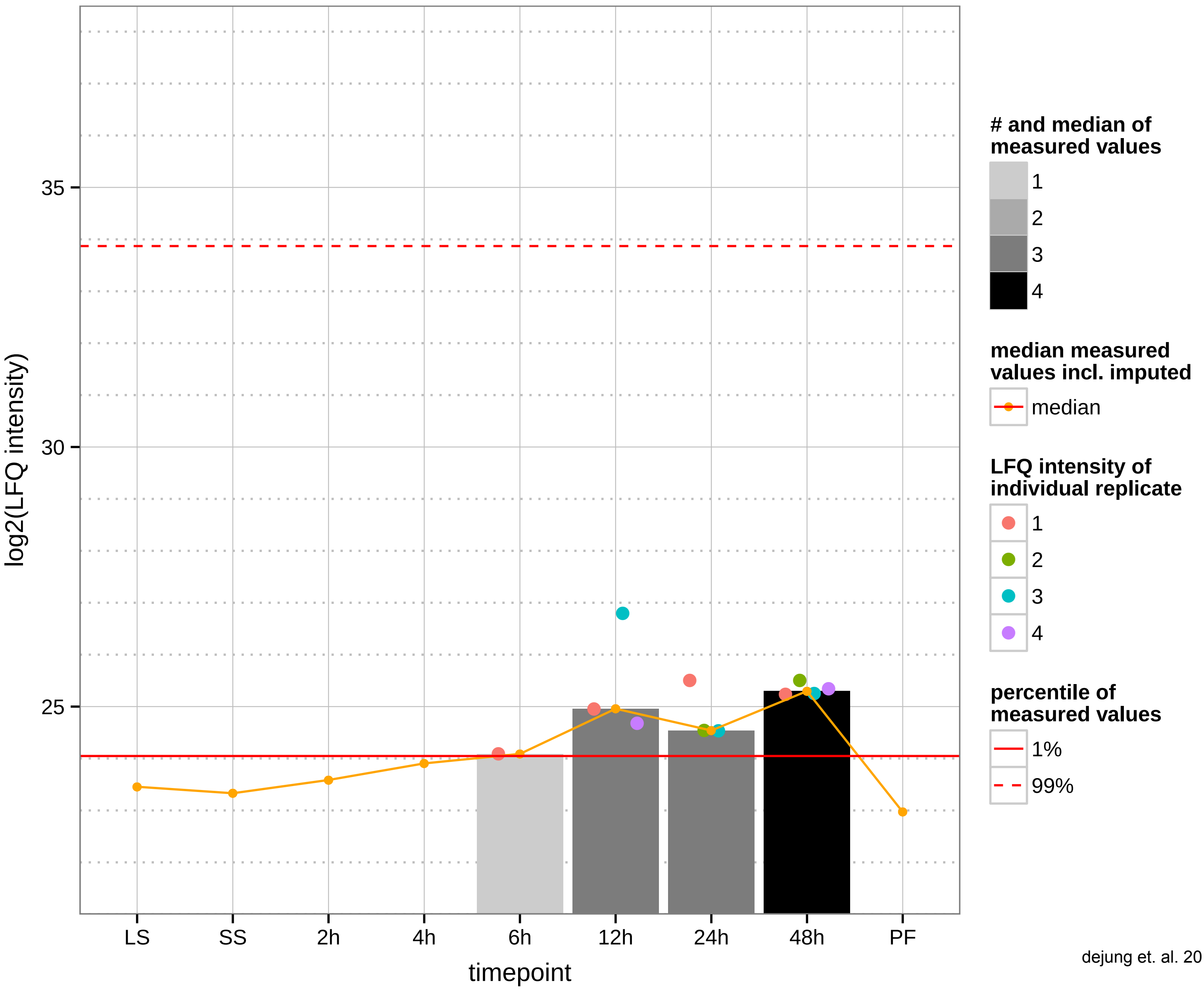

dejung et. al. 2015

hypothetical protein, conserved
Tb927.8.3540
AGOF: null
AGOC: null
AGOP: null
PGOF: null
PGOC: null
PGOP: null

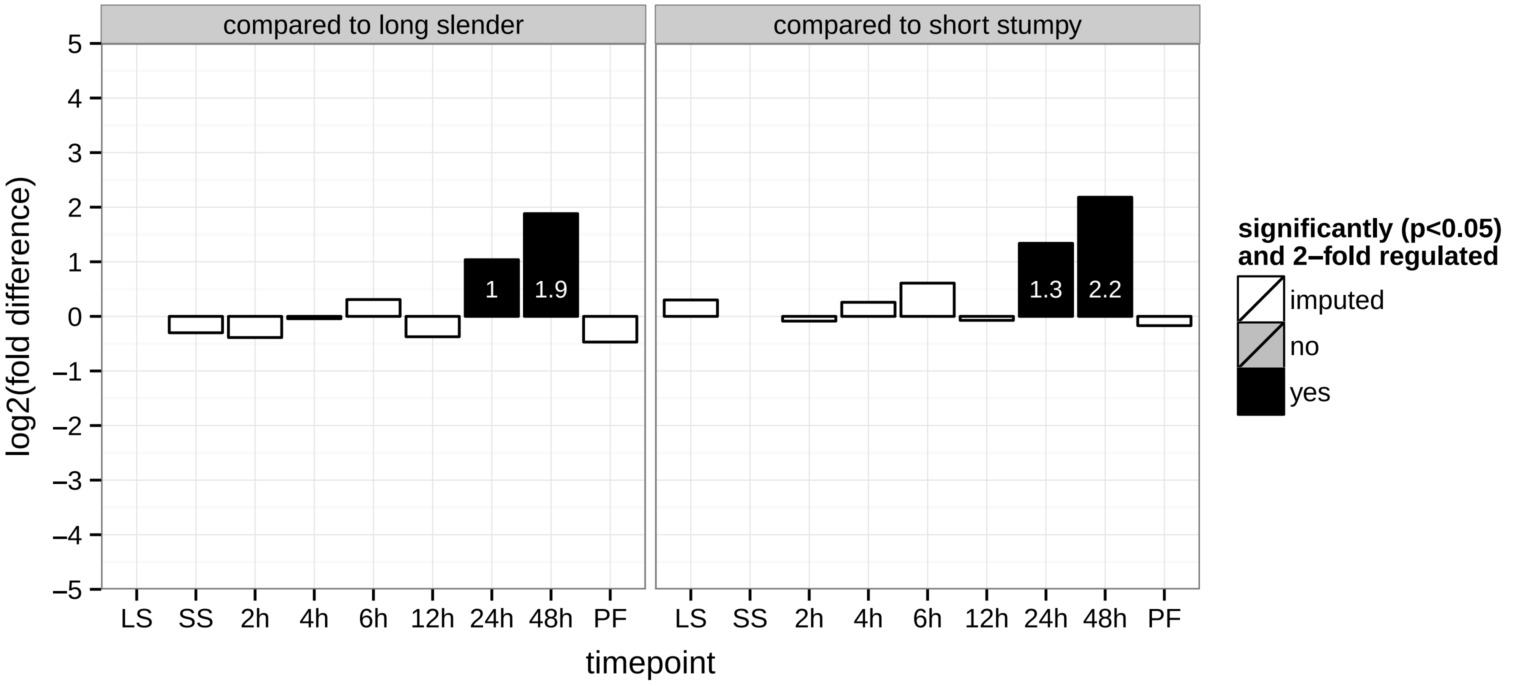

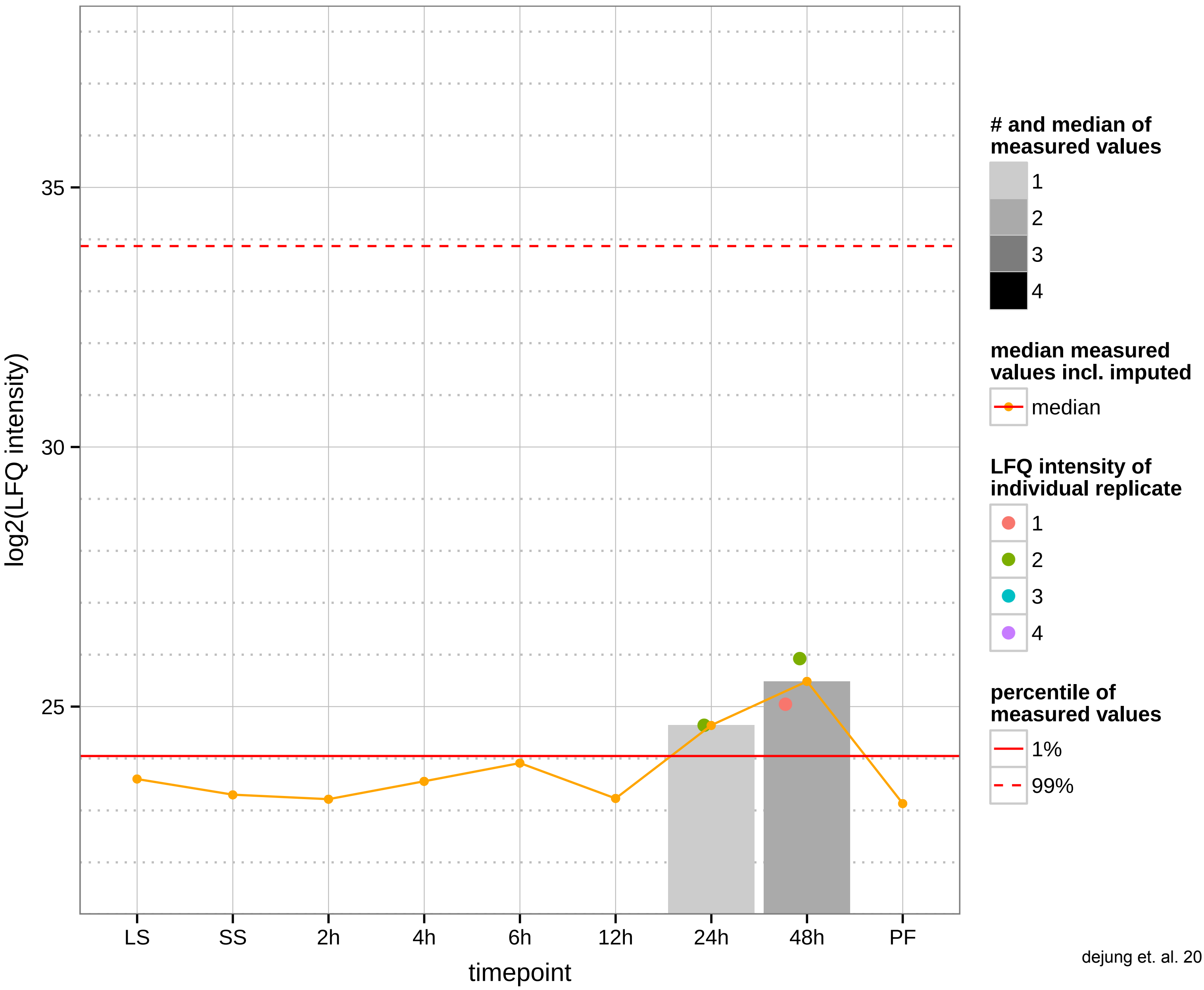

dejung et. al. 2015

hypothetical protein, conserved
Tb927.8.4550
AGOF: null
AGOC: mitochondrion
AGOP: null
PGOF: null
PGOC: null
PGOP: null

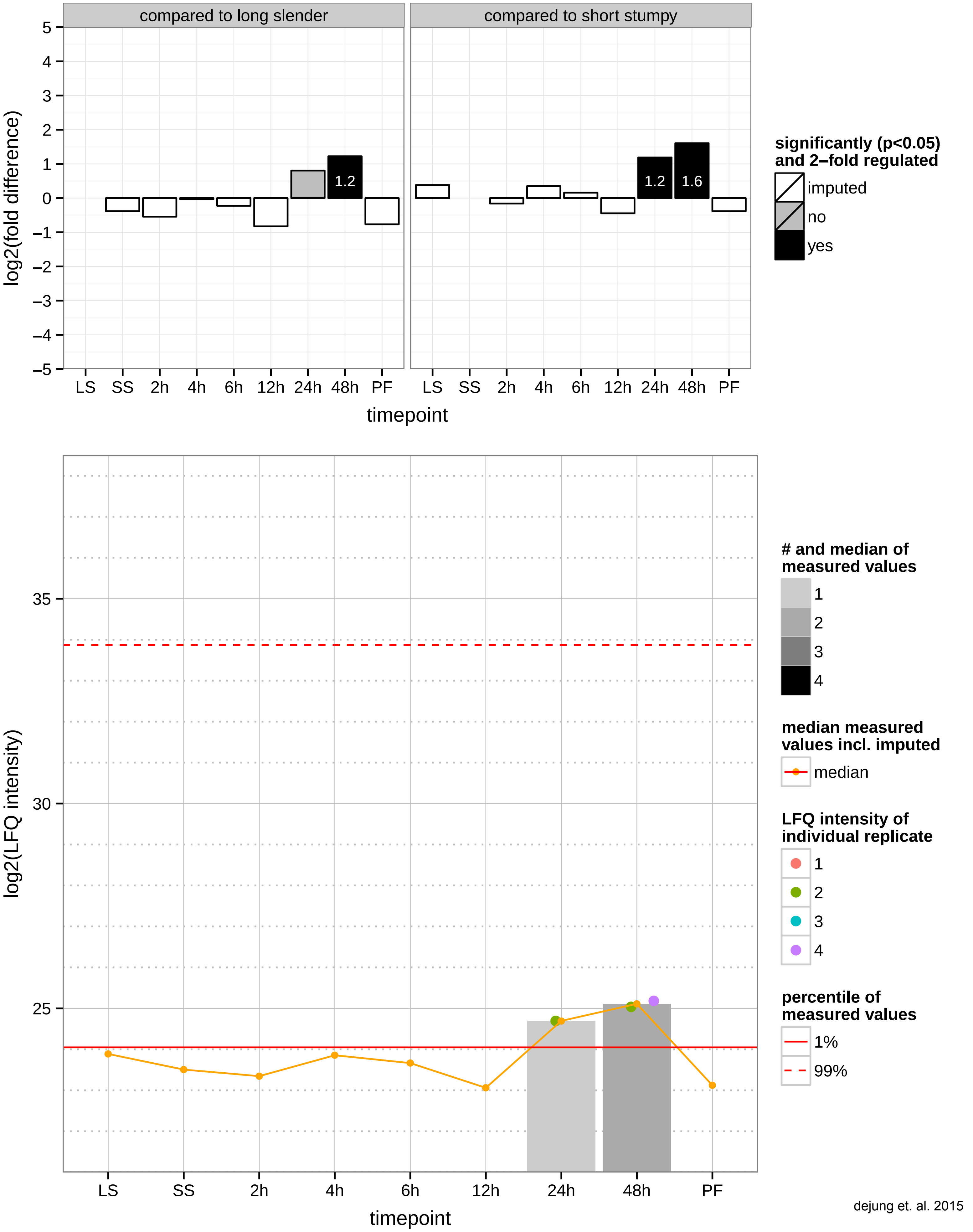

dejung et. al. 2015

prohibitin 1 (PHB1)
Tb927.8.4810
AGOF: null
AGOC: membrane, mitochondrion
AGOP: mitochondrion organization, regulation of cell cycle, regulation of mitochondrial membrane potential, regulation of mito
PGOF: null
PGOC: null
PGOP: null

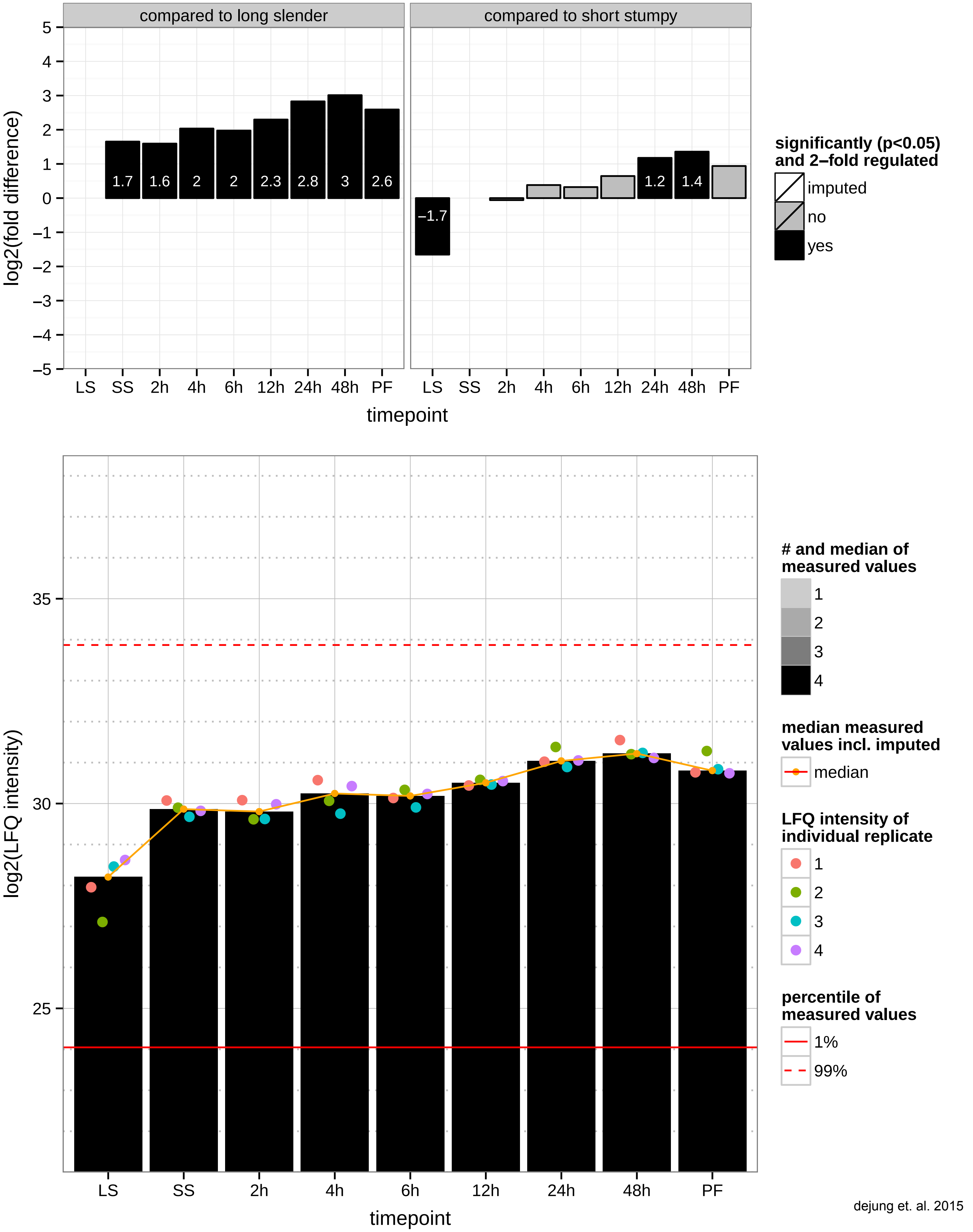

dejung et. al. 2015

50S ribosomal protein L17, putative
Tb927.8.5860
AGOF: structural constituent of ribosome
AGOC: intracellular, mitochondrion, organellar large ribosomal subunit, ribosome
AGOP: translation
PGOF: structural constituent of ribosome
PGOC: intracellular, ribosome
PGOP: translation

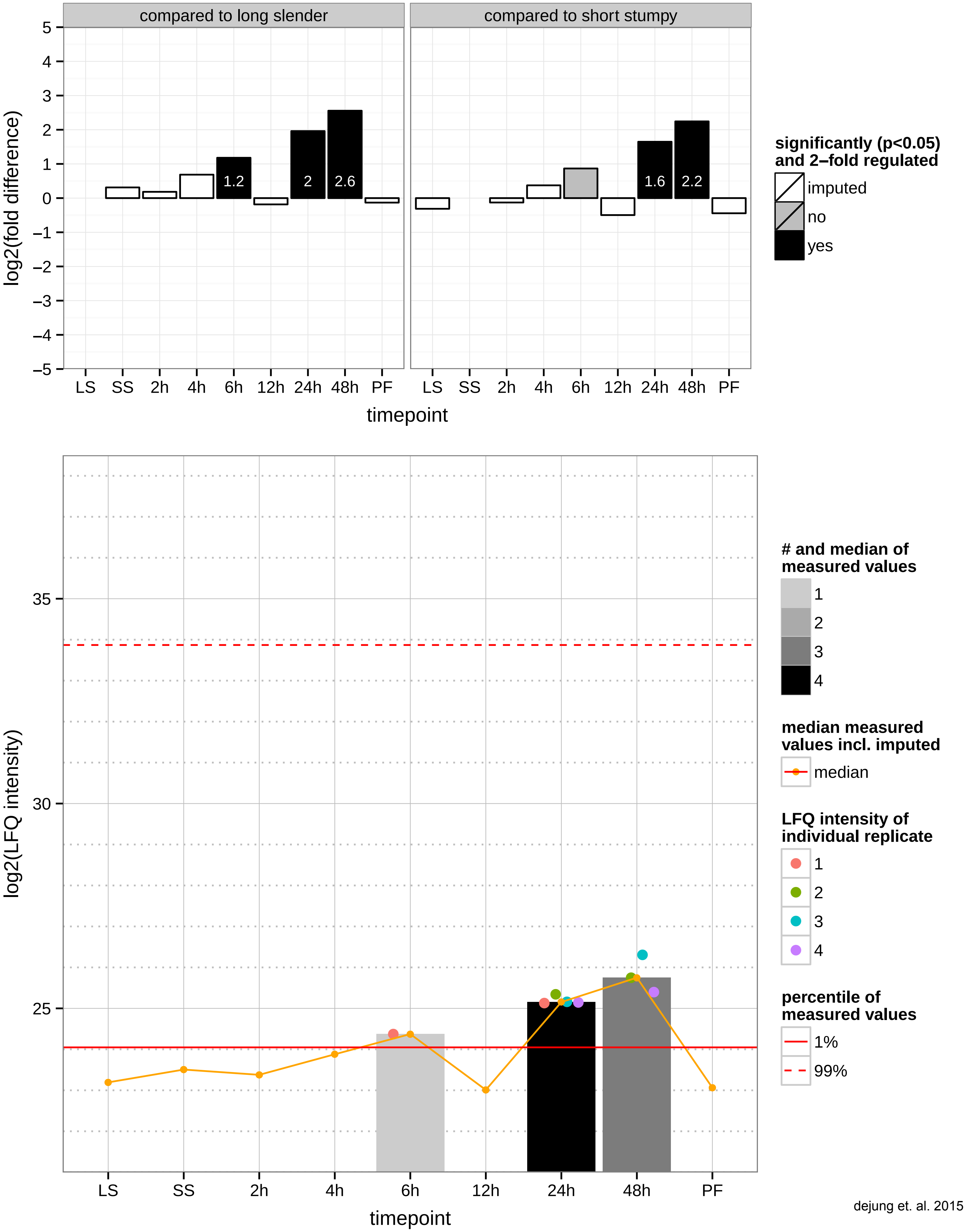

dejung et. al. 2015

TFIIH basal transcription factor complex helicase subunit, putative, DNA excision repair protein, Transcription factor II H comp
Tb927.8.5980
AGOF: 5'−3' DNA helicase activity, ATP binding, ATP−dependent DNA helicase activity, sequence−specific DNA binding trans
AGOC: holo TFIIH complex
AGOP: DNA repair, mRNA transcription from RNA polymerase II promoter, nucleotide−excision repair
PGOF: ATP binding, ATP−dependent DNA helicase activity, ATP−dependent helicase activity, DNA binding, hydrolase activity,
PGOC: nucleus
PGOP: nucleobase−containing compound metabolic process

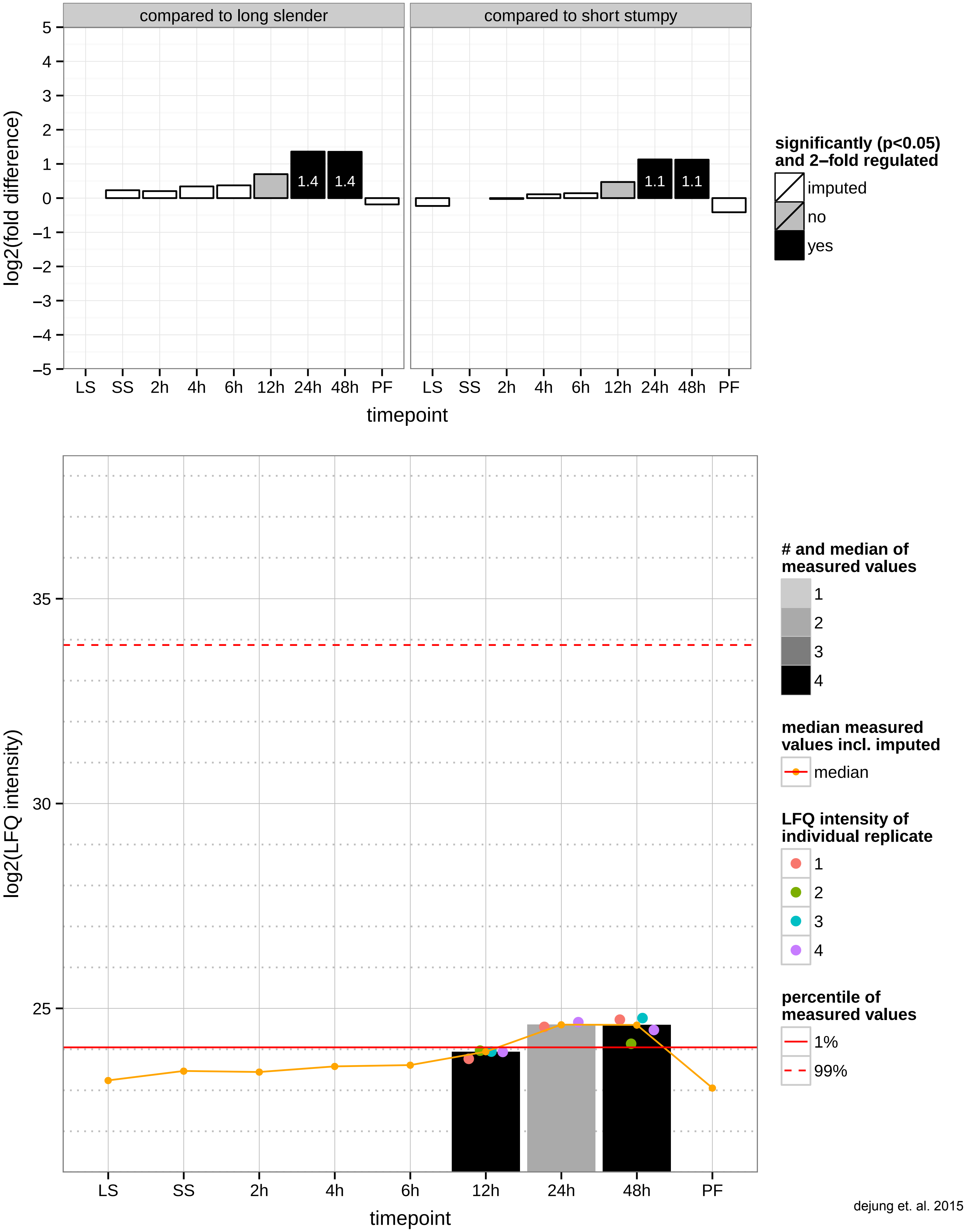

dejung et. al. 2015

DNA polymerase epsilon catalytic subunit, putative
Tb927.9.10440
AGOF: DNA binding, DNA−directed DNA polymerase activity, nucleotide binding, zinc ion binding
AGOC: nucleus
AGOP: DNA−dependent DNA replication
PGOF: DNA binding, DNA−directed DNA polymerase activity, nucleic acid binding, nucleotide binding, zinc ion binding
PGOC: nucleus
PGOP: DNA replication, nucleobase−containing compound metabolic process

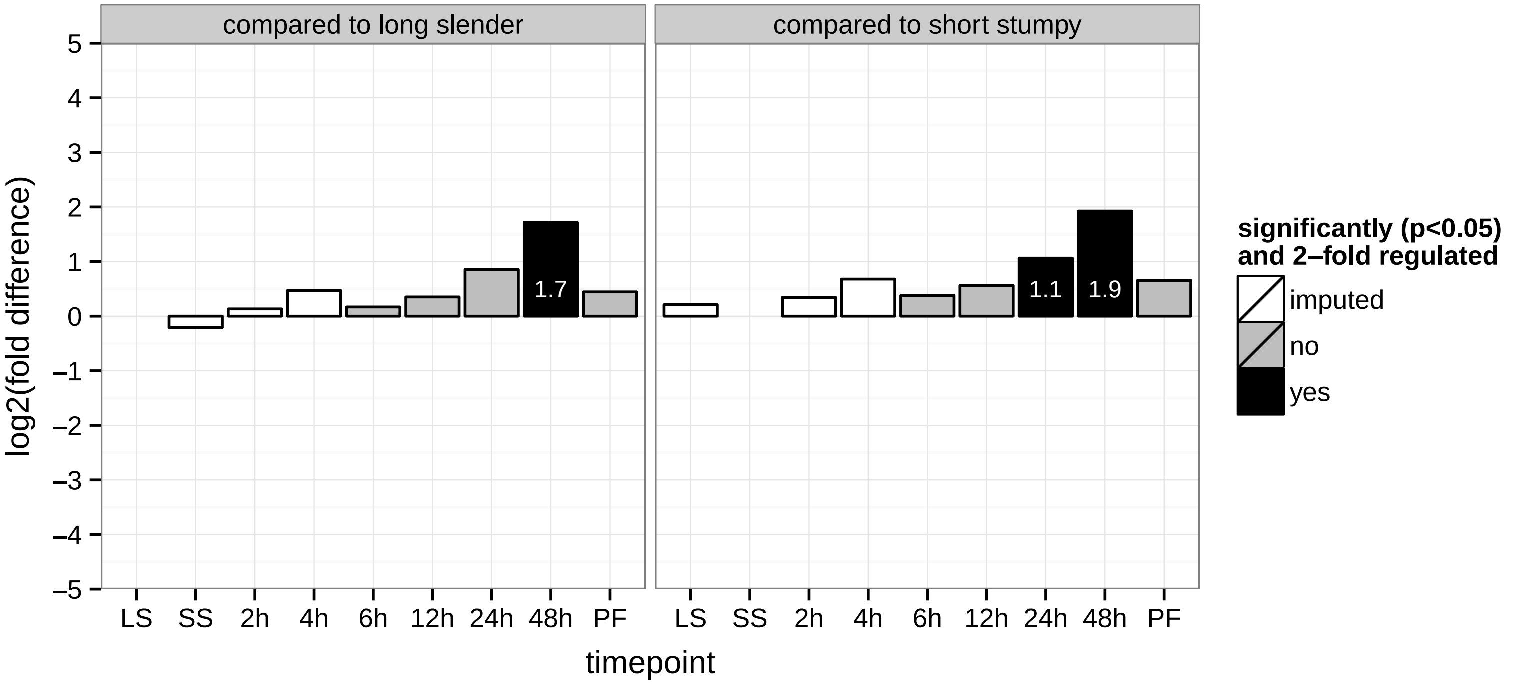

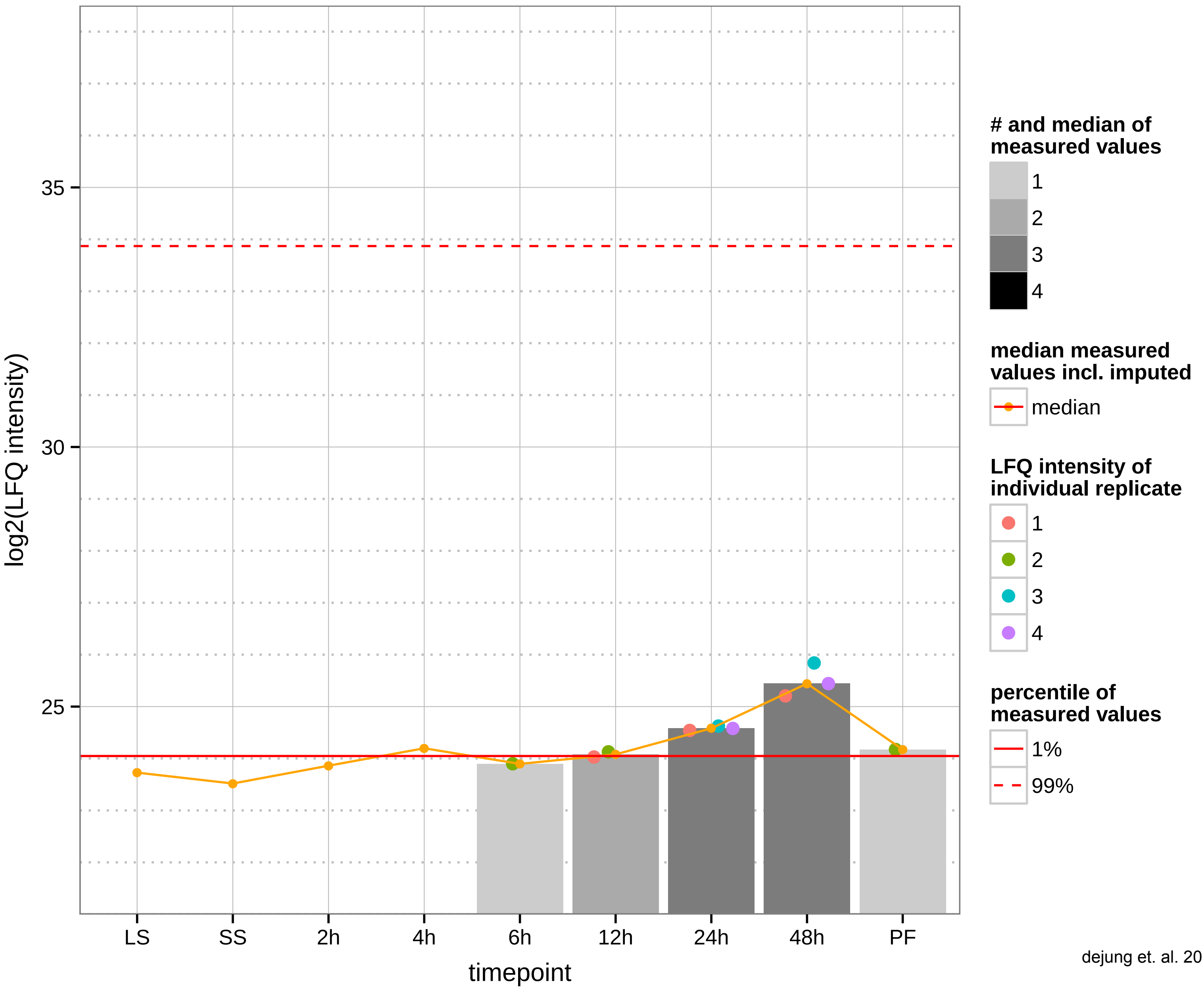

dejung et. al. 2015

calmodulin–like protein, putative, EF hand containing protein
Tb927.9.11230
AGOF: calcium ion binding
AGOC: null
AGOP: null
PGOF: calcium ion binding
PGOC: null
PGOP: null

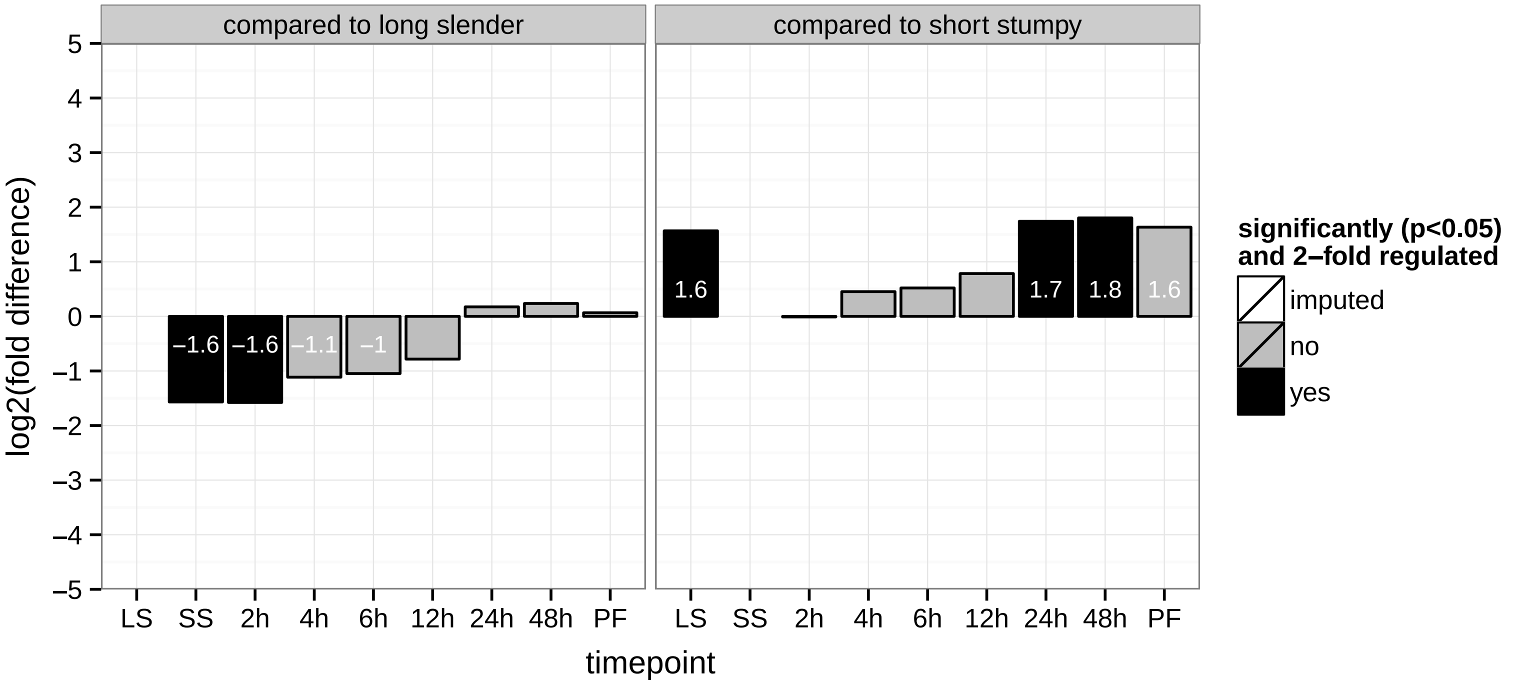

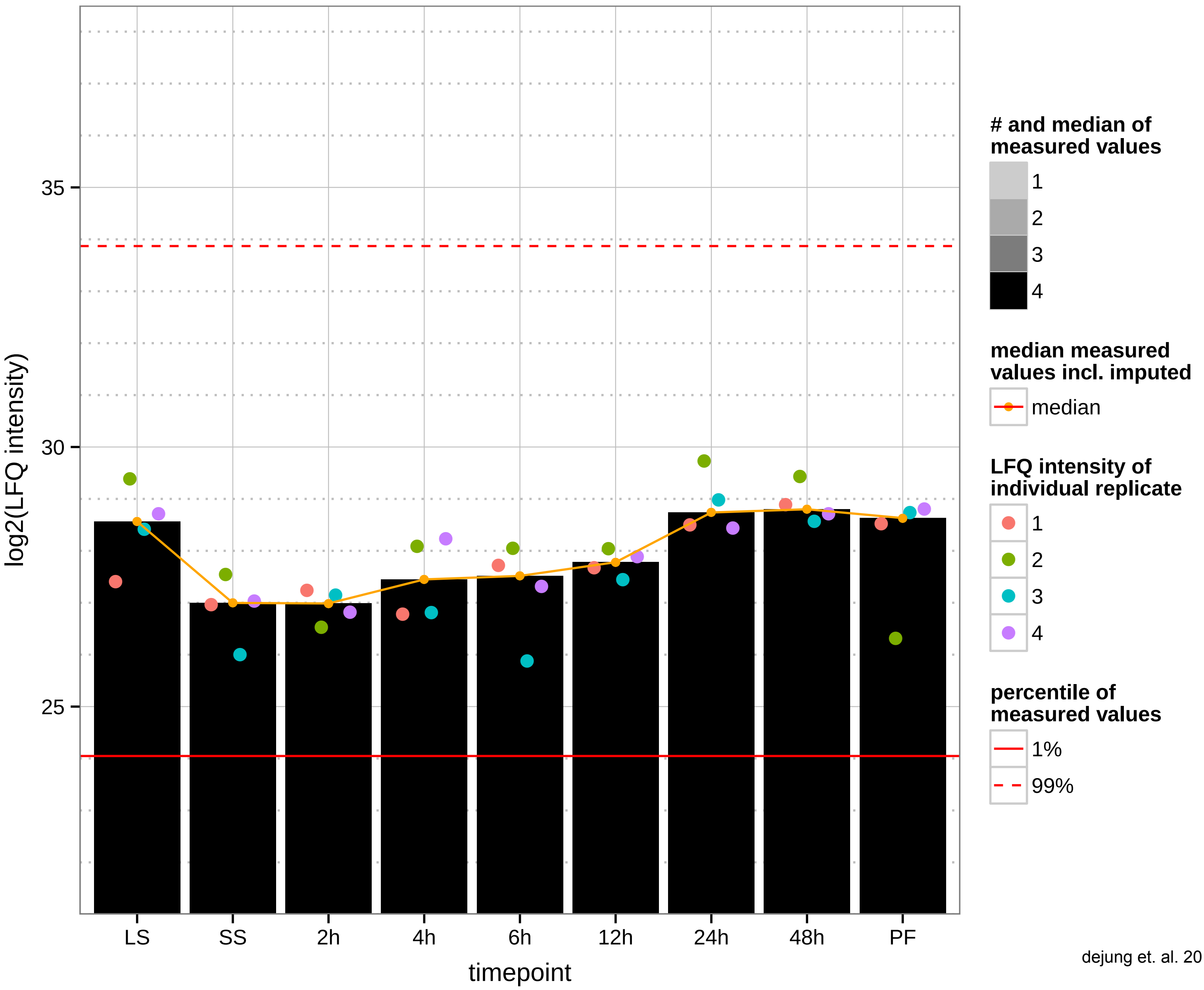

dejung et. al. 2015

hypothetical protein, conserved
Tb927.9.2760
AGOF: microtubule binding
AGOC: null
AGOP: null
PGOF: microtubule binding, protein binding
PGOC: null
PGOP: null

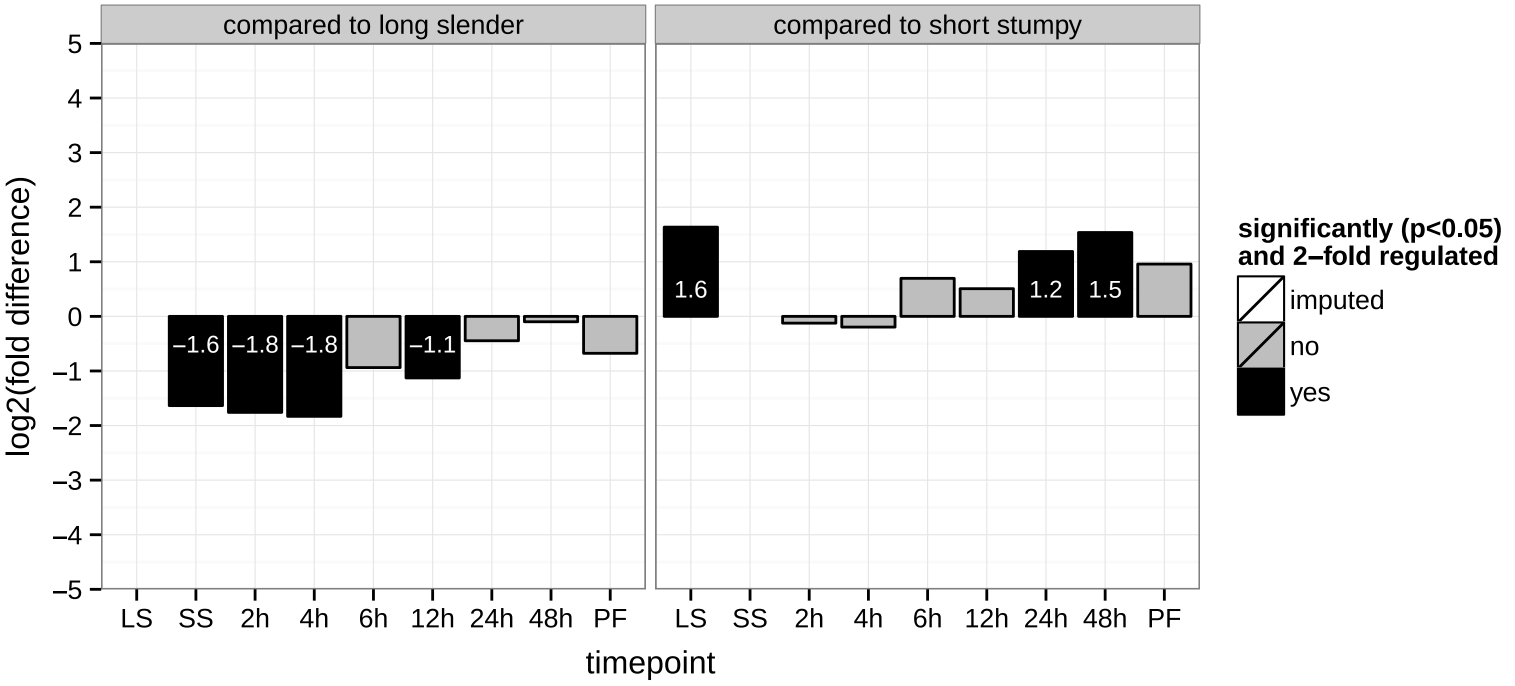

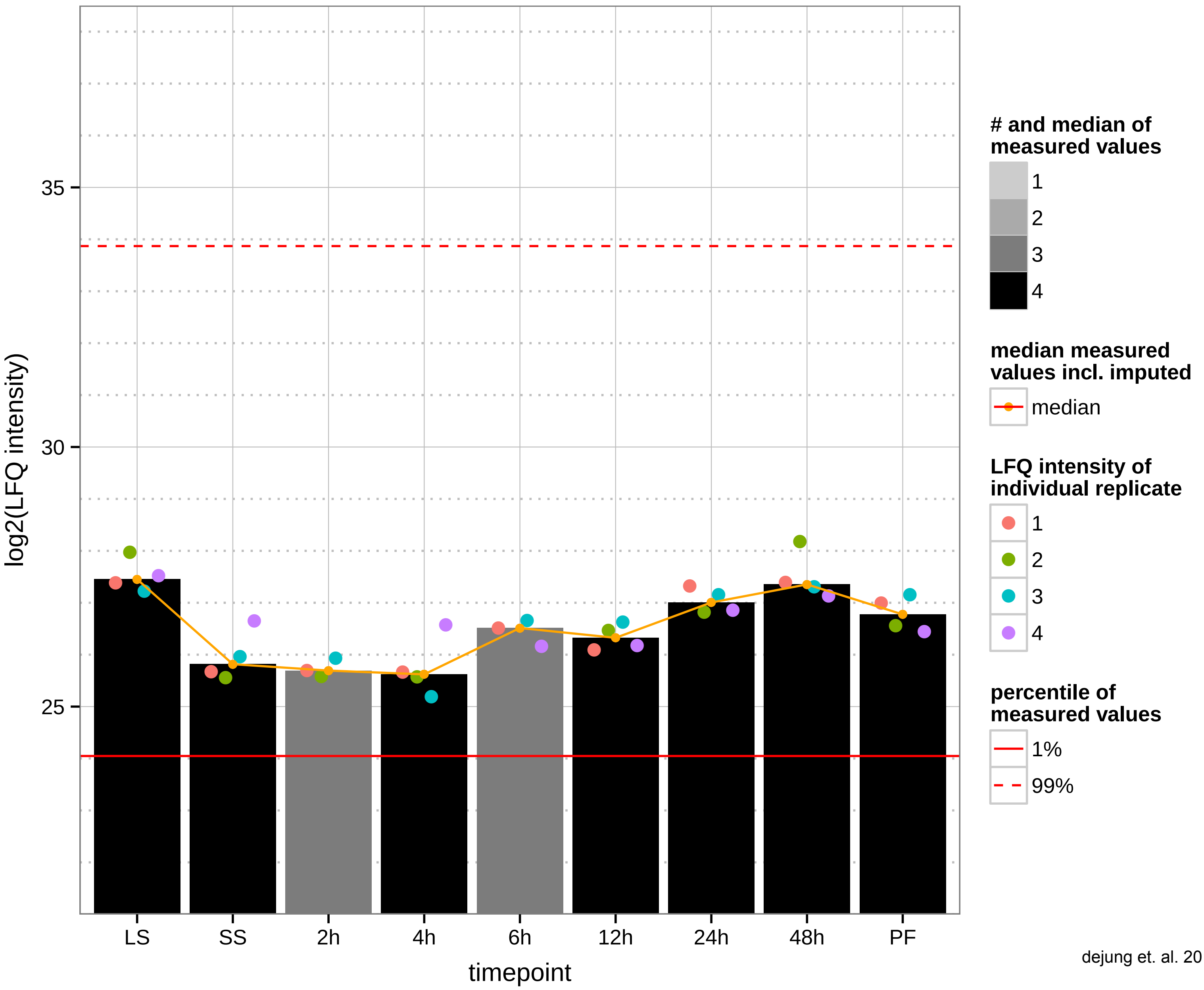

dejung et. al. 2015

cation transporter, putative
Tb927.9.5490
AGOF: metal ion transmembrane transporter activity
AGOC: membrane
AGOP: metal ion transport
PGOF: metal ion transmembrane transporter activity
PGOC: membrane
PGOP: metal ion transport, transmembrane transport

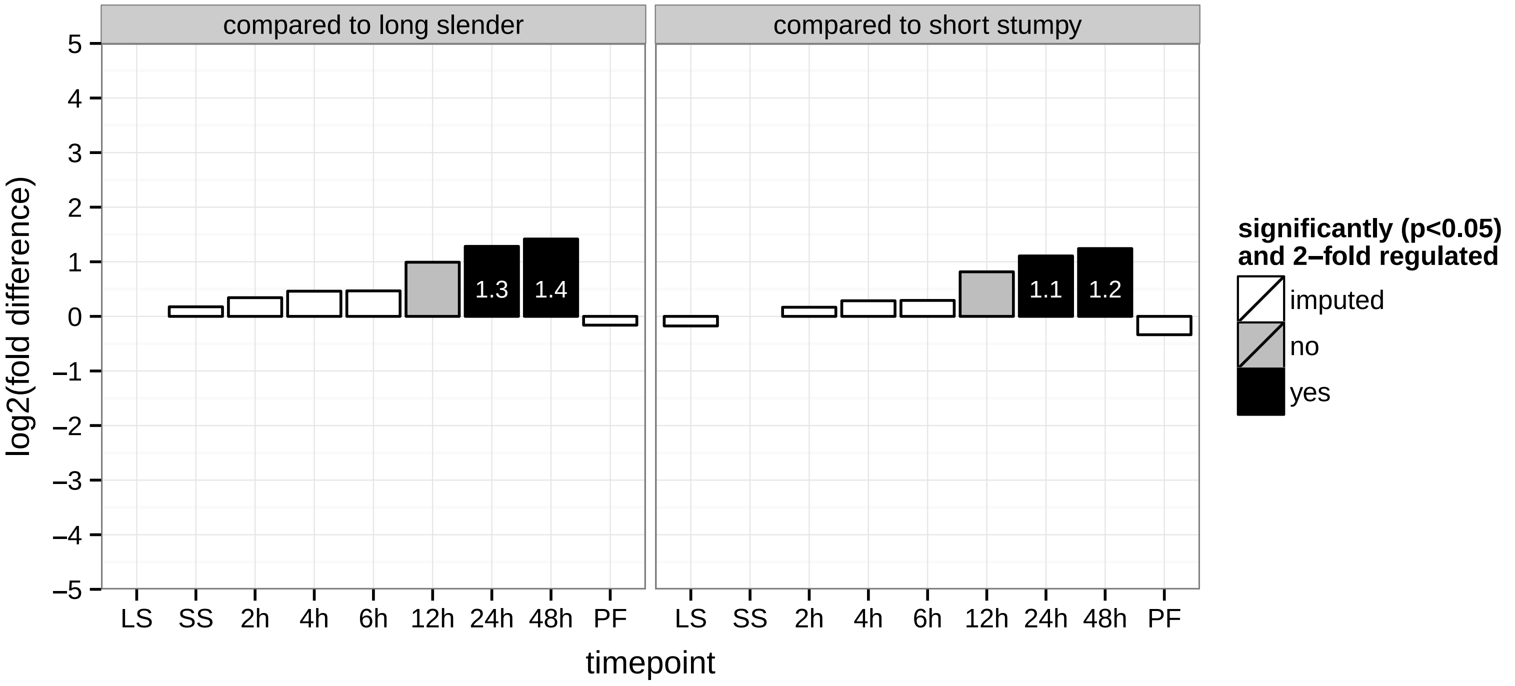

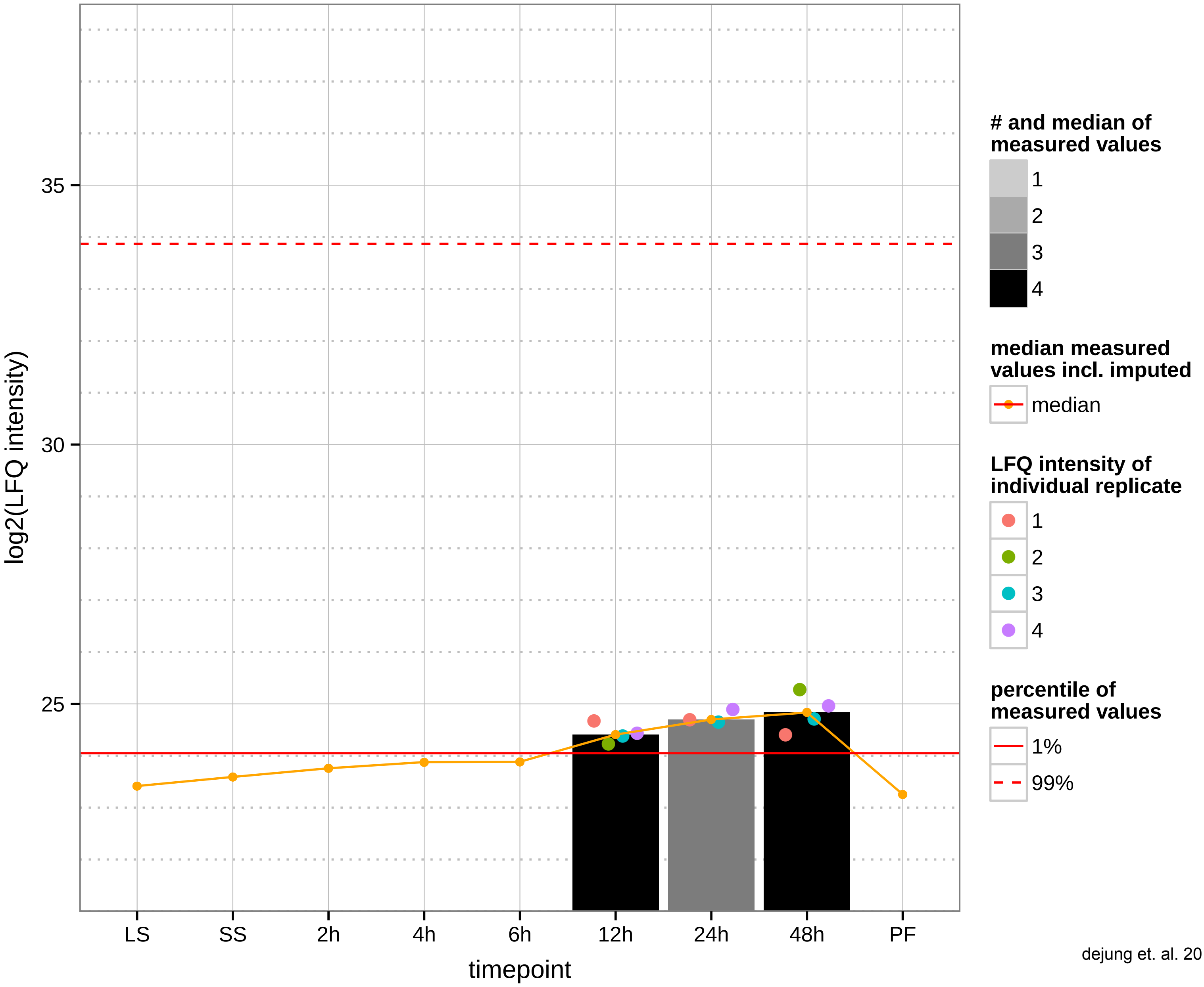

dejung et. al. 2015

hypothetical protein, conserved
Tb927.9.7670
AGOF: null
AGOC: null
AGOP: null
PGOF: null
PGOC: null
PGOP: null

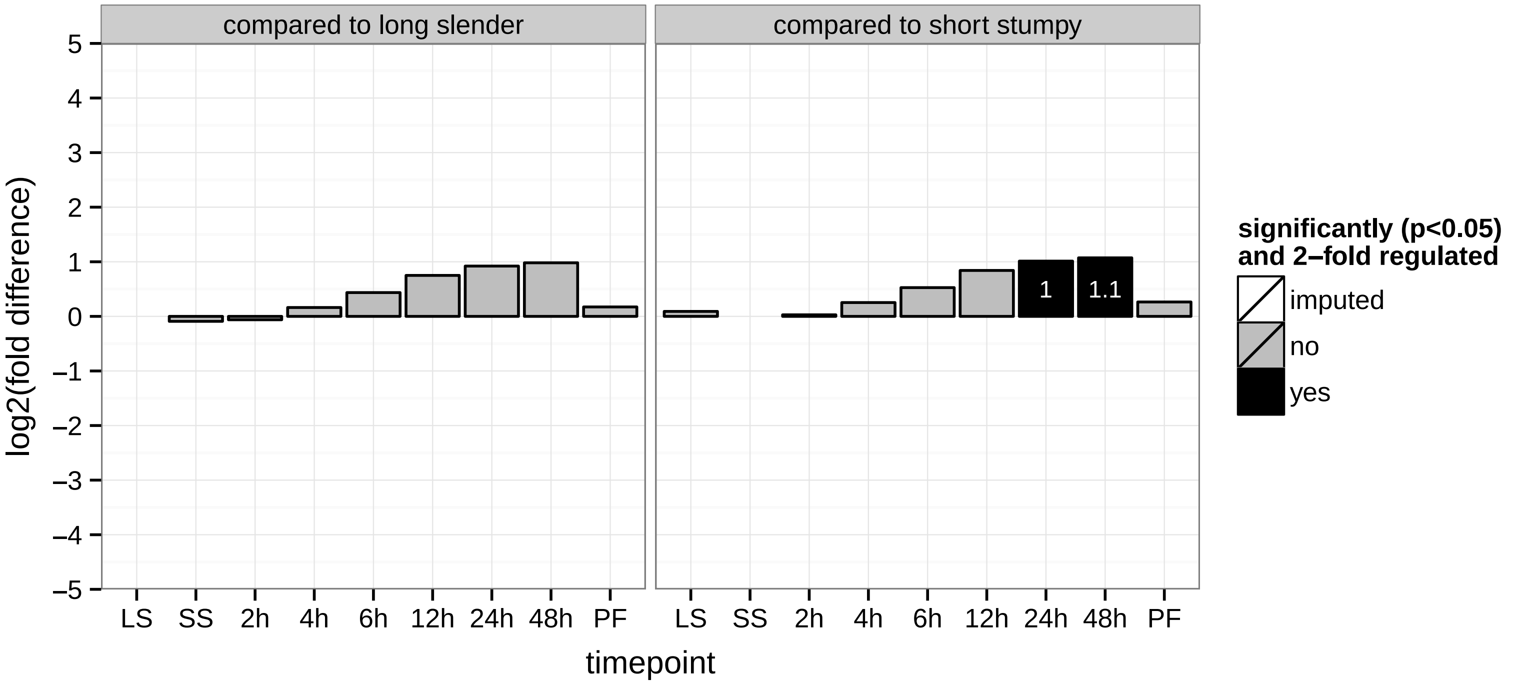

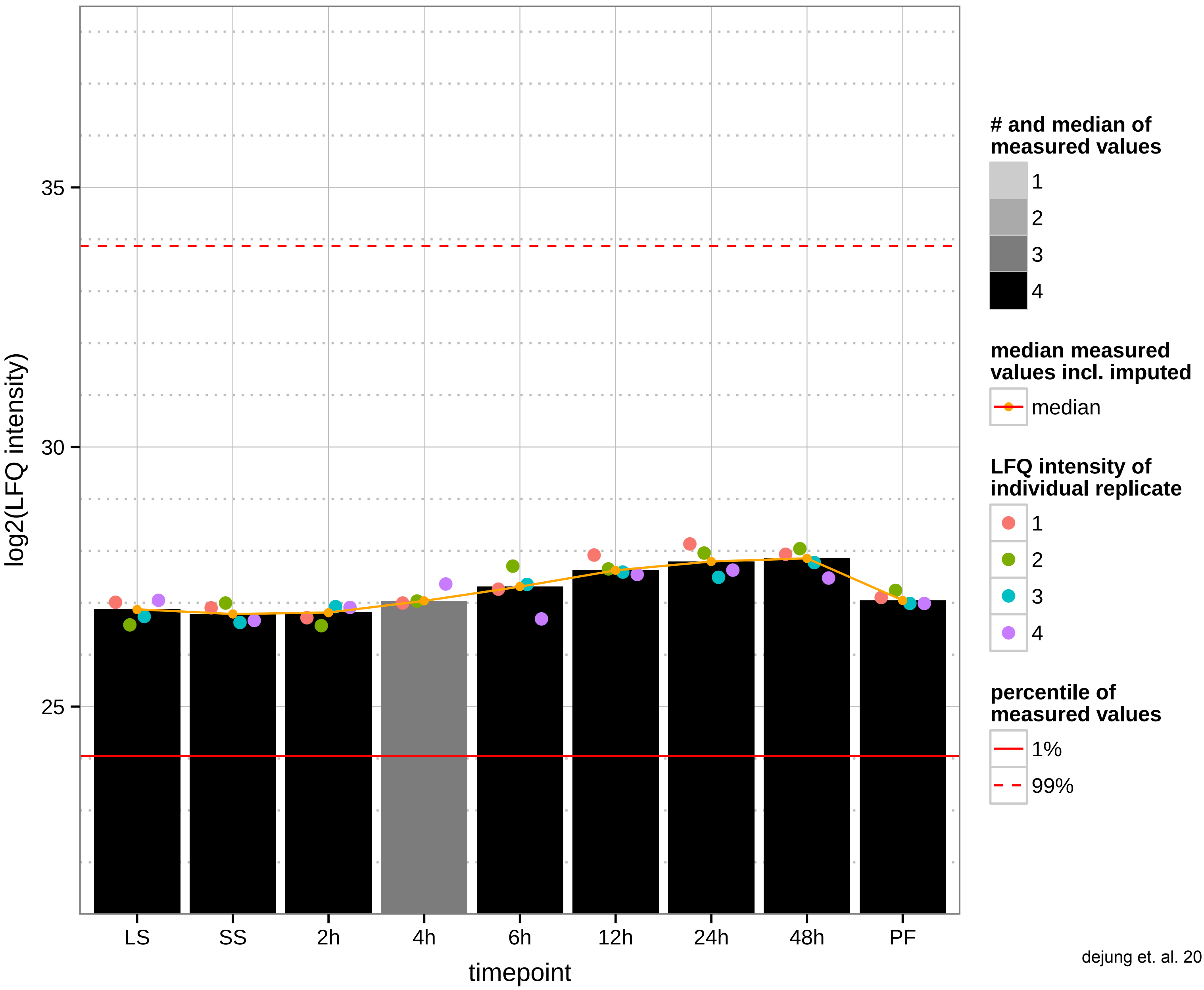

dejung et. al. 2015

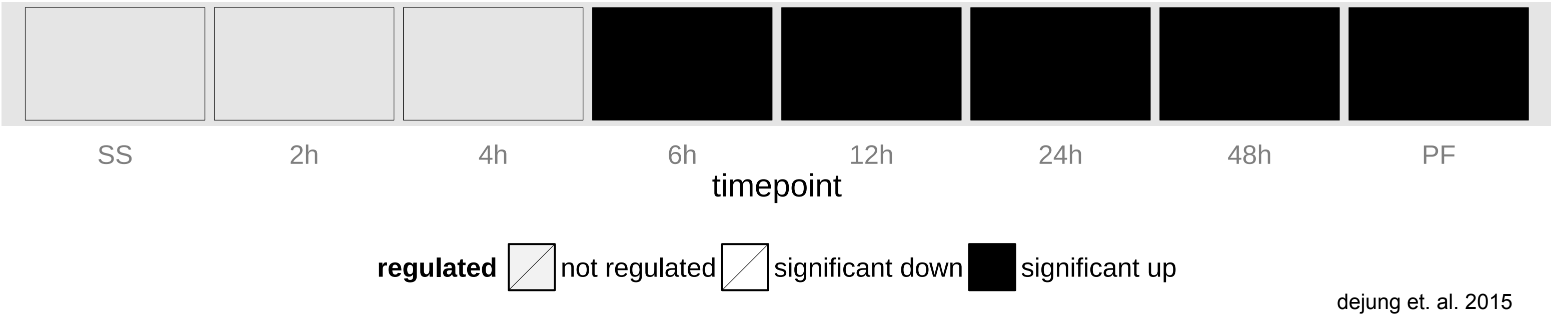

SS
2h
4h
6h
12h
24h
48h
PF
timepoint
regulated
not regulated
significant down
significant up
dejung et. al. 2015

zinc finger protein family member, putative (ZC3H18), hypothetical protein
Tb927.7.2140;Tb07.11L3.90
AGOF: nucleic acid binding, zinc ion binding, RNA binding
AGOC: null
AGOP: null
PGOF: nucleic acid binding, zinc ion binding
PGOC: null
PGOP: null

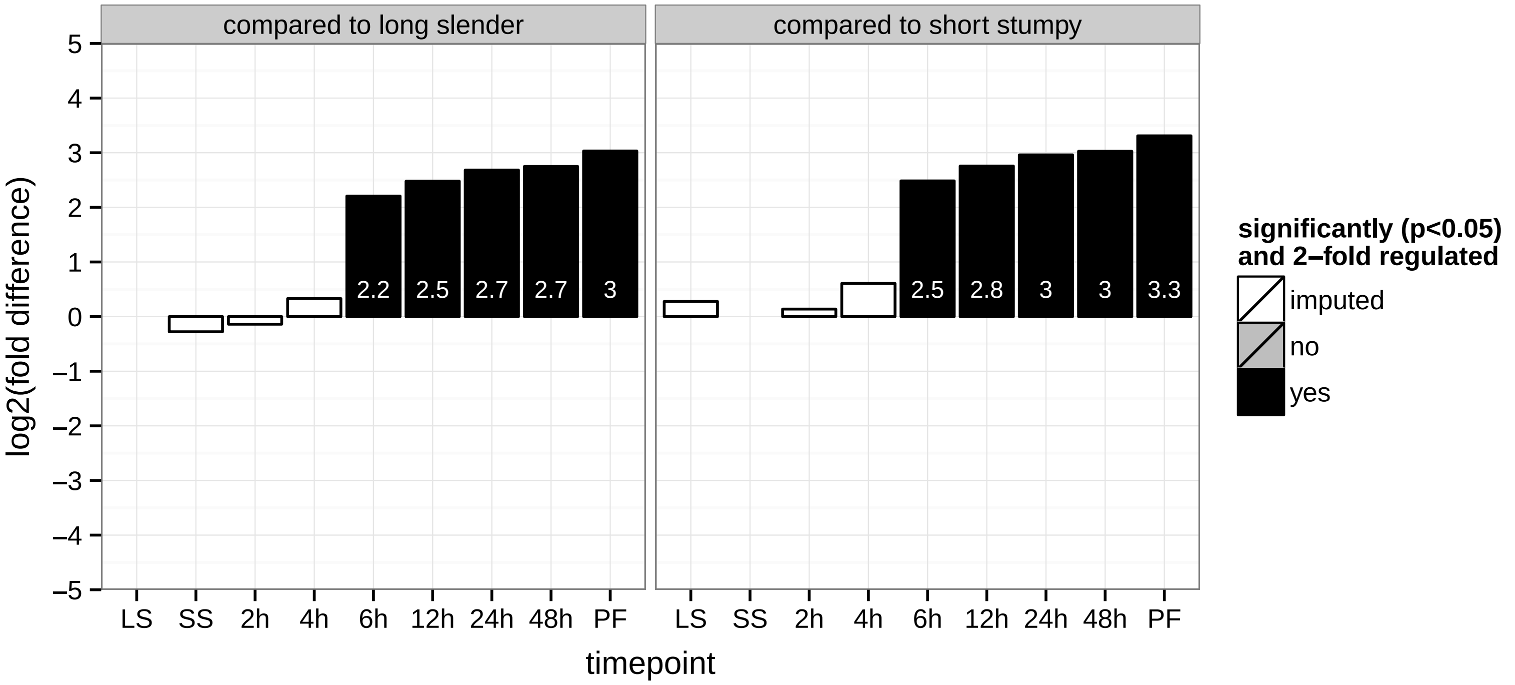

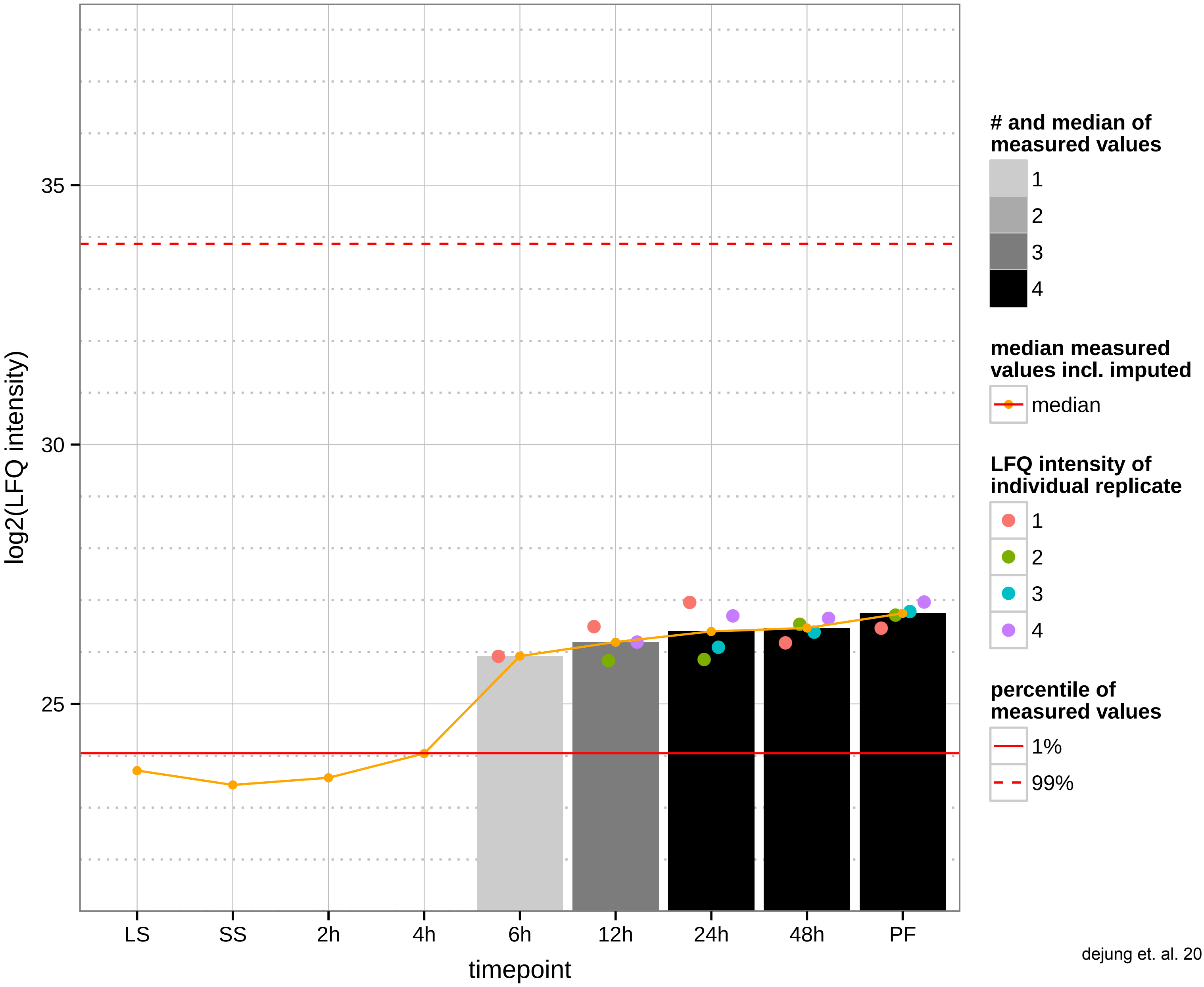

dejung et. al. 2015

s–adenosyl–L–methionine–c–24–delta–sterol–methyl transferase a, putative, sterol 24–c–methyltransferase, Sterol methyltra
Tb927.10.6950;Tb927.10.6910;Tb11.v5.0496;Tb10.v4.0247
AGOF: methyltransferase activity, null
AGOC: null
AGOP: steroid biosynthetic process, null
PGOF: methyltransferase activity, sterol 24–C–methyltransferase activity
PGOC: null
PGOP: metabolic process, steroid biosynthetic process

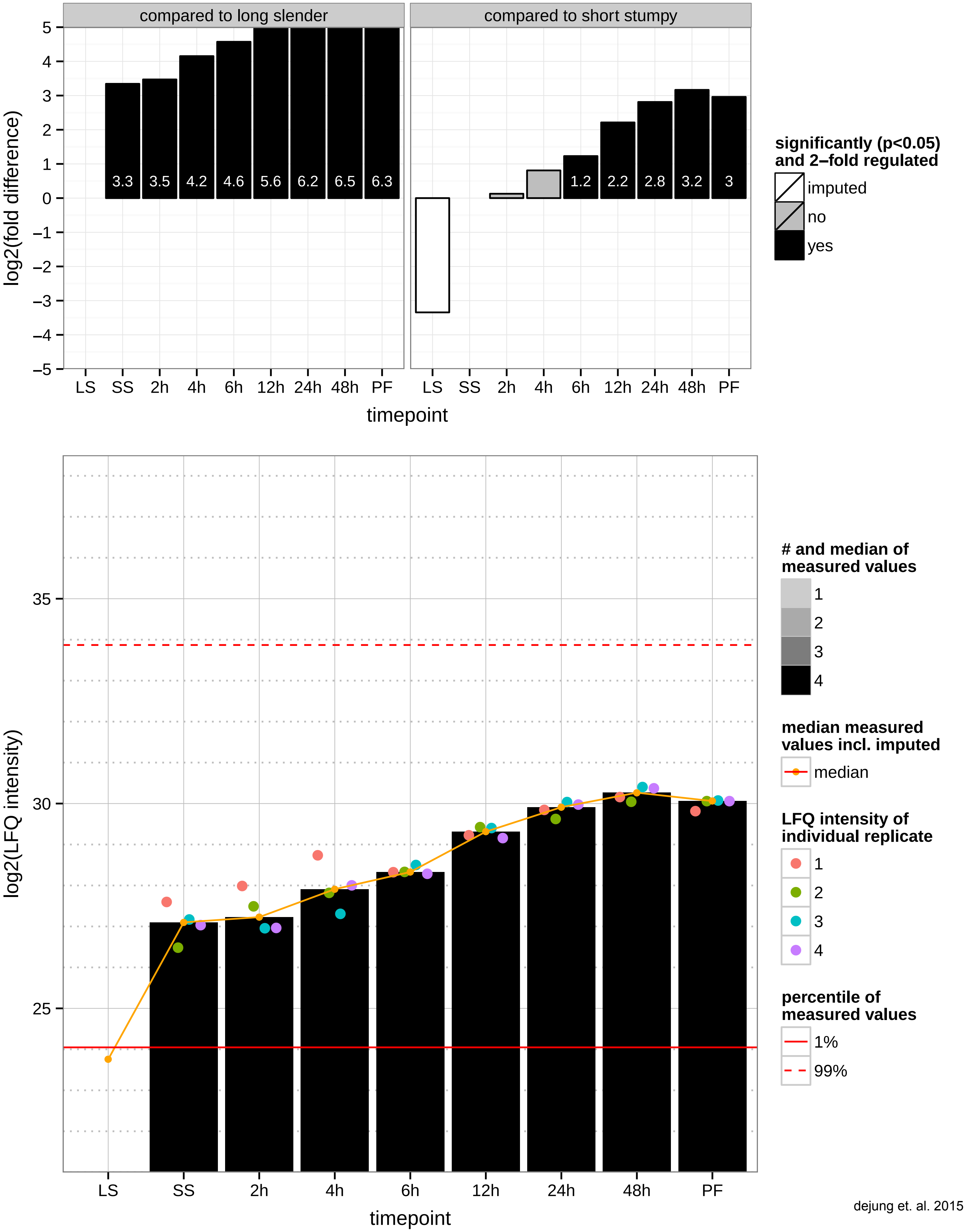

dejung et. al. 2015

hypothetical protein, conserved
Tb927.10.5220;Tb11.v5.0235
AGOF: null
AGOC: null, mitochondrion
AGOP: null
PGOF: binding
PGOC: null
PGOP: null

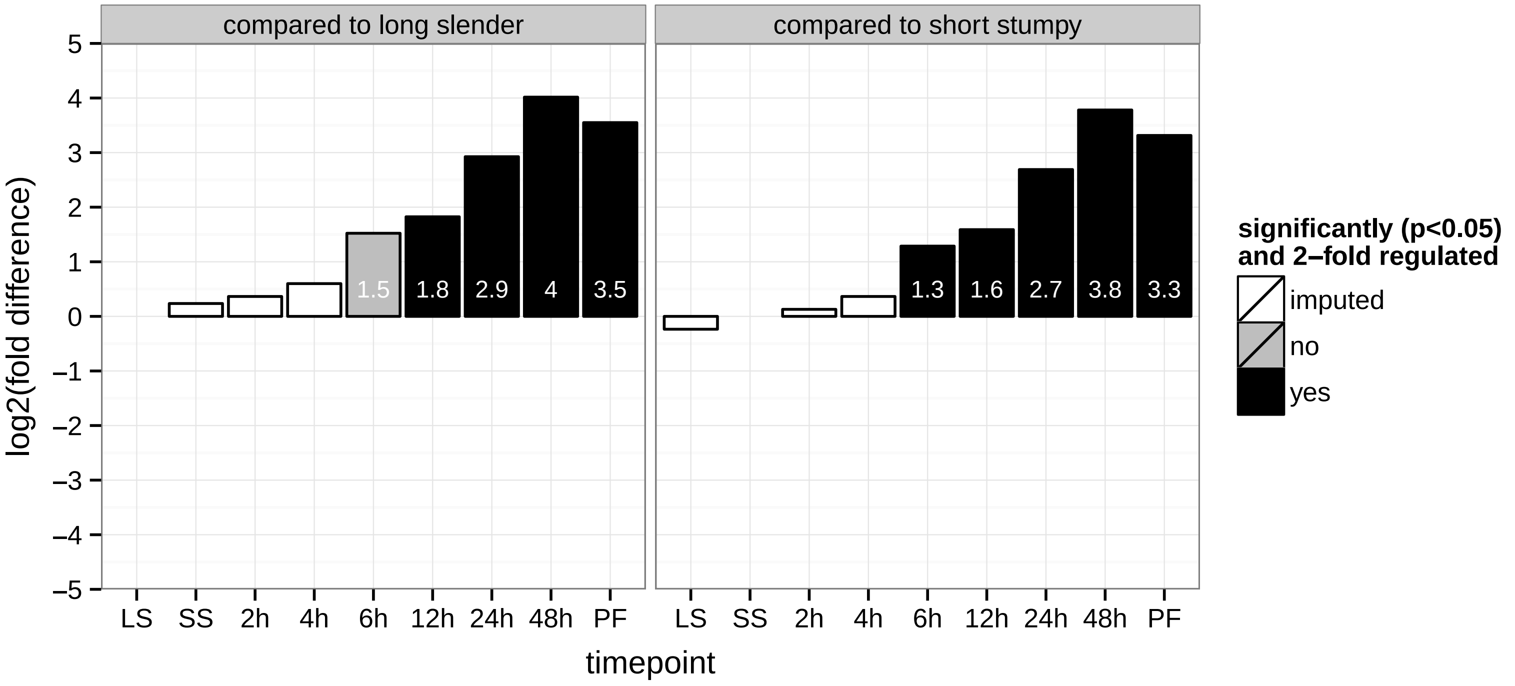

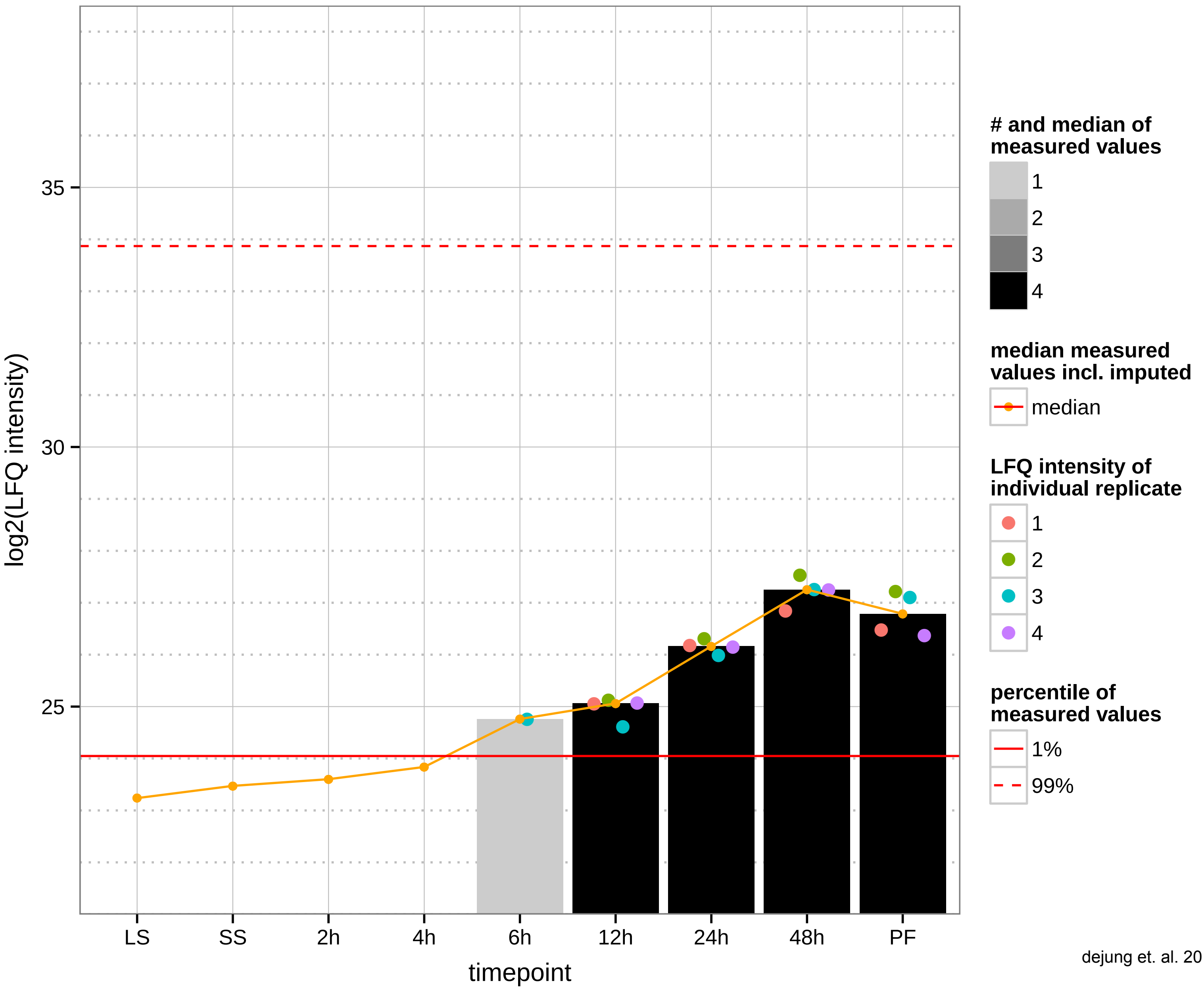

dejung et. al. 2015

receptor–type adenylate cyclase GRESAG 4, putative, adenylyl cyclase, ATP pyrophosphate–lyase
Tb927.5.330;Tb11.v5.0281
AGOF: null, adenylate cyclase activity
AGOC: null, integral to membrane
AGOP: null, cyclic nucleotide biosynthetic process, intracellular signal transduction
PGOF: phosphorus–oxygen lyase activity
PGOC: null
PGOP: cyclic nucleotide biosynthetic process, intracellular signal transduction

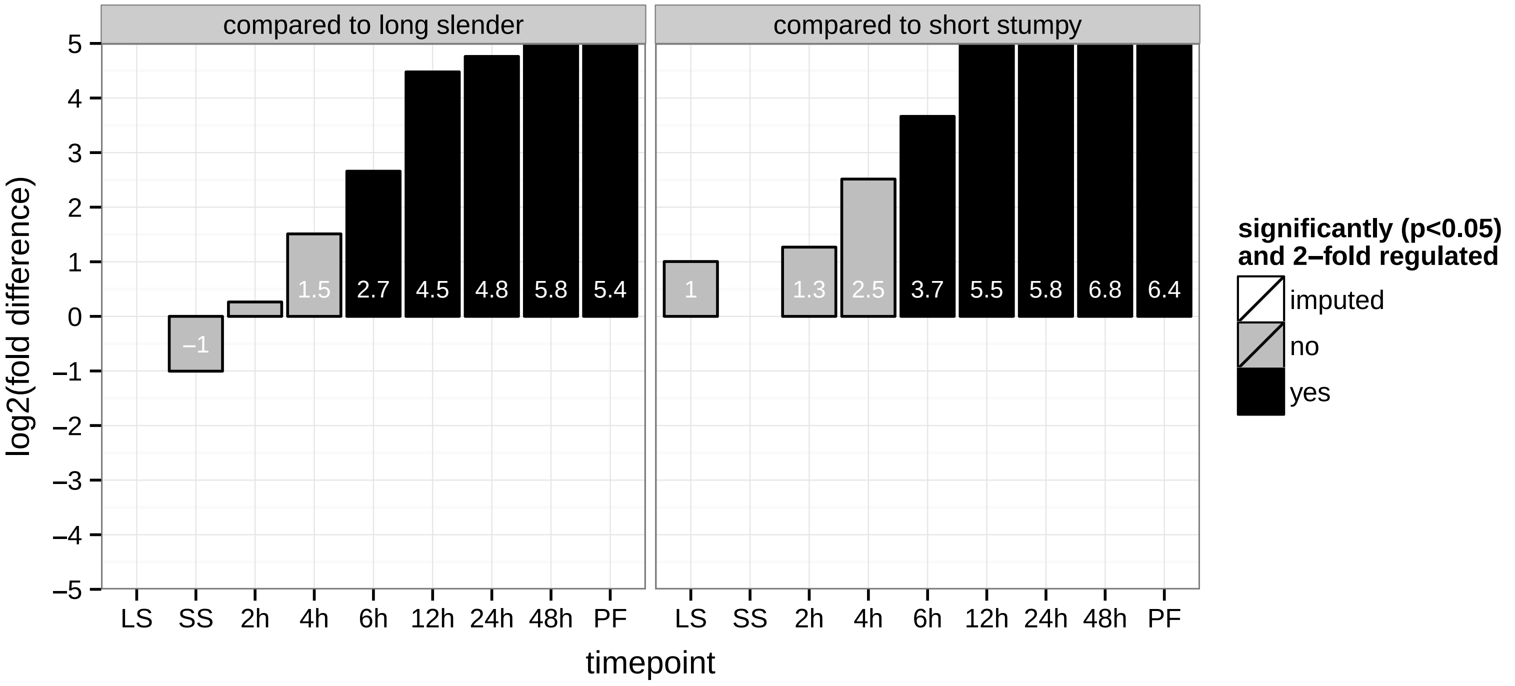

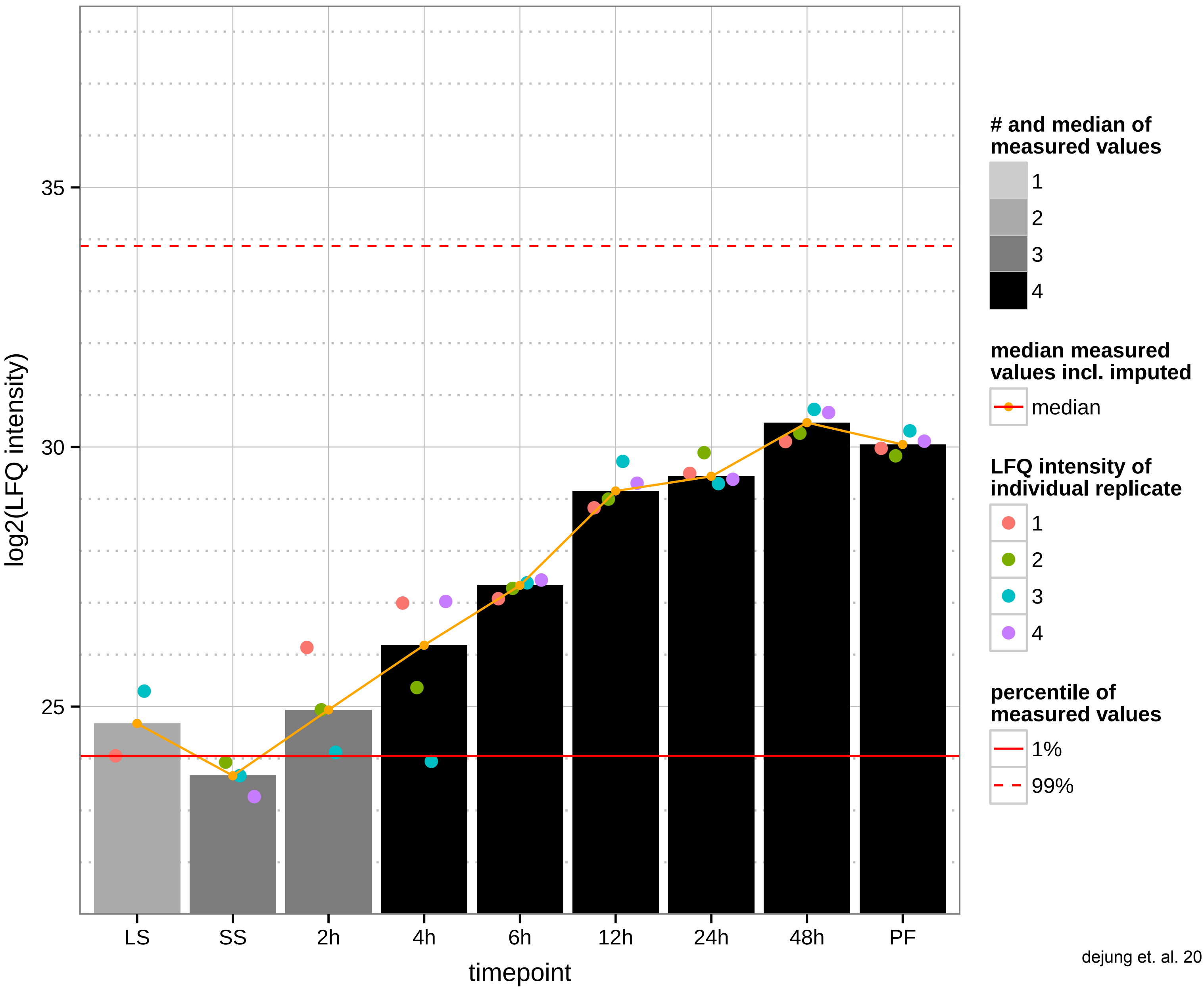

dejung et. al. 2015

hypothetical protein, conserved, predicted SET domain protein
Tb927.10.4600;Tb11.v5.0422
AGOF: null
AGOC: null
AGOP: null
PGOF: protein binding
PGOC: null
PGOP: null

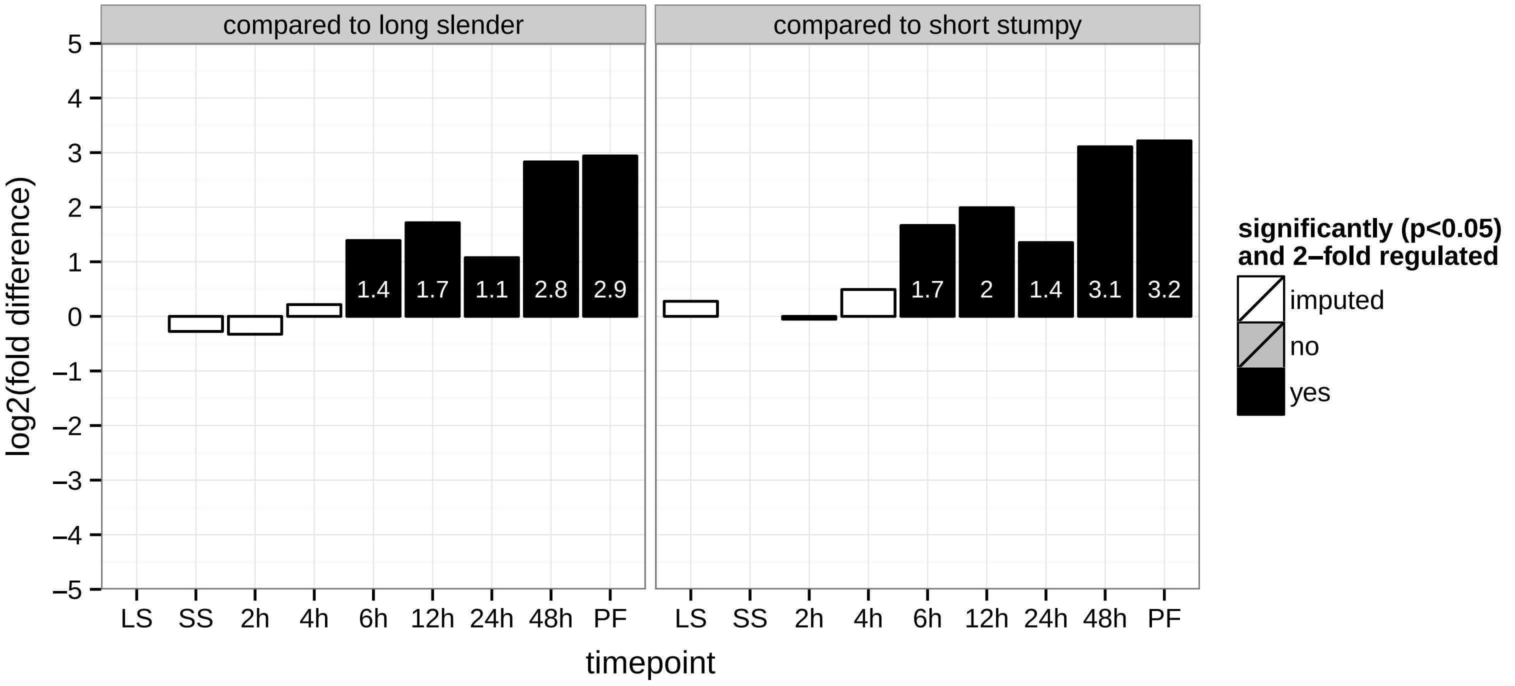

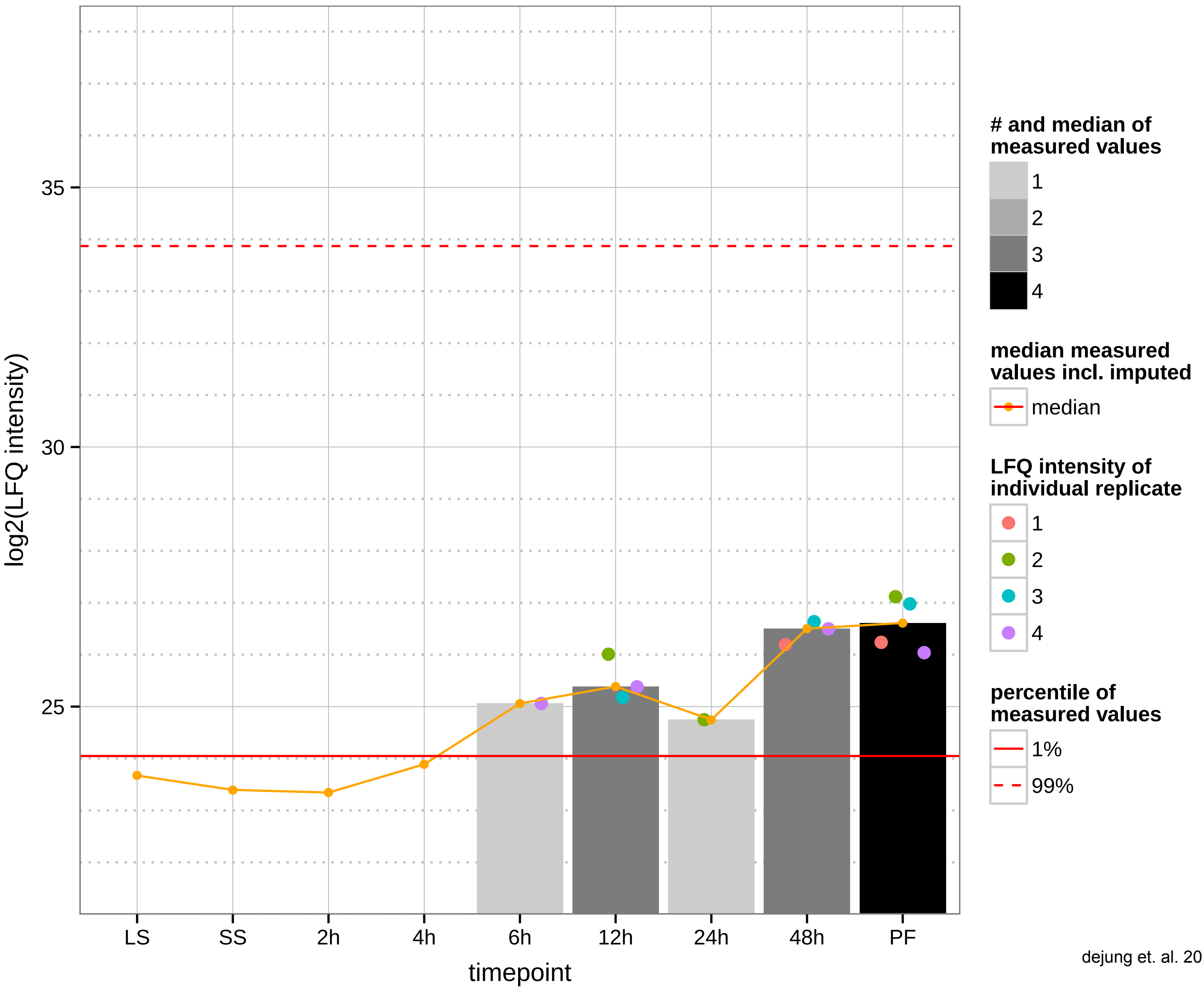

dejung et. al. 2015

delta–6 fatty acid desaturase, putative, delta–4 fatty acid desaturase
Tb927.10.7100;Tb11.v5.0580
AGOF: null, heme binding, oxidoreductase activity, stearoyl–CoA 9–desaturase activity
AGOC: null
AGOP: null, fatty acid biosynthetic process
PGOF: heme binding
PGOC: null
PGOP: lipid metabolic process

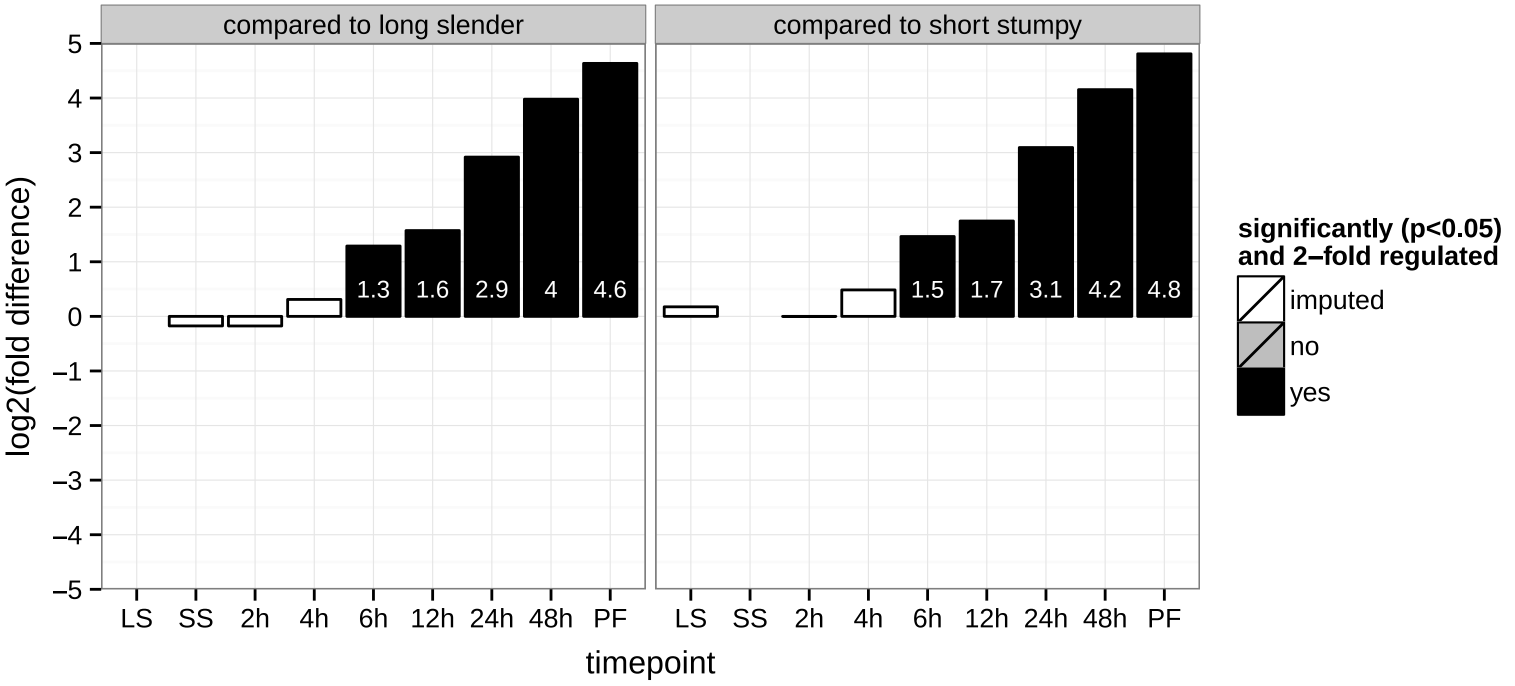

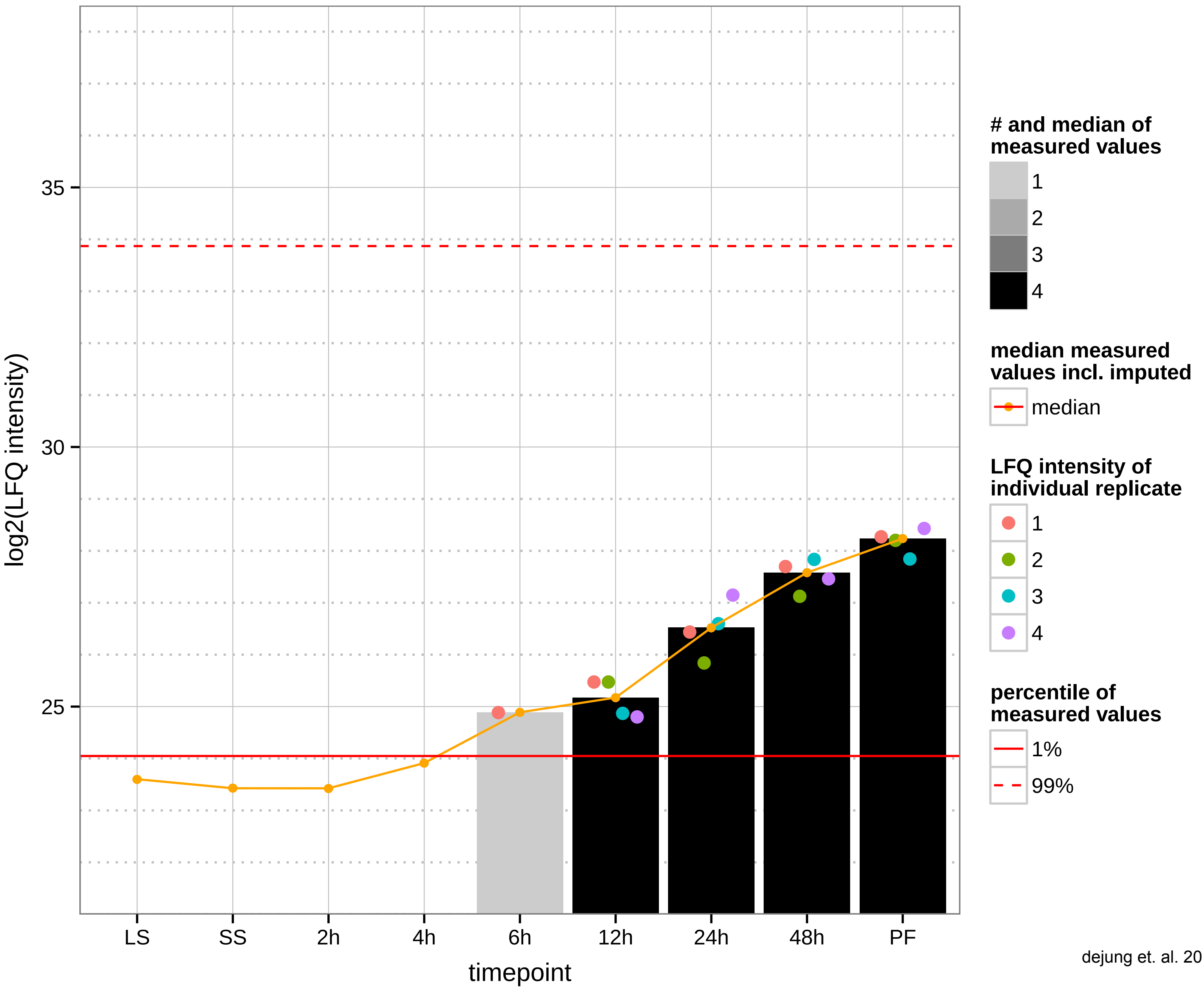

dejung et. al. 2015

transcription elongation factor 1 homolog (ELOF1)
Tb927.1.1630
AGOF: null
AGOC: null
AGOP: null
PGOF: null
PGOC: null
PGOP: null

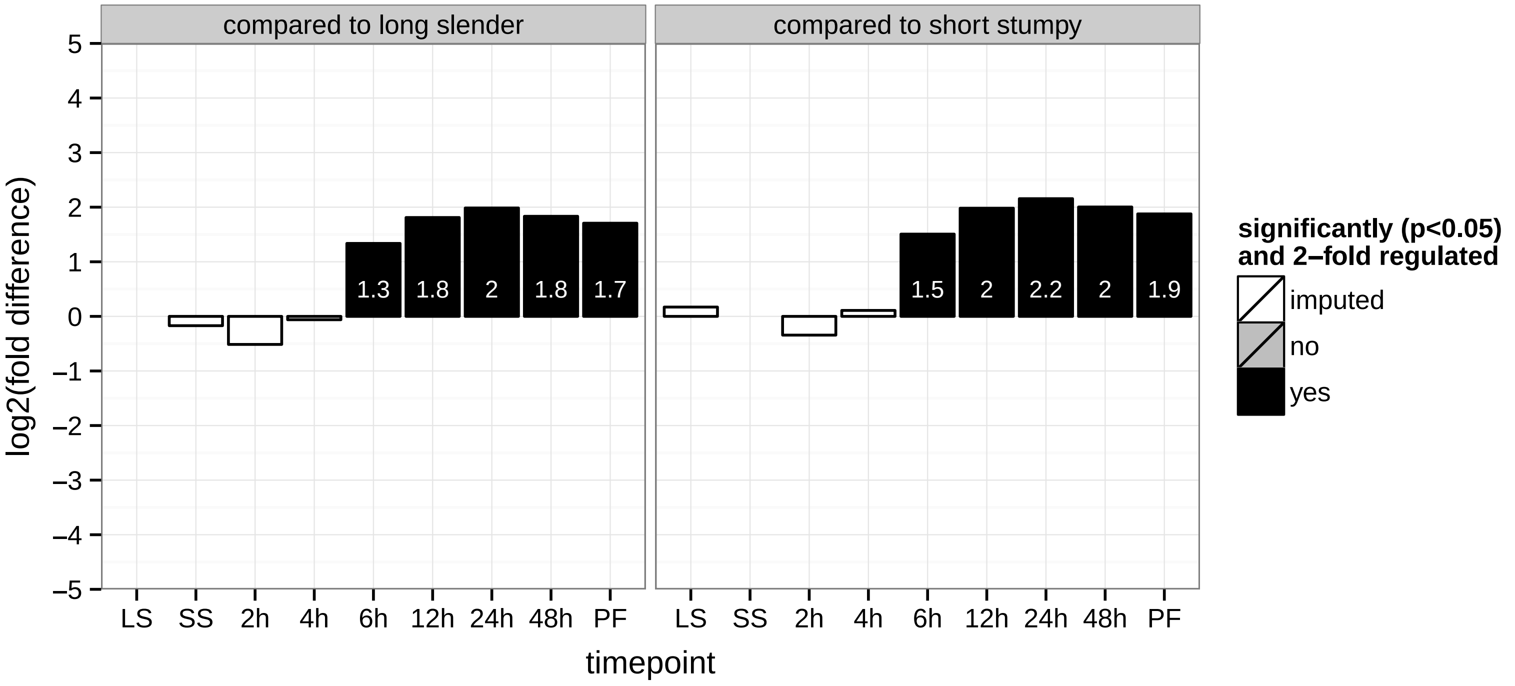

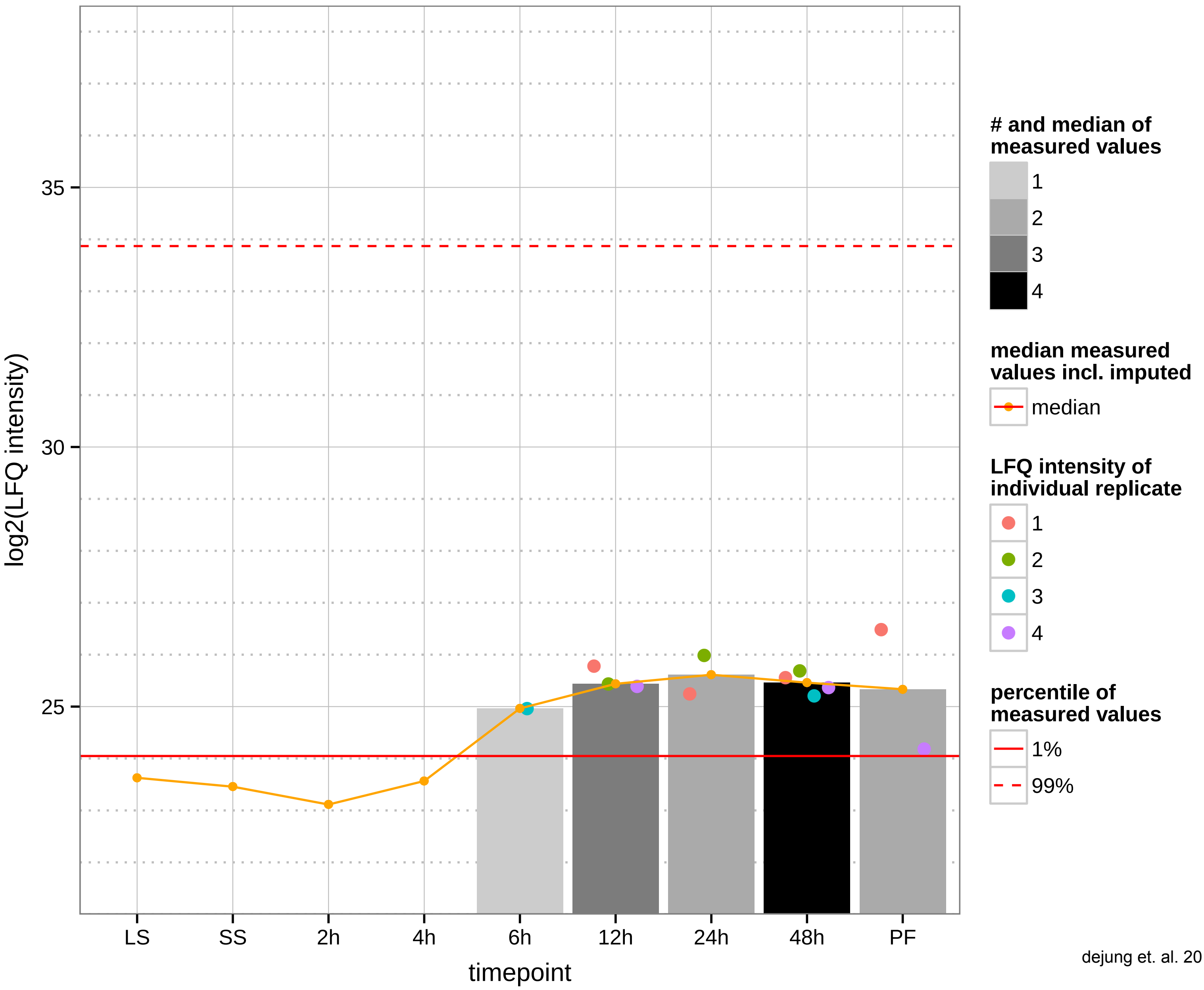

dejung et. al. 2015

transmembrane protein, putative
Tb927.1.840
AGOF: null
AGOC: mitochondrion
AGOP: null
PGOF: null
PGOC: null
PGOP: null

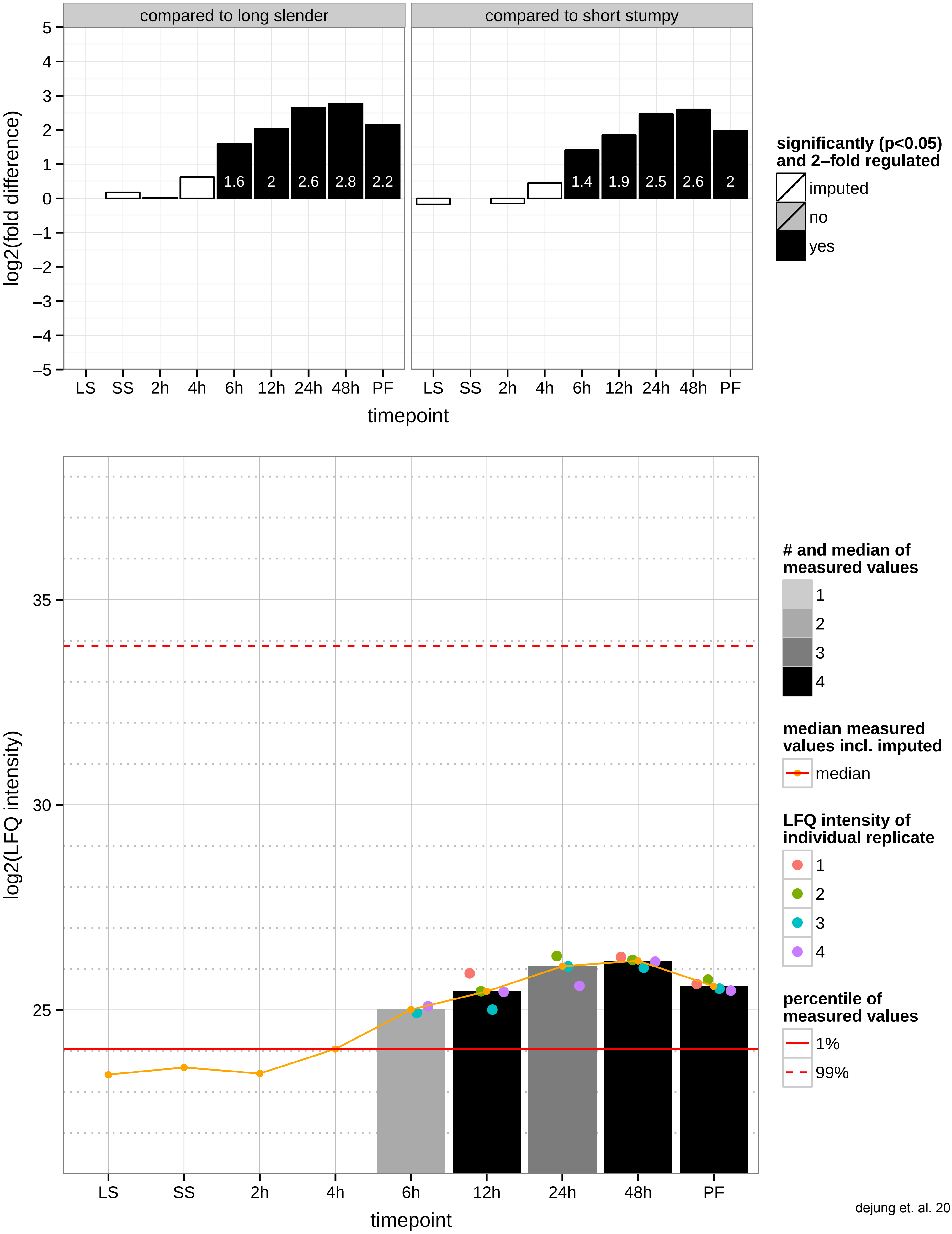

dejung et. al. 2015

hypothetical protein, conserved
Tb927.10.10120
AGOF: null
AGOC: mitochondrion
AGOP: null
PGOF: null
PGOC: null
PGOP: null

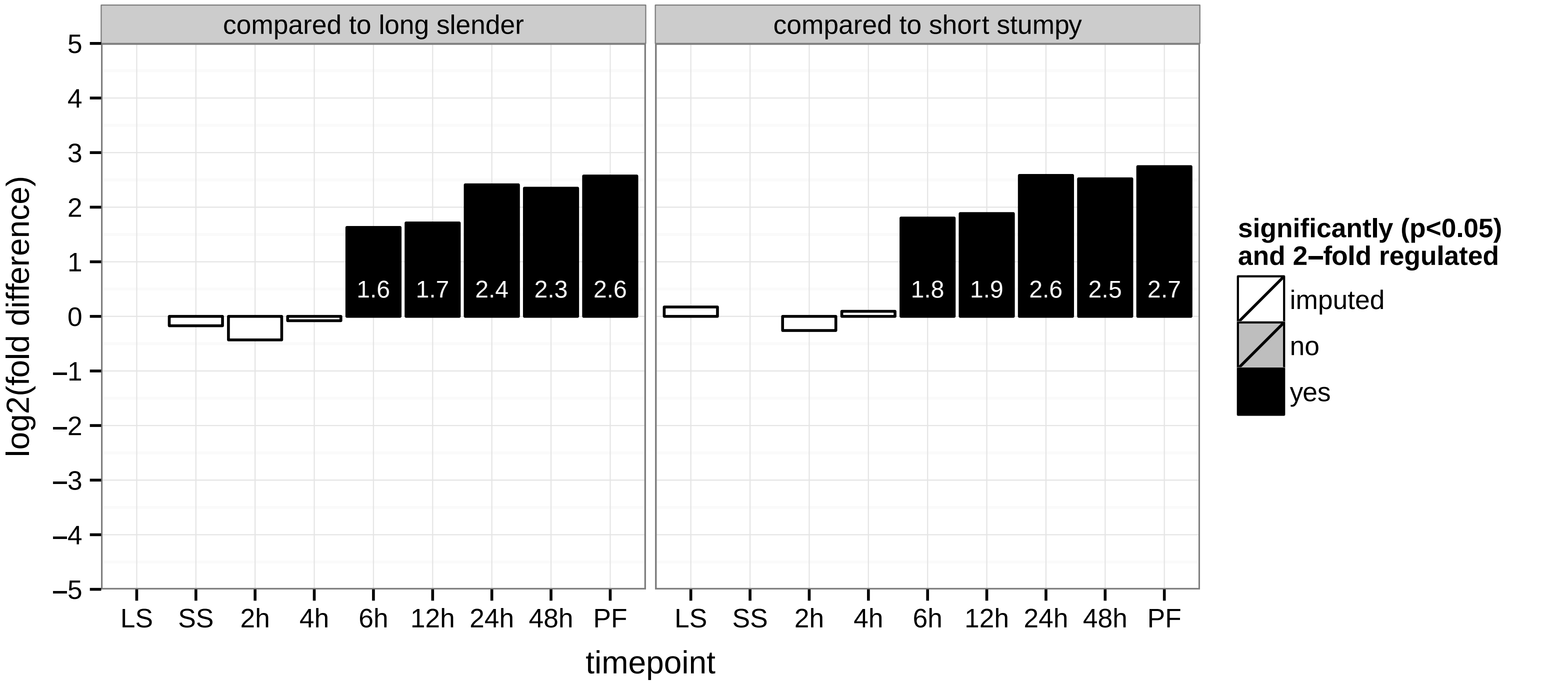

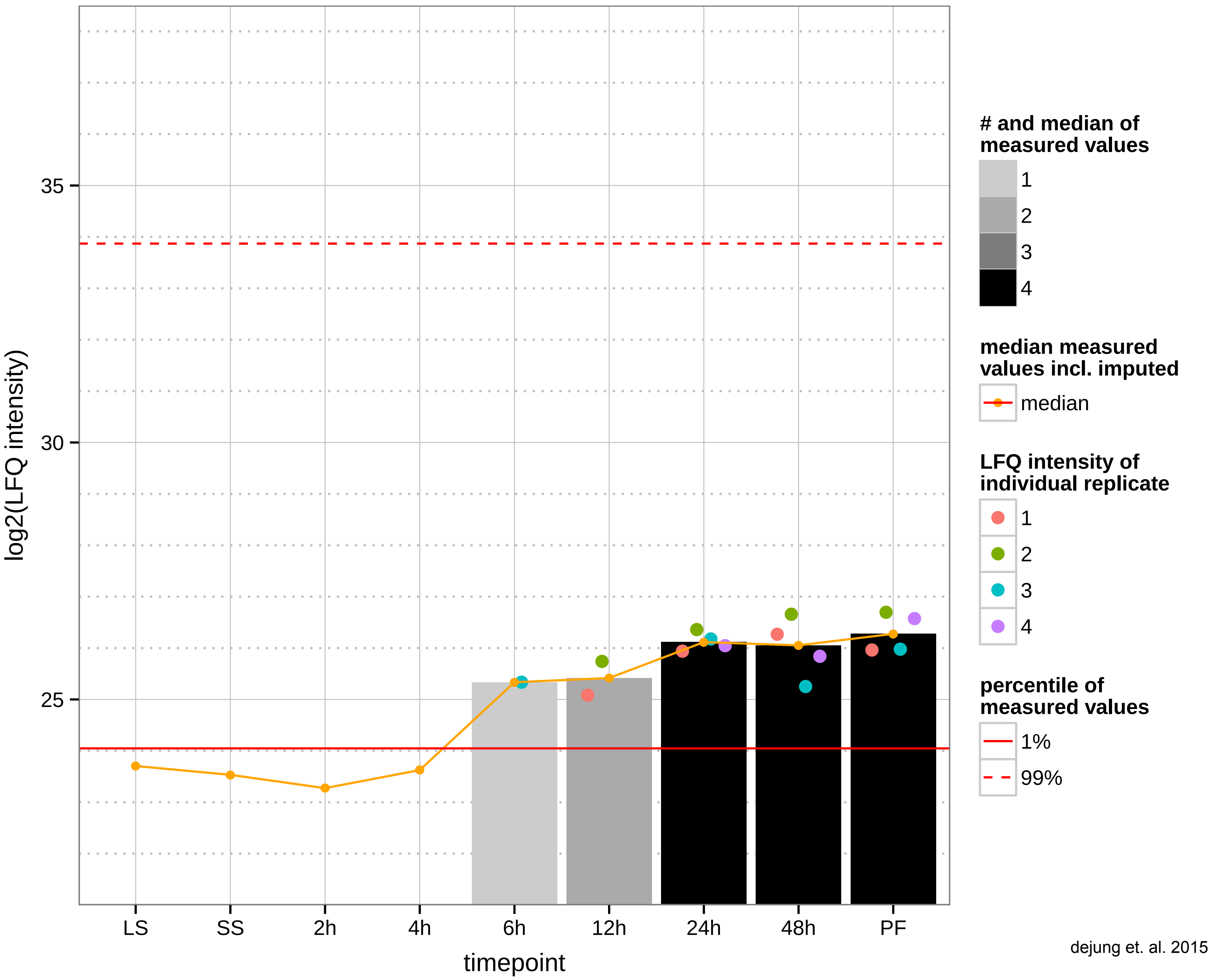

dejung et. al. 2015

mitochondrial malate dehydrogenase (mMDH)
Tb927.10.2560
AGOF: L−malate dehydrogenase activity, malate dehydrogenase activity, oxidoreductase activity
AGOC: mitochondrial inner membrane, mitochondrion
AGOP: glycolysis, malate metabolic process, oxidation−reduction process, tricarboxylic acid cycle
PGOF: L−malate dehydrogenase activity, catalytic activity, oxidoreductase activity, acting on the CH−OH group of donors, NAI
PGOC: null
PGOP: carbohydrate metabolic process, cellular carbohydrate metabolic process, malate metabolic process, oxidation−reduct

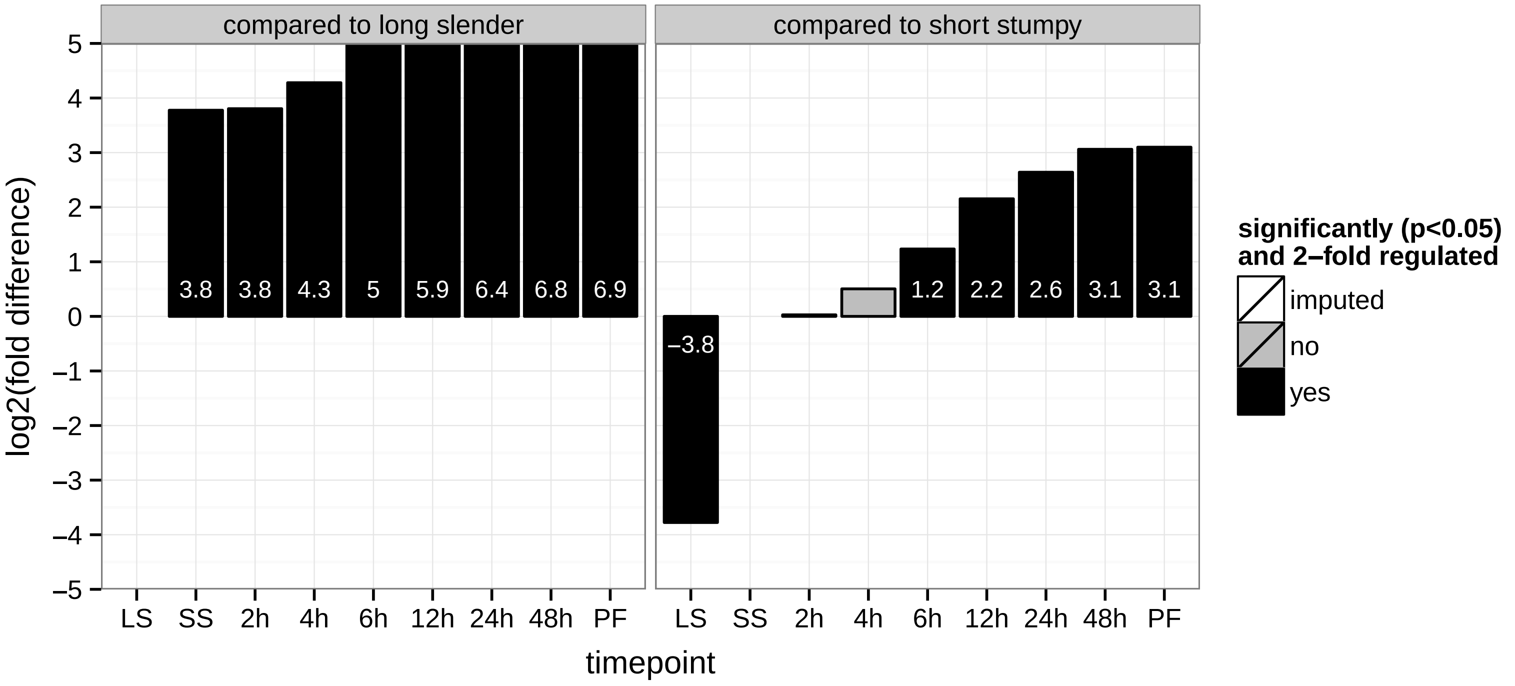

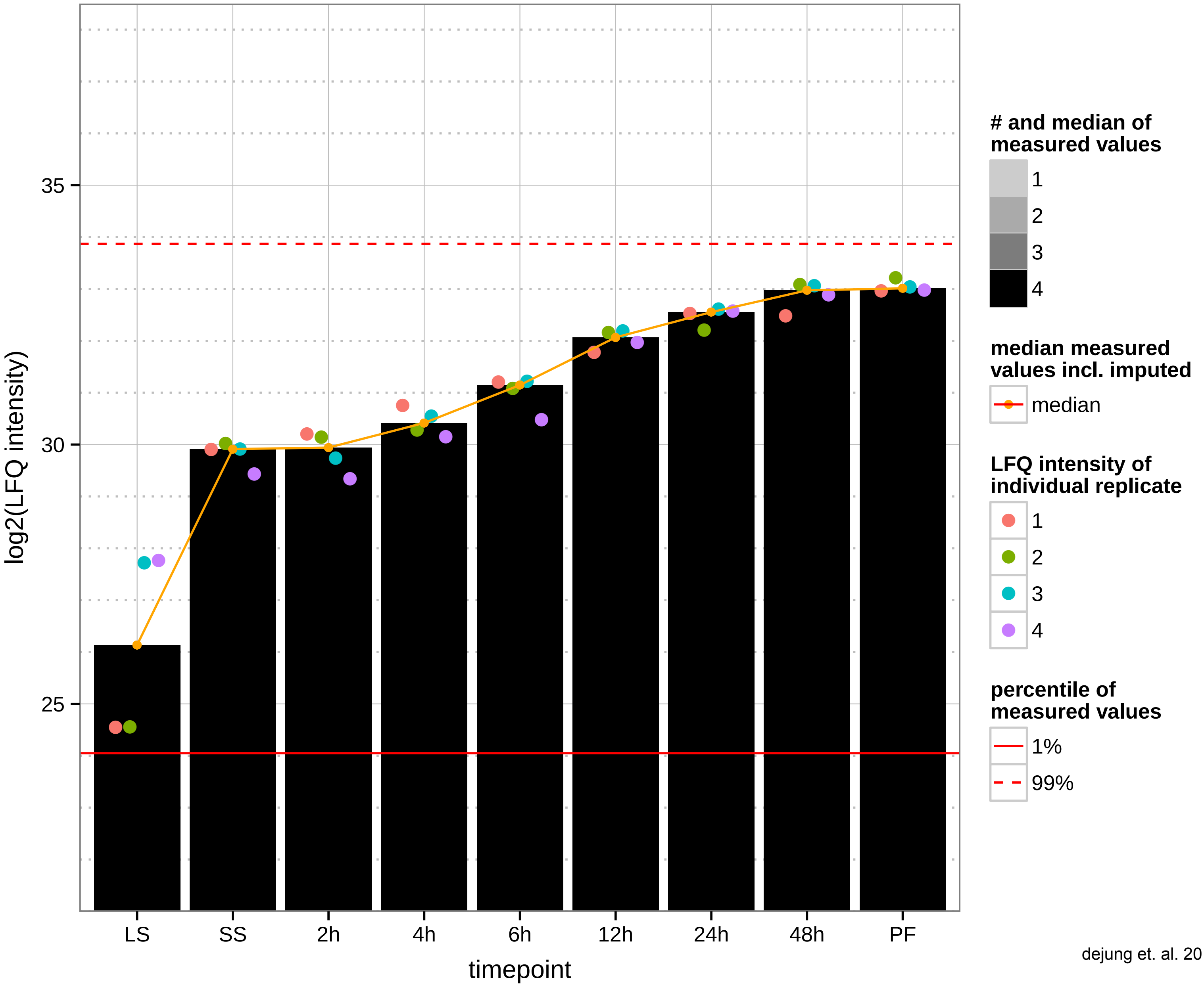

dejung et. al. 2015

choline dehydrogenase, putative
Tb927.10.470
AGOF: choline dehydrogenase activity, oxidoreductase activity, acting on paired donors, with incorporation or reduction of mol
AGOC: mitochondrion
AGOP: carotenoid biosynthetic process, glycine betaine biosynthetic process from choline
PGOF: flavin adenine dinucleotide binding, oxidoreductase activity, acting on CH–OH group of donors
PGOC: null
PGOP: oxidation–reduction process

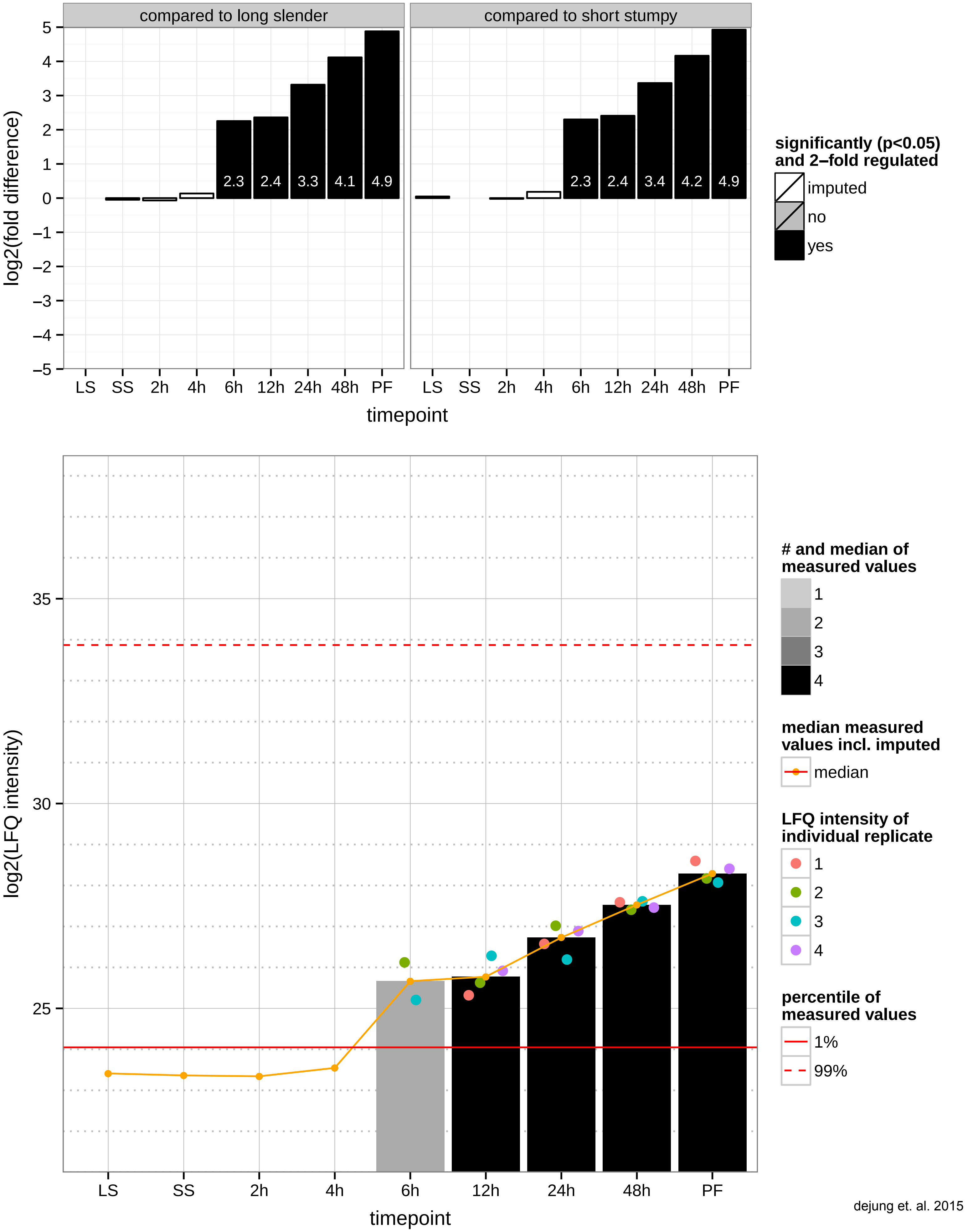

dejung et. al. 2015

protein kinase, putative, (OTHER) NEK family, HsNEK1–like
Tb927.10.5940;Tb927.10.5930
AGOF: ATP binding, protein serine/threonine kinase activity, protein tyrosine kinase activity
AGOC: null
AGOP: modulation of development of symbiont involved in interaction with host, protein phosphorylation, quorum sensing invo
PGOF: ATP binding, phospholipid binding, protein binding, protein kinase activity, transferase activity, transferring phosphorus
PGOC: null
PGOP: protein phosphorylation

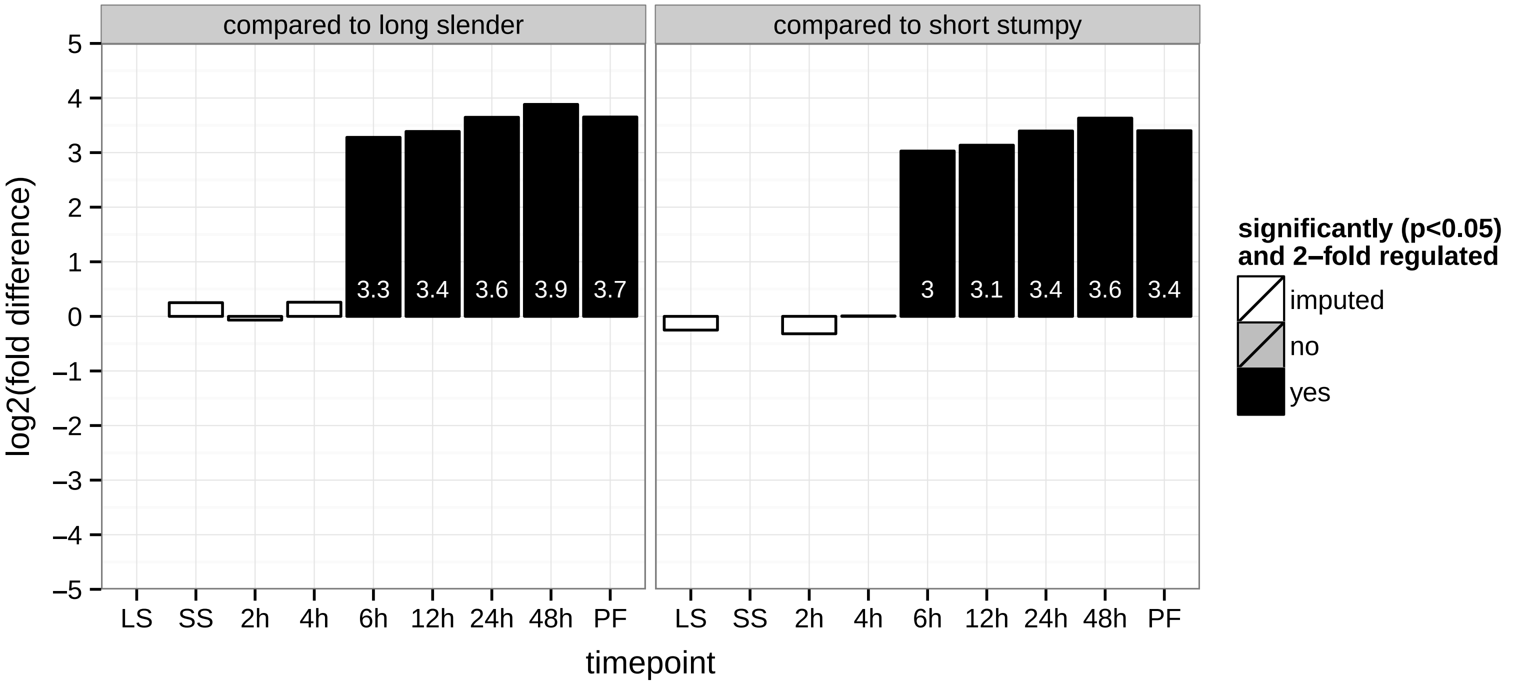

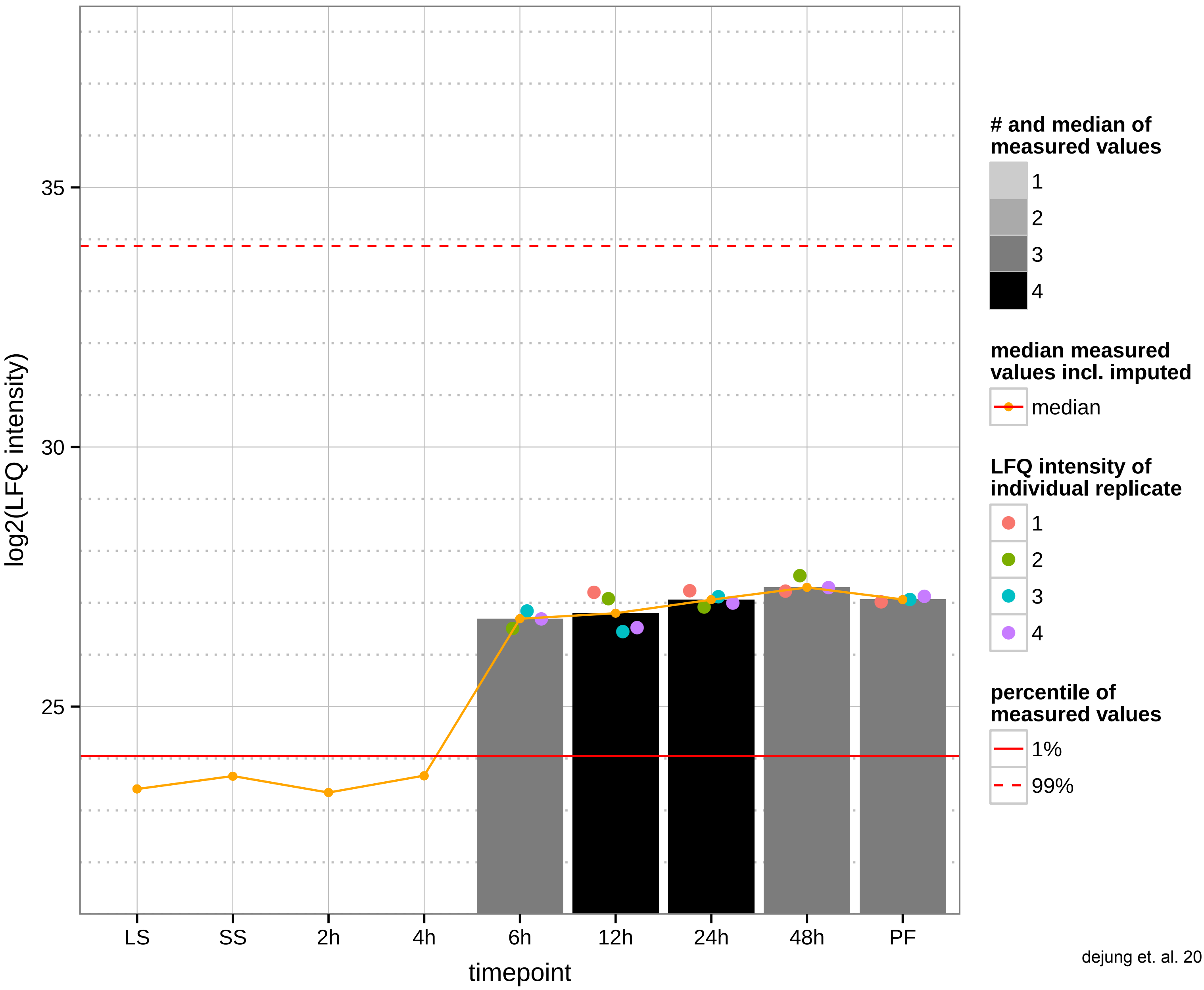

dejung et. al. 2015

ATP−dependent DEAD/H RNA helicase HEL64, putative (HEL64)
Tb927.10.6630
AGOF: ATP binding, ATP−dependent helicase activity, helicase activity, nucleic acid binding
AGOC: cytosol
AGOP: nucleobase−containing compound metabolic process
PGOF: ATP binding, ATP−dependent helicase activity, helicase activity, nucleic acid binding
PGOC: null
PGOP: null

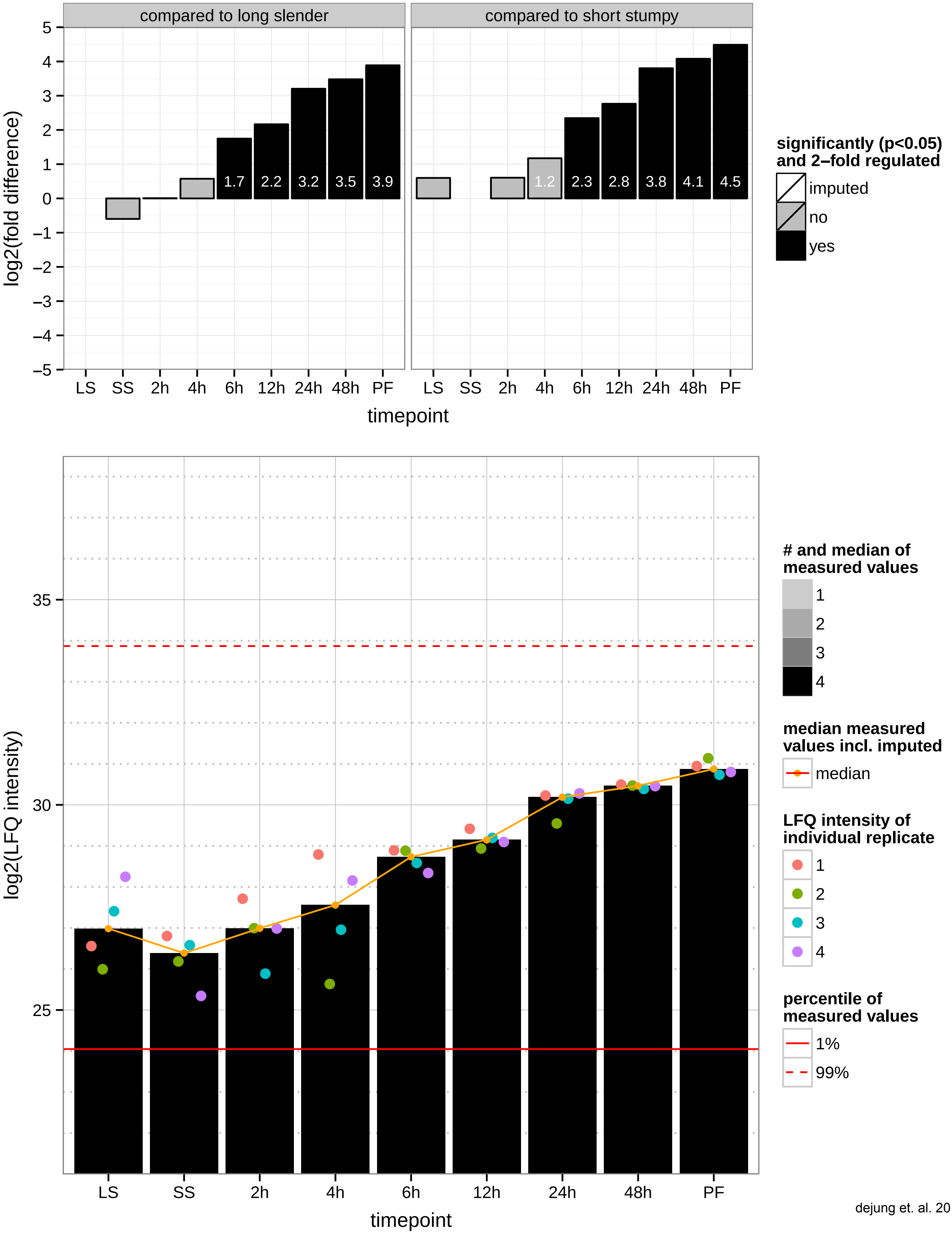

dejung et. al. 2015

hypothetical protein, conserved
Tb927.10.9000
AGOF: null
AGOC: mitochondrion
AGOP: null
PGOF: null
PGOC: null
PGOP: null

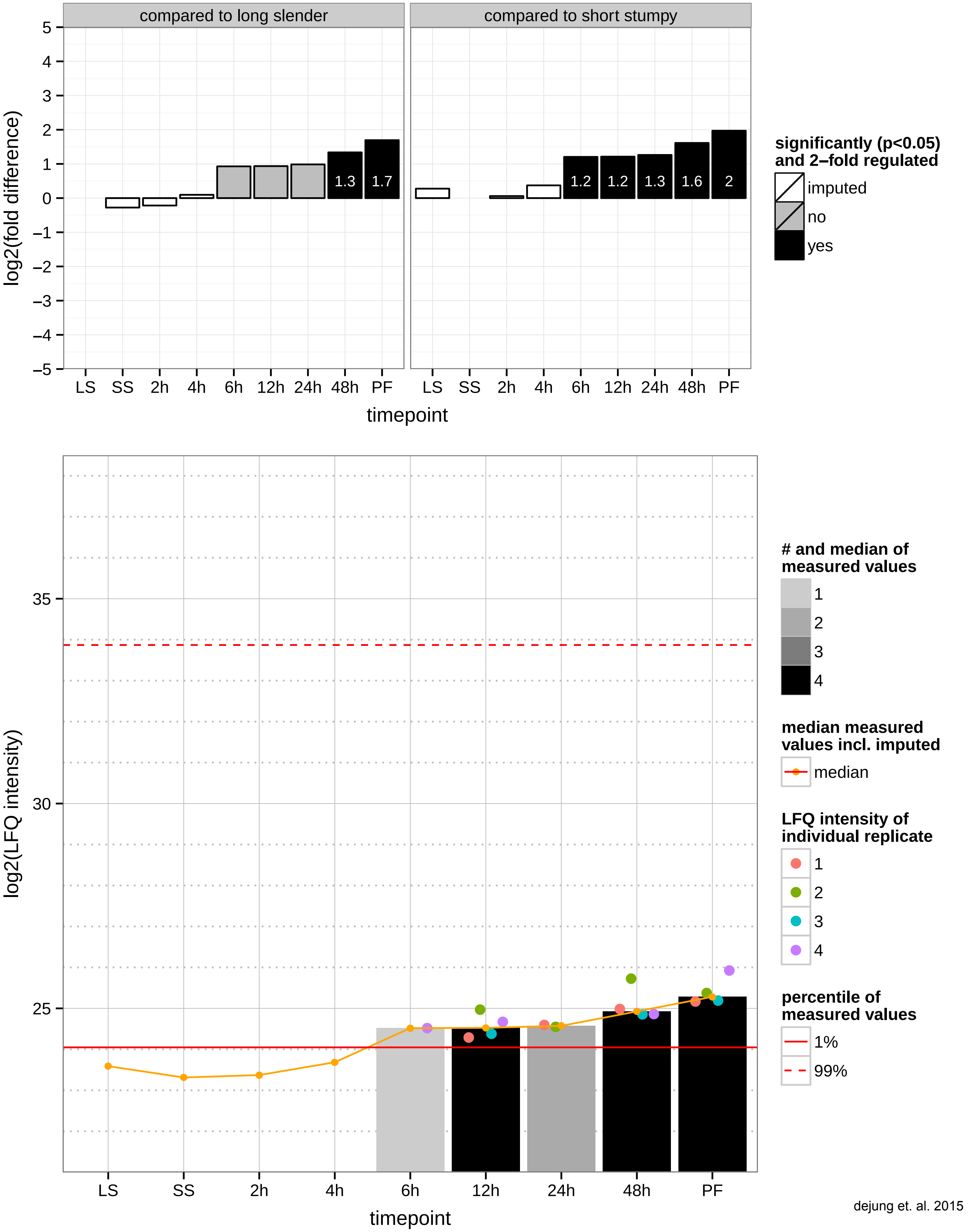

dejung et. al. 2015

hypothetical protein, conserved
Tb927.11.10150
AGOF: catalytic activity
AGOC: mitochondrion
AGOP: metabolic process
PGOF: catalytic activity
PGOC: null
PGOP: metabolic process

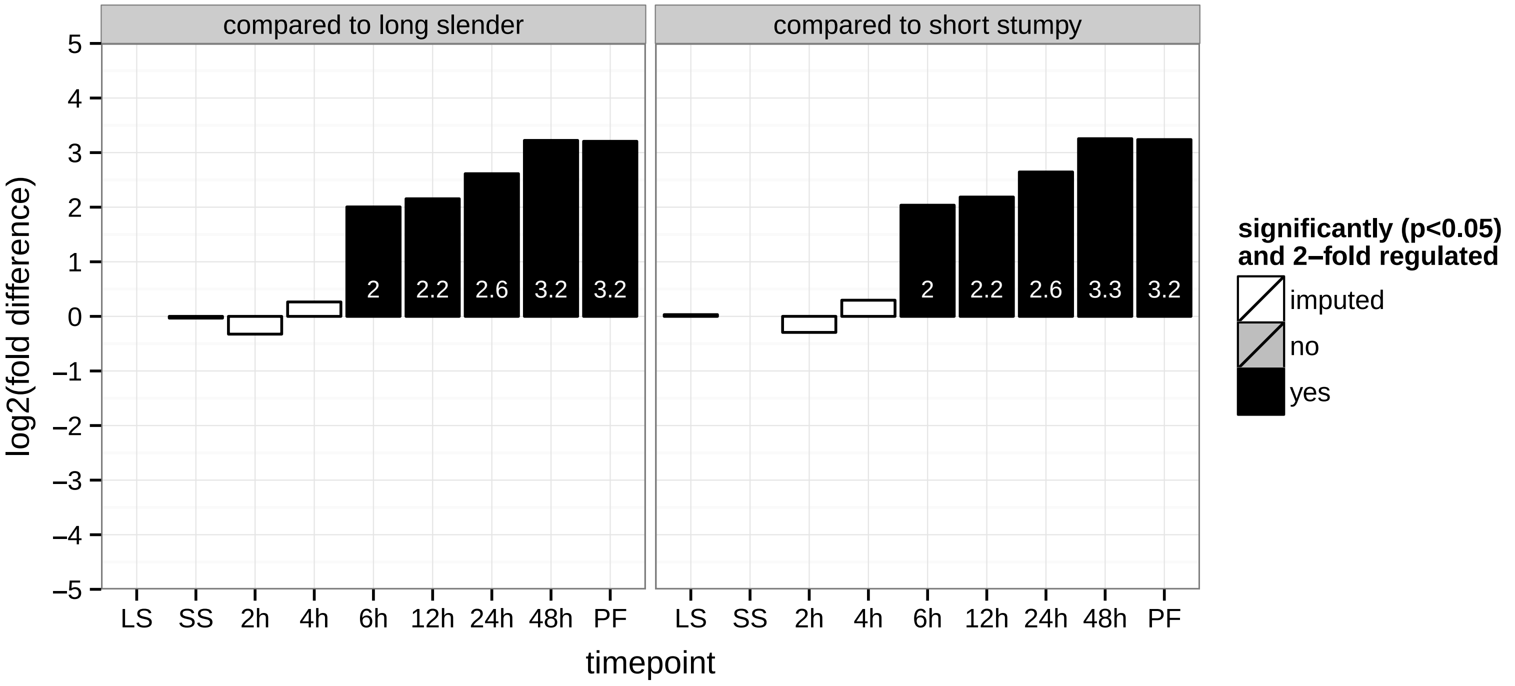

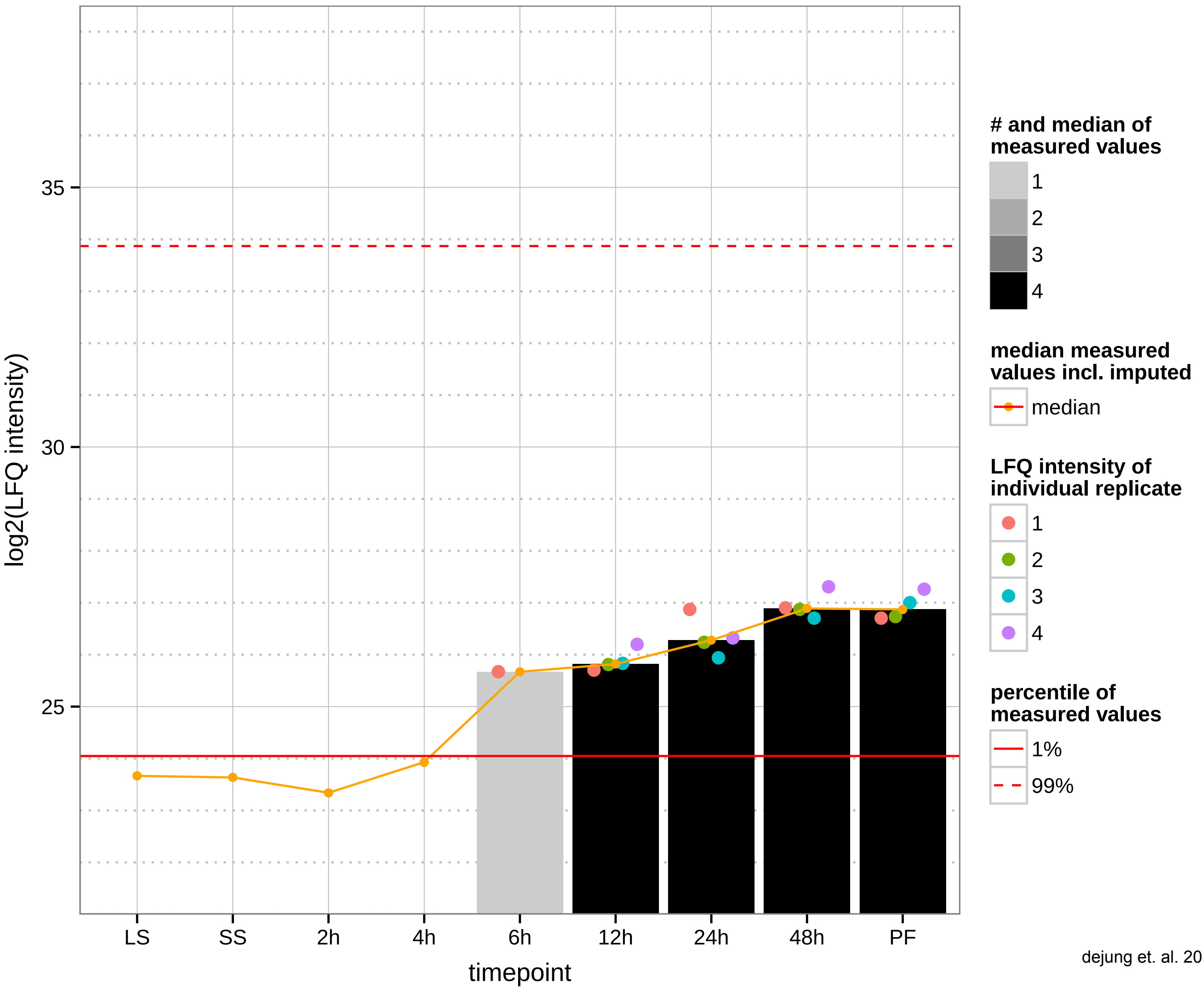

dejung et. al. 2015

glycosyl hydrolase–like protein
Tb927.11.10370
AGOF: hydrolase activity, hydrolyzing O–glycosyl compounds
AGOC: null
AGOP: carbohydrate metabolic process
PGOF: hydrolase activity, hydrolyzing O–glycosyl compounds
PGOC: null
PGOP: carbohydrate metabolic process

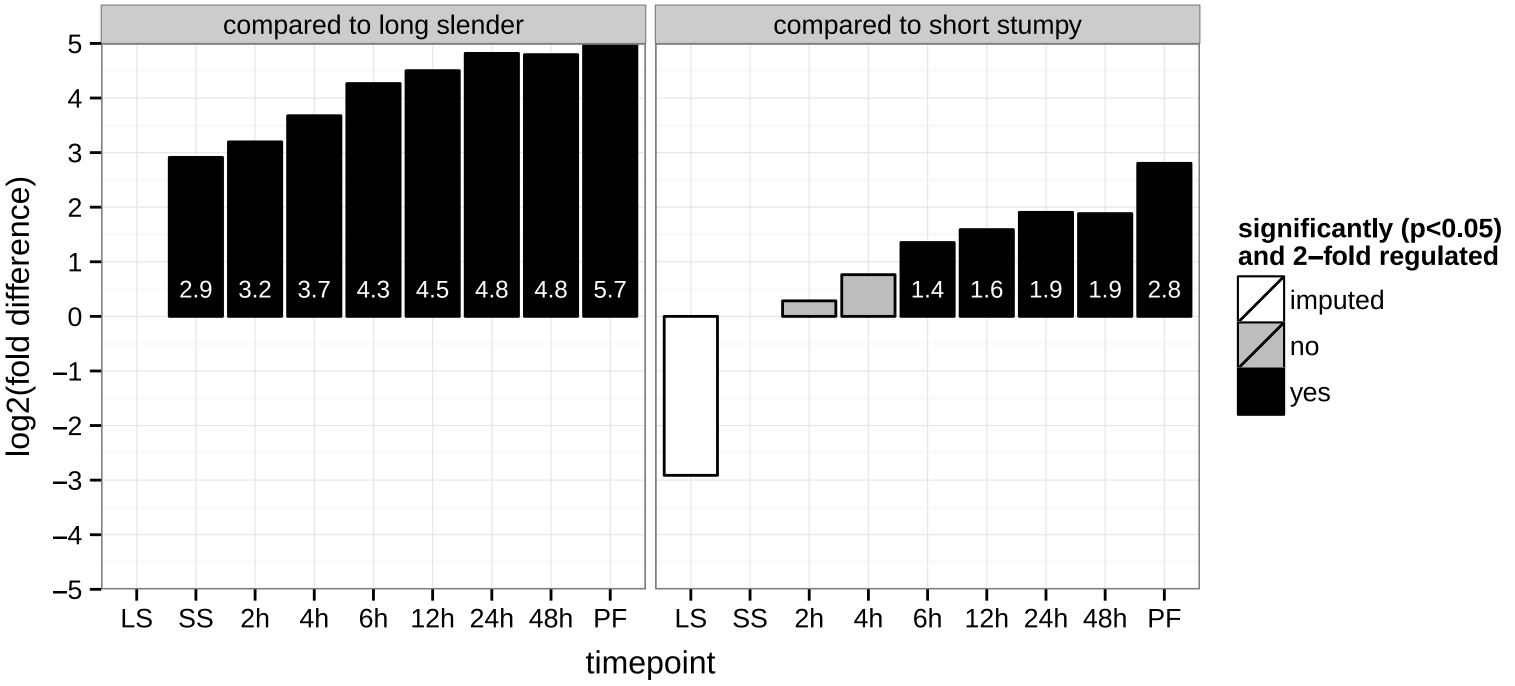

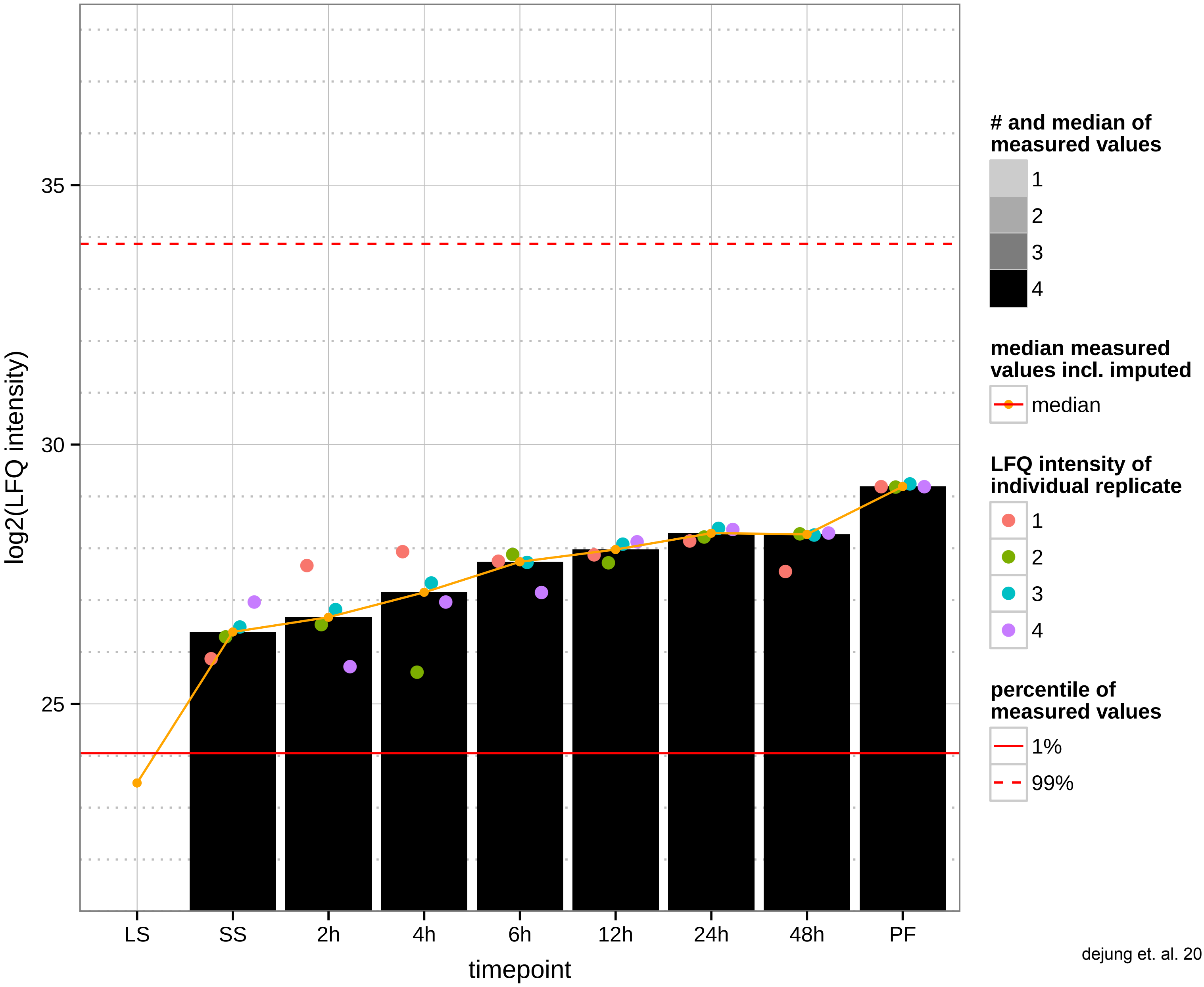

dejung et. al. 2015

hypothetical protein, conserved
Tb927.11.10770
AGOF: null
AGOC: null
AGOP: intracellular protein transport
PGOF: null
PGOC: null
PGOP: intracellular protein transport

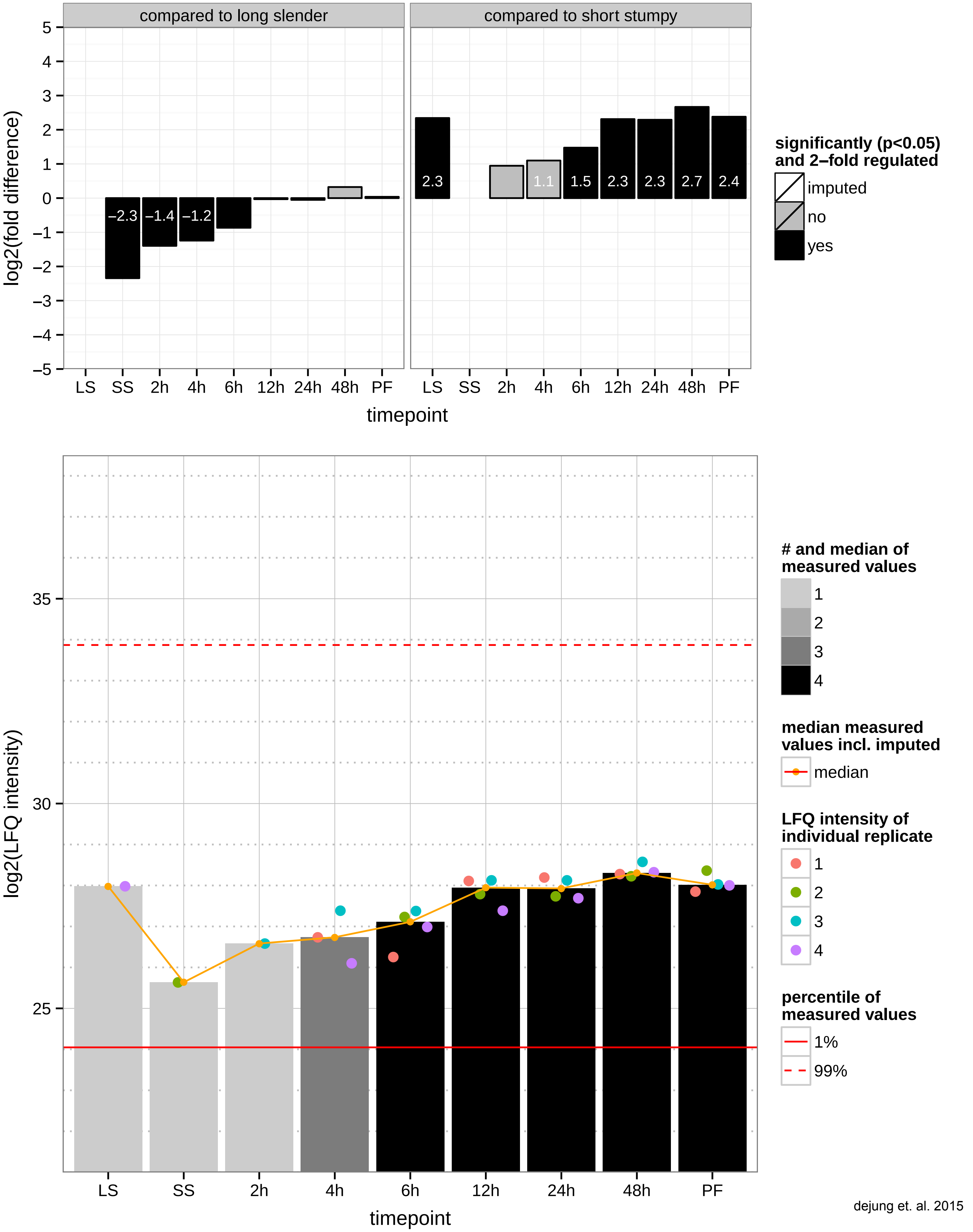

dejung et. al. 2015

hypothetical protein, conserved
Tb927.11.12440
AGOF: DNA binding, zinc ion binding
AGOC: nucleus
AGOP: DNA–dependent transcription, initiation, histone modification
PGOF: DNA binding, protein binding, zinc ion binding
PGOC: nucleus
PGOP: DNA–dependent transcription, initiation, histone modification

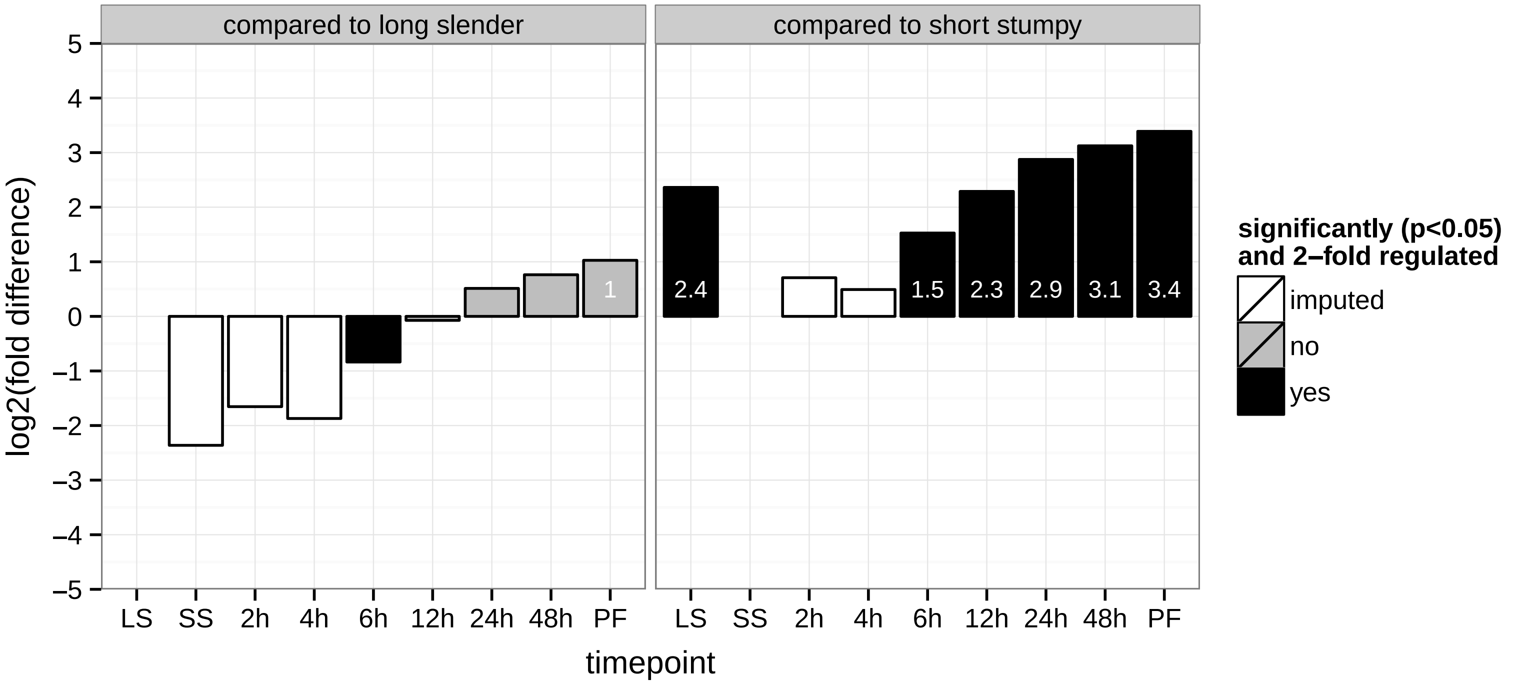

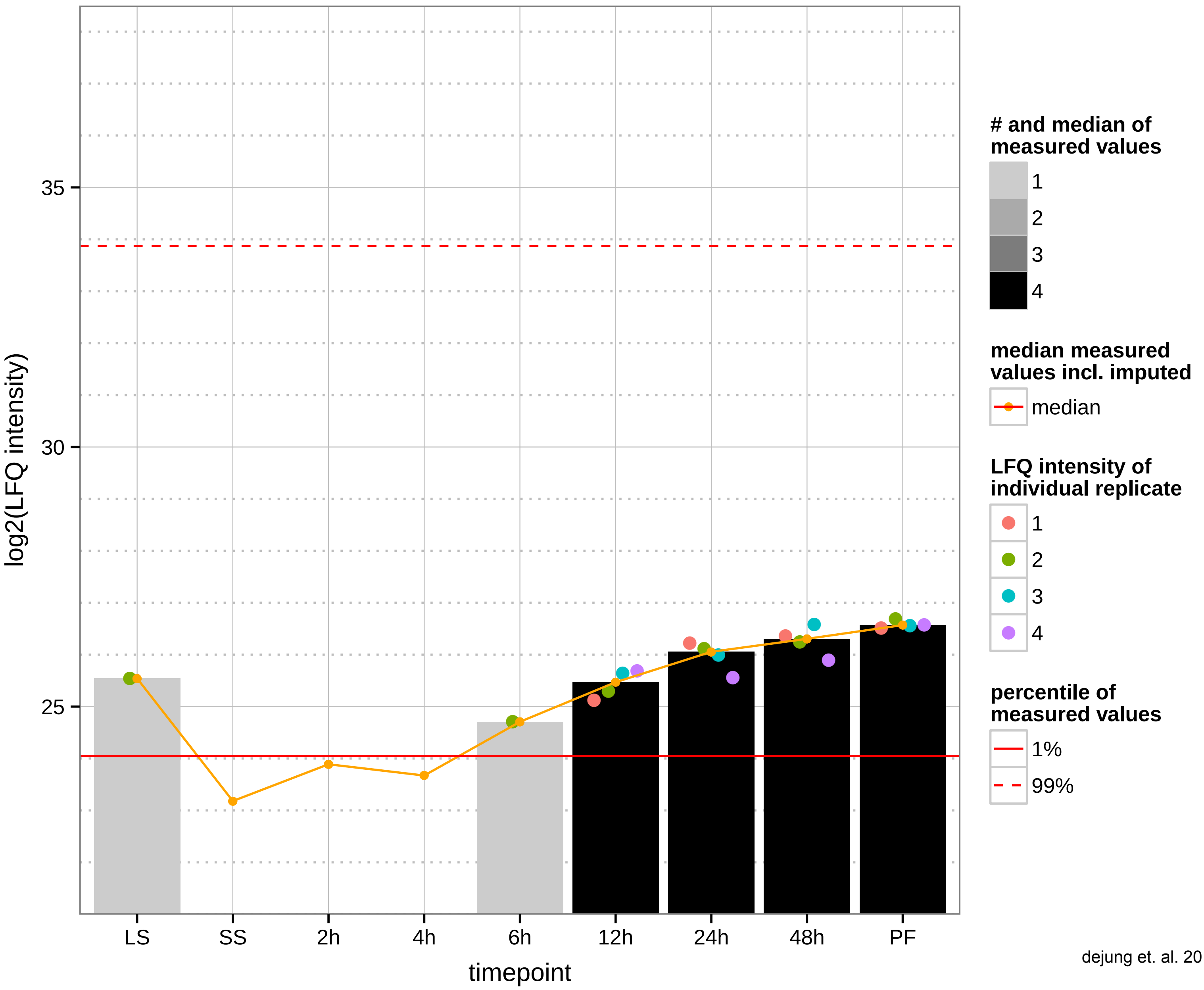

dejung et. al. 2015

cytochrome b5, putative (CYB5)
Tb927.11.13650
AGOF: glutathione−disulfide reductase activity, heme binding
AGOC: integral to membrane
AGOP: oxidation−reduction process
PGOF: heme binding
PGOC: null
PGOP: null

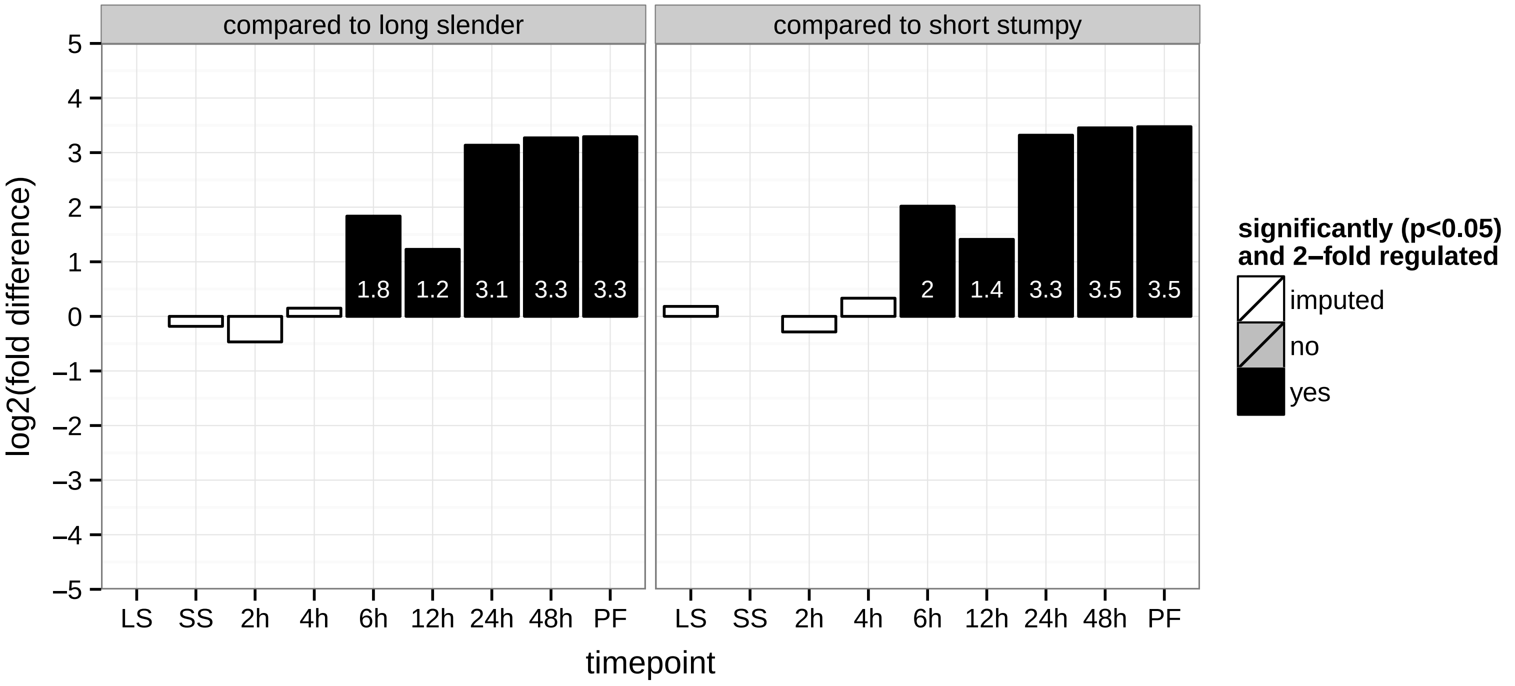

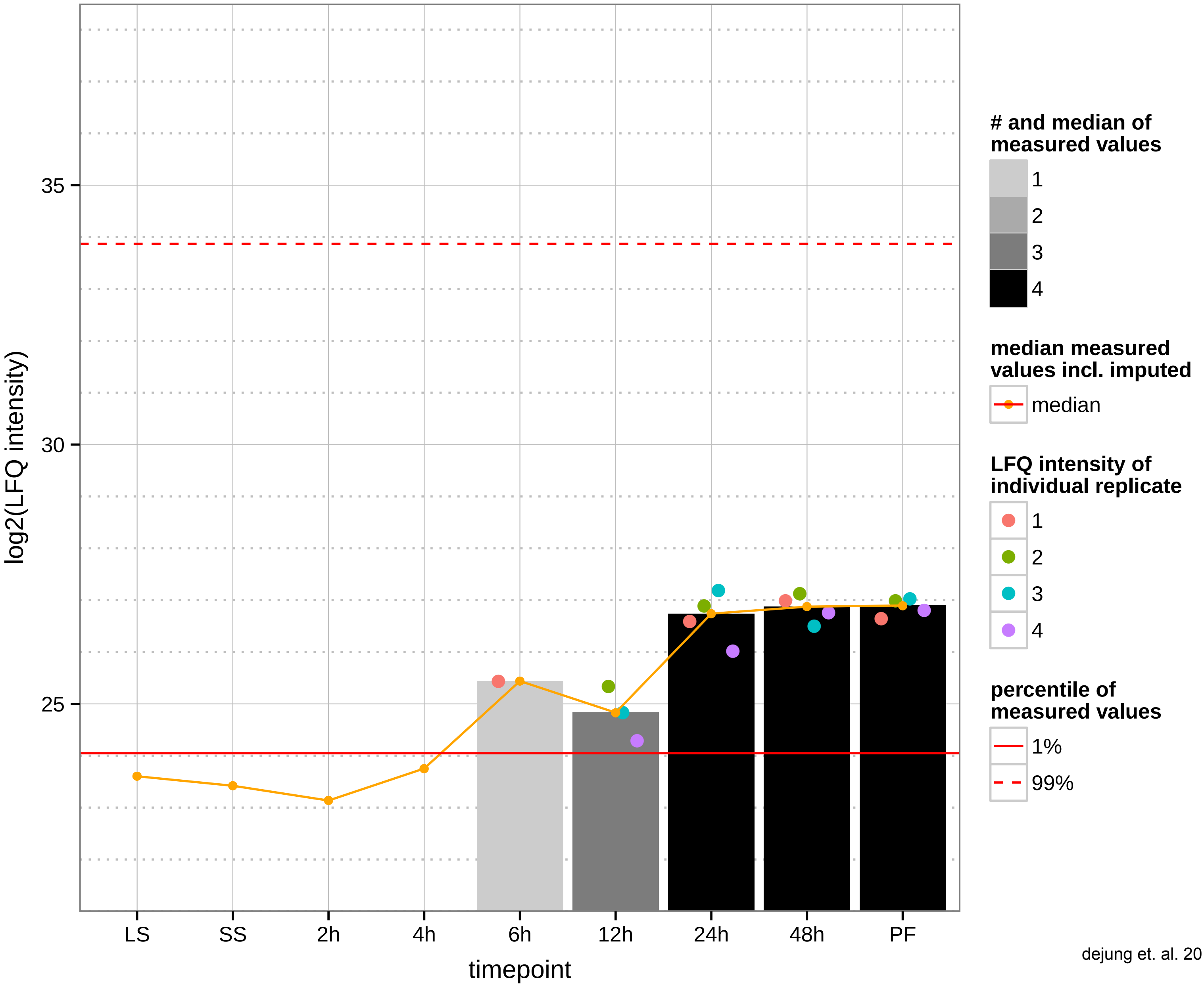

dejung et. al. 2015

hypothetical protein, conserved
Tb927.11.14160
AGOF: null
AGOC: null
AGOP: null
PGOF: carboxylesterase activity
PGOC: null
PGOP: null

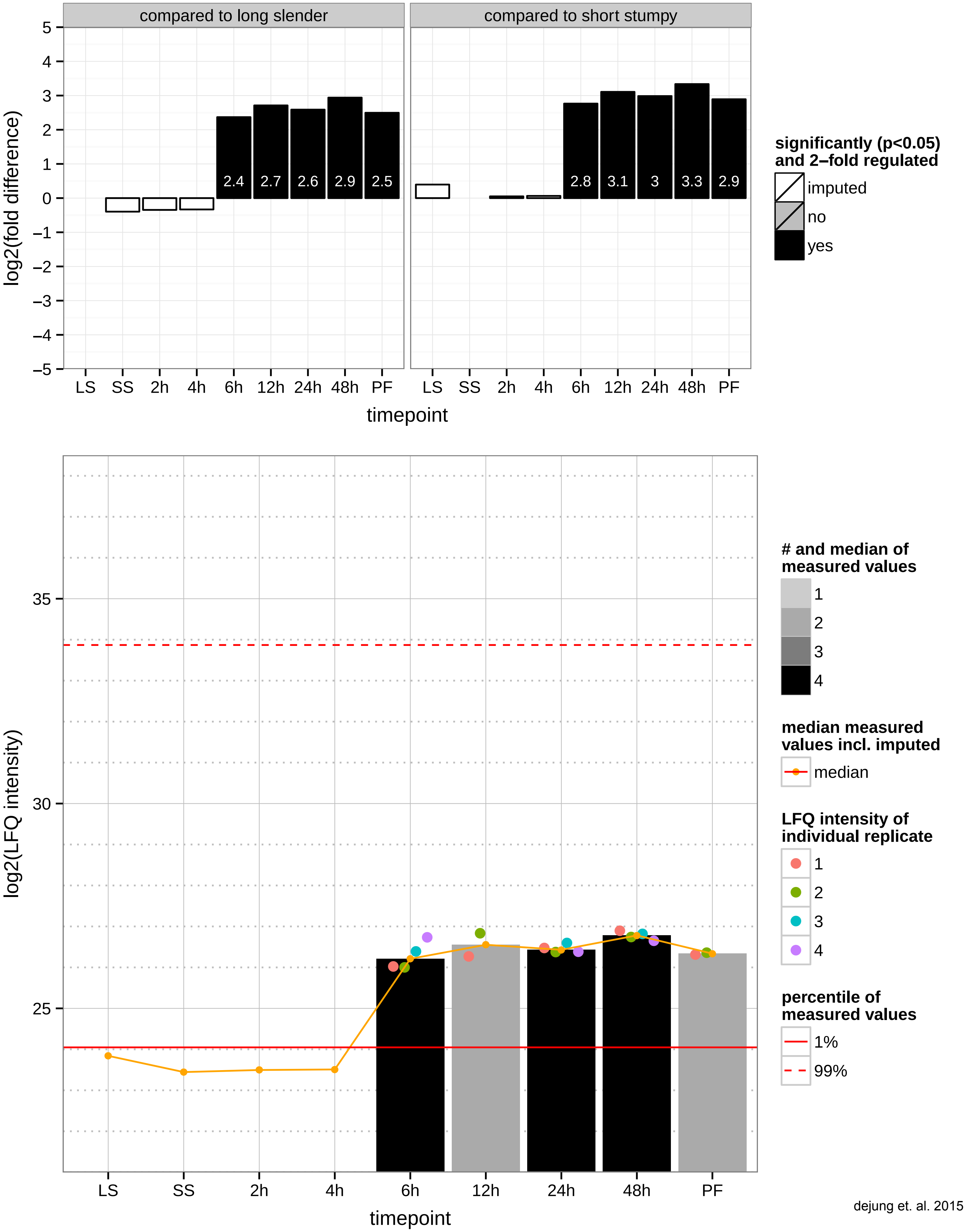

dejung et. al. 2015

hypothetical protein, conserved
Tb927.11.15630
AGOF: null
AGOC: null
AGOP: null
PGOF: null
PGOC: null
PGOP: null

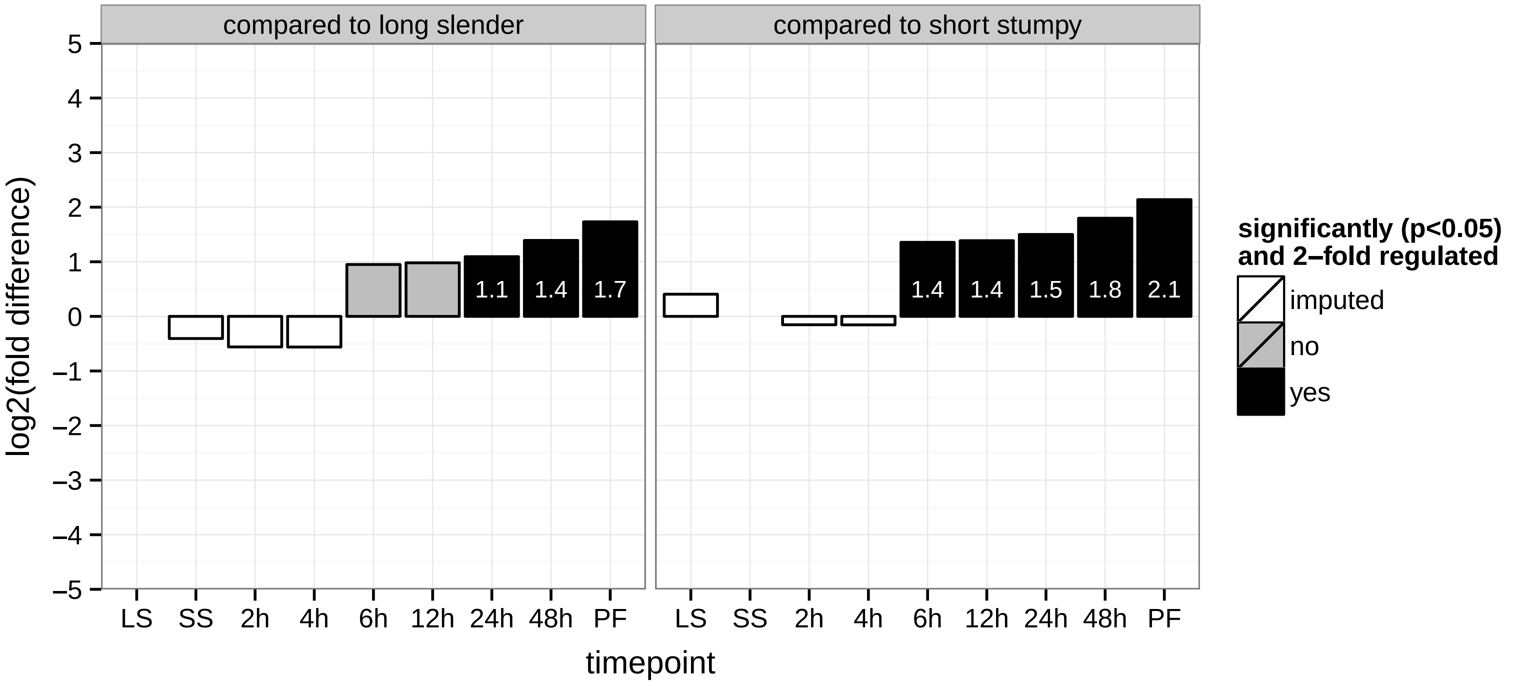

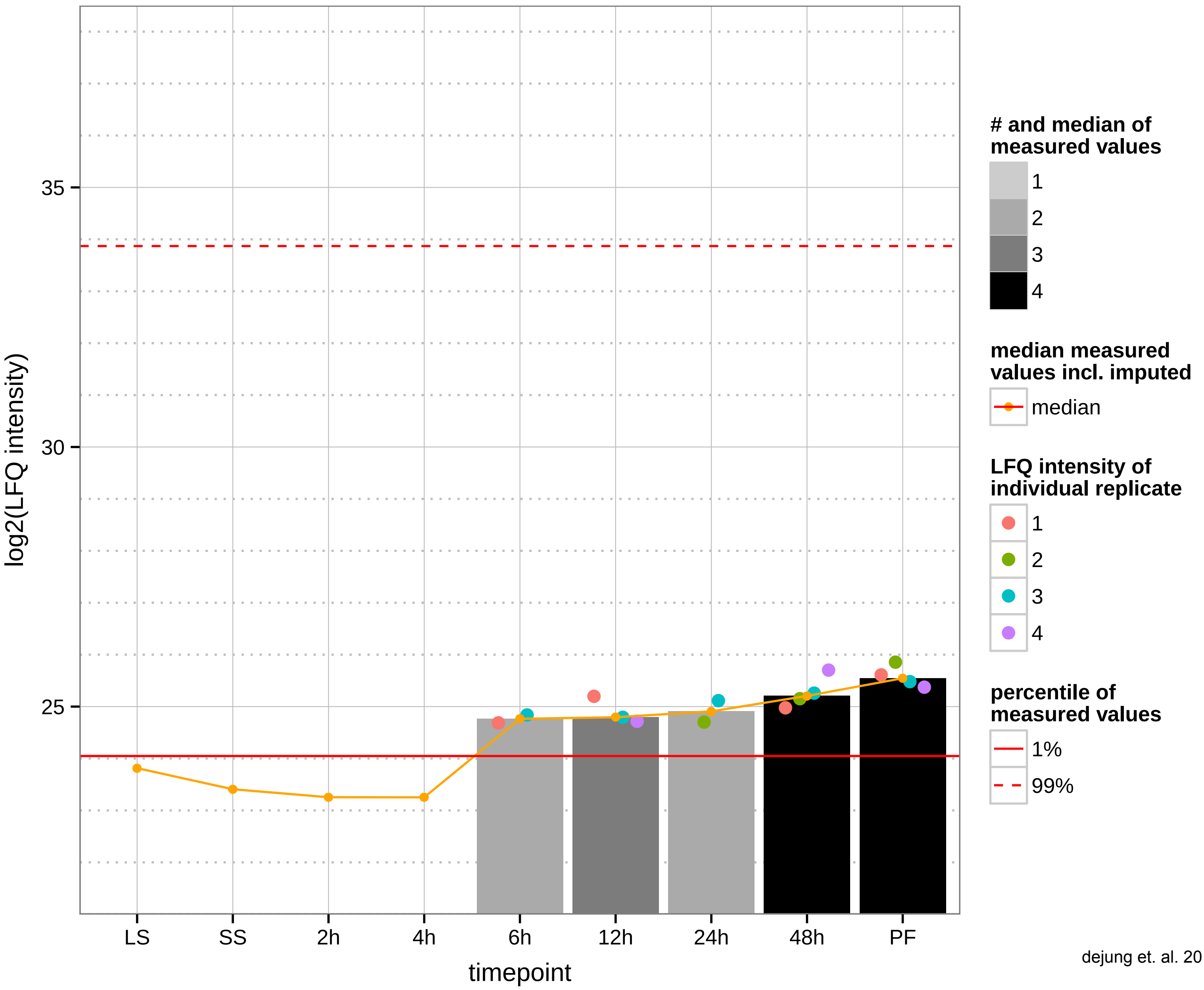

dejung et. al. 2015

nucleolar GTP−binding protein 1 (NOG1)
Tb927.11.3120
AGOF: GTP binding
AGOC: nucleolus
AGOP: ribosome biogenesis
PGOF: GTP binding
PGOC: nucleolus
PGOP: null

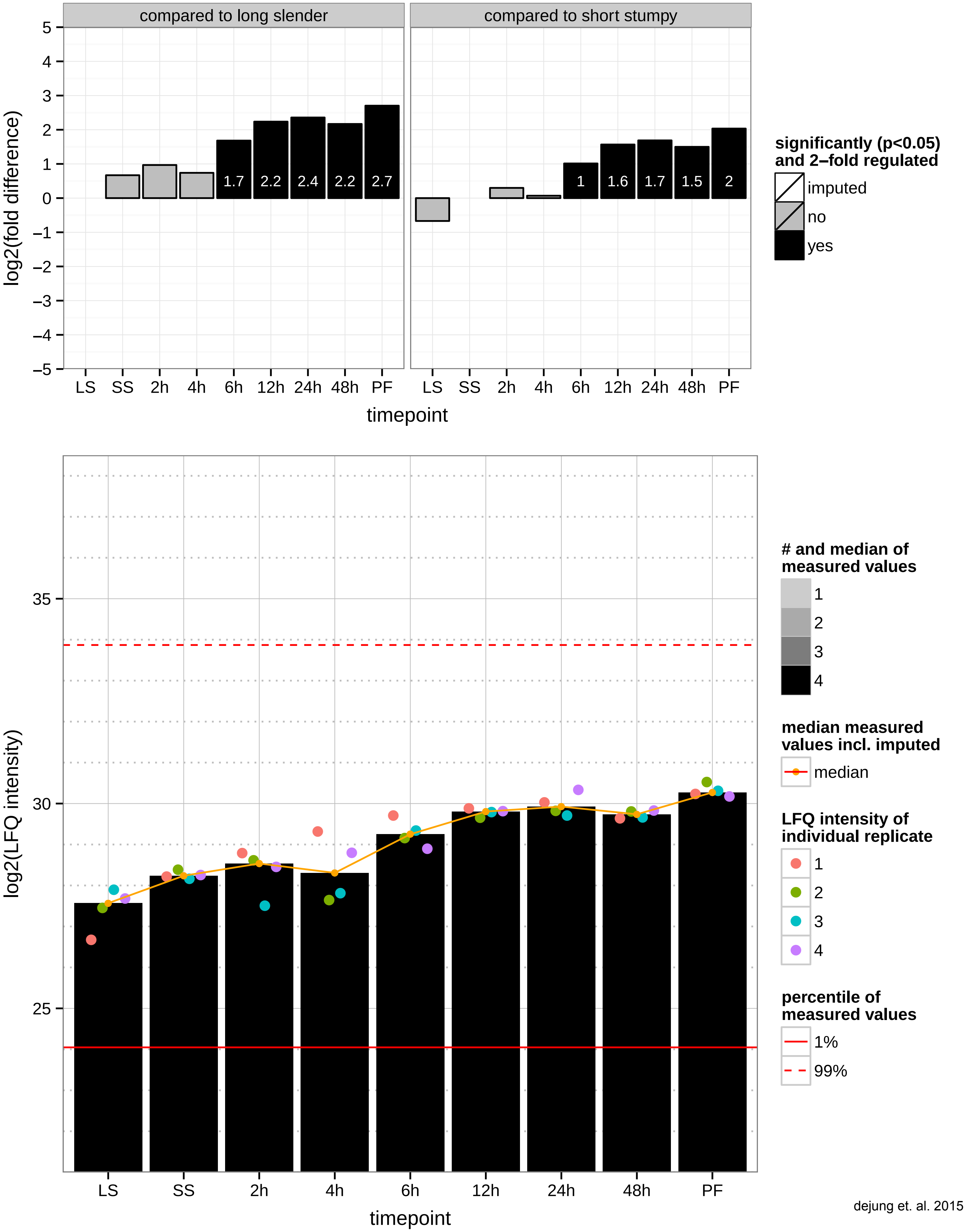

dejung et. al. 2015

nucleoporin 48 (TbNup48)
Tb927.11.4540
AGOF: null
AGOC: nuclear pore
AGOP: null
PGOF: protein binding
PGOC: null
PGOP: null

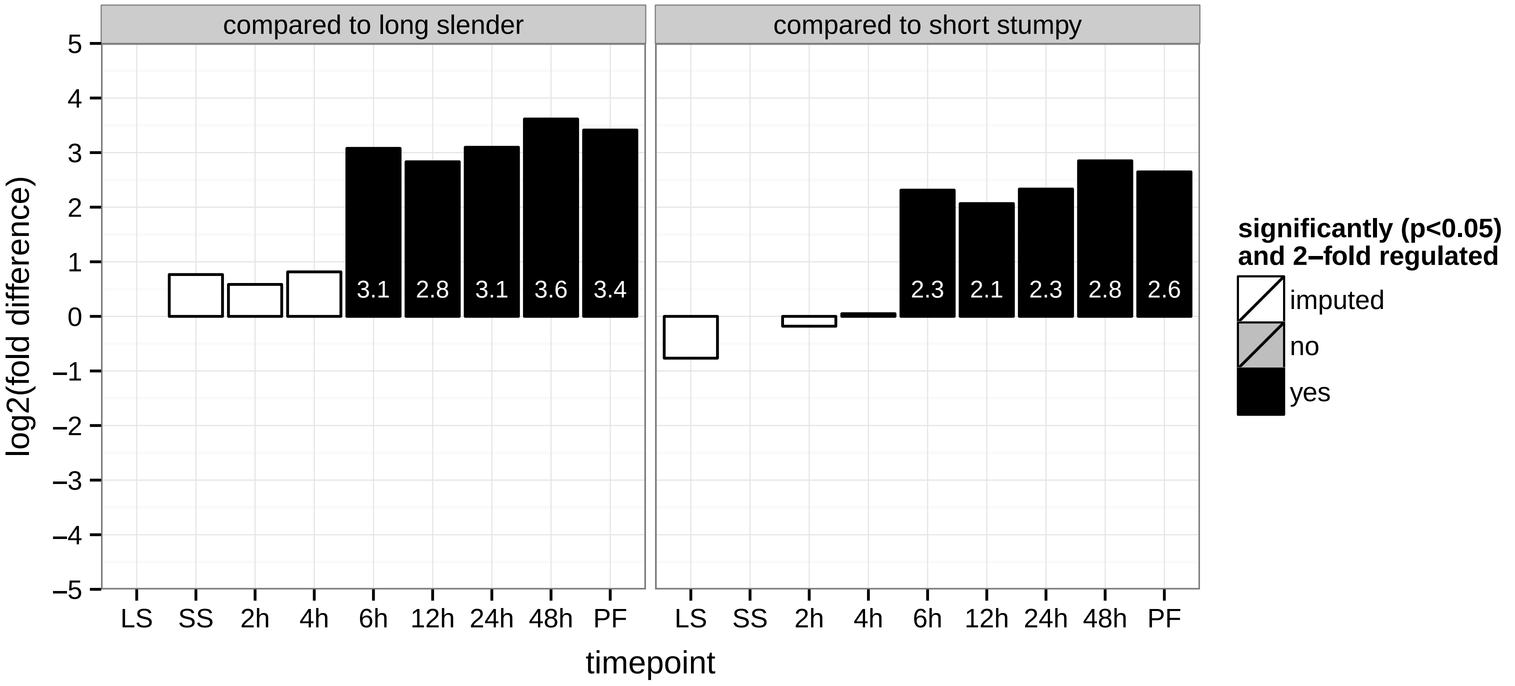

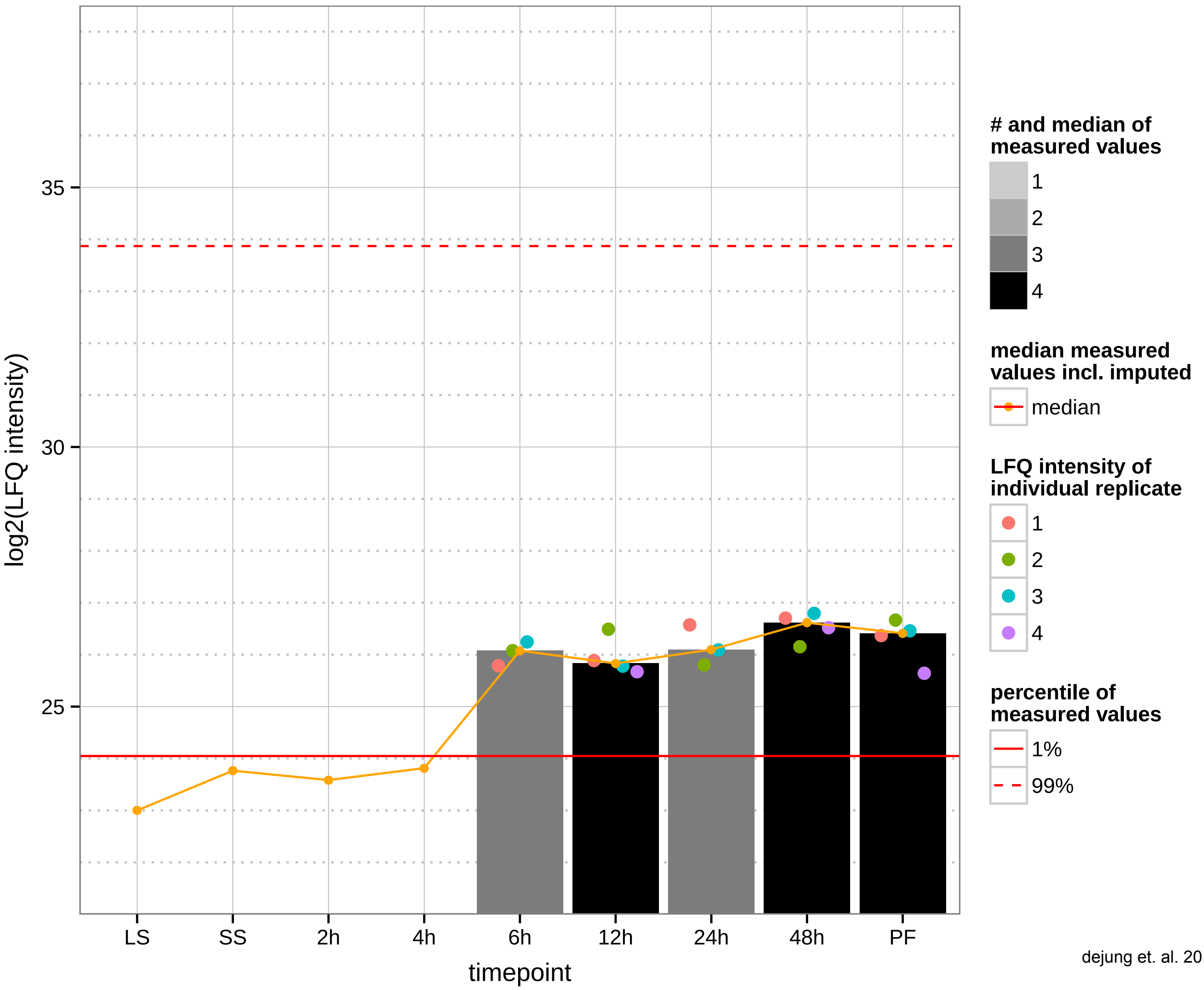

dejung et. al. 2015

sterol 14−alpha−demethylase, cytochrome P450 51A1, lanosterol 14−alpha−demethylase (CYP51)
Tb927.11.6210
AGOF: electron carrier activity, heme binding, iron ion binding, monooxygenase activity, oxidoreductase activity
AGOC: null
AGOP: metabolic process
PGOF: electron carrier activity, heme binding, iron ion binding, oxidoreductase activity, acting on paired donors, with incorpora [illegible]
PGOC: null
PGOP: oxidation−reduction process

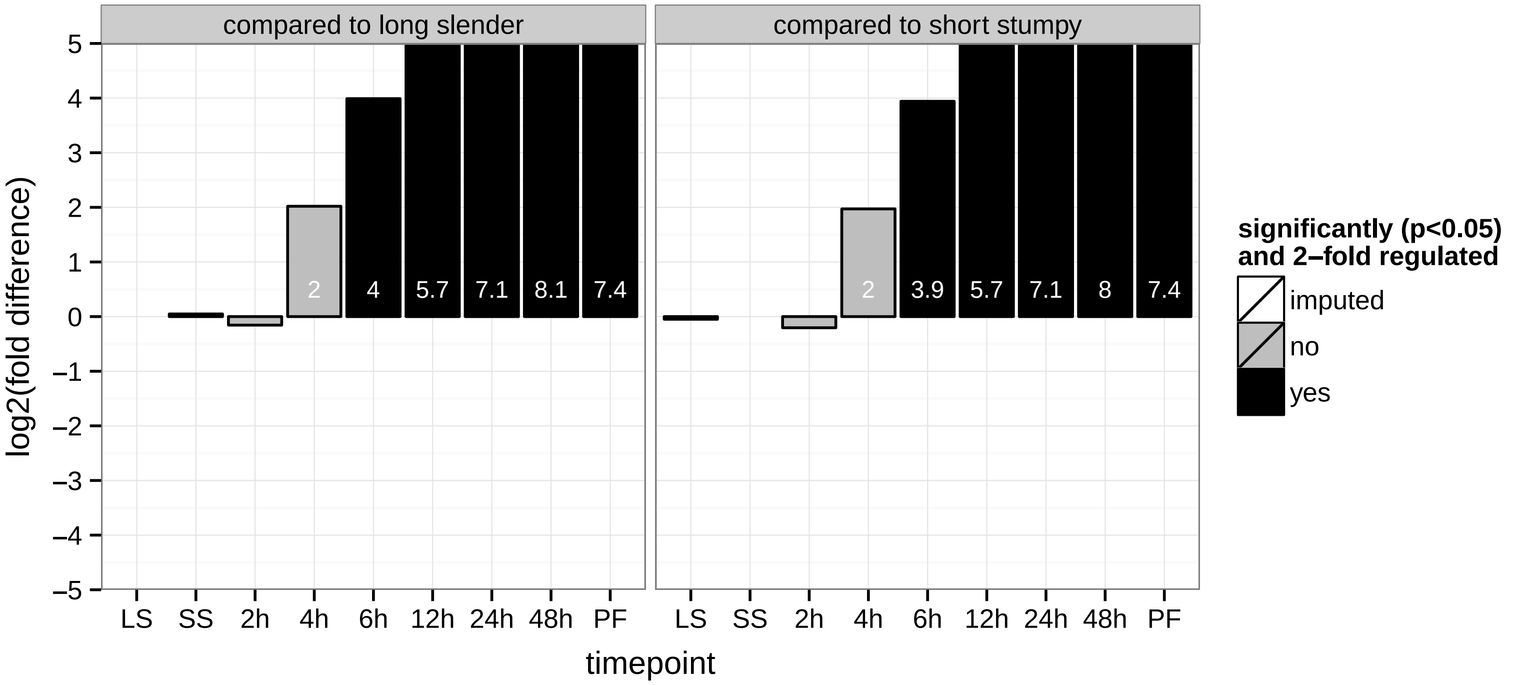

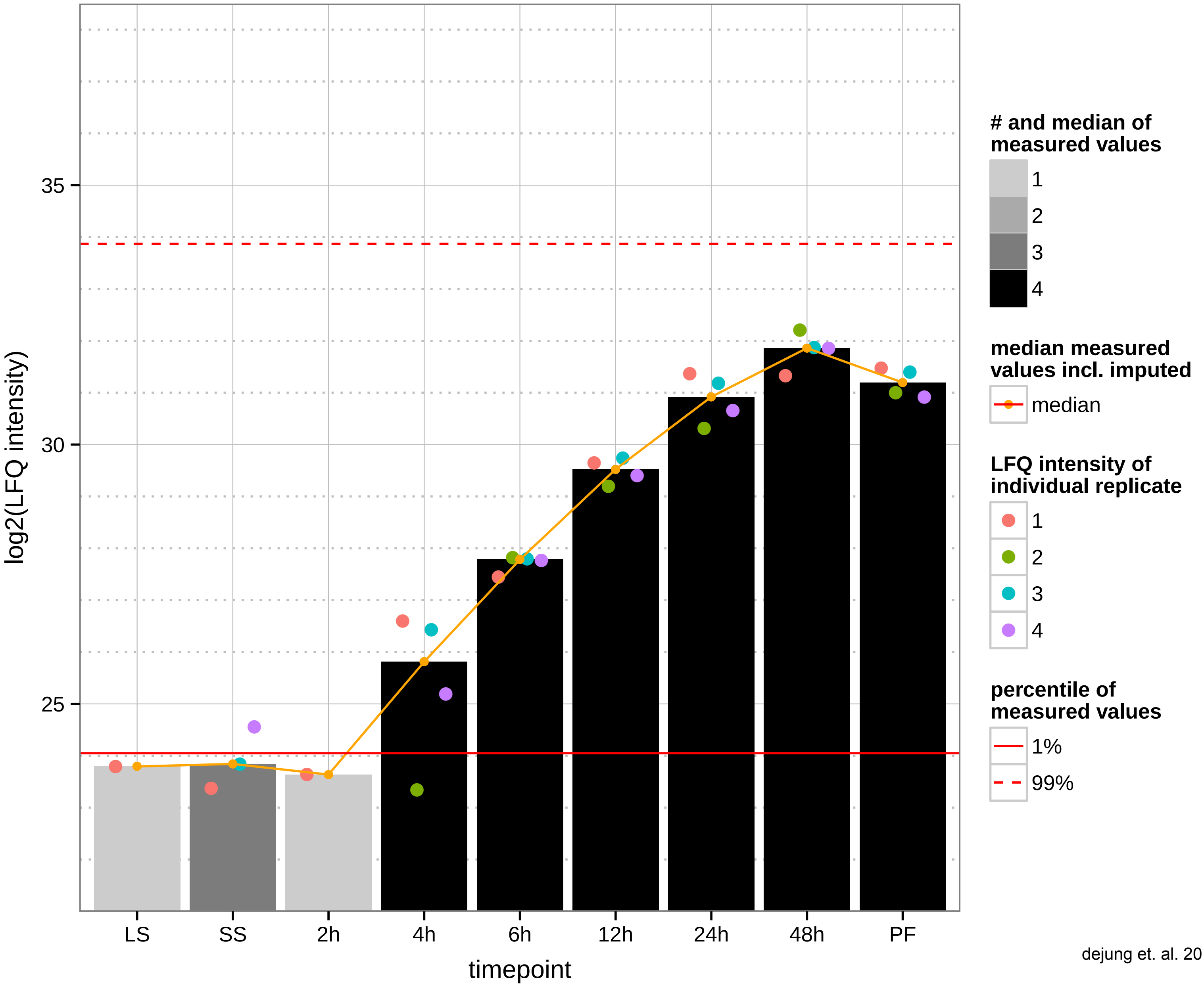

dejung et. al. 2015

hypothetical protein, conserved
Tb927.11.6670
AGOF: RNA binding, pseudouridine synthase activity
AGOC: null
AGOP: pseudouridine synthesis
PGOF: RNA binding, pseudouridine synthase activity
PGOC: null
PGOP: RNA modification, pseudouridine synthesis

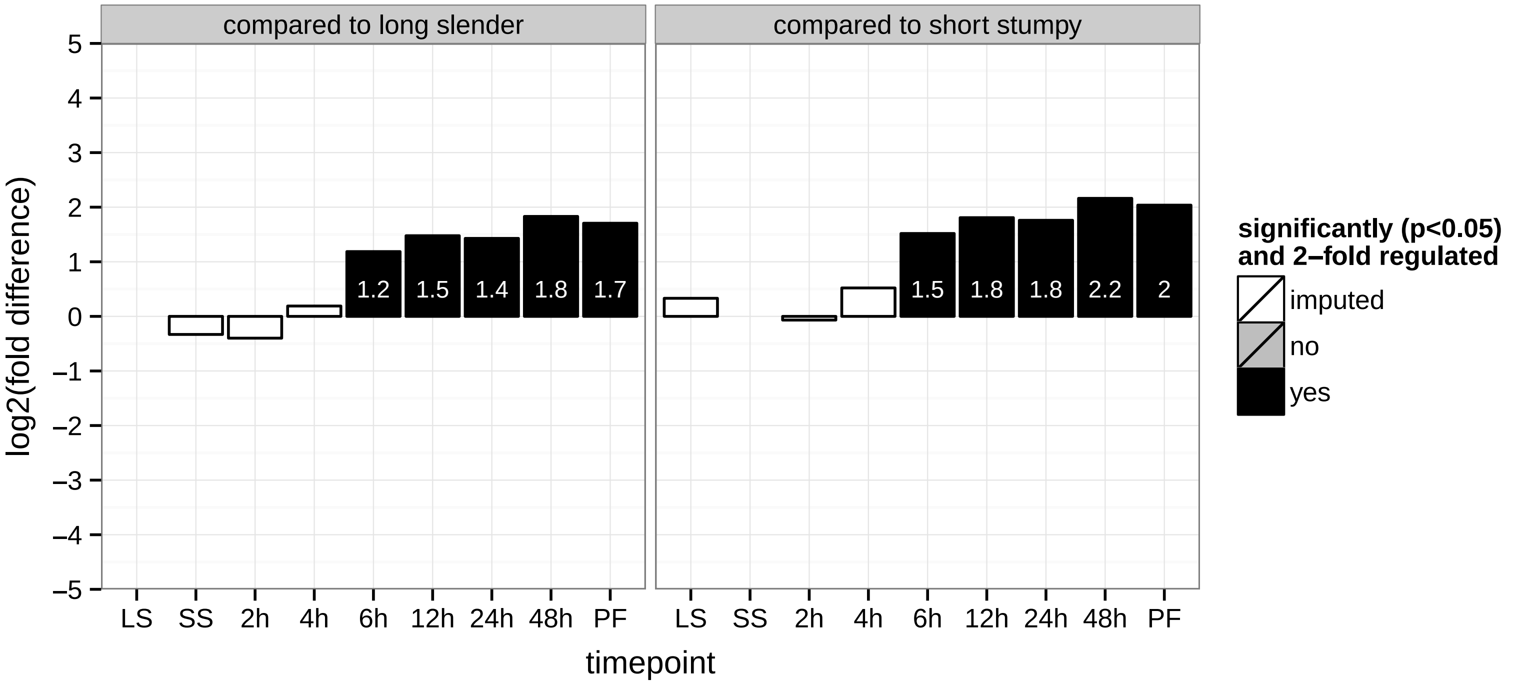

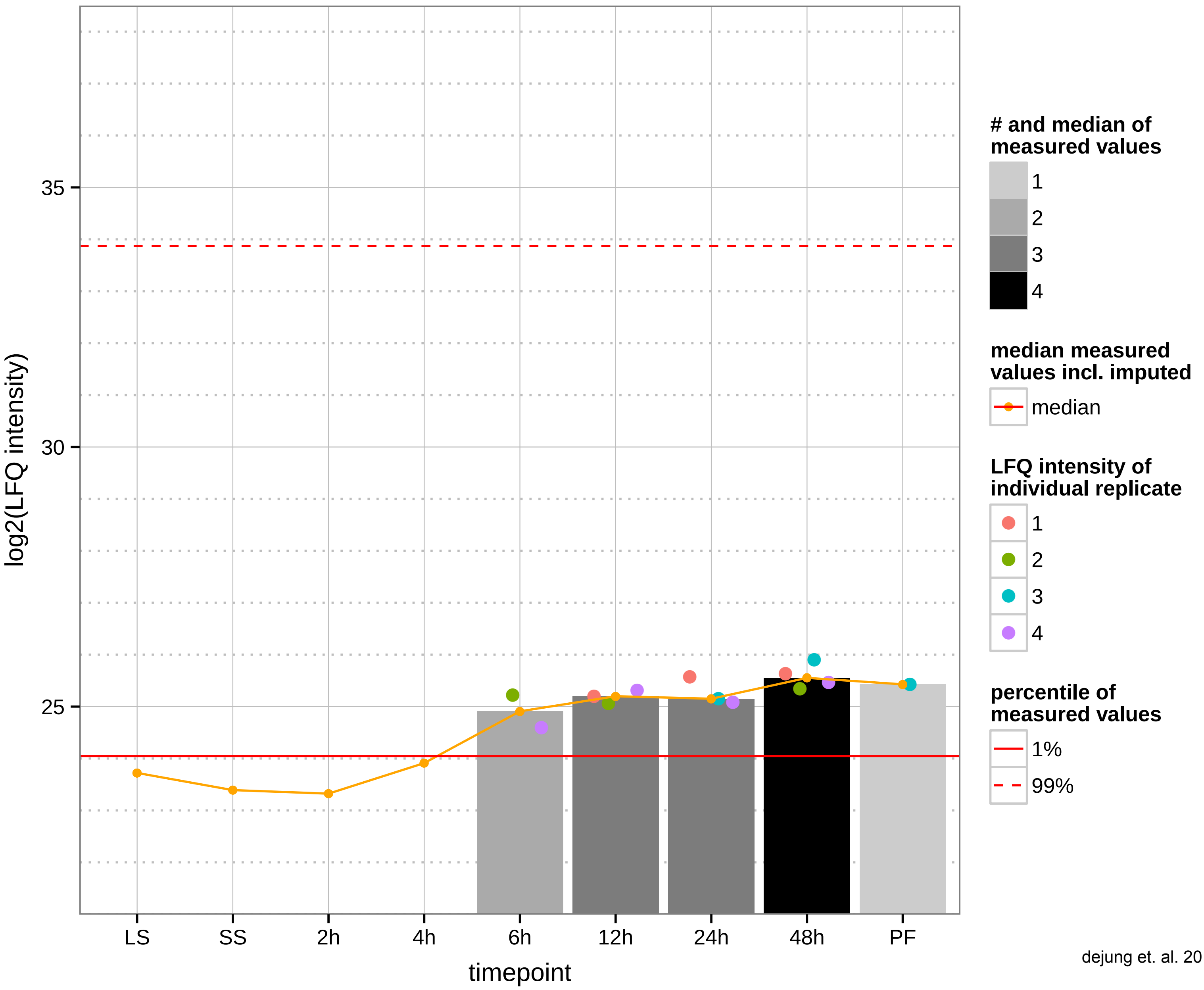

dejung et. al. 2015

phosphoadenosine phosphosulfate reductase–like protein
Tb927.11.7420
AGOF: transferase activity
AGOC: null
AGOP: metabolic process
PGOF: catalytic activity
PGOC: null
PGOP: metabolic process

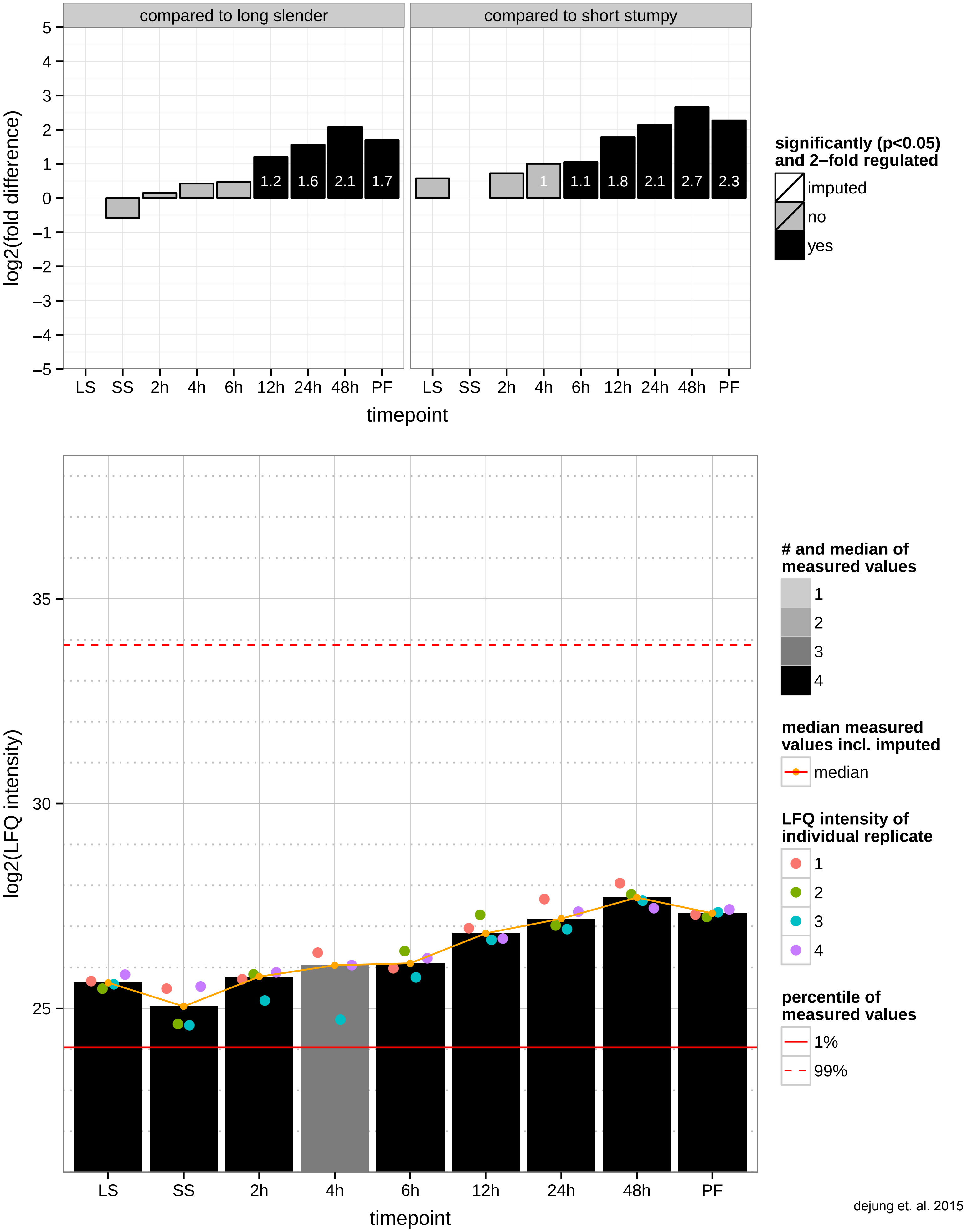

dejung et. al. 2015

oxidoreductase–like protein
Tb927.11.7770
AGOF: oxidoreductase activity
AGOC: null
AGOP: metabolic process
PGOF: null
PGOC: null
PGOP: null

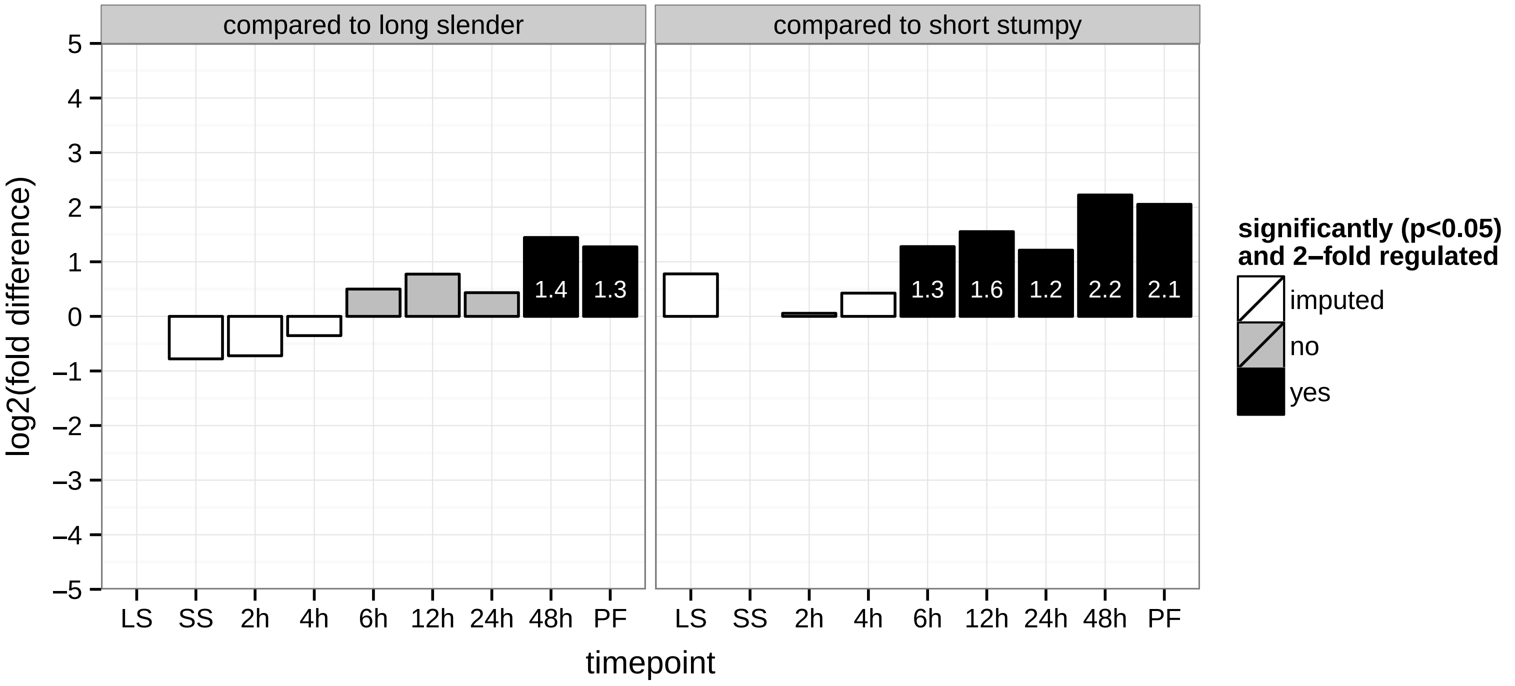

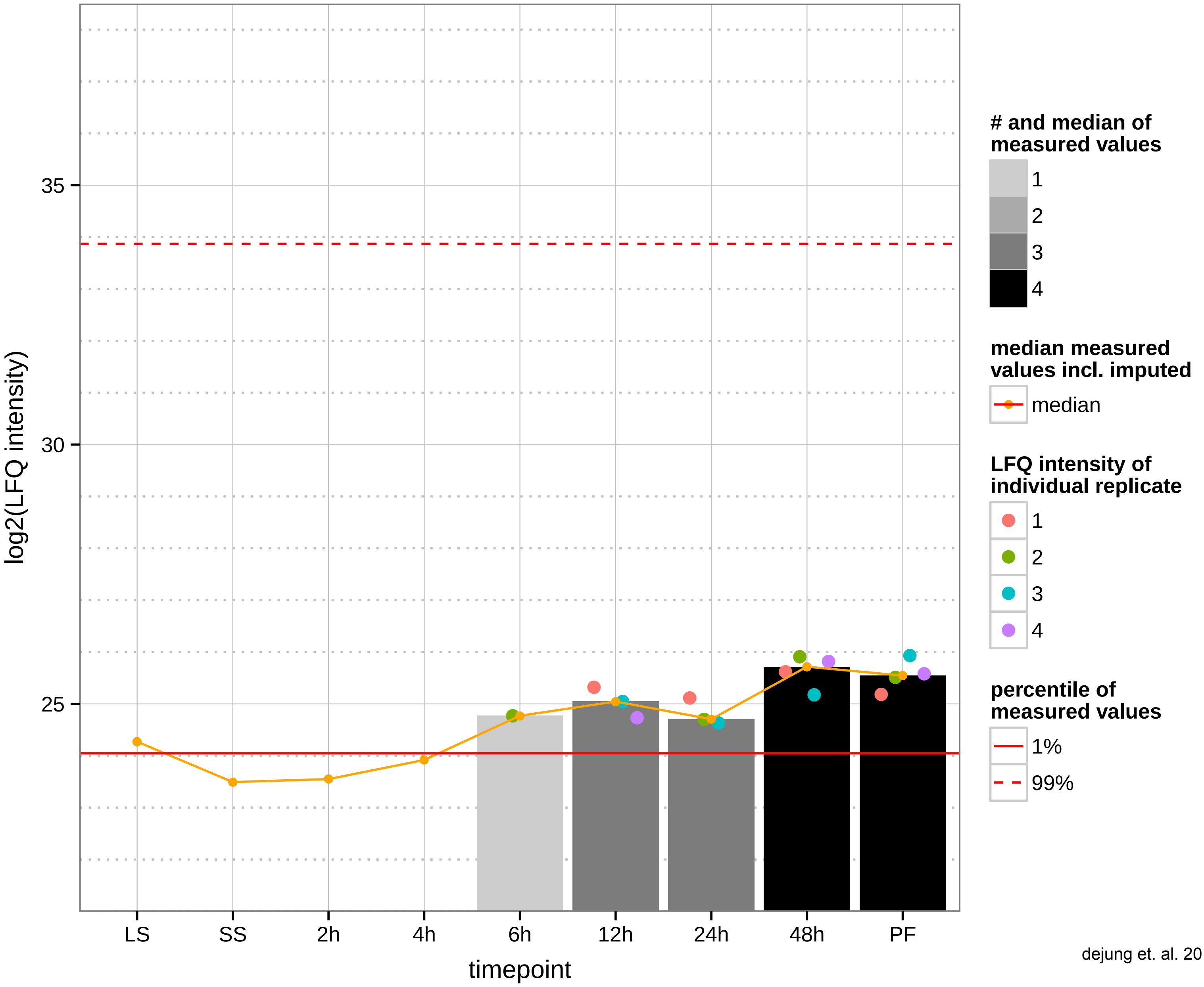

dejung et. al. 2015

RAD50 DNA repair−like protein, putative
Tb927.11.8210
AGOF: null, ATP binding, double−stranded DNA binding
AGOC: null, chromosome, nucleus
AGOP: null, double−strand break repair via nonhomologous end joining
PGOF: null, ATP binding
PGOC: null, Mre11 complex
PGOP: null, DNA repair

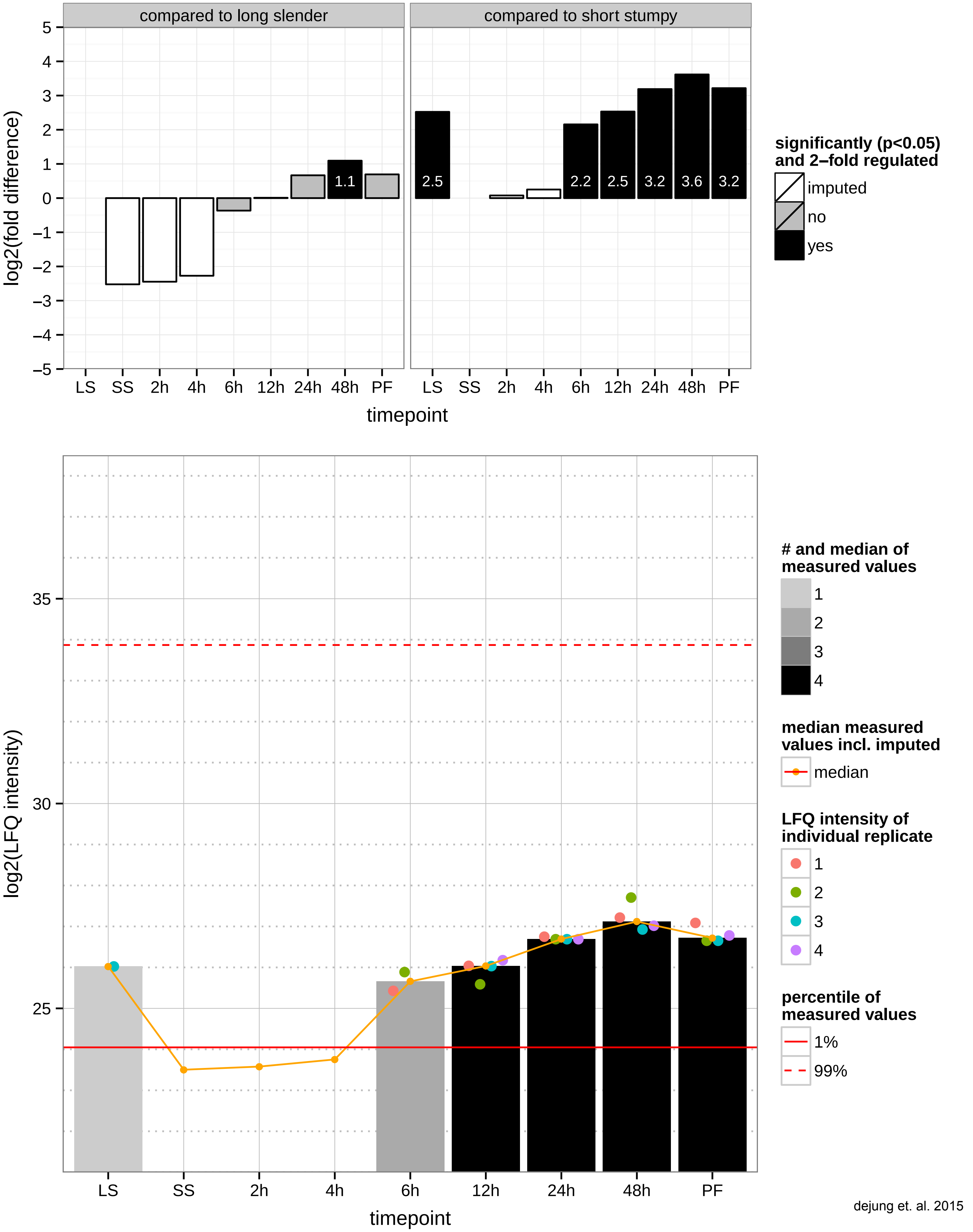

dejung et. al. 2015

cation transporter, putative
Tb927.11.8990
AGOF: metal ion transmembrane transporter activity
AGOC: mitochondrial membrane
AGOP: metal ion transport
PGOF: metal ion transmembrane transporter activity
PGOC: membrane
PGOP: metal ion transport, transmembrane transport

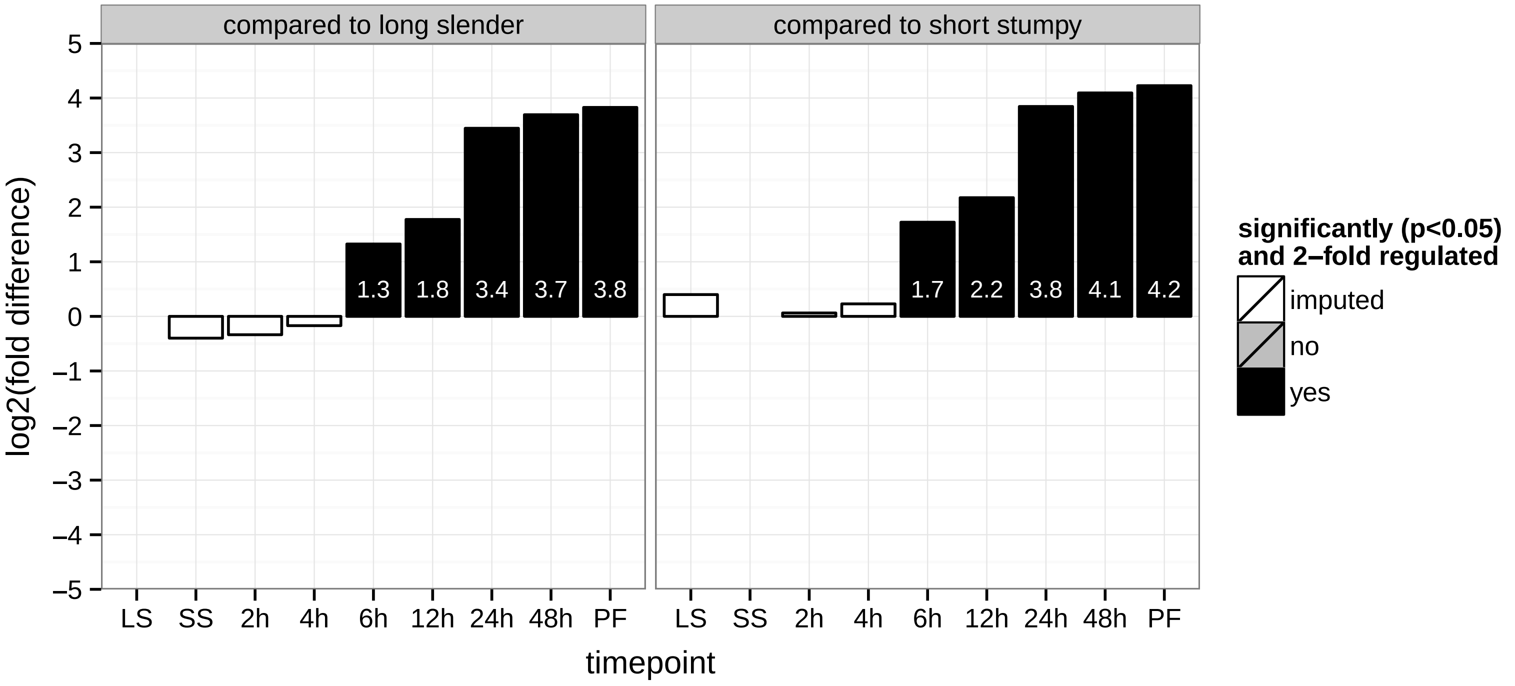

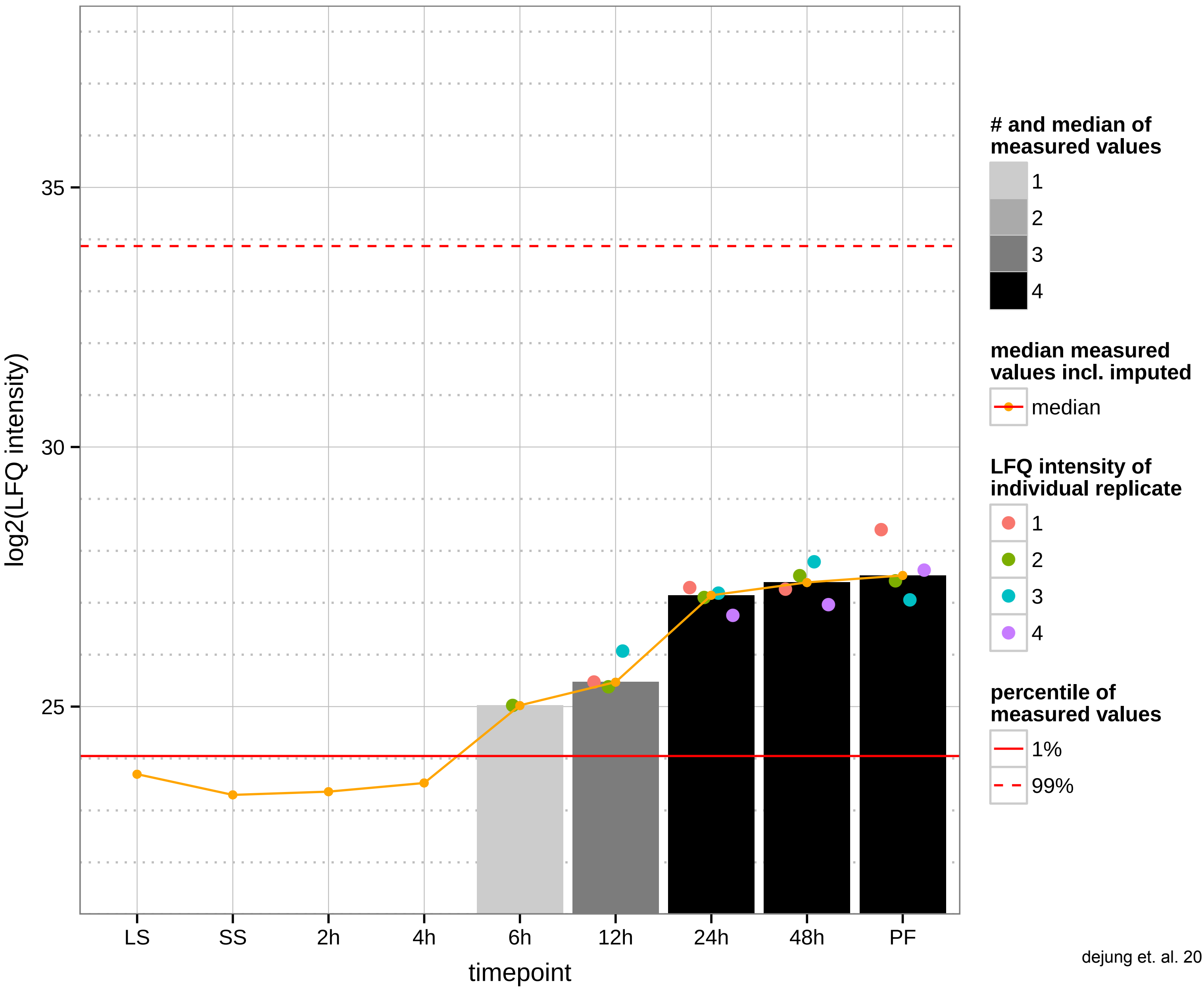

dejung et. al. 2015

hypothetical protein, conserved
Tb927.3.2680
AGOF: null
AGOC: null
AGOP: null
PGOF: null
PGOC: null
PGOP: null

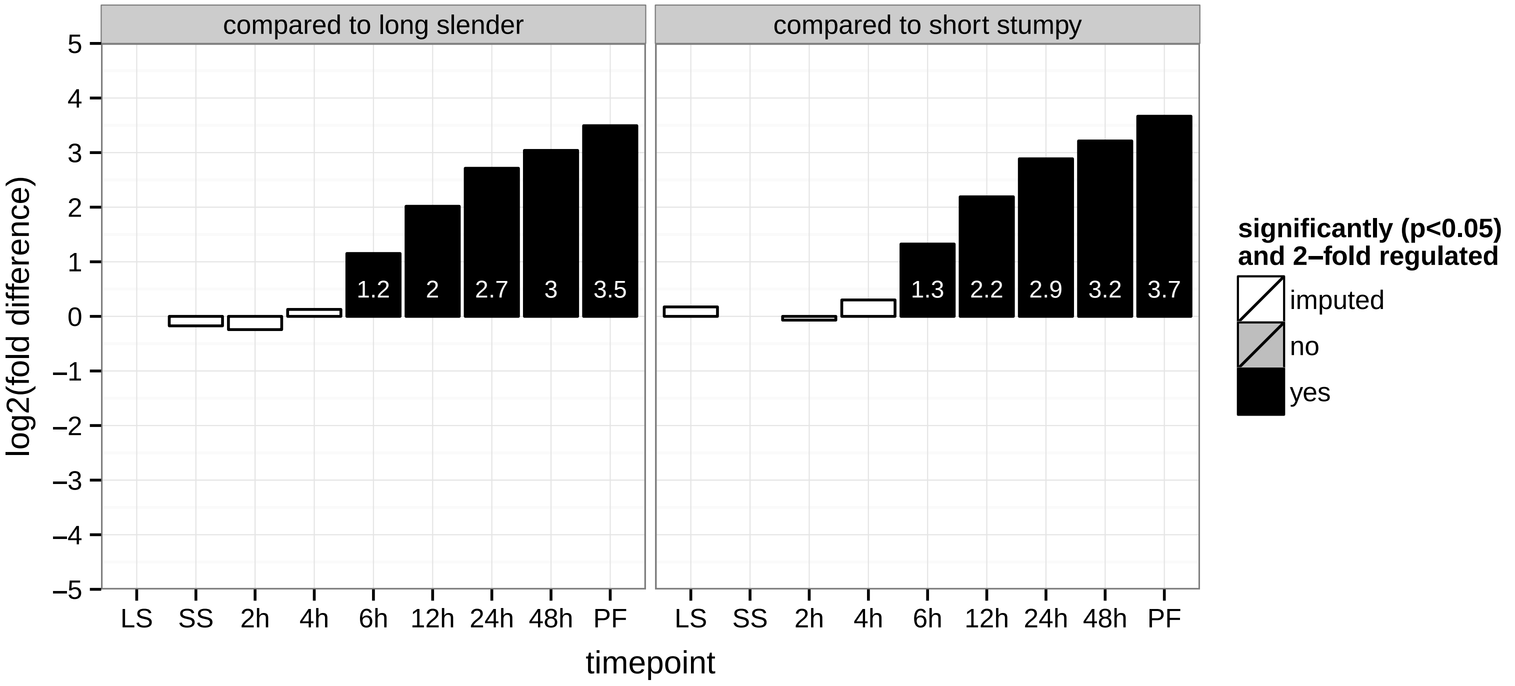

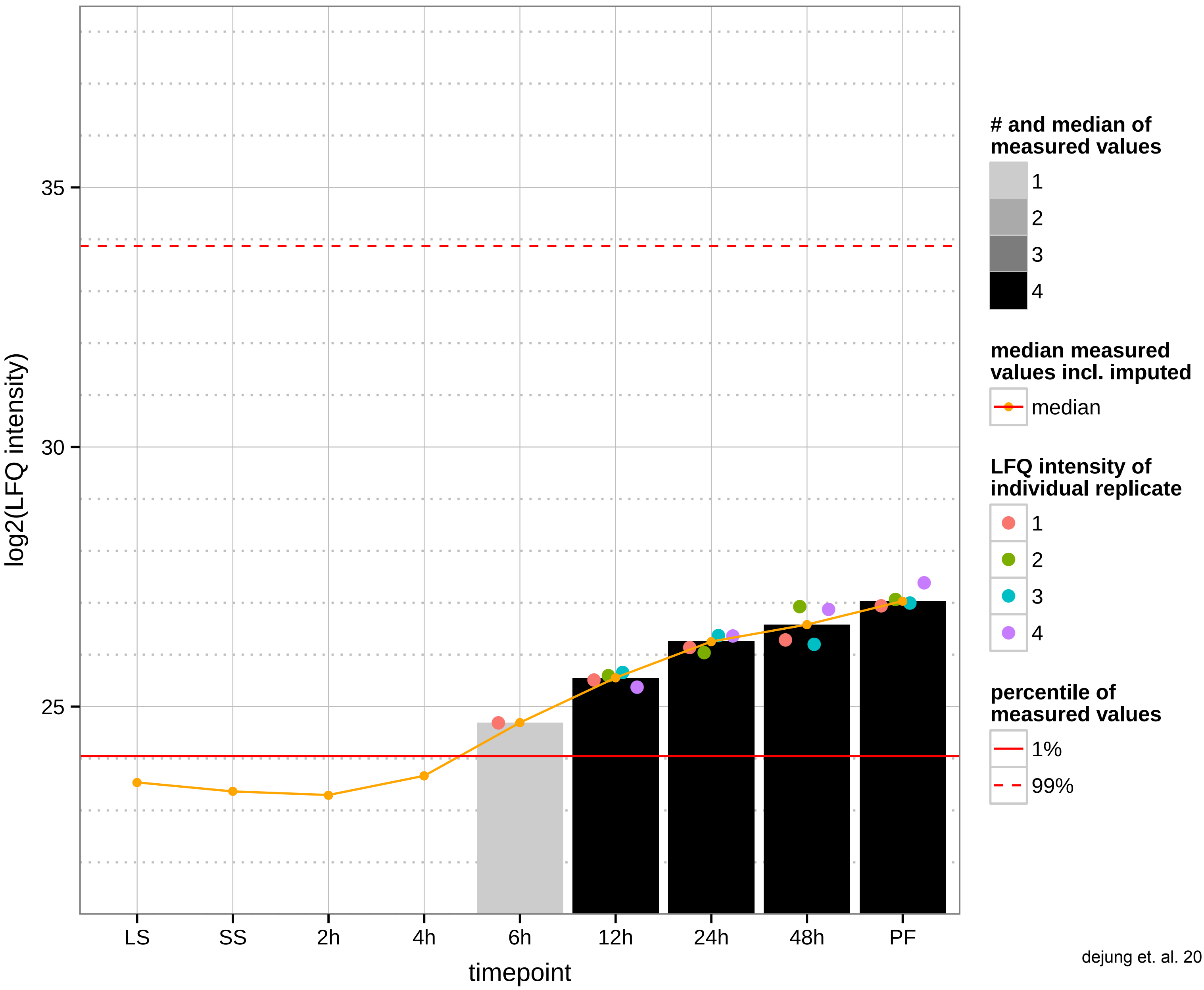

dejung et. al. 2015

Elongation factor Ts, mitochondrial, putative (EF−Ts)
Tb927.3.3630
AGOF: protein binding, translation elongation factor activity
AGOC: intracellular, mitochondrion
AGOP: growth, oxidative phosphorylation, translational elongation
PGOF: protein binding, translation elongation factor activity
PGOC: intracellular
PGOP: translational elongation

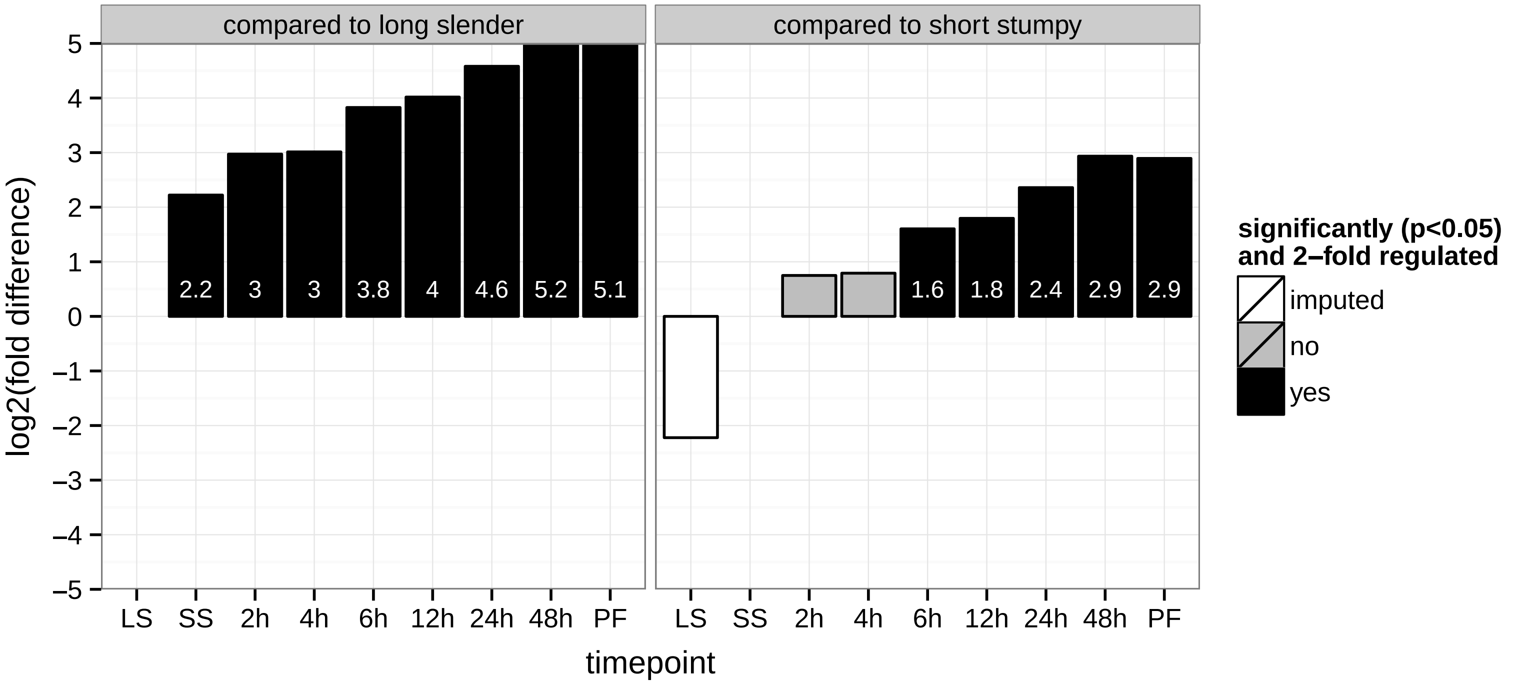

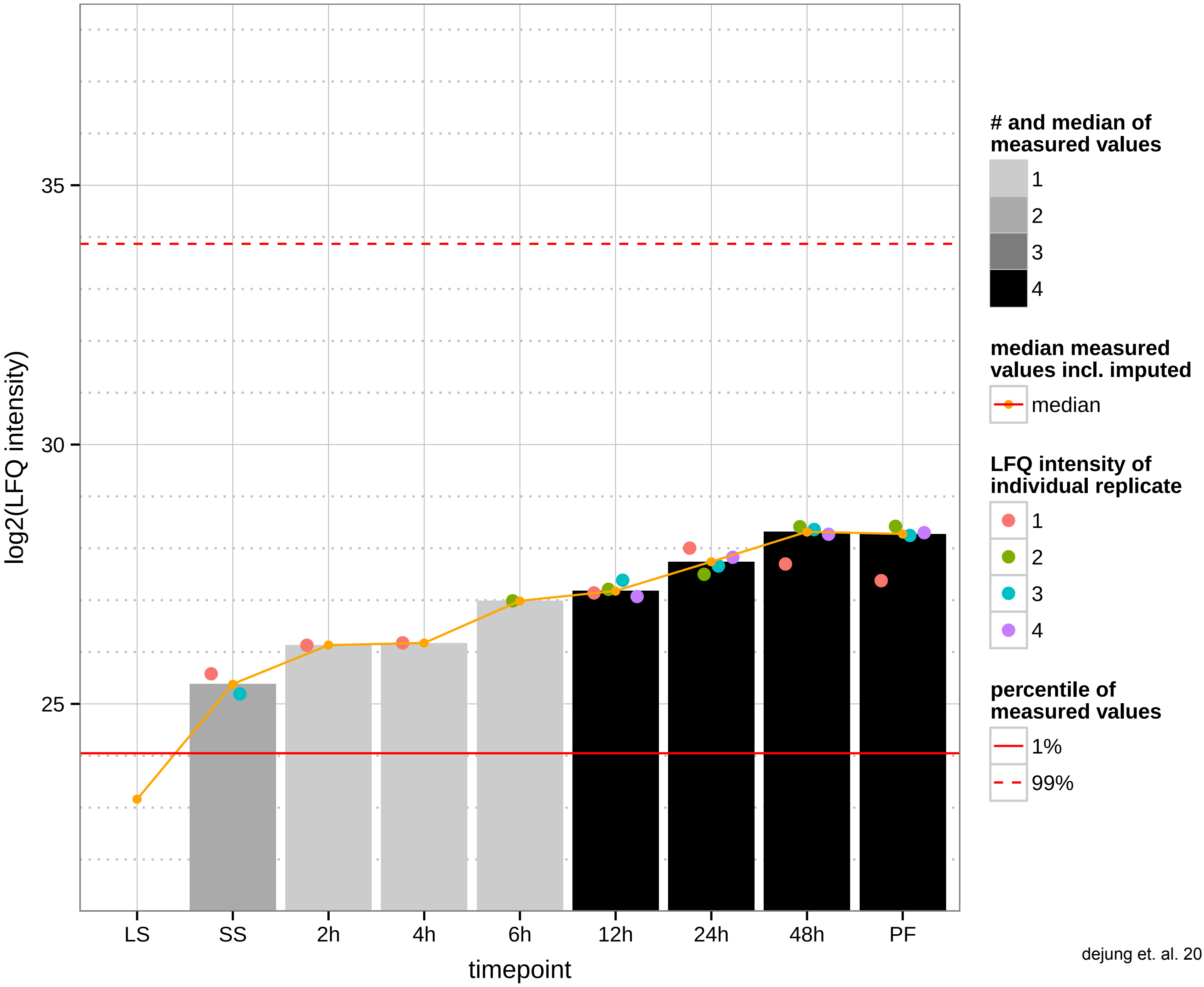

dejung et. al. 2015

fumarate hydratase, putative, class I (FHc)
Tb927.3.4500;Tb11.v5.0629
AGOF: null, fumarate hydratase activity
AGOC: null, cytosol
AGOP: null, generation of precursor metabolites and energy
PGOF: hydro–lyase activity, lyase activity, fumarate hydratase activity, hydro–lyase activity
PGOC: null
PGOP: null, generation of precursor metabolites and energy

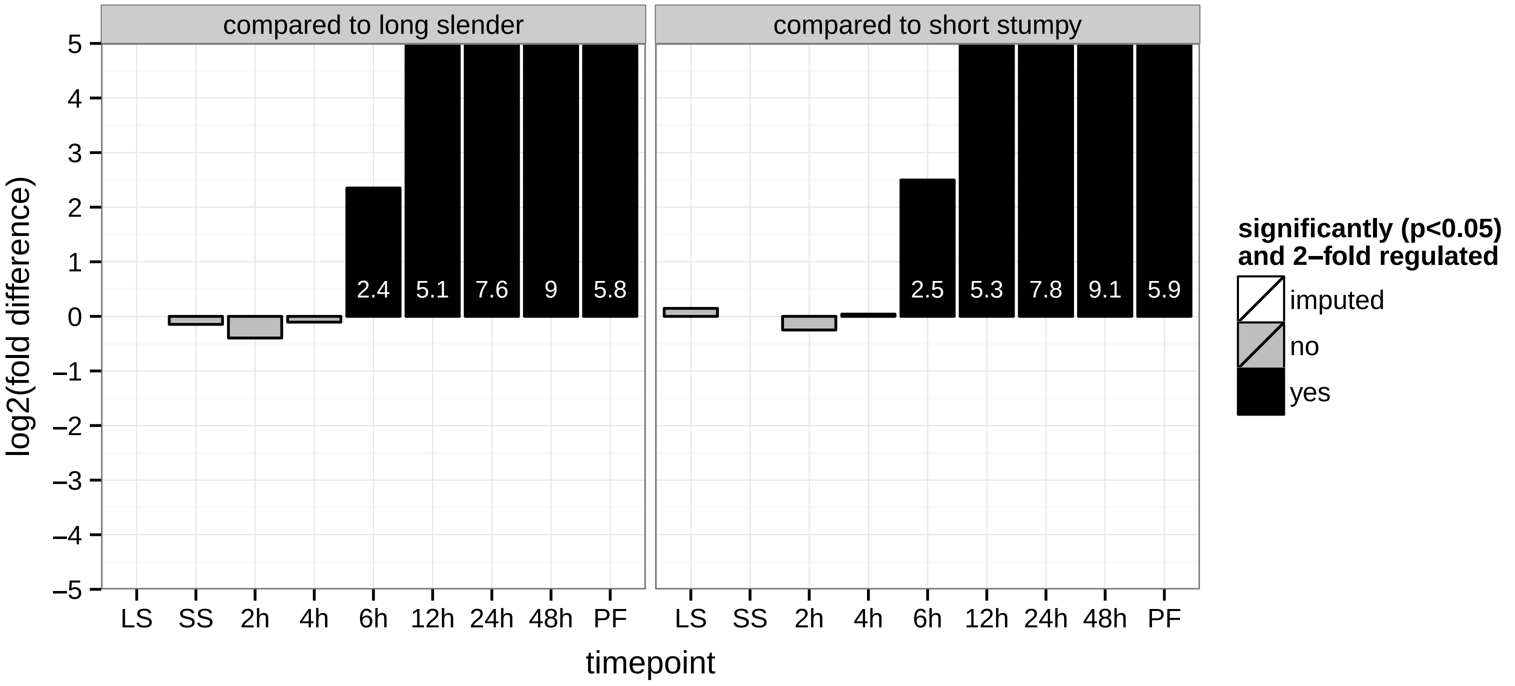

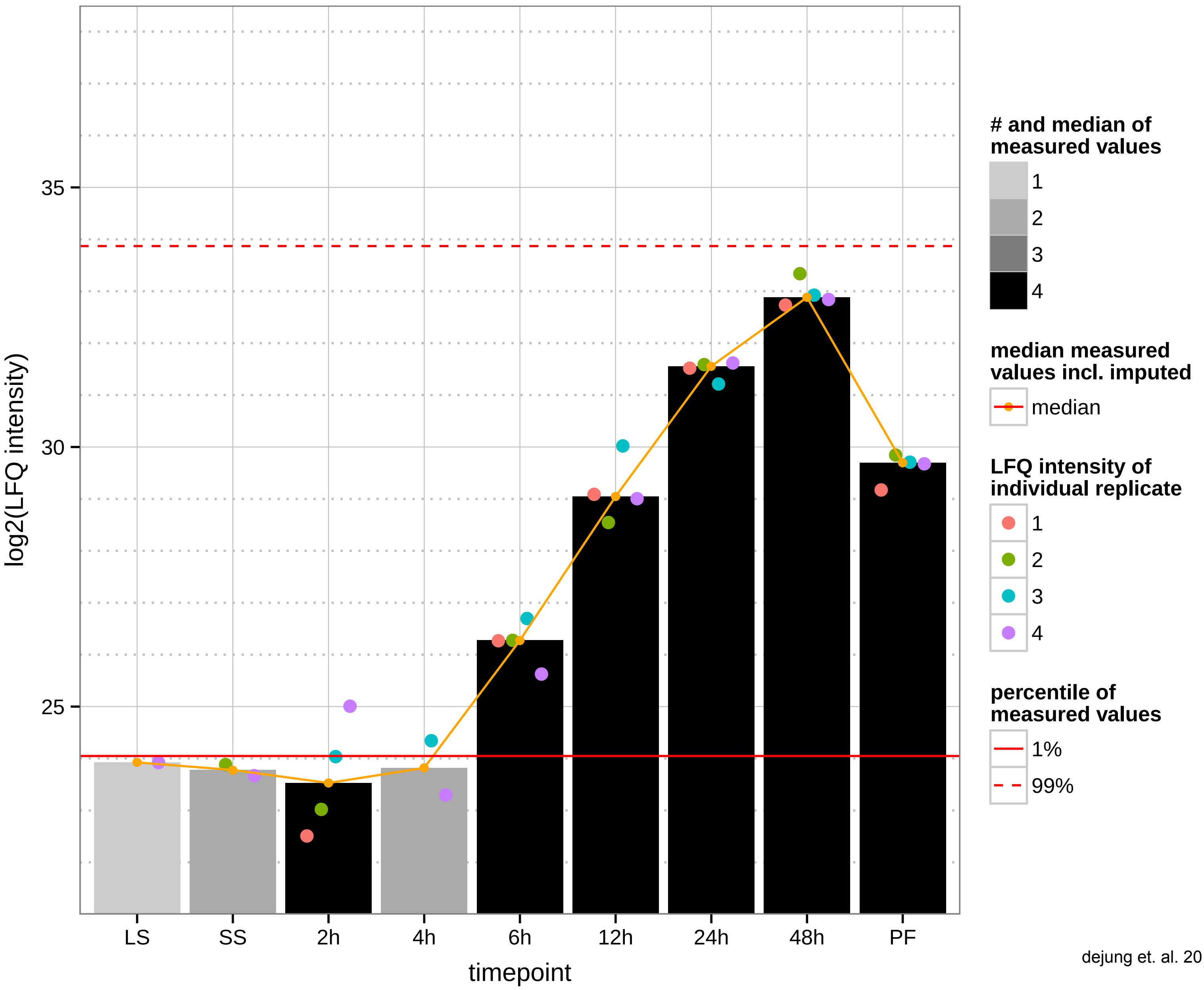

dejung et. al. 2015

hypothetical protein, conserved
Tb927.3.640
AGOF: null
AGOC: null
AGOP: null
PGOF: null
PGOC: null
PGOP: null

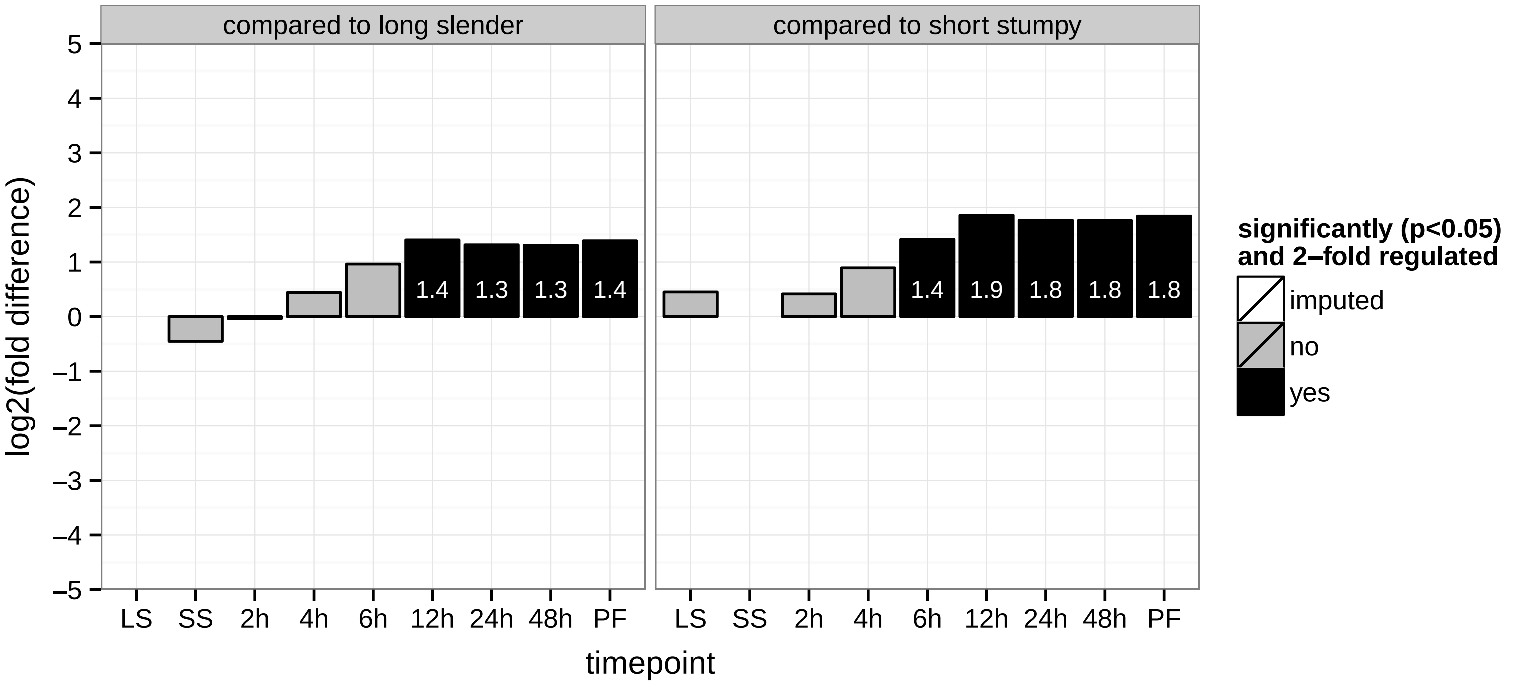

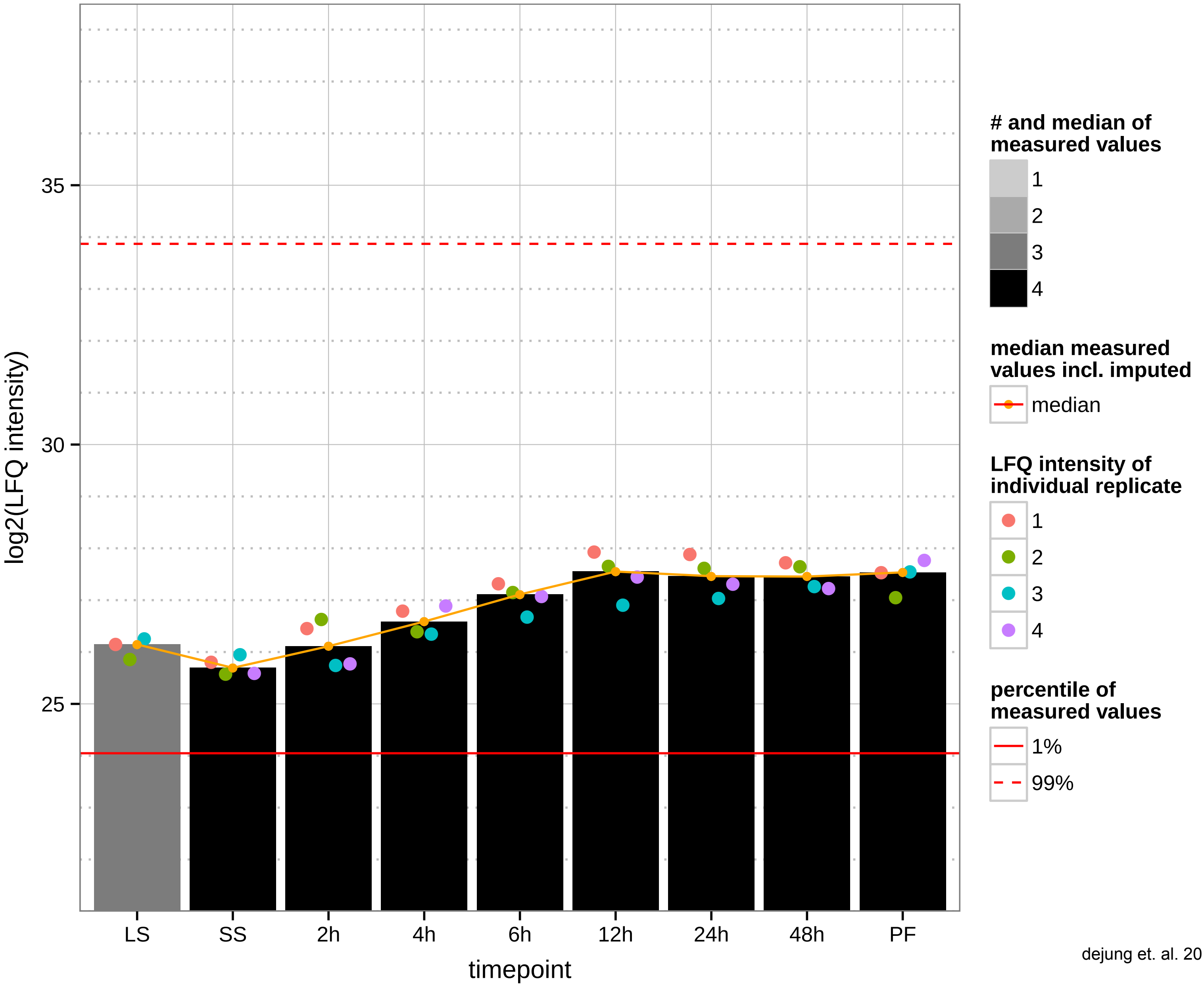

dejung et. al. 2015

hypothetical protein, conserved
Tb927.4.3670
AGOF: null
AGOC: null
AGOP: null
PGOF: null
PGOC: null
PGOP: null

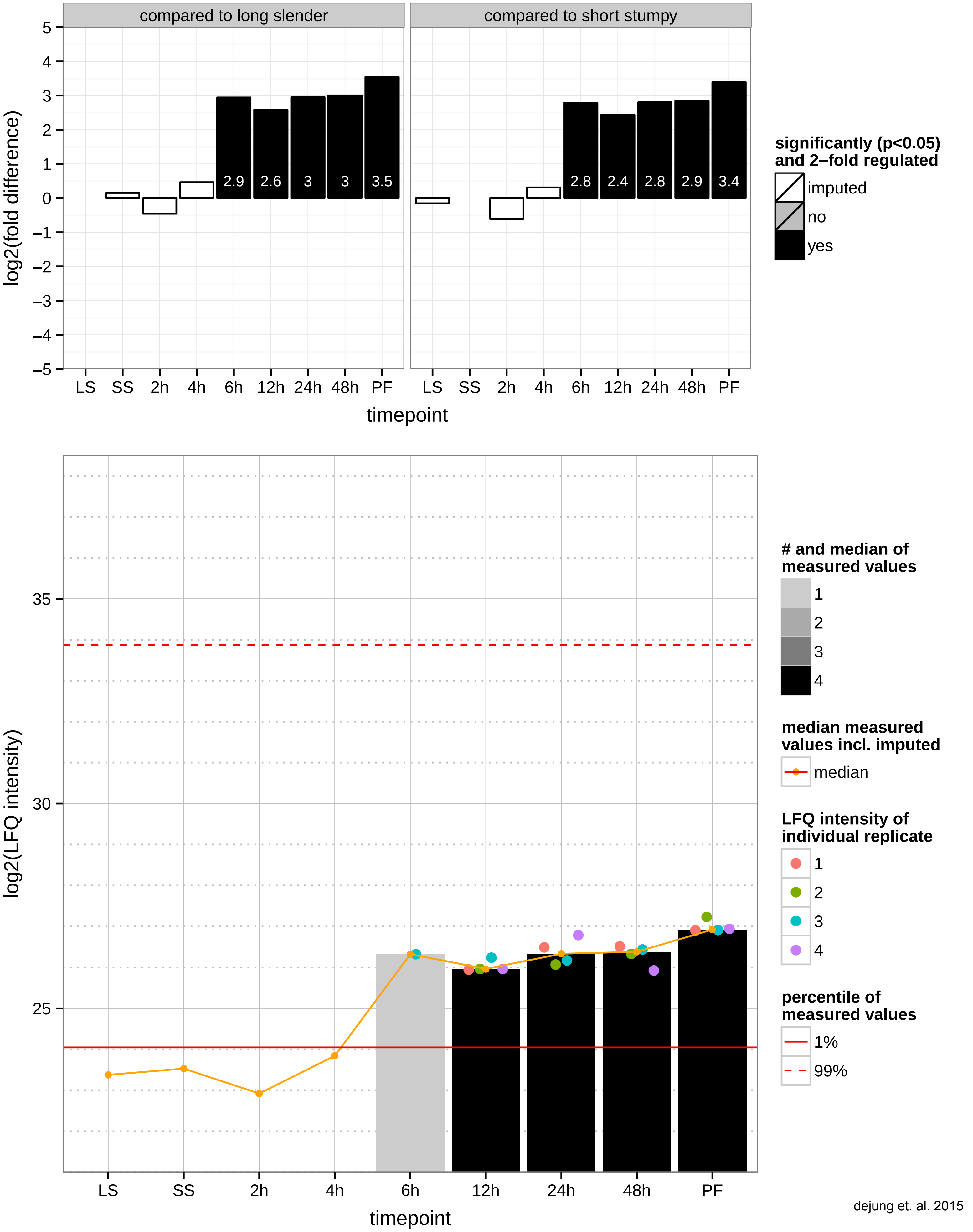

dejung et. al. 2015

glycosomal transporter (GAT1), ABC transporter, putative (GAT1)
Tb927.4.4050
AGOF: ATP binding, ATPase activity, coupled to transmembrane movement of substances, transporter activity
AGOC: glycosome membrane, integral to membrane
AGOP: fatty acid transport
PGOF: ATP binding, ATPase activity, coupled to transmembrane movement of substances, nucleoside–triphosphatase activity
PGOC: integral to membrane, membrane
PGOP: transmembrane transport, transport

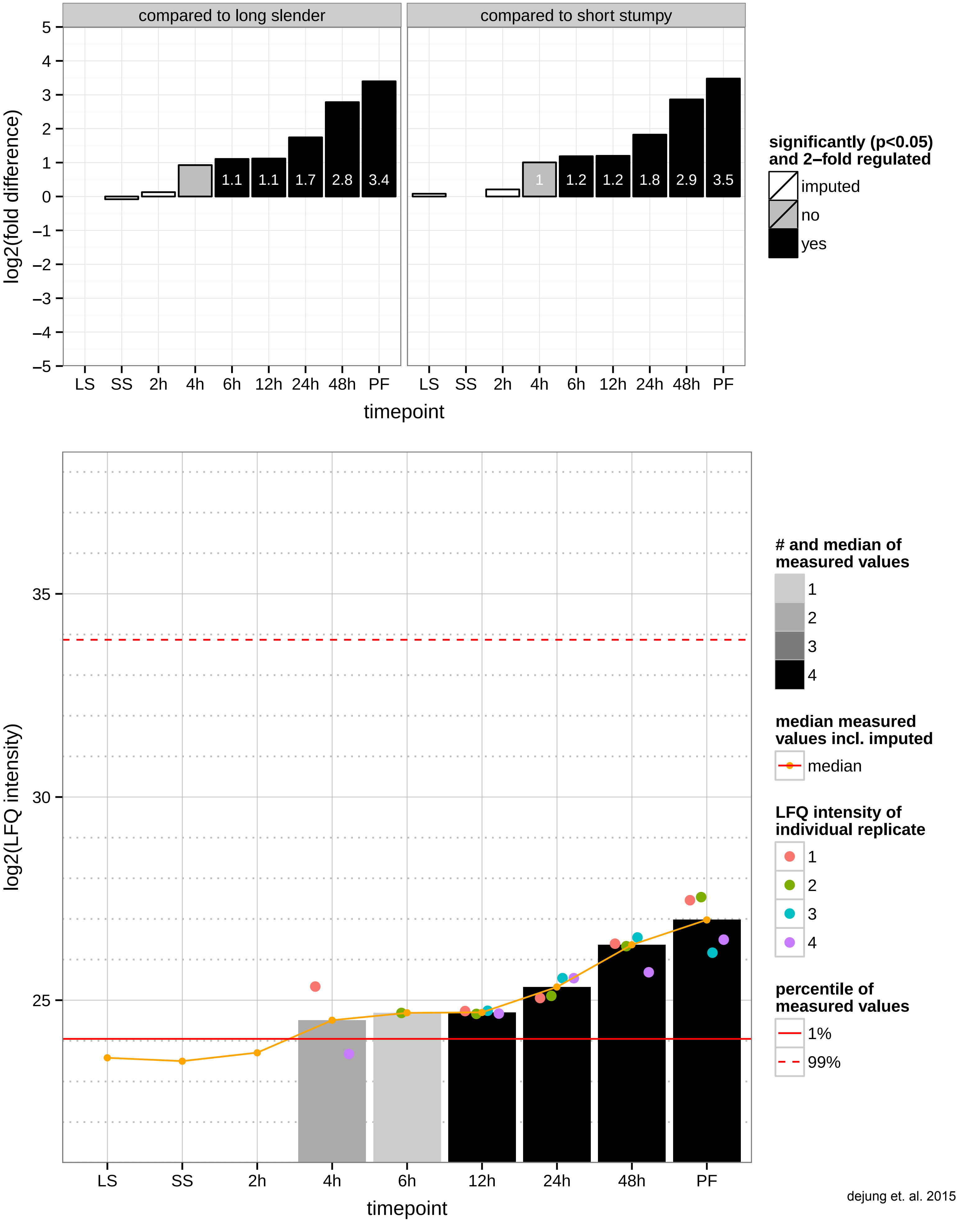

dejung et. al. 2015

Eukaryotic translation initiation factor 4 gamma type 1 (eif4g1)
Tb927.5.1490
AGOF: protein binding
AGOC: eukaryotic translation initiation factor 4F complex
AGOP: RNA metabolic process
PGOF: DNA binding, RNA binding, binding, protein binding
PGOC: null
PGOP: null

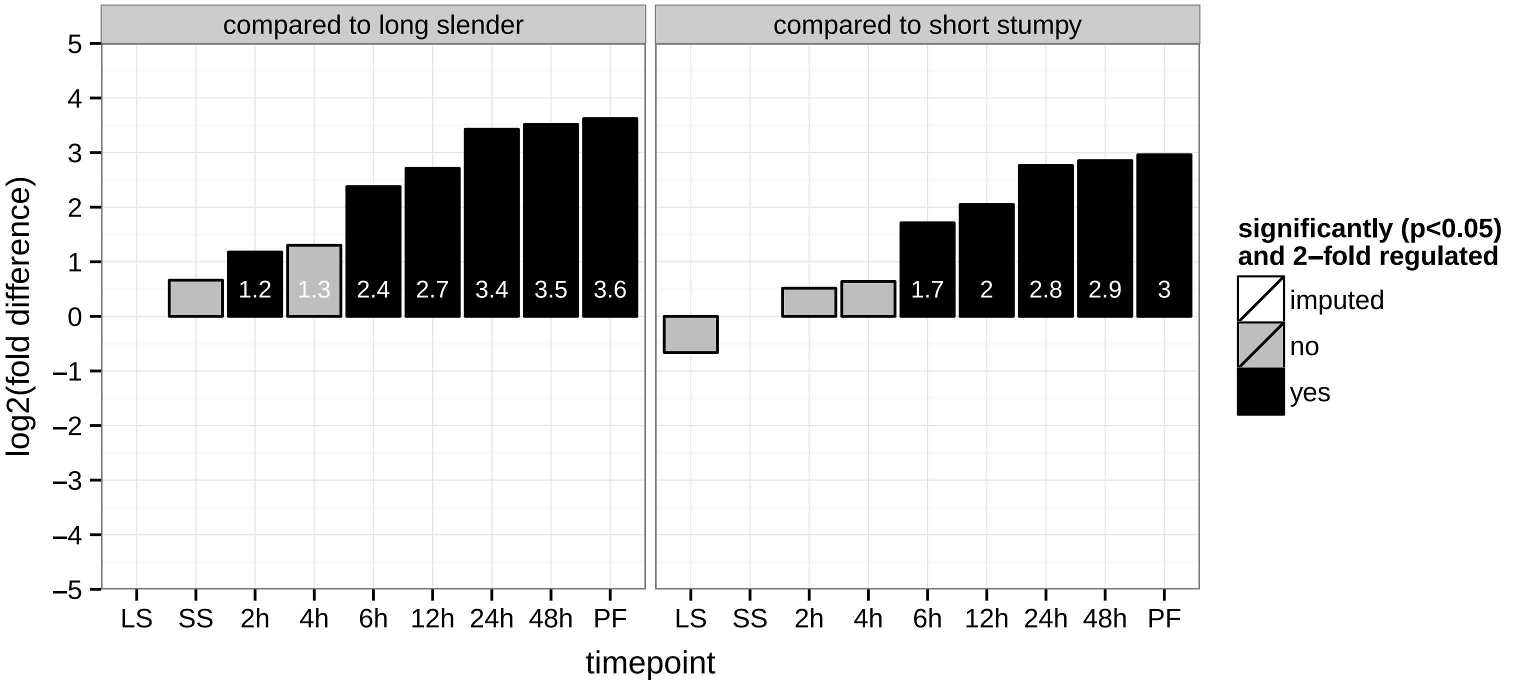

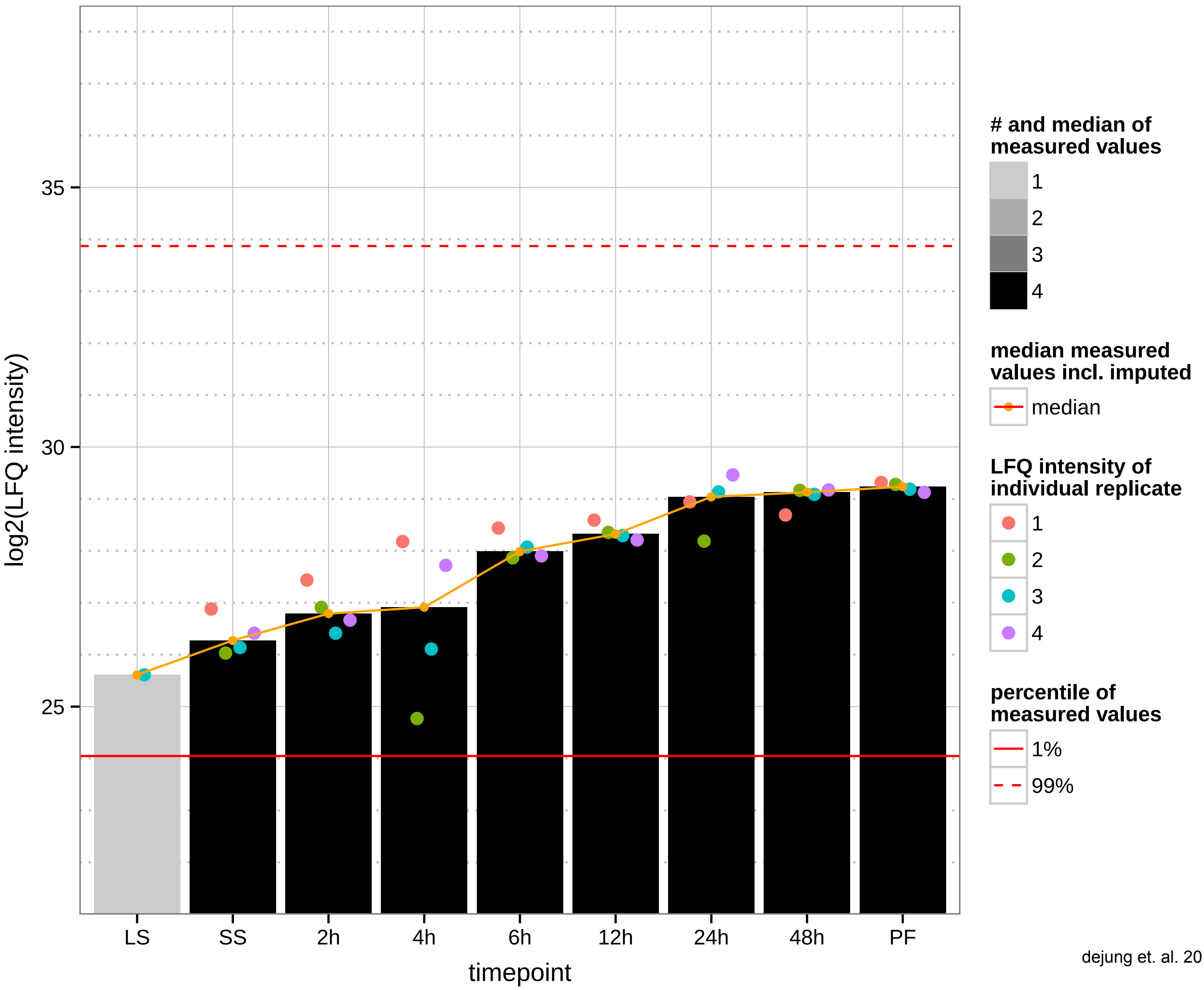

dejung et. al. 2015

protein phosphatase 2C, putative
Tb927.5.1660
AGOF: phosphatase activity
AGOC: null
AGOP: dephosphorylation
PGOF: catalytic activity
PGOC: null
PGOP: null

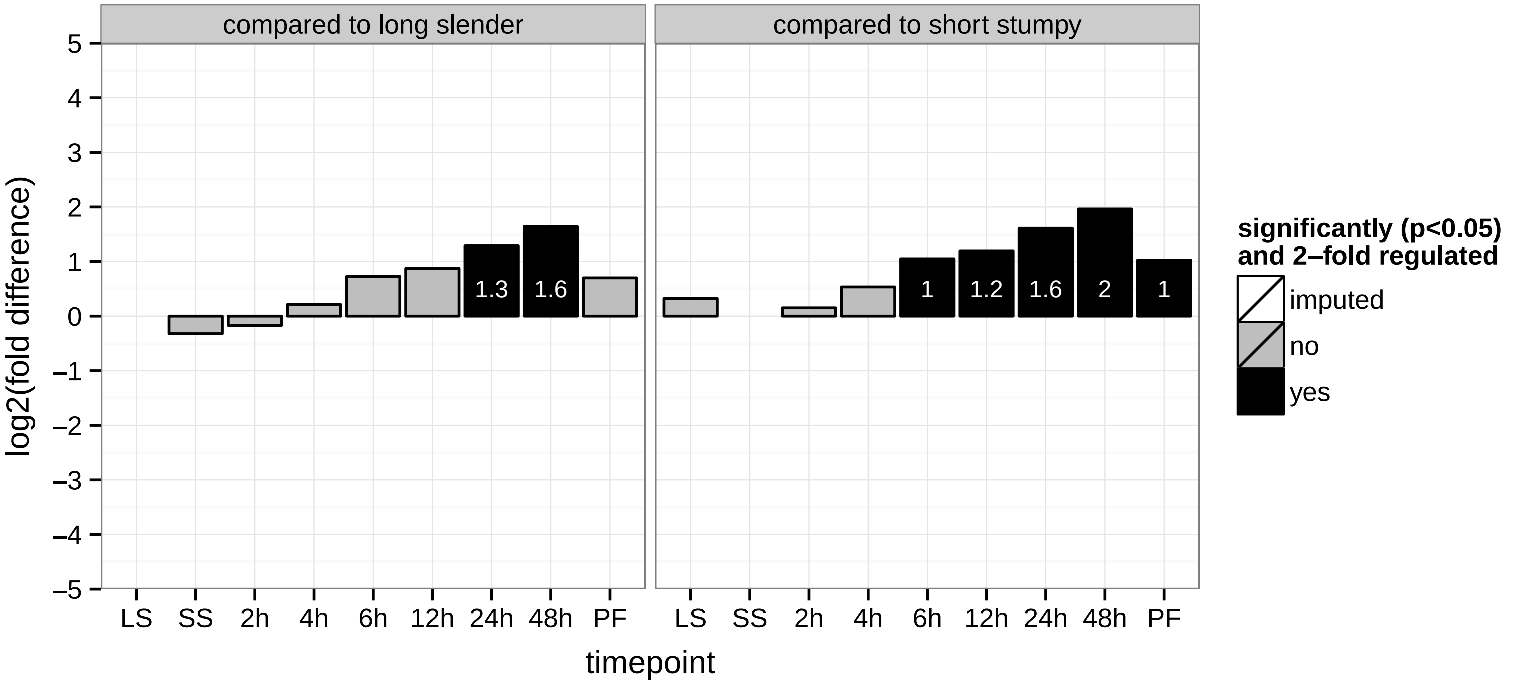

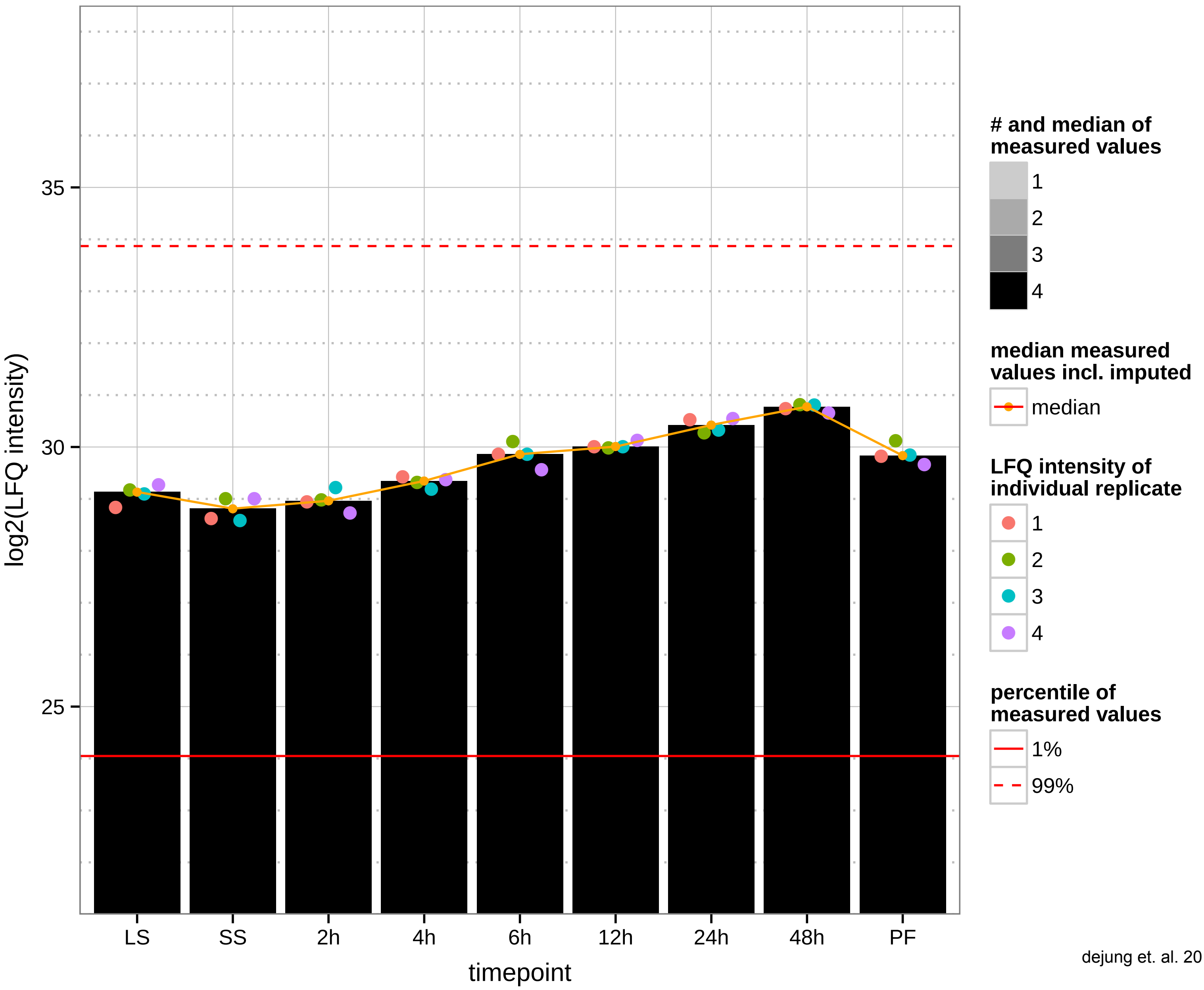

dejung et. al. 2015

GTP–binding protein, putative
Tb927.5.4310
AGOF: GTP binding
AGOC: intracellular
AGOP: null
PGOF: GTP binding
PGOC: null
PGOP: null

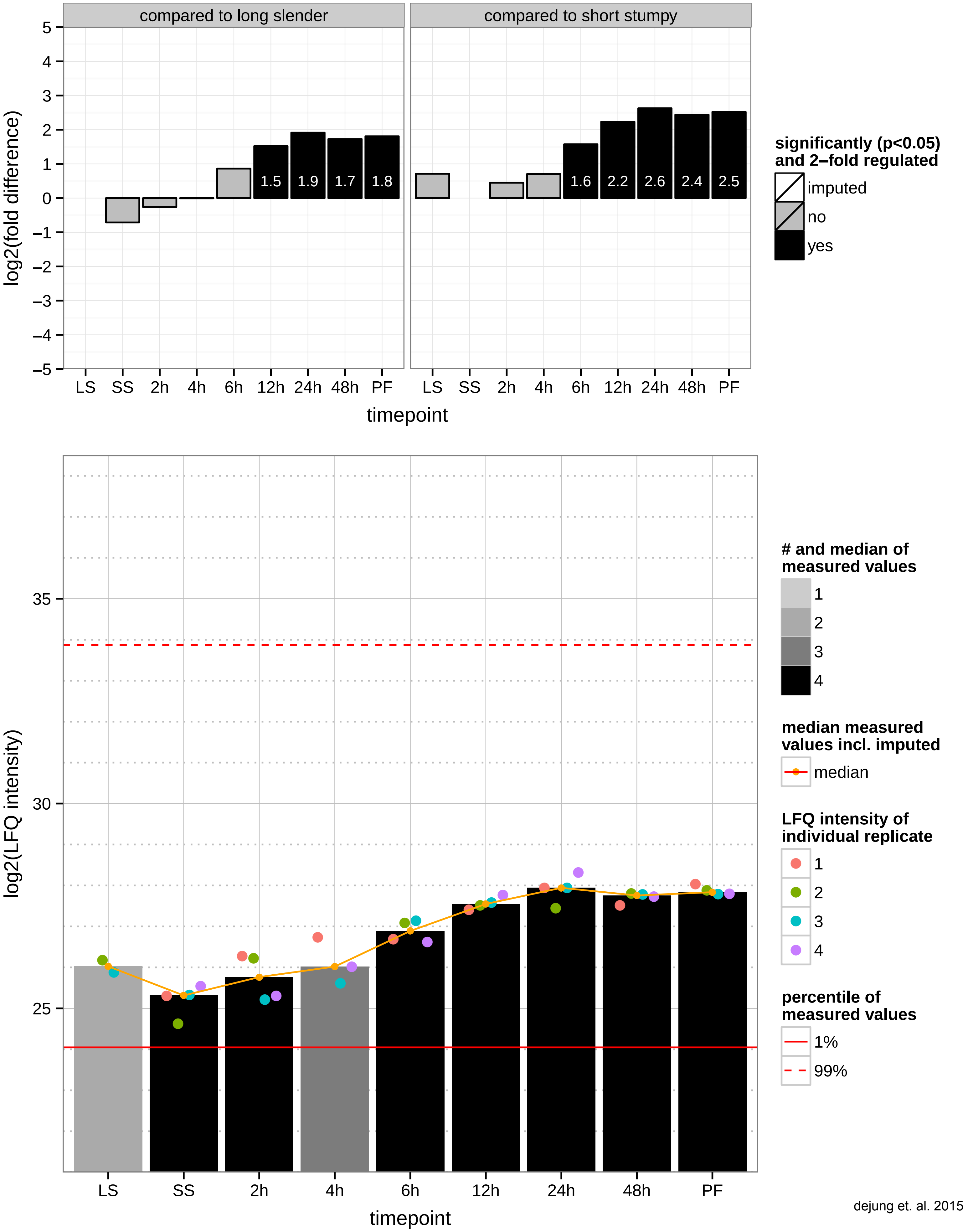

dejung et. al. 2015

hypothetical protein, conserved
Tb927.6.3030
AGOF: nucleoside−triphosphatase activity, nucleotide binding
AGOC: null
AGOP: null
PGOF: ATP binding, nucleoside−triphosphatase activity, nucleotide binding
PGOC: null
PGOP: null

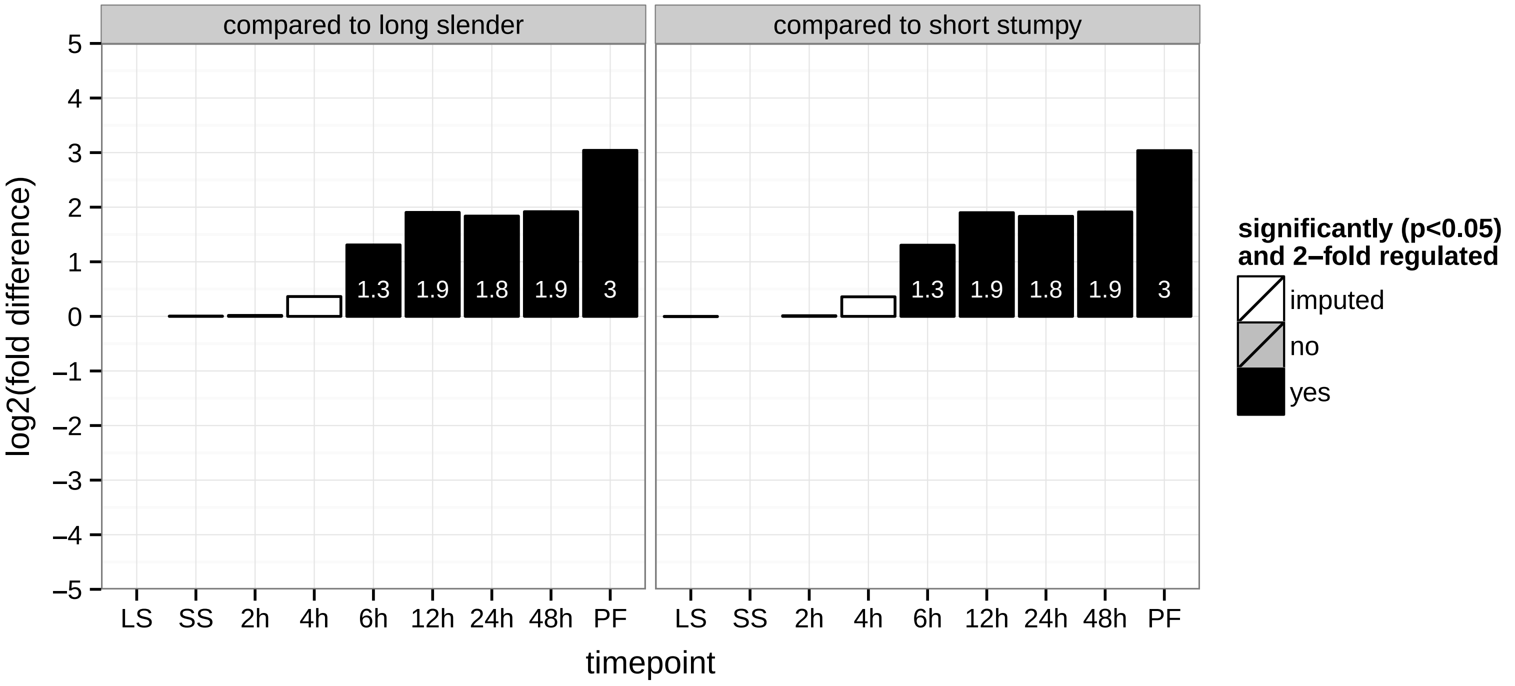

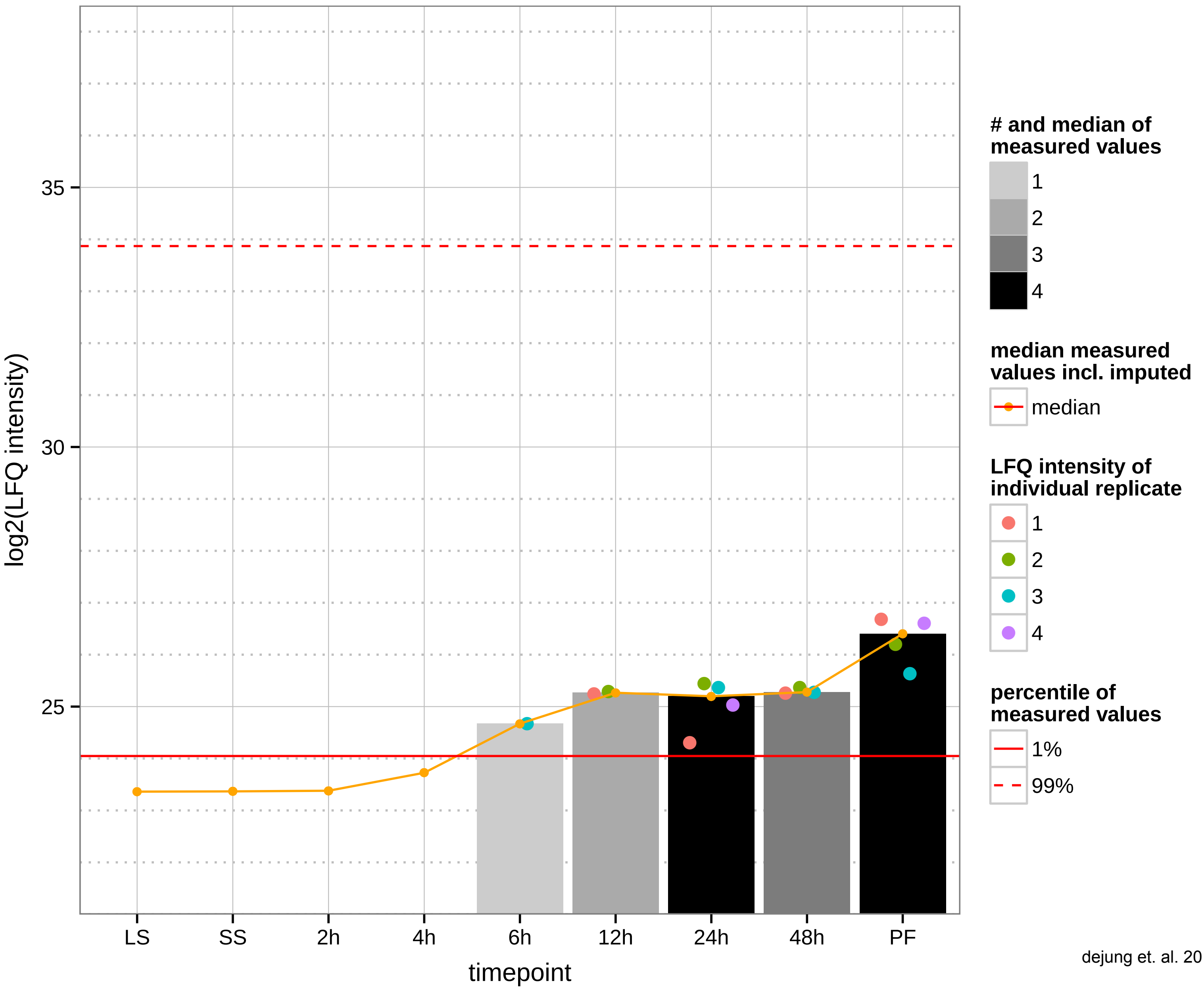

dejung et. al. 2015

homoserine kinase, putative (HK)
Tb927.6.4430
AGOF: ATP binding, homoserine kinase activity
AGOC: null
AGOP: phosphorylation, threonine metabolic process
PGOF: ATP binding, homoserine kinase activity
PGOC: null
PGOP: threonine metabolic process

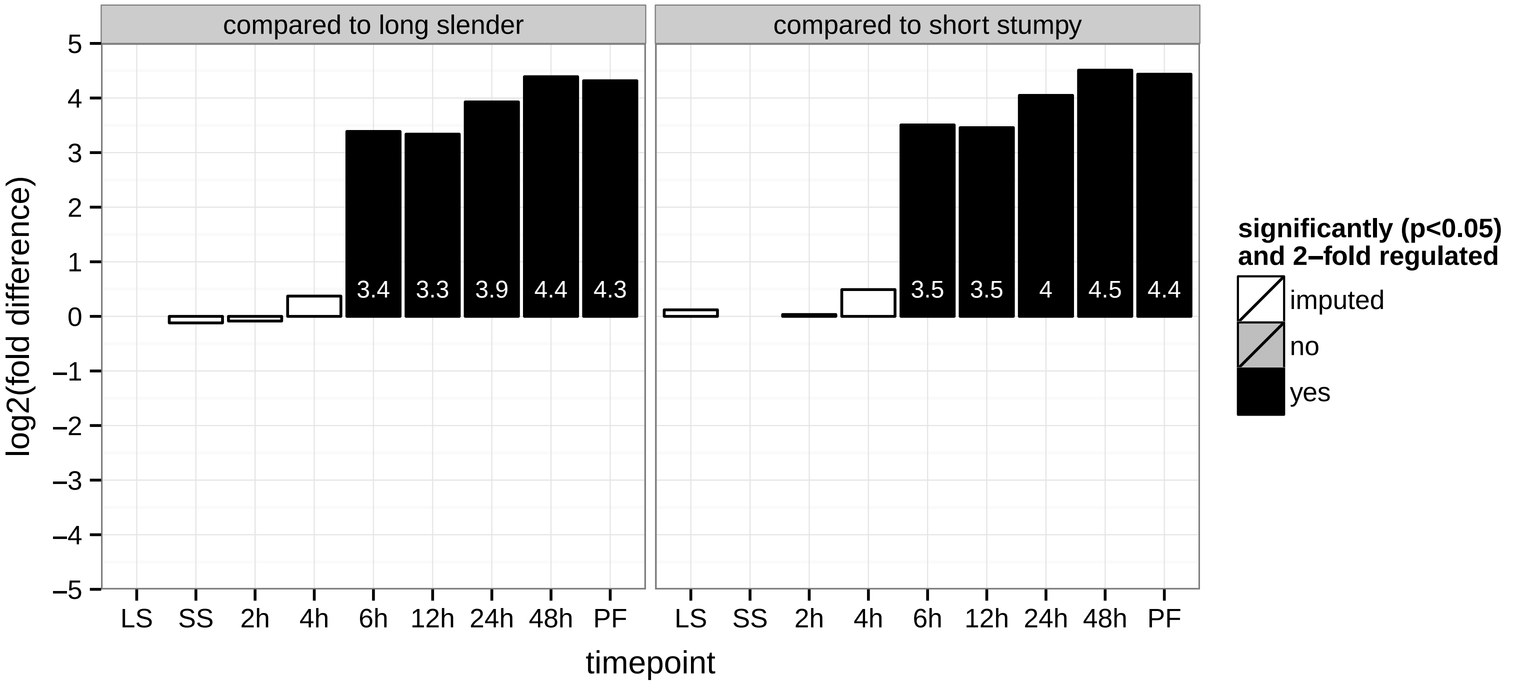

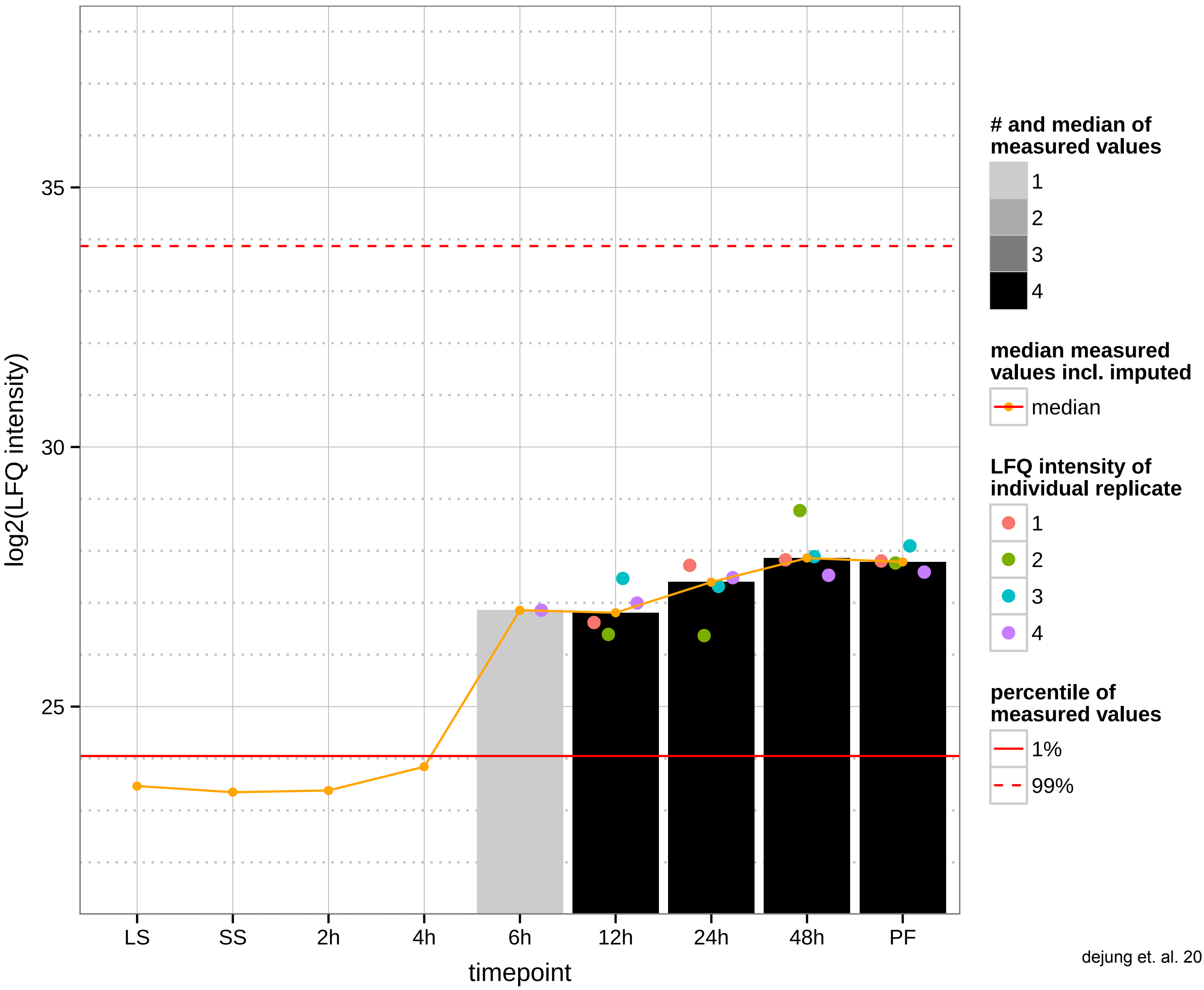

dejung et. al. 2015

hypothetical protein, conserved
Tb927.7.2640
AGOF: null
AGOC: null
AGOP: null
PGOF: null
PGOC: null
PGOP: null

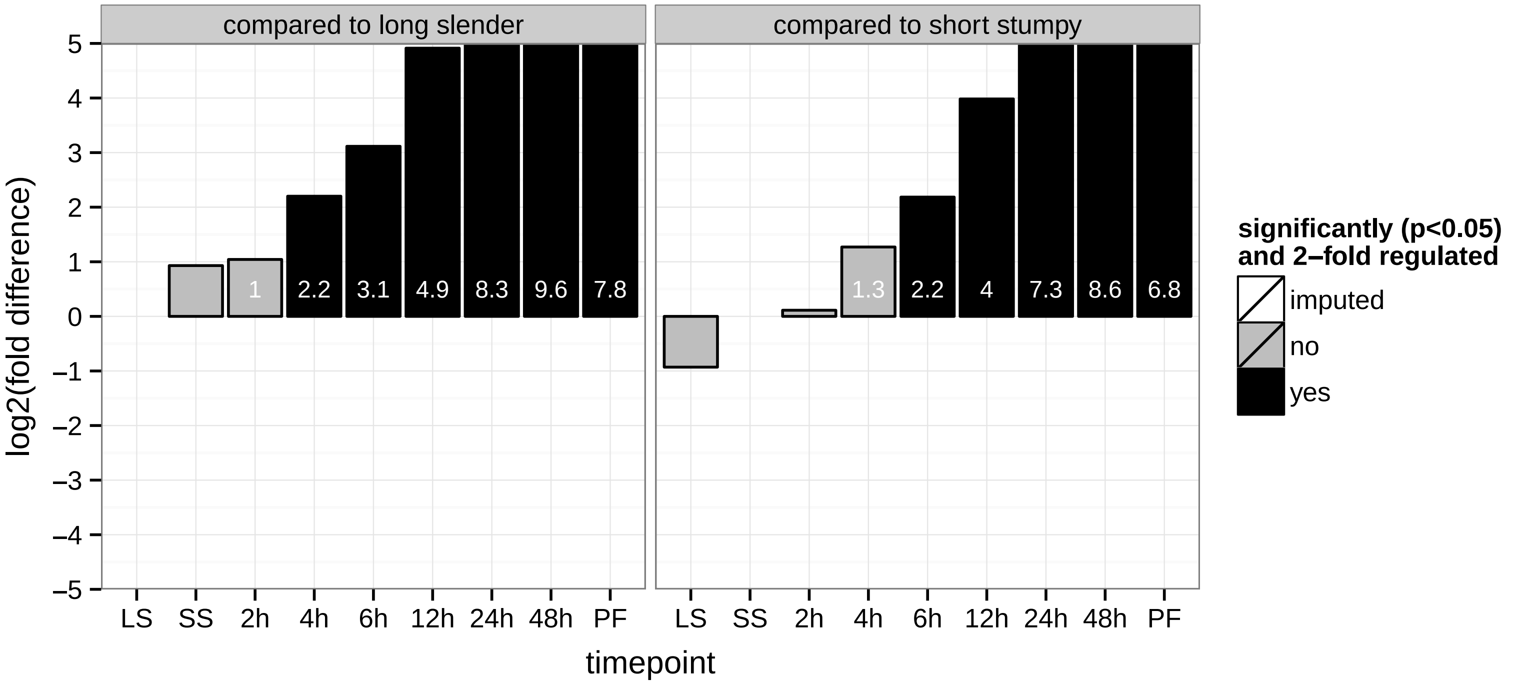

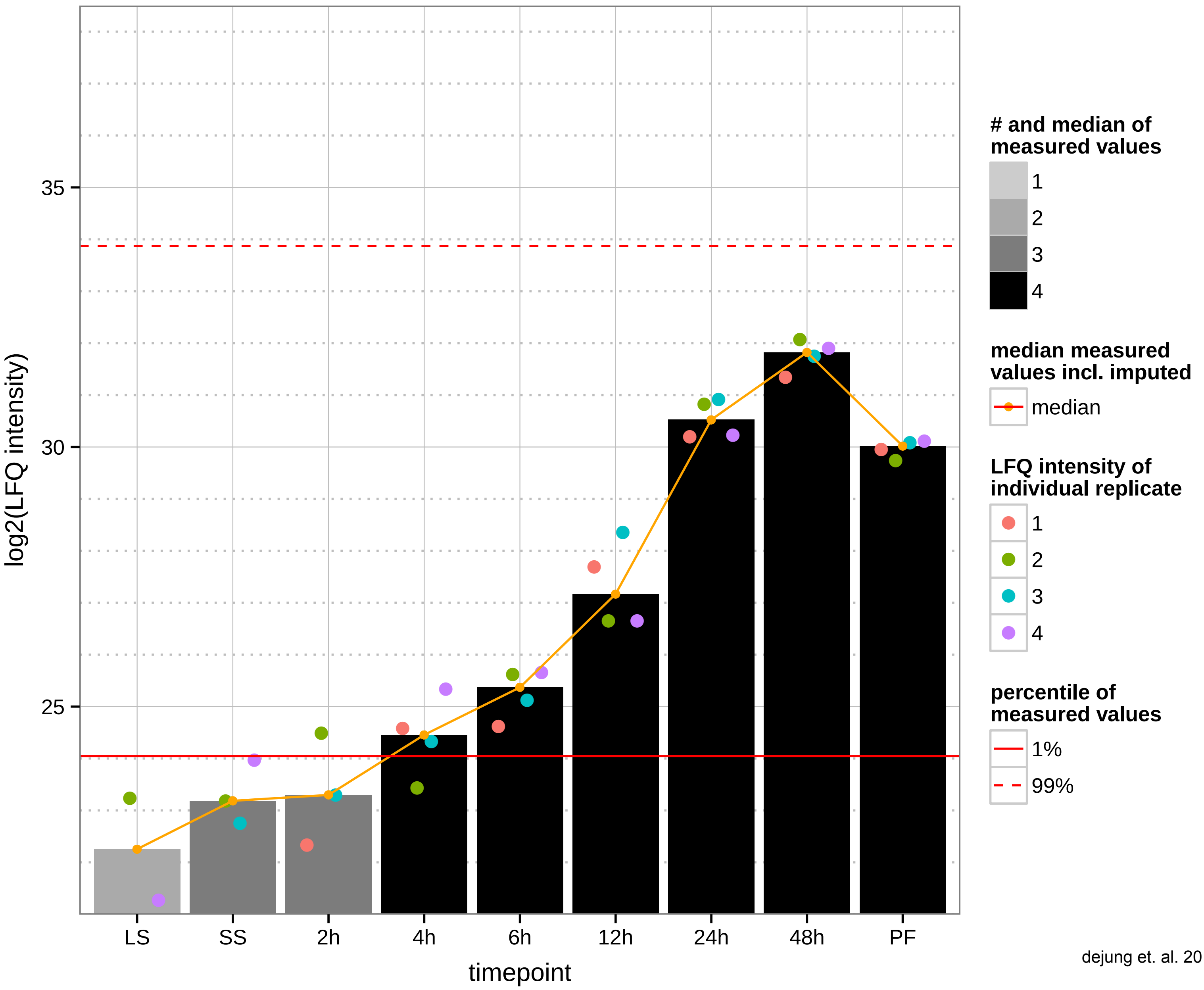

dejung et. al. 2015

hypothetical protein, conserved
Tb927.7.2740
AGOF: null
AGOC: null
AGOP: null
PGOF: null
PGOC: null
PGOP: null

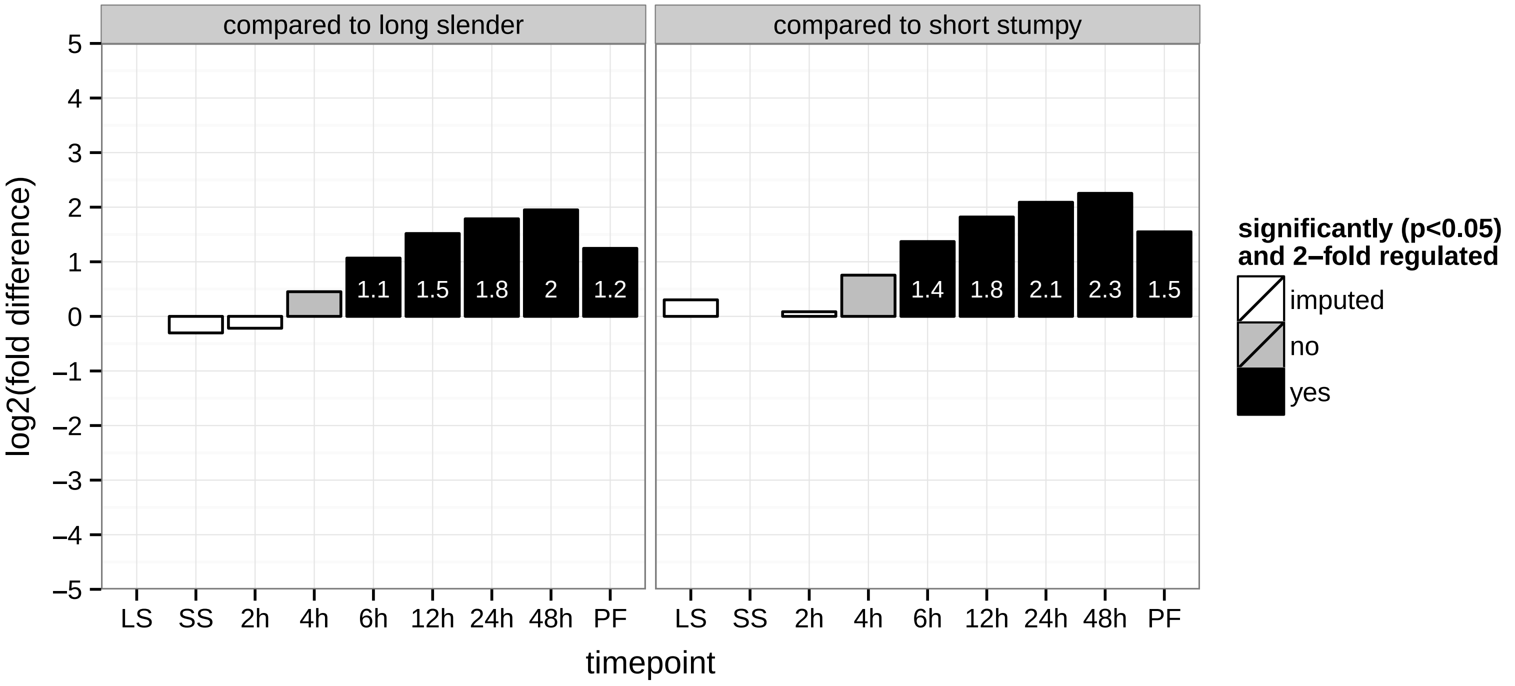

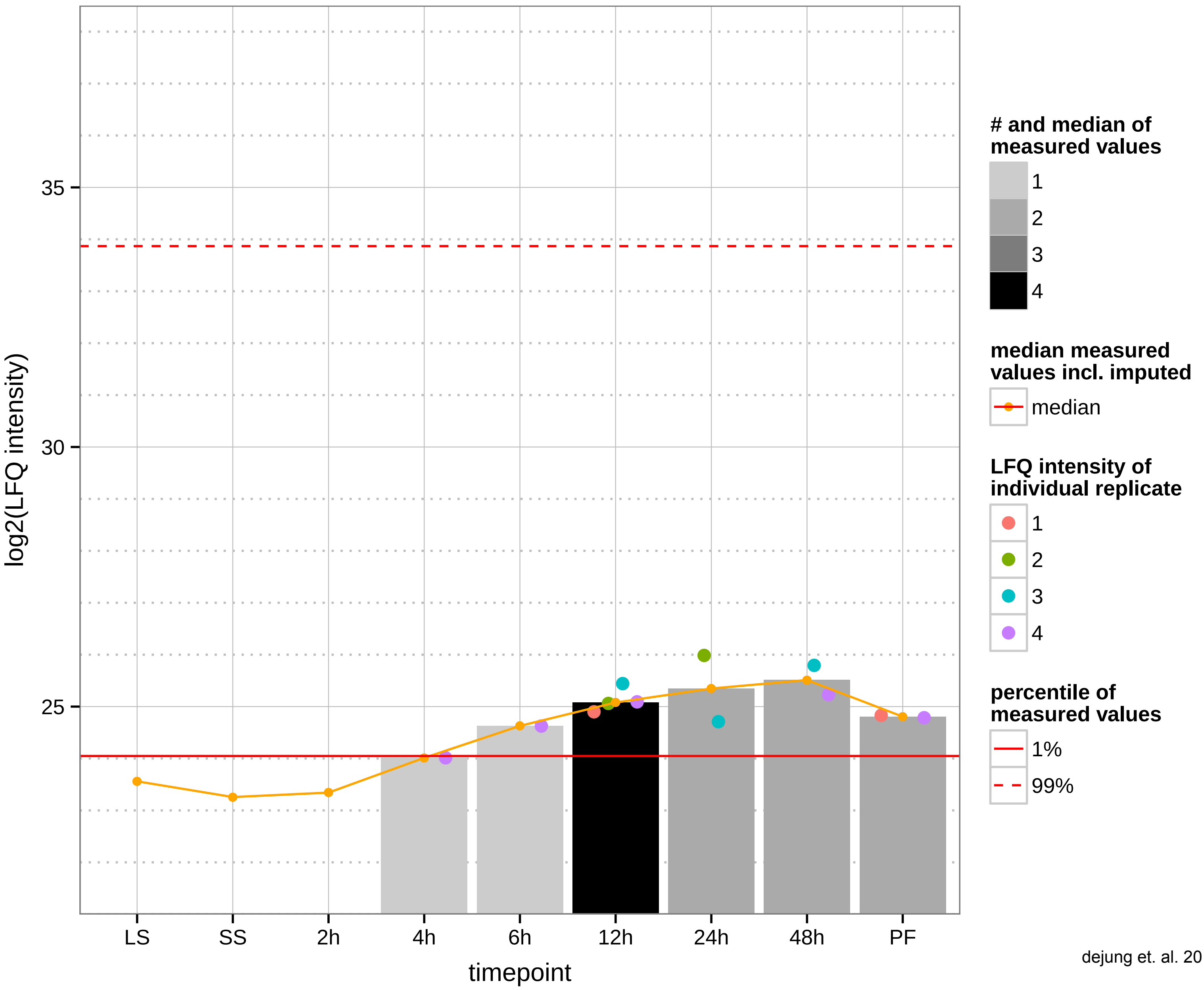

dejung et. al. 2015

hypothetical protein, conserved (TbRBP8)
Tb927.7.320
AGOF: RNA binding
AGOC: null
AGOP: RNA processing
PGOF: nucleic acid binding
PGOC: null
PGOP: null

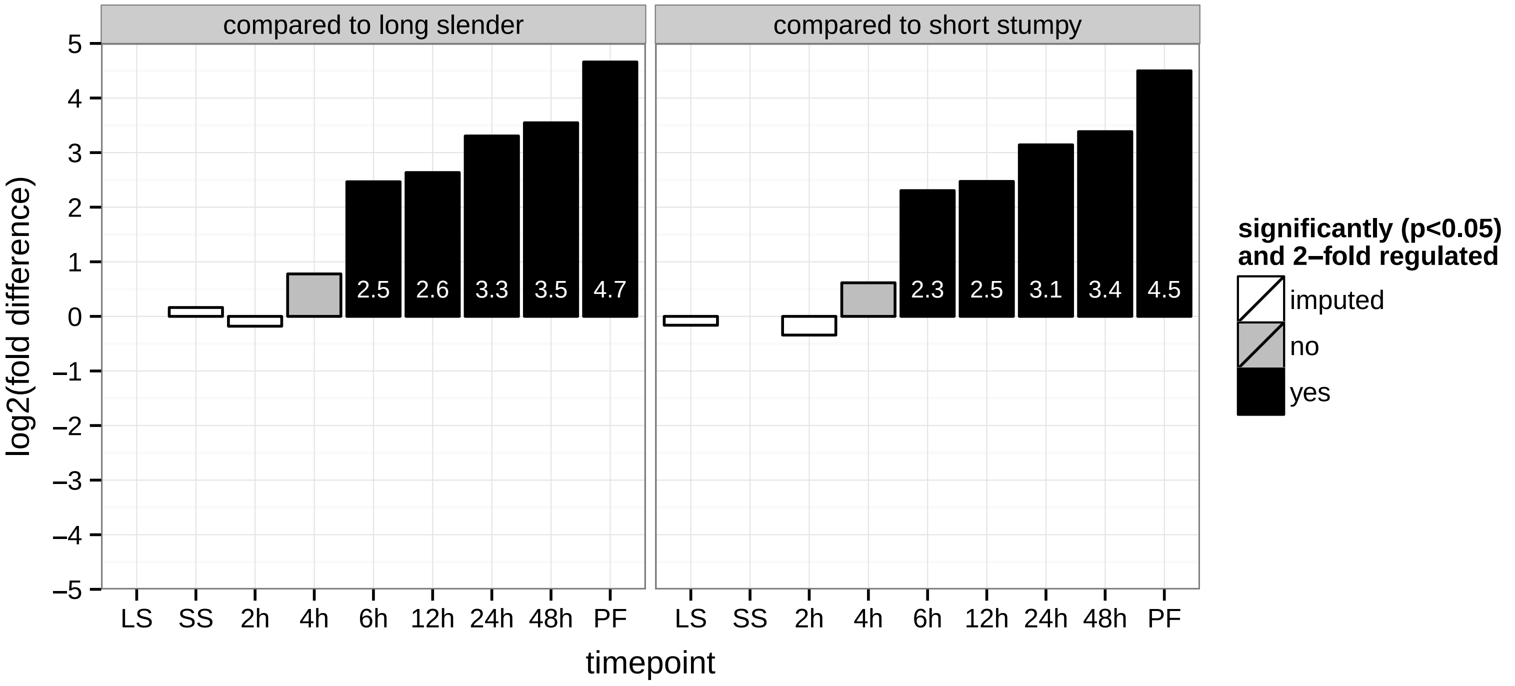

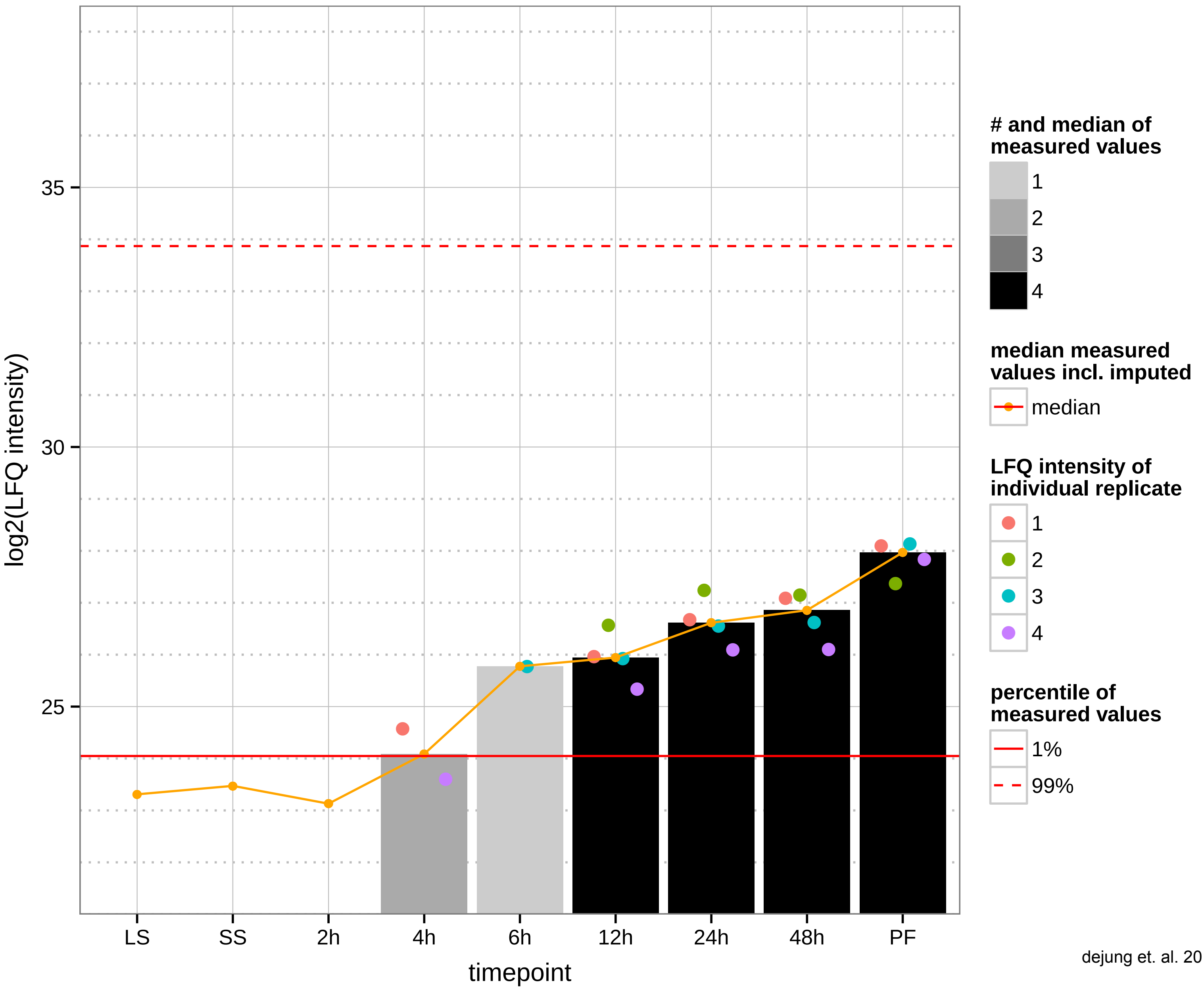

dejung et. al. 2015

translation initiation factor IF−2, putative
Tb927.7.3280
AGOF: GTP binding, GTPase activity, translation factor activity, nucleic acid binding
AGOC: null
AGOP: translation
PGOF: GTP binding, GTPase activity
PGOC: null
PGOP: null

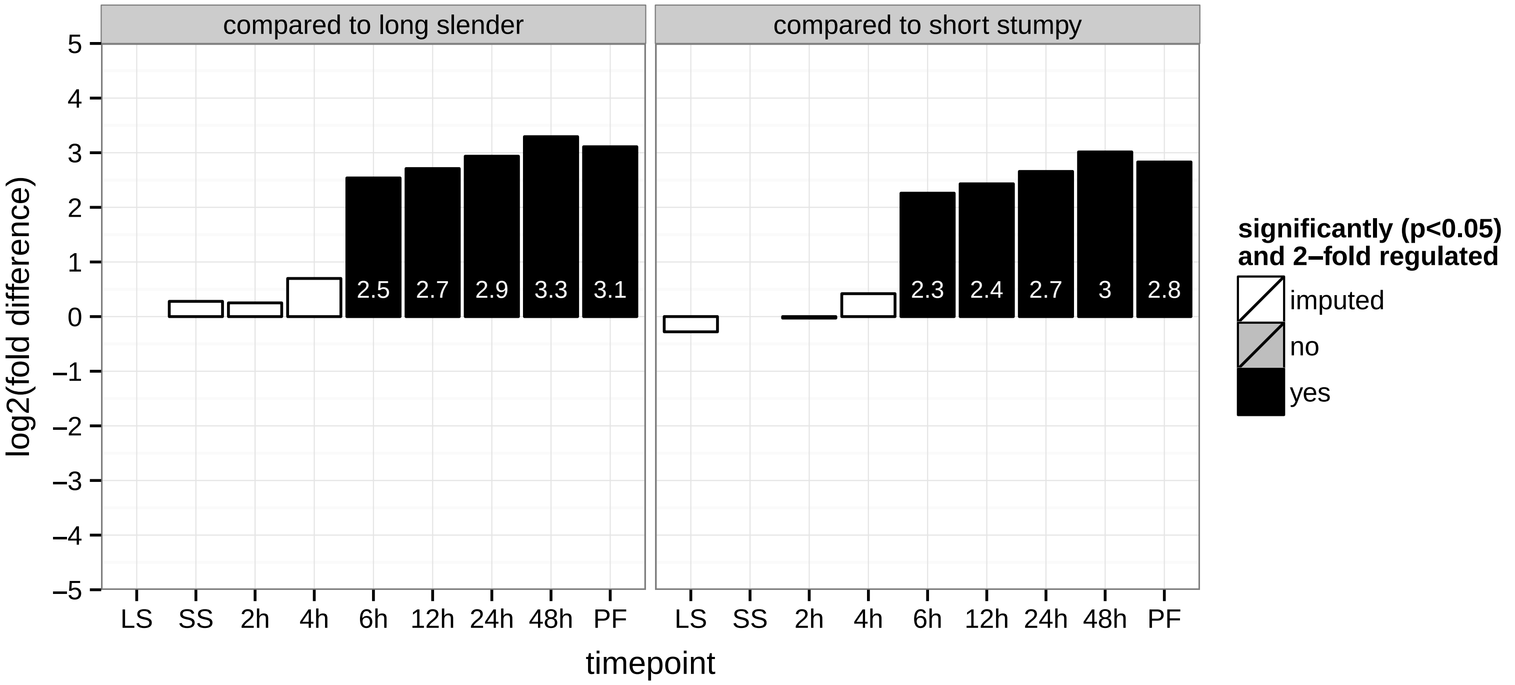

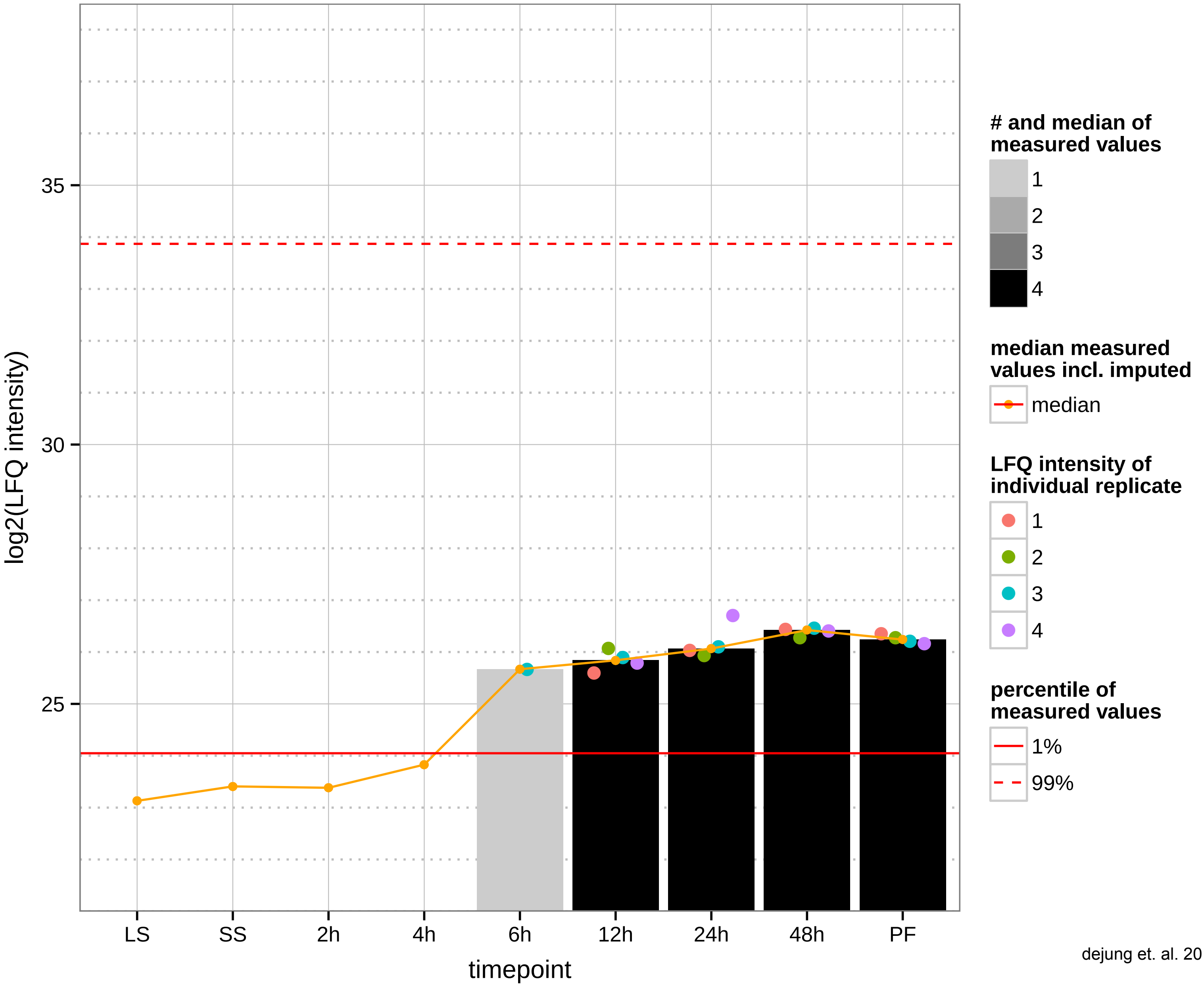

dejung et. al. 2015

hypothetical protein, conserved
Tb927.7.3890
AGOF: null
AGOC: null
AGOP: null
PGOF: null
PGOC: null
PGOP: null

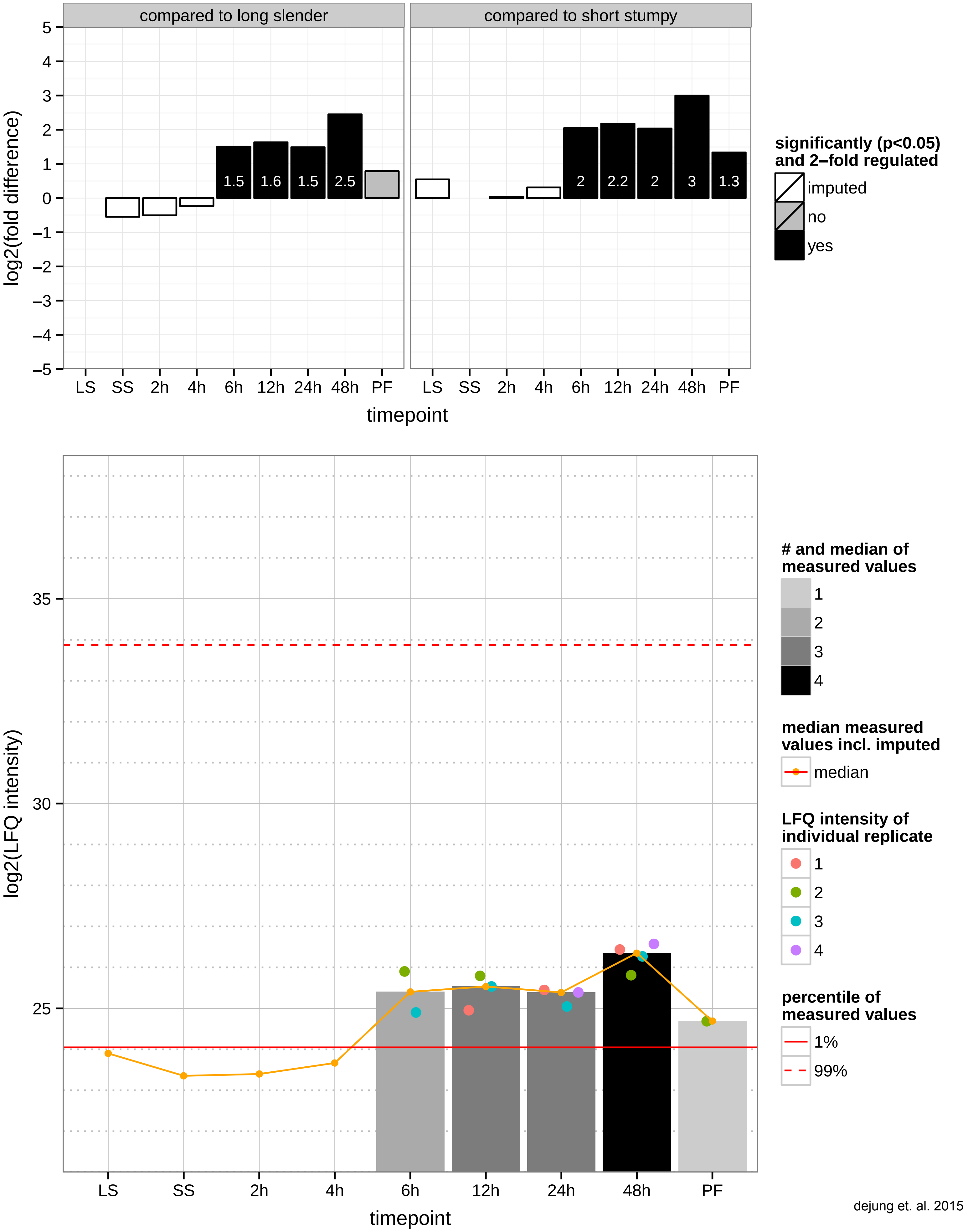

dejung et. al. 2015

deoxyribose–phosphate aldolase, putative
Tb927.7.5680
AGOF: deoxyribose–phosphate aldolase activity
AGOC: cytoplasm, mitochondrial membrane
AGOP: deoxyribonucleotide catabolic process
PGOF: deoxyribose–phosphate aldolase activity, lyase activity
PGOC: cytoplasm
PGOP: deoxyribonucleotide catabolic process

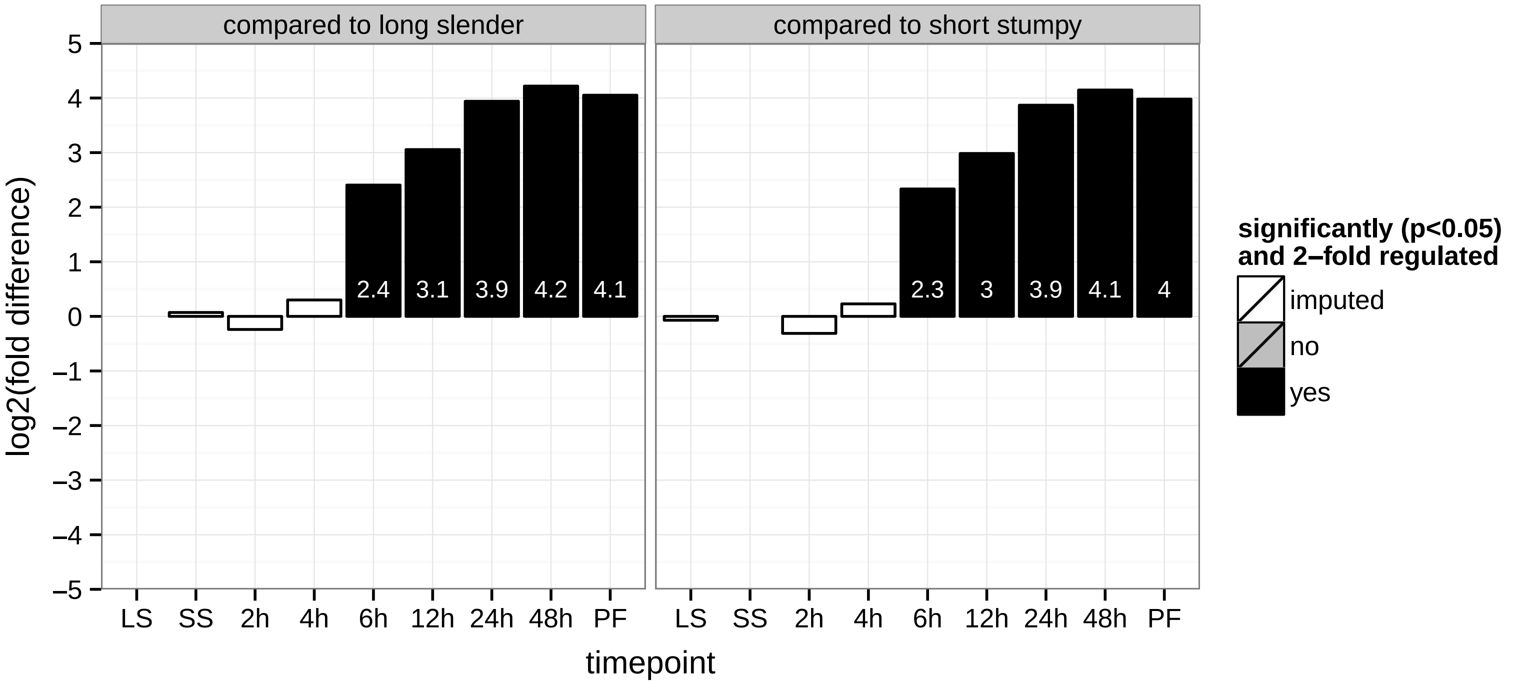

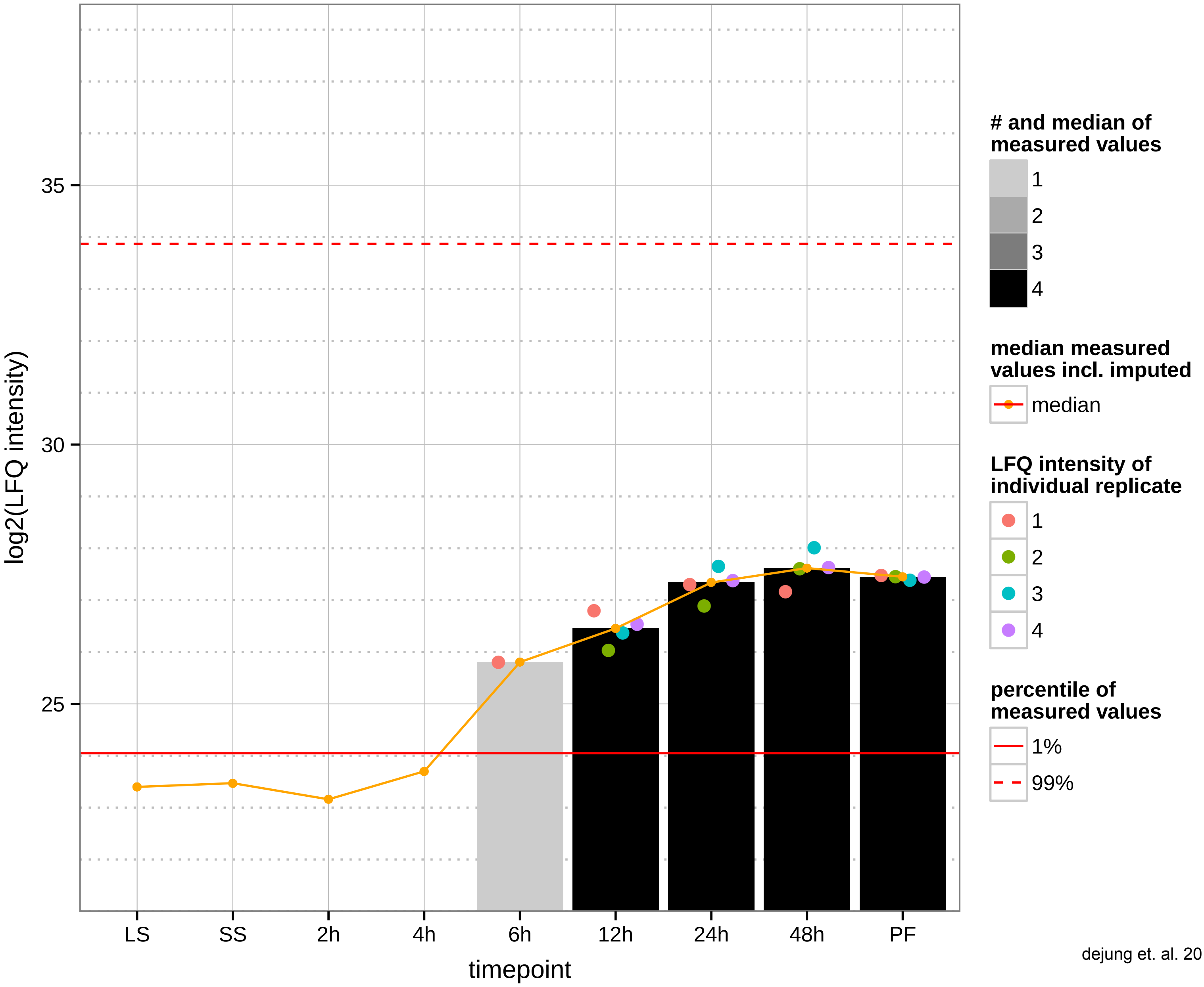

dejung et. al. 2015

protein associated with differentiation 5, putative (PAD5), protein associated with differentiation 7, putative (PAD7)
Tb927.7.5990;Tb927.7.5970
AGOF: null
AGOC: null
AGOP: null
PGOF: null
PGOC: integral to membrane
PGOP: transmembrane transport

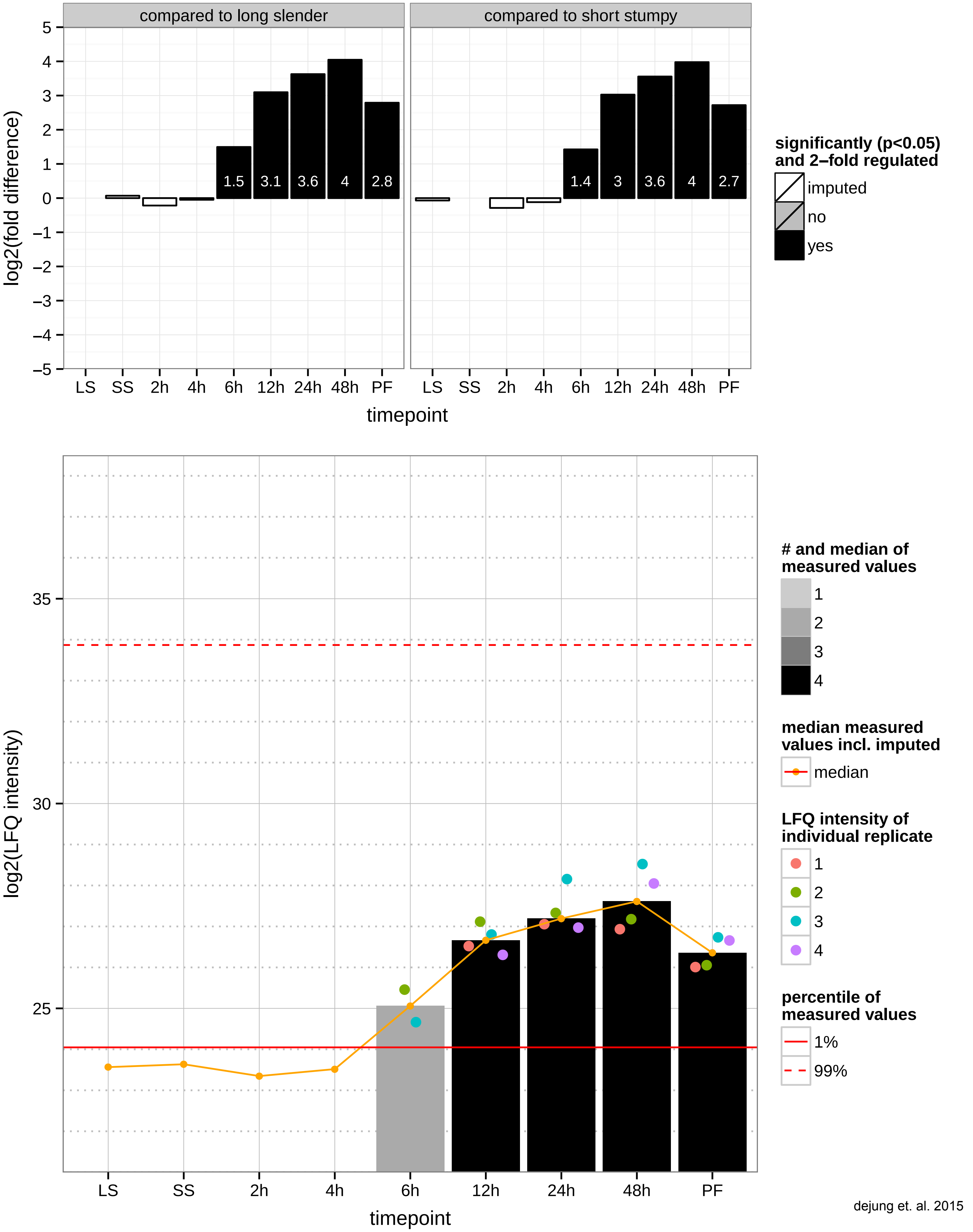

dejung et. al. 2015

tRNA–dihydrouridine synthase 3, putative
Tb927.7.6450
AGOF: flavin adenine dinucleotide binding, tRNA dihydrouridine synthase activity
AGOC: null
AGOP: oxidation–reduction process, tRNA processing
PGOF: flavin adenine dinucleotide binding, tRNA dihydrouridine synthase activity
PGOC: null
PGOP: oxidation–reduction process, tRNA processing

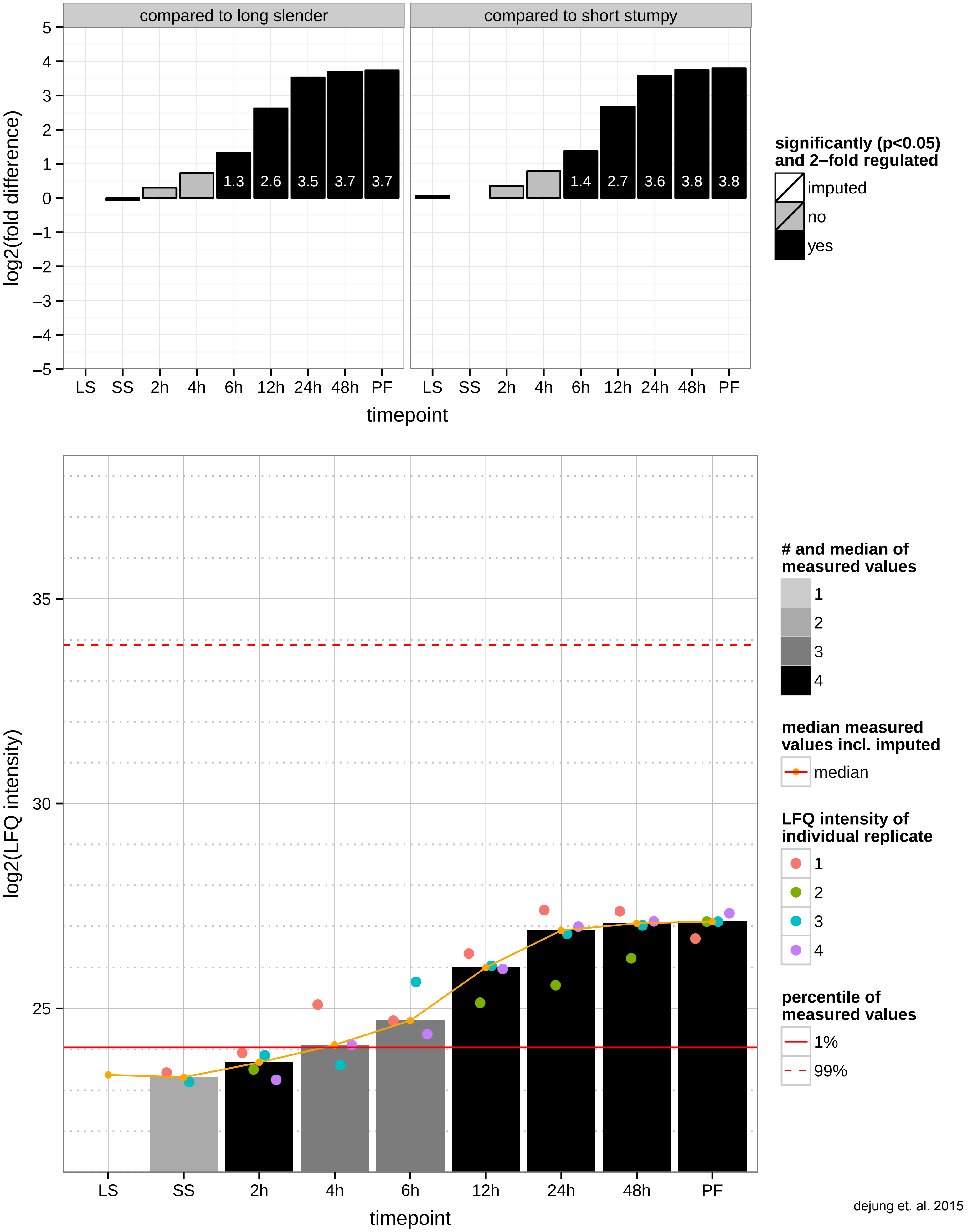

dejung et. al. 2015

hypothetical protein, conserved
Tb927.7.6990
AGOF: null
AGOC: mitochondrial inner membrane, mitochondrion
AGOP: null
PGOF: null
PGOC: null
PGOP: null

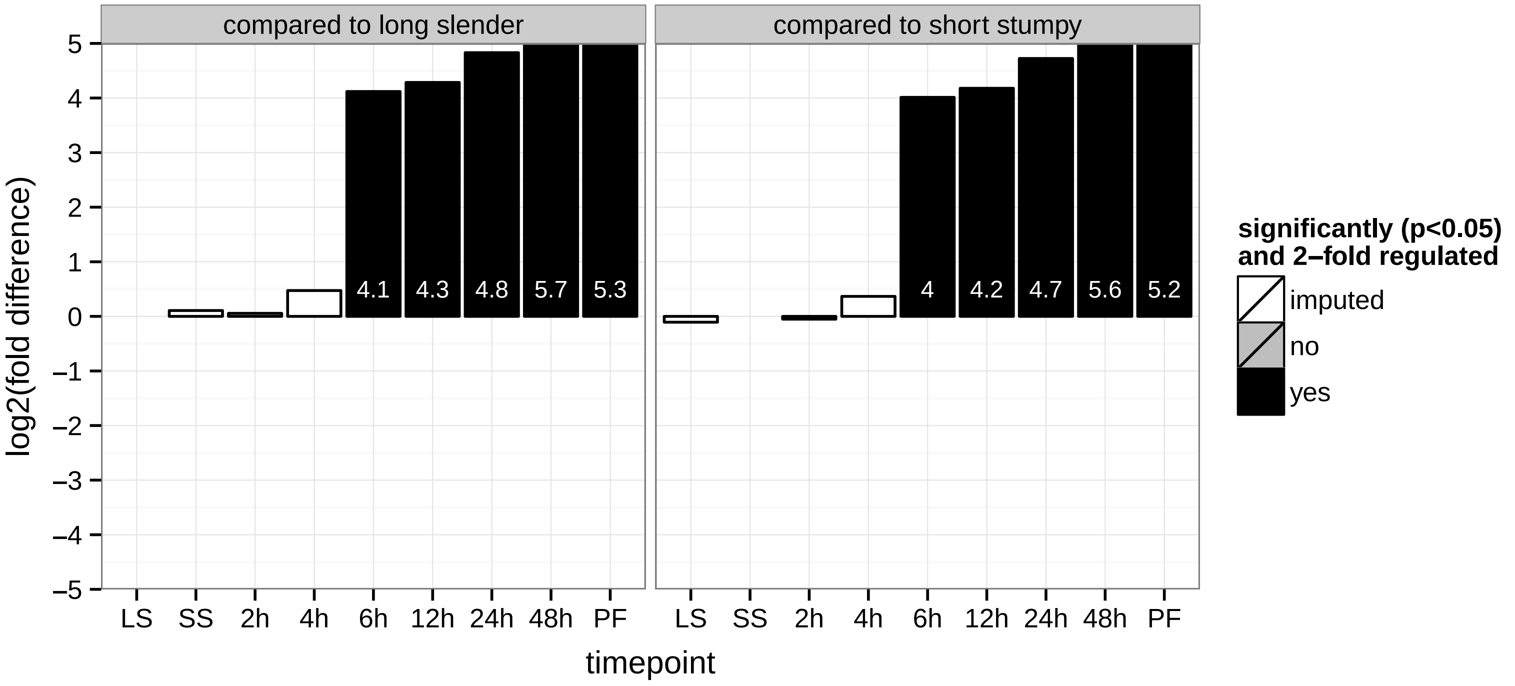

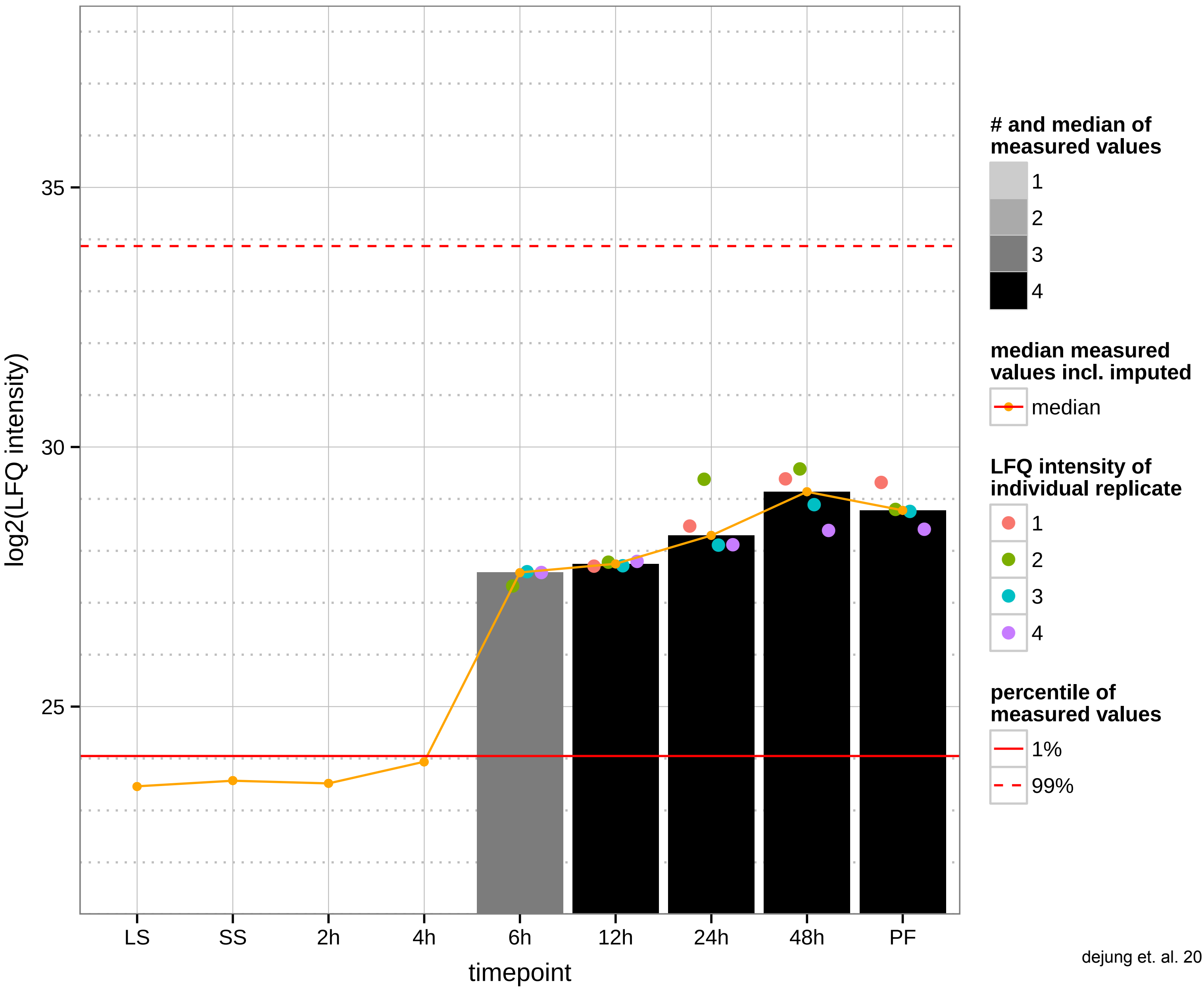

dejung et. al. 2015

acetylornithine deacetylase, putative, metallo–peptidase, Clan MH, Family M18 (ArgE)
Tb927.8.1910
AGOF: acetylornithine deacetylase activity, cobalt ion binding, metallopeptidase activity, protein dimerization activity, zinc ion b
AGOC: cytoplasm
AGOP: arginine biosynthetic process, proteolysis
PGOF: acetylornithine deacetylase activity, cobalt ion binding, hydrolase activity, zinc ion binding
PGOC: cytoplasm
PGOP: arginine biosynthetic process, metabolic process

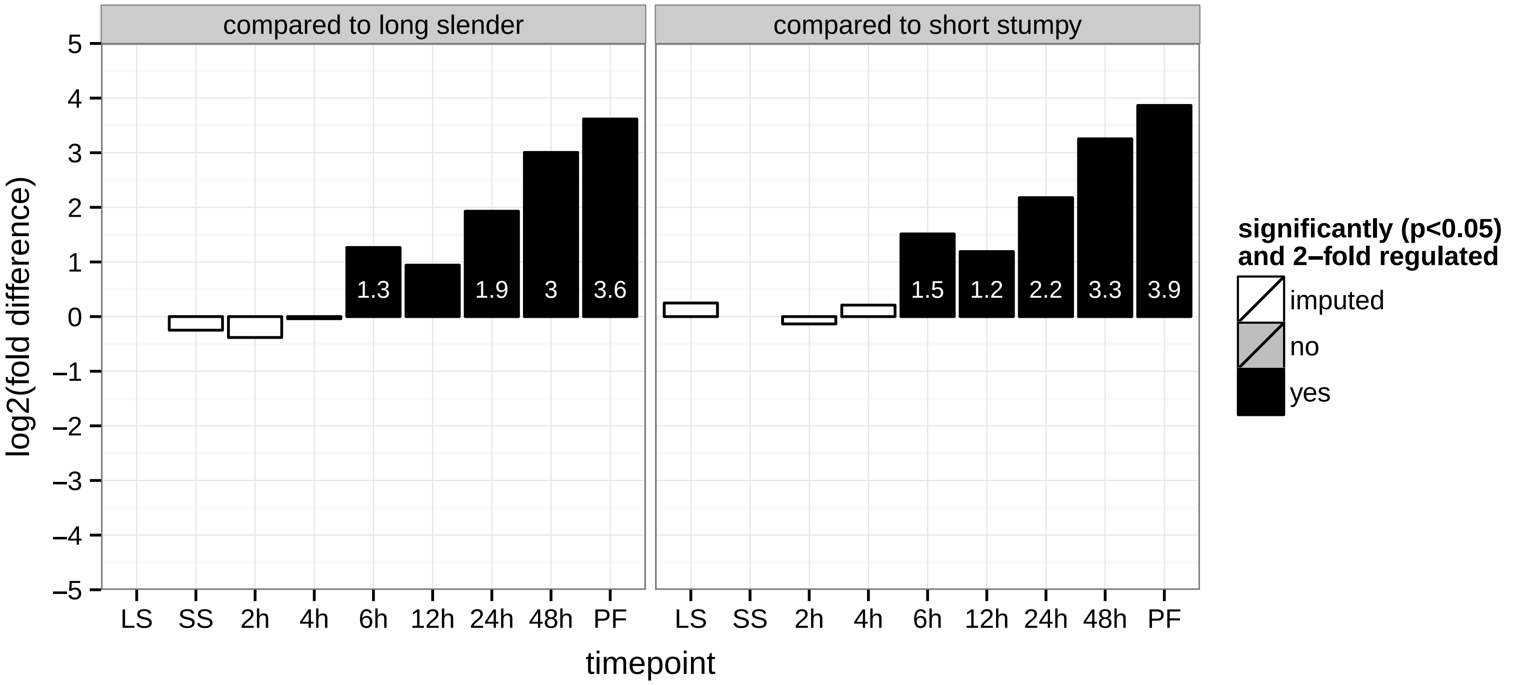

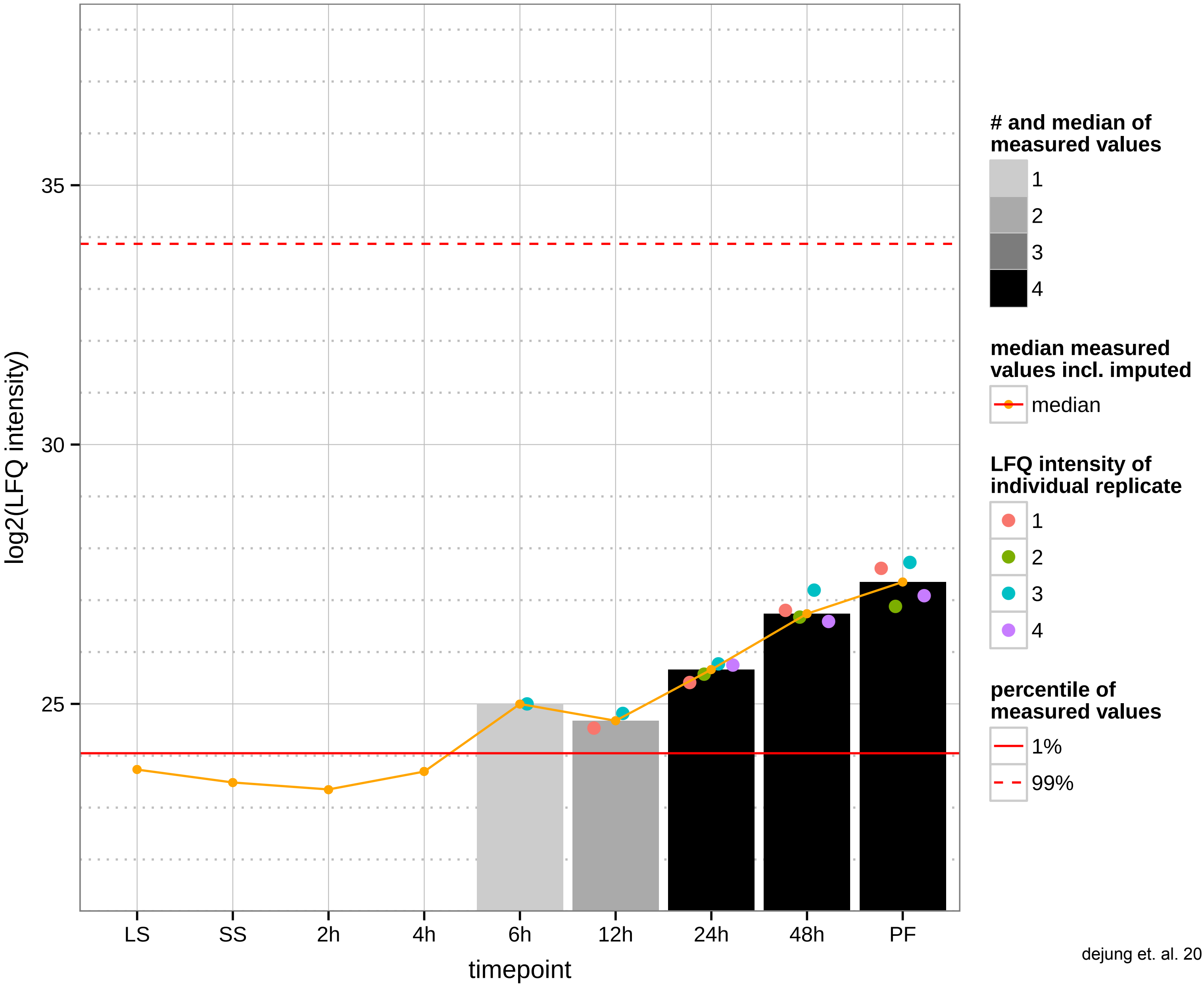

dejung et. al. 2015

Tryptophanyl–tRNA synthetase 2
Tb927.8.2240
AGOF: ATP binding, aminoacyl–tRNA ligase activity, tryptophan–tRNA ligase activity
AGOC: cytoplasm, mitochondrial part
AGOP: tRNA aminoacylation for protein translation, translation, tryptophanyl–tRNA aminoacylation
PGOF: ATP binding, aminoacyl–tRNA ligase activity, nucleotide binding, tryptophan–tRNA ligase activity
PGOC: cytoplasm
PGOP: tRNA aminoacylation for protein translation, tryptophanyl–tRNA aminoacylation

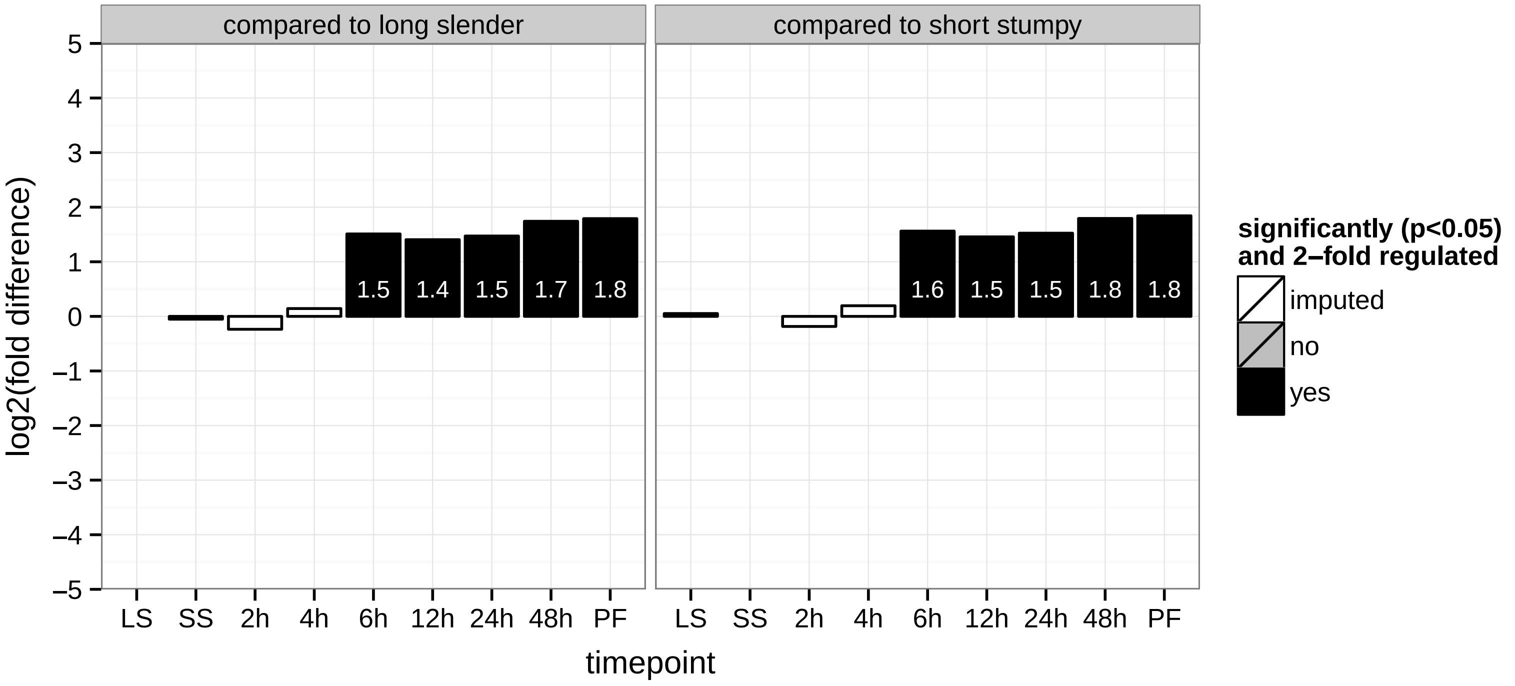

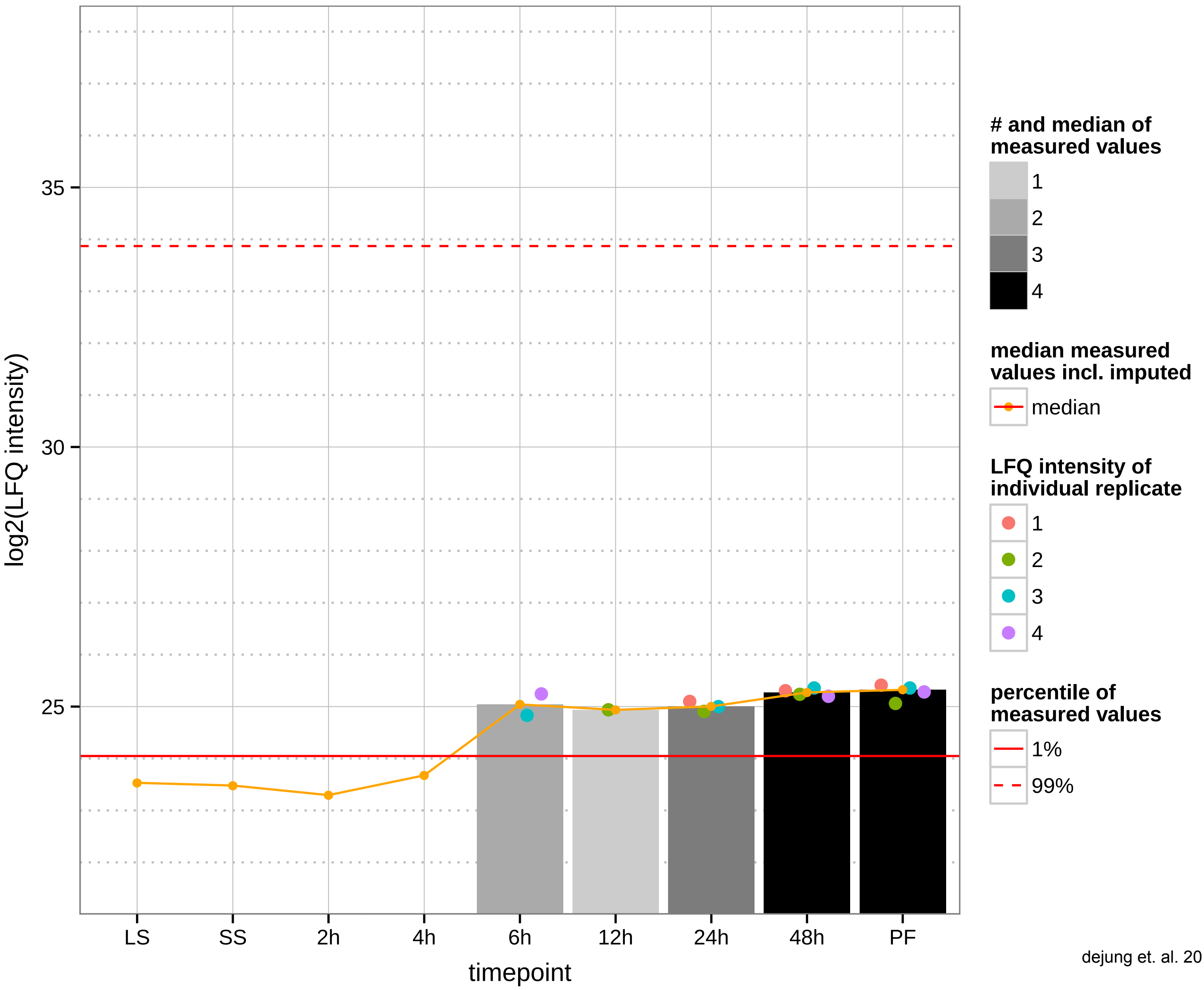

dejung et. al. 2015

hypothetical protein, conserved
Tb927.8.5990
AGOF: null
AGOC: null
AGOP: null
PGOF: protein binding
PGOC: null
PGOP: null

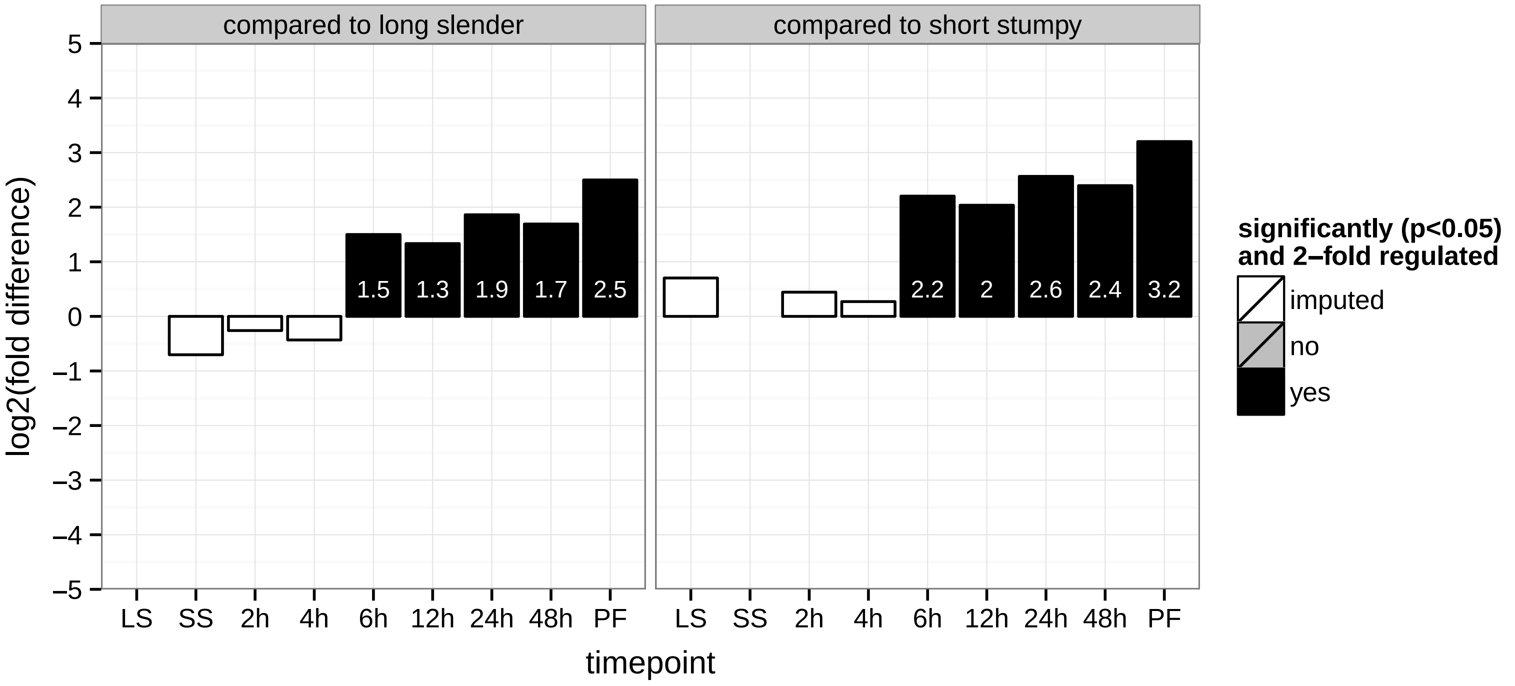

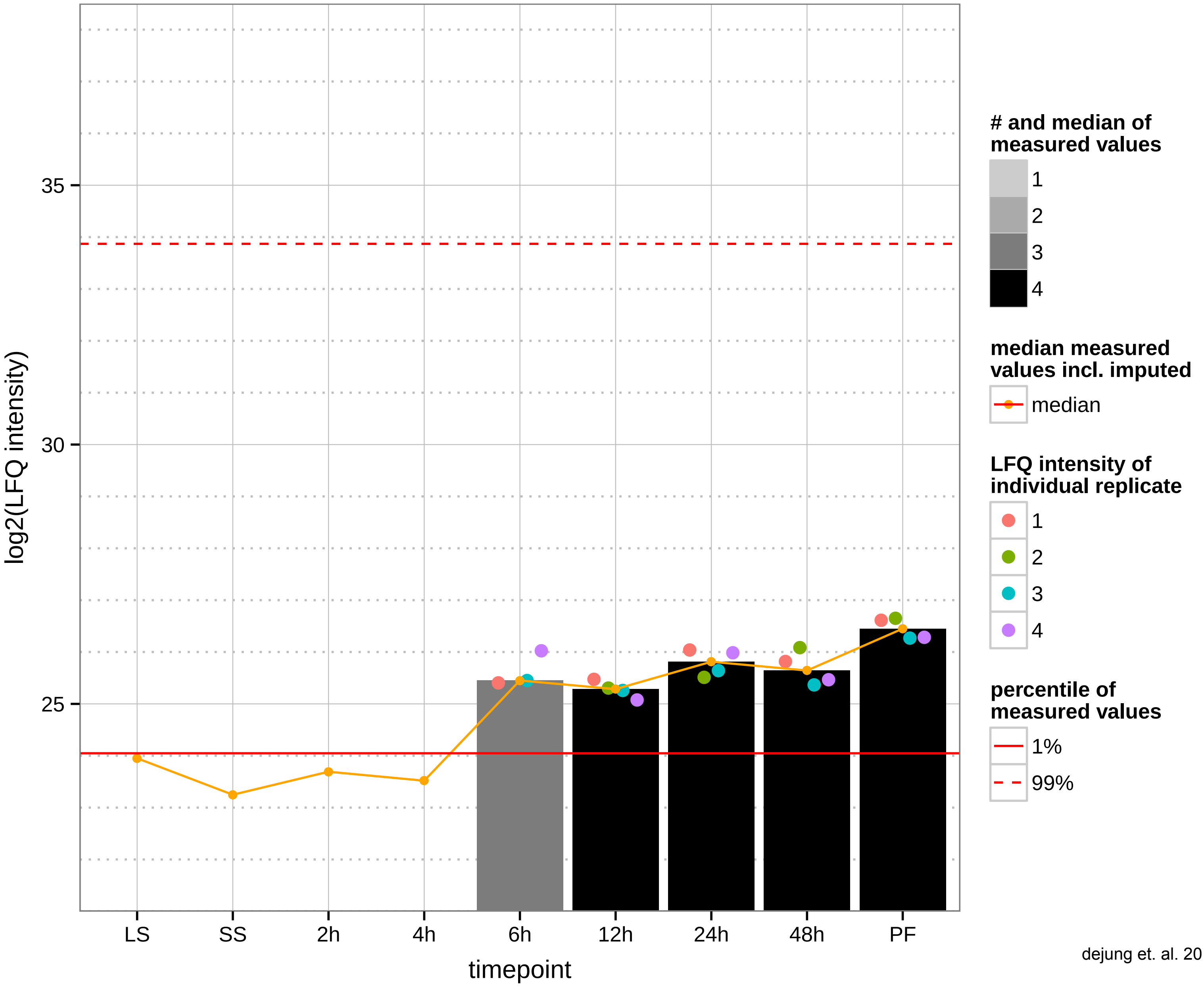

dejung et. al. 2015

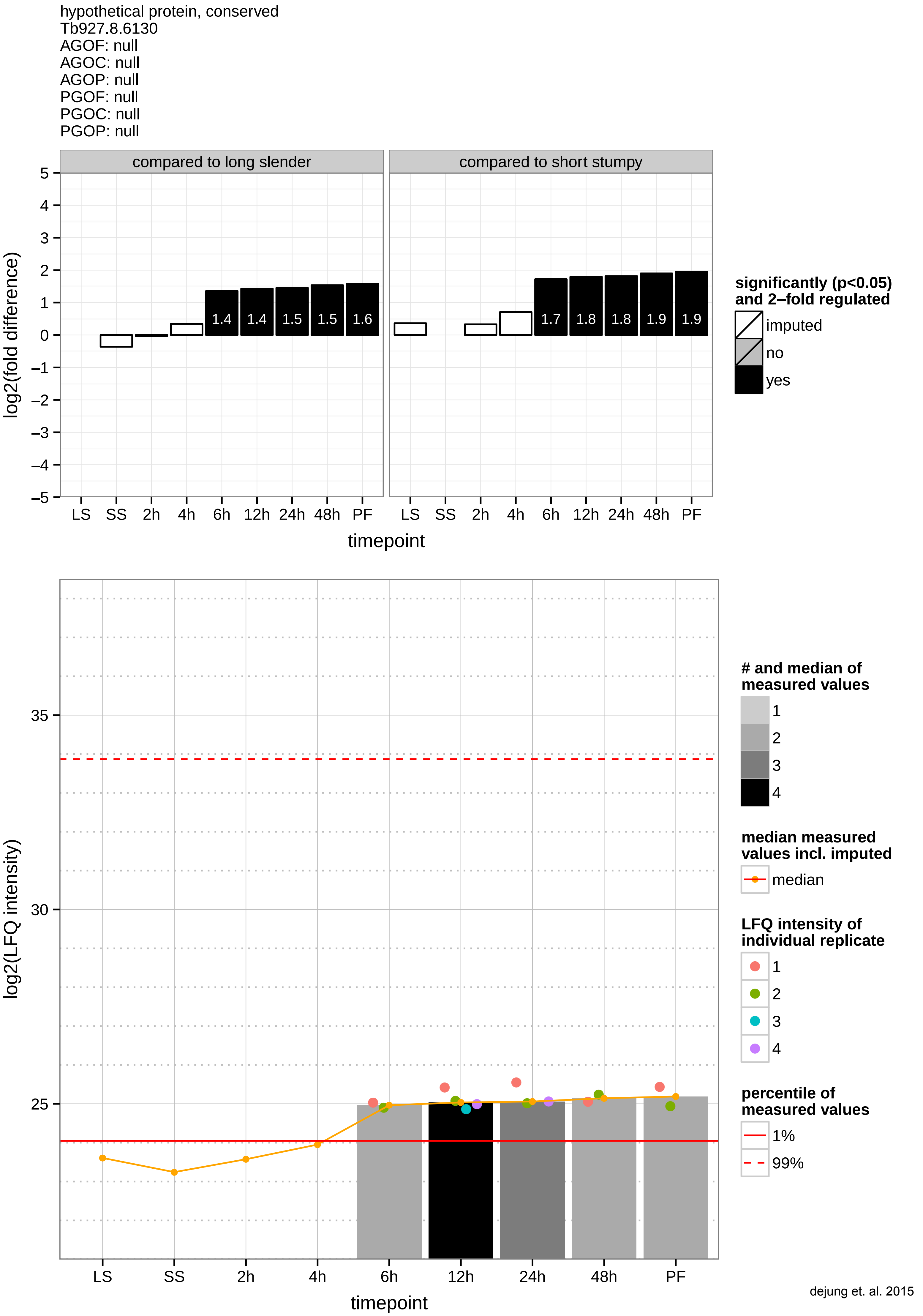
hypothetical protein, conserved
Tb927.8.6130
AGOF: null
AGOC: null
AGOP: null
PGOF: null
PGOC: null
PGOP: null
compared to long slender
compared to short stumpy
log2(fold difference)
1.4
1.4
1.5
1.5
1.6
1.7
1.8
1.8
1.9
1.9
LS SS 2h 4h 6h 12h 24h 48h PF
timepoint
significantly (p<0.05) and 2−fold regulated
imputed
no
yes
log2(LFQ intensity)
# and median of measured values
median measured values incl. imputed
median
LFQ intensity of individual replicate
percentile of measured values
1%
99%
dejung et. al. 2015

amino acid transporter, putative
Tb927.8.7700;Tb927.8.7680
AGOF: amine transmembrane transporter activity
AGOC: integral to membrane
AGOP: amino acid transport
PGOF: null
PGOC: null
PGOP: null

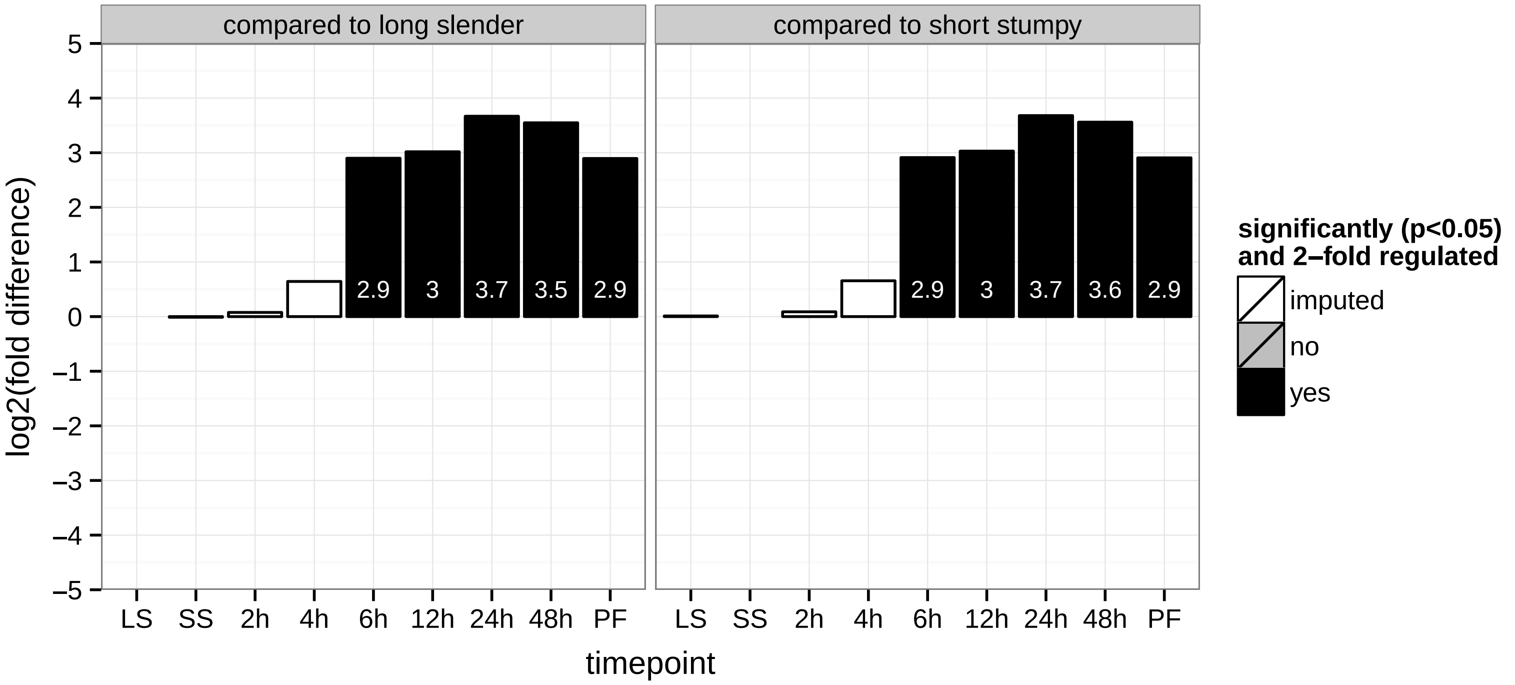

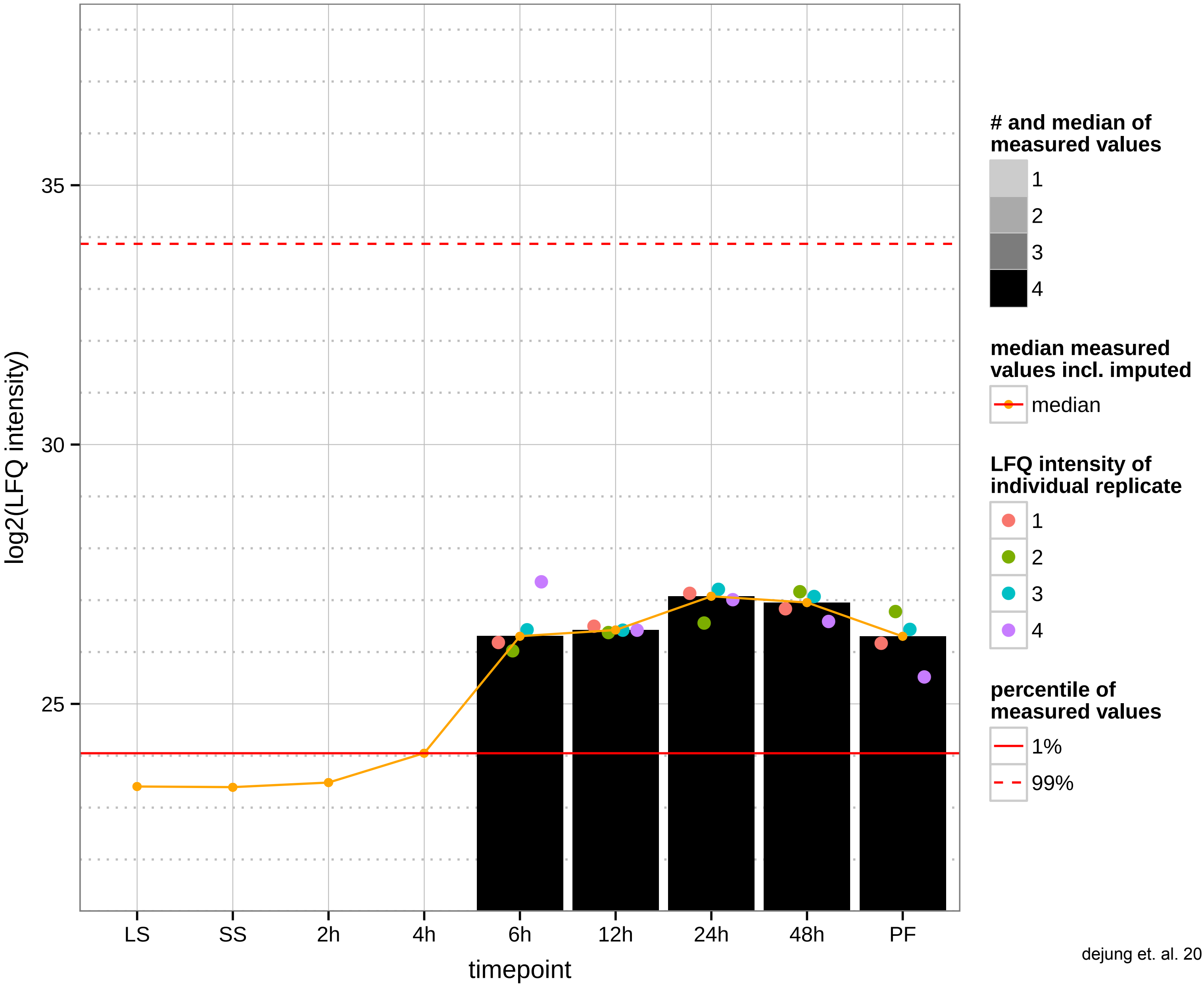

dejung et. al. 2015

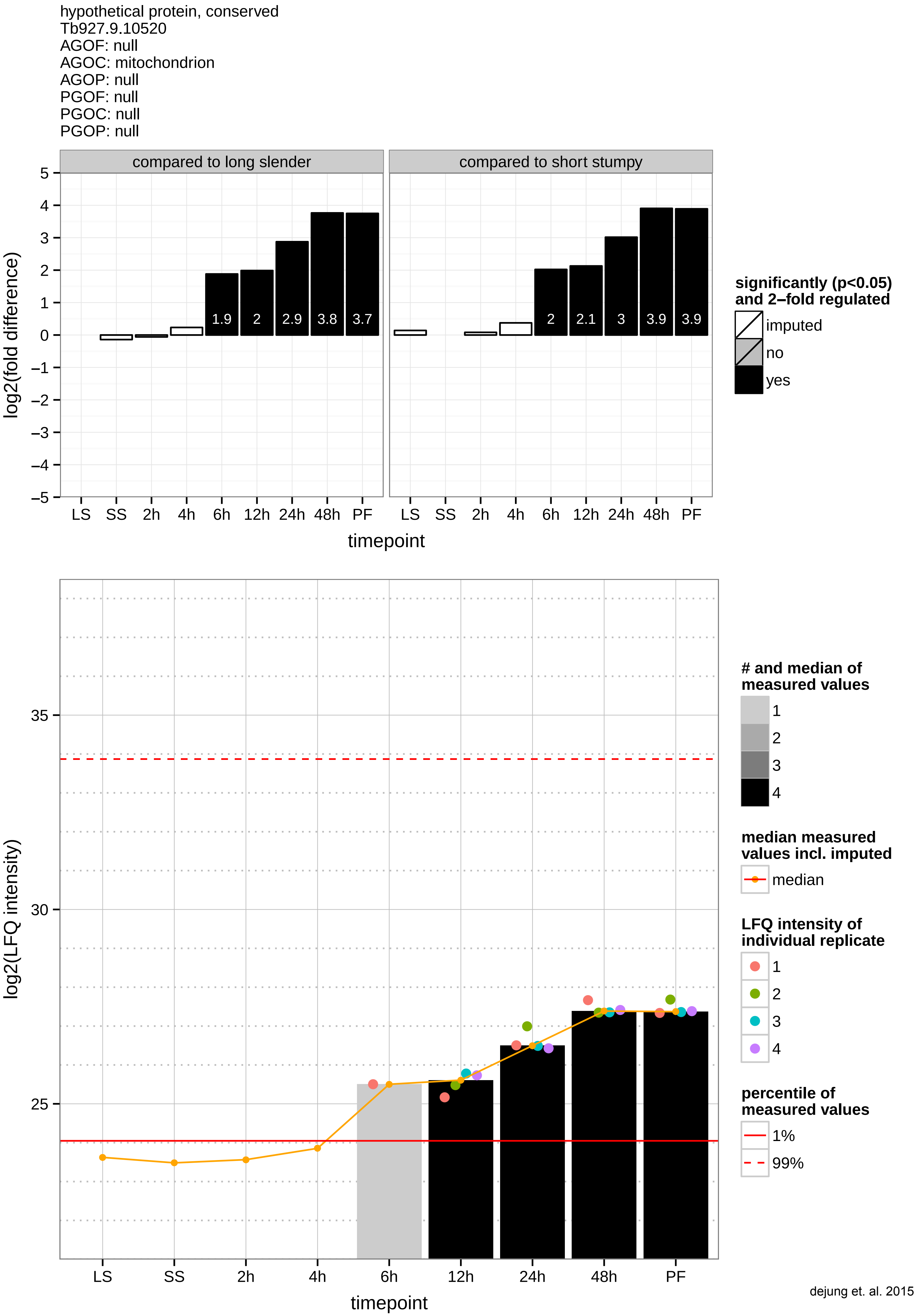

hypothetical protein, conserved
Tb927.9.10520
AGOF: null
AGOC: mitochondrion
AGOP: null
PGOF: null
PGOC: null
PGOP: null
compared to long slender
compared to short stumpy
log2(fold difference)
1.9
2
2.9
3.8
3.7
2
2.1
3
3.9
3.9
LS
SS
2h
4h
6h
12h
24h
48h
PF
timepoint
significantly (p<0.05) and 2–fold regulated
imputed
no
yes
log2(LFQ intensity)
# and median of measured values
1
2
3
4
median measured values incl. imputed
median
LFQ intensity of individual replicate
percentile of measured values
1%
99%
dejung et. al. 2015

Iron–sulfur cluster assembly protein
Tb927.9.11720
AGOF: iron ion binding, iron–sulfur cluster binding, metallochaperone activity
AGOC: mitochondrion
AGOP: iron–sulfur cluster assembly
PGOF: iron ion binding, iron–sulfur cluster binding
PGOC: null
PGOP: iron–sulfur cluster assembly

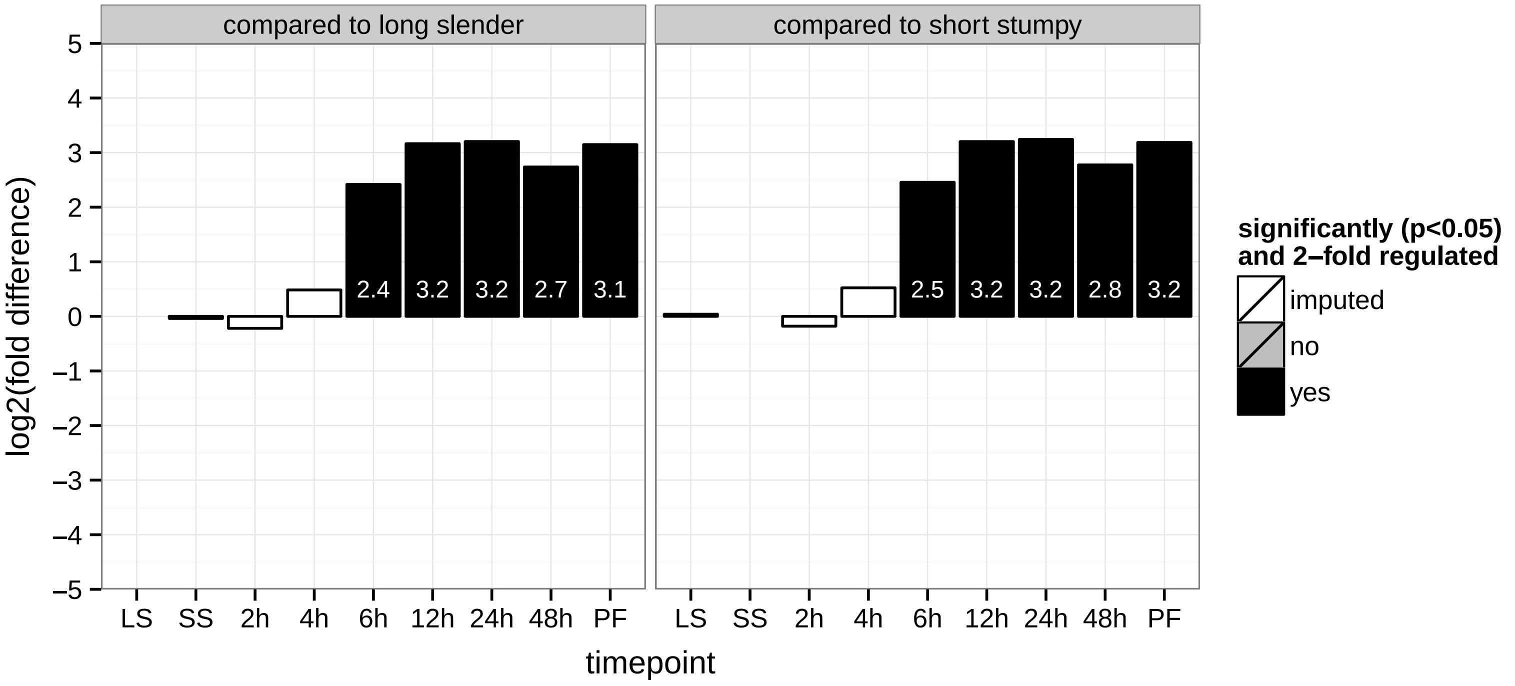

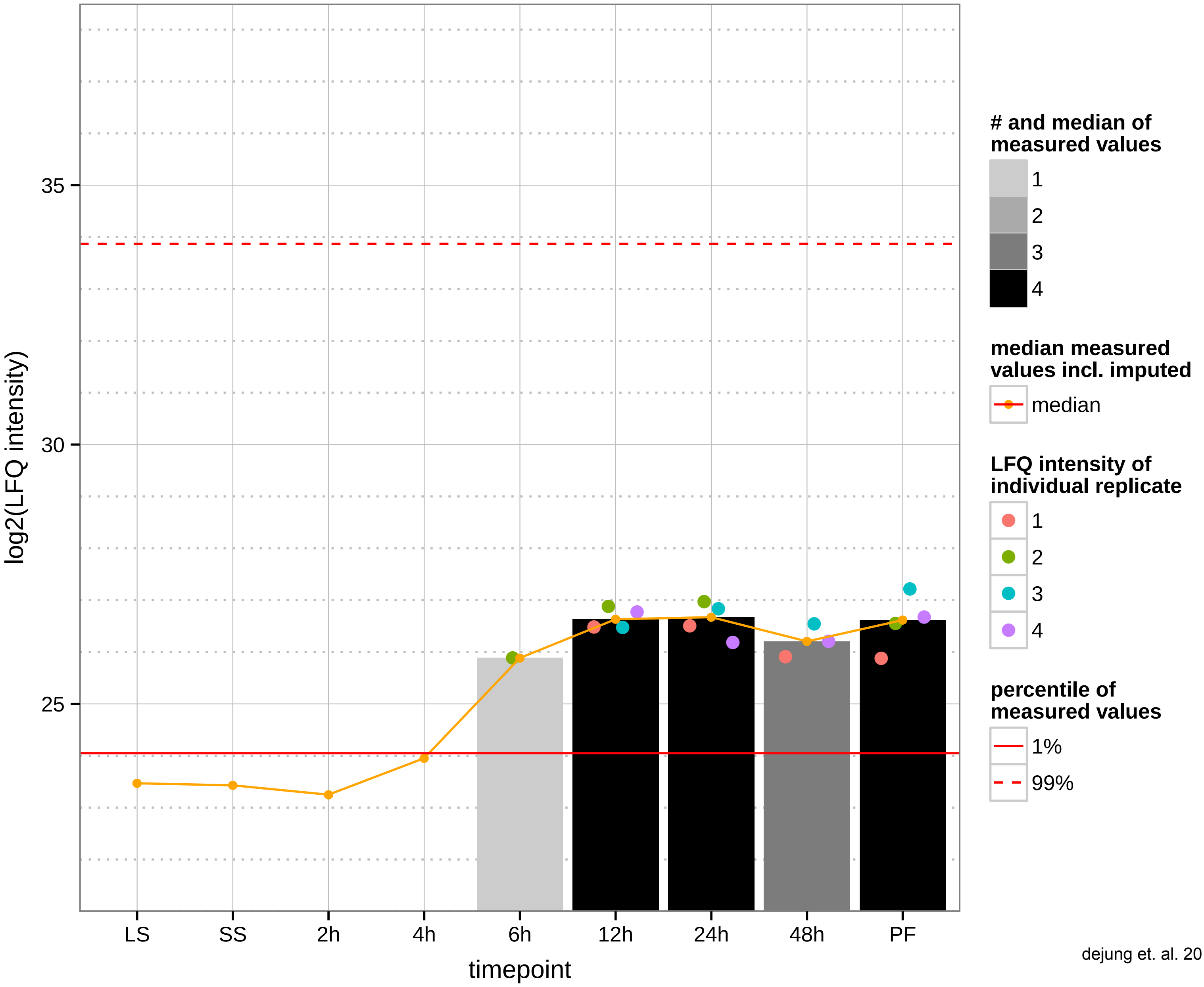

dejung et. al. 2015

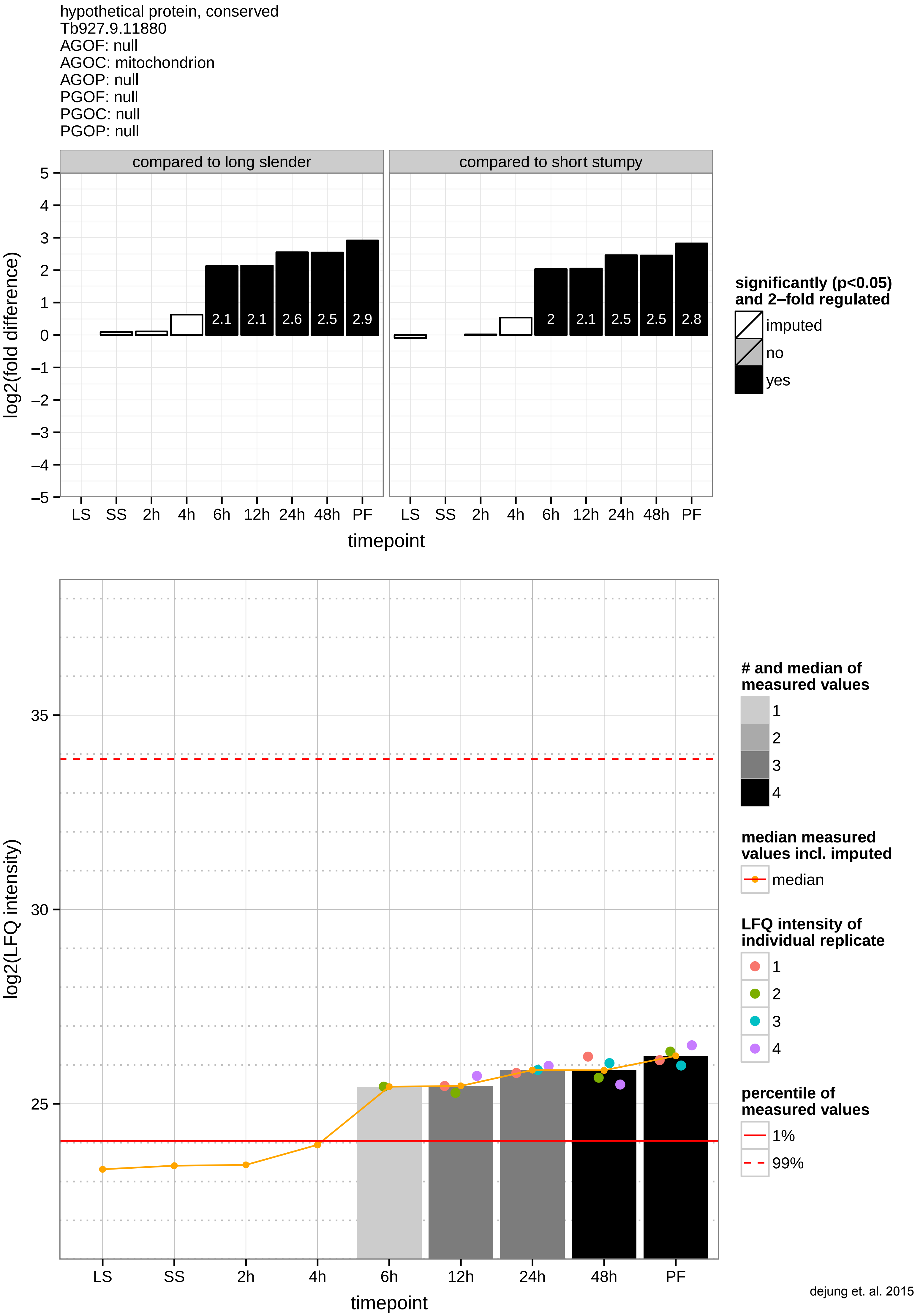

dejung et. al. 2015

cystathione gamma lyase, putative
Tb927.9.12320
AGOF: null, cystathionine gamma–lyase activity, pyridoxal phosphate binding
AGOC: null
AGOP: null, sulfur amino acid metabolic process
PGOF: pyridoxal phosphate binding
PGOC: null
PGOP: cellular amino acid metabolic process

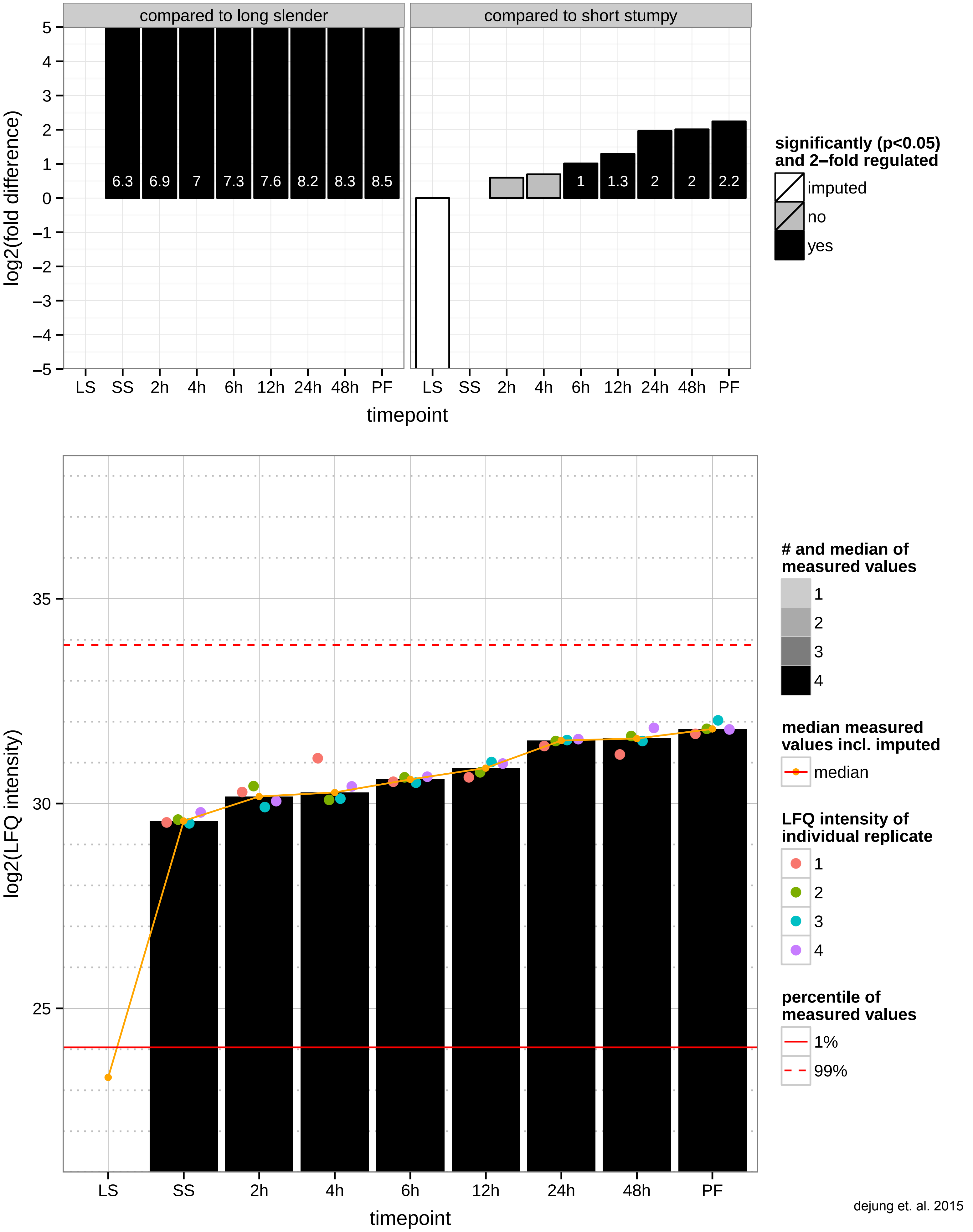

dejung et. al. 2015

hypothetical protein, conserved
Tb927.9.13780
AGOF: null
AGOC: mitochondrion
AGOP: null
PGOF: null
PGOC: null
PGOP: null

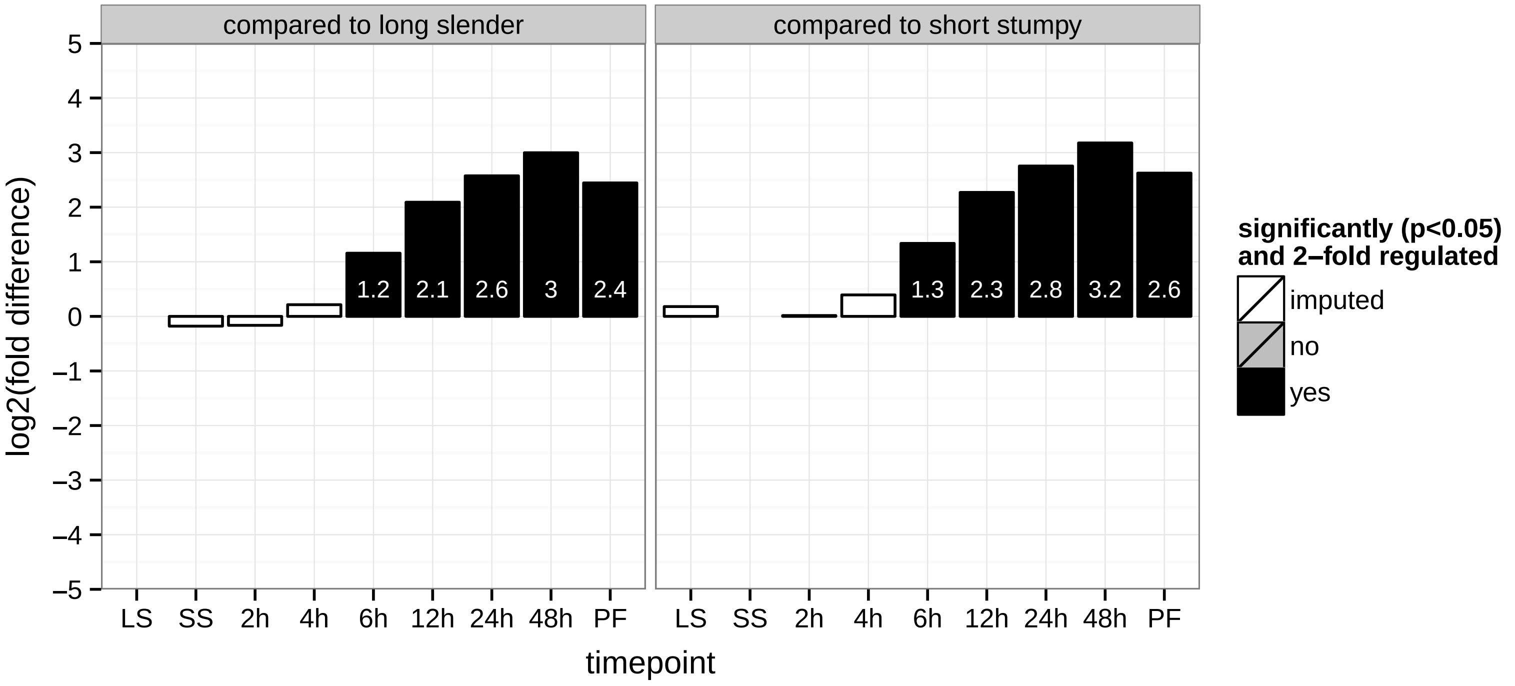

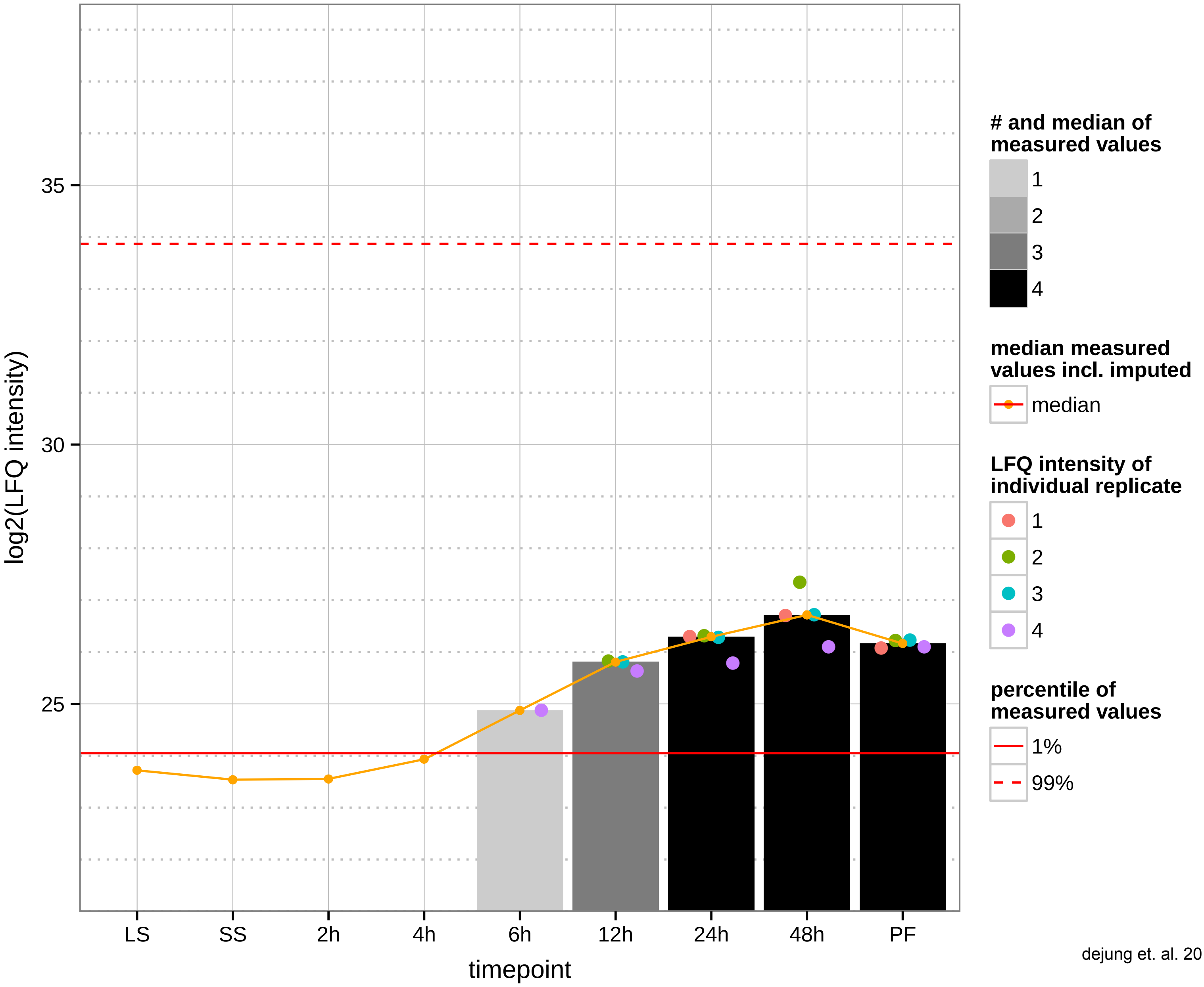

dejung et. al. 2015

hypothetical protein, conserved
Tb927.9.15290
AGOF: null
AGOC: null
AGOP: null
PGOF: null
PGOC: null
PGOP: null

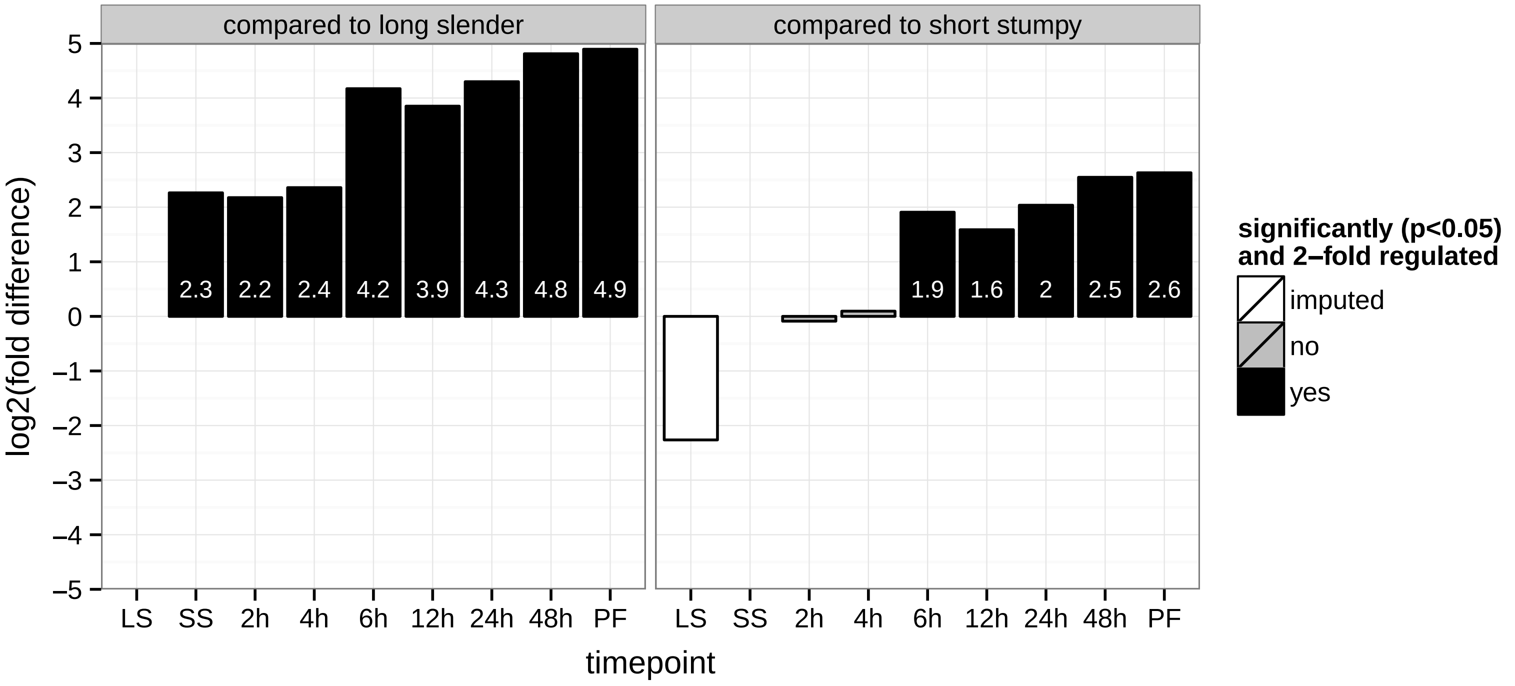

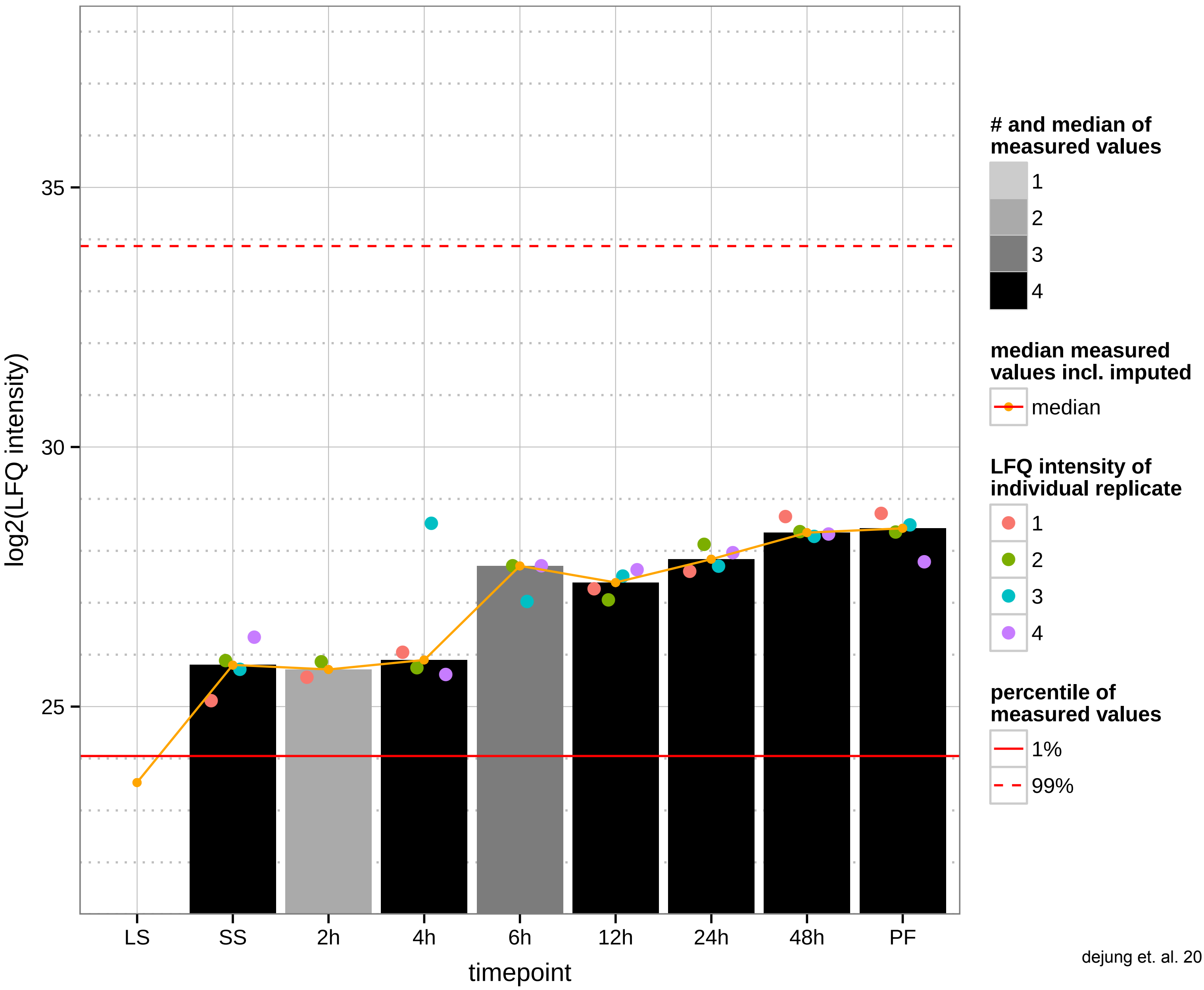

dejung et. al. 2015

hypothetical protein, conserved
Tb927.9.2270
AGOF: null
AGOC: mitochondrion
AGOP: null
PGOF: null
PGOC: null
PGOP: null

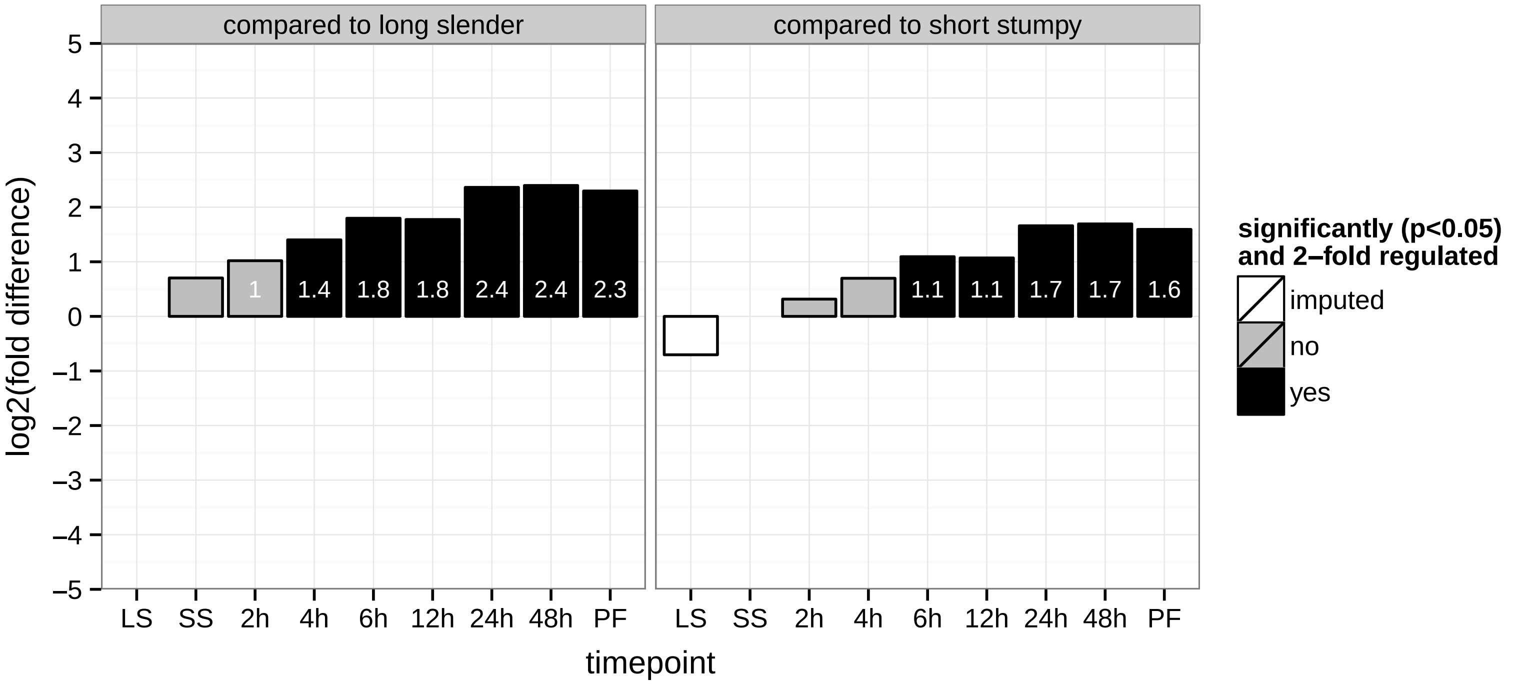

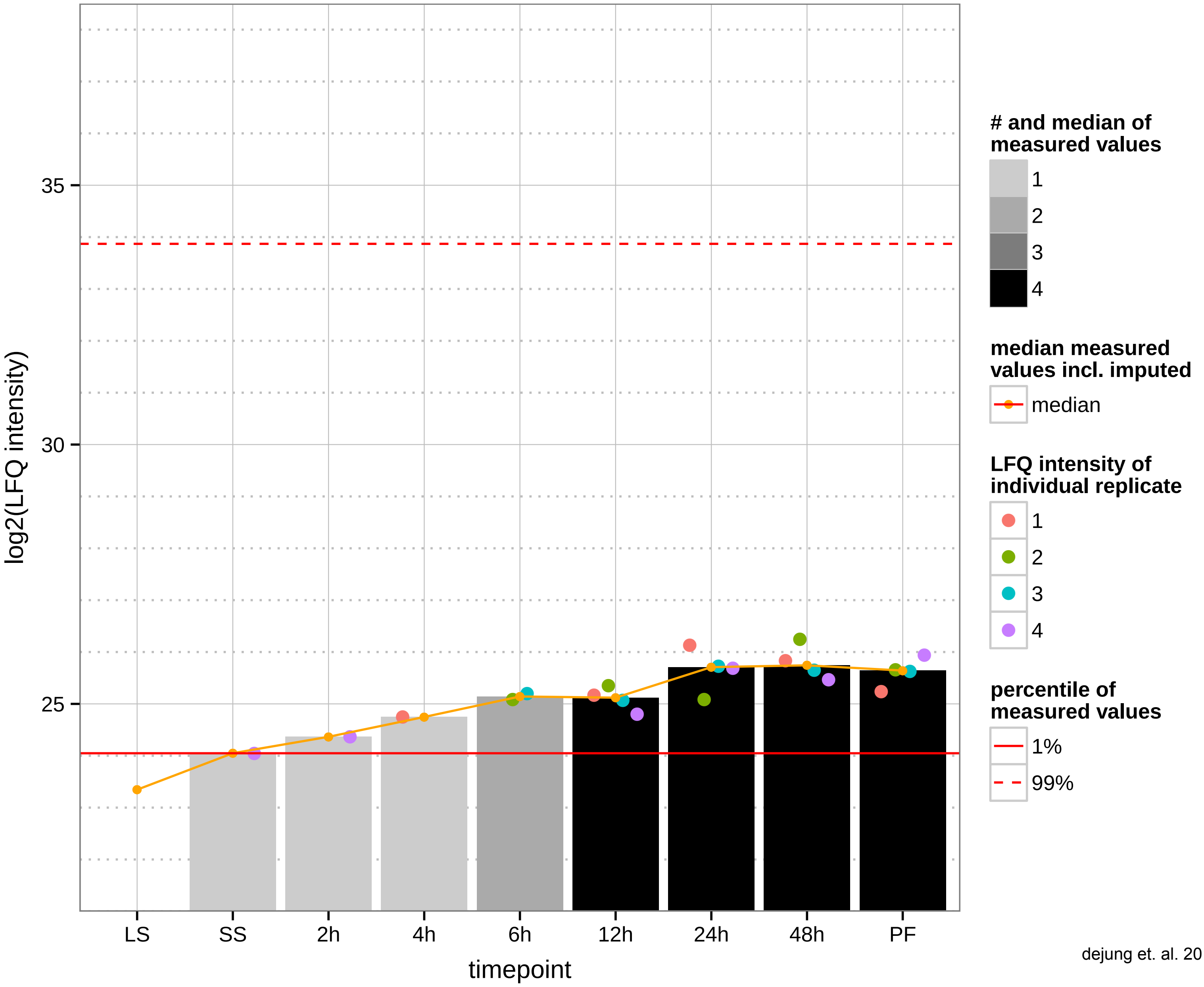

dejung et. al. 2015

protein kinase, putative
Tb927.9.3120
AGOF: ATP binding, protein serine/threonine kinase activity, protein tyrosine kinase activity
AGOC: integral to membrane
AGOP: protein phosphorylation
PGOF: ATP binding, protein kinase activity, transferase activity, transferring phosphorus–containing groups
PGOC: null
PGOP: protein phosphorylation

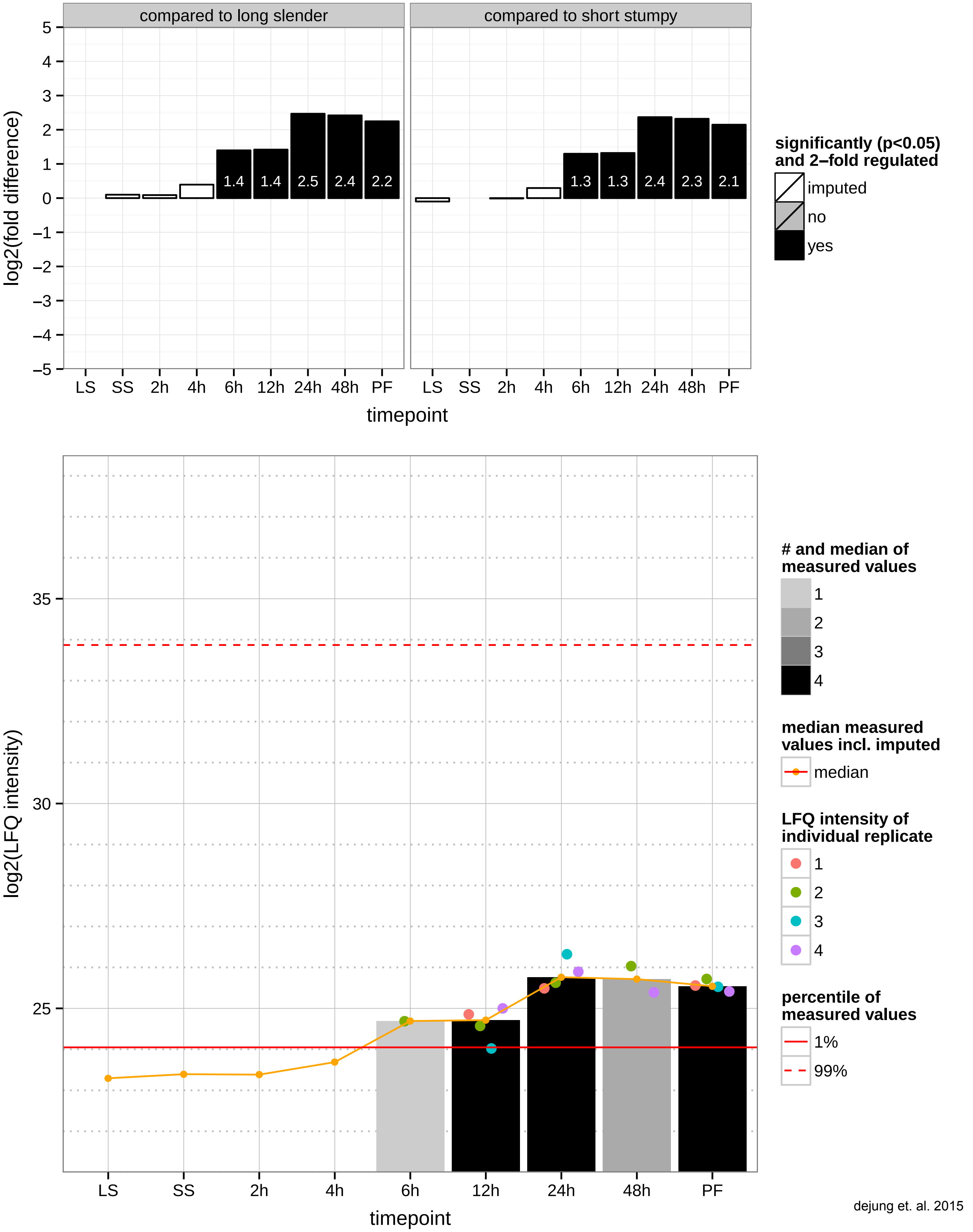

dejung et. al. 2015

tricarboxylate carrier, putative
Tb927.9.4310
AGOF: cation transmembrane transporter activity
AGOC: mitochondrial inner membrane
AGOP: cation transport
PGOF: cation transmembrane transporter activity
PGOC: membrane
PGOP: cation transport, transmembrane transport

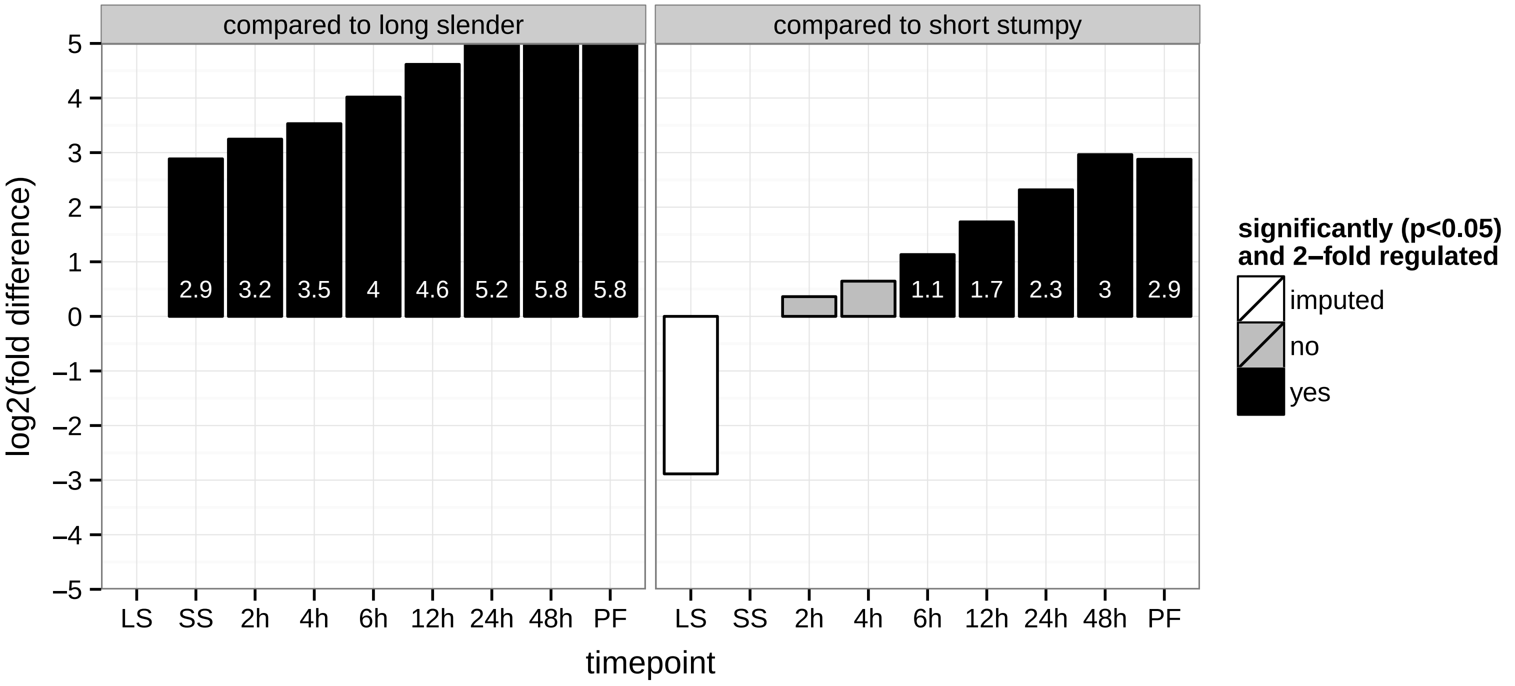

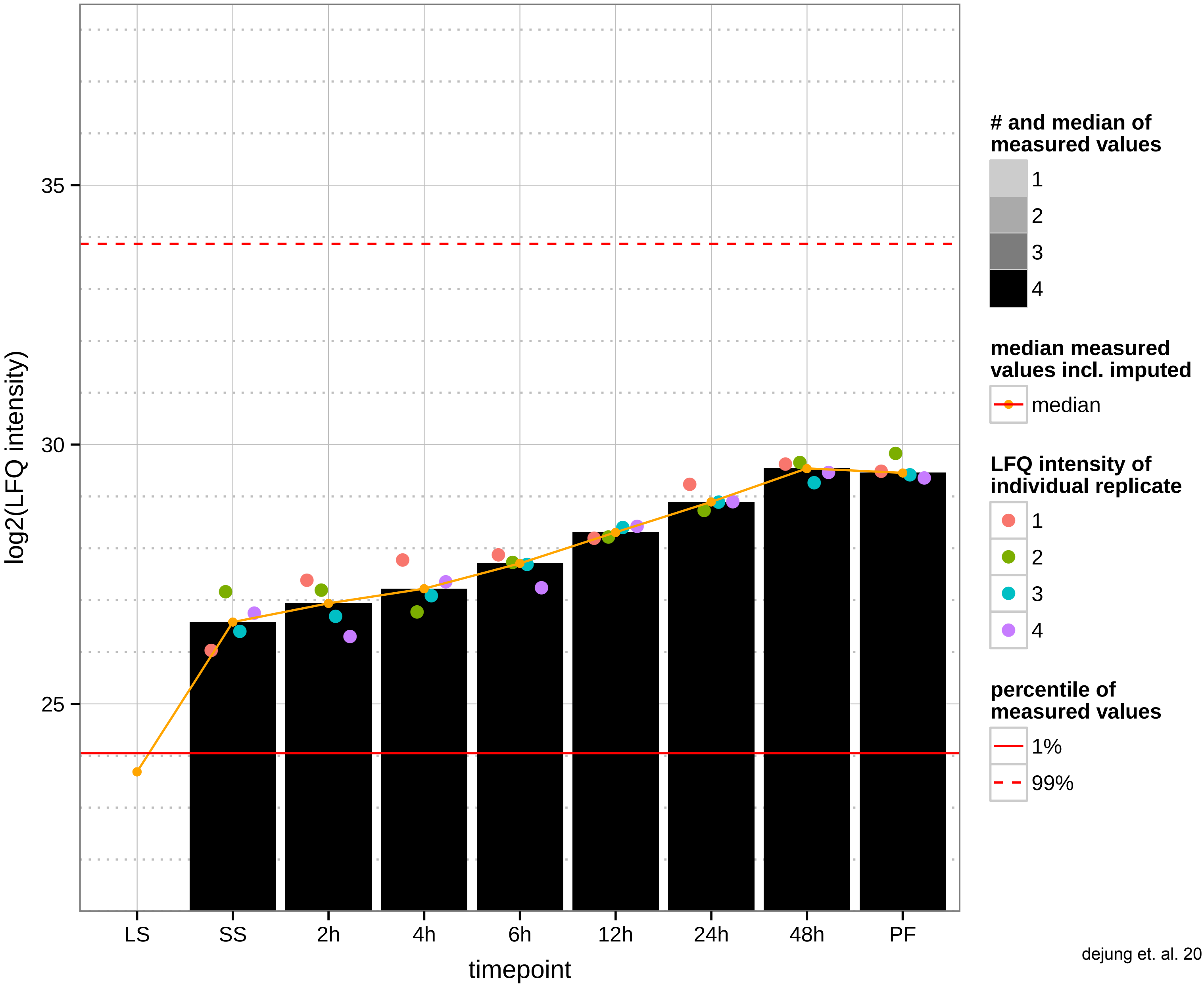

dejung et. al. 2015

exosome complex exonuclease RRP40 (RRP40)
Tb927.9.7070
AGOF: 3'–5'–exoribonuclease activity
AGOC: null
AGOP: rRNA processing
PGOF: null
PGOC: null
PGOP: null

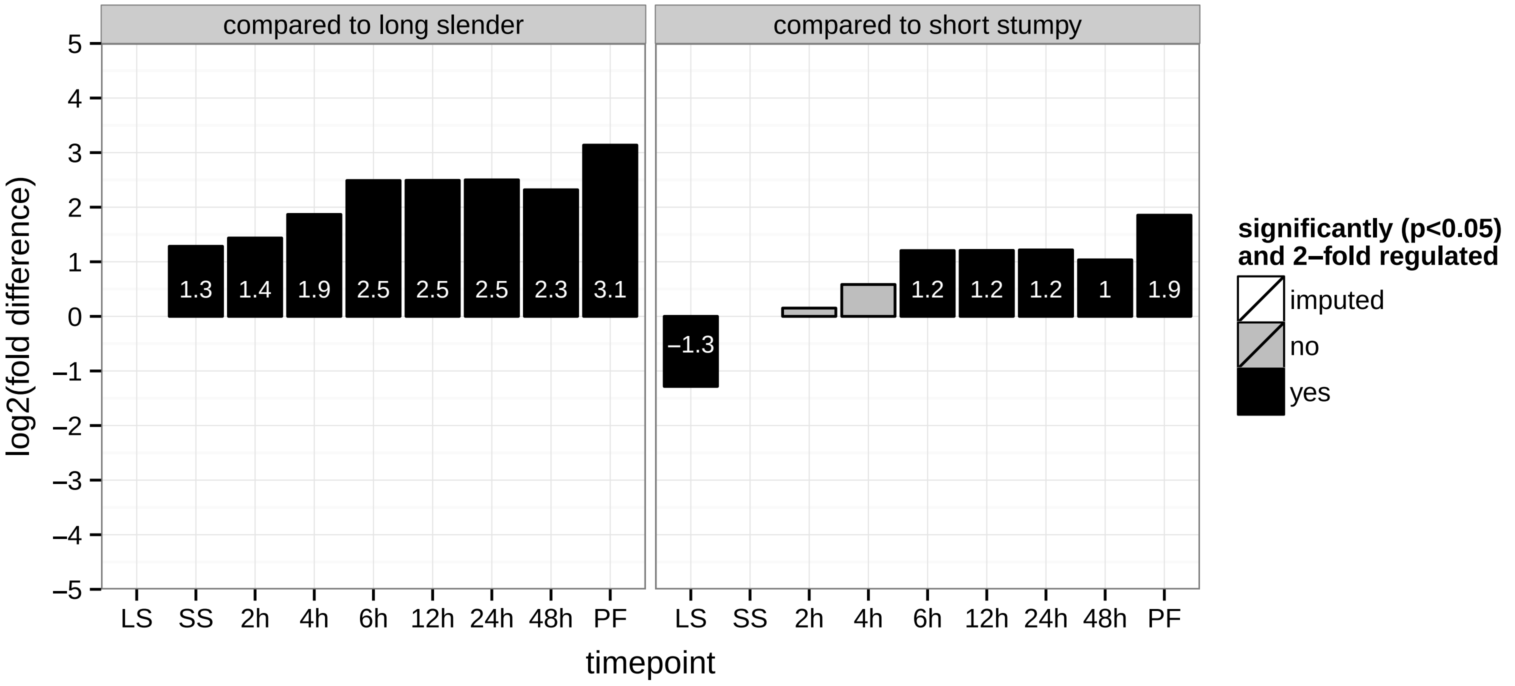

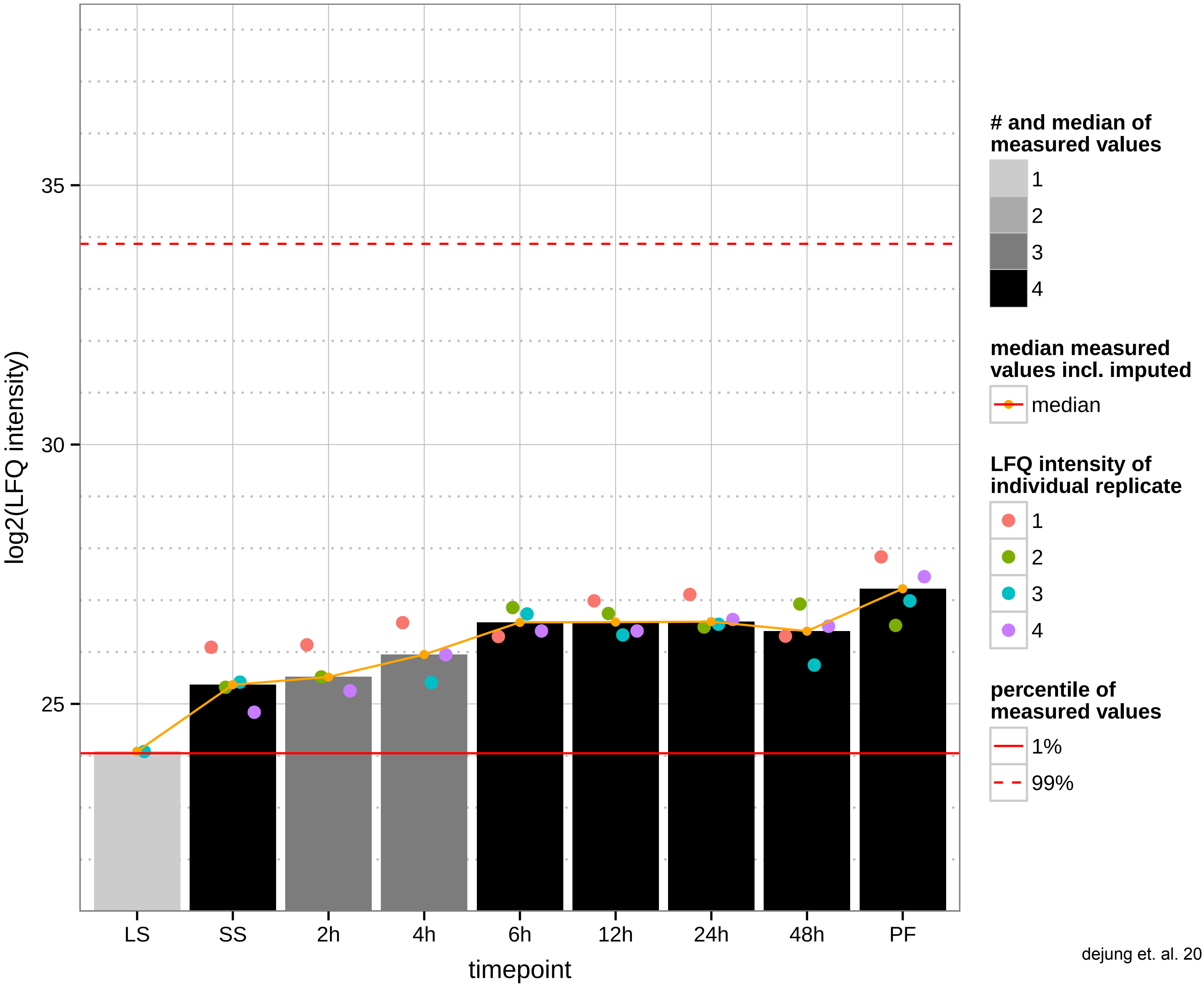

dejung et. al. 2015

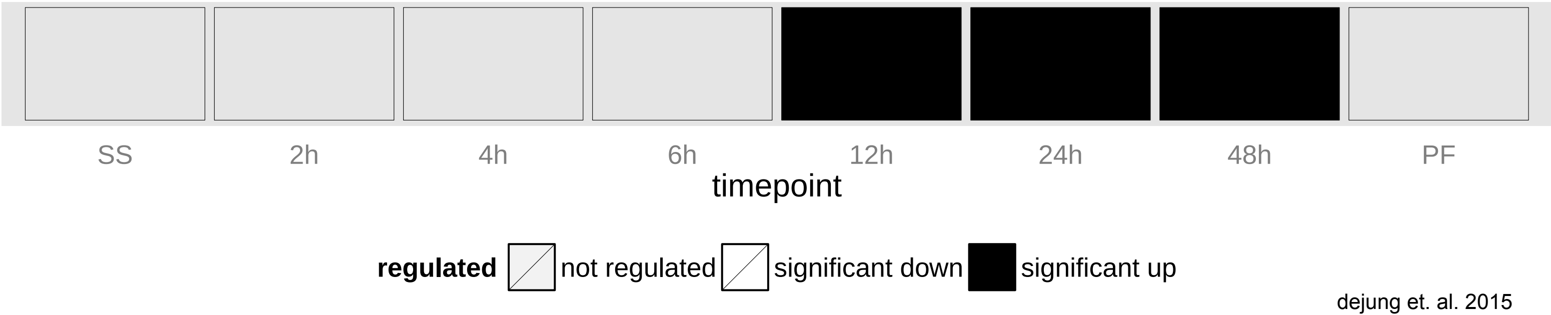
SS
2h
4h
6h
12h
24h
48h
PF
timepoint
regulated
not regulated
significant down
significant up
dejung et. al. 2015

DNA–directed RNA polymerase II, subunit 9, putative, DNA–direcetd RNA polymerase II, putative (RPB9)
Tb927.11.7280;Tb11.v5.0255
AGOF: null, DNA binding, DNA–directed RNA polymerase activity
AGOC: null, nucleus
AGOP: null, transcription, DNA–dependent
PGOF: DNA binding, DNA–directed RNA polymerase activity
PGOC: null
PGOP: transcription, DNA–dependent

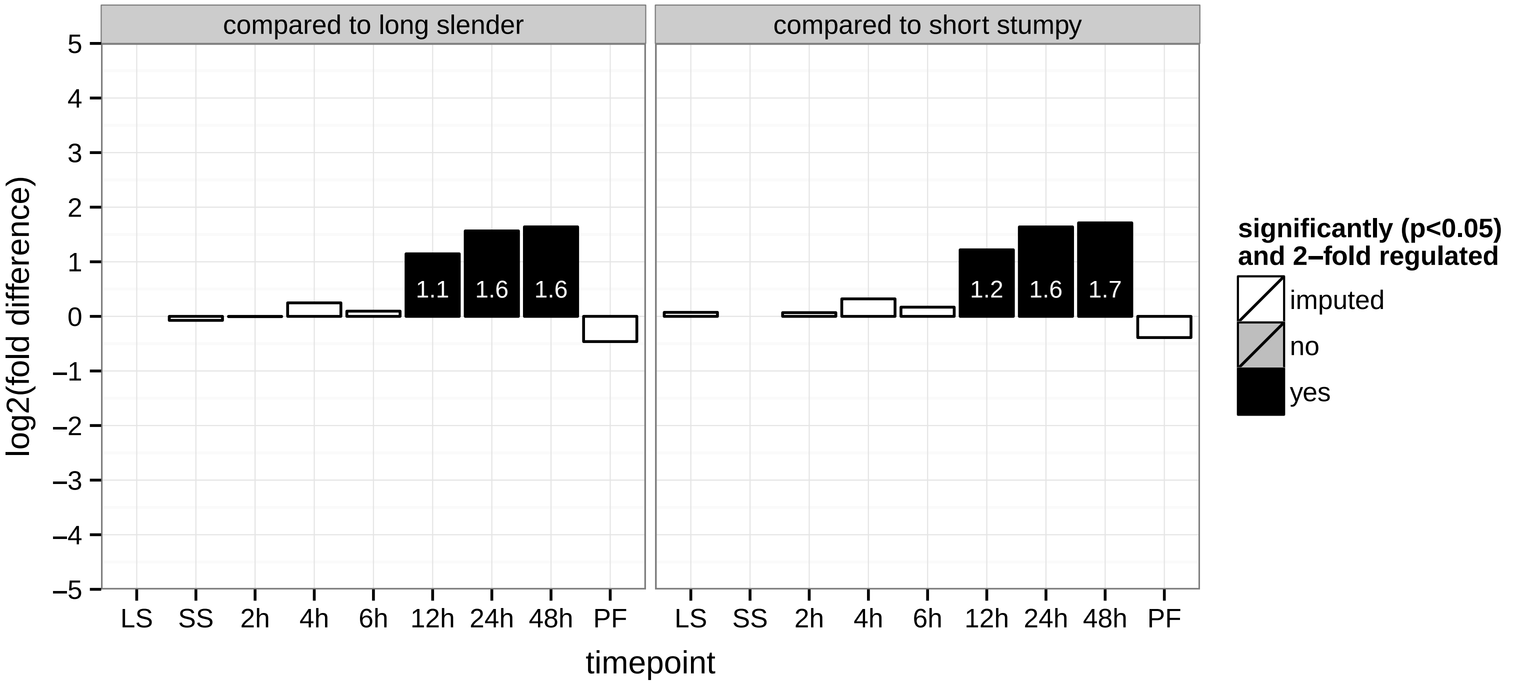

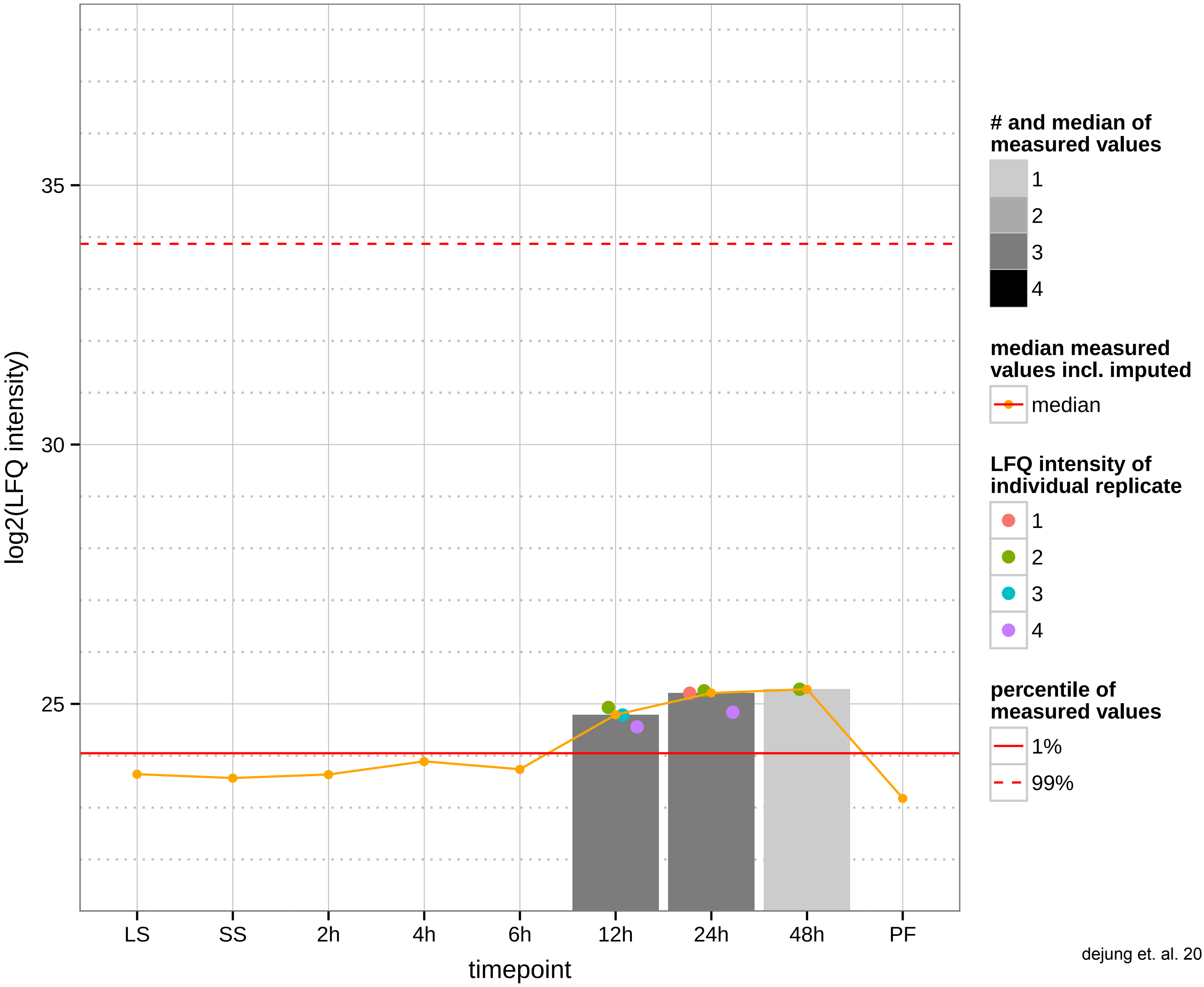

dejung et. al. 2015

conserved protein, unknown function
Tb927.1.1750
AGOF: null
AGOC: null
AGOP: null
PGOF: null
PGOC: null
PGOP: null

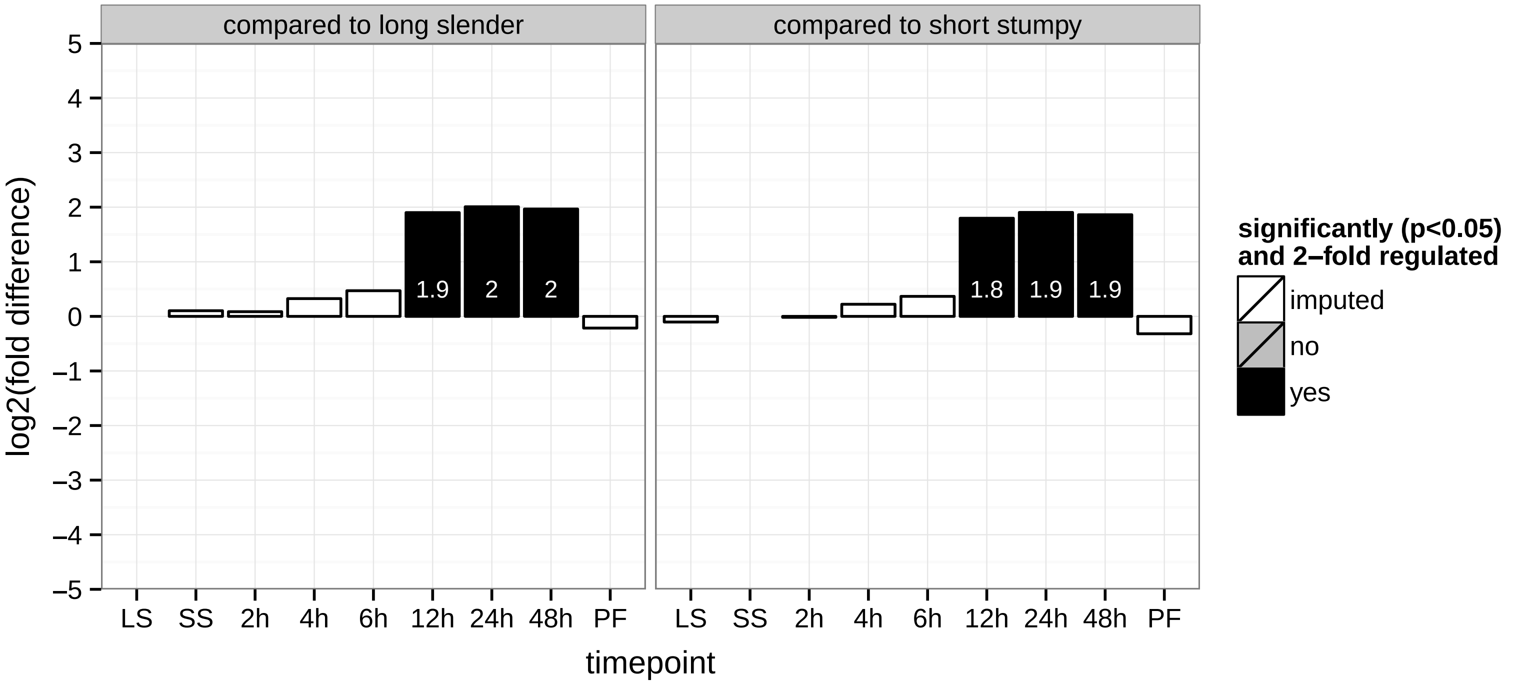

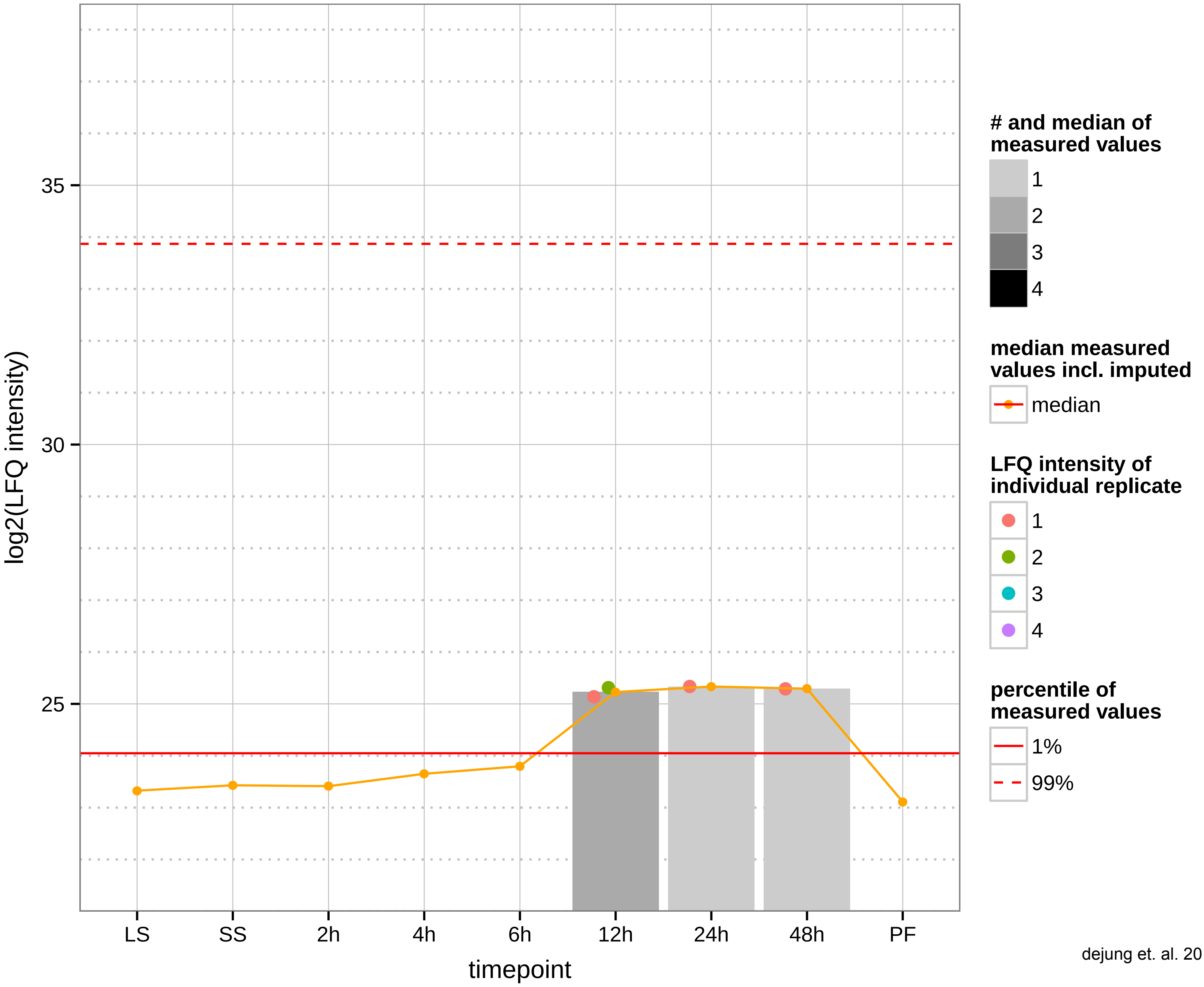

dejung et. al. 2015

hypothetical protein, conserved
Tb927.1.2730
AGOF: null
AGOC: mitochondrion
AGOP: null
PGOF: null
PGOC: null
PGOP: null

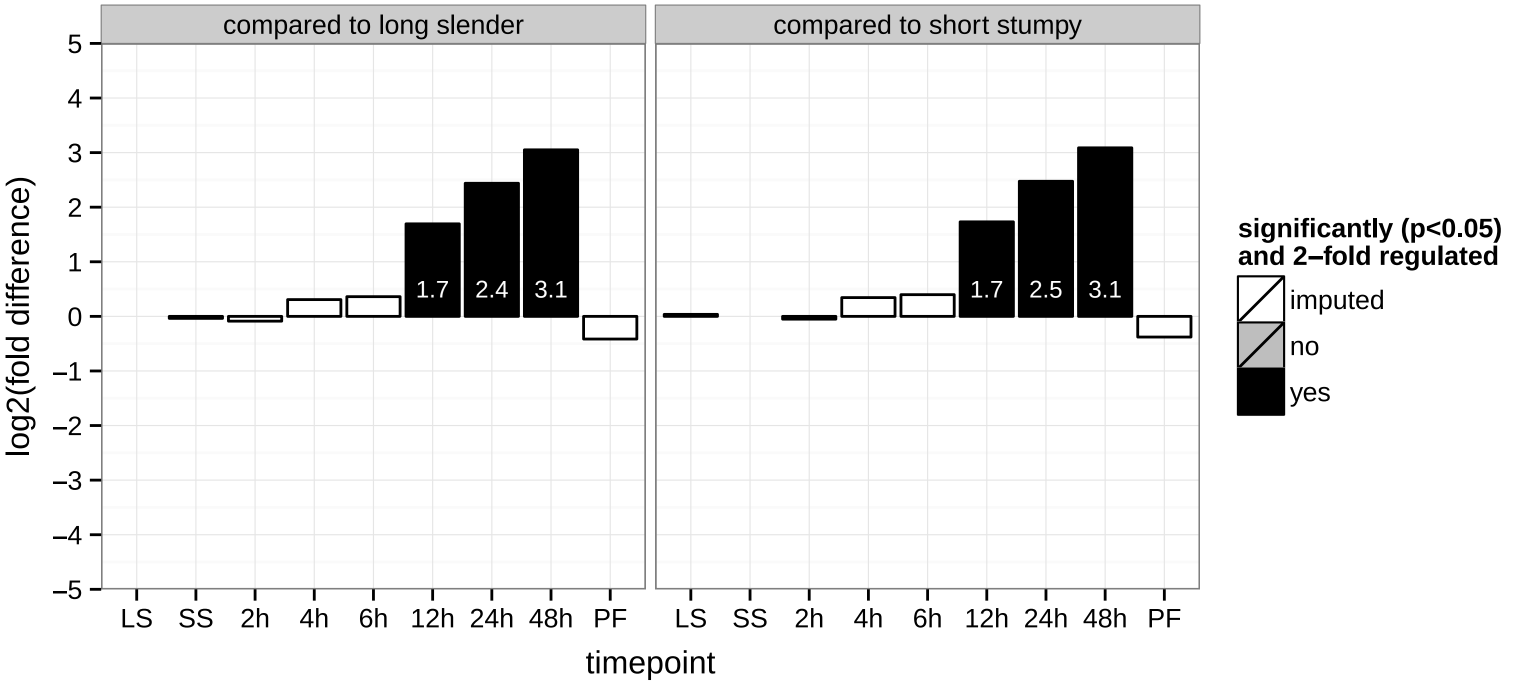

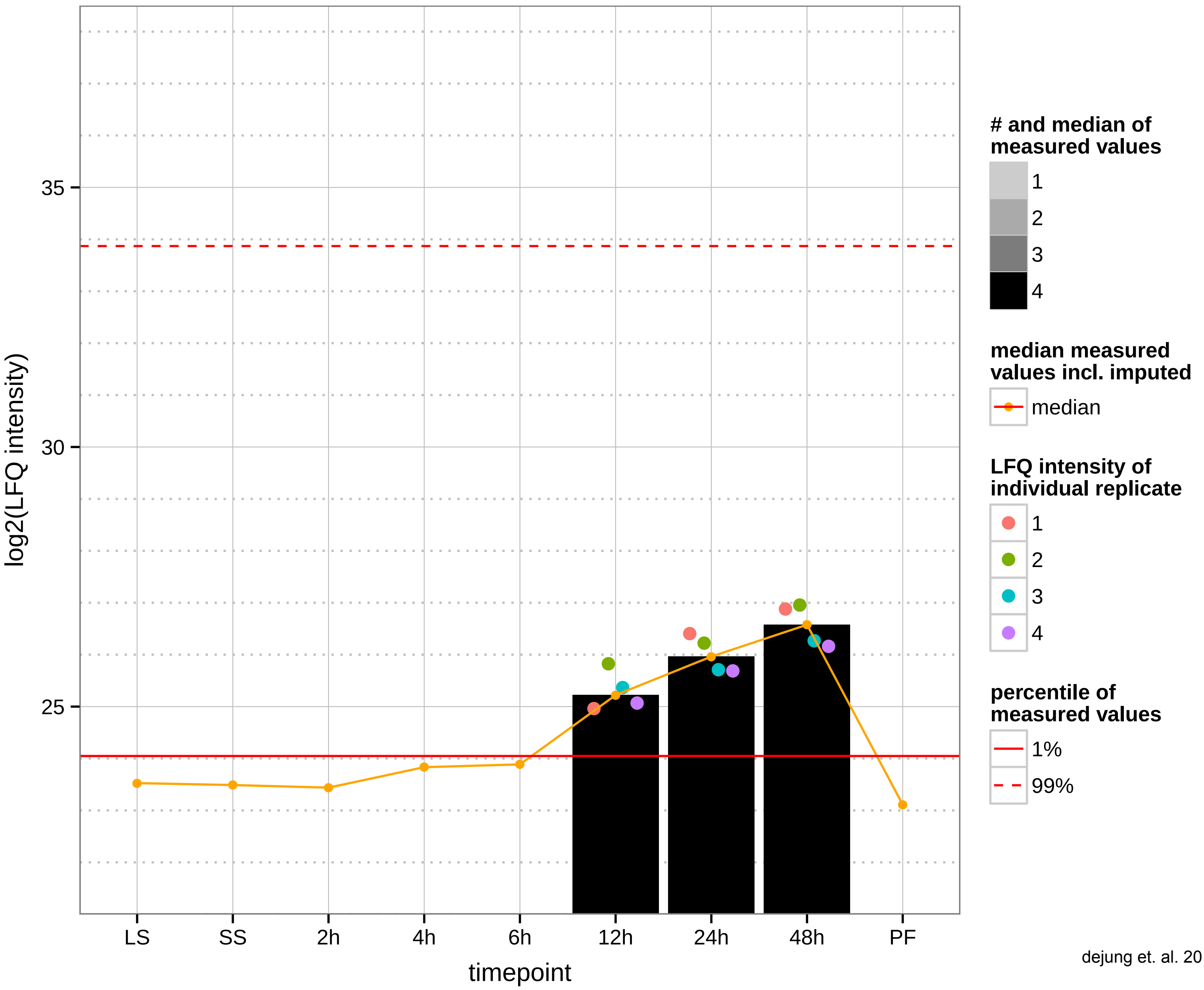

dejung et. al. 2015

hypothetical protein, conserved
Tb927.1.2990
AGOF: null
AGOC: mitochondrion
AGOP: null
PGOF: null
PGOC: null
PGOP: null

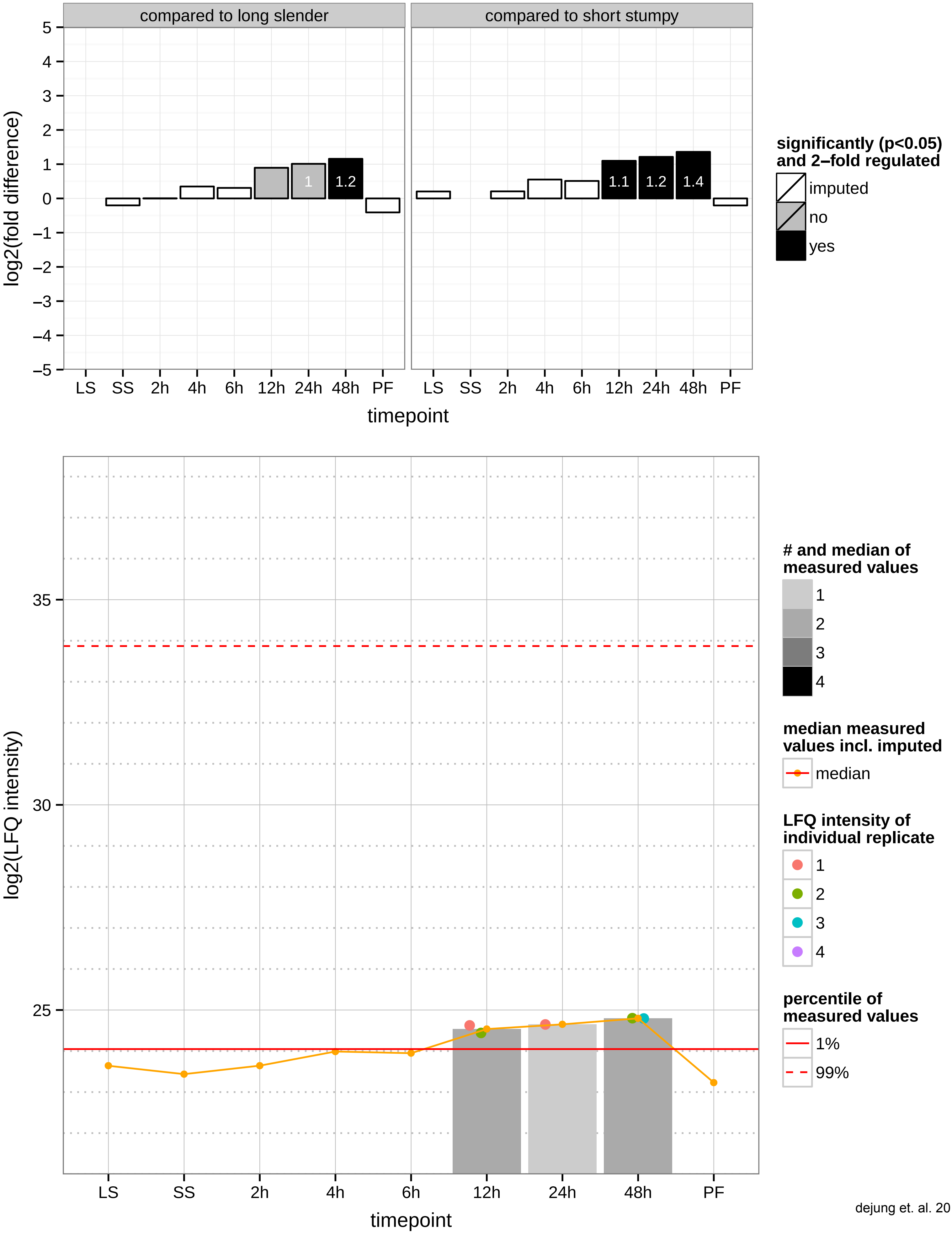

dejung et. al. 2015

hypothetical protein, conserved
Tb927.10.1930
AGOF: null
AGOC: mitochondrion
AGOP: null
PGOF: null
PGOC: null
PGOP: null

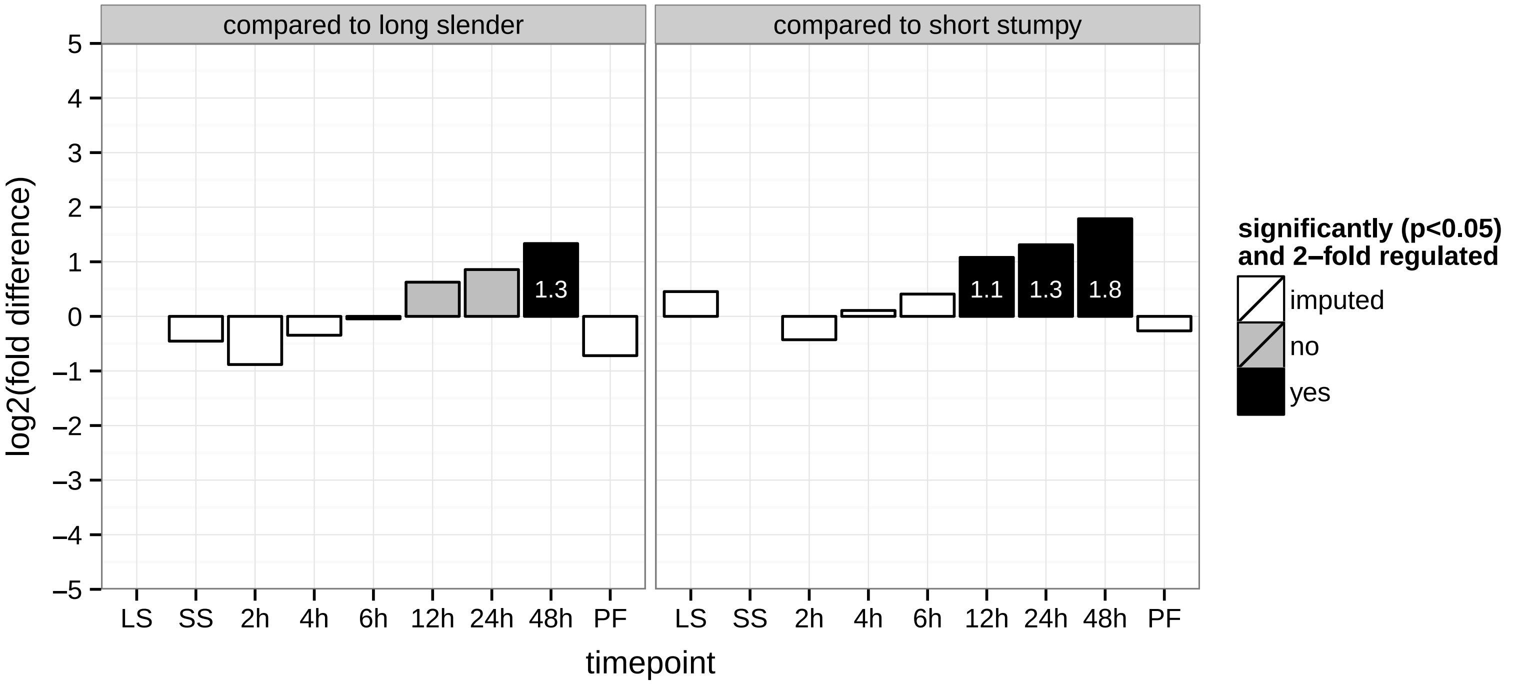

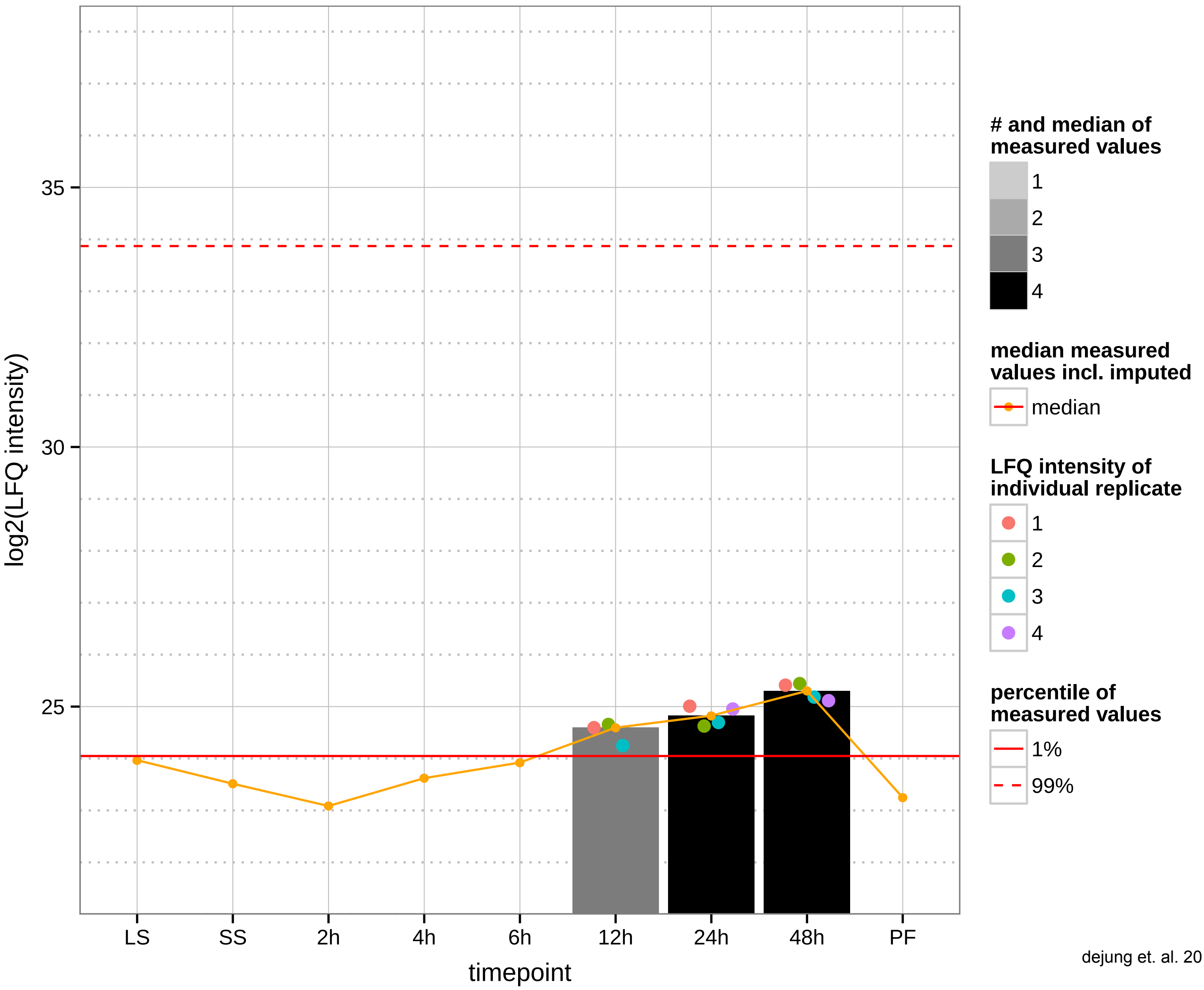

dejung et. al. 2015

actin–like protein, putative
Tb927.10.2000
AGOF: structural constituent of cytoskeleton
AGOC: mitochondrion
AGOP: cytoskeleton organization
PGOF: null
PGOC: null
PGOP: null

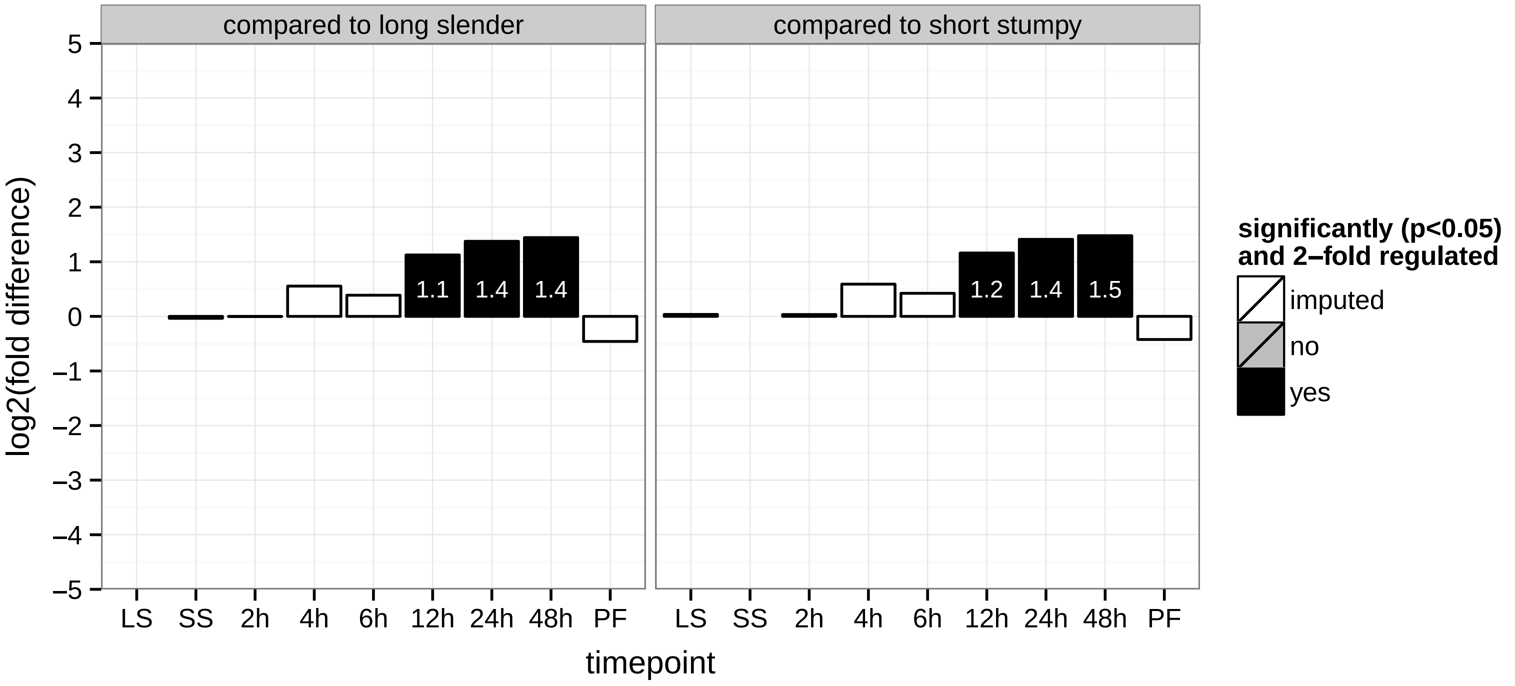

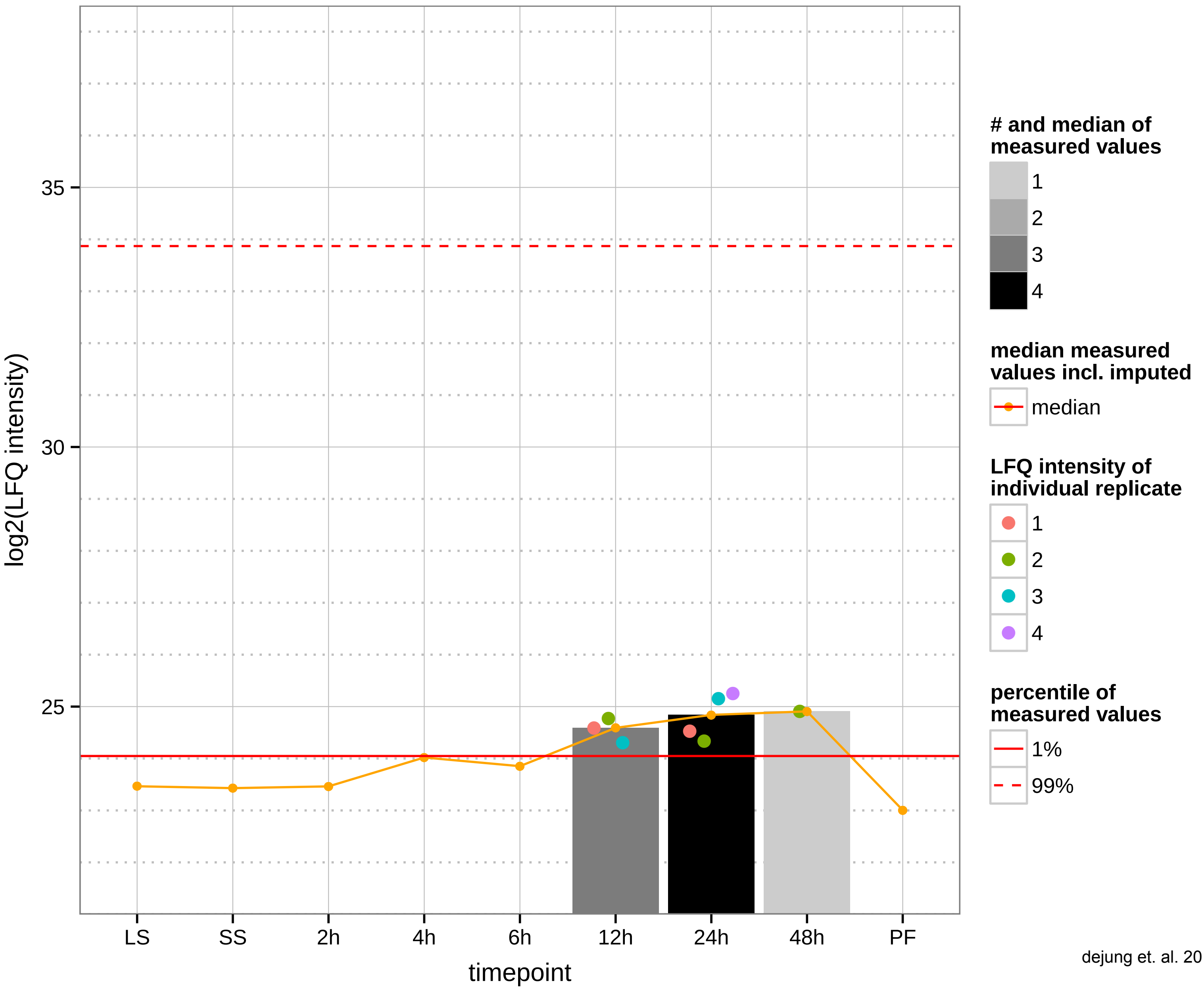

dejung et. al. 2015

ubiquitin–conjugating enzyme variant Kua homologue, putative
Tb927.10.2250
AGOF: null
AGOC: null
AGOP: null
PGOF: null
PGOC: null
PGOP: null

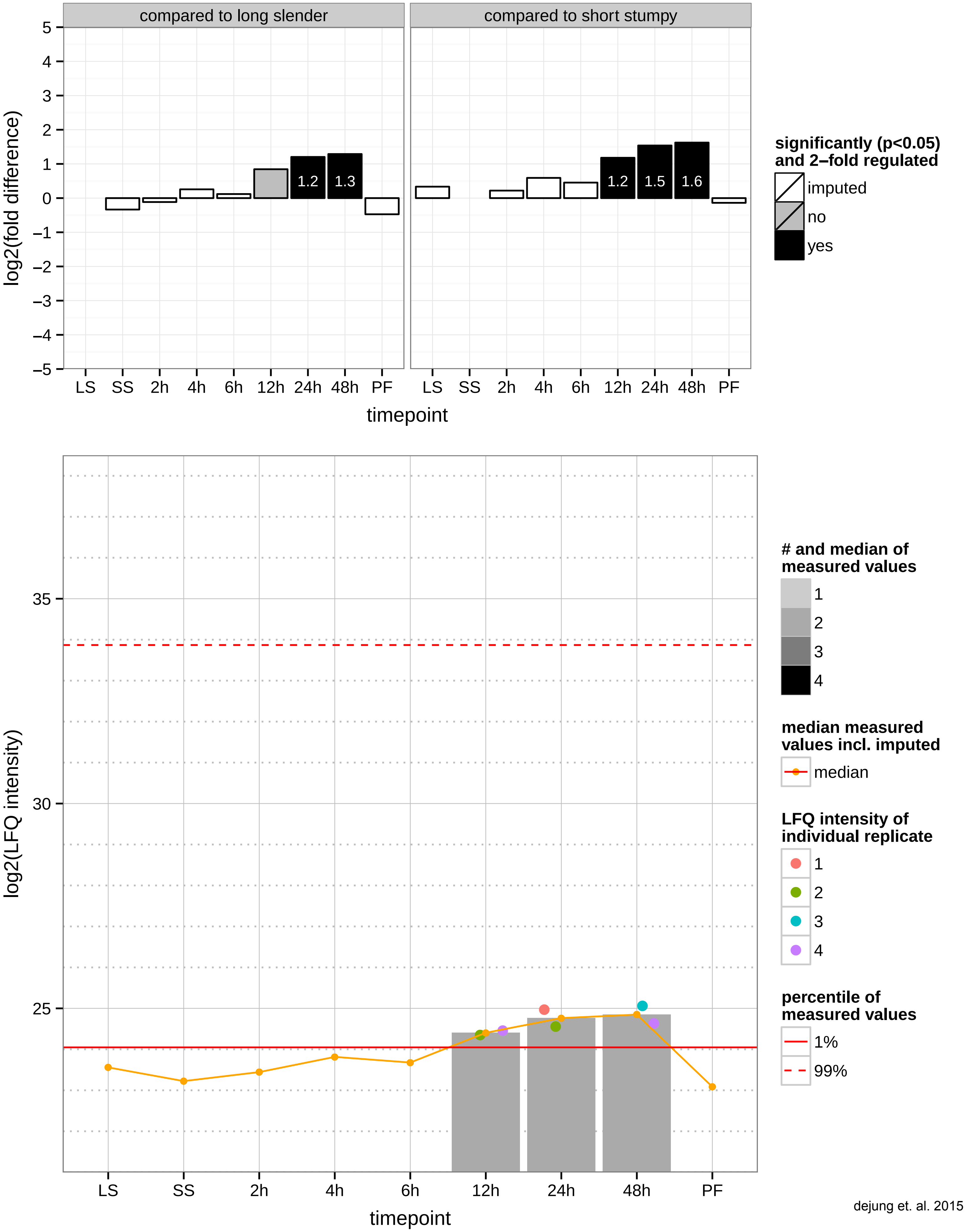

dejung et. al. 2015

hypothetical protein, conserved
Tb927.10.2260
AGOF: null
AGOC: null
AGOP: null
PGOF: protein binding
PGOC: null
PGOP: null

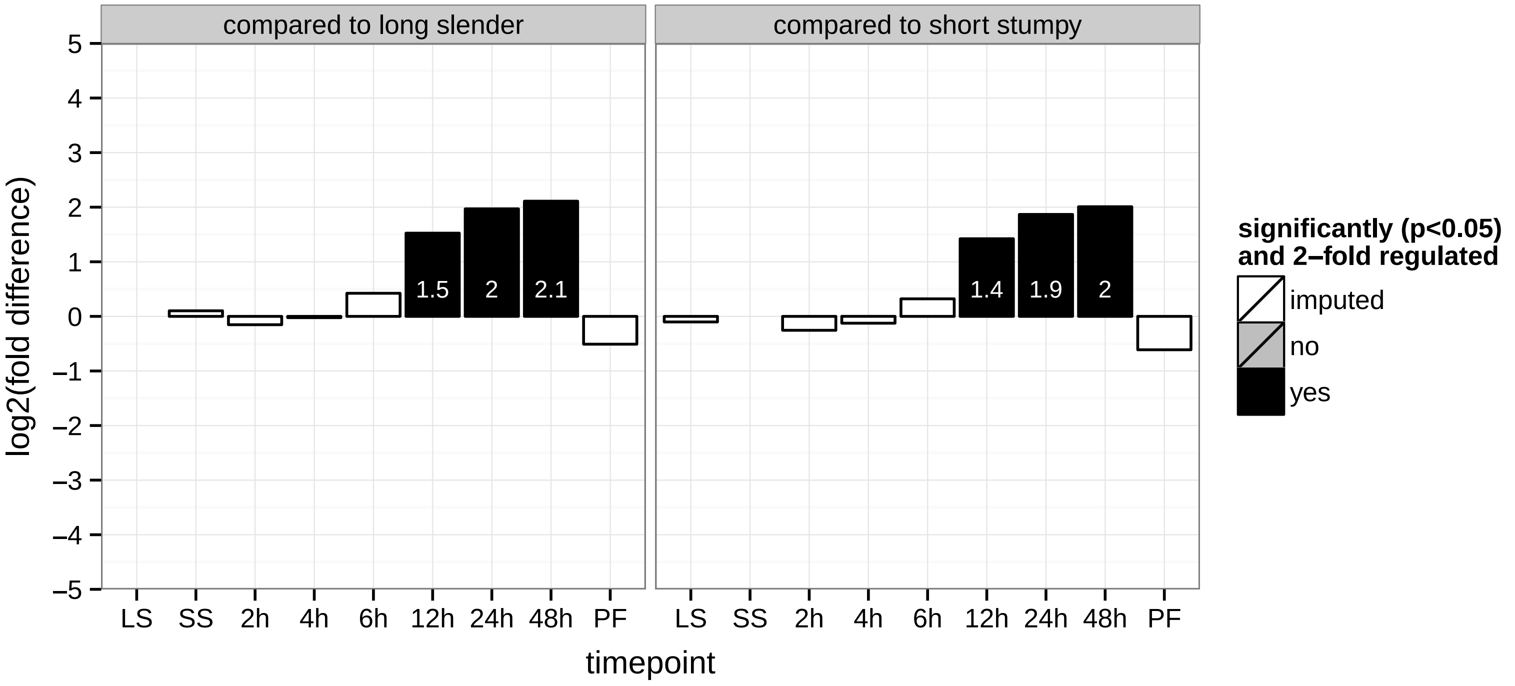

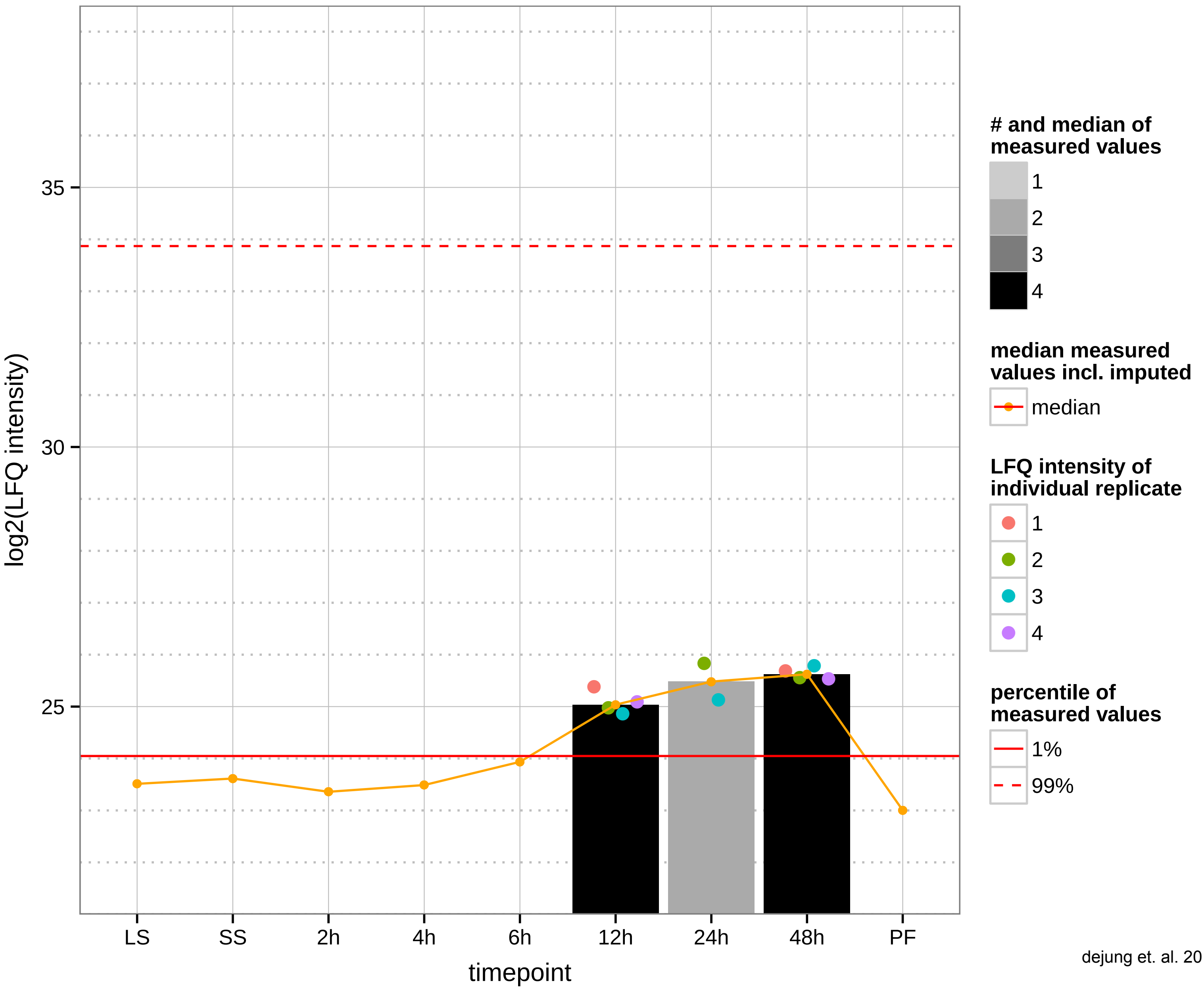

dejung et. al. 2015

hypothetical protein, conserved
Tb927.10.2970
AGOF: null
AGOC: null
AGOP: null
PGOF: null
PGOC: null
PGOP: null

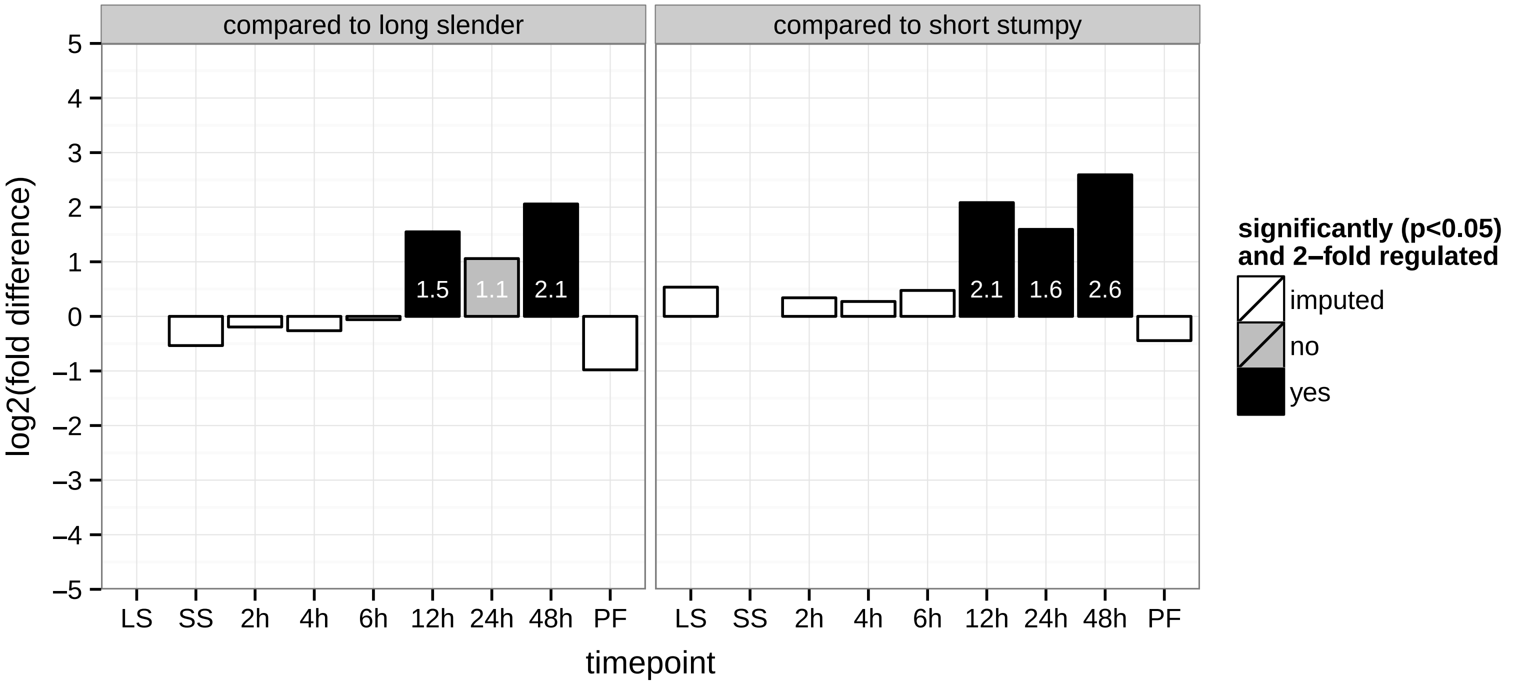

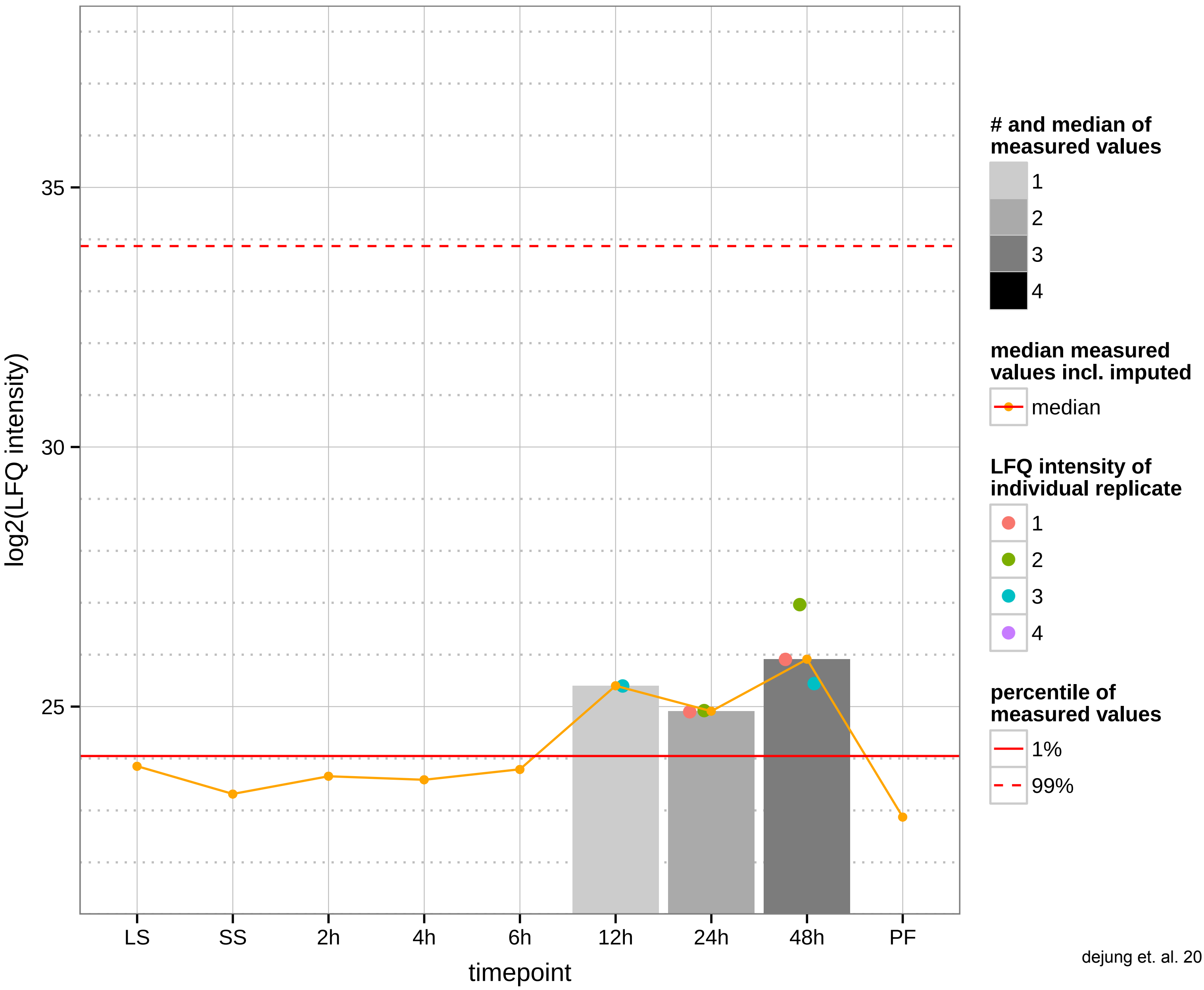

dejung et. al. 2015

hypothetical protein, conserved
Tb927.10.3630
AGOF: null
AGOC: null
AGOP: null
PGOF: null
PGOC: null
PGOP: null

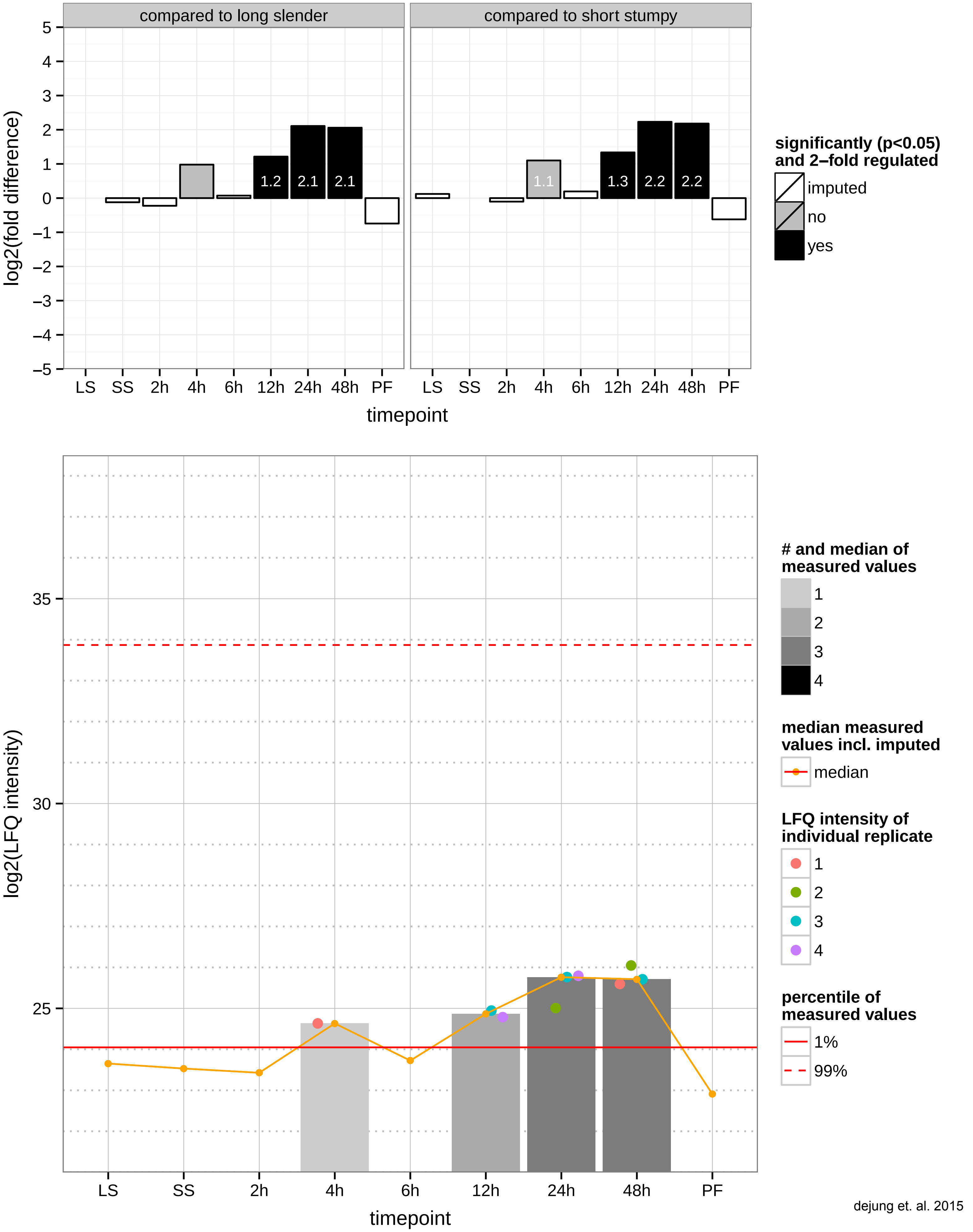

dejung et. al. 2015

ARP2/3 complex subunit, putative
Tb927.10.4540
AGOF: actin binding
AGOC: Arp2/3 protein complex, cytoskeleton
AGOP: cytoskeleton organization, regulation of actin filament polymerization
PGOF: null
PGOC: cytoskeleton
PGOP: regulation of actin filament polymerization

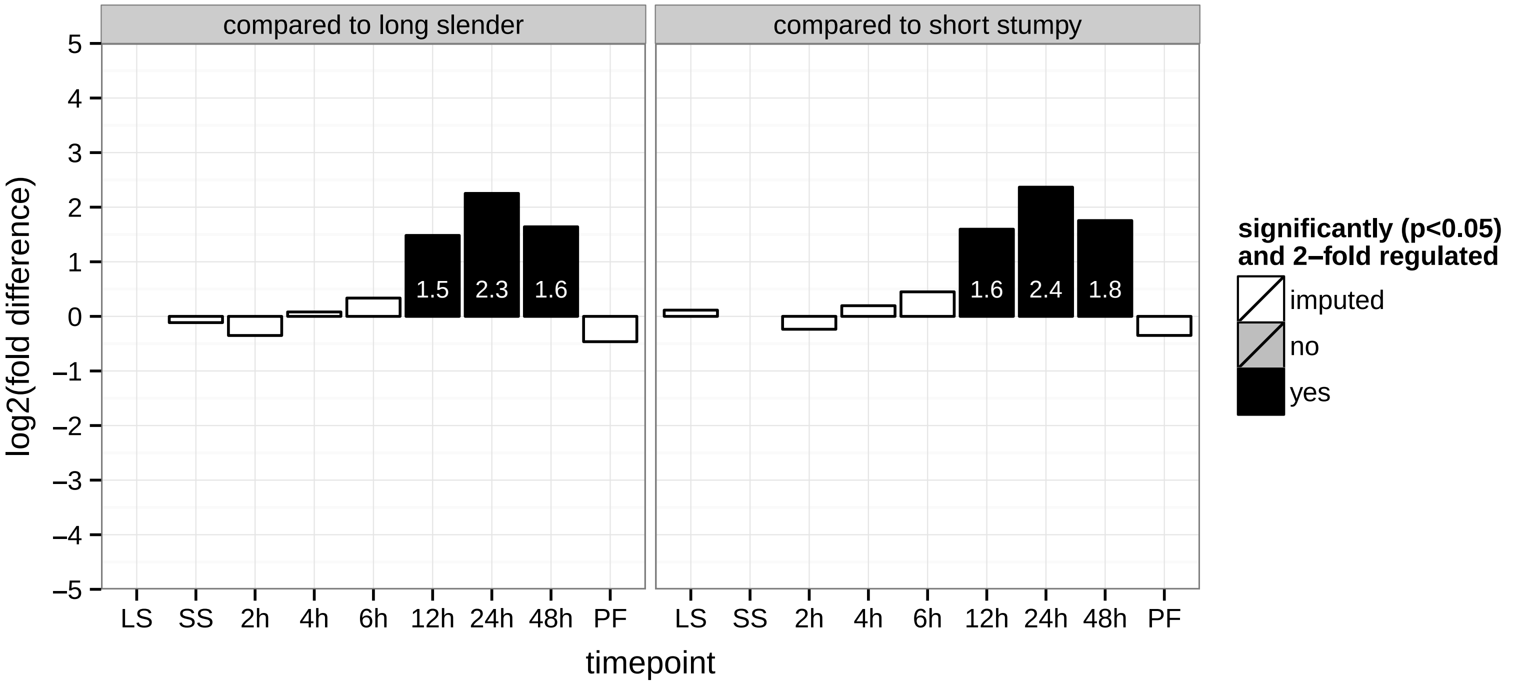

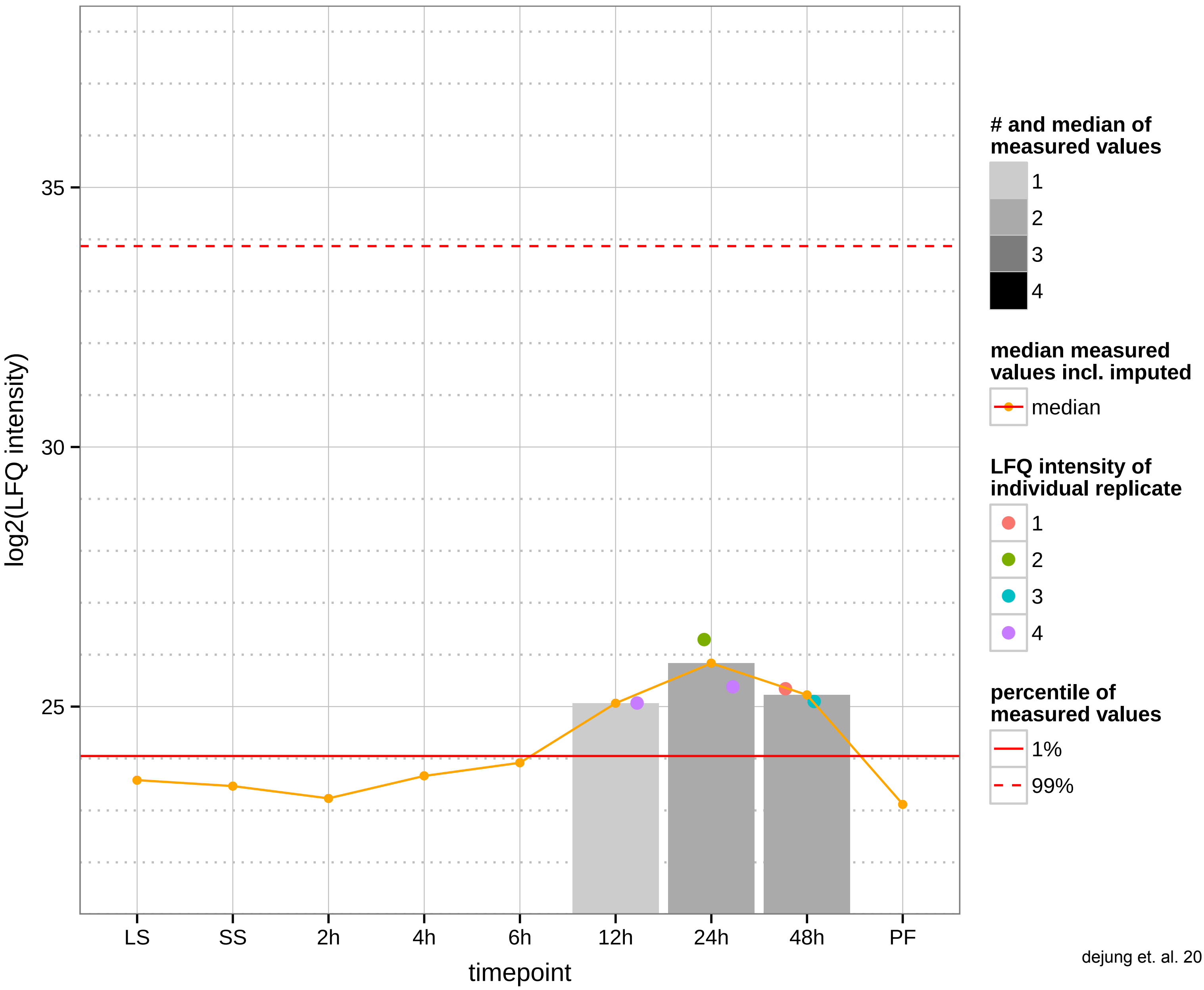

dejung et. al. 2015

DNA–directed RNA polymerase I subunit, putative (RPA12)
Tb927.11.10420
AGOF: DNA binding, sequence–specific DNA binding transcription factor activity, zinc ion binding
AGOC: null
AGOP: DNA–dependent transcription, elongation, regulation of transcription, DNA–dependent
PGOF: nucleic acid binding, zinc ion binding
PGOC: null
PGOP: transcription, DNA–dependent

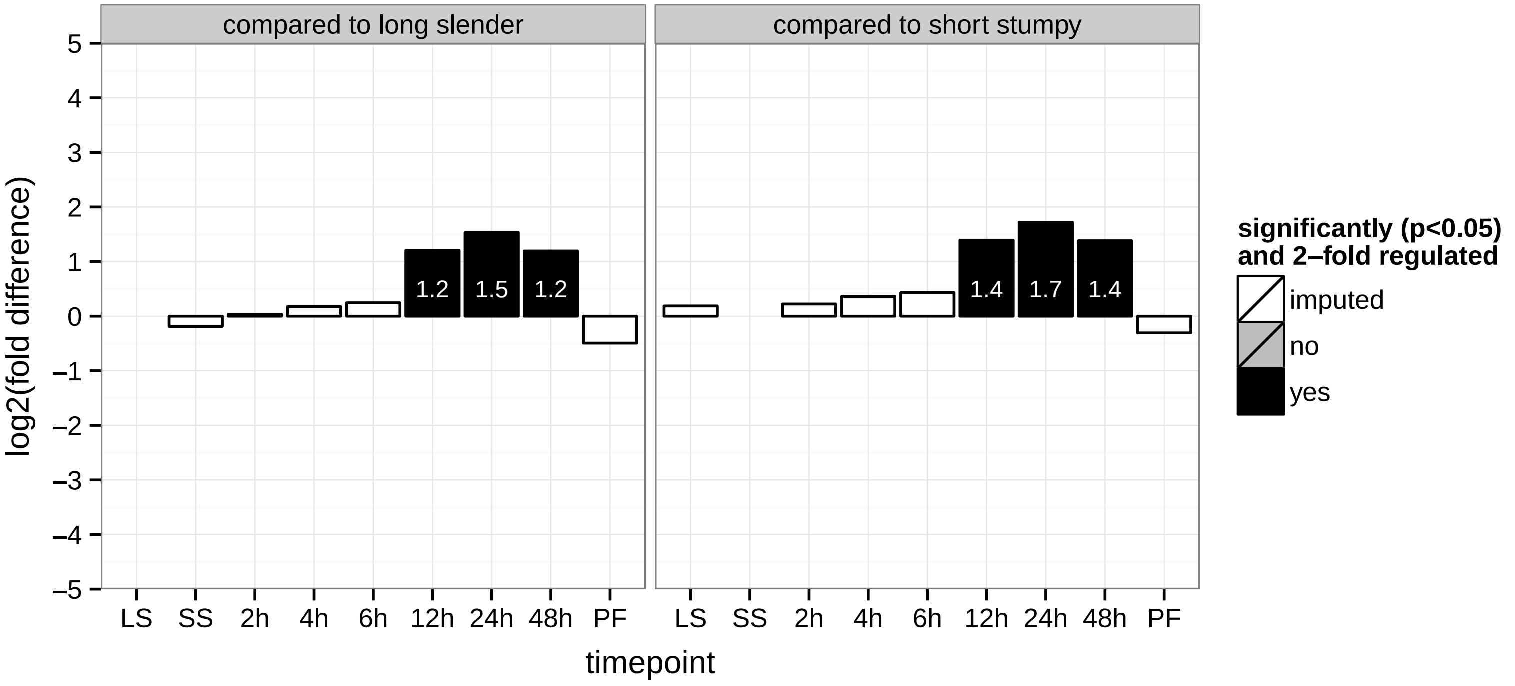

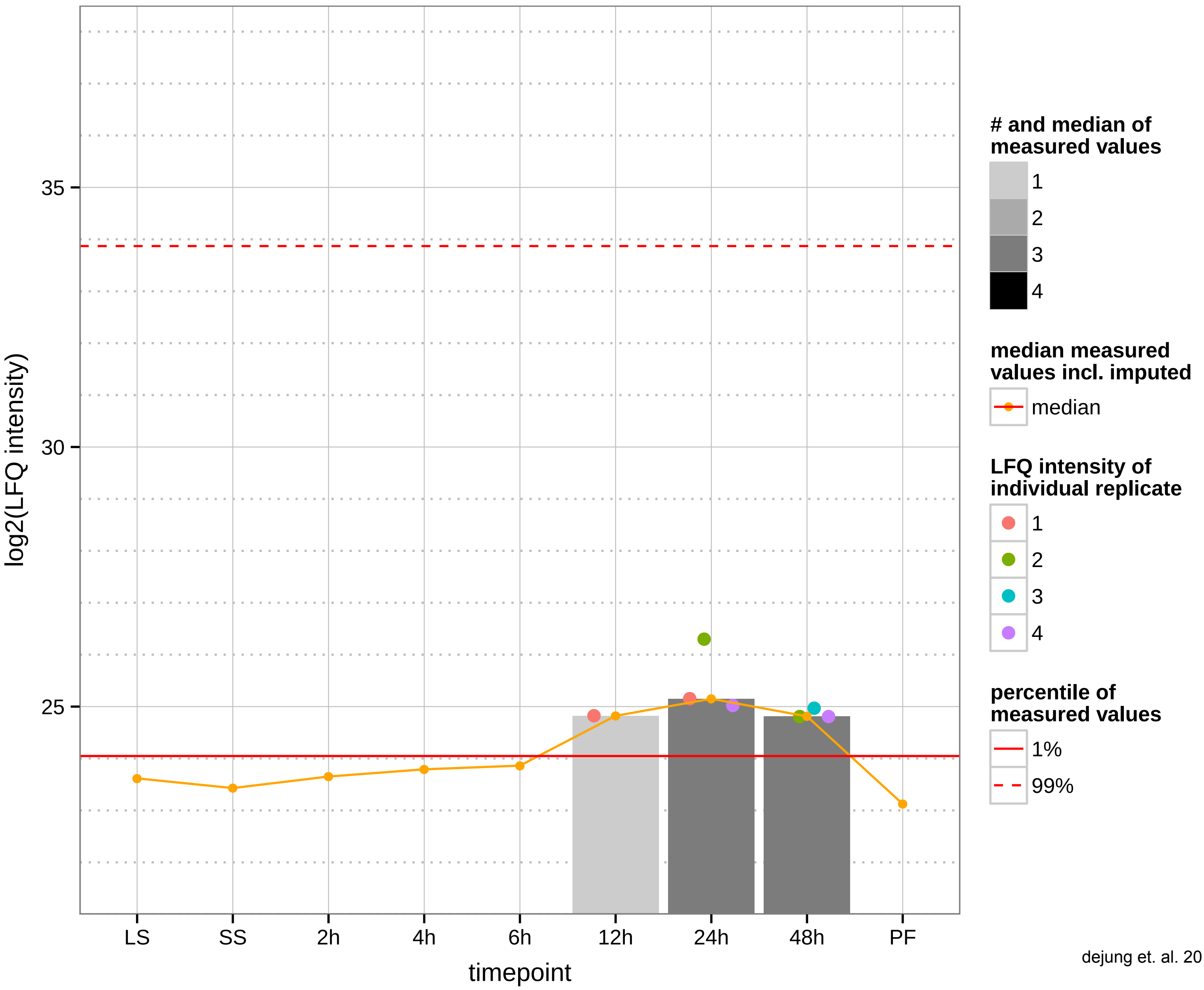

dejung et. al. 2015

Nucleoporin (TbNup149)
Tb927.11.11080
AGOF: null
AGOC: nuclear pore
AGOP: null
PGOF: null
PGOC: null
PGOP: null

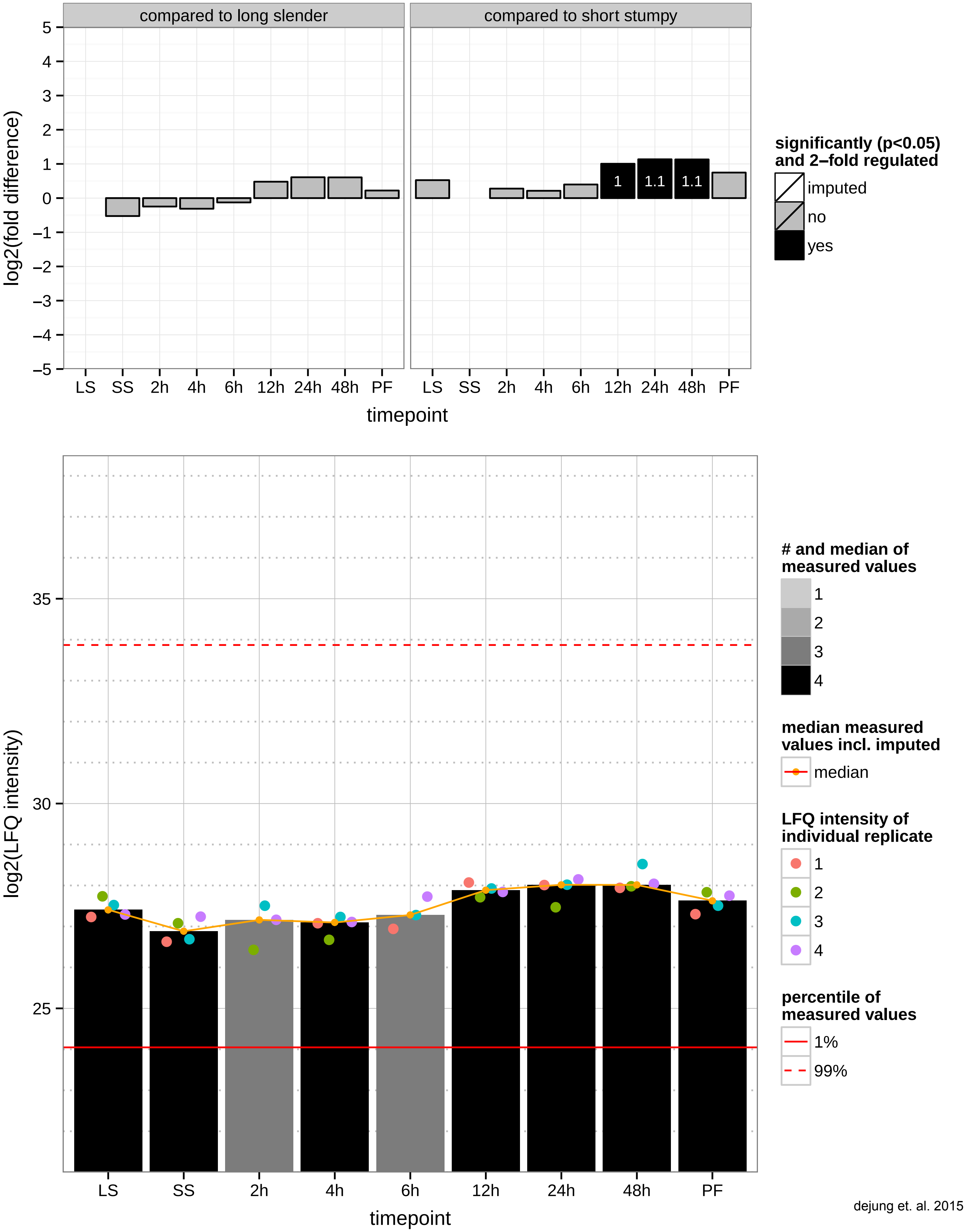

dejung et. al. 2015

histone acetyltransferase, putative (HAT2)
Tb927.11.11530
AGOF: histone acetyltransferase activity
AGOC: null
AGOP: histone acetylation, regulation of transcription, DNA–dependent
PGOF: transferase activity, transferring acyl groups other than amino–acyl groups
PGOC: nucleus
PGOP: regulation of transcription, DNA–dependent

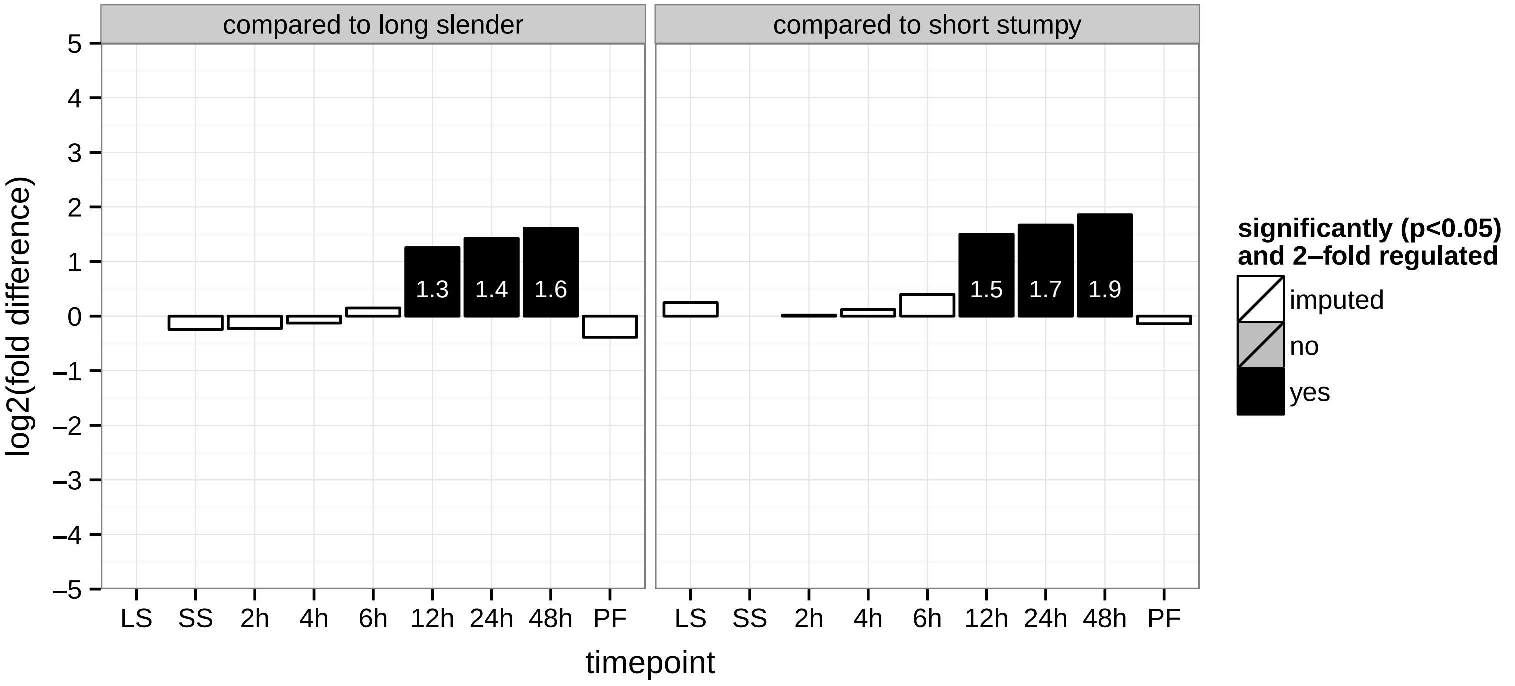

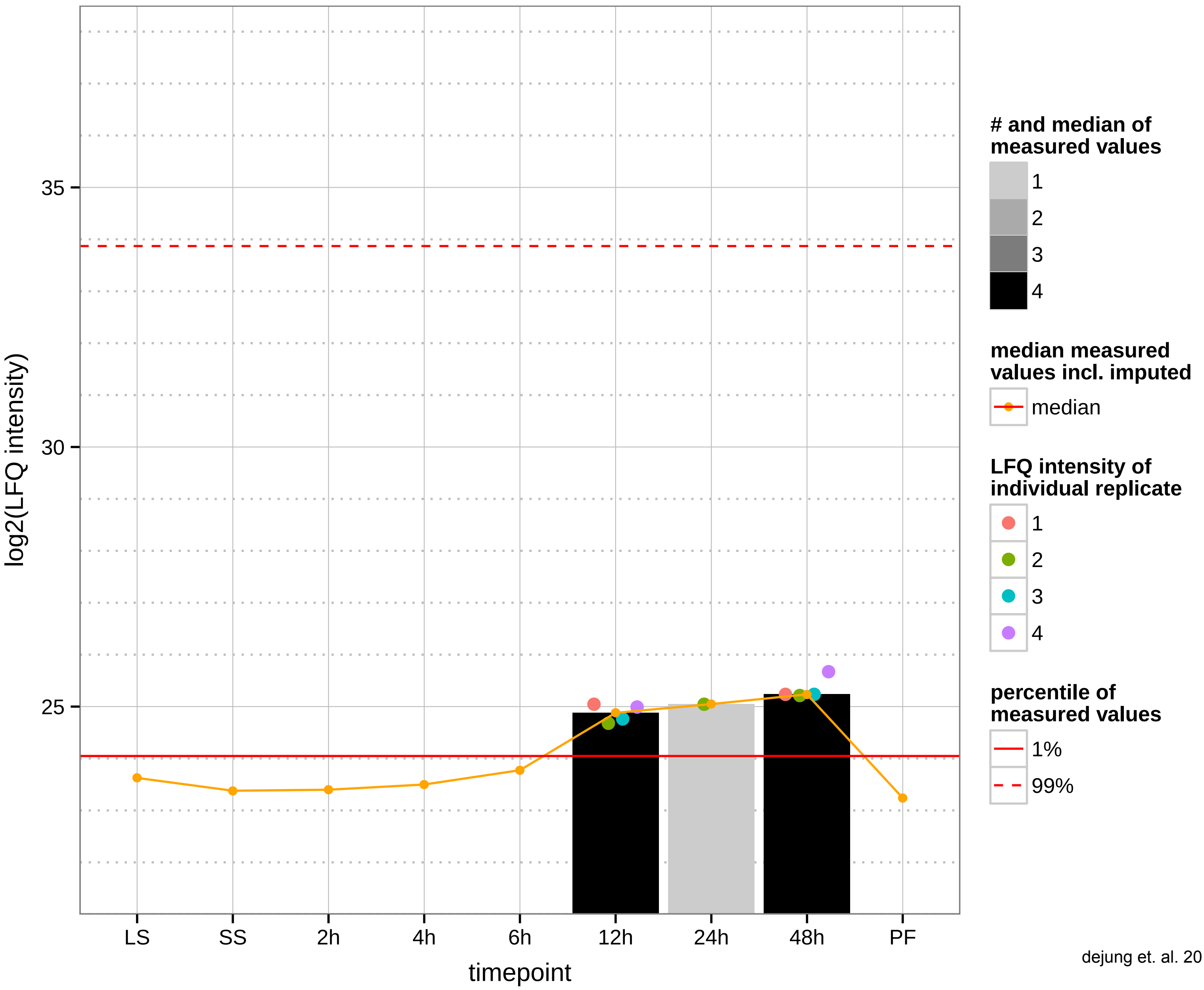

dejung et. al. 2015

hypothetical protein, conserved
Tb927.11.13600
AGOF: heat shock protein binding
AGOC: integral to membrane
AGOP: null
PGOF: heat shock protein binding
PGOC: null
PGOP: null

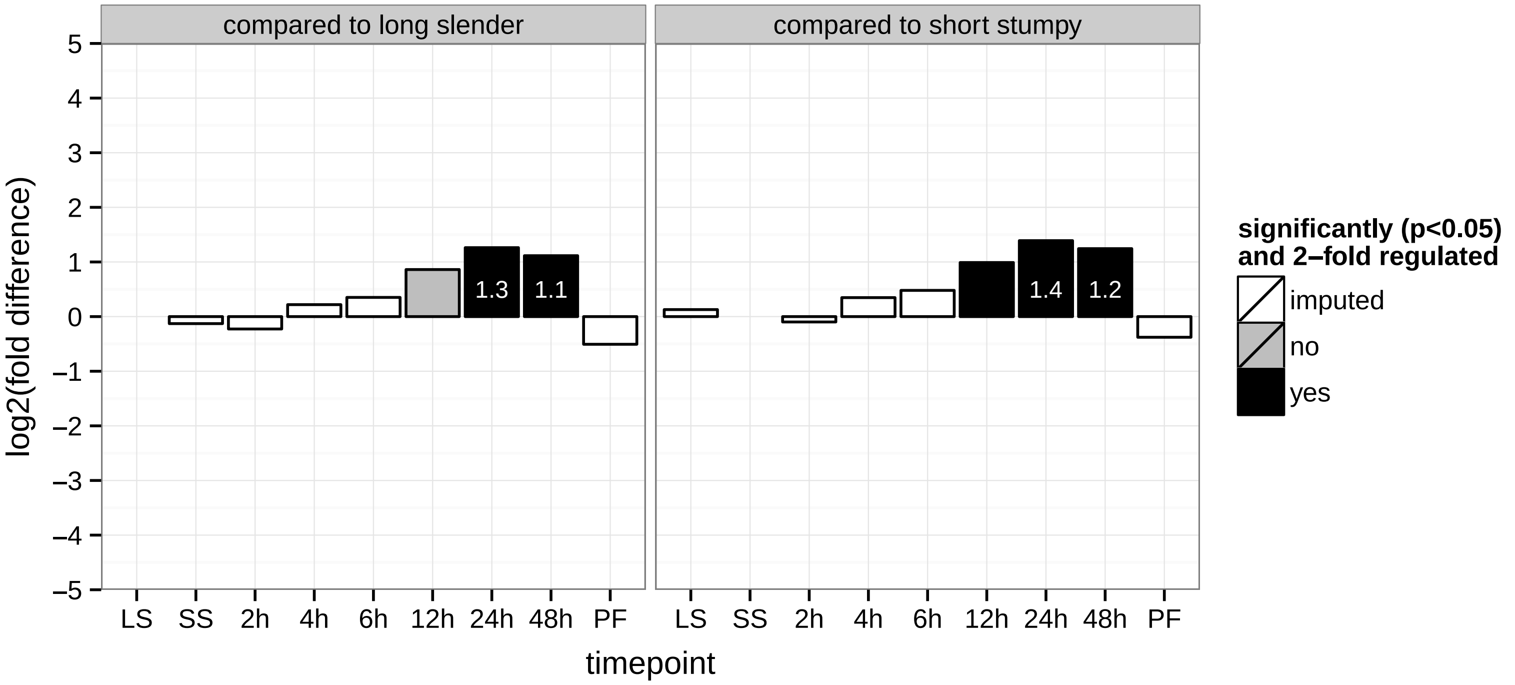

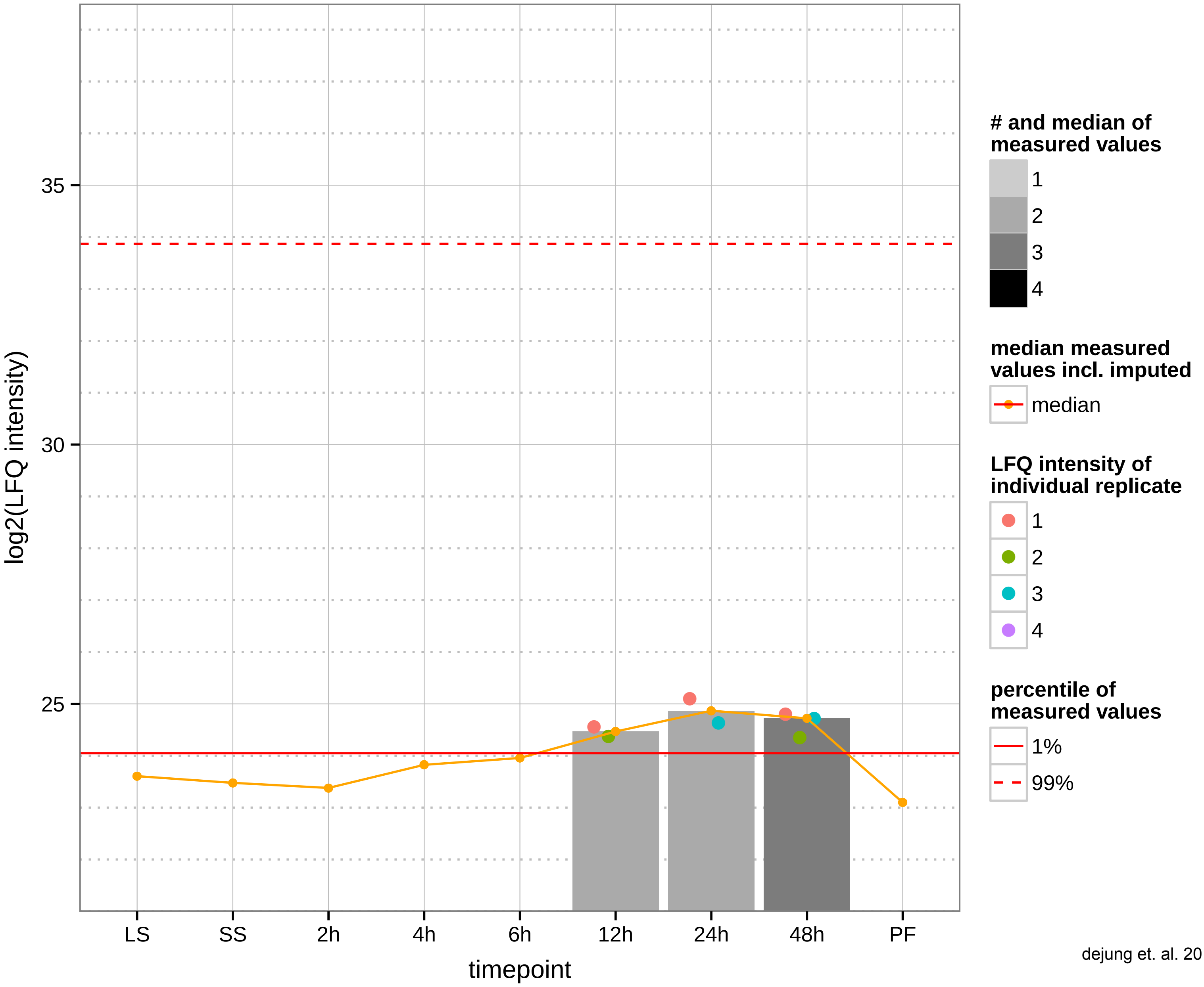

dejung et. al. 2015

C–14 sterol reductase, putative
Tb927.11.15530
AGOF: delta14–sterol reductase activity
AGOC: integral to membrane, membrane
AGOP: ergosterol biosynthetic process
PGOF: null
PGOC: membrane
PGOP: null

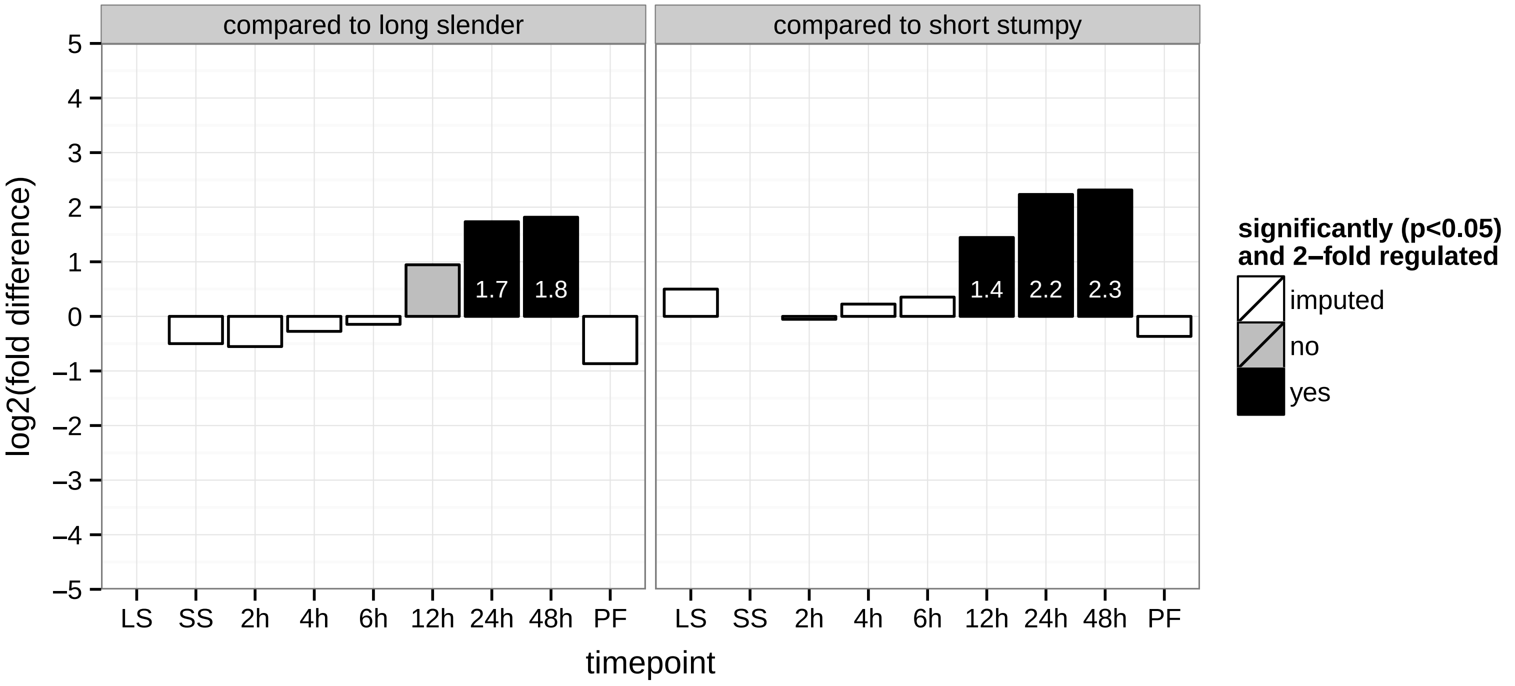

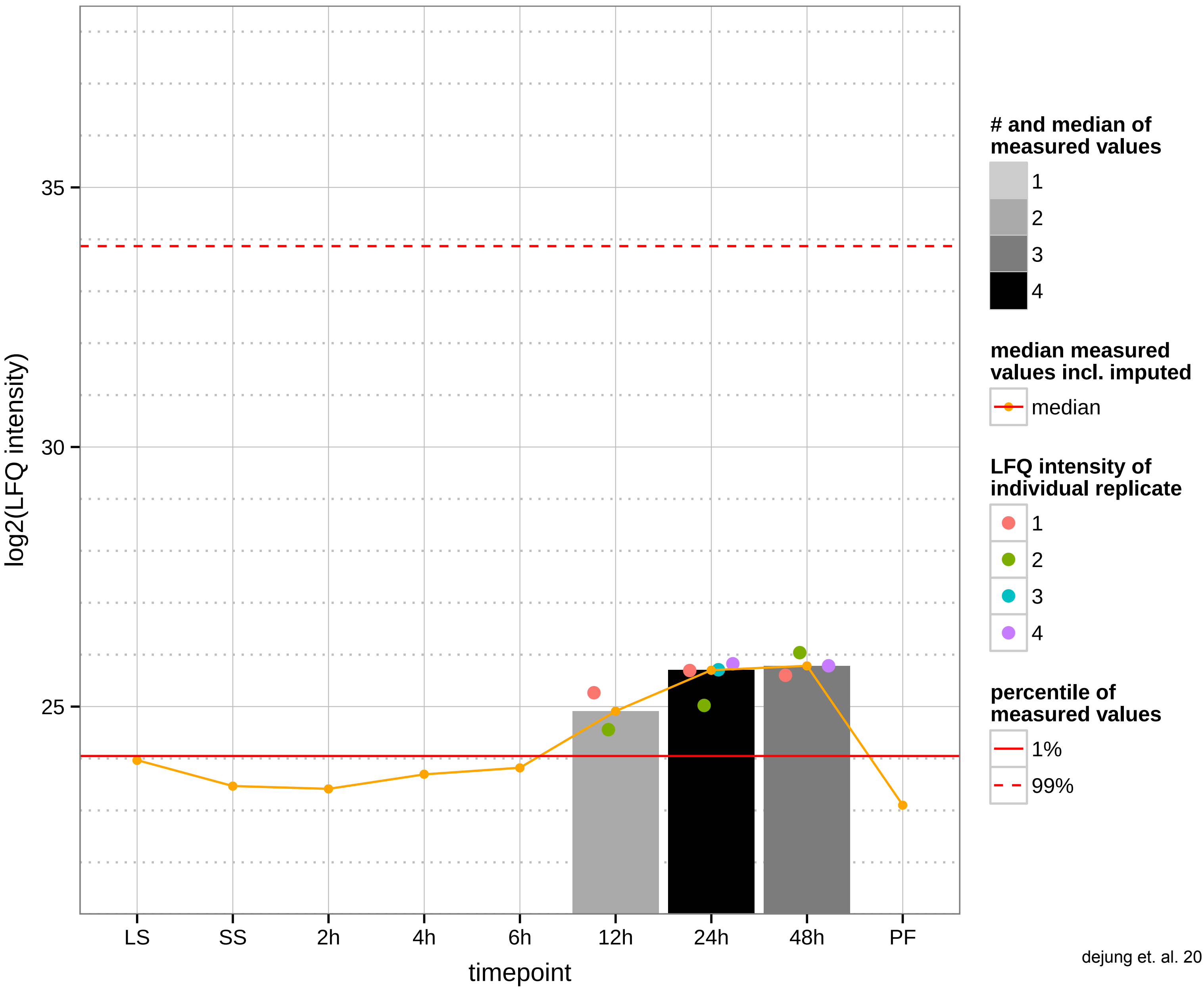

dejung et. al. 2015

hypothetical protein, conserved
Tb927.11.16010
AGOF: null
AGOC: null
AGOP: null
PGOF: null
PGOC: null
PGOP: null

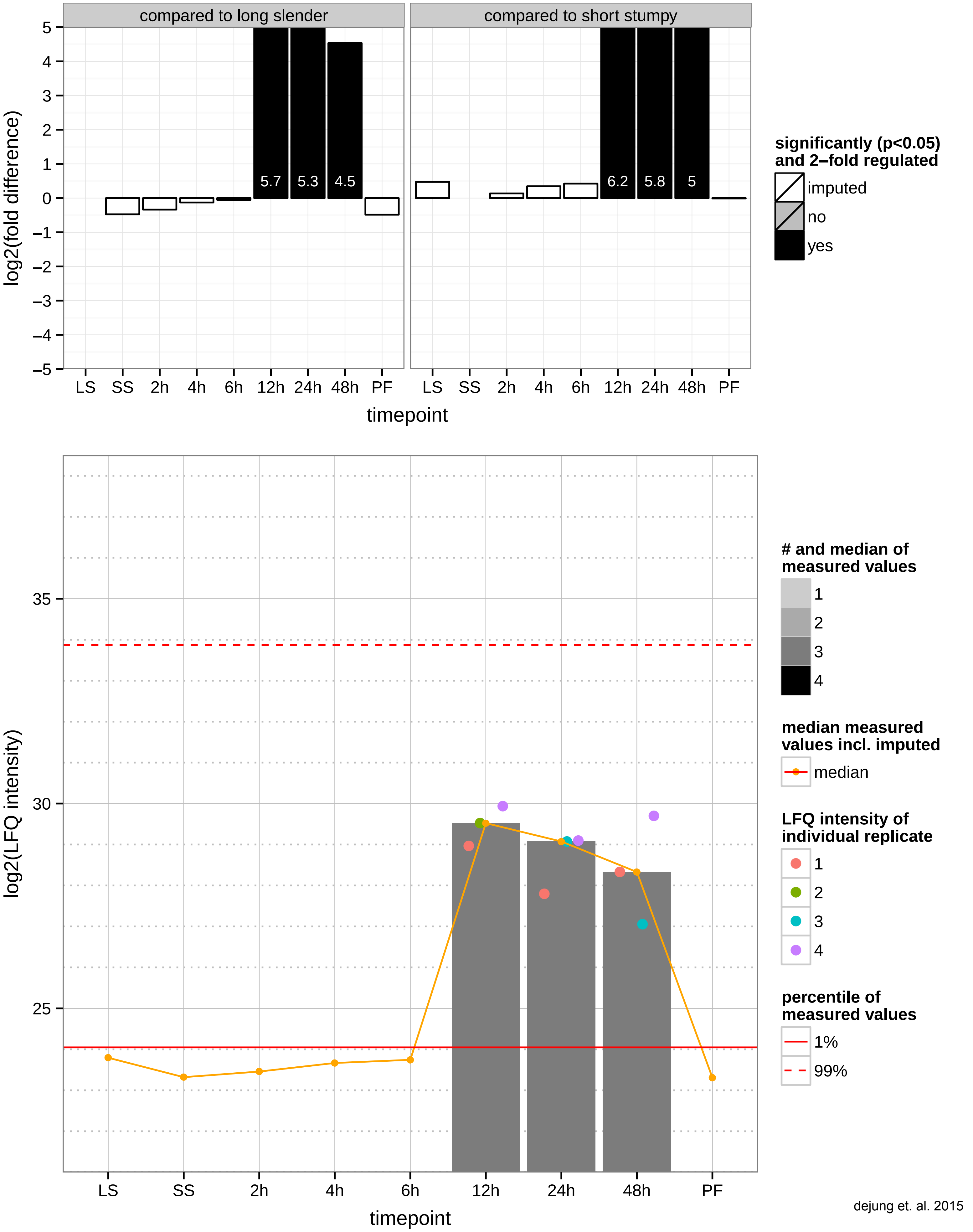

dejung et. al. 2015

tubulin binding cofactor A, putative
Tb927.11.16150
AGOF: unfolded protein binding
AGOC: microtubule
AGOP: protein folding, tubulin complex assembly
PGOF: unfolded protein binding
PGOC: microtubule
PGOP: tubulin complex assembly

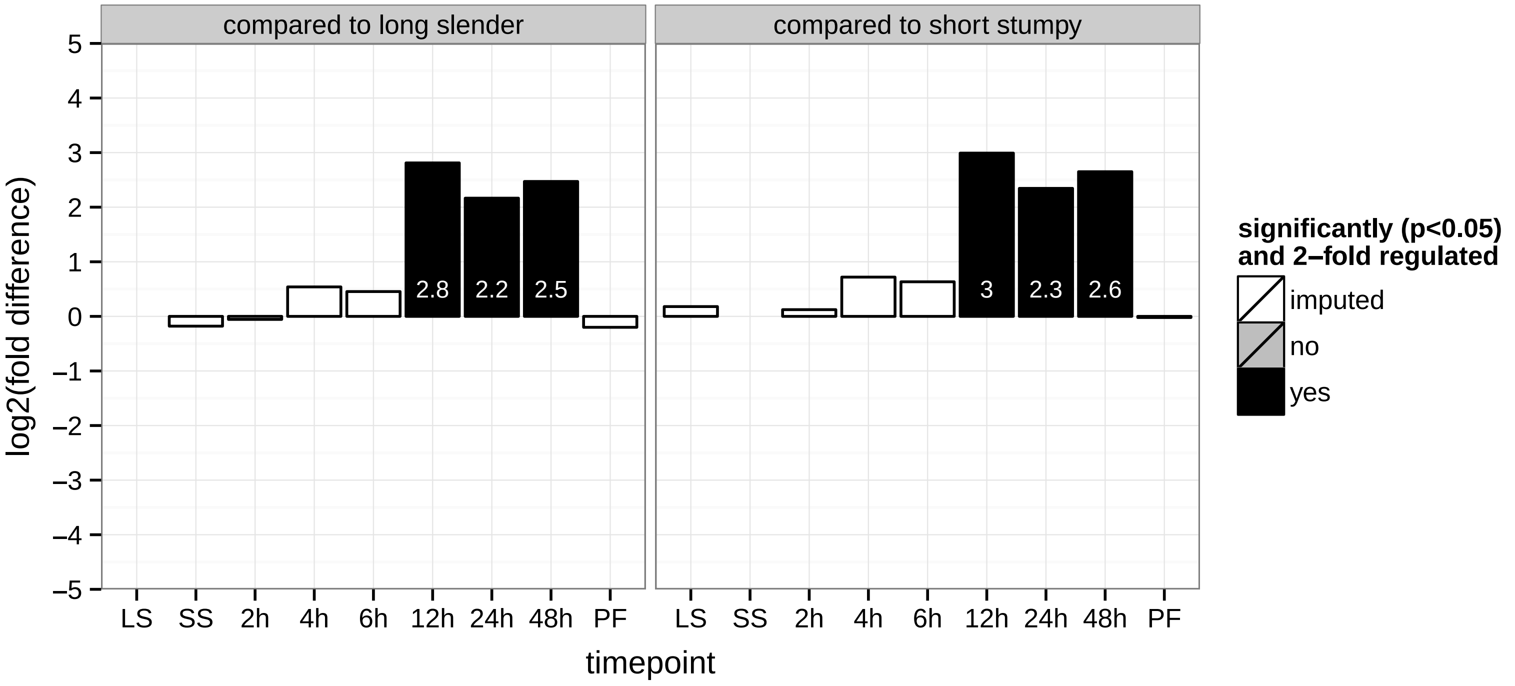

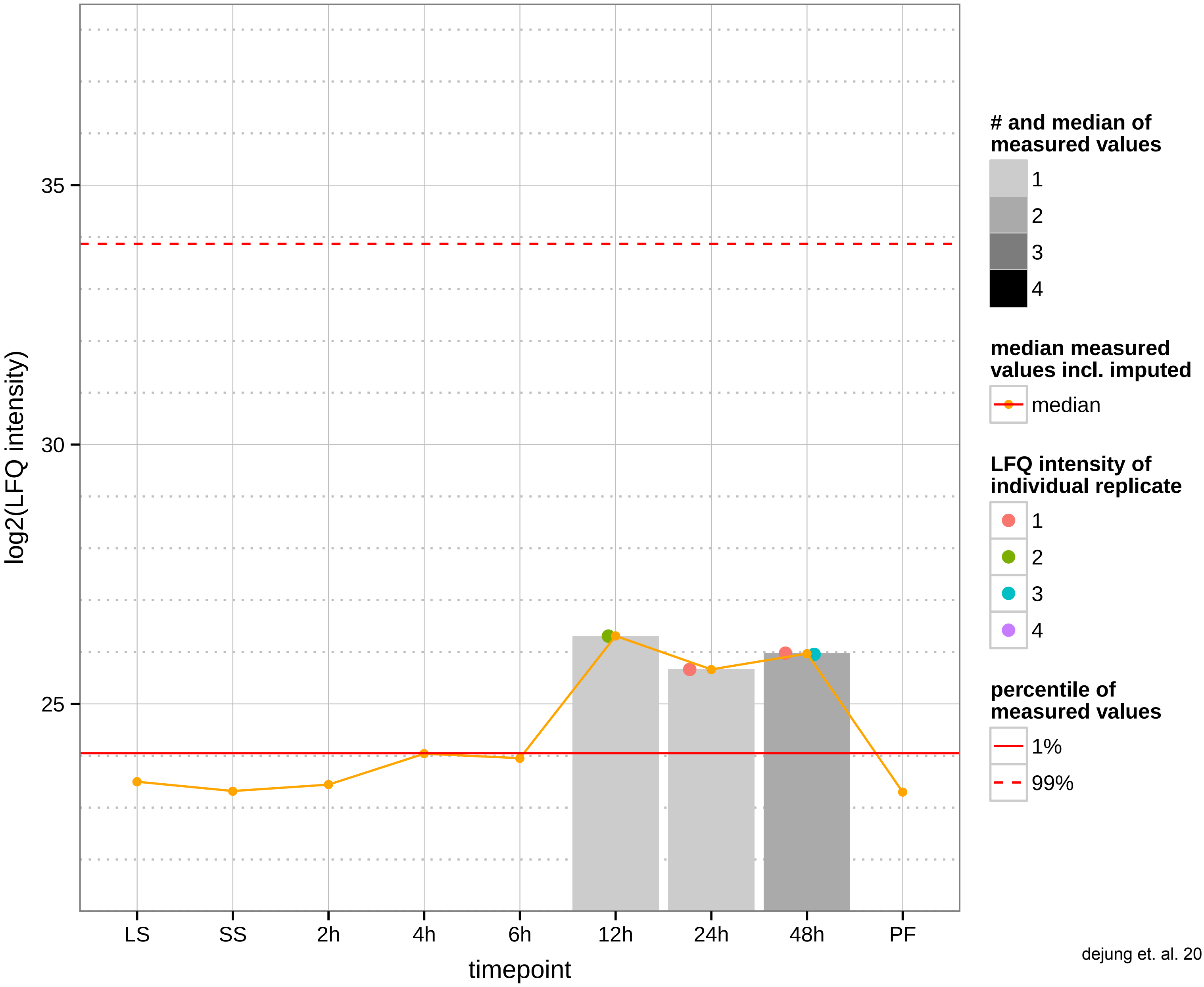

dejung et. al. 2015

hypothetical protein, conserved
Tb927.11.4400
AGOF: null
AGOC: null
AGOP: null
PGOF: null
PGOC: null
PGOP: null

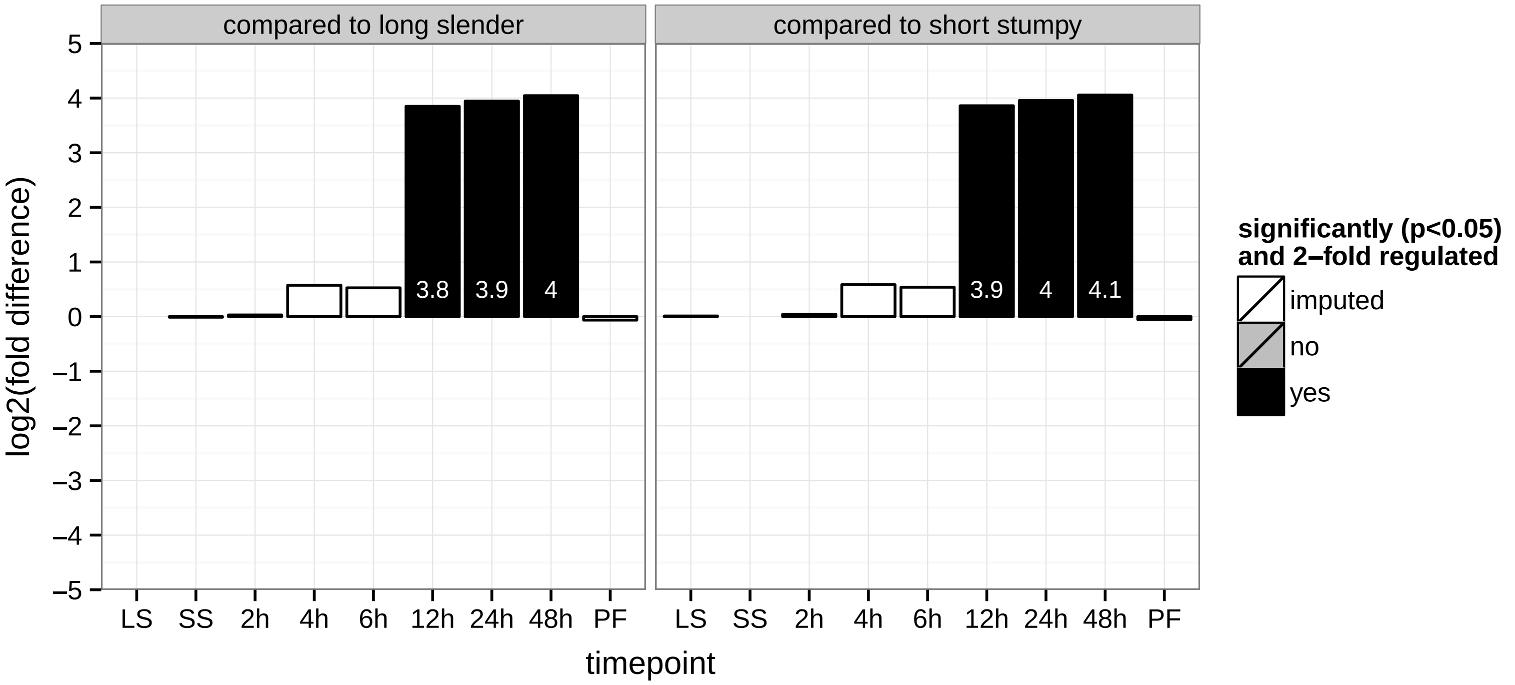

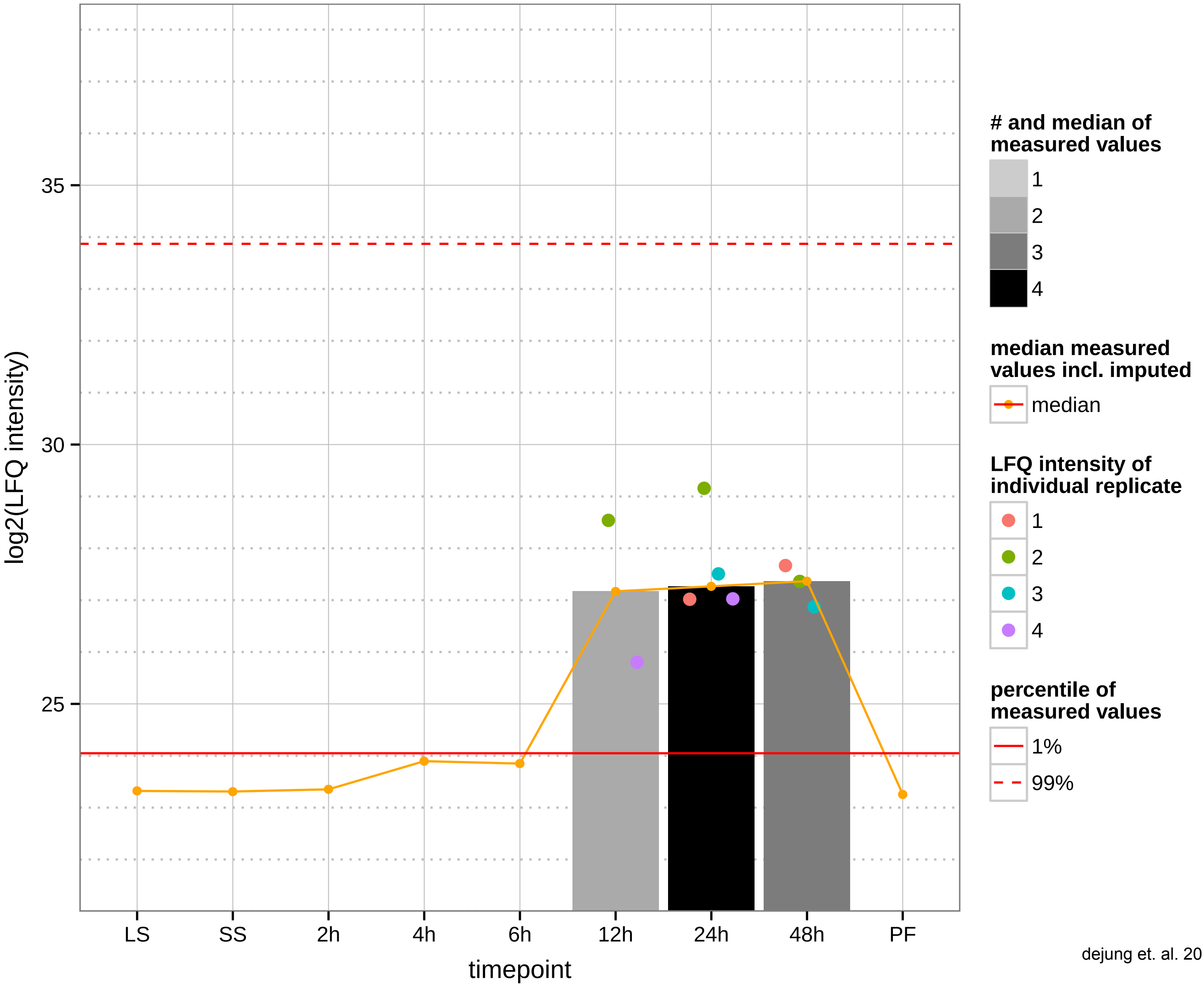

dejung et. al. 2015

hypothetical protein, conserved
Tb927.11.4850
AGOF: heat shock protein binding
AGOC: integral to membrane, mitochondrion
AGOP: null
PGOF: heat shock protein binding
PGOC: null
PGOP: null

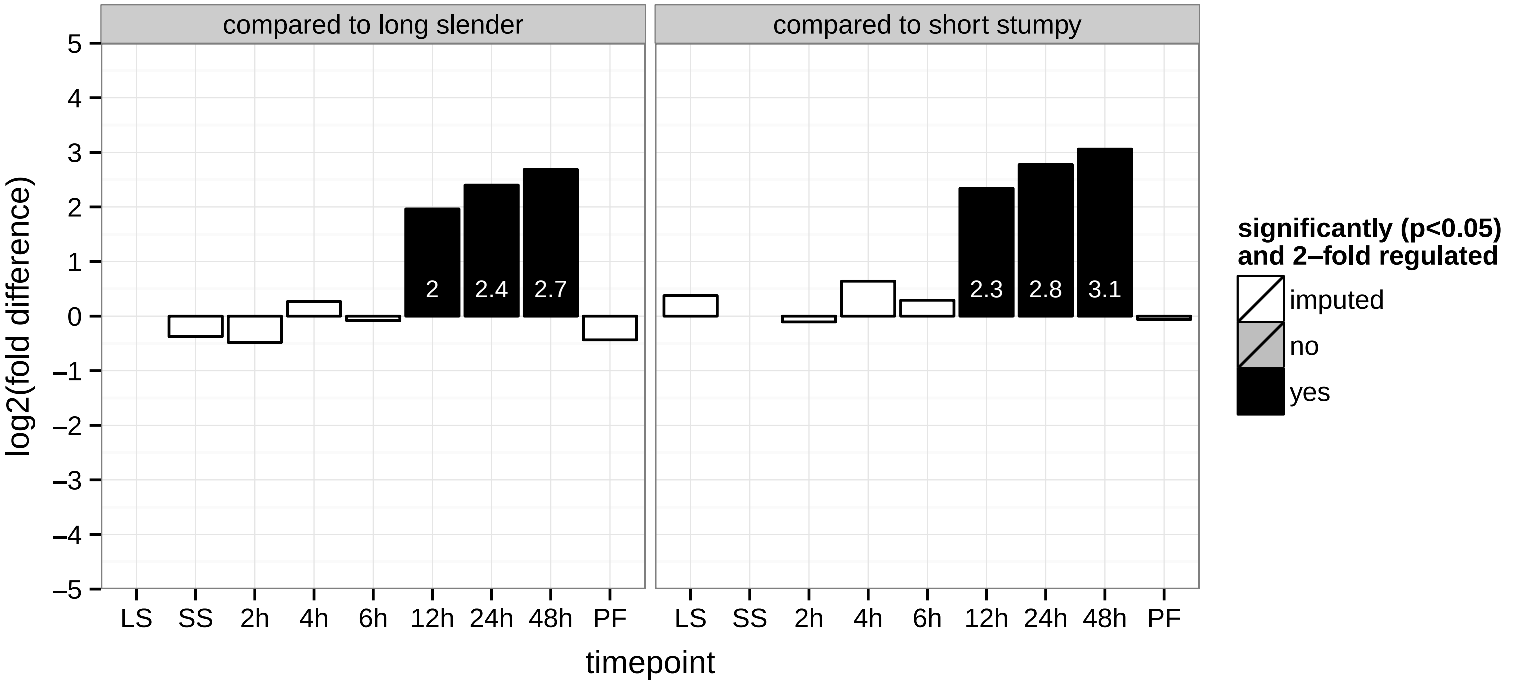

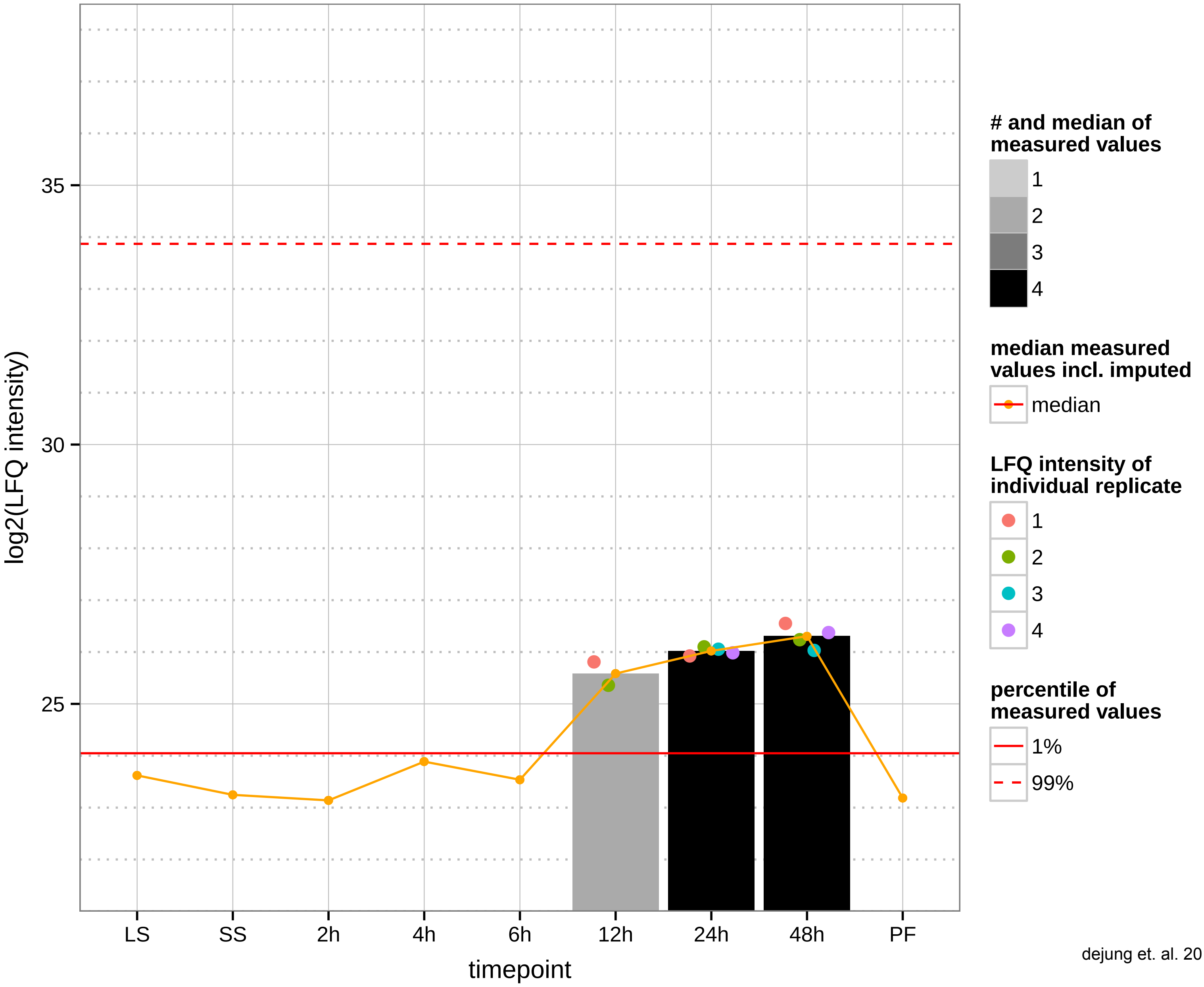

dejung et. al. 2015

hypothetical protein, conserved
Tb927.11.5960;Tb927.11.5950
AGOF: null
AGOC: null
AGOP: null
PGOF: null
PGOC: null
PGOP: null

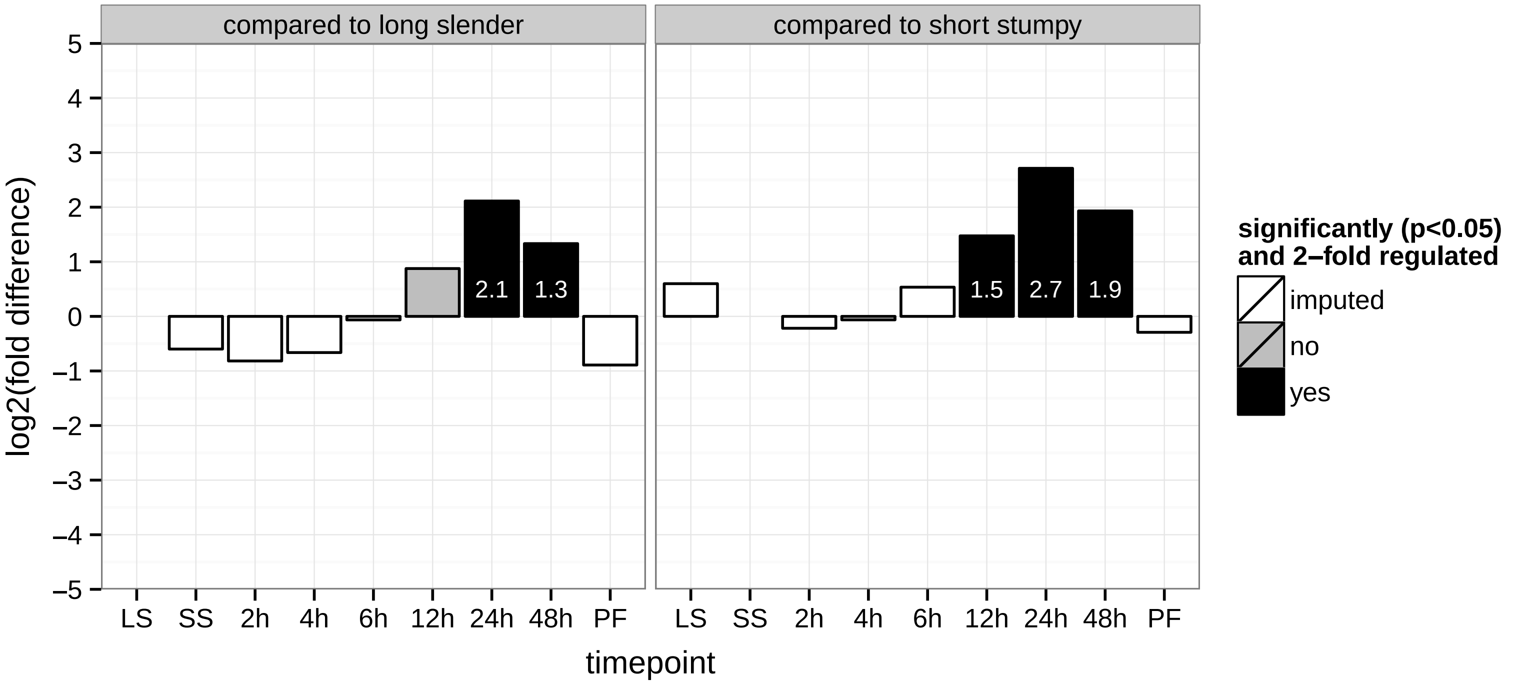

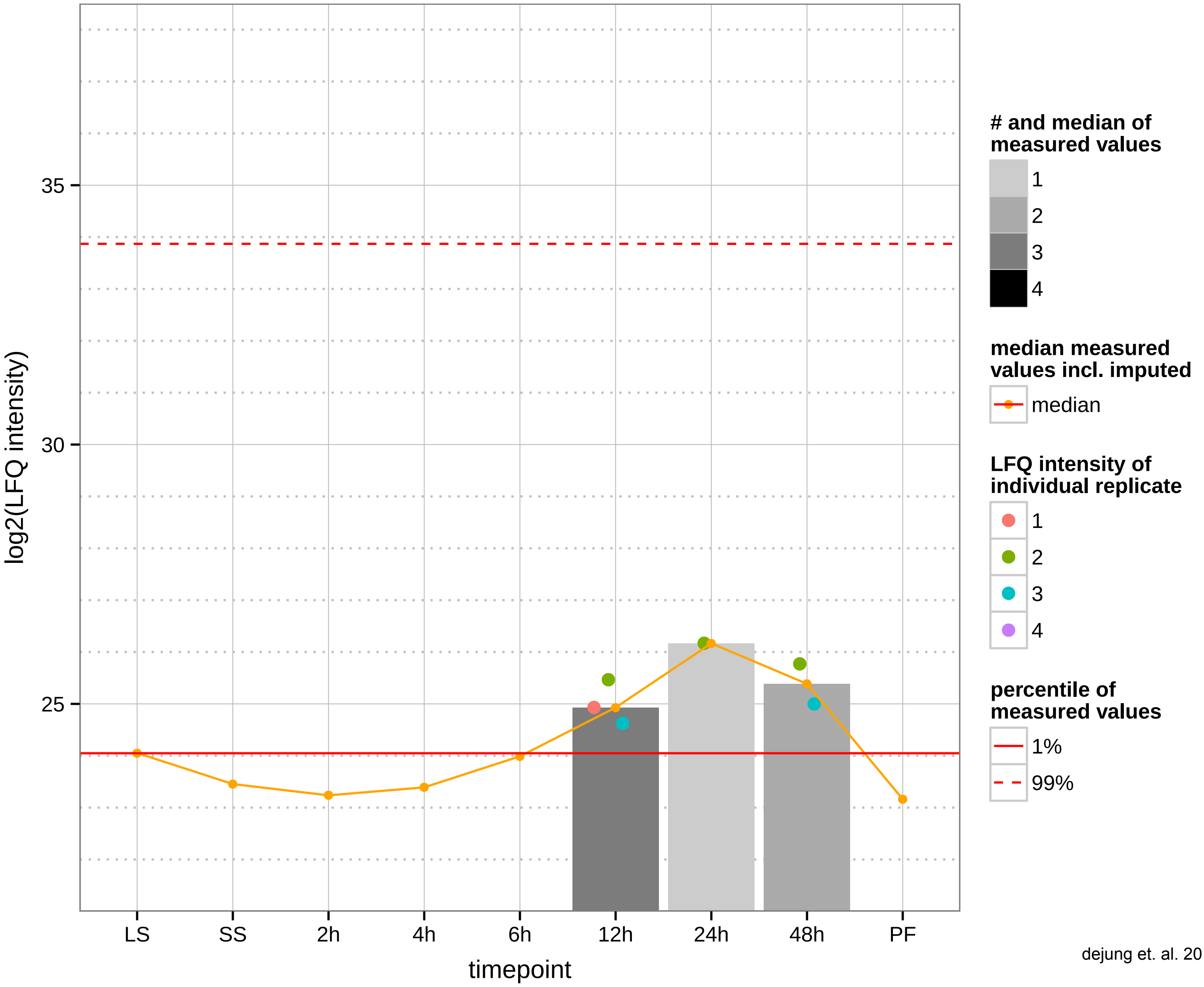

dejung et. al. 2015

hypothetical protein, conserved
Tb927.11.800
AGOF: unfolded protein binding
AGOC: prefoldin complex
AGOP: protein folding
PGOF: chaperone binding, unfolded protein binding
PGOC: prefoldin complex
PGOP: protein folding

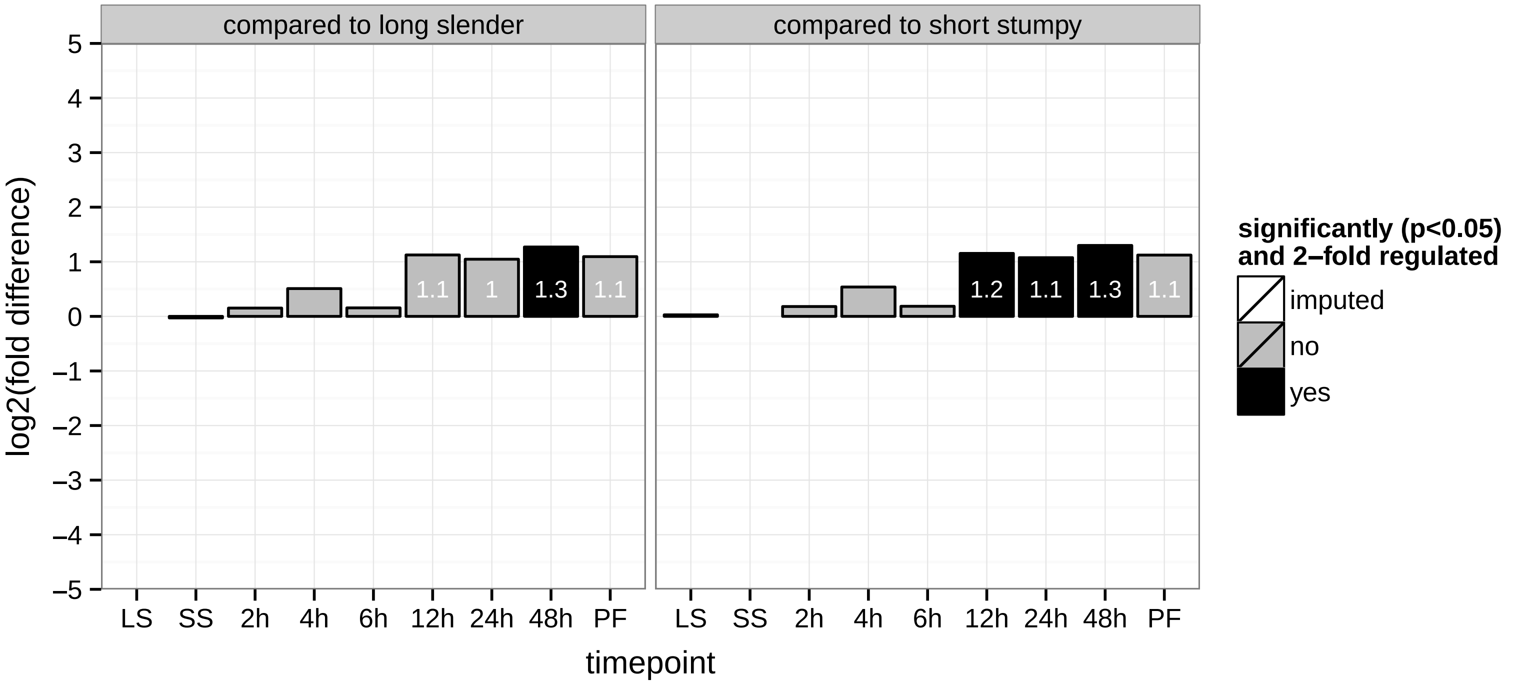

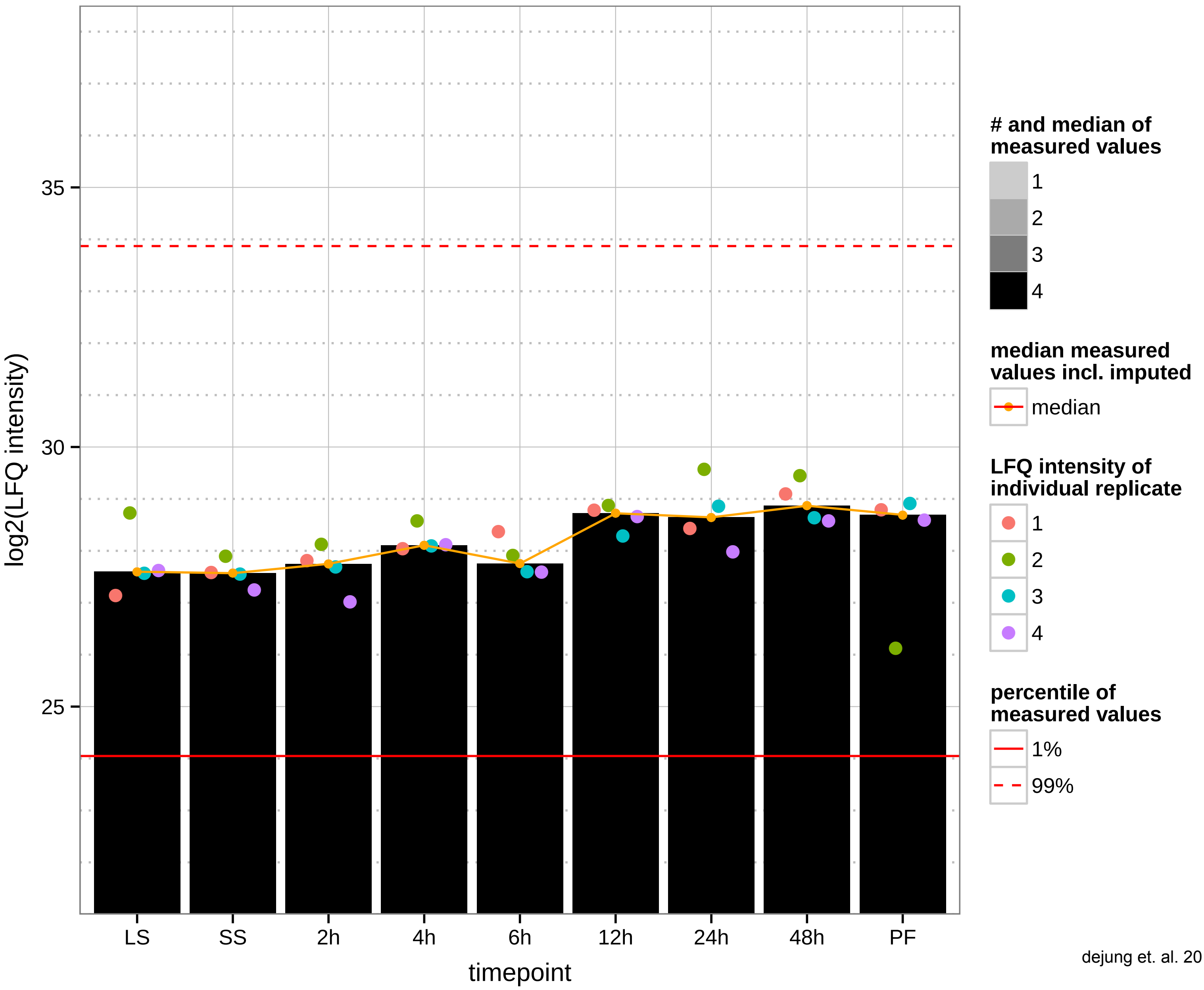

dejung et. al. 2015

hypothetical protein, conserved
Tb927.11.8100
AGOF: null
AGOC: null
AGOP: null
PGOF: protein binding
PGOC: null
PGOP: null

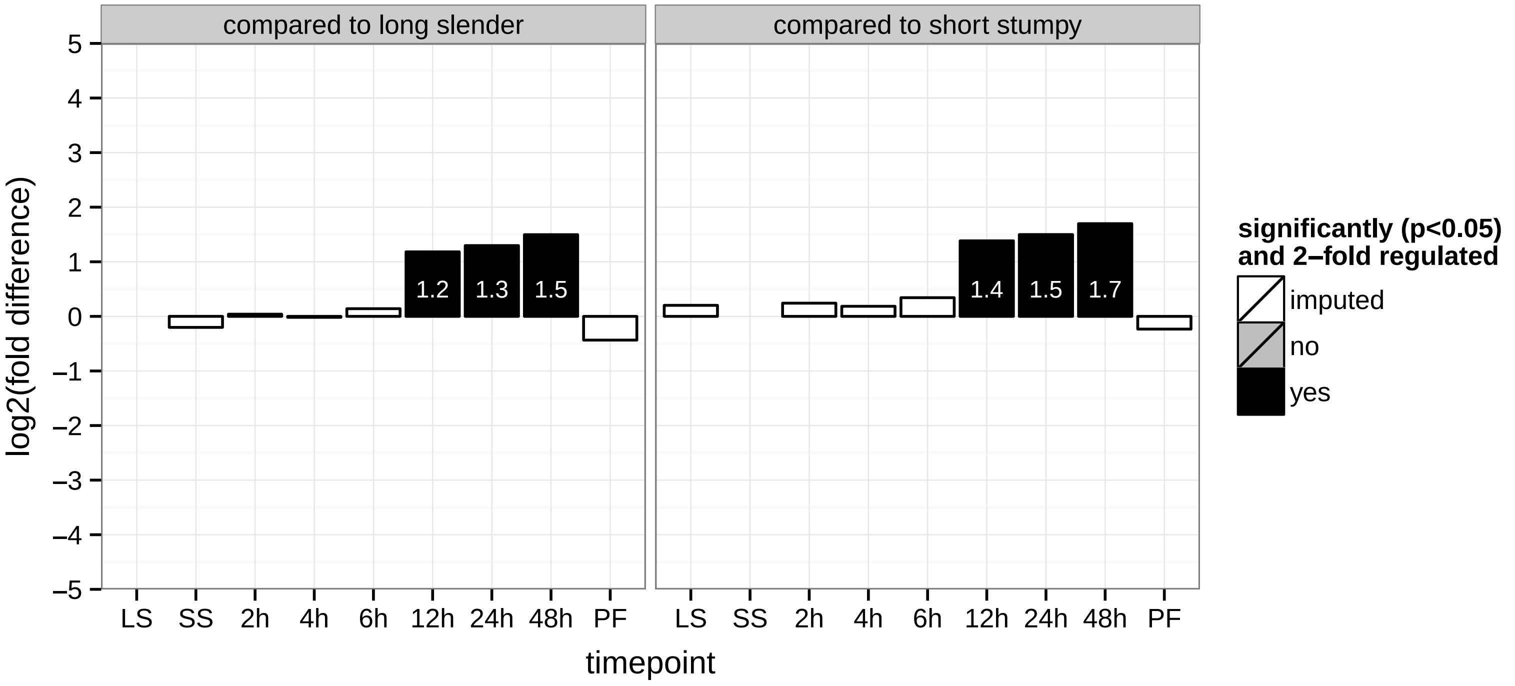

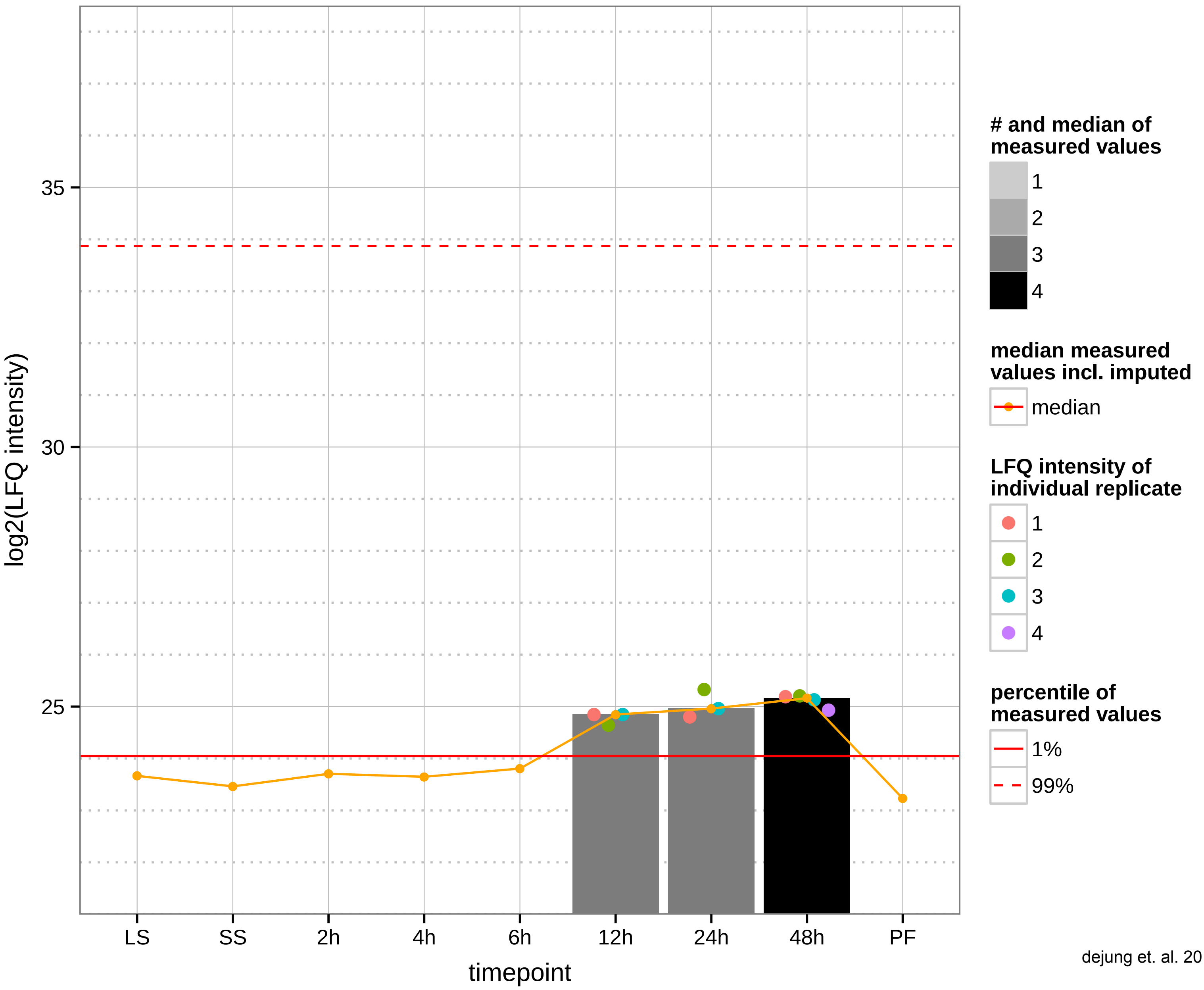

dejung et. al. 2015

NADH dehydrogenase subunit NB6M, putative, NB6M, Complex 1, NADH–ubiquinone oxidoreductase chain NB6M
Tb927.11.8910
AGOF: NADH dehydrogenase (ubiquinone) activity, NADH dehydrogenase activity
AGOC: NADH dehydrogenase complex, mitochondrial membrane, mitochondrion
AGOP: NADH regeneration
PGOF: null
PGOC: null
PGOP: null

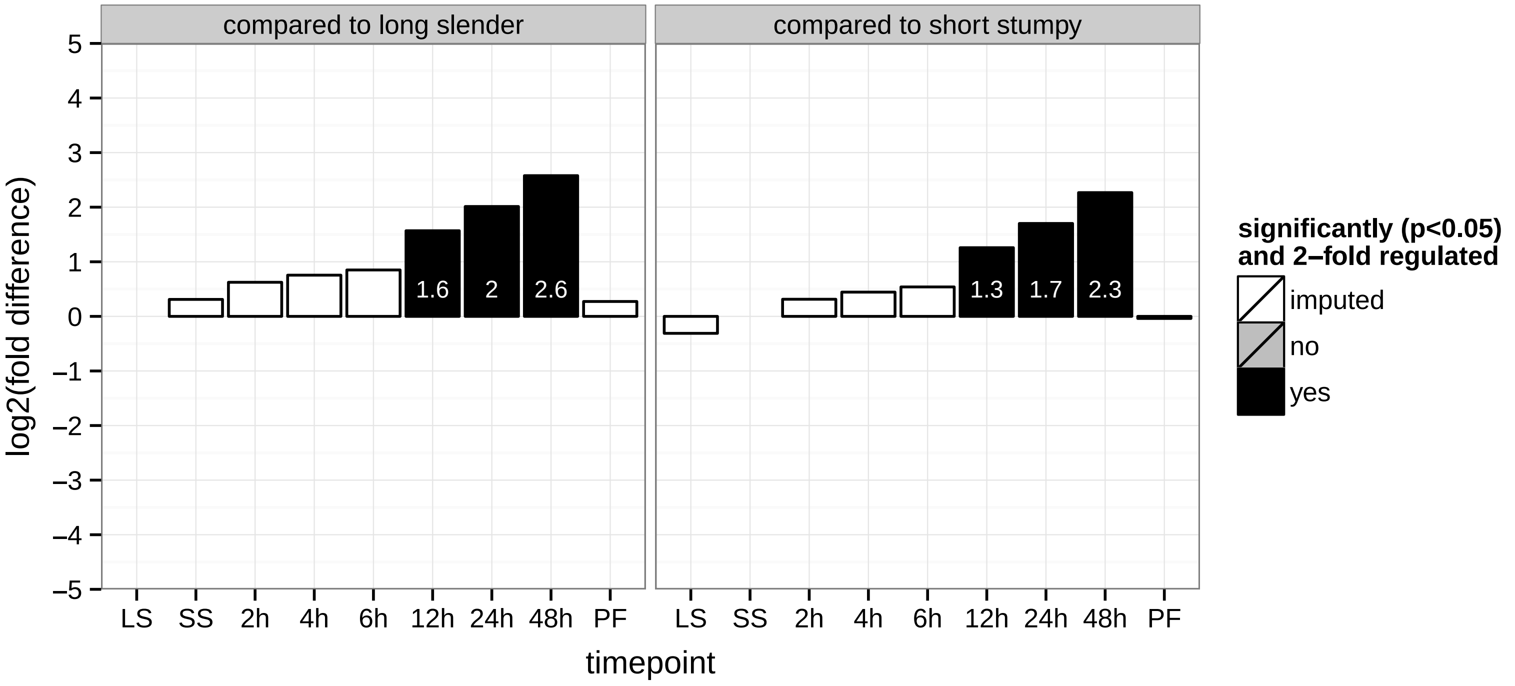

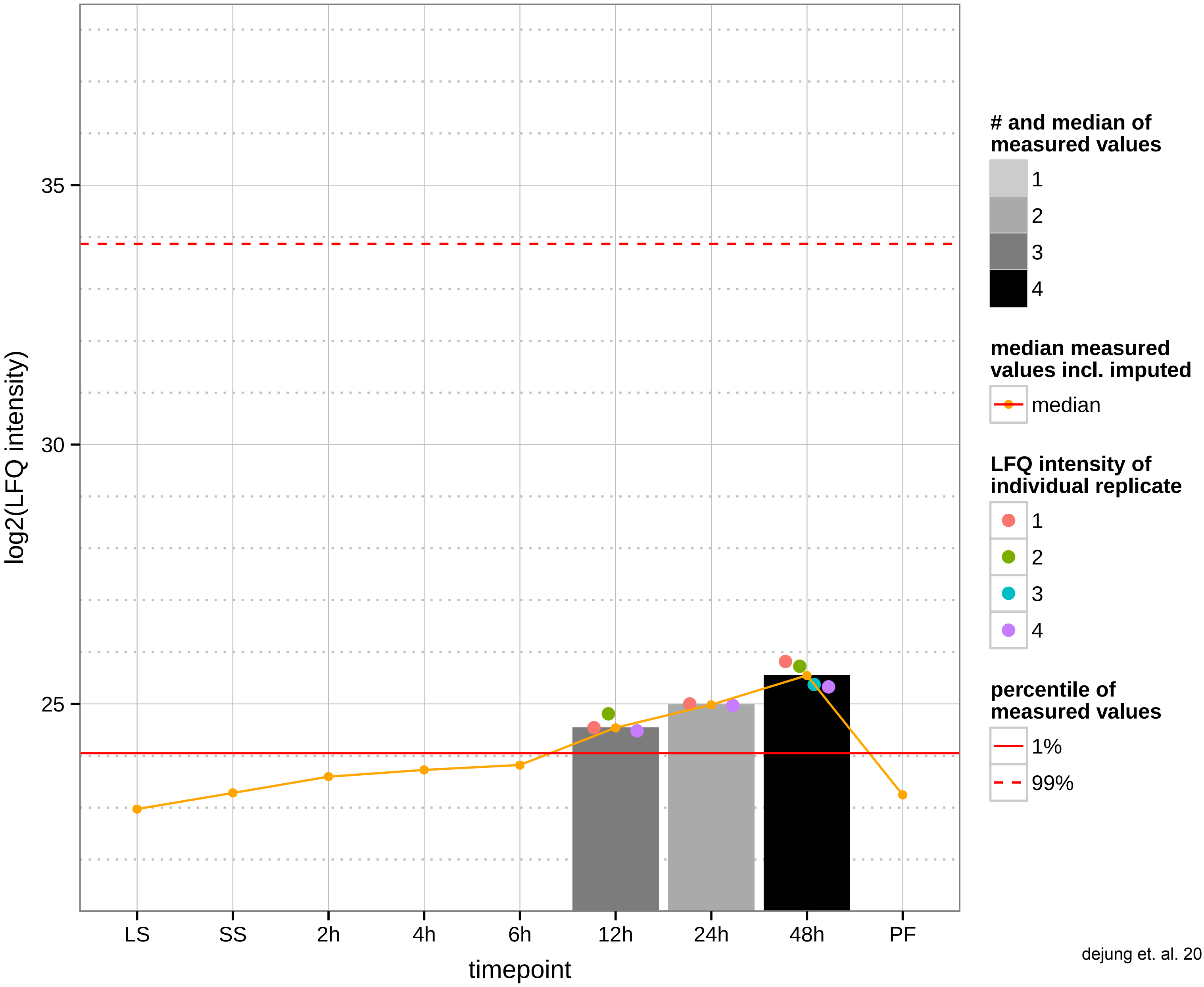

dejung et. al. 2015

hypothetical protein, conserved
Tb927.11.9830
AGOF: null
AGOC: mitochondrion
AGOP: null
PGOF: null
PGOC: null
PGOP: null

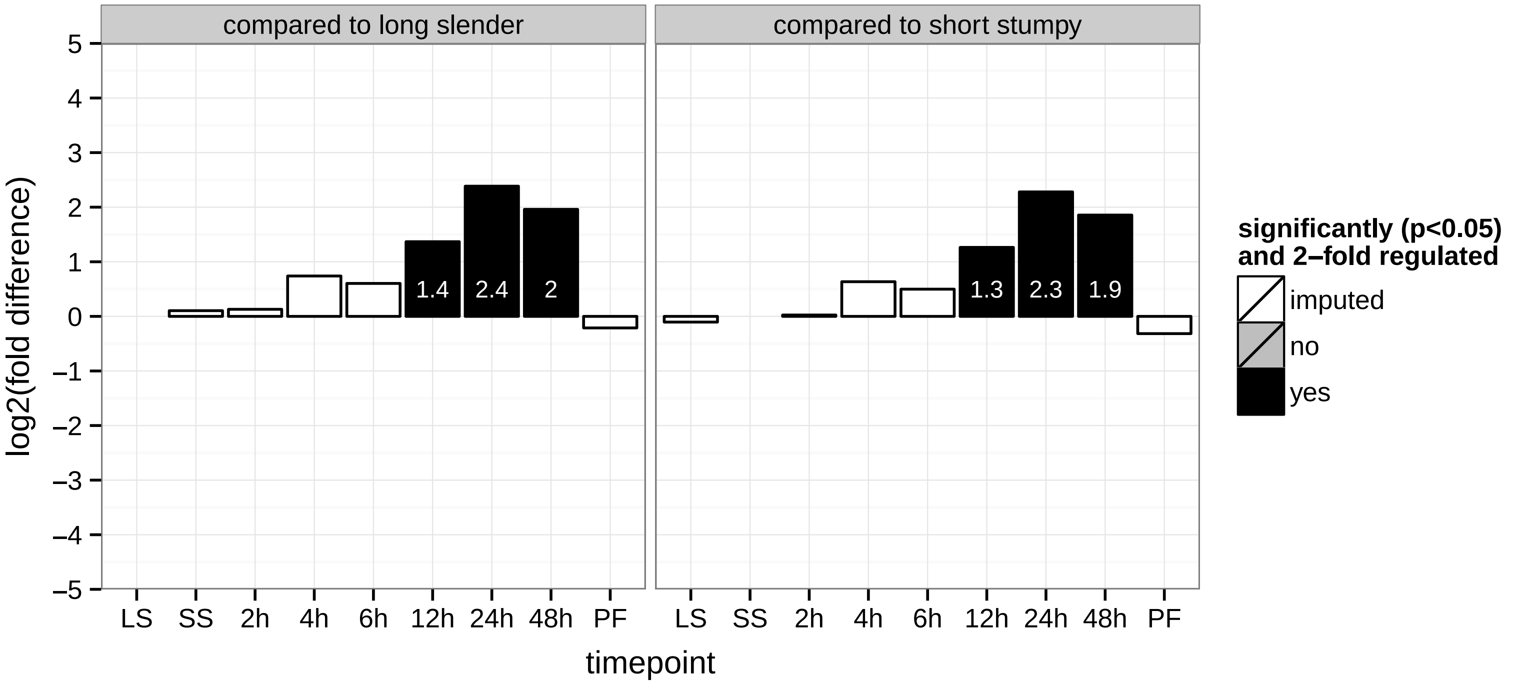

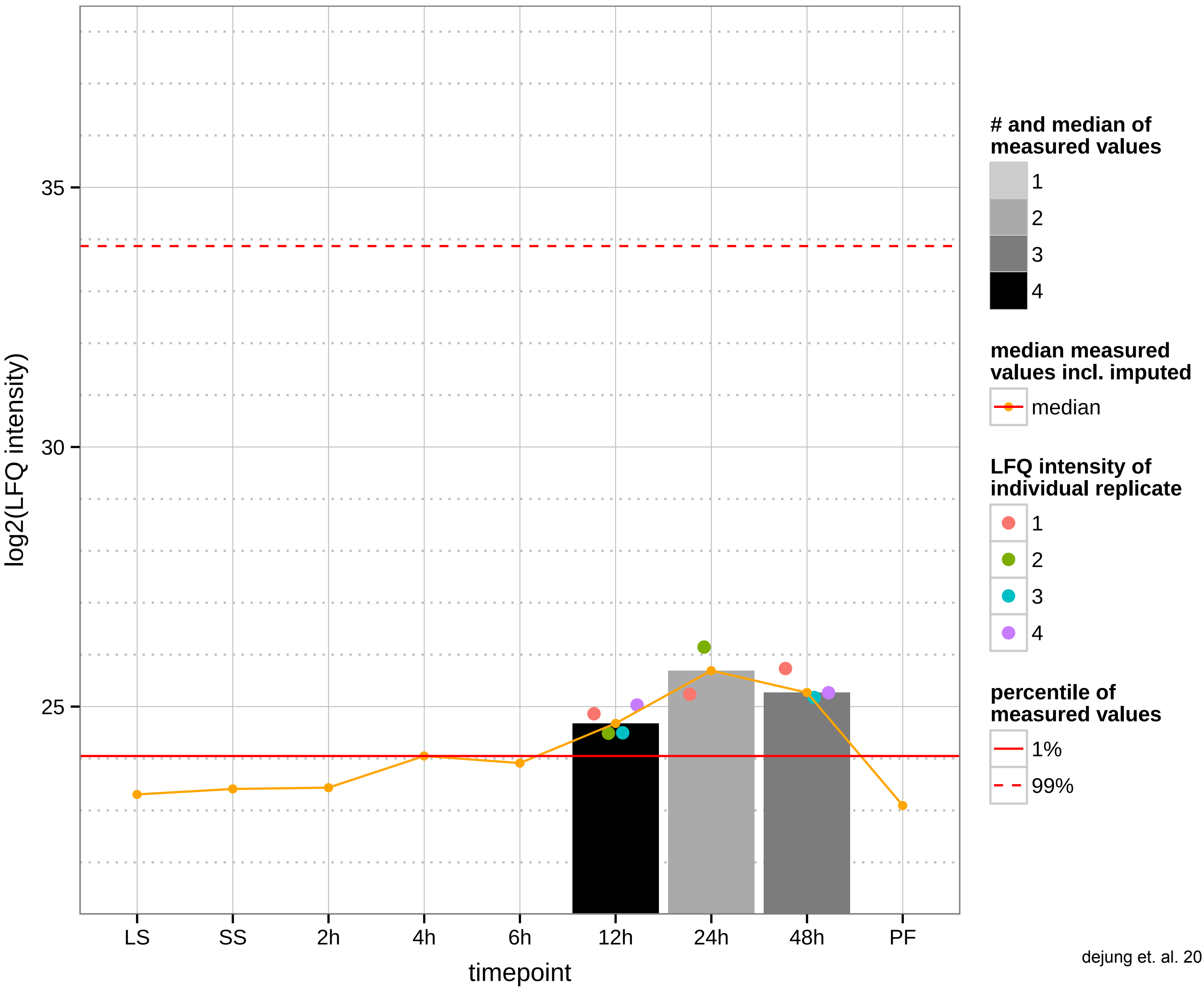

dejung et. al. 2015

hypothetical protein, conserved
Tb927.11.9940
AGOF: null
AGOC: null
AGOP: null
PGOF: null
PGOC: null
PGOP: null

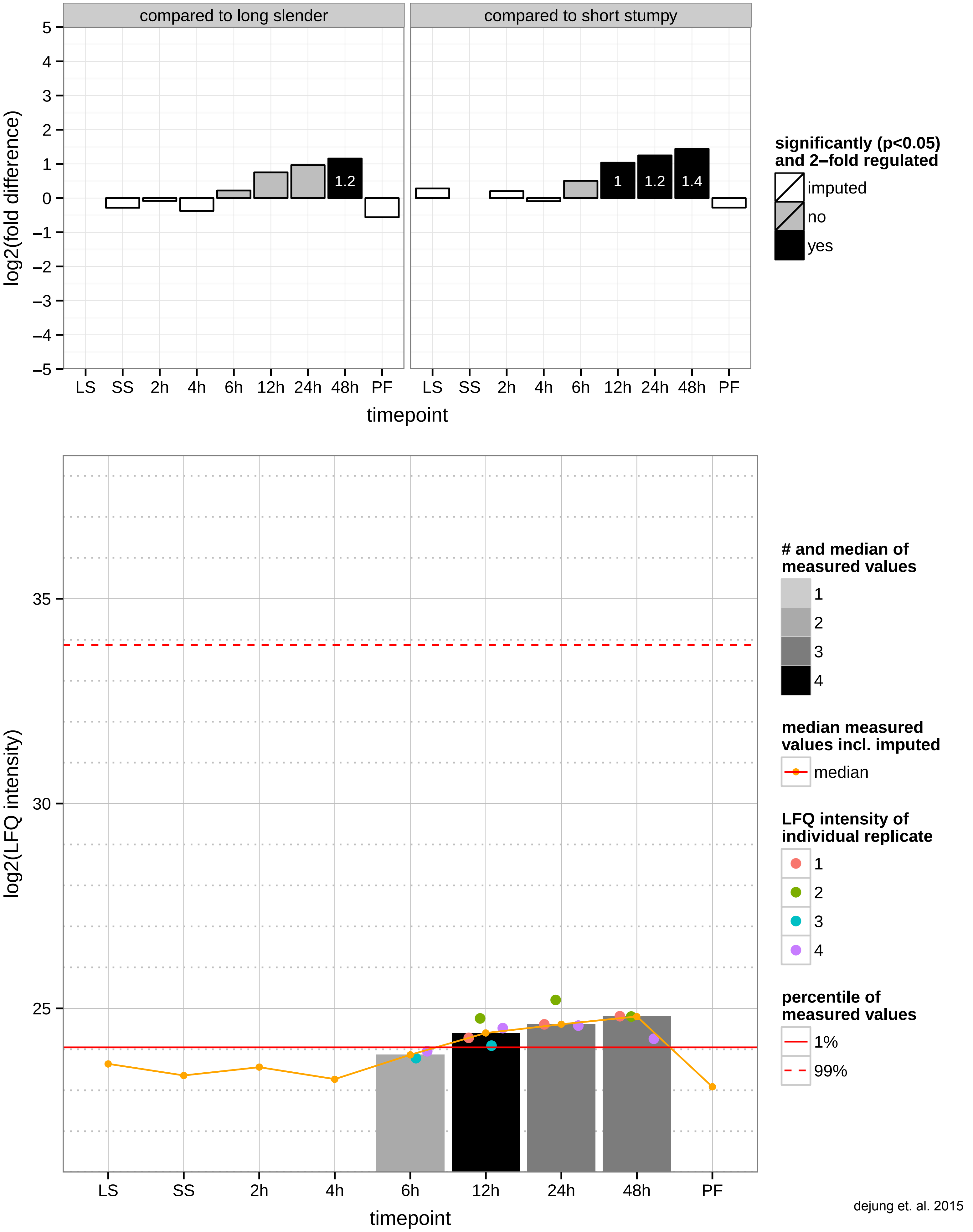

dejung et. al. 2015

mitochondrial RNA binding complex 1 subunit (MRB1860)
Tb927.2.1860
AGOF: RNA binding
AGOC: mitochondrion
AGOP: cytidine to uridine editing, mRNA stabilization
PGOF: null
PGOC: null
PGOP: null

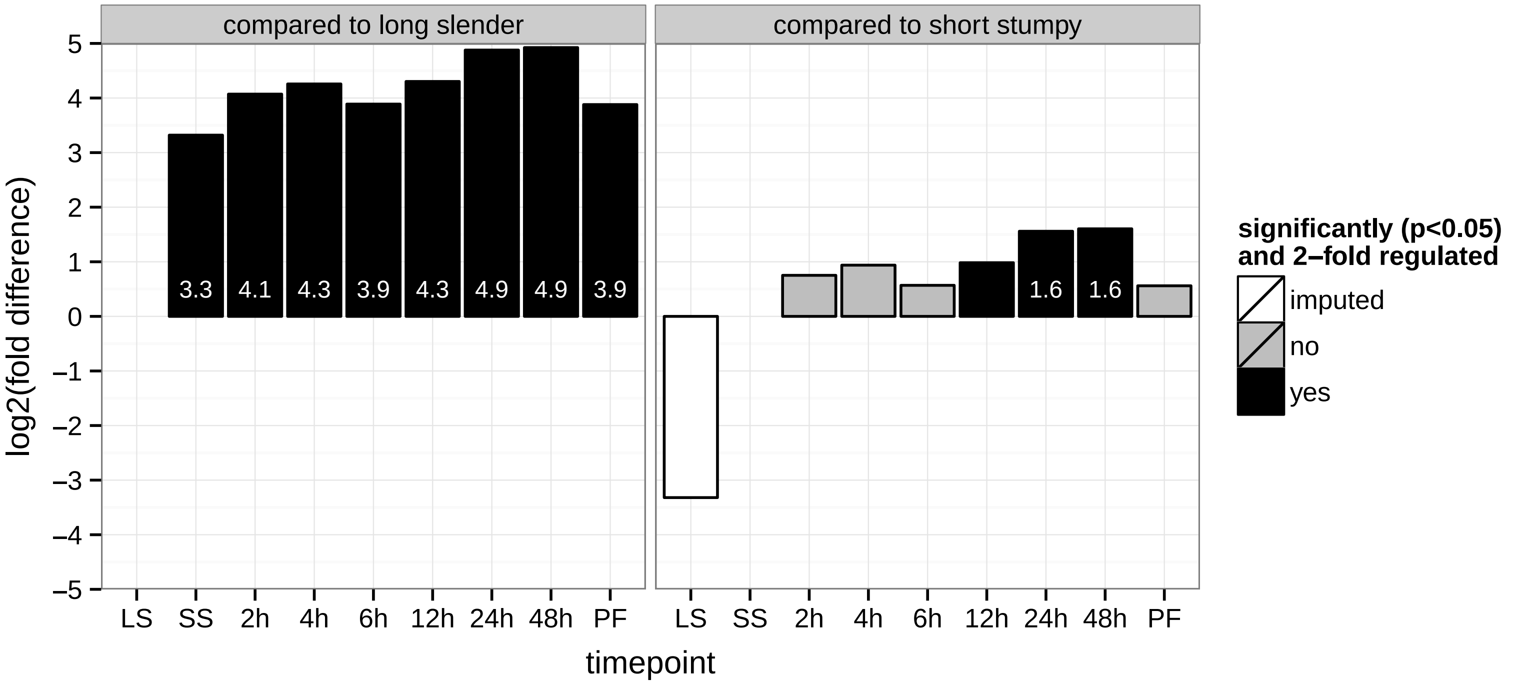

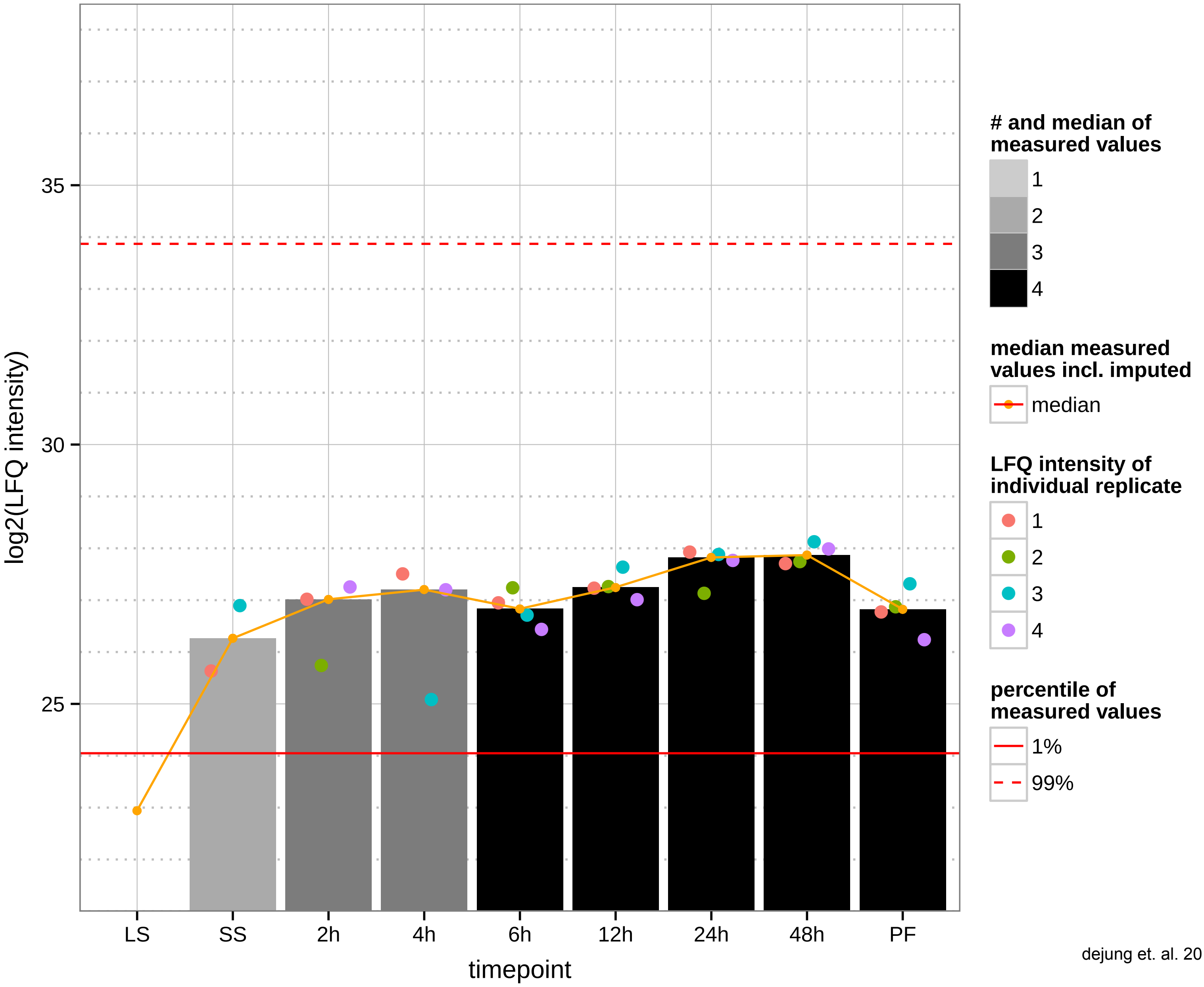

dejung et. al. 2015

hypothetical protein, conserved
Tb927.2.3160
AGOF: null
AGOC: null
AGOP: null
PGOF: snoRNA binding
PGOC: null
PGOP: ribosome biogenesis, snRNA pseudouridine synthesis

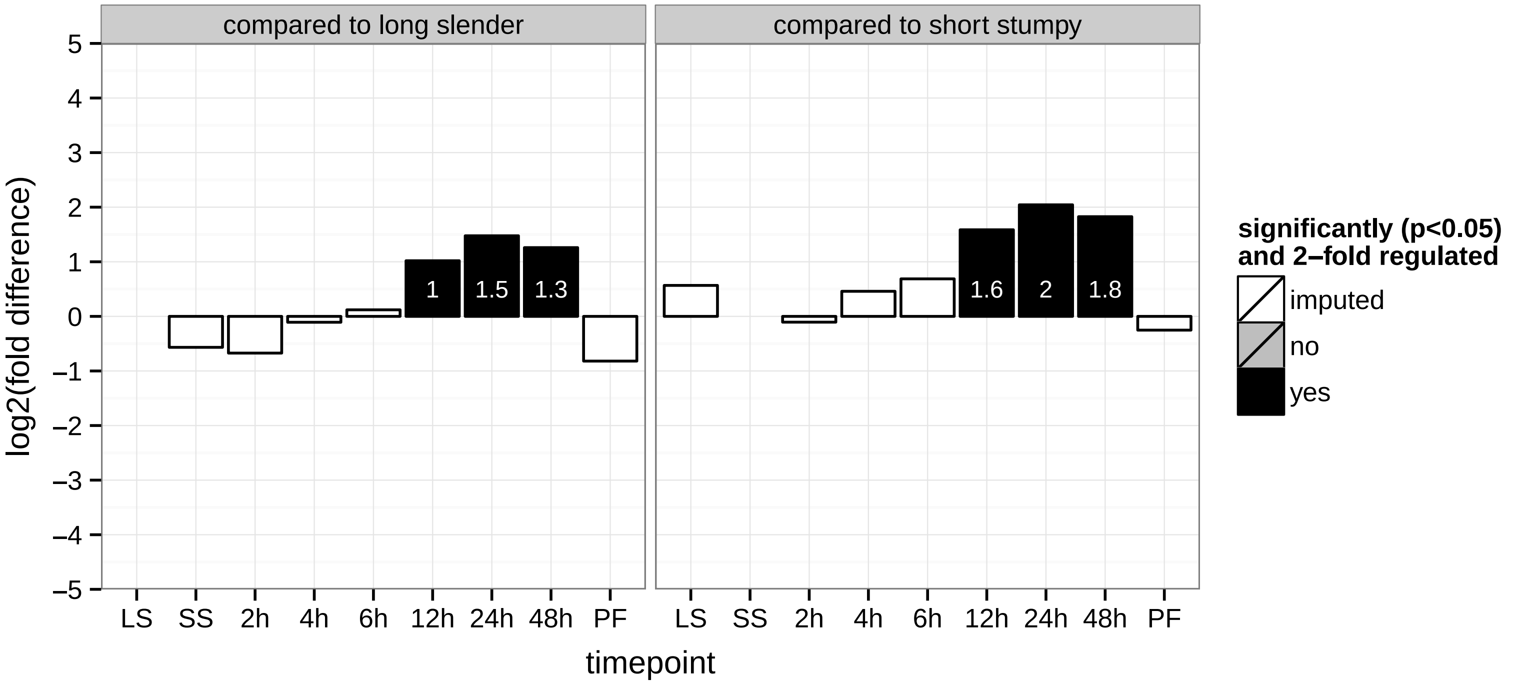

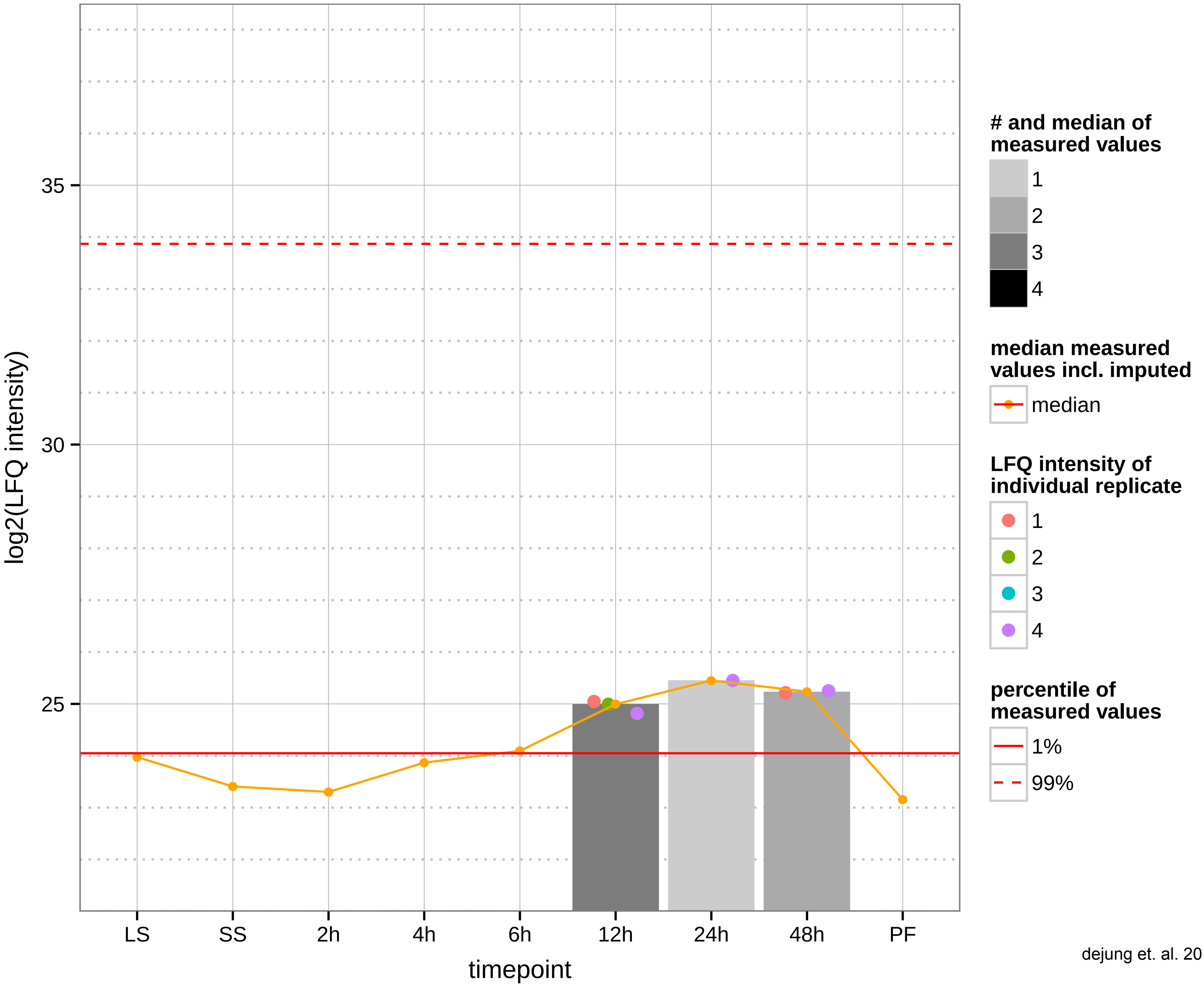

dejung et. al. 2015

aldo–keto reductase, putative
Tb927.2.5180
AGOF: alditol:NADP+ 1–oxidoreductase activity
AGOC: mitochondrion
AGOP: oxidation–reduction process
PGOF: oxidoreductase activity
PGOC: null
PGOP: oxidation–reduction process

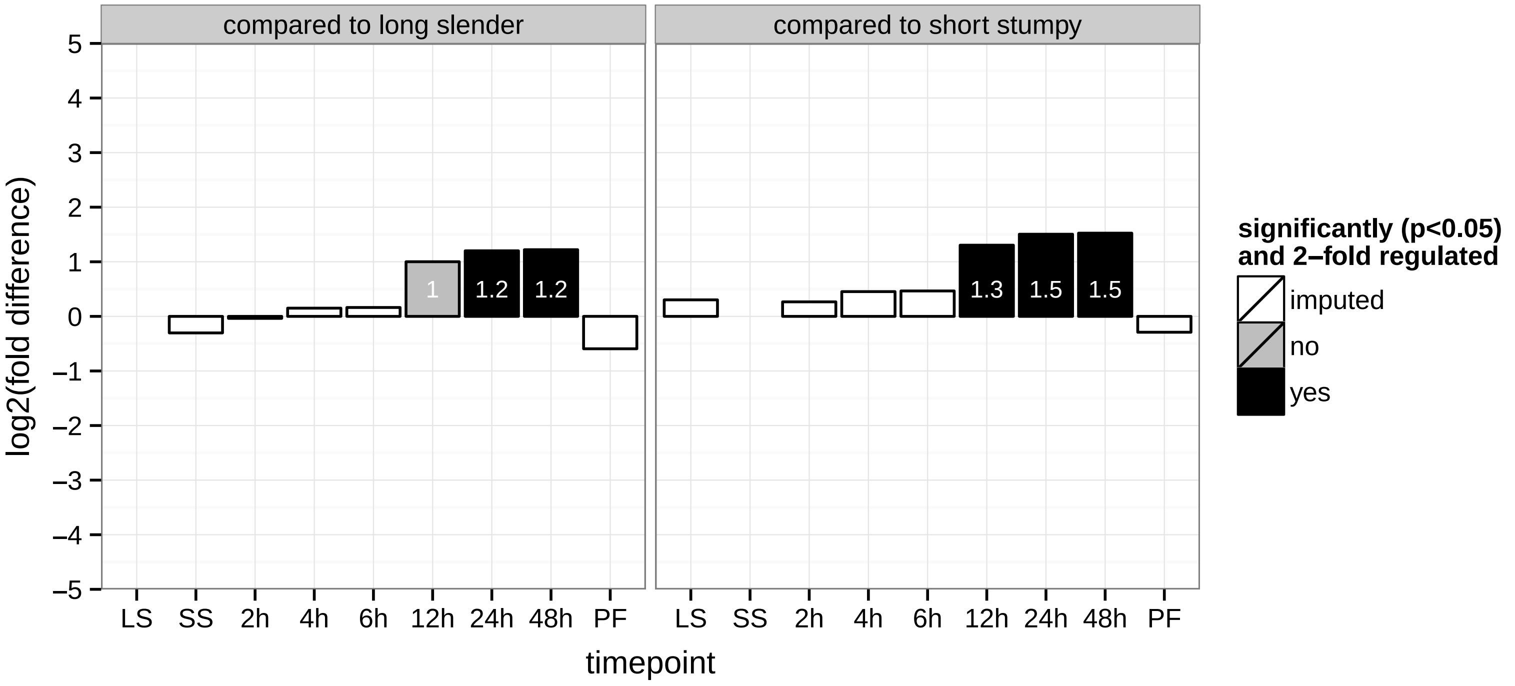

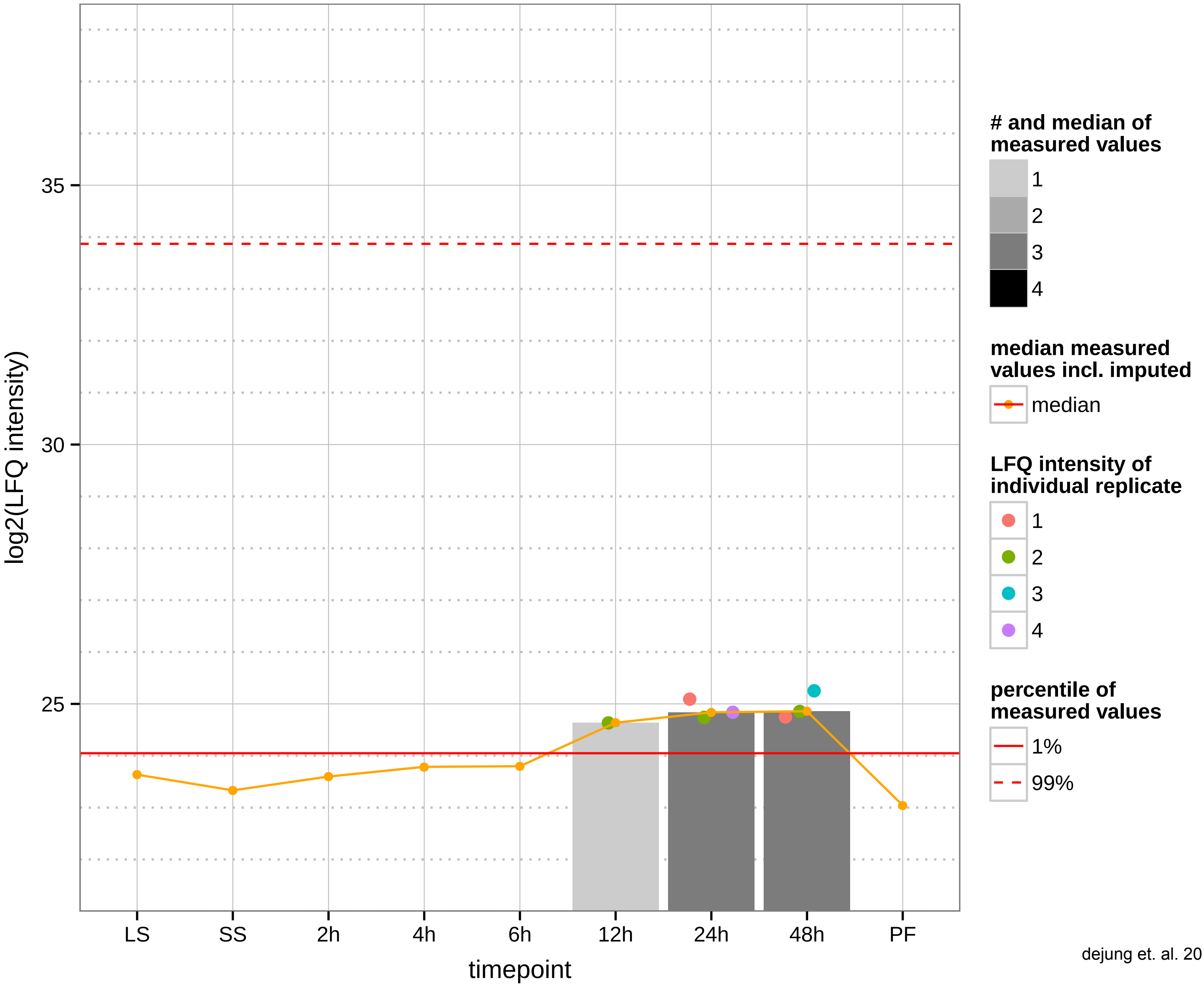

dejung et. al. 2015

hypothetical protein, conserved
Tb927.3.2010
AGOF: null
AGOC: mitochondrion
AGOP: null
PGOF: null
PGOC: null
PGOP: null

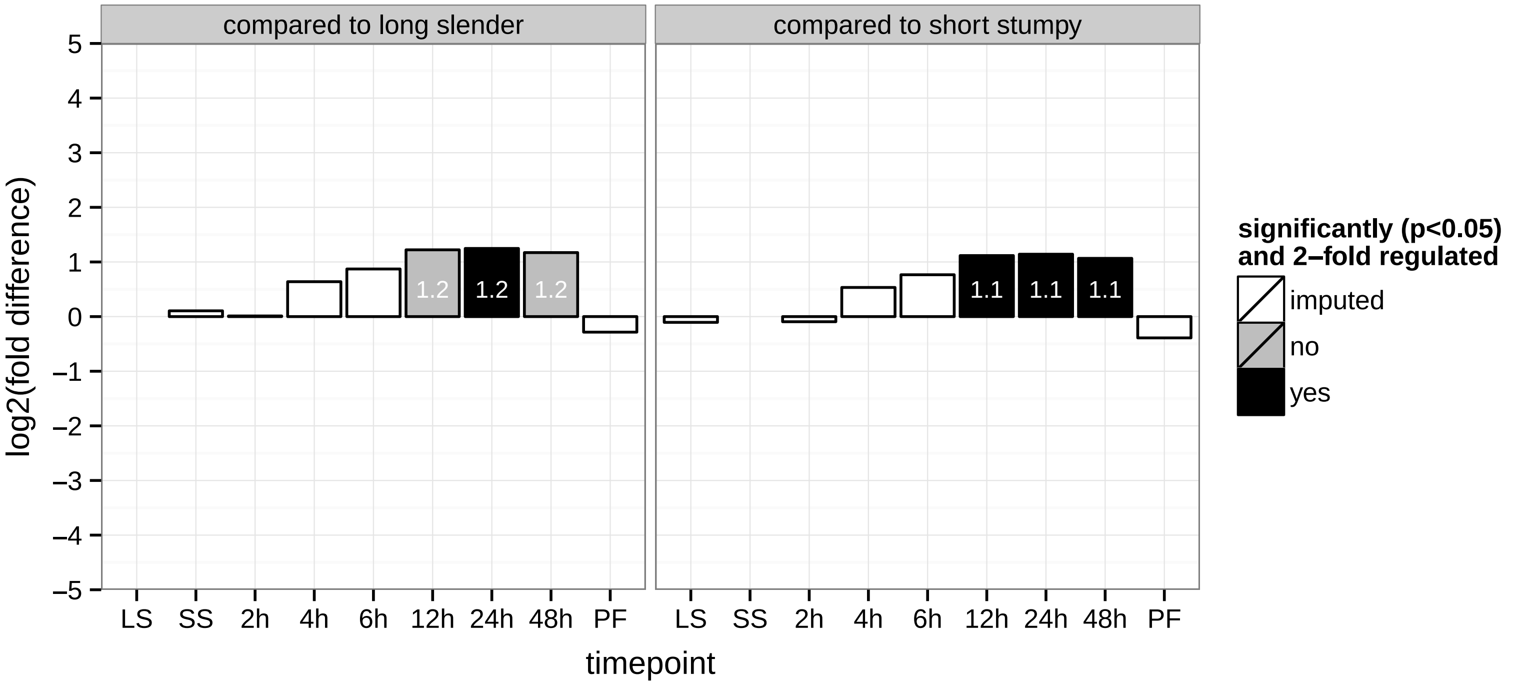

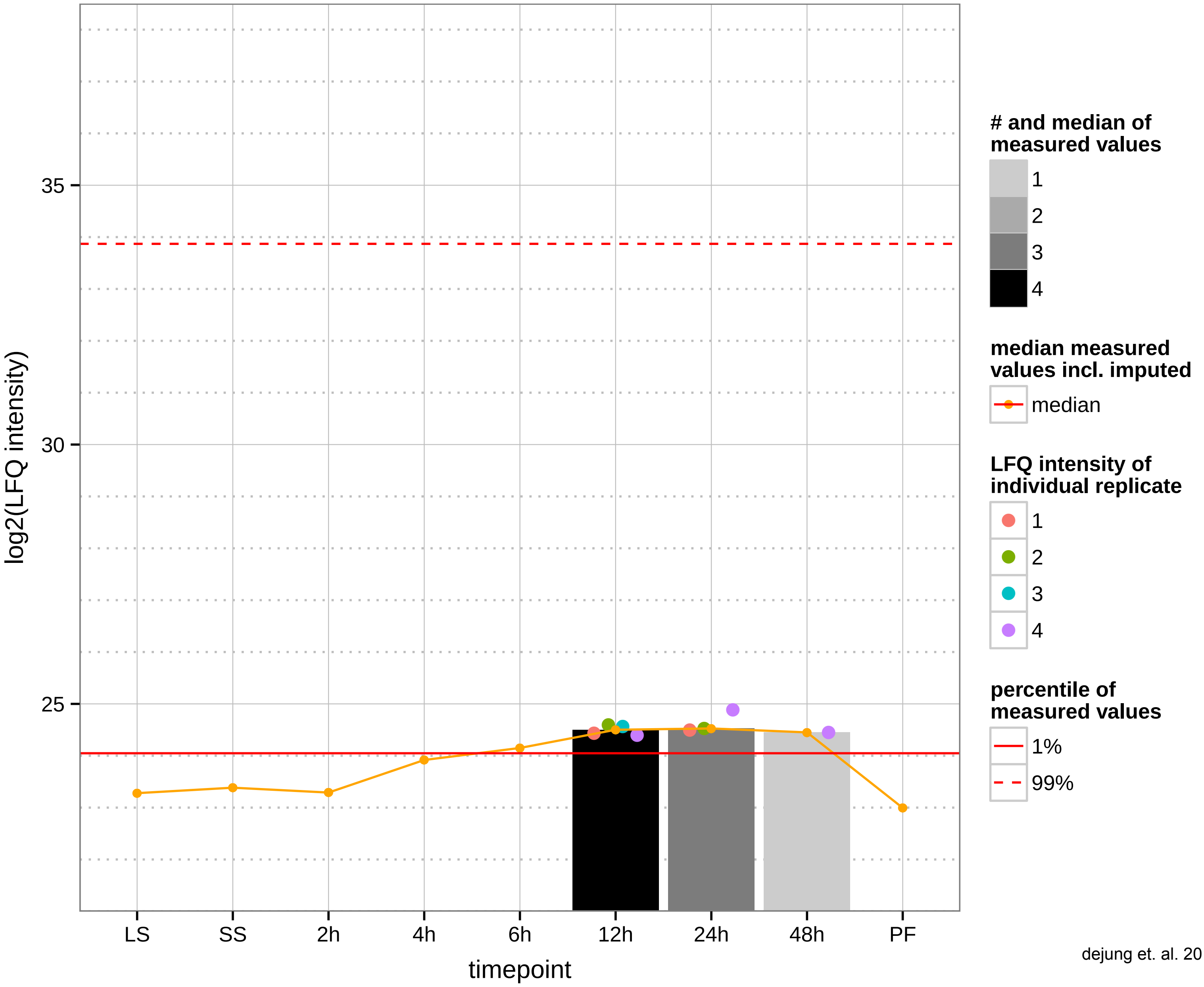

dejung et. al. 2015

chaperone protein DNAj, putative
Tb927.3.2290
AGOF: heat shock protein binding, unfolded protein binding
AGOC: null
AGOP: protein folding
PGOF: heat shock protein binding
PGOC: null
PGOP: null

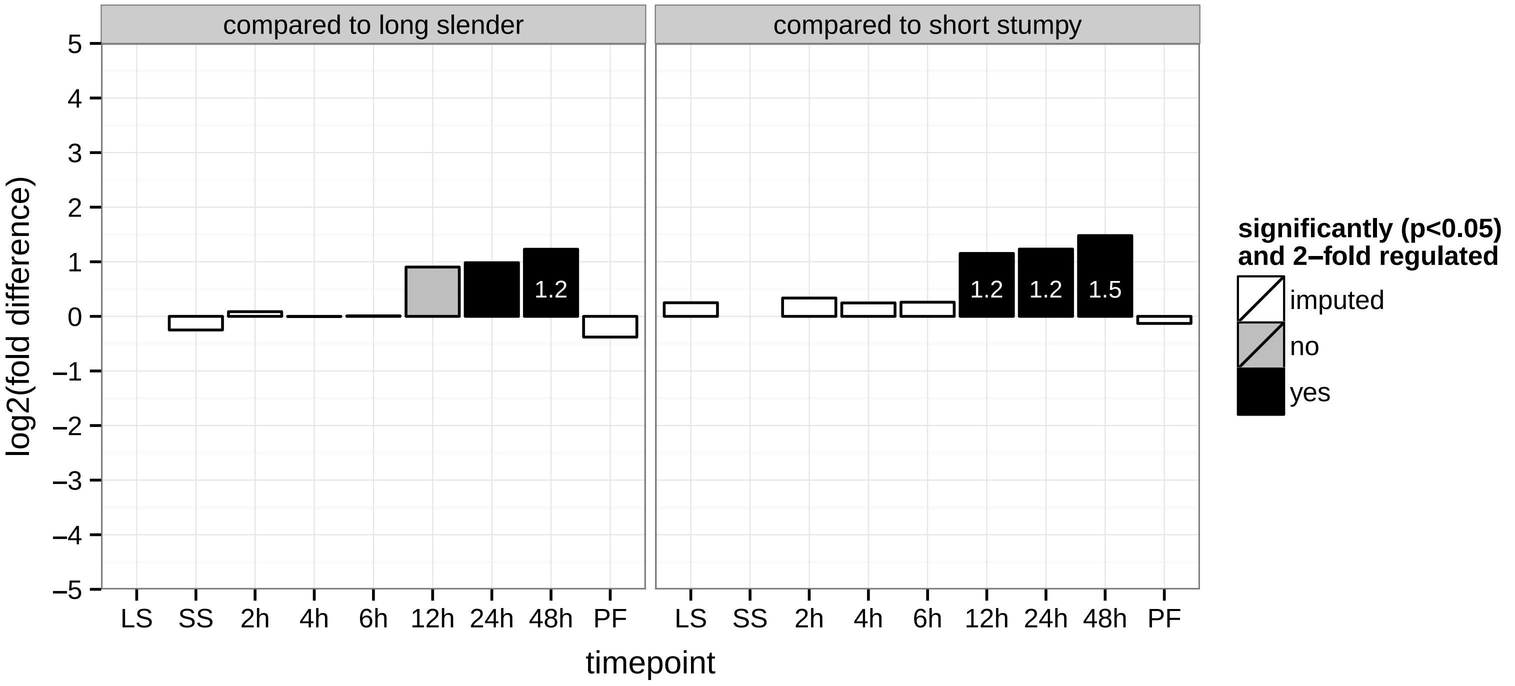

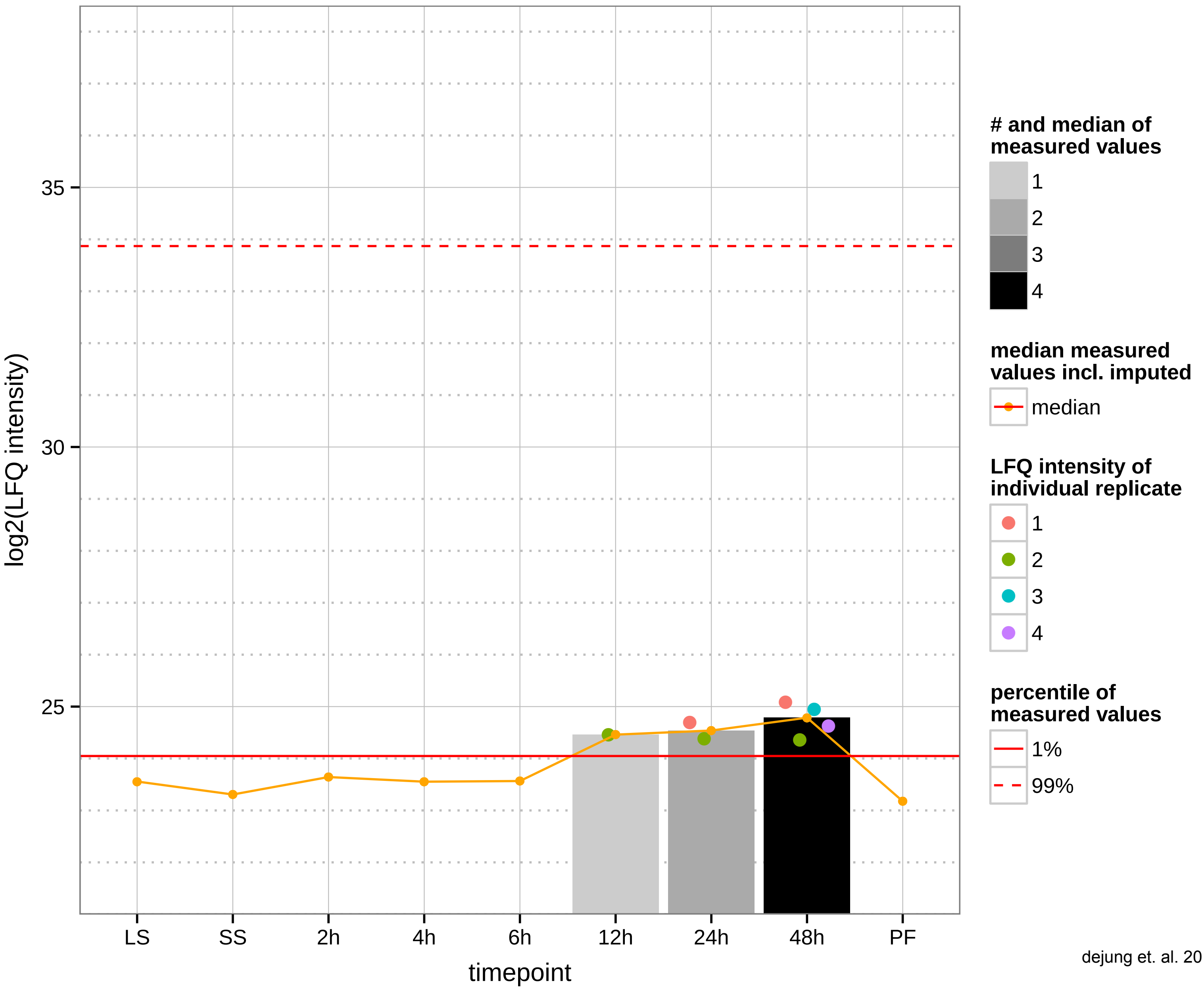

dejung et. al. 2015

hypothetical protein, conserved
Tb927.3.3950
AGOF: null
AGOC: null
AGOP: null
PGOF: null
PGOC: null
PGOP: null

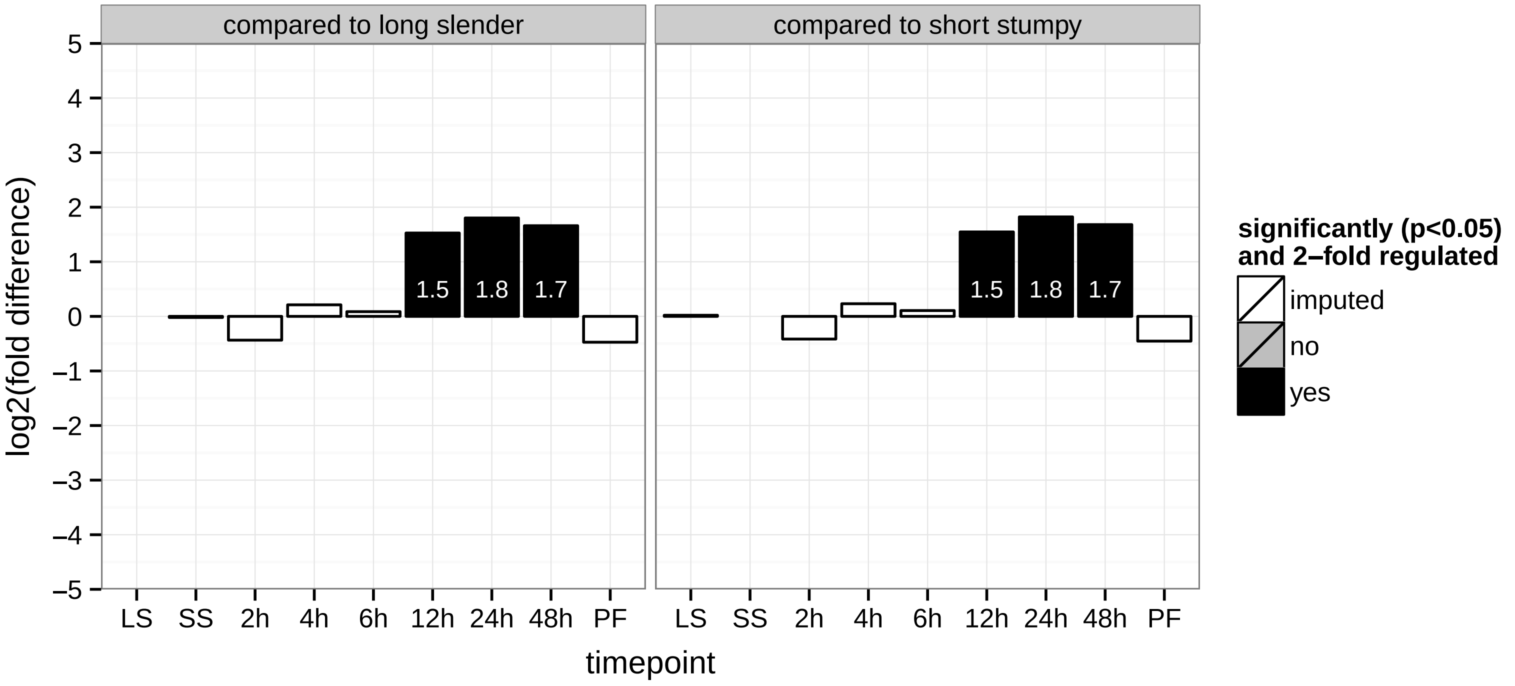

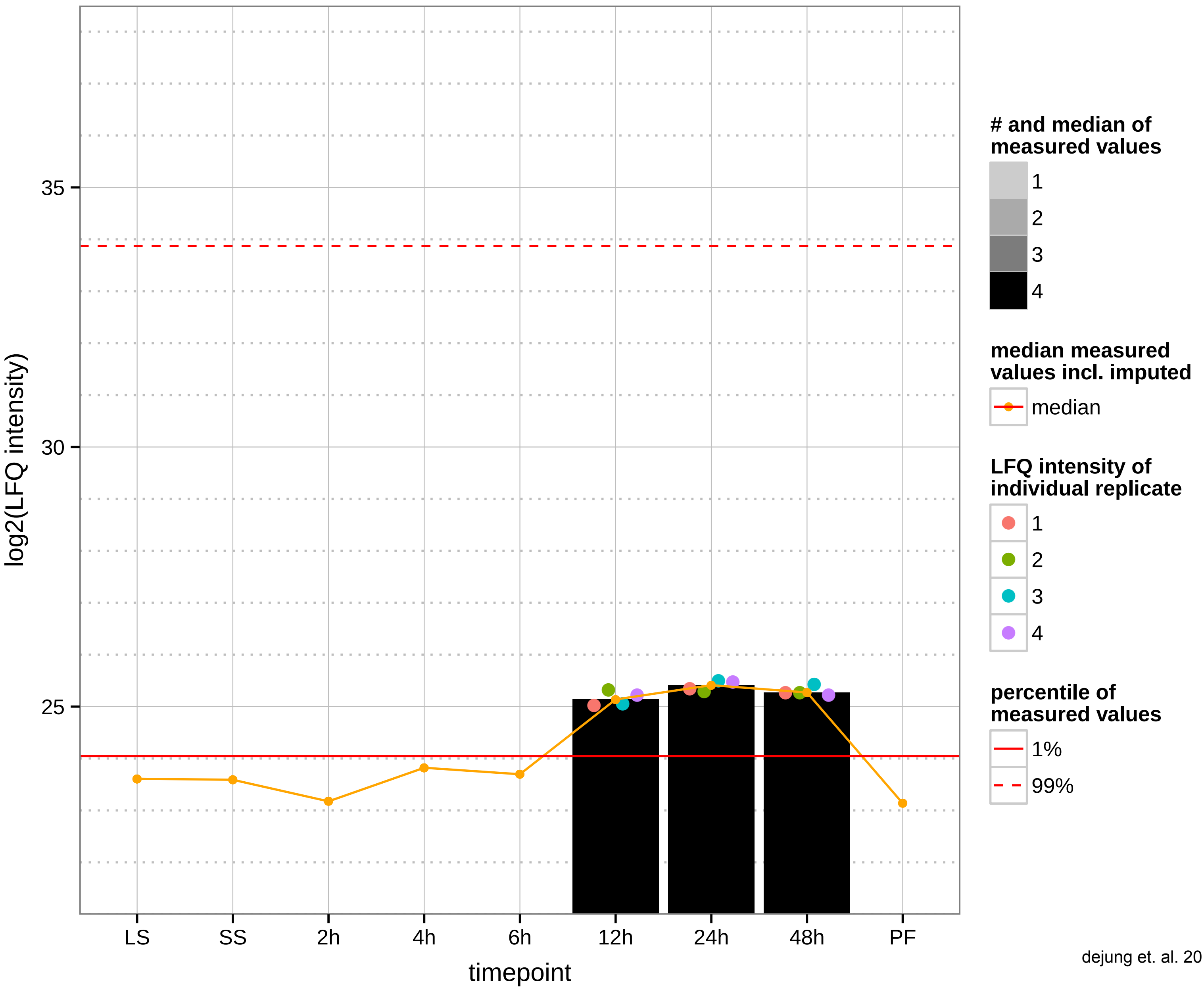

dejung et. al. 2015

hypothetical protein, conserved, leucine−rich repeat protein (LRRP), putative
Tb927.3.4770;Tb927.3.4730
AGOF: null
AGOC: null
AGOP: null
PGOF: protein binding
PGOC: null
PGOP: null

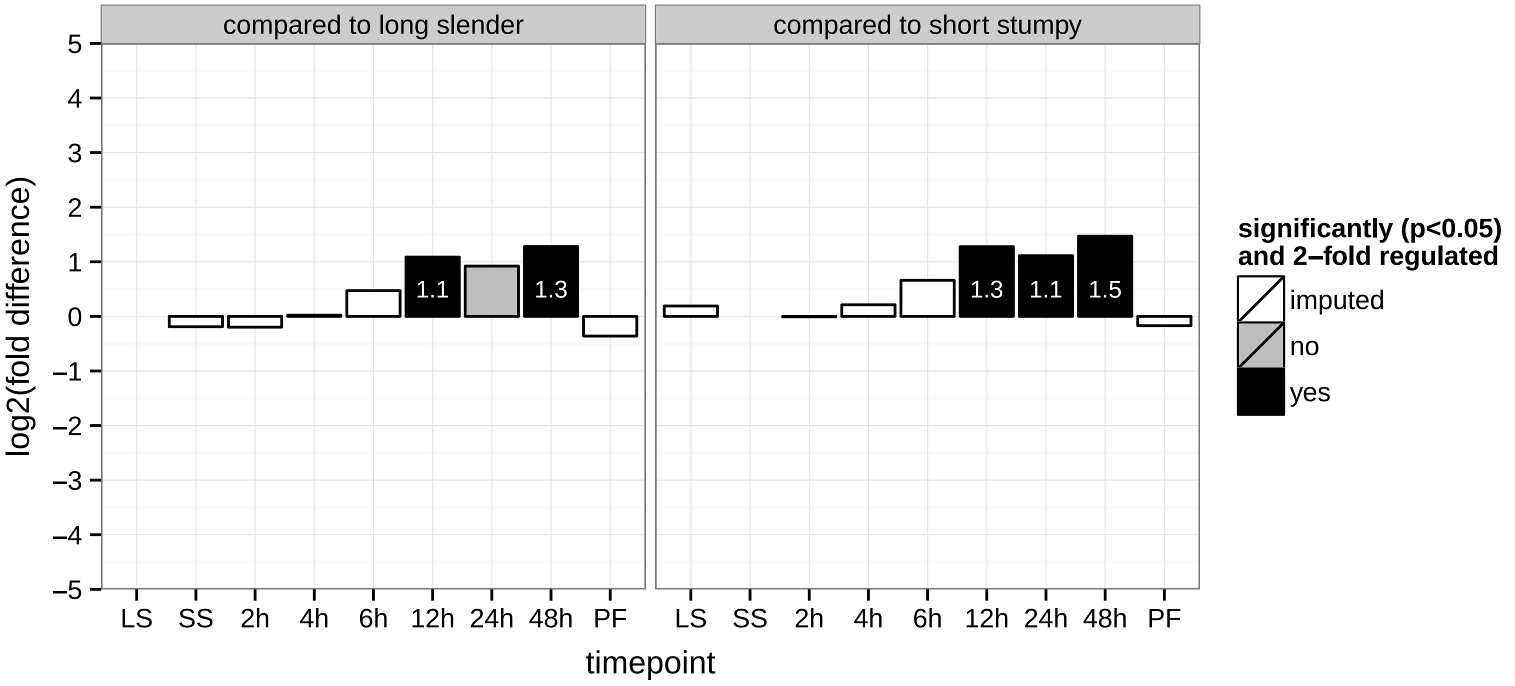

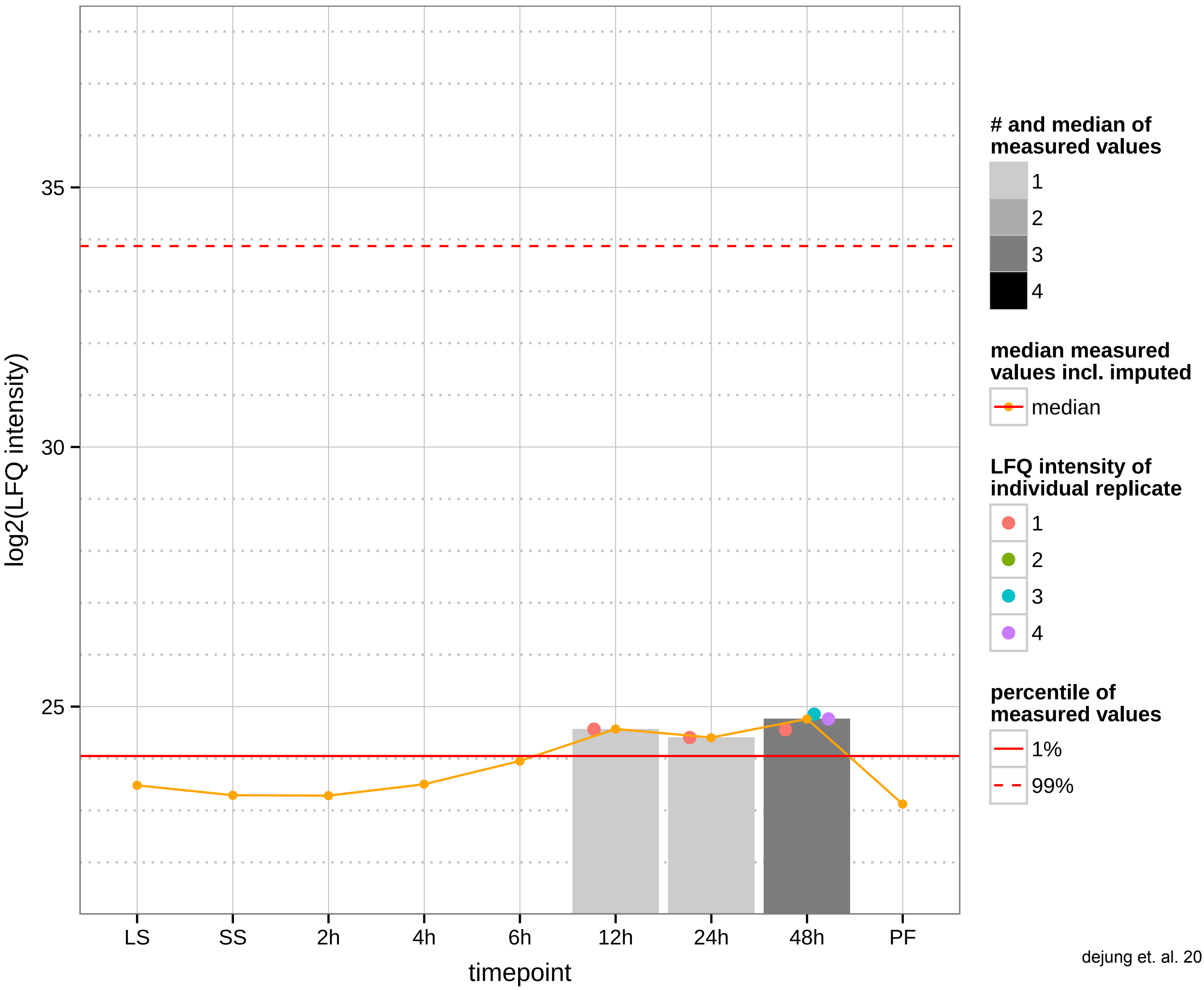

dejung et. al. 2015

hypothetical protein, conserved
Tb927.3.4870
AGOF: null
AGOC: null
AGOP: null
PGOF: null
PGOC: null
PGOP: null

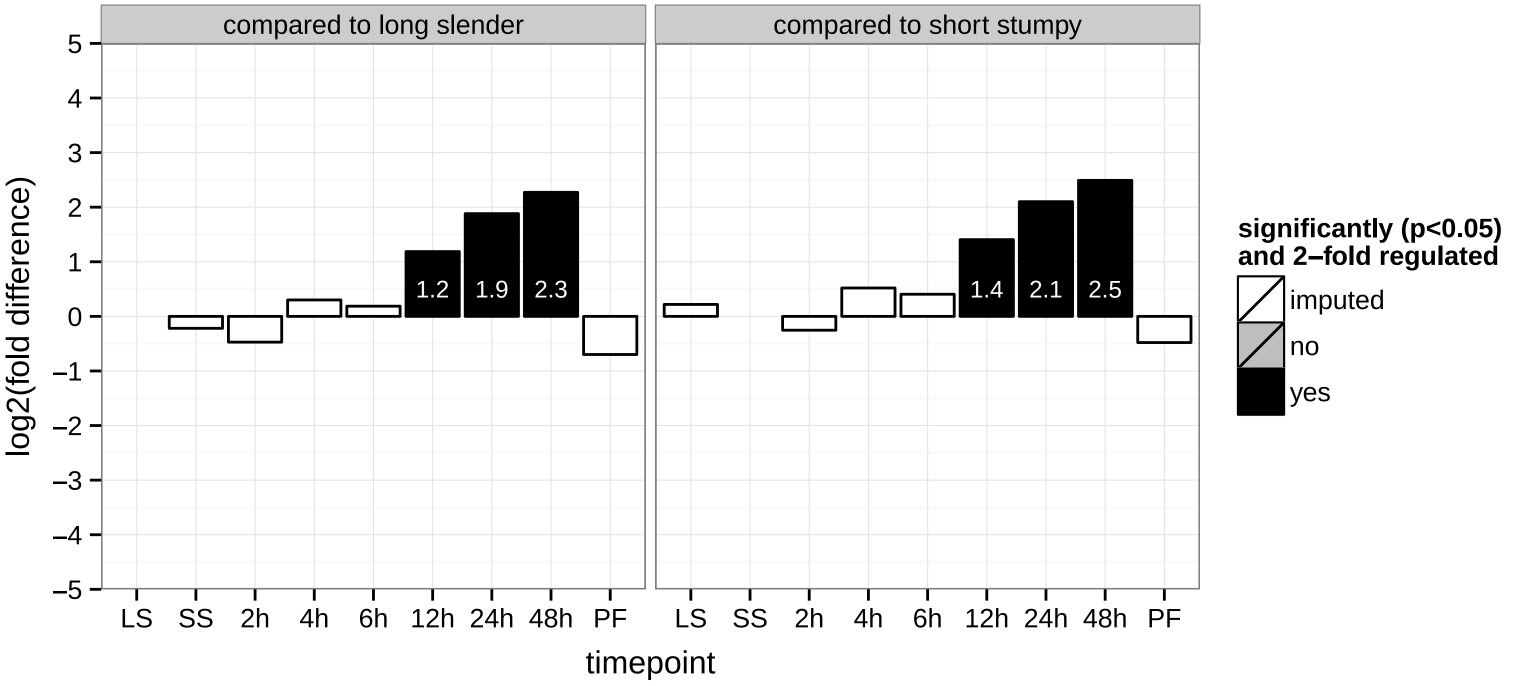

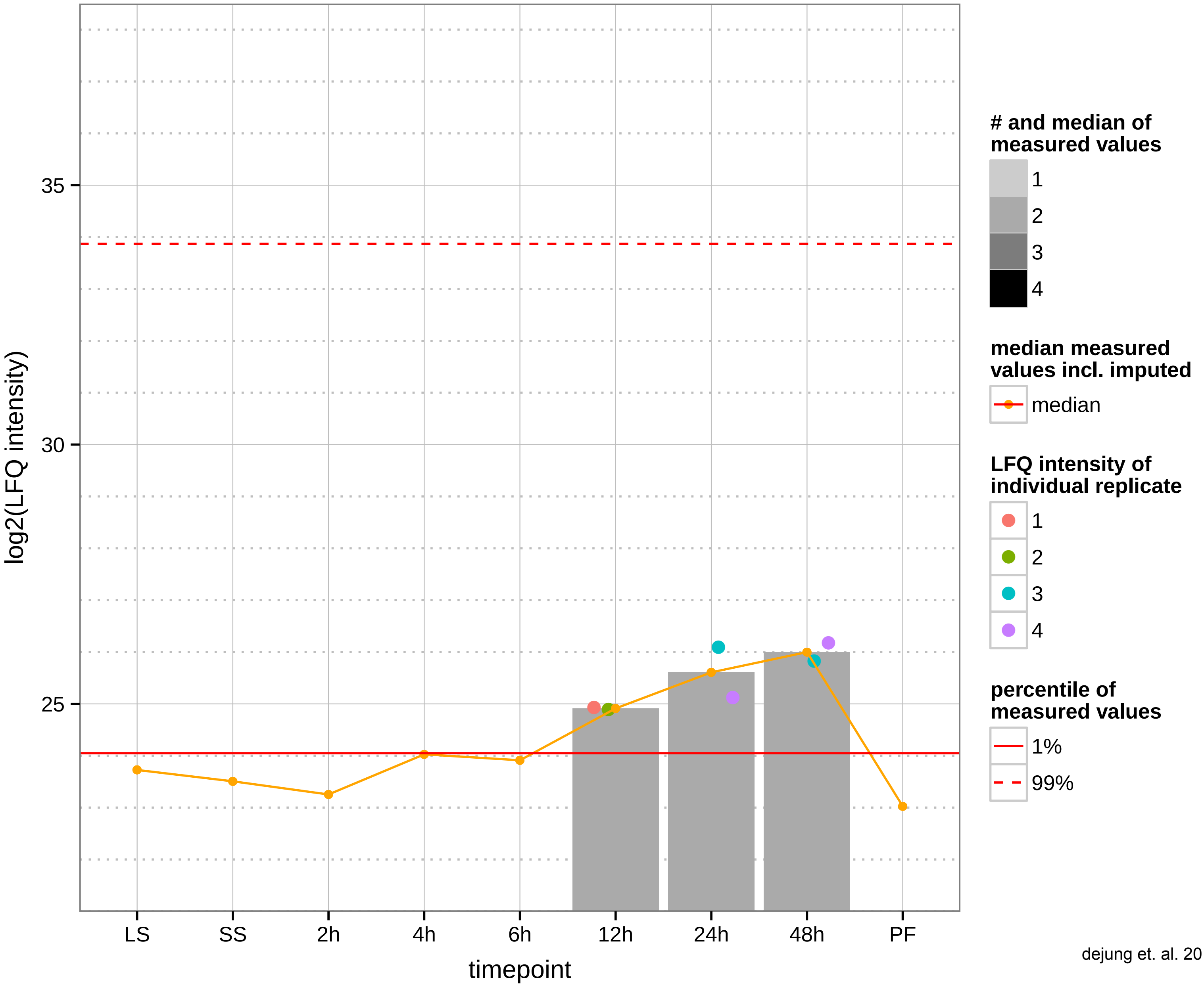

dejung et. al. 2015

hypothetical protein, conserved
Tb927.3.5210
AGOF: null
AGOC: null
AGOP: null
PGOF: null
PGOC: null
PGOP: null

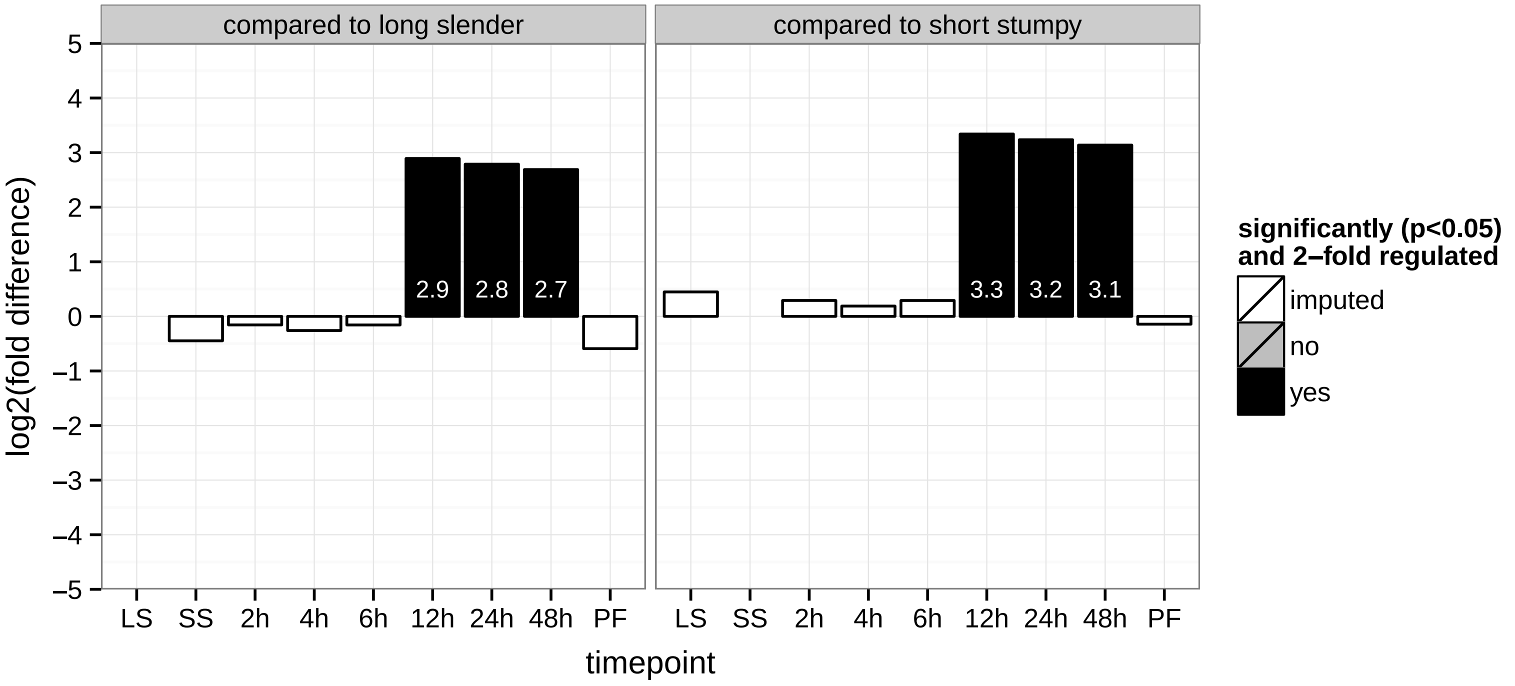

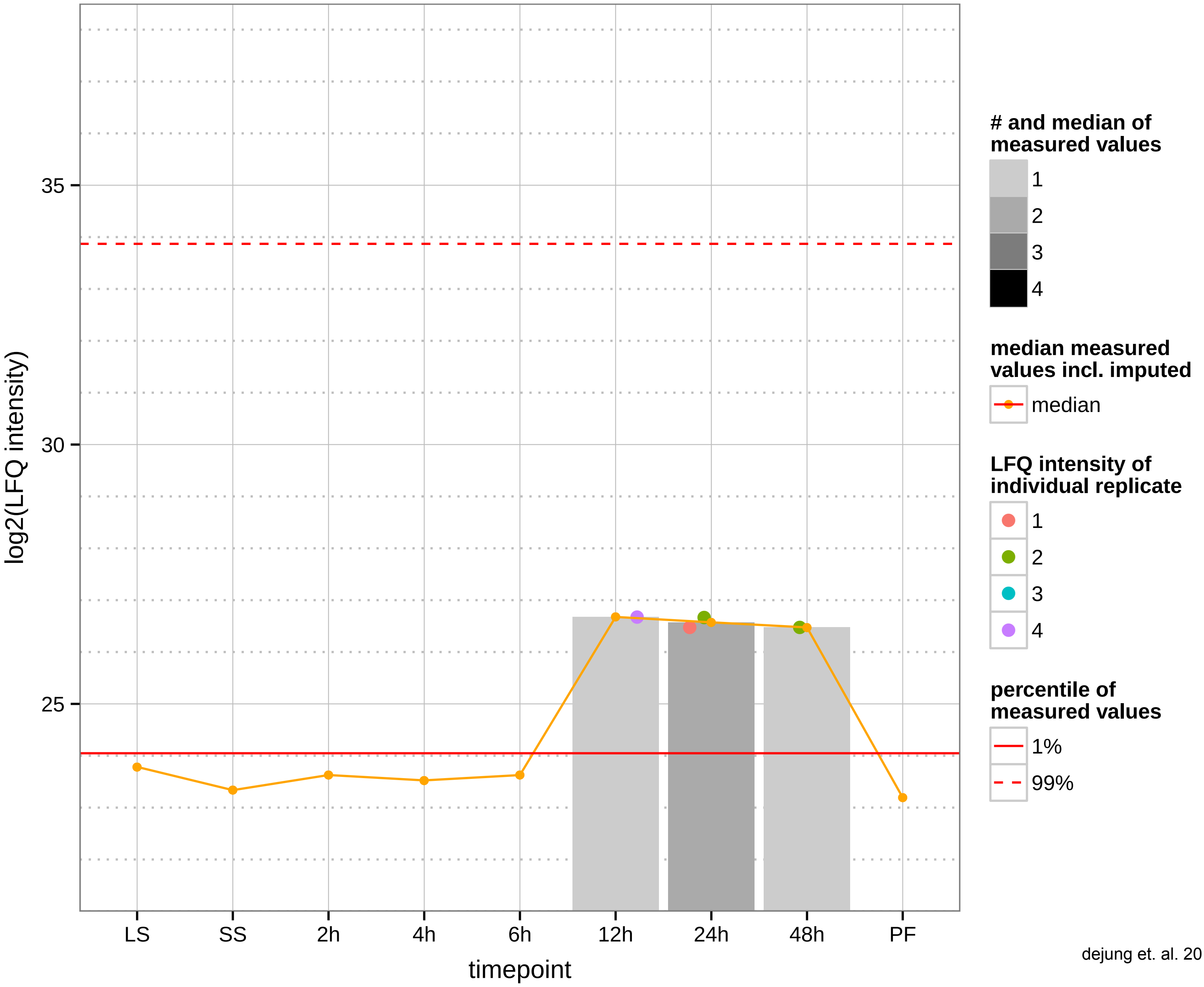

dejung et. al. 2015

adenosine transporter, putative
Tb927.3.590
AGOF: nucleoside transmembrane transporter activity
AGOC: integral to membrane, membrane
AGOP: nucleoside transport
PGOF: nucleoside transmembrane transporter activity
PGOC: integral to membrane
PGOP: transport

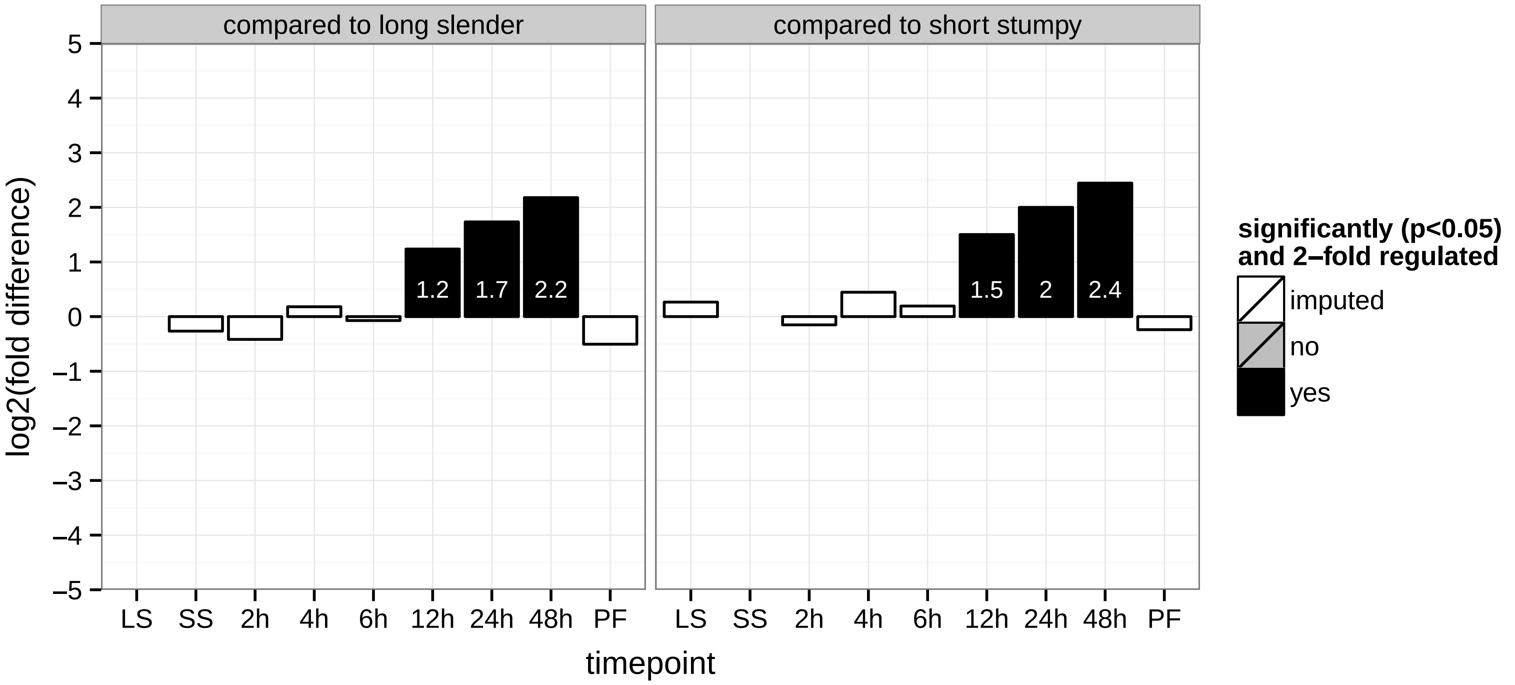

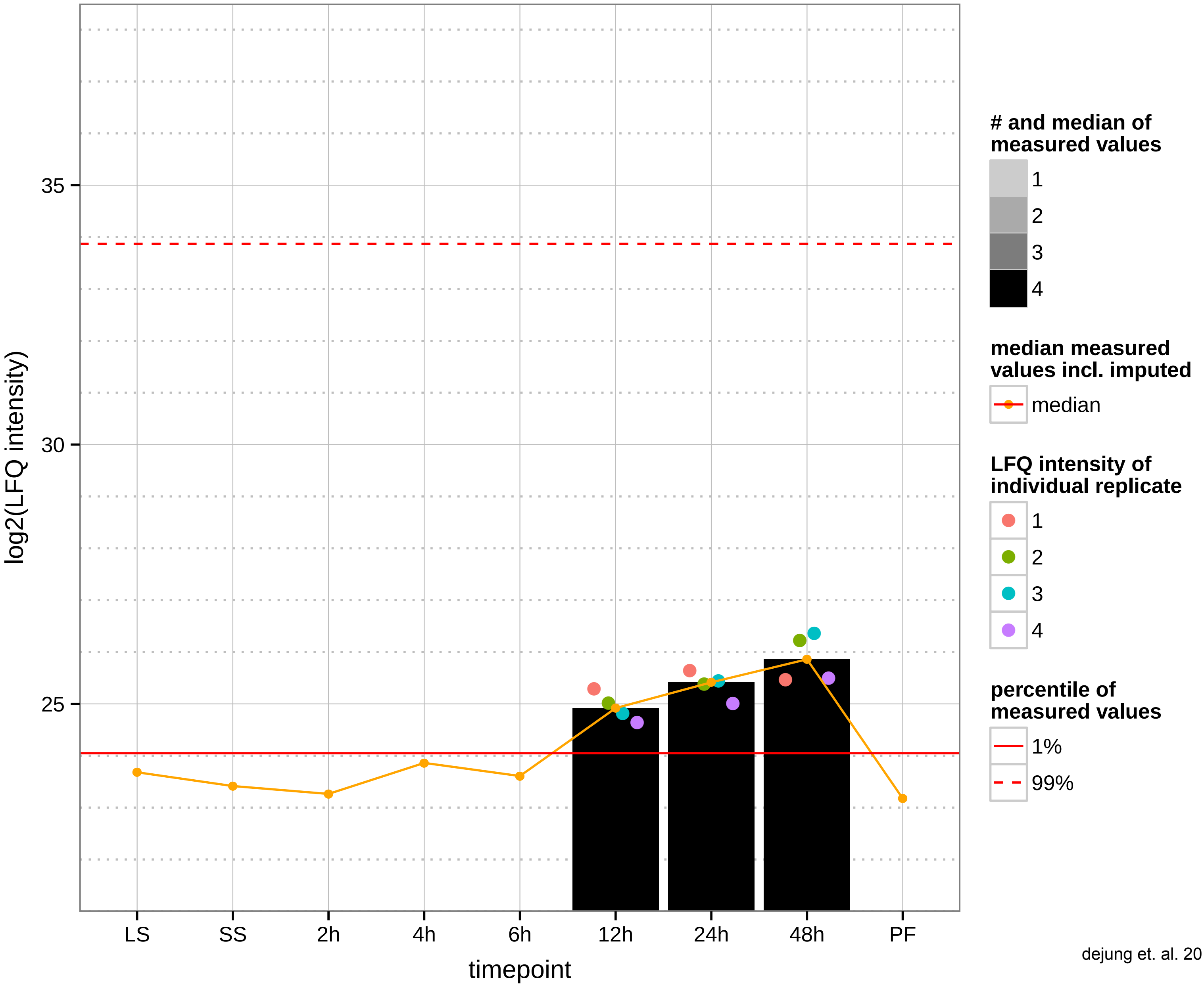

dejung et. al. 2015

hypothetical protein, conserved
Tb927.4.1760
AGOF: null
AGOC: mitochondrion
AGOP: null
PGOF: null
PGOC: null
PGOP: null

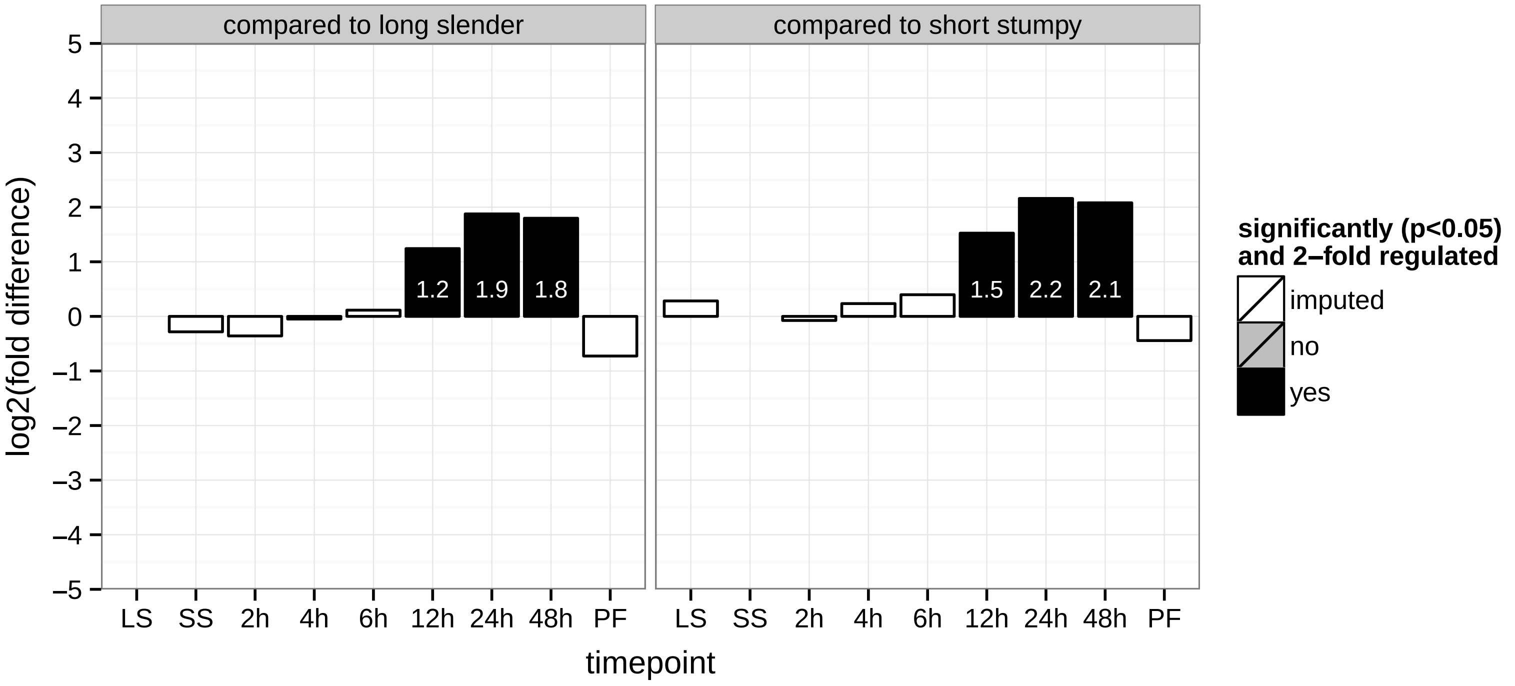

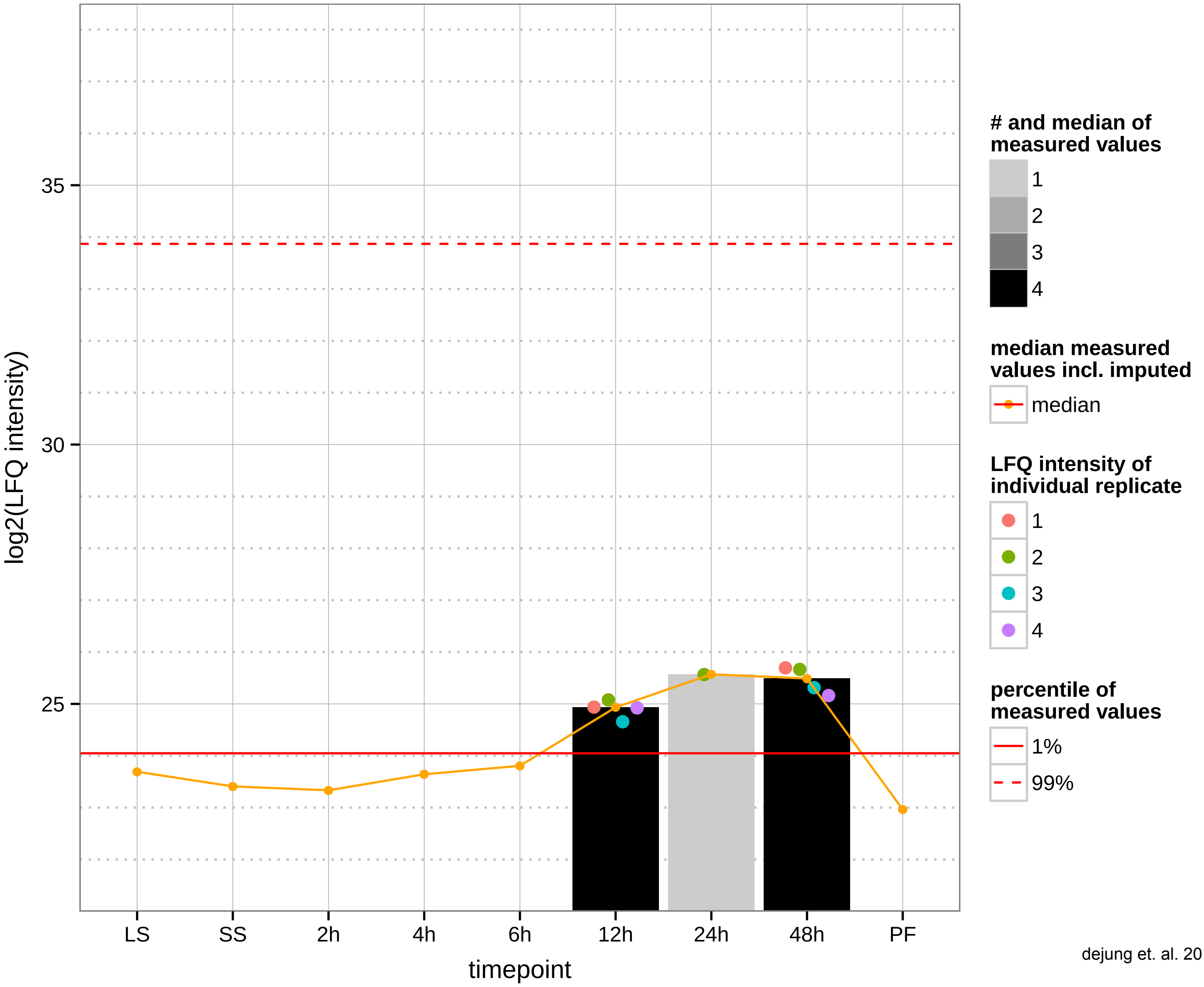

dejung et. al. 2015

hypothetical protein, conserved
Tb927.4.4110
AGOF: null
AGOC: null
AGOP: null
PGOF: null
PGOC: null
PGOP: null

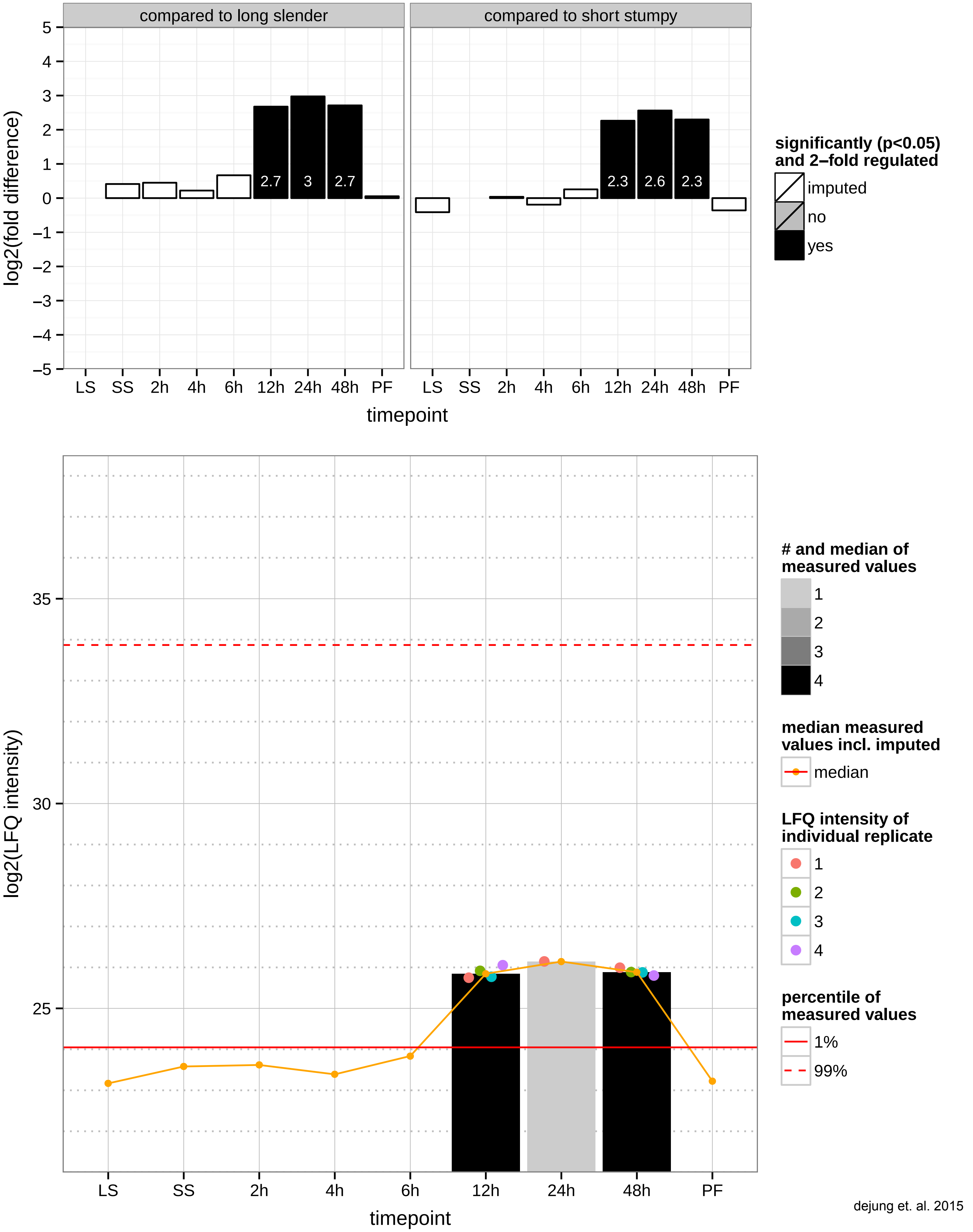

dejung et. al. 2015

hypothetical protein, conserved
Tb927.4.940
AGOF: null
AGOC: mitochondrion
AGOP: null
PGOF: null
PGOC: null
PGOP: null

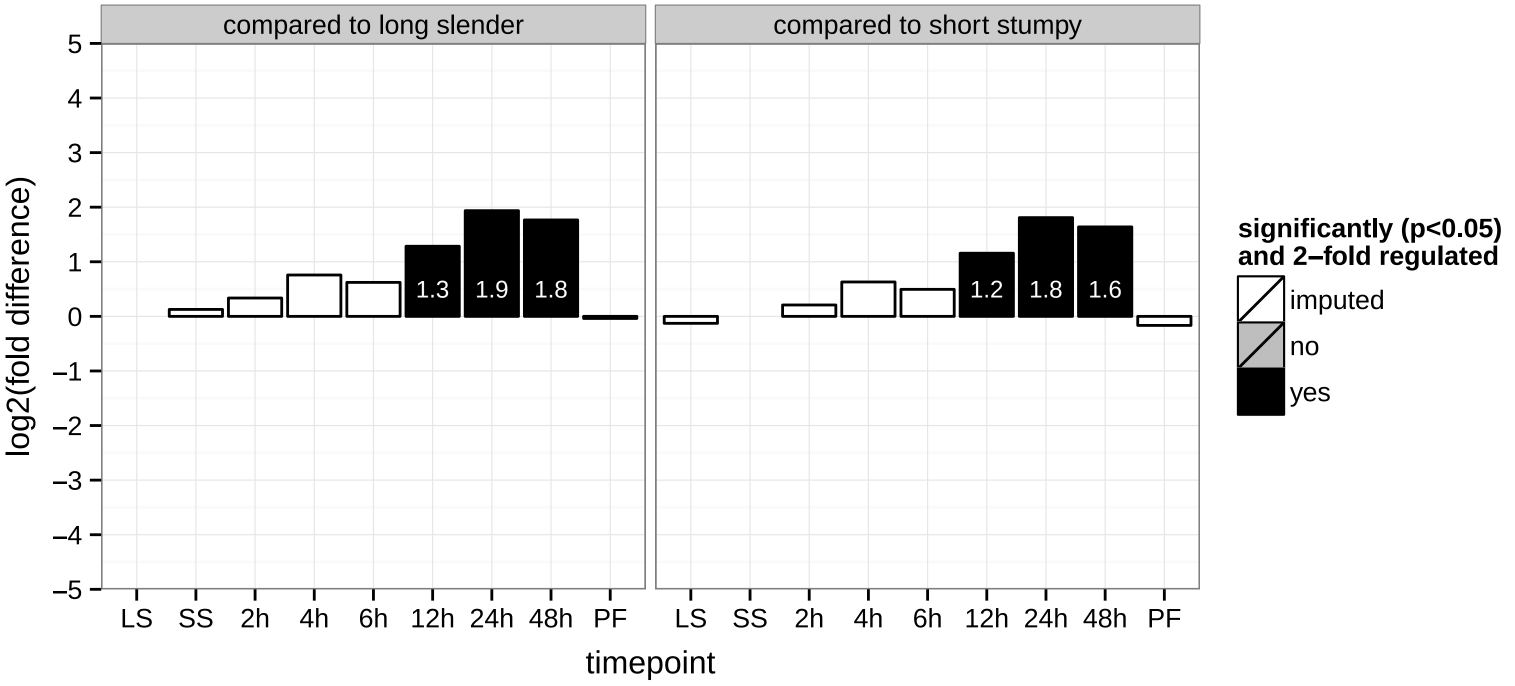

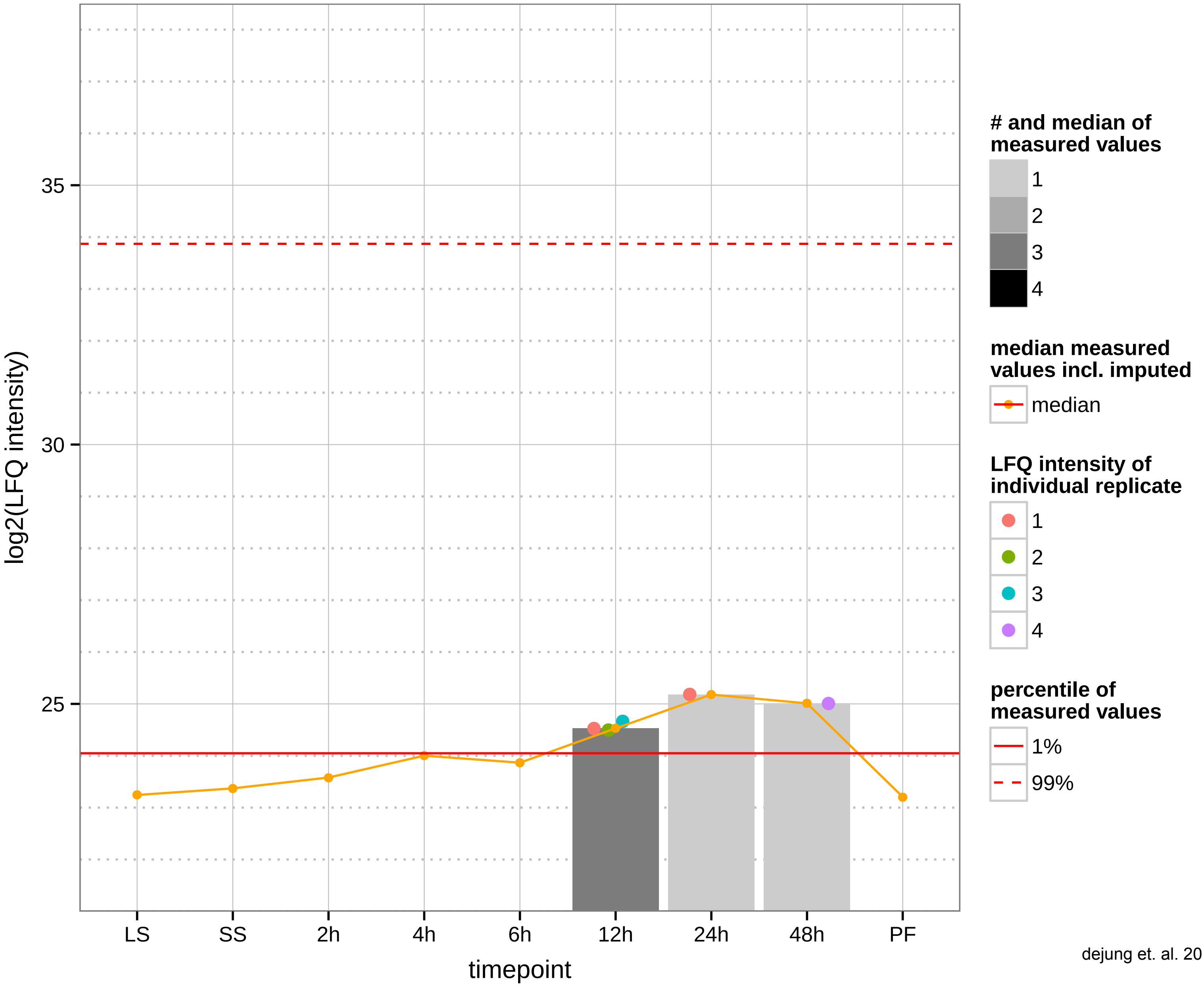

dejung et. al. 2015

hypothetical protein, conserved
Tb927.5.2240;Tb927.5.2210;Tb927.5.2180
AGOF: null
AGOC: null
AGOP: null
PGOF: null
PGOC: null
PGOP: null

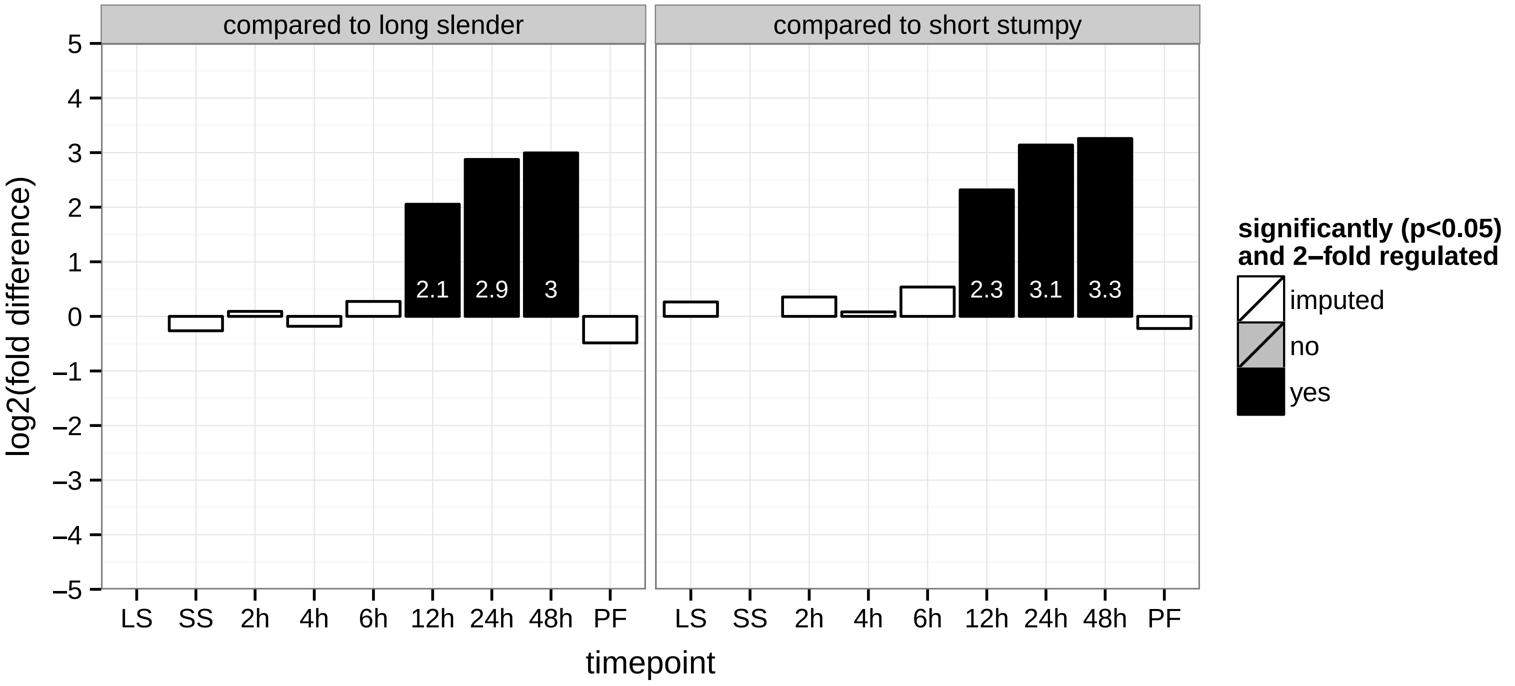

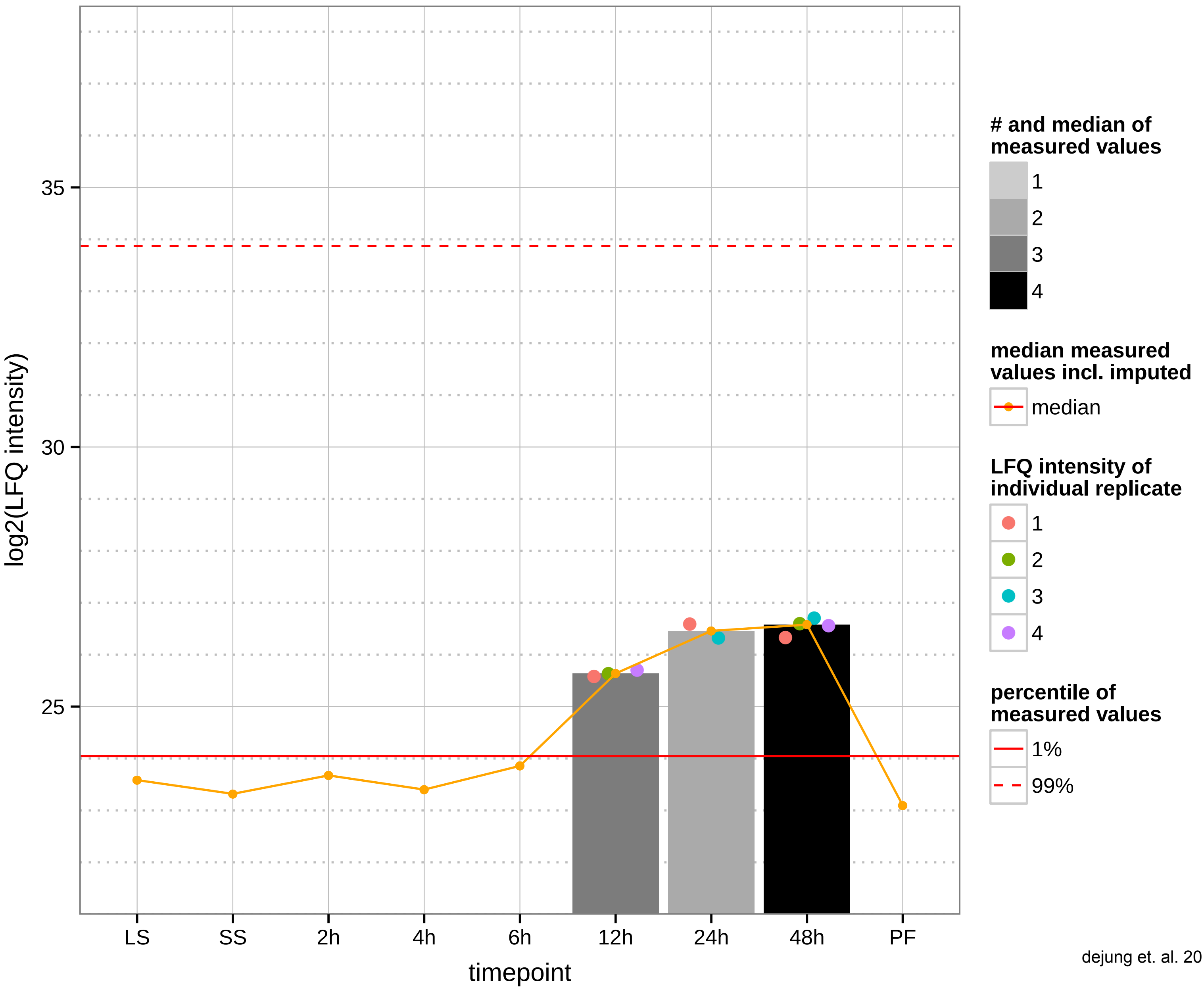

dejung et. al. 2015

hypothetical protein, conserved
Tb927.5.680
AGOF: null
AGOC: mitochondrion
AGOP: null
PGOF: null
PGOC: null
PGOP: null

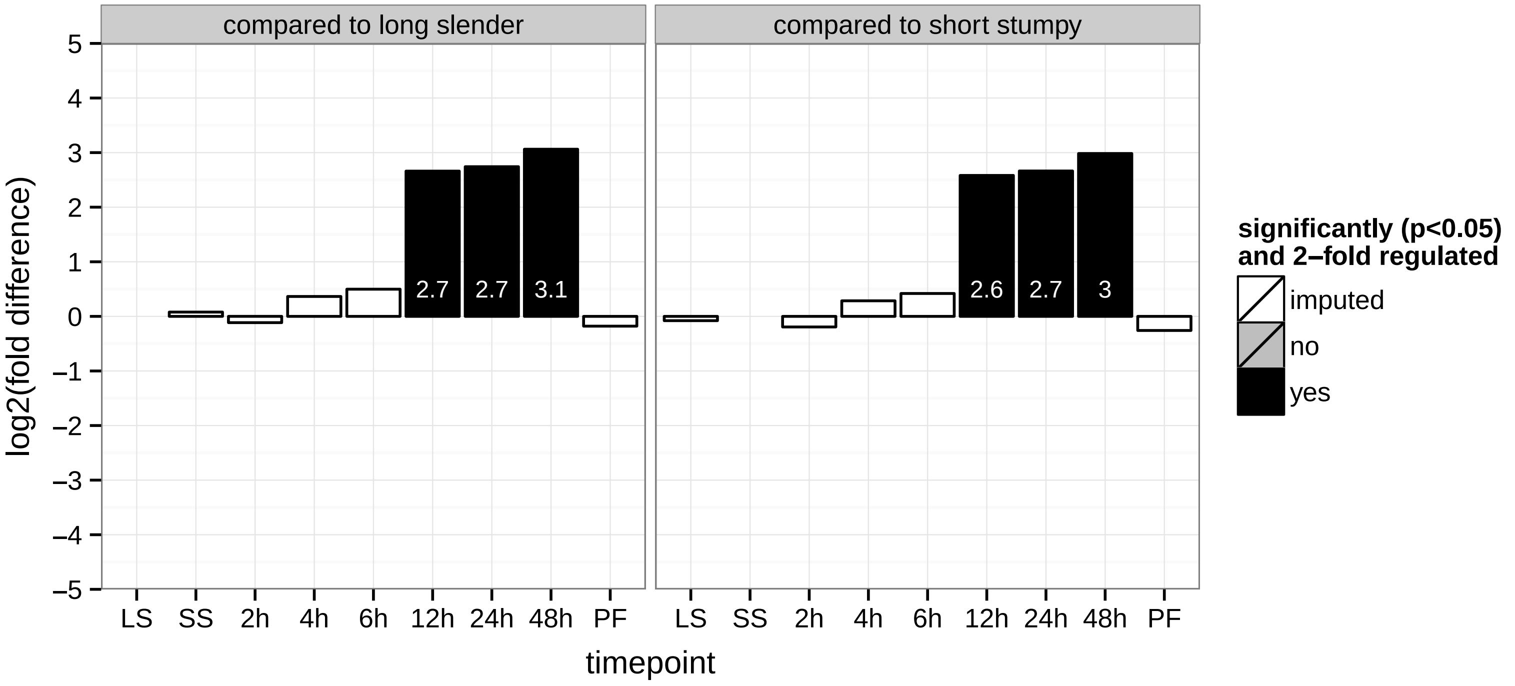

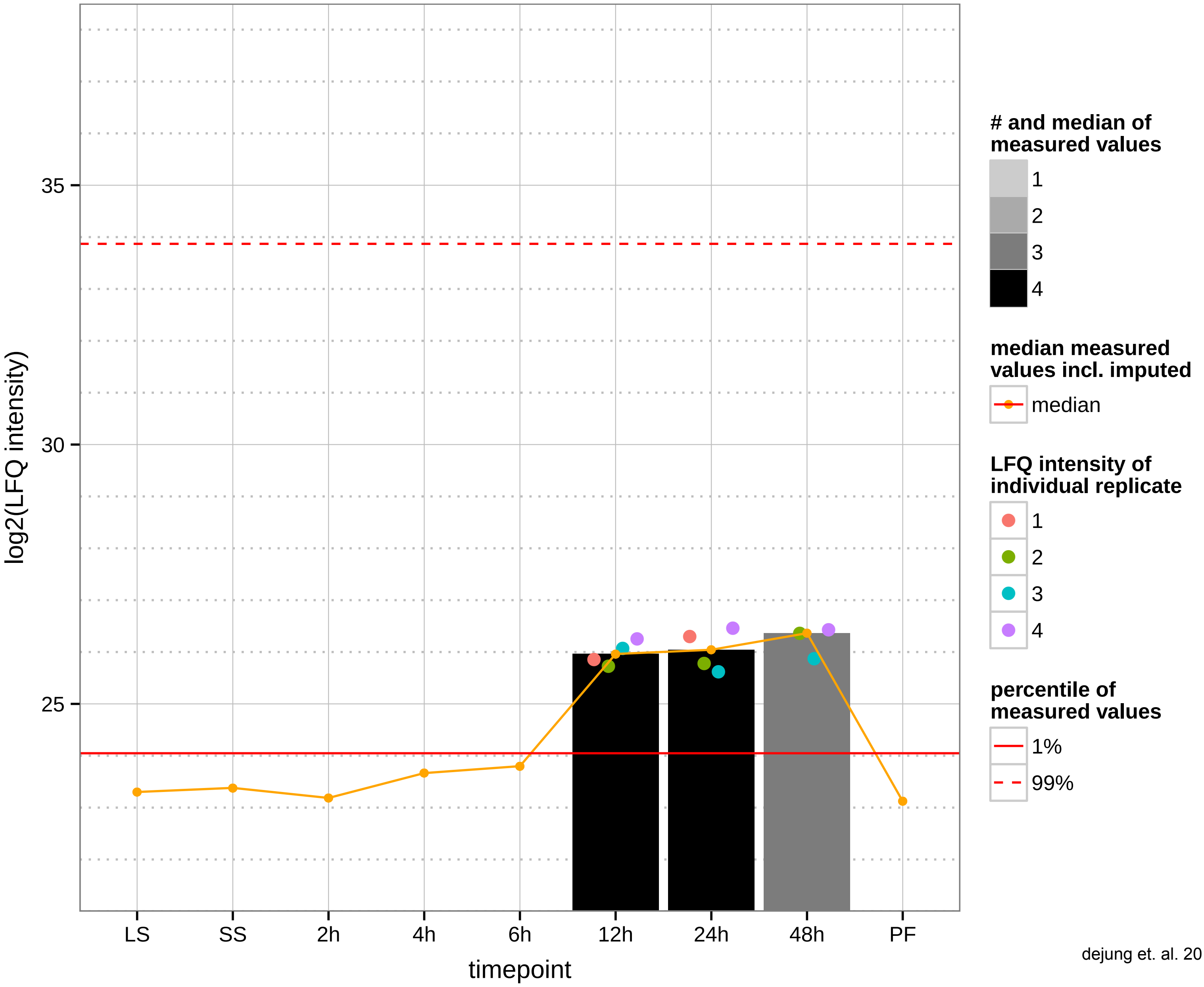

dejung et. al. 2015

hypothetical protein, conserved
Tb927.6.1560
AGOF: null
AGOC: null
AGOP: null
PGOF: null
PGOC: null
PGOP: null

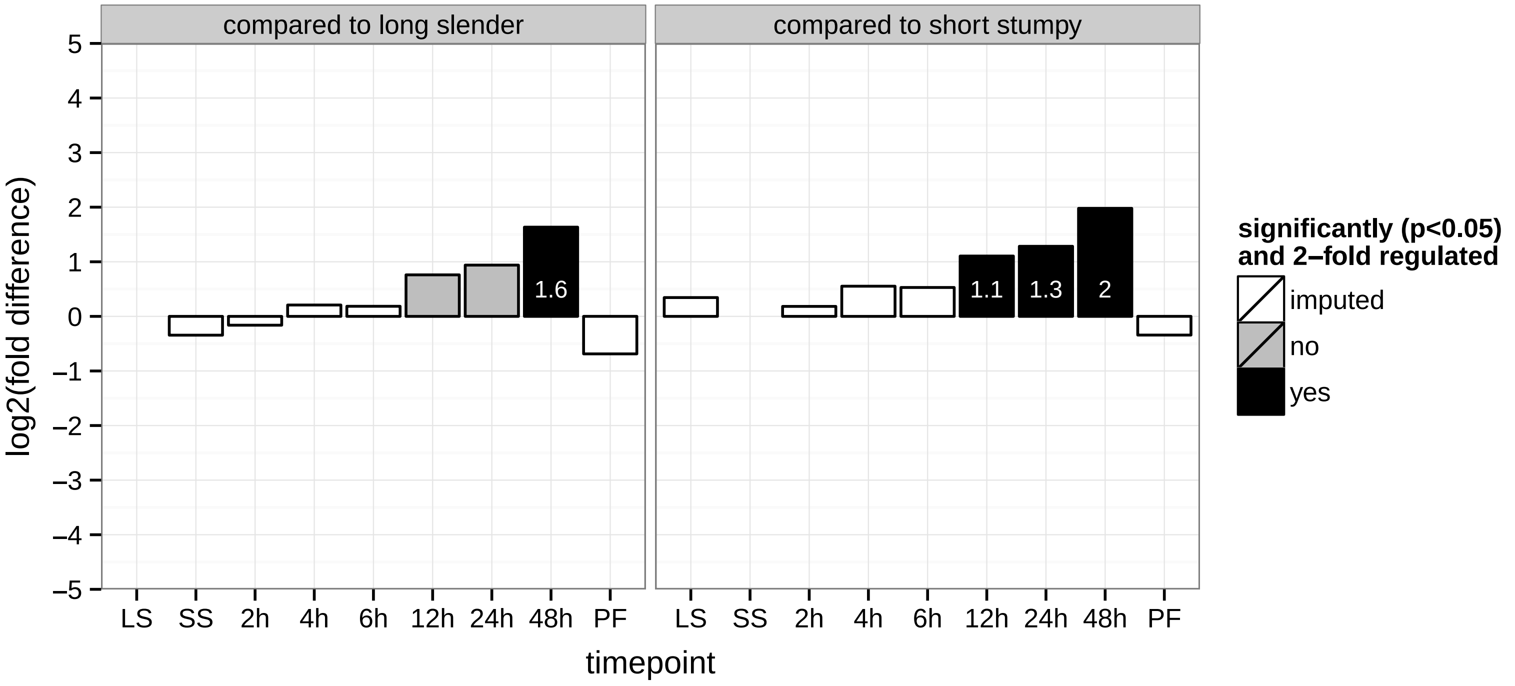

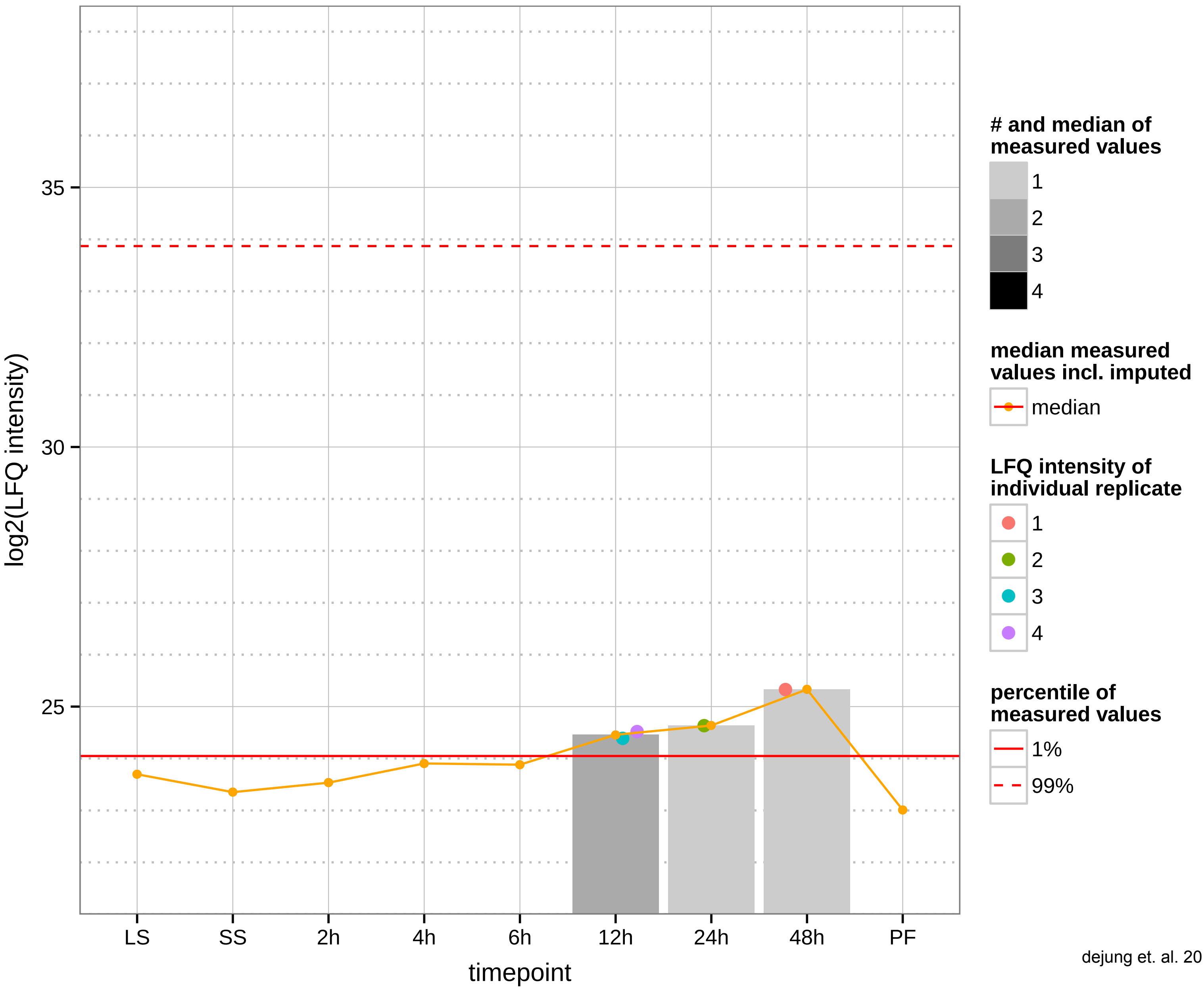

dejung et. al. 2015

heat shock 70 kDa protein, putative (HSP70)
Tb927.7.1030
AGOF: ATP binding
AGOC: null
AGOP: null
PGOF: null
PGOC: null
PGOP: null

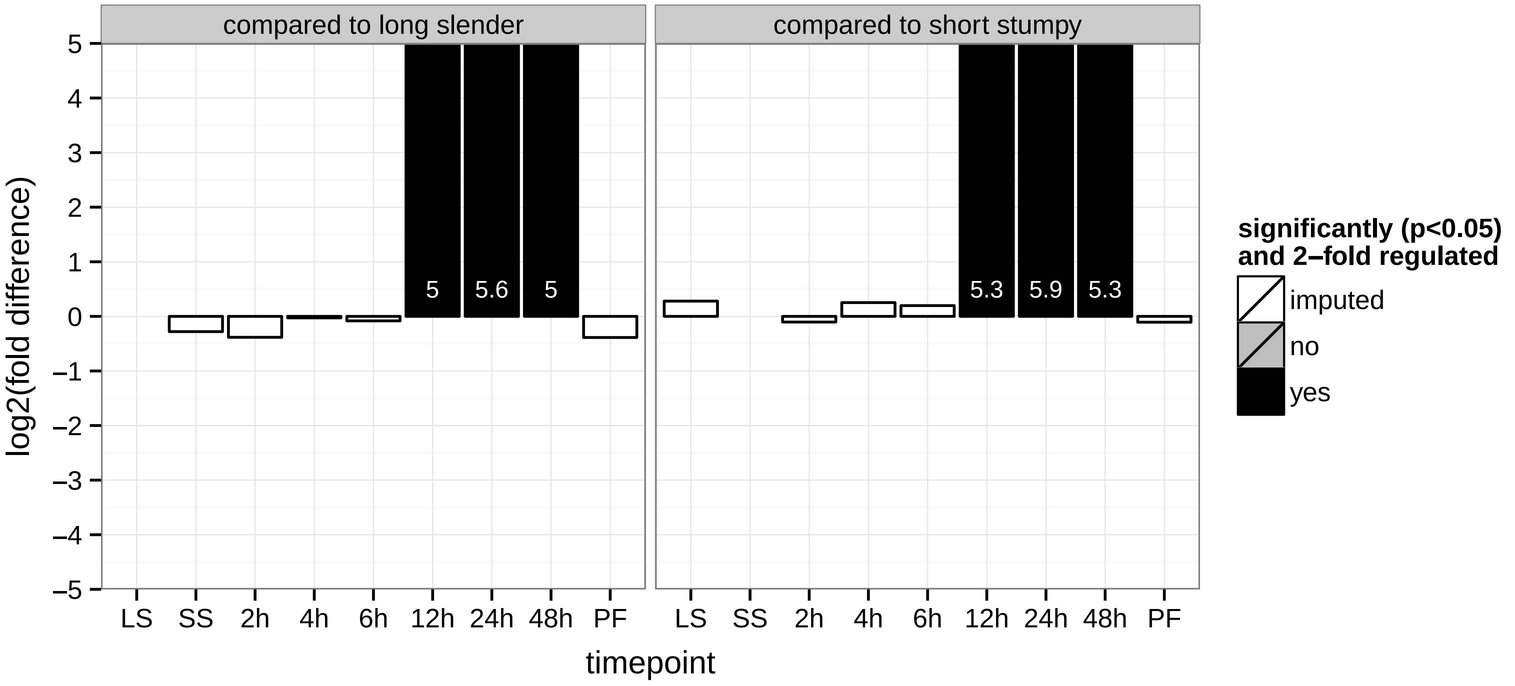

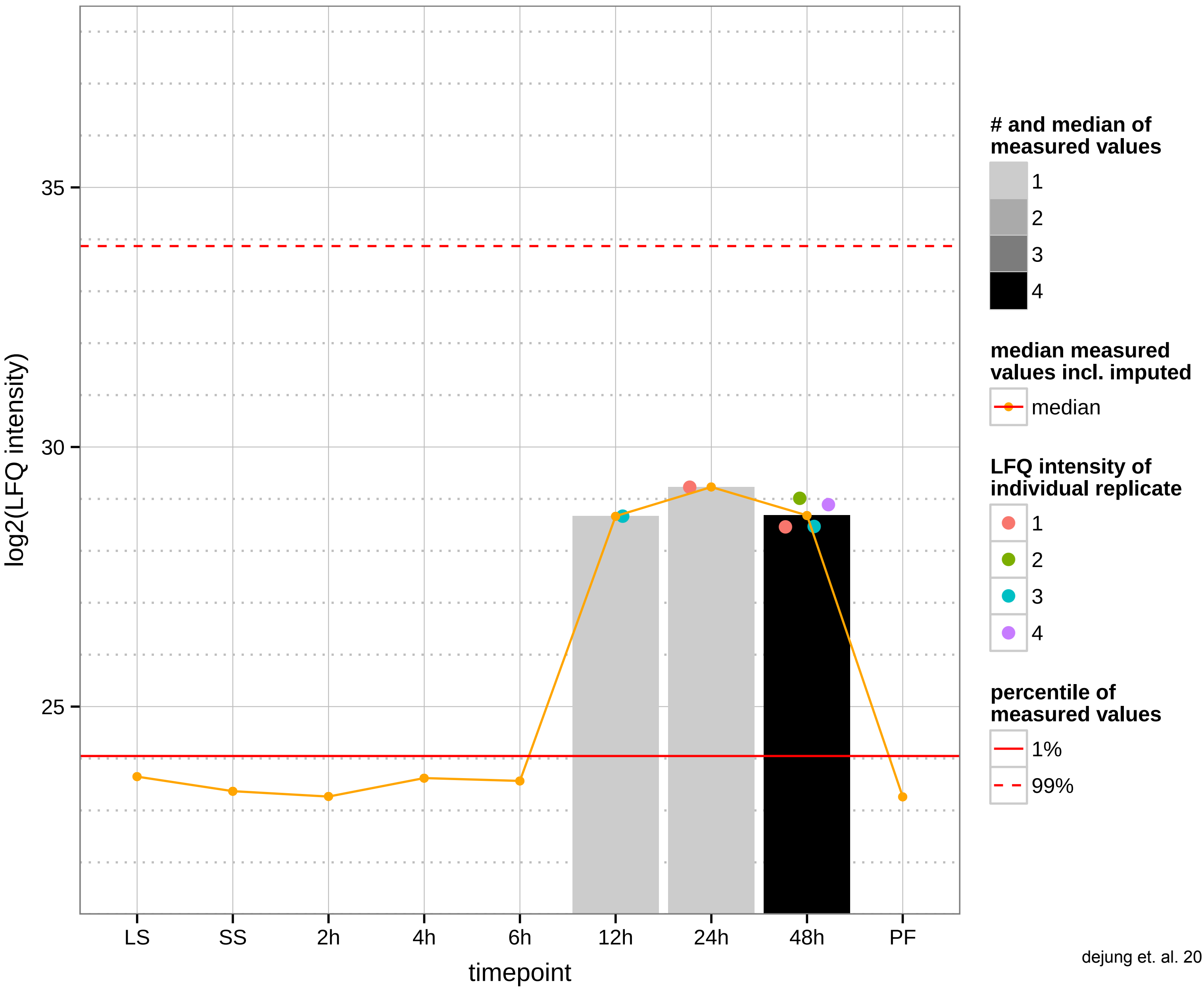

dejung et. al. 2015

eukaryotic peptide chain release factor subunit 1, putative
Tb927.7.1710
AGOF: null
AGOC: nucleus
AGOP: translation
PGOF: null
PGOC: null
PGOP: null

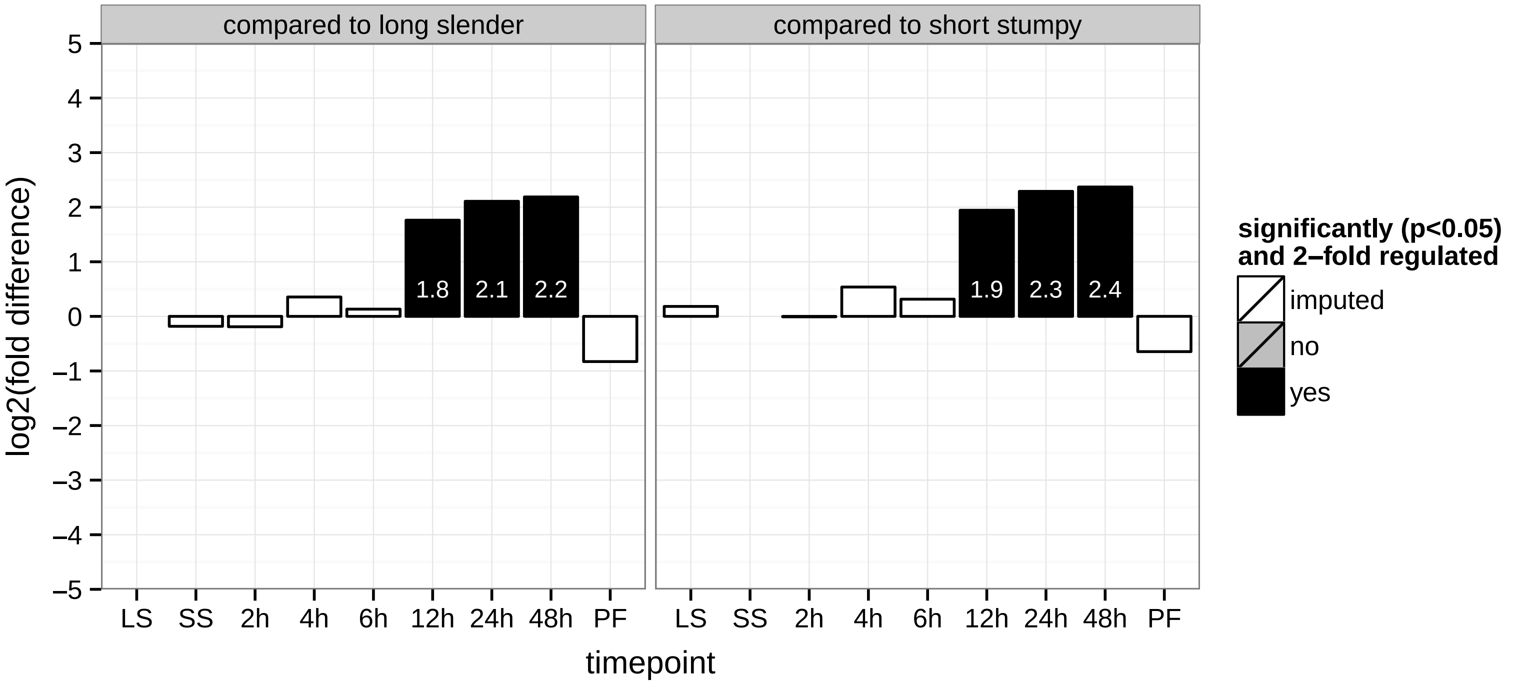

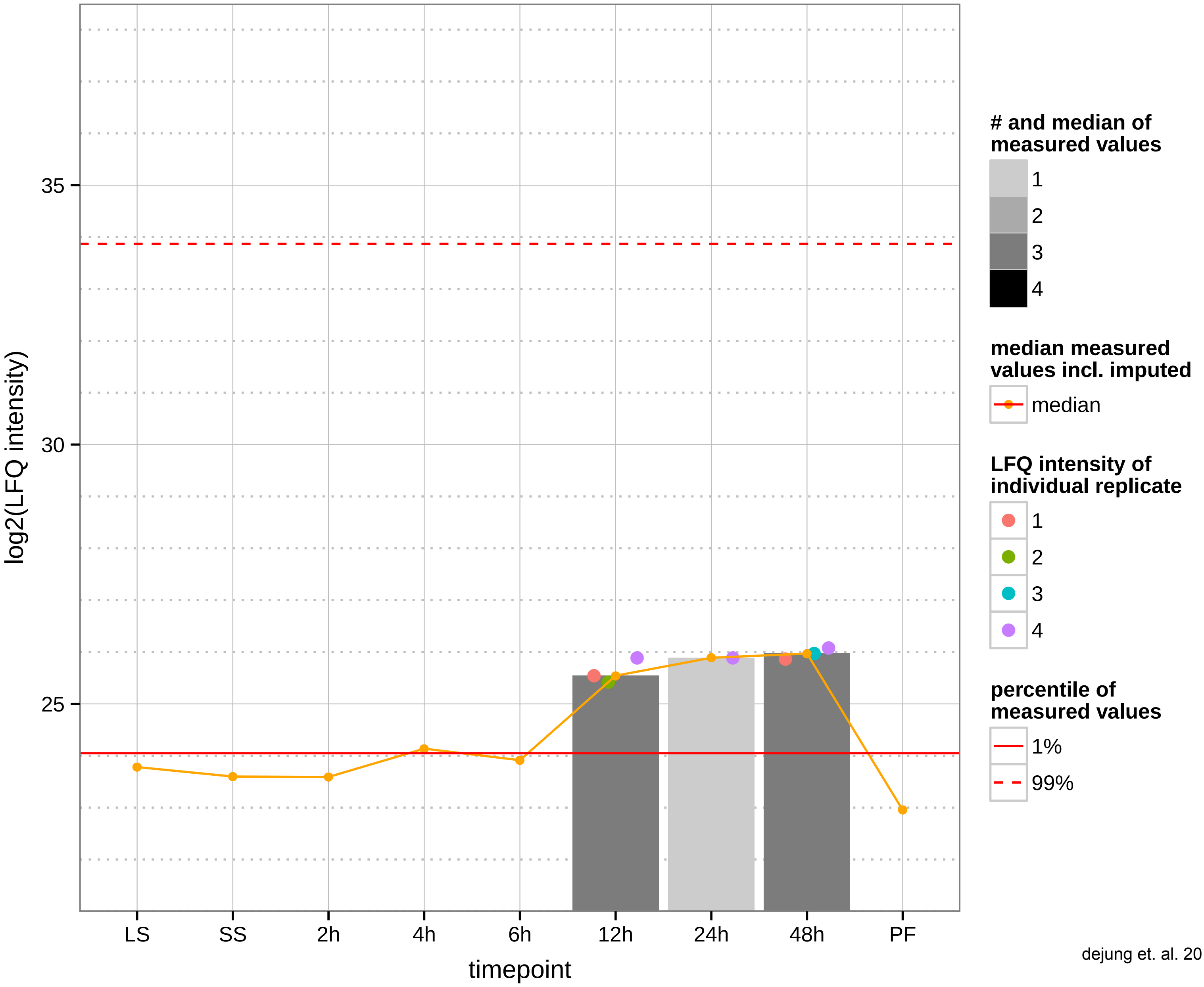

dejung et. al. 2015

hypothetical protein, conserved
Tb927.7.3140
AGOF: null
AGOC: null
AGOP: null
PGOF: null
PGOC: null
PGOP: null

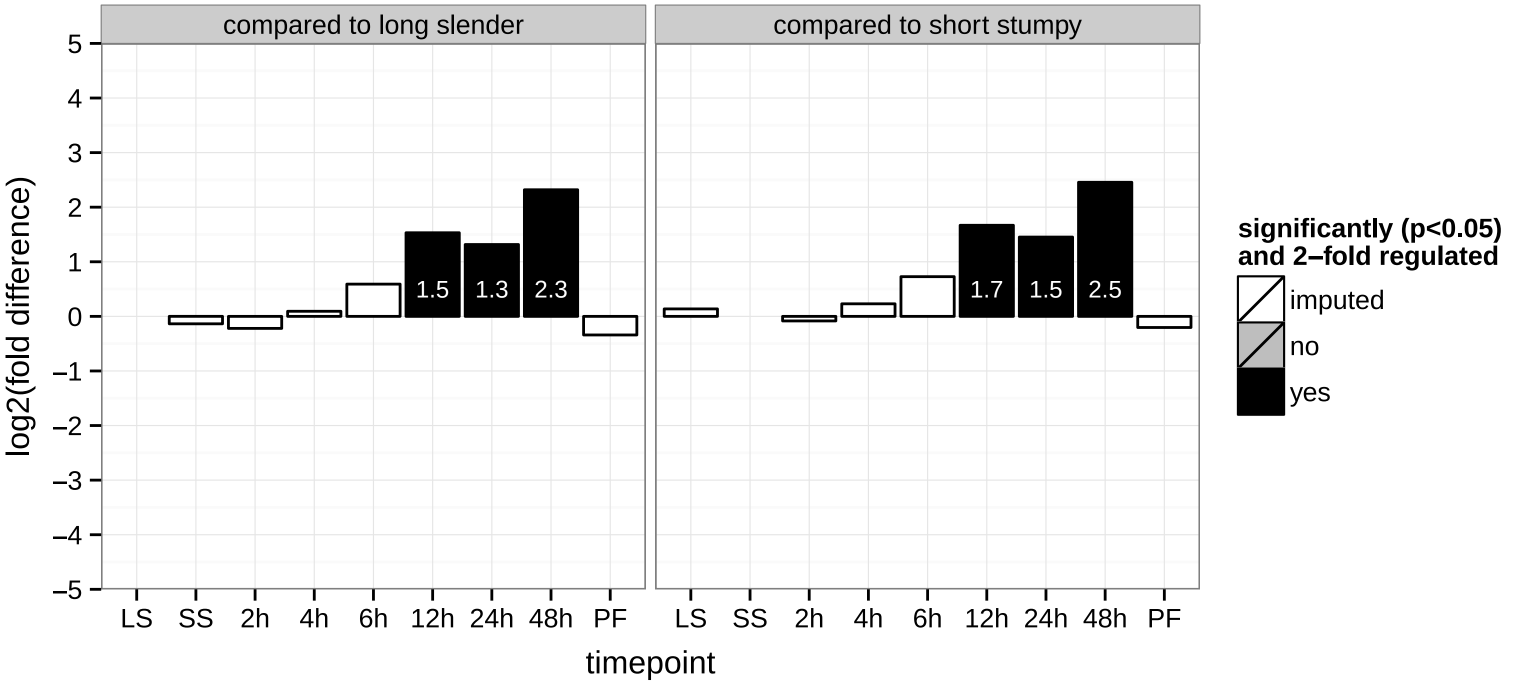

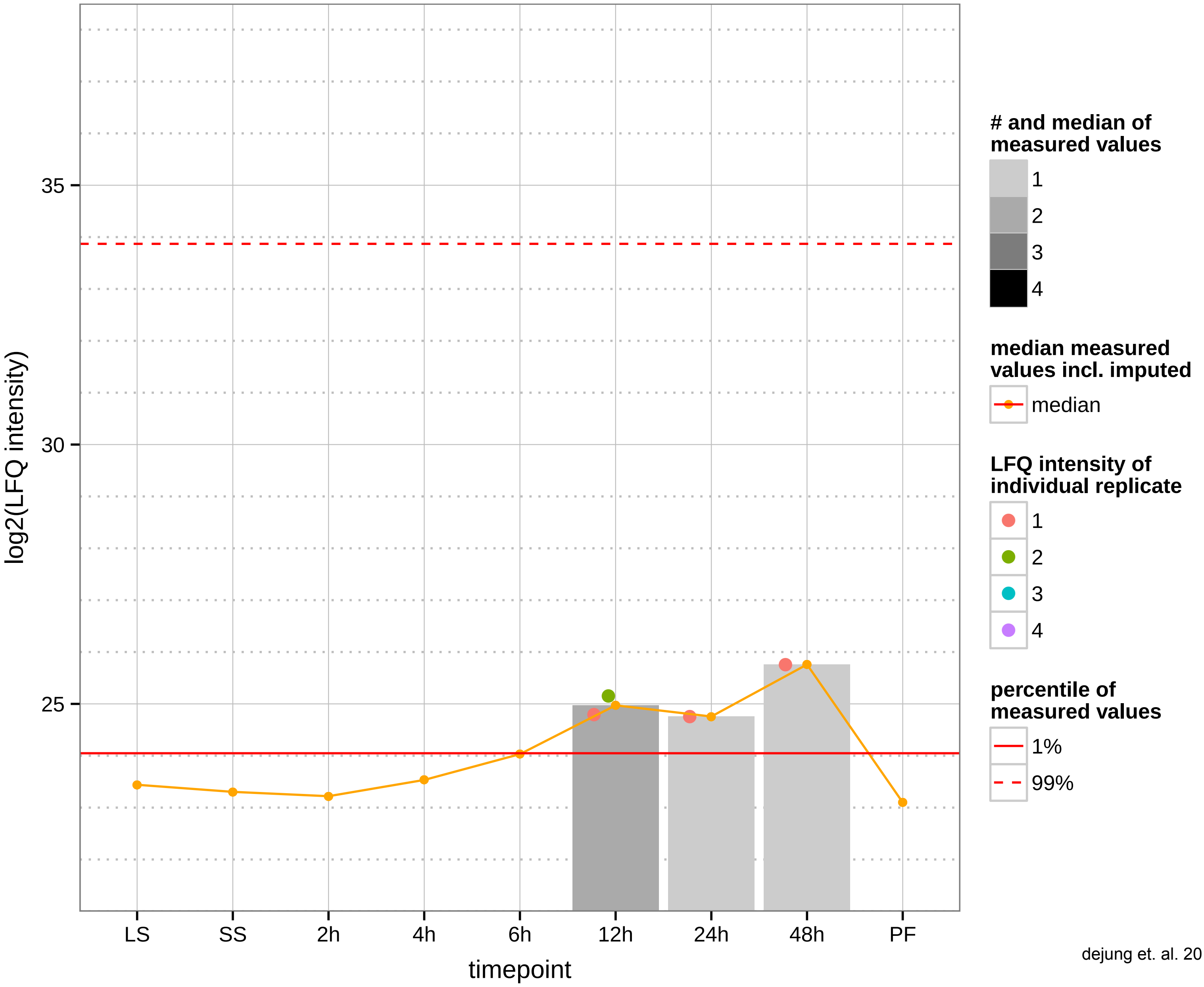

dejung et. al. 2015

centrin, putative
Tb927.7.3410
AGOF: calcium ion binding
AGOC: cilium basal body
AGOP: null
PGOF: calcium ion binding
PGOC: null
PGOP: null

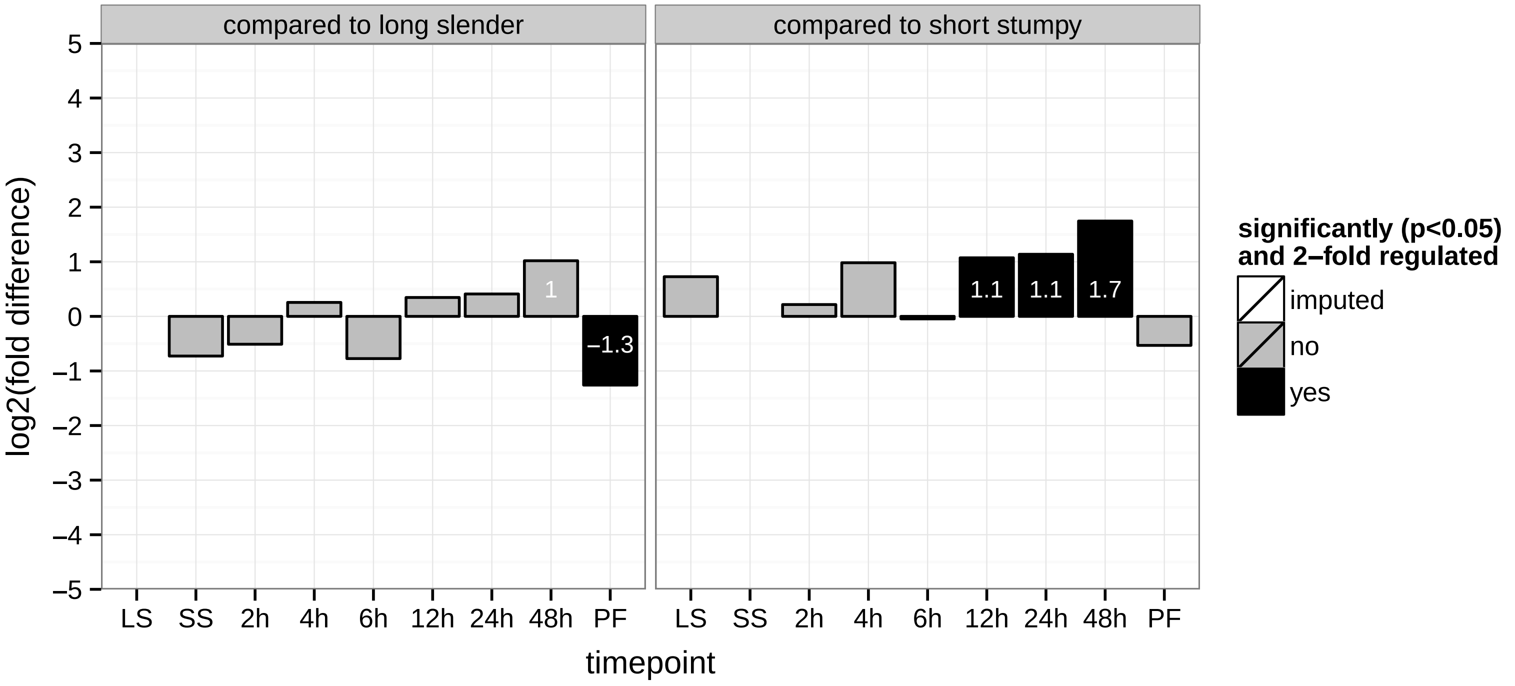

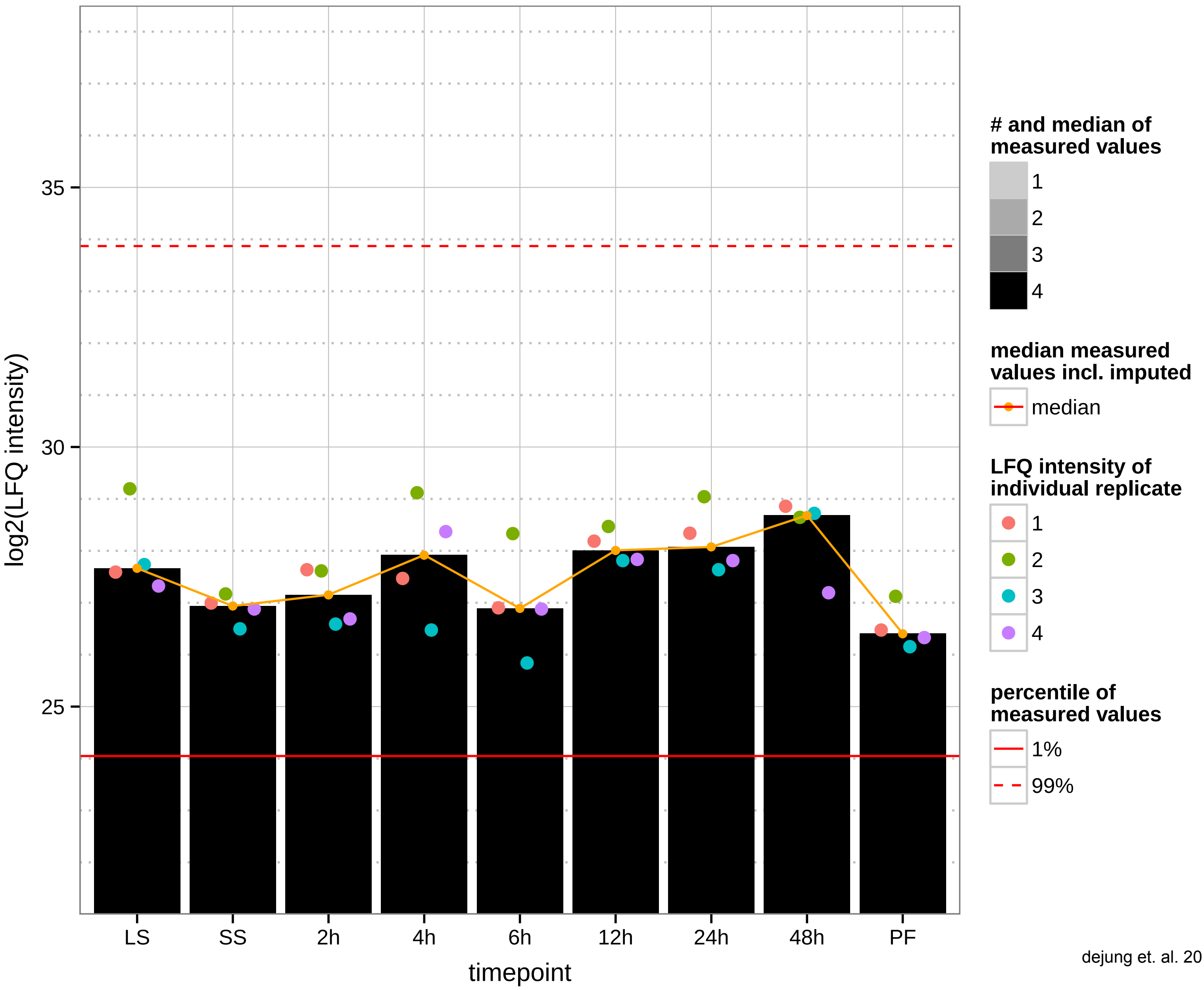

dejung et. al. 2015

50S ribosomal protein L16, putative
Tb927.7.3960
AGOF: structural constituent of ribosome
AGOC: intracellular, mitochondrion, ribosome
AGOP: translation
PGOF: structural constituent of ribosome
PGOC: ribosome
PGOP: translation

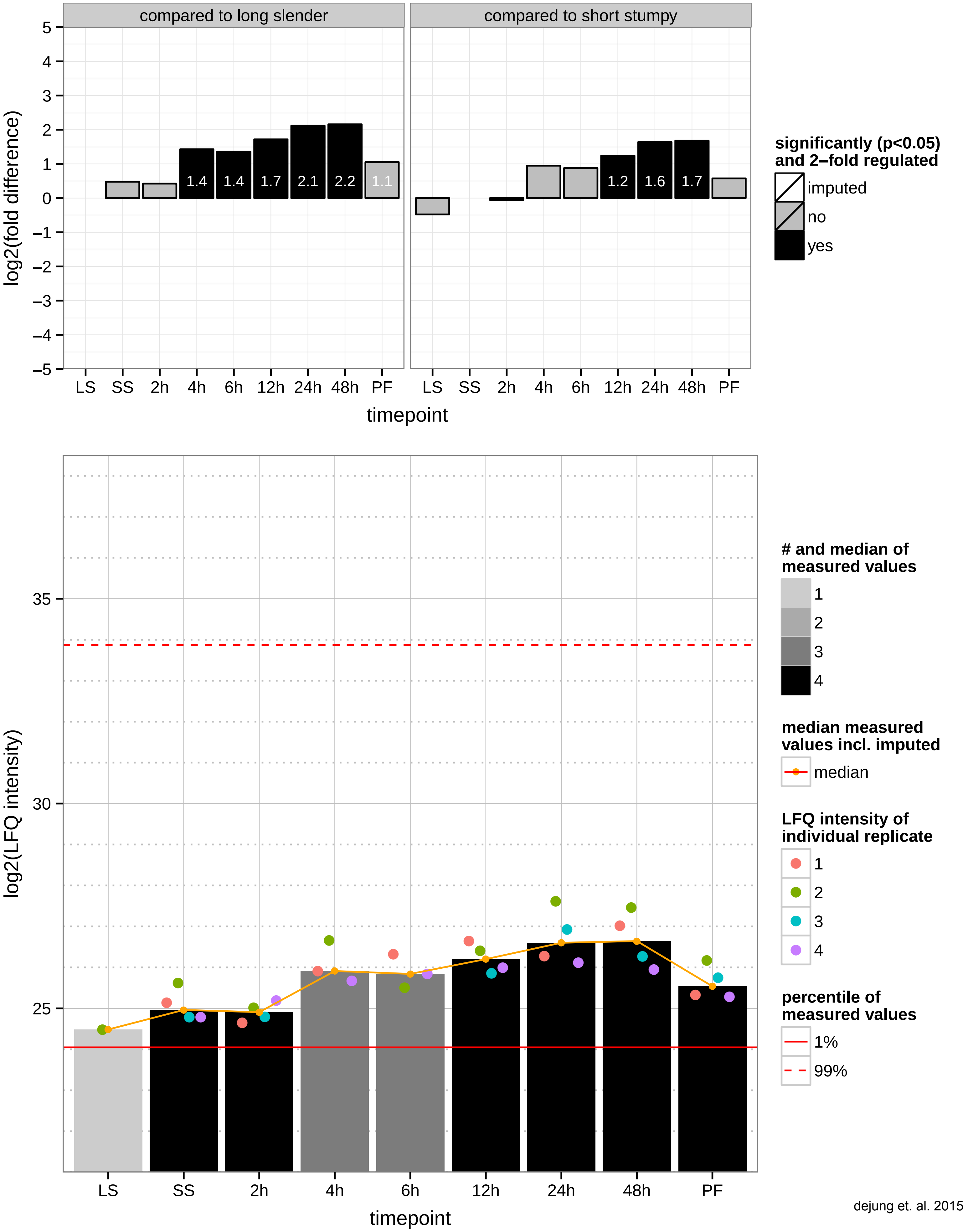

dejung et. al. 2015

hypothetical protein, conserved
Tb927.7.5280
AGOF: null
AGOC: mitochondrion
AGOP: null
PGOF: null
PGOC: null
PGOP: null

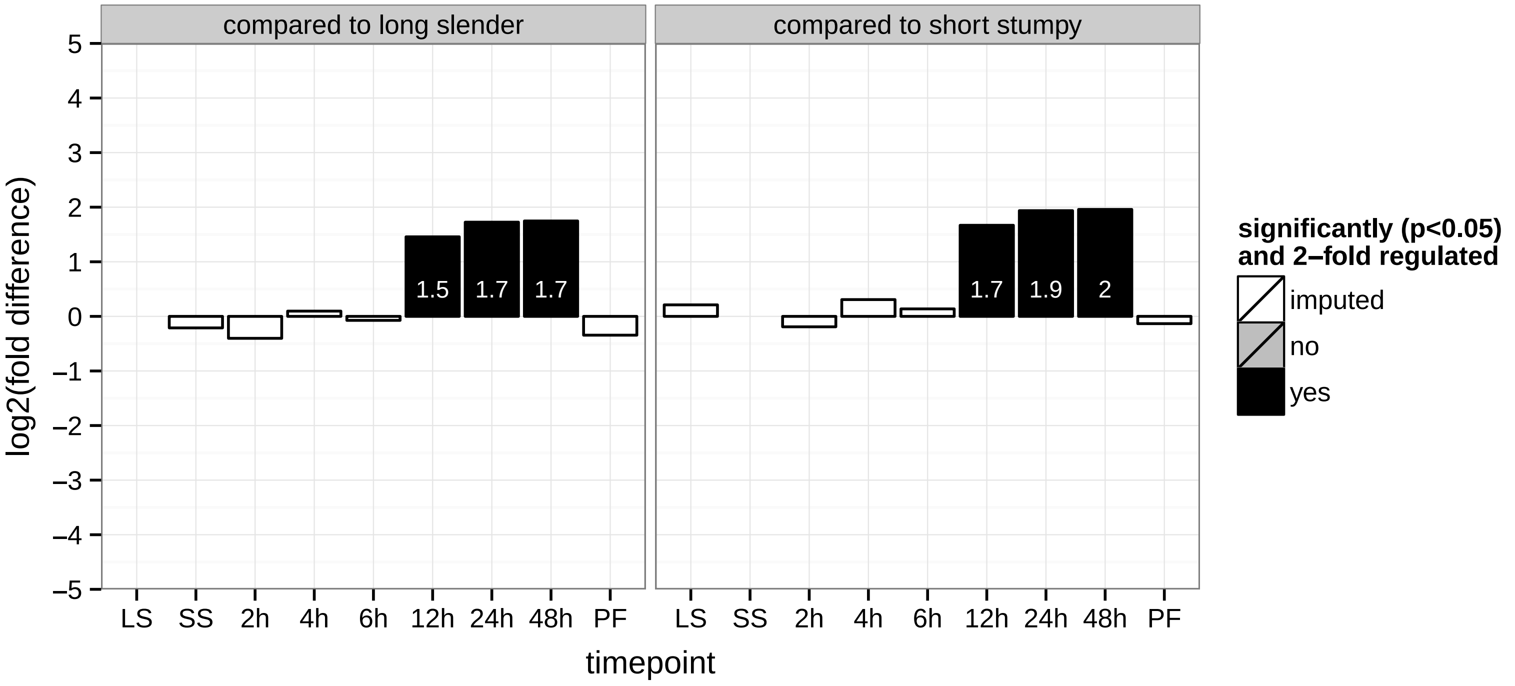

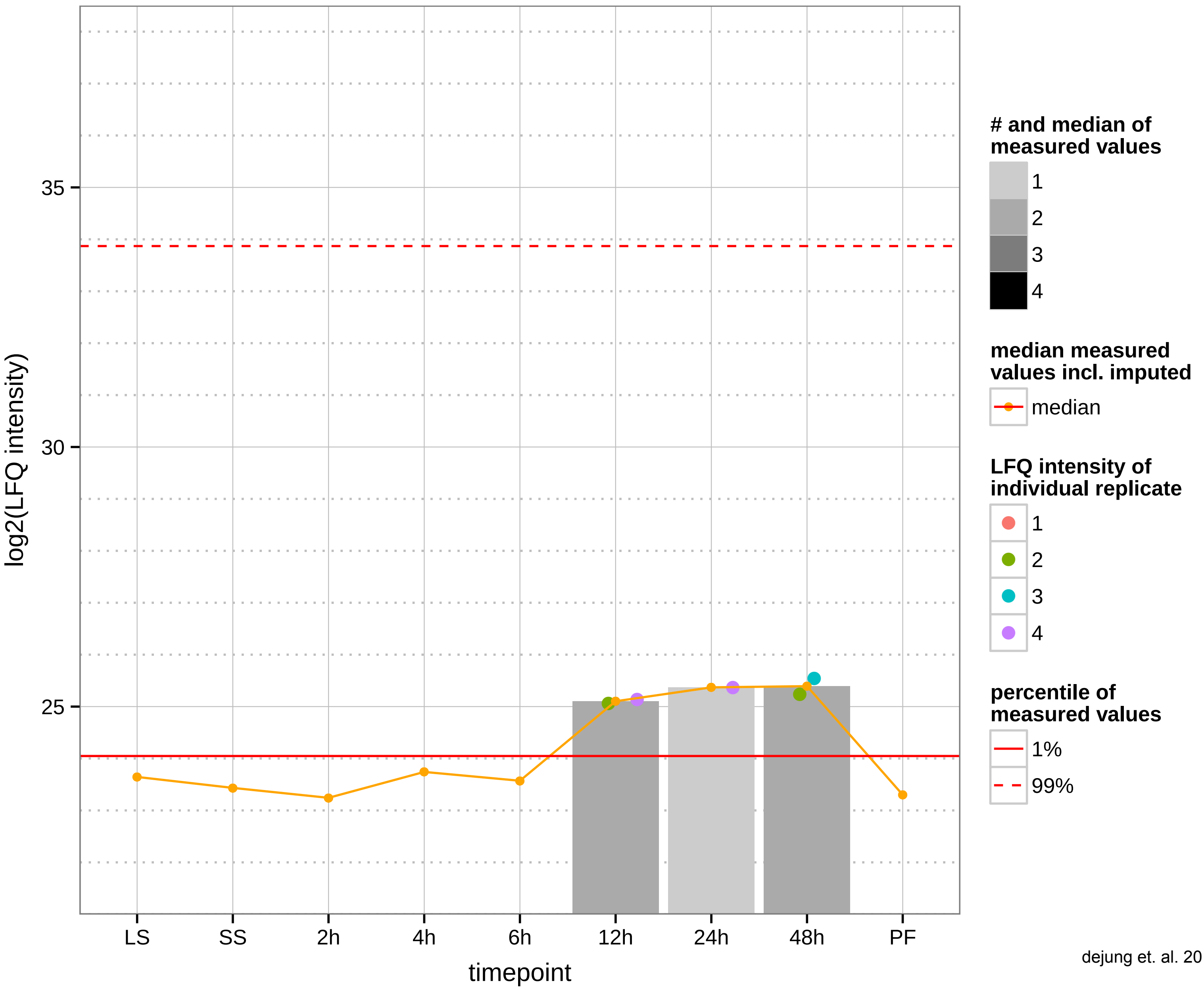

dejung et. al. 2015

hypothetical protein, conserved
Tb927.7.5320
AGOF: null
AGOC: null
AGOP: null
PGOF: null
PGOC: null
PGOP: null

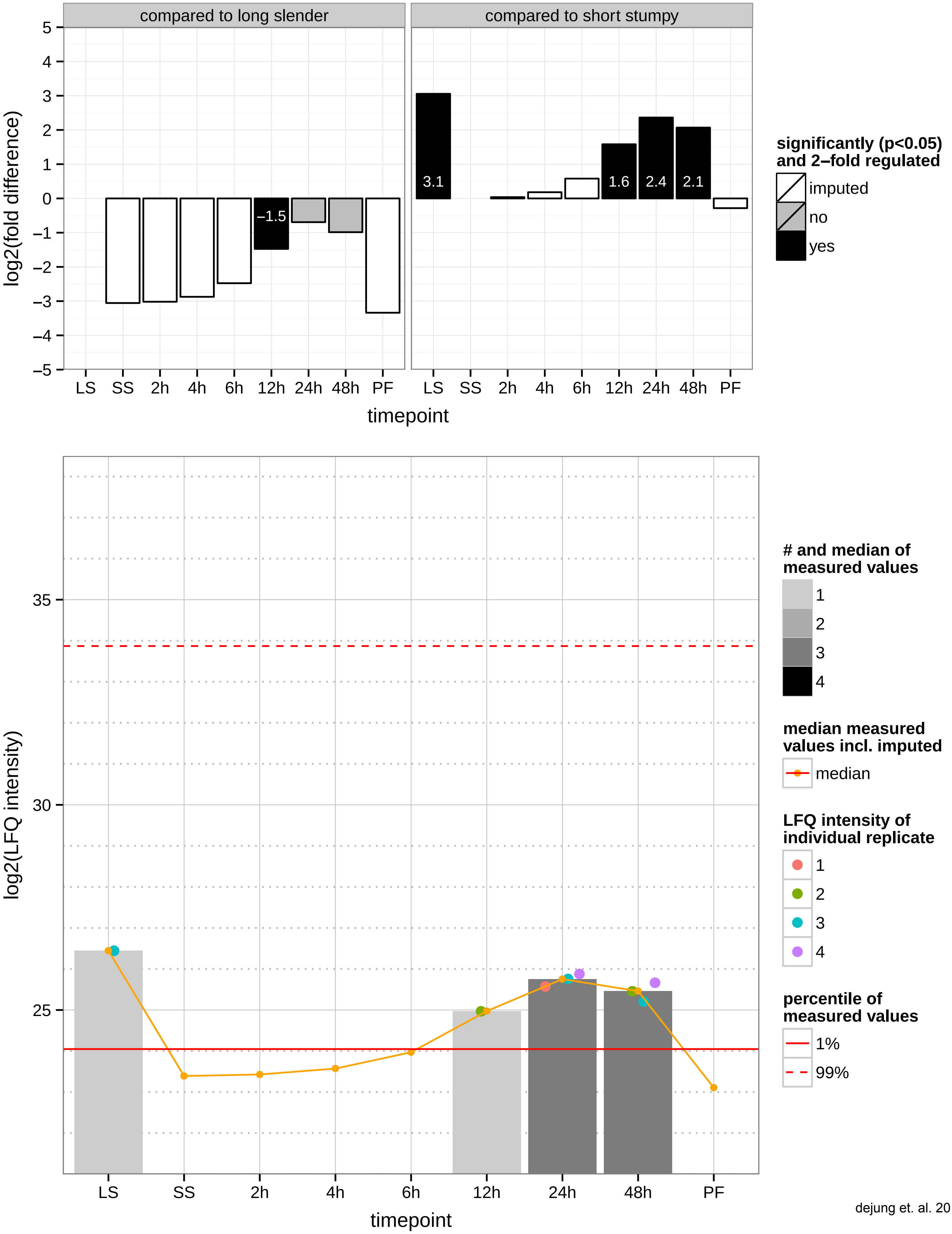

dejung et. al. 2015

hypothetical protein, conserved
Tb927.7.5610
AGOF: null
AGOC: null
AGOP: null
PGOF: protein binding
PGOC: null
PGOP: null

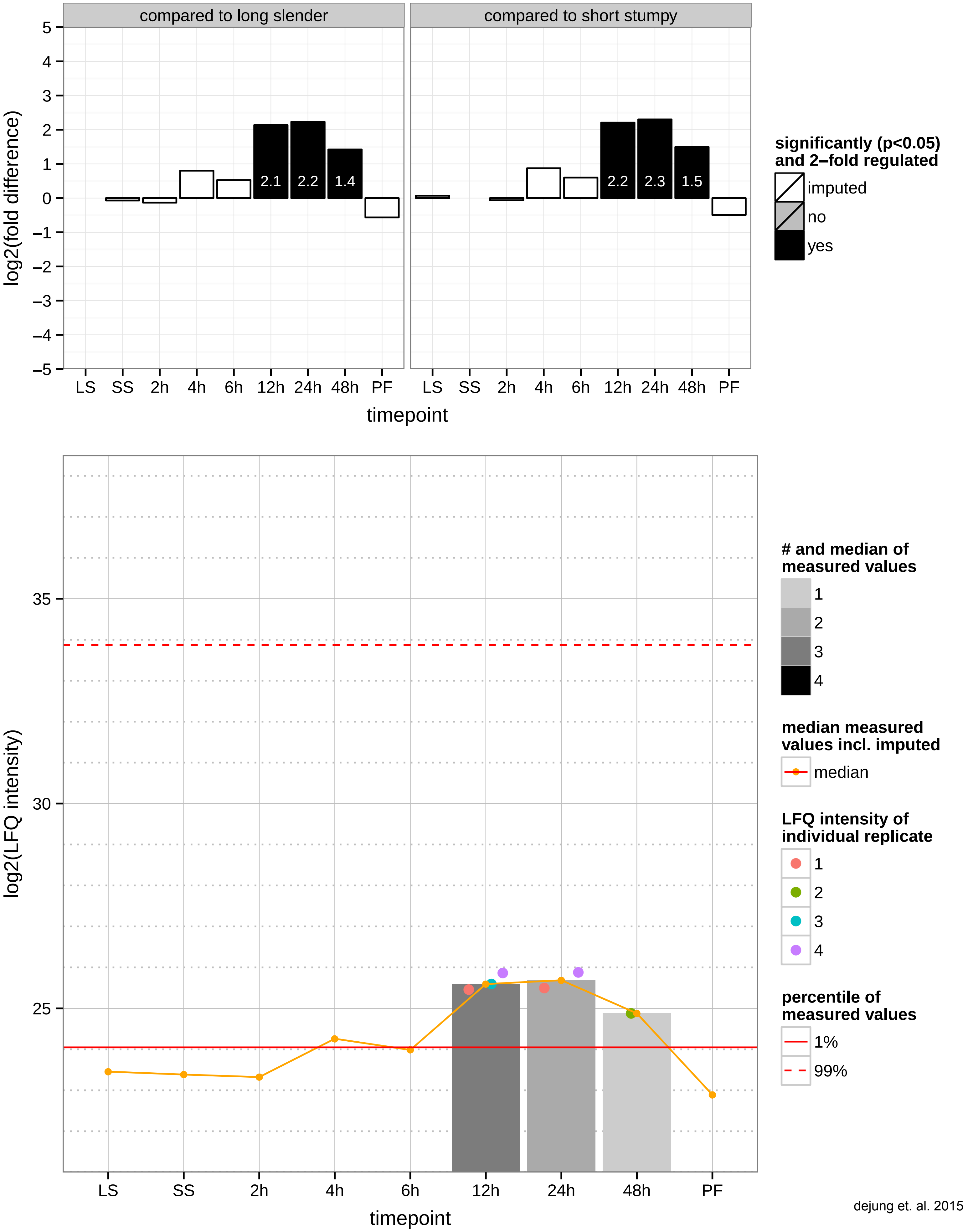

dejung et. al. 2015

protein associated with differentiation 3, putative (PAD3)
Tb927.7.5950
AGOF: null
AGOC: null
AGOP: null
PGOF: null
PGOC: integral to membrane
PGOP: transmembrane transport

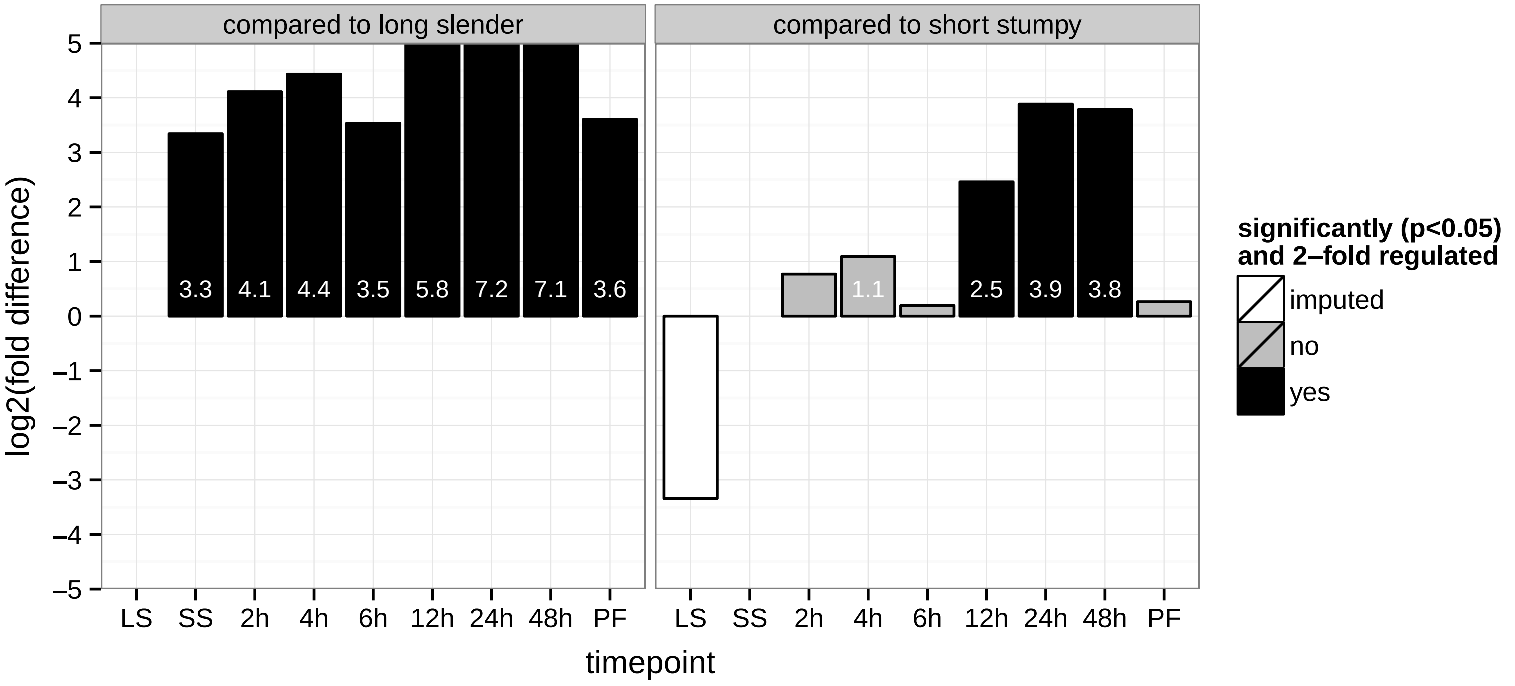

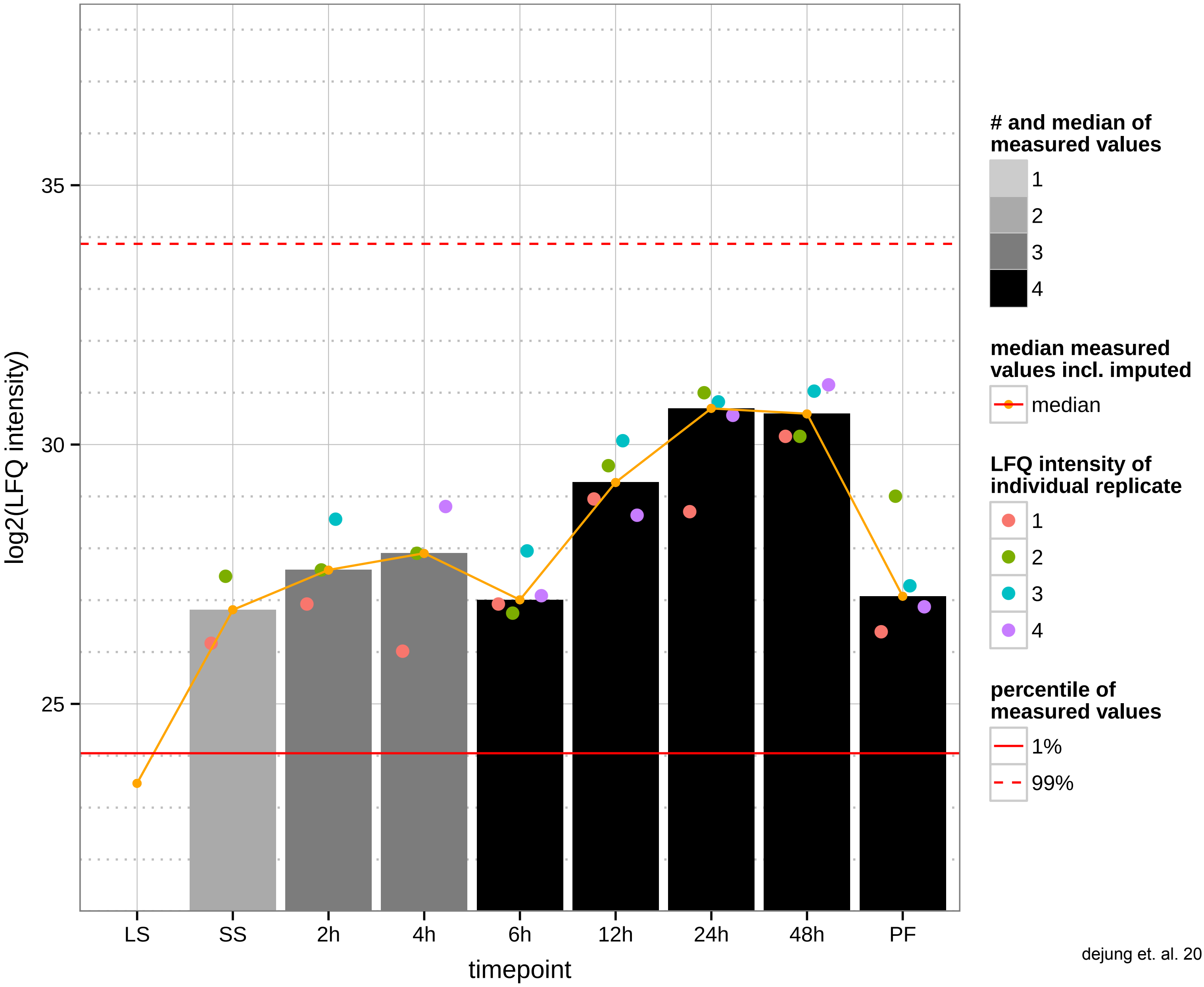

dejung et. al. 2015

hypothetical protein, conserved
Tb927.8.4220
AGOF: null
AGOC: null
AGOP: null
PGOF: null
PGOC: null
PGOP: null

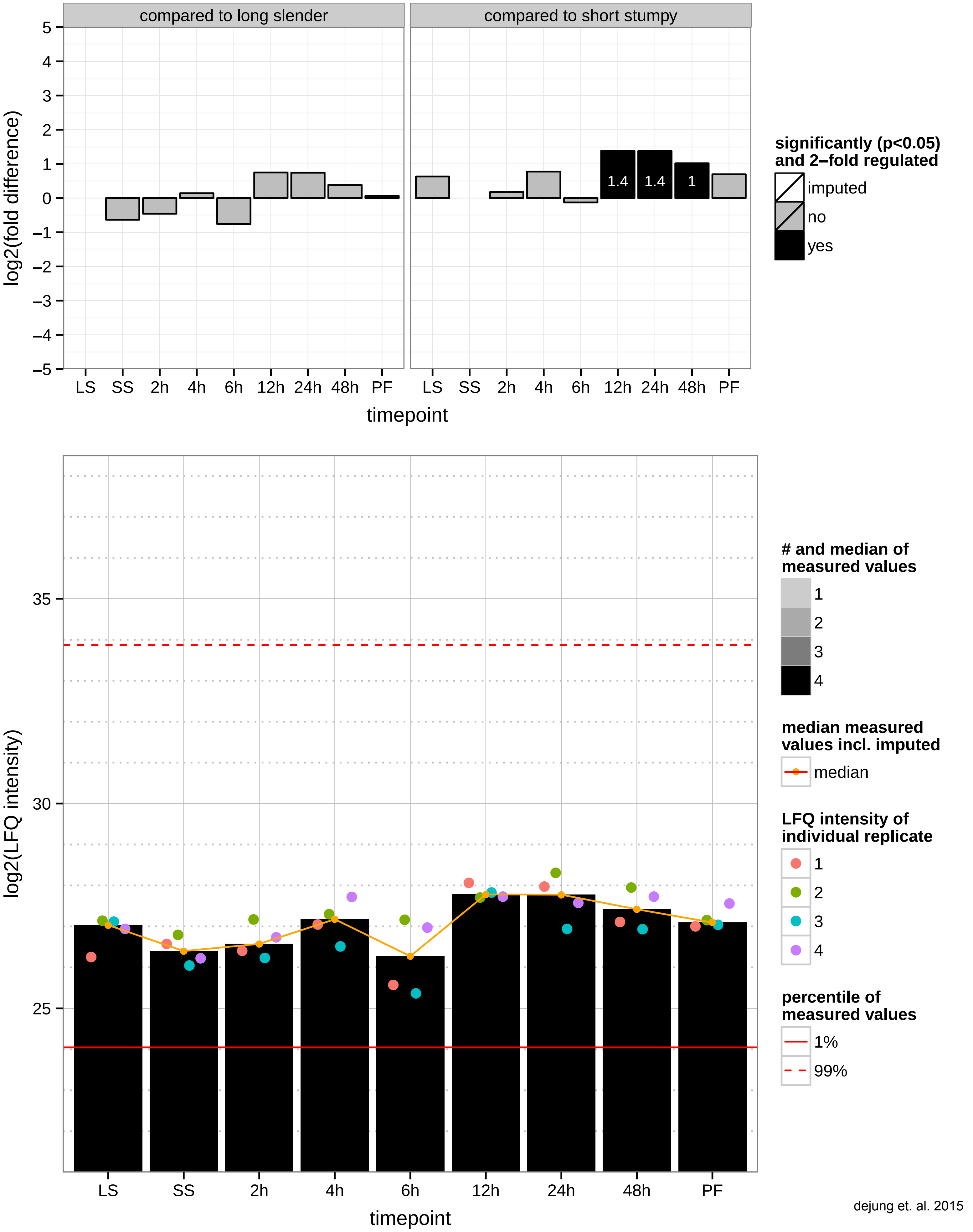

dejung et. al. 2015

hypothetical protein, conserved
Tb927.8.5370
AGOF: null
AGOC: null
AGOP: null
PGOF: null
PGOC: null
PGOP: null

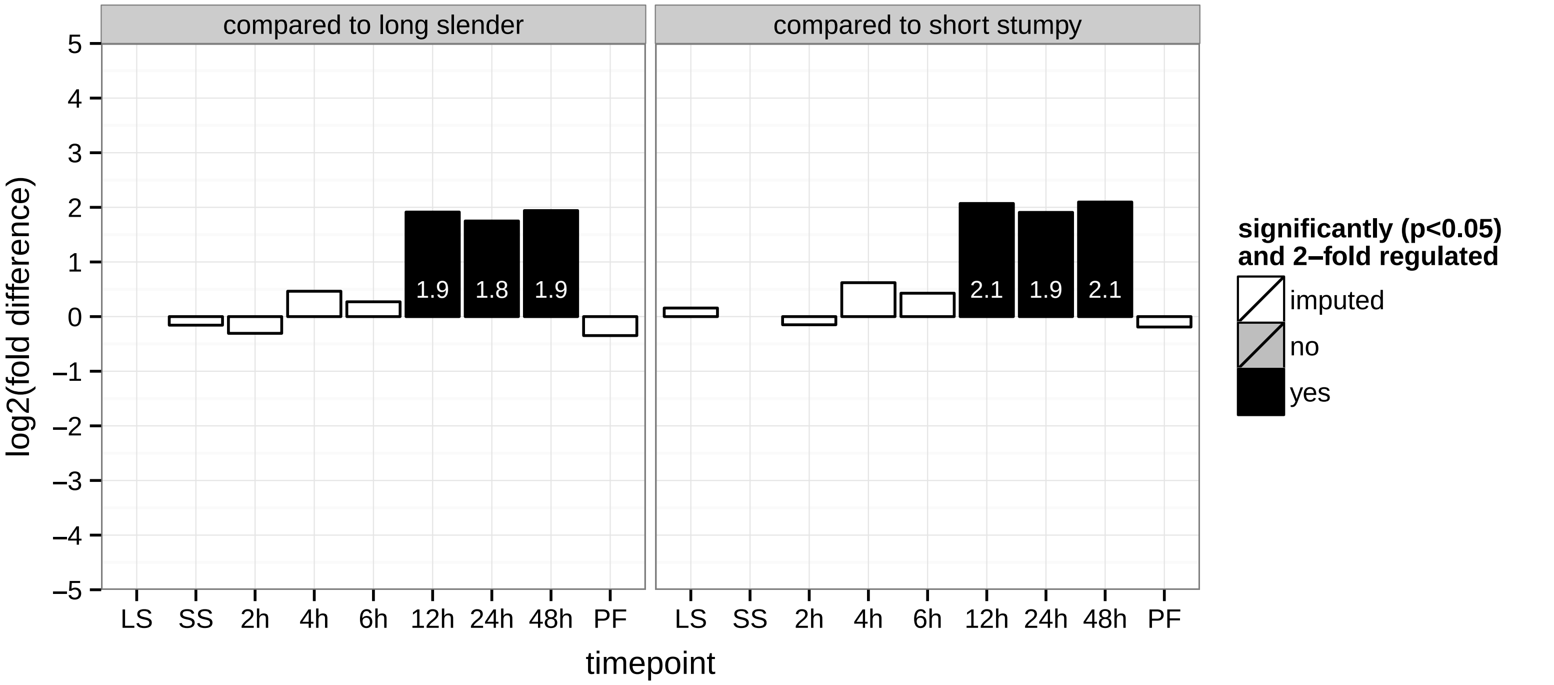

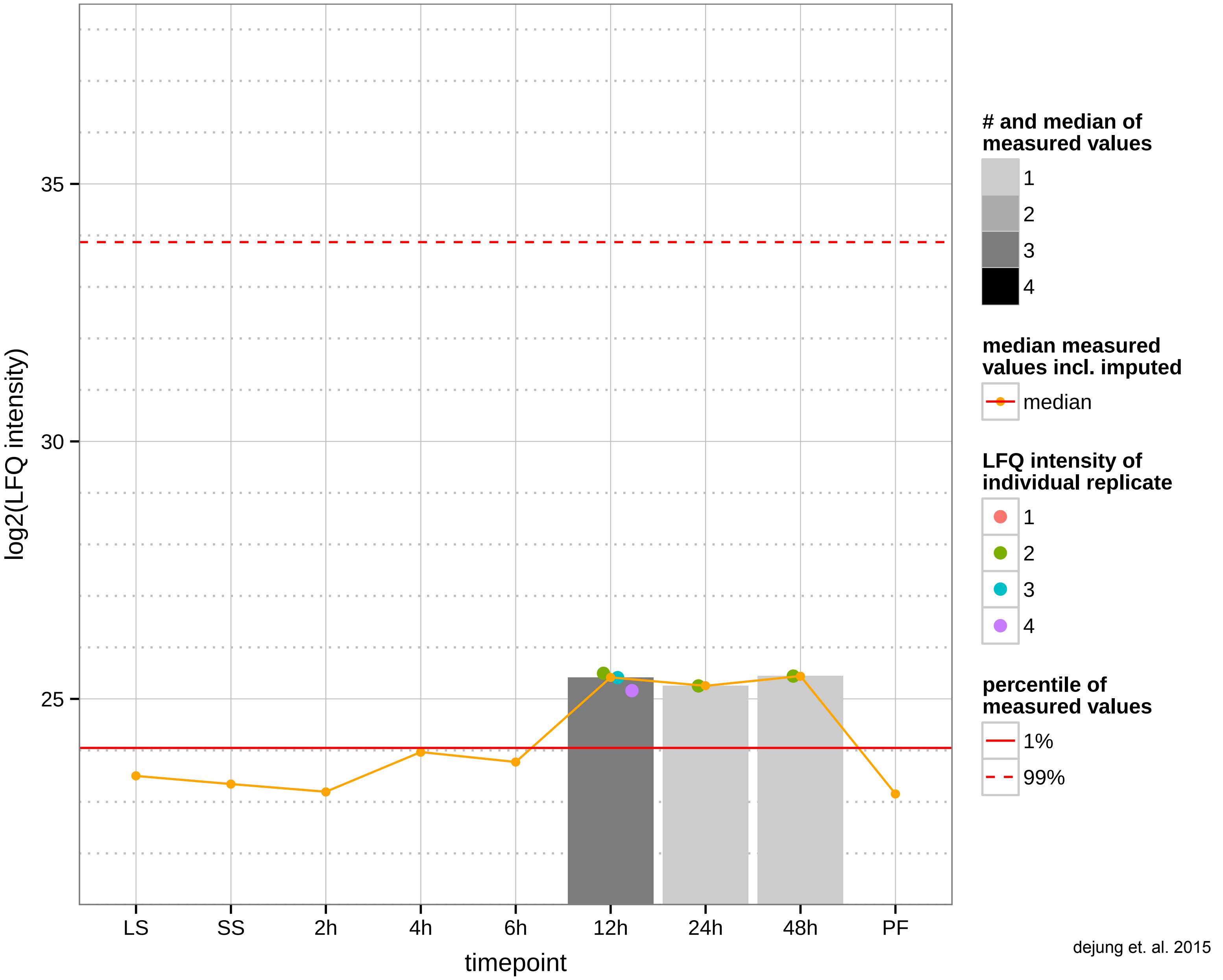

dejung et. al. 2015

hypothetical protein, conserved
Tb927.8.5660
AGOF: null
AGOC: mitochondrial inner membrane, mitochondrial membrane, mitochondrion
AGOP: null
PGOF: null
PGOC: null
PGOP: null

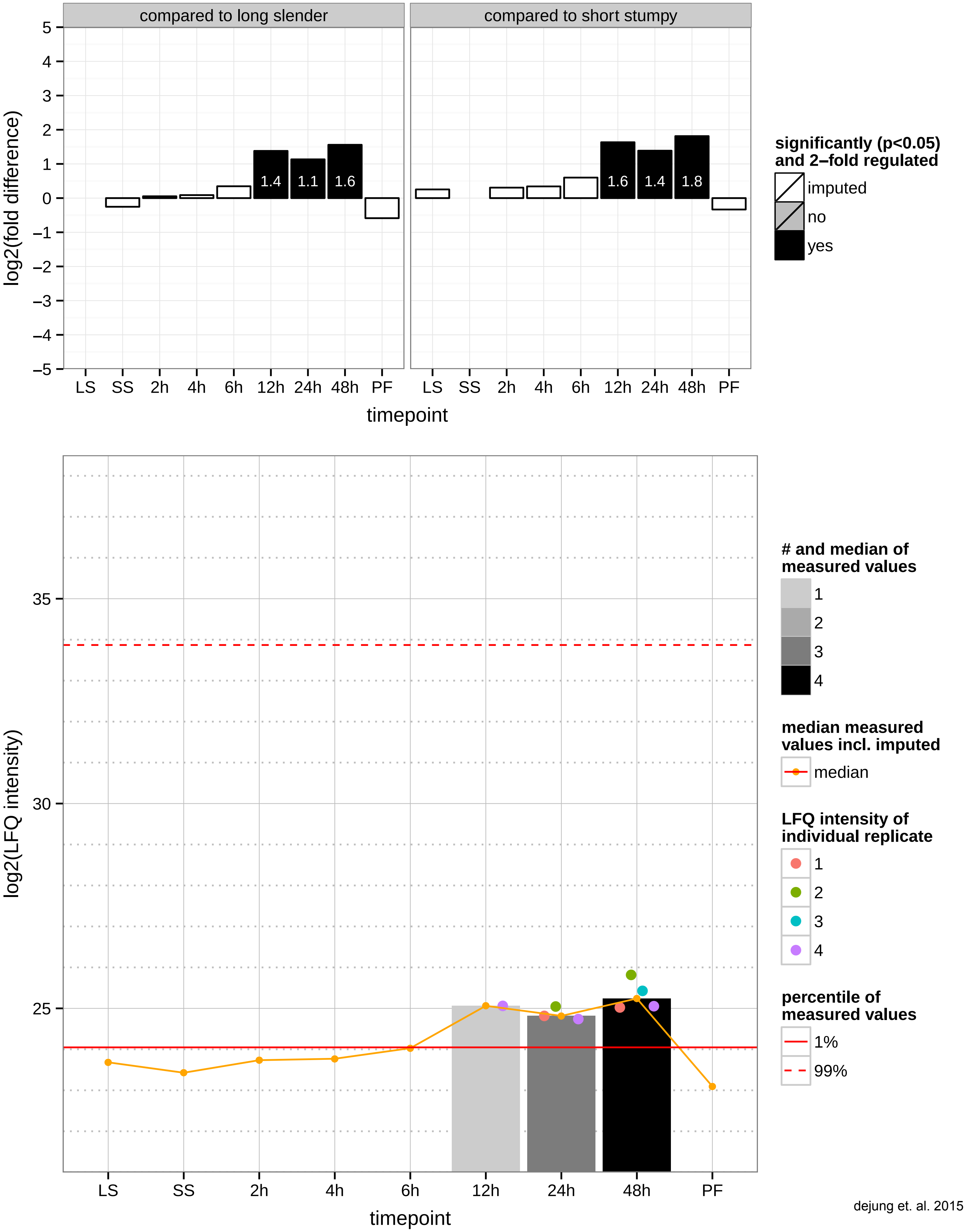

dejung et. al. 2015

Elongator–like Protein 3a
Tb927.8.5770
AGOF: N–acetyltransferase activity, iron–sulfur cluster binding
AGOC: nucleolus
AGOP: null
PGOF: N–acetyltransferase activity, catalytic activity, iron–sulfur cluster binding
PGOC: null
PGOP: null

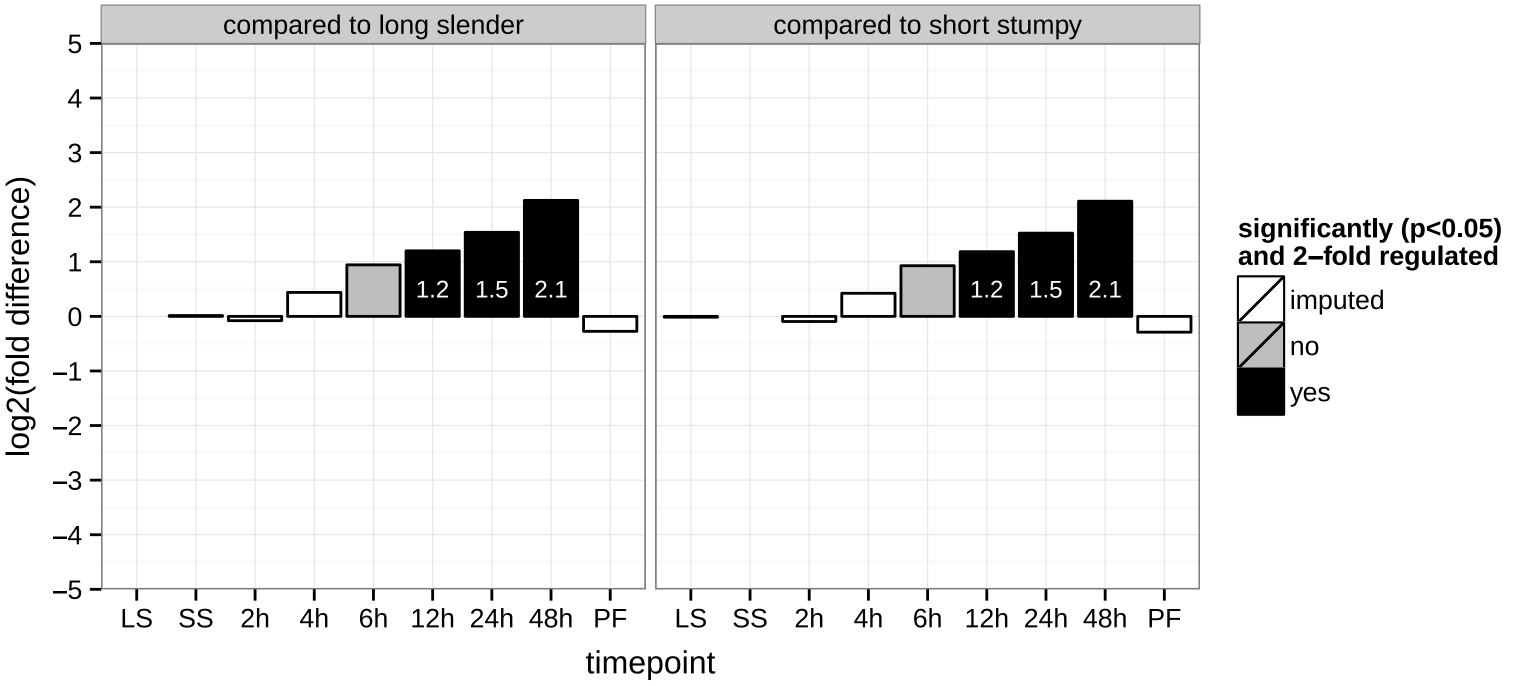

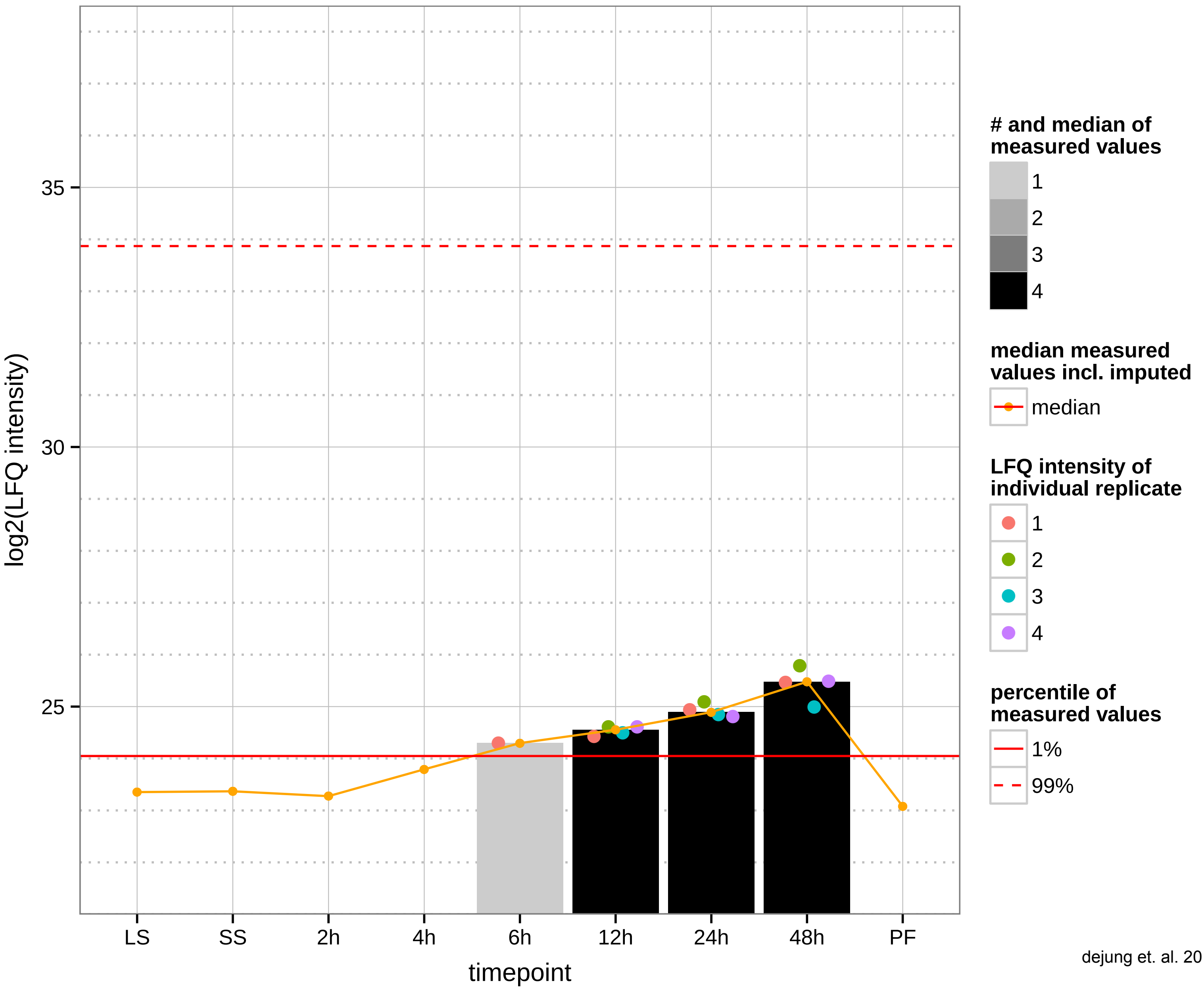

dejung et. al. 2015

hypothetical protein, conserved
Tb927.8.6670
AGOF: null
AGOC: null
AGOP: null
PGOF: null
PGOC: null
PGOP: null

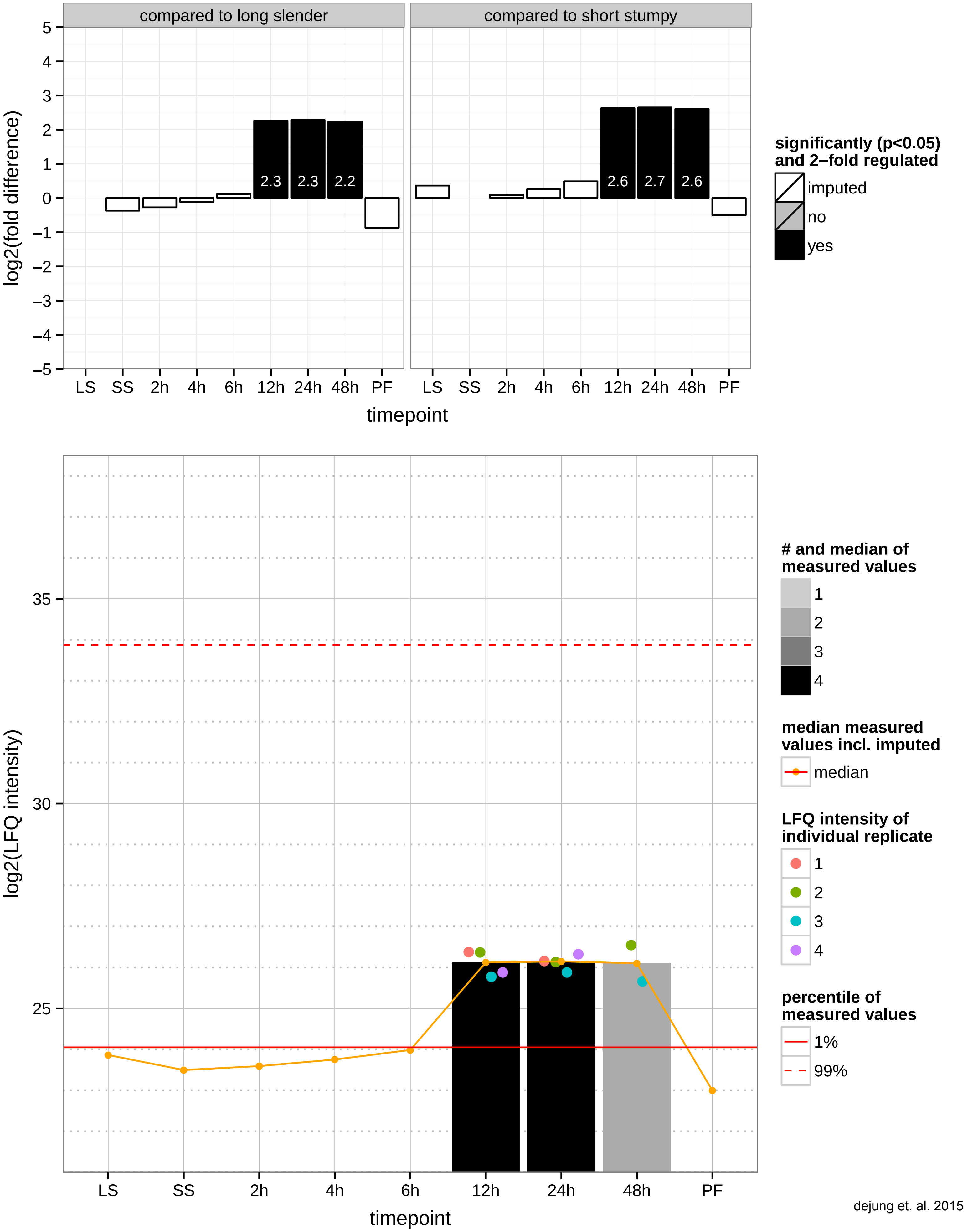

dejung et. al. 2015

hypothetical protein, conserved
Tb927.8.6850
AGOF: calcium ion binding, zinc ion binding
AGOC: null
AGOP: null
PGOF: calcium ion binding, protein binding, zinc ion binding
PGOC: null
PGOP: null

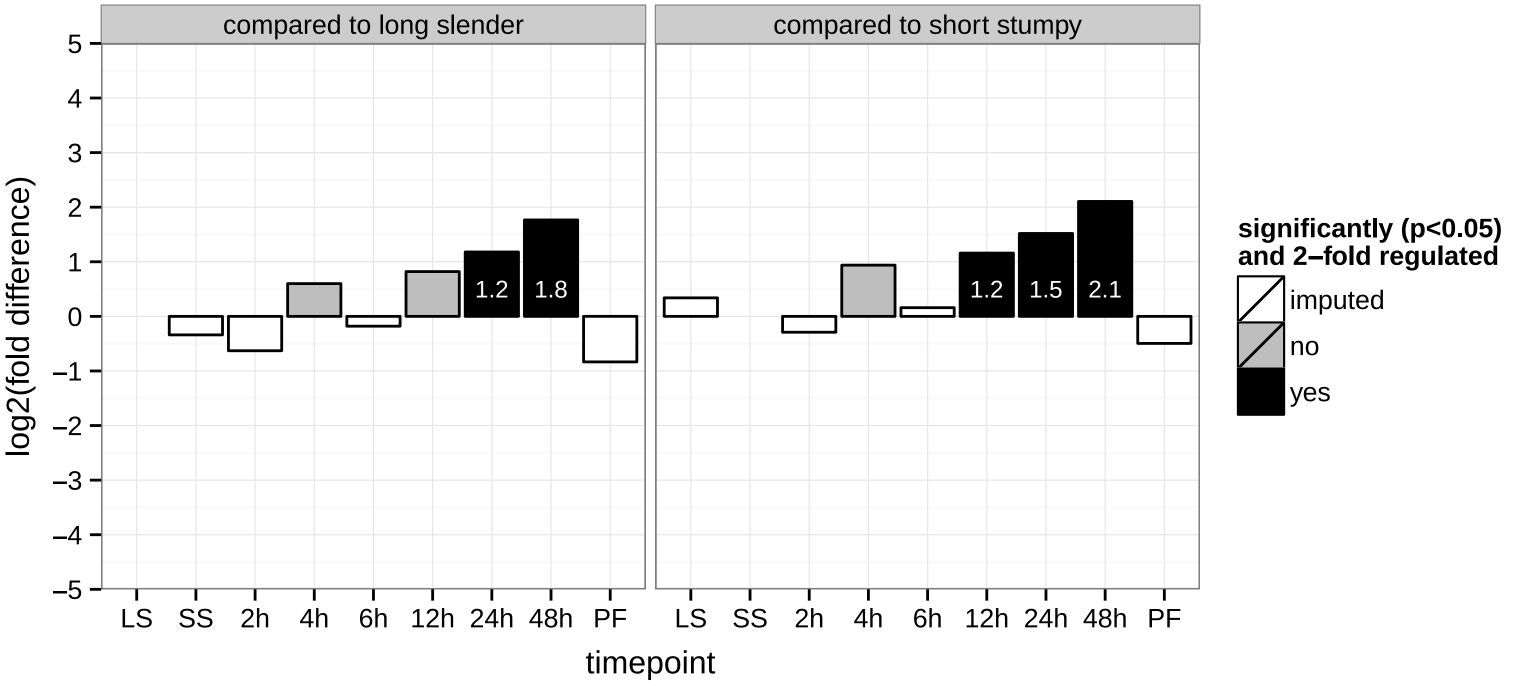

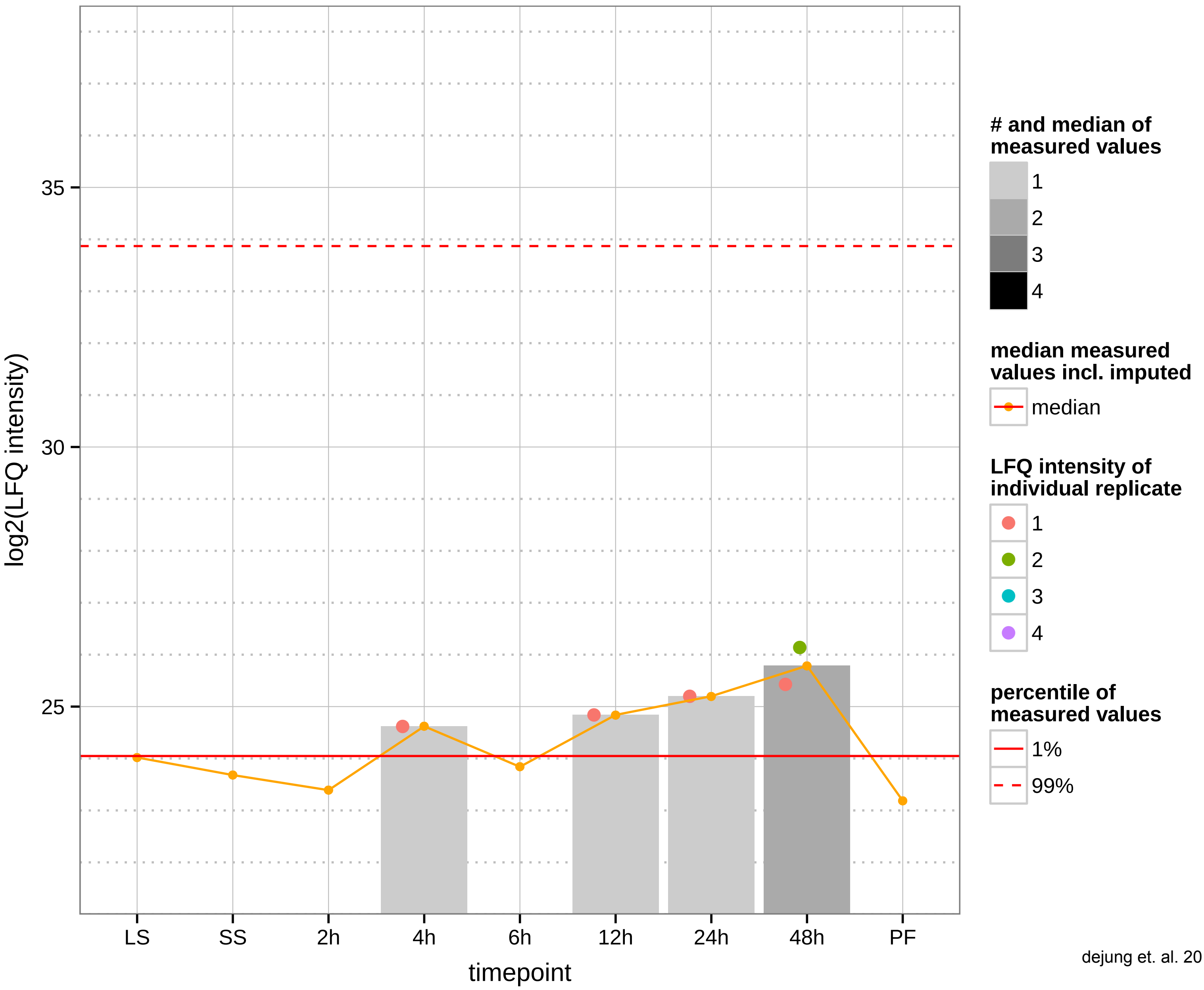

dejung et. al. 2015

hypothetical protein, conserved
Tb927.9.12800
AGOF: null
AGOC: null
AGOP: null
PGOF: null
PGOC: null
PGOP: null

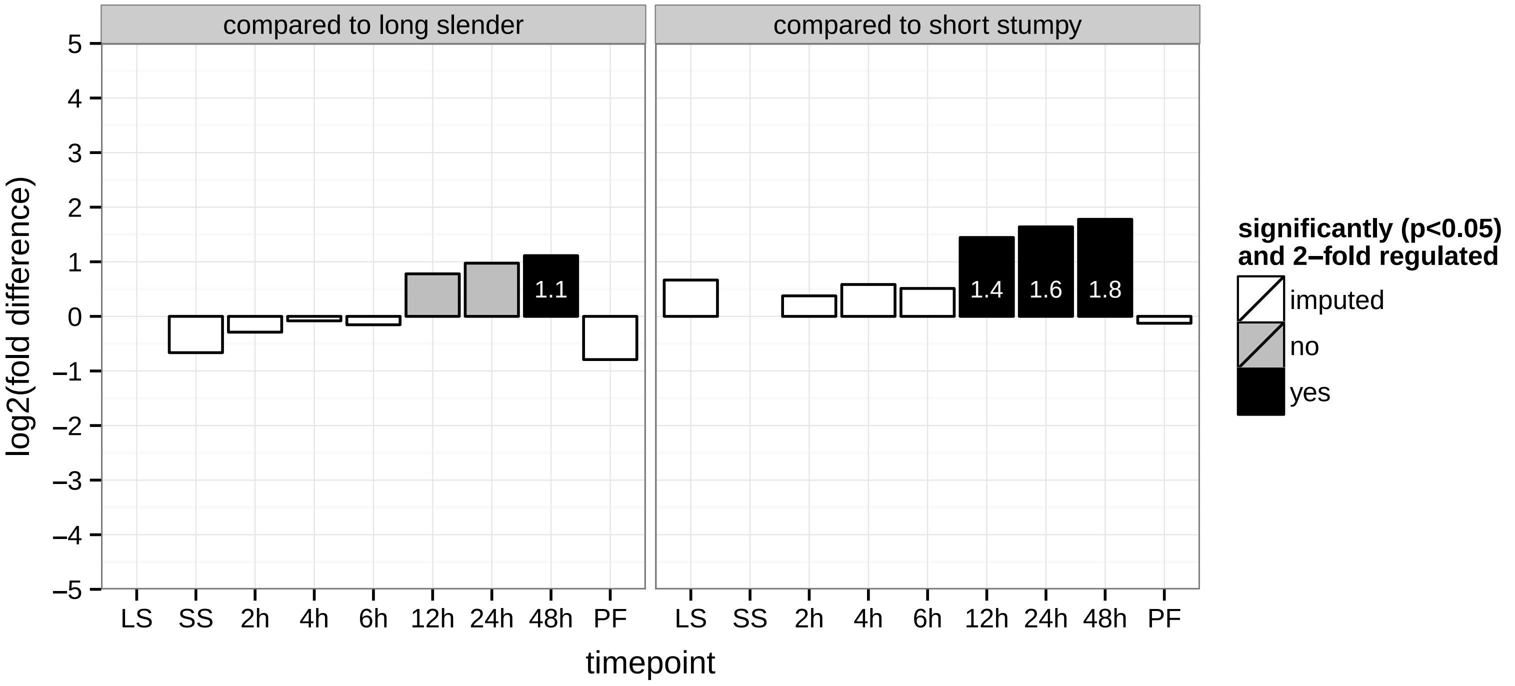

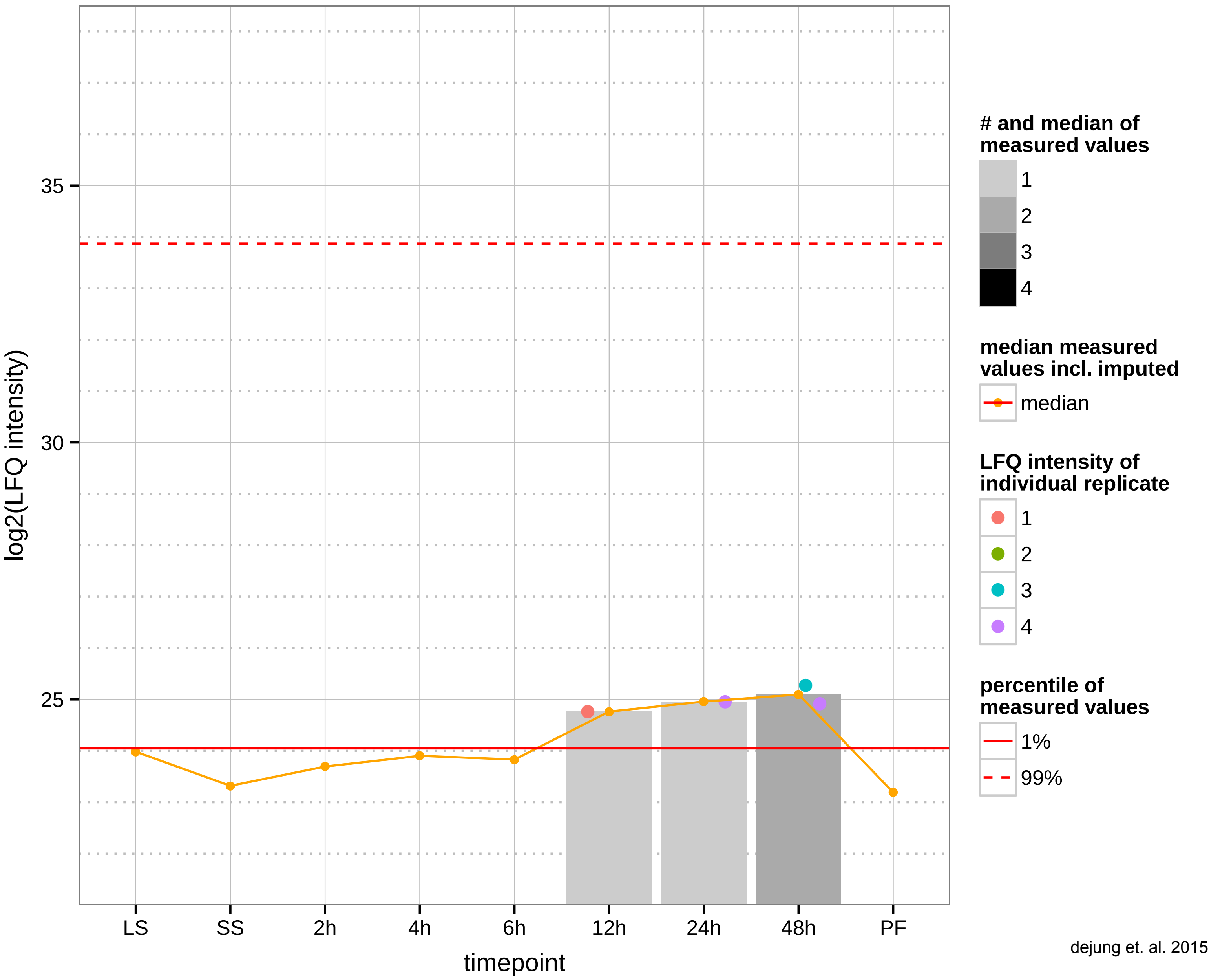

dejung et. al. 2015

calcium motive p−type ATPase, putative
Tb927.9.15460
AGOF: ATP binding, ATPase activity, coupled to transmembrane movement of ions, phosphorylative mechanism, calcium−tra
AGOC: integral to membrane, plasma membrane
AGOP: ATP biosynthetic process, cation transport, metabolic process
PGOF: ATP binding, ATPase activity, coupled to transmembrane movement of ions, phosphorylative mechanism, catalytic acti
PGOC: membrane
PGOP: cation transport, metabolic process

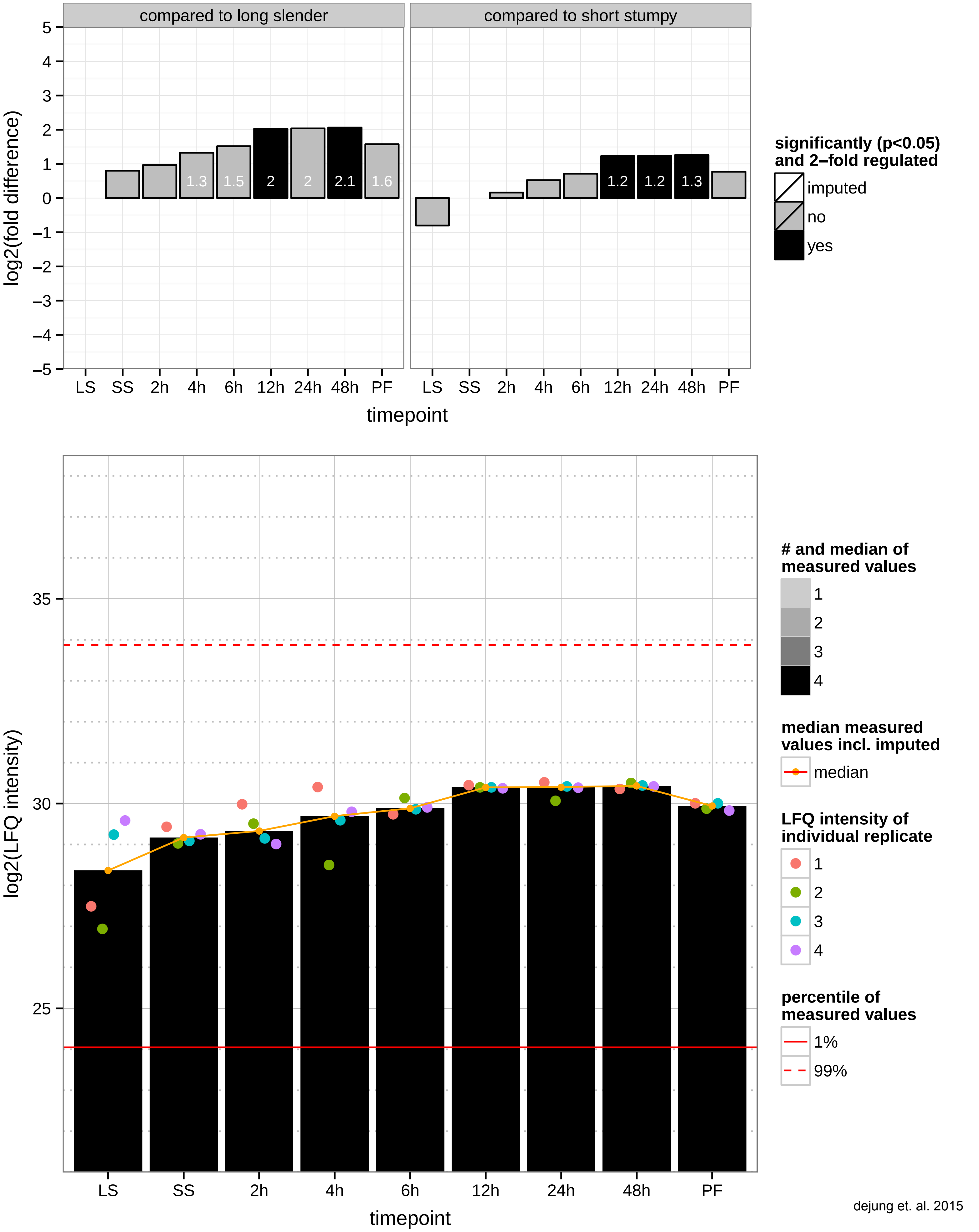

dejung et. al. 2015

hypothetical protein, conserved
Tb927.9.6650
AGOF: ATP binding, alanine–tRNA ligase activity, nucleic acid binding
AGOC: cytoplasm
AGOP: alanyl–tRNA aminoacylation, translation
PGOF: ATP binding, alanine–tRNA ligase activity, ligase activity, forming aminoacyl–tRNA and related compounds, nucleic ac
PGOC: cytoplasm
PGOP: alanyl–tRNA aminoacylation, tRNA aminoacylation

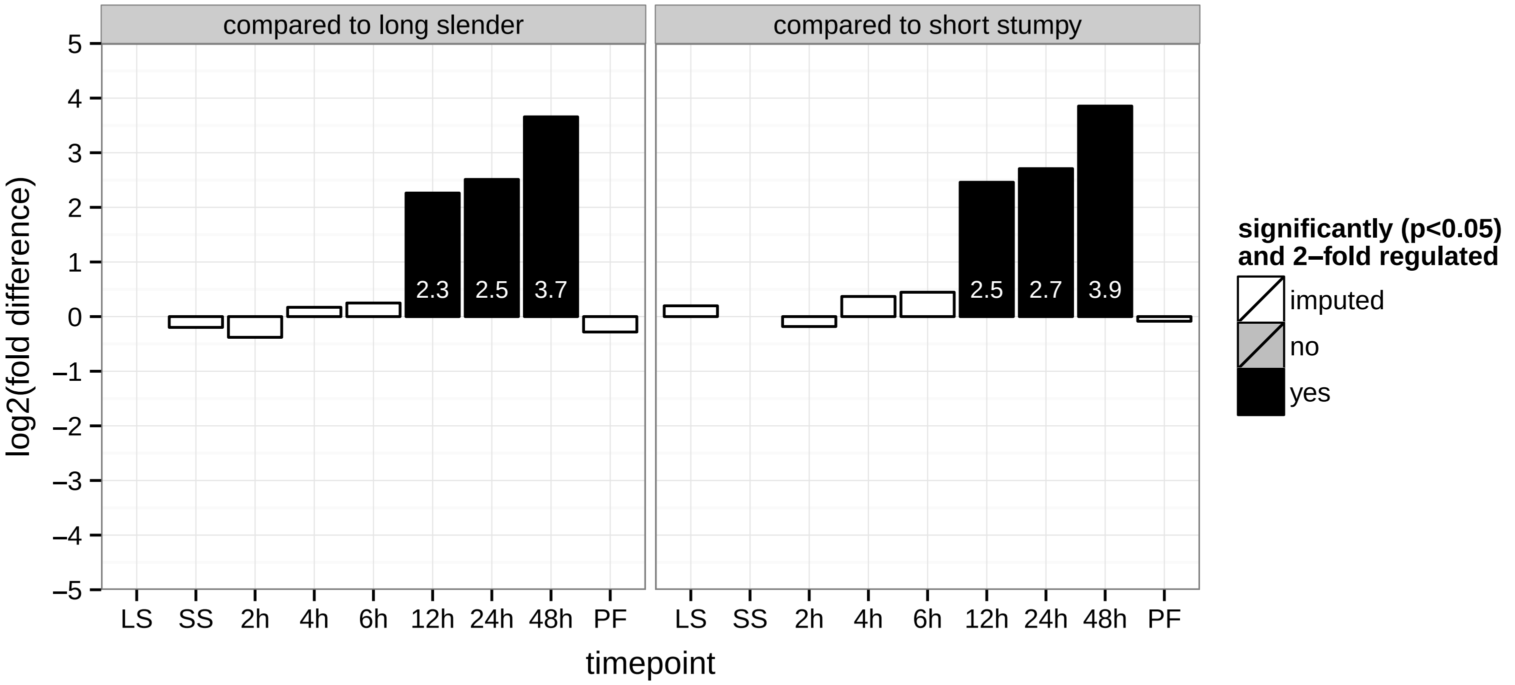

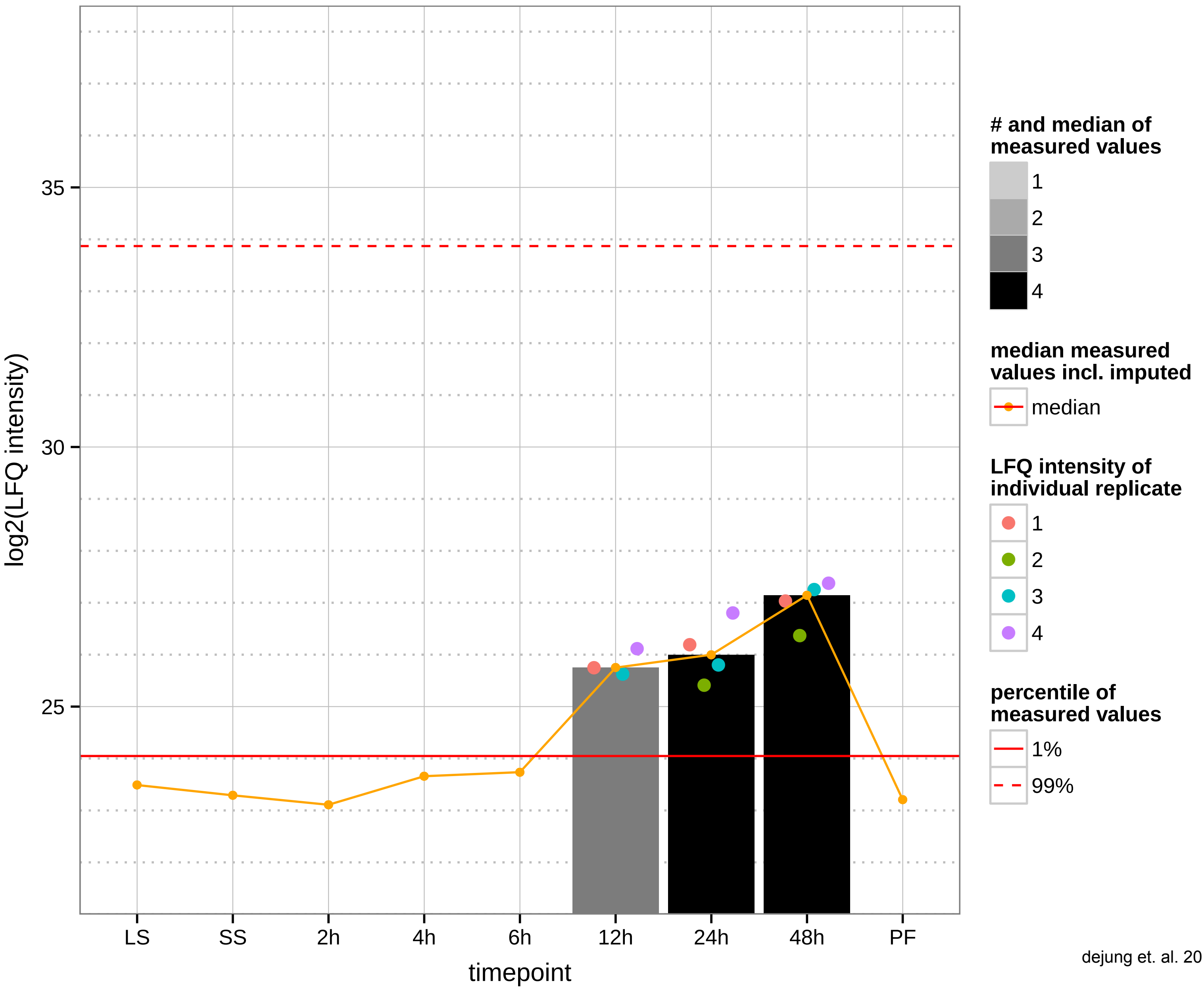

dejung et. al. 2015

hypothetical protein, conserved
Tb927.9.6720
AGOF: null
AGOC: null
AGOP: null
PGOF: null
PGOC: null
PGOP: null

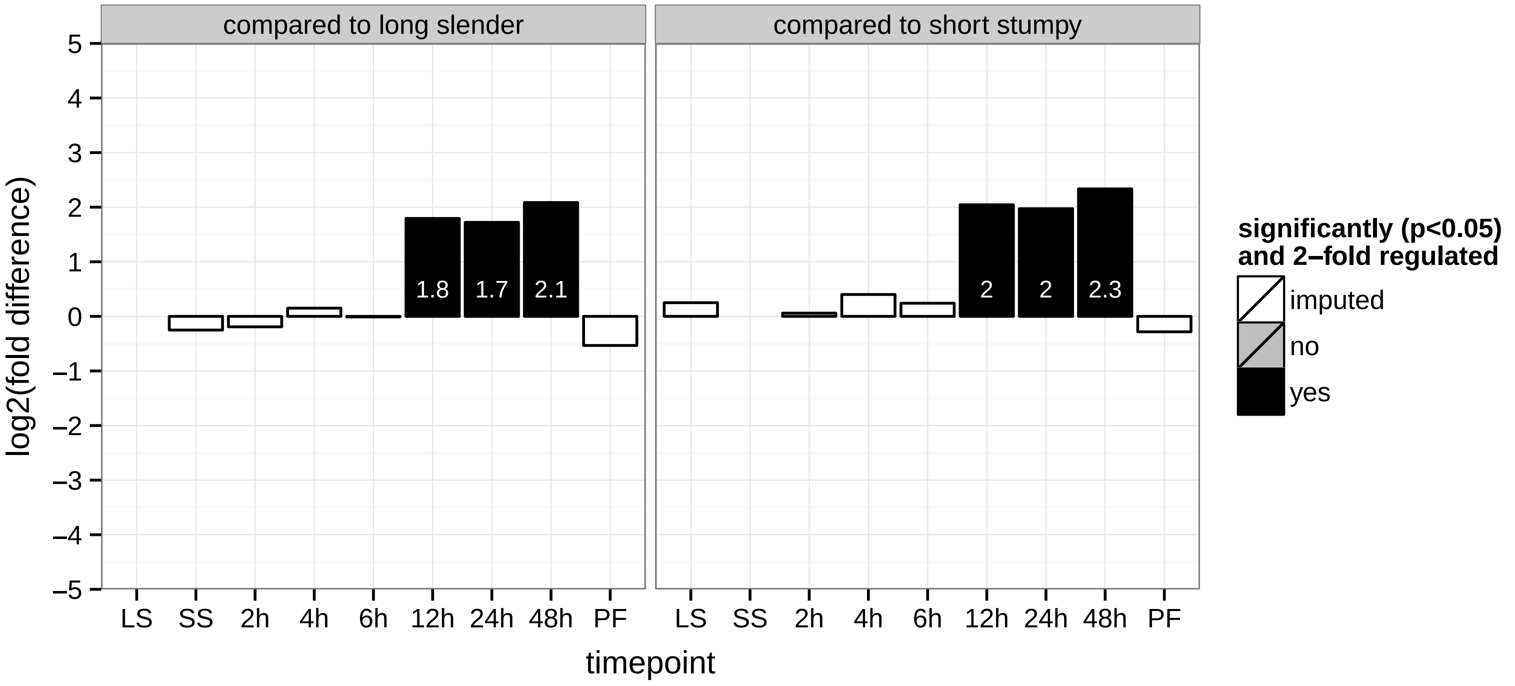

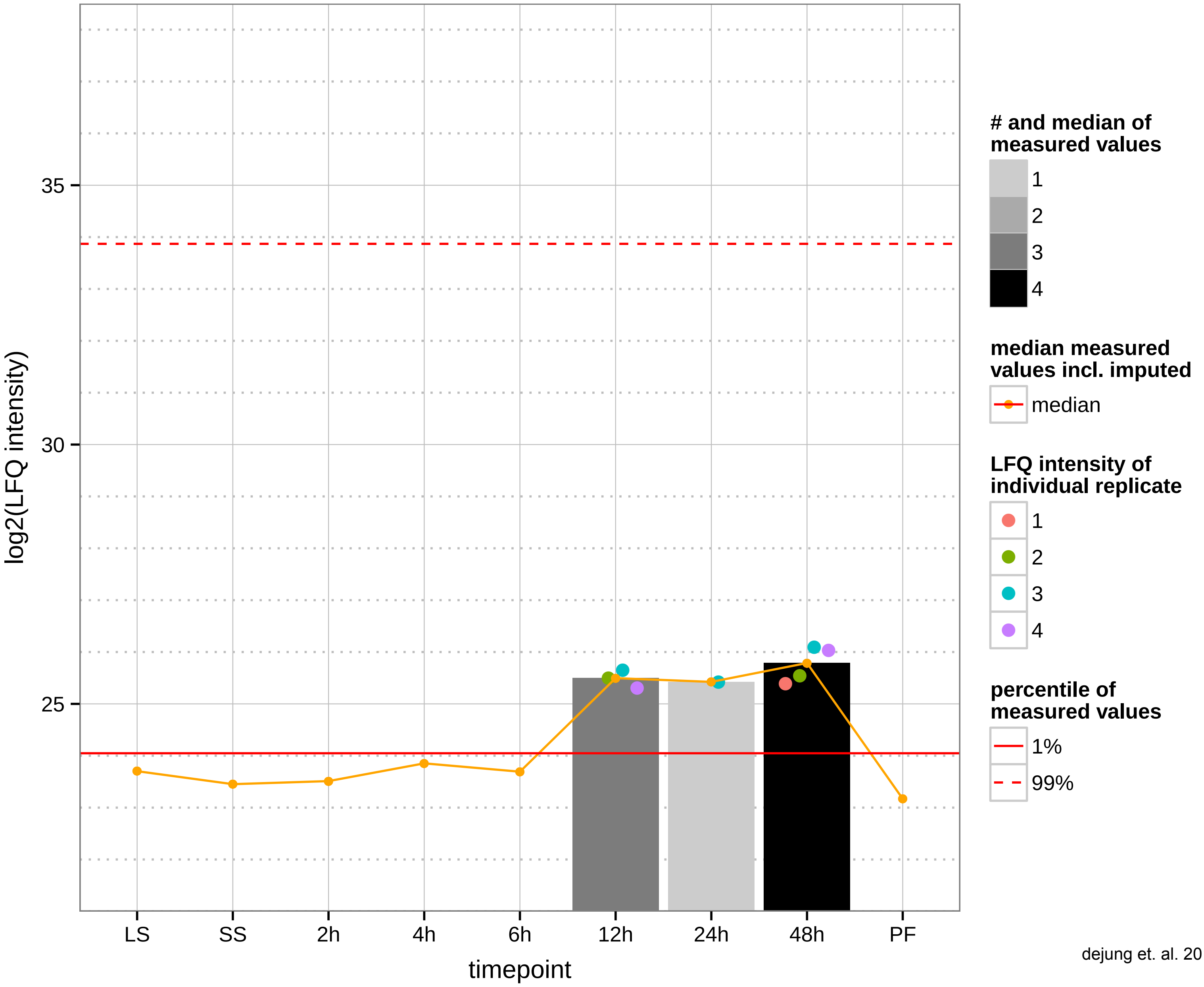

dejung et. al. 2015

cytochrome c oxidase assembly factor, putative
Tb927.9.8680
AGOF: copper ion binding
AGOC: mitochondrial inner membrane, mitochondrion
AGOP: cellular copper ion homeostasis, copper ion transport, oxidation–reduction process, respiratory chain complex IV asse
PGOF: copper ion binding
PGOC: mitochondrial inner membrane
PGOP: cellular copper ion homeostasis, copper ion transport, respiratory chain complex IV assembly

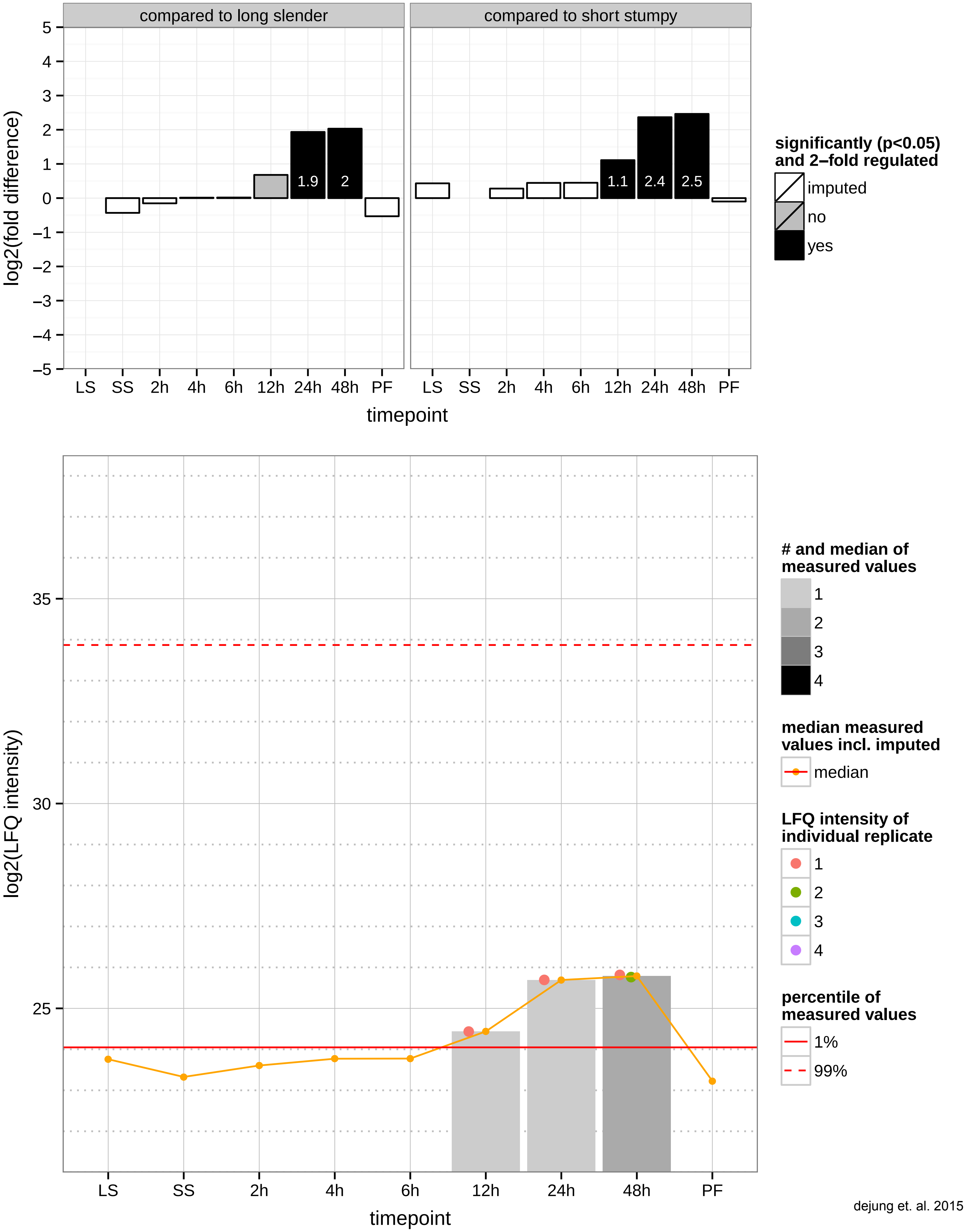

dejung et. al. 2015

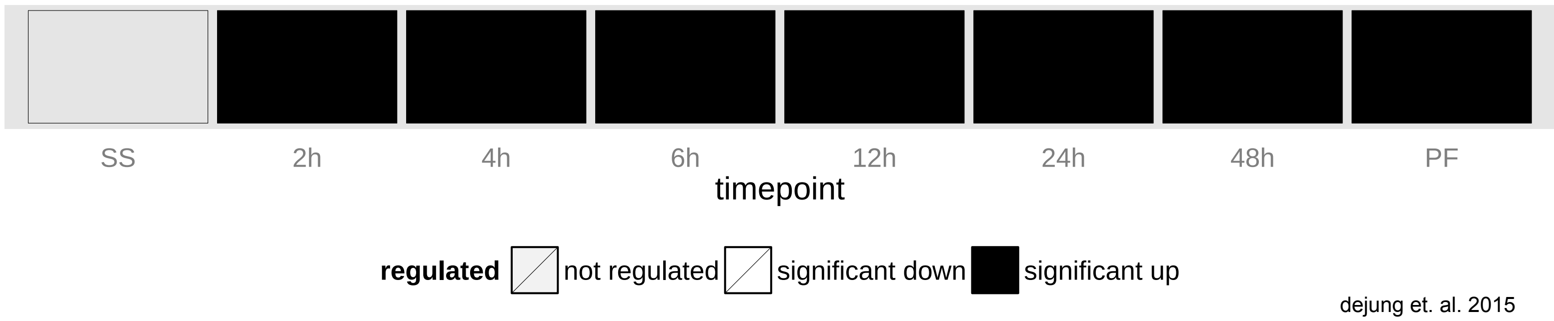
SS
2h
4h
6h
12h
24h
48h
PF
timepoint
regulated
not regulated
significant down
significant up
dejung et. al. 2015

NA, cystathionine beta−synthase, putative
Tb11.02.5400b;Tb11.02.5400
AGOF: NA, cystathionine beta−synthase activity, pyridoxal phosphate binding
AGOC: NA, null
AGOP: NA, cysteine biosynthetic process
PGOF: NA, catalytic activity, pyridoxal phosphate binding
PGOC: NA, null
PGOP: NA, metabolic process

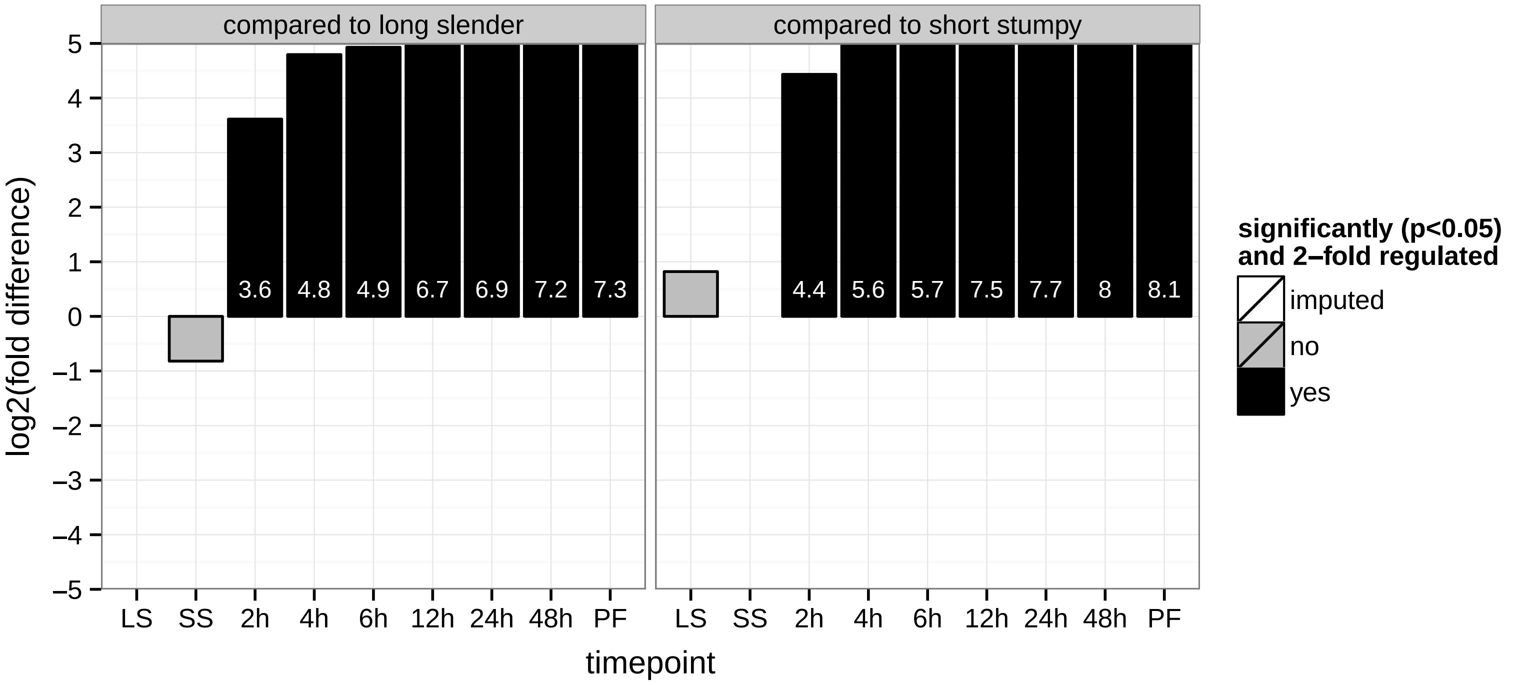

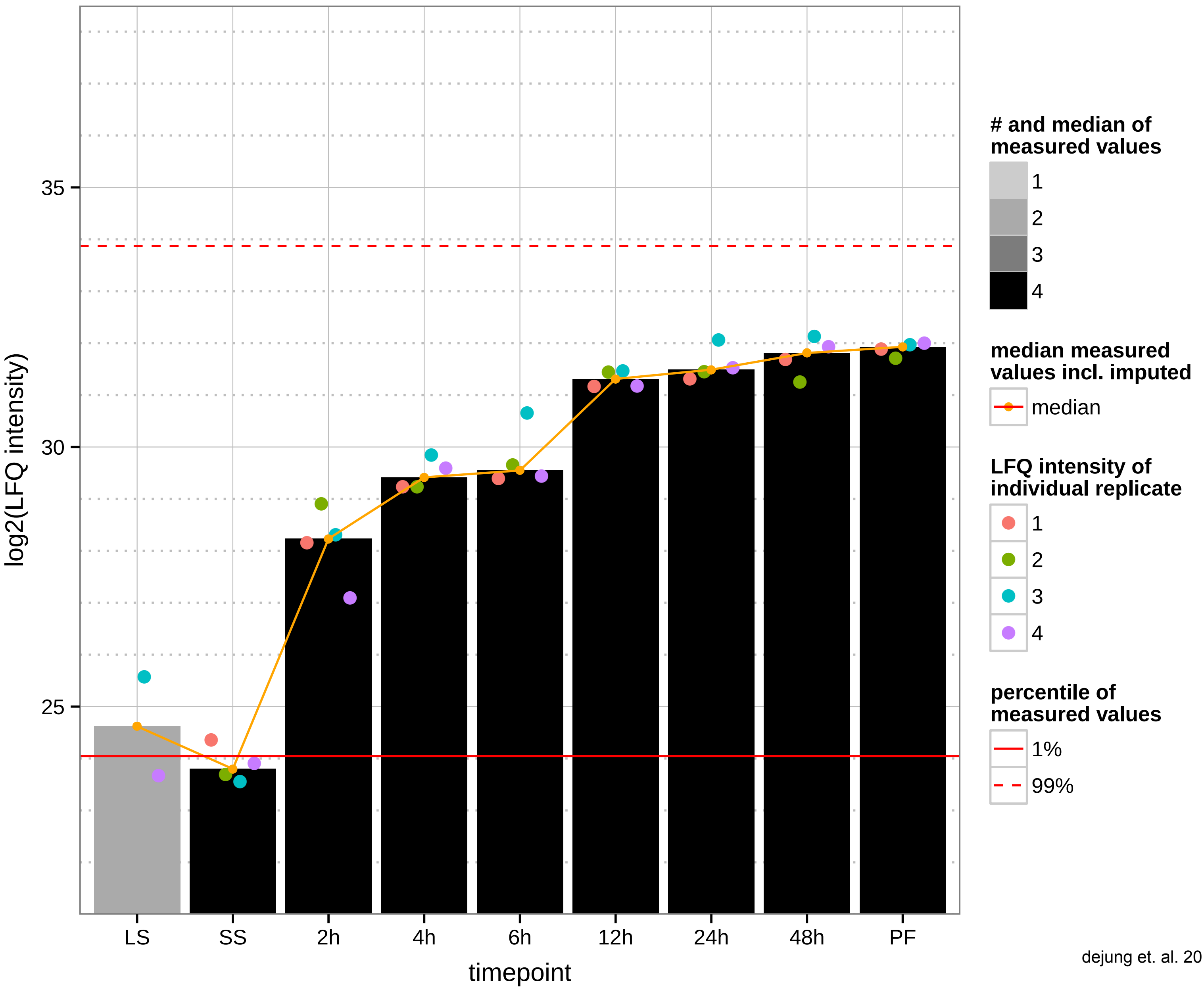

dejung et. al. 2015

NAD(p)−dependent steroid dehydrogenase−like protein, NAD(P)−dependent steroid dehydrogenase protein, putative
Tb927.7.4950;Tb11.v5.0176
AGOF: null, 3−beta−hydroxy−delta5−steroid dehydrogenase activity, catalytic activity
AGOC: null
AGOP: null, steroid biosynthetic process
PGOF: 3−beta−hydroxy−delta5−steroid dehydrogenase activity, oxidoreductase activity, acting on the CH−OH group of donors
PGOC: null
PGOP: oxidation−reduction process, steroid biosynthetic process

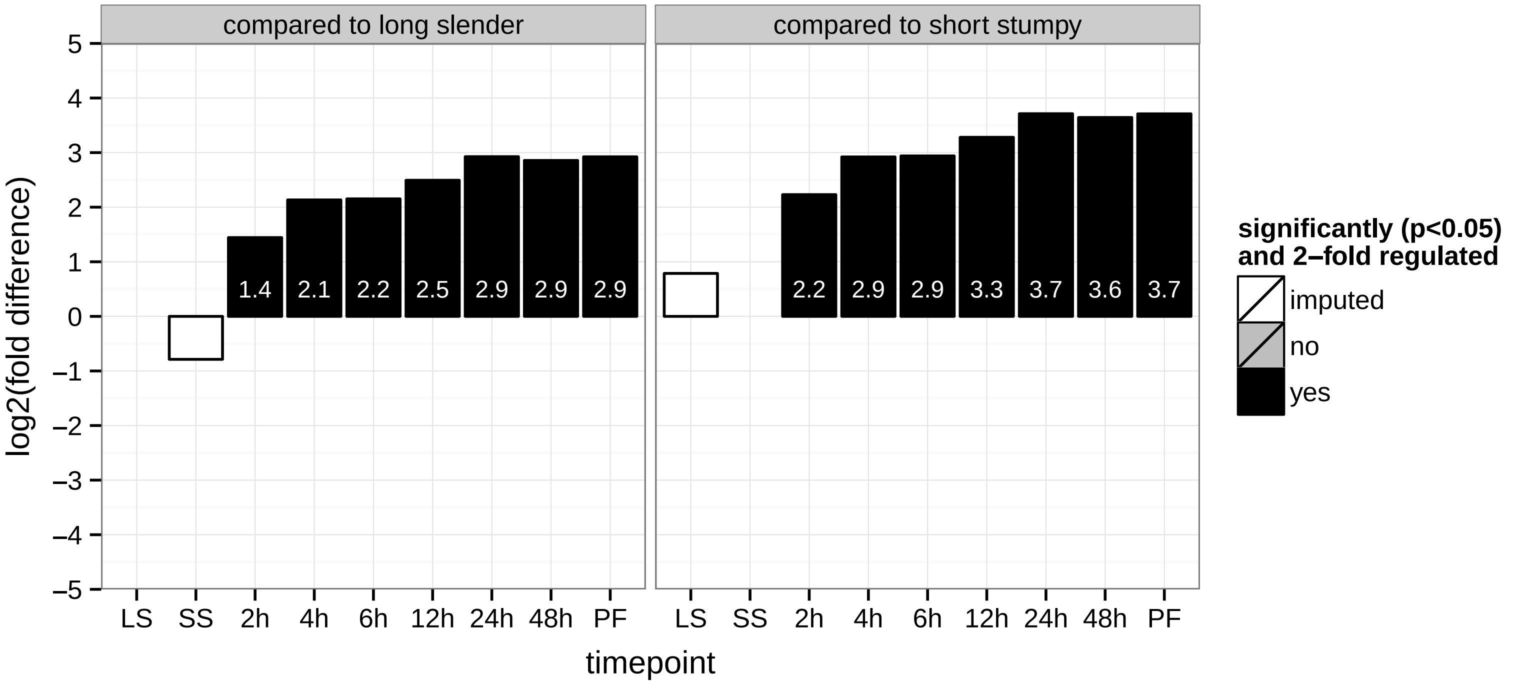

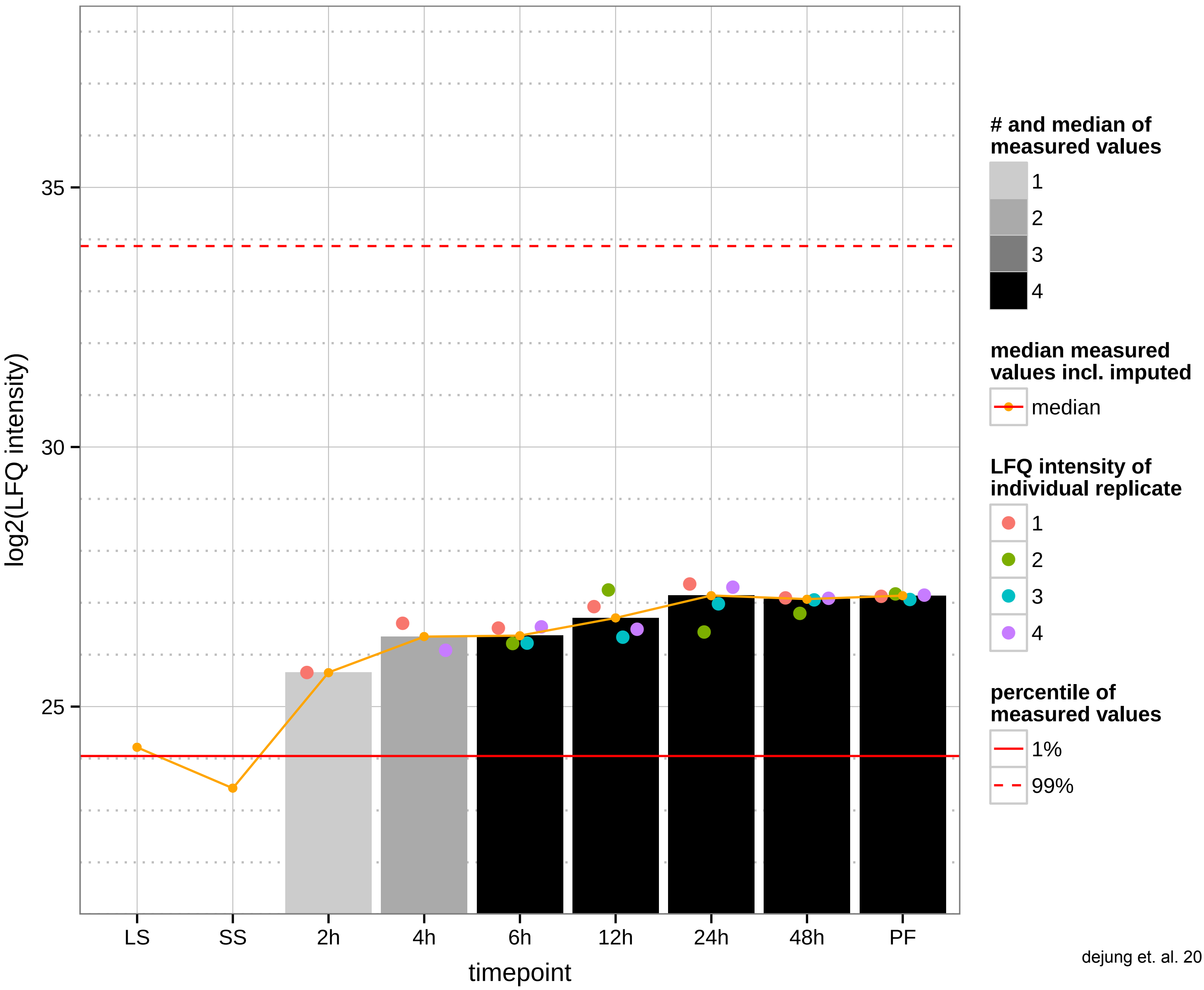

dejung et. al. 2015

hypothetical protein, conserved, conserved protein, unknown function
Tb927.1.1470;Tb11.v5.0315
AGOF: null
AGOC: null
AGOP: null
PGOF: null
PGOC: null
PGOP: null

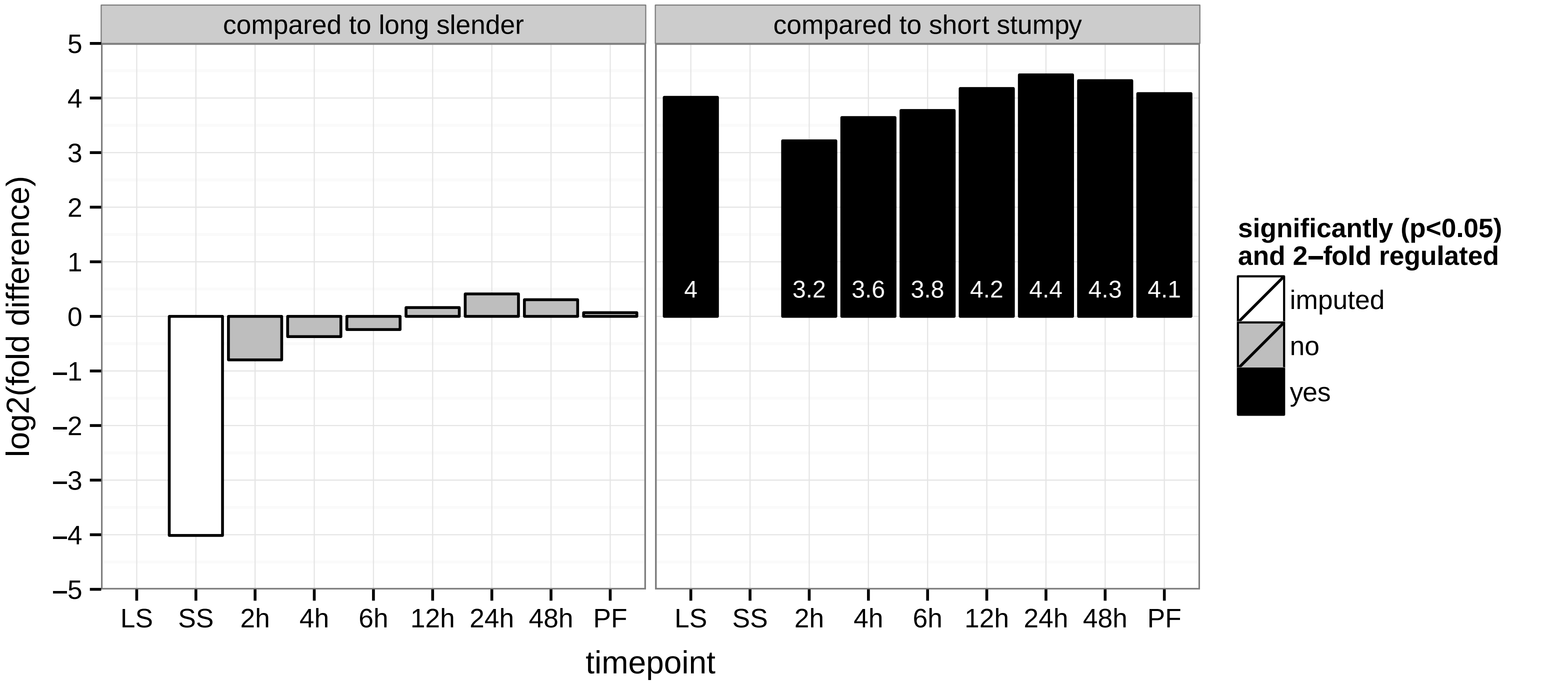

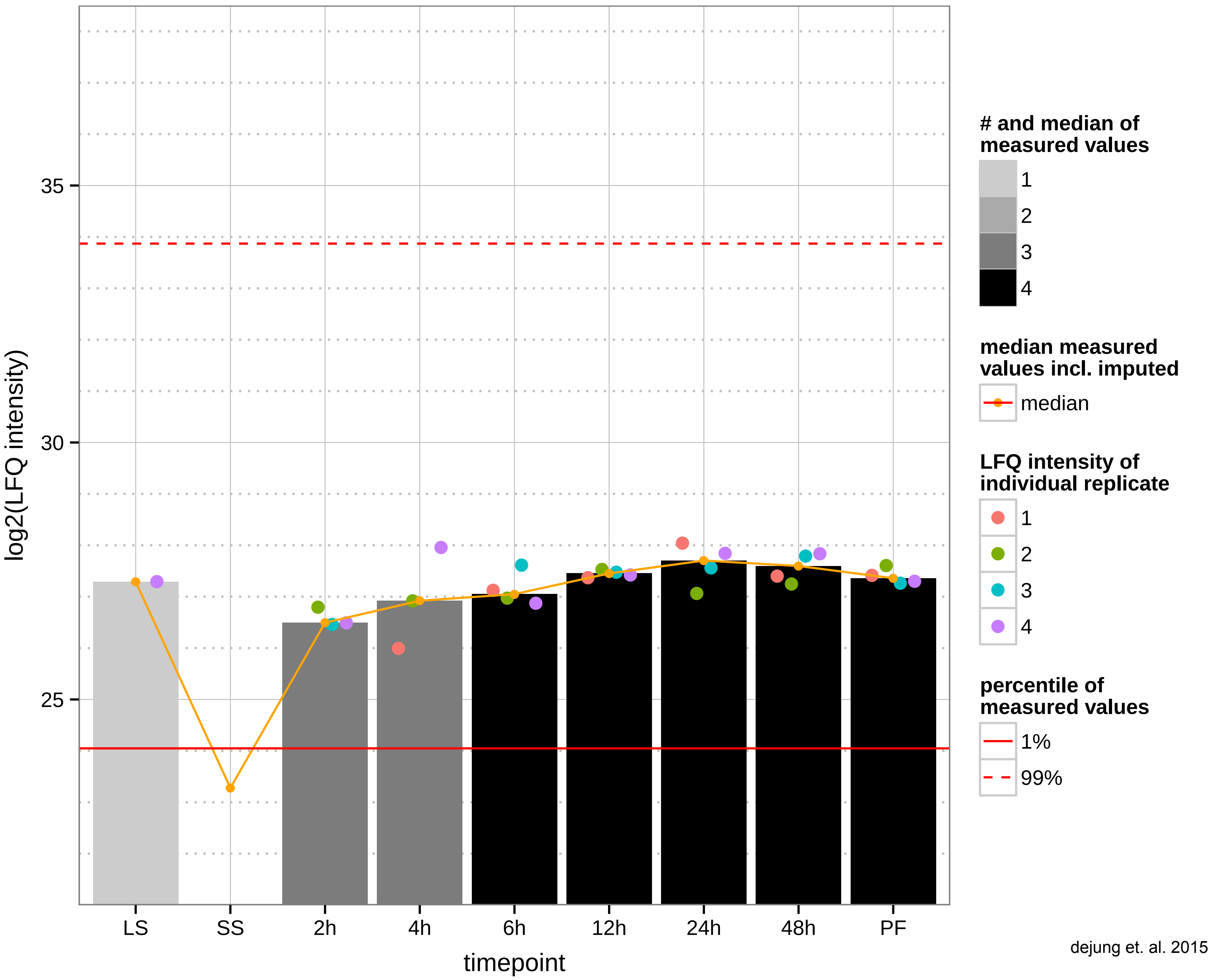

dejung et. al. 2015

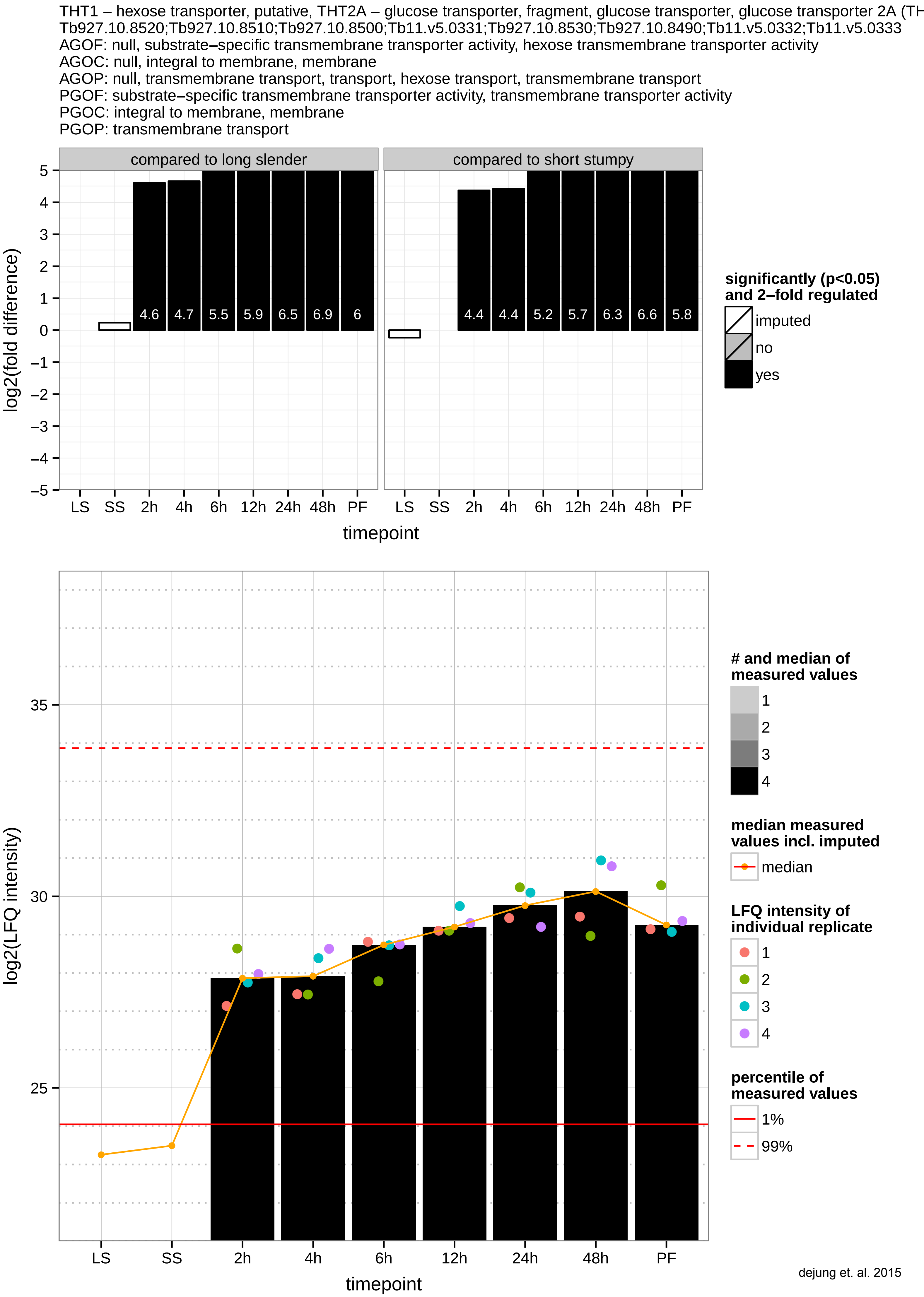
THT1 – hexose transporter, putative, THT2A – glucose transporter, fragment, glucose transporter, glucose transporter 2A (TH
Tb927.10.8520;Tb927.10.8510;Tb927.10.8500;Tb11.v5.0331;Tb927.10.8530;Tb927.10.8490;Tb11.v5.0332;Tb11.v5.0333
AGOF: null, substrate–specific transmembrane transporter activity, hexose transmembrane transporter activity
AGOC: null, integral to membrane, membrane
AGOP: null, transmembrane transport, transport, hexose transport, transmembrane transport
PGOF: substrate–specific transmembrane transporter activity, transmembrane transporter activity
PGOC: integral to membrane, membrane
PGOP: transmembrane transport
compared to long slender
compared to short stumpy
log2(fold difference)
4.6
4.7
5.5
5.9
6.5
6.9
6
4.4
4.4
5.2
5.7
6.3
6.6
5.8
LS SS 2h 4h 6h 12h 24h 48h PF
timepoint
significantly (p<0.05) and 2–fold regulated
imputed
no
yes
log2(LFQ intensity)
# and median of measured values
1
2
3
4
median measured values incl. imputed
median
LFQ intensity of individual replicate
1
2
3
4
percentile of measured values
1%
99%
dejung et. al. 2015

major surface protease gp63, putative, MSP−B
Tb927.8.1630;Tb927.8.1620;Tb927.8.1610;Tb11.v5.0601
AGOF: null, metalloendopeptidase activity, zinc ion binding
AGOC: null, membrane
AGOP: null, cell adhesion, proteolysis
PGOF: metalloendopeptidase activity, zinc ion binding, null
PGOC: membrane, null
PGOP: cell adhesion, proteolysis, null

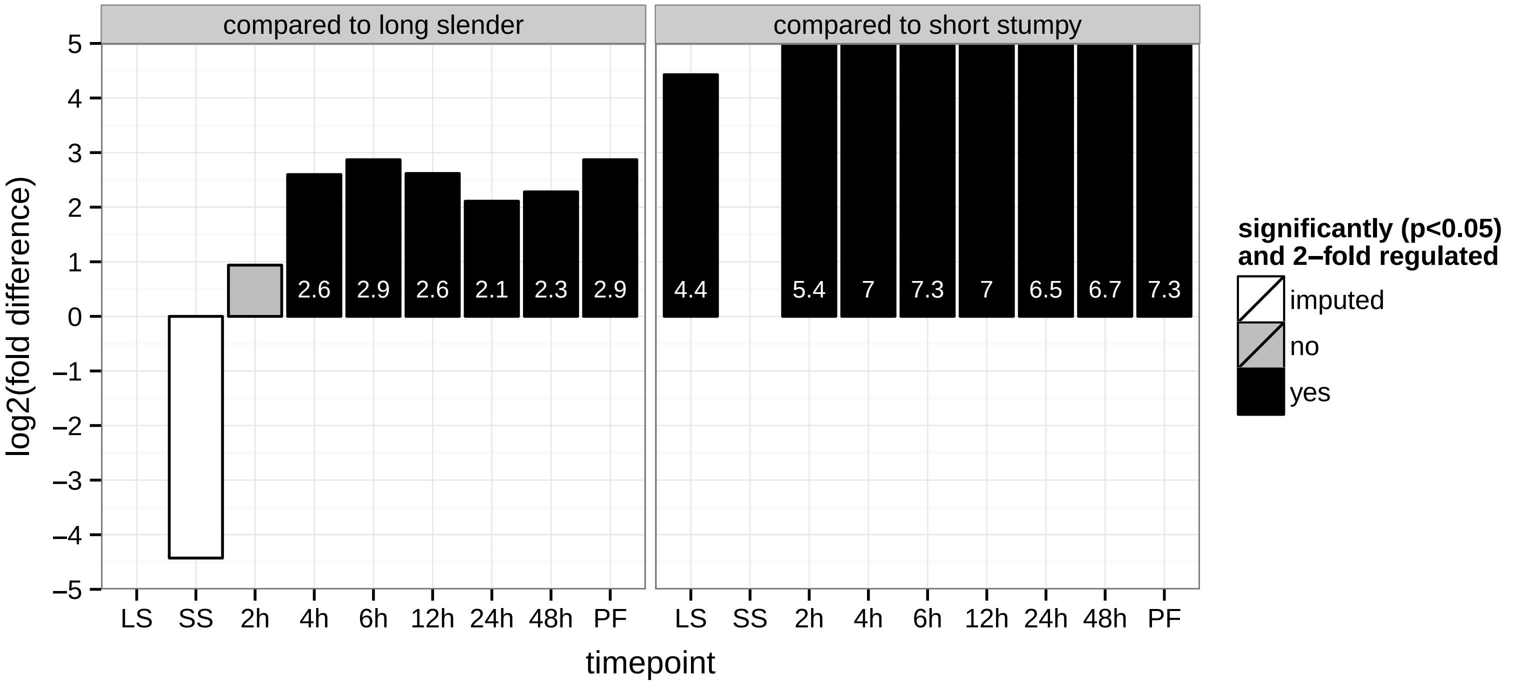

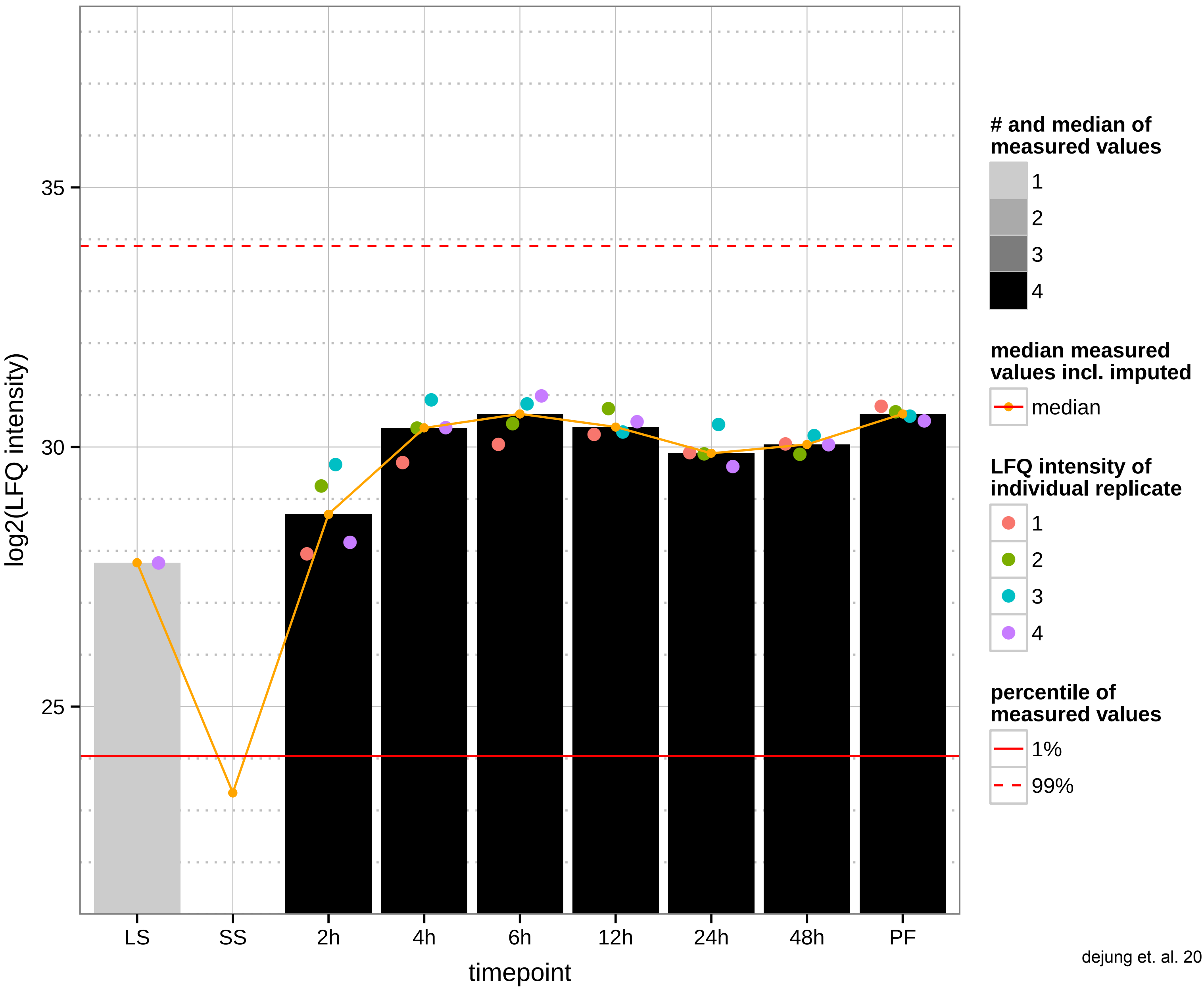

dejung et. al. 2015

phosphatidylinositol 3−kinase, putative
Tb927.1.1930
AGOF: 1−phosphatidylinositol−3−kinase activity, phosphotransferase activity, alcohol group as acceptor
AGOC: null
AGOP: cell proliferation, phosphatidylinositol phosphorylation
PGOF: binding, phosphotransferase activity, alcohol group as acceptor, protein binding, transferase activity, transferring phosp
PGOC: null
PGOP: null

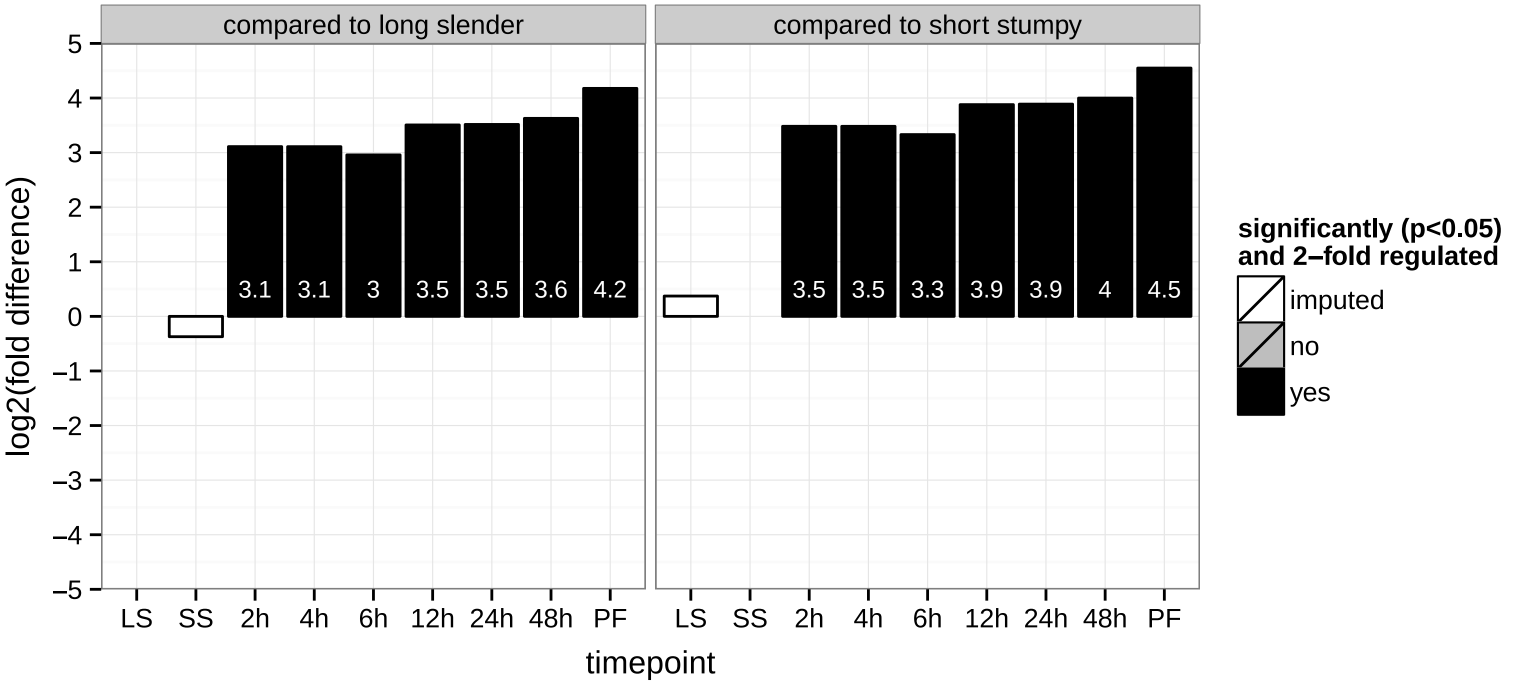

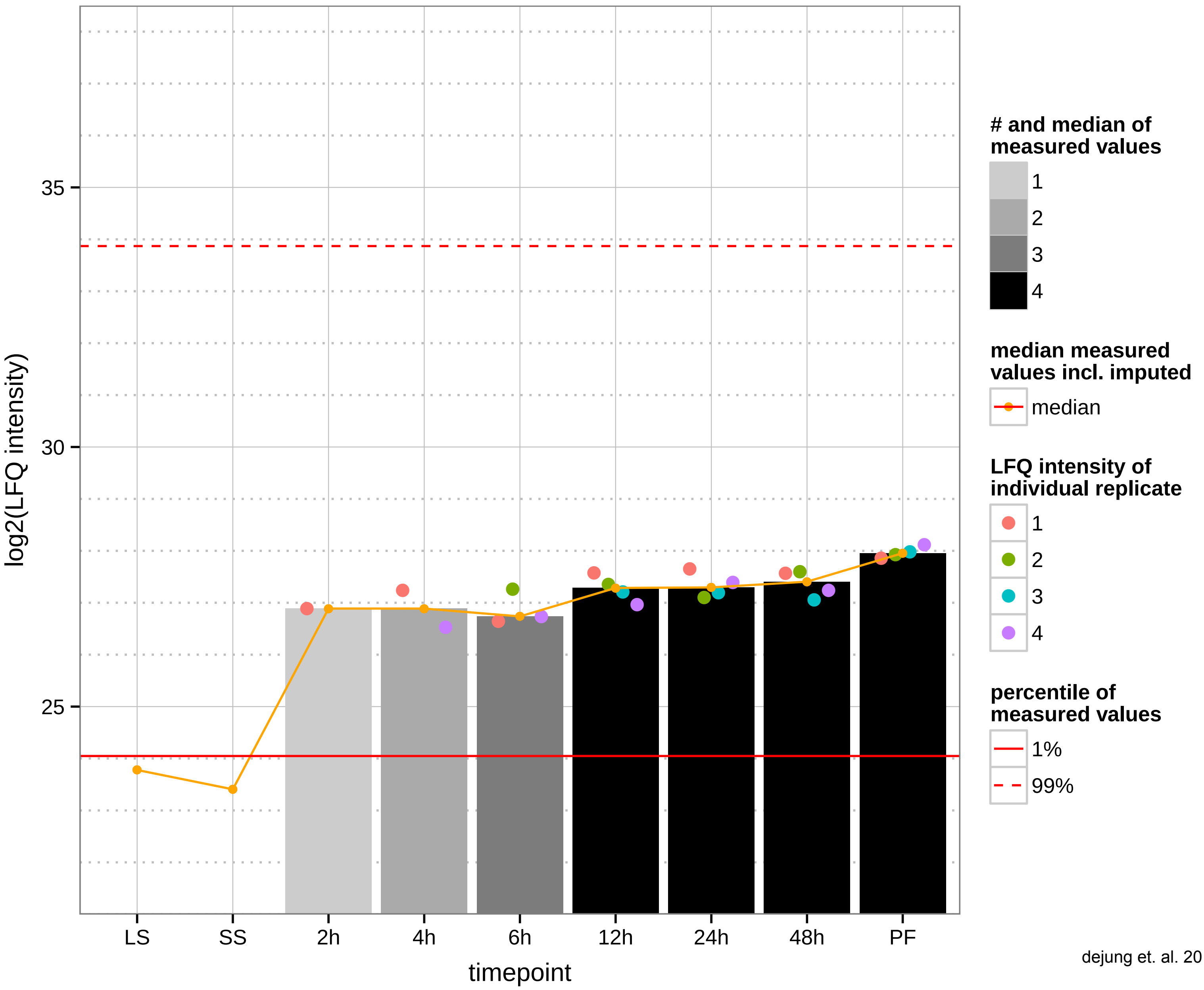

dejung et. al. 2015

hypothetical protein, conserved
Tb927.10.10000
AGOF: null
AGOC: null
AGOP: null
PGOF: null
PGOC: null
PGOP: null

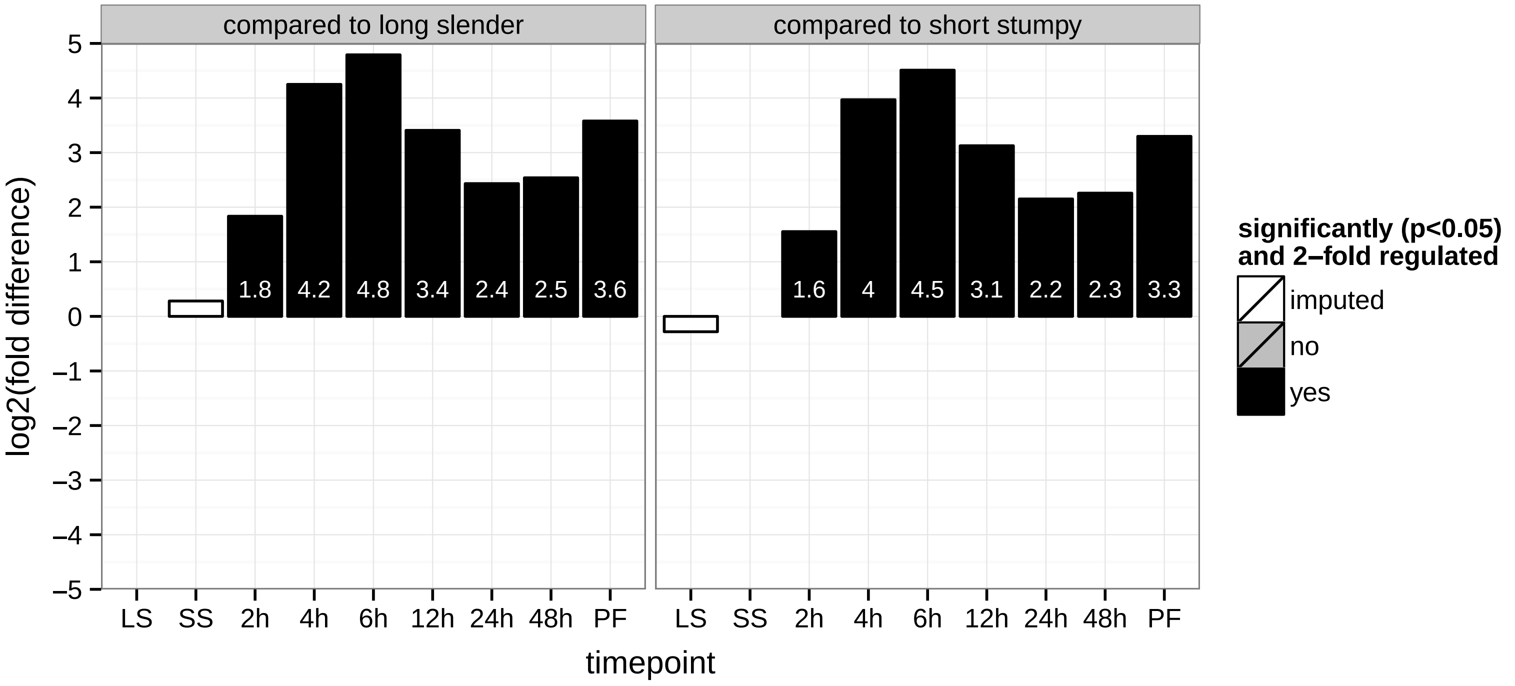

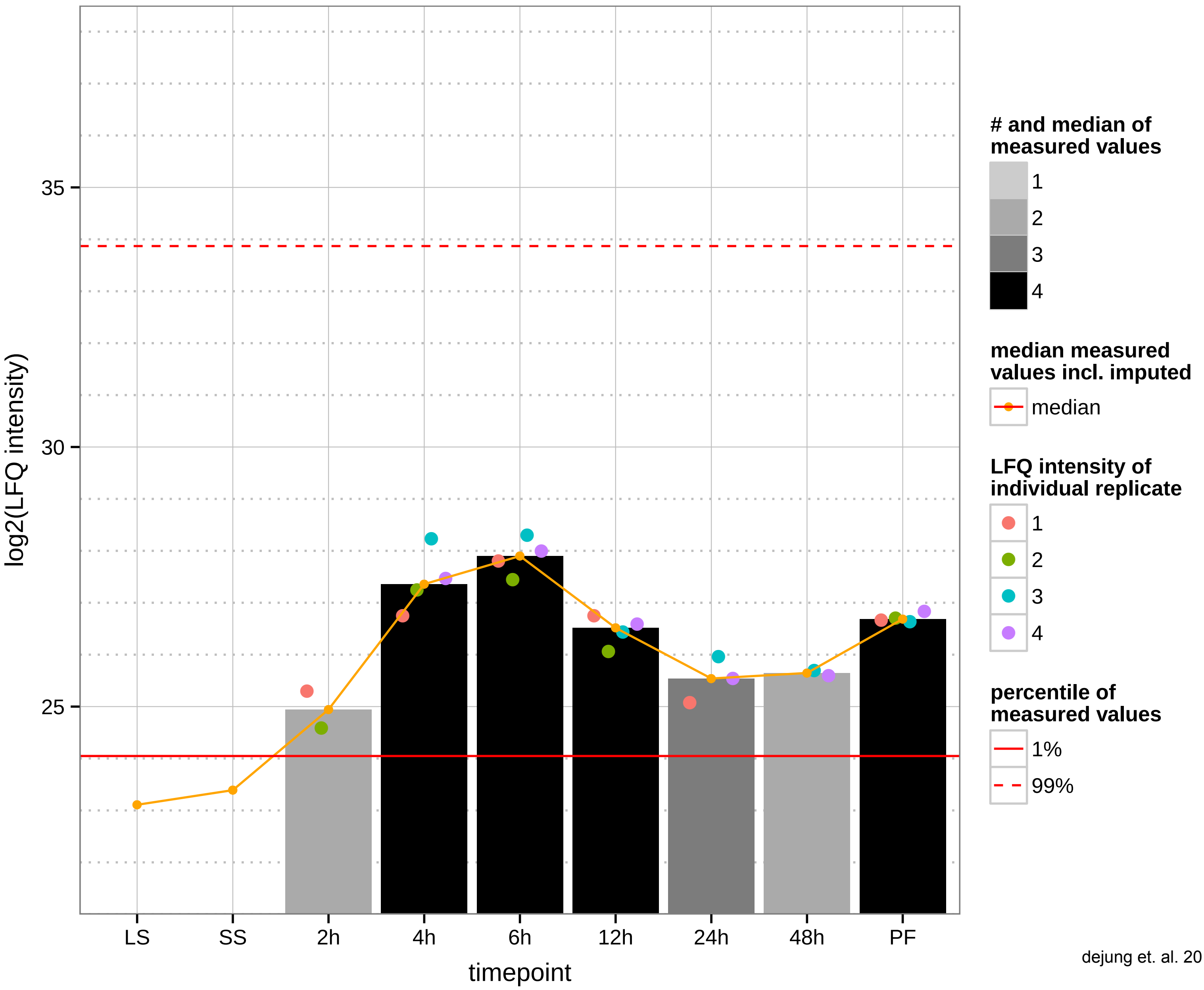

dejung et. al. 2015

procyclic form surface phosphoprotein (PSSA–2)
Tb927.10.11220
AGOF: structural constituent of cell wall
AGOC: cell surface
AGOP: plant–type cell wall organization
PGOF: structural constituent of cell wall
PGOC: null
PGOP: plant–type cell wall organization

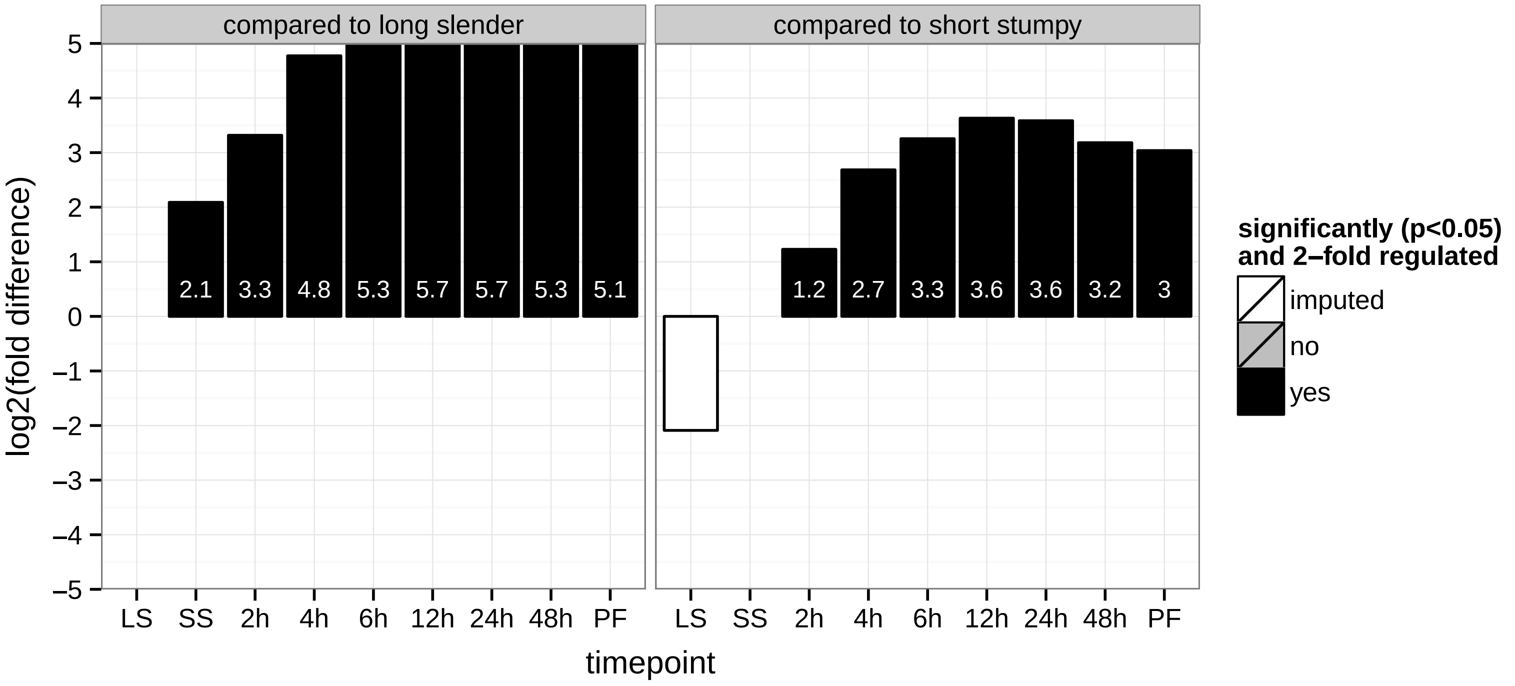

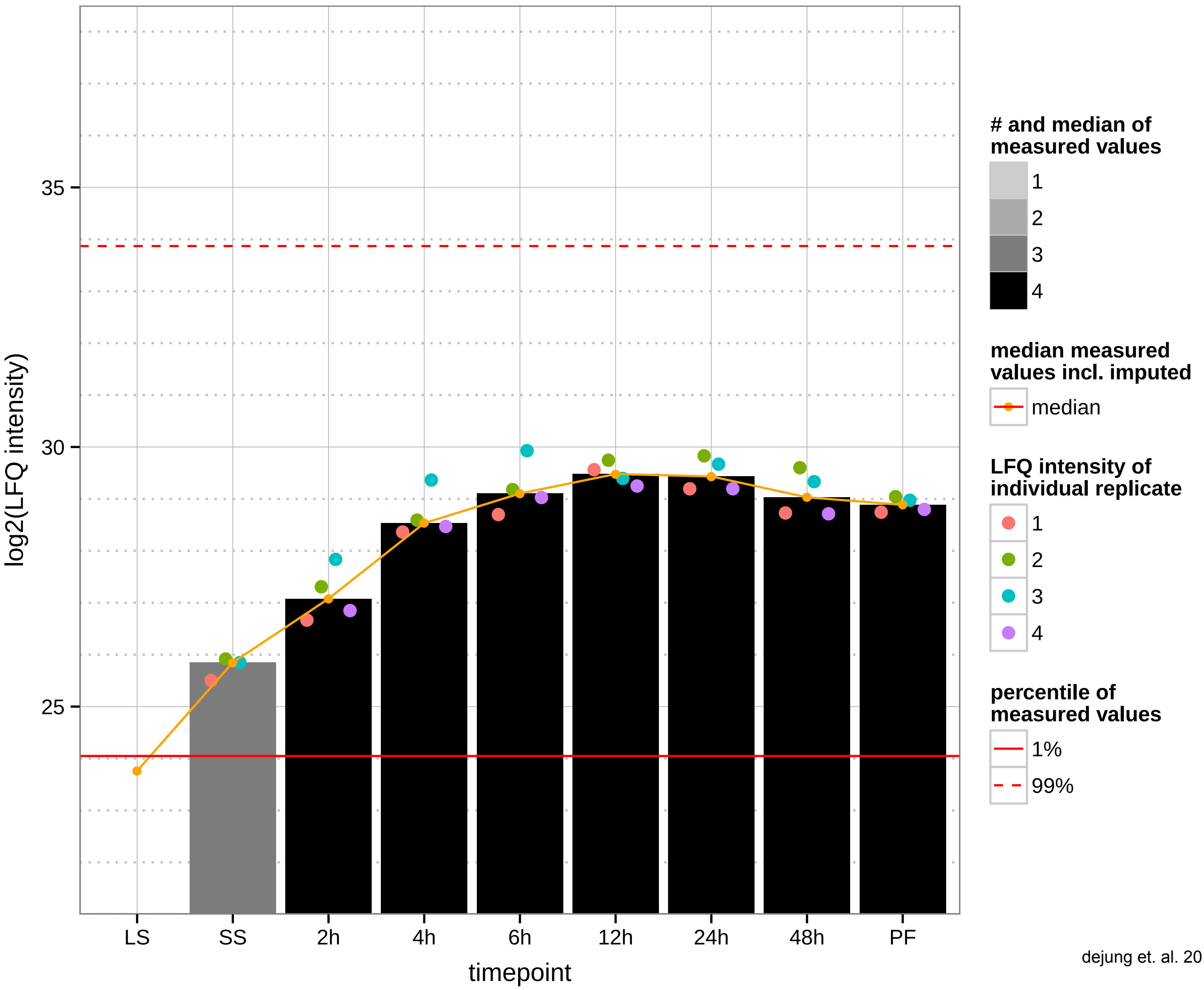

dejung et. al. 2015

NADH–ubiquinone oxidoreductase complex I subunit, FT putative, NDUFA9 subunit, putative
Tb927.10.13620
AGOF: NAD binding, oxidoreductase activity, acting on the aldehyde or oxo group of donors, NAD or NADP as acceptor
AGOC: cytoplasm, mitochondrion
AGOP: cellular amino acid metabolic process
PGOF: 3–beta–hydroxy–delta5–steroid dehydrogenase activity, oxidoreductase activity, acting on the CH–OH group of donors
PGOC: null
PGOP: oxidation–reduction process, steroid biosynthetic process

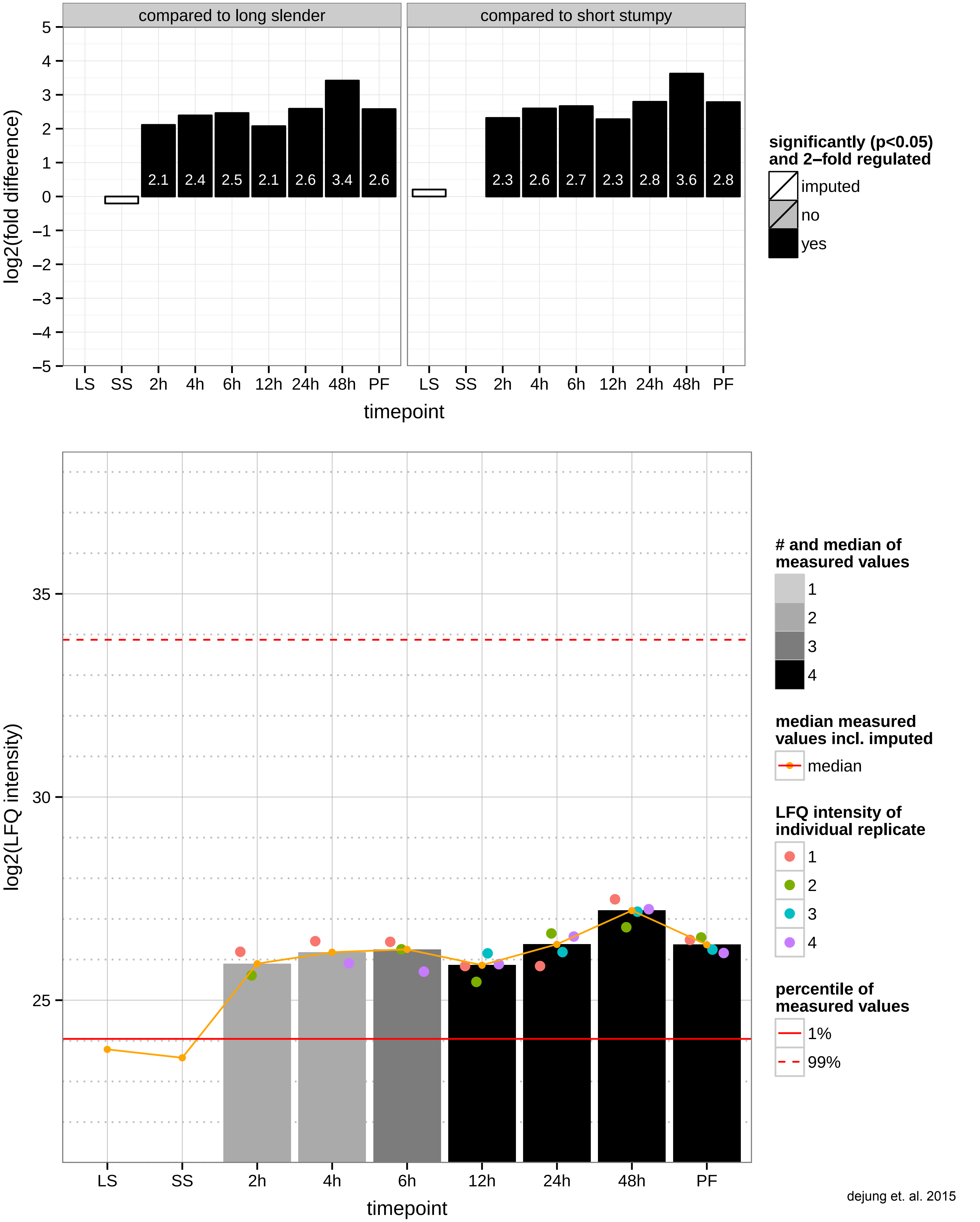

dejung et. al. 2015

chaperone protein DNAj, putative
Tb927.10.13830
AGOF: heat shock protein binding, unfolded protein binding
AGOC: integral to membrane, mitochondrial inner membrane, mitochondrion
AGOP: protein folding
PGOF: heat shock protein binding
PGOC: null
PGOP: null

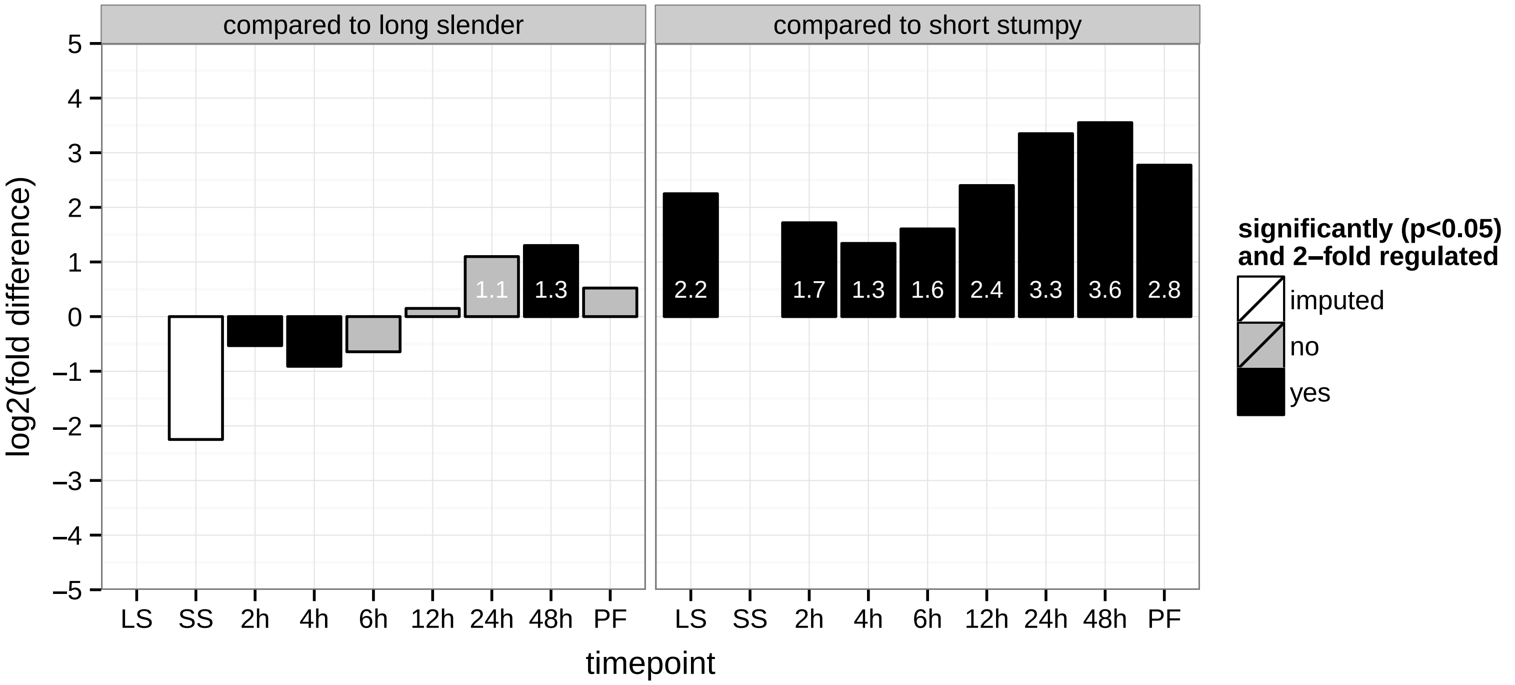

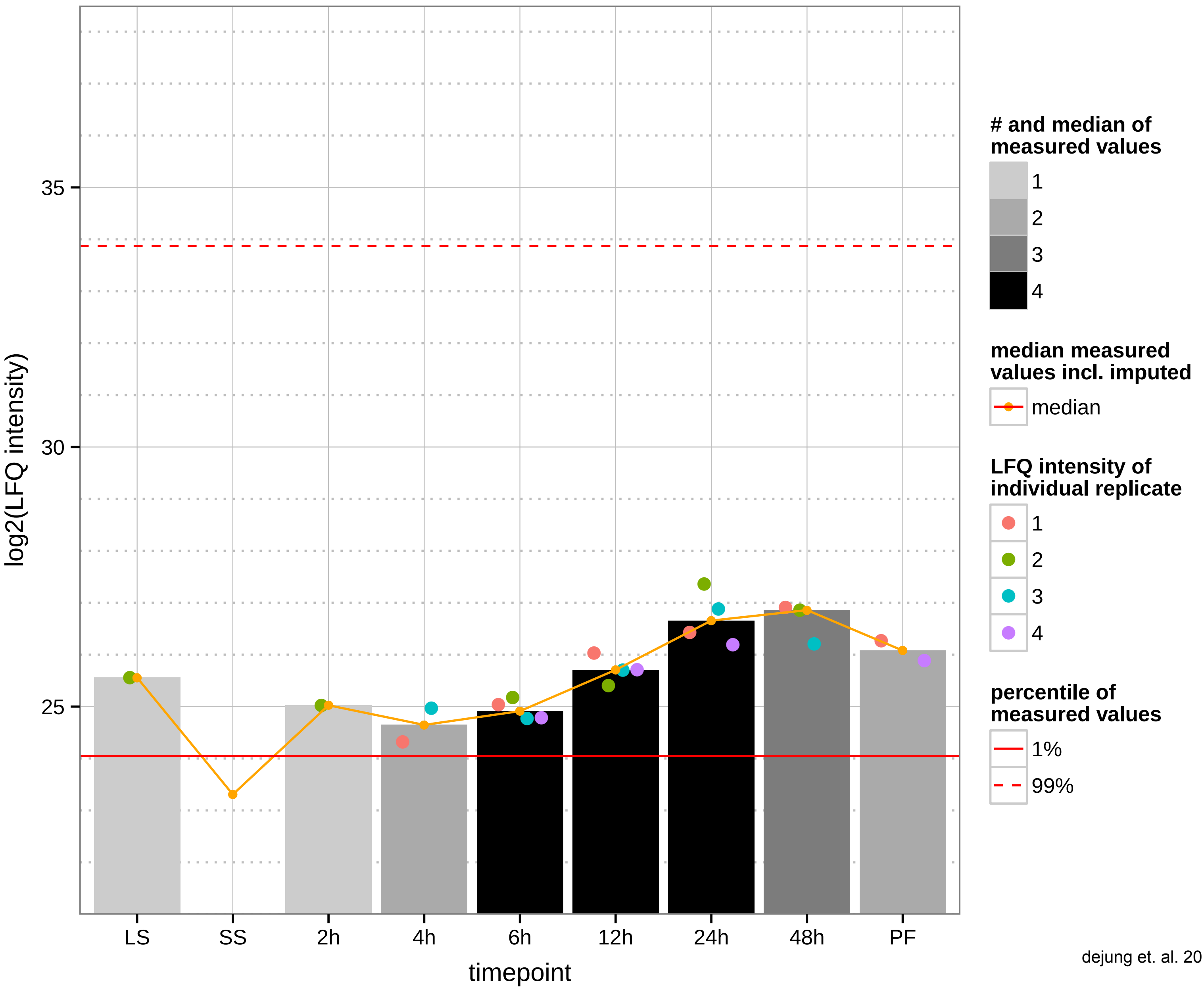

dejung et. al. 2015

hypothetical protein, conserved
Tb927.10.13850
AGOF: null
AGOC: mitochondrion
AGOP: proton–transporting ATP synthase complex assembly
PGOF: null
PGOC: null
PGOP: proton–transporting ATP synthase complex assembly

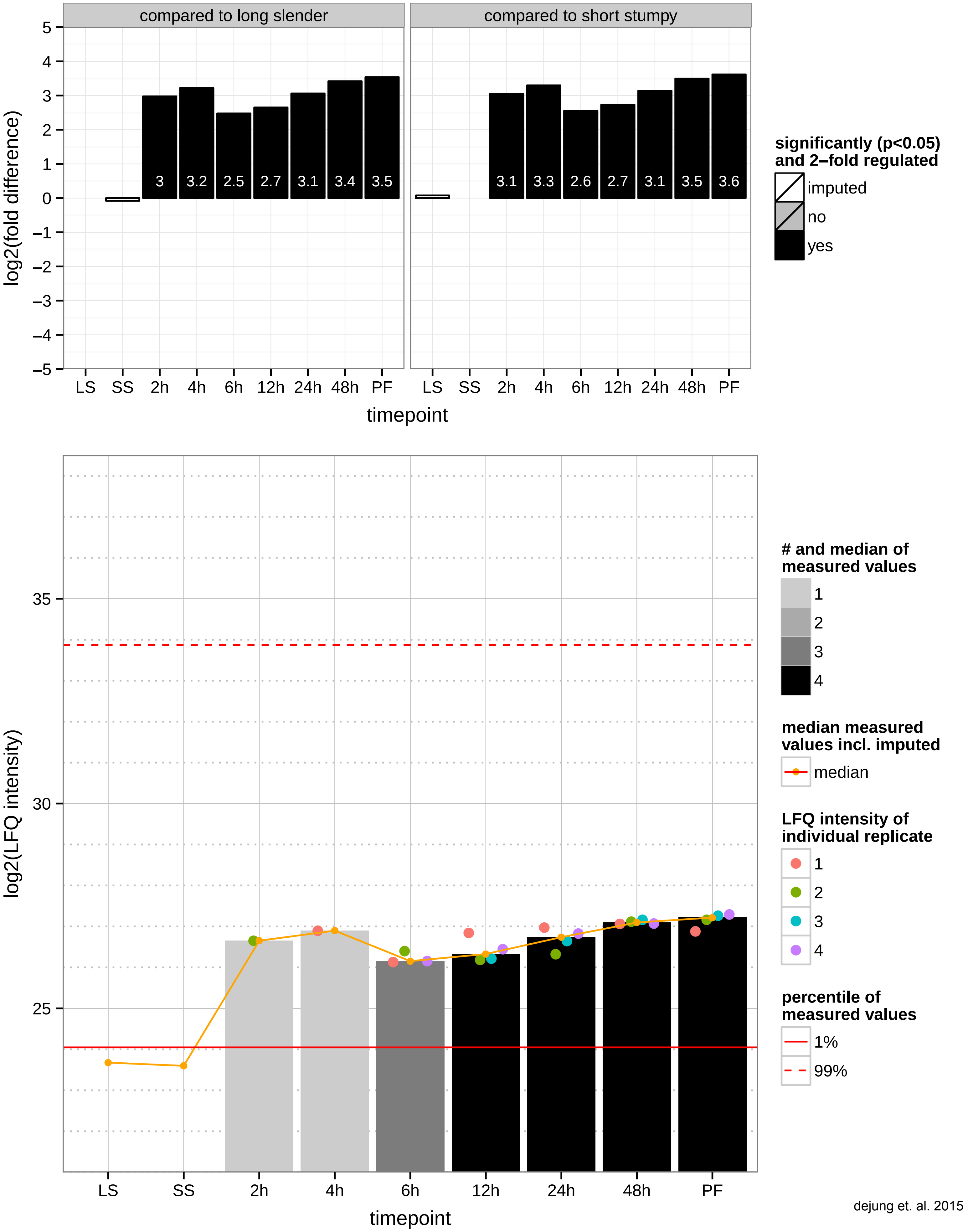

dejung et. al. 2015

hypothetical protein, conserved
Tb927.10.15280;Tb11.v5.0999
AGOF: null
AGOC: null
AGOP: null
PGOF: protein binding
PGOC: null
PGOP: null

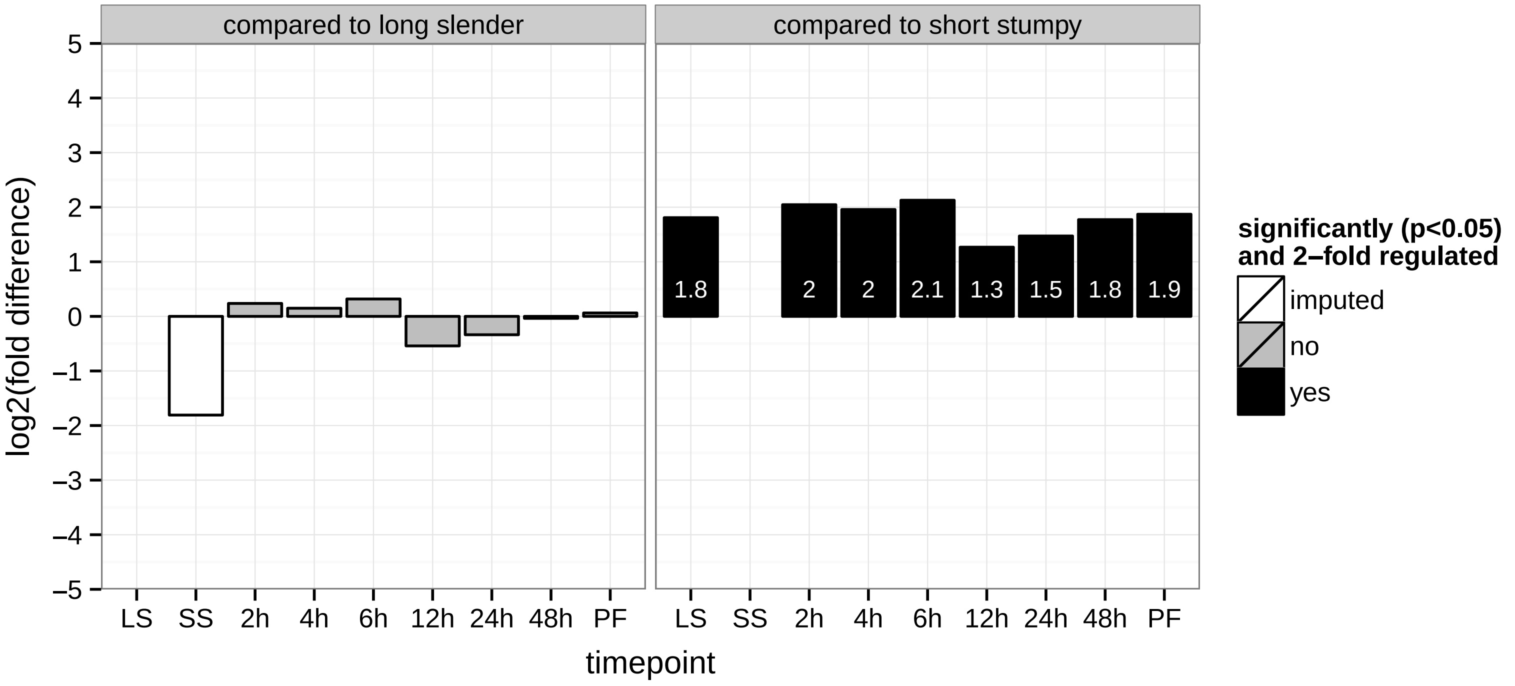

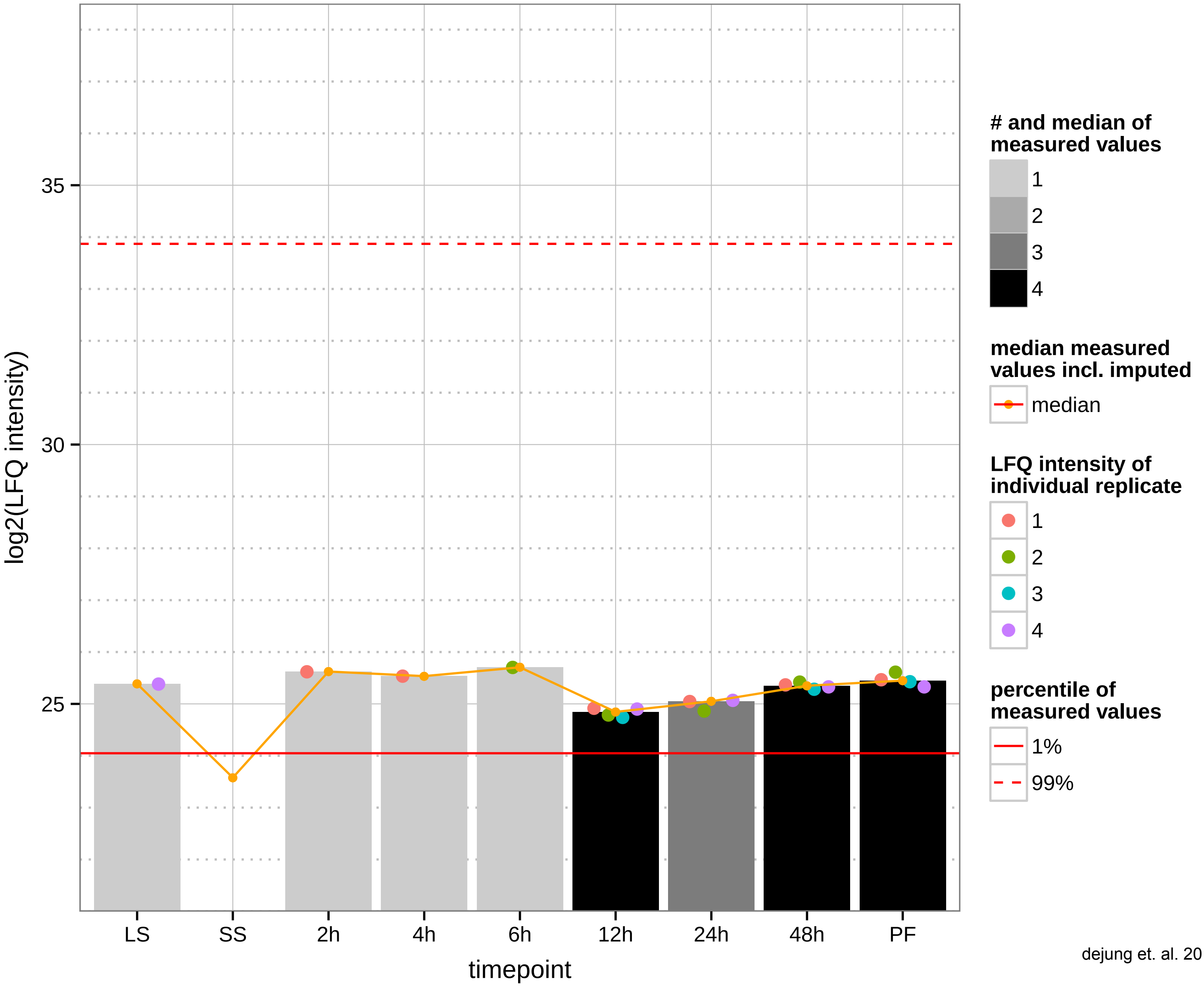

dejung et. al. 2015

glycosomal malate dehydrogenase (gMDH)
Tb927.10.15410
AGOF: L−malate dehydrogenase activity, malate dehydrogenase activity, oxidoreductase activity
AGOC: glycosome
AGOP: glycolysis, malate metabolic process, oxidation−reduction process, tricarboxylic acid cycle
PGOF: L−malate dehydrogenase activity, catalytic activity, oxidoreductase activity, acting on the CH−OH group of donors, NAD
PGOC: null
PGOP: carbohydrate metabolic process, cellular carbohydrate metabolic process, malate metabolic process, oxidation−reduct

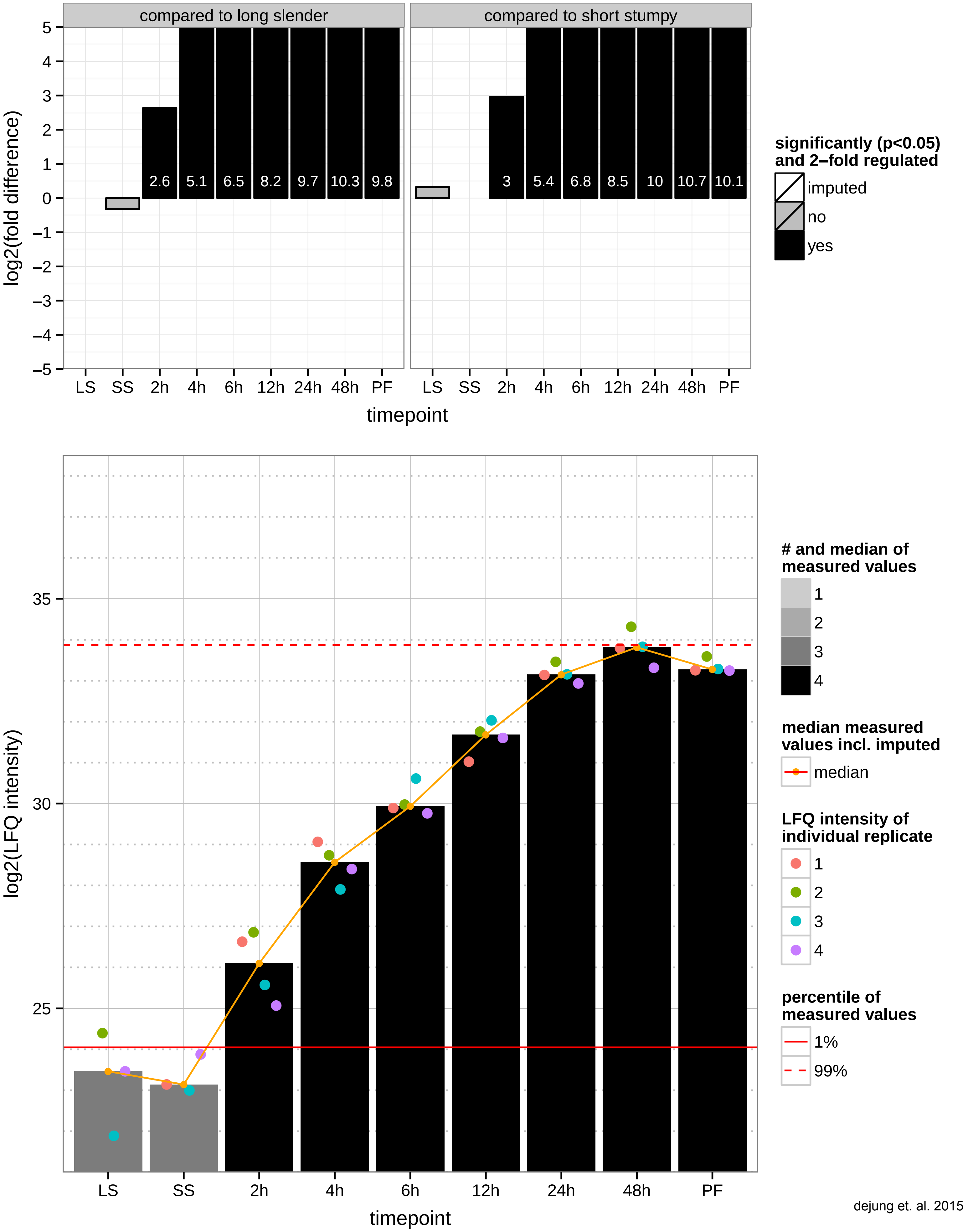

dejung et. al. 2015

hypothetical protein, conserved
Tb927.10.1960
AGOF: null
AGOC: null
AGOP: null
PGOF: binding
PGOC: null
PGOP: null

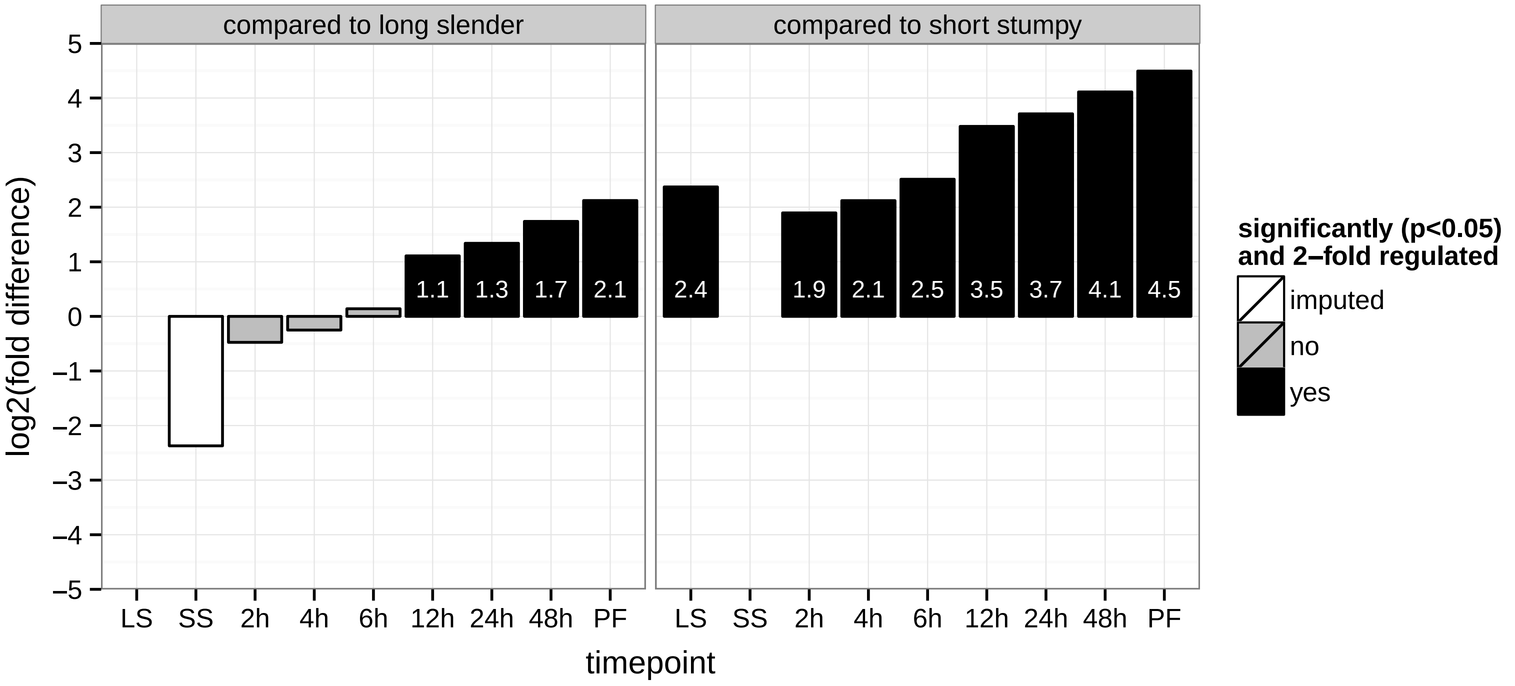

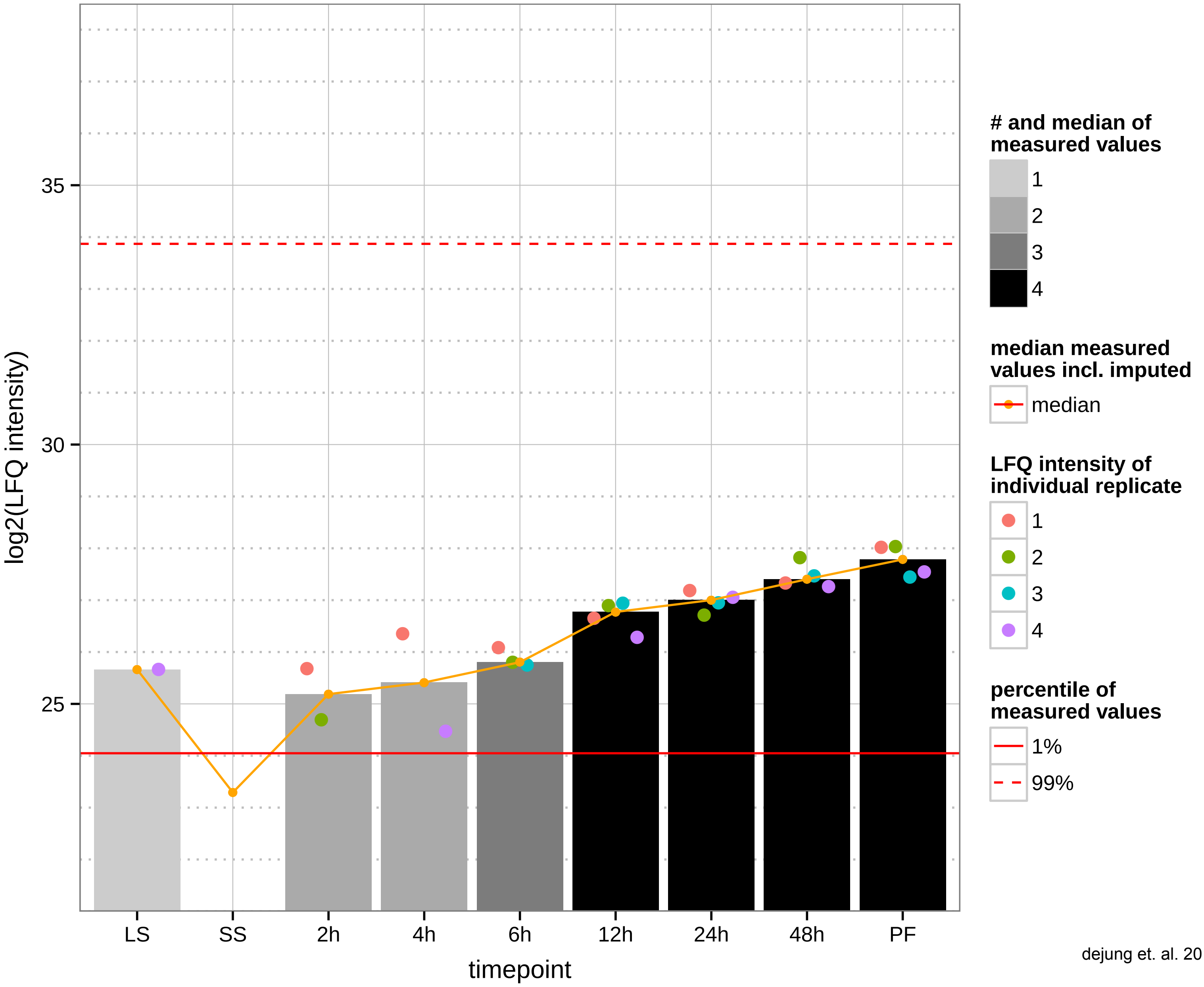

dejung et. al. 2015

ubiquitin carboxyl–terminal hydrolase, putative, Peptidase C19, ubiquitin carboxyl–terminal hydrolase
Tb927.10.2210
AGOF: null, cysteine–type endopeptidase activity, ubiquitin thiolesterase activity
AGOC: null
AGOP: null, ubiquitin–dependent protein catabolic process
PGOF: ubiquitin thiolesterase activity
PGOC: null
PGOP: ubiquitin–dependent protein catabolic process

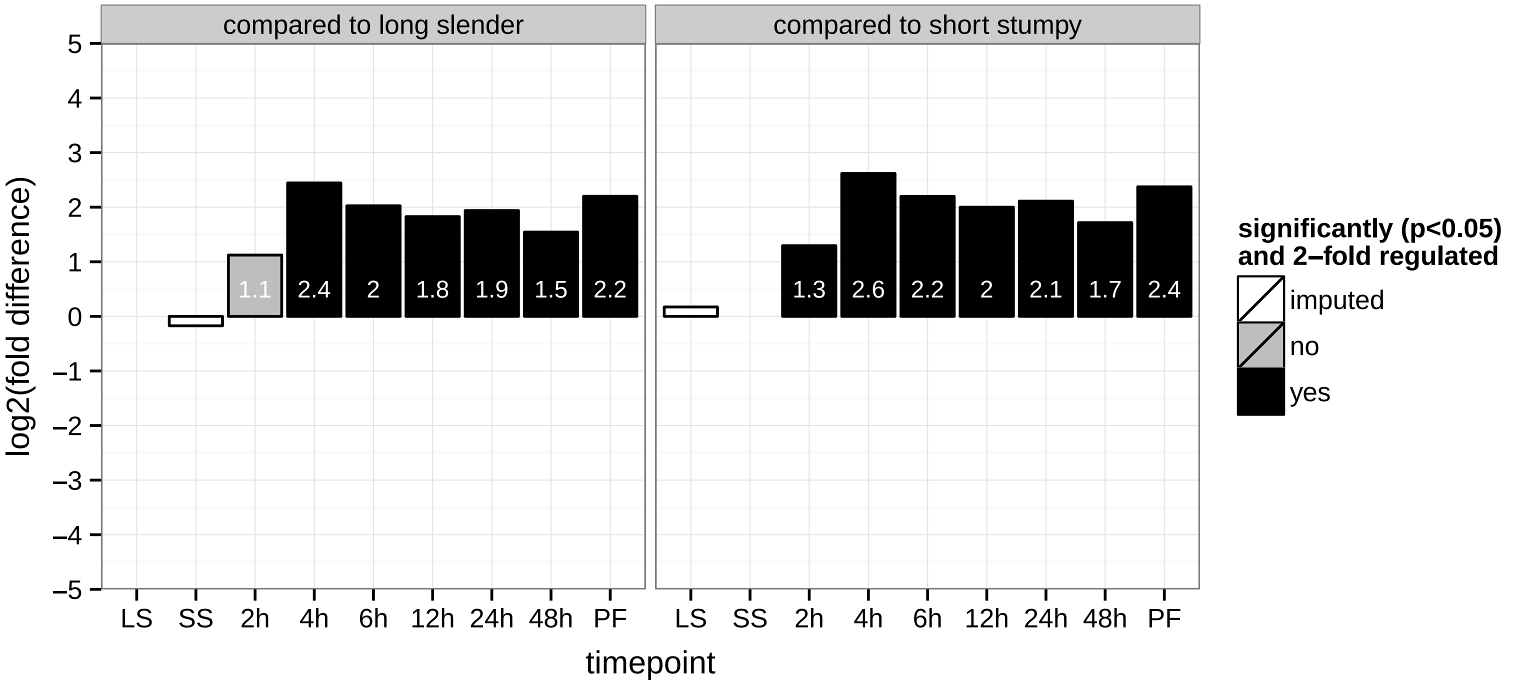

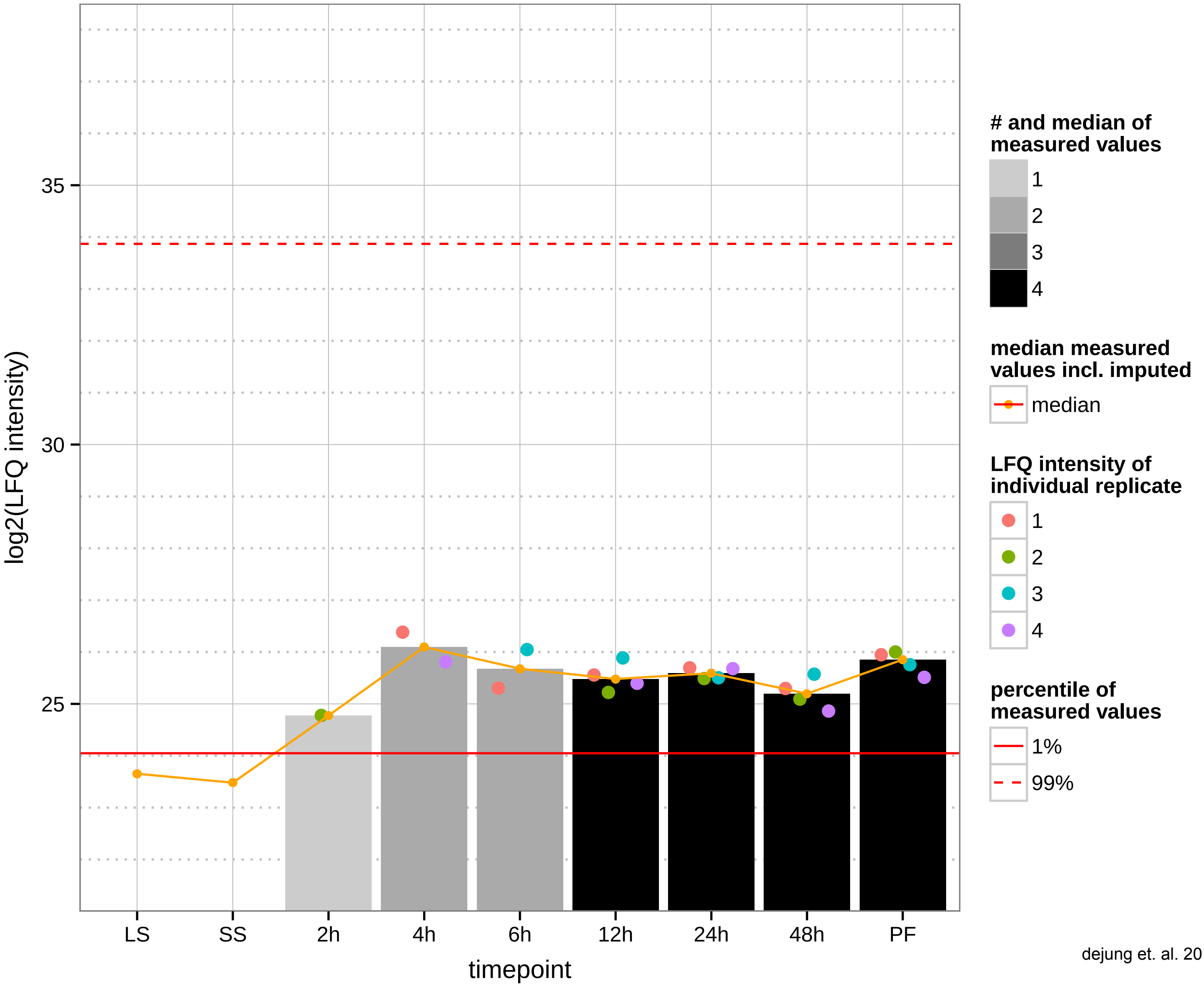

dejung et. al. 2015

hypothetical protein, conserved
Tb927.10.3040
AGOF: null
AGOC: mitochondrial inner membrane, mitochondrion
AGOP: null
PGOF: null
PGOC: null
PGOP: null

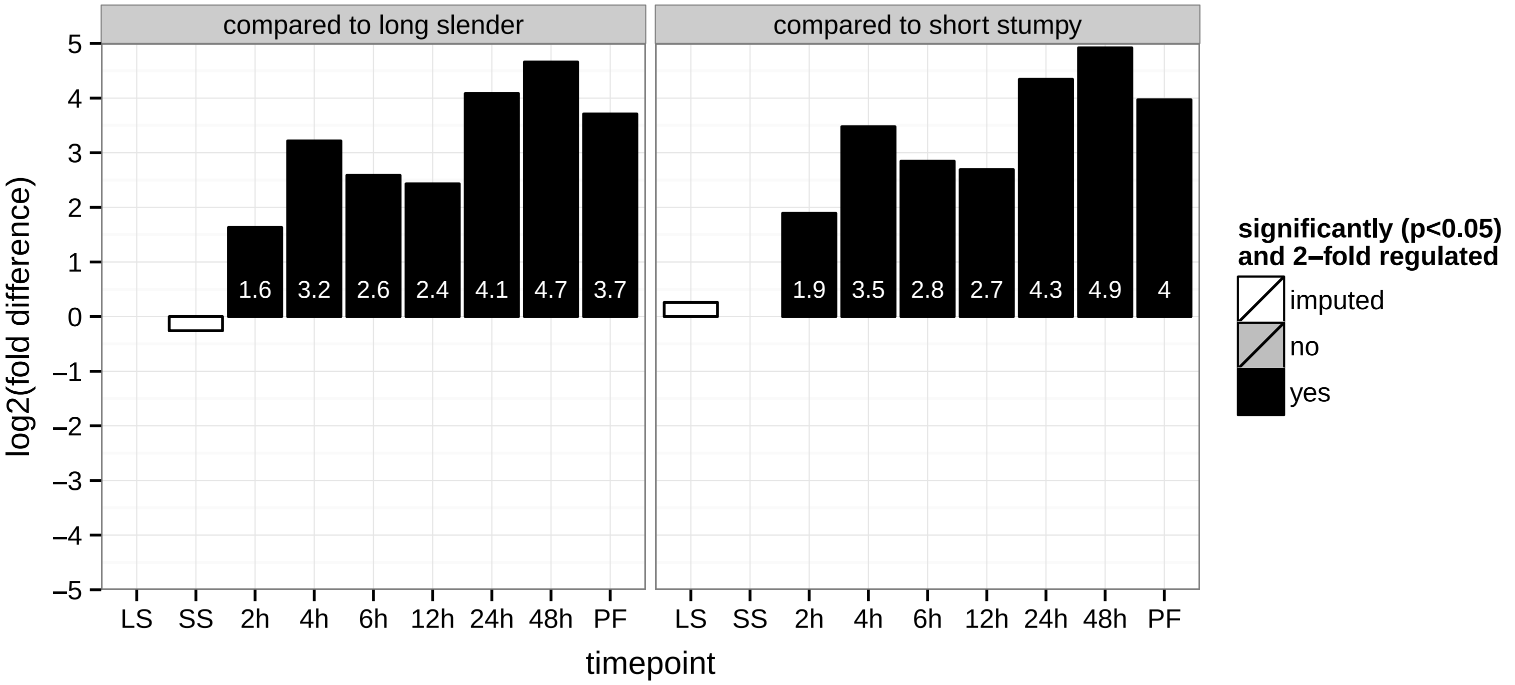

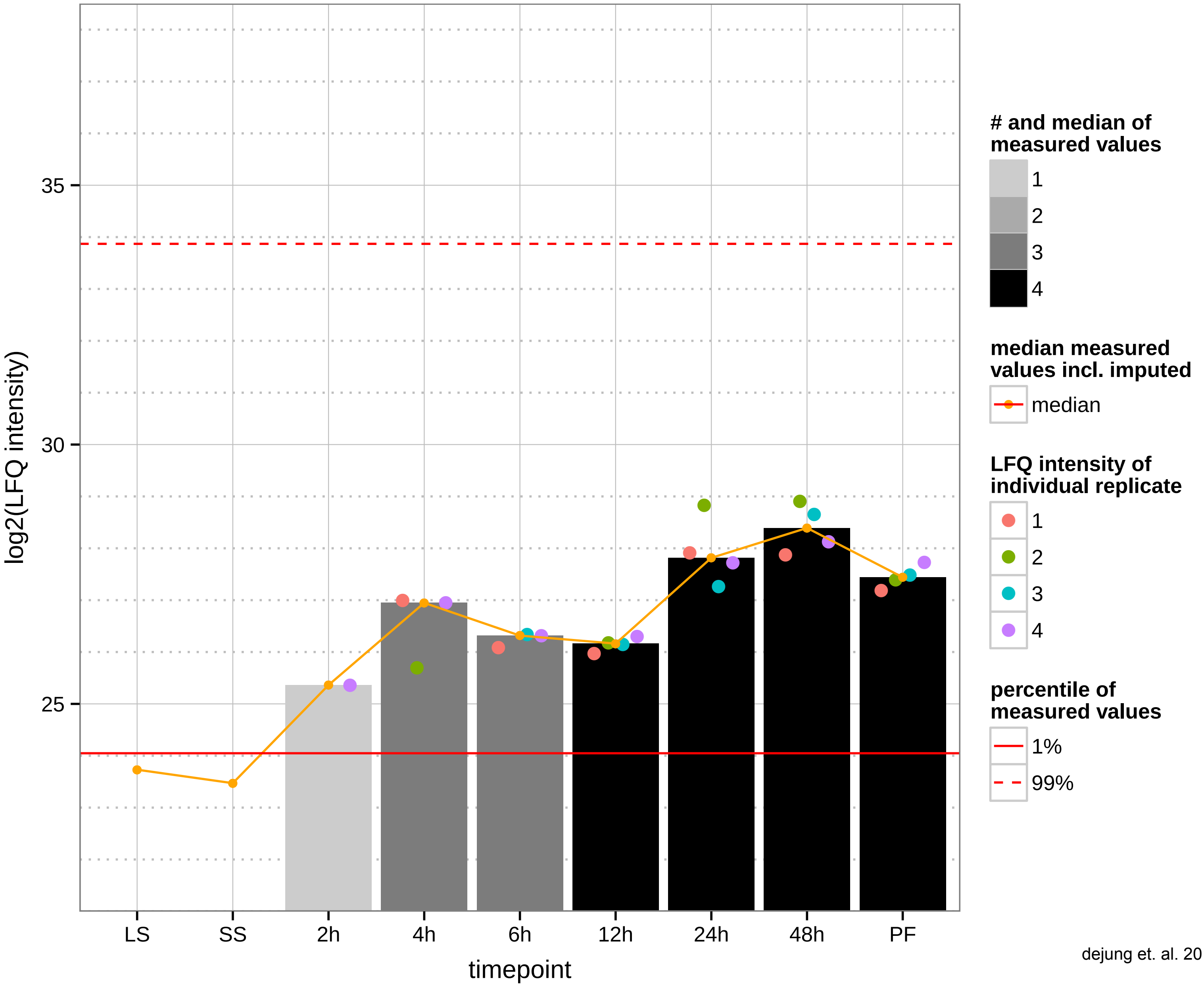

dejung et. al. 2015

hypothetical protein, conserved
Tb927.10.3160
AGOF: null
AGOC: null
AGOP: null
PGOF: null
PGOC: null
PGOP: null

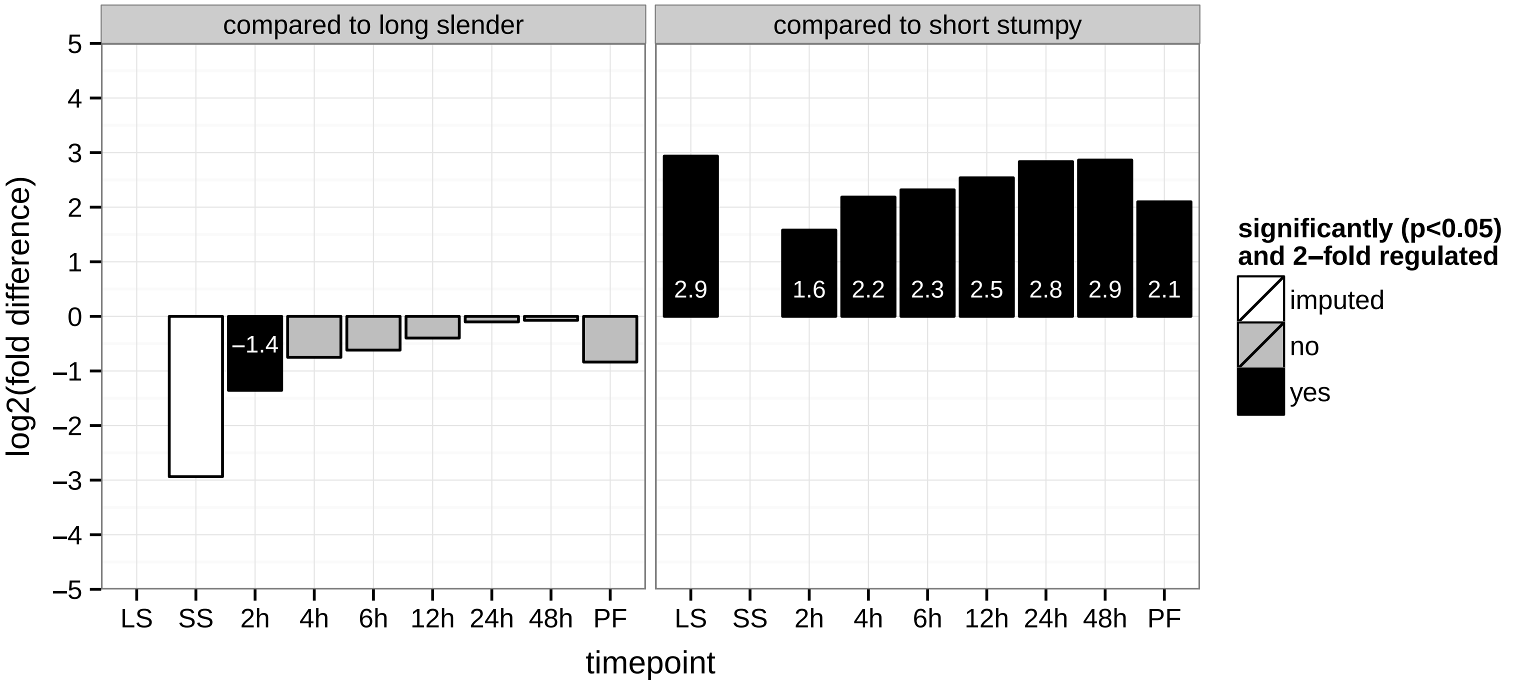

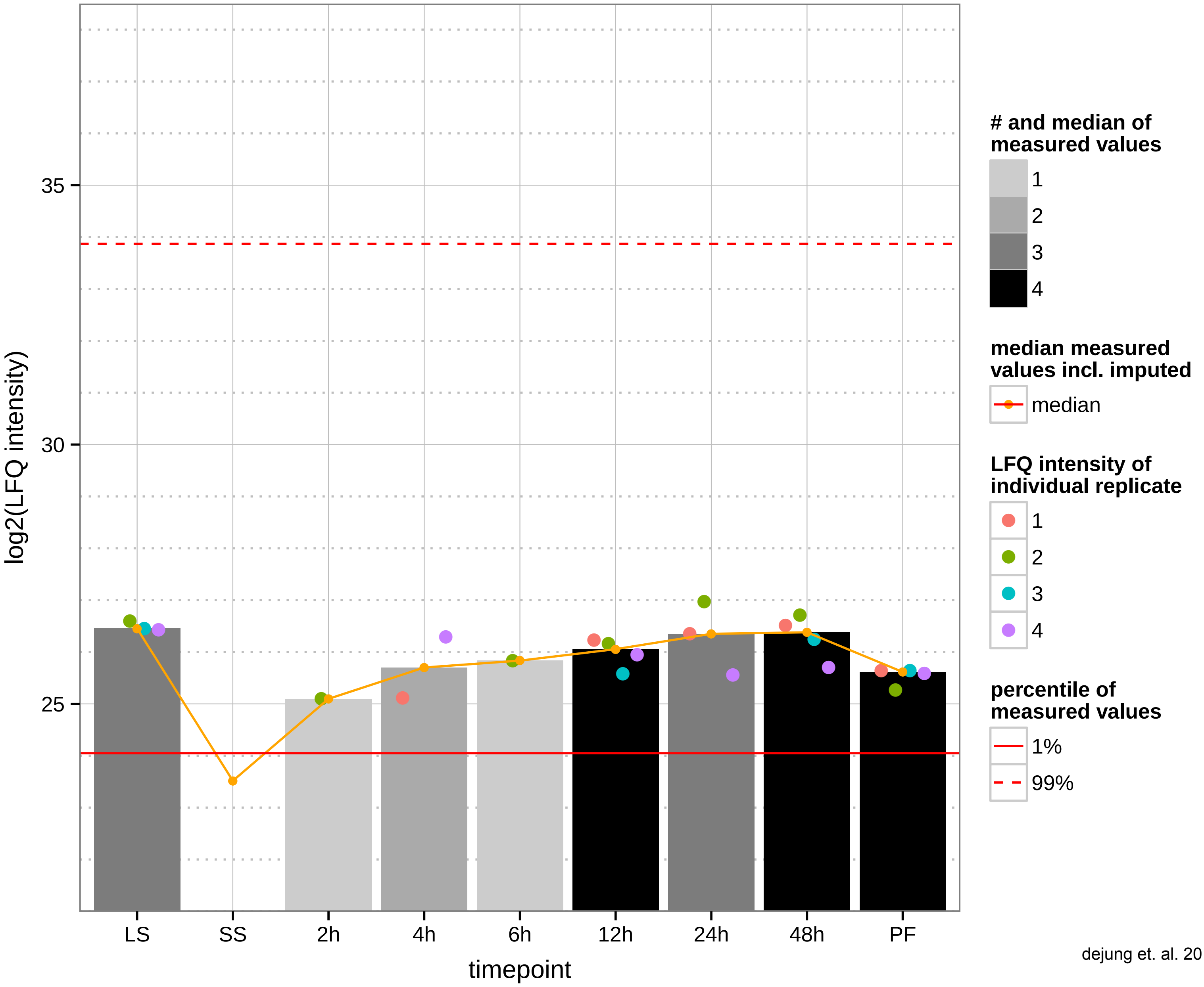

dejung et. al. 2015

hypothetical protein, conserved
Tb927.10.6550
AGOF: DNA binding, zinc ion binding
AGOC: null
AGOP: null
PGOF: DNA binding, zinc ion binding
PGOC: null
PGOP: null

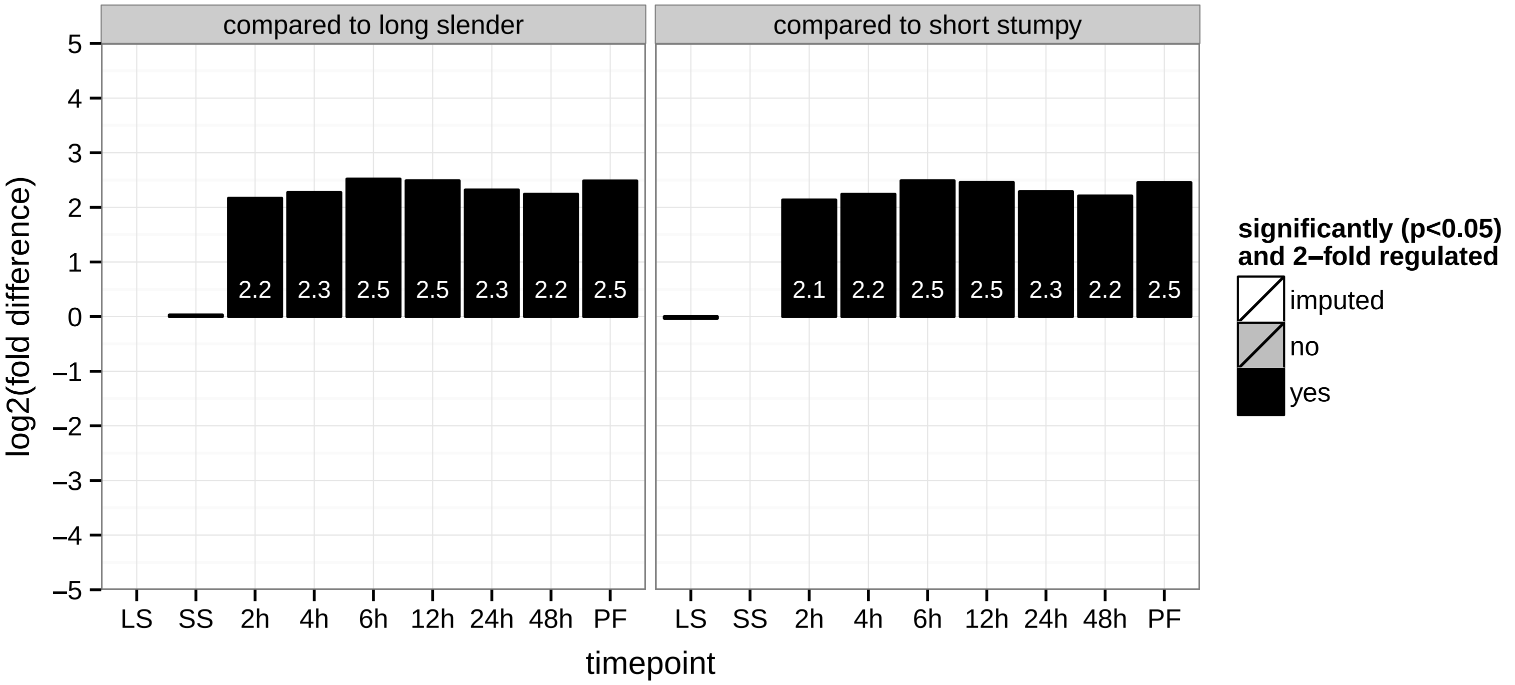

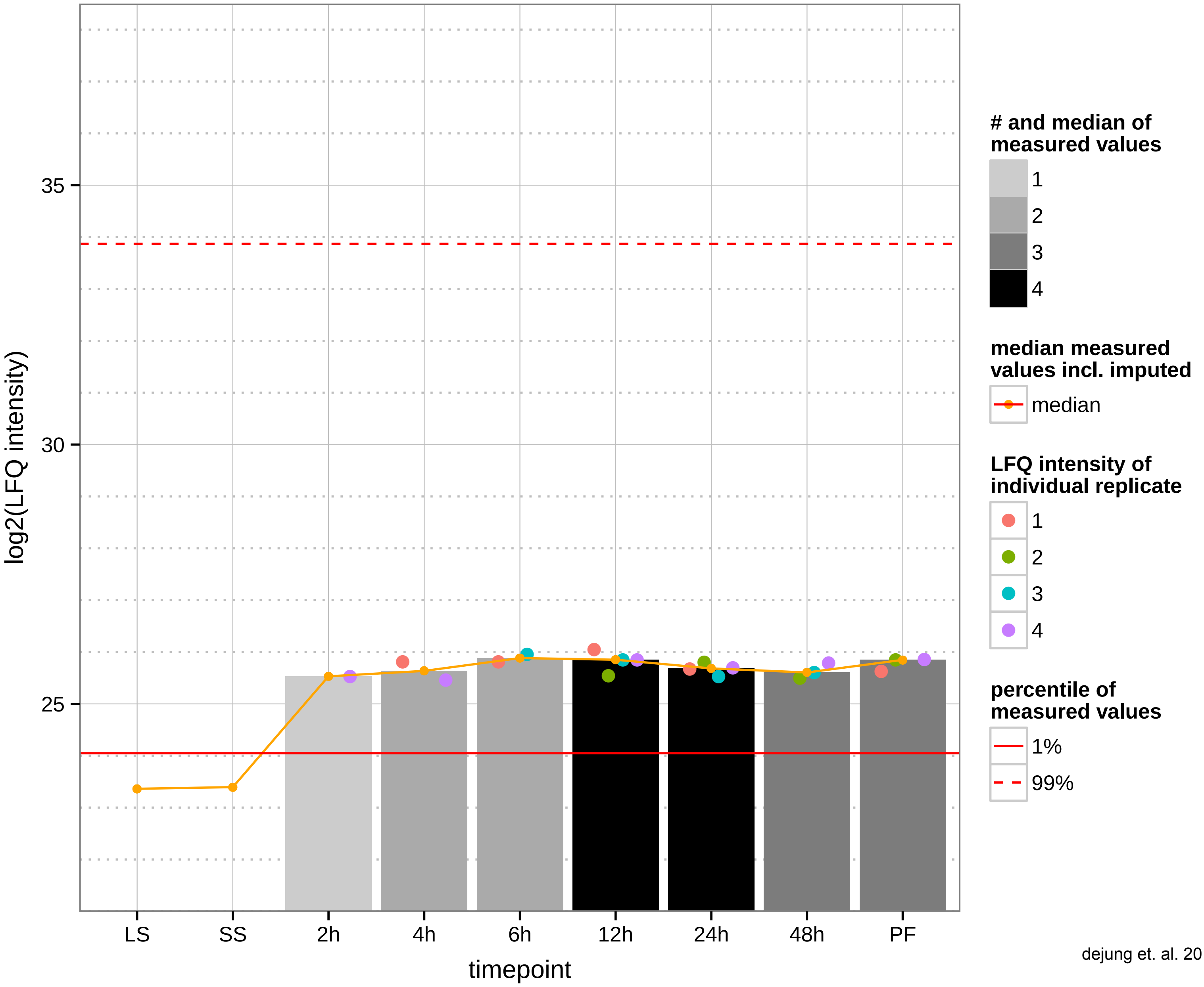

dejung et. al. 2015

tRNA pseudouridine synthase TruD, putative, pseudouridine synthase TruD, putative (pus7)
Tb927.10.9050
AGOF: RNA binding, pseudouridine synthase activity
AGOC: null
AGOP: pseudouridine synthesis
PGOF: RNA binding, pseudouridine synthase activity
PGOC: null
PGOP: RNA modification, pseudouridine synthesis

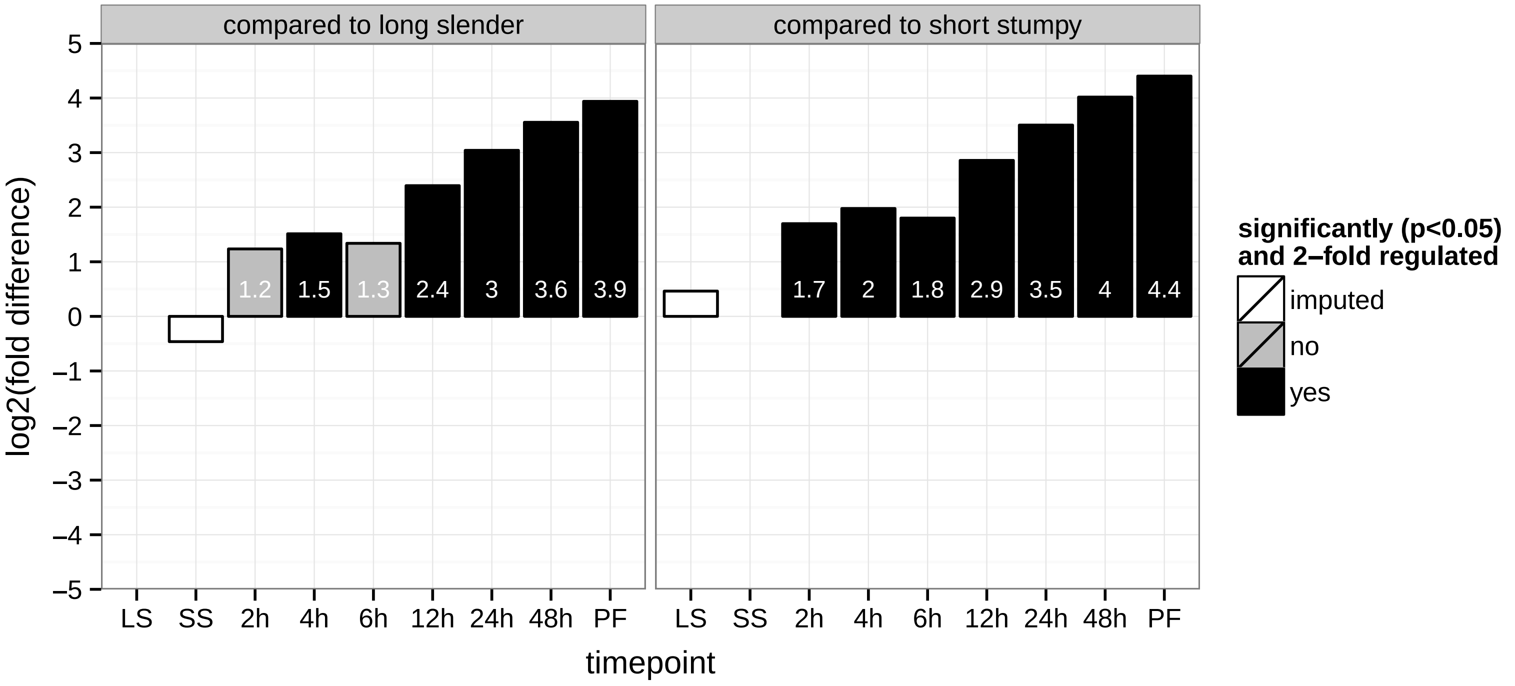

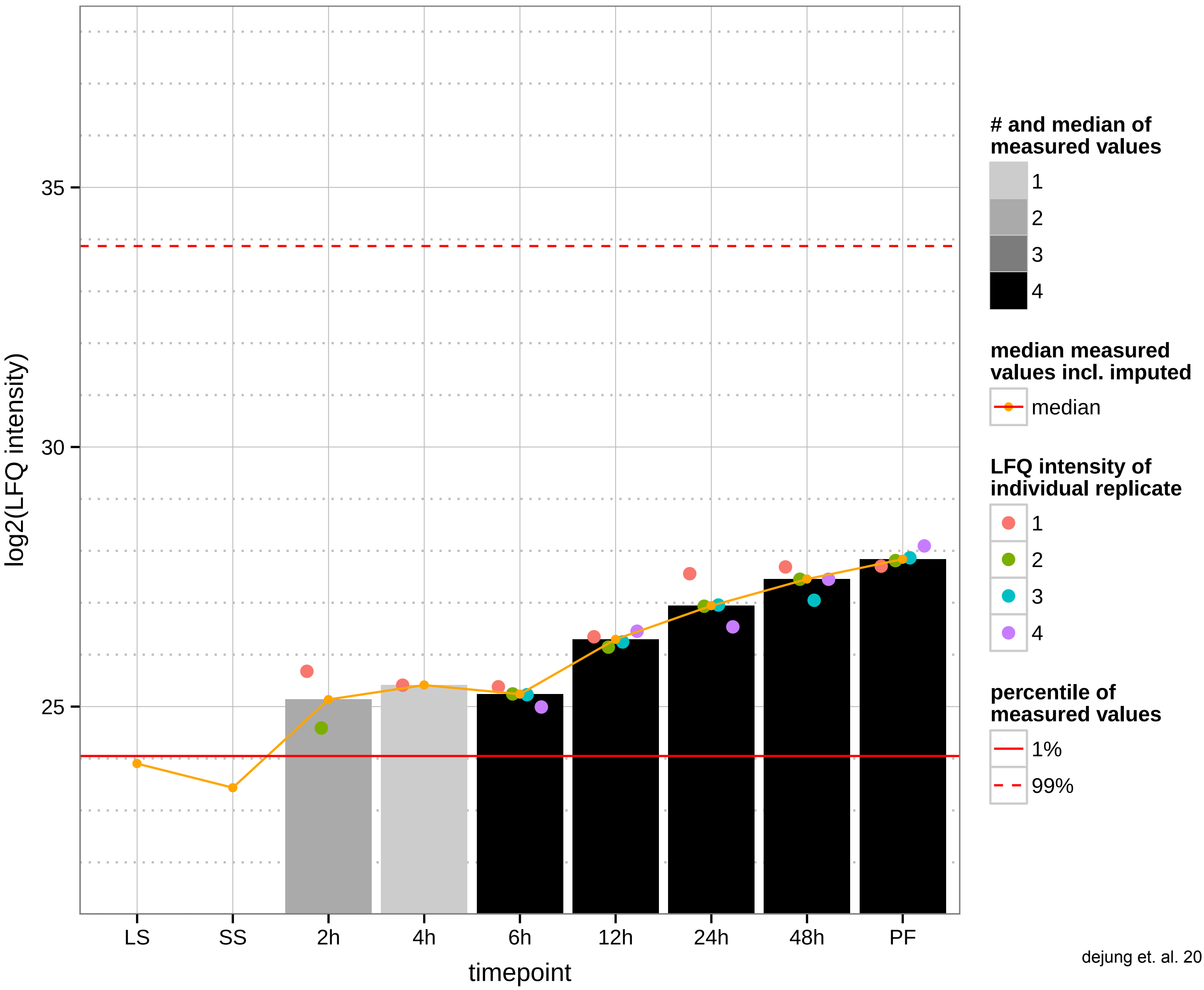

dejung et. al. 2015

pteridine transporter, putative
Tb927.10.9080
AGOF: biopterin transporter activity
AGOC: null
AGOP: biopterin transport
PGOF: null
PGOC: null
PGOP: null

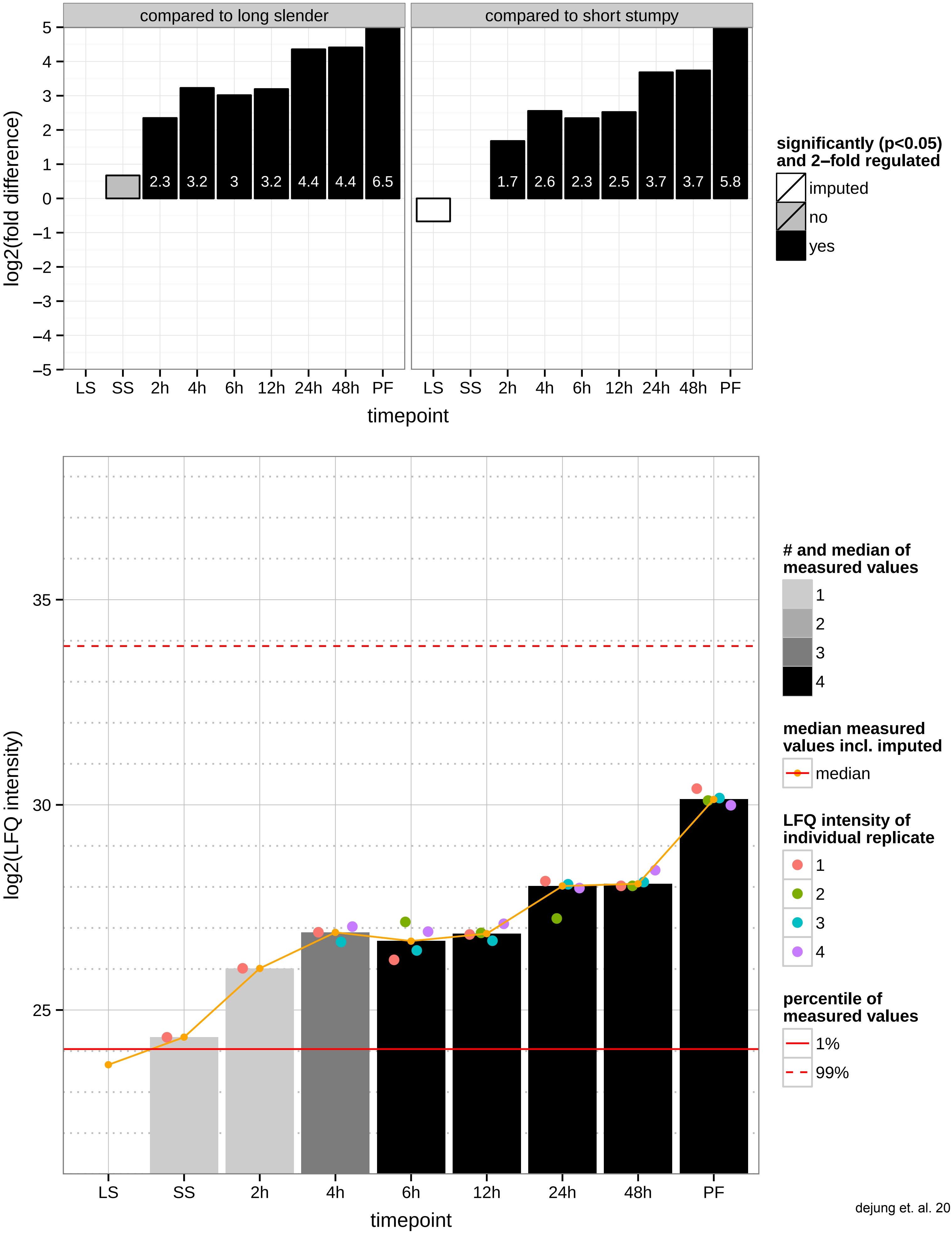

dejung et. al. 2015

hypothetical protein, conserved
Tb927.11.10880
AGOF: transferase activity
AGOC: null
AGOP: null
PGOF: protein binding, transferase activity
PGOC: null
PGOP: null

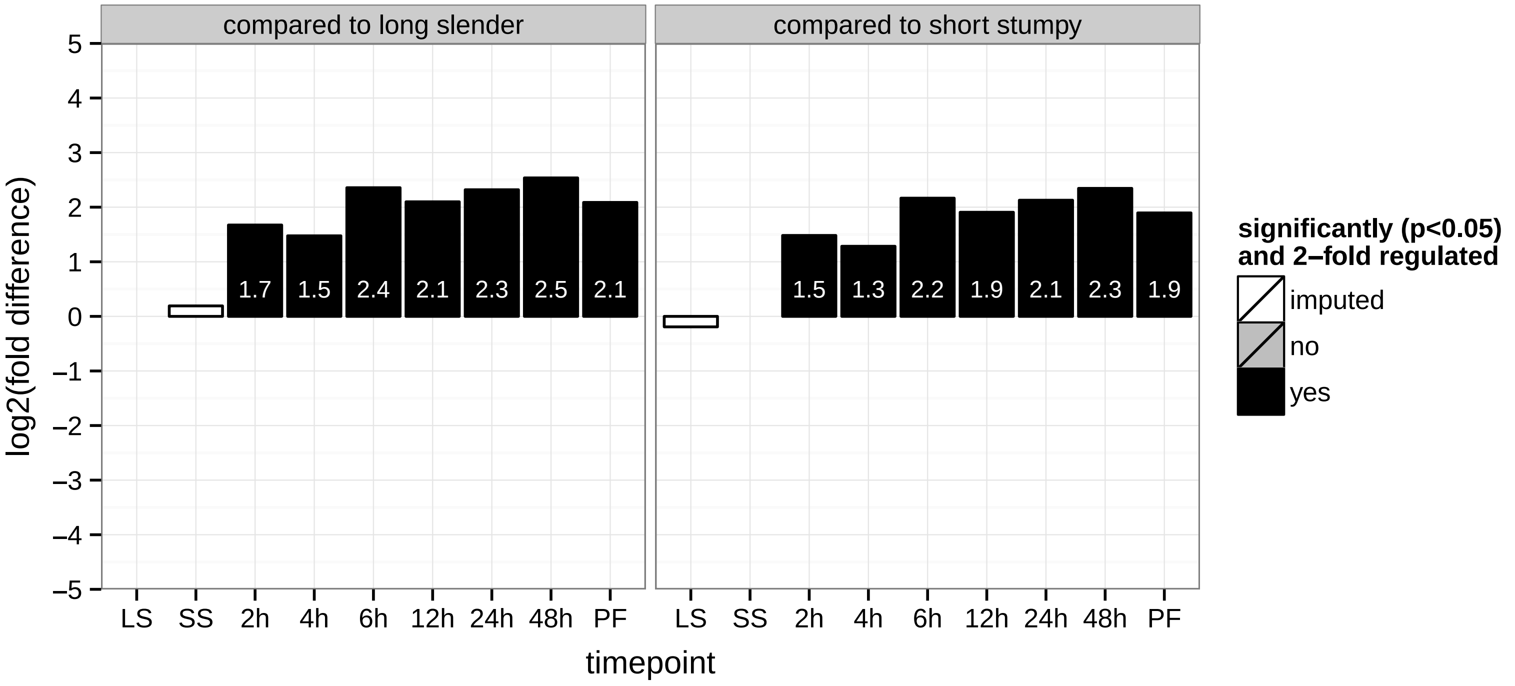

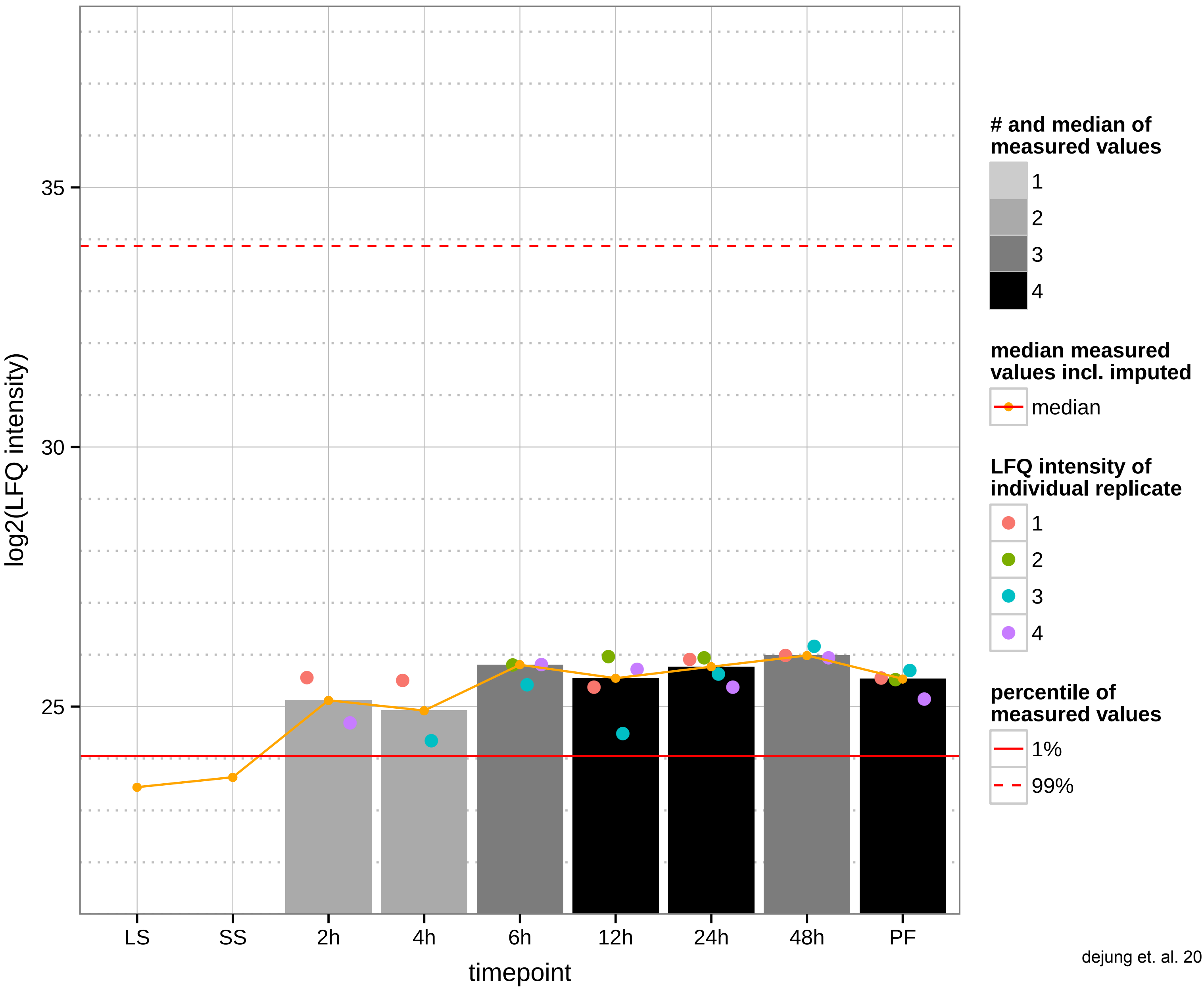

dejung et. al. 2015

membrane–bound acid phosphatase, putative
Tb927.11.11740;Tb927.11.11750
AGOF: acid phosphatase activity
AGOC: null
AGOP: null
PGOF: null
PGOC: null
PGOP: null

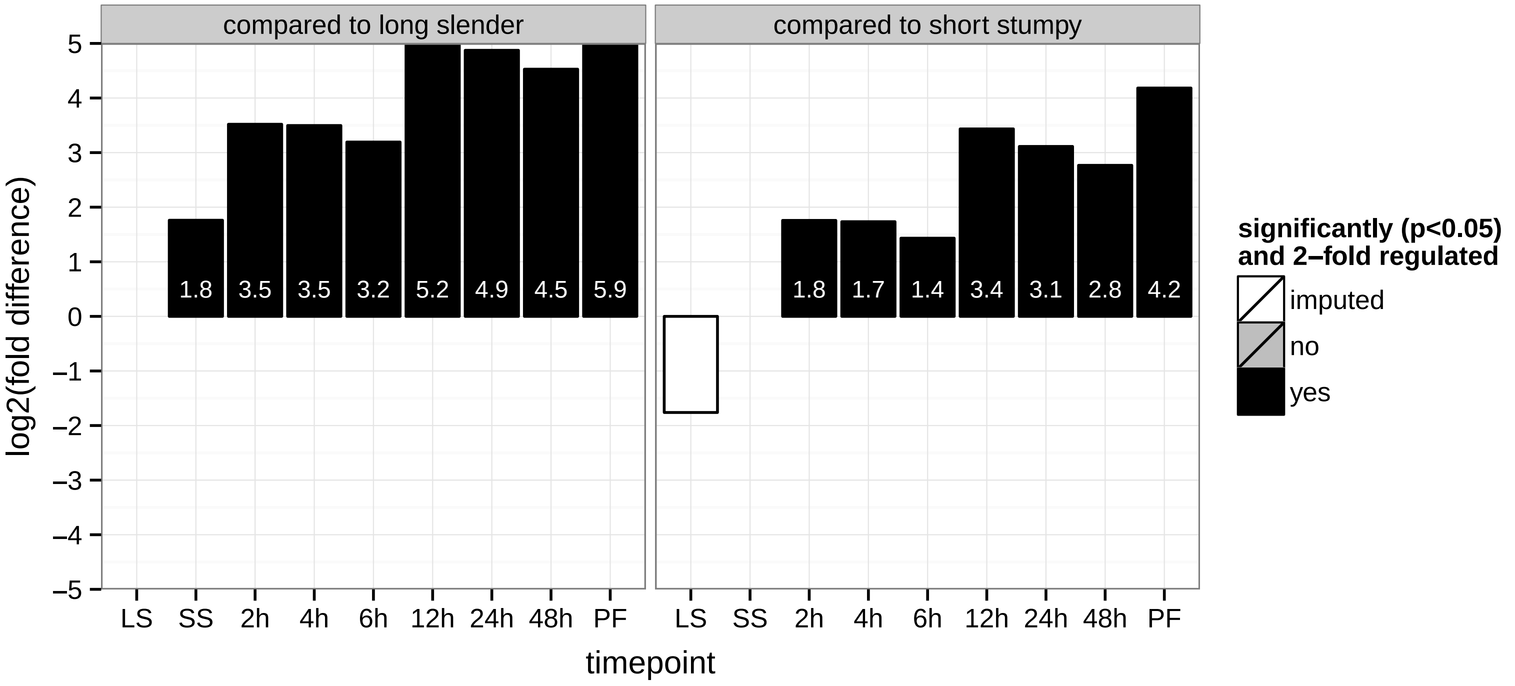

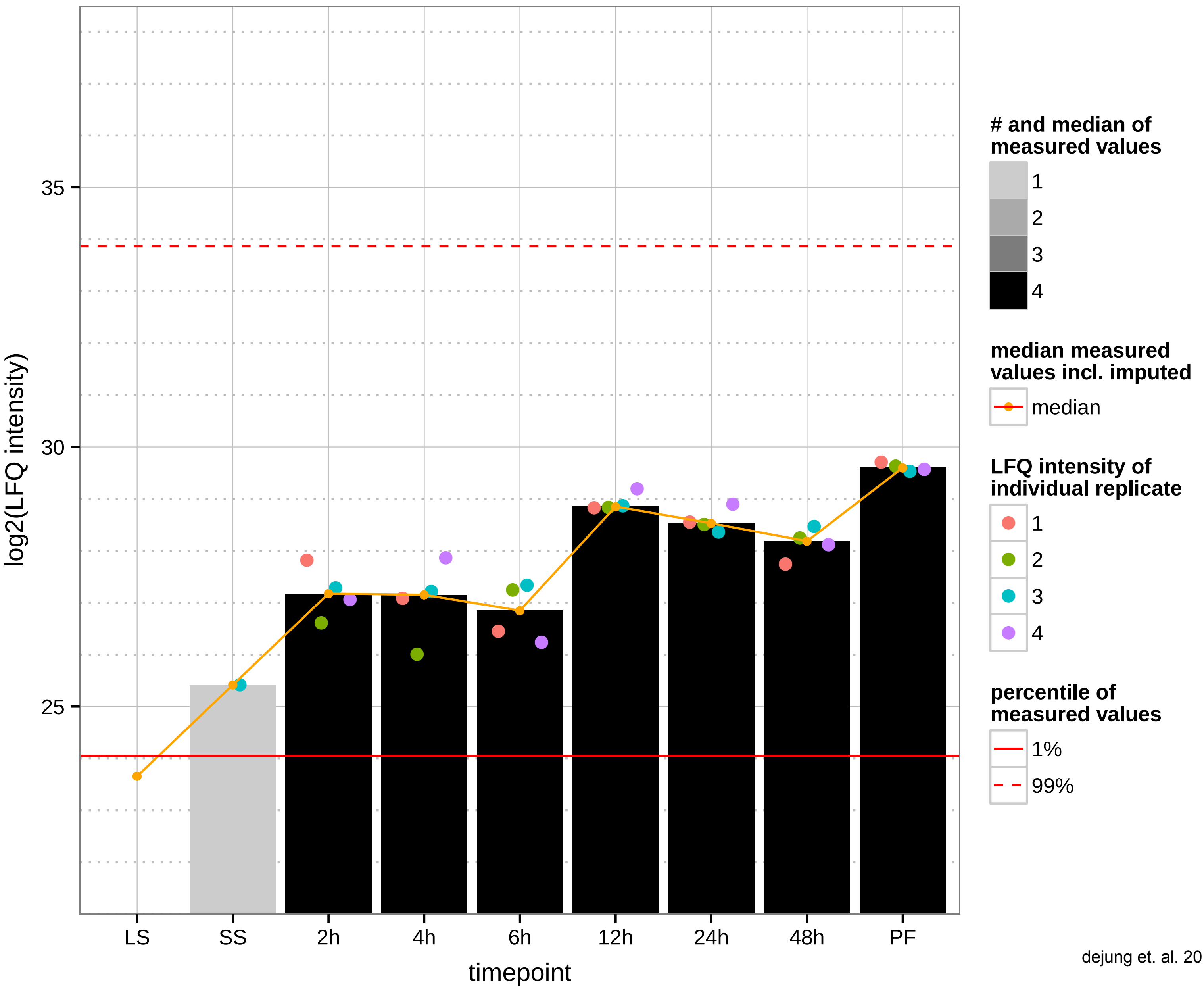

dejung et. al. 2015

serine/threonine–protein kinase, putative
Tb927.11.15010
AGOF: ATP binding, protein serine/threonine kinase activity, protein tyrosine kinase activity
AGOC: null
AGOP: protein phosphorylation
PGOF: ATP binding, phospholipid binding, protein binding, protein kinase activity, transferase activity, transferring phosphorus
PGOC: null
PGOP: protein phosphorylation

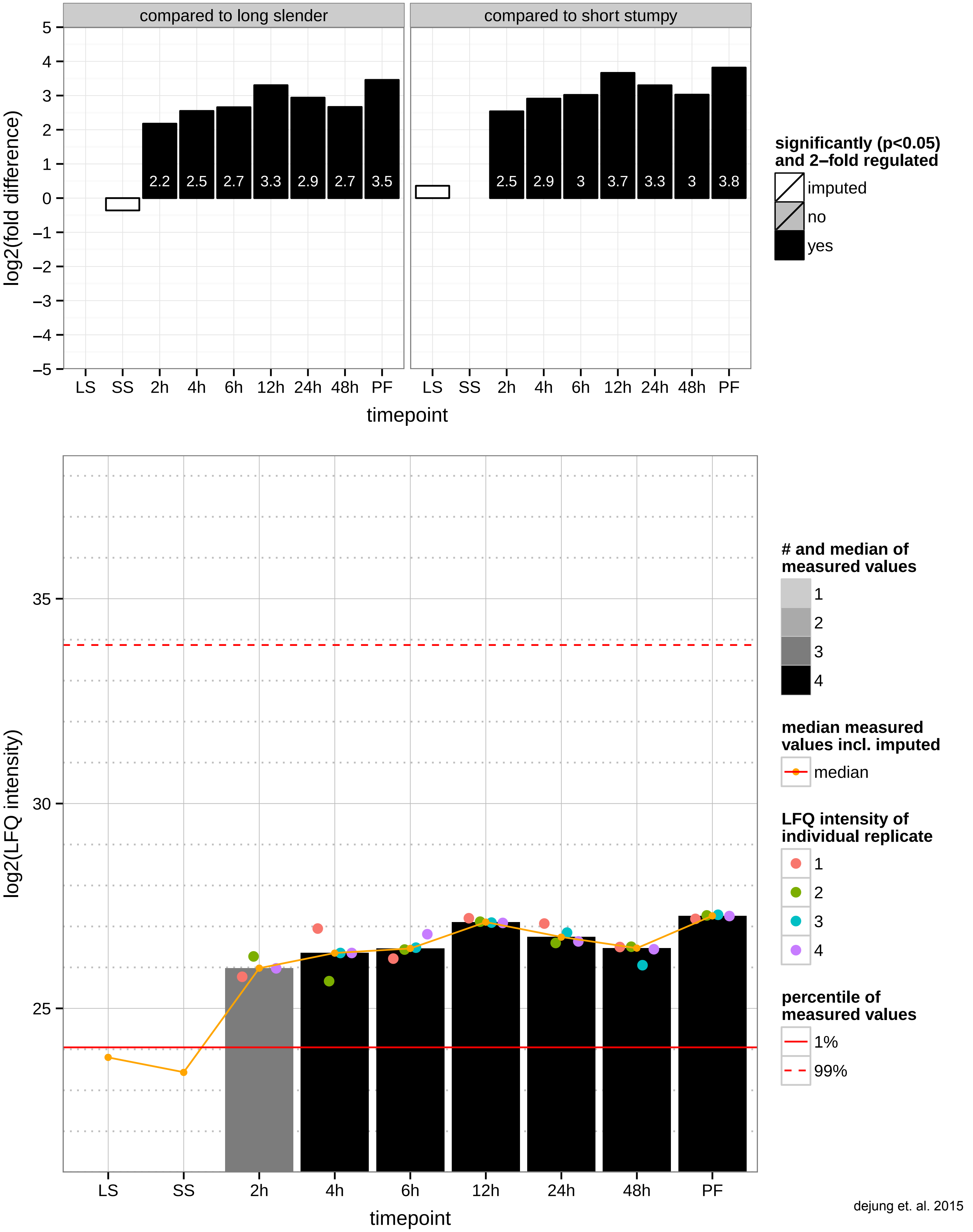

dejung et. al. 2015

amino acid transporter, putative
Tb927.11.15950
AGOF: amine transmembrane transporter activity
AGOC: integral to membrane
AGOP: amino acid transport
PGOF: null
PGOC: null
PGOP: null

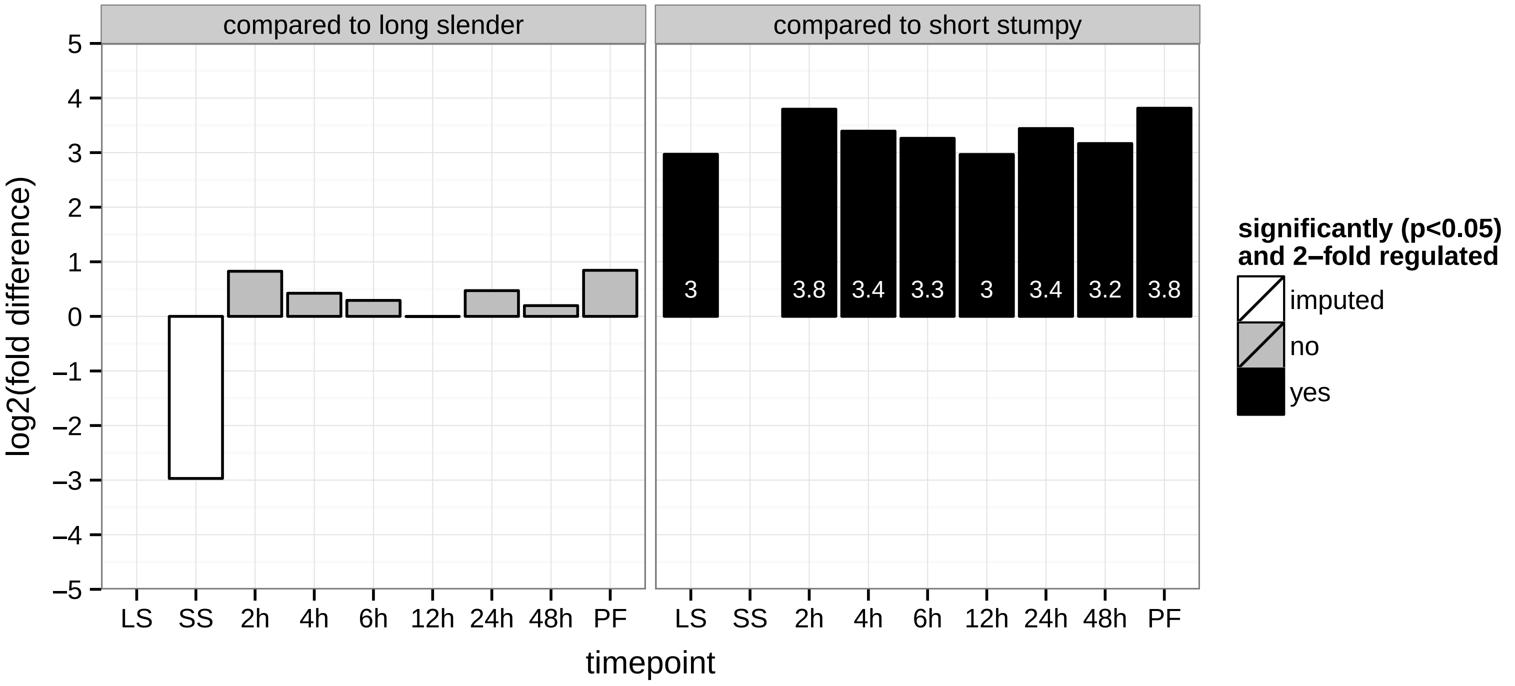

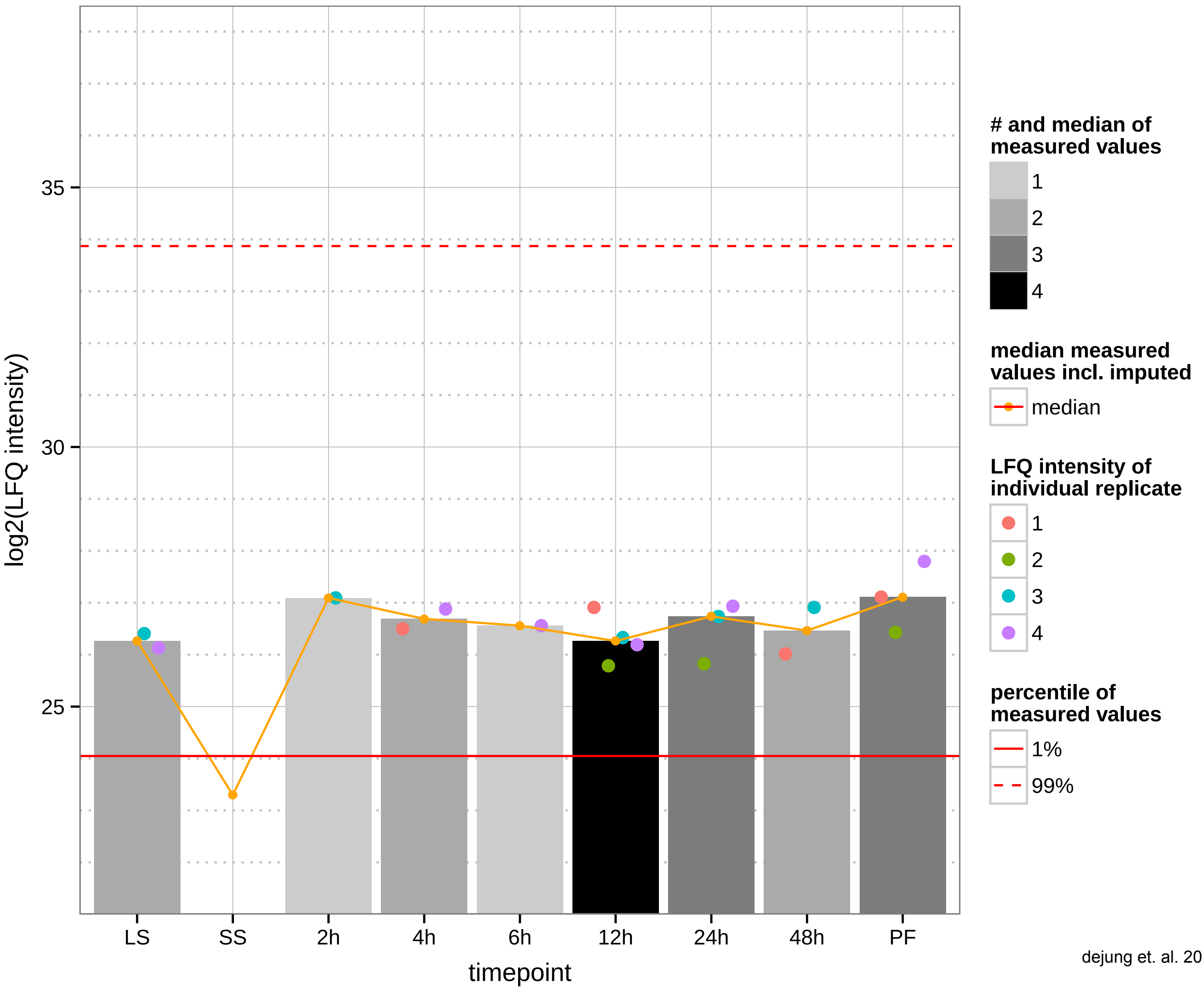

dejung et. al. 2015

hypothetical protein, conserved
Tb927.11.2100
AGOF: null
AGOC: null
AGOP: null
PGOF: ATP binding
PGOC: null
PGOP: null

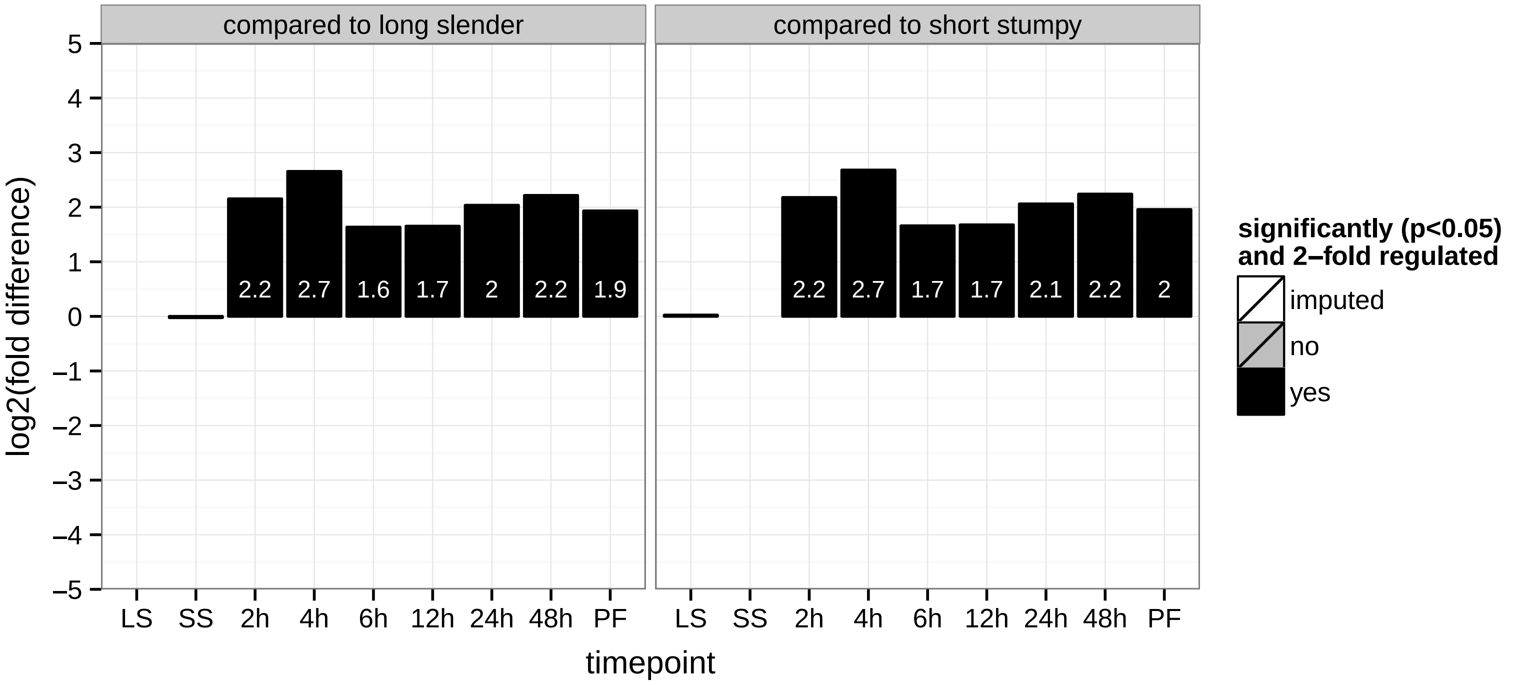

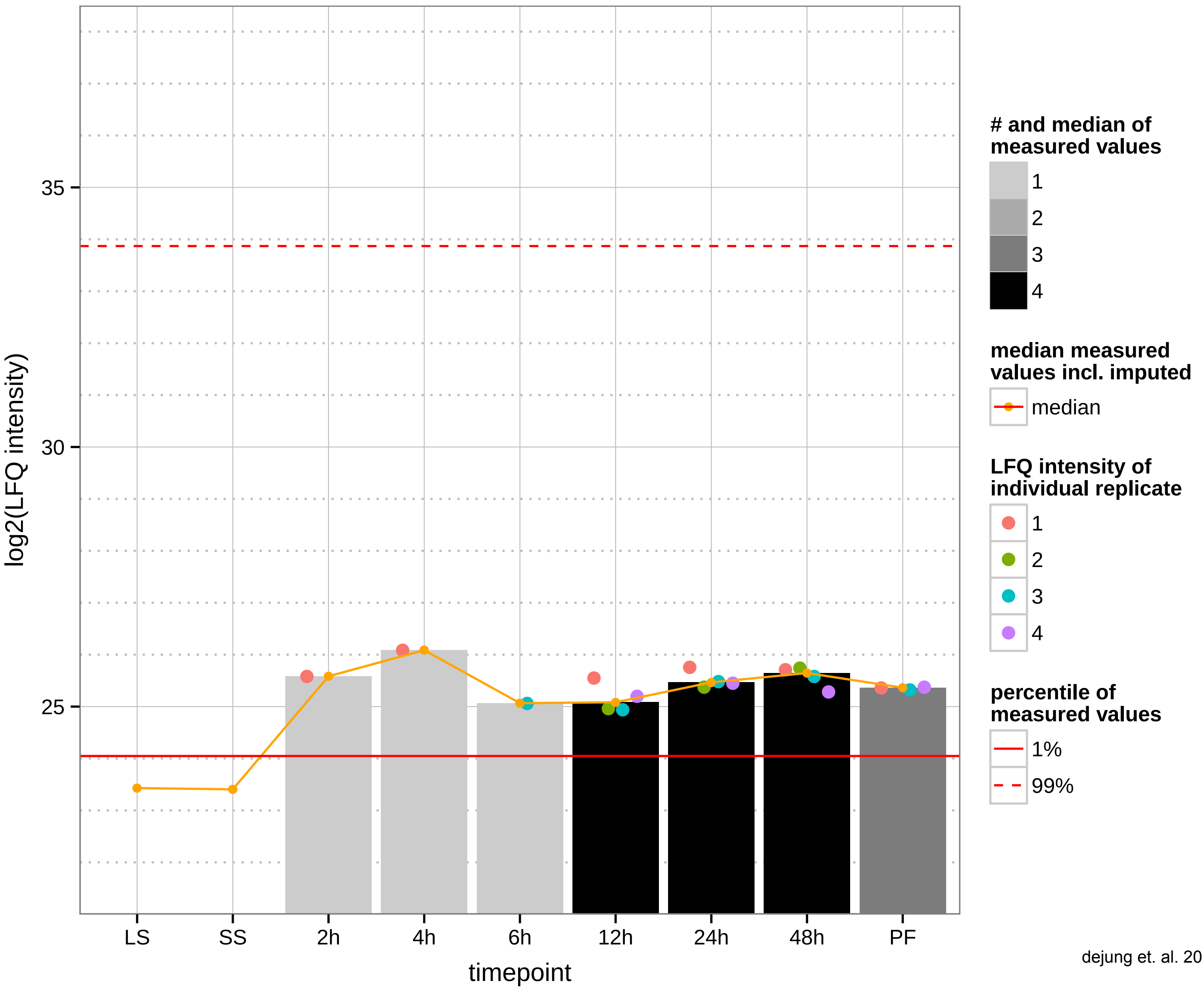

dejung et. al. 2015

hypothetical protein, conserved
Tb927.11.2410
AGOF: null
AGOC: null
AGOP: null
PGOF: null
PGOC: null
PGOP: null

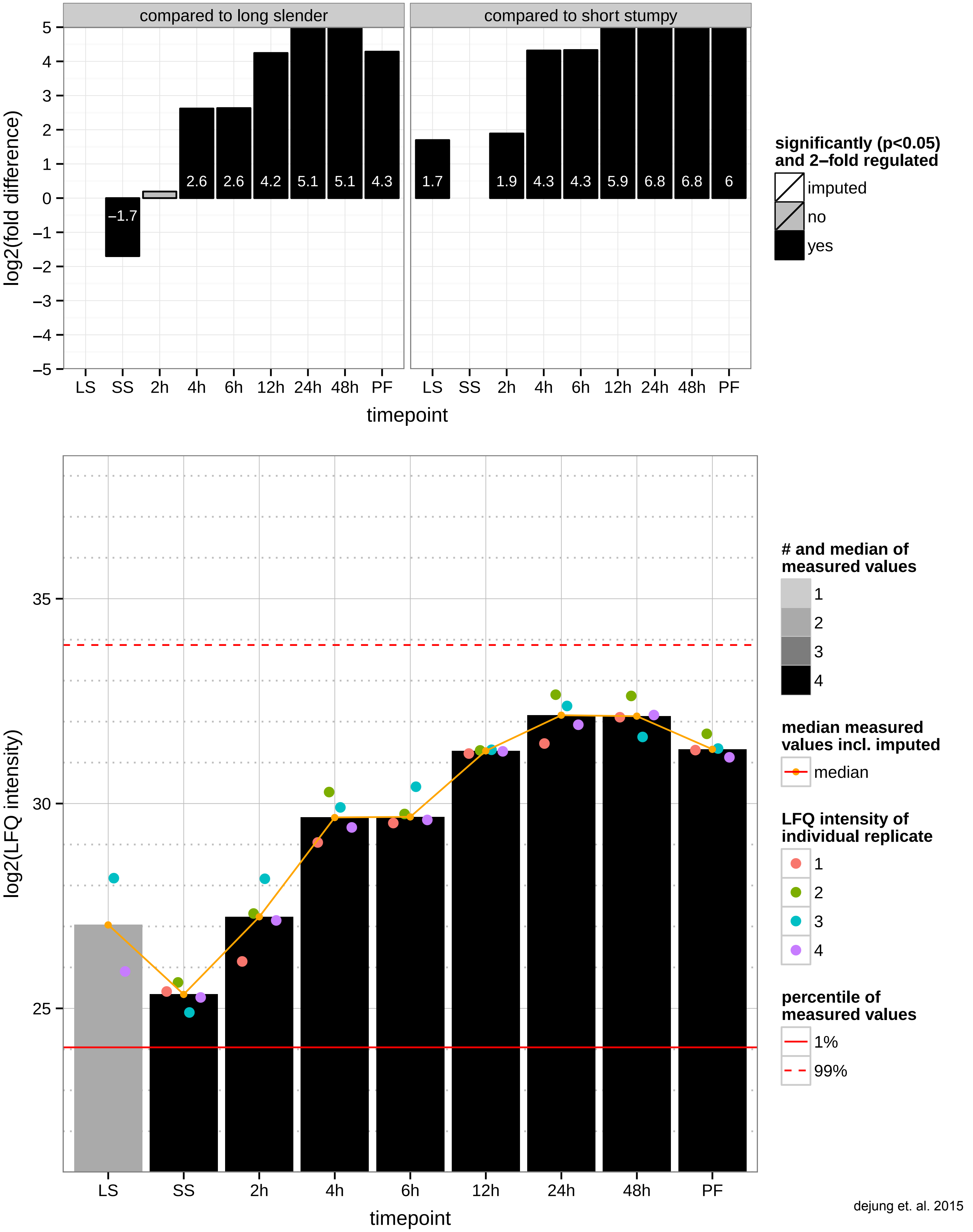

dejung et. al. 2015

hypothetical protein, conserved (p166)
Tb927.11.3290
AGOF: null
AGOC: kinetoplast
AGOP: null
PGOF: null
PGOC: null
PGOP: null

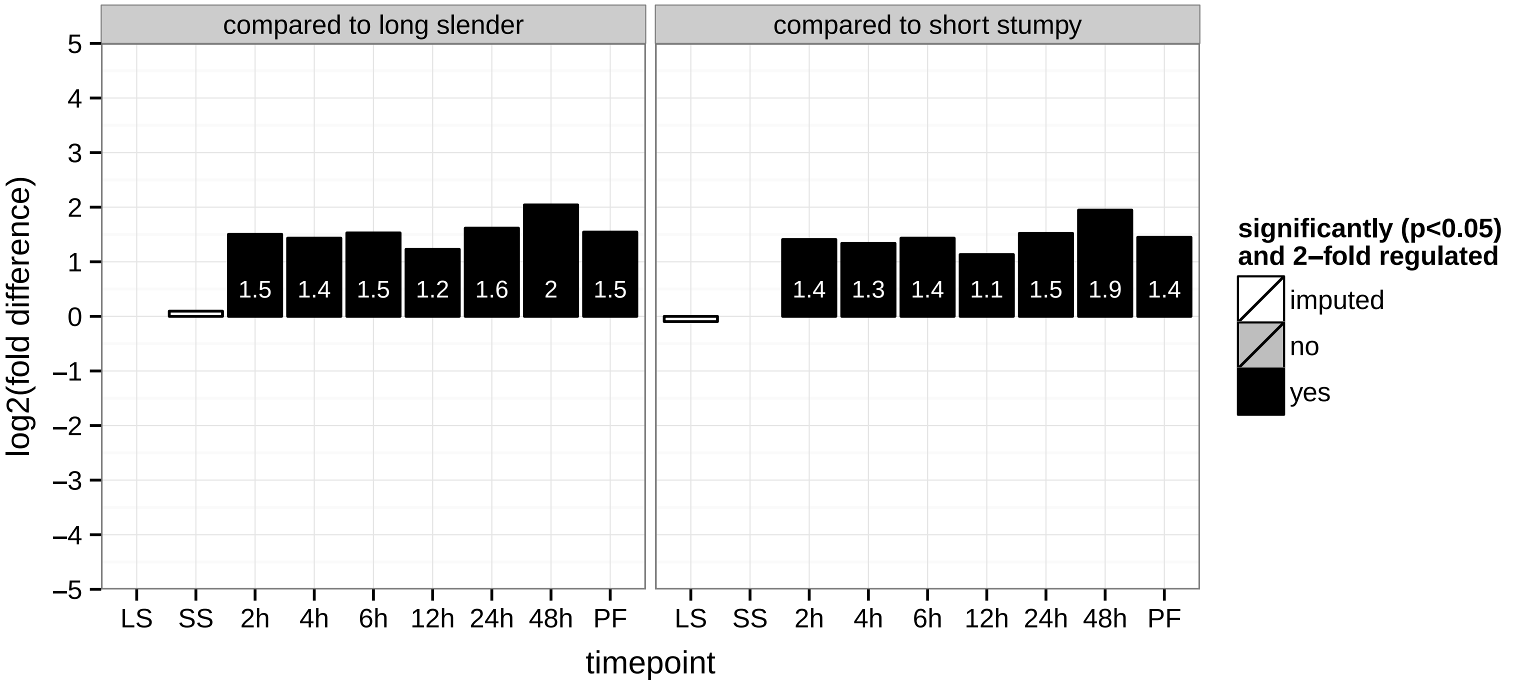

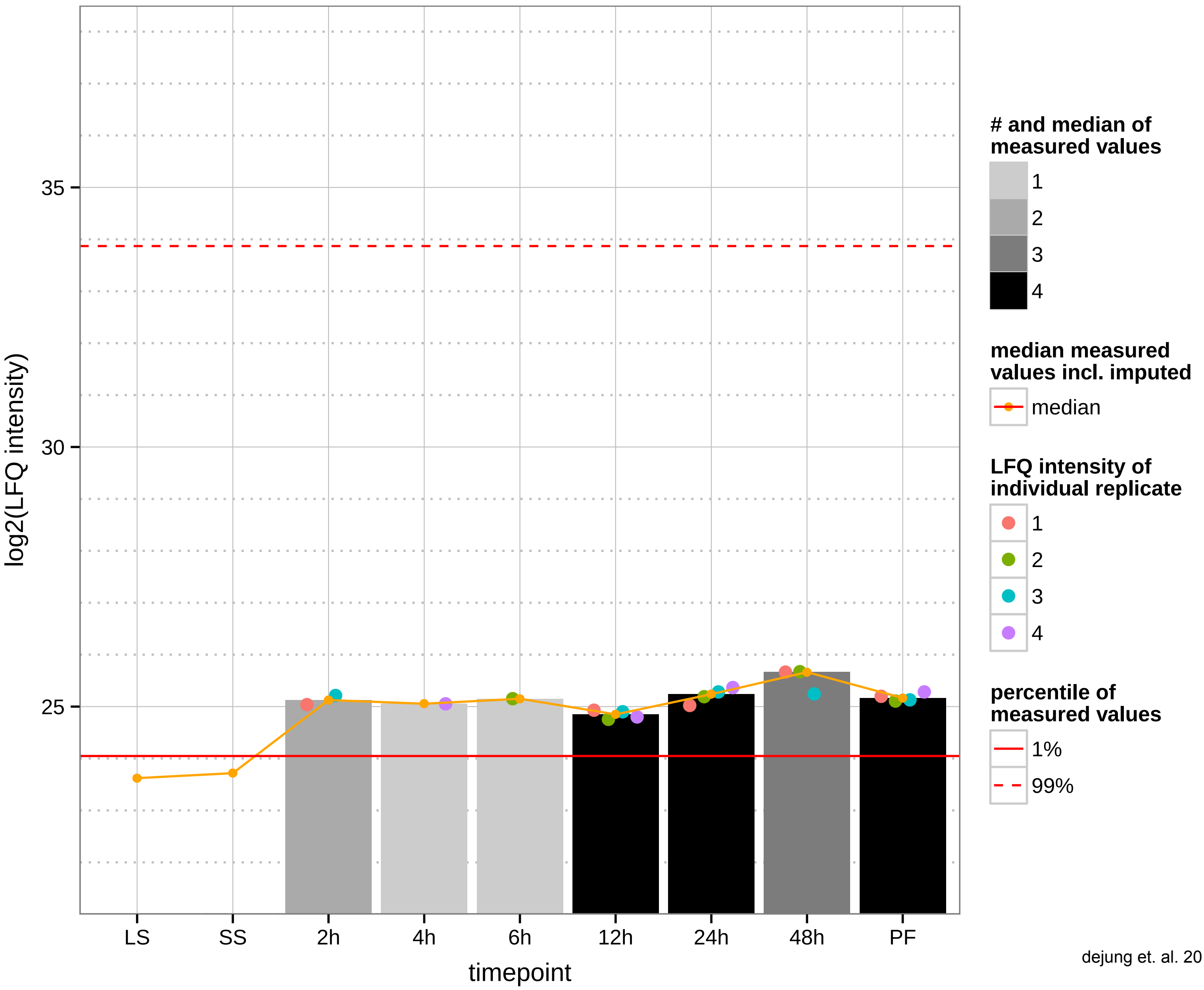

dejung et. al. 2015

hypothetical protein, conserved
Tb927.11.4680
AGOF: null
AGOC: mitochondrion
AGOP: null
PGOF: null
PGOC: membrane
PGOP: null

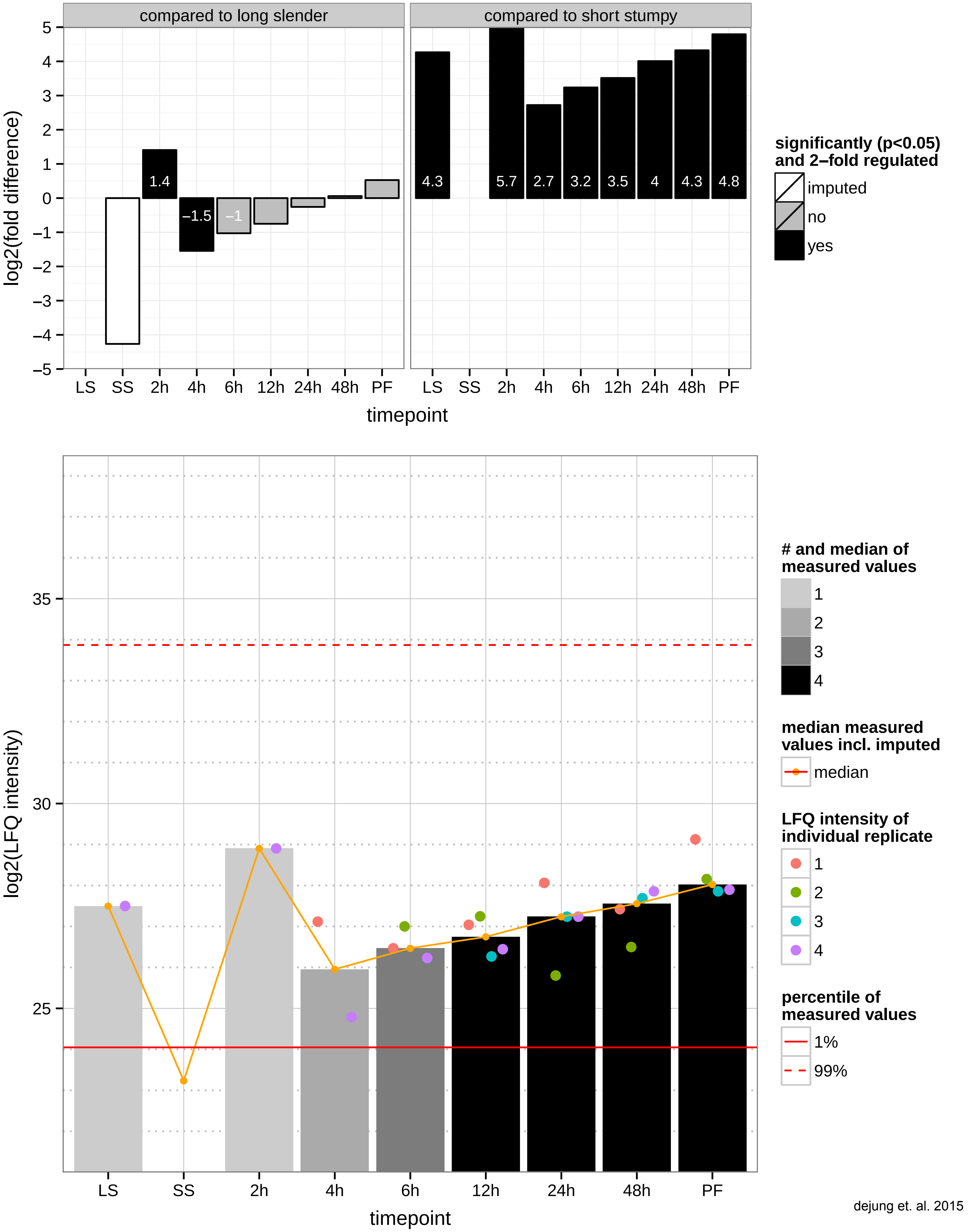

dejung et. al. 2015

Mitochondrial SSU ribosomal protein, putative, kinetoplast ribosomal PPR−repeat containing protein 1 (KRIPP1)
Tb927.11.5500
AGOF: null
AGOC: mitochondrion
AGOP: null
PGOF: null
PGOC: null
PGOP: null

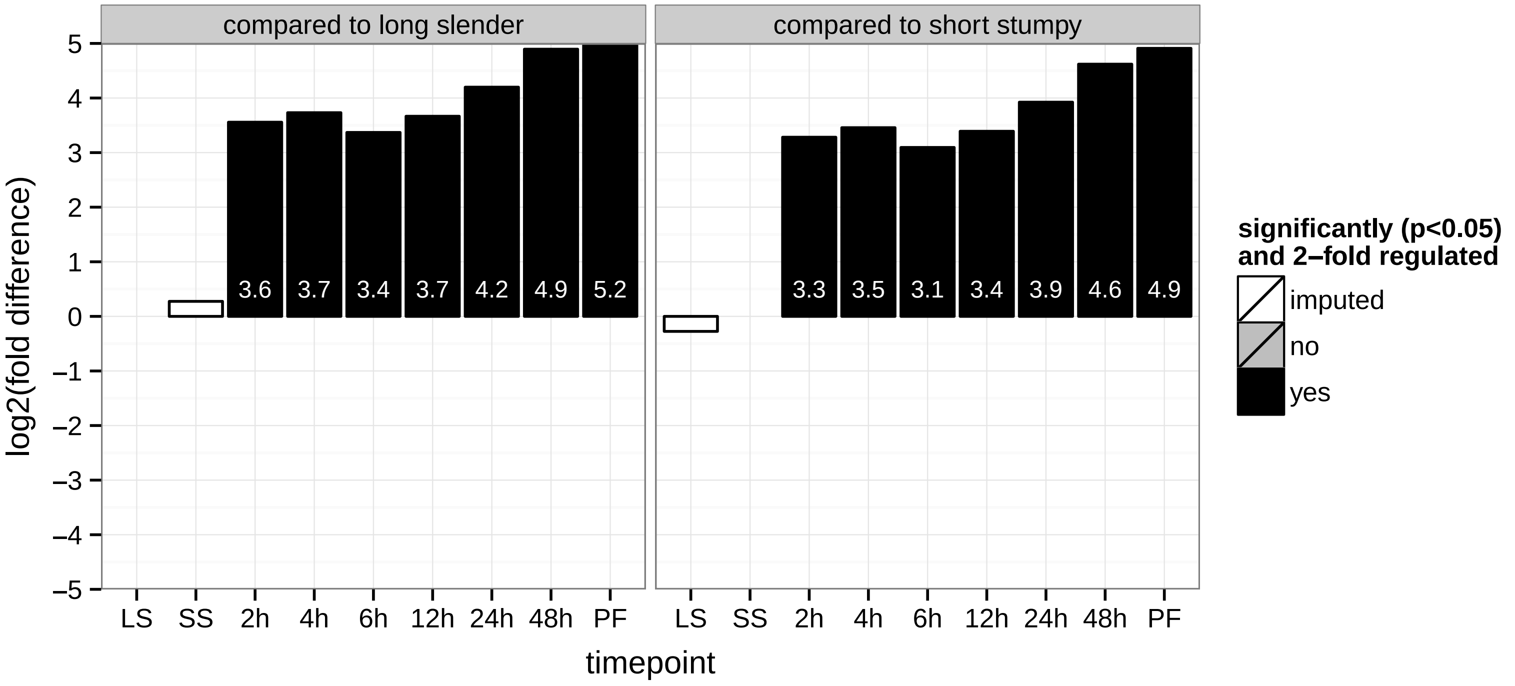

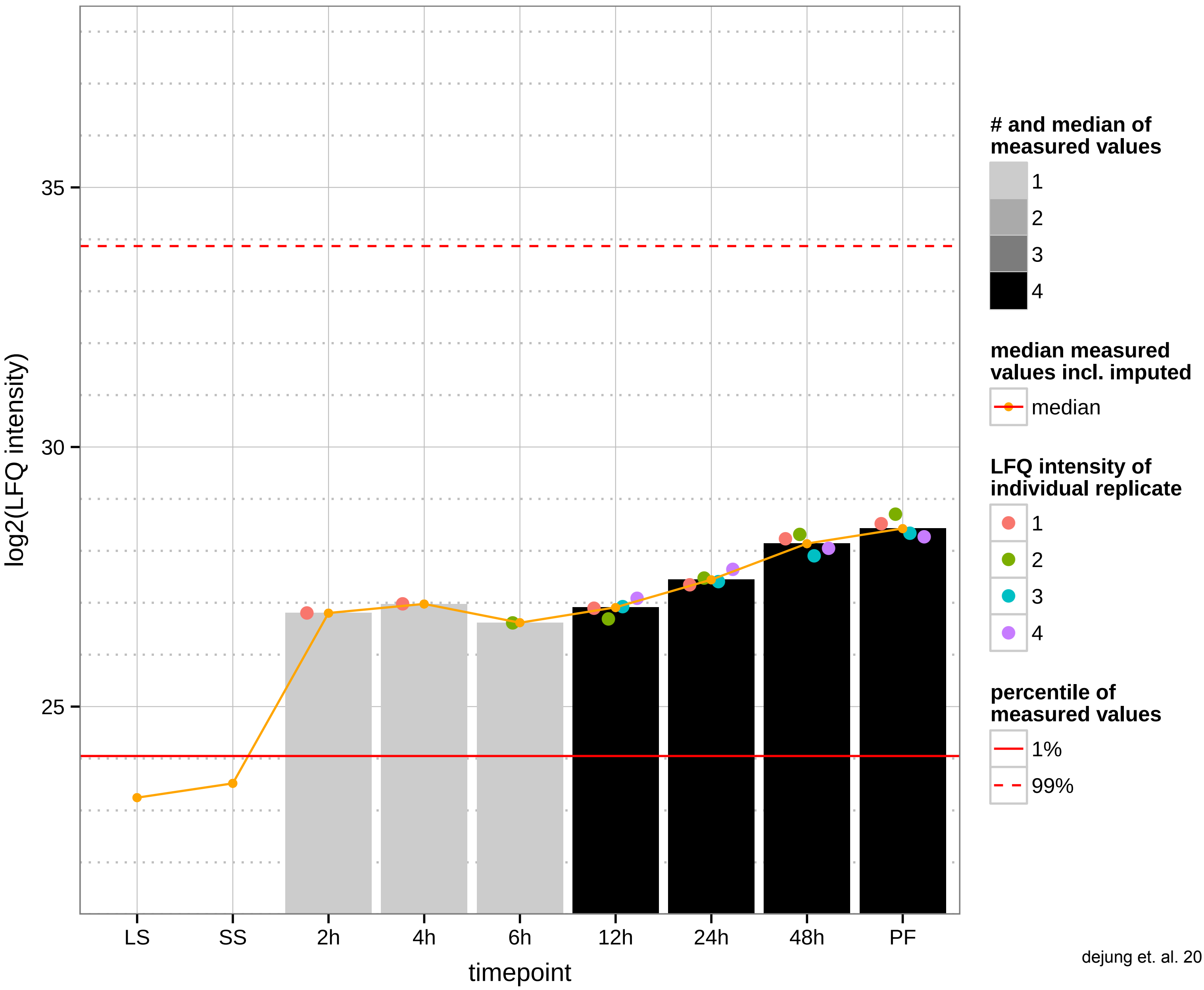

dejung et. al. 2015

RNA–binding protein, putative (RBP38)
Tb927.11.5850
AGOF: RNA binding
AGOC: cytosol
AGOP: null
PGOF: null
PGOC: null
PGOP: null

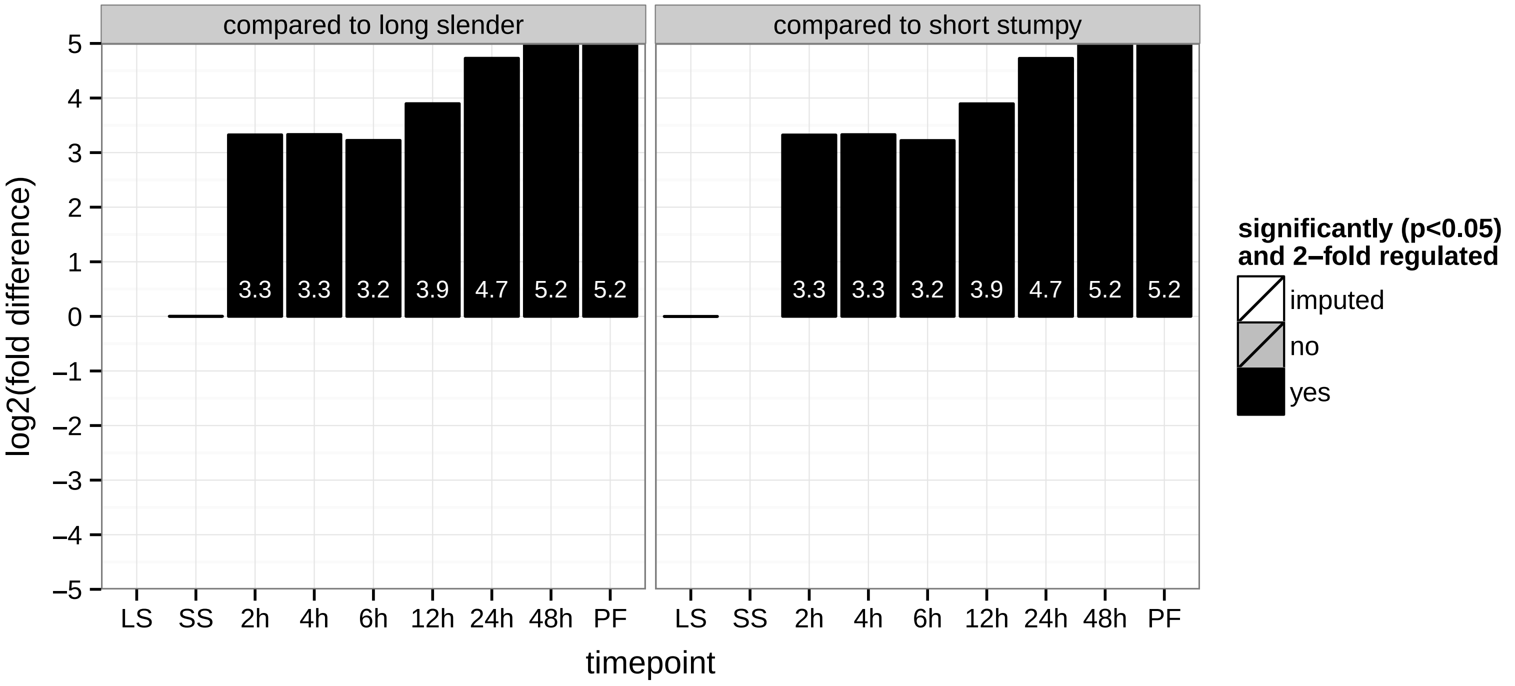

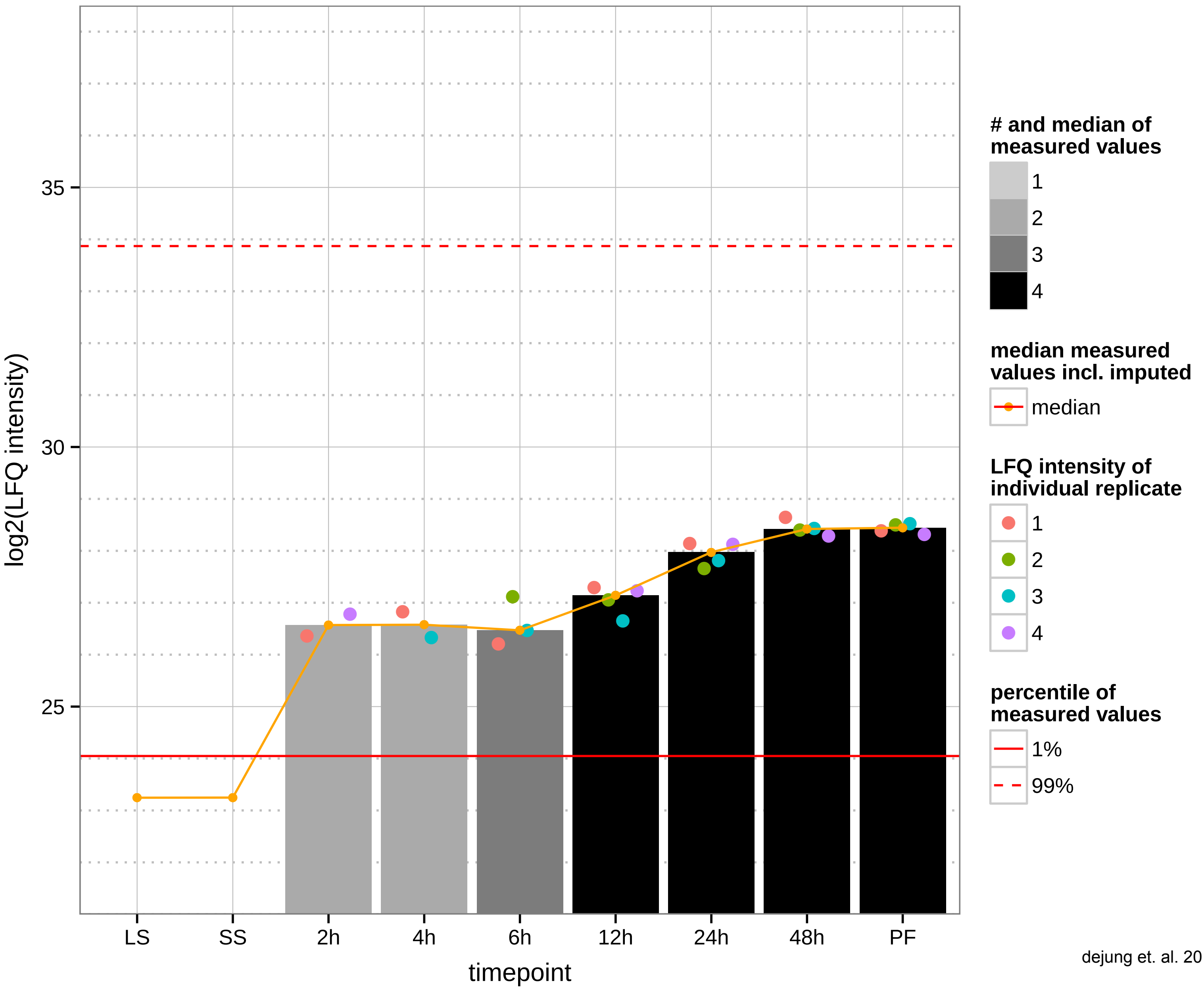

dejung et. al. 2015

phosphoinositide−specific phospholipase C, putative
Tb927.11.5970
AGOF: phosphatidylinositol phospholipase C activity, phospholipase C activity
AGOC: membrane
AGOP: intracellular signal transduction, lipid metabolic process, signal transduction
PGOF: phosphatidylinositol phospholipase C activity, phospholipase C activity, phosphoric diester hydrolase activity, protein b
PGOC: null
PGOP: intracellular signal transduction, lipid metabolic process, signal transduction

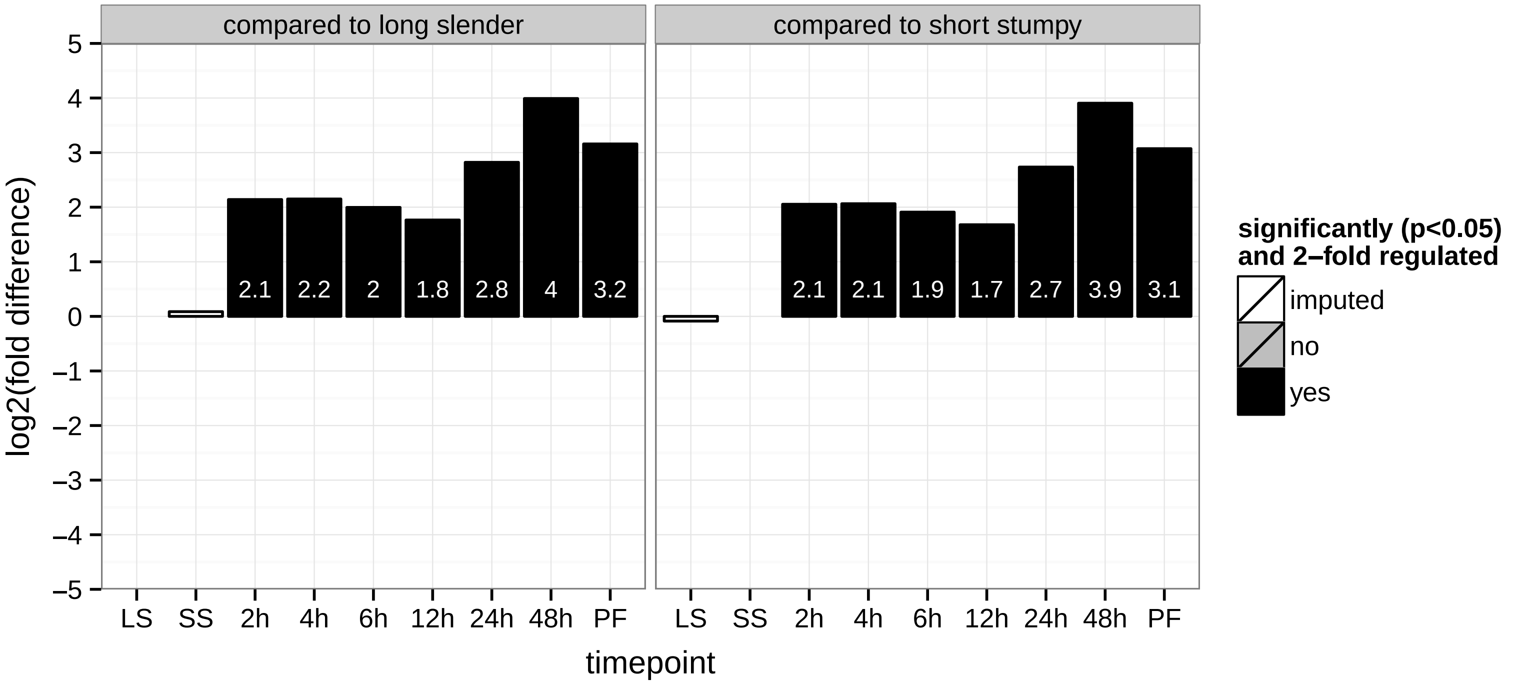

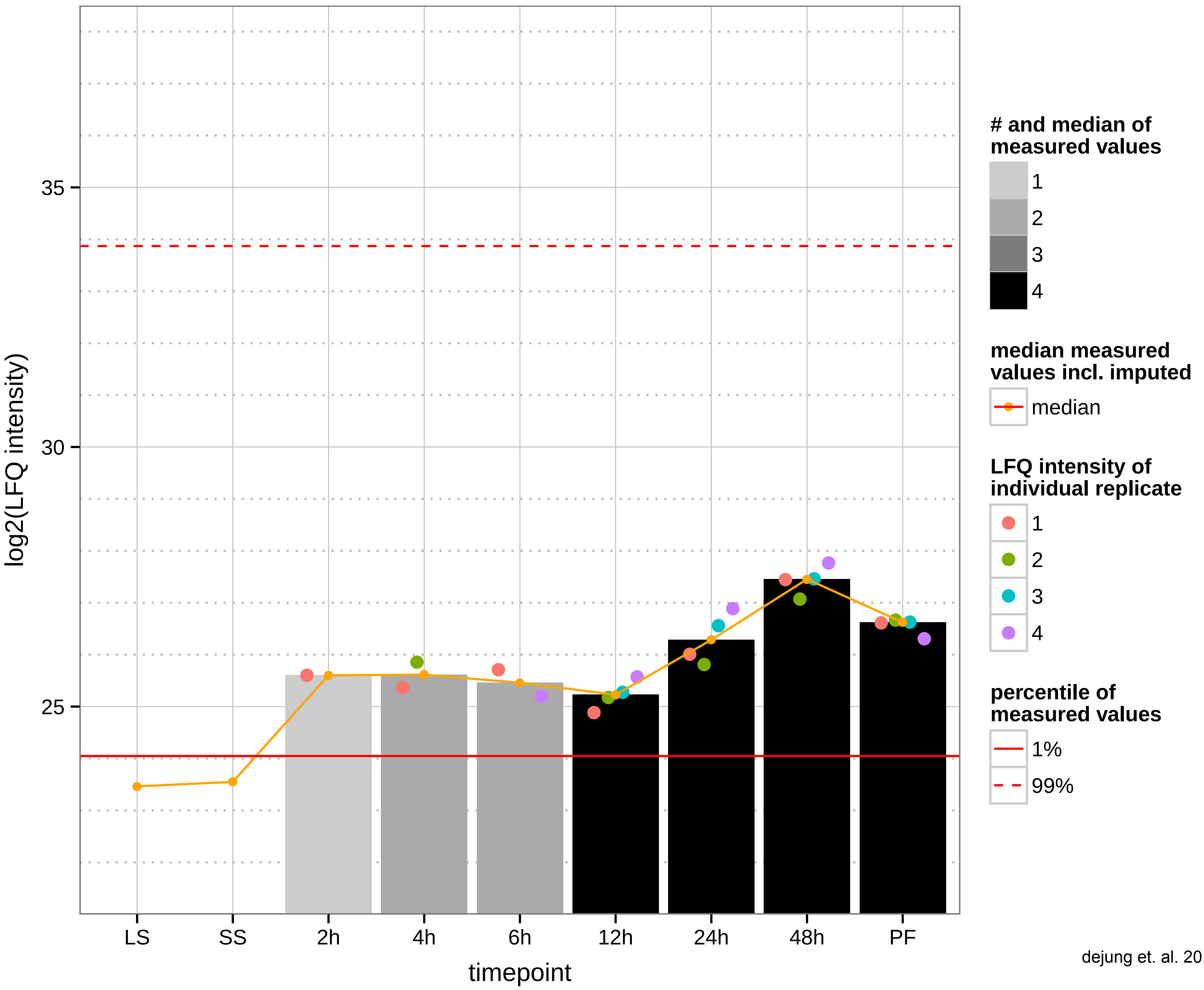

dejung et. al. 2015

hypothetical protein, conserved
Tb927.11.6660
AGOF: hydrolase activity, acting on ester bonds
AGOC: mitochondrion
AGOP: nucleobase–containing compound metabolic process
PGOF: hydrolase activity, acting on ester bonds, nucleic acid binding
PGOC: null
PGOP: nucleobase–containing compound metabolic process

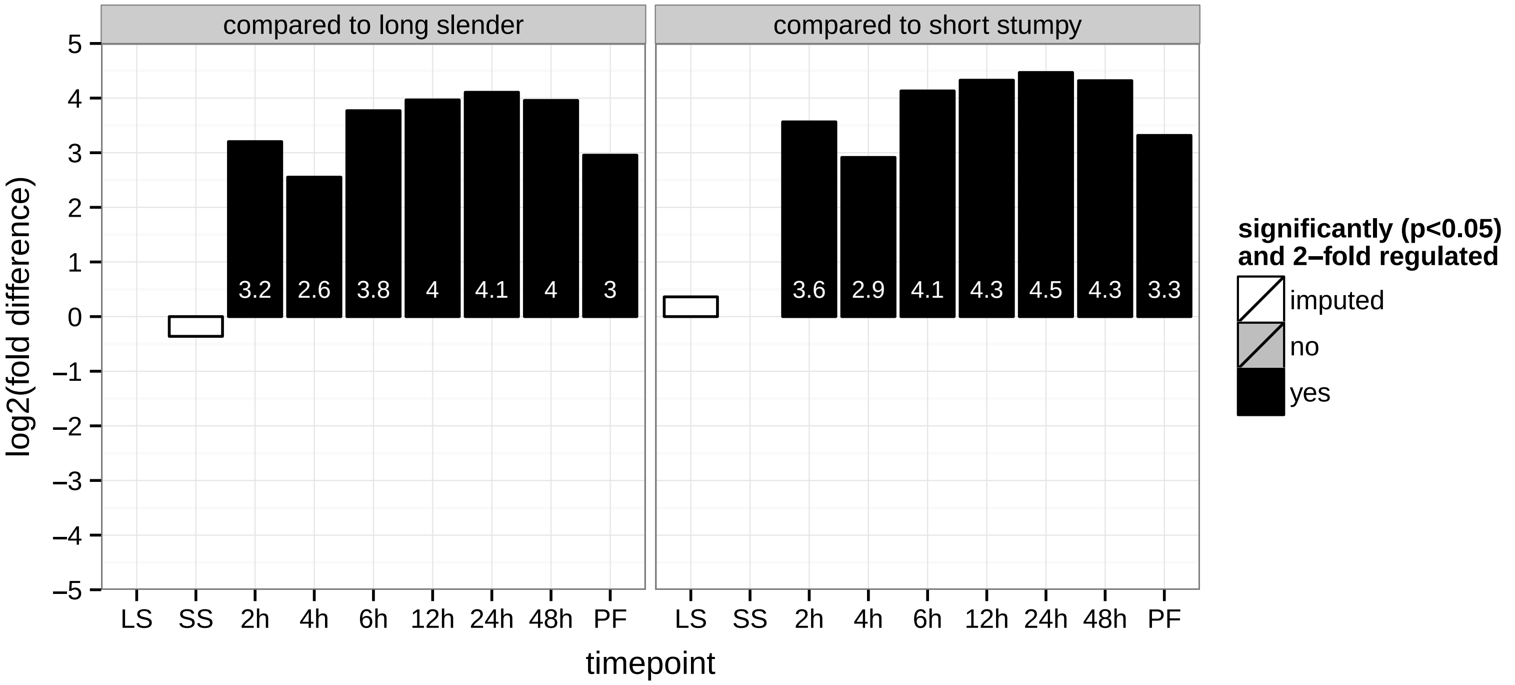

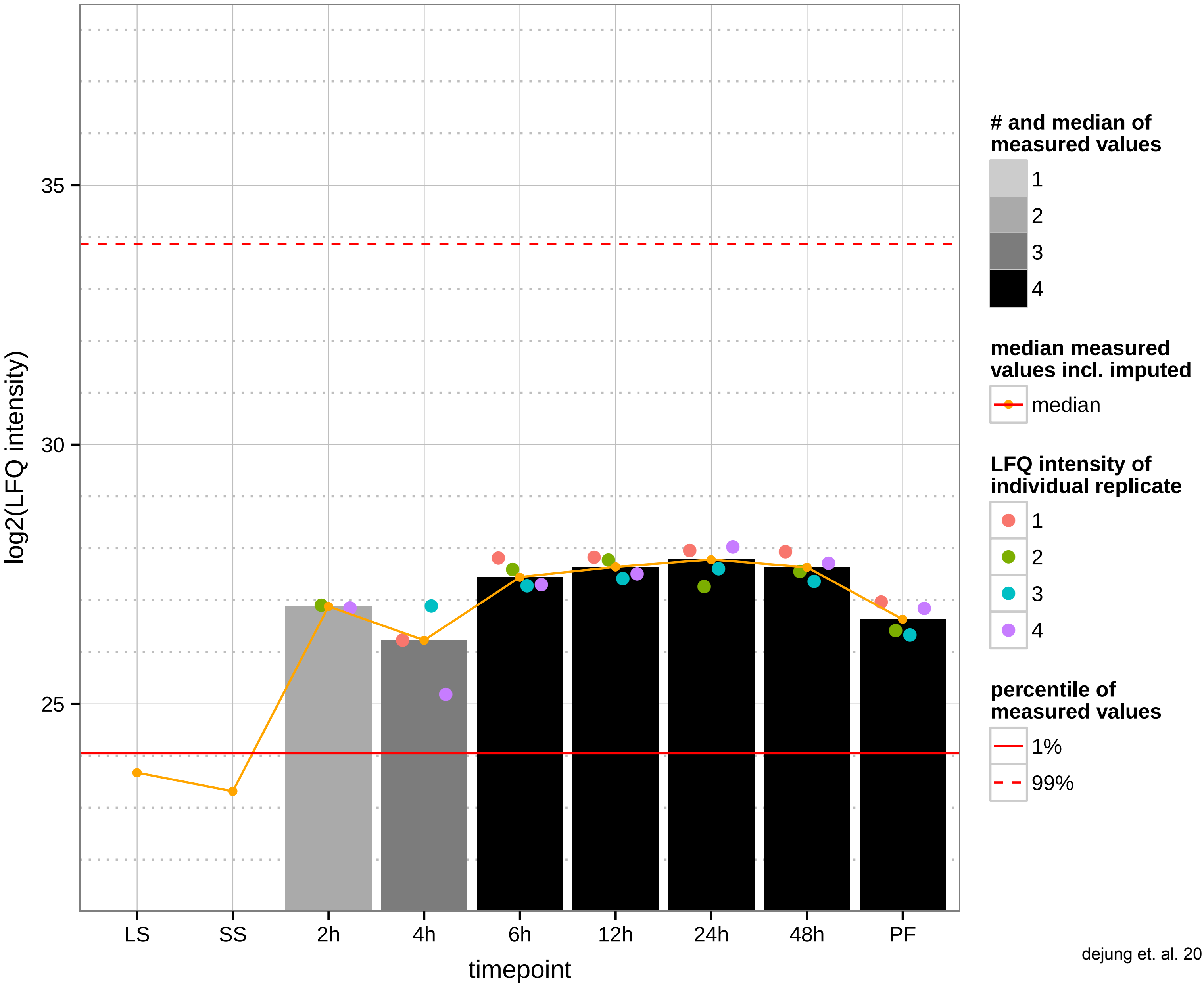

dejung et. al. 2015

hypothetical protein, conserved
Tb927.11.840.1;Tb927.11.840.2
AGOF: solute:hydrogen antiporter activity
AGOC: integral to membrane
AGOP: cation transport
PGOF: solute:hydrogen antiporter activity
PGOC: integral to membrane
PGOP: cation transport, transmembrane transport

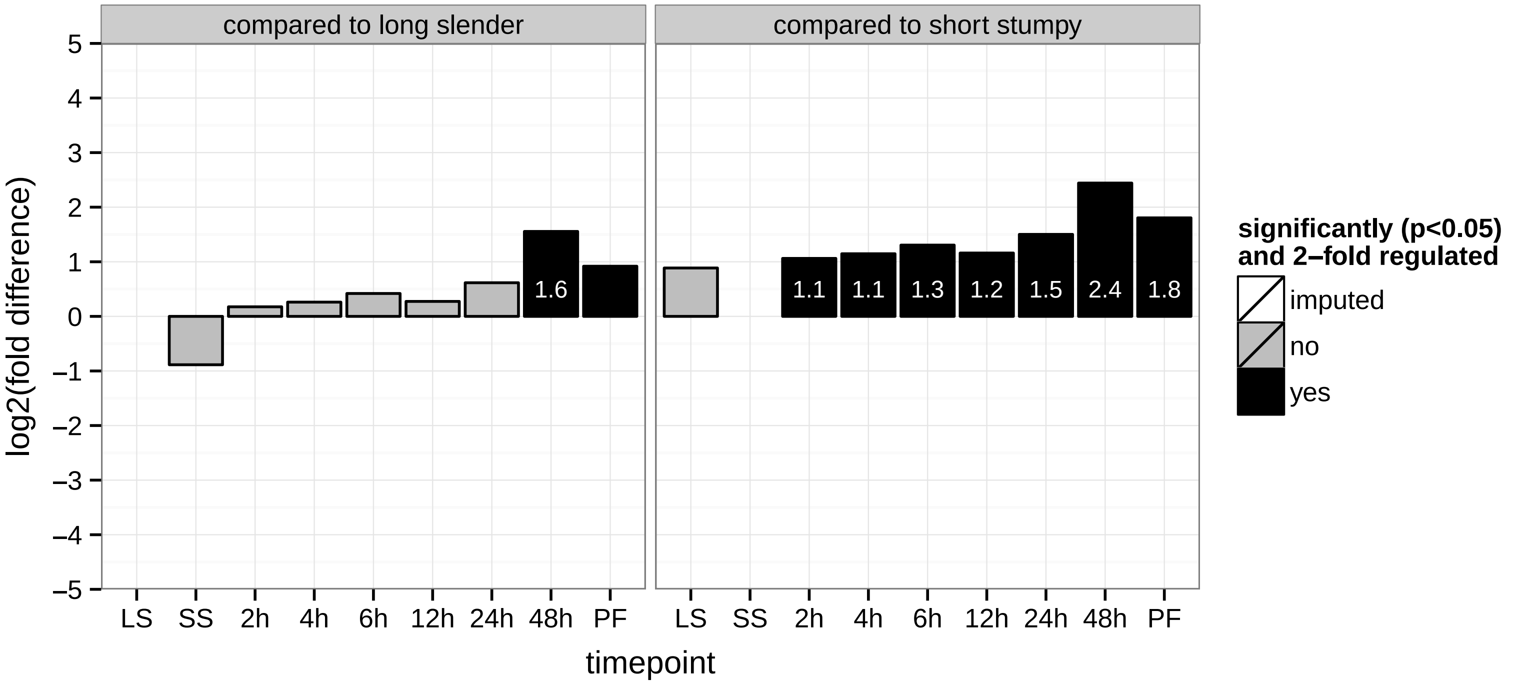

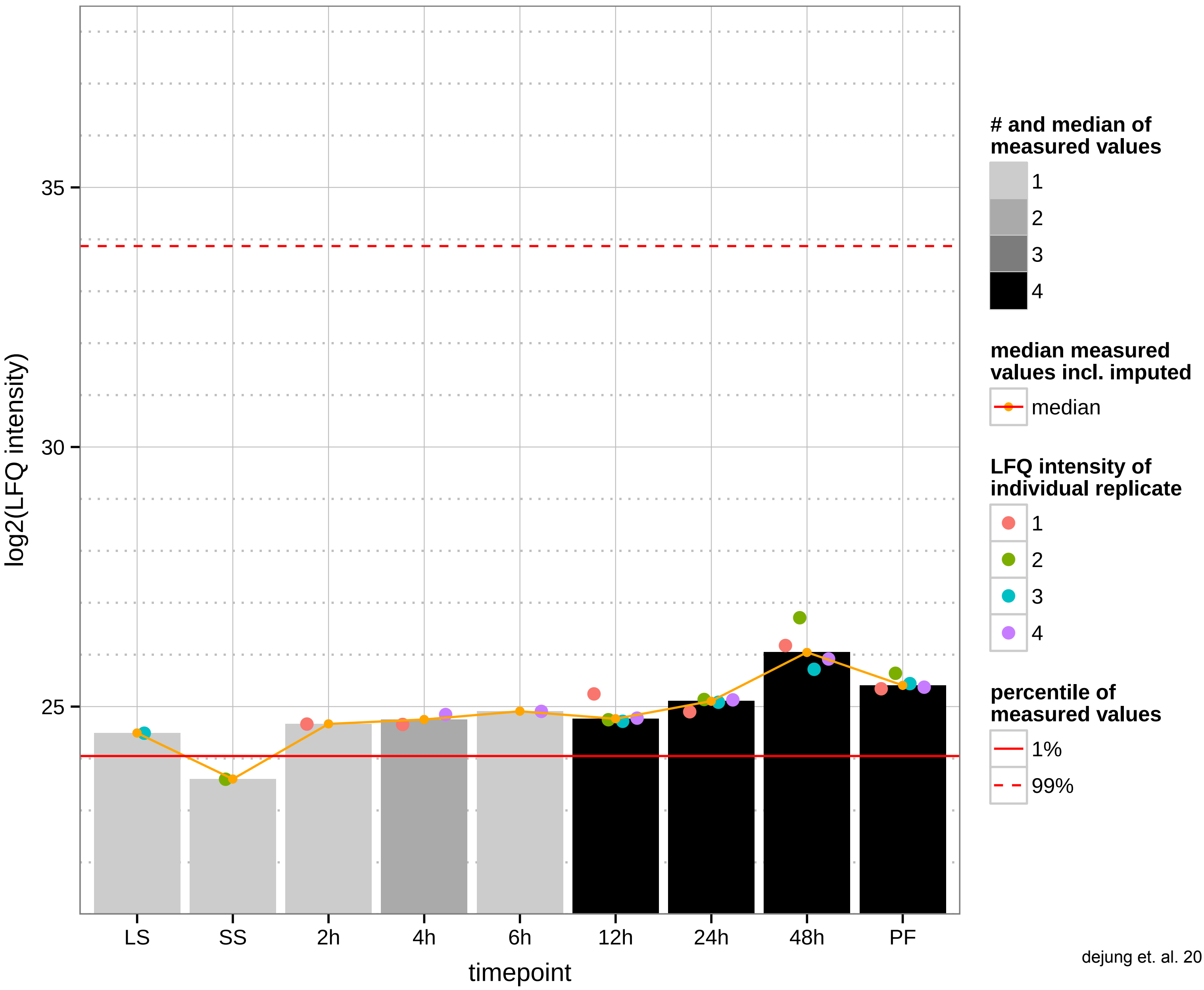

dejung et. al. 2015

retrotransposon hot spot protein (RHS, pseudogene), putative, retrotransposon hot spot protein 6 (RHS6), degenerate
Tb927.2.1330
AGOF: null
AGOC: null
AGOP: null
PGOF: null
PGOC: null
PGOP: null

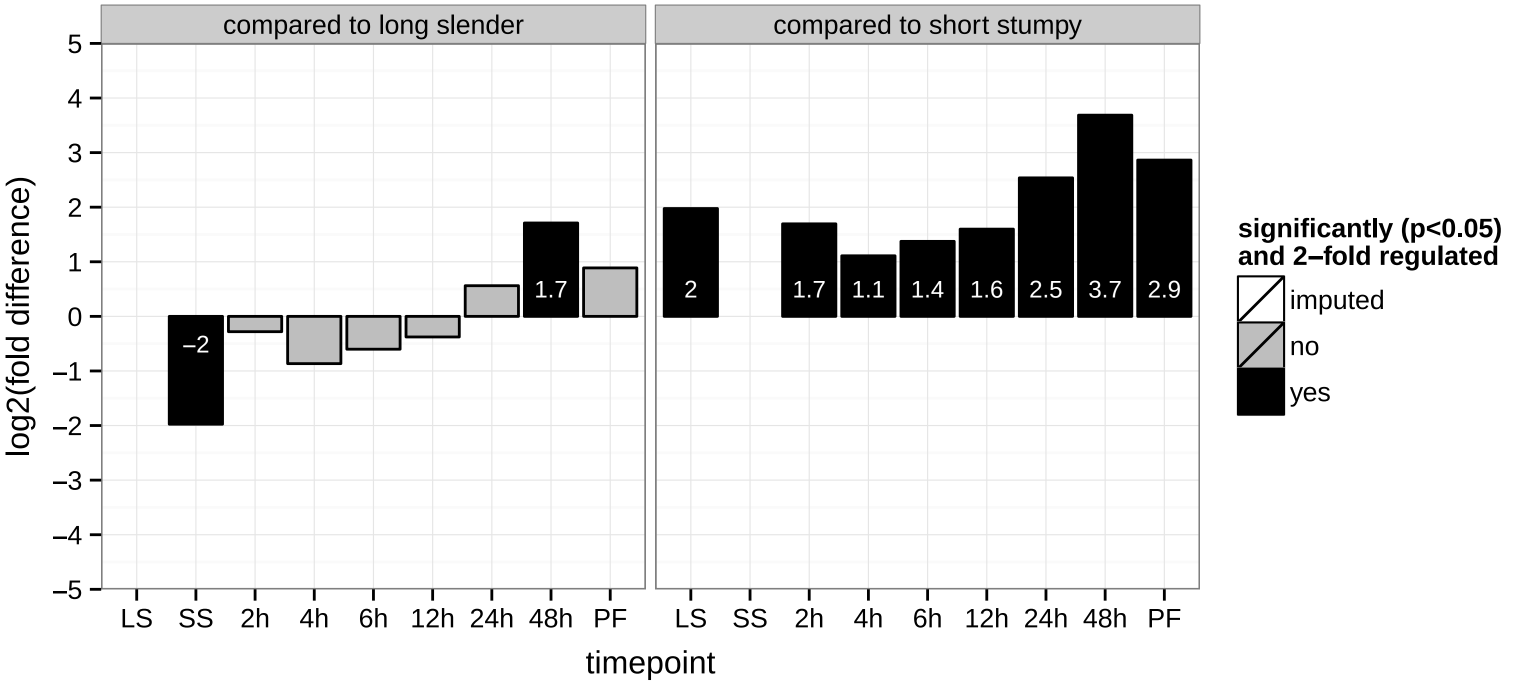

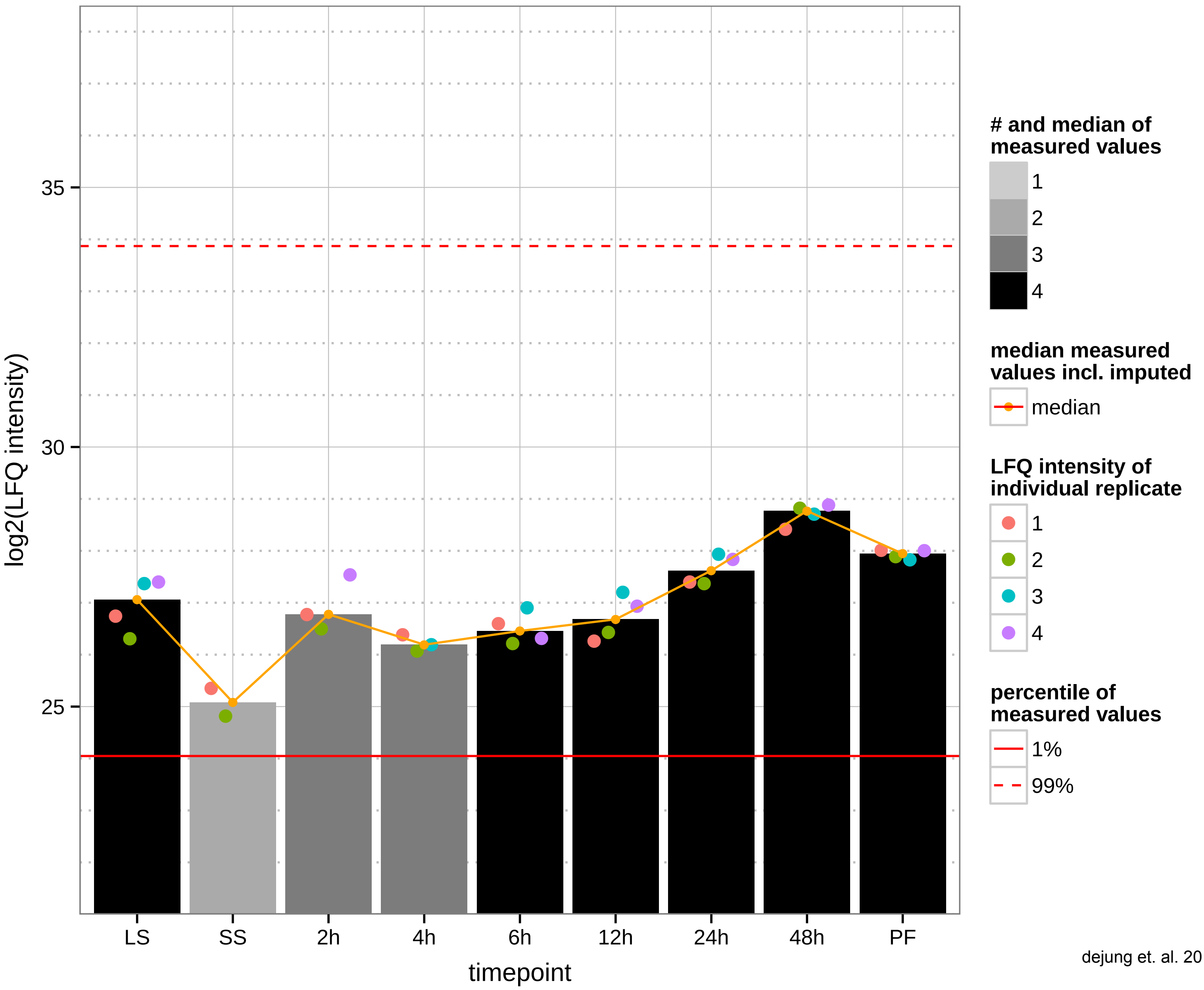

dejung et. al. 2015

hypothetical protein, conserved
Tb927.3.1970
AGOF: null
AGOC: null
AGOP: null
PGOF: null
PGOC: null
PGOP: null

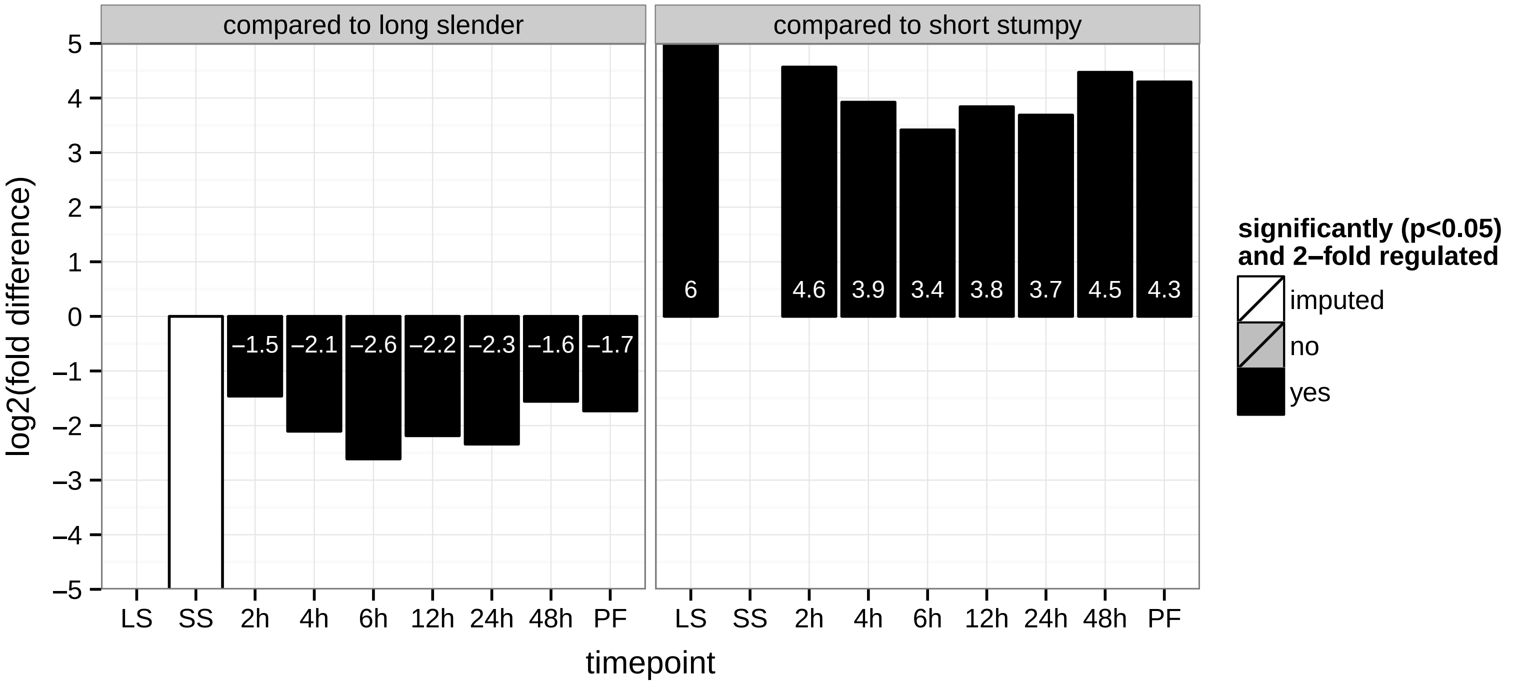

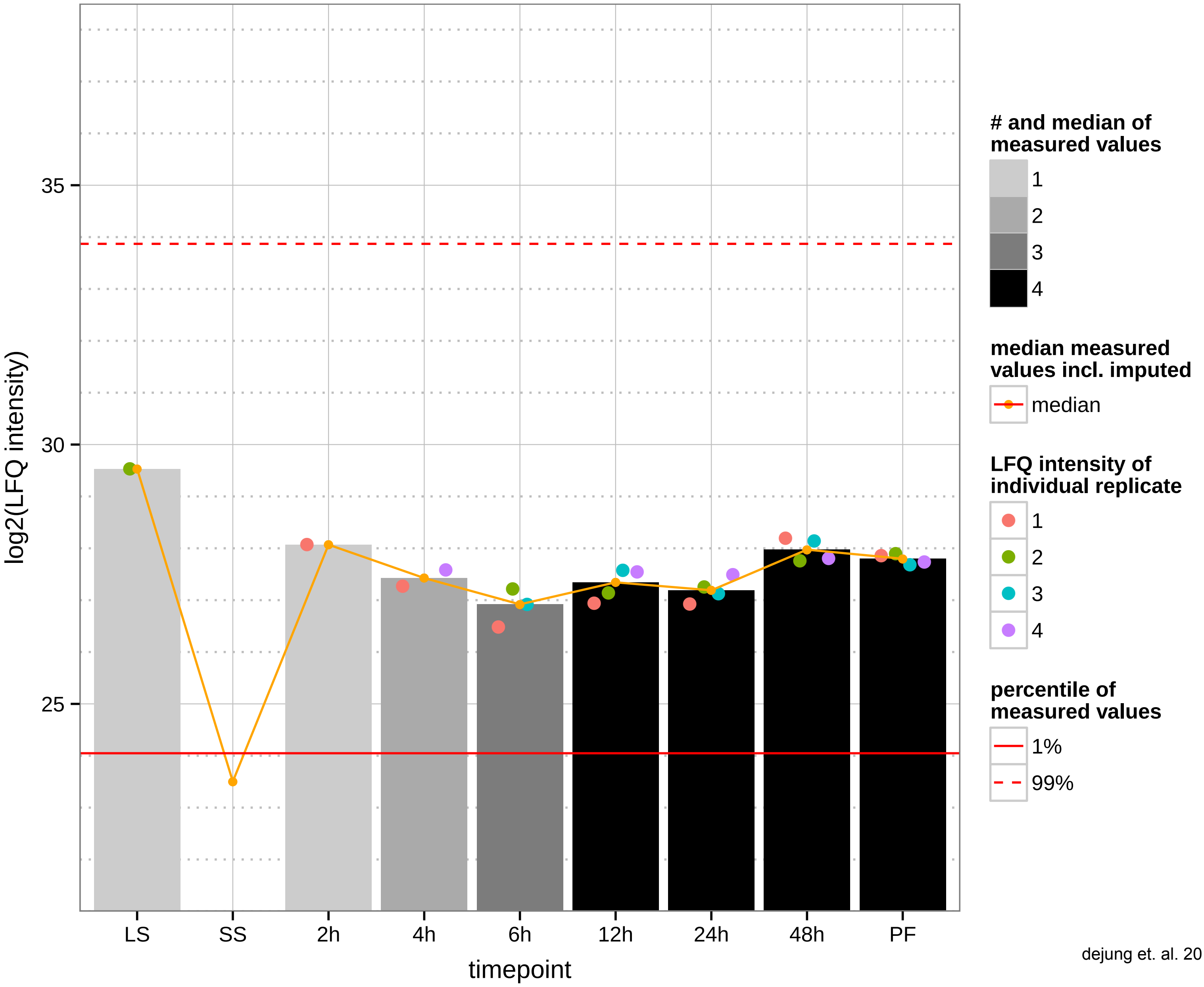

dejung et. al. 2015

hypothetical protein, conserved
Tb927.3.750
AGOF: null
AGOC: mitochondrial inner membrane, mitochondrion
AGOP: null
PGOF: protein binding
PGOC: null
PGOP: null

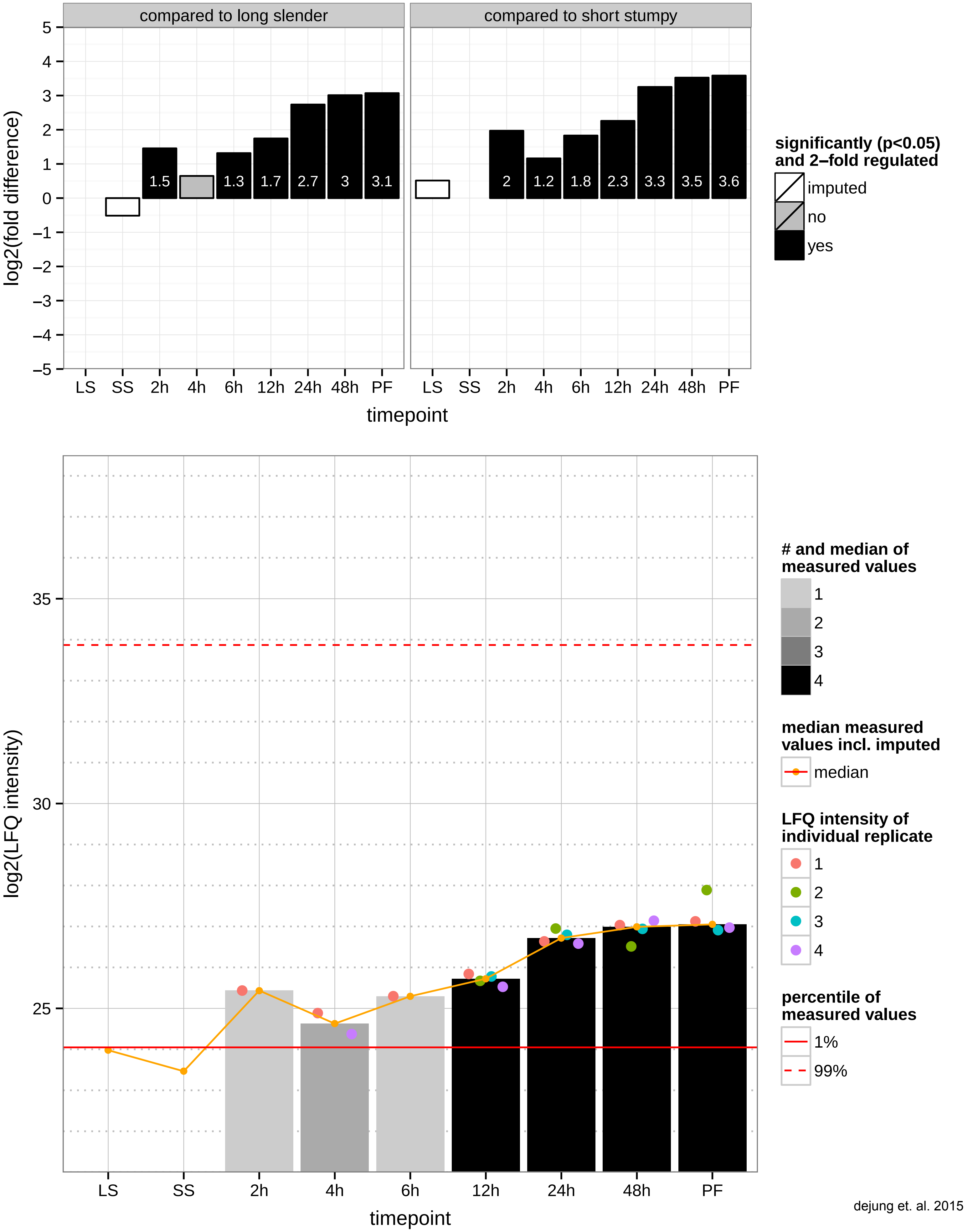

dejung et. al. 2015

hypothetical protein, conserved
Tb927.4.1670
AGOF: heme binding
AGOC: null
AGOP: null
PGOF: heme binding
PGOC: null
PGOP: null

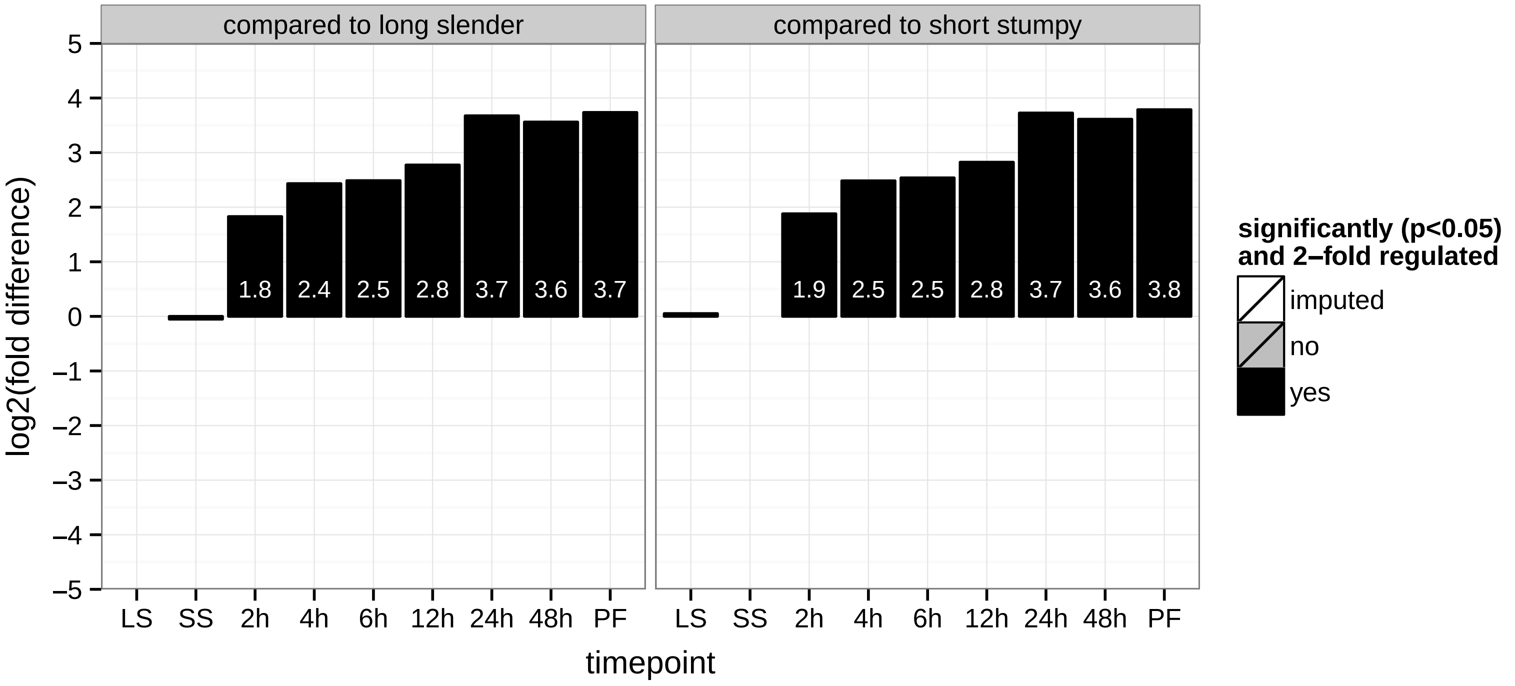

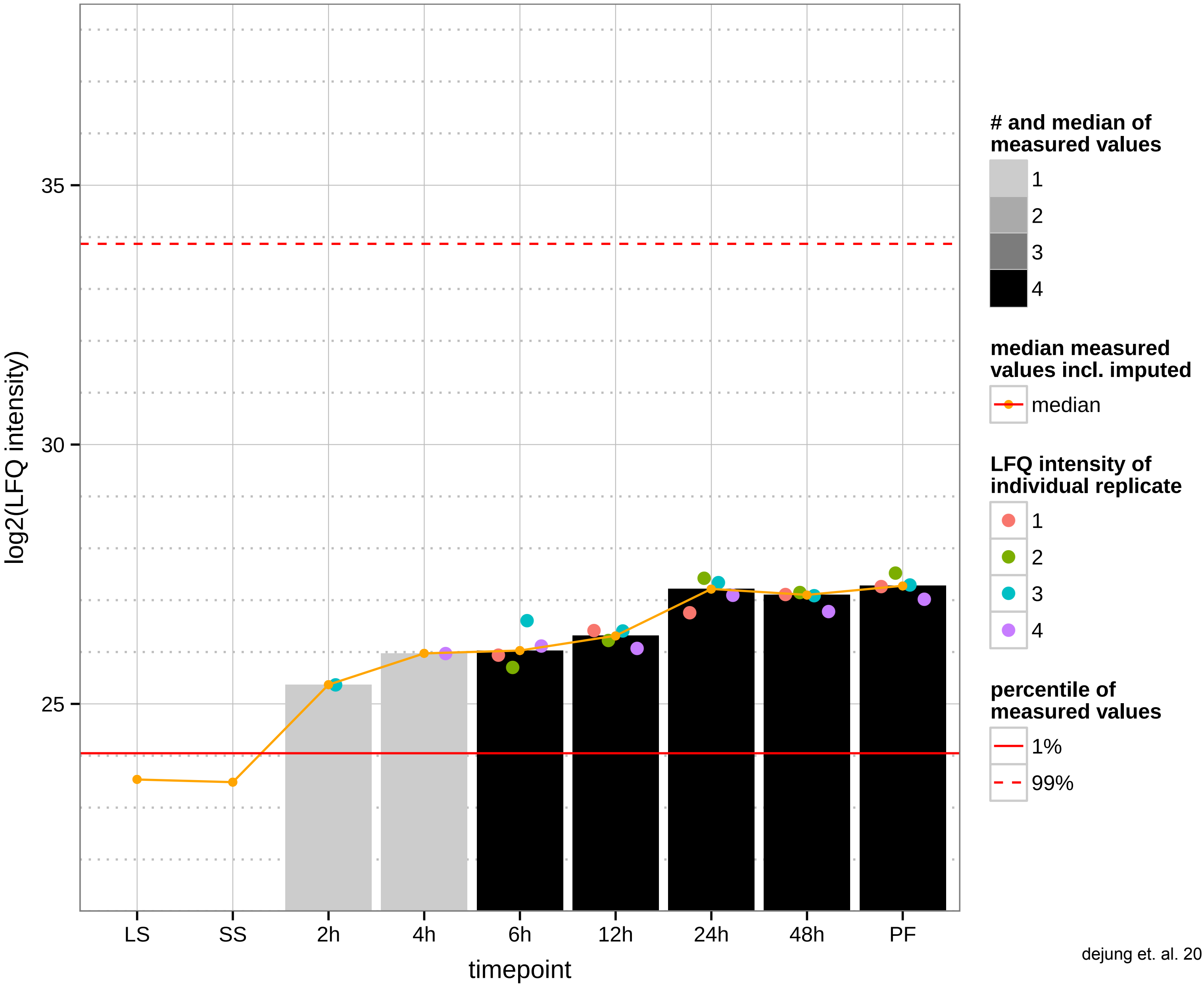

dejung et. al. 2015

L–galactonolactone oxidase, D–arabinonolactone oxidase (GAL/ALO)
Tb927.5.2650
AGOF: D–arabinono–1, 4–lactone oxidase activity, FMN binding, L–galactonolactone oxidase activity, flavin adenine dinucleot
AGOC: glycosome, membrane
AGOP: L–ascorbic acid biosynthetic process
PGOF: D–arabinono–1, 4–lactone oxidase activity, UDP–N–acetylmuramate dehydrogenase activity, catalytic activity, flavin a
PGOC: membrane
PGOP: oxidation–reduction process

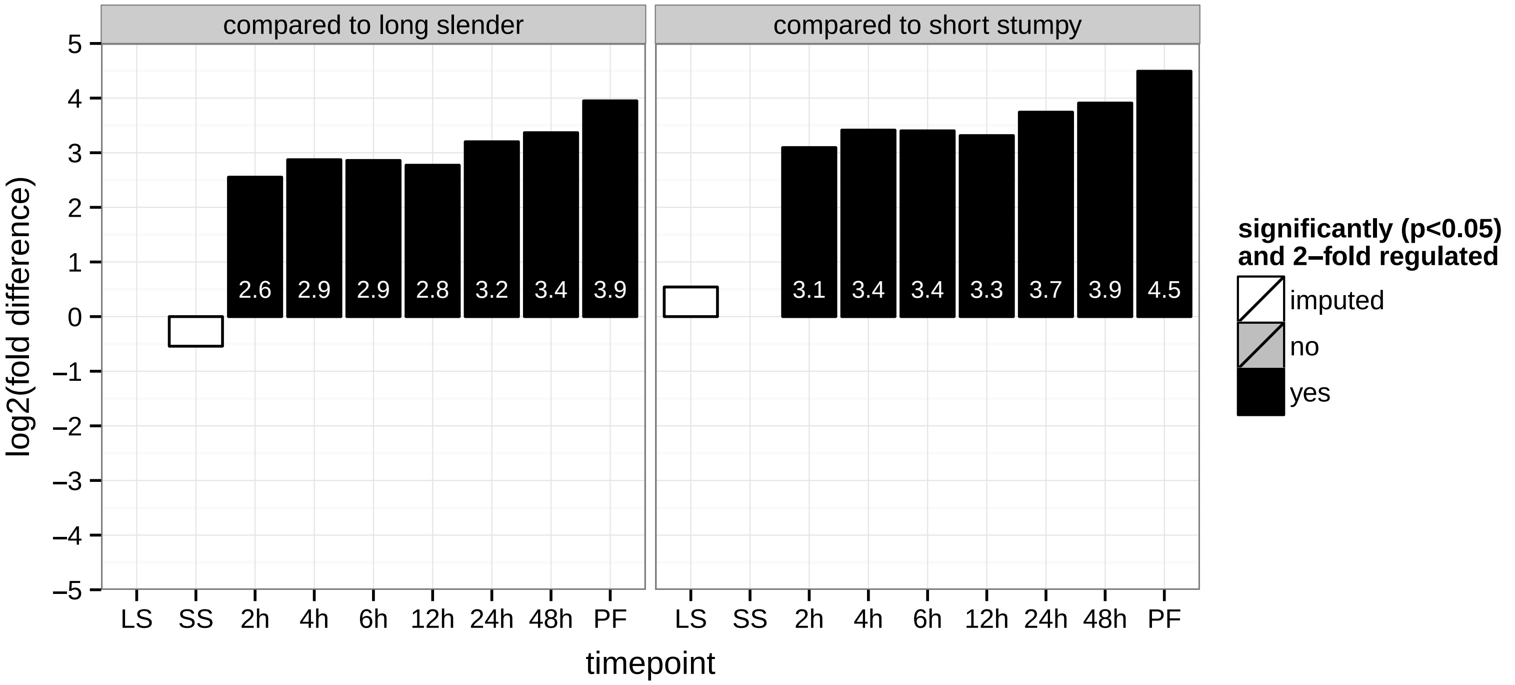

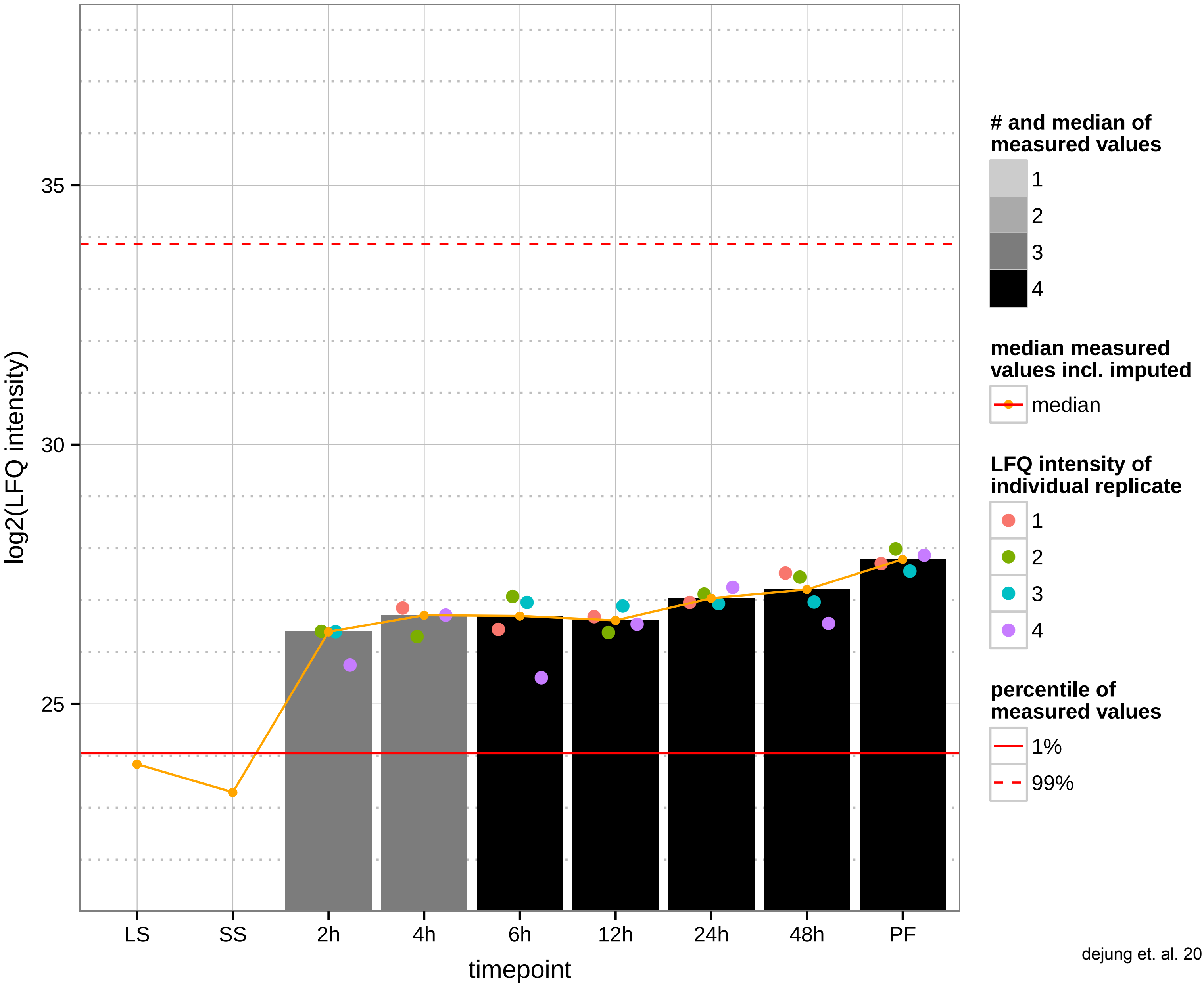

dejung et. al. 2015

cell cycle sequence binding phosphoprotein (RBP33), putative
Tb927.5.760
AGOF: phosphoprotein binding
AGOC: null
AGOP: null
PGOF: null
PGOC: null
PGOP: null

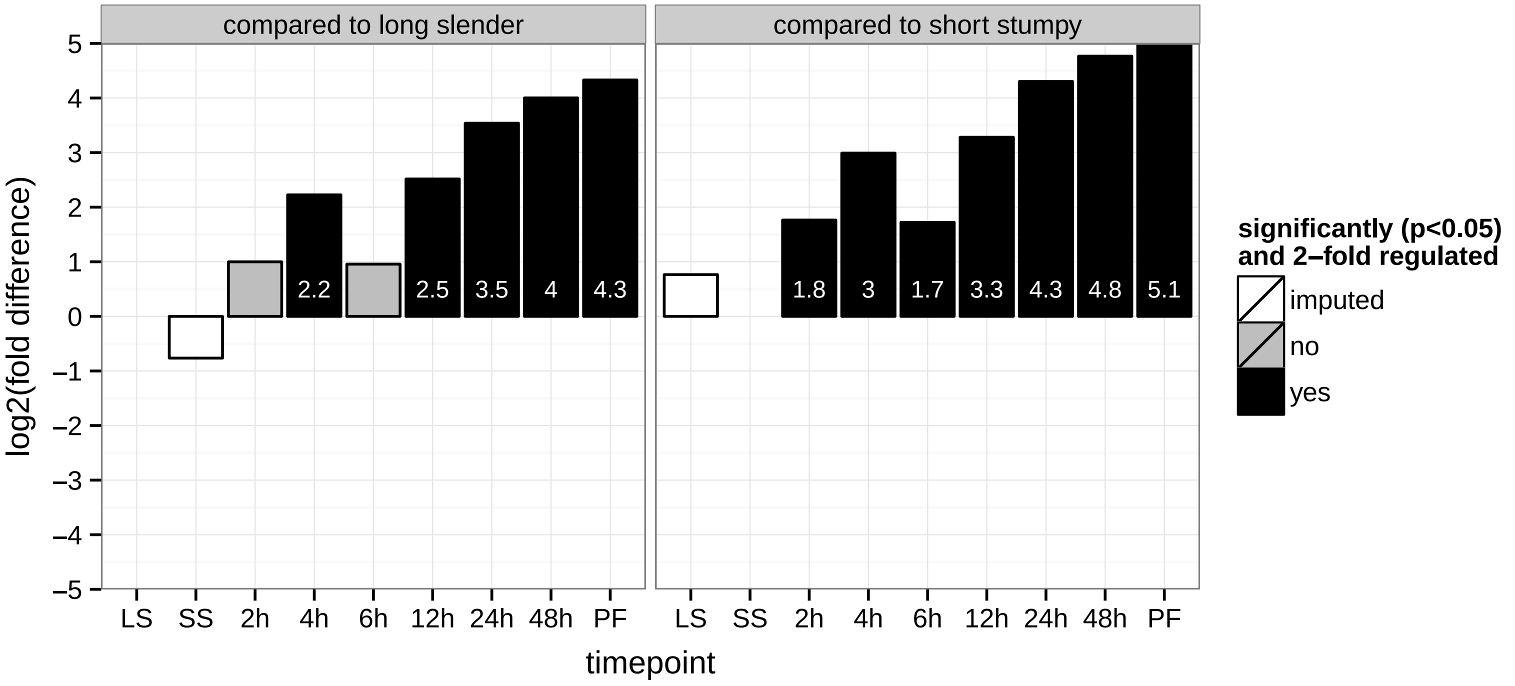

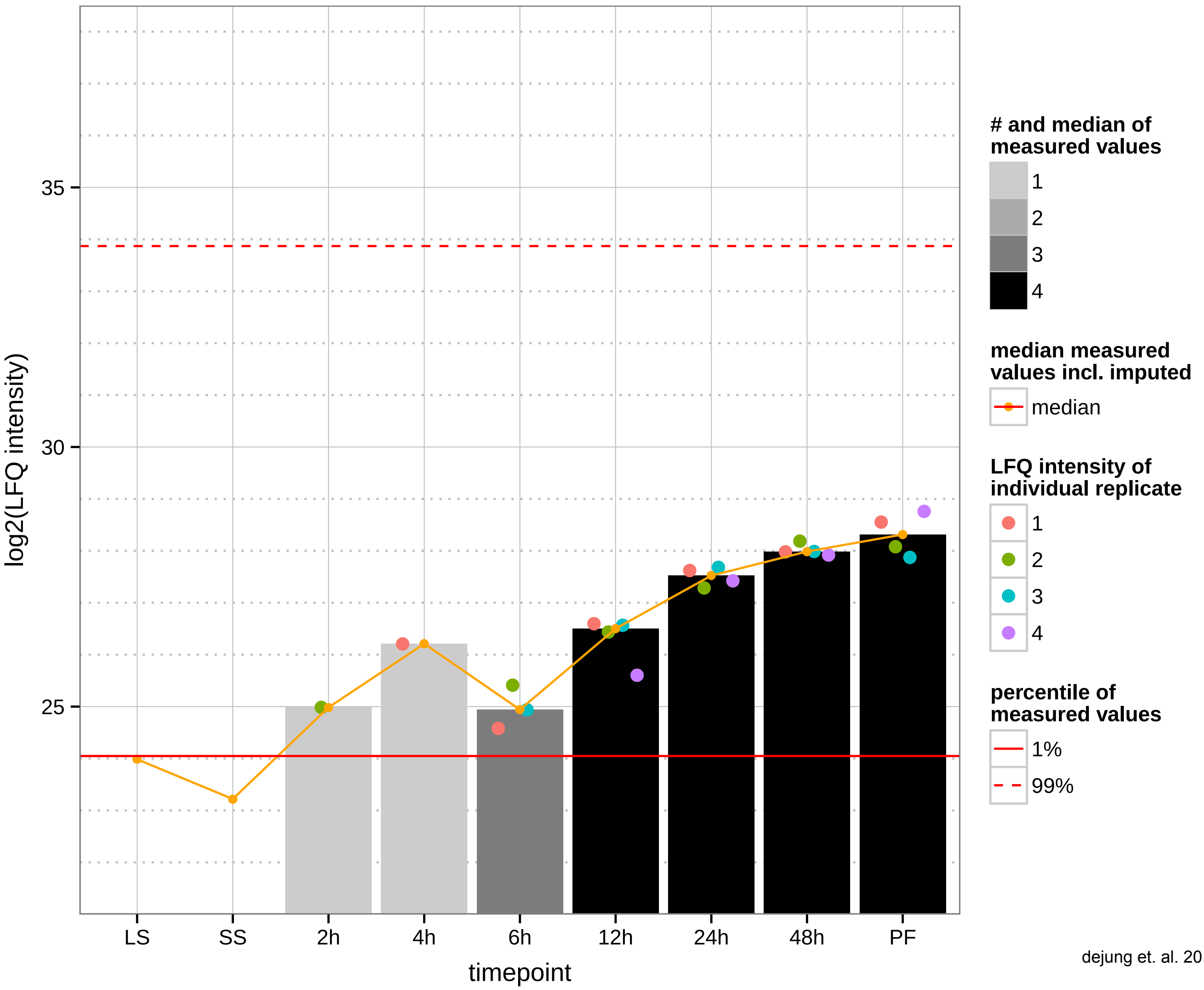

dejung et. al. 2015

single strand–specific nuclease, putative
Tb927.6.1650
AGOF: endonuclease activity, nucleic acid binding
AGOC: null
AGOP: DNA catabolic process
PGOF: endonuclease activity, hydrolase activity, acting on ester bonds, nucleic acid binding
PGOC: null
PGOP: DNA catabolic process

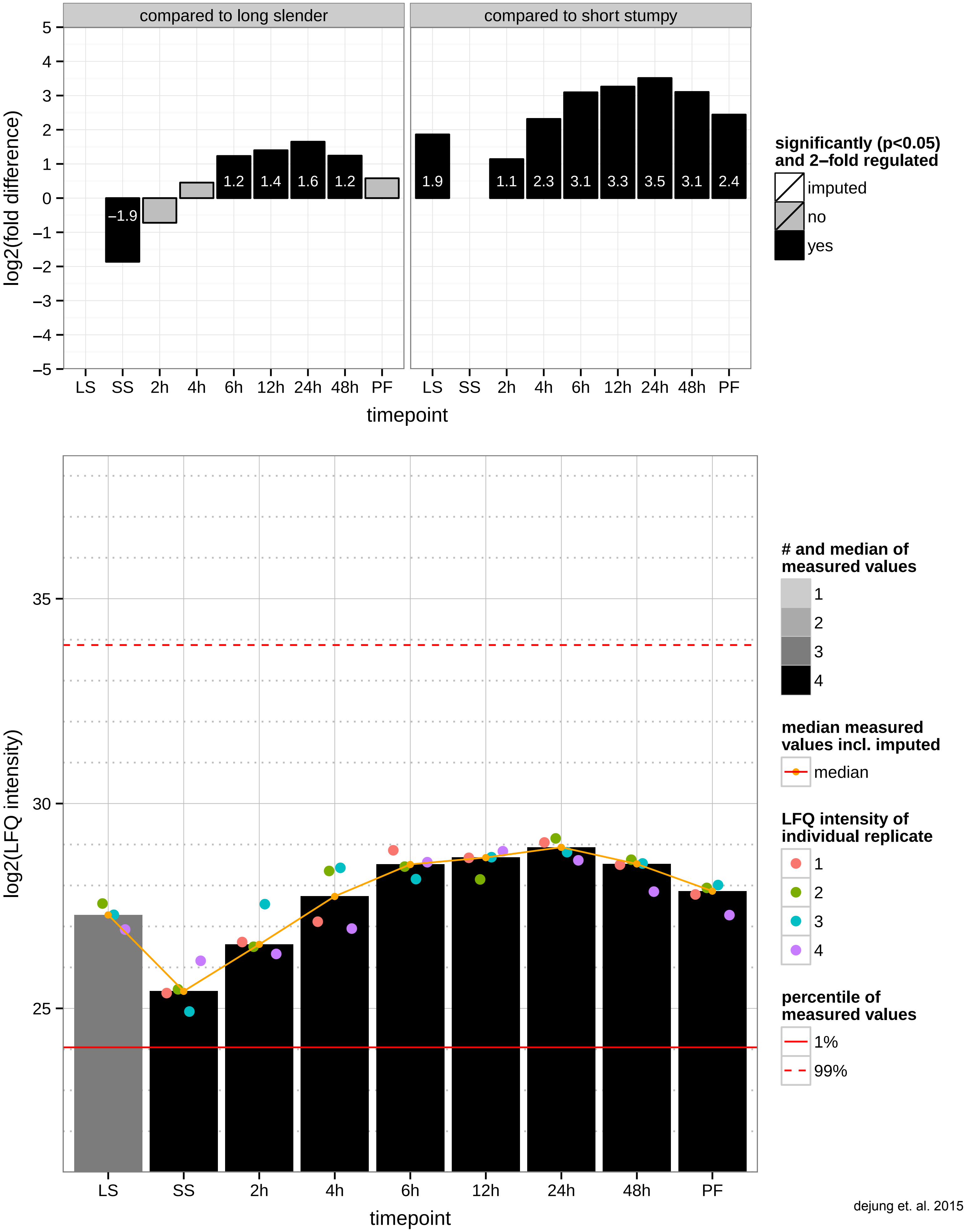

dejung et. al. 2015

single strand–specific nuclease, putative
Tb927.6.2890
AGOF: endonuclease activity, nucleic acid binding
AGOC: null
AGOP: DNA catabolic process
PGOF: endonuclease activity, hydrolase activity, acting on ester bonds, nucleic acid binding
PGOC: null
PGOP: DNA catabolic process

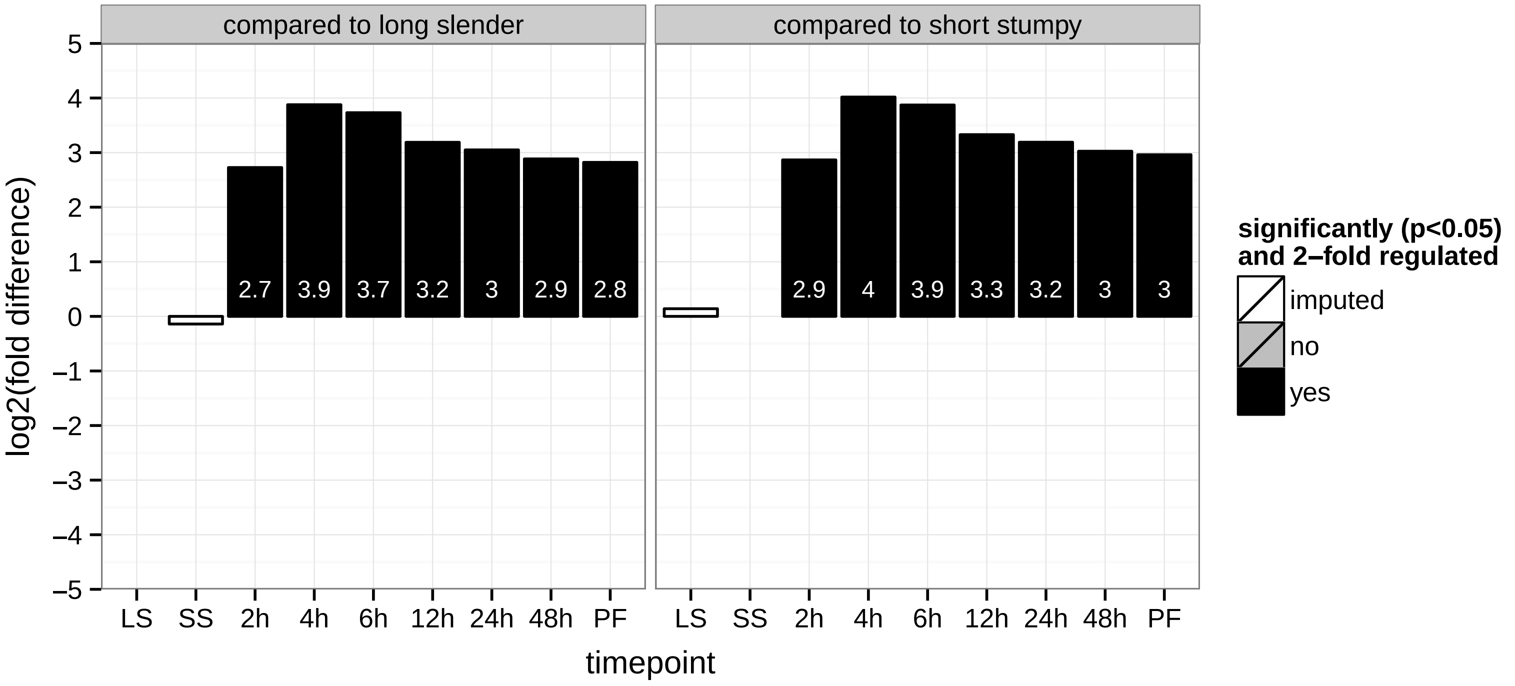

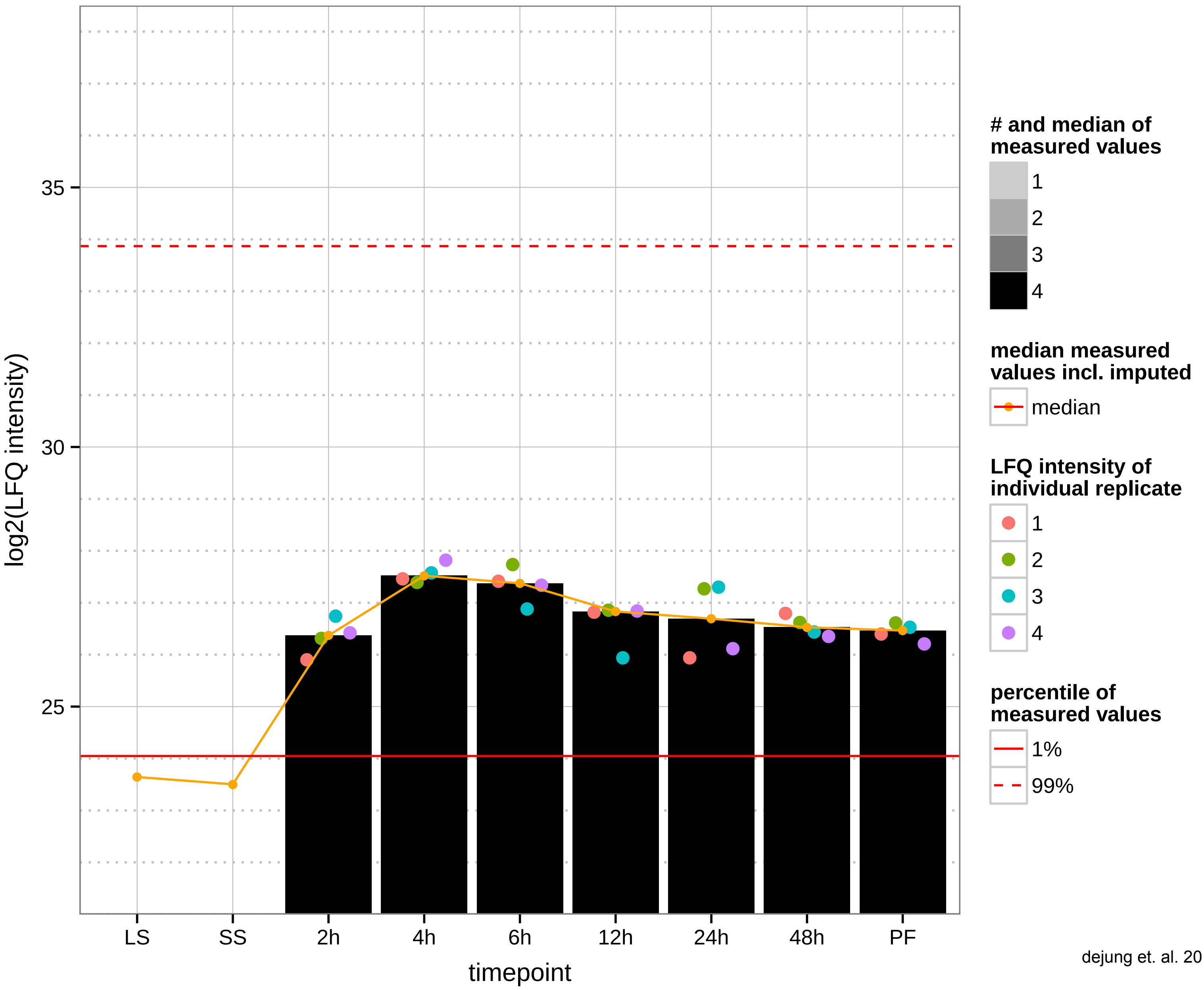

dejung et. al. 2015

hypothetical protein, conserved
Tb927.6.4080
AGOF: null
AGOC: mitochondrion
AGOP: null
PGOF: null
PGOC: null
PGOP: null

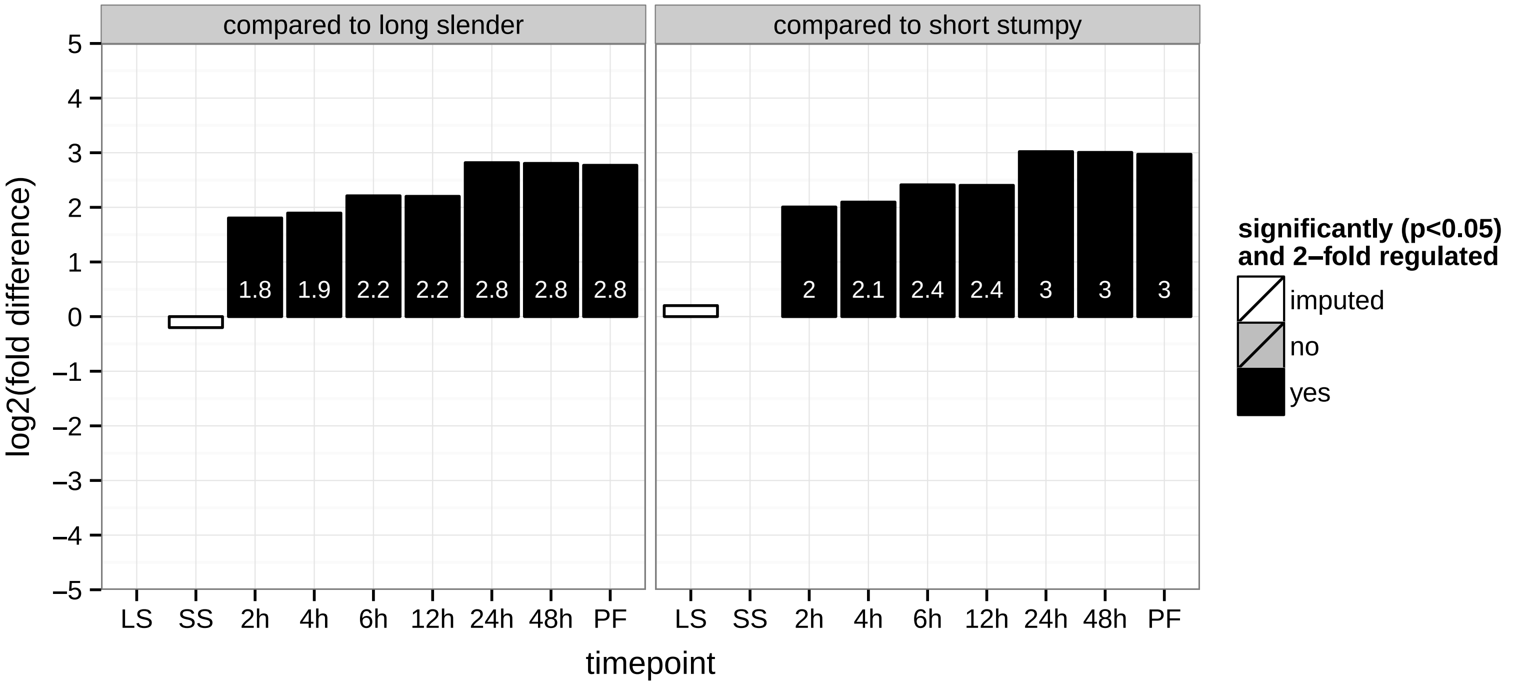

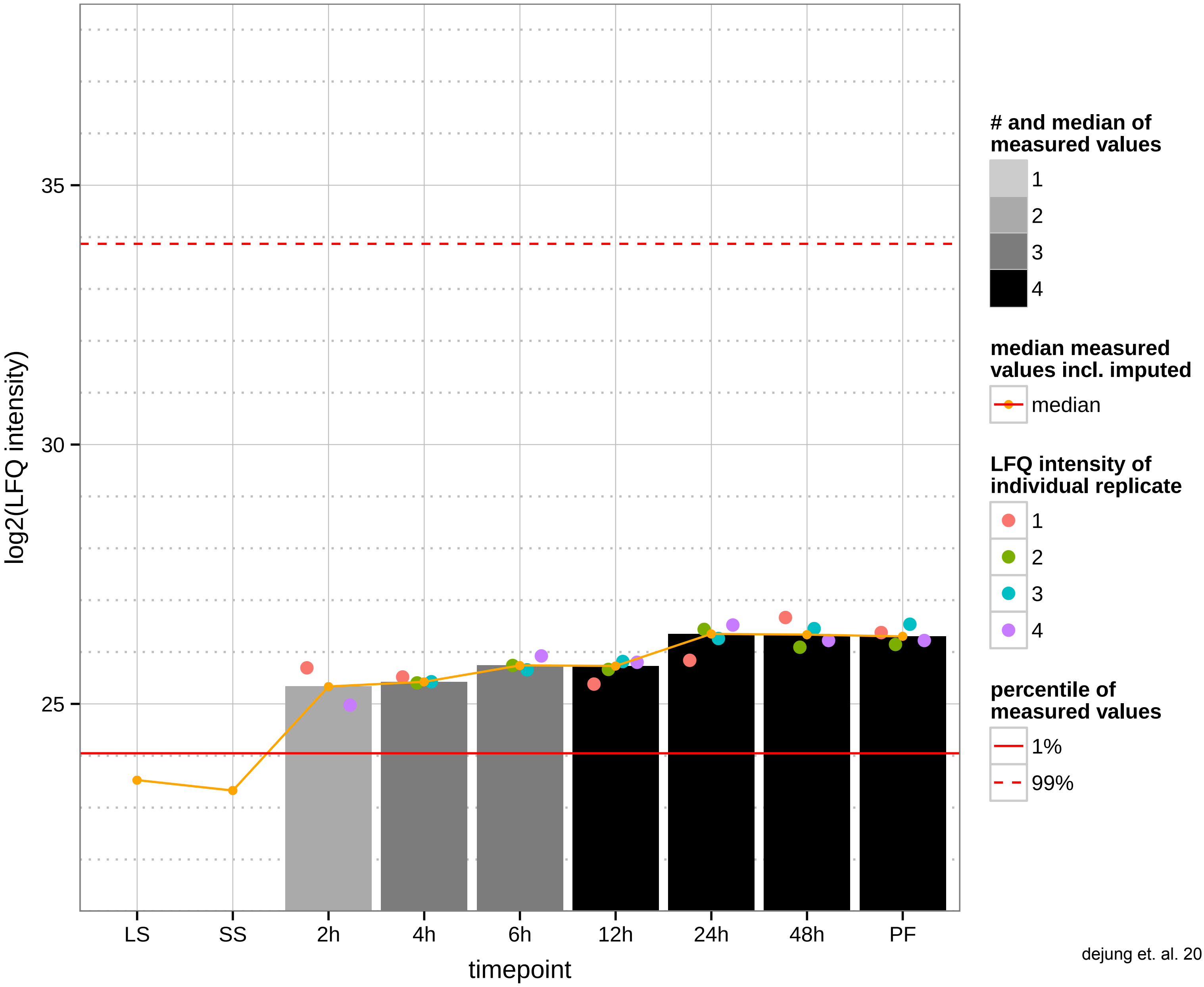

dejung et. al. 2015

zinc finger protein family member, putative (ZC3H22)
Tb927.7.2680
AGOF: RNA binding, zinc ion binding
AGOC: null
AGOP: null
PGOF: nucleic acid binding, zinc ion binding
PGOC: null
PGOP: null

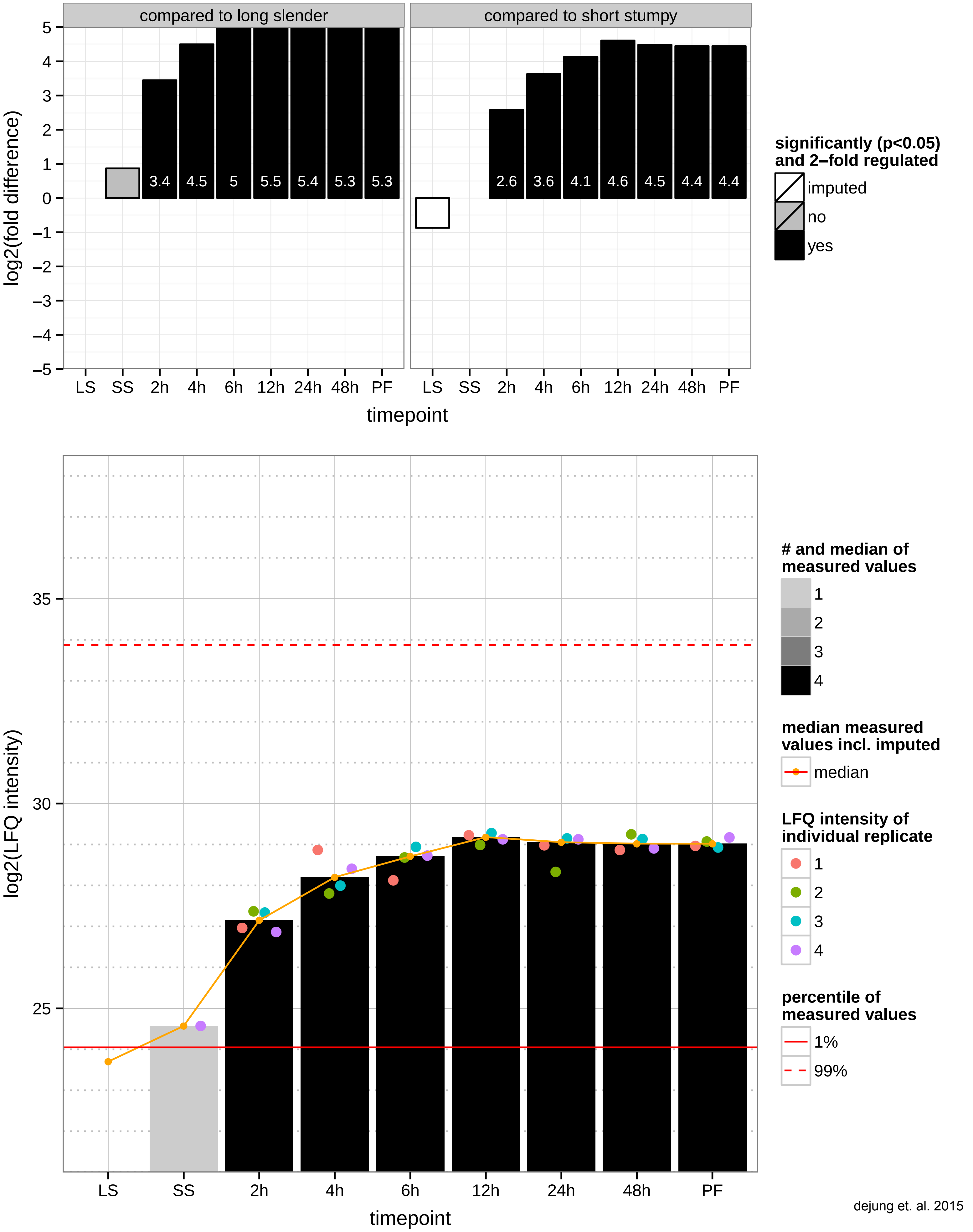

dejung et. al. 2015

hypothetical protein, conserved
Tb927.7.3090
AGOF: null
AGOC: null
AGOP: null
PGOF: protein binding
PGOC: null
PGOP: null

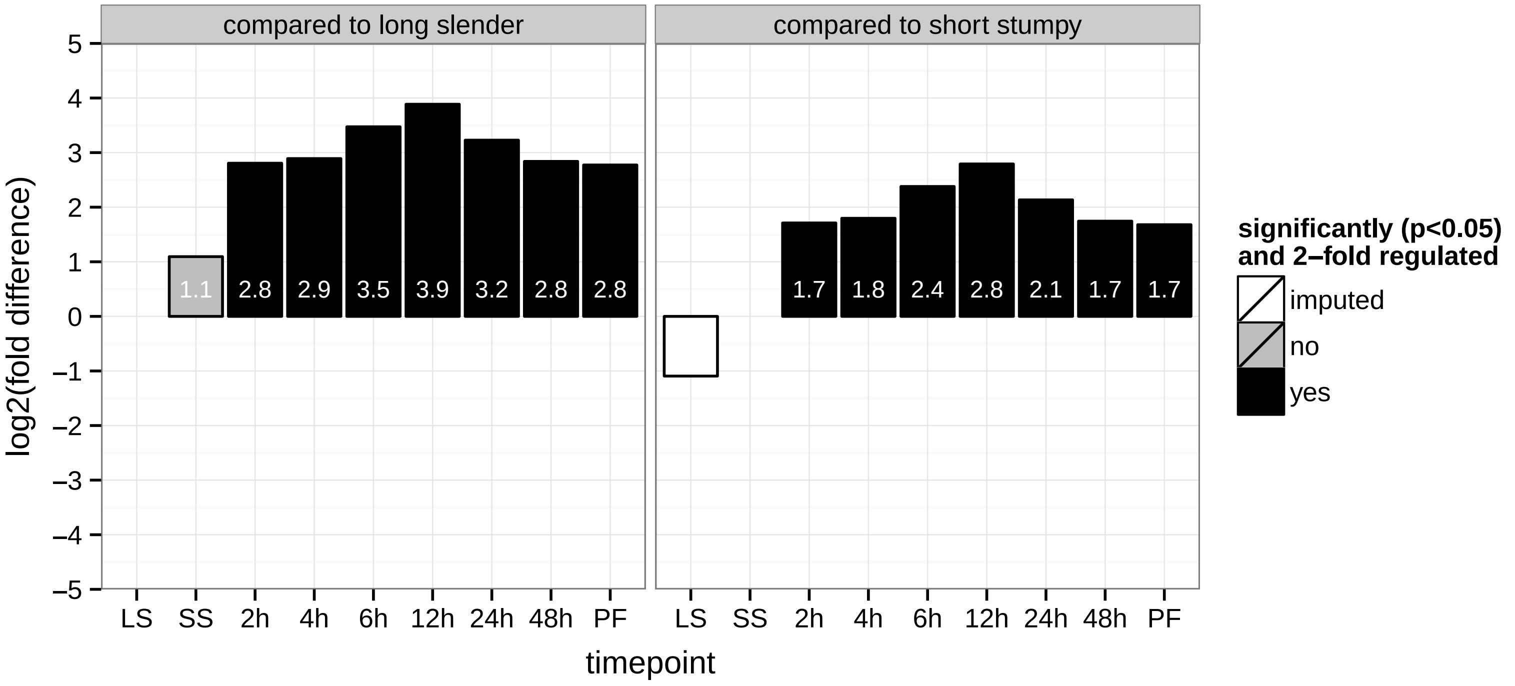

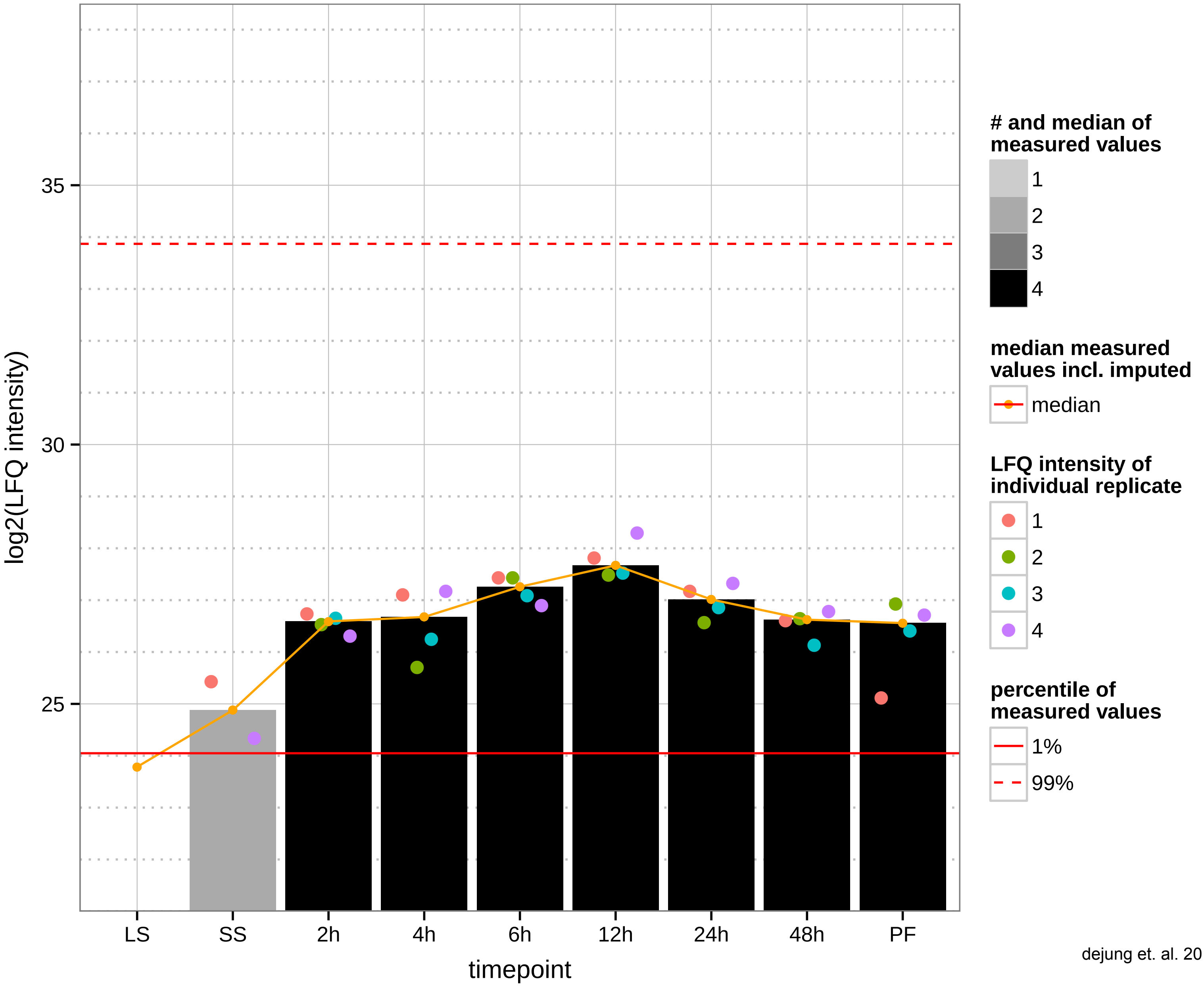

dejung et. al. 2015

glutamine synthetase, putative, putative (GS)
Tb927.7.4970;Tb11.v5.0178
AGOF: null, glutamate–ammonia ligase activity
AGOC: null, cytoplasm, mitochondrion
AGOP: null, glutamine biosynthetic process, nitrogen compound metabolic process
PGOF: glutamate–ammonia ligase activity
PGOC: null
PGOP: glutamine biosynthetic process, nitrogen compound metabolic process

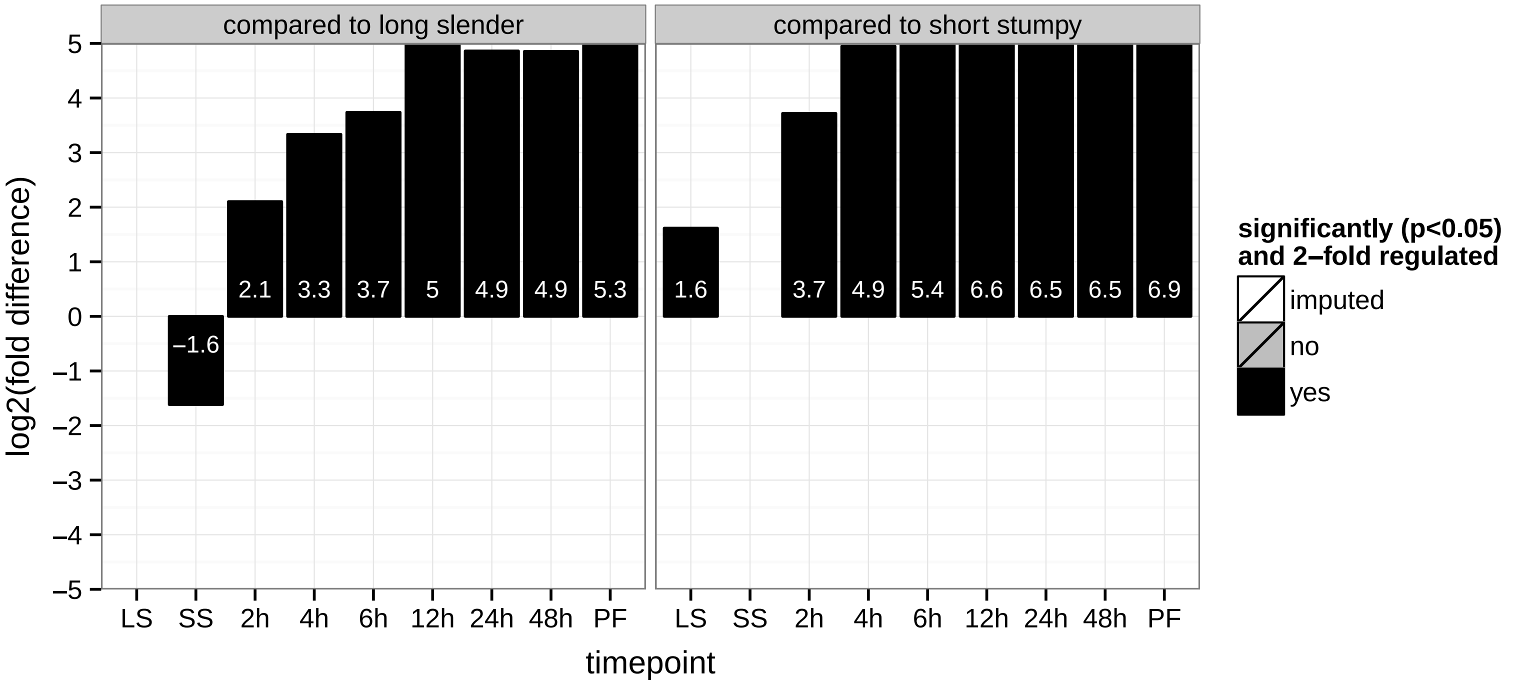

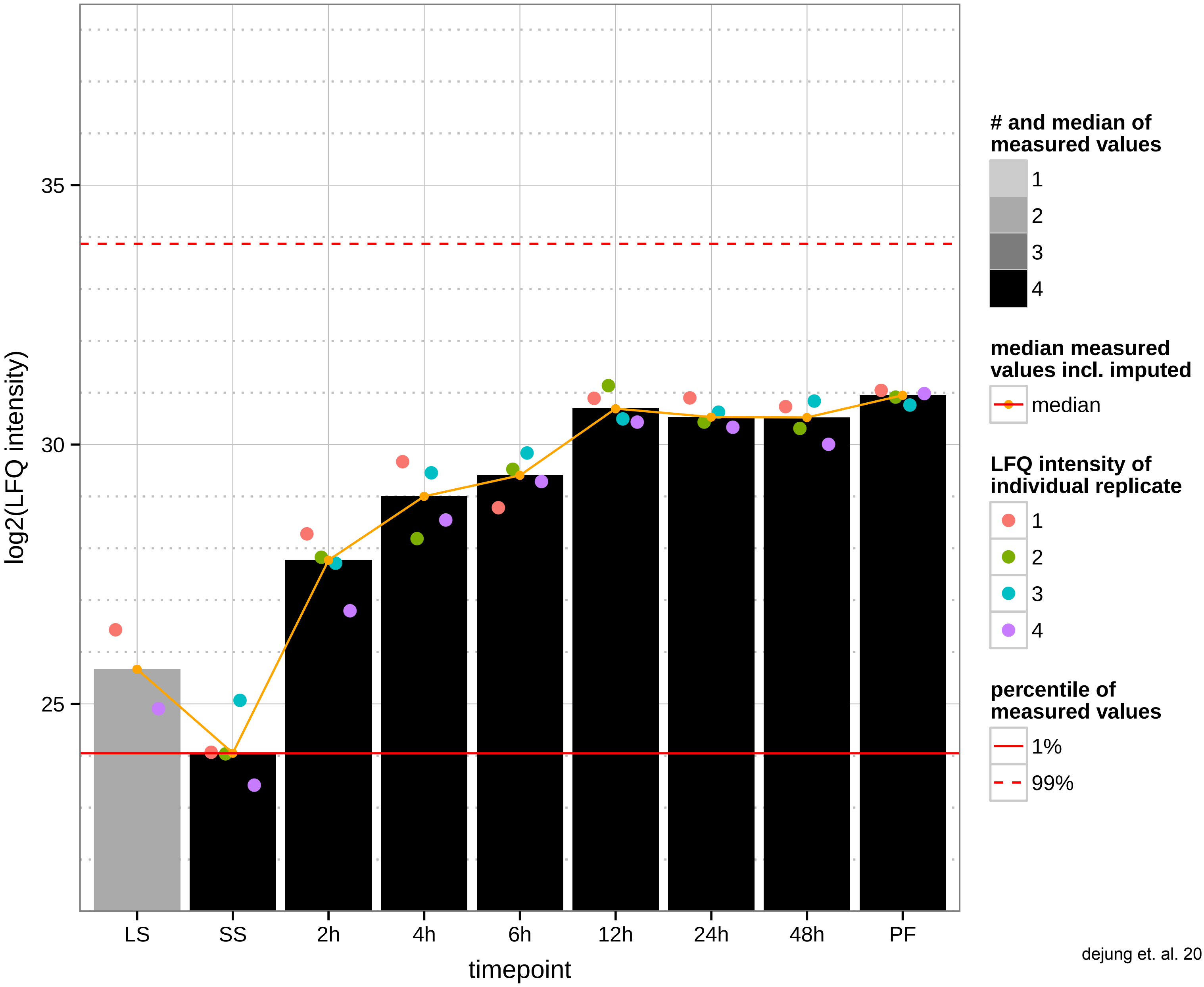

dejung et. al. 2015

trans−sialidase (TS)
Tb927.7.6850
AGOF: exo−alpha−sialidase activity, sialyltransferase activity
AGOC: cell surface
AGOP: pathogenesis, sialic acid transport
PGOF: null
PGOC: null
PGOP: null

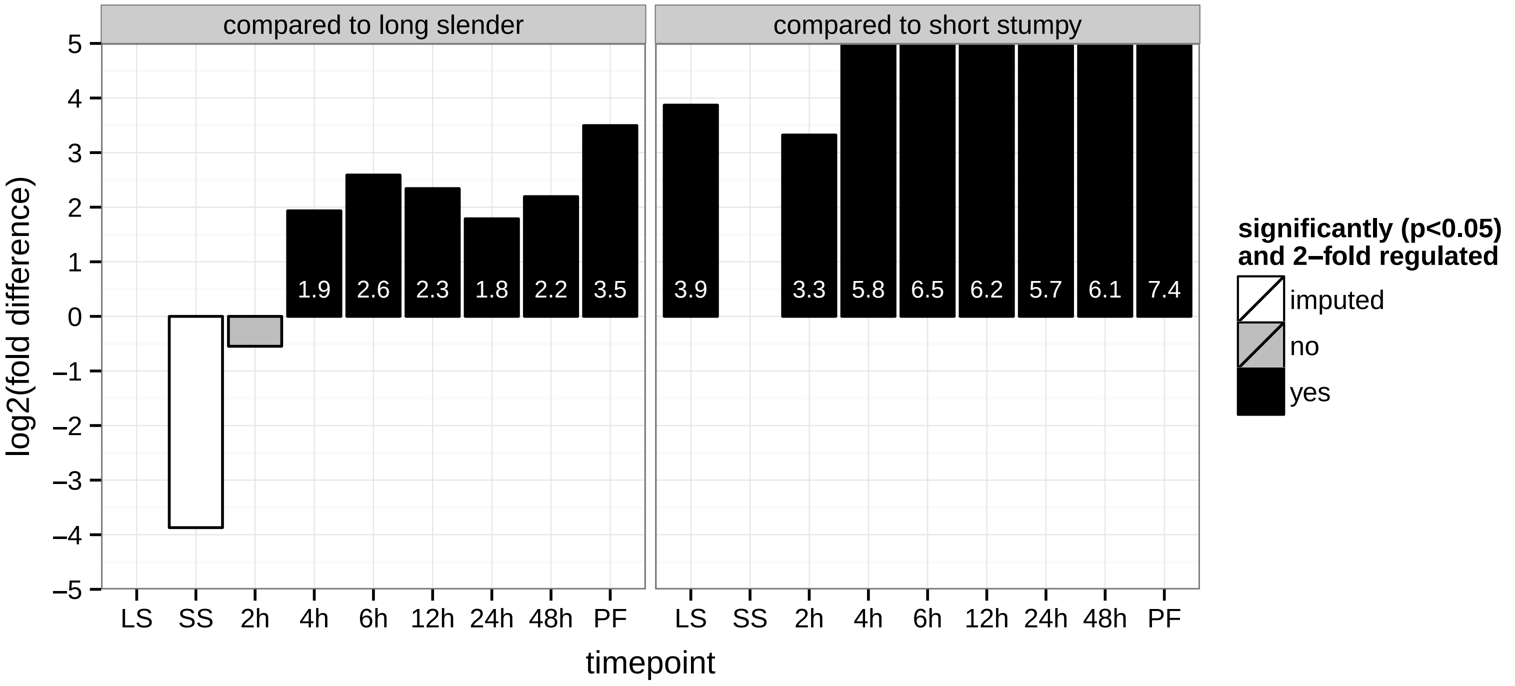

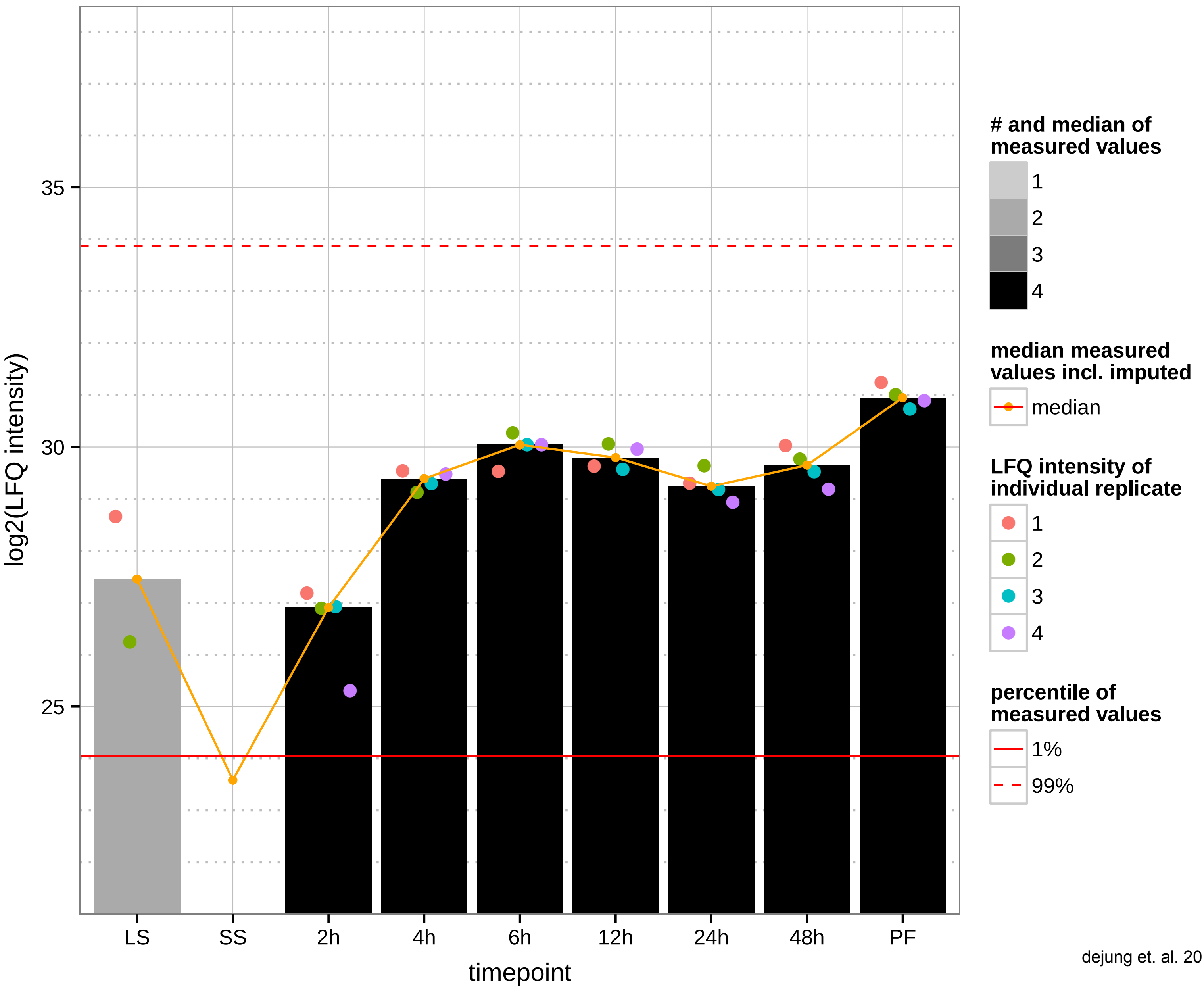

dejung et. al. 2015

kinesin, putative
Tb927.8.4950
AGOF: ATP binding, microtubule motor activity, motor activity
AGOC: kinesin complex, microtubule associated complex
AGOP: microtubule−based movement
PGOF: ATP binding, microtubule motor activity
PGOC: null
PGOP: microtubule−based movement

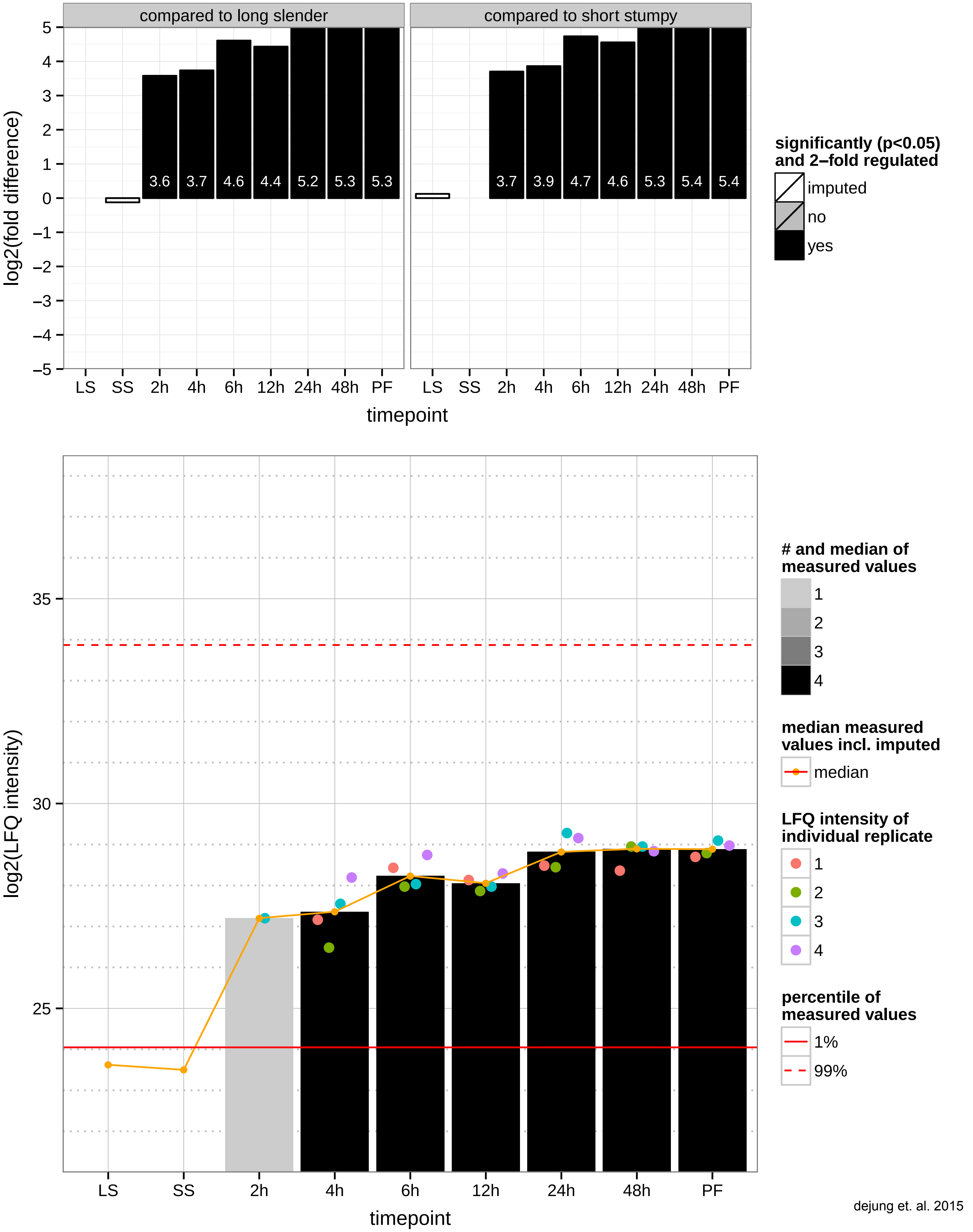

dejung et. al. 2015

amino acid transporter, putative
Tb927.8.8300
AGOF: amine transmembrane transporter activity
AGOC: integral to membrane
AGOP: amino acid transport
PGOF: null
PGOC: null
PGOP: null

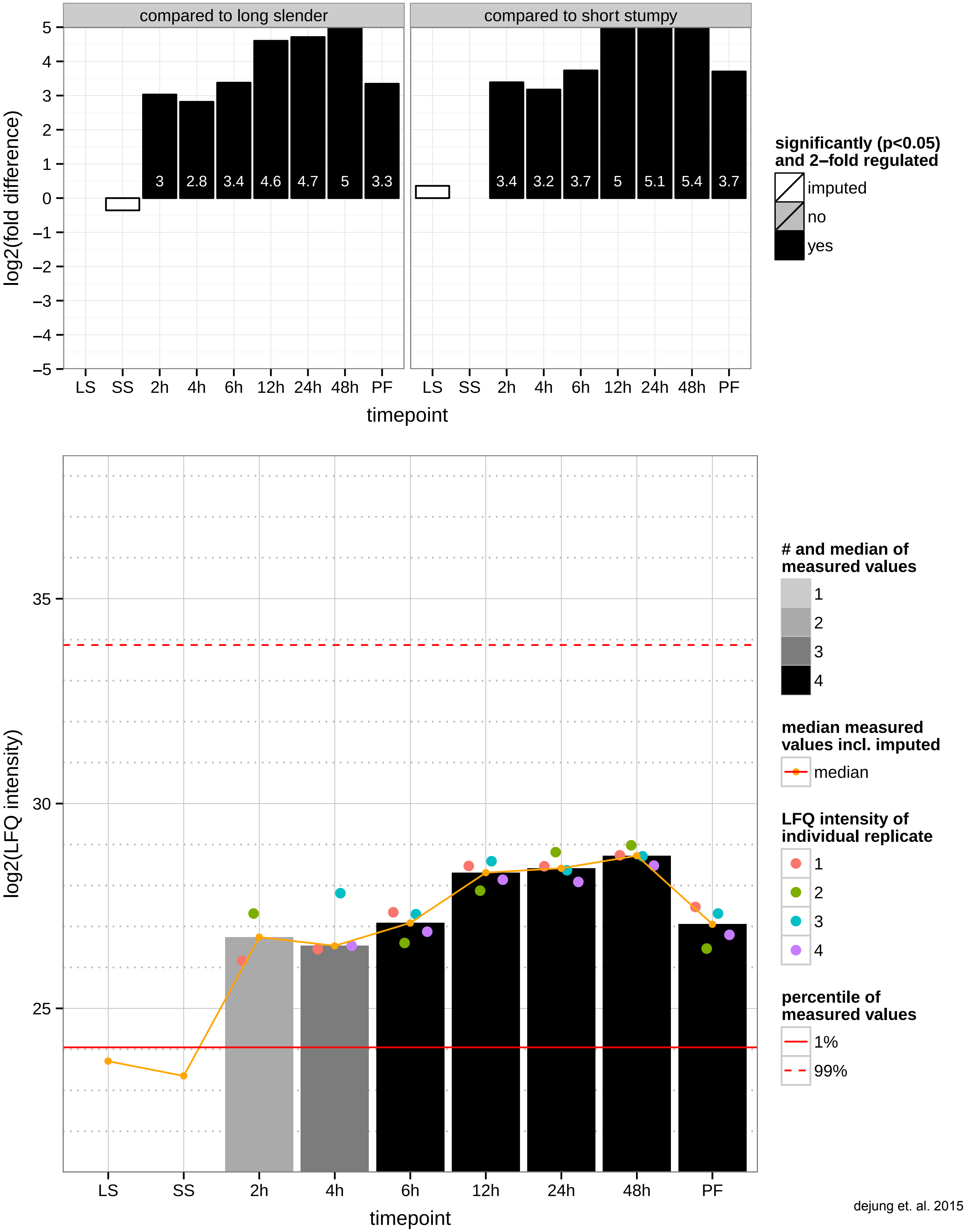

dejung et. al. 2015

hypothetical protein, conserved
Tb927.9.13240
AGOF: null
AGOC: null
AGOP: null
PGOF: null
PGOC: null
PGOP: null

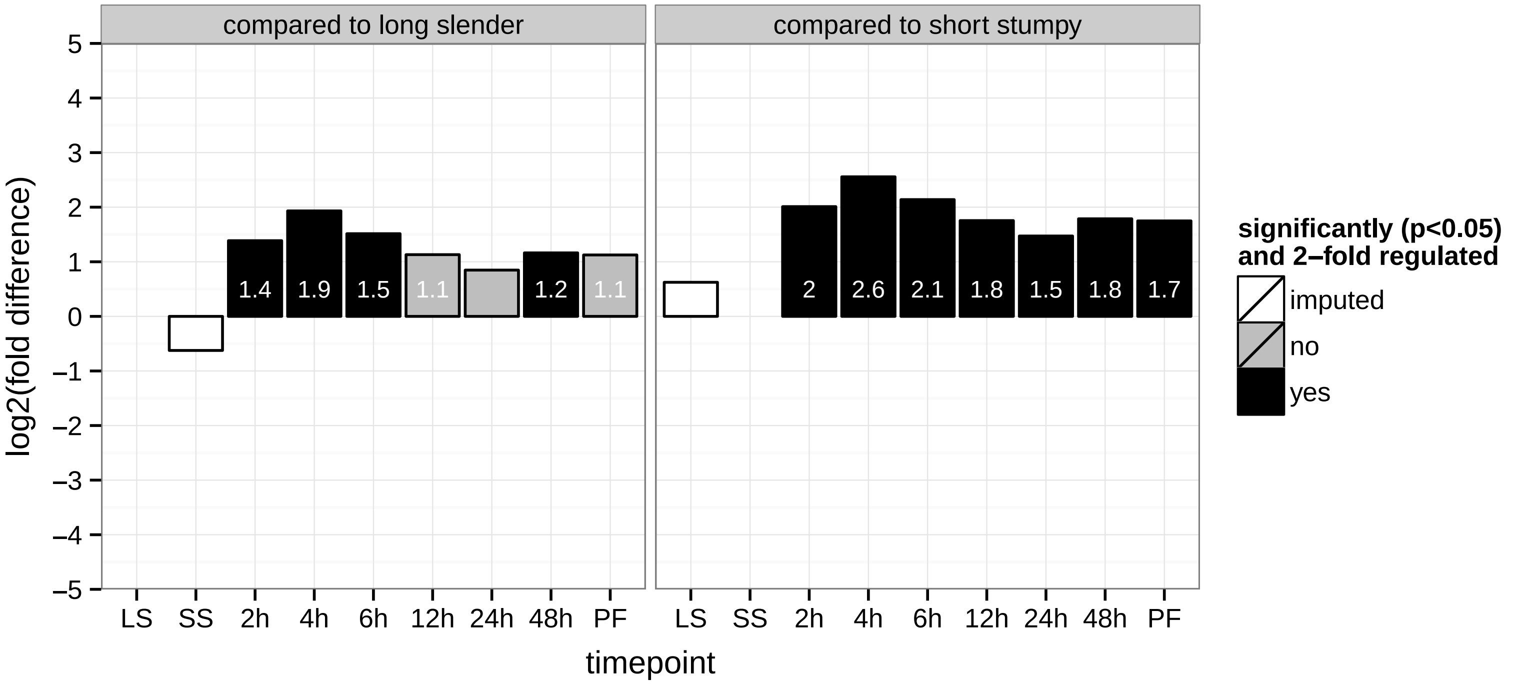

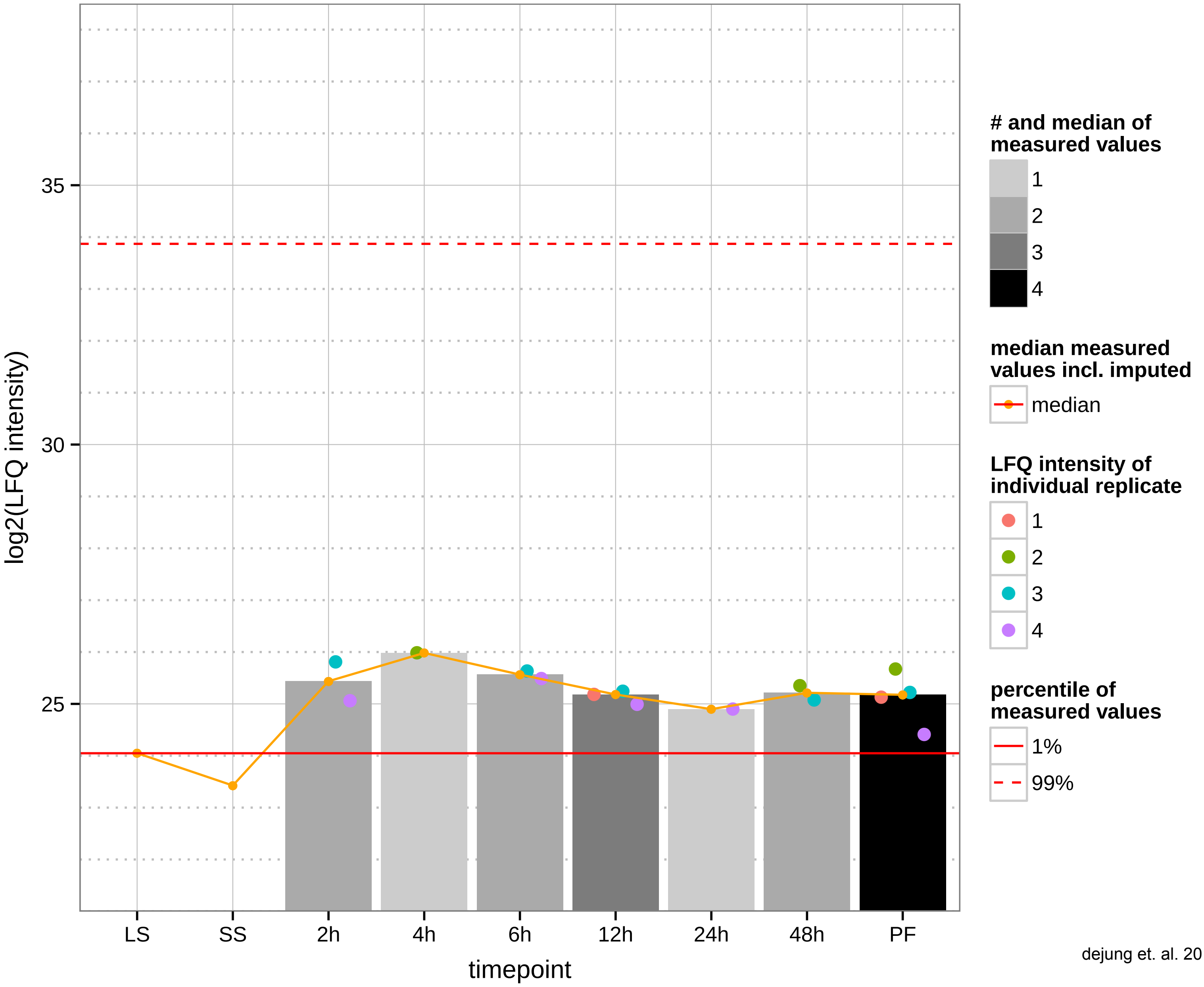

dejung et. al. 2015

hypothetical protein, conserved
Tb927.9.14120
AGOF: RNA binding
AGOC: null
AGOP: null
PGOF: protein binding
PGOC: null
PGOP: null

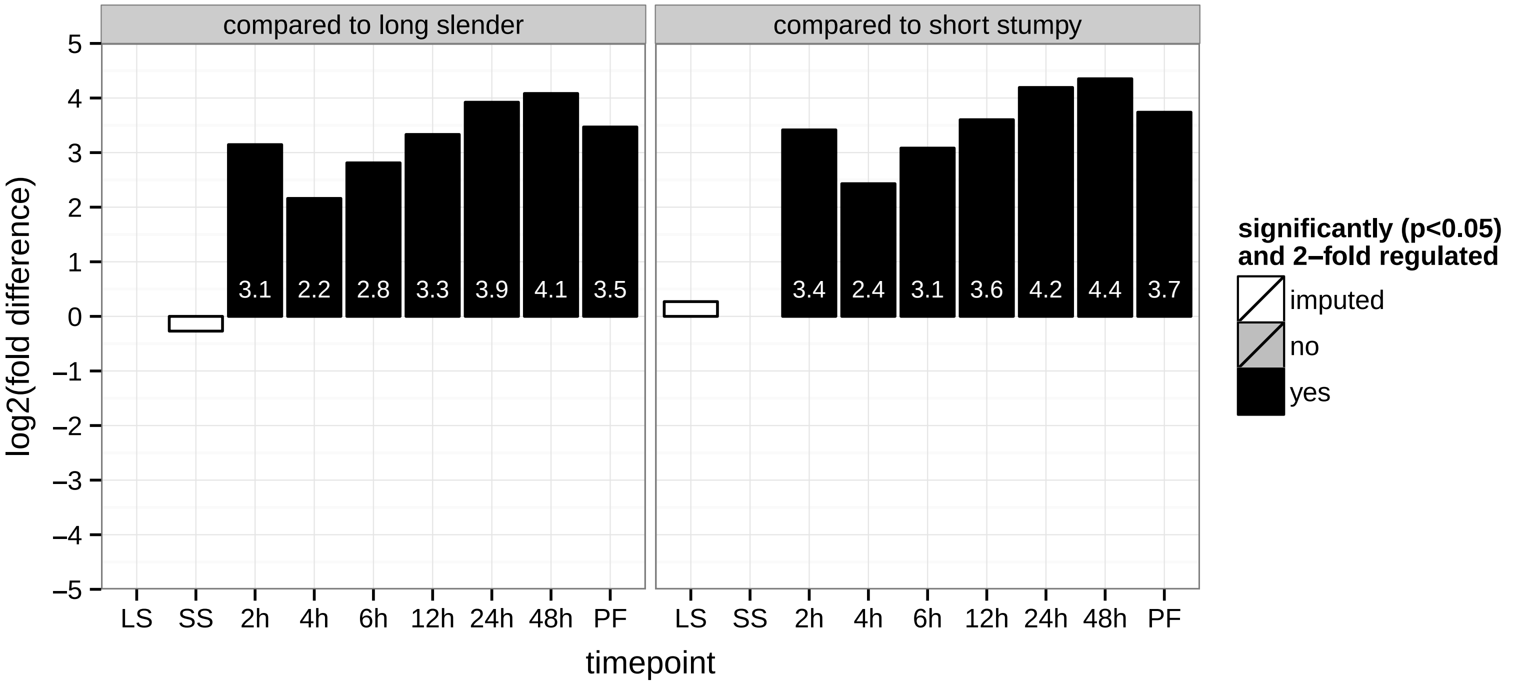

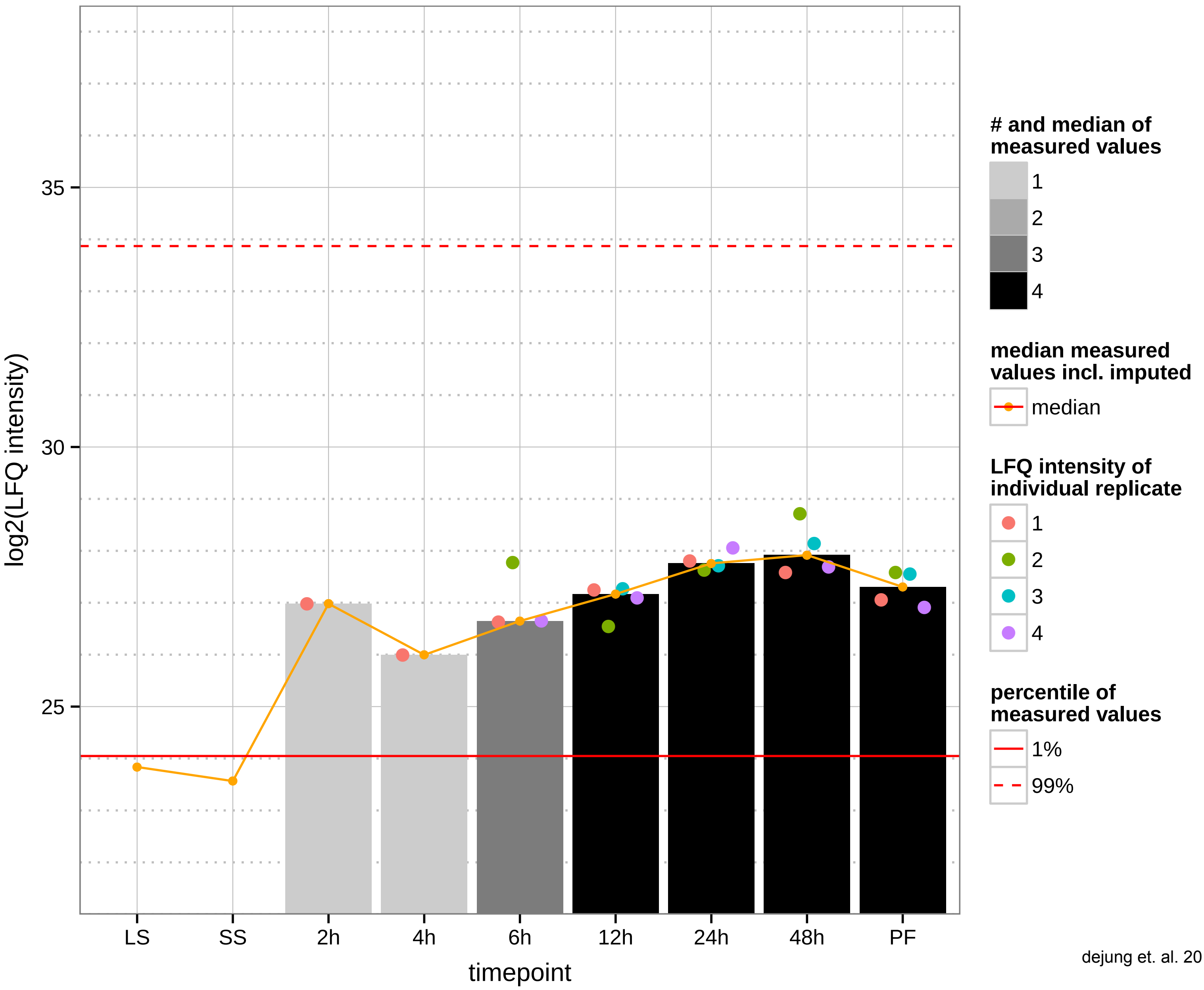

dejung et. al. 2015

rieske iron–sulfur protein, mitochondrial precursor (RISP)
Tb927.9.14160
AGOF: 2 iron, 2 sulfur cluster binding, electron carrier activity, glutathione–disulfide reductase activity, ubiquinol–cytochrome–
AGOC: mitochondrial inner membrane, mitochondrion, respiratory chain complex III
AGOP: aerobic respiration, oxidation–reduction process
PGOF: 2 iron, 2 sulfur cluster binding, oxidoreductase activity, ubiquinol–cytochrome–c reductase activity
PGOC: null
PGOP: oxidation–reduction process

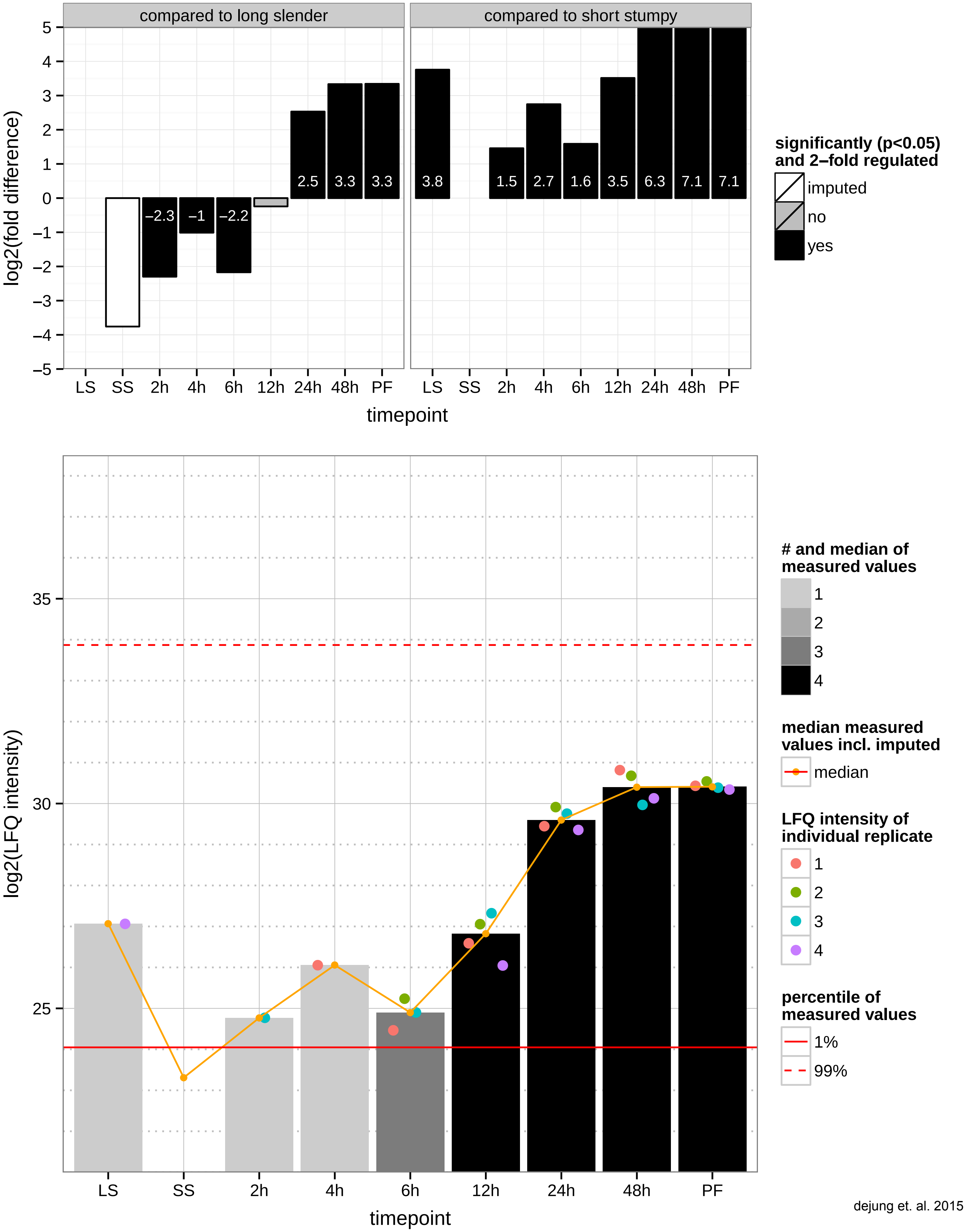

dejung et. al. 2015

hypothetical protein, conserved
Tb927.9.15240
AGOF: null
AGOC: mitochondrion
AGOP: protein complex assembly
PGOF: null
PGOC: mitochondrion
PGOP: protein complex assembly

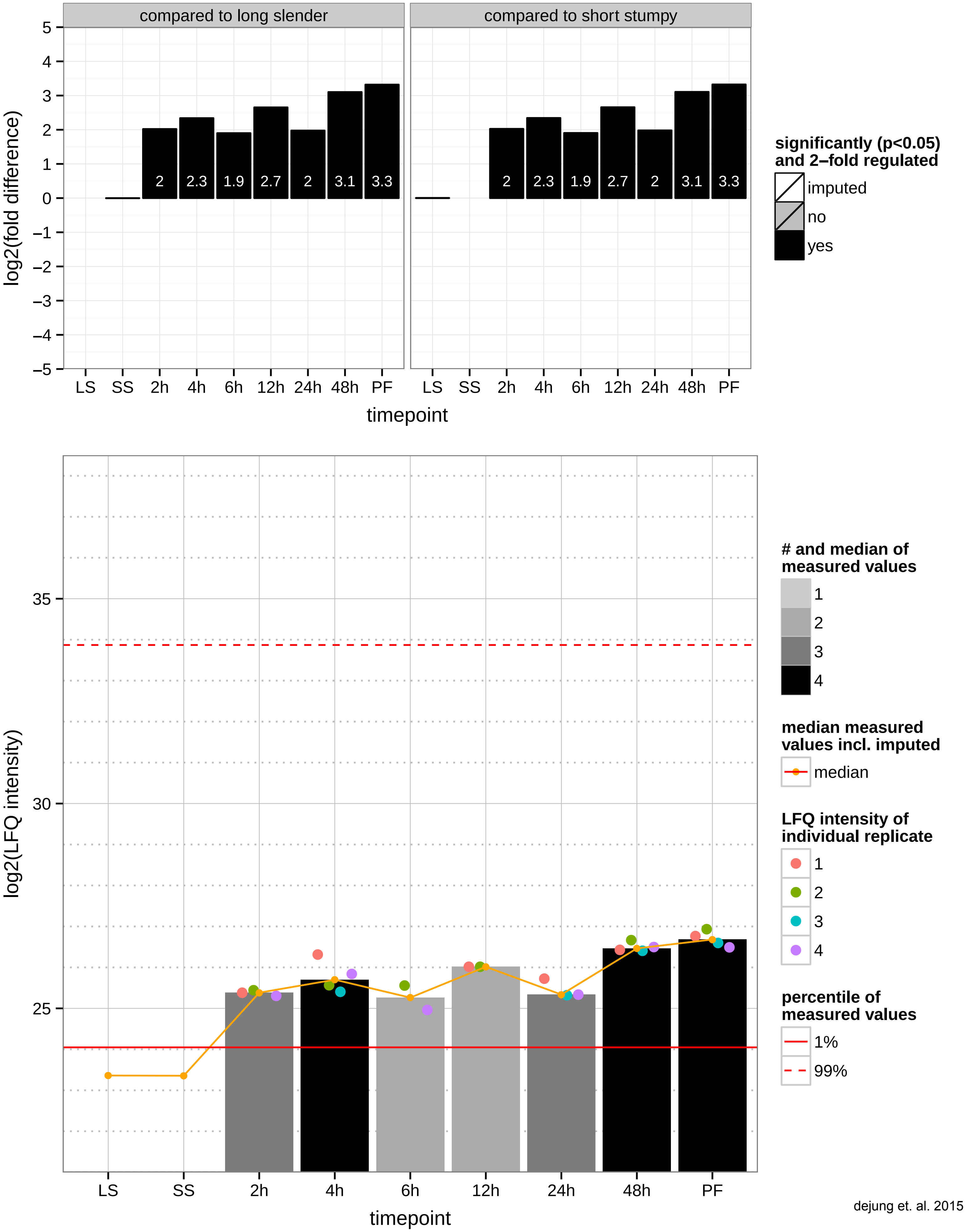

dejung et. al. 2015

hypothetical protein, conserved, PTP1–interacting protein, 39 kDa
Tb927.9.6090
AGOF: null, magnesium–dependent protein serine/threonine phosphatase activity
AGOC: null, cytosol, glycosome, mitochondrion
AGOP: null, protein dephosphorylation
PGOF: protein binding
PGOC: null
PGOP: null

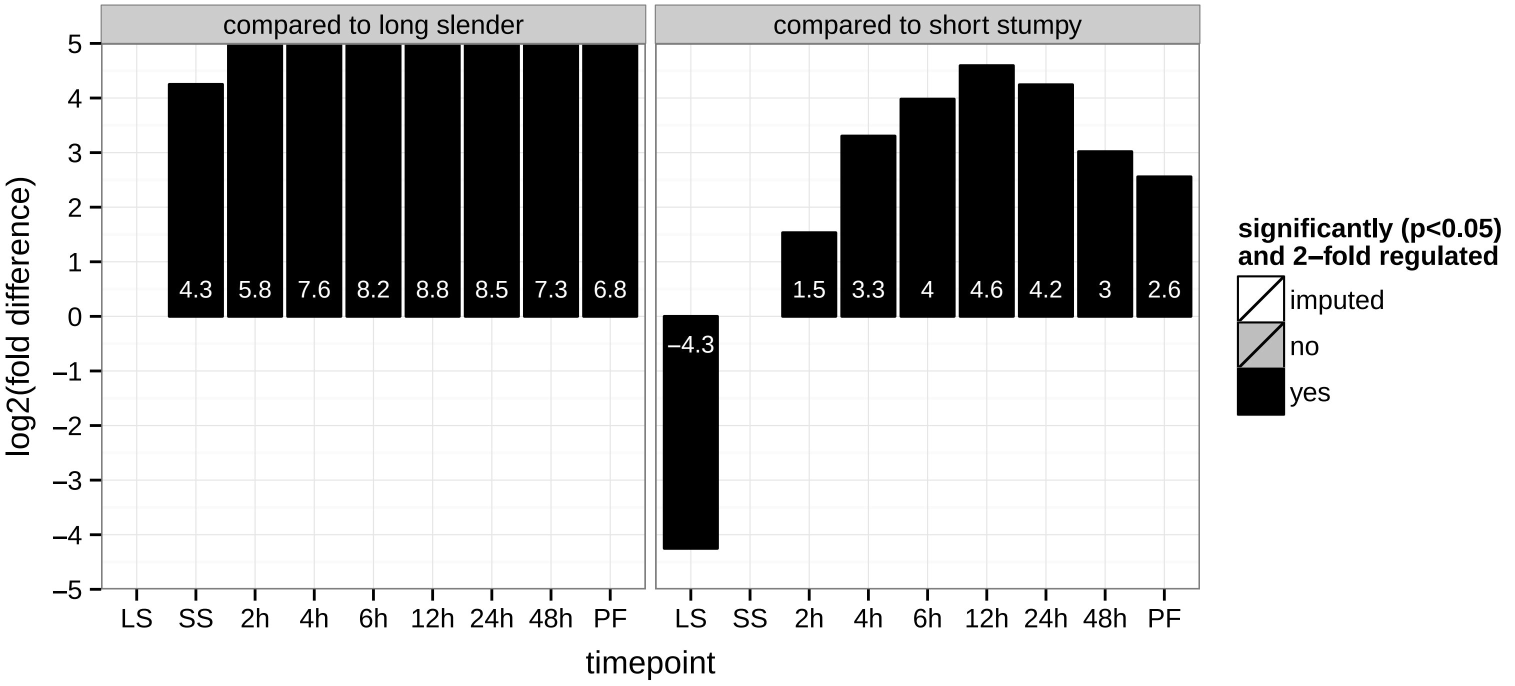

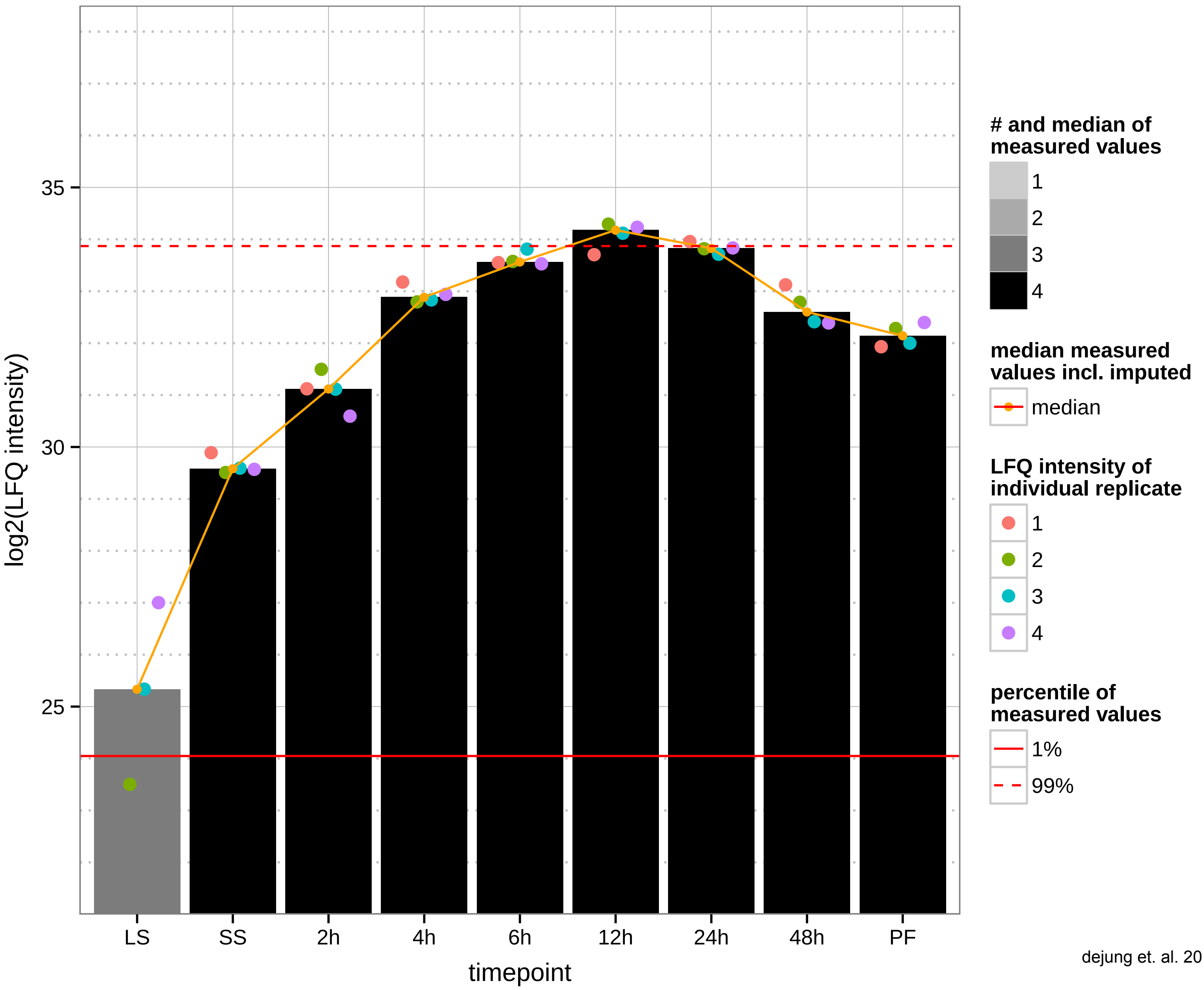

dejung et. al. 2015

hypothetical protein, conserved
Tb927.9.9730
AGOF: null
AGOC: intracellular
AGOP: null
PGOF: null
PGOC: null
PGOP: null

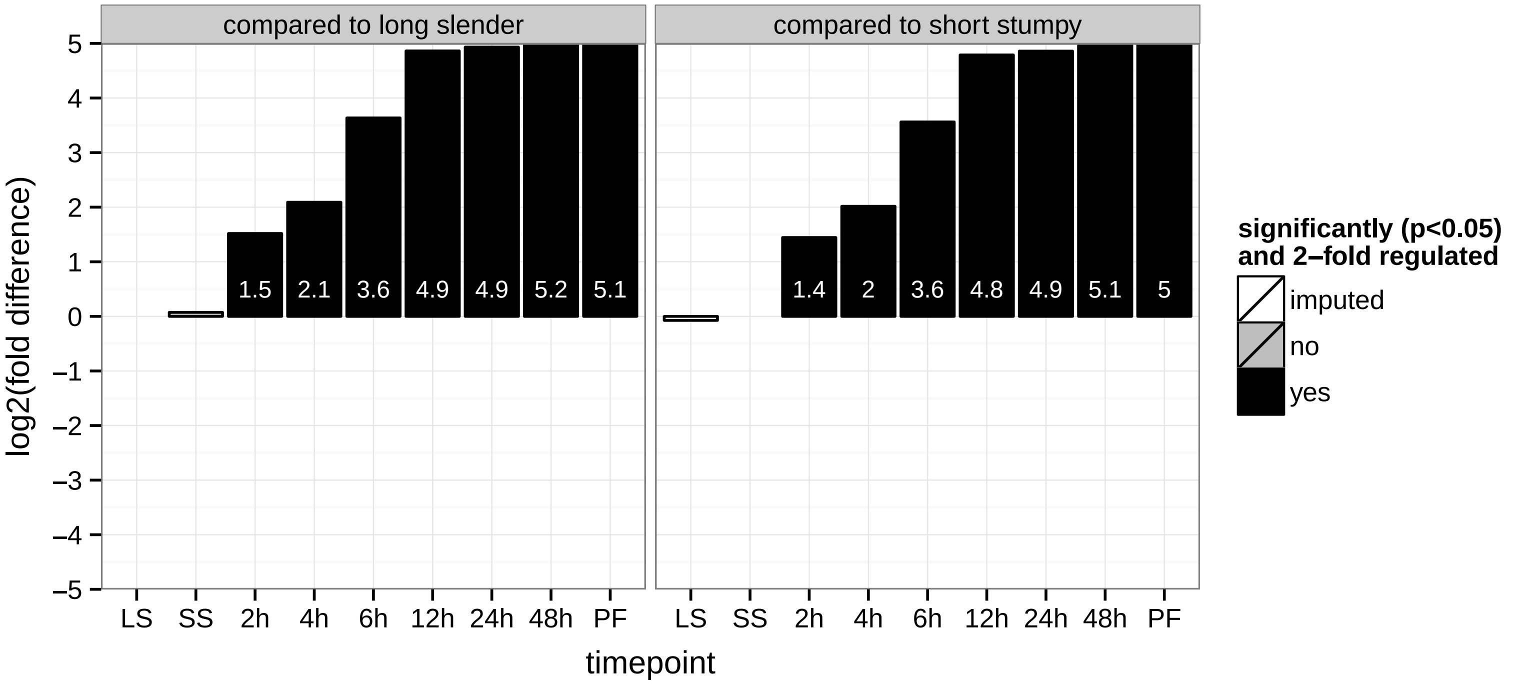

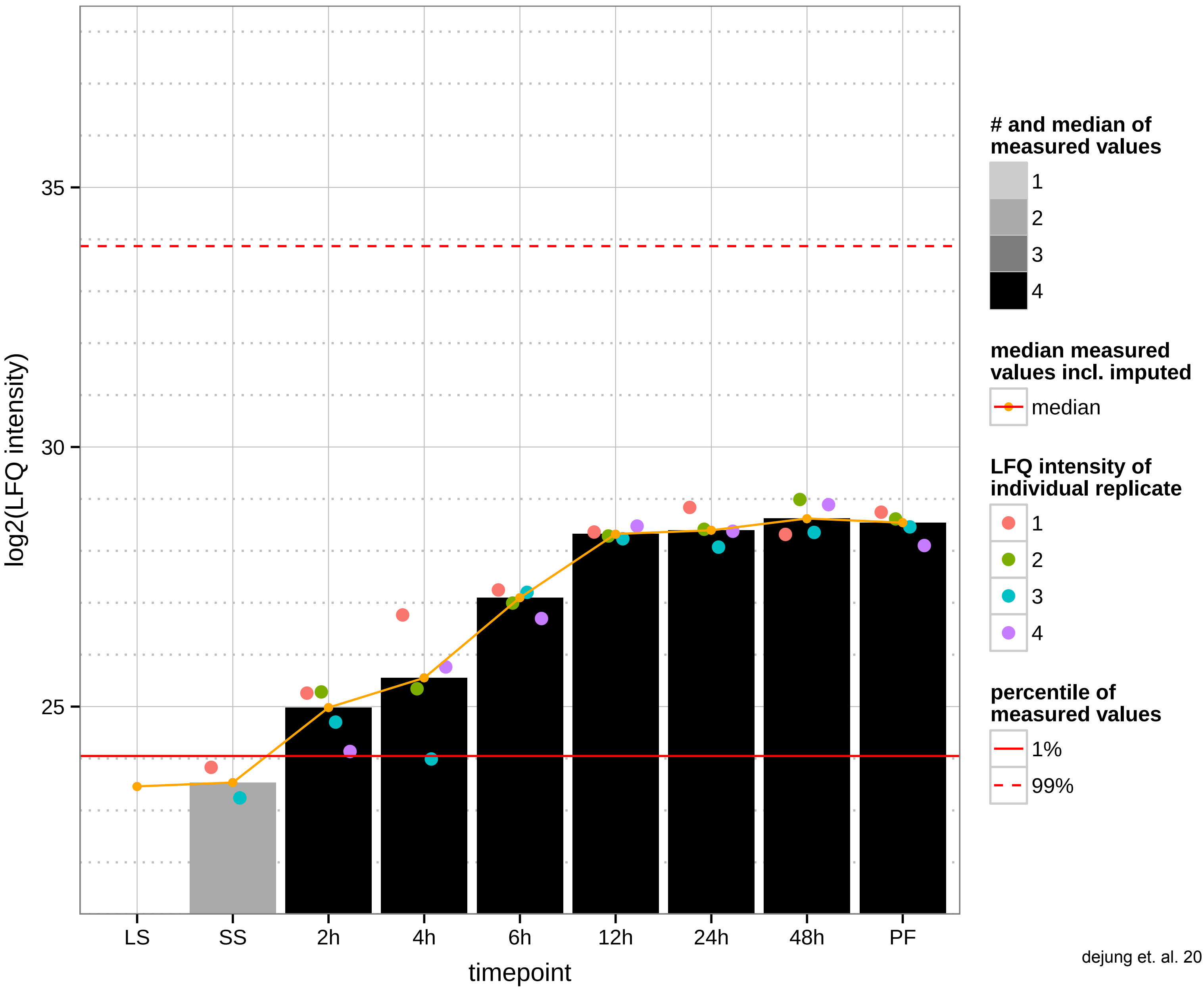

dejung et. al. 2015

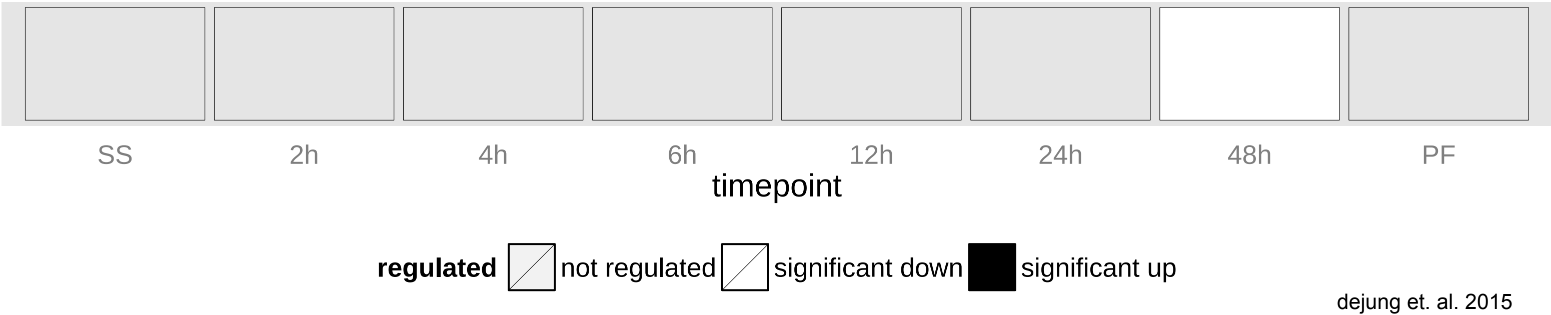
SS
2h
4h
6h
12h
24h
48h
PF
timepoint
regulated
not regulated
significant down
significant up
dejung et. al. 2015

hypothetical protein, conserved
Tb927.7.4750;Tb11.v5.0158
AGOF: null
AGOC: null
AGOP: null
PGOF: null
PGOC: null
PGOP: null

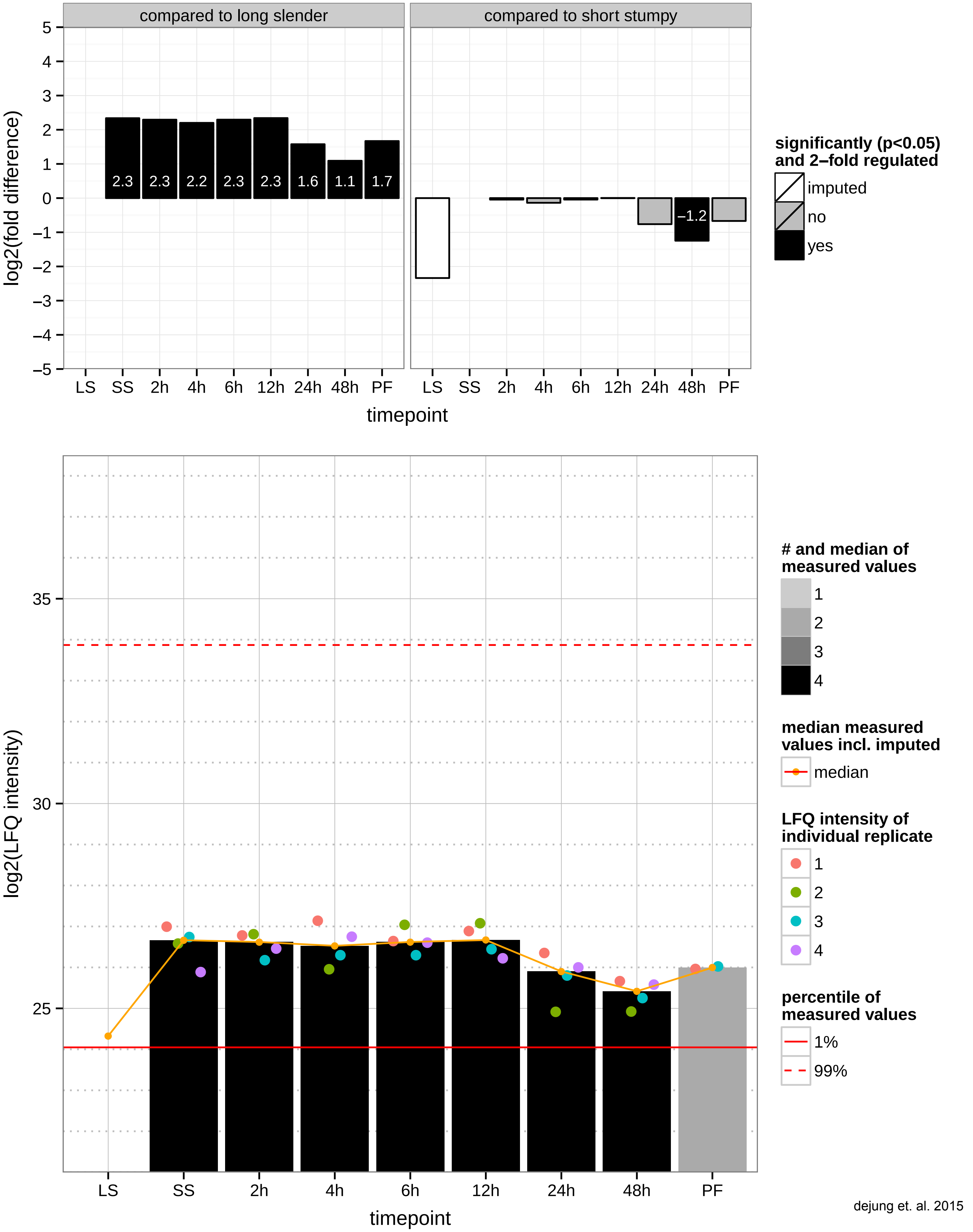

hypothetical protein, conserved
Tb927.7.4490;Tb11.v5.0262
AGOF: null
AGOC: null
AGOP: null
PGOF: null
PGOC: null
PGOP: null

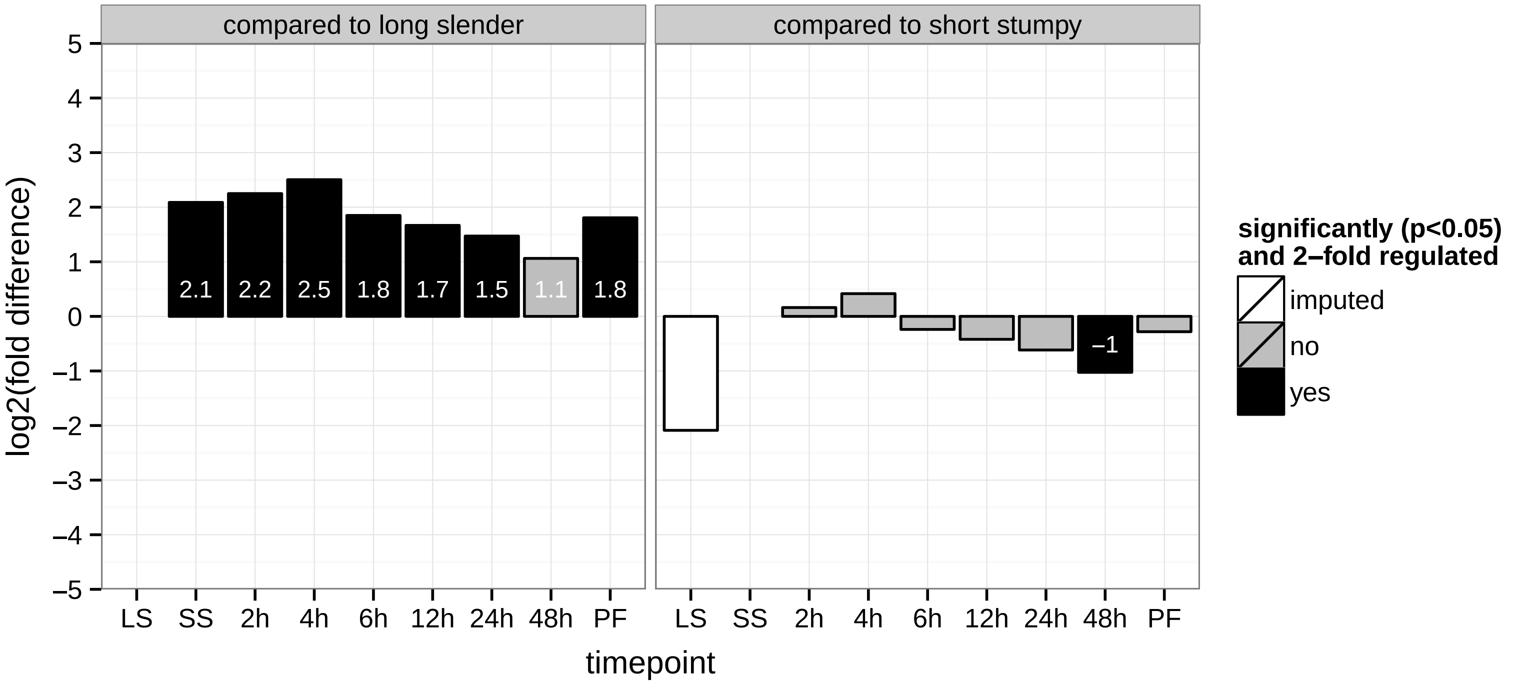

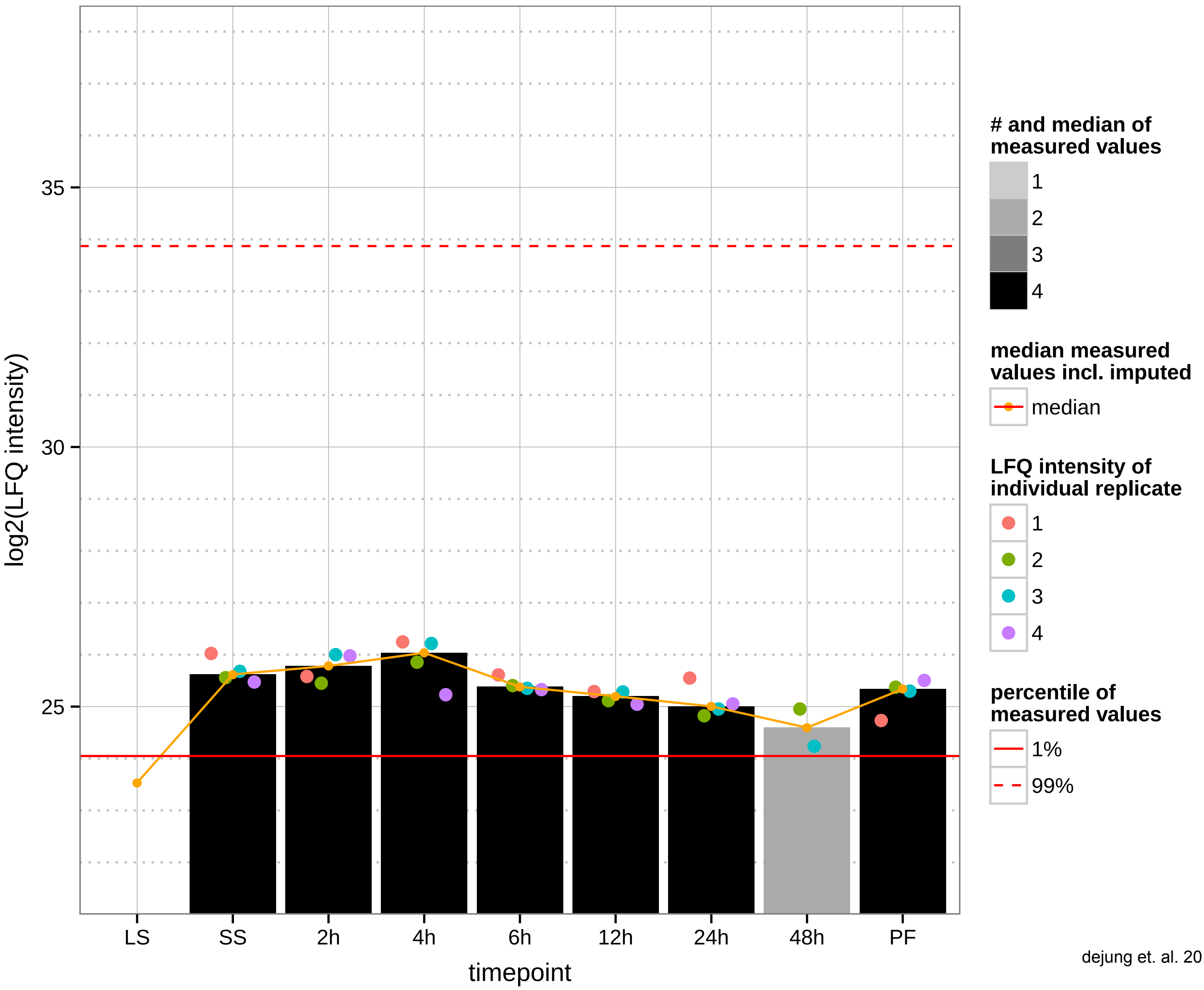

dejung et. al. 2015

nicotinate phosphoribosyltransferase, putative
Tb927.10.11590
AGOF: nicotinate phosphoribosyltransferase activity
AGOC: null
AGOP: nicotinate nucleotide biosynthetic process, nicotinate nucleotide salvage, pyridine nucleotide biosynthetic process
PGOF: nicotinate phosphoribosyltransferase activity, nicotinate–nucleotide diphosphorylase (carboxylating) activity
PGOC: cytoplasm
PGOP: NAD biosynthetic process, nicotinate nucleotide biosynthetic process

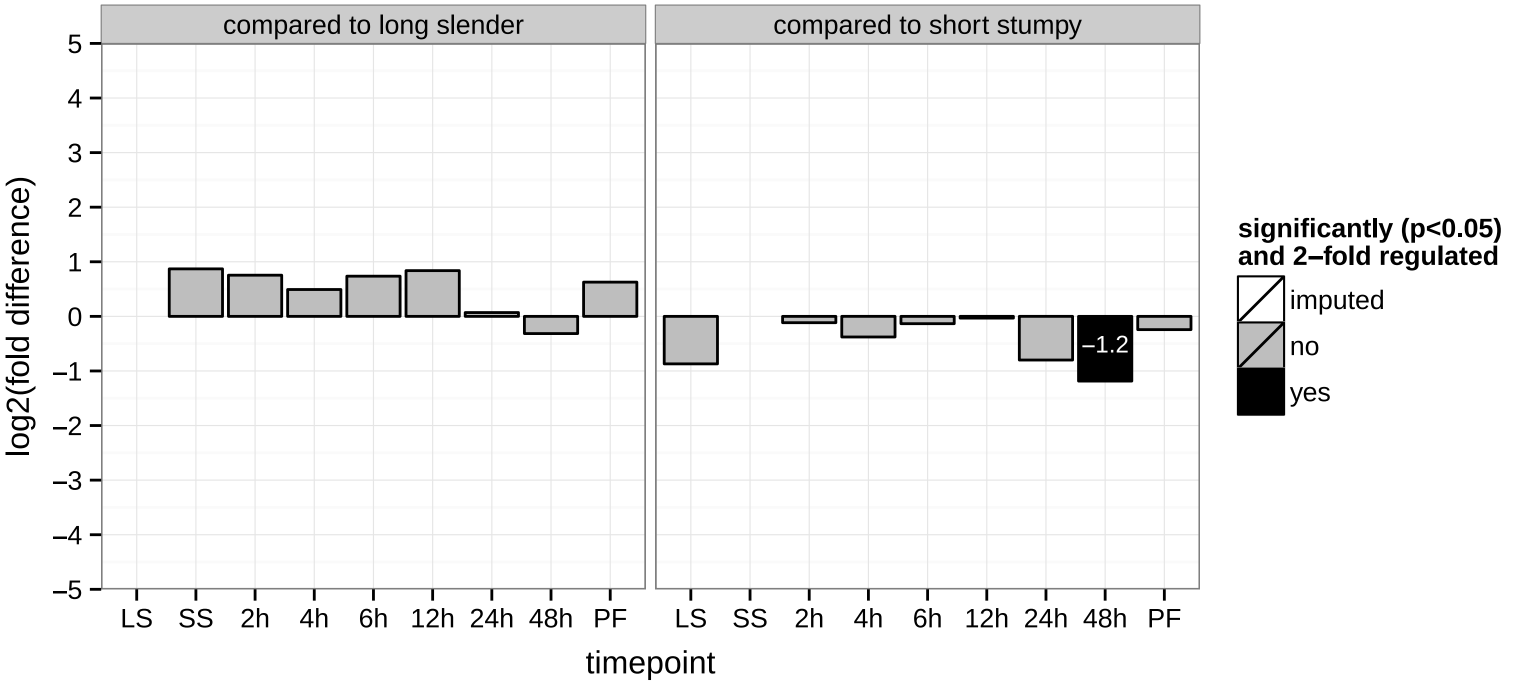

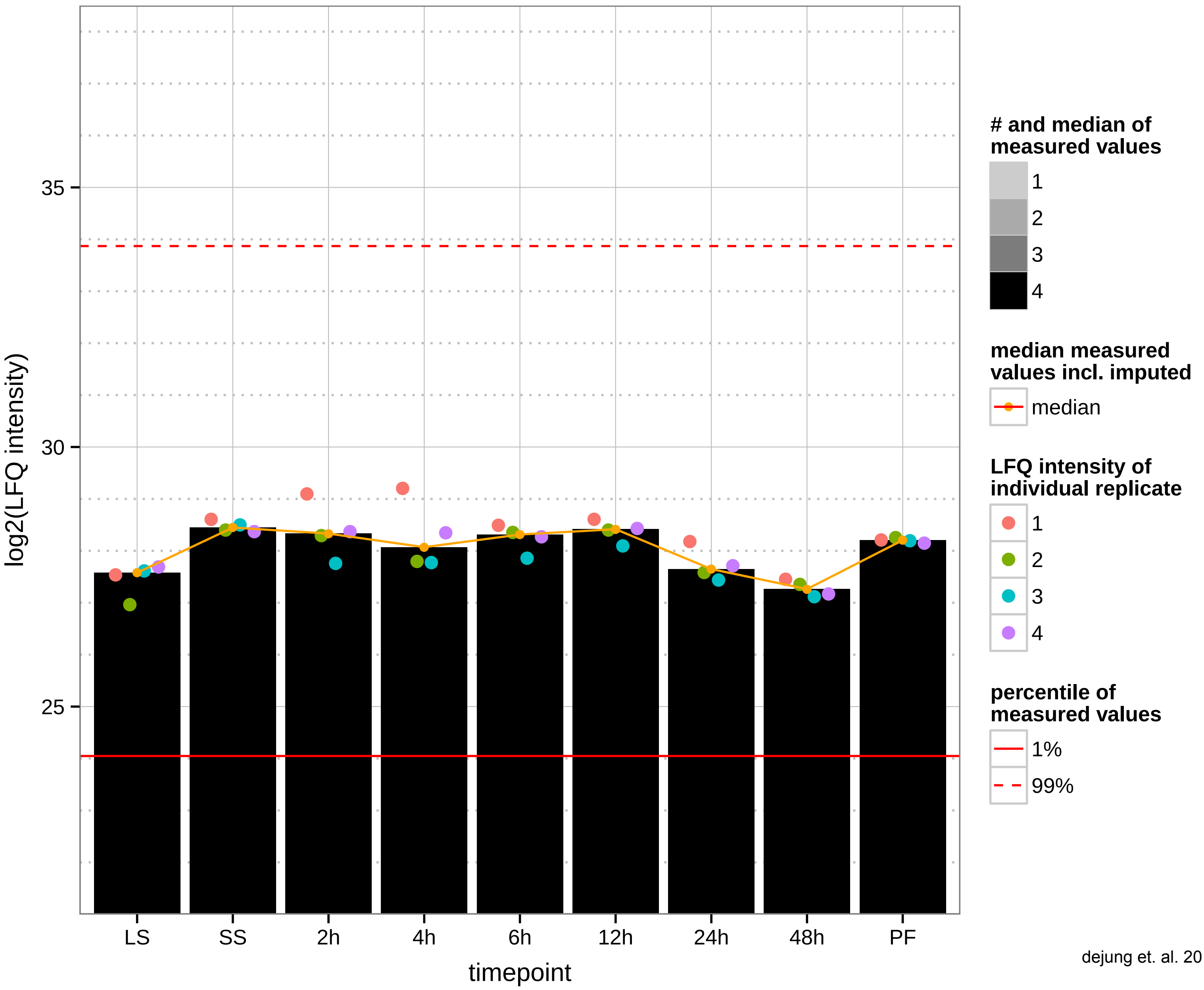

dejung et. al. 2015

chrX additional, unordered contigs, hypothetical protein, conserved, intraflagellar transport protein IFT172, putative (IFT172)
Tb927.10.1170;Tb11.0560
AGOF: null
AGOC: null
AGOP: null
PGOF: null, protein binding
PGOC: null
PGOP: null

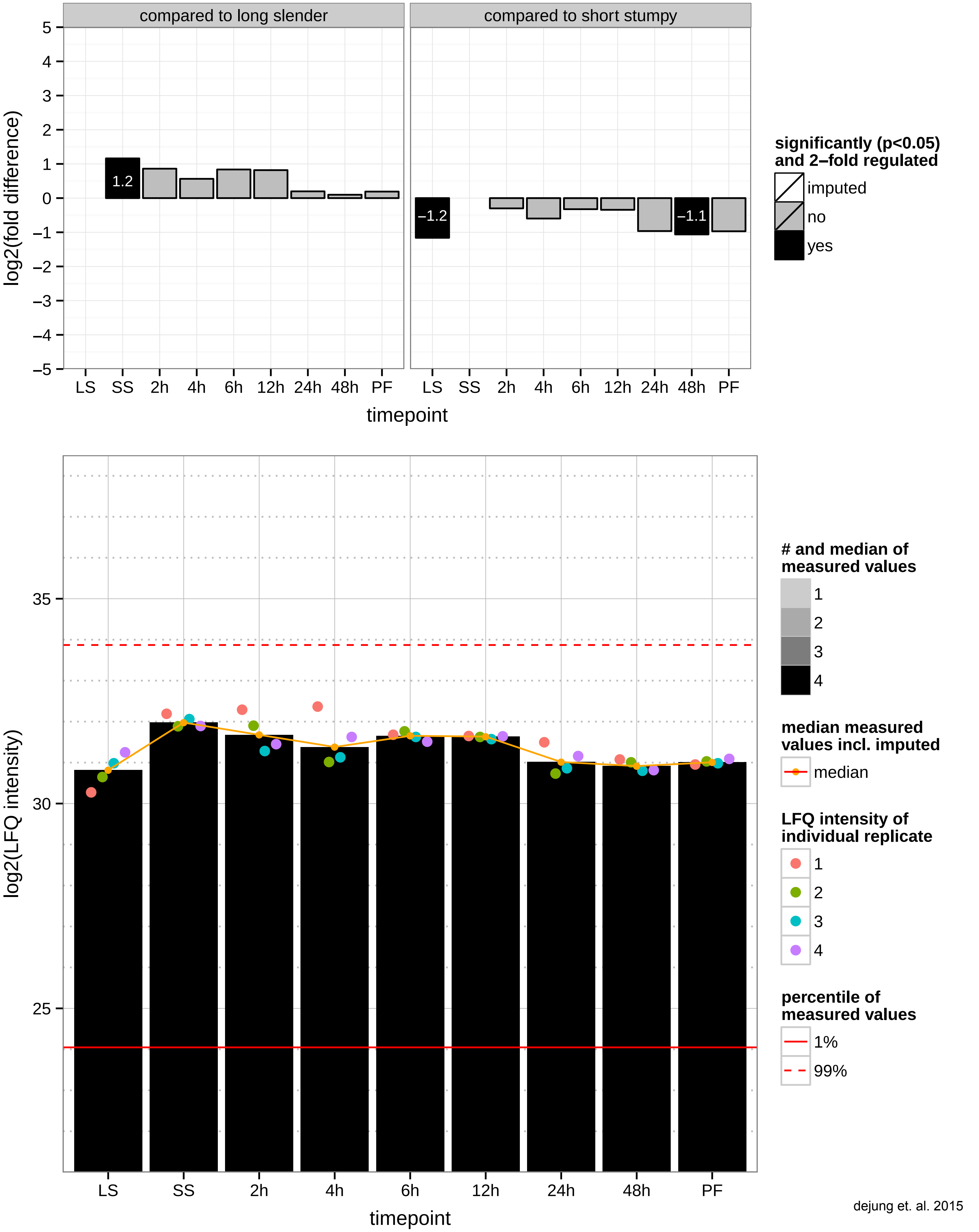

dejung et. al. 2015

P−type H+−ATPase, putative
Tb927.10.12510
AGOF: ATP binding, ATPase activity, coupled to transmembrane movement of ions, phosphorylative mechanism
AGOC: integral to membrane, plasma membrane
AGOP: ATP biosynthetic process, metabolic process, proton transport
PGOF: ATP binding, ATPase activity, coupled to transmembrane movement of ions, phosphorylative mechanism, catalytic acti
PGOC: integral to membrane, membrane
PGOP: ATP biosynthetic process, ATP catabolic process, cation transport, metabolic process

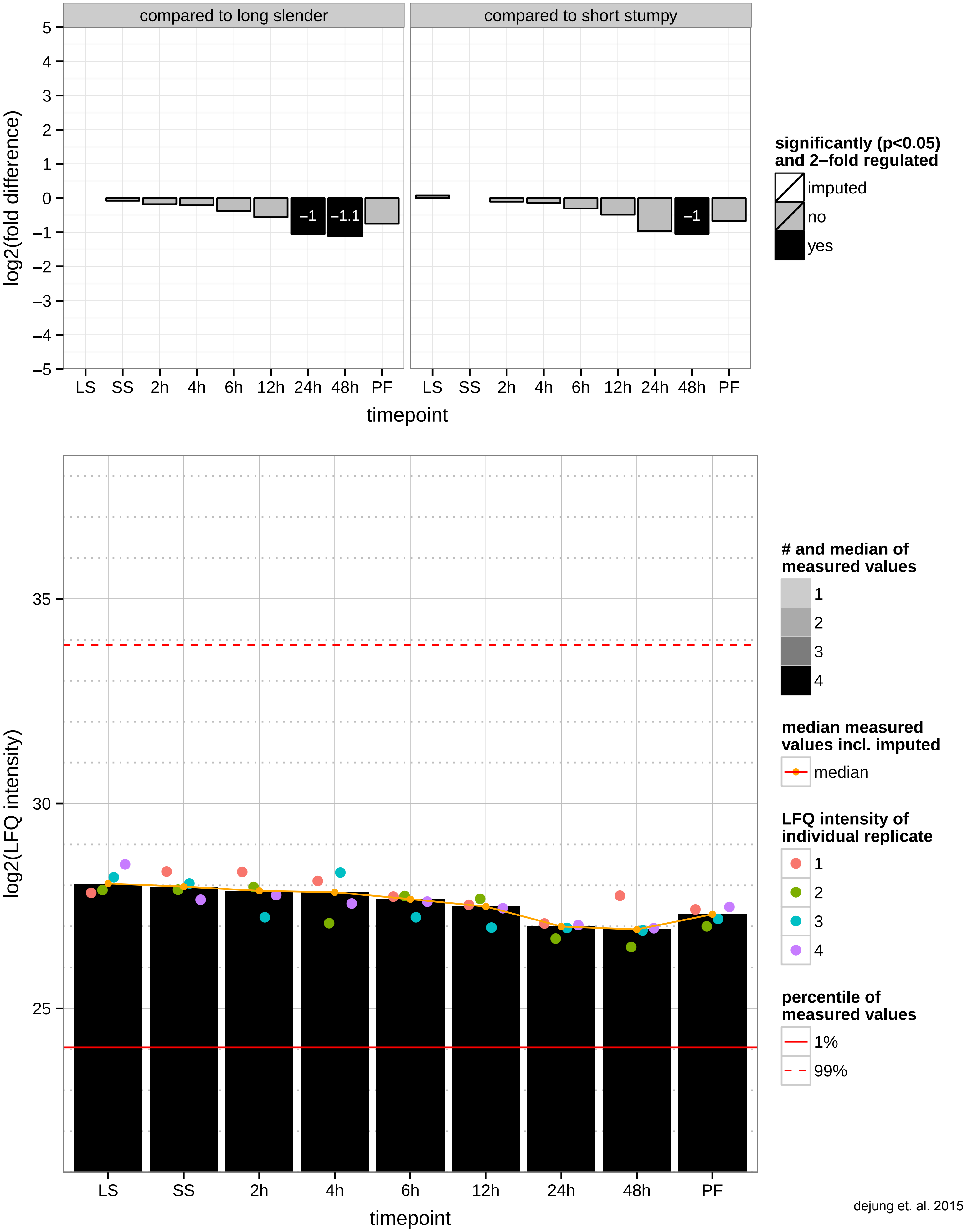

dejung et. al. 2015

ethanolamine phosphotransferase (EPT)
Tb927.10.13290
AGOF: ethanolaminephosphotransferase activity
AGOC: membrane
AGOP: phosphatidylethanolamine biosynthetic process, phospholipid biosynthetic process
PGOF: phosphotransferase activity, for other substituted phosphate groups
PGOC: membrane
PGOP: phospholipid biosynthetic process

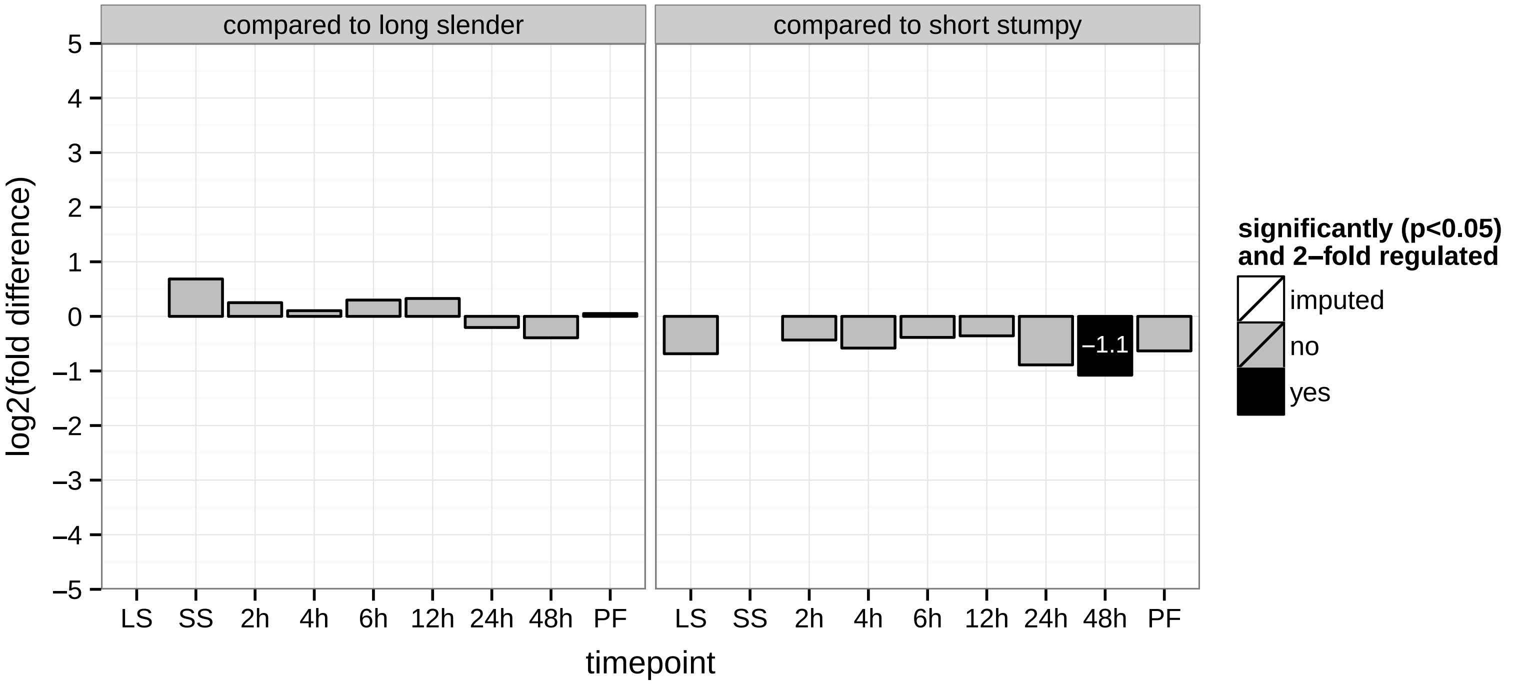

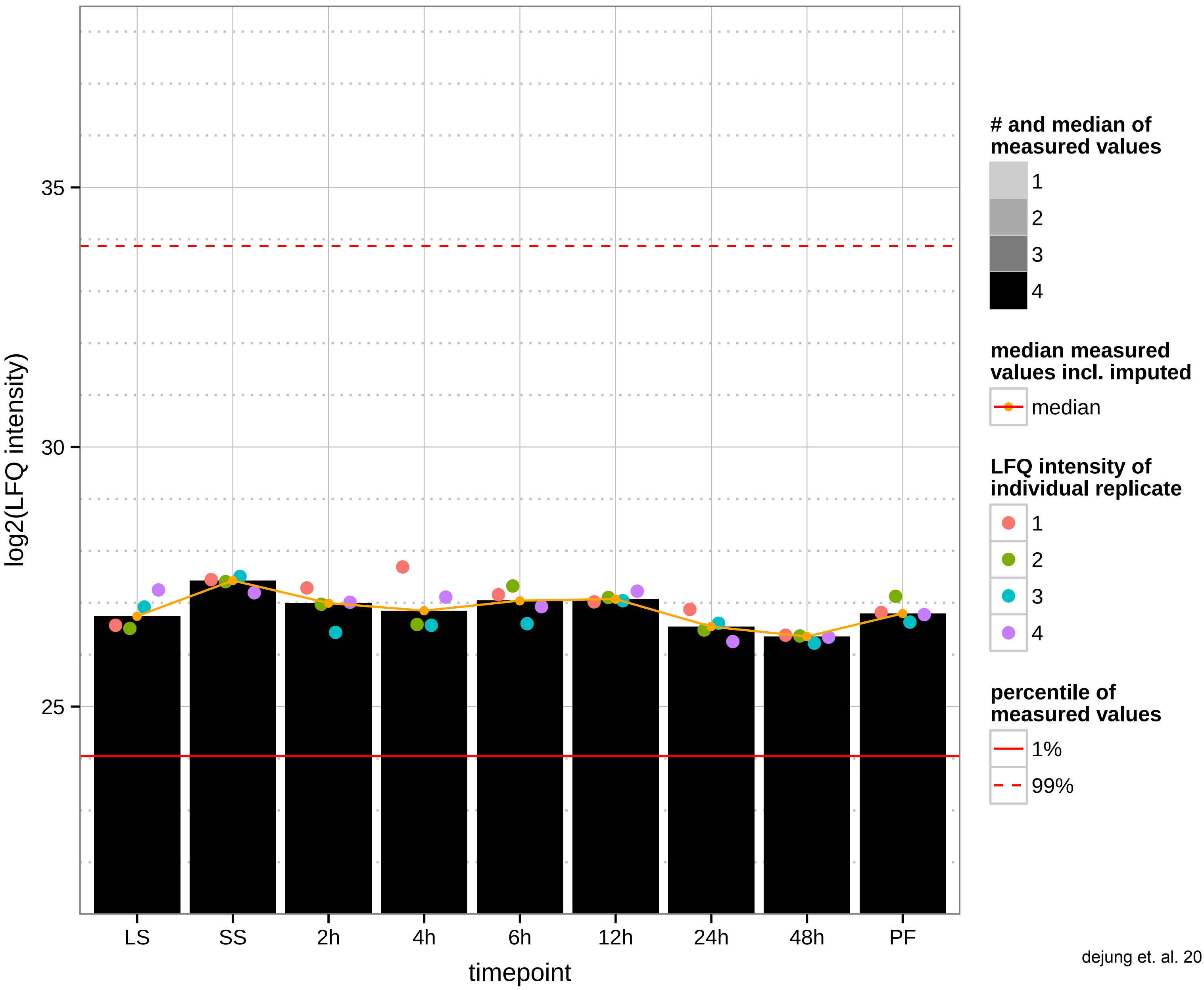

dejung et. al. 2015

hypothetical protein, conserved, variant surface glycoprotein (VSG), pseudogene
Tb927.10.14740;Tb927.10.14620;Tb11.v5.1063
AGOF: null, heat shock protein binding
AGOC: null
AGOP: null
PGOF: heat shock protein binding, null
PGOC: null
PGOP: null

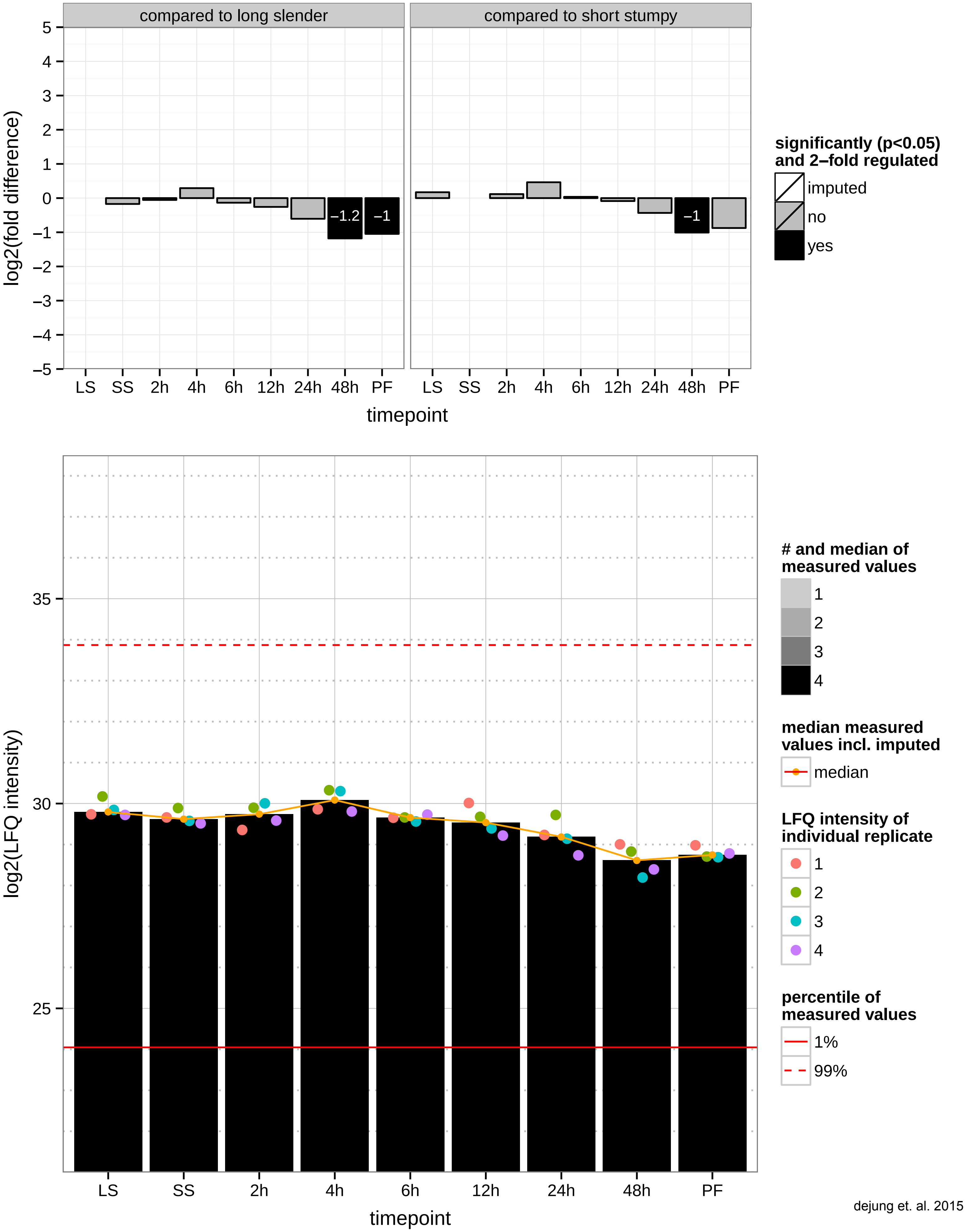

dejung et. al. 2015

ATP synthase, putative
Tb927.10.730;Tb11.v5.0822
AGOF: null, proton−transporting ATPase activity, rotational mechanism
AGOC: null, proton−transporting two−sector ATPase complex, vacuolar proton−transporting V−type ATPase, V1 domain
AGOP: null, ATP synthesis coupled proton transport
PGOF: binding, hydrolase activity, acting on acid anhydrides, catalyzing transmembrane movement of substances, proton−tra
PGOC: vacuolar proton−transporting V−type ATPase, V1 domain
PGOP: ATP hydrolysis coupled proton transport

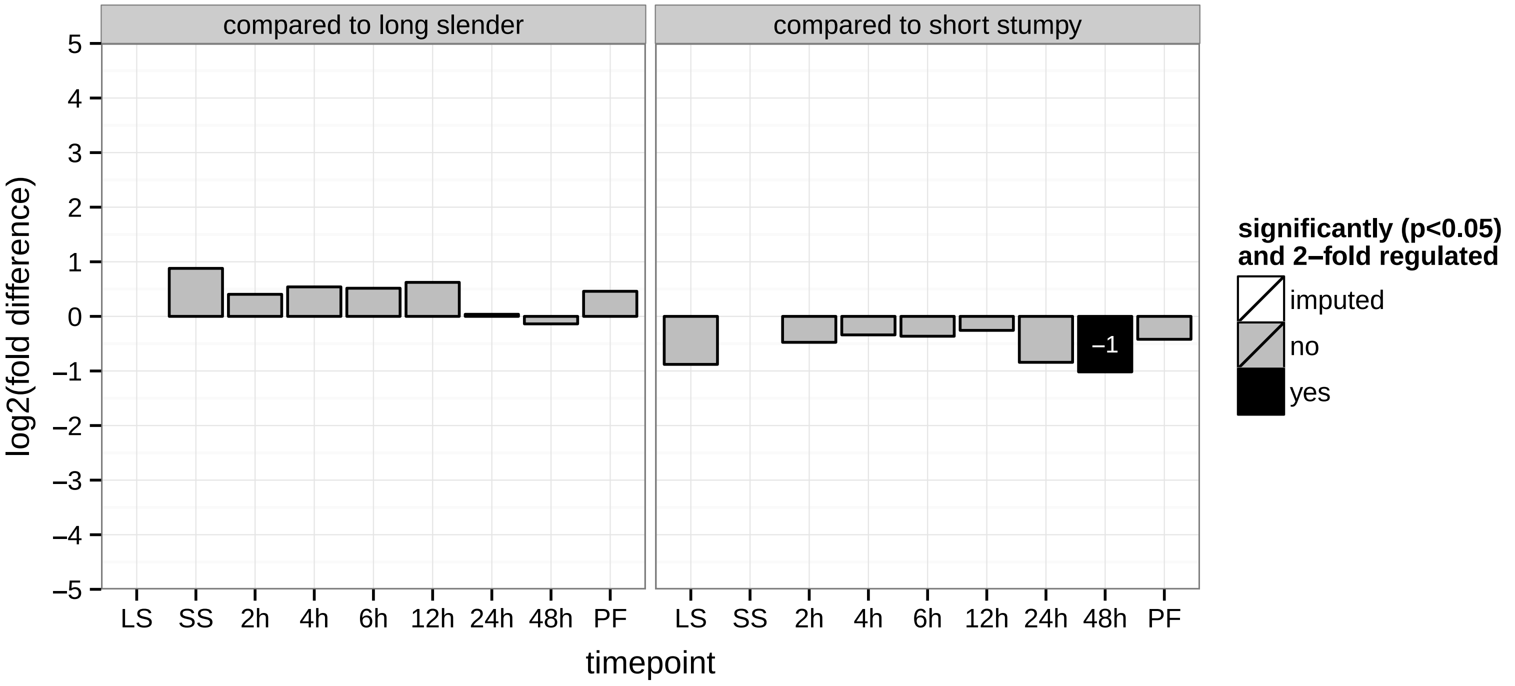

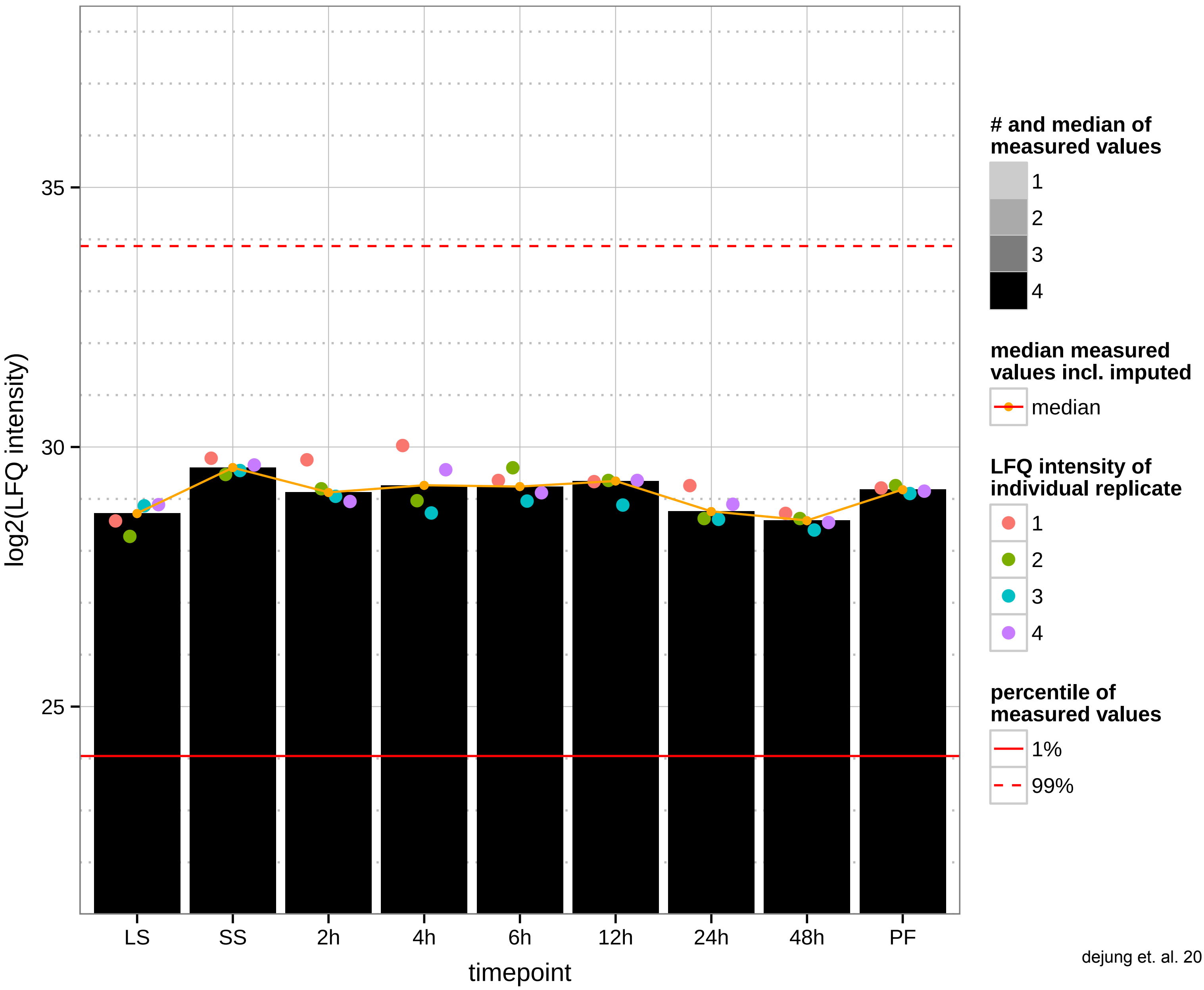

dejung et. al. 2015

vesicle–associated membrane protein, putative, synaptobrevin, putative (VAMP)
Tb927.10.790
AGOF: SNAP receptor activity
AGOC: integral to membrane
AGOP: synaptic vesicle exocytosis
PGOF: protein binding
PGOC: integral to membrane
PGOP: vesicle–mediated transport

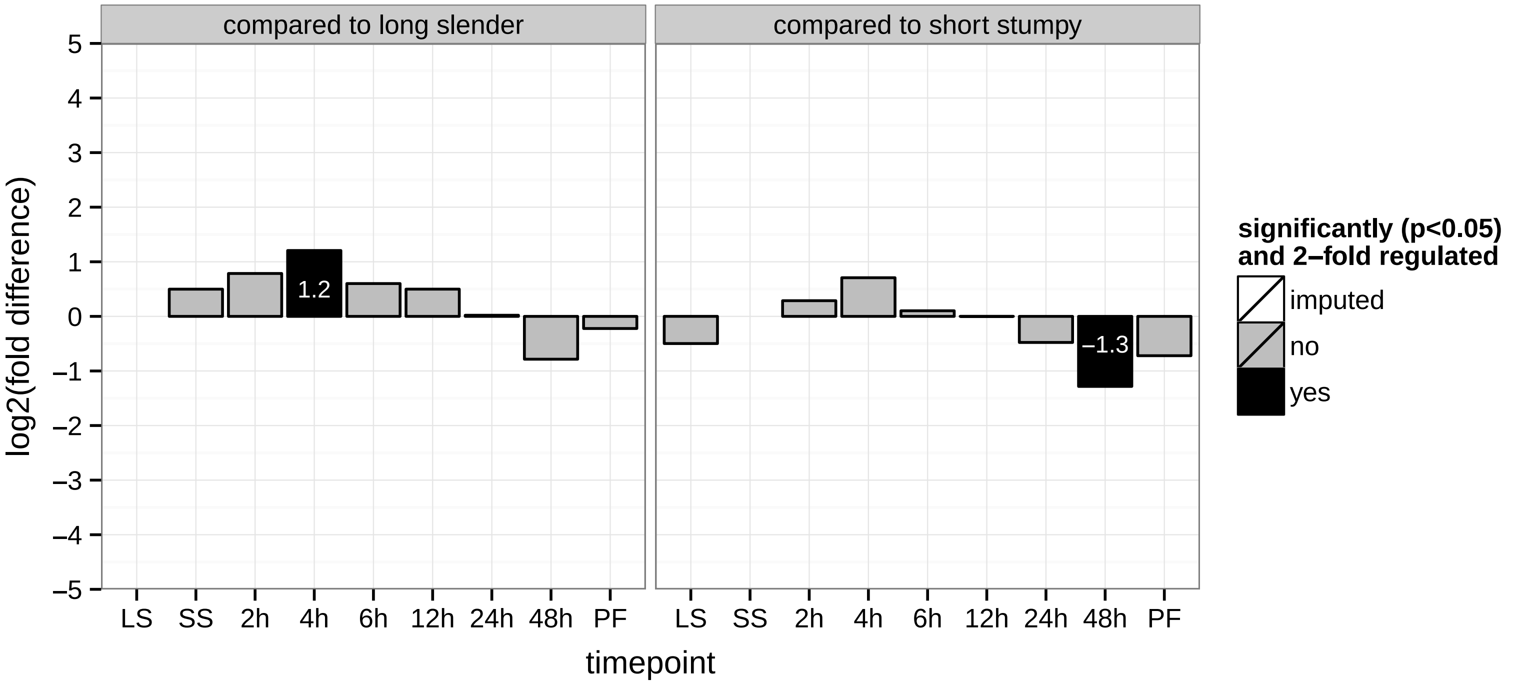

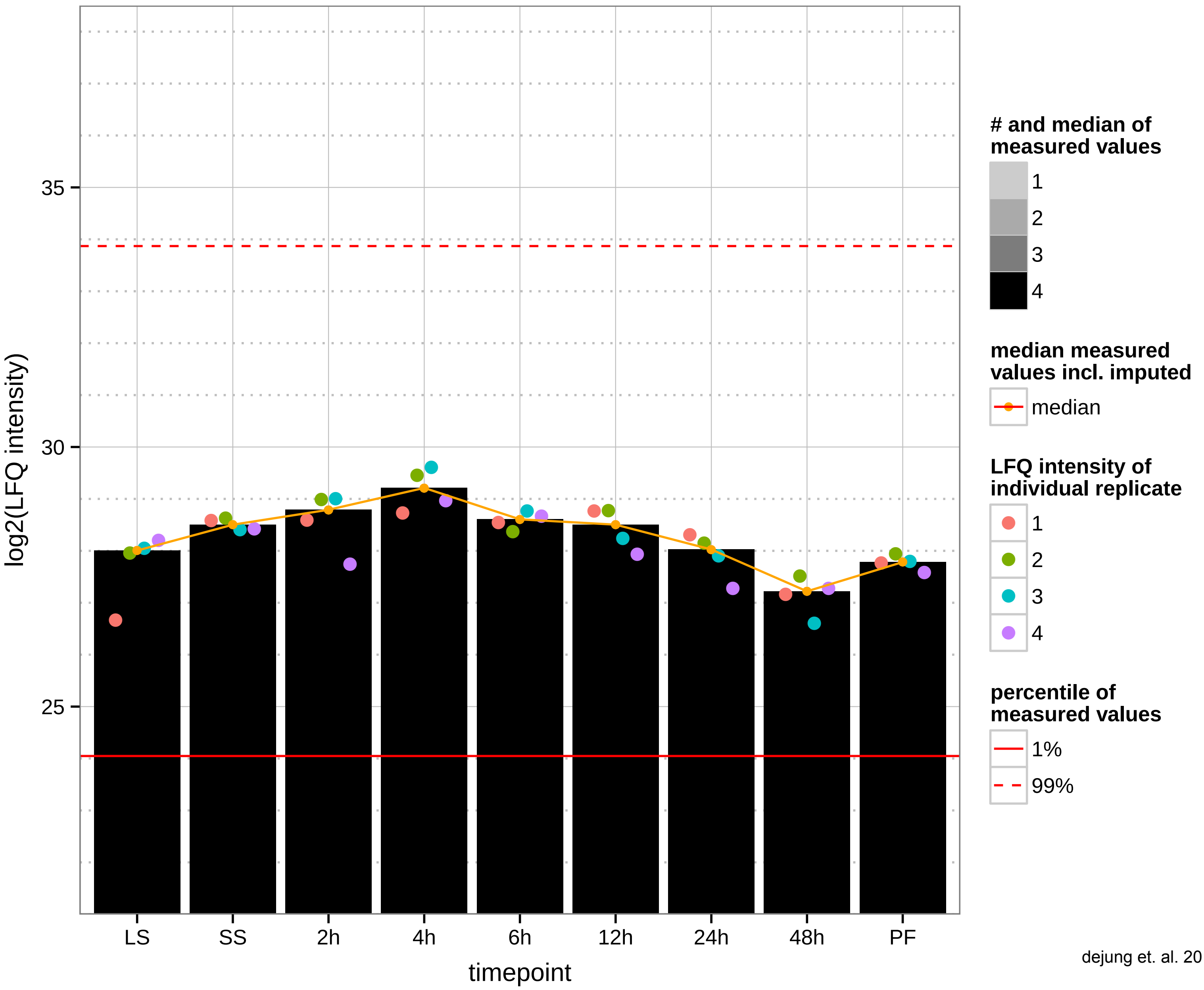

dejung et. al. 2015

vacuolar sorting–associated protein–like, putative
Tb927.11.10010
AGOF: null
AGOC: retromer complex
AGOP: retrograde transport, endosome to Golgi, vacuolar transport
PGOF: null
PGOC: retromer complex
PGOP: vacuolar transport

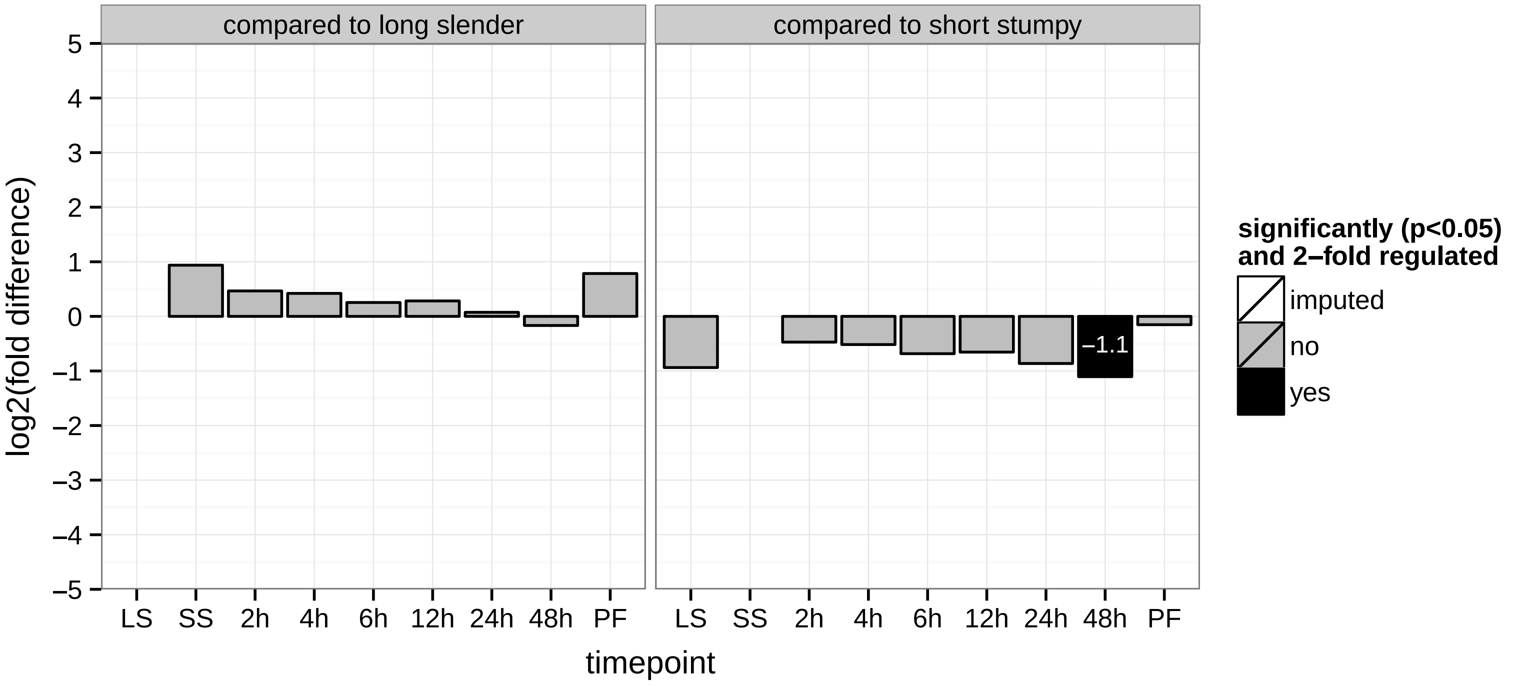

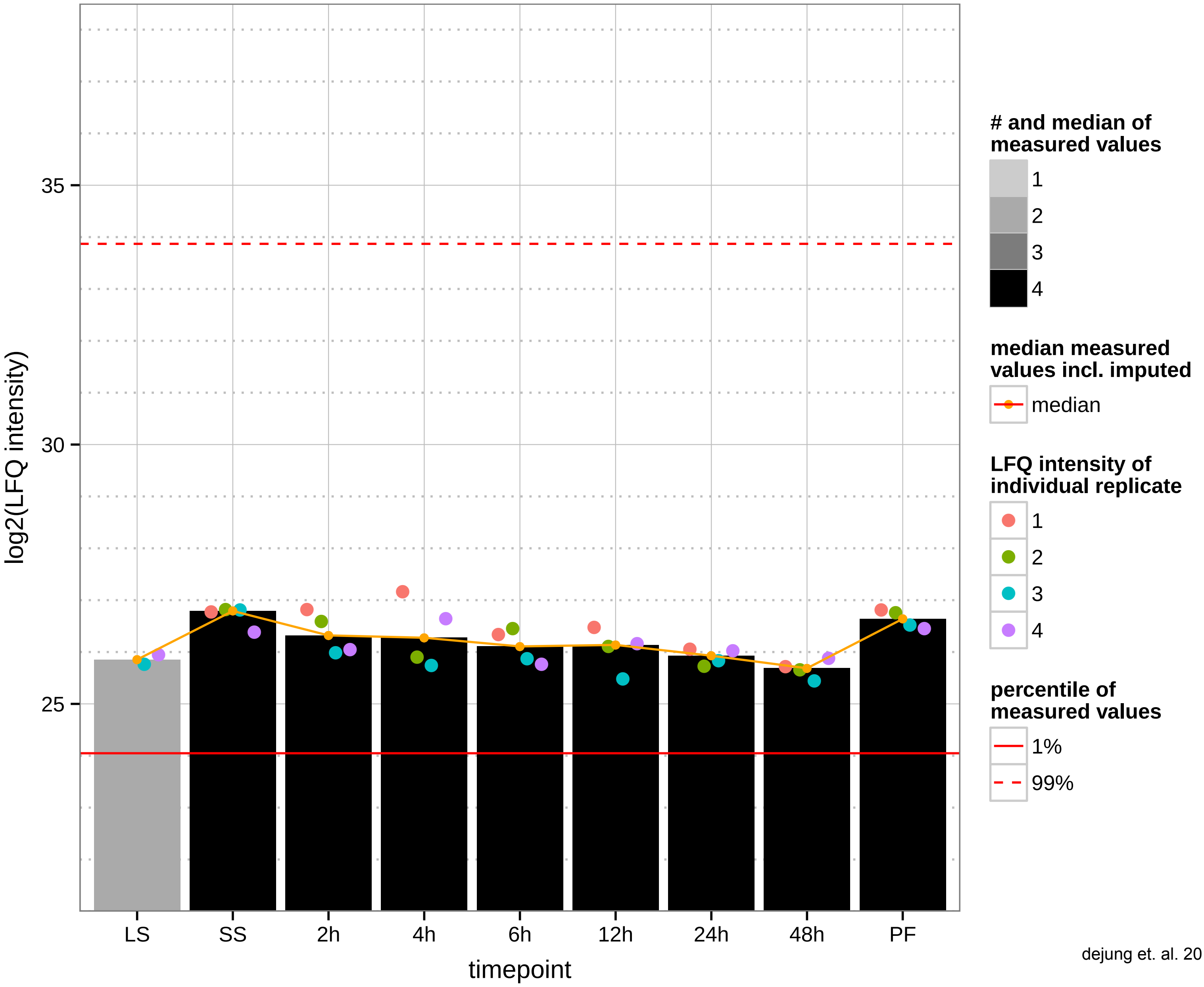

dejung et. al. 2015

vacuolar ATP synthase subunit b, putative, v– ATPase B subunit, vacuolar proton pump B subunit
Tb927.11.11690
AGOF: hydrogen ion transporting ATP synthase activity, rotational mechanism, hydrogen–exporting ATPase activity, phosphor
AGOC: contractile vacuole, lysosomal proton–transporting V–type ATPase complex, proton–transporting V–type ATPase, V1 d
AGOP: ATP hydrolysis coupled proton transport, ATP synthesis coupled proton transport
PGOF: ATP binding, hydrogen ion transporting ATP synthase activity, rotational mechanism, hydrolase activity, acting on acid
PGOC: proton–transporting V–type ATPase, V1 domain, proton–transporting two–sector ATPase complex, catalytic domain
PGOP: ATP hydrolysis coupled proton transport, ATP metabolic process, proton transport

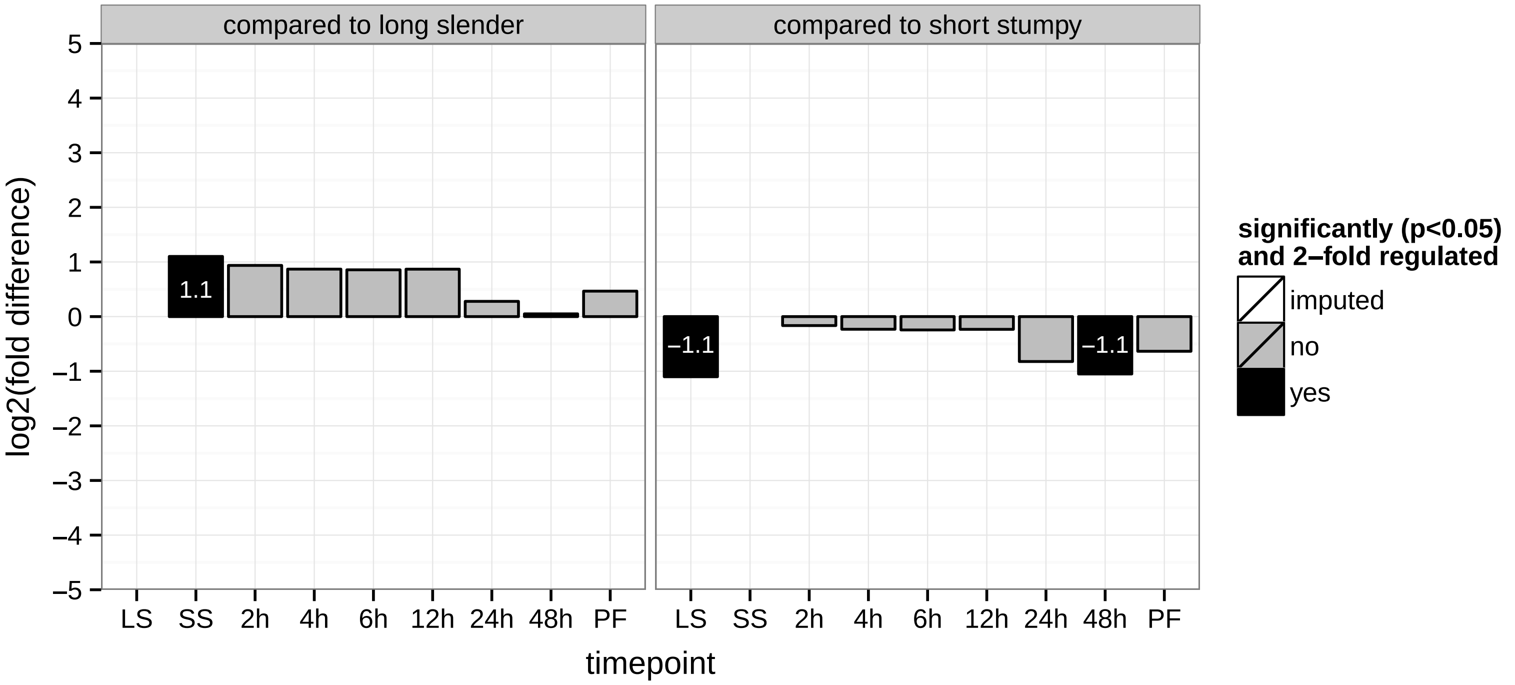

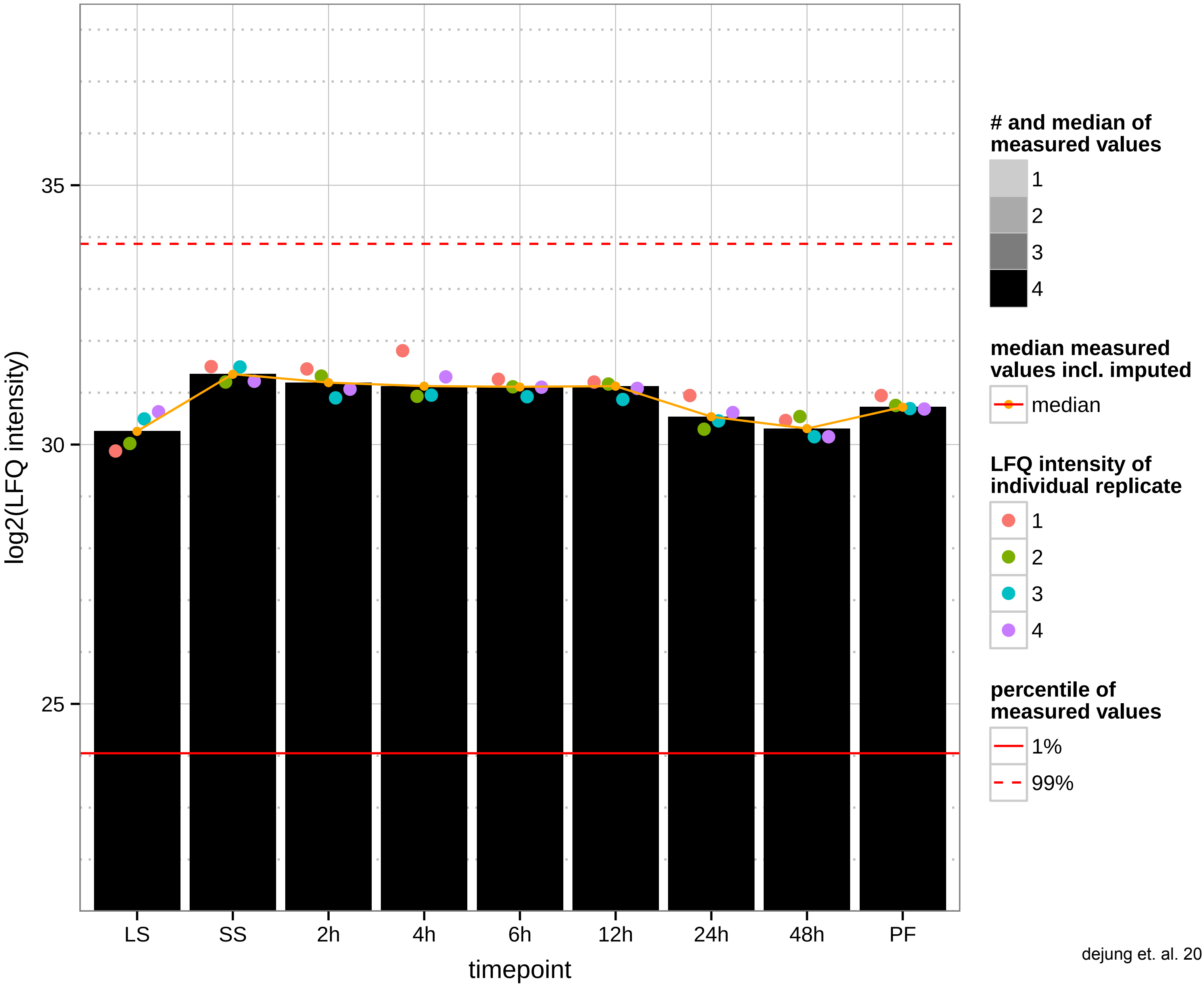

dejung et. al. 2015

vacuolar sorting protein, putative
Tb927.11.14290
AGOF: hydrolase activity, acting on ester bonds
AGOC: cytoplasm, membrane
AGOP: protein transport, vesicle–mediated transport
PGOF: hydrolase activity, acting on ester bonds
PGOC: null
PGOP: null

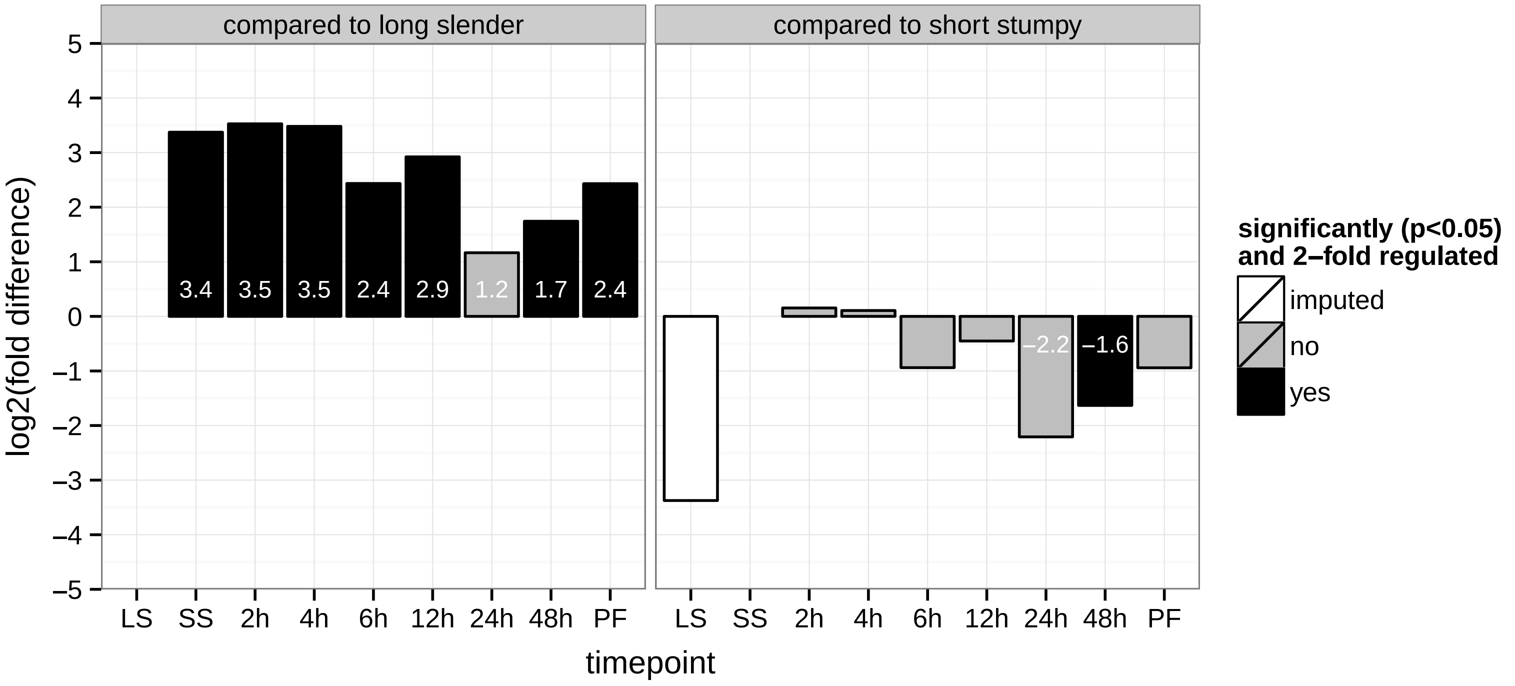

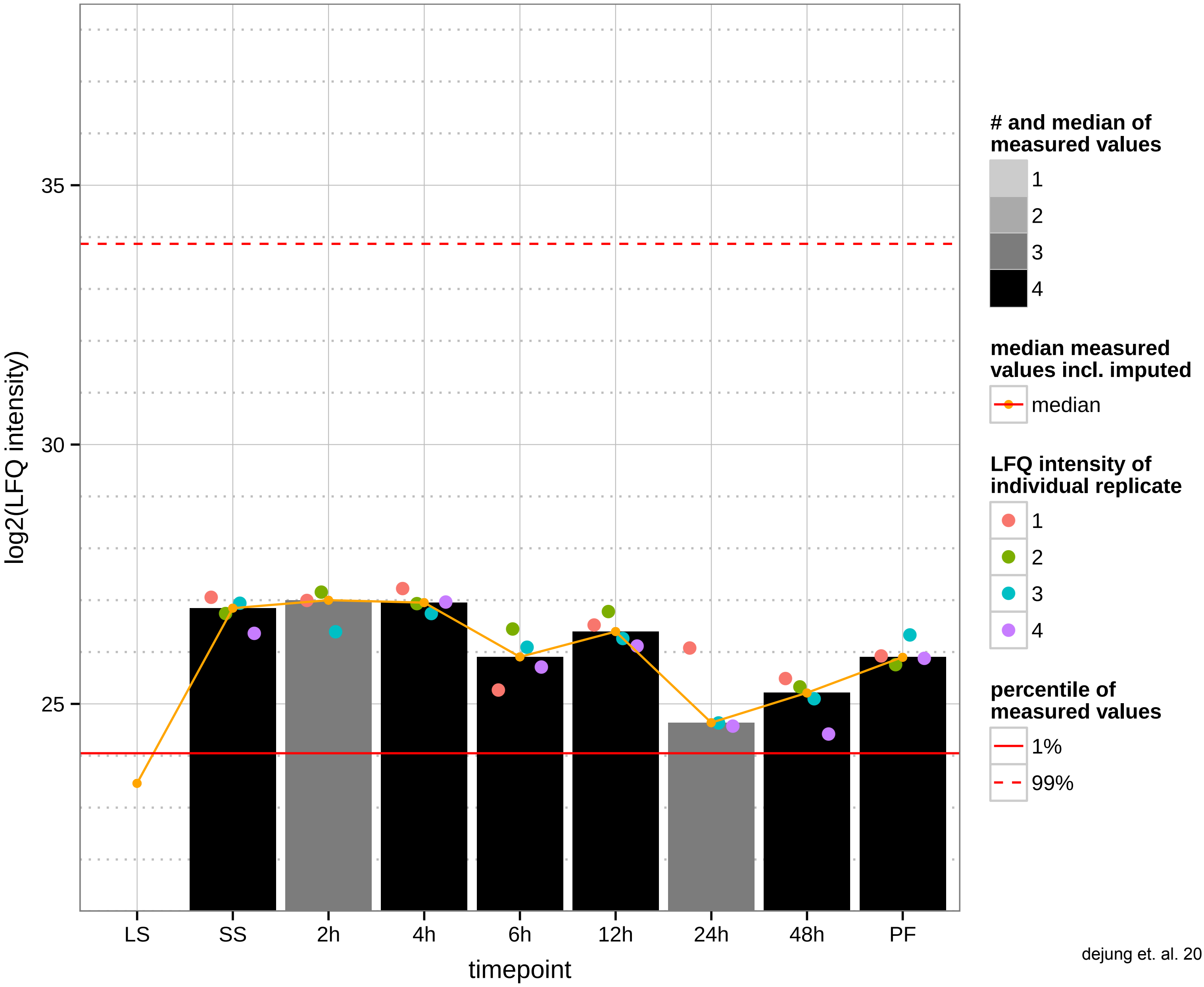

dejung et. al. 2015

mitochondrial carrier protein (MCP2)
Tb927.11.14360
AGOF: null
AGOC: integral to membrane, mitochondrial part
AGOP: transport
PGOF: null
PGOC: null
PGOP: null

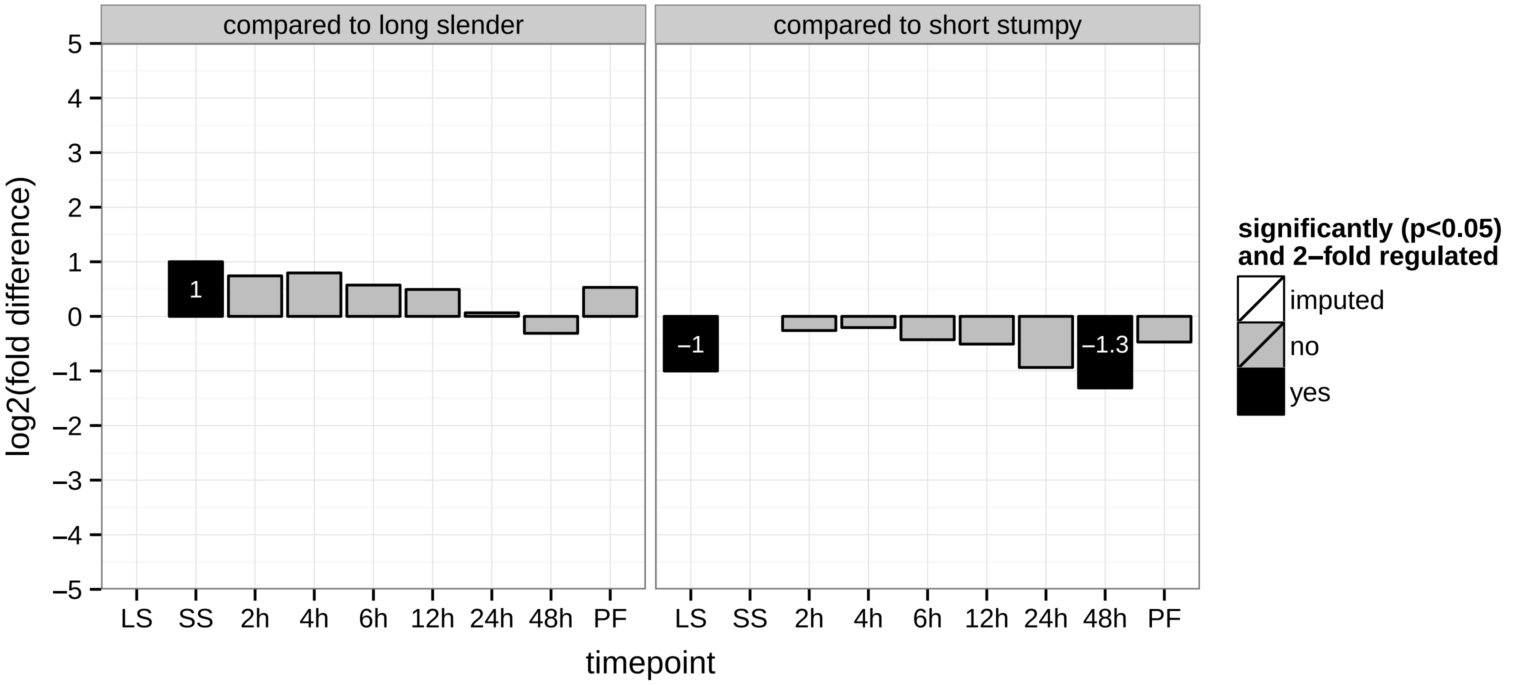

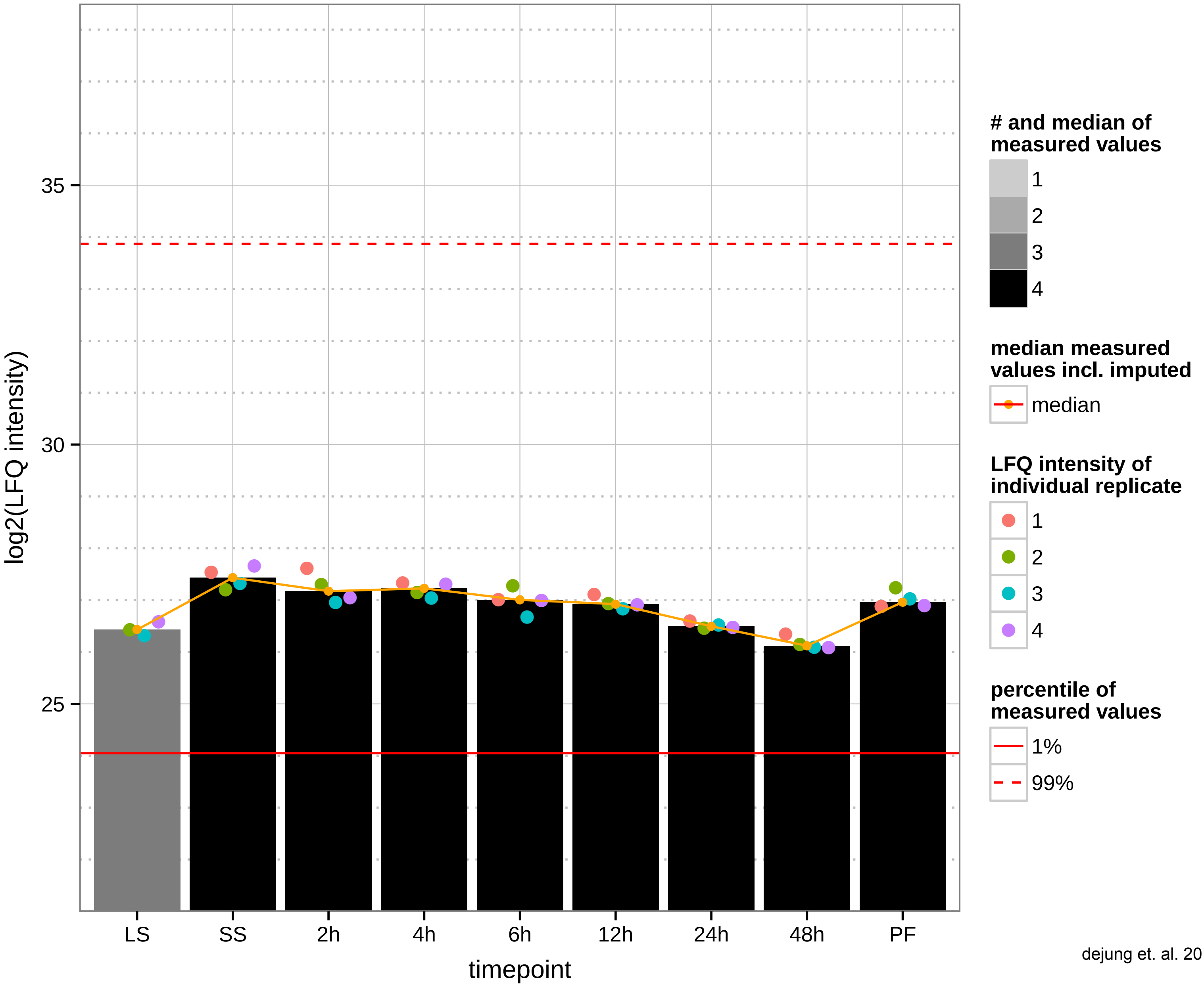

dejung et. al. 2015

Ras–related protein Rab2 (RAB2)
Tb927.11.15240
AGOF: GTP binding, GTPase activity
AGOC: intracellular
AGOP: intracellular protein transport, nucleocytoplasmic transport, small GTPase mediated signal transduction
PGOF: GTP binding, GTPase activity
PGOC: intracellular, membrane
PGOP: GTP catabolic process, intracellular protein transport, nucleocytoplasmic transport, protein transport, signal transducti

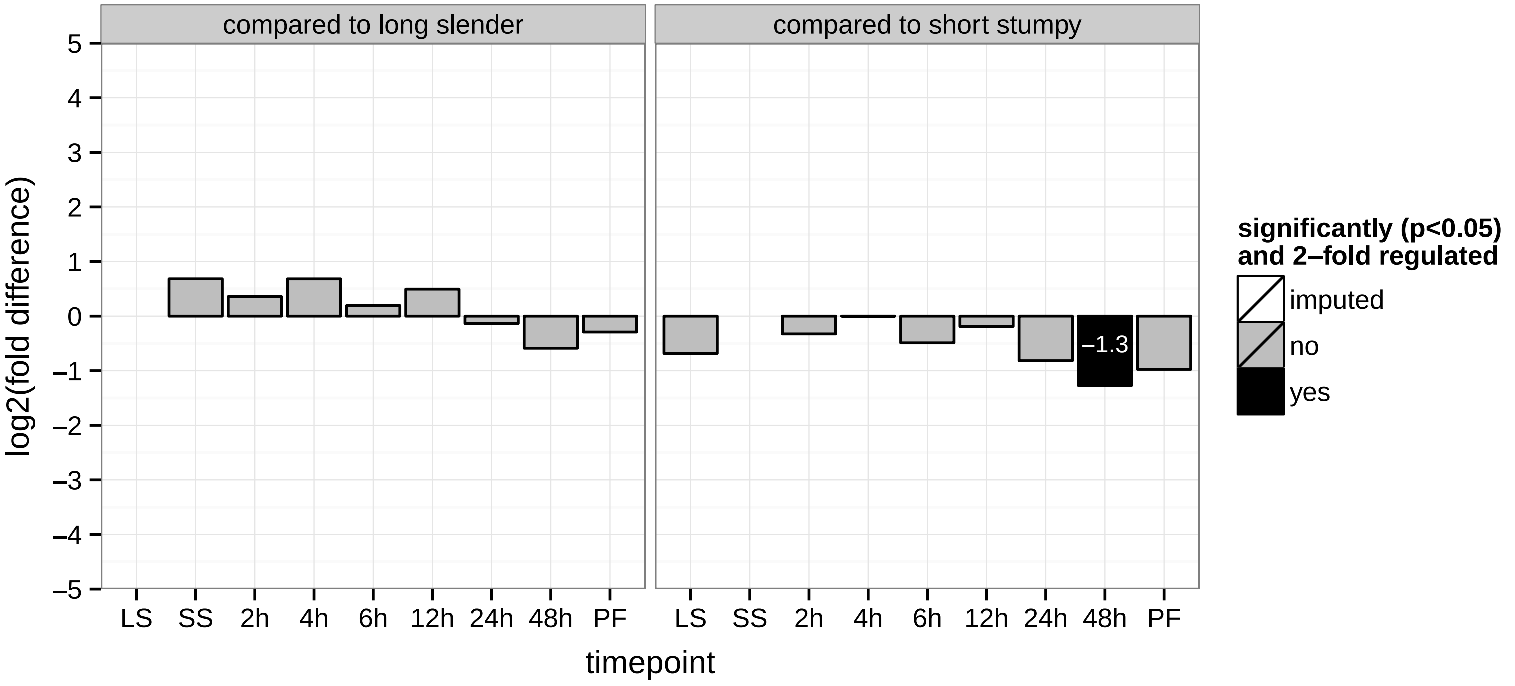

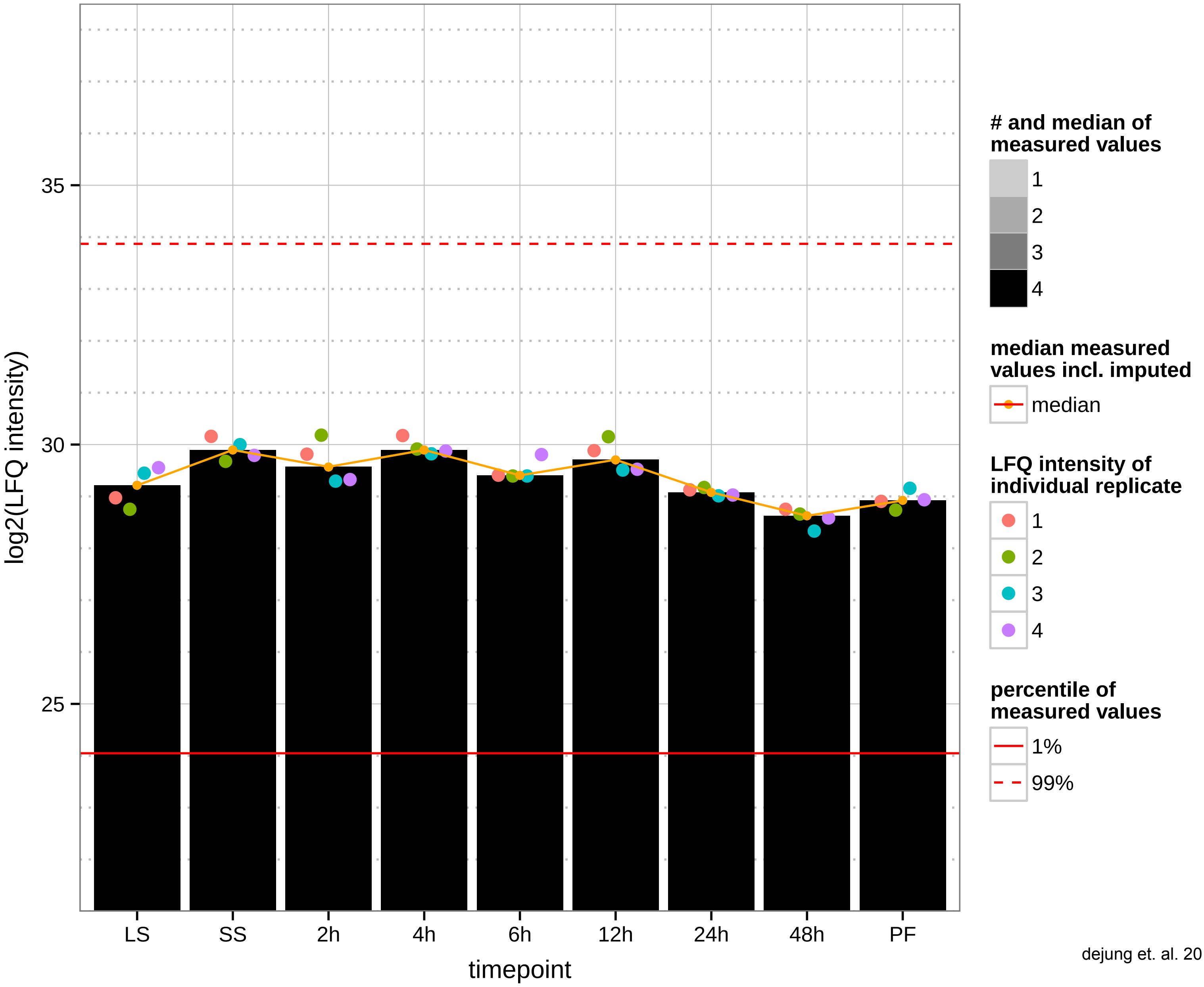

dejung et. al. 2015

GPI transamidase subunit Tta1 (TTA1)
Tb927.11.15760
AGOF: GPI–anchor transamidase activity
AGOC: GPI–anchor transamidase complex, integral to membrane
AGOP: null
PGOF: null
PGOC: null
PGOP: null

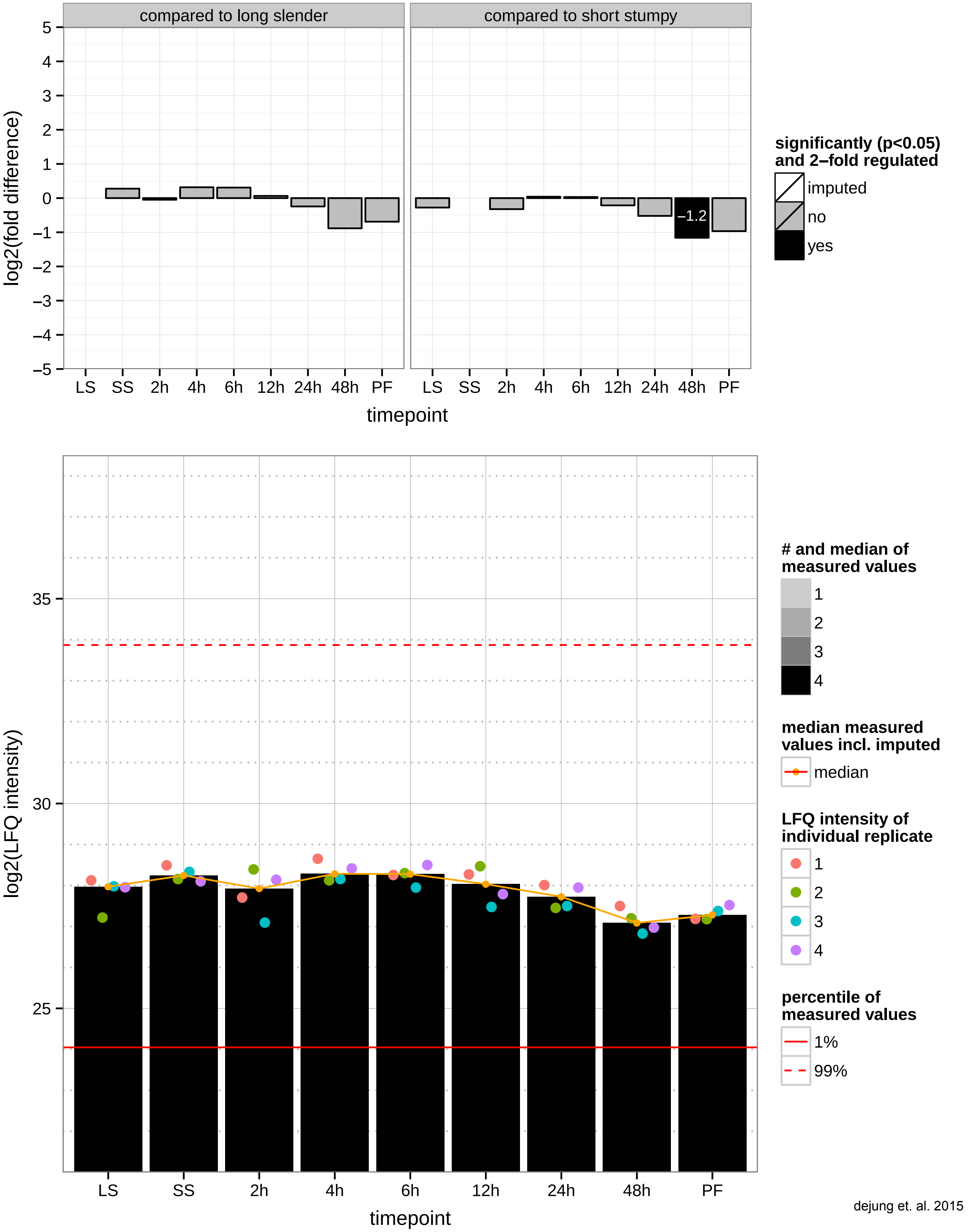

dejung et. al. 2015

hypothetical protein, conserved
Tb927.11.15970
AGOF: ARF guanyl−nucleotide exchange factor activity
AGOC: intracellular
AGOP: regulation of ARF protein signal transduction
PGOF: ARF guanyl−nucleotide exchange factor activity
PGOC: intracellular
PGOP: regulation of ARF protein signal transduction

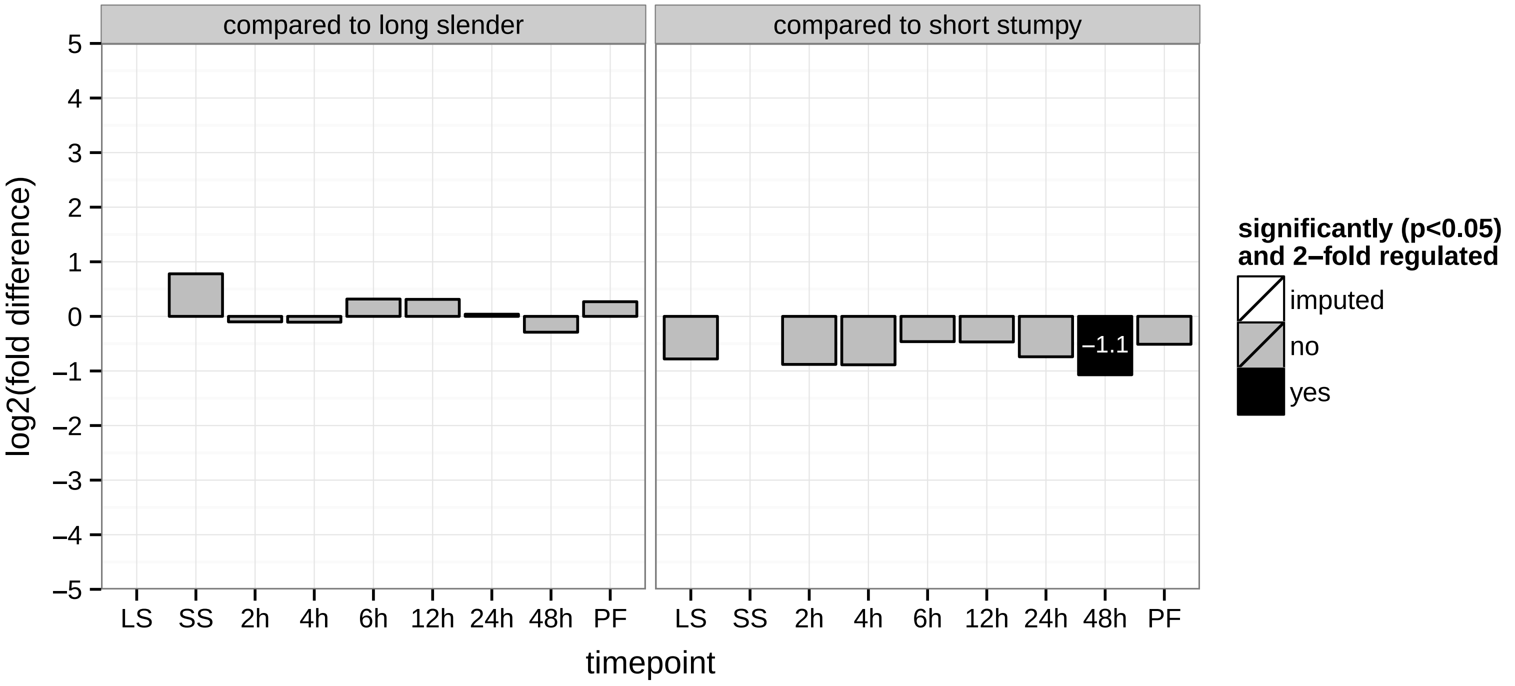

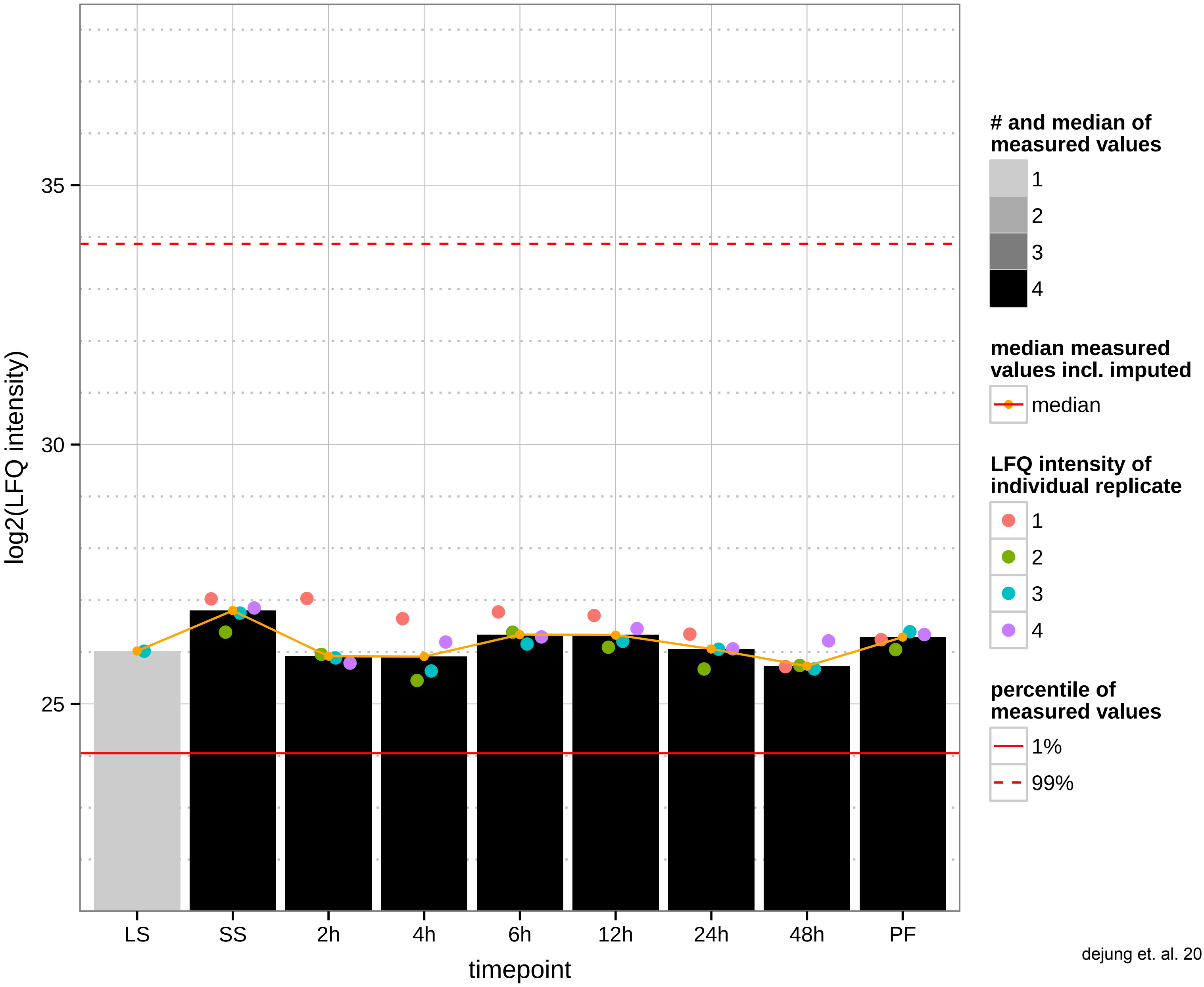

dejung et. al. 2015

hypothetical protein, conserved
Tb927.11.16490
AGOF: null
AGOC: null
AGOP: null
PGOF: protein binding
PGOC: null
PGOP: null

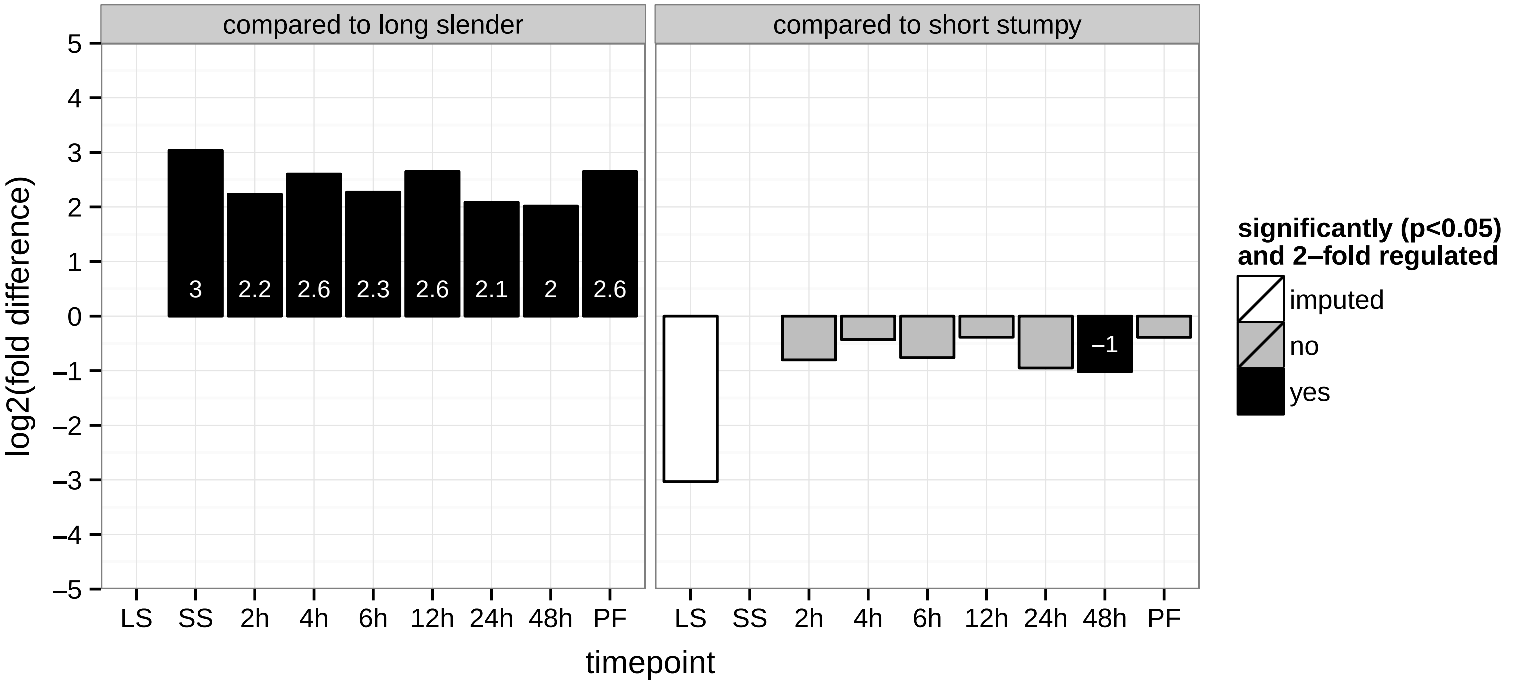

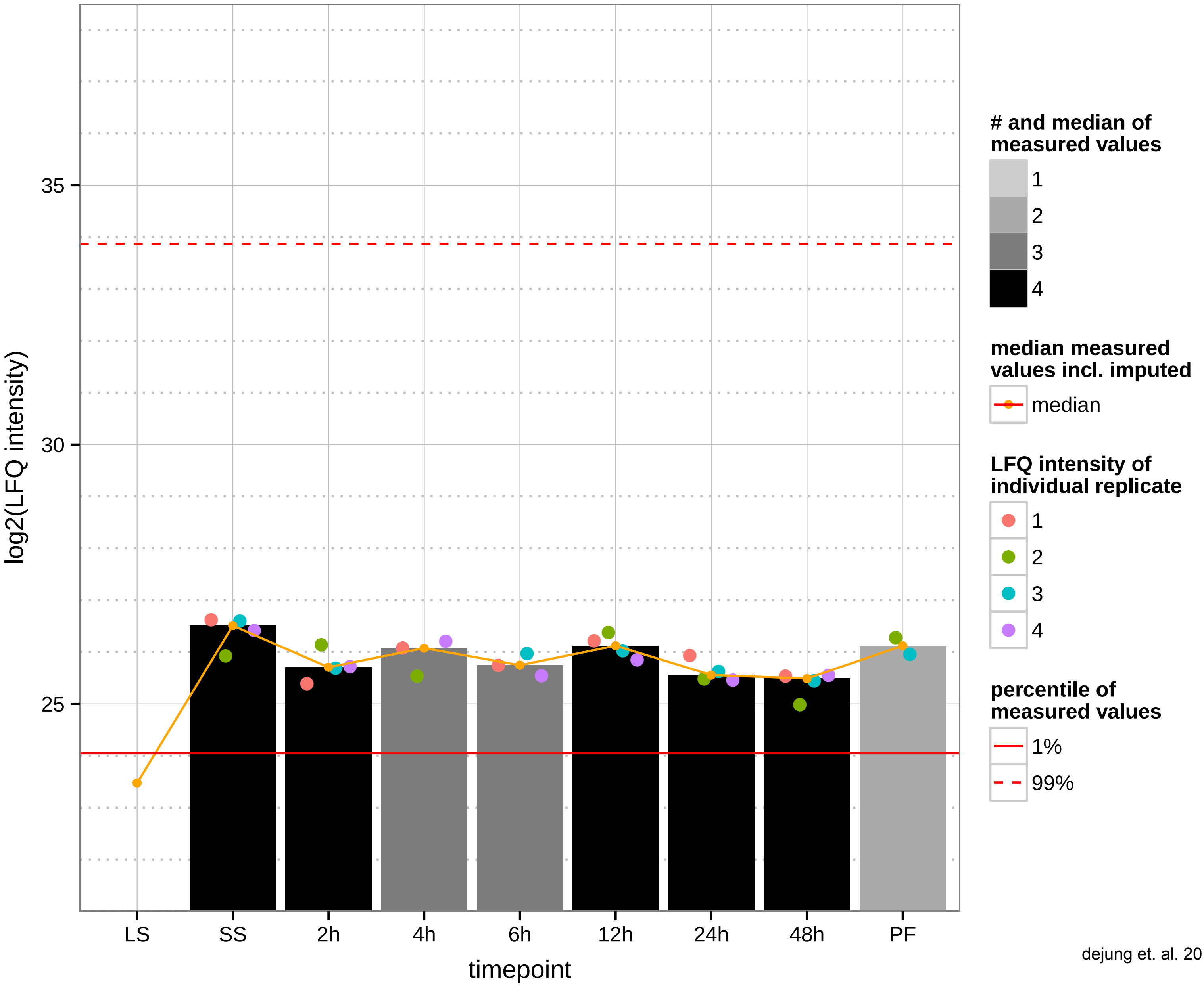

dejung et. al. 2015

hypothetical protein, conserved
Tb927.11.8060
AGOF: null
AGOC: integral to membrane
AGOP: null
PGOF: null
PGOC: null
PGOP: null

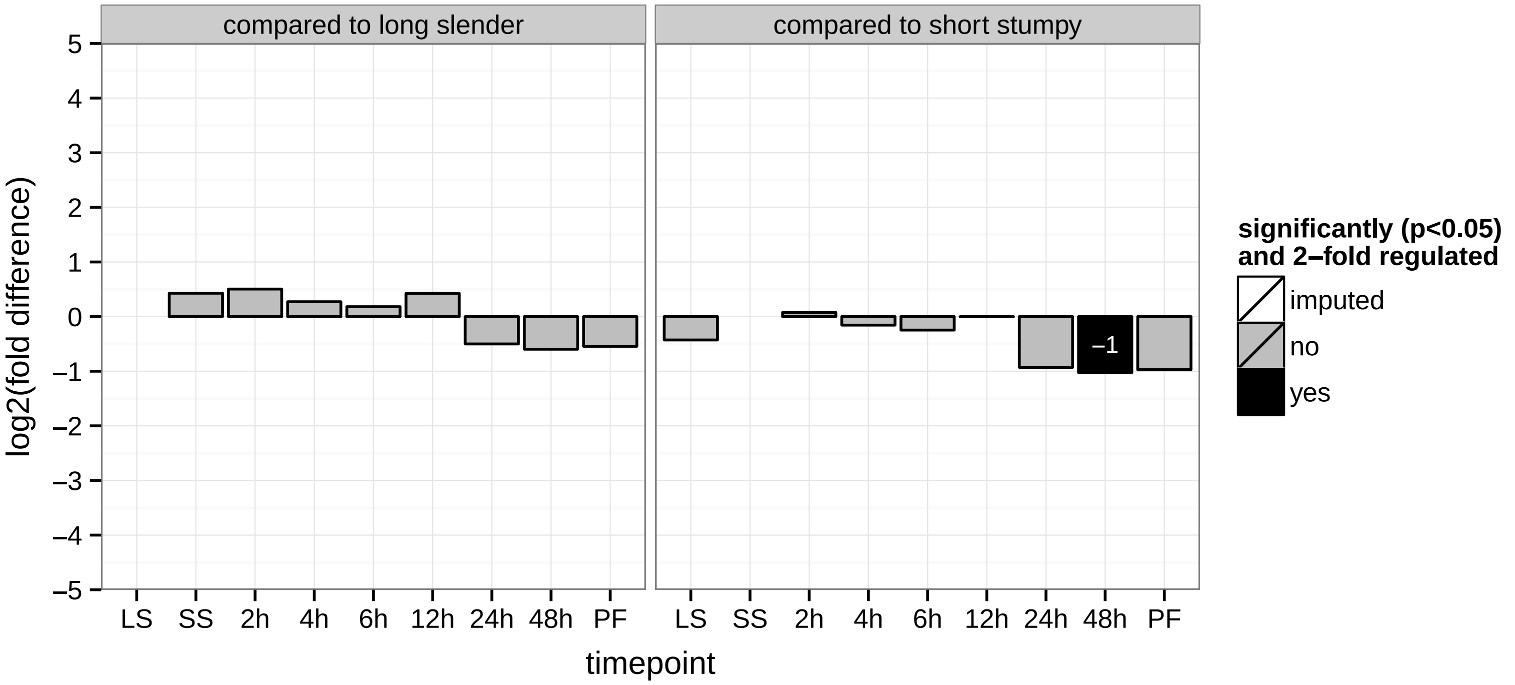

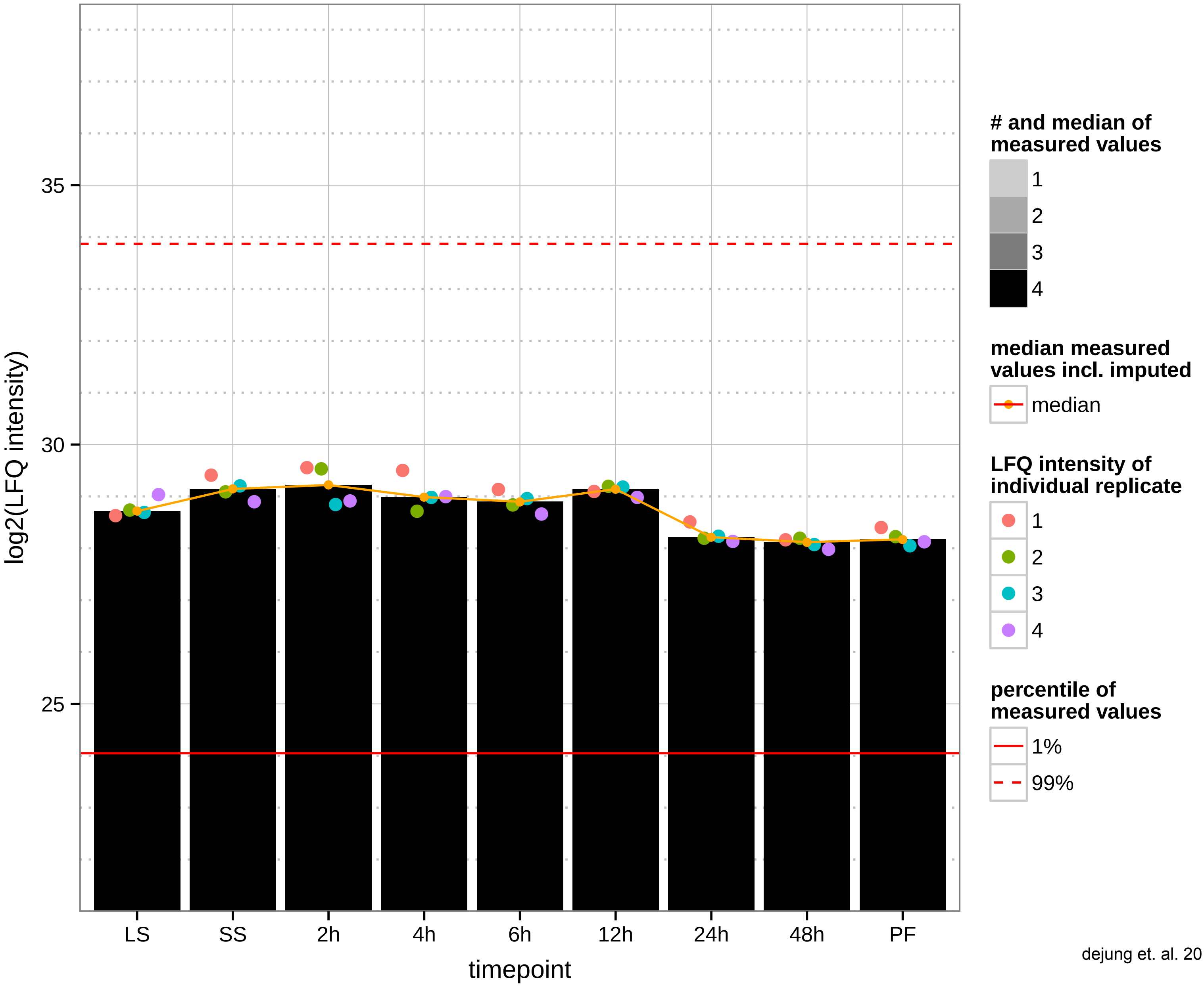

dejung et. al. 2015

hypothetical protein, conserved
Tb927.11.9650
AGOF: null
AGOC: null
AGOP: null
PGOF: null
PGOC: null
PGOP: null

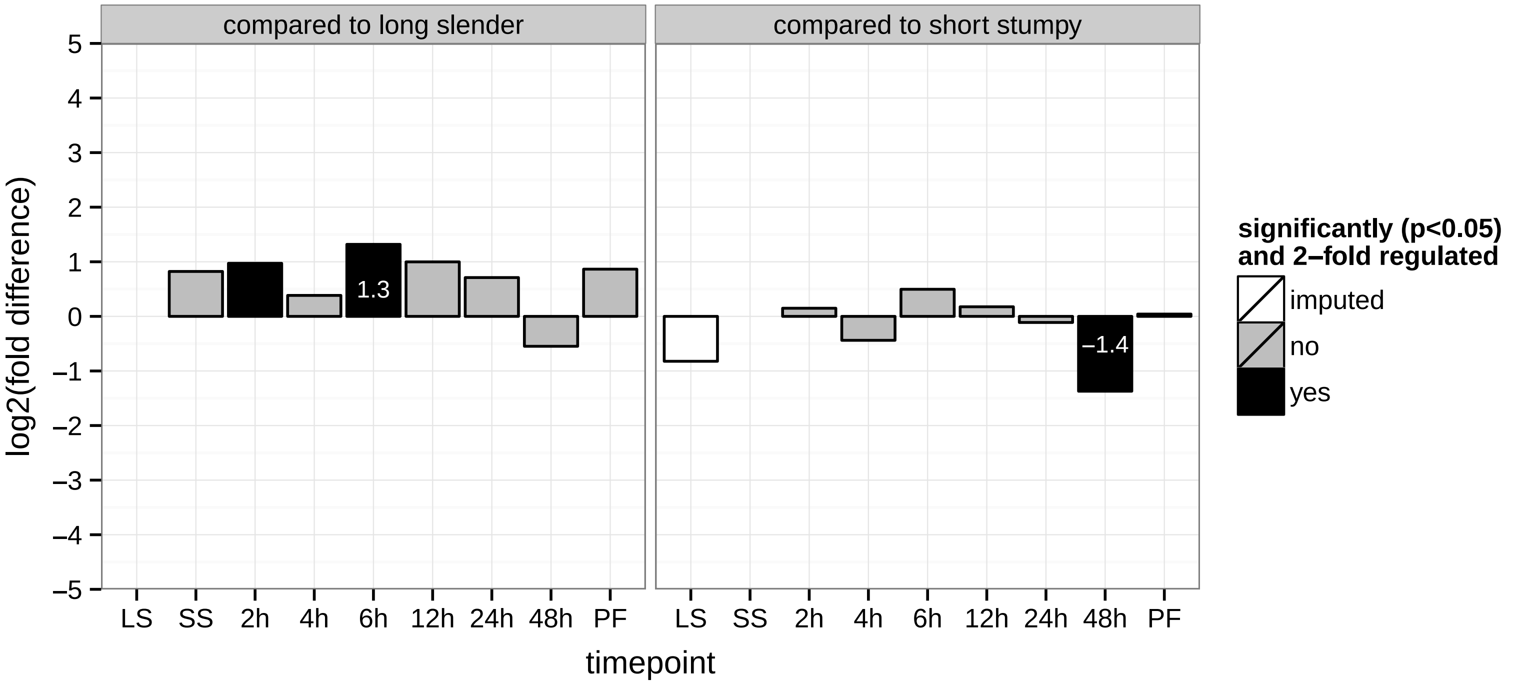

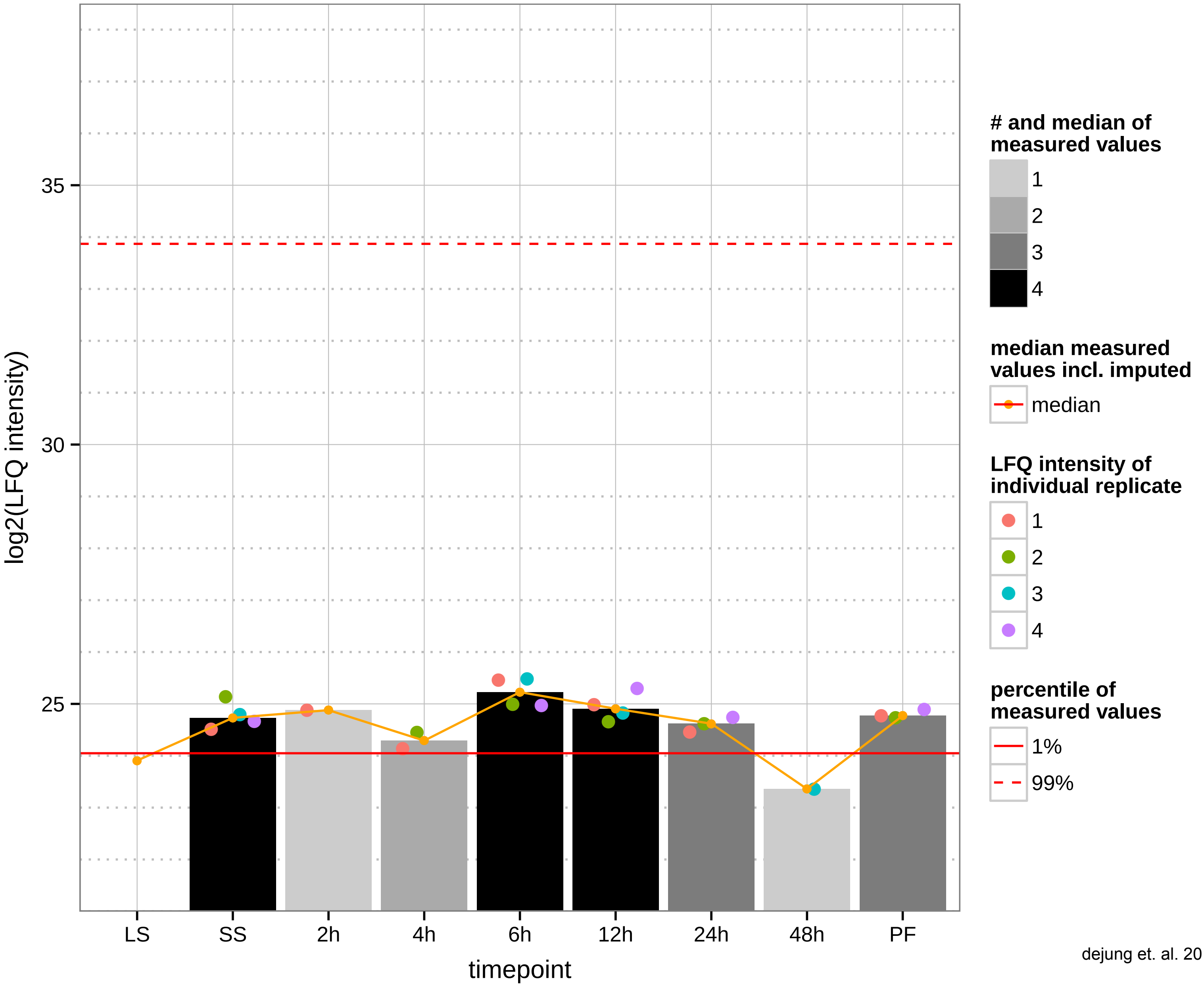

dejung et. al. 2015

G−actin binding protein, putative, twinfilin
Tb927.4.2350
AGOF: actin binding
AGOC: intracellular
AGOP: null
PGOF: actin binding
PGOC: intracellular
PGOP: null

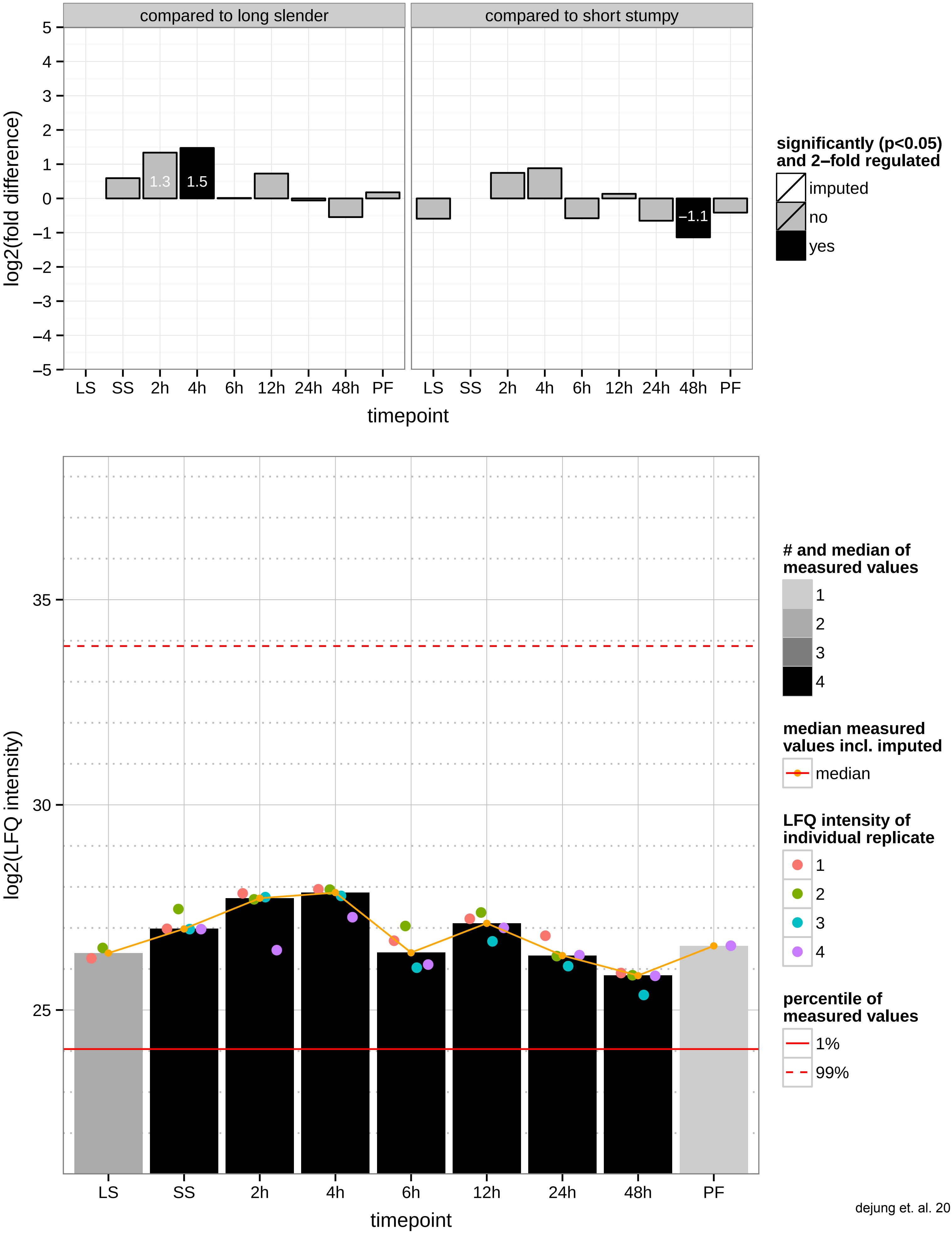

dejung et. al. 2015

hypothetical protein, conserved
Tb927.4.3350
AGOF: null
AGOC: null
AGOP: null
PGOF: null
PGOC: null
PGOP: null

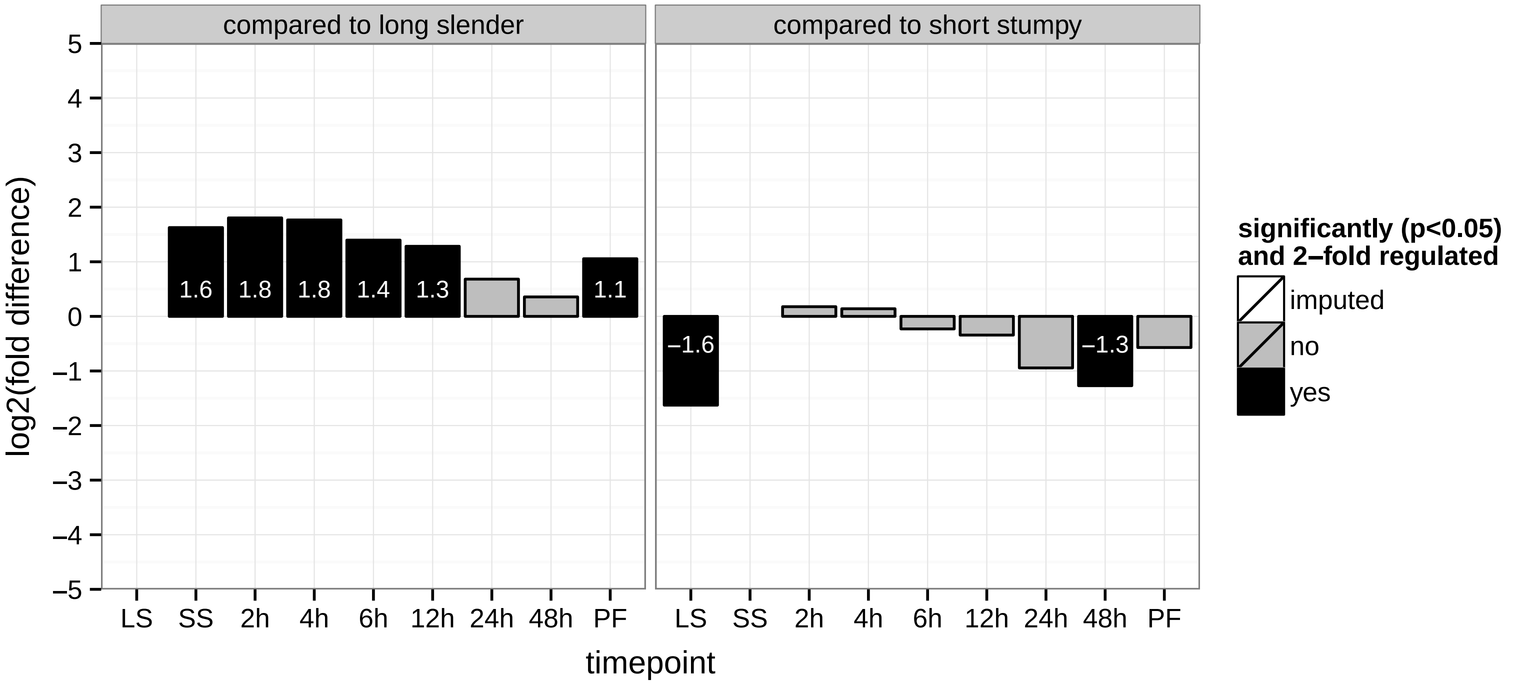

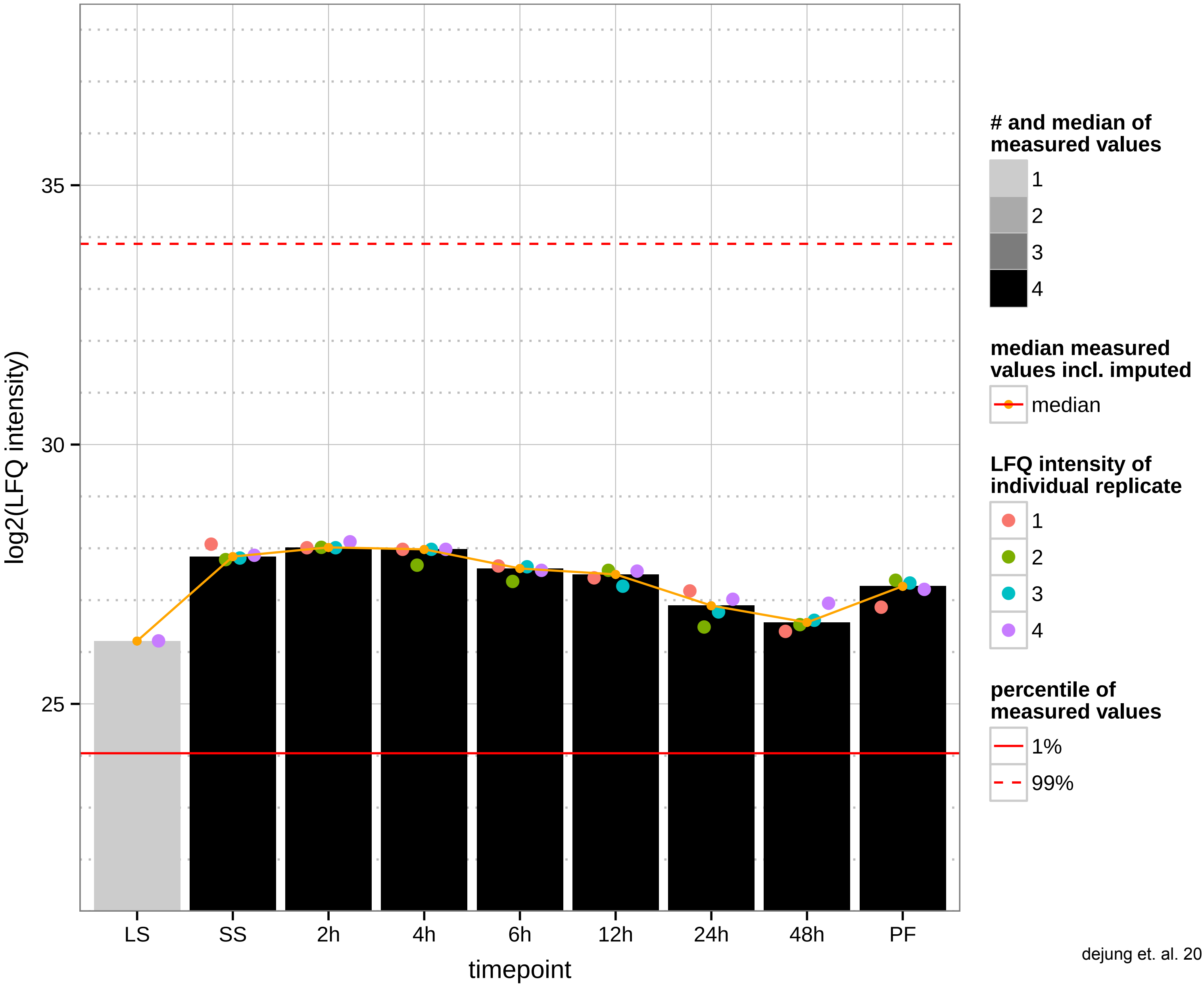

dejung et. al. 2015

vacuolar proton translocating ATPase subunit A, putative
Tb927.5.1300
AGOF: hydrogen ion transmembrane transporter activity
AGOC: proton–transporting two–sector ATPase complex, proton–transporting domain
AGOP: ATP synthesis coupled proton transport
PGOF: hydrogen ion transmembrane transporter activity
PGOC: proton–transporting two–sector ATPase complex, proton–transporting domain, vacuolar proton–transporting V–type A
PGOP: ATP hydrolysis coupled proton transport

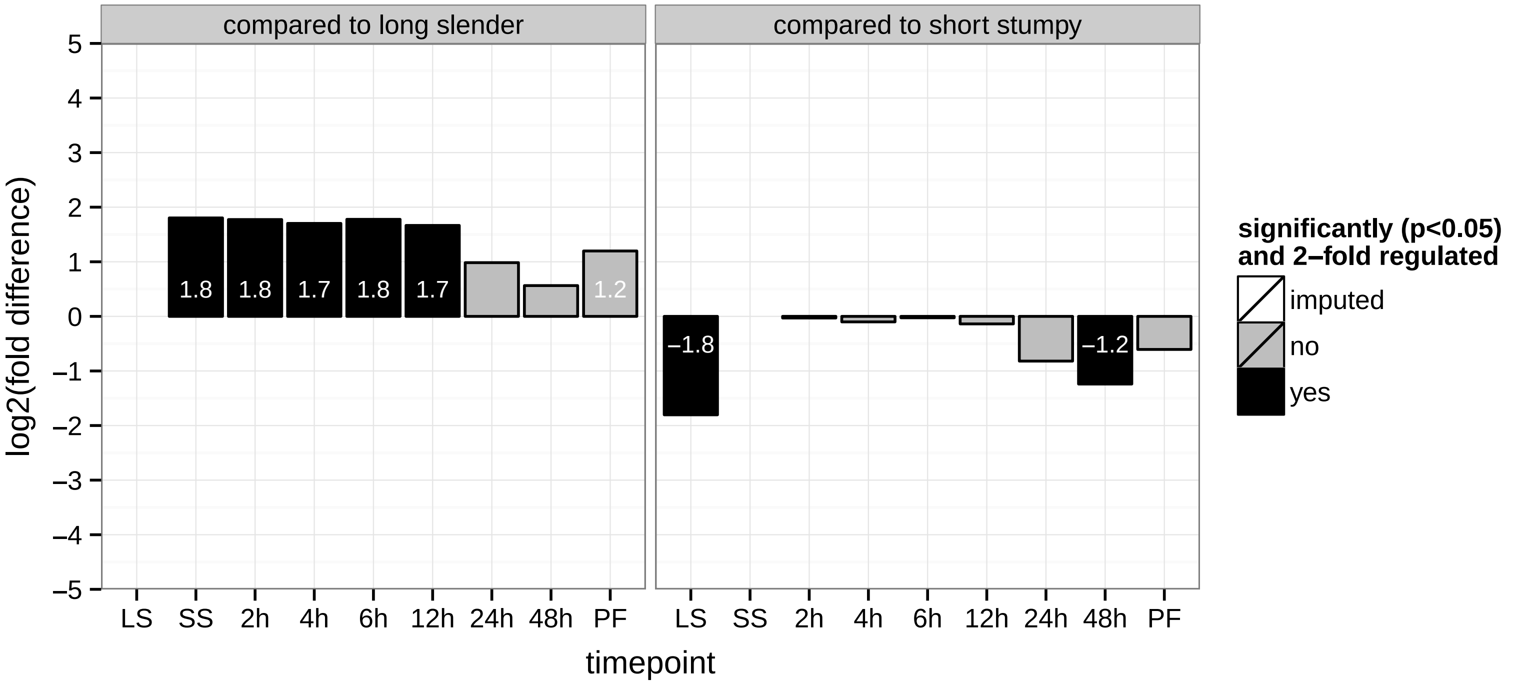

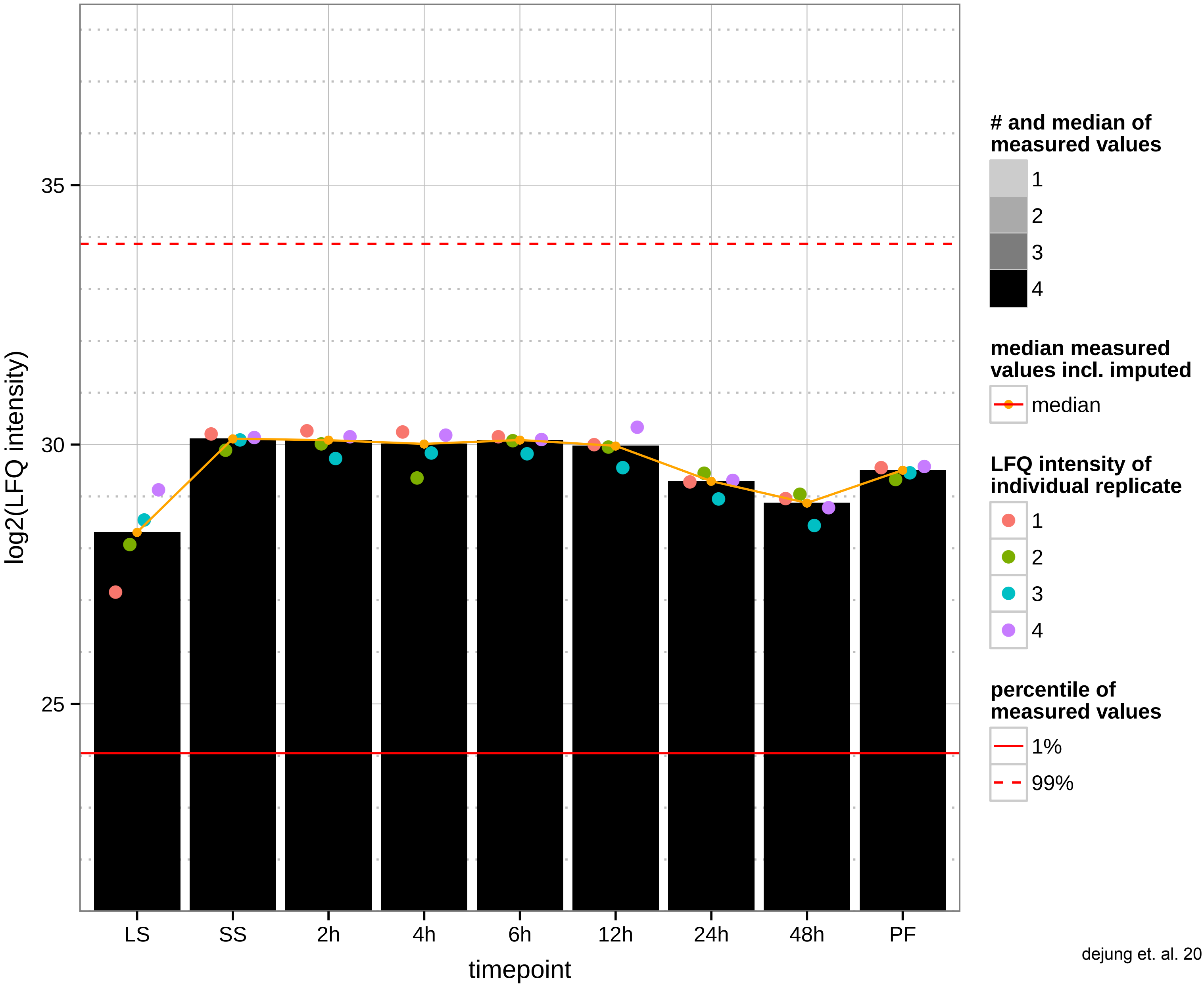

dejung et. al. 2015

signal peptidase type I, putative, serine peptidase, Clan SF, Family S26A
Tb927.5.3220
AGOF: peptidase activity
AGOC: membrane
AGOP: proteolysis, signal peptide processing
PGOF: peptidase activity
PGOC: membrane
PGOP: signal peptide processing

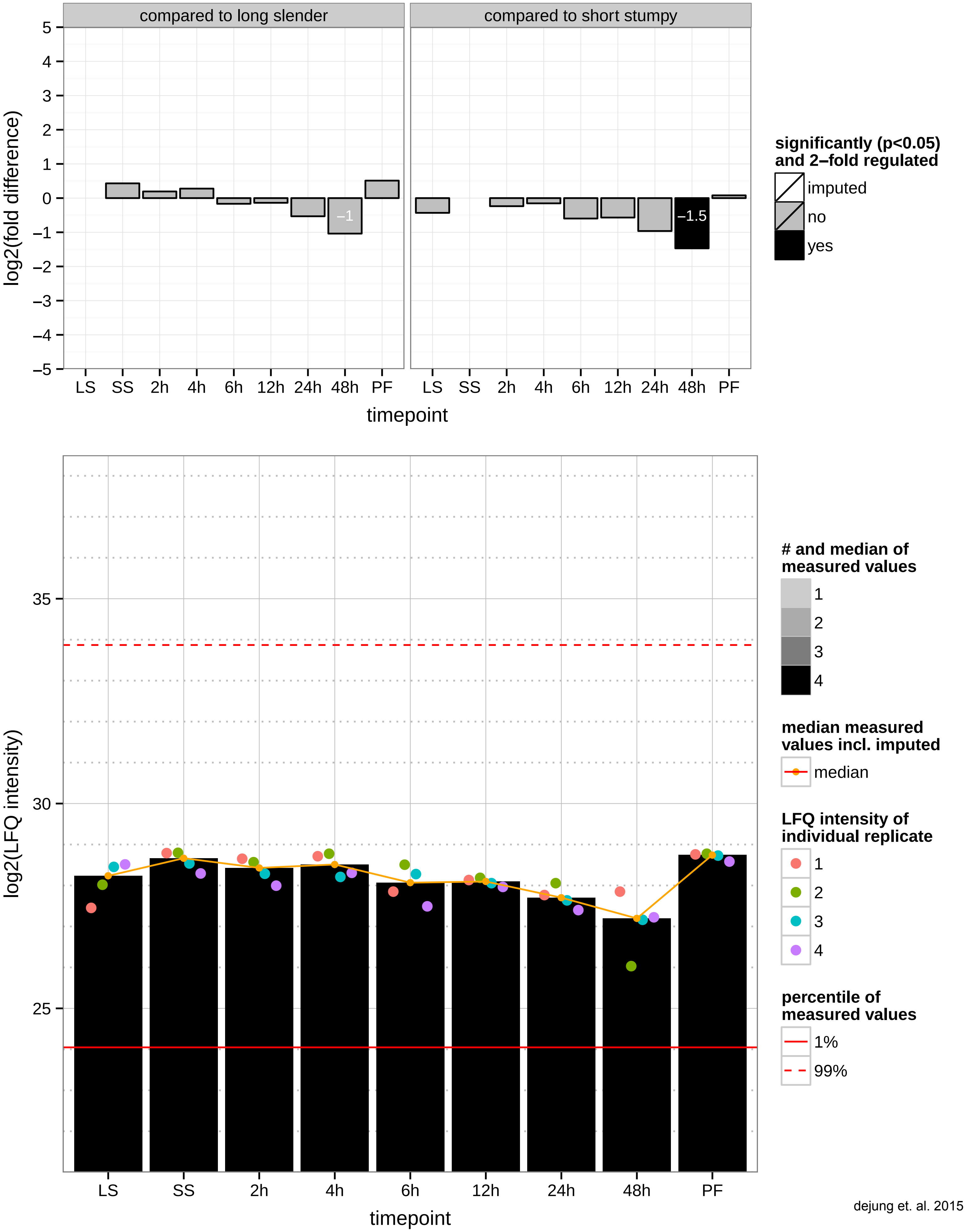

dejung et. al. 2015

hypothetical protein, conserved
Tb927.6.3610
AGOF: null
AGOC: mitochondrion
AGOP: null
PGOF: protein binding
PGOC: null
PGOP: null

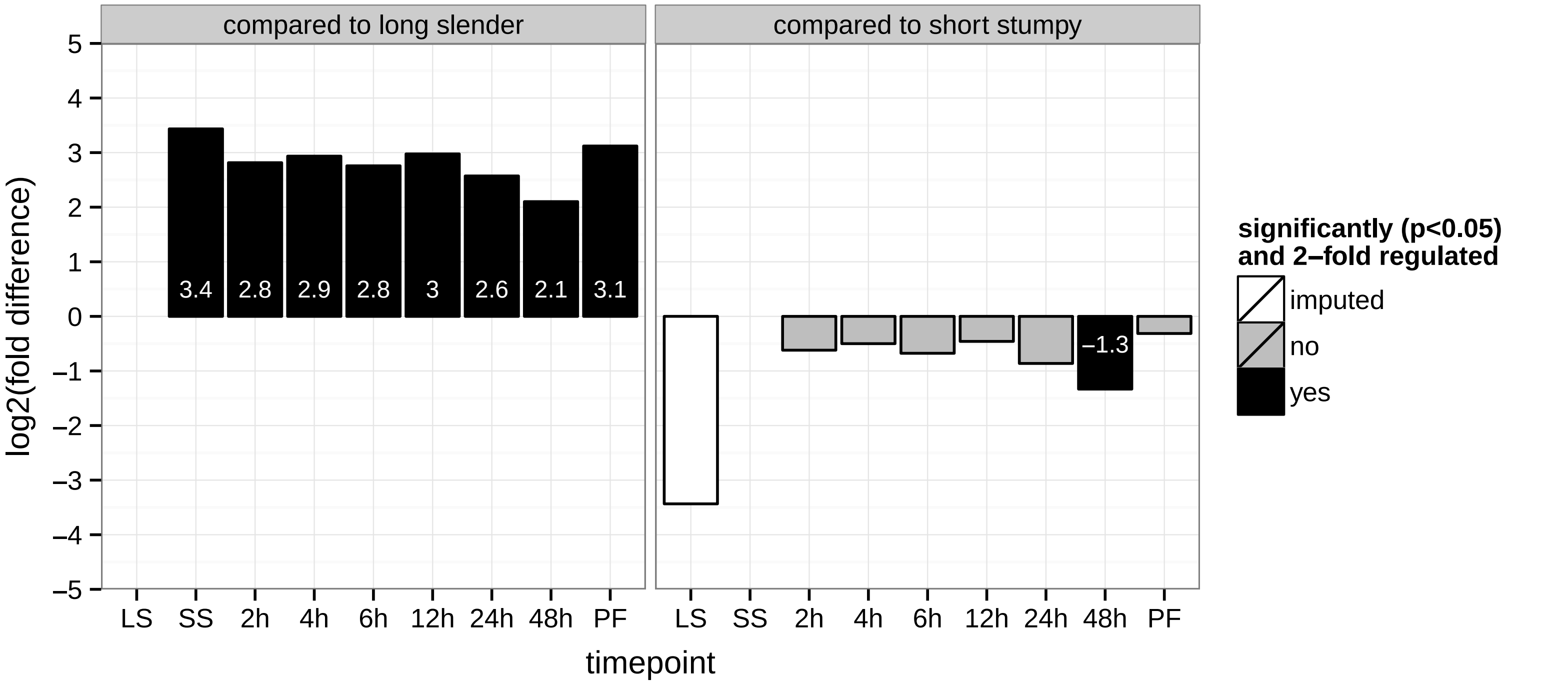

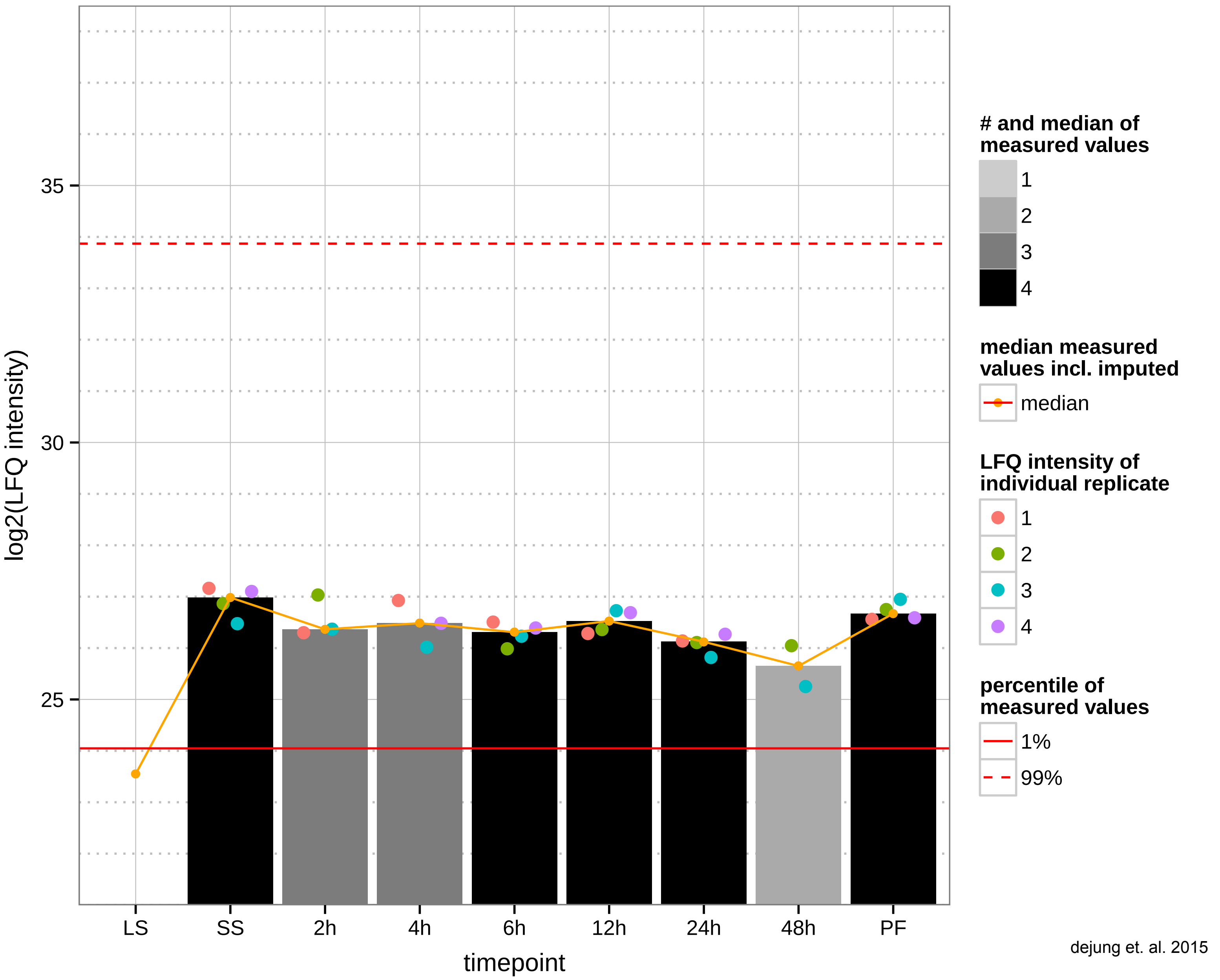

dejung et. al. 2015

peptidase M20/M25/M40, putative
Tb927.6.400
AGOF: metallopeptidase activity
AGOC: null
AGOP: proteolysis
PGOF: hydrolase activity
PGOC: null
PGOP: metabolic process

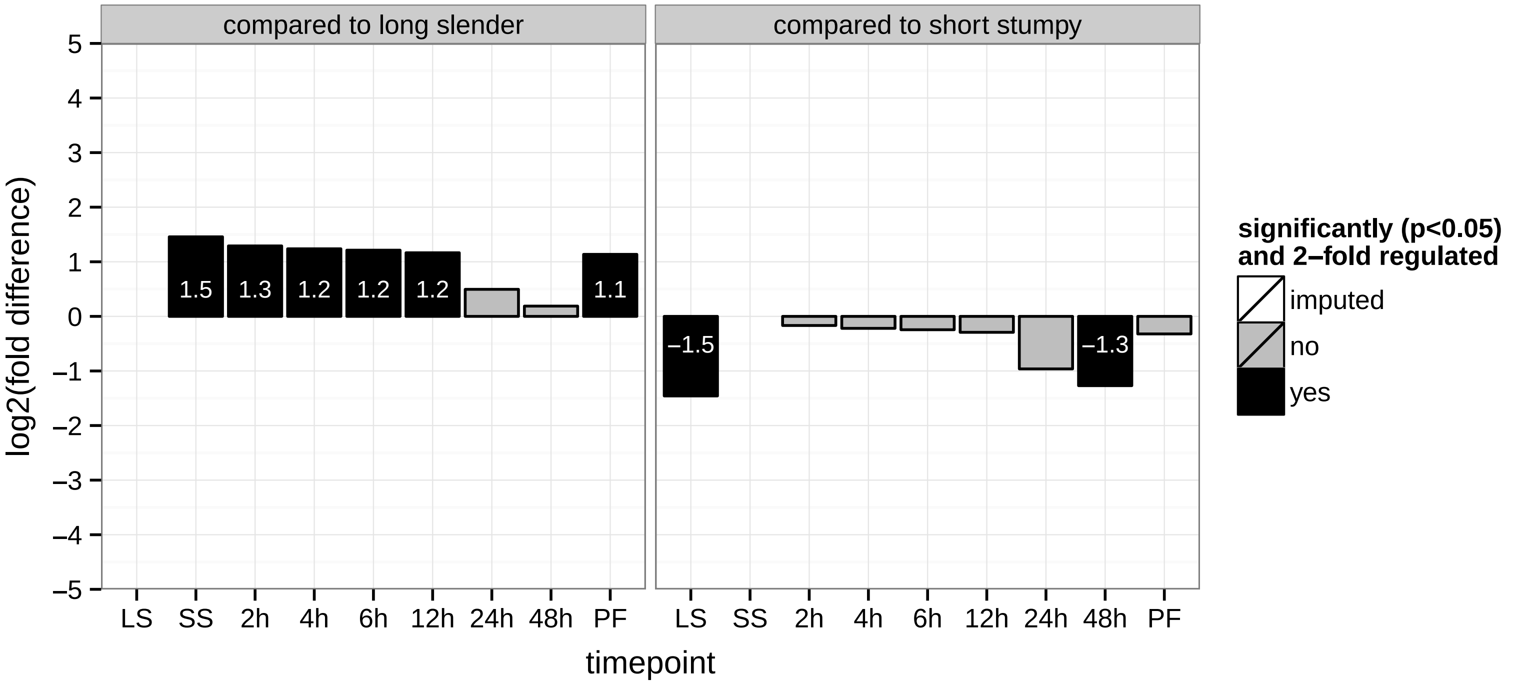

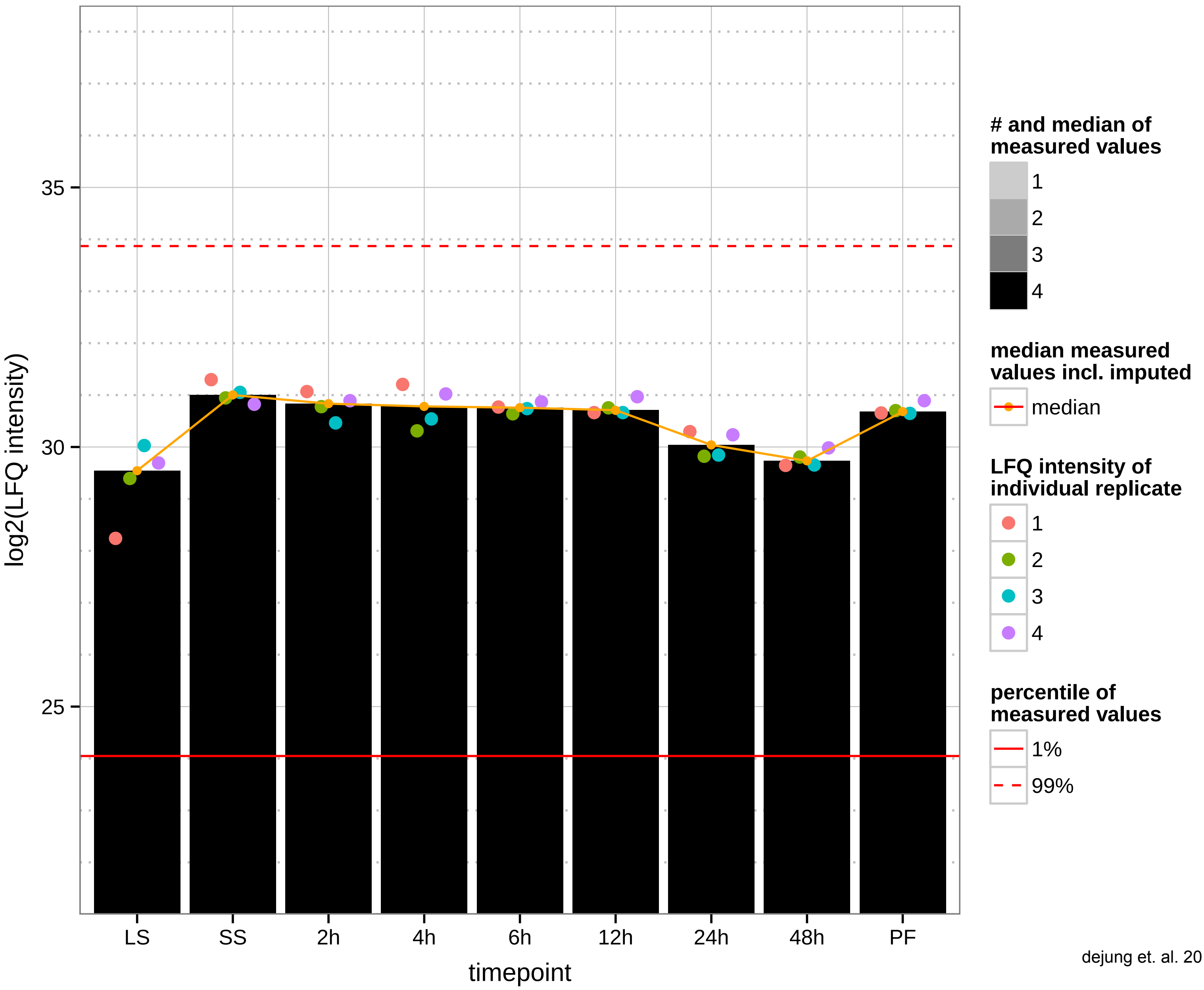

dejung et. al. 2015

hypothetical protein, conserved
Tb927.6.5010
AGOF: null
AGOC: null
AGOP: null
PGOF: null
PGOC: null
PGOP: null

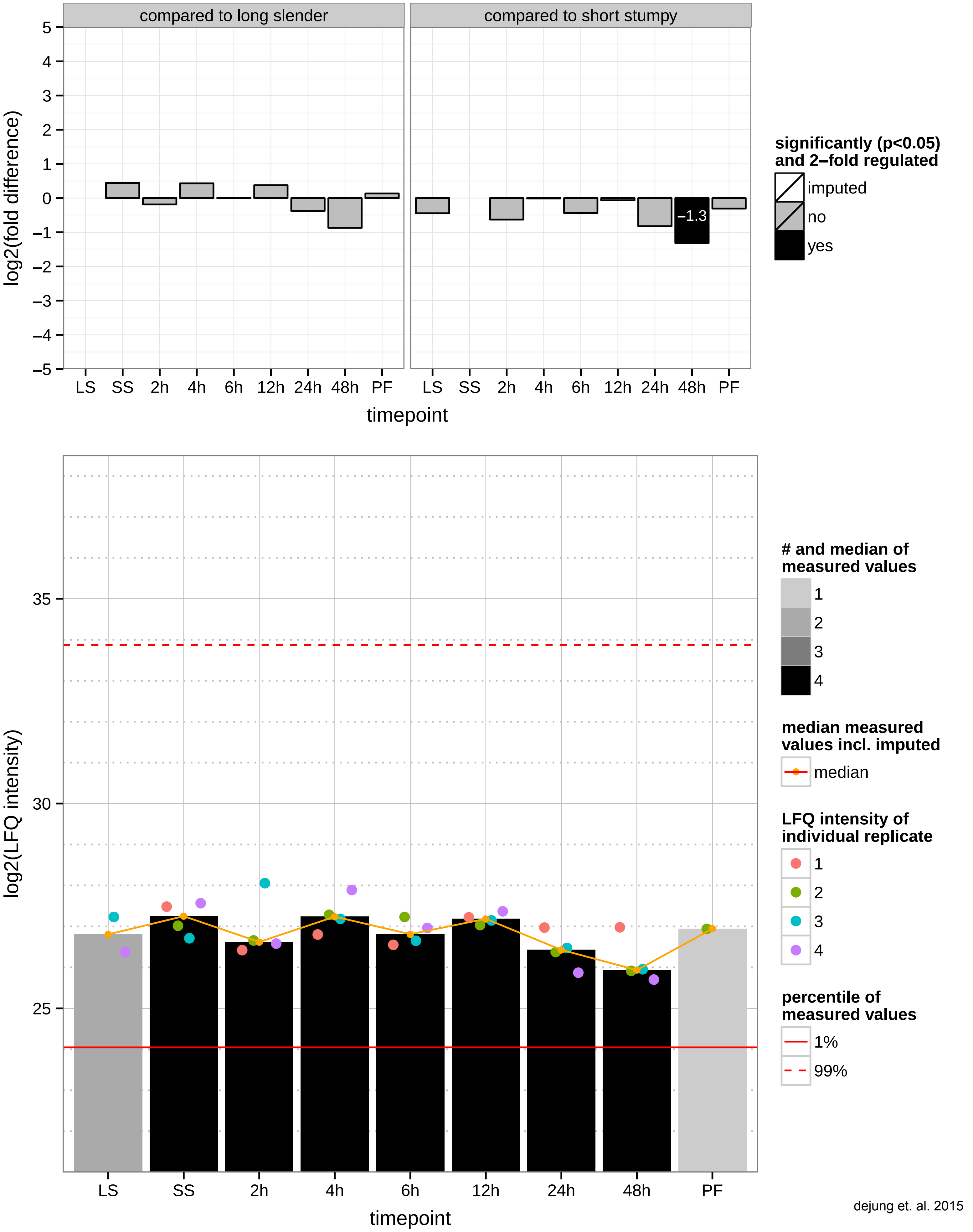

dejung et. al. 2015

hypothetical protein, conserved
Tb927.7.2950
AGOF: ATP binding, nucleoside−triphosphatase activity
AGOC: null
AGOP: null
PGOF: ATP binding, nucleoside−triphosphatase activity, nucleotide binding
PGOC: null
PGOP: null

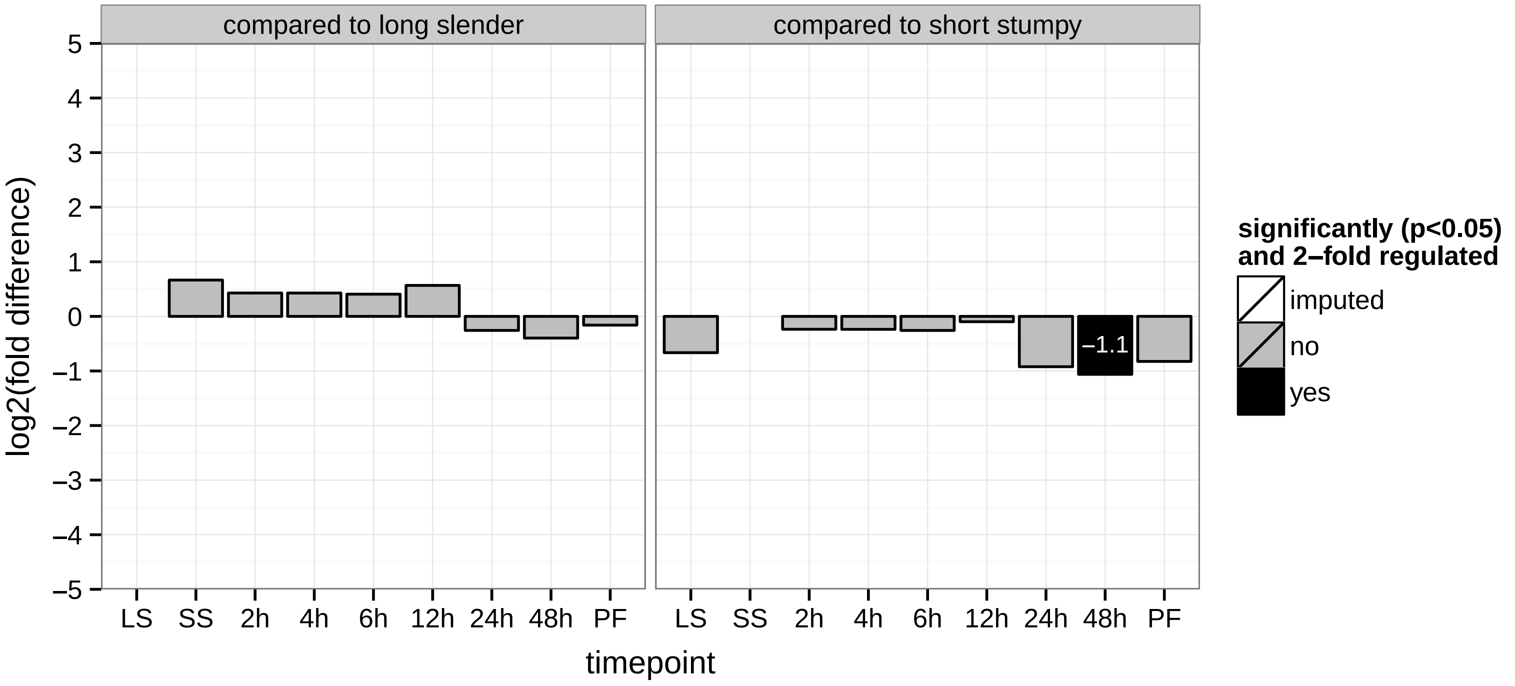

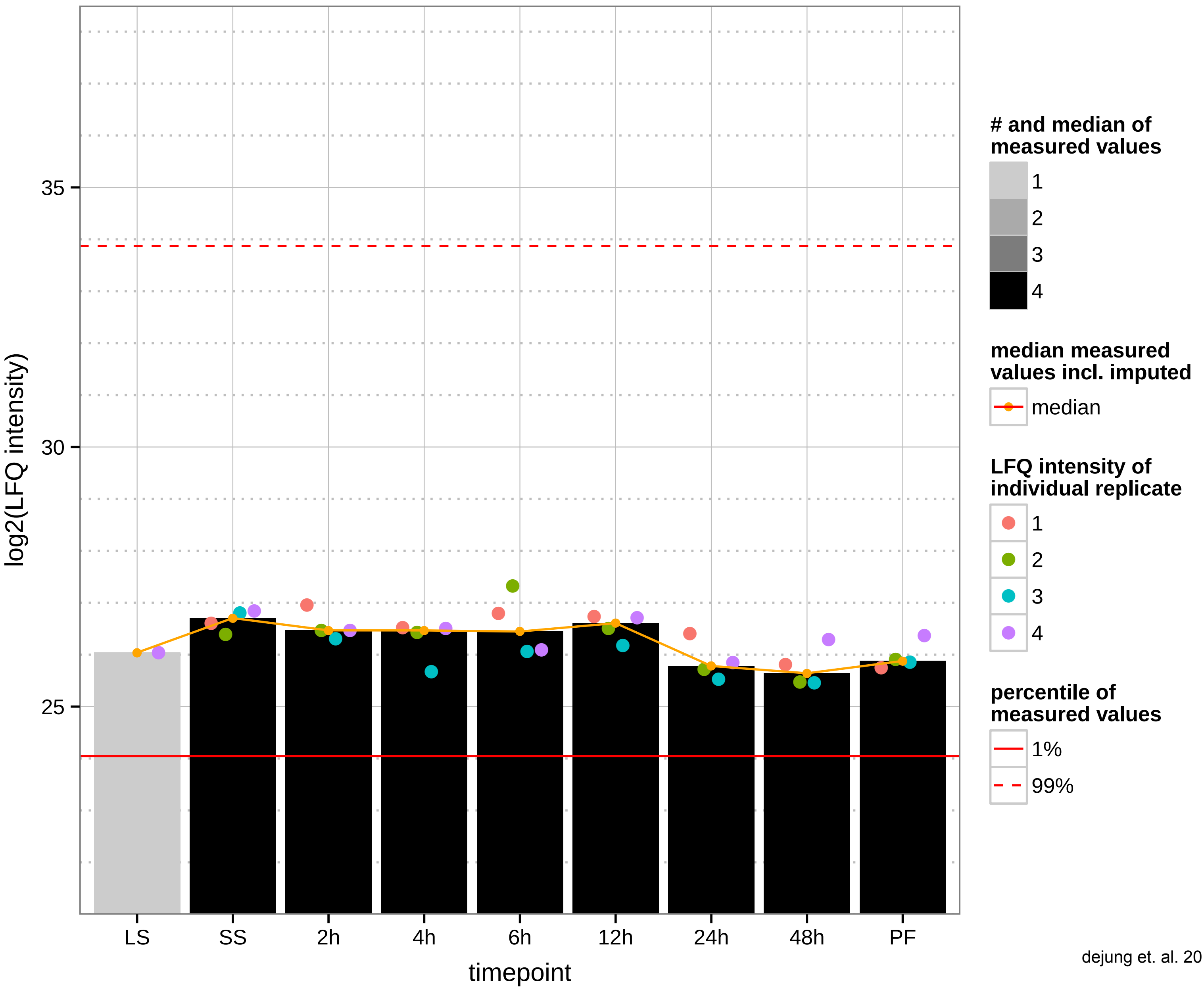

dejung et. al. 2015

I/6 autoantigen
Tb927.7.3450
AGOF: calcium ion binding, structural constituent of cytoskeleton
AGOC: microtubule cytoskeleton
AGOP: null
PGOF: null
PGOC: null
PGOP: null

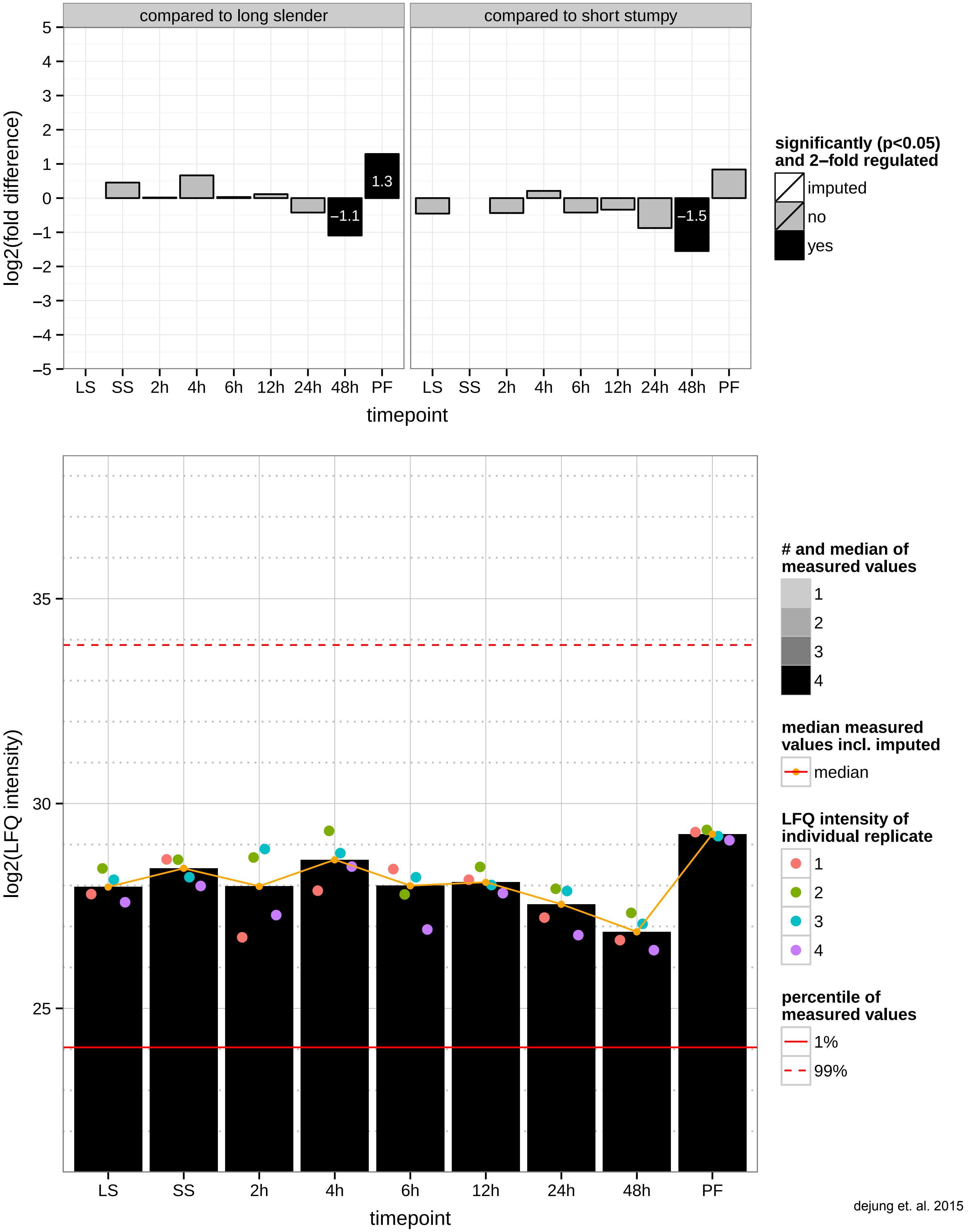

dejung et. al. 2015

hypothetical protein, conserved
Tb927.7.590
AGOF: null
AGOC: null
AGOP: null
PGOF: null
PGOC: null
PGOP: null

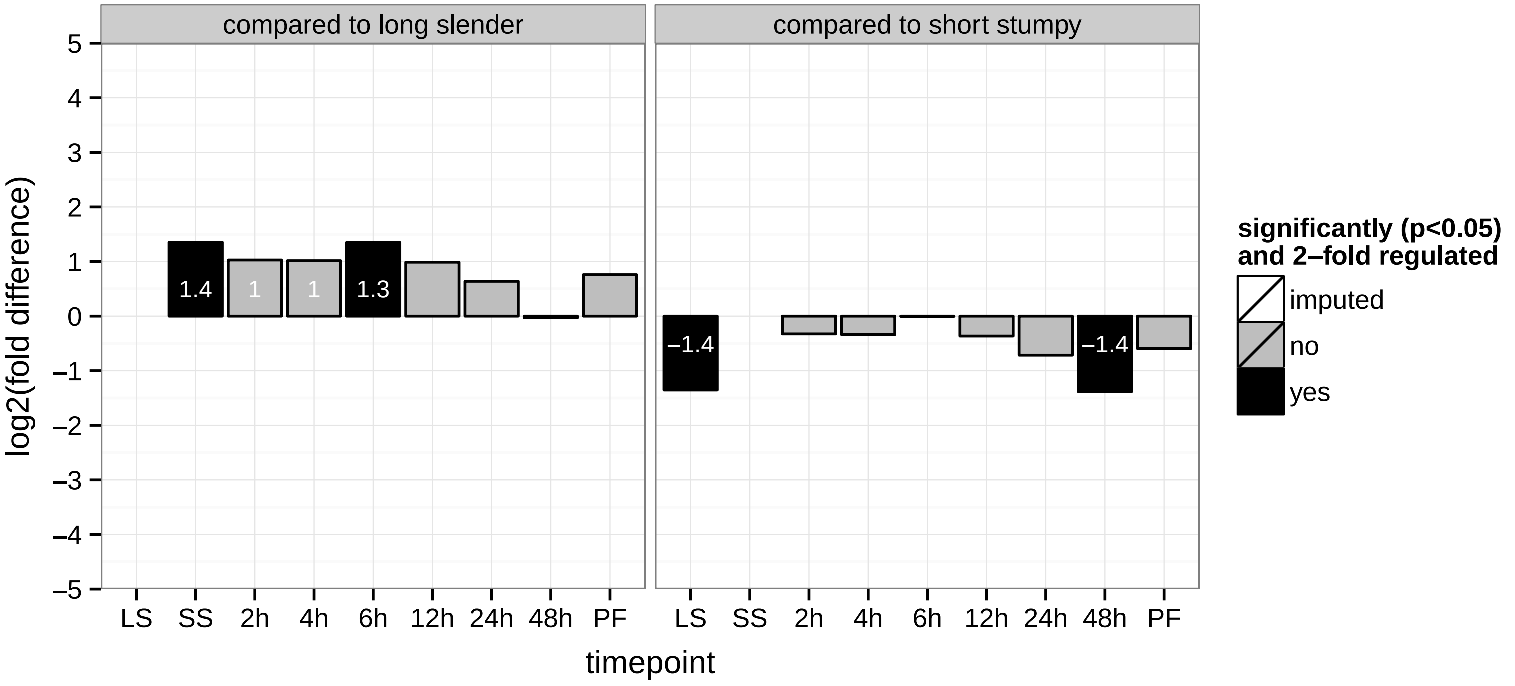

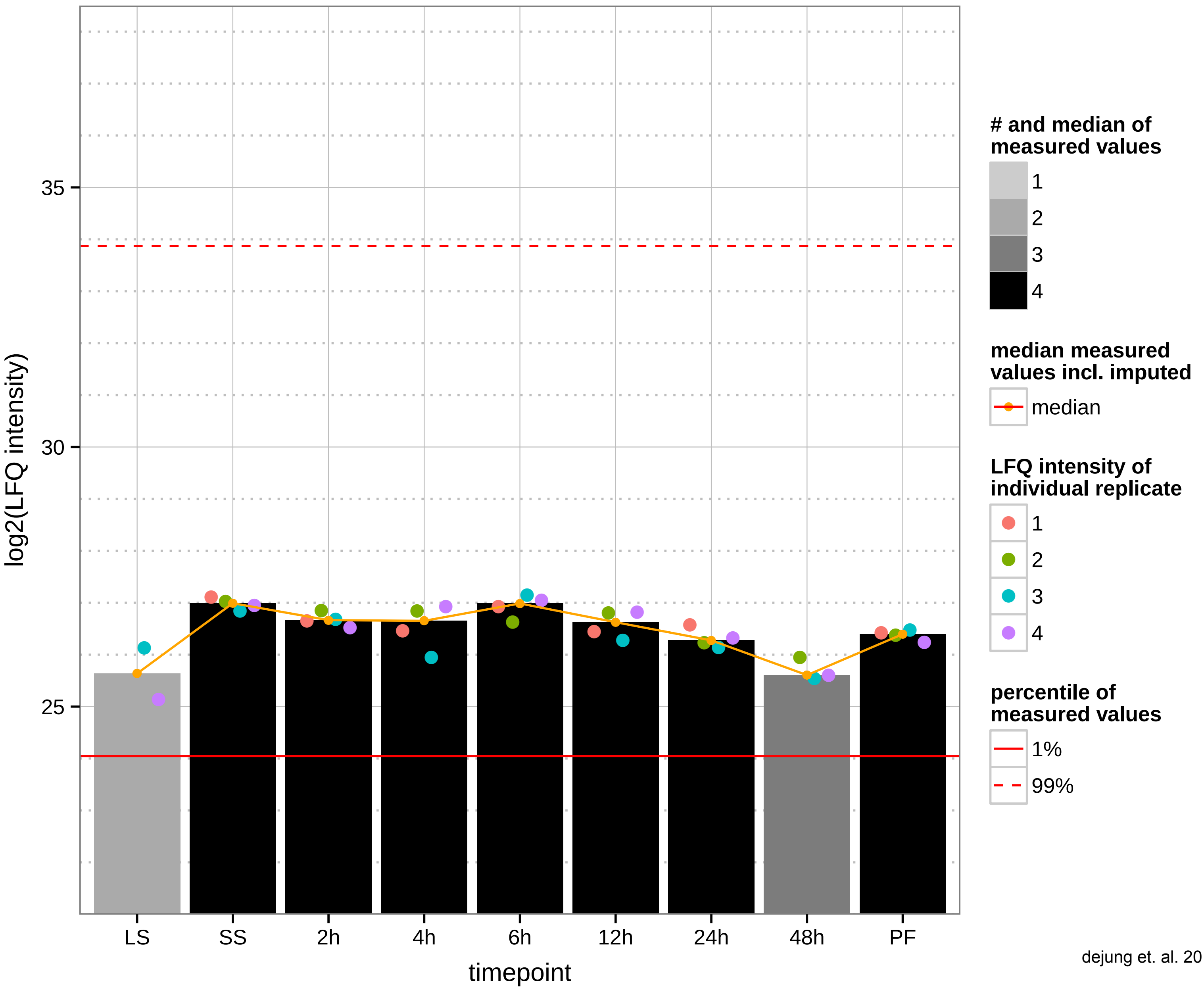

dejung et. al. 2015

hypothetical protein, conserved
Tb927.8.1210
AGOF: DNA topoisomerase type I activity
AGOC: chromosome
AGOP: DNA topological change
PGOF: null
PGOC: null
PGOP: null

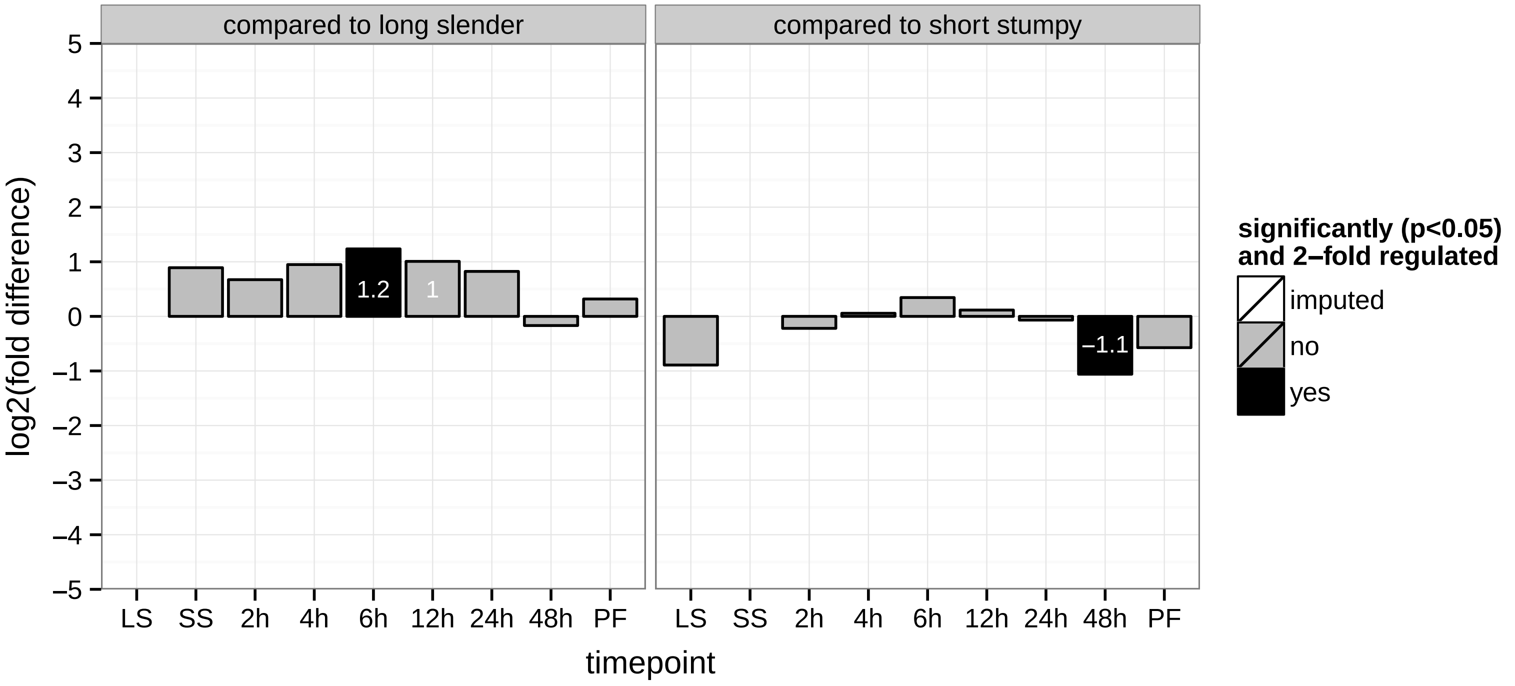

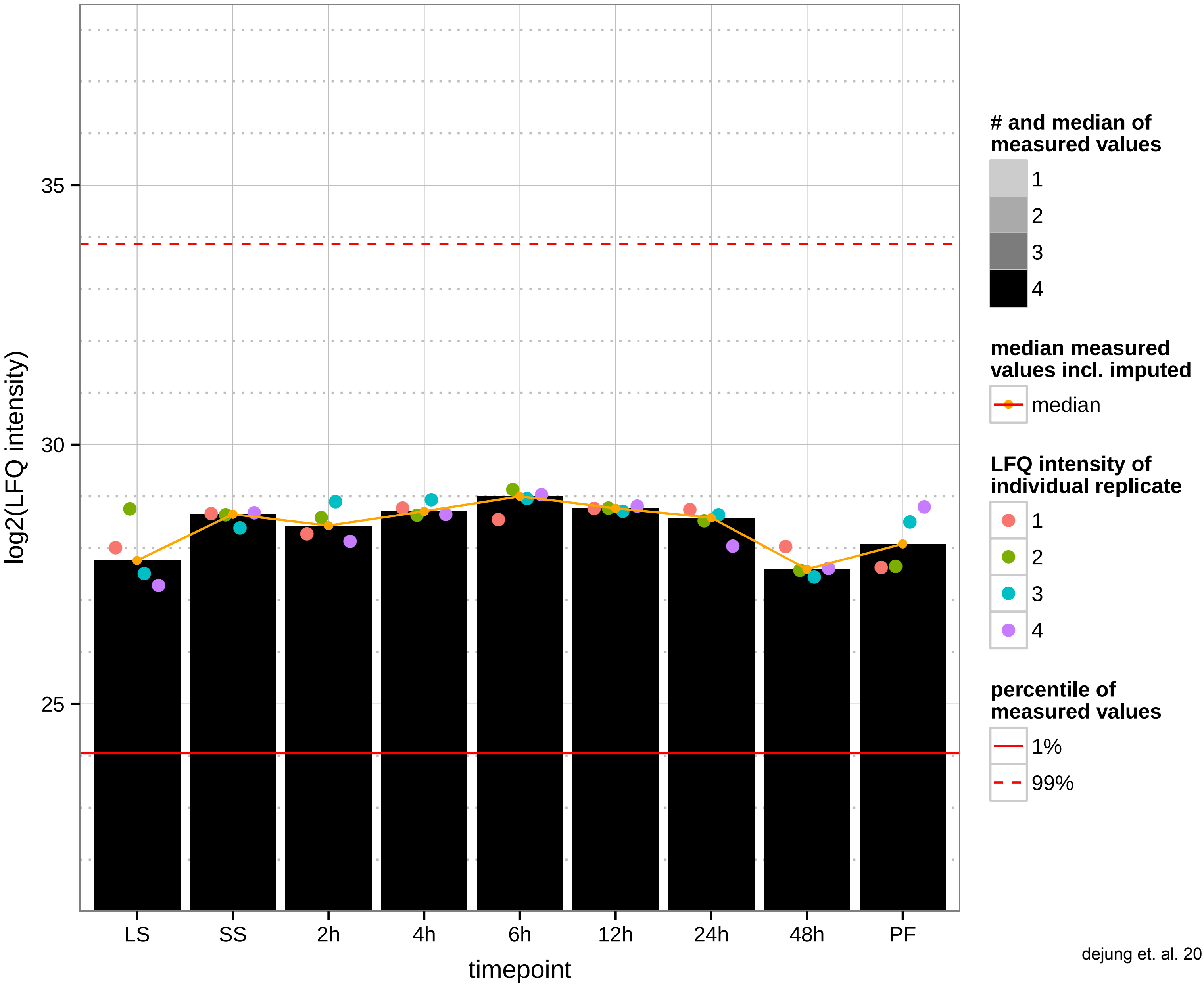

dejung et. al. 2015

Golgi/lysosome glycoprotein 1 (tGLP1)
Tb927.8.1870
AGOF: null
AGOC: Golgi apparatus, lysosomal membrane
AGOP: null
PGOF: null
PGOC: null
PGOP: null

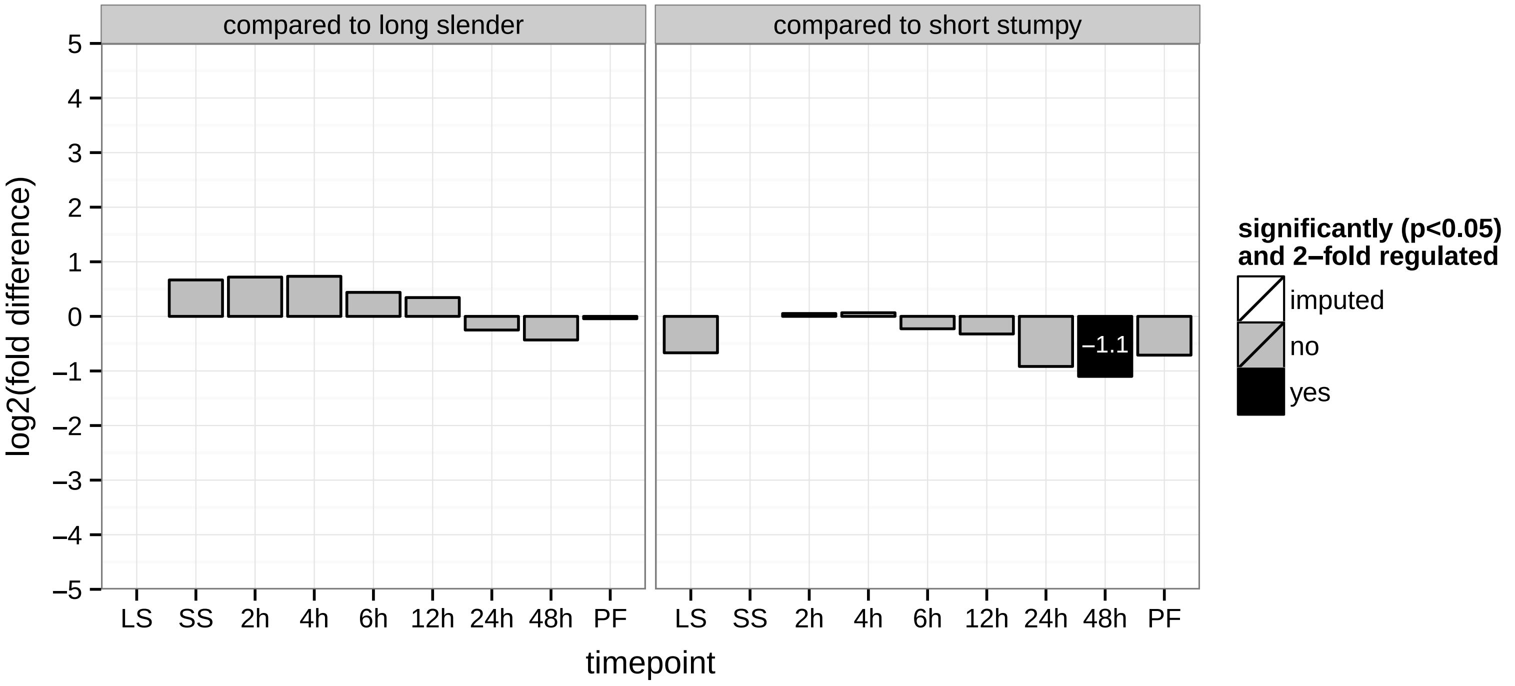

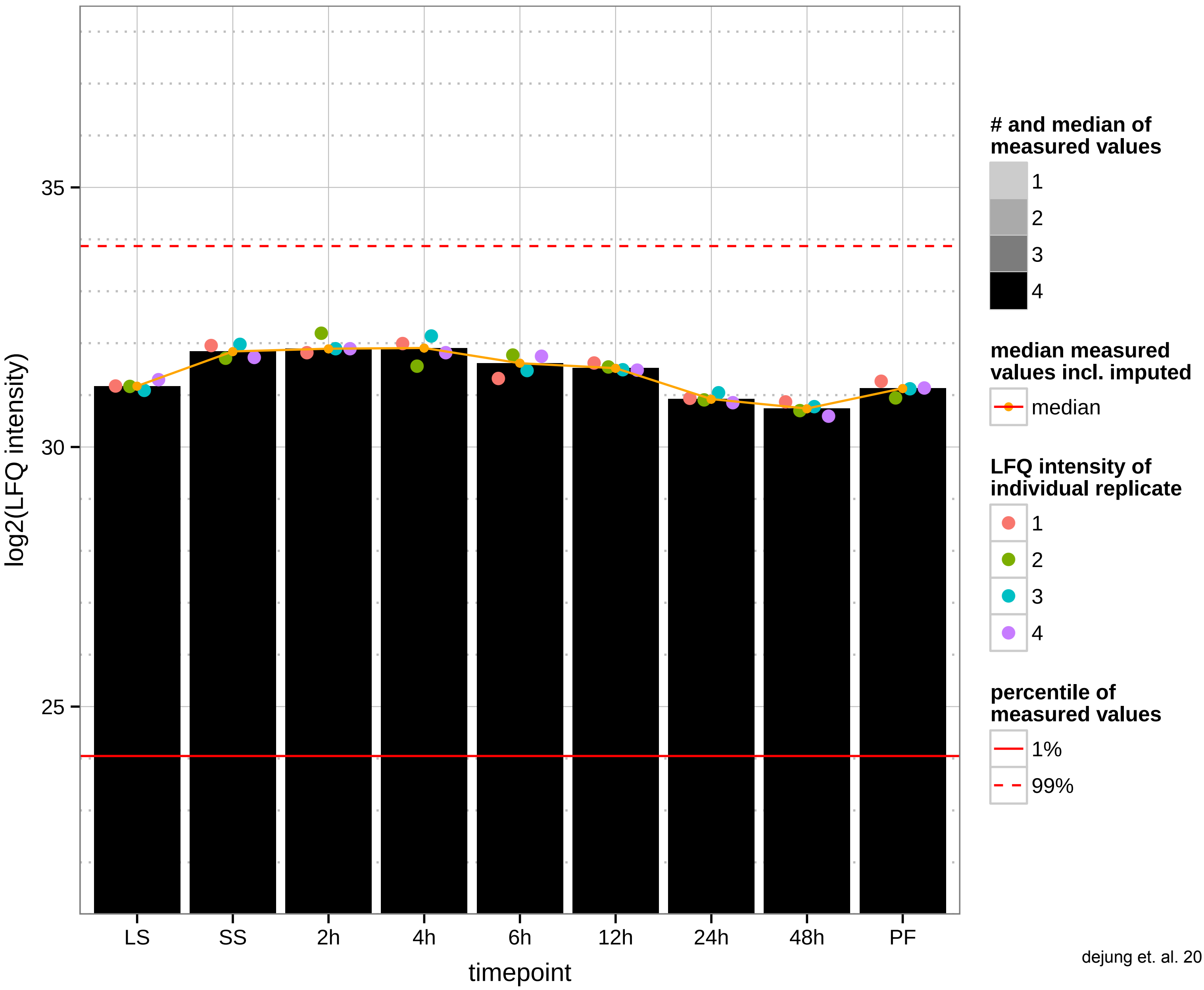

dejung et. al. 2015

coronin, putative (CRN12)
Tb927.8.3100
AGOF: actin binding, kinesin binding, microtubule binding
AGOC: cell surface, cilium basal body, flagellar pocket
AGOP: microtubule cytoskeleton organization, microtubule−based process
PGOF: protein binding
PGOC: null
PGOP: null

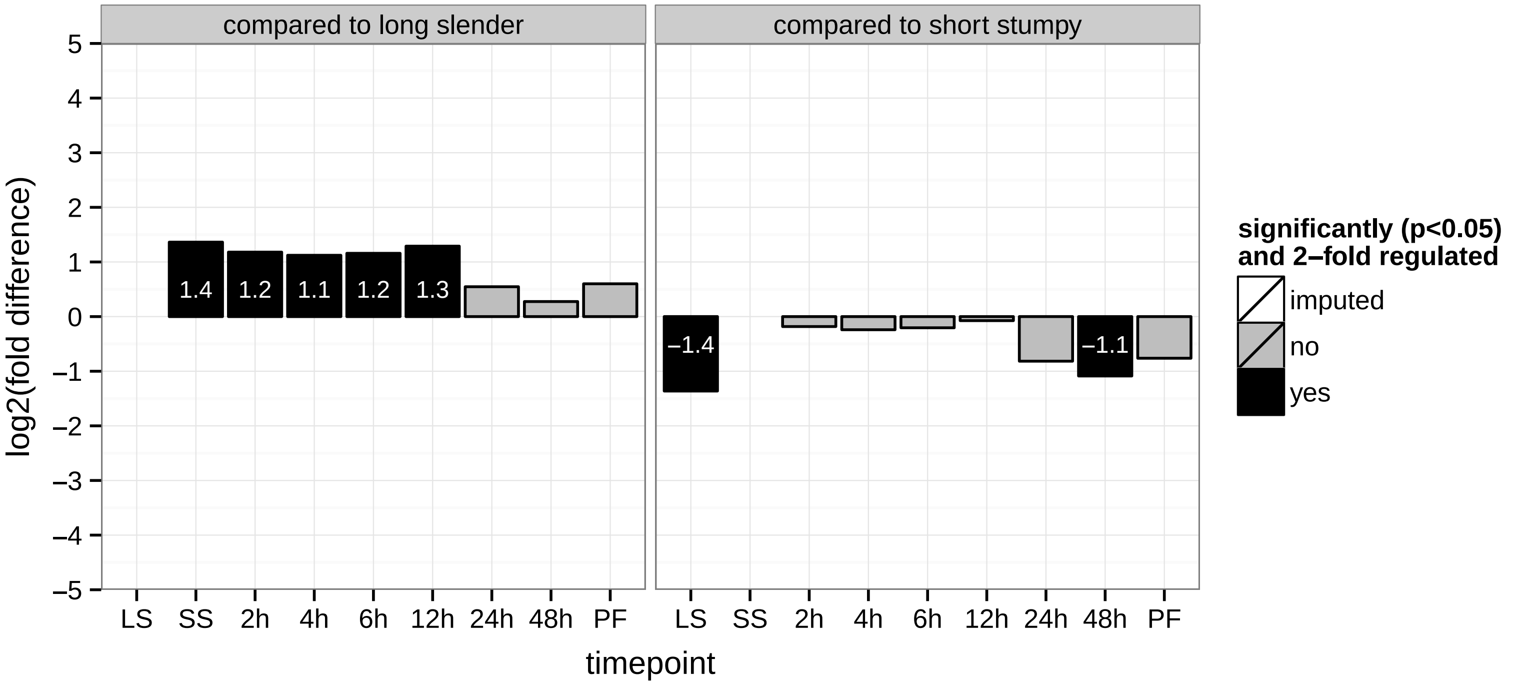

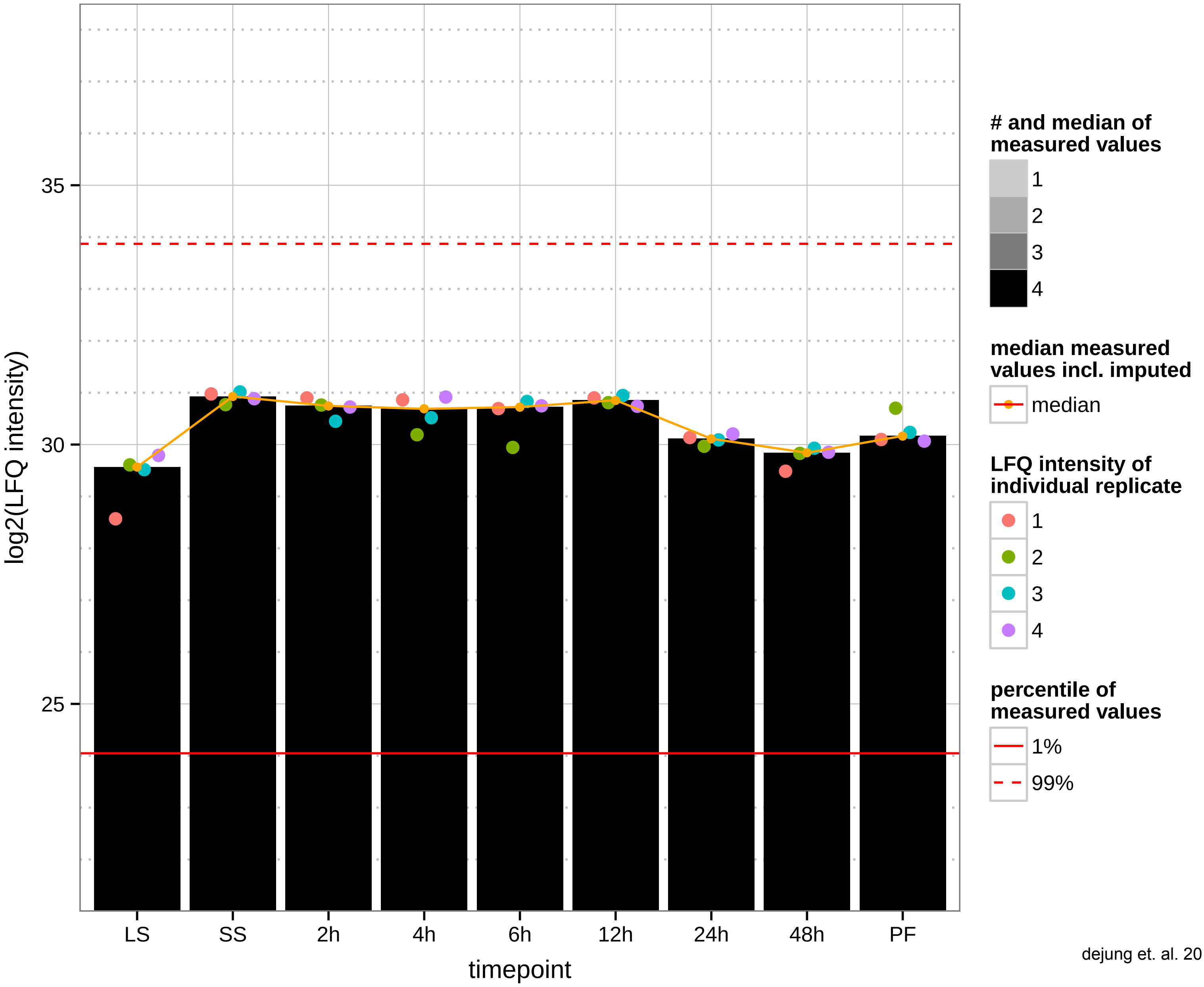

dejung et. al. 2015

nucleoside phosphatase, putative, guanosine diphosphatase
Tb927.8.3800
AGOF: guanosine–diphosphatase activity, nucleoside–diphosphatase activity
AGOC: Golgi membrane, extracellular region
AGOP: cellular protein modification process, nucleoside metabolic process
PGOF: hydrolase activity
PGOC: null
PGOP: null

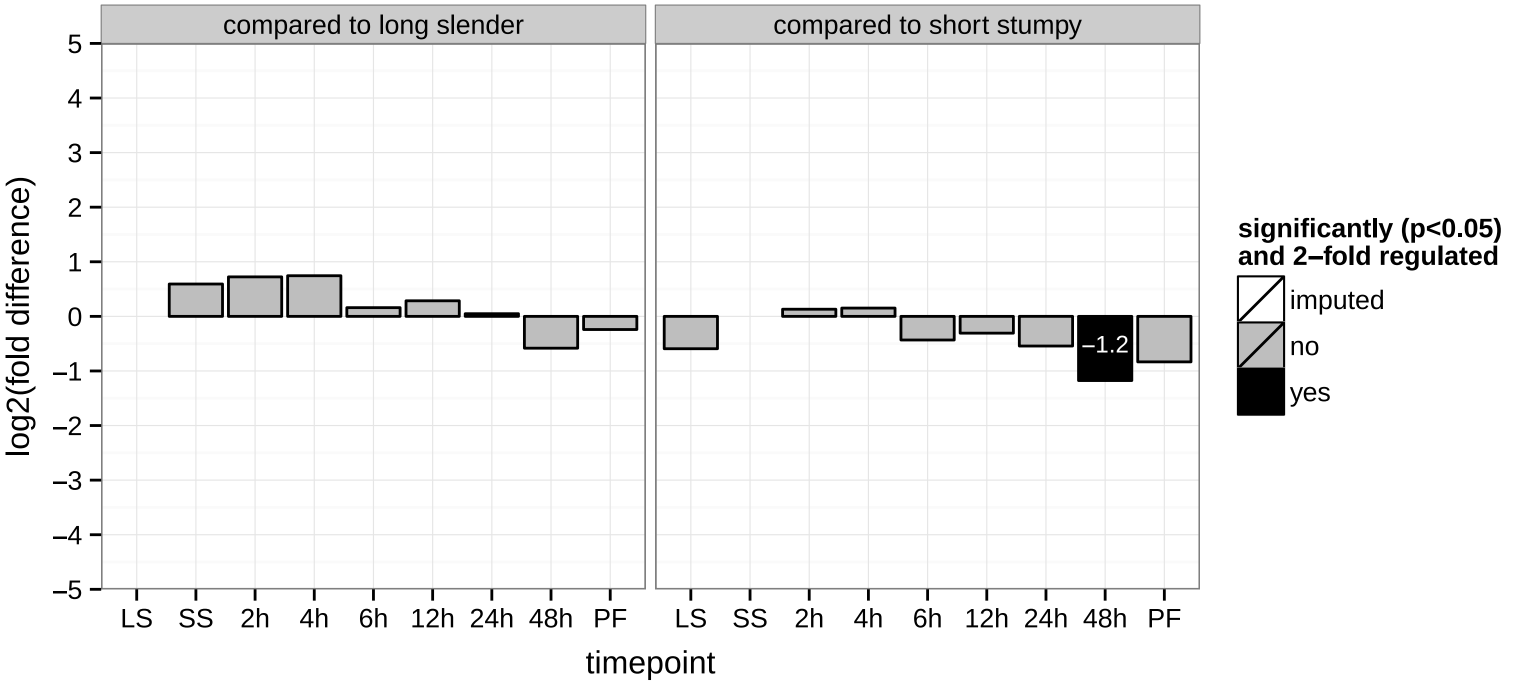

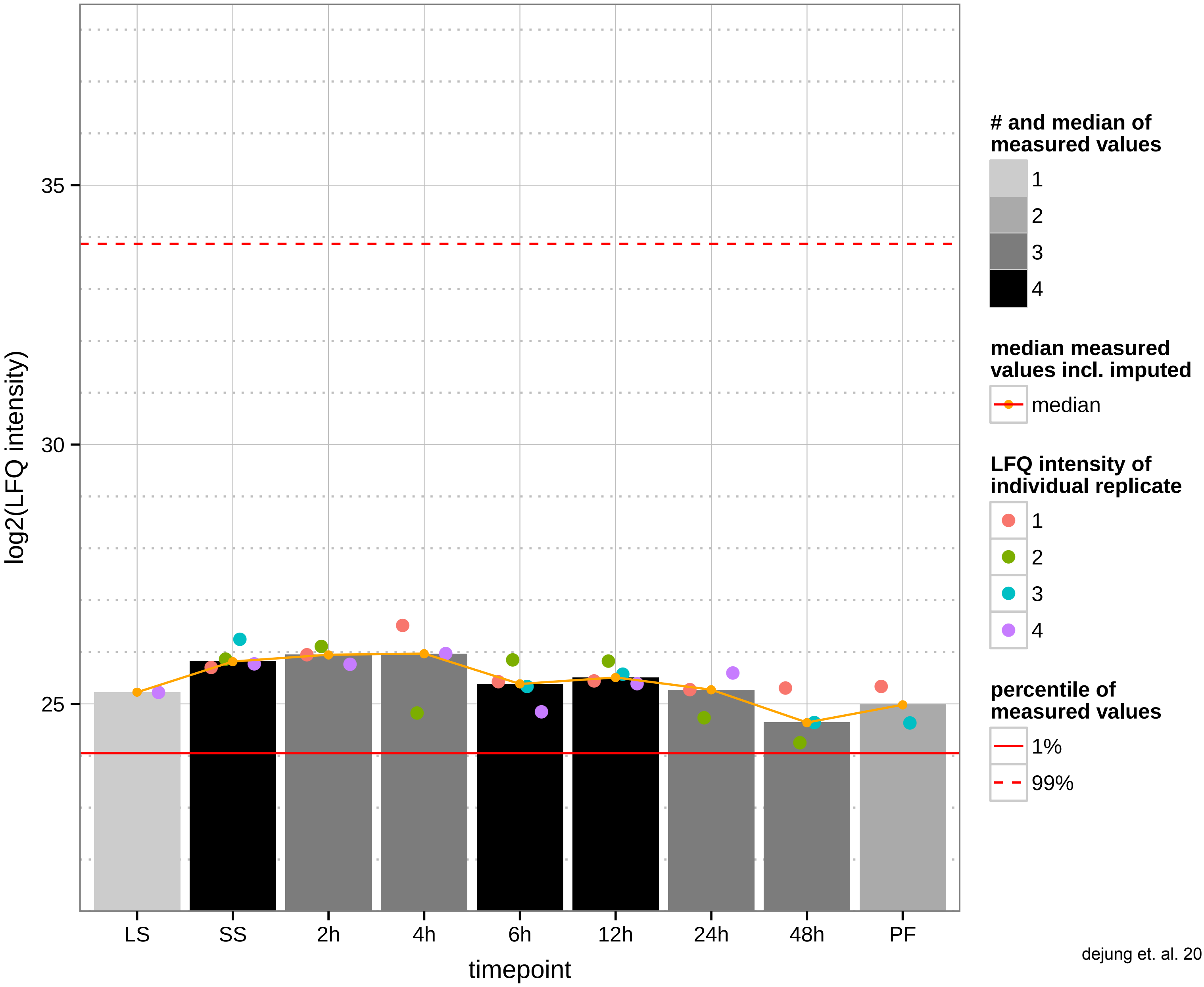

dejung et. al. 2015

hypothetical protein, conserved
Tb927.8.4570
AGOF: zinc ion binding
AGOC: null
AGOP: null
PGOF: protein binding, zinc ion binding
PGOC: null
PGOP: null

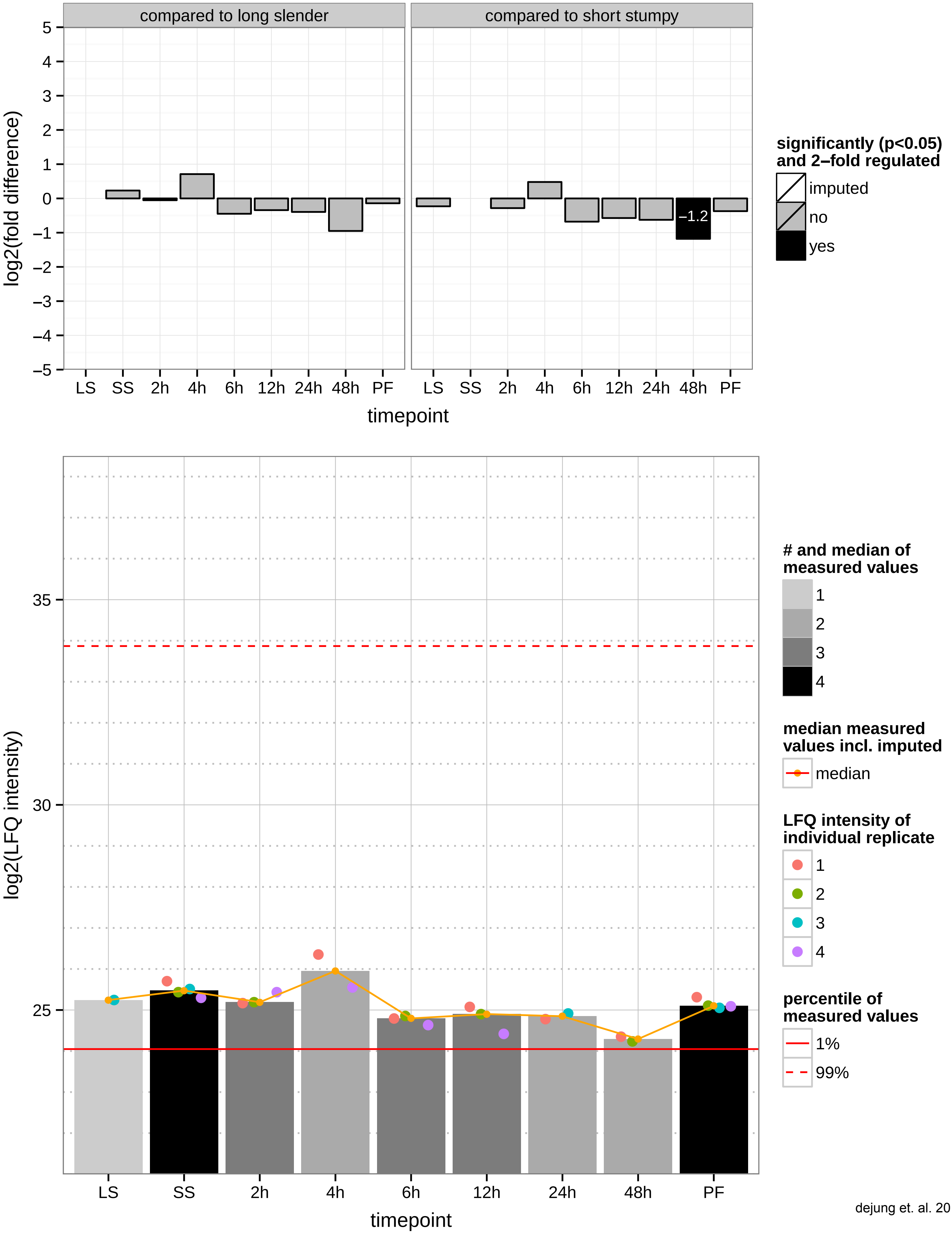

dejung et. al. 2015

hypothetical protein, conserved
Tb927.8.5760
AGOF: null
AGOC: null
AGOP: null
PGOF: protein binding
PGOC: null
PGOP: null

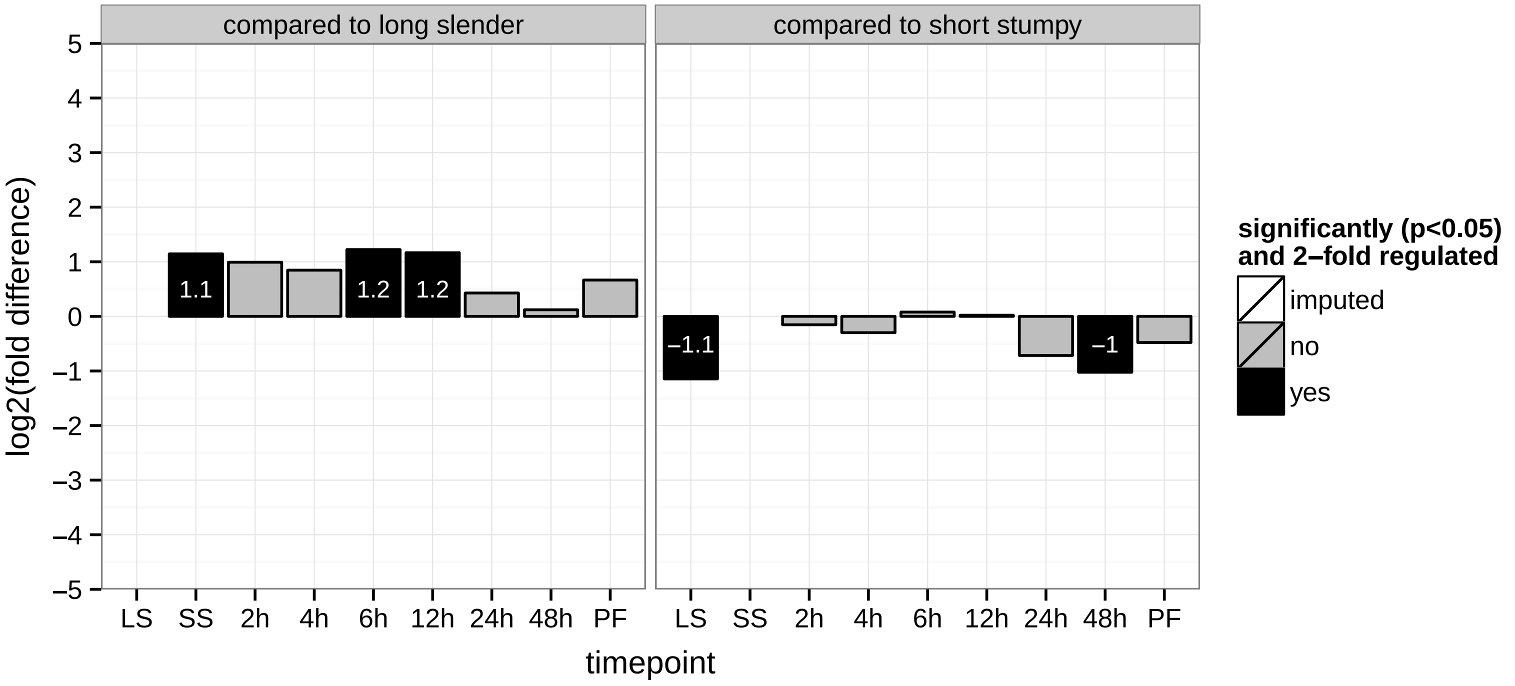

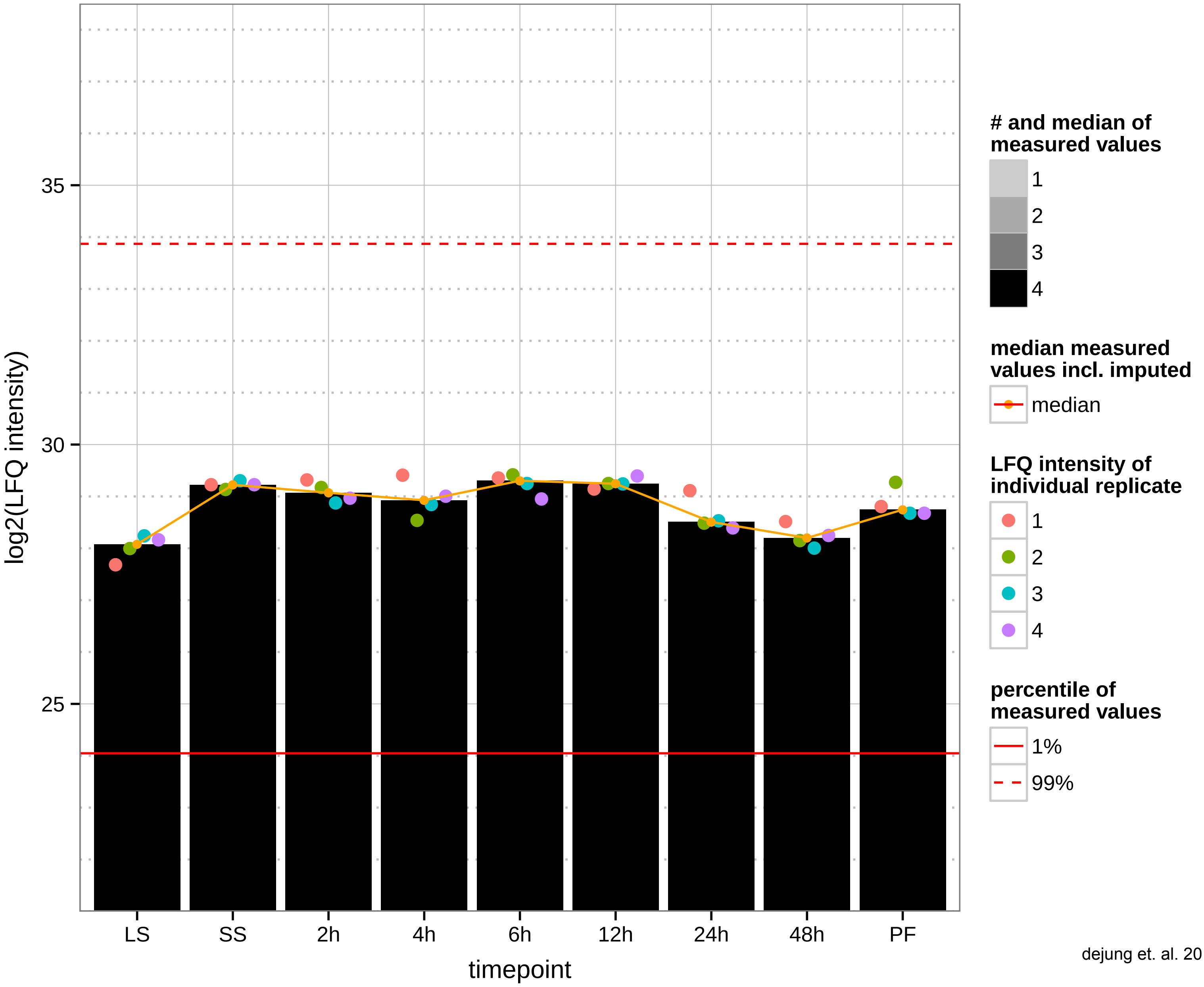

dejung et. al. 2015

hypothetical protein, conserved
Tb927.8.7270
AGOF: null
AGOC: null
AGOP: null
PGOF: null
PGOC: null
PGOP: null

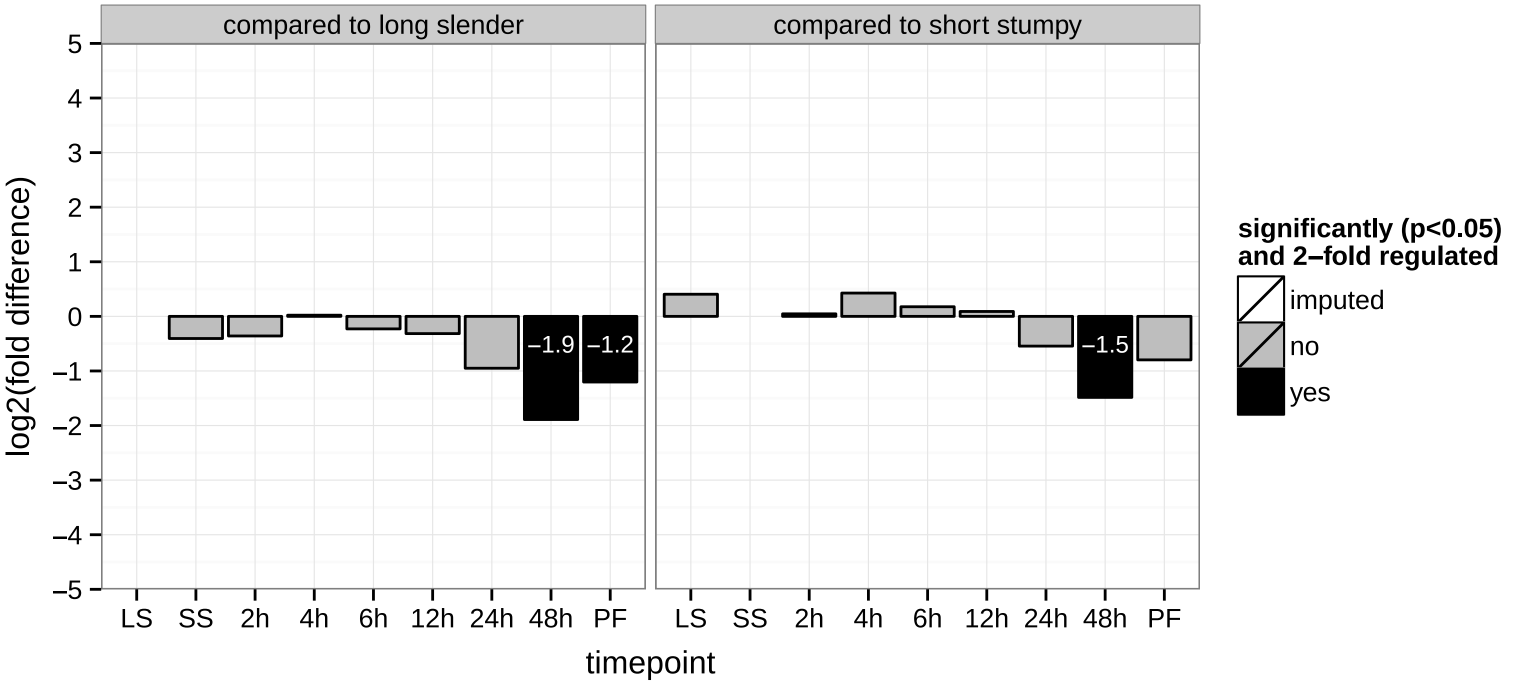

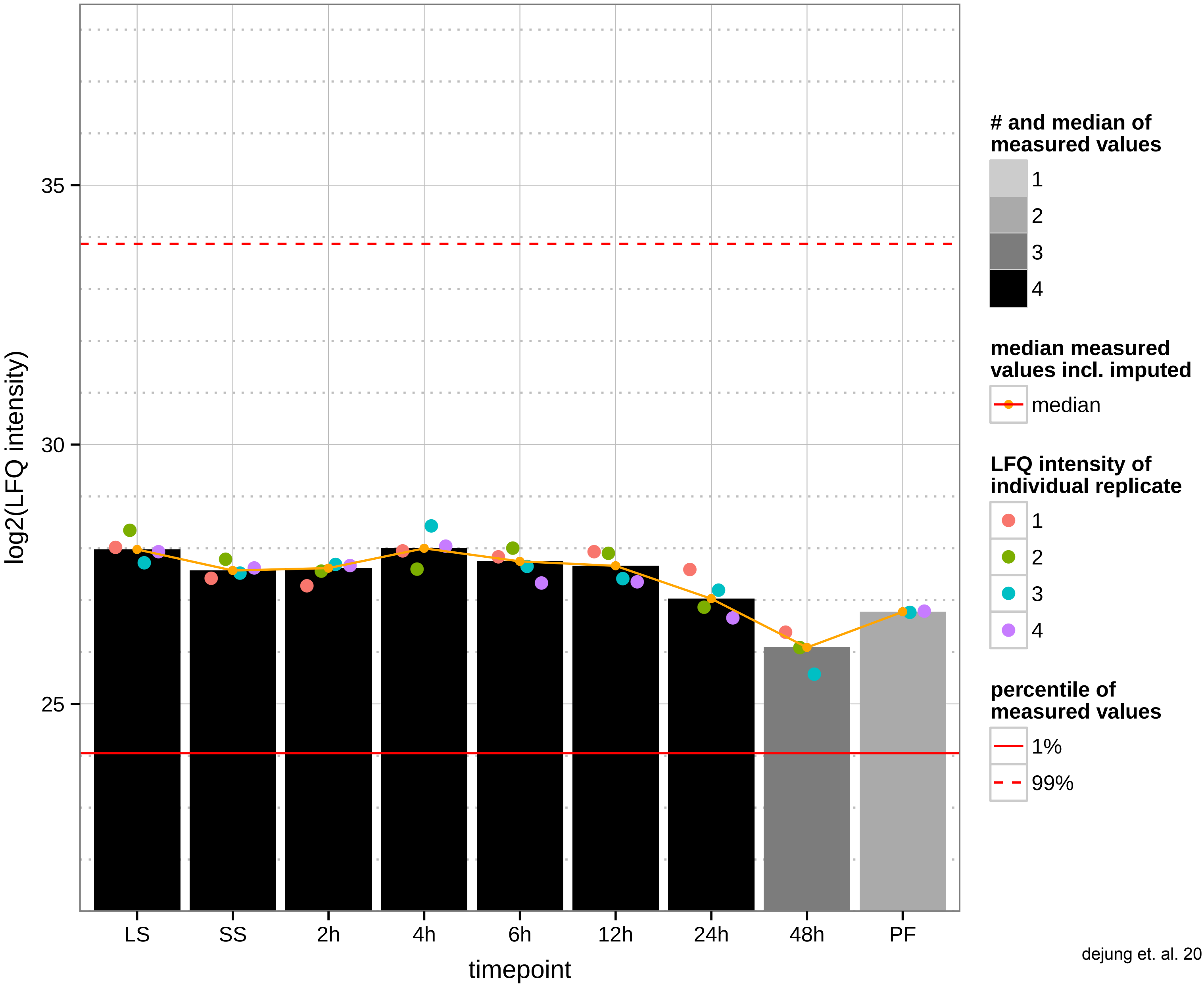

dejung et. al. 2015

calpain, putative, cysteine peptidase
Tb927.8.8330
AGOF: calcium−dependent cysteine−type endopeptidase activity
AGOC: intracellular
AGOP: proteolysis
PGOF: calcium−dependent cysteine−type endopeptidase activity
PGOC: intracellular
PGOP: proteolysis

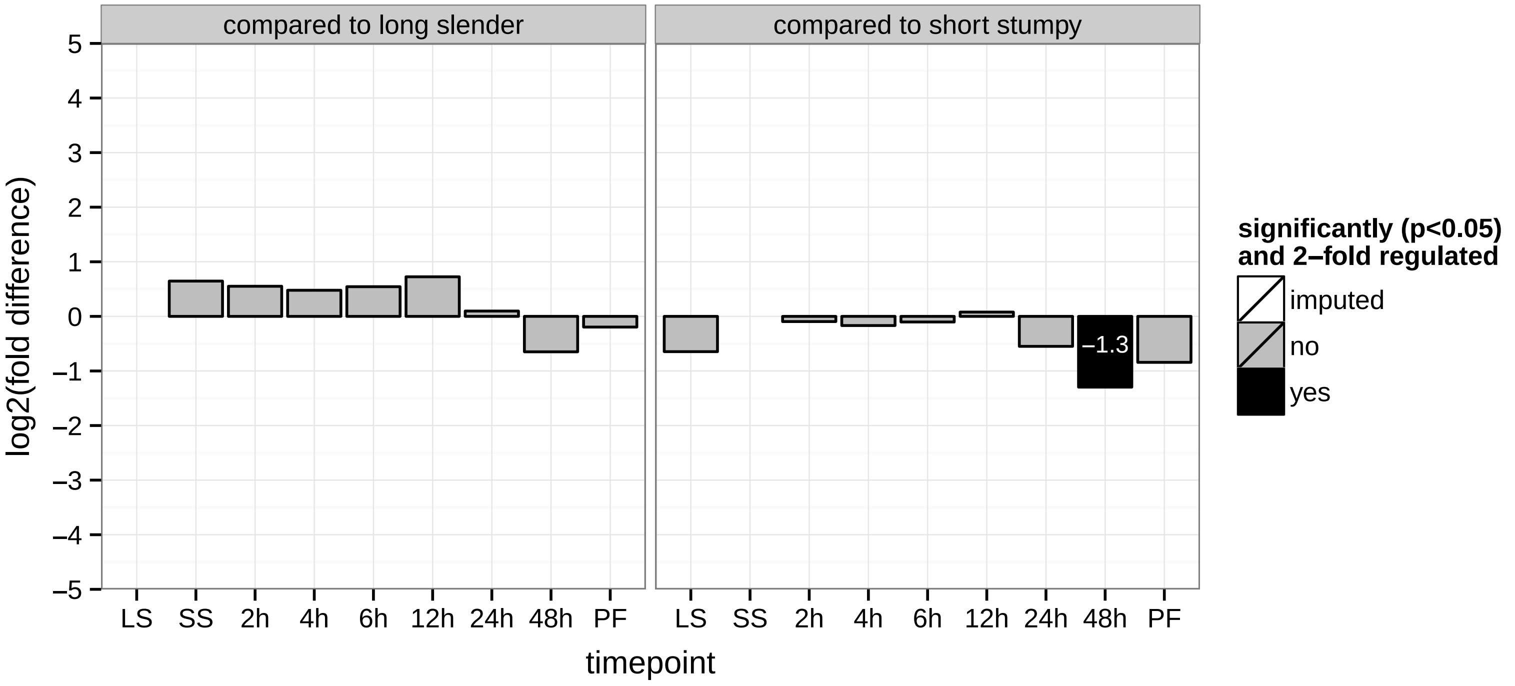

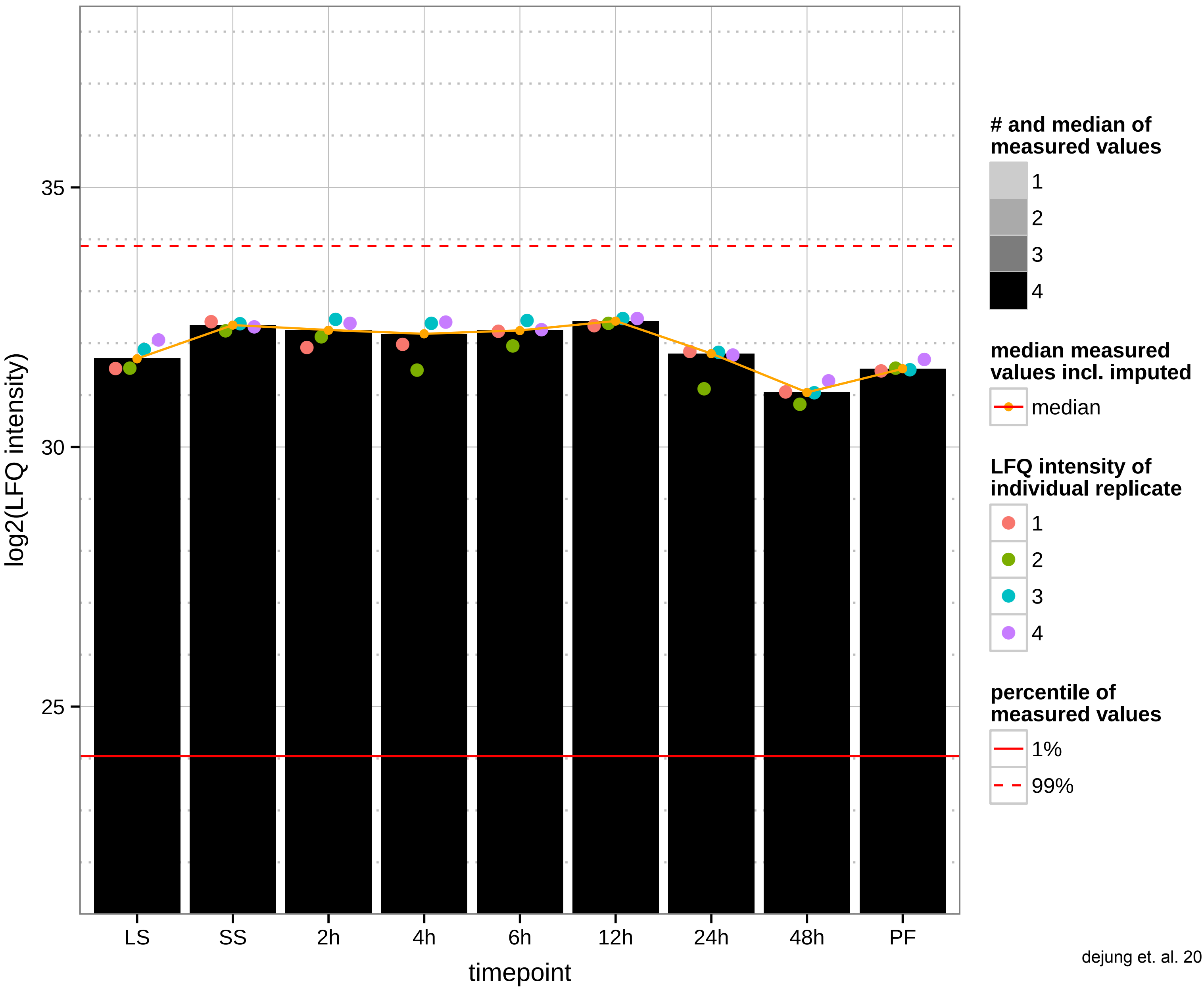

dejung et. al. 2015

hypothetical protein, conserved
Tb927.9.11640
AGOF: null
AGOC: null
AGOP: null
PGOF: null
PGOC: null
PGOP: null

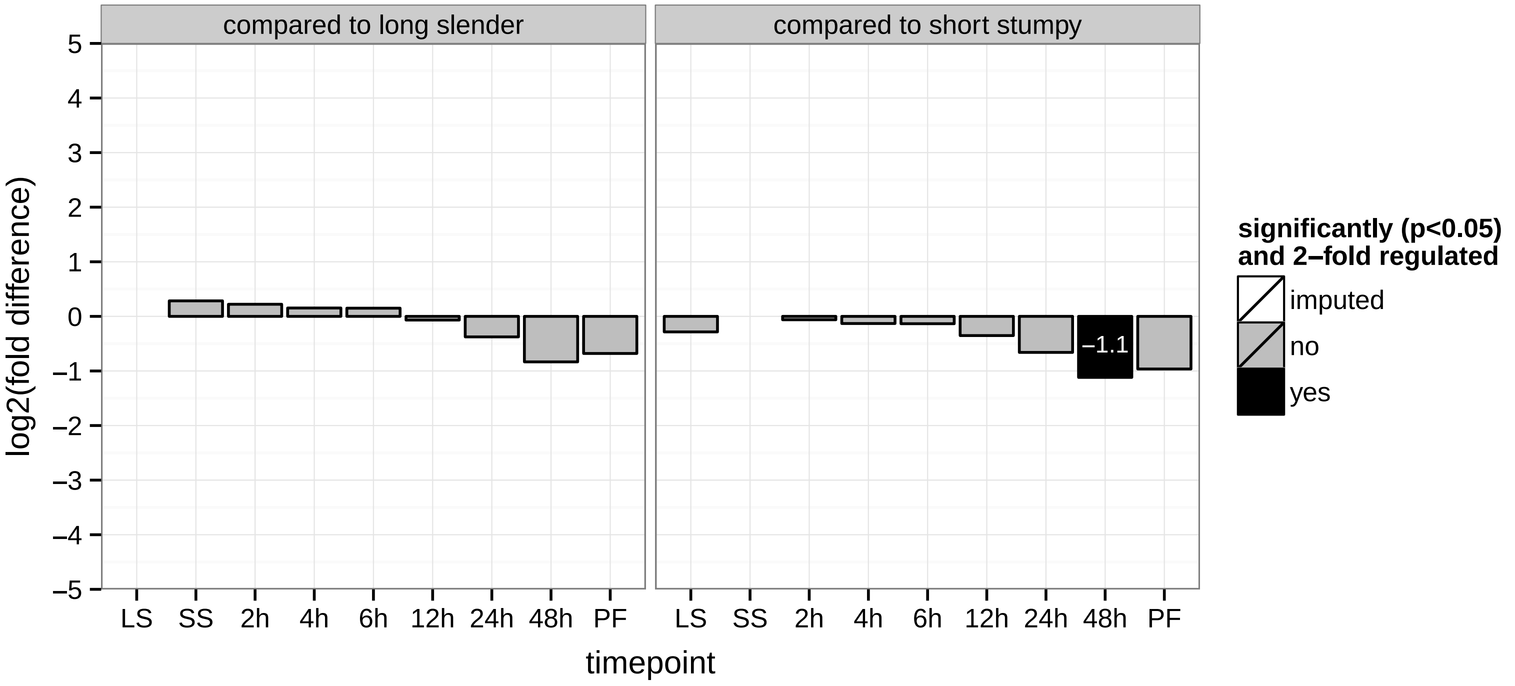

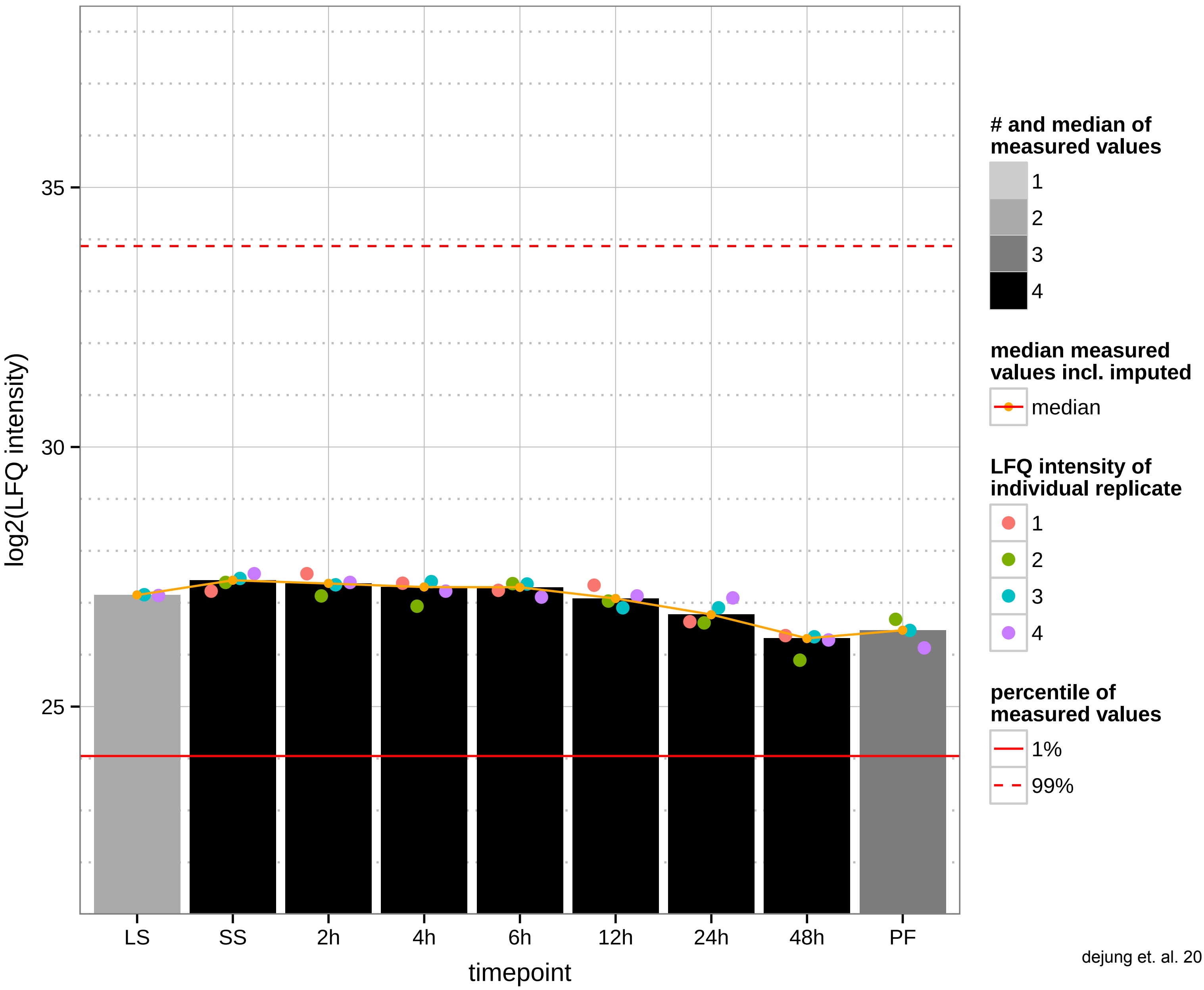

dejung et. al. 2015

6–phosphogluconate dehydrogenase, decarboxylating (gnD)
Tb927.9.12110
AGOF: NADP binding, phosphogluconate dehydrogenase (decarboxylating) activity
AGOC: cytoplasm
AGOP: oxidation–reduction process, pentose–phosphate shunt
PGOF: NADP binding, phosphogluconate dehydrogenase (decarboxylating) activity
PGOC: null
PGOP: oxidation–reduction process, pentose–phosphate shunt

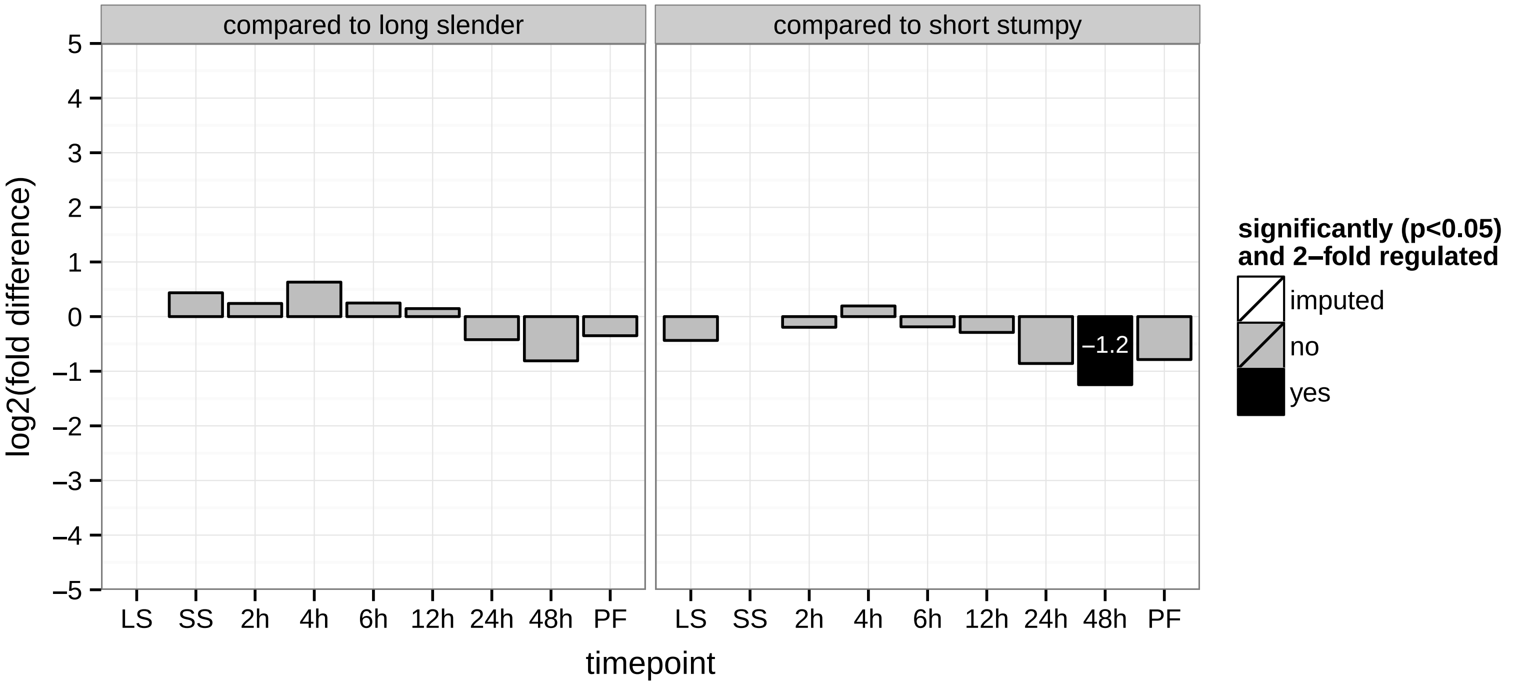

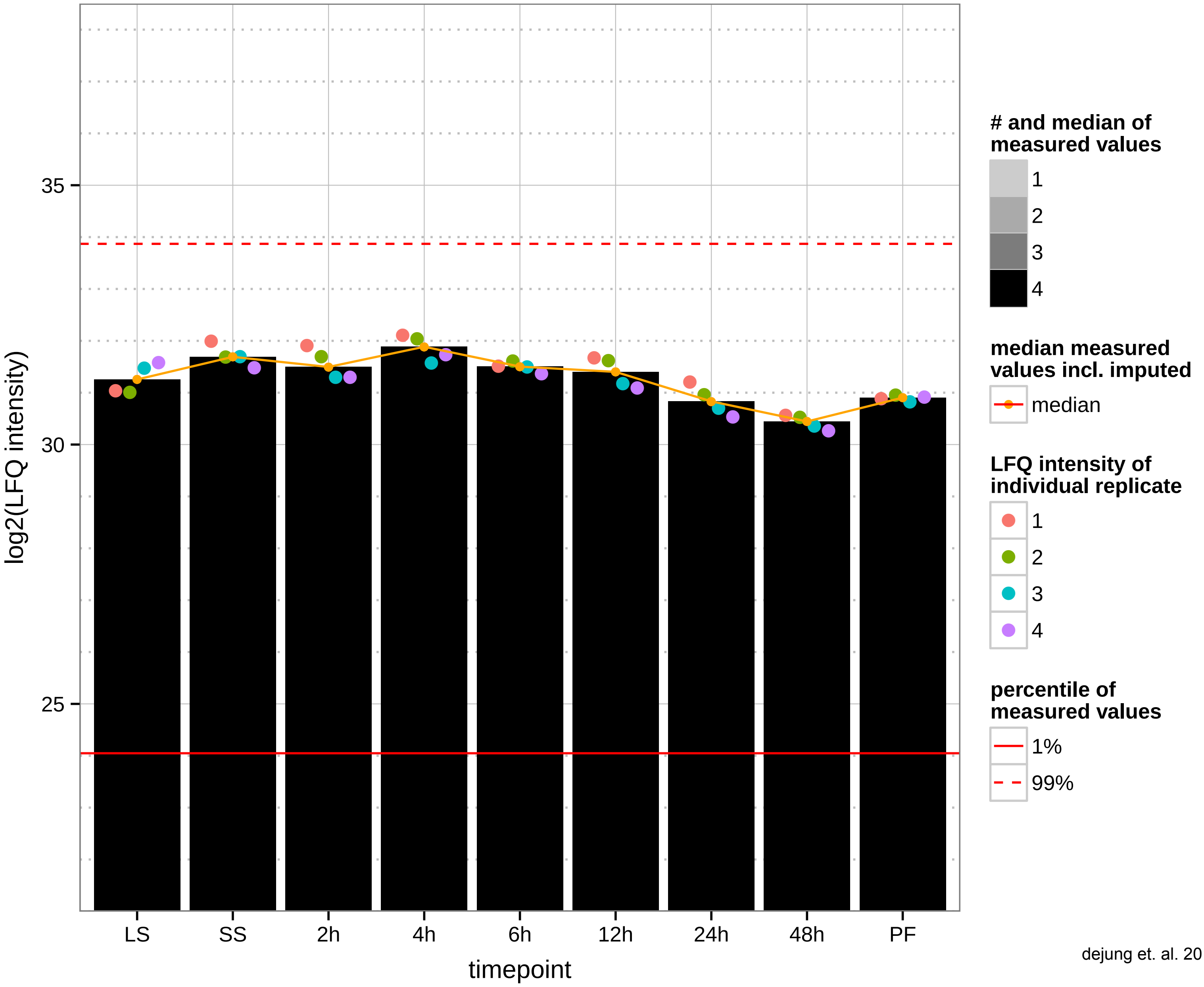

dejung et. al. 2015

QA–SNARE protein putative
Tb927.9.13030
AGOF: SNAP receptor activity
AGOC: integral to membrane, membrane
AGOP: membrane fusion, vesicle–mediated transport
PGOF: protein binding
PGOC: membrane
PGOP: vesicle–mediated transport

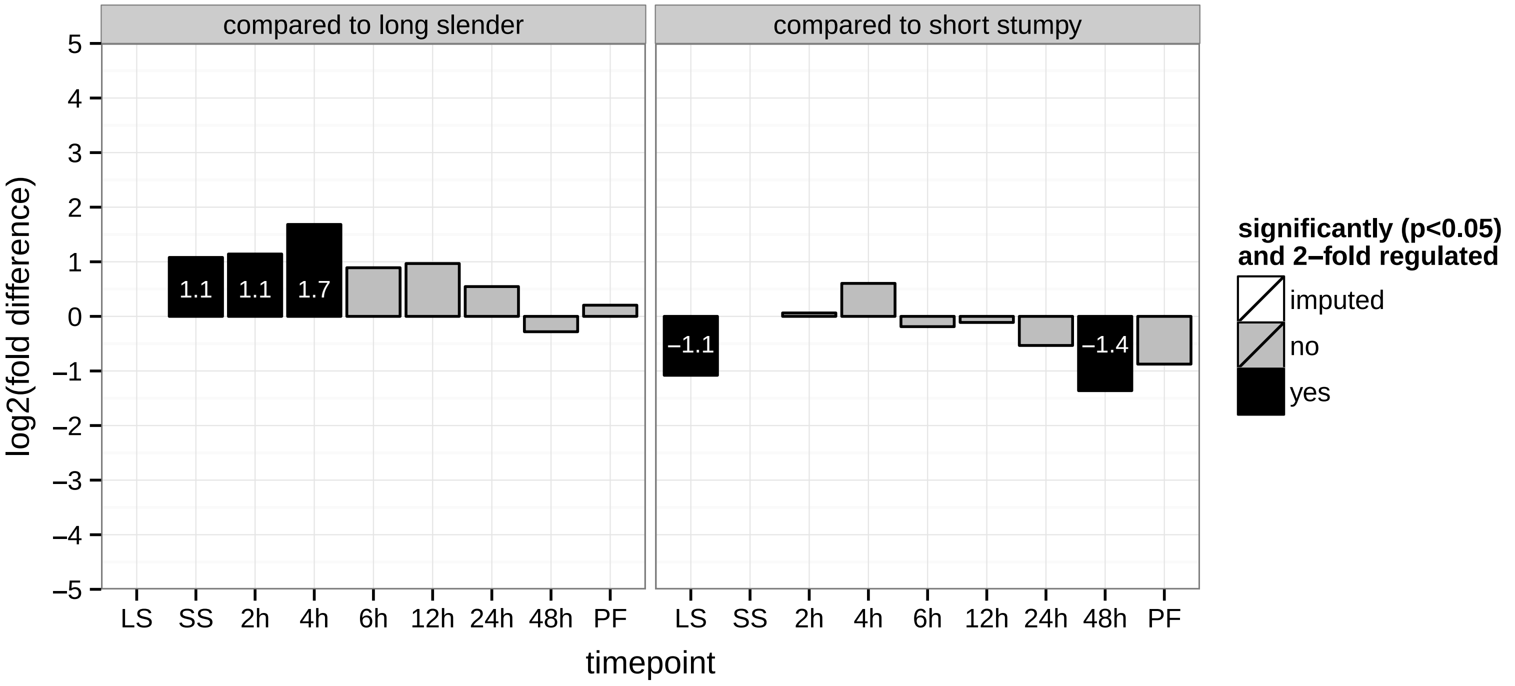

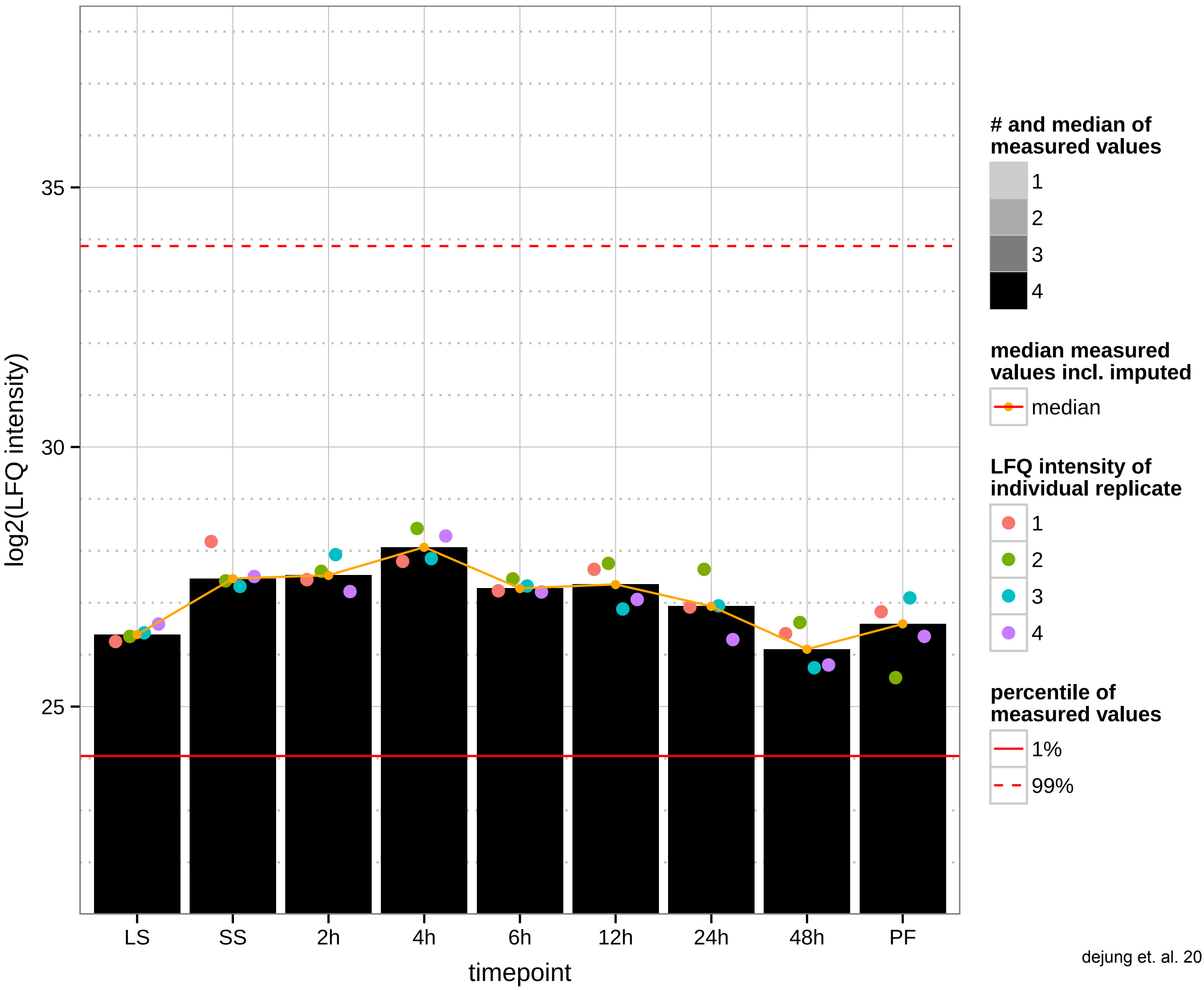

dejung et. al. 2015

hypothetical protein, conserved
Tb927.9.3770
AGOF: null
AGOC: null
AGOP: null
PGOF: null
PGOC: null
PGOP: null

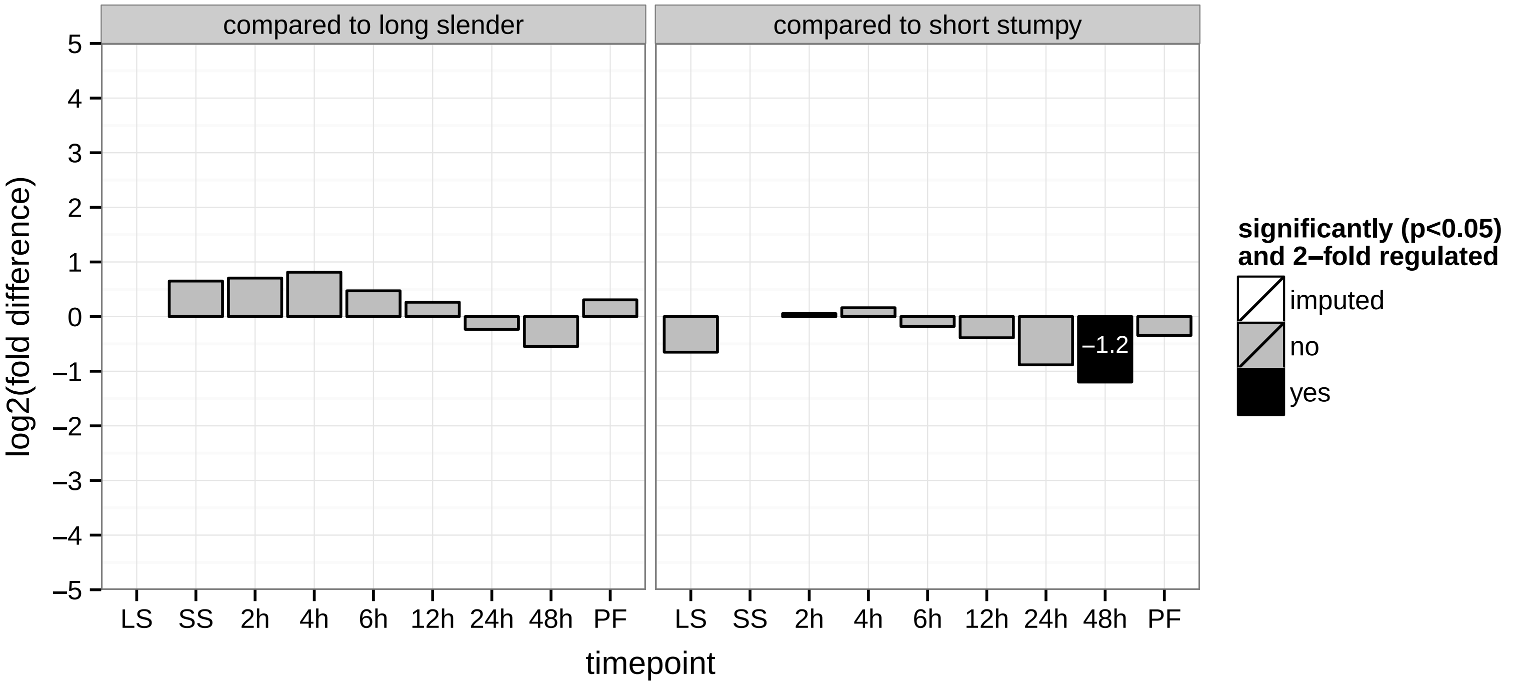

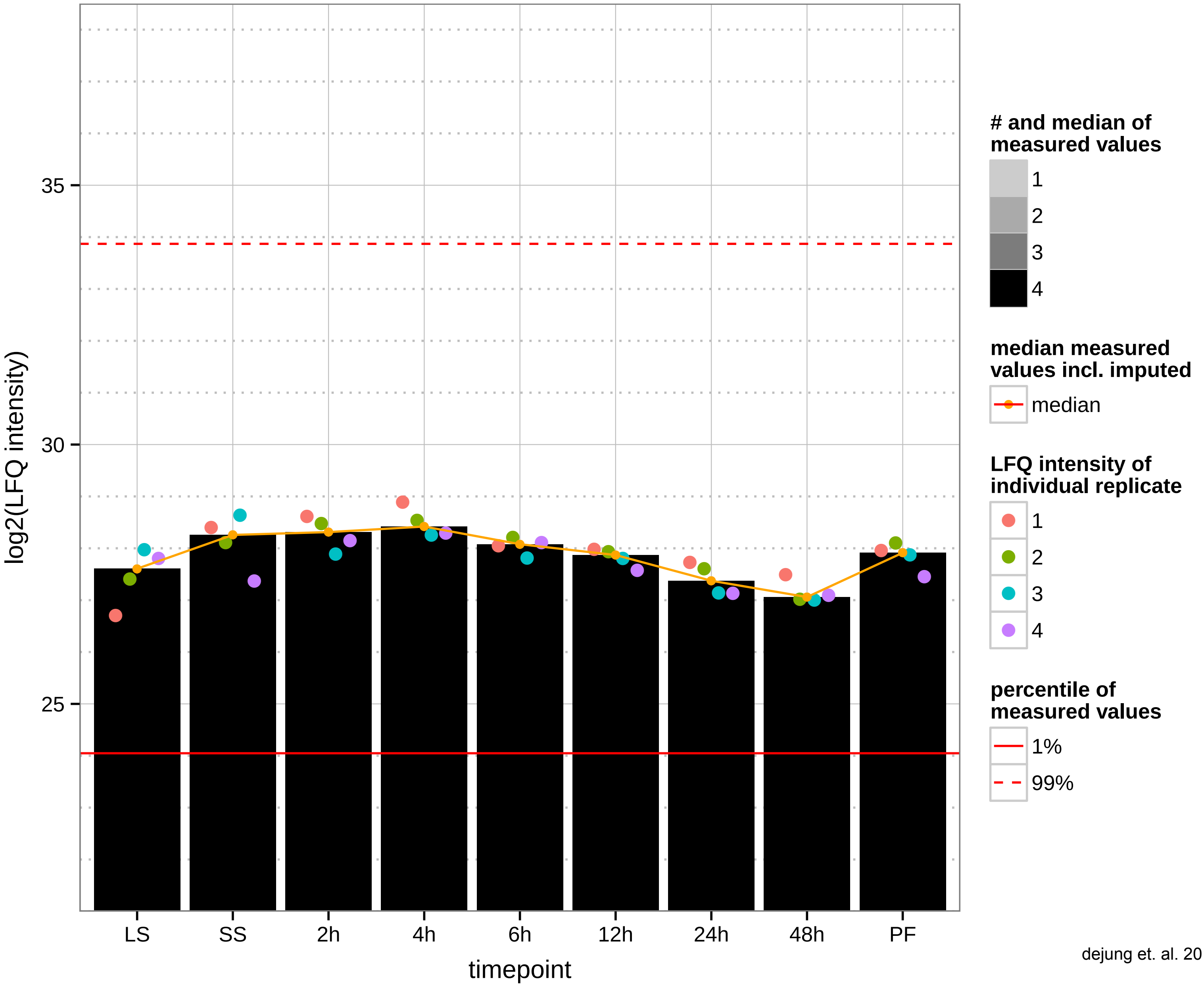

dejung et. al. 2015

heat shock protein, putative, HSP70–like protein
Tb927.9.4500
AGOF: ATP binding
AGOC: mitochondrial inner membrane
AGOP: protein folding
PGOF: null
PGOC: null
PGOP: null

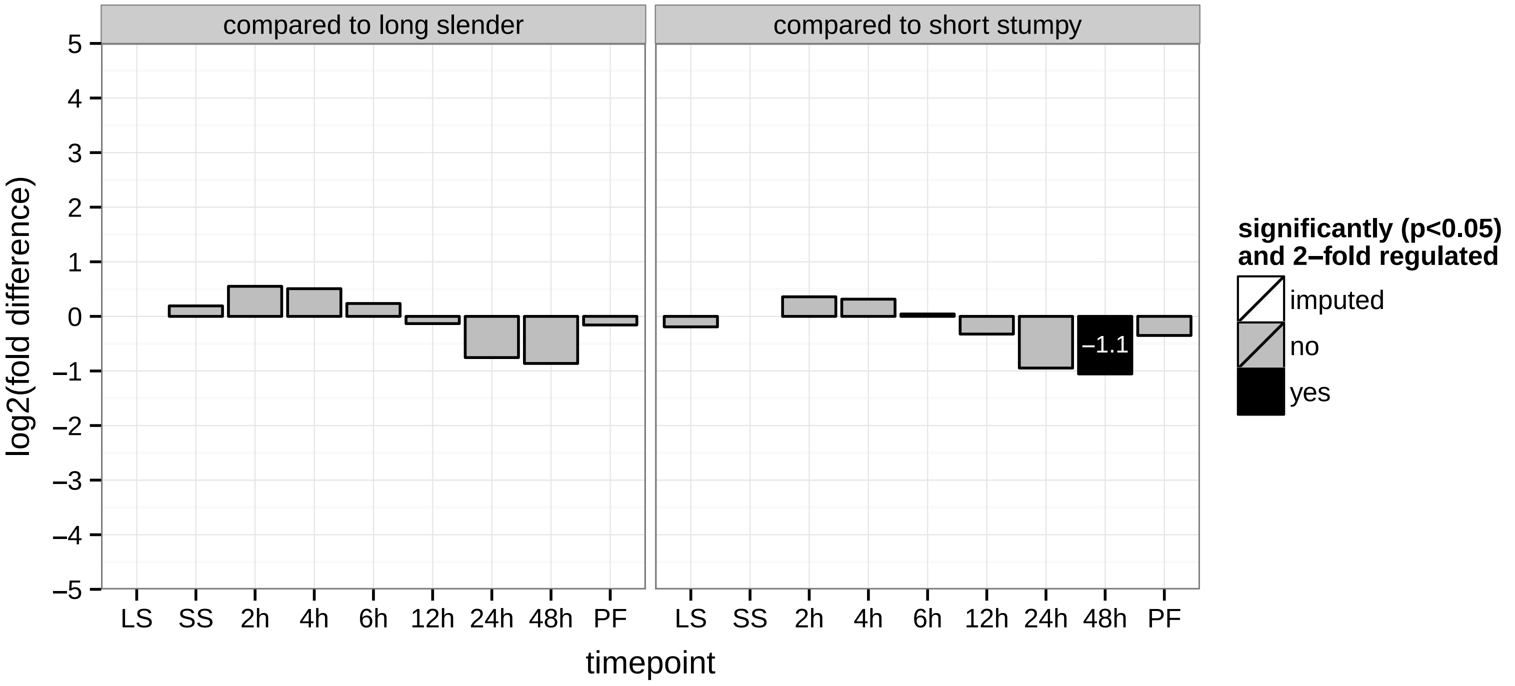

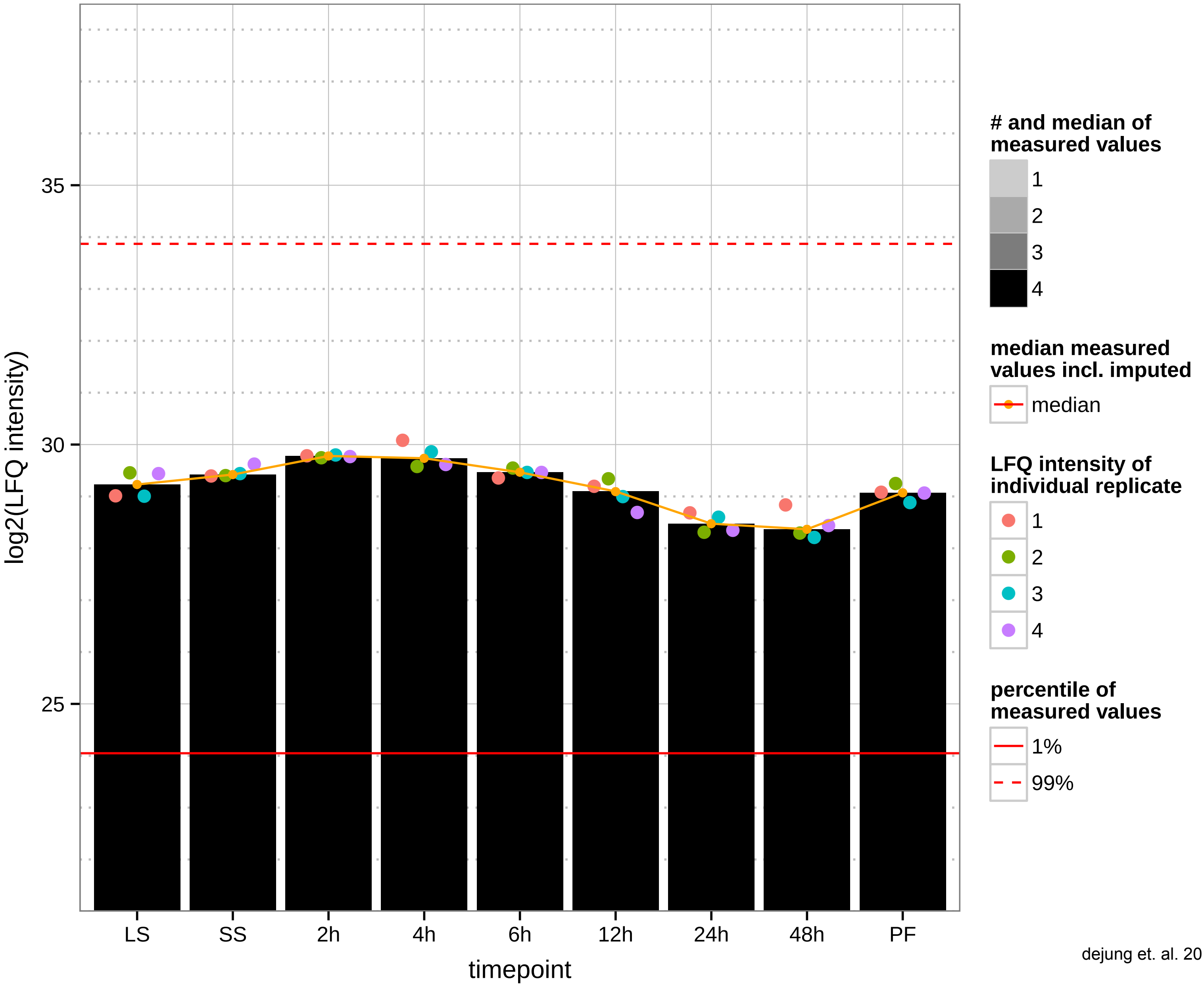

dejung et. al. 2015

inositol–1(or 4)–monophosphatase, putative (IMPase)
Tb927.9.6350
AGOF: inositol monophosphate 1–phosphatase activity
AGOC: null
AGOP: phosphate–containing compound metabolic process, signal transduction
PGOF: null
PGOC: null
PGOP: phosphatidylinositol phosphorylation

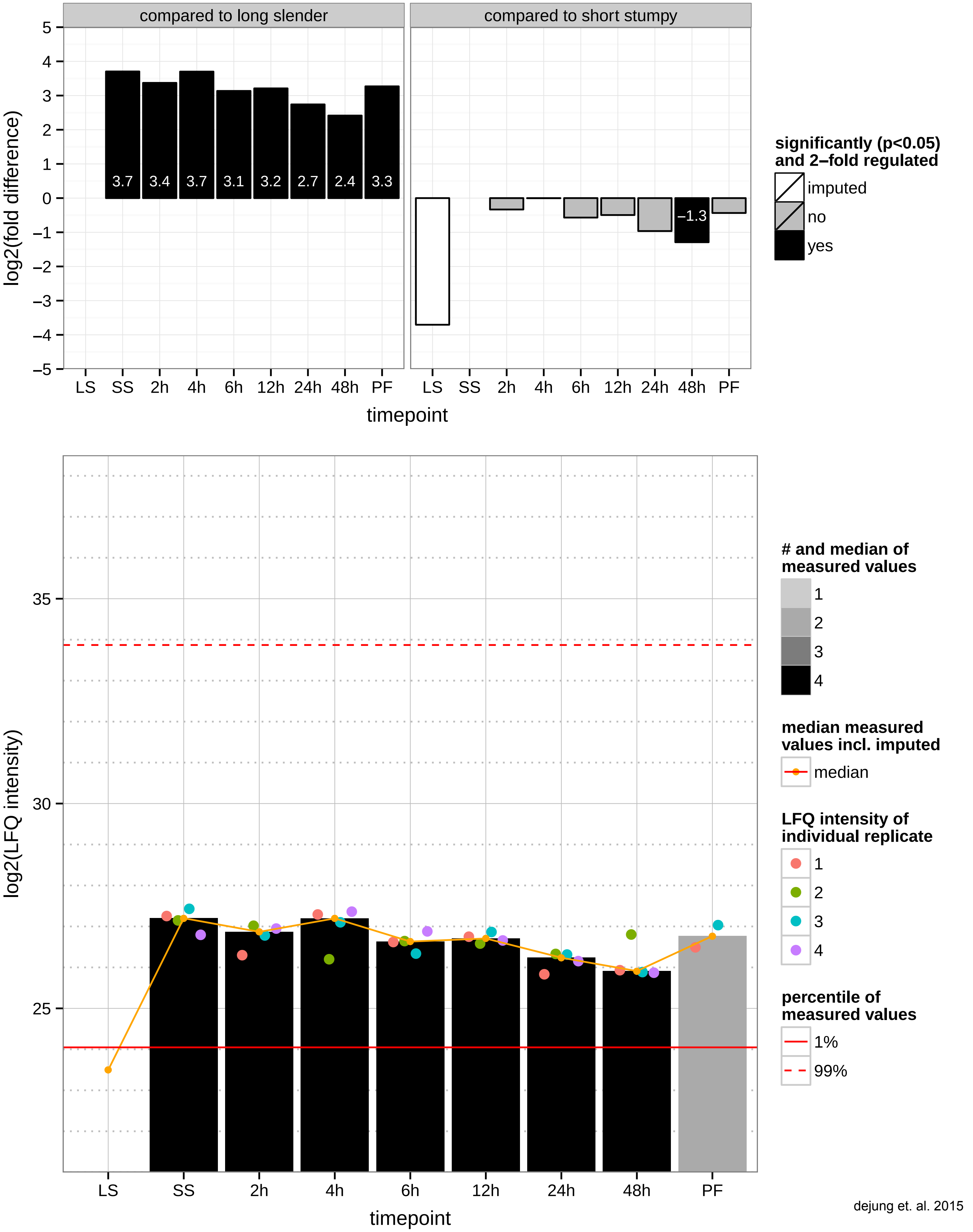

dejung et. al. 2015

hypothetical protein, conserved
Tb927.9.7110
AGOF: null
AGOC: null
AGOP: null
PGOF: null
PGOC: null
PGOP: null

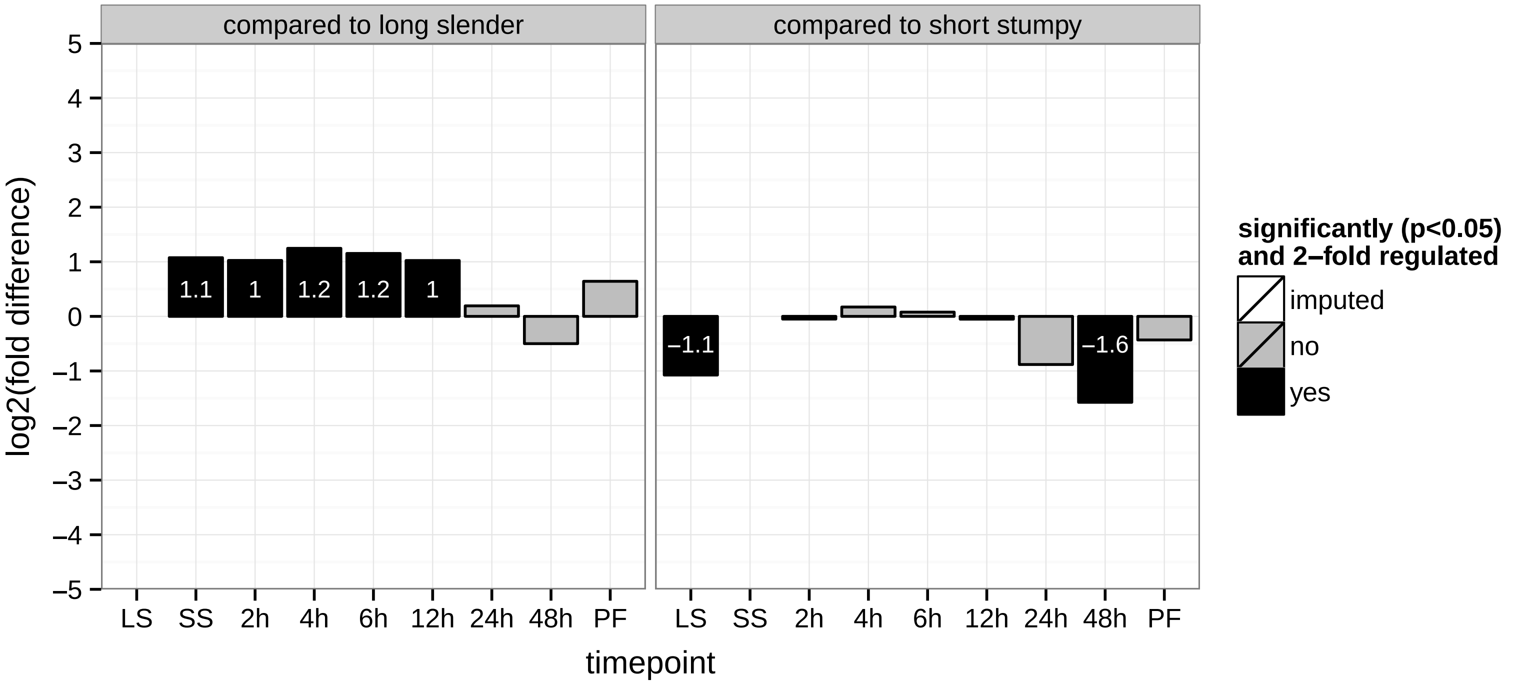

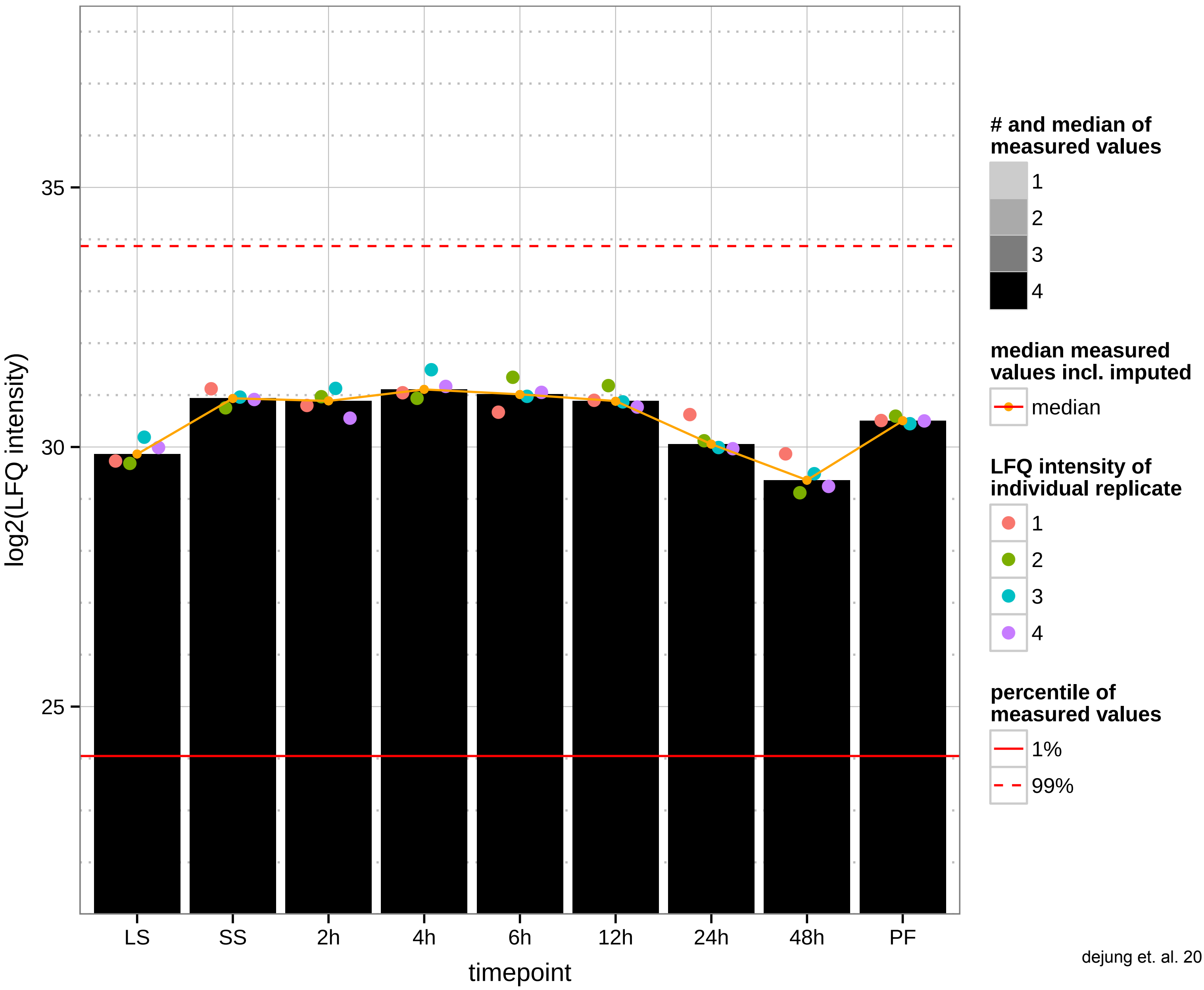

dejung et. al. 2015

peptidyl–prolyl cis–trans isomerase (cyclophilin– 40), putative, cyclophilin–40
Tb927.9.9780
AGOF: peptidyl–prolyl cis–trans isomerase activity
AGOC: null
AGOP: null
PGOF: peptidyl–prolyl cis–trans isomerase activity, protein binding
PGOC: null
PGOP: protein folding

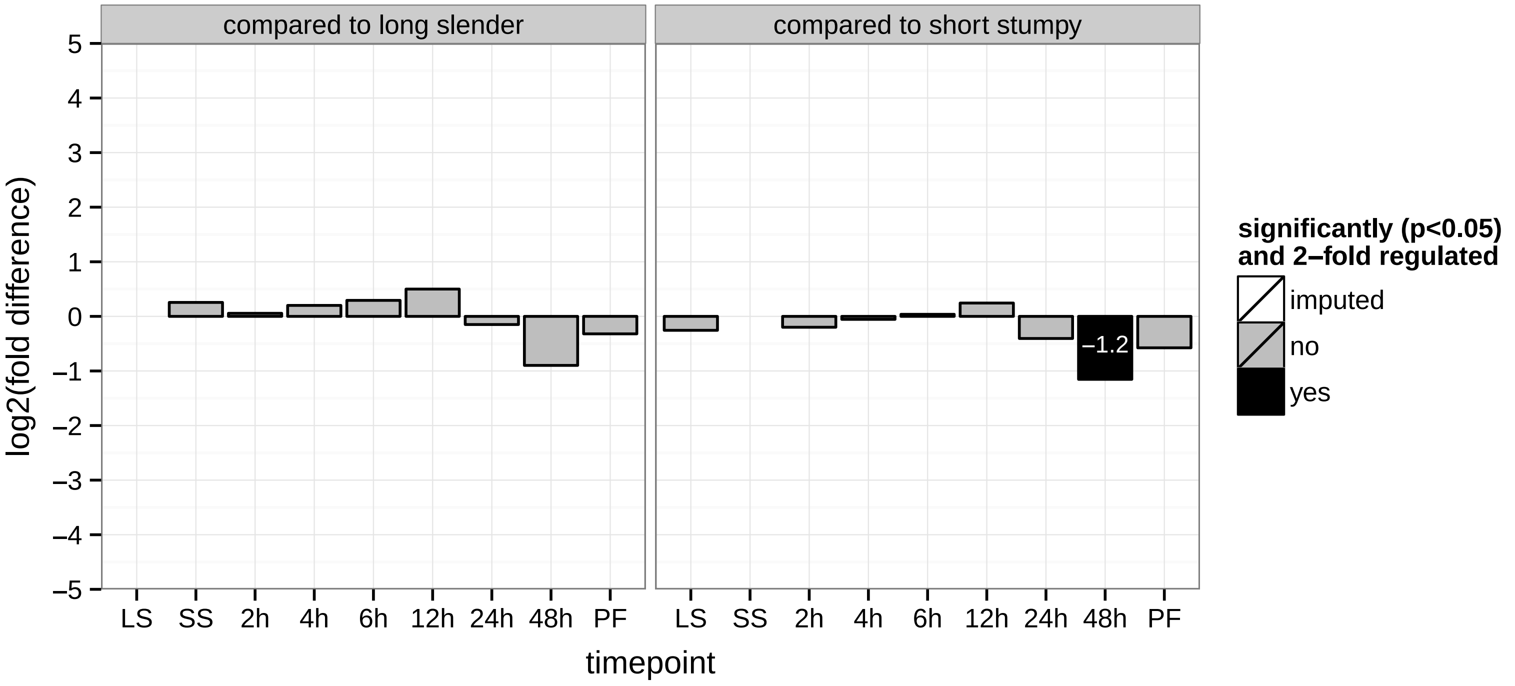

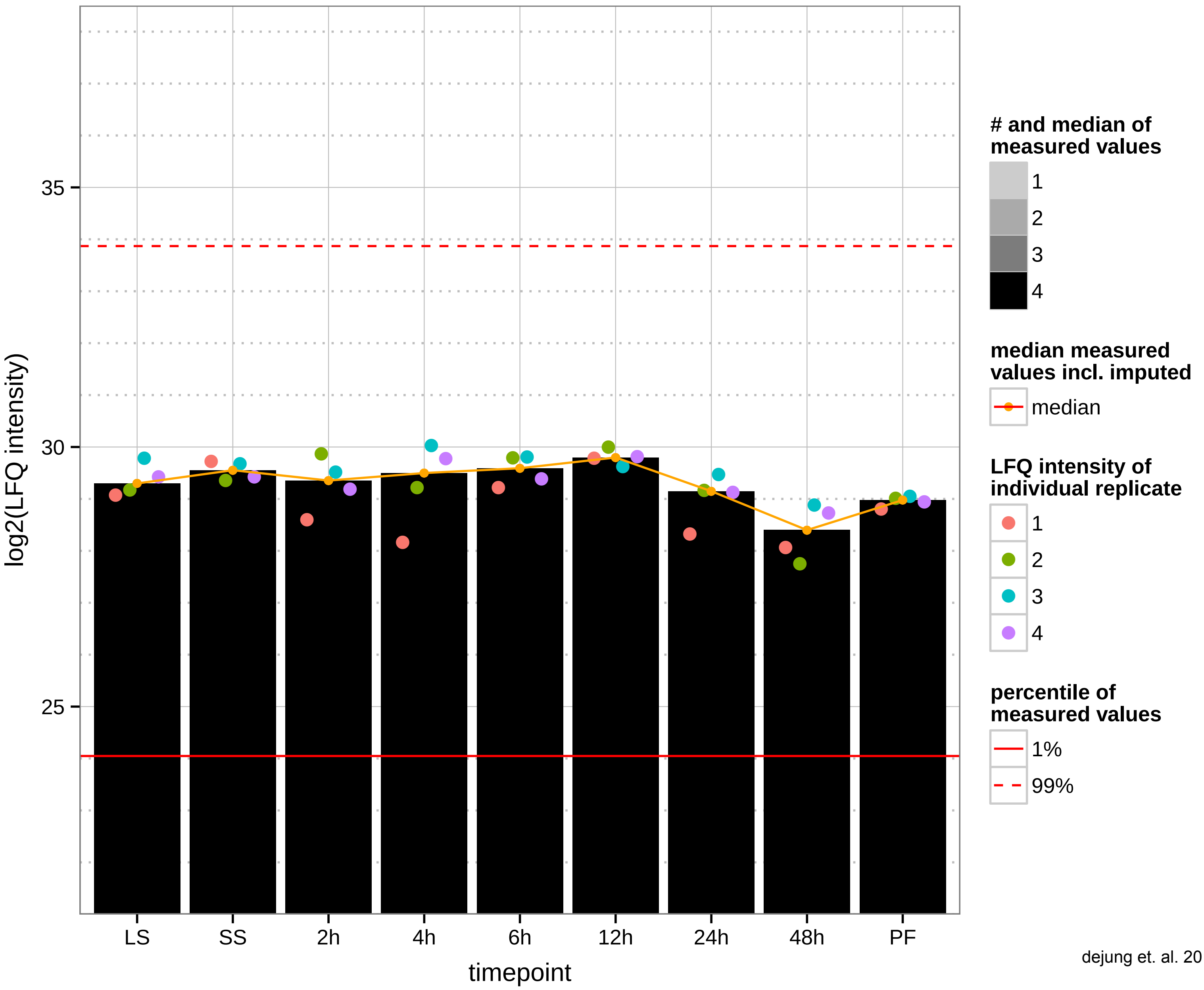

dejung et. al. 2015

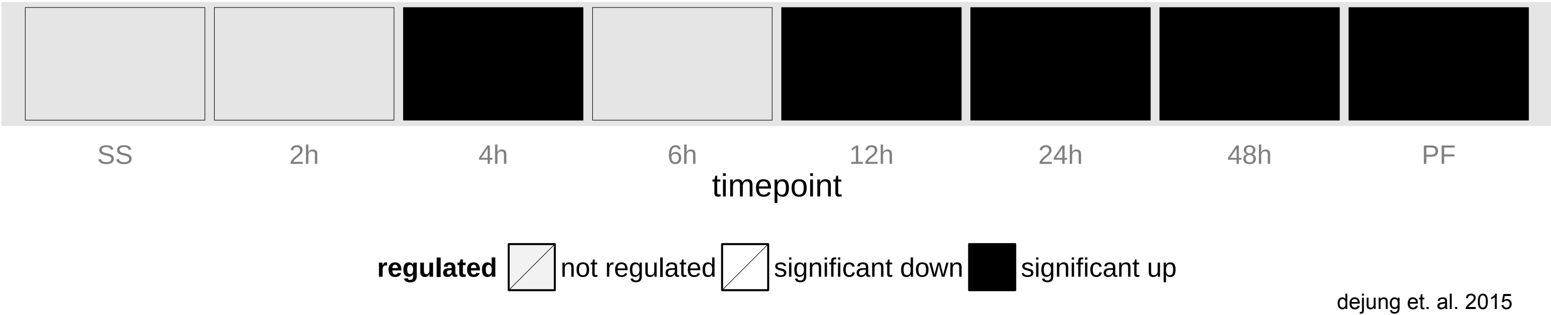
SS
2h
4h
6h
12h
24h
48h
PF
timepoint
regulated
not regulated
significant down
significant up
dejung et. al. 2015

branched−chain alpha−keto acid dehydrogenase e1−beta subunit precursor, putative, chrX additional, unordered contigs, 2−o
Tb927.10.4330;Tb11.v5.1012;Tb10.v4.0043
AGOF: catalytic activity, null, 3−methyl−2−oxobutanoate dehydrogenase (2−methylpropanoyl−transferring) activity
AGOC: null, mitochondrial alpha−ketoglutarate dehydrogenase complex, mitochondrion
AGOP: metabolic process, null
PGOF: catalytic activity
PGOC: null
PGOP: metabolic process

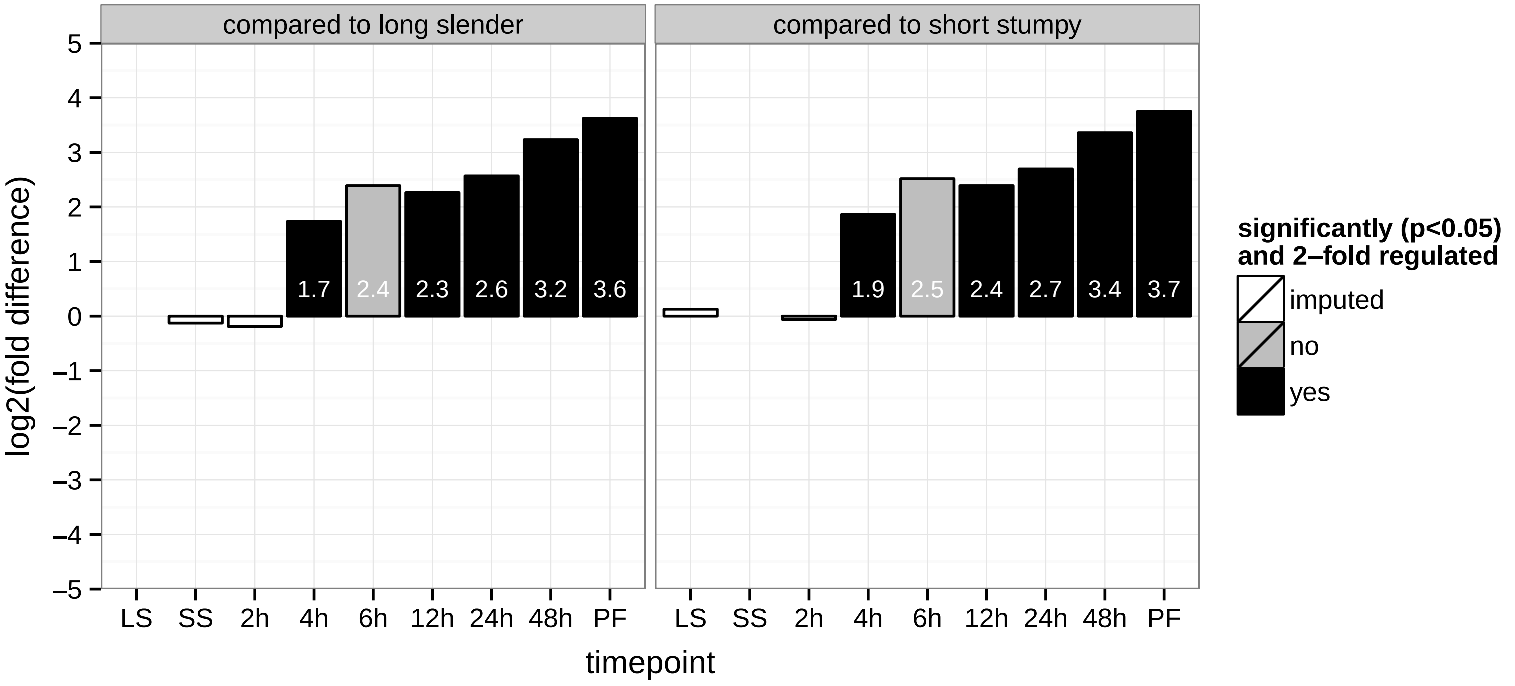

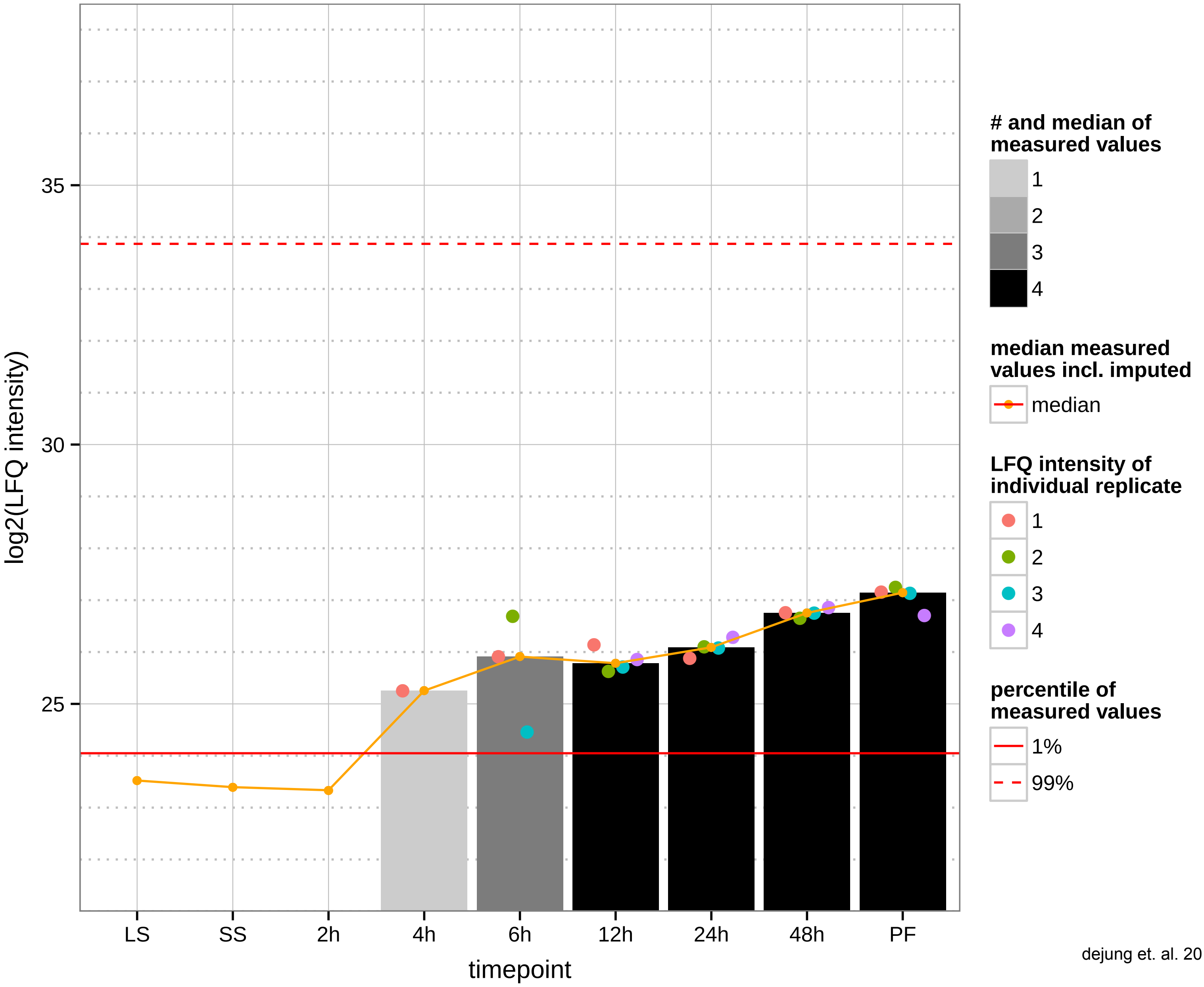

dejung et. al. 2015

chrX additional, unordered contigs, epsilon tubulin, epsilon tubulin (TUBE1)
Tb927.10.1140;Tb11.0530
AGOF: GTP binding, GTPase activity, structural molecule activity
AGOC: microtubule, protein complex
AGOP: microtubule–based movement, protein polymerization, microtubule polymerization, microtubule–based movement
PGOF: GTP binding, GTPase activity
PGOC: protein complex
PGOP: GTP catabolic process, protein polymerization

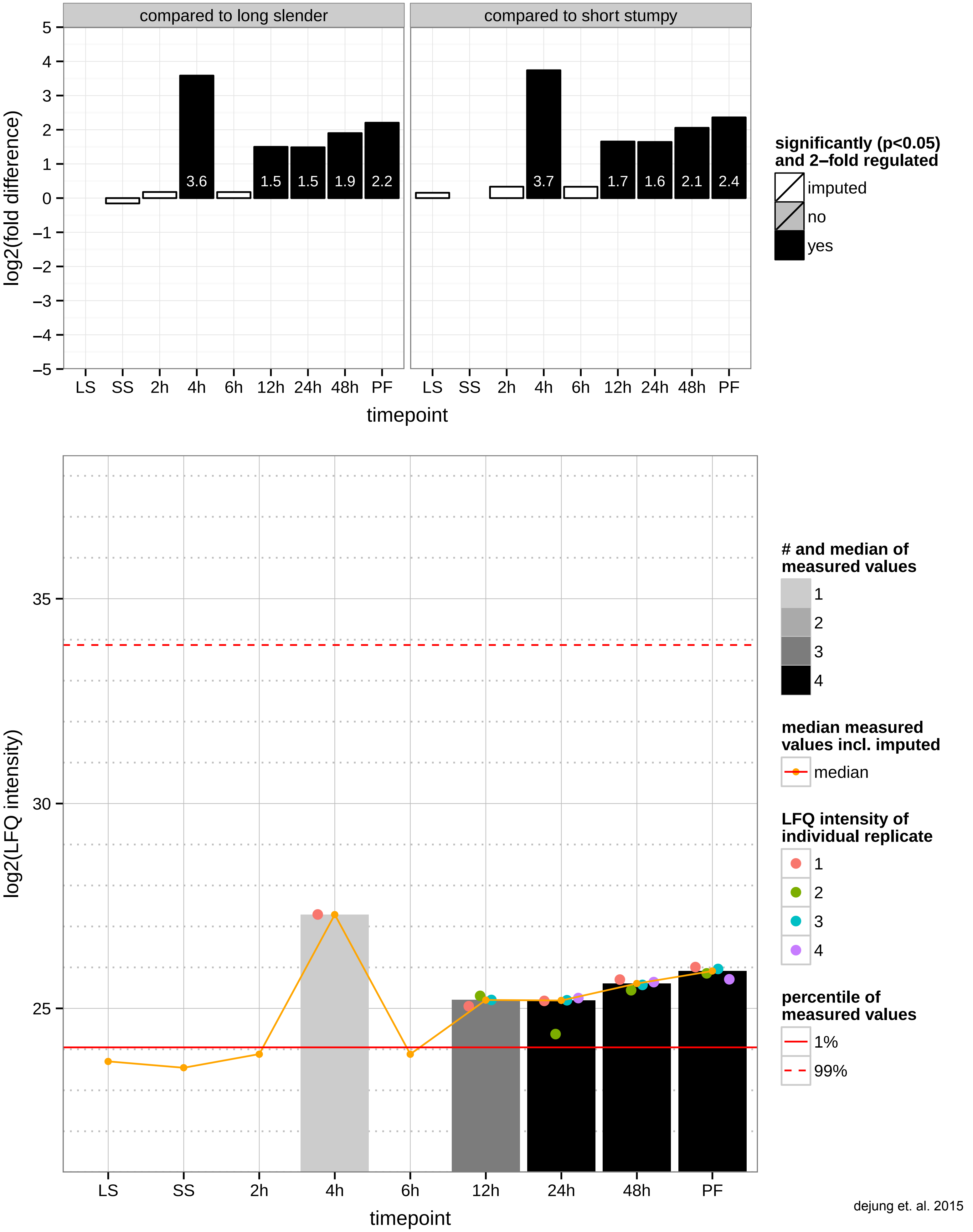

dejung et. al. 2015

amino acid transporter, putative, putative (AATP6)
Tb927.8.4740;Tb927.8.4730;Tb927.8.4720;Tb927.8.4710;Tb927.8.4700;Tb11.v5.0569
AGOF: null, amine transmembrane transporter activity
AGOC: null, integral to membrane, membrane
AGOP: null, amino acid transport
PGOF: null
PGOC: null
PGOP: null

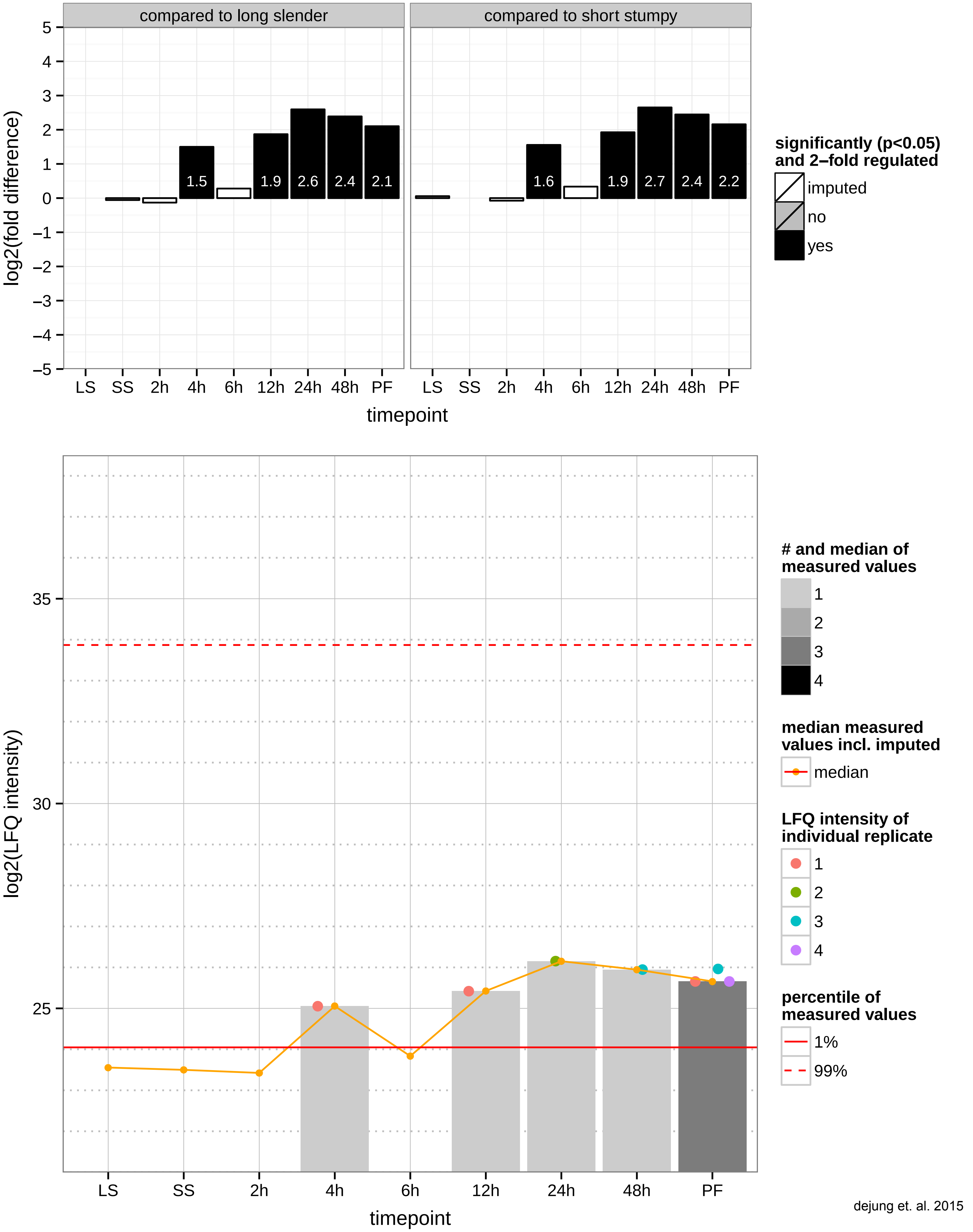

dejung et. al. 2015

cytochrome c oxidase assembly factor, putative, electron transport protein SCO1/2
Tb927.1.1580
AGOF: copper ion binding
AGOC: mitochondrion
AGOP: respiratory chain complex IV assembly
PGOF: null
PGOC: null
PGOP: null

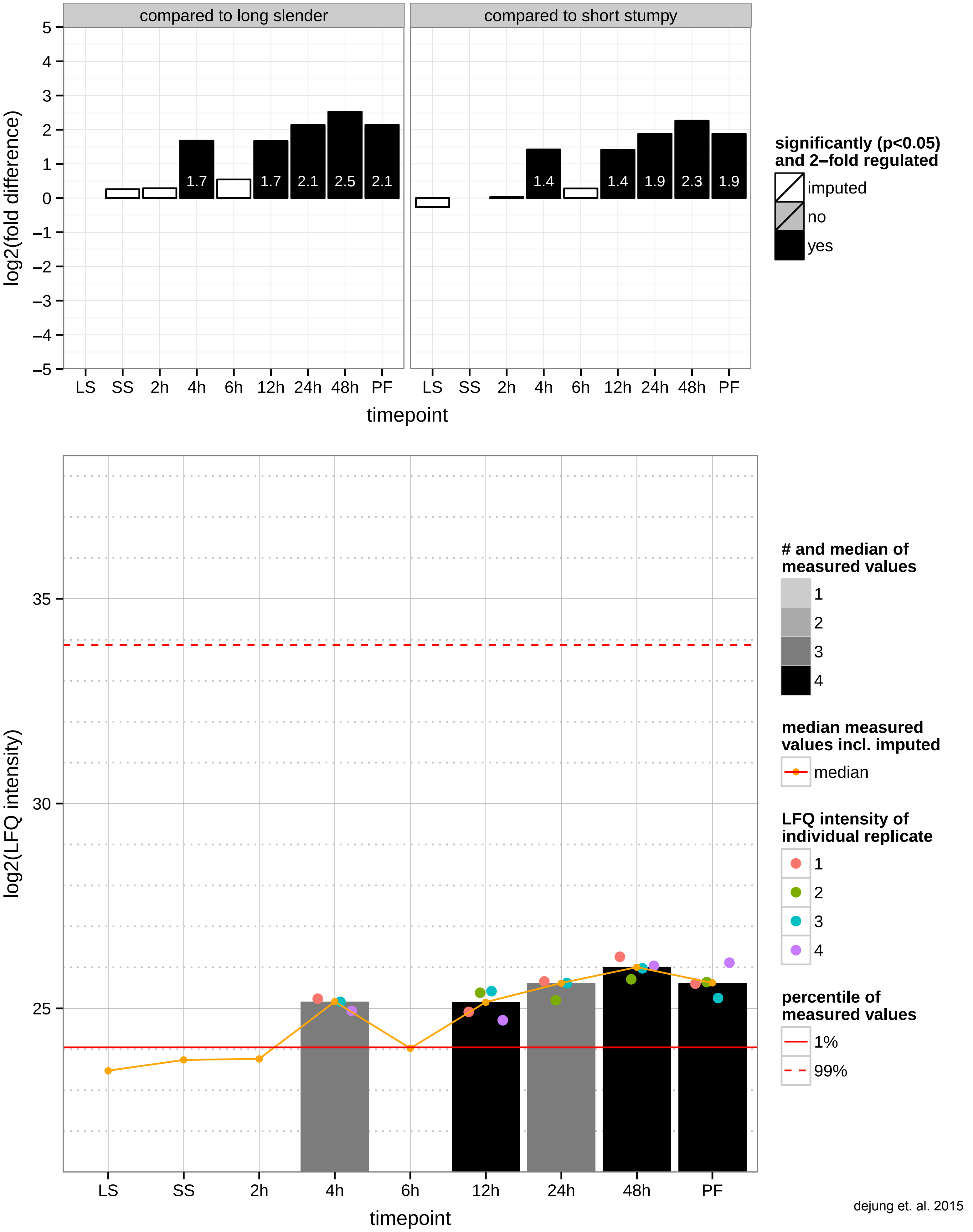

dejung et. al. 2015

palmitoyl acyltransferase 2, putative
Tb927.10.10800
AGOF: zinc ion binding
AGOC: integral to membrane
AGOP: endocytosis, protein palmitoylation, transport
PGOF: zinc ion binding
PGOC: null
PGOP: null

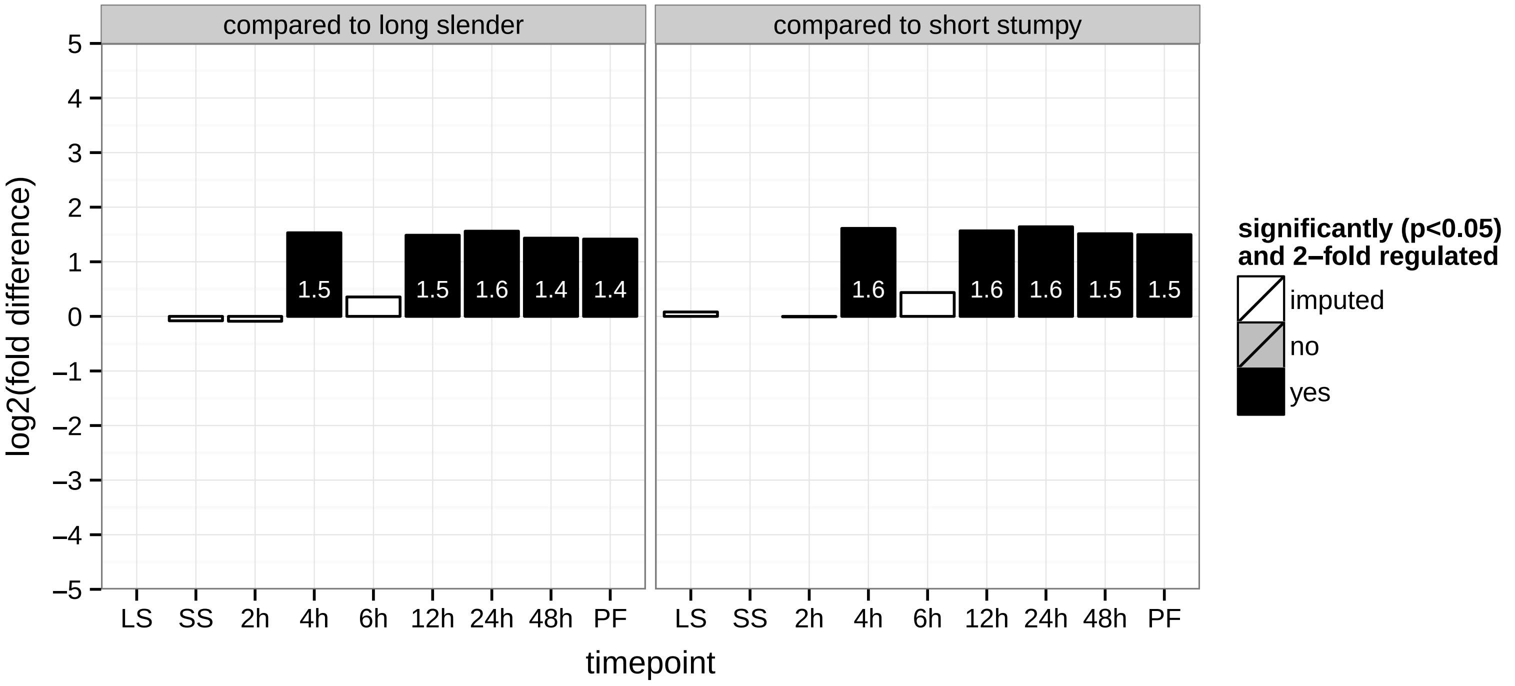

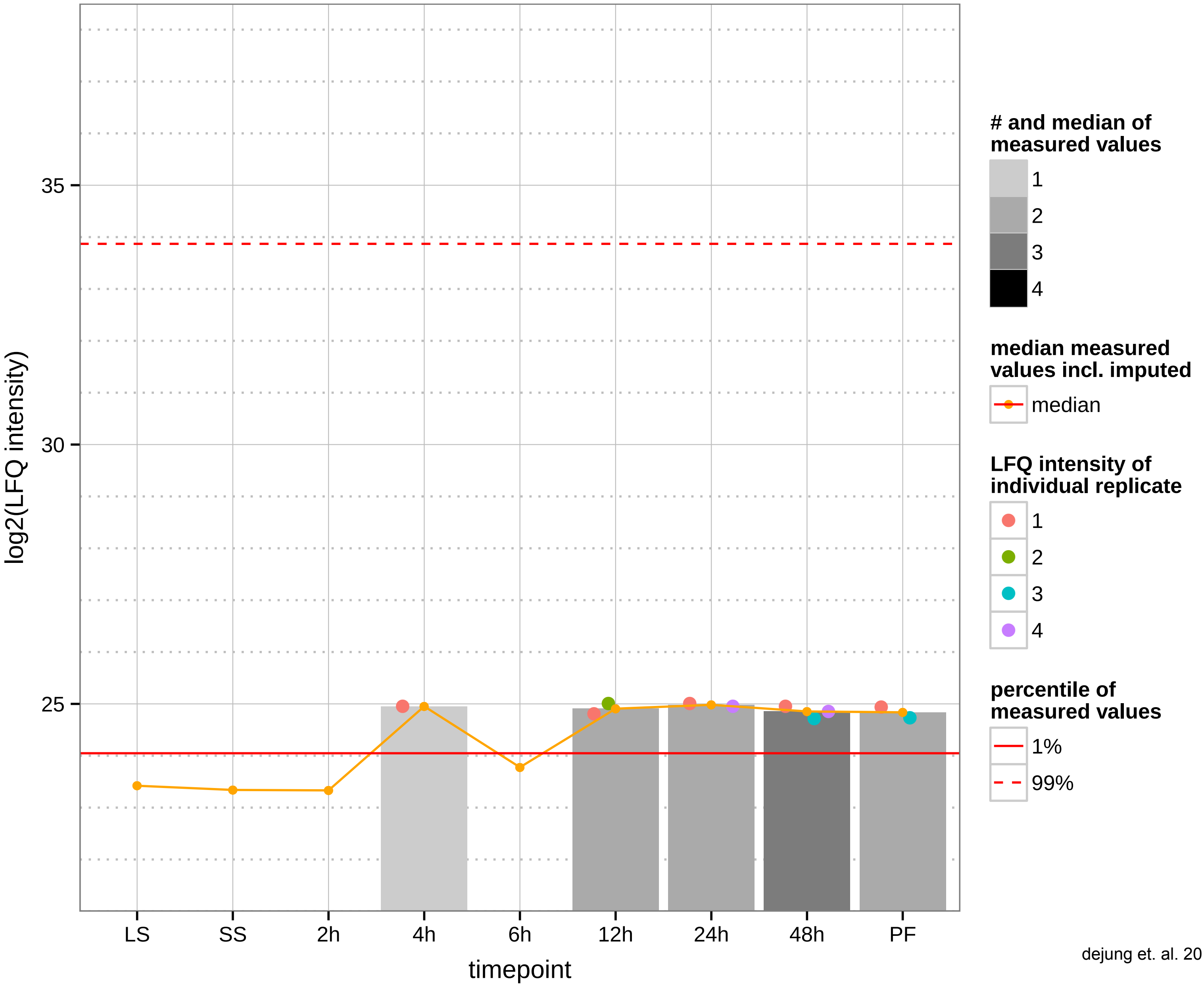

dejung et. al. 2015

hypothetical protein, conserved
Tb927.10.13600
AGOF: null
AGOC: mitochondrion
AGOP: null
PGOF: null
PGOC: null
PGOP: null

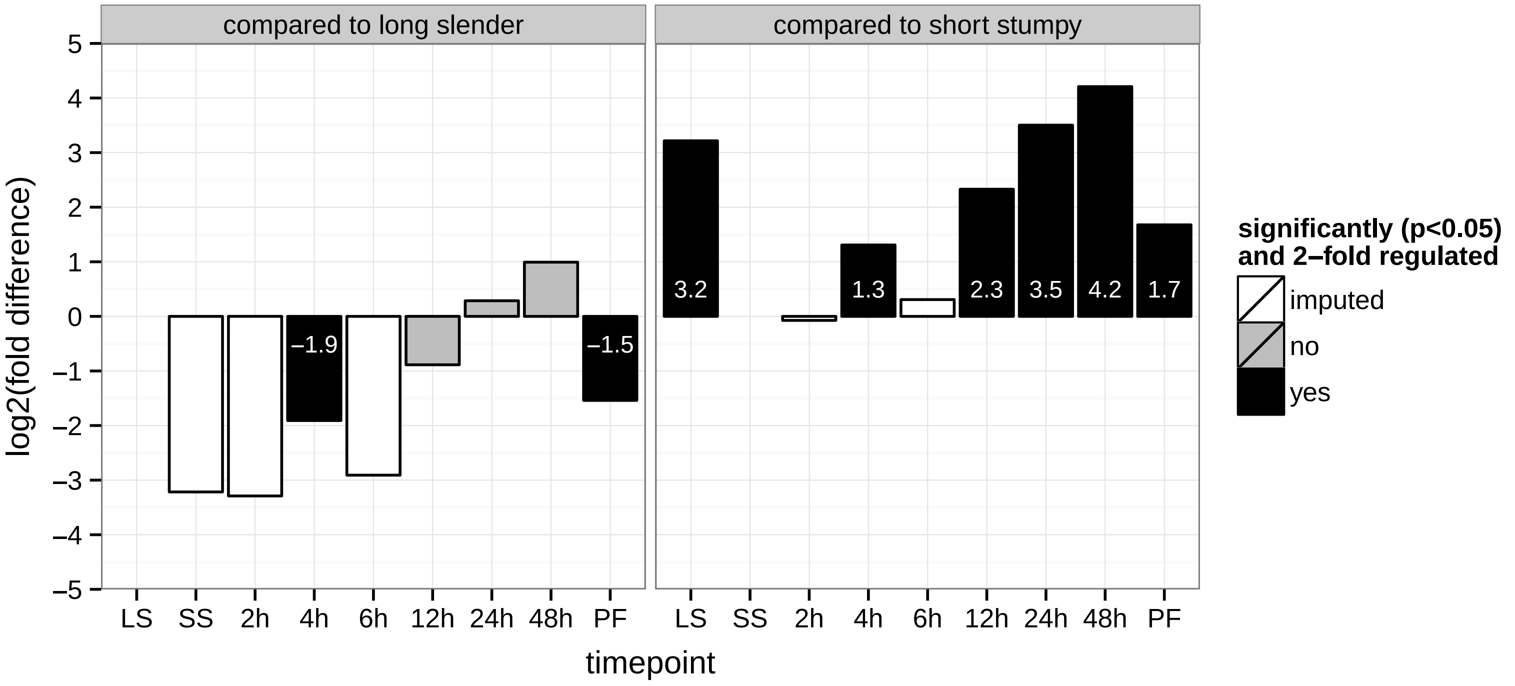

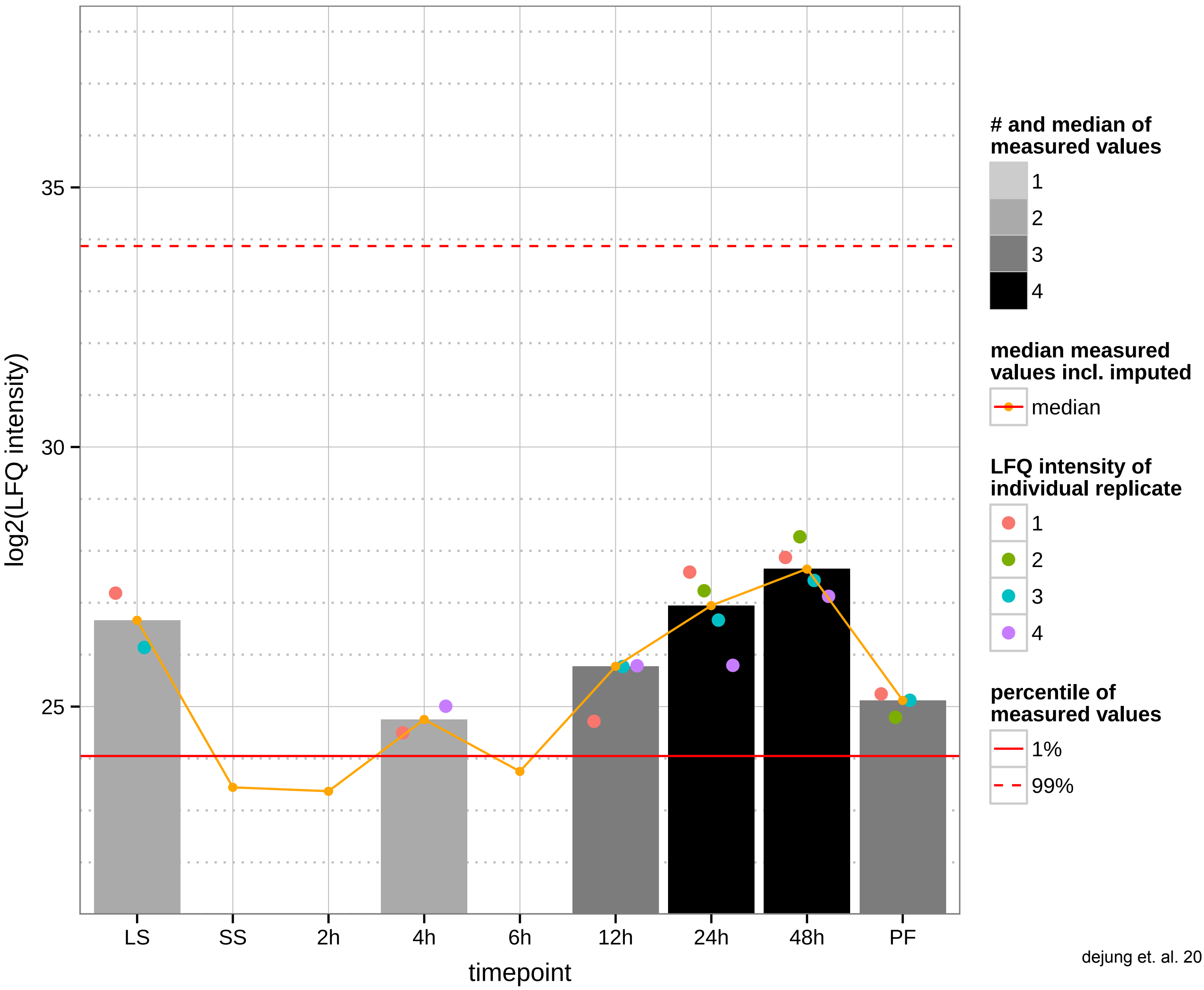

dejung et. al. 2015

Zinc finger CCCH domain–containing protein 40, RNA binding protein, putative (ZC3H40)
Tb927.10.14950
AGOF: RNA binding, zinc ion binding
AGOC: null
AGOP: null
PGOF: nucleic acid binding, zinc ion binding
PGOC: null
PGOP: null

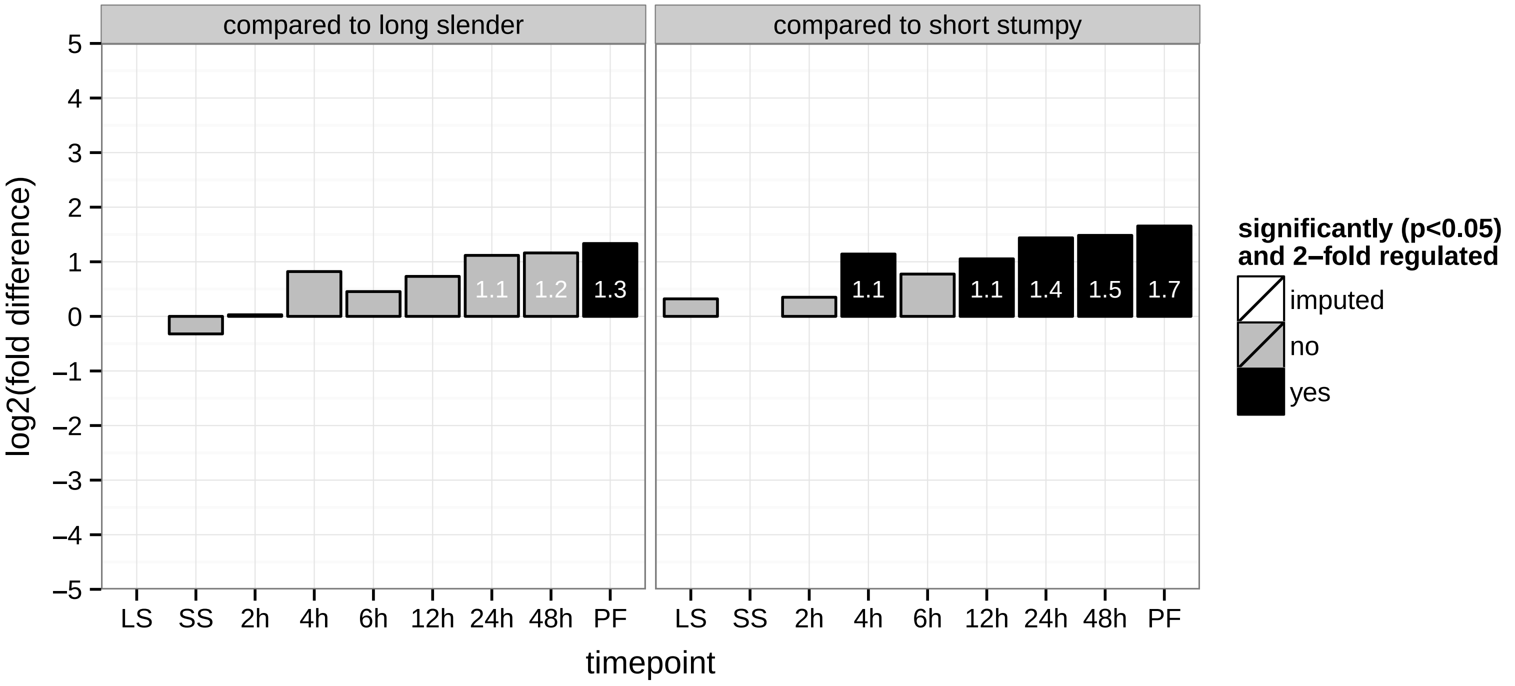

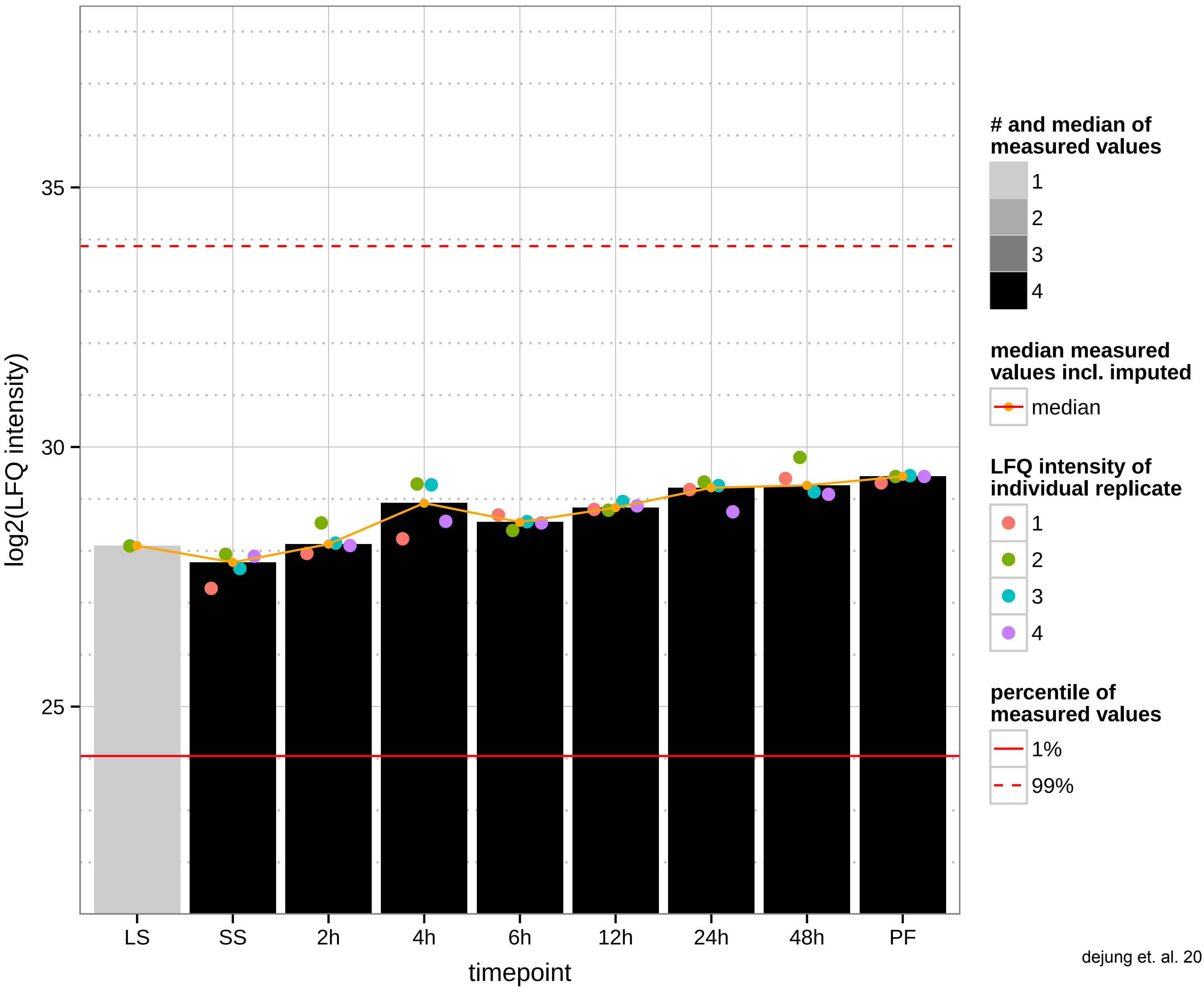

dejung et. al. 2015

hypothetical protein, conserved
Tb927.10.2310
AGOF: null
AGOC: null
AGOP: null
PGOF: null
PGOC: null
PGOP: null

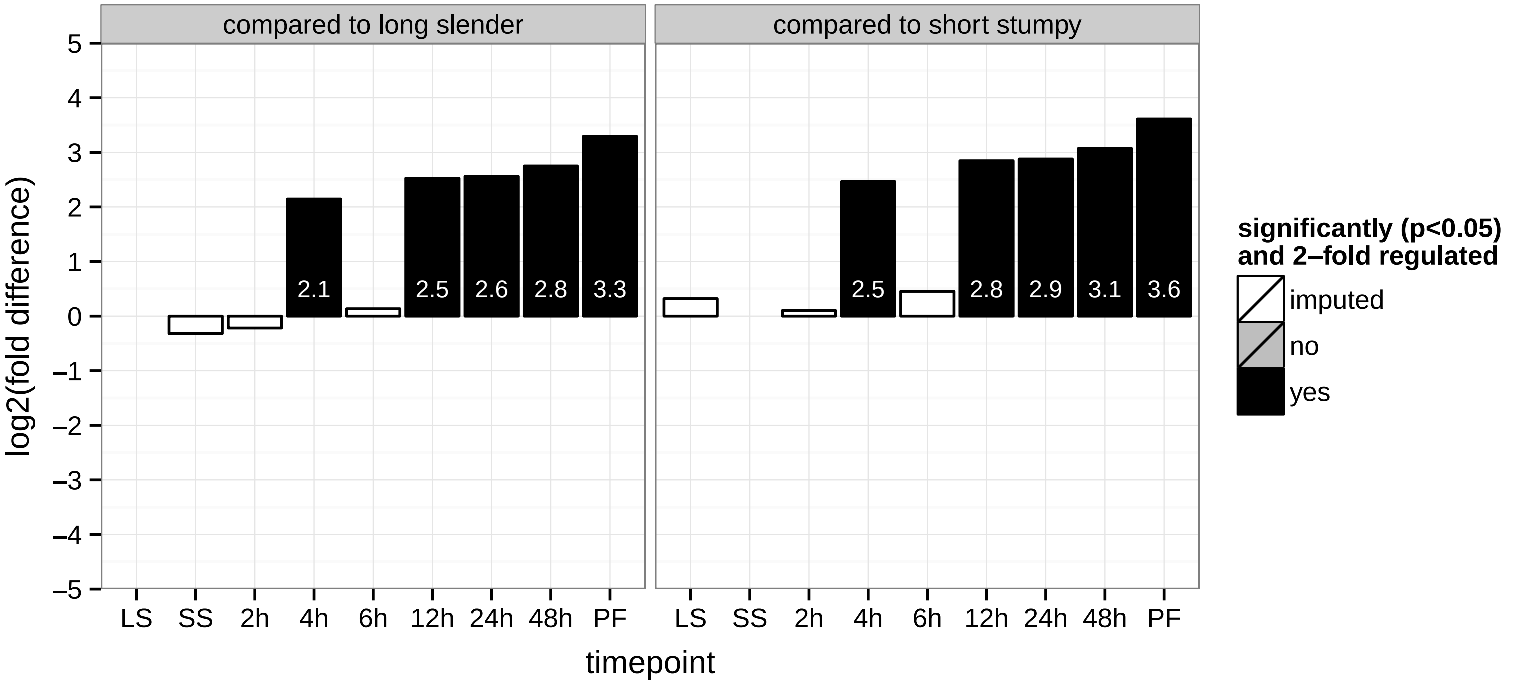

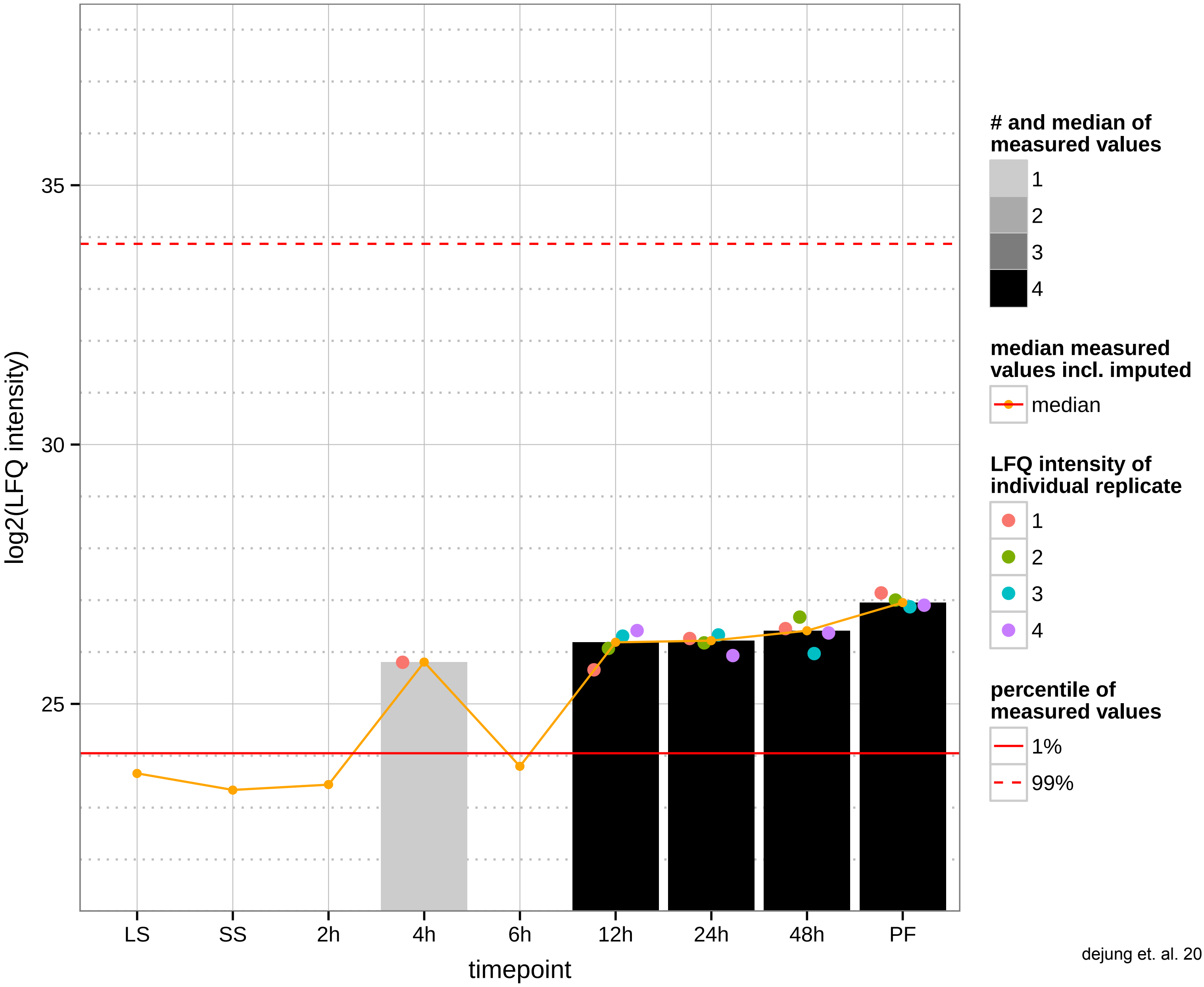

dejung et. al. 2015

hypothetical protein, conserved
Tb927.10.5050
AGOF: hydrogen ion transporting ATP synthase activity, rotational mechanism, proton–transporting ATPase activity
AGOC: mitochondrial proton–transporting ATP synthase complex, catalytic core F(1), mitochondrion
AGOP: ATP synthesis coupled proton transport
PGOF: hydrogen ion transporting ATP synthase activity, rotational mechanism, proton–transporting ATPase activity
PGOC: mitochondrial proton–transporting ATP synthase complex, catalytic core F(1)
PGOP: ATP synthesis coupled proton transport

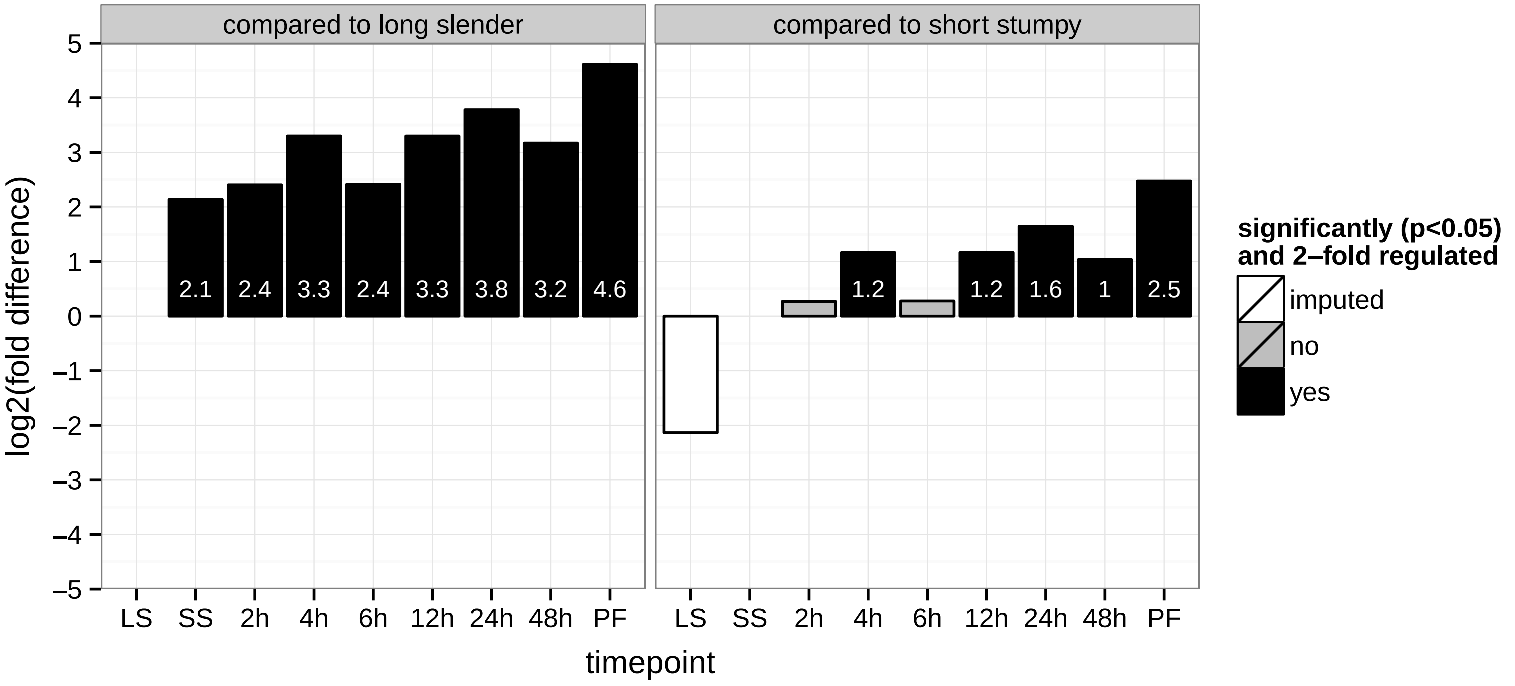

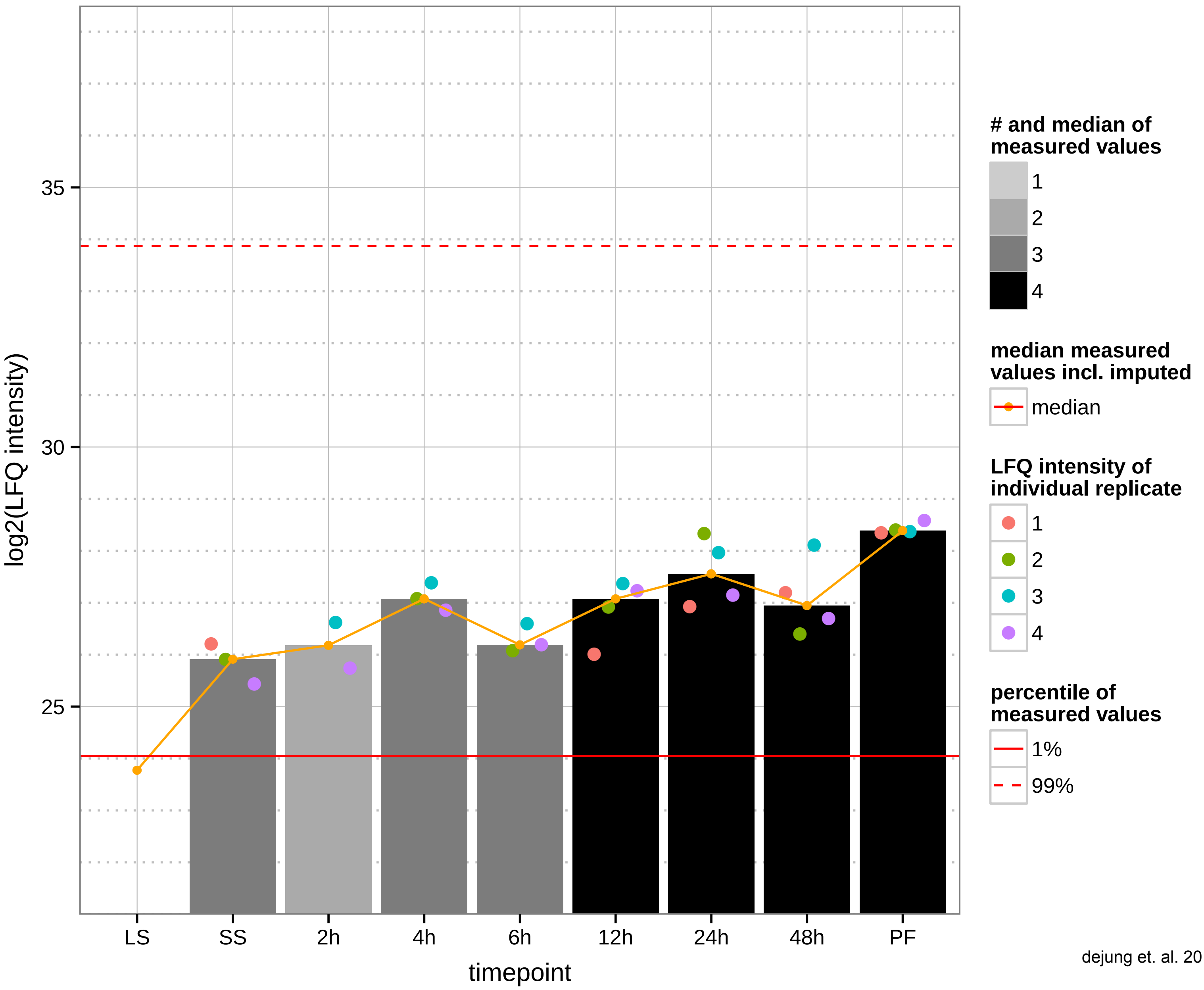

dejung et. al. 2015

hypothetical protein, conserved
Tb927.10.5870
AGOF: null
AGOC: null
AGOP: null
PGOF: null
PGOC: null
PGOP: null

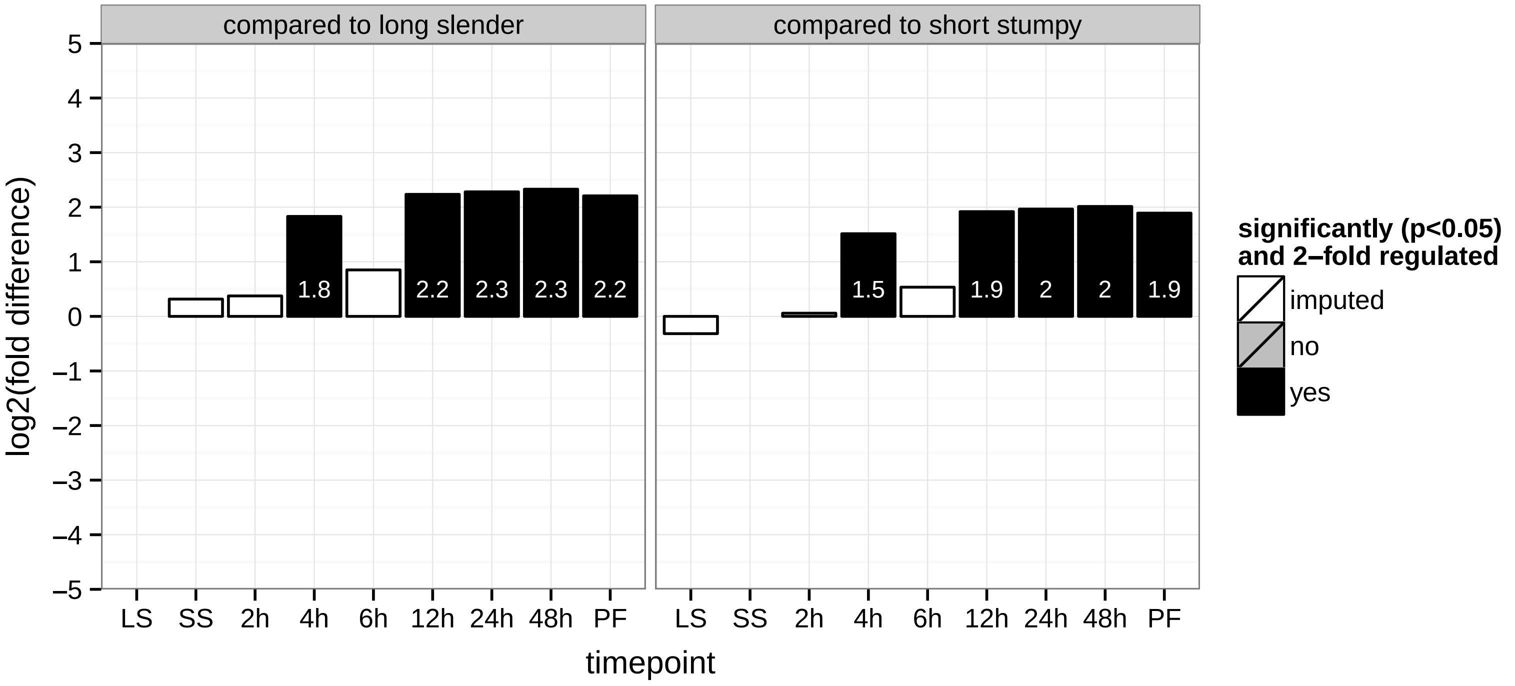

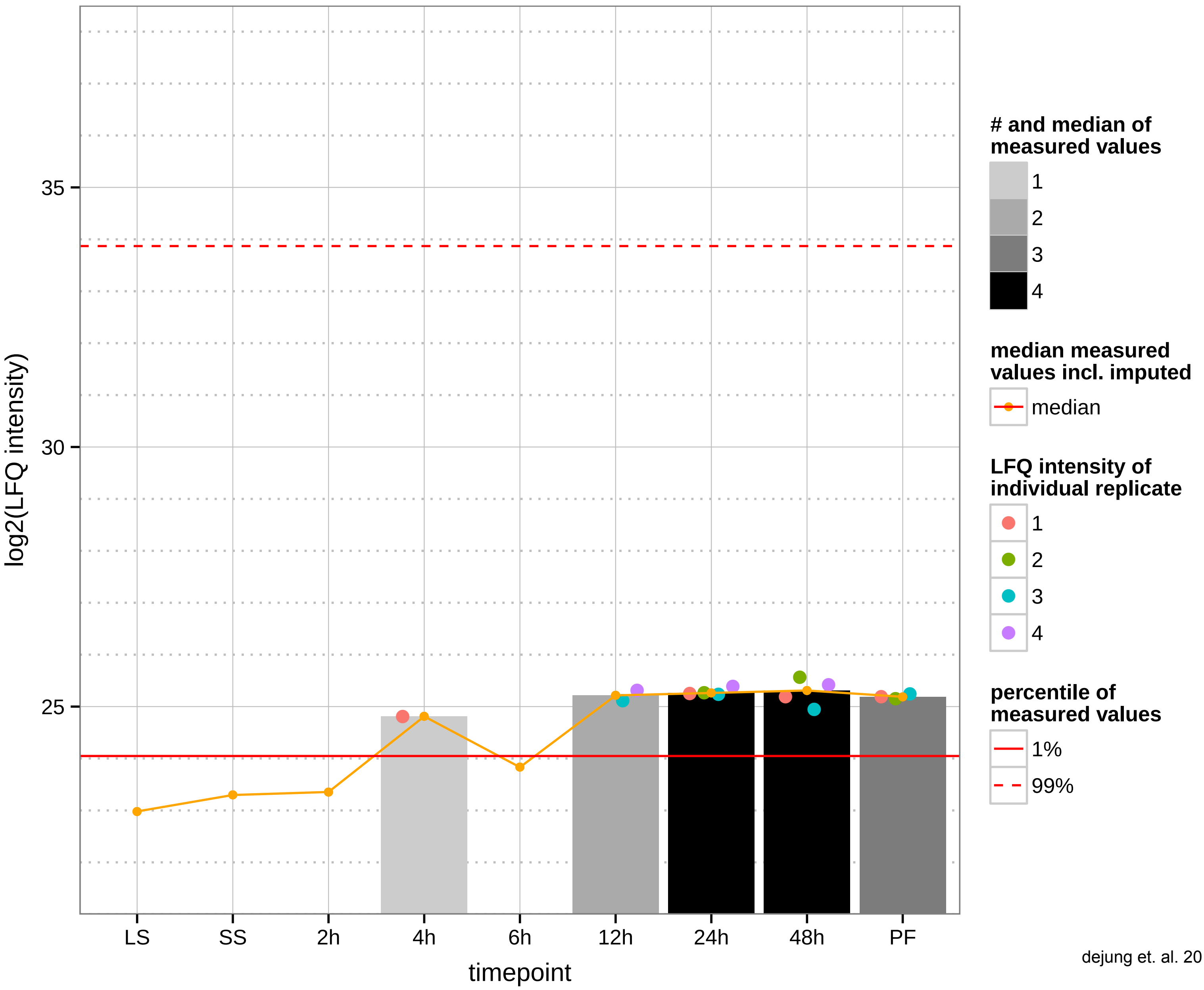

dejung et. al. 2015

hypothetical protein, conserved
Tb927.10.8030
AGOF: null
AGOC: mitochondrion
AGOP: null
PGOF: hydrogen ion transporting ATP synthase activity, rotational mechanism
PGOC: null
PGOP: ATP synthesis coupled proton transport

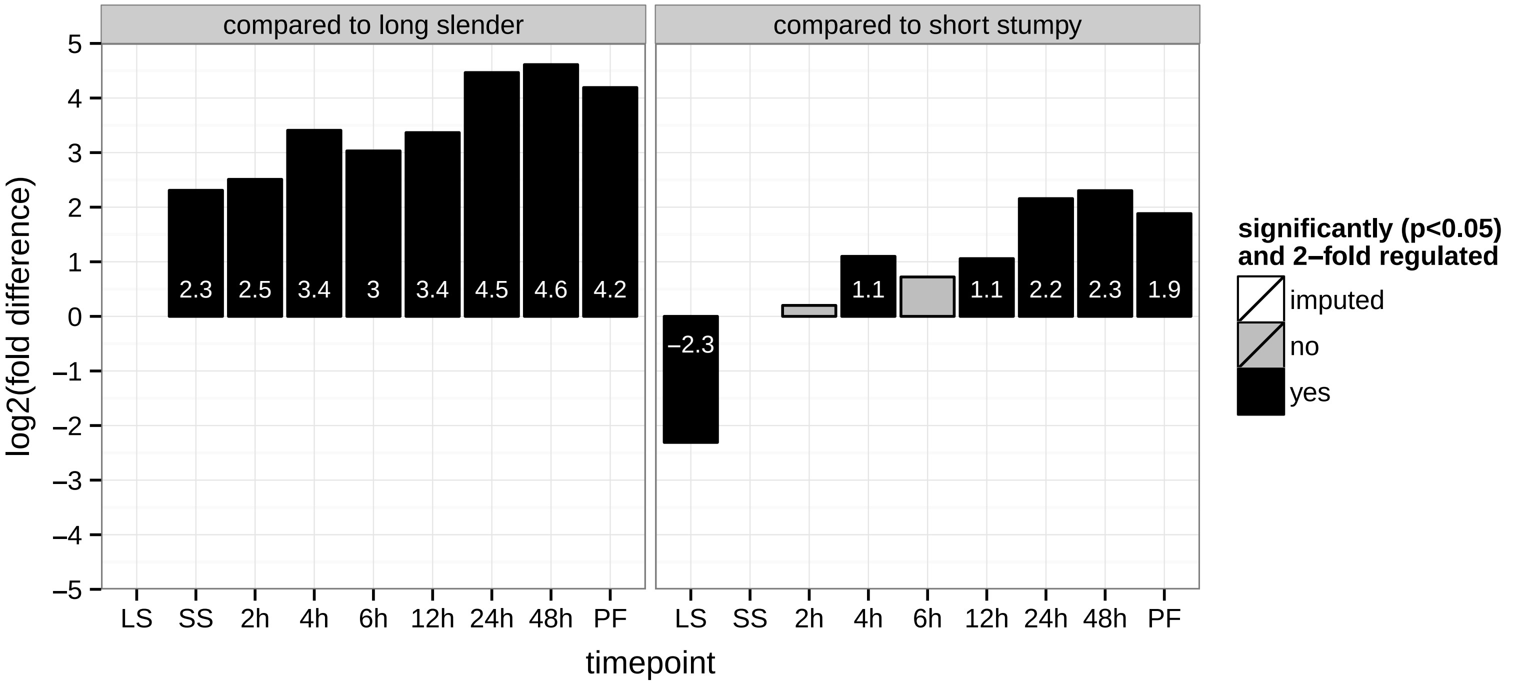

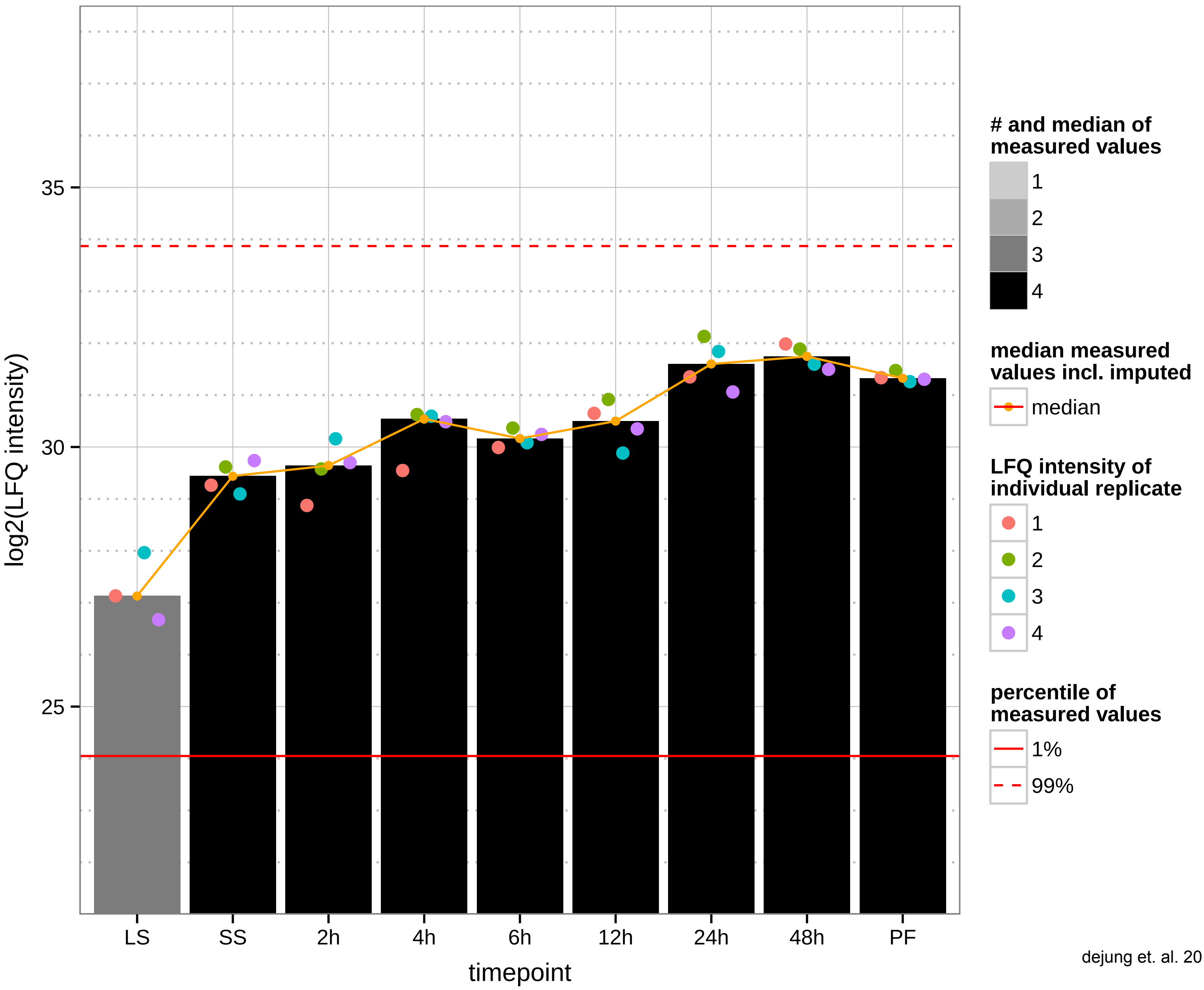

dejung et. al. 2015

hypothetical protein, conserved
Tb927.10.8240
AGOF: null
AGOC: null
AGOP: null
PGOF: null
PGOC: null
PGOP: null

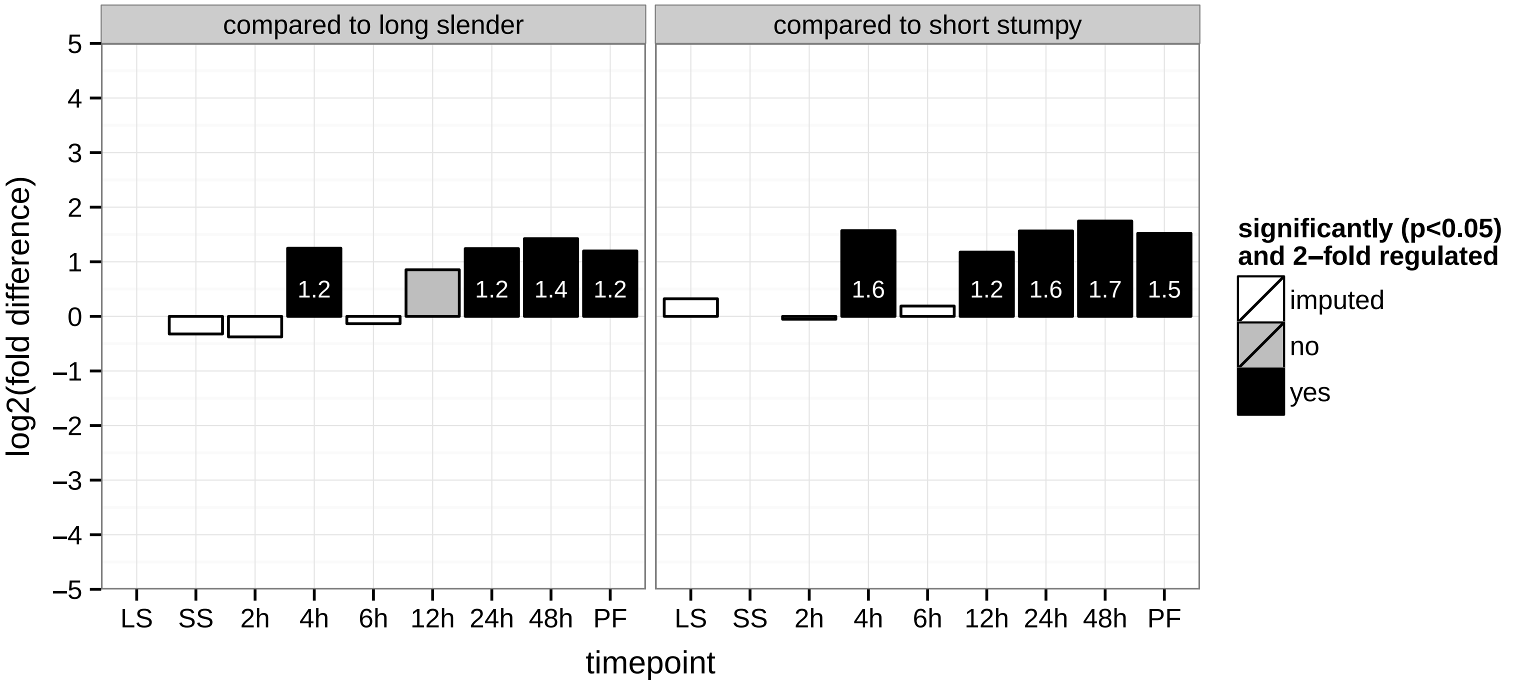

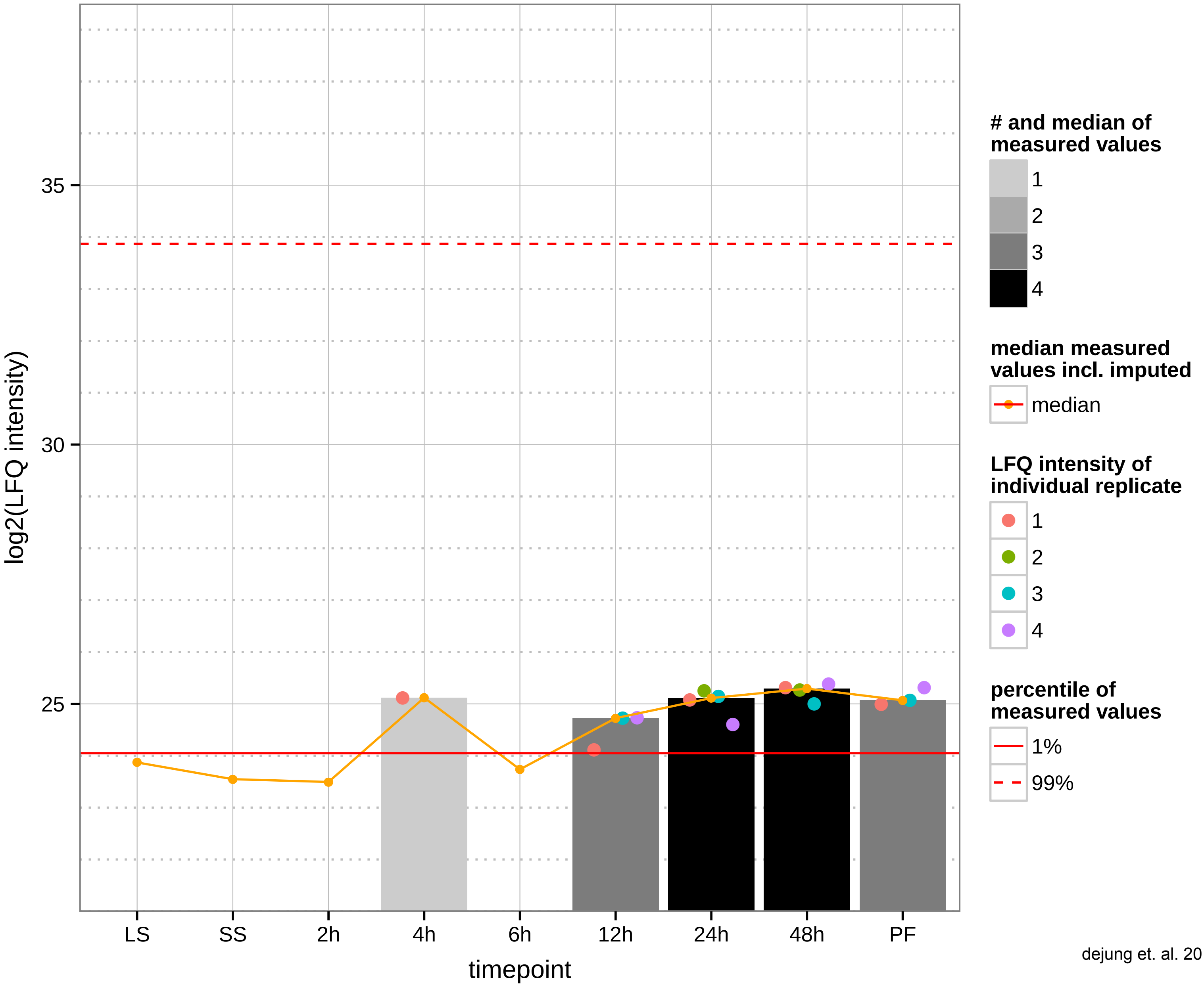

dejung et. al. 2015

hypothetical protein, conserved
Tb927.11.10040
AGOF: null
AGOC: null
AGOP: null
PGOF: null
PGOC: null
PGOP: null

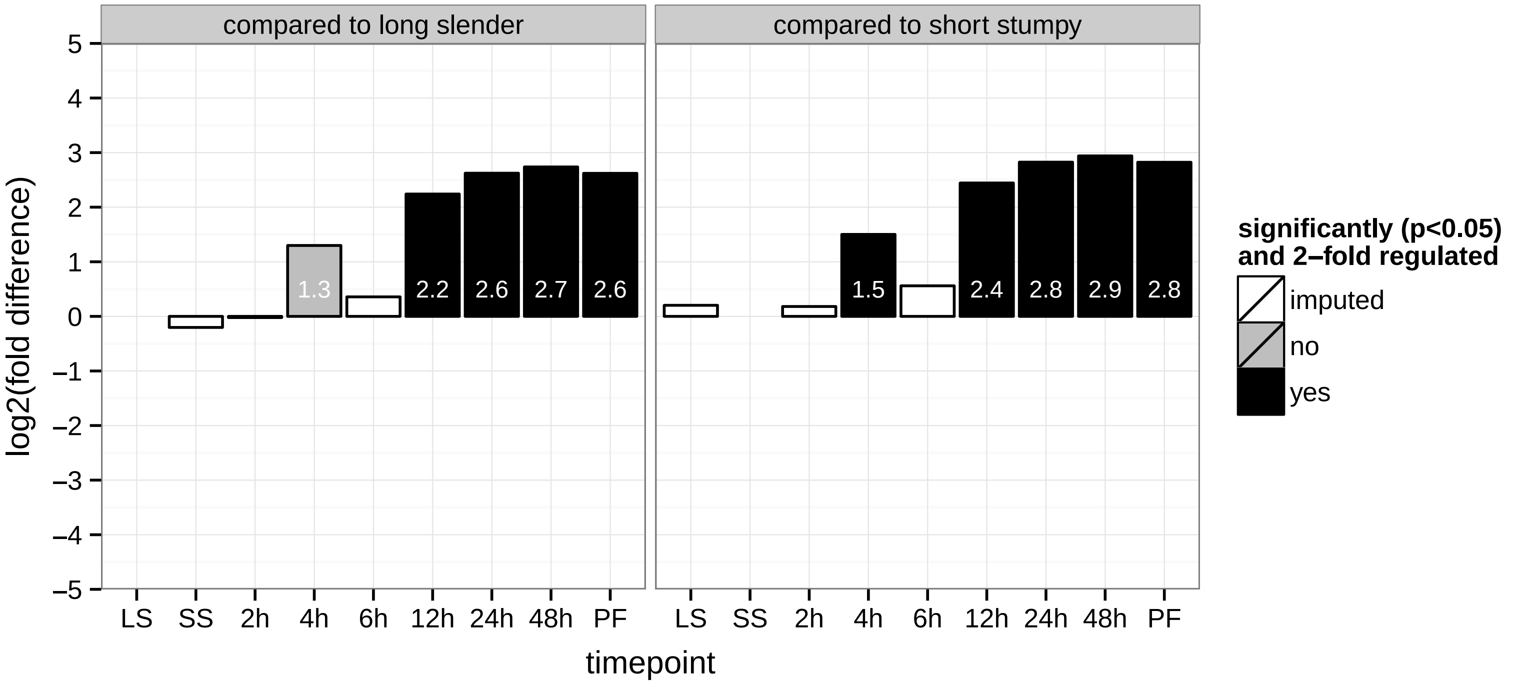

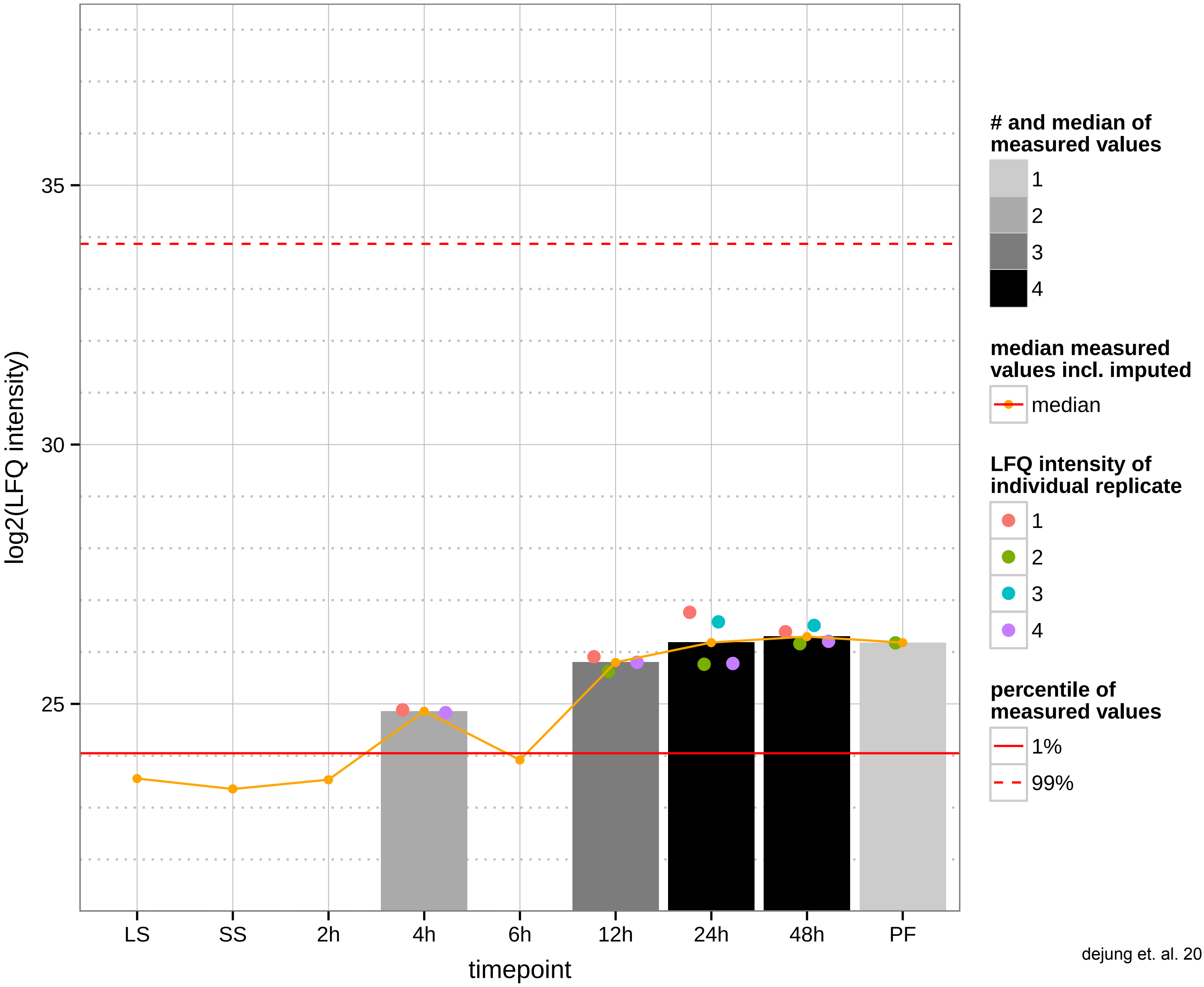

dejung et. al. 2015

RNA processing factor 1, putative, ribosome biogenesis protein
Tb927.11.1050
AGOF: rRNA primary transcript binding
AGOC: nucleolus
AGOP: RNA processing
PGOF: null
PGOC: null
PGOP: null

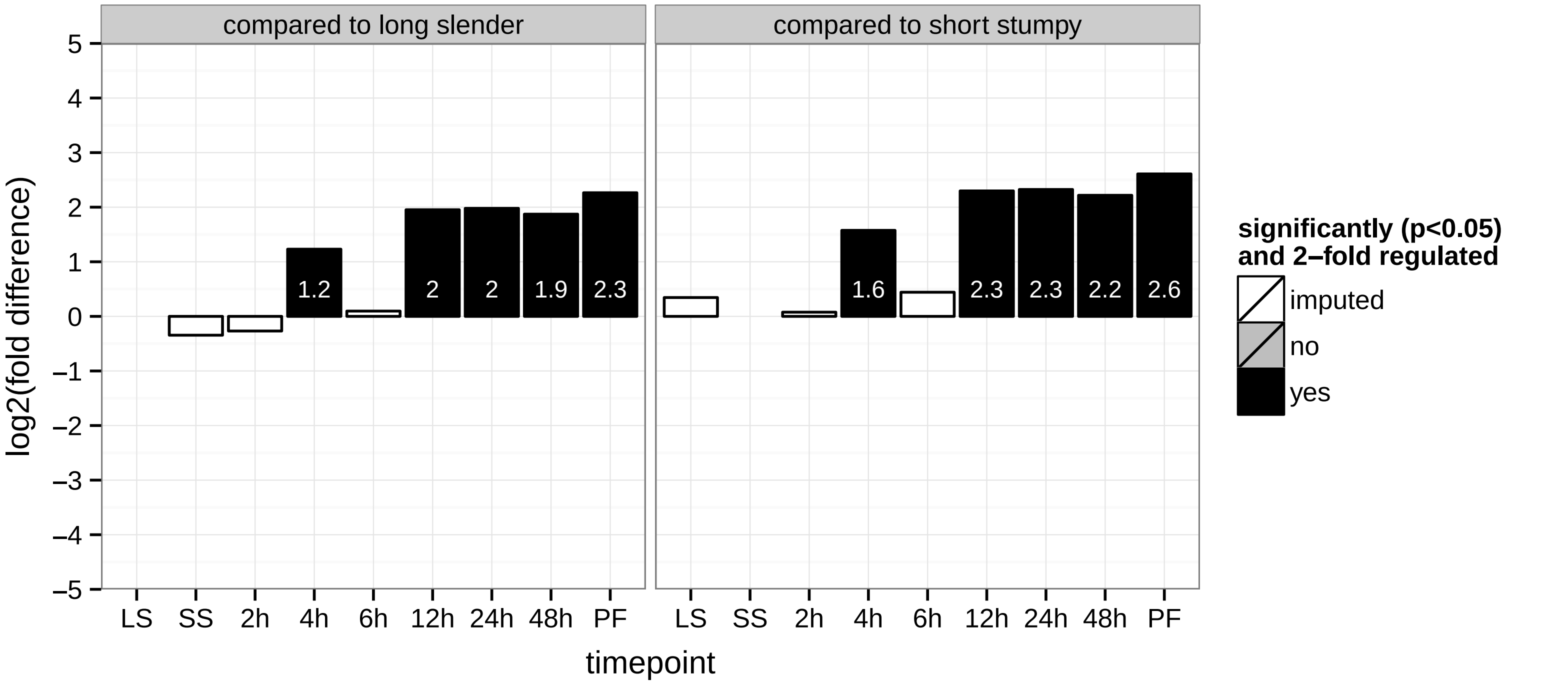

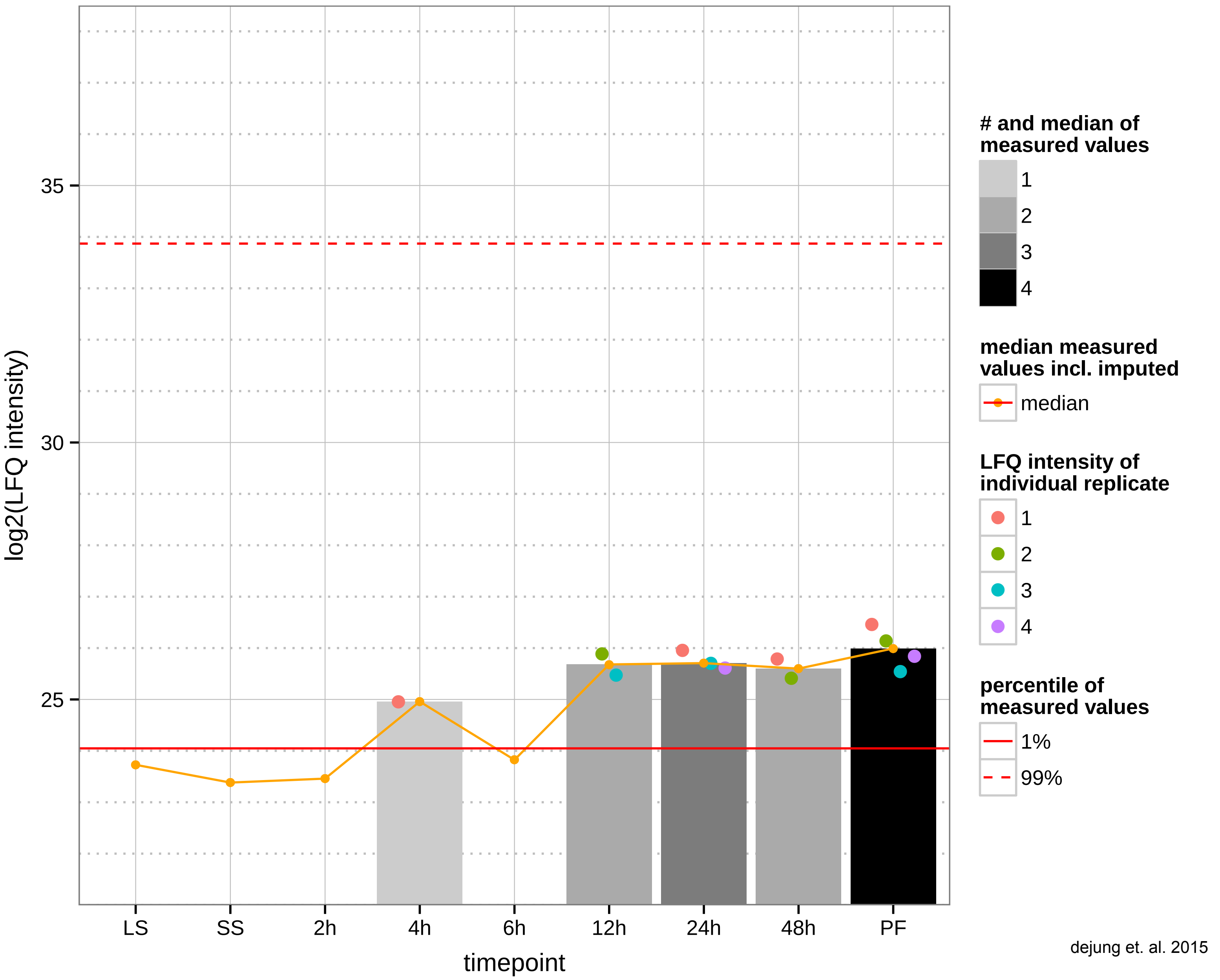

dejung et. al. 2015

DNA–directed RNA polymerase II, putative, RNA polymerase subunit, putative (RPB11)
Tb927.11.2200
AGOF: DNA binding, DNA–directed RNA polymerase activity, protein dimerization activity
AGOC: nucleus
AGOP: transcription, DNA–dependent
PGOF: DNA binding, DNA–directed RNA polymerase activity
PGOC: null
PGOP: transcription, DNA–dependent

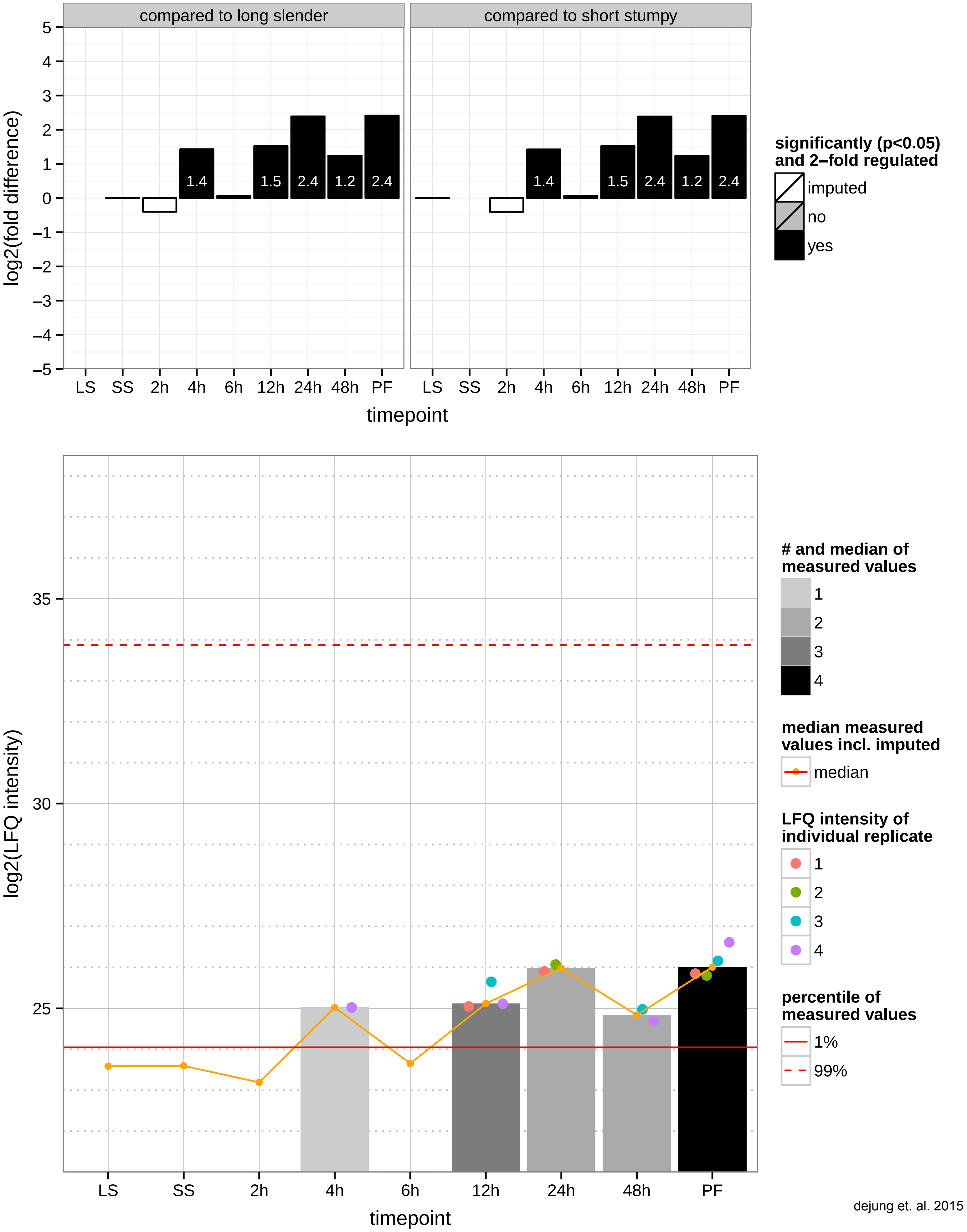

dejung et. al. 2015

glycosomal transporter (GAT2) (GAT2)
Tb927.11.3130
AGOF: ATP binding, ATPase activity, coupled to transmembrane movement of substances
AGOC: glycosome membrane, integral to membrane
AGOP: fatty acid transport
PGOF: ATP binding, ATPase activity, coupled to transmembrane movement of substances, nucleoside–triphosphatase activity
PGOC: integral to membrane, membrane
PGOP: transmembrane transport, transport

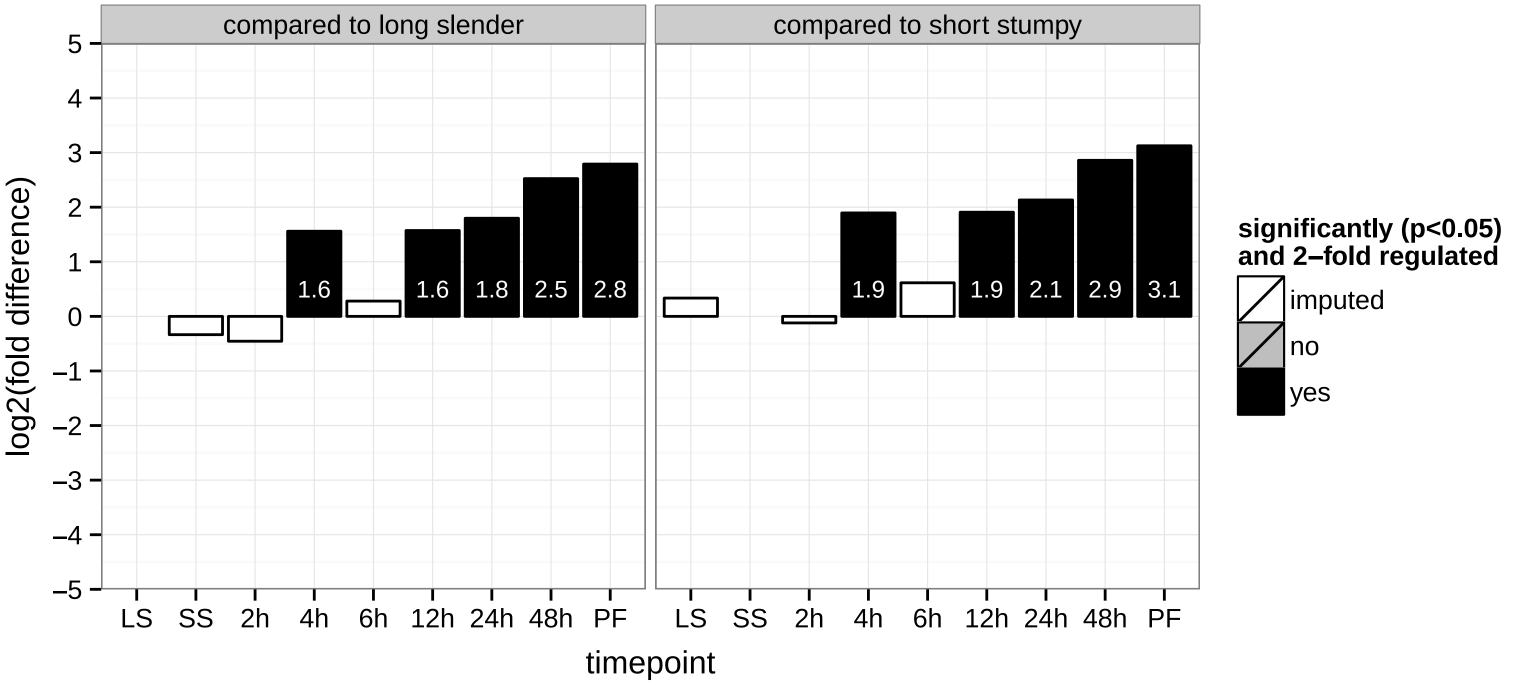

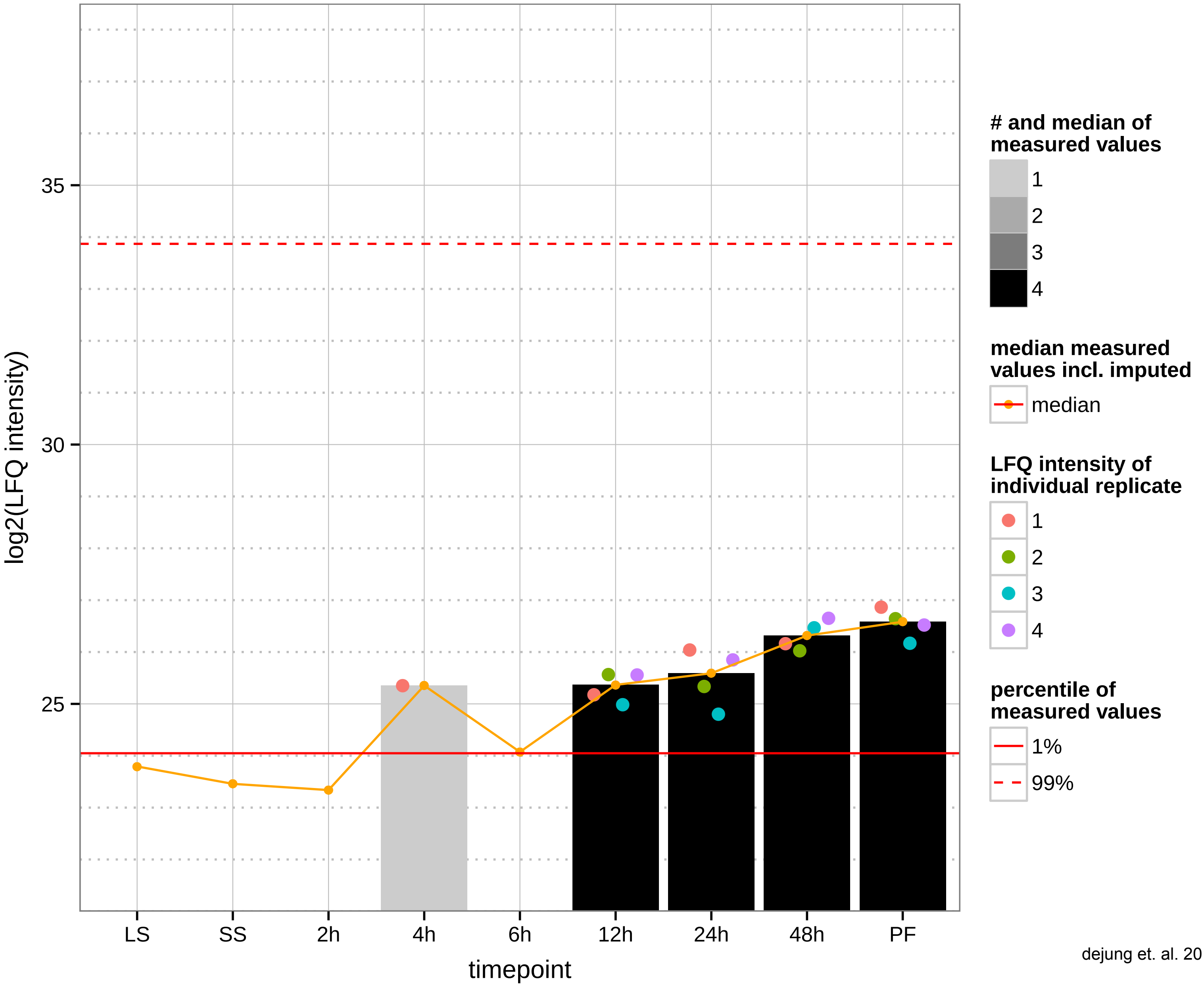

dejung et. al. 2015

hypothetical protein
Tb927.11.3370
AGOF: null
AGOC: null
AGOP: null
PGOF: null
PGOC: null
PGOP: null

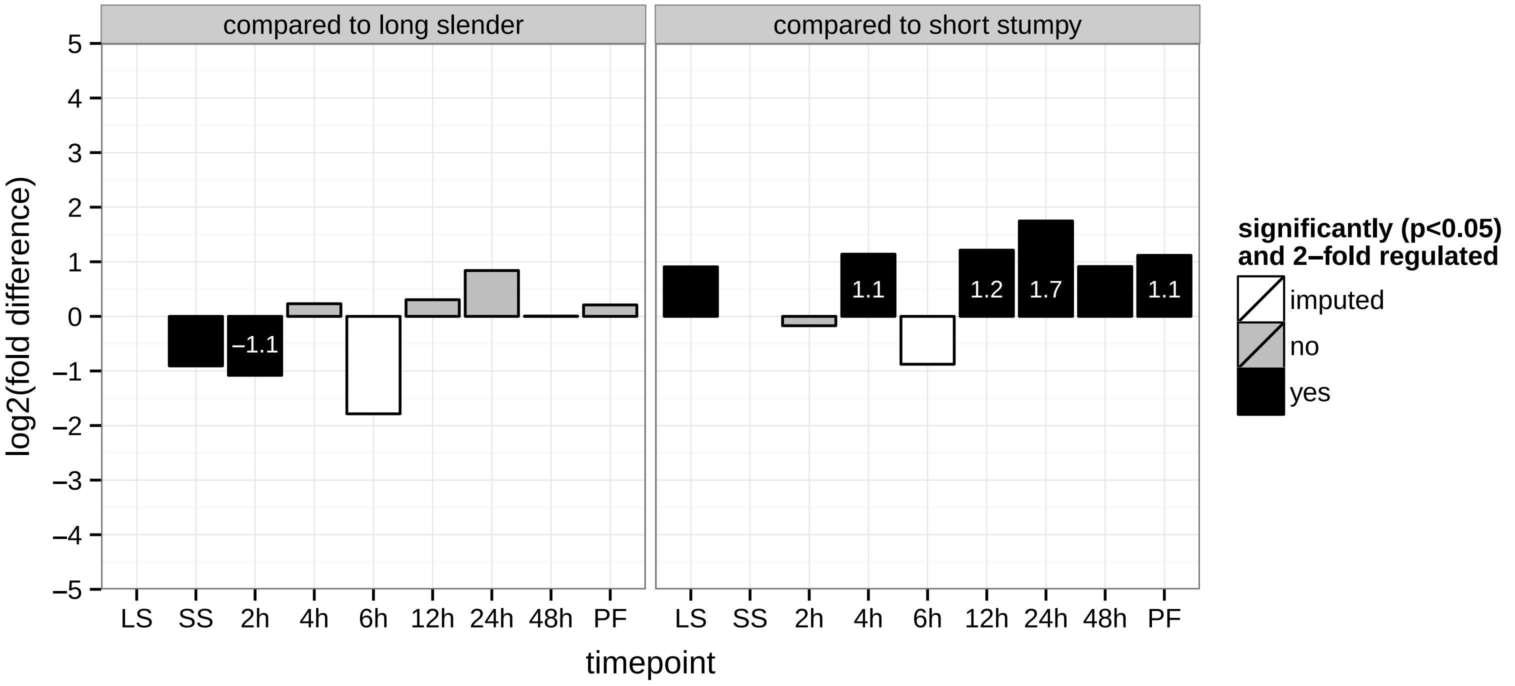

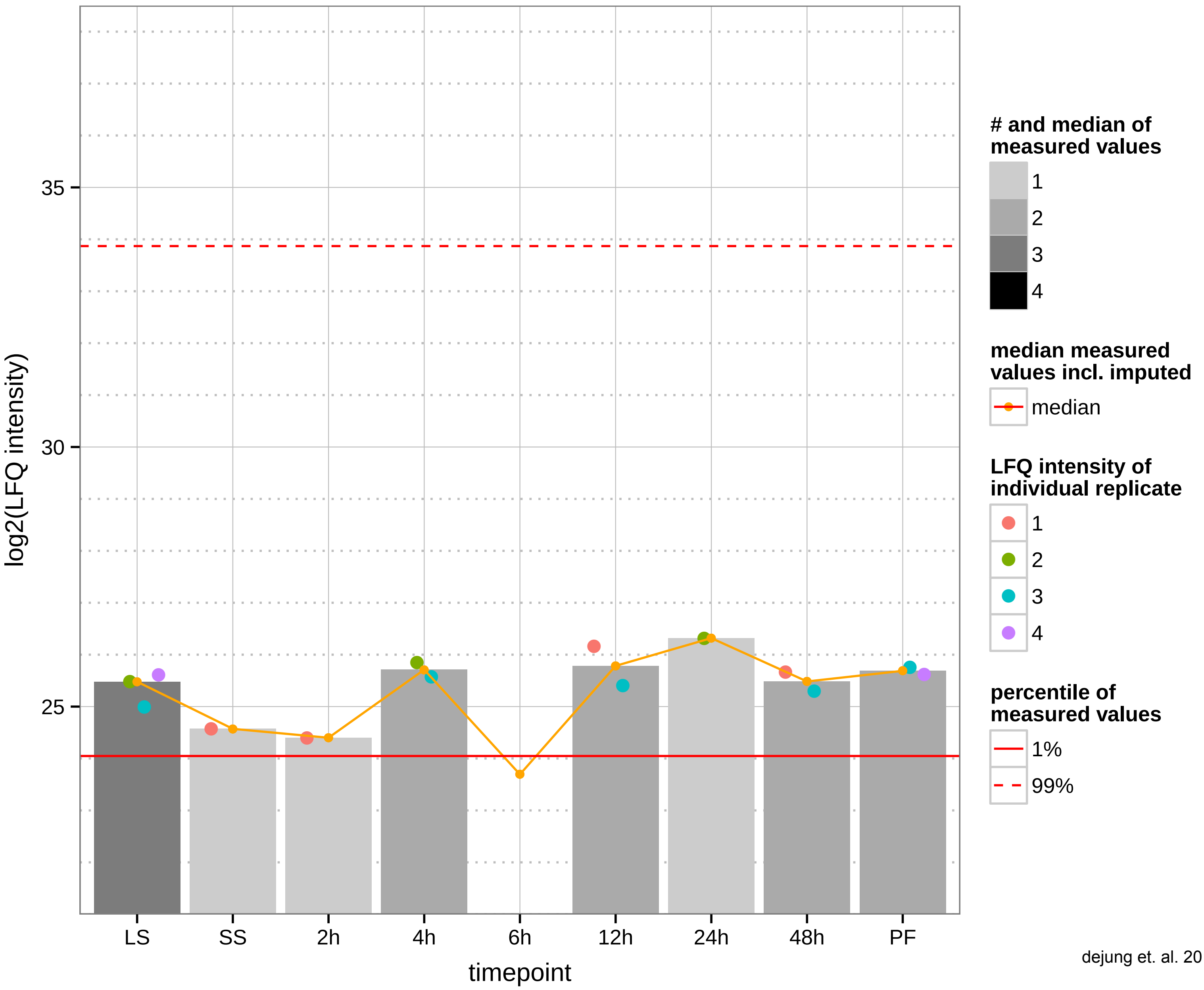

dejung et. al. 2015

nucleic acid binding protein, putative
Tb927.11.6620
AGOF: nucleic acid binding, zinc ion binding
AGOC: null
AGOP: null
PGOF: nucleic acid binding, zinc ion binding
PGOC: null
PGOP: null

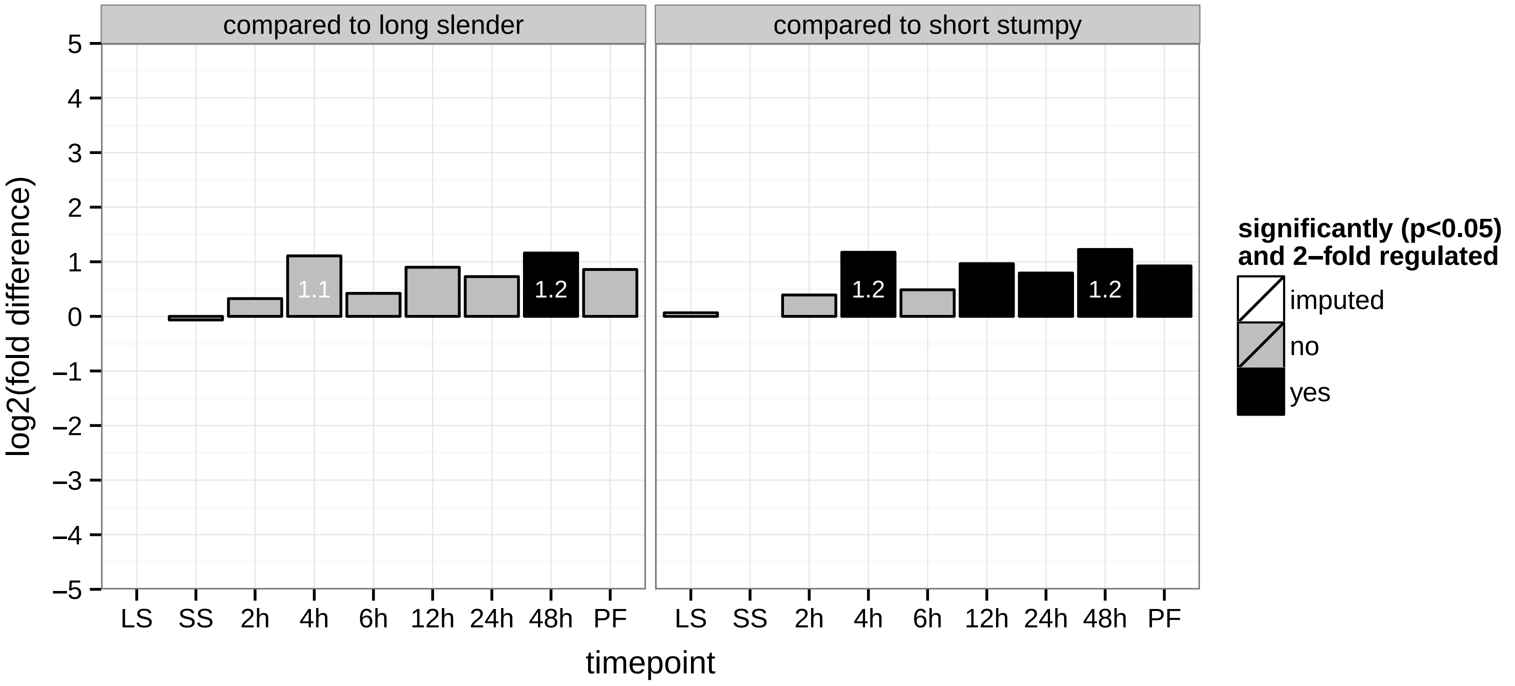

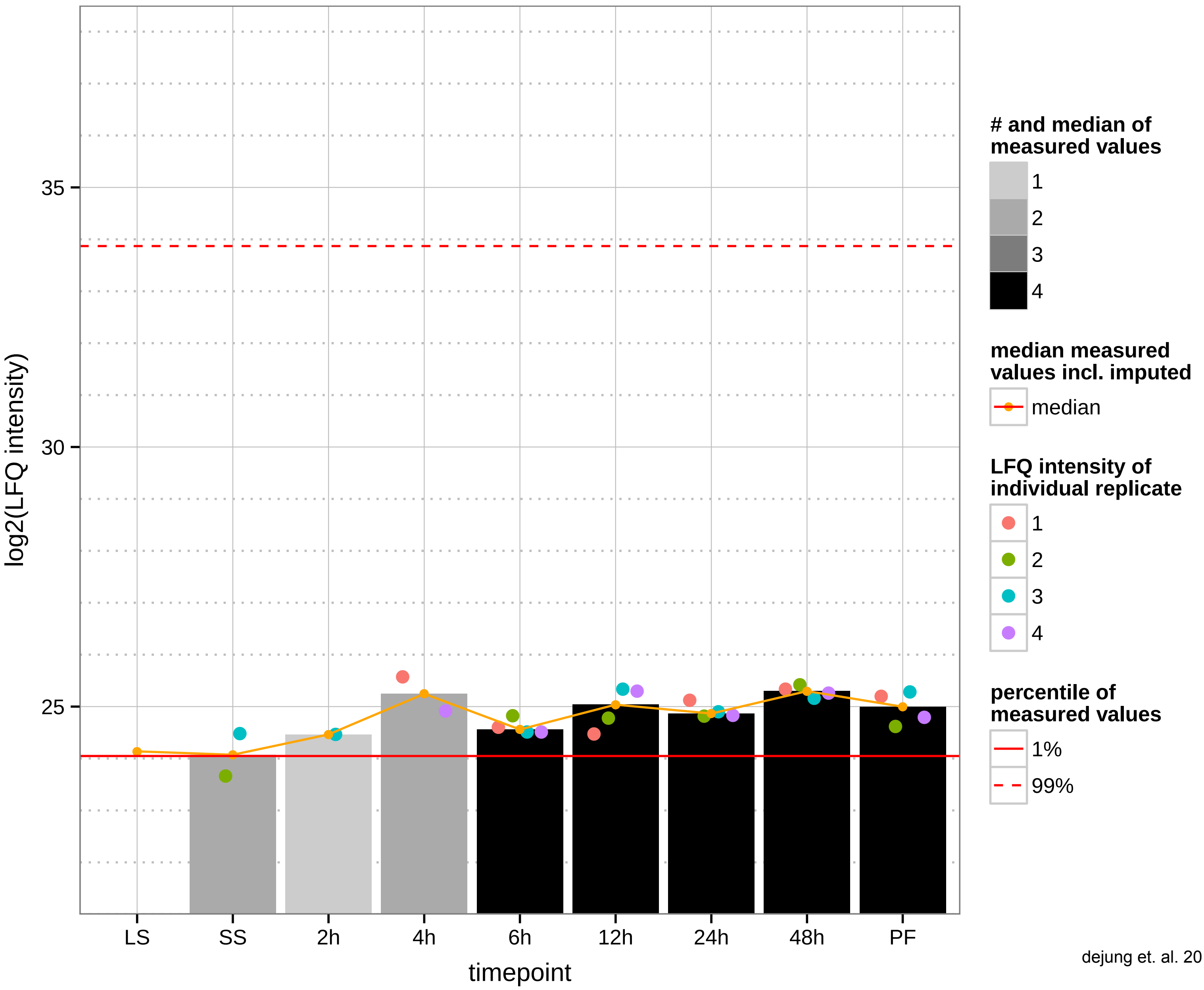

dejung et. al. 2015

cytochrome b5, putative (CYB5)
Tb927.11.6640
AGOF: glutathione−disulfide reductase activity, heme binding
AGOC: integral to membrane, mitochondrial inner membrane
AGOP: oxidation−reduction process
PGOF: heme binding
PGOC: null
PGOP: null

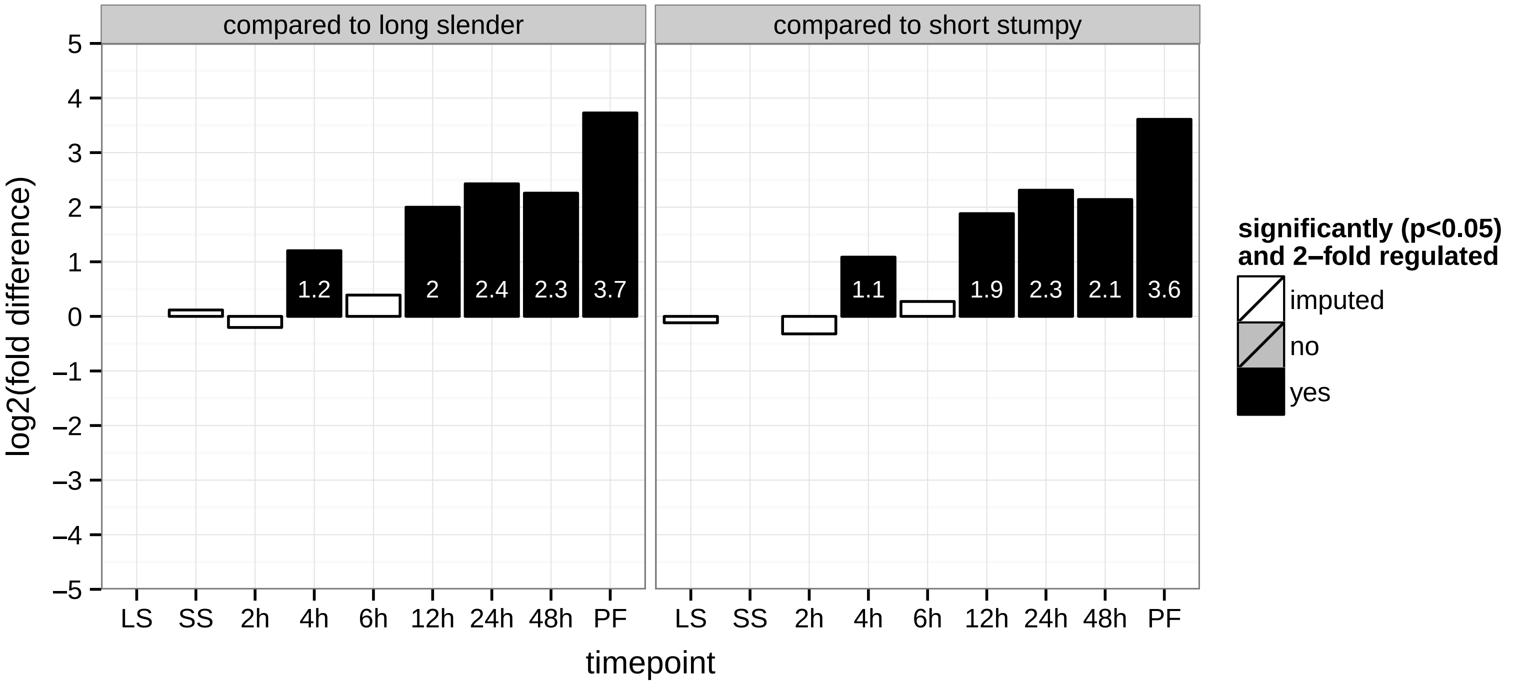

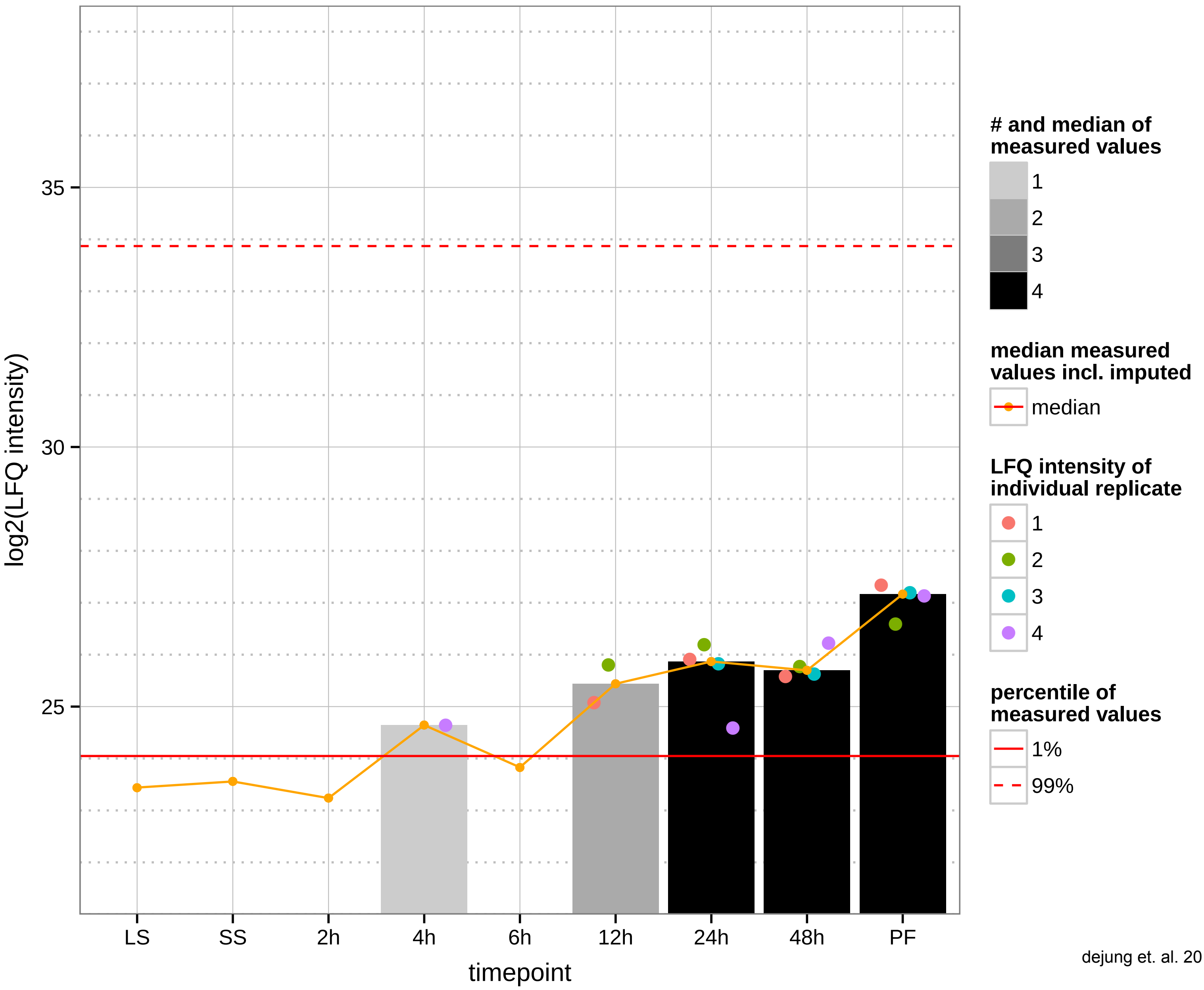

dejung et. al. 2015

hypothetical protein, conserved
Tb927.11.6830
AGOF: null
AGOC: mitochondrion
AGOP: null
PGOF: null
PGOC: null
PGOP: null

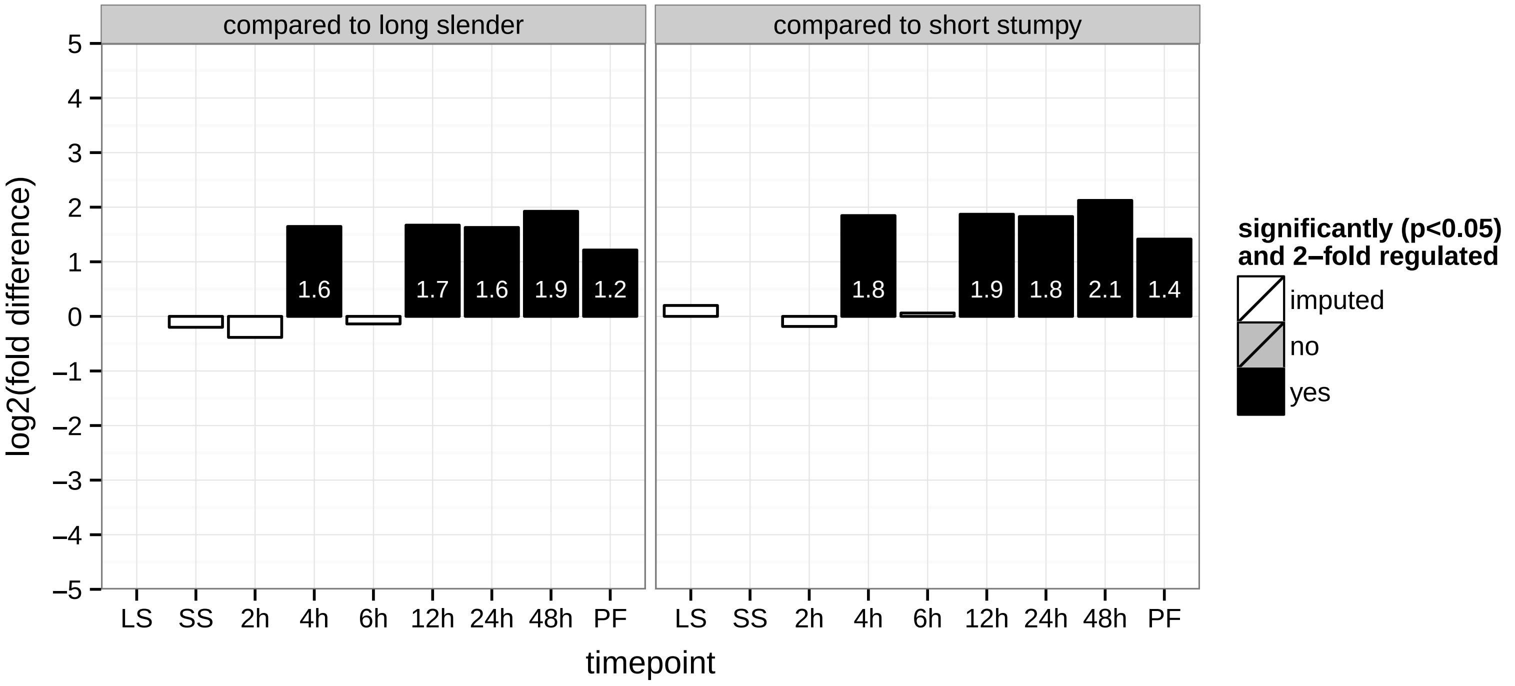

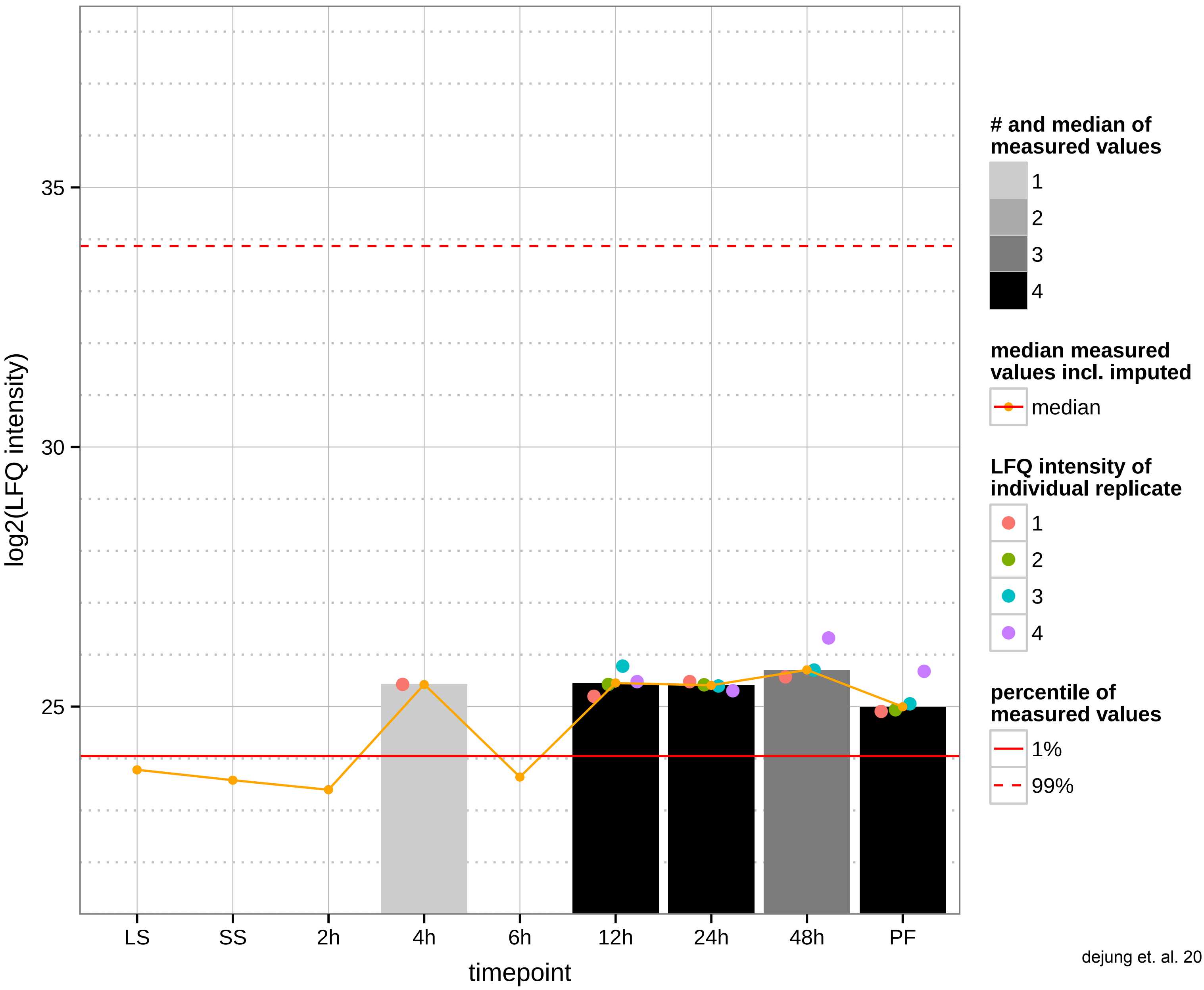

dejung et. al. 2015

hypothetical protein, conserved
Tb927.11.9600
AGOF: null
AGOC: mitochondrial matrix, mitochondrion
AGOP: null
PGOF: null
PGOC: mitochondrial matrix
PGOP: null

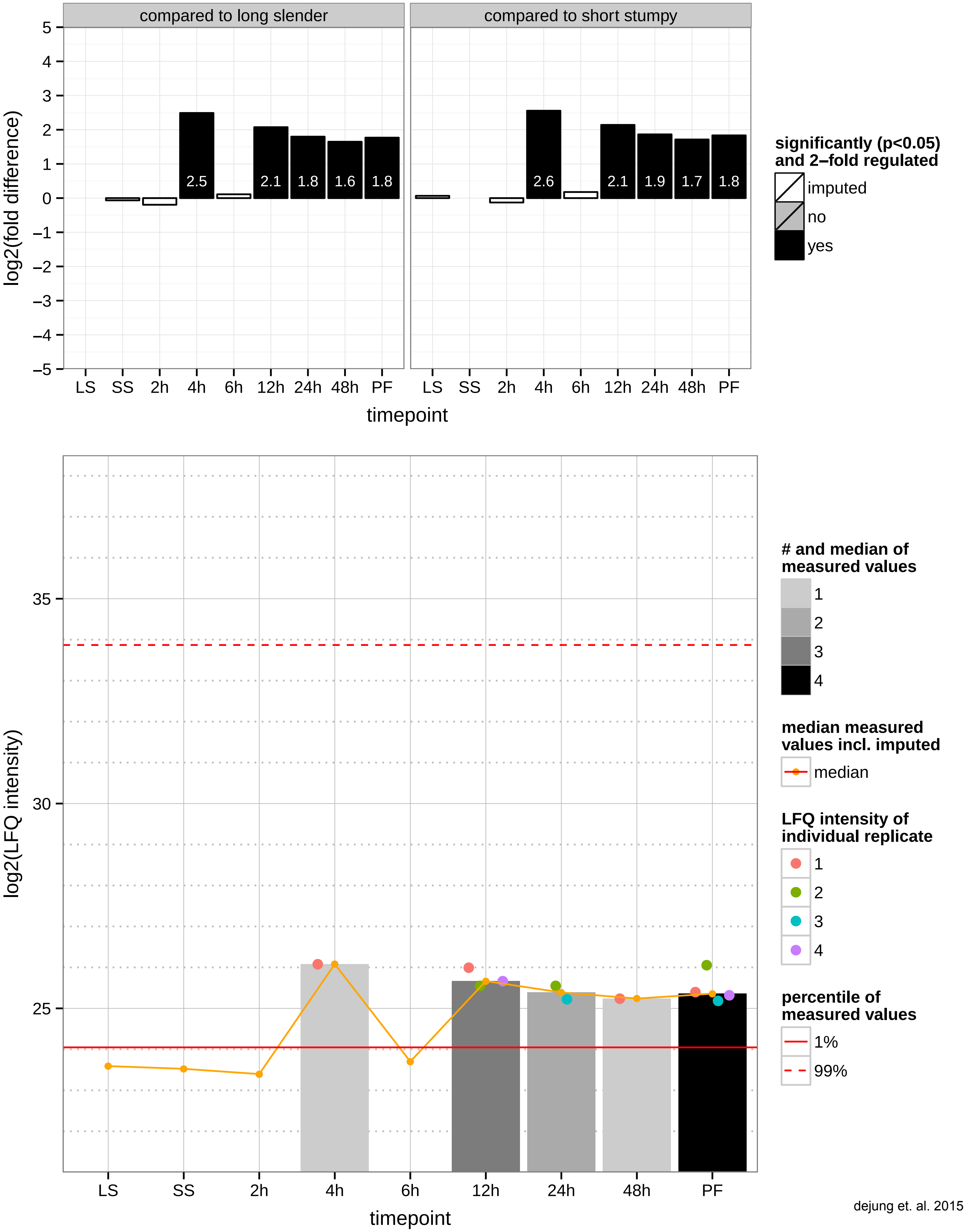

dejung et. al. 2015

hypothetical protein, conserved
Tb927.11.9750
AGOF: null
AGOC: mitochondrion
AGOP: null
PGOF: null
PGOC: null
PGOP: null

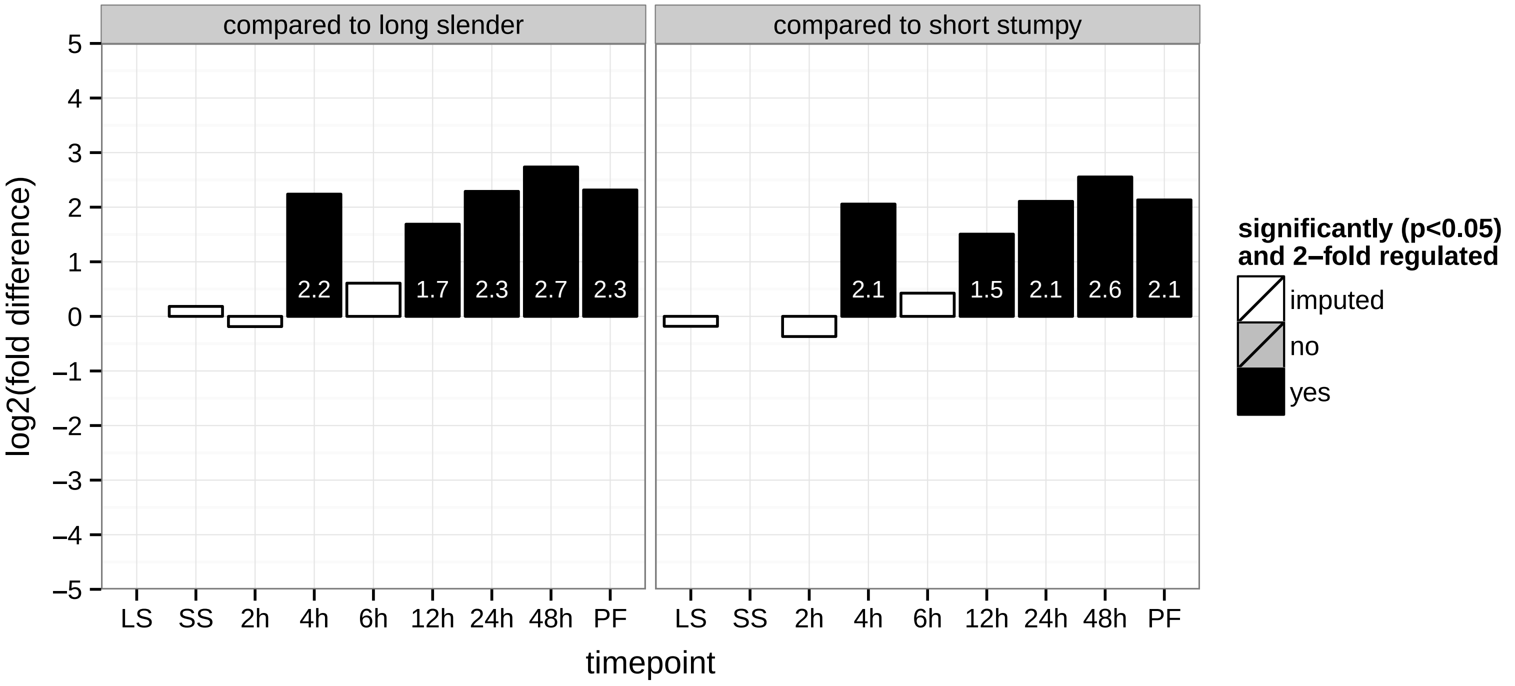

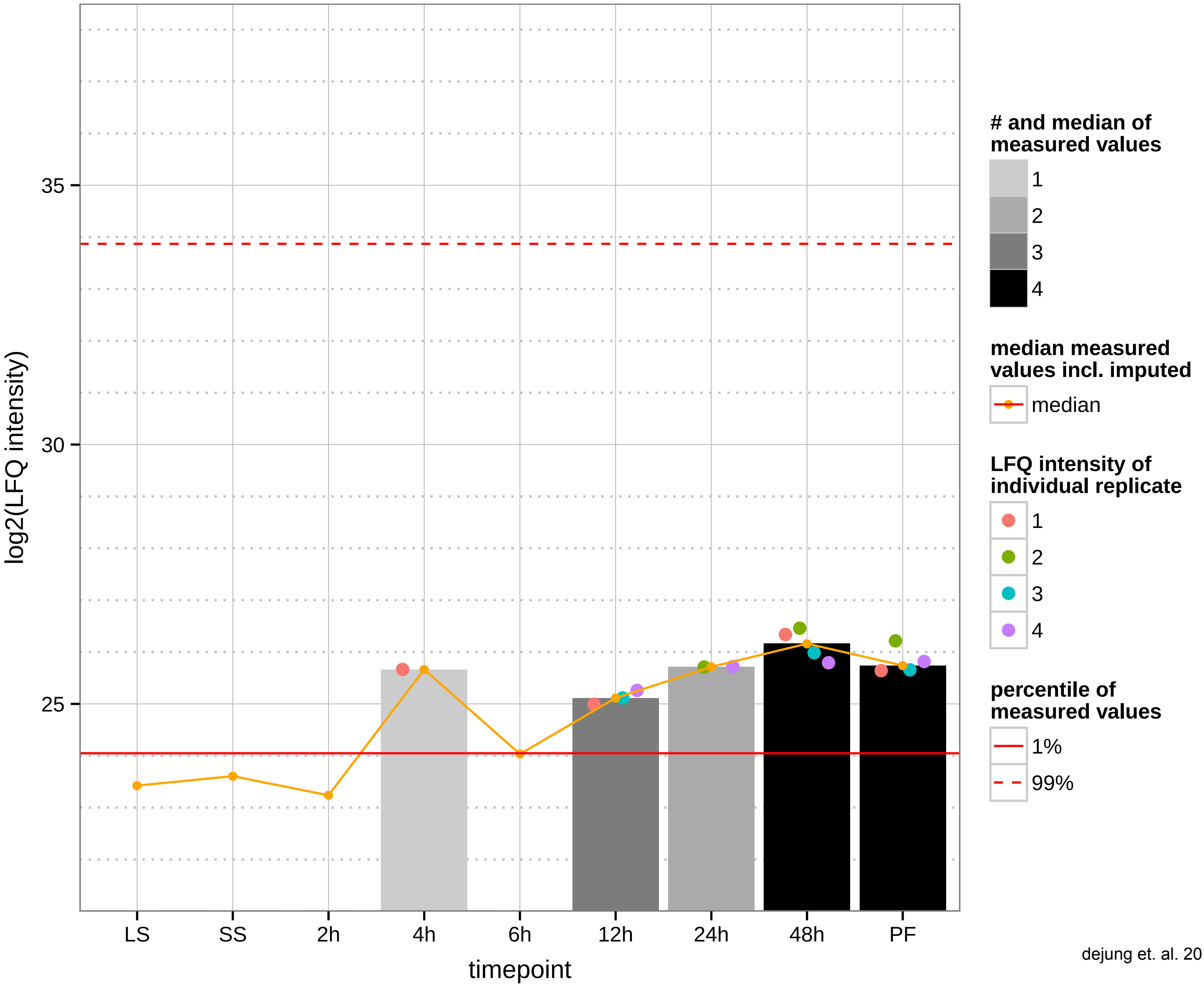

dejung et. al. 2015

endo/exonuclease Mre11 (MRE11)
Tb927.2.4390
AGOF: damaged DNA binding, endonuclease activity, exonuclease activity, hydrolase activity, manganese ion binding
AGOC: nucleus
AGOP: double−strand break repair
PGOF: endonuclease activity, exonuclease activity, hydrolase activity, manganese ion binding
PGOC: nucleus
PGOP: DNA metabolic process, double−strand break repair

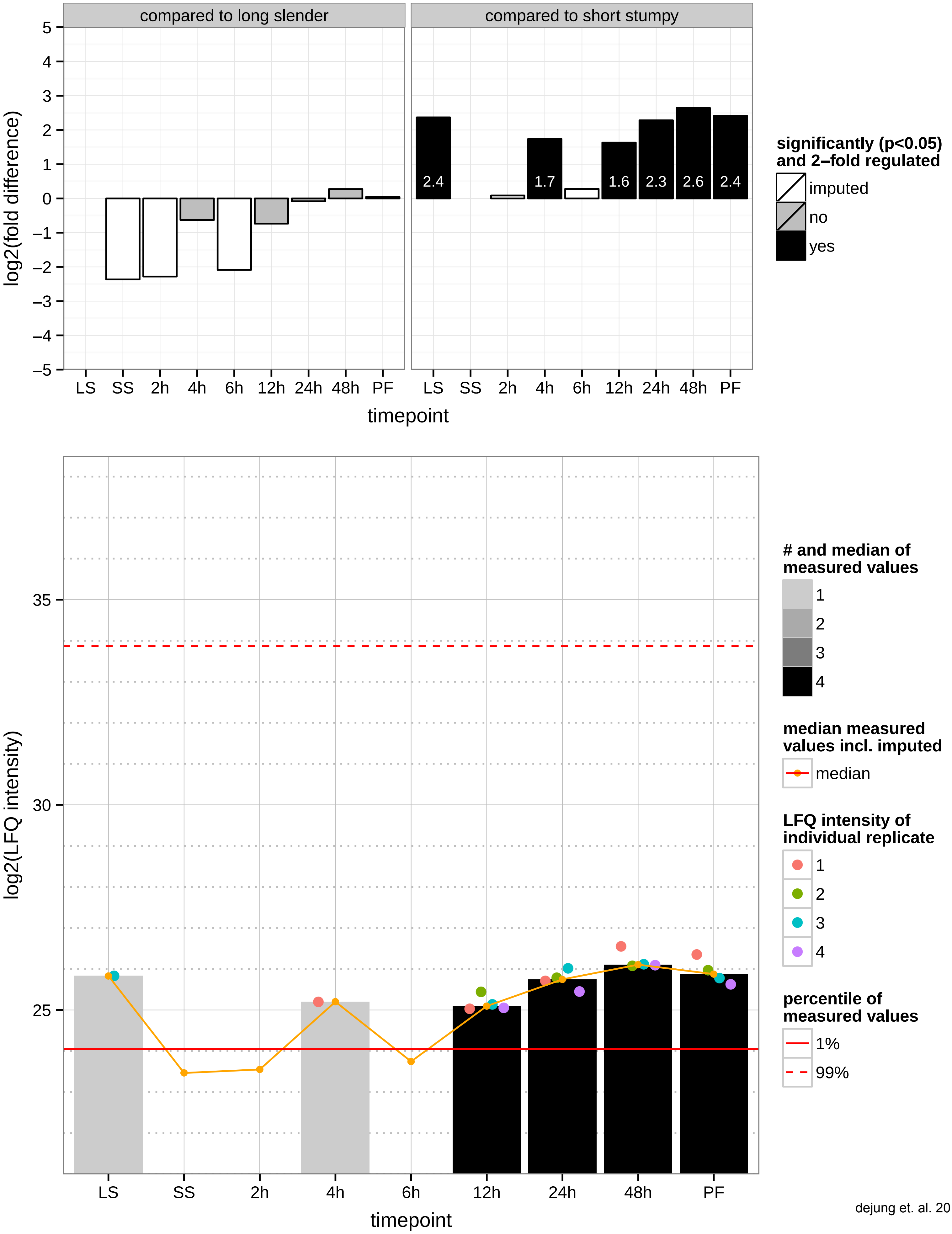

dejung et. al. 2015

hypothetical protein, conserved
Tb927.2.4850
AGOF: null
AGOC: null
AGOP: null
PGOF: null
PGOC: null
PGOP: null

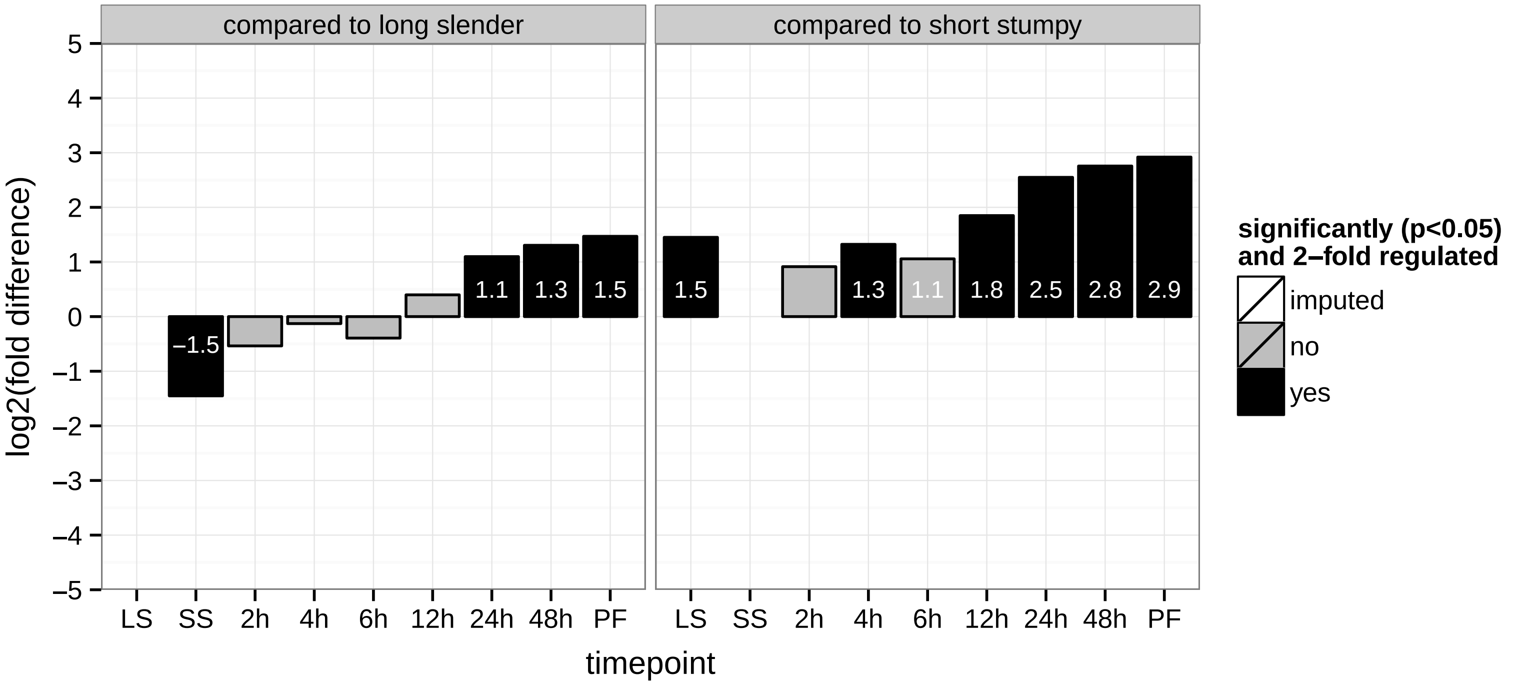

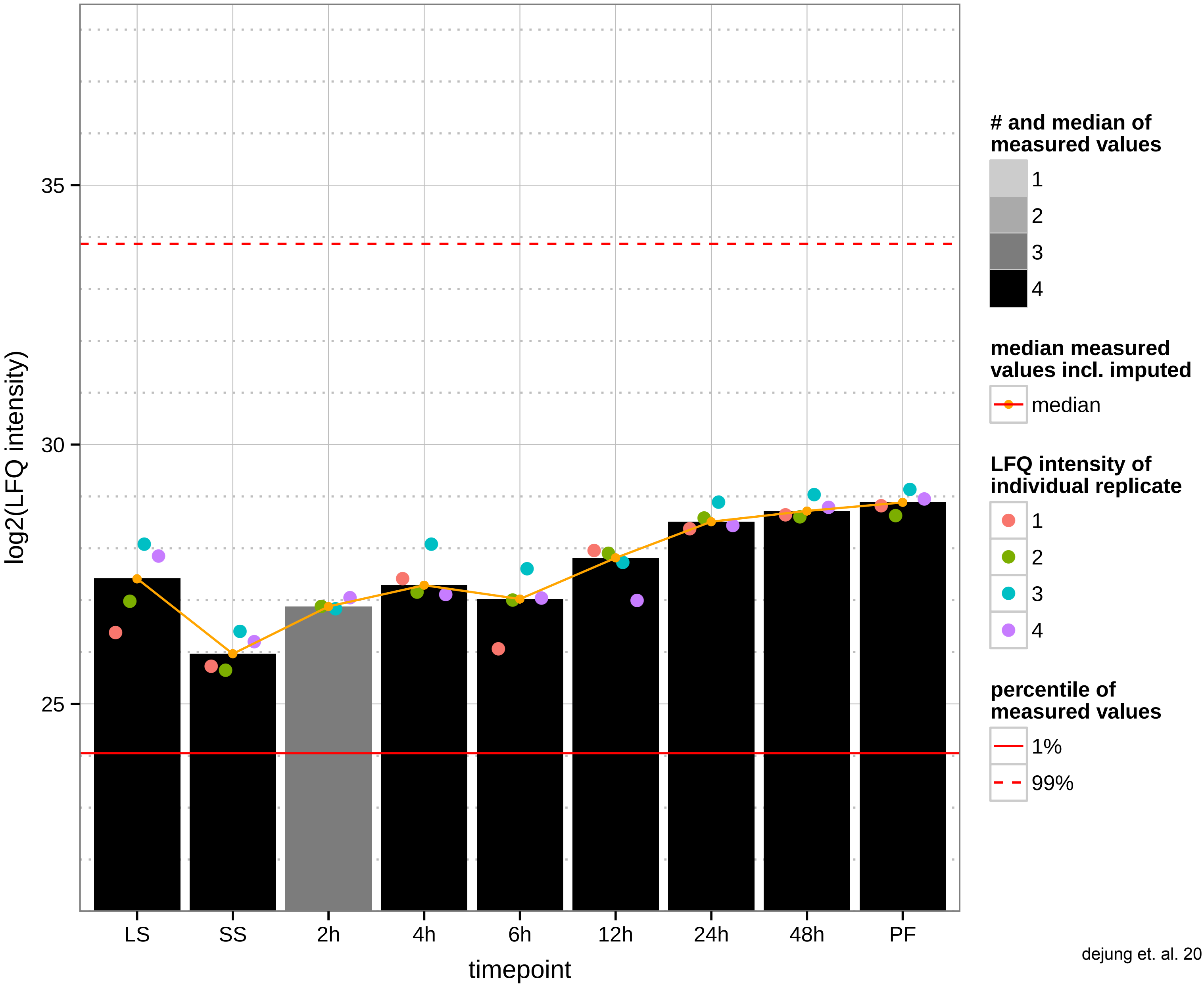

dejung et. al. 2015

hypothetical protein, conserved
Tb927.2.5970
AGOF: null
AGOC: null
AGOP: null
PGOF: null
PGOC: null
PGOP: null

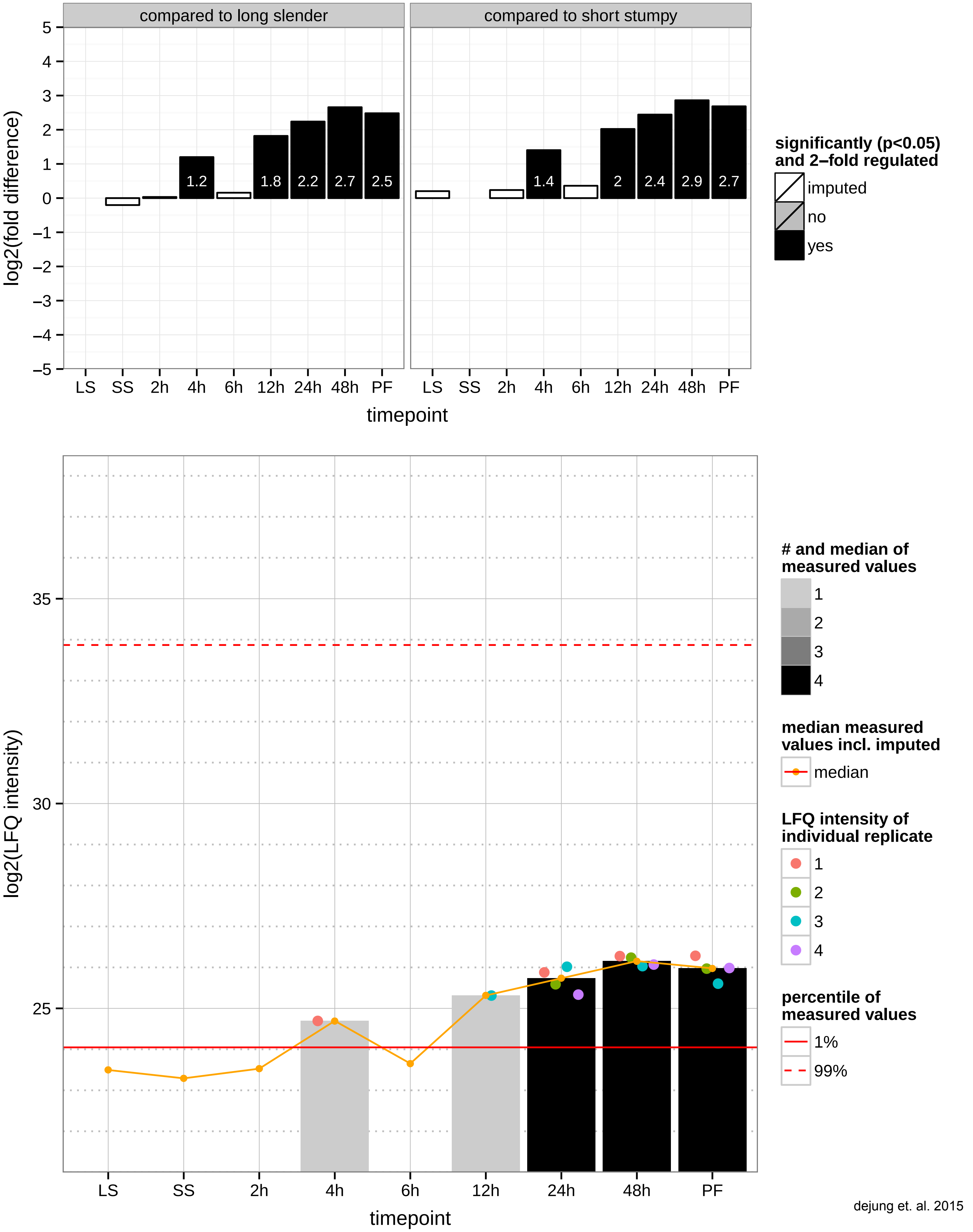

dejung et. al. 2015

hypothetical protein, conserved
Tb927.3.2080
AGOF: null
AGOC: mitochondrion
AGOP: null
PGOF: null
PGOC: null
PGOP: null

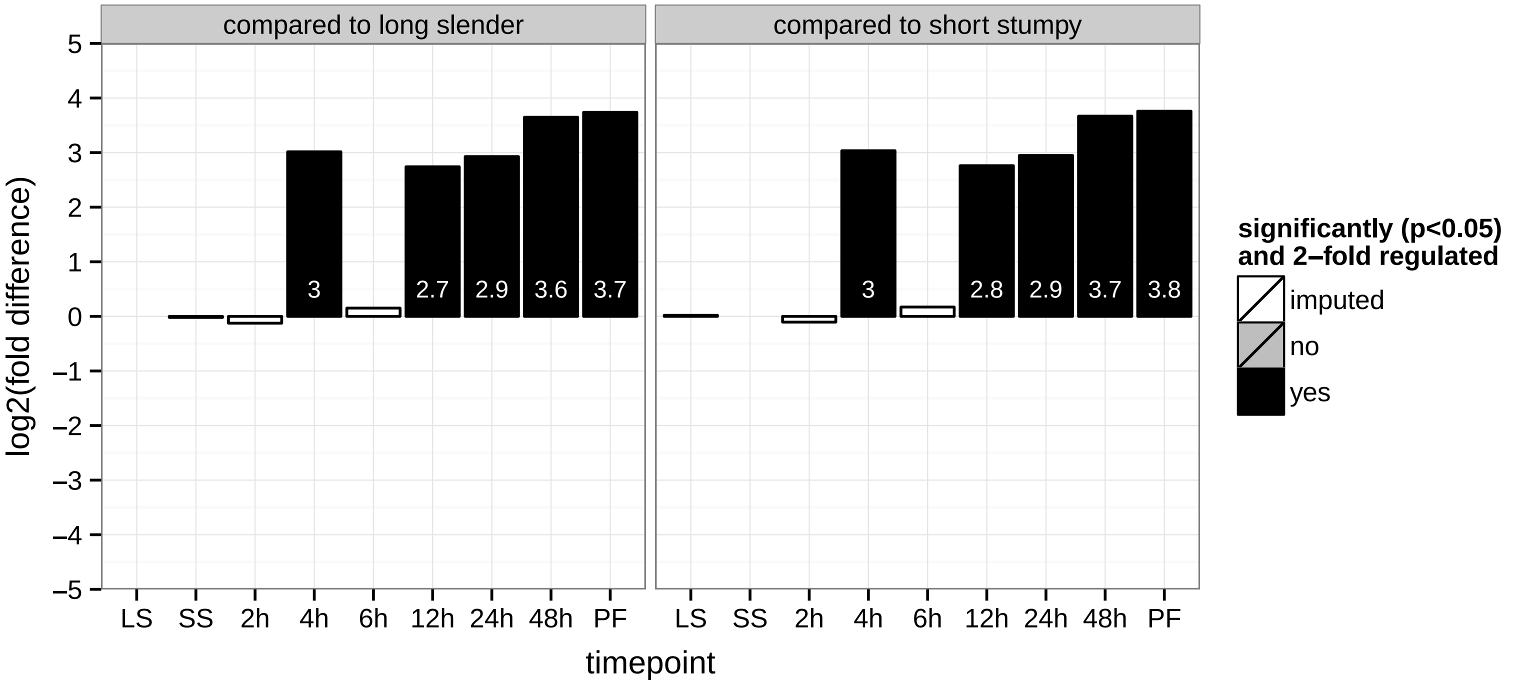

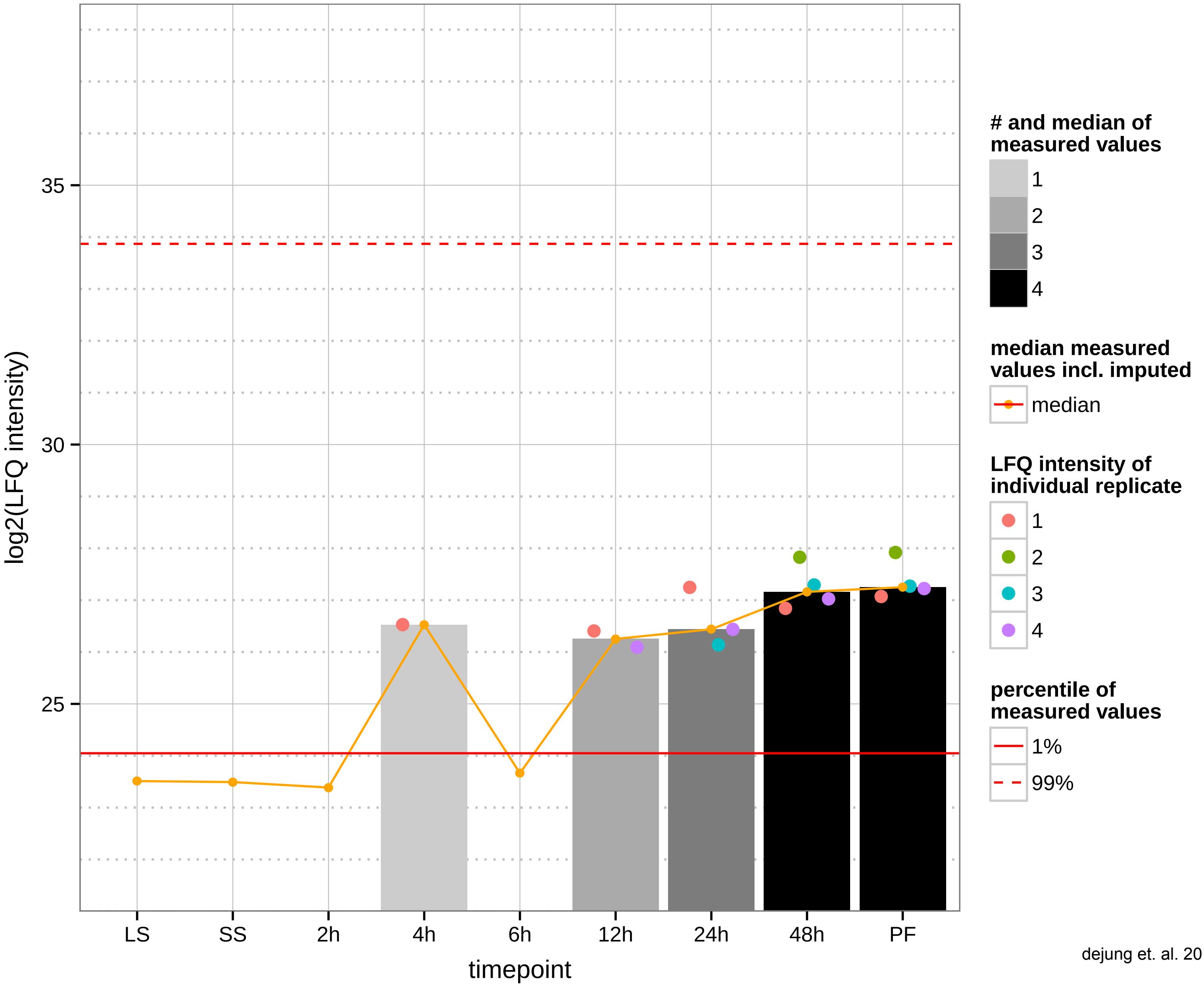

dejung et. al. 2015

hypothetical protein, conserved
Tb927.3.2130
AGOF: RNA binding, pseudouridine synthase activity
AGOC: mitochondrion
AGOP: pseudouridine synthesis
PGOF: RNA binding, pseudouridine synthase activity
PGOC: null
PGOP: RNA modification, pseudouridine synthesis

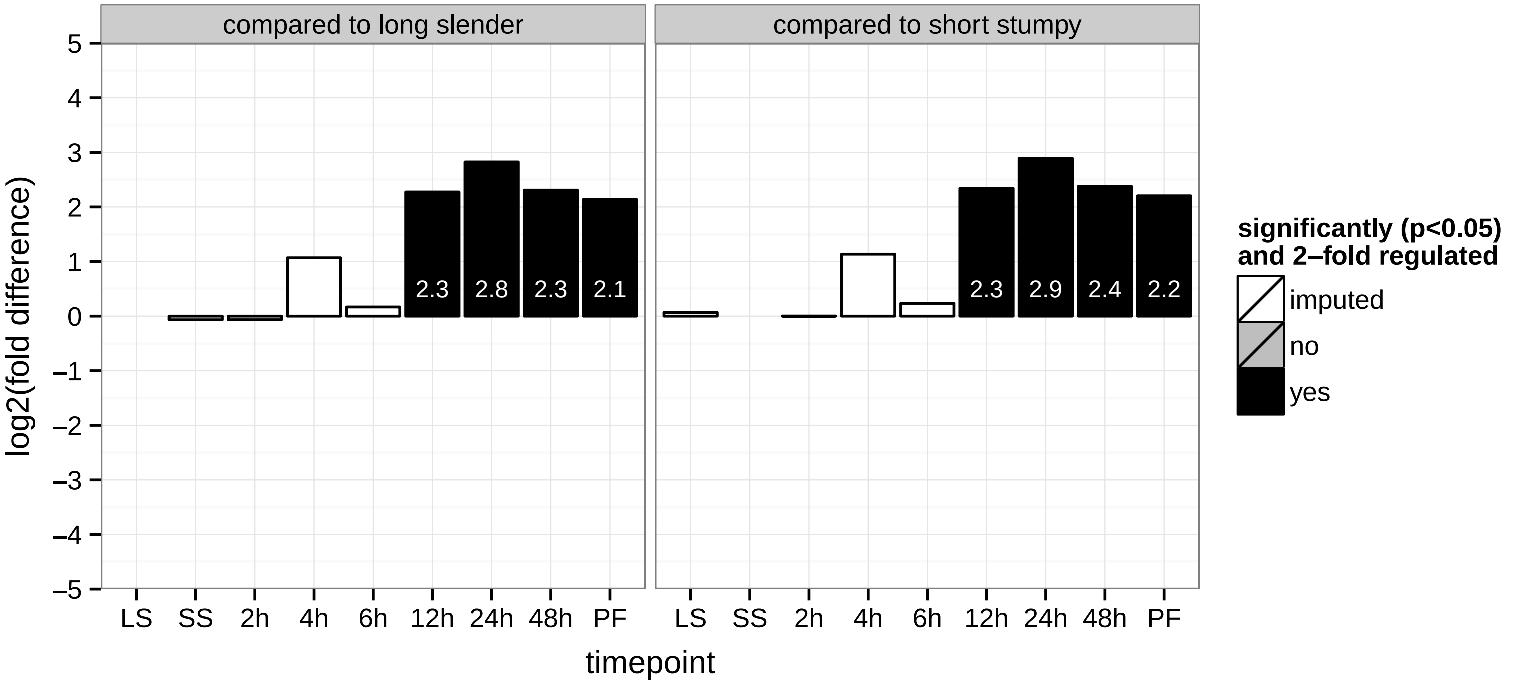

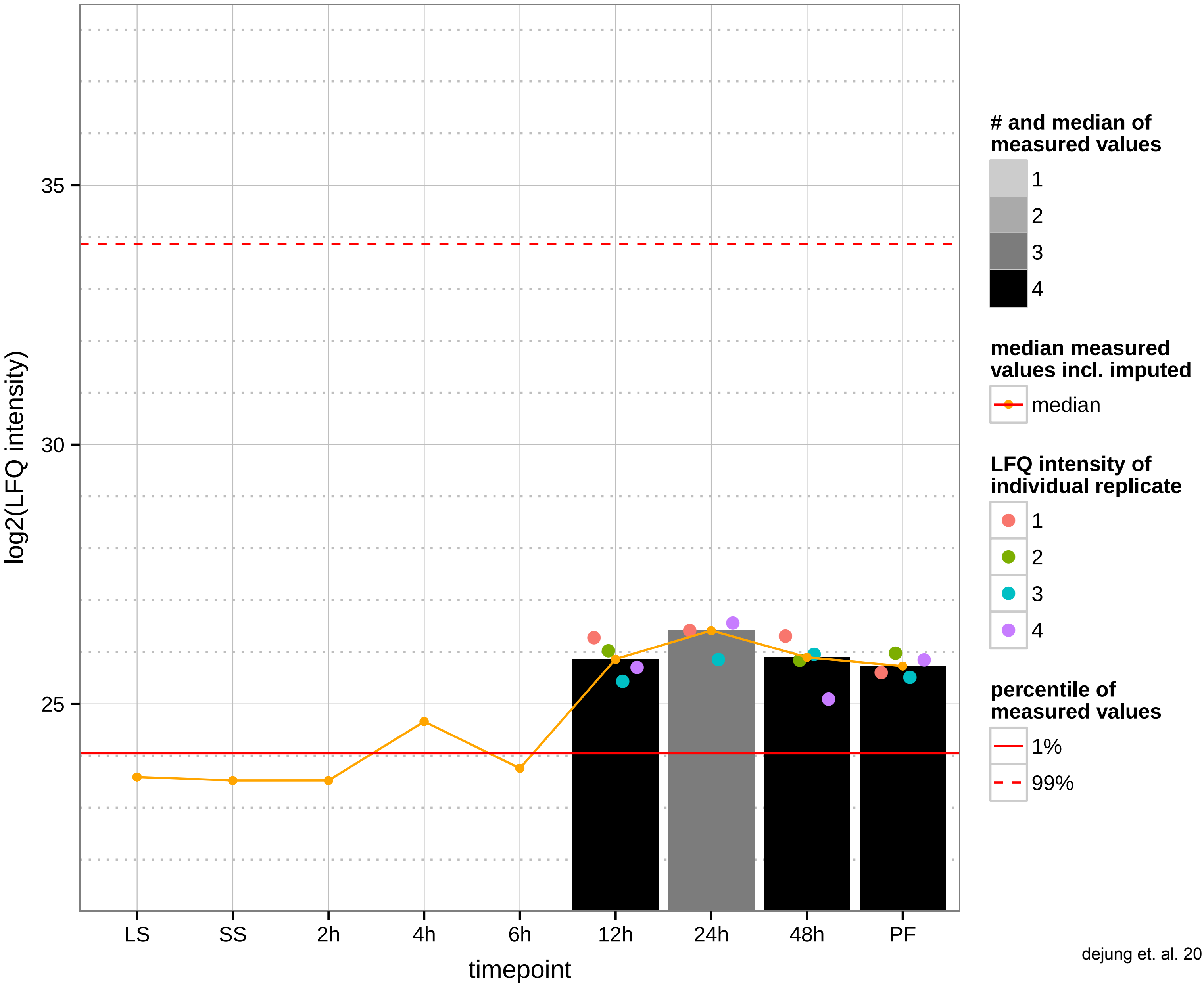

dejung et. al. 2015

C–8 sterol isomerase, putative
Tb927.3.4650
AGOF: C–8 sterol isomerase activity
AGOC: endoplasmic reticulum
AGOP: ergosterol biosynthetic process
PGOF: C–8 sterol isomerase activity
PGOC: endoplasmic reticulum
PGOP: ergosterol biosynthetic process

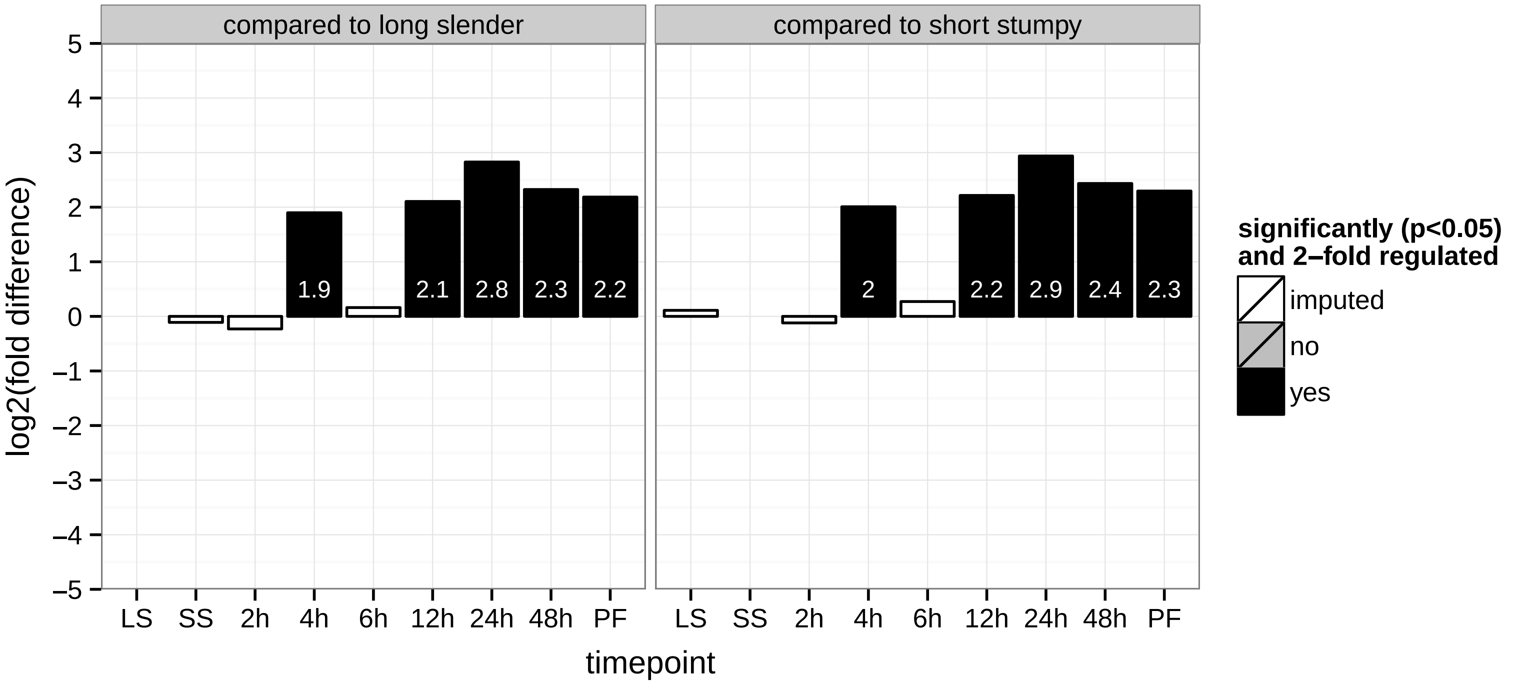

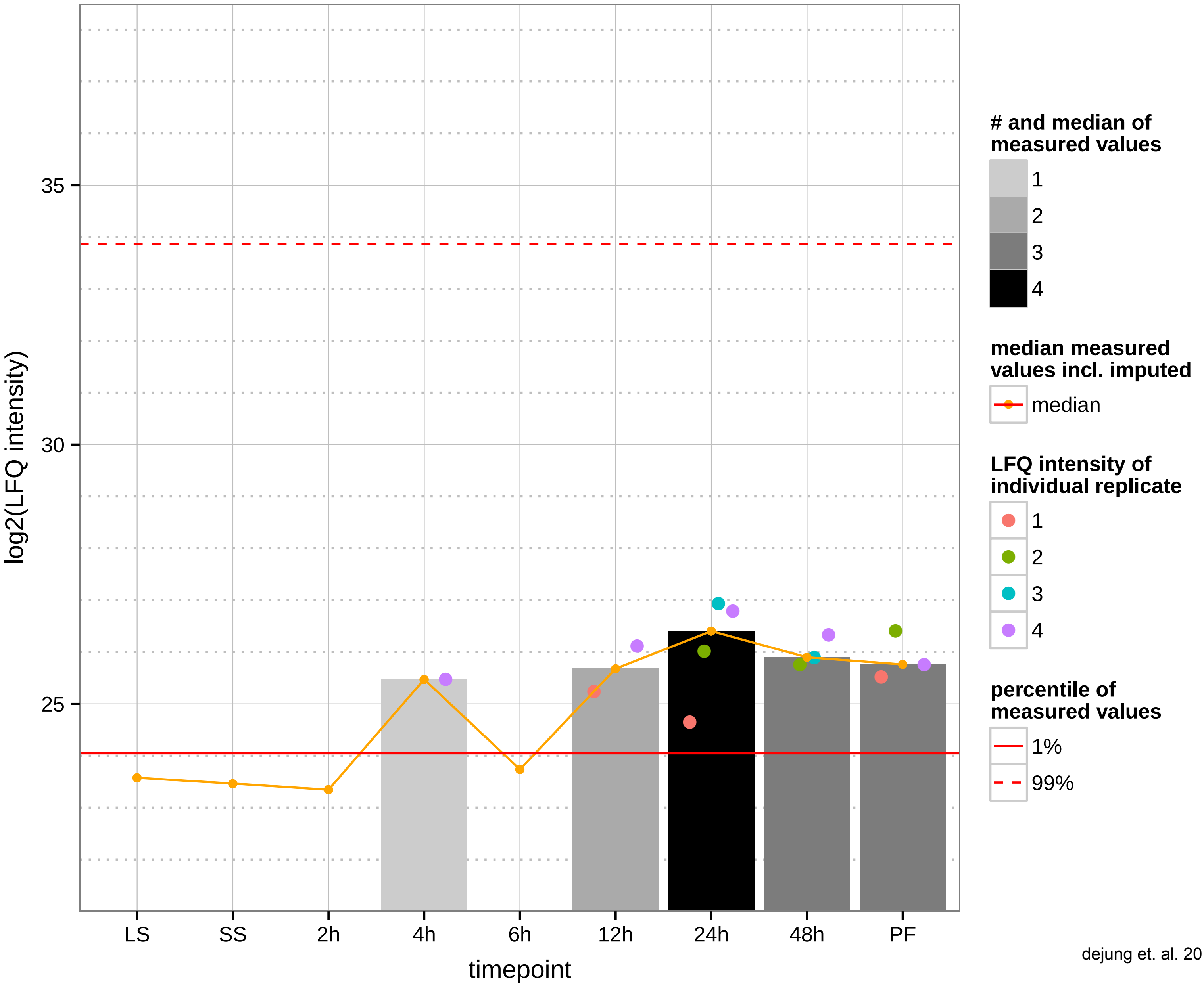

dejung et. al. 2015

hypothetical protein, conserved
Tb927.5.1120
AGOF: null
AGOC: null
AGOP: null
PGOF: null
PGOC: null
PGOP: null

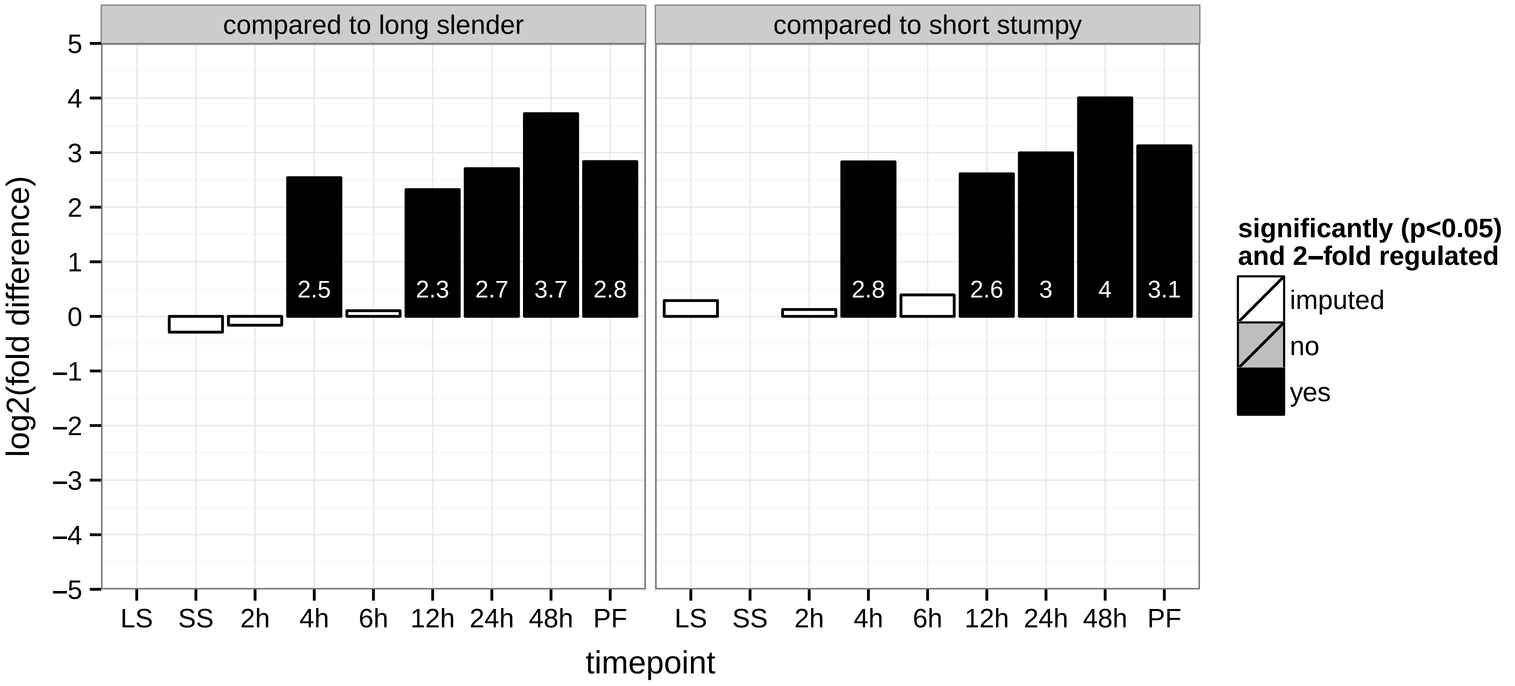

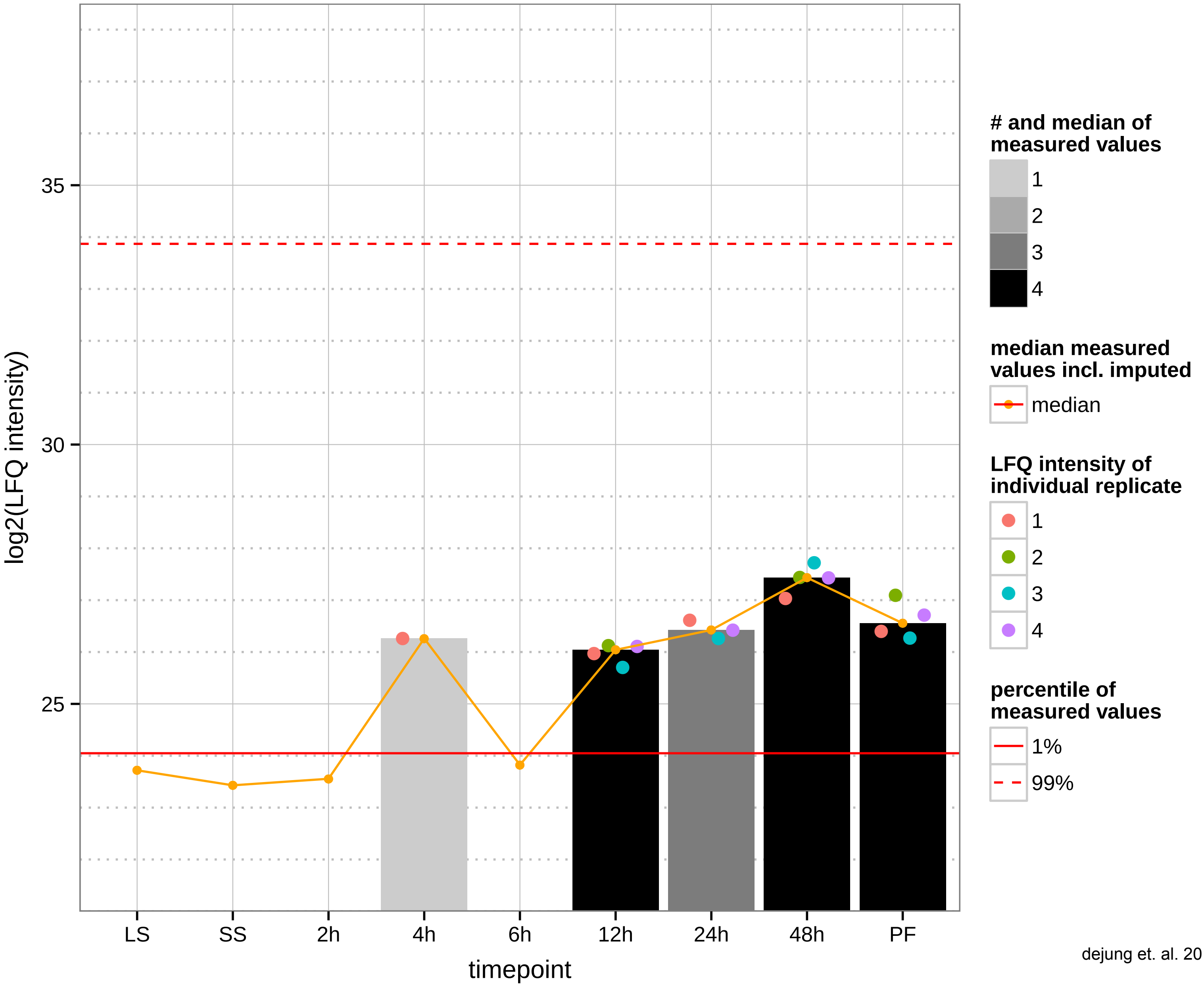

dejung et. al. 2015

conserved protein
Tb927.5.2260
AGOF: null
AGOC: null
AGOP: null
PGOF: null
PGOC: null
PGOP: null

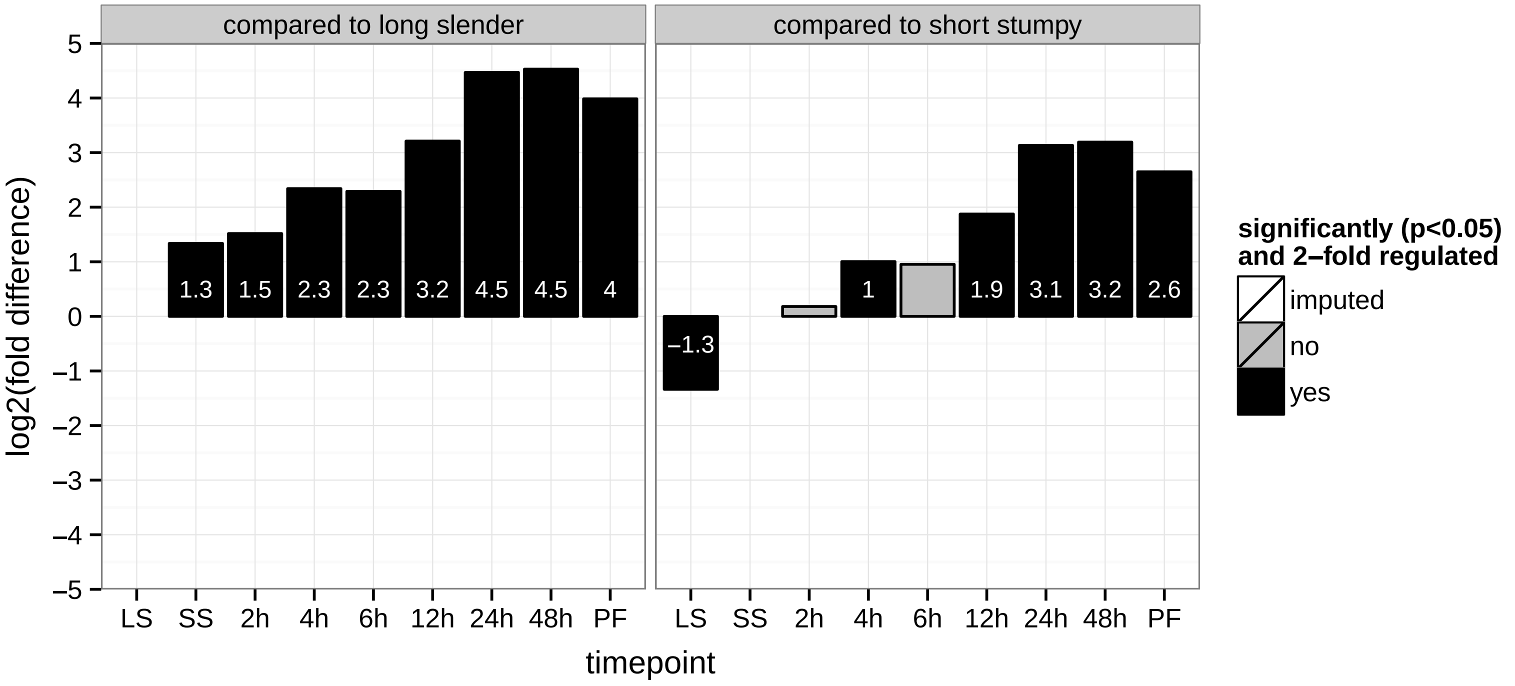

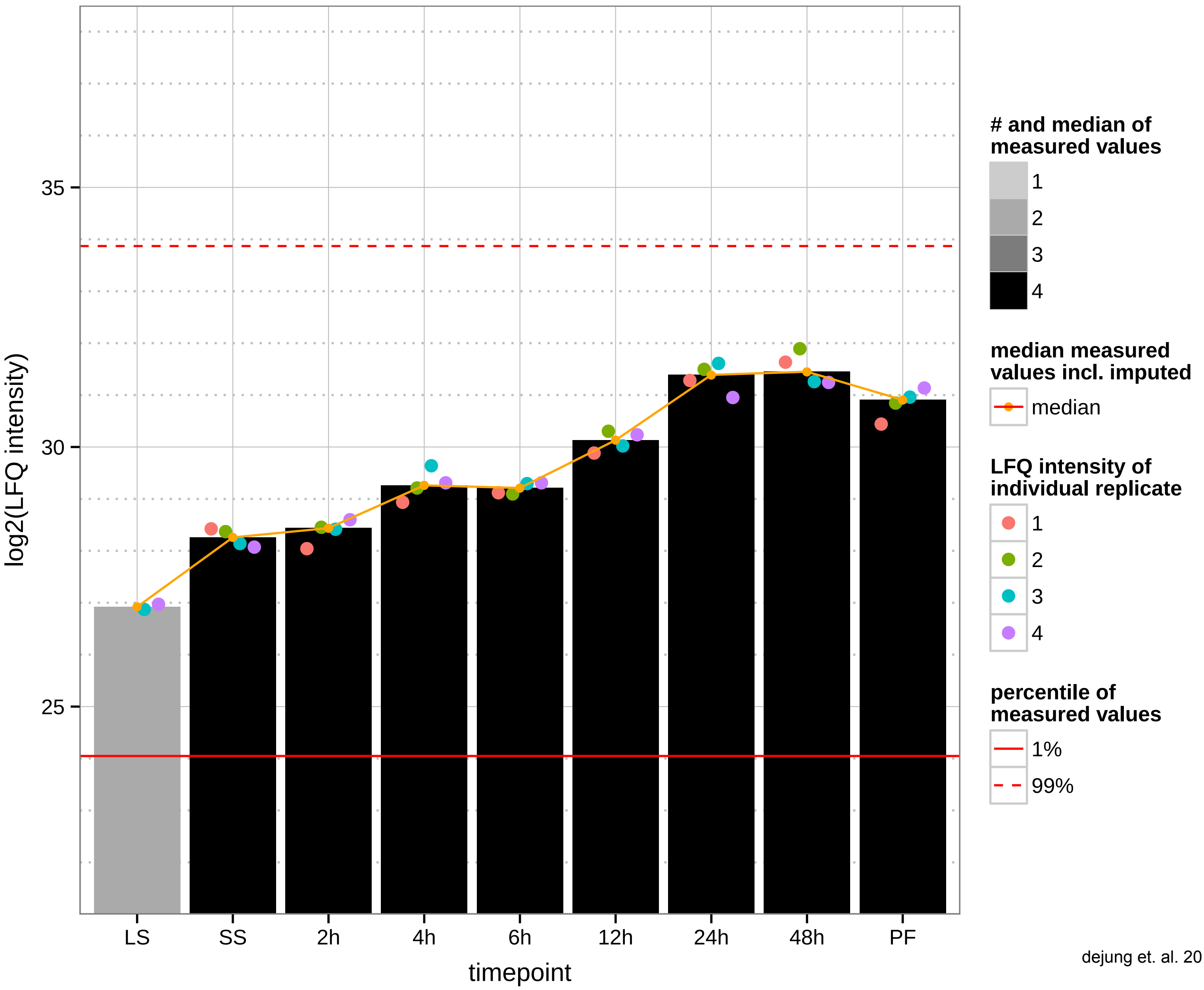

dejung et. al. 2015

co–chaperone GrpE, putative
Tb927.6.2170
AGOF: adenyl–nucleotide exchange factor activity, chaperone binding, protein homodimerization activity
AGOC: mitochondrion
AGOP: protein folding, protein import into mitochondrial matrix
PGOF: adenyl–nucleotide exchange factor activity, chaperone binding, protein homodimerization activity
PGOC: null
PGOP: protein folding

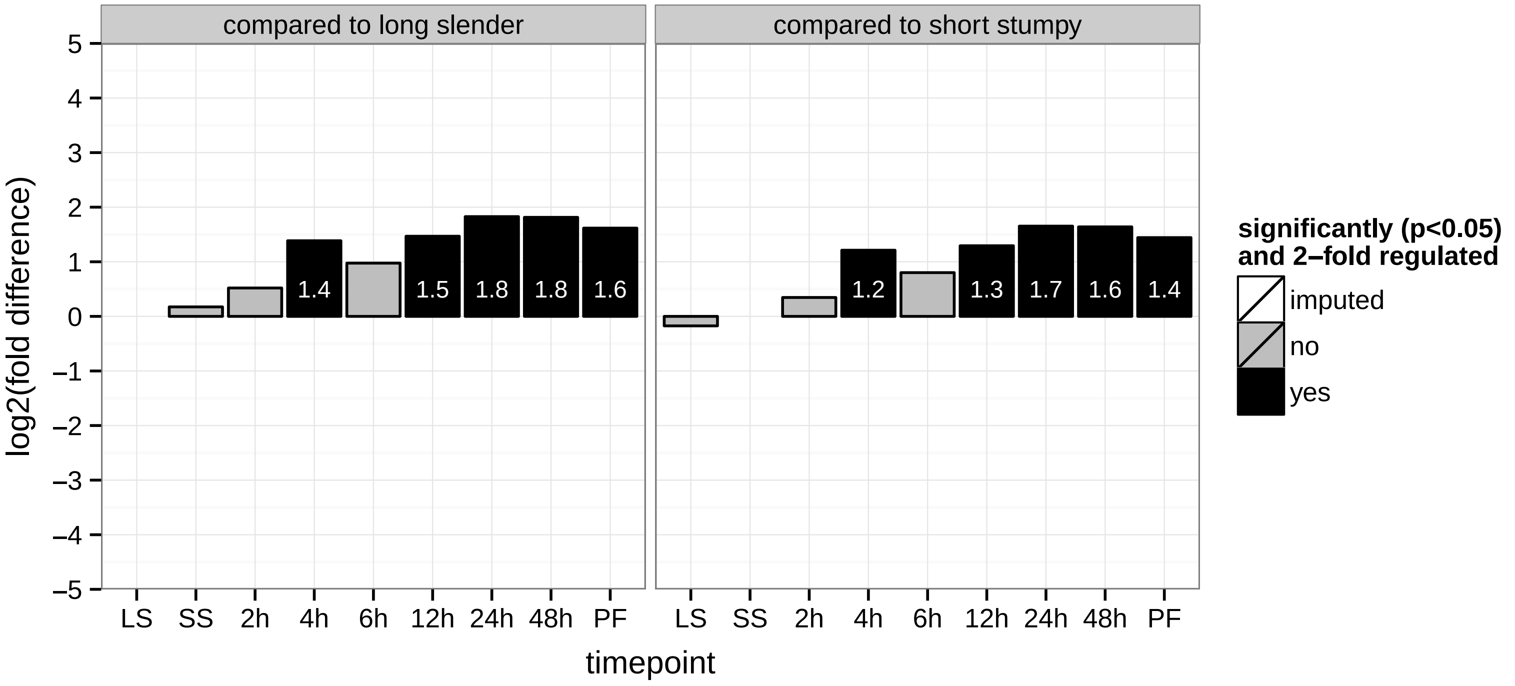

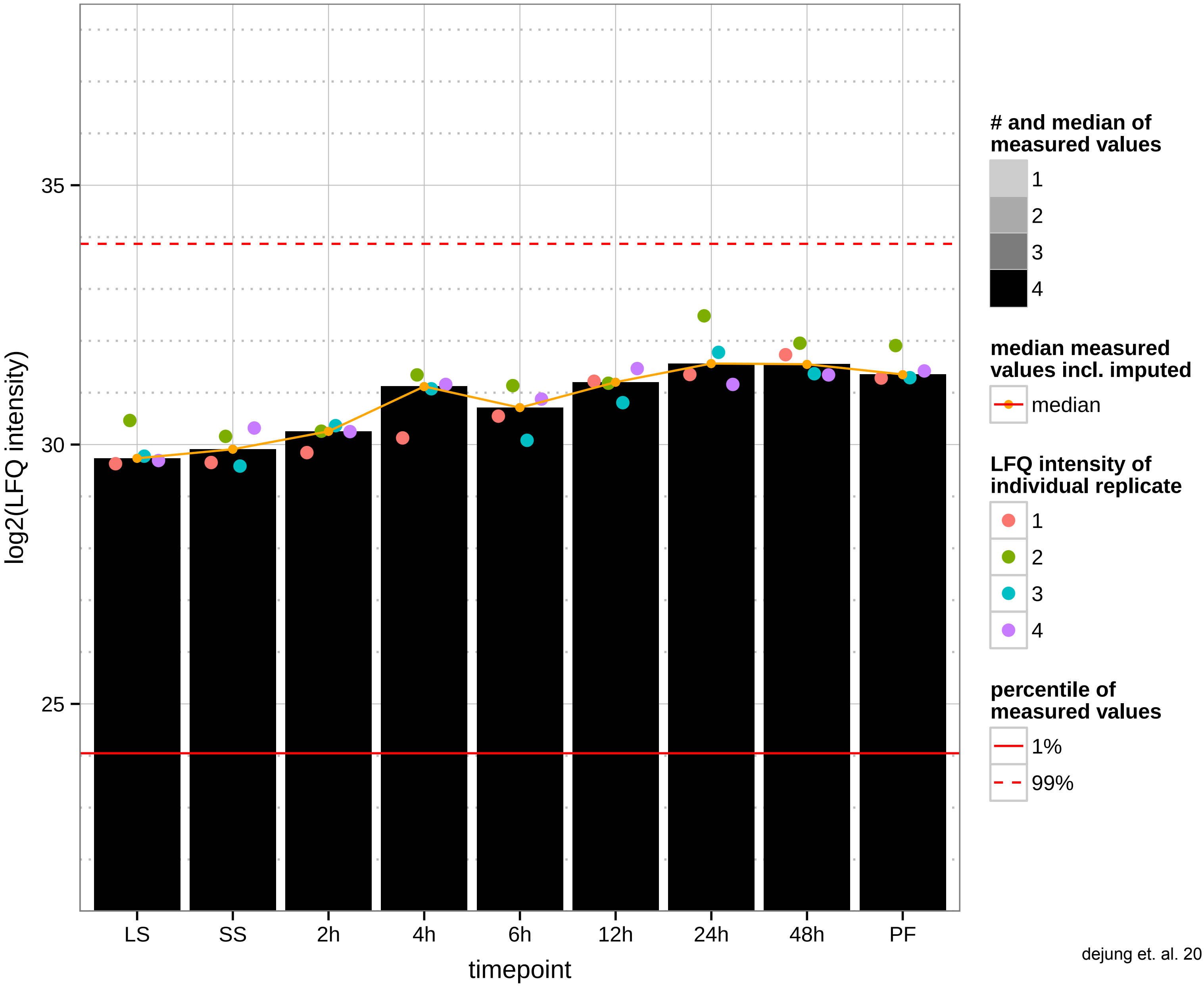

dejung et. al. 2015

tRNA modification enzyme, putative
Tb927.6.3510
AGOF: null, catalytic activity, iron−sulfur cluster binding
AGOC: null
AGOP: null
PGOF: 4 iron, 4 sulfur cluster binding, catalytic activity, iron−sulfur cluster binding, transferase activity
PGOC: null
PGOP: RNA modification, macromolecule modification

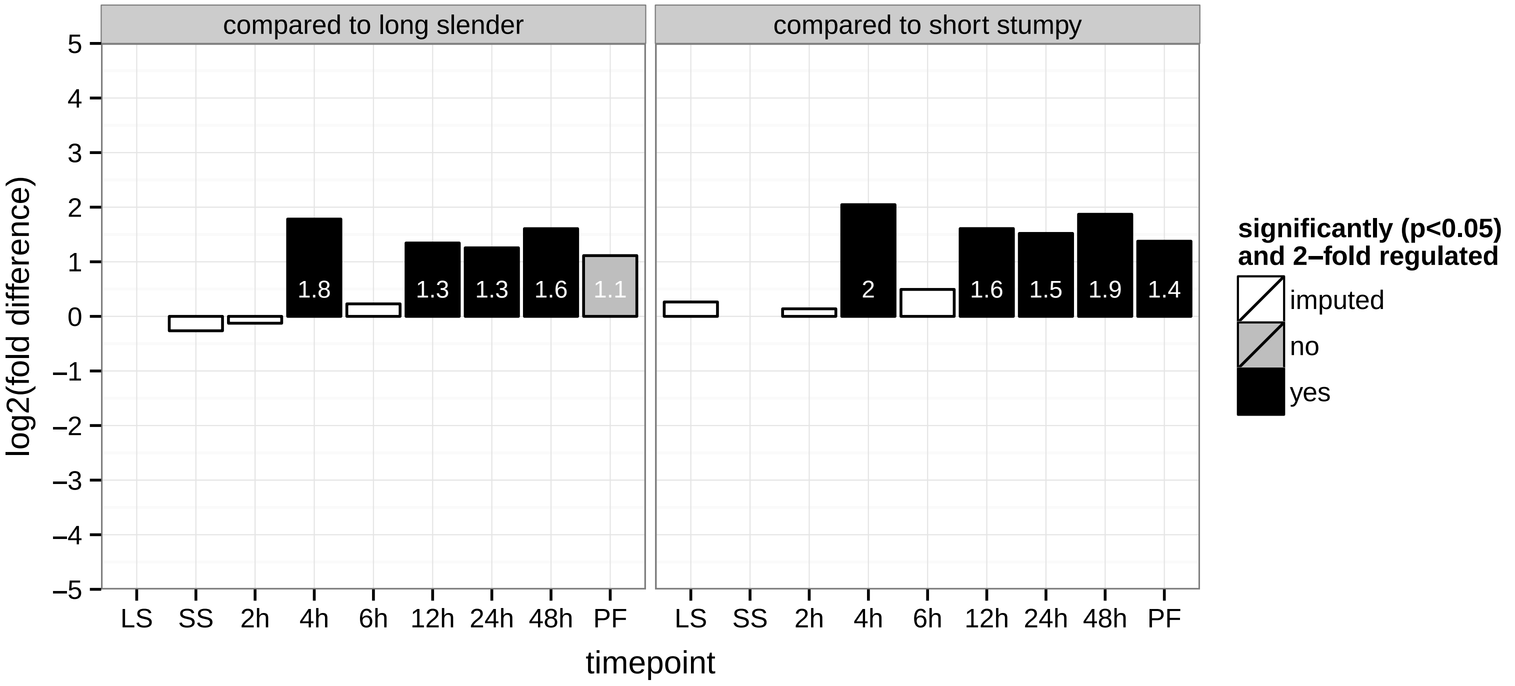

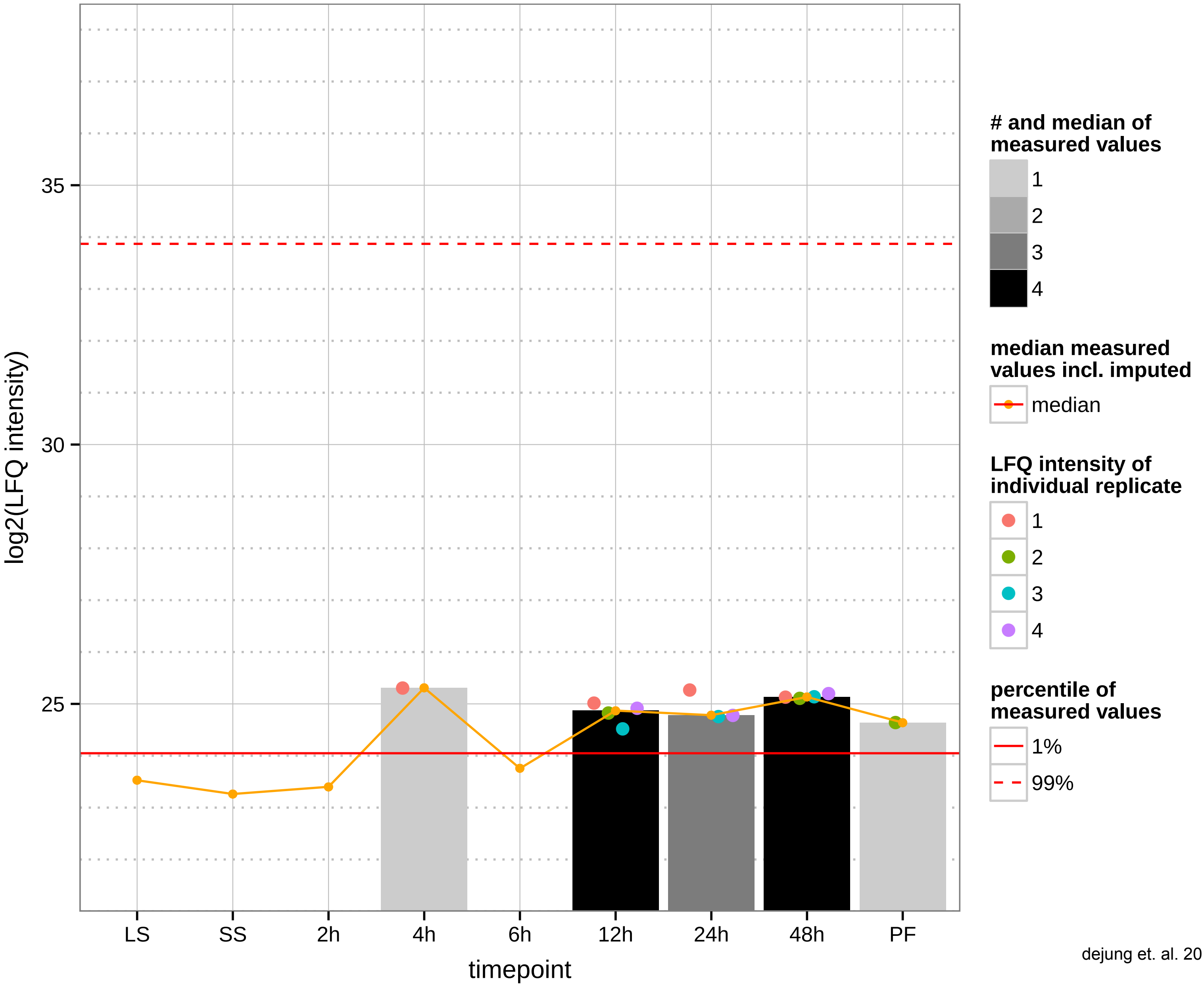

dejung et. al. 2015

UV excision repair RAD23 protein, putative
Tb927.6.4650
AGOF: damaged DNA binding
AGOC: null
AGOP: nucleotide–excision repair, proteasomal ubiquitin–dependent protein catabolic process
PGOF: damaged DNA binding, protein binding
PGOC: null
PGOP: nucleotide–excision repair, proteasomal ubiquitin–dependent protein catabolic process

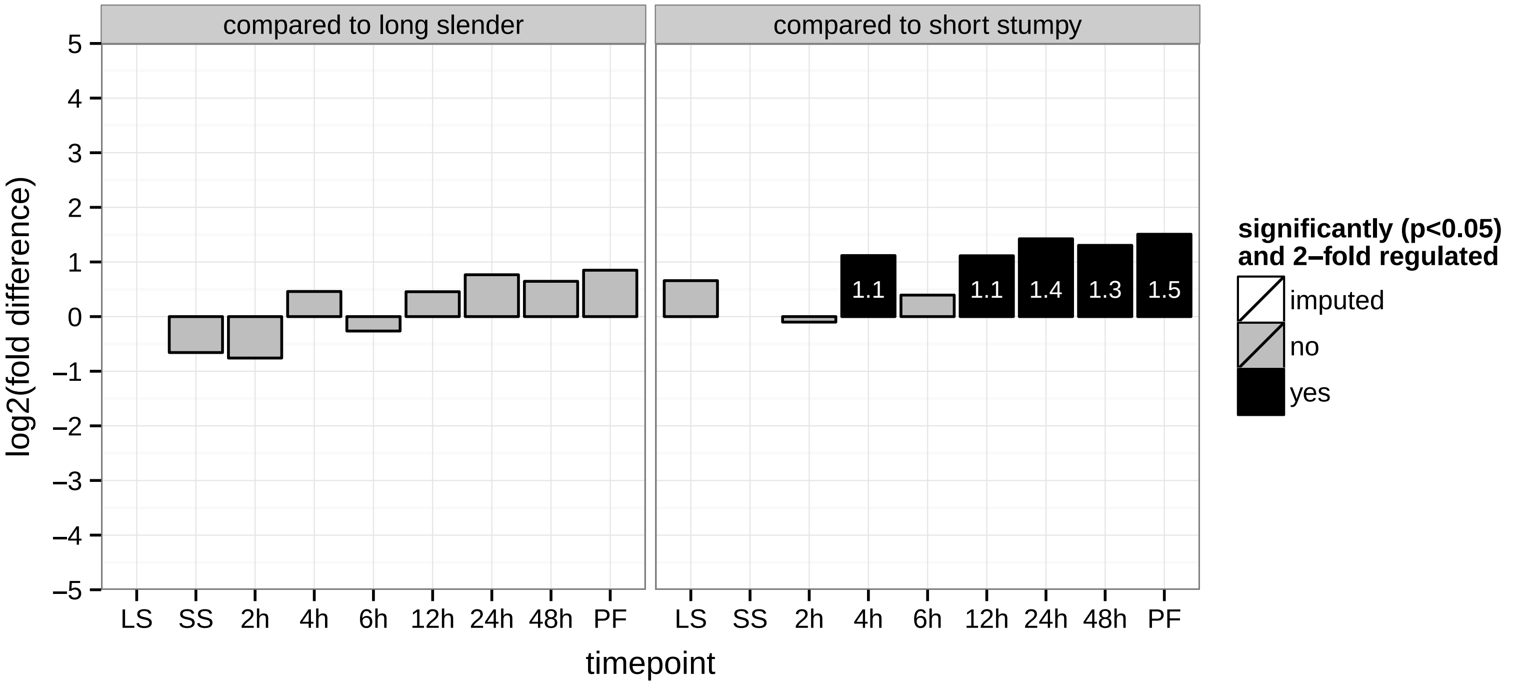

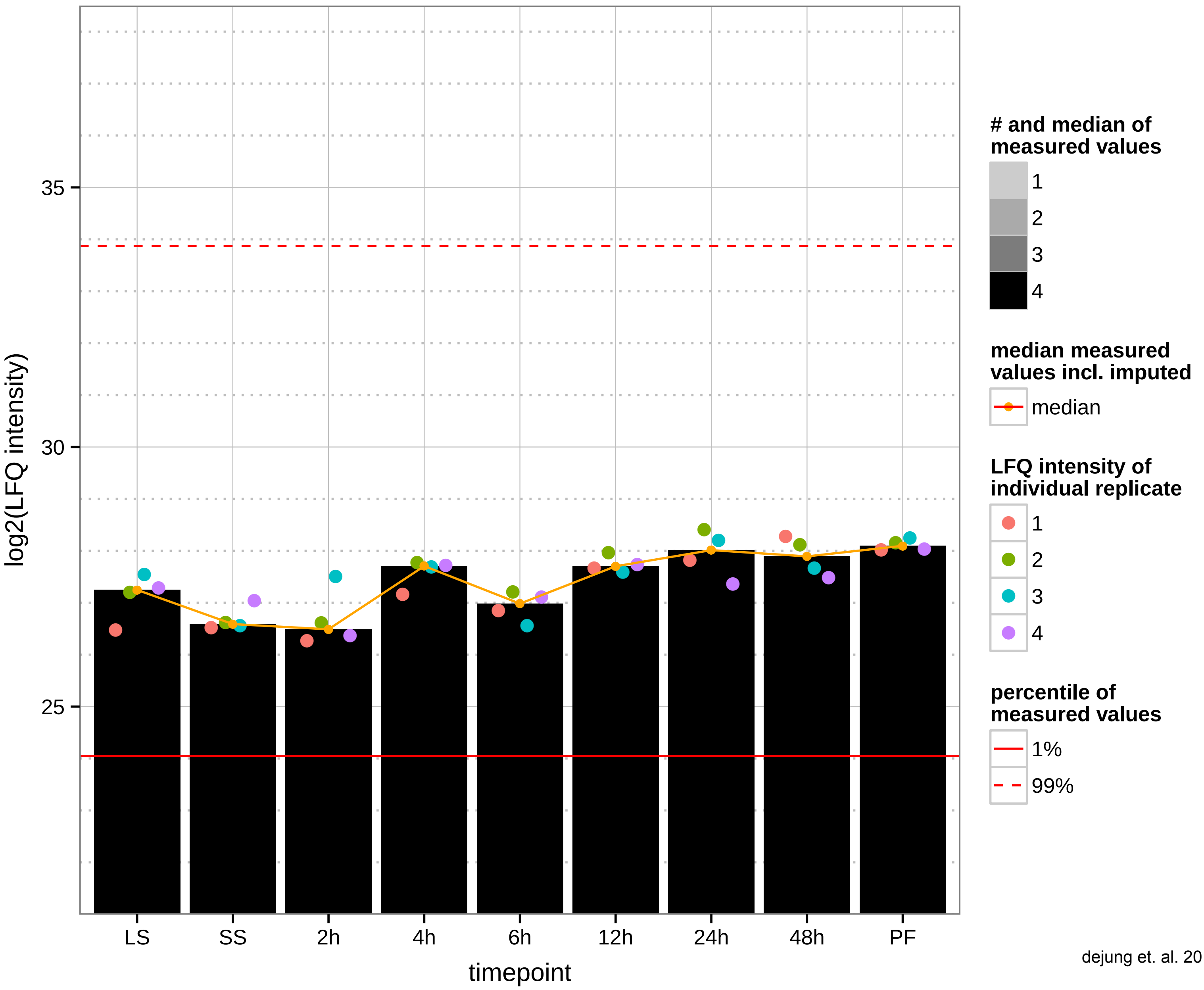

dejung et. al. 2015

ras–like small GTPase, putative (TbEAR)
Tb927.7.1640
AGOF: GTP binding
AGOC: intracellular, mitochondrion
AGOP: null
PGOF: GTP binding
PGOC: null
PGOP: null

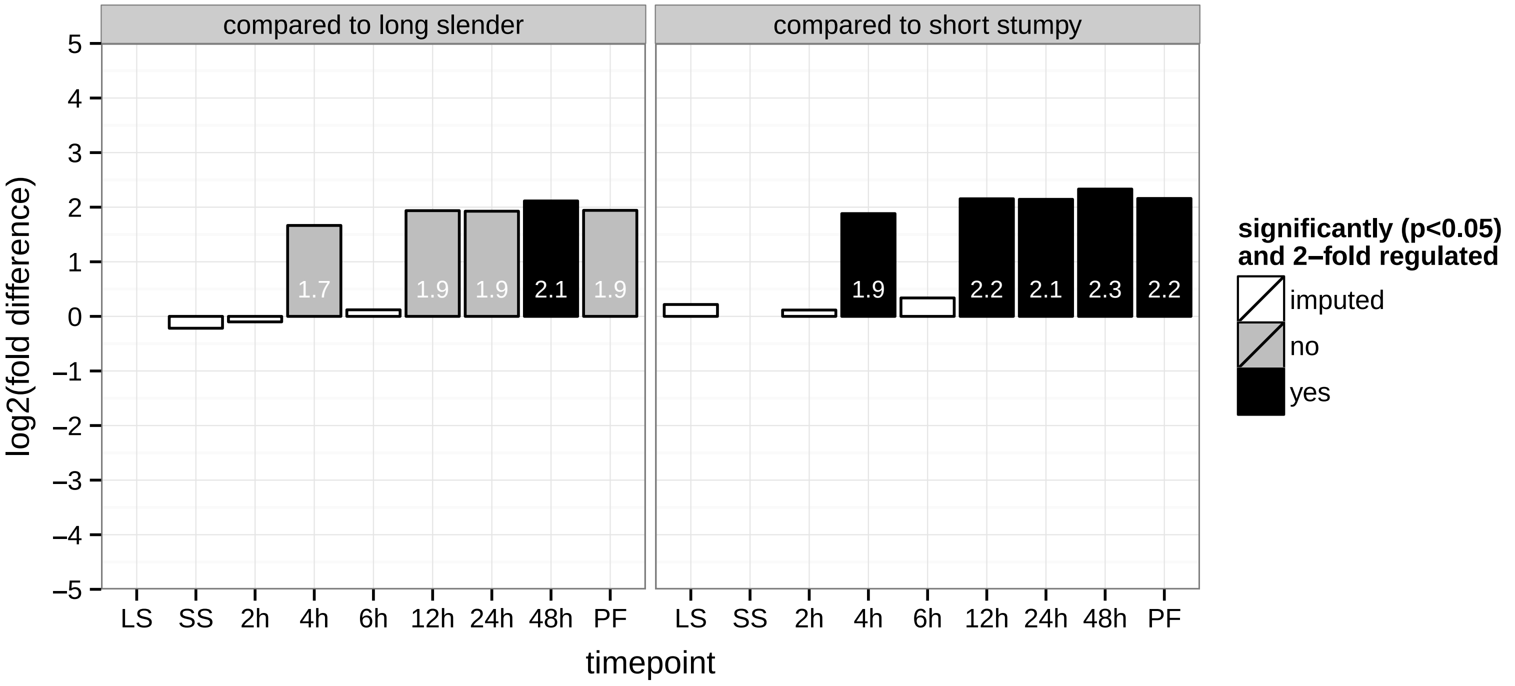

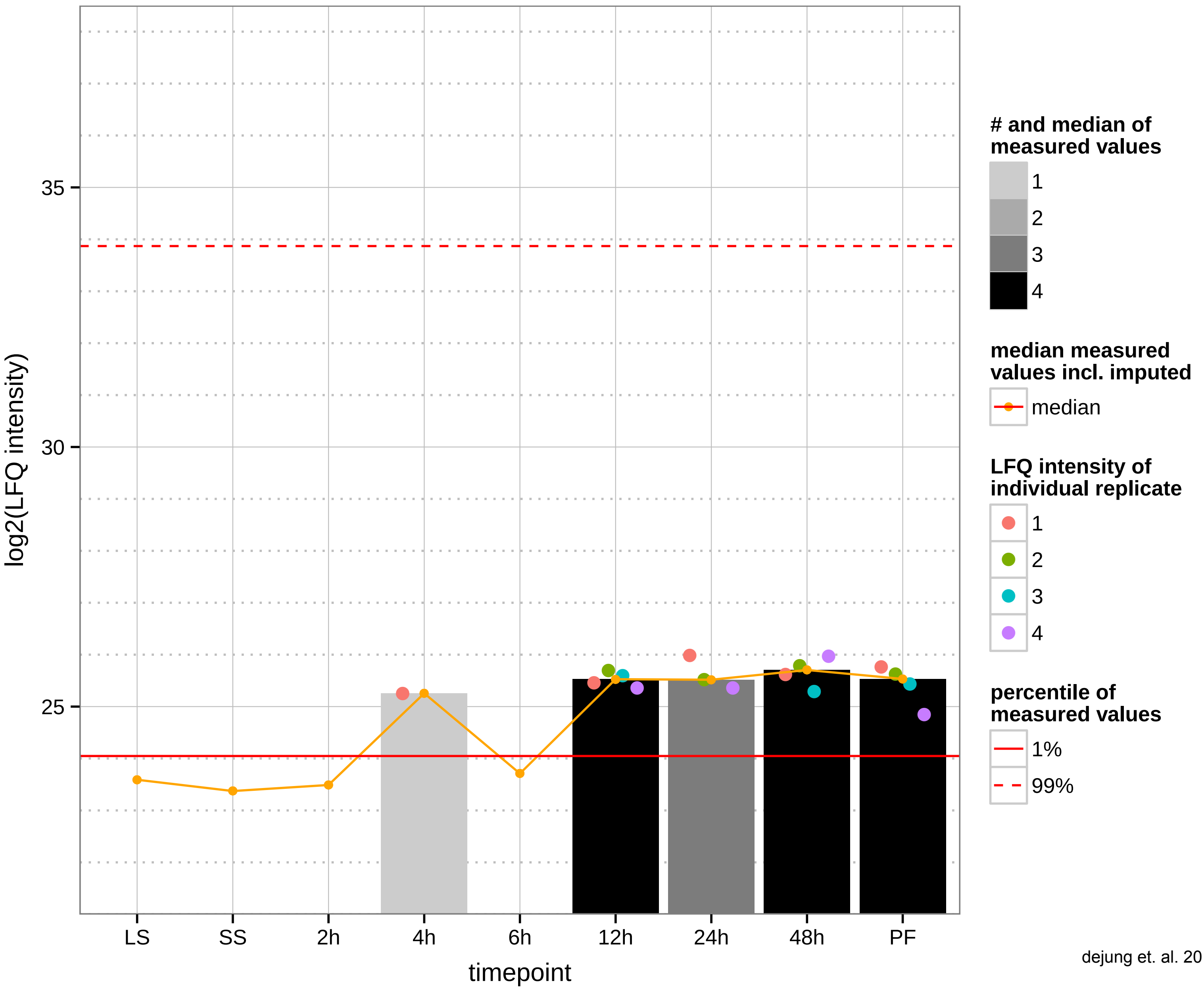

dejung et. al. 2015

calpain–like cysteine peptidase, putative, cysteine peptidase, Clan CA, family C2
Tb927.7.4070
AGOF: null
AGOC: null
AGOP: null
PGOF: null
PGOC: null
PGOP: null

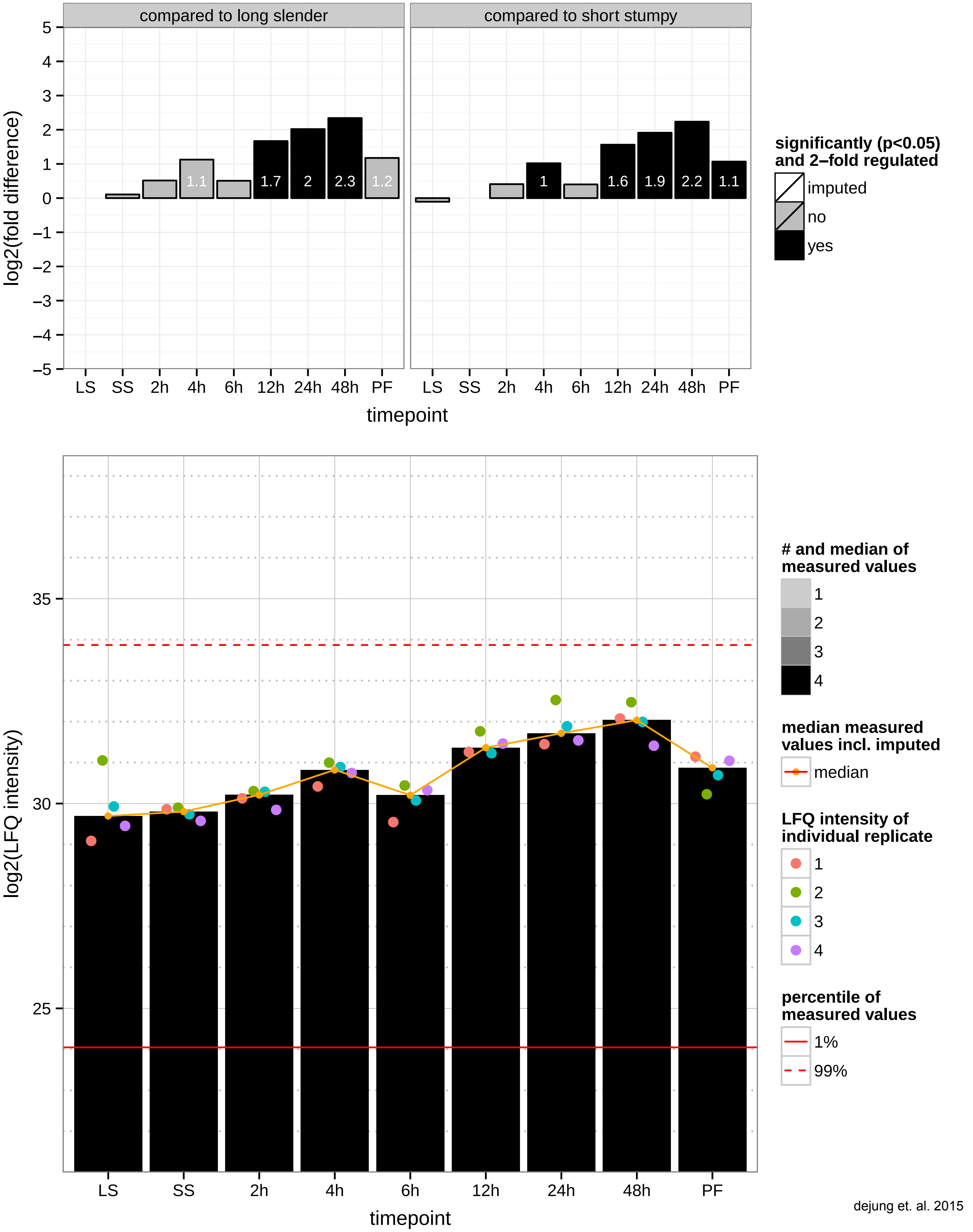

dejung et. al. 2015

zinc finger CCCH domain containing protein 17 (ZC3H17)
Tb927.7.930
AGOF: RNA binding
AGOC: null
AGOP: null
PGOF: null
PGOC: null
PGOP: null

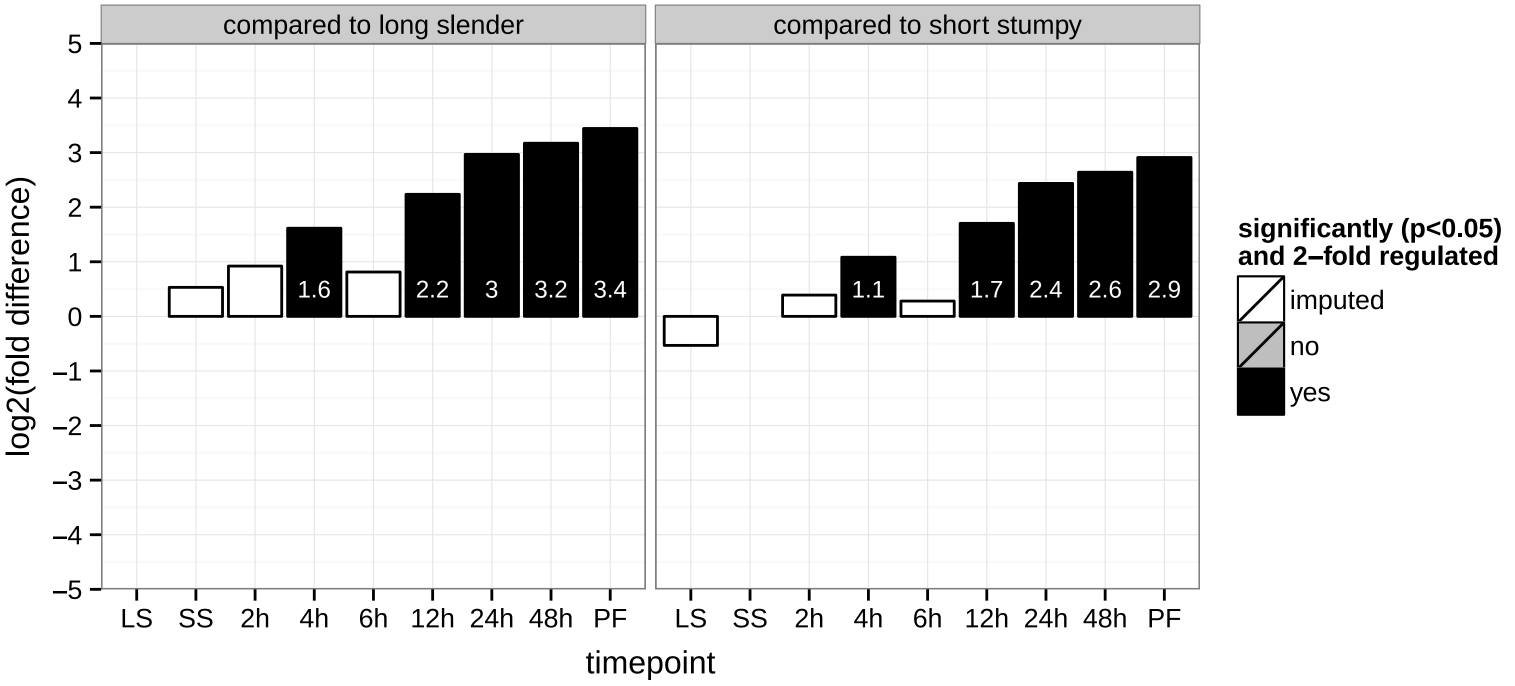

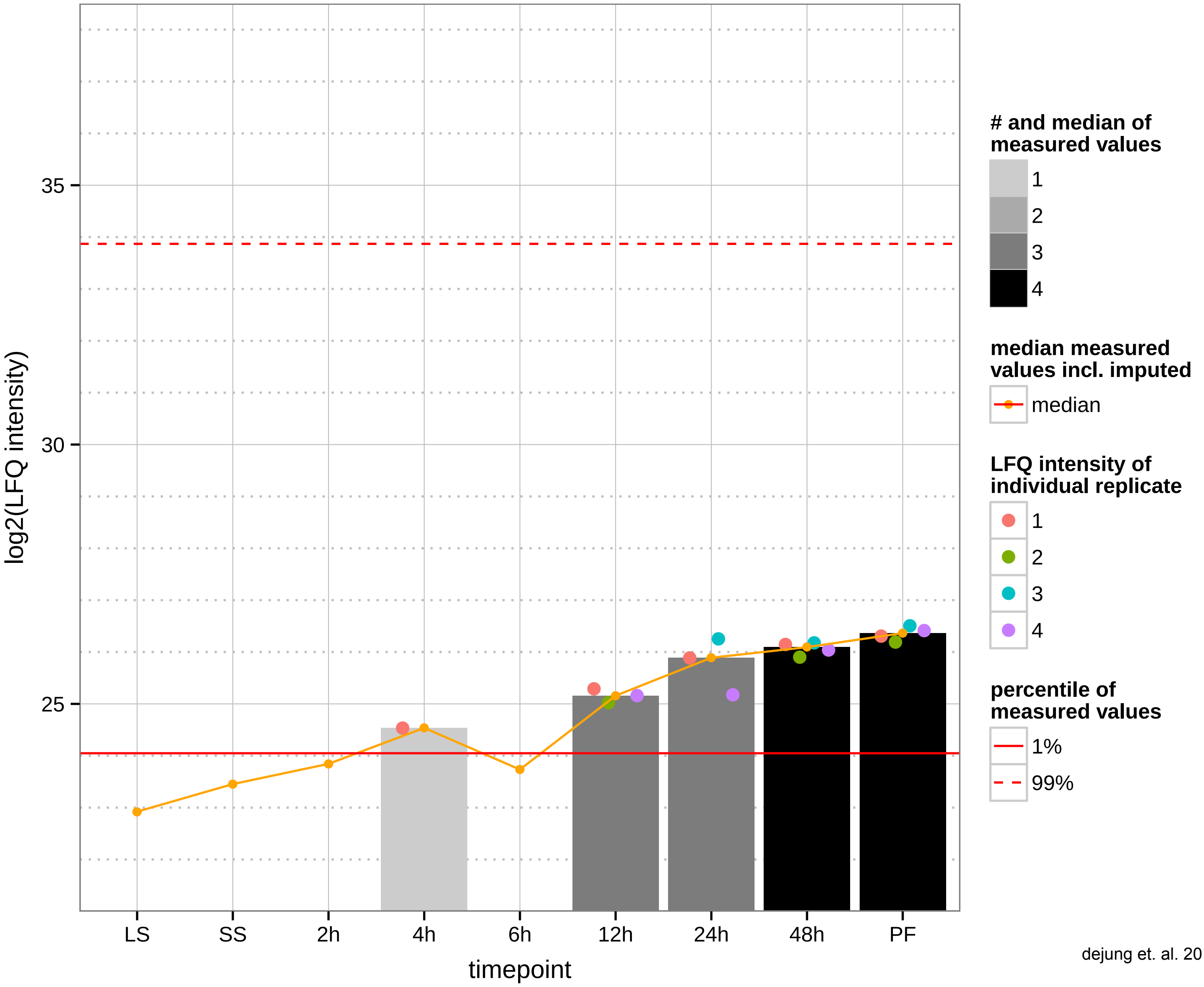

dejung et. al. 2015

hypothetical protein, conserved
Tb927.8.5300
AGOF: null
AGOC: null
AGOP: null
PGOF: null
PGOC: null
PGOP: null

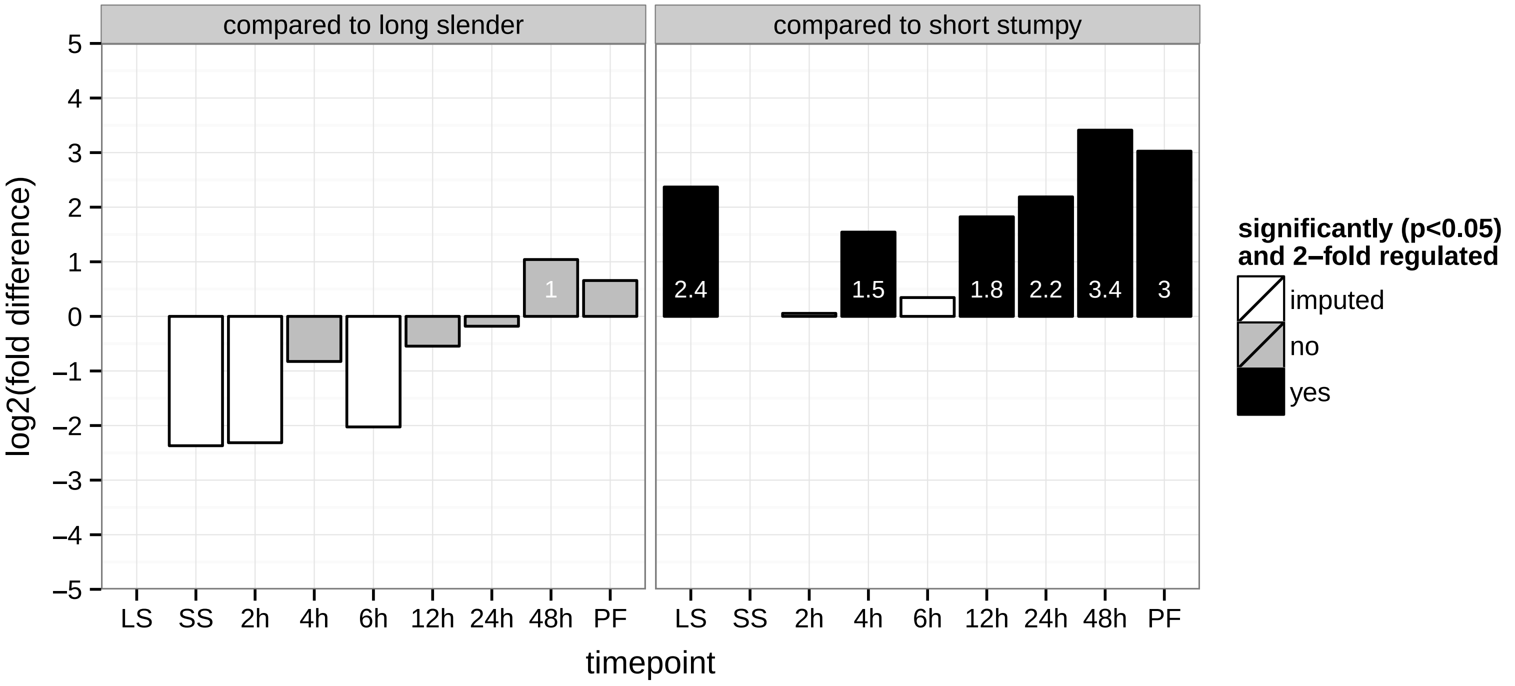

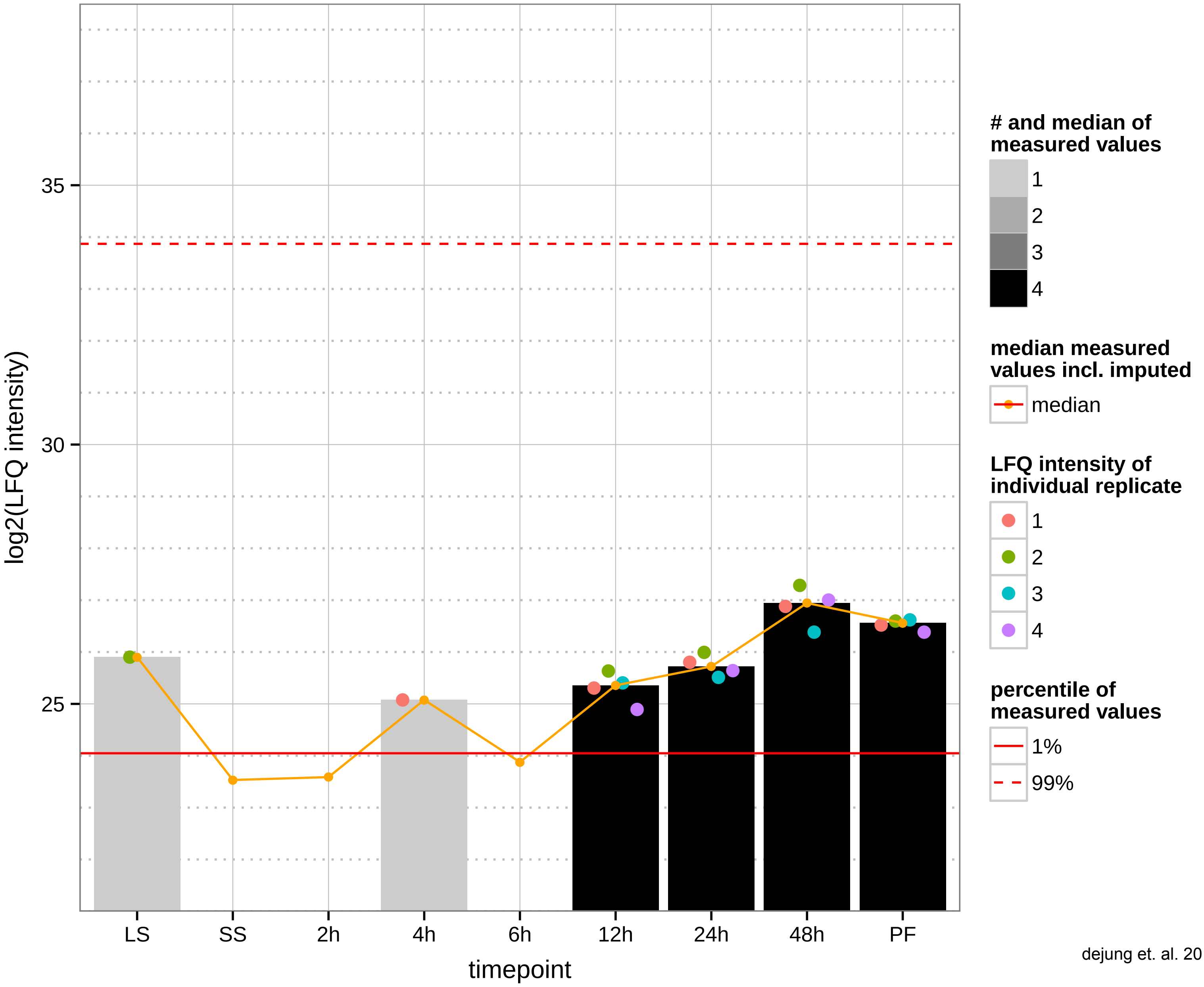

dejung et. al. 2015

hypothetical protein, conserved
Tb927.8.6380
AGOF: null
AGOC: null
AGOP: null
PGOF: null
PGOC: null
PGOP: null

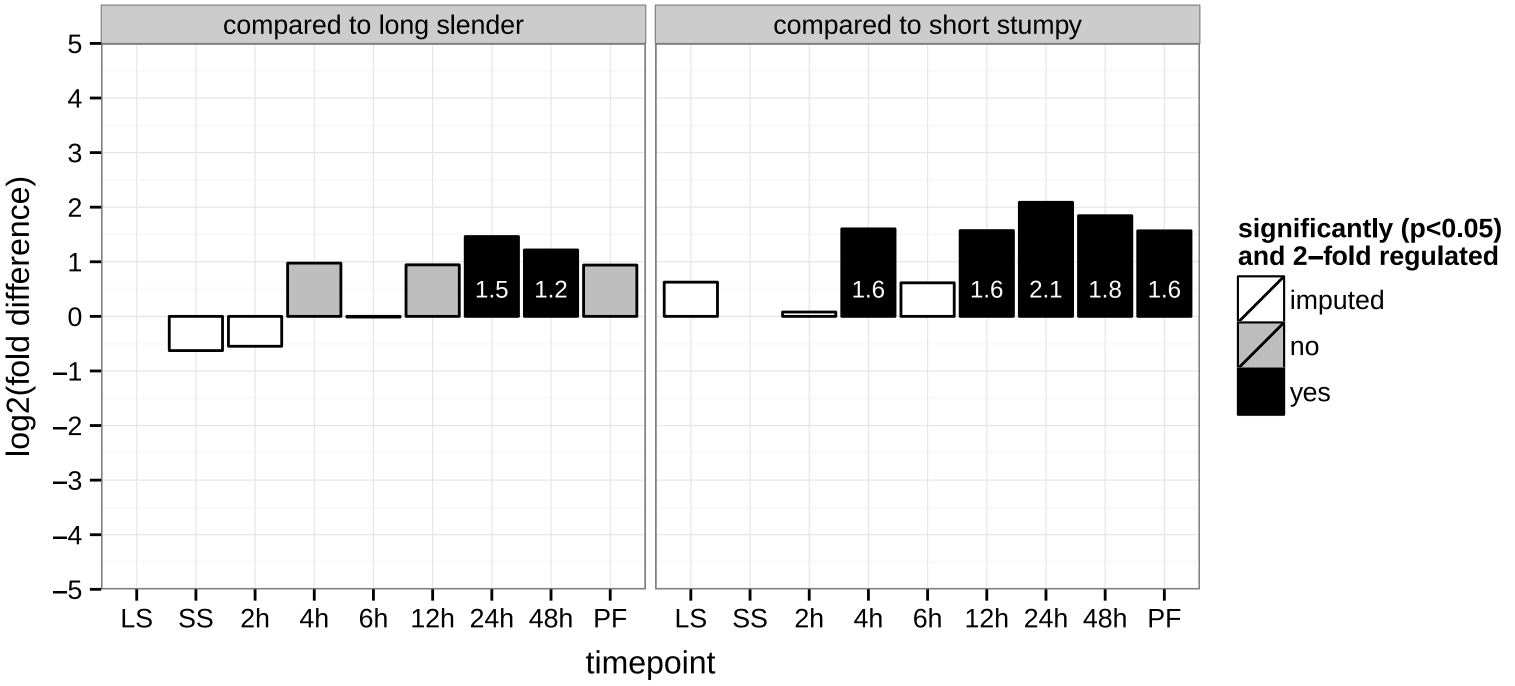

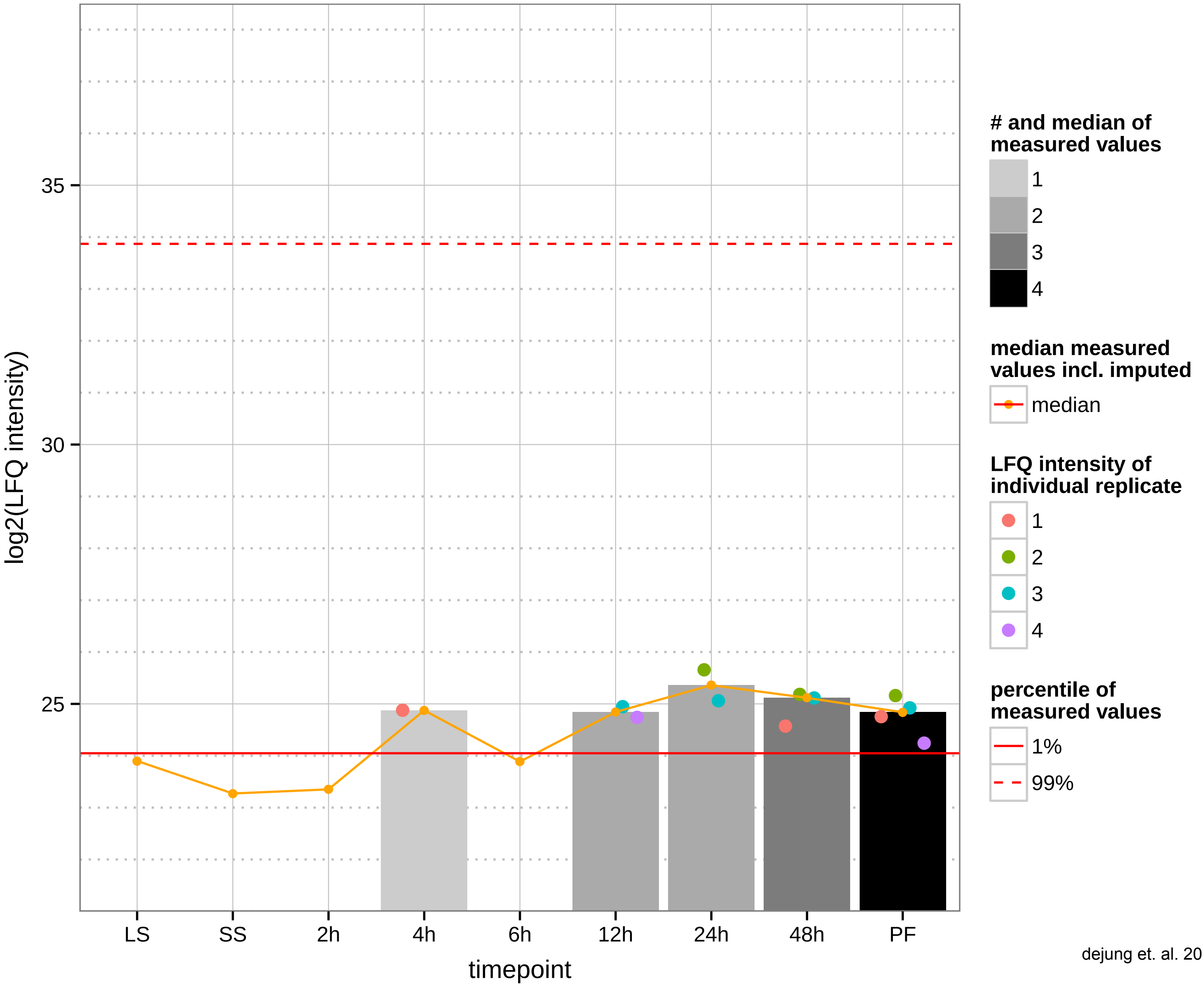

dejung et. al. 2015

dihydrolipoamide dehydrogenase, pseudogene, putative, acetoin dehydrogenase e3 component, point mutation
Tb927.8.7380;Tb927.4.5050
AGOF: dihydrolipoyl dehydrogenase activity, flavin adenine dinucleotide binding, disulfide oxidoreductase activity
AGOC: cytoplasm, mitochondrion
AGOP: cell redox homeostasis, oxidation–reduction process
PGOF: flavin adenine dinucleotide binding, oxidoreductase activity
PGOC: cytoplasm
PGOP: cell redox homeostasis, oxidation–reduction process

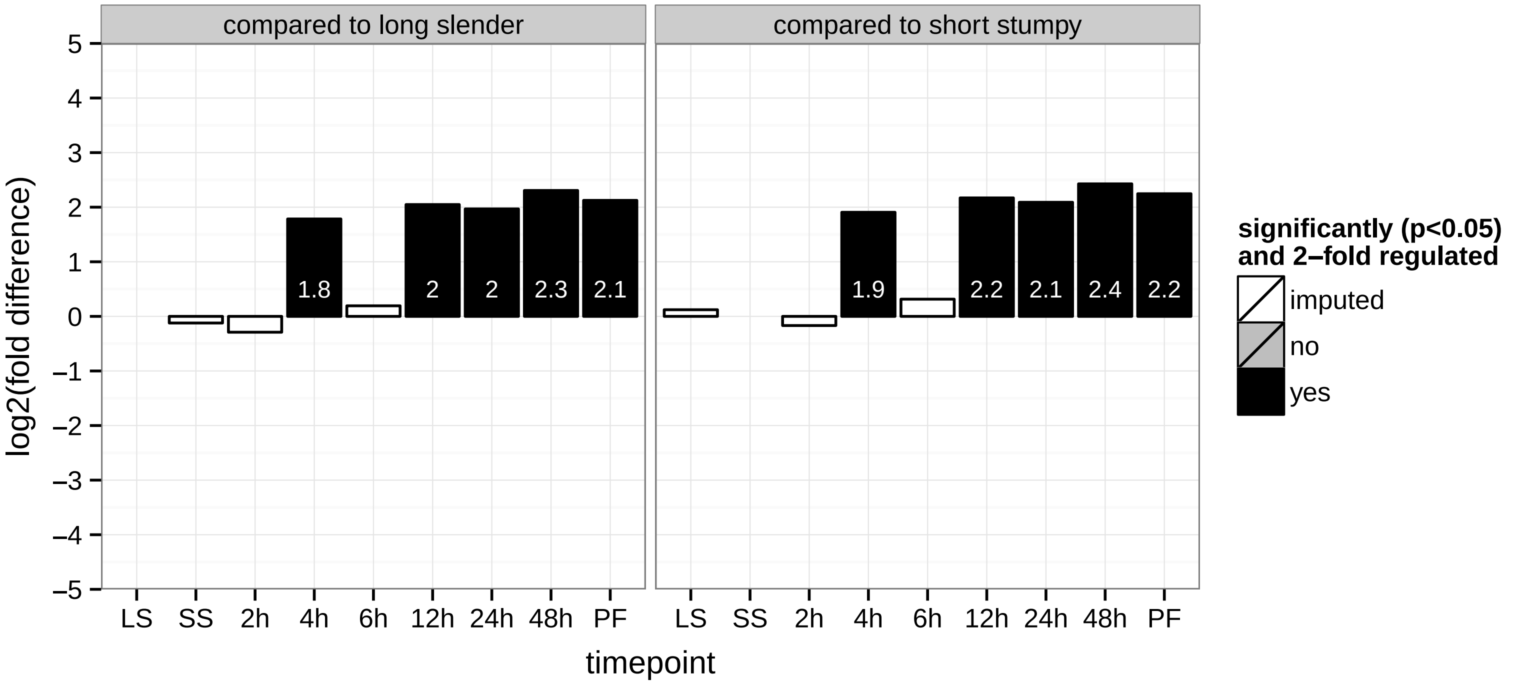

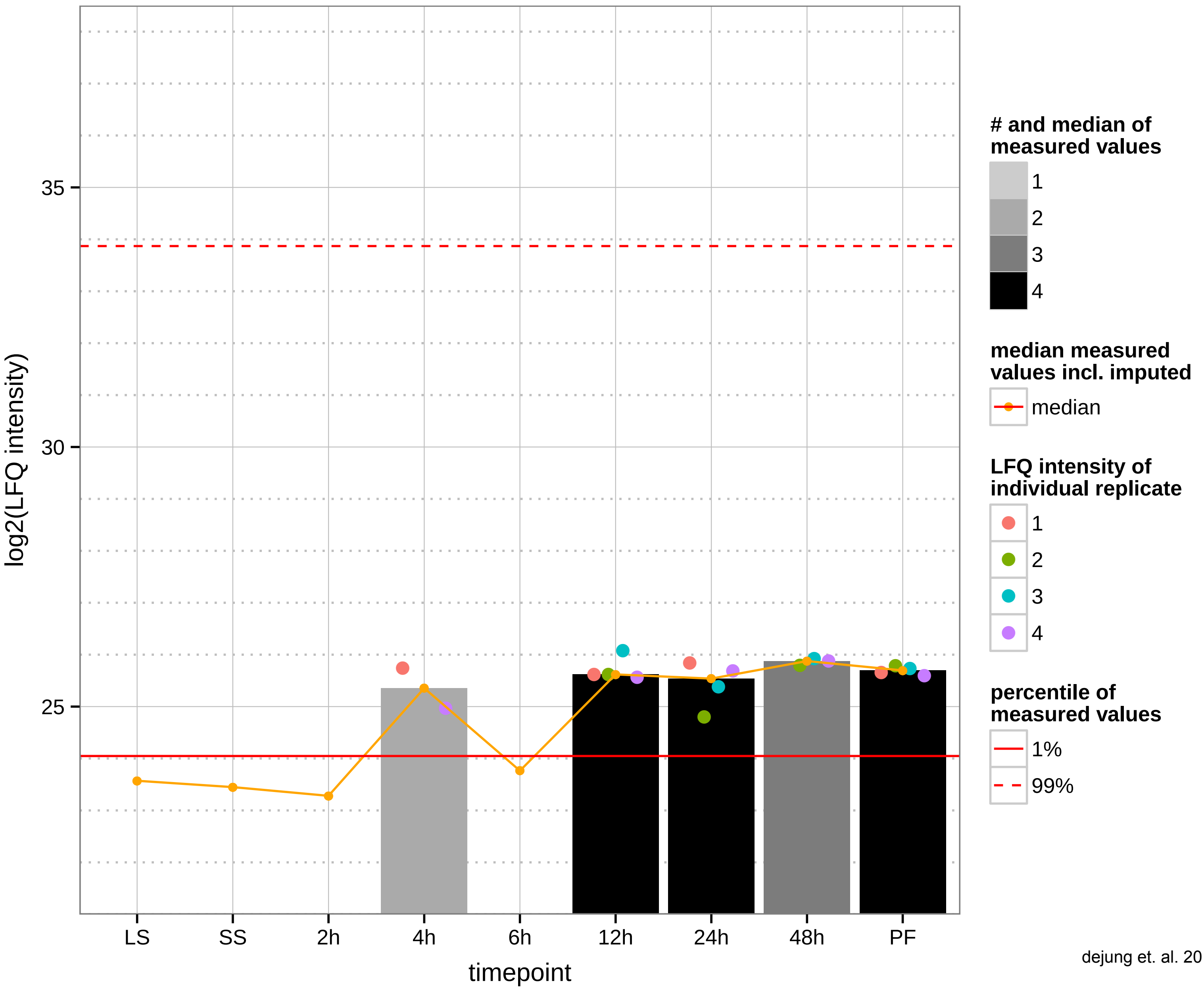

dejung et. al. 2015

hypothetical protein, conserved
Tb927.9.10470
AGOF: null
AGOC: null
AGOP: null
PGOF: null
PGOC: null
PGOP: null

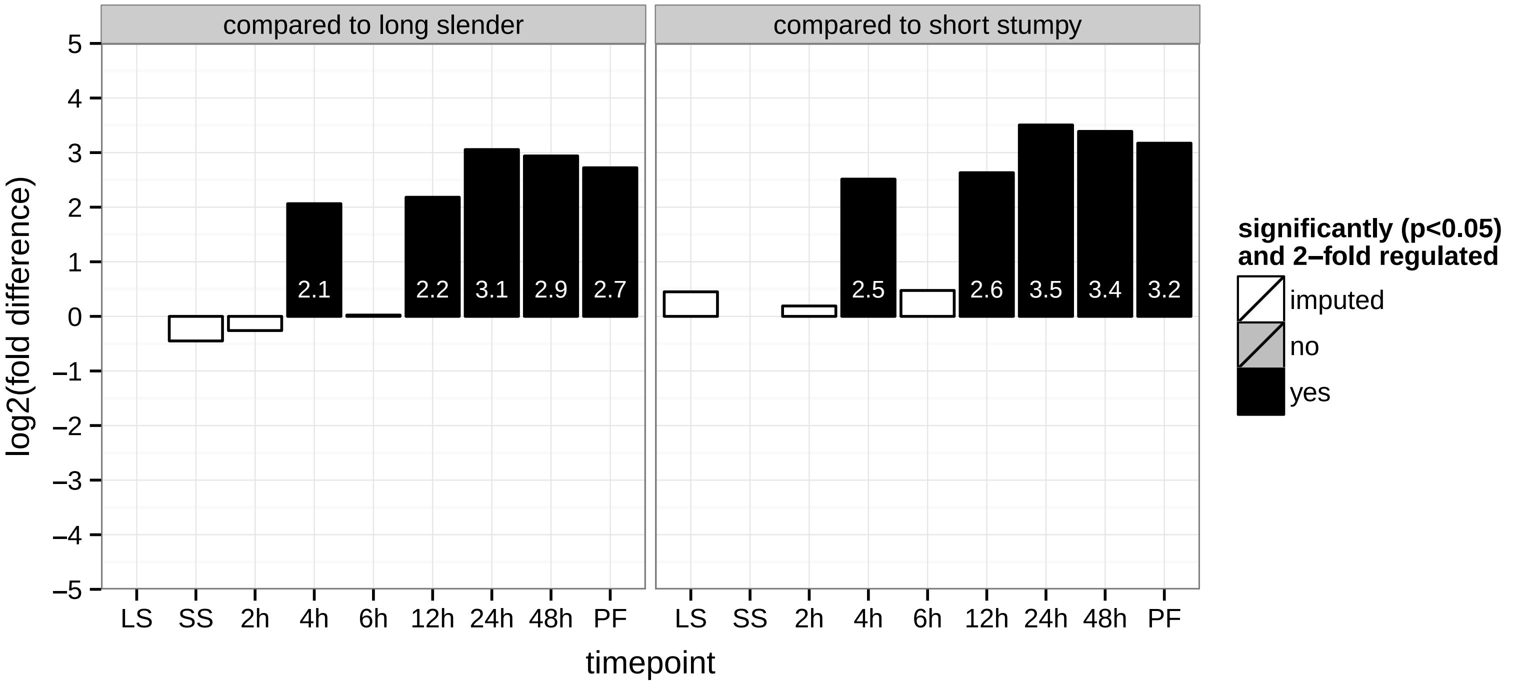

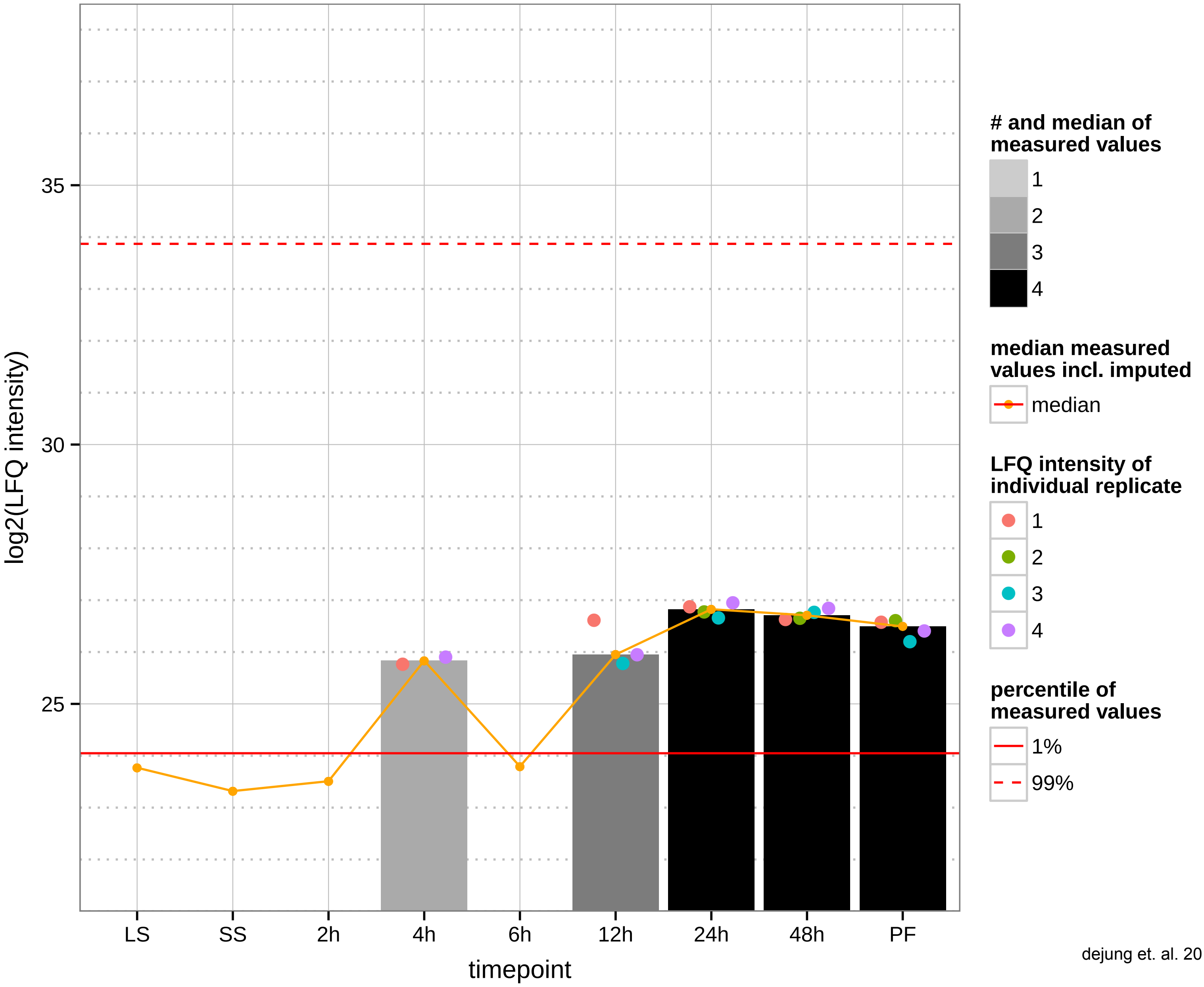

dejung et. al. 2015

hypothetical protein, conserved
Tb927.9.12750
AGOF: null
AGOC: null
AGOP: null
PGOF: null
PGOC: null
PGOP: null

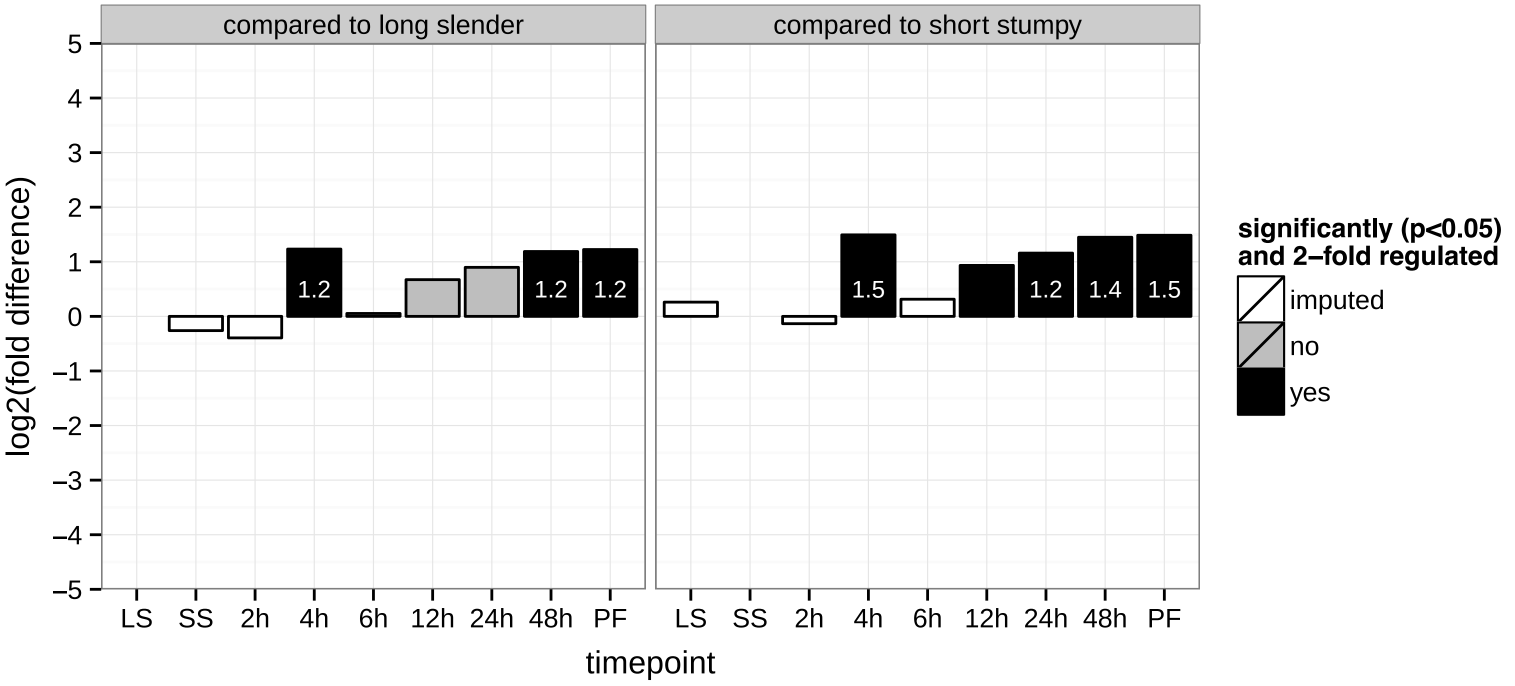

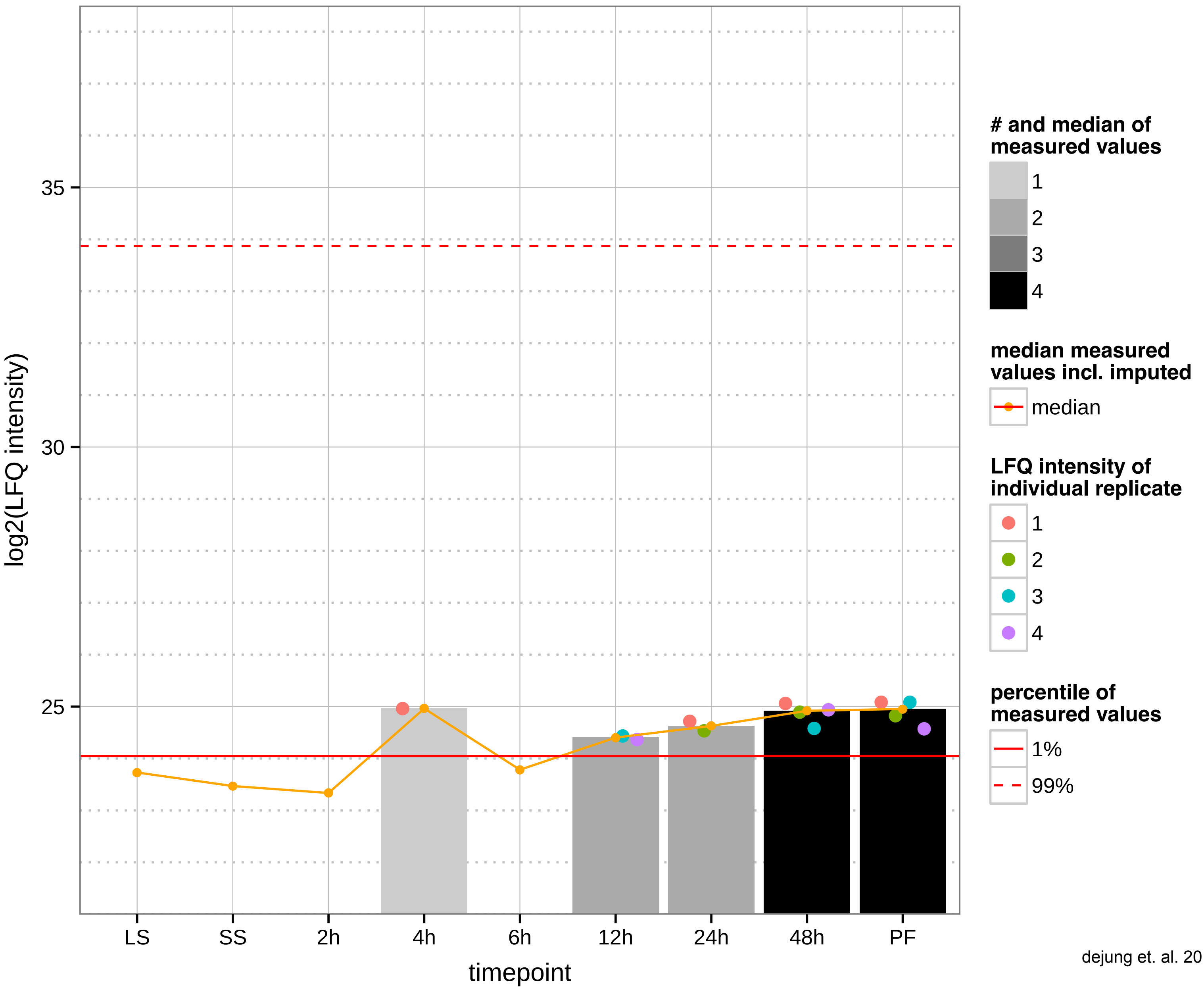

dejung et. al. 2015

hypothetical protein, conserved
Tb927.9.7000
AGOF: null
AGOC: null
AGOP: null
PGOF: null
PGOC: null
PGOP: null

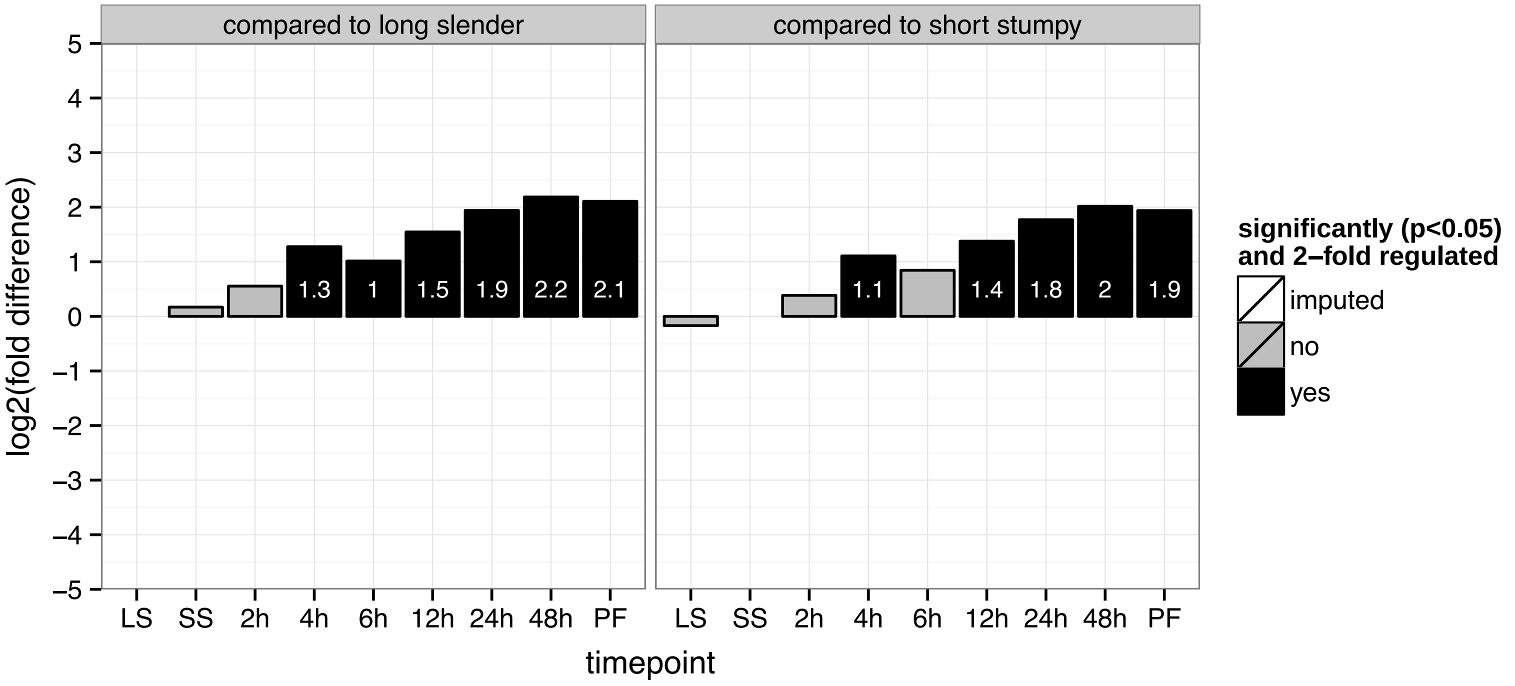

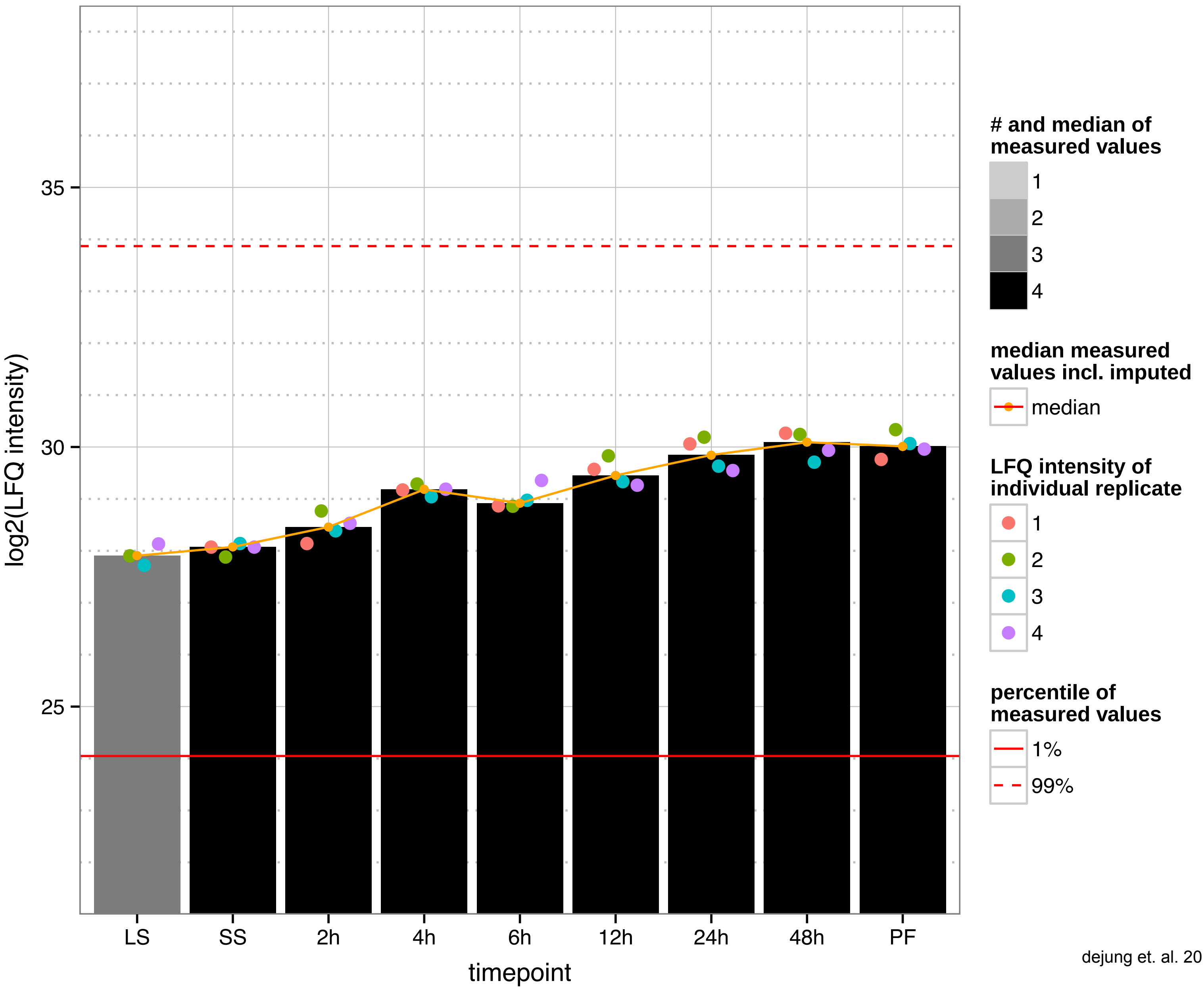

dejung et. al. 2015

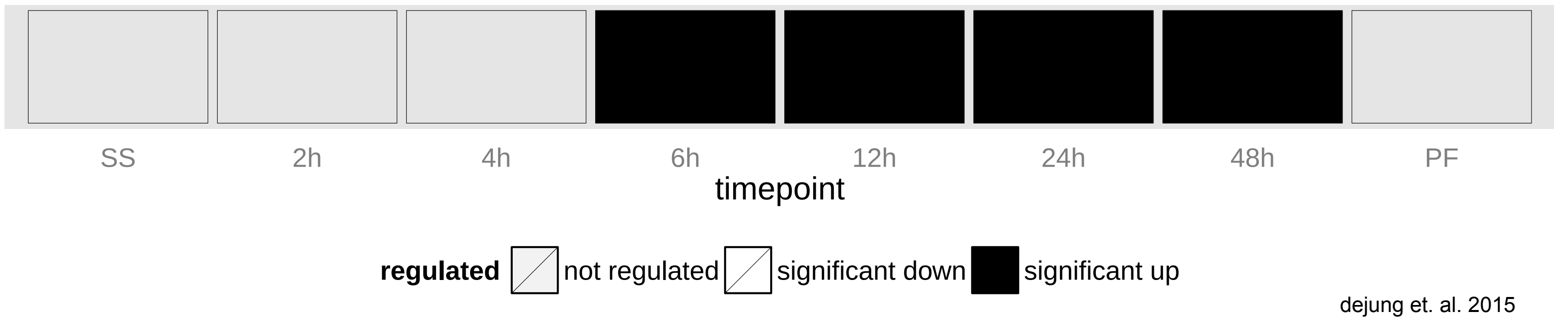
SS
2h
4h
6h
12h
24h
48h
PF
timepoint
regulated
not regulated
significant down
significant up
dejung et. al. 2015

hypothetical protein, conserved
Tb927.1.3800
AGOF: null
AGOC: mitochondrion
AGOP: response to stress
PGOF: null
PGOC: null
PGOP: response to stress

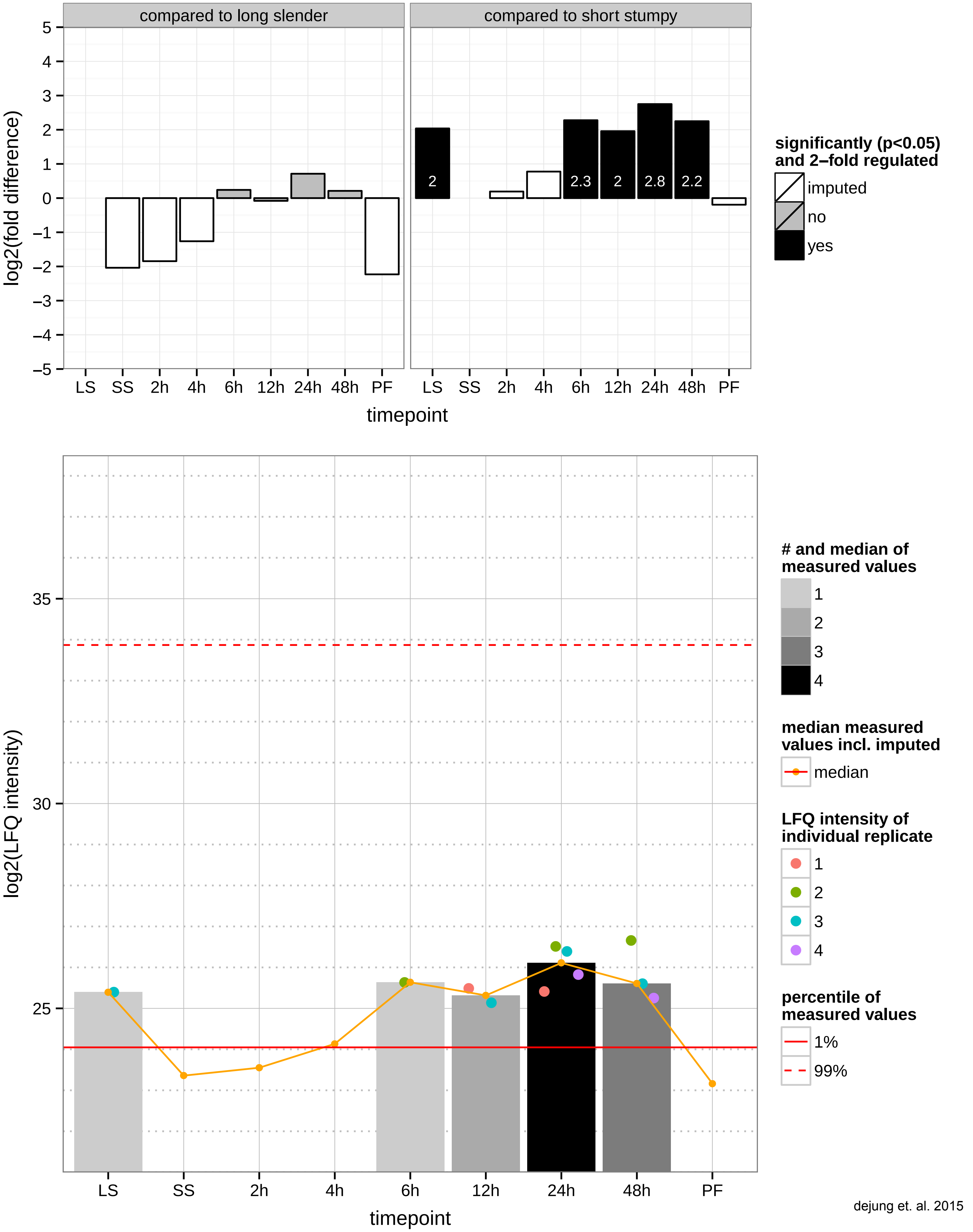

dejung et. al. 2015

hypothetical protein, conserved
Tb927.10.12050
AGOF: null
AGOC: mitochondrion
AGOP: null
PGOF: null
PGOC: null
PGOP: null

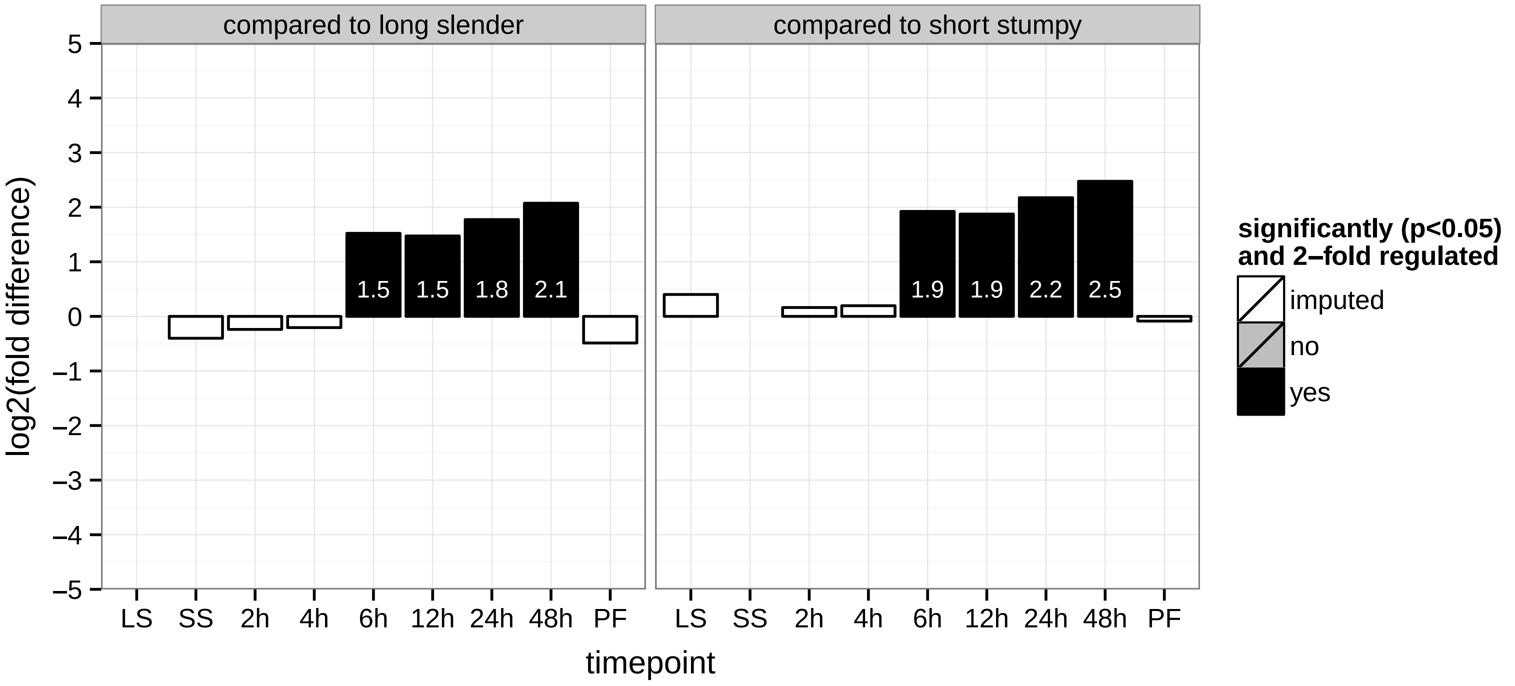

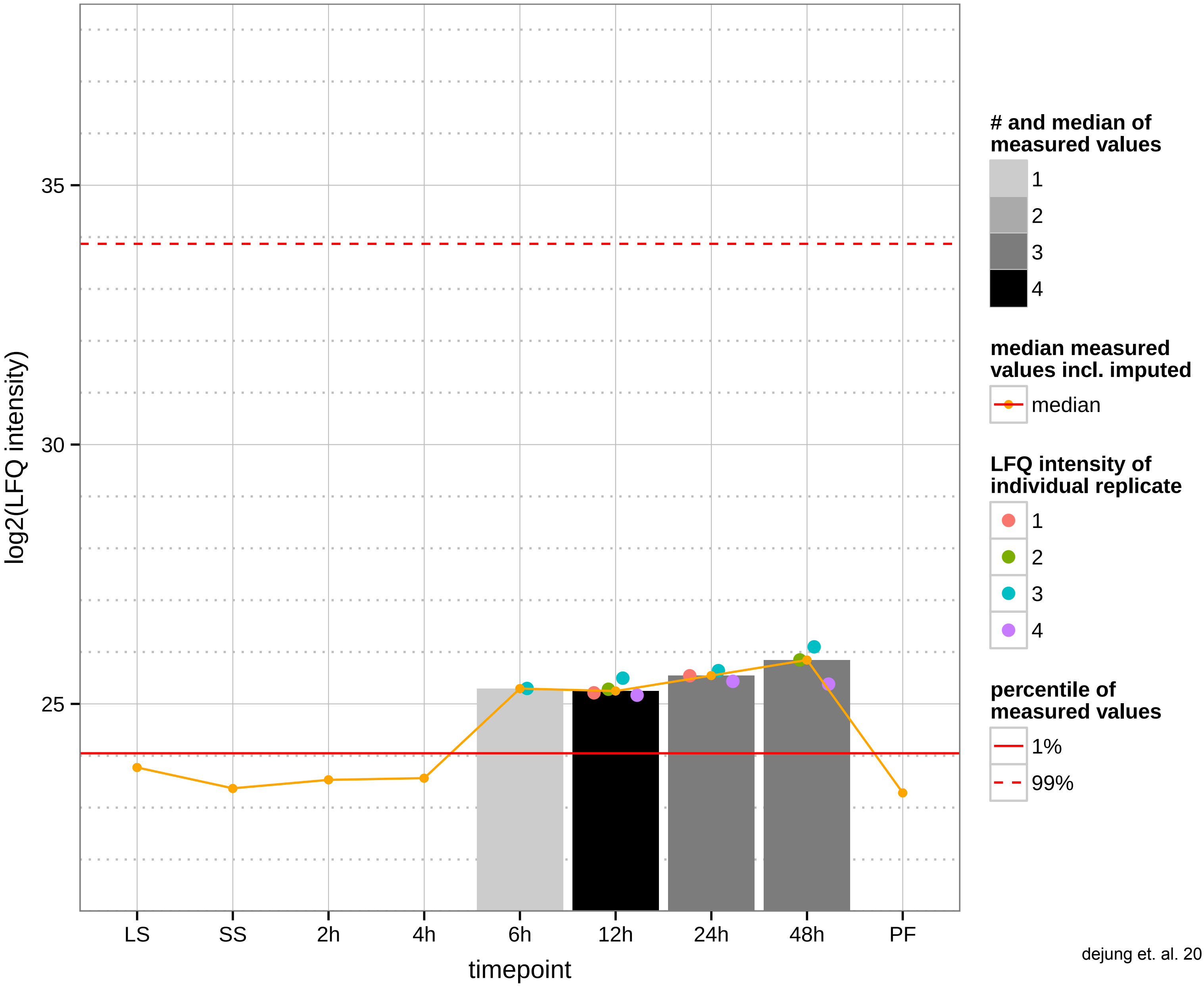

dejung et. al. 2015

Zinc finger CCCH domain–containing protein 37 (ZC3H37), Zinc finger CCCH domain–containing protein 38 (ZC3H38)
Tb927.10.12780
AGOF: RNA binding, nucleic acid binding, zinc ion binding, nucleic acid binding
AGOC: null
AGOP: null
PGOF: nucleic acid binding, zinc ion binding
PGOC: null
PGOP: null

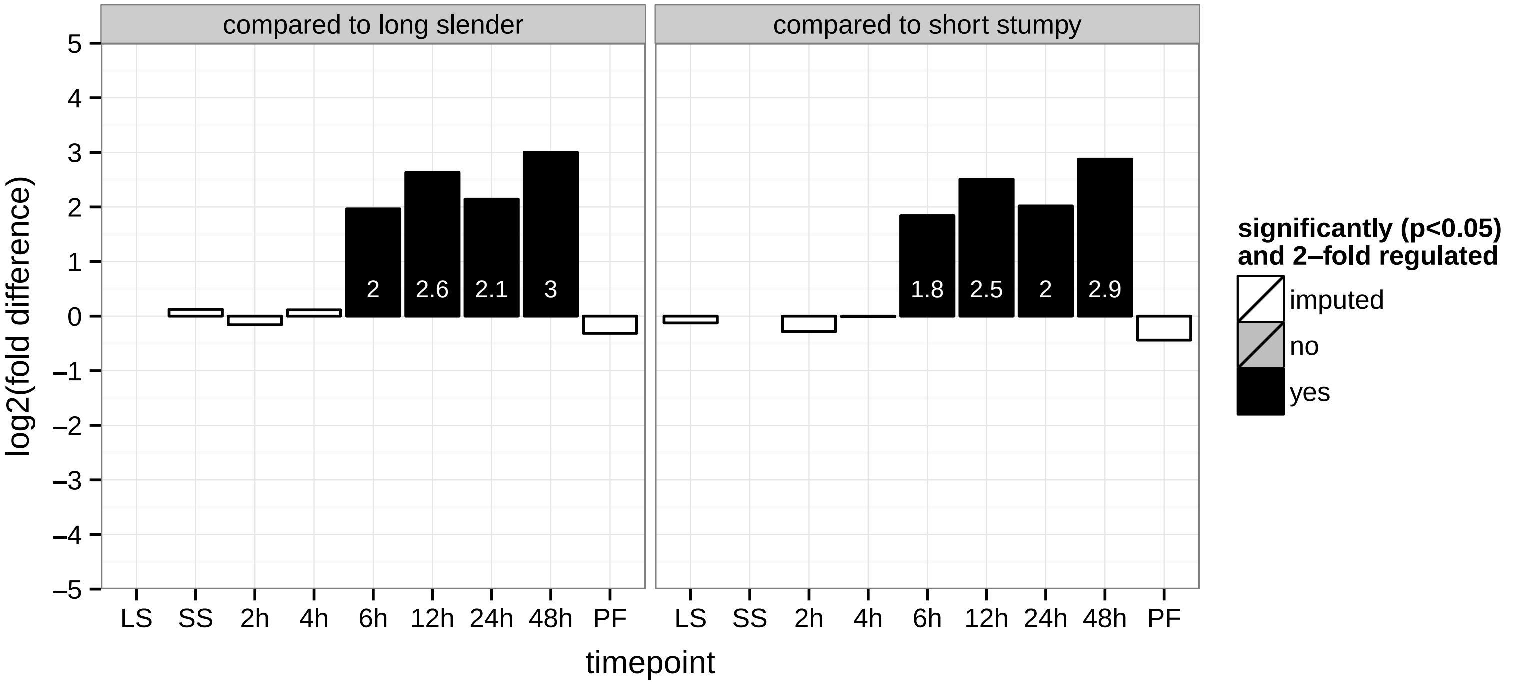

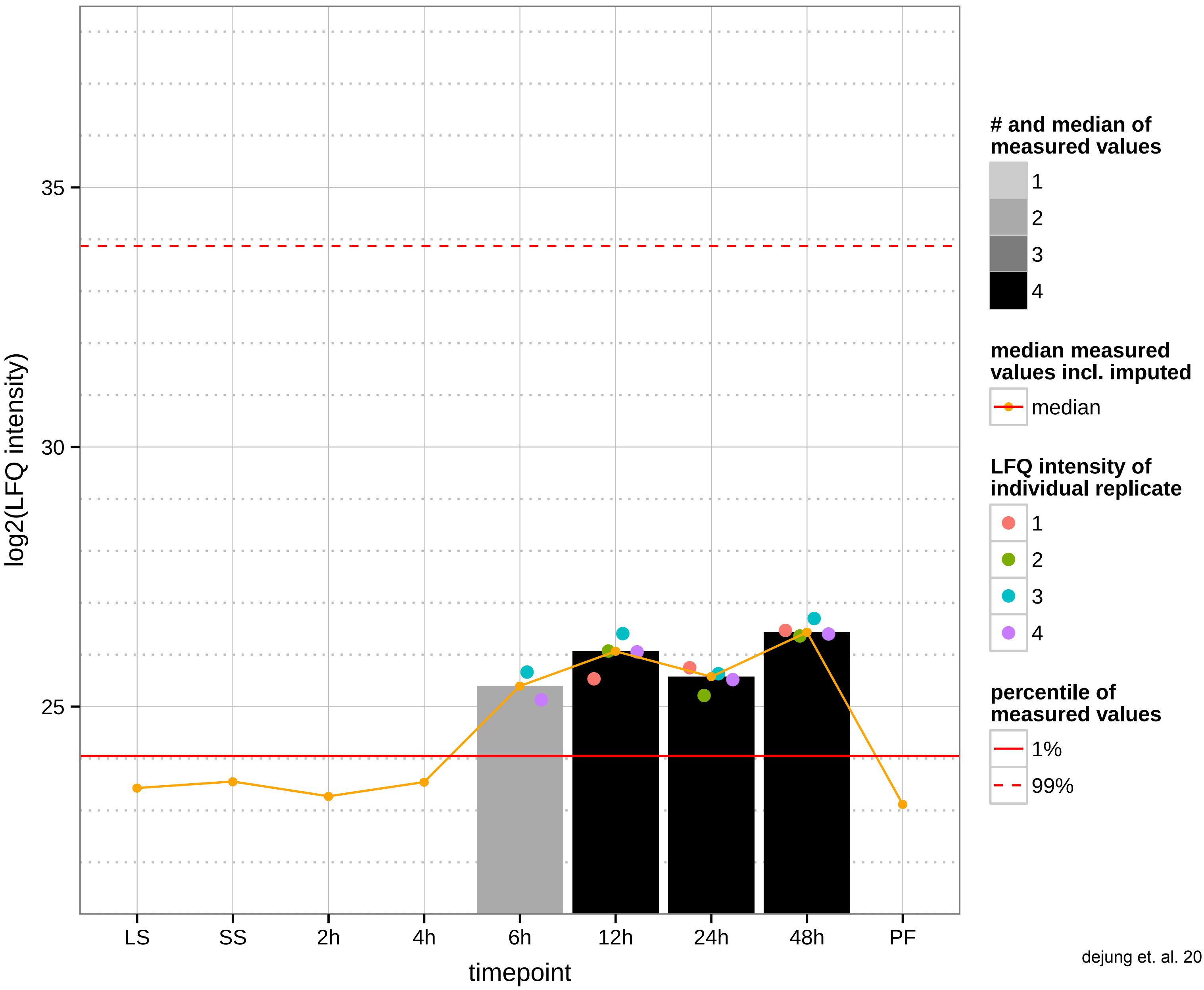

dejung et. al. 2015

hypothetical protein, conserved
Tb927.10.5860
AGOF: null
AGOC: null
AGOP: null
PGOF: cAMP–dependent protein kinase regulator activity
PGOC: null
PGOP: signal transduction

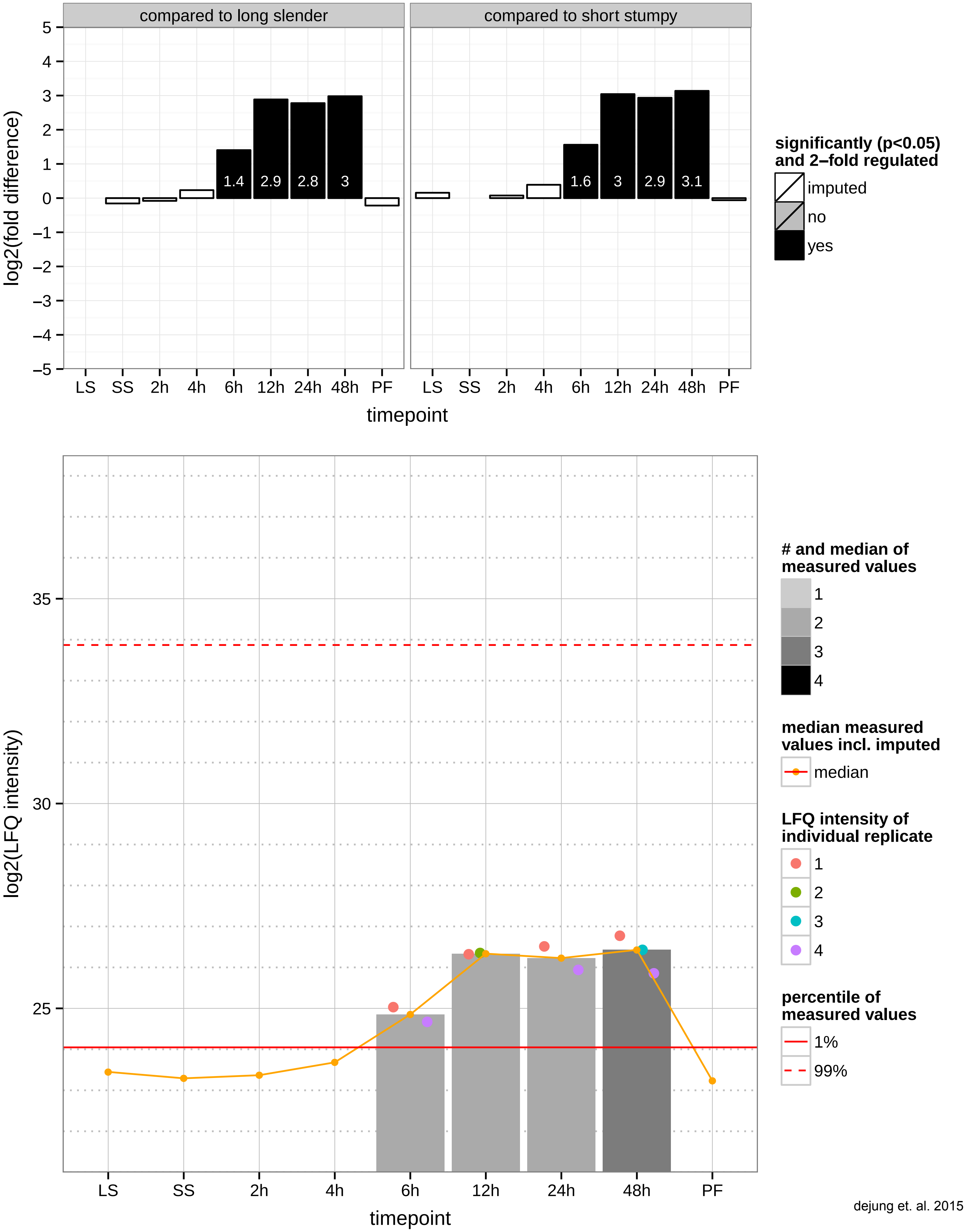

dejung et. al. 2015

ras–like small GTPase, putative (TbRHP)
Tb927.10.6240
AGOF: GTP binding, GTPase activity
AGOC: intracellular
AGOP: protein transport
PGOF: GTP binding, GTPase activity
PGOC: intracellular, membrane
PGOP: GTP catabolic process, protein transport, signal transduction, small GTPase mediated signal transduction

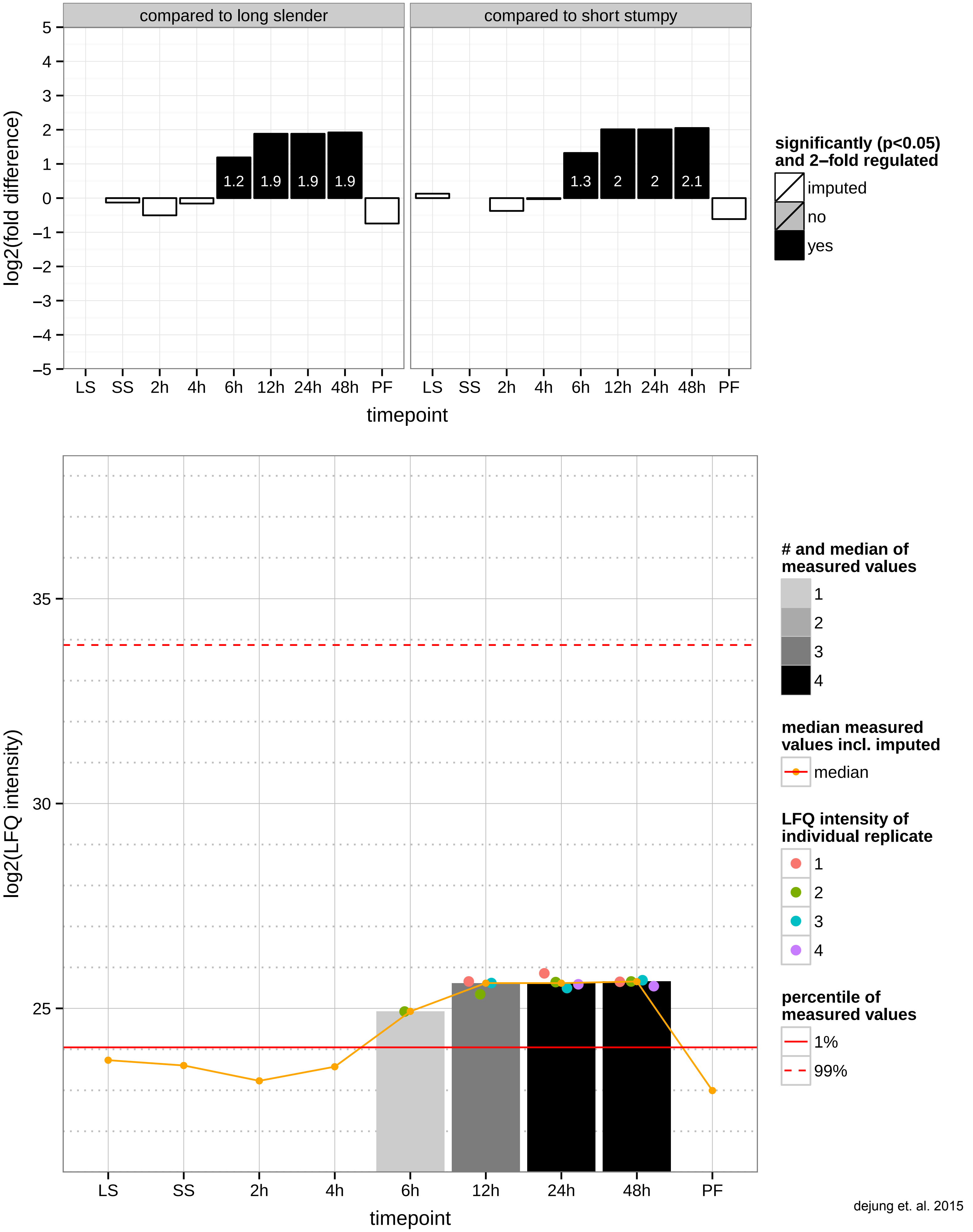

dejung et. al. 2015

hypothetical protein, conserved
Tb927.10.6360
AGOF: null
AGOC: null
AGOP: null
PGOF: null
PGOC: null
PGOP: null

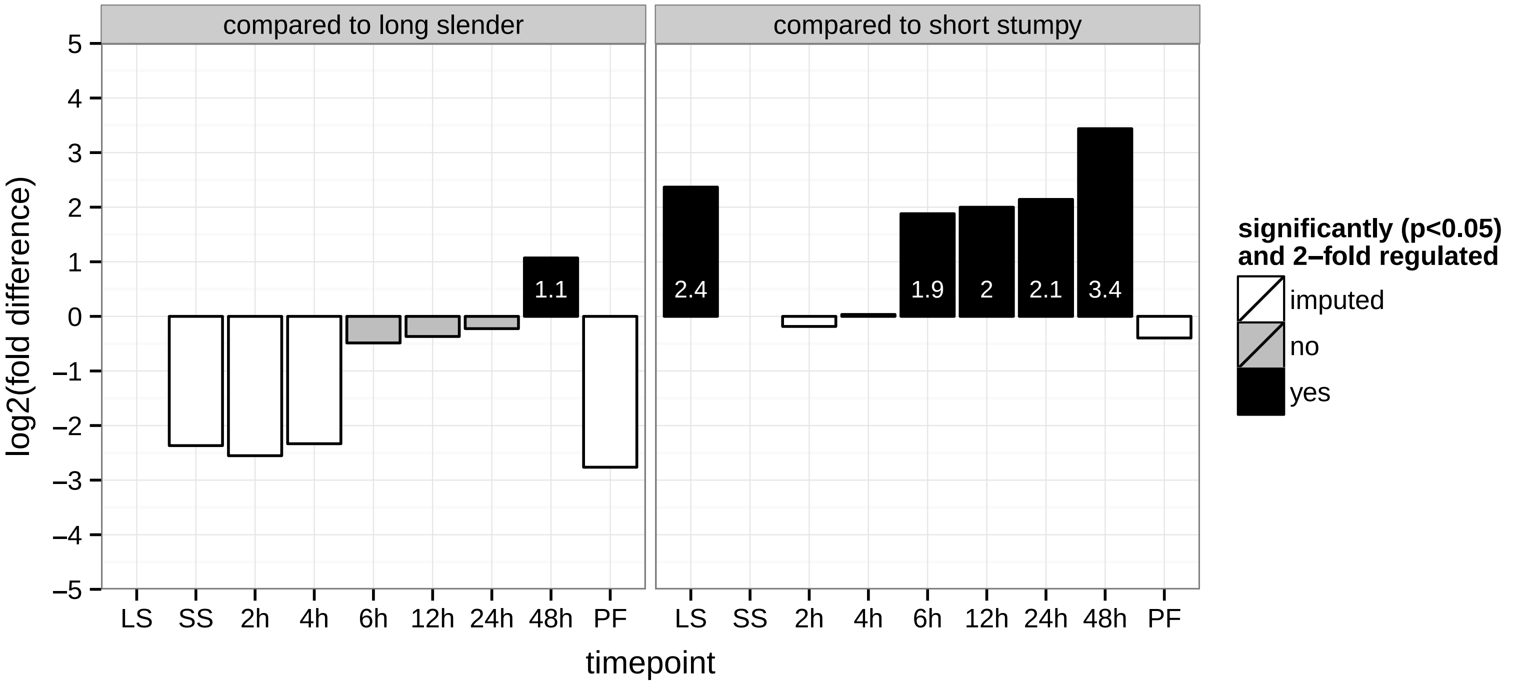

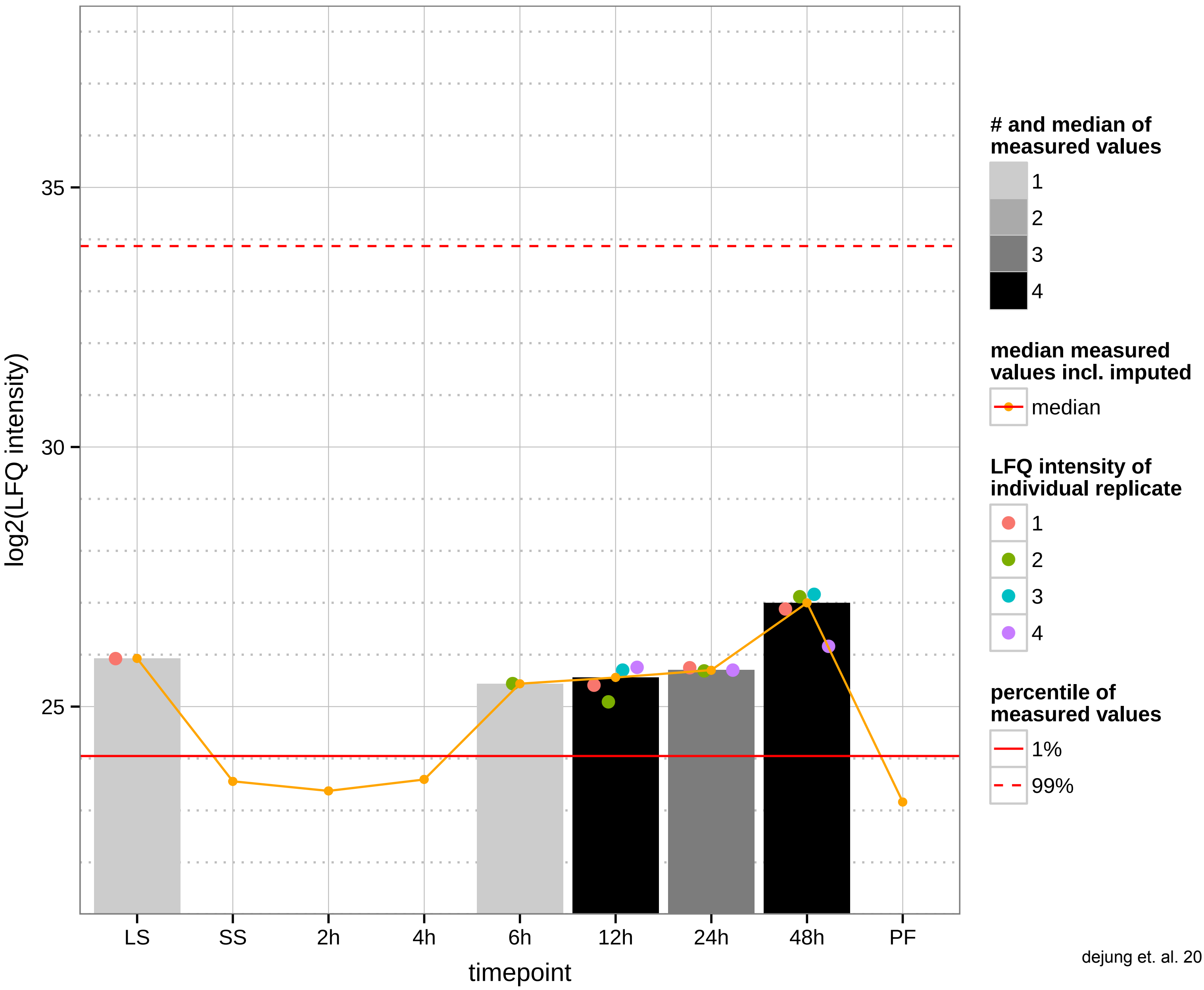

dejung et. al. 2015

hypothetical protein, conserved
Tb927.10.9280;Tb11.v5.0485
AGOF: null
AGOC: null, mitochondrion
AGOP: null
PGOF: null
PGOC: null
PGOP: null

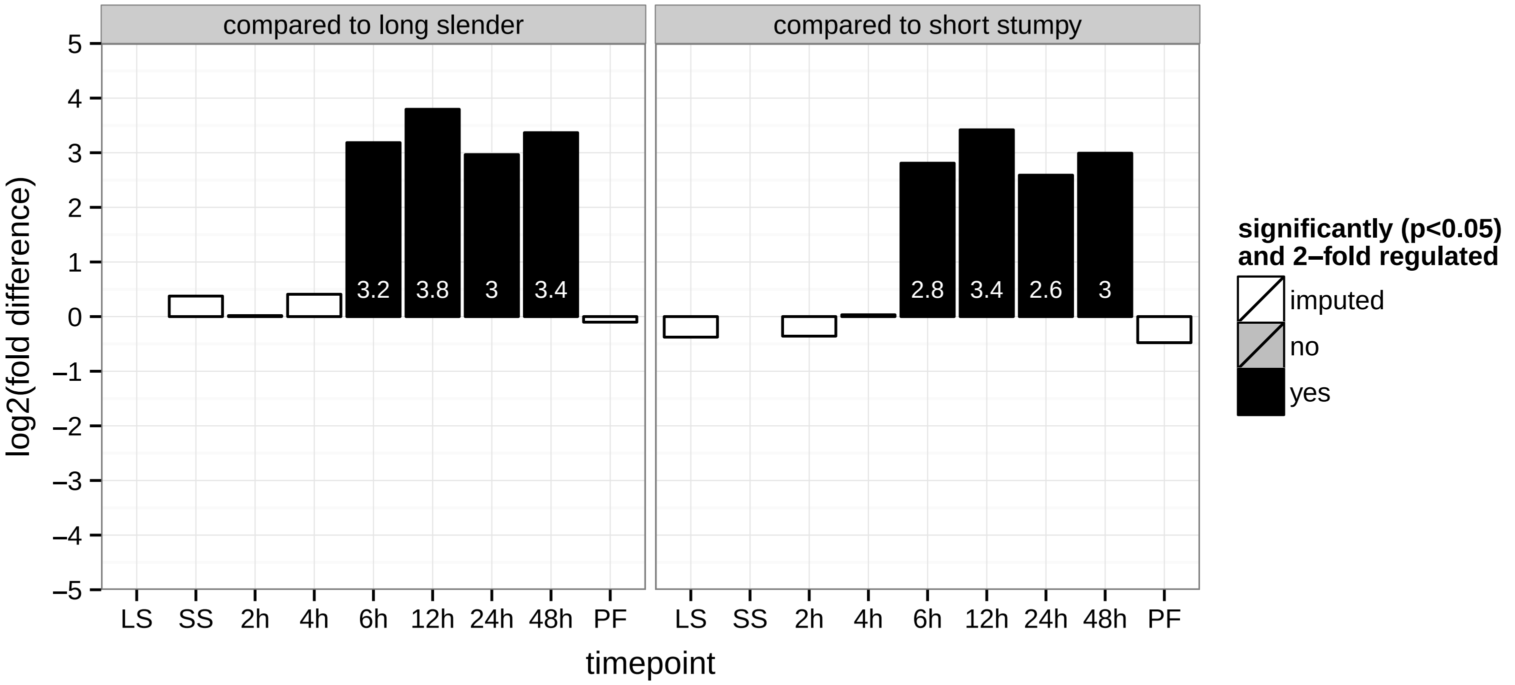

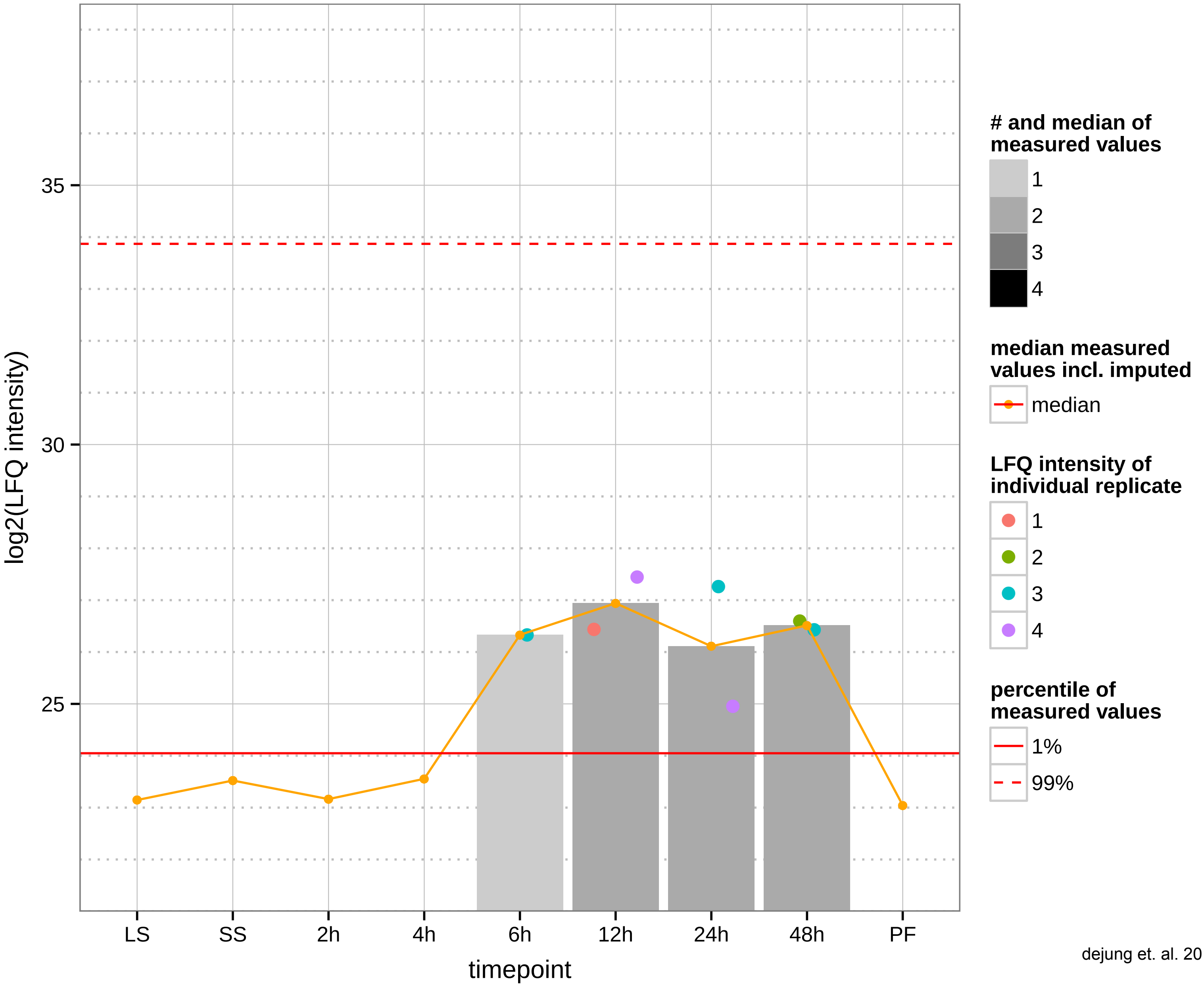

dejung et. al. 2015

actin–like protein, putative
Tb927.11.10110
AGOF: structural constituent of cytoskeleton
AGOC: actin cytoskeleton
AGOP: cytoskeleton organization
PGOF: null
PGOC: null
PGOP: null

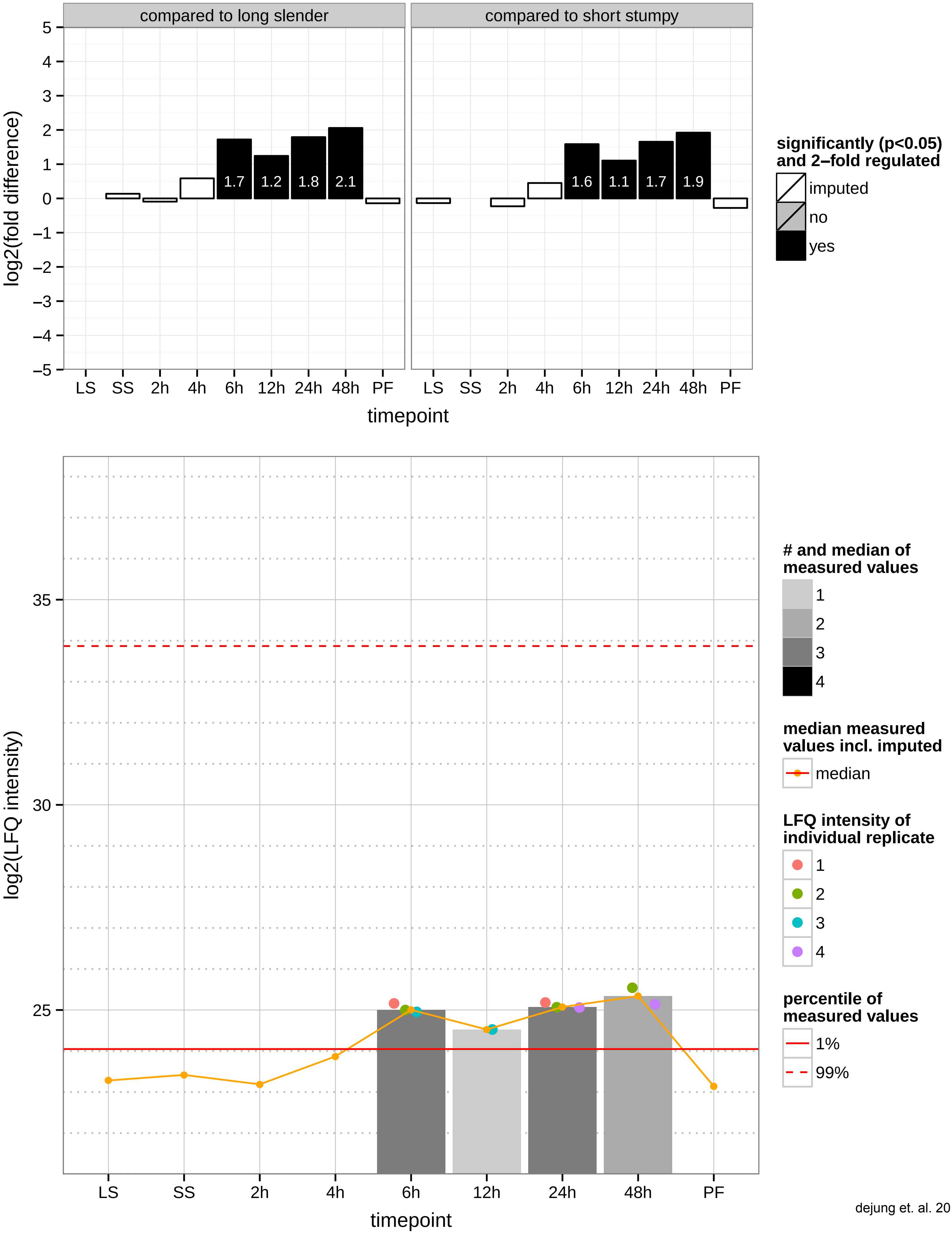

dejung et. al. 2015

hypothetical protein, conserved
Tb927.11.1300
AGOF: null
AGOC: integral to membrane
AGOP: null
PGOF: protein binding
PGOC: null
PGOP: null

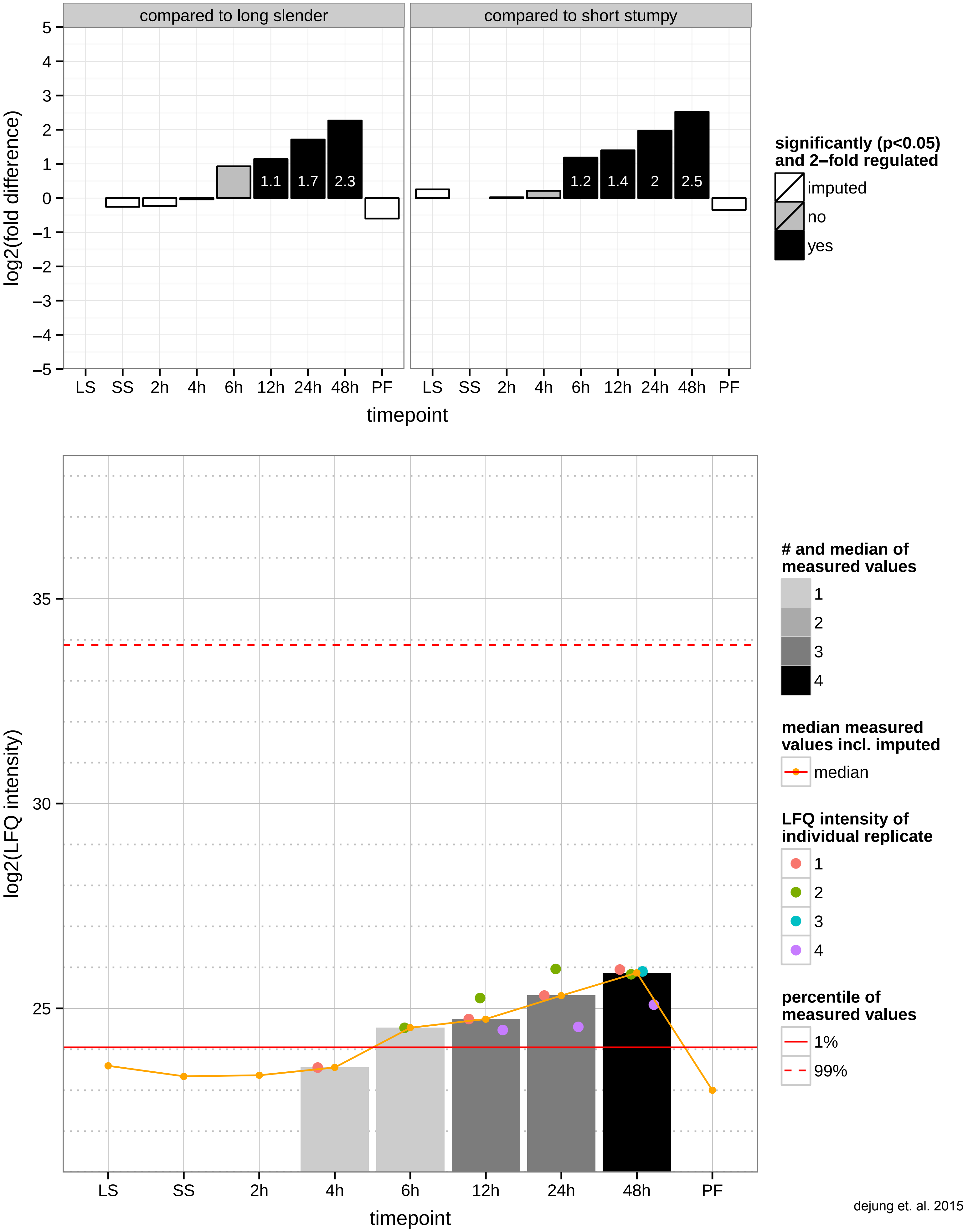

dejung et. al. 2015

hypothetical protein, conserved
Tb927.11.13840
AGOF: null
AGOC: mitochondrion
AGOP: null
PGOF: null
PGOC: null
PGOP: null

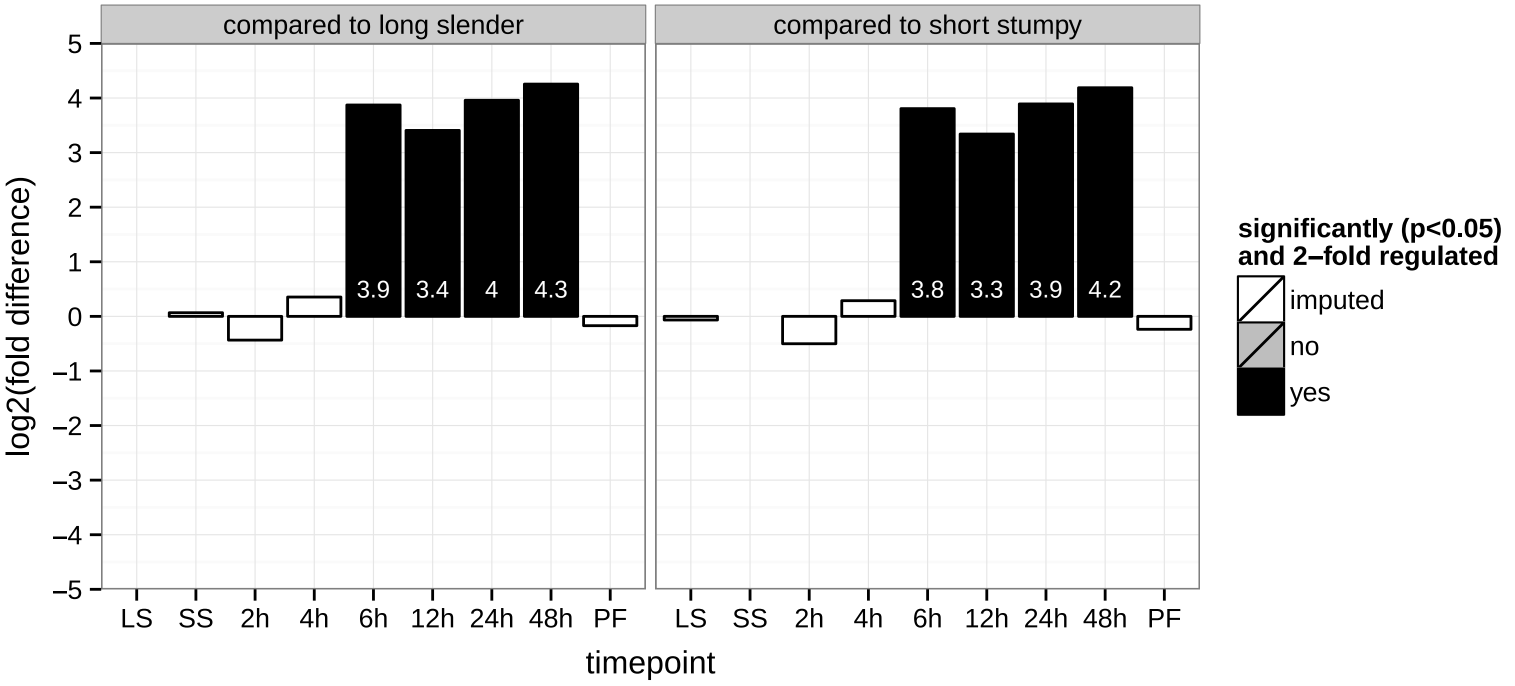

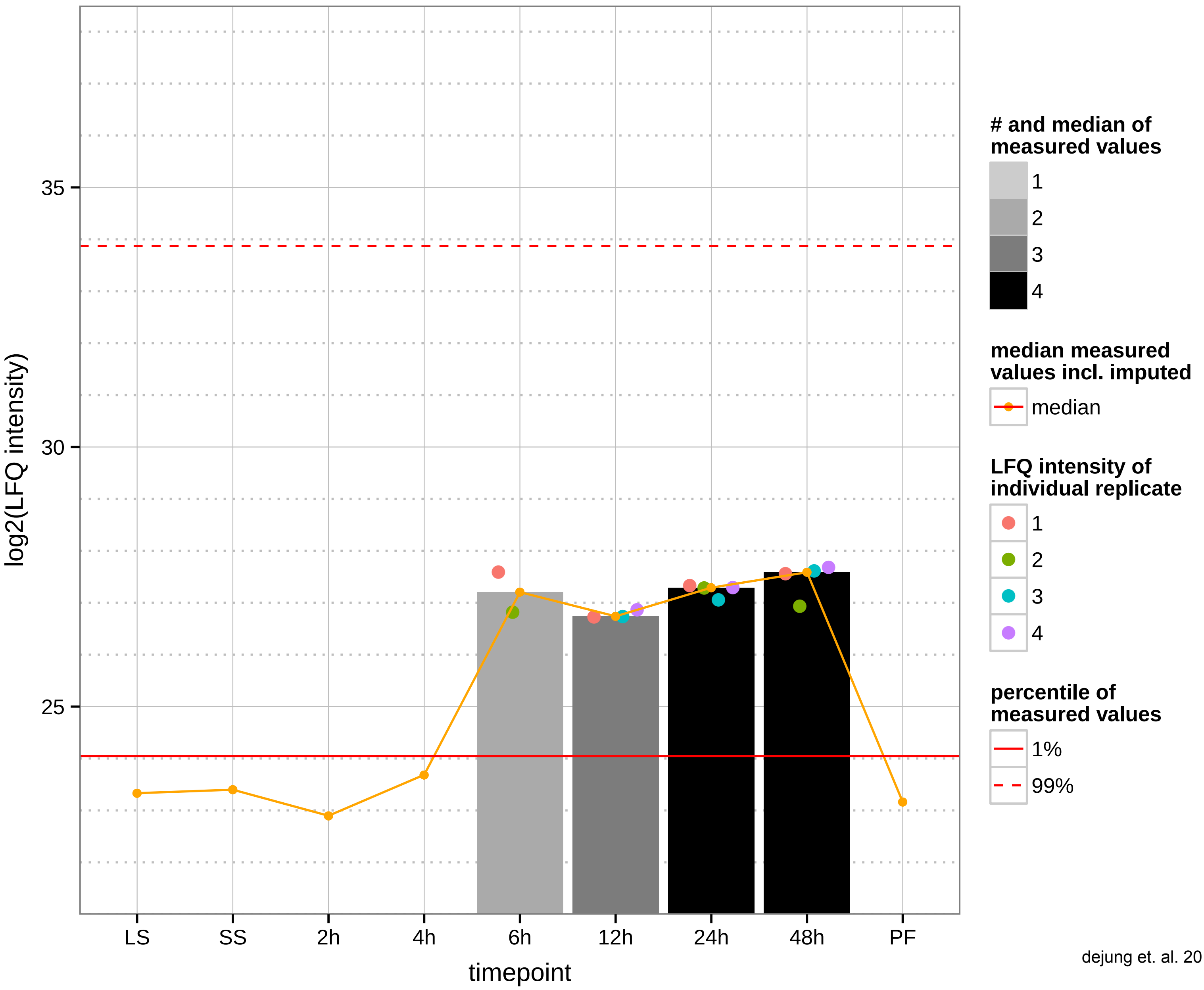

dejung et. al. 2015

hypothetical protein, conserved
Tb927.11.15440
AGOF: null
AGOC: mitochondrion
AGOP: null
PGOF: null
PGOC: null
PGOP: null

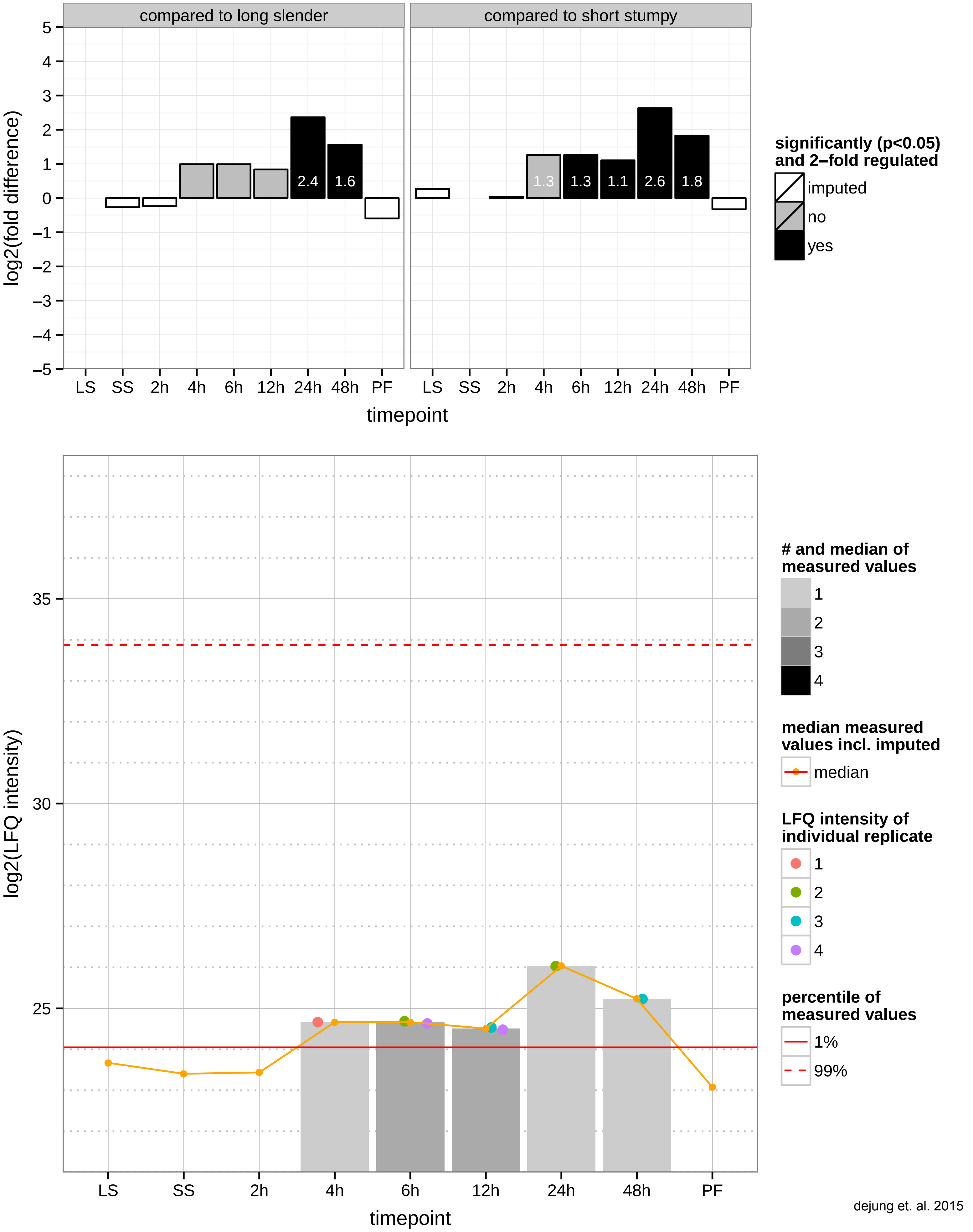

dejung et. al. 2015

hypothetical protein, conserved
Tb927.11.15580
AGOF: null
AGOC: null
AGOP: null
PGOF: null
PGOC: null
PGOP: null

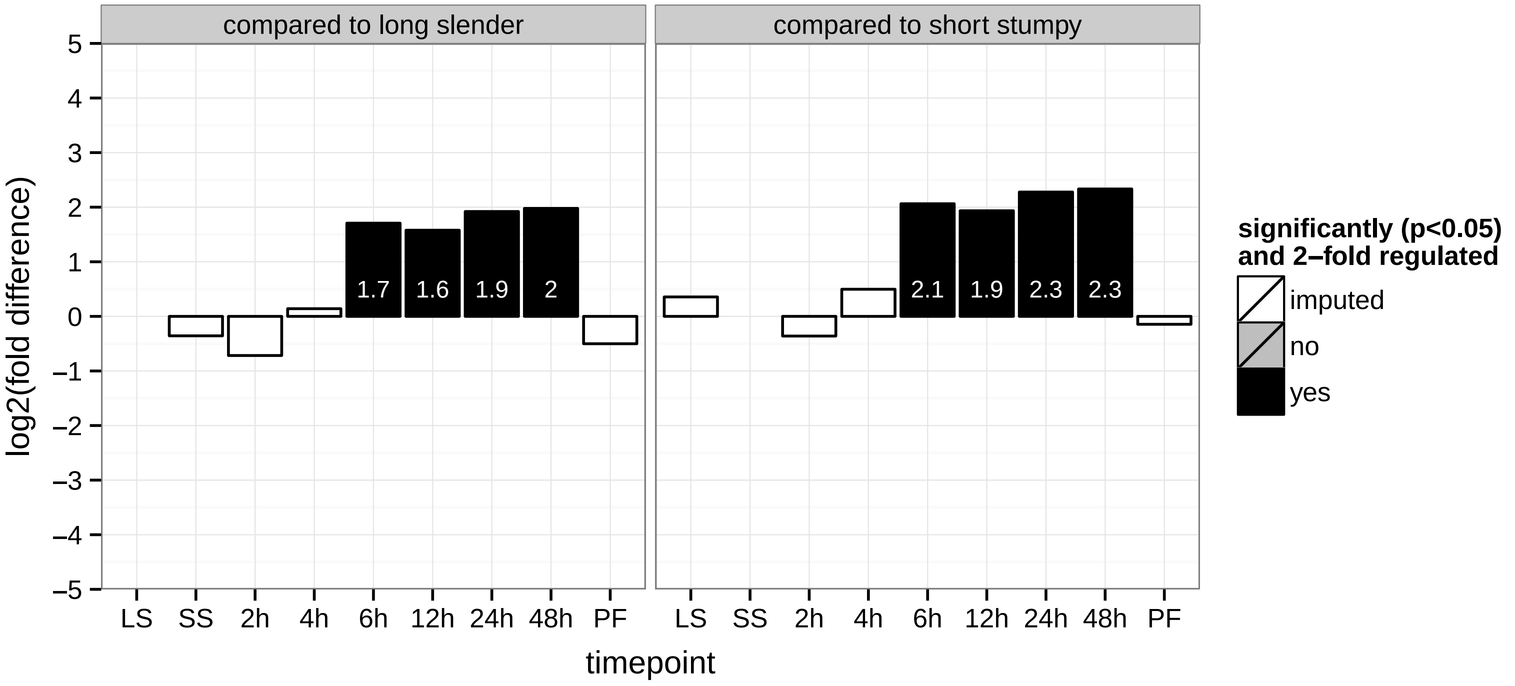

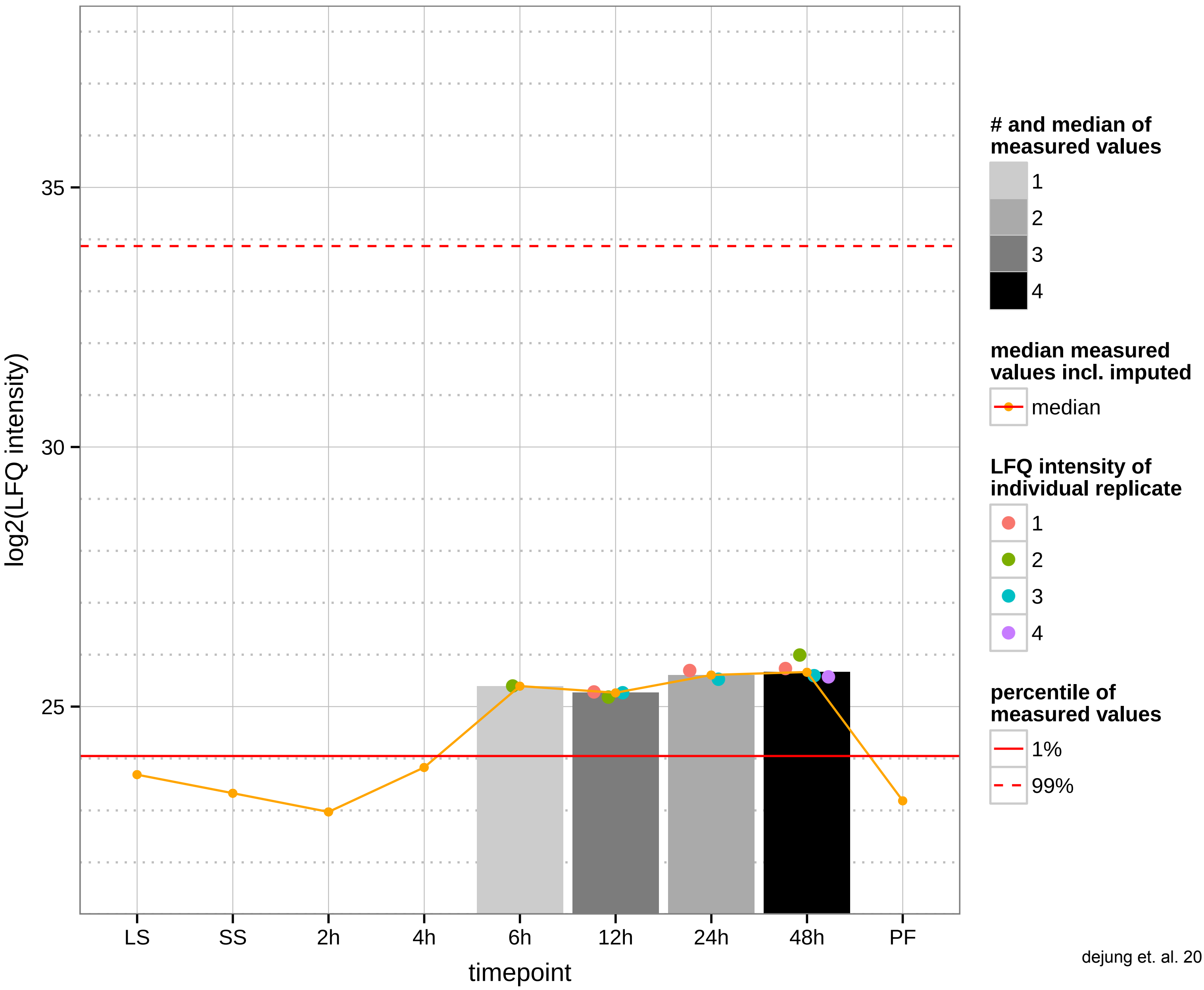

dejung et. al. 2015

hypothetical protein, conserved
Tb927.11.17020
AGOF: null
AGOC: null
AGOP: null
PGOF: null
PGOC: null
PGOP: null

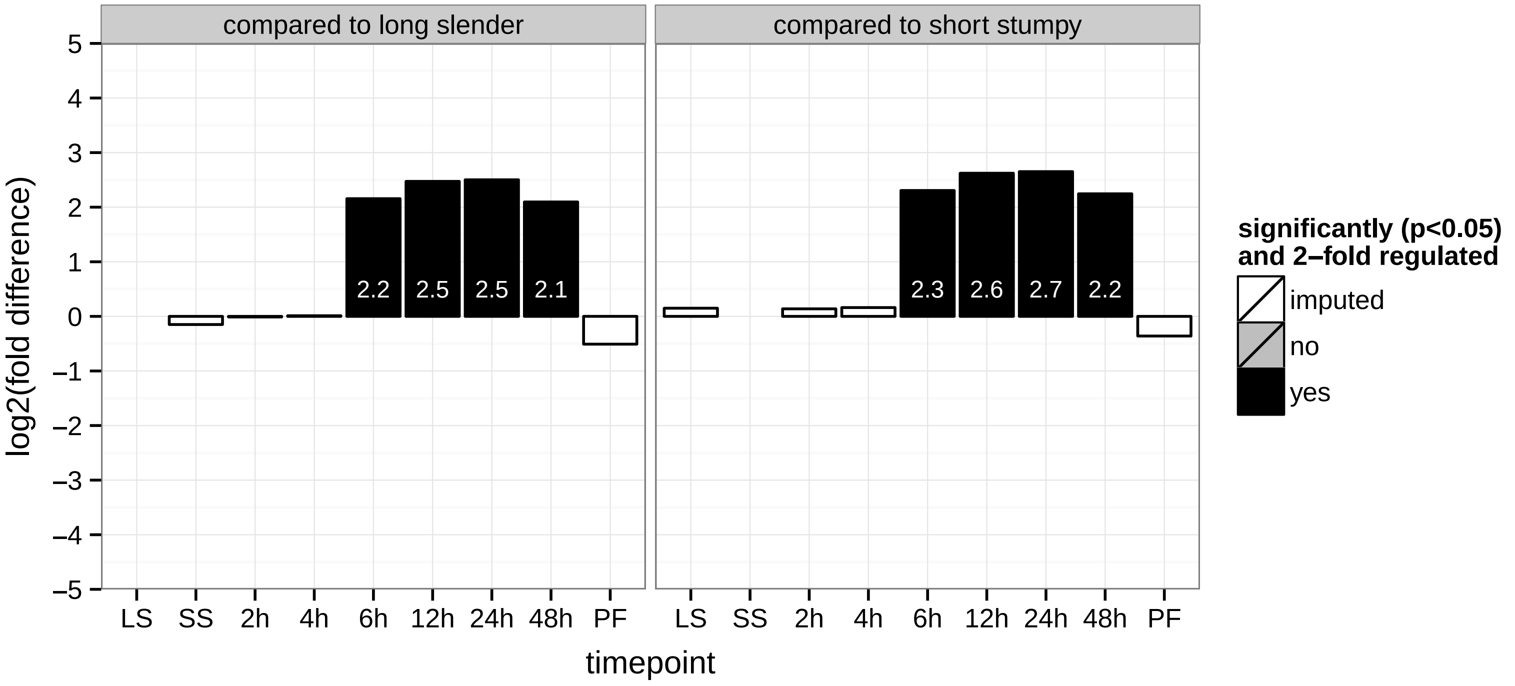

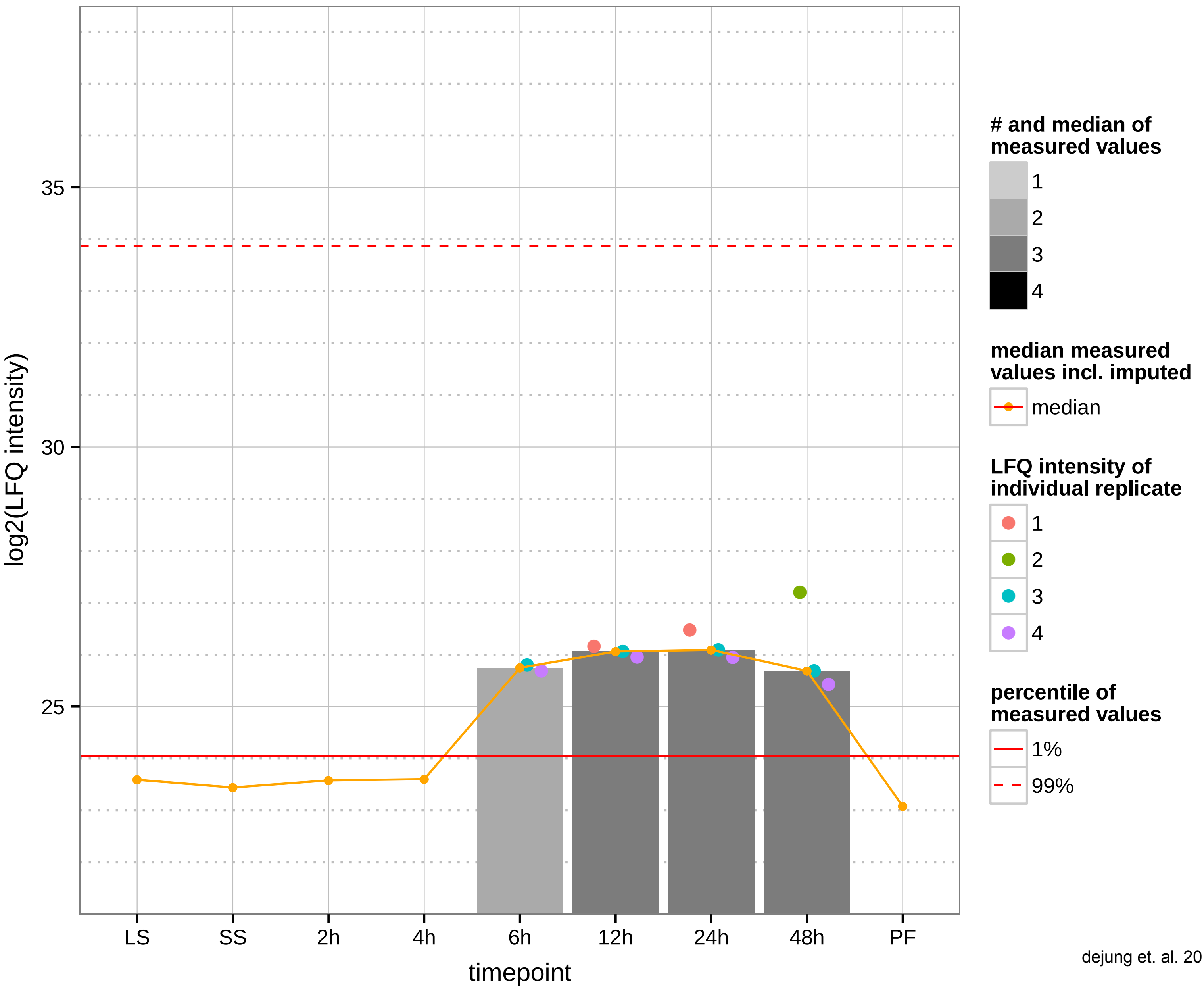

dejung et. al. 2015

hypothetical protein, conserved
Tb927.11.1770
AGOF: null
AGOC: null
AGOP: null
PGOF: null
PGOC: null
PGOP: null

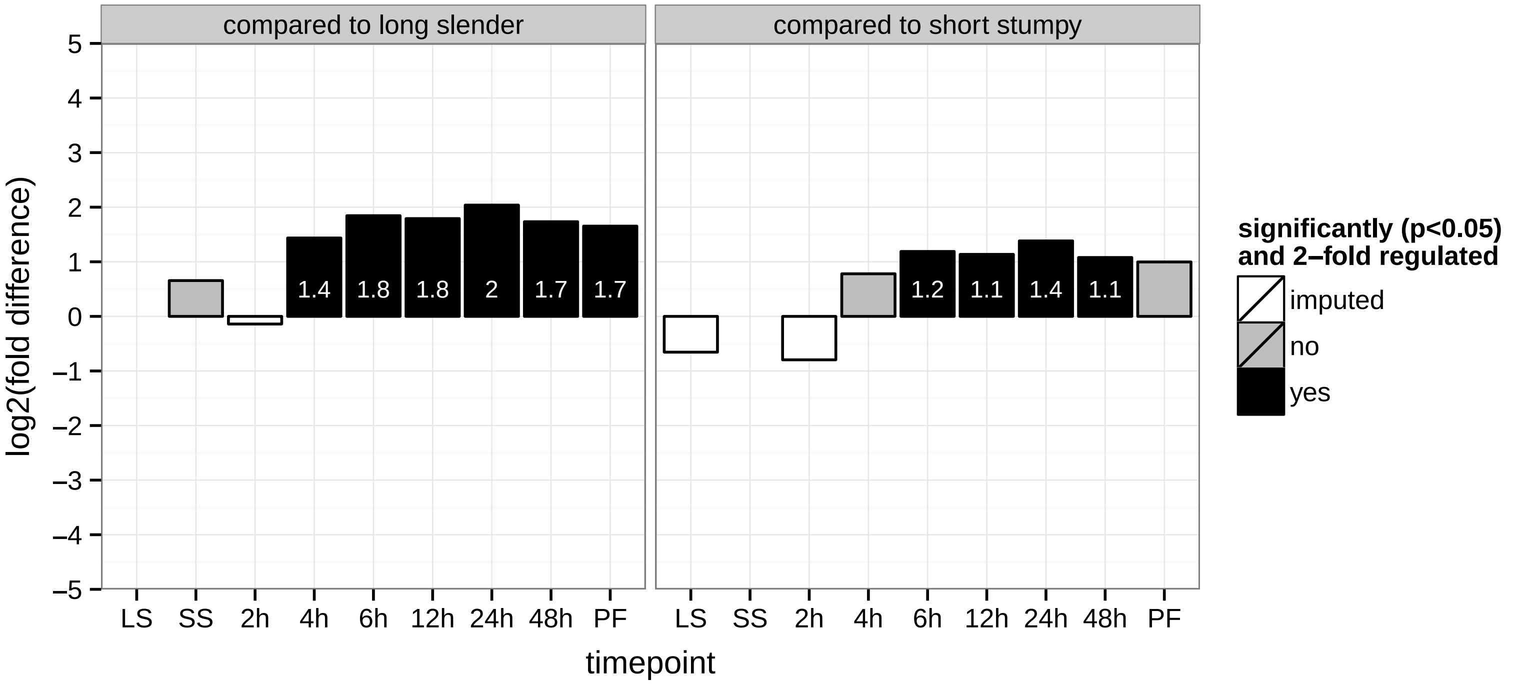

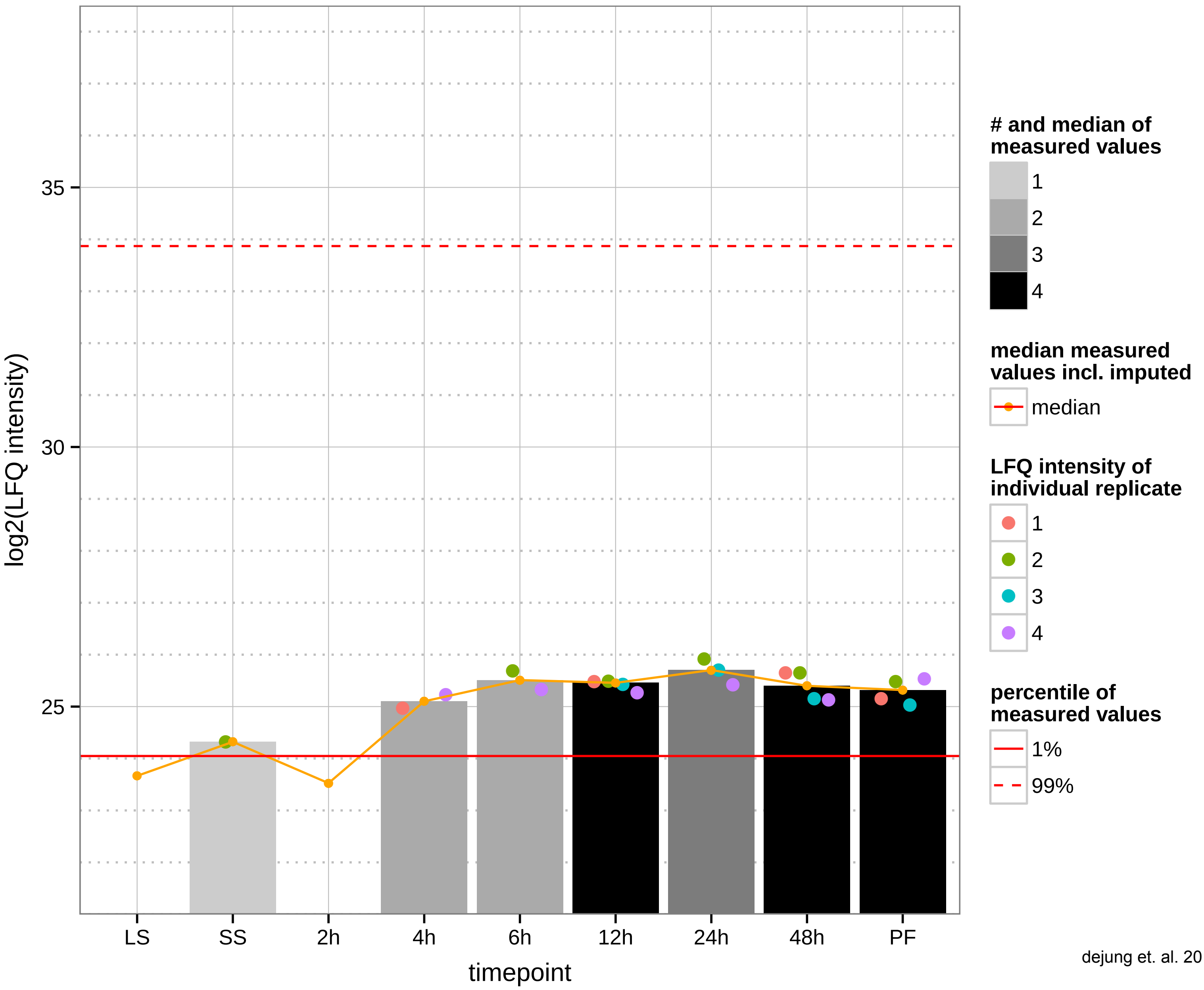

dejung et. al. 2015

hypothetical protein, conserved
Tb927.11.2070
AGOF: null
AGOC: null
AGOP: null
PGOF: null
PGOC: null
PGOP: null

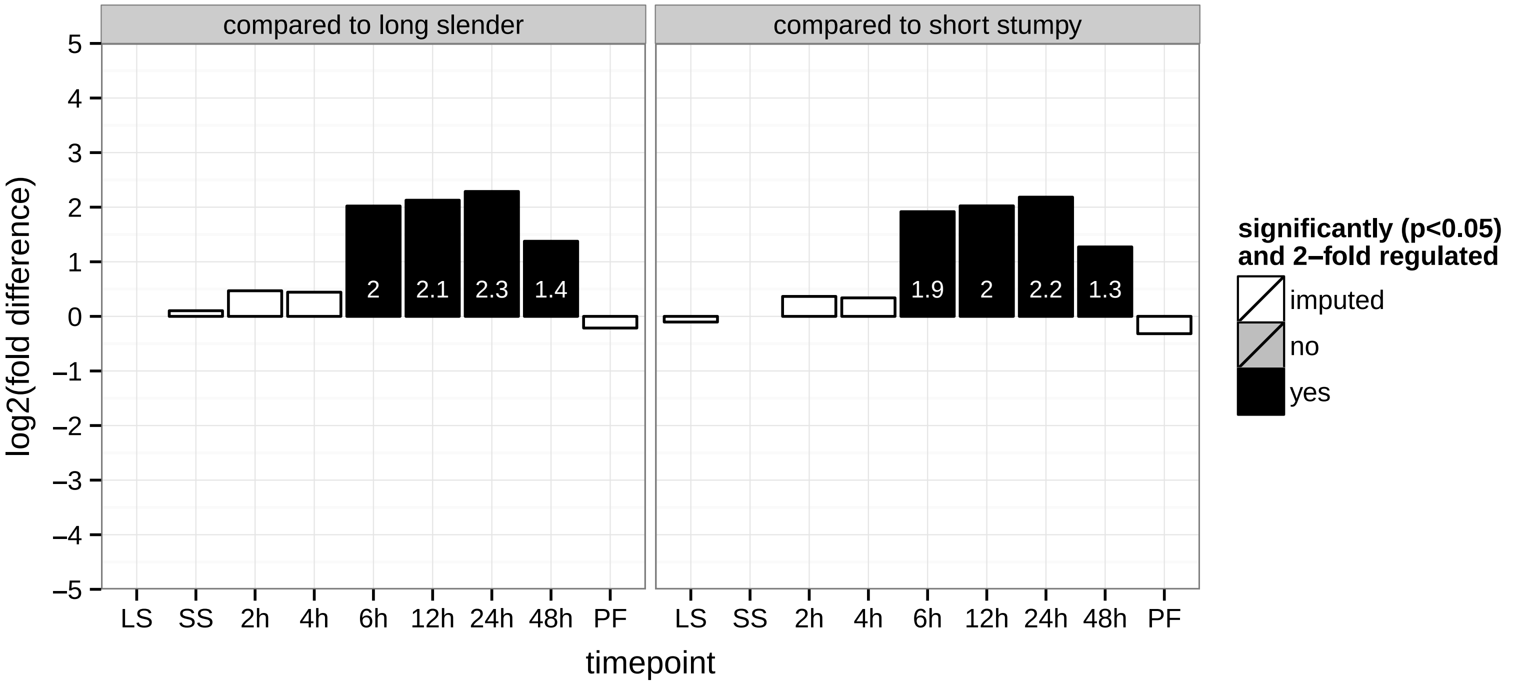

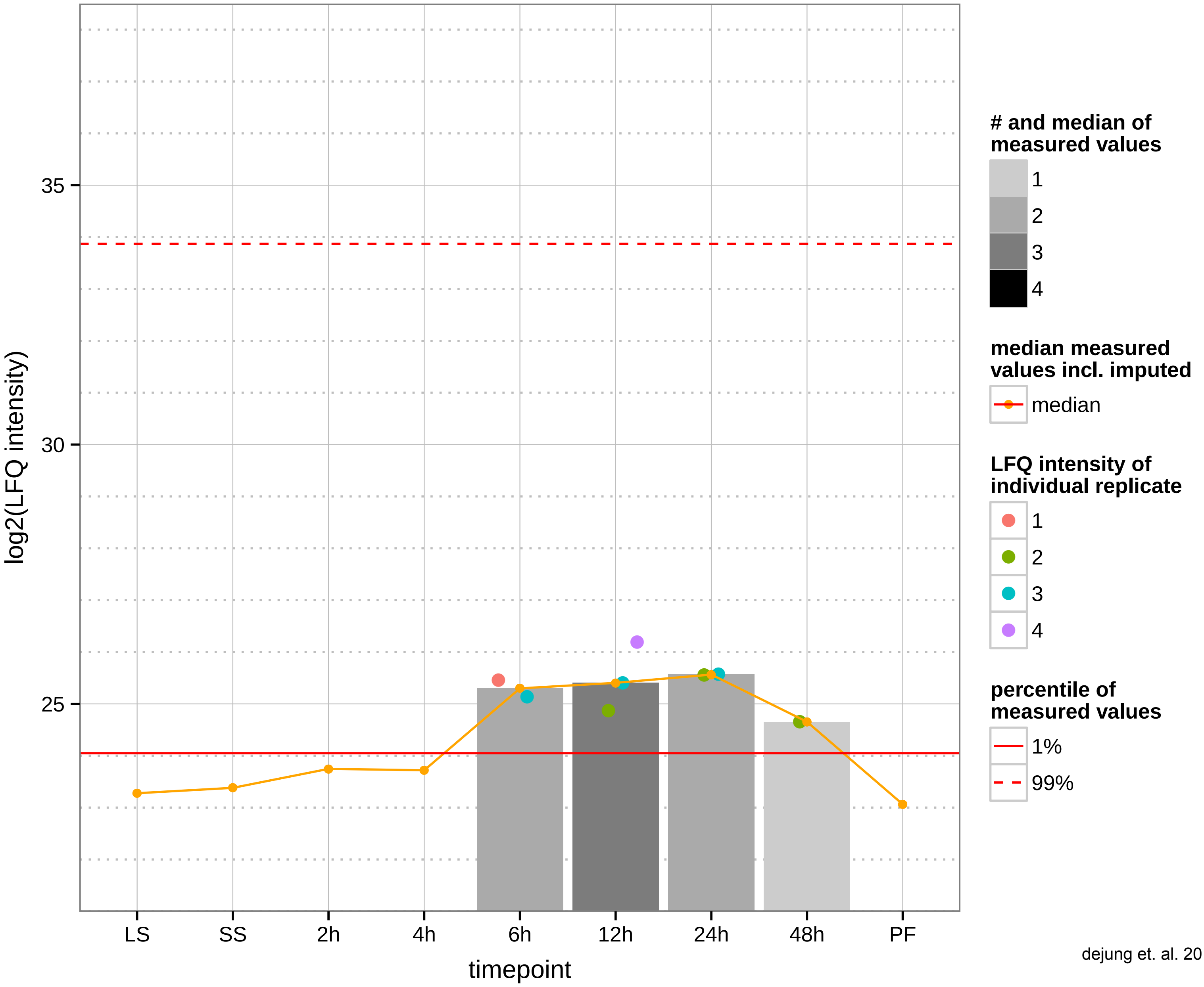

dejung et. al. 2015

hypothetical protein, conserved
Tb927.11.2150
AGOF: null
AGOC: null
AGOP: null
PGOF: null
PGOC: null
PGOP: null

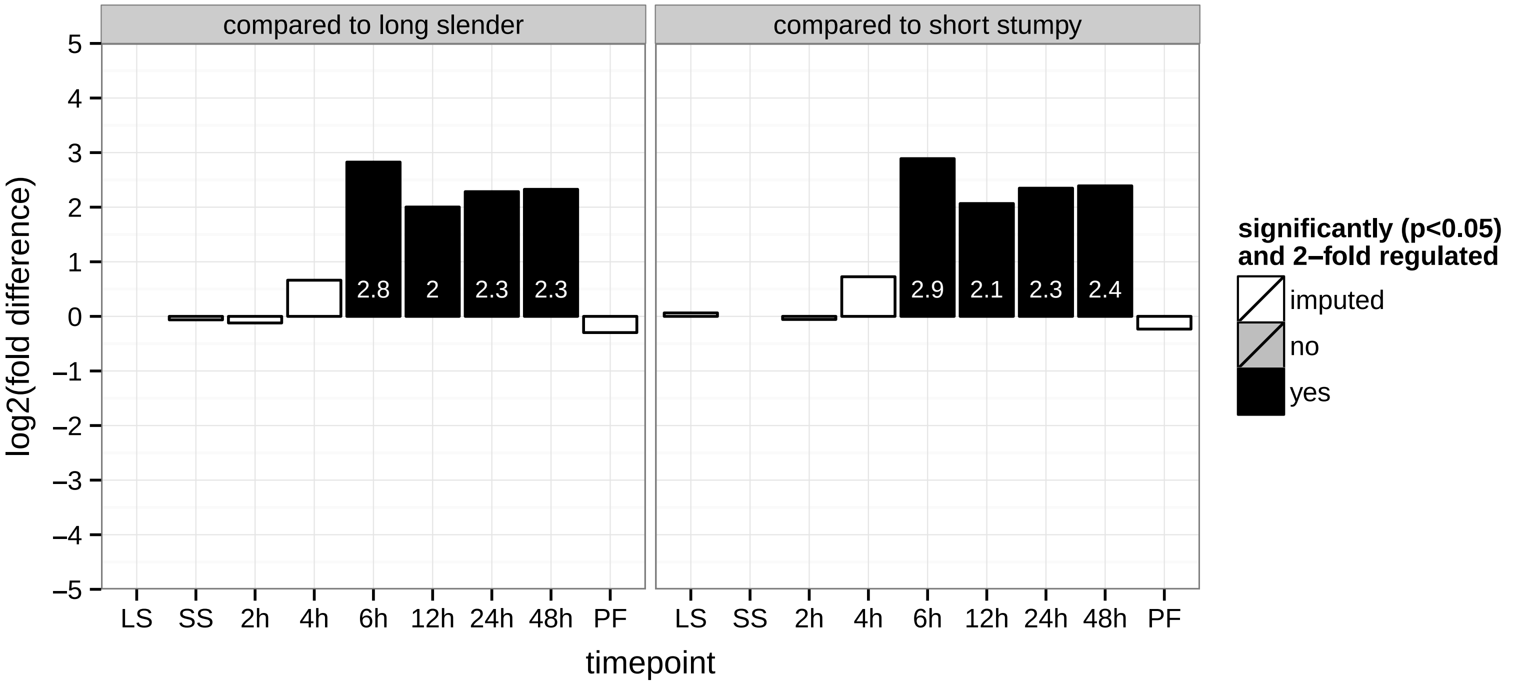

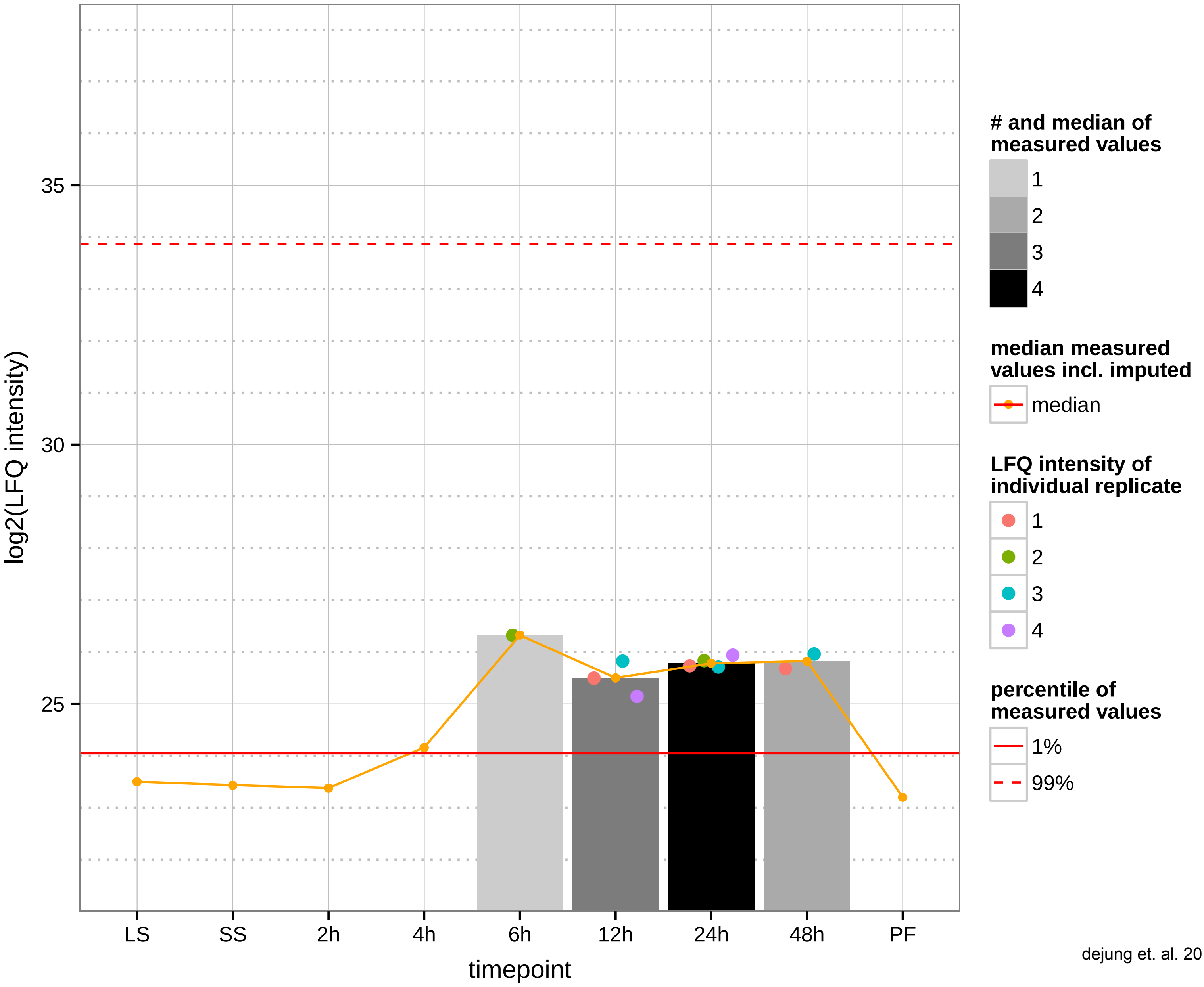

dejung et. al. 2015

molybdopterin synthase sulphurylase protein, putative
Tb927.11.2330
AGOF: catalytic activity
AGOC: integral to membrane
AGOP: null
PGOF: catalytic activity
PGOC: null
PGOP: null

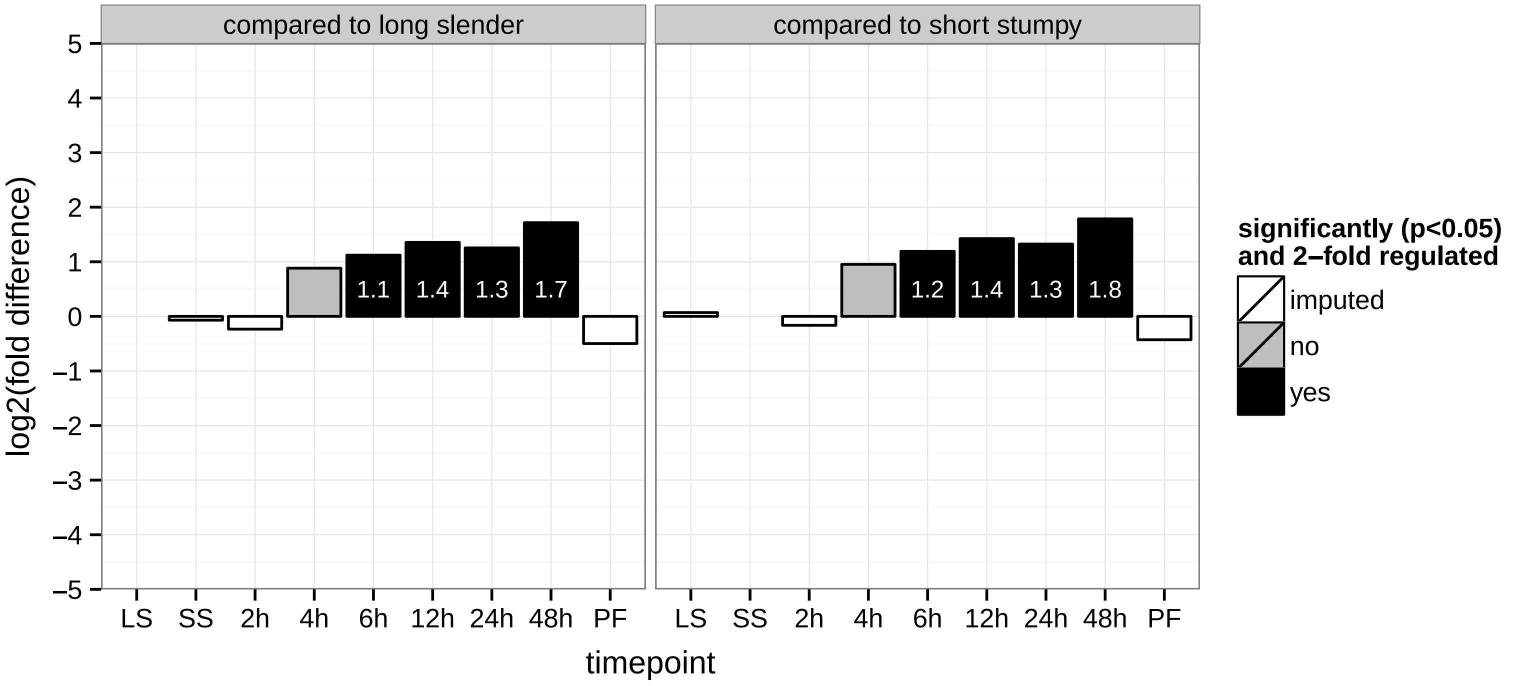

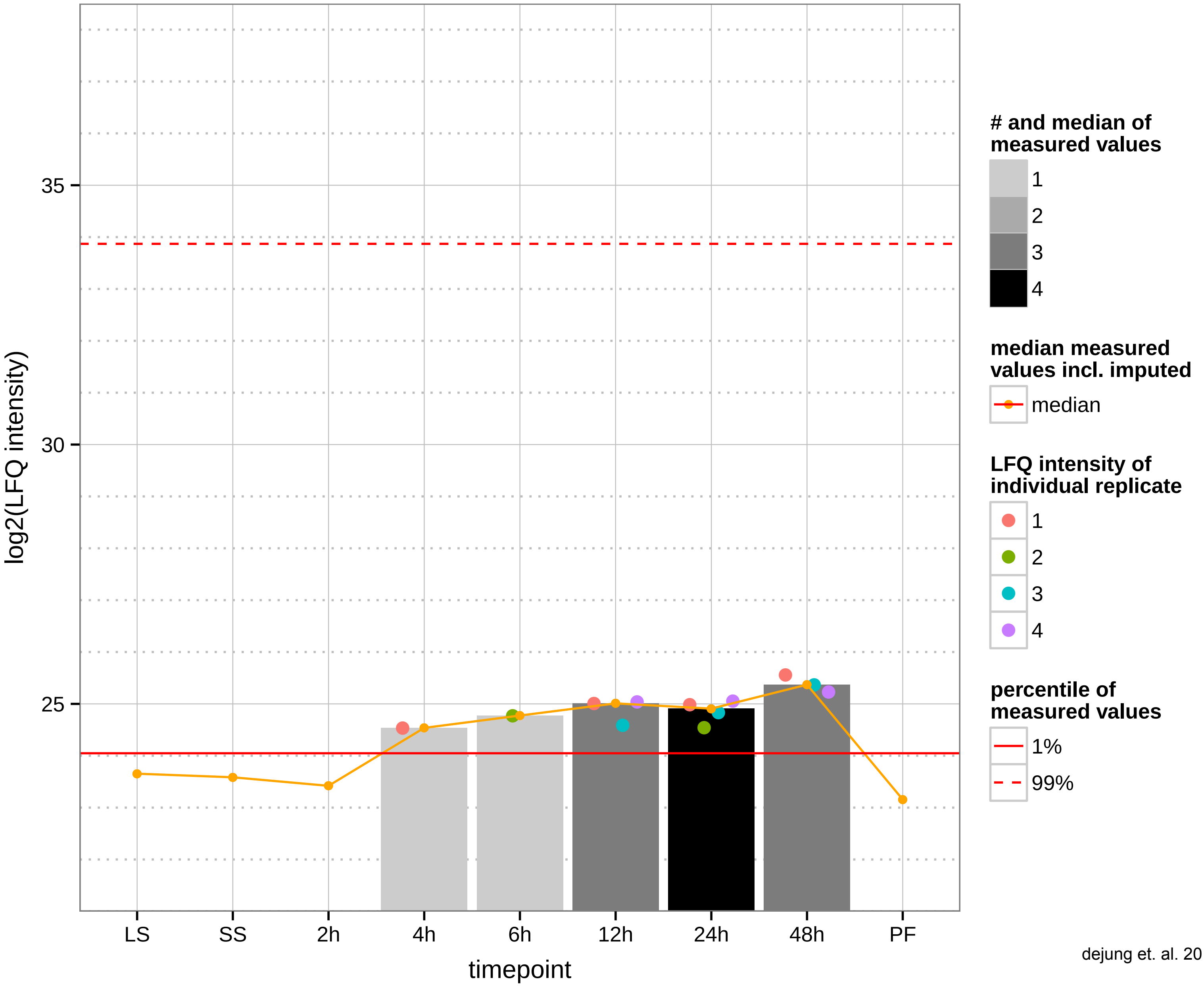

dejung et. al. 2015

NUDIX hydrolase, conserved
Tb927.11.9810
AGOF: hydrolase activity
AGOC: null
AGOP: metabolic process
PGOF: hydrolase activity
PGOC: null
PGOP: null

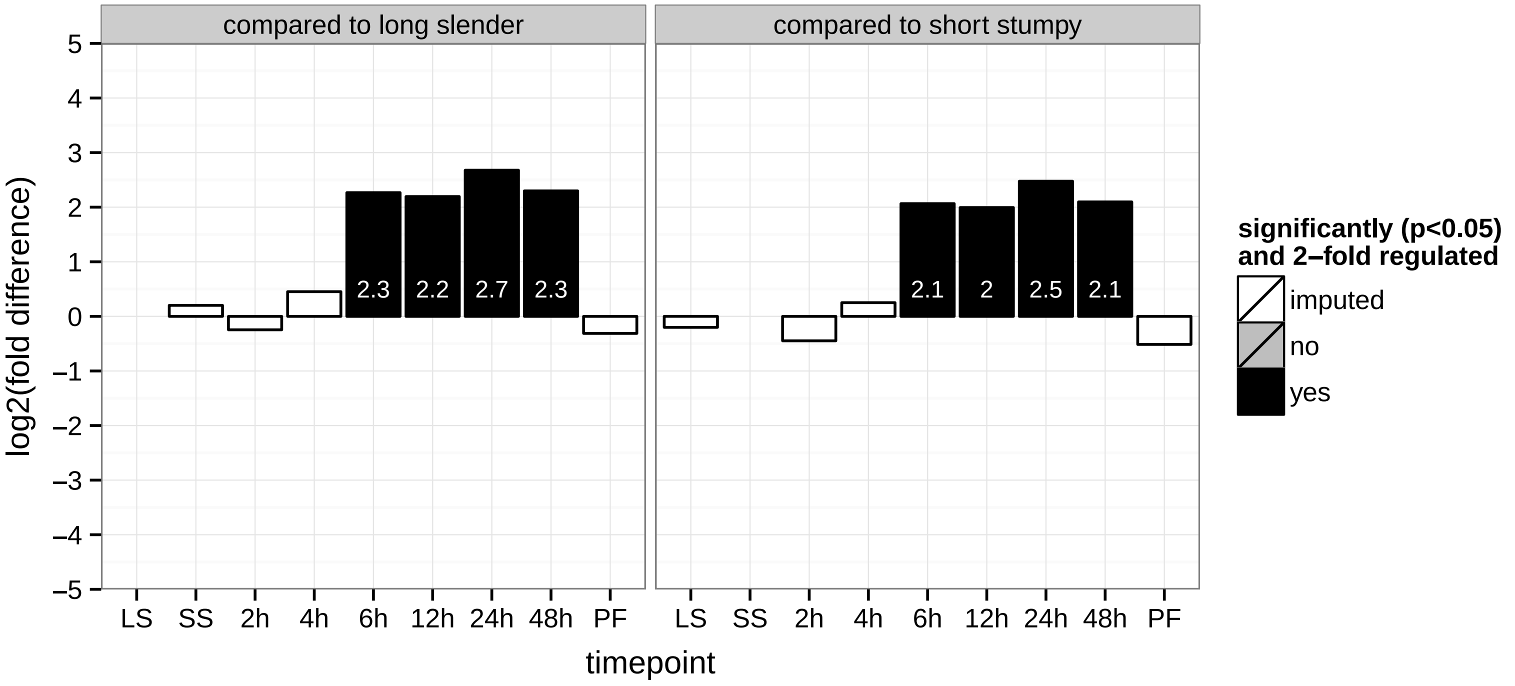

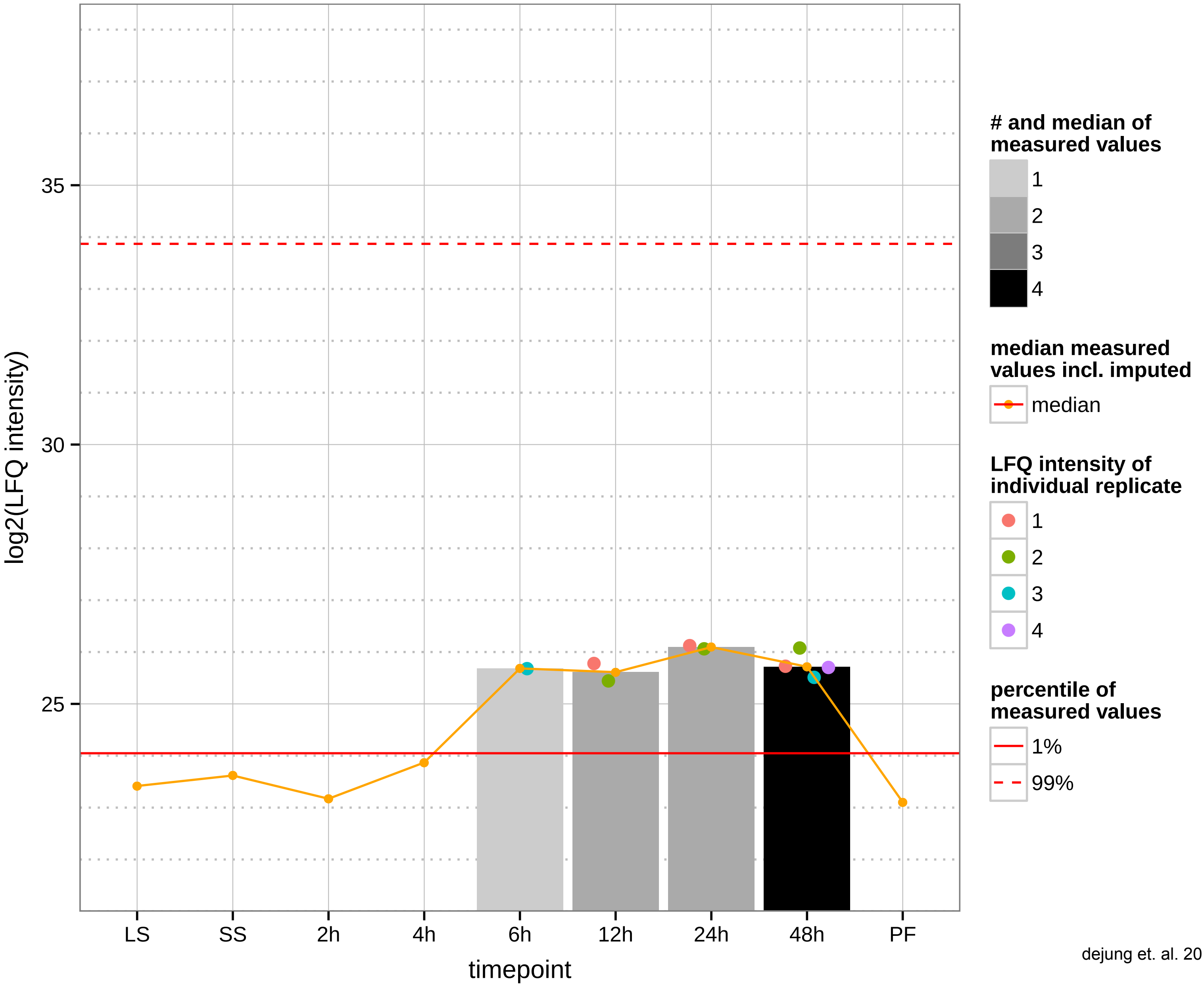

dejung et. al. 2015

minichromosome maintenance (MCM) complex subunit, putative, DNA replication licensing factor MCM3
Tb927.2.3930;Tb11.v5.0640
AGOF: null, ATP binding, DNA binding
AGOC: null, nucleus
AGOP: null, DNA replication, DNA–dependent DNA replication initiation
PGOF: ATP binding, DNA binding
PGOC: null
PGOP: DNA replication

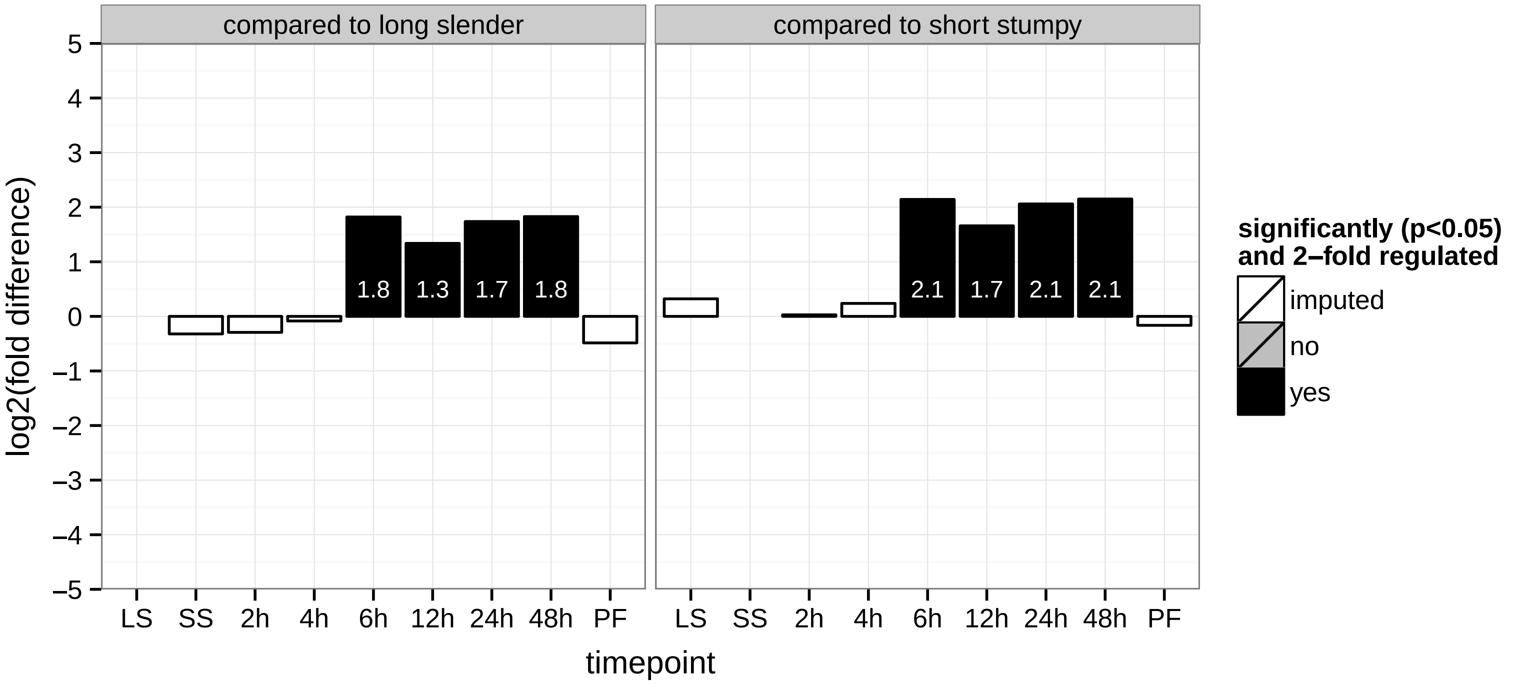

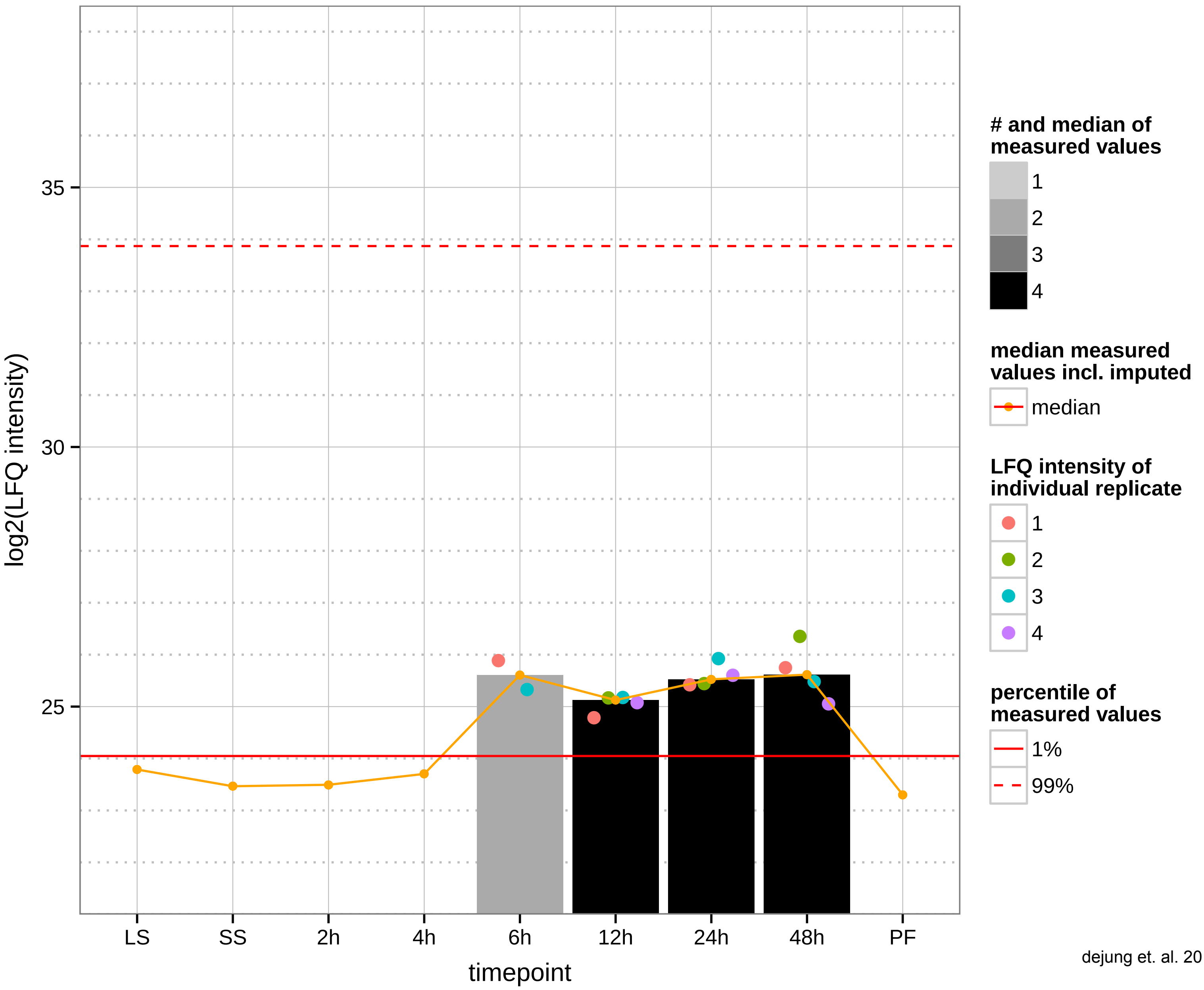

dejung et. al. 2015

hypothetical protein, conserved
Tb927.2.5870
AGOF: null
AGOC: null
AGOP: null
PGOF: null
PGOC: null
PGOP: null

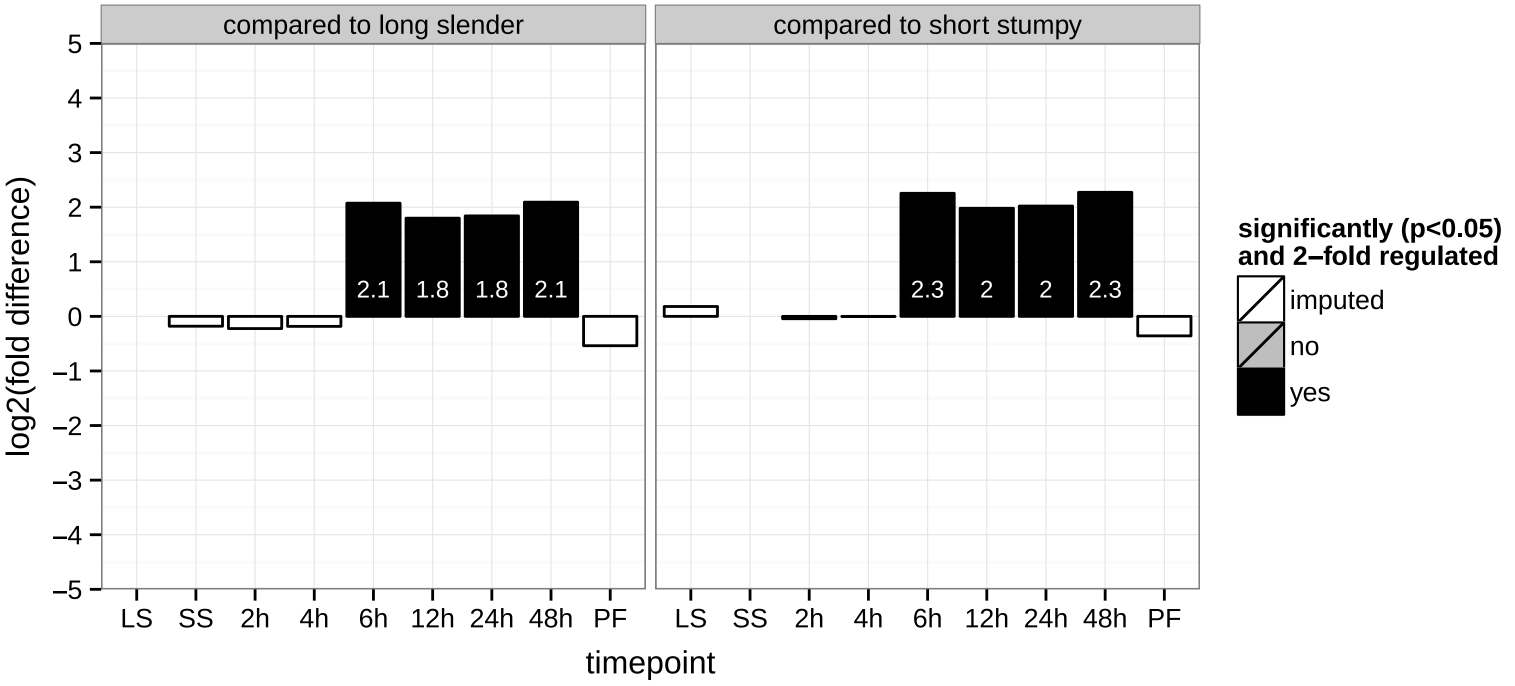

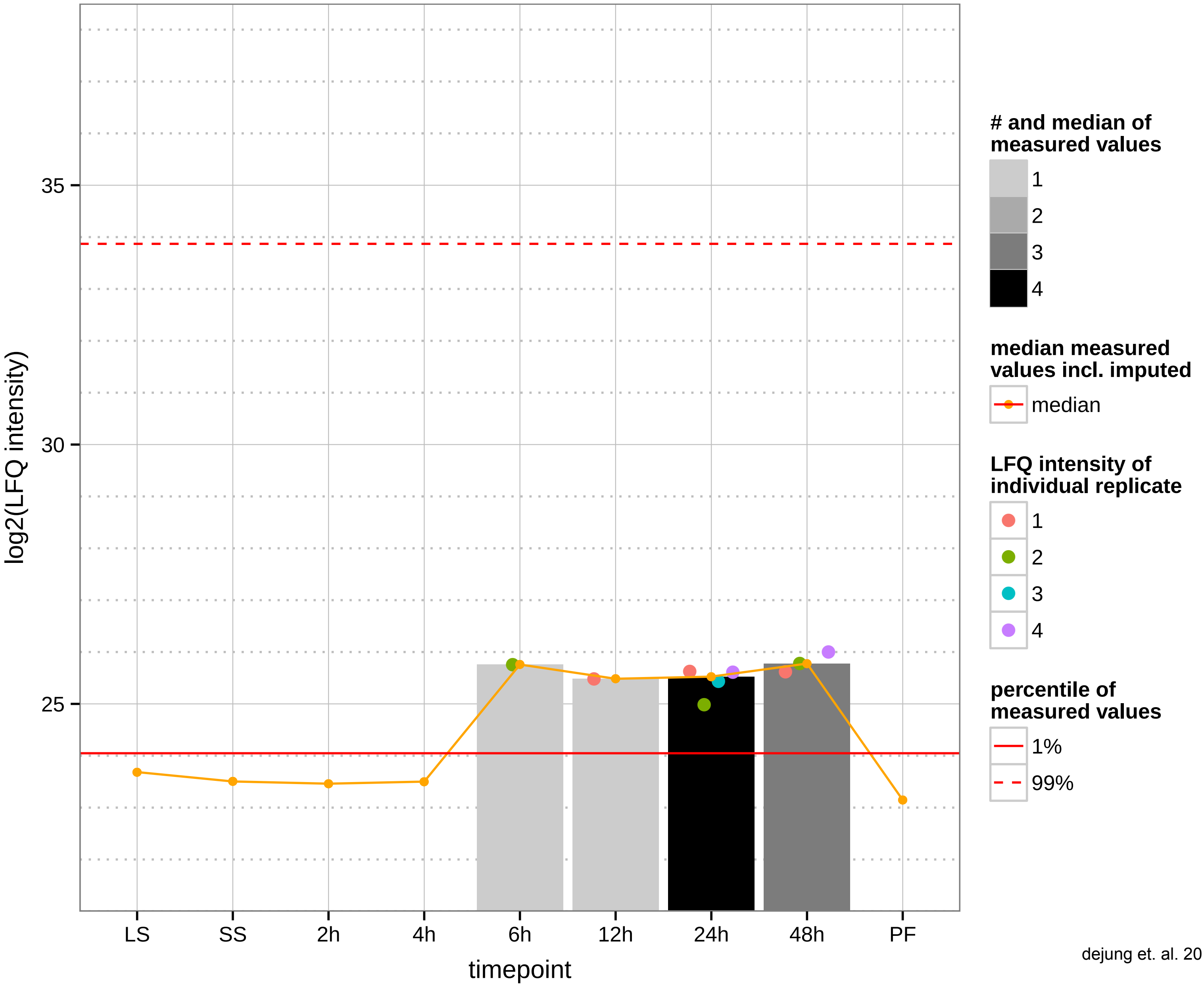

dejung et. al. 2015

peroxisome biogenesis factor 1, putative
Tb927.4.1250
AGOF: ATP binding, nucleoside–triphosphatase activity
AGOC: peroxisome
AGOP: peroxisome organization, protein import into peroxisome membrane
PGOF: ATP binding, nucleoside–triphosphatase activity, nucleotide binding
PGOC: peroxisome
PGOP: peroxisome organization

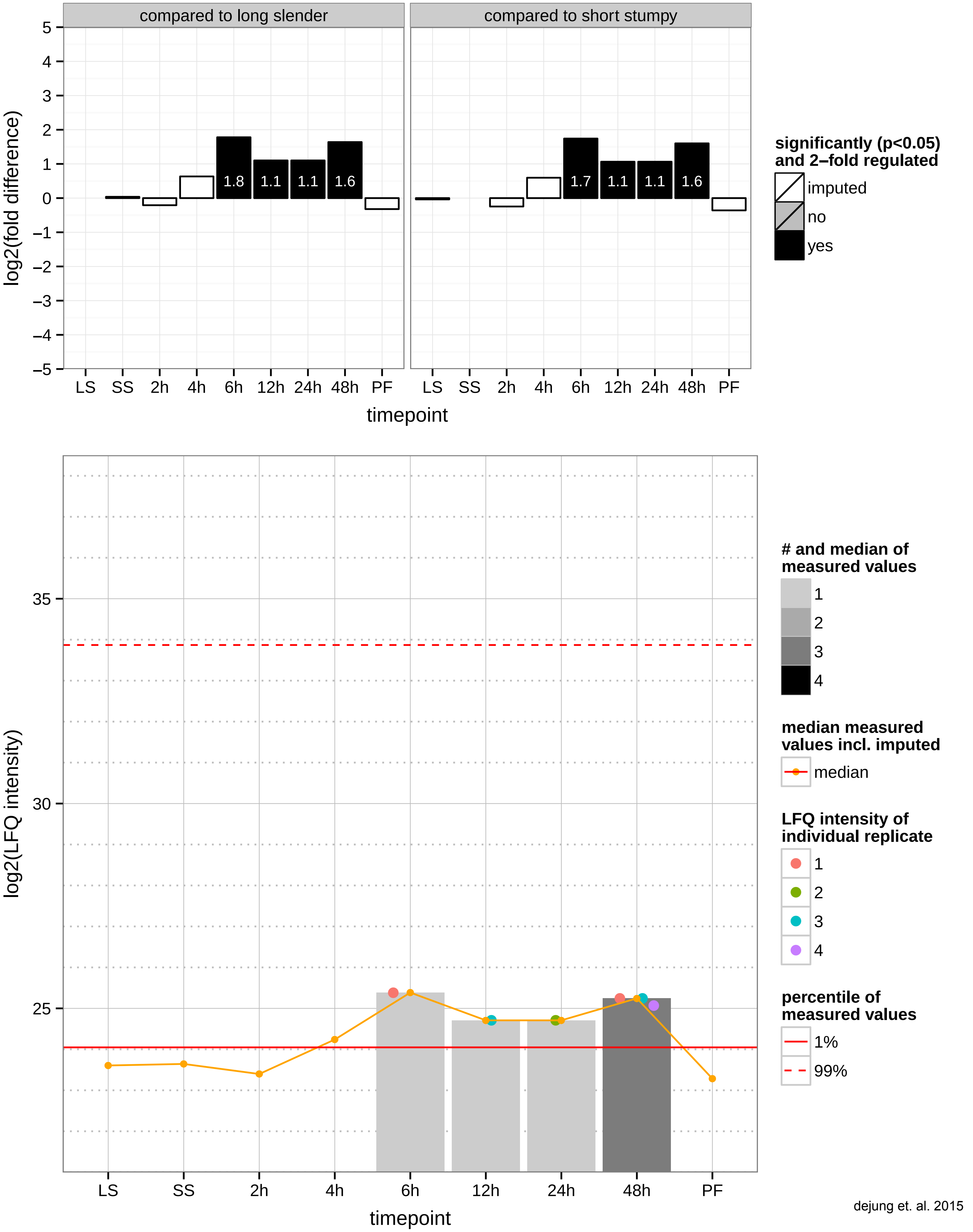

dejung et. al. 2015

leucine−rich repeat protein (LRRP, pseudogene), putative, leucine−rich repeat protein 1 (LRRP1), frameshift
Tb927.4.160
AGOF: null
AGOC: null
AGOP: null
PGOF: null
PGOC: null
PGOP: null

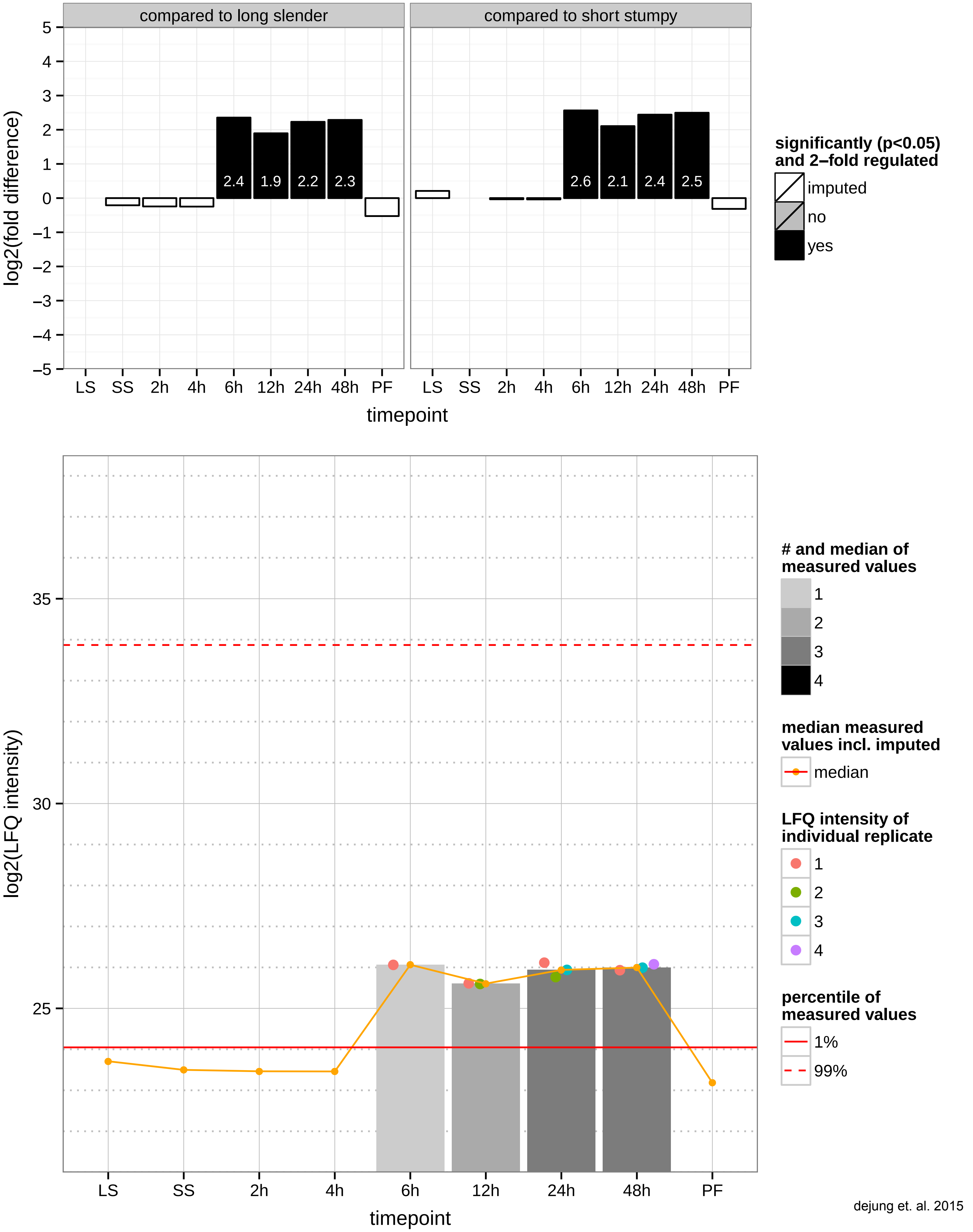

dejung et. al. 2015

hypothetical protein, conserved
Tb927.4.5190
AGOF: null
AGOC: null
AGOP: null
PGOF: null
PGOC: null
PGOP: null

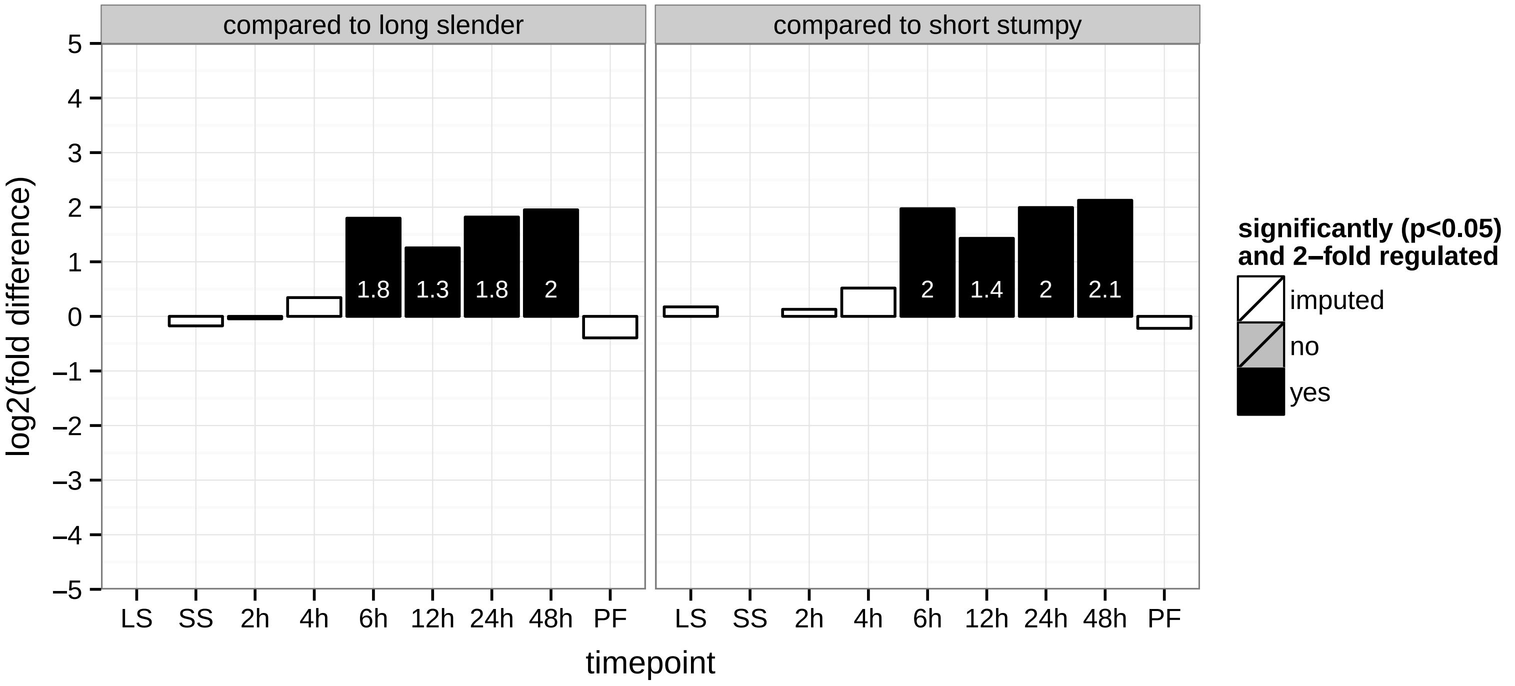

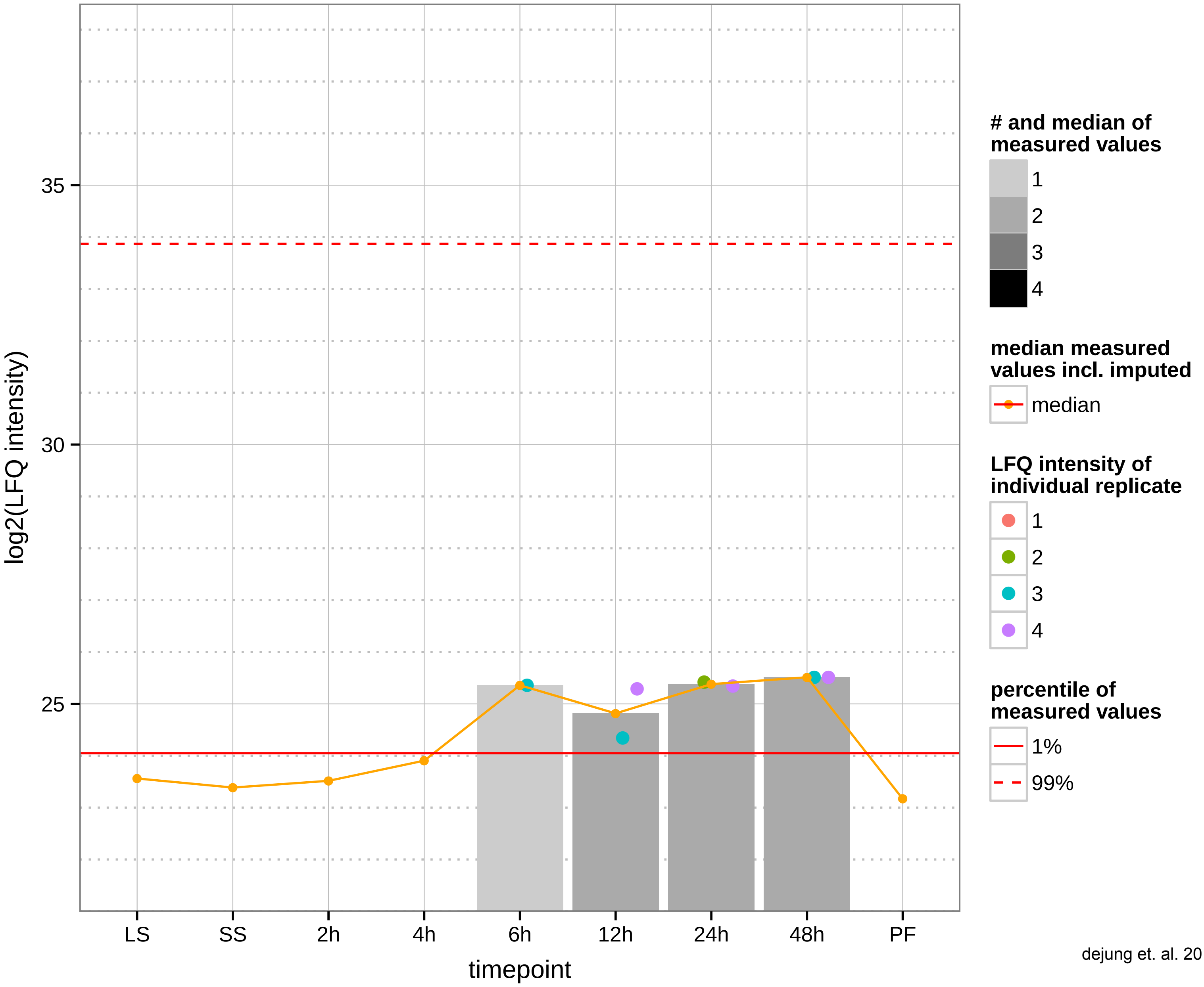

dejung et. al. 2015

hypothetical protein, conserved
Tb927.7.3380
AGOF: null
AGOC: null
AGOP: null
PGOF: null
PGOC: null
PGOP: null

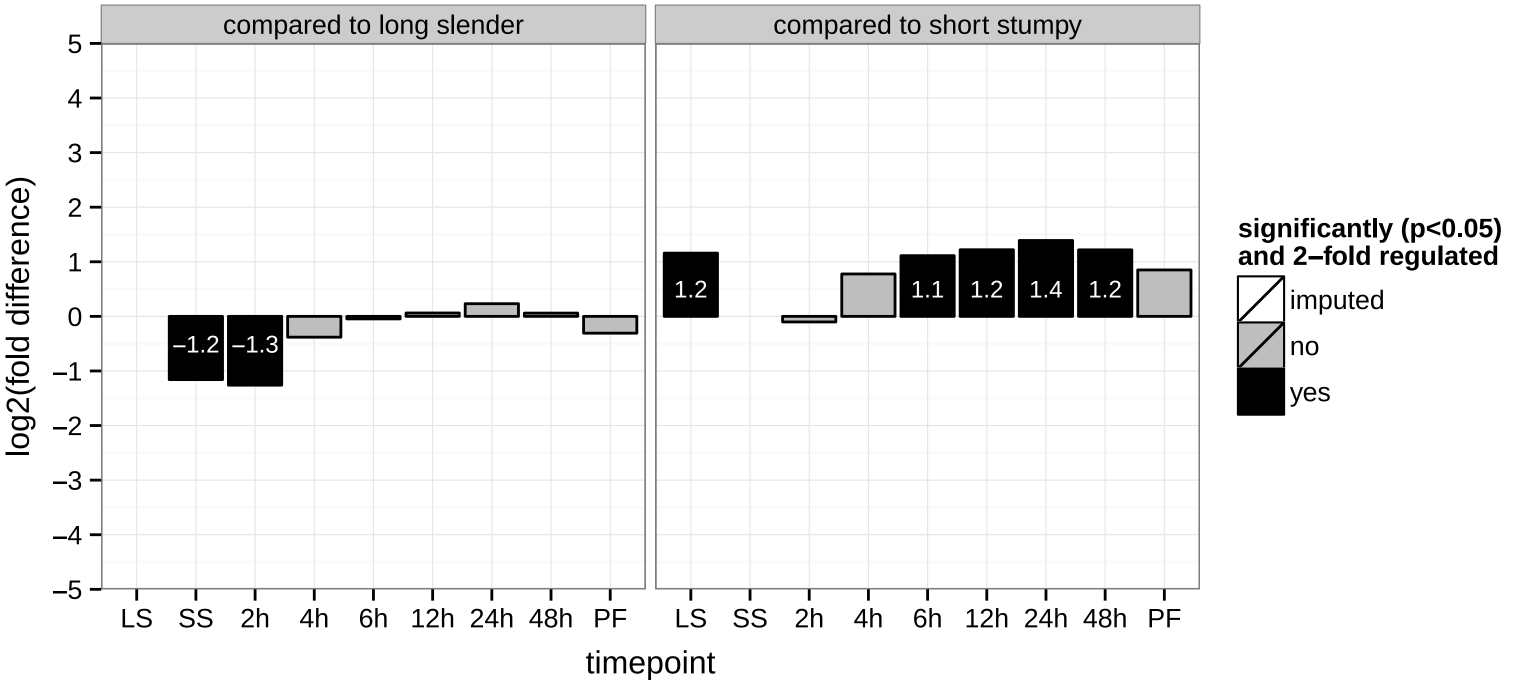

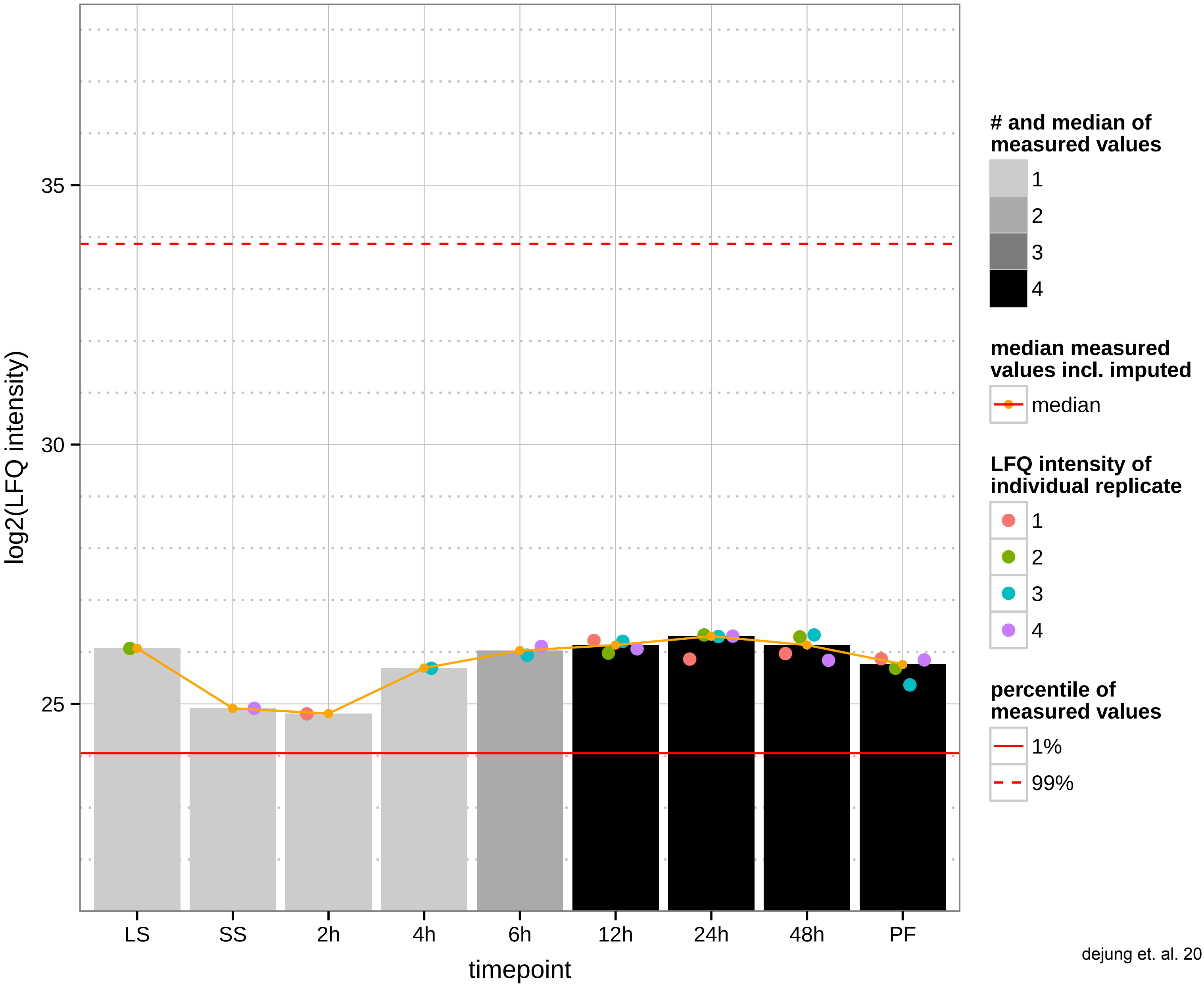

dejung et. al. 2015

Protein Associated with Differentiation (PAD2)
Tb927.7.5940
AGOF: null
AGOC: flagellar pocket, plasma membrane
AGOP: citrate transport, regulation of developmental process, response to temperature stimulus
PGOF: null
PGOC: integral to membrane
PGOP: transmembrane transport

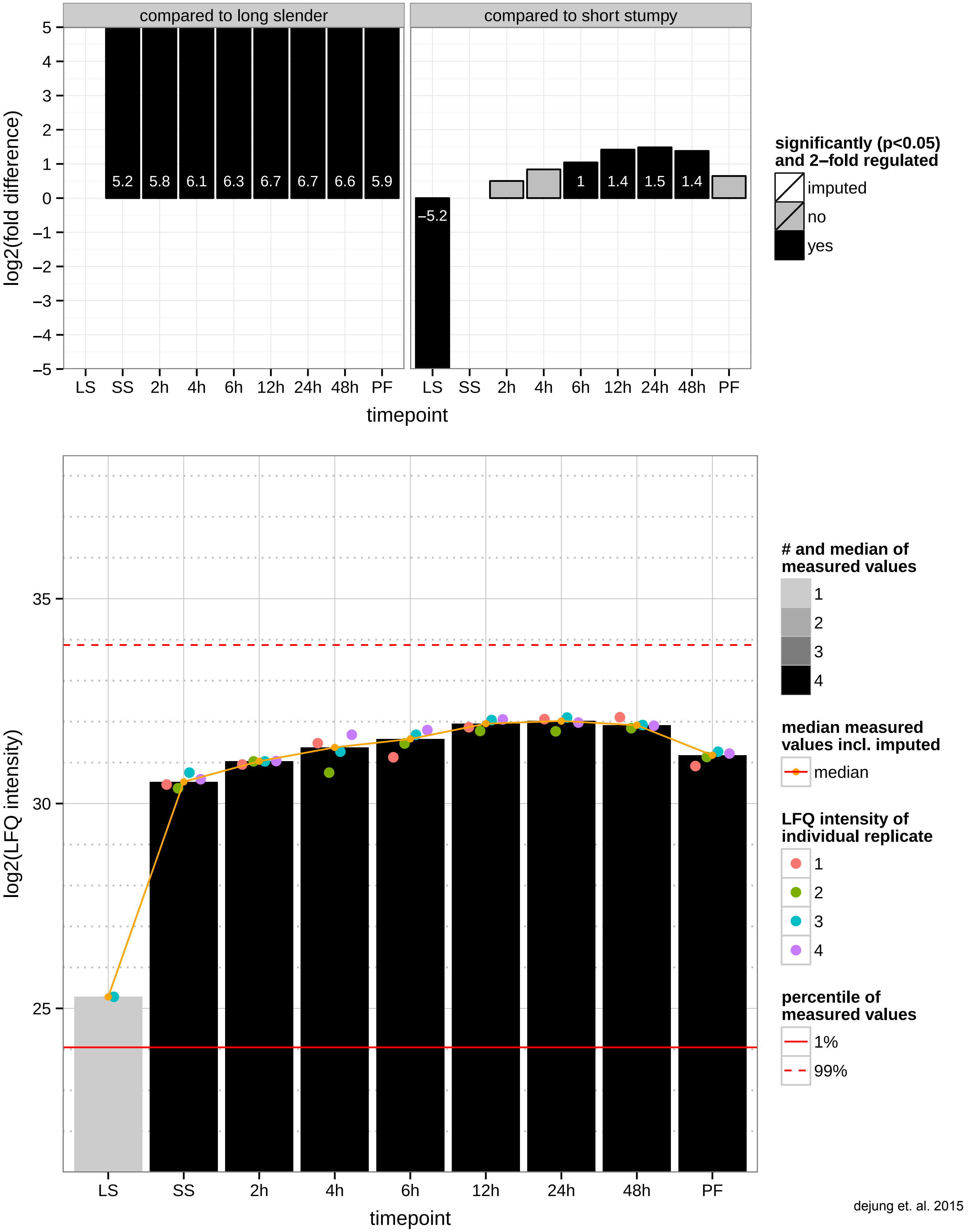

dejung et. al. 2015

nitroreductase, NADH dehydrogenase, putative (NTR)
Tb927.7.7230
AGOF: oxidoreductase activity
AGOC: mitochondrion
AGOP: drug metabolic process
PGOF: oxidoreductase activity
PGOC: null
PGOP: null

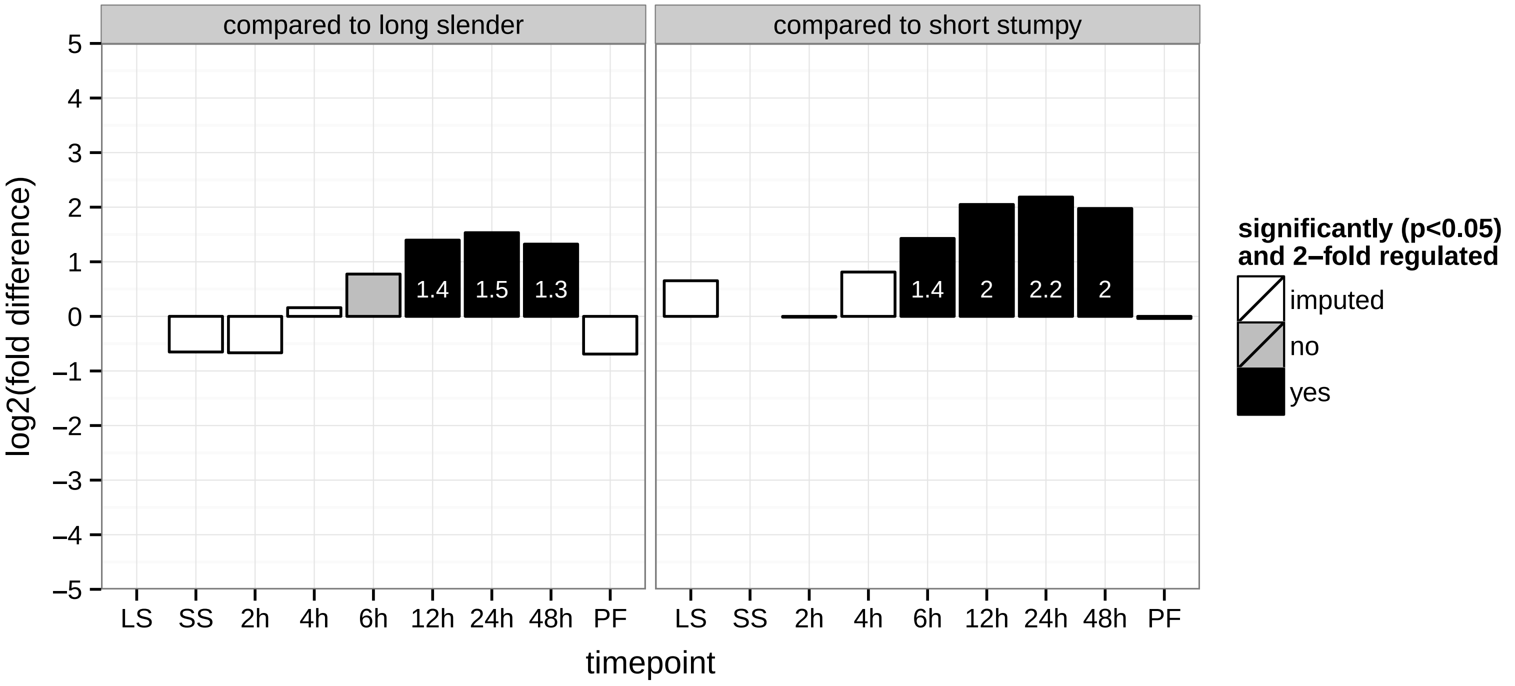

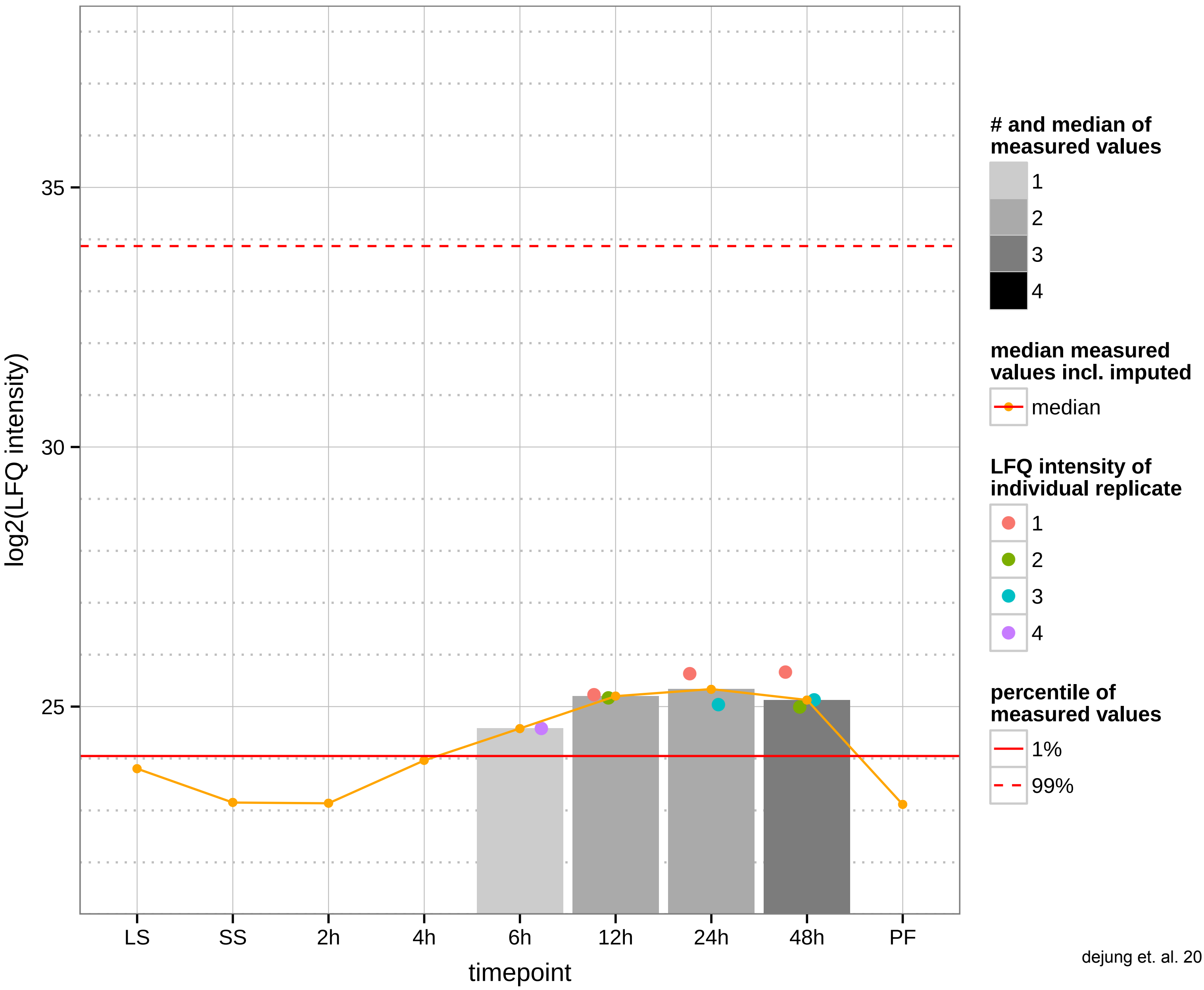

dejung et. al. 2015

hypothetical protein, conserved
Tb927.8.4860
AGOF: null
AGOC: mitochondrion
AGOP: null
PGOF: null
PGOC: null
PGOP: null

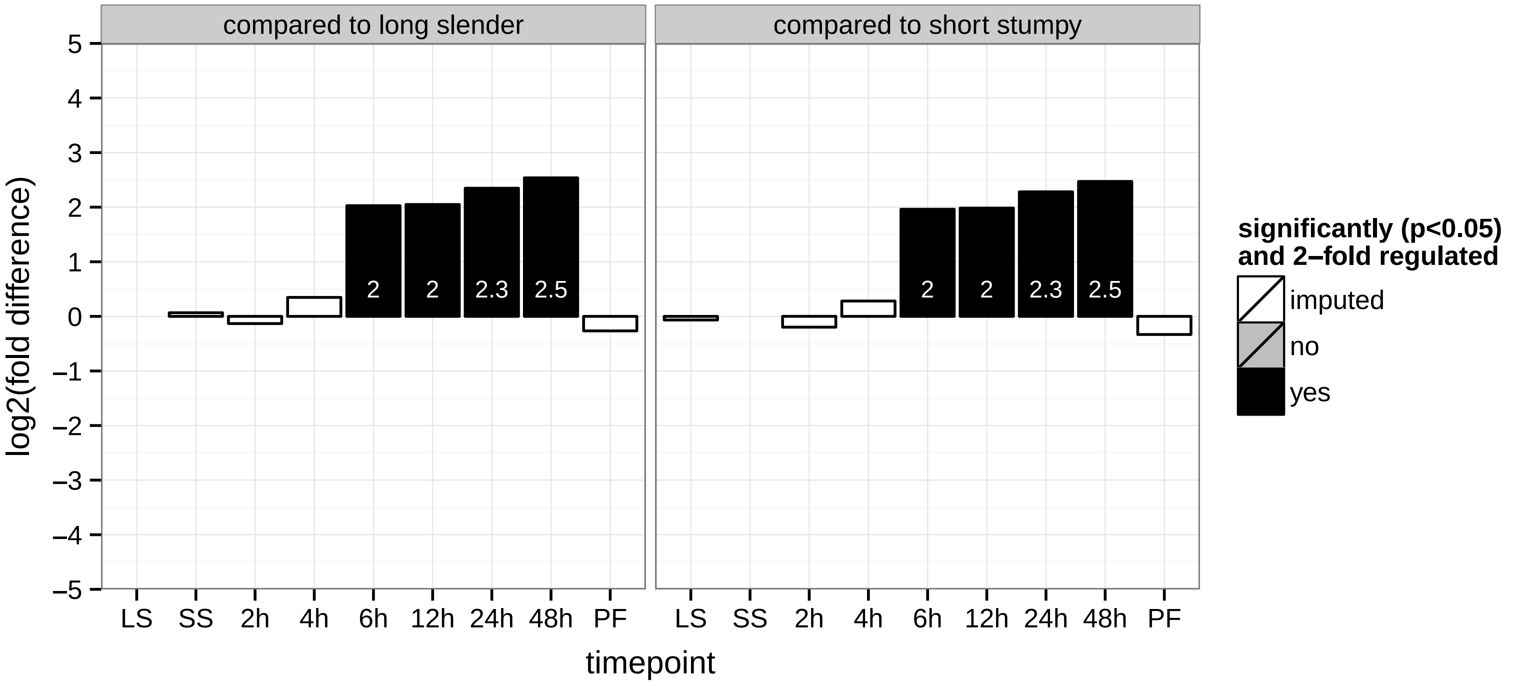

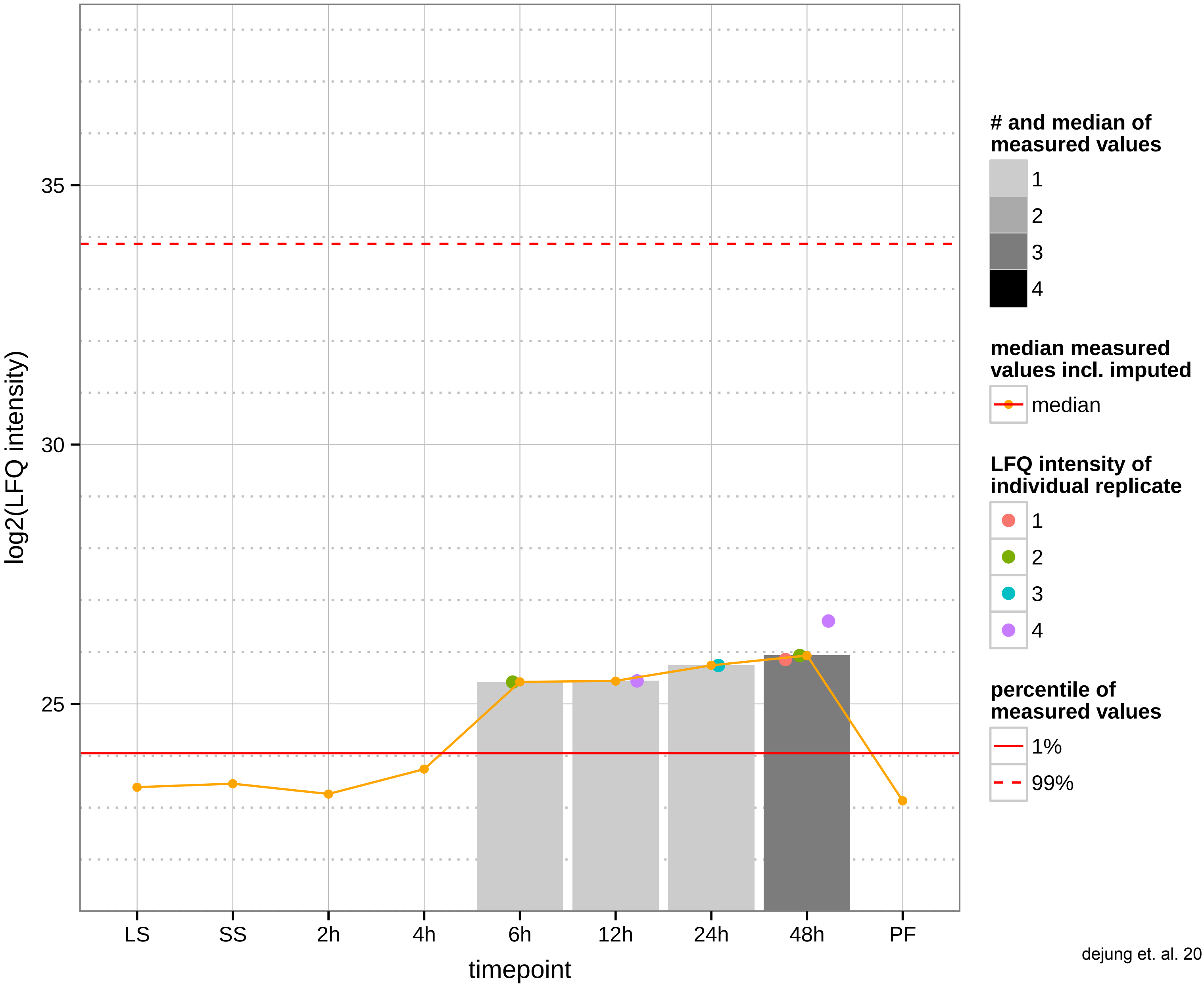

dejung et. al. 2015

hypothetical protein, conserved
Tb927.9.11820
AGOF: null
AGOC: null
AGOP: null
PGOF: null
PGOC: null
PGOP: null

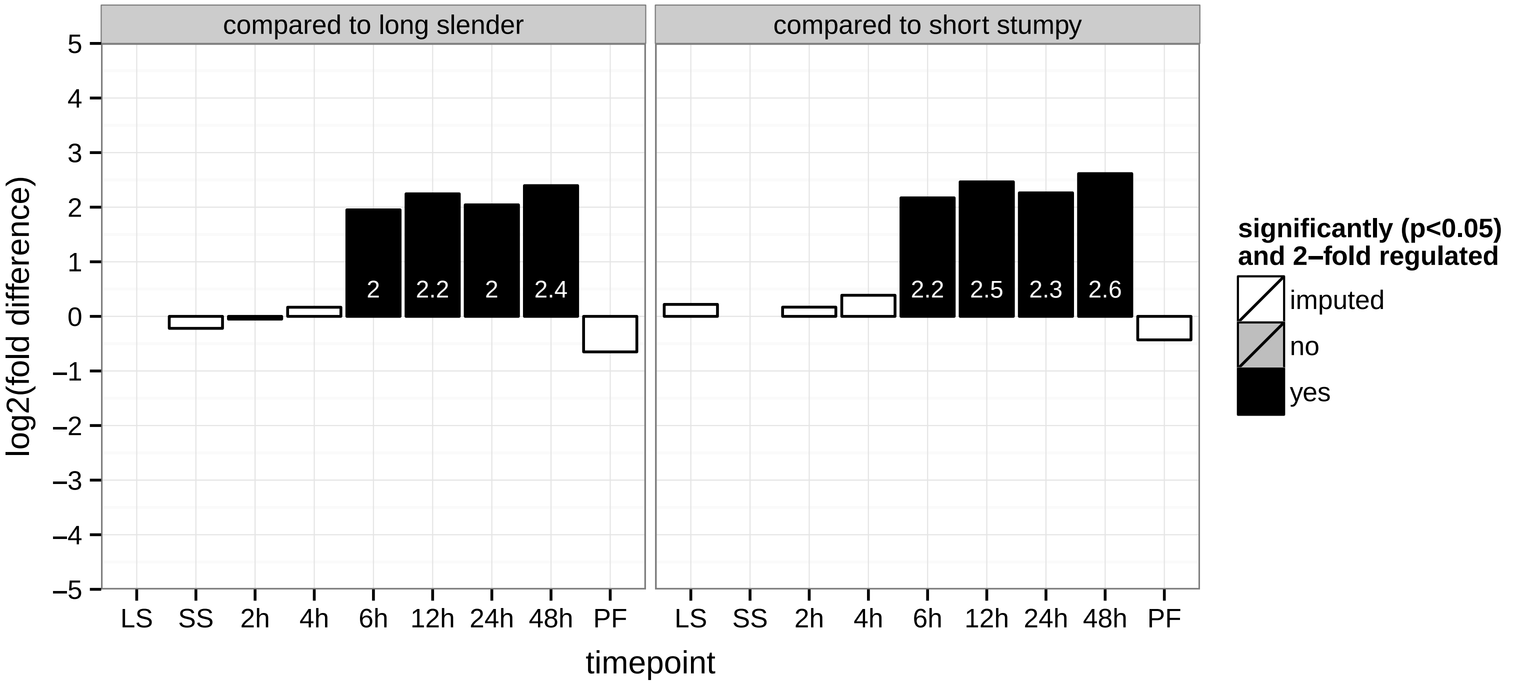

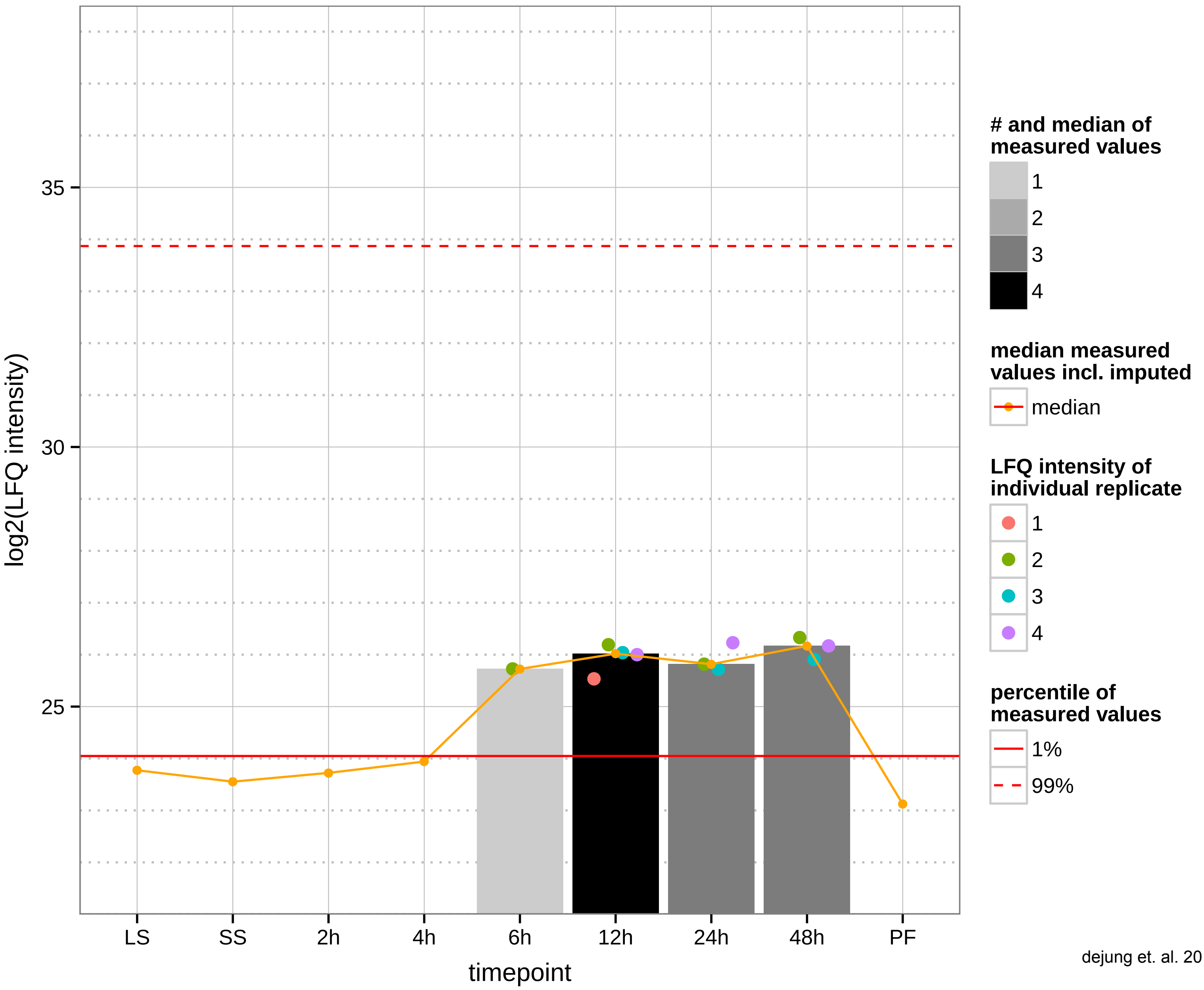

dejung et. al. 2015

ankyrin repeat protein, putative
Tb927.9.13100
AGOF: null
AGOC: null
AGOP: null
PGOF: protein binding
PGOC: null
PGOP: null

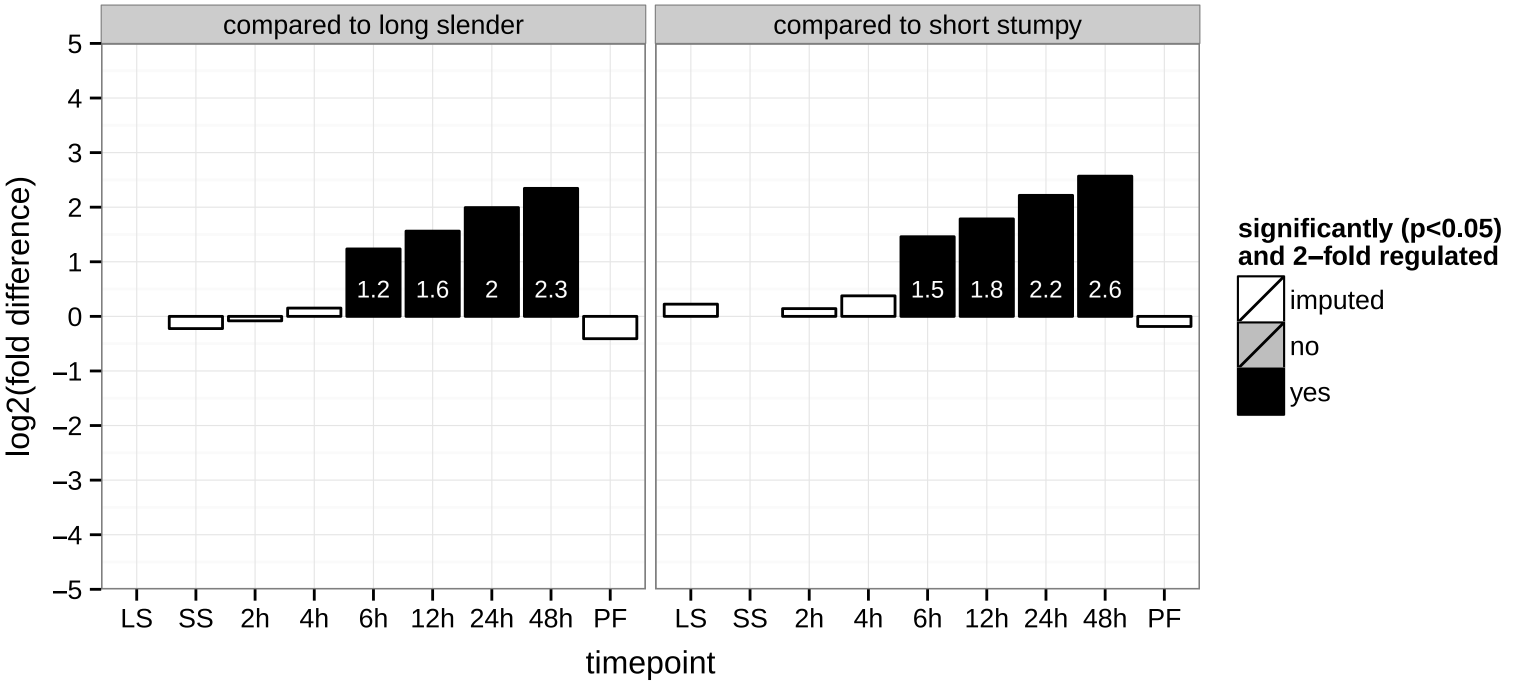

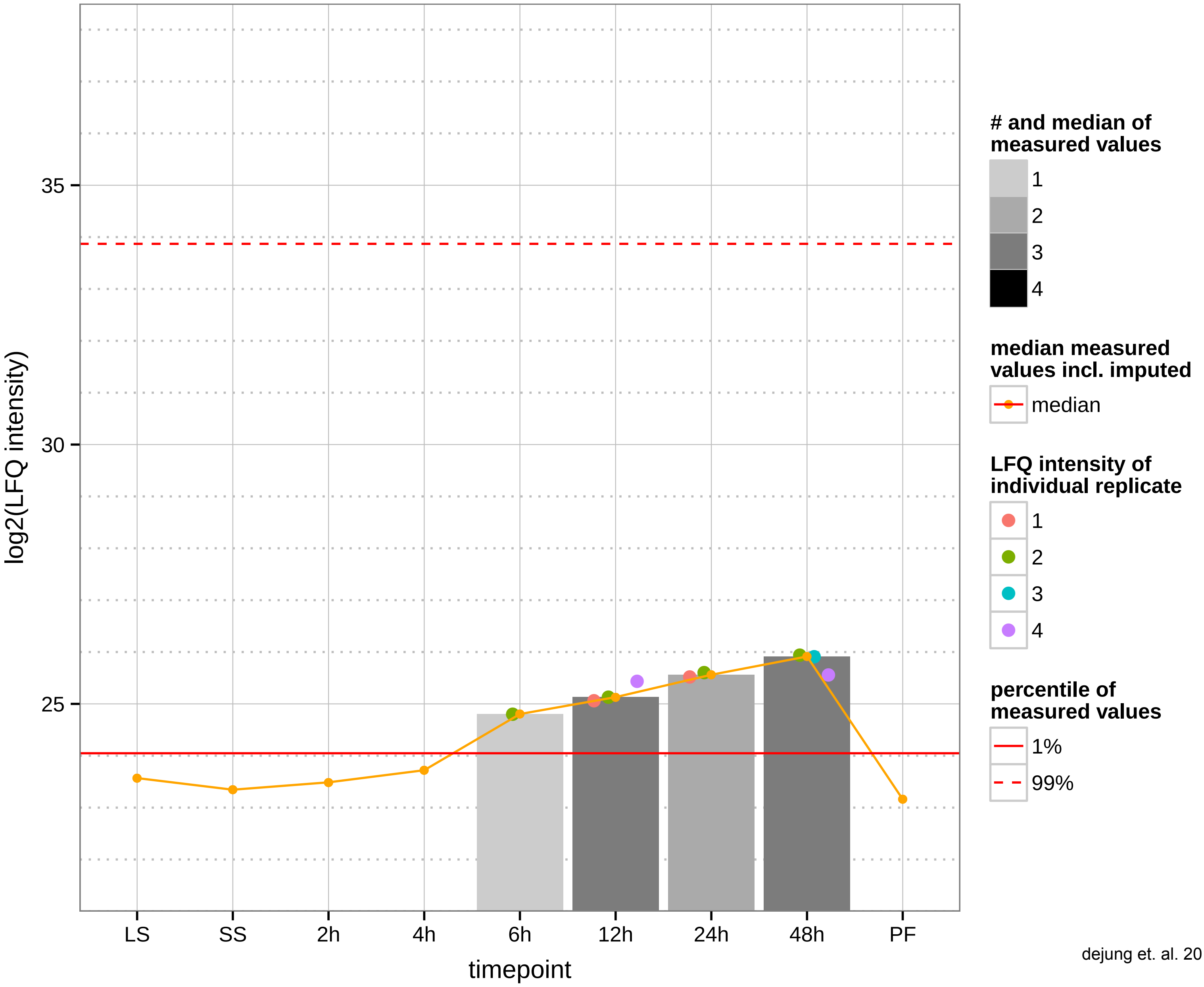

dejung et. al. 2015

hypothetical protein, conserved
Tb927.9.13120
AGOF: null
AGOC: null
AGOP: null
PGOF: null
PGOC: null
PGOP: null

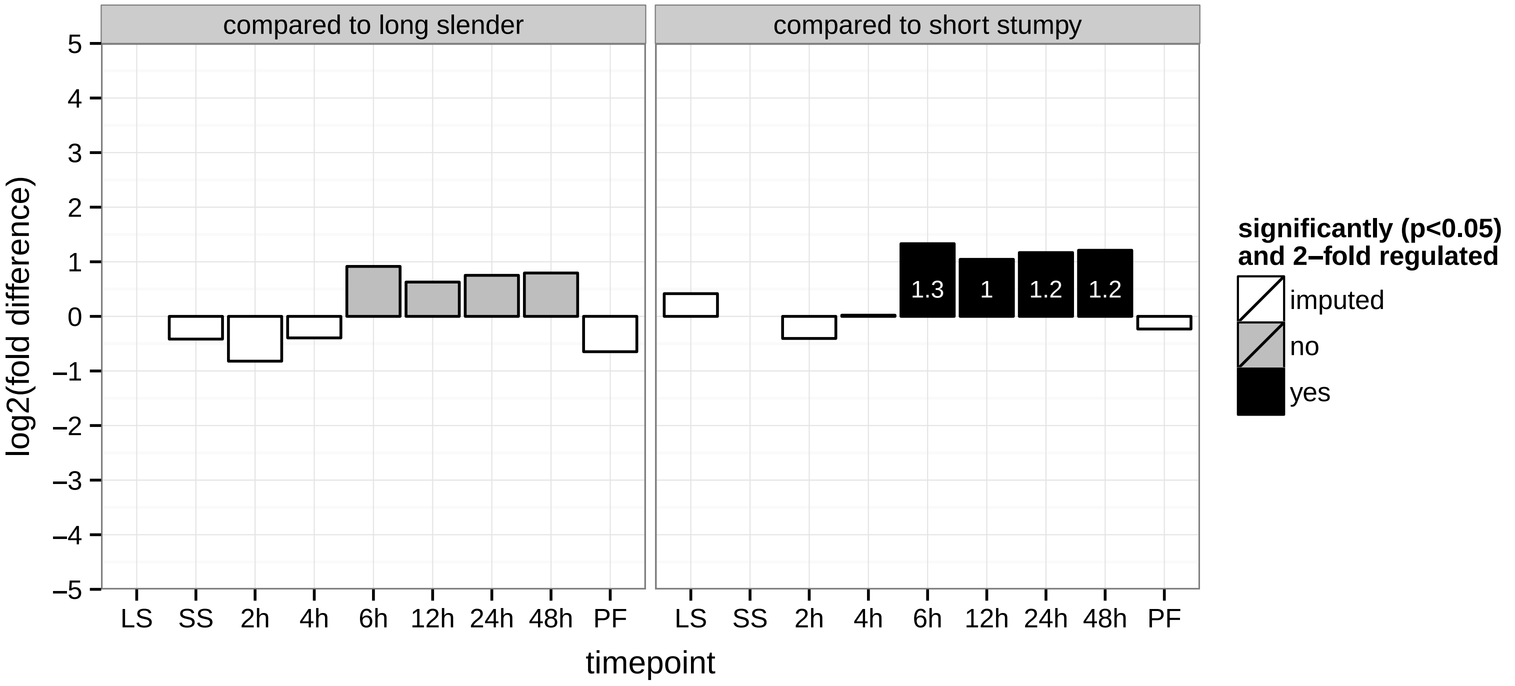

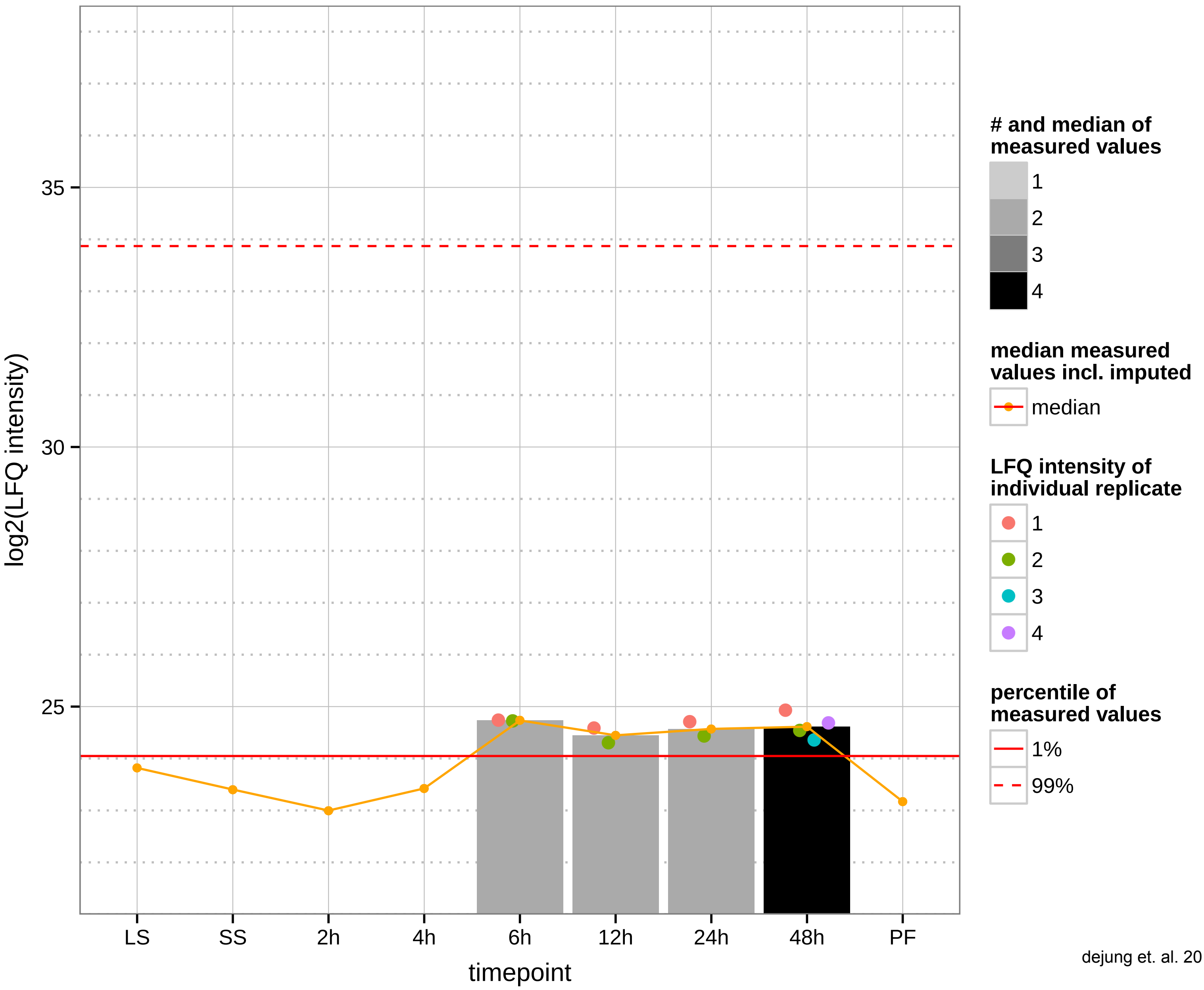

dejung et. al. 2015

hypothetical protein, conserved
Tb927.9.1440
AGOF: null
AGOC: null
AGOP: null
PGOF: null
PGOC: null
PGOP: null

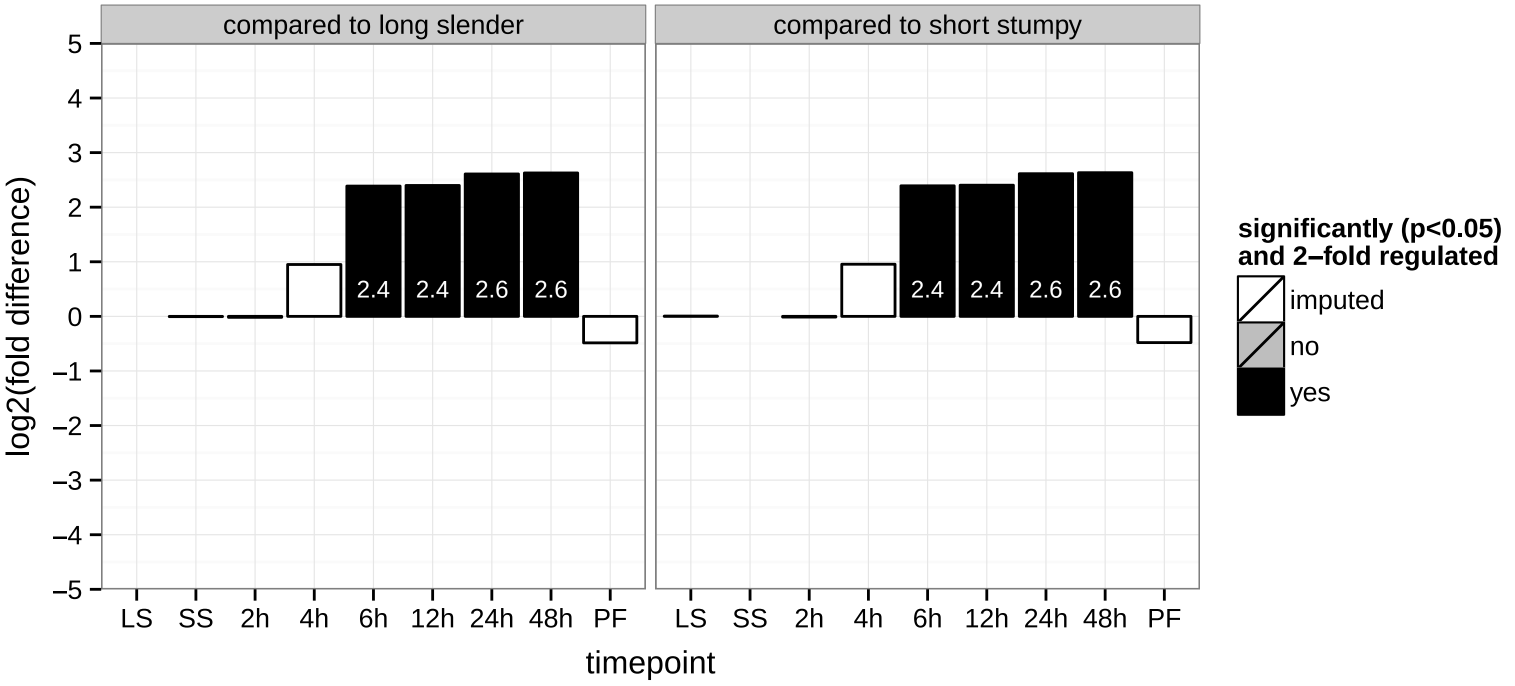

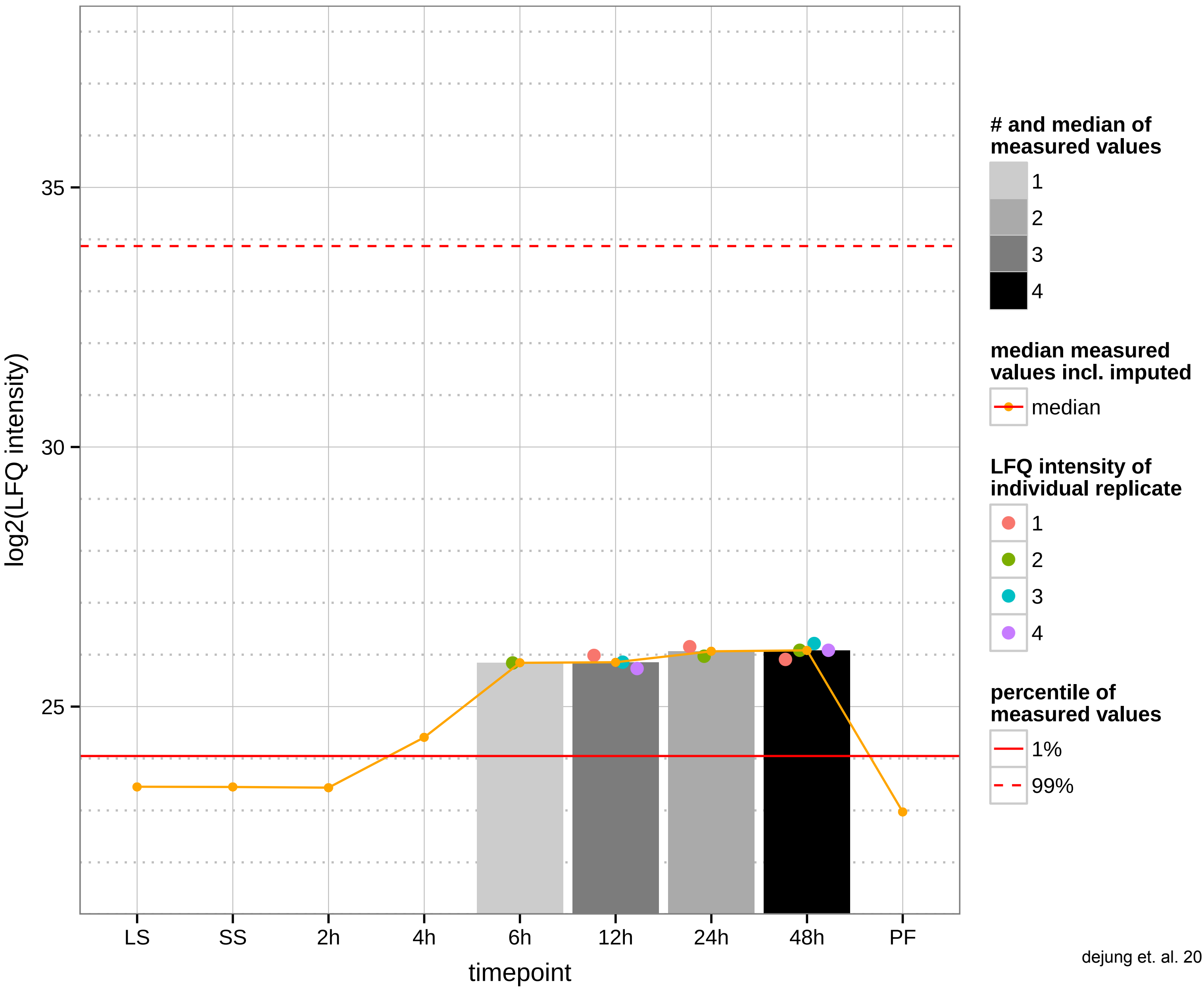

dejung et. al. 2015

hypothetical protein, conserved
Tb927.9.15040
AGOF: null
AGOC: null
AGOP: null
PGOF: null
PGOC: null
PGOP: null

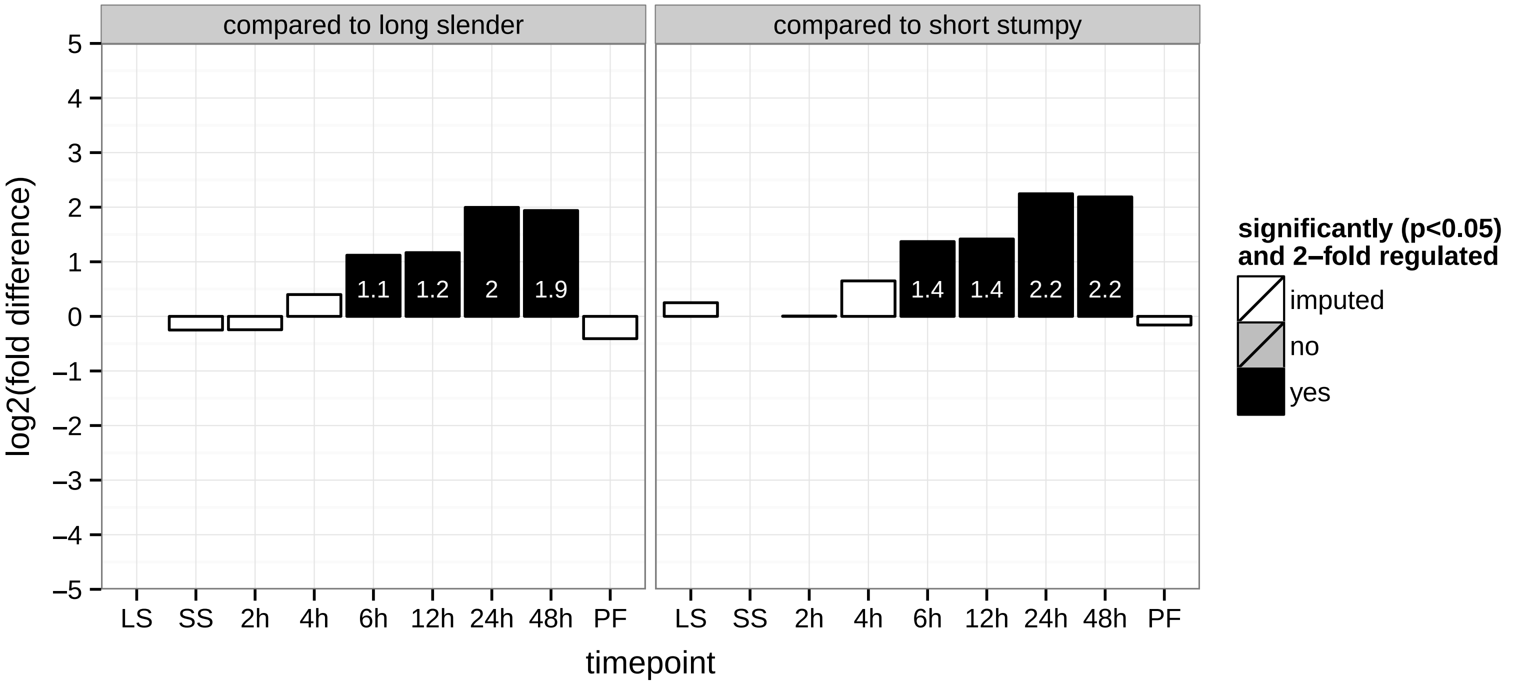

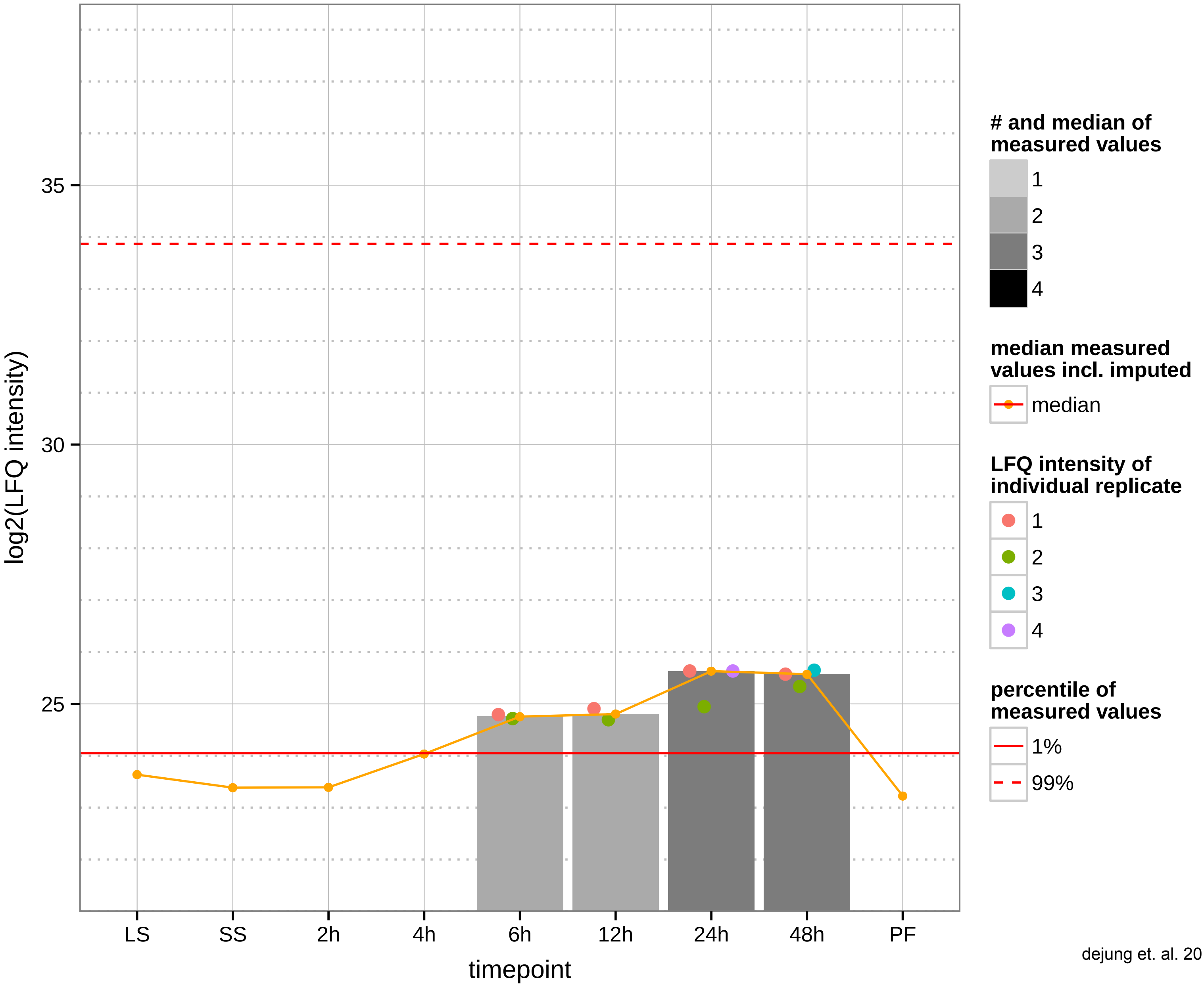

dejung et. al. 2015

hypothetical protein, conserved
Tb927.9.2060
AGOF: GTP binding
AGOC: null
AGOP: null
PGOF: GTP binding
PGOC: intracellular
PGOP: small GTPase mediated signal transduction

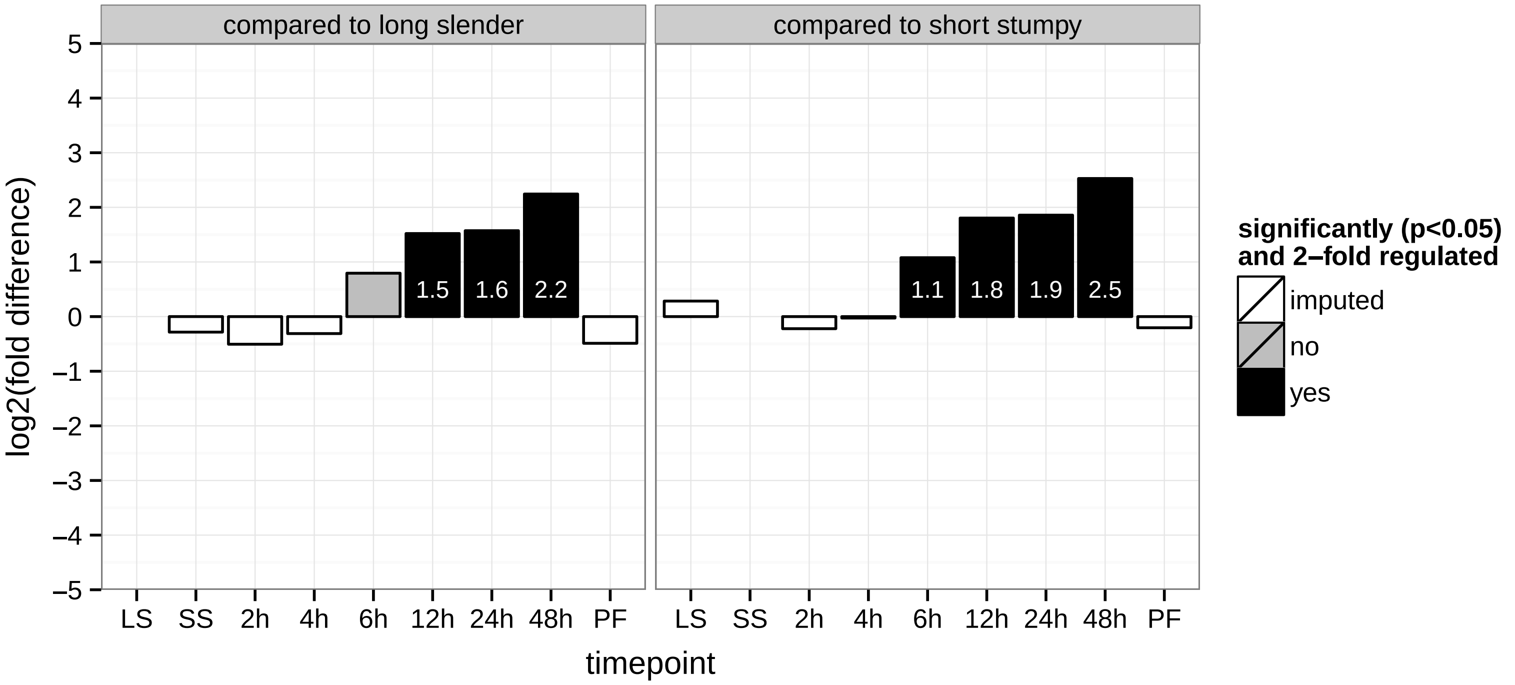

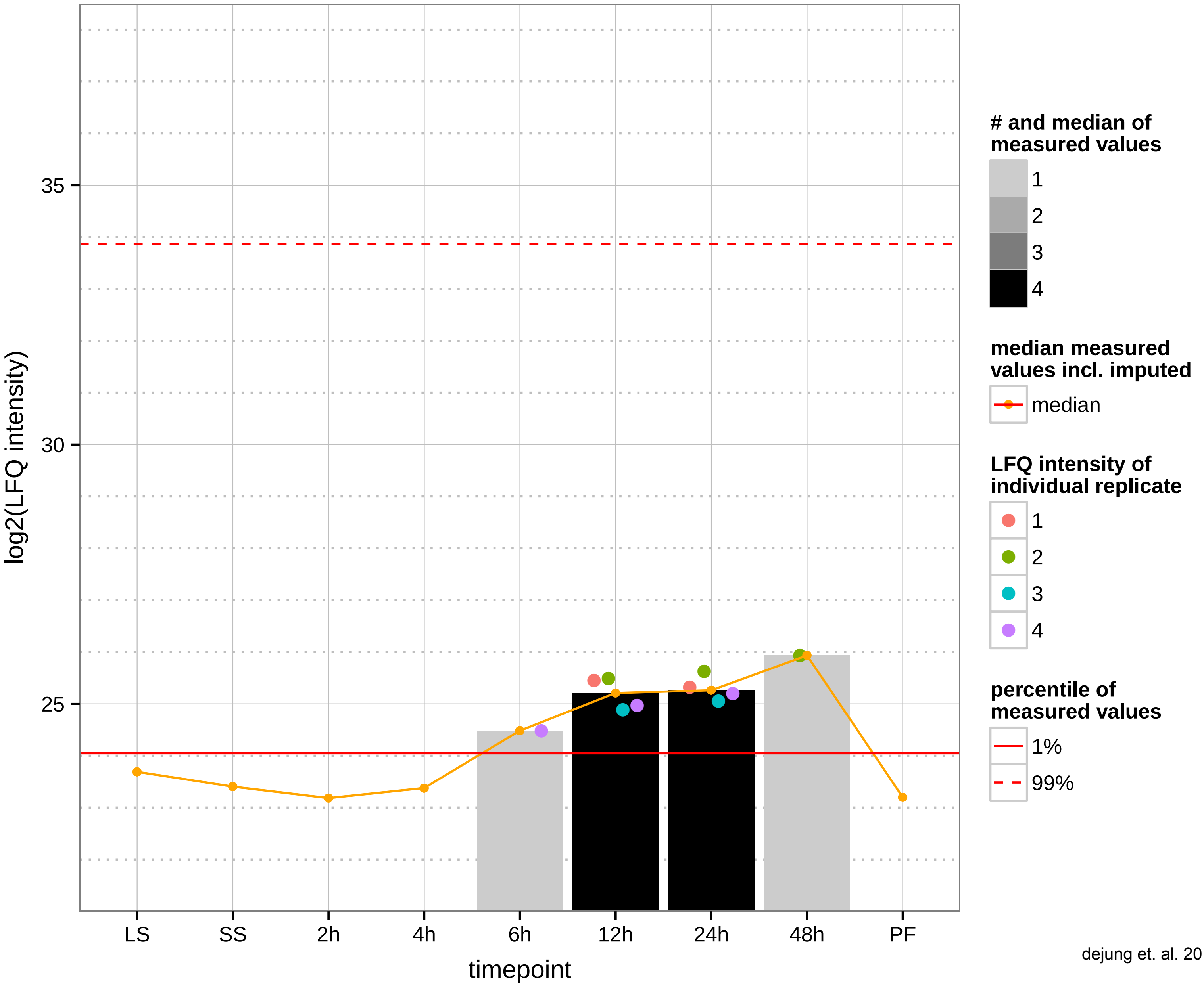

dejung et. al. 2015

hypothetical protein, conserved
Tb927.9.7980
AGOF: null
AGOC: mitochondrion
AGOP: null
PGOF: null
PGOC: null
PGOP: null

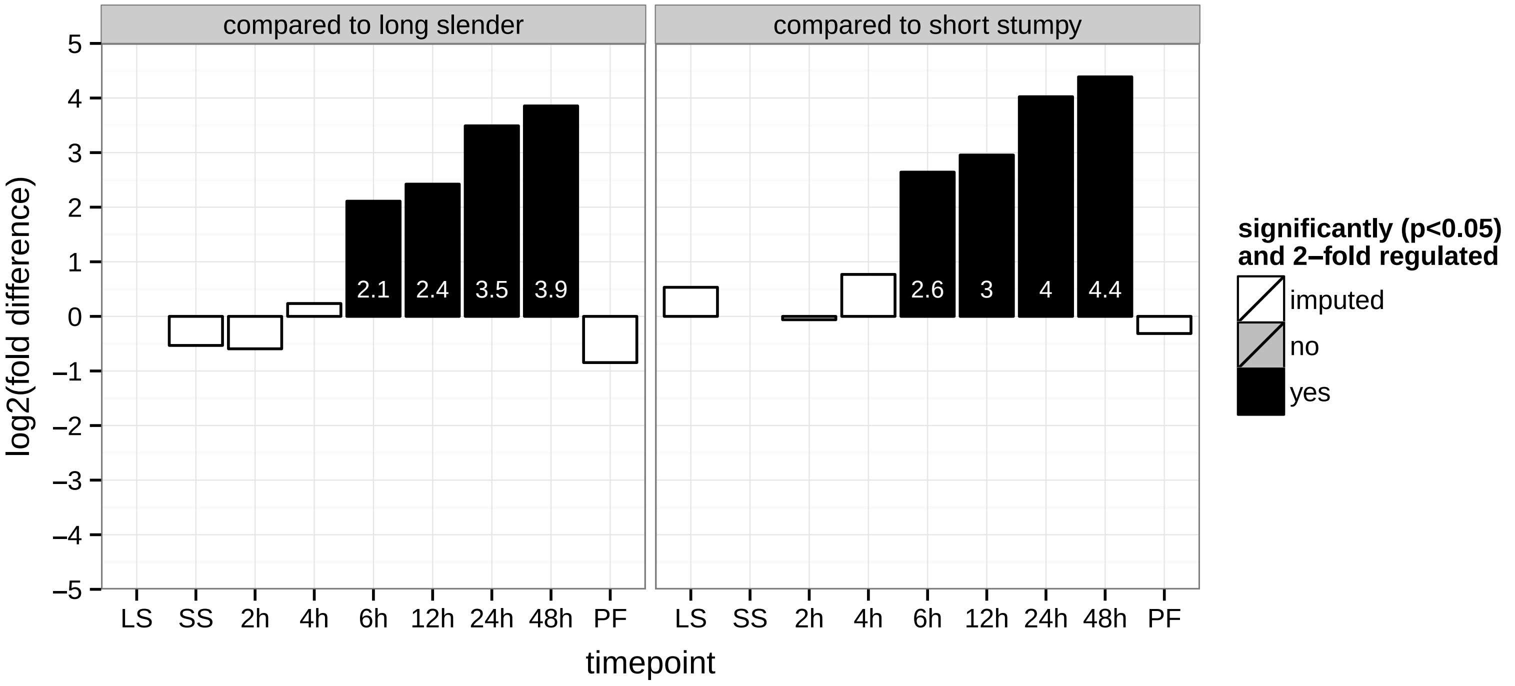

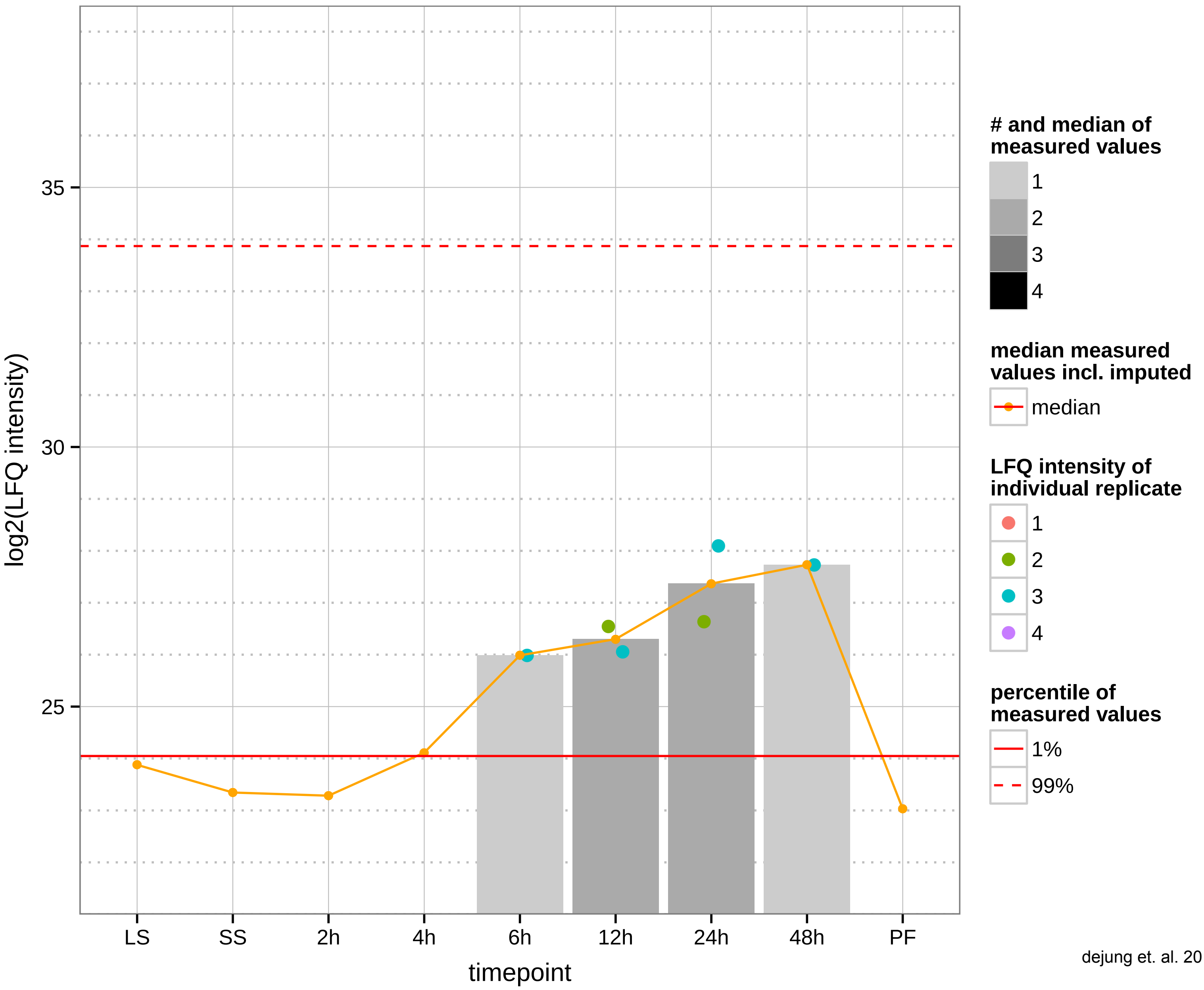

dejung et. al. 2015

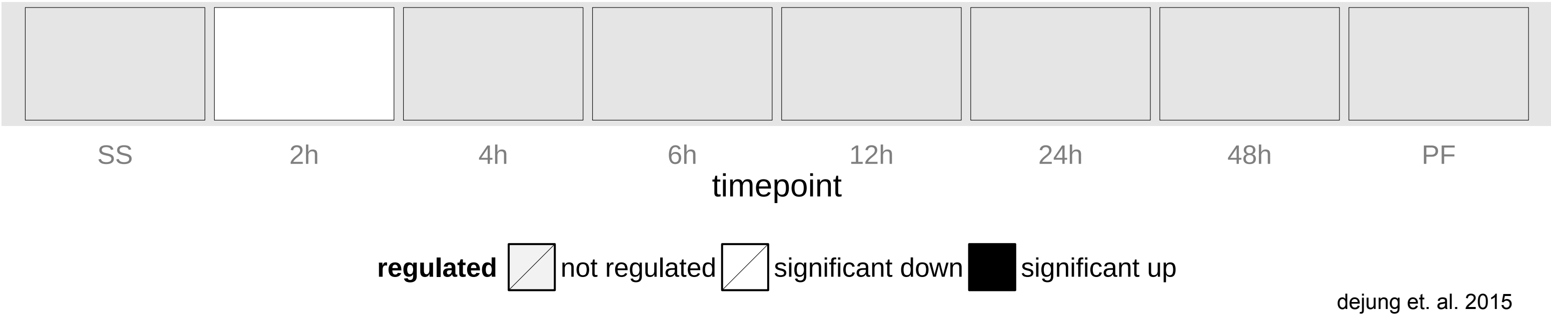
SS
2h
4h
6h
12h
24h
48h
PF
timepoint
regulated
not regulated
significant down
significant up
dejung et. al. 2015

hypothetical protein, conserved, chrX additional, unordered contigs
Tb927.10.4470;Tb10.v4.0037
AGOF: null
AGOC: null
AGOP: null
PGOF: binding
PGOC: null
PGOP: null

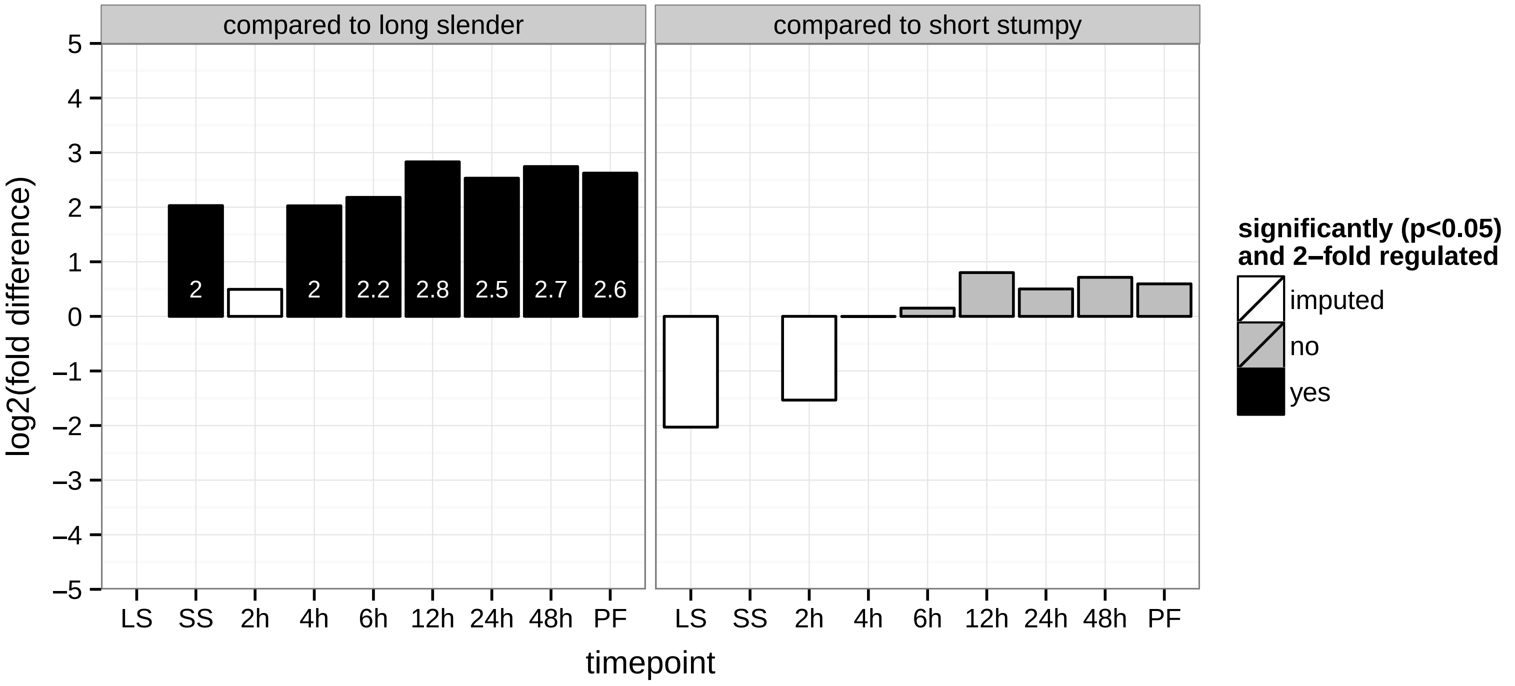

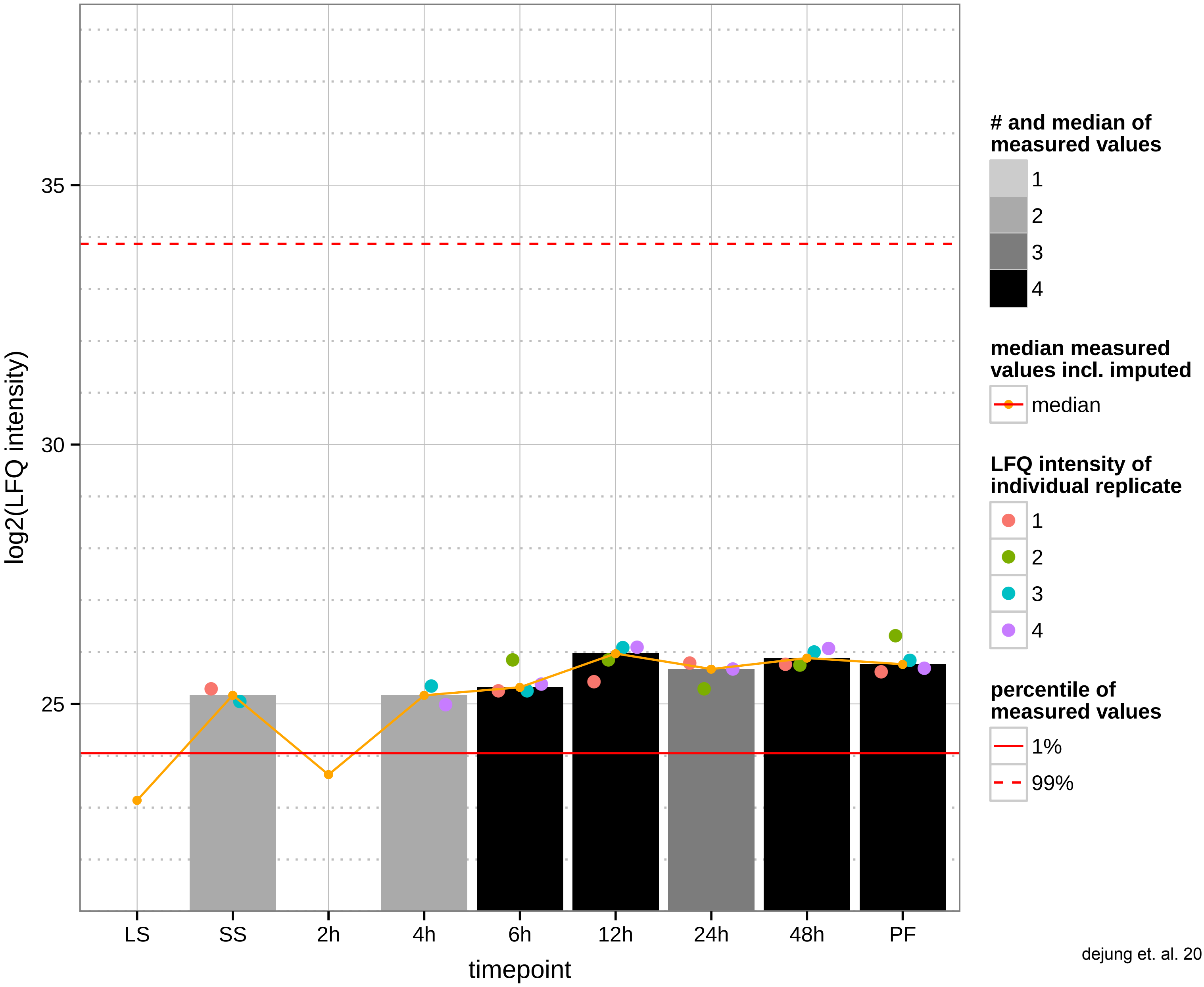

dejung et. al. 2015

hypothetical protein, conserved
Tb927.7.4840;Tb11.v5.0166
AGOF: null
AGOC: null
AGOP: null
PGOF: null
PGOC: null
PGOP: null

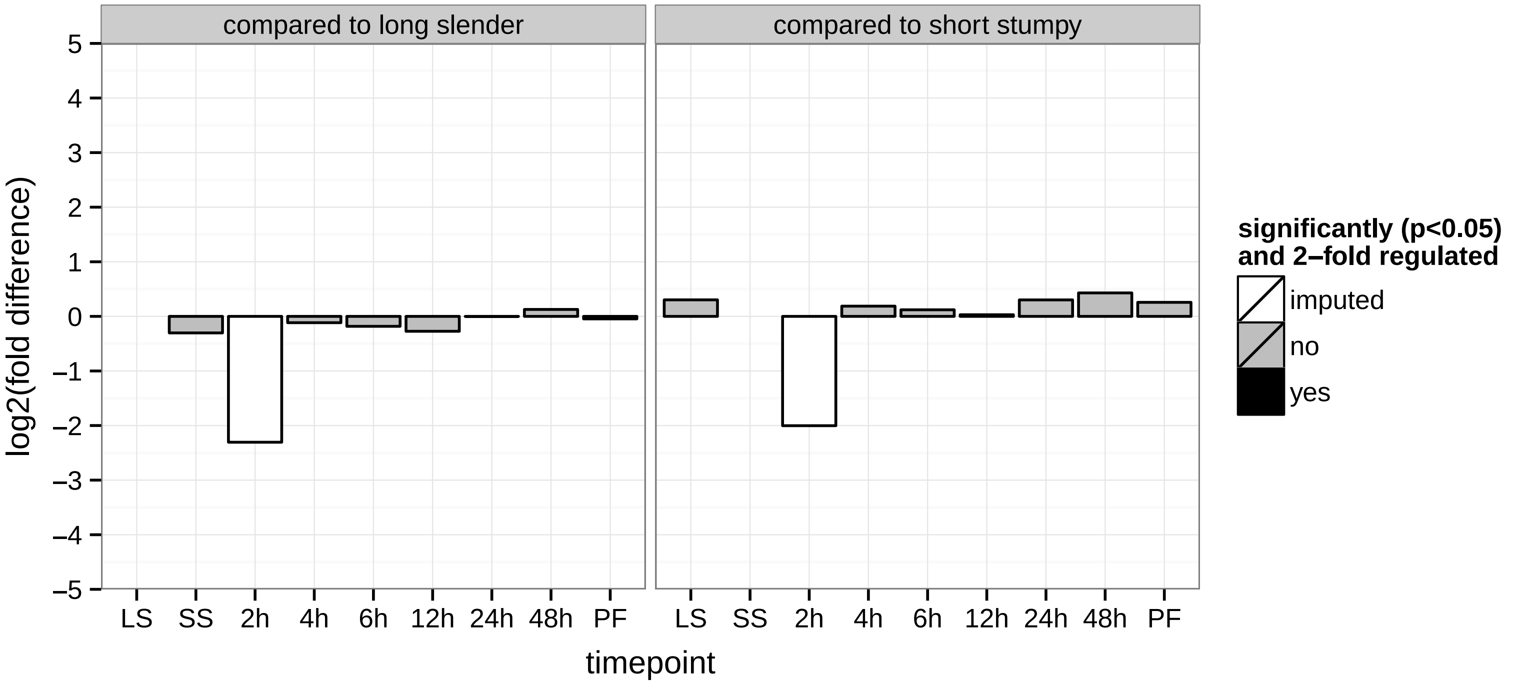

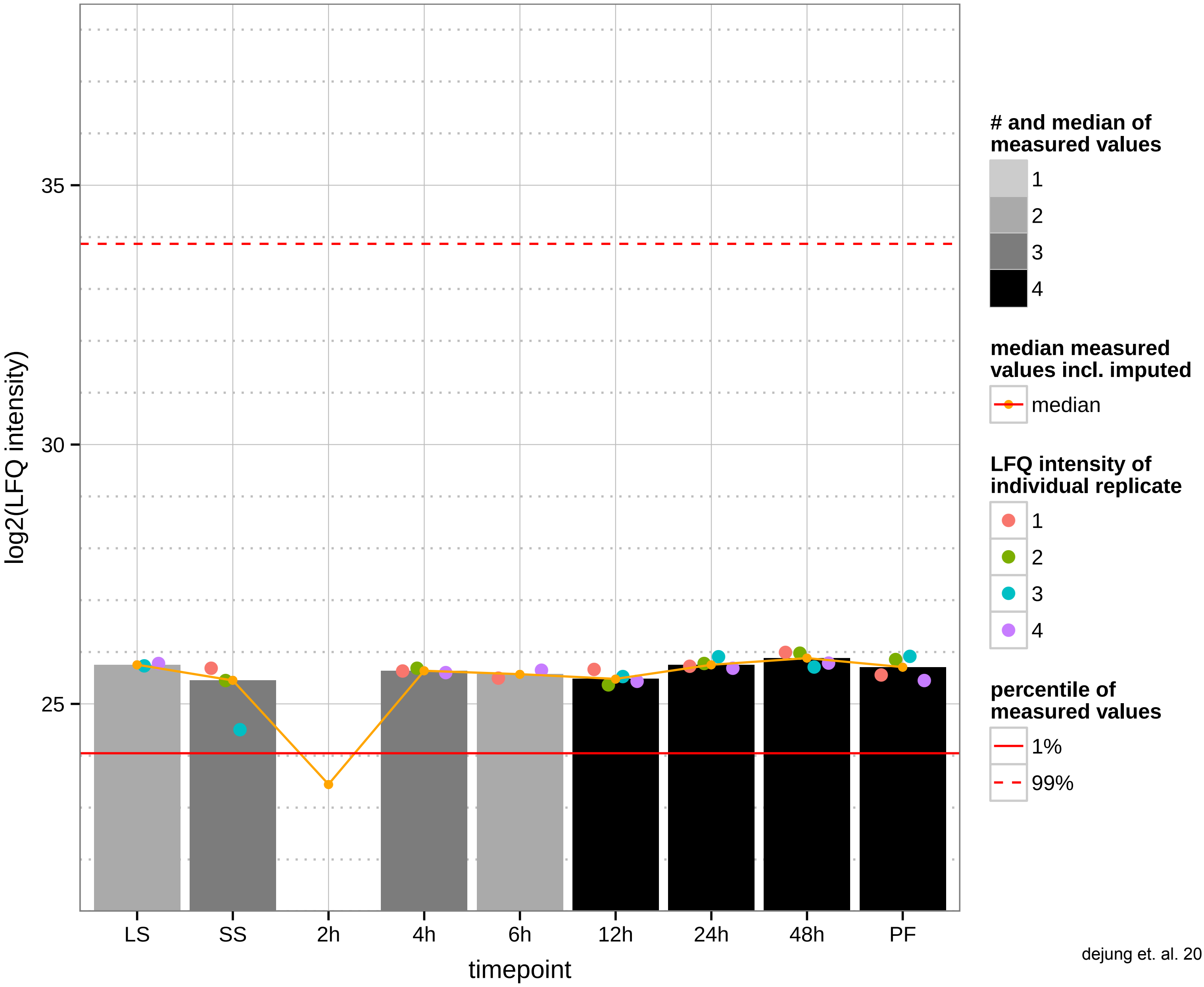

dejung et. al. 2015

hypothetical protein, conserved
Tb927.11.7250;Tb11.v5.0257
AGOF: null
AGOC: null, mitochondrion
AGOP: null
PGOF: null
PGOC: null
PGOP: null

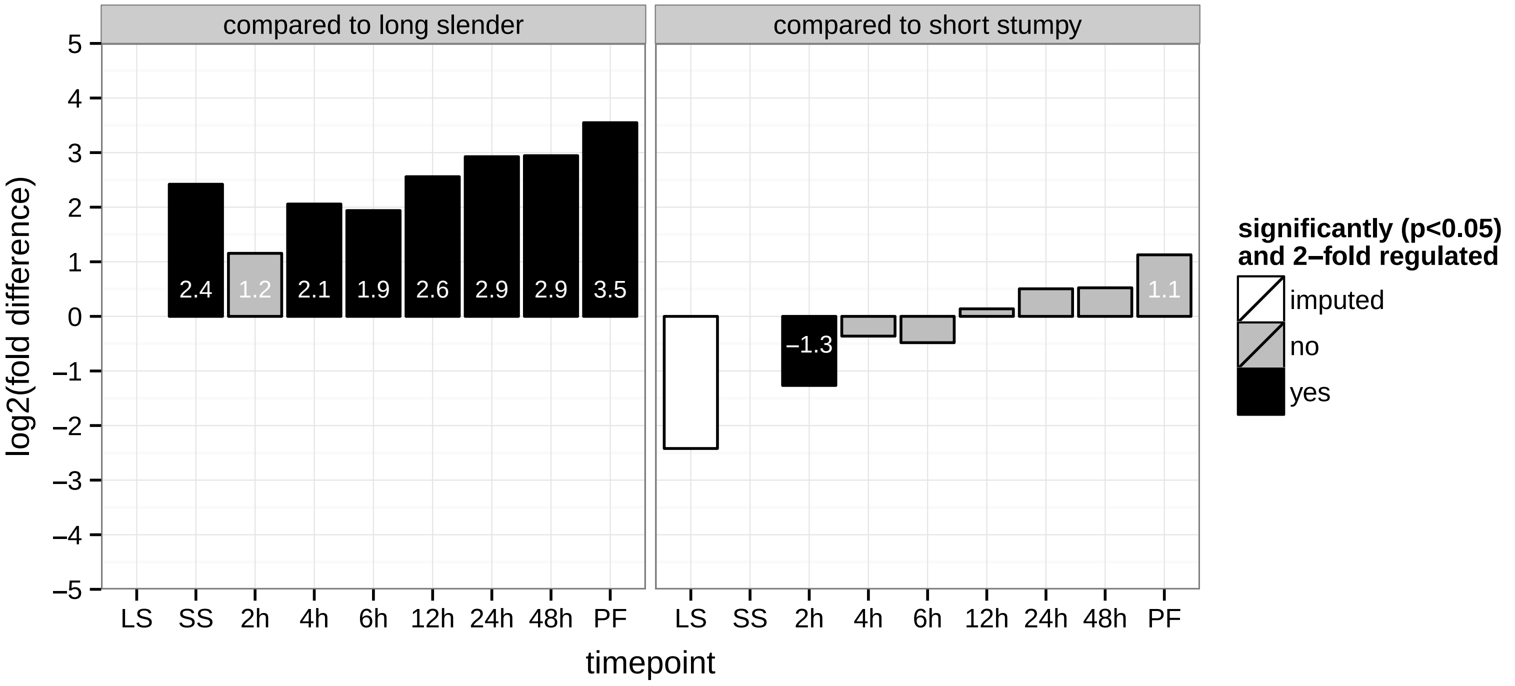

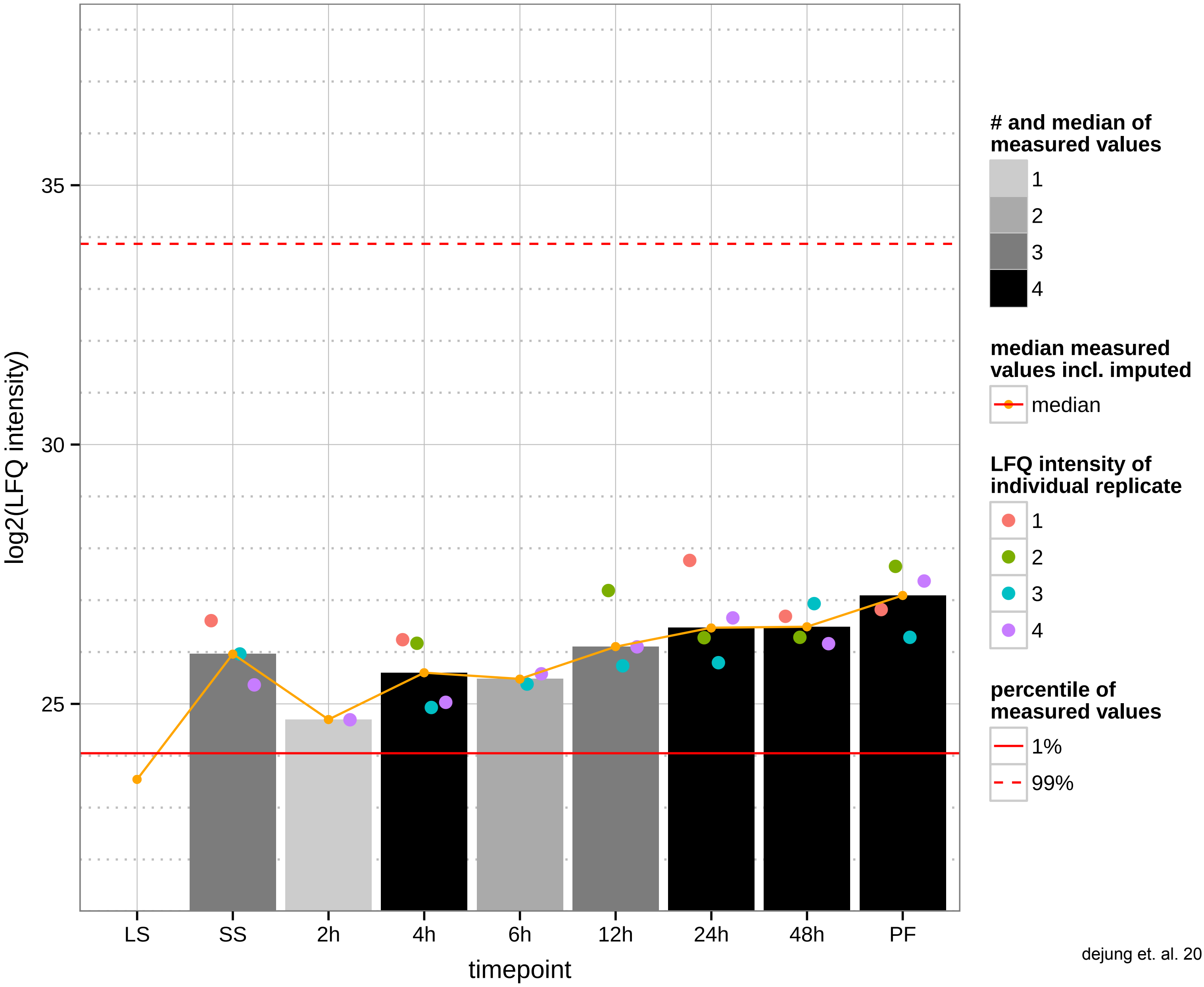

dejung et. al. 2015

hypothetical protein, conserved
Tb11.v5.0562;Tb927.8.1260
AGOF: null
AGOC: null
AGOP: null
PGOF: null
PGOC: null
PGOP: null

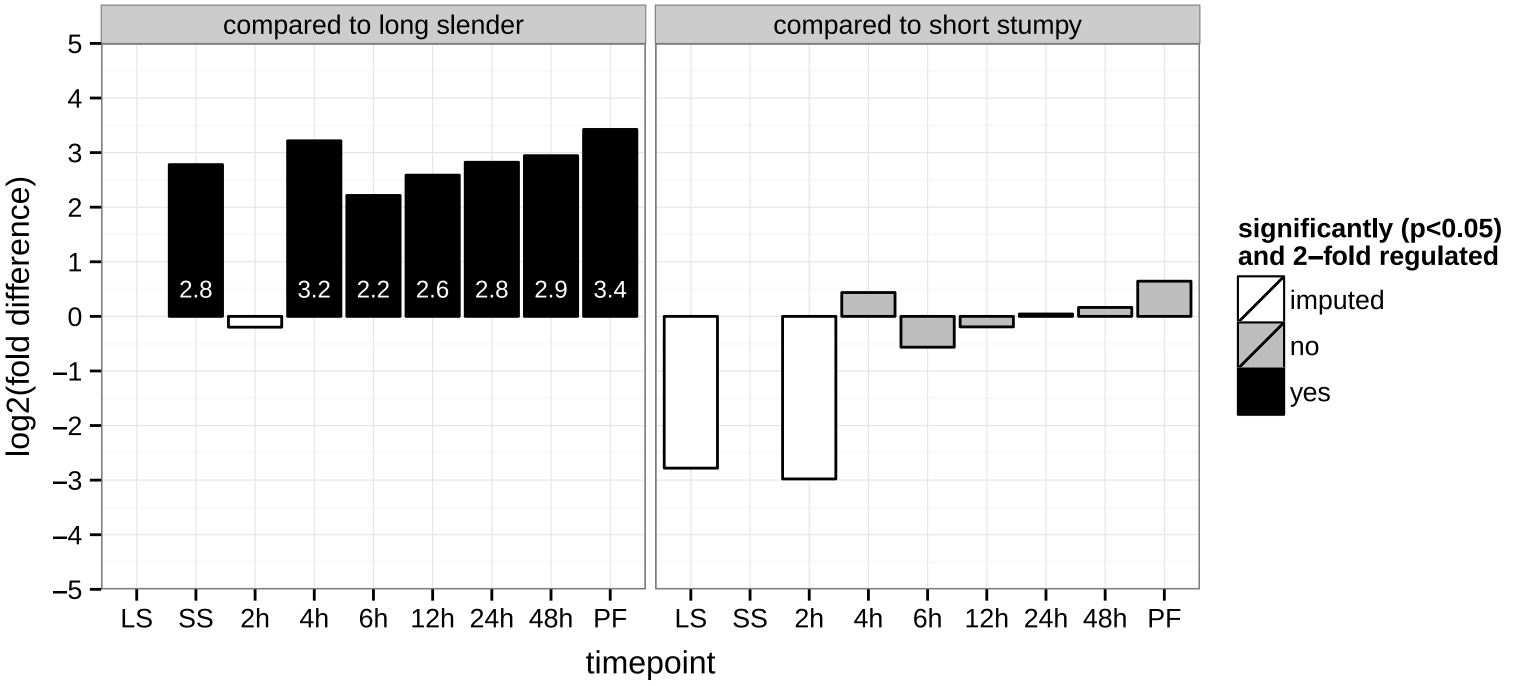

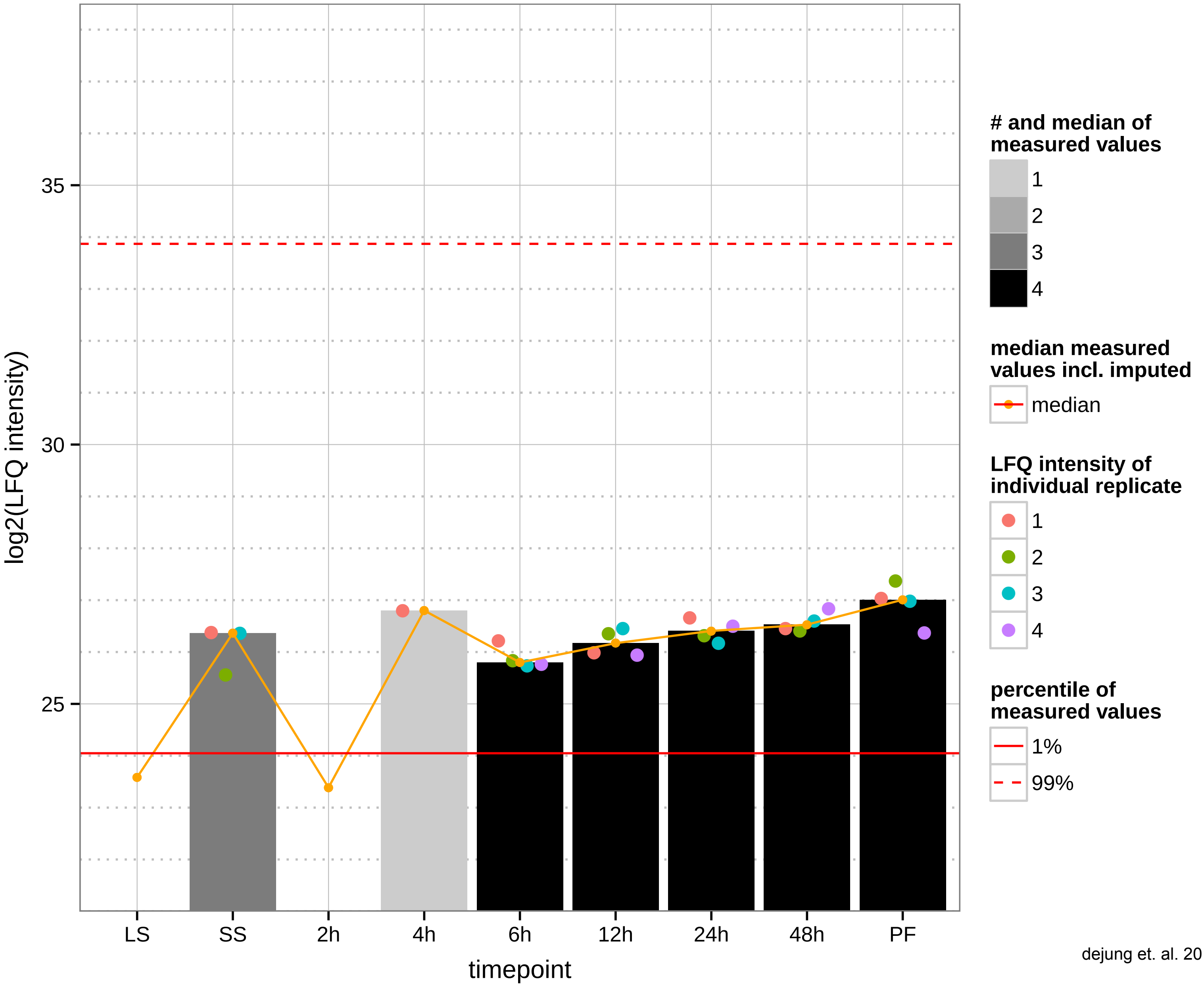

dejung et. al. 2015

hypothetical protein, conserved
Tb927.10.3470
AGOF: null
AGOC: null
AGOP: null
PGOF: protein binding
PGOC: null
PGOP: null

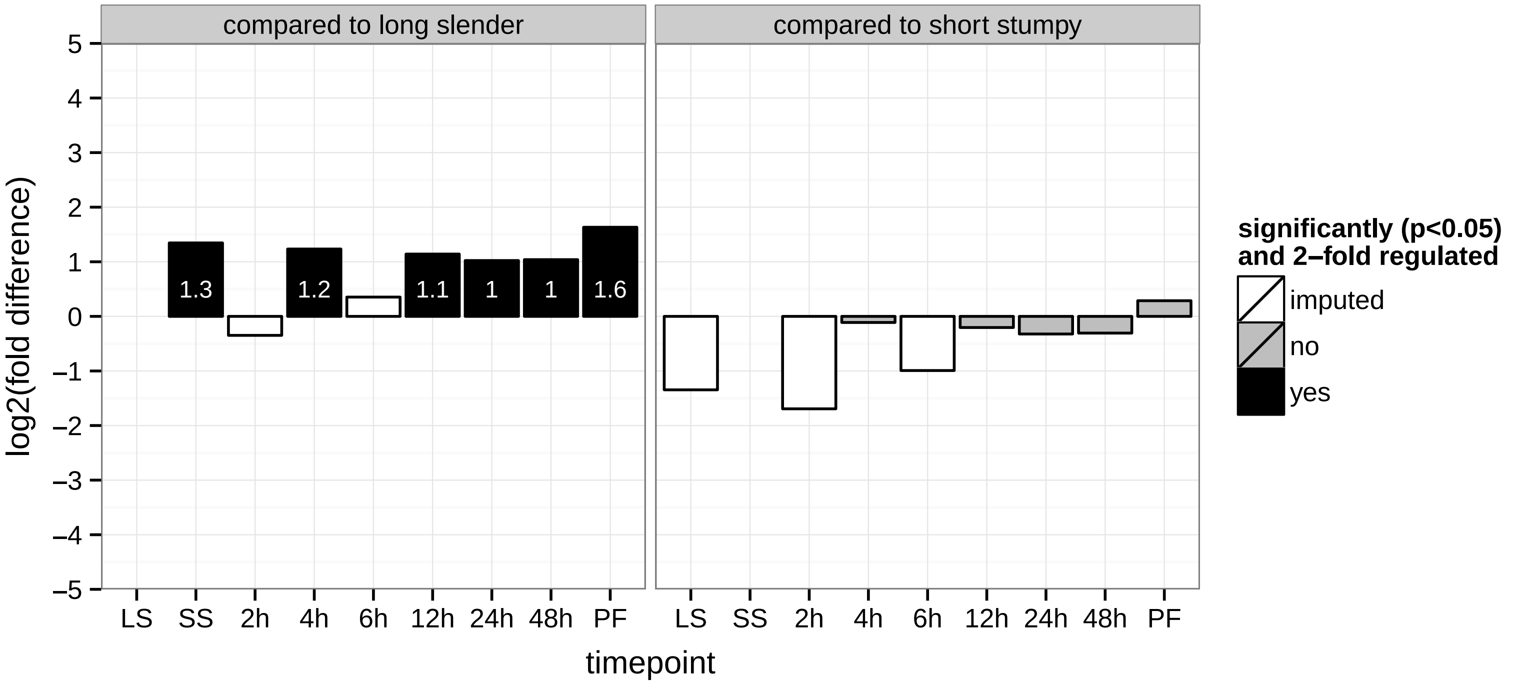

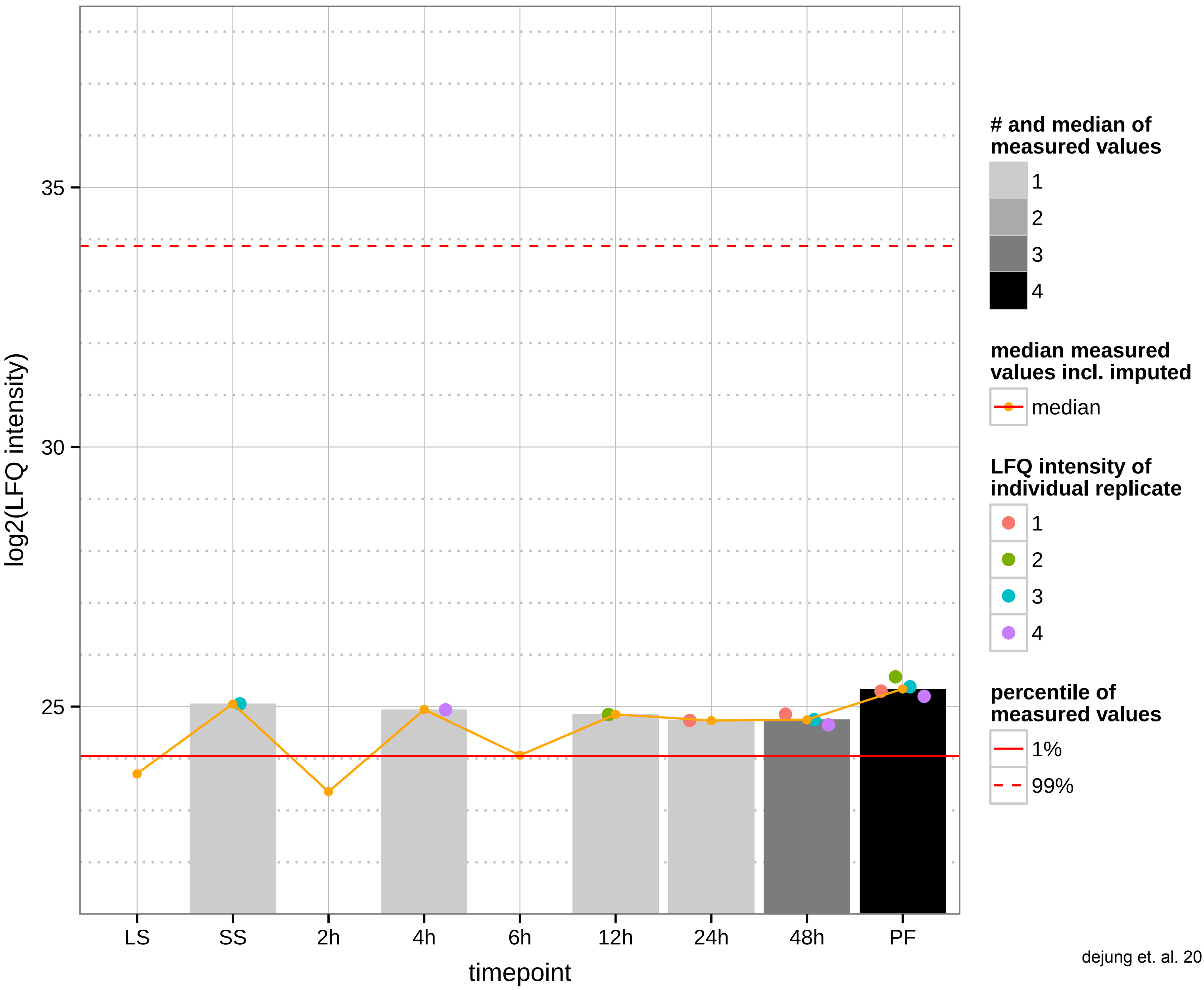

dejung et. al. 2015

hypothetical protein, conserved
Tb927.10.3580
AGOF: null
AGOC: mitochondrion
AGOP: null
PGOF: null
PGOC: null
PGOP: null

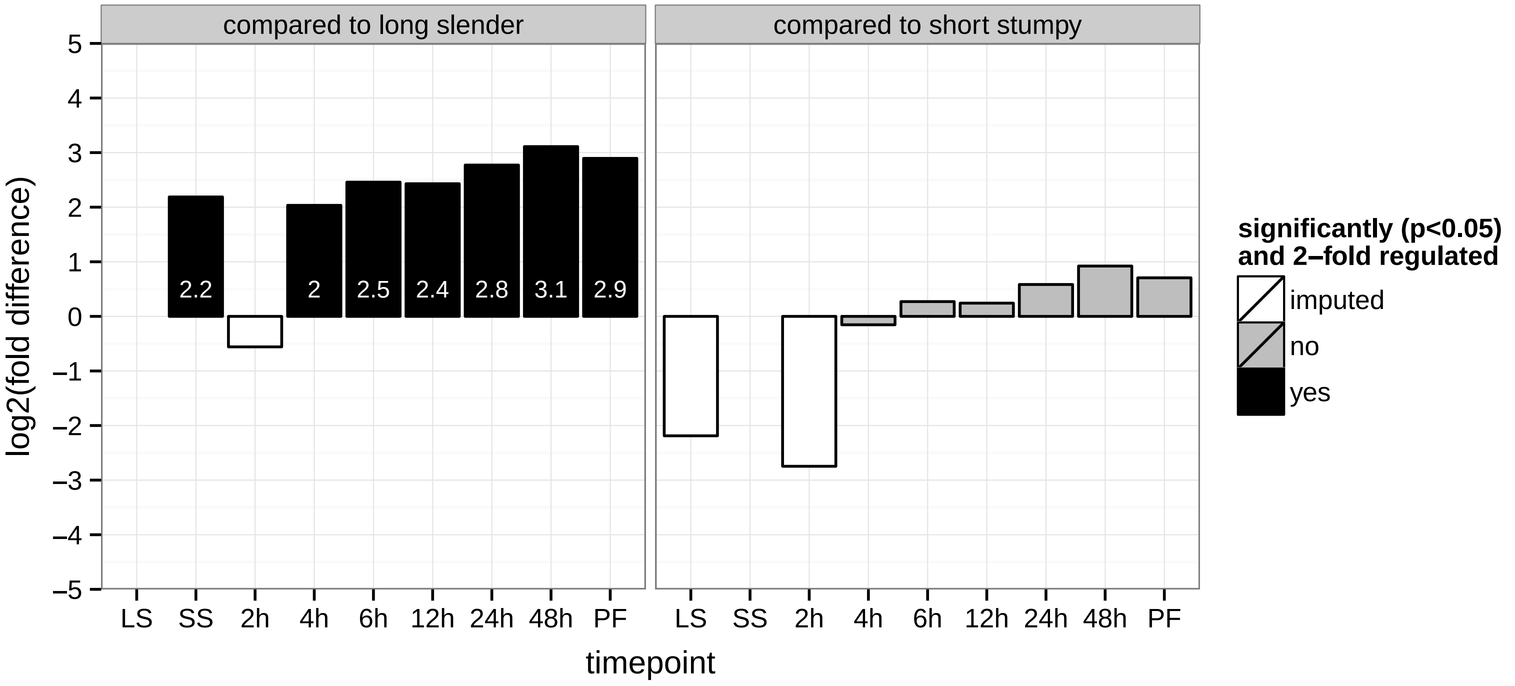

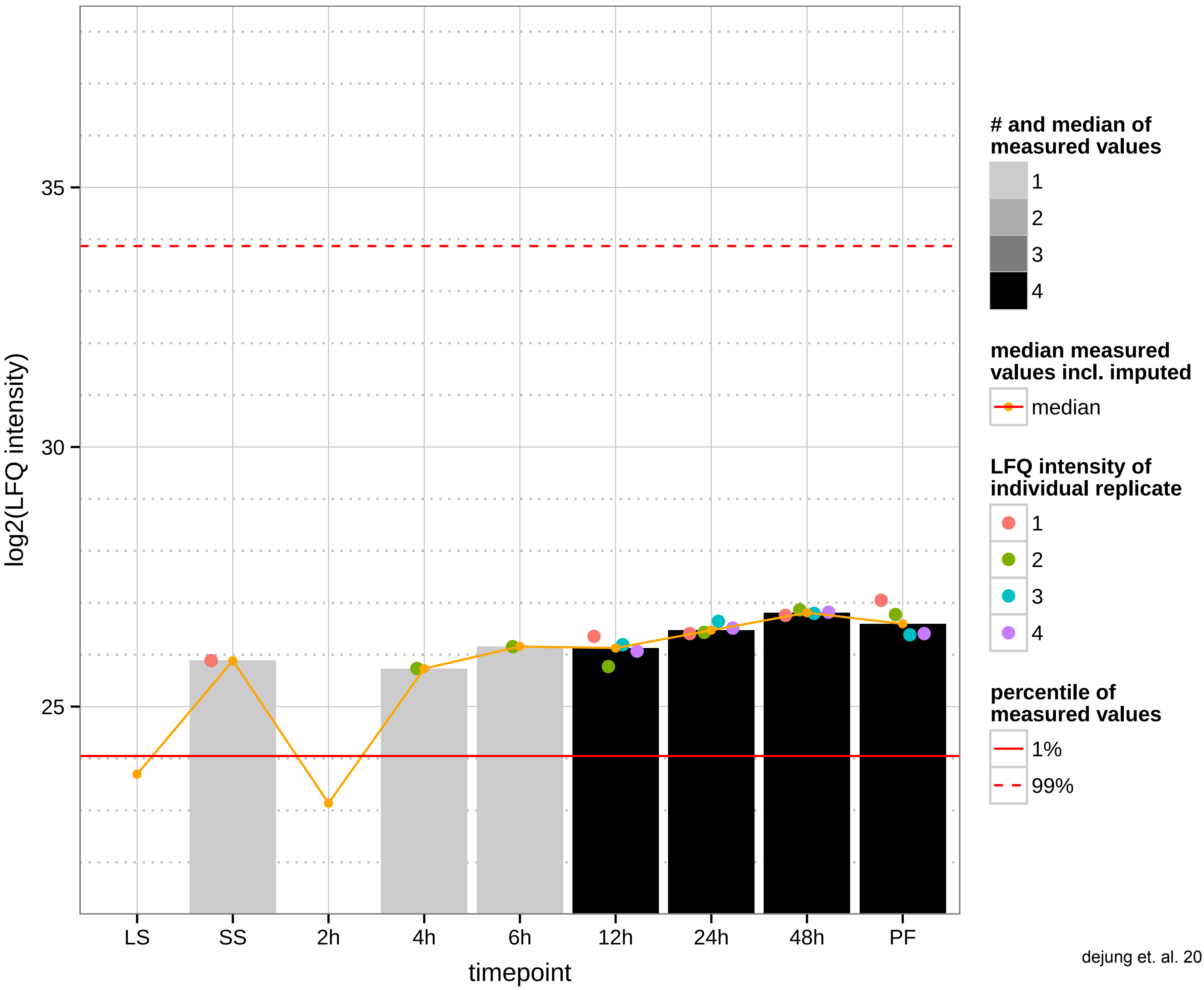

dejung et. al. 2015

hypothetical protein, conserved
Tb927.10.4220
AGOF: null
AGOC: null
AGOP: null
PGOF: null
PGOC: null
PGOP: null

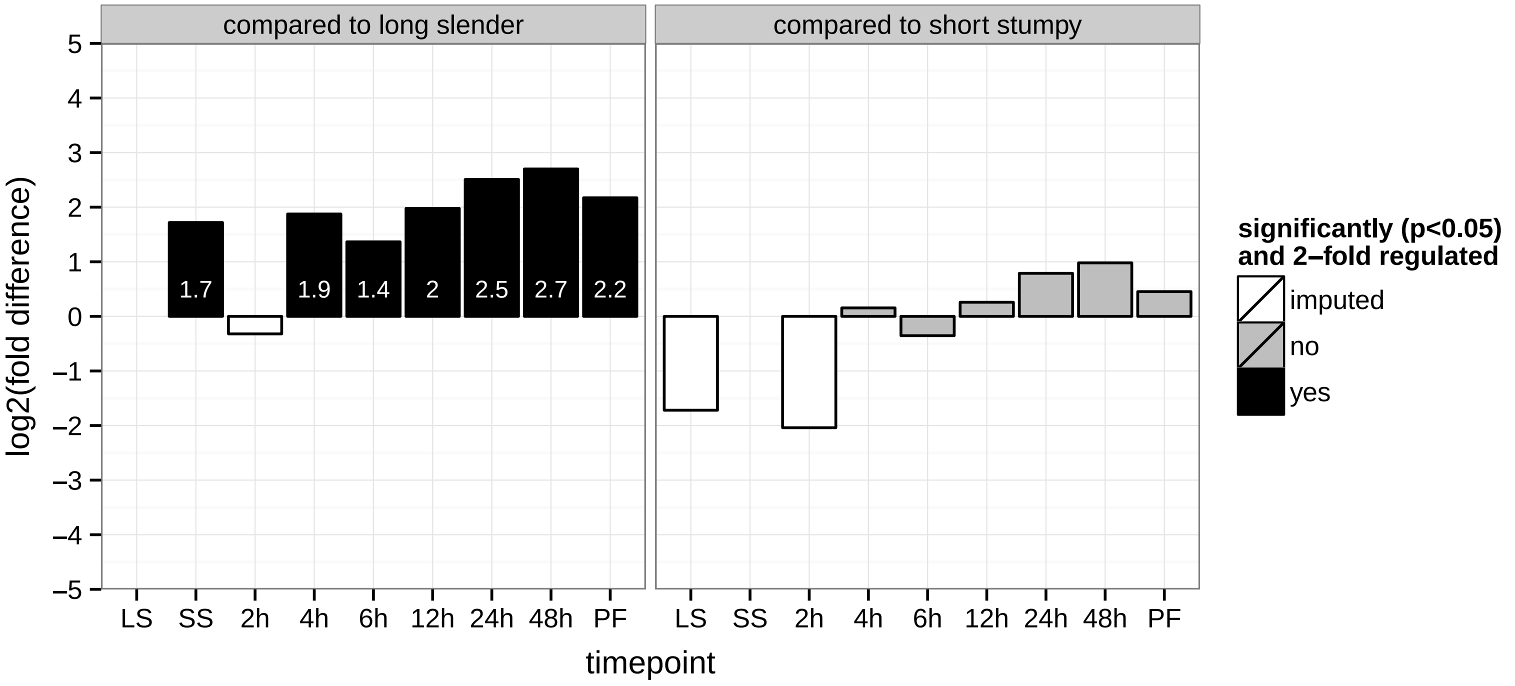

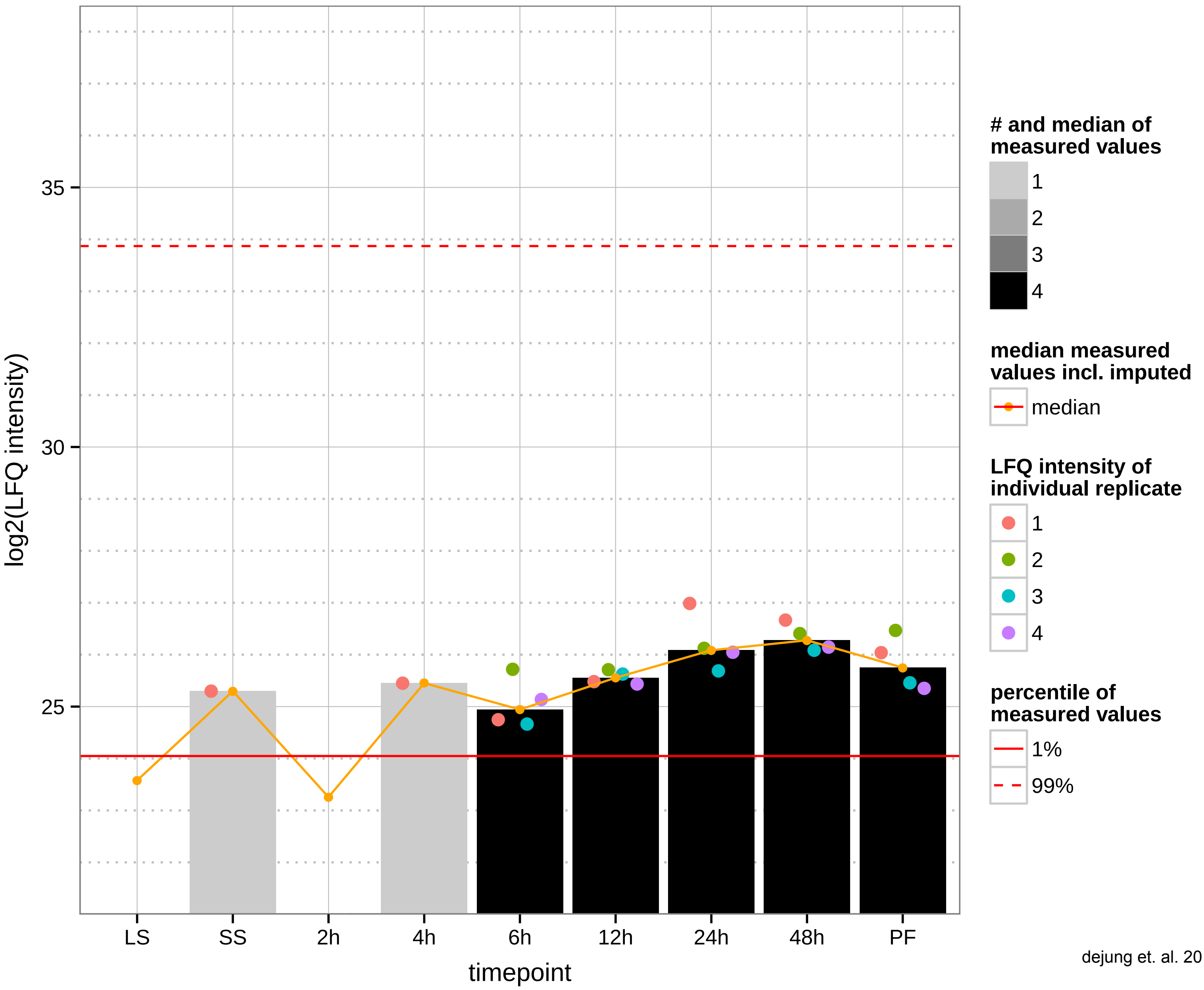

dejung et. al. 2015

caltractin, putative
Tb927.10.6980
AGOF: calcium ion binding
AGOC: null
AGOP: null
PGOF: calcium ion binding
PGOC: null
PGOP: null

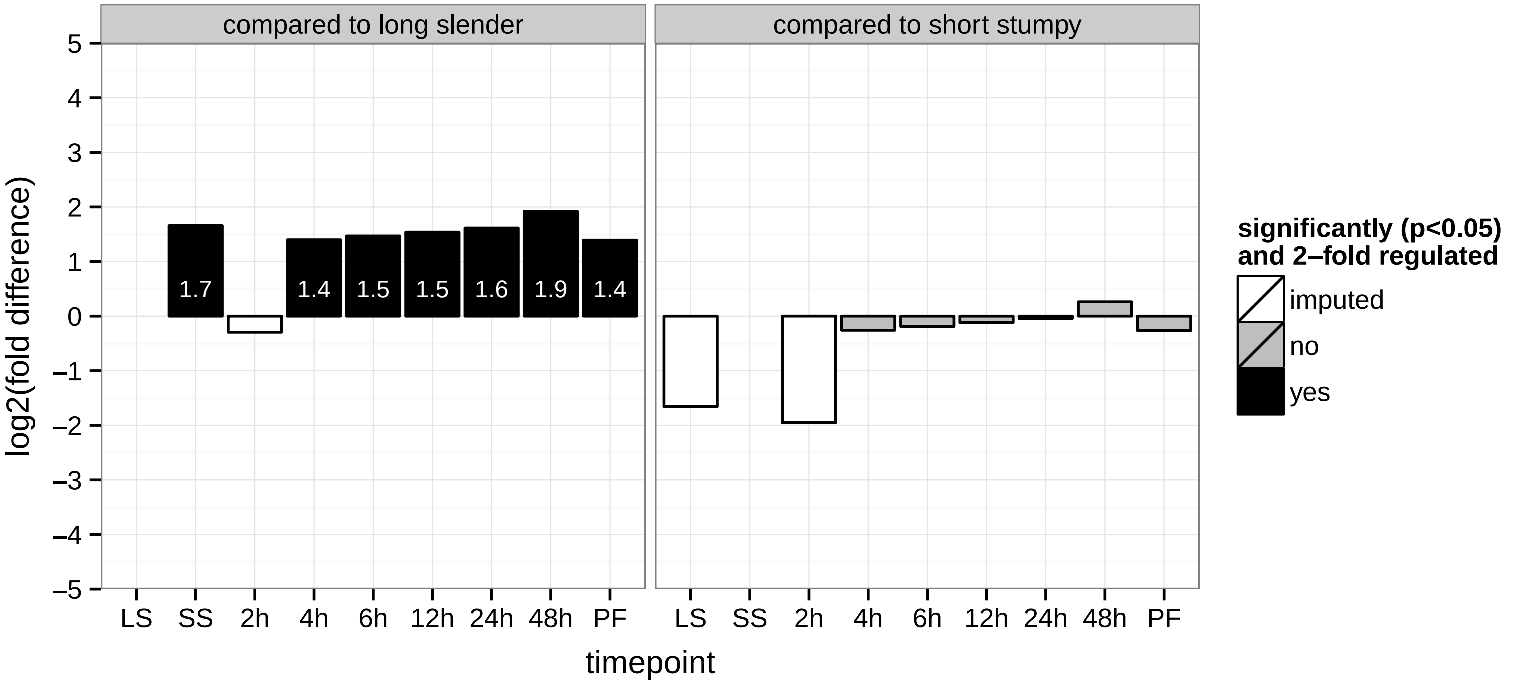

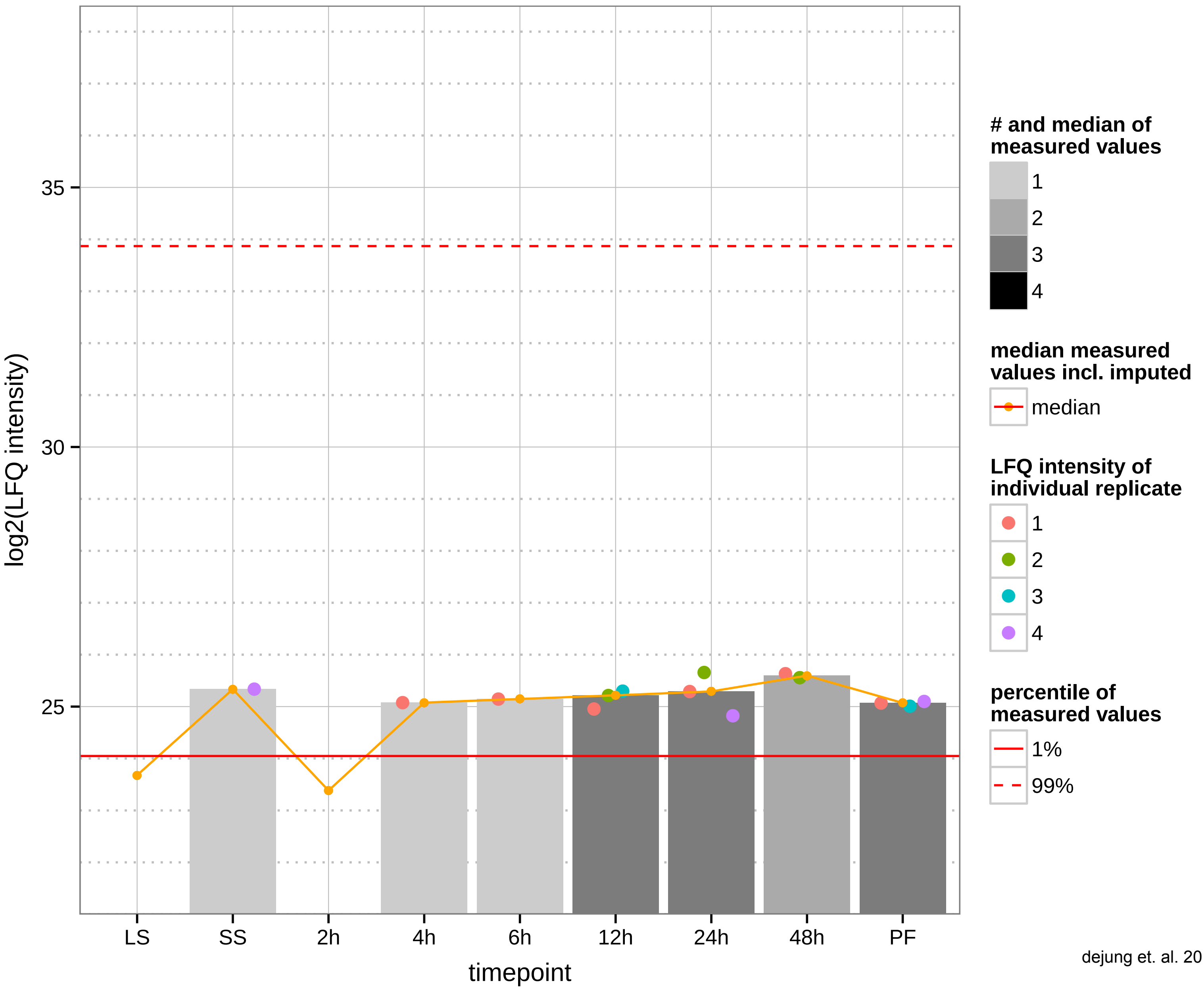

dejung et. al. 2015

beta–adaptin 3, putative, adaptin complex 3 subunit
Tb927.11.10650
AGOF: null
AGOC: Golgi apparatus, coated pit, membrane coat
AGOP: intracellular protein transport, vesicle–mediated transport
PGOF: binding
PGOC: membrane coat
PGOP: intracellular protein transport, vesicle–mediated transport

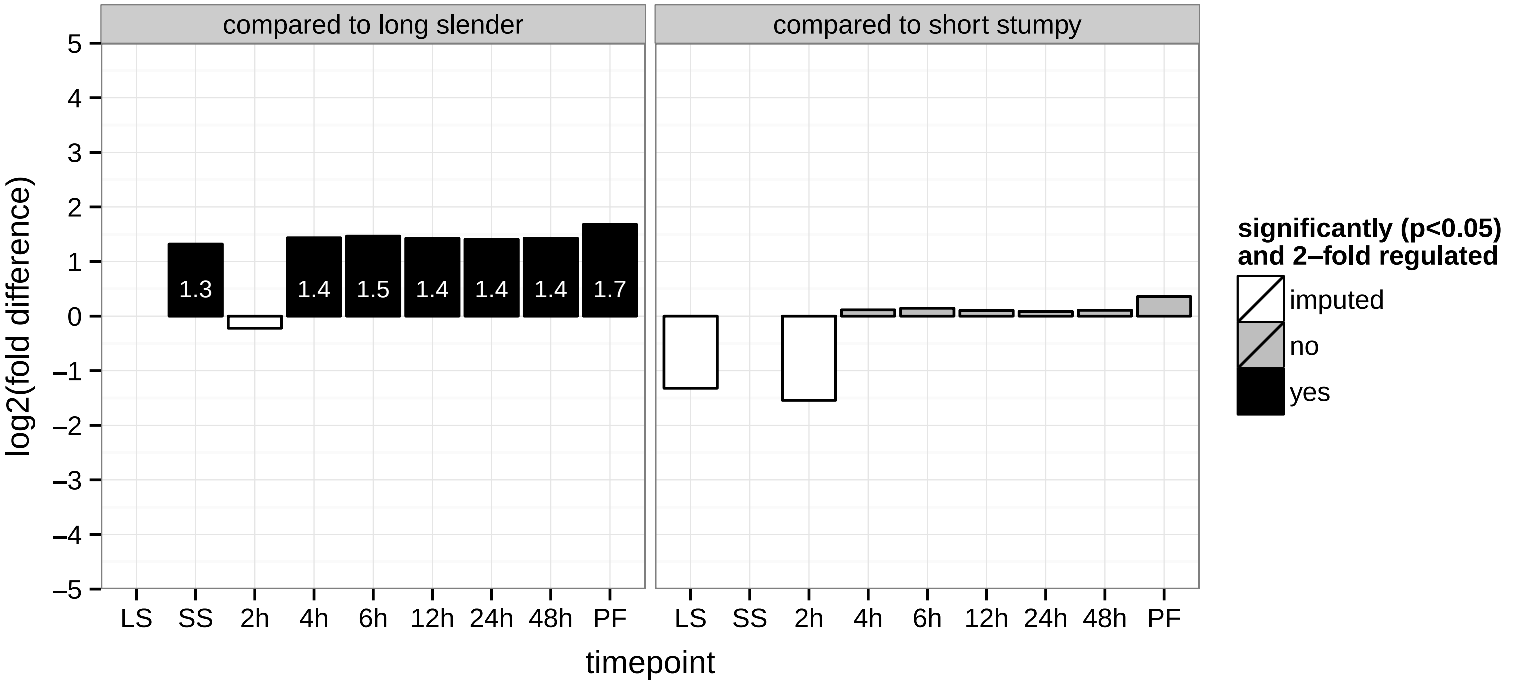

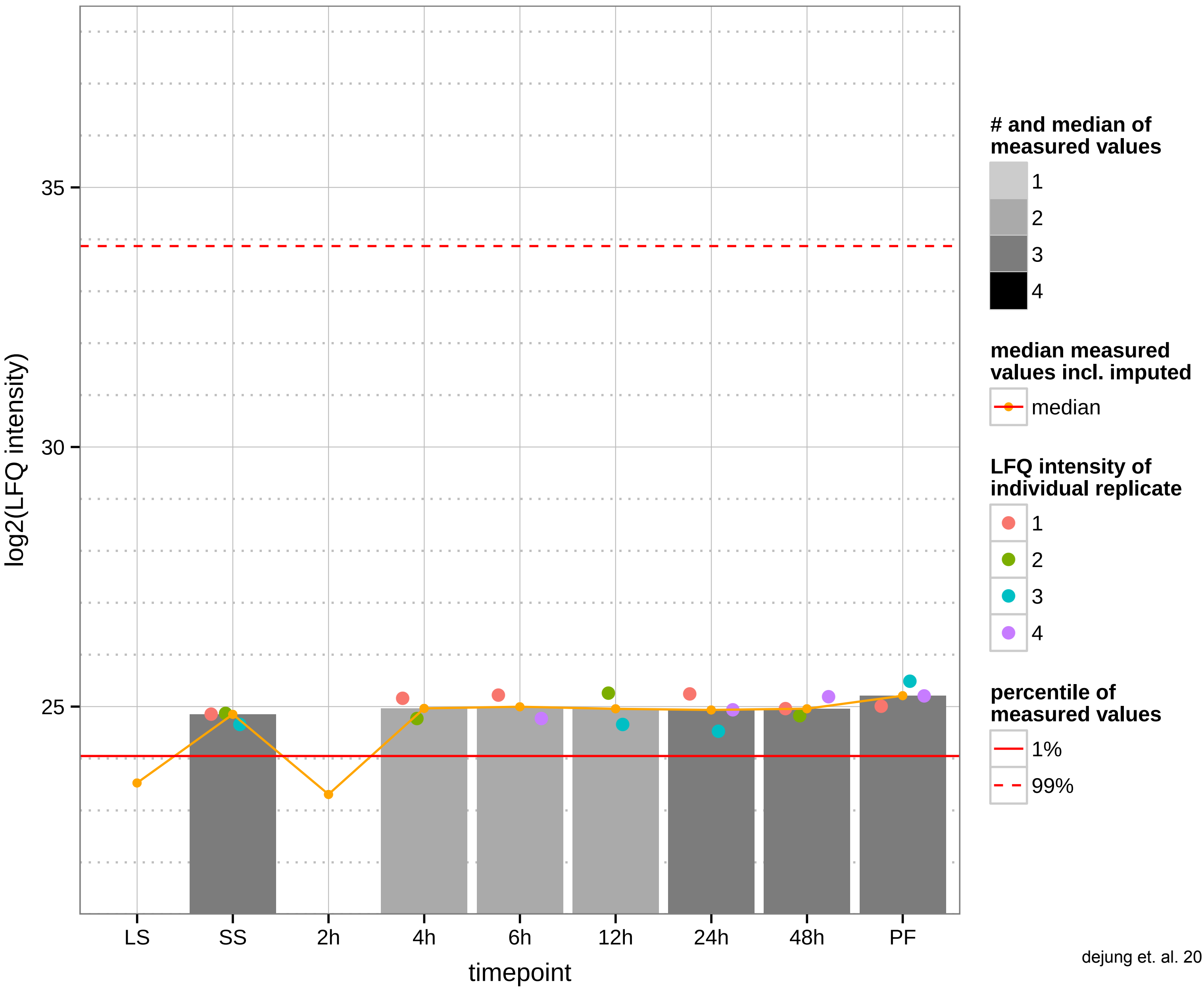

dejung et. al. 2015

RNA helicase, putative
Tb927.11.12110
AGOF: ATP binding, ATP−dependent helicase activity, helicase activity, nucleic acid binding, zinc ion binding
AGOC: intracellular
AGOP: nucleobase−containing compound metabolic process, transport
PGOF: ATP binding, ATP−dependent helicase activity, helicase activity, nucleic acid binding, protein binding, zinc ion binding
PGOC: null
PGOP: null

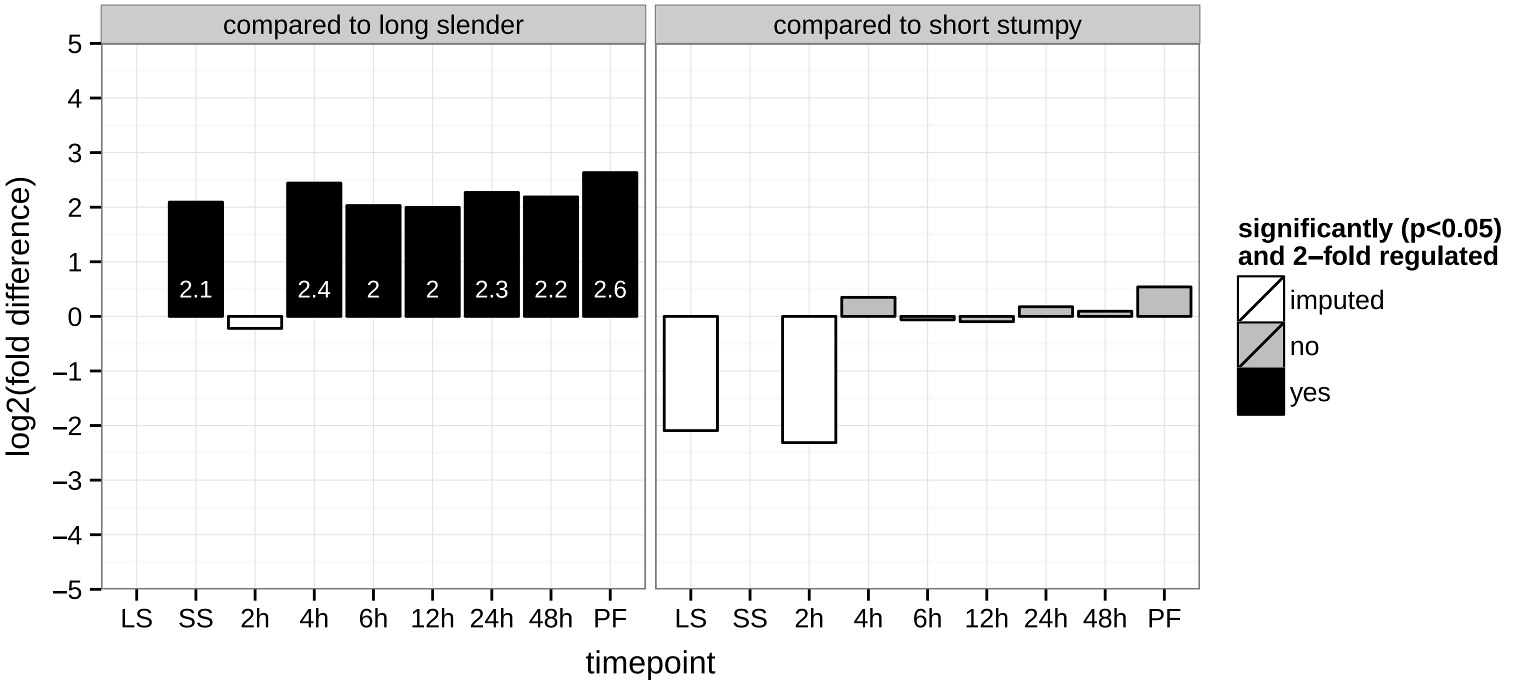

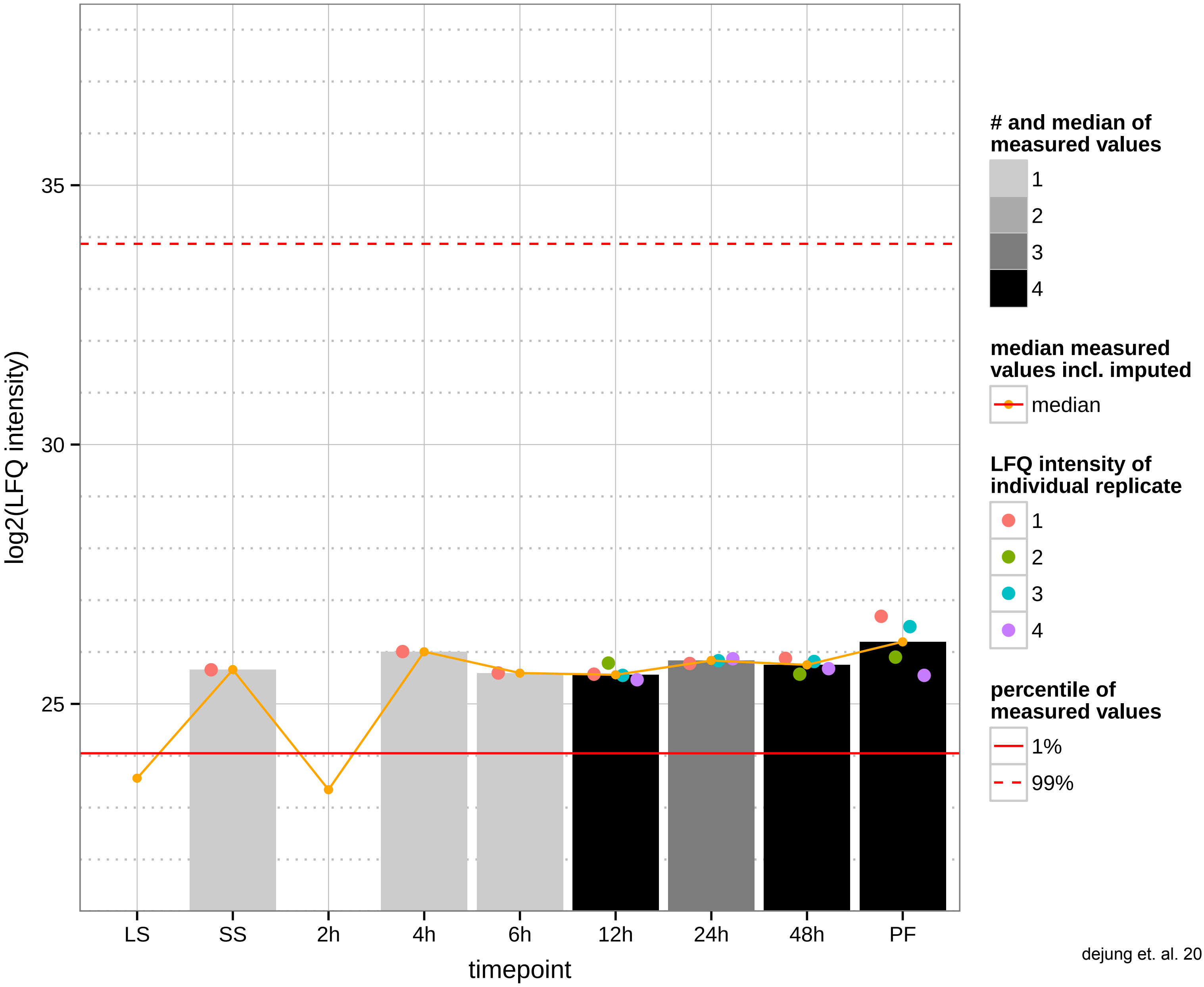

dejung et. al. 2015

phospholipid:diacylglycerol acyltransferase−like protein
Tb927.11.13220
AGOF: phosphatidylcholine−sterol O−acyltransferase activity
AGOC: null
AGOP: lipid metabolic process
PGOF: O−acyltransferase activity
PGOC: null
PGOP: lipid metabolic process

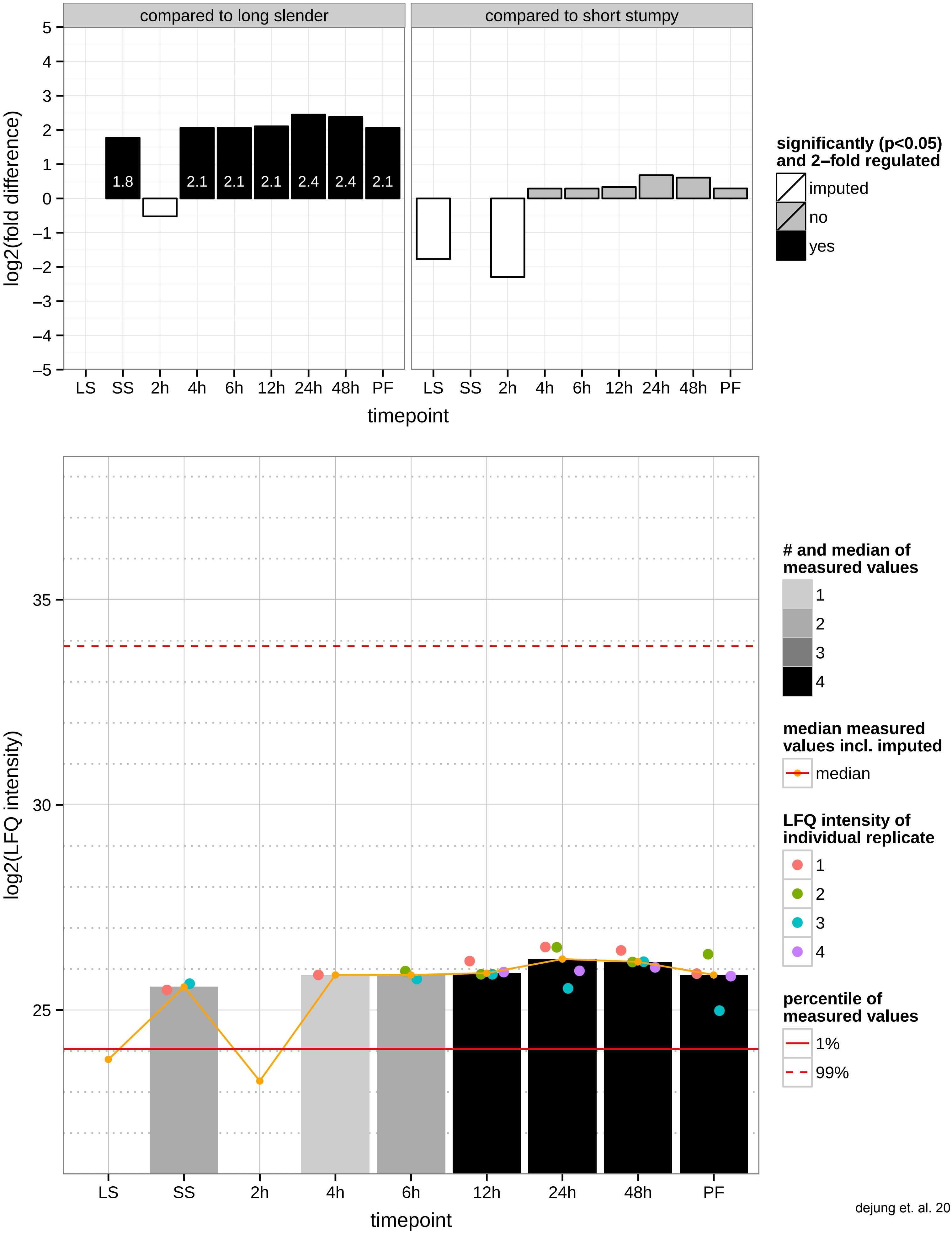

dejung et. al. 2015

hypothetical protein, conserved
Tb927.11.16230
AGOF: null
AGOC: null
AGOP: null
PGOF: null
PGOC: null
PGOP: null

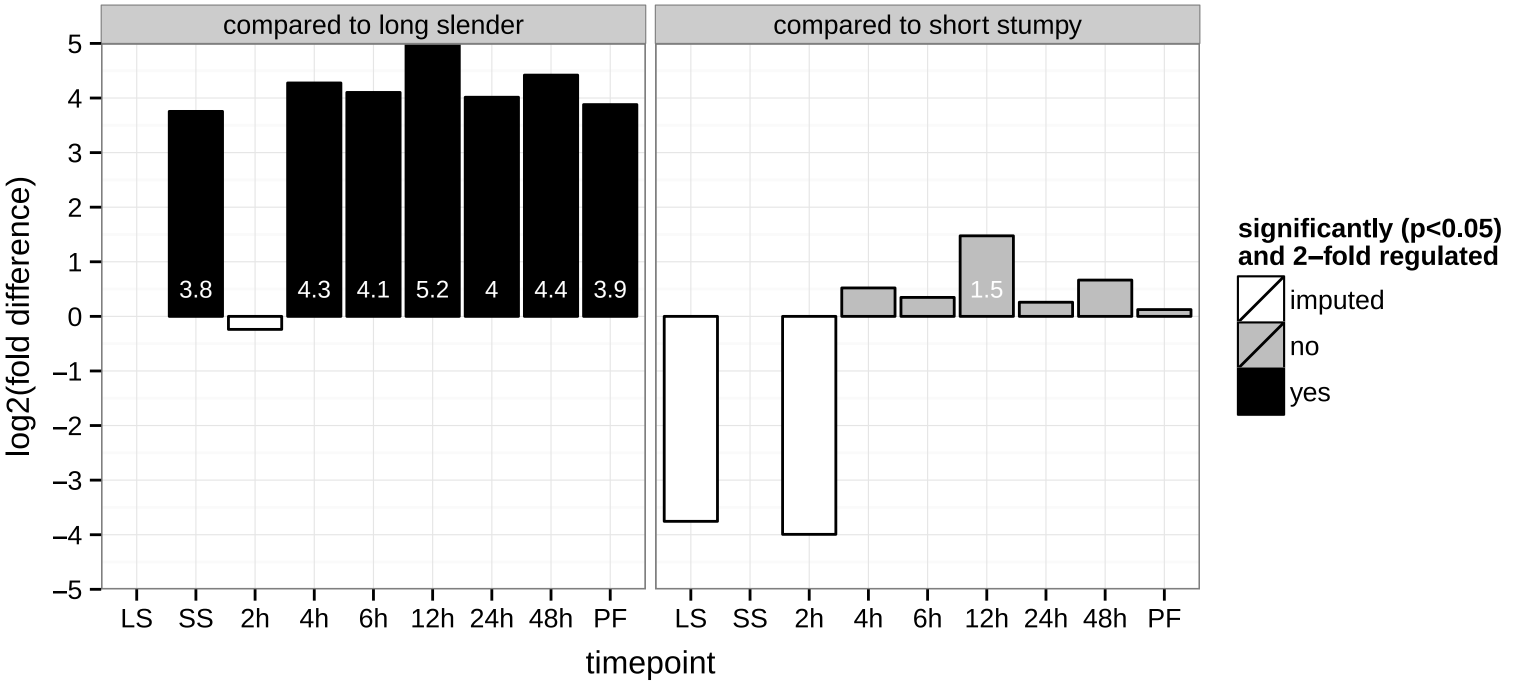

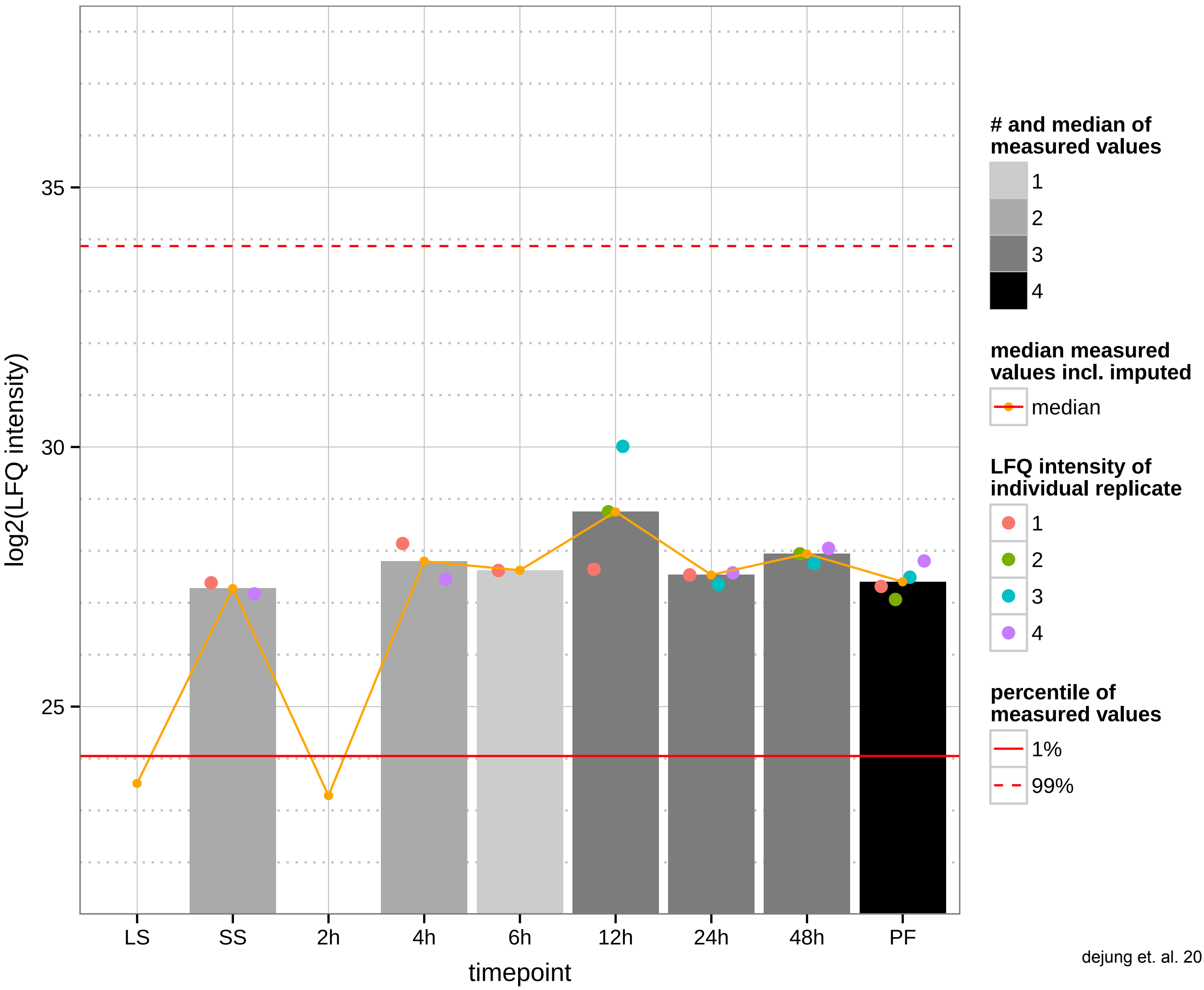

dejung et. al. 2015

KREPB4, RNA–editing complex protein (KREPB4)
Tb927.11.2990
AGOF: RNA binding
AGOC: mRNA editing complex, mitochondrial mRNA editing complex, mitochondrion
AGOP: RNA modification
PGOF: null
PGOC: null
PGOP: null

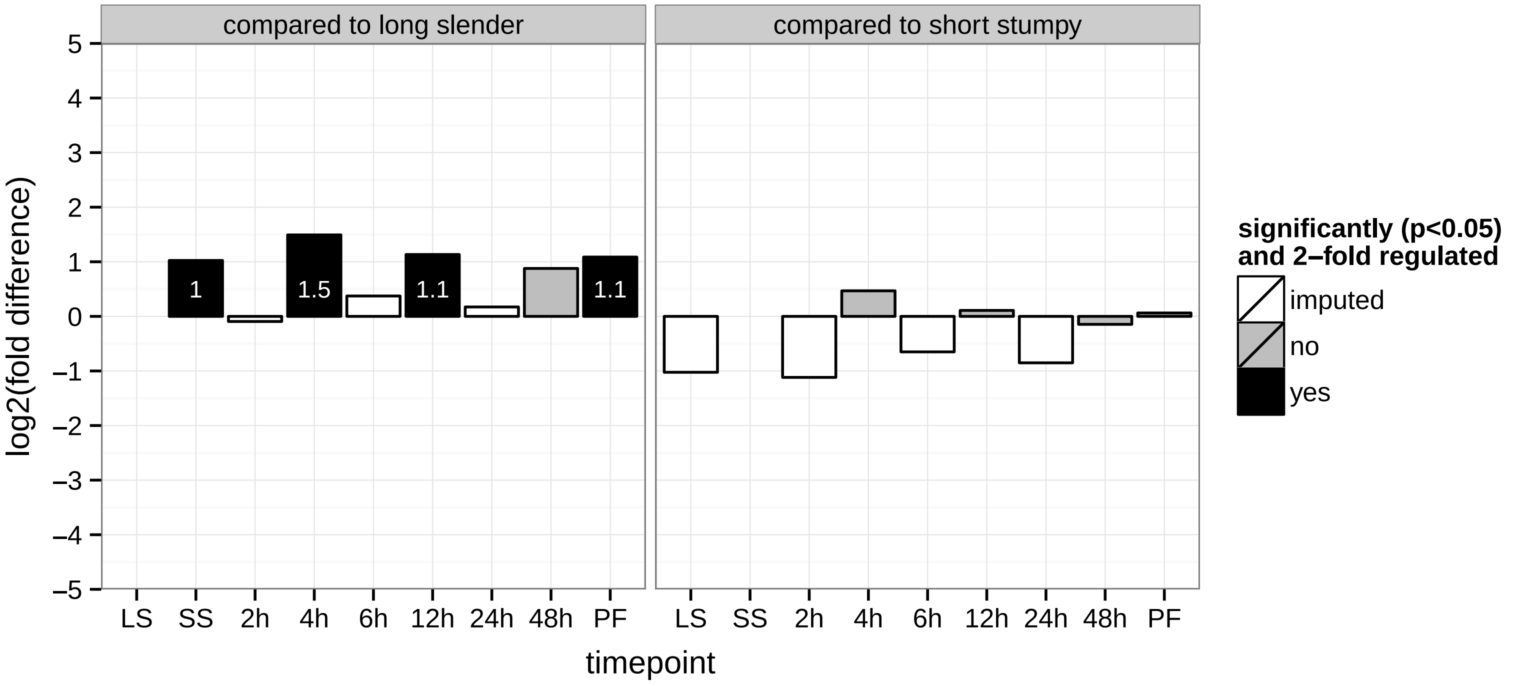

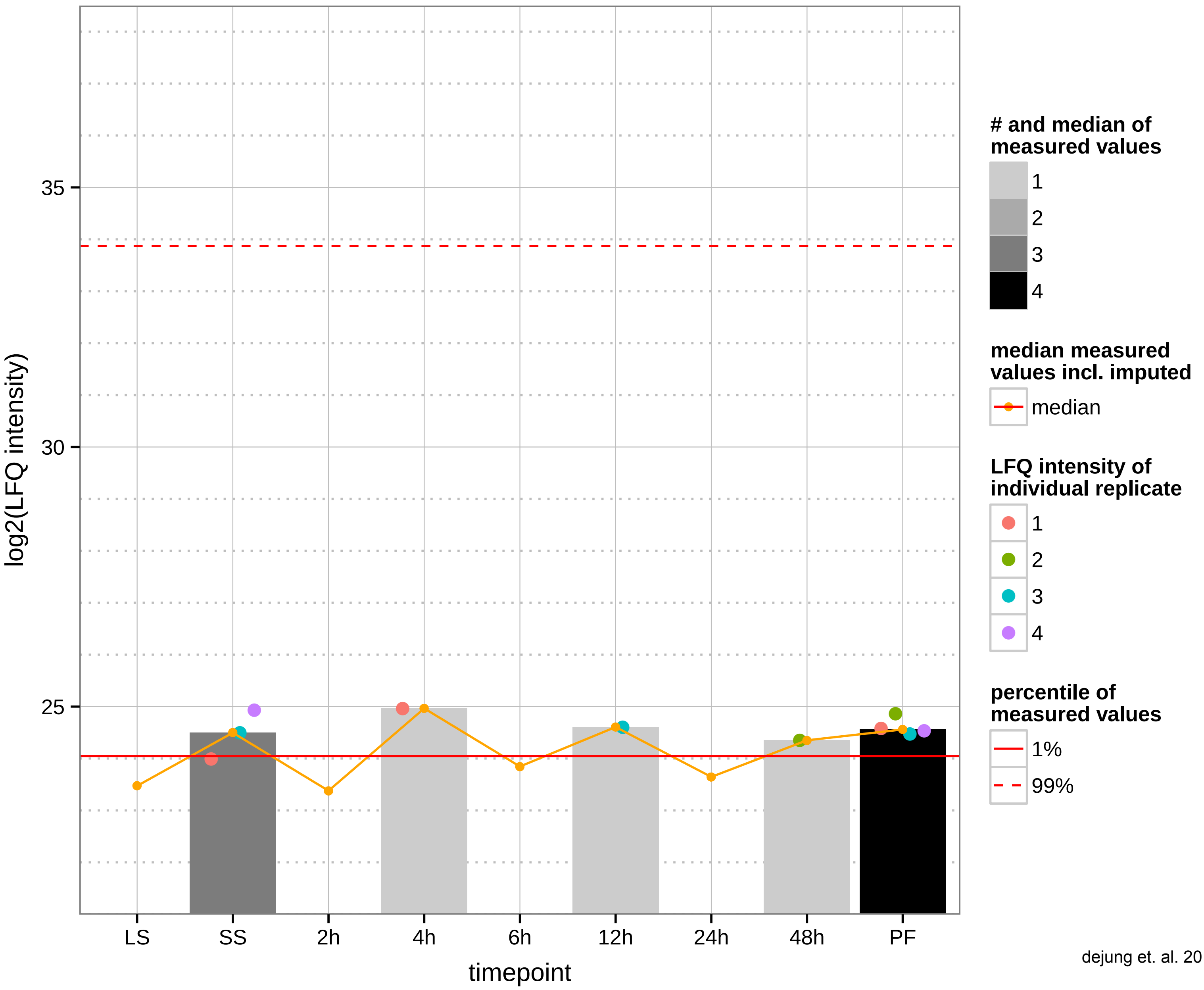

dejung et. al. 2015

glycosyltransferase family–like protein, putative
Tb927.11.3900
AGOF: null
AGOC: null
AGOP: lipopolysaccharide biosynthetic process
PGOF: null
PGOC: null
PGOP: lipopolysaccharide biosynthetic process

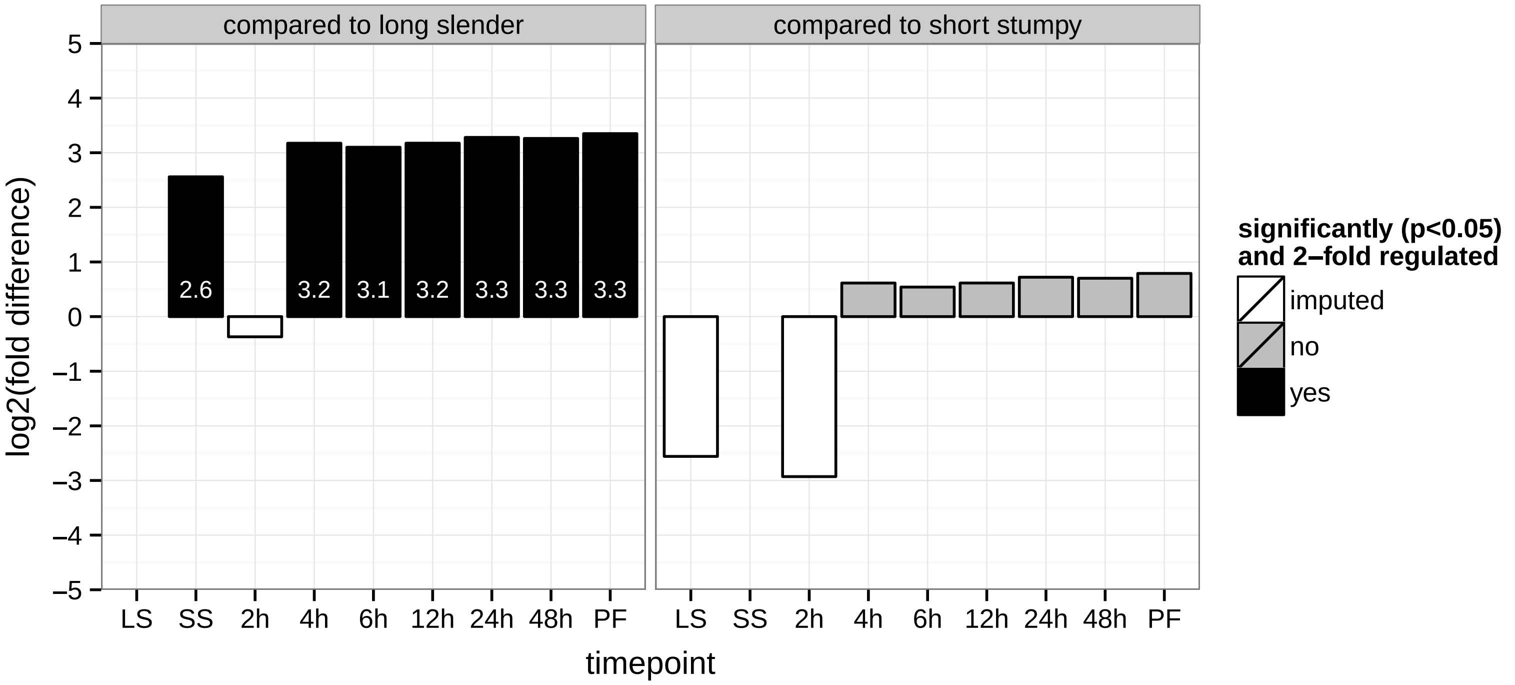

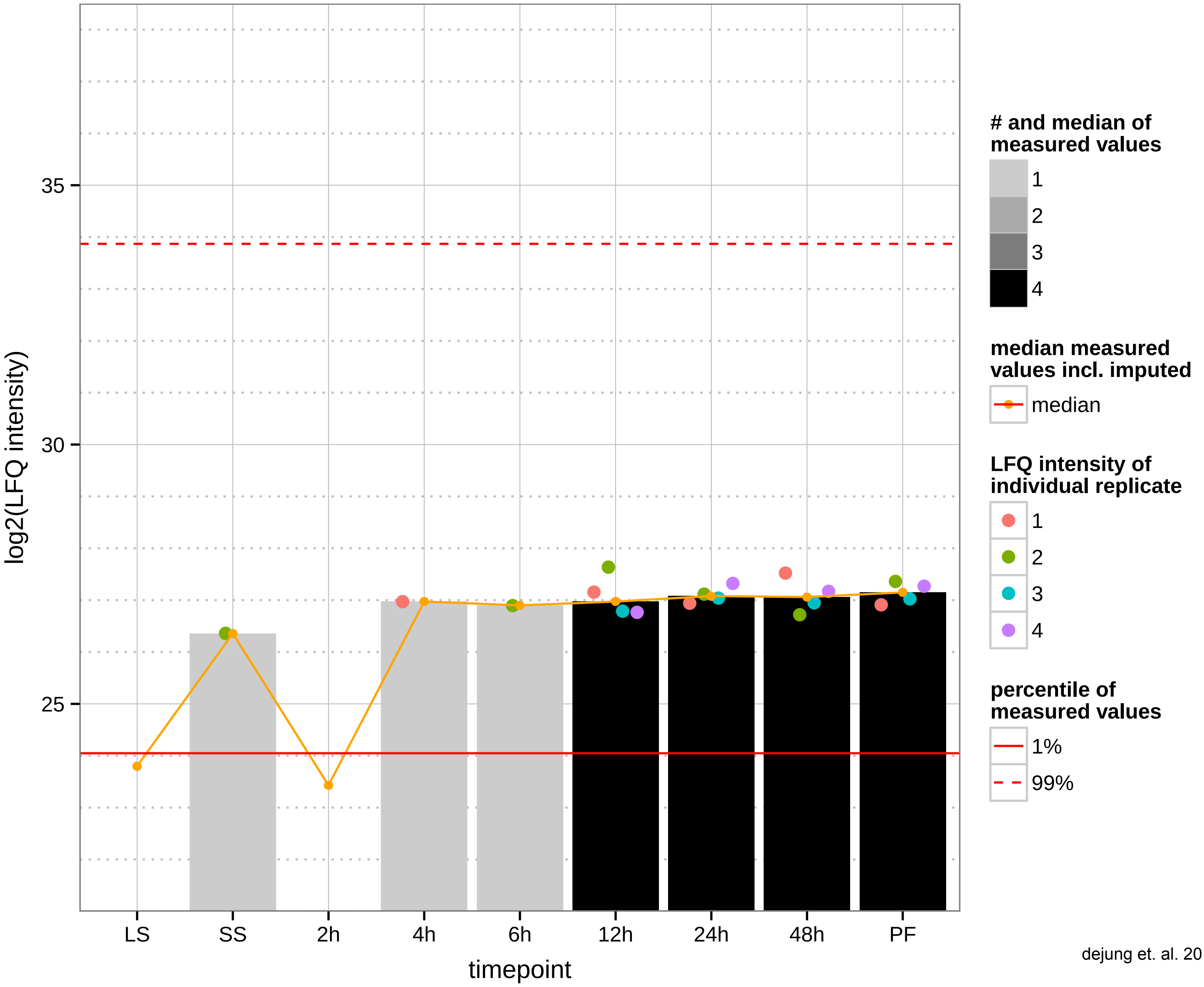

dejung et. al. 2015

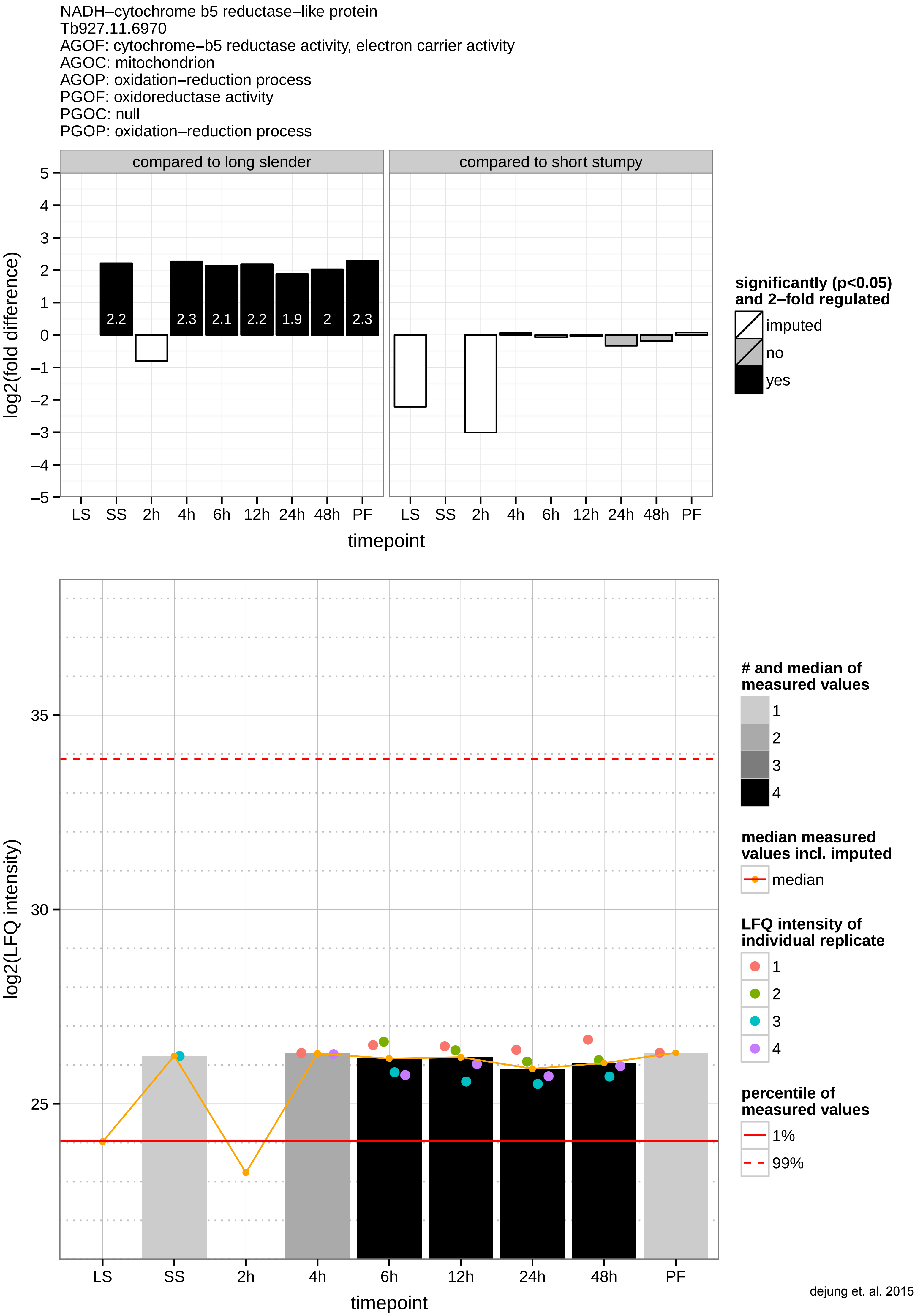

NADH−cytochrome b5 reductase−like protein
Tb927.11.6970
AGOF: cytochrome−b5 reductase activity, electron carrier activity
AGOC: mitochondrion
AGOP: oxidation−reduction process
PGOF: oxidoreductase activity
PGOC: null
PGOP: oxidation−reduction process
compared to long slender
compared to short stumpy
log2(fold difference)
2.2
2.3
2.1
2.2
1.9
2
2.3
LS
SS
2h
4h
6h
12h
24h
48h
PF
timepoint
significantly (p<0.05) and 2−fold regulated
imputed
no
yes
log2(LFQ intensity)
# and median of measured values
1
2
3
4
median measured values incl. imputed
median
LFQ intensity of individual replicate
percentile of measured values
1%
99%
dejung et. al. 2015

prenyltransferase, putative
Tb927.11.9360
AGOF: 4−hydroxybenzoate octaprenyltransferase activity
AGOC: integral to membrane, mitochondrion
AGOP: ubiquinone biosynthetic process
PGOF: prenyltransferase activity
PGOC: integral to membrane
PGOP: null

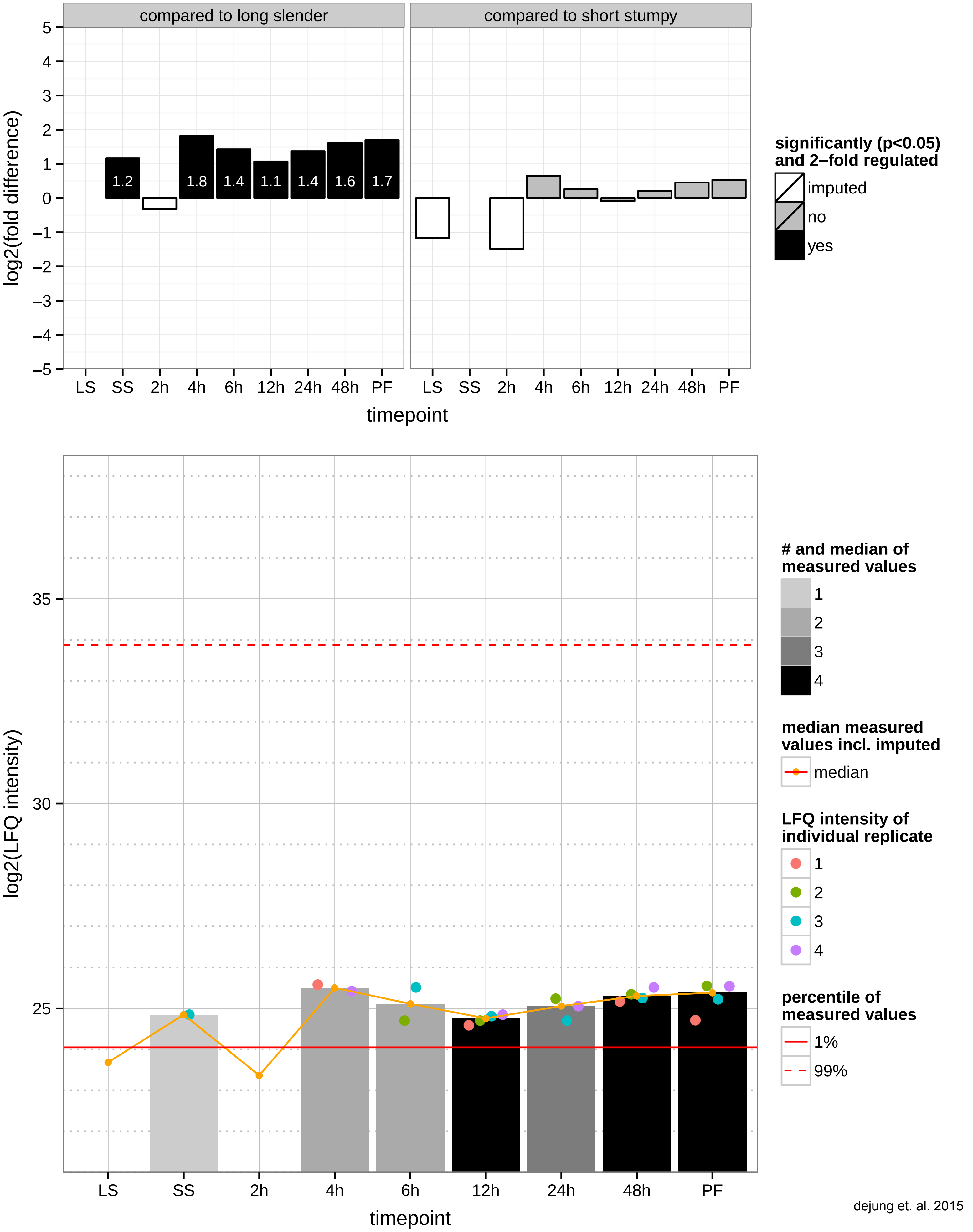

dejung et. al. 2015

DNA polymerase delta catalytic subunit, putative
Tb927.2.1800
AGOF: DNA binding, DNA−directed DNA polymerase activity, nucleotide binding
AGOC: null
AGOP: DNA replication
PGOF: DNA binding, DNA−directed DNA polymerase activity, nucleic acid binding, nucleotide binding
PGOC: null
PGOP: DNA replication, nucleobase−containing compound metabolic process

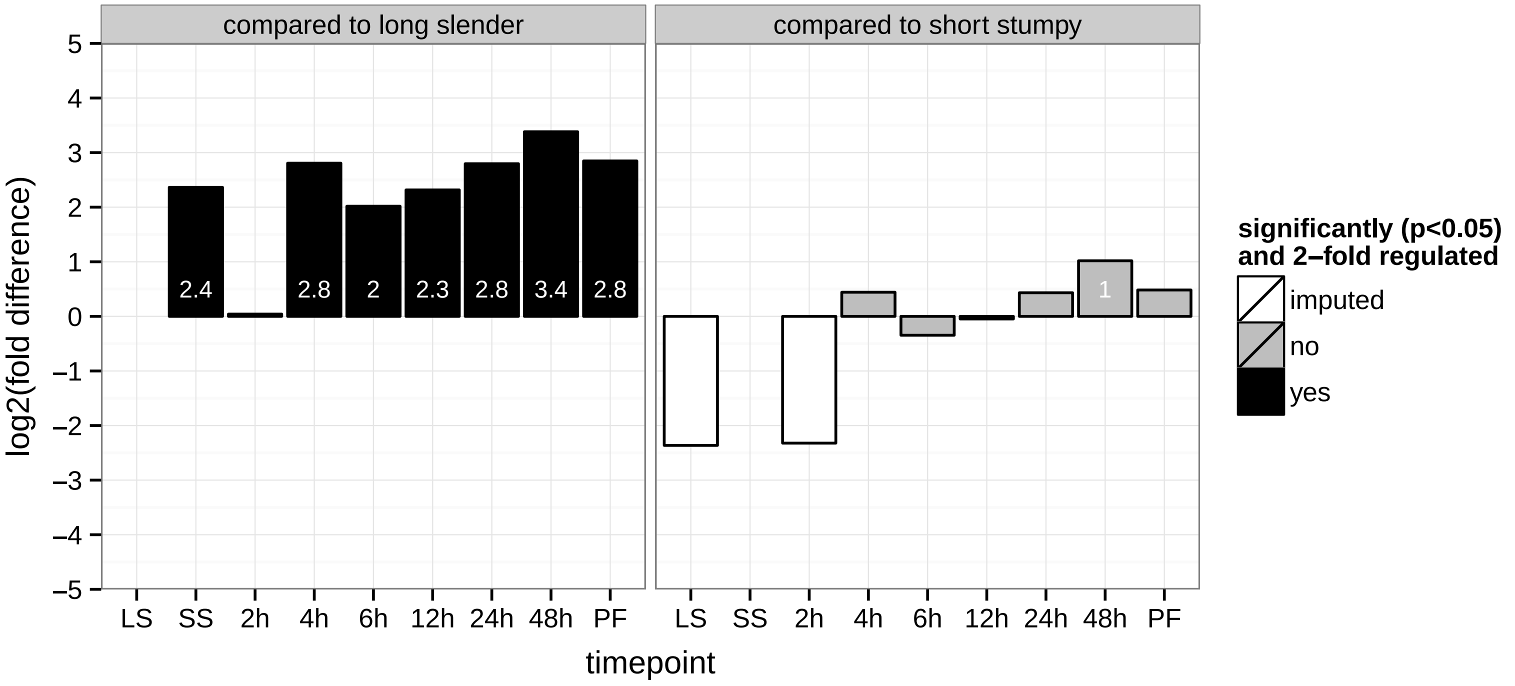

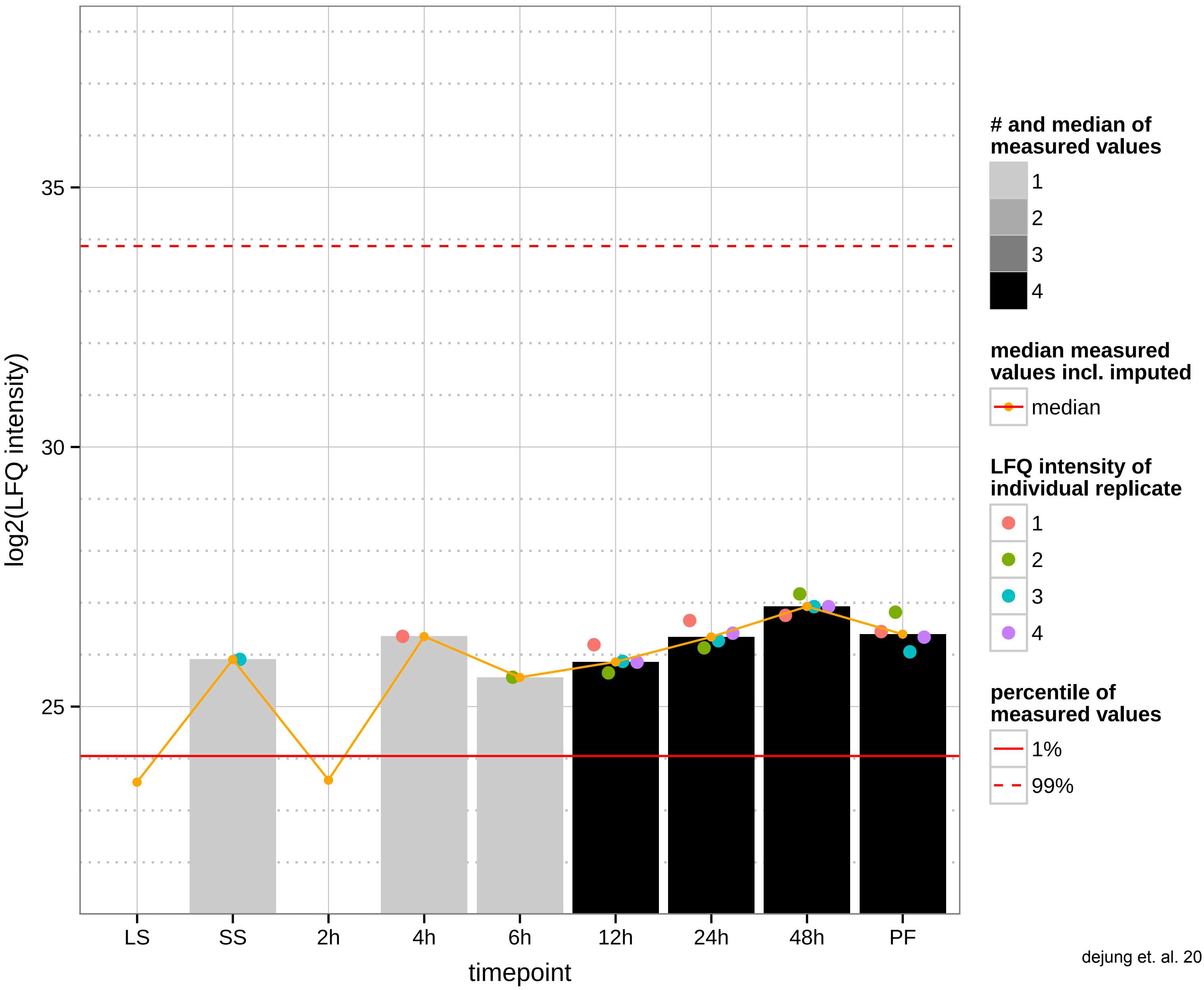

dejung et. al. 2015

hypothetical protein, conserved
Tb927.2.2370
AGOF: null
AGOC: null
AGOP: null
PGOF: null
PGOC: null
PGOP: null

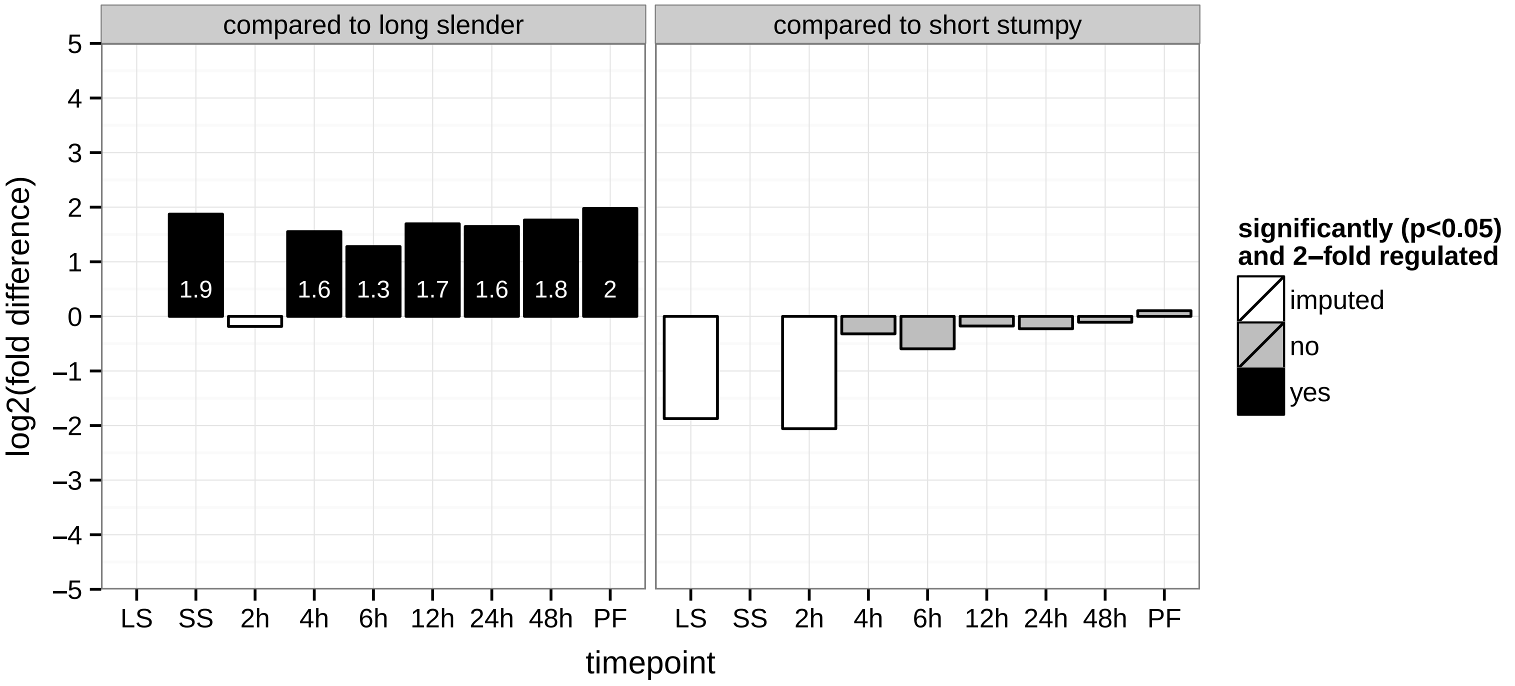

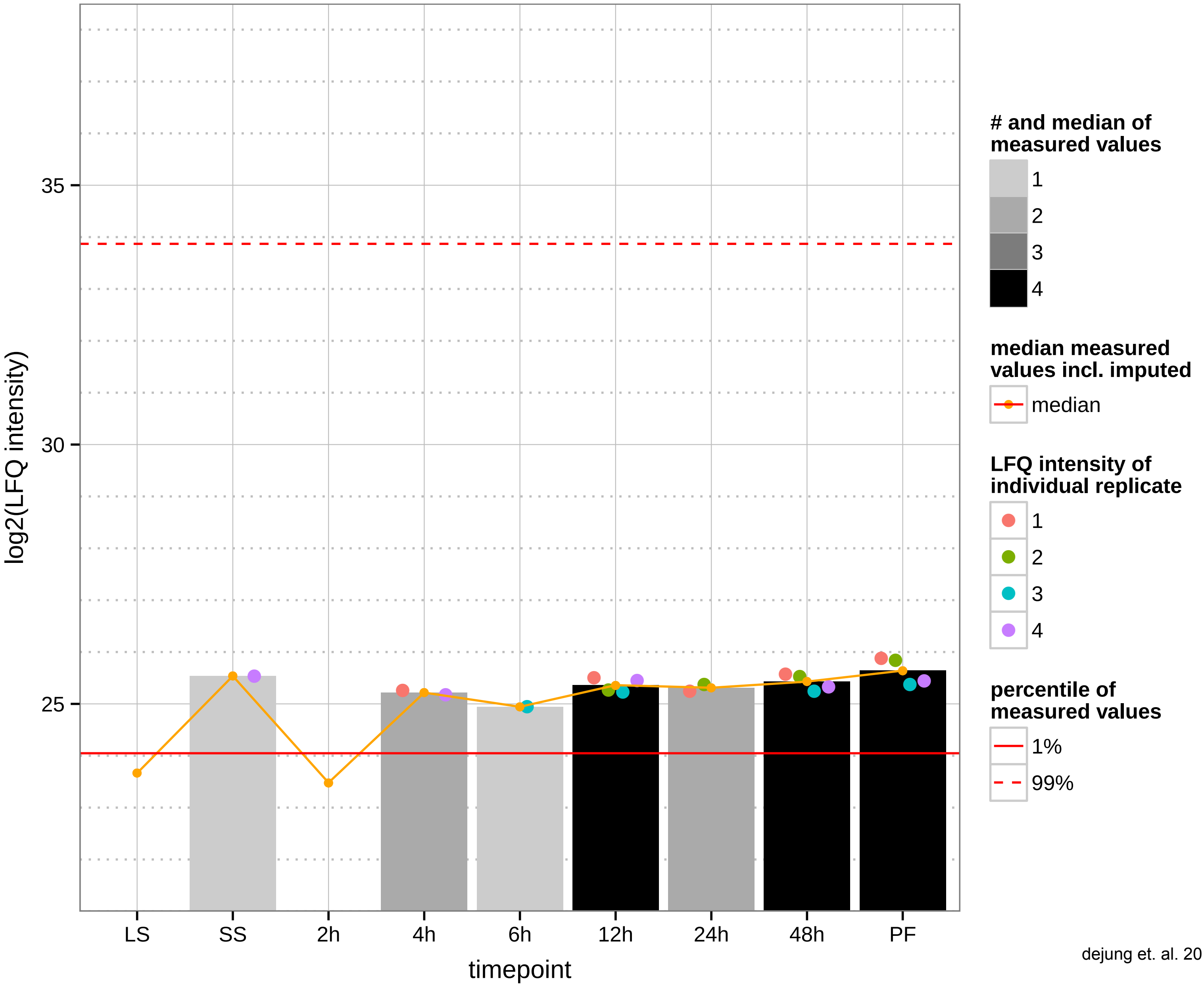

dejung et. al. 2015

hypothetical protein, conserved
Tb927.2.6080
AGOF: null
AGOC: null
AGOP: null
PGOF: null
PGOC: null
PGOP: null

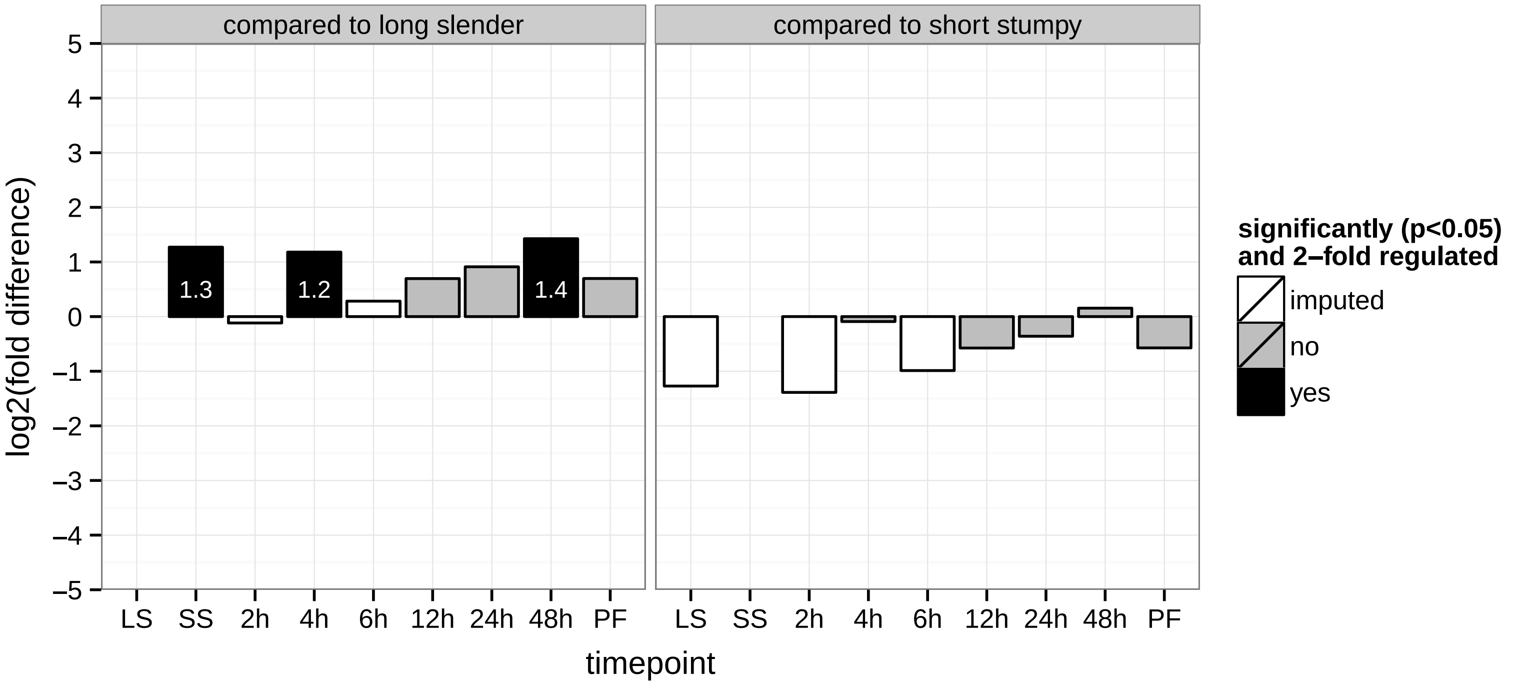

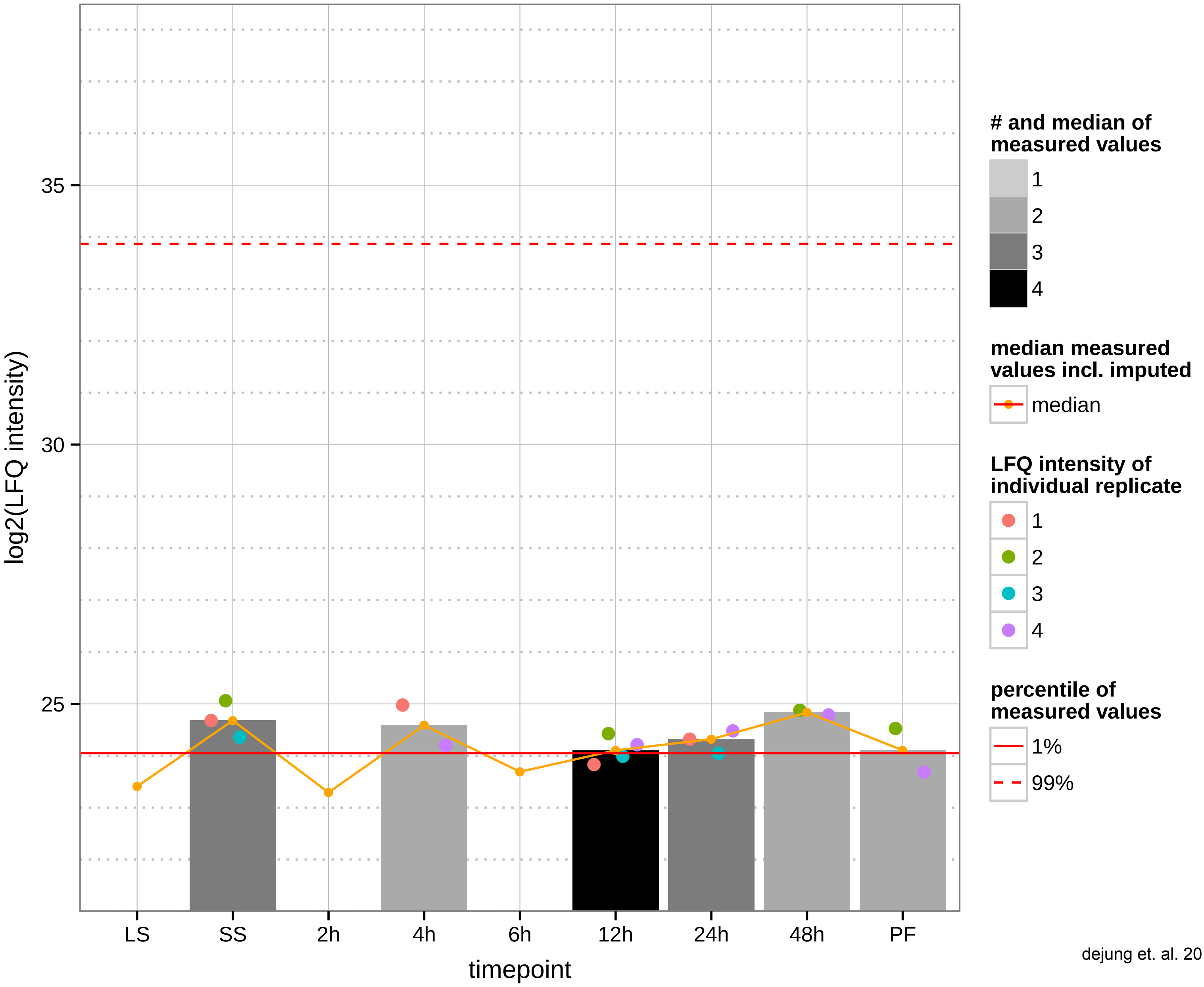

dejung et. al. 2015

hypothetical protein, conserved
Tb927.3.1100
AGOF: exonuclease activity, nucleic acid binding
AGOC: intracellular, mitochondrion
AGOP: null
PGOF: exonuclease activity, nucleic acid binding
PGOC: intracellular
PGOP: null

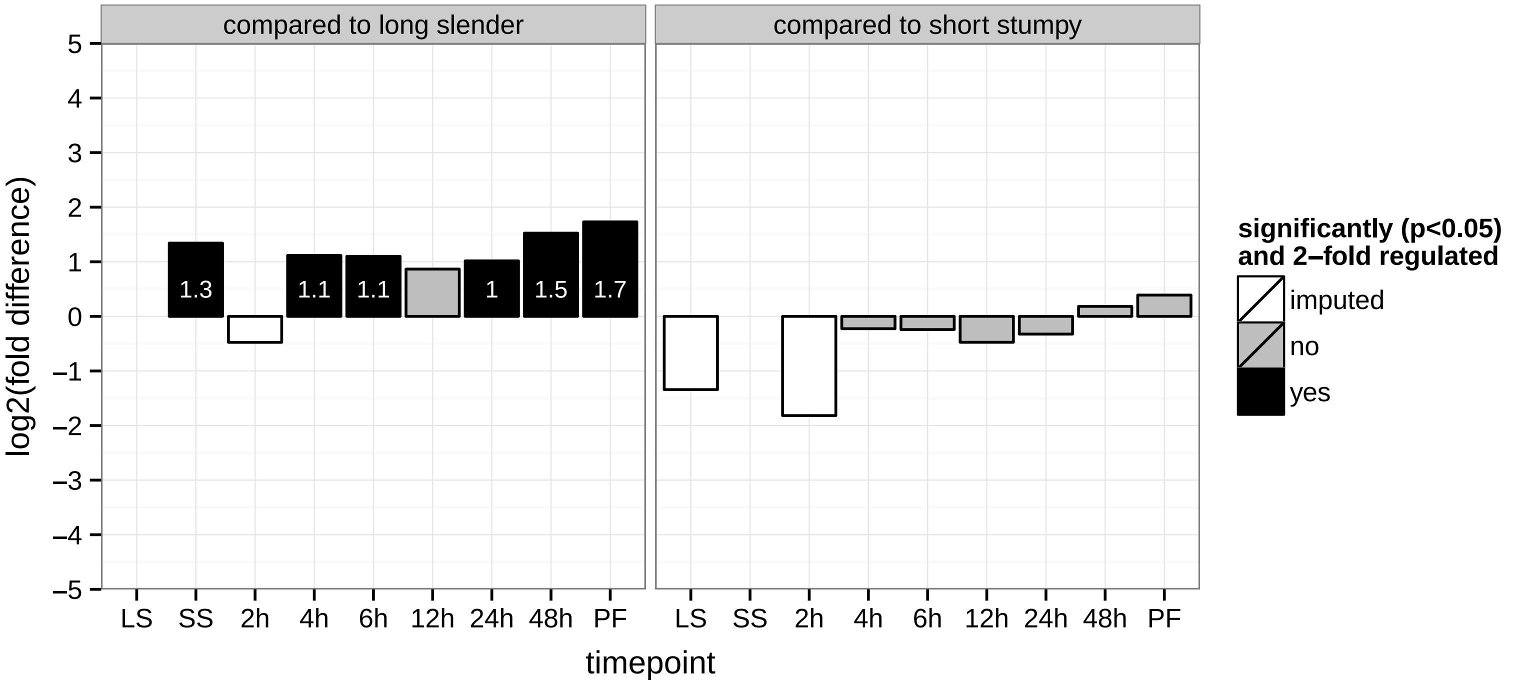

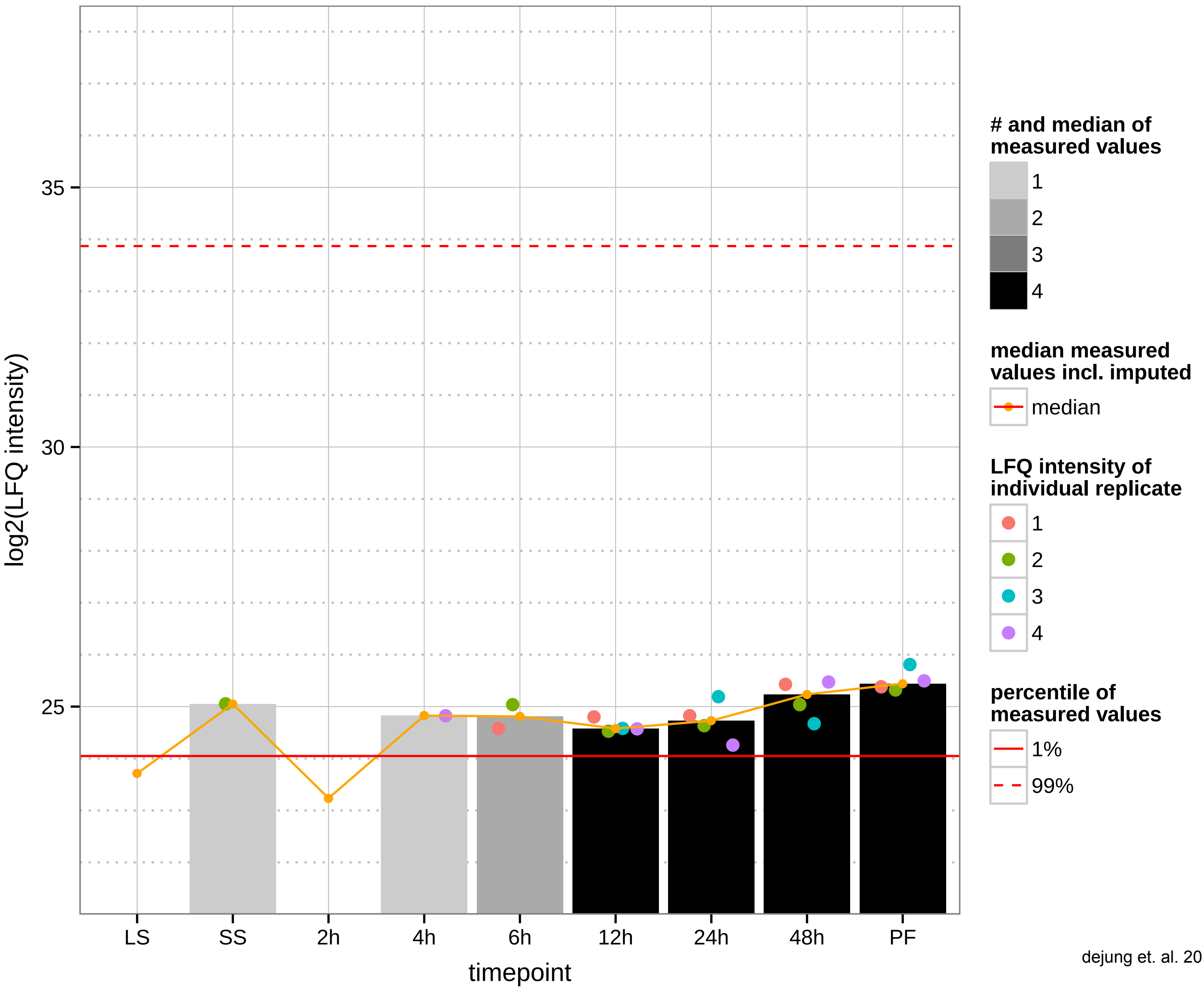

dejung et. al. 2015

hypothetical protein, conserved
Tb927.3.2300
AGOF: null
AGOC: mitochondrion
AGOP: null
PGOF: zinc ion binding
PGOC: null
PGOP: null

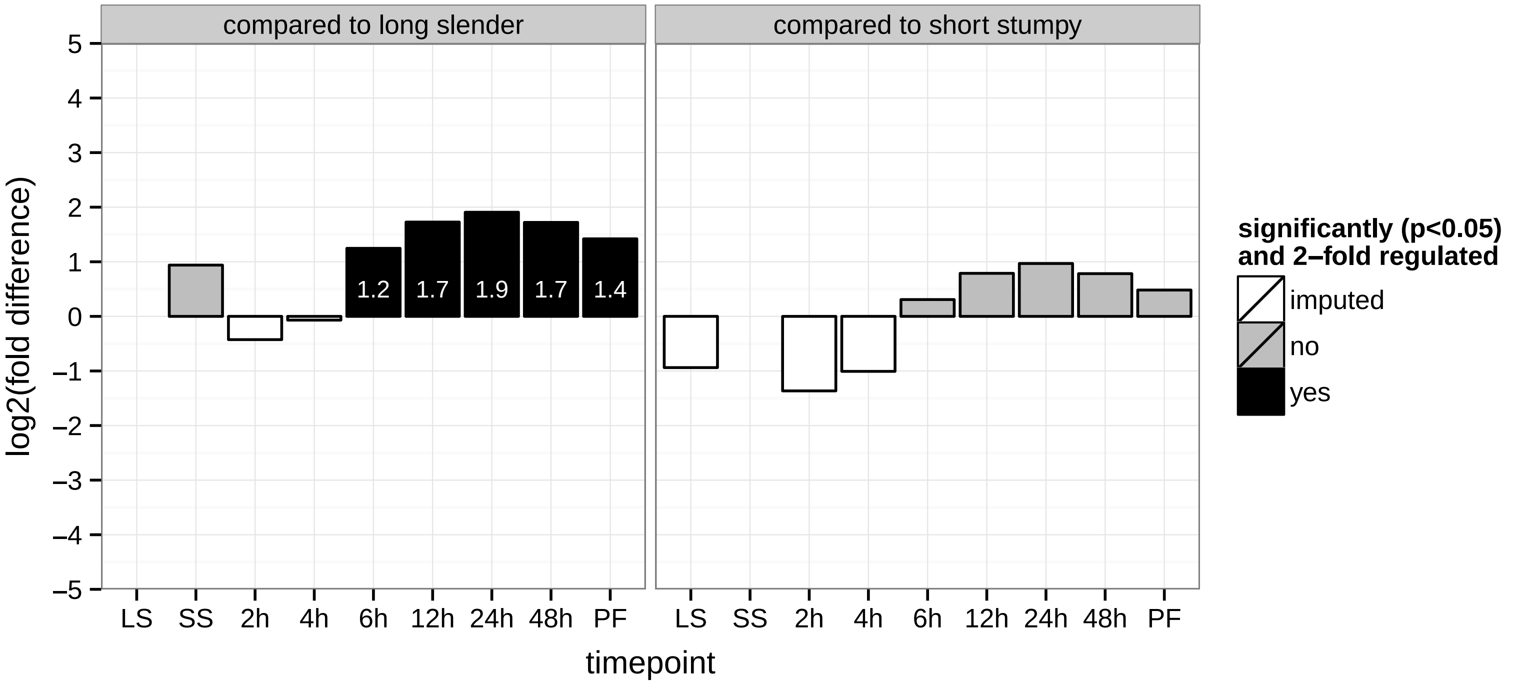

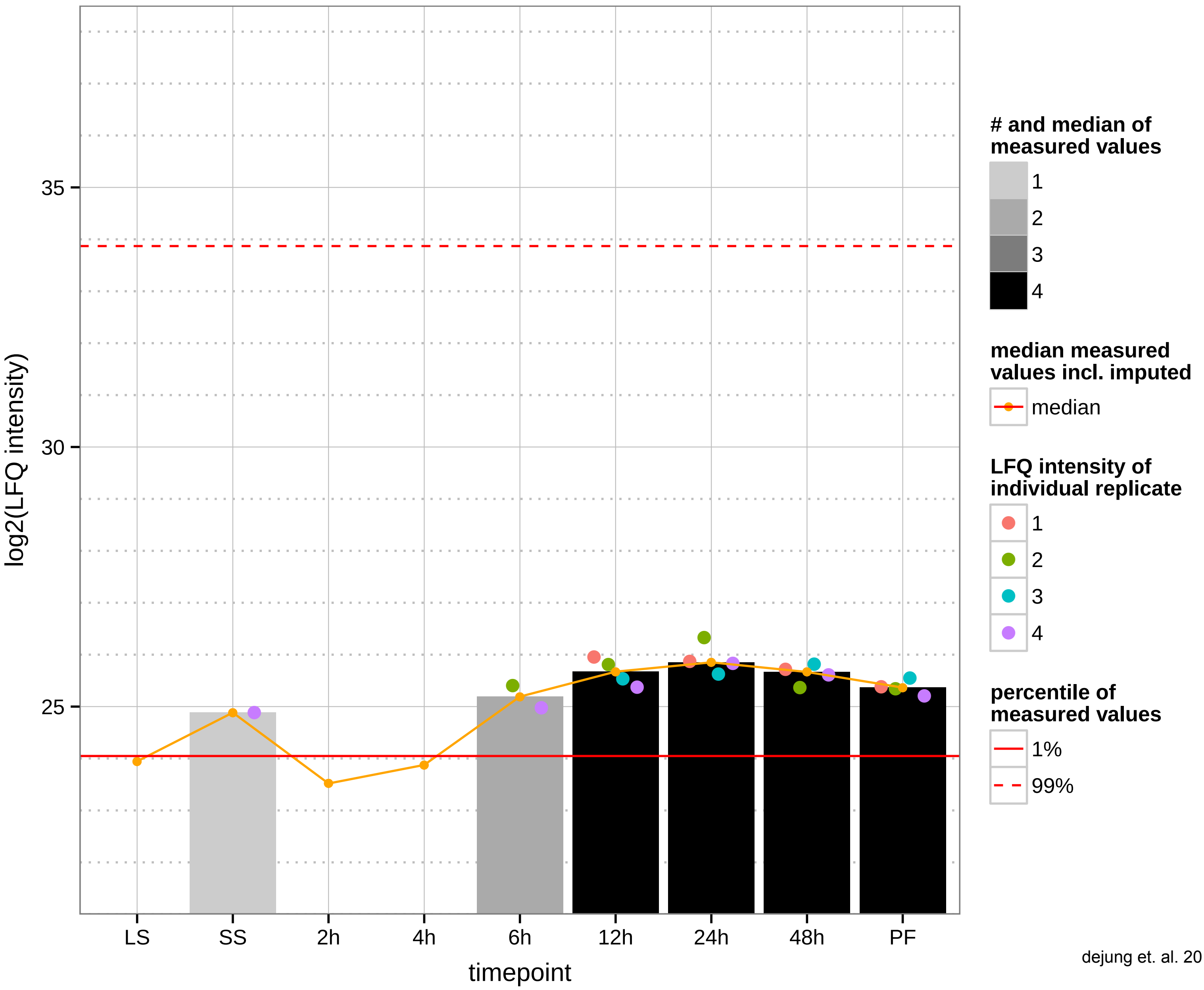

dejung et. al. 2015

ubiquinone biosynthesis protein COQ7 homolog, putative
Tb927.3.4890
AGOF: null
AGOC: mitochondrion
AGOP: protein metabolic process, ubiquinone biosynthetic process
PGOF: null
PGOC: null
PGOP: oxidation−reduction process, ubiquinone biosynthetic process

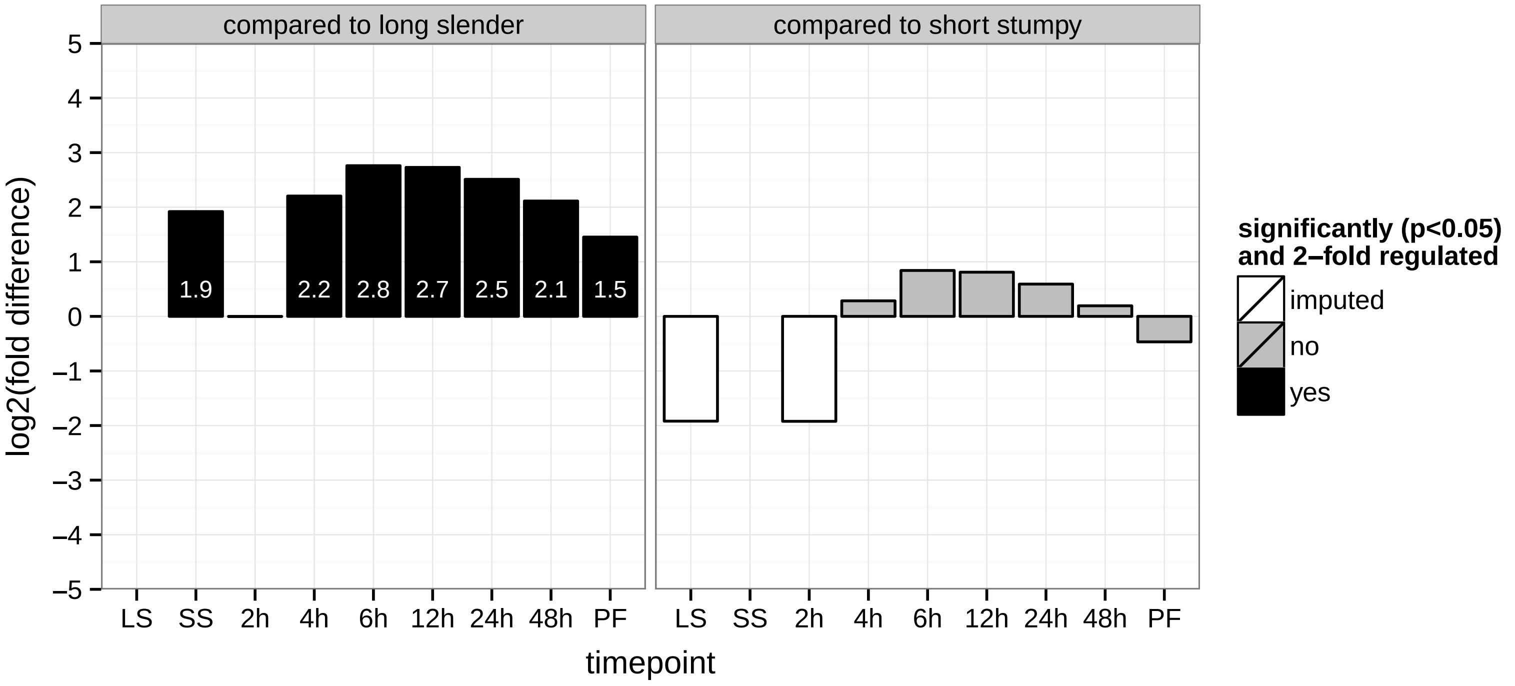

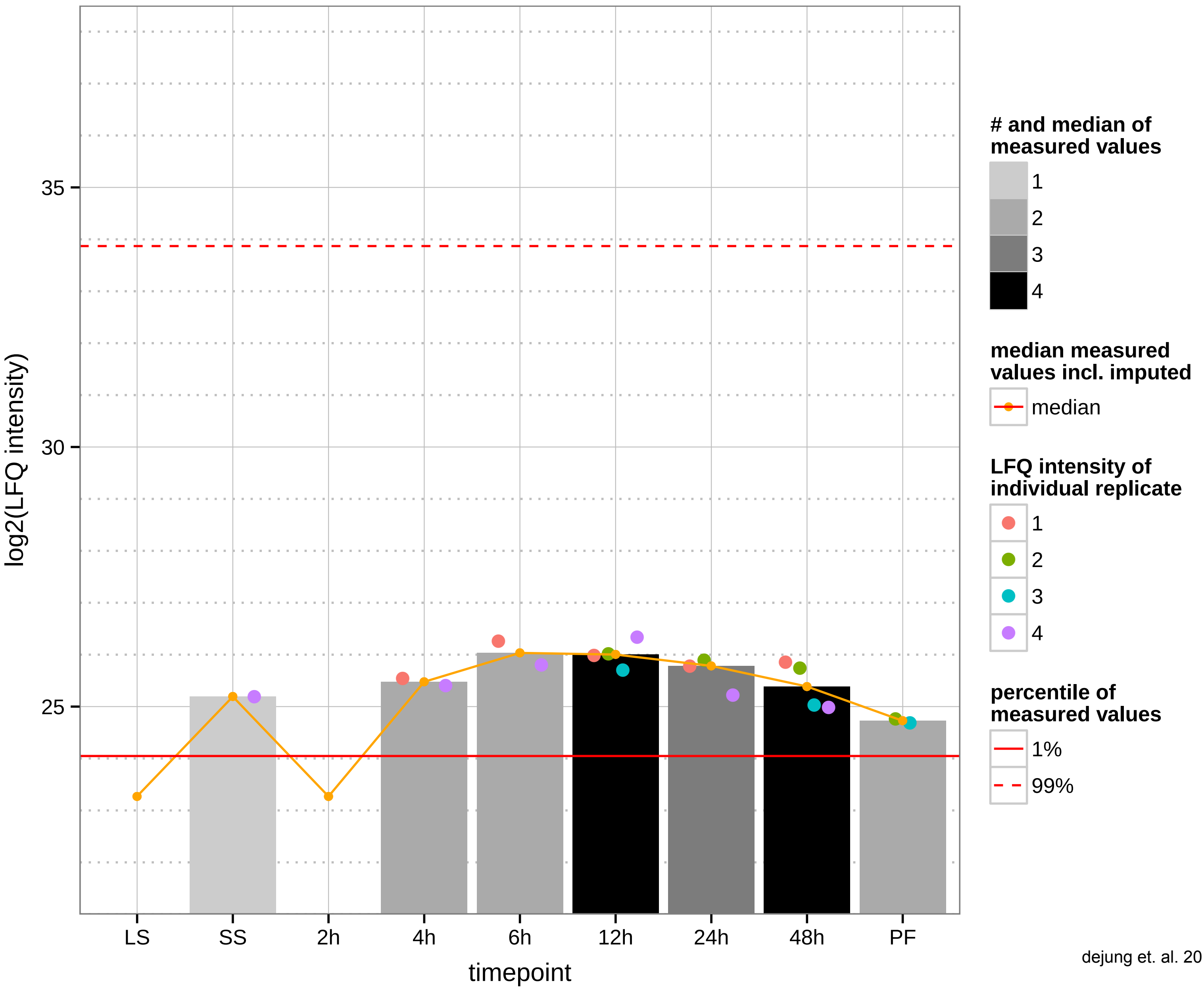

dejung et. al. 2015

centrin, putative (centrin1)
Tb927.4.2260
AGOF: calcium ion binding
AGOC: centrosome, mitochondrion
AGOP: null
PGOF: calcium ion binding
PGOC: null
PGOP: null

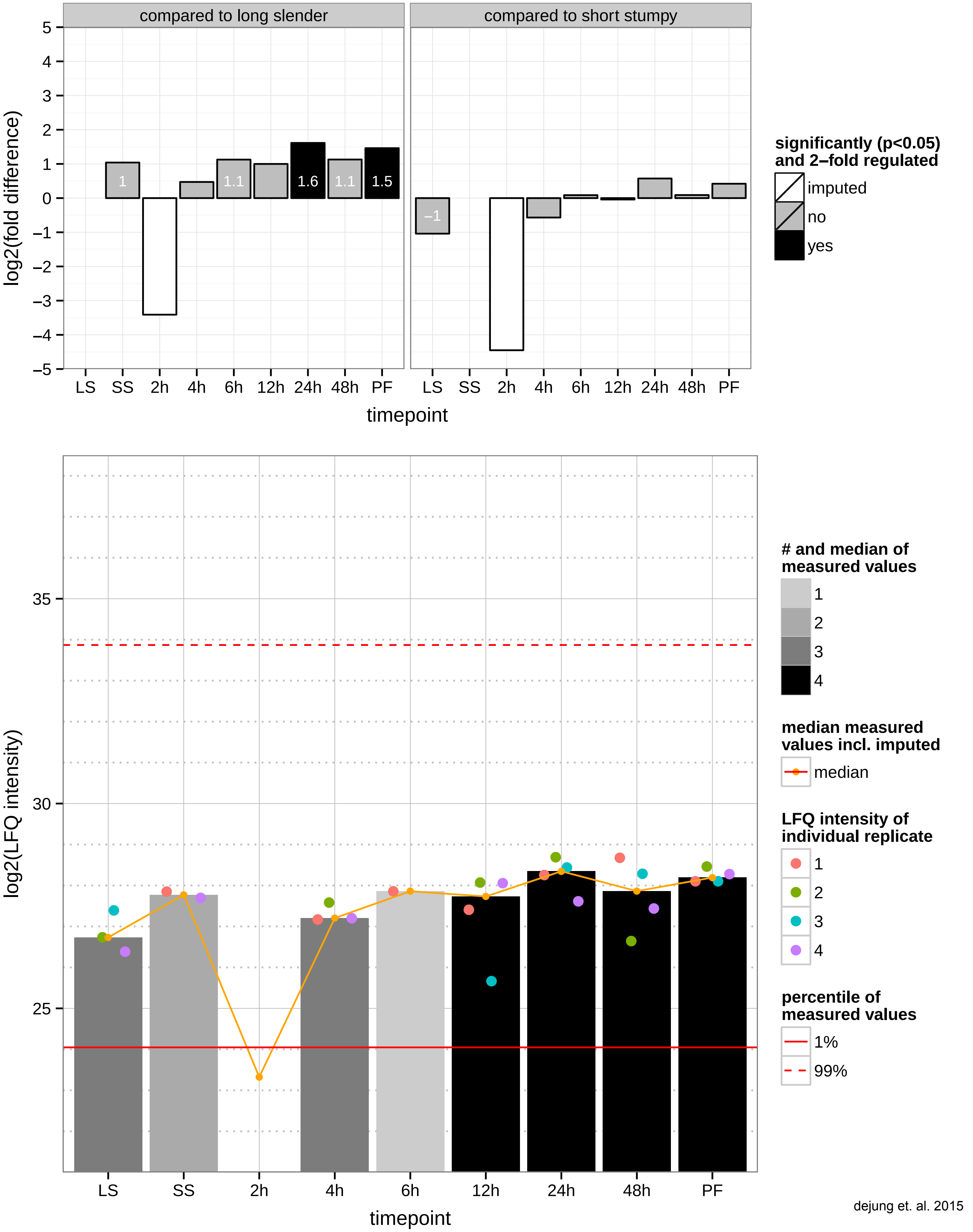

dejung et. al. 2015

protein phosphatase 1 (PP1)
Tb927.4.5030
AGOF: protein serine/threonine phosphatase activity
AGOC: nucleus
AGOP: null
PGOF: hydrolase activity
PGOC: null
PGOP: null

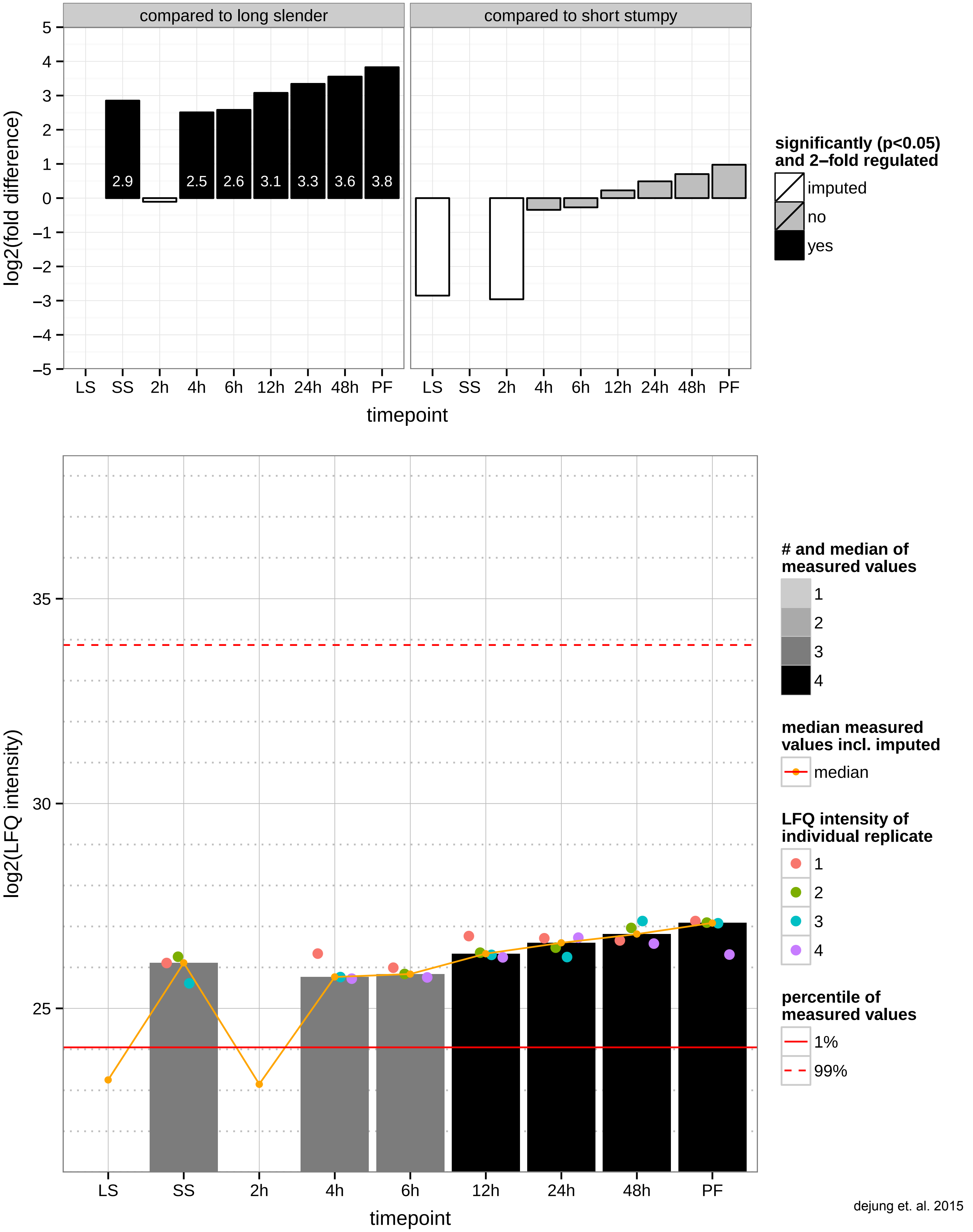

dejung et. al. 2015

geranylgeranyl transferase type II beta subunit, putative
Tb927.4.690
AGOF: catalytic activity
AGOC: null
AGOP: null
PGOF: catalytic activity
PGOC: null
PGOP: null

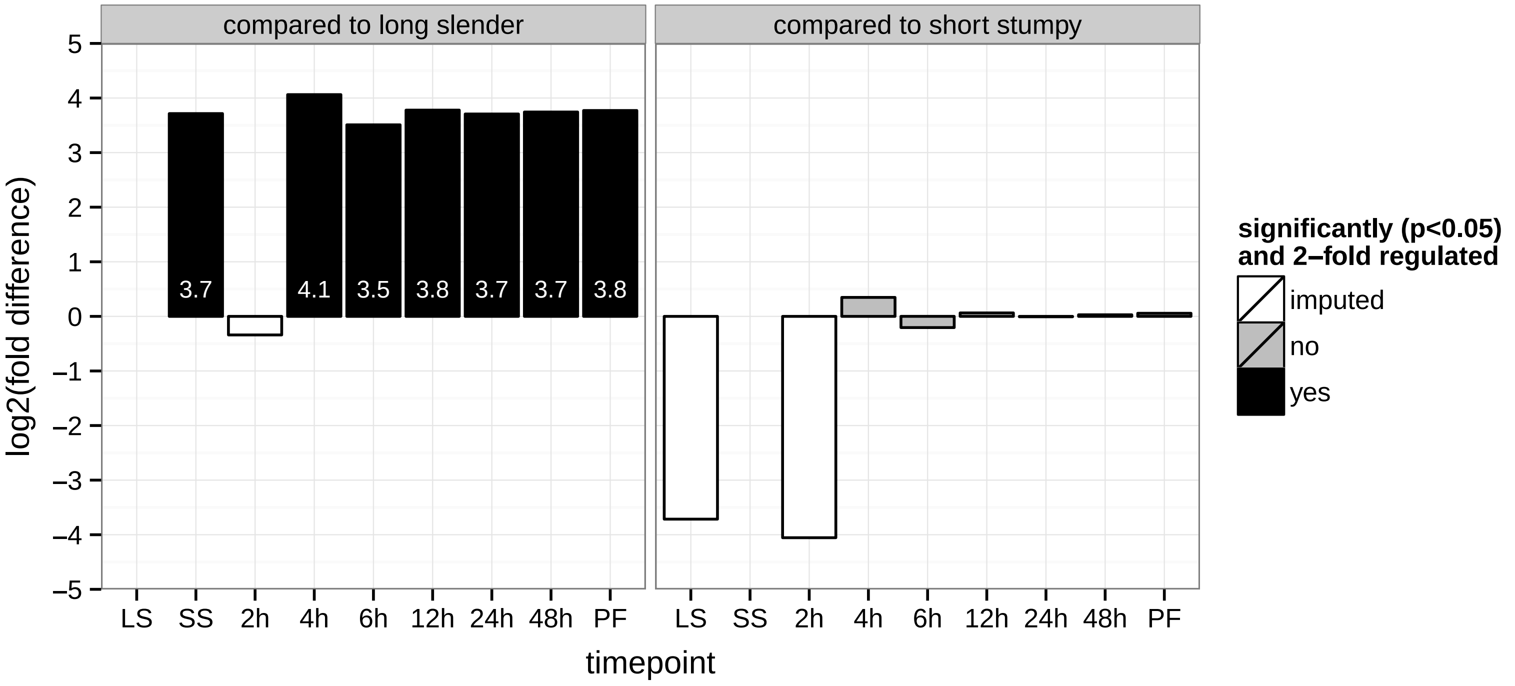

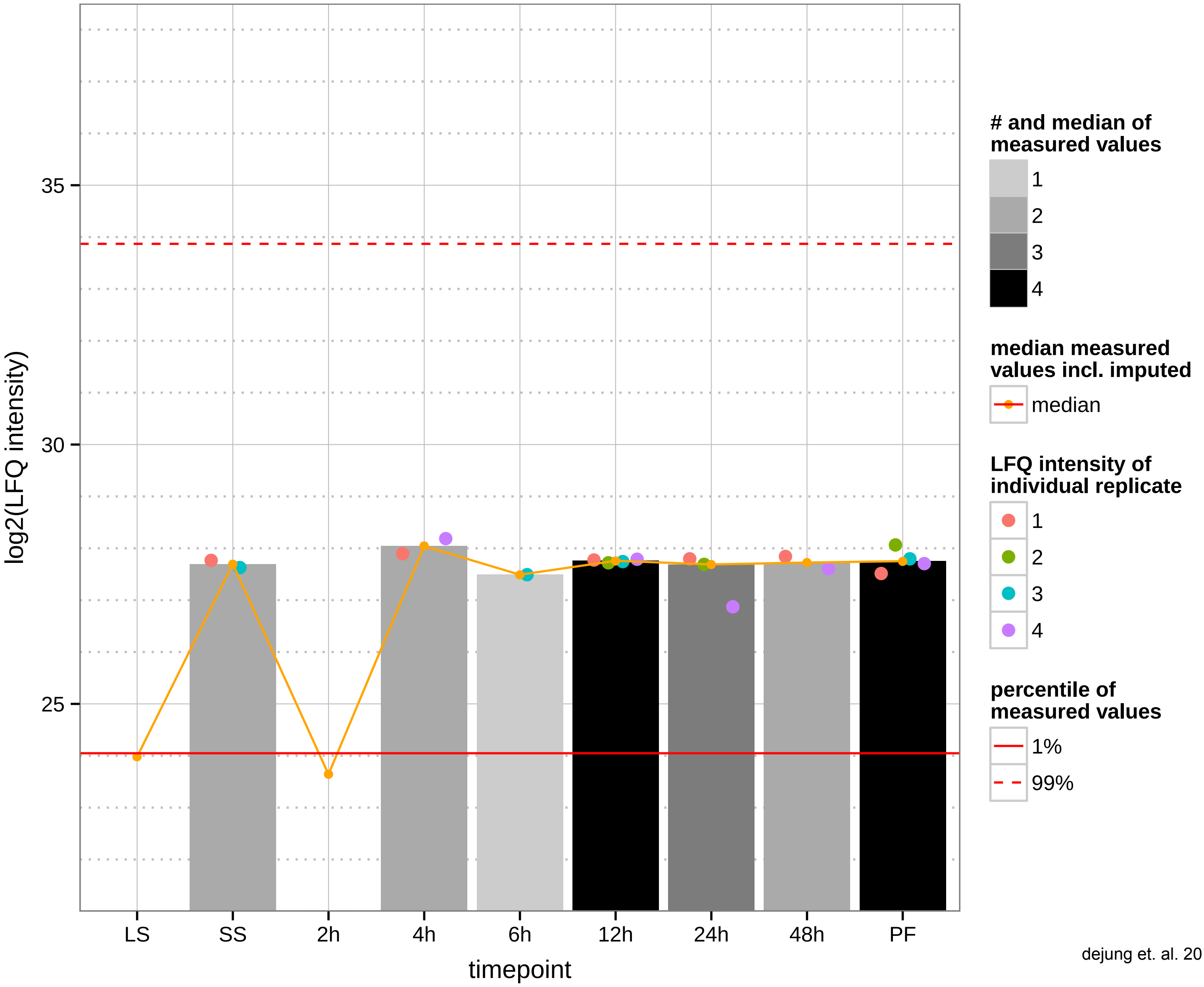

dejung et. al. 2015

hypothetical protein, conserved
Tb927.5.2070
AGOF: null
AGOC: mitochondrion
AGOP: null
PGOF: null
PGOC: null
PGOP: null

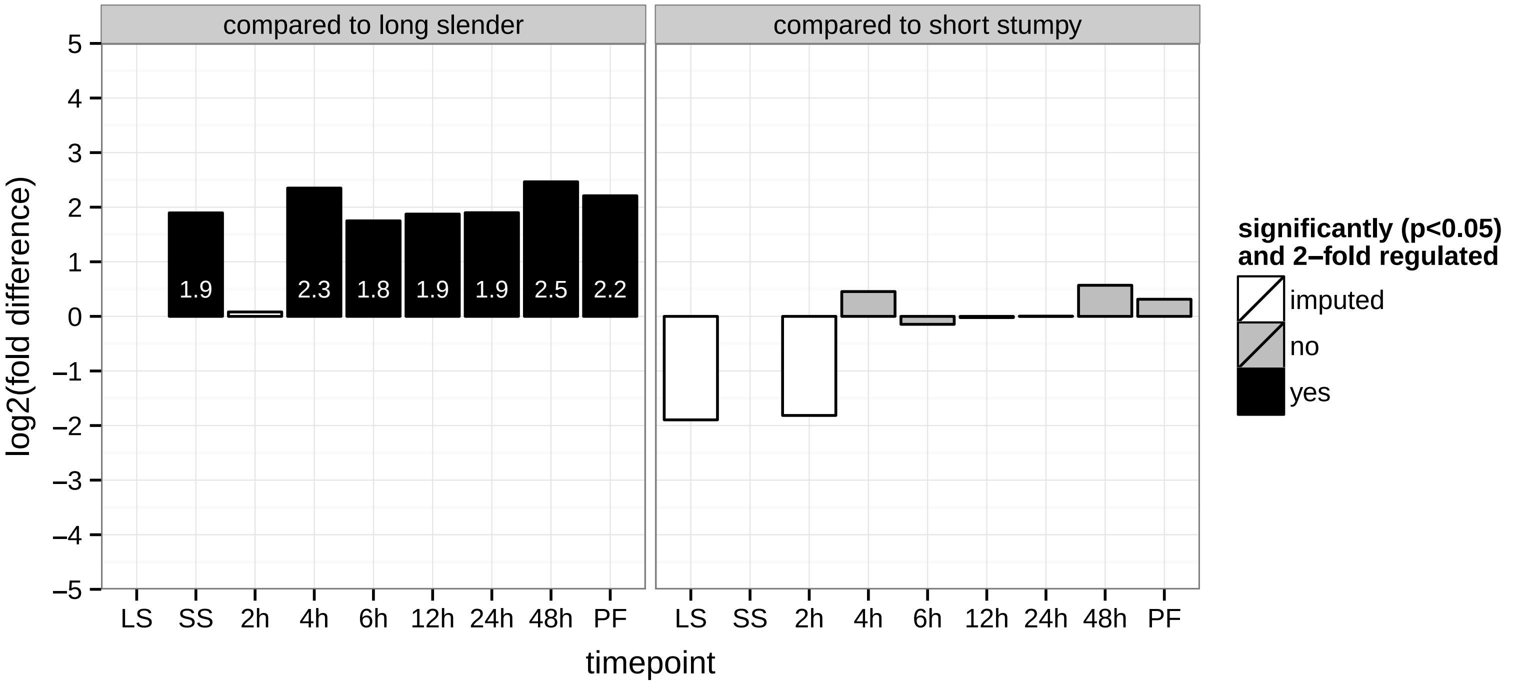

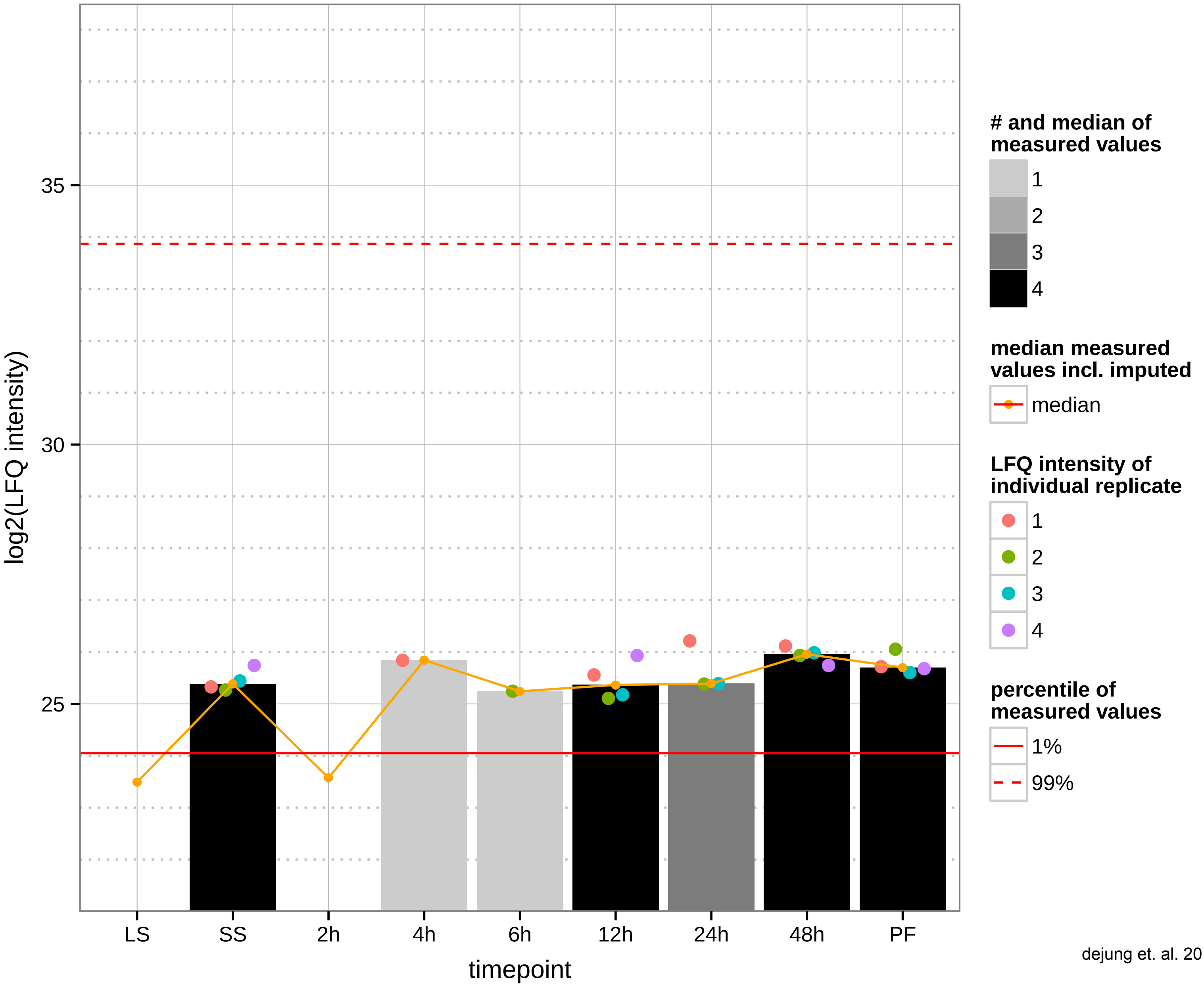

dejung et. al. 2015

hypothetical protein, conserved
Tb927.5.3240
AGOF: null
AGOC: null
AGOP: null
PGOF: null
PGOC: null
PGOP: null

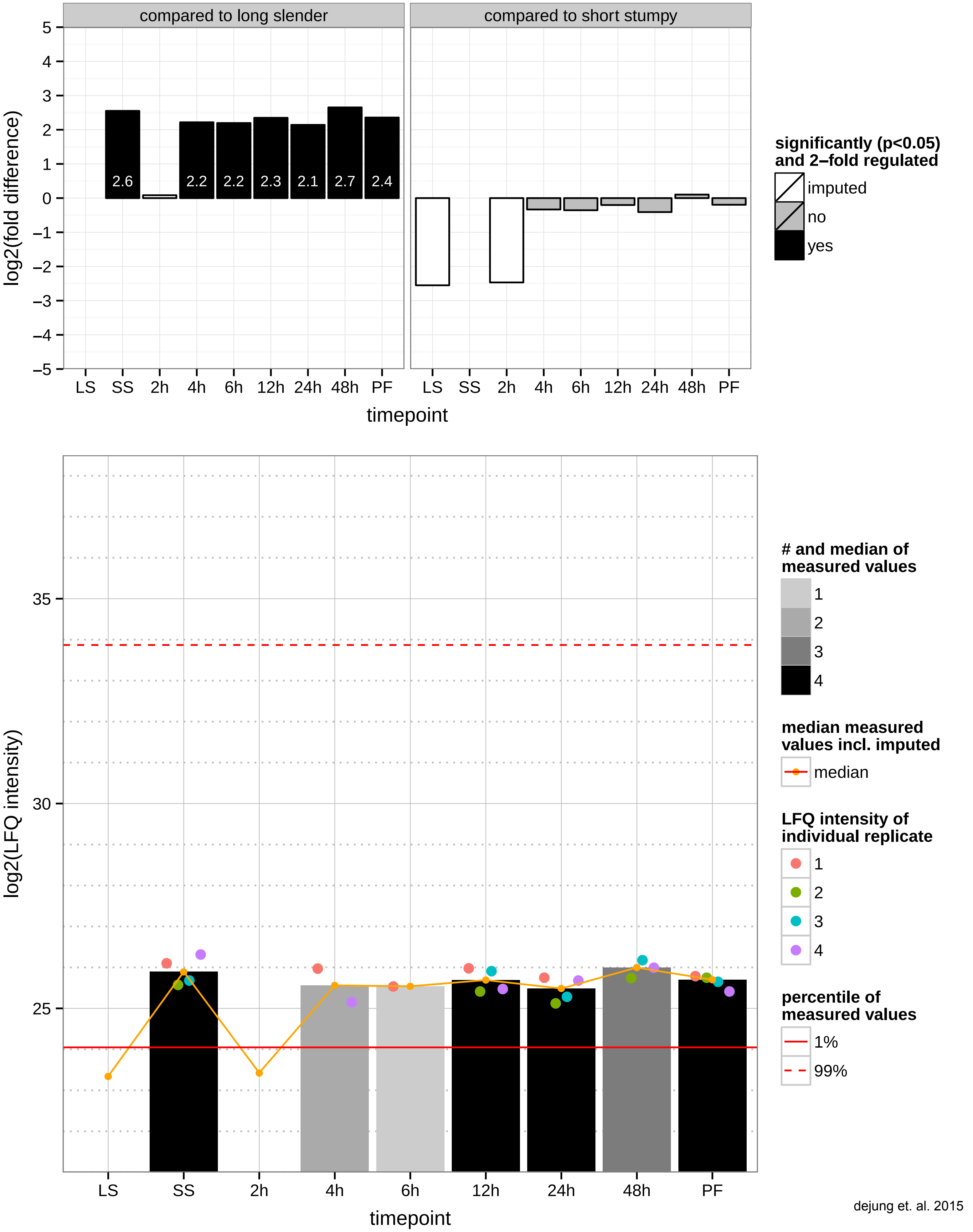

dejung et. al. 2015

hypothetical protein, conserved
Tb927.6.1830
AGOF: null
AGOC: null
AGOP: null
PGOF: protein binding
PGOC: null
PGOP: null

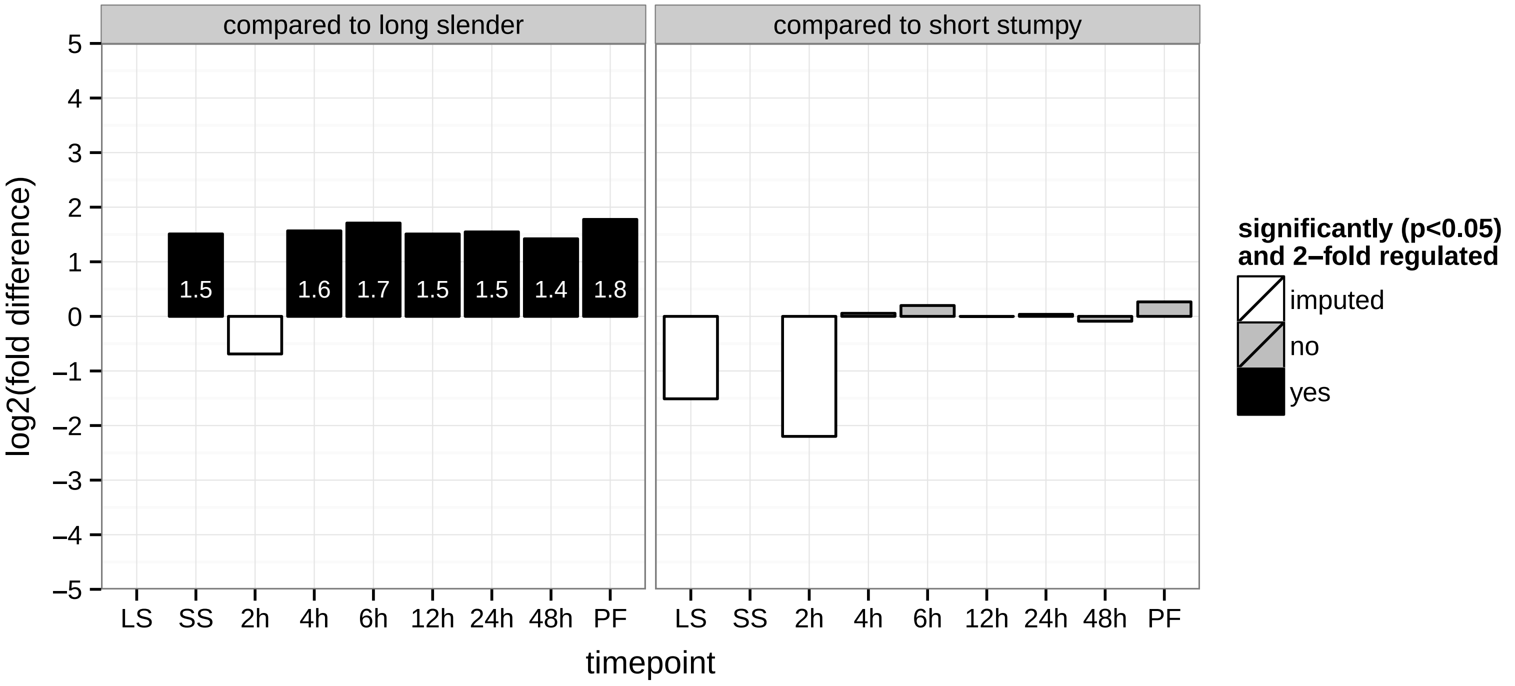

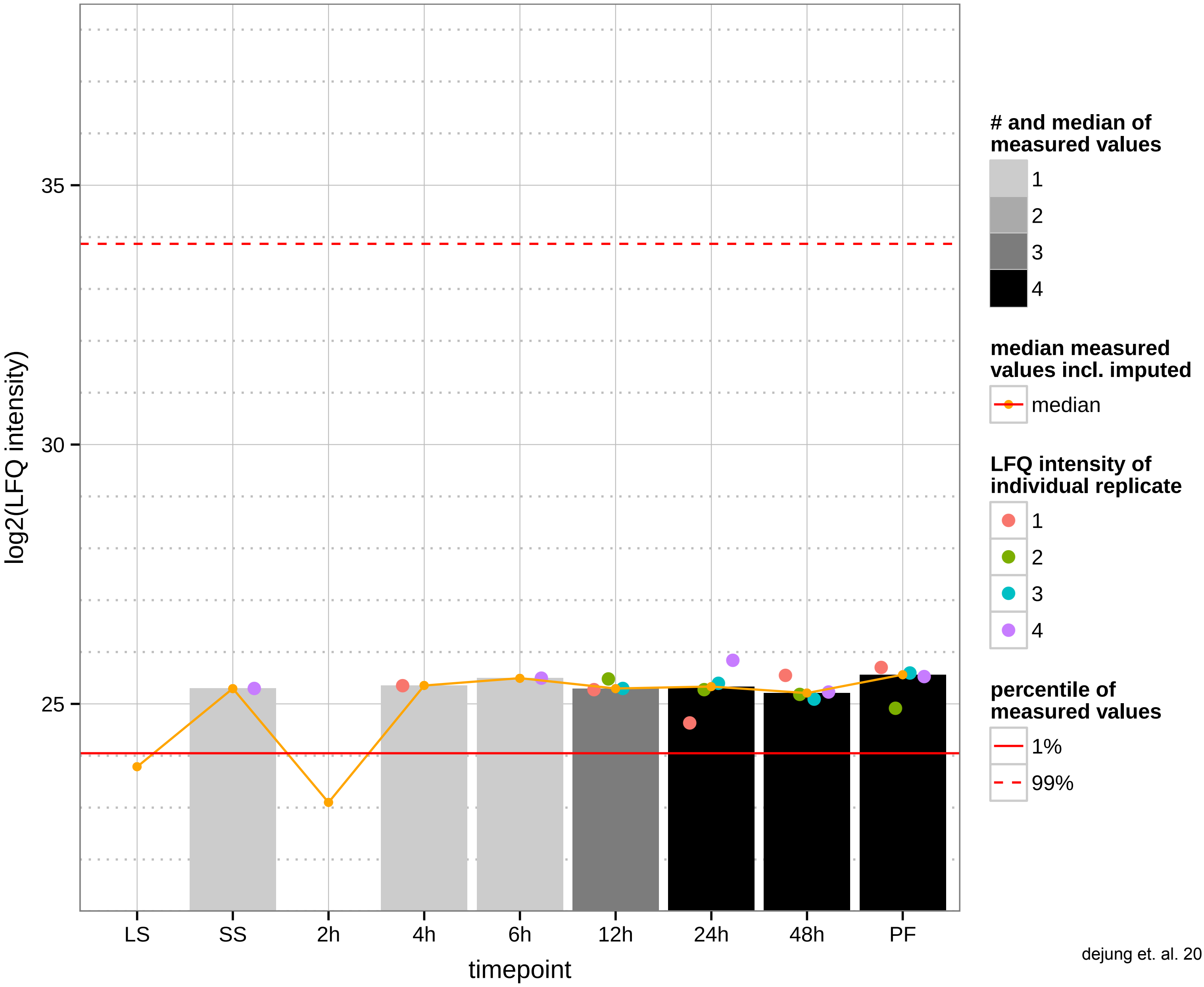

dejung et. al. 2015

hypothetical protein, conserved
Tb927.7.1530
AGOF: oxidoreductase activity
AGOC: null
AGOP: null
PGOF: oxidoreductase activity, oxidoreductase activity, acting on paired donors, with incorporation or reduction of molecular o
PGOC: null
PGOP: oxidation–reduction process

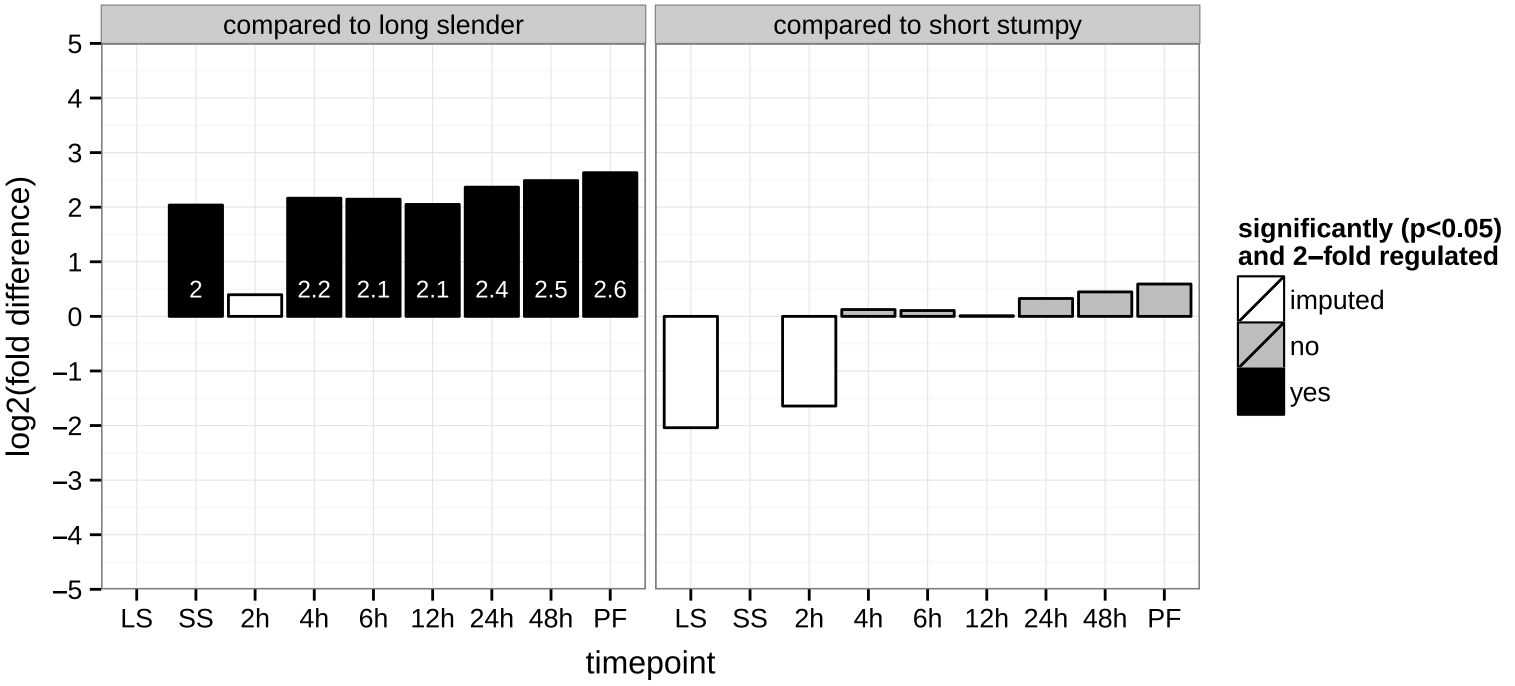

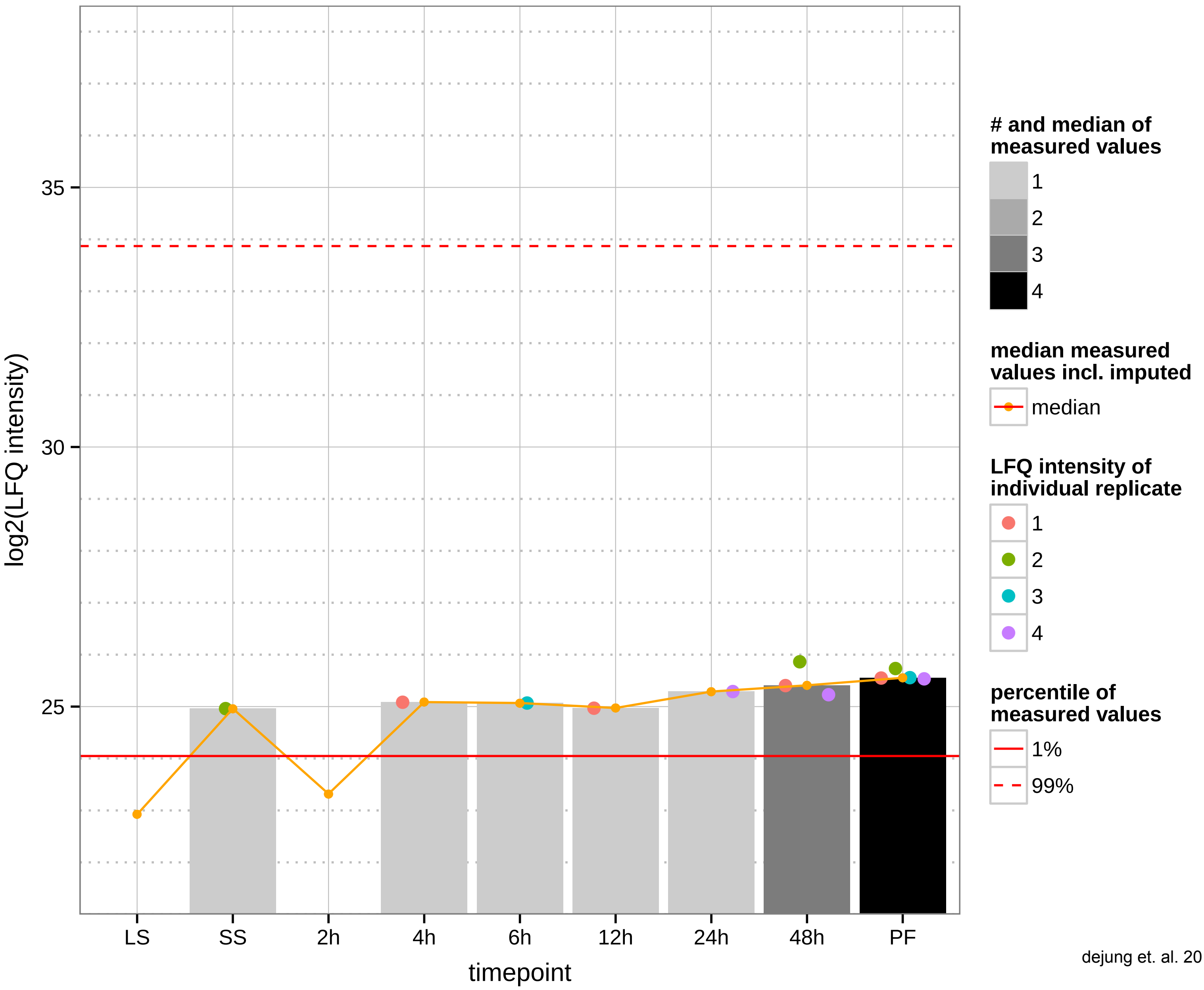

dejung et. al. 2015

hypothetical protein, conserved
Tb927.7.5750
AGOF: null
AGOC: null
AGOP: null
PGOF: null
PGOC: null
PGOP: null

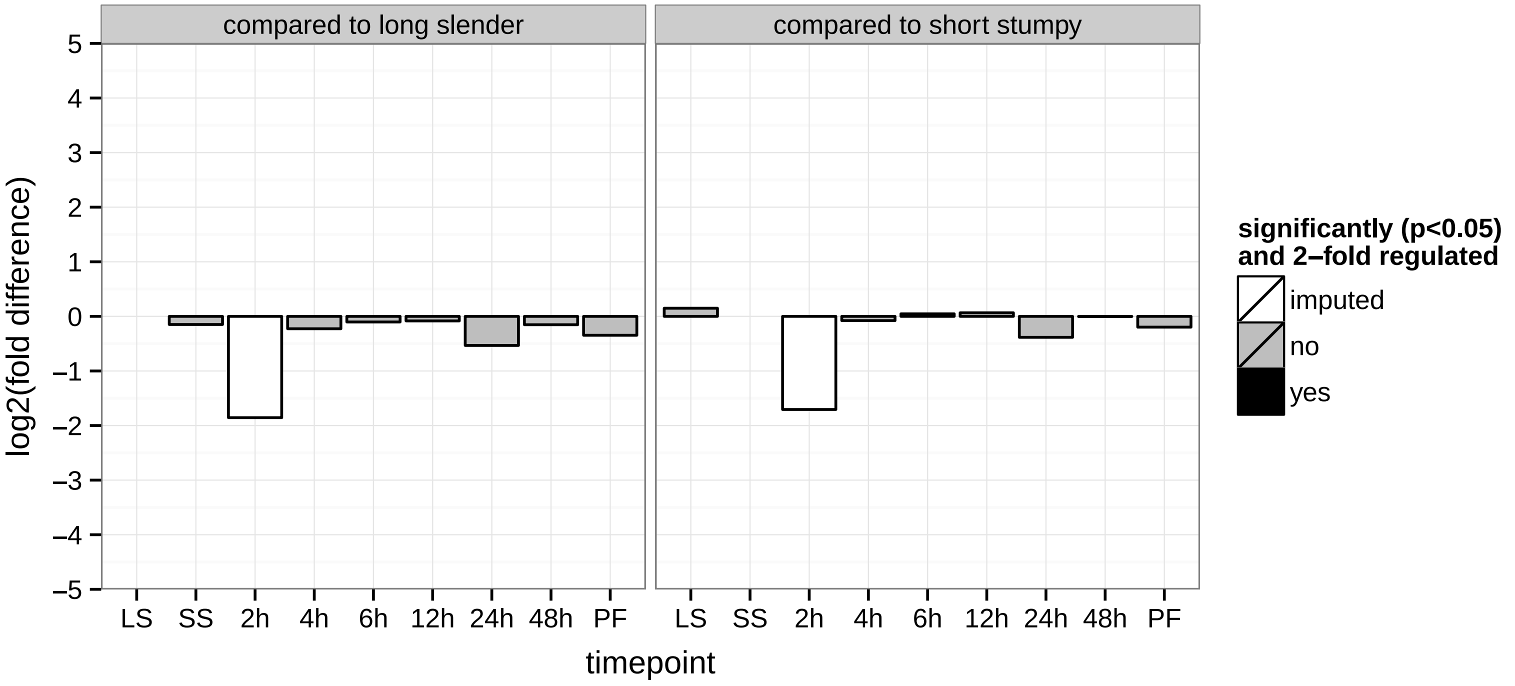

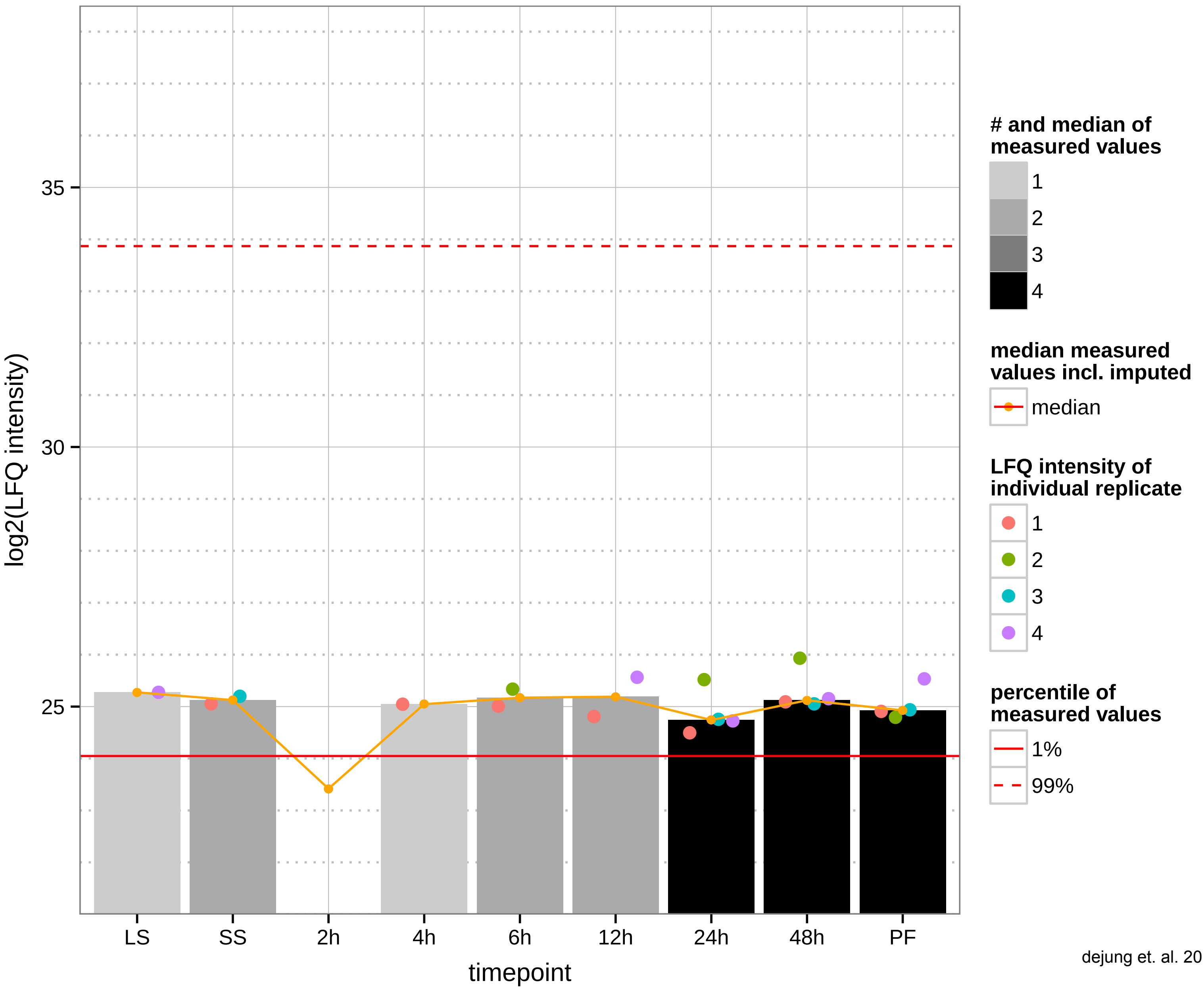

dejung et. al. 2015

telomerase–associated protein, putative
Tb927.7.830
AGOF: RNA binding
AGOC: ribonucleoprotein complex
AGOP: null
PGOF: RNA binding, protein binding
PGOC: ribonucleoprotein complex
PGOP: null

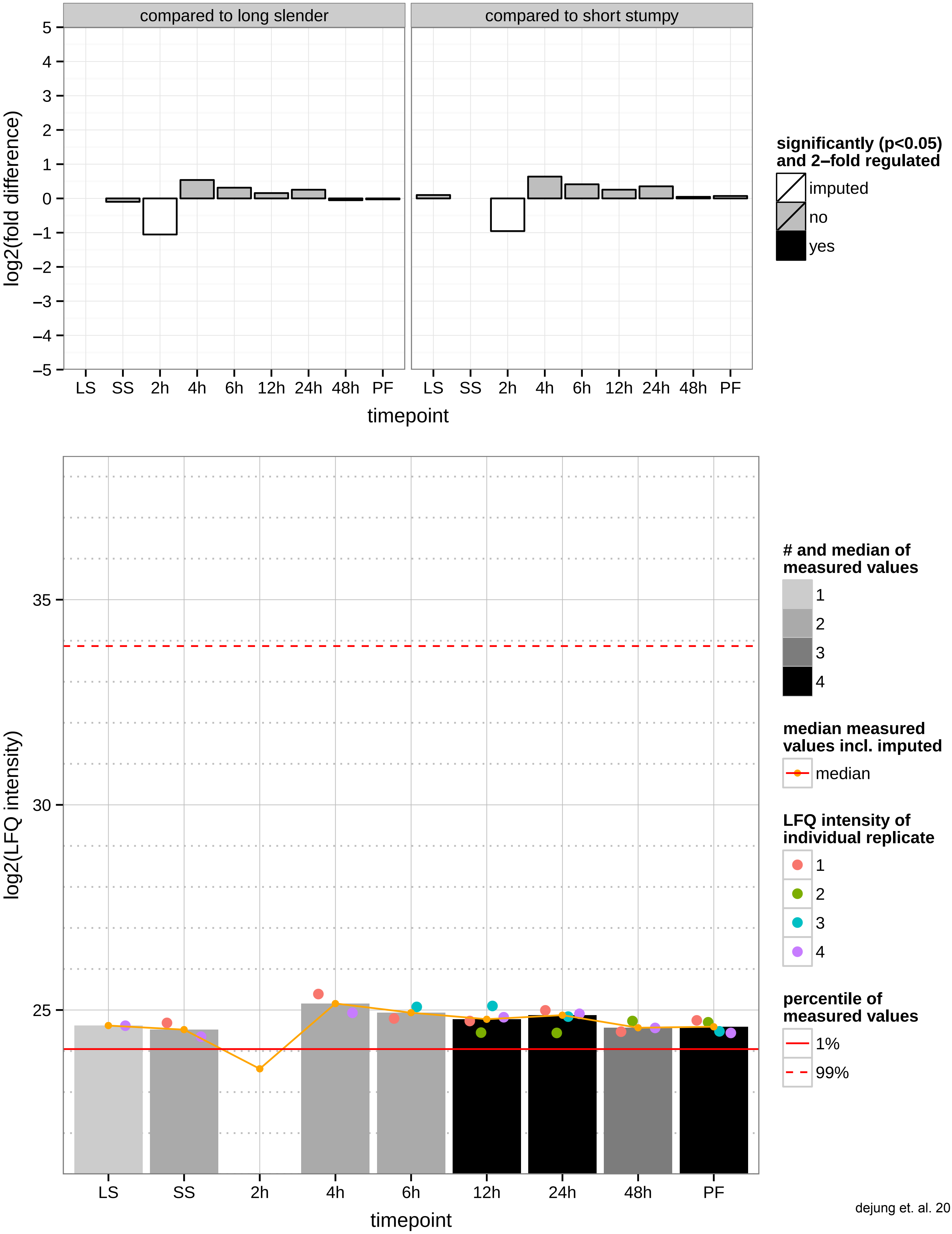

dejung et. al. 2015

hypothetical protein, conserved
Tb927.8.5650
AGOF: null
AGOC: null
AGOP: null
PGOF: null
PGOC: null
PGOP: null

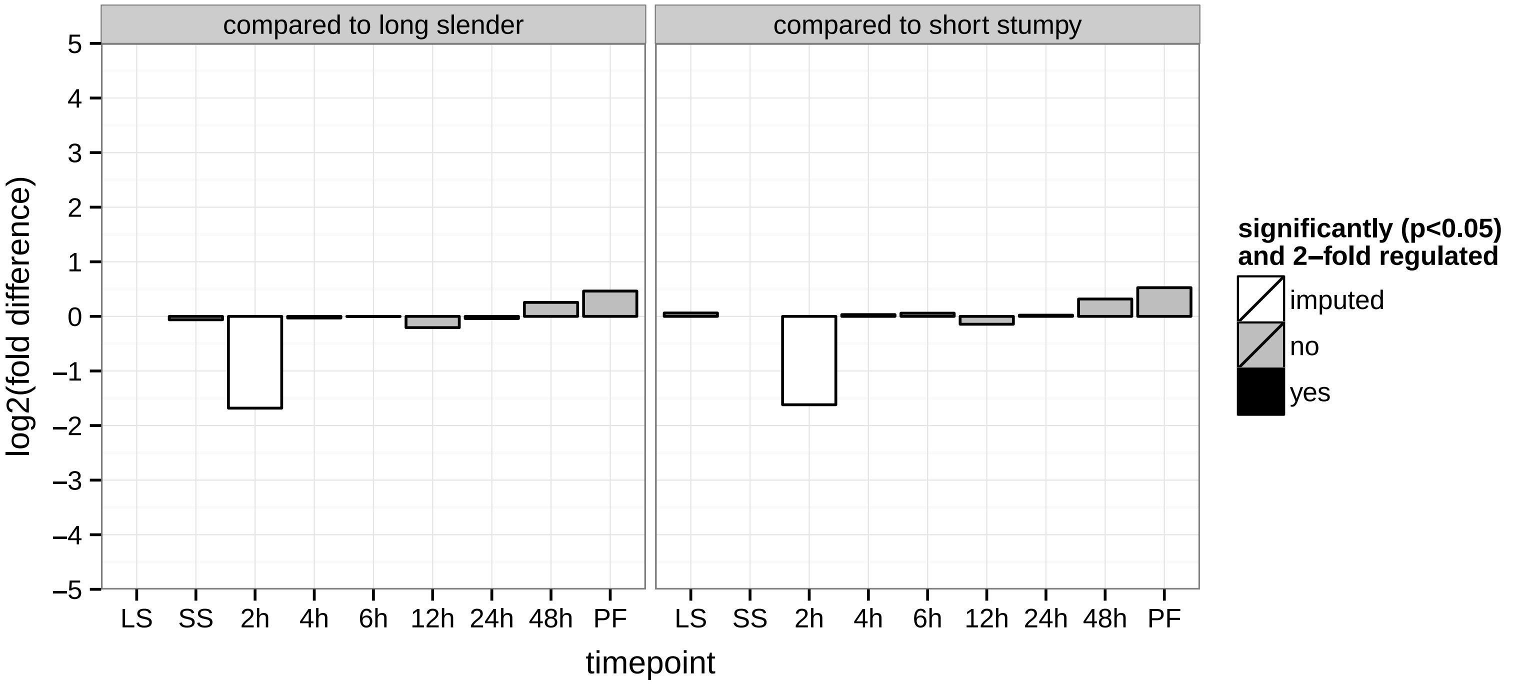

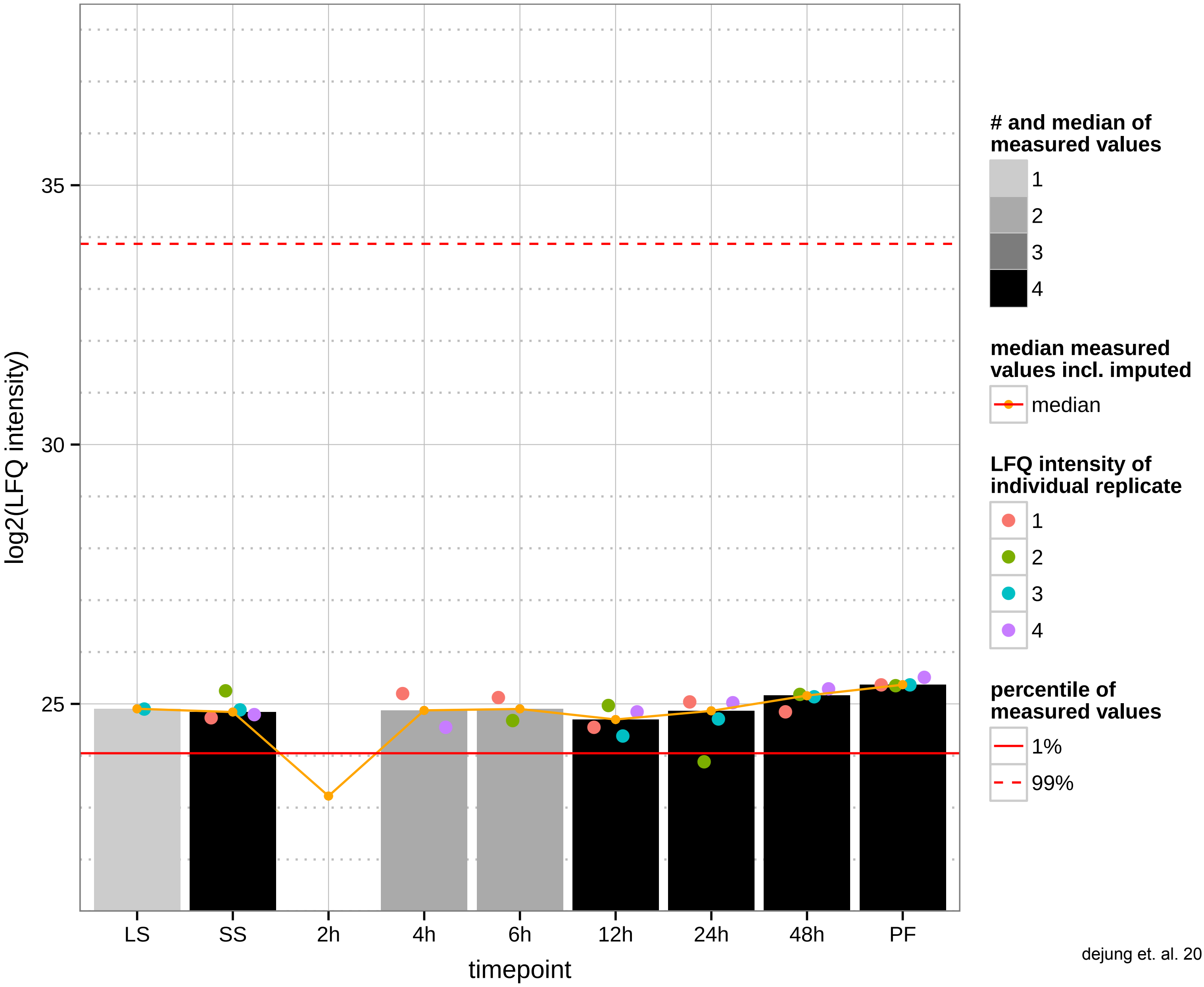

dejung et. al. 2015

hypothetical protein, conserved
Tb927.8.6460
AGOF: null
AGOC: null
AGOP: null
PGOF: null
PGOC: null
PGOP: null

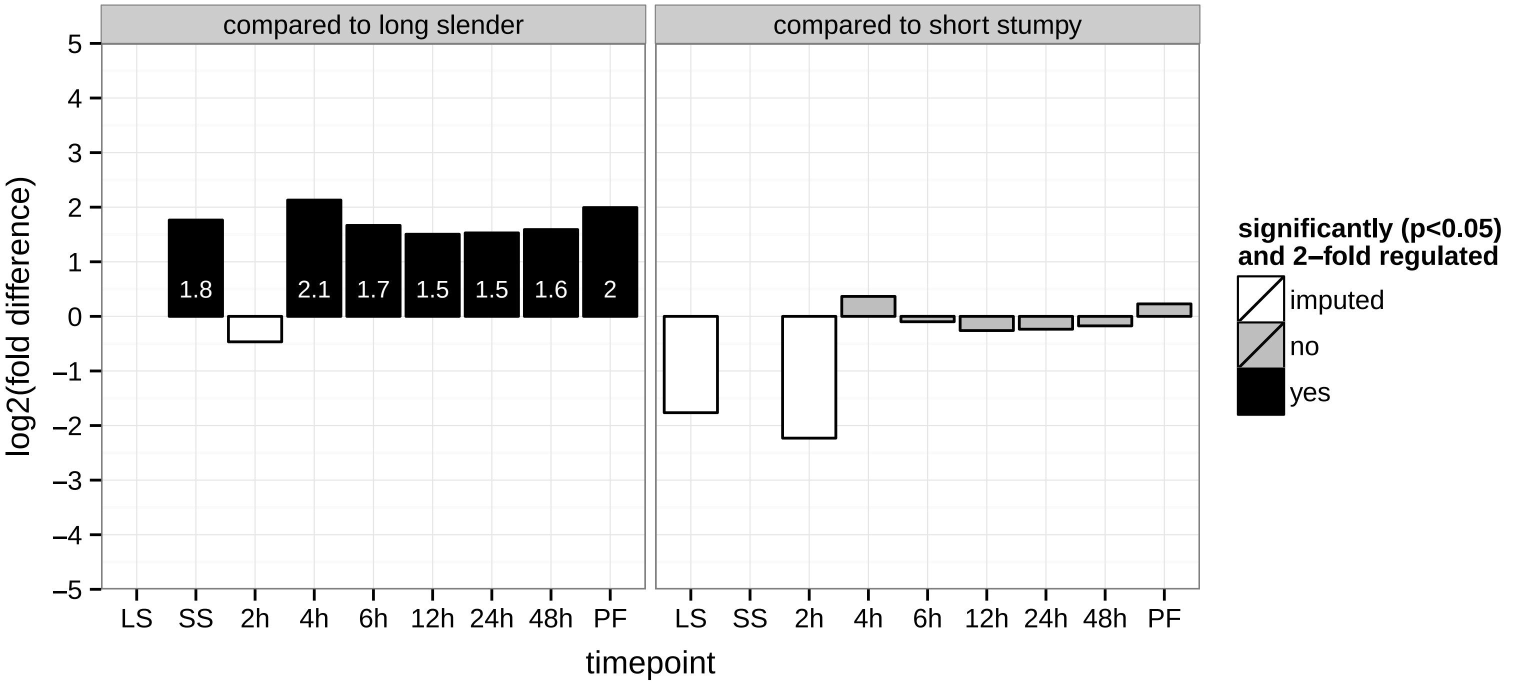

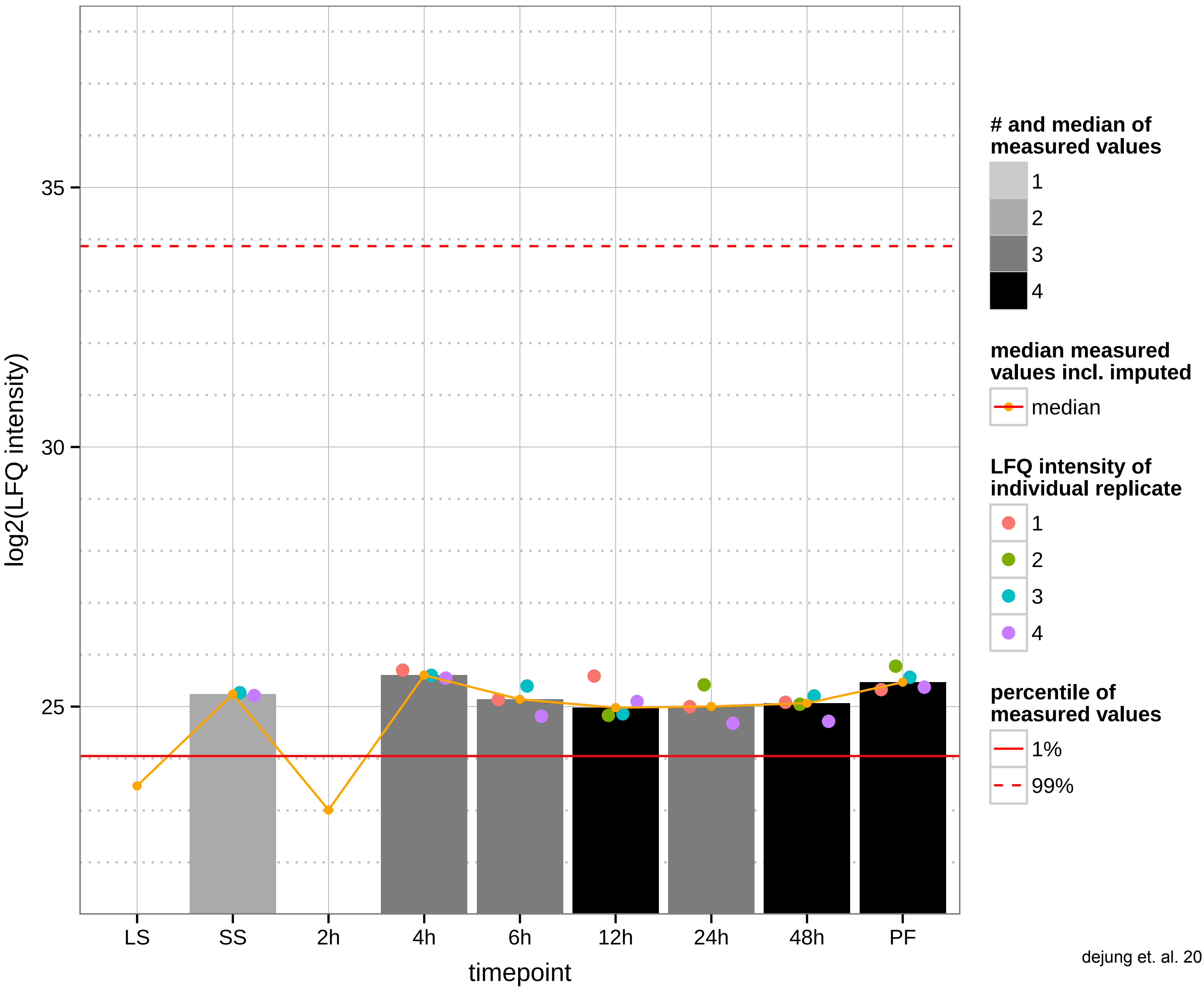

dejung et. al. 2015

kinetoplast–associated protein, putative
Tb927.8.7260
AGOF: structural molecule activity
AGOC: kinetoplast, mitochondrion
AGOP: cell morphogenesis
PGOF: null
PGOC: null
PGOP: null

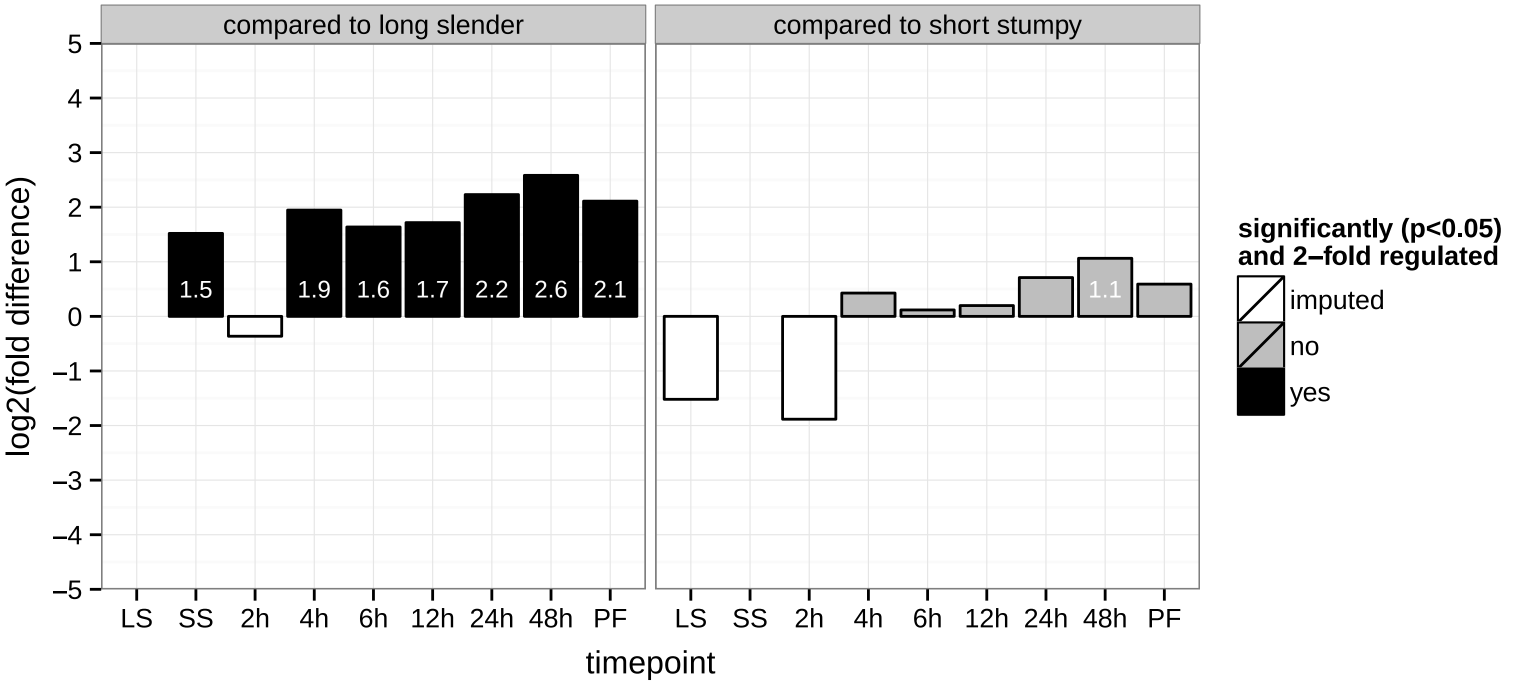

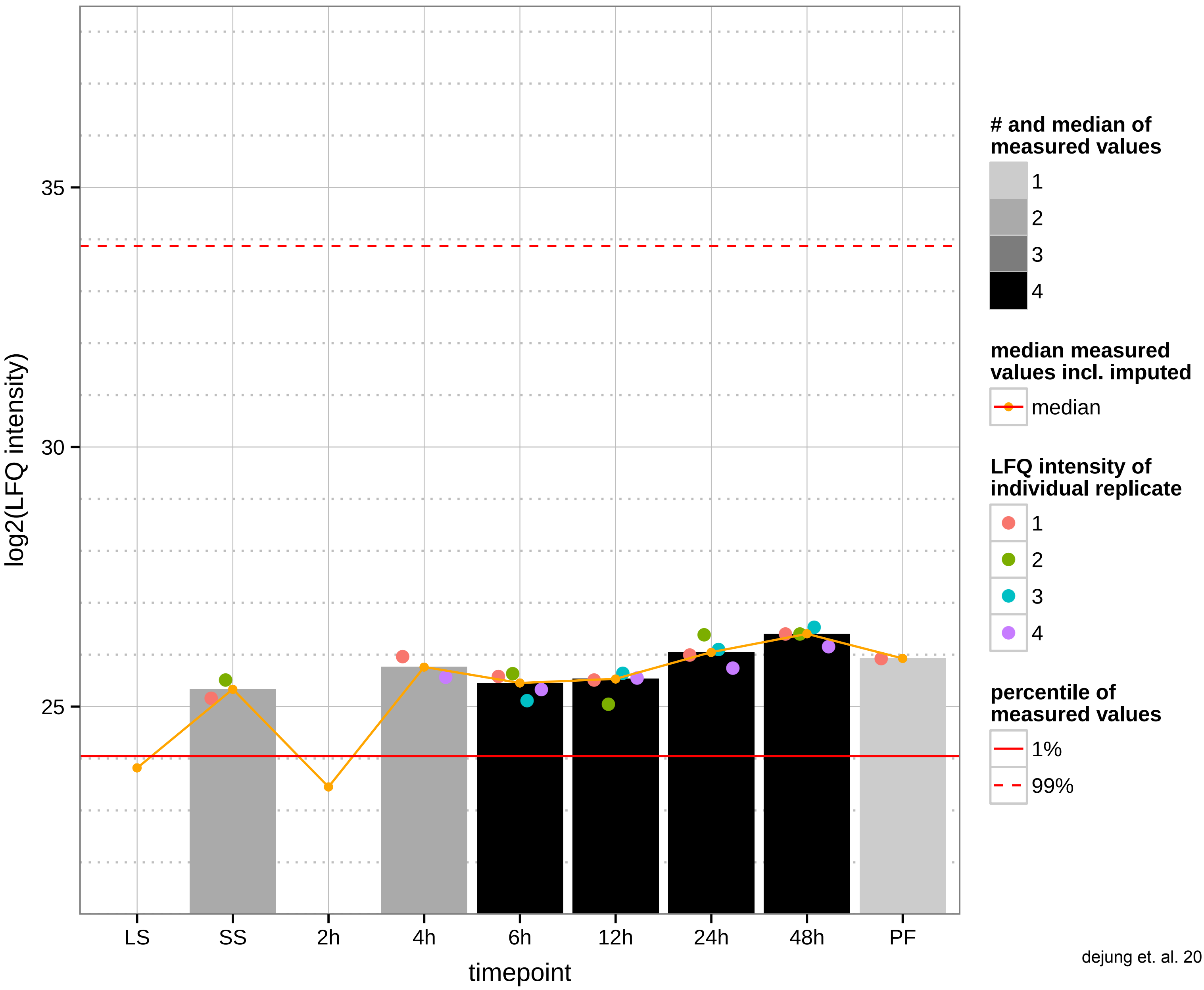

dejung et. al. 2015

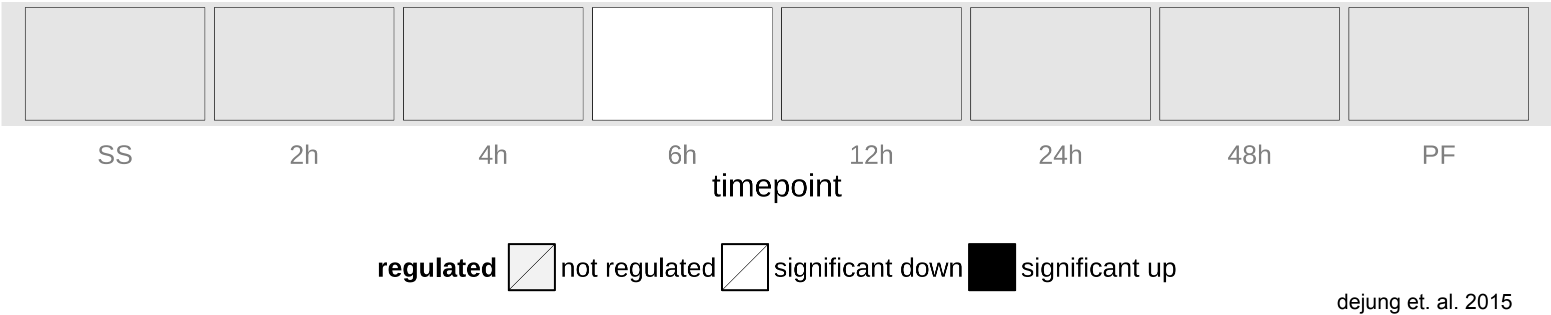
SS
2h
4h
6h
12h
24h
48h
PF
timepoint
regulated
not regulated
significant down
significant up
dejung et. al. 2015

tRNA pseudouridine synthase A–like protein, putative
Tb927.10.15650;Tb11.v5.0465
AGOF: null, RNA binding, pseudouridine synthase activity
AGOC: null, mitochondrion
AGOP: null, pseudouridine synthesis, tRNA modification
PGOF: RNA binding, pseudouridine synthase activity
PGOC: null
PGOP: RNA modification, pseudouridine synthesis

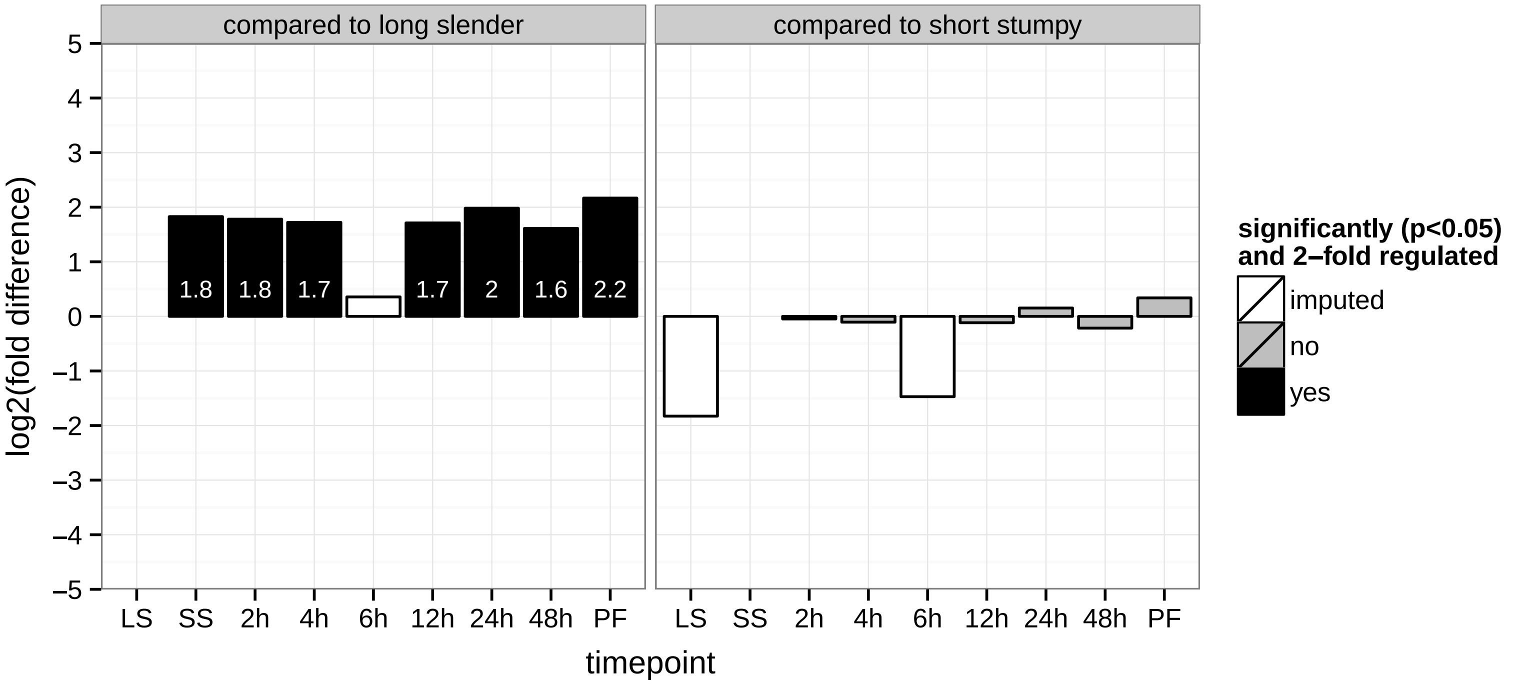

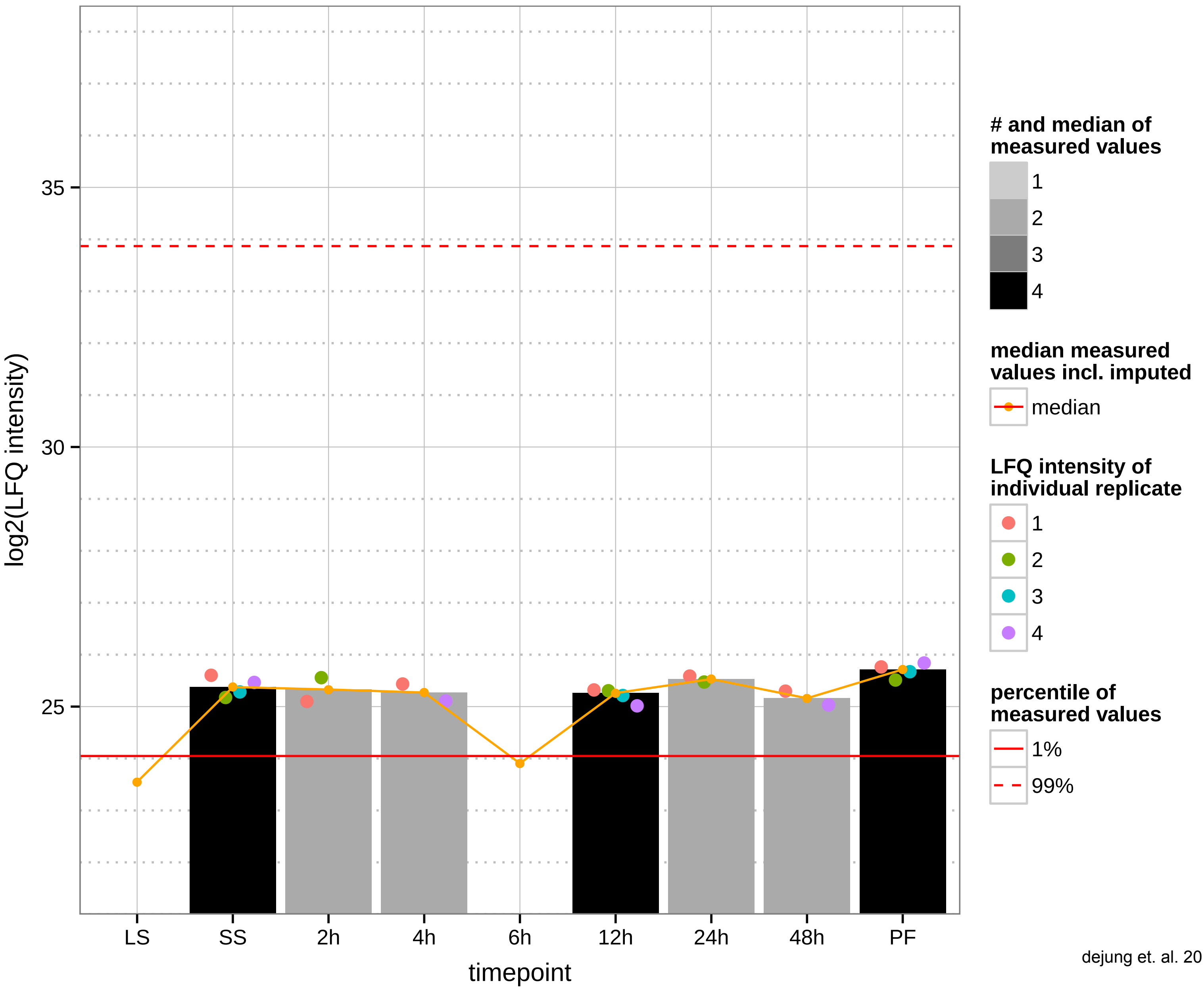

dejung et. al. 2015

hypothetical protein, conserved
Tb927.10.1800;Tb11.v5.0832
AGOF: null, methyltransferase activity
AGOC: null
AGOP: null
PGOF: methyltransferase activity
PGOC: null
PGOP: null

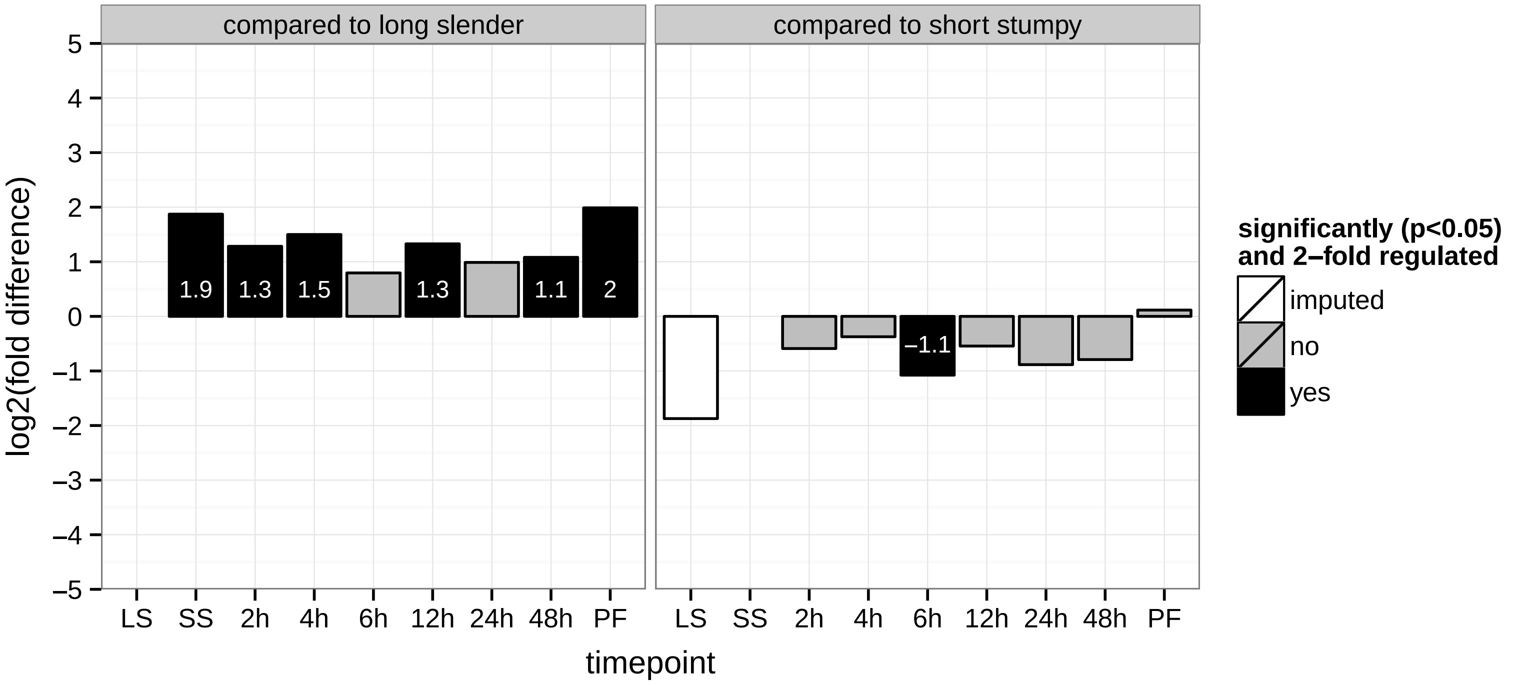

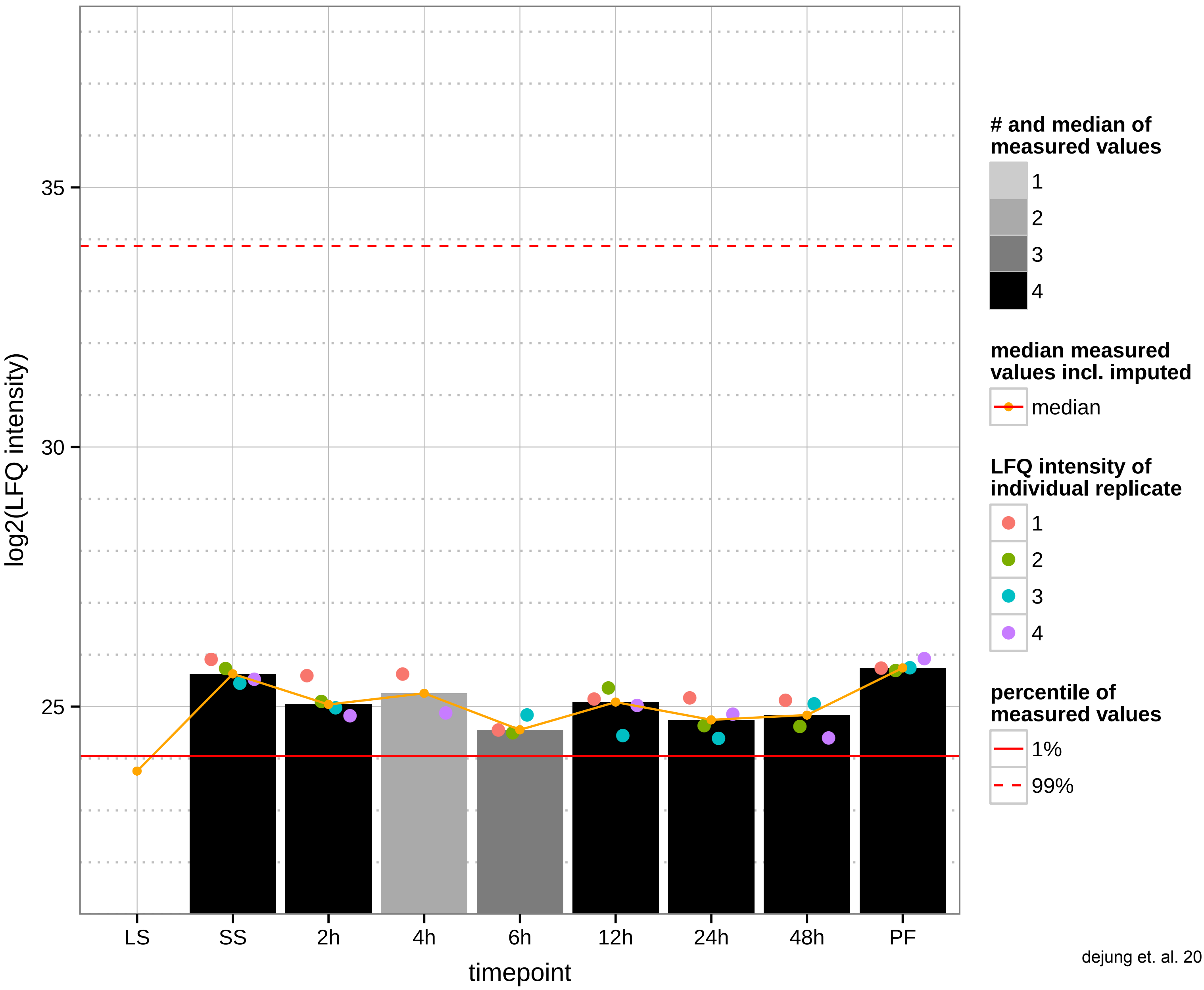

dejung et. al. 2015

hypothetical protein, conserved
Tb927.10.2930
AGOF: null
AGOC: null
AGOP: null
PGOF: null
PGOC: null
PGOP: null

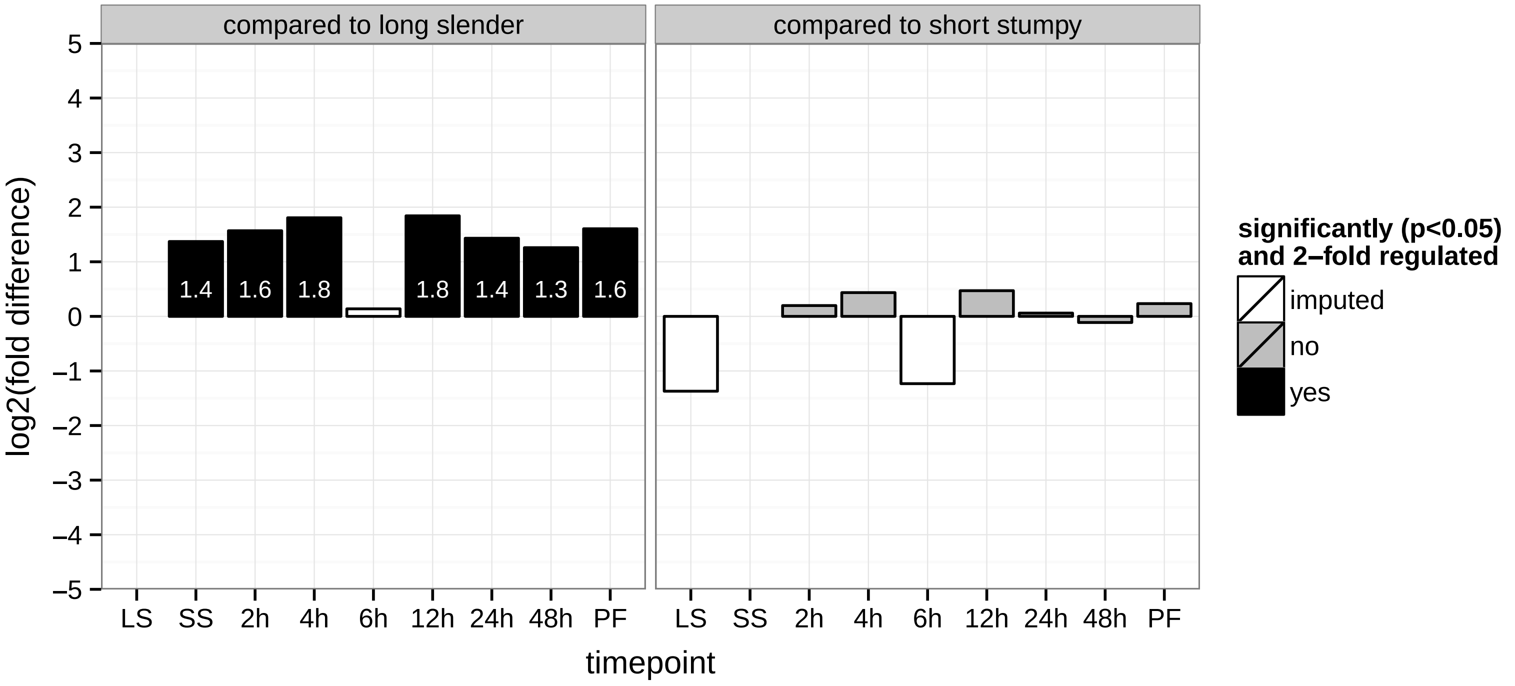

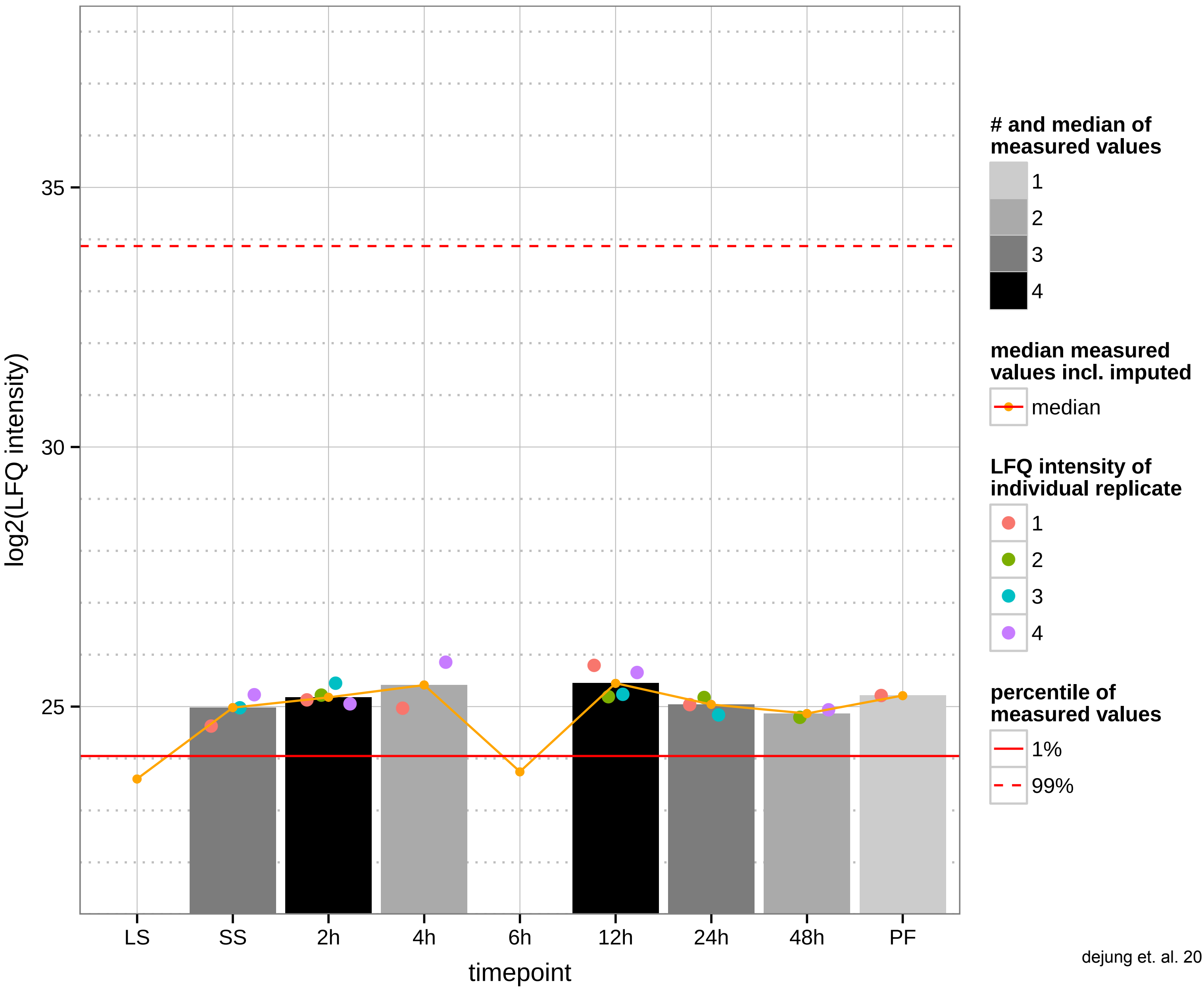

dejung et. al. 2015

TPR–repeat–containing chaperone protein DNAJ, putative, TPR repeat protein
Tb927.10.4900
AGOF: heat shock protein binding
AGOC: null
AGOP: protein folding
PGOF: heat shock protein binding, protein binding
PGOC: null
PGOP: null

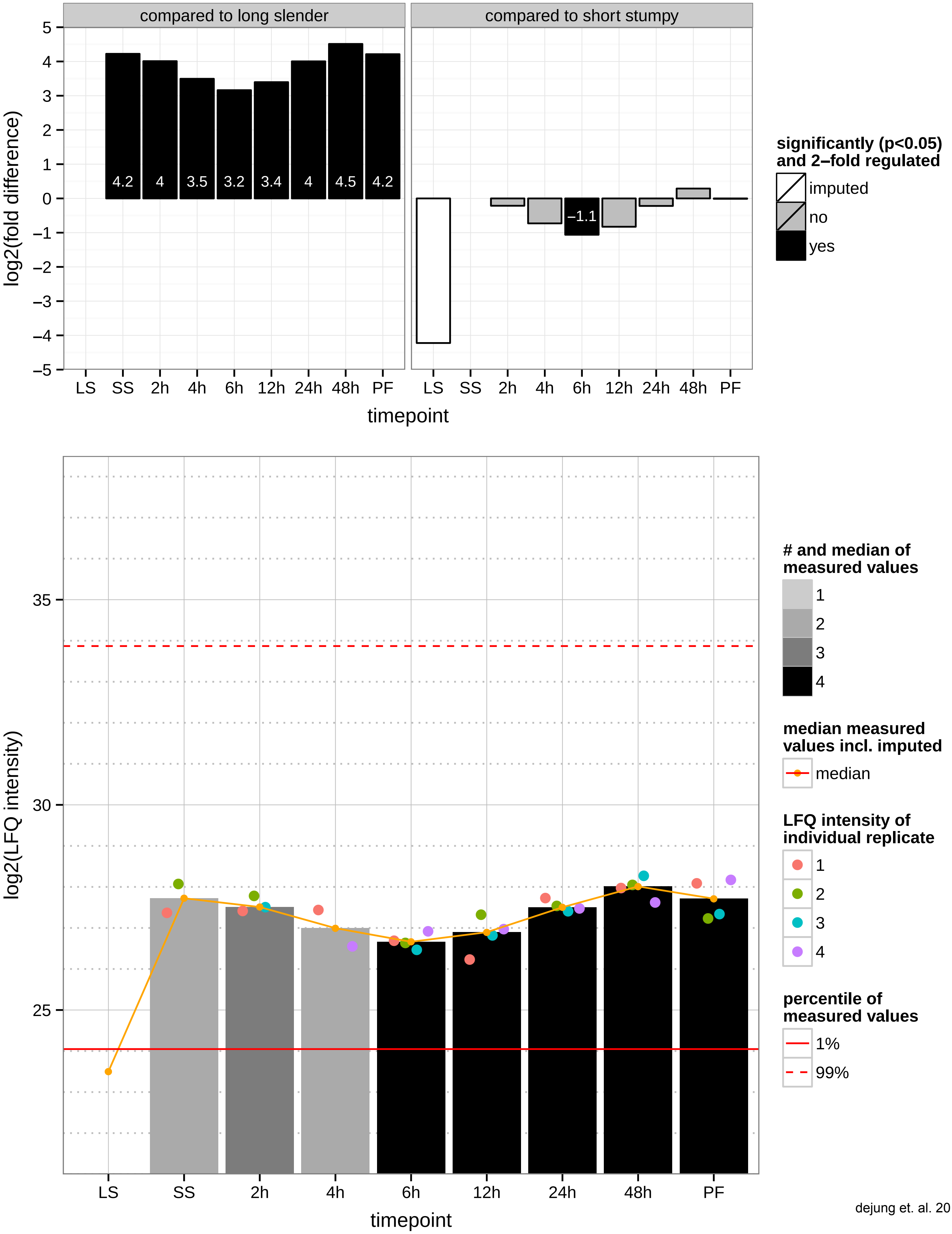

dejung et. al. 2015

hypothetical protein, conserved
Tb927.10.7380
AGOF: null
AGOC: mitochondrion
AGOP: null
PGOF: null
PGOC: null
PGOP: null

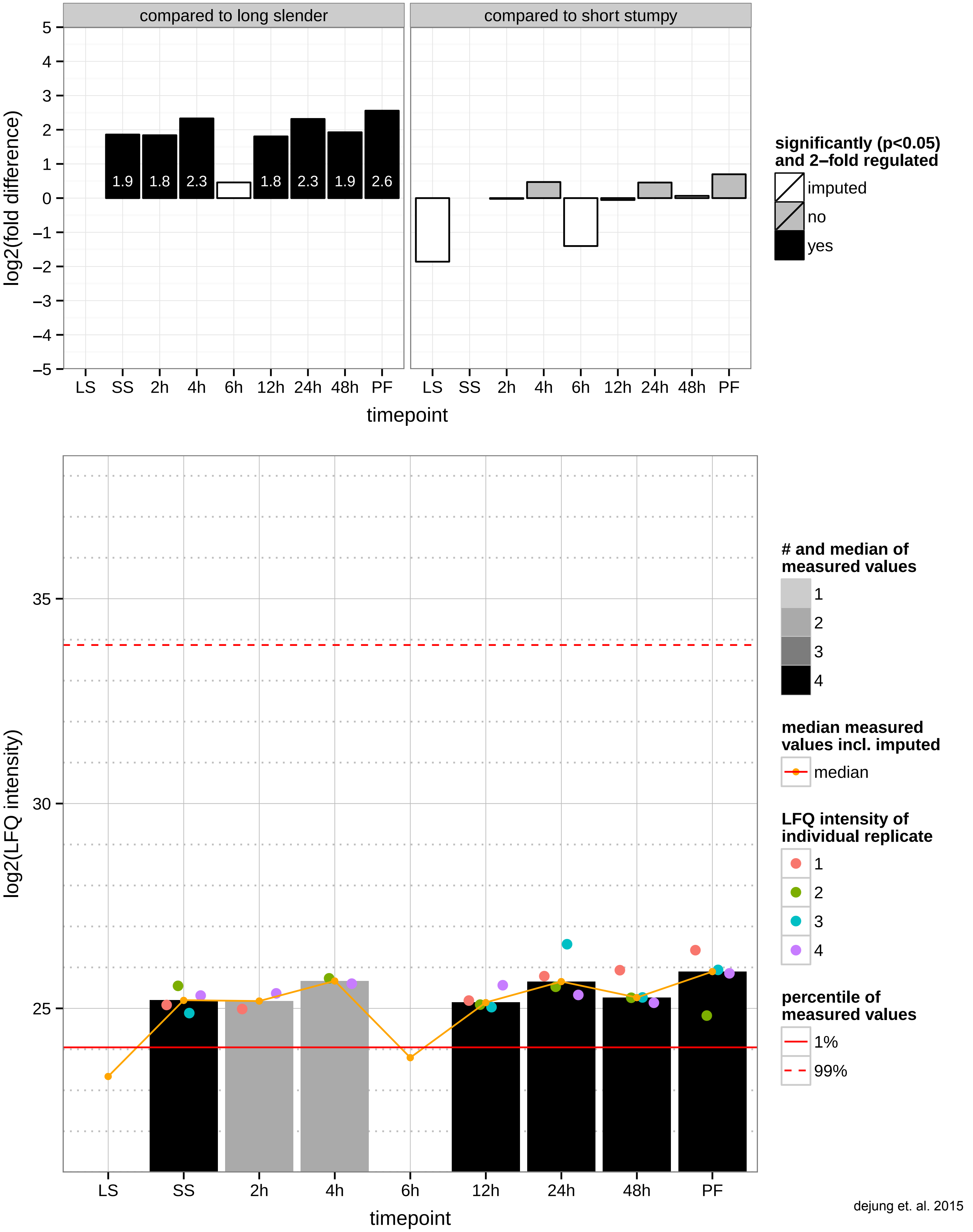

dejung et. al. 2015

gamma–tubulin complex subunit, putative, spindle pole body protein, putative (GCP2)
Tb927.10.9770;Tb11.v5.0848
AGOF: null
AGOC: null, microtubule organizing center, spindle pole
AGOP: null, microtubule cytoskeleton organization
PGOF: null
PGOC: microtubule organizing center, spindle pole
PGOP: microtubule cytoskeleton organization

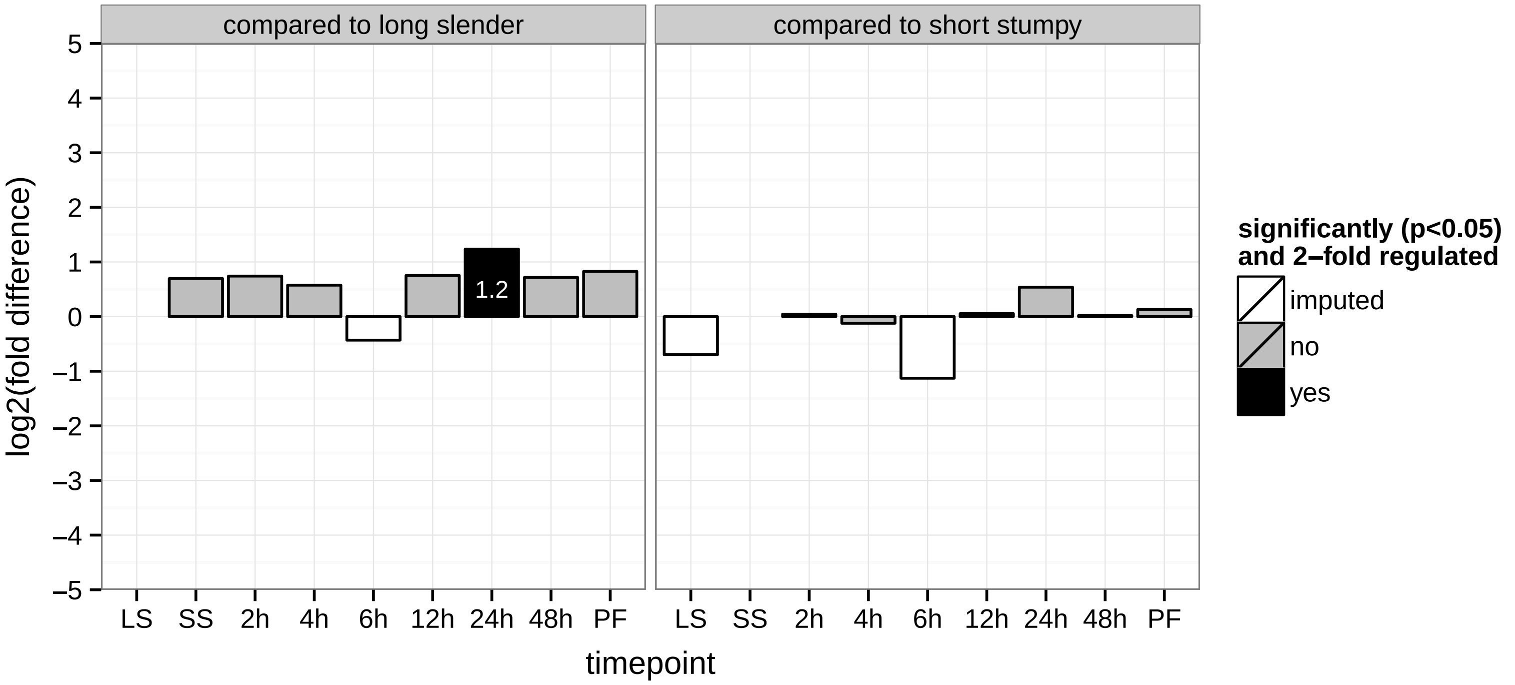

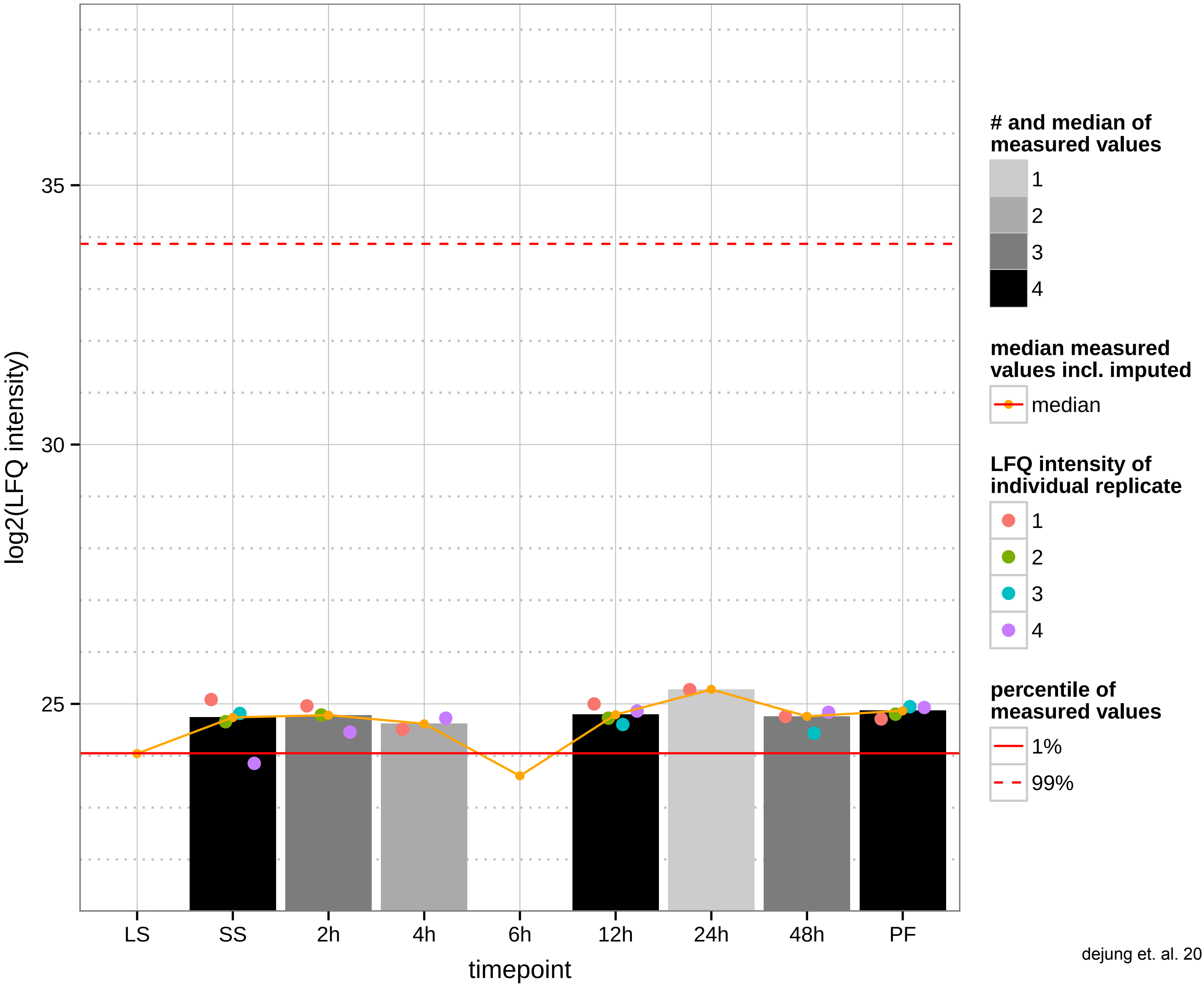

dejung et. al. 2015

calpain–like cysteine peptidase, putative, antigen
Tb927.11.1130
AGOF: null, calcium–dependent cysteine–type endopeptidase activity
AGOC: null, intracellular
AGOP: null, proteolysis
PGOF: null, calcium–dependent cysteine–type endopeptidase activity
PGOC: null, intracellular
PGOP: null, proteolysis

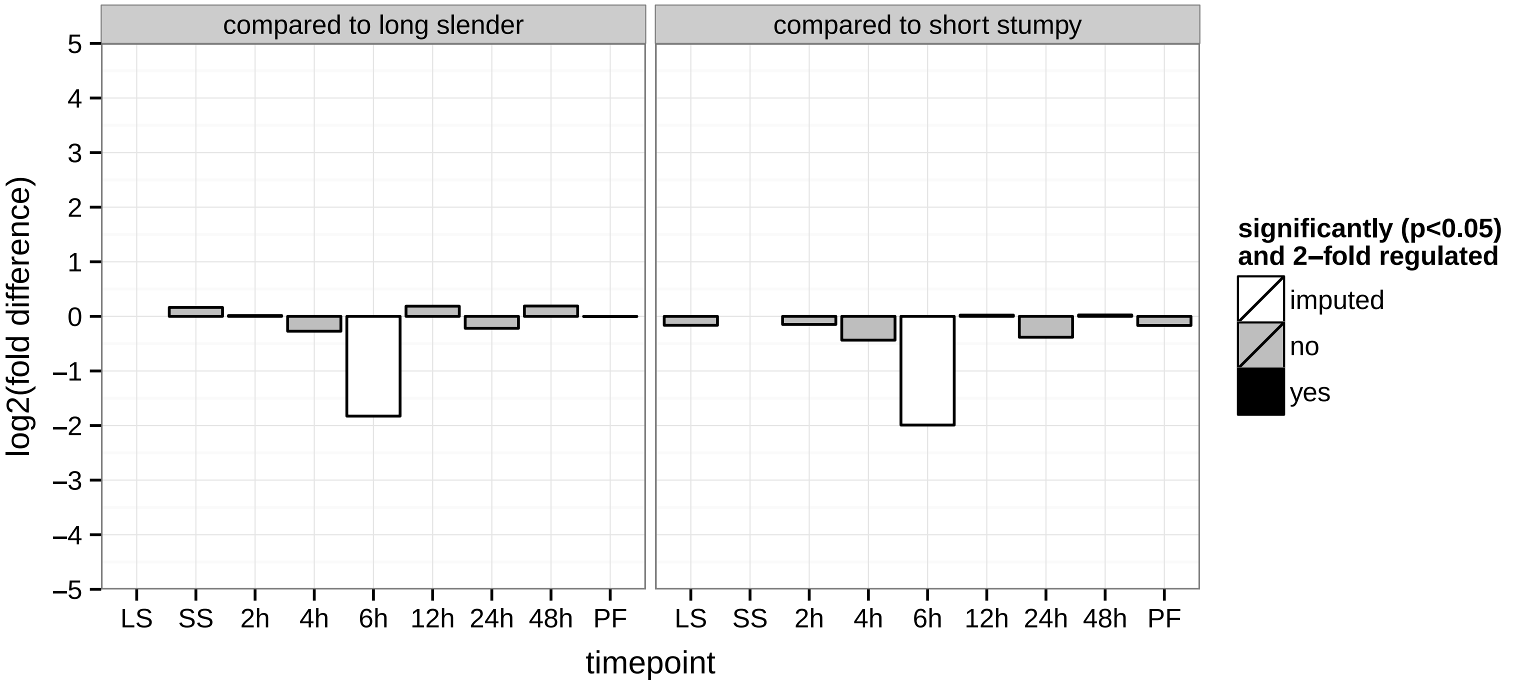

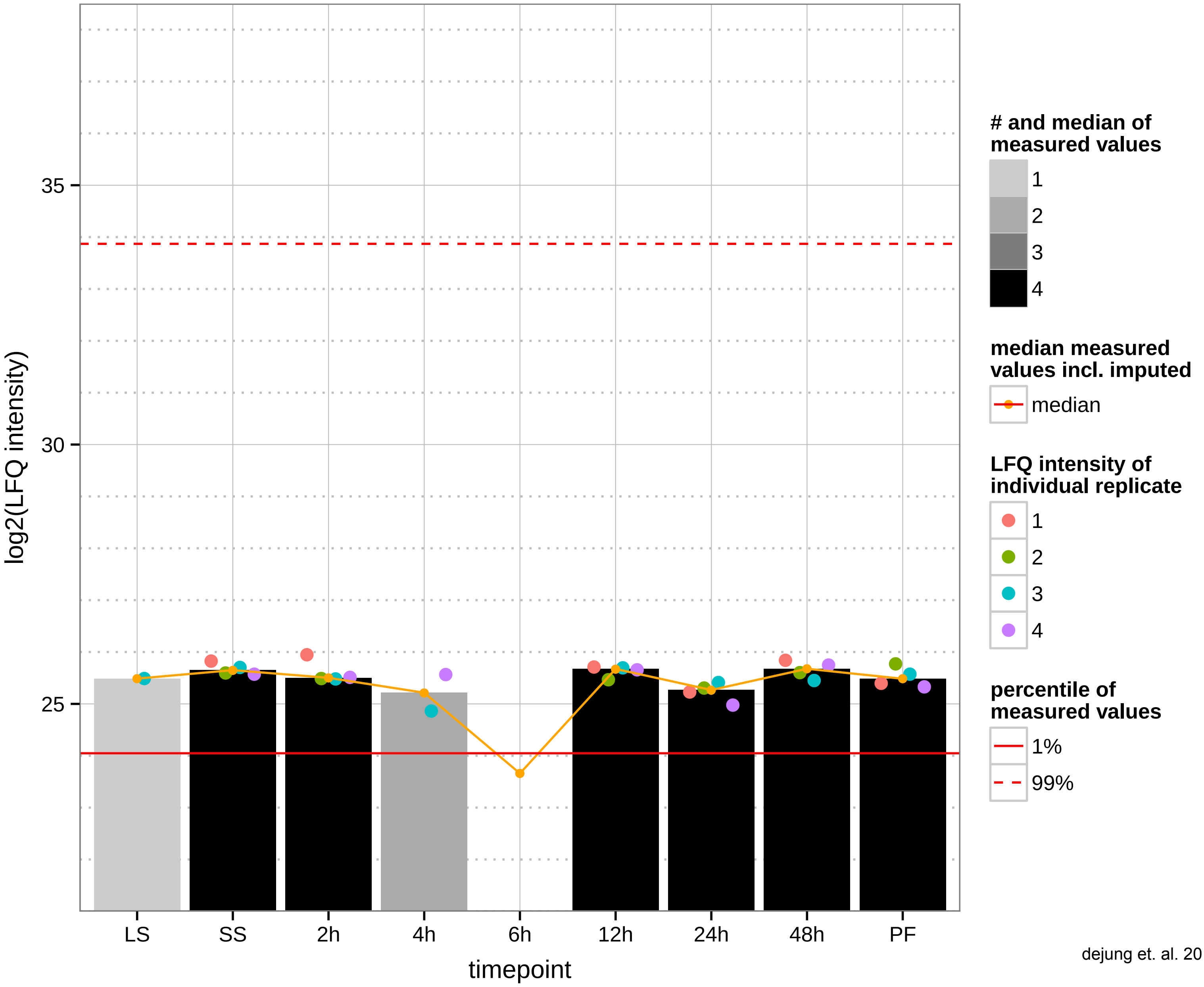

dejung et. al. 2015

ornithine decarboxylase (ODC)
Tb927.11.13730
AGOF: ornithine decarboxylase activity
AGOC: null
AGOP: polyamine biosynthetic process
PGOF: catalytic activity
PGOC: null
PGOP: null

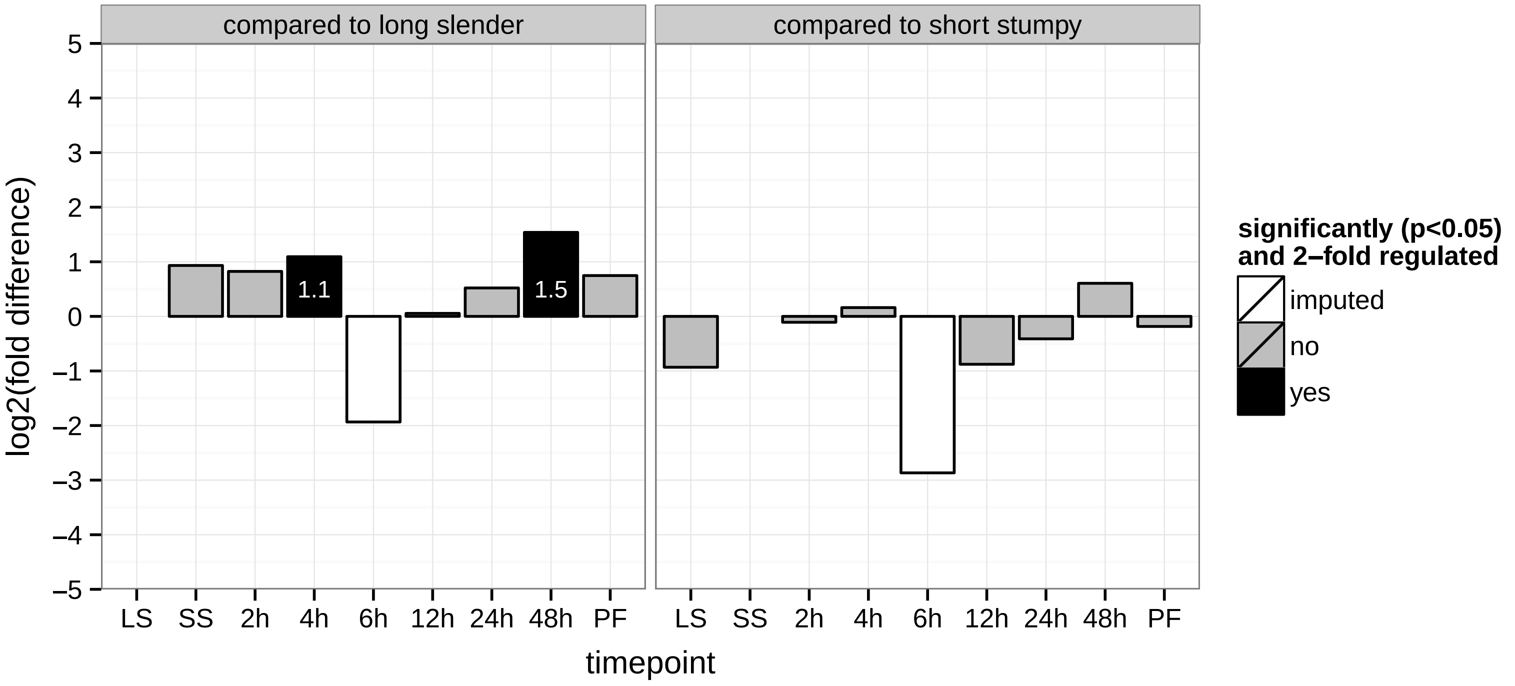

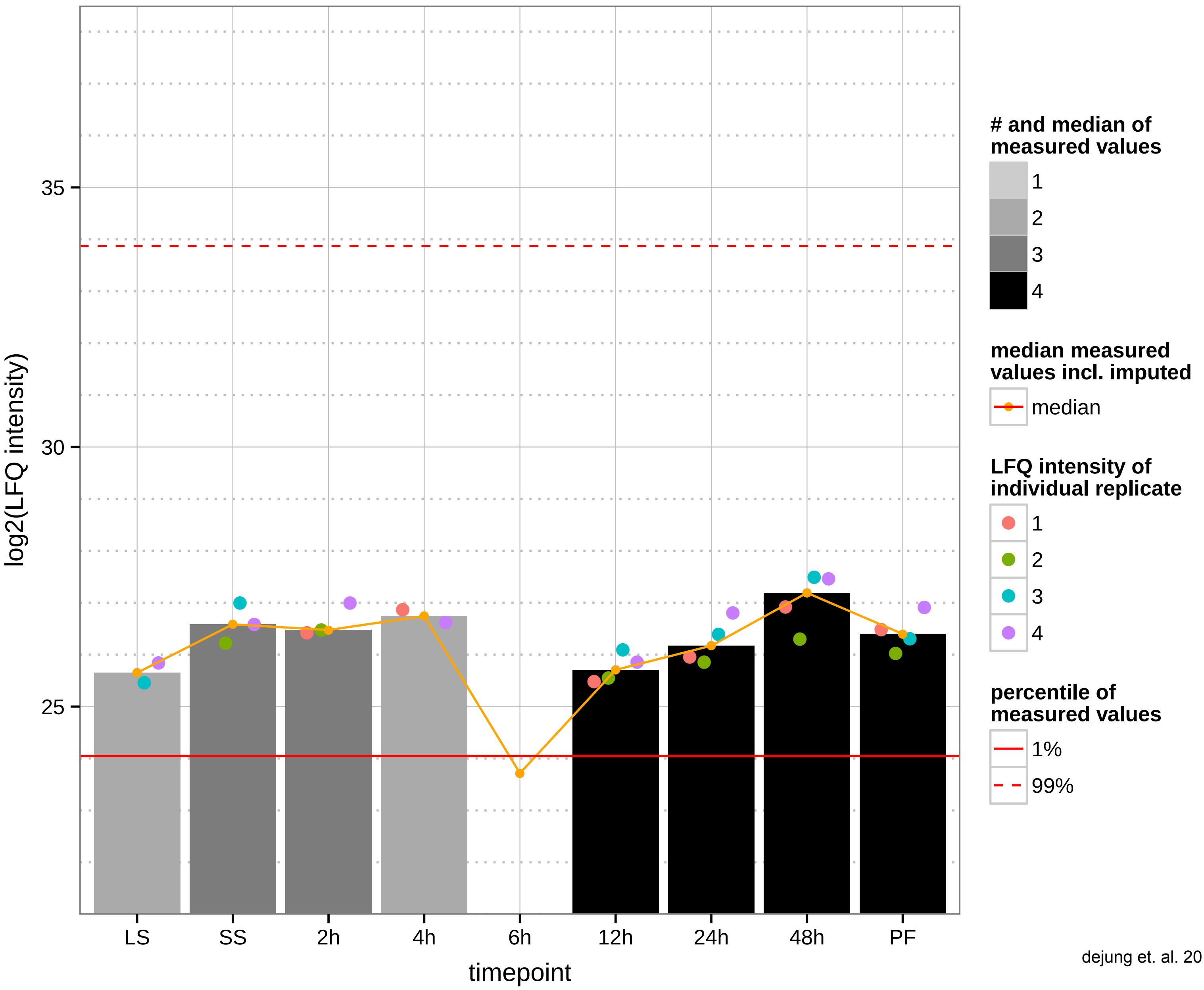

dejung et. al. 2015

hypothetical protein, conserved
Tb927.11.15940
AGOF: null
AGOC: null
AGOP: null
PGOF: null
PGOC: null
PGOP: null

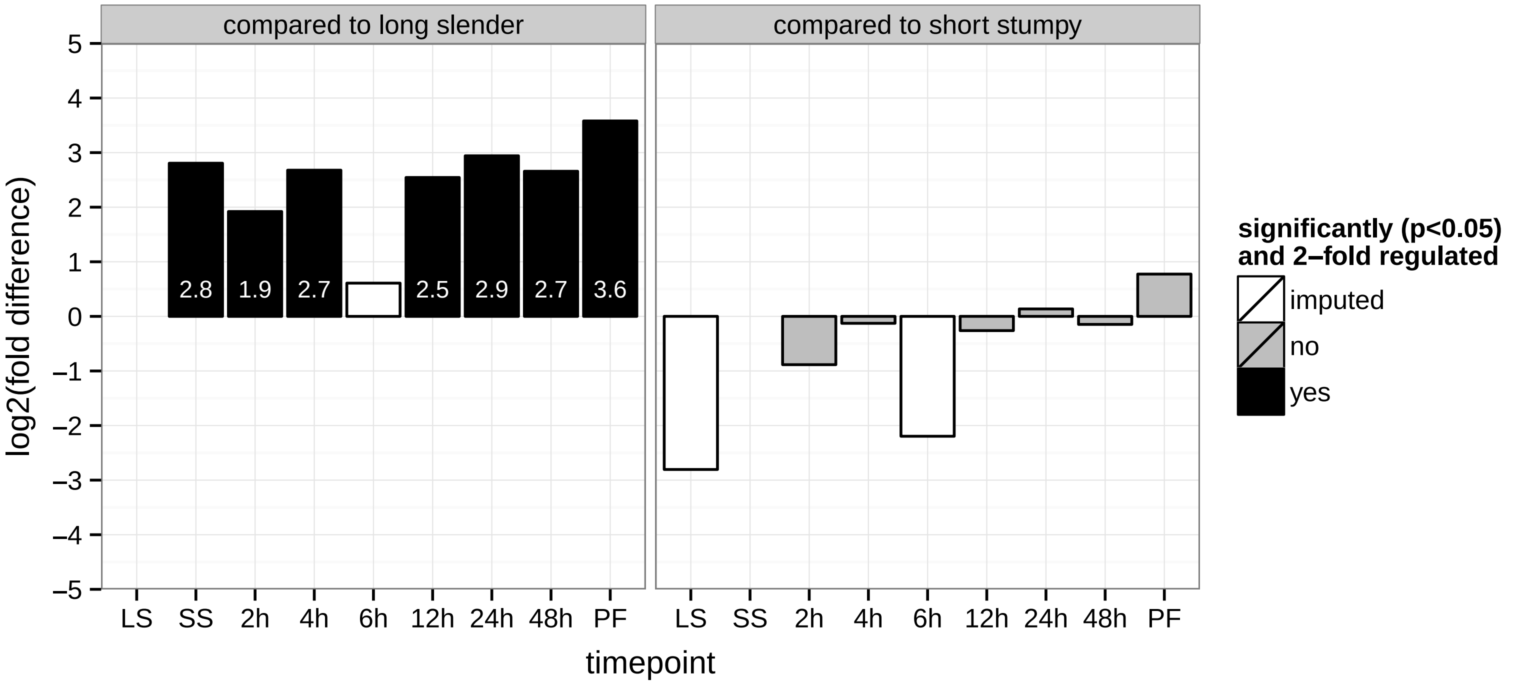

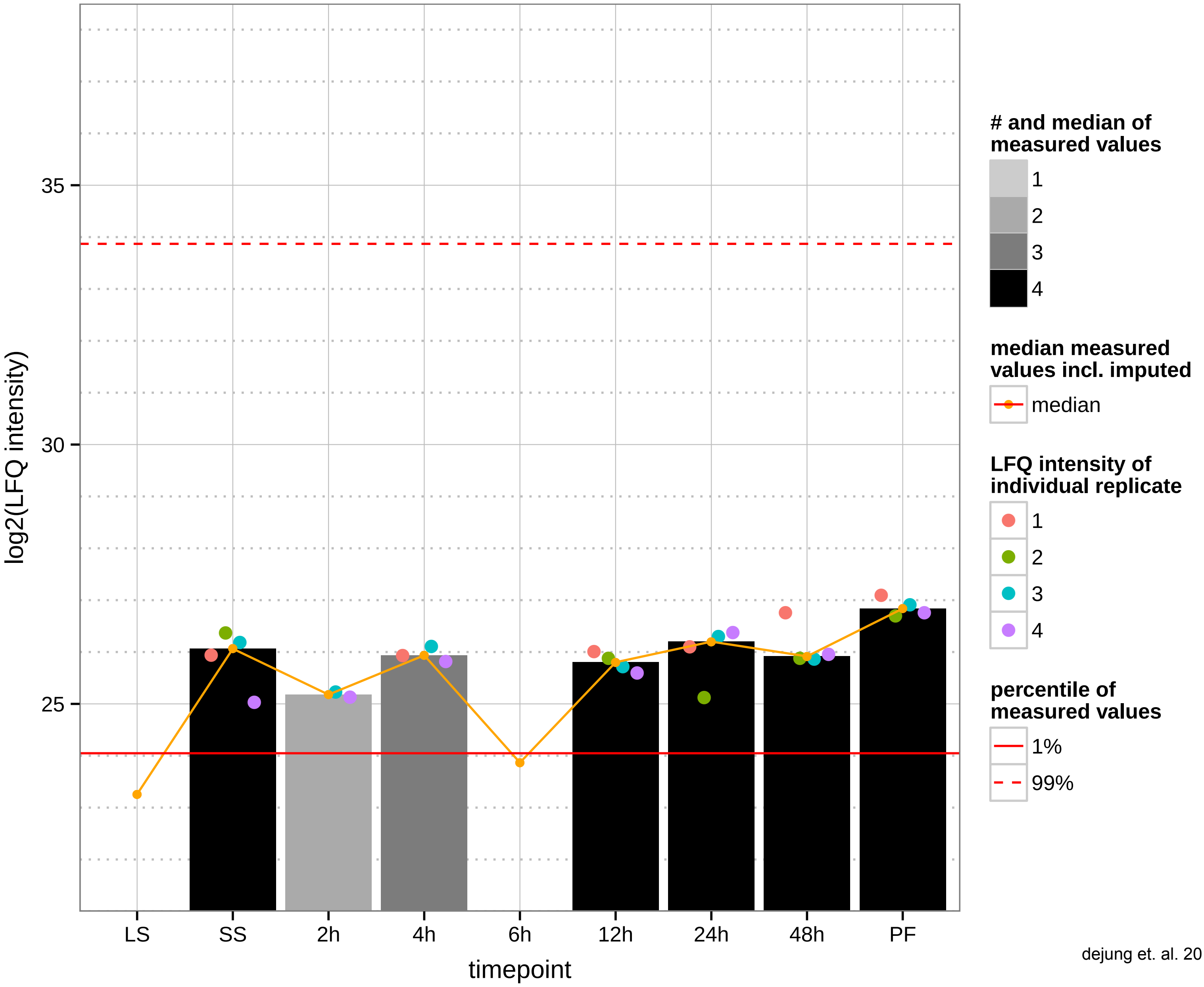

dejung et. al. 2015

hypothetical protein, conserved
Tb927.11.2580
AGOF: null
AGOC: mitochondrion
AGOP: null
PGOF: null
PGOC: null
PGOP: null

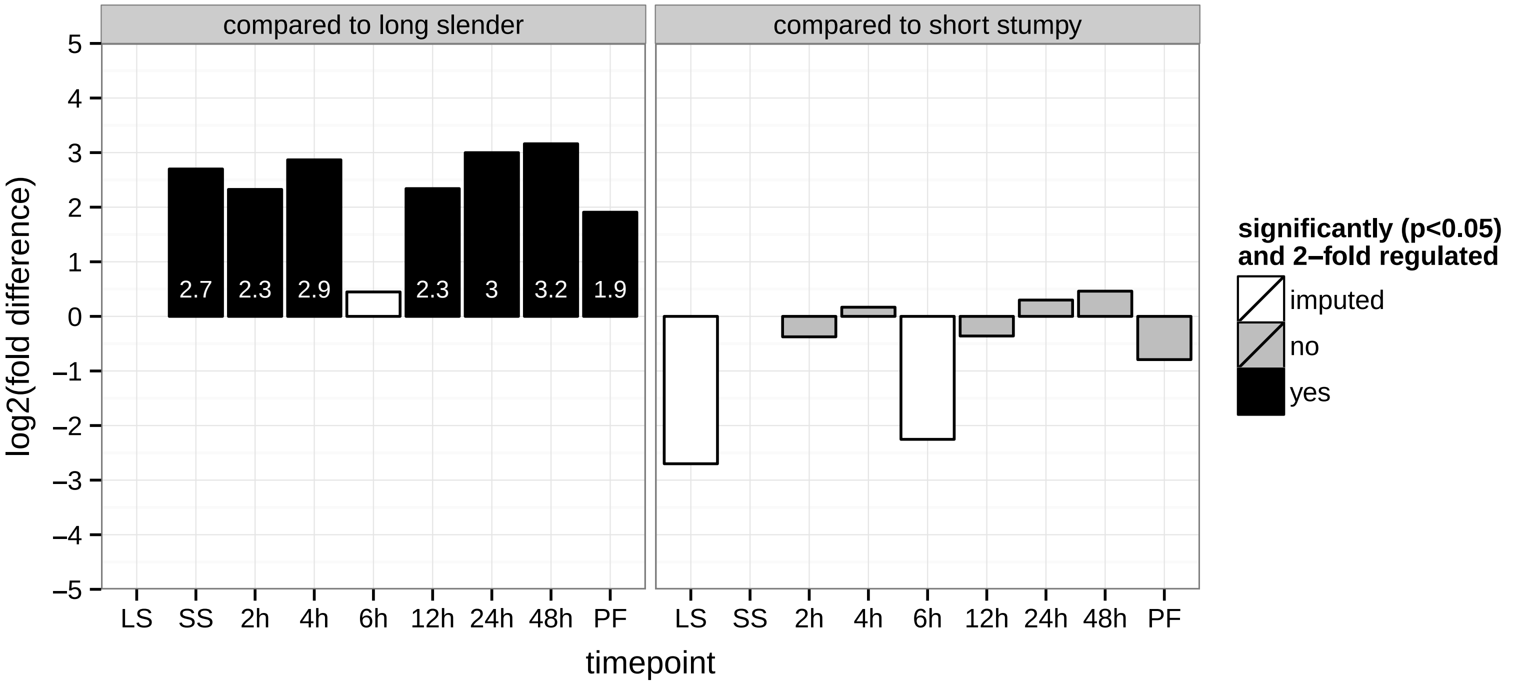

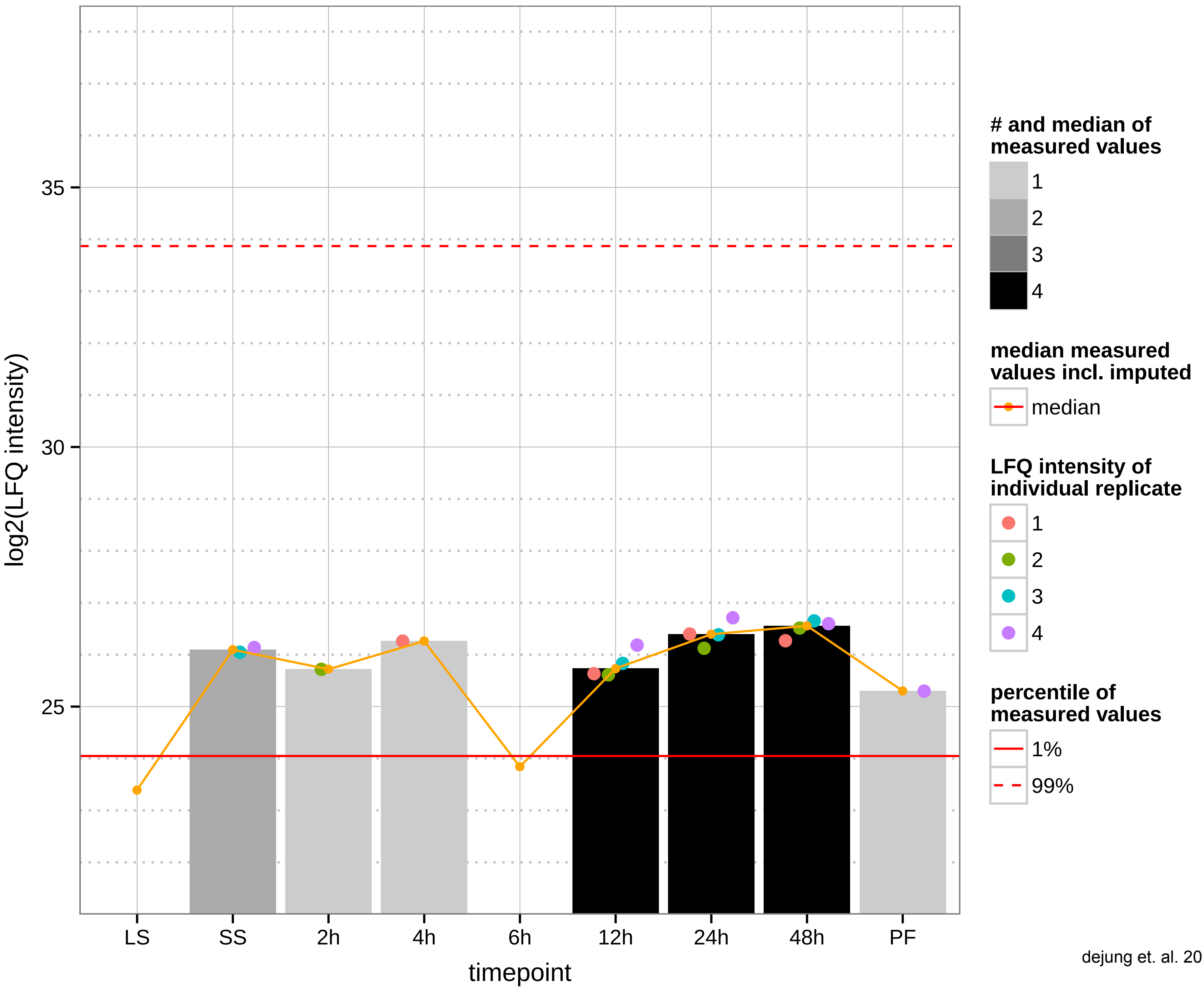

dejung et. al. 2015

long–chain–fatty–acid–CoA ligase, putative, acyl–CoA synthetase, fatty acid thiokinase (long chain)
Tb927.11.4490
AGOF: catalytic activity
AGOC: mitochondrion
AGOP: metabolic process
PGOF: catalytic activity
PGOC: null
PGOP: metabolic process

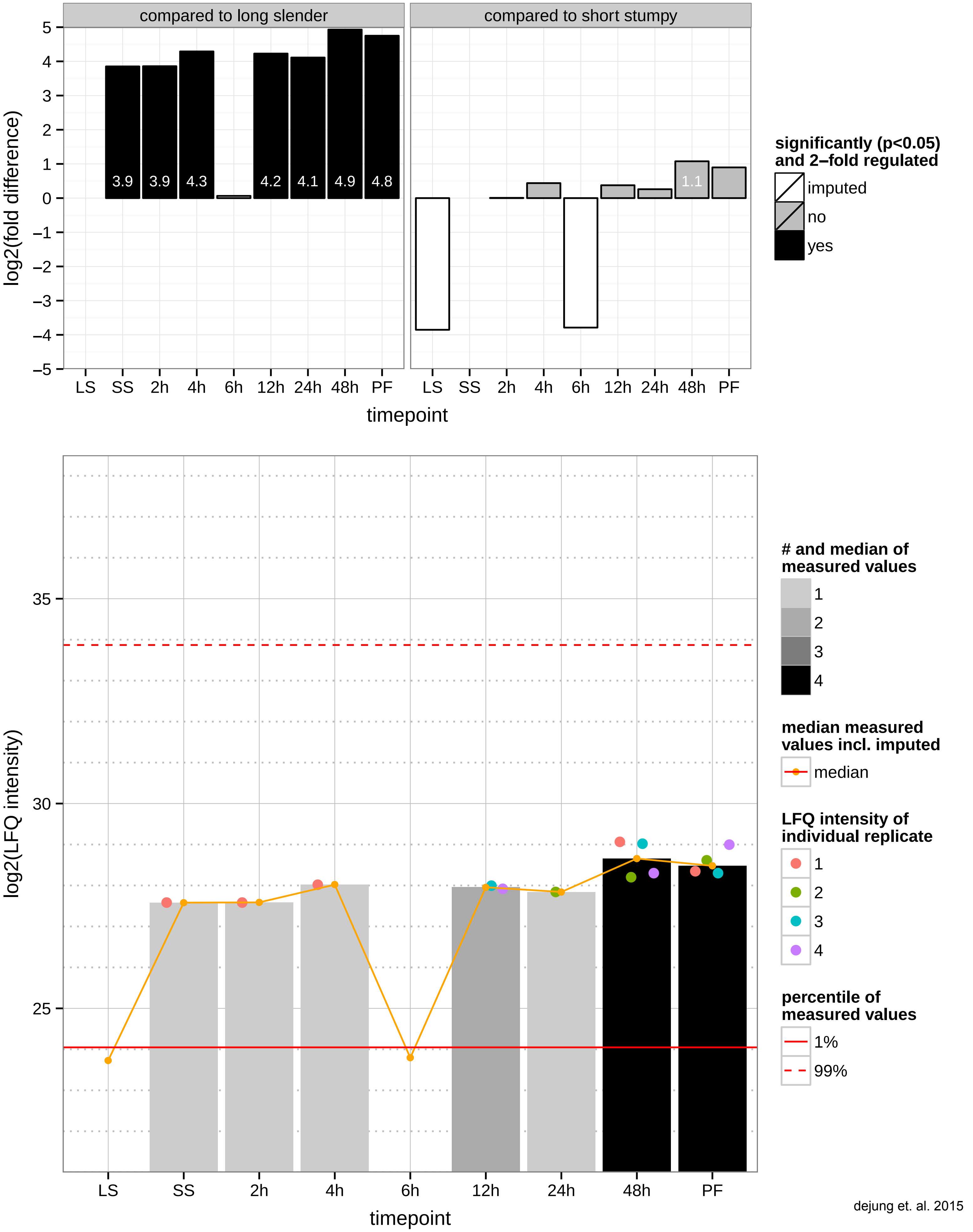

dejung et. al. 2015

protein kinase, putative
Tb927.11.7210
AGOF: ATP binding, protein serine/threonine kinase activity, protein tyrosine kinase activity, histone serine kinase activity
AGOC: null
AGOP: protein phosphorylation
PGOF: ATP binding, protein binding, protein kinase activity, transferase activity, transferring phosphorus−containing groups
PGOC: null
PGOP: protein phosphorylation

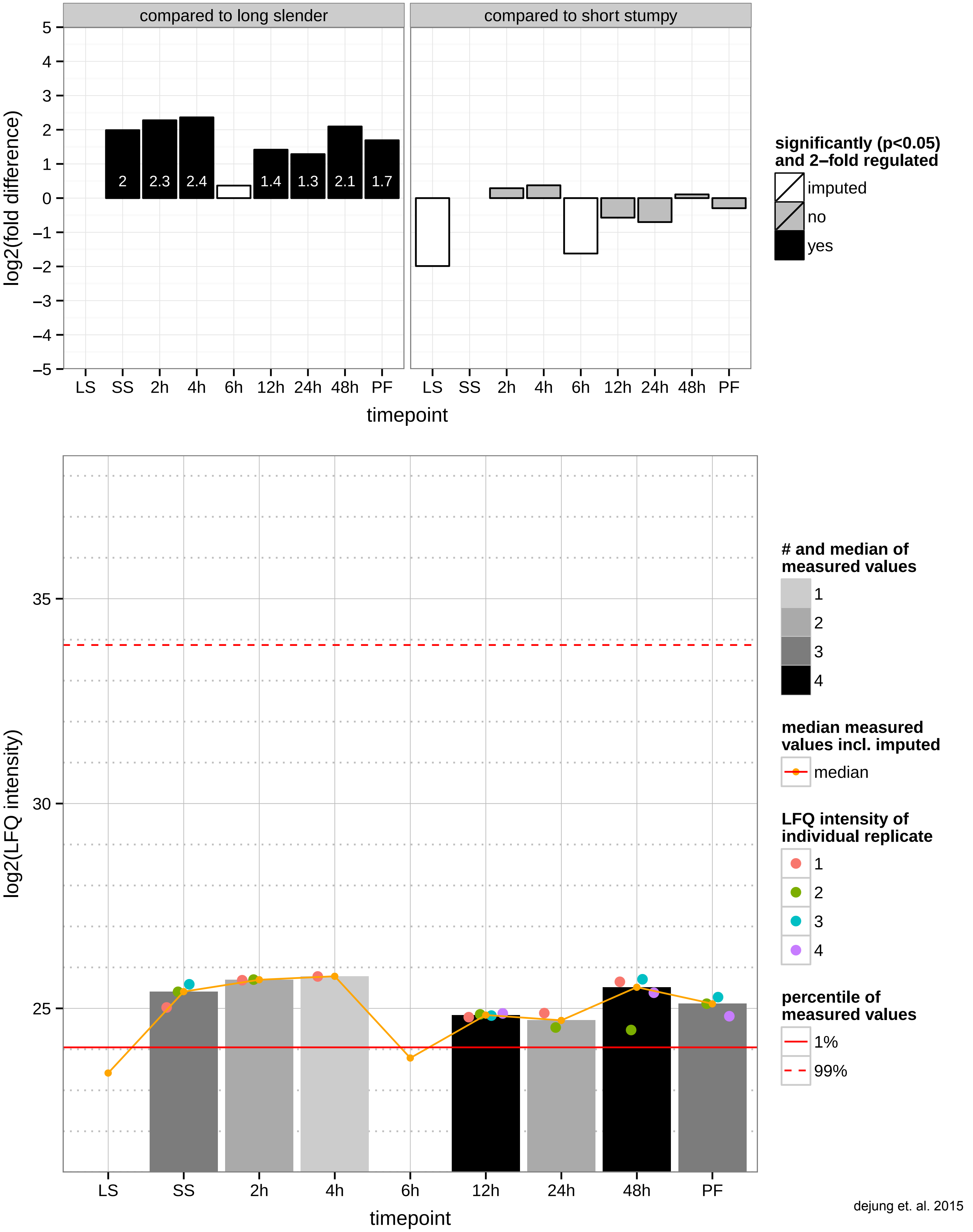

dejung et. al. 2015

hypothetical protein, conserved
Tb927.11.7330
AGOF: null
AGOC: null
AGOP: null
PGOF: null
PGOC: null
PGOP: null

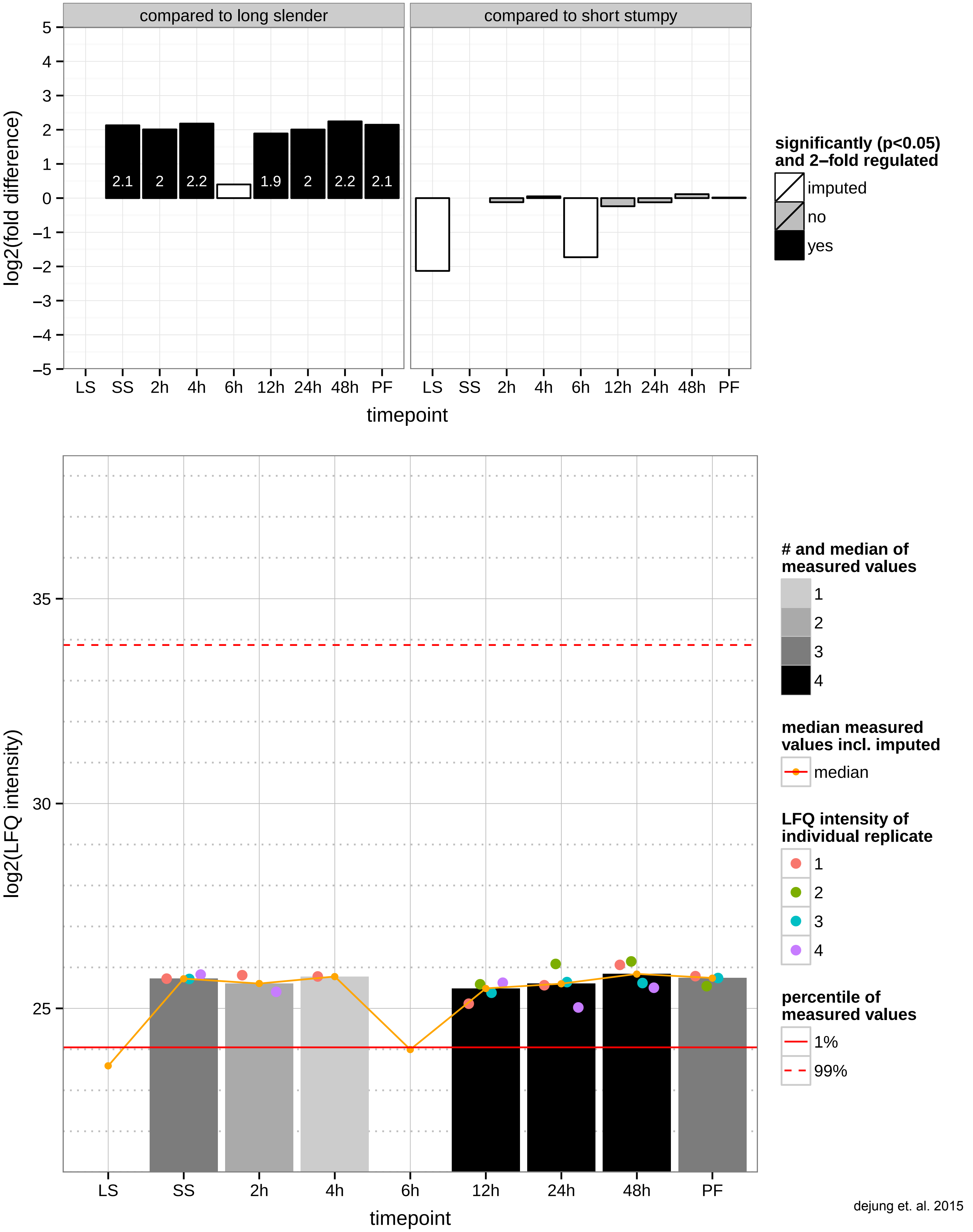

dejung et. al. 2015

hypothetical protein, conserved
Tb927.2.2200
AGOF: null
AGOC: null
AGOP: null
PGOF: null
PGOC: null
PGOP: null

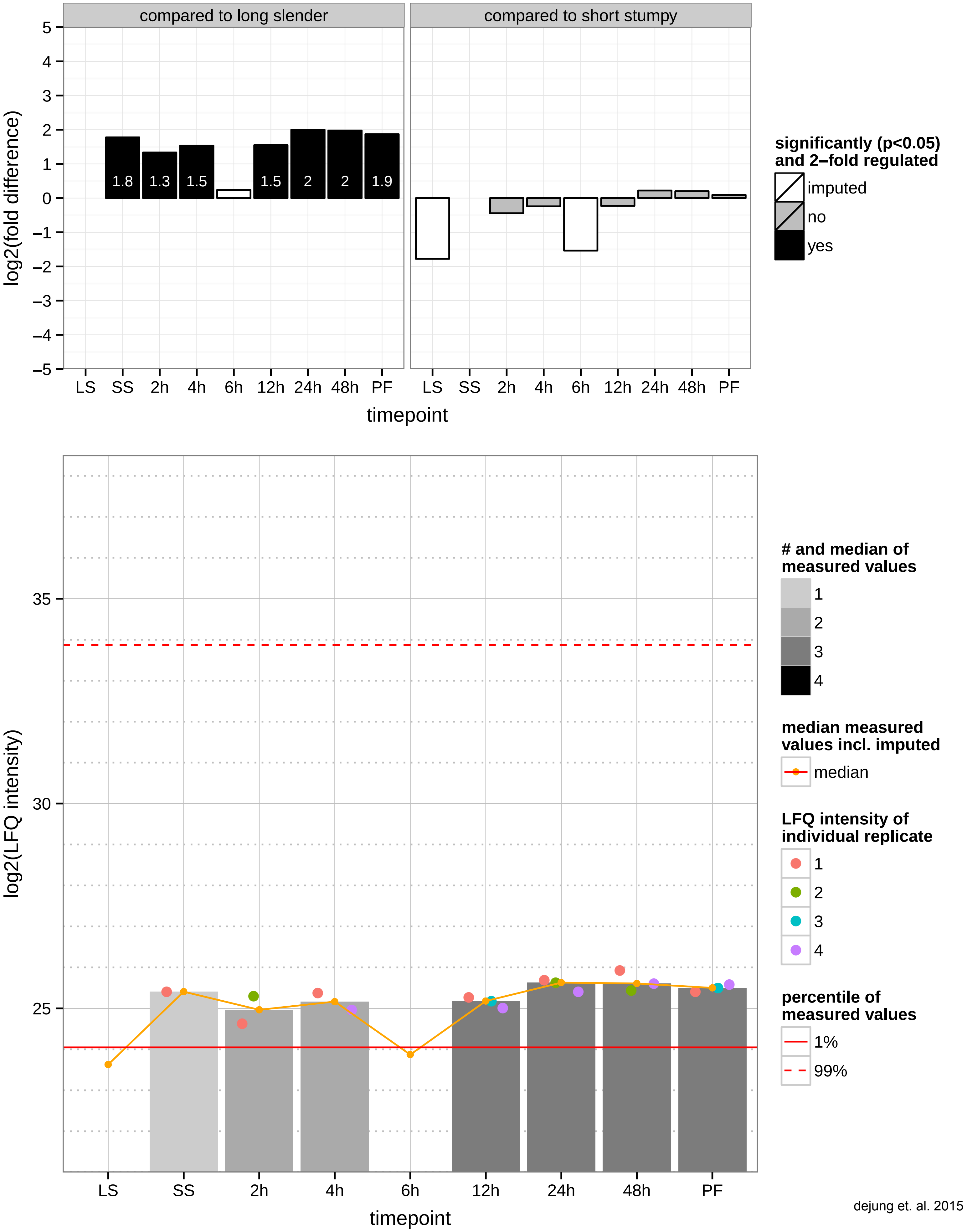

dejung et. al. 2015

protein kinase, putative
Tb927.2.5230
AGOF: ATP binding, protein kinase activity, protein serine/threonine kinase activity, protein tyrosine kinase activity
AGOC: null
AGOP: protein phosphorylation
PGOF: ATP binding, protein kinase activity, transferase activity, transferring phosphorus–containing groups
PGOC: null
PGOP: protein phosphorylation

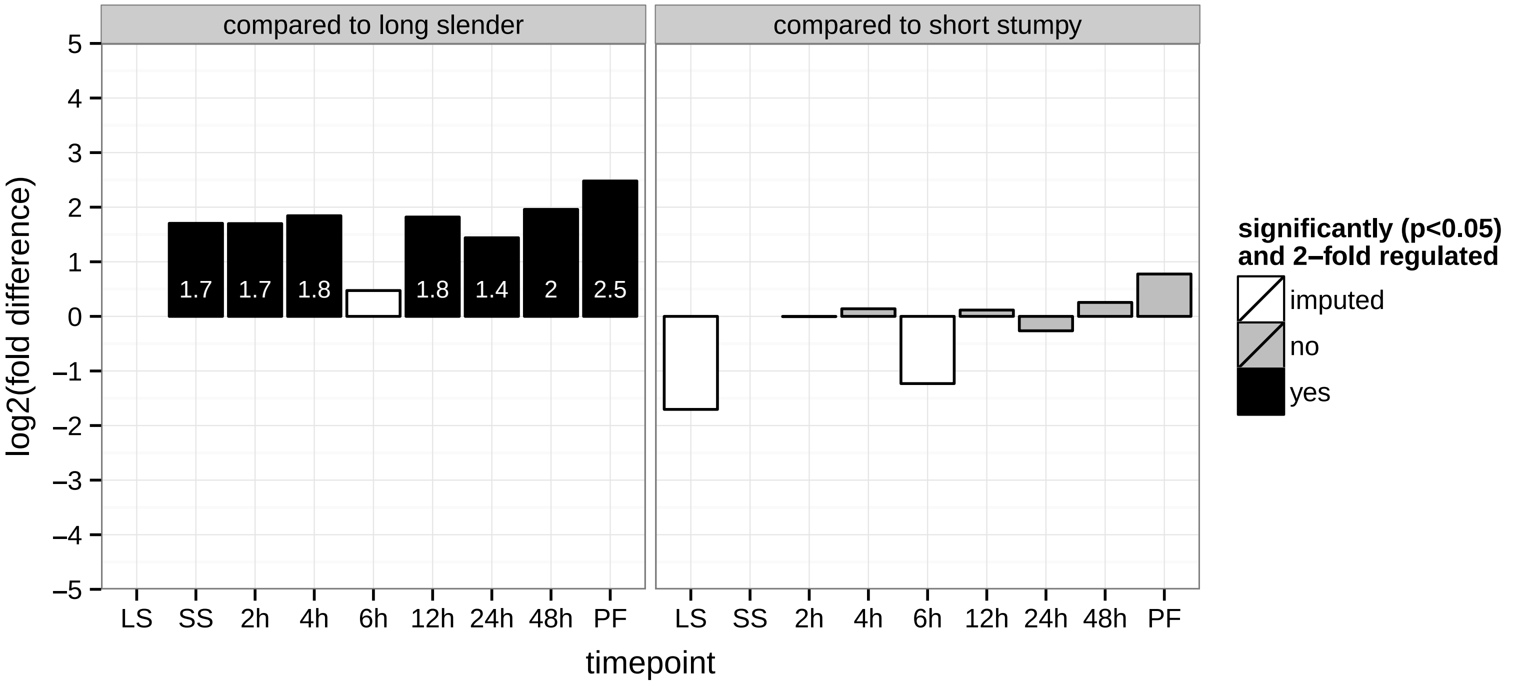

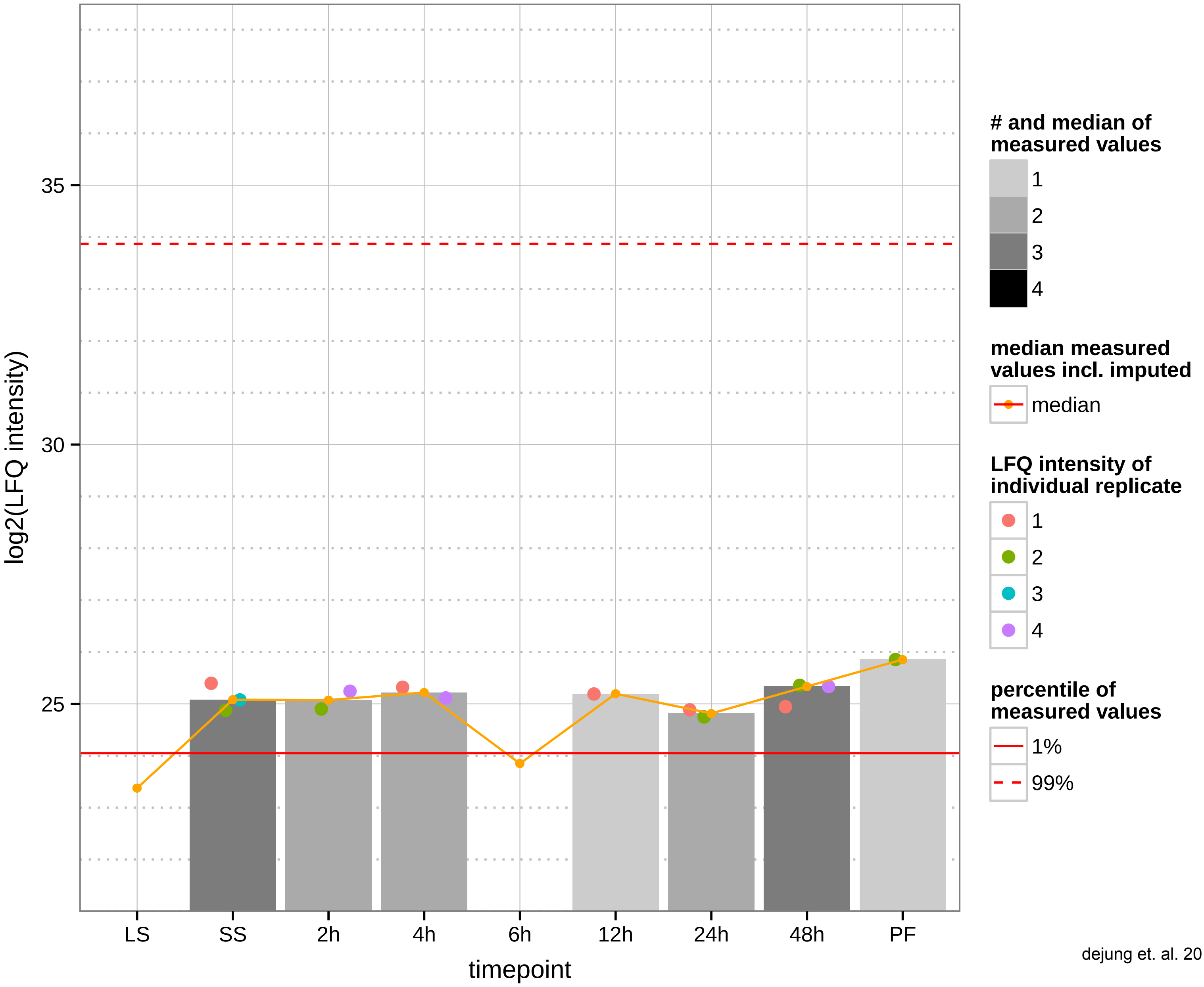

dejung et. al. 2015

hypothetical protein, conserved
Tb927.3.2210
AGOF: null
AGOC: null
AGOP: null
PGOF: null
PGOC: null
PGOP: null

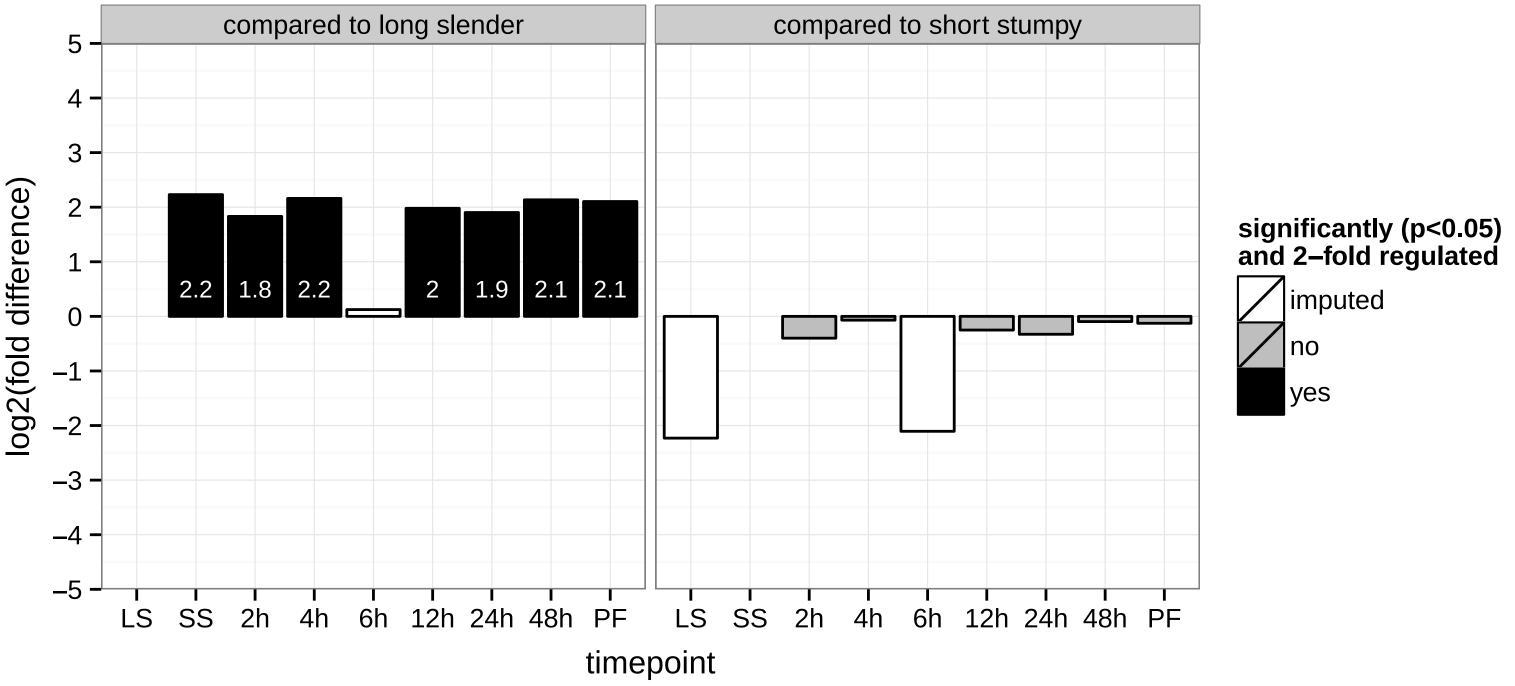

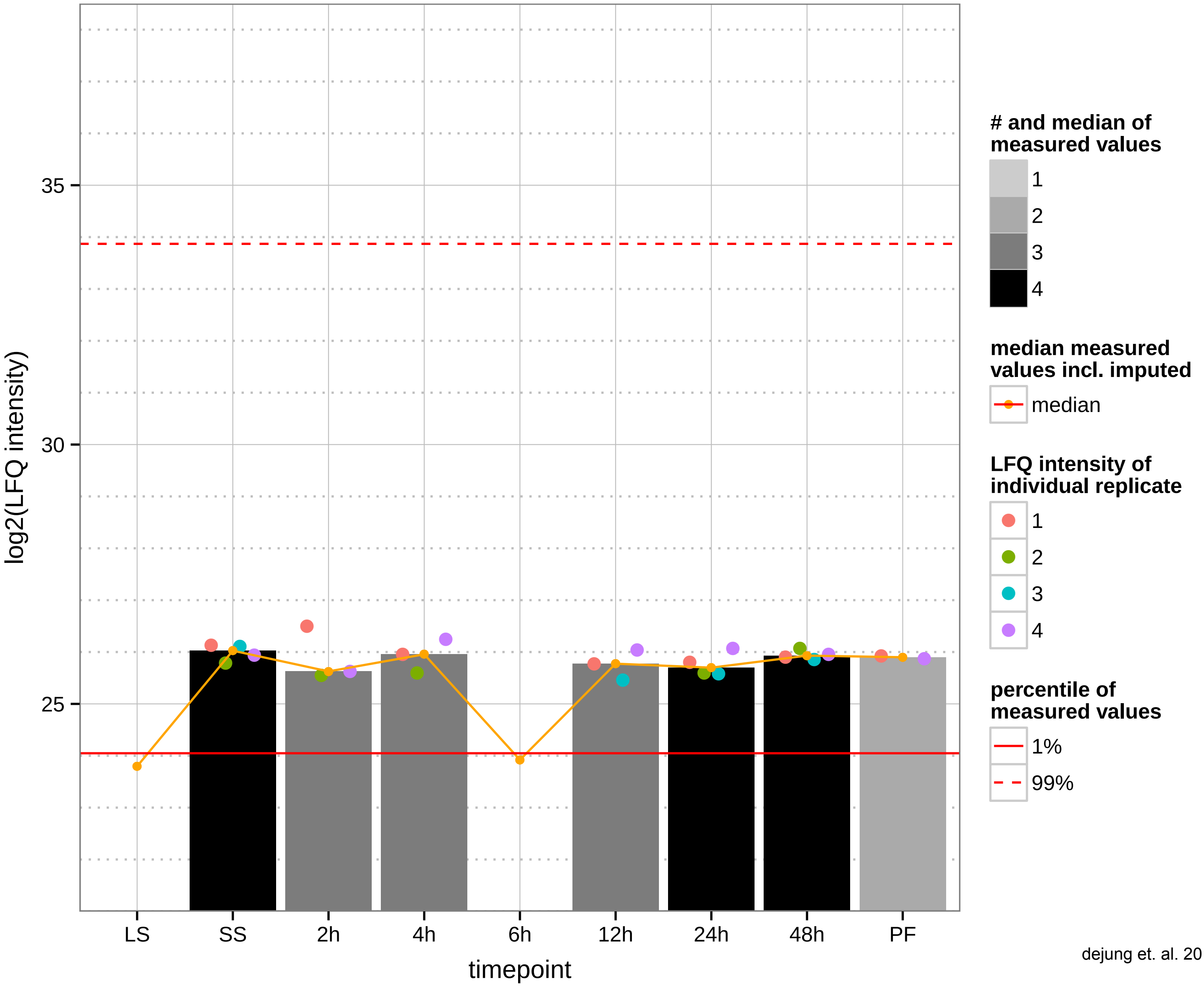

dejung et. al. 2015

hypothetical protein, conserved
Tb927.3.5350
AGOF: null
AGOC: null
AGOP: null
PGOF: null
PGOC: null
PGOP: null

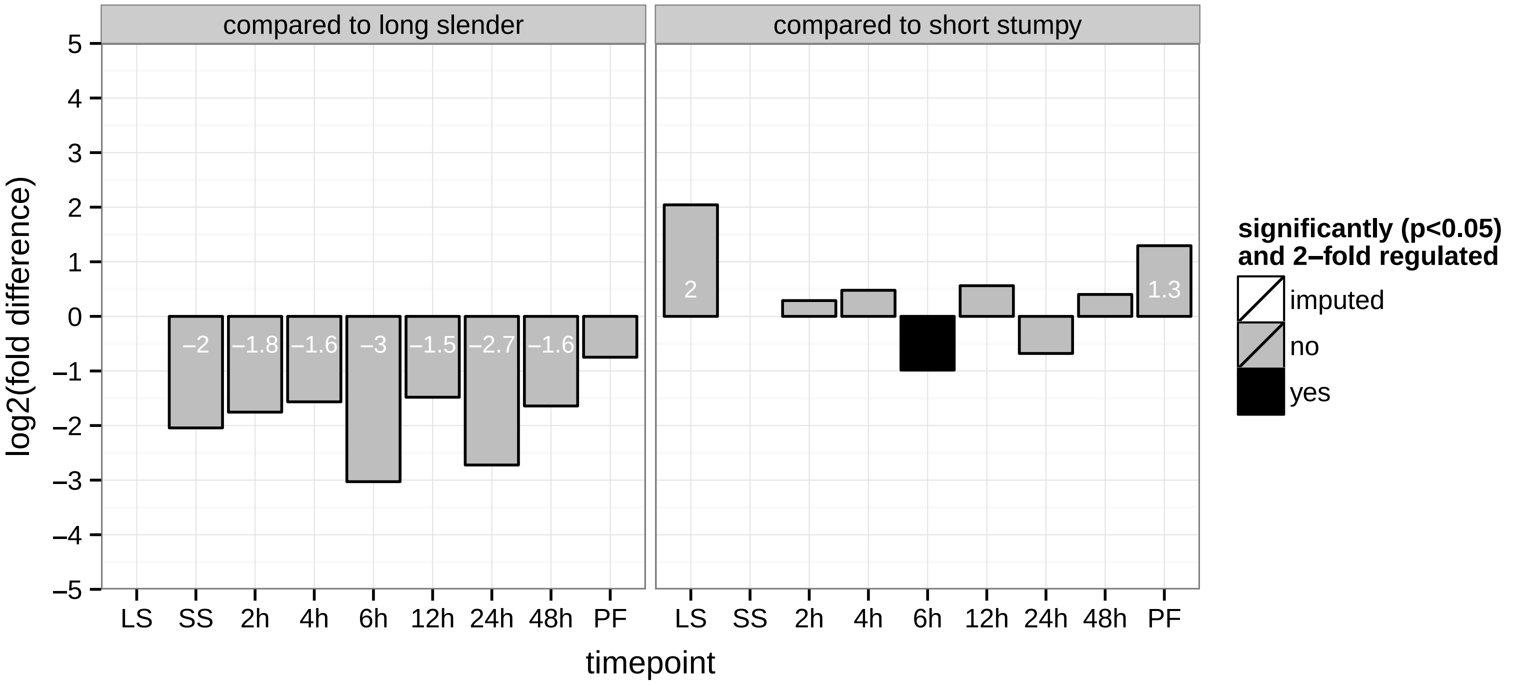

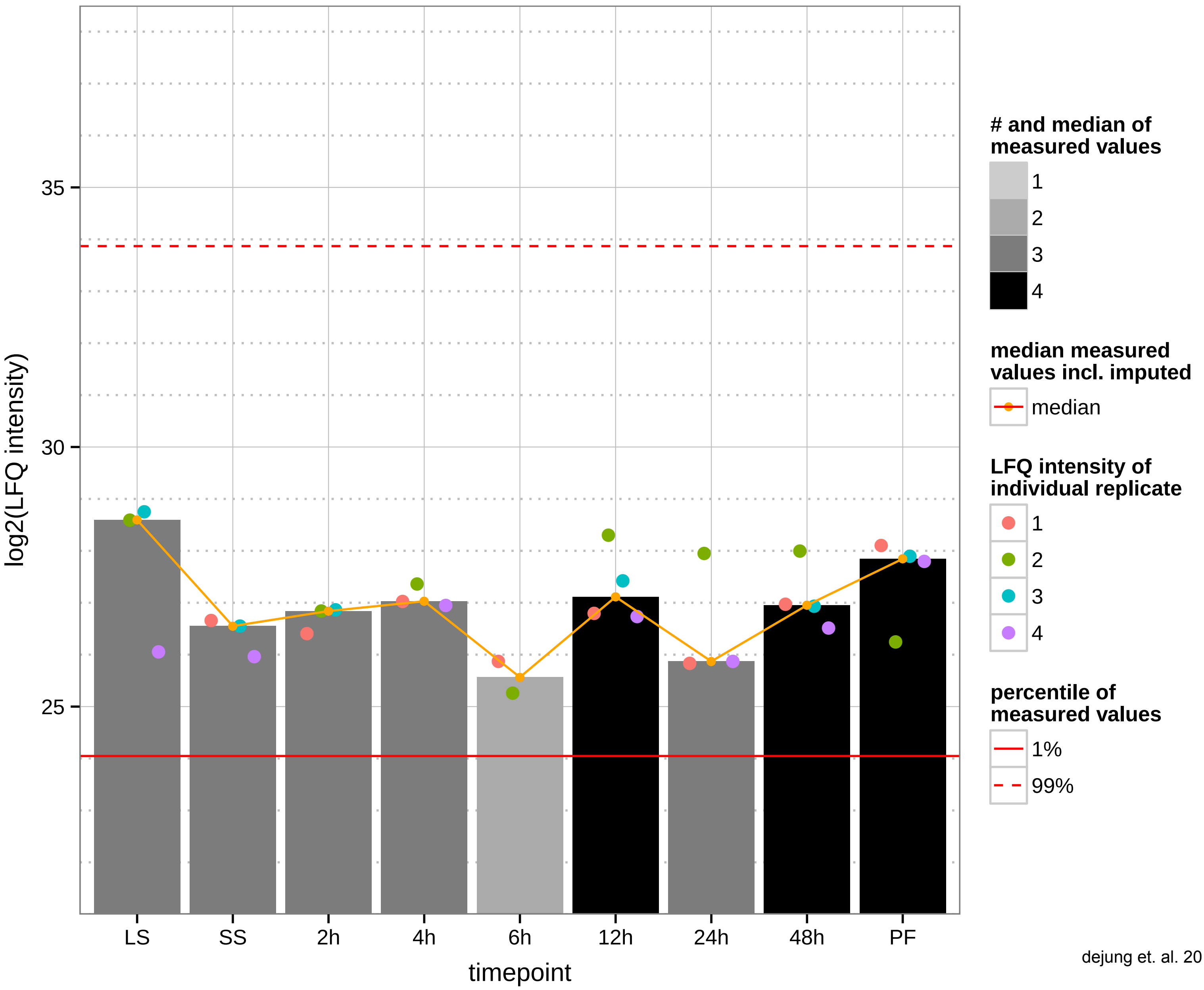

dejung et. al. 2015

hypothetical protein, conserved
Tb927.4.4560
AGOF: null
AGOC: null
AGOP: null
PGOF: null
PGOC: null
PGOP: null

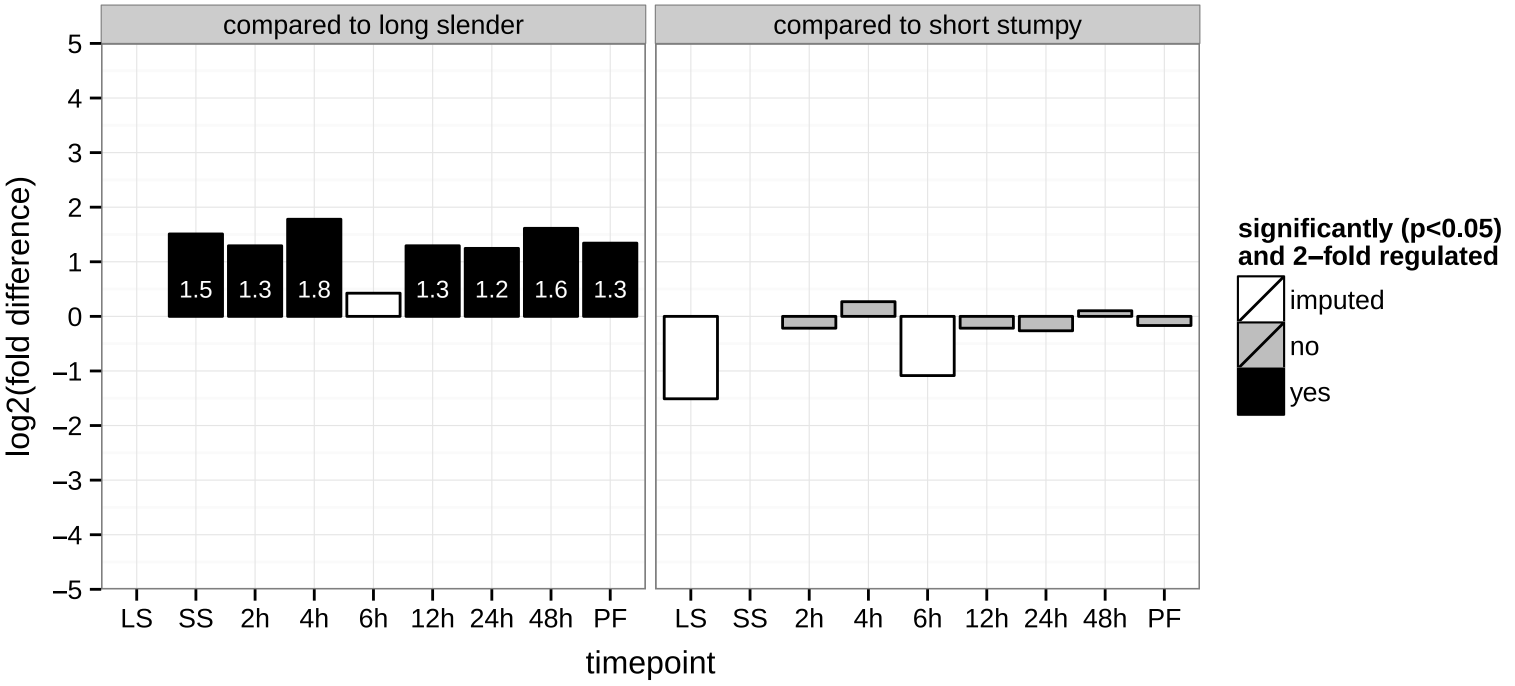

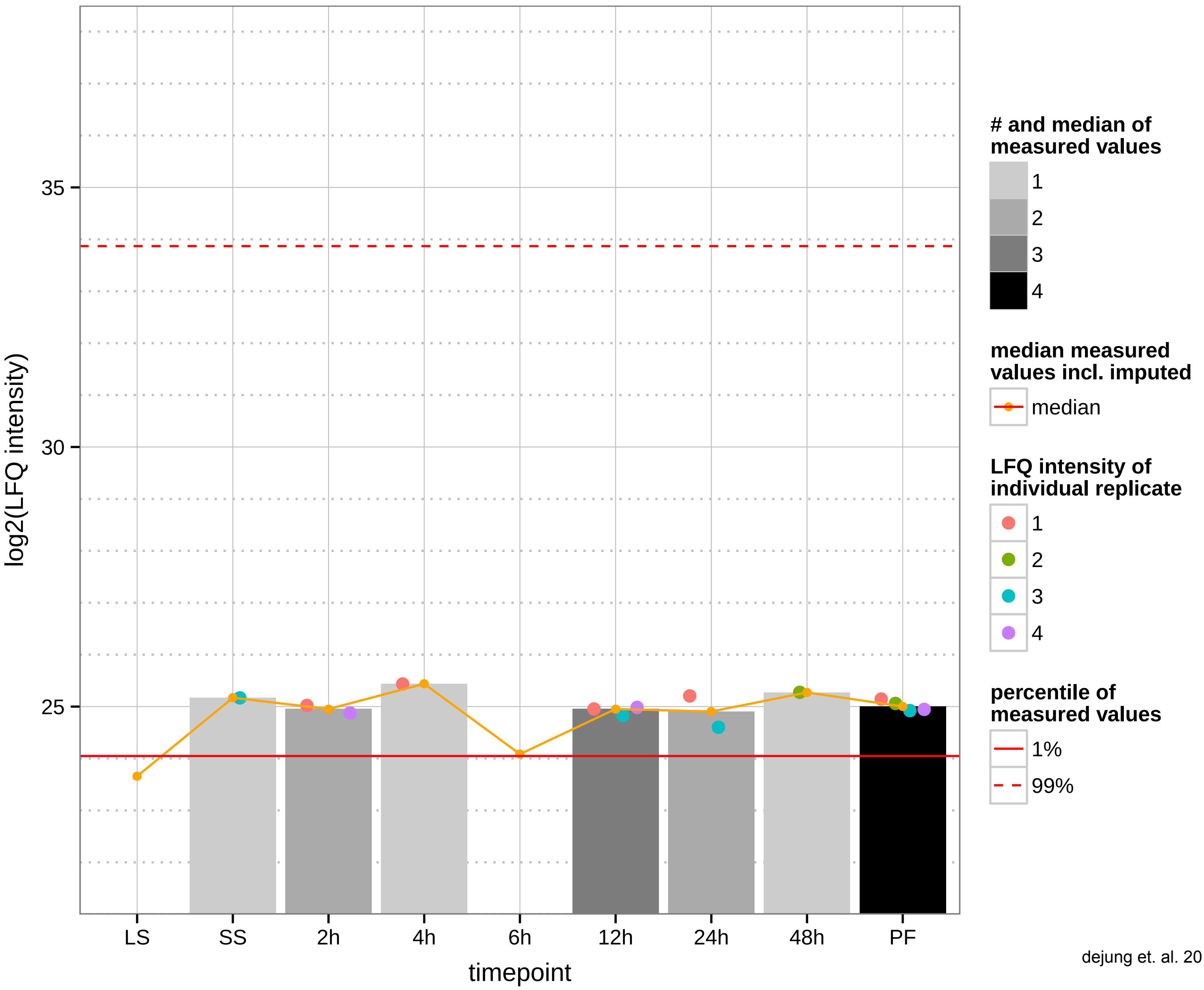

dejung et. al. 2015

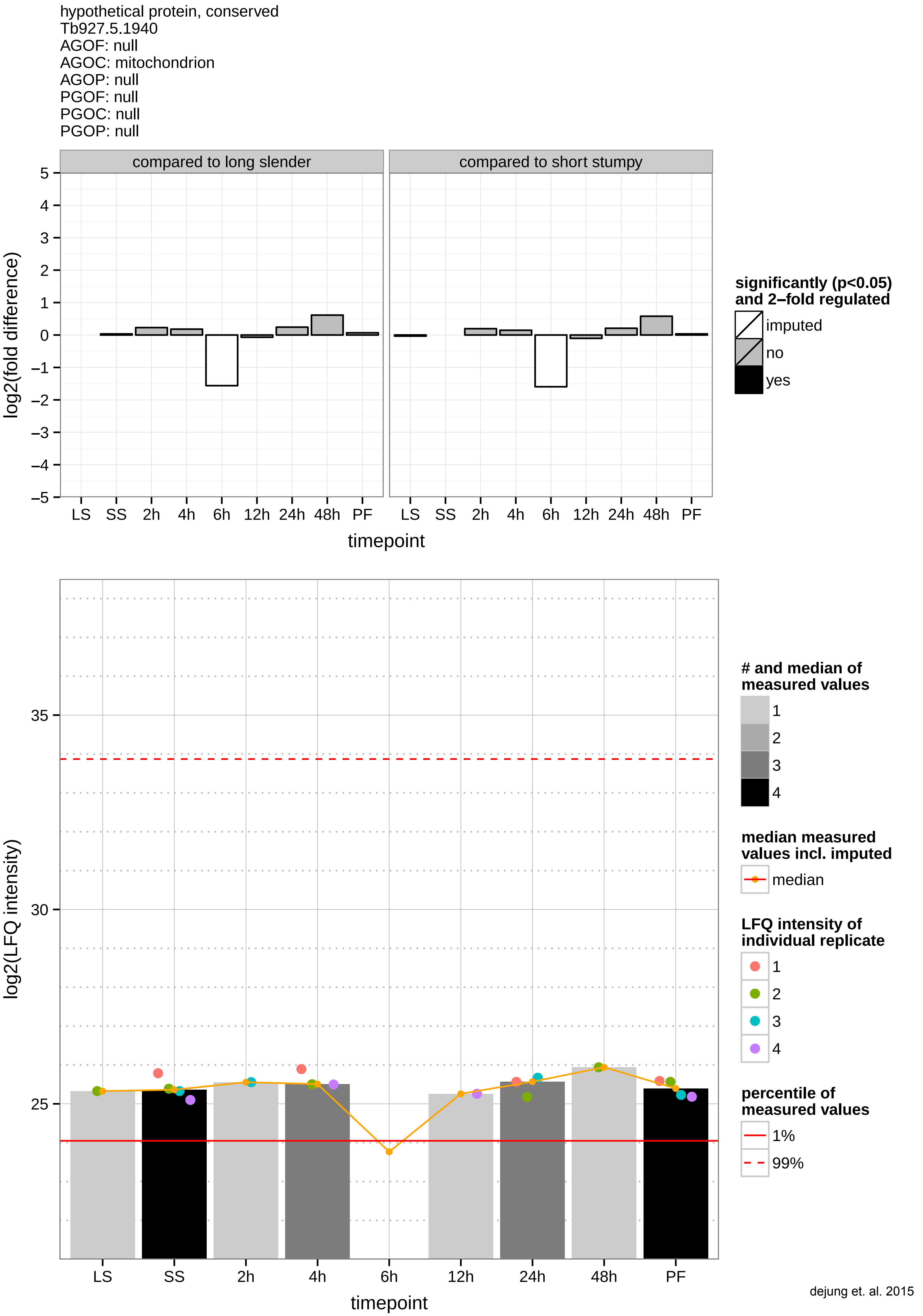

dejung et. al. 2015

hypothetical protein, conserved
Tb927.6.1590
AGOF: null
AGOC: mitochondrion
AGOP: null
PGOF: null
PGOC: null
PGOP: null

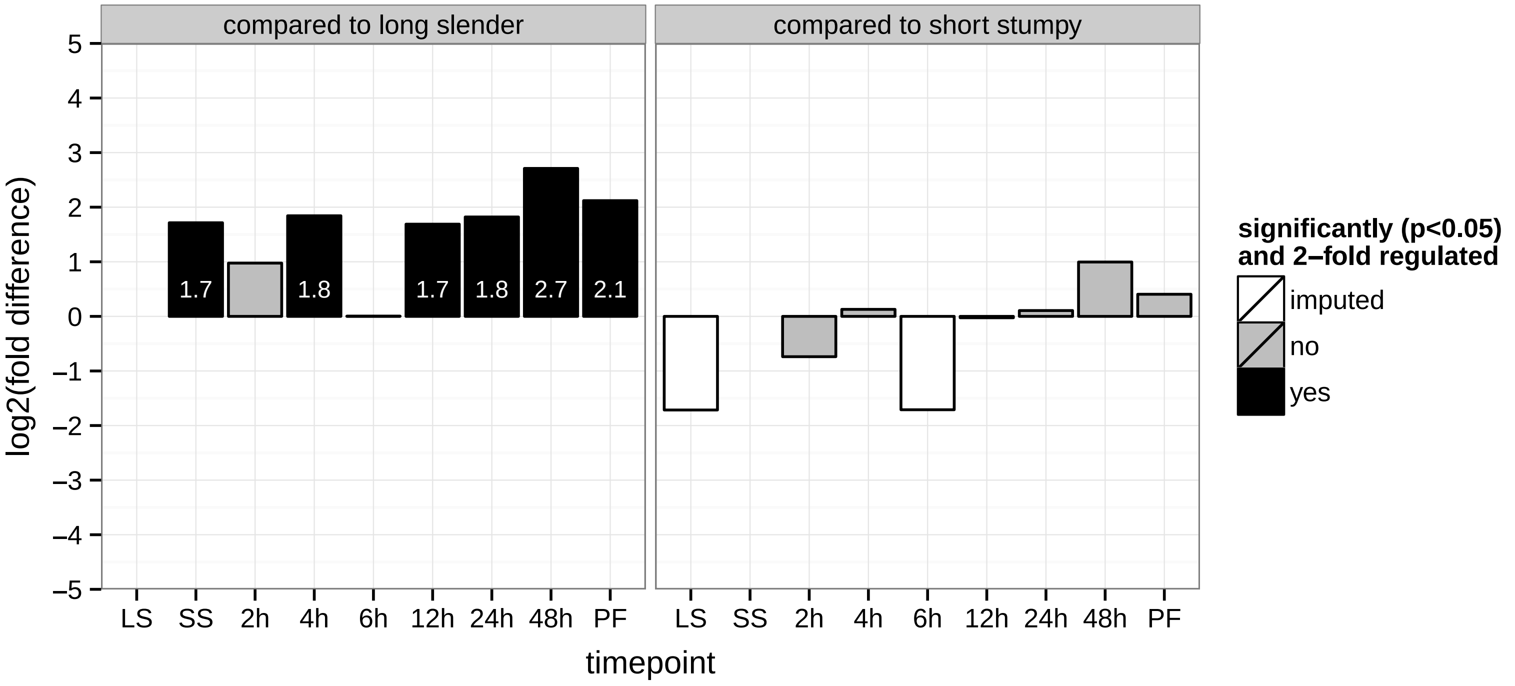

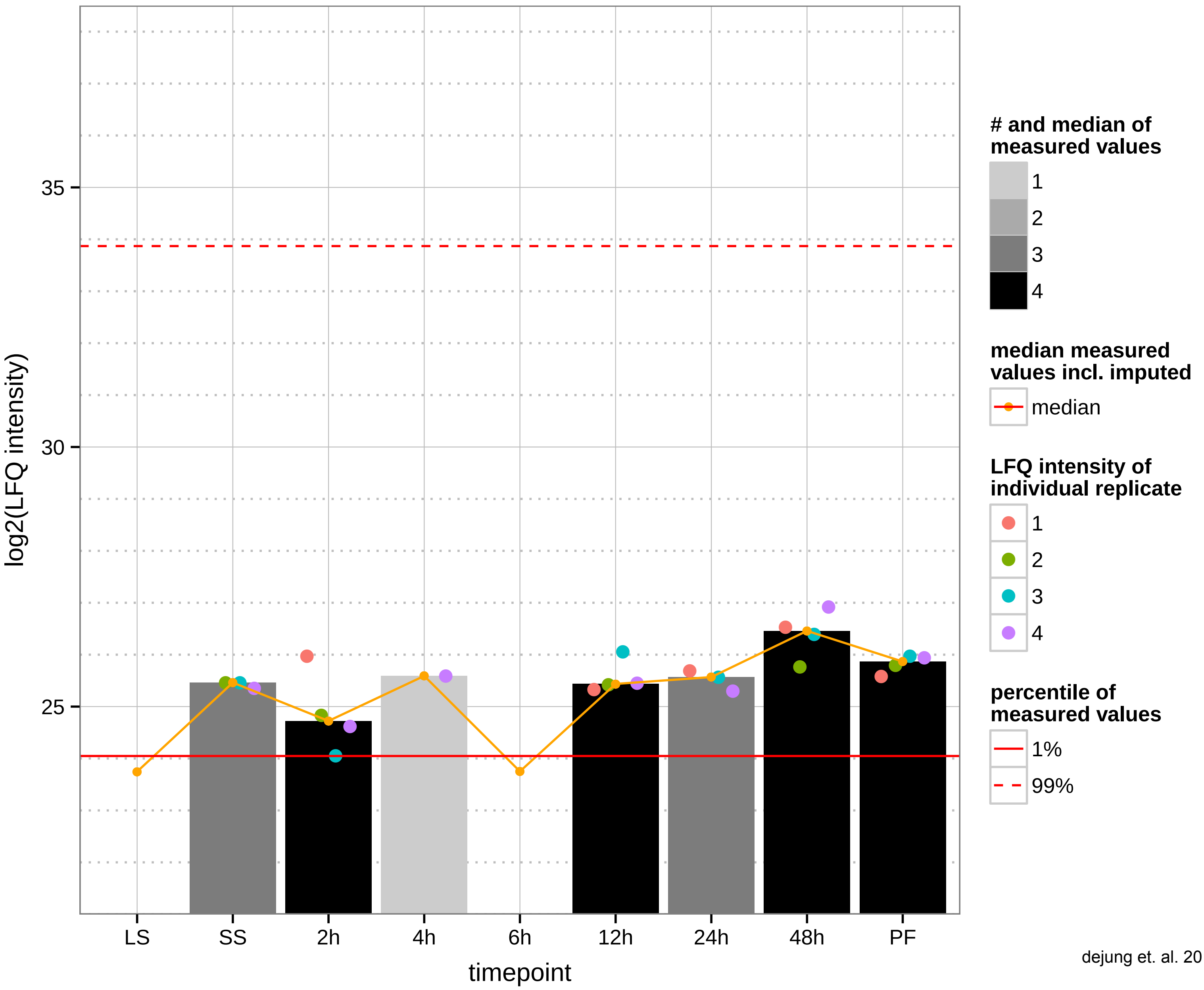

dejung et. al. 2015

mitochondrial RNA binding protein 1 (MRB1680)
Tb927.6.1680
AGOF: zinc ion binding
AGOC: intracellular, mitochondrion
AGOP: RNA processing
PGOF: zinc ion binding
PGOC: intracellular
PGOP: null

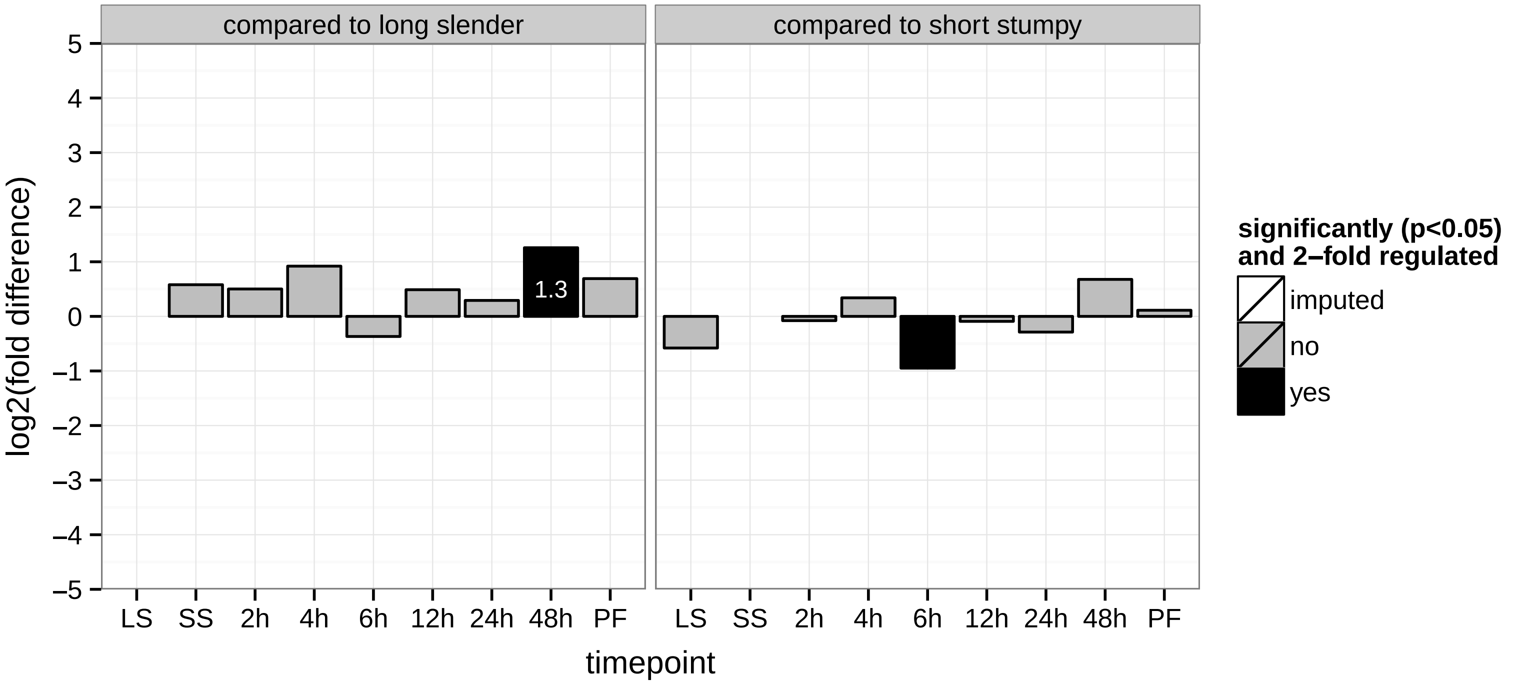

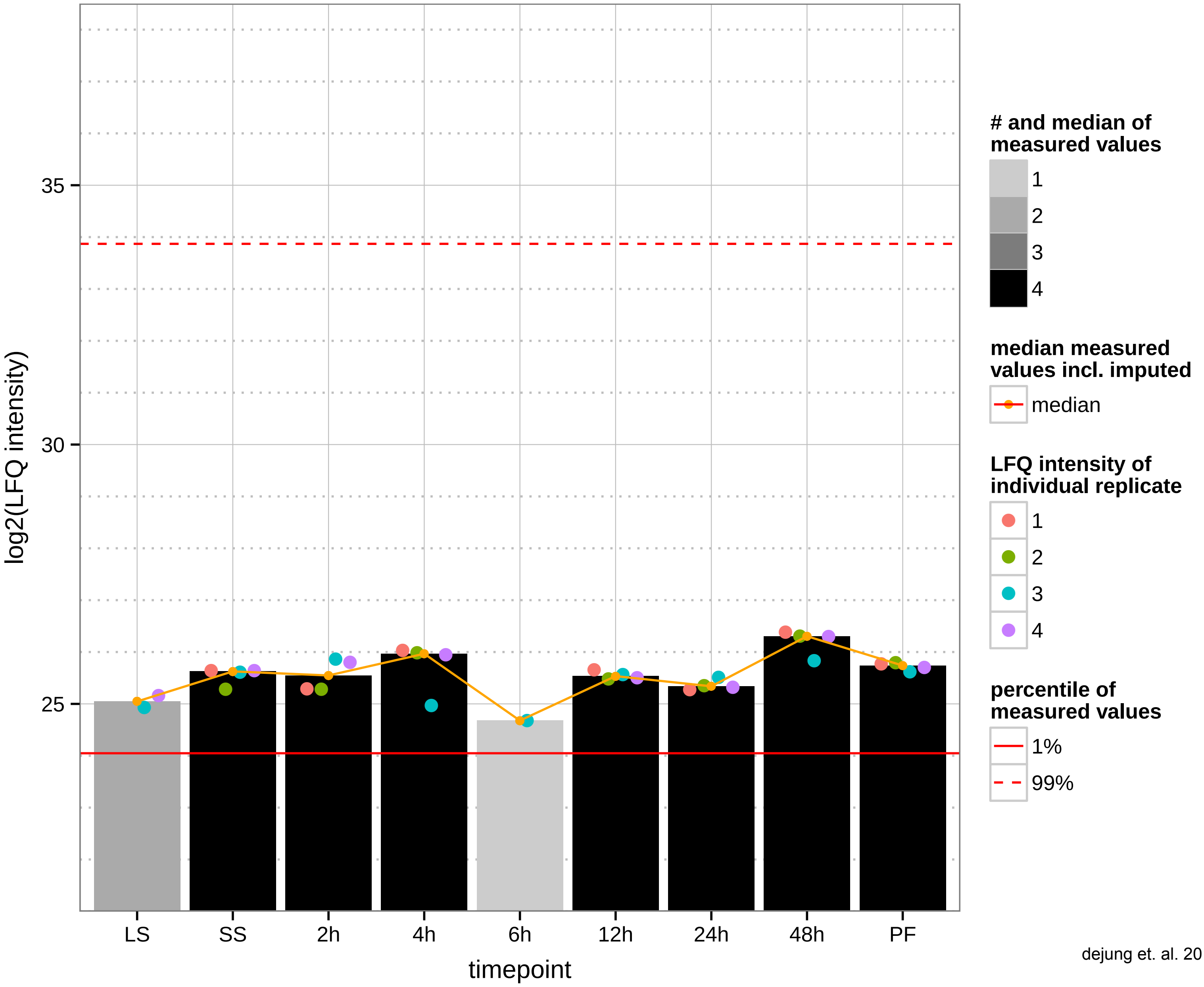

dejung et. al. 2015

prozyme, S–adenosylmethionine decarboxylaseregulator
Tb927.6.4470
AGOF: adenosylmethionine decarboxylase activity
AGOC: null
AGOP: spermidine biosynthetic process, spermine biosynthetic process
PGOF: adenosylmethionine decarboxylase activity
PGOC: null
PGOP: spermidine biosynthetic process, spermine biosynthetic process

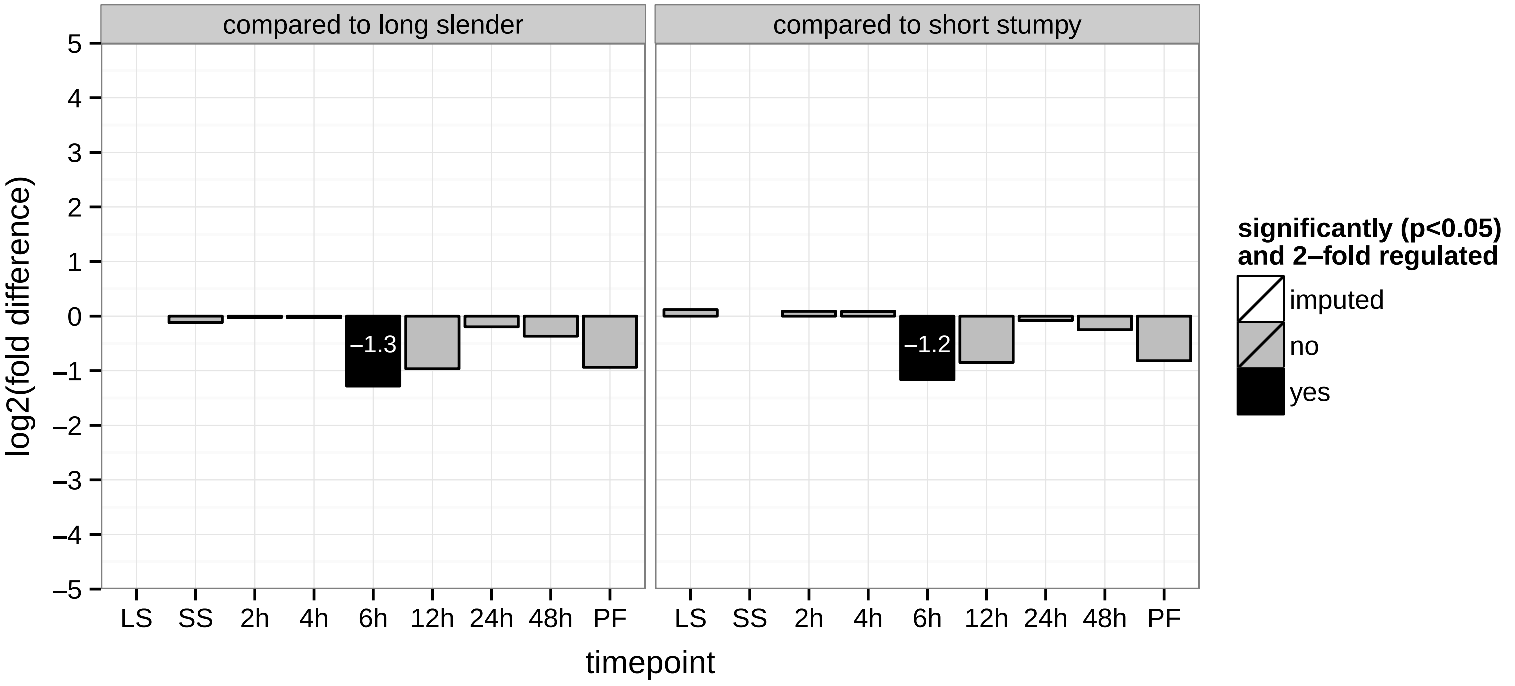

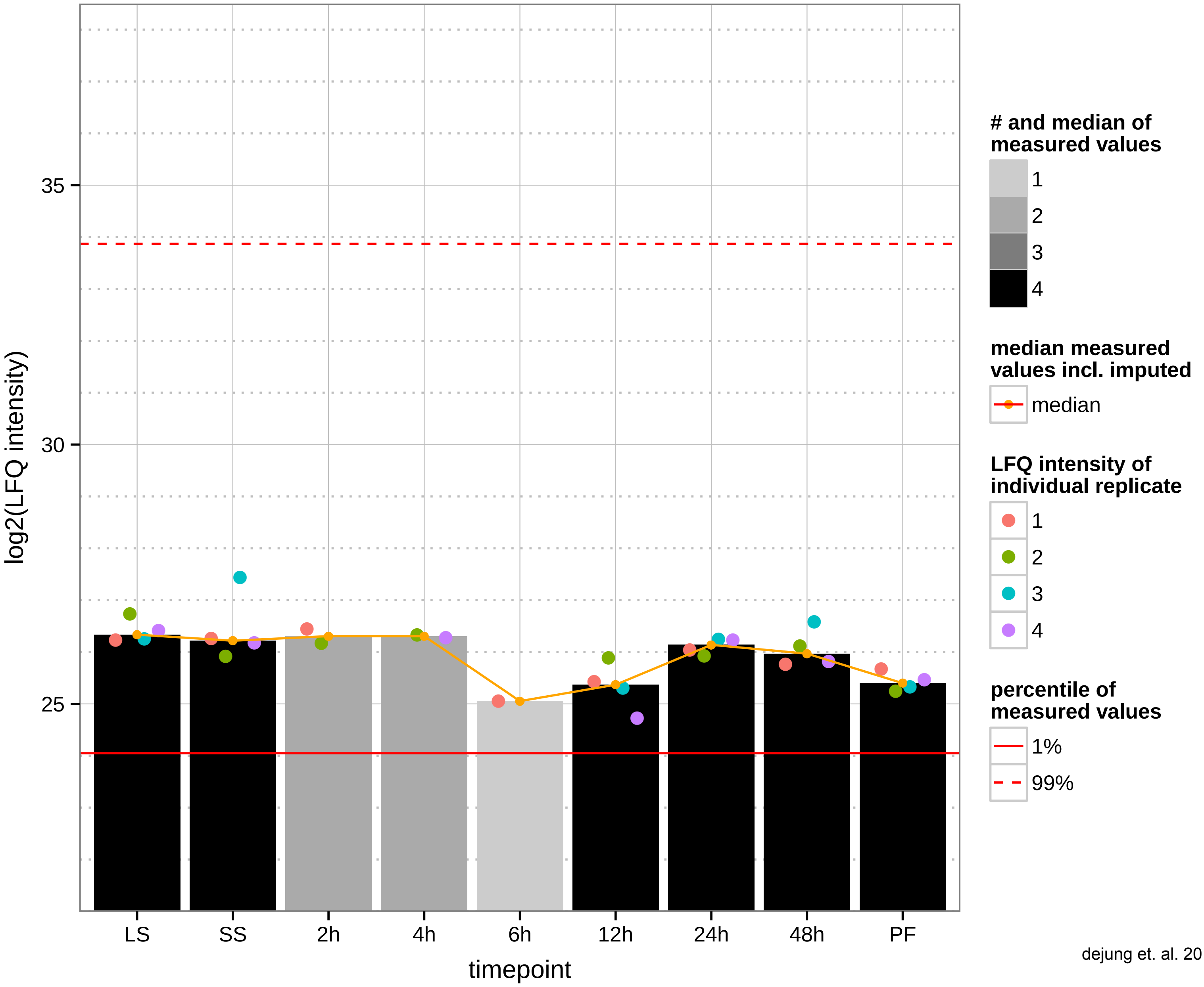

dejung et. al. 2015

RNA–binding protein, putative (Y14)
Tb927.7.1170
AGOF: RNA binding, protein binding
AGOC: exon–exon junction complex, nucleus
AGOP: RNA processing
PGOF: nucleic acid binding
PGOC: null
PGOP: null

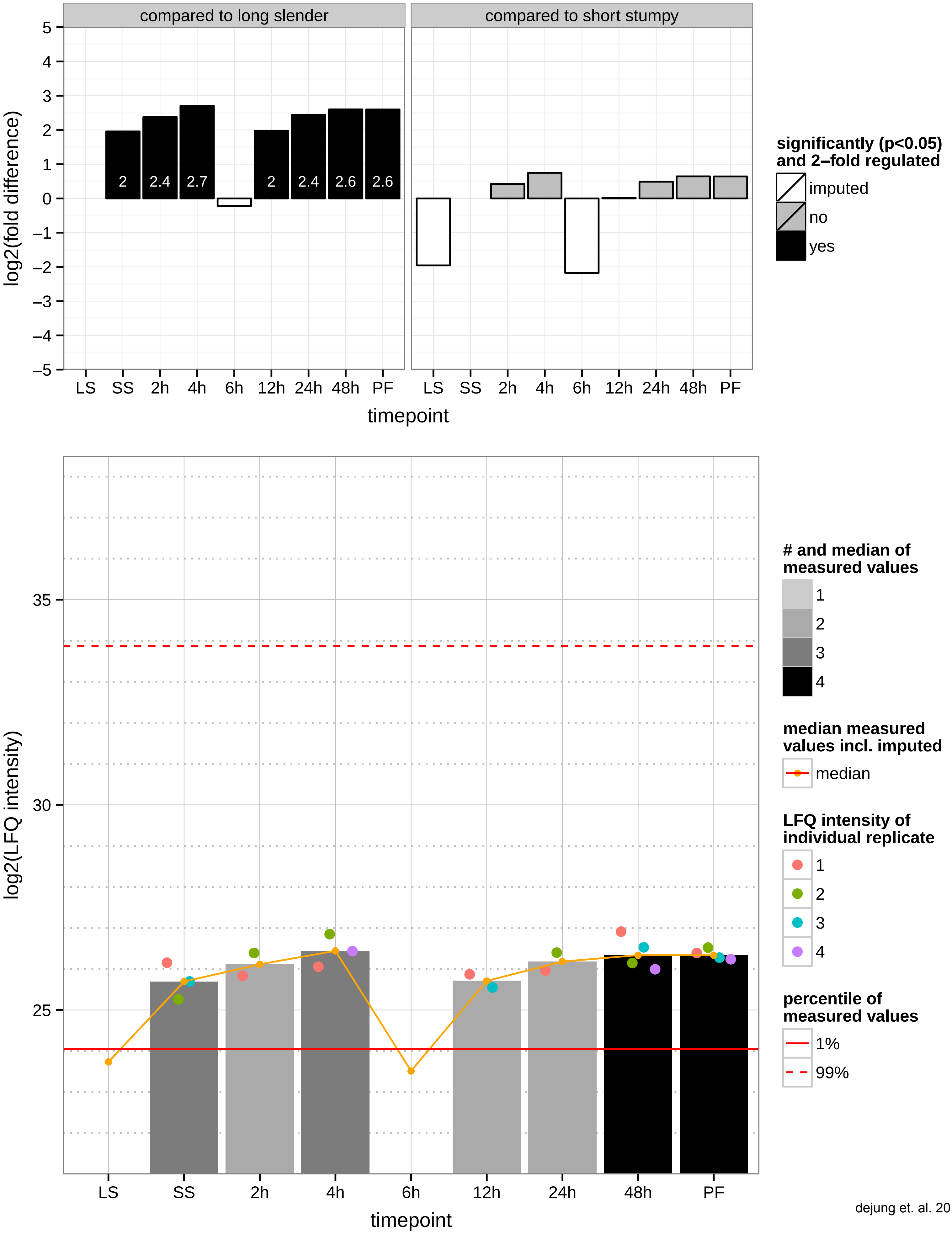

dejung et. al. 2015

zinc finger protein family member, putative (ZC3H21)
Tb927.7.2670
AGOF: RNA binding
AGOC: null
AGOP: null
PGOF: nucleic acid binding, zinc ion binding
PGOC: null
PGOP: null

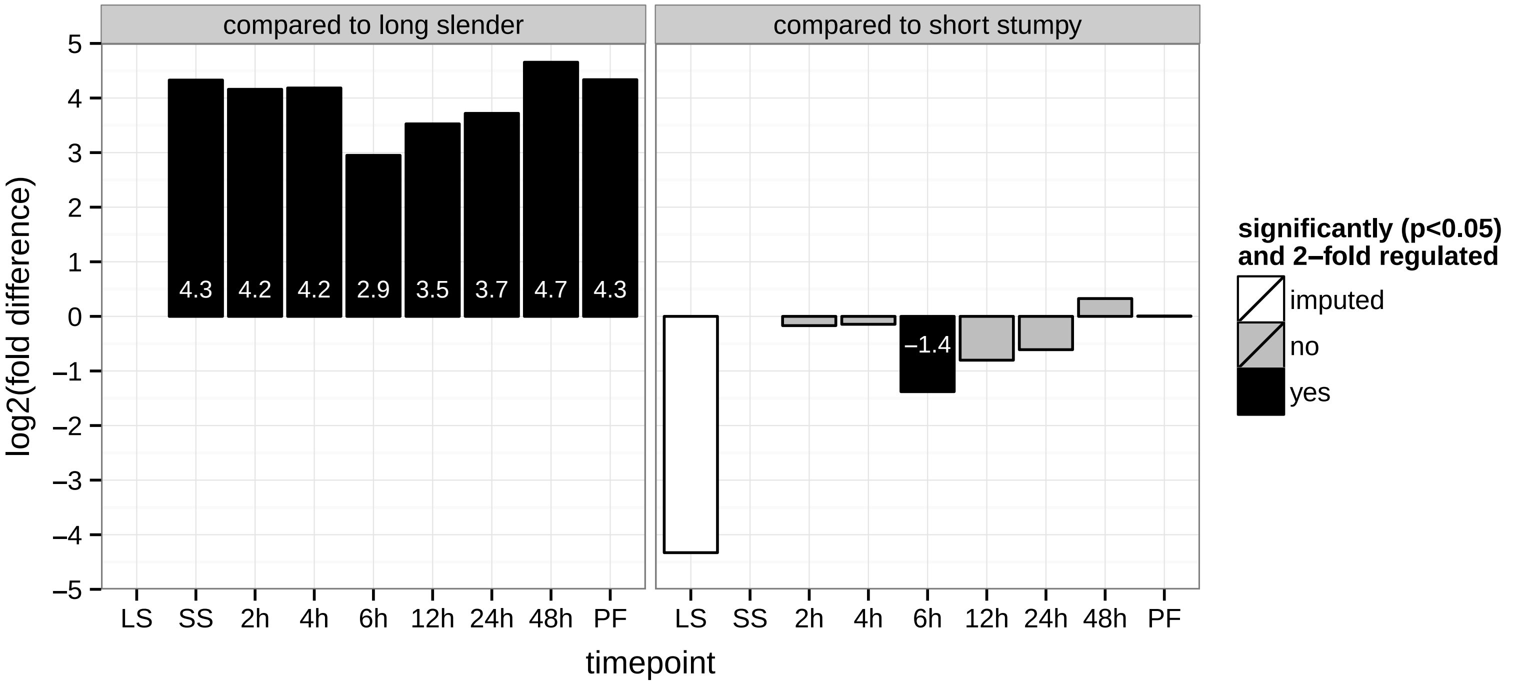

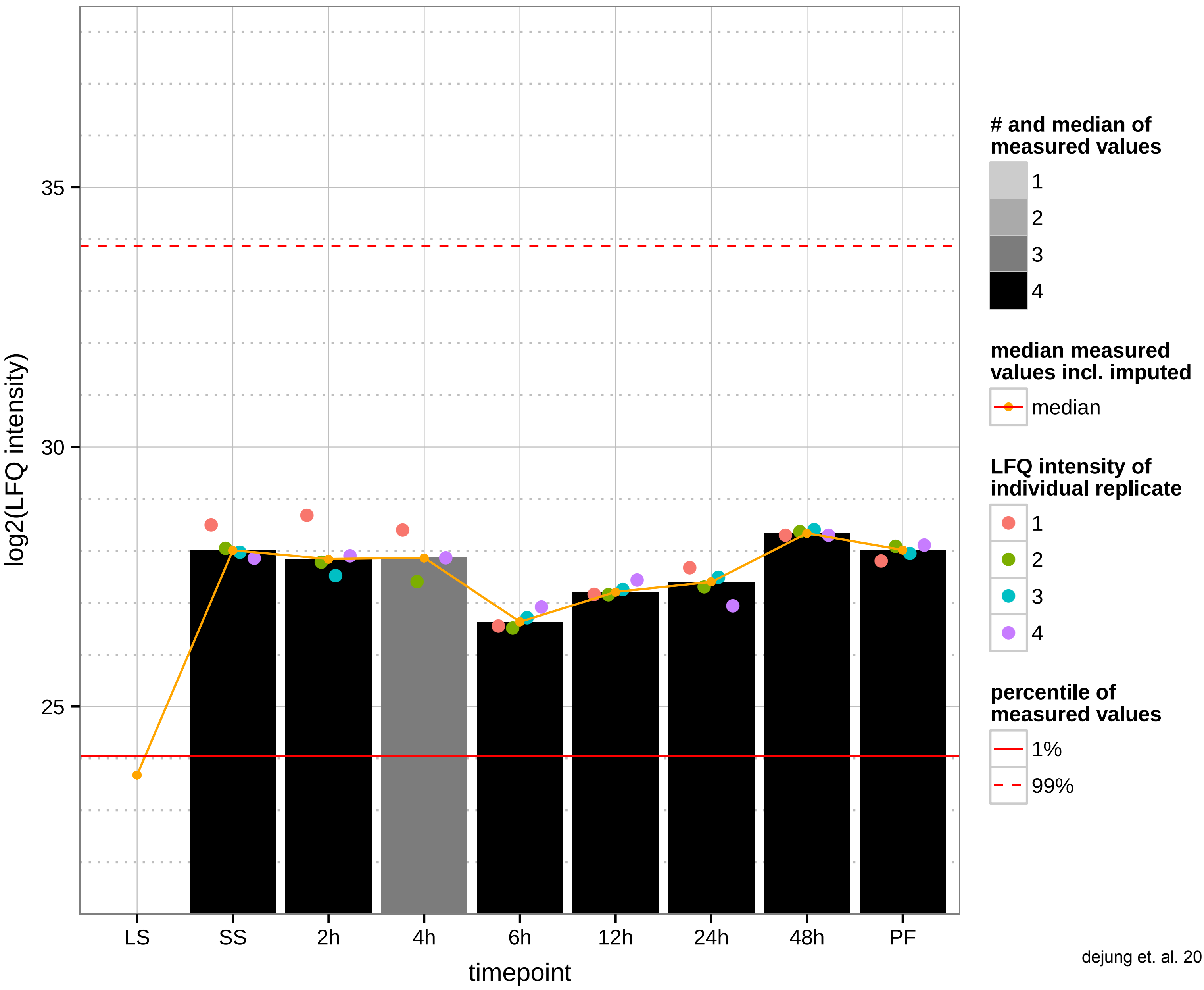

dejung et. al. 2015

hypothetical protein, conserved
Tb927.8.1770
AGOF: null
AGOC: mitochondrion
AGOP: null
PGOF: null
PGOC: null
PGOP: null

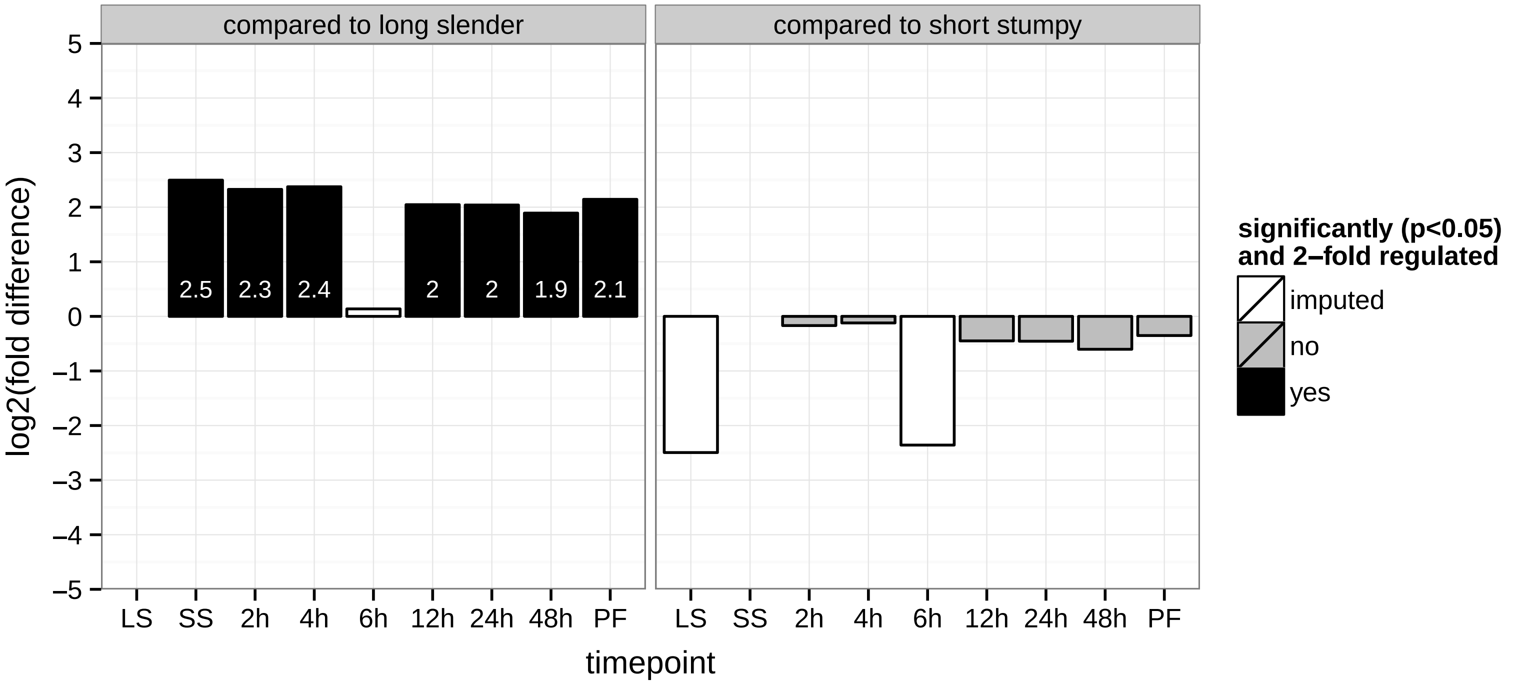

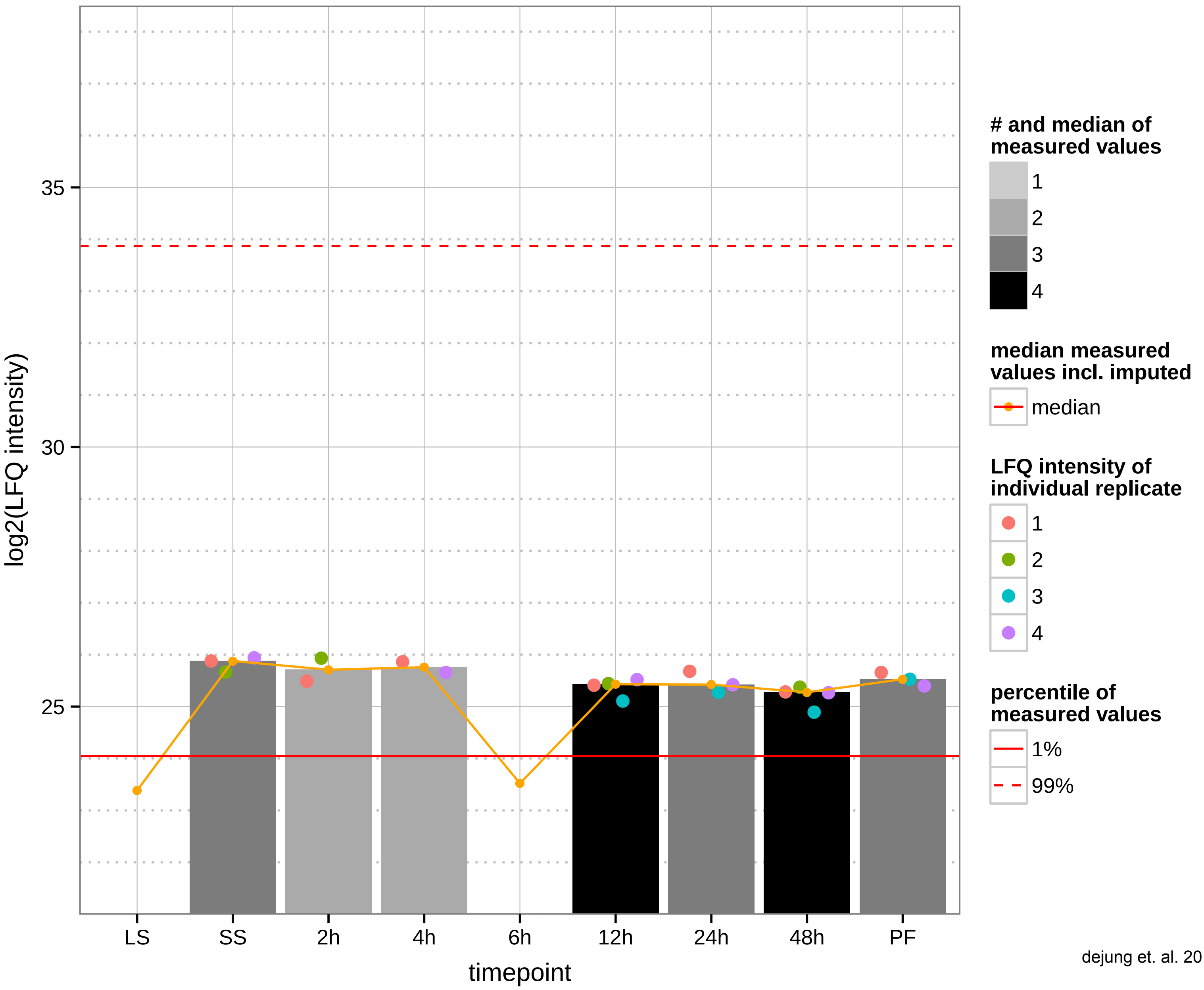

dejung et. al. 2015

cullin, putative
Tb927.8.5970
AGOF: ubiquitin protein ligase binding
AGOC: cullin–RING ubiquitin ligase complex
AGOP: cell cycle, ubiquitin–dependent protein catabolic process
PGOF: ubiquitin protein ligase binding
PGOC: cullin–RING ubiquitin ligase complex
PGOP: ubiquitin–dependent protein catabolic process

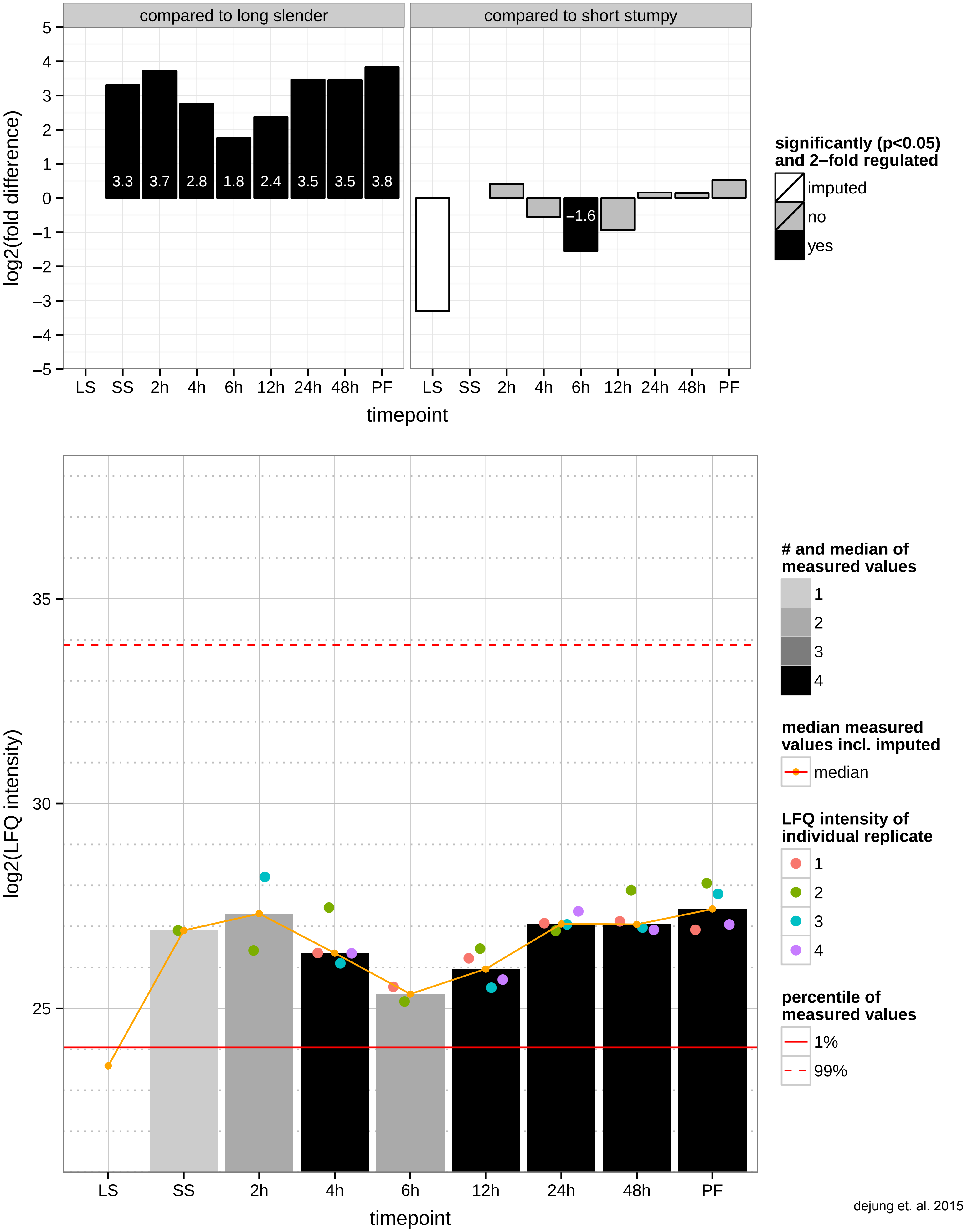

dejung et. al. 2015

hypothetical protein, conserved
Tb927.8.6050
AGOF: null
AGOC: null
AGOP: null
PGOF: null
PGOC: null
PGOP: null

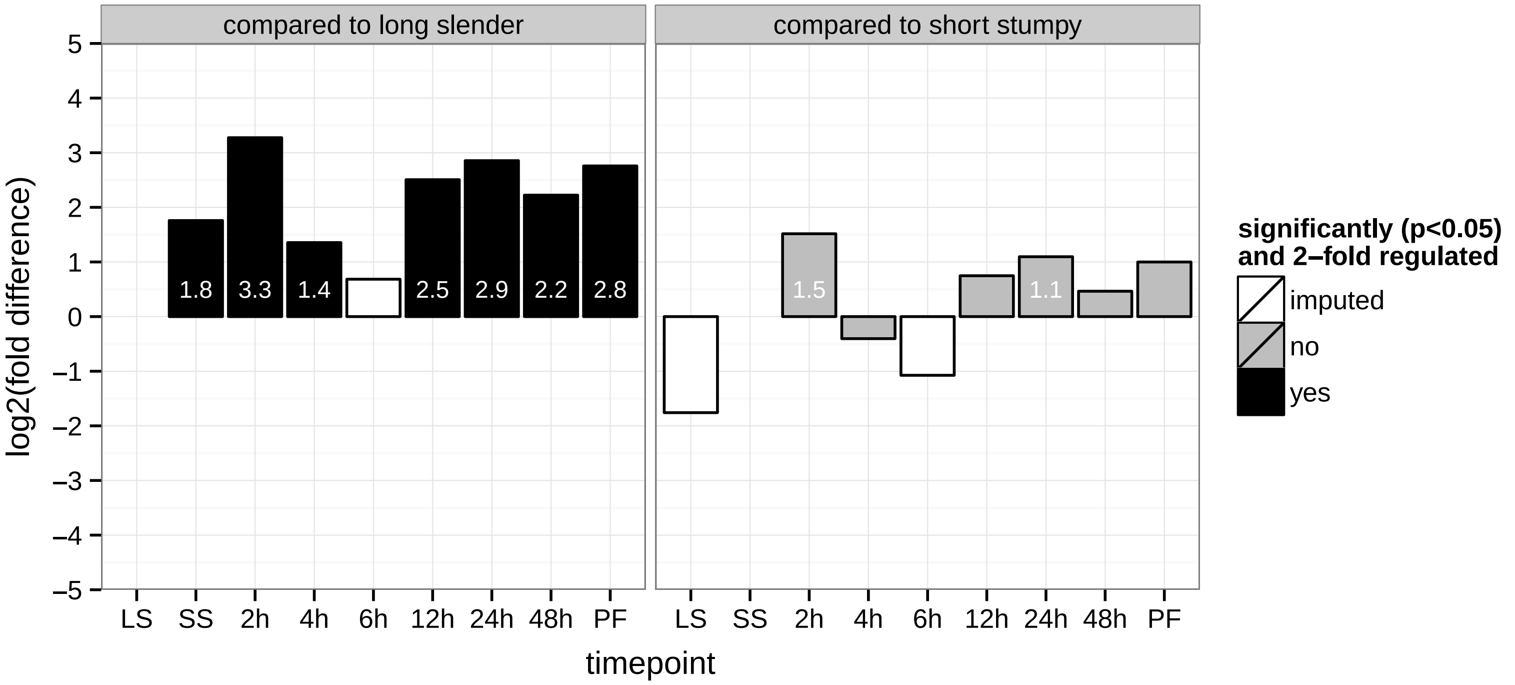

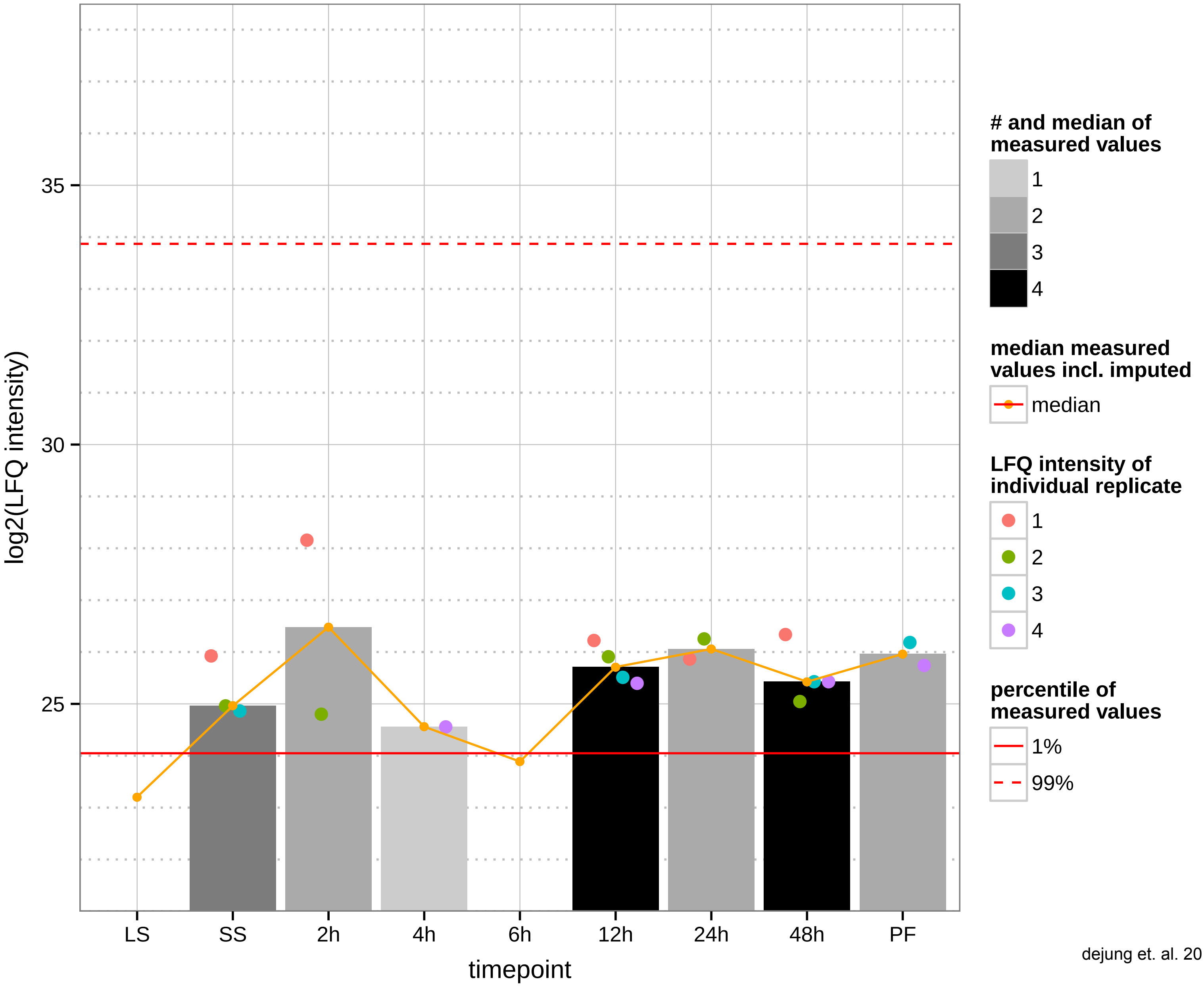

dejung et. al. 2015

hypothetical protein, conserved
Tb927.8.610
AGOF: null
AGOC: null
AGOP: null
PGOF: protein binding
PGOC: null
PGOP: null

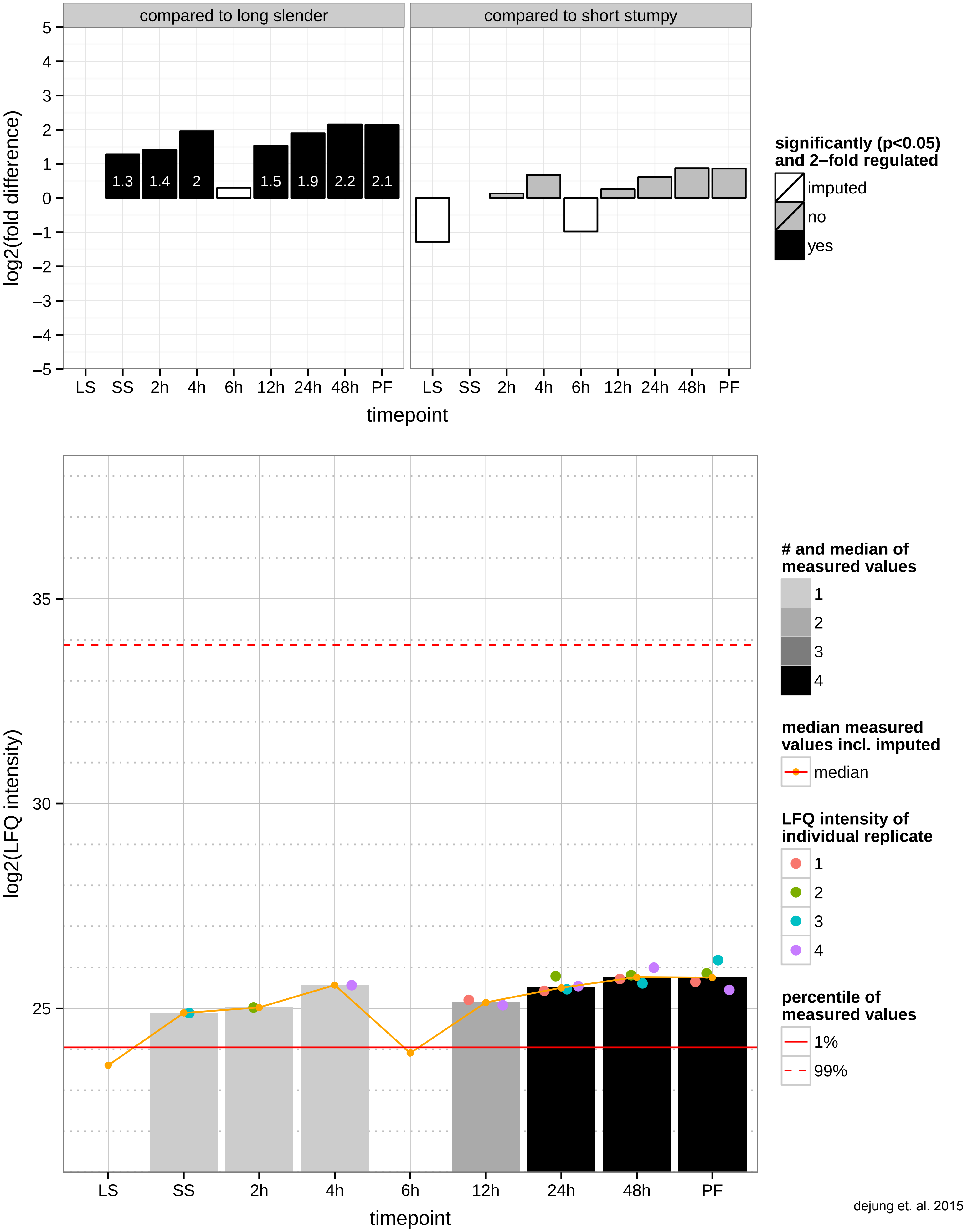

dejung et. al. 2015

kinetoplastid kinetochore protein 3, protein kinase, putative (kkt3)
Tb927.9.10920
AGOF: ATP binding, protein serine/threonine kinase activity, protein tyrosine kinase activity, zinc ion binding
AGOC: intracellular, kinetochore
AGOP: cell proliferation, chromosome segregation, protein phosphorylation
PGOF: ATP binding, protein kinase activity, transferase activity, transferring phosphorus–containing groups, zinc ion binding
PGOC: intracellular
PGOP: protein phosphorylation

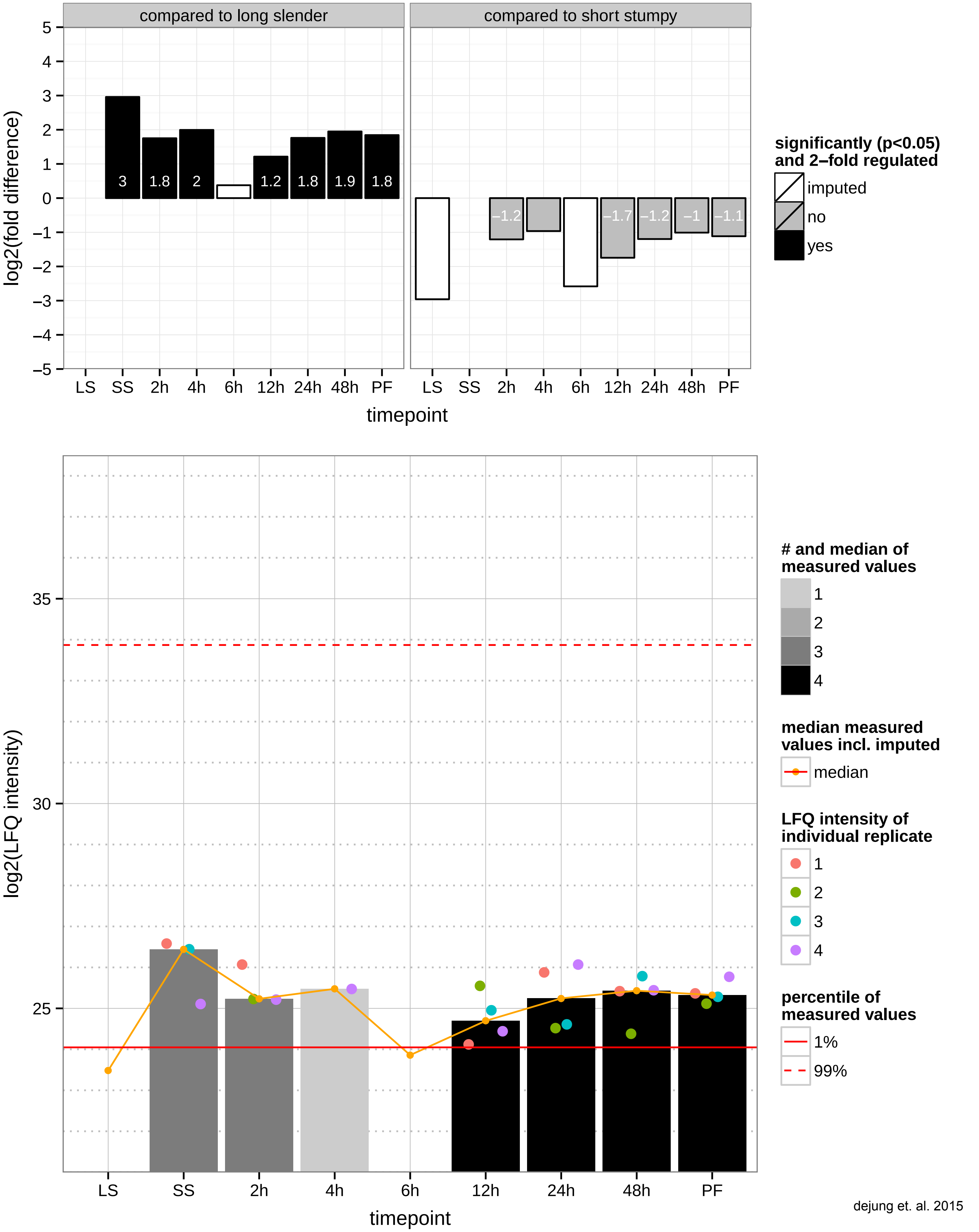

dejung et. al. 2015

hypothetical protein, conserved
Tb927.9.8180
AGOF: null
AGOC: null
AGOP: null
PGOF: null
PGOC: null
PGOP: null

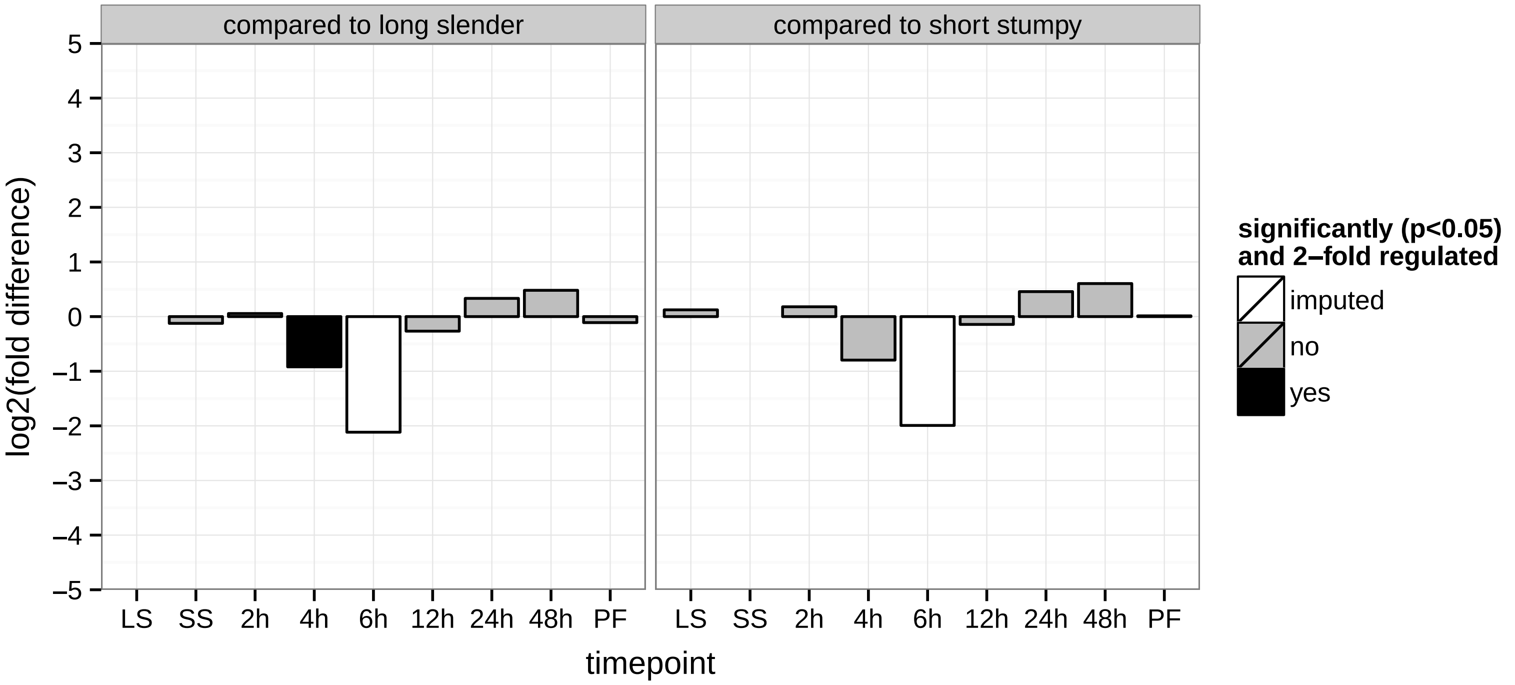

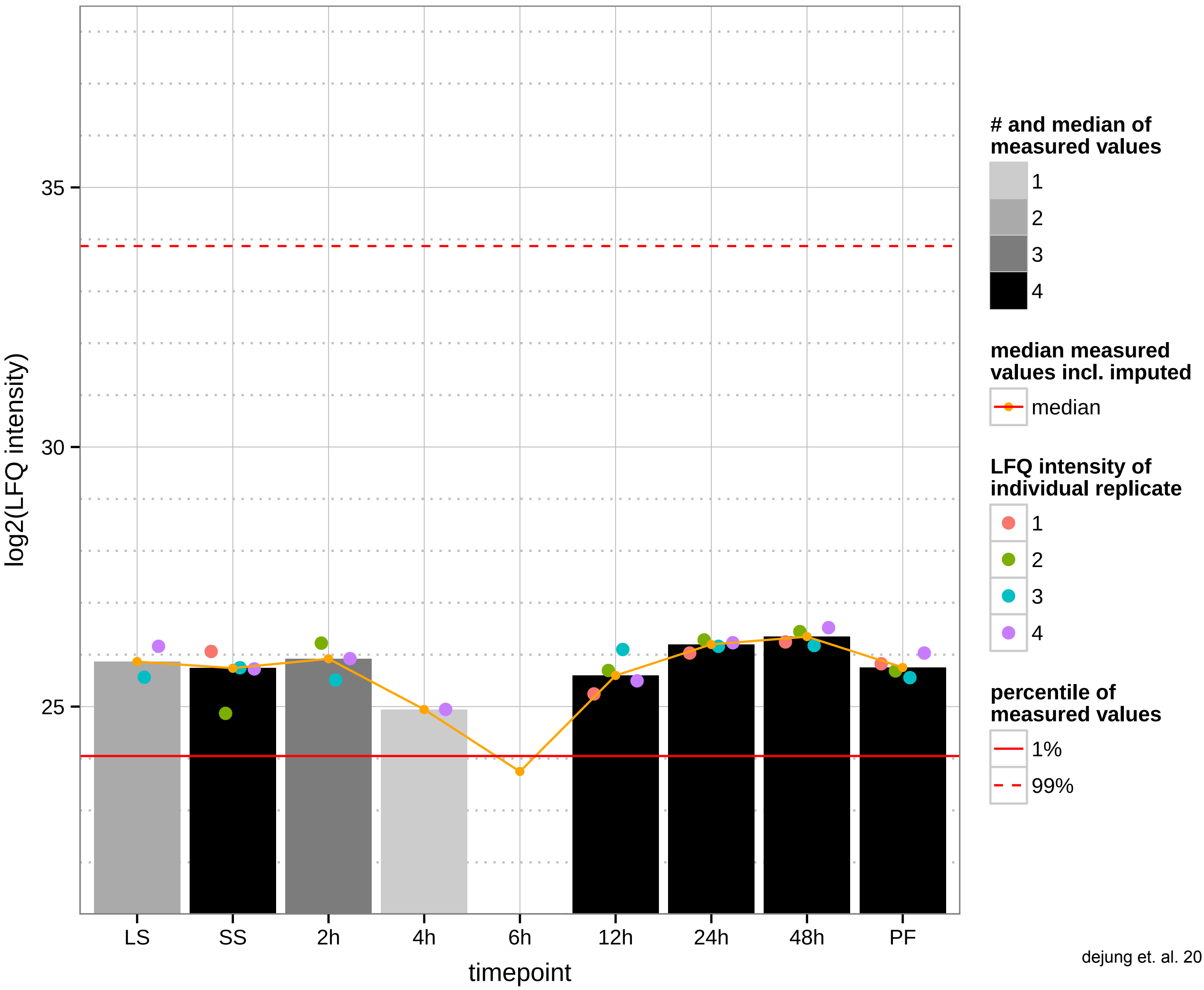

dejung et. al. 2015

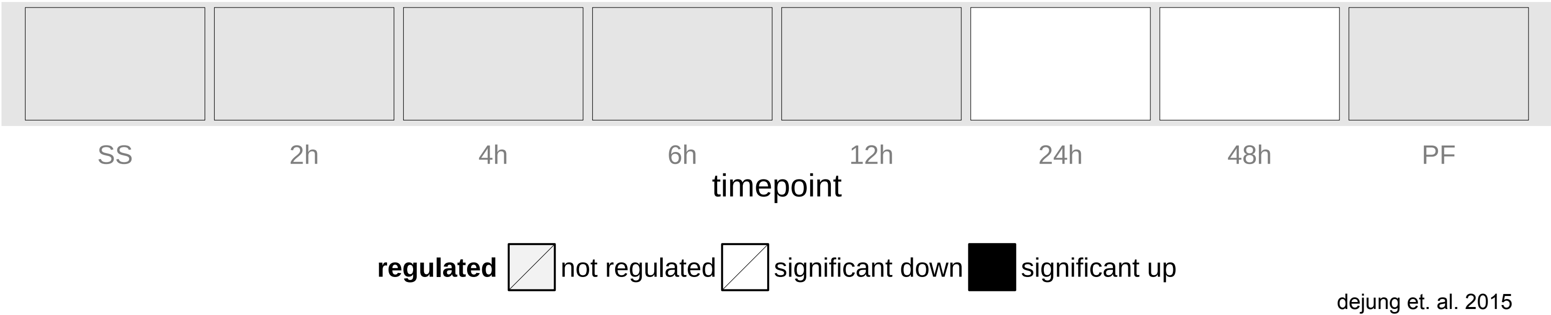
SS
2h
4h
6h
12h
24h
48h
PF
timepoint
regulated
not regulated
significant down
significant up
dejung et. al. 2015

protein kinase, putative, mitogen–activated protein kinase 9, putative (MPK9)
Tb927.10.14800;Tb11.v5.0626
AGOF: null, ATP binding, protein serine/threonine kinase activity, protein tyrosine kinase activity
AGOC: null
AGOP: null, protein phosphorylation
PGOF: ATP binding, protein kinase activity, transferase activity, transferring phosphorus–containing groups
PGOC: null
PGOP: protein phosphorylation

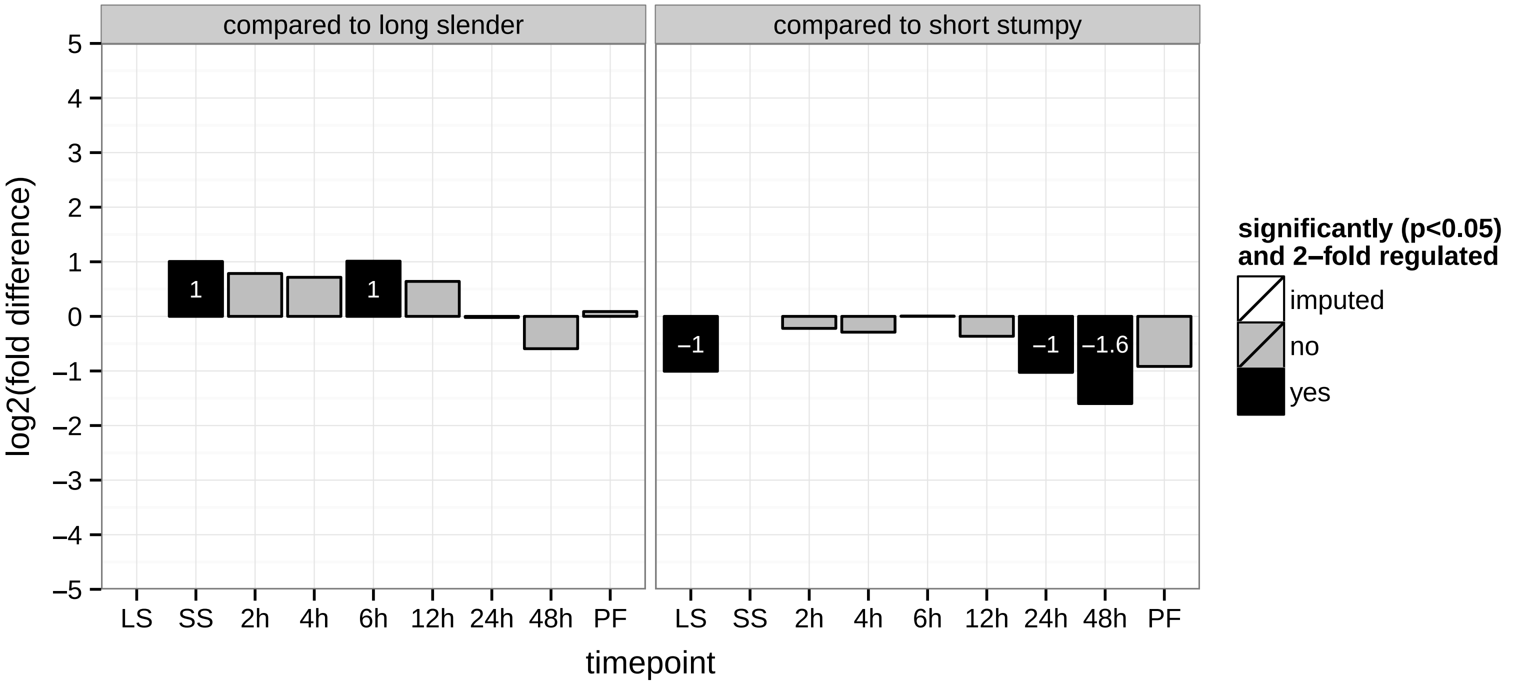

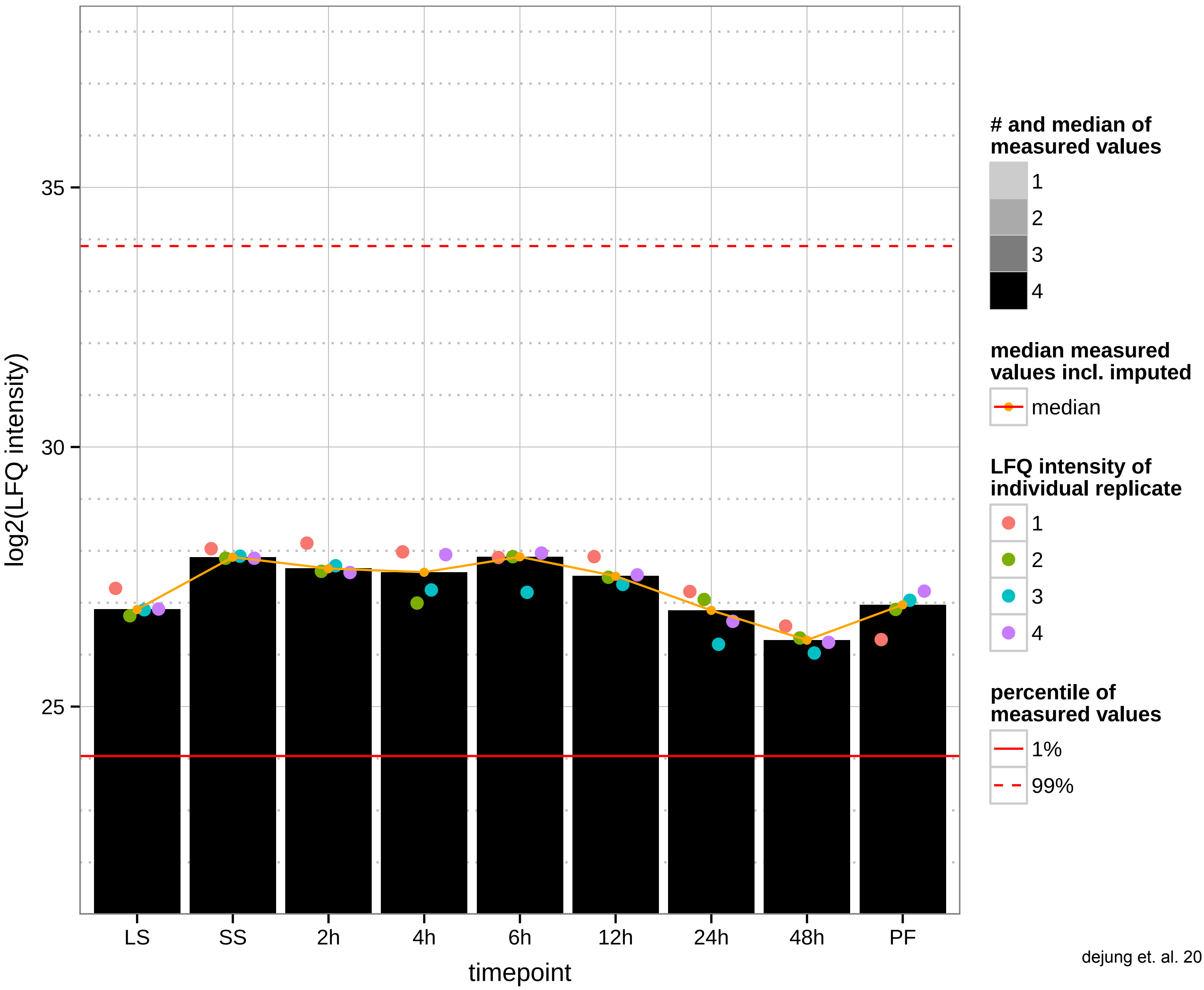

dejung et. al. 2015

hypothetical protein, conserved
Tb927.1.4500
AGOF: null
AGOC: null
AGOP: null
PGOF: null
PGOC: null
PGOP: null

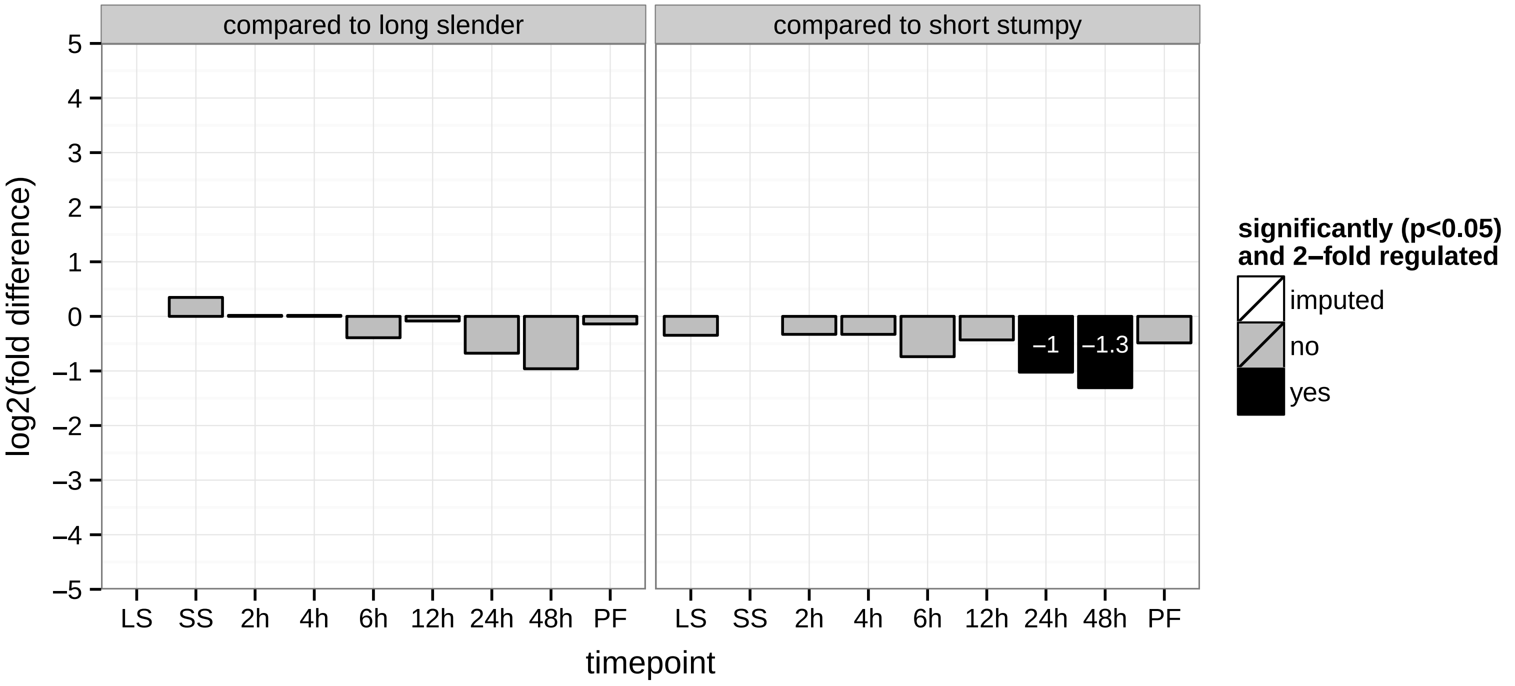

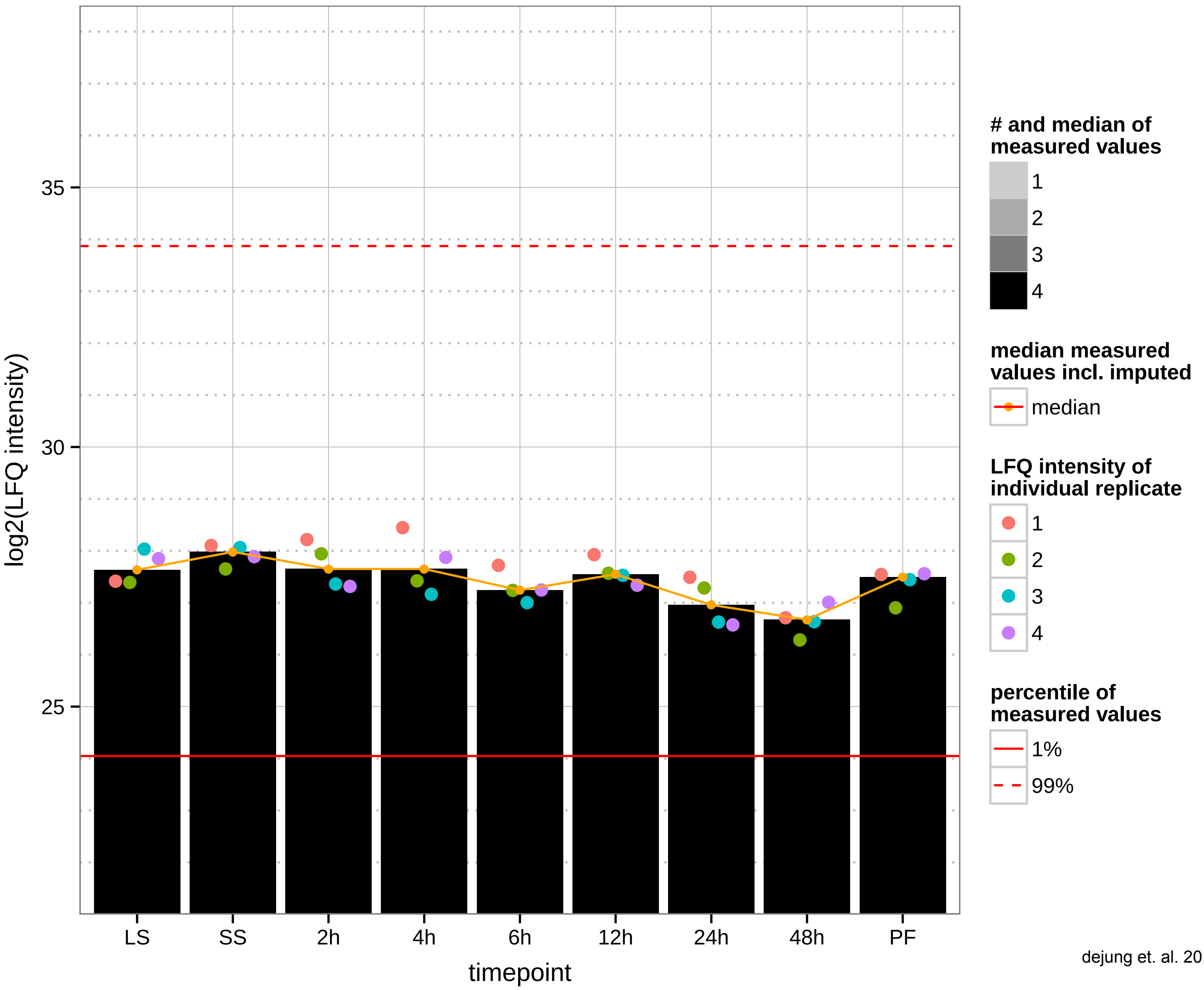

dejung et. al. 2015

vacuolar ATP synthase, putative
Tb927.10.200
AGOF: hydrogen−exporting ATPase activity, phosphorylative mechanism
AGOC: proton−transporting V−type ATPase, V0 domain, vacuolar proton−transporting V−type ATPase complex
AGOP: ATP hydrolysis coupled proton transport, ATP synthesis coupled proton transport
PGOF: hydrogen ion transmembrane transporter activity
PGOC: proton−transporting V−type ATPase, V0 domain, proton−transporting two−sector ATPase complex, proton−transportin
PGOP: ATP hydrolysis coupled proton transport

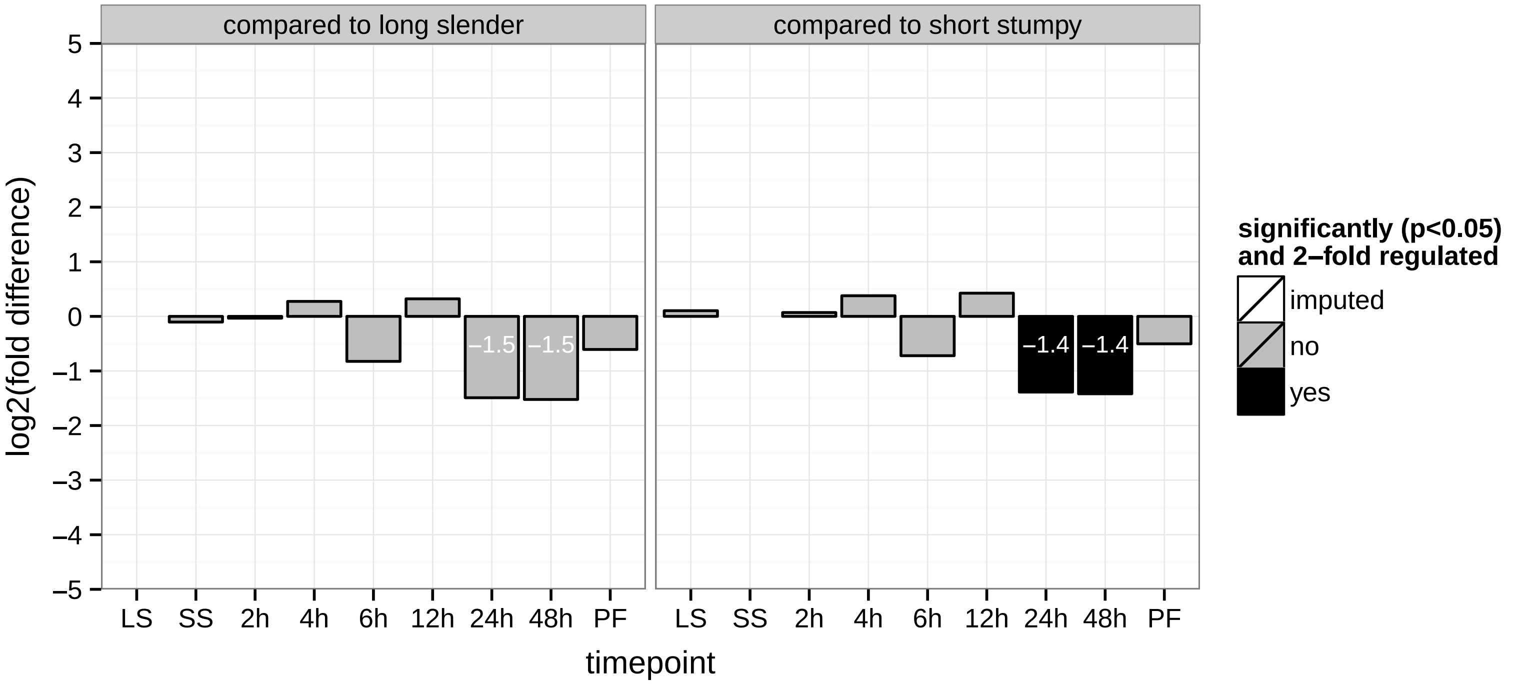

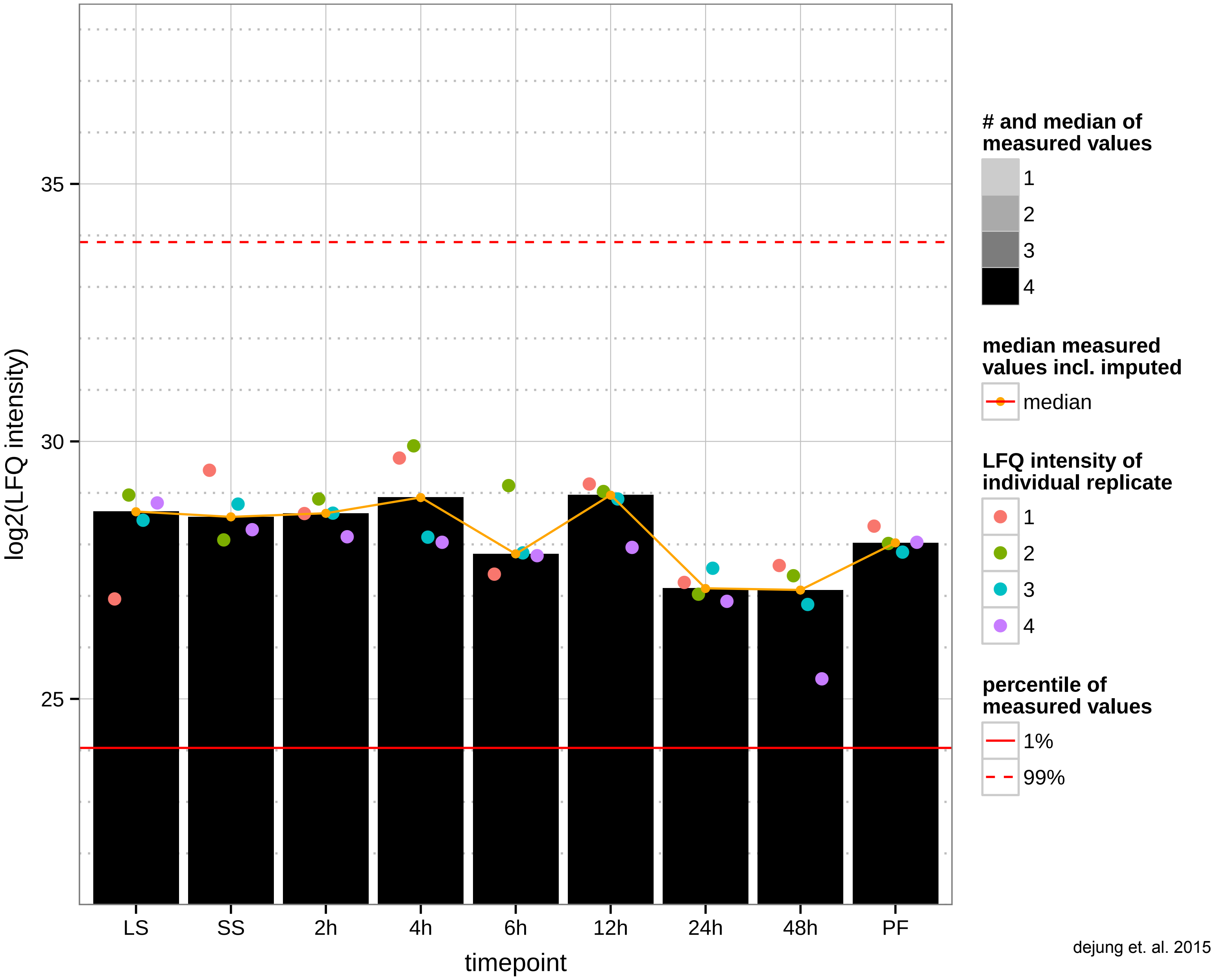

dejung et. al. 2015

hypothetical protein, conserved
Tb927.11.15140
AGOF: null
AGOC: null
AGOP: null
PGOF: null
PGOC: null
PGOP: null

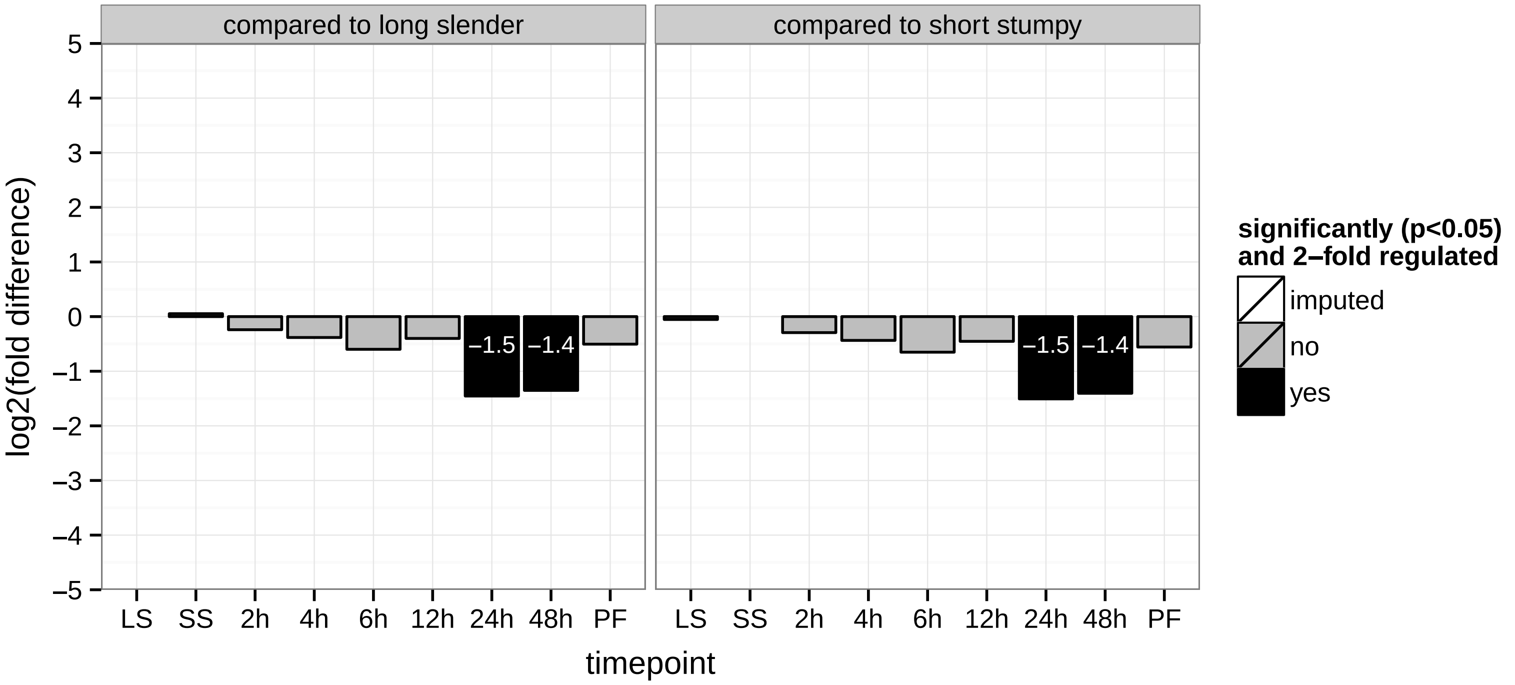

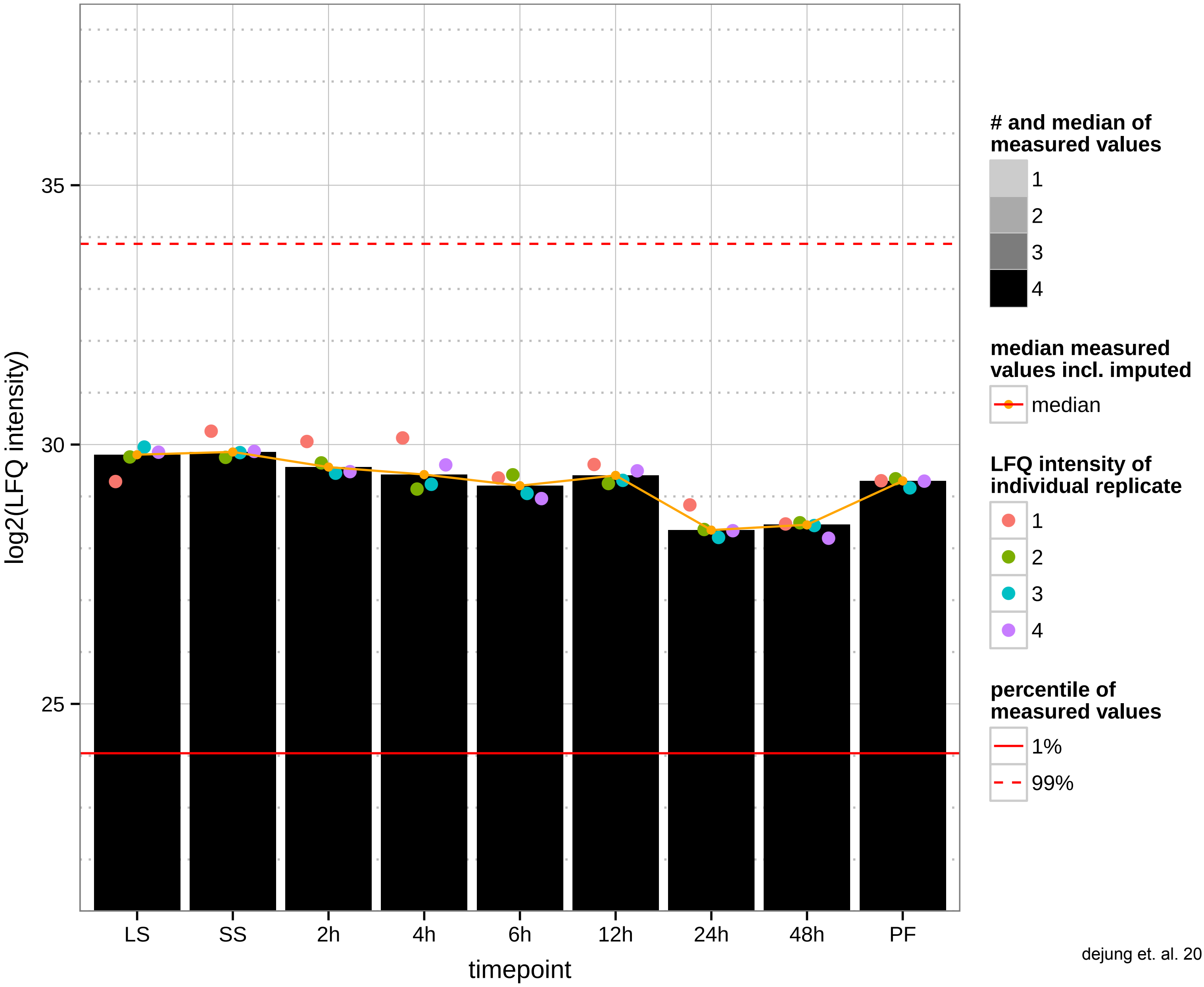

dejung et. al. 2015

6–phosphogluconolactonase (6PGL)
Tb927.11.6330
AGOF: 6–phosphogluconolactonase activity
AGOC: cytoplasm, glycosome
AGOP: pentose–phosphate shunt
PGOF: 6–phosphogluconolactonase activity
PGOC: null
PGOP: carbohydrate metabolic process, pentose–phosphate shunt

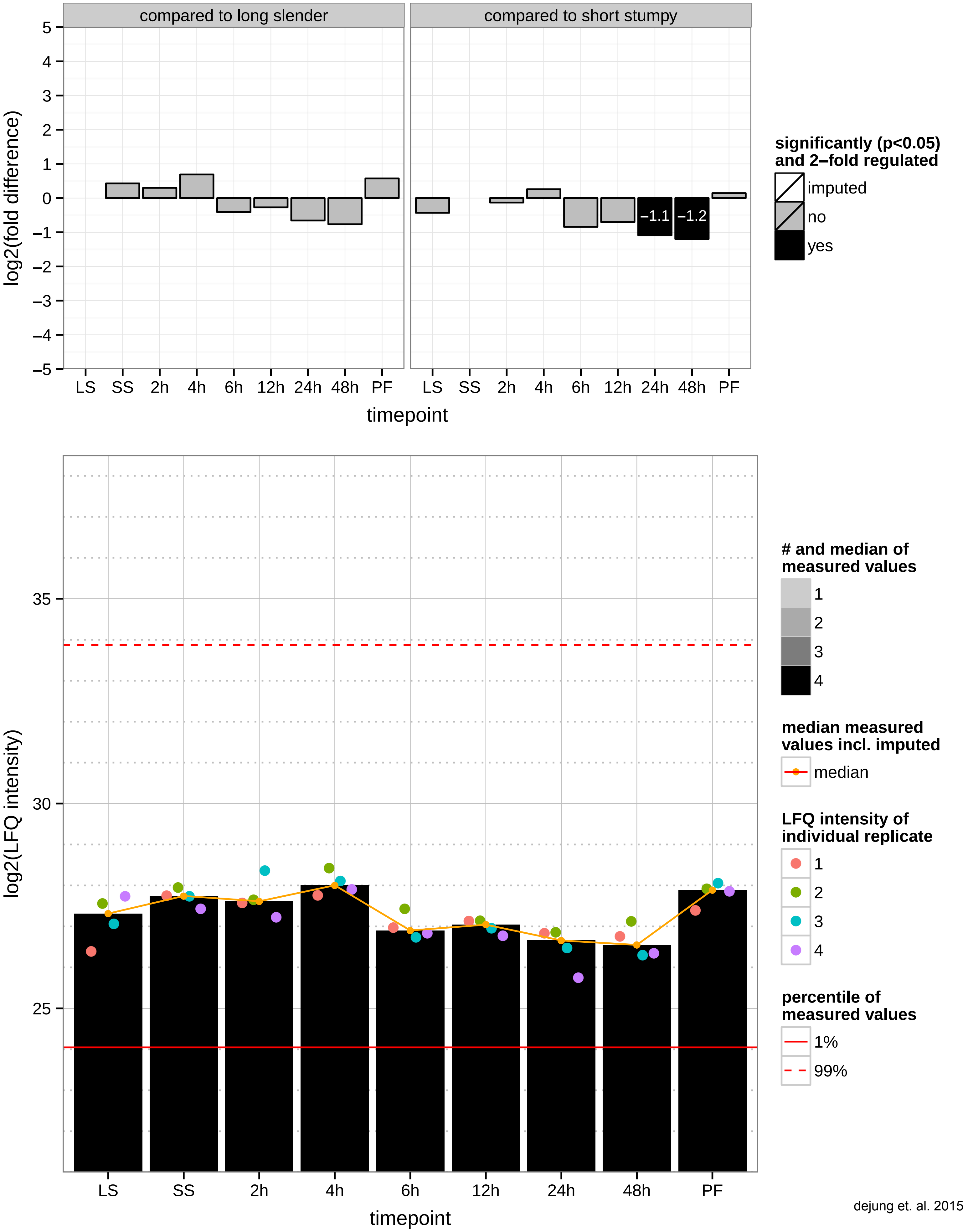

dejung et. al. 2015

branched−chain amino acid aminotransferase, putative
Tb927.2.4590;Tb11.v5.0820;Tb927.2.4610
AGOF: null, branched−chain−amino−acid transaminase activity
AGOC: null, mitochondrion
AGOP: null, branched chain family amino acid metabolic process, cellular amino acid metabolic process, branched chain fami
PGOF: branched−chain−amino−acid transaminase activity, catalytic activity
PGOC: null
PGOP: branched chain family amino acid metabolic process, metabolic process

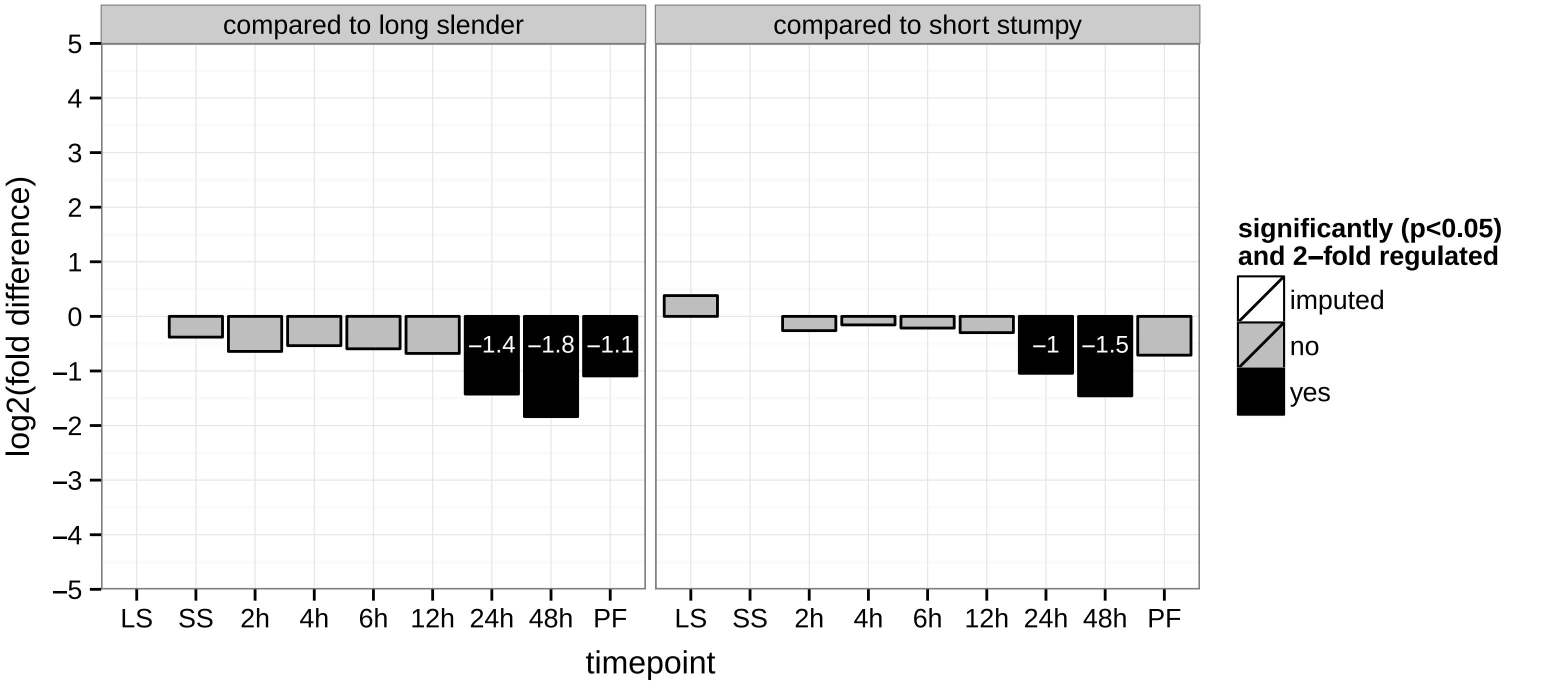

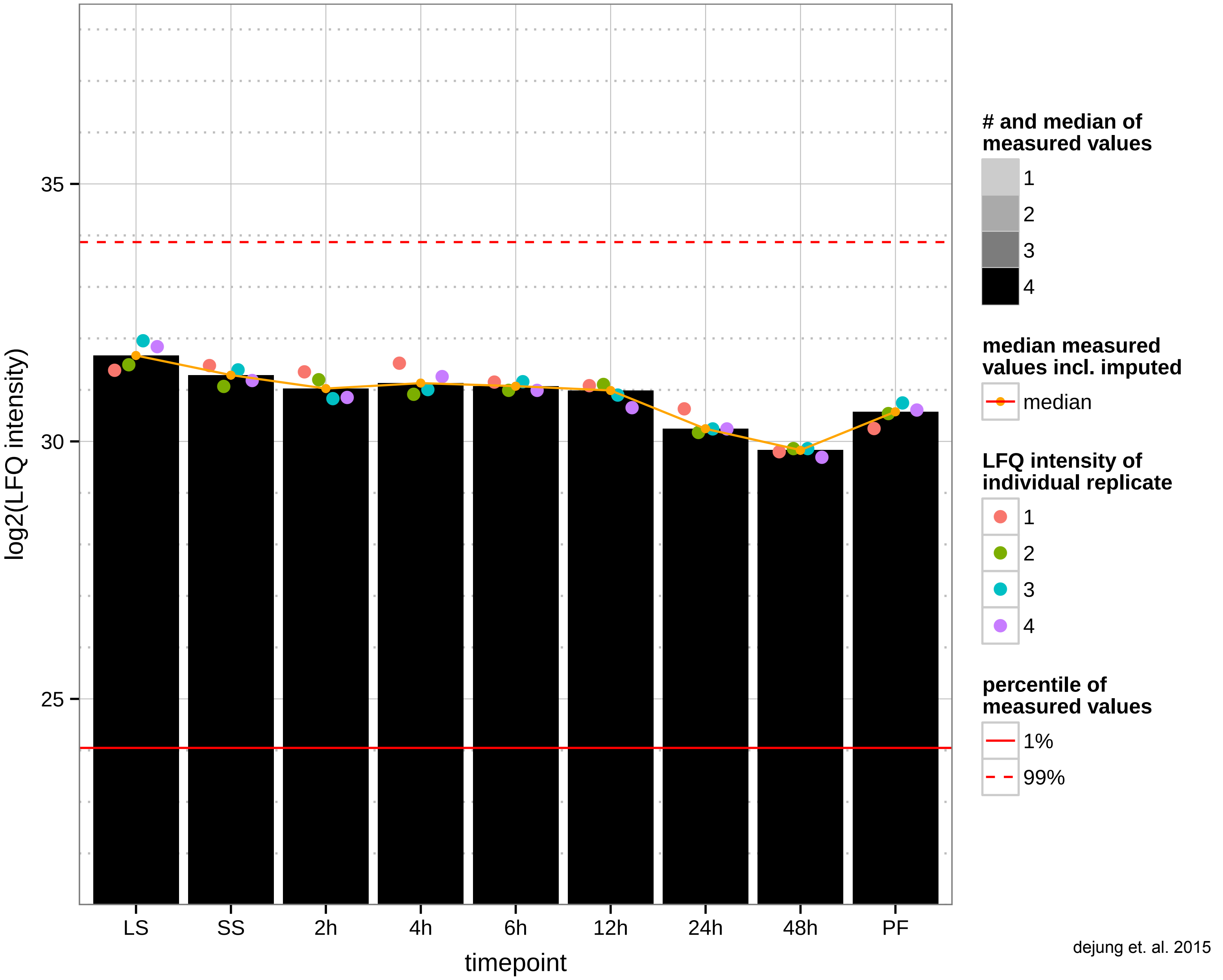

dejung et. al. 2015

hypothetical protein, conserved, flagellar transport protein, putative (PIFTA1)
Tb927.3.1110;Tb11.v5.0589
AGOF: null
AGOC: null
AGOP: null
PGOF: null
PGOC: null
PGOP: null

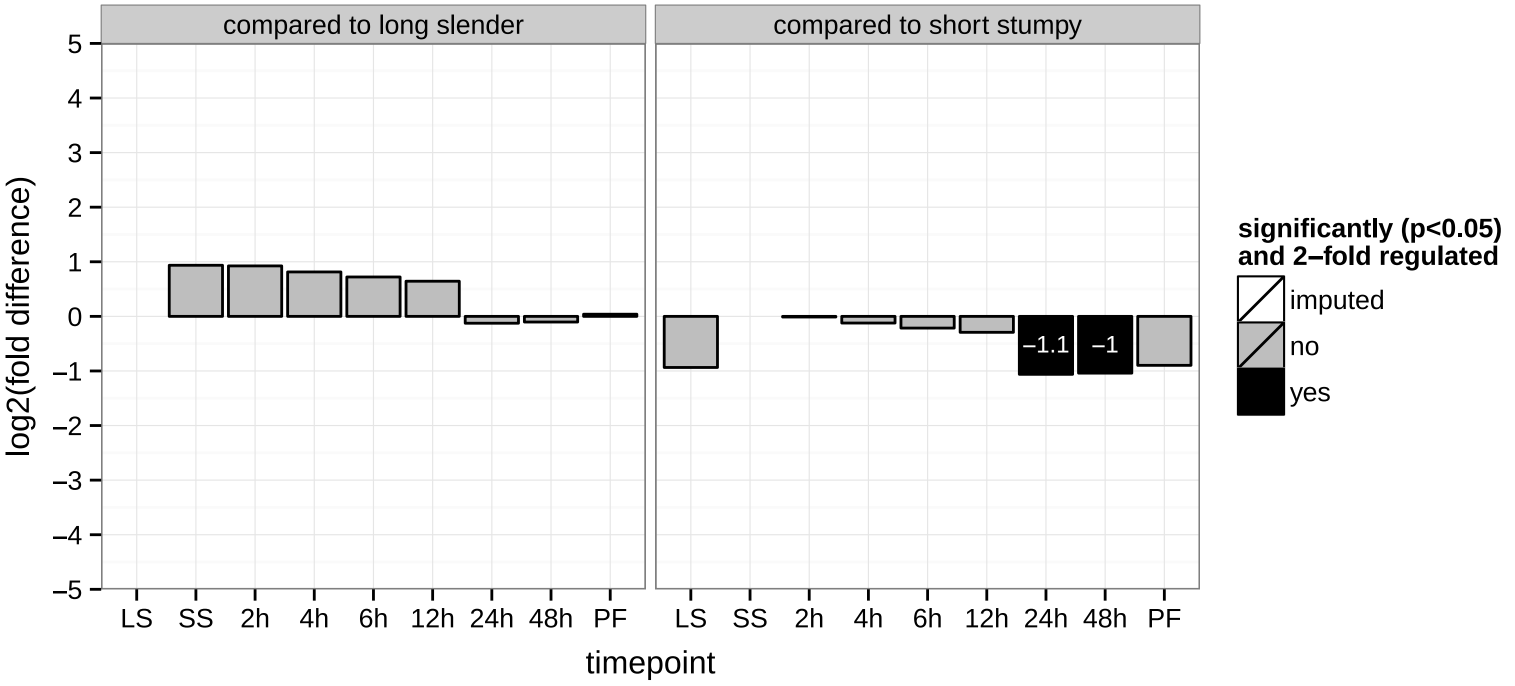

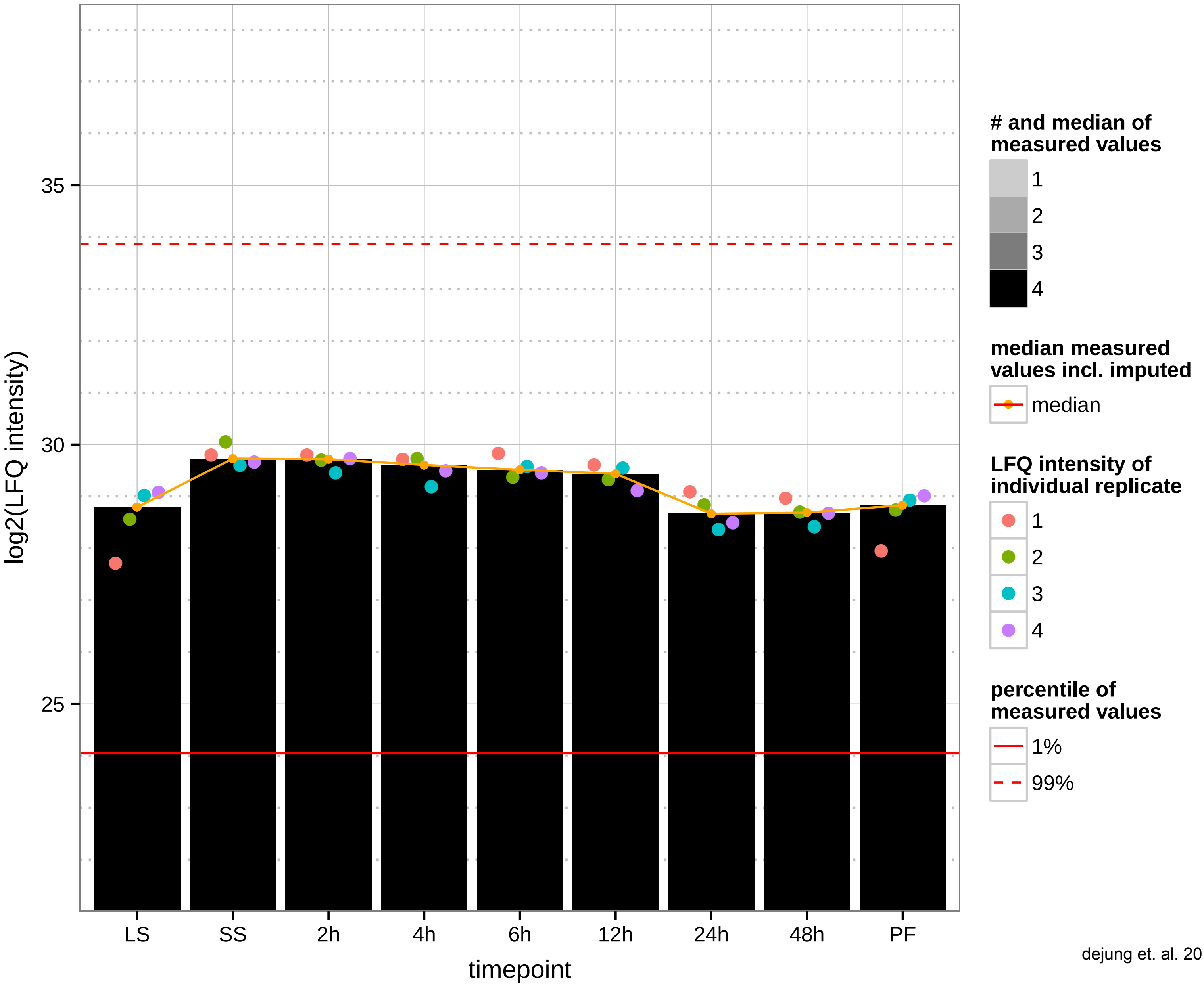

dejung et. al. 2015

flagellar transport protein, putative (PIFTC3)
Tb927.3.3000
AGOF: null
AGOC: null
AGOP: null
PGOF: protein binding
PGOC: null
PGOP: null

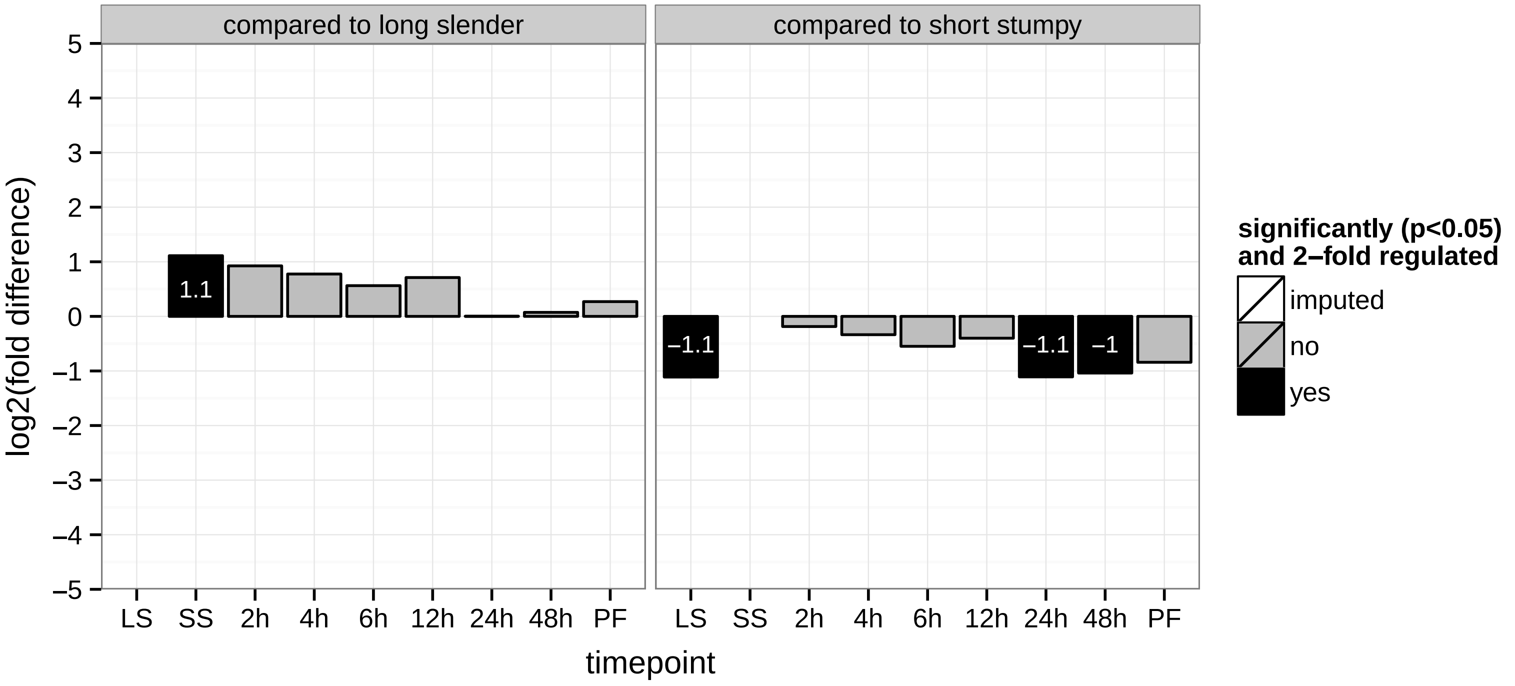

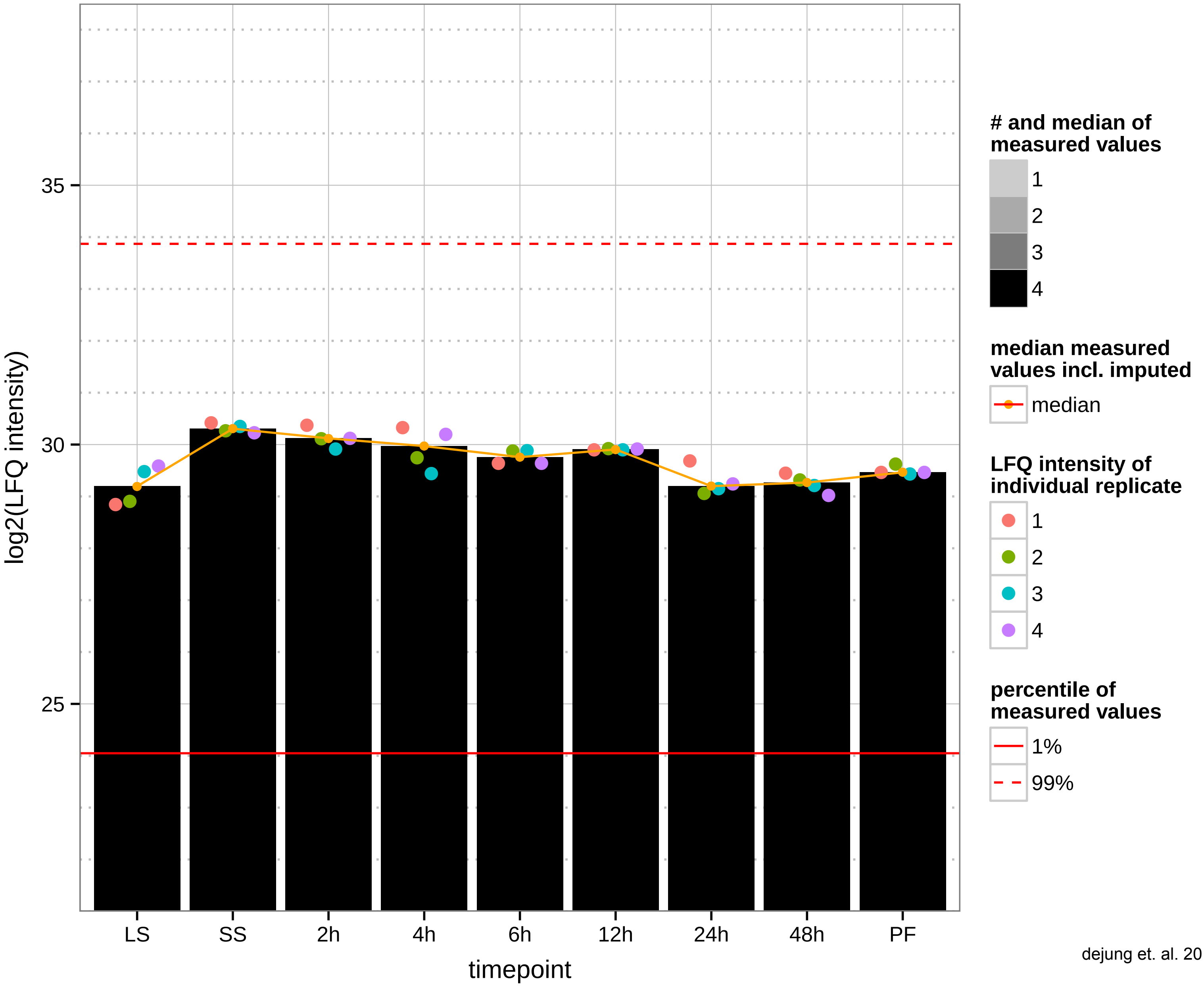

dejung et. al. 2015

hypothetical protein, conserved
Tb927.3.3560
AGOF: ubiquitin–protein ligase activity
AGOC: ubiquitin ligase complex
AGOP: protein ubiquitination
PGOF: ubiquitin–protein ligase activity
PGOC: ubiquitin ligase complex
PGOP: protein ubiquitination

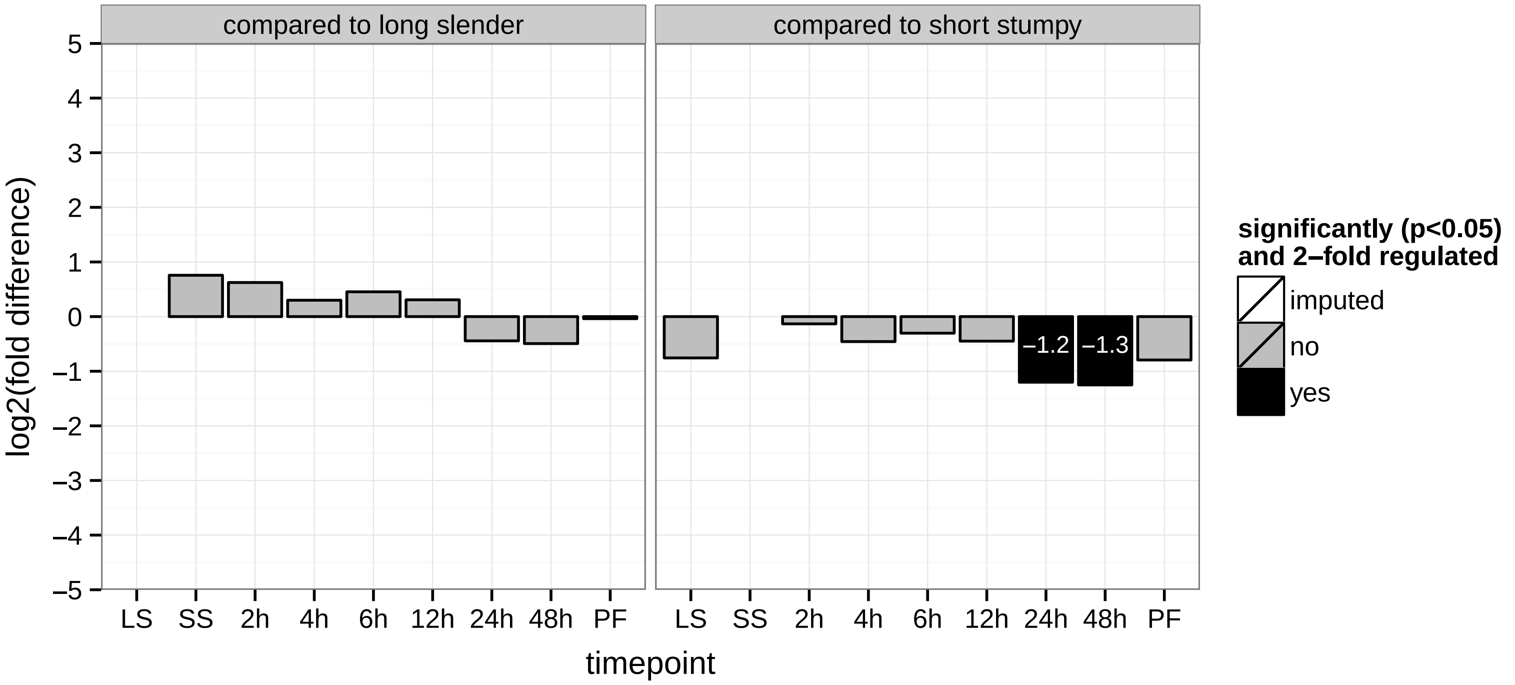

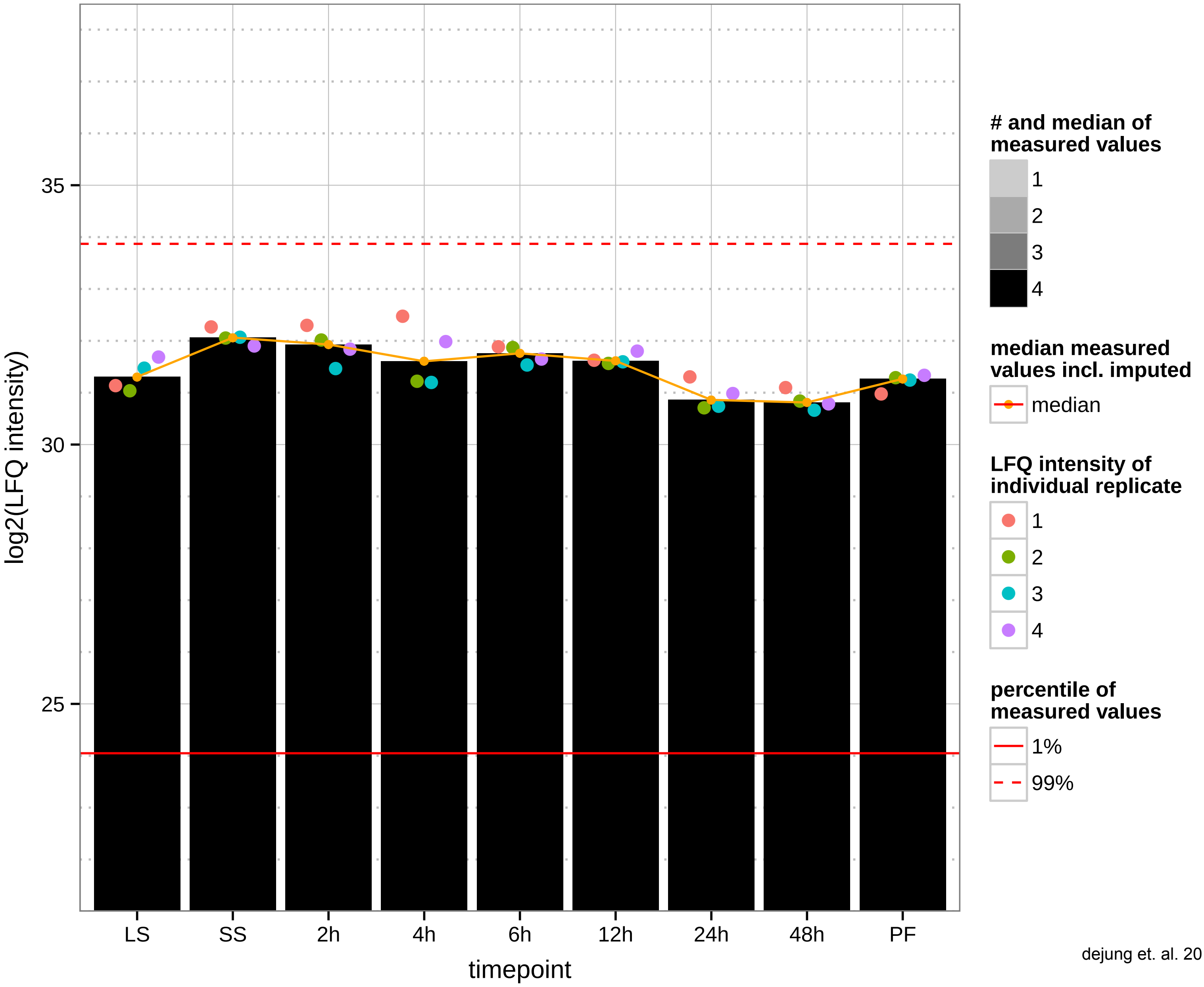

dejung et. al. 2015

dynein intermediate chain WDRD34, putative
Tb927.3.5540
AGOF: null
AGOC: null
AGOP: ciliary or flagellar motility
PGOF: protein binding
PGOC: null
PGOP: null

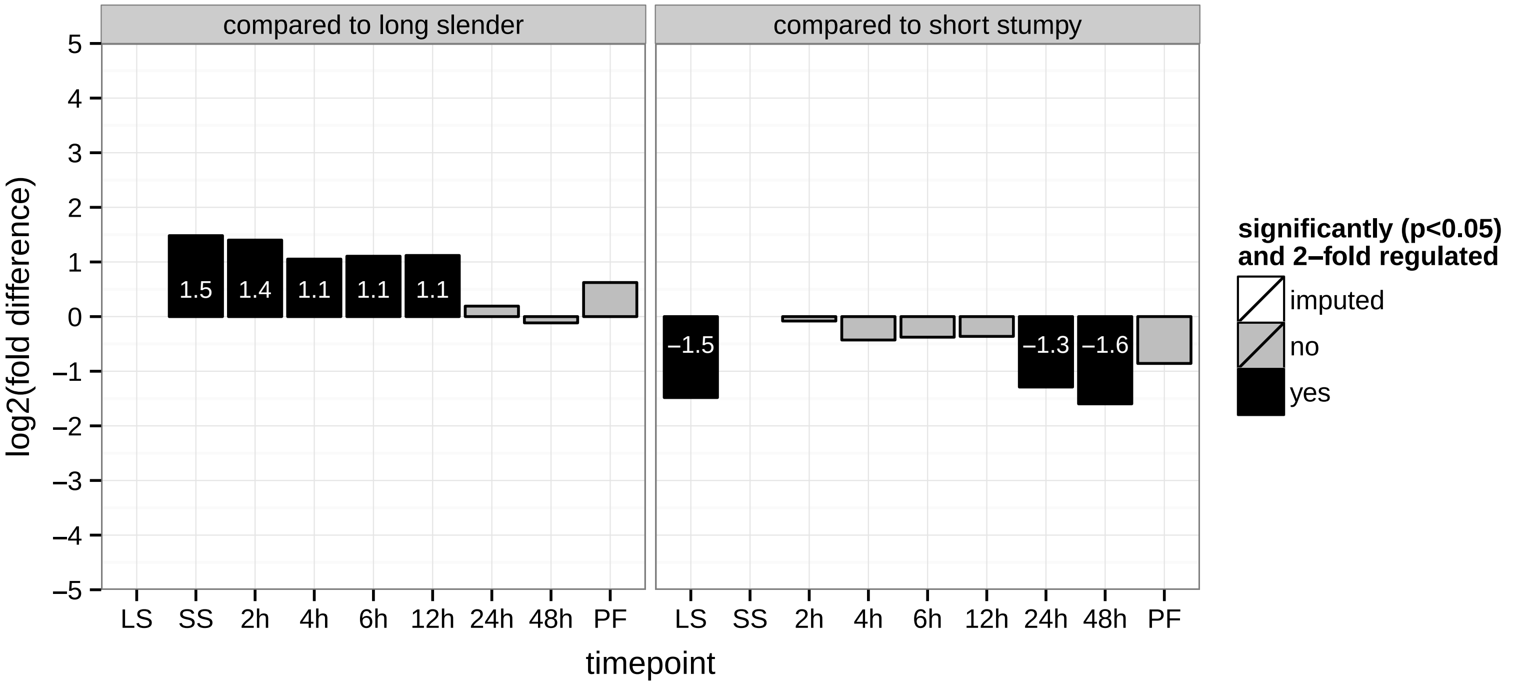

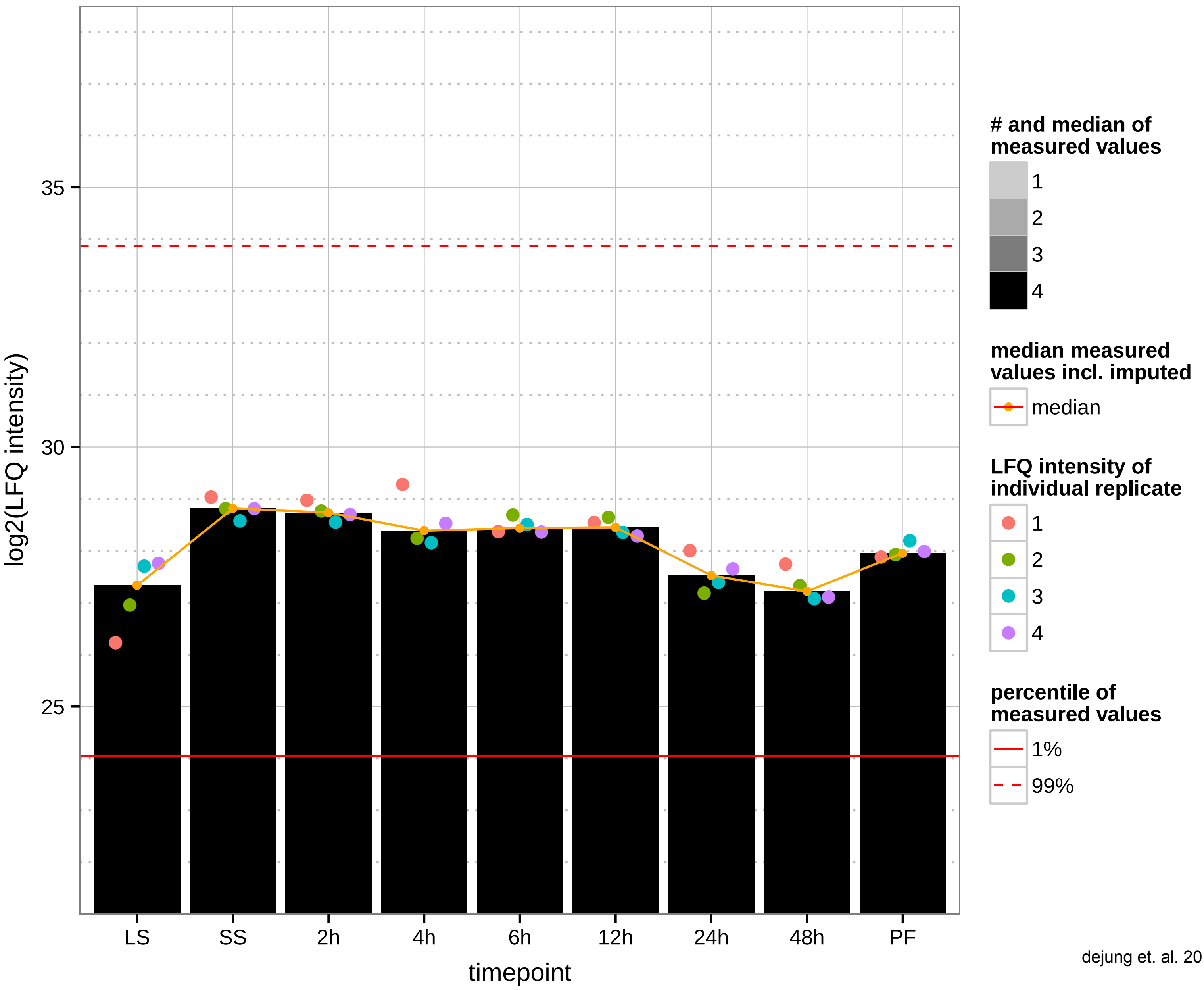

dejung et. al. 2015

thioredoxin, putative
Tb927.4.2450
AGOF: glutathione−disulfide reductase activity
AGOC: null
AGOP: cell redox homeostasis, oxidation−reduction process
PGOF: null
PGOC: null
PGOP: cell redox homeostasis

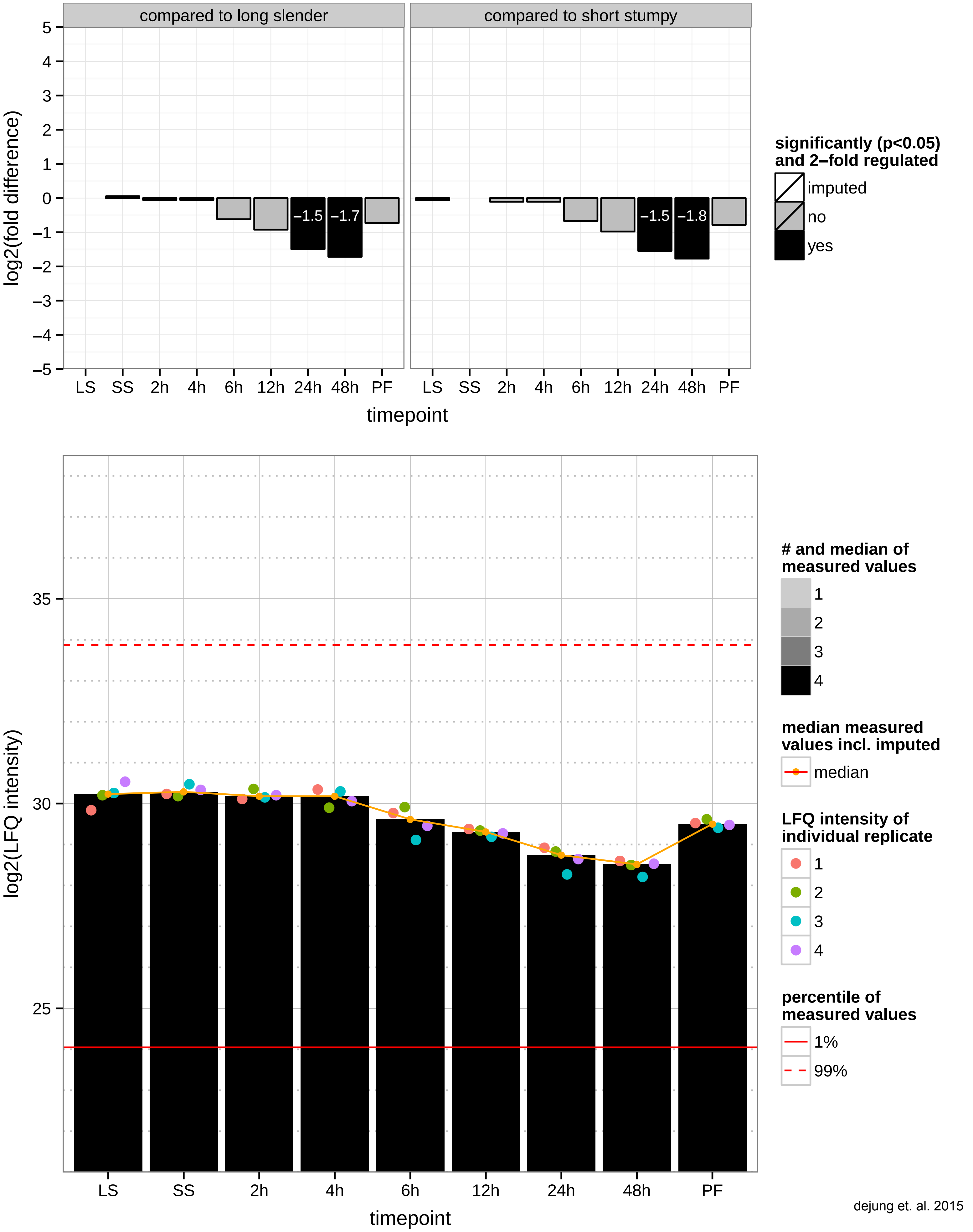

dejung et. al. 2015

choline/ethanolamine kinase (C/EK1)
Tb927.5.1140
AGOF: choline kinase activity, ethanolamine kinase activity
AGOC: cytoplasm
AGOP: phosphatidylcholine biosynthetic process, phosphatidylethanolamine biosynthetic process
PGOF: phosphotransferase activity, alcohol group as acceptor, transferase activity, transferring phosphorus–containing groups
PGOC: null
PGOP: null

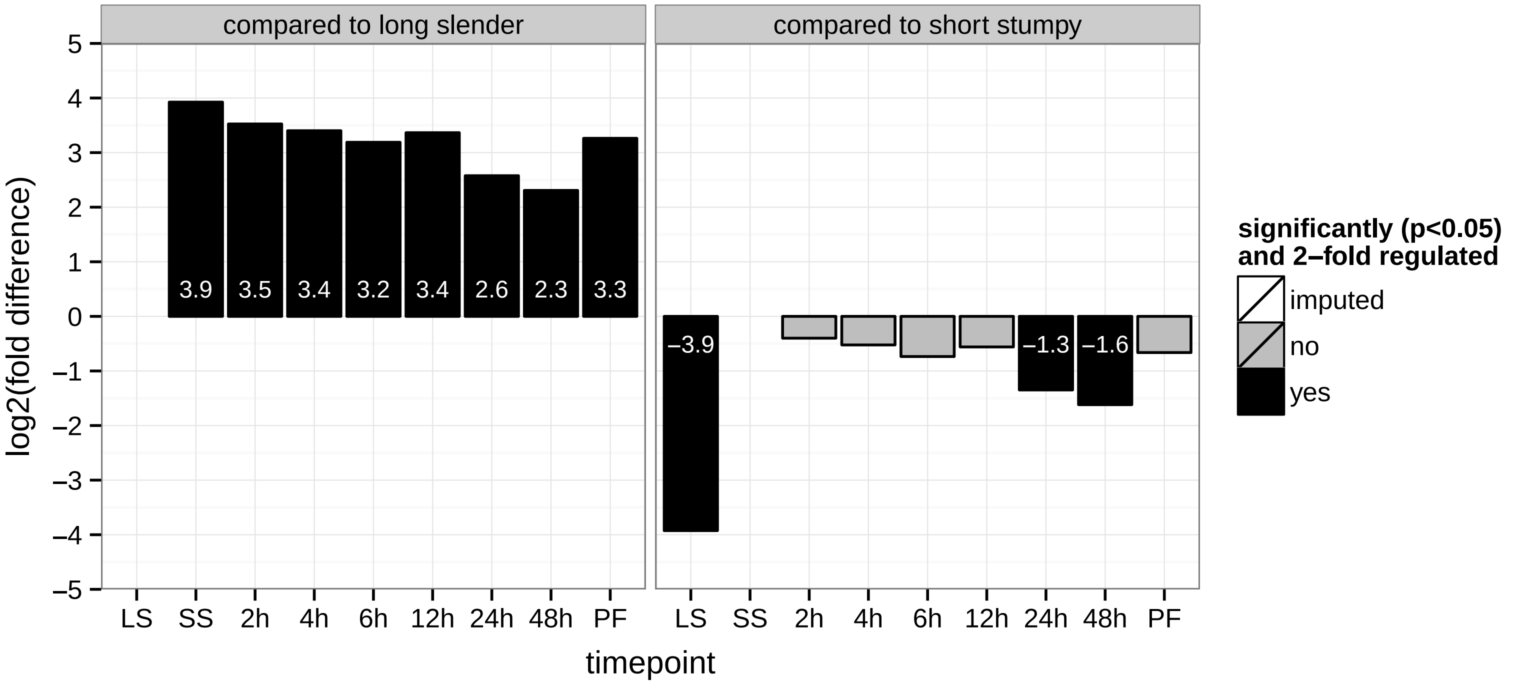

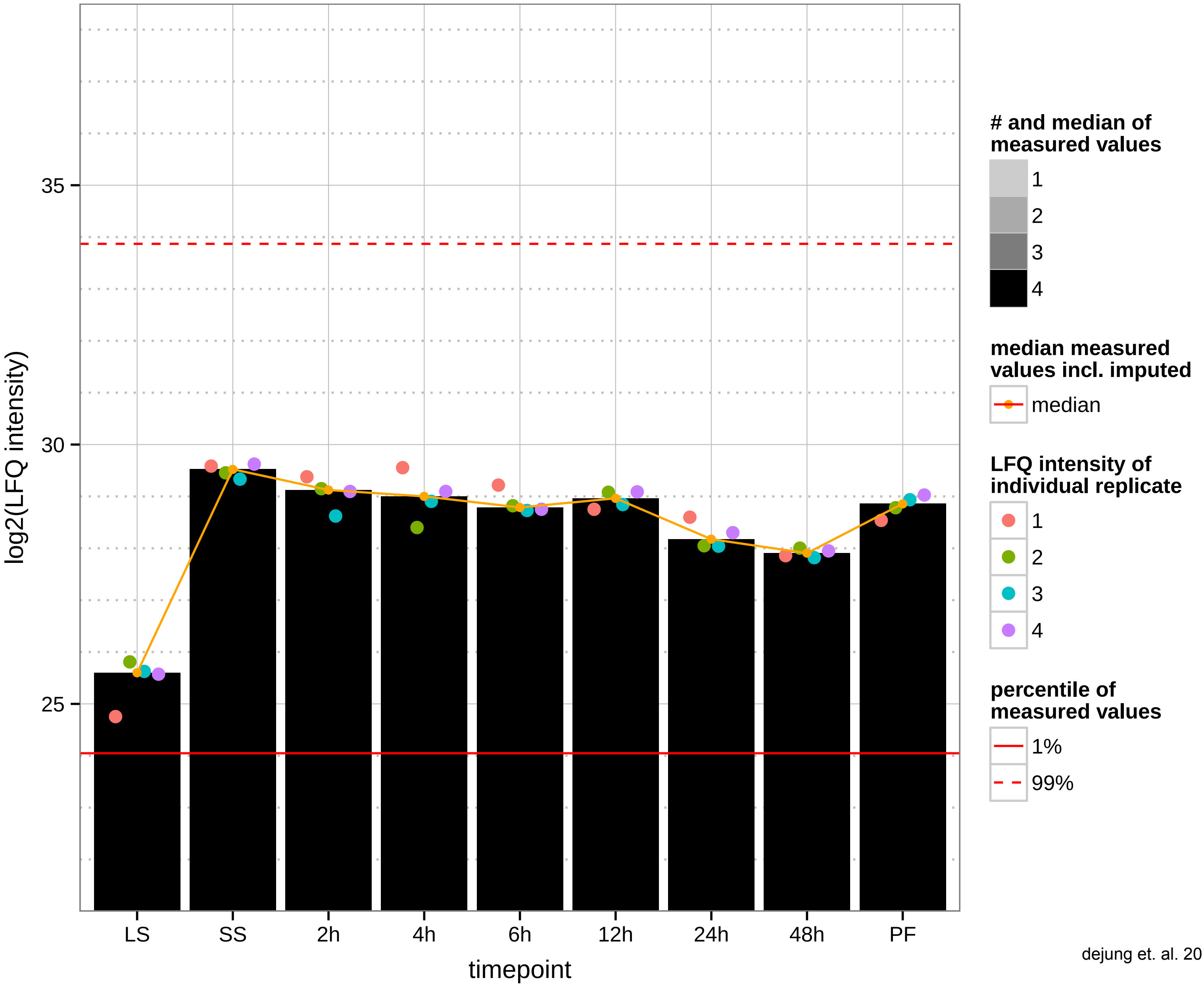

dejung et. al. 2015

hypothetical protein, conserved
Tb927.5.1460
AGOF: null
AGOC: null
AGOP: null
PGOF: null
PGOC: null
PGOP: null

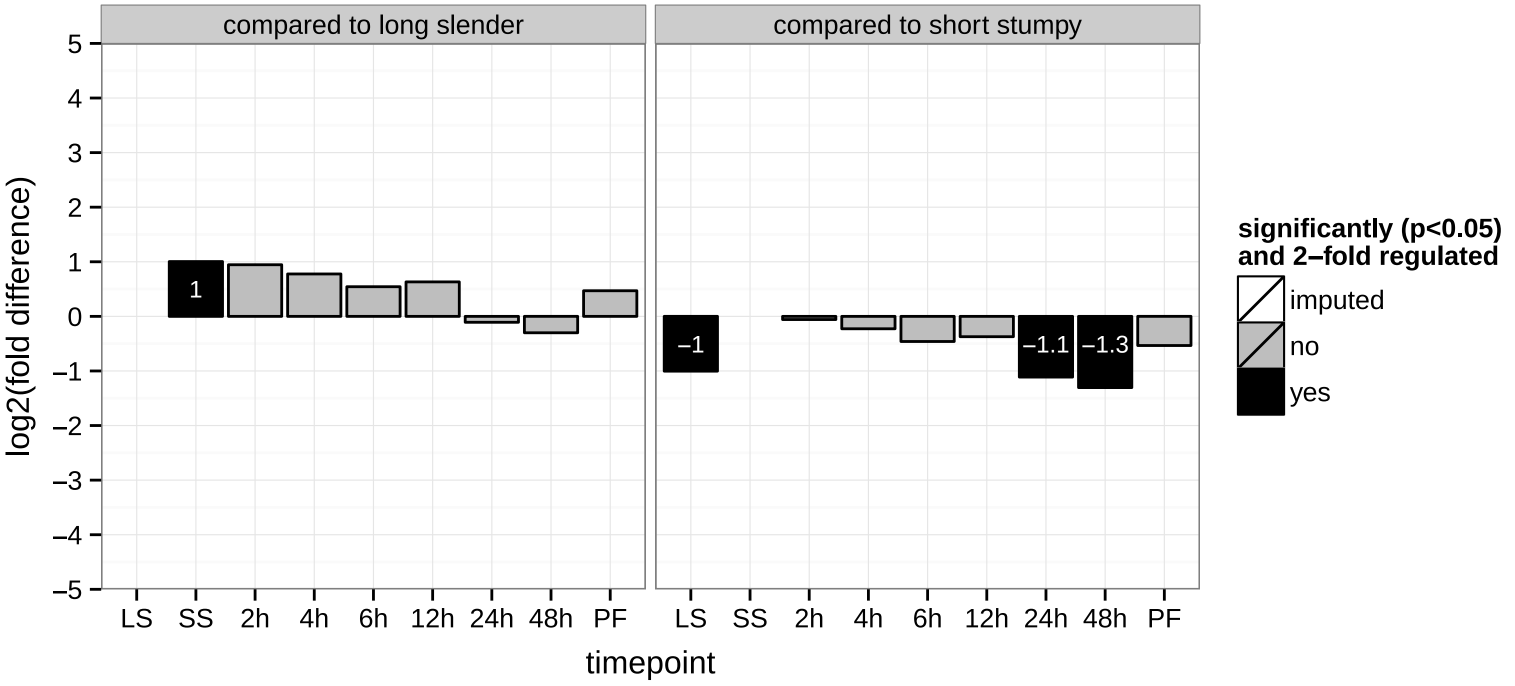

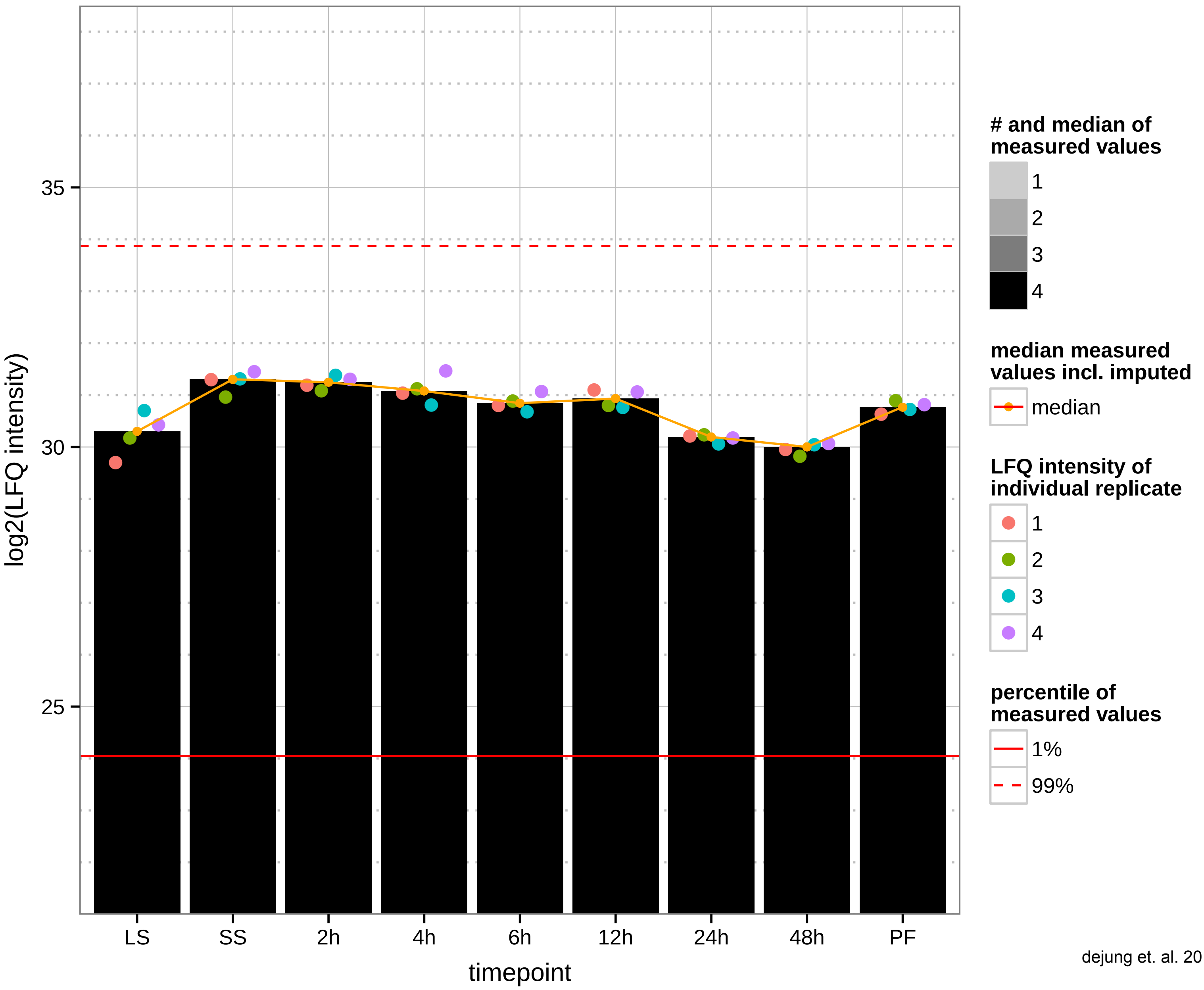

dejung et. al. 2015

protein kinase, putative
Tb927.5.2820
AGOF: ATP binding, protein serine/threonine kinase activity, protein tyrosine kinase activity
AGOC: null
AGOP: protein phosphorylation
PGOF: ATP binding, protein kinase activity, transferase activity, transferring phosphorus–containing groups
PGOC: null
PGOP: protein phosphorylation

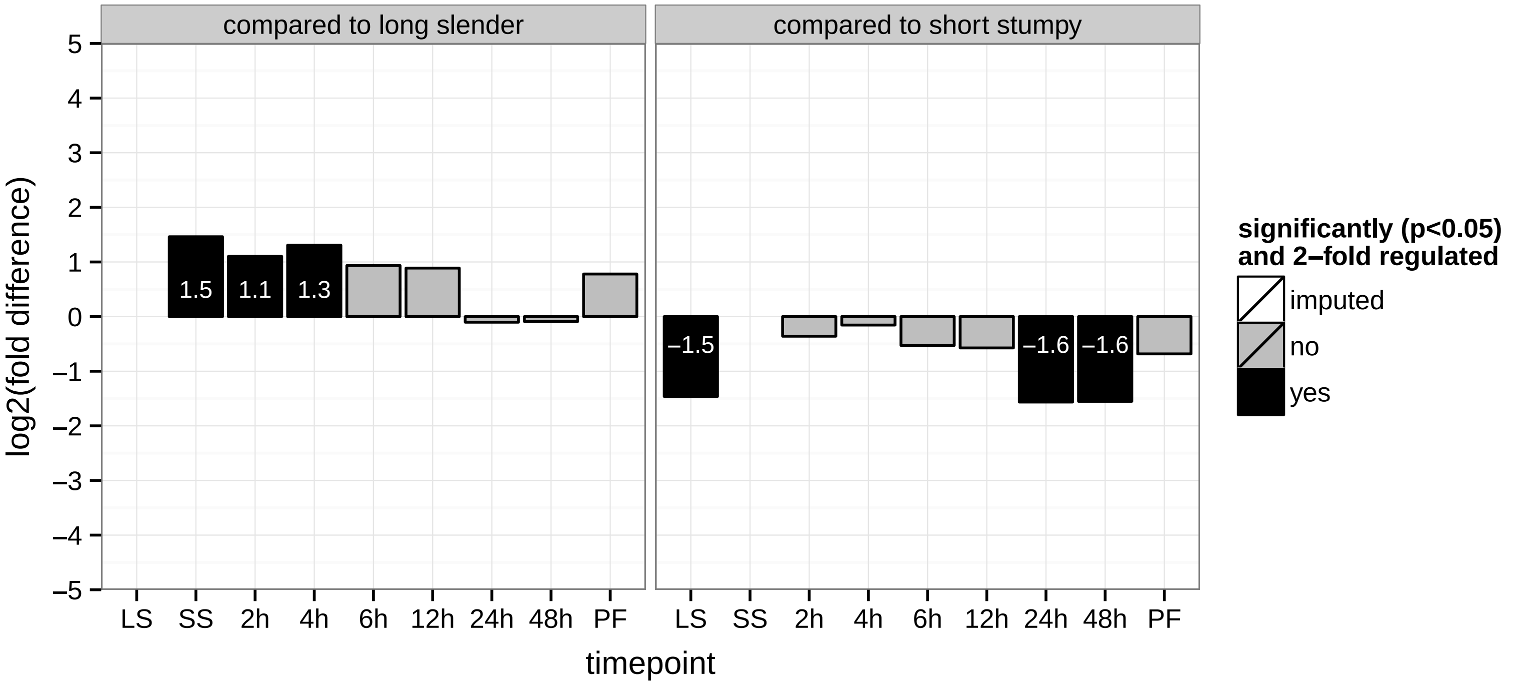

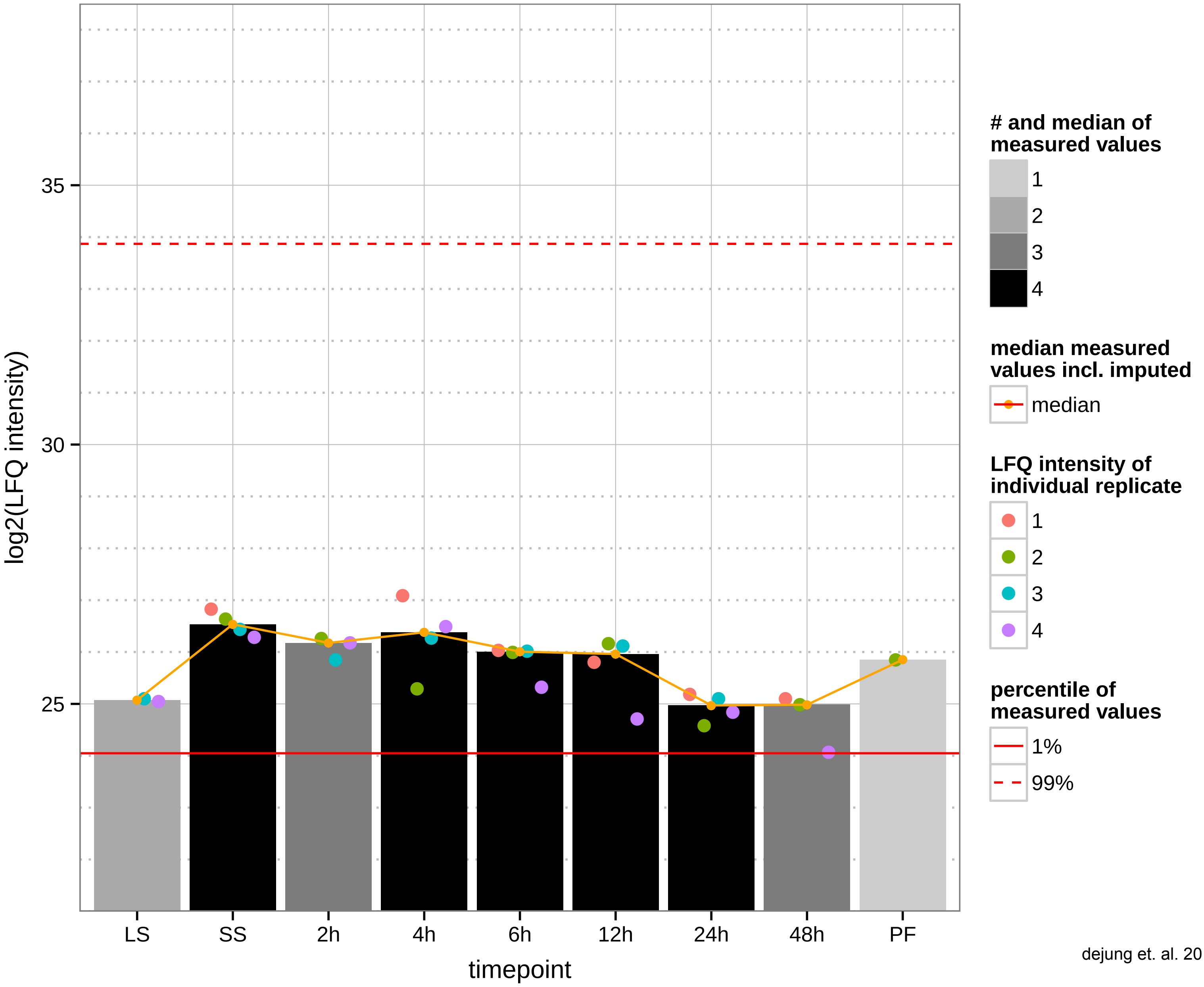

dejung et. al. 2015

NADH−dependent fumarate reductase, putative
Tb927.5.940
AGOF: electron carrier activity, succinate dehydrogenase activity
AGOC: null
AGOP: null
PGOF: oxidoreductase activity, succinate dehydrogenase activity
PGOC: null
PGOP: oxidation−reduction process

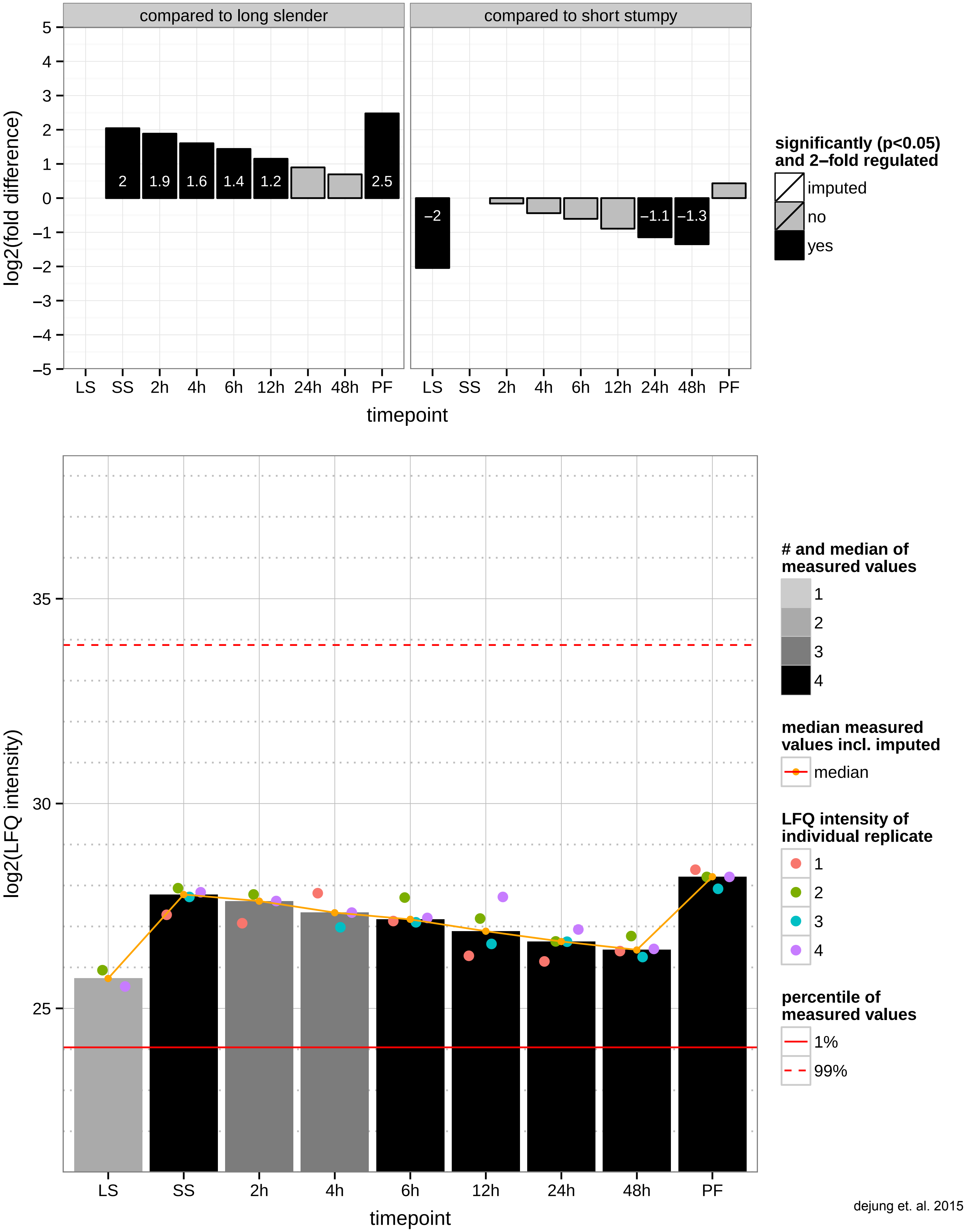

endosomal trafficking protein RME−8, putative
Tb927.6.3500
AGOF: heat shock protein binding
AGOC: null
AGOP: endocytosis
PGOF: binding, heat shock protein binding
PGOC: null
PGOP: null

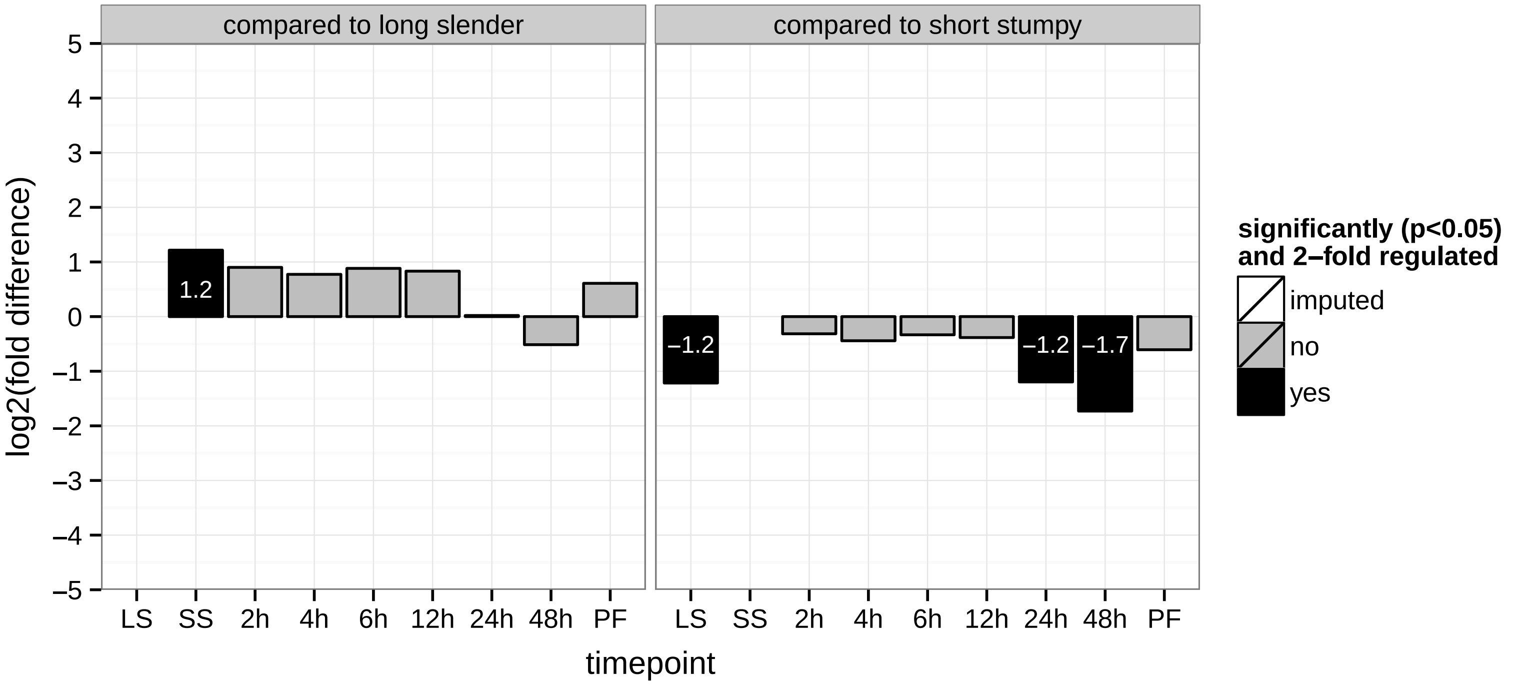

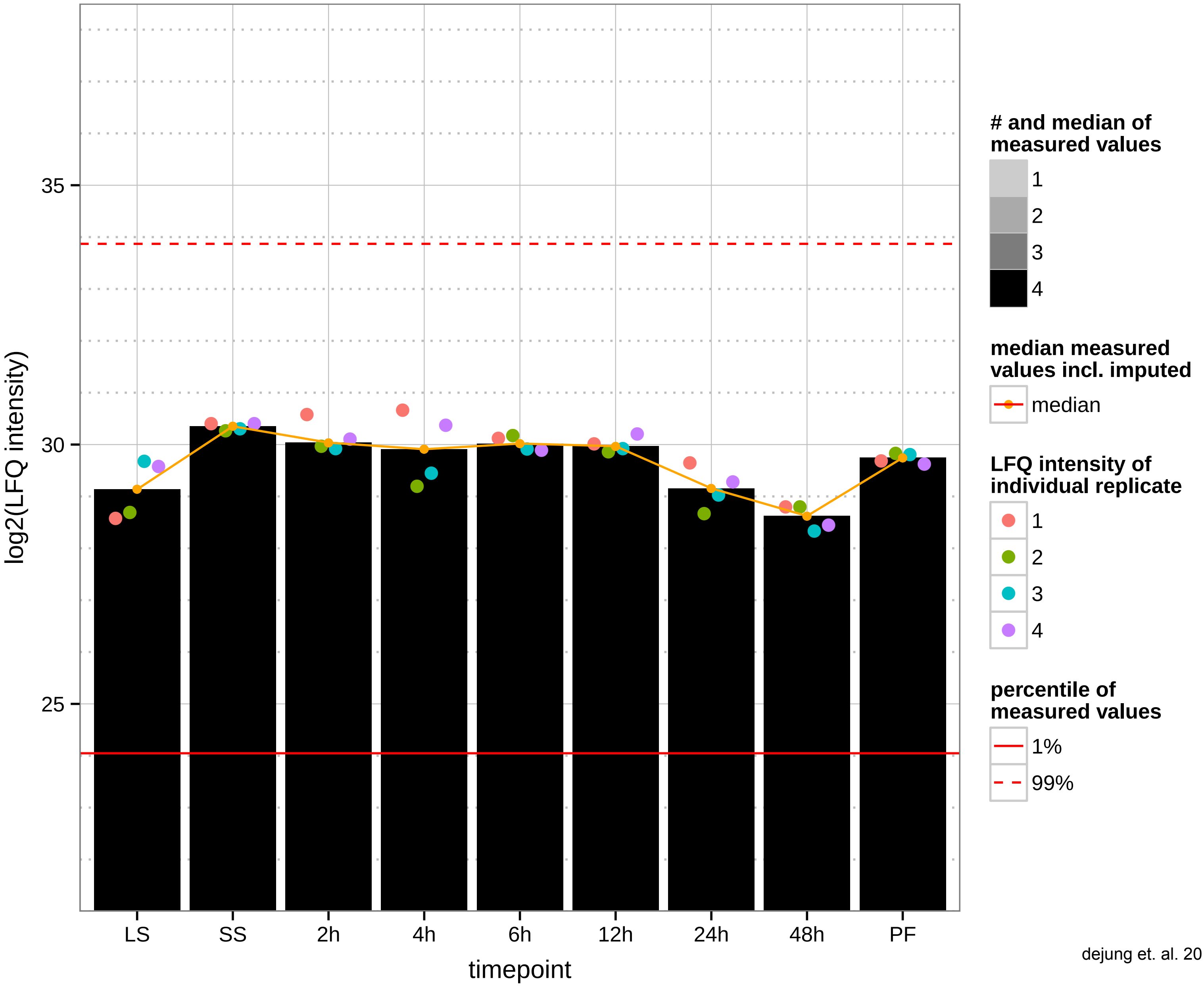

dejung et. al. 2015

pre–mRNA splicing factor ATP–dependent RNA helicase, putative, ATP–dependent RNA helicase
Tb927.6.4600
AGOF: ATP binding, helicase activity, nucleic acid binding
AGOC: null
AGOP: null
PGOF: ATP binding, ATP–dependent helicase activity, helicase activity, nucleic acid binding
PGOC: null
PGOP: null

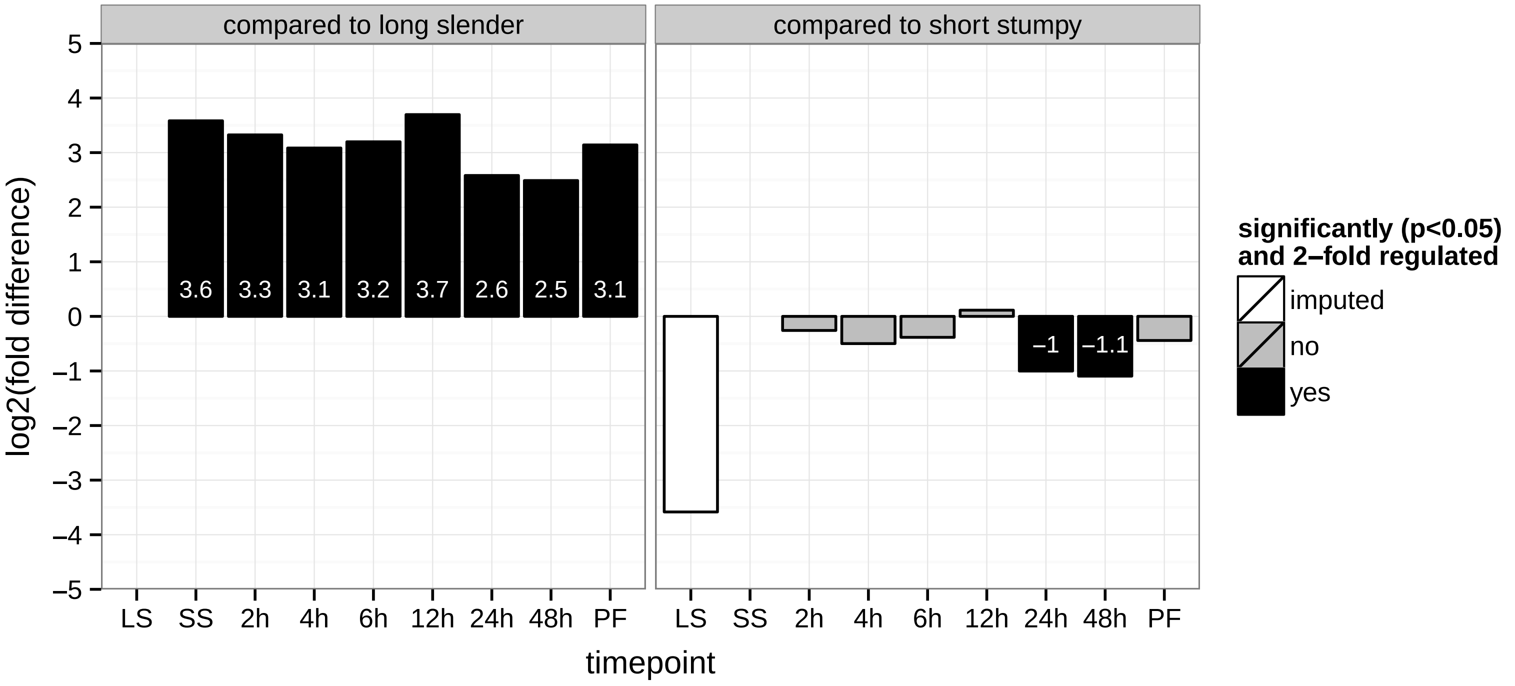

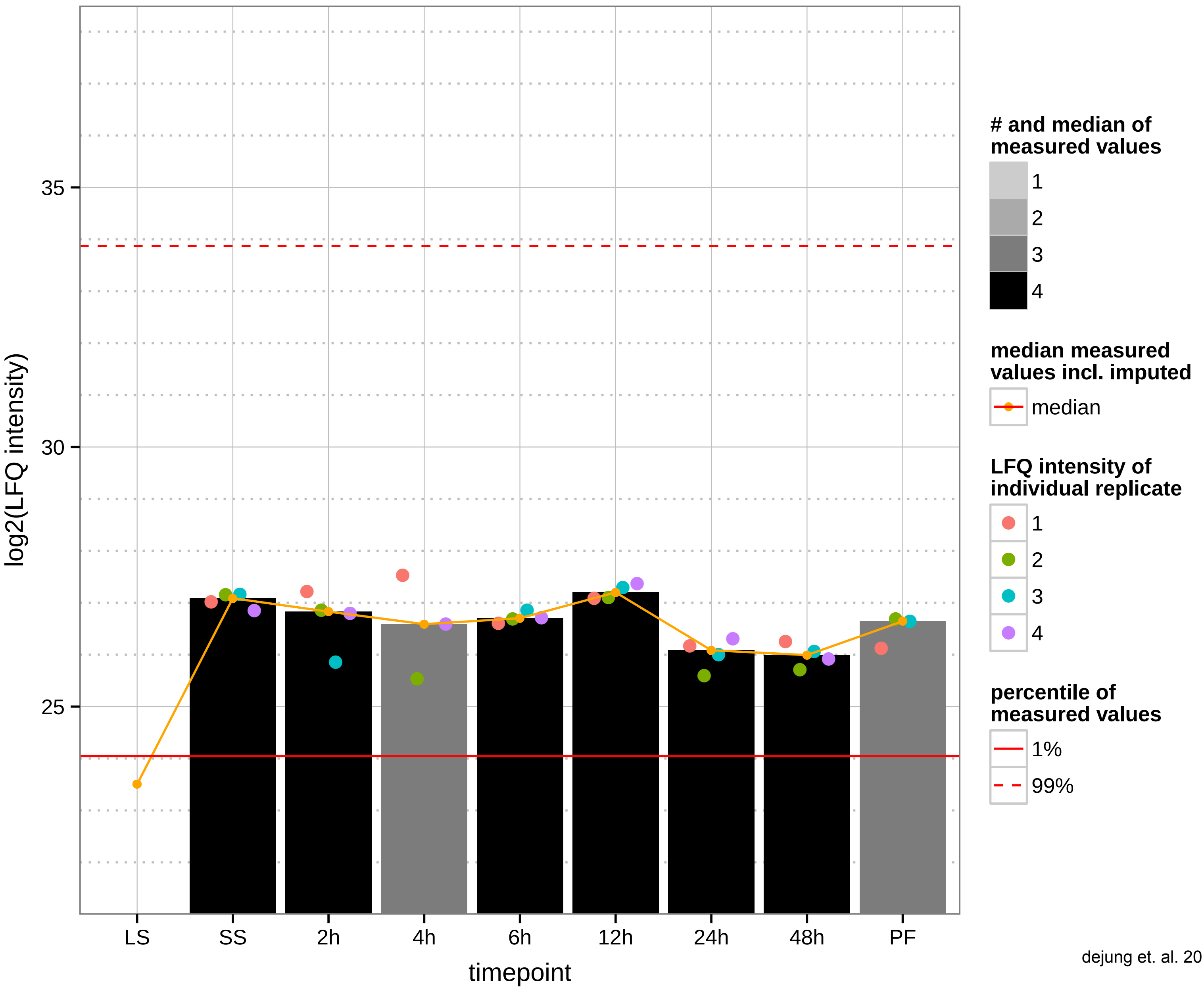

dejung et. al. 2015

cysteine peptidase C (CPC), CPC cysteine peptidase, Clan CA, family C1, Cathepsin B–like (TbcatB)
Tb927.6.560
AGOF: cysteine–type peptidase activity
AGOC: null
AGOP: proteolysis
PGOF: cysteine–type peptidase activity
PGOC: null
PGOP: proteolysis

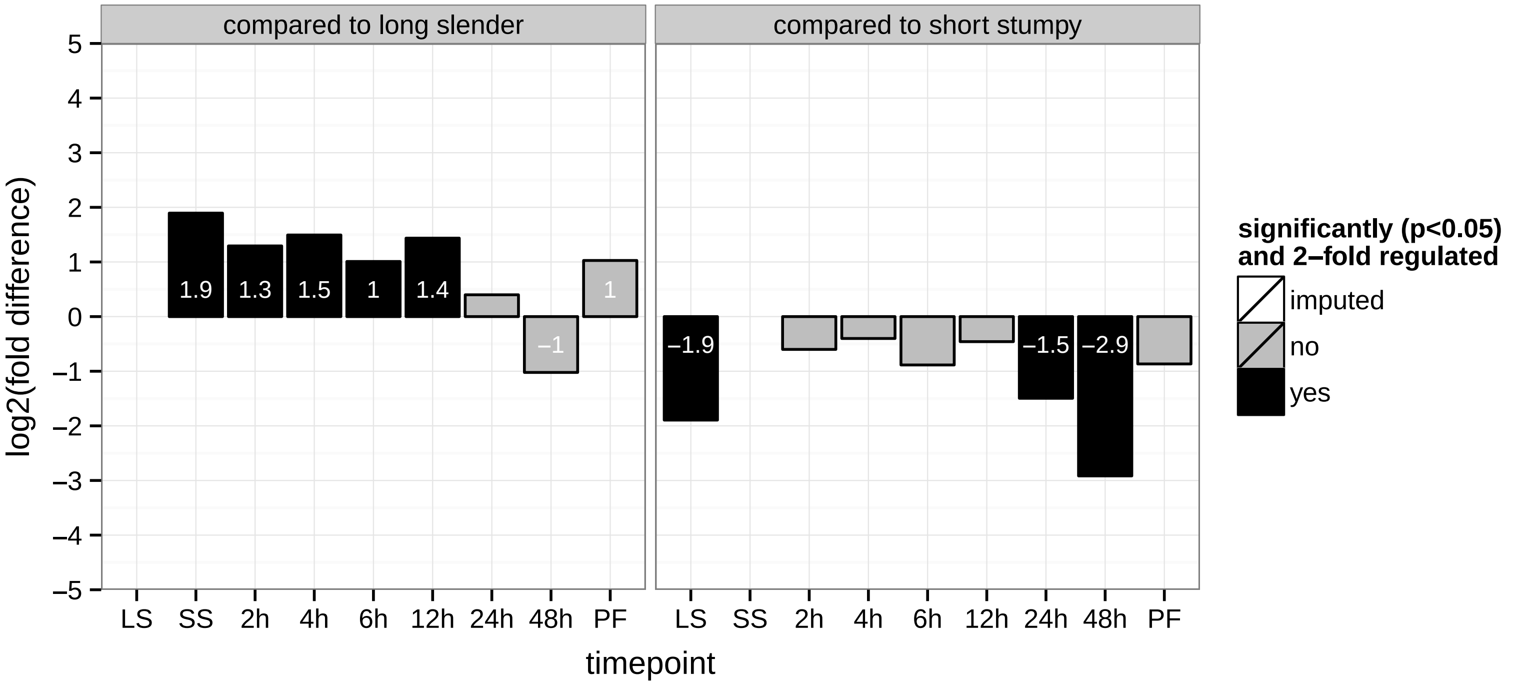

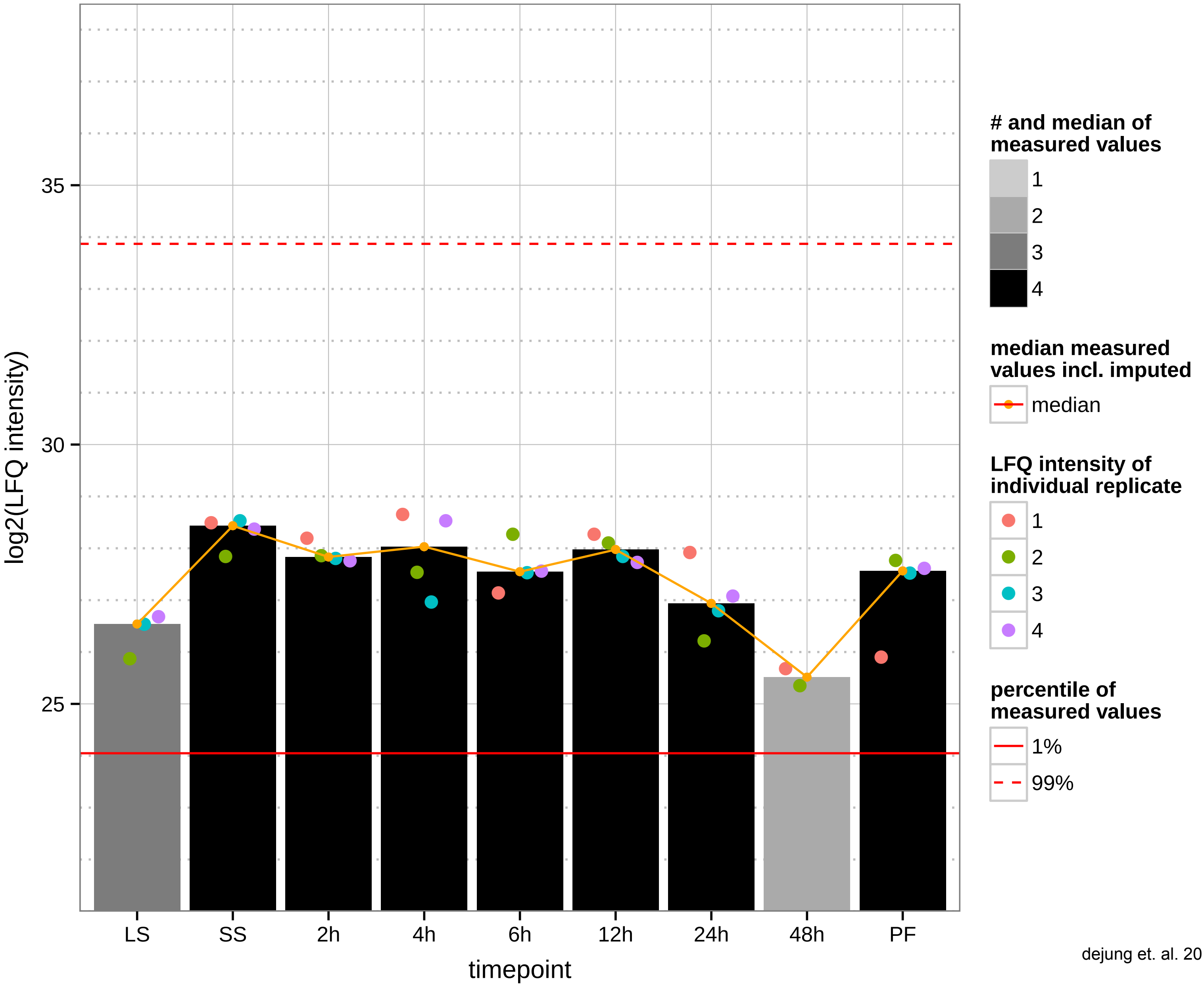

dejung et. al. 2015

Mu–adaptin 1, putative, adaptor complex AP–1 medium subunit
Tb927.7.3180
AGOF: null
AGOC: clathrin adaptor complex
AGOP: intracellular protein transport, vesicle–mediated transport
PGOF: protein binding
PGOC: clathrin adaptor complex
PGOP: intracellular protein transport, transport, vesicle–mediated transport

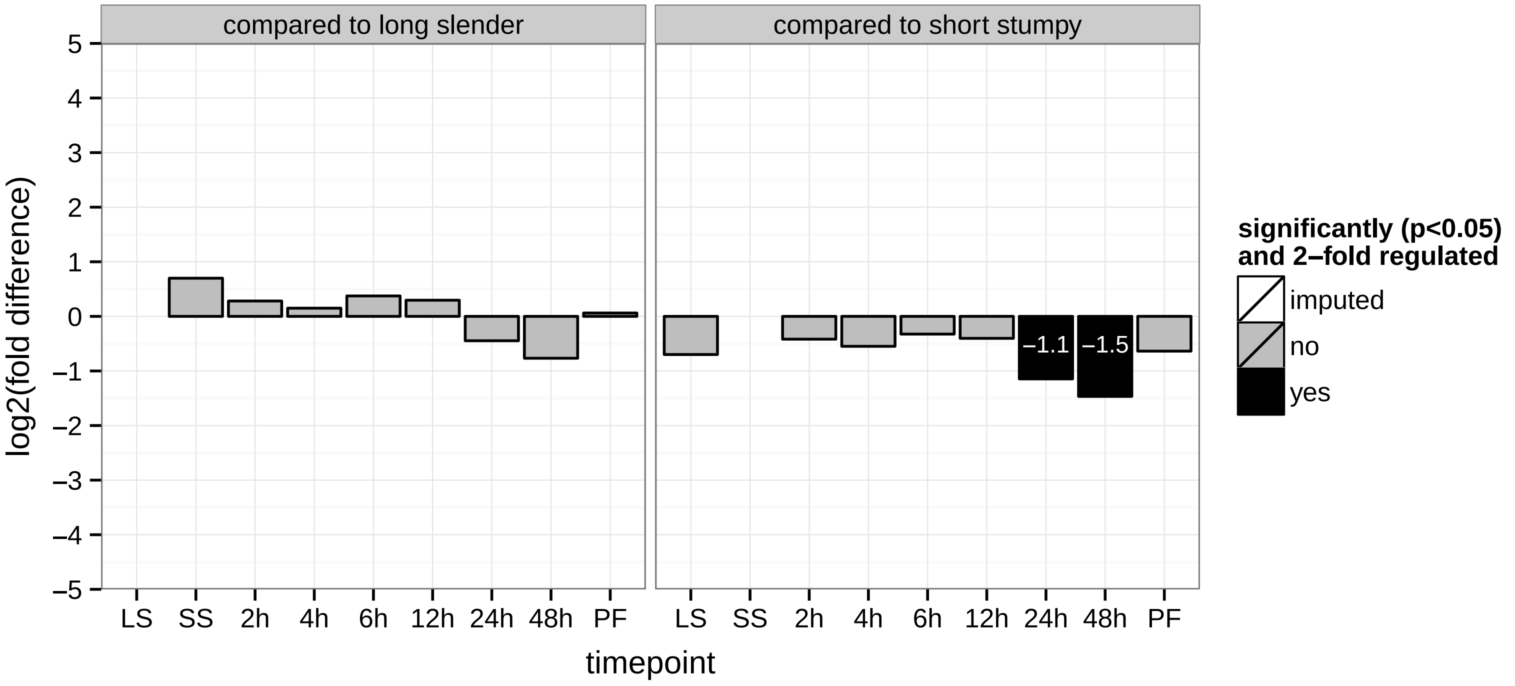

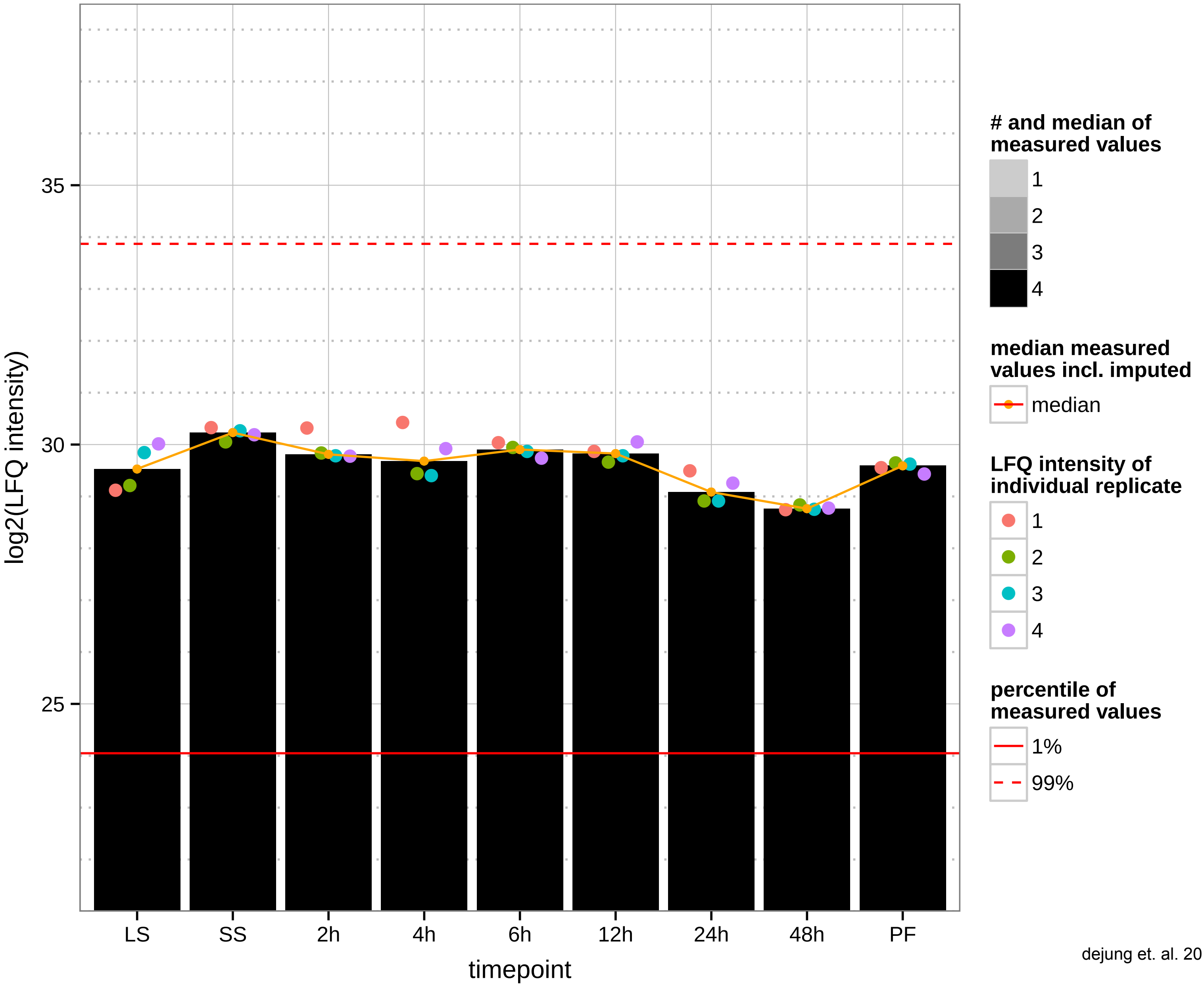

dejung et. al. 2015

hypothetical protein, conserved
Tb927.7.550
AGOF: null
AGOC: null
AGOP: null
PGOF: protein binding
PGOC: null
PGOP: null

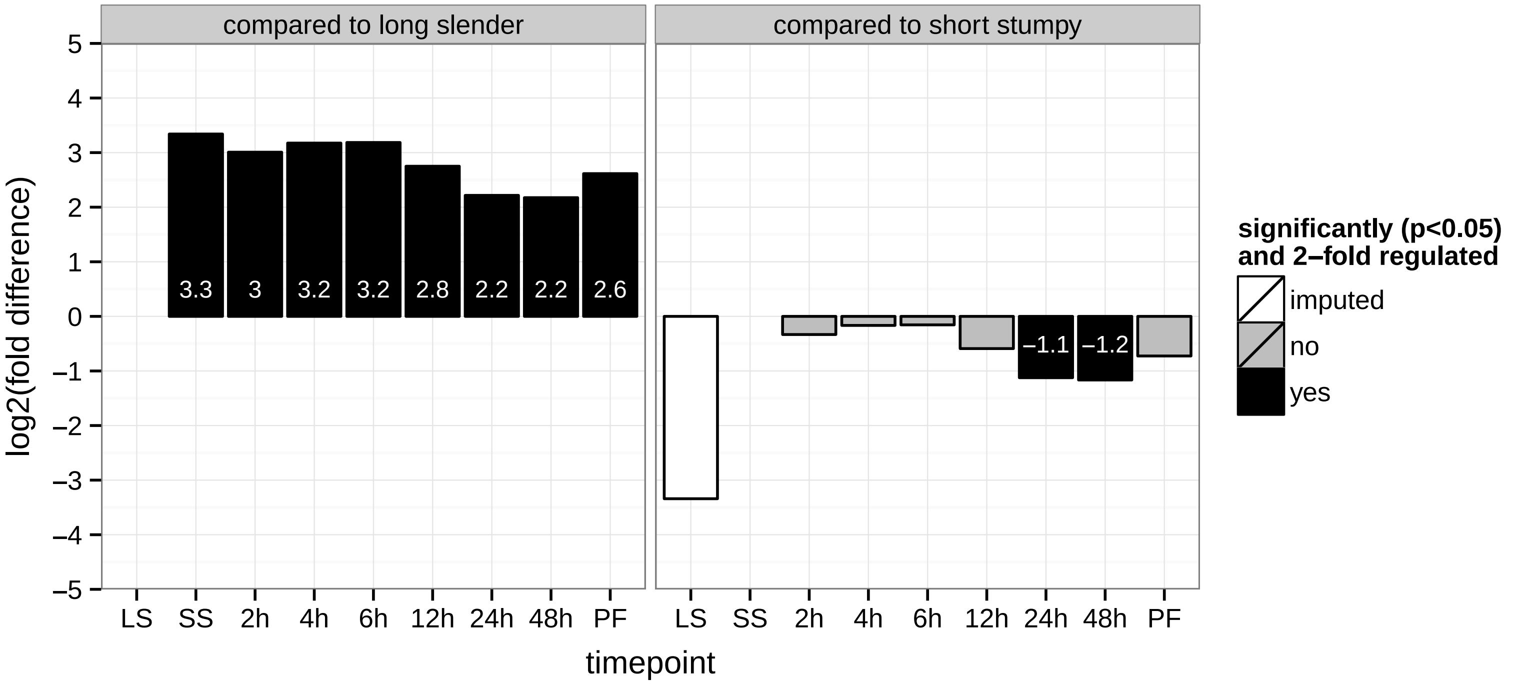

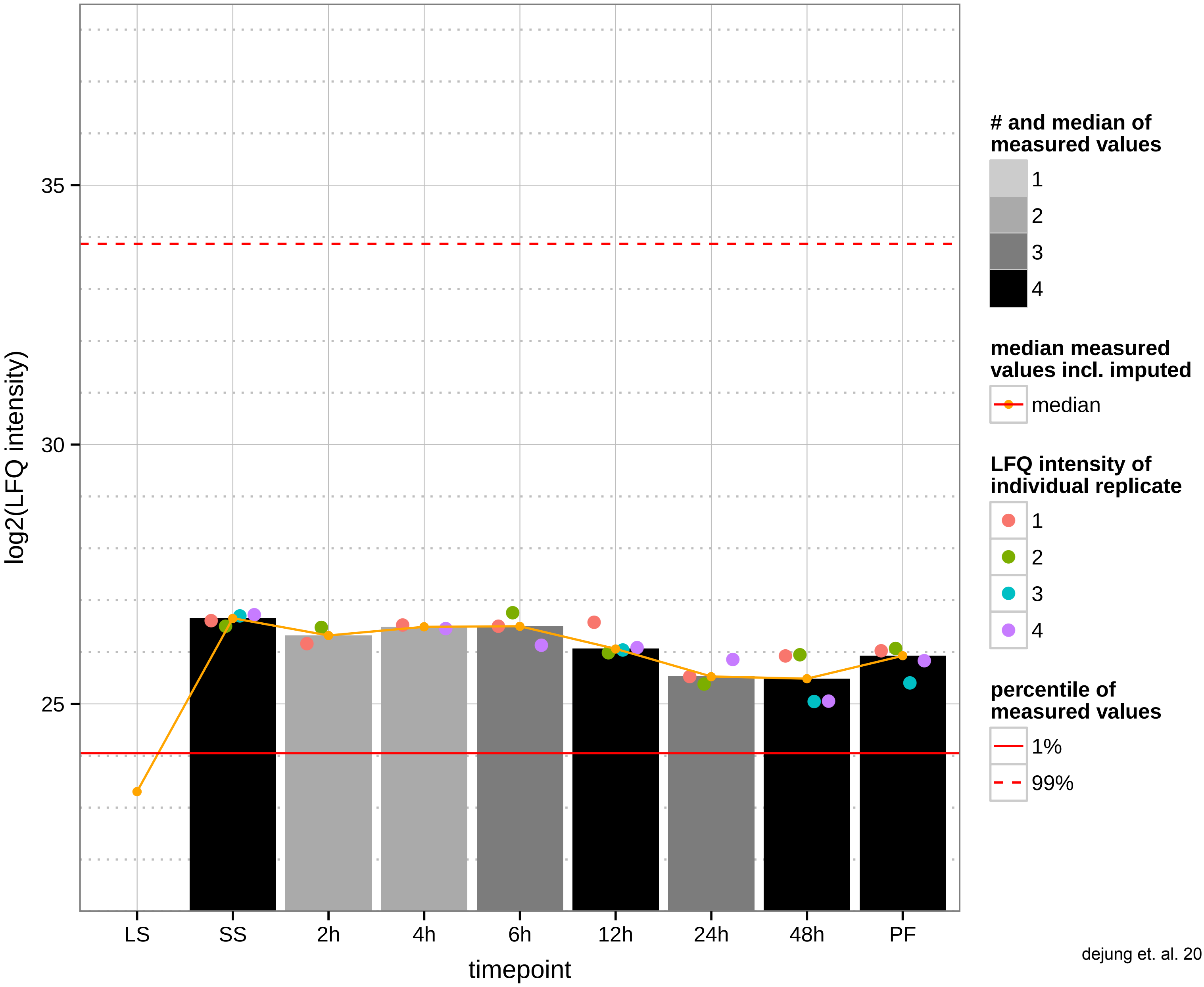

dejung et. al. 2015

hypothetical protein, conserved
Tb927.8.3470
AGOF: null
AGOC: membrane
AGOP: intracellular protein transport
PGOF: null
PGOC: membrane
PGOP: intracellular protein transport, vesicle−mediated transport

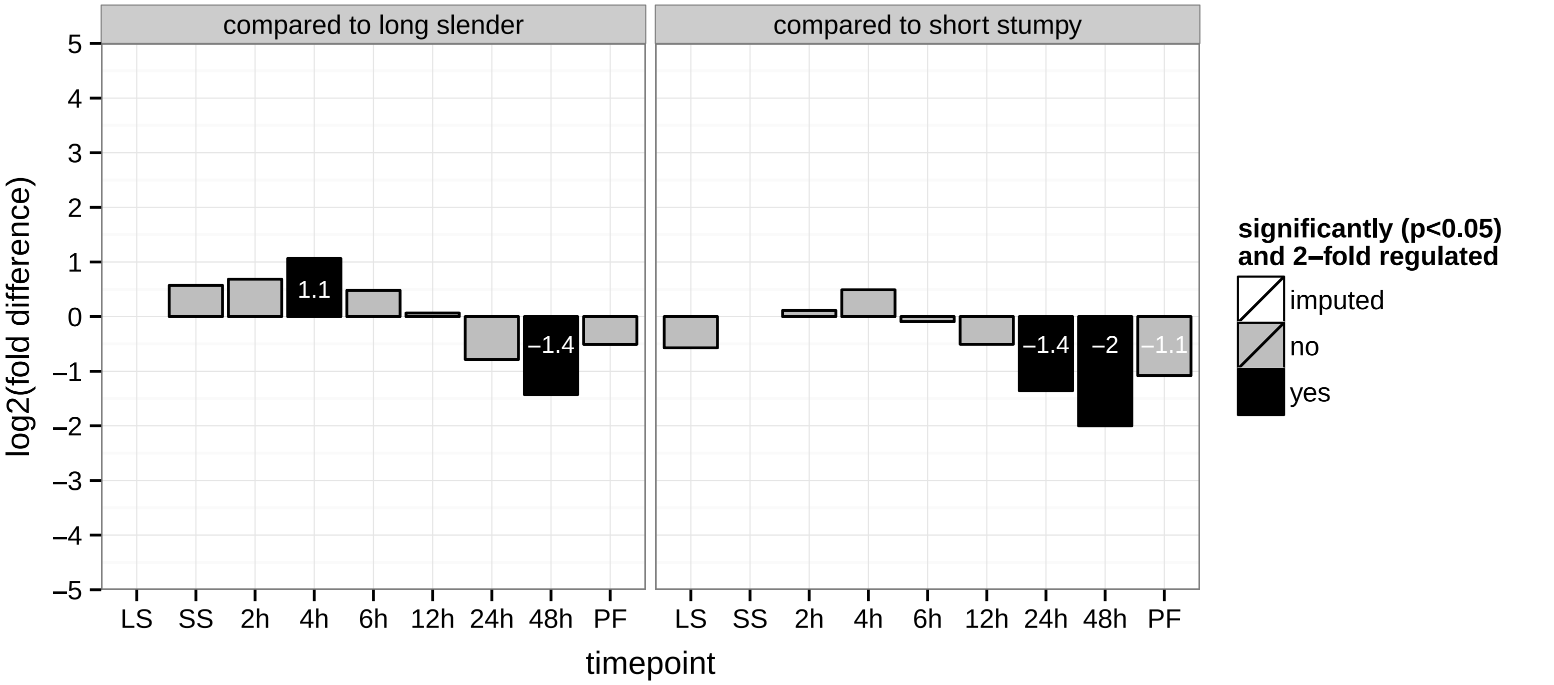

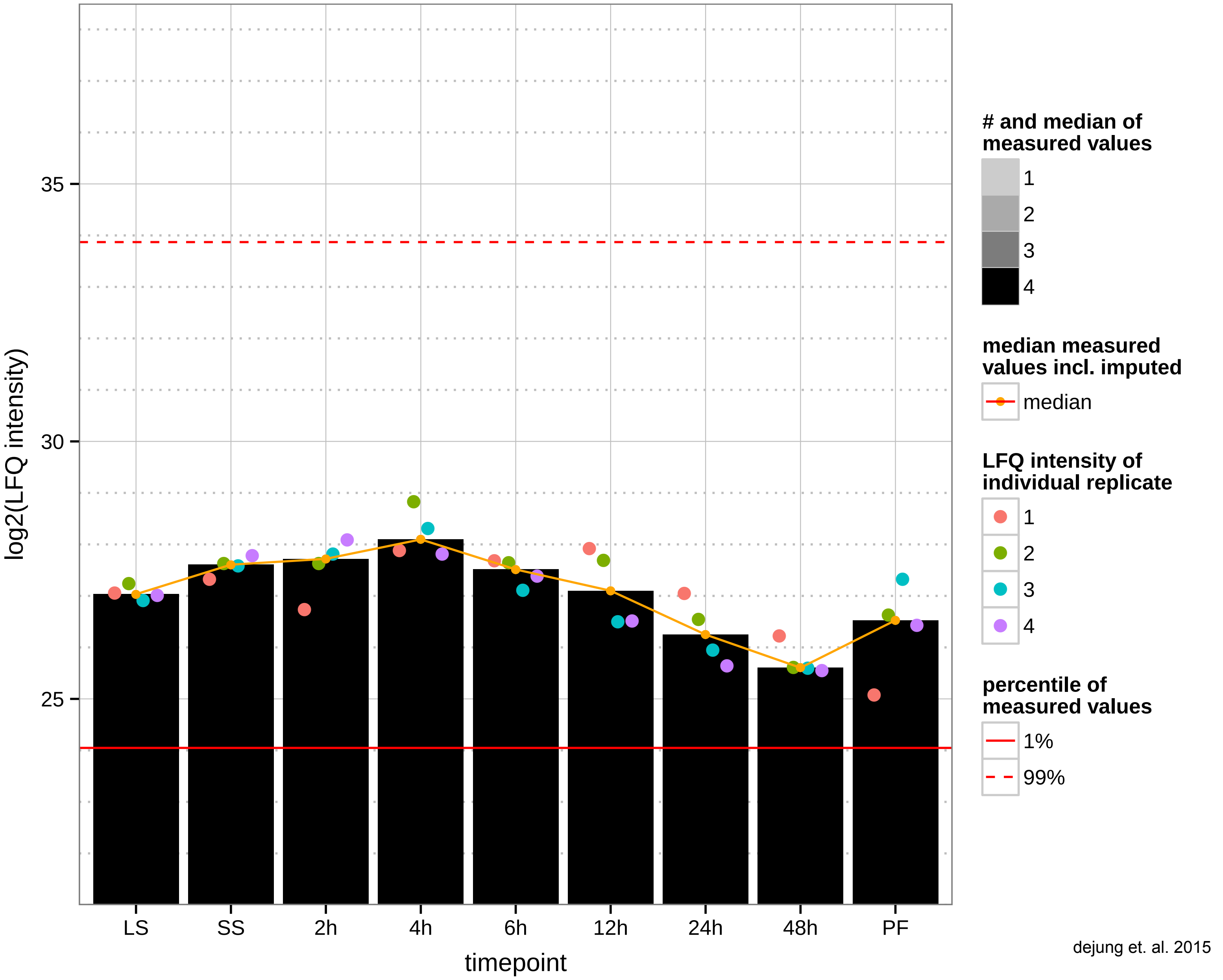

dejung et. al. 2015

uridine phosphorylase
Tb927.8.4430
AGOF: purine−nucleoside phosphorylase activity, uridine phosphorylase activity
AGOC: null
AGOP: nucleoside metabolic process
PGOF: catalytic activity
PGOC: null
PGOP: nucleoside metabolic process

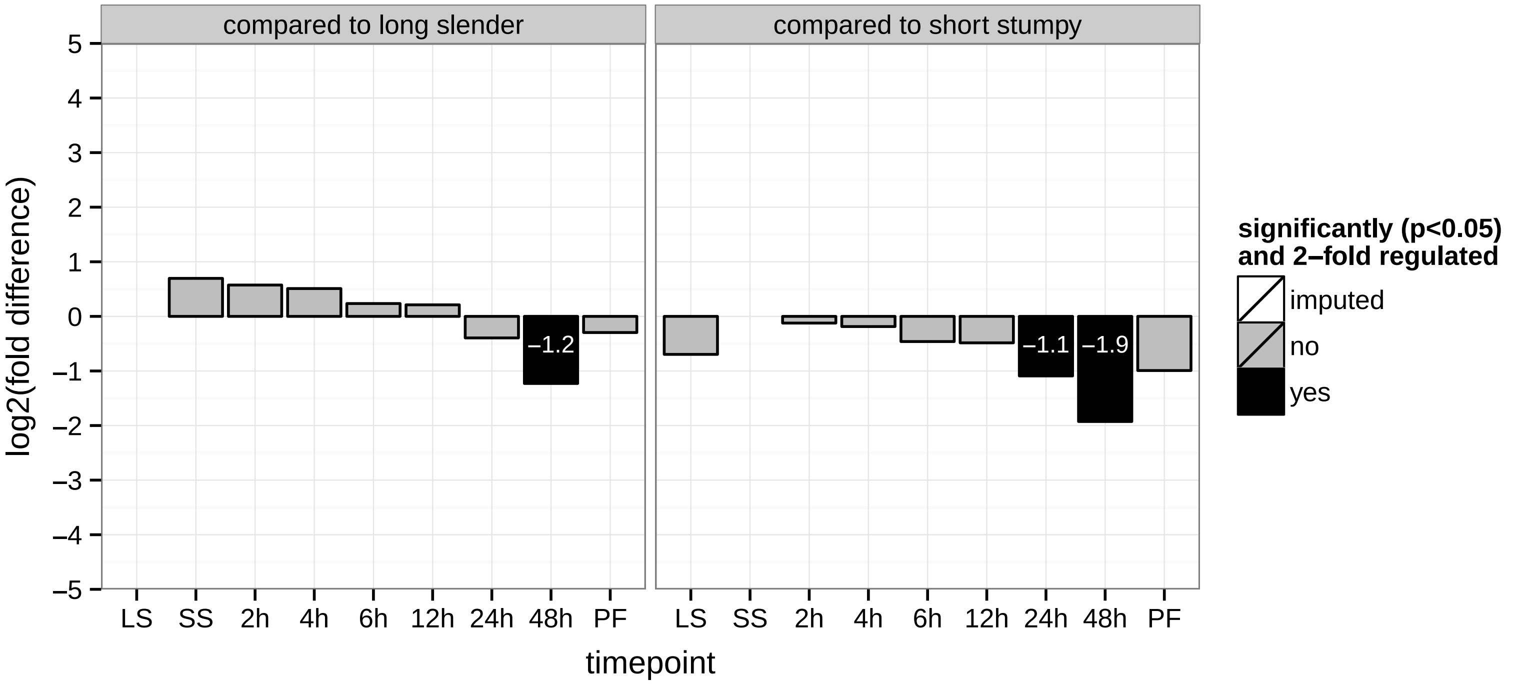

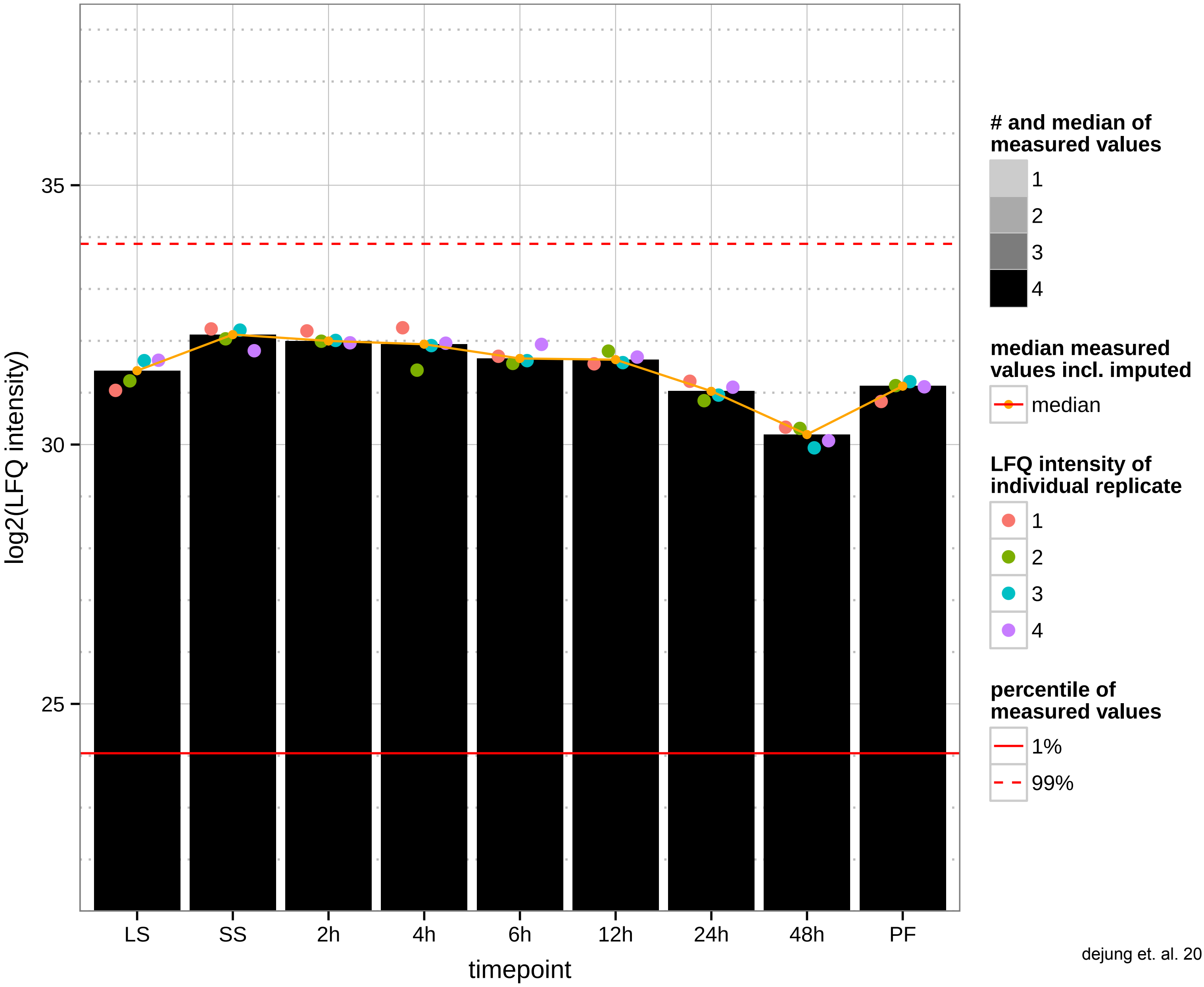

dejung et. al. 2015

hypothetical protein, conserved
Tb927.9.6380;Tb927.9.6360;Tb927.9.6370
AGOF: null
AGOC: null
AGOP: null
PGOF: null
PGOC: null, integral to membrane
PGOP: null, transmembrane transport

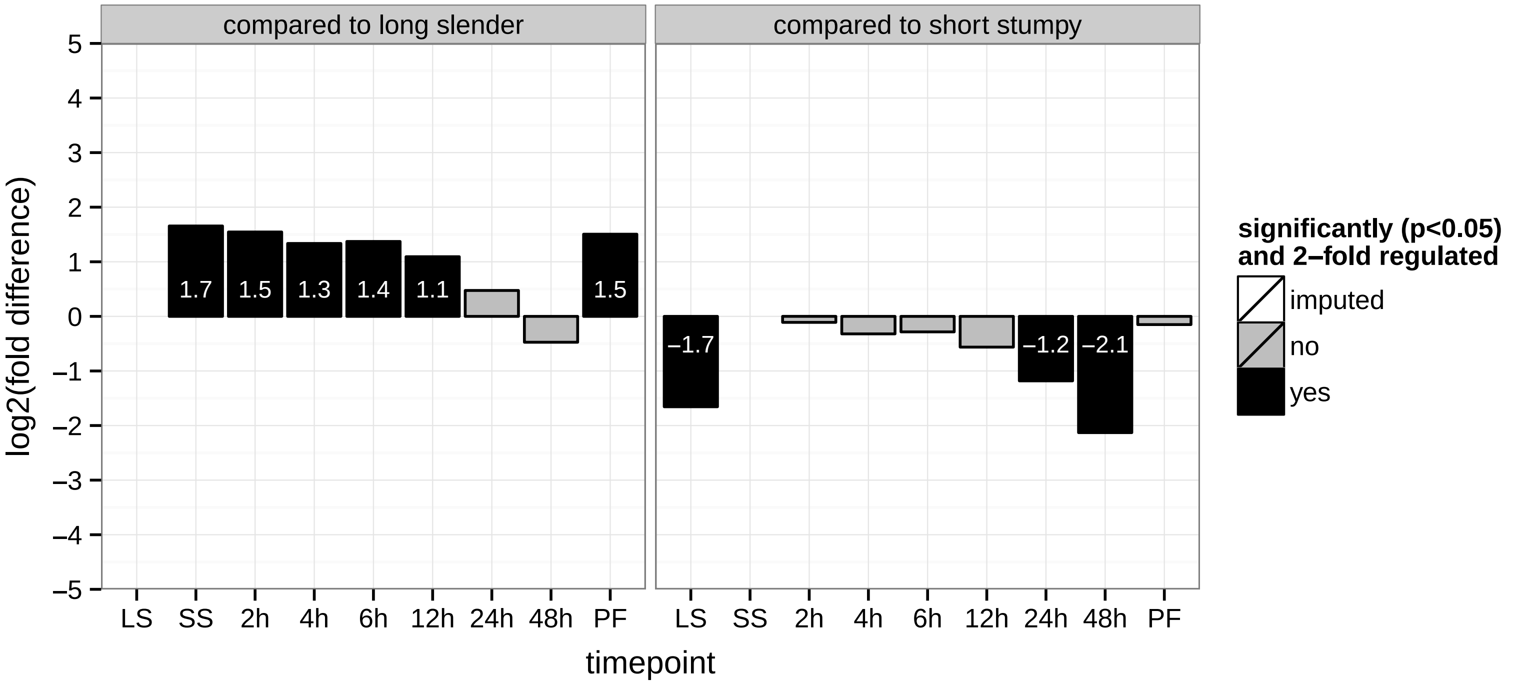

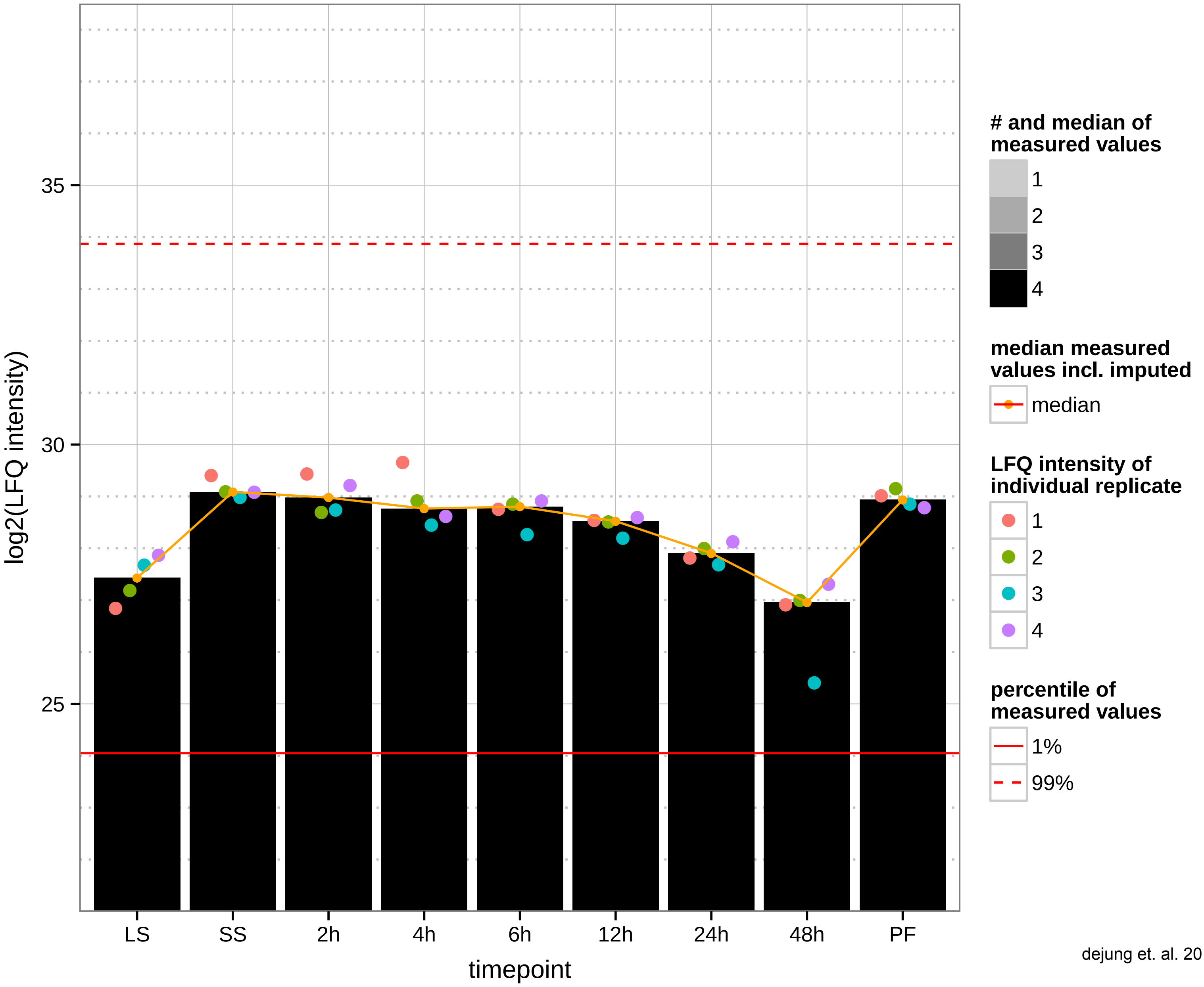

dejung et. al. 2015

protein kinase, putative
Tb927.9.6560
AGOF: ATP binding, protein kinase activity, protein serine/threonine kinase activity
AGOC: null
AGOP: growth, protein phosphorylation
PGOF: ATP binding, protein kinase activity, transferase activity, transferring phosphorus–containing groups
PGOC: null
PGOP: protein phosphorylation

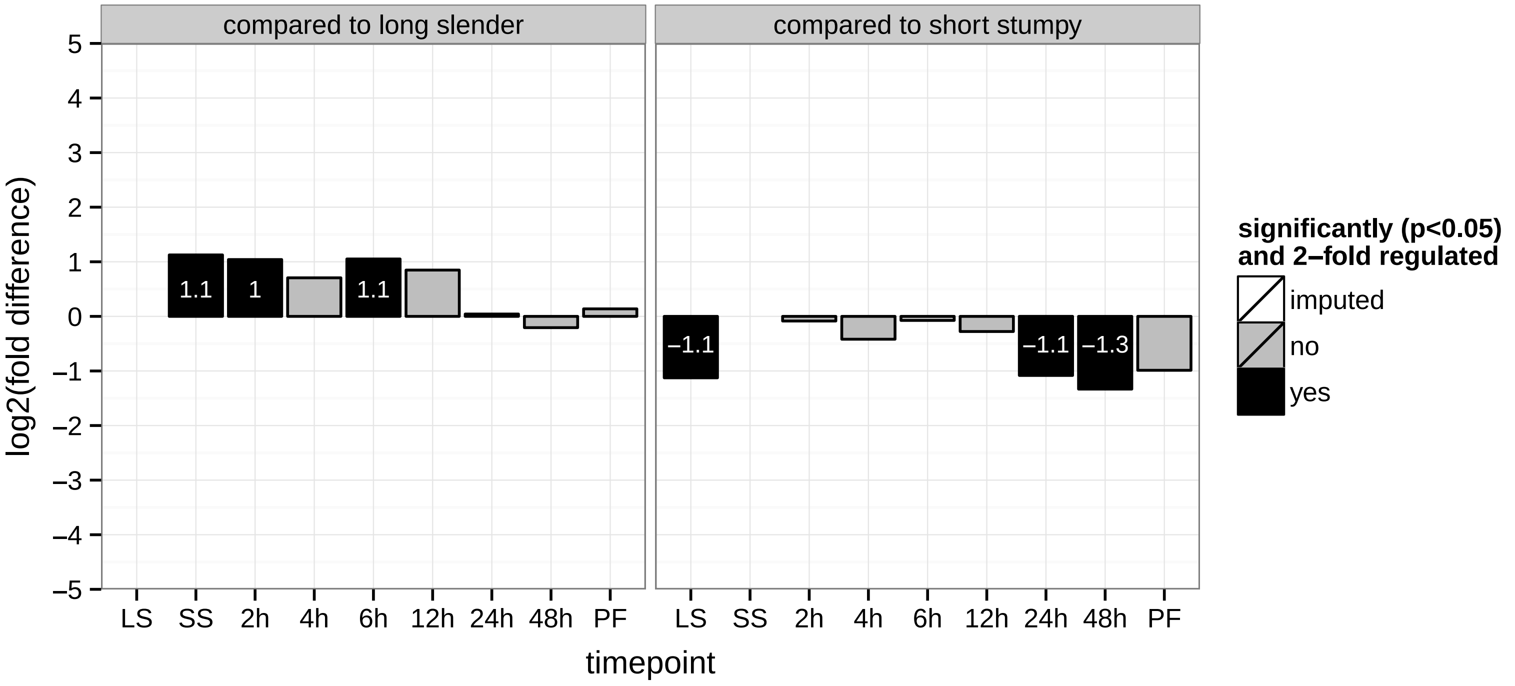

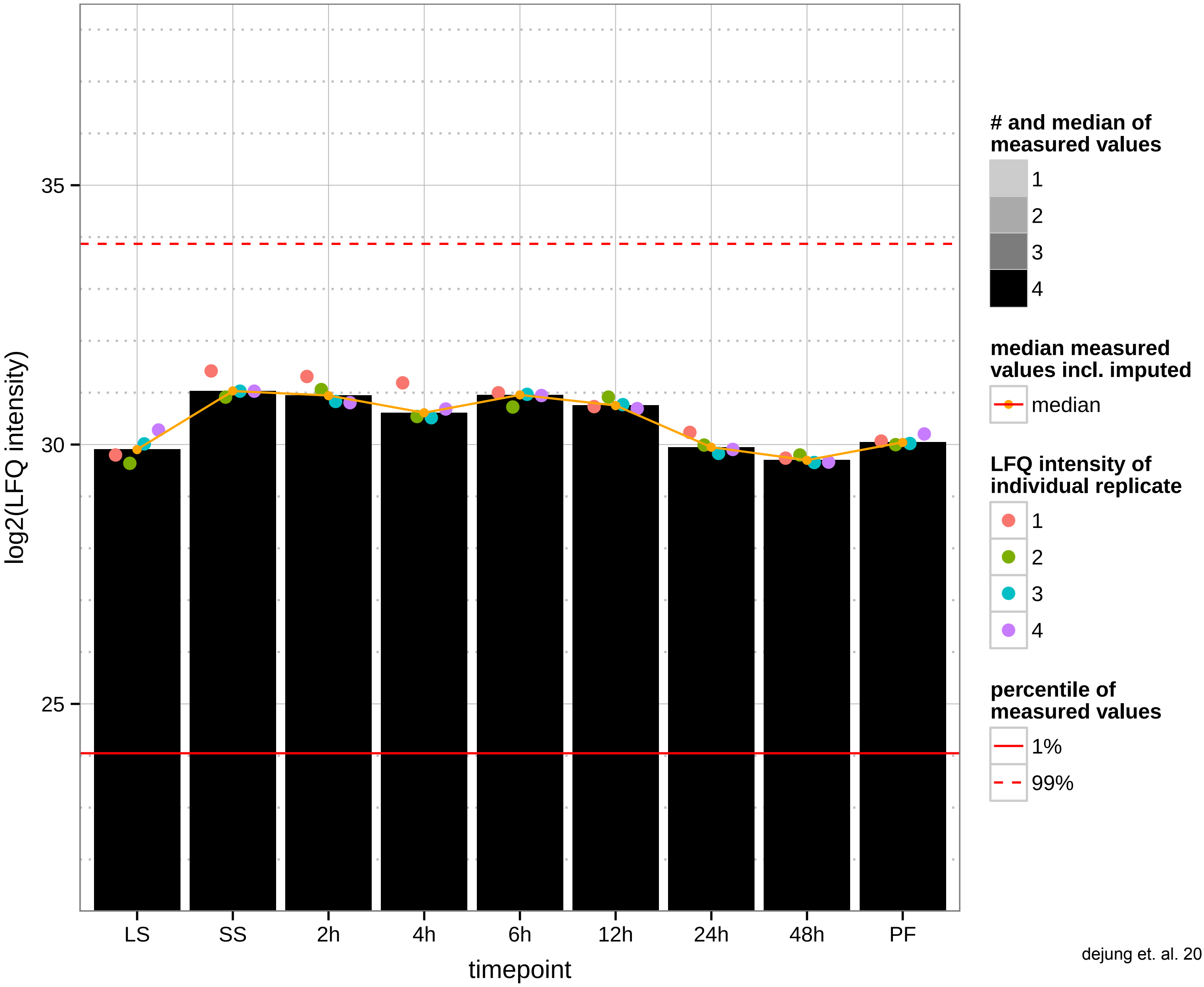

dejung et. al. 2015

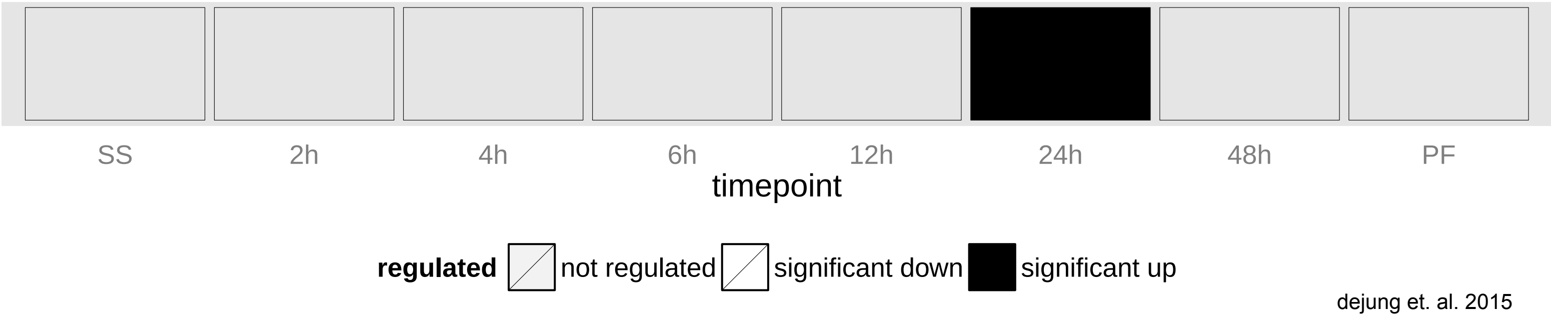
SS
2h
4h
6h
12h
24h
48h
PF
timepoint
regulated
not regulated
significant down
significant up
dejung et. al. 2015

hypothetical protein, conserved
Tb11.v5.0685;Tb927.8.3110
AGOF: null
AGOC: null, mitochondrion
AGOP: null
PGOF: null, structural constituent of ribosome
PGOC: null, intracellular, ribosome
PGOP: null, translation

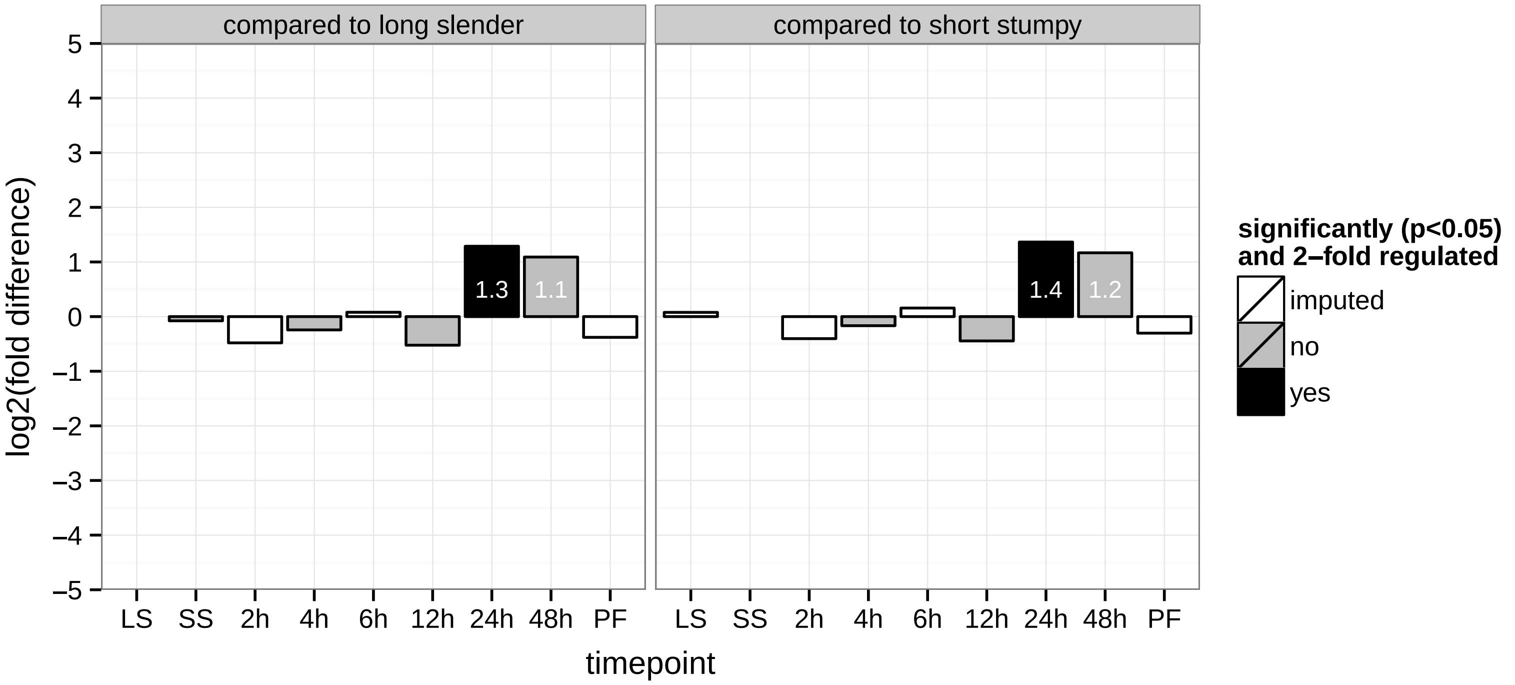

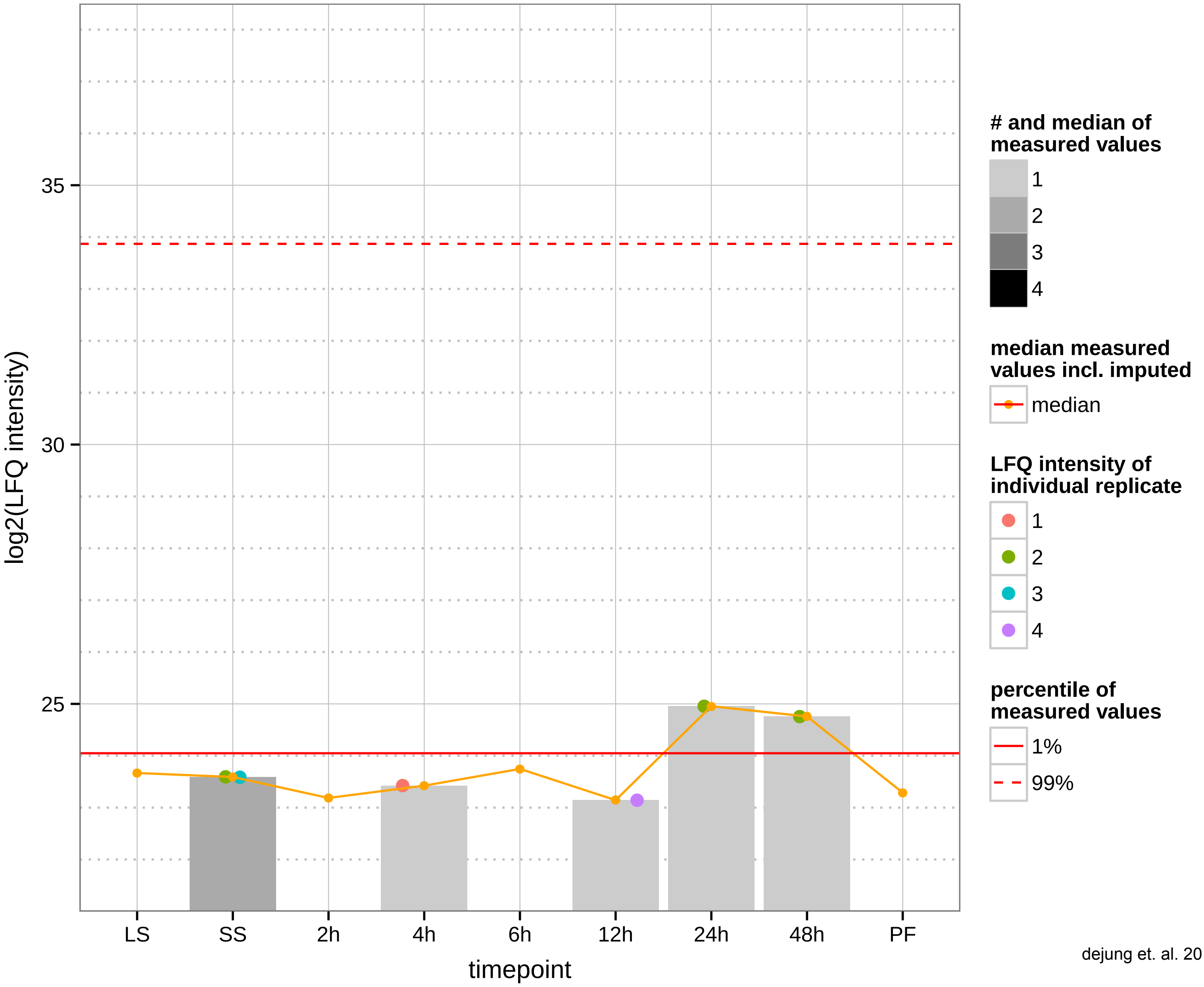

dejung et. al. 2015

hypothetical protein, conserved
Tb927.10.11750
AGOF: null
AGOC: null
AGOP: null
PGOF: null
PGOC: null
PGOP: null

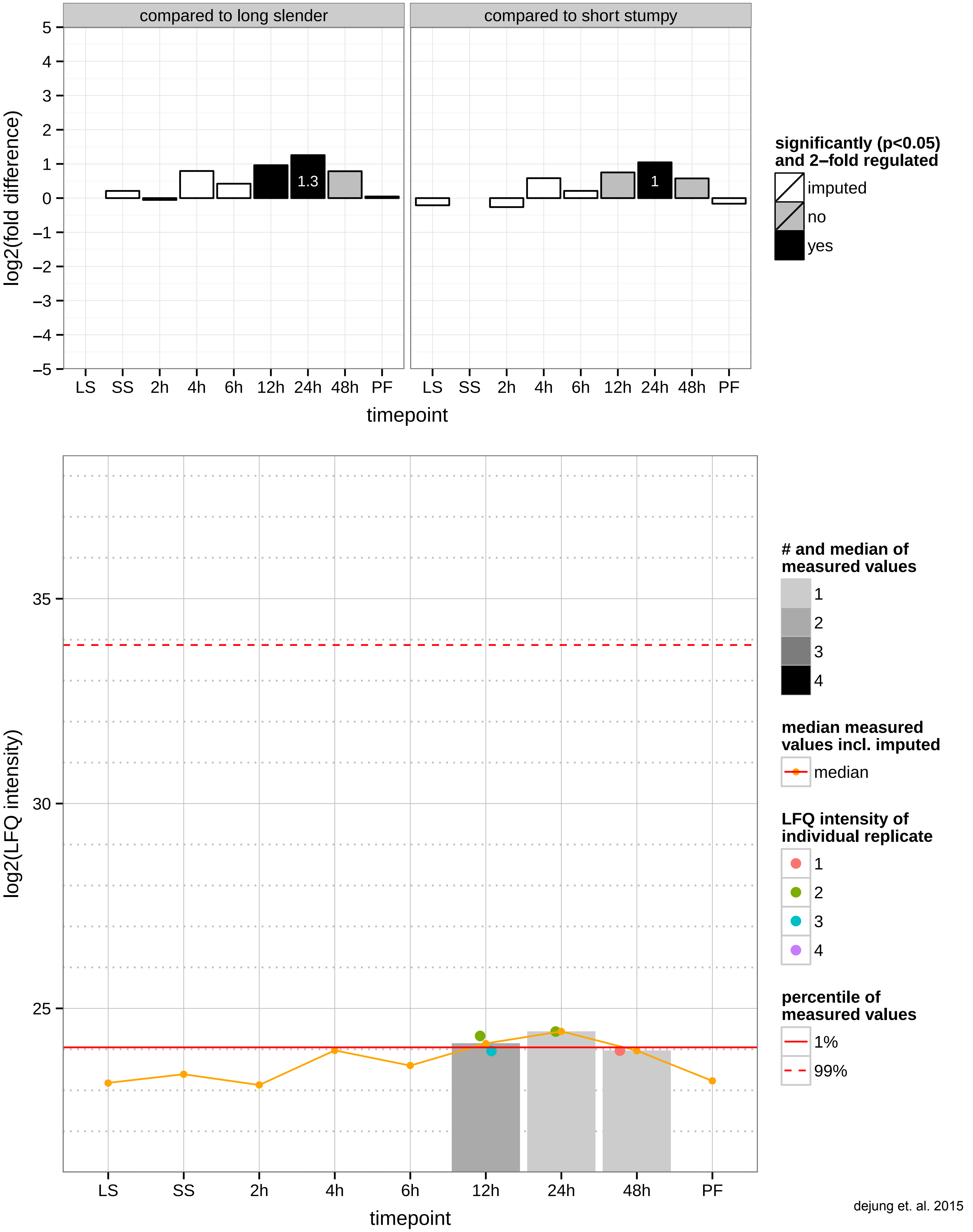

dejung et. al. 2015

nucleosome assembly protein, putative
Tb927.10.15180
AGOF: histone binding
AGOC: nucleus
AGOP: nucleosome assembly
PGOF: null
PGOC: nucleus
PGOP: nucleosome assembly

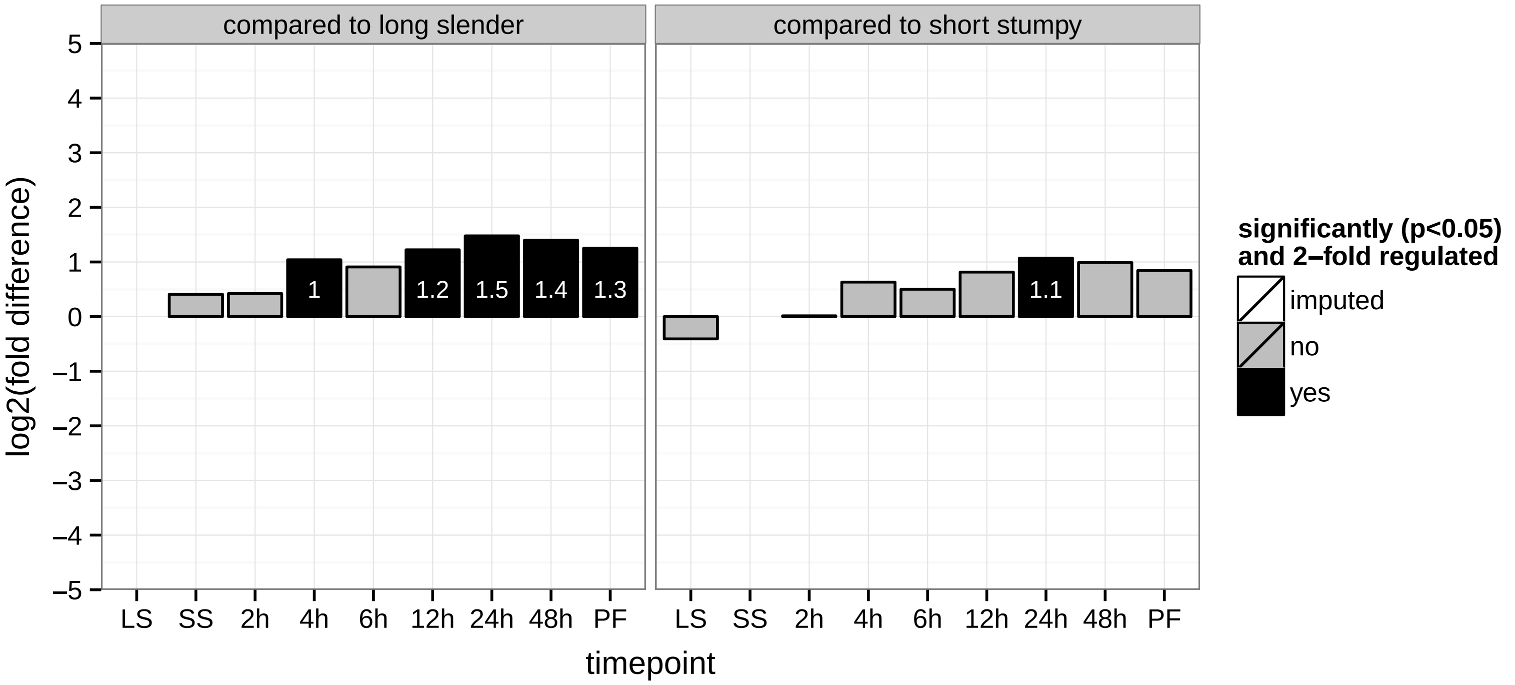

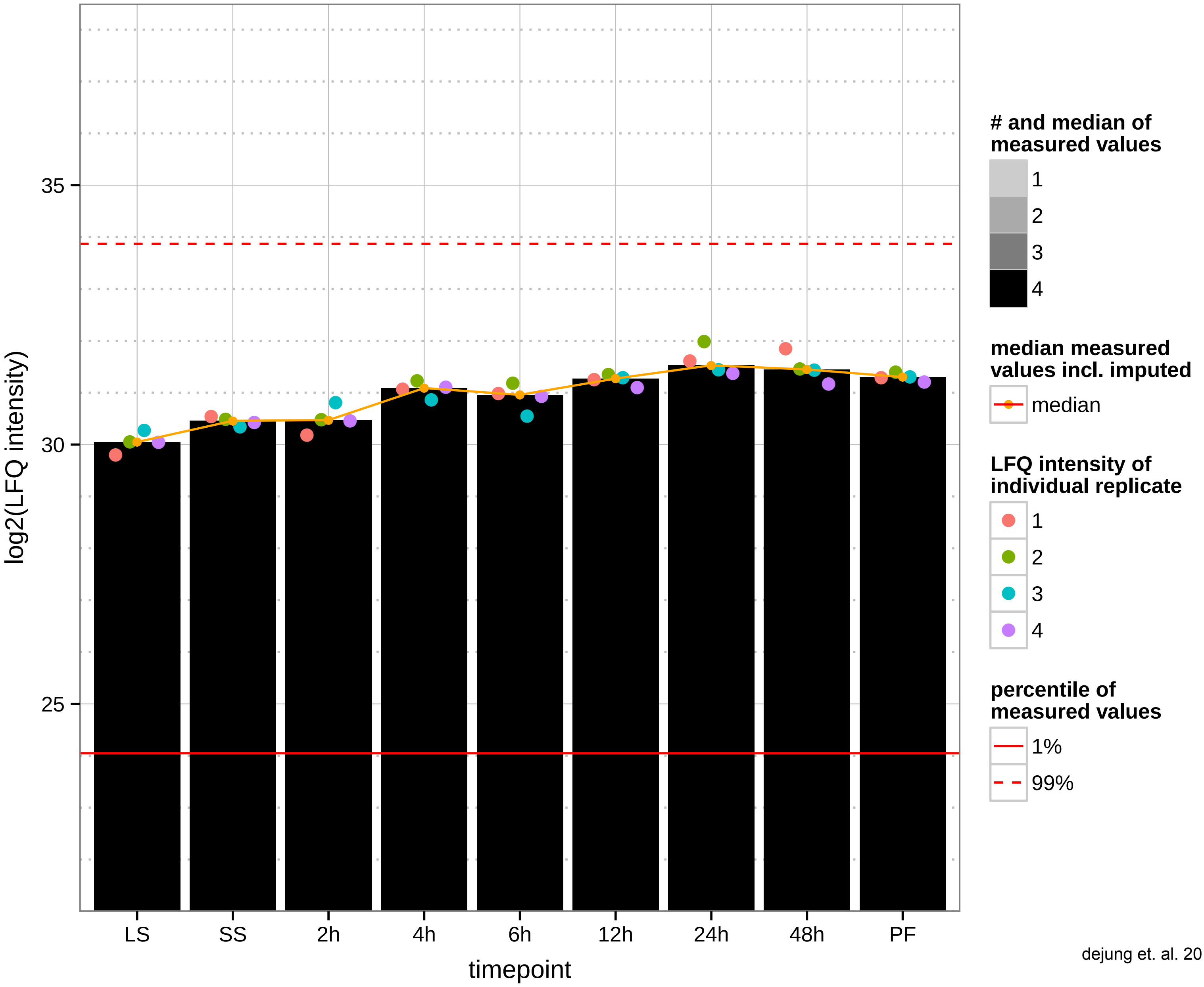

dejung et. al. 2015

hypothetical protein, conserved
Tb927.10.7750
AGOF: null
AGOC: null
AGOP: null
PGOF: null
PGOC: null
PGOP: null

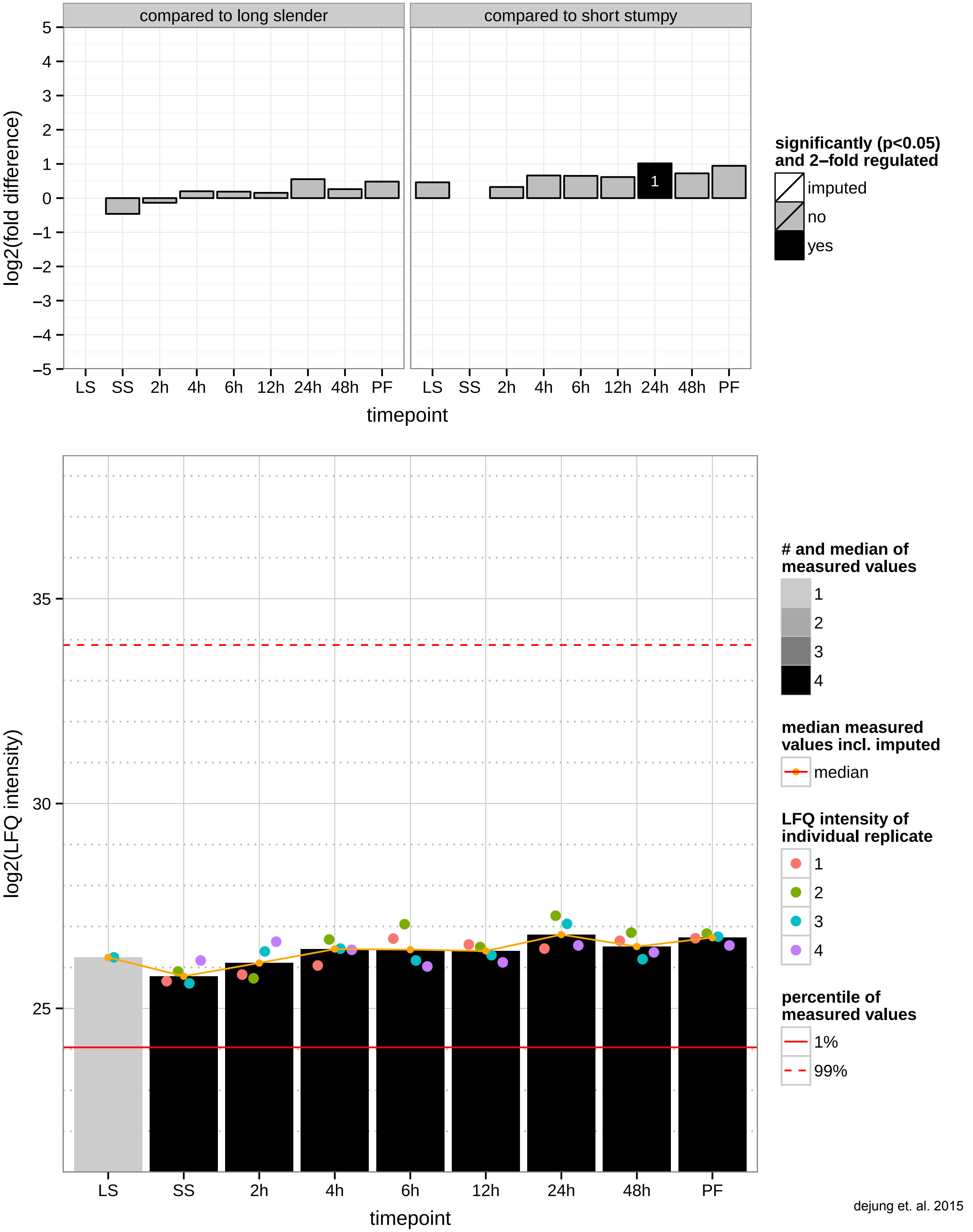

hslVU complex proteolytic subunit, putative, threonine peptidase, Clan T(1), family T1B
Tb927.11.10240
AGOF: endopeptidase activity, threonine−type endopeptidase activity
AGOC: HslUV protease complex, mitochondrion, proteasome core complex
AGOP: protein catabolic process, proteolysis involved in cellular protein catabolic process, rolling circle DNA replication
PGOF: threonine−type endopeptidase activity
PGOC: HslUV protease complex, proteasome core complex
PGOP: proteolysis, proteolysis involved in cellular protein catabolic process

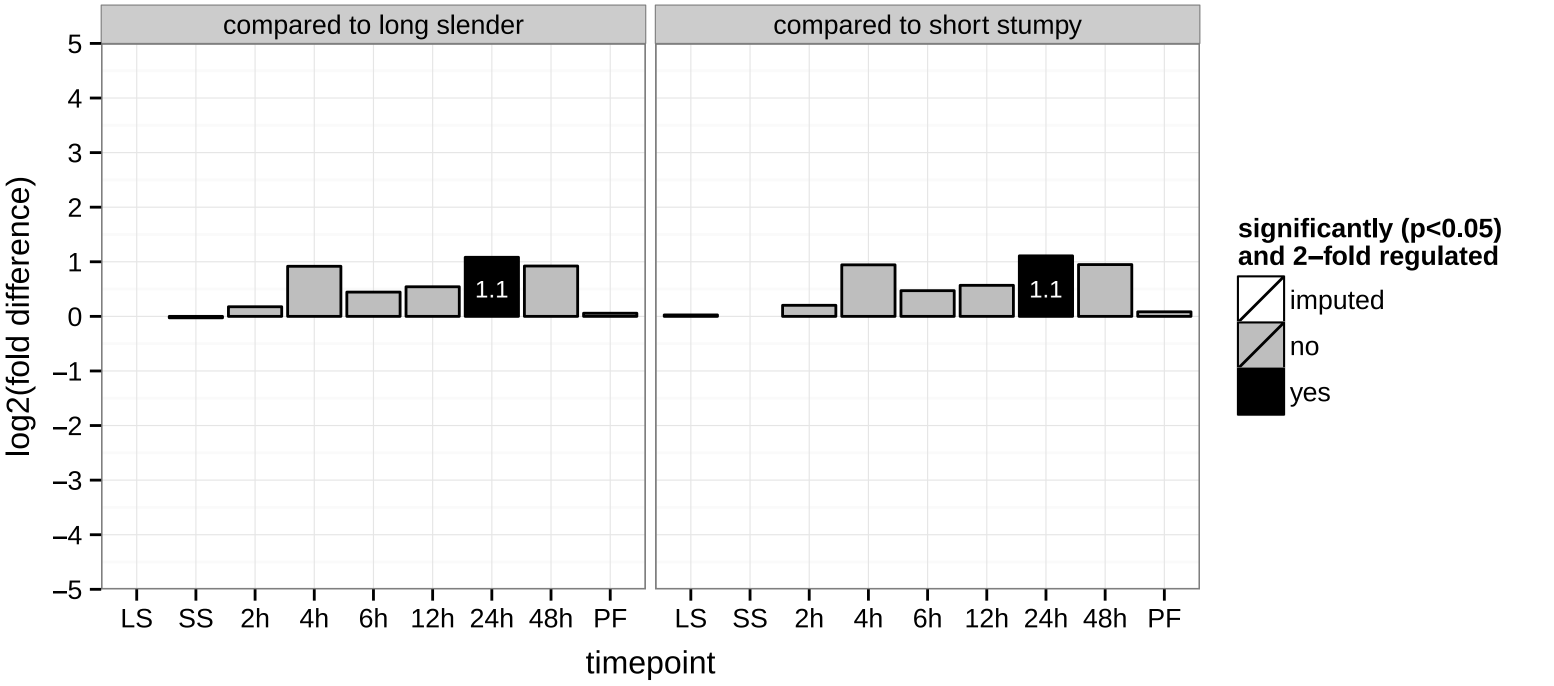

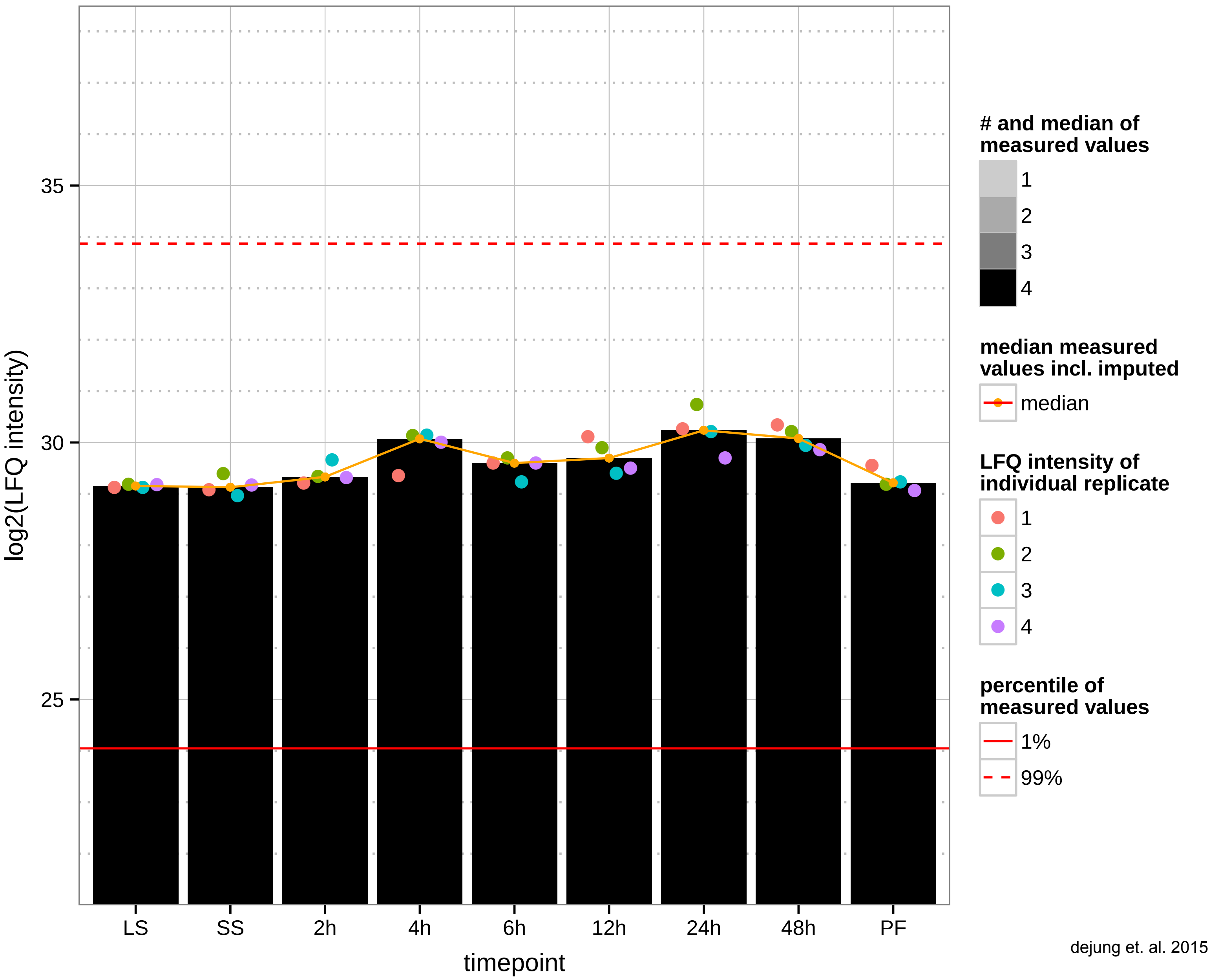

dejung et. al. 2015

hypothetical protein, conserved
Tb927.11.10570
AGOF: null
AGOC: mitochondrion
AGOP: null
PGOF: null
PGOC: null
PGOP: null

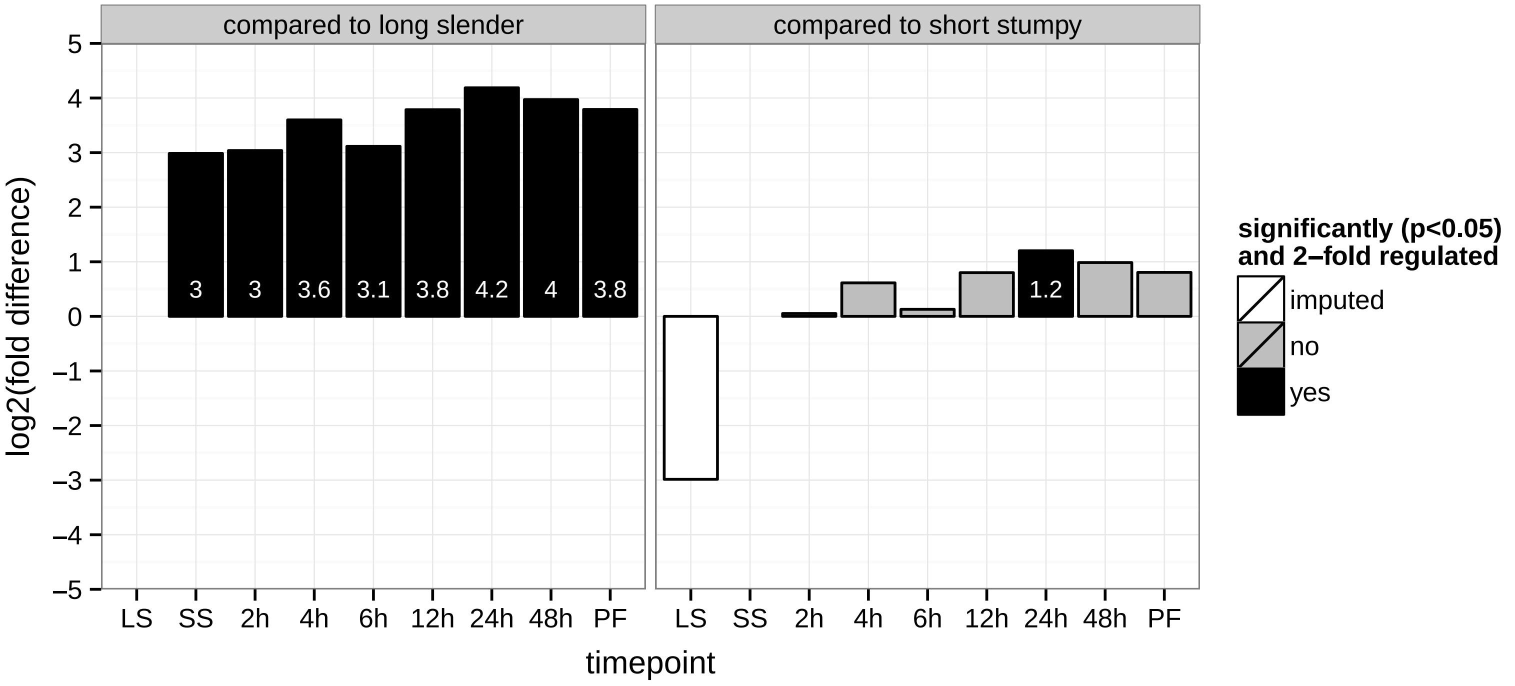

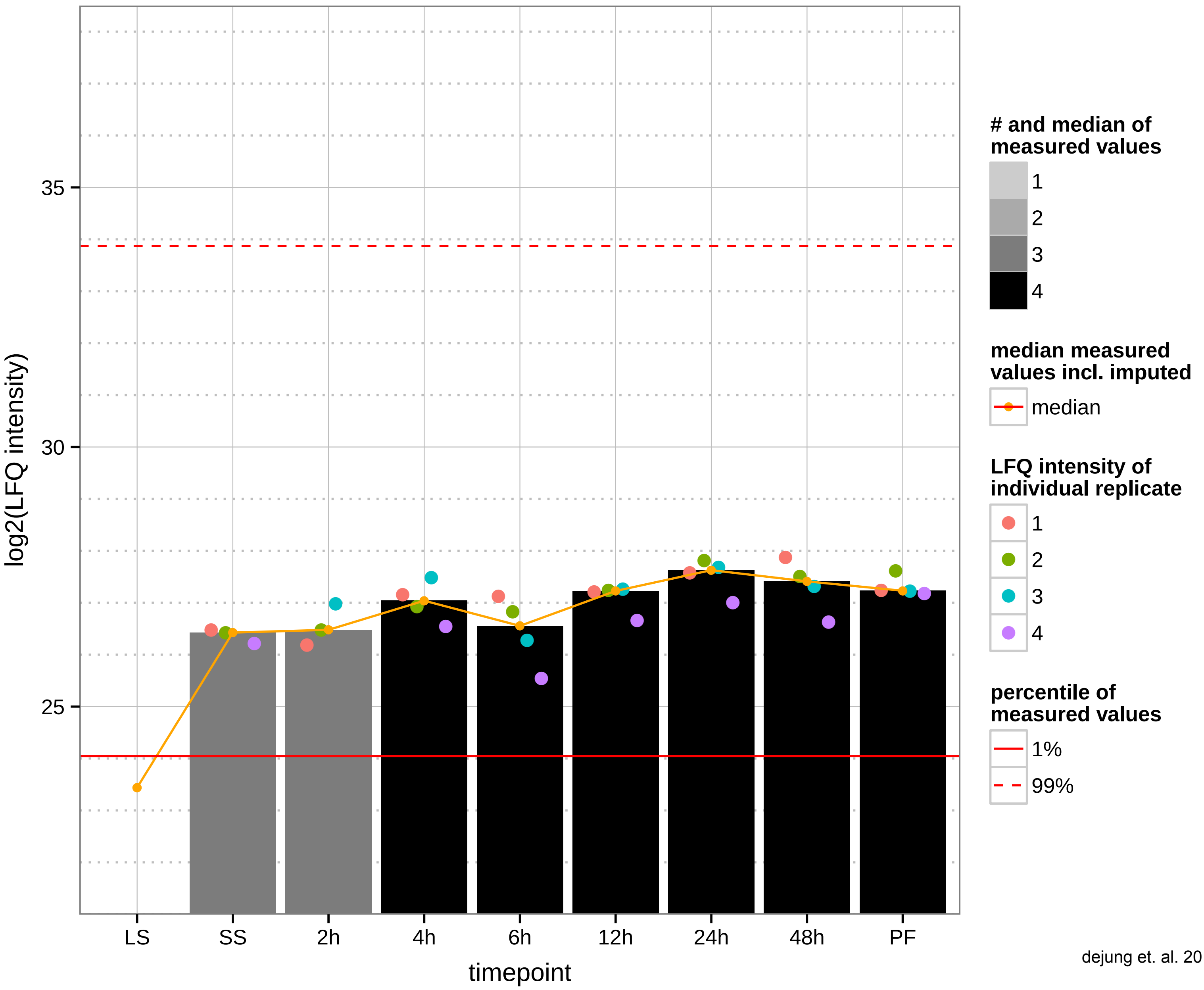

dejung et. al. 2015

mitochondrial import inner membrane translocase subunit tim17 (Tim17)
Tb927.11.13290
AGOF: protein transporter activity
AGOC: integral to mitochondrial inner membrane, mitochondrial inner membrane, mitochondrial inner membrane presequence
AGOP: protein targeting to mitochondrion, stabilization of membrane potential
PGOF: null
PGOC: null
PGOP: null

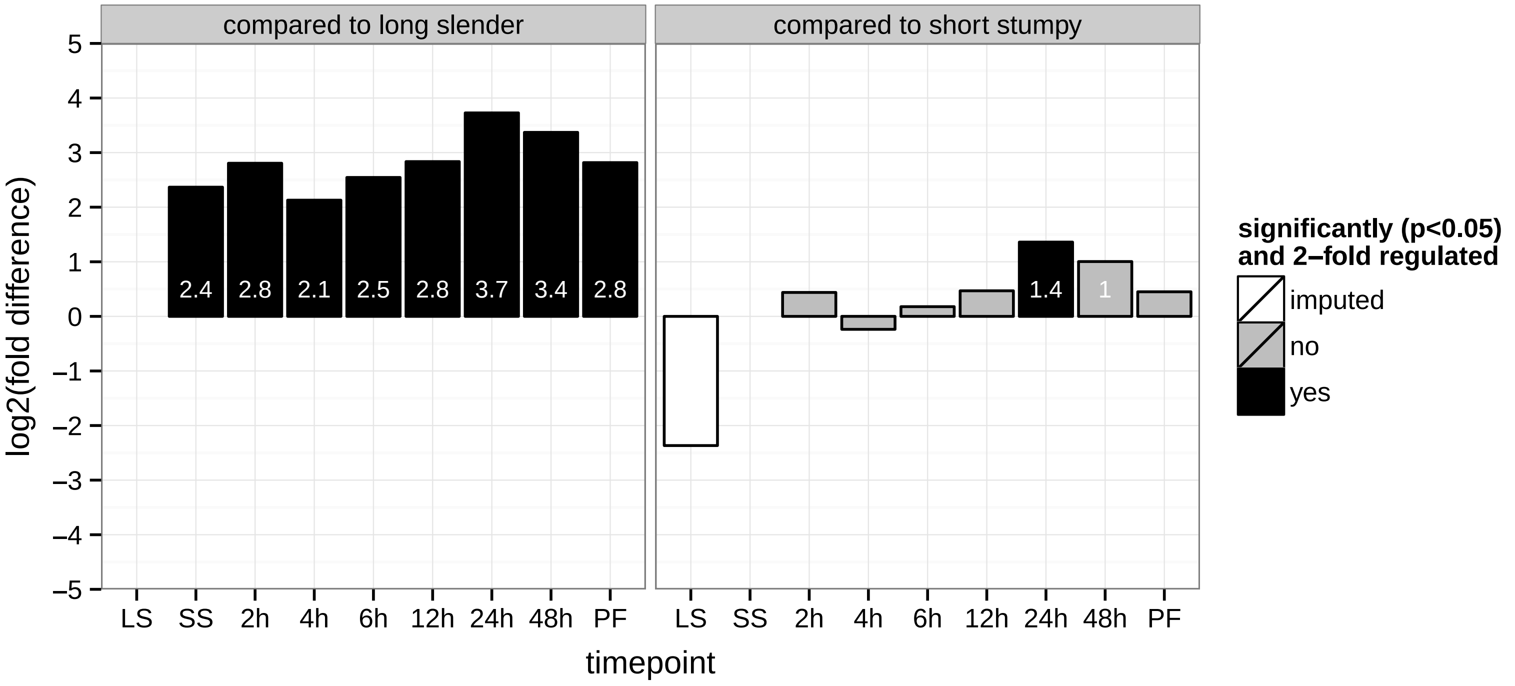

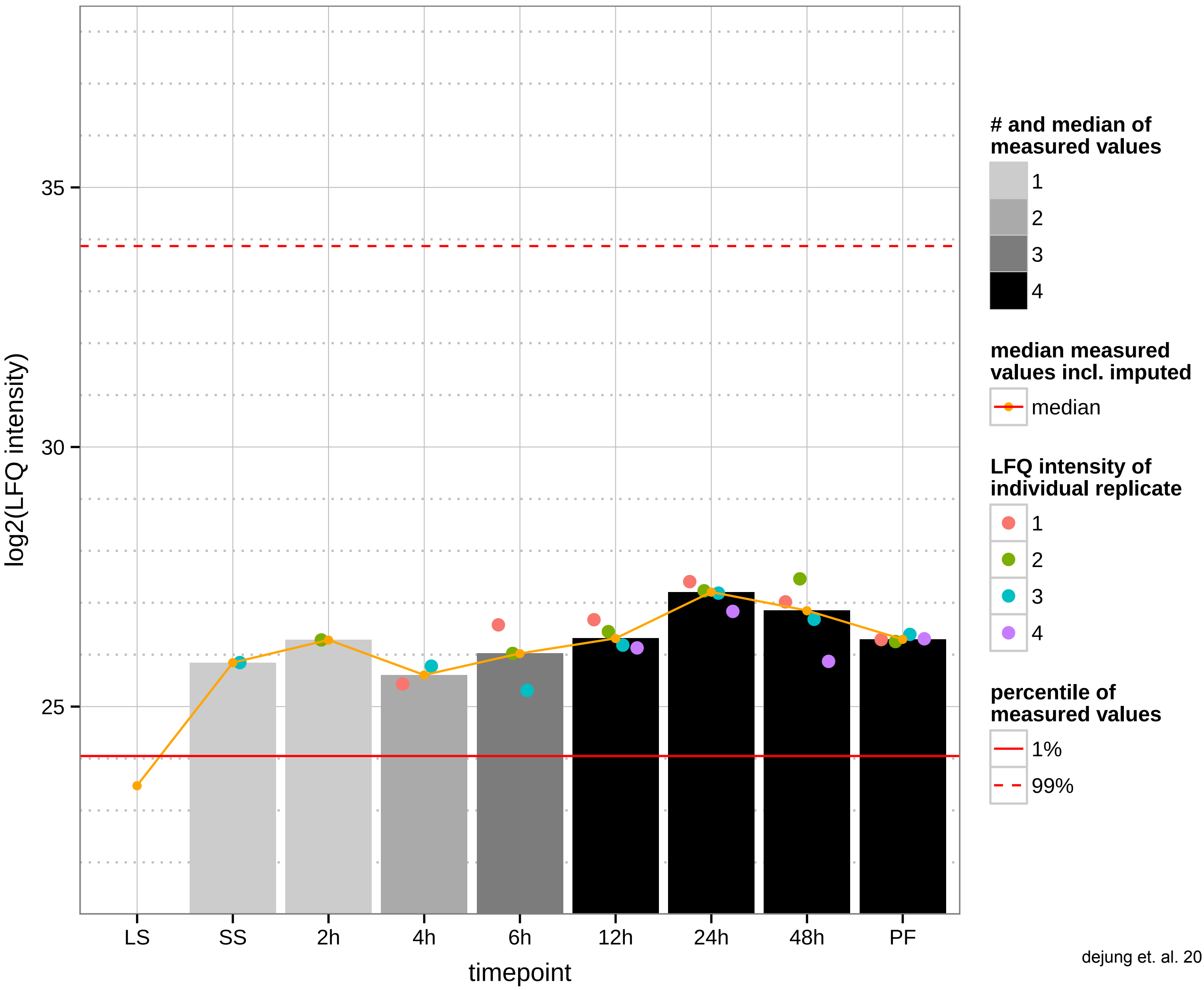

dejung et. al. 2015

hypothetical protein, conserved
Tb927.11.13950
AGOF: null
AGOC: null
AGOP: null
PGOF: null
PGOC: null
PGOP: null

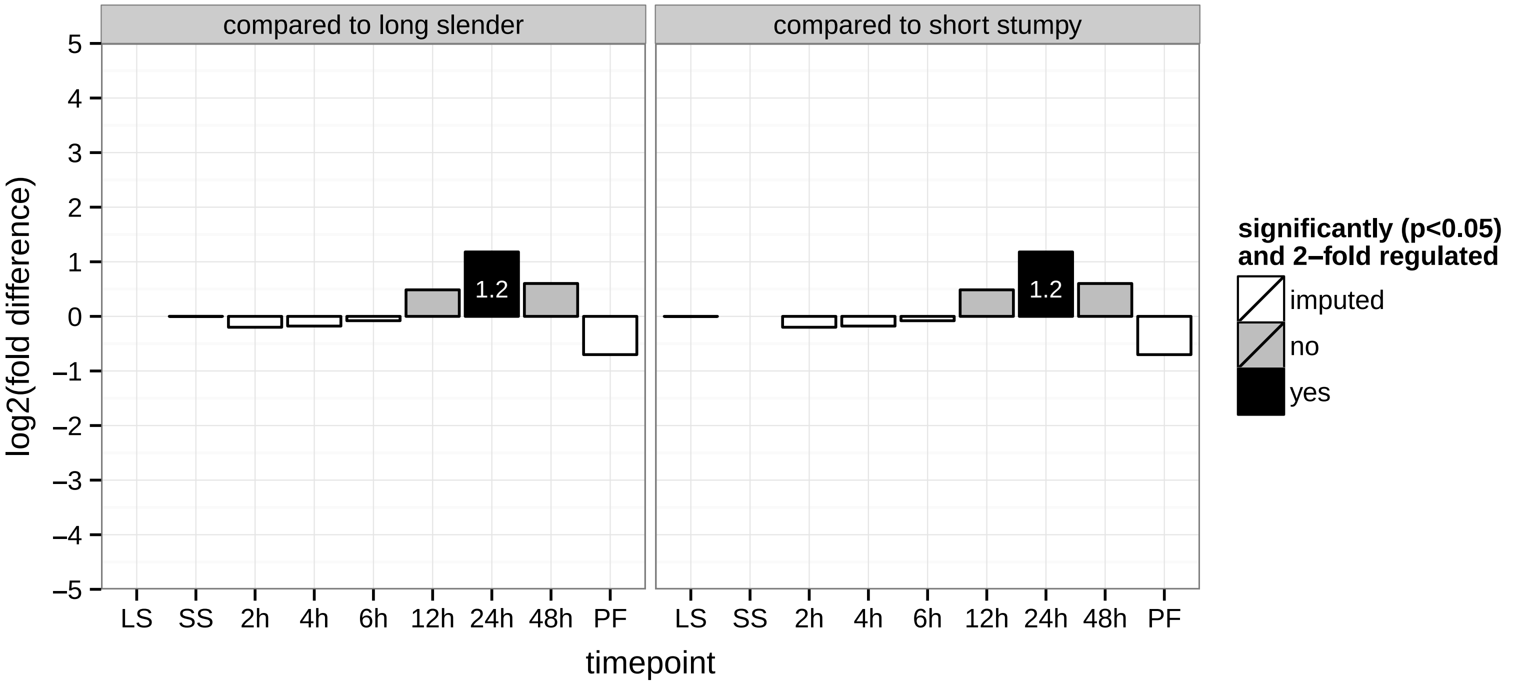

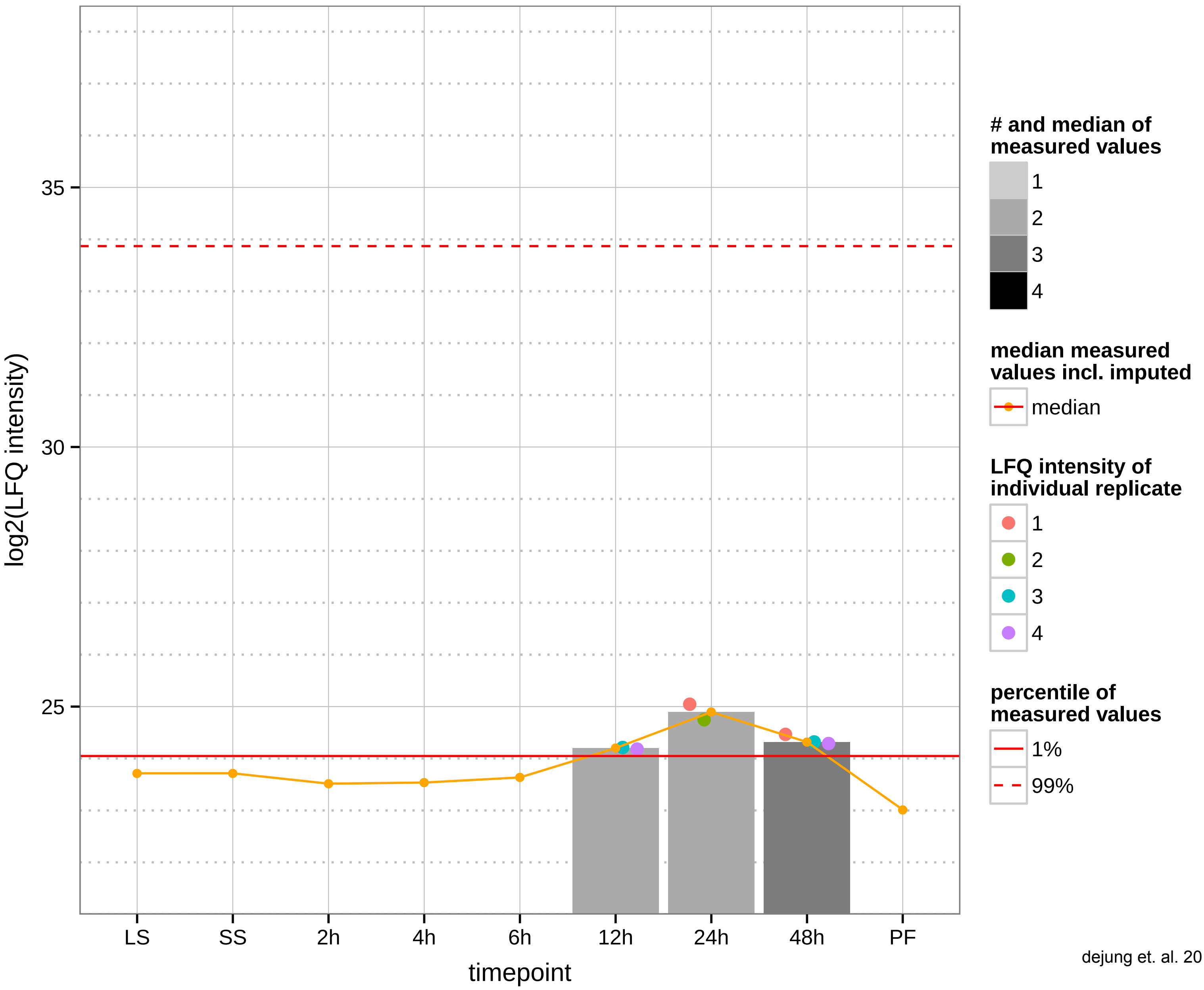

dejung et. al. 2015

hypothetical protein, conserved
Tb927.11.16040
AGOF: null
AGOC: null
AGOP: null
PGOF: unfolded protein binding
PGOC: prefoldin complex
PGOP: protein folding

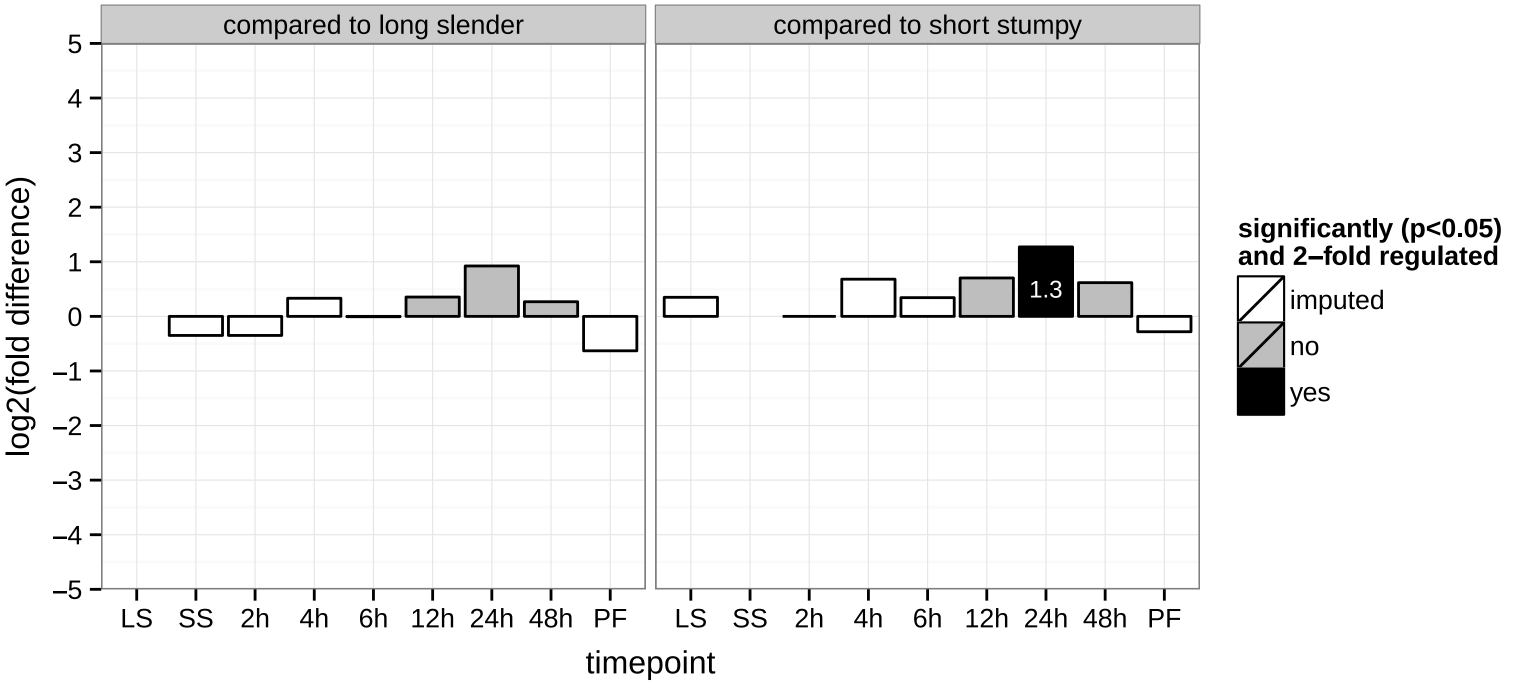

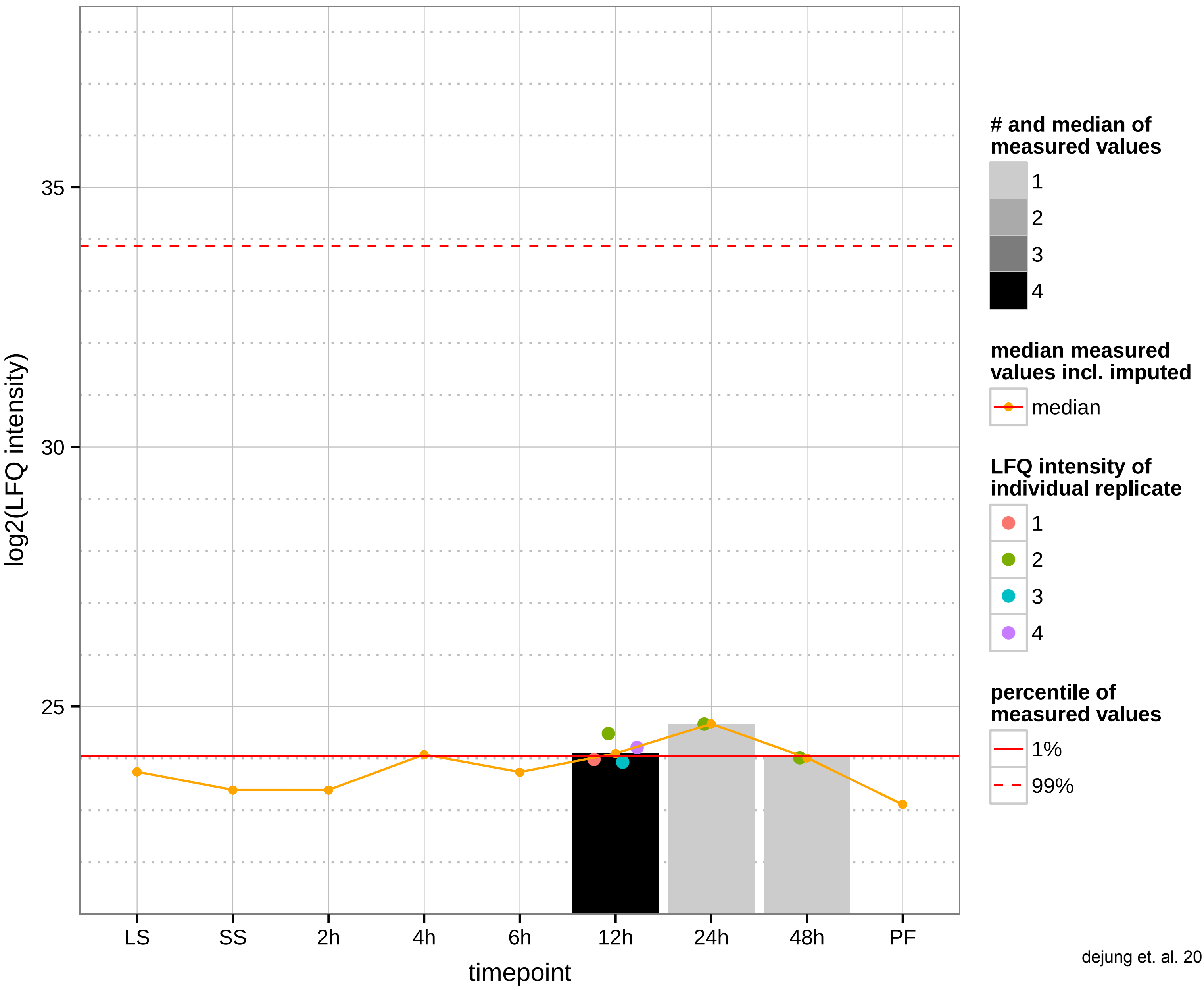

dejung et. al. 2015

glycerolphosphate mutase, putative
Tb927.11.2920
AGOF: catalytic activity
AGOC: mitochondrion
AGOP: metabolic process
PGOF: null
PGOC: null
PGOP: null

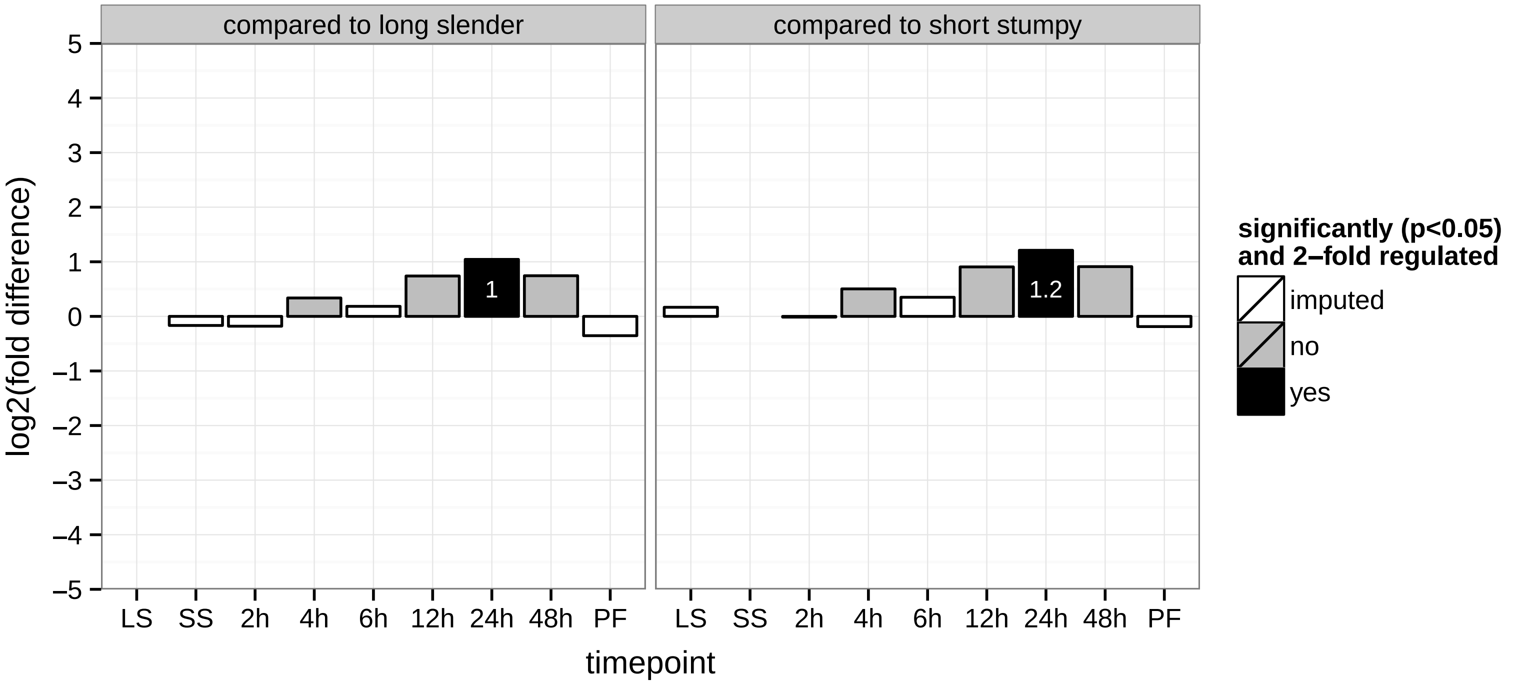

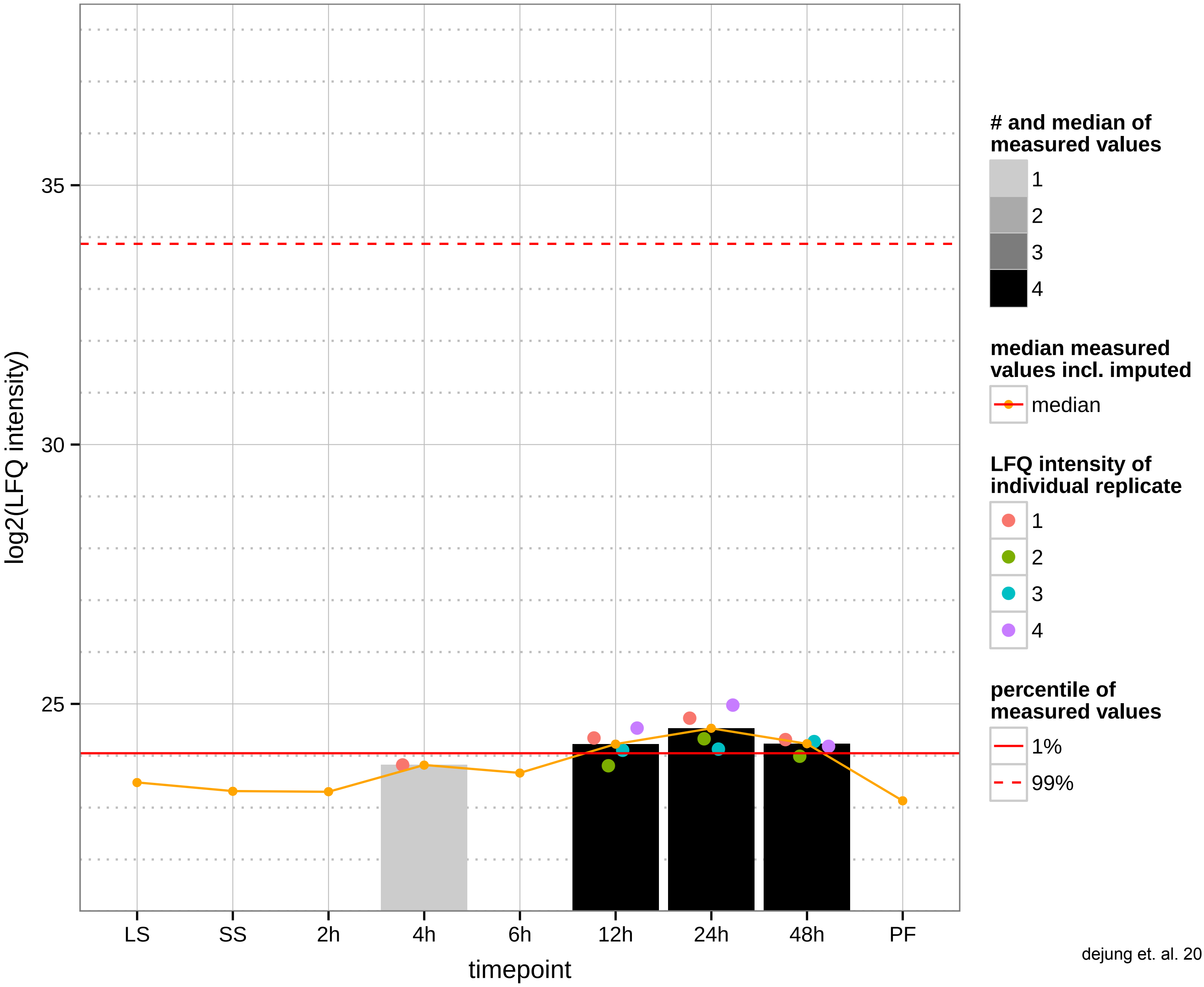

dejung et. al. 2015

hypothetical protein, conserved
Tb927.11.5200
AGOF: null
AGOC: null
AGOP: null
PGOF: null
PGOC: null
PGOP: null

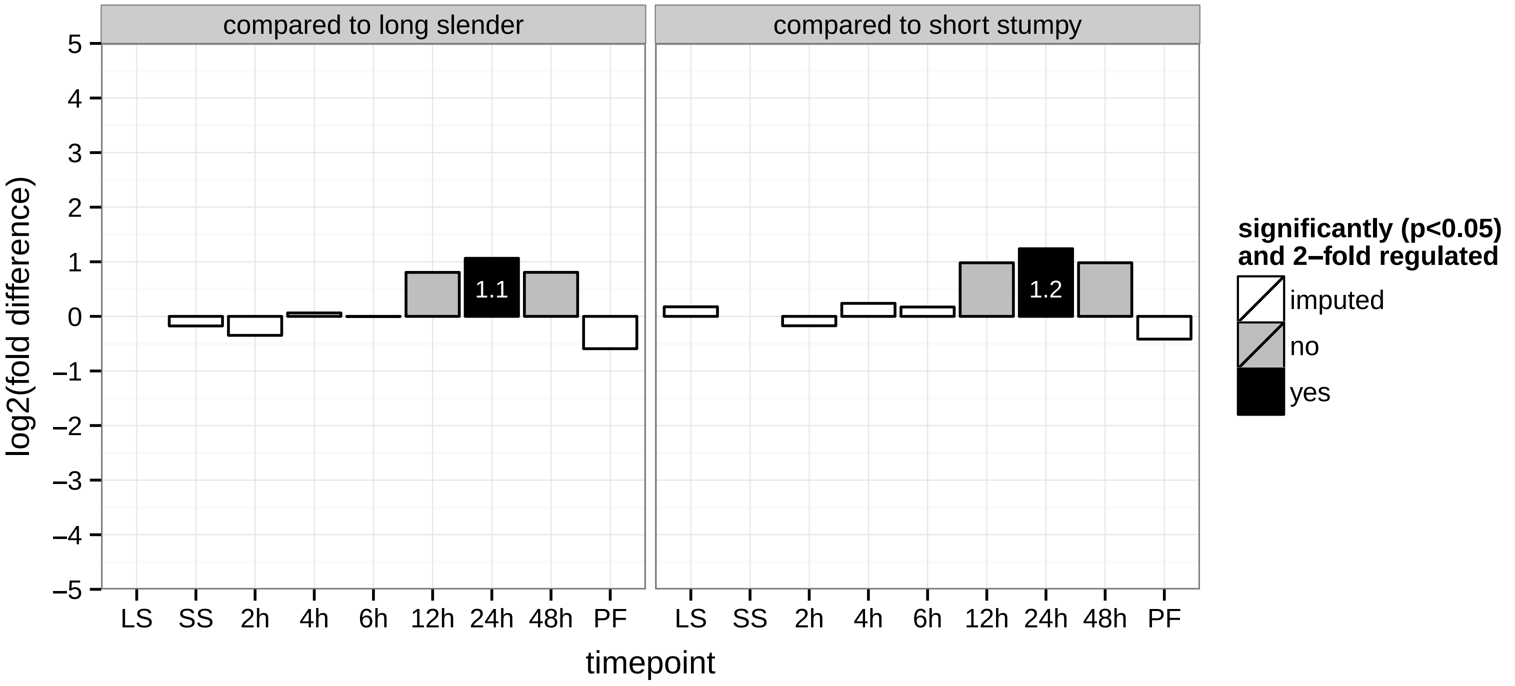

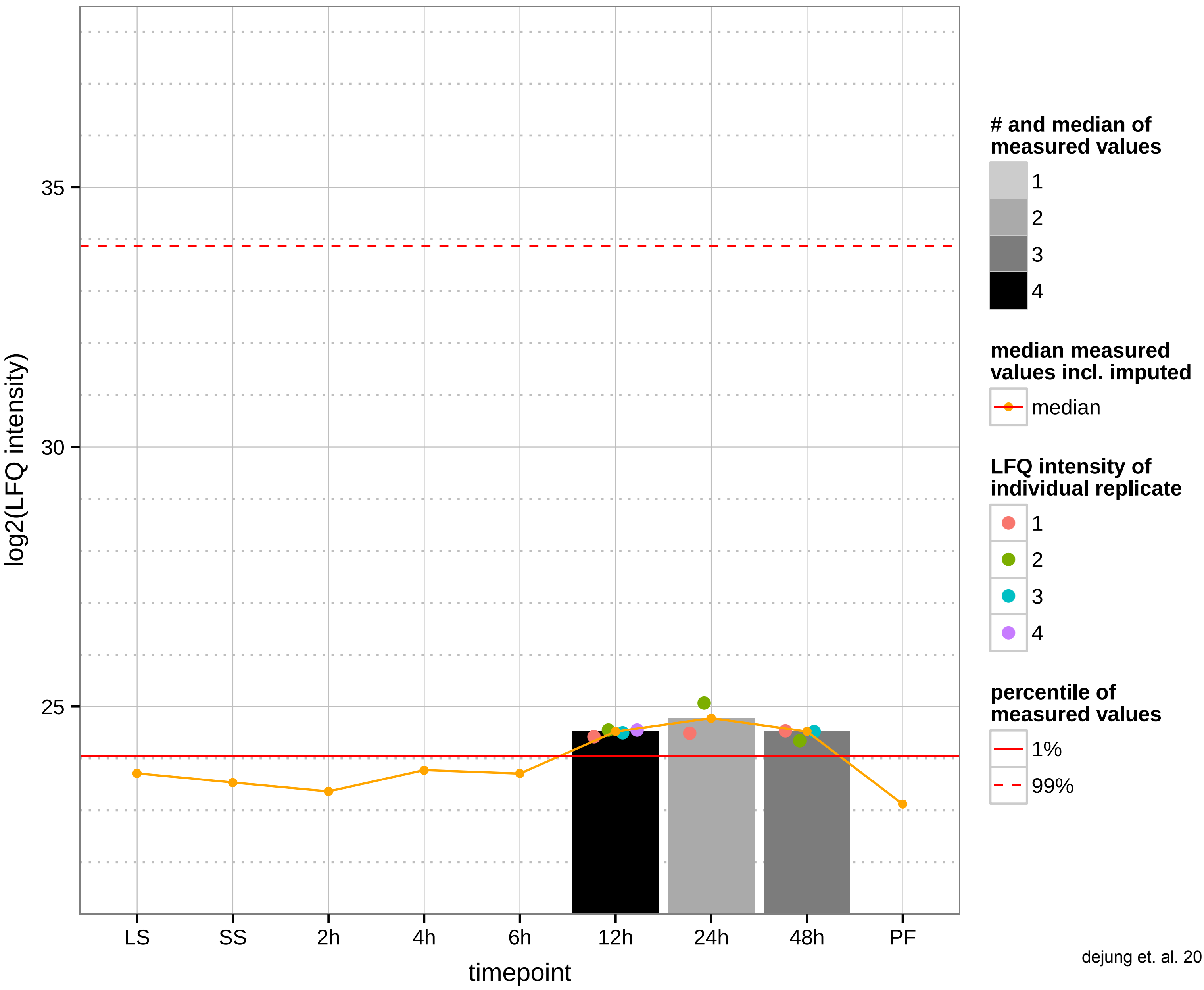

dejung et. al. 2015

hypothetical protein, conserved
Tb927.3.4450
AGOF: null
AGOC: null
AGOP: null
PGOF: null
PGOC: null
PGOP: null

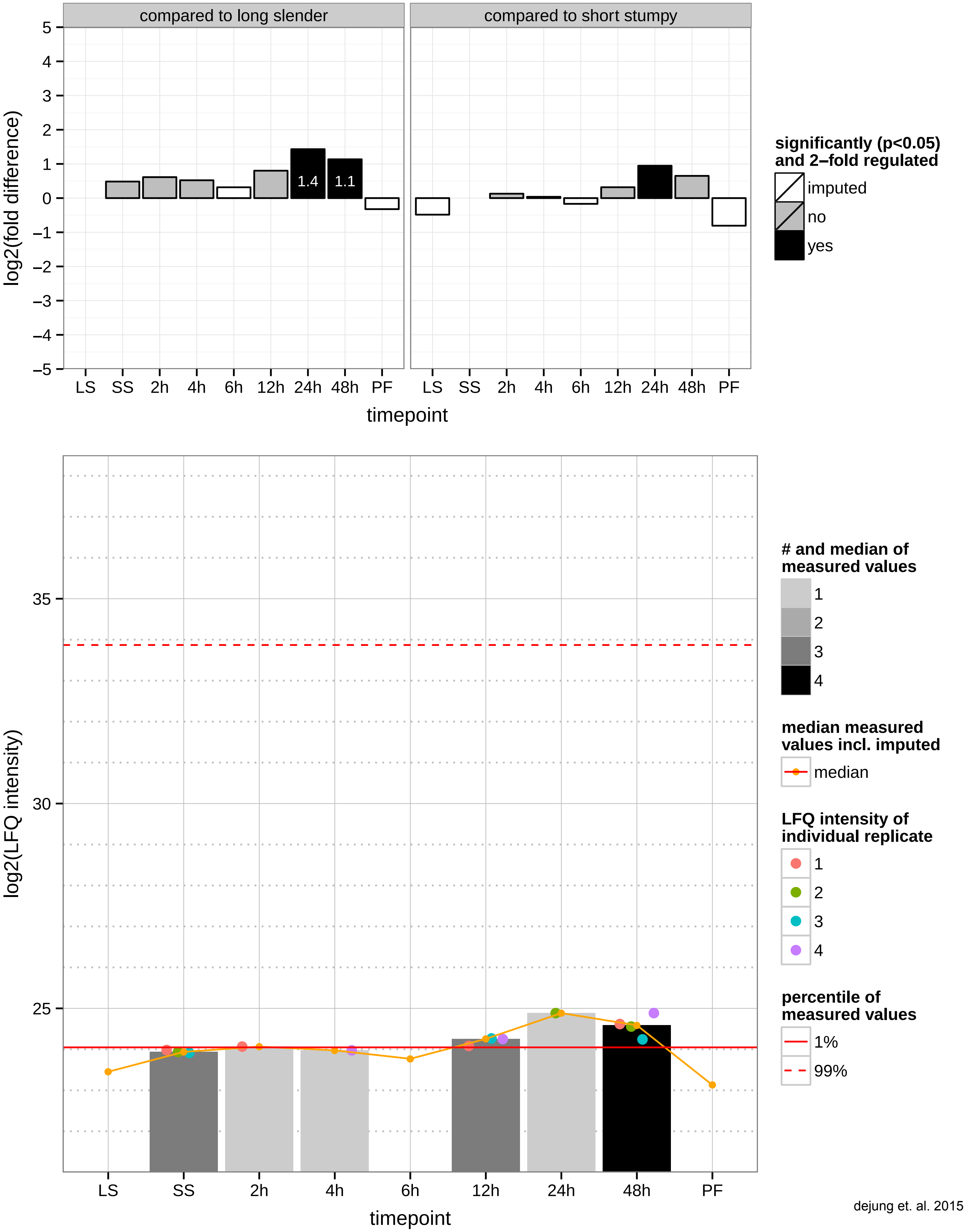

dejung et. al. 2015

Spef1, flagellar component
Tb927.4.3130
AGOF: null
AGOC: null
AGOP: ciliary or flagellar motility
PGOF: protein binding
PGOC: null
PGOP: null

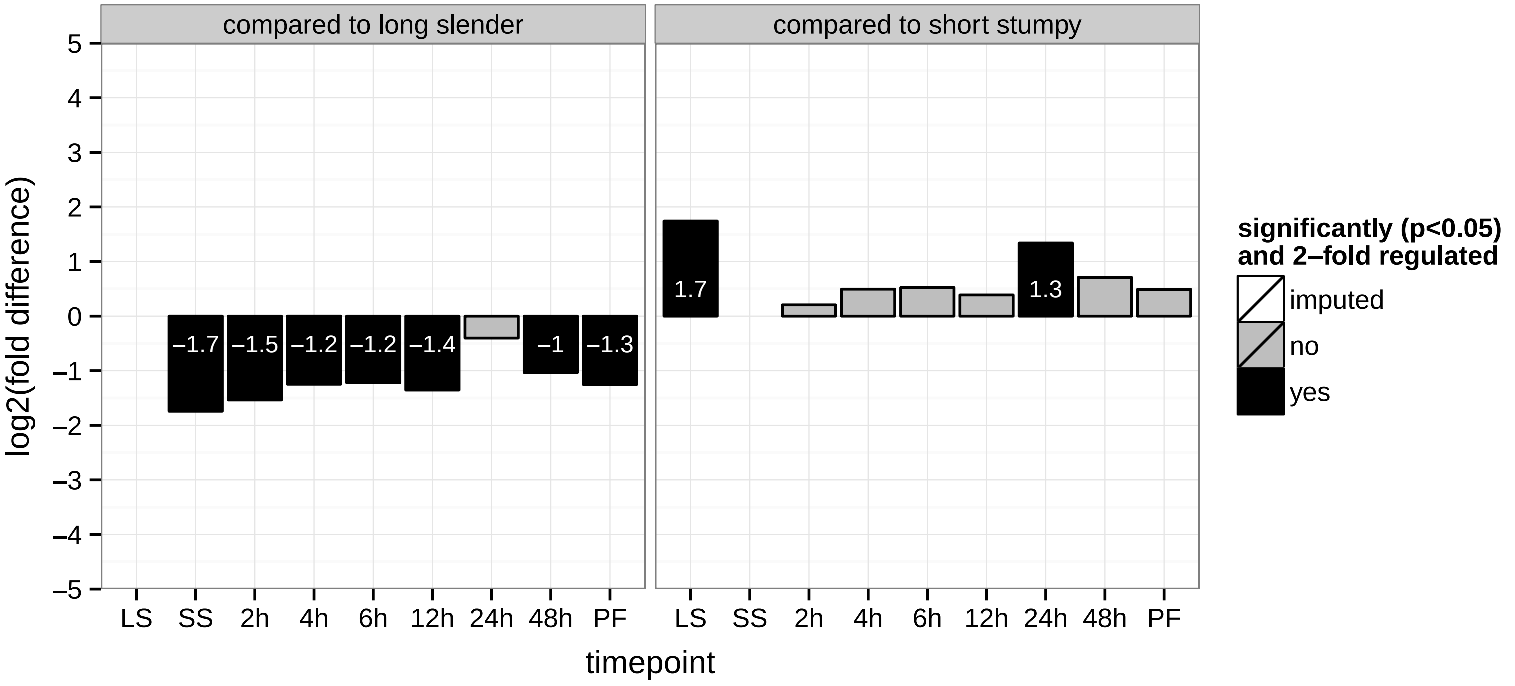

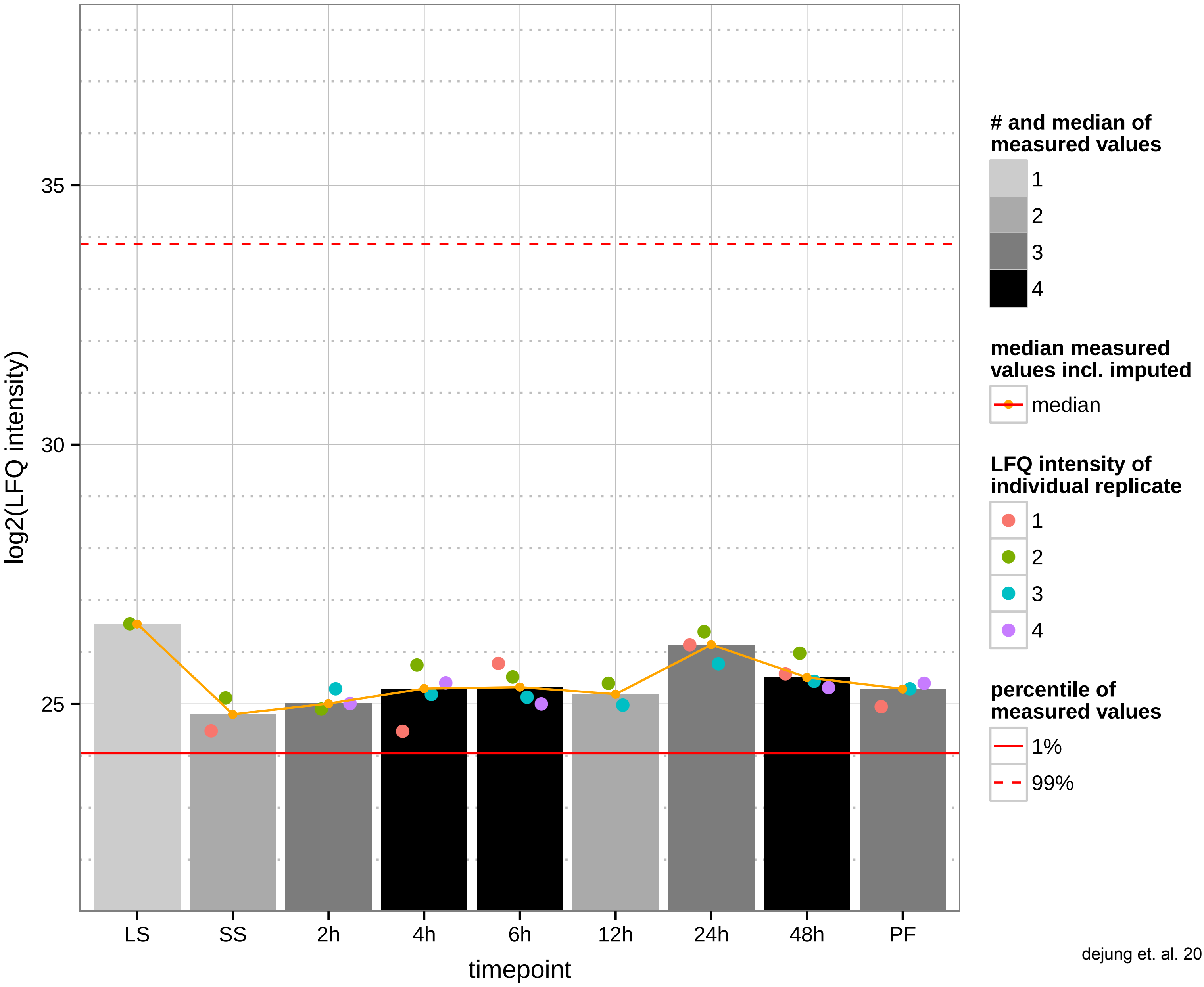

dejung et. al. 2015

hypothetical protein, conserved
Tb927.5.1340
AGOF: null
AGOC: null, intracellular
AGOP: null, RNA processing
PGOF: null
PGOC: null, intracellular
PGOP: null, RNA processing

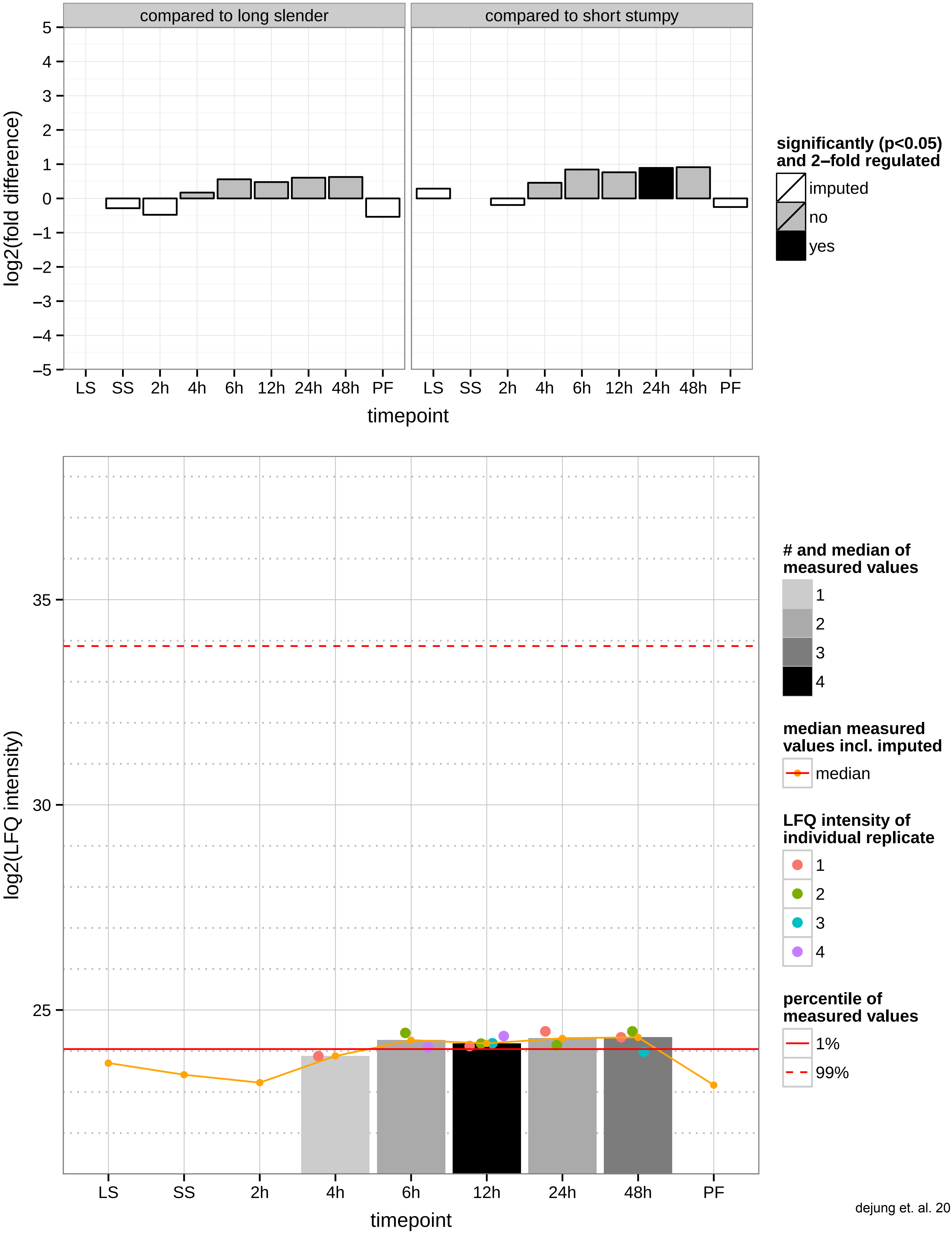

dejung et. al. 2015

DNA repair and recombination helicase protein PIF1, putative (TbPIF8)
Tb927.7.1000
AGOF: null
AGOC: mitochondrion
AGOP: null
PGOF: null
PGOC: null
PGOP: null

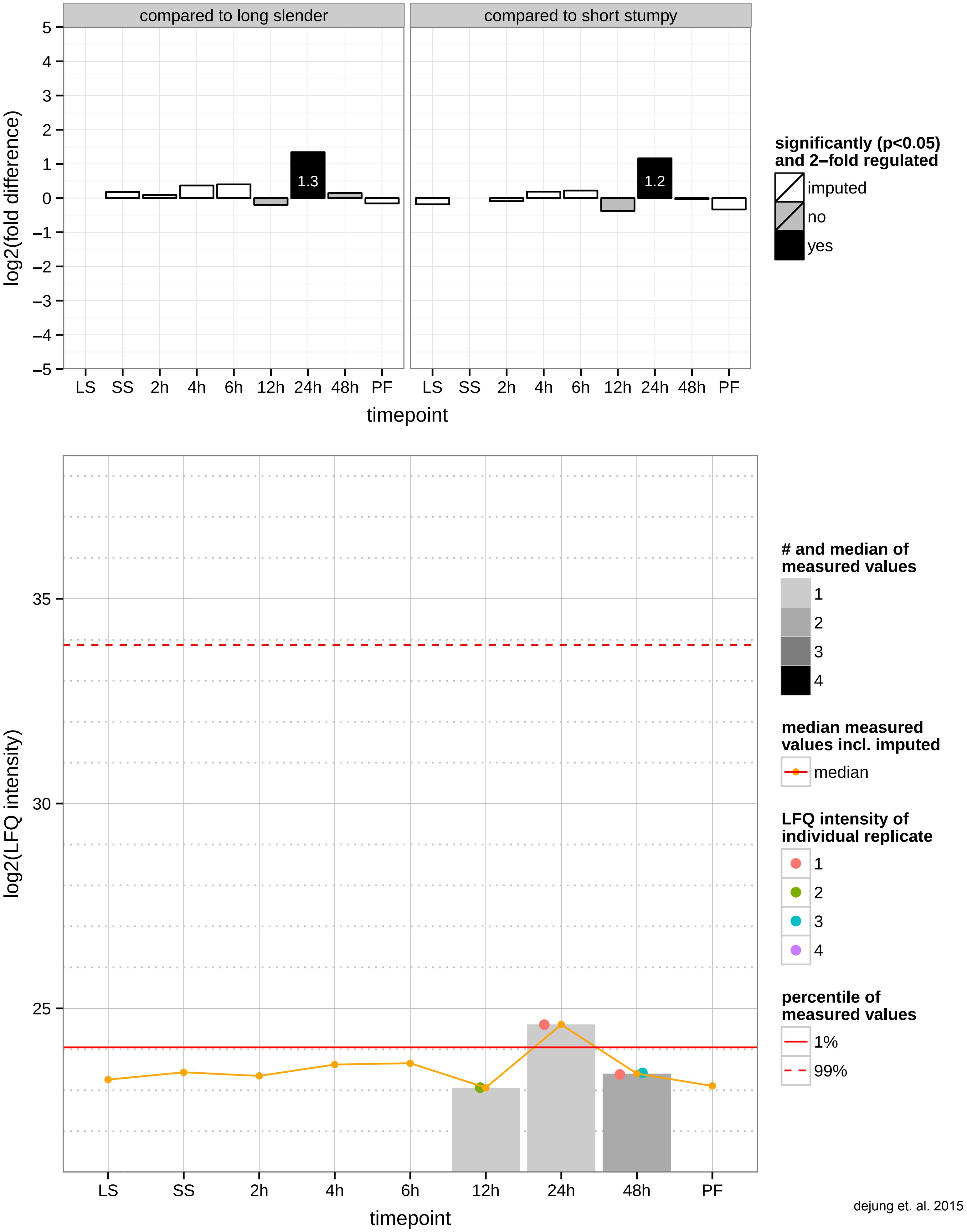

dejung et. al. 2015

Eukaryotic translation initiation factor 4E type 6 (eif4e6)
Tb927.7.1670
AGOF: RNA 7–methylguanosine cap binding, RNA cap 4 binding, protein binding, translation initiation factor activity
AGOC: cytoplasm, eukaryotic translation initiation factor 4F complex
AGOP: cell motility, motile cilium assembly, translational initiation
PGOF: RNA binding, translation initiation factor activity
PGOC: cytoplasm
PGOP: translational initiation

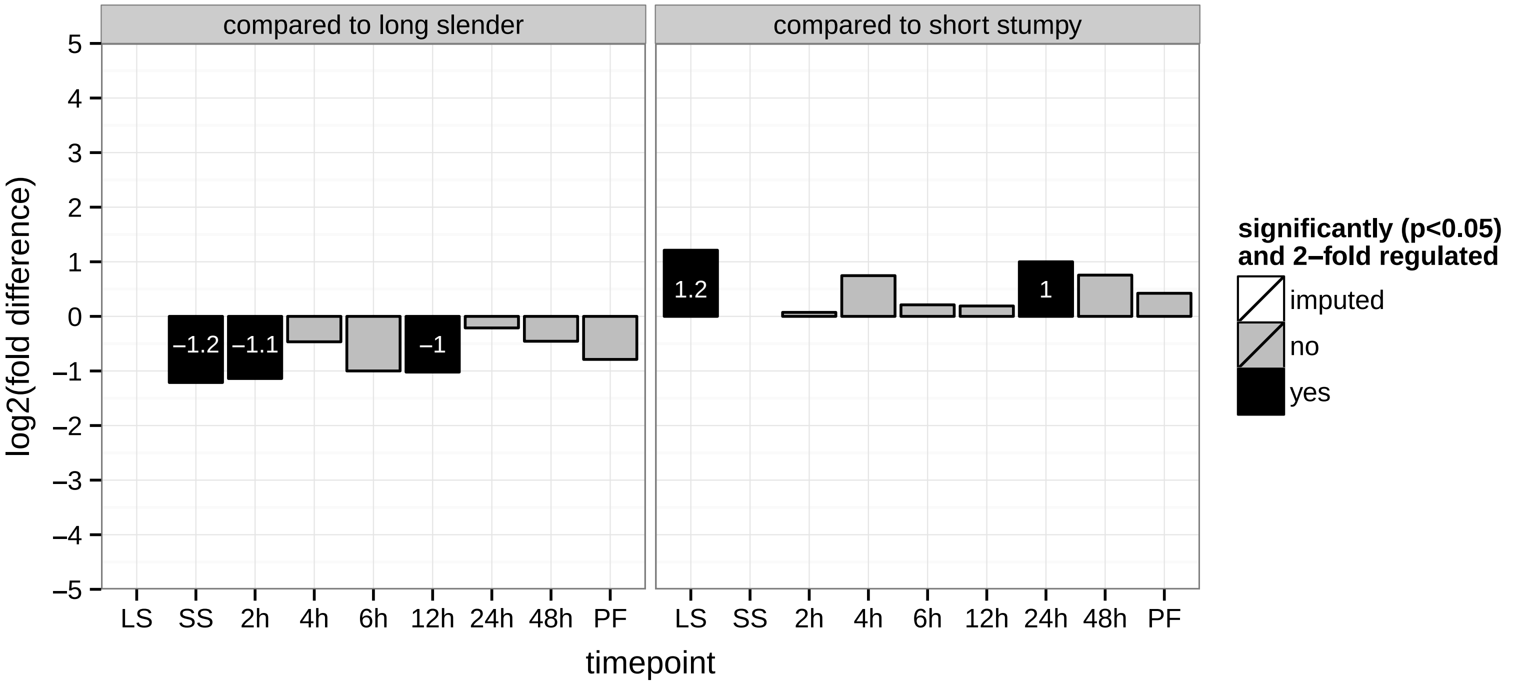

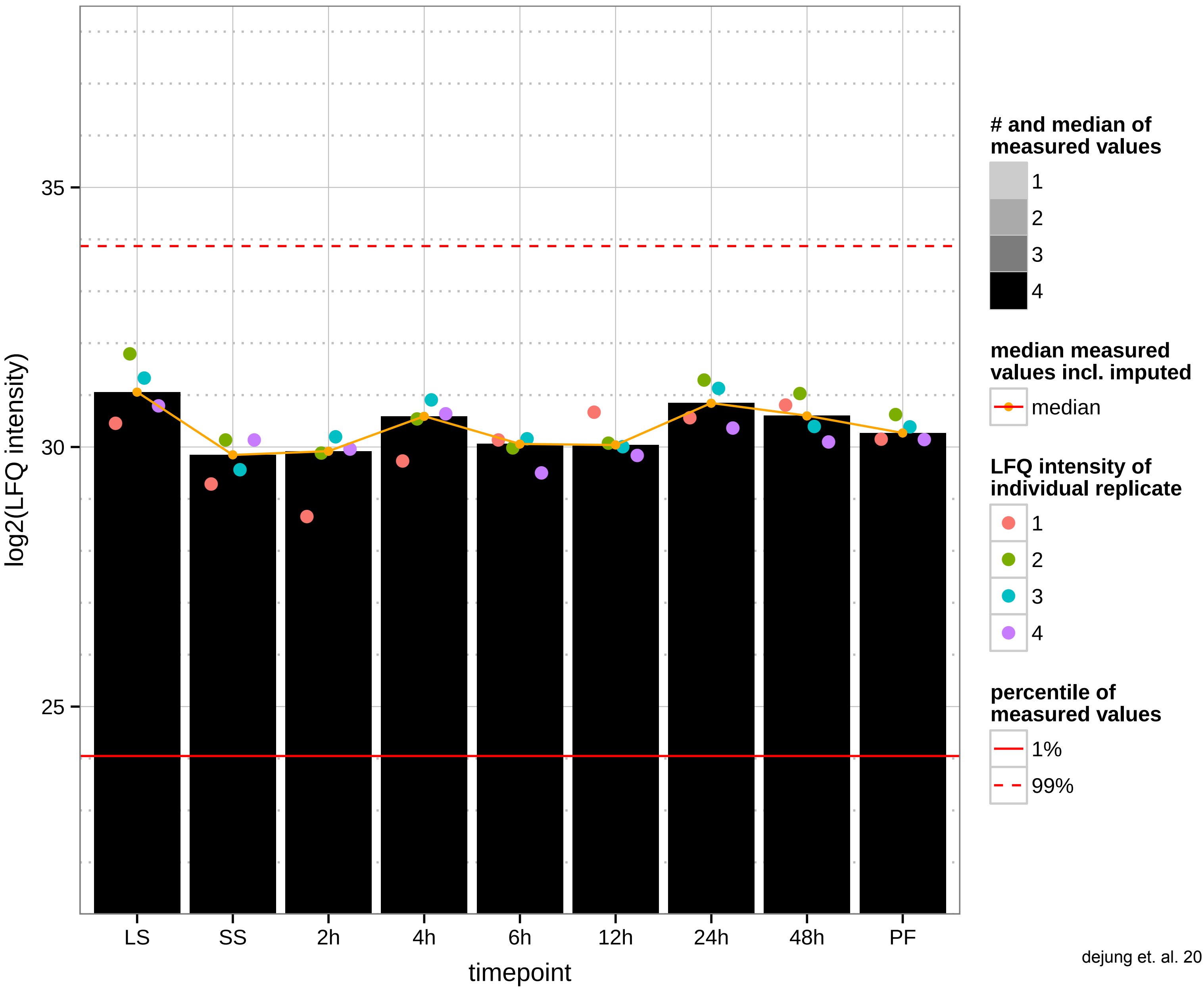

dejung et. al. 2015

prefoldin, putative
Tb927.7.2590
AGOF: unfolded protein binding
AGOC: prefoldin complex
AGOP: protein folding
PGOF: unfolded protein binding
PGOC: prefoldin complex
PGOP: protein folding

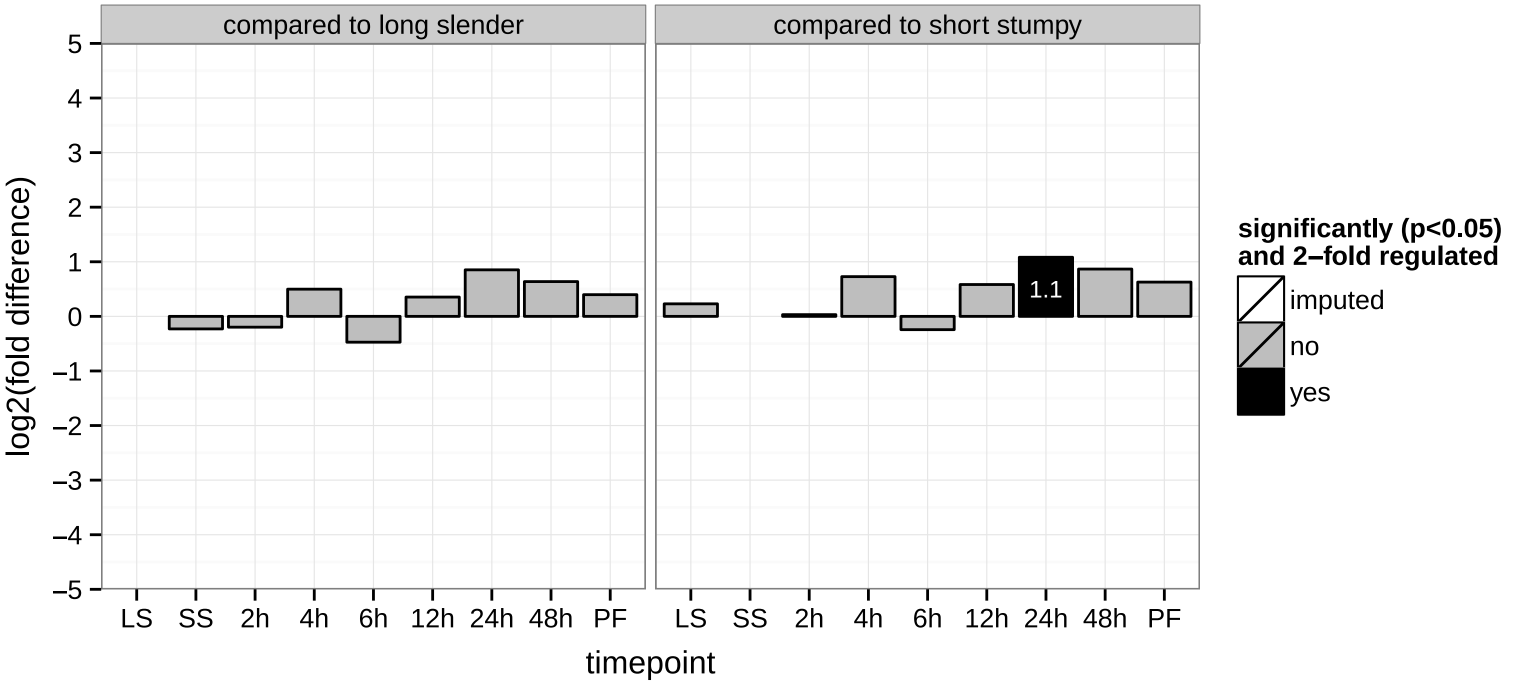

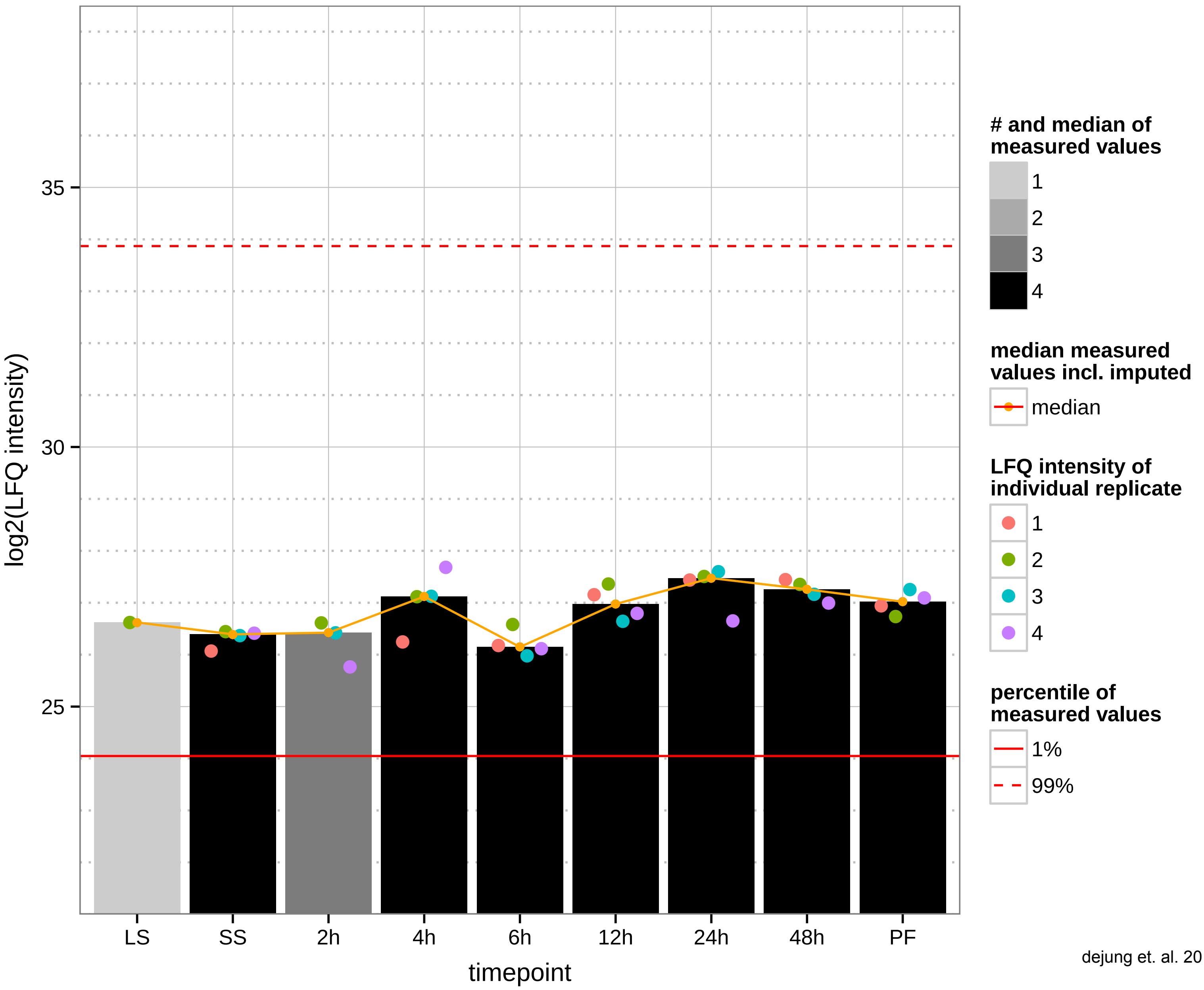

dejung et. al. 2015

hypothetical protein, conserved
Tb927.7.4470
AGOF: null
AGOC: null
AGOP: null
PGOF: null
PGOC: null
PGOP: null

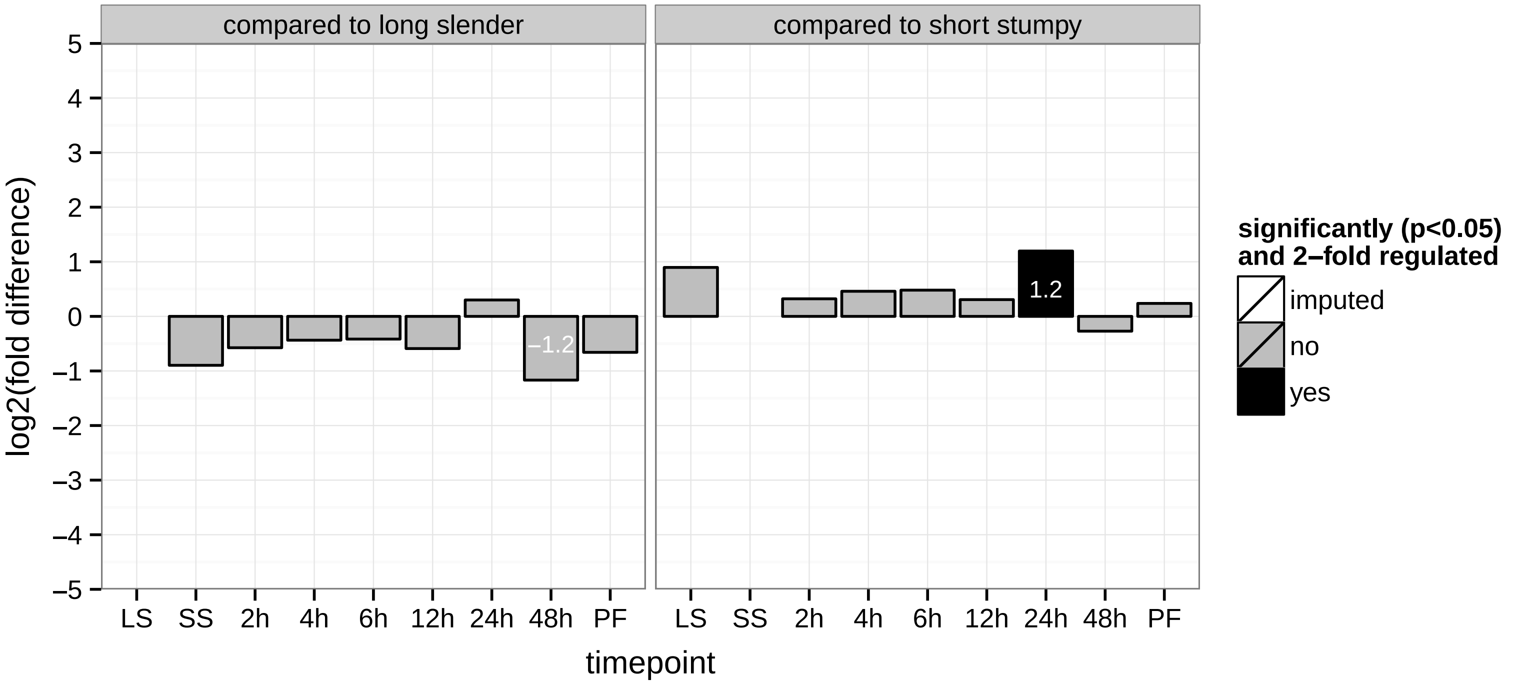

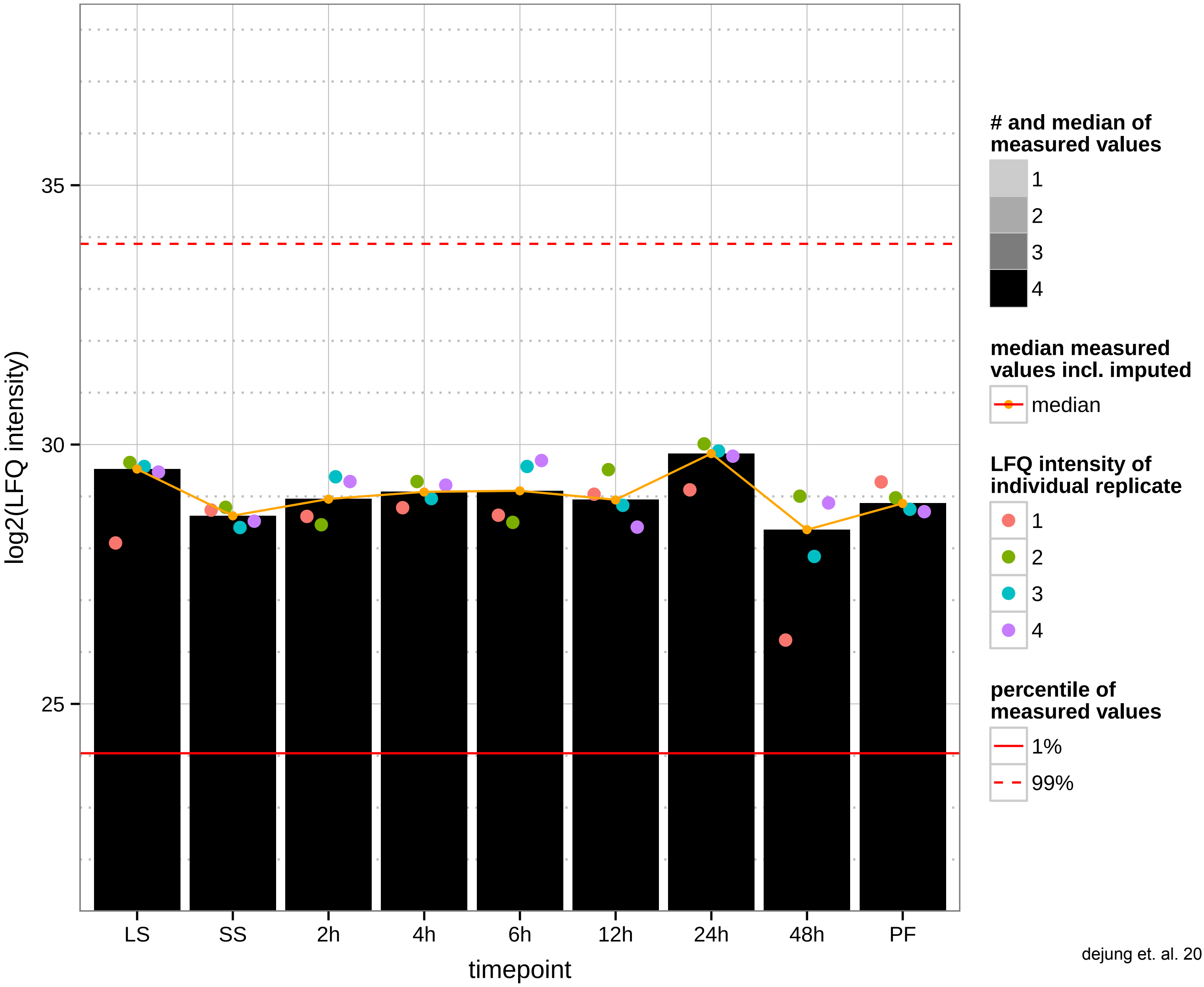

dejung et. al. 2015

exosome–associated protein 3, 3' exoribonuclease, putative (EAP3)
Tb927.7.5460
AGOF: null
AGOC: null
AGOP: null
PGOF: null
PGOC: null
PGOP: null

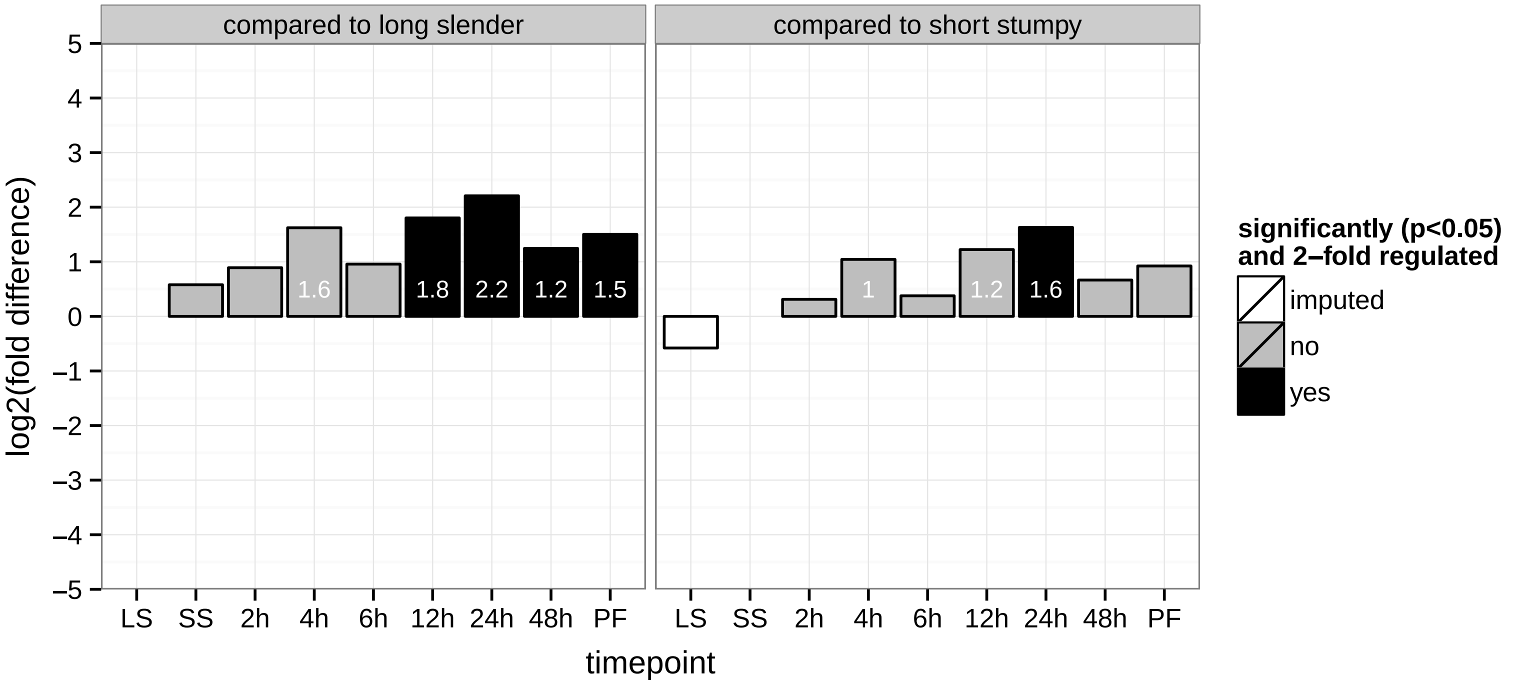

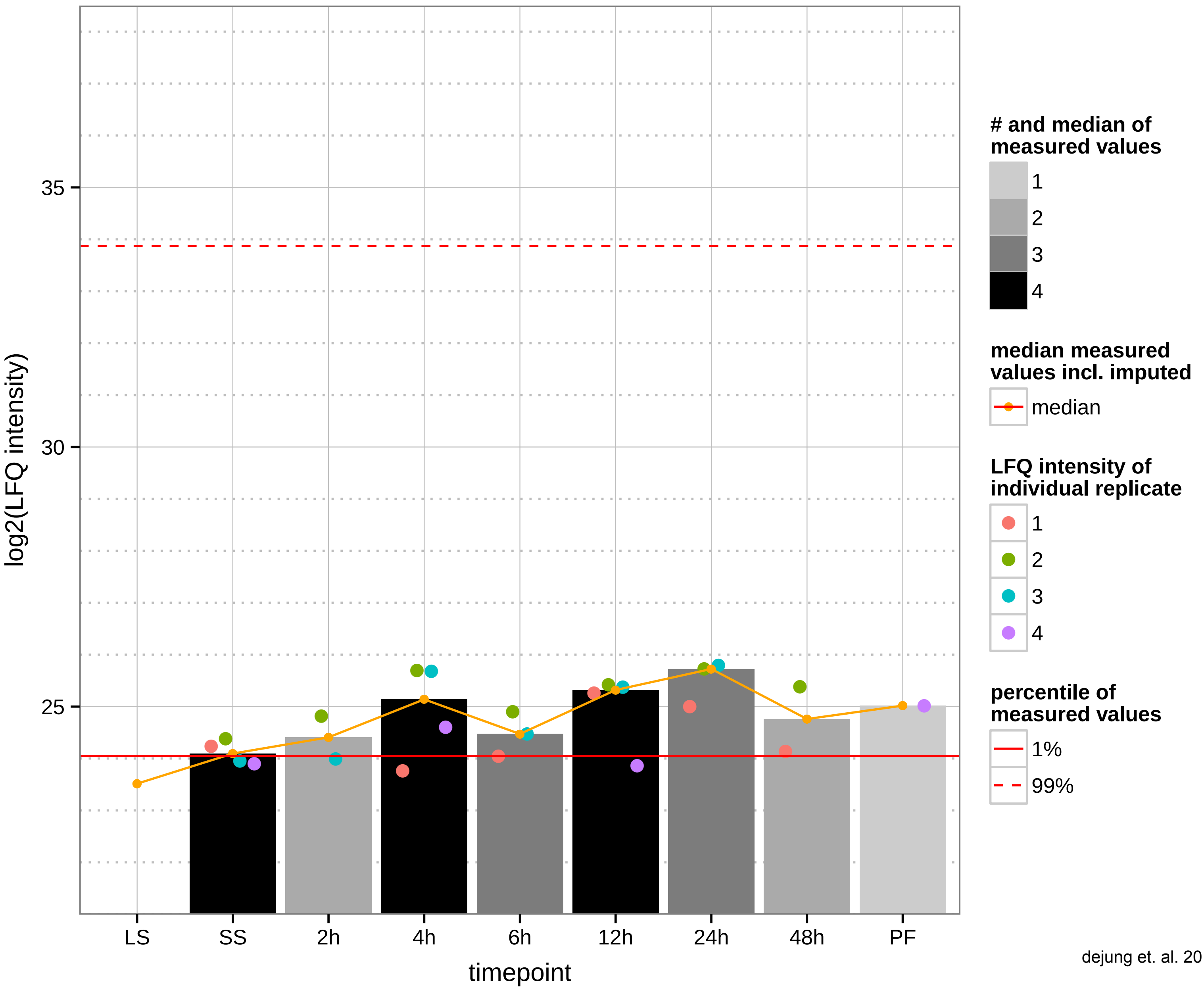

dejung et. al. 2015

hypothetical protein, conserved
Tb927.8.6520
AGOF: null
AGOC: null
AGOP: null
PGOF: null
PGOC: null
PGOP: null

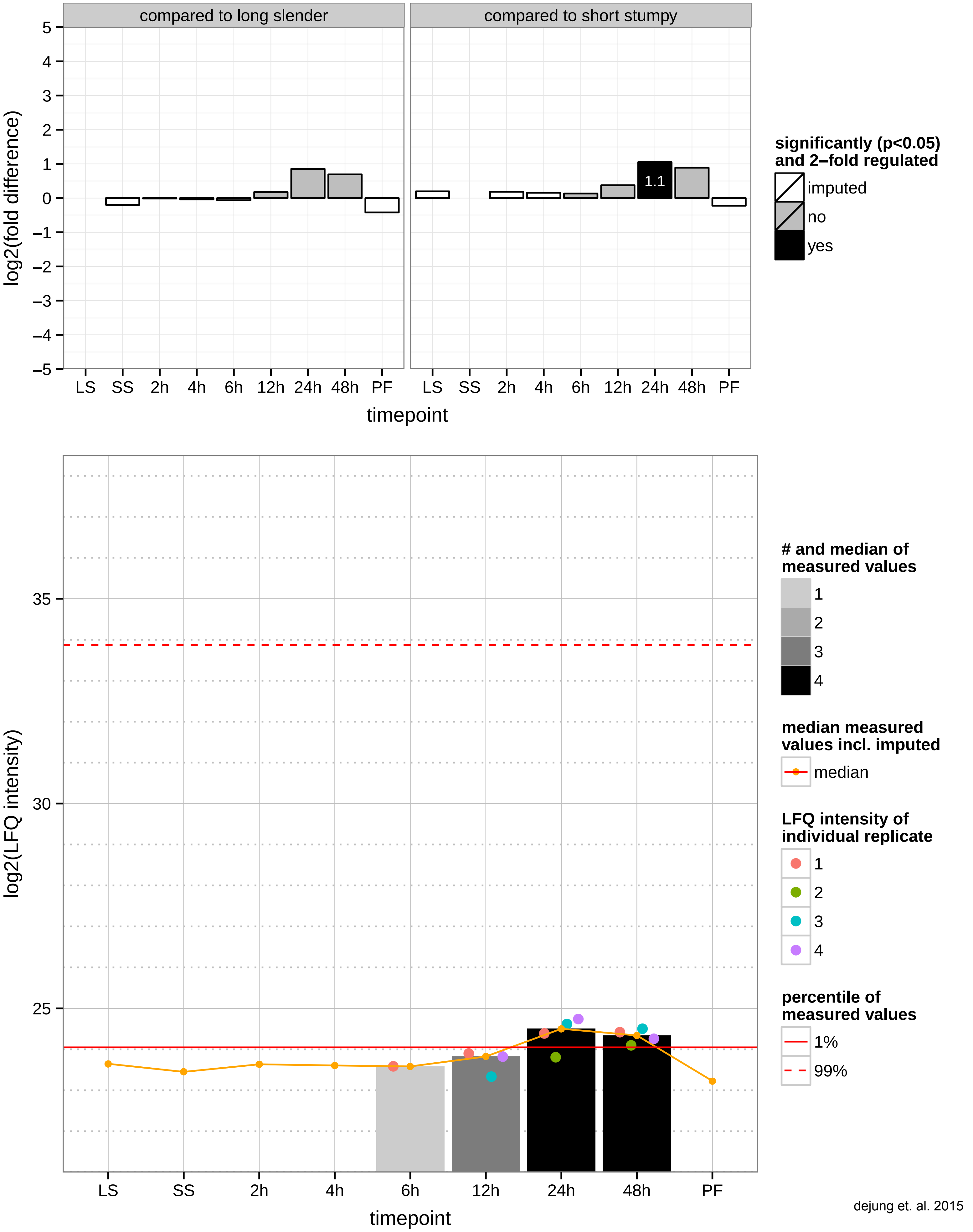

dejung et. al. 2015

hypothetical protein, conserved
Tb927.9.11220
AGOF: null
AGOC: mitochondrion
AGOP: null
PGOF: null
PGOC: null
PGOP: null

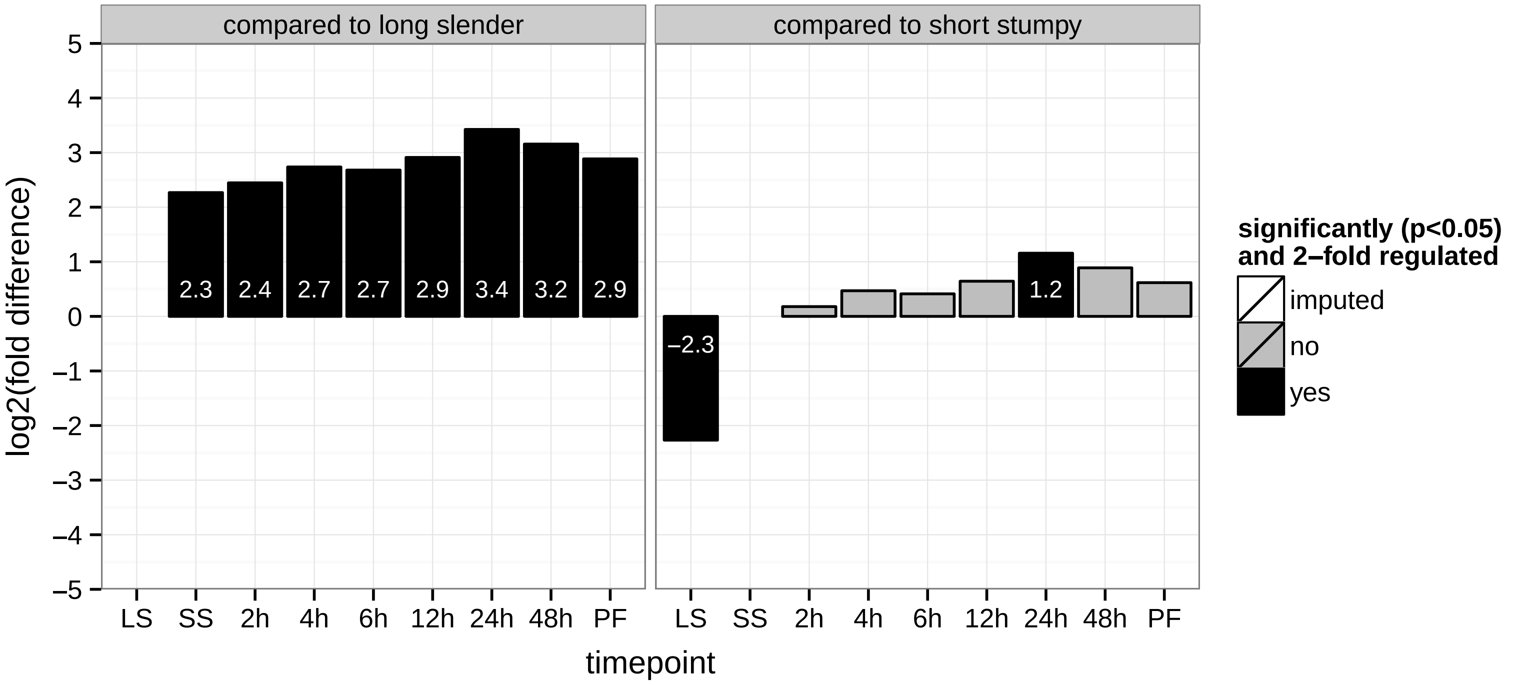

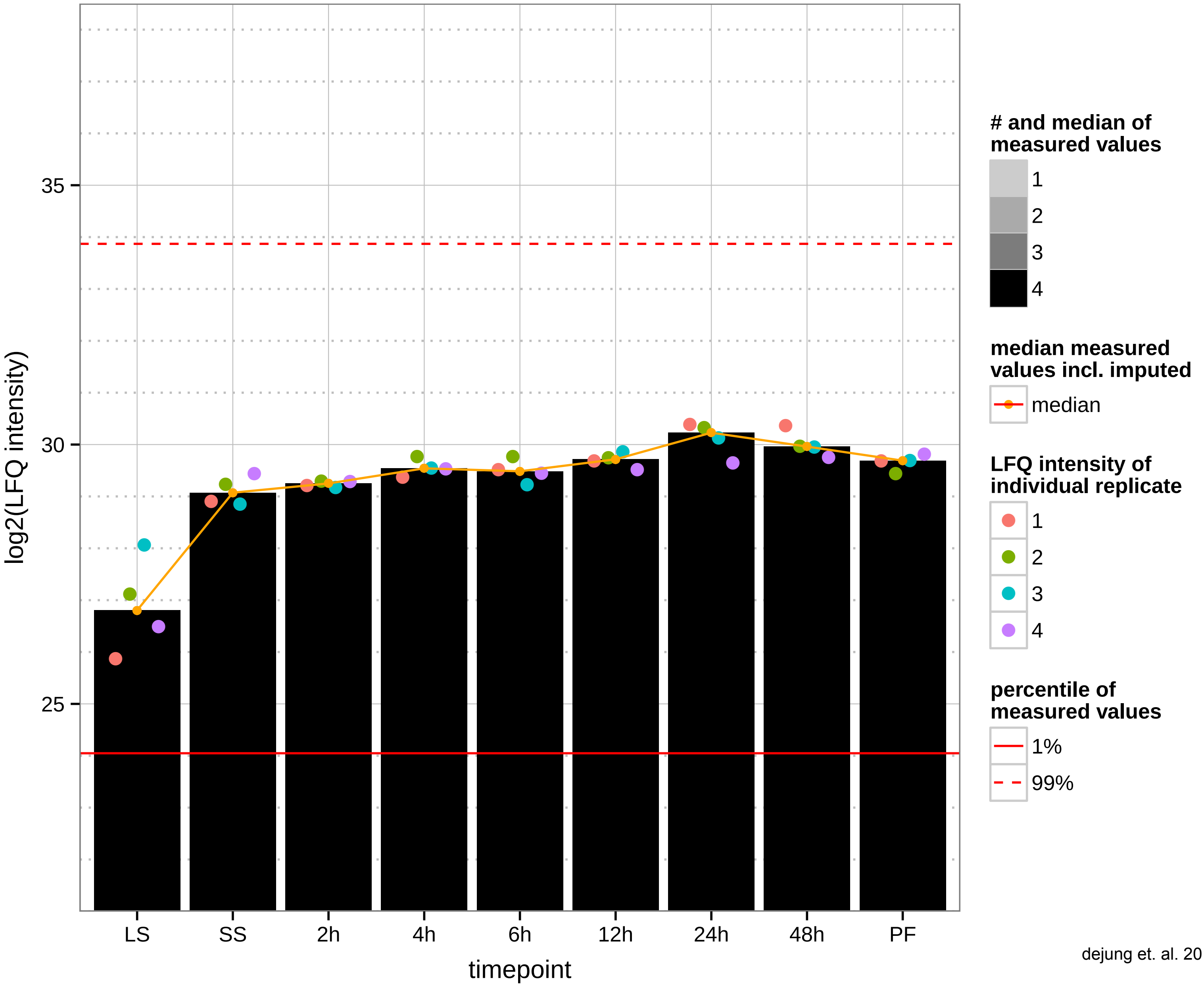

dejung et. al. 2015

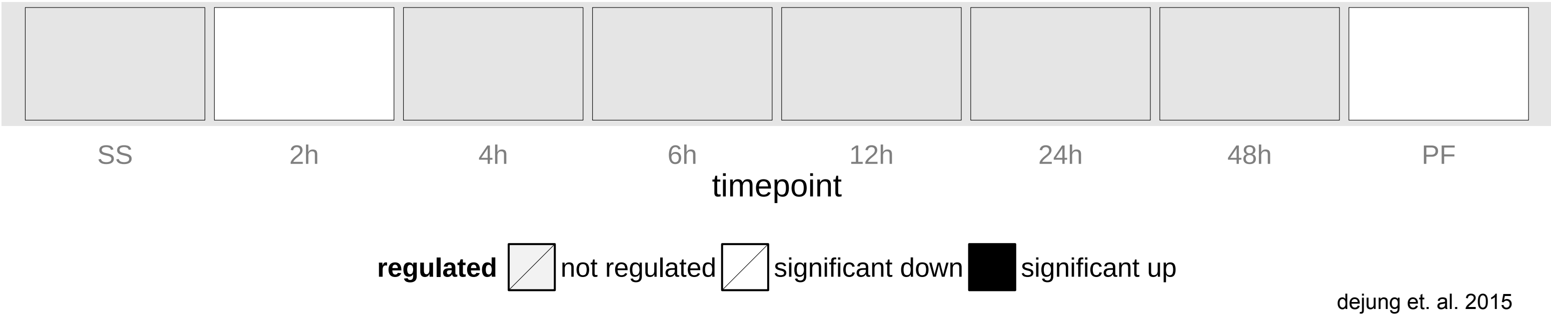
SS
2h
4h
6h
12h
24h
48h
PF
timepoint
regulated
not regulated
significant down
significant up
dejung et. al. 2015

kinesin, putative
Tb927.10.15390;Tb11.v5.0434
AGOF: null, microtubule motor activity, motor activity
AGOC: null, kinesin complex, microtubule associated complex
AGOP: null, microtubule–based movement
PGOF: ATP binding, microtubule motor activity
PGOC: null
PGOP: microtubule–based movement

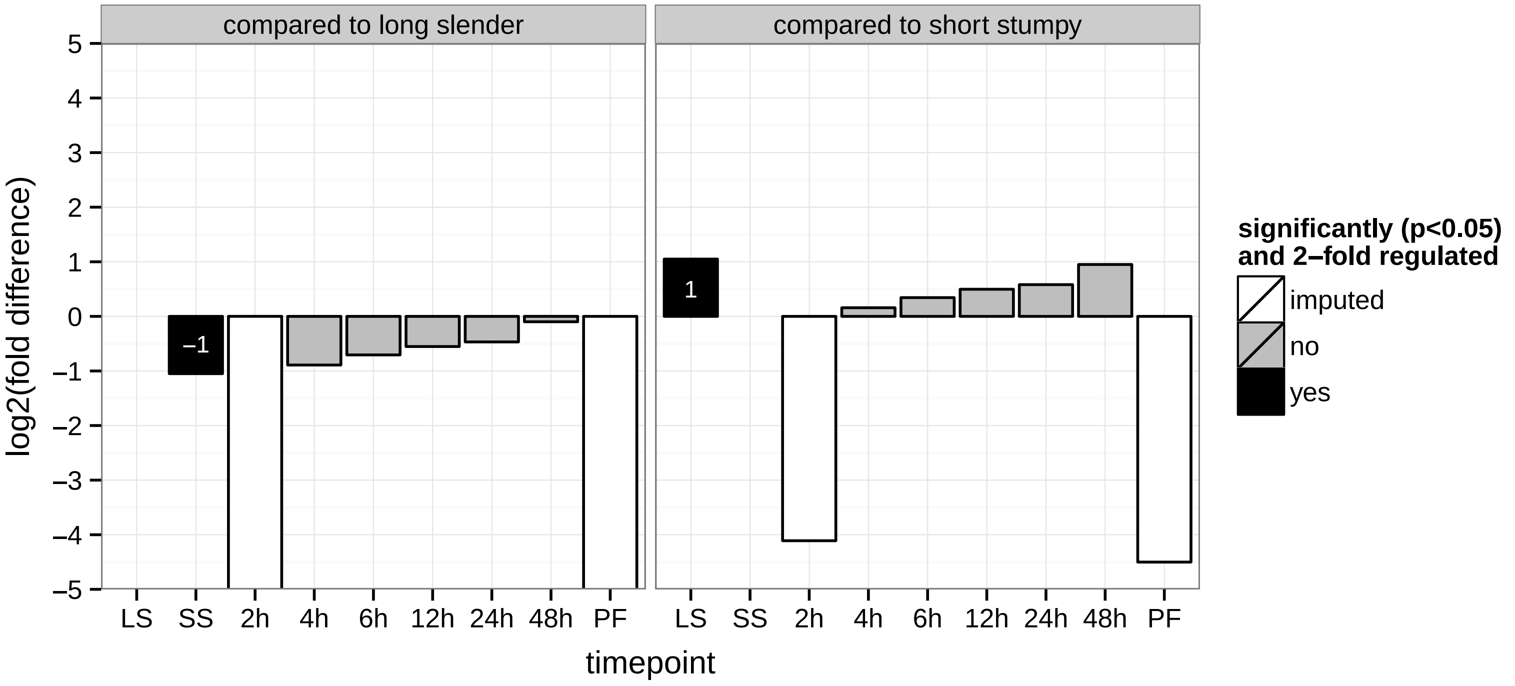

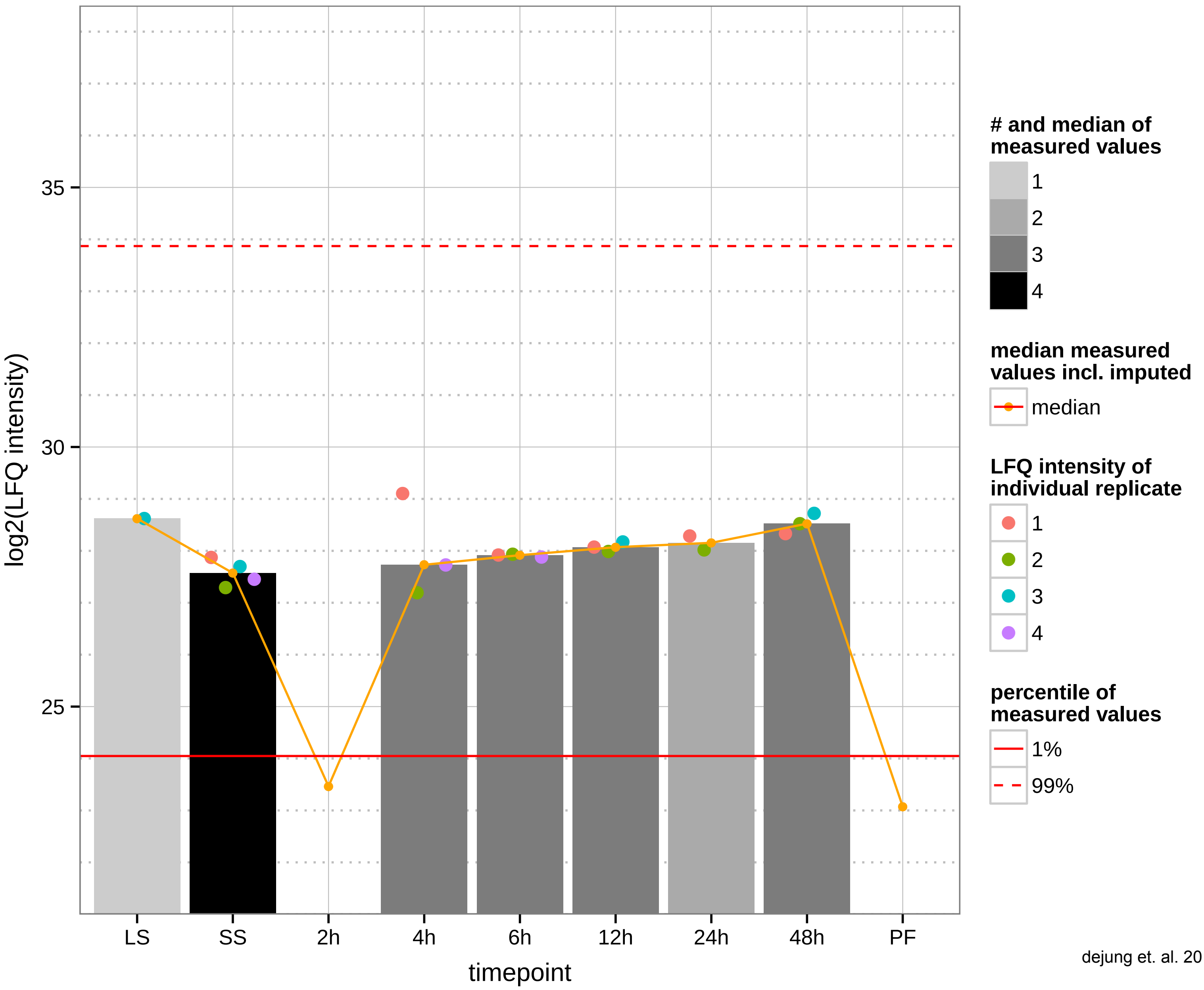

dejung et. al. 2015

hypothetical protein, conserved
Tb11.v5.0736;Tb927.5.3420
AGOF: null
AGOC: null, mitochondrion
AGOP: null
PGOF: transferase activity, transferring phosphorus–containing groups
PGOC: null
PGOP: null

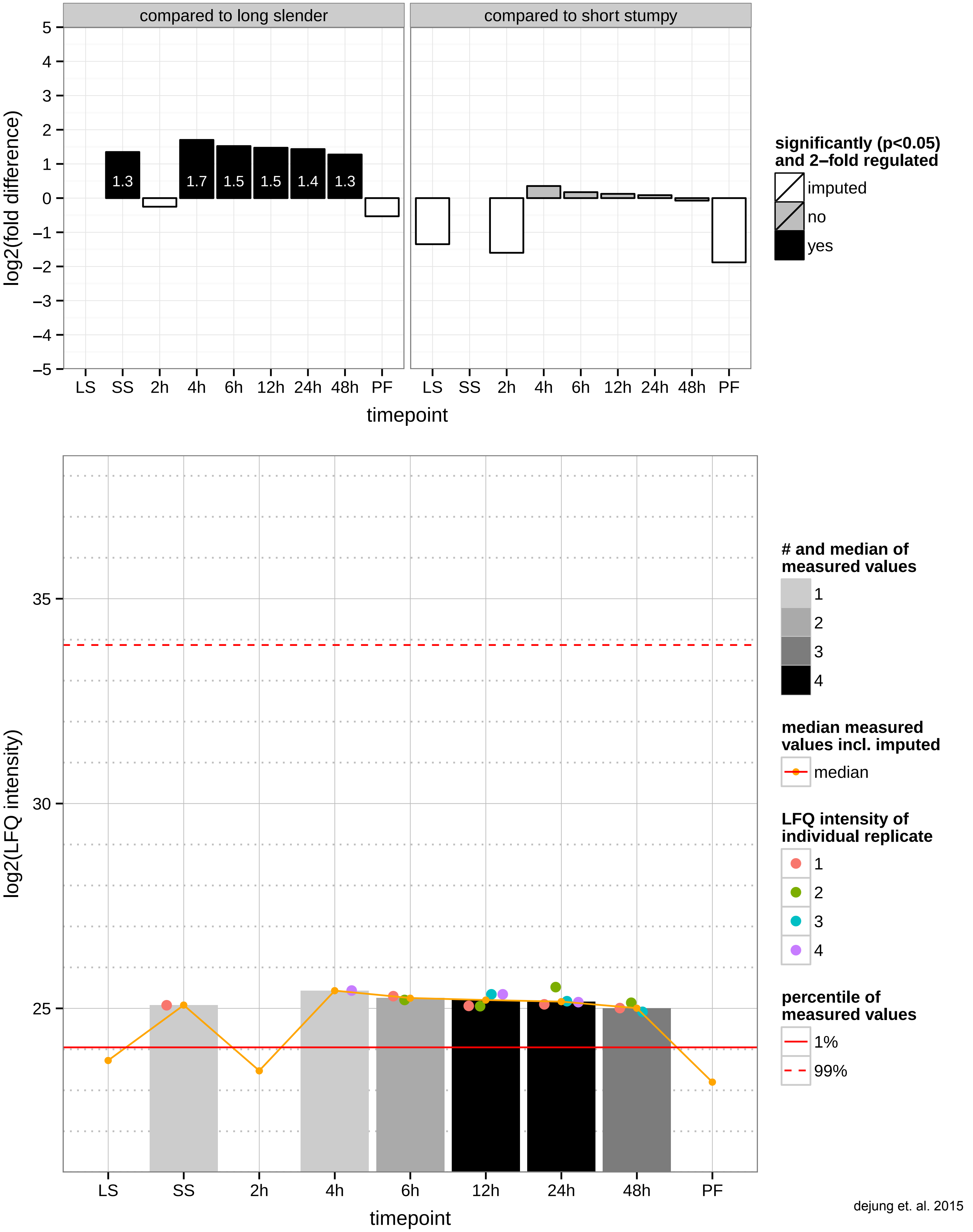

dejung et. al. 2015

hypothetical protein, conserved
Tb927.10.15430
AGOF: null
AGOC: null
AGOP: null
PGOF: protein binding
PGOC: null
PGOP: null

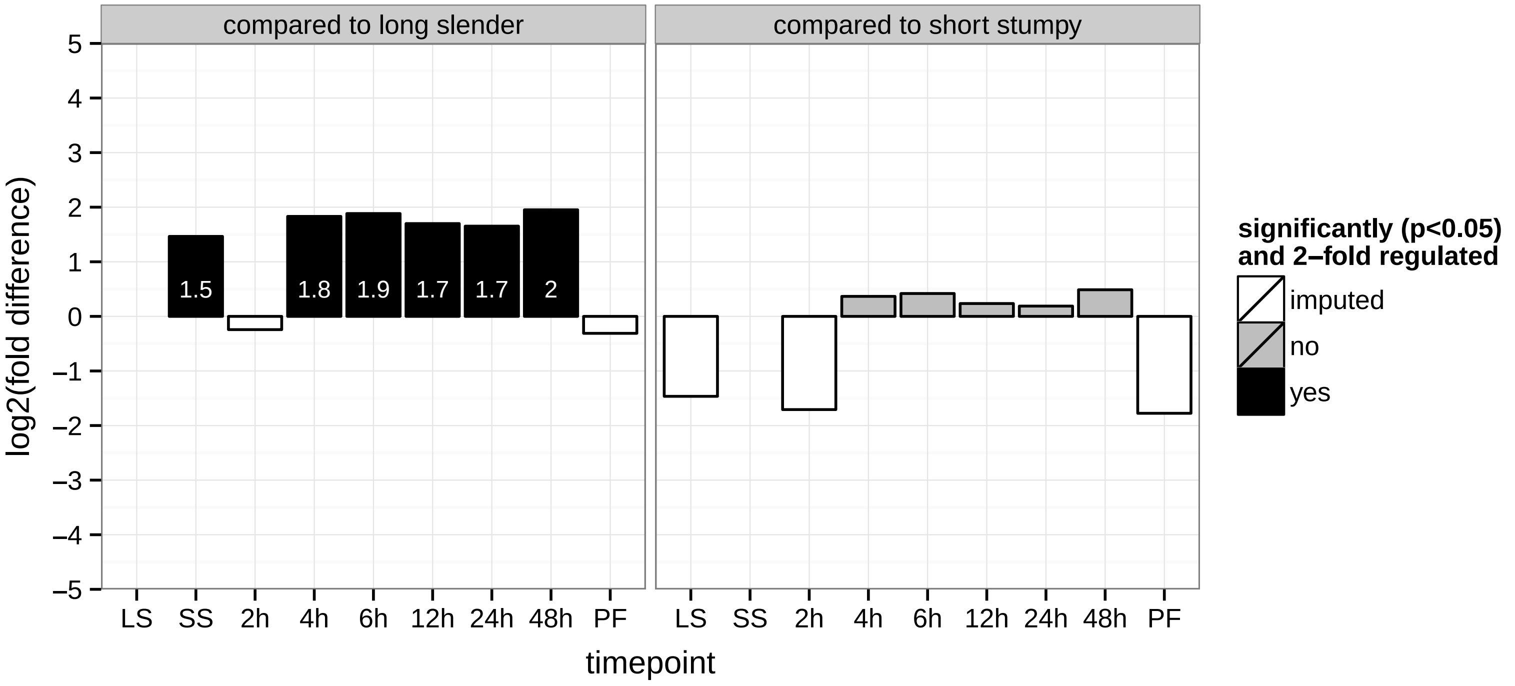

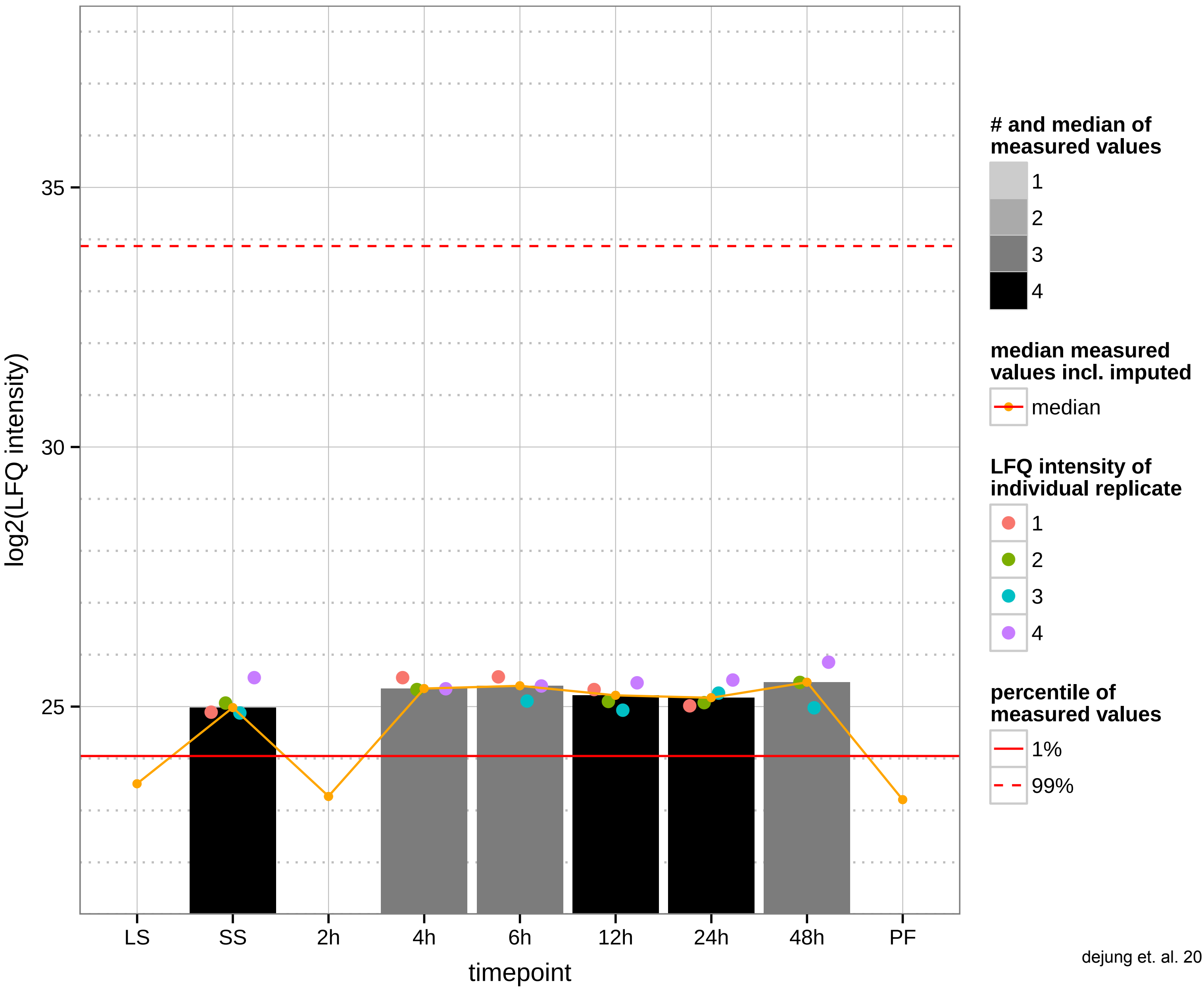

dejung et. al. 2015

Pre–mRNA–splicing factor CWC22, putative (TbCWC22)
Tb927.11.10750
AGOF: RNA binding
AGOC: null
AGOP: RNA metabolic process, translation
PGOF: DNA binding, RNA binding, binding, protein binding
PGOC: null
PGOP: null

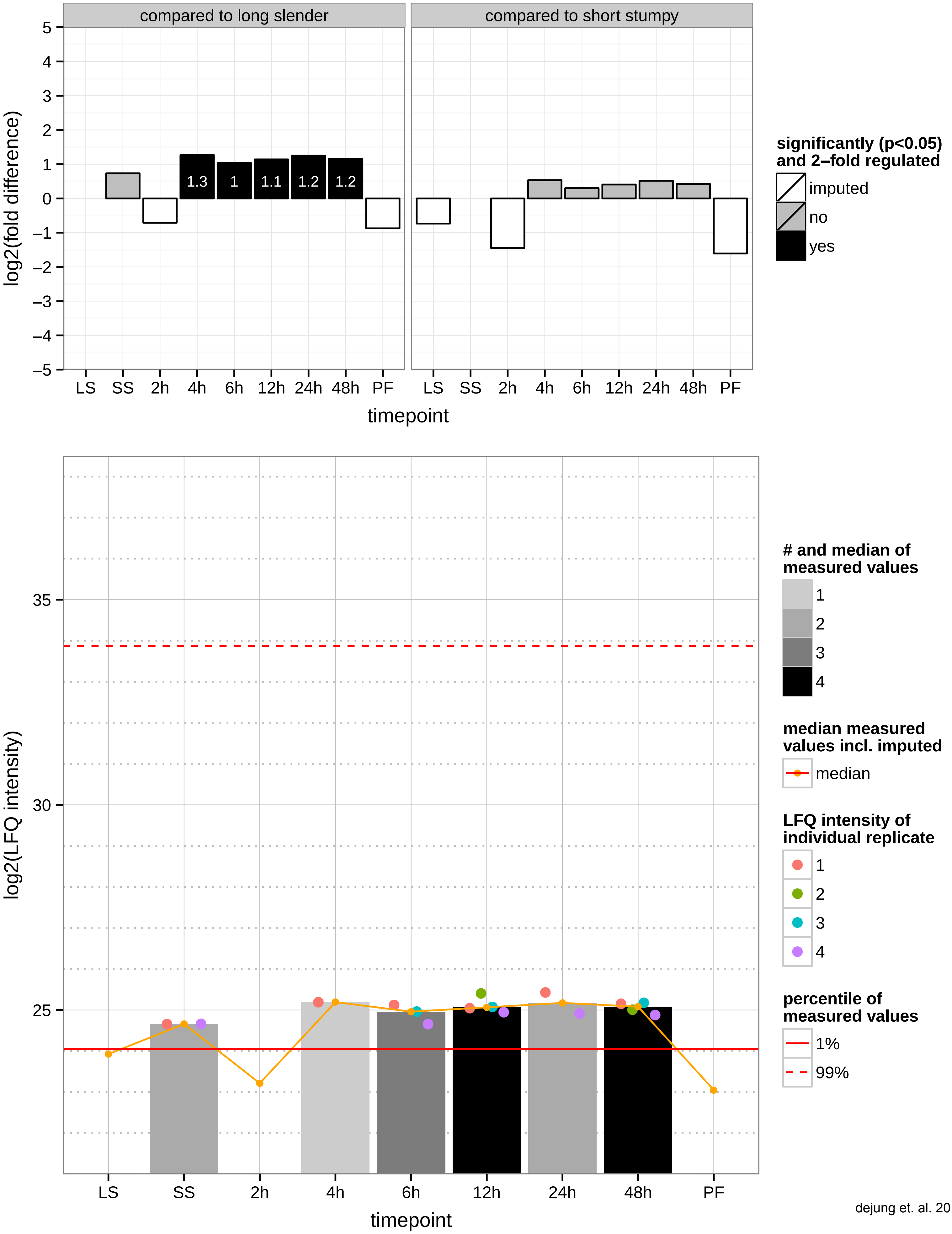

dejung et. al. 2015

hypothetical protein, conserved
Tb927.11.14450
AGOF: null
AGOC: null
AGOP: null
PGOF: null
PGOC: null
PGOP: null

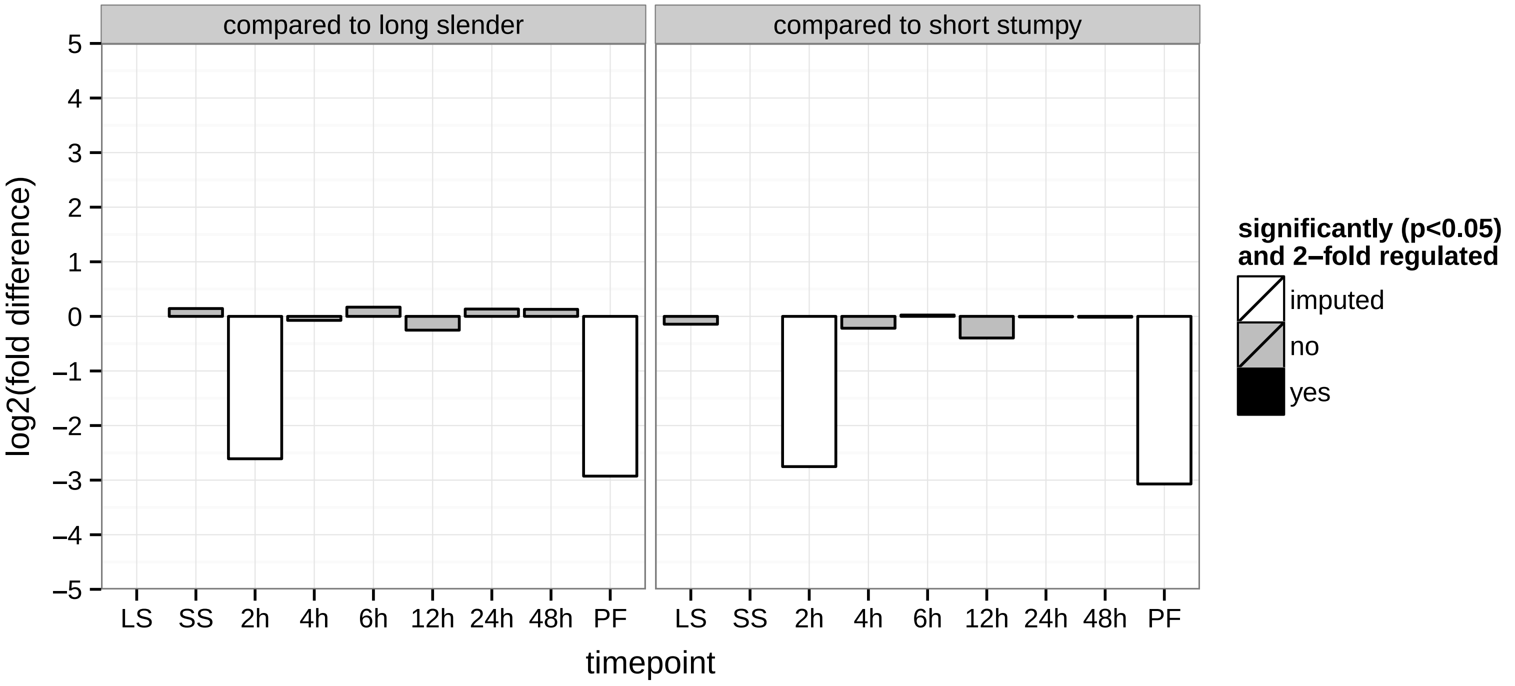

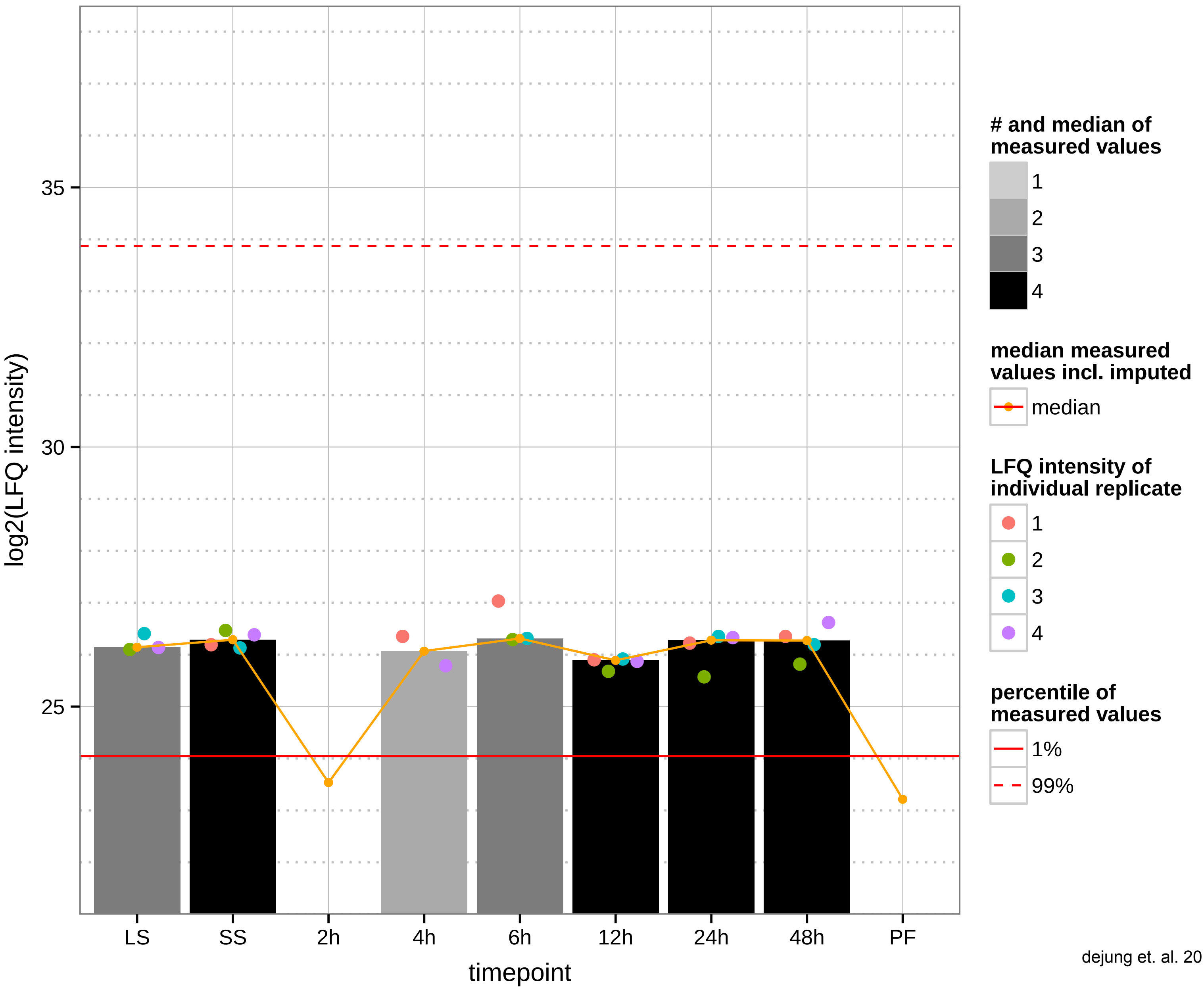

dejung et. al. 2015

tatD related deoxyribonuclease, putative
Tb927.11.1600
AGOF: deoxyribonuclease activity, endodeoxyribonuclease activity, producing 5'−phosphomonoesters
AGOC: null
AGOP: metabolic process
PGOF: endodeoxyribonuclease activity, producing 5'−phosphomonoesters
PGOC: null
PGOP: null

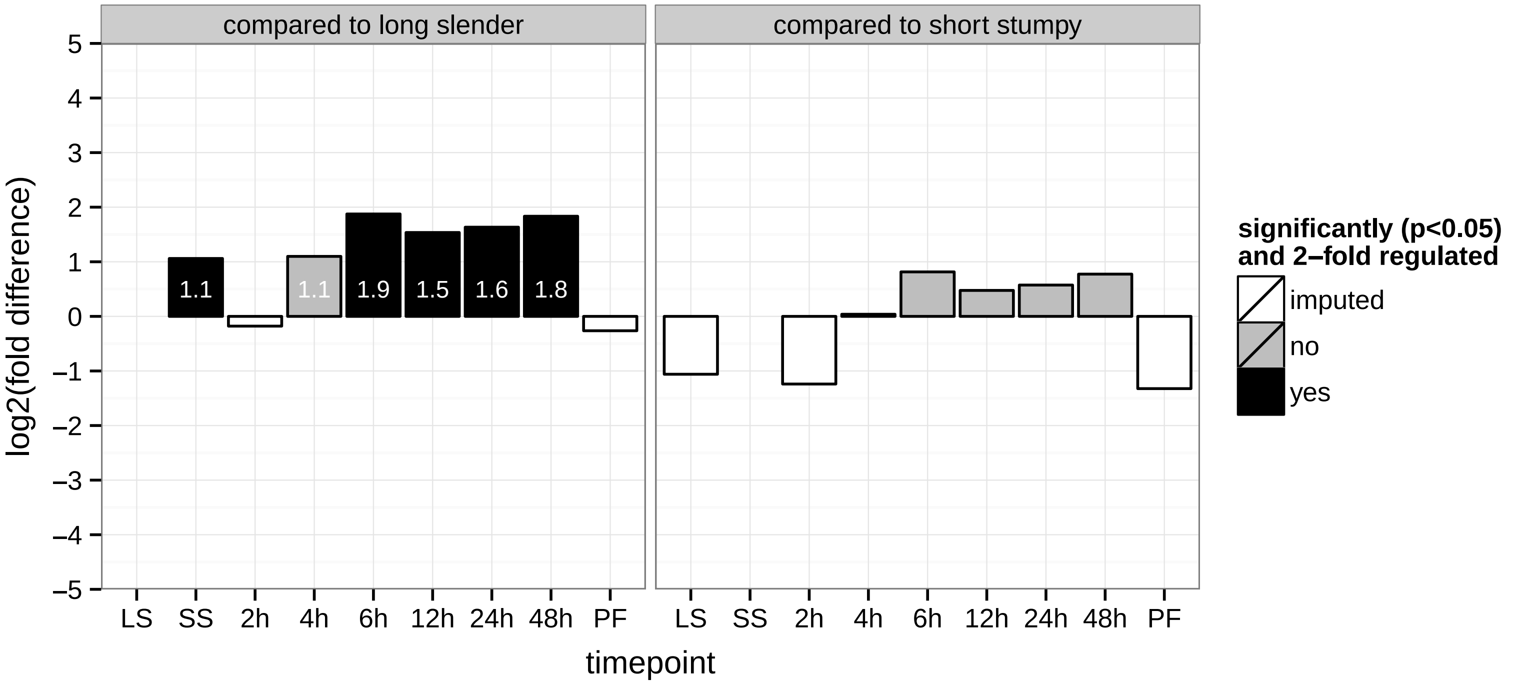

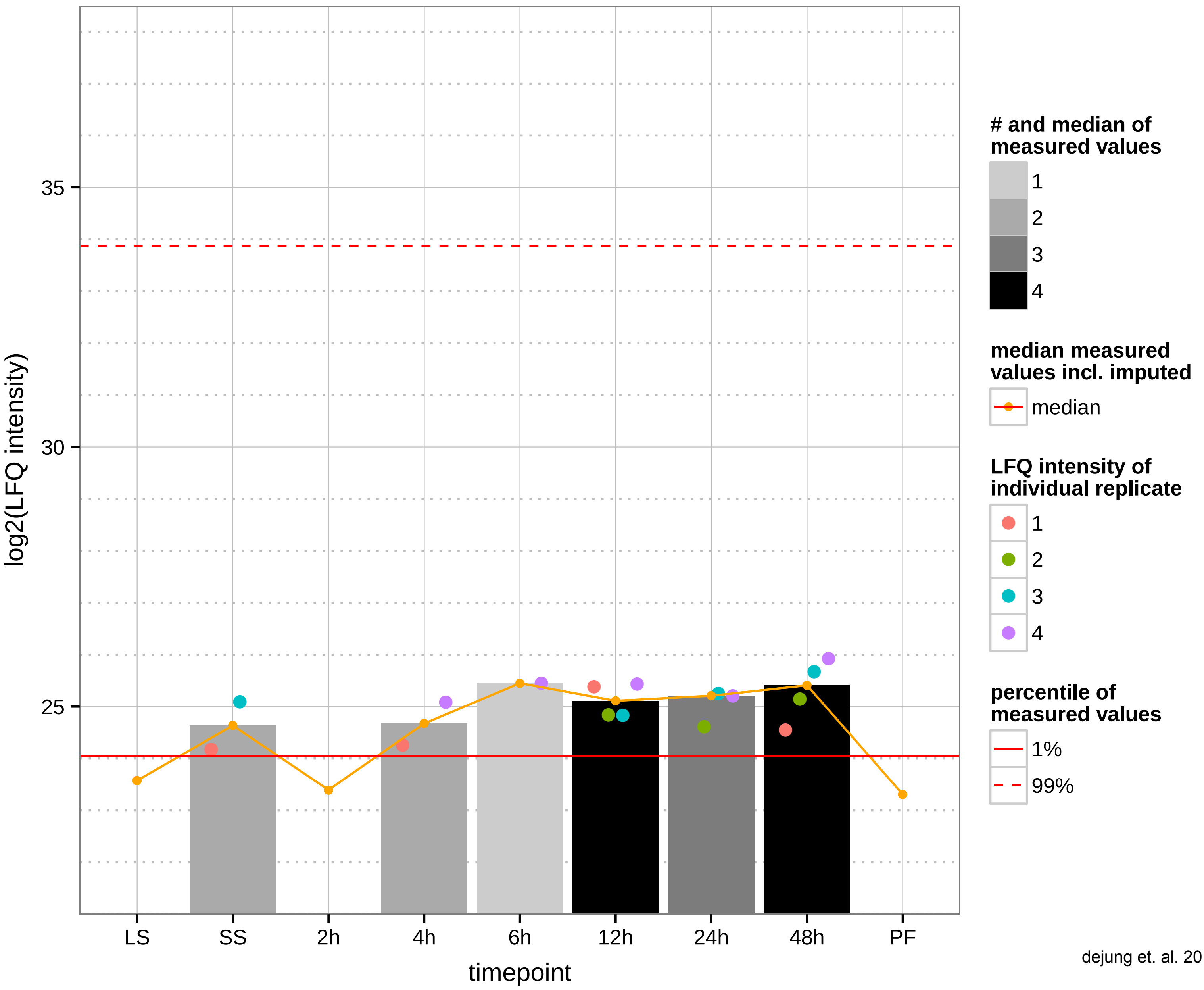

dejung et. al. 2015

ras–like small GTPase, putative (TbGTR)
Tb927.11.3320
AGOF: GTP binding
AGOC: cytoplasm, nucleus
AGOP: null
PGOF: GTP binding
PGOC: cytoplasm, nucleus
PGOP: null

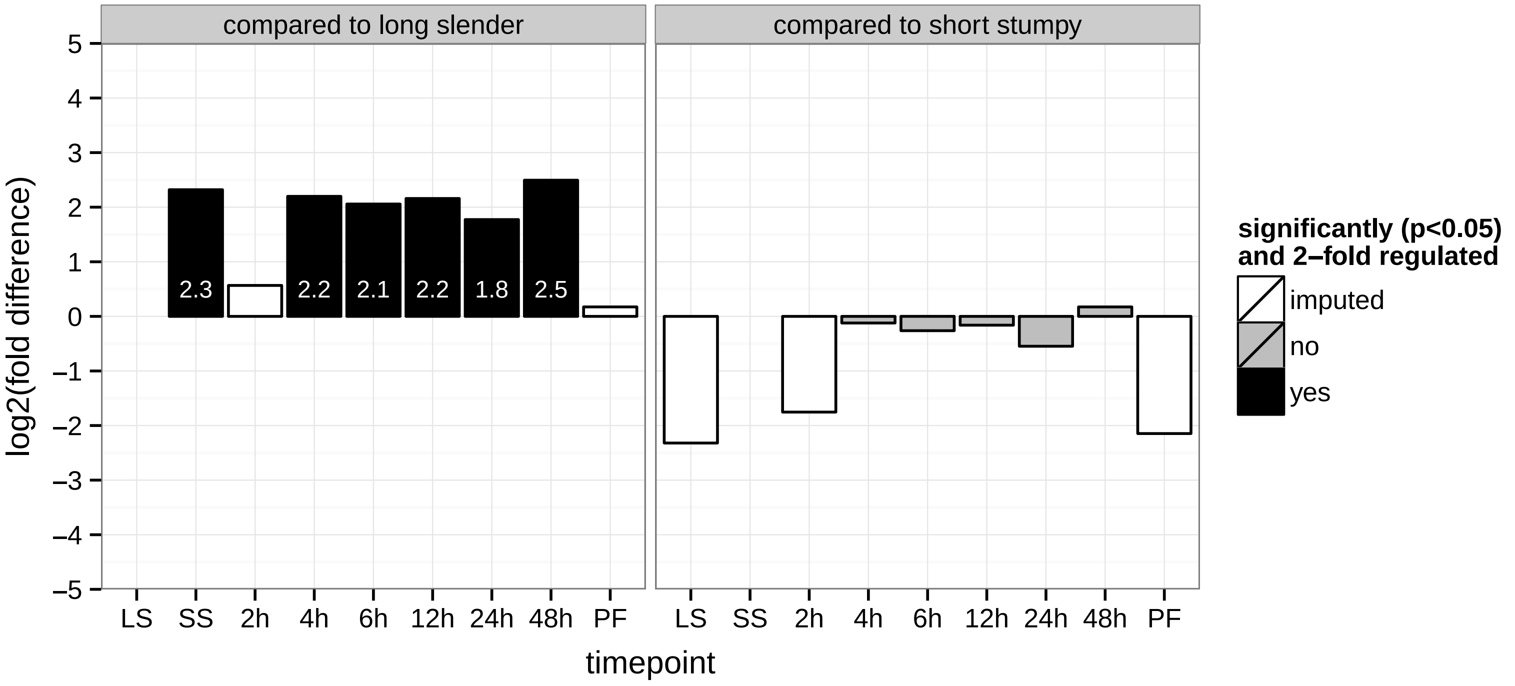

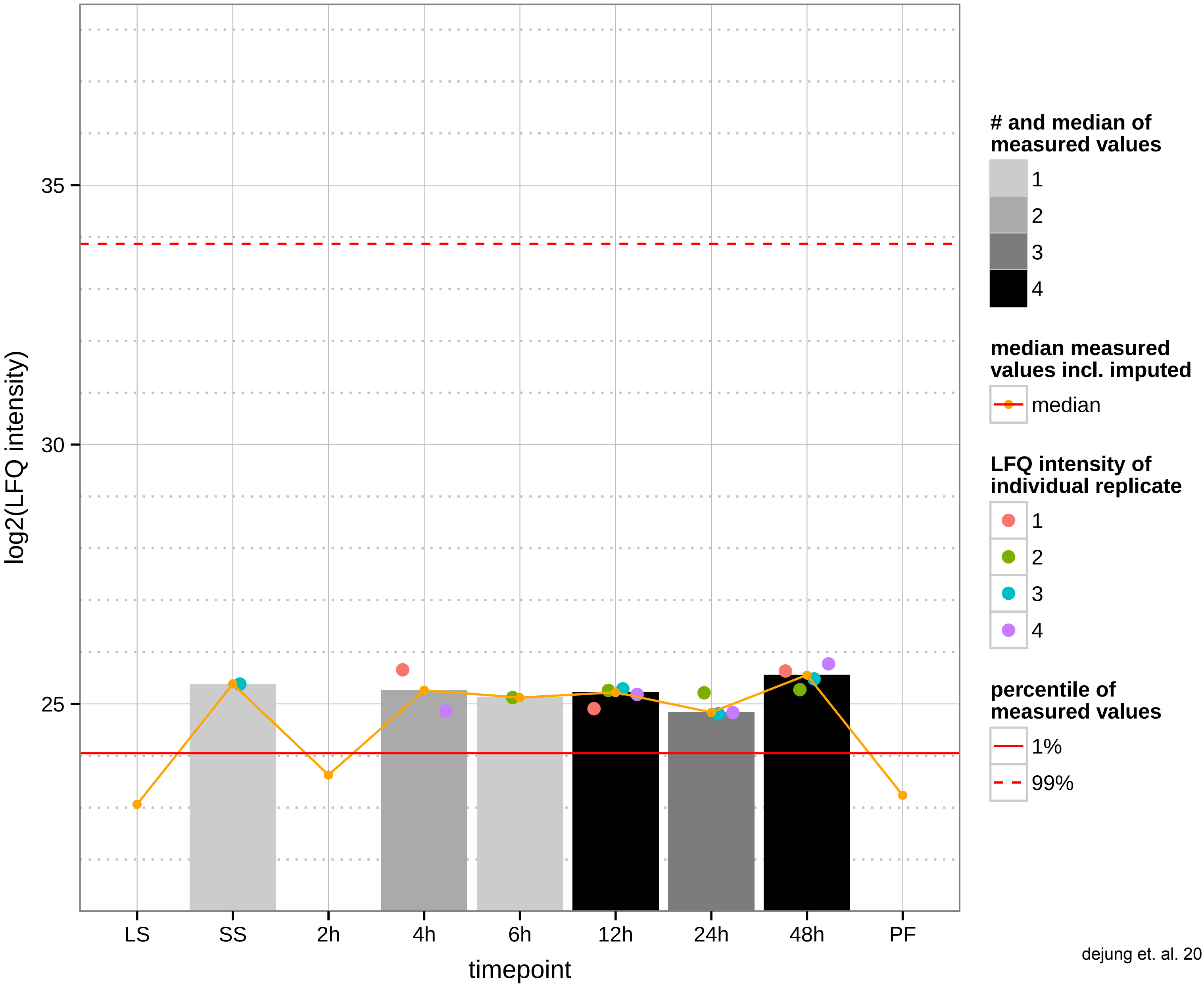

dejung et. al. 2015

leucine−rich repeat protein (LRRP), putative
Tb927.11.5190
AGOF: null
AGOC: null
AGOP: null
PGOF: protein binding
PGOC: null
PGOP: null

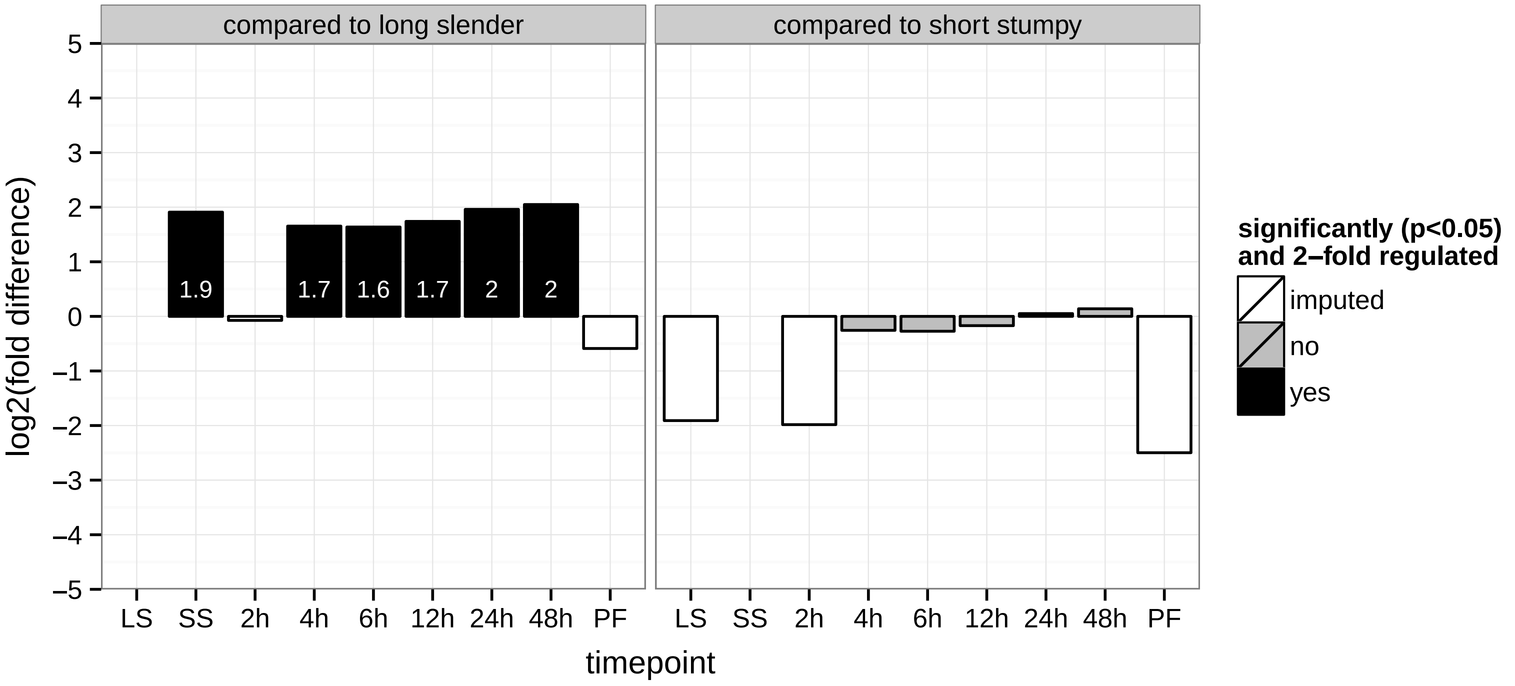

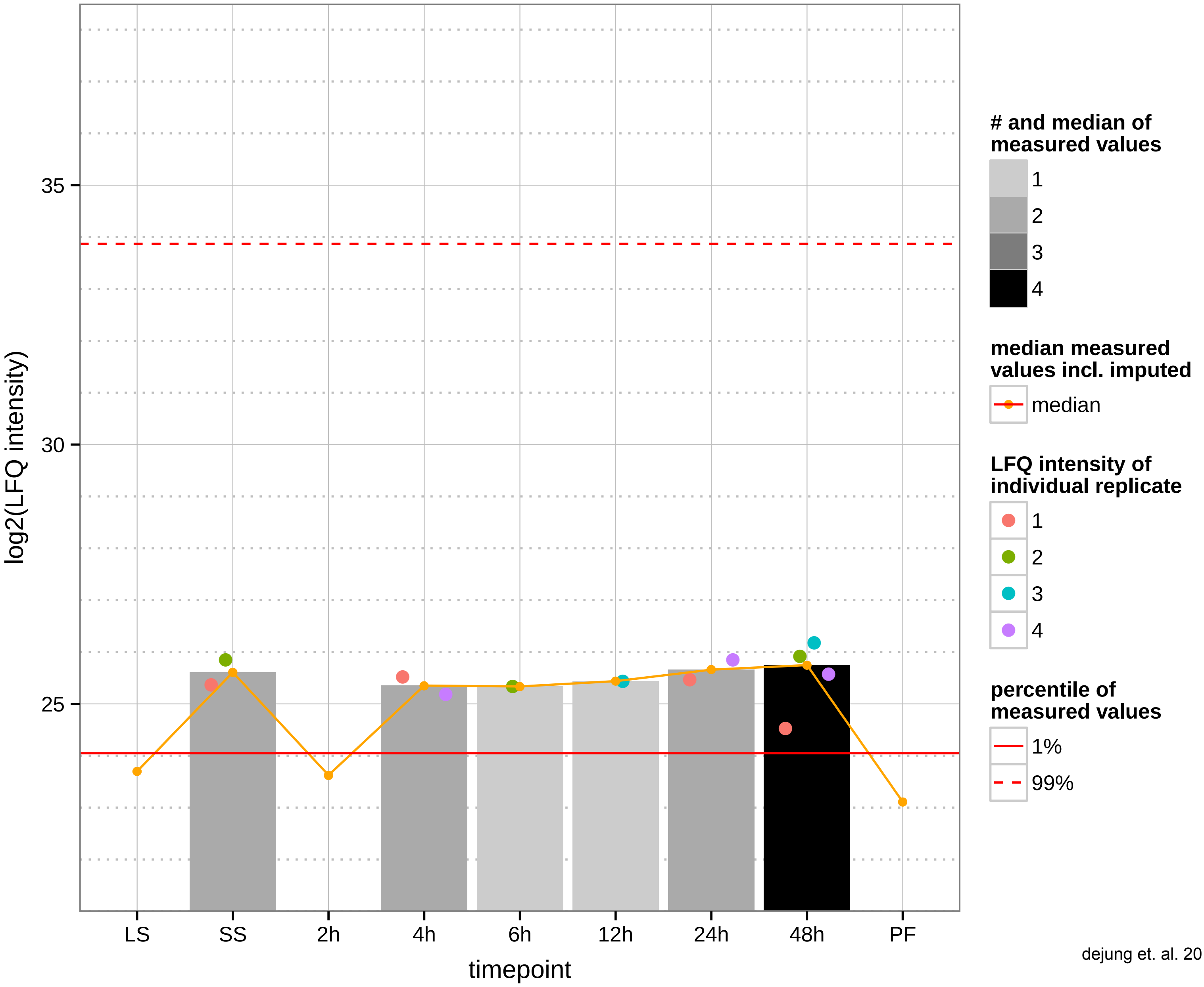

dejung et. al. 2015

hypothetical protein, conserved
Tb927.11.6190
AGOF: null
AGOC: null
AGOP: null
PGOF: null
PGOC: null
PGOP: null

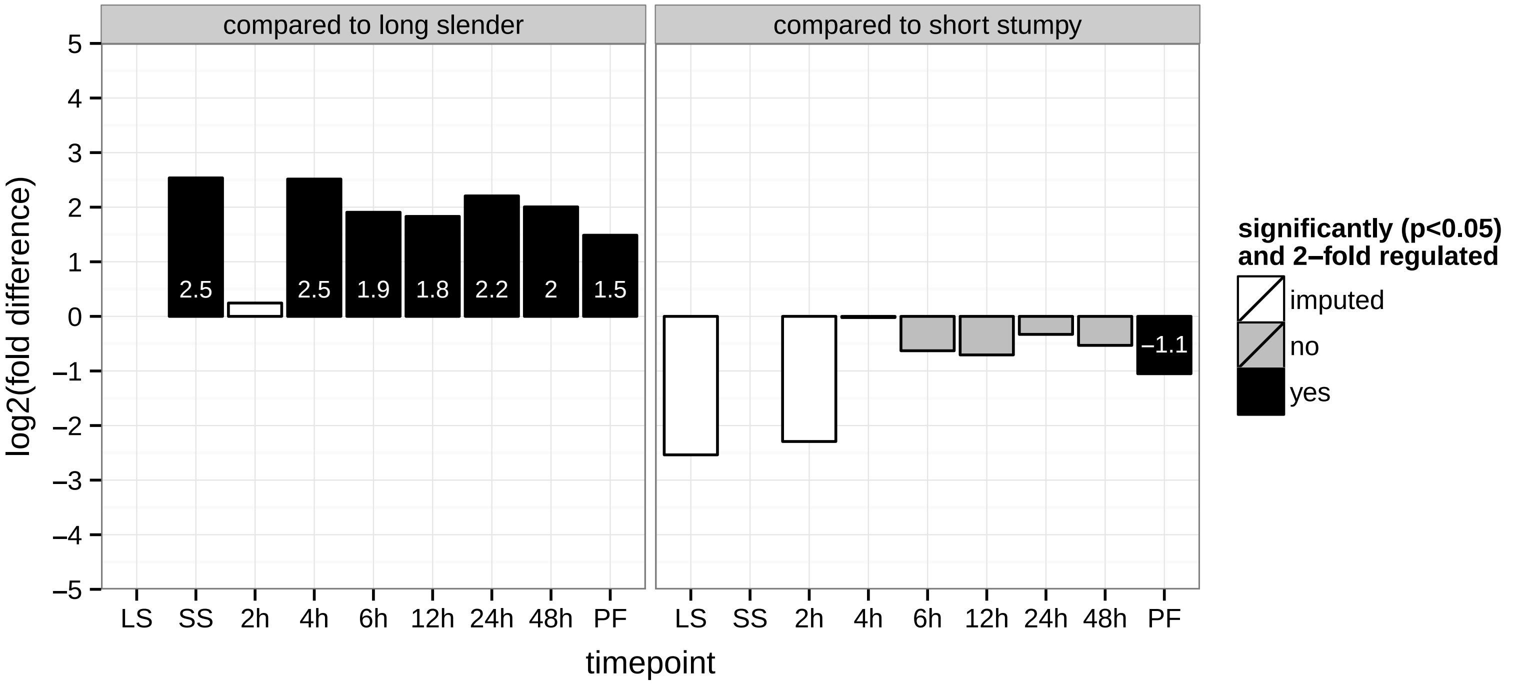

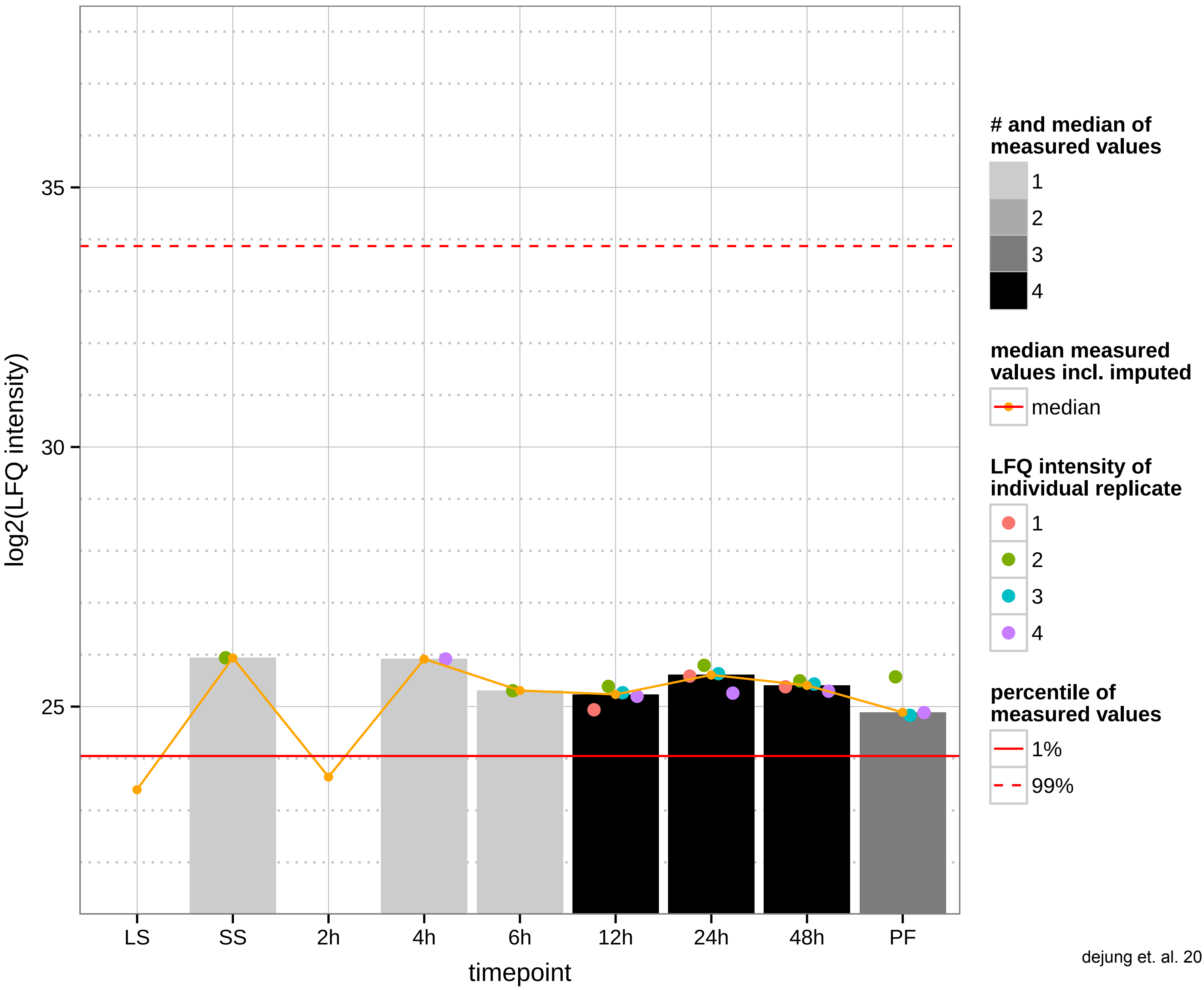

dejung et. al. 2015

hypothetical protein, conserved
Tb927.11.8700
AGOF: null
AGOC: null
AGOP: null
PGOF: null
PGOC: null
PGOP: null

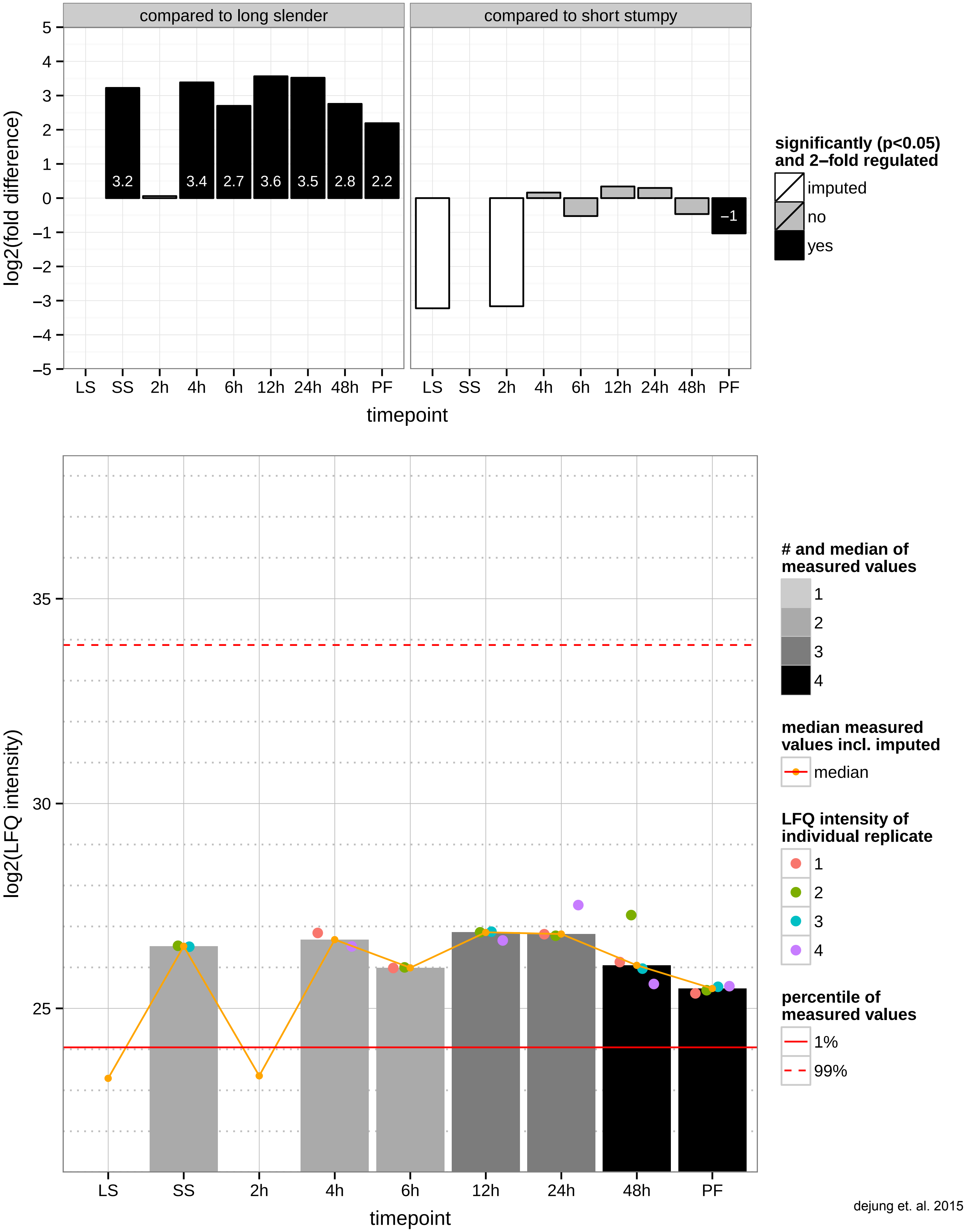

dejung et. al. 2015

acyl–CoA binding protein, putative (ACBP)
Tb927.4.2010
AGOF: fatty–acyl–CoA binding
AGOC: null
AGOP: fatty acid metabolic process
PGOF: fatty–acyl–CoA binding
PGOC: null
PGOP: null

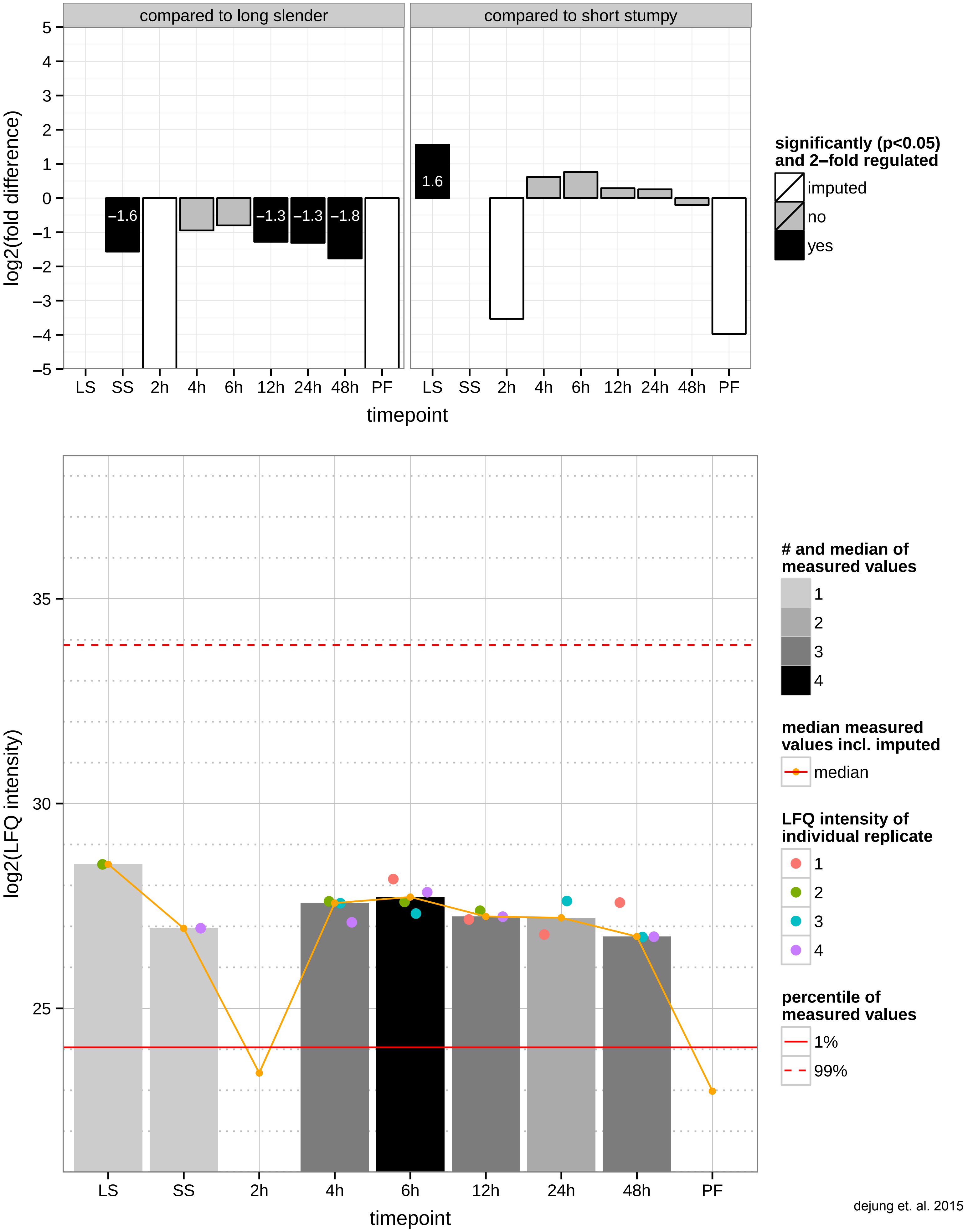

dejung et. al. 2015

hypothetical protein, conserved
Tb927.4.2130
AGOF: null
AGOC: null
AGOP: null
PGOF: null
PGOC: null
PGOP: null

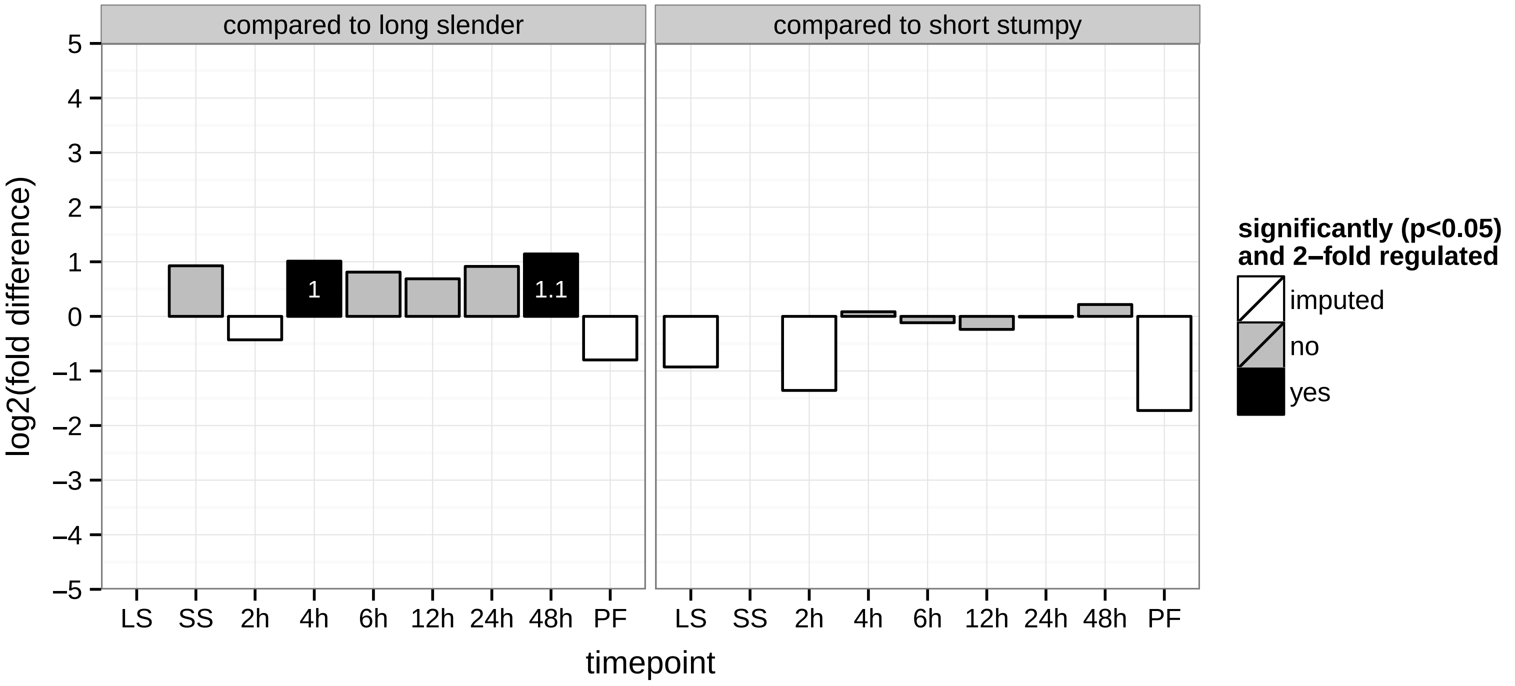

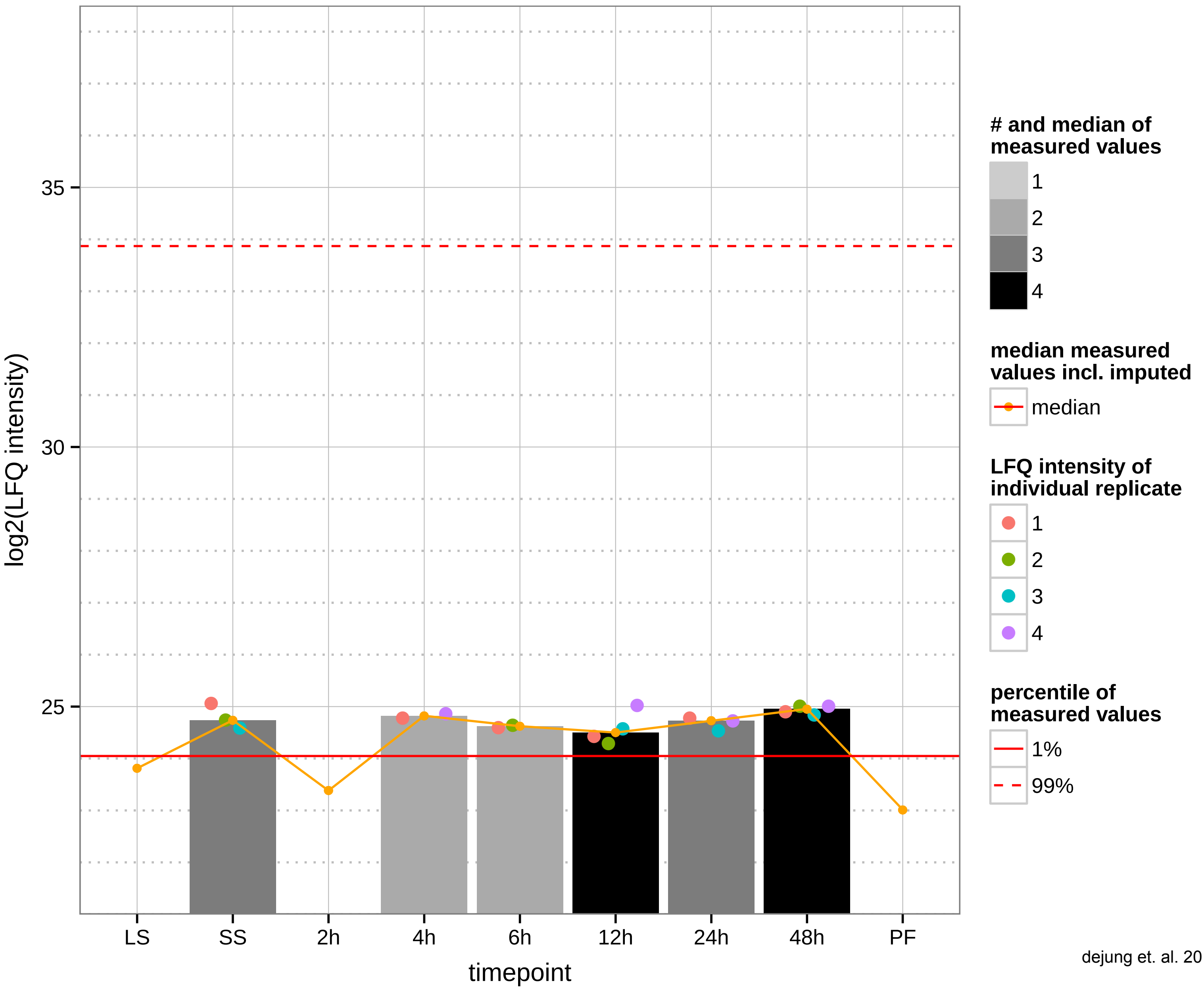

dejung et. al. 2015

regulator of chromosome condensation, putative
Tb927.7.1190
AGOF: null
AGOC: null
AGOP: null
PGOF: null
PGOC: null
PGOP: null

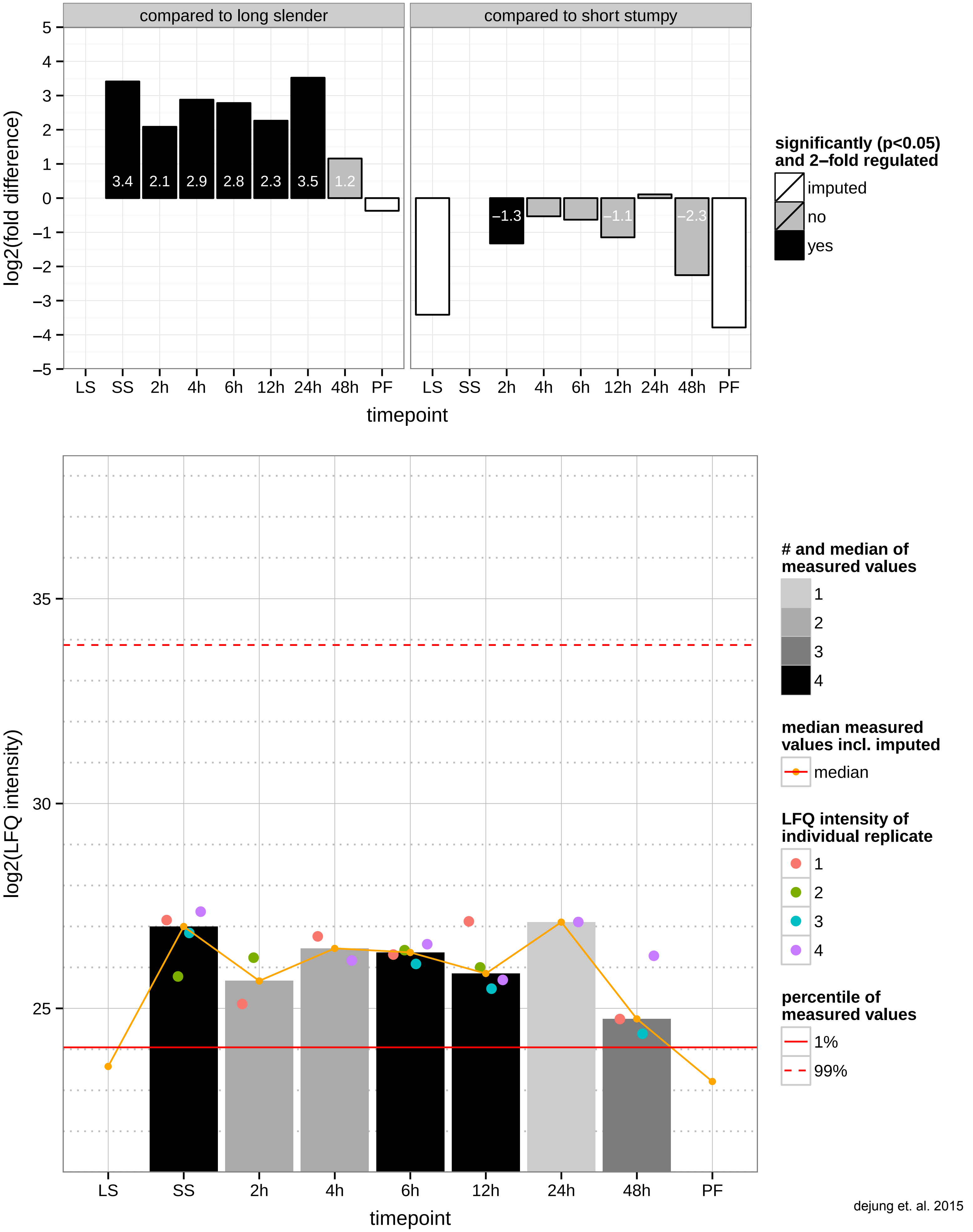

dejung et. al. 2015

hypothetical protein, conserved
Tb927.7.4140
AGOF: RNA binding, structural constituent of ribosome
AGOC: intracellular, mitochondrion, ribosome
AGOP: translation
PGOF: RNA binding, structural constituent of ribosome
PGOC: intracellular, ribosome
PGOP: translation

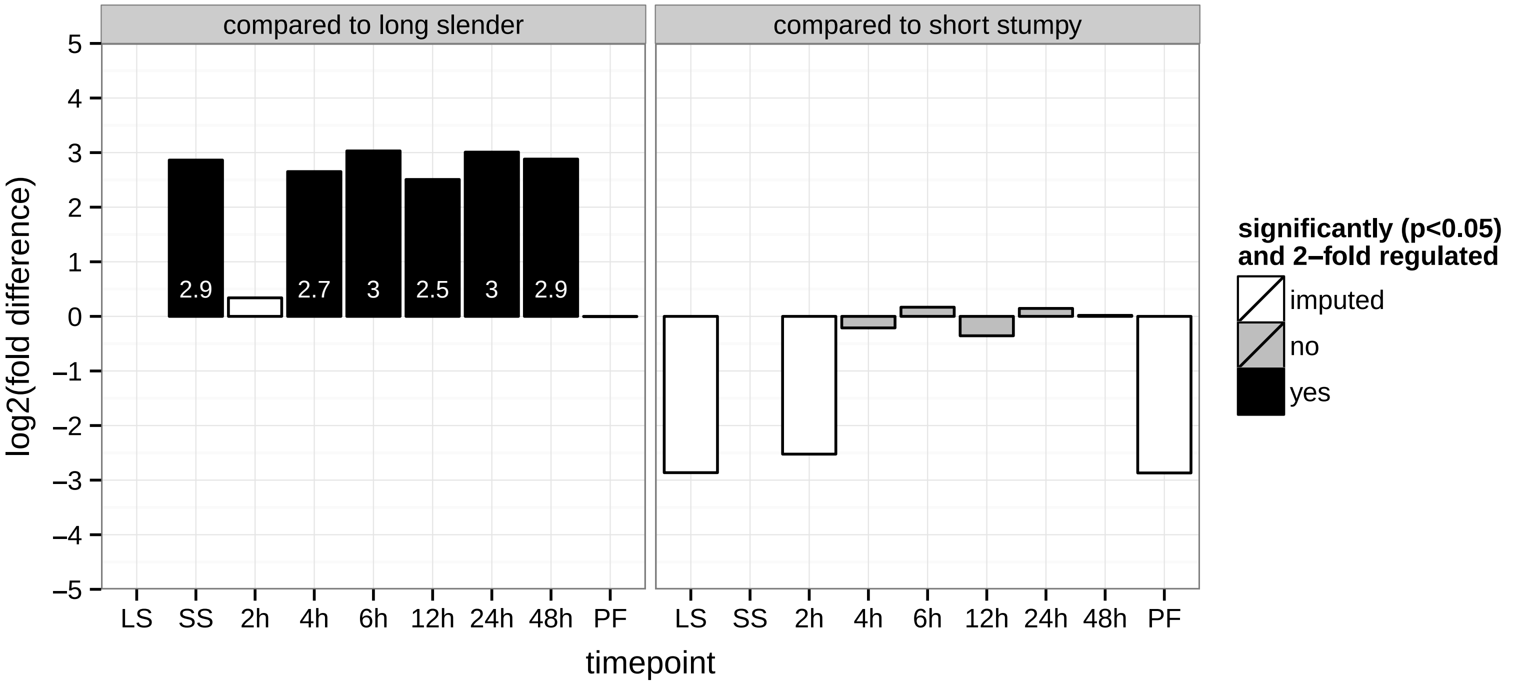

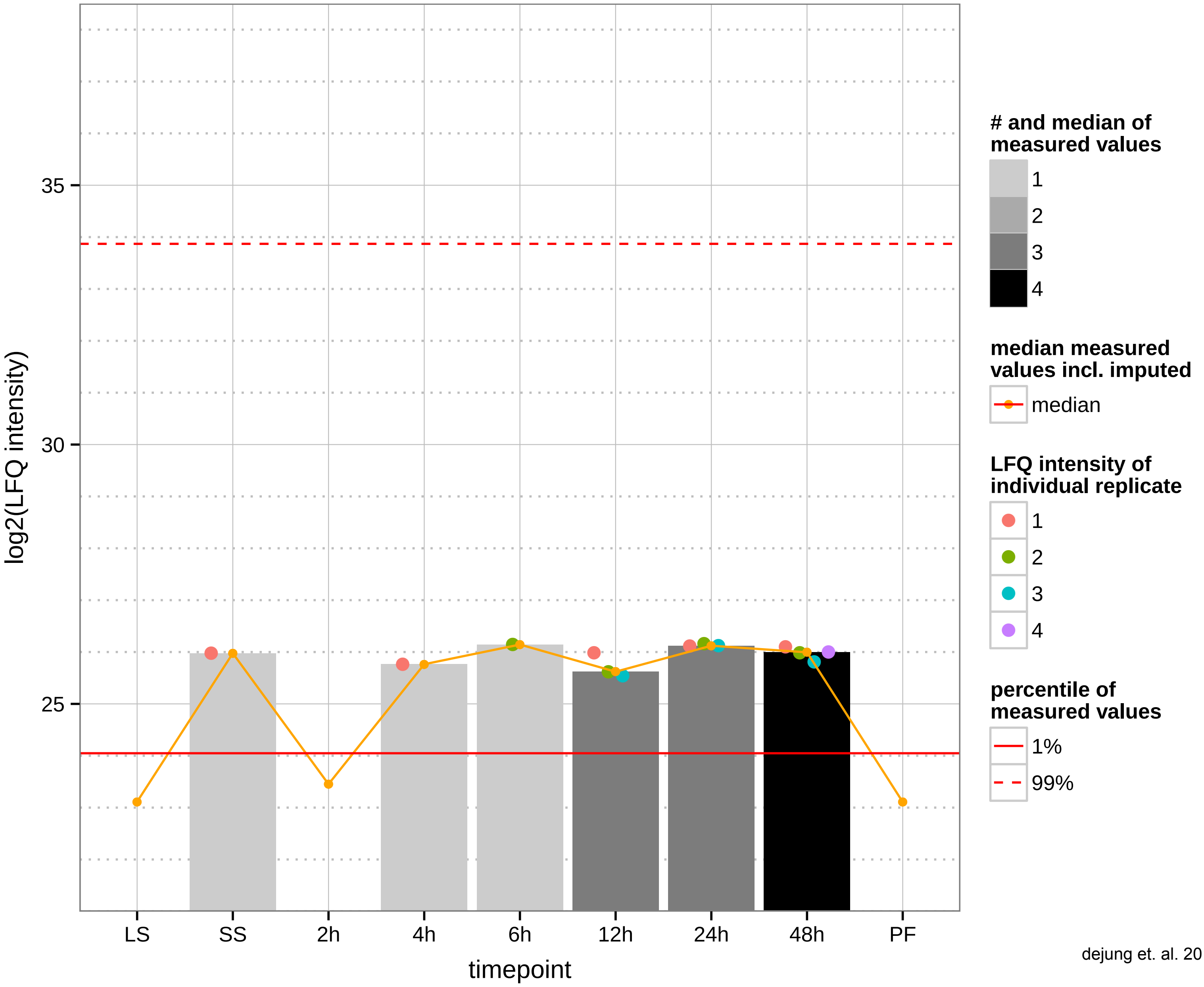

dejung et. al. 2015

protein kinase, putative
Tb927.7.6220
AGOF: ATP binding, protein kinase activity, protein serine/threonine kinase activity, protein tyrosine kinase activity
AGOC: null
AGOP: cytokinesis after mitosis, growth, protein phosphorylation
PGOF: ATP binding, protein binding, protein kinase activity, transferase activity, transferring phosphorus–containing groups
PGOC: null
PGOP: protein phosphorylation

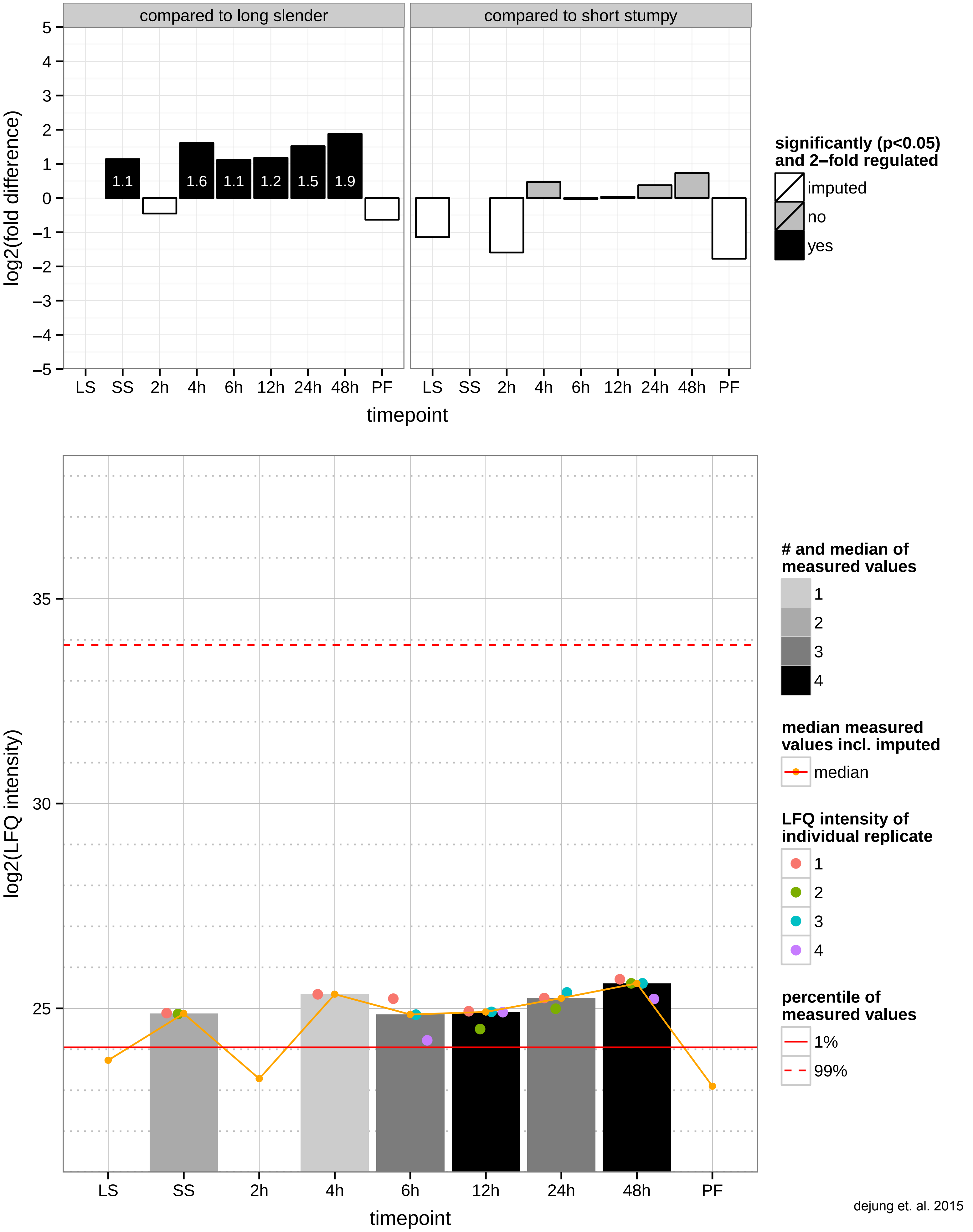

dejung et. al. 2015

hypothetical protein, conserved
Tb927.8.1050
AGOF: null
AGOC: null
AGOP: null
PGOF: protein binding
PGOC: null
PGOP: null

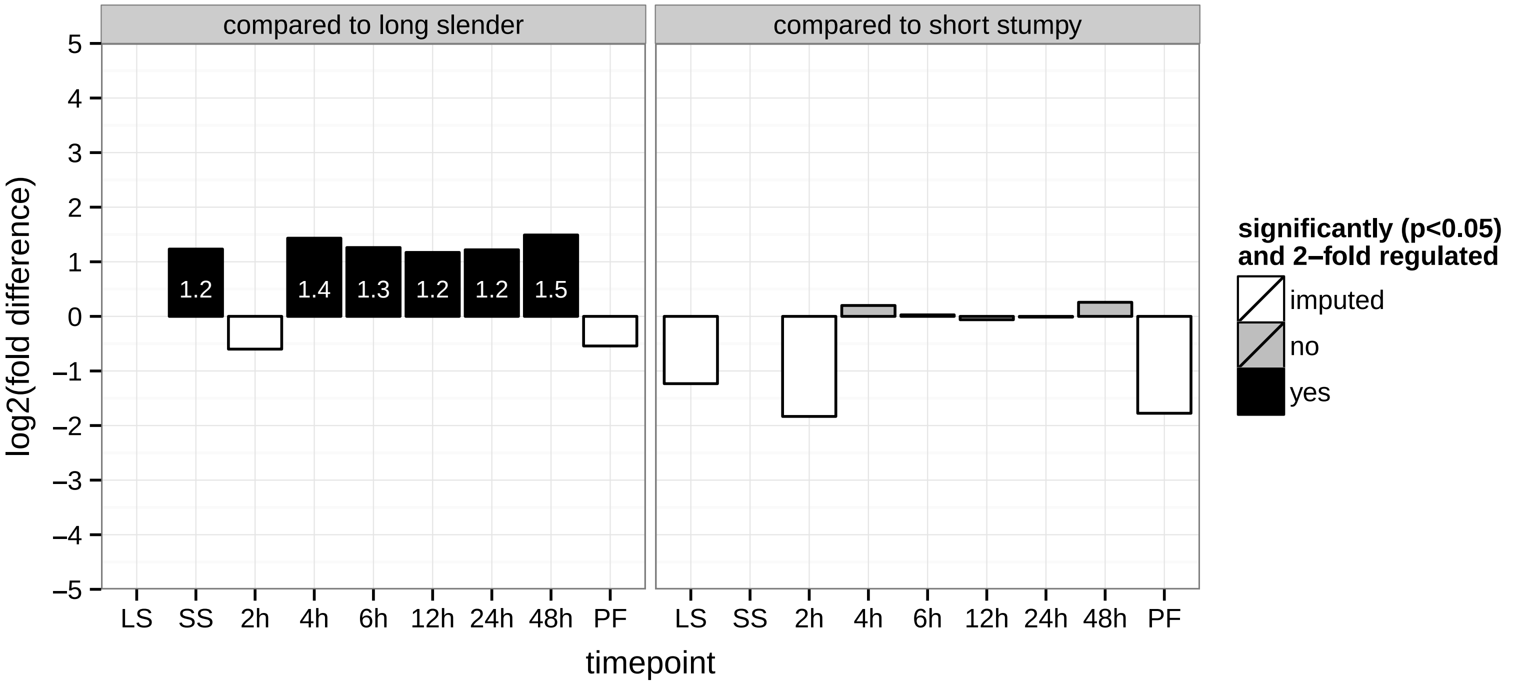

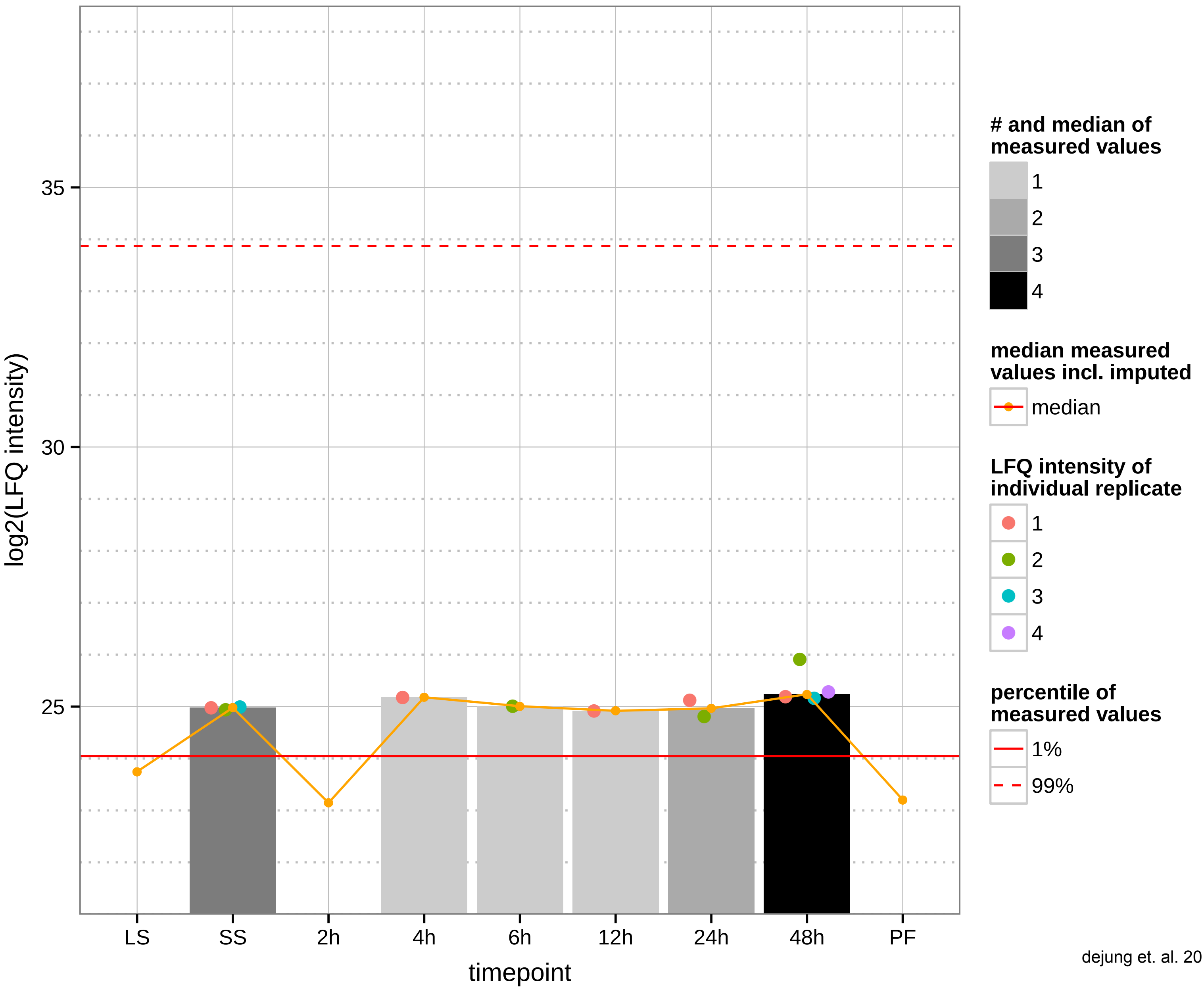

dejung et. al. 2015

vacuolar−type Ca2+−ATPase, putative
Tb927.8.1160
AGOF: ATP binding, calcium−transporting ATPase activity
AGOC: acidocalcisome, integral to membrane, membrane
AGOP: ATP biosynthetic process, calcium ion transport, cell growth, cellular calcium ion homeostasis, metabolic process
PGOF: ATP binding, ATPase activity, coupled to transmembrane movement of ions, phosphorylative mechanism, calcium−trar
PGOC: membrane
PGOP: ATP biosynthetic process, calcium ion transmembrane transport, cation transport, metabolic process

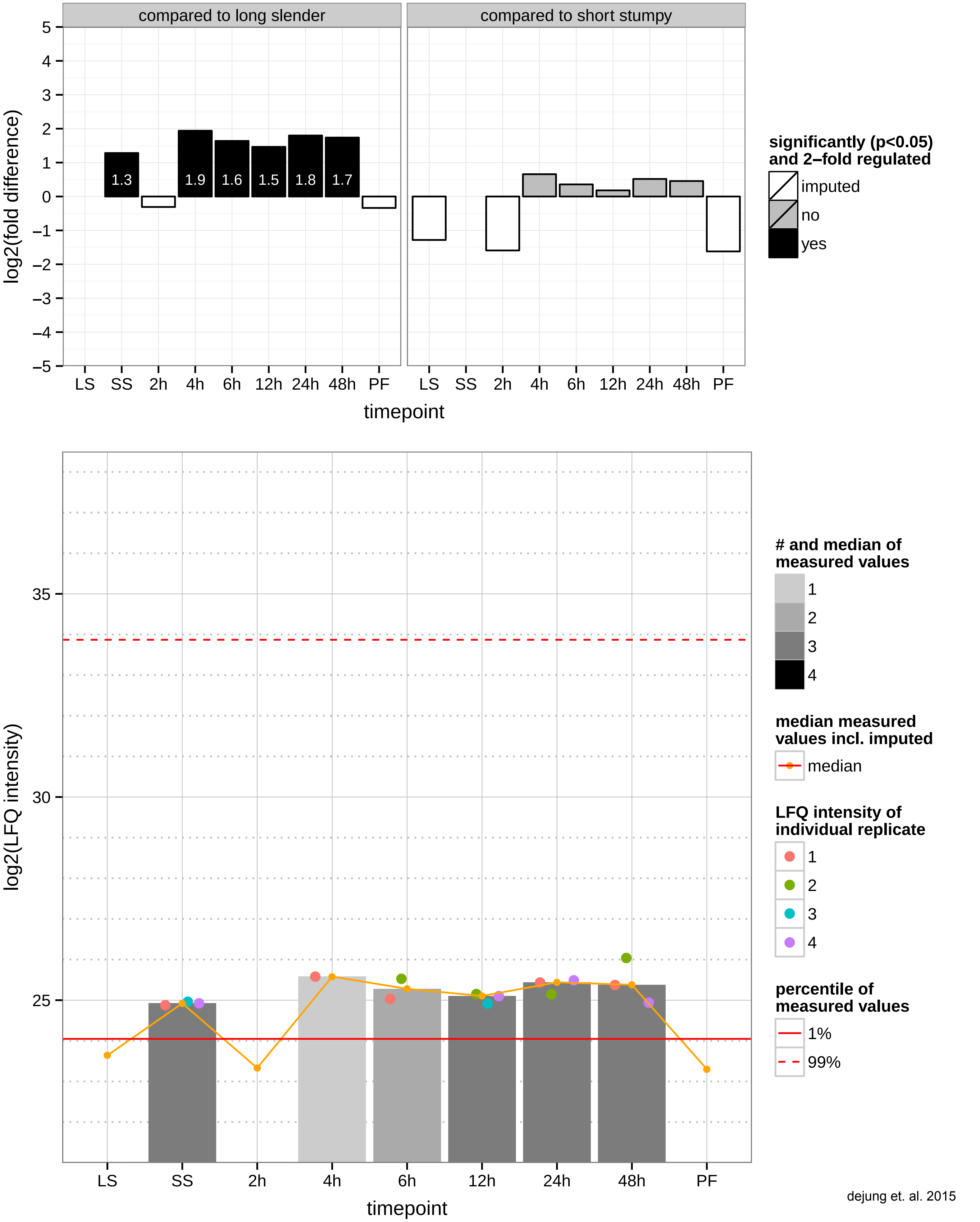

dejung et. al. 2015

electron transfer flavoprotein–ubiquinone oxidoreductase, putative
Tb927.8.1240
AGOF: electron–transferring–flavoprotein dehydrogenase activity
AGOC: mitochondrion
AGOP: oxidation–reduction process
PGOF: electron–transferring–flavoprotein dehydrogenase activity, iron–sulfur cluster binding
PGOC: null
PGOP: oxidation–reduction process

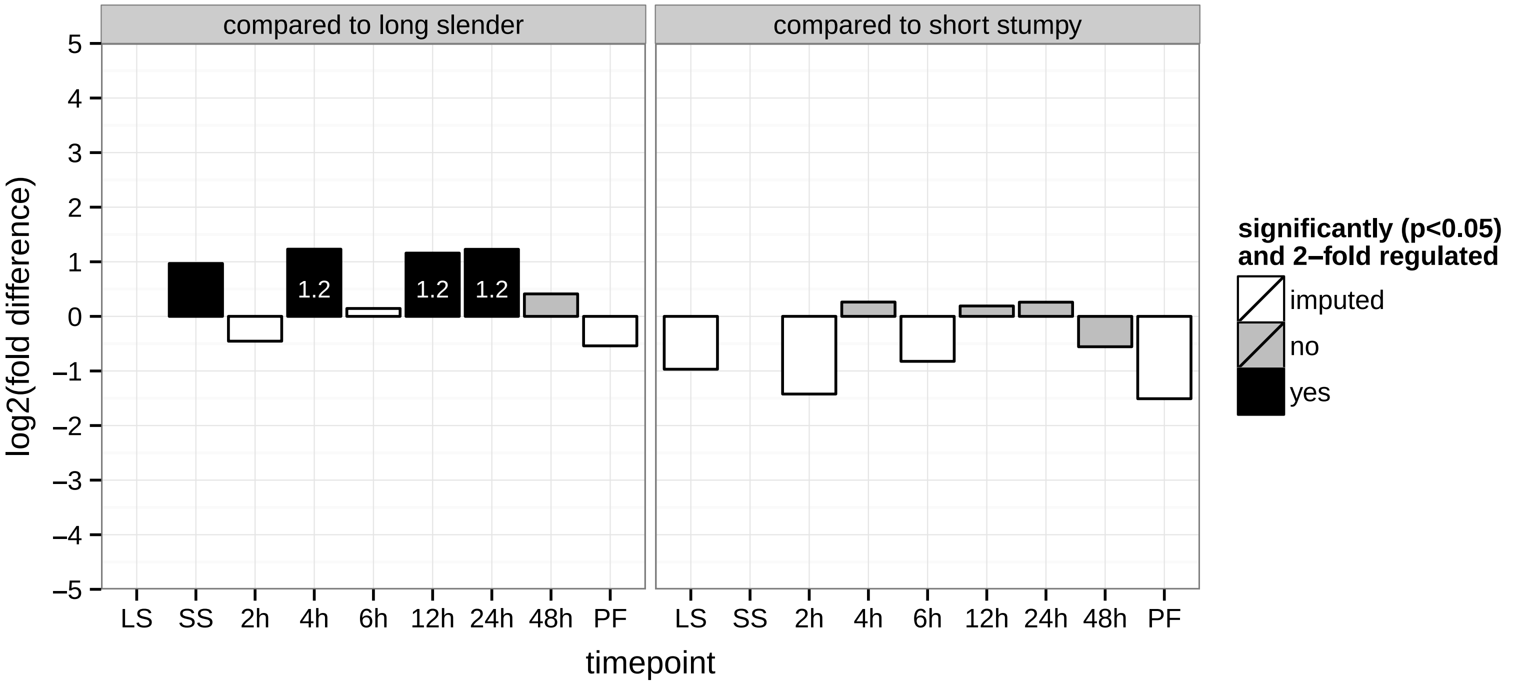

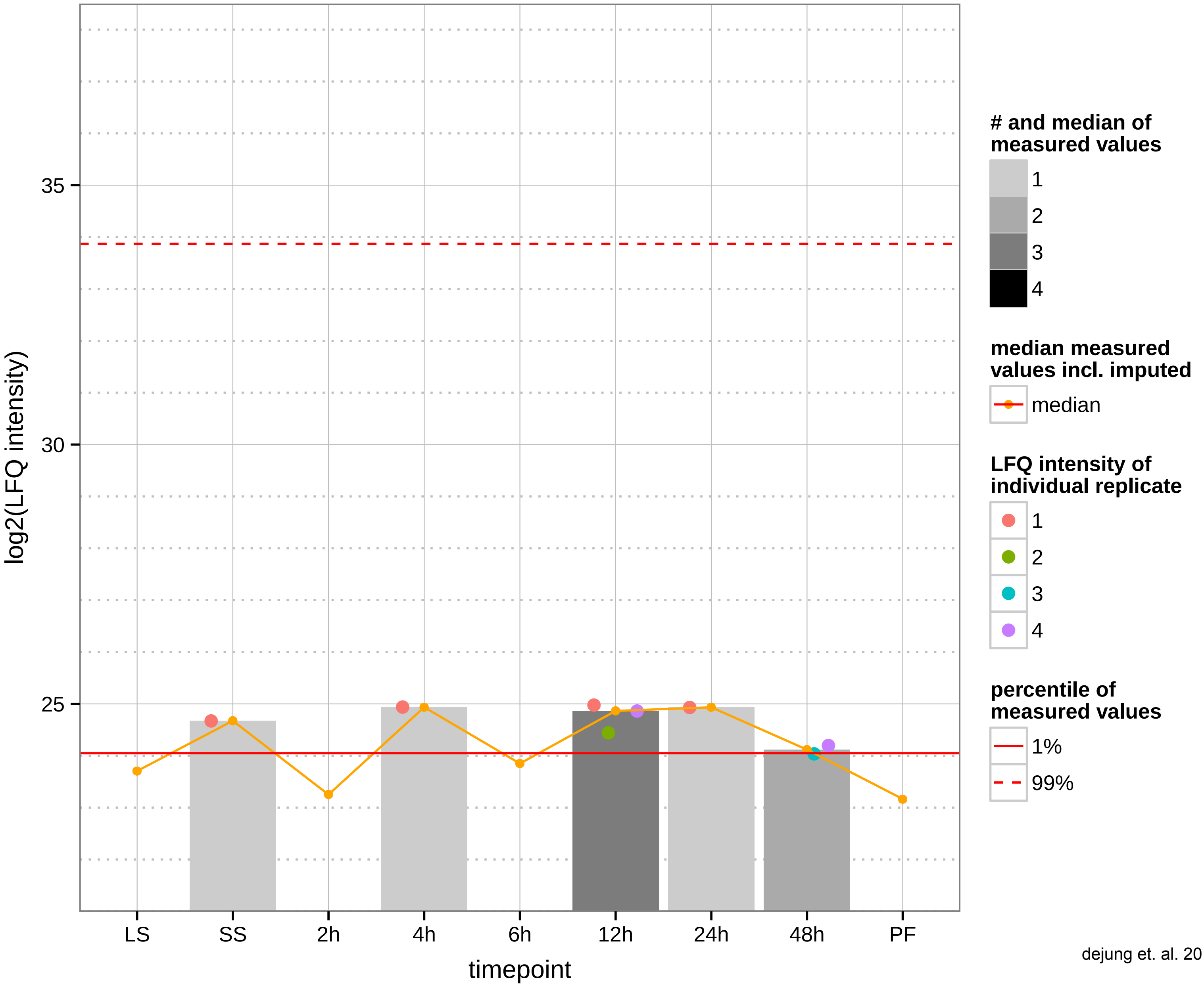

dejung et. al. 2015

phosphatidylinositol 3–kinase, putative, PI3–kinase, putative (PI3K)
Tb927.8.6210
AGOF: 1–phosphatidylinositol–3–kinase activity, phosphotransferase activity, alcohol group as acceptor
AGOC: phosphatidylinositol 3–kinase complex
AGOP: phosphatidylinositol phosphorylation, phosphatidylinositol–mediated signaling
PGOF: binding, phosphotransferase activity, alcohol group as acceptor, protein binding, transferase activity, transferring phosp
PGOC: null
PGOP: null

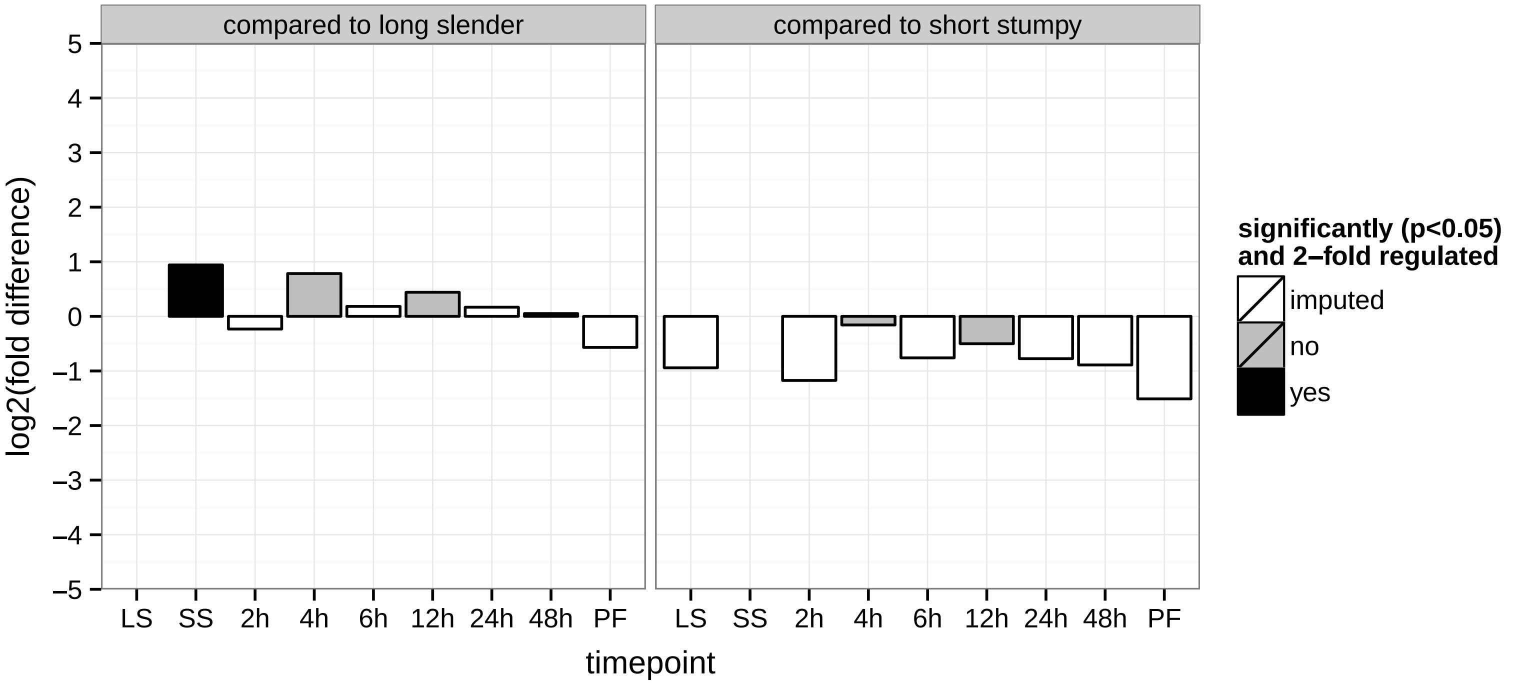

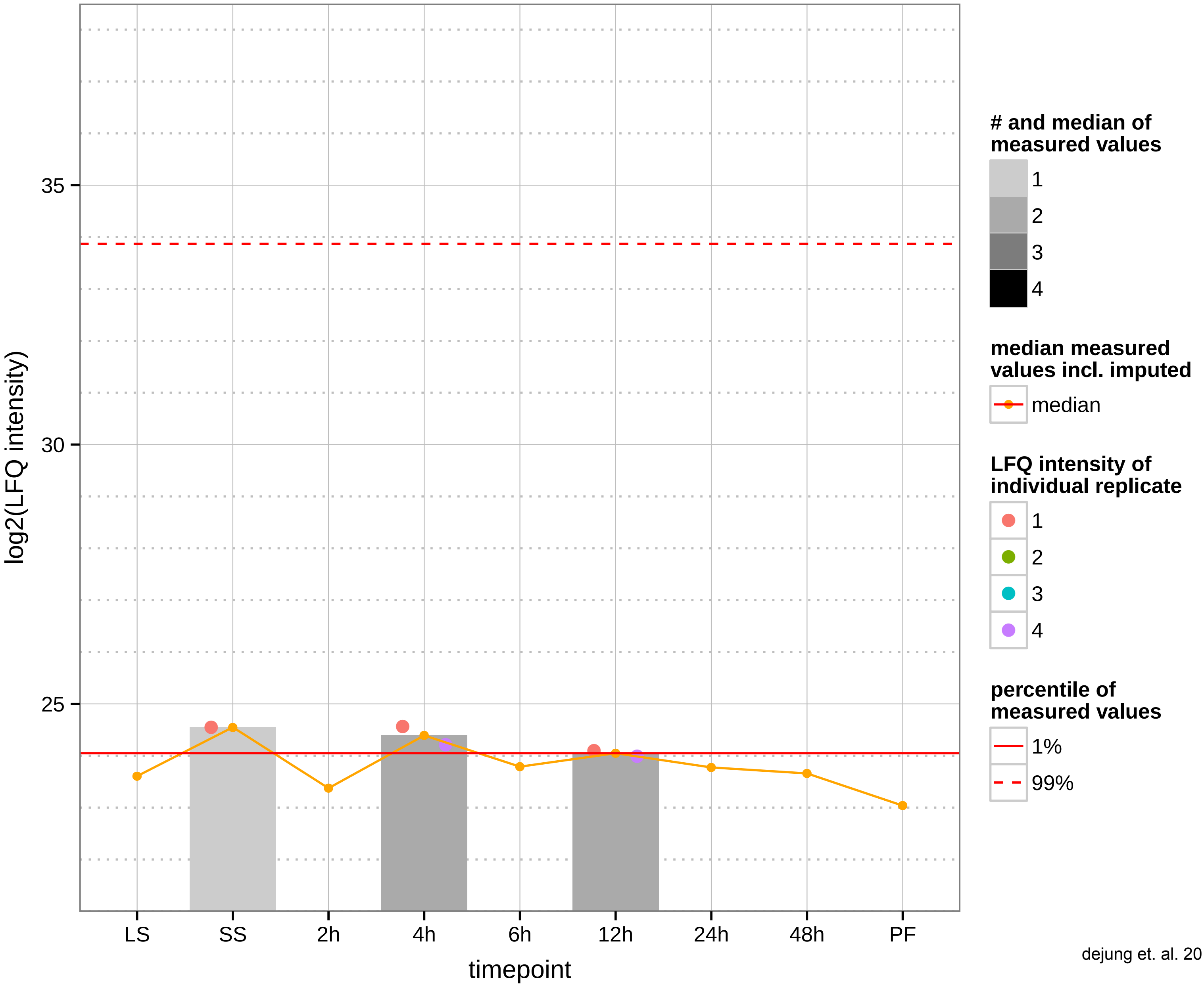

dejung et. al. 2015

hypothetical protein, conserved
Tb927.9.13060
AGOF: voltage−gated potassium channel activity
AGOC: membrane, voltage−gated potassium channel complex
AGOP: potassium ion transport
PGOF: protein binding
PGOC: null
PGOP: protein homooligomerization

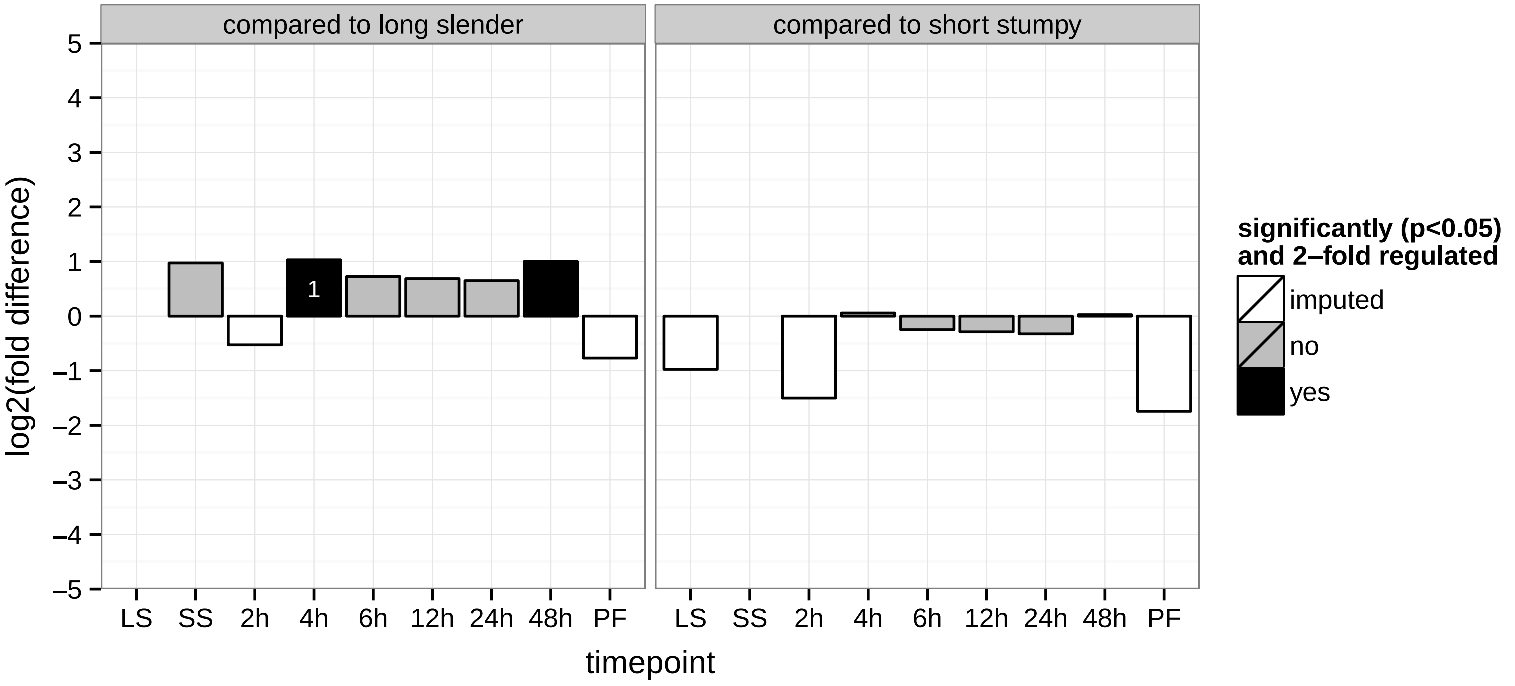

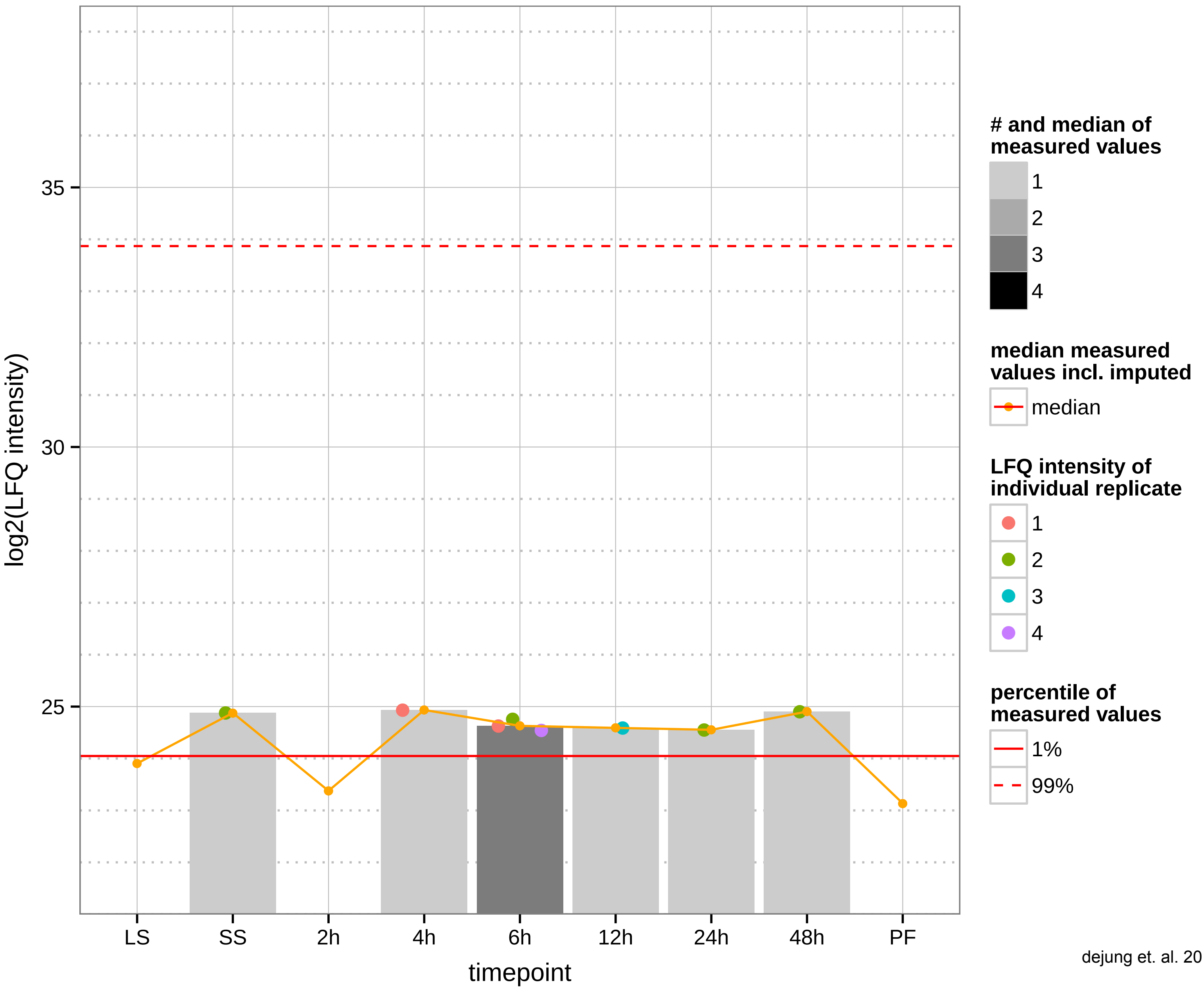

dejung et. al. 2015

hypothetical protein, conserved
Tb927.9.9420
AGOF: null
AGOC: null
AGOP: null
PGOF: null
PGOC: null
PGOP: null

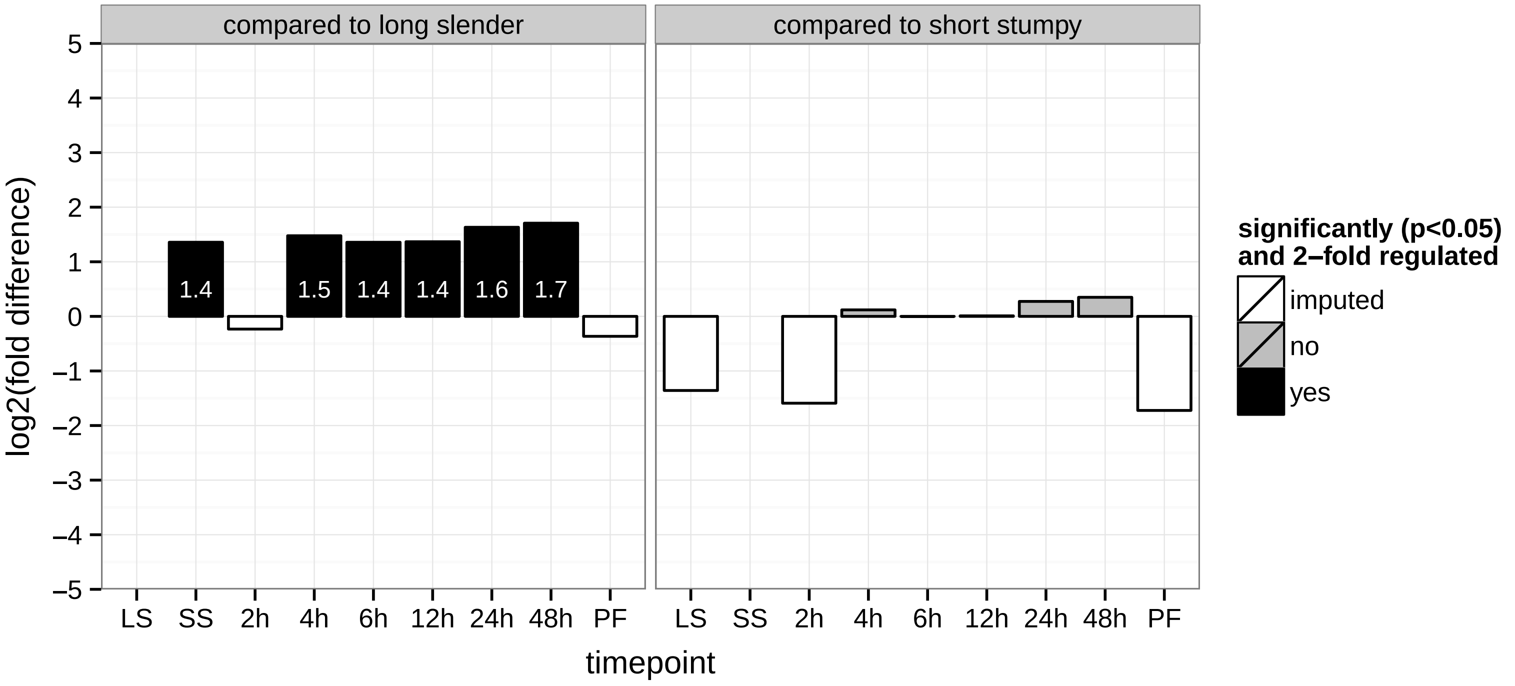

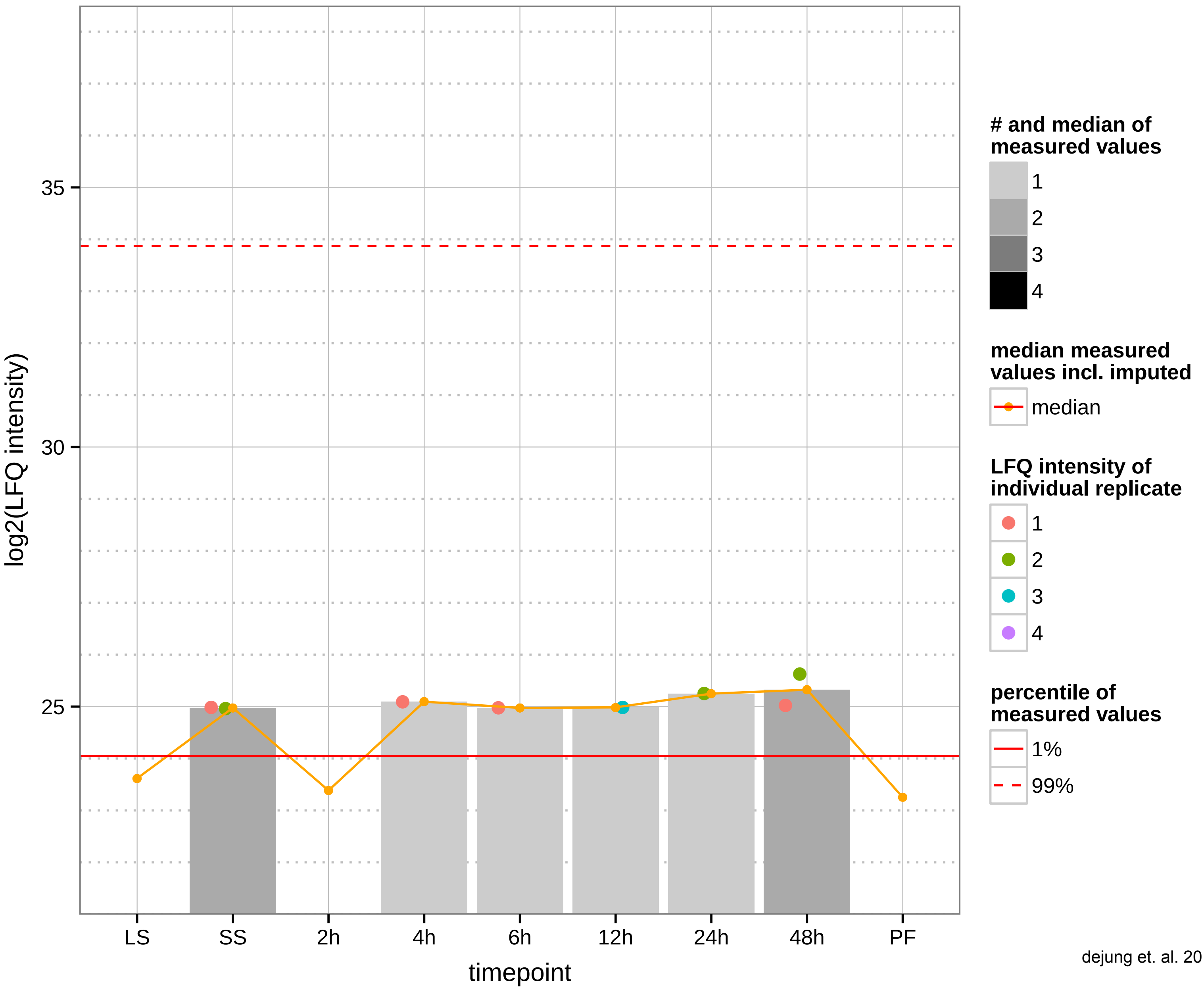

dejung et. al. 2015

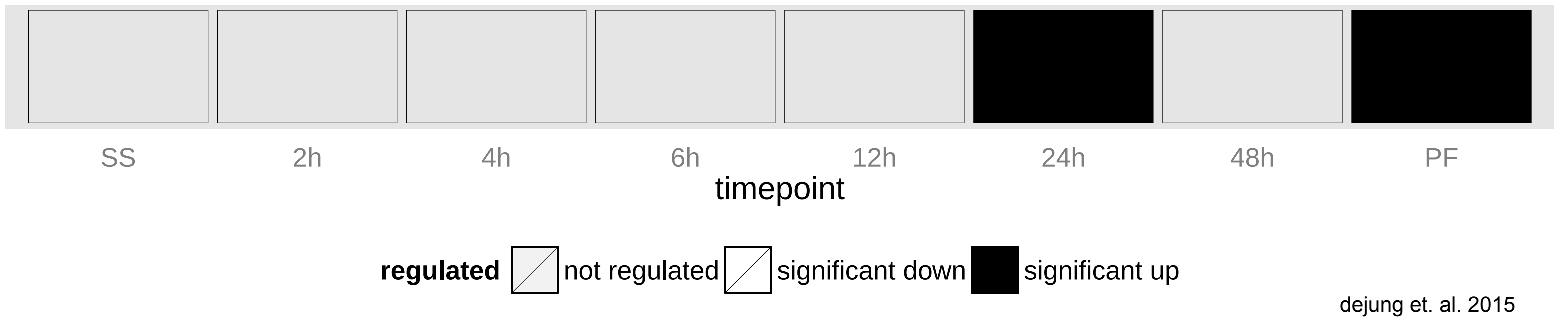
SS
2h
4h
6h
12h
24h
48h
PF
timepoint
regulated
not regulated
significant down
significant up
dejung et. al. 2015

dynein light chain LC8, putative, chrXI additional, unordered contigs (DYNLL2), putative (DYNLL1)
Tb927.11.18680;Tb11.0845
AGOF: microtubule motor activity
AGOC: microtubule associated complex
AGOP: microtubule–based process
PGOF: null
PGOC: microtubule associated complex
PGOP: microtubule–based process

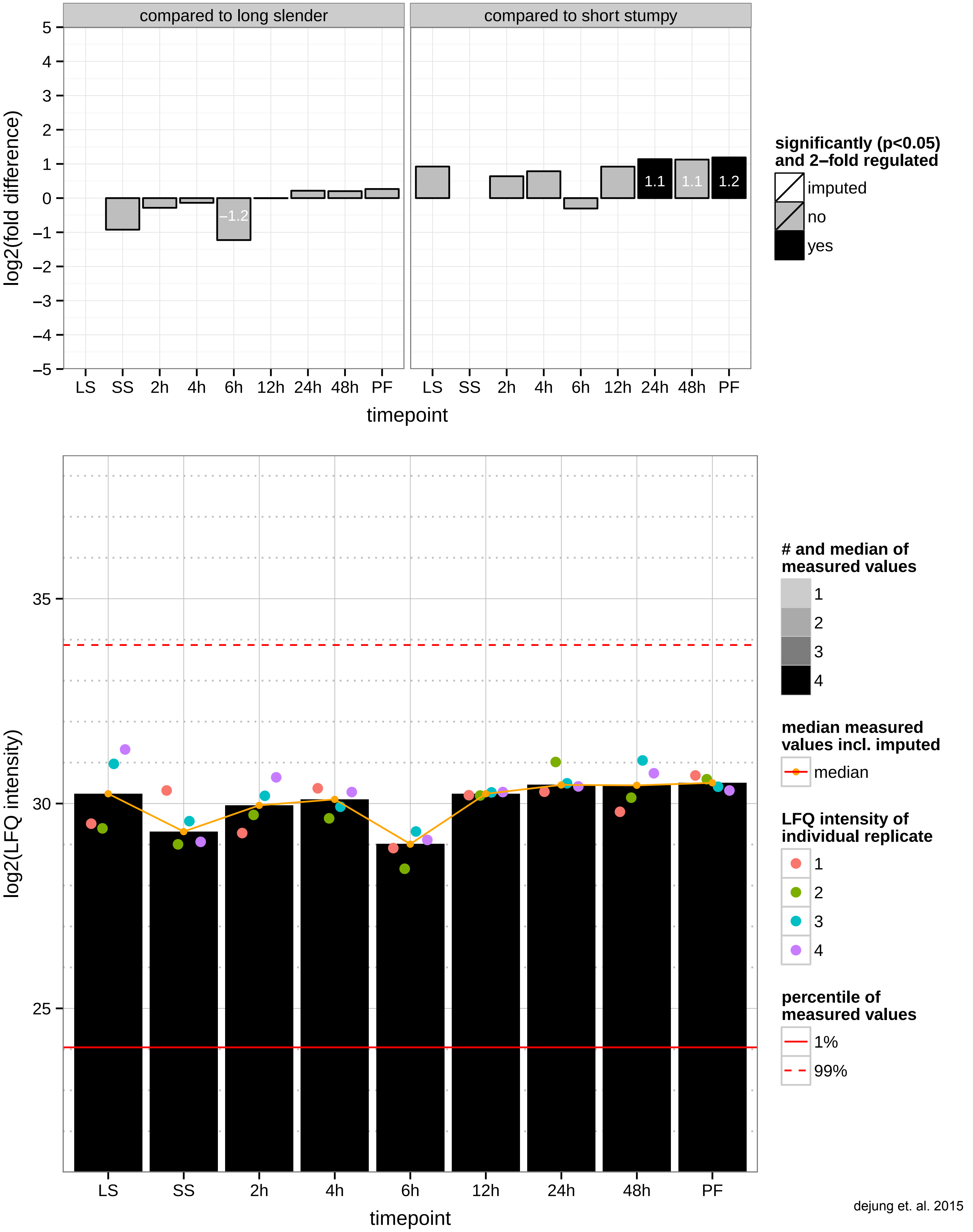

dejung et. al. 2015

60S ribosomal protein L7, putative
Tb927.10.13730
AGOF: structural constituent of ribosome
AGOC: ribosome
AGOP: translation
PGOF: null
PGOC: null
PGOP: null

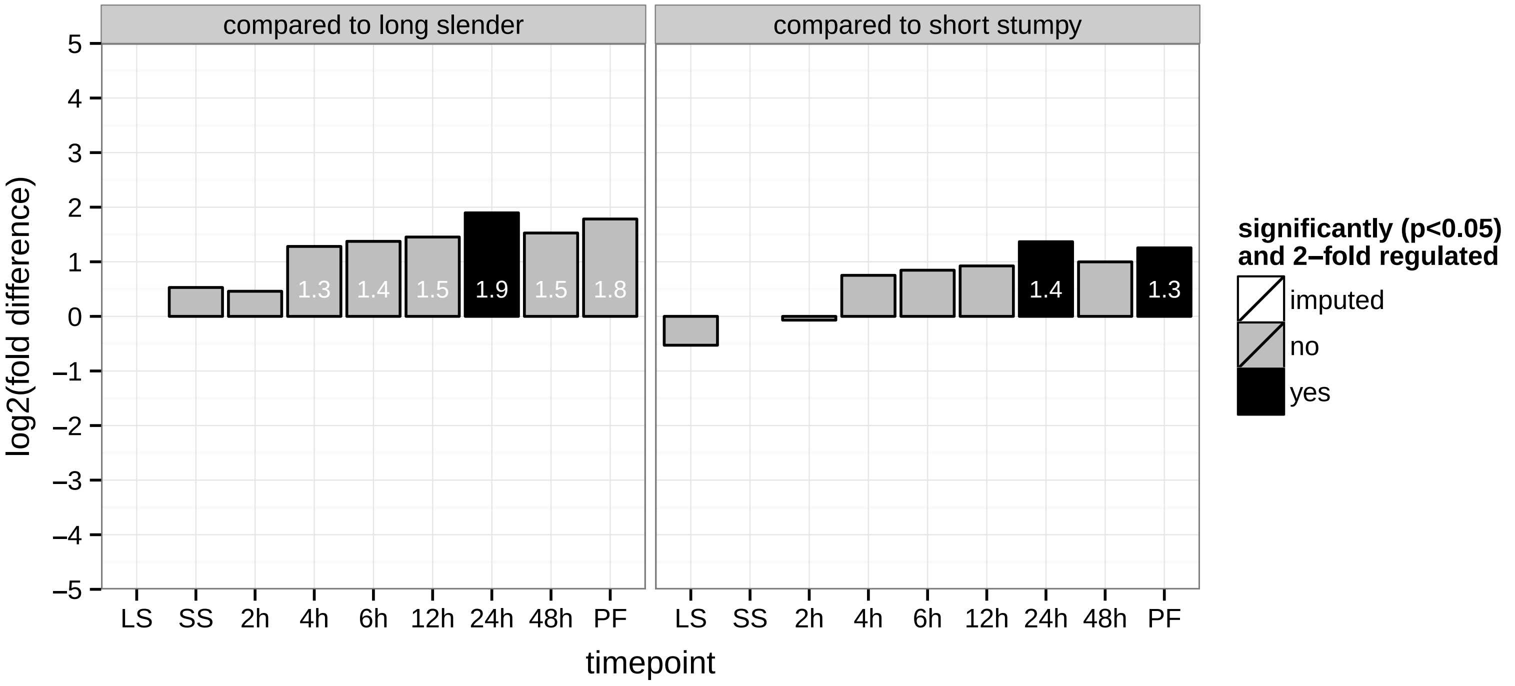

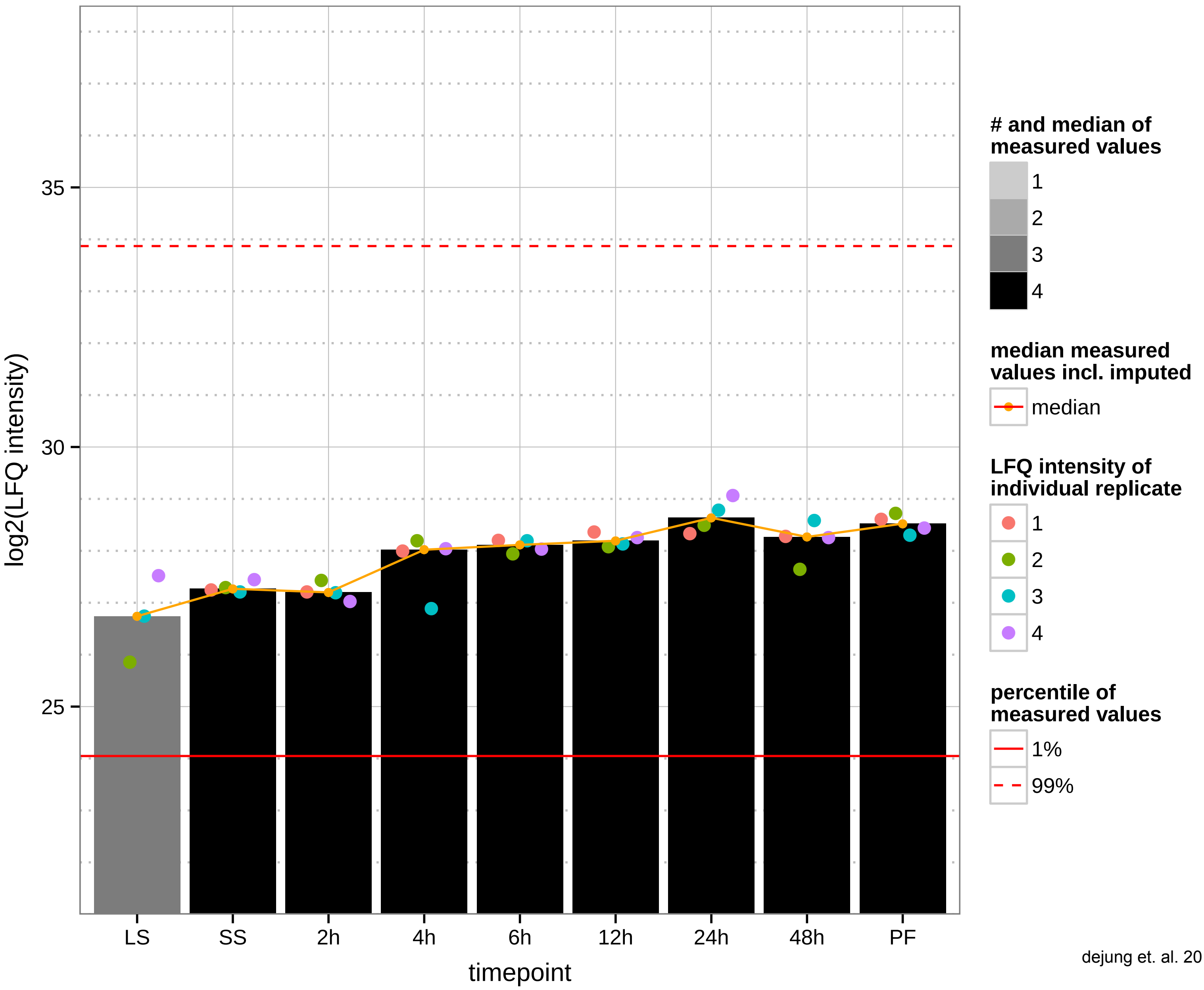

dejung et. al. 2015

cell division related protein kinase 2, putative, CDC2−related protein kinase (CRK3)
Tb927.10.4990
AGOF: ATP binding, protein binding, protein serine/threonine kinase activity, protein tyrosine kinase activity
AGOC: mitochondrion
AGOP: cell cycle, protein phosphorylation
PGOF: ATP binding, protein kinase activity, transferase activity, transferring phosphorus−containing groups
PGOC: null
PGOP: protein phosphorylation

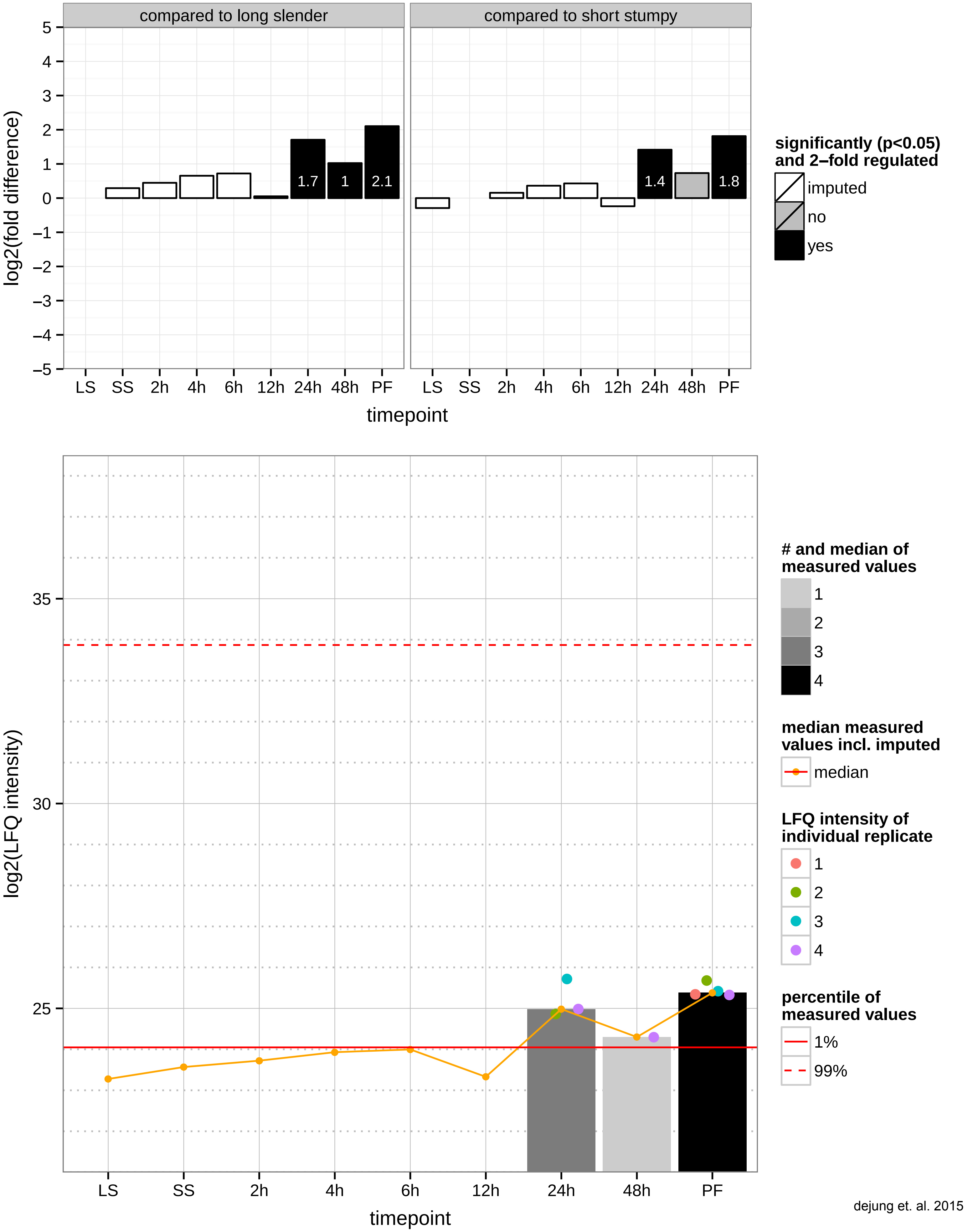

dejung et. al. 2015

hypothetical protein, conserved
Tb927.10.7010;Tb11.v5.0806
AGOF: null
AGOC: null
AGOP: null
PGOF: null
PGOC: null
PGOP: null

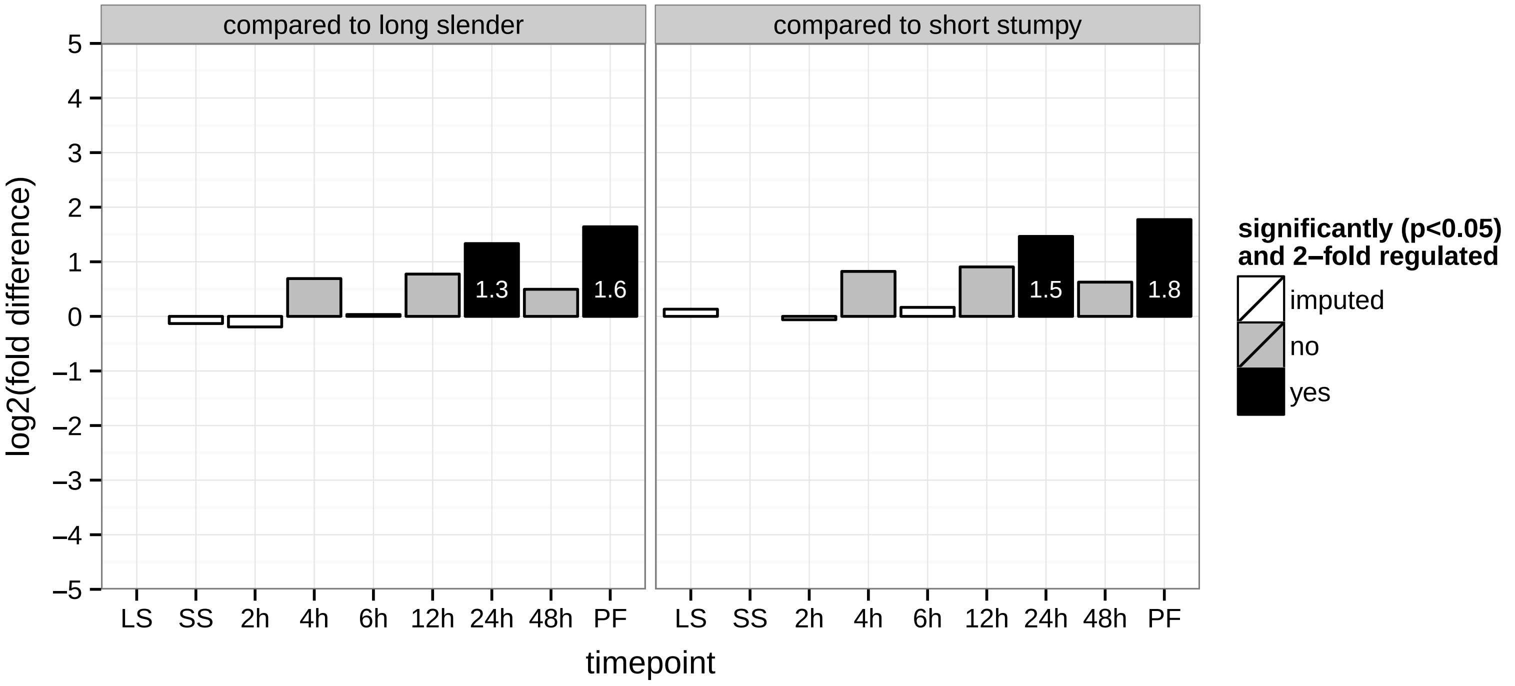

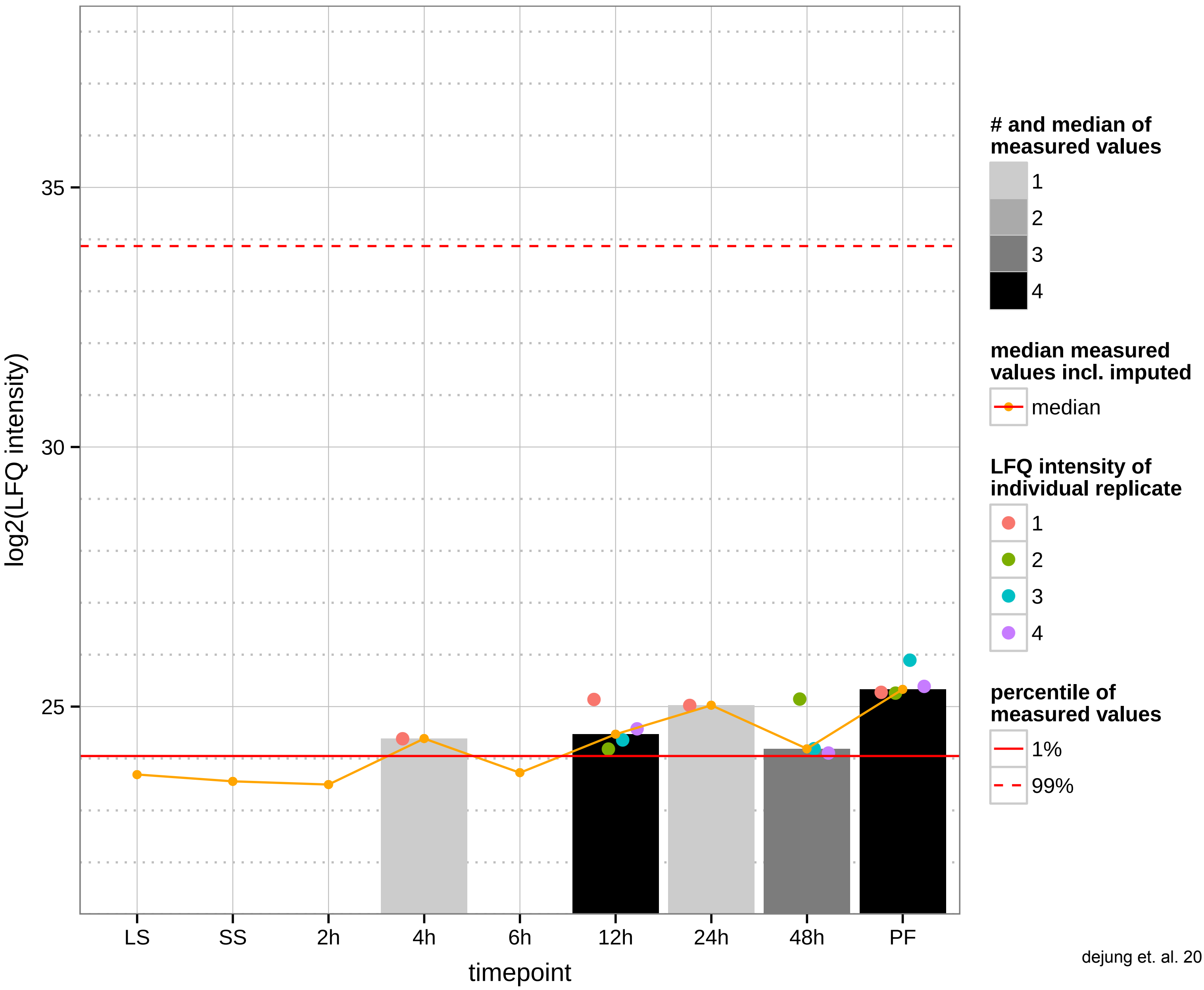

dejung et. al. 2015

hypothetical protein, conserved
Tb927.10.9870
AGOF: null
AGOC: null
AGOP: ciliary or flagellar motility
PGOF: null
PGOC: null
PGOP: null

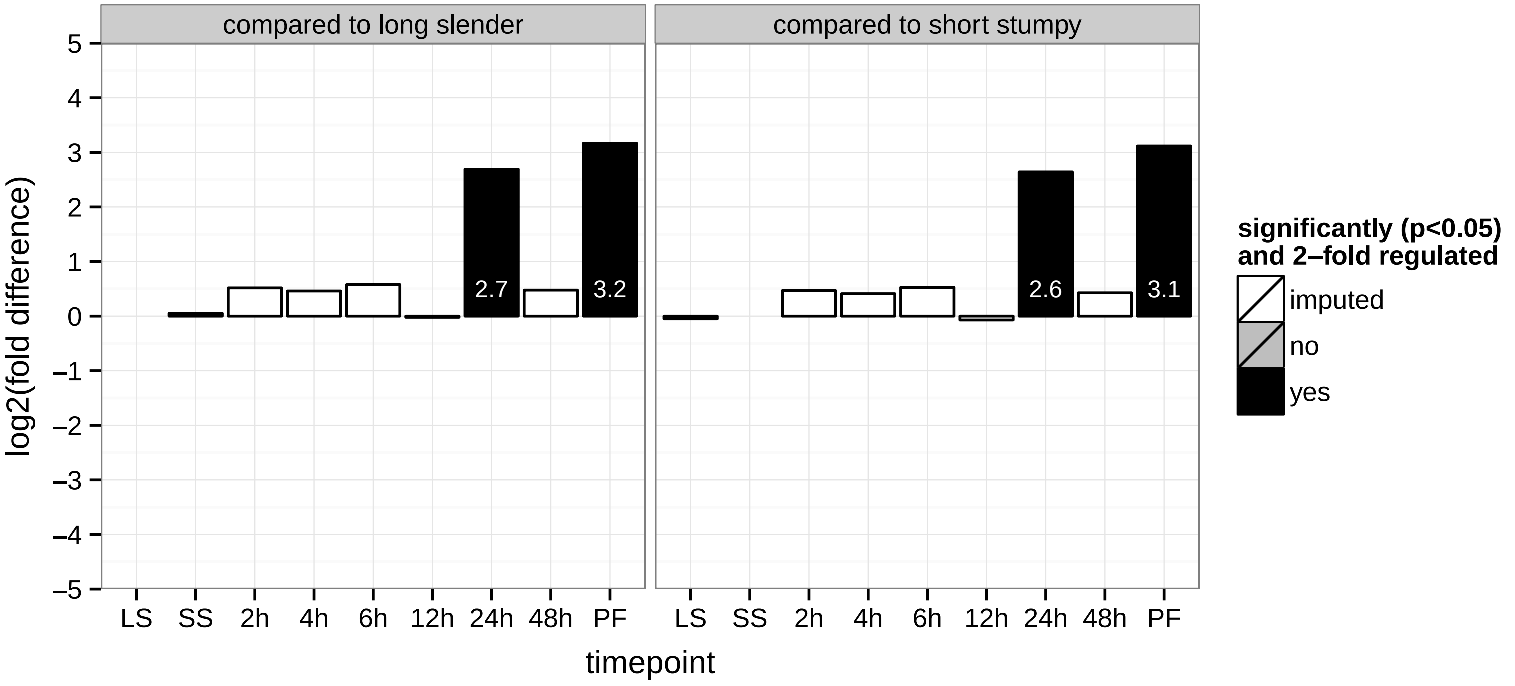

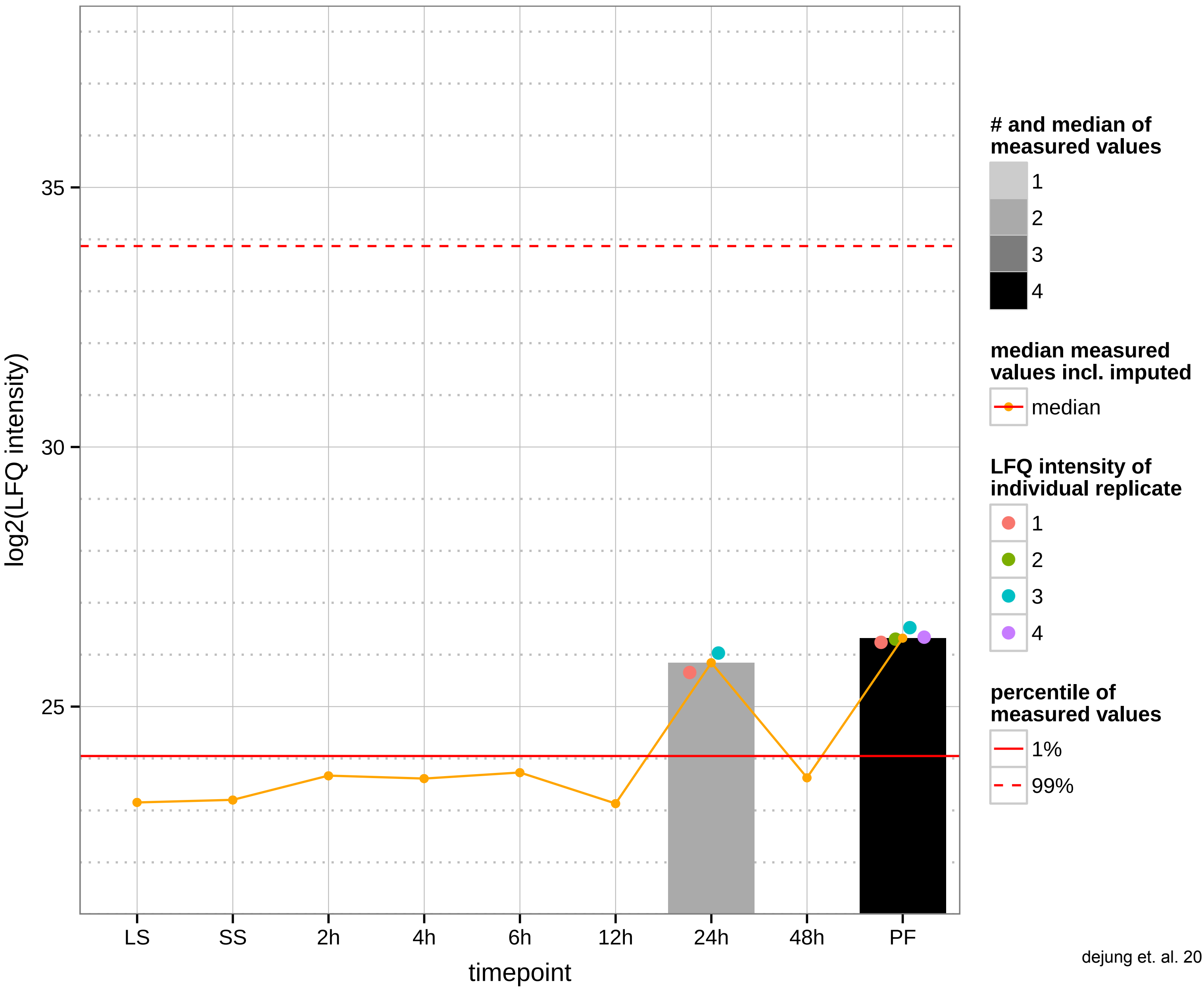

dejung et. al. 2015

hypothetical protein, conserved
Tb927.11.1690
AGOF: cholestenol delta–isomerase activity
AGOC: endoplasmic reticulum, integral to membrane
AGOP: sterol metabolic process
PGOF: cholestenol delta–isomerase activity
PGOC: endoplasmic reticulum, integral to membrane
PGOP: sterol metabolic process

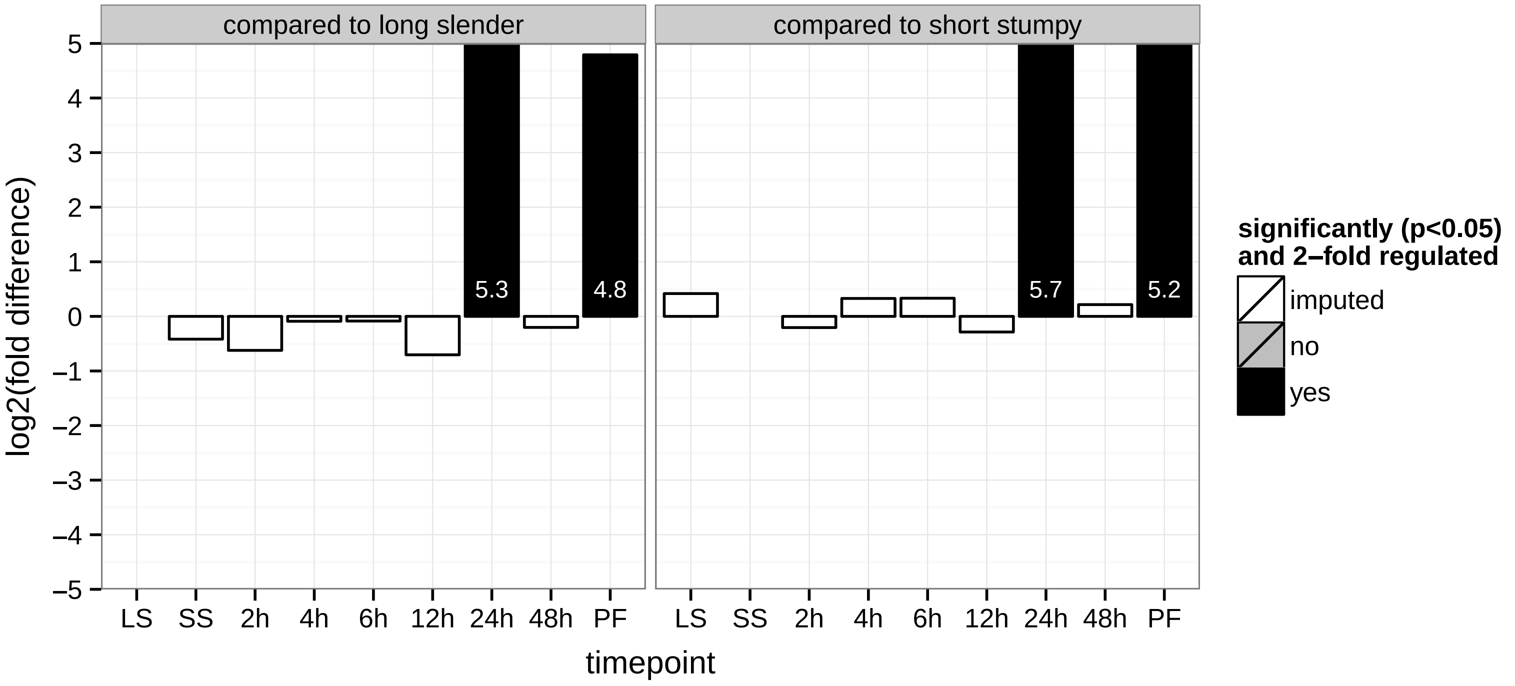

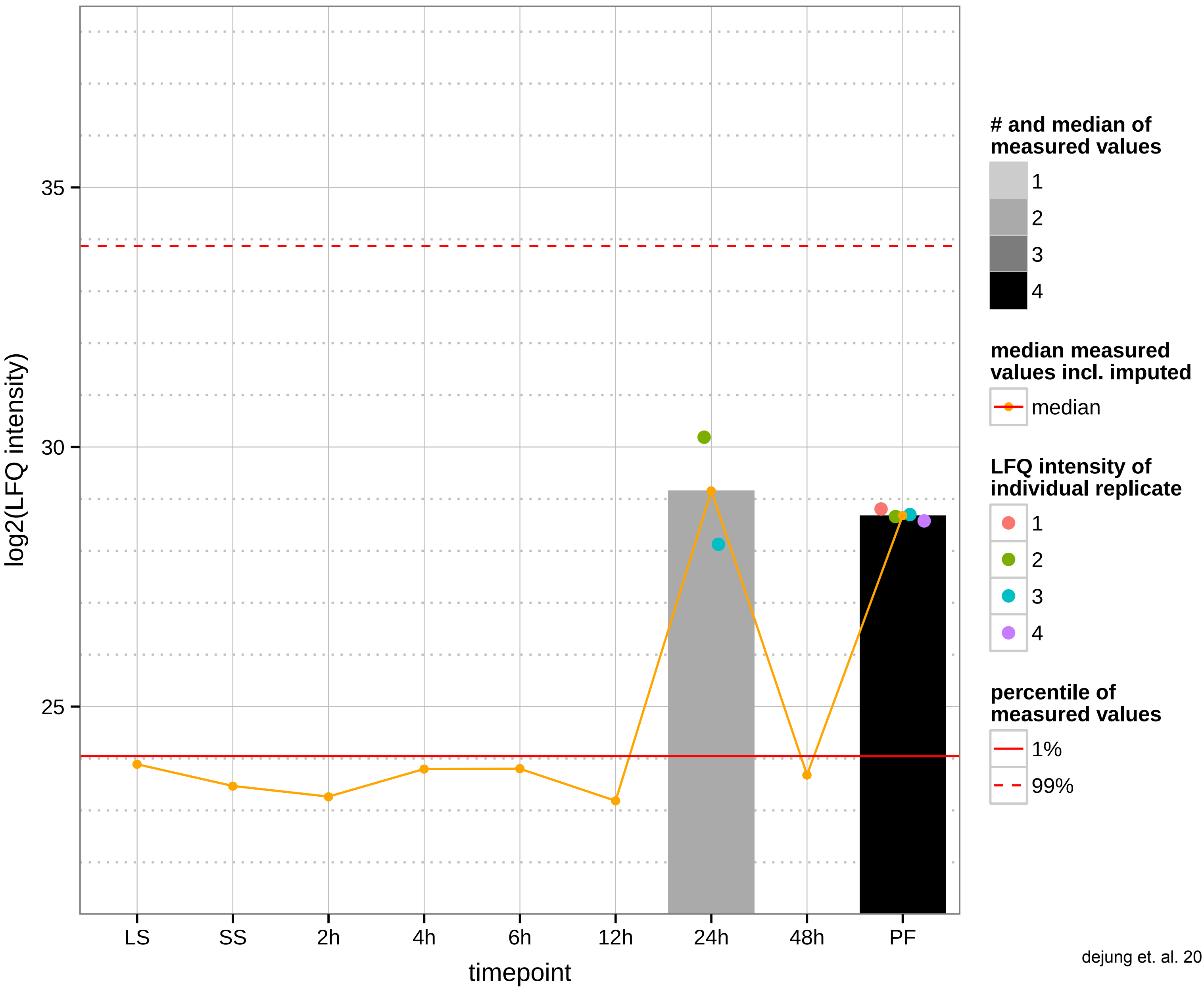

dejung et. al. 2015

hypothetical protein, conserved
Tb927.11.2510
AGOF: null
AGOC: null
AGOP: null
PGOF: binding
PGOC: null
PGOP: null

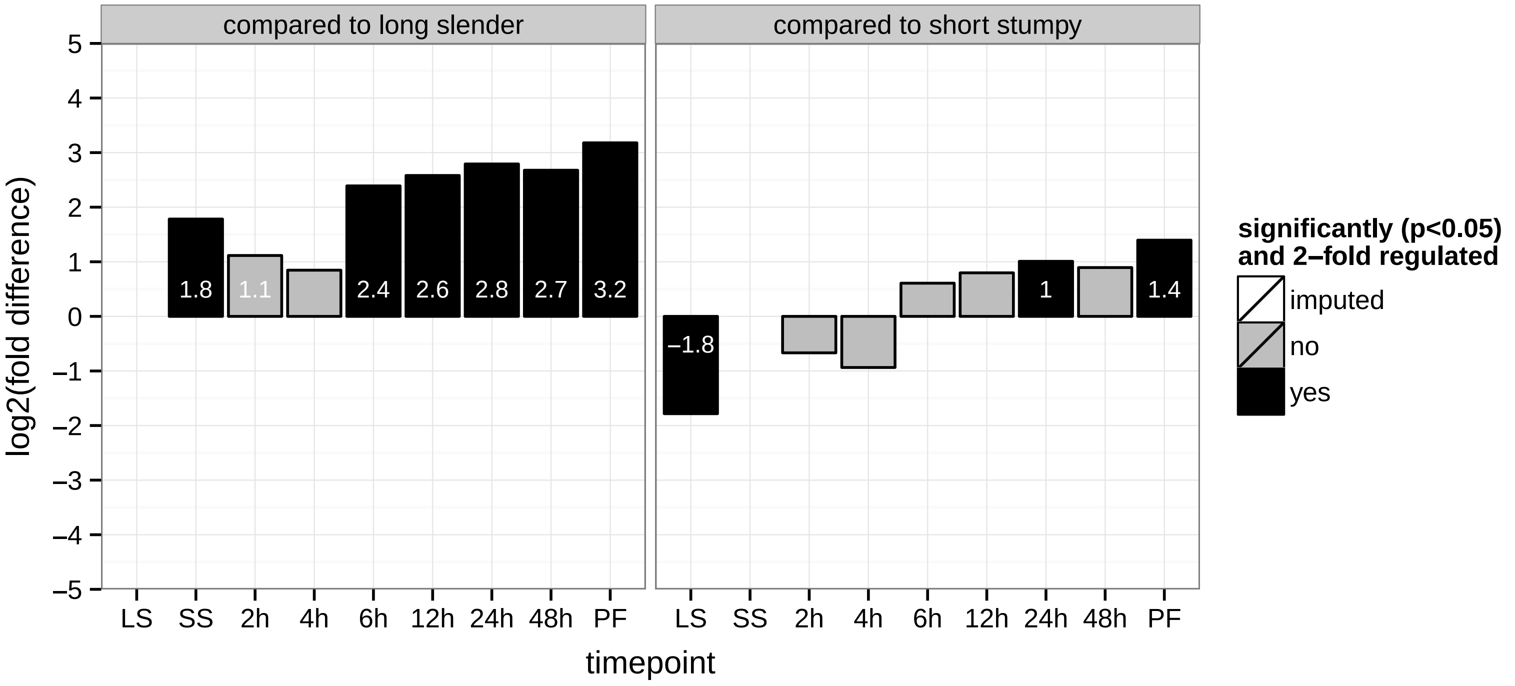

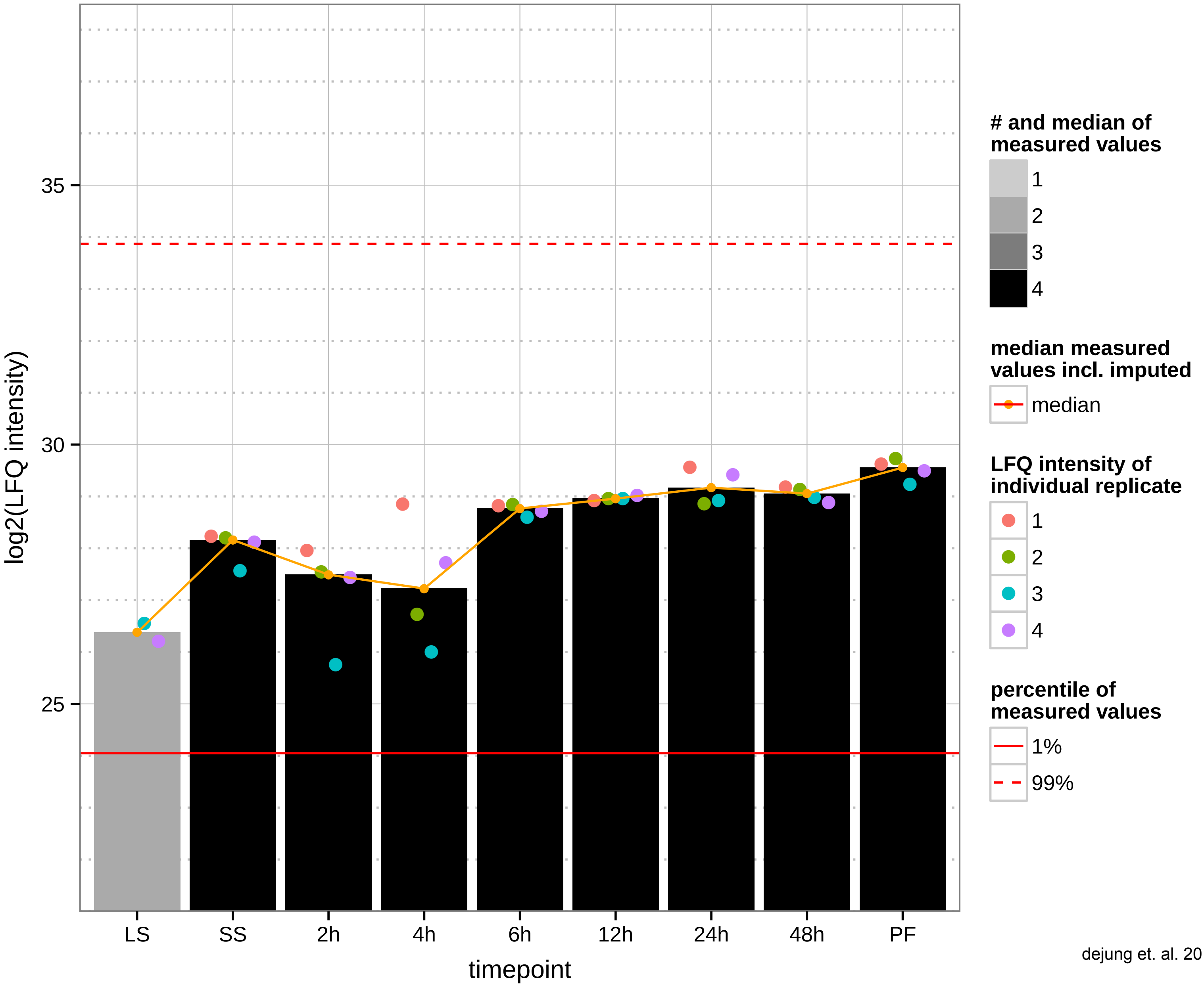

dejung et. al. 2015

60S ribosomal protein L44, ribosomal protein L36a.e (RPL44)
Tb927.2.6090;Tb927.11.3230
AGOF: structural constituent of ribosome
AGOC: intracellular, ribosome
AGOP: translation
PGOF: structural constituent of ribosome
PGOC: intracellular, ribosome
PGOP: translation

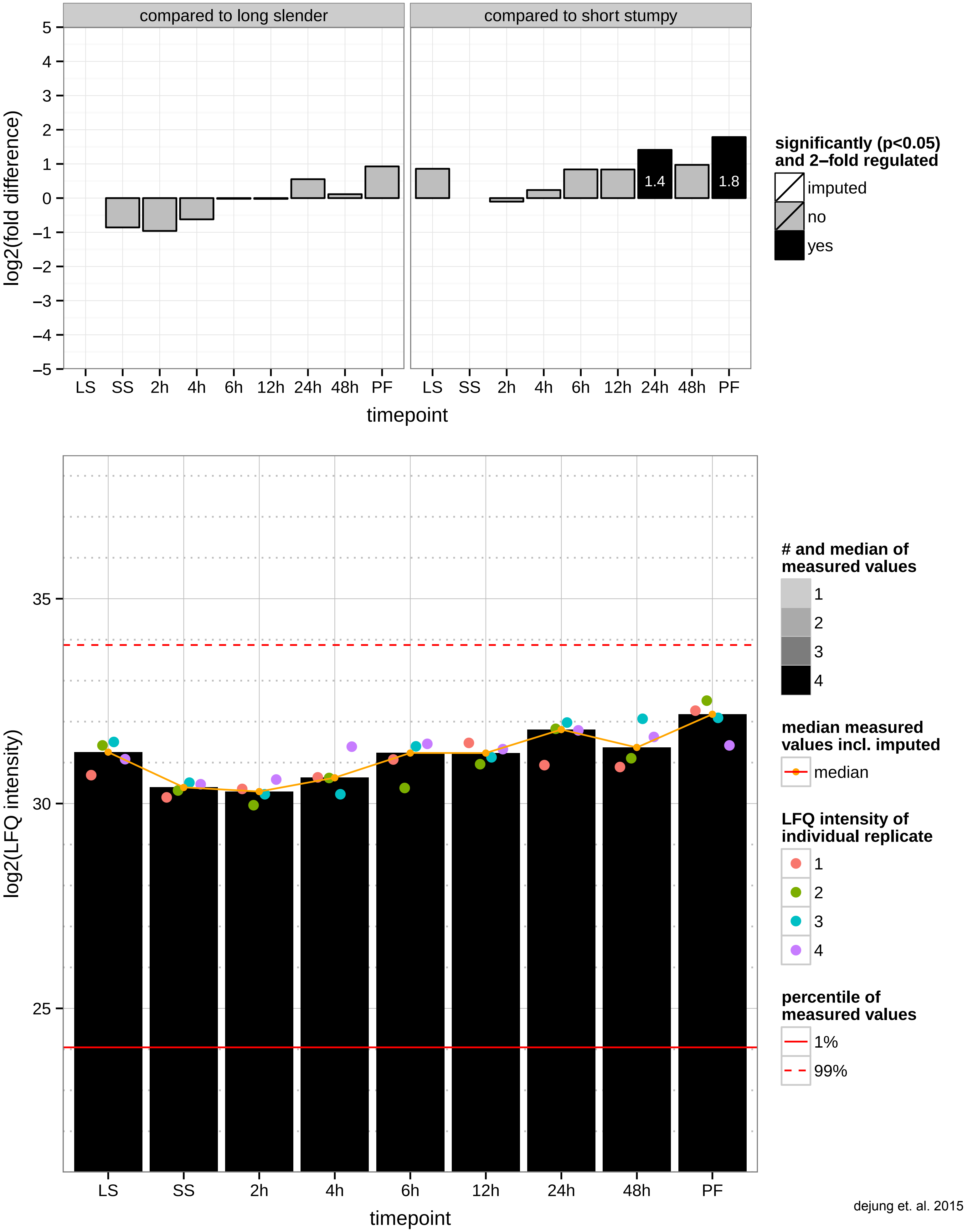

dejung et. al. 2015

hypothetical protein, conserved
Tb927.11.4190
AGOF: null
AGOC: null
AGOP: cellular process
PGOF: null
PGOC: null
PGOP: cellular process

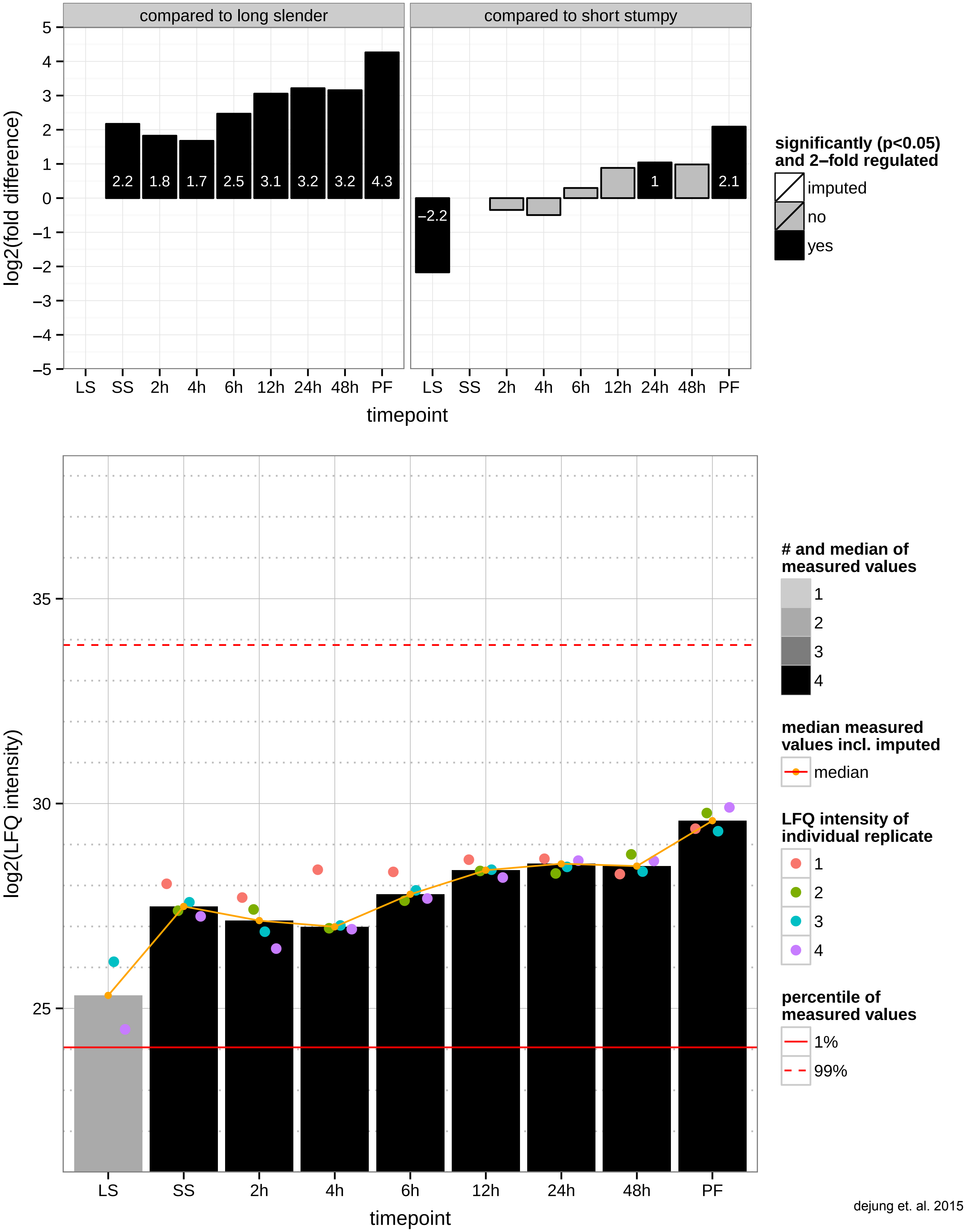

dejung et. al. 2015

hypothetical protein, conserved
Tb927.11.5180
AGOF: null
AGOC: null
AGOP: null
PGOF: null
PGOC: null
PGOP: null

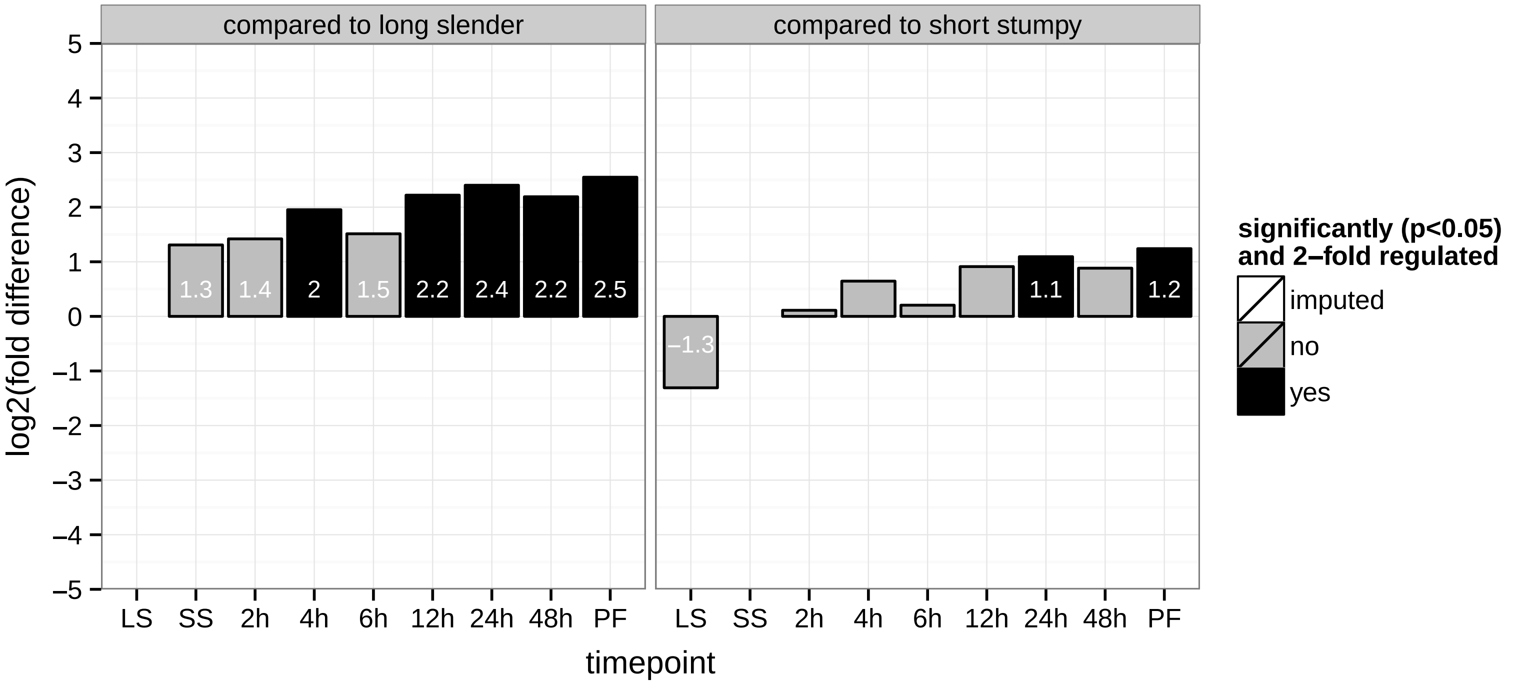

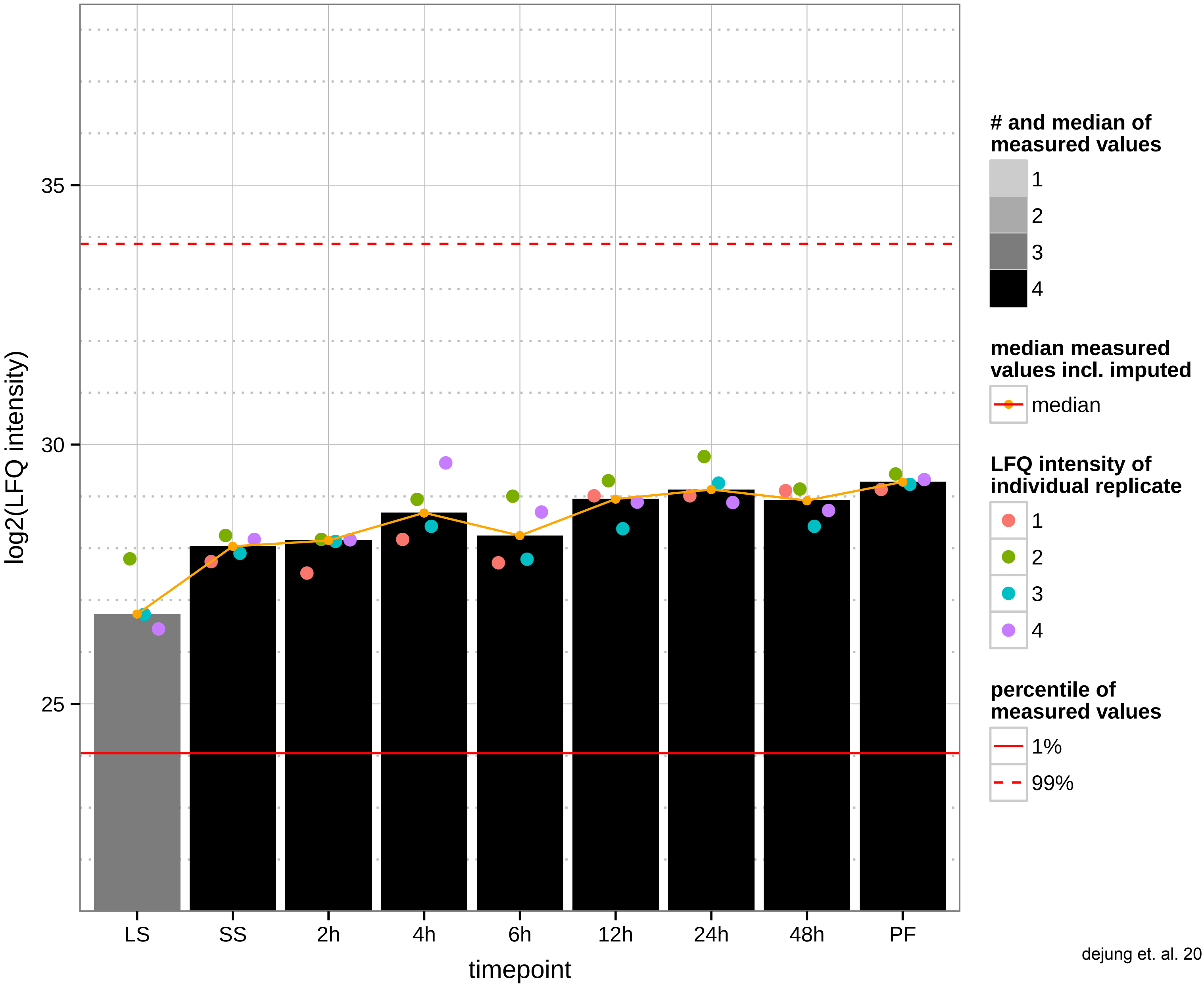

dejung et. al. 2015

hypothetical protein, conserved
Tb927.11.7780
AGOF: null
AGOC: mitochondrial inner membrane, mitochondrion
AGOP: null
PGOF: binding
PGOC: null
PGOP: null

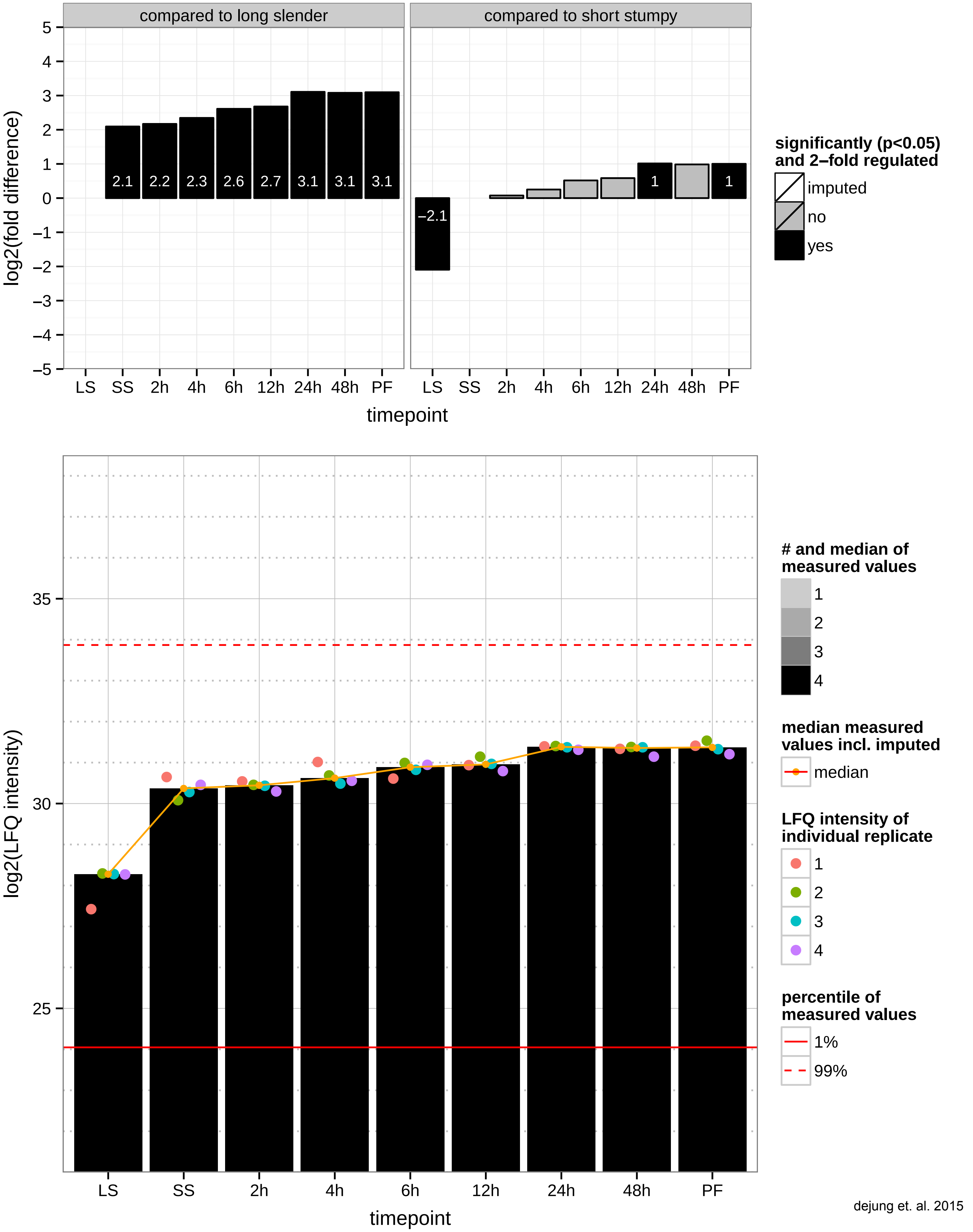

dejung et. al. 2015

retrotransposon hot spot (RHS) protein, putative, retrotransposon hot spot protein (RHS, pseudogene), retrotransposon hot sp
Tb927.2.1100;Tb11.v5.0800
AGOF: null
AGOC: null
AGOP: null
PGOF: null
PGOC: null
PGOP: null

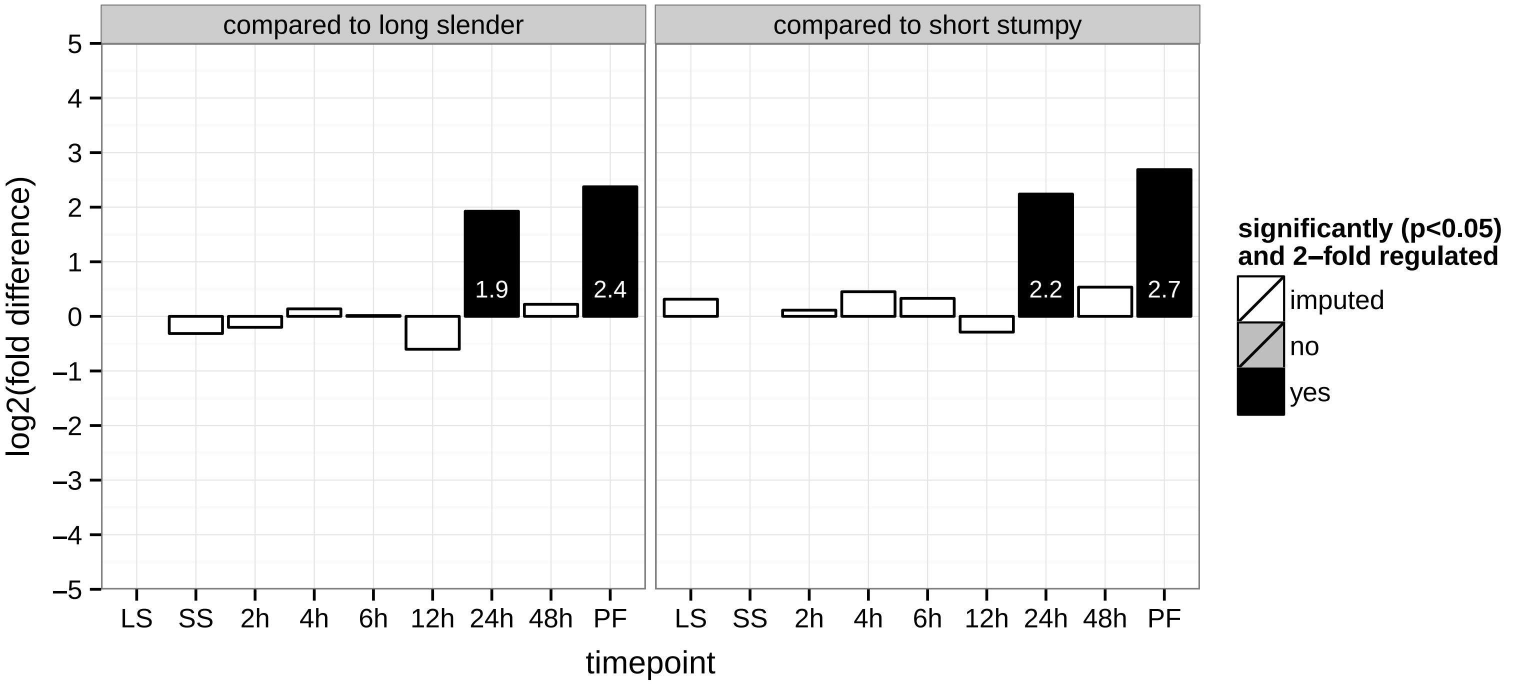

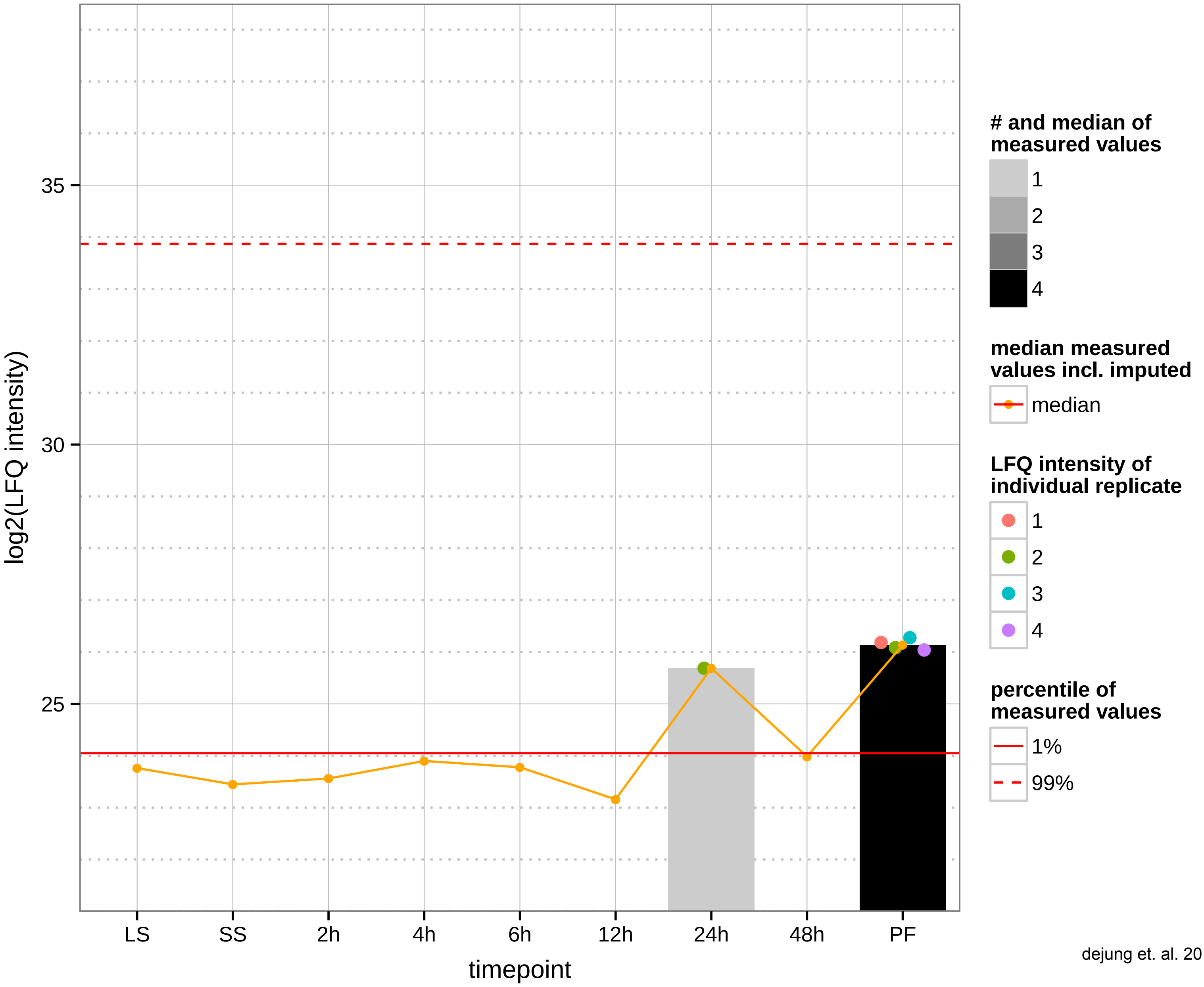

dejung et. al. 2015

hypothetical protein, conserved
Tb927.6.1410
AGOF: null
AGOC: mitochondrion
AGOP: null
PGOF: null
PGOC: null
PGOP: null

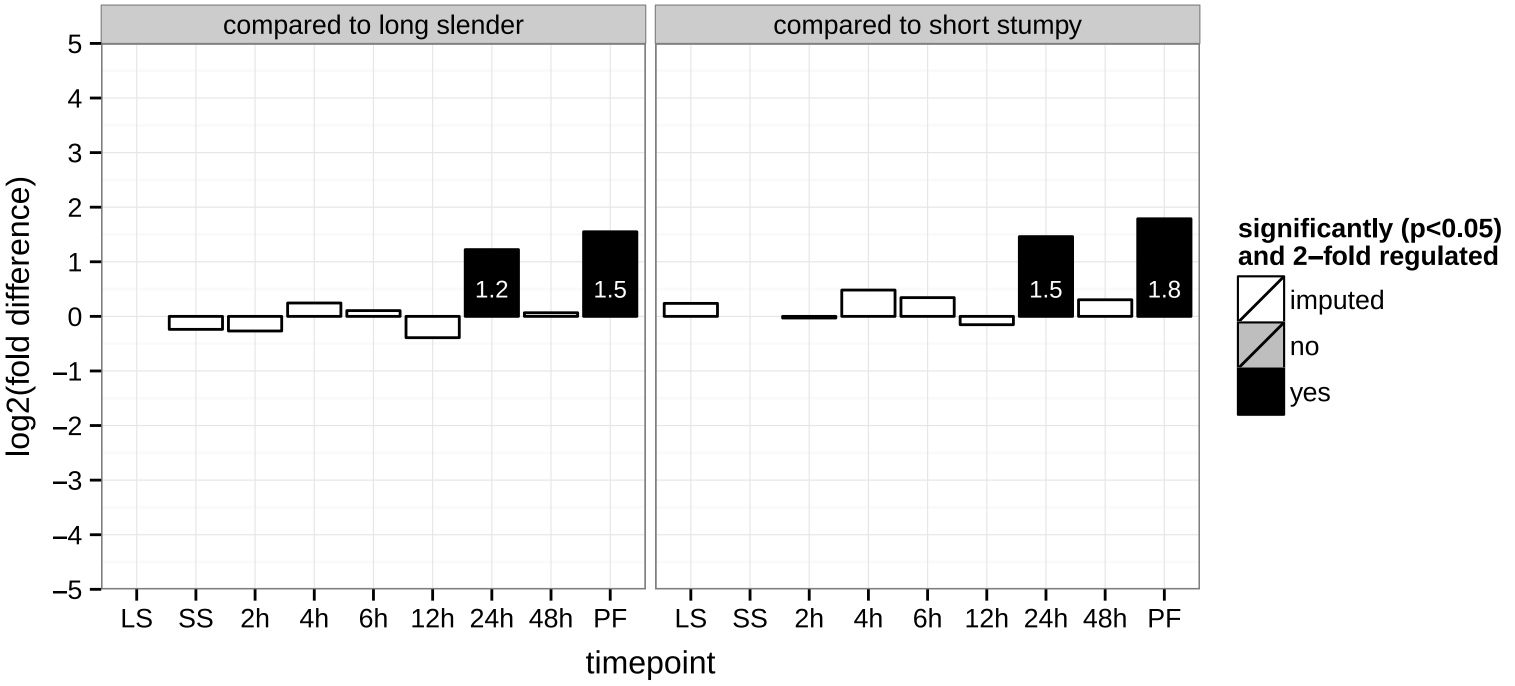

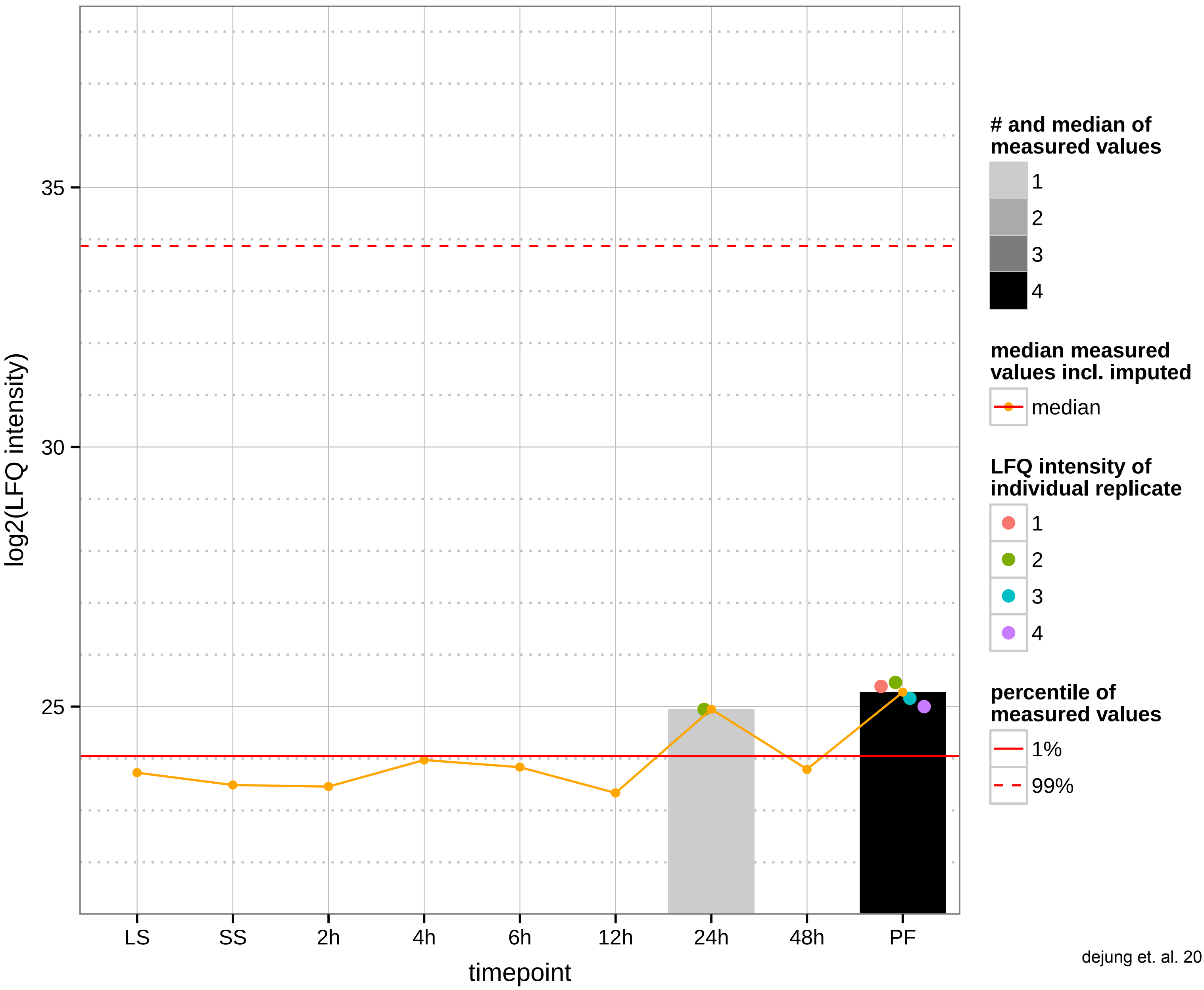

dejung et. al. 2015

hypothetical protein, conserved
Tb927.6.2340;Tb927.6.2280
AGOF: unfolded protein binding
AGOC: prefoldin complex
AGOP: protein folding
PGOF: unfolded protein binding
PGOC: prefoldin complex
PGOP: protein folding

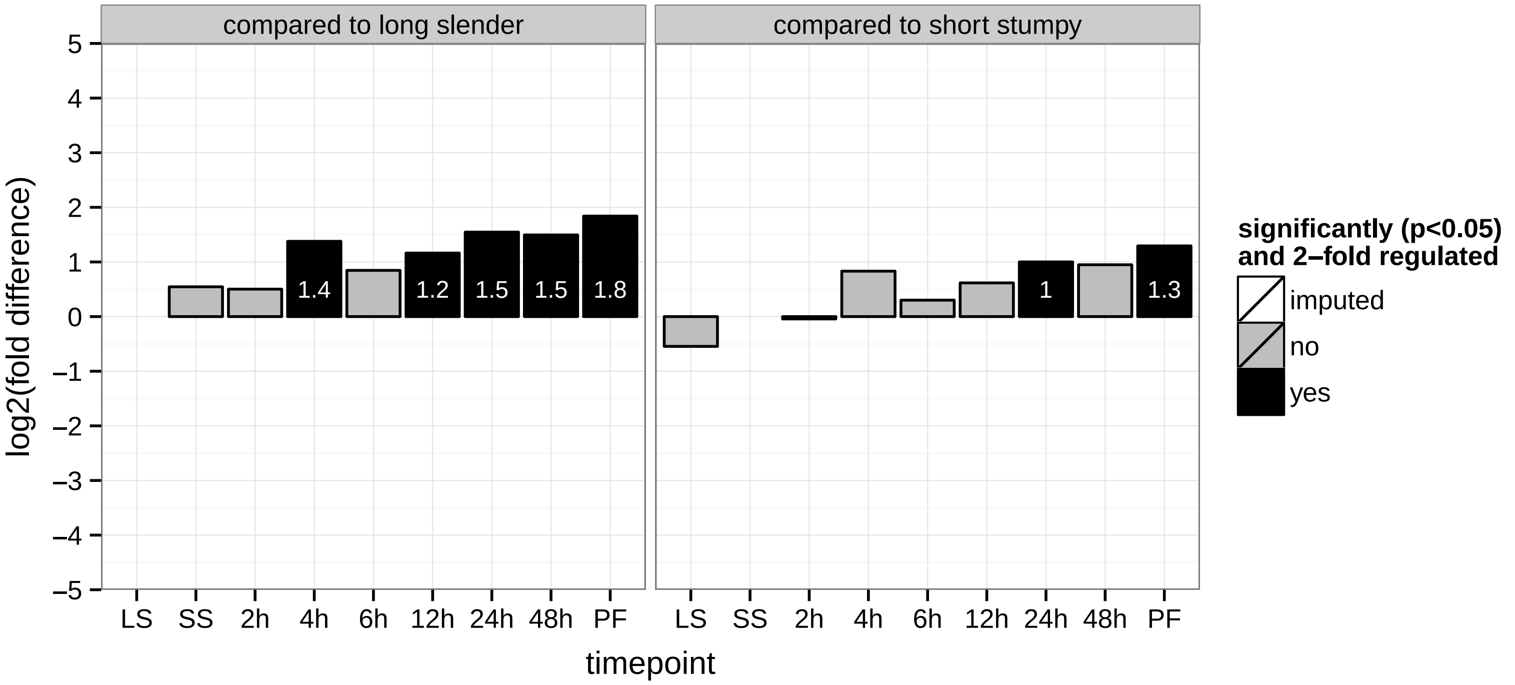

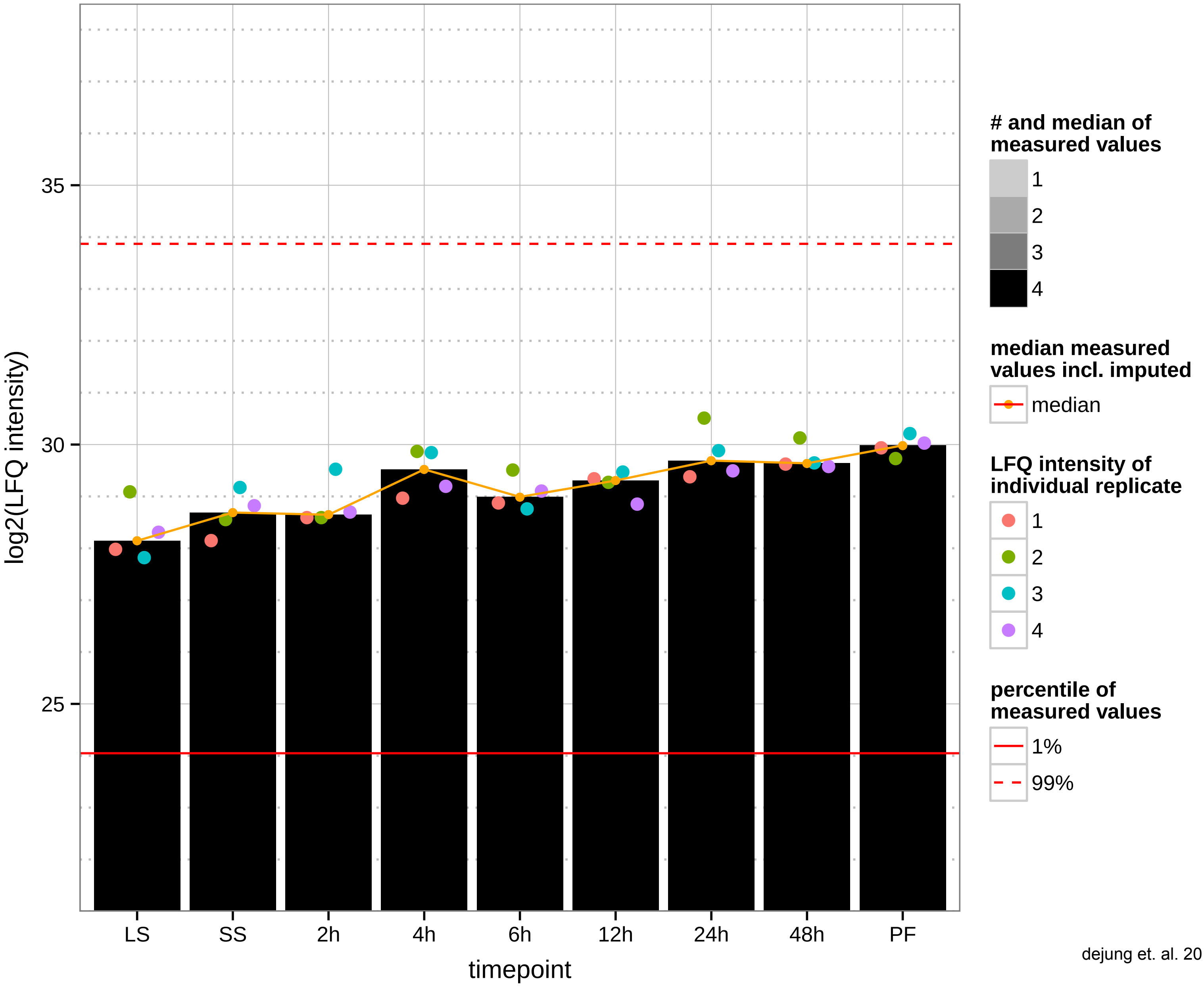

dejung et. al. 2015

hypothetical protein, conserved
Tb927.7.2620
AGOF: null
AGOC: mitochondrion
AGOP: null
PGOF: null
PGOC: null
PGOP: null

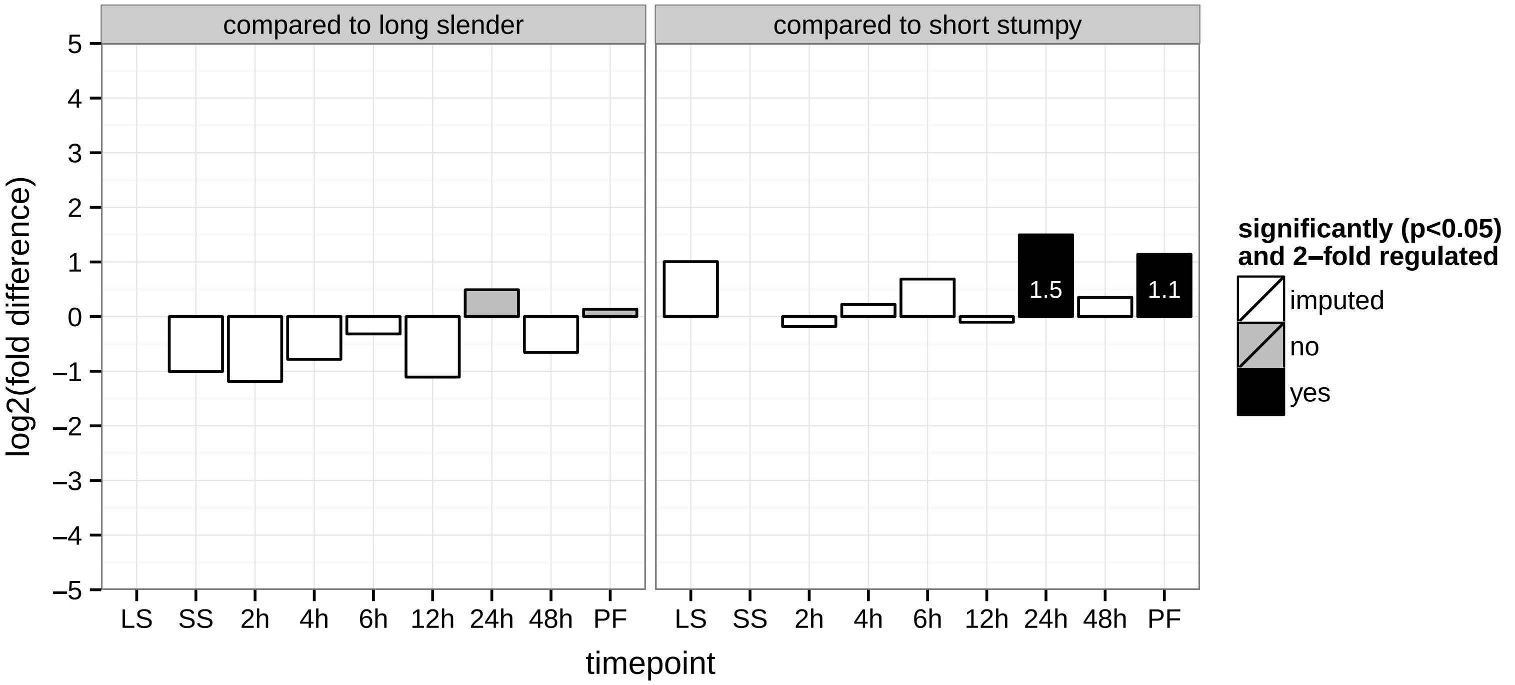

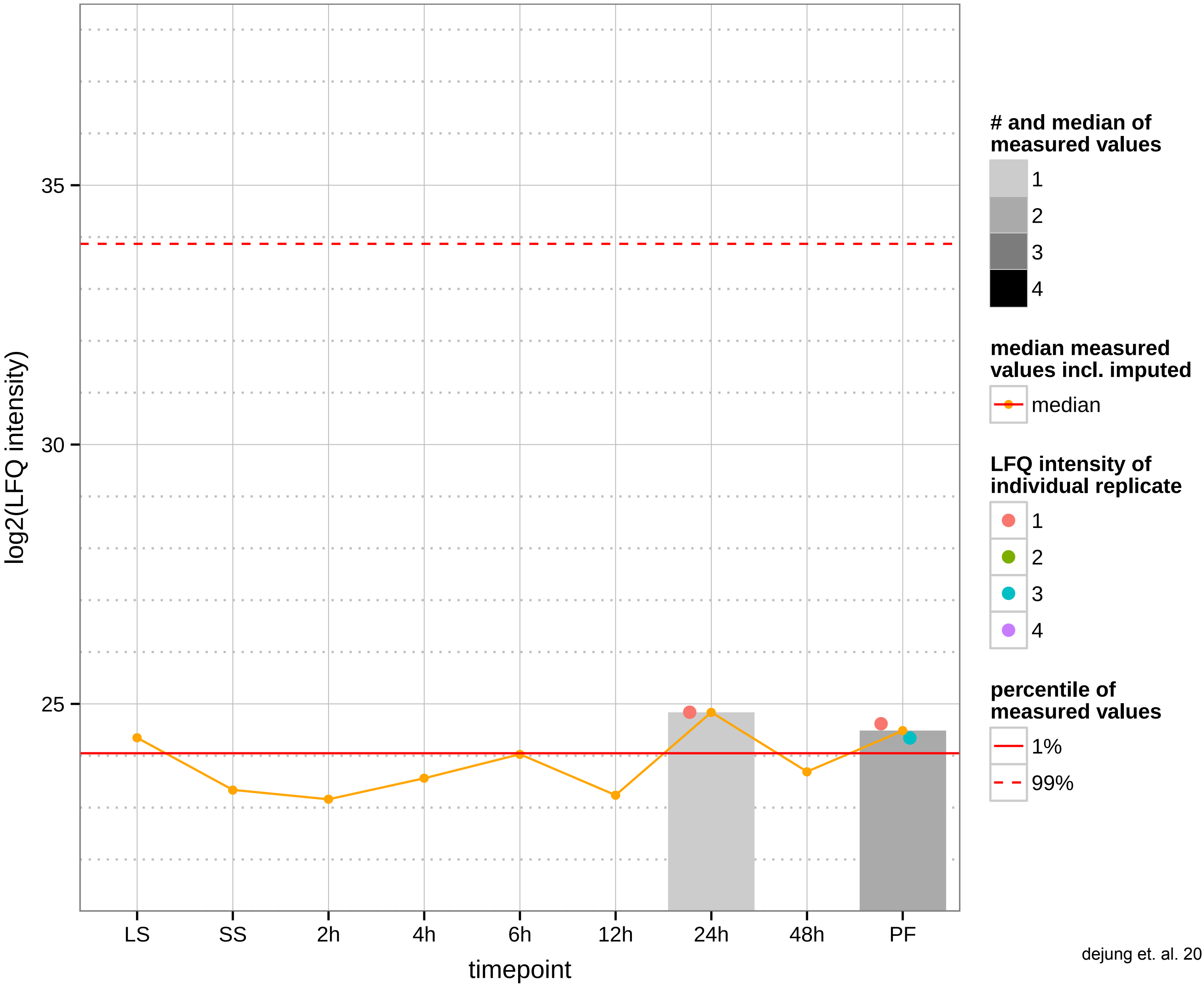

dejung et. al. 2015

hypothetical protein, conserved
Tb927.7.3700
AGOF: null
AGOC: null
AGOP: null
PGOF: null
PGOC: null
PGOP: null

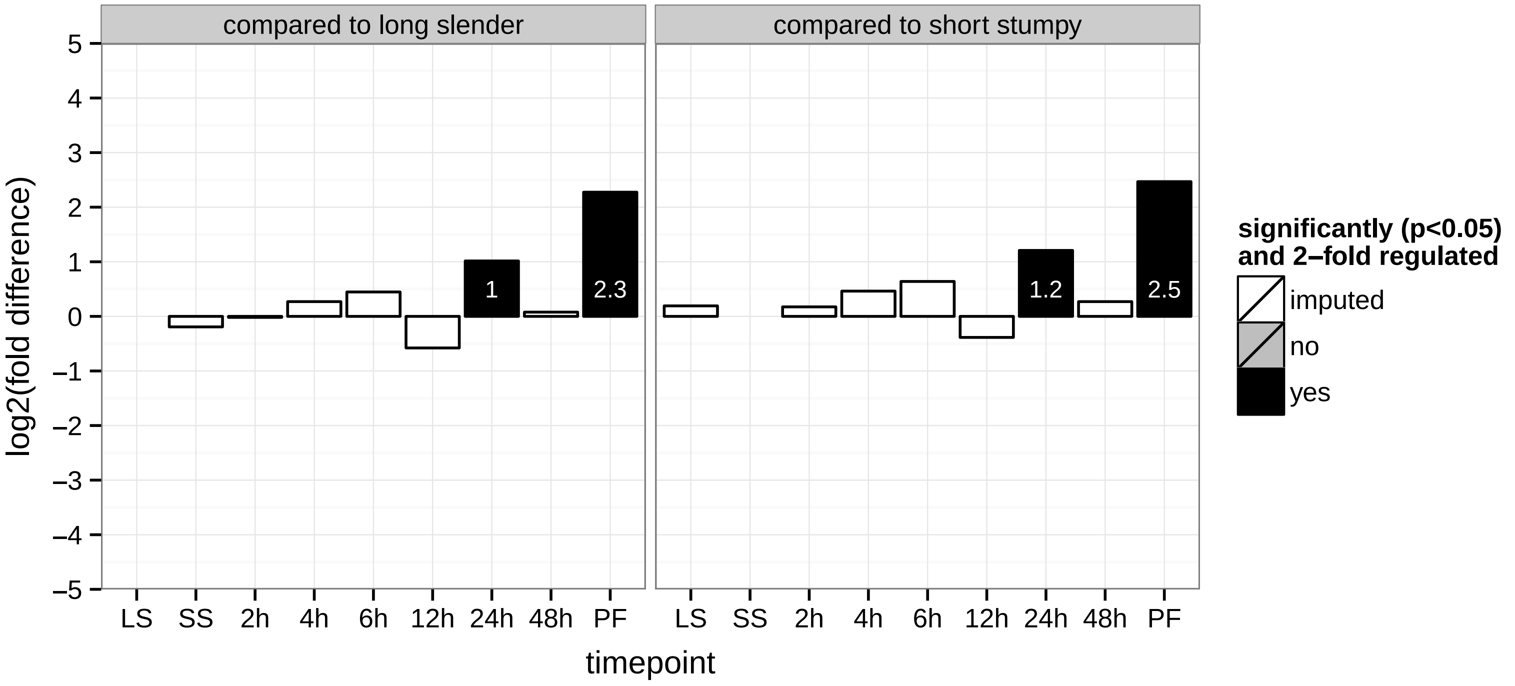

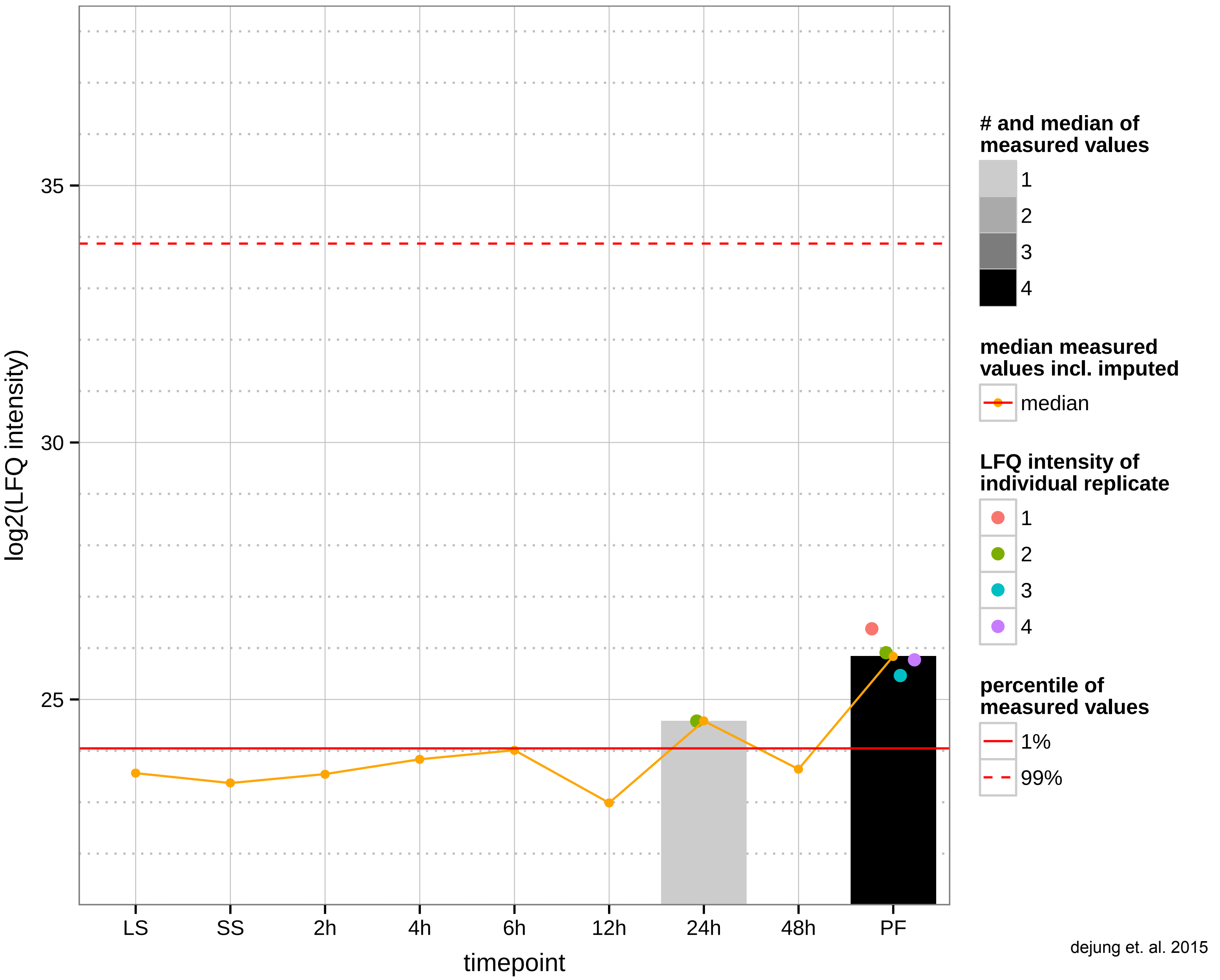

dejung et. al. 2015

lanosterol synthase
Tb927.7.5230
AGOF: lanosterol synthase activity
AGOC: integral to membrane
AGOP: sterol biosynthetic process
PGOF: catalytic activity, intramolecular transferase activity
PGOC: null
PGOP: null

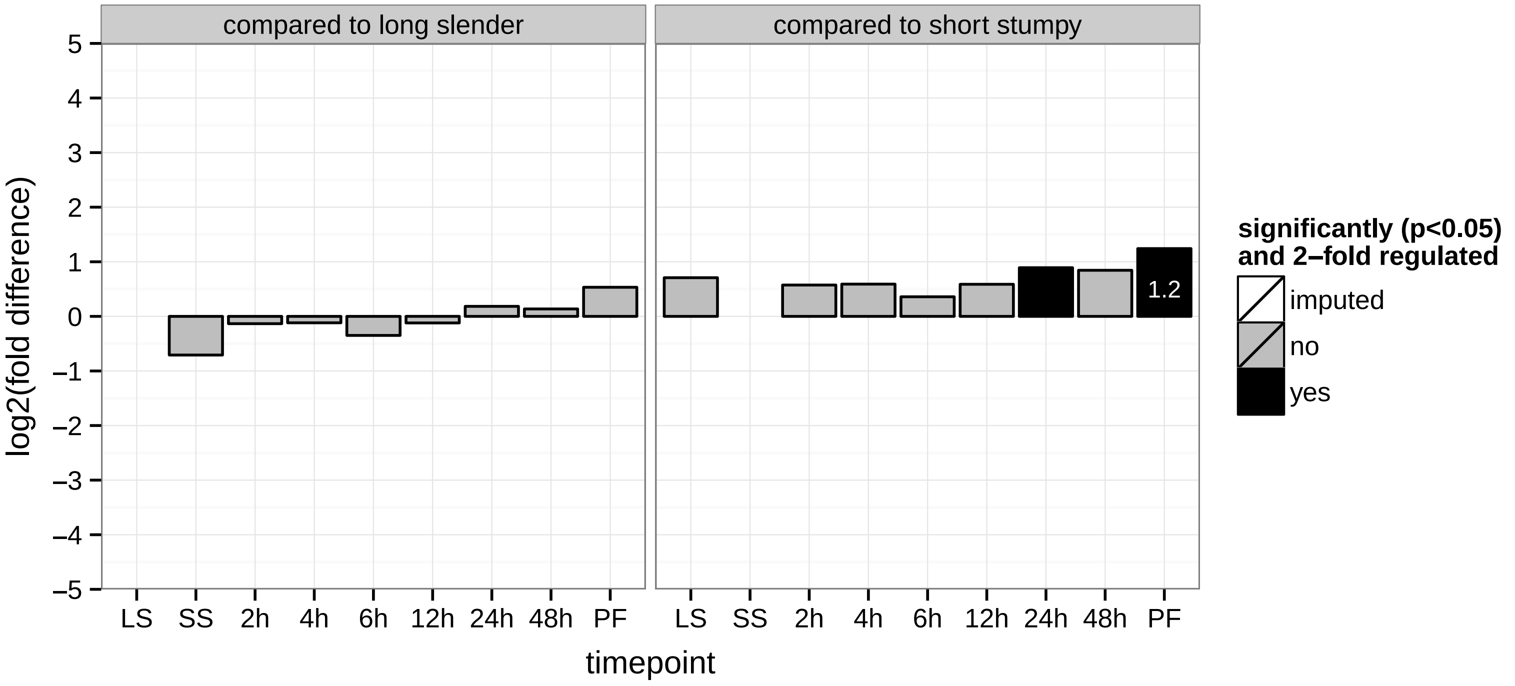

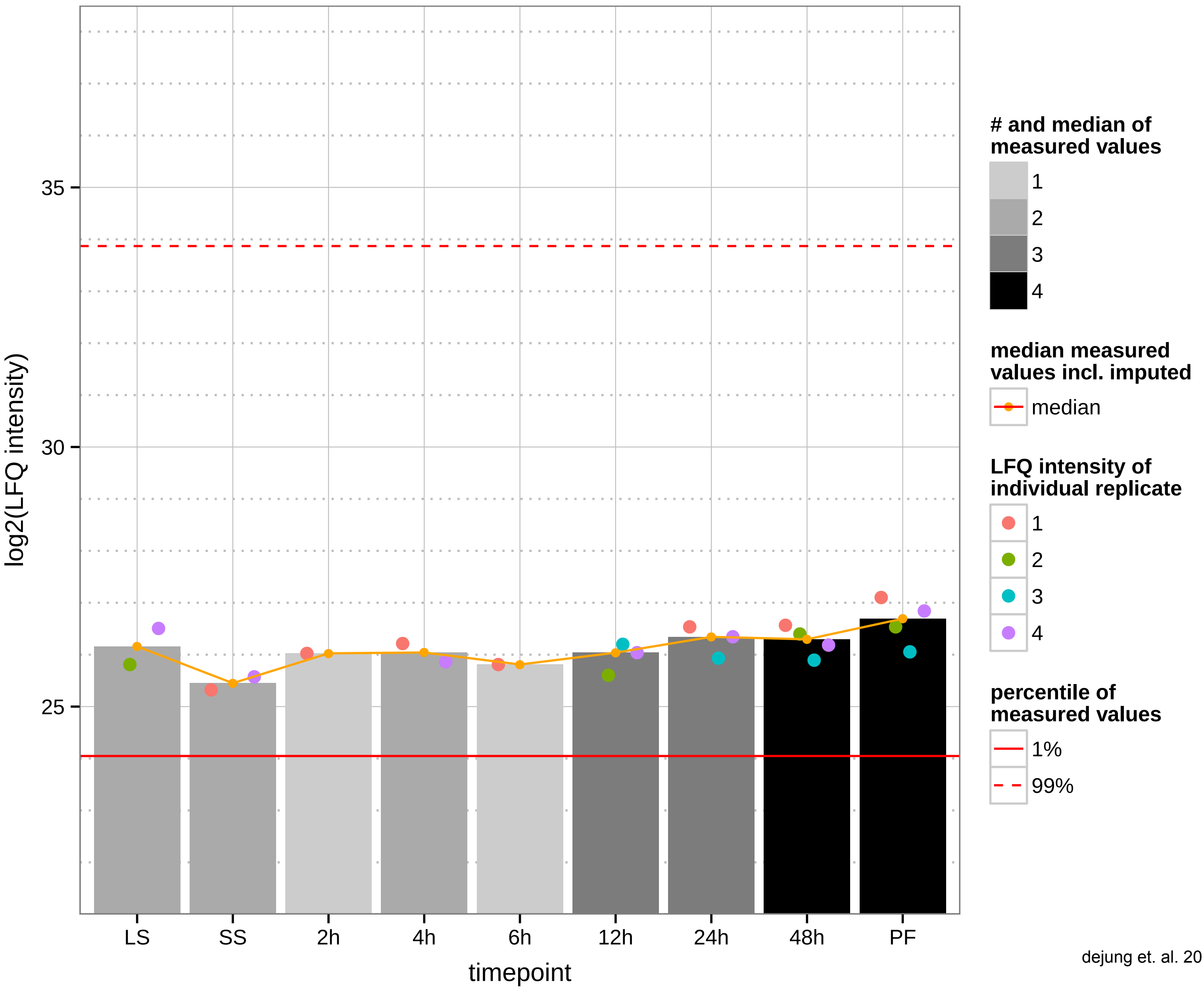

dejung et. al. 2015

5'–3' exonuclease XRNC, putative, exoribonuclease 2, putative (XRNC)
Tb927.8.2810
AGOF: exonuclease activity, nucleic acid binding
AGOC: intracellular, nucleus
AGOP: RNA processing
PGOF: exonuclease activity, nucleic acid binding
PGOC: intracellular
PGOP: null

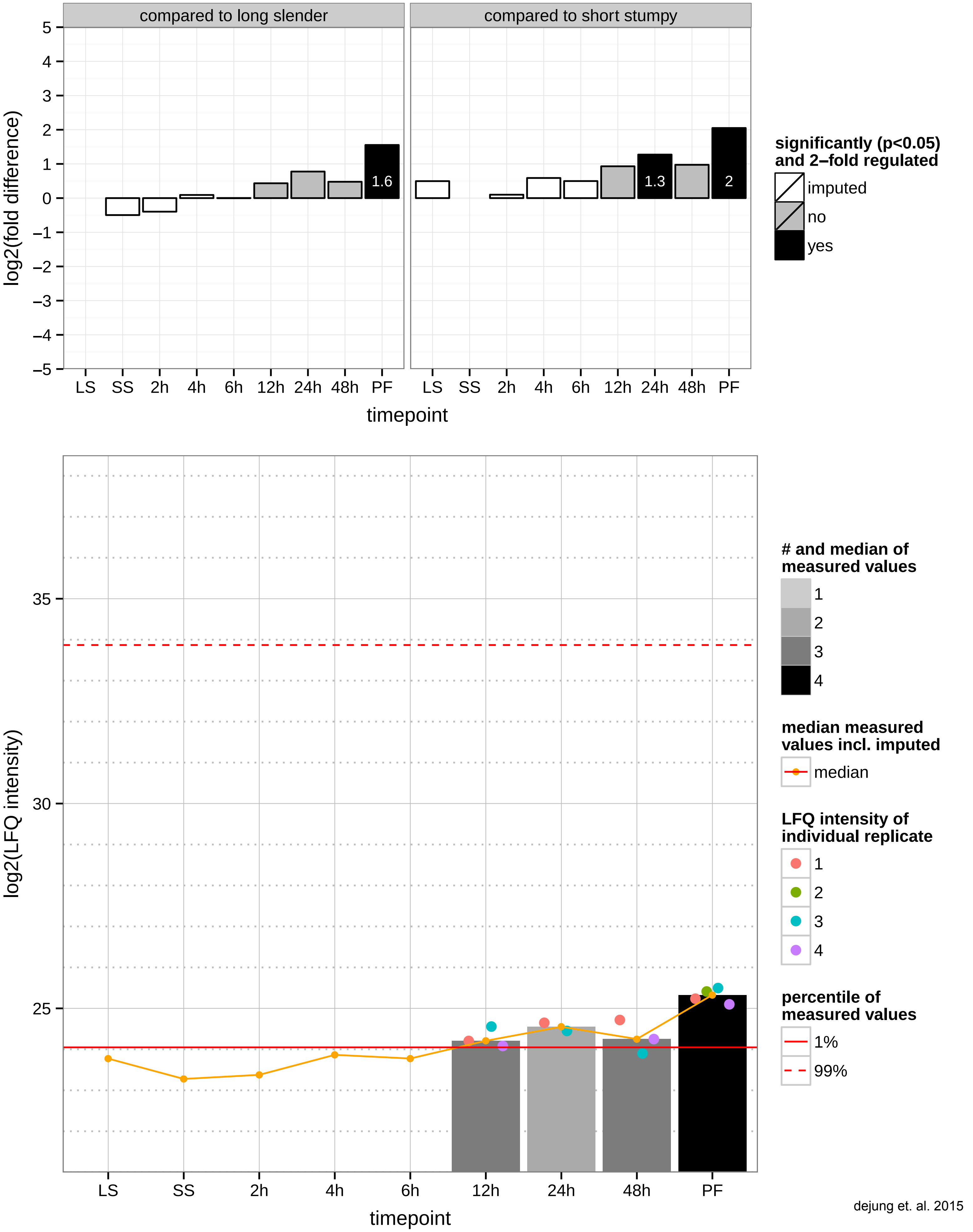

dejung et. al. 2015

hypothetical protein, conserved
Tb927.8.5220
AGOF: null
AGOC: mitochondrion
AGOP: null
PGOF: null
PGOC: null
PGOP: null

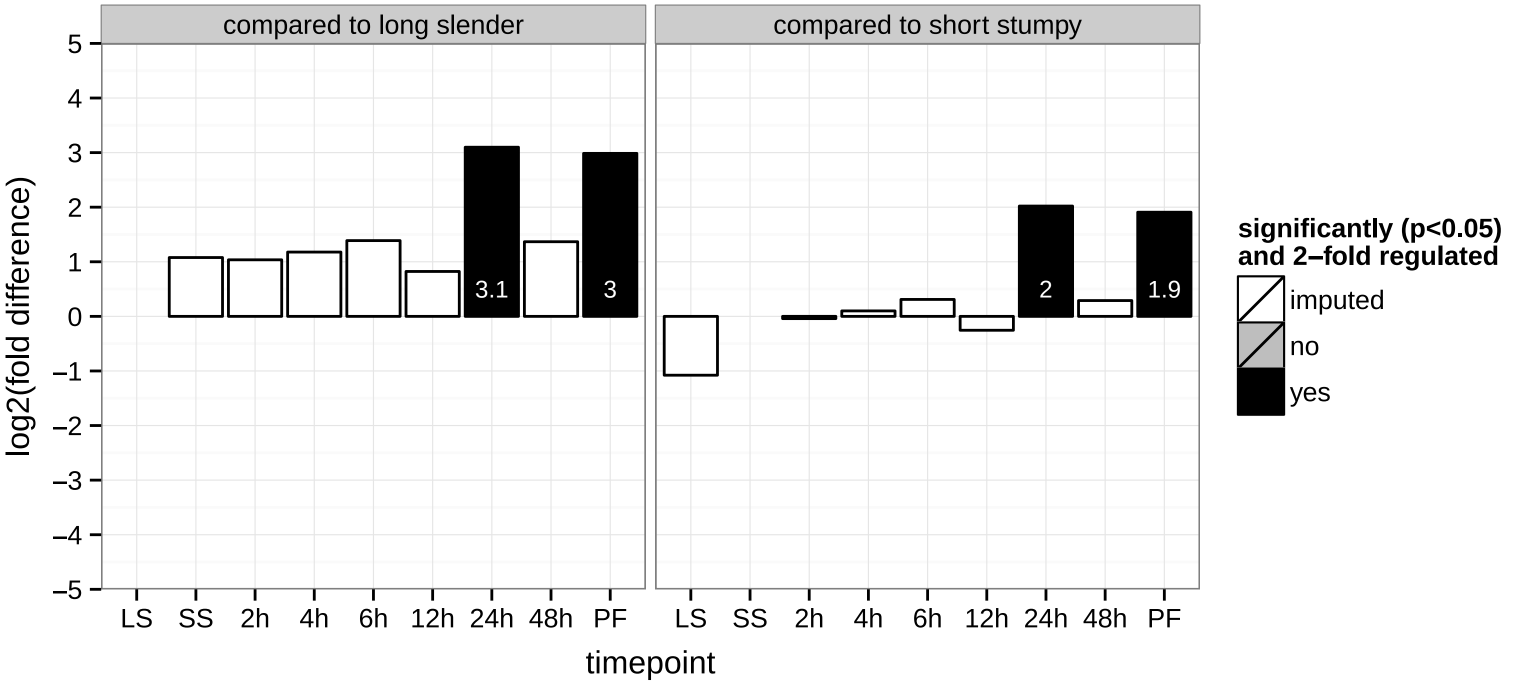

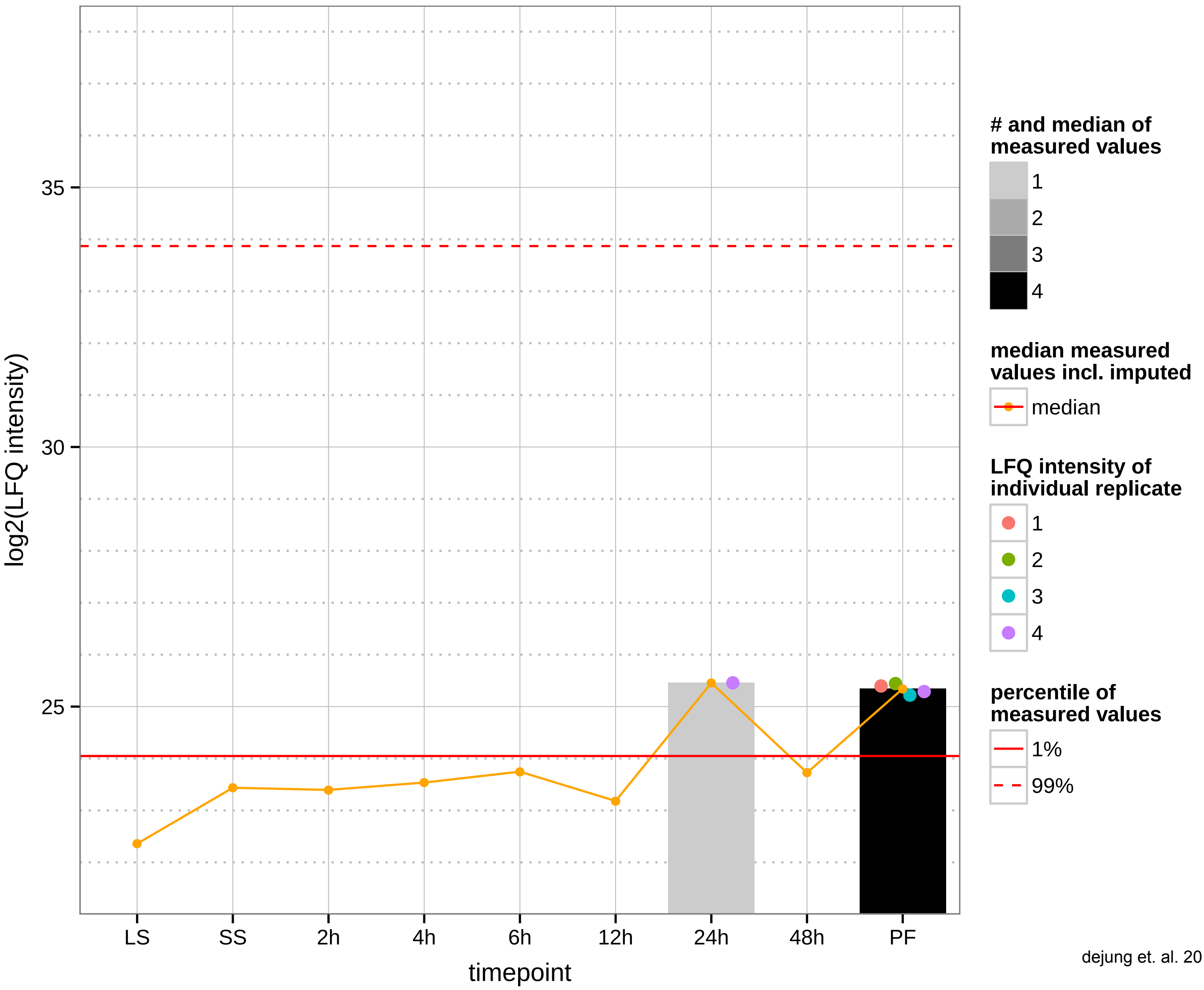

dejung et. al. 2015

hypothetical protein, conserved
Tb927.9.9630
AGOF: null
AGOC: null
AGOP: null
PGOF: protein binding
PGOC: null
PGOP: null

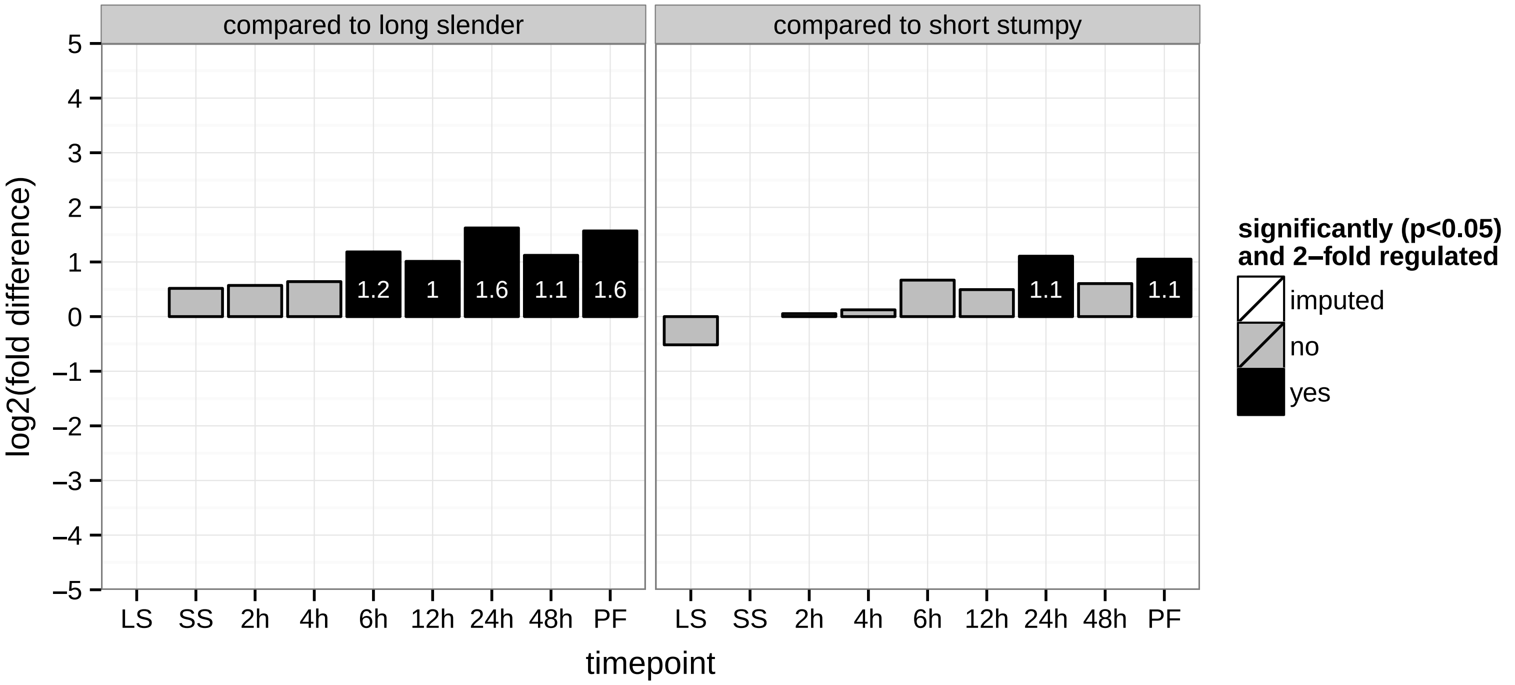

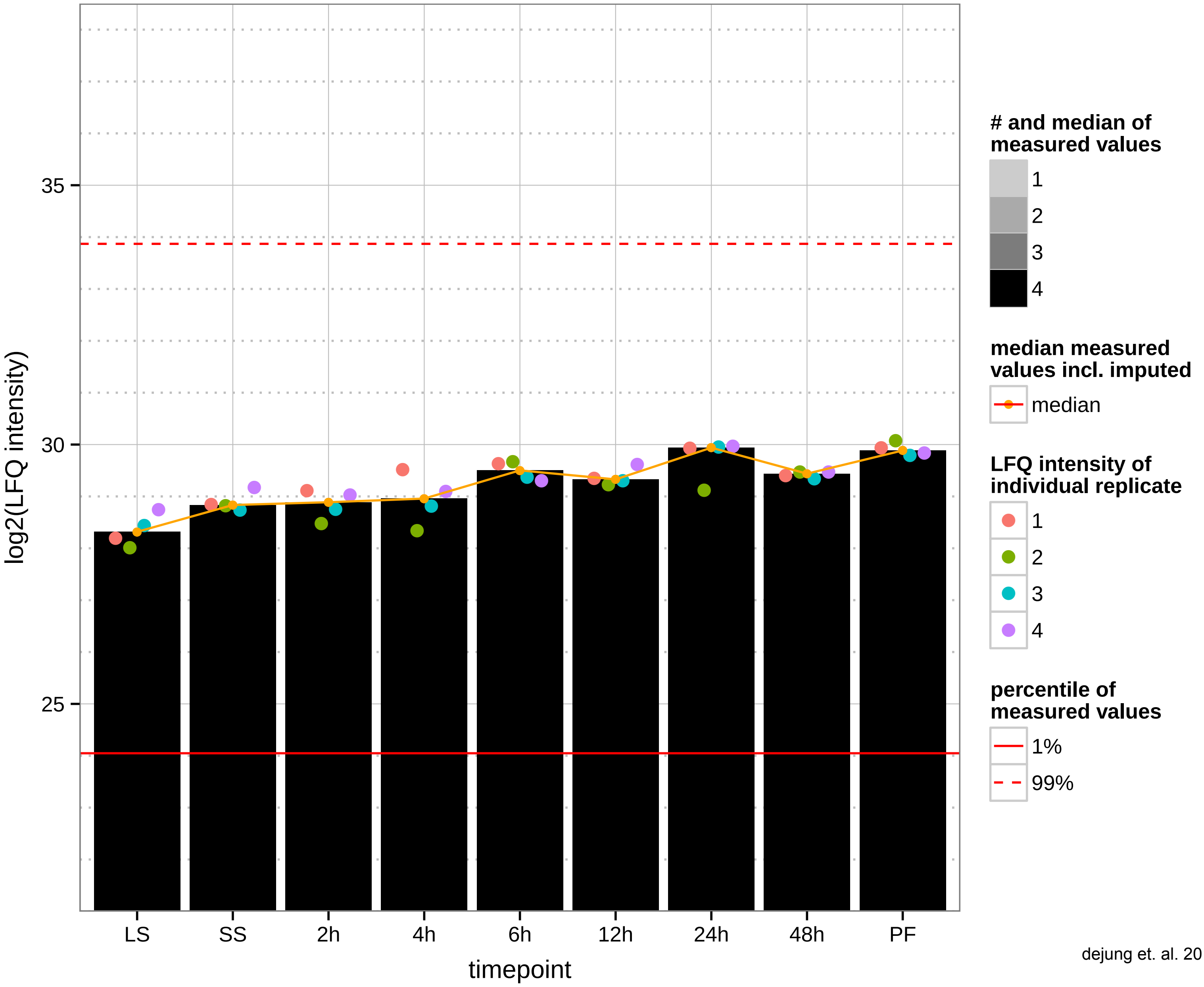

dejung et. al. 2015

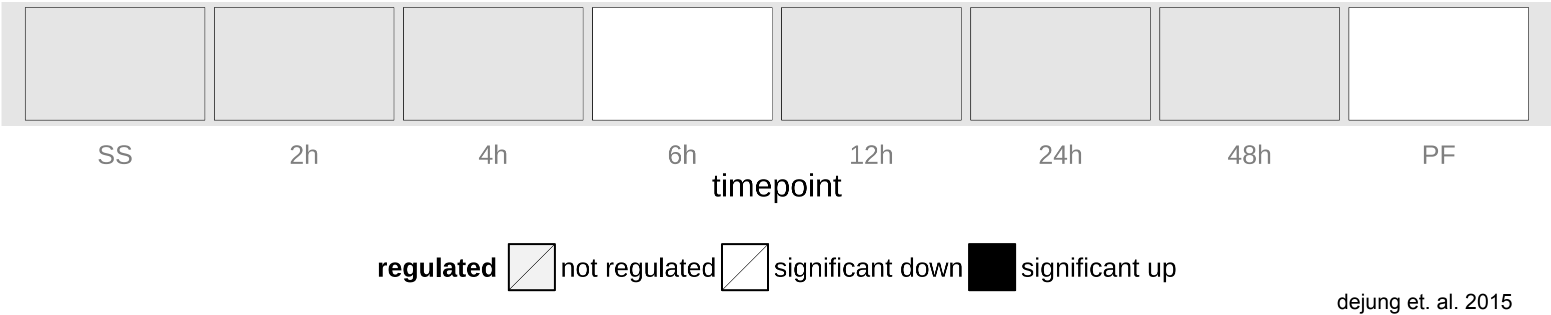
SS
2h
4h
6h
12h
24h
48h
PF
timepoint
regulated
not regulated
significant down
significant up
dejung et. al. 2015

hypothetical protein, conserved, hypothetical transmembrane protein
Tb927.1.790;Tb11.v5.0524
AGOF: null
AGOC: null, mitochondrion
AGOP: null
PGOF: null
PGOC: null
PGOP: null

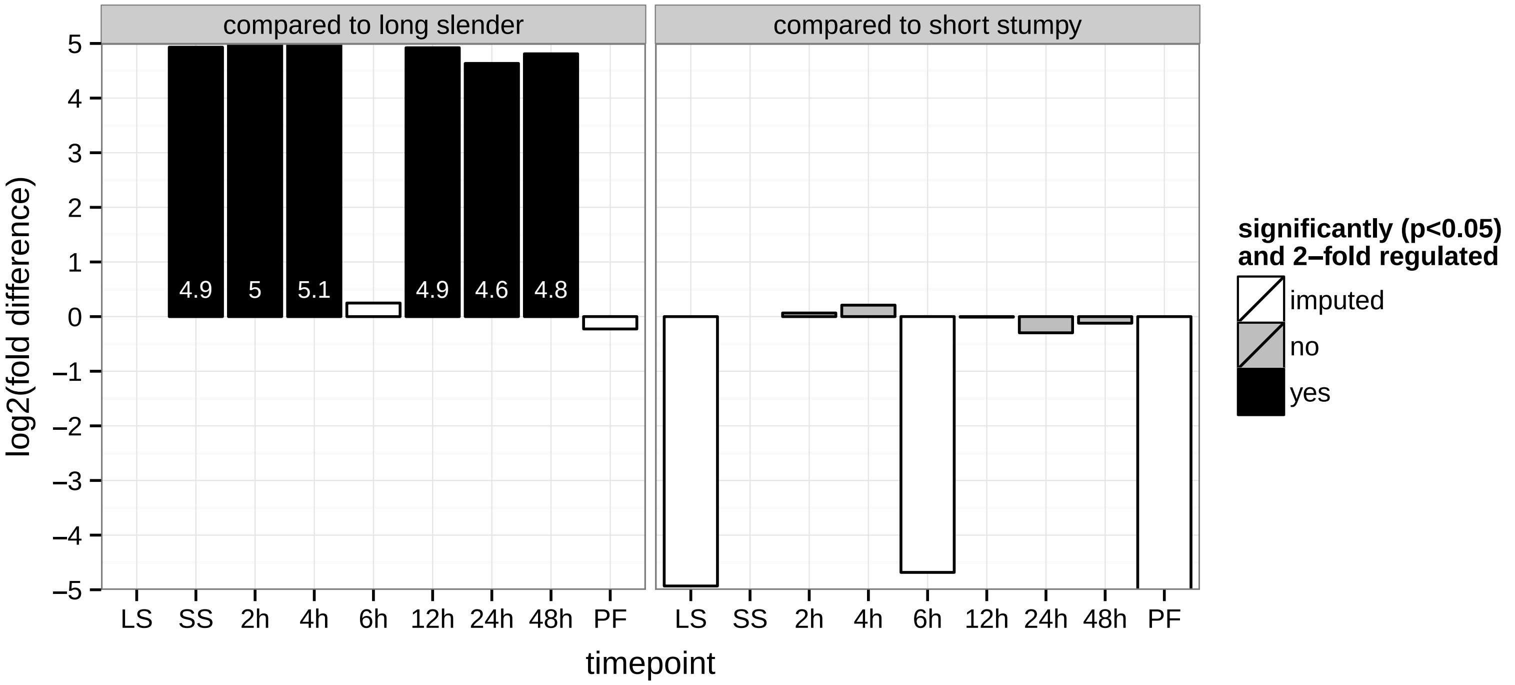

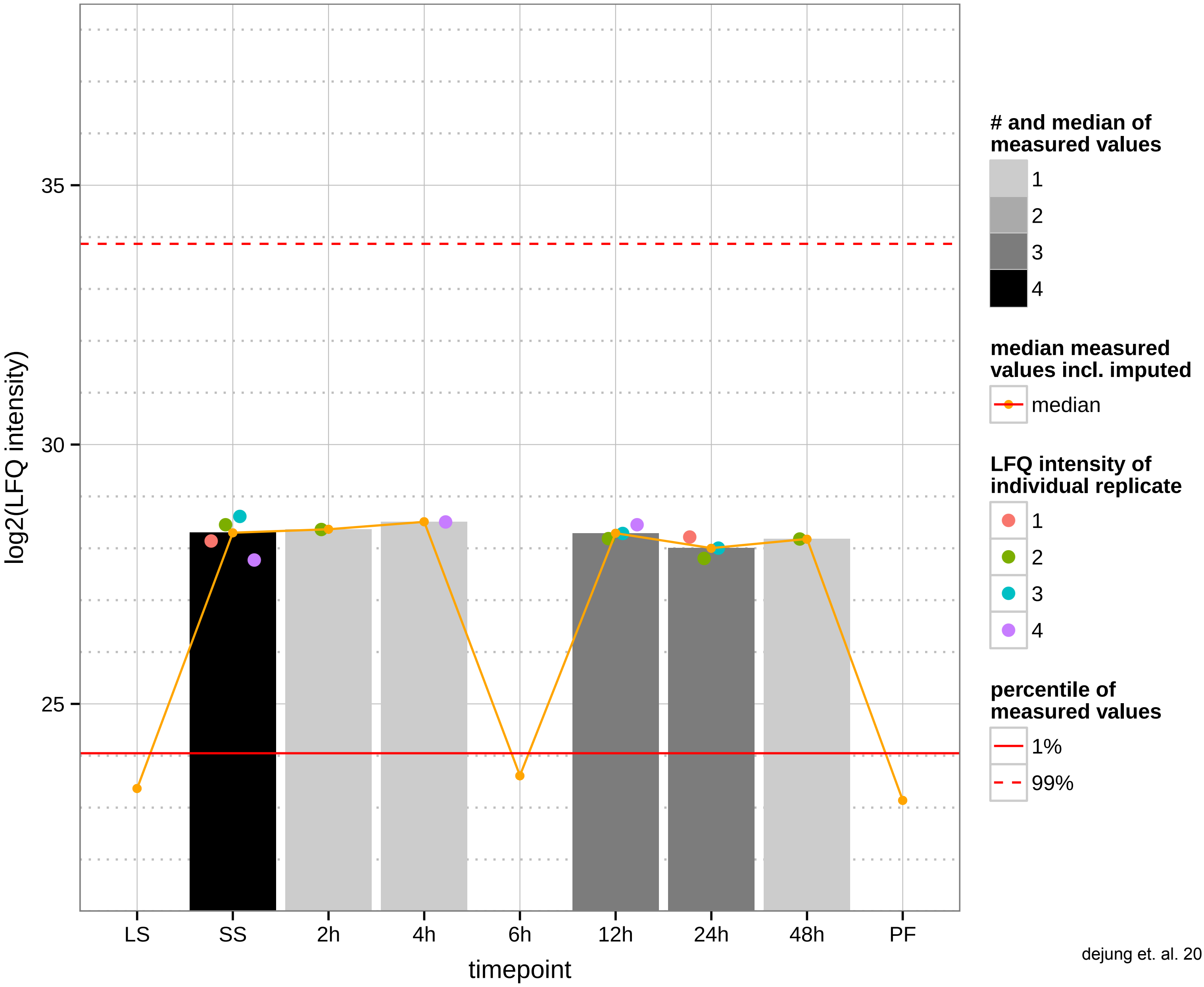

dejung et. al. 2015

RNA–binding protein, putative (RBP27), RNA–binding protein 27
Tb927.10.14080;Tb927.10.13570
AGOF: RNA binding, nucleic acid binding
AGOC: null
AGOP: null
PGOF: null, nucleic acid binding
PGOC: null
PGOP: null

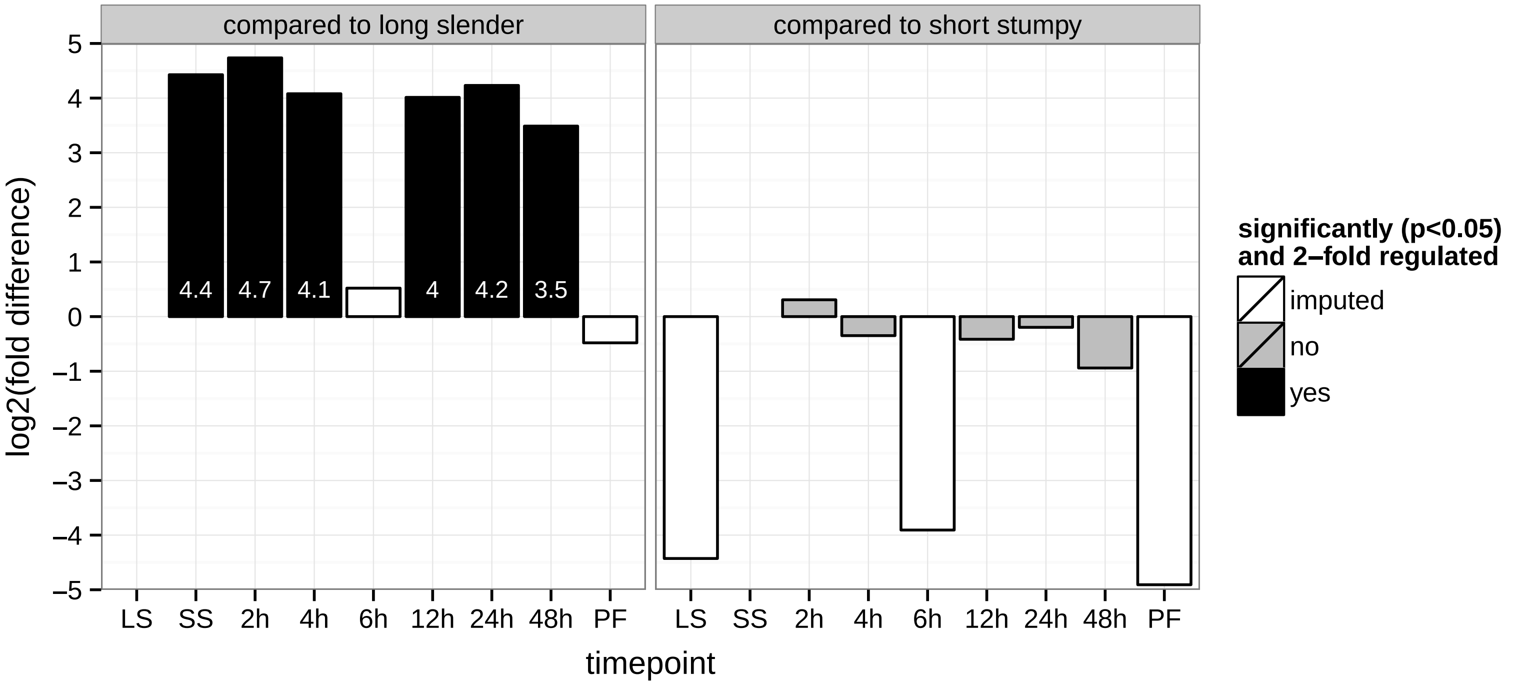

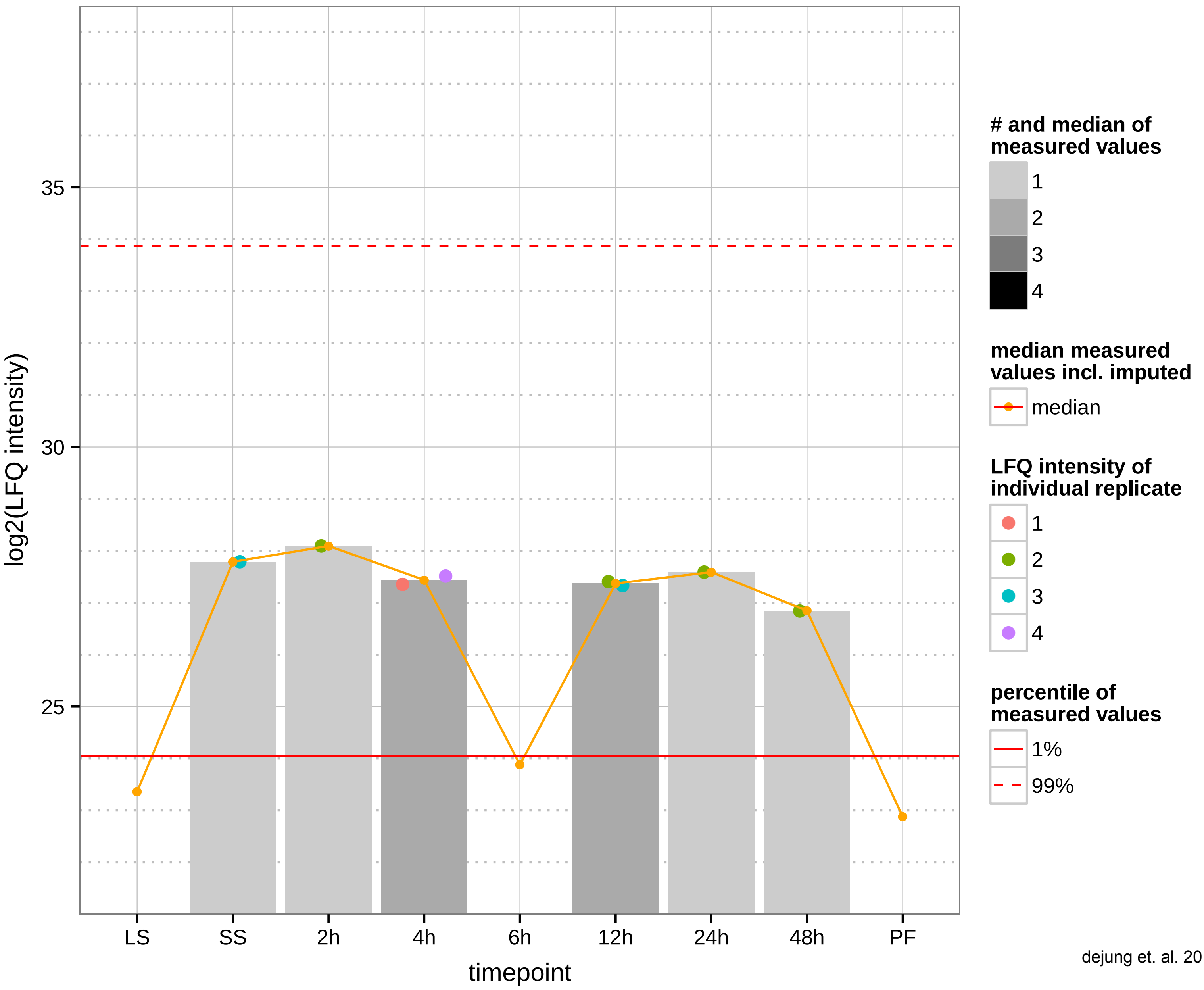

dejung et. al. 2015

uracil–DNA glycosylase, putative
Tb927.10.13970
AGOF: uracil DNA N–glycosylase activity
AGOC: null
AGOP: base–excision repair
PGOF: uracil DNA N–glycosylase activity
PGOC: null
PGOP: base–excision repair

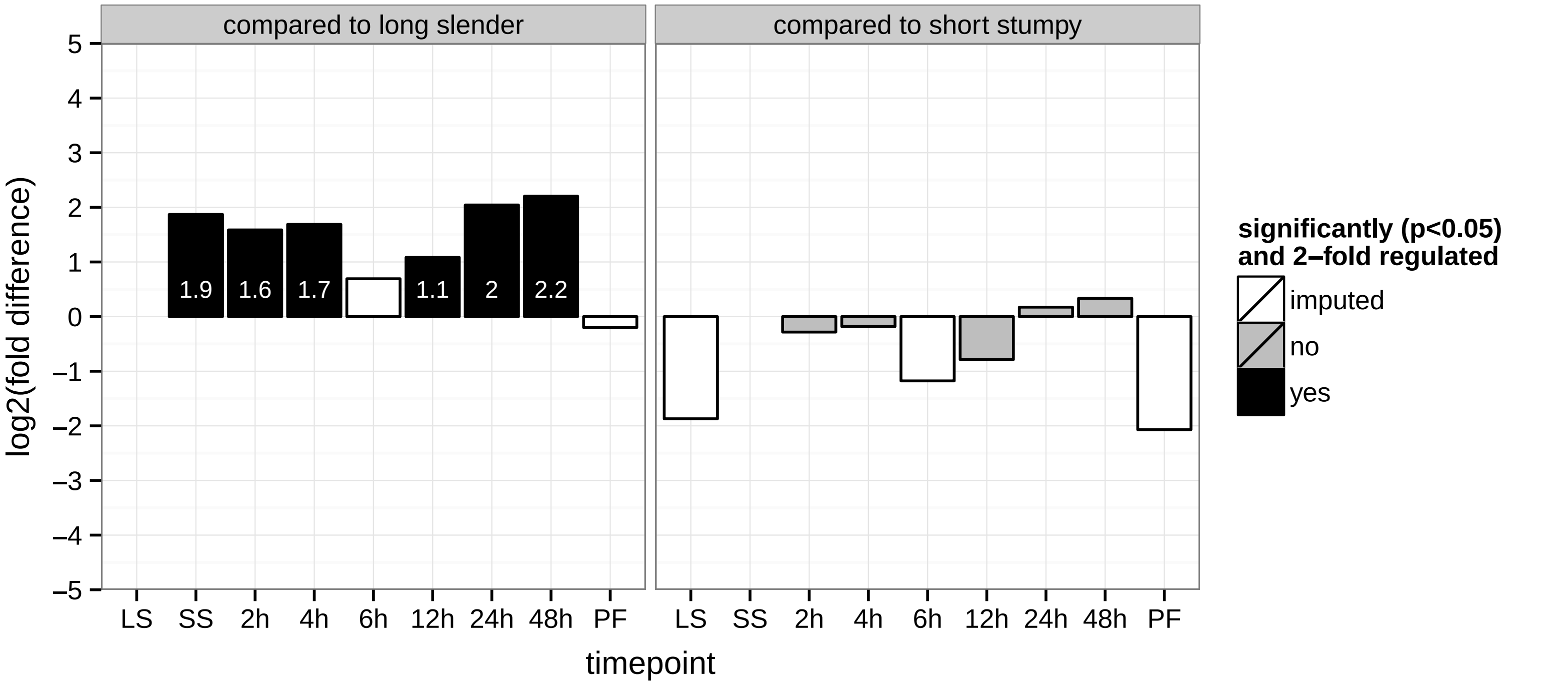

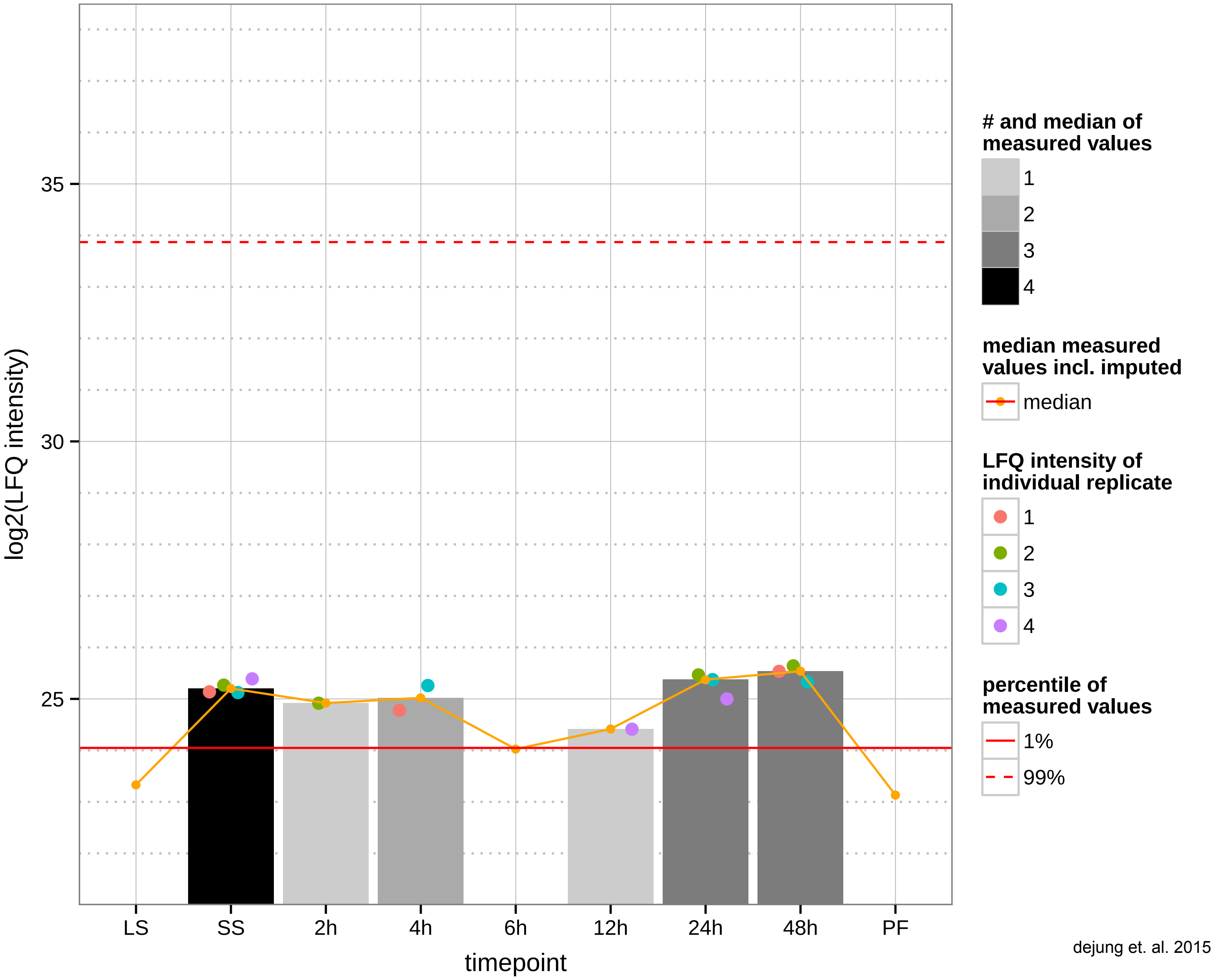

dejung et. al. 2015

G10 protein homologue, putative
Tb927.10.6650
AGOF: null
AGOC: nucleus
AGOP: null
PGOF: null
PGOC: nucleus
PGOP: null

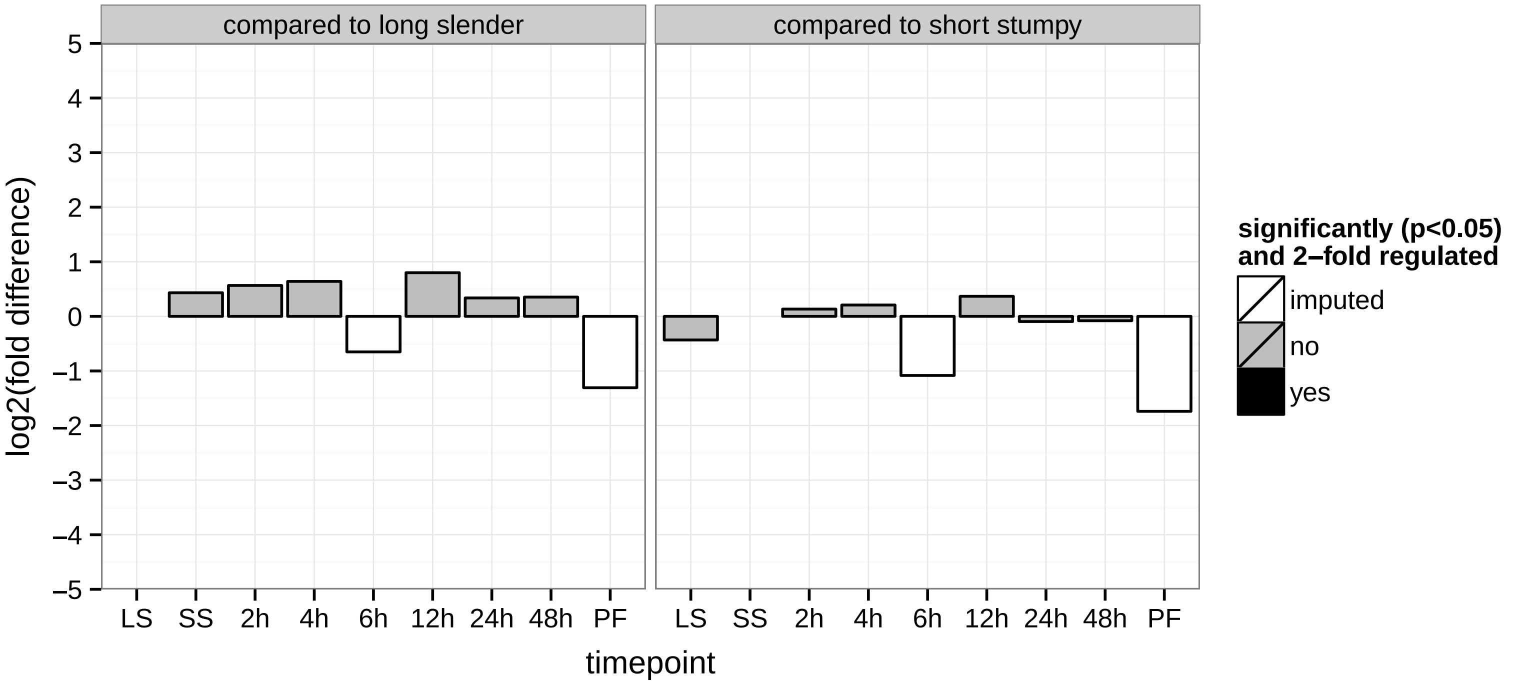

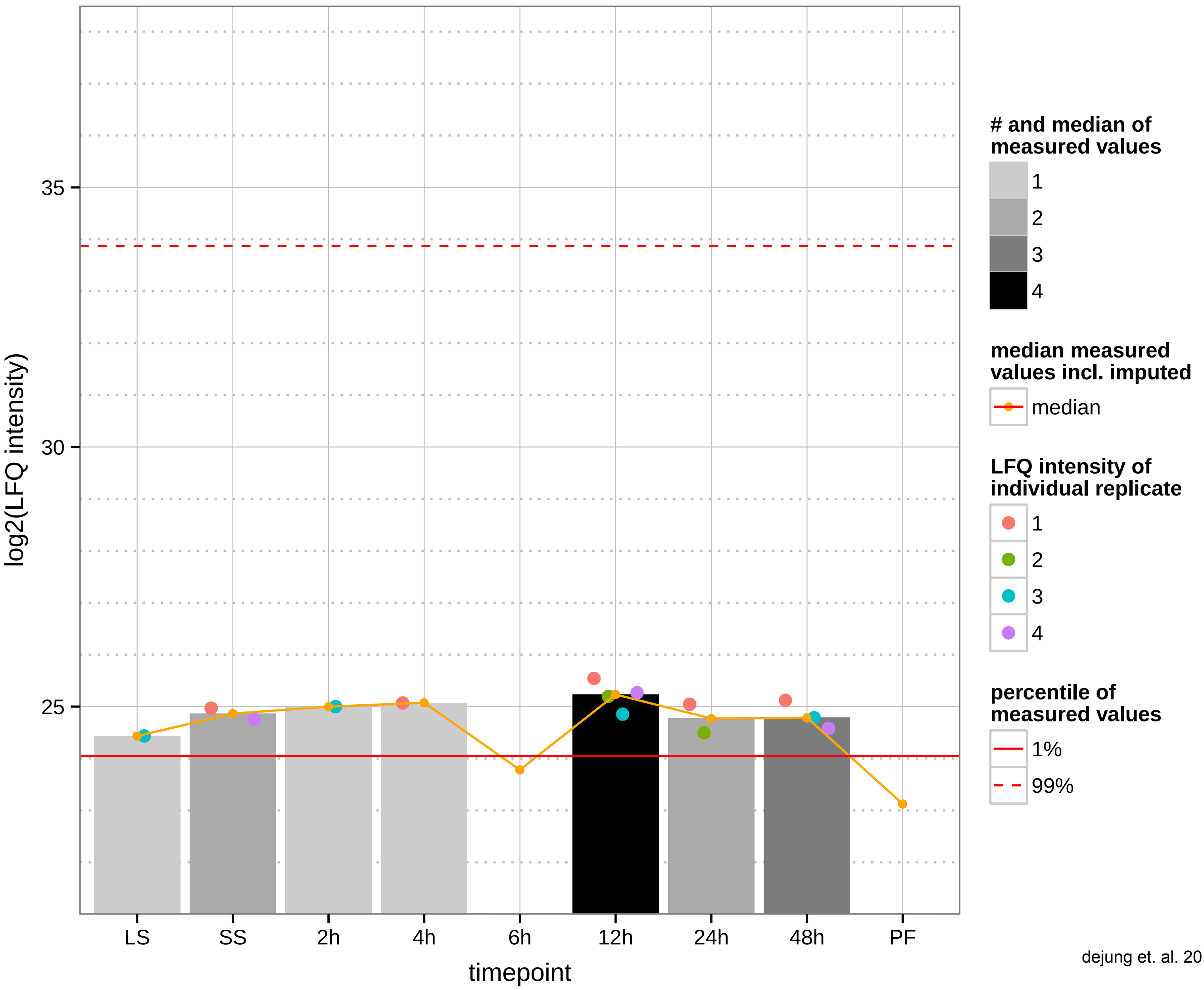

dejung et. al. 2015

Poly(A)–specific ribonuclease PARN–2
Tb927.10.8360
AGOF: nuclease activity, nucleic acid binding
AGOC: nucleus
AGOP: null
PGOF: nucleic acid binding
PGOC: nucleus
PGOP: null

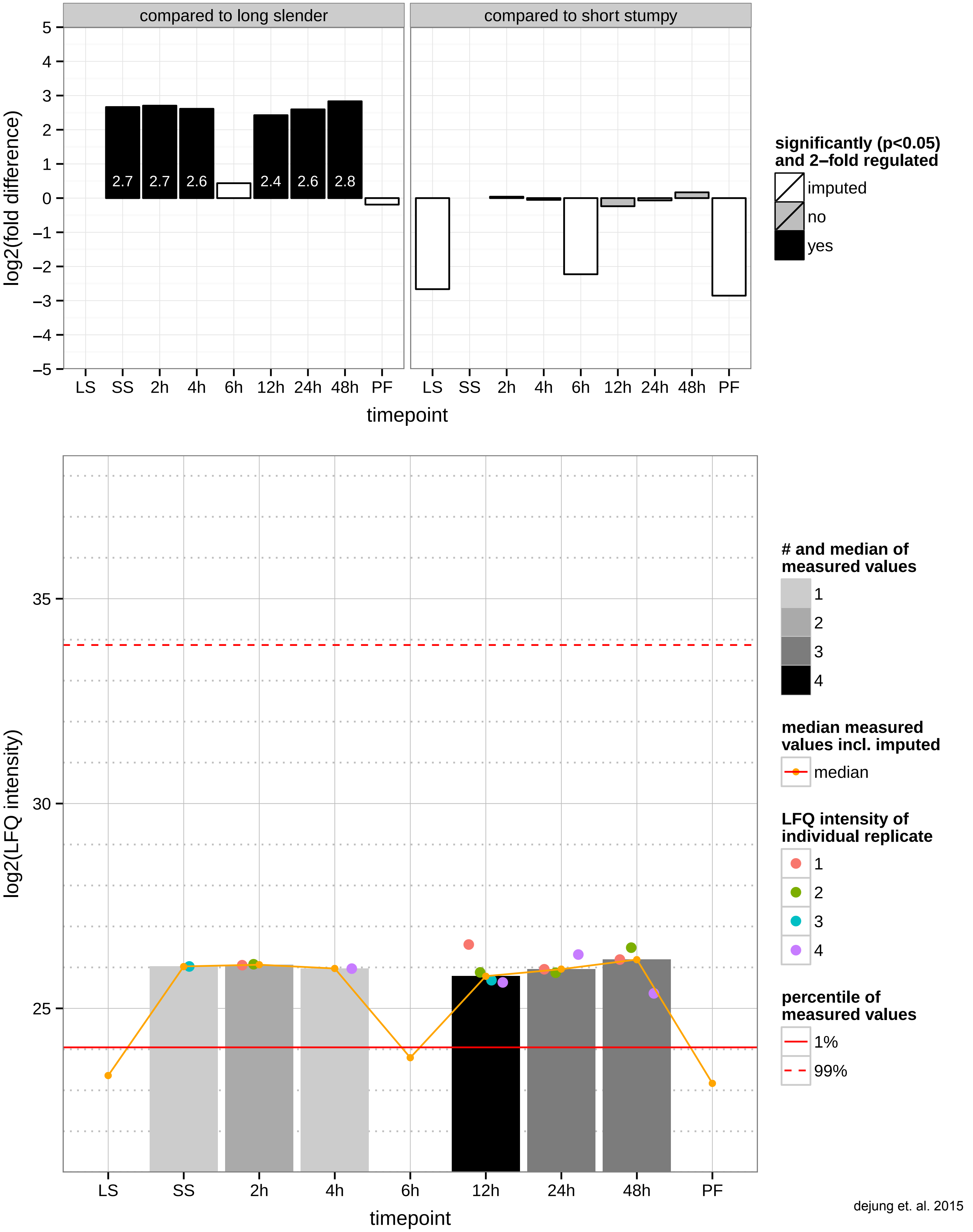

dejung et. al. 2015

hypothetical protein, conserved
Tb927.11.13460
AGOF: null
AGOC: null
AGOP: null
PGOF: null
PGOC: null
PGOP: null

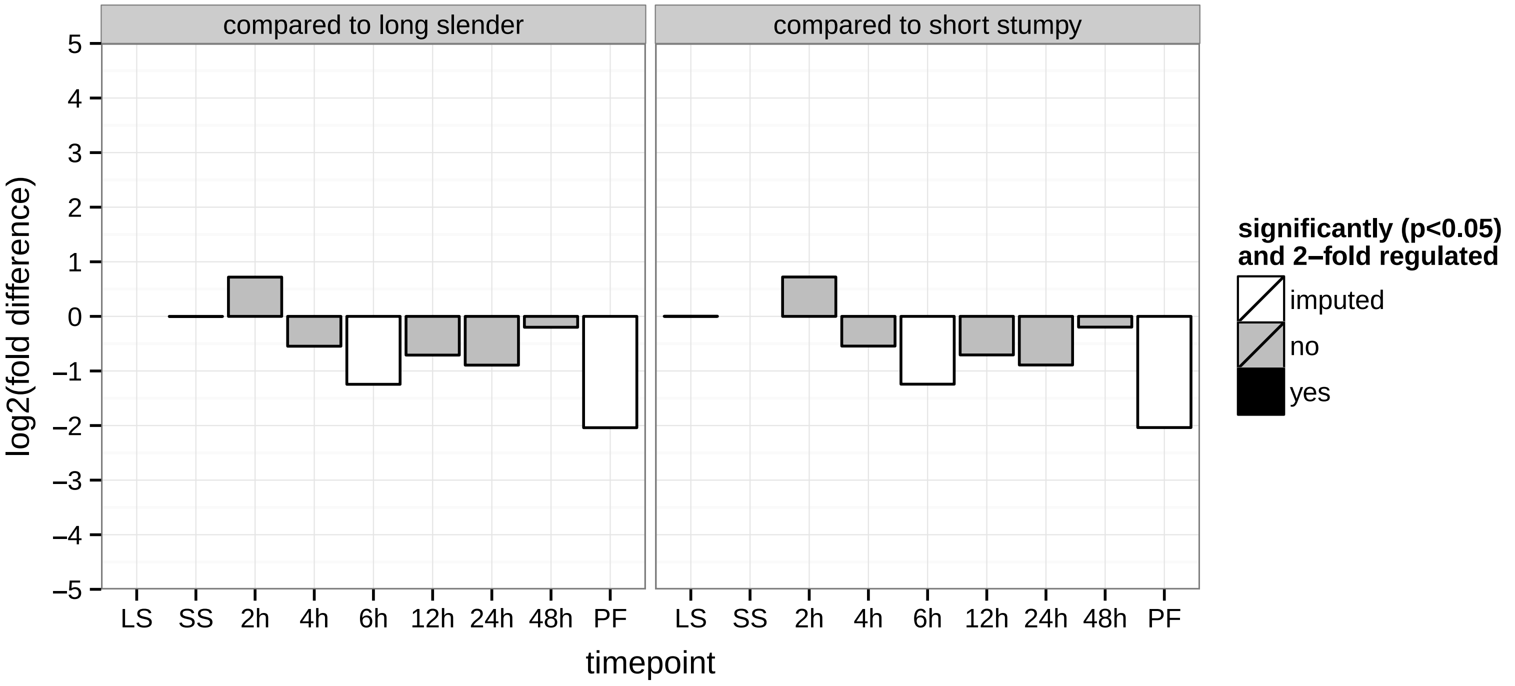

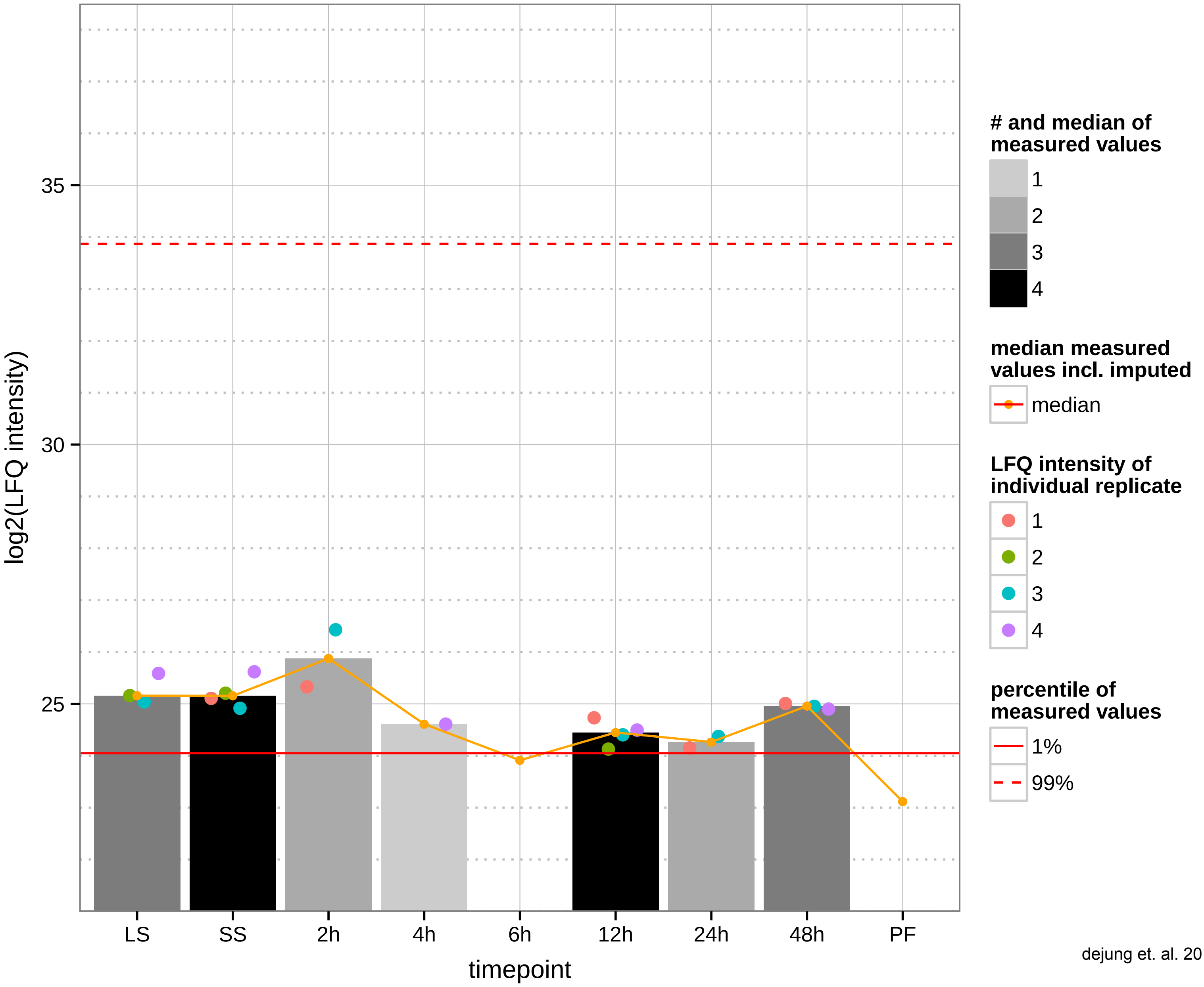

dejung et. al. 2015

hypothetical protein, conserved
Tb927.11.620
AGOF: null
AGOC: null
AGOP: null
PGOF: binding, protein binding
PGOC: null
PGOP: null

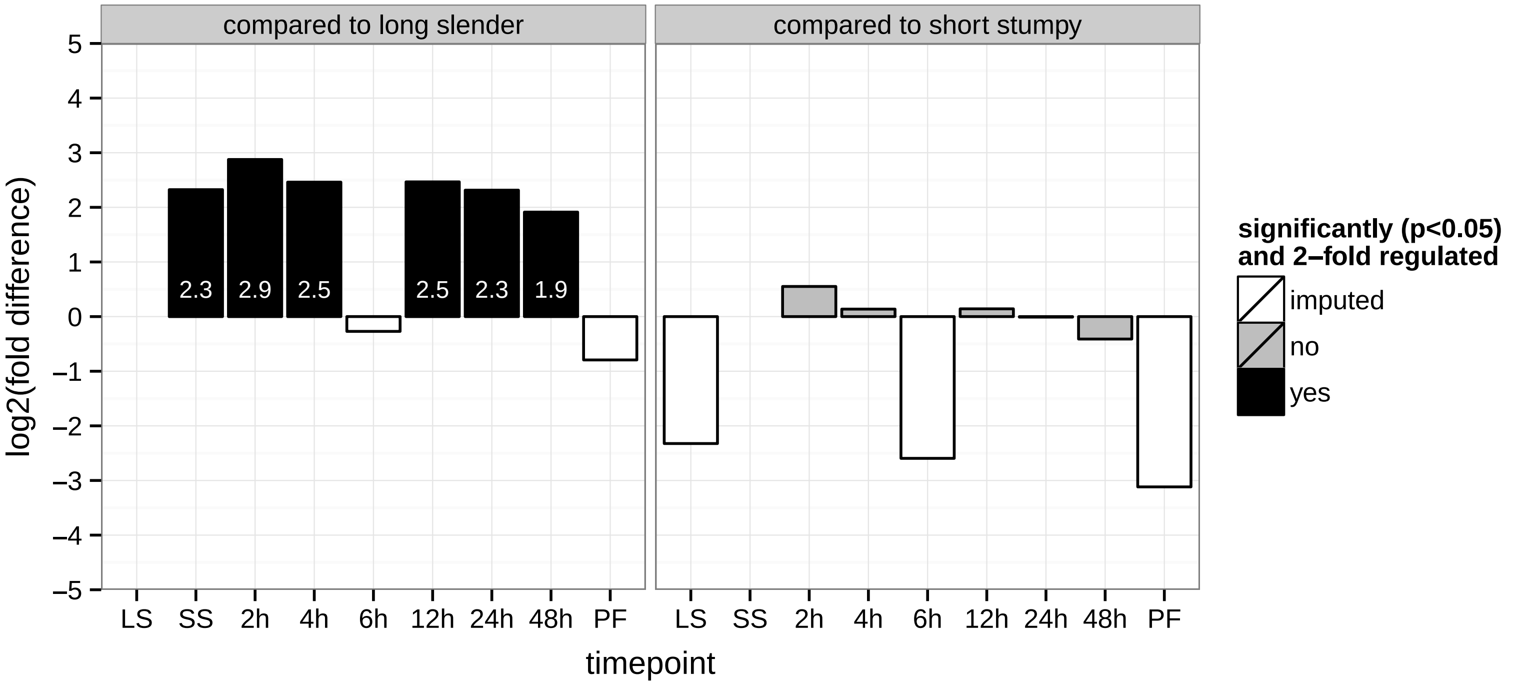

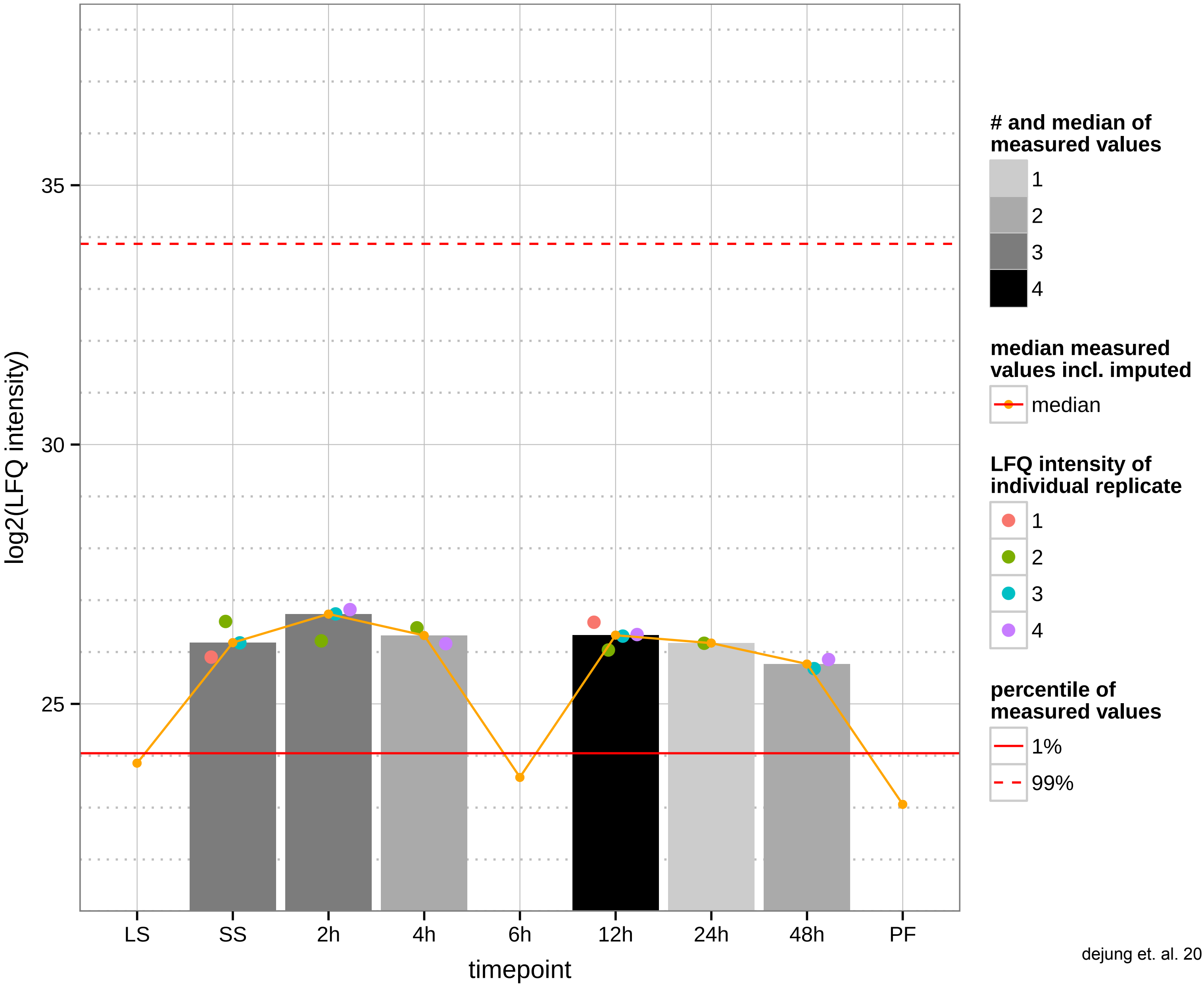

dejung et. al. 2015

protein phosphatase 2C, putative
Tb927.2.5050
AGOF: phosphatase activity
AGOC: null
AGOP: dephosphorylation
PGOF: catalytic activity
PGOC: null
PGOP: null

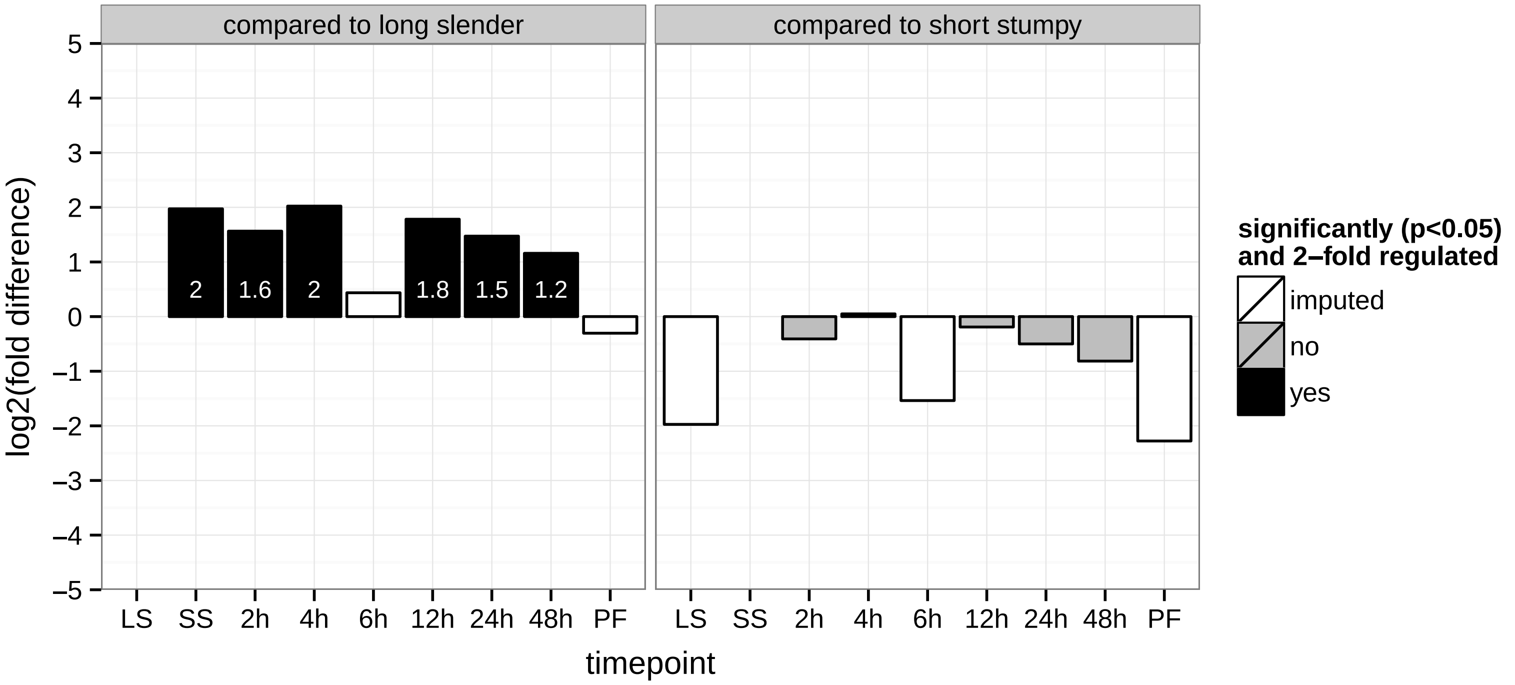

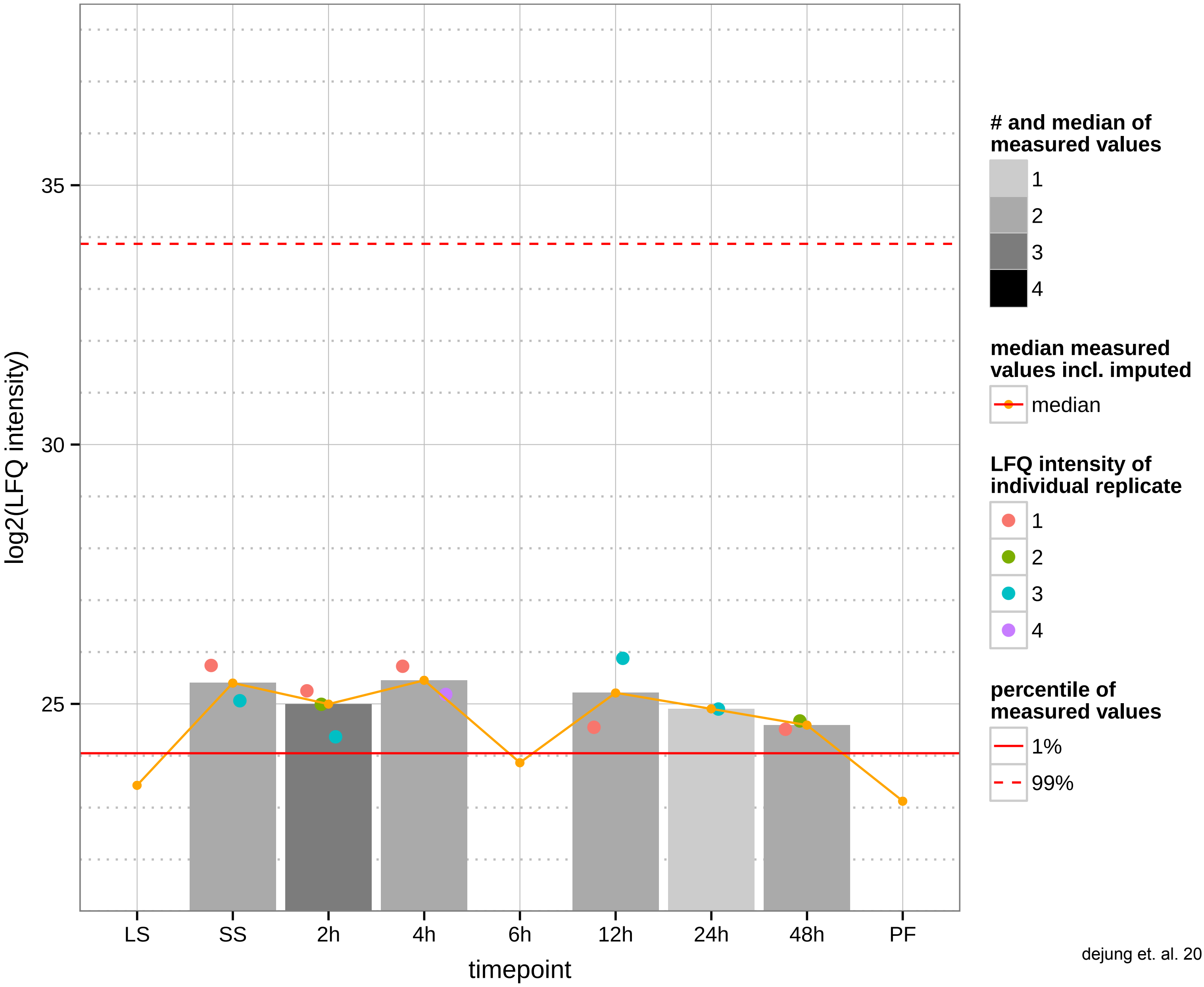

dejung et. al. 2015

vesicle–associated membrane protein, putative, synaptobrevin
Tb927.2.5120
AGOF: null
AGOC: integral to membrane
AGOP: membrane fusion, vesicle–mediated transport
PGOF: null
PGOC: integral to membrane
PGOP: transport, vesicle–mediated transport

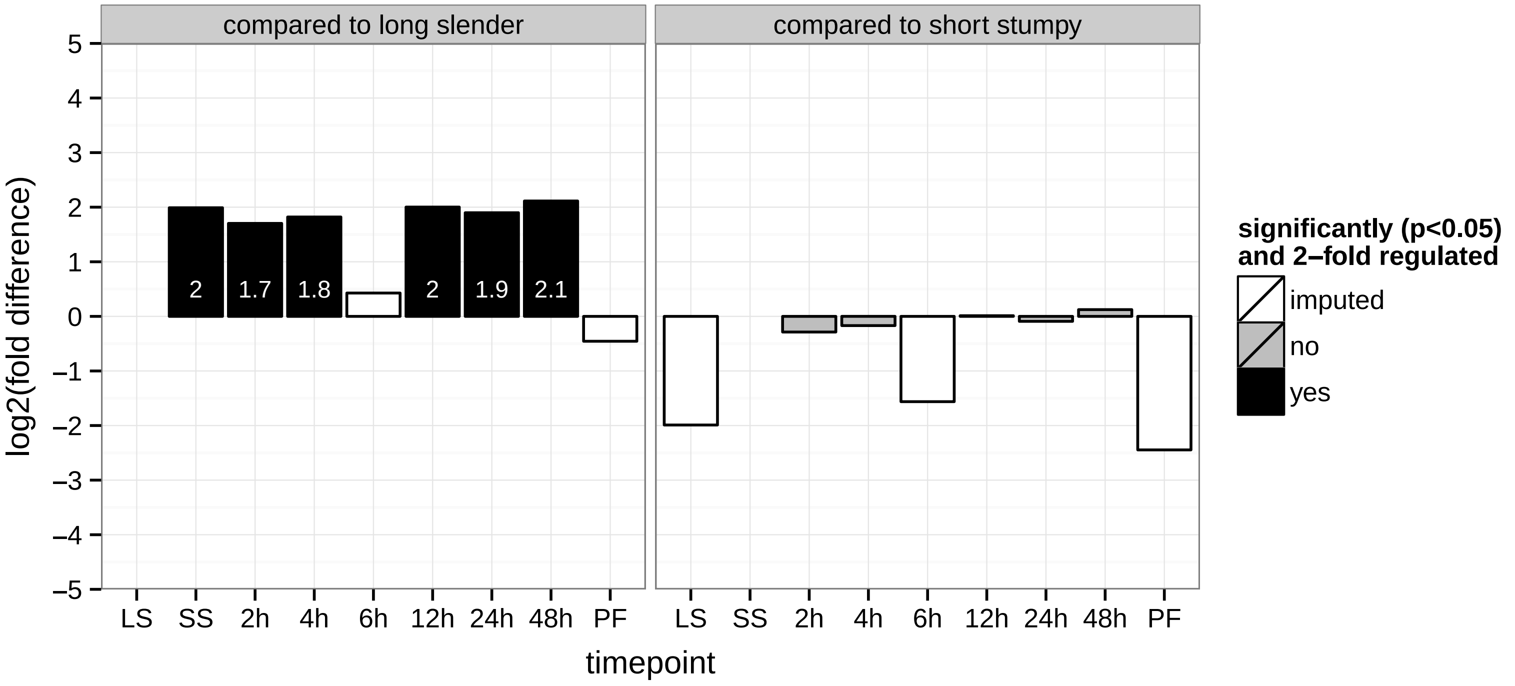

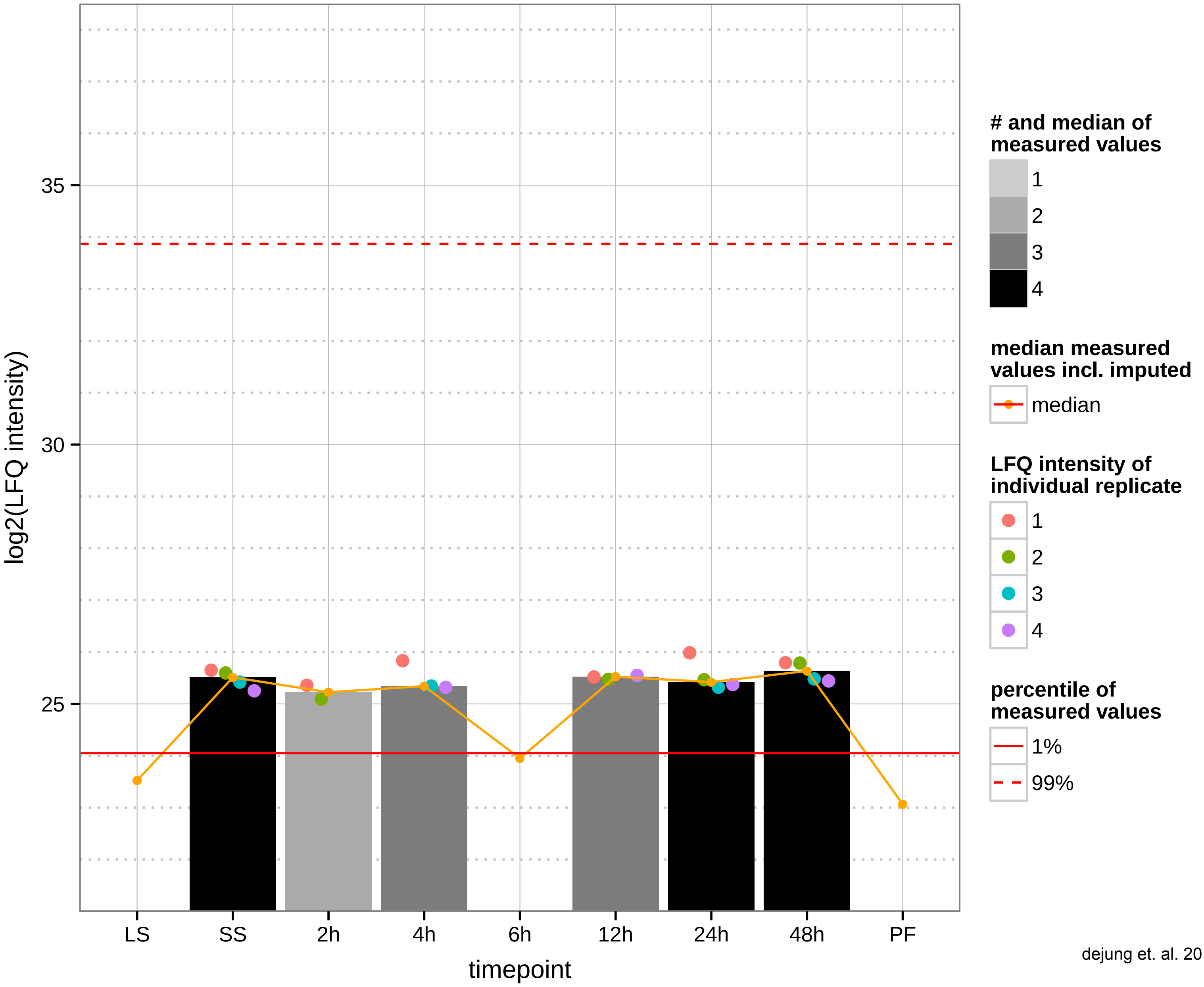

dejung et. al. 2015

casein kinase 1, putative (CK1)
Tb927.3.1630
AGOF: ATP binding, protein kinase activity, protein serine/threonine kinase activity, protein tyrosine kinase activity
AGOC: cytoplasm, cytoskeleton, integral to membrane, nucleus
AGOP: protein phosphorylation
PGOF: ATP binding, protein kinase activity, transferase activity, transferring phosphorus–containing groups
PGOC: null
PGOP: protein phosphorylation

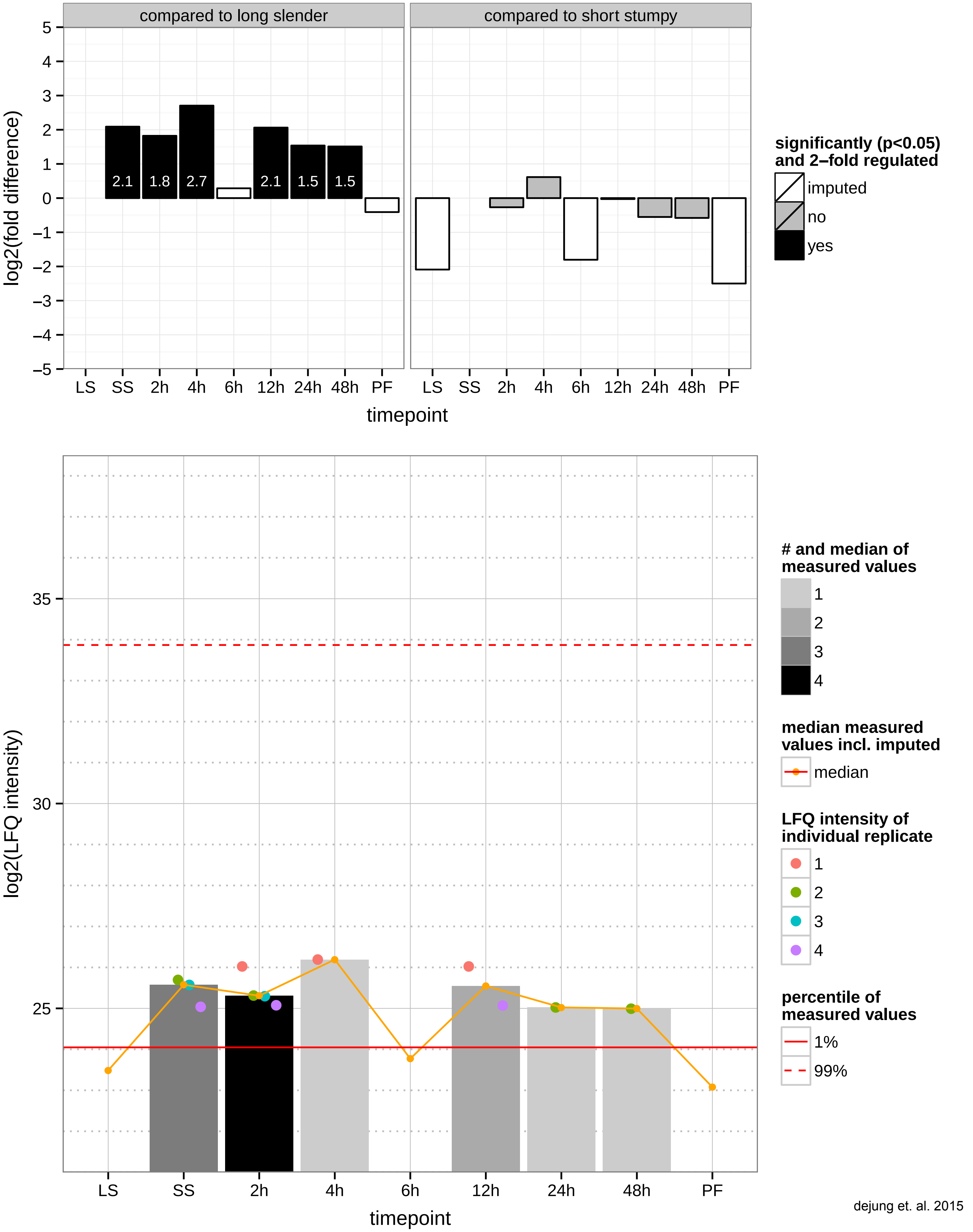

dejung et. al. 2015

hypothetical protein, conserved
Tb927.3.4070
AGOF: null
AGOC: null
AGOP: null
PGOF: null
PGOC: null
PGOP: null

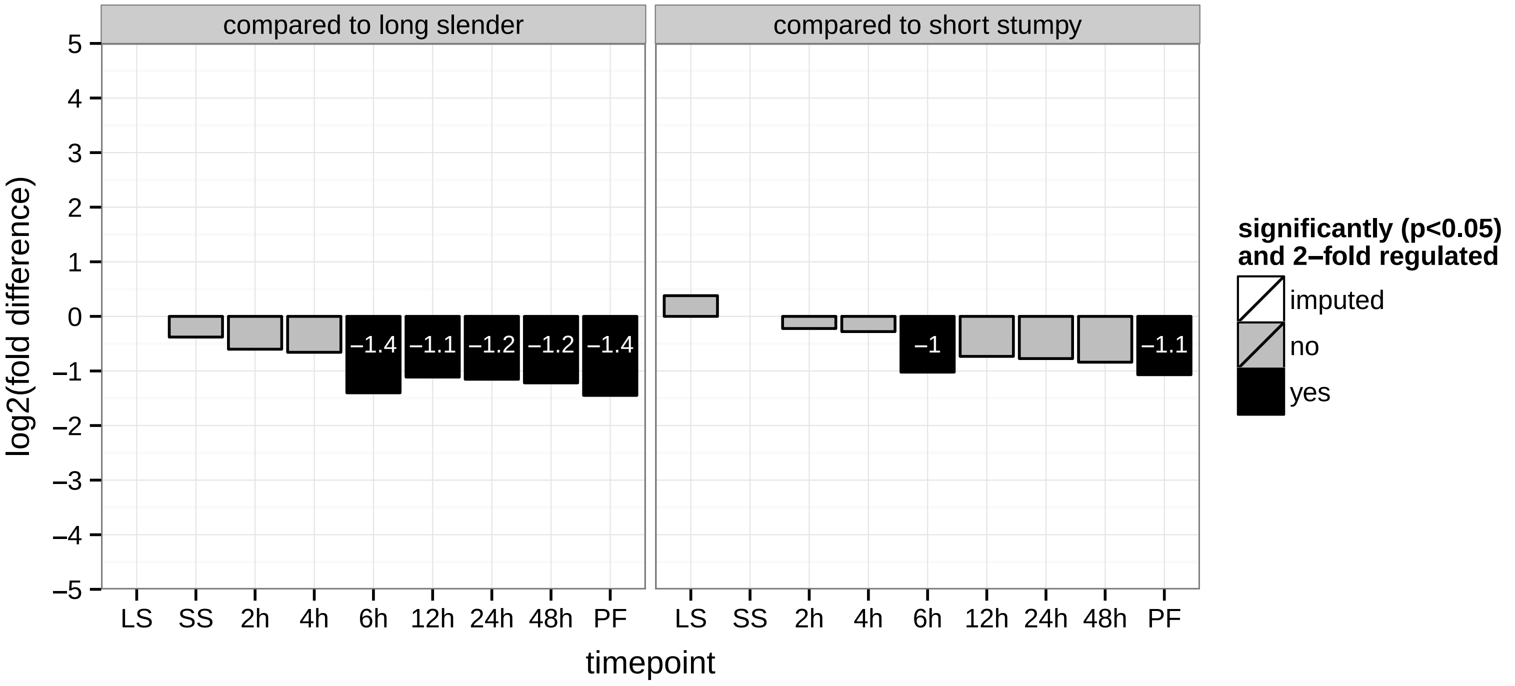

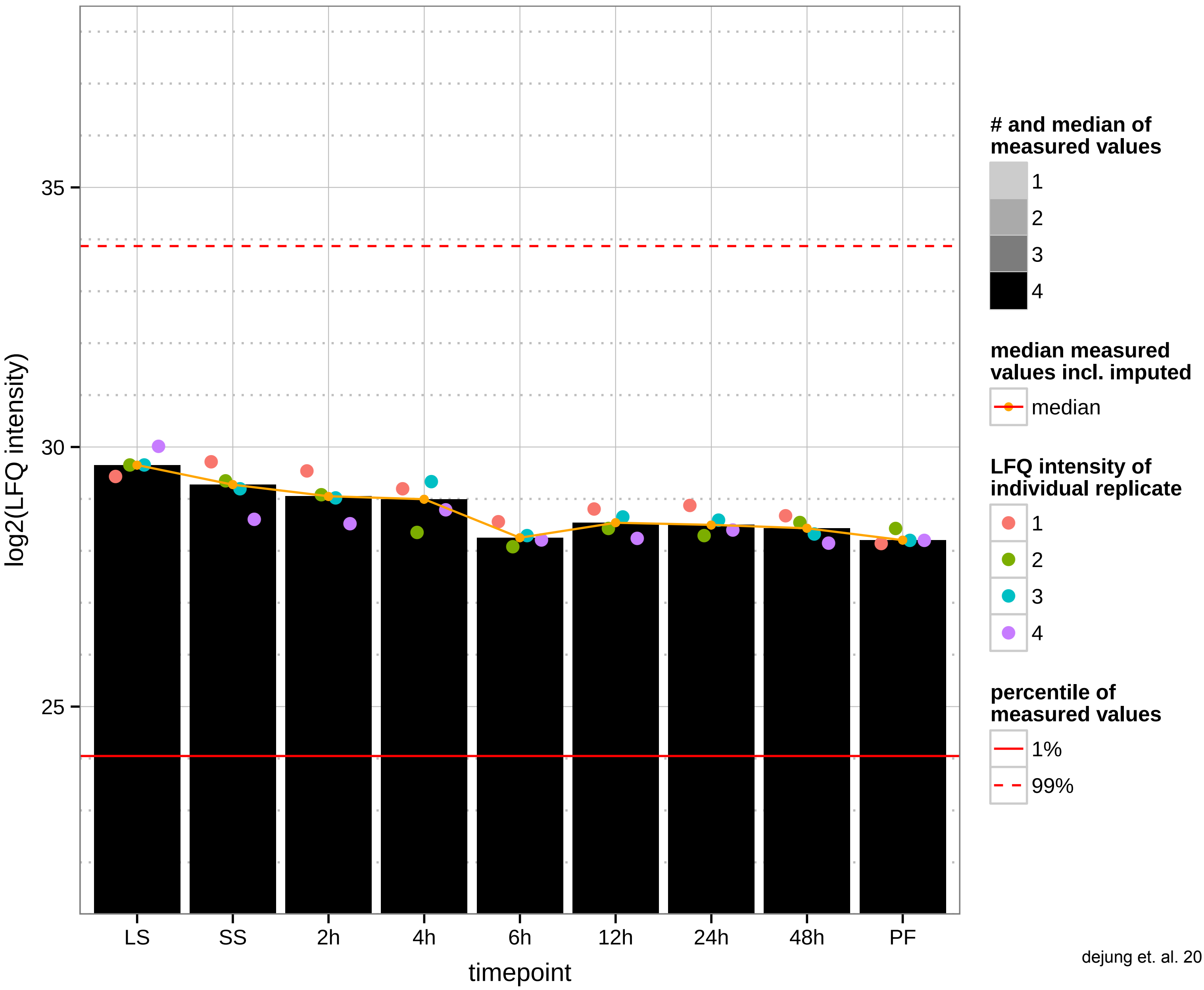

dejung et. al. 2015

hypothetical protein, conserved
Tb927.4.3310
AGOF: null
AGOC: null
AGOP: null
PGOF: protein binding
PGOC: null
PGOP: null

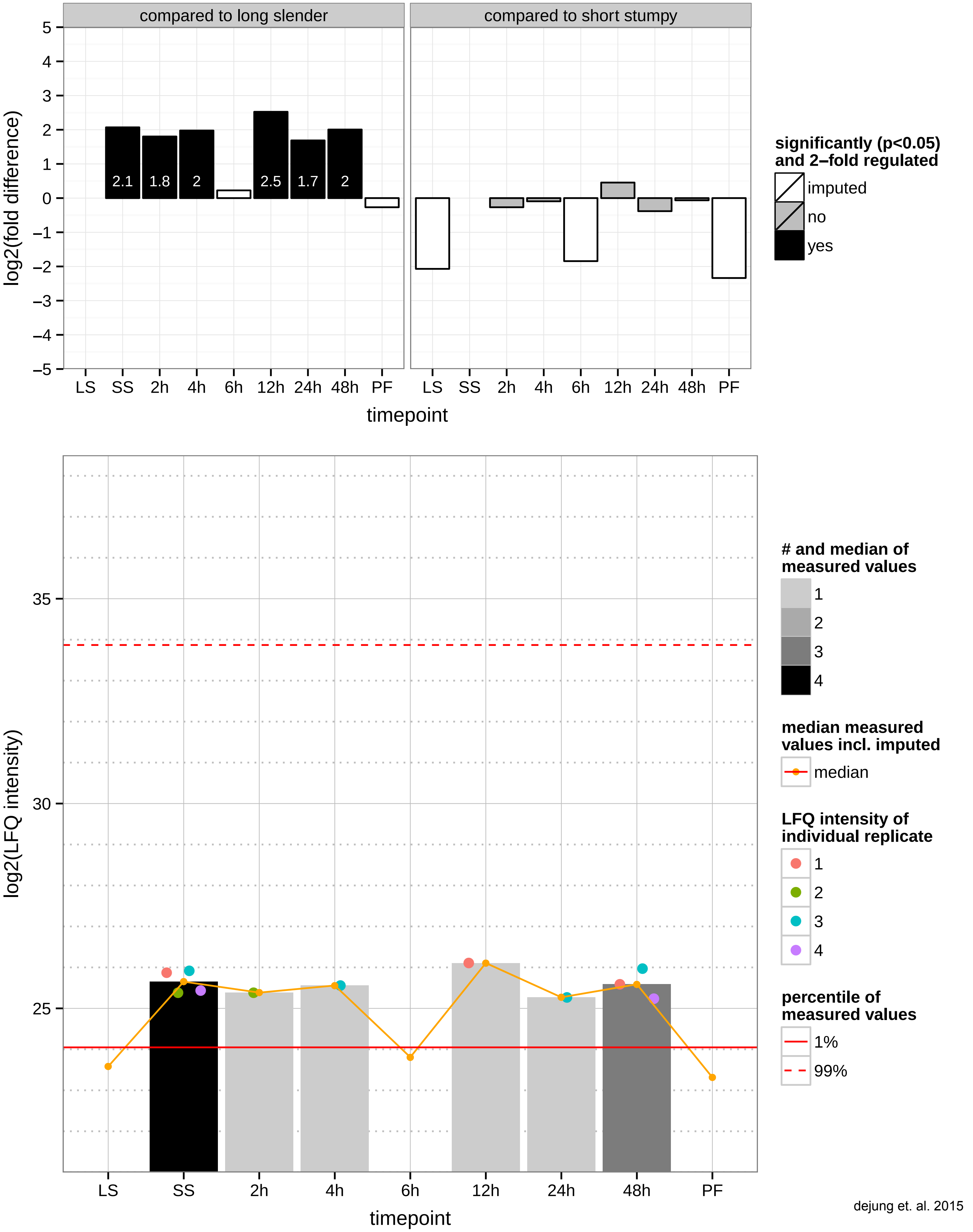

dejung et. al. 2015

hypothetical protein, conserved
Tb927.5.3550
AGOF: null
AGOC: null
AGOP: null
PGOF: null
PGOC: null
PGOP: null

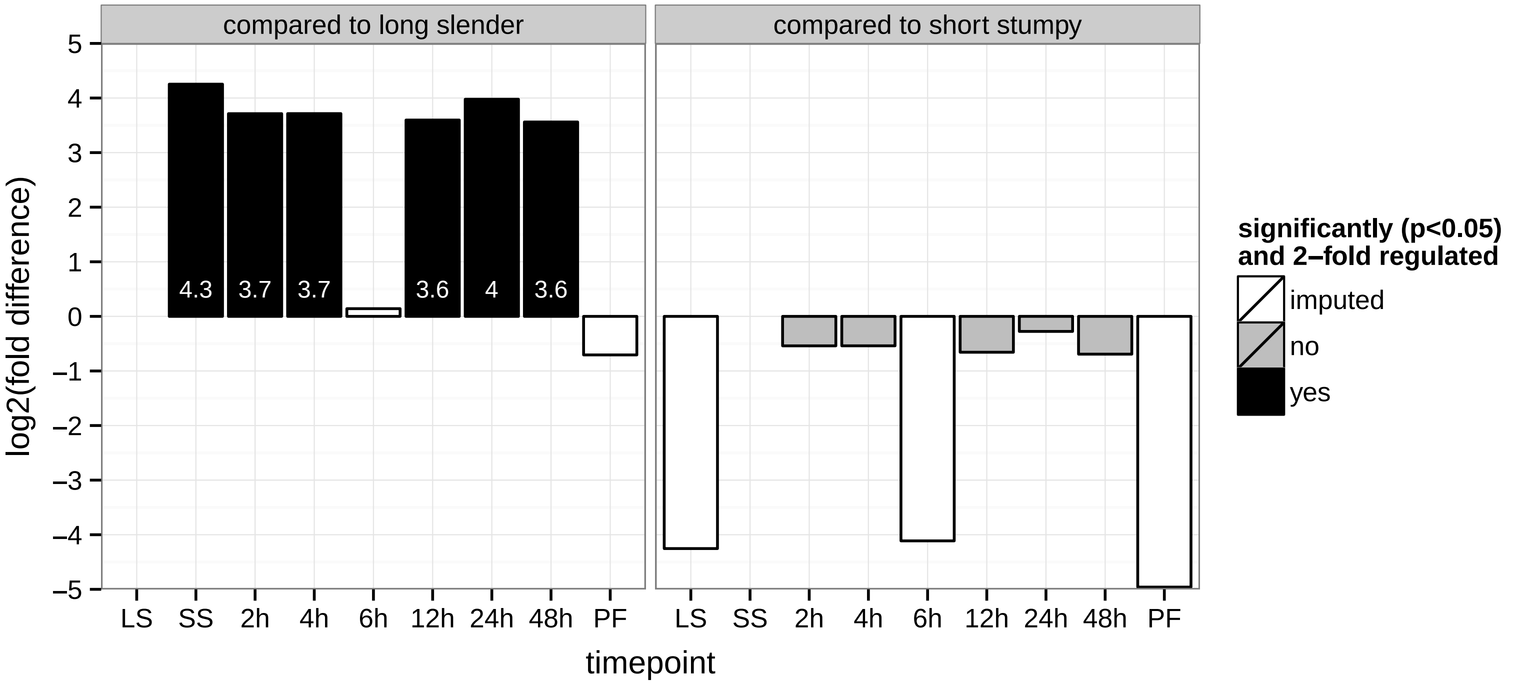

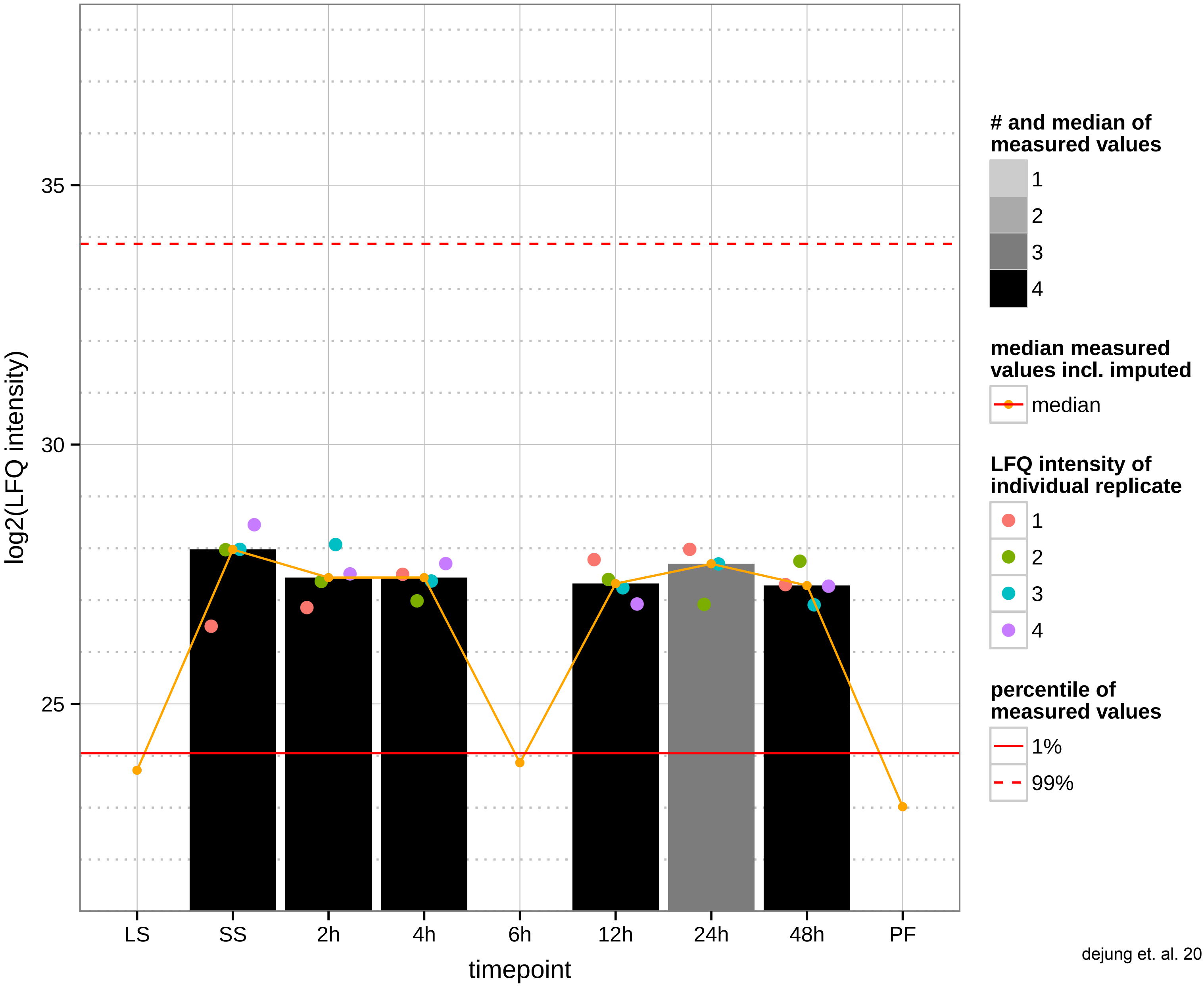

dejung et. al. 2015

hypothetical protein, conserved
Tb927.6.1270
AGOF: null
AGOC: null
AGOP: null
PGOF: null
PGOC: null
PGOP: null

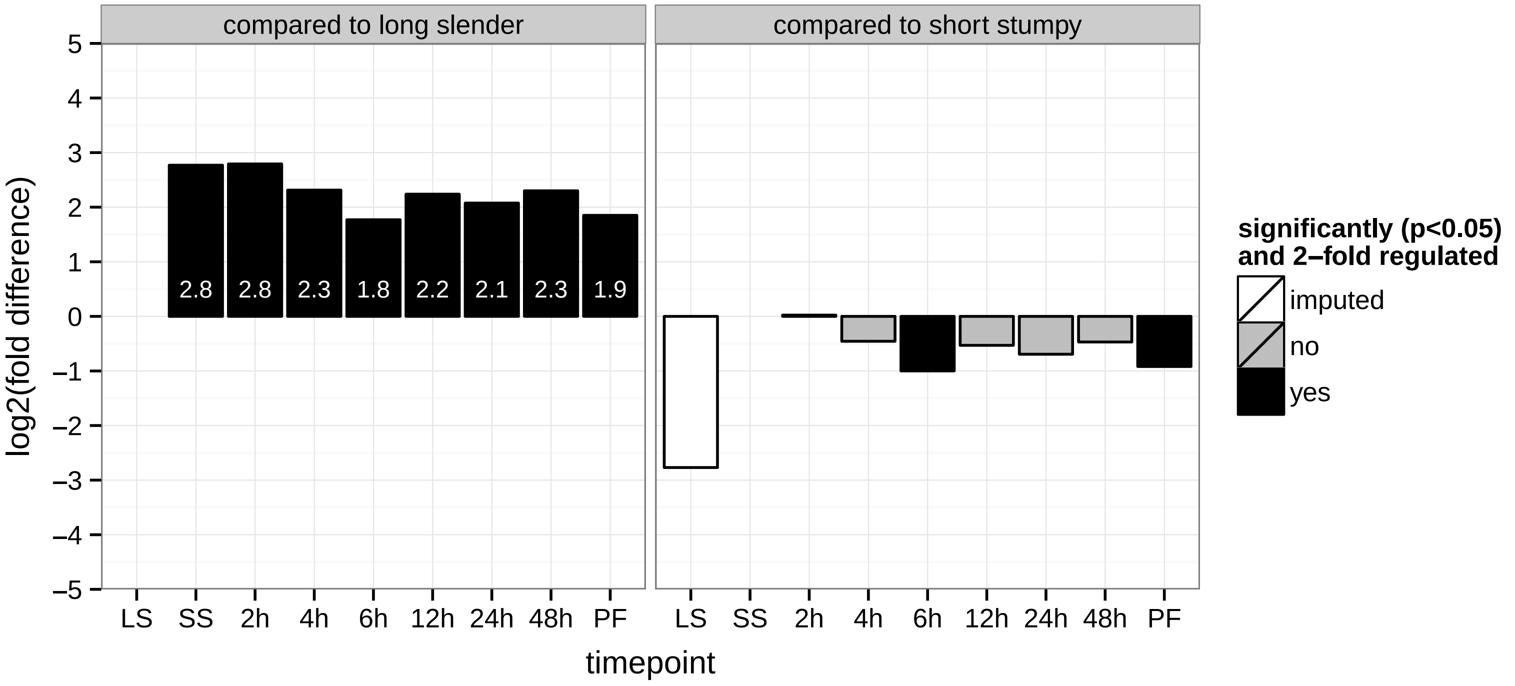

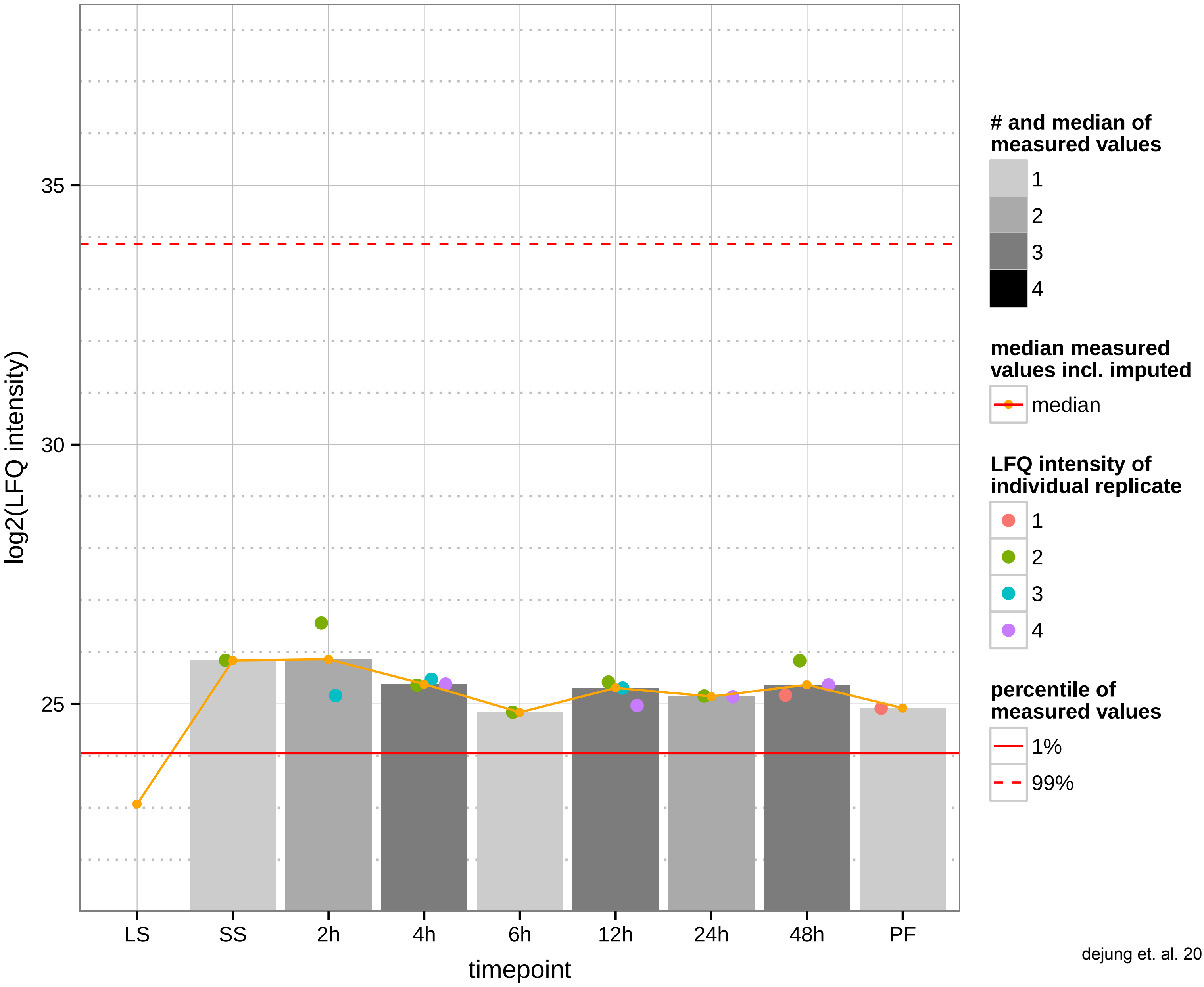

dejung et. al. 2015

hypothetical protein, conserved
Tb927.6.1930
AGOF: null
AGOC: null
AGOP: null
PGOF: protein binding
PGOC: null
PGOP: null

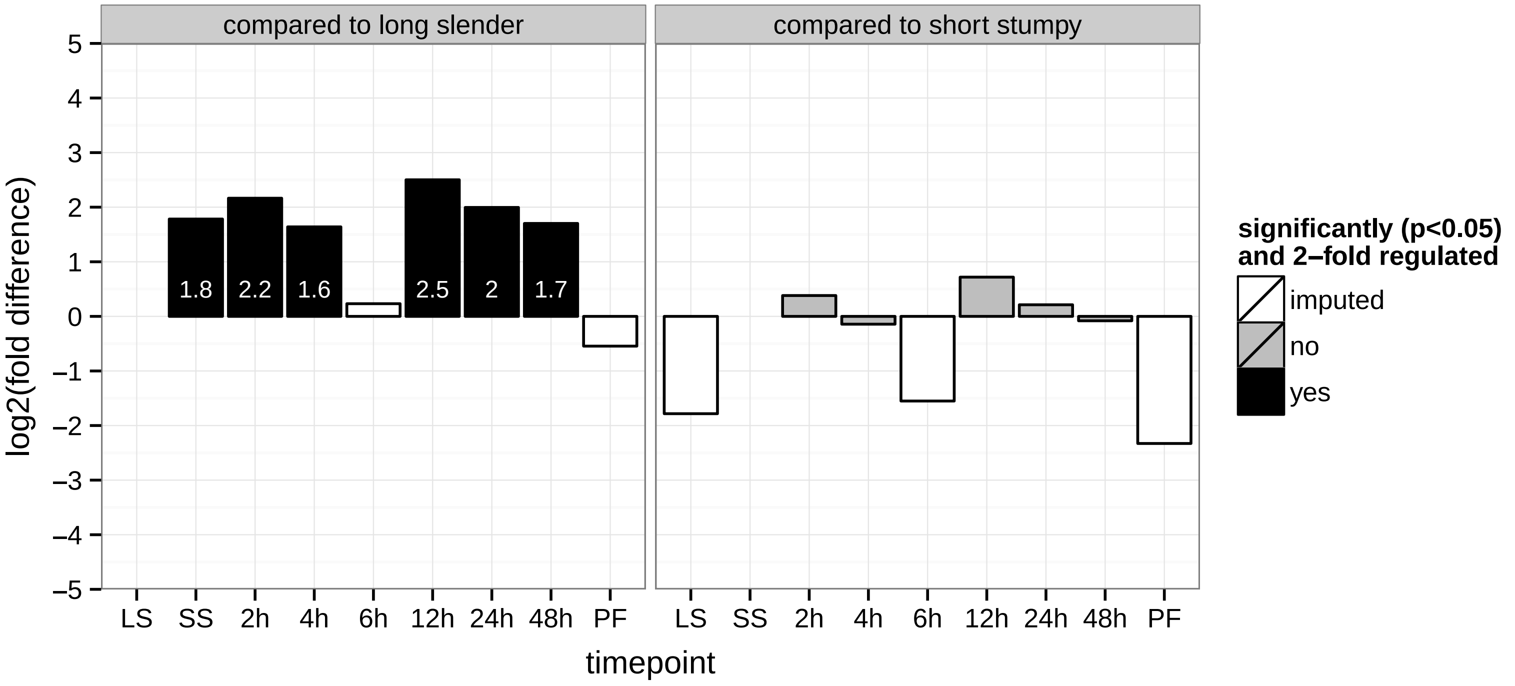

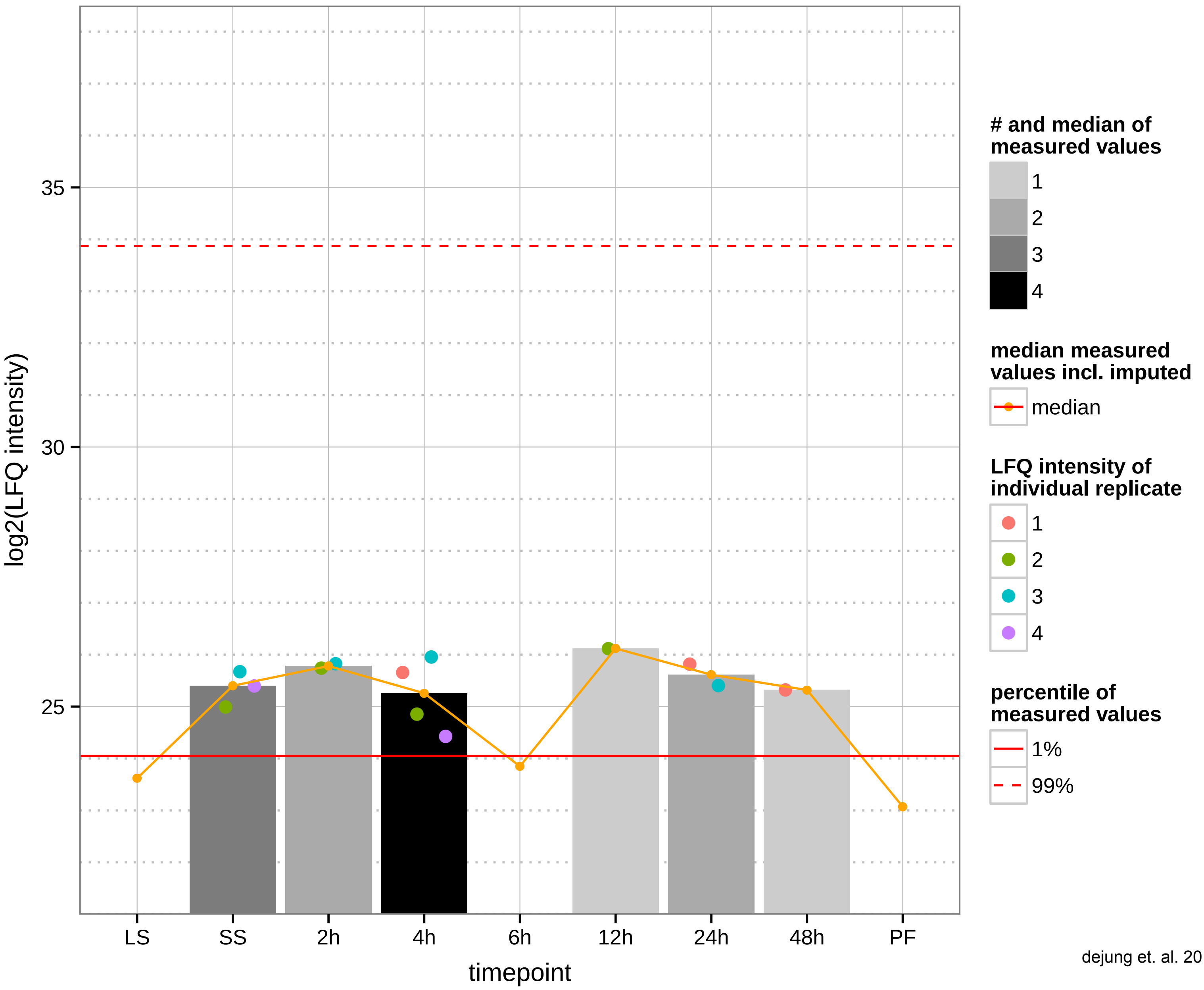

dejung et. al. 2015

acetyltransferase, putative, Elongator–like Protein 3b (ELP3b)
Tb927.8.3310;Tb11.v5.0520
AGOF: null, N–acetyltransferase activity, iron–sulfur cluster binding
AGOC: null, nucleolus
AGOP: null, histone acetylation, maintenance of rDNA, negative regulation of DNA–dependent transcription, elongation
PGOF: N–acetyltransferase activity, catalytic activity, iron–sulfur cluster binding
PGOC: null
PGOP: null

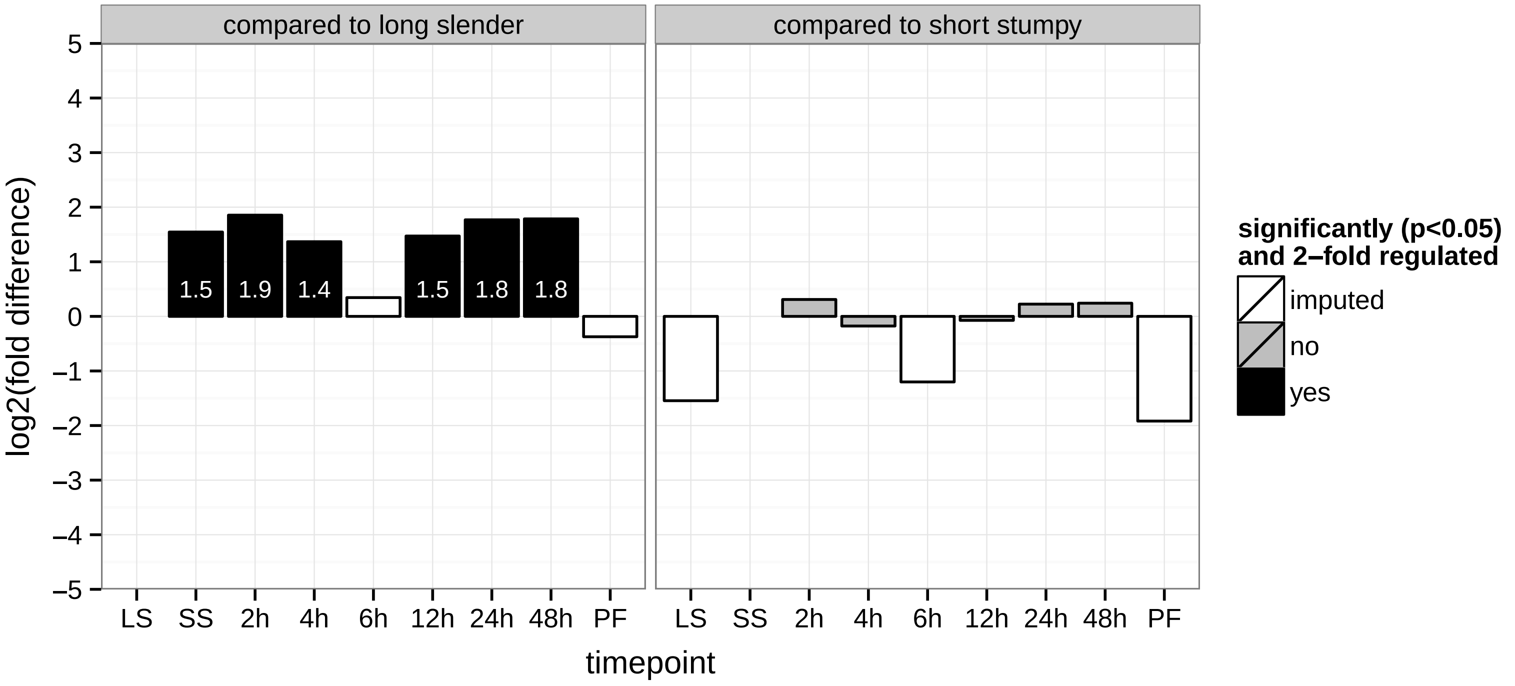

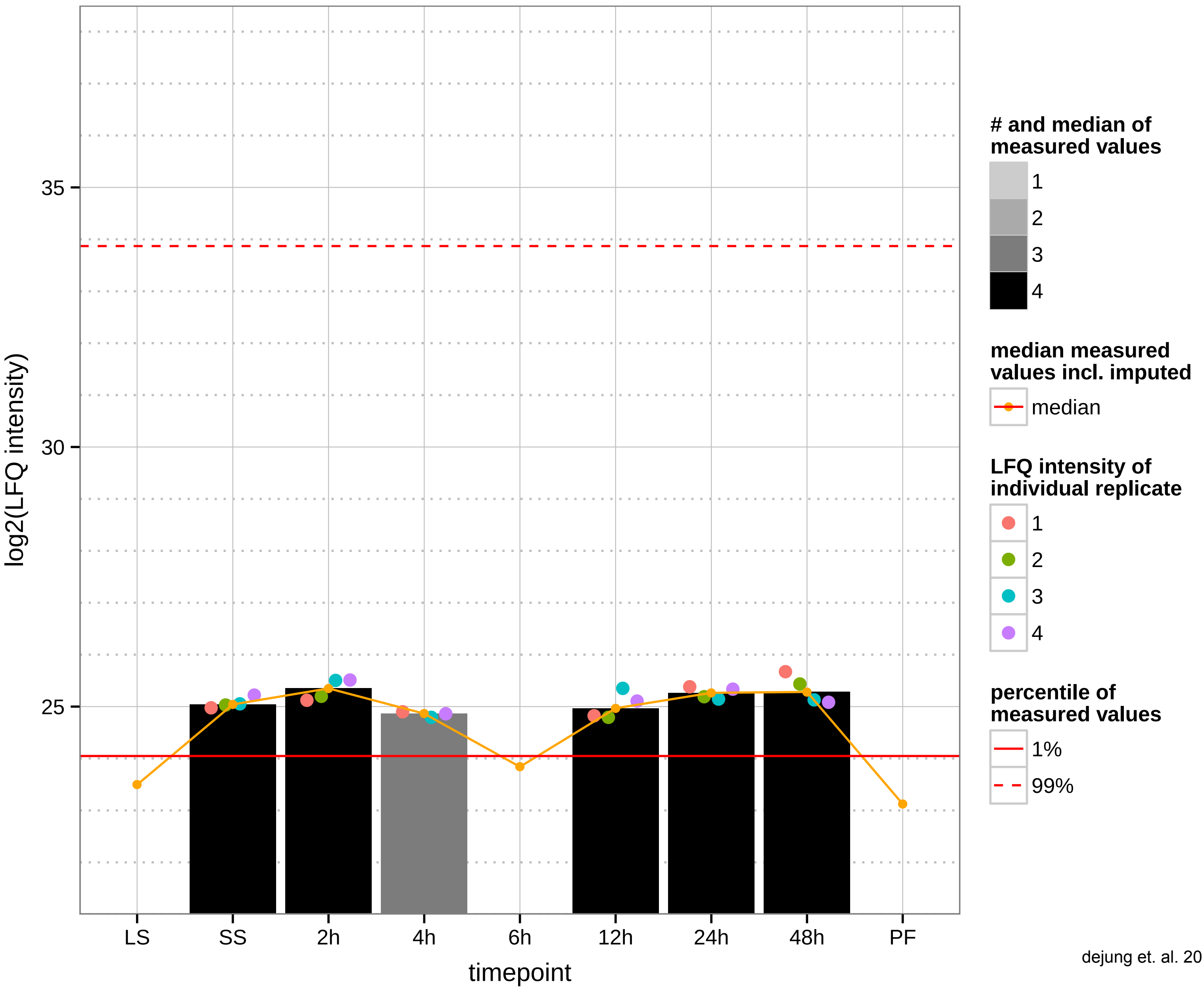

dejung et. al. 2015

hypothetical protein, conserved
Tb927.8.4480
AGOF: null
AGOC: null
AGOP: null
PGOF: null
PGOC: null
PGOP: null

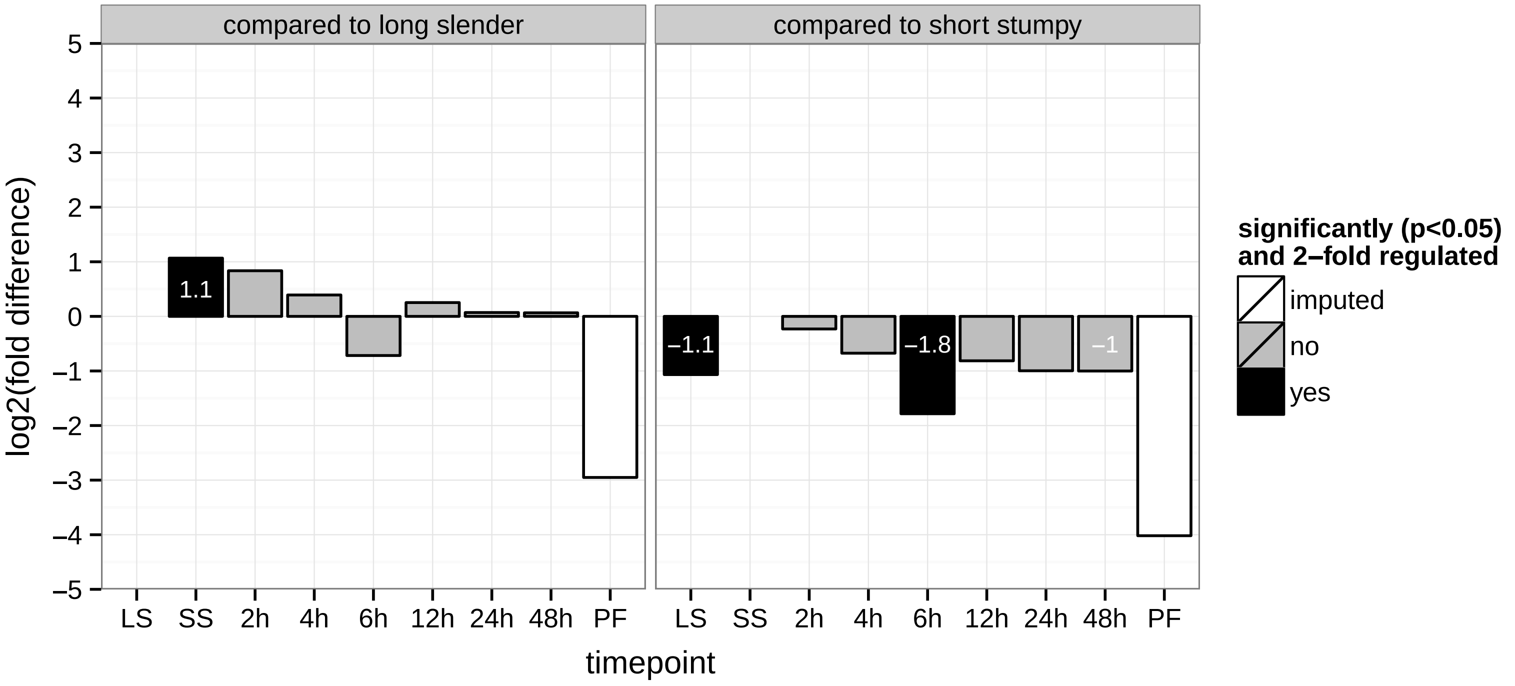

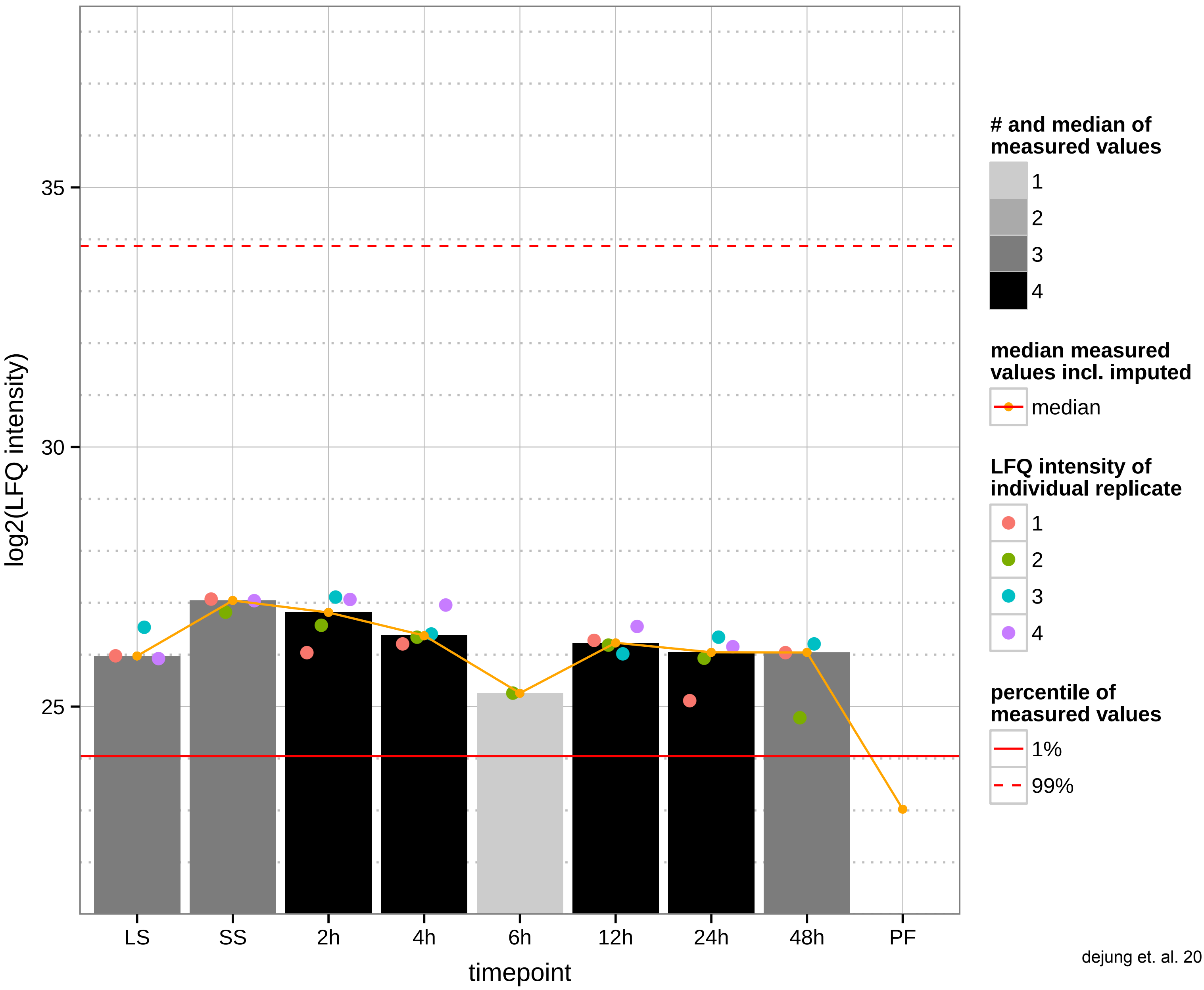

dejung et. al. 2015

60S ribosomal protein L38, putative
Tb927.9.2020
AGOF: structural constituent of ribosome
AGOC: intracellular, ribosome
AGOP: translation
PGOF: structural constituent of ribosome
PGOC: intracellular, ribosome
PGOP: translation

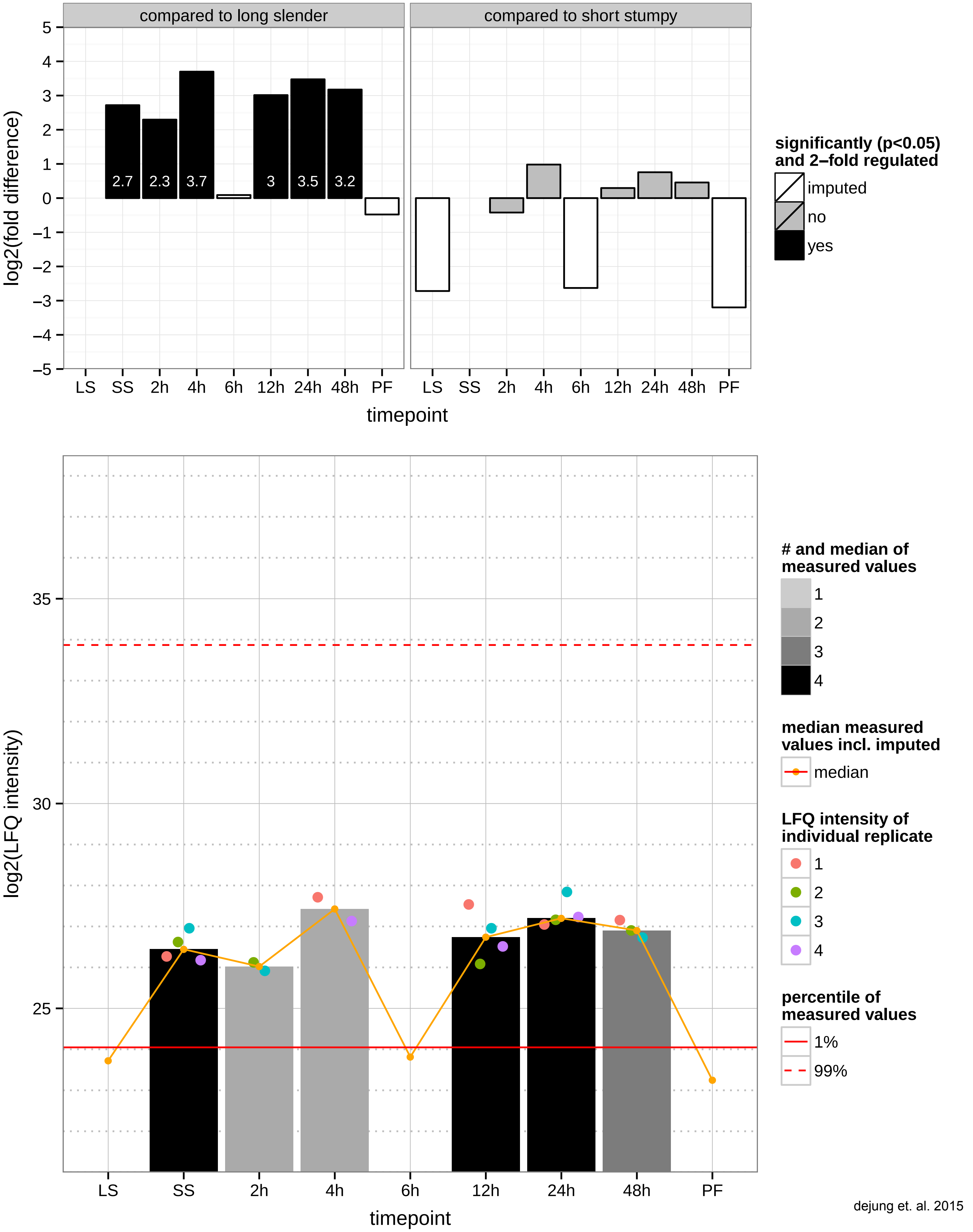

dejung et. al. 2015

hypothetical protein, conserved
Tb927.9.9090
AGOF: null
AGOC: null
AGOP: null
PGOF: protein binding
PGOC: null
PGOP: null

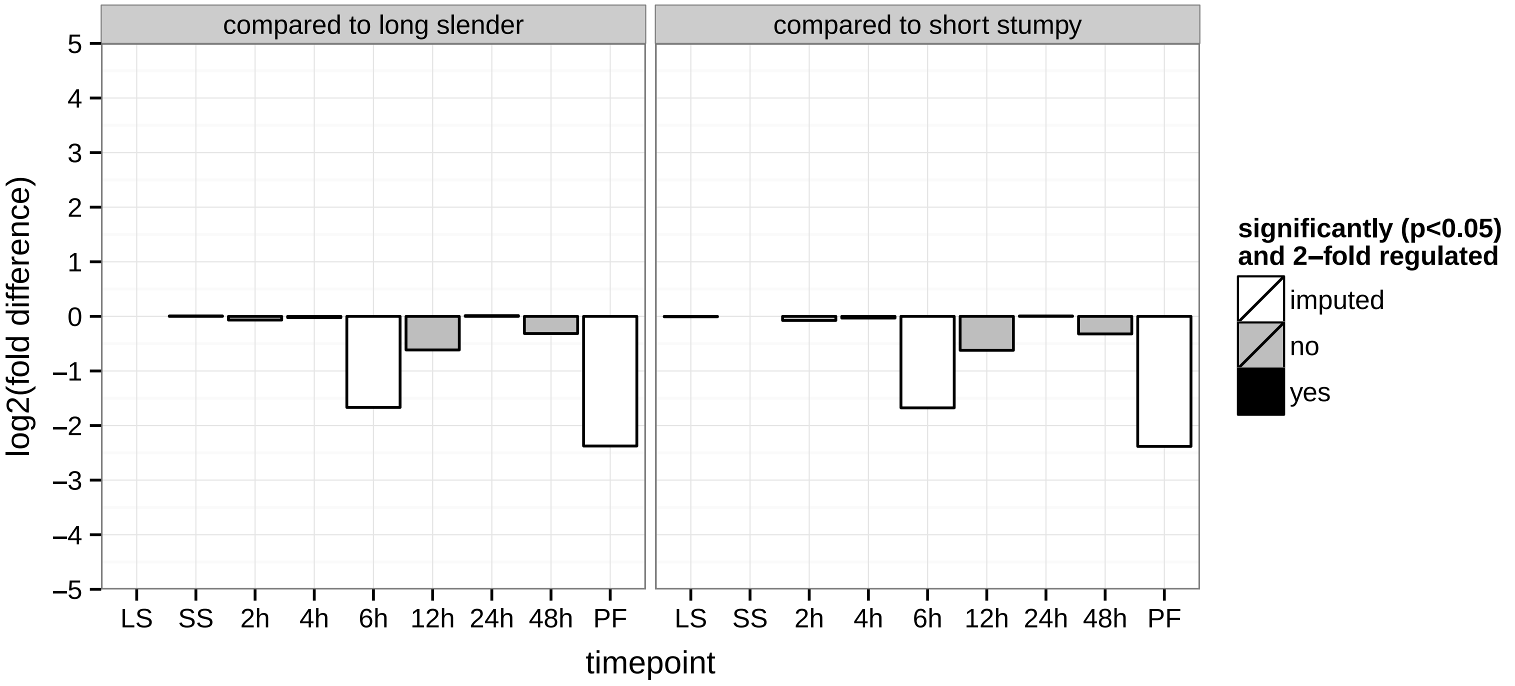

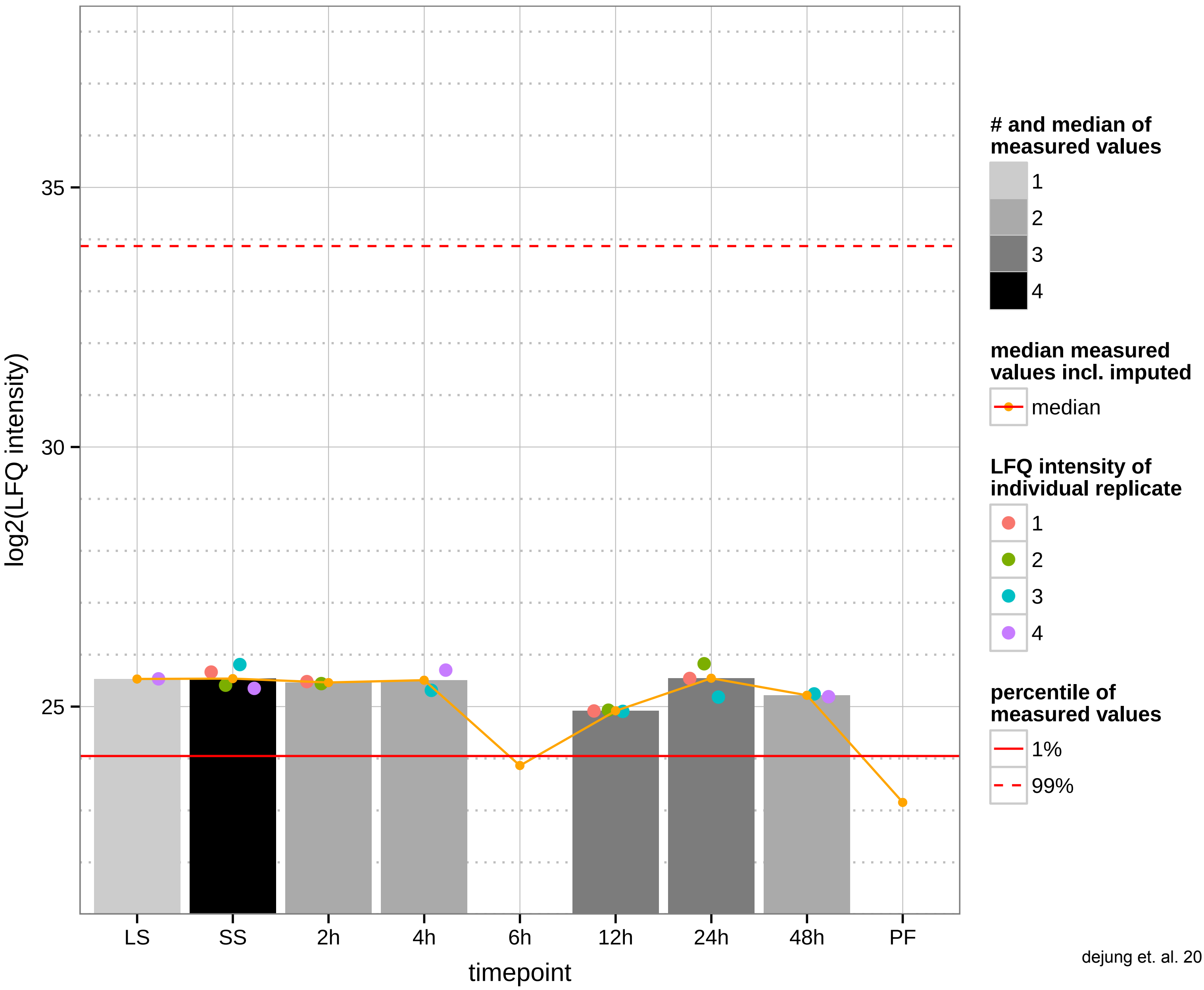

dejung et. al. 2015

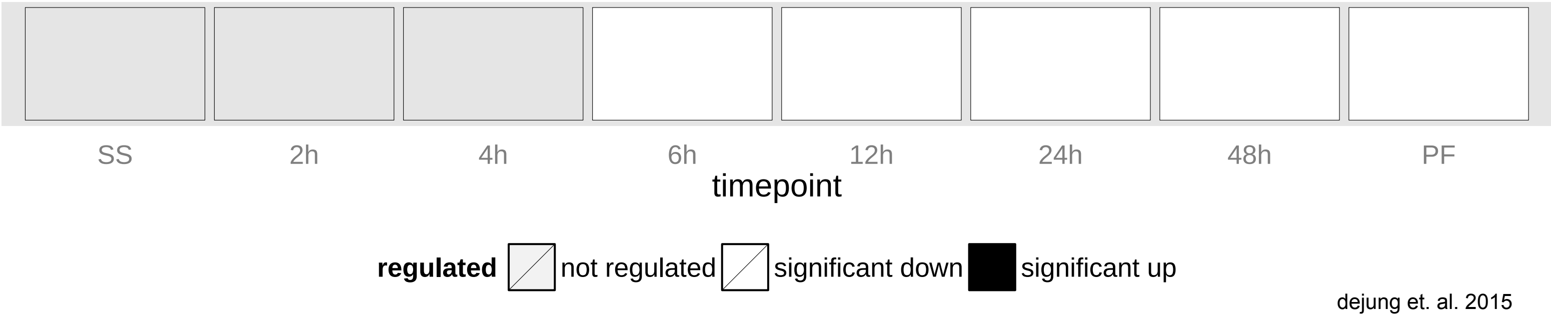
SS
2h
4h
6h
12h
24h
48h
PF
timepoint
regulated
not regulated
significant down
significant up
dejung et. al. 2015

hypothetical protein, conserved, BAC from homologous region on chr5
Tb927.5.4390;Tb05.5K5.40
AGOF: null
AGOC: null
AGOP: null
PGOF: null
PGOC: null
PGOP: null

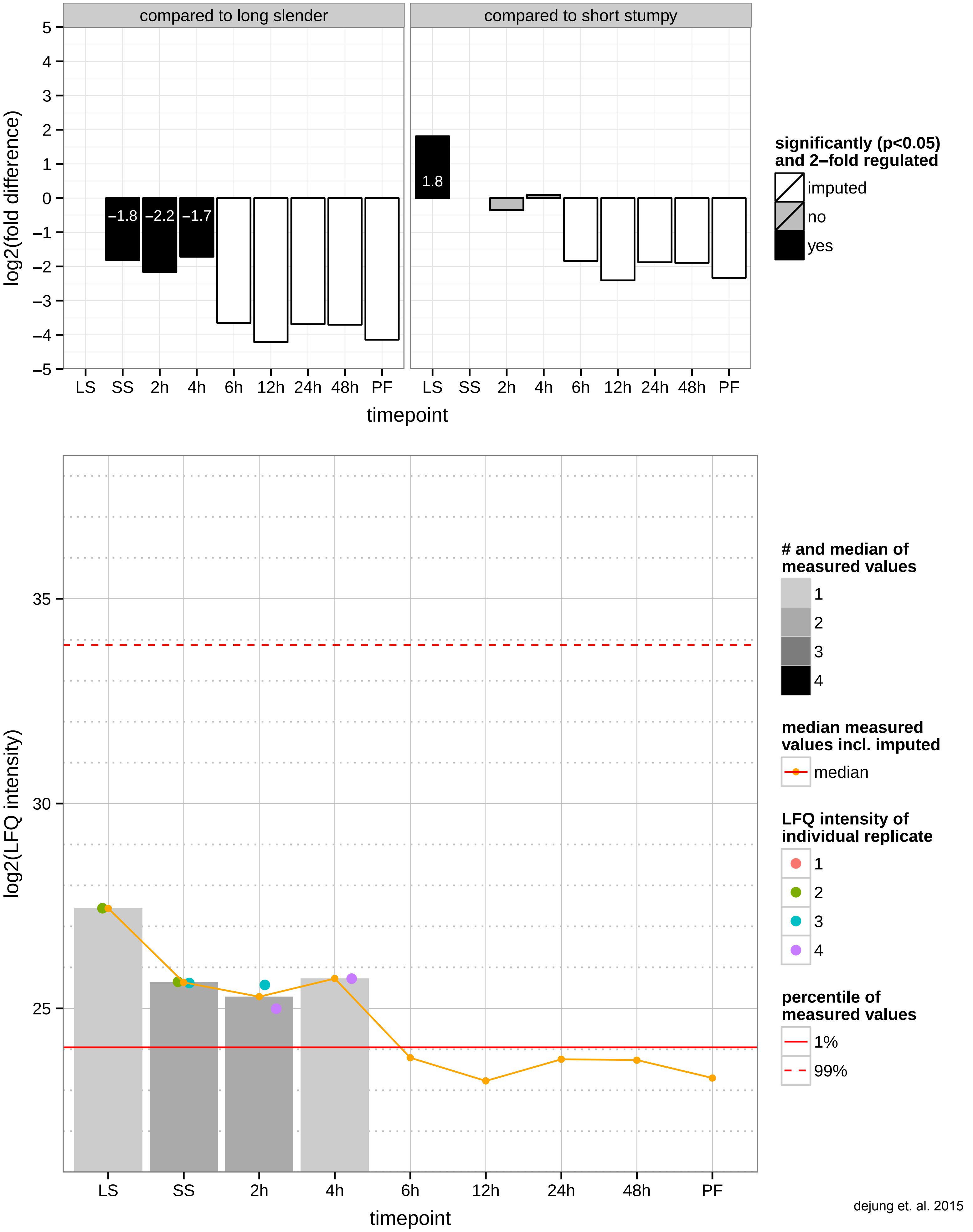

dejung et. al. 2015

retrotransposon hot spot protein (RHS, pseudogene), putative, retrotransposon hot spot protein 4 (RHS4, chrXI additional, un
Tb11.0820;Tb11.v5.0295
AGOF: null
AGOC: null
AGOP: null
PGOF: null
PGOC: null
PGOP: null

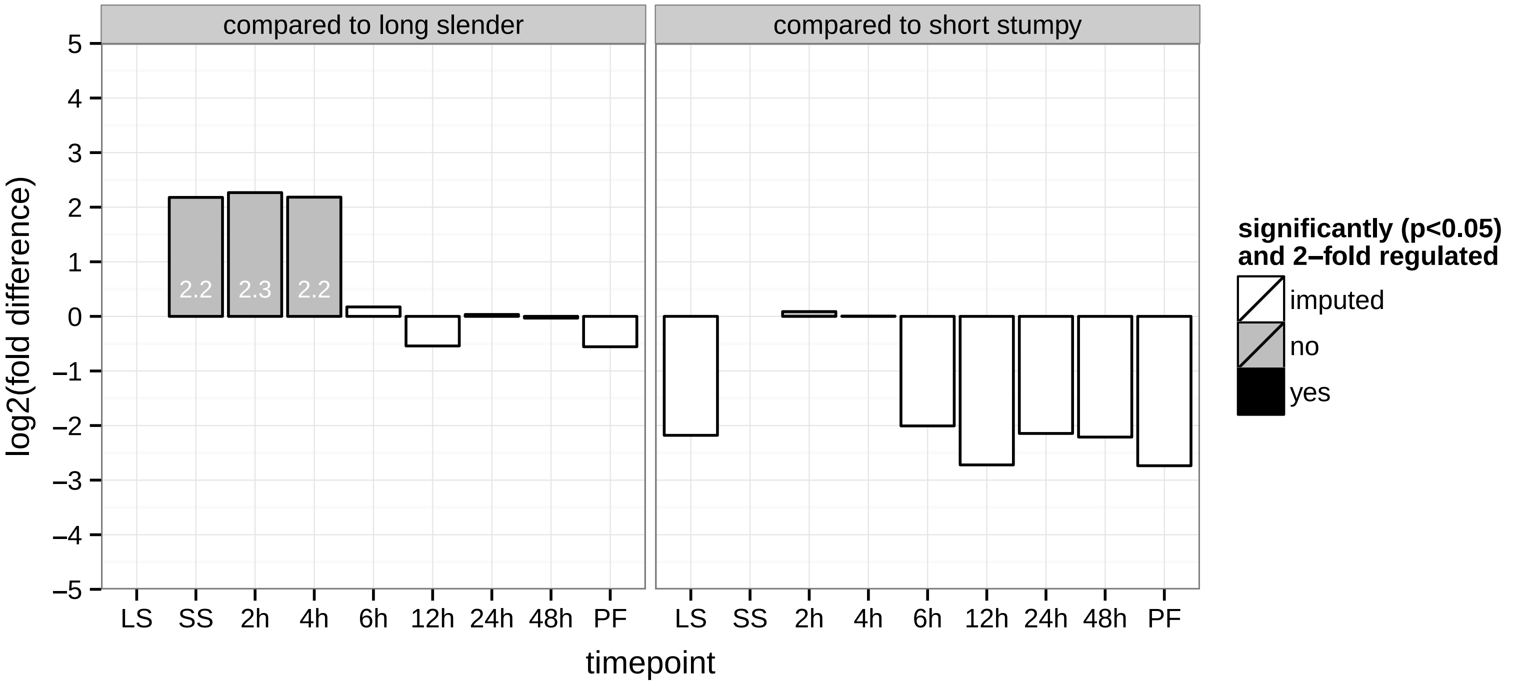

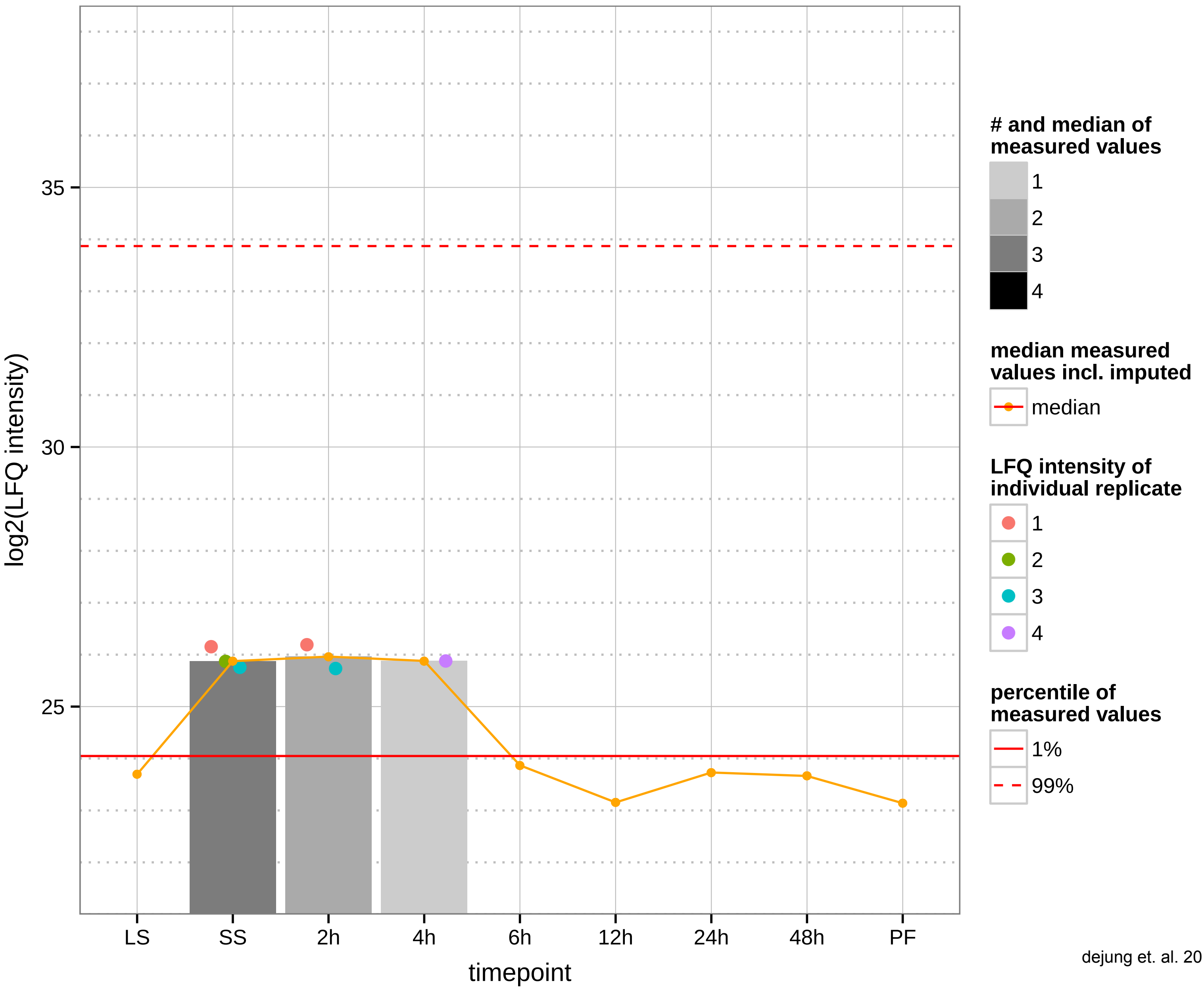

dejung et. al. 2015

BarP protein, putative, hypothetical protein
Tb927.7.4690;Tb11.v5.0153
AGOF: null
AGOC: null
AGOP: null
PGOF: null
PGOC: null
PGOP: null

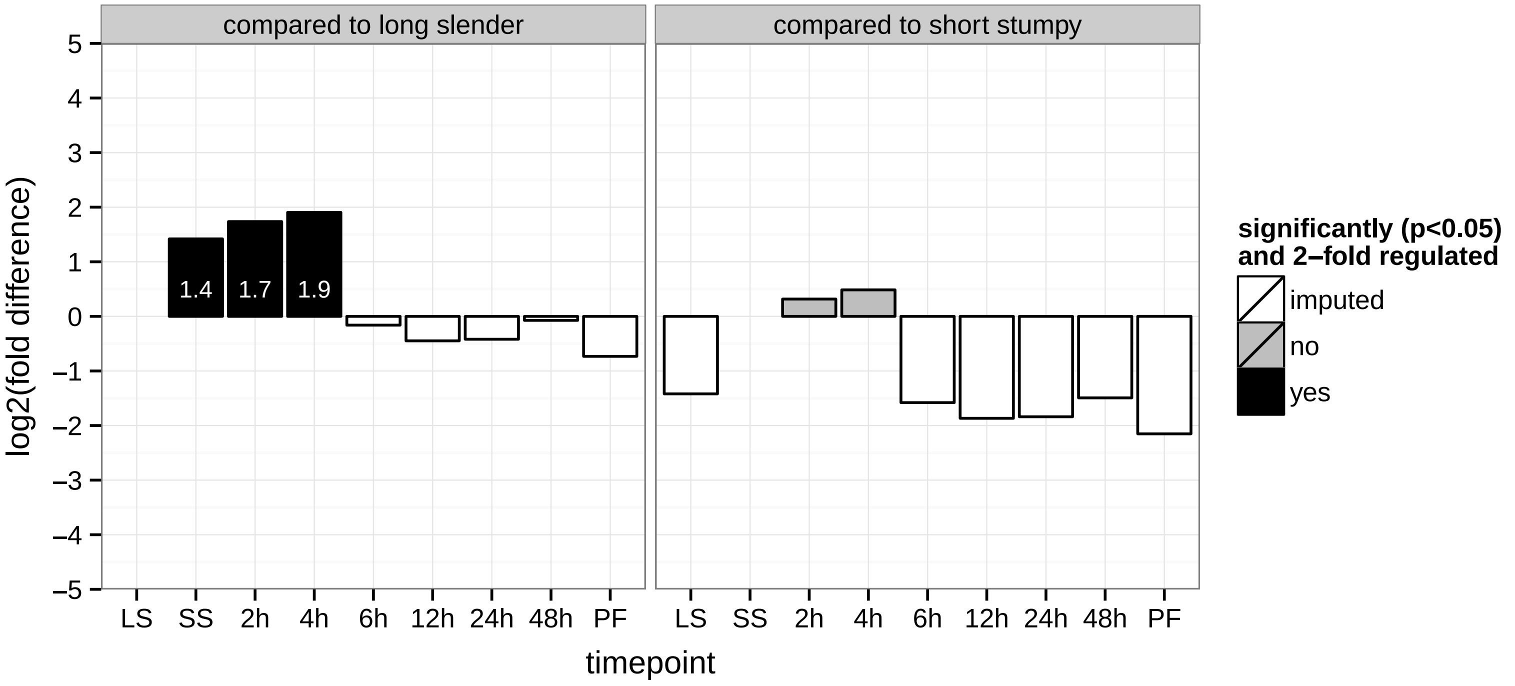

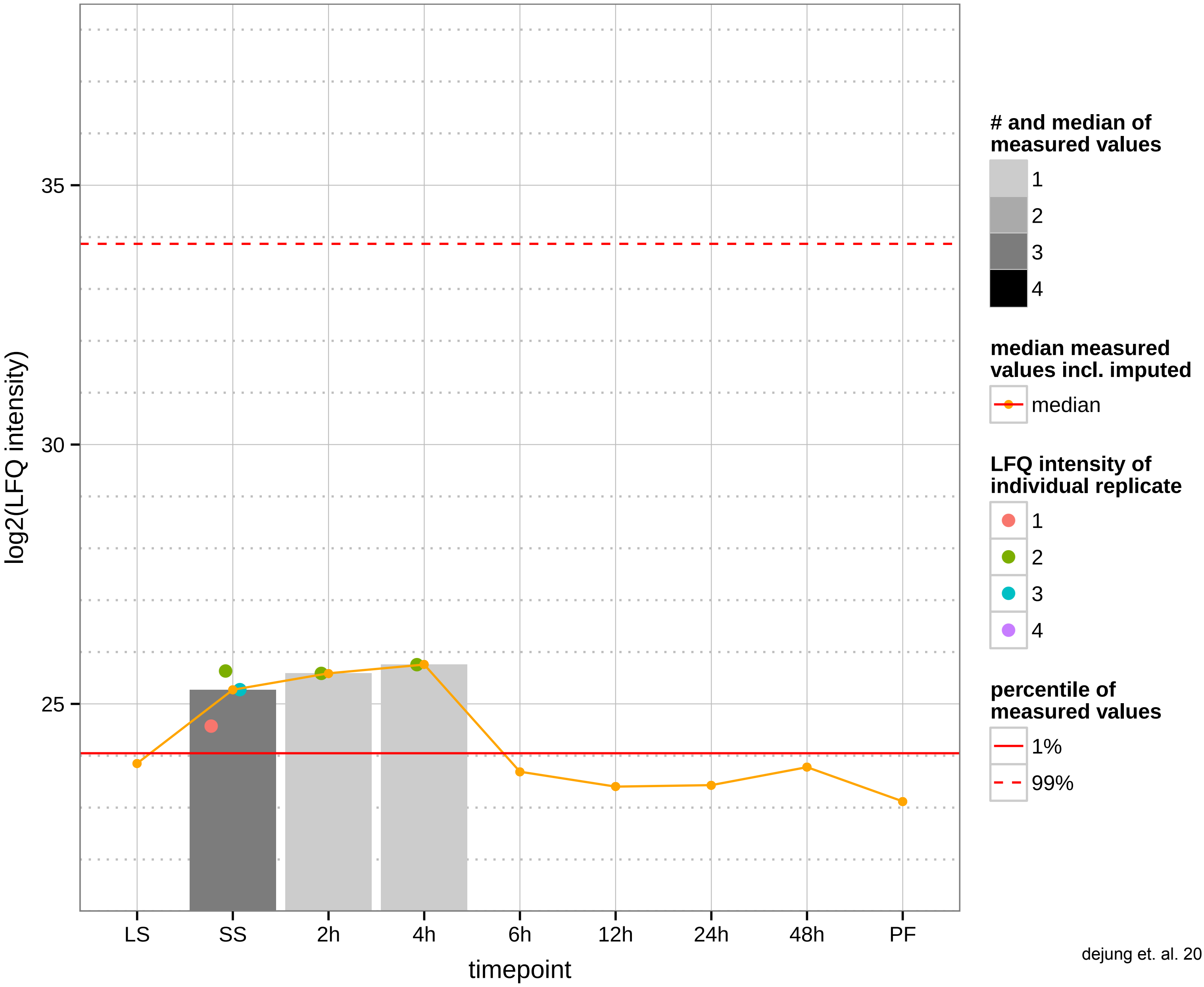

dejung et. al. 2015

75 kDa invariant surface glycoprotein, putative, putative (ISG75)
Tb927.5.370;Tb11.v5.0230
AGOF: null
AGOC: null, plasma membrane
AGOP: null
PGOF: null
PGOC: null
PGOP: null

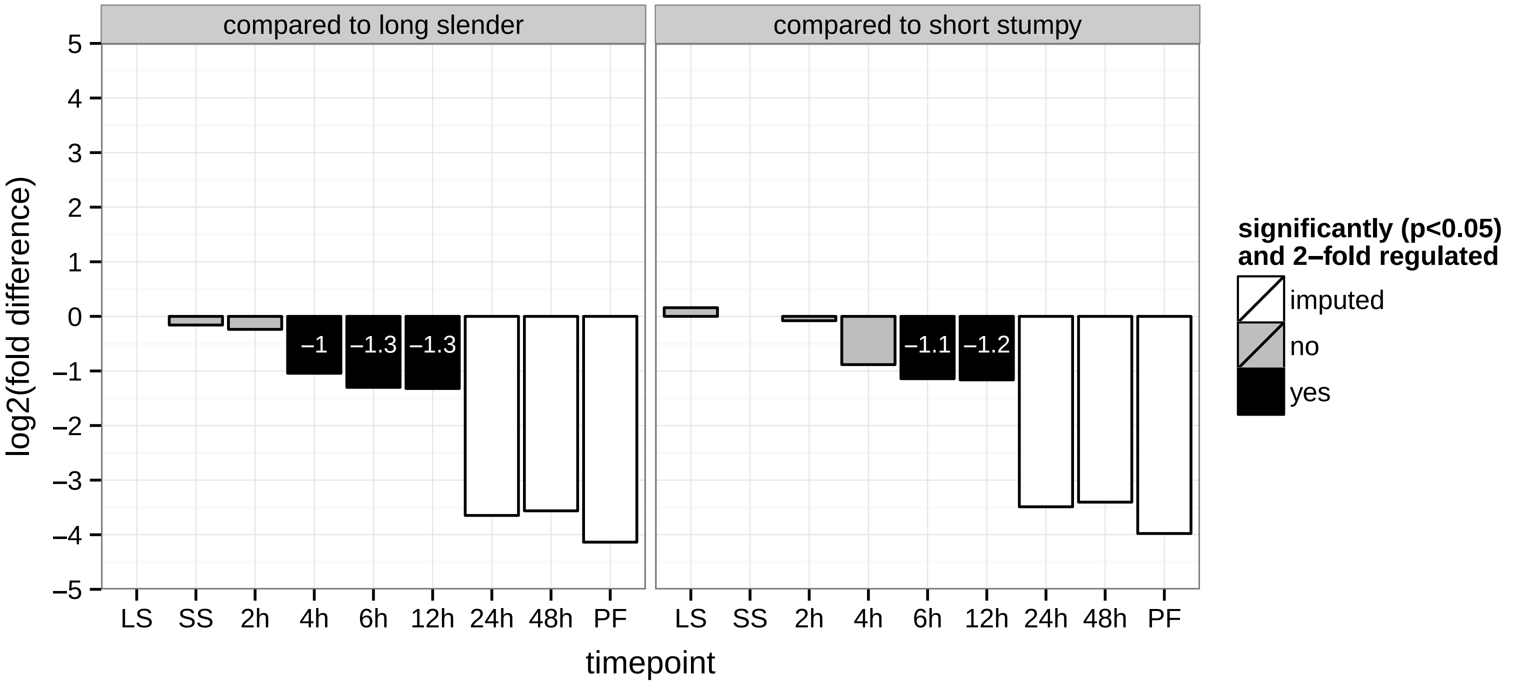

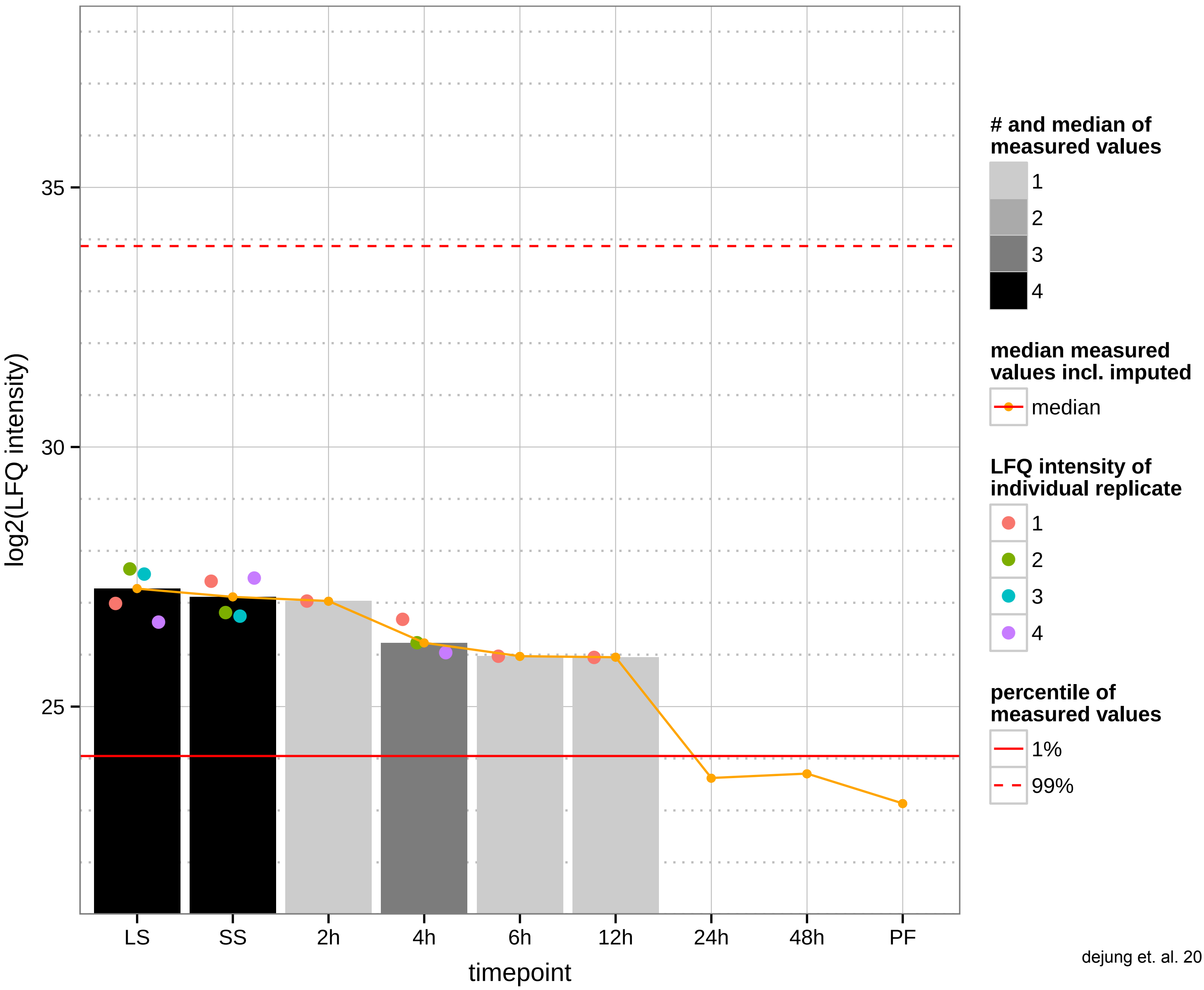

dejung et. al. 2015

hypothetical protein, conserved
Tb11.v5.0365
AGOF: null
AGOC: null
AGOP: null
PGOF: null
PGOC: null
PGOP: null

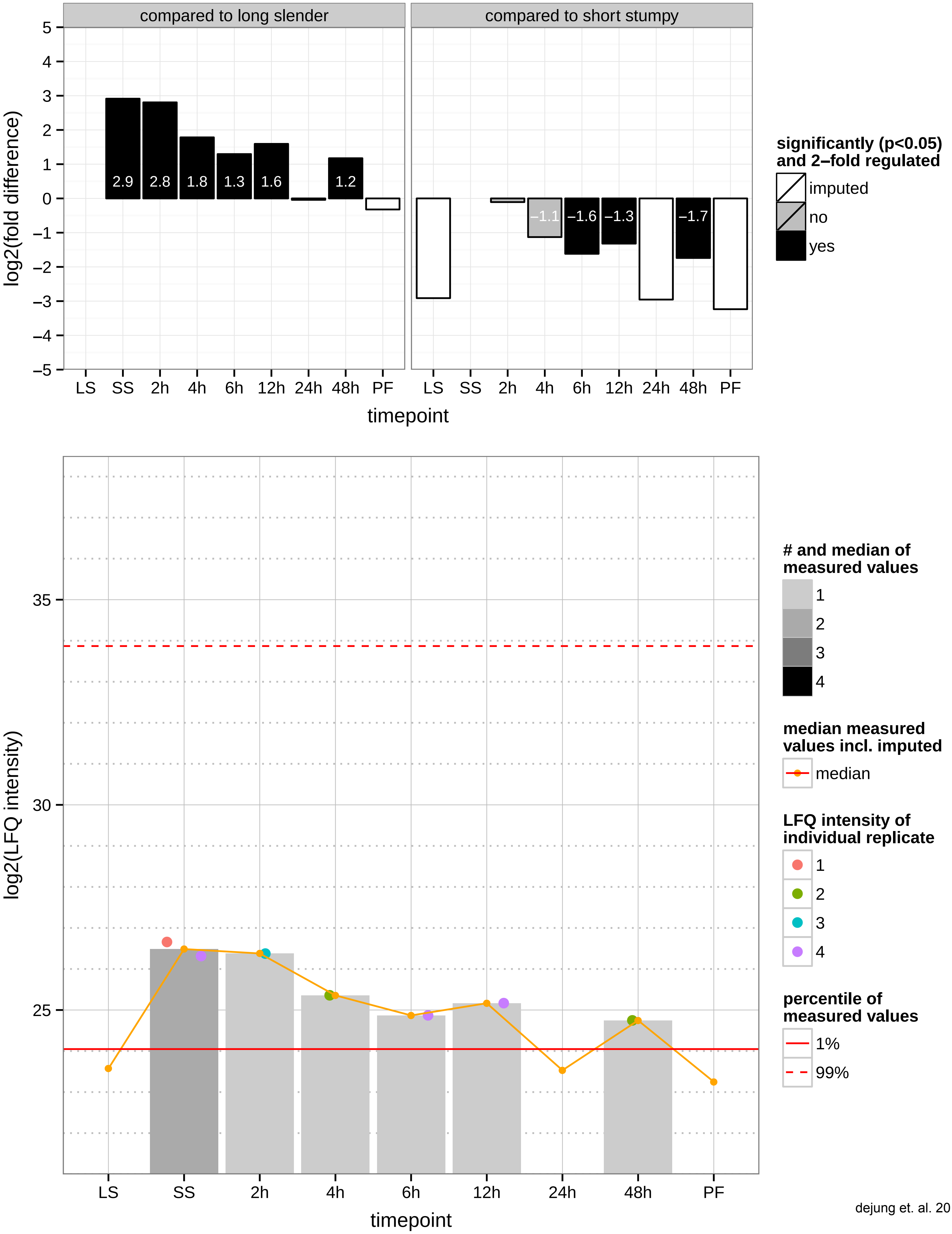

dejung et. al. 2015

65 kDa invariant surface glycoprotein, putative
Tb11.v5.0731
AGOF: null
AGOC: null
AGOP: null
PGOF: null
PGOC: null
PGOP: null

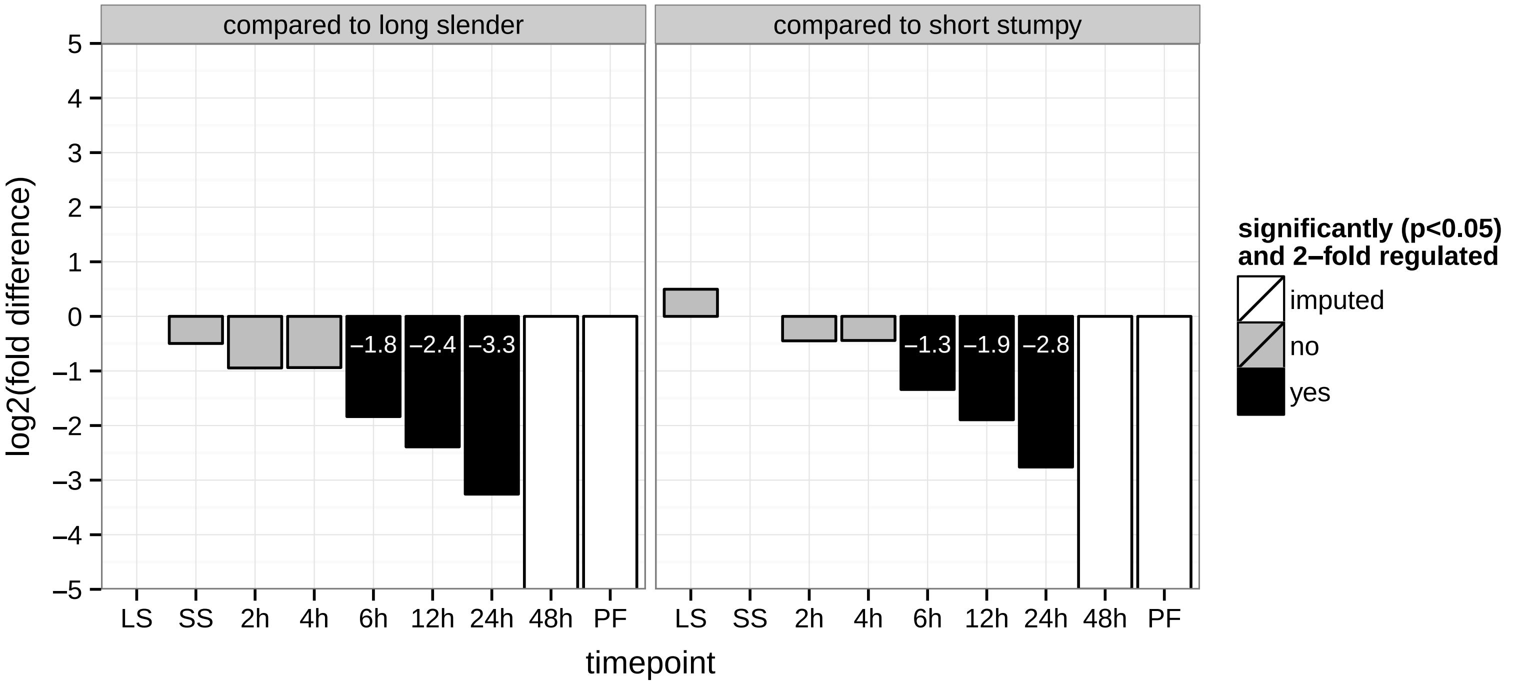

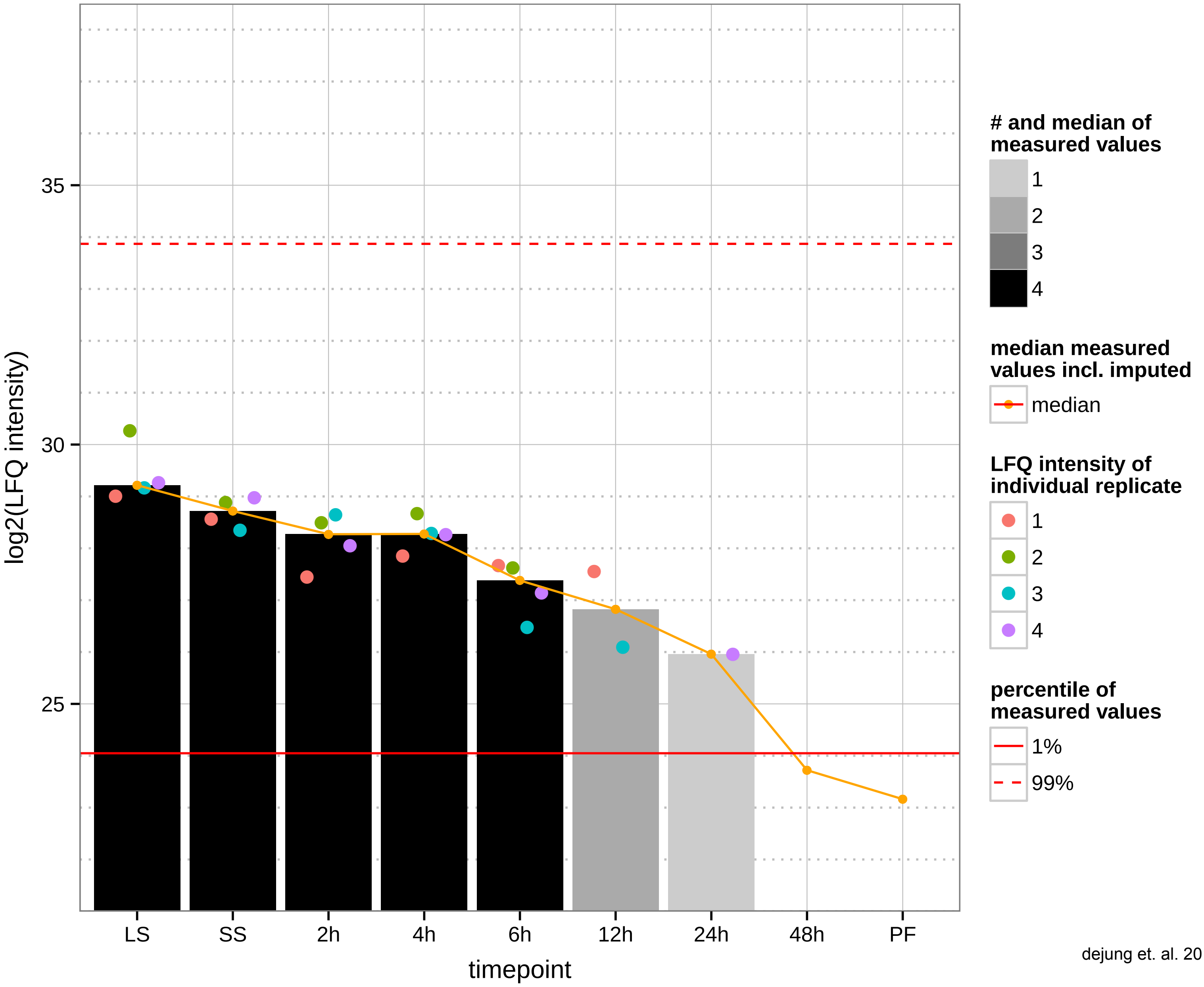

dejung et. al. 2015

Sarcalumenin, putative, sarcoplasmic reticulum glycoprotein
Tb927.10.14910
AGOF: GTP binding, calcium ion binding
AGOC: intracellular, sarcoplasmic reticulum
AGOP: endosomal transport
PGOF: GTP binding, GTPase activity
PGOC: null
PGOP: null

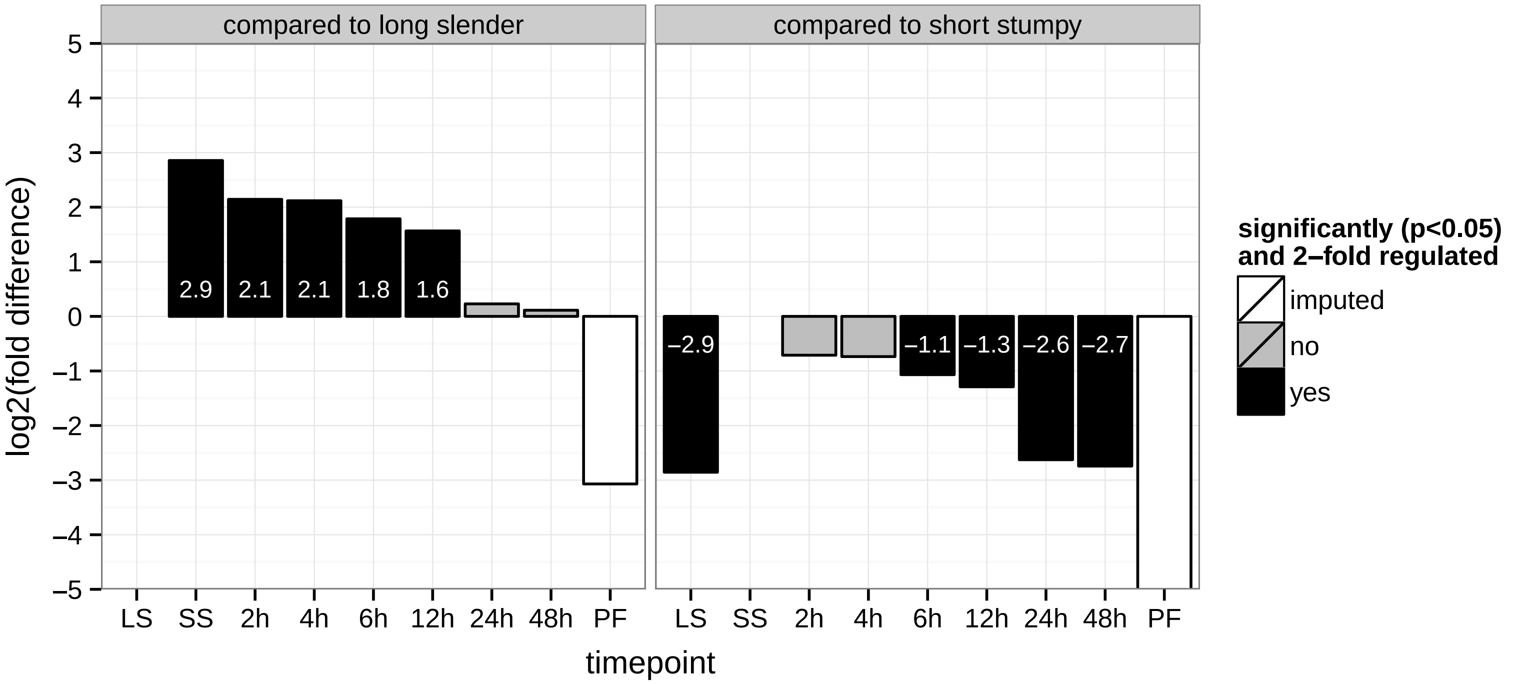

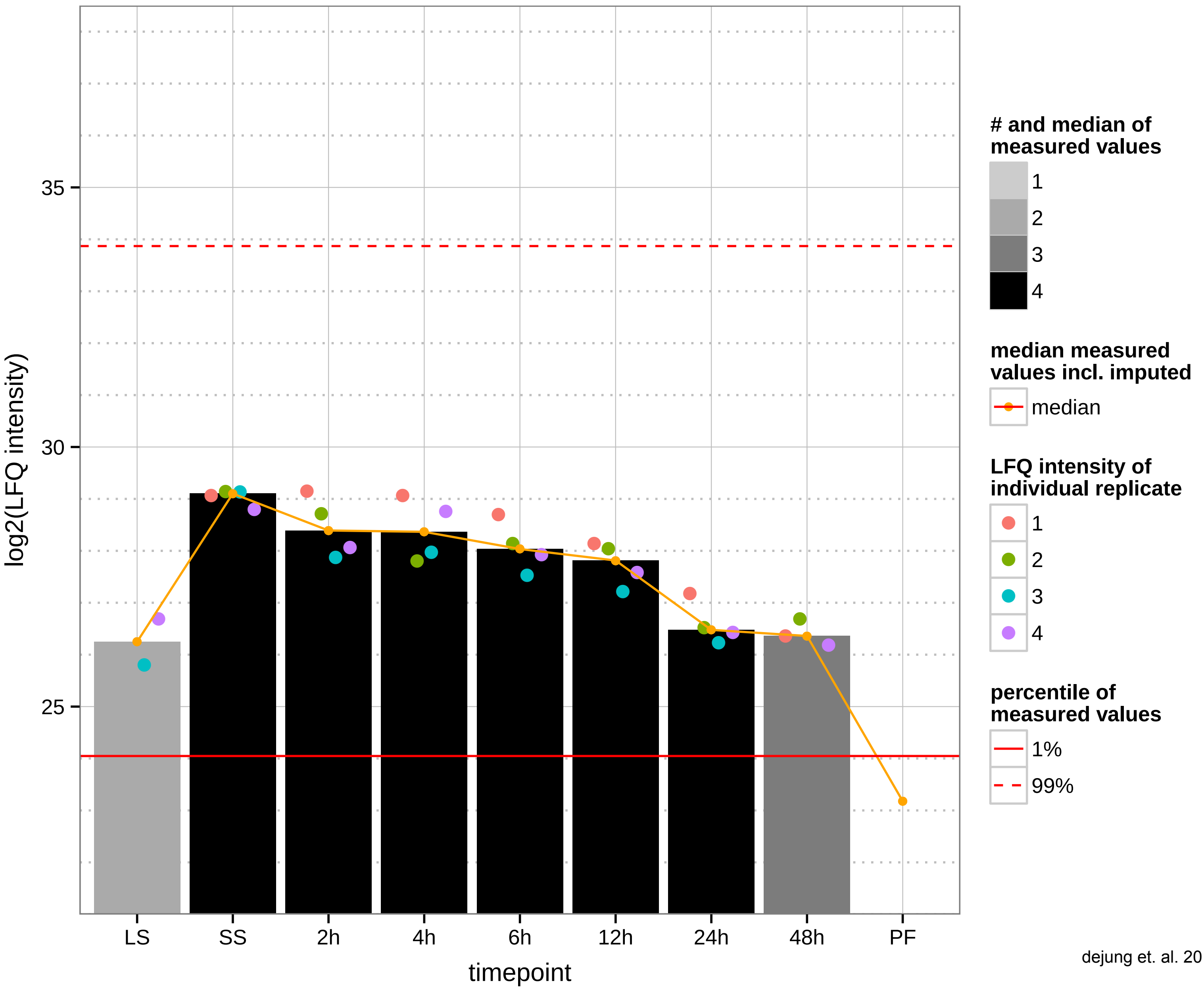

dejung et. al. 2015

protein–L–isoaspartate, putative
Tb927.10.7850
AGOF: protein–L–isoaspartate (D–aspartate) O–methyltransferase activity
AGOC: null
AGOP: cellular protein modification process
PGOF: protein–L–isoaspartate (D–aspartate) O–methyltransferase activity
PGOC: null
PGOP: cellular protein modification process

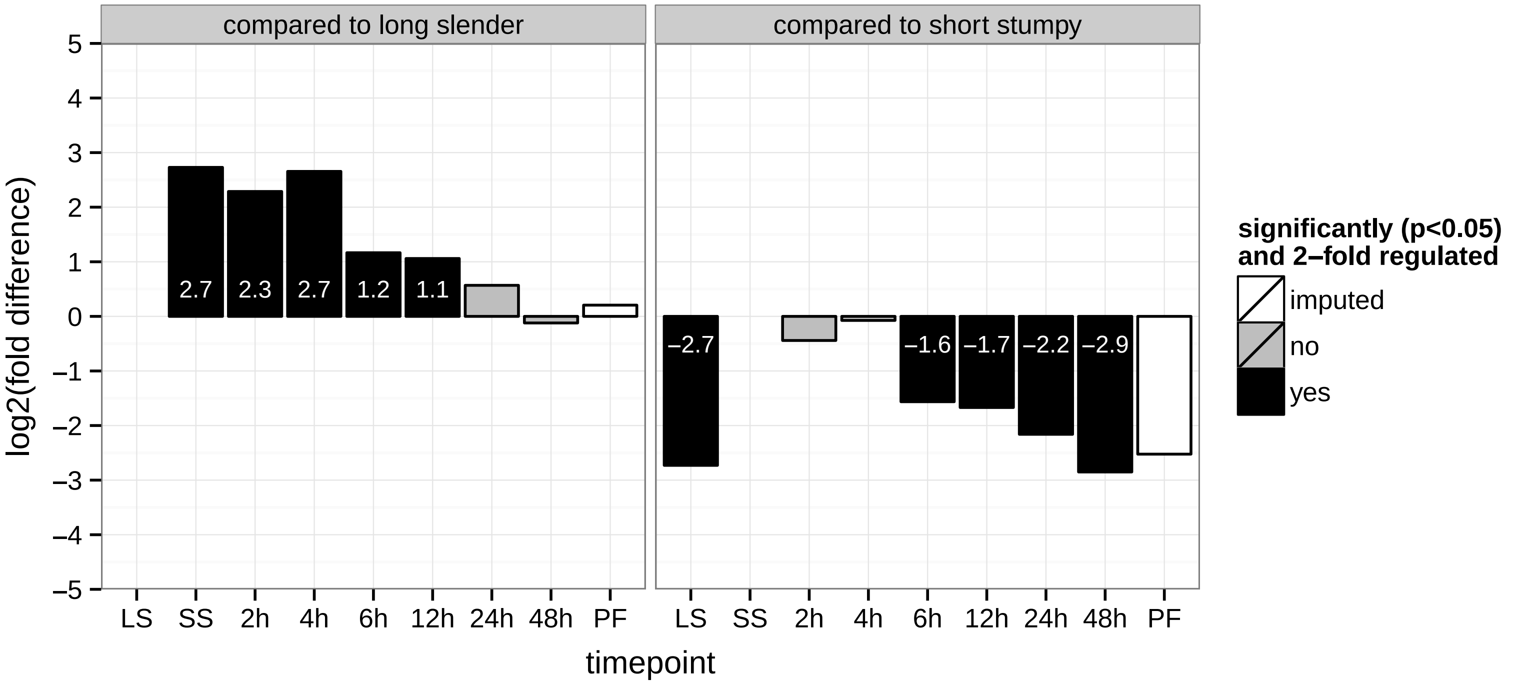

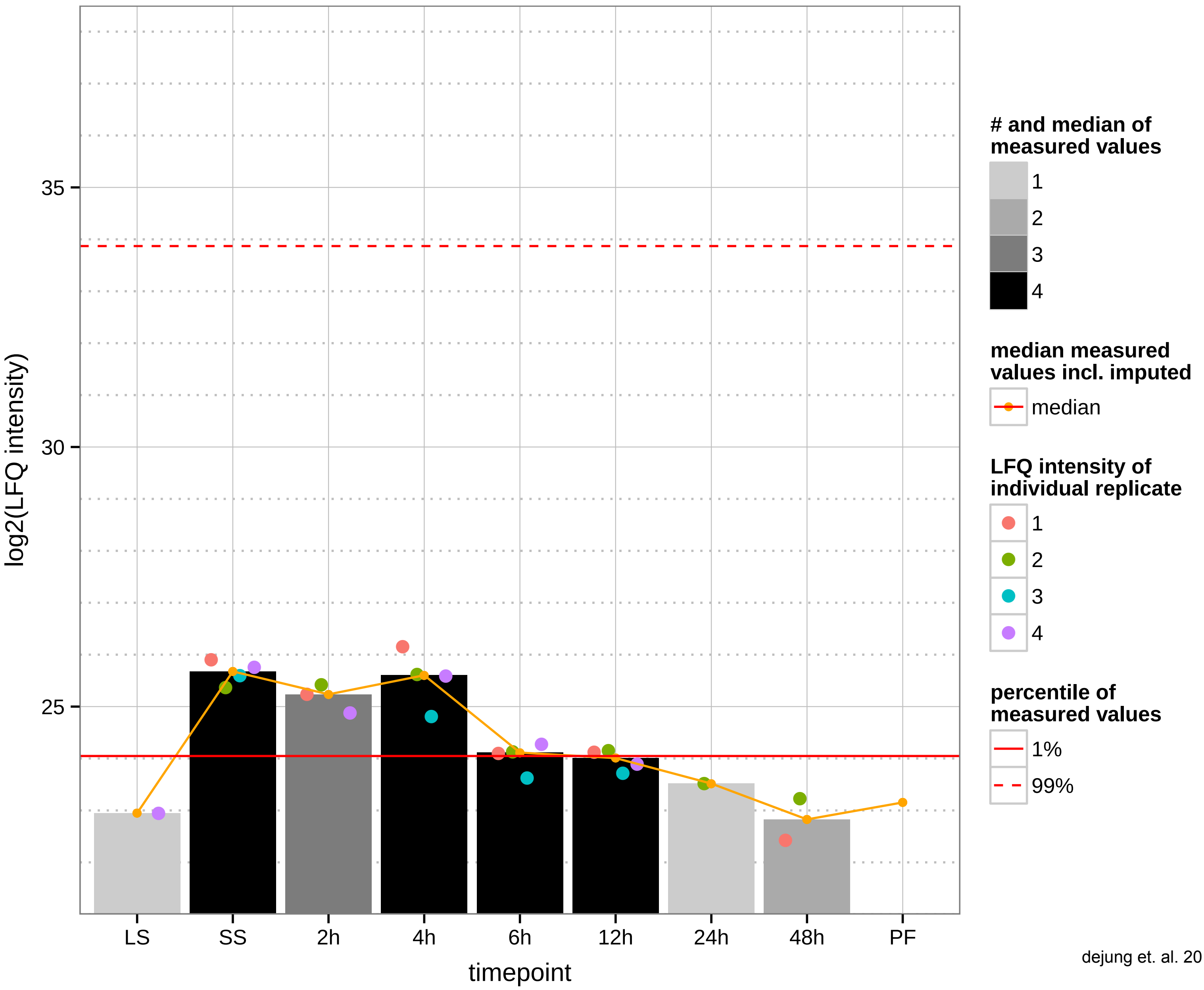

dejung et. al. 2015

hypothetical protein, conserved
Tb927.11.7550
AGOF: null
AGOC: null
AGOP: null
PGOF: null
PGOC: null
PGOP: null

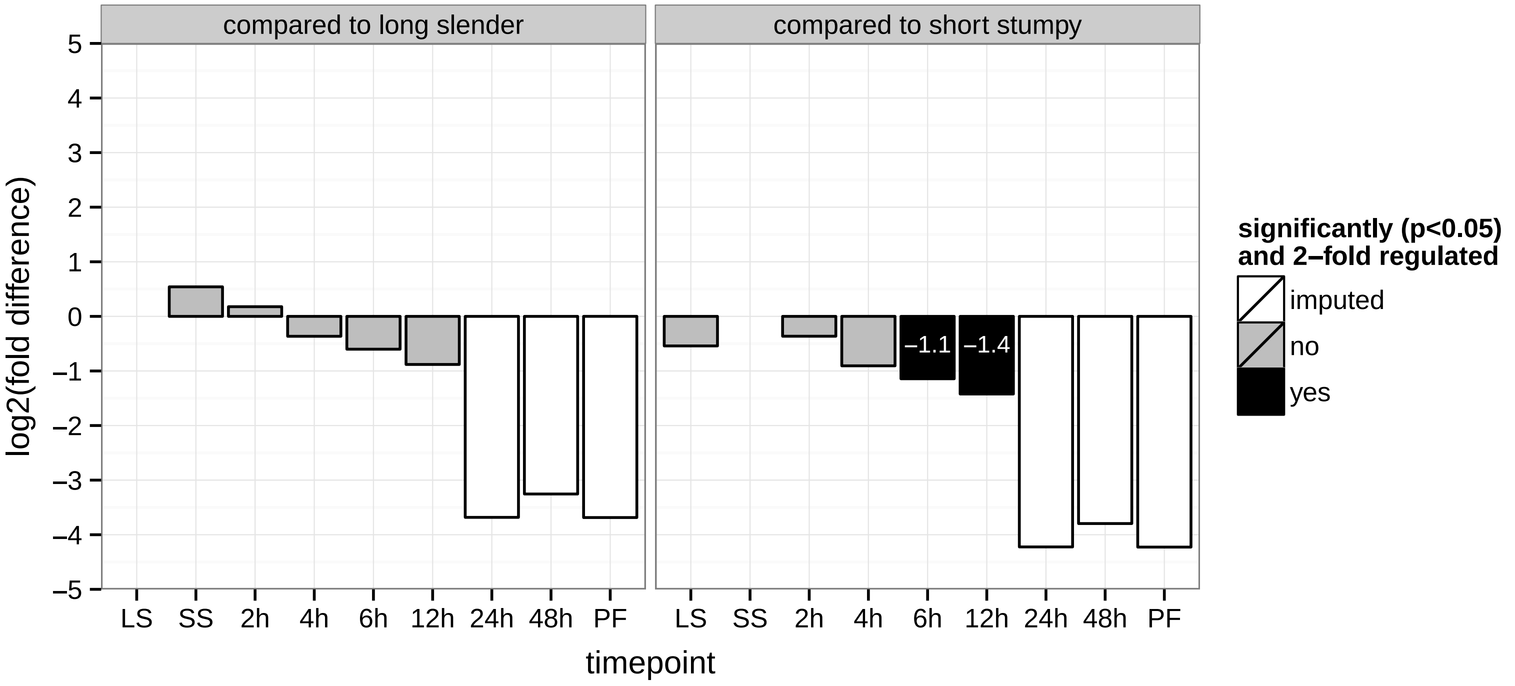

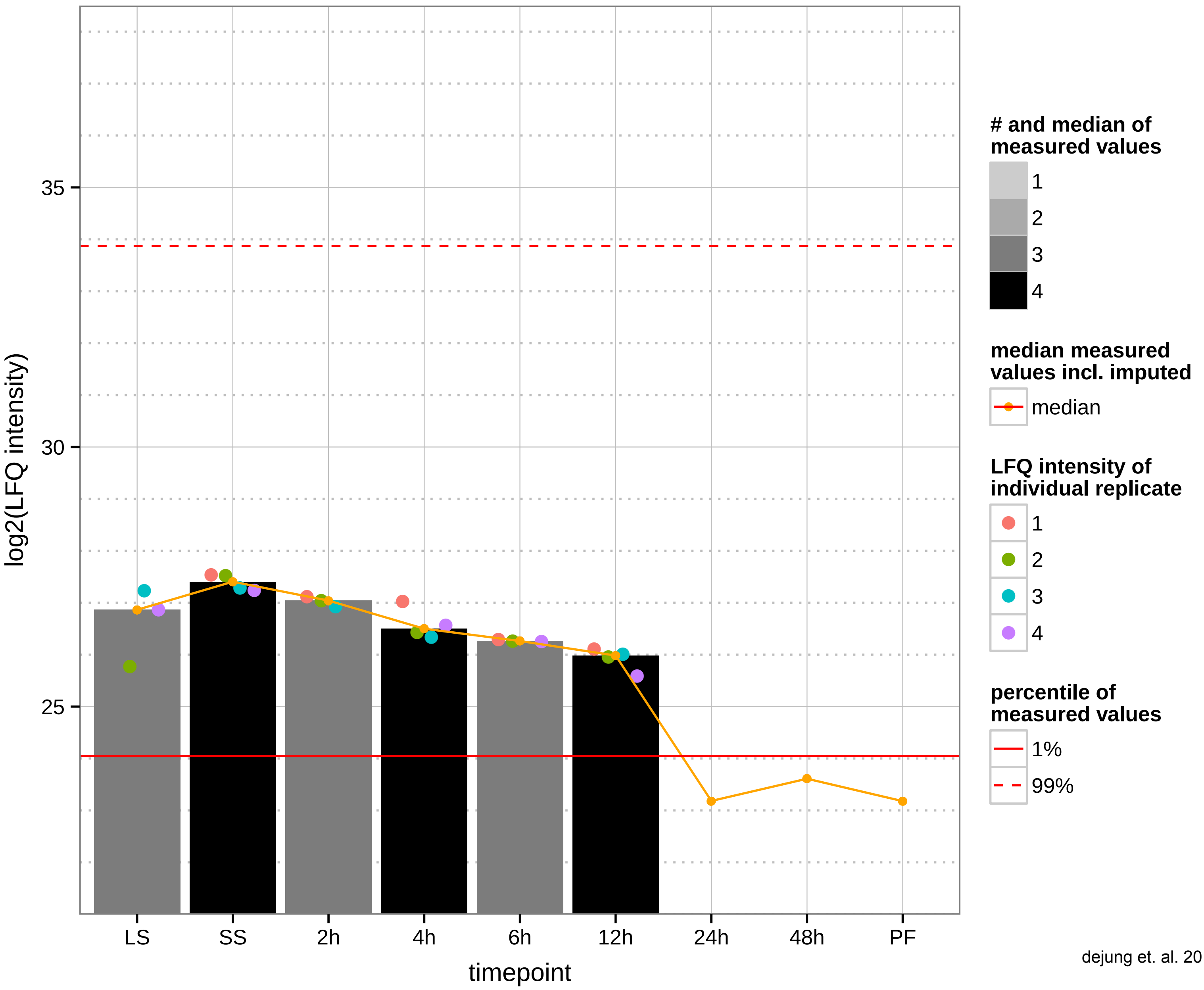

dejung et. al. 2015

hypothetical protein, conserved
Tb927.11.9150
AGOF: null
AGOC: mitochondrion
AGOP: null
PGOF: null
PGOC: null
PGOP: null

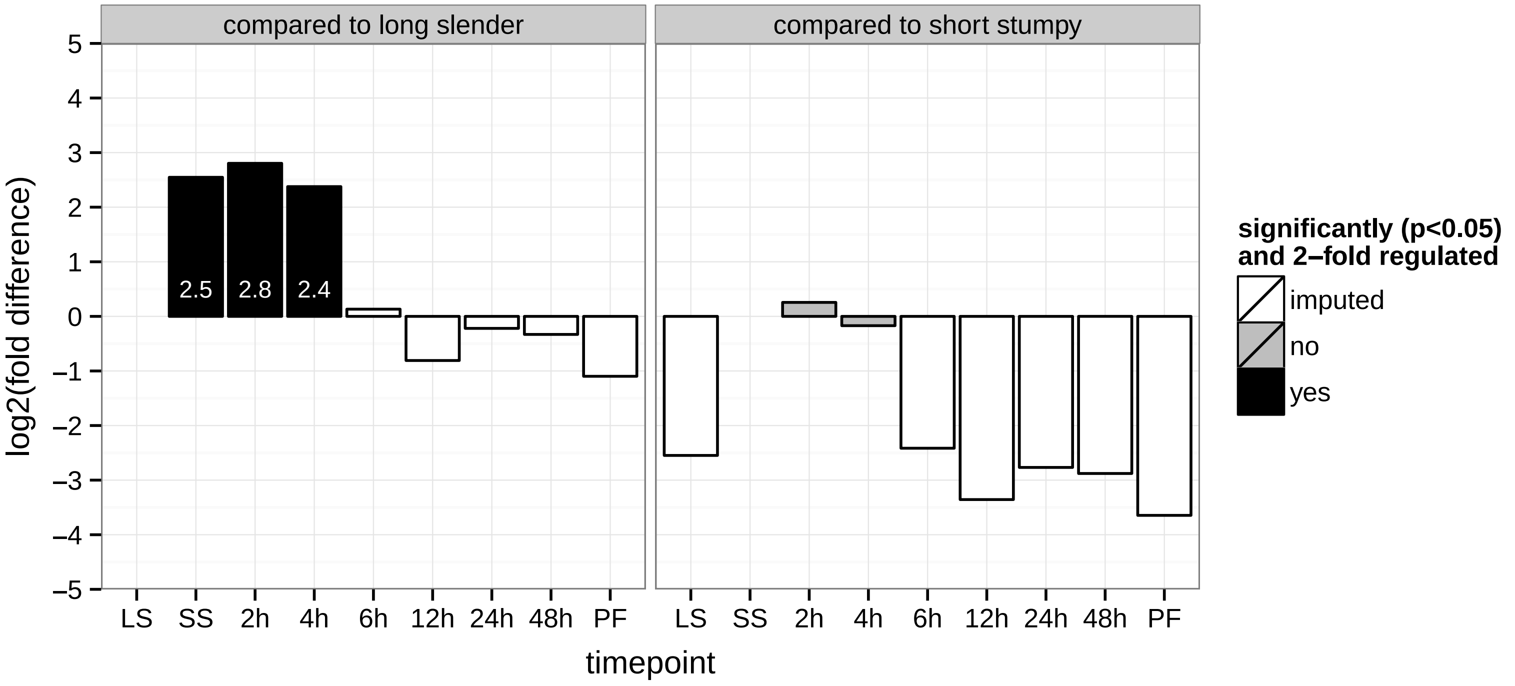

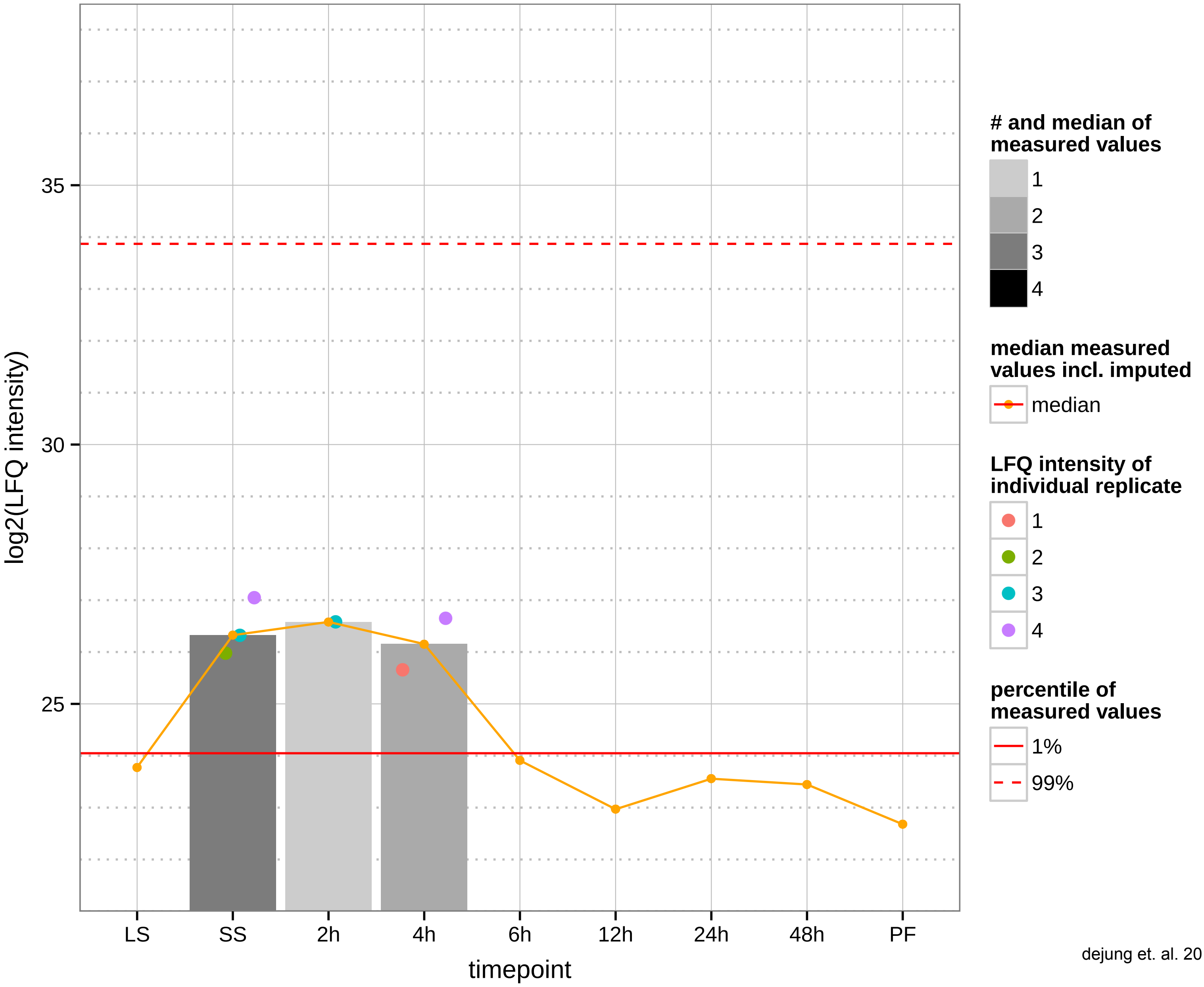

dejung et. al. 2015

65 kDa invariant surface glycoprotein
Tb927.2.3320
AGOF: null
AGOC: plasma membrane
AGOP: plasma membrane organization
PGOF: null
PGOC: null
PGOP: null

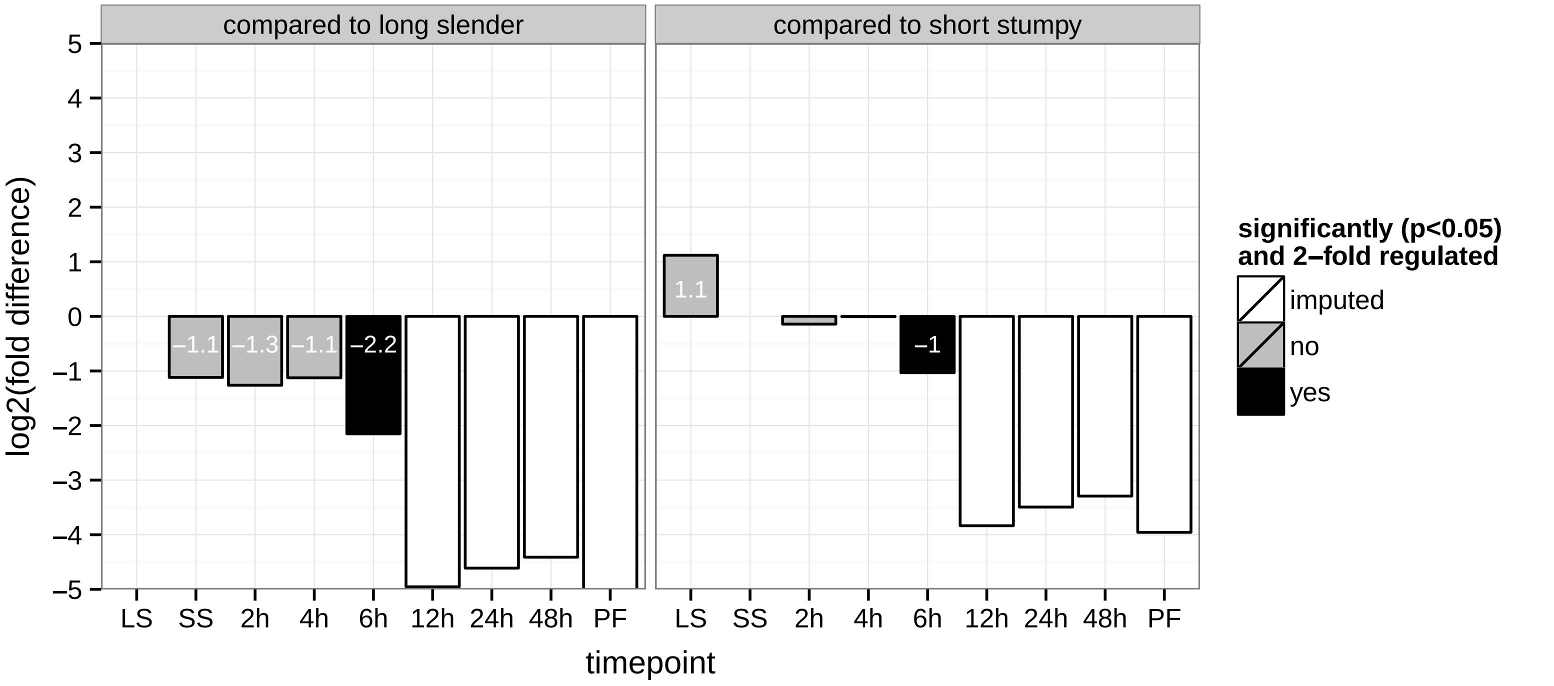

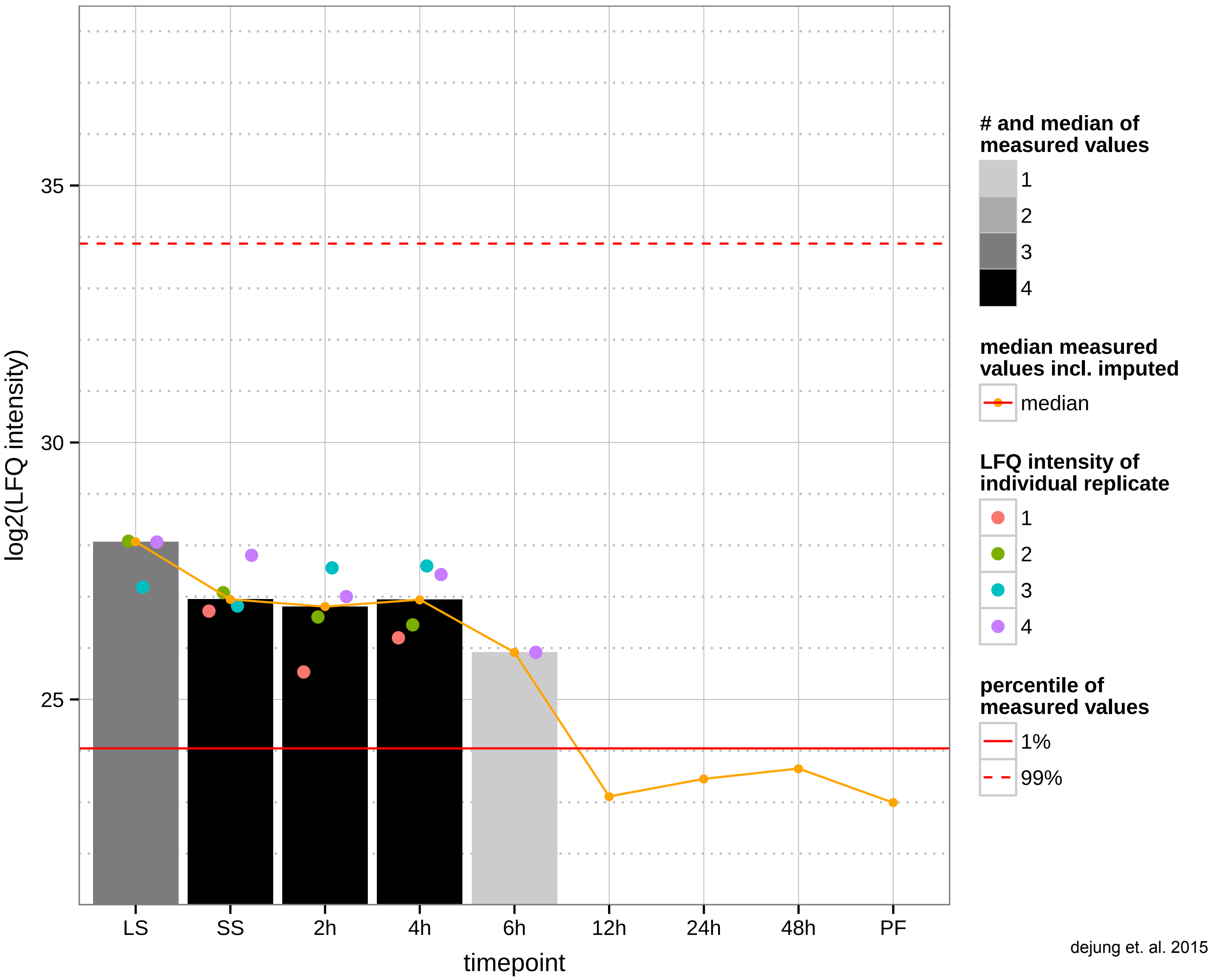

dejung et. al. 2015

hypothetical protein, conserved
Tb927.2.5200
AGOF: null
AGOC: null
AGOP: null
PGOF: null
PGOC: null
PGOP: null

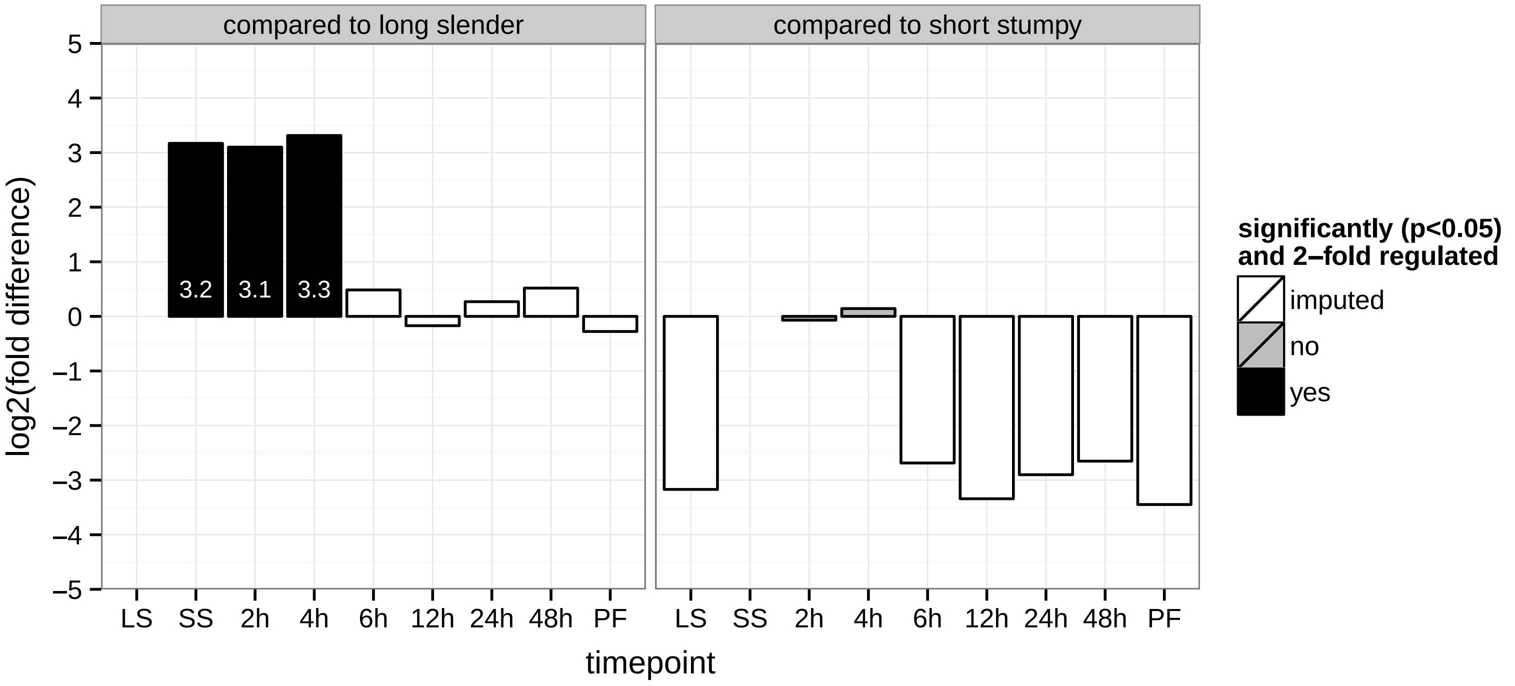

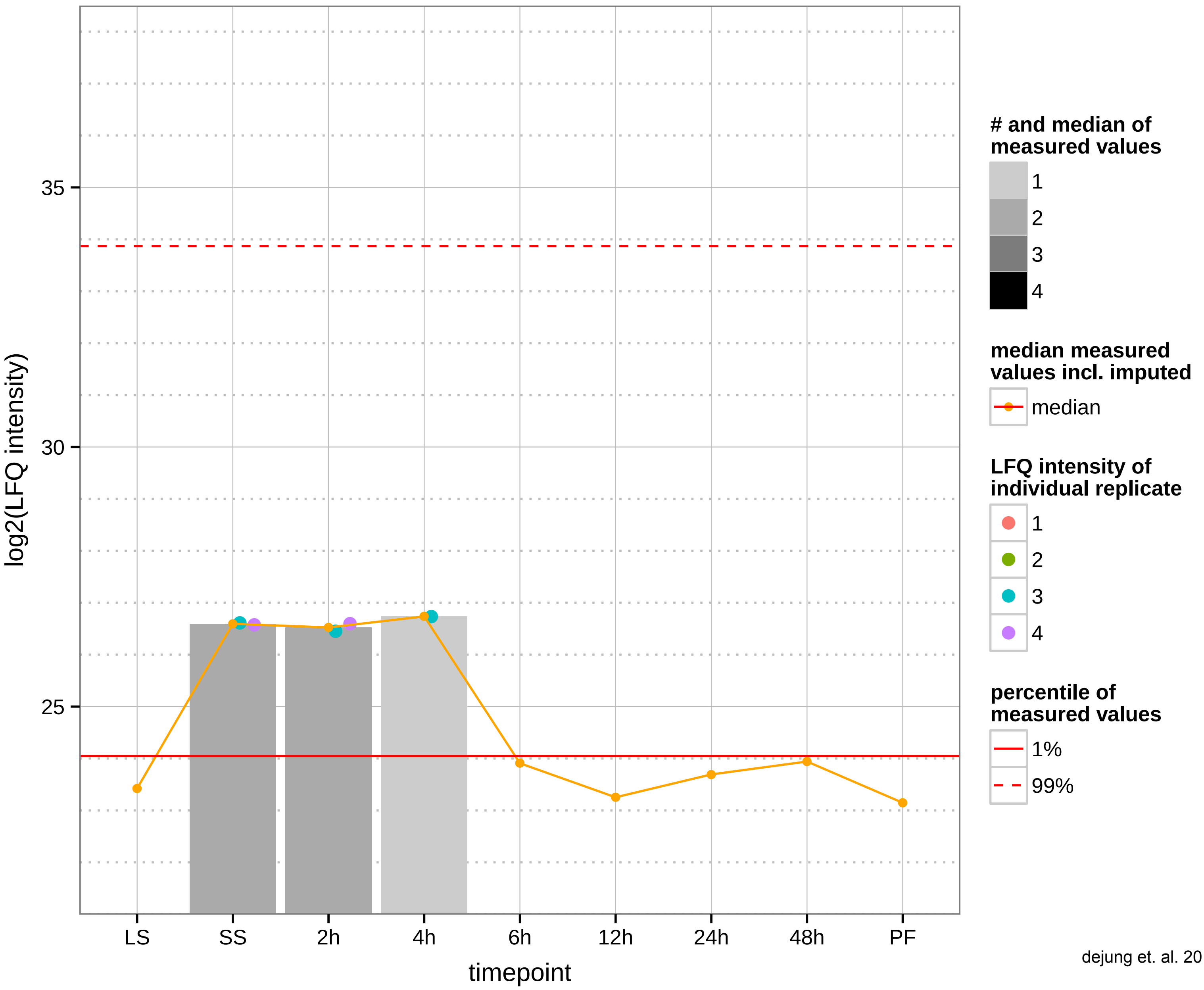

dejung et. al. 2015

zinc finger protein family member, putative (ZC3H20)
Tb927.7.2660
AGOF: RNA binding
AGOC: null
AGOP: null
PGOF: nucleic acid binding, zinc ion binding
PGOC: null
PGOP: null

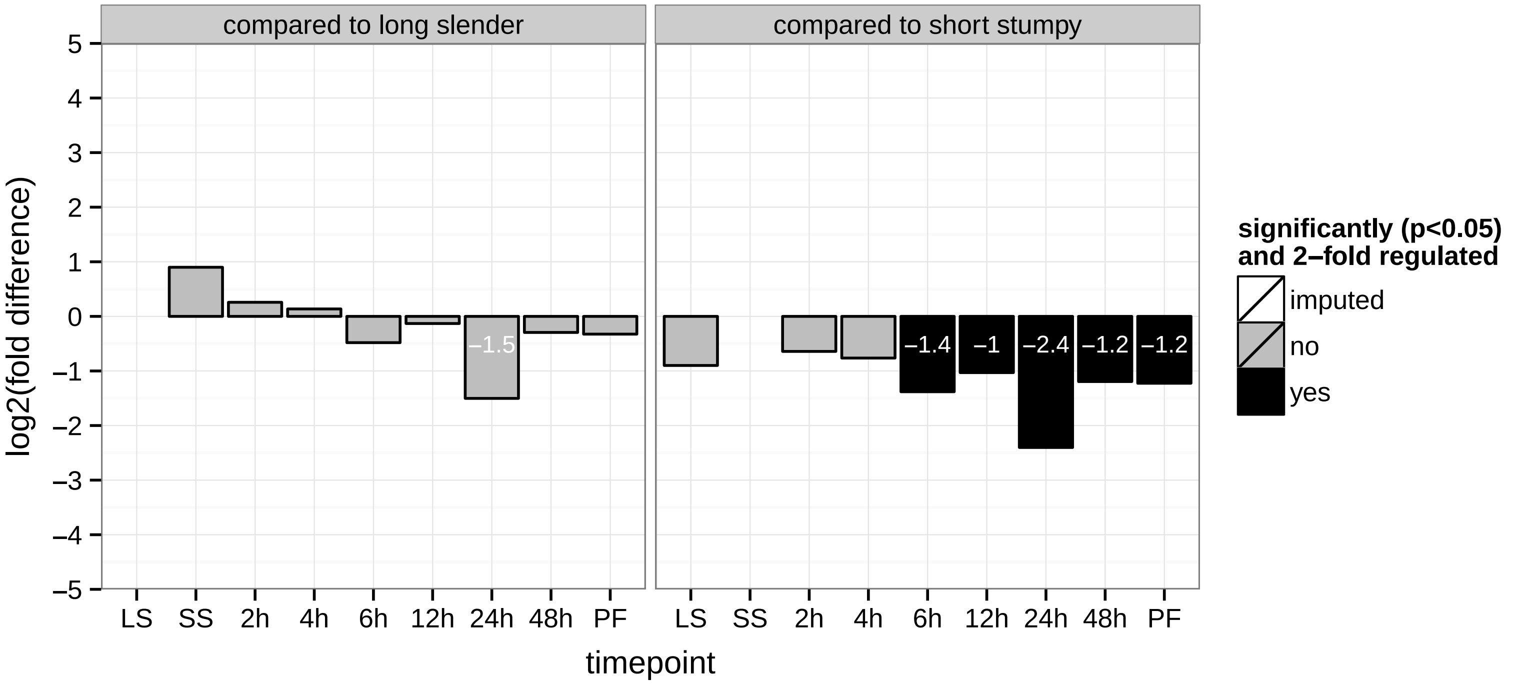

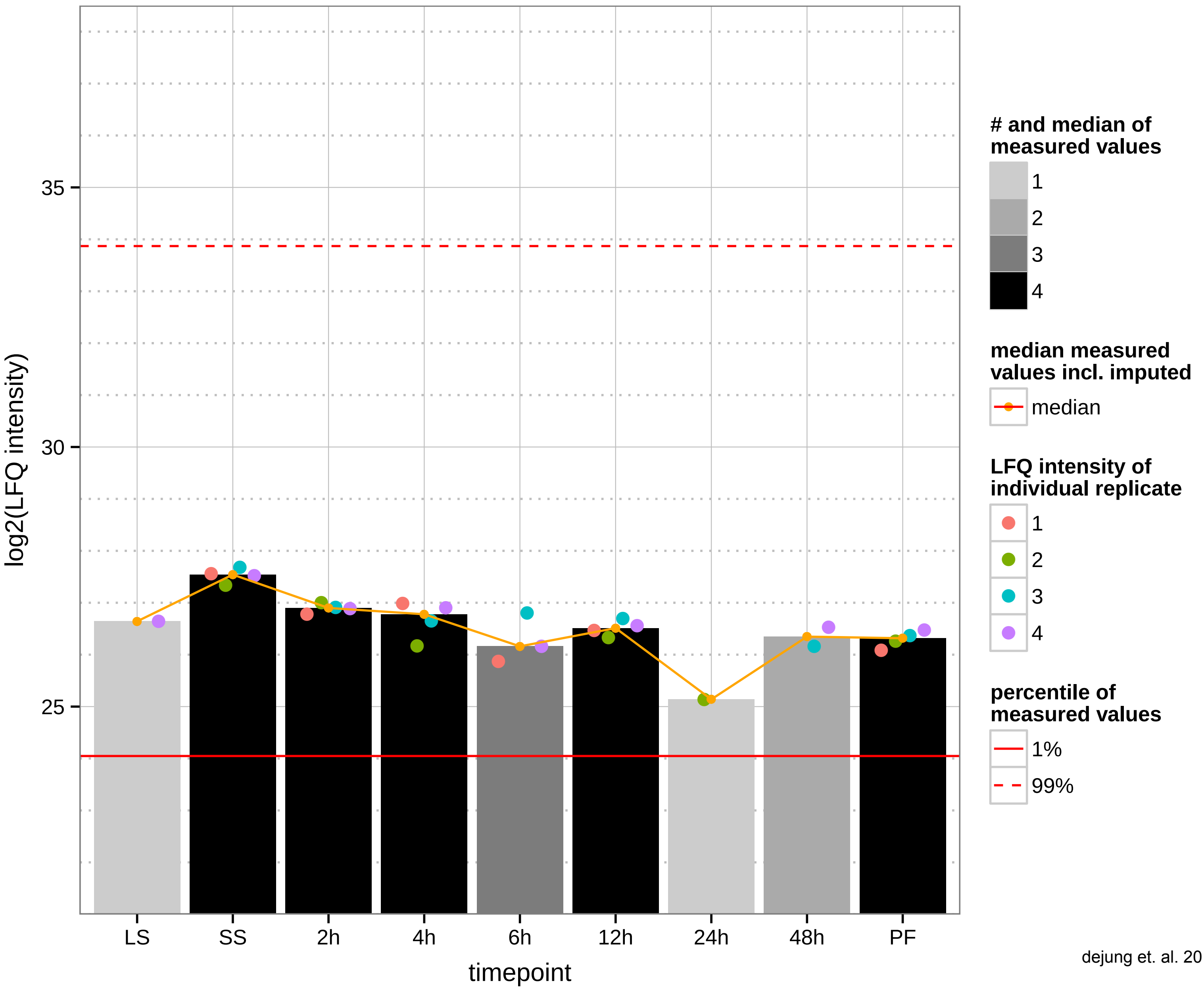

dejung et. al. 2015

ras–like small GTPase, putative (TbFRP)
Tb927.7.3790
AGOF: GTP binding, zinc ion binding
AGOC: intracellular
AGOP: protein transport, small GTPase mediated signal transduction
PGOF: GTP binding, metal ion binding, protein binding
PGOC: null
PGOP: protein transport, small GTPase mediated signal transduction

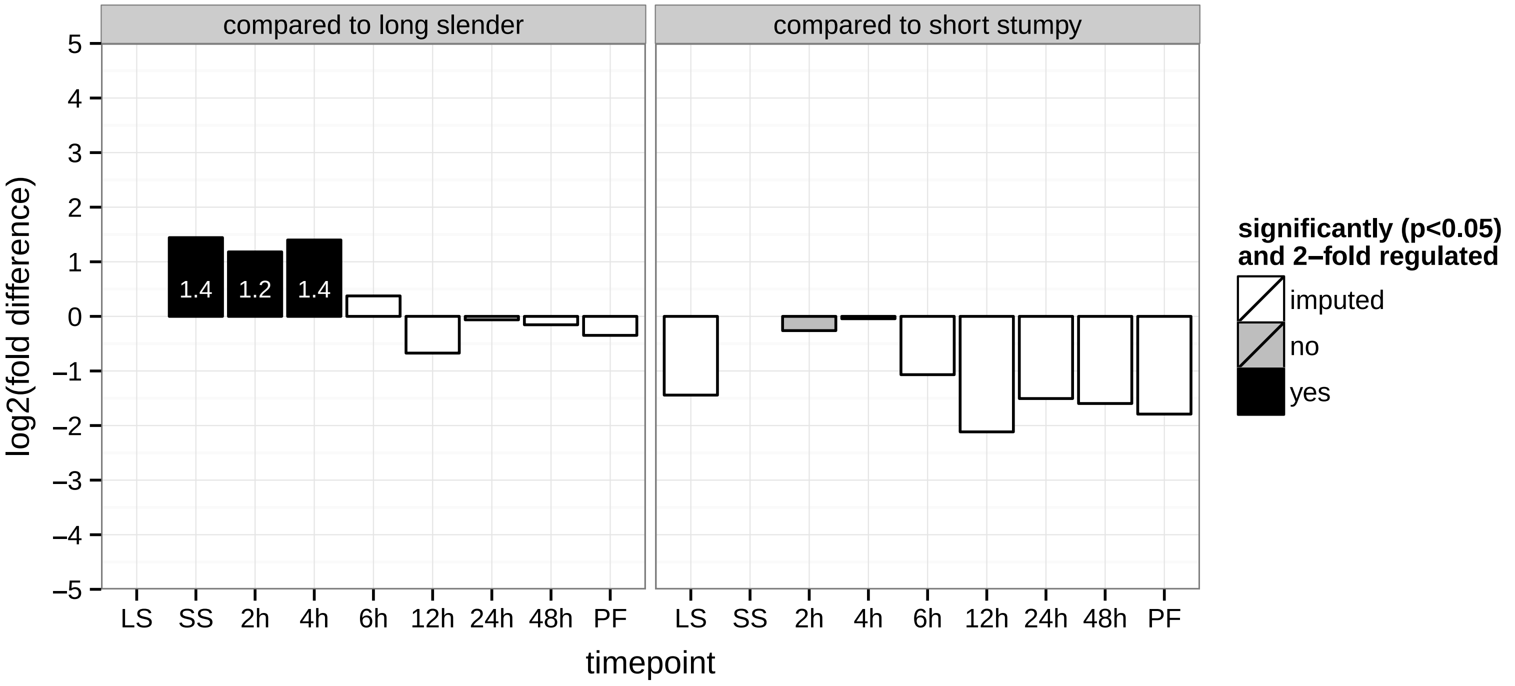

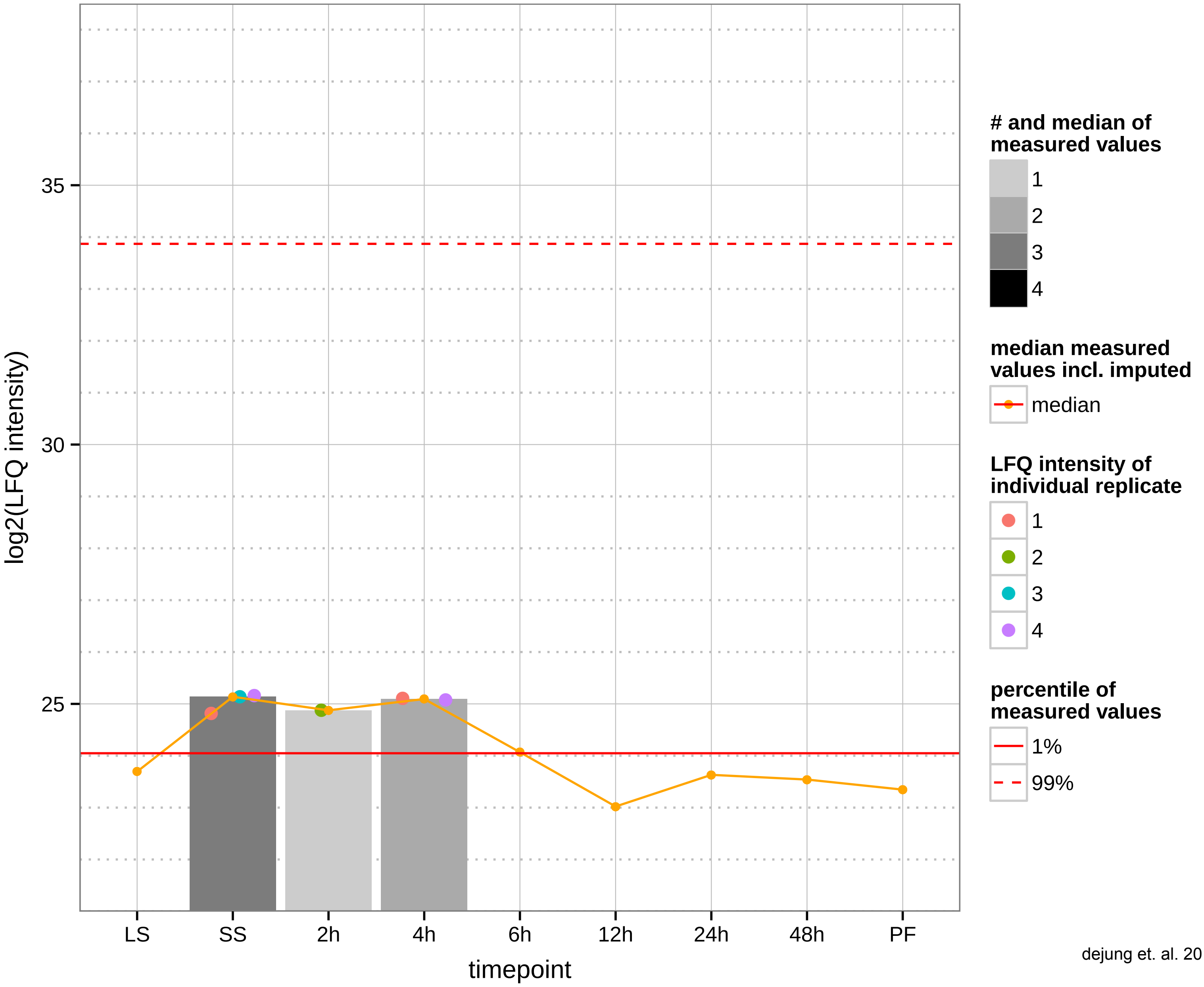

dejung et. al. 2015

hypothetical protein, conserved
Tb927.7.4410
AGOF: null
AGOC: null
AGOP: null
PGOF: null
PGOC: null
PGOP: null

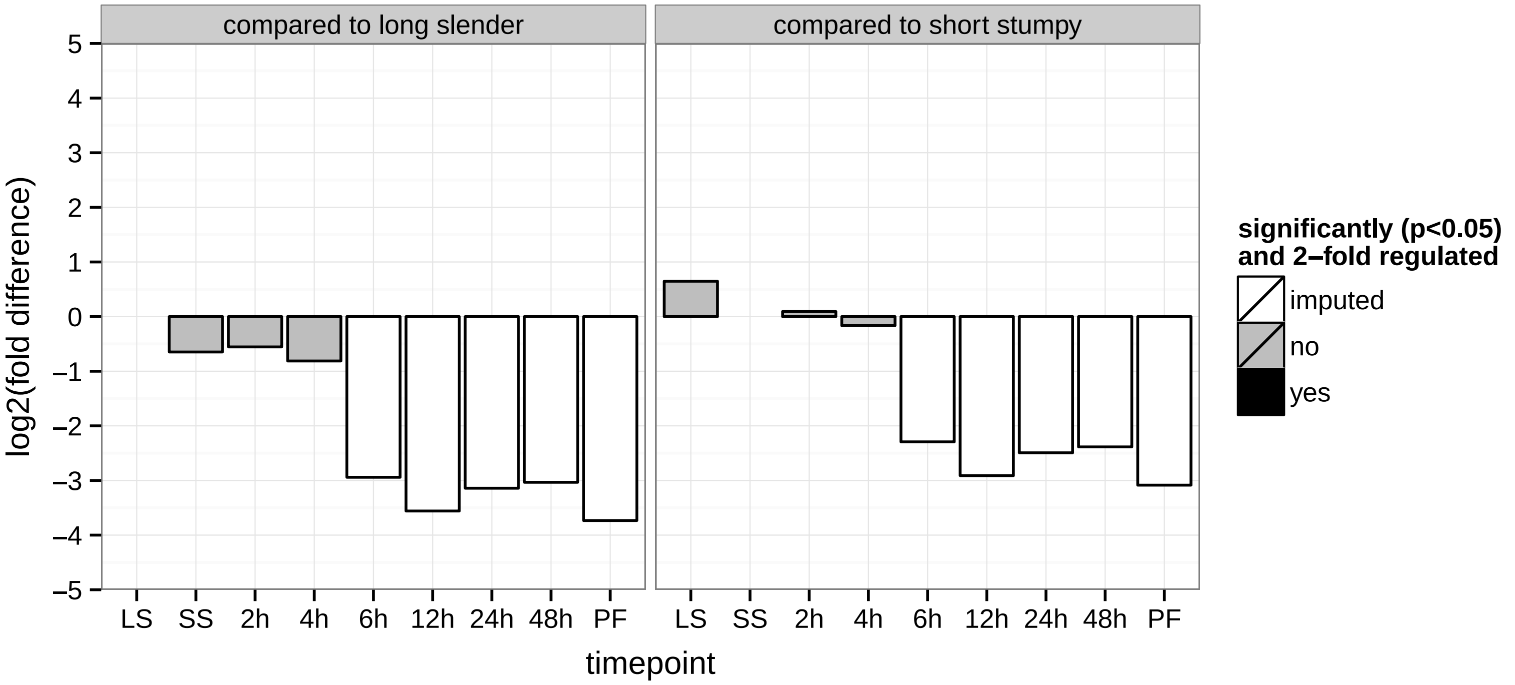

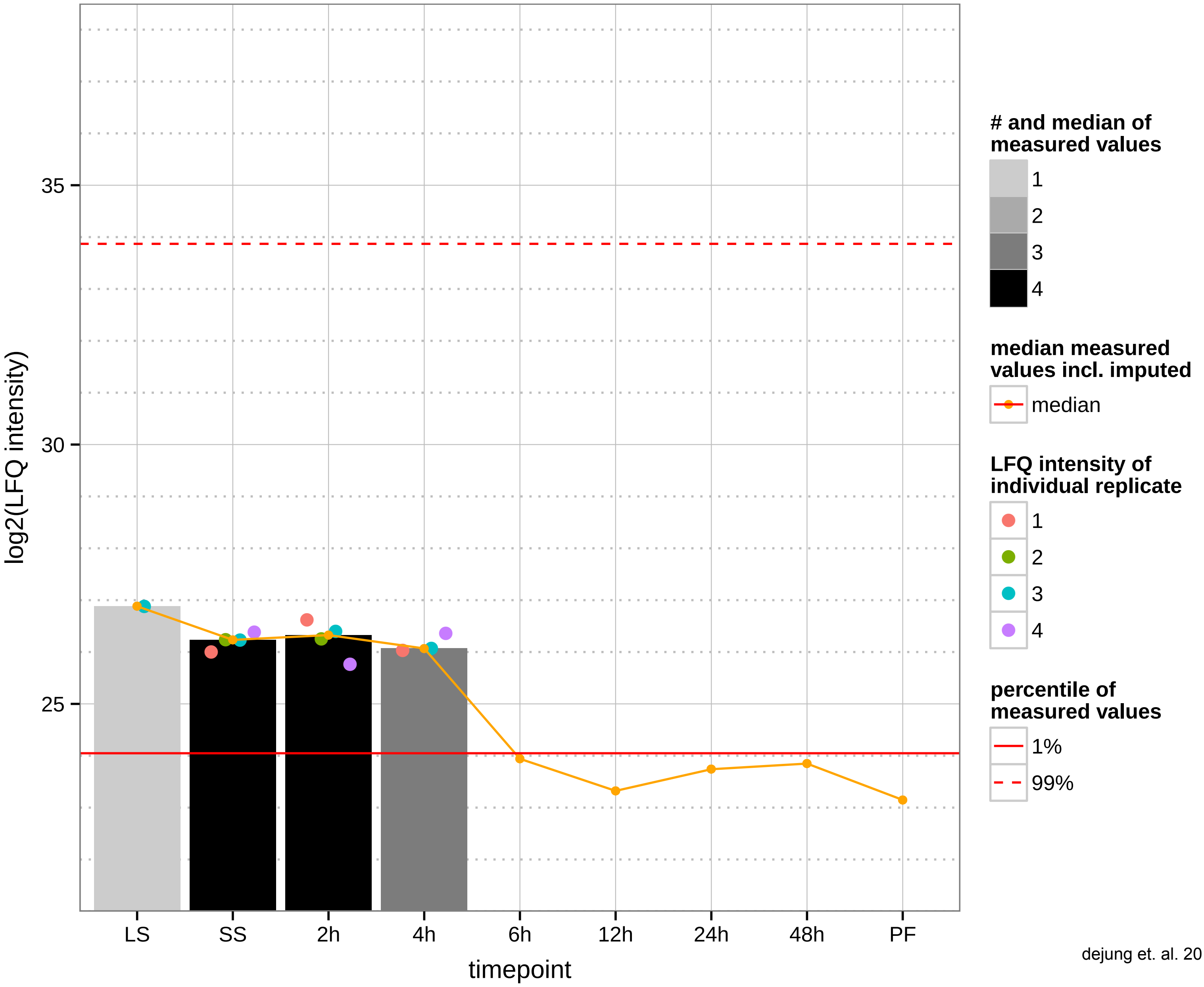

dejung et. al. 2015

hypothetical protein
Tb927.7.470
AGOF: null
AGOC: null
AGOP: null
PGOF: null
PGOC: null
PGOP: null

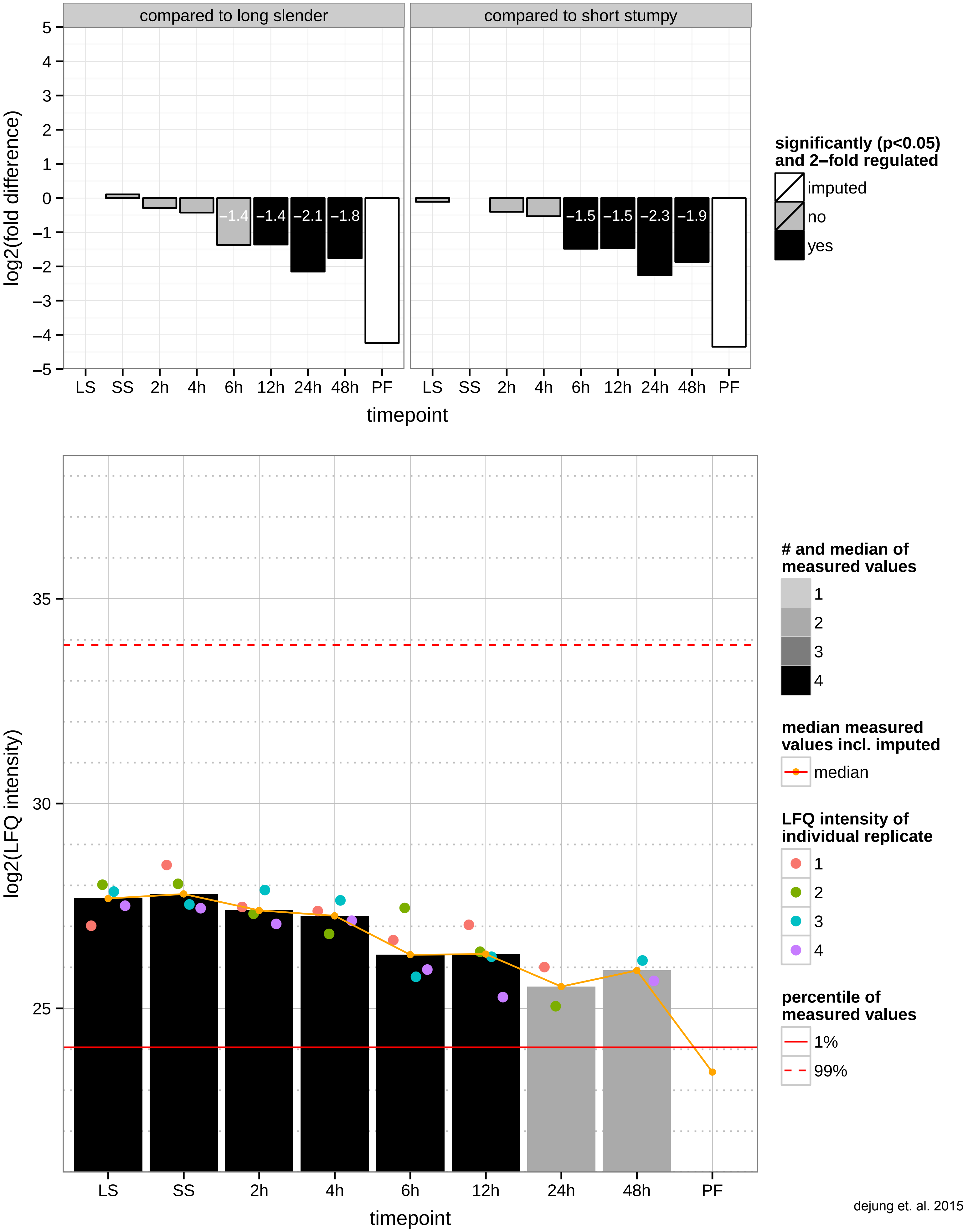

dejung et. al. 2015

hypothetical protein, conserved
Tb927.7.7000
AGOF: null
AGOC: null
AGOP: null
PGOF: null
PGOC: null
PGOP: null

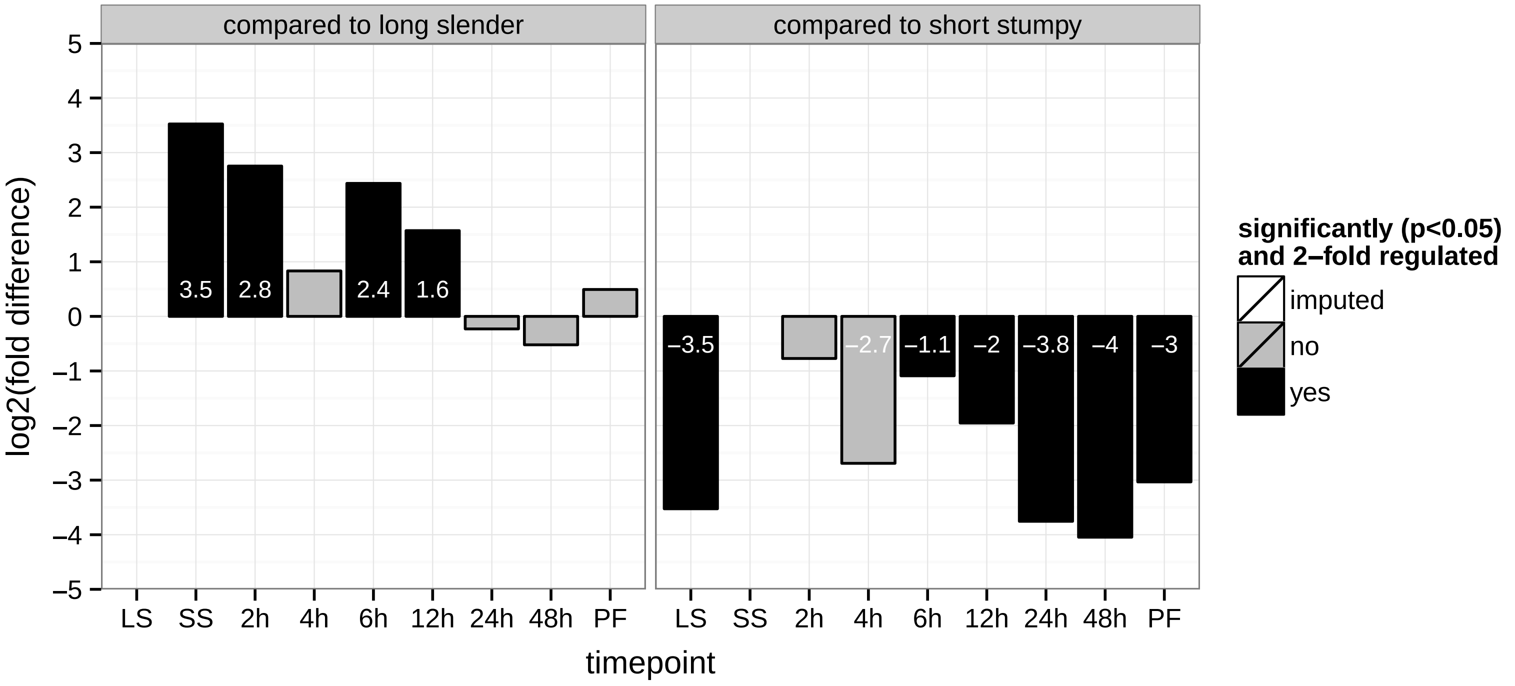

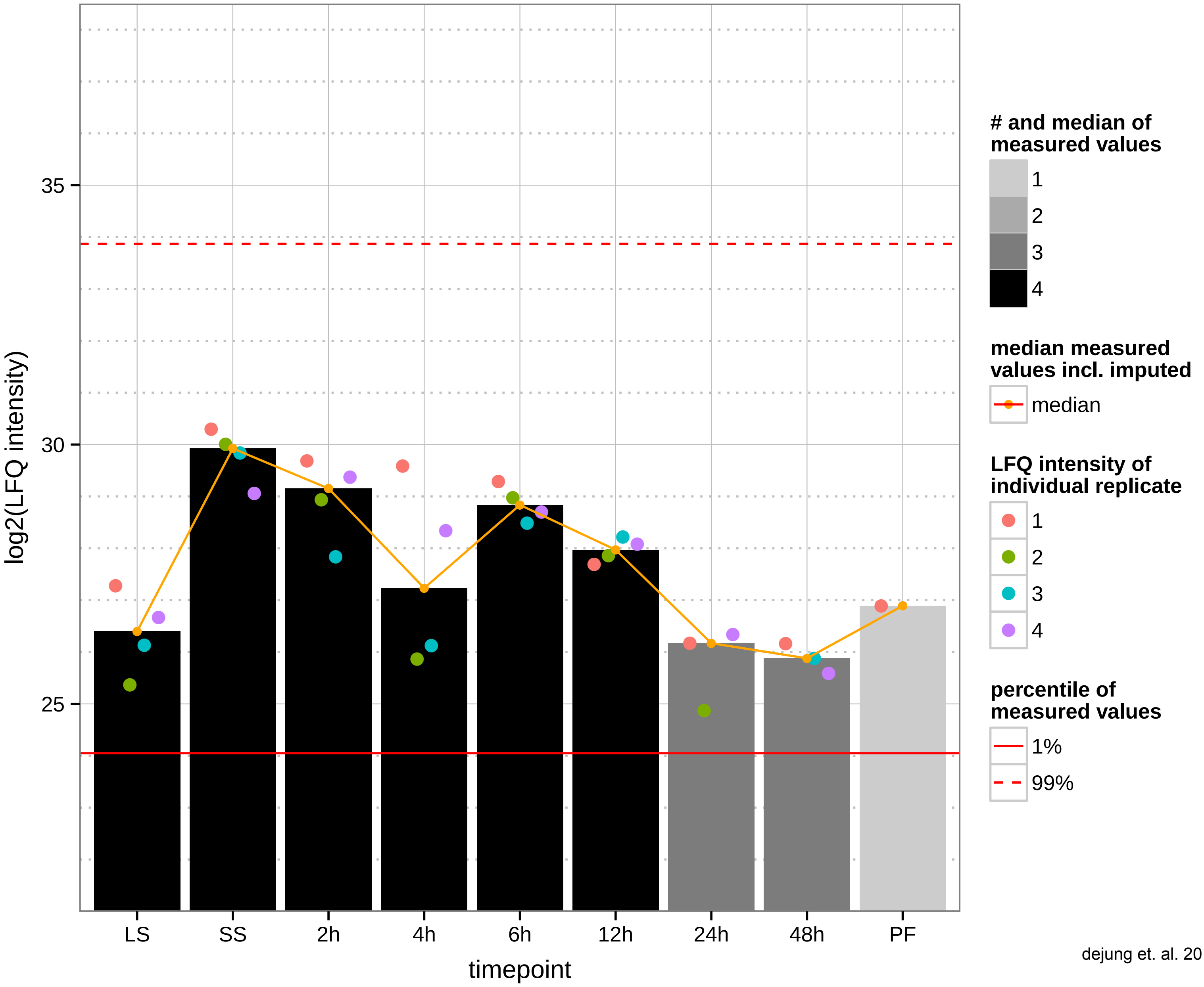

dejung et. al. 2015

hypothetical protein, conserved
Tb927.9.11480
AGOF: null
AGOC: null
AGOP: null
PGOF: null
PGOC: null
PGOP: null

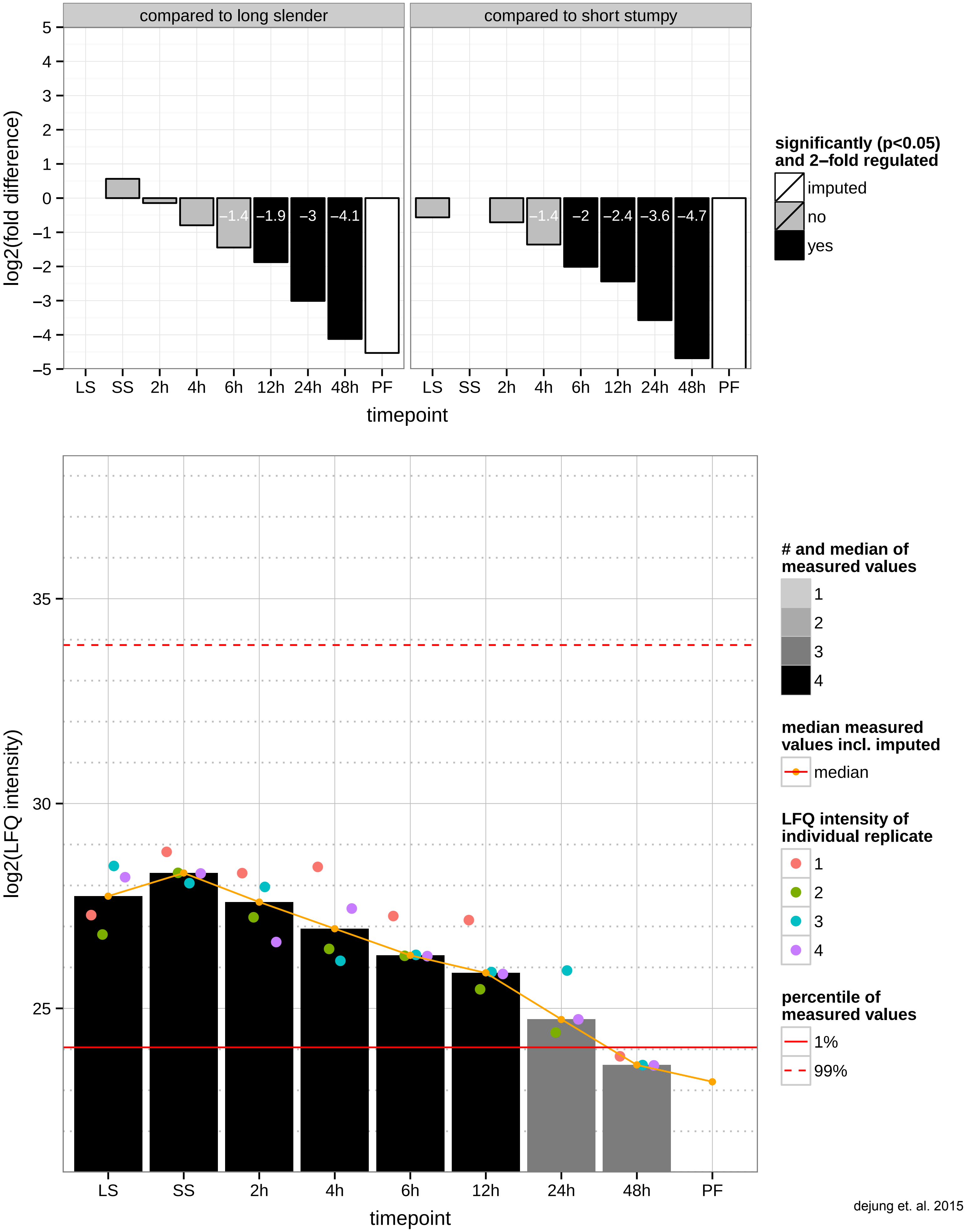

dejung et. al. 2015

hypothetical protein, conserved
Tb927.9.3130
AGOF: null
AGOC: null
AGOP: null
PGOF: null
PGOC: null
PGOP: null

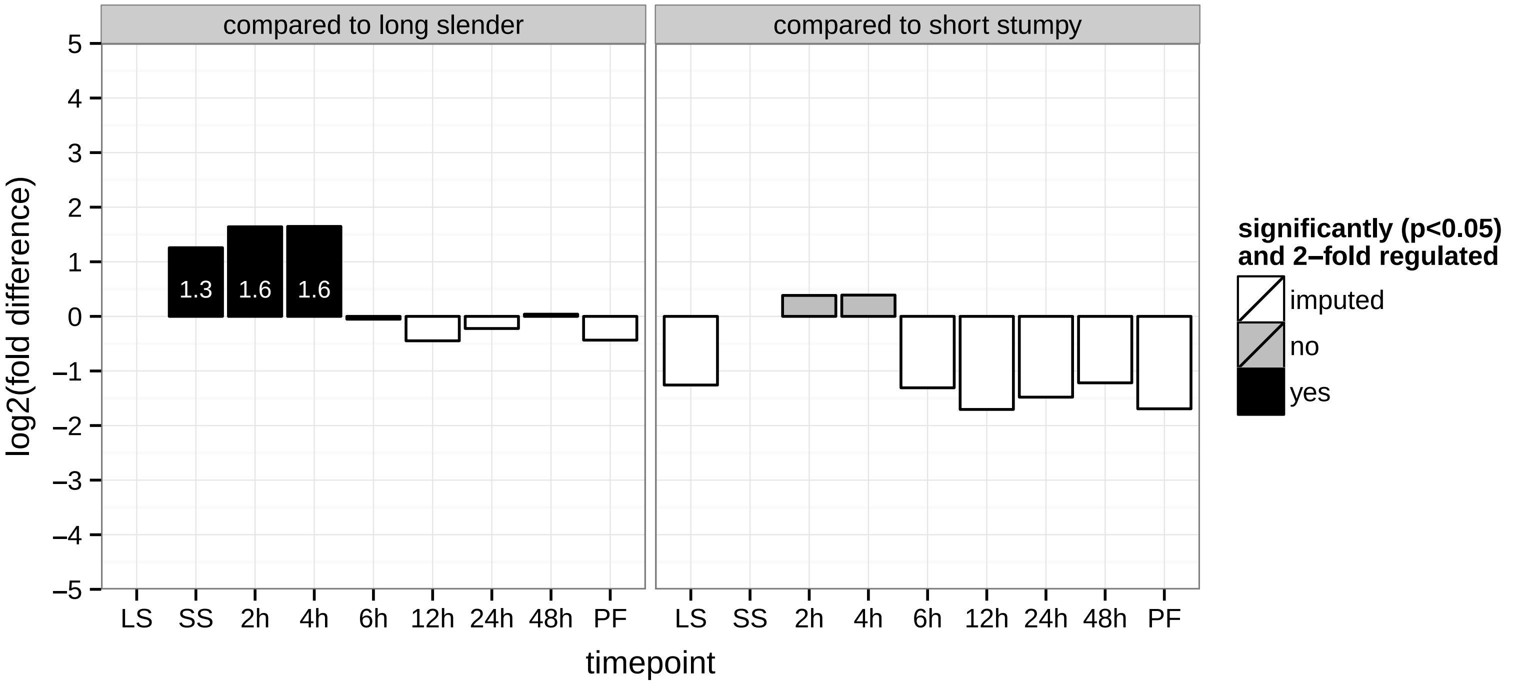

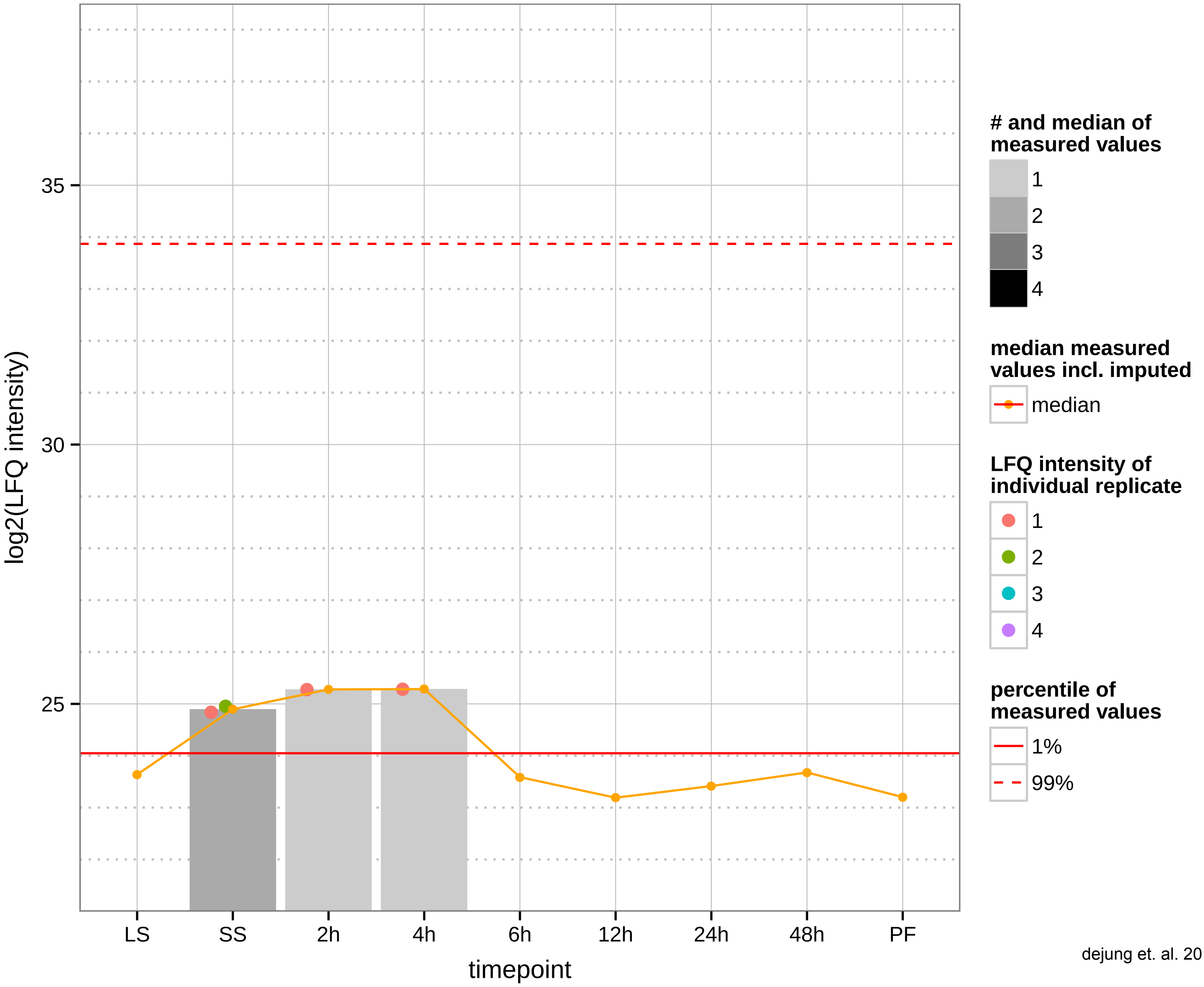

dejung et. al. 2015

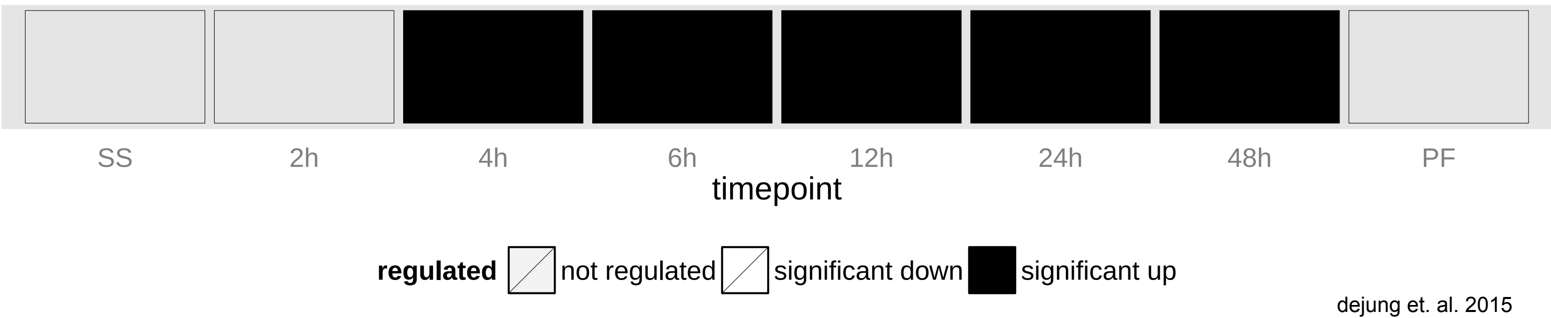
SS
2h
4h
6h
12h
24h
48h
PF
timepoint
regulated
not regulated
significant down
significant up
dejung et. al. 2015

hypothetical protein, conserved
Tb927.7.4740;Tb11.v5.0157
AGOF: null
AGOC: null
AGOP: null
PGOF: null
PGOC: null
PGOP: null

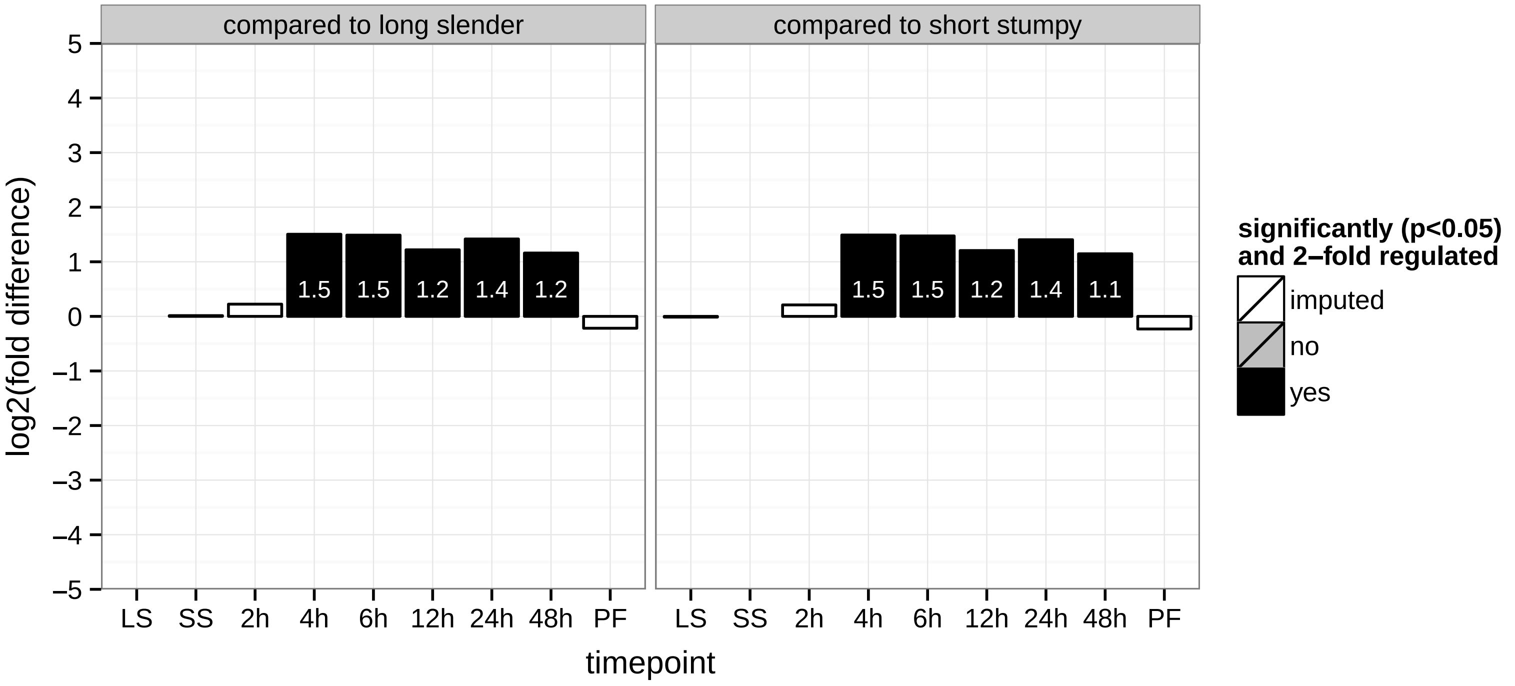

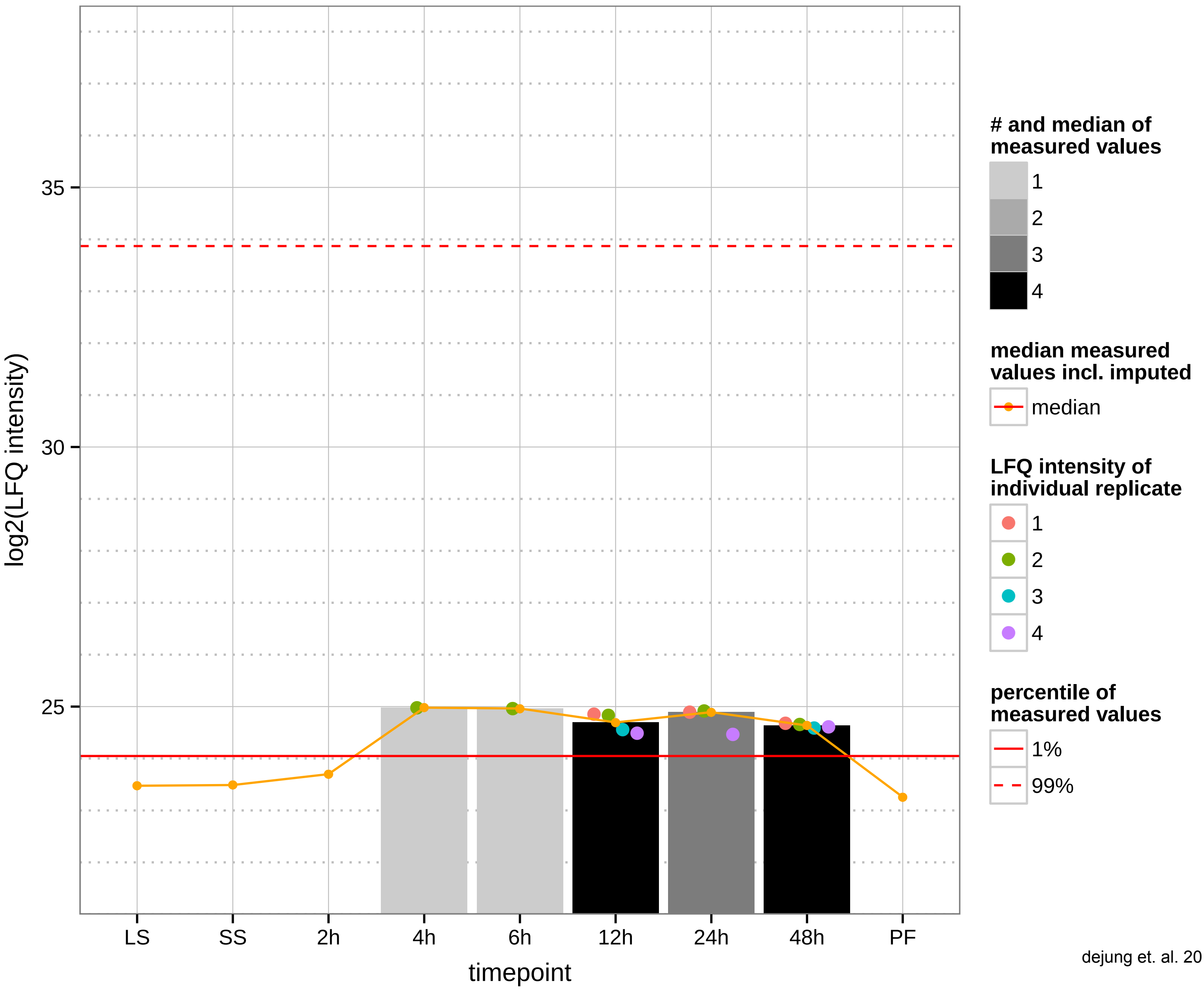

dejung et. al. 2015

protein kinase, putative
Tb927.10.3230
AGOF: ATP binding, protein serine/threonine kinase activity, protein tyrosine kinase activity
AGOC: null
AGOP: protein phosphorylation
PGOF: ATP binding, protein kinase activity, transferase activity, transferring phosphorus–containing groups
PGOC: null
PGOP: protein phosphorylation

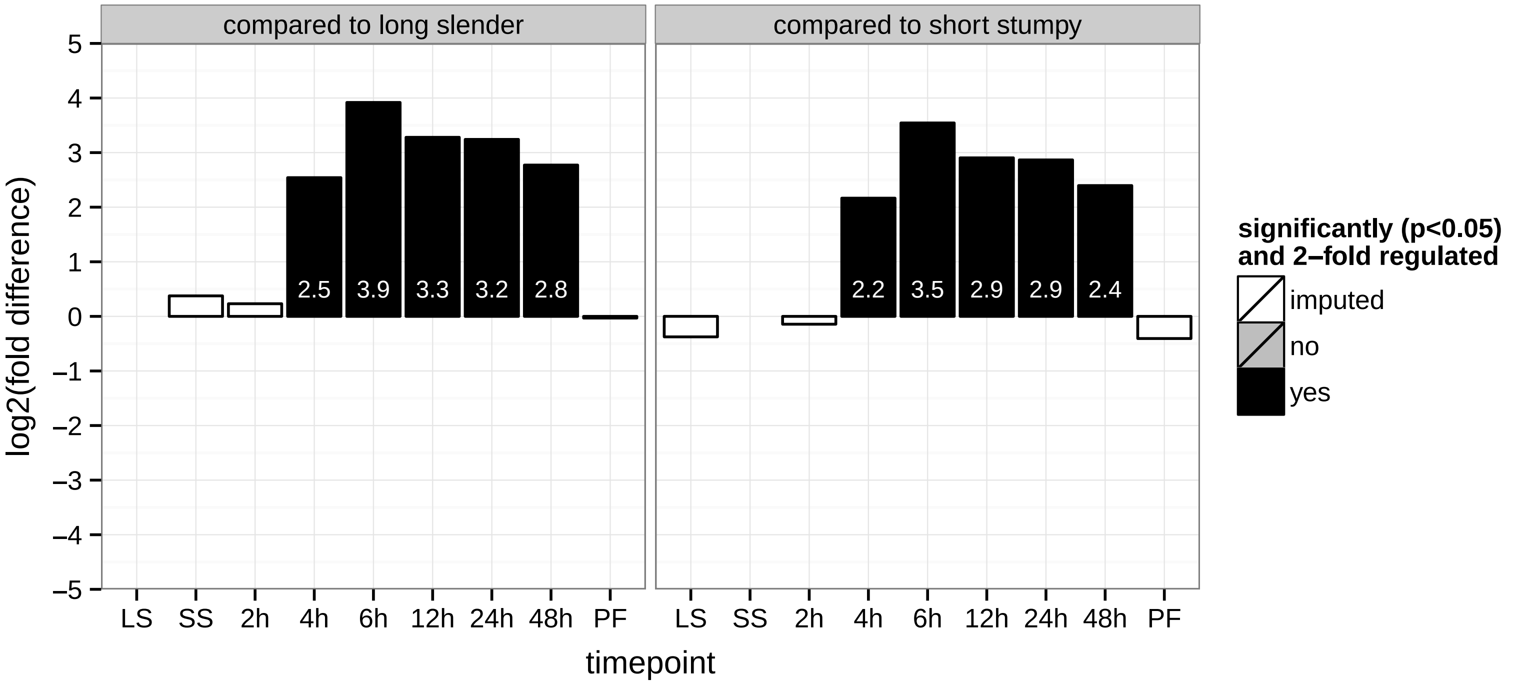

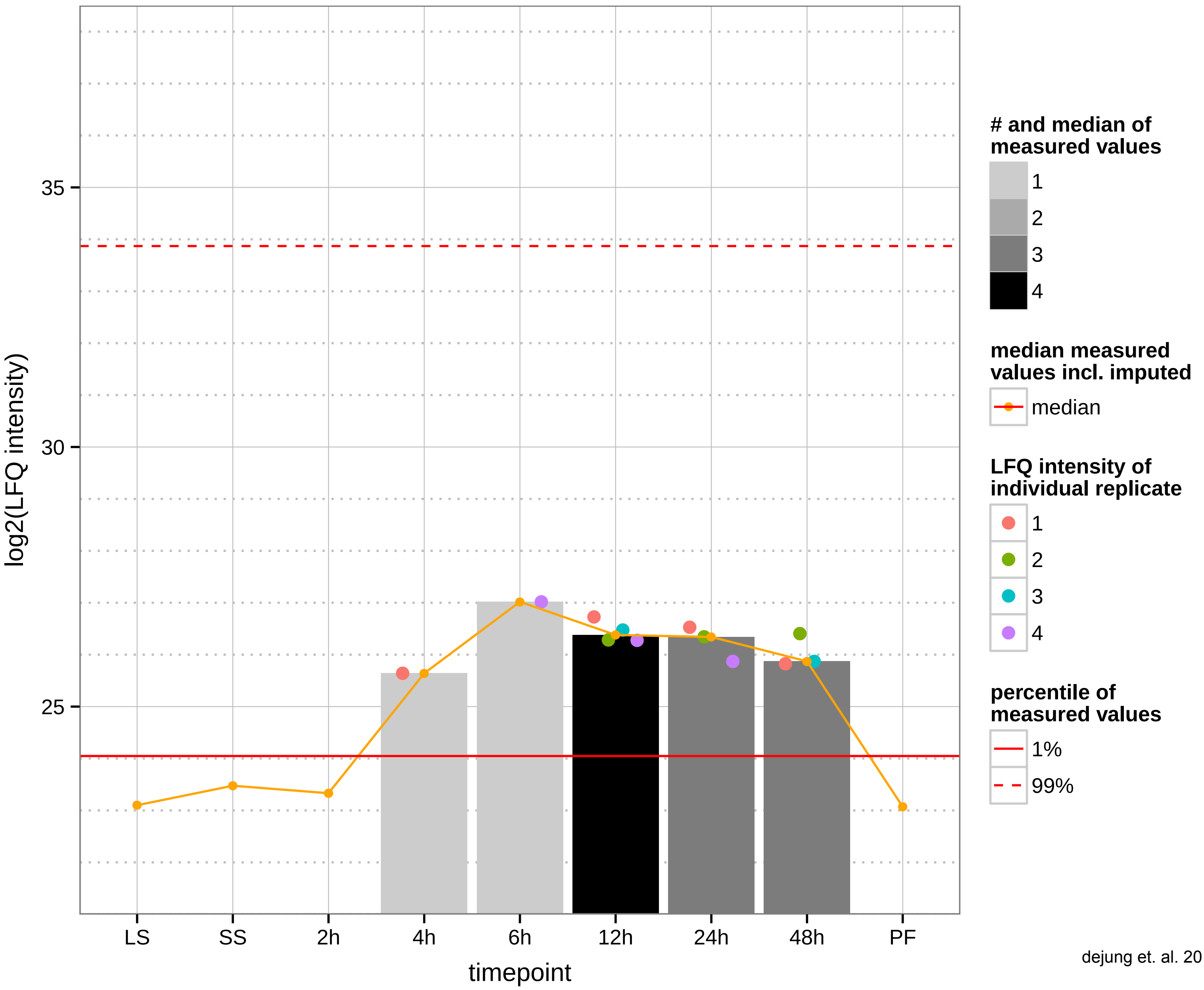

dejung et. al. 2015

hypothetical protein, conserved
Tb927.10.7910
AGOF: null
AGOC: mitochondrion
AGOP: null
PGOF: null
PGOC: null
PGOP: null

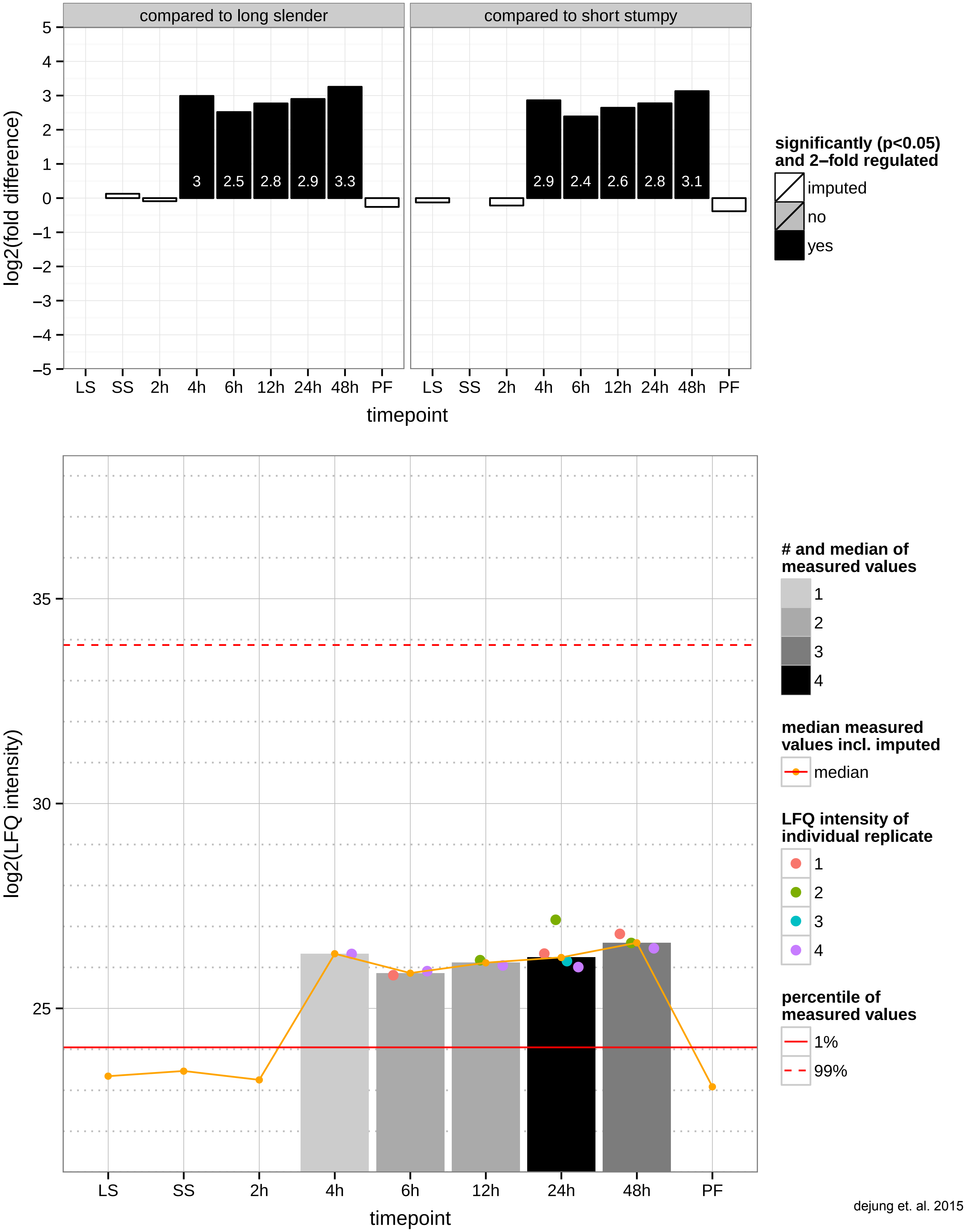

dejung et. al. 2015

hypothetical protein, conserved
Tb927.11.12320
AGOF: null
AGOC: null
AGOP: null
PGOF: null
PGOC: null
PGOP: null

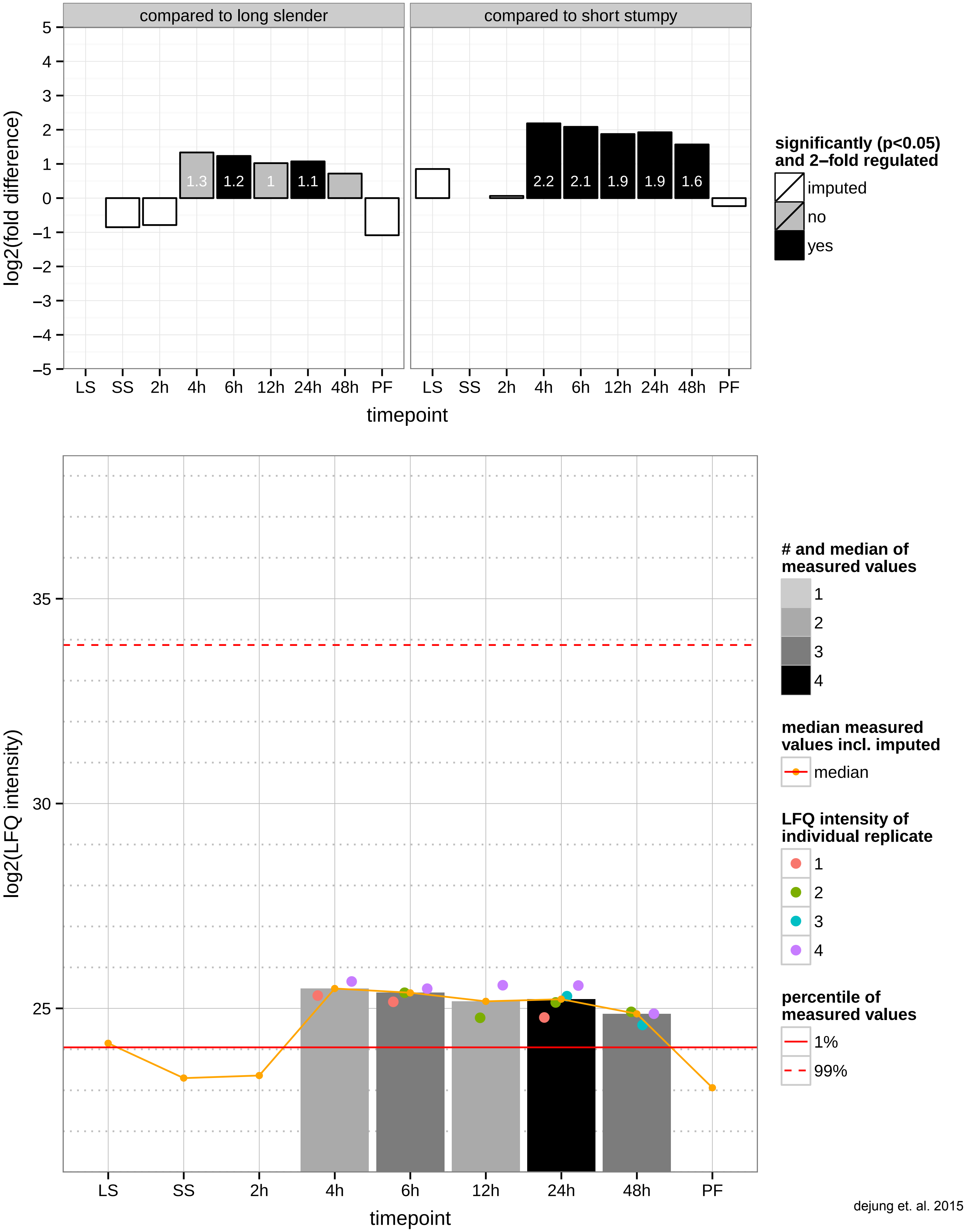

dejung et. al. 2015

hypothetical protein, conserved
Tb927.11.2590
AGOF: null
AGOC: null
AGOP: null
PGOF: null
PGOC: null
PGOP: null

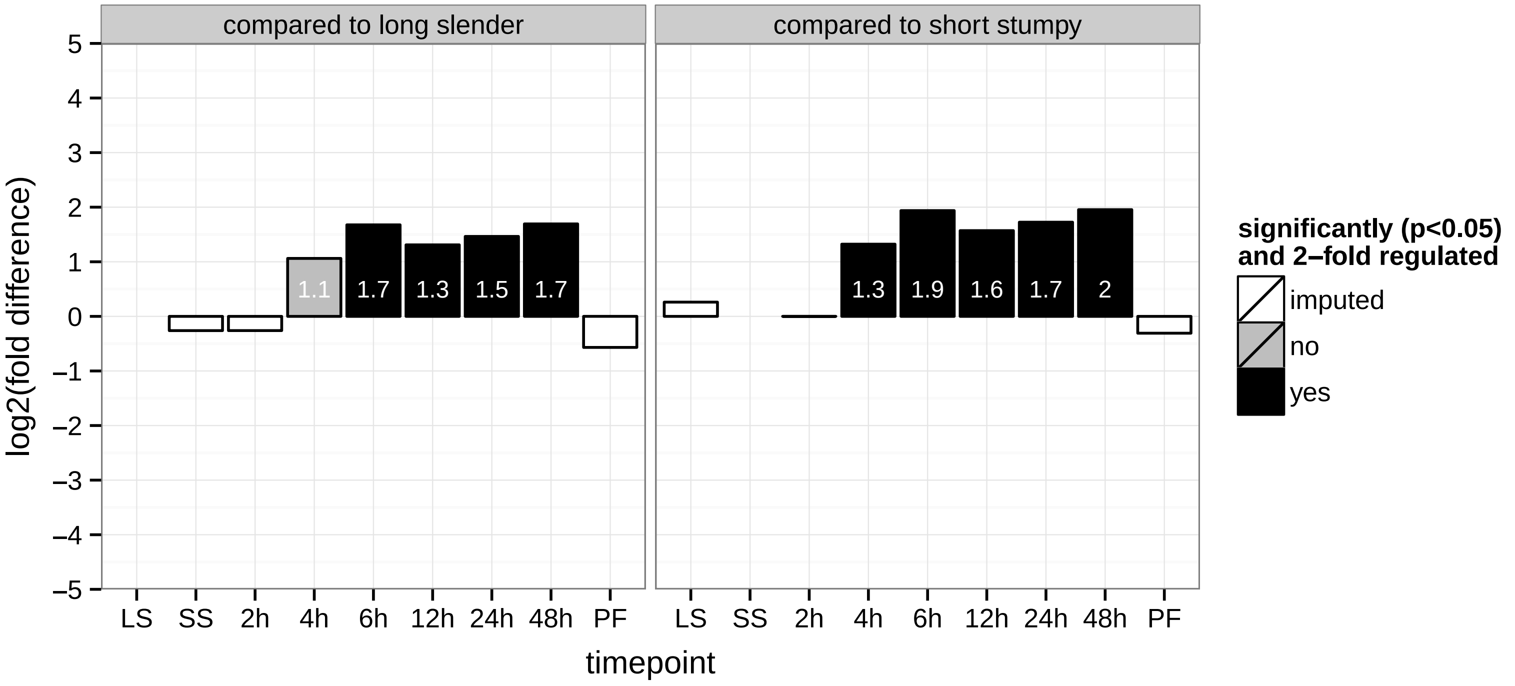

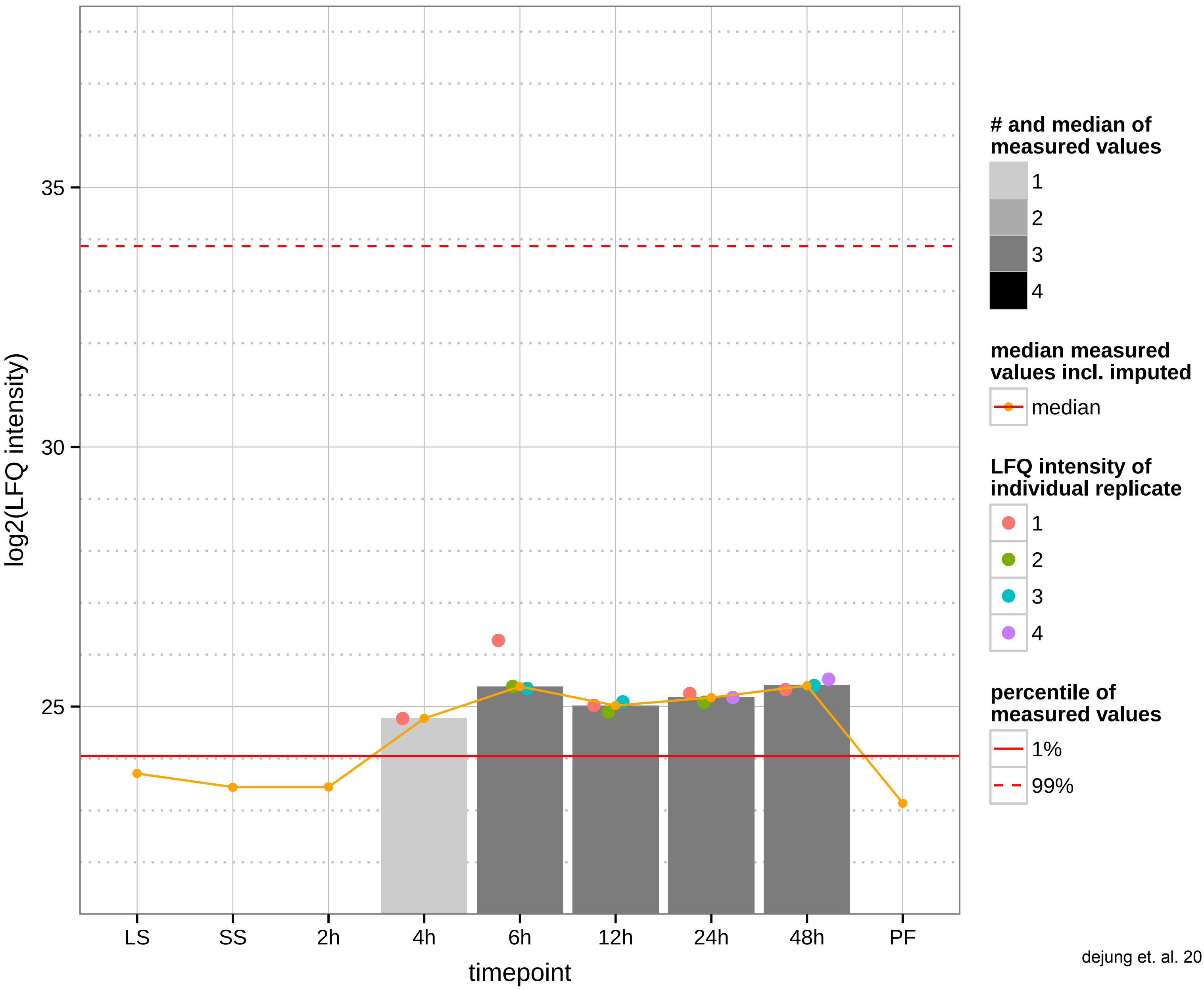

dejung et. al. 2015

hypothetical protein, conserved
Tb927.11.7500;Tb927.11.7490
AGOF: null
AGOC: integral to membrane
AGOP: null
PGOF: null
PGOC: null
PGOP: null

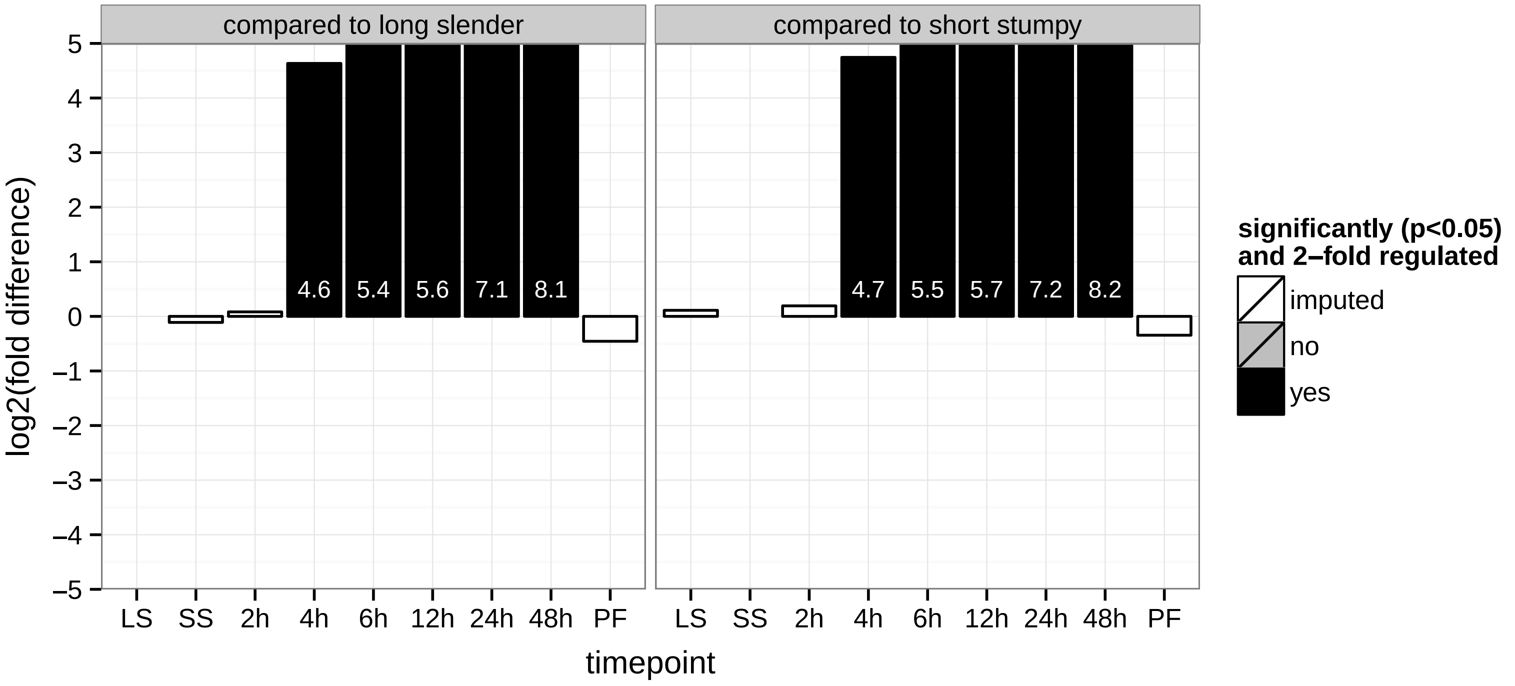

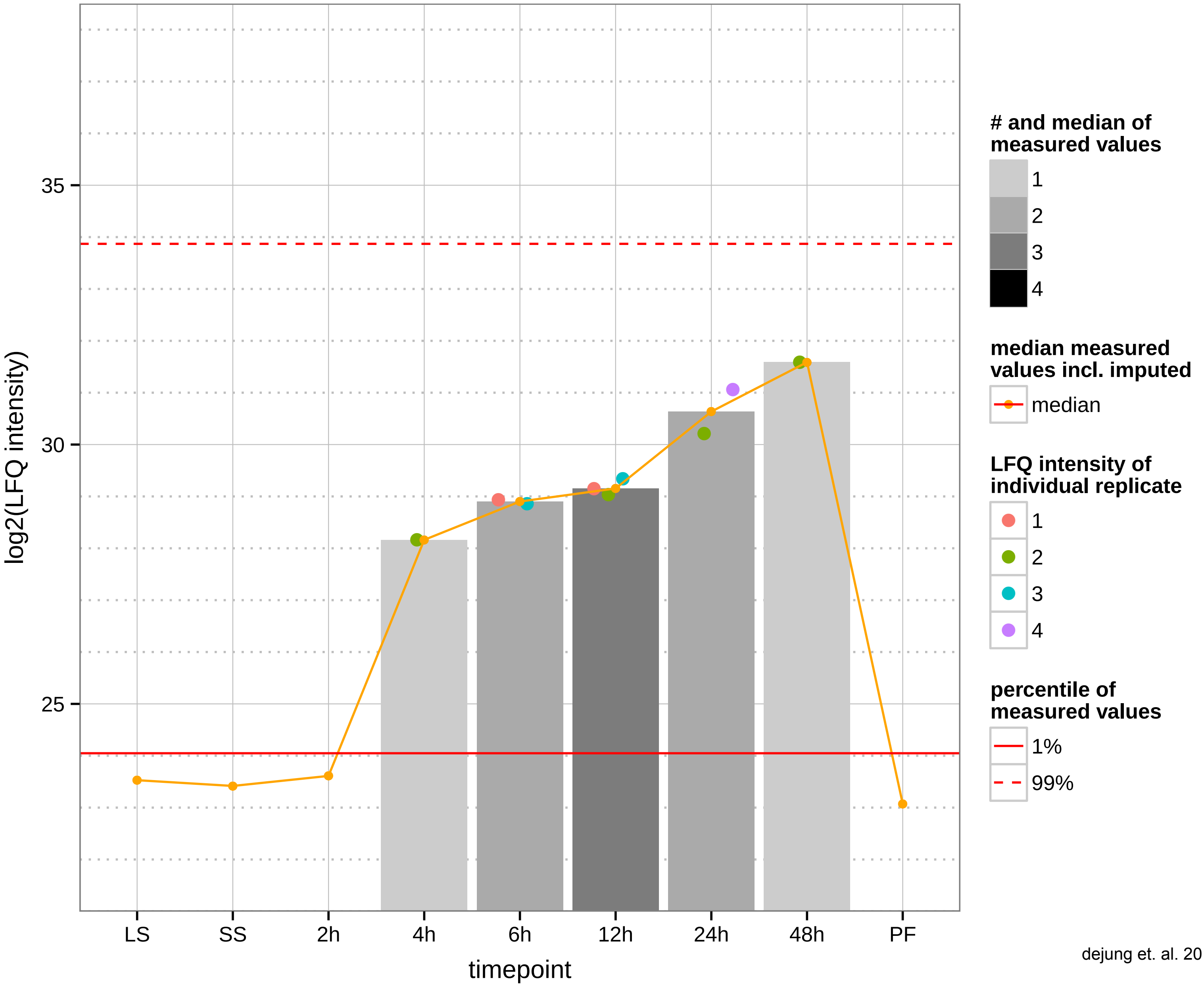

dejung et. al. 2015

calmodulin, putative
Tb927.11.7940
AGOF: calcium ion binding
AGOC: null
AGOP: null
PGOF: null
PGOC: null
PGOP: null

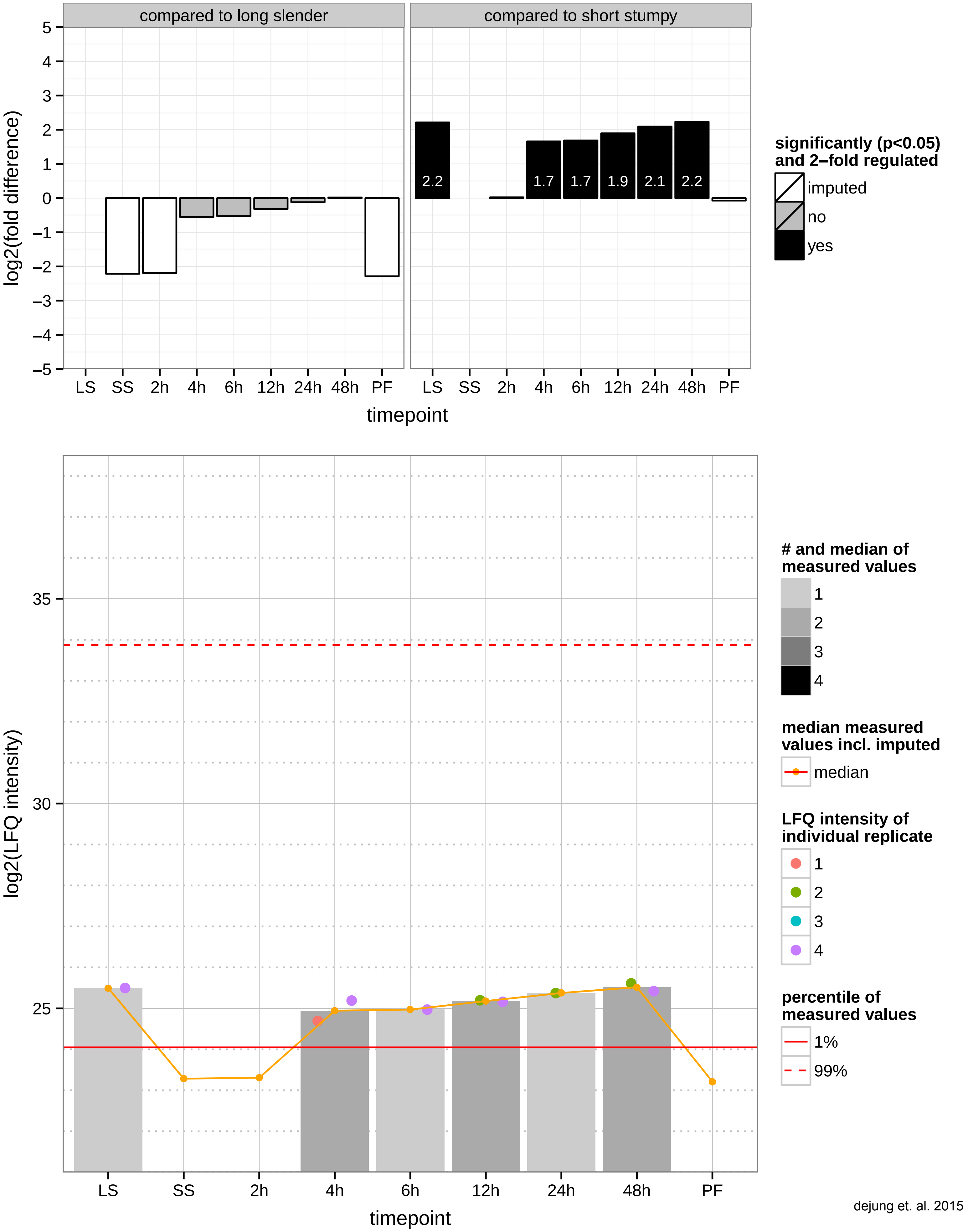

dejung et. al. 2015

hypothetical protein, conserved
Tb927.11.9320
AGOF: null
AGOC: null
AGOP: null
PGOF: null
PGOC: null
PGOP: null

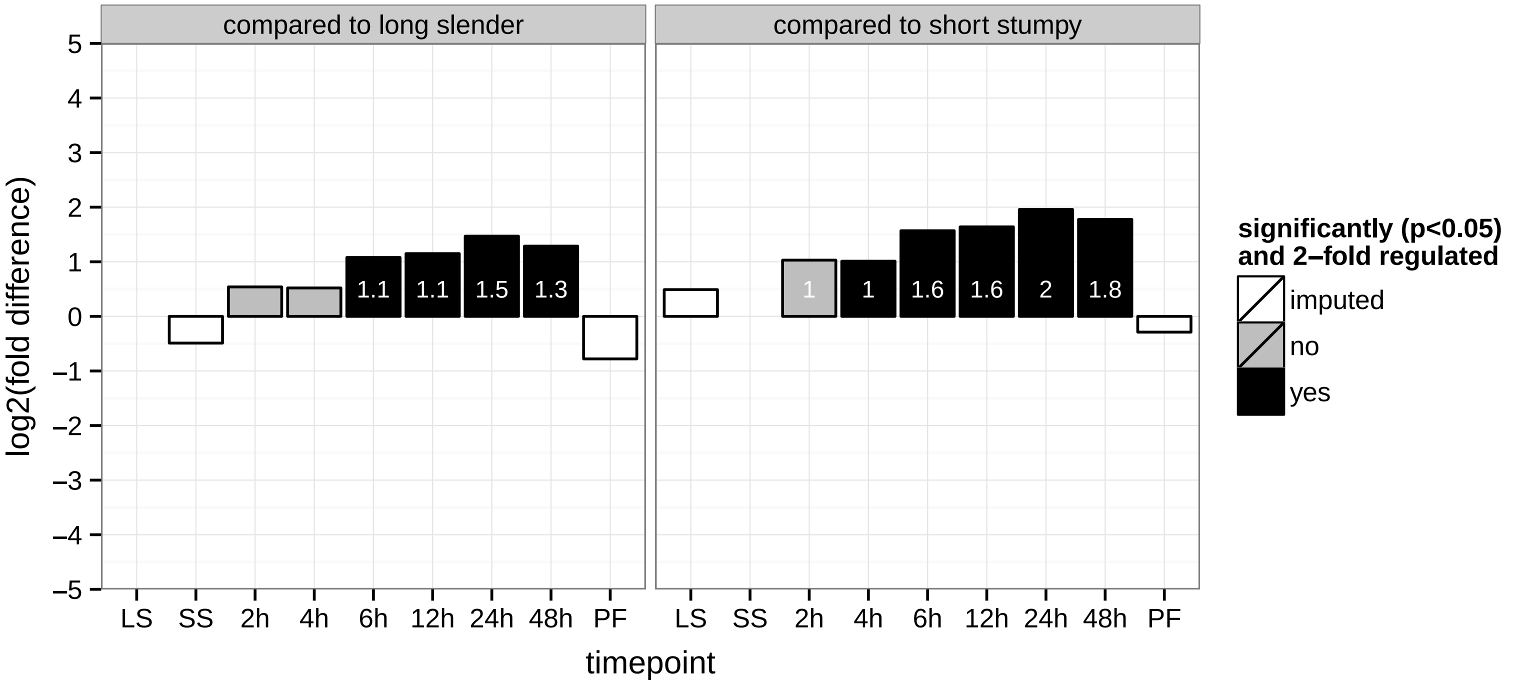

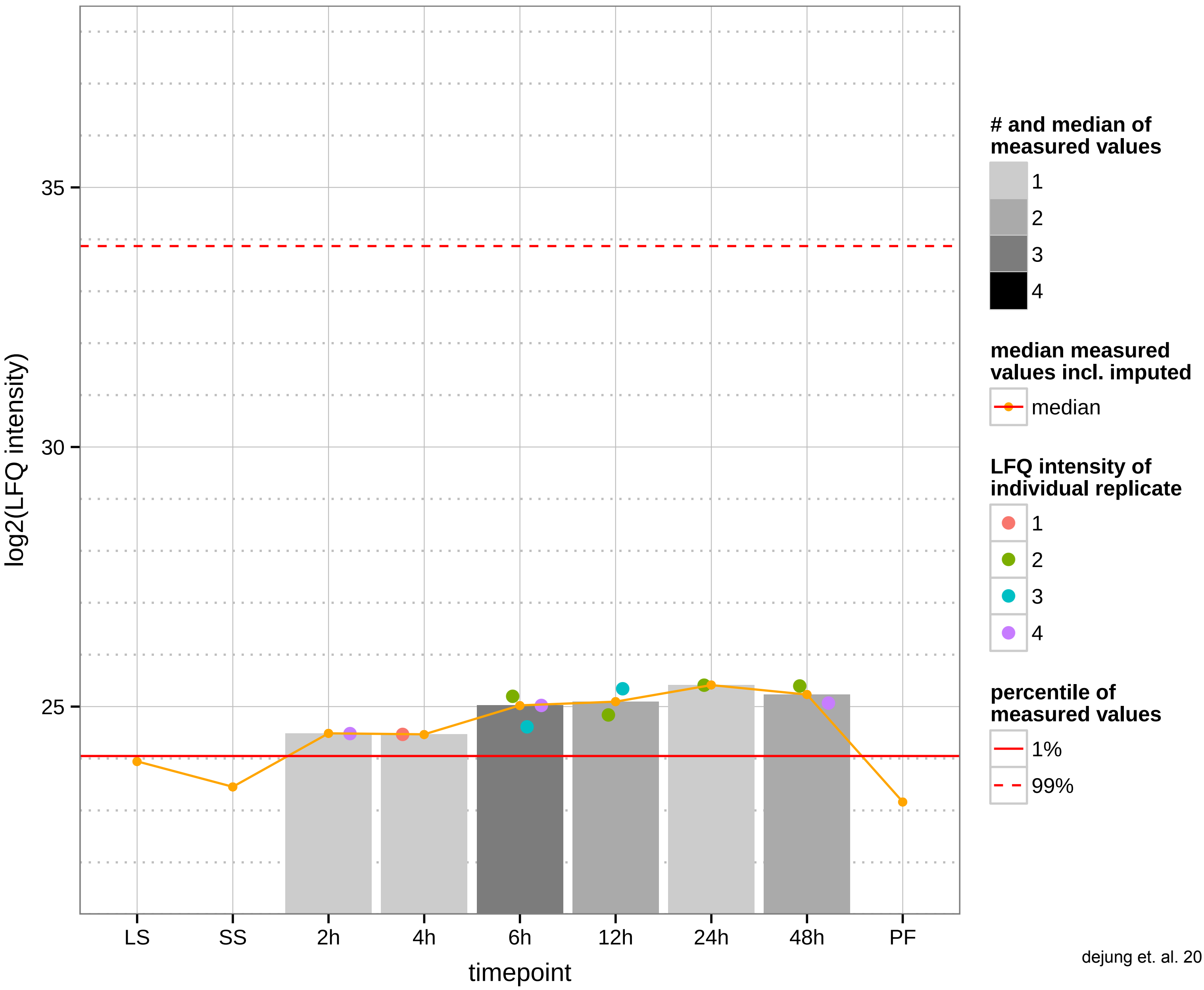

dejung et. al. 2015

calreticulin, putative
Tb927.4.5010
AGOF: calcium ion binding, unfolded protein binding
AGOC: endoplasmic reticulum
AGOP: protein folding
PGOF: calcium ion binding, unfolded protein binding
PGOC: endoplasmic reticulum
PGOP: protein folding

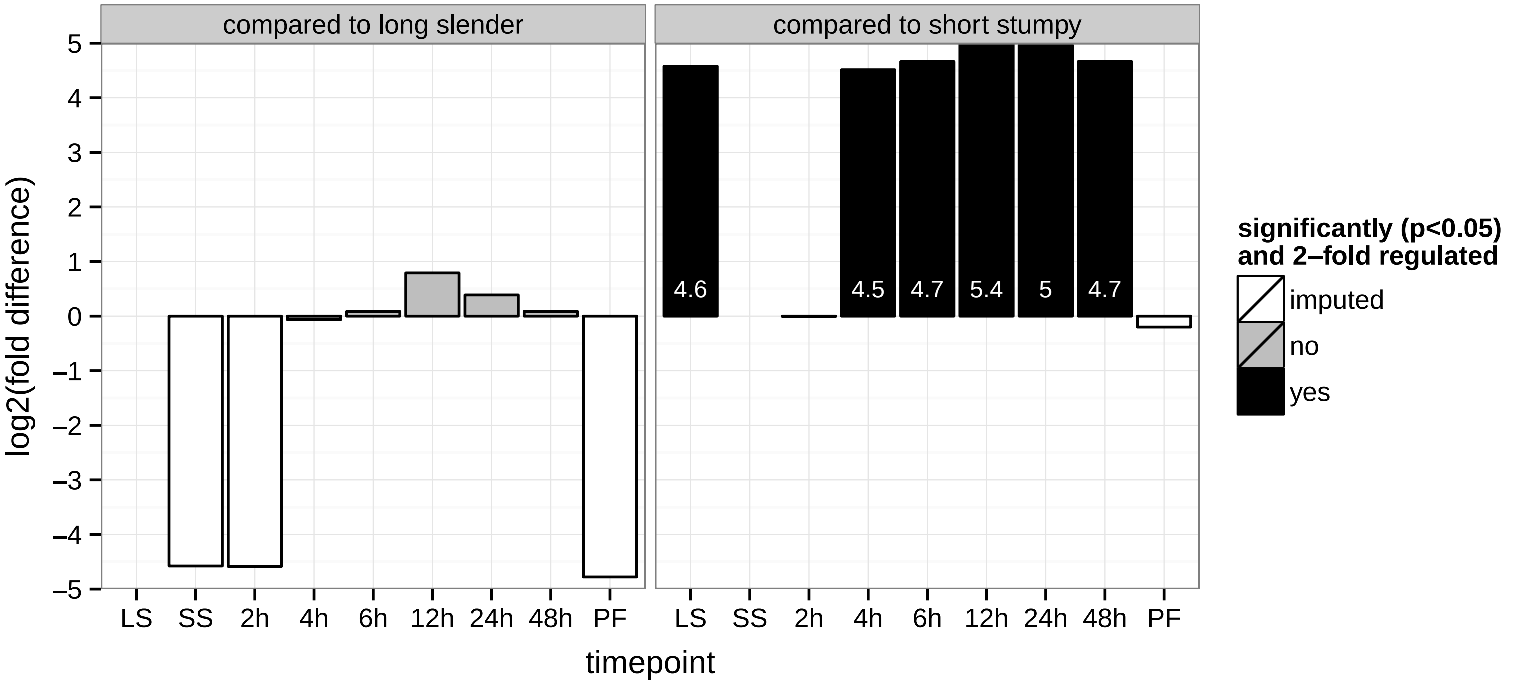

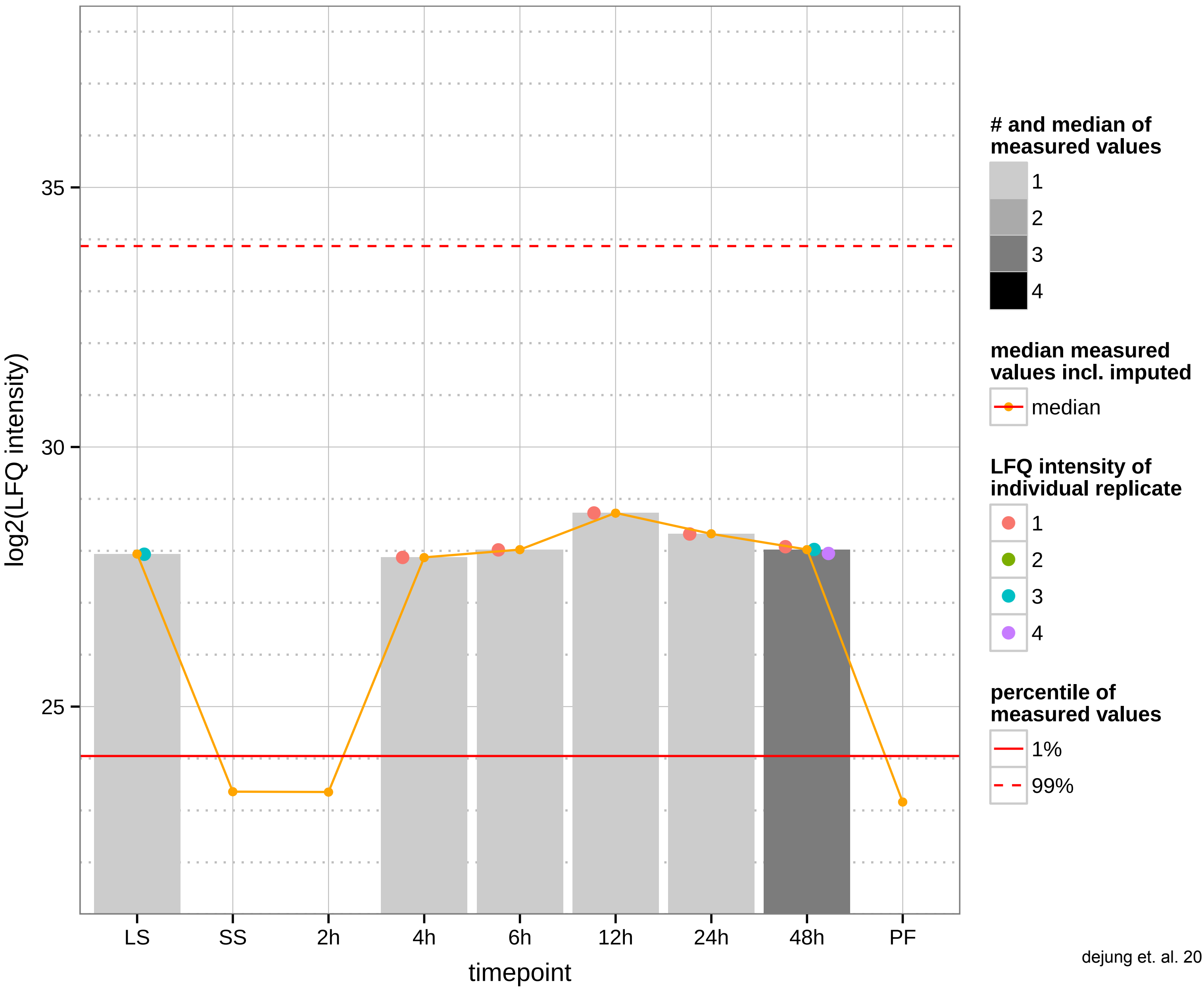

dejung et. al. 2015

hypothetical protein, conserved
Tb927.5.840
AGOF: null
AGOC: null
AGOP: null
PGOF: null
PGOC: null
PGOP: null

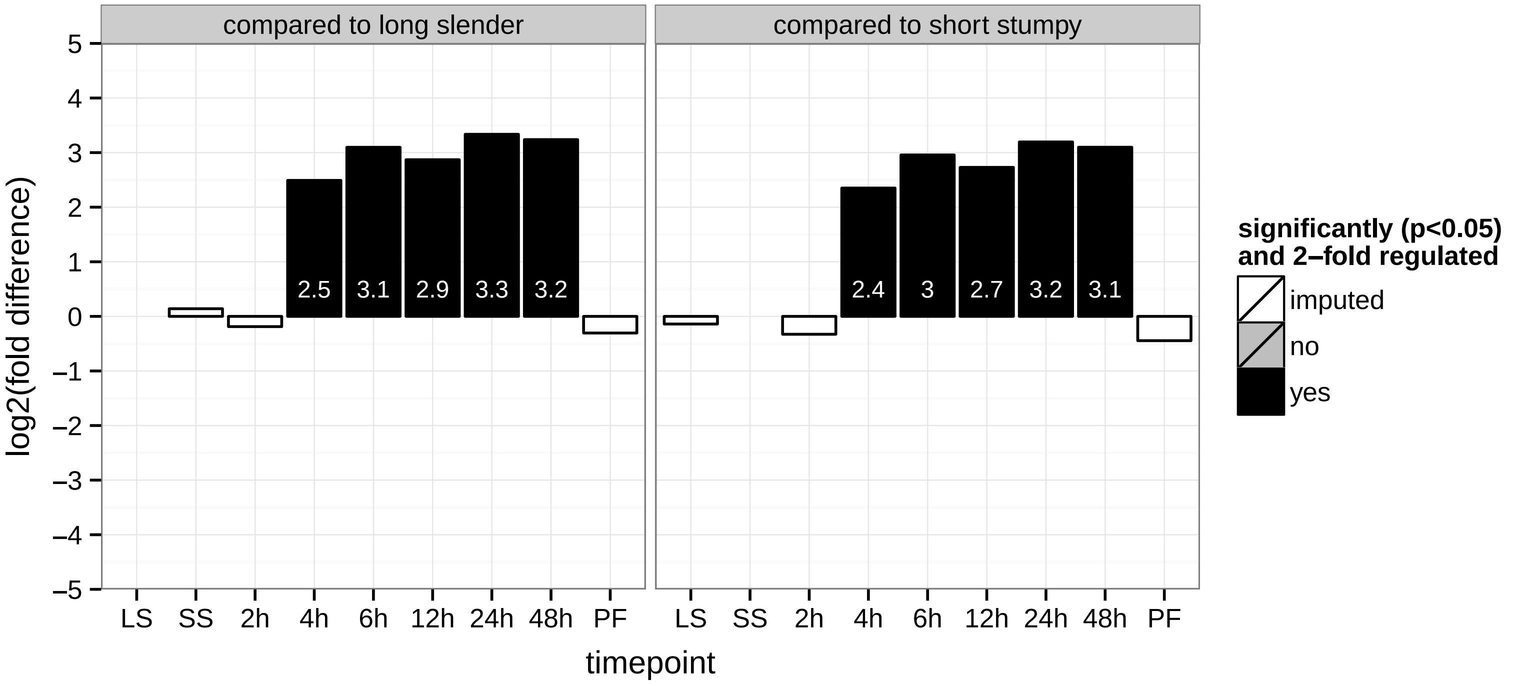

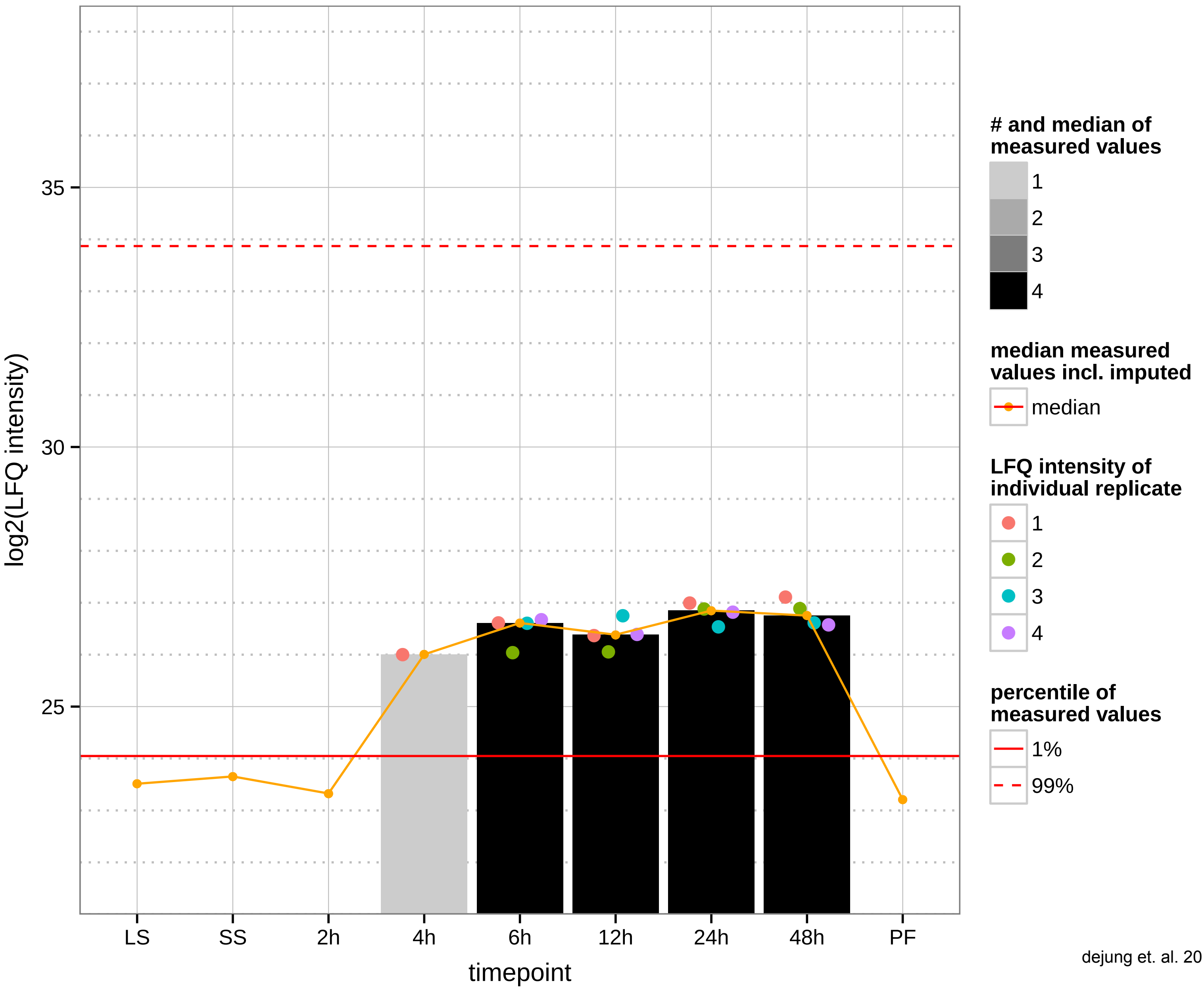

dejung et. al. 2015

leucine−rich repeat protein (LRRP), putative
Tb927.6.1940
AGOF: null
AGOC: null
AGOP: null
PGOF: null
PGOC: null
PGOP: null

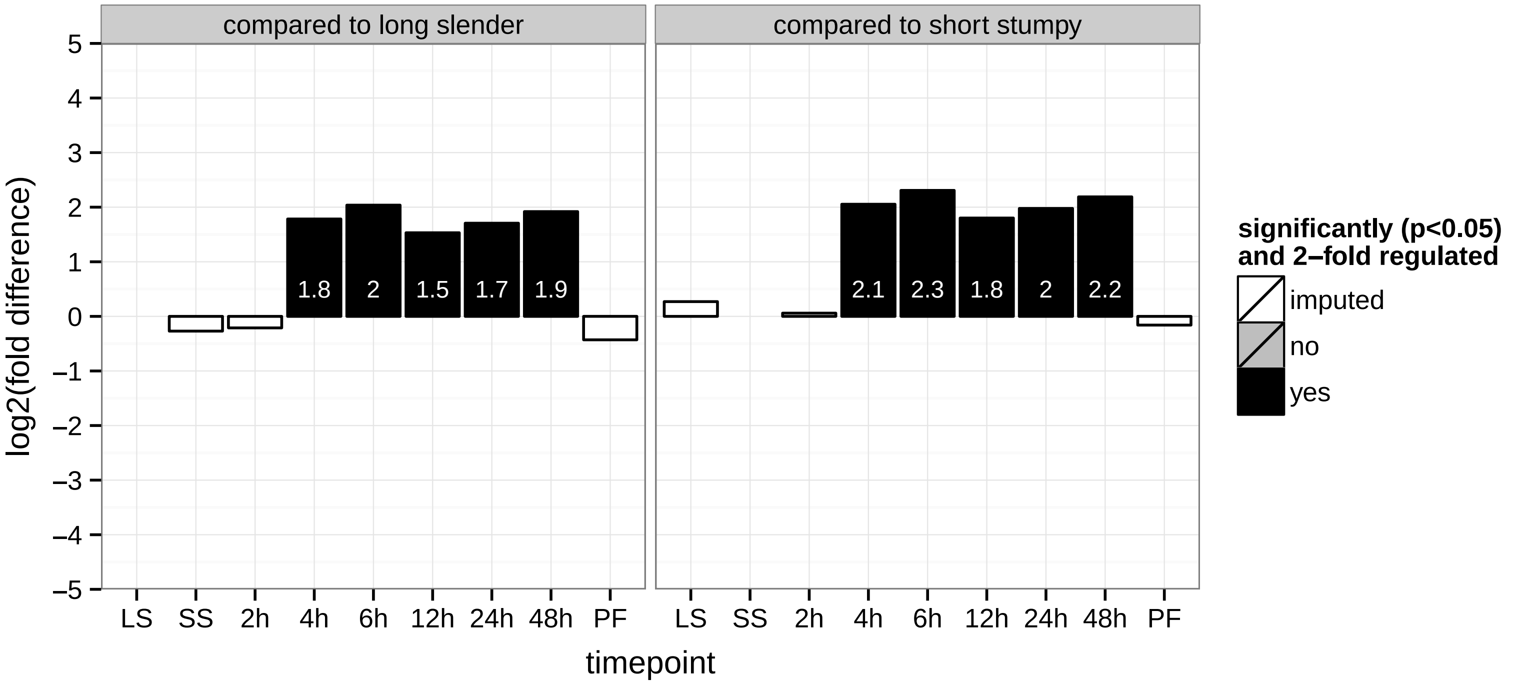

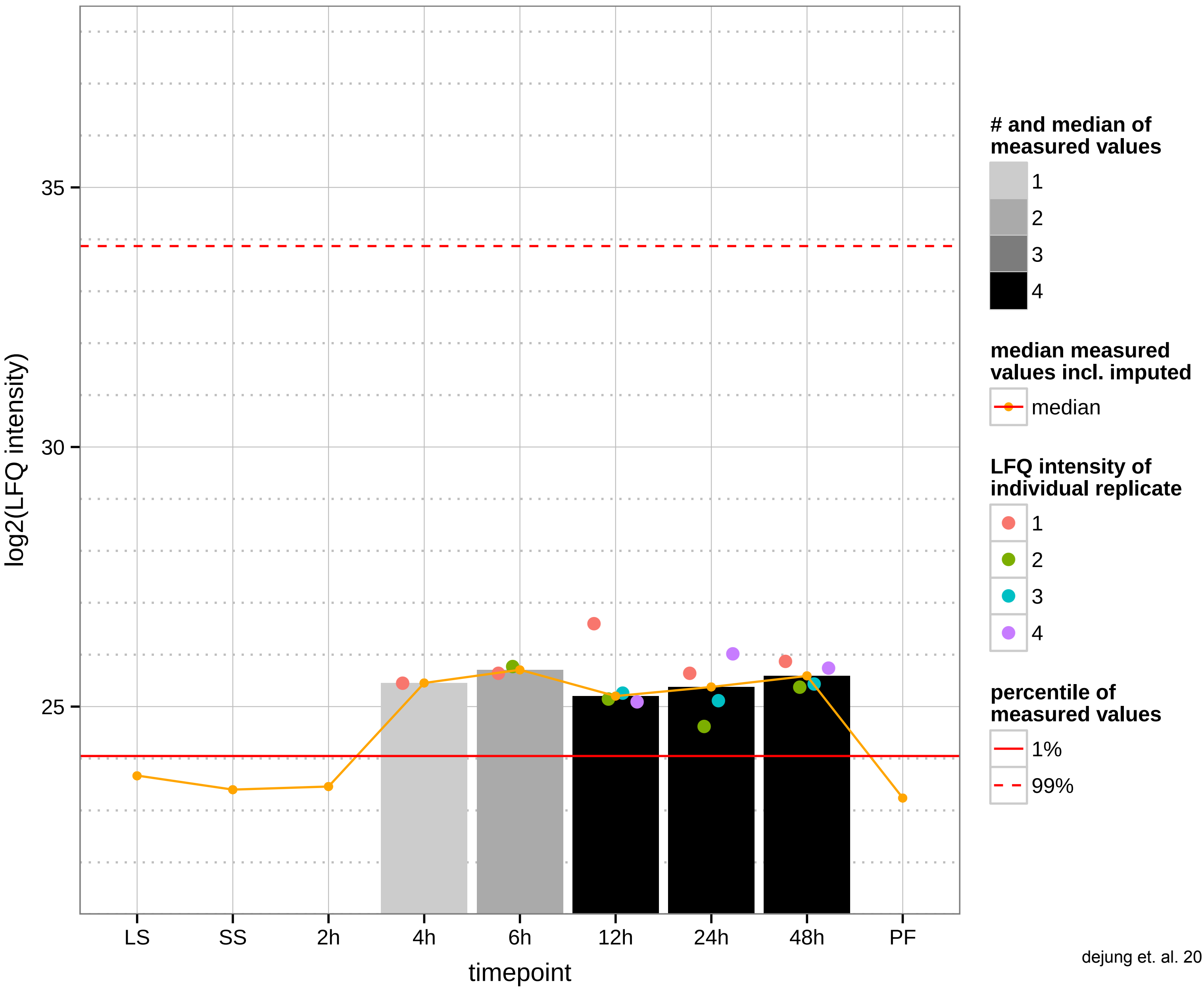

dejung et. al. 2015

hypothetical protein, conserved (YCF45)
Tb927.6.3350
AGOF: nucleoside–triphosphatase activity, nucleotide binding
AGOC: mitochondrion
AGOP: null
PGOF: nucleoside–triphosphatase activity, nucleotide binding
PGOC: null
PGOP: null

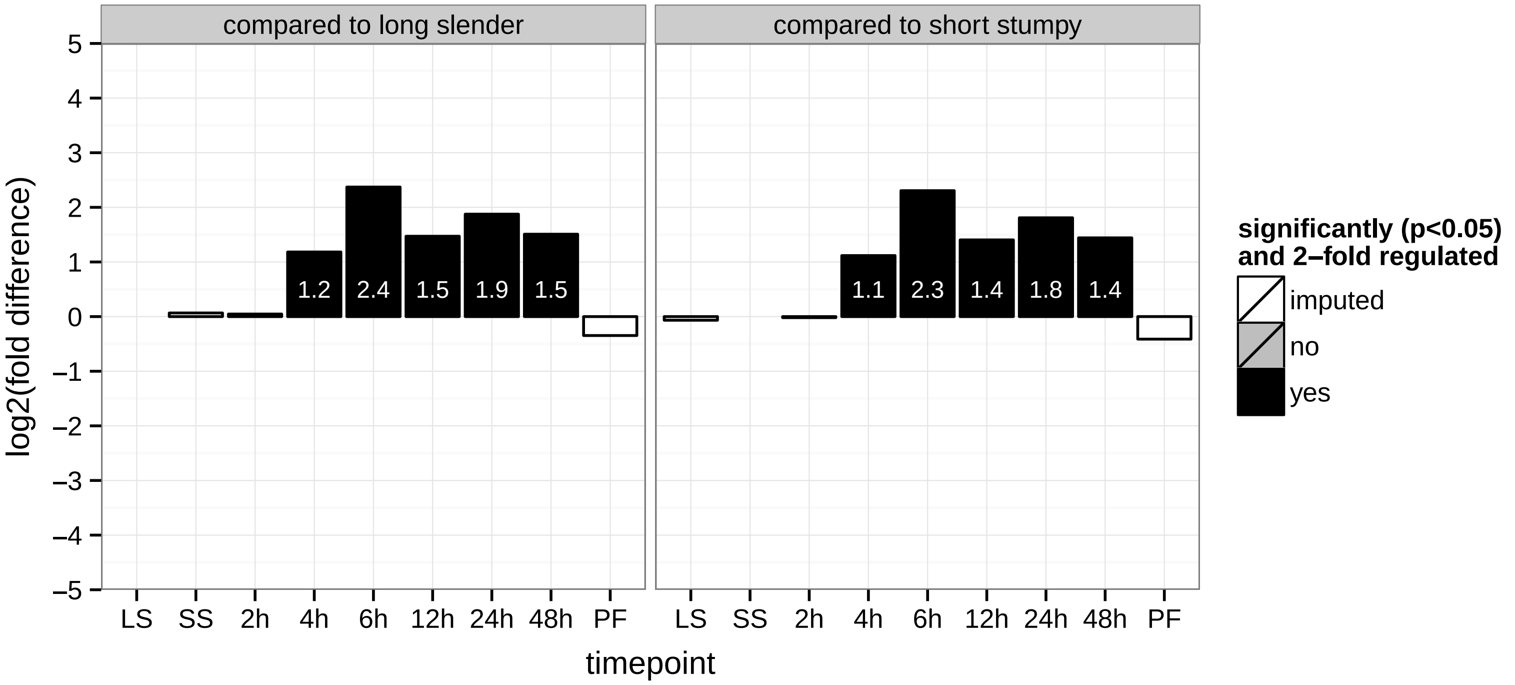

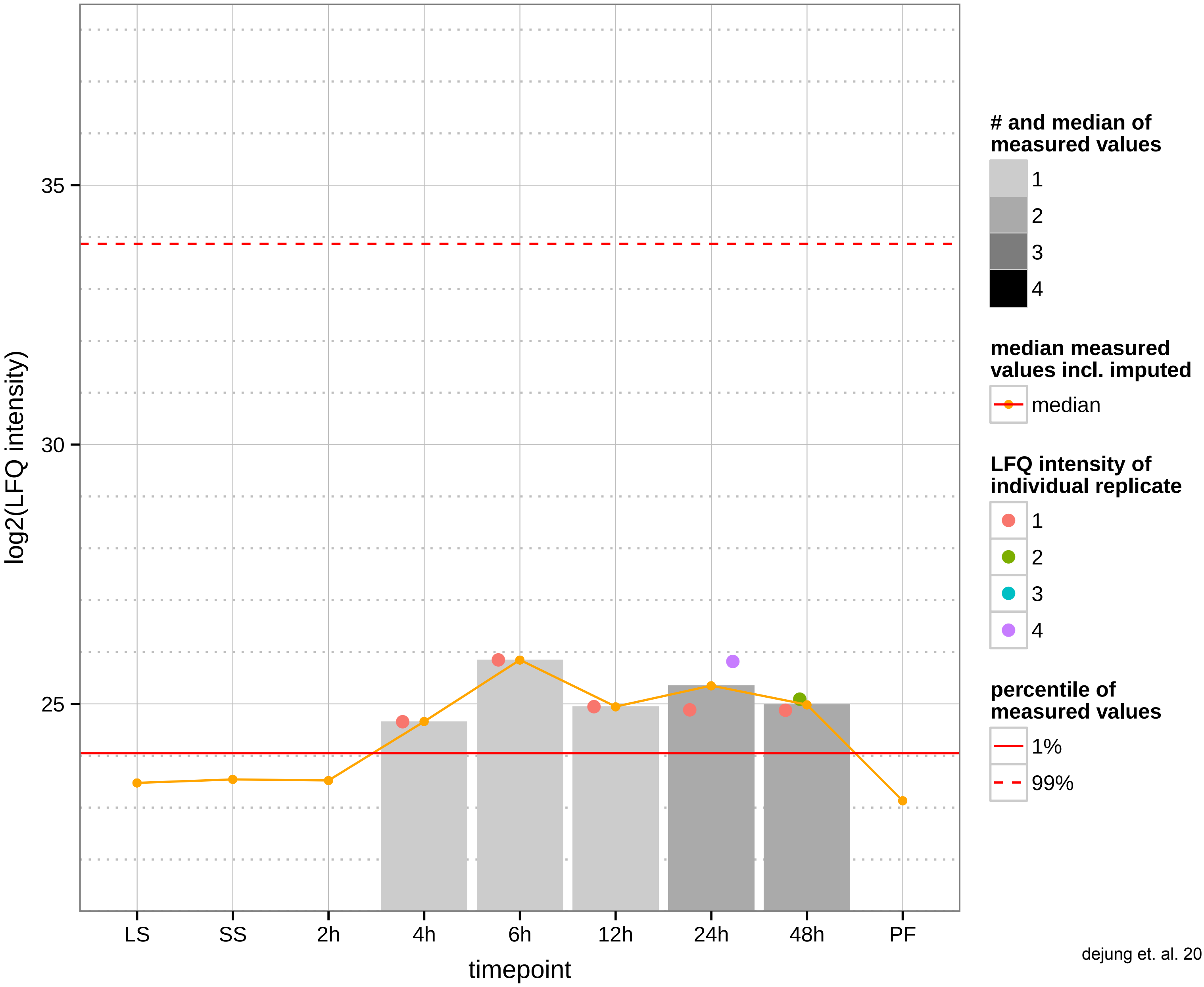

dejung et. al. 2015

hypothetical protein, conserved
Tb927.7.5240
AGOF: null
AGOC: null
AGOP: null
PGOF: null
PGOC: null
PGOP: null

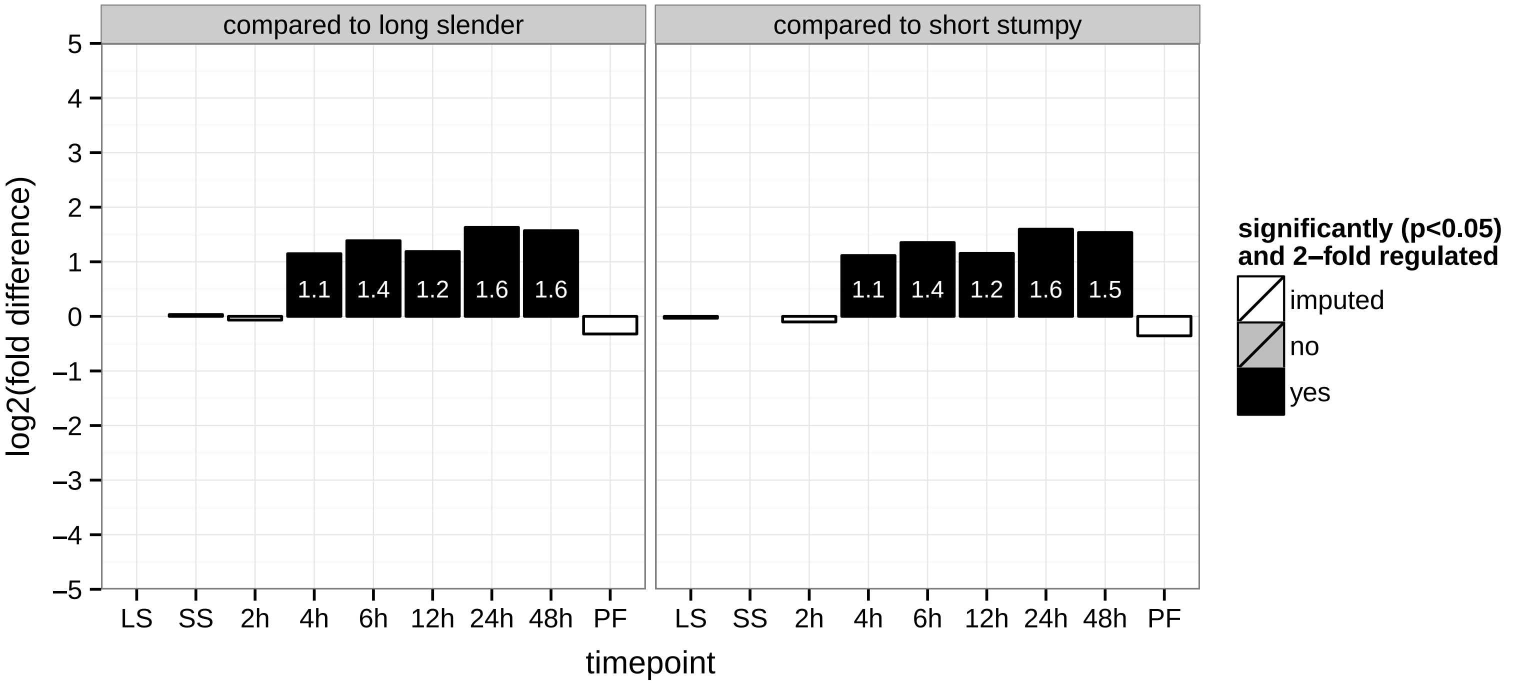

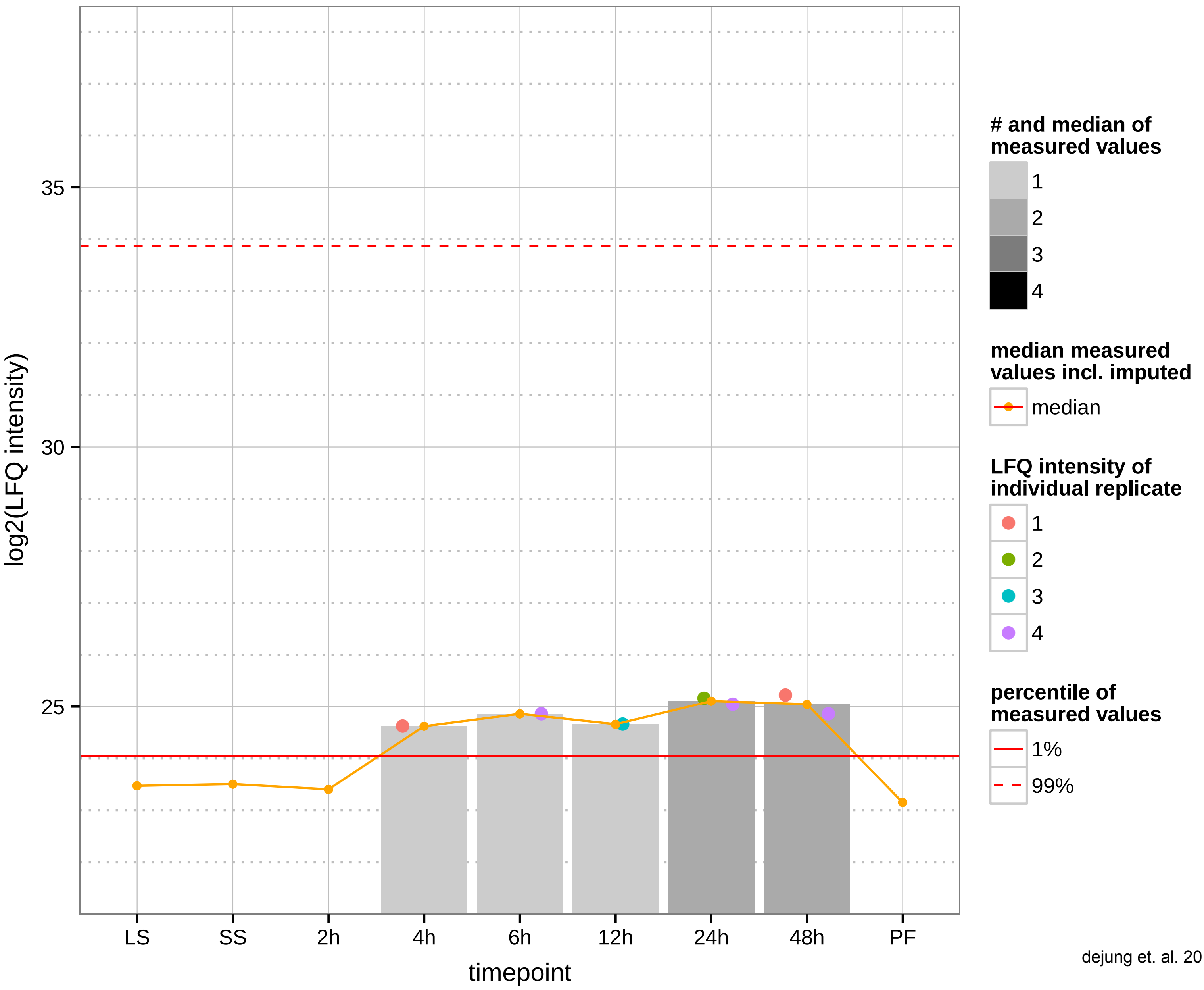

dejung et. al. 2015

hypothetical protein, conserved
Tb927.8.1120
AGOF: null
AGOC: null
AGOP: null
PGOF: null
PGOC: null
PGOP: null

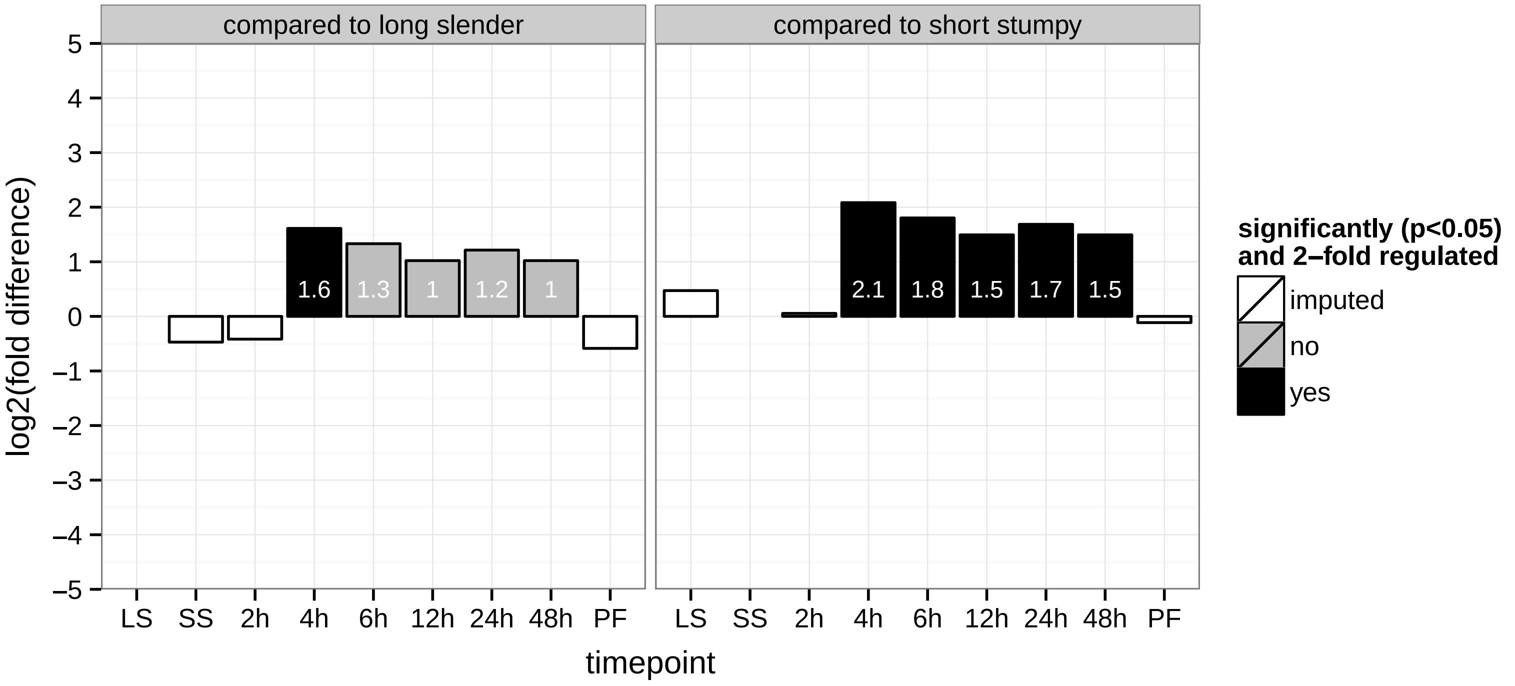

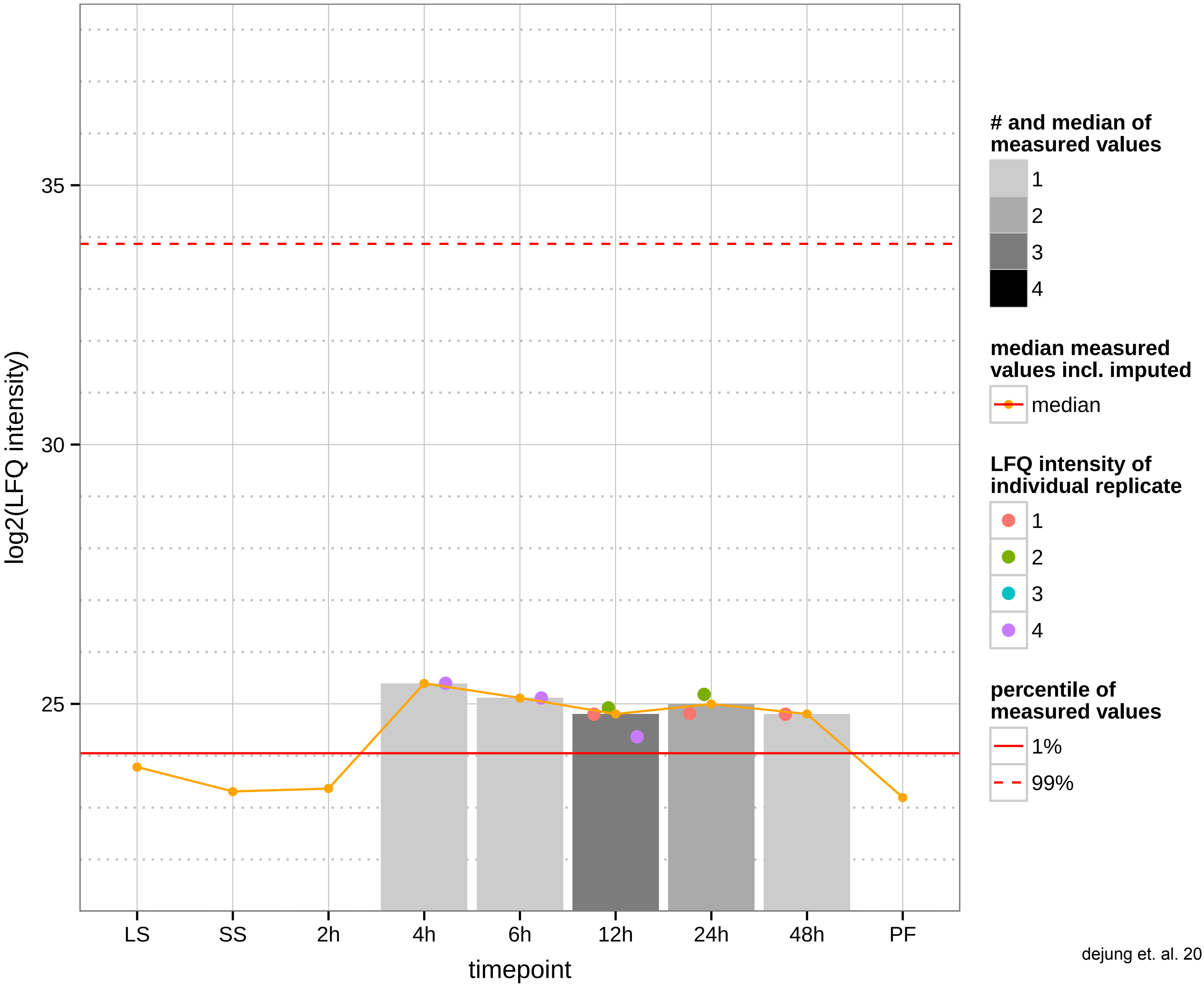

dejung et. al. 2015

hypothetical protein, conserved
Tb927.9.12890
AGOF: null
AGOC: null
AGOP: null
PGOF: null
PGOC: null
PGOP: null

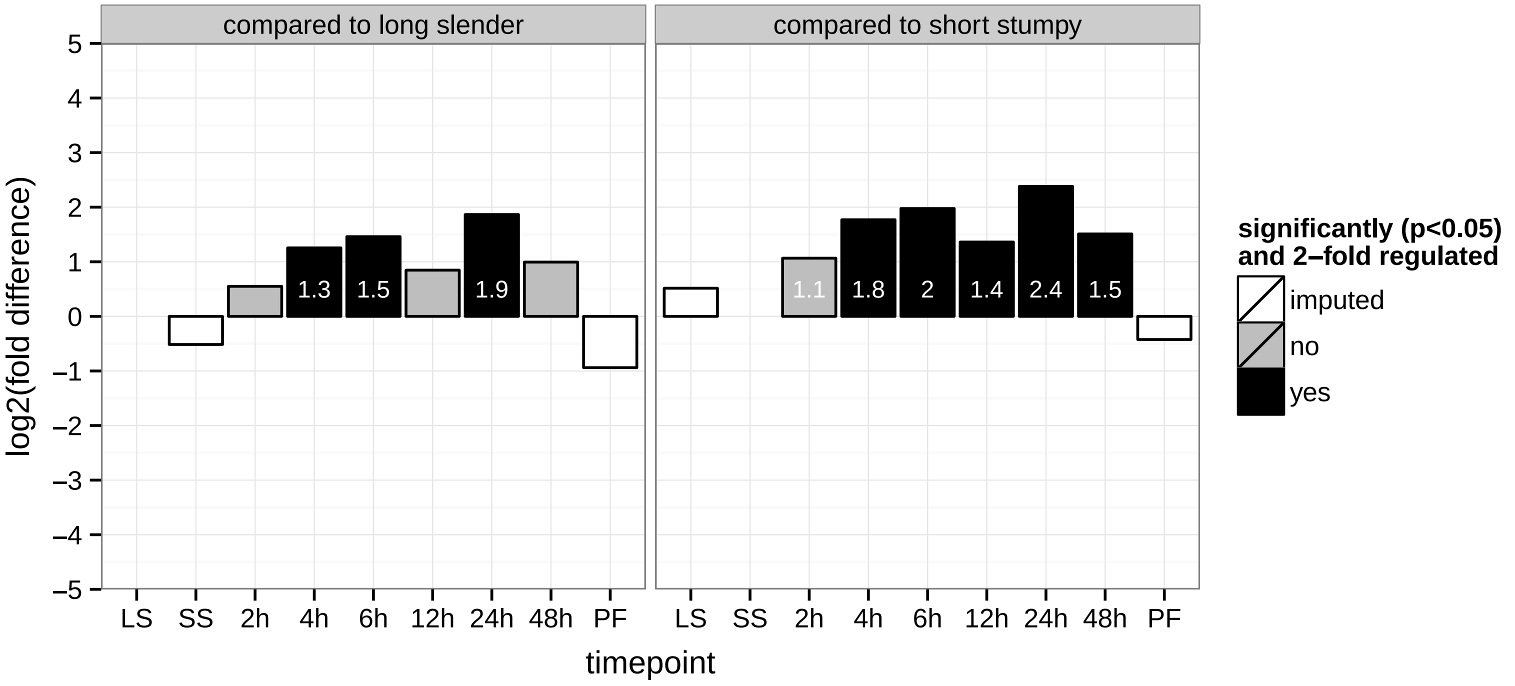

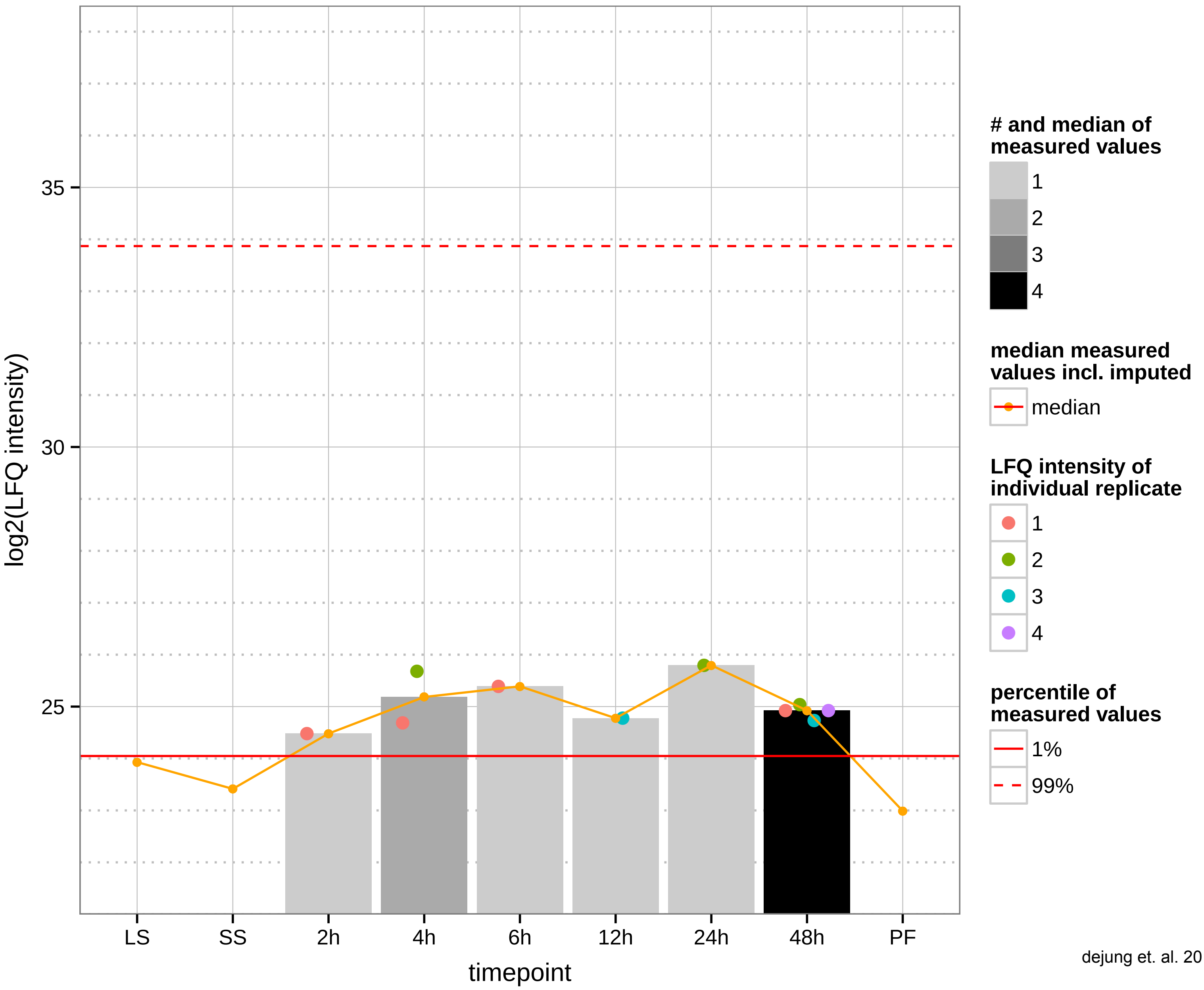

dejung et. al. 2015

hypothetical protein, conserved
Tb927.9.2160
AGOF: null
AGOC: null
AGOP: null
PGOF: null
PGOC: null
PGOP: null

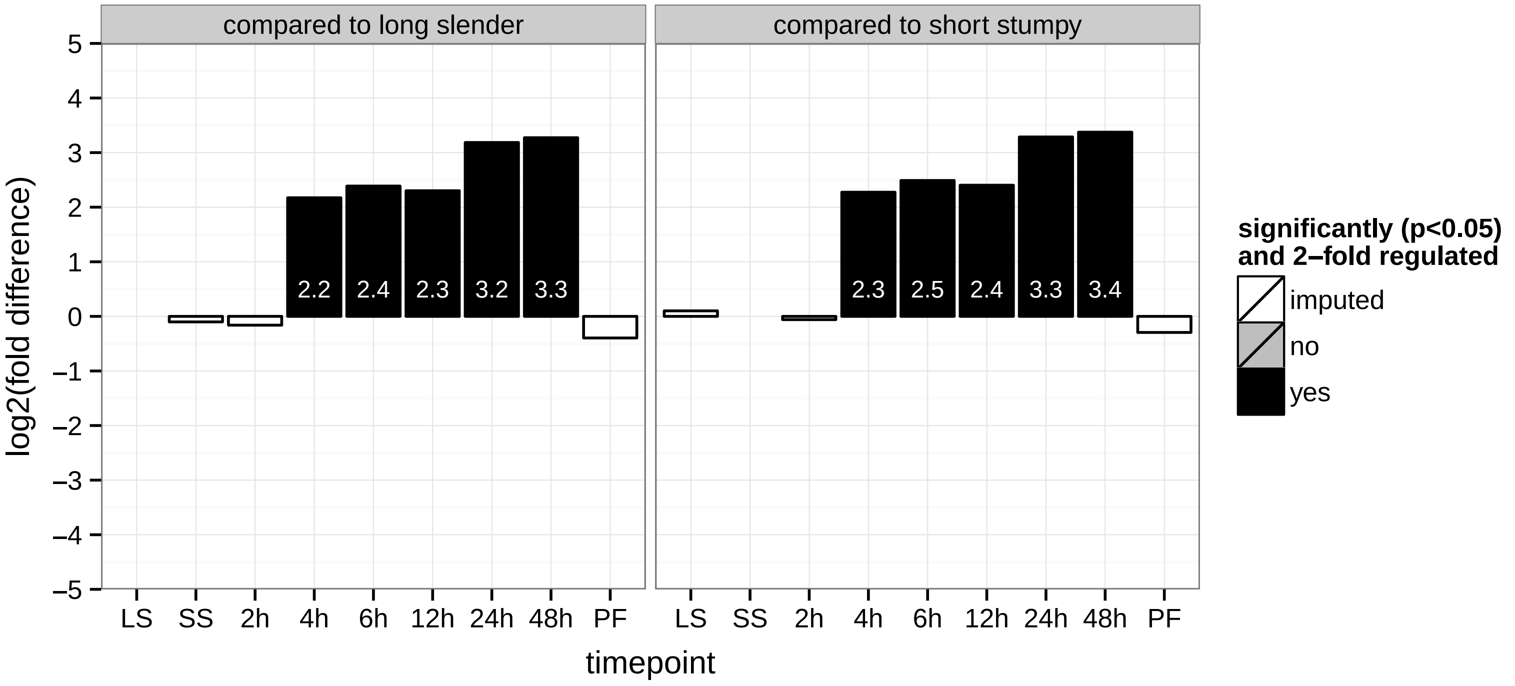

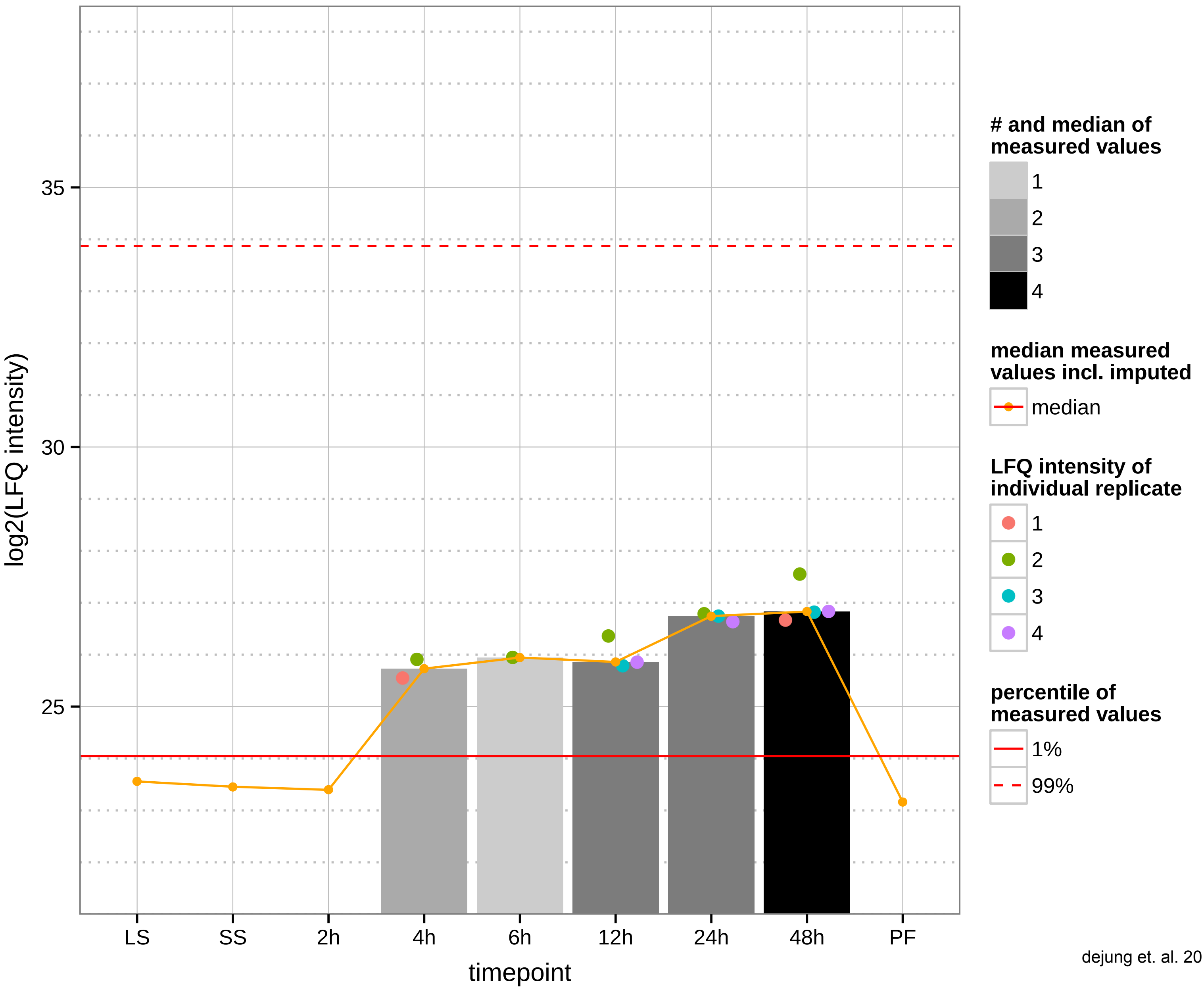

dejung et. al. 2015

thioredoxin (trx)
Tb927.9.3370
AGOF: disulfide oxidoreductase activity, protein disulfide isomerase activity
AGOC: null
AGOP: cell redox homeostasis
PGOF: electron carrier activity, protein disulfide oxidoreductase activity
PGOC: null
PGOP: cell redox homeostasis, glycerol ether metabolic process

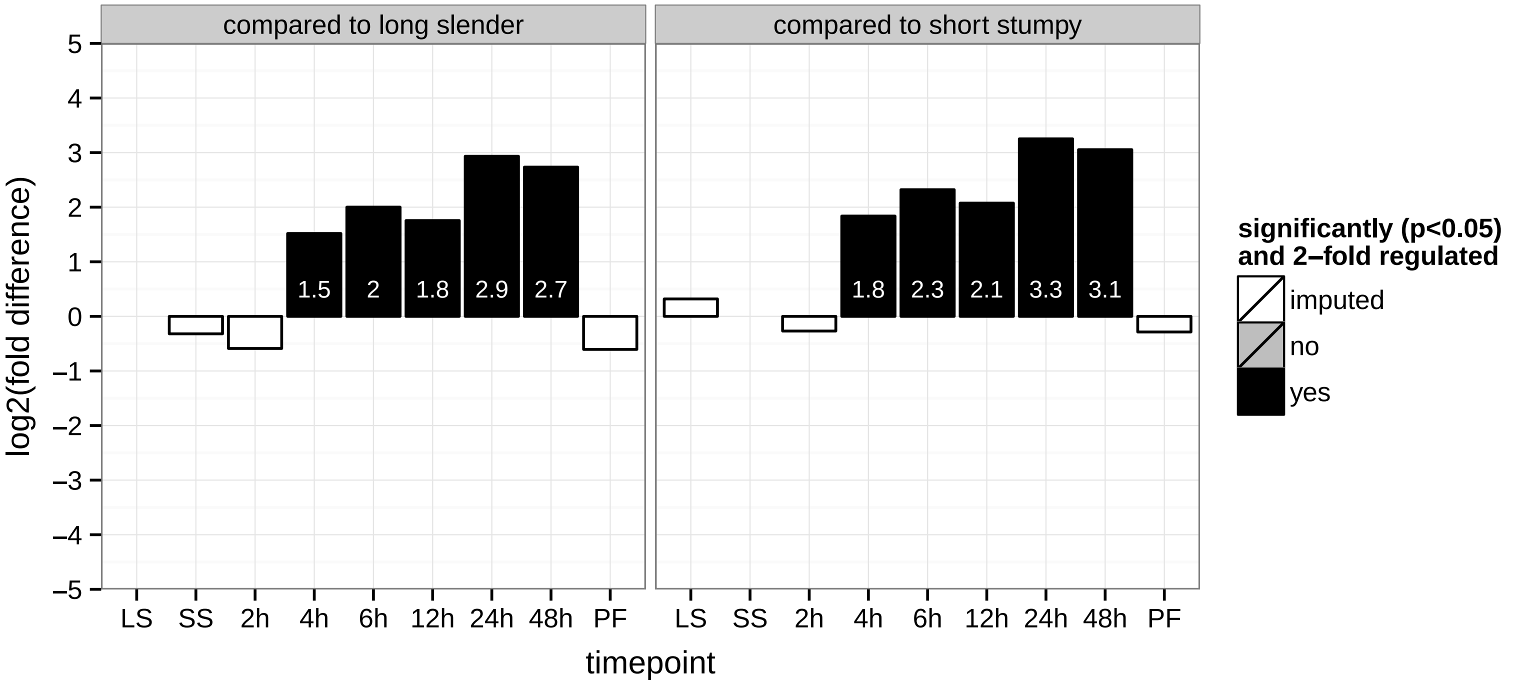

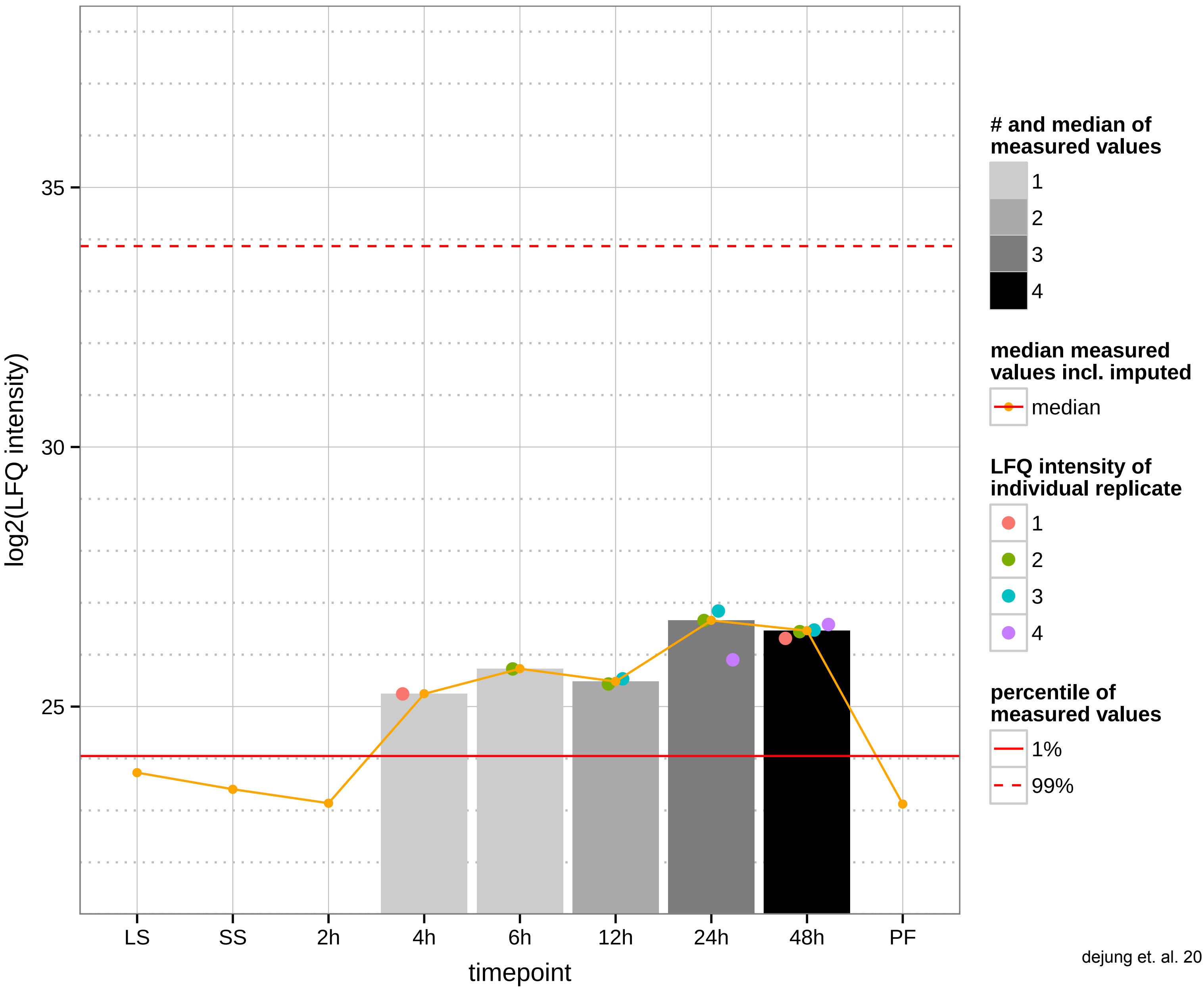

dejung et. al. 2015

hypothetical protein, conserved
Tb927.9.4350
AGOF: null
AGOC: null
AGOP: null
PGOF: protein binding
PGOC: null
PGOP: null

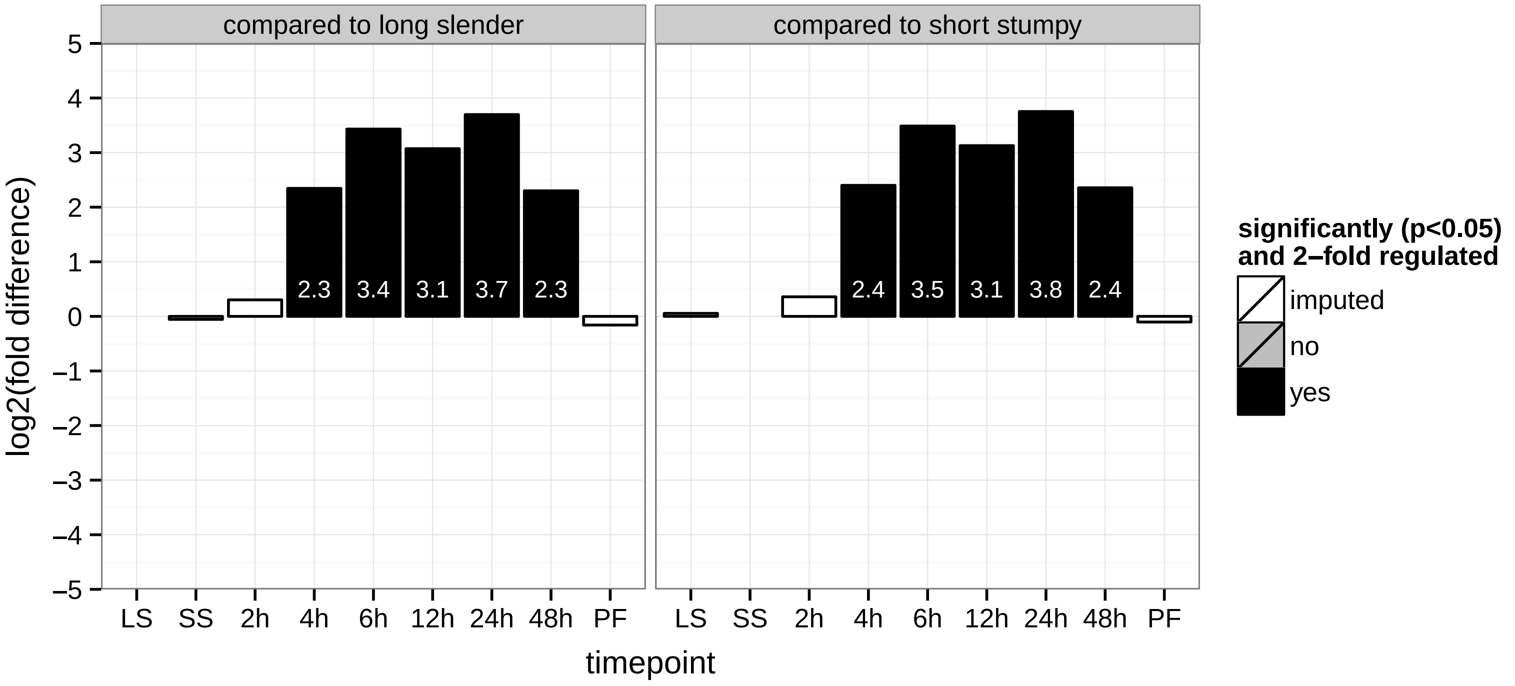

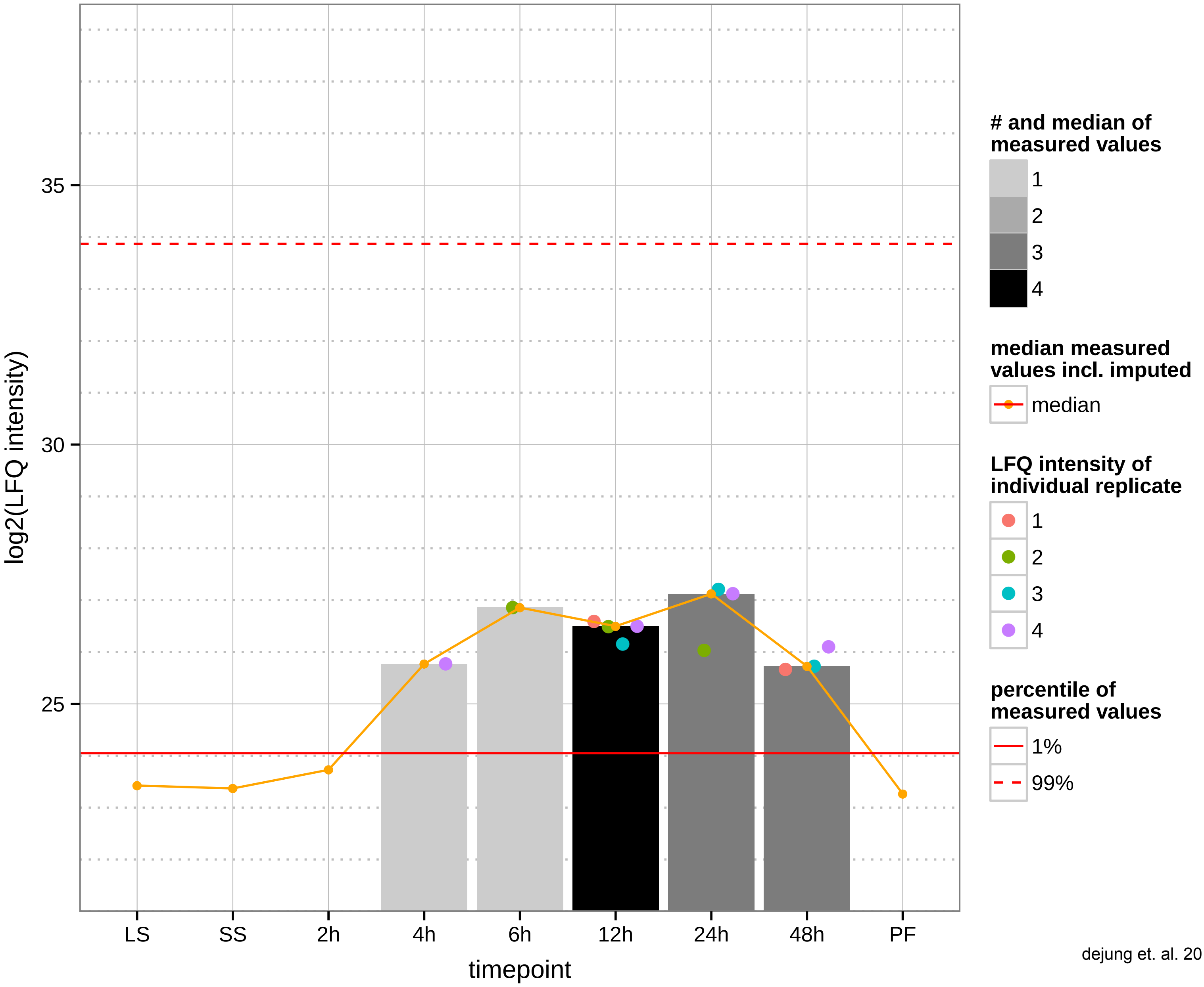

dejung et. al. 2015

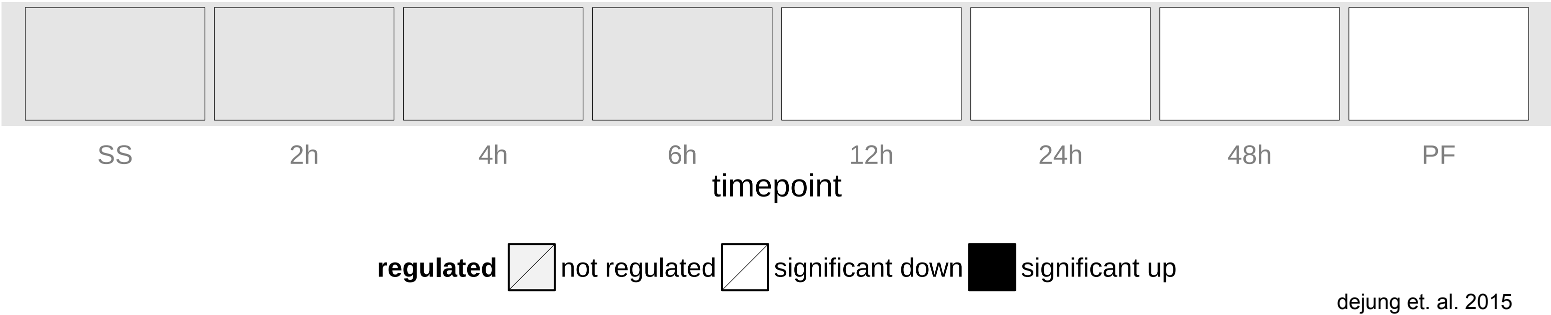
SS
2h
4h
6h
12h
24h
48h
PF
timepoint
regulated
not regulated
significant down
significant up
dejung et. al. 2015

MSP–A, putative, chrXI additional, unordered contigs, Gp63–1 surface protease homolog
Tb927.11.7640;Tb11.0360;Tb927.11.7620
AGOF: metalloendopeptidase activity, zinc ion binding
AGOC: membrane
AGOP: cell adhesion, proteolysis
PGOF: metalloendopeptidase activity, zinc ion binding
PGOC: membrane
PGOP: cell adhesion, proteolysis

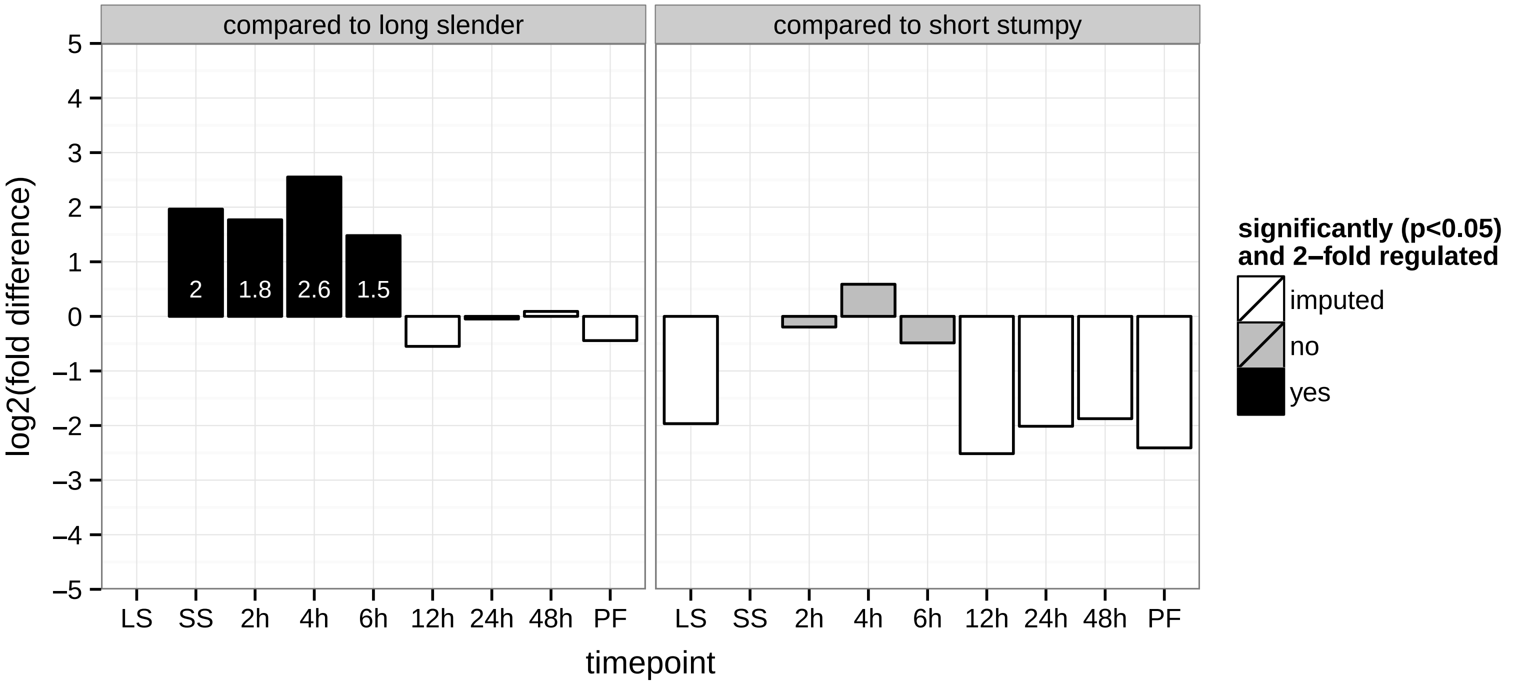

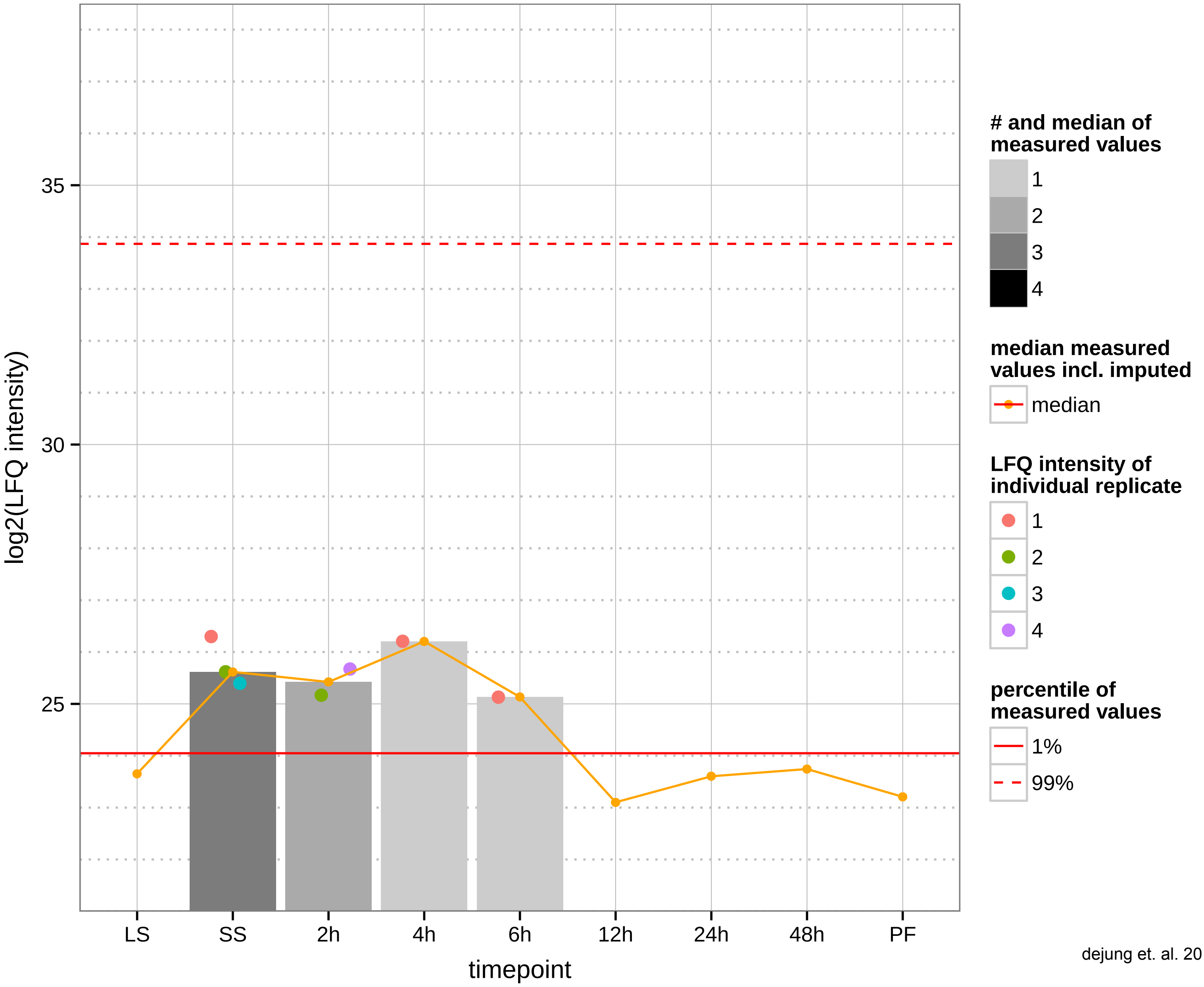

dejung et. al. 2015

variant surface glycoprotein (VSG, pseudogene), putative, chrXI additional, unordered contigs, variant surface protein (VSG)
Tb11.1690;Tb11.v5.0142
AGOF: null
AGOC: null
AGOP: evasion or tolerance of host immune response, null
PGOF: null
PGOC: null
PGOP: null

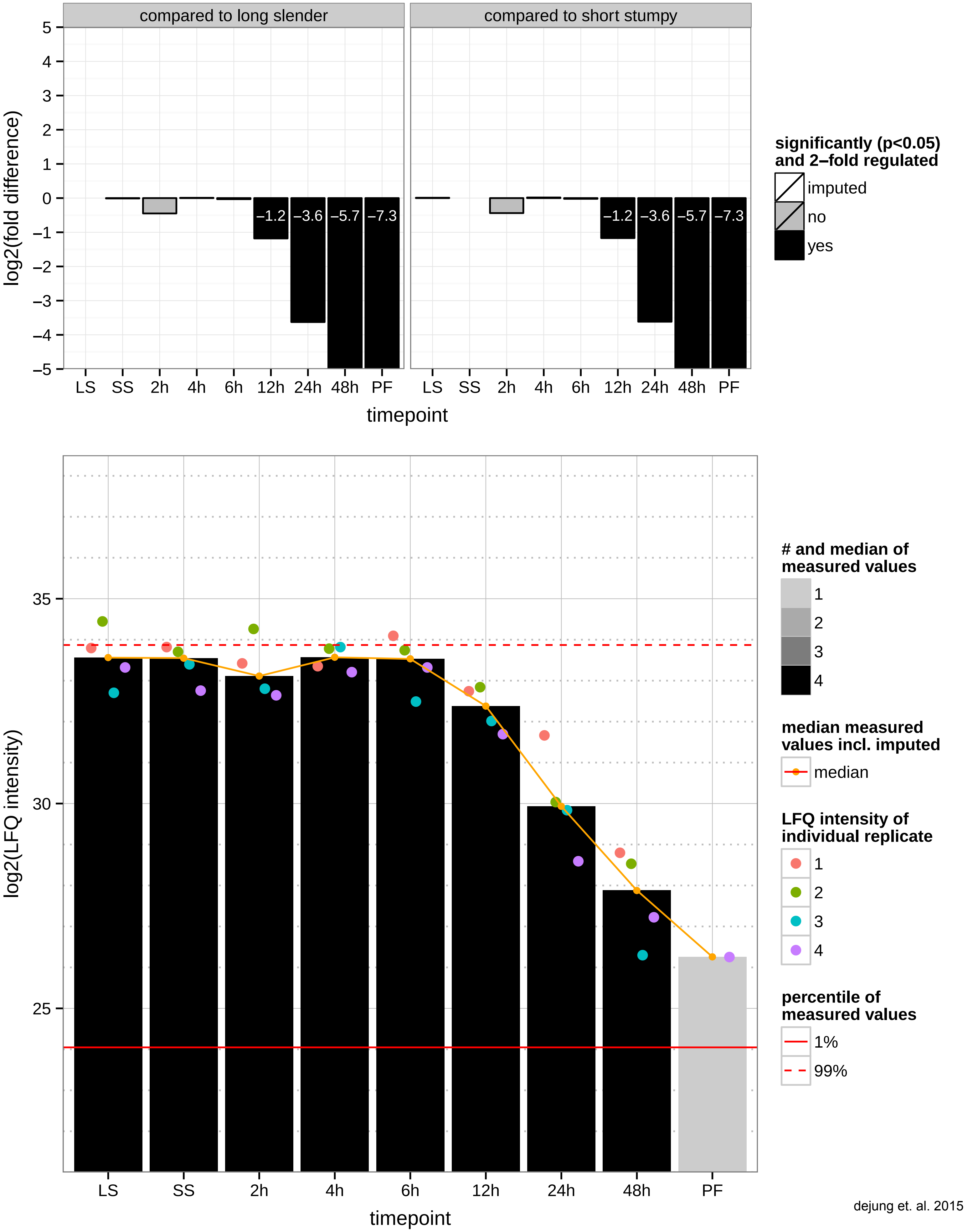

dejung et. al. 2015

75 kDa invariant surface glycoprotein, putative, 75 kDa invariant surface glycoprotein (ISG75)
Tb927.5.360;Tb11.v5.0231
AGOF: null
AGOC: null, plasma membrane
AGOP: null
PGOF: null
PGOC: null
PGOP: null

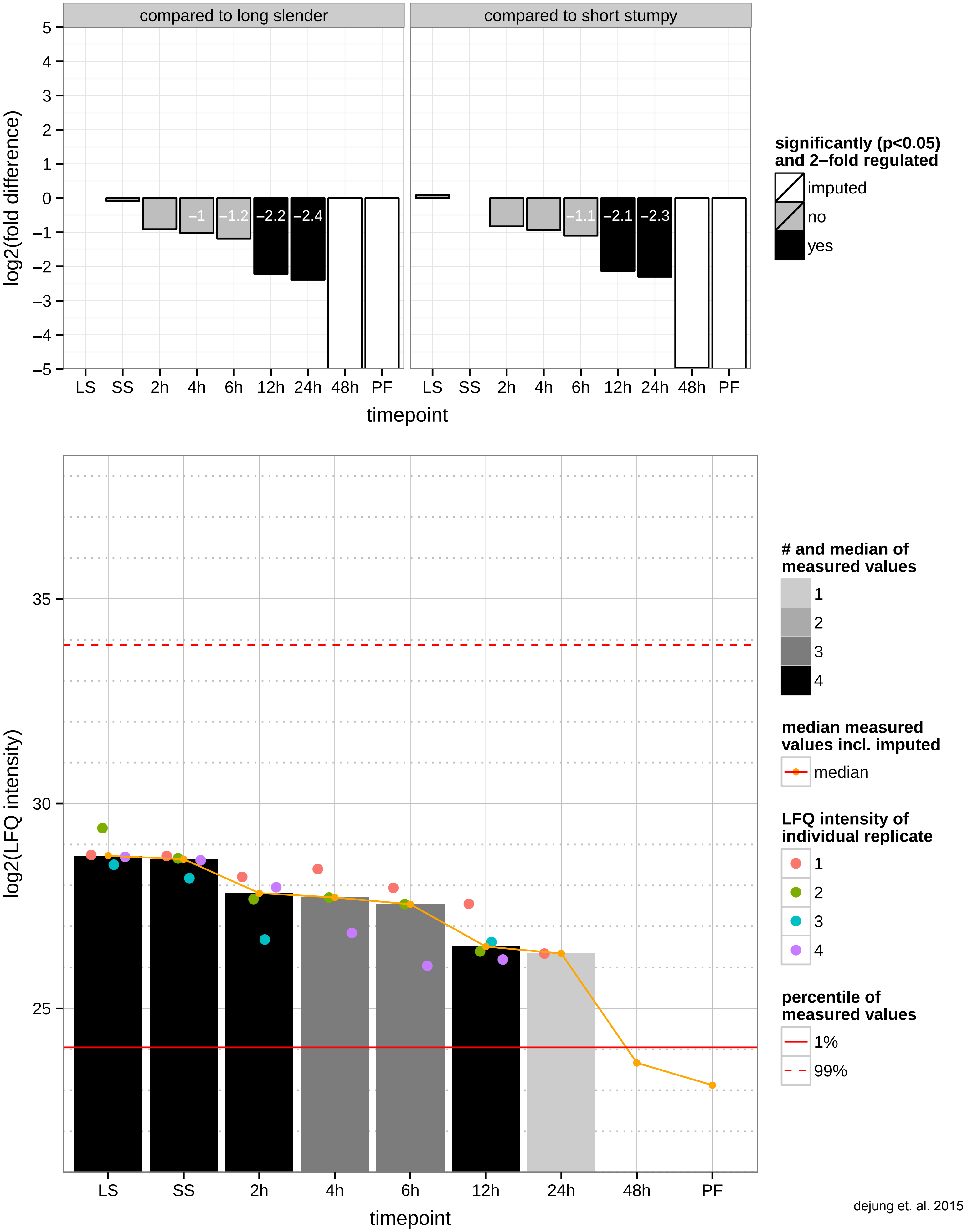

dejung et. al. 2015

variant surface glycoprotein (VSG), putative
Tb927.1.05
AGOF: null
AGOC: integral to membrane
AGOP: antigenic variation
PGOF: null
PGOC: null
PGOP: null

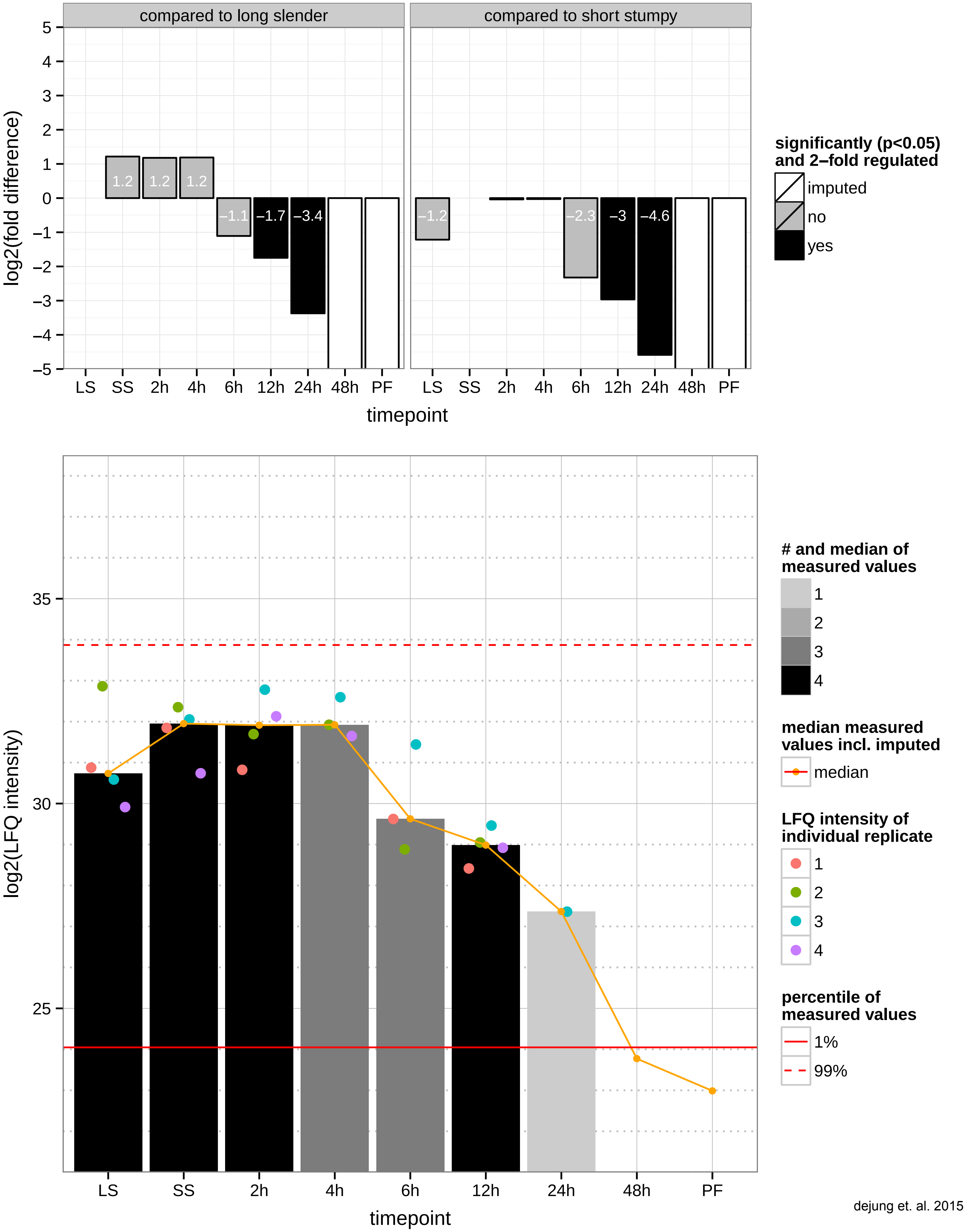

dejung et. al. 2015

actin related protein 2, putative
Tb927.10.15800
AGOF: structural constituent of cytoskeleton
AGOC: actin cytoskeleton
AGOP: cytoskeleton organization
PGOF: null
PGOC: null
PGOP: null

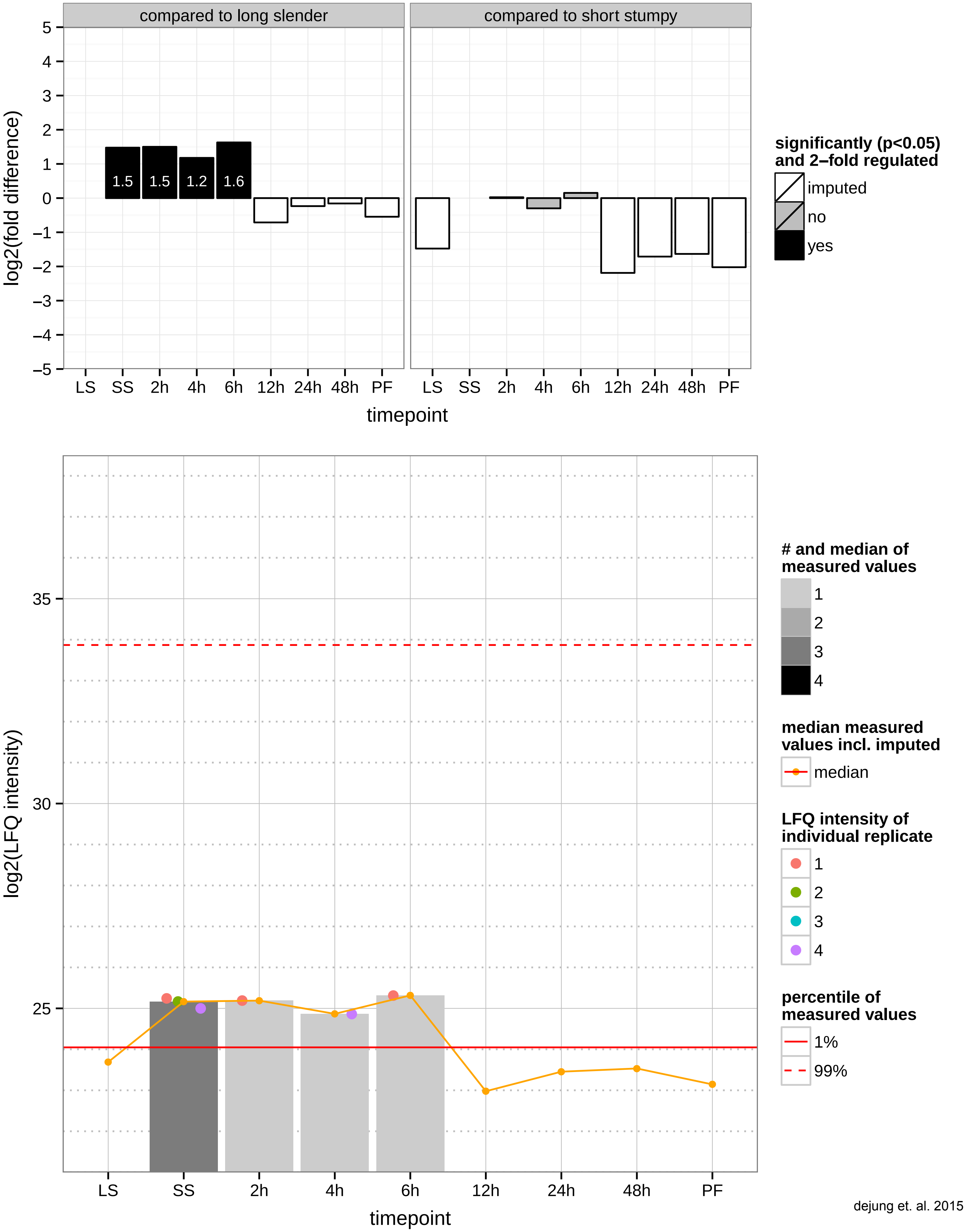

dejung et. al. 2015

ribosomal RNA processing protein 41B, RNase PH–like protein, putative, exosome complex exonuclease 1, putative (RRP41B
Tb927.2.2180
AGOF: 3'–5' exonuclease activity, 3'–5'–exoribonuclease activity, RNA binding
AGOC: cellular_component
AGOP: RNA processing, rRNA metabolic process
PGOF: null
PGOC: null
PGOP: null

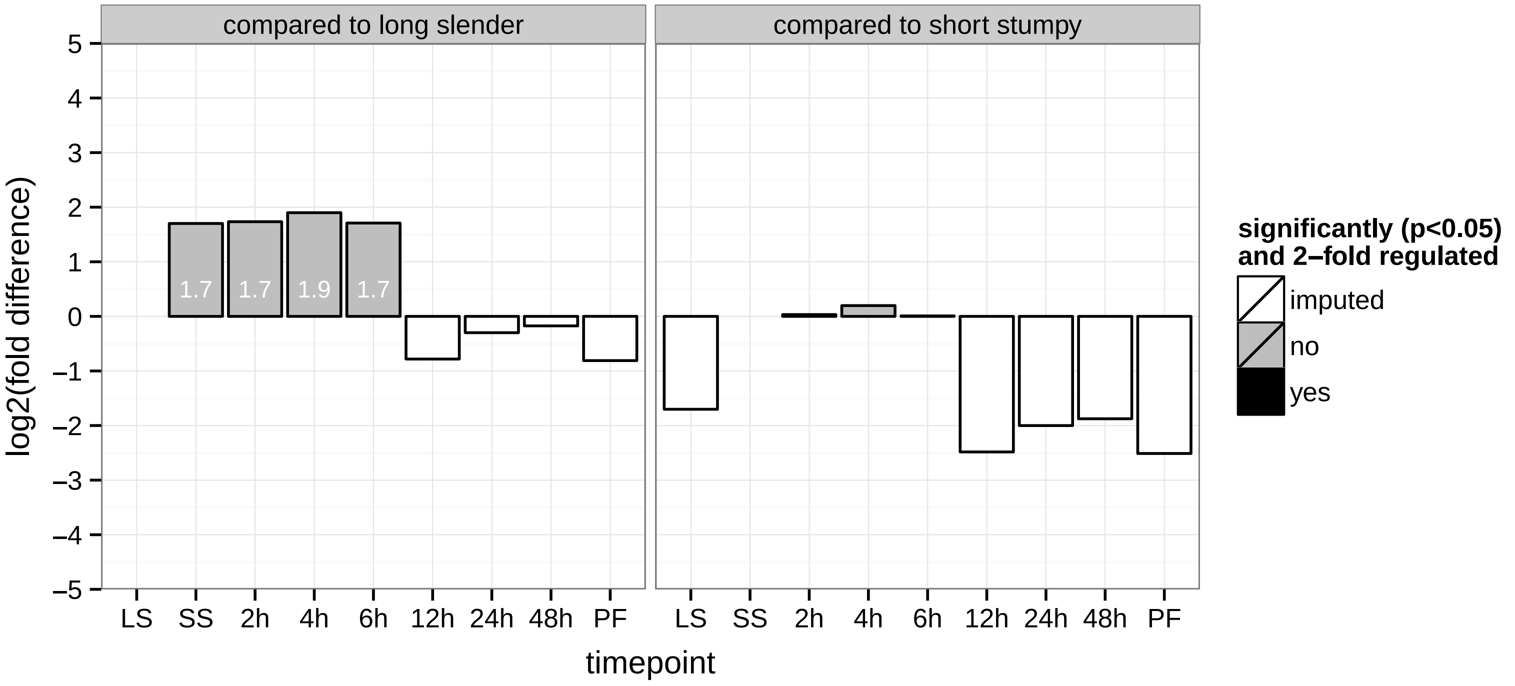

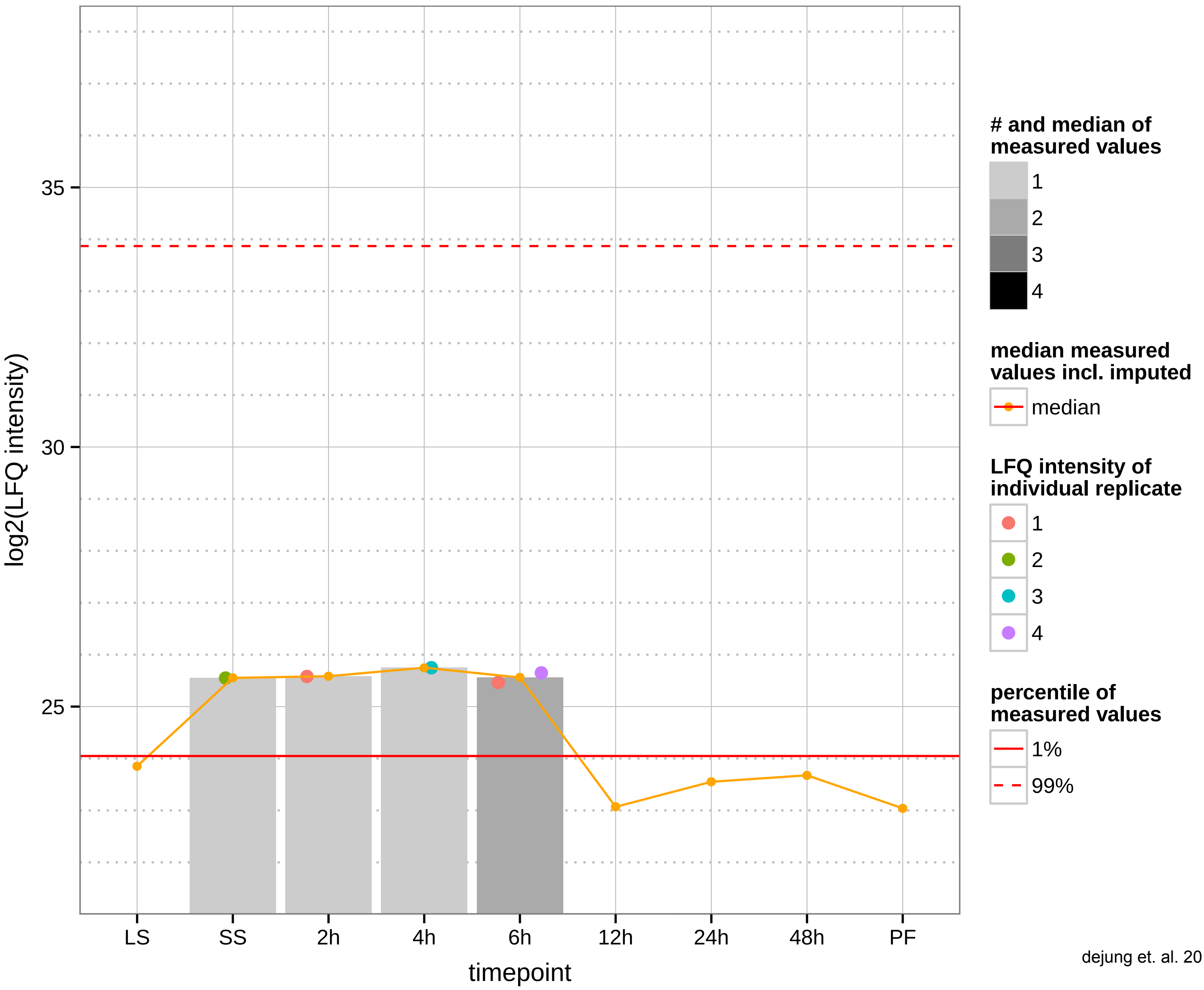

dejung et. al. 2015

kinesin, putative
Tb927.3.3390;Tb927.3.3400
AGOF: ATP binding, microtubule motor activity, motor activity
AGOC: microtubule associated complex
AGOP: microtubule–based movement
PGOF: ATP binding, microtubule motor activity
PGOC: null
PGOP: microtubule–based movement

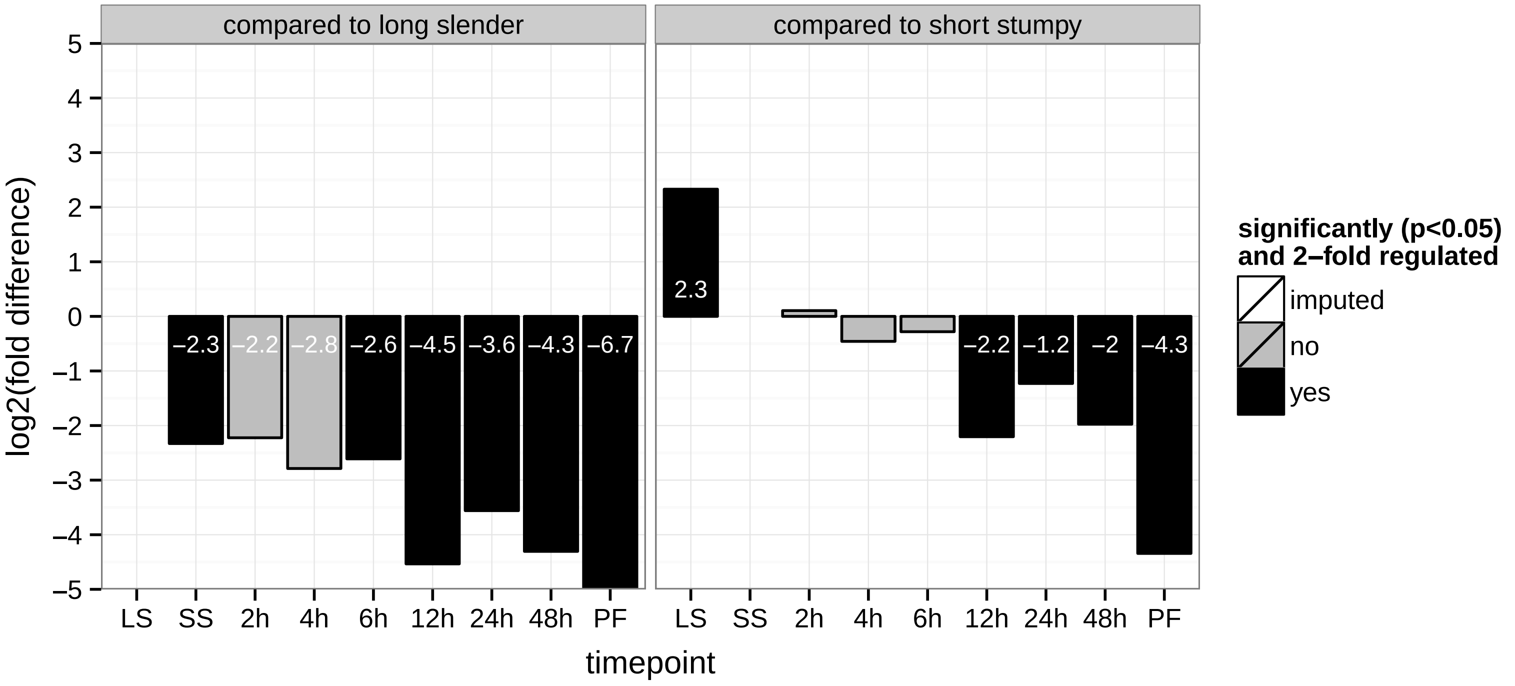

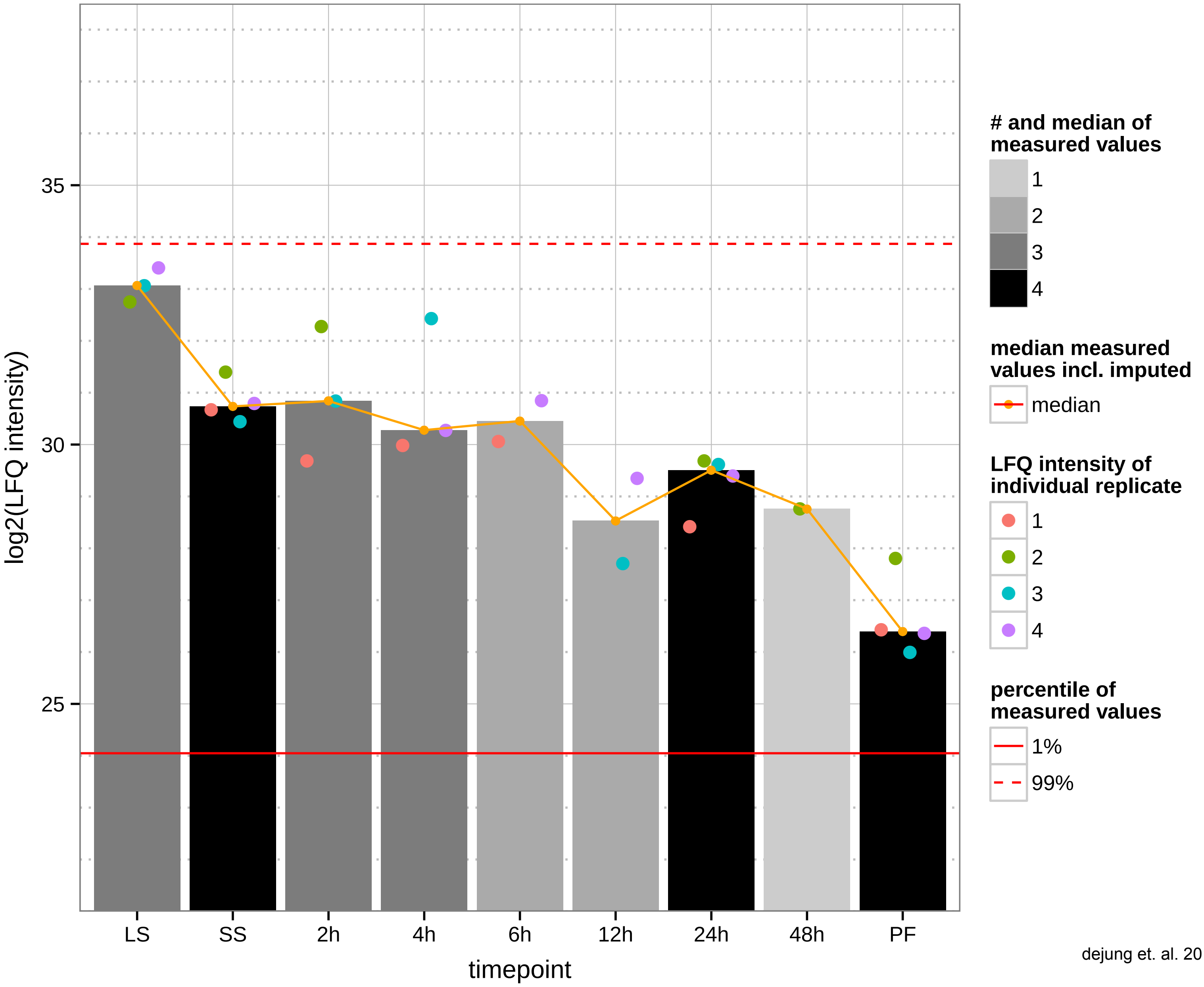

dejung et. al. 2015

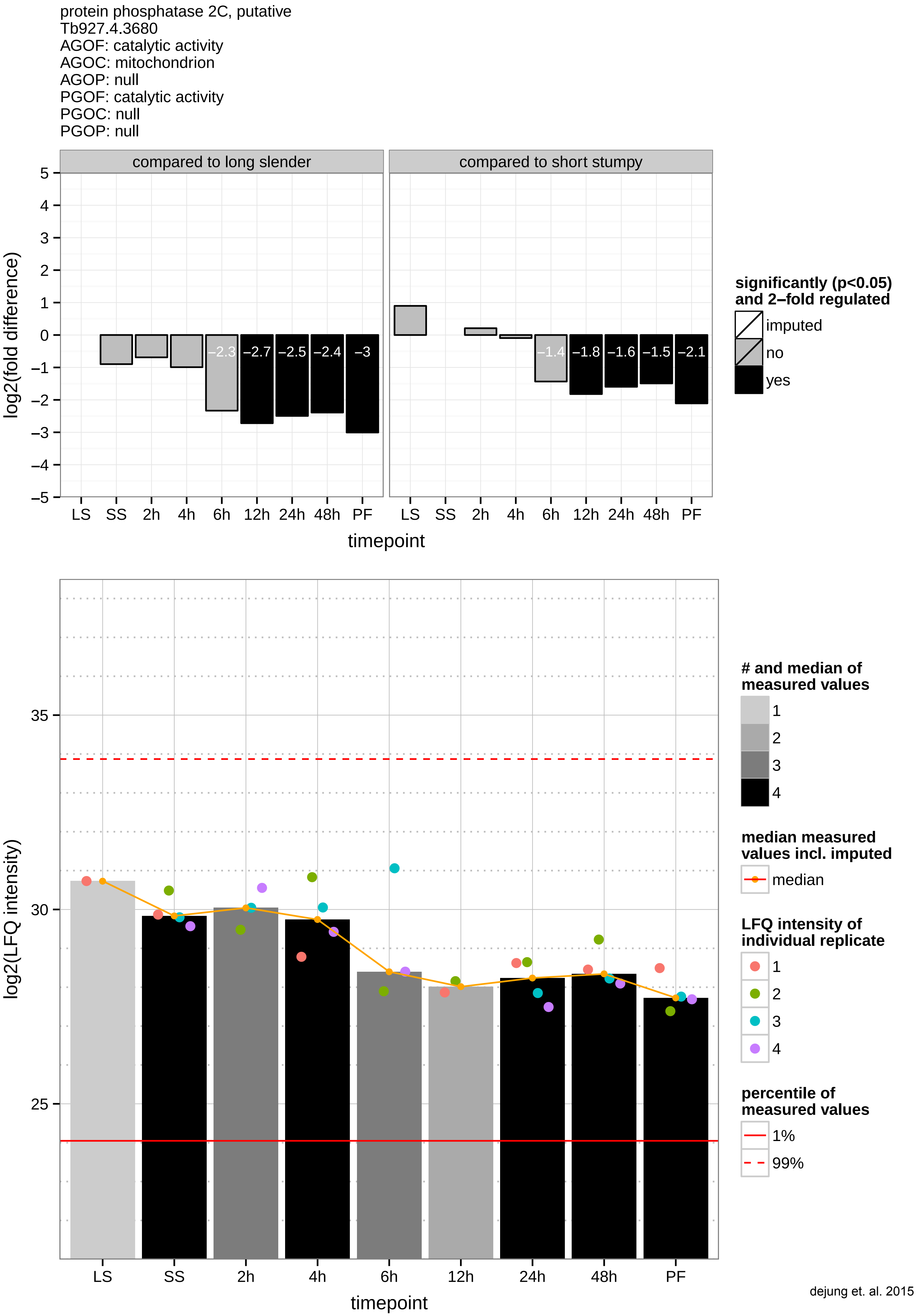
protein phosphatase 2C, putative
Tb927.4.3680
AGOF: catalytic activity
AGOC: mitochondrion
AGOP: null
PGOF: catalytic activity
PGOC: null
PGOP: null
compared to long slender
compared to short stumpy
log2(fold difference)
5
4
3
2
1
0
−1
−2
−3
−4
−5
−2.3
−2.7
−2.5
−2.4
−3
−1.4
−1.8
−1.6
−1.5
−2.1
LS
SS
2h
4h
6h
12h
24h
48h
PF
timepoint
significantly (p<0.05) and 2−fold regulated
imputed
no
yes
log2(LFQ intensity)
35
30
25
and median of measured values
1
2
3
4
median measured values incl. imputed
median
LFQ intensity of individual replicate
1
2
3
4
percentile of measured values
1%
99%
dejung et. al. 2015

64 kDa invariant surface glycoprotein (ISG64)
Tb927.5.1430
AGOF: null
AGOC: null
AGOP: null
PGOF: null
PGOC: null
PGOP: null

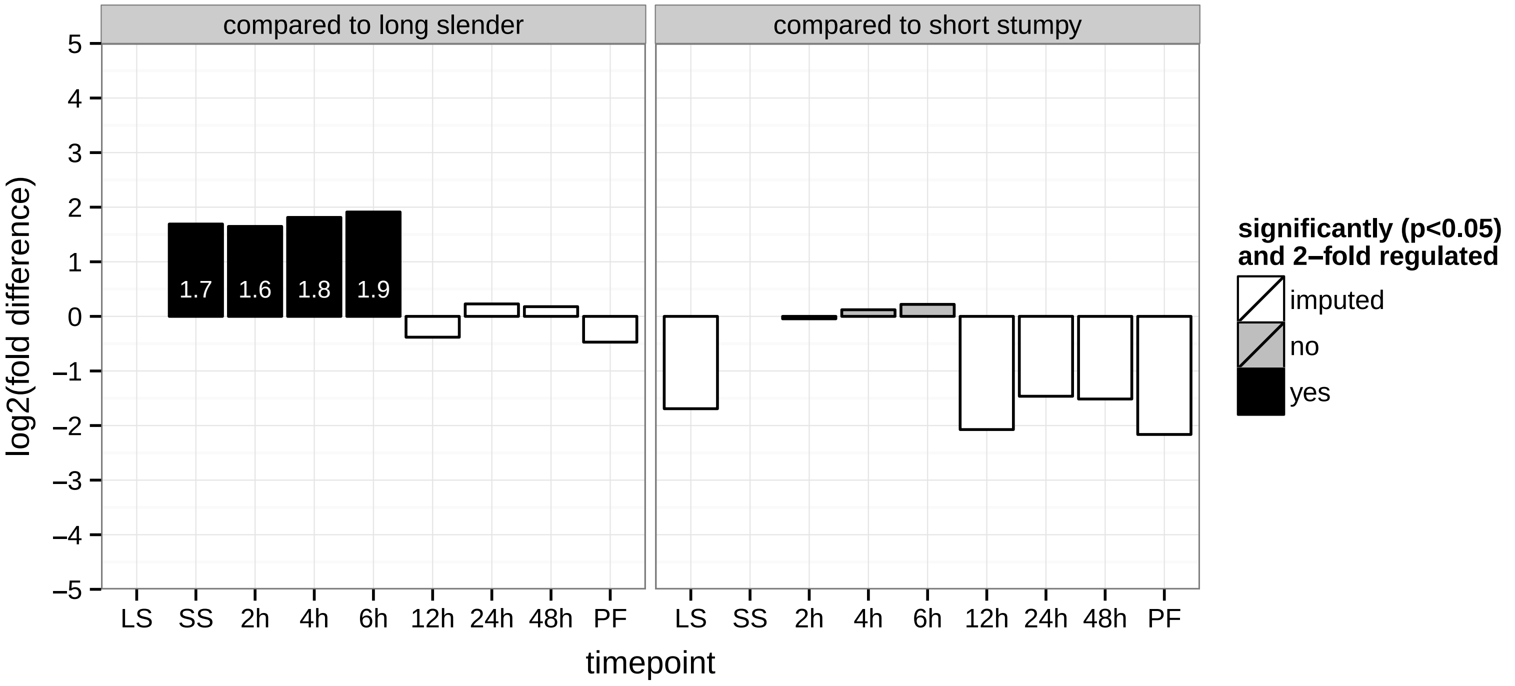

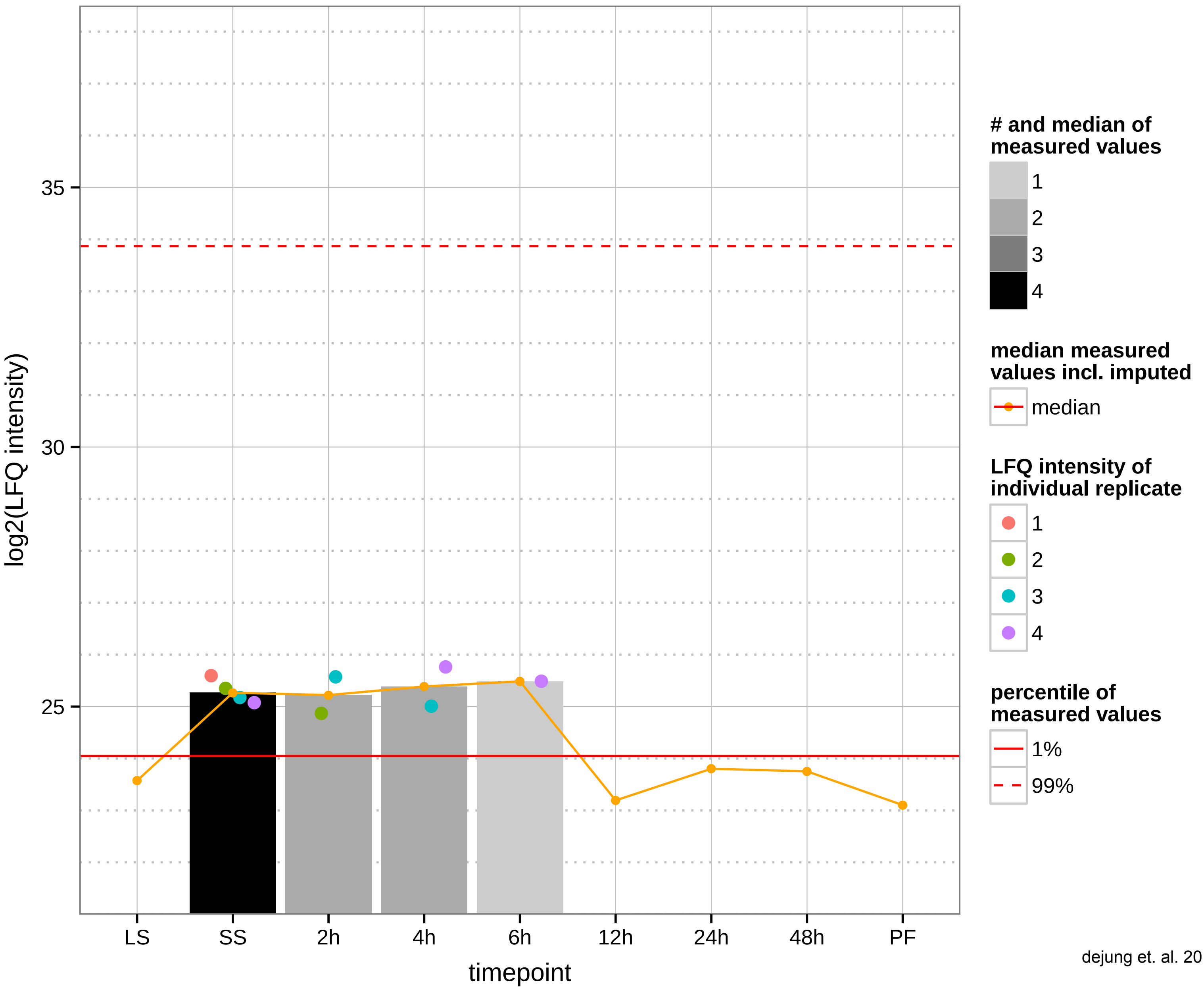

dejung et. al. 2015

hypothetical protein
Tb927.5.3100
AGOF: null
AGOC: null
AGOP: null
PGOF: null
PGOC: null
PGOP: null

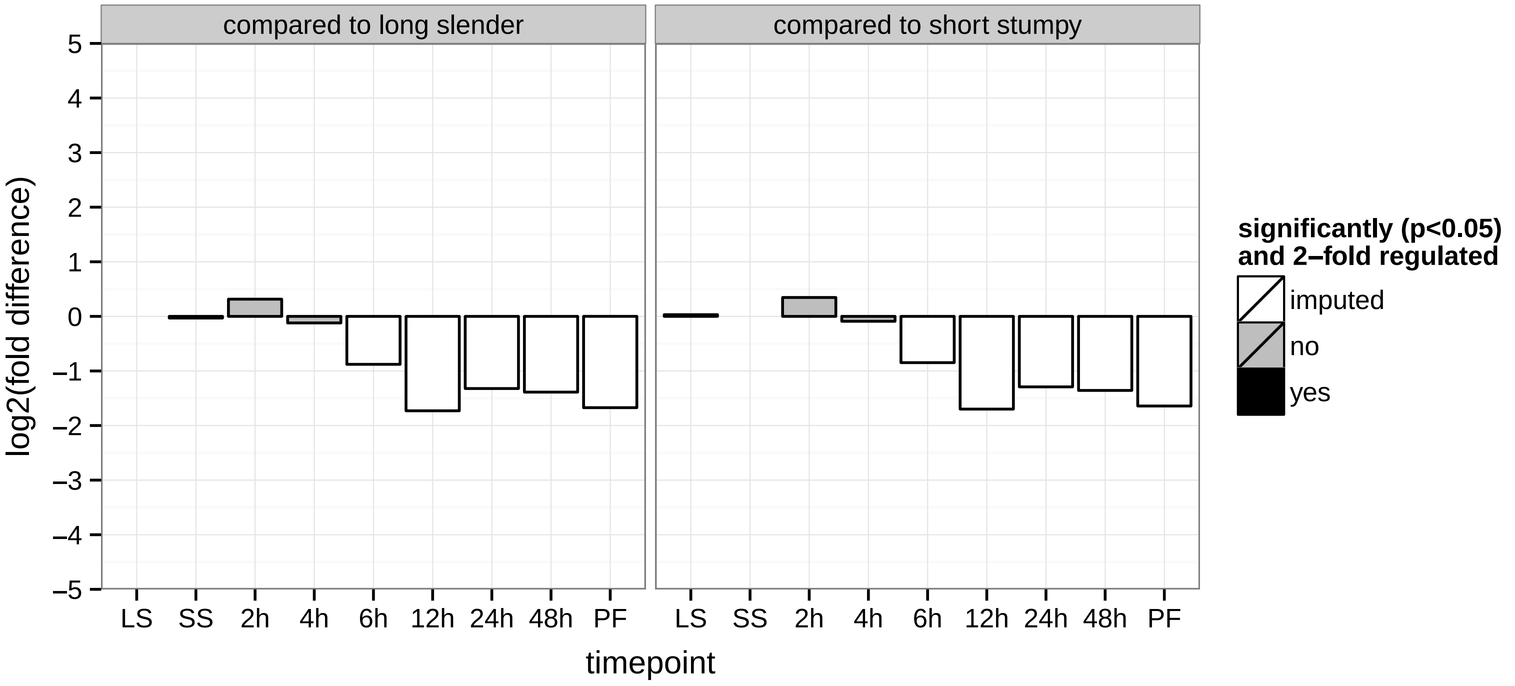

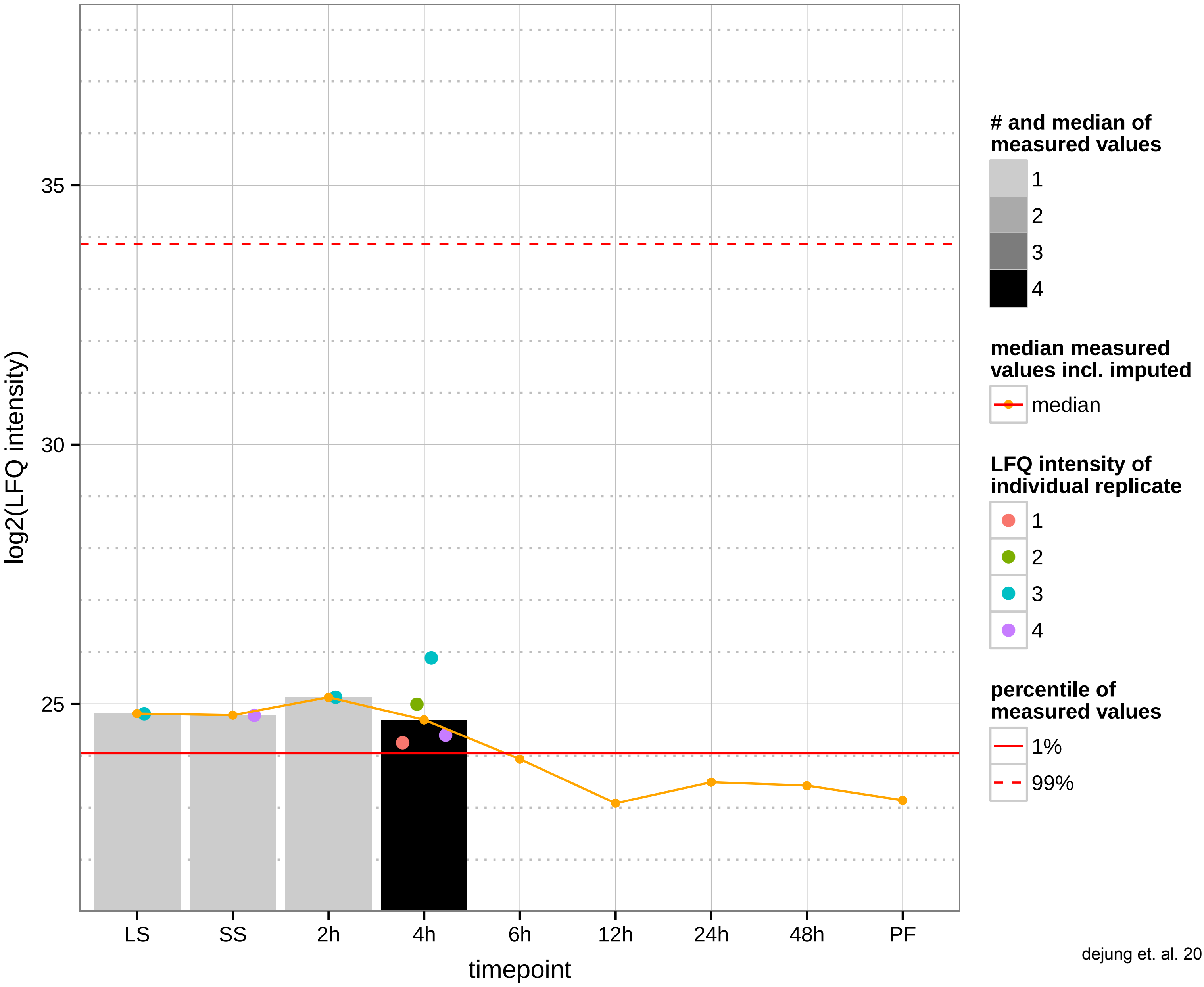

dejung et. al. 2015

hypothetical protein, conserved
Tb927.6.1750
AGOF: Rab GTPase activator activity
AGOC: intracellular
AGOP: regulation of Rab GTPase activity
PGOF: Rab GTPase activator activity
PGOC: intracellular
PGOP: regulation of Rab GTPase activity

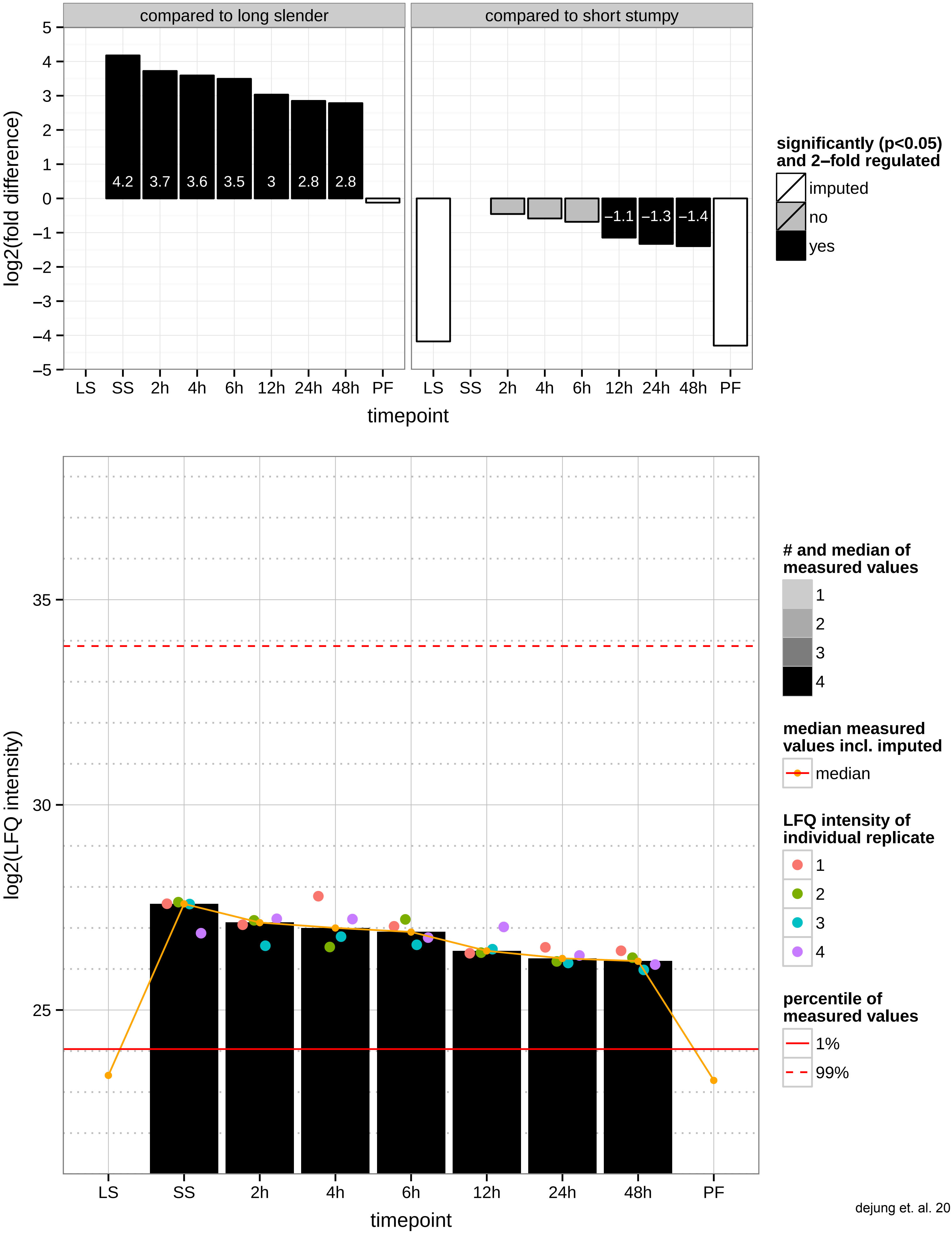

dejung et. al. 2015

hypothetical protein, conserved
Tb927.6.1980
AGOF: null
AGOC: null
AGOP: null
PGOF: null
PGOC: null
PGOP: null

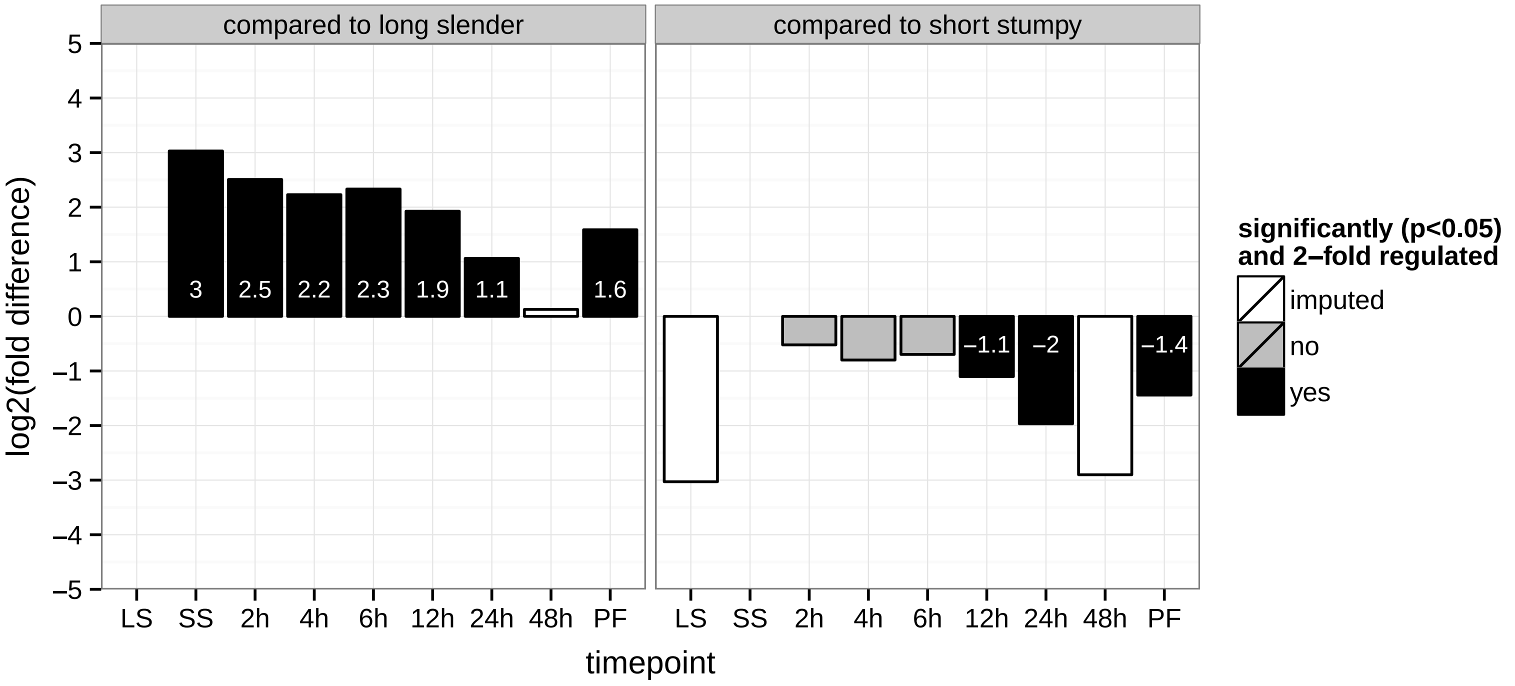

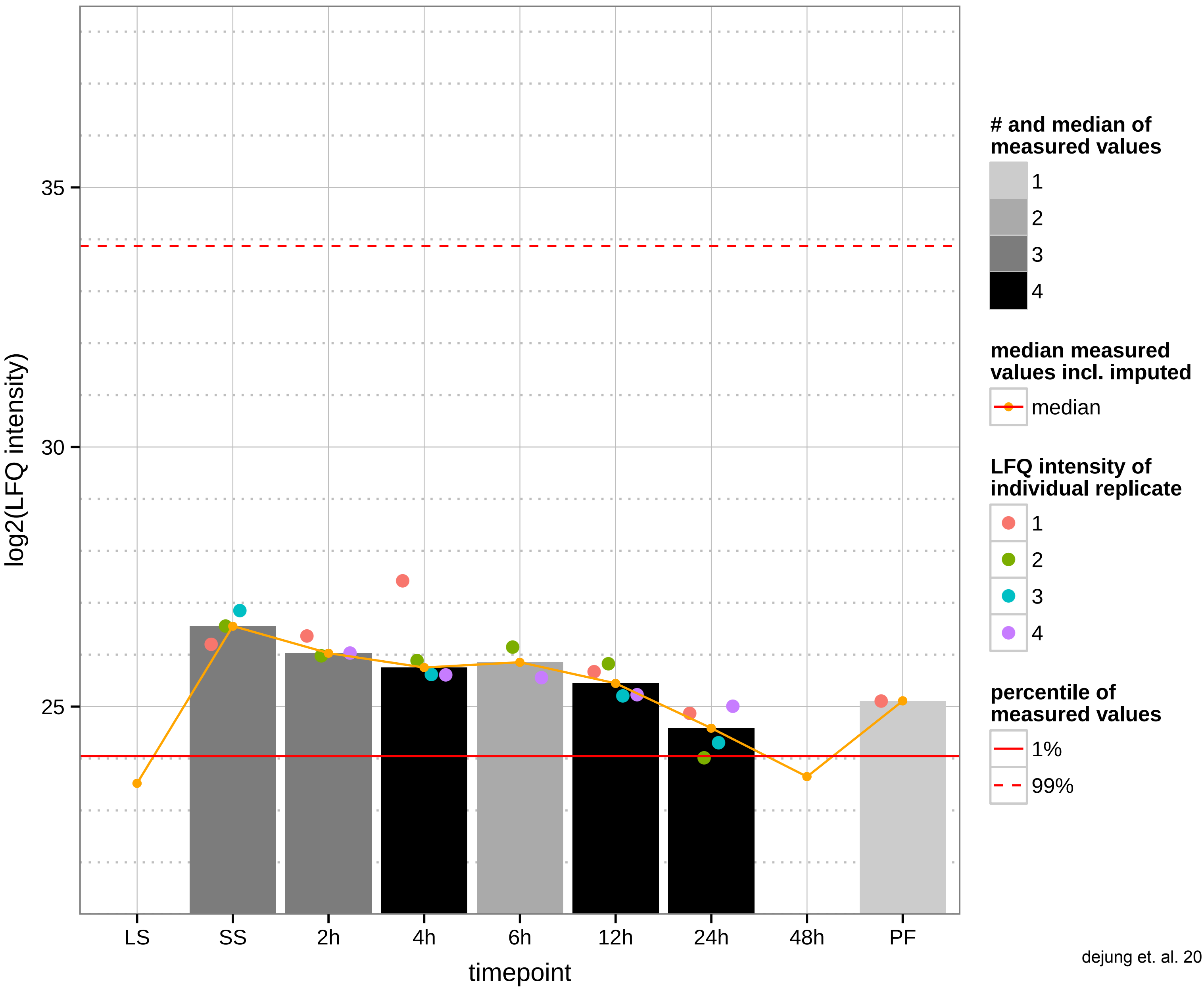

dejung et. al. 2015

palmitoyl acyltransferase 11, putative
Tb927.7.3350
AGOF: zinc ion binding
AGOC: integral to membrane
AGOP: endocytosis, protein palmitoylation, transport
PGOF: zinc ion binding
PGOC: null
PGOP: null

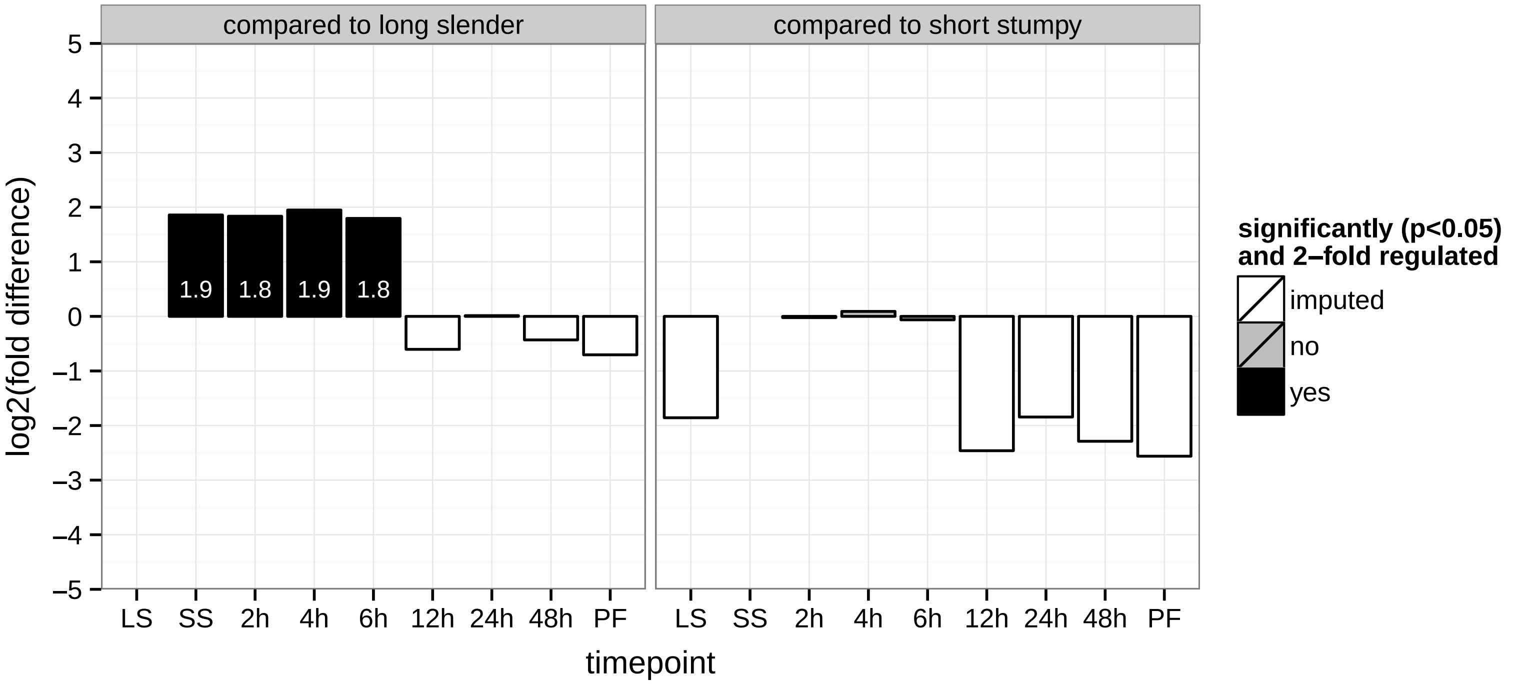

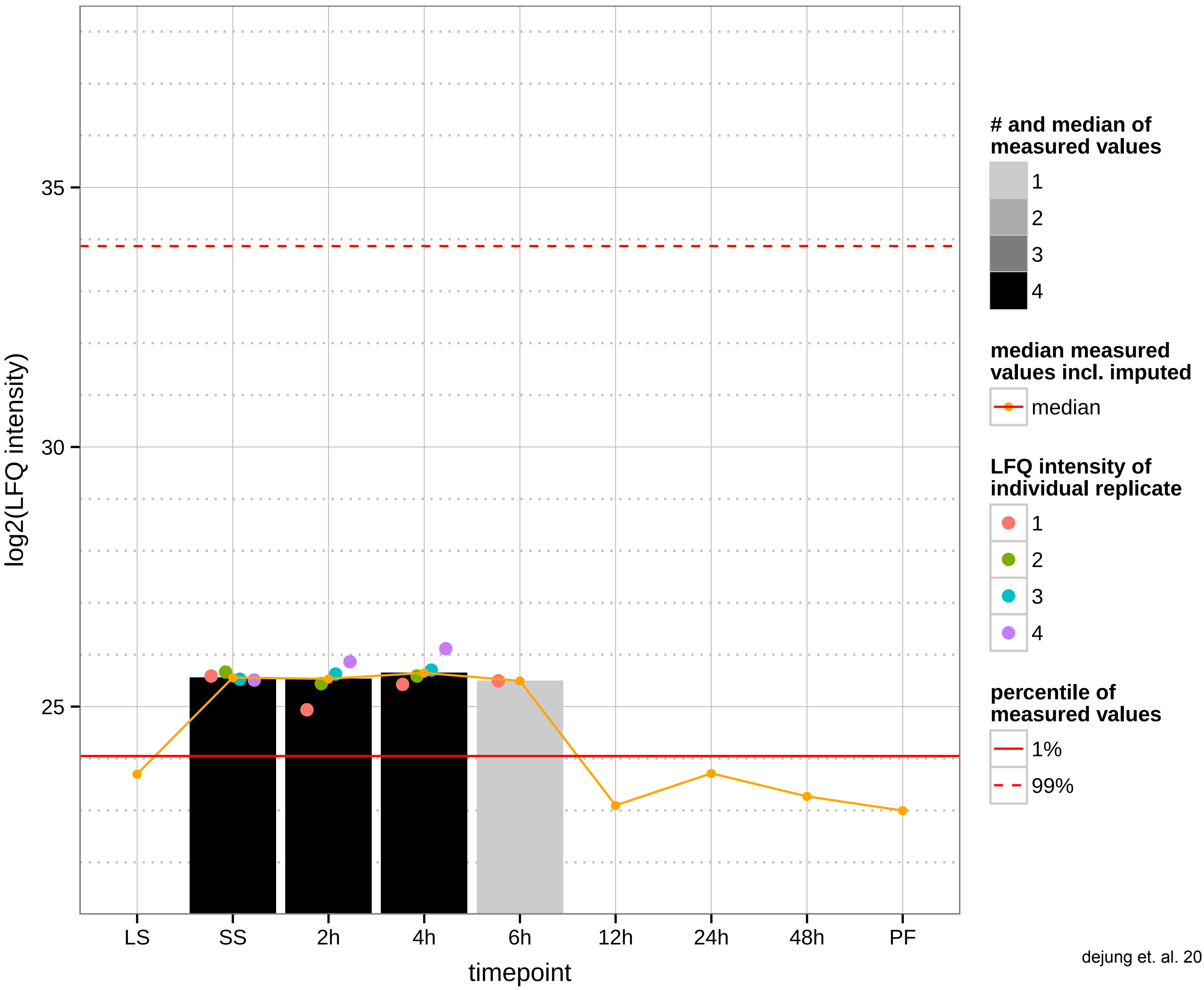

dejung et. al. 2015

hypothetical protein, conserved
Tb927.7.3970
AGOF: null
AGOC: mitochondrion
AGOP: null
PGOF: null
PGOC: null
PGOP: null

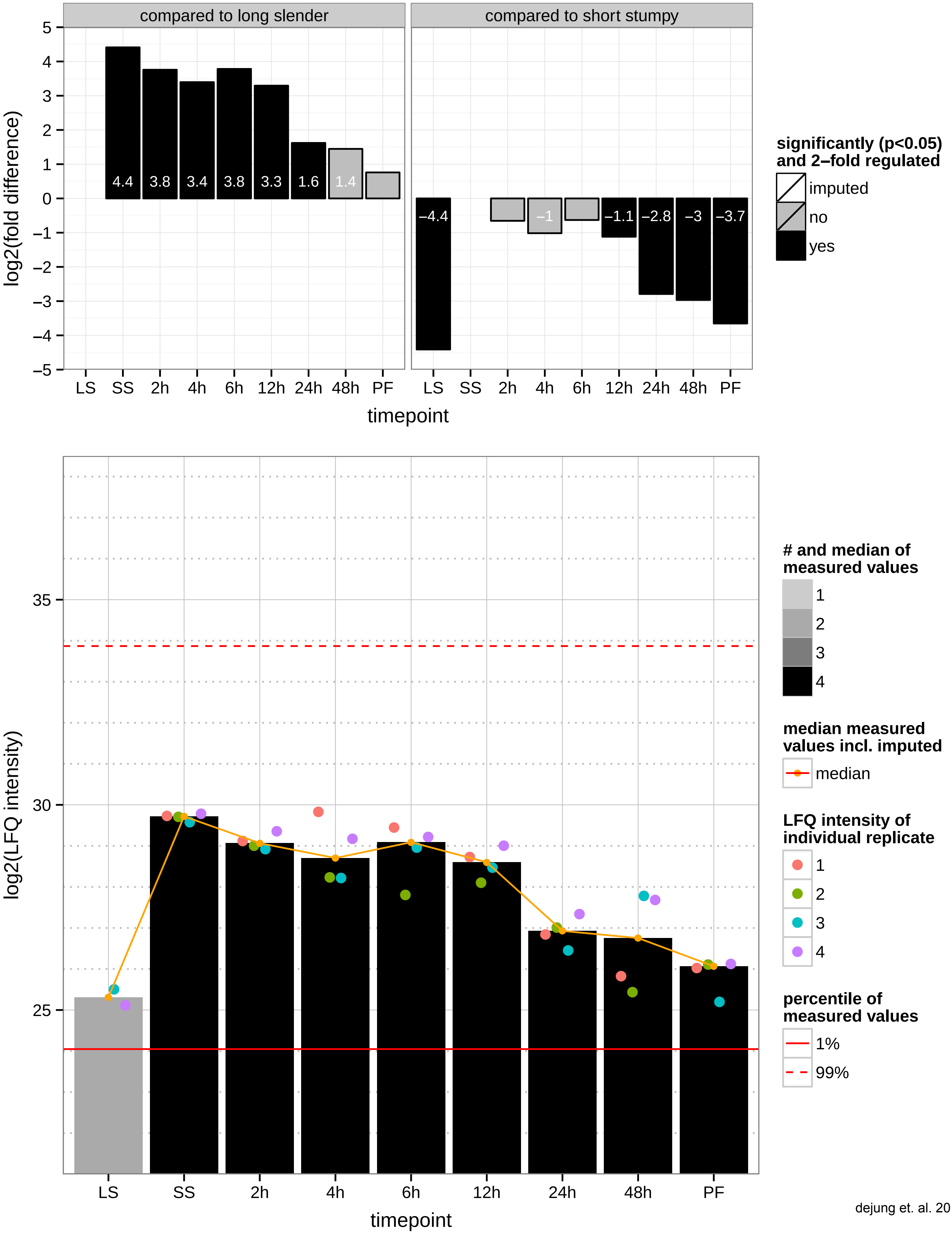

dejung et. al. 2015

hypothetical protein, conserved
Tb927.7.520
AGOF: heme binding
AGOC: null
AGOP: null
PGOF: heme binding
PGOC: null
PGOP: null

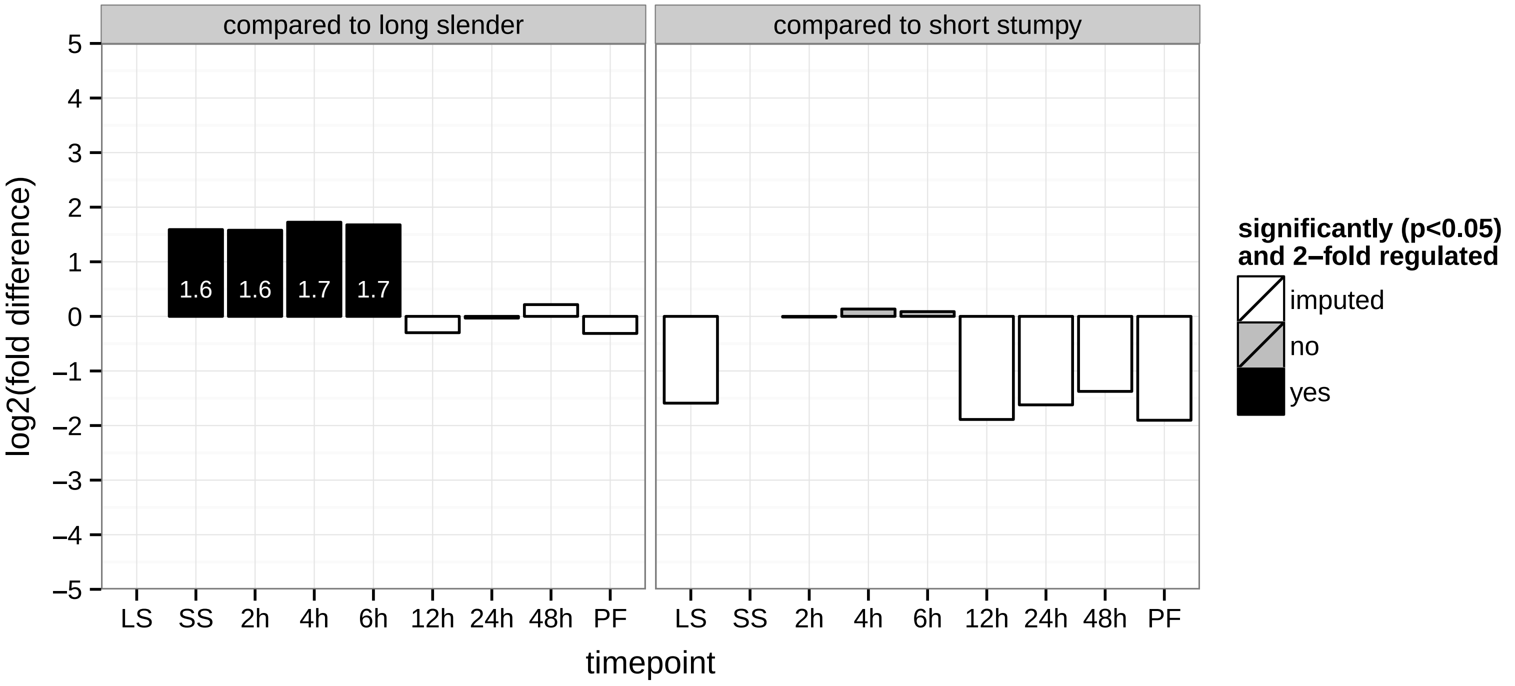

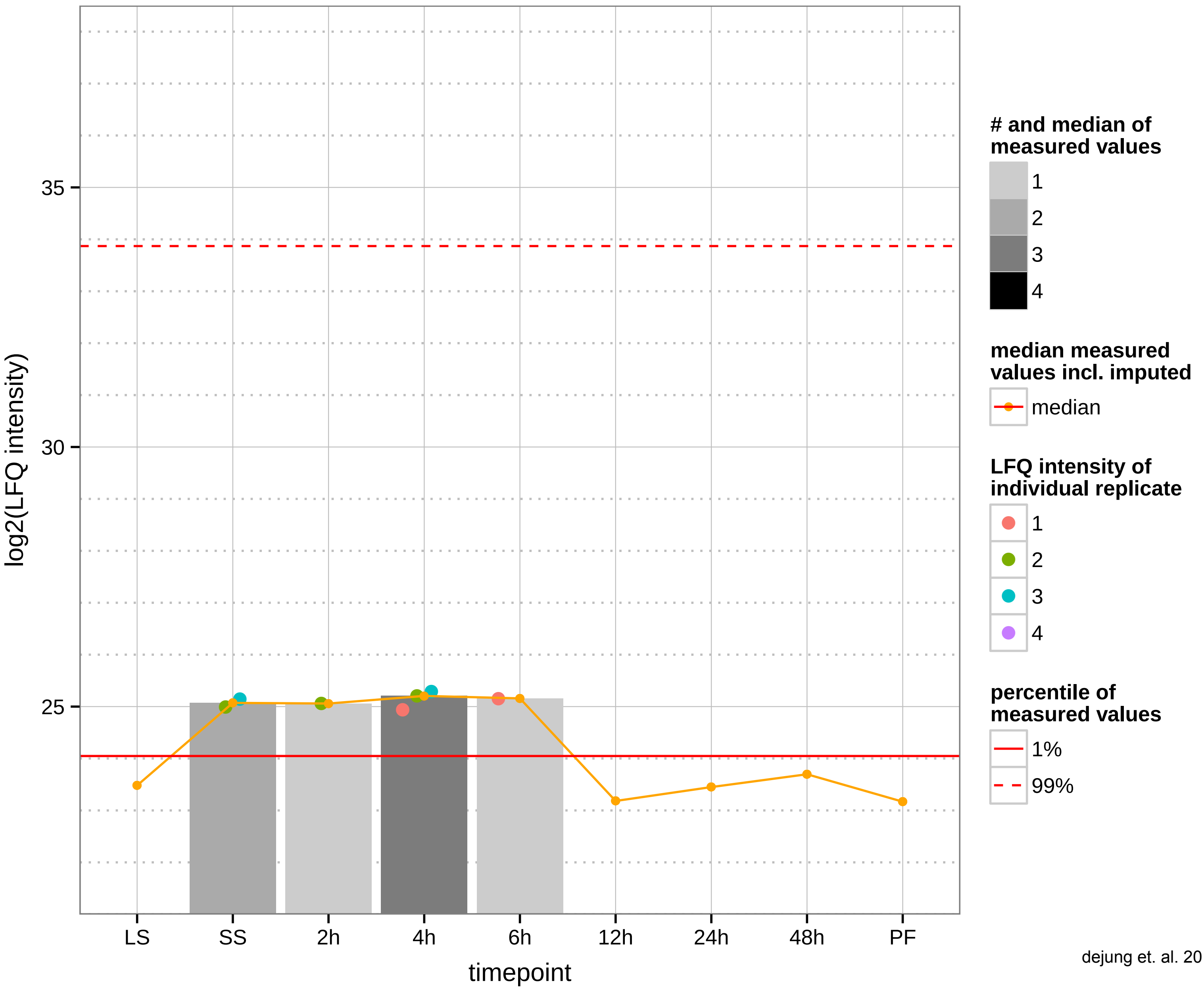

dejung et. al. 2015

lipin, putative
Tb927.7.5450
AGOF: null
AGOC: null
AGOP: null
PGOF: null
PGOC: null
PGOP: null

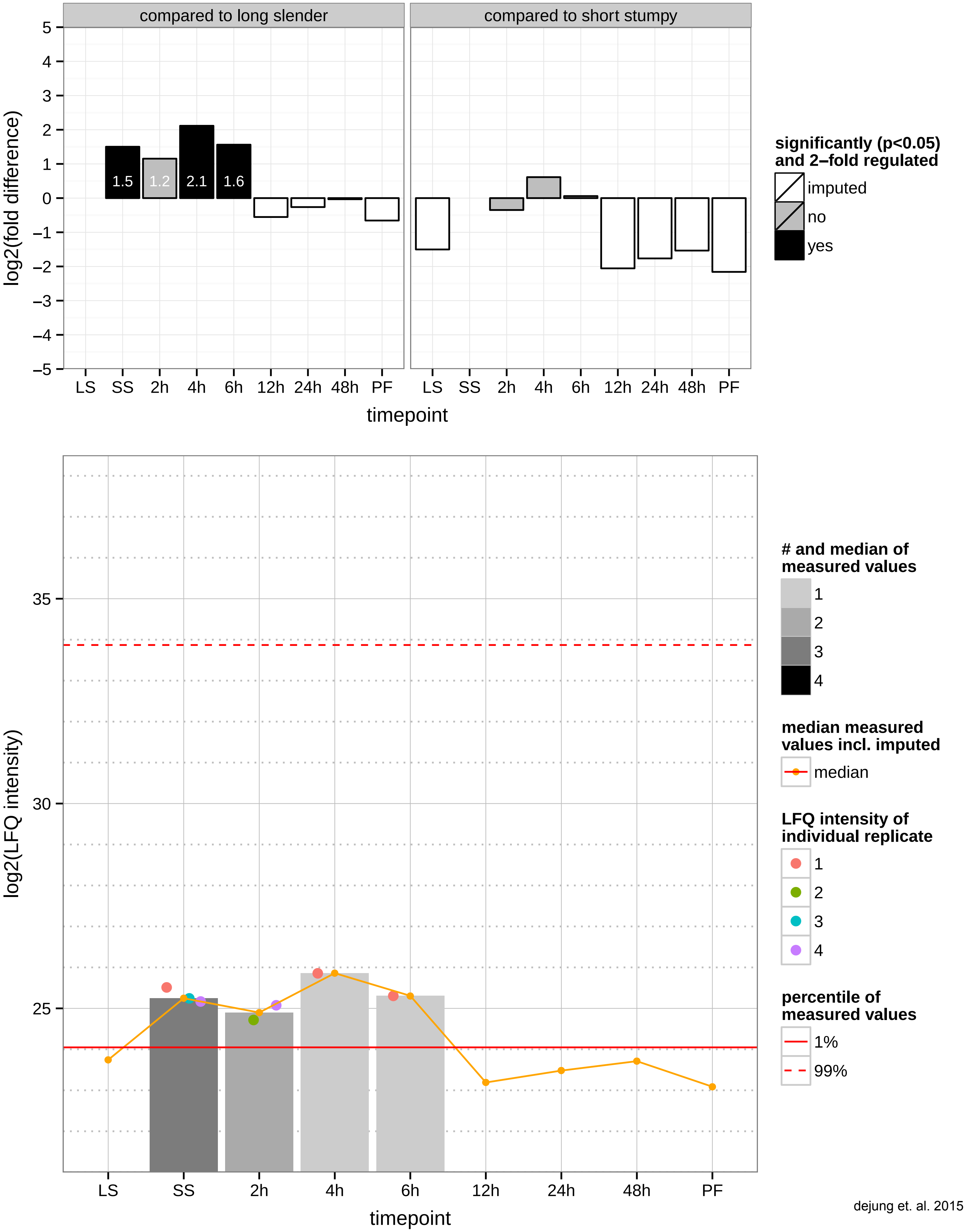

dejung et. al. 2015

hypothetical protein, conserved
Tb927.7.6600
AGOF: null
AGOC: null
AGOP: null
PGOF: null
PGOC: null
PGOP: null

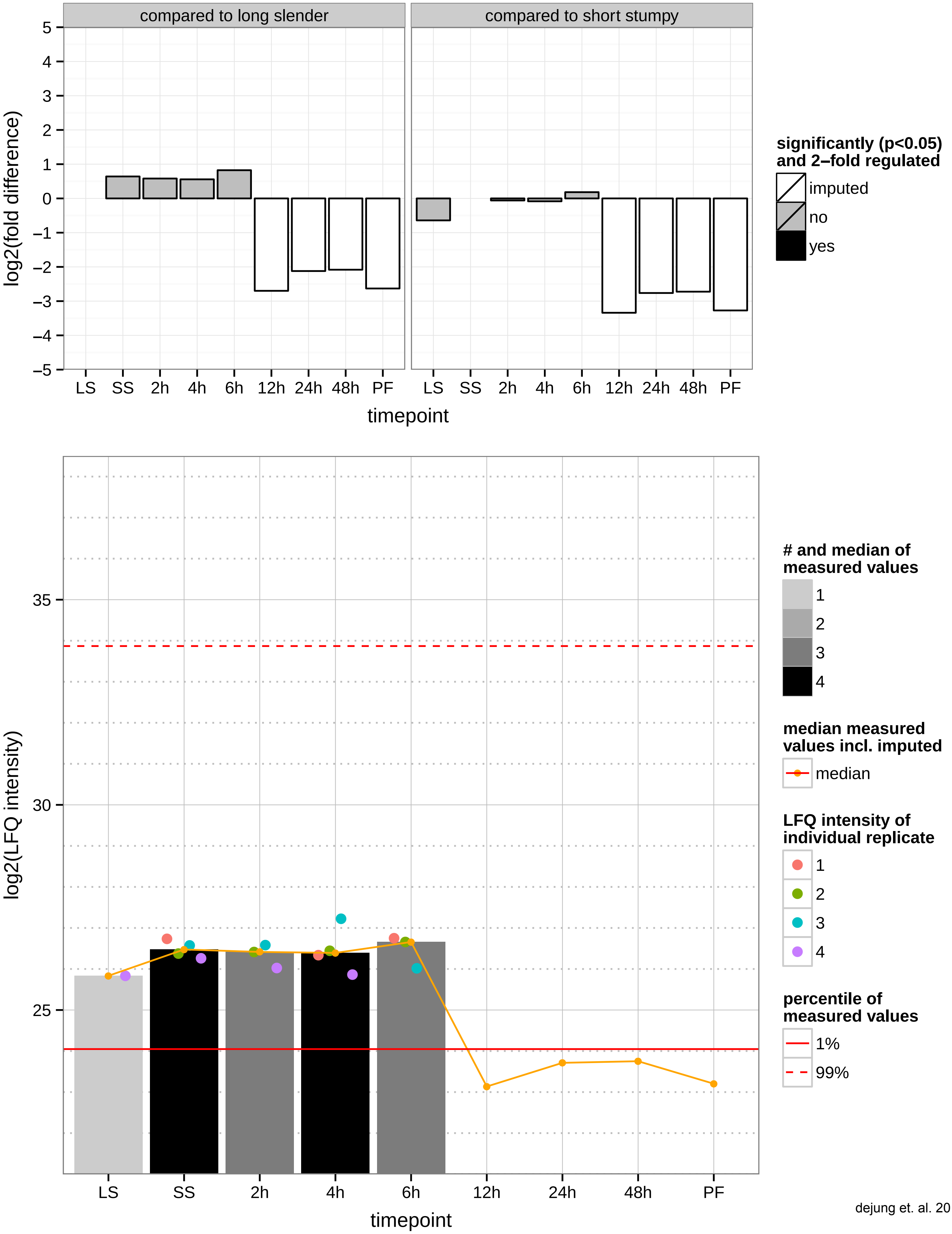

dejung et. al. 2015

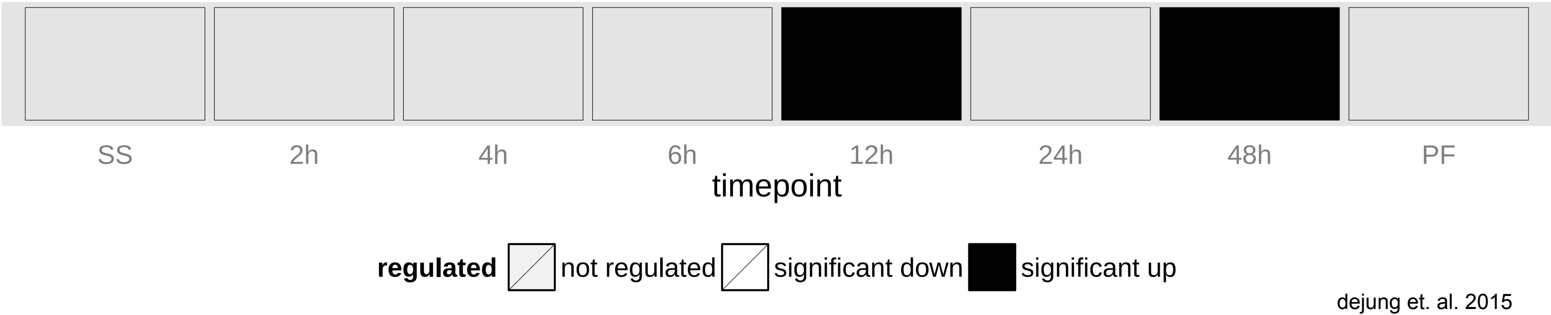

SS
2h
4h
6h
12h
24h
48h
PF
timepoint
regulated
not regulated
significant down
significant up
dejung et. al. 2015

retrotransposon hot spot protein (RHS, pseudogene), putative, chrXI additional, unordered contigs, retrotransposon hot spot p
Tb11.v5.0442;Tb11.1800
AGOF: null
AGOC: null
AGOP: null
PGOF: null
PGOC: null
PGOP: null

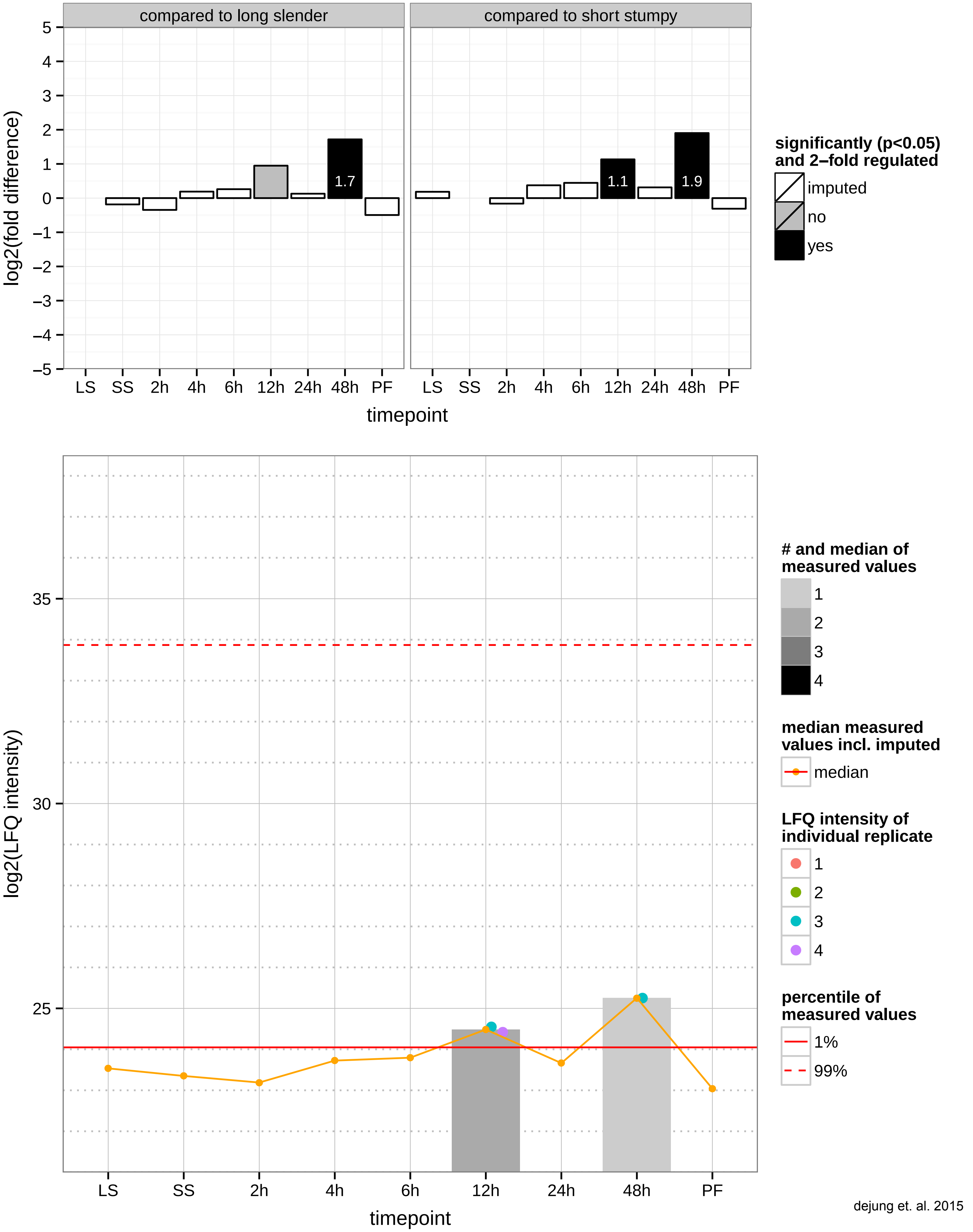

dejung et. al. 2015

hypothetical protein, conserved
Tb927.7.4510;Tb11.v5.0264
AGOF: null
AGOC: null
AGOP: null
PGOF: null
PGOC: null
PGOP: null

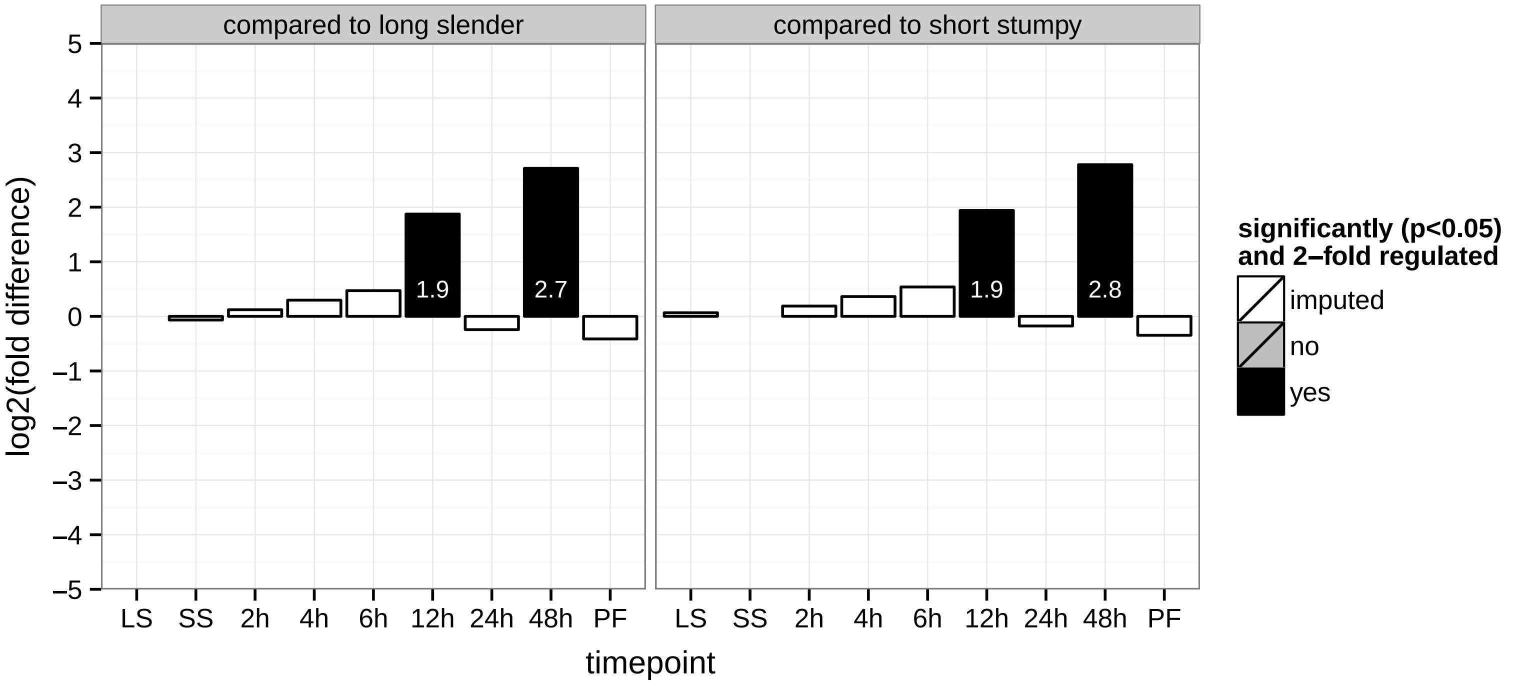

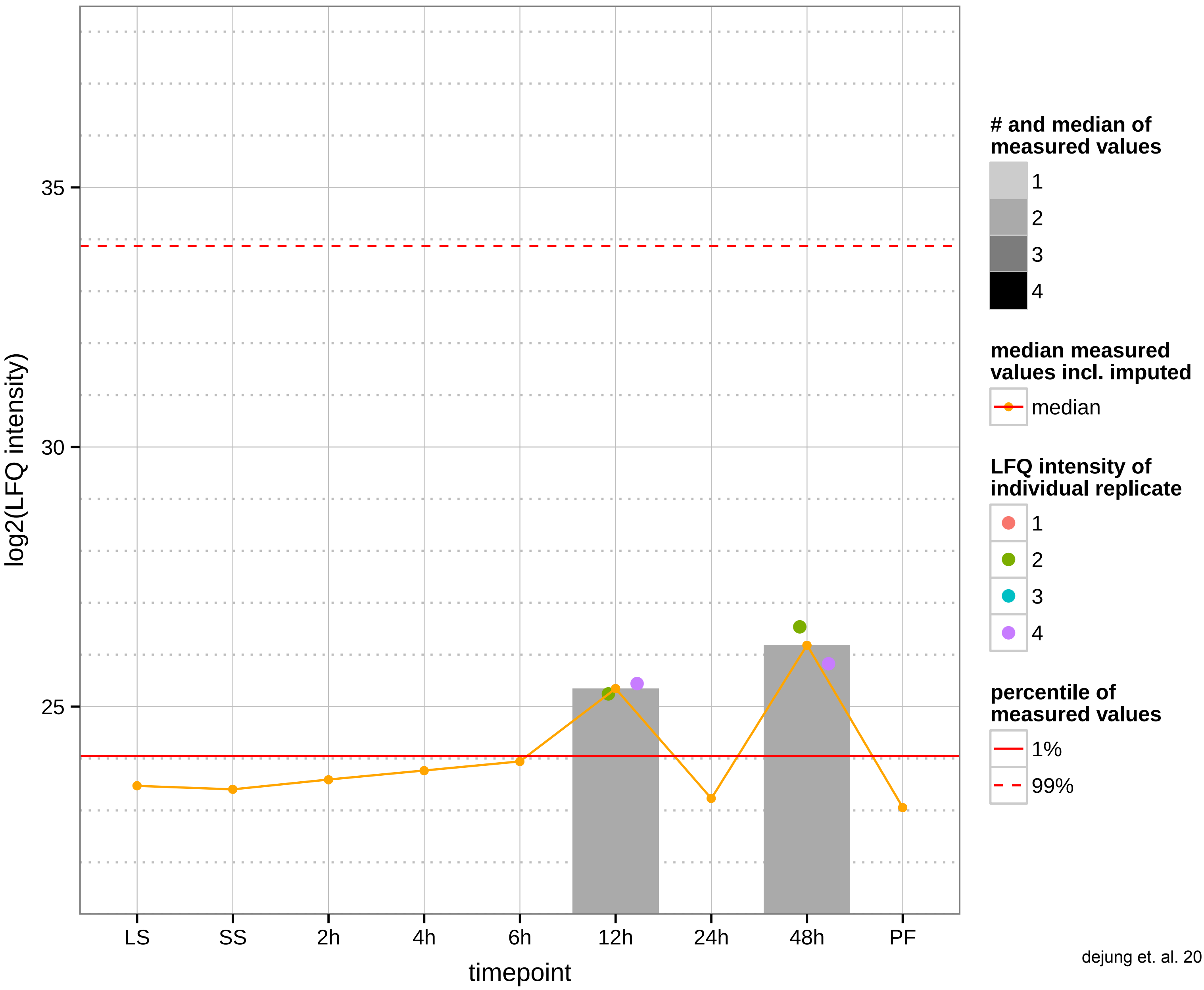

dejung et. al. 2015

hypothetical protein, conserved
Tb927.1.4220
AGOF: null
AGOC: nucleus
AGOP: null
PGOF: null
PGOC: nucleus
PGOP: null

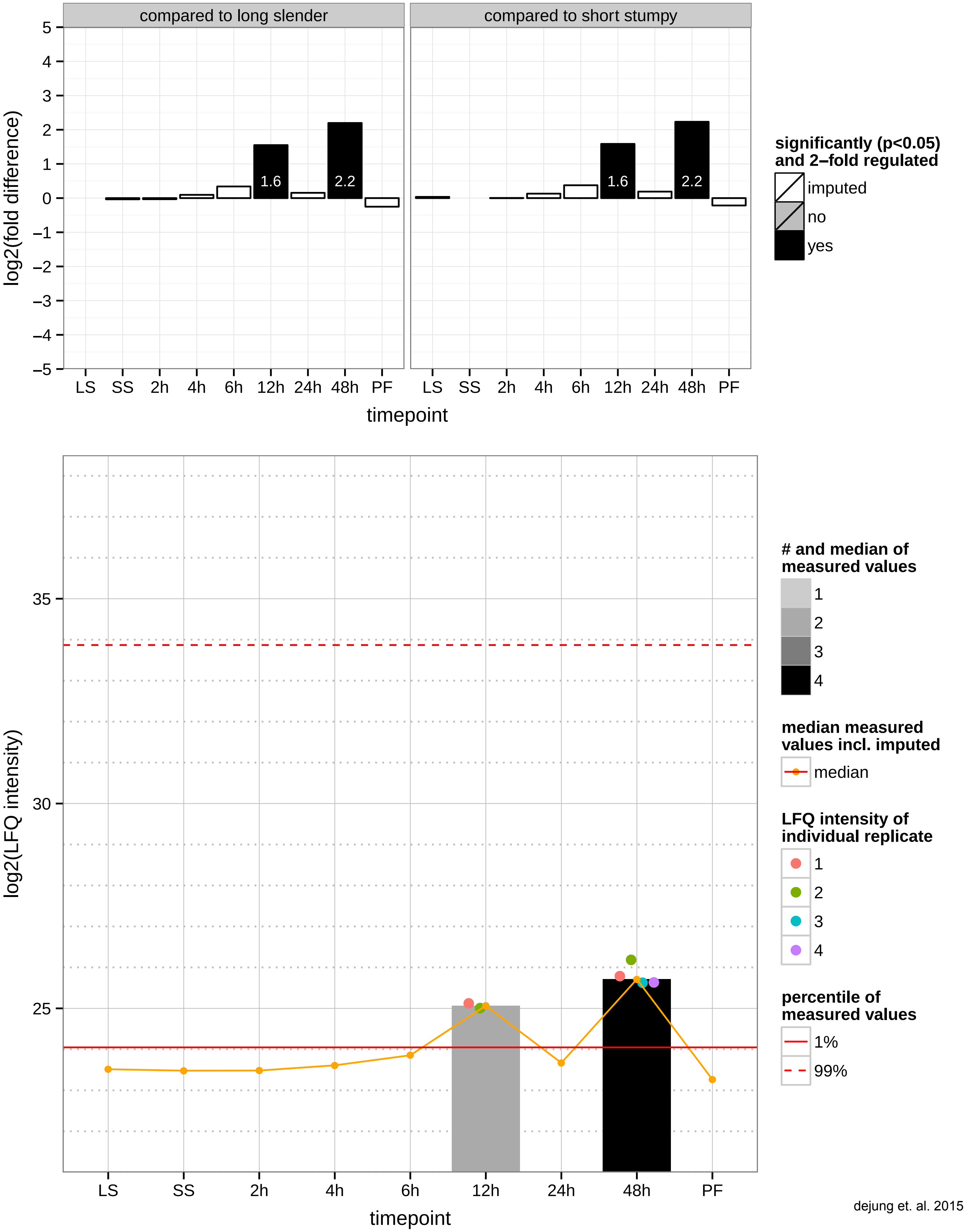

dejung et. al. 2015

Zinc finger CCCH domain–containing protein 35 (ZC3H35)
Tb927.10.12740
AGOF: RNA binding, nucleic acid binding, zinc ion binding
AGOC: null
AGOP: null
PGOF: nucleic acid binding, zinc ion binding
PGOC: null
PGOP: null

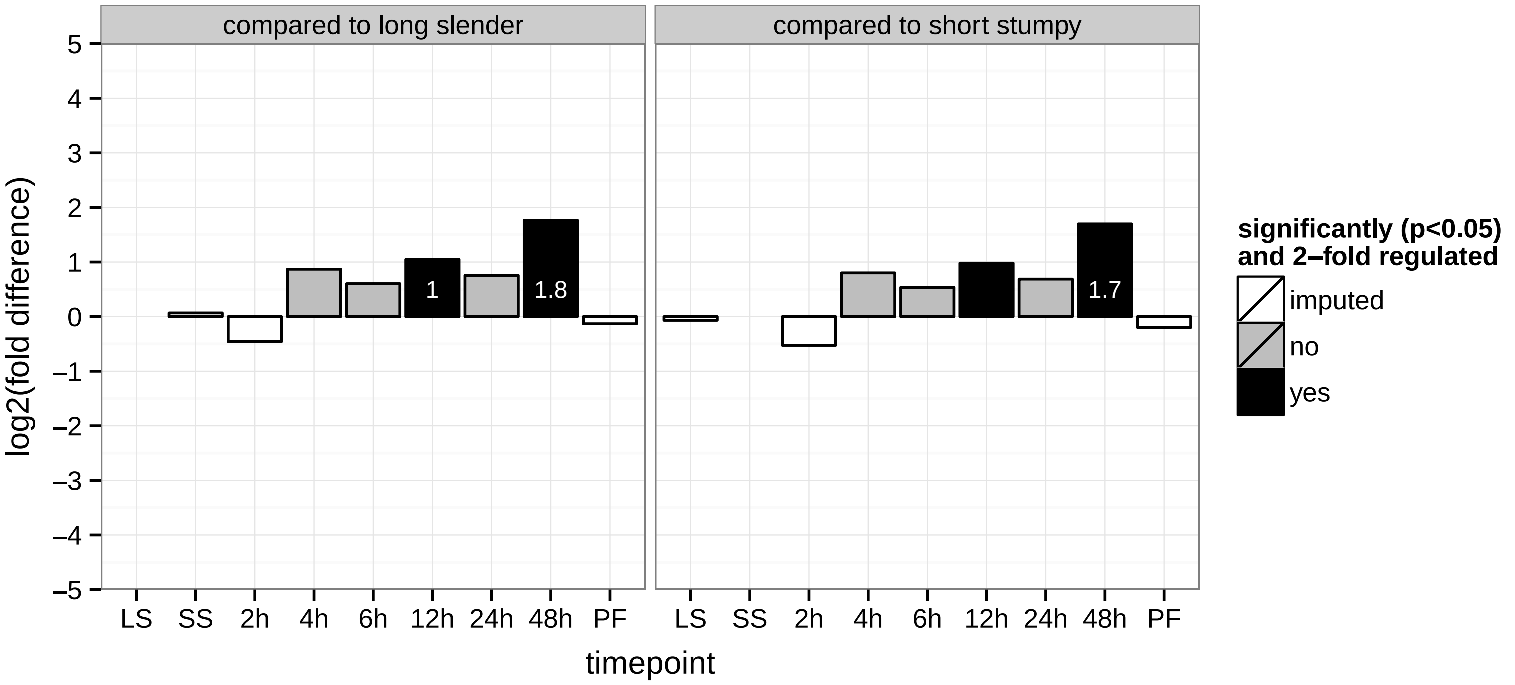

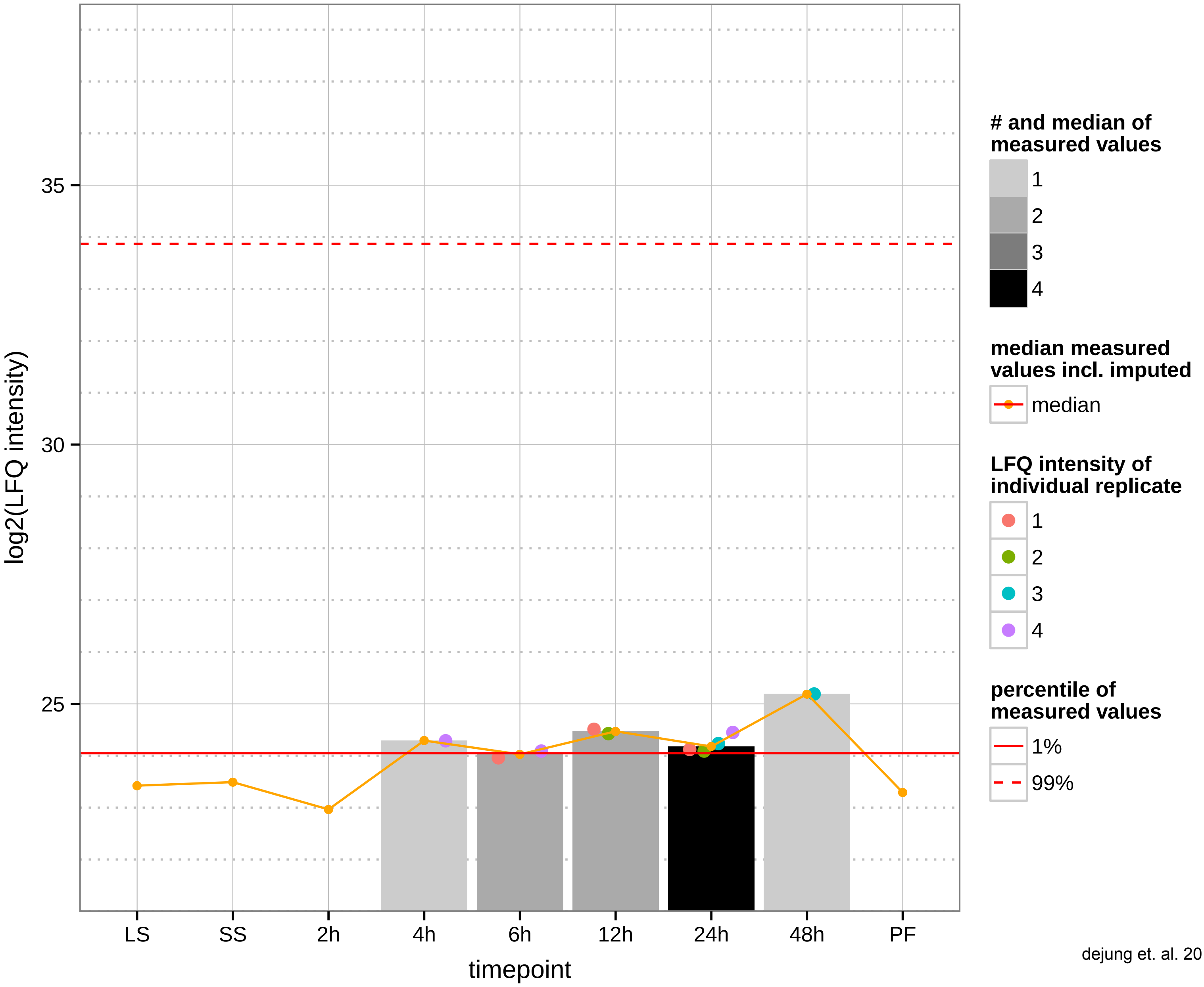

dejung et. al. 2015

sjogren s syndrome nuclear autoantigen 1, putative
Tb927.10.14110
AGOF: null
AGOC: null
AGOP: null
PGOF: null
PGOC: null
PGOP: null

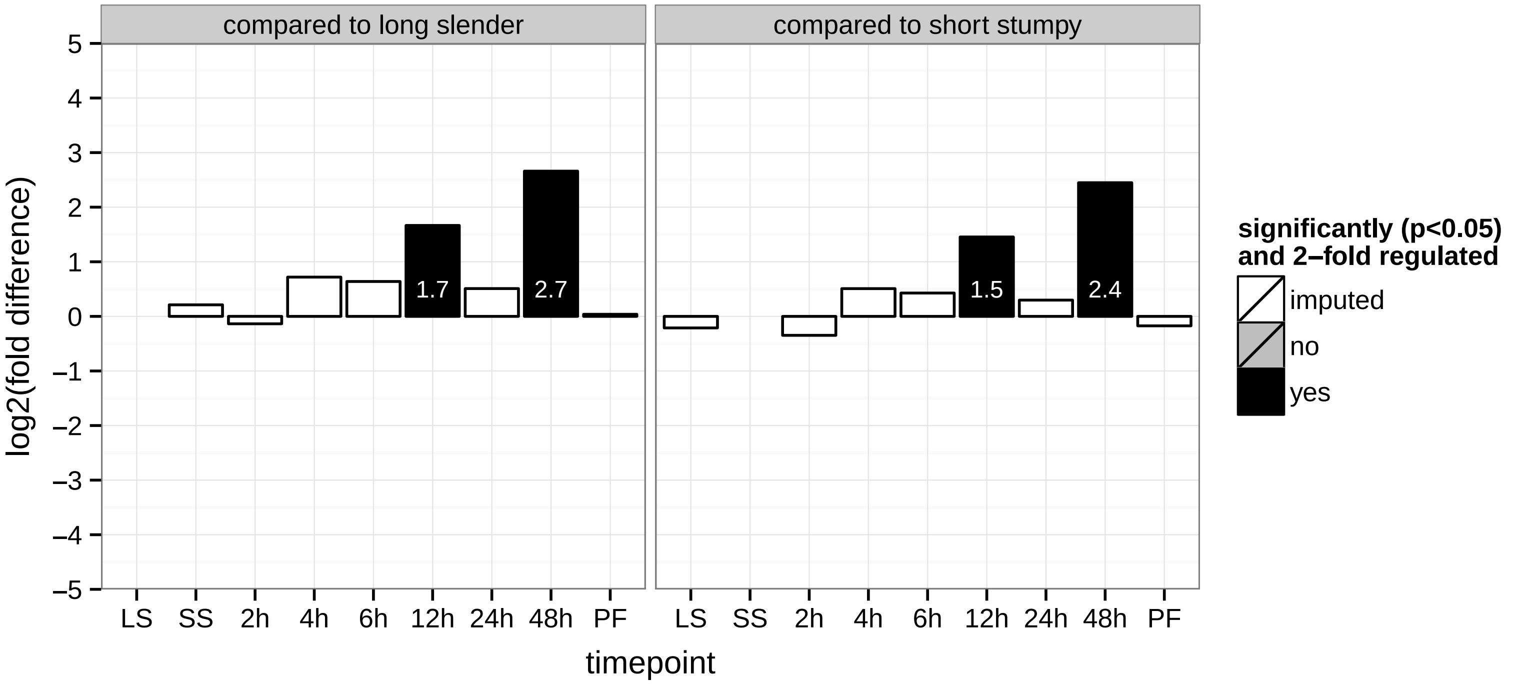

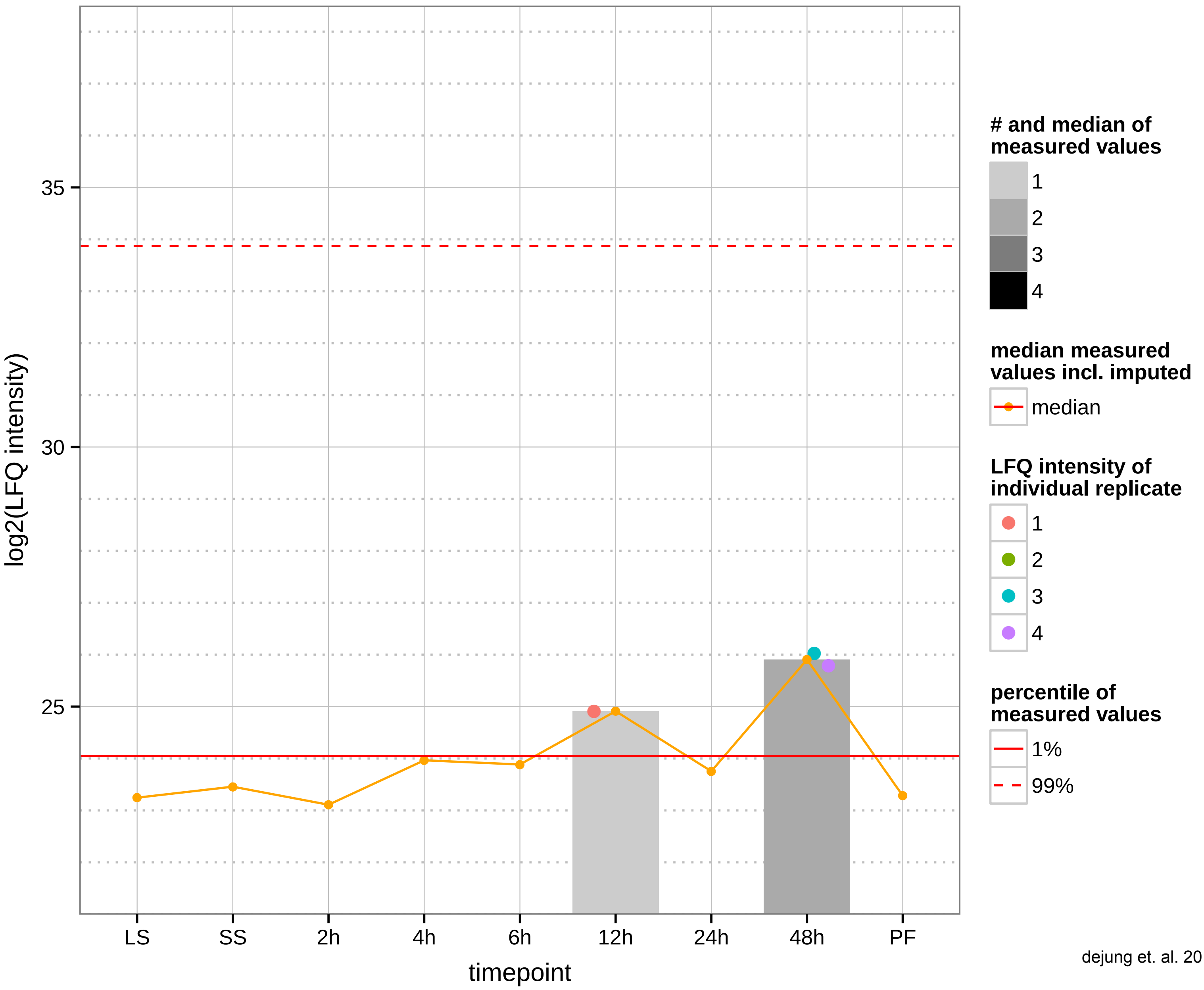

dejung et. al. 2015

hypothetical protein, conserved
Tb927.11.11790
AGOF: nucleic acid binding
AGOC: null
AGOP: null
PGOF: nucleic acid binding
PGOC: null
PGOP: null

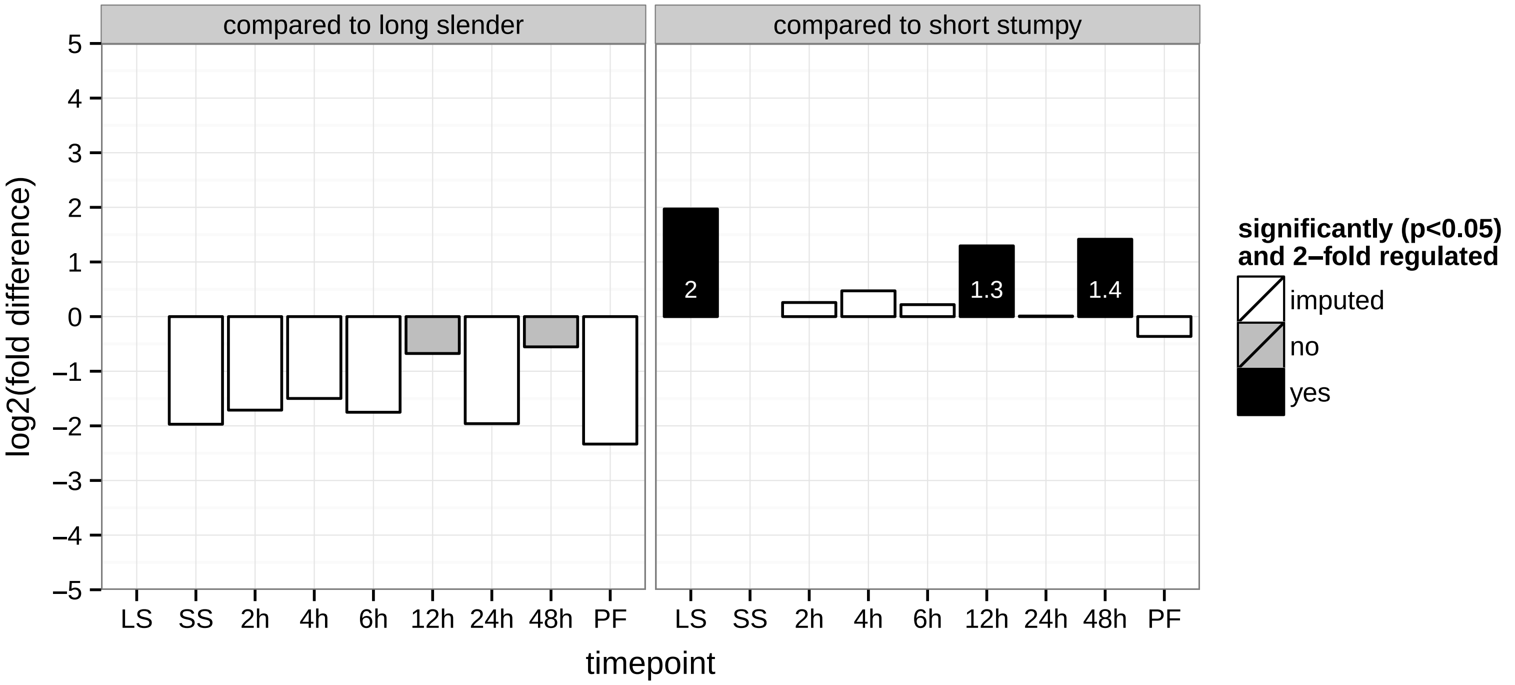

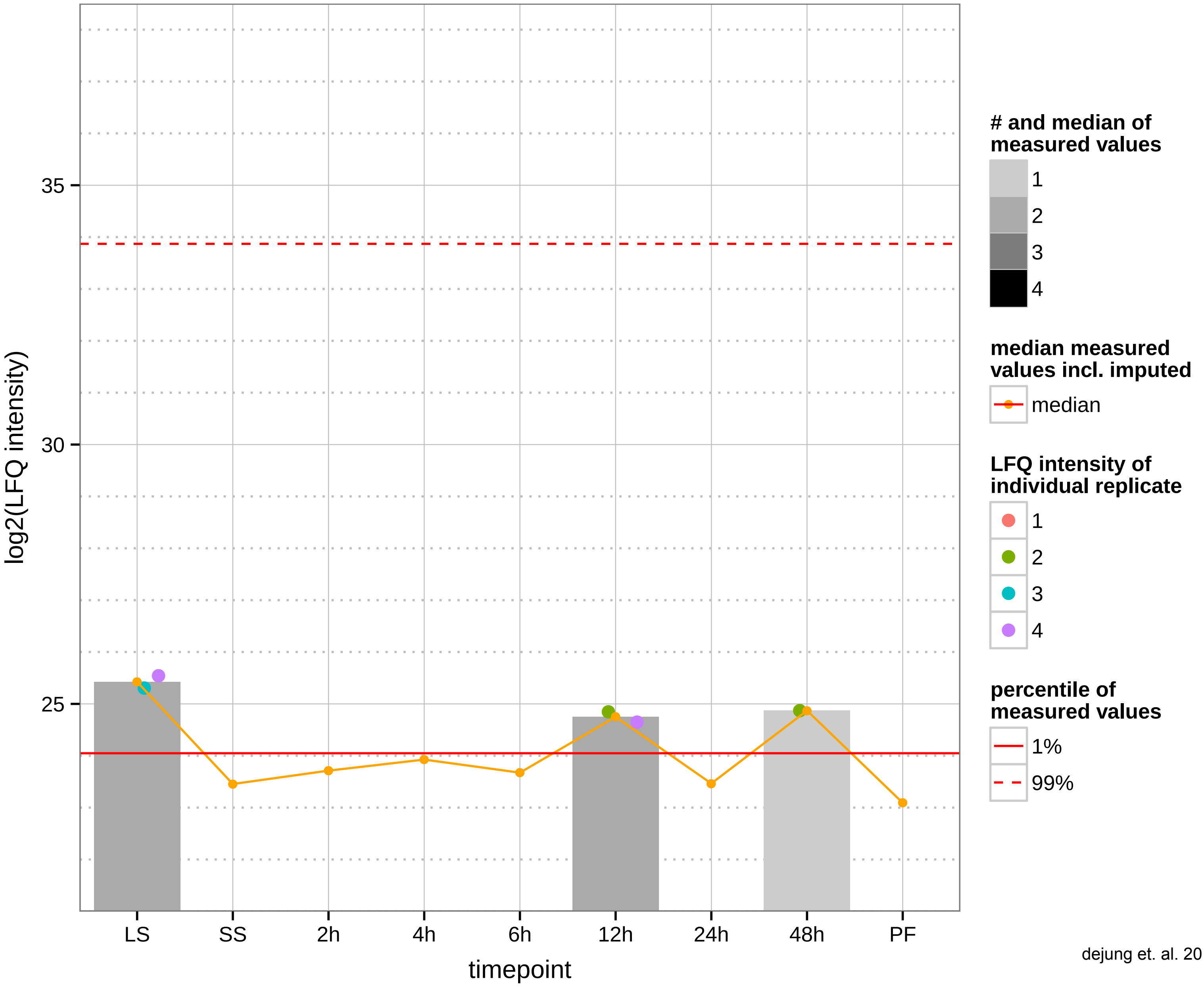

dejung et. al. 2015

Polypeptide deformylase 1
Tb927.11.14520
AGOF: formylmethionine deformylase activity, iron ion binding, peptide deformylase activity
AGOC: null
AGOP: cellular protein modification process, translation
PGOF: null
PGOC: null
PGOP: null

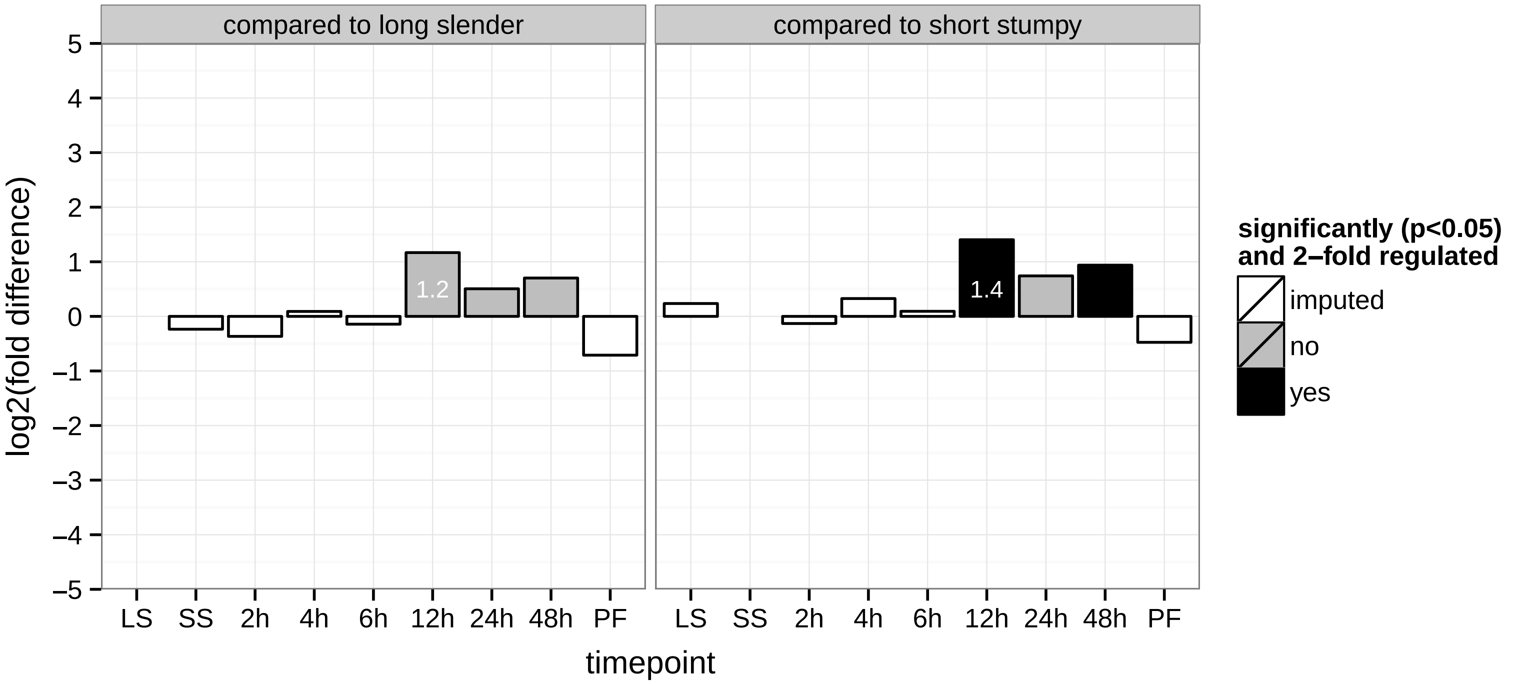

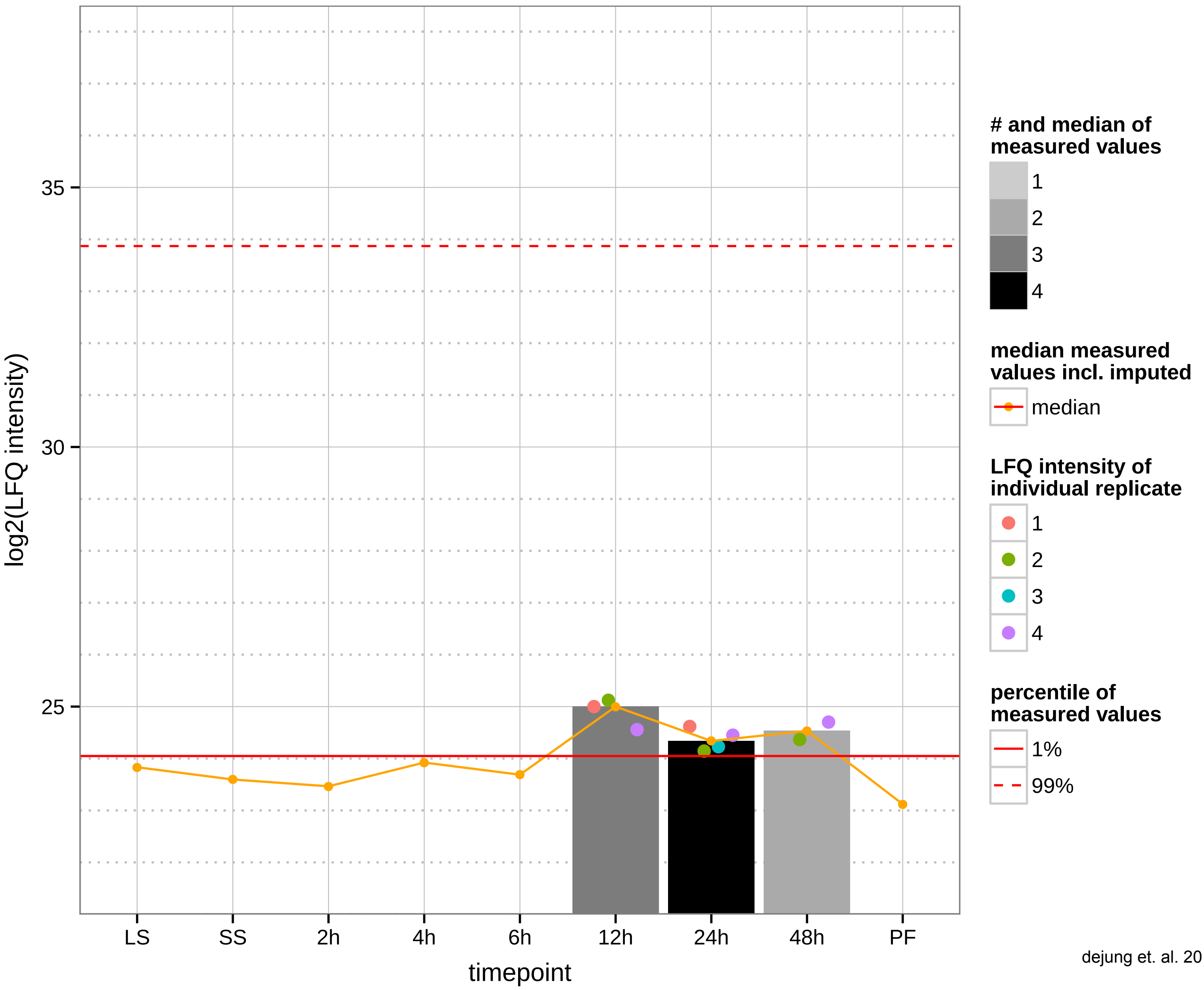

dejung et. al. 2015

aldo/keto reductase, putative
Tb927.11.5360
AGOF: aldo–keto reductase (NADP) activity
AGOC: cytoplasm, nucleus
AGOP: metabolic process, oxidation–reduction process
PGOF: null
PGOC: null
PGOP: null

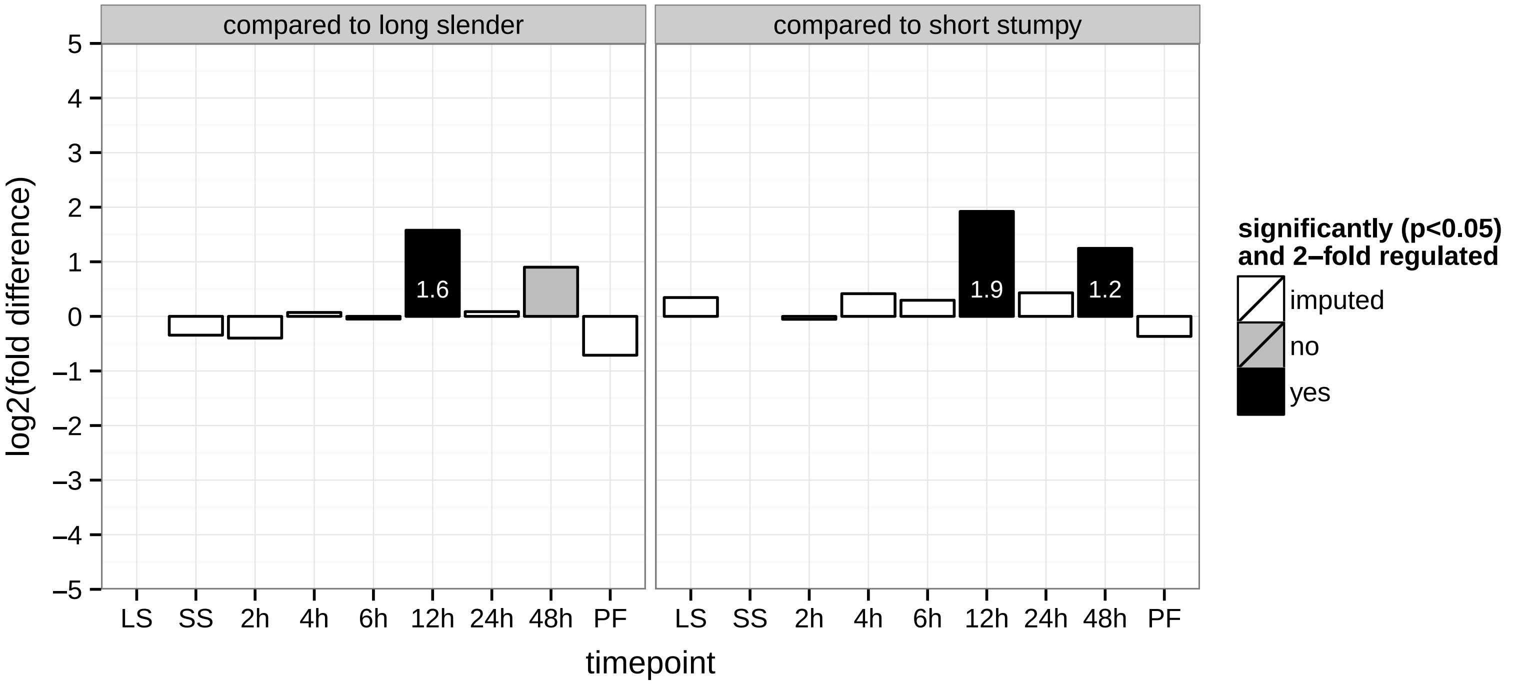

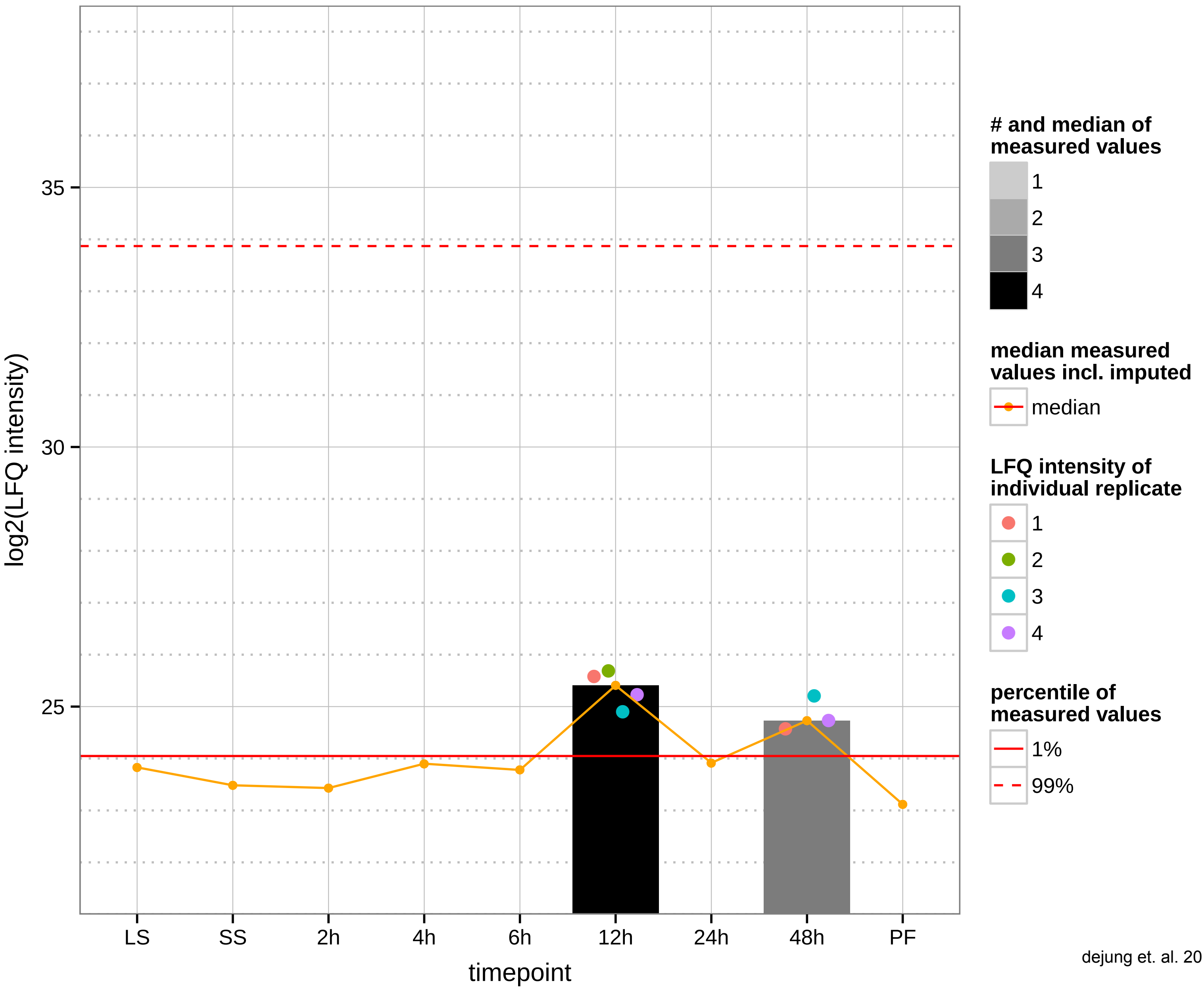

dejung et. al. 2015

hypothetical protein, conserved
Tb927.11.8860
AGOF: null
AGOC: null
AGOP: null
PGOF: null
PGOC: null
PGOP: null

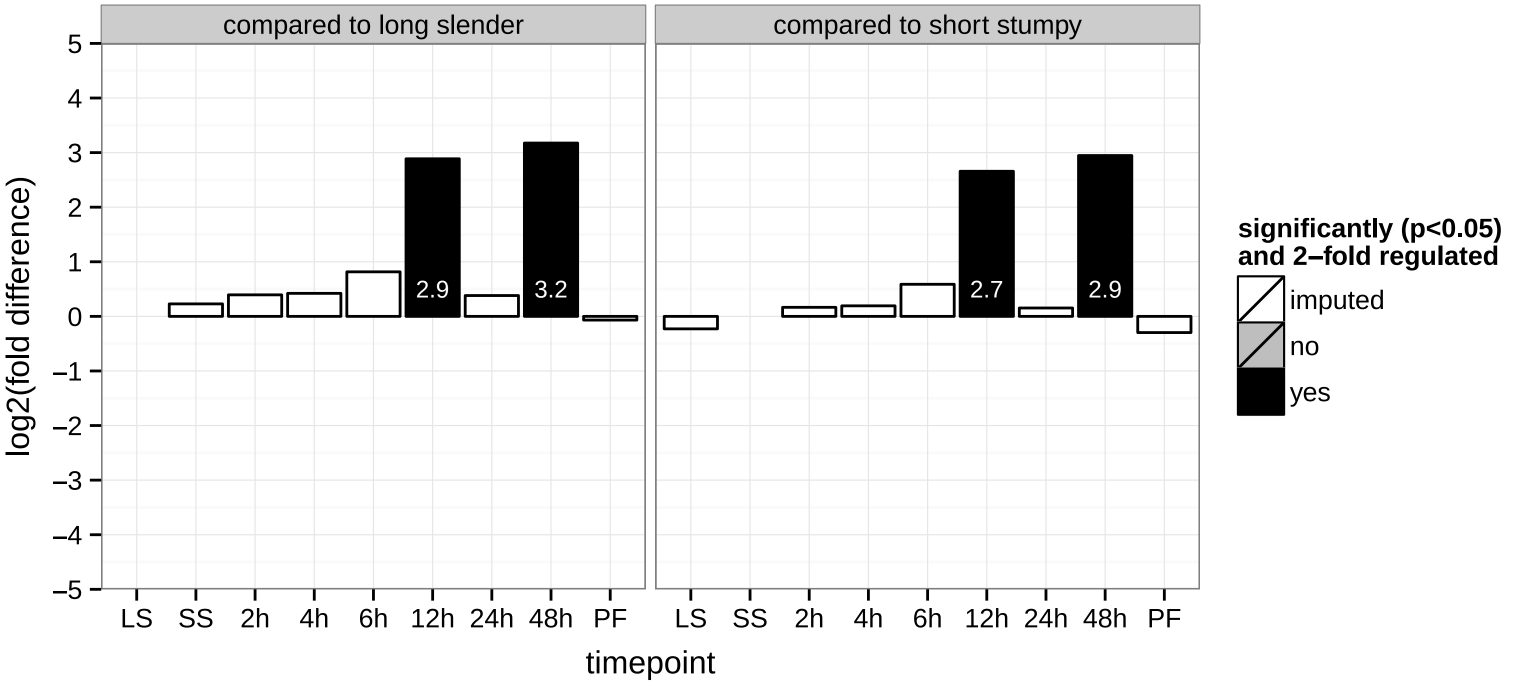

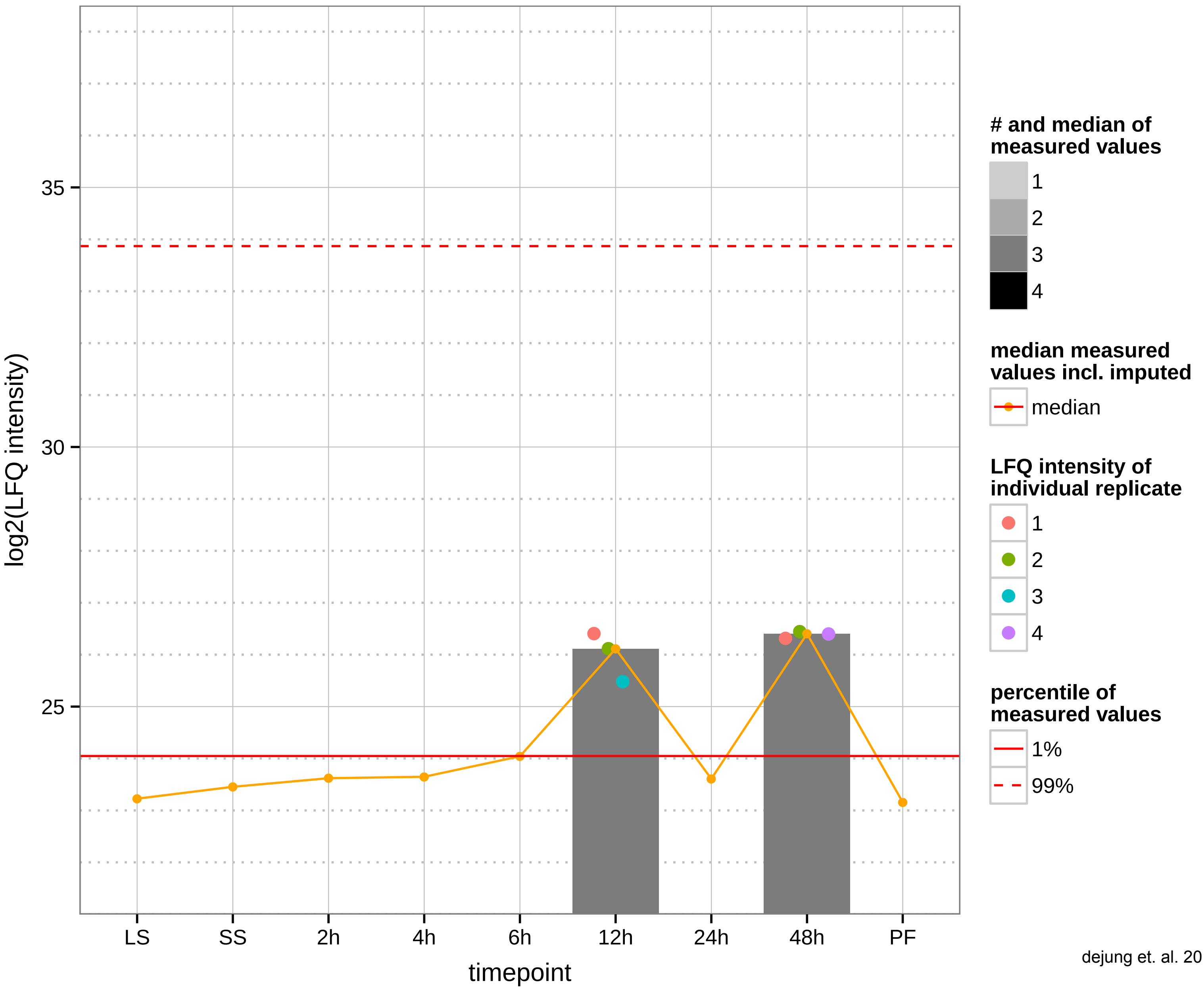

dejung et. al. 2015

hypothetical protein, conserved
Tb927.2.2270
AGOF: null
AGOC: null
AGOP: null
PGOF: null
PGOC: null
PGOP: null

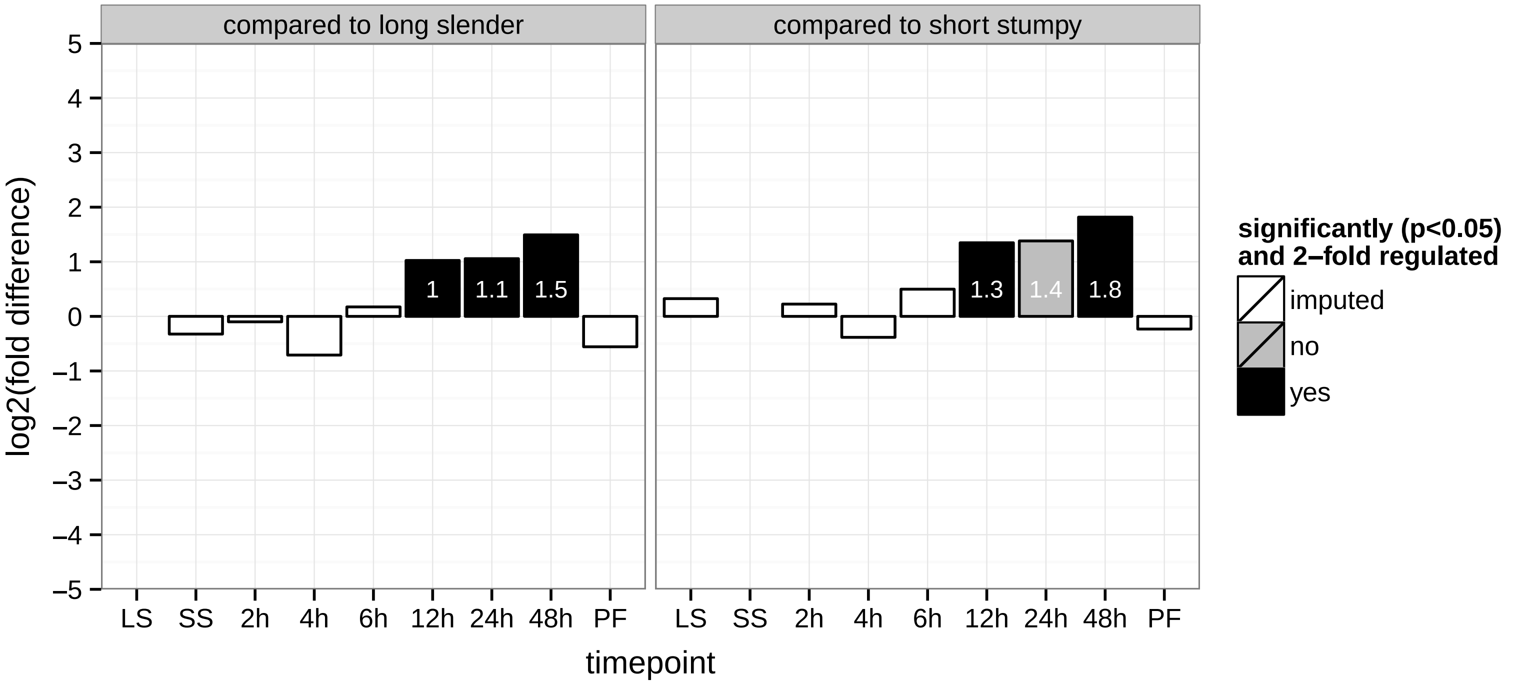

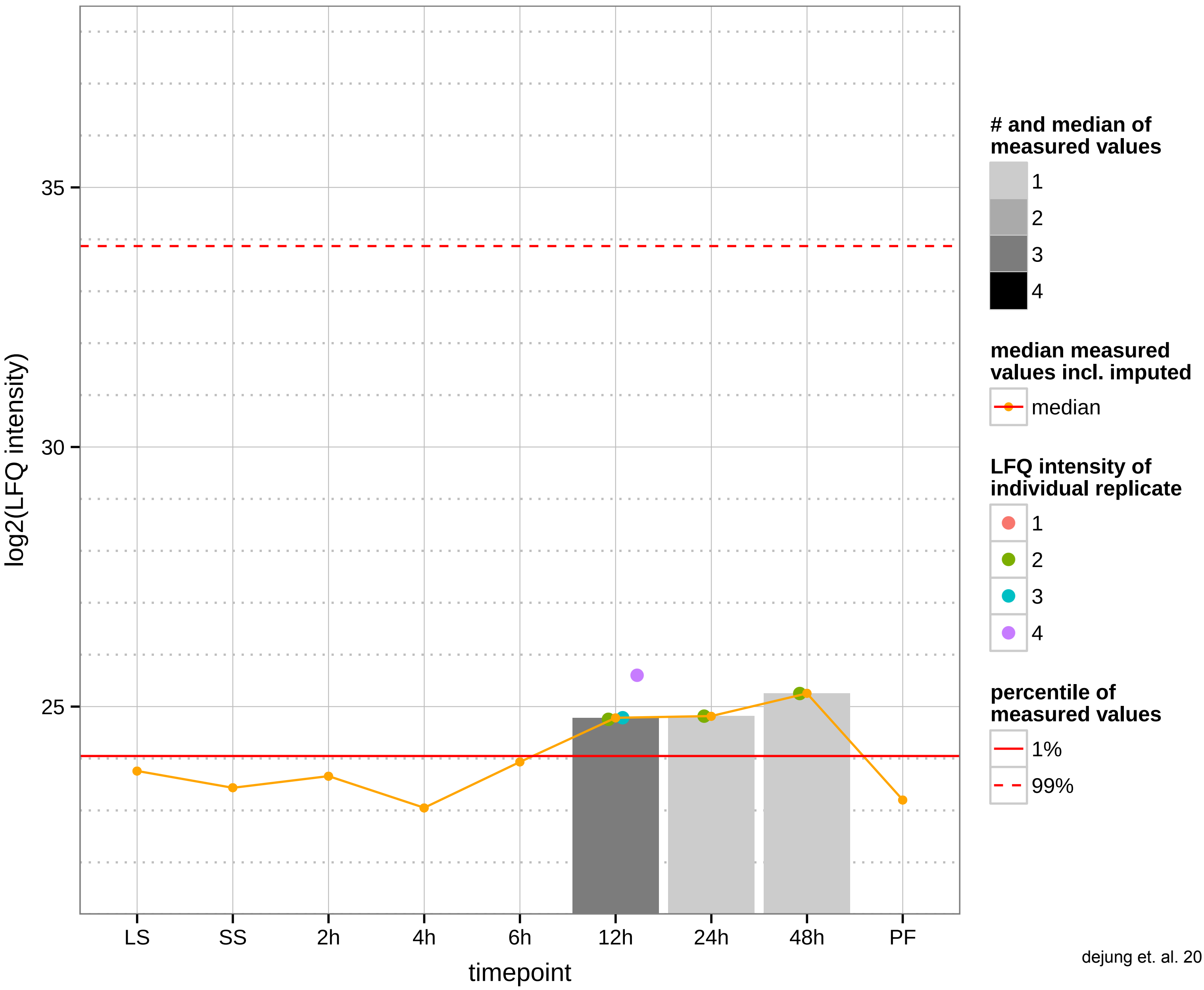

dejung et. al. 2015

hypothetical protein, conserved
Tb927.2.4000
AGOF: nucleic acid binding, zinc ion binding
AGOC: null
AGOP: null
PGOF: nucleic acid binding, zinc ion binding
PGOC: null
PGOP: null

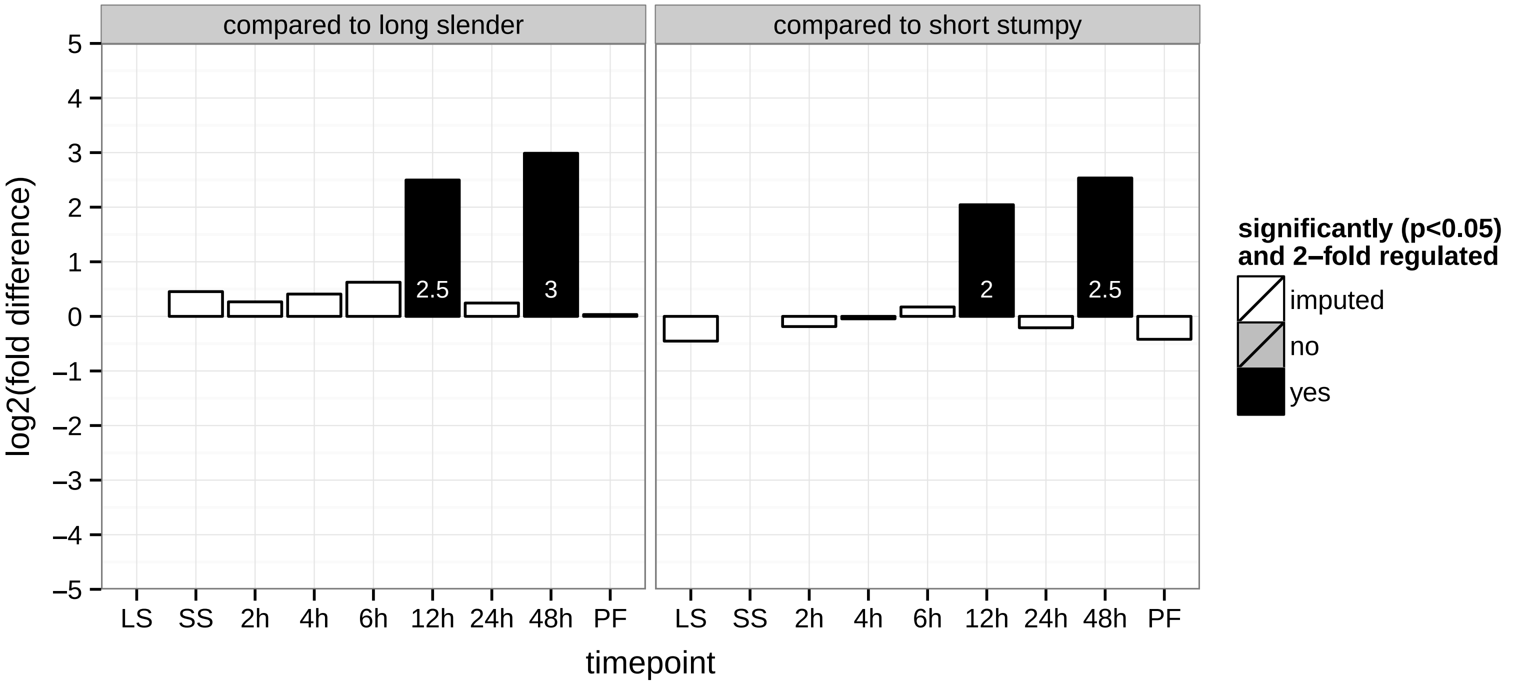

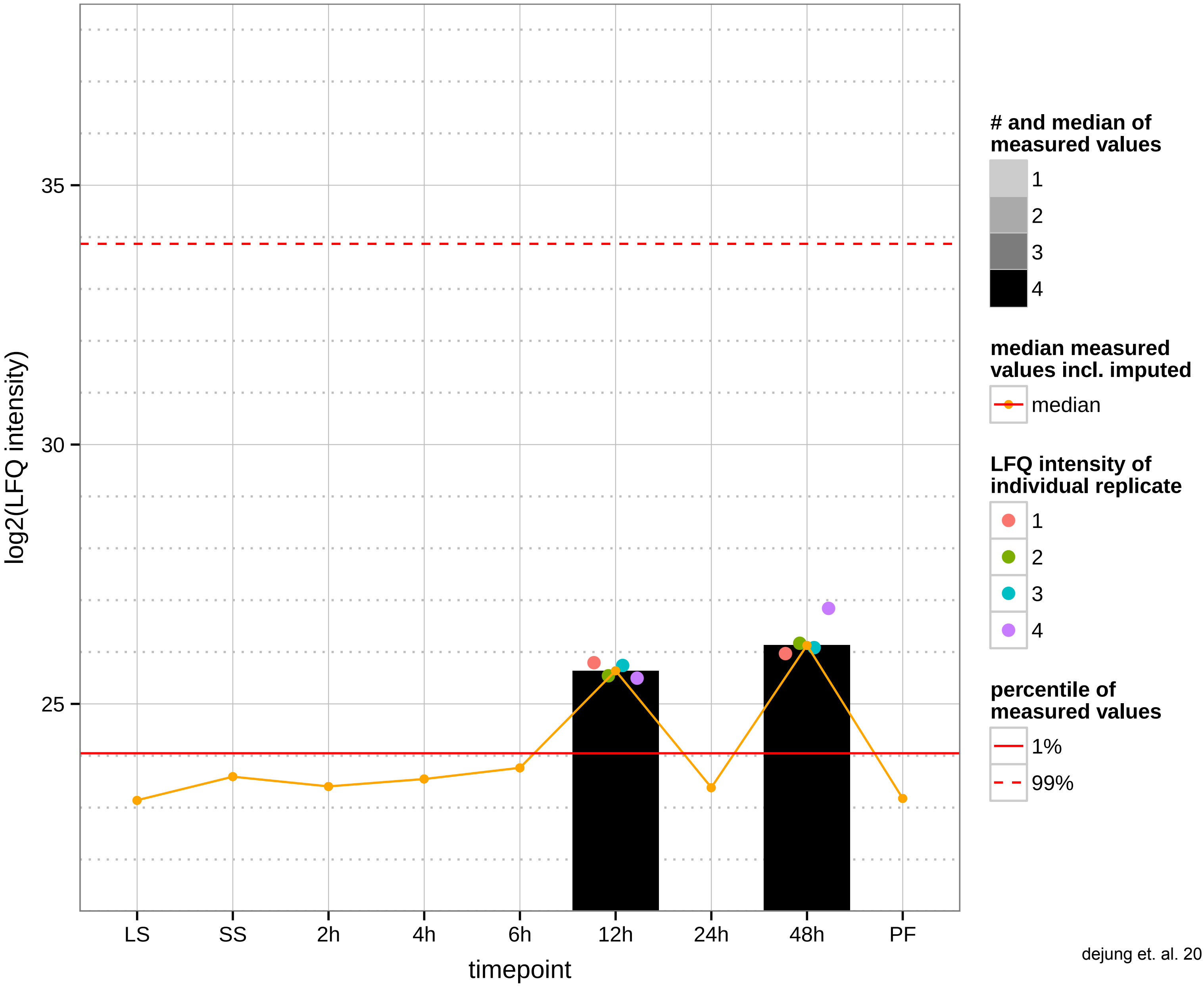

dejung et. al. 2015

DNA polymerase delta subunit 2, putative
Tb927.3.1130
AGOF: DNA binding, DNA−directed DNA polymerase activity
AGOC: nucleus
AGOP: DNA replication
PGOF: DNA binding, DNA−directed DNA polymerase activity
PGOC: null
PGOP: DNA replication

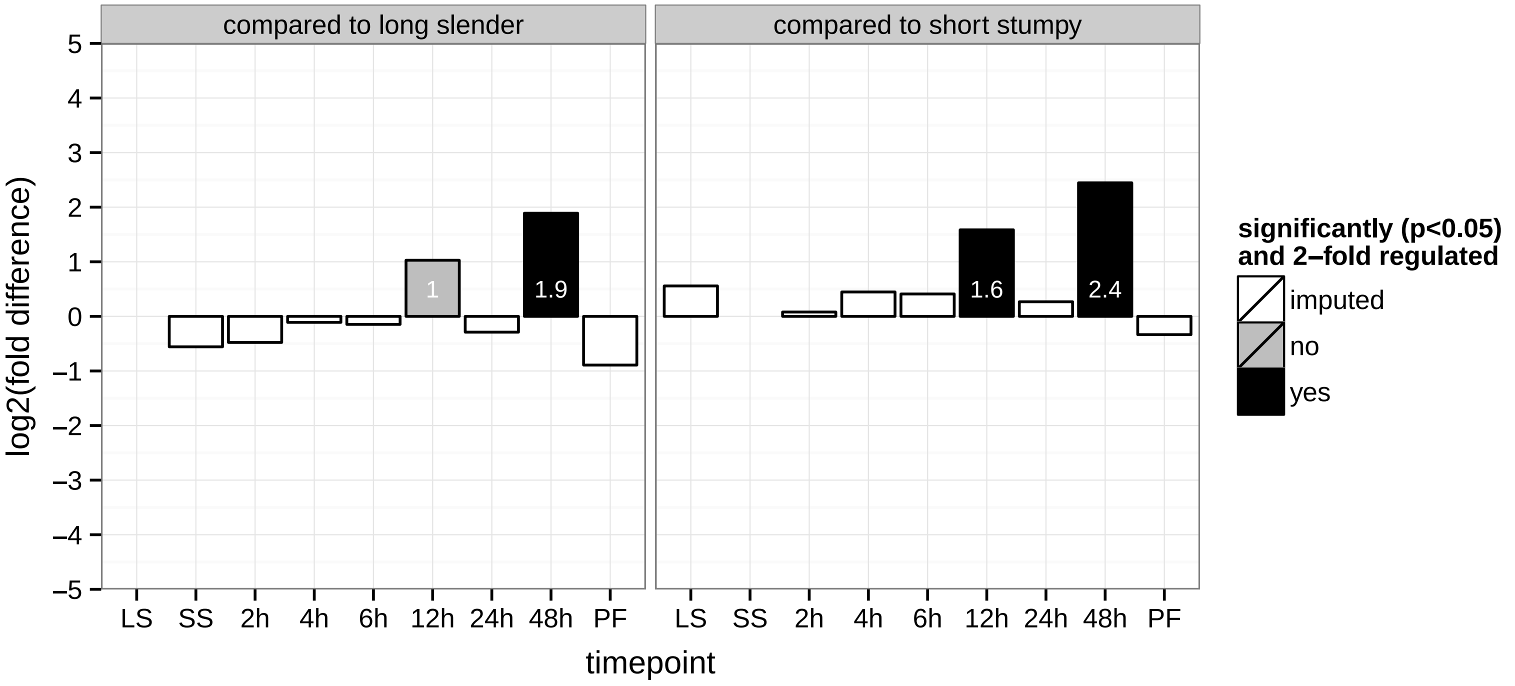

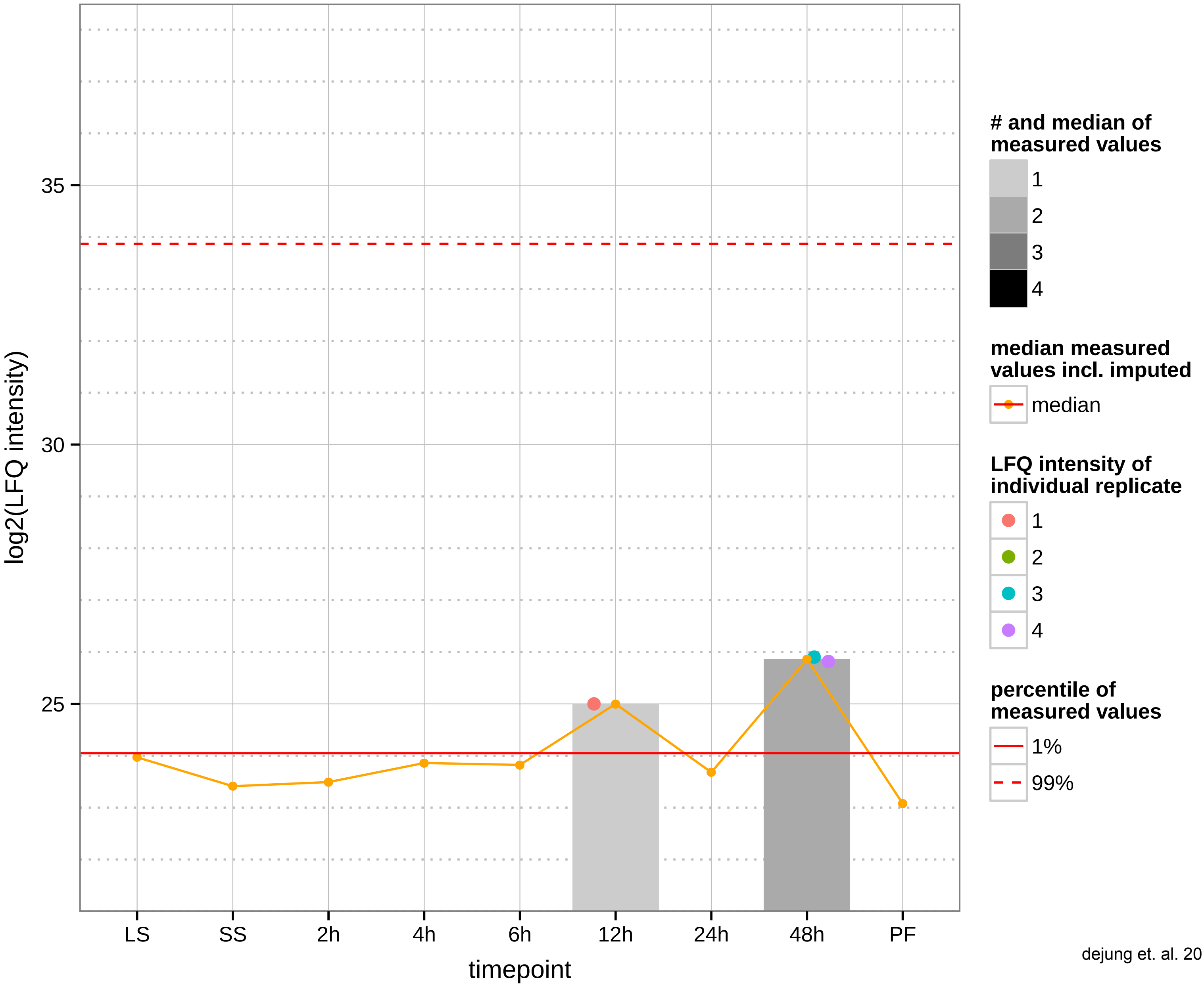

dejung et. al. 2015

hypothetical protein, conserved
Tb927.3.4060
AGOF: asparagine synthase (glutamine–hydrolyzing) activity
AGOC: null
AGOP: asparagine biosynthetic process
PGOF: asparagine synthase (glutamine–hydrolyzing) activity
PGOC: null
PGOP: asparagine biosynthetic process

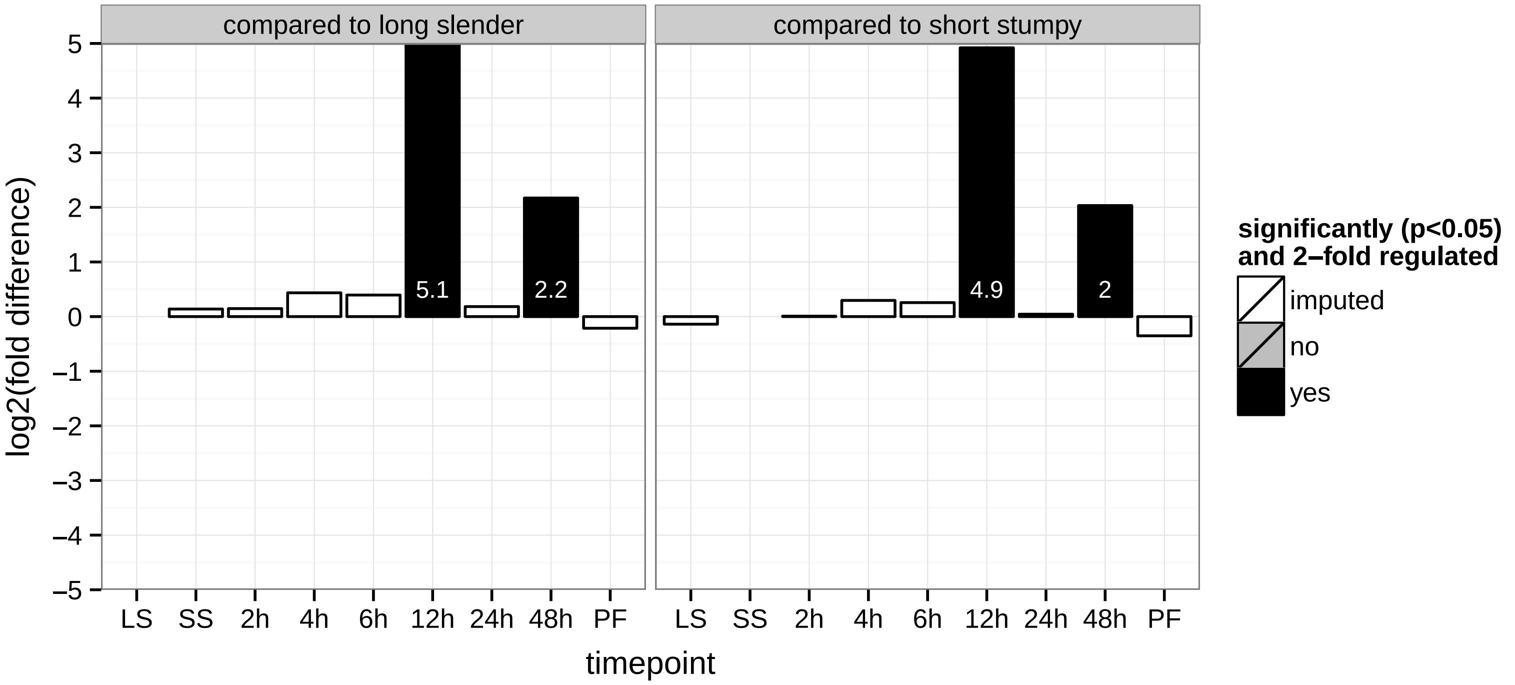

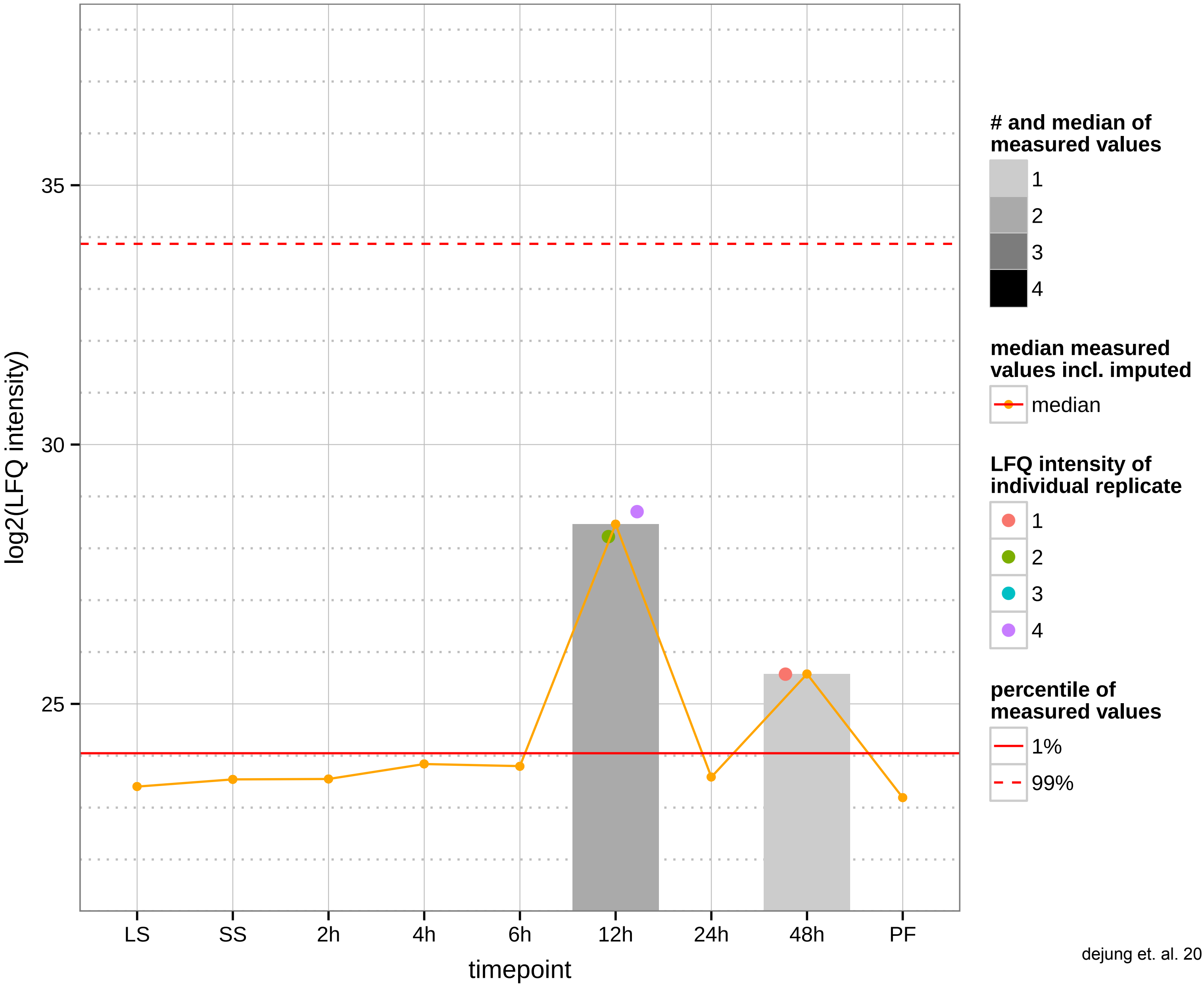

dejung et. al. 2015

hypothetical protein, conserved
Tb927.4.2280
AGOF: null
AGOC: null
AGOP: null
PGOF: null
PGOC: null
PGOP: null

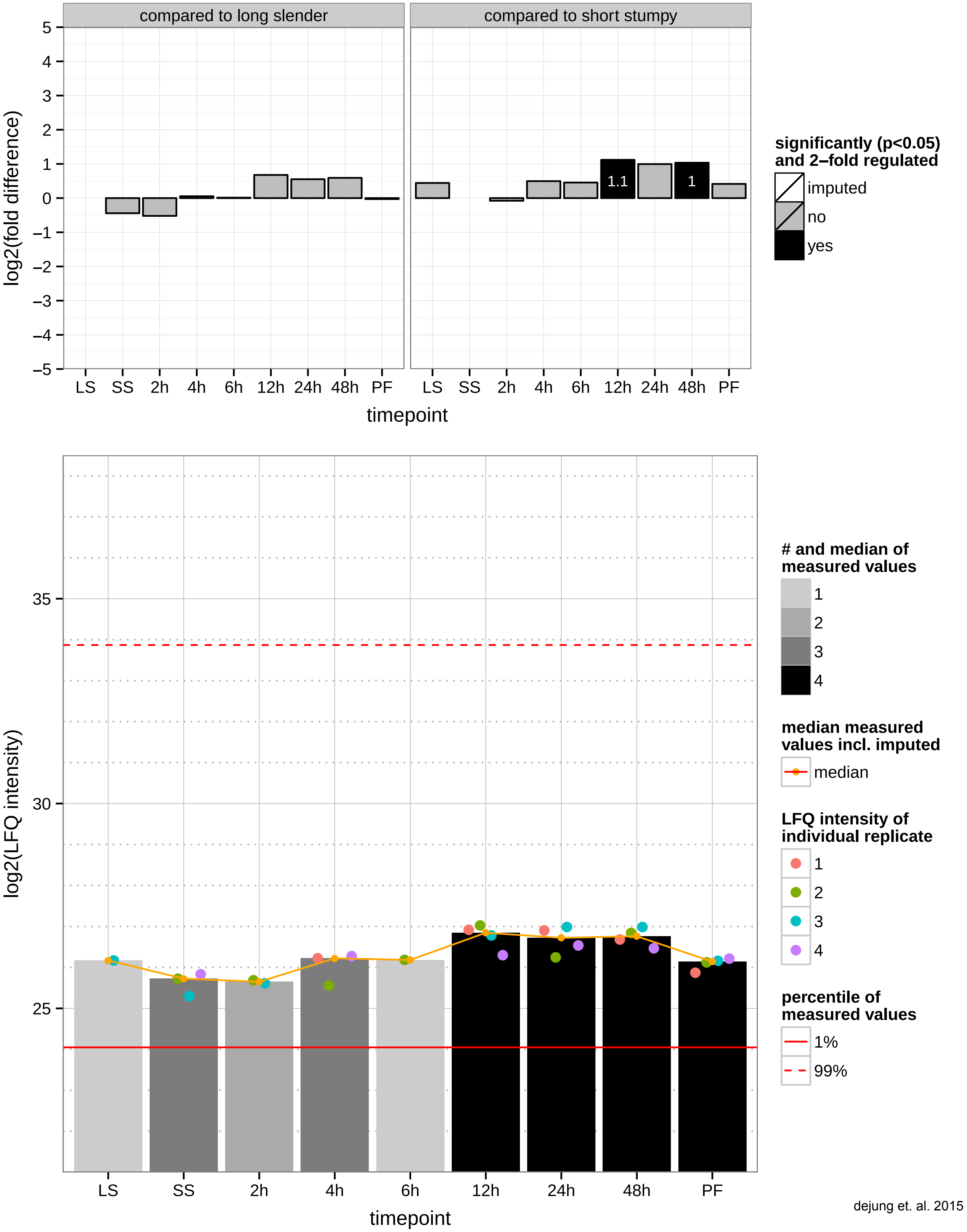

hypothetical protein, conserved
Tb927.6.2390
AGOF: null
AGOC: null
AGOP: null
PGOF: null
PGOC: null
PGOP: null

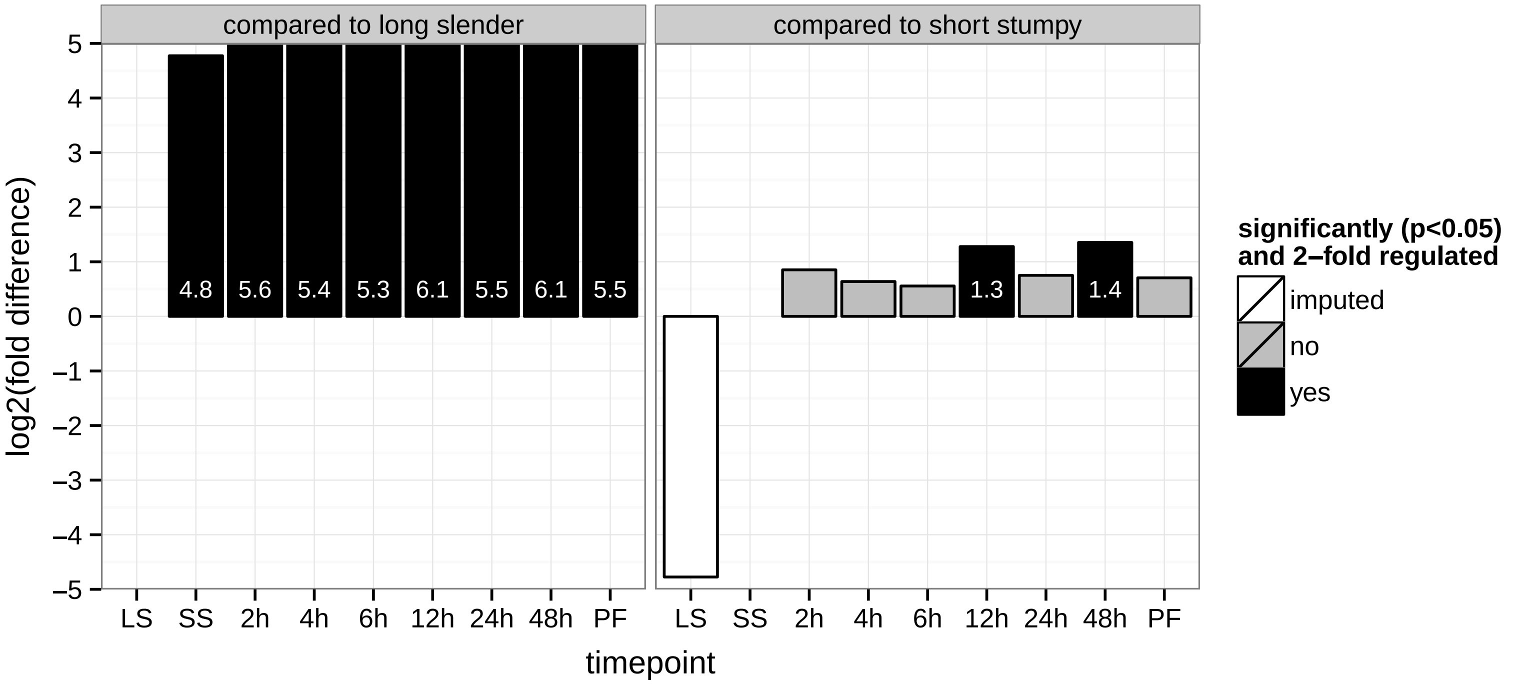

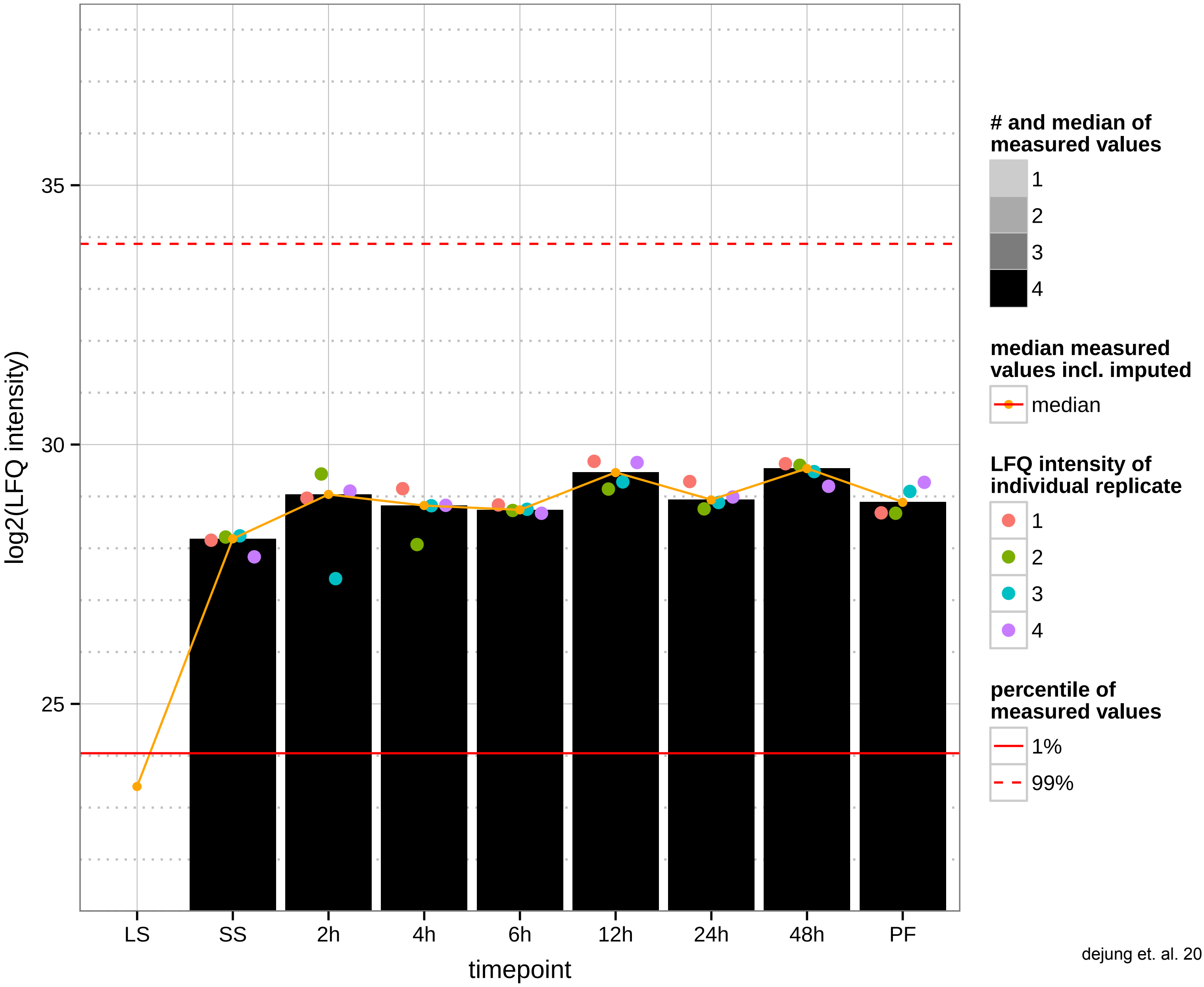

dejung et. al. 2015

hypothetical protein, conserved
Tb927.7.6180
AGOF: null
AGOC: null
AGOP: null
PGOF: null
PGOC: null
PGOP: null

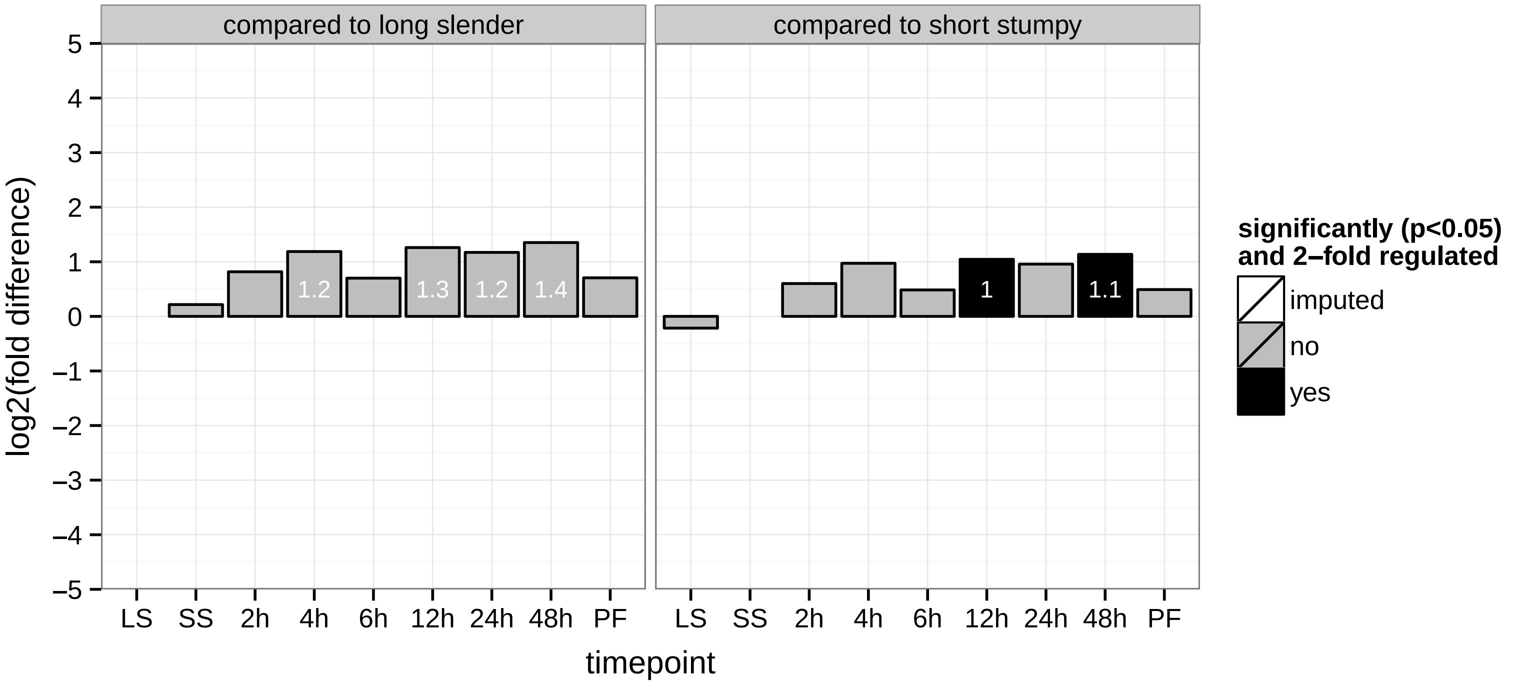

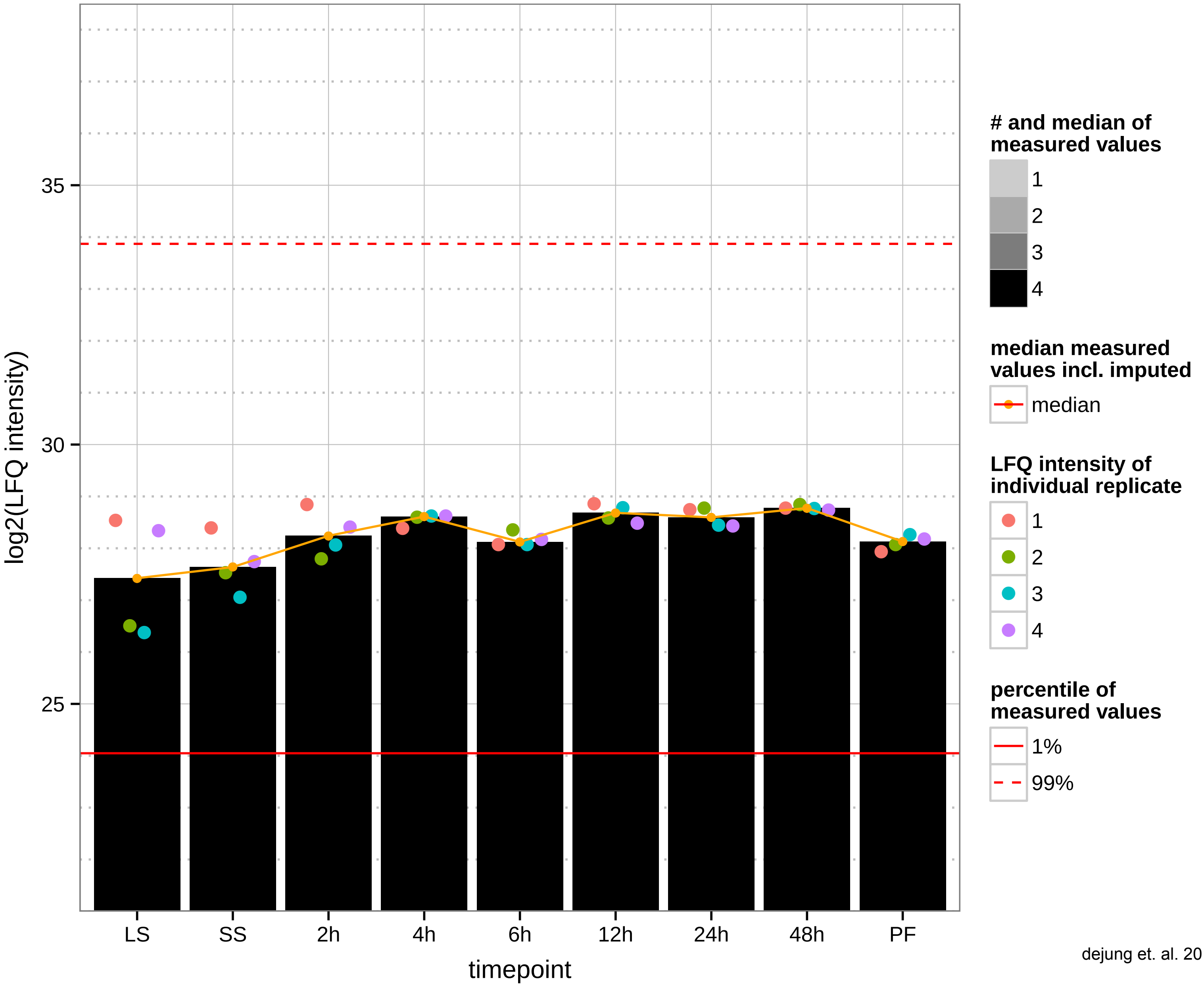

dejung et. al. 2015

hypothetical protein, conserved
Tb927.8.8200
AGOF: null
AGOC: null
AGOP: null
PGOF: null
PGOC: null
PGOP: null

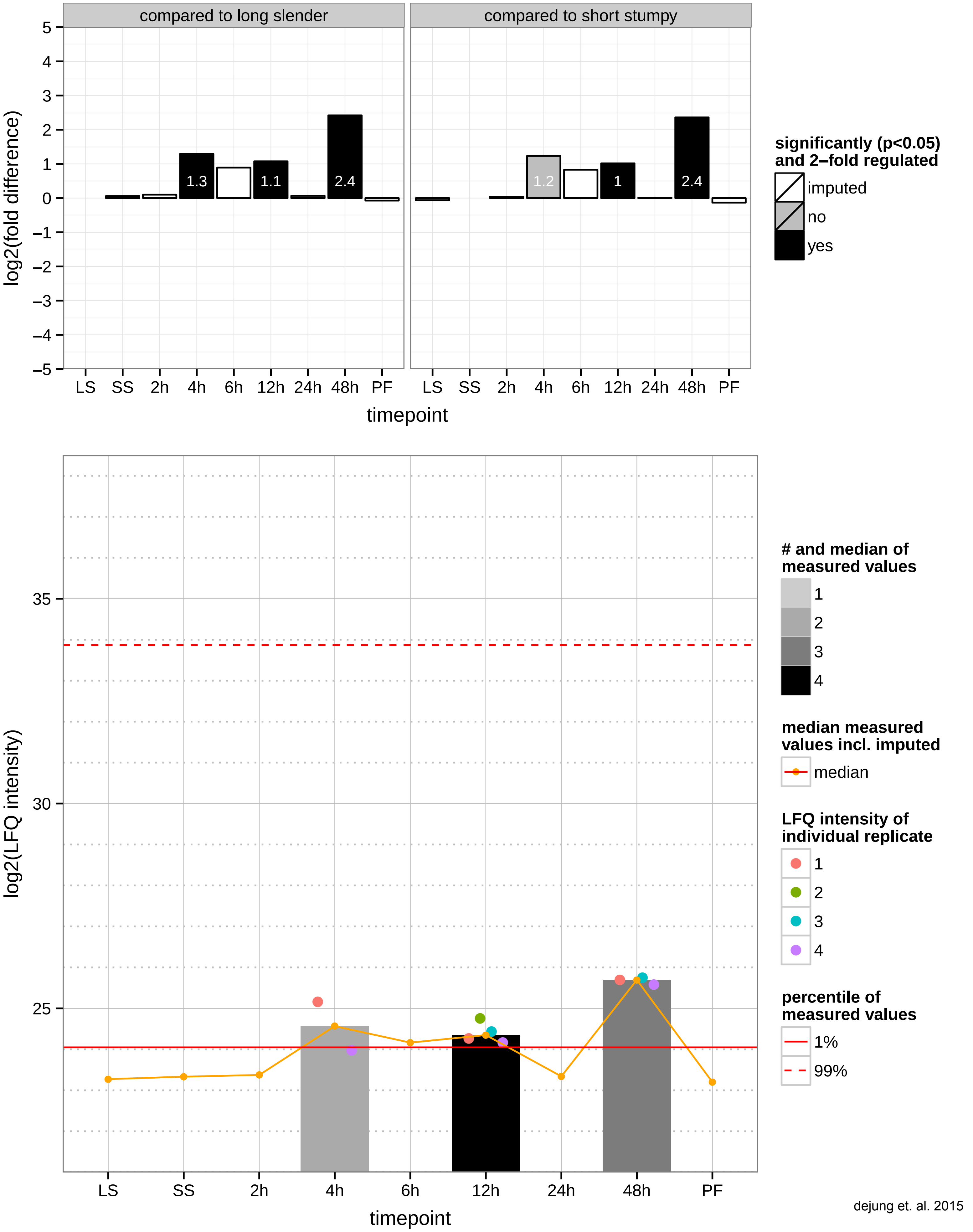

dejung et. al. 2015

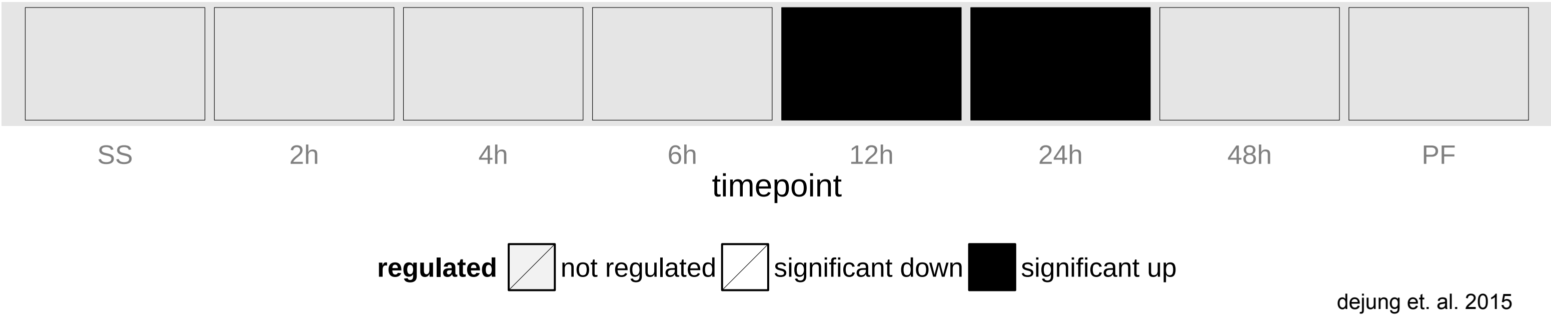

SS
2h
4h
6h
12h
24h
48h
PF
timepoint
regulated
not regulated
significant down
significant up
dejung et. al. 2015

hypothetical protein, conserved
Tb11.v5.0581;Tb927.10.570
AGOF: null
AGOC: null
AGOP: null
PGOF: null
PGOC: exocyst
PGOP: protein transport, vesicle docking involved in exocytosis

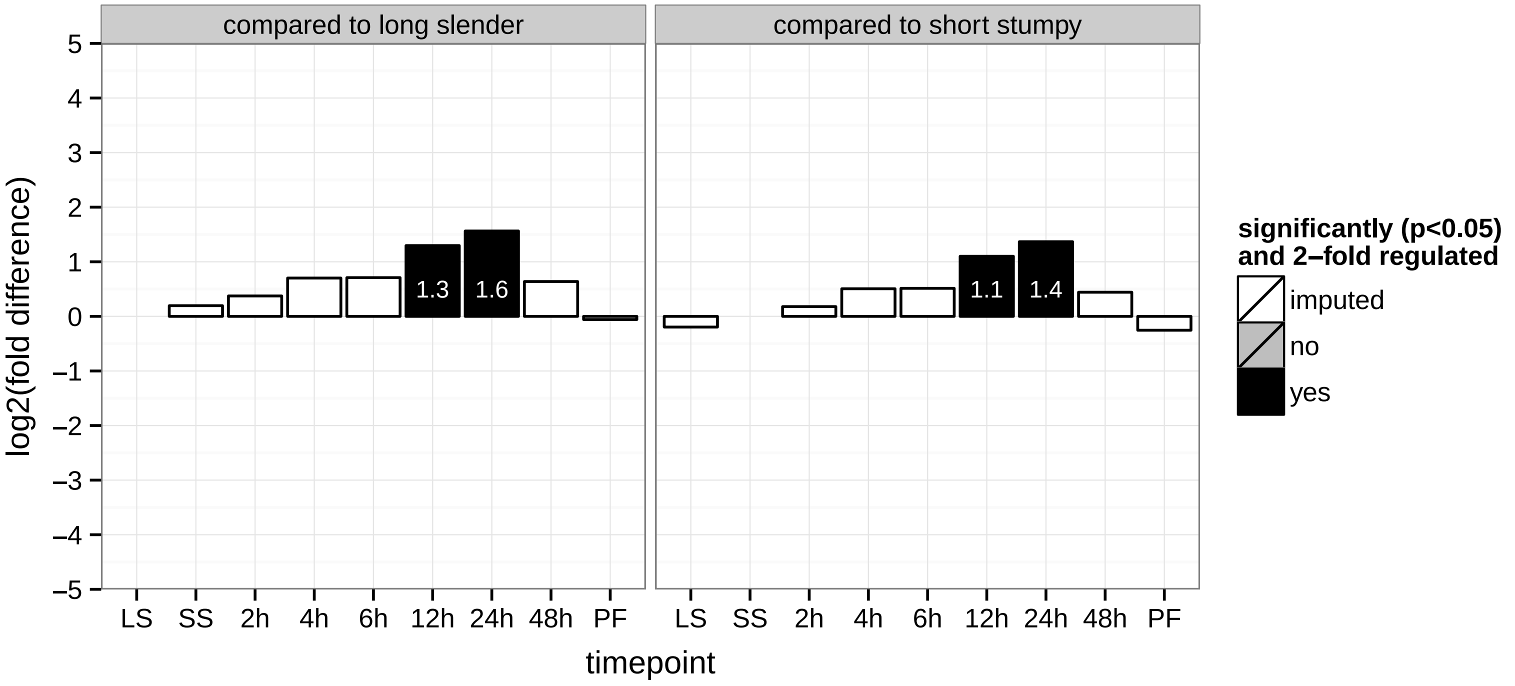

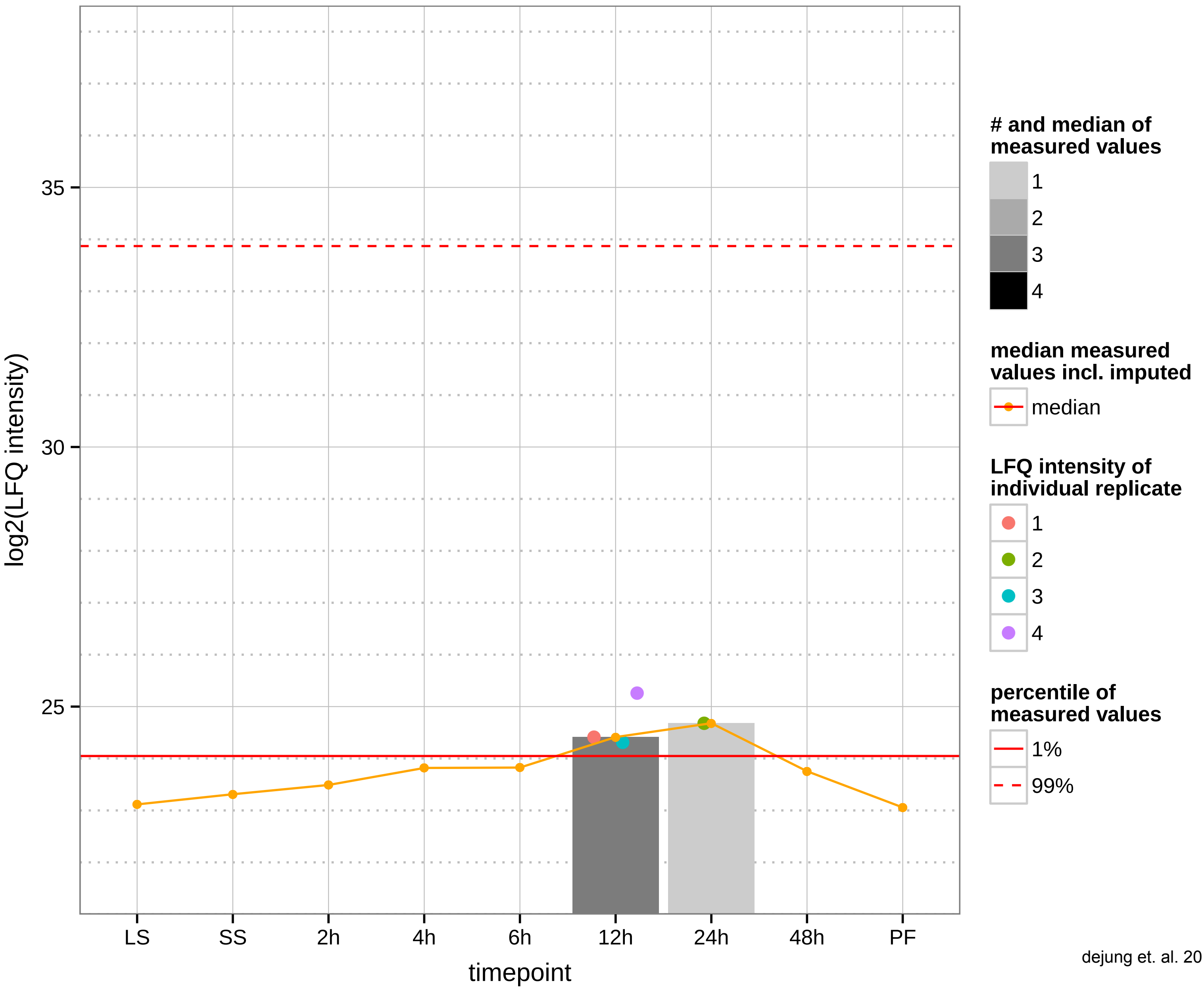

dejung et. al. 2015

hypothetical protein, conserved
Tb927.11.10860
AGOF: null
AGOC: null
AGOP: null
PGOF: null
PGOC: null
PGOP: null

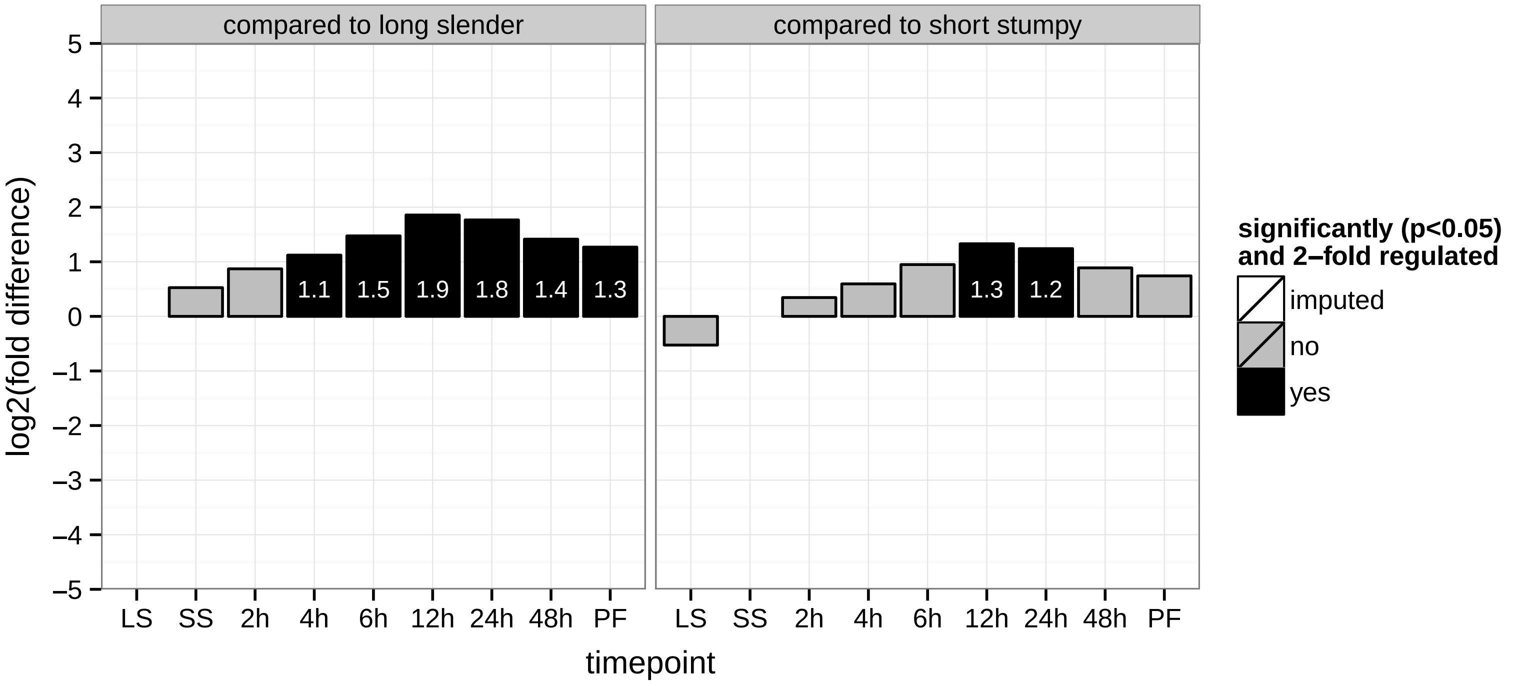

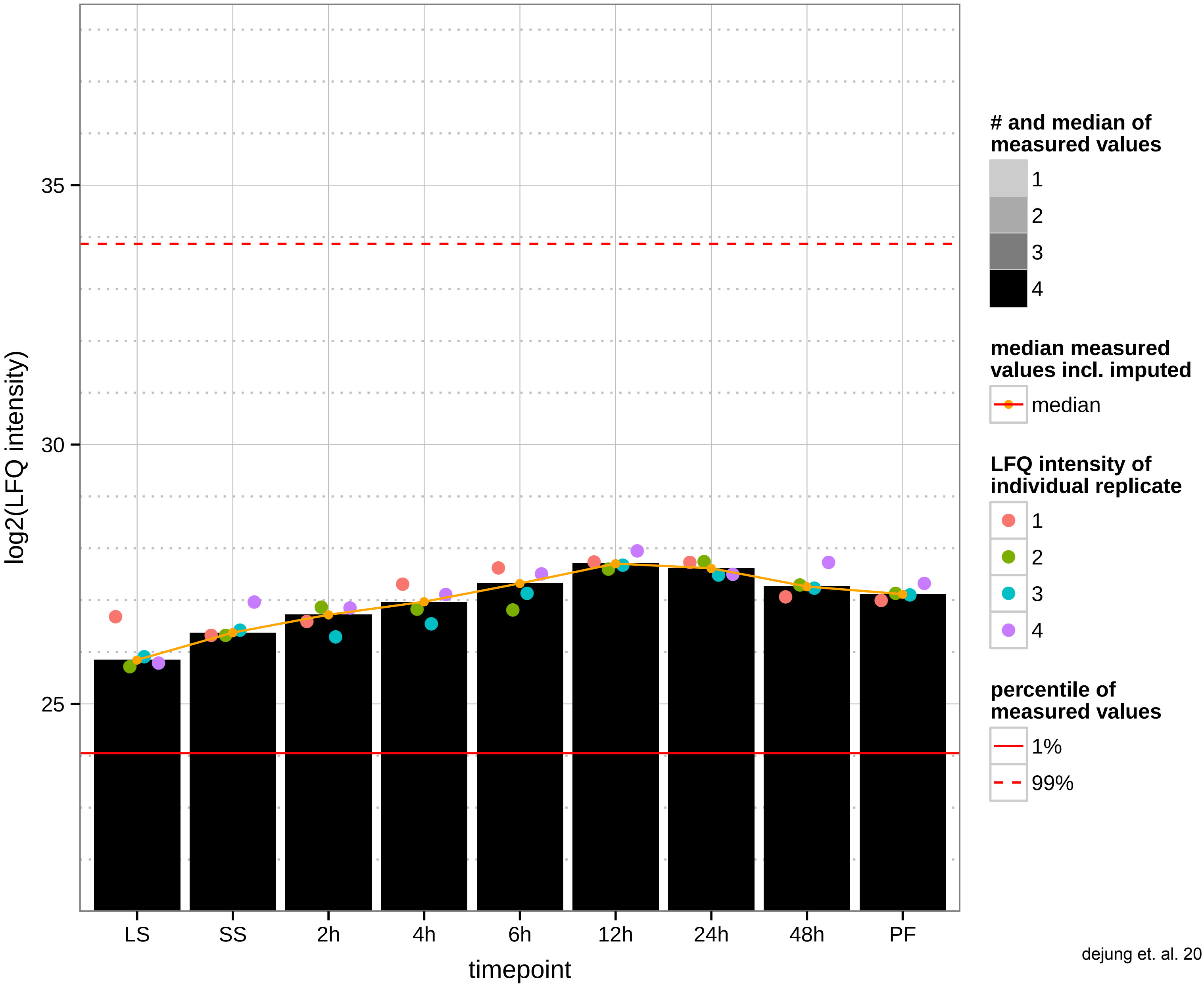

dejung et. al. 2015

hypothetical protein, conserved
Tb927.11.13590
AGOF: null
AGOC: mitochondrion
AGOP: null
PGOF: null
PGOC: null
PGOP: null

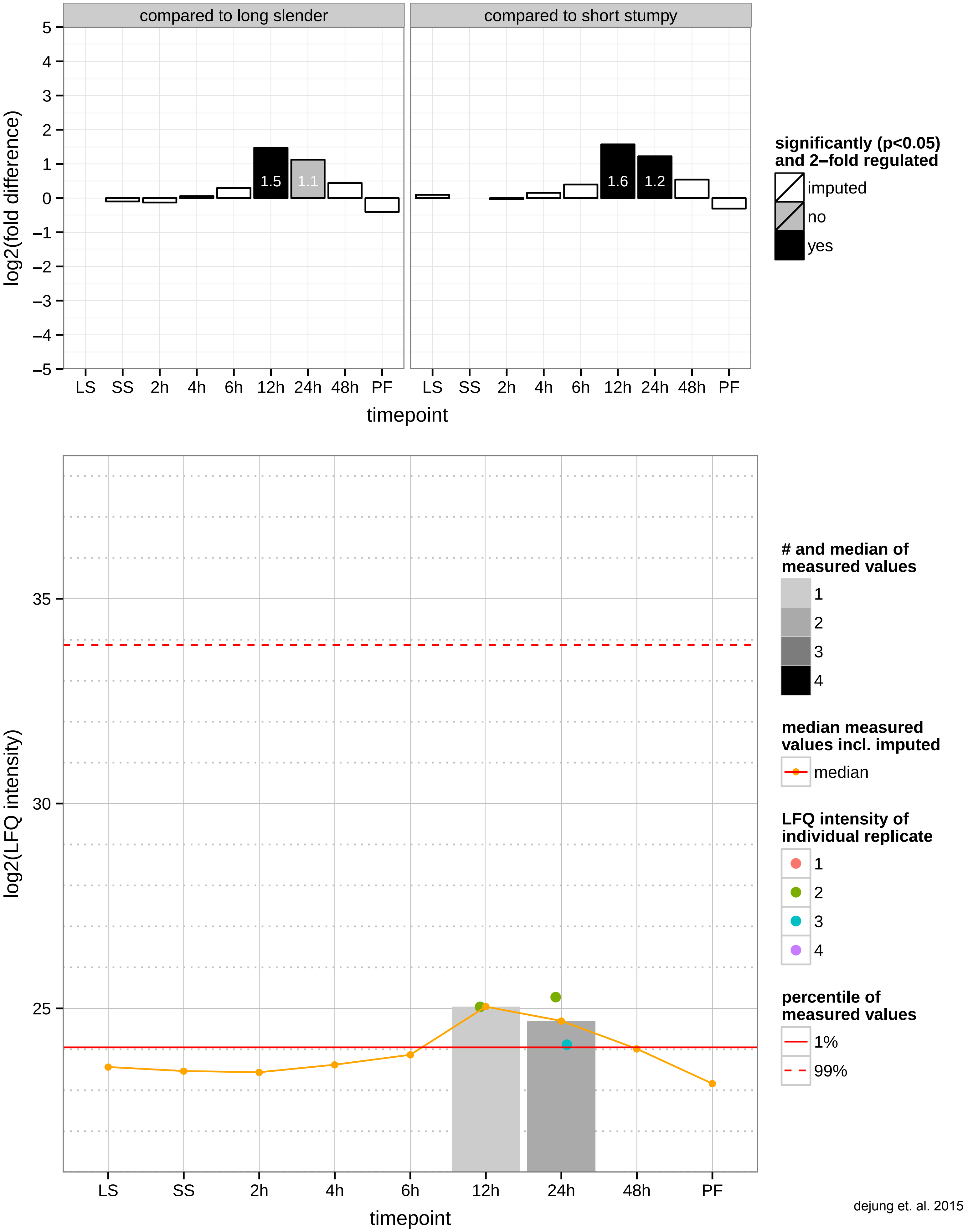

dejung et. al. 2015

hypothetical protein, conserved
Tb927.11.5760
AGOF: null
AGOC: mitochondrion
AGOP: null
PGOF: null
PGOC: null
PGOP: null

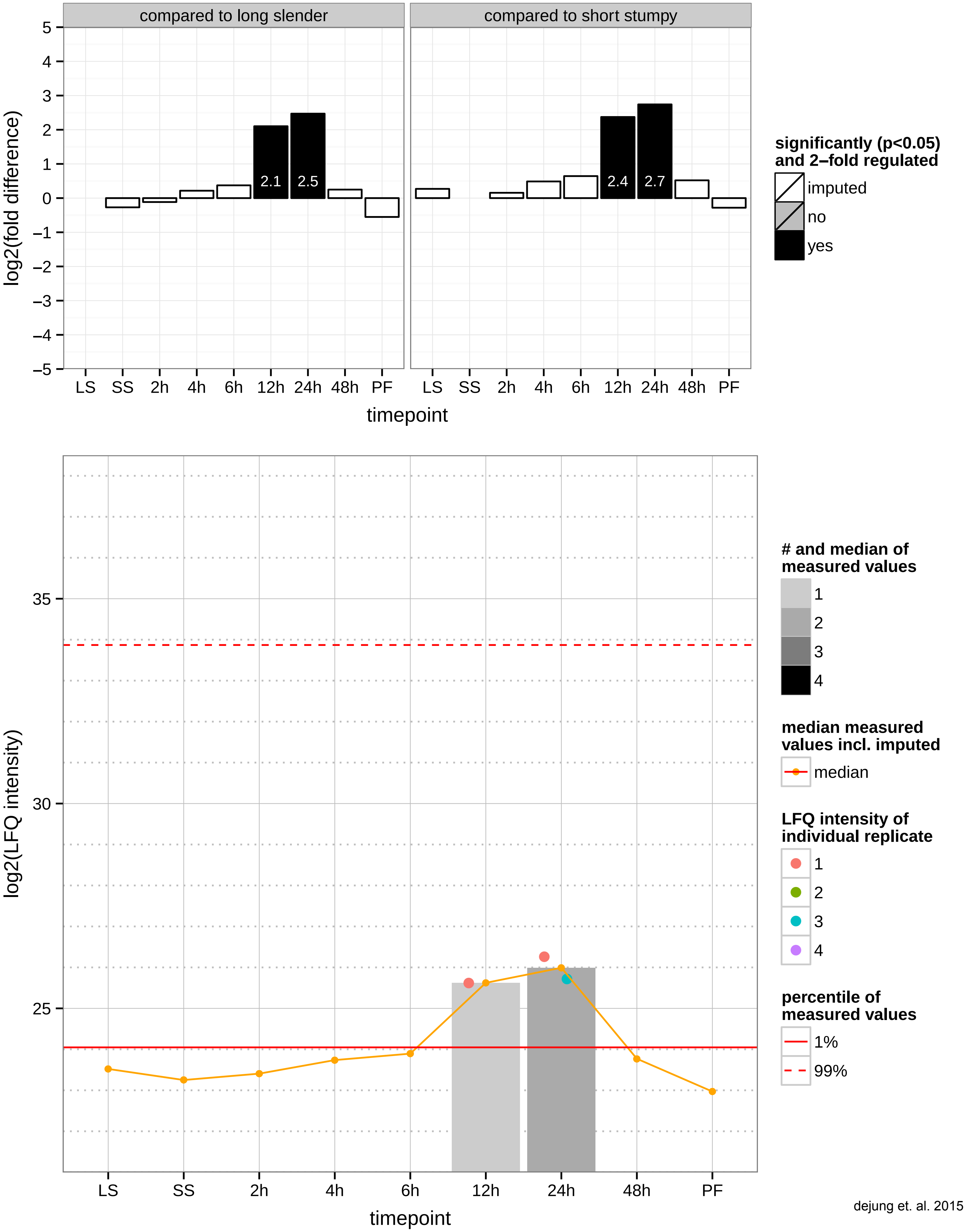

dejung et. al. 2015

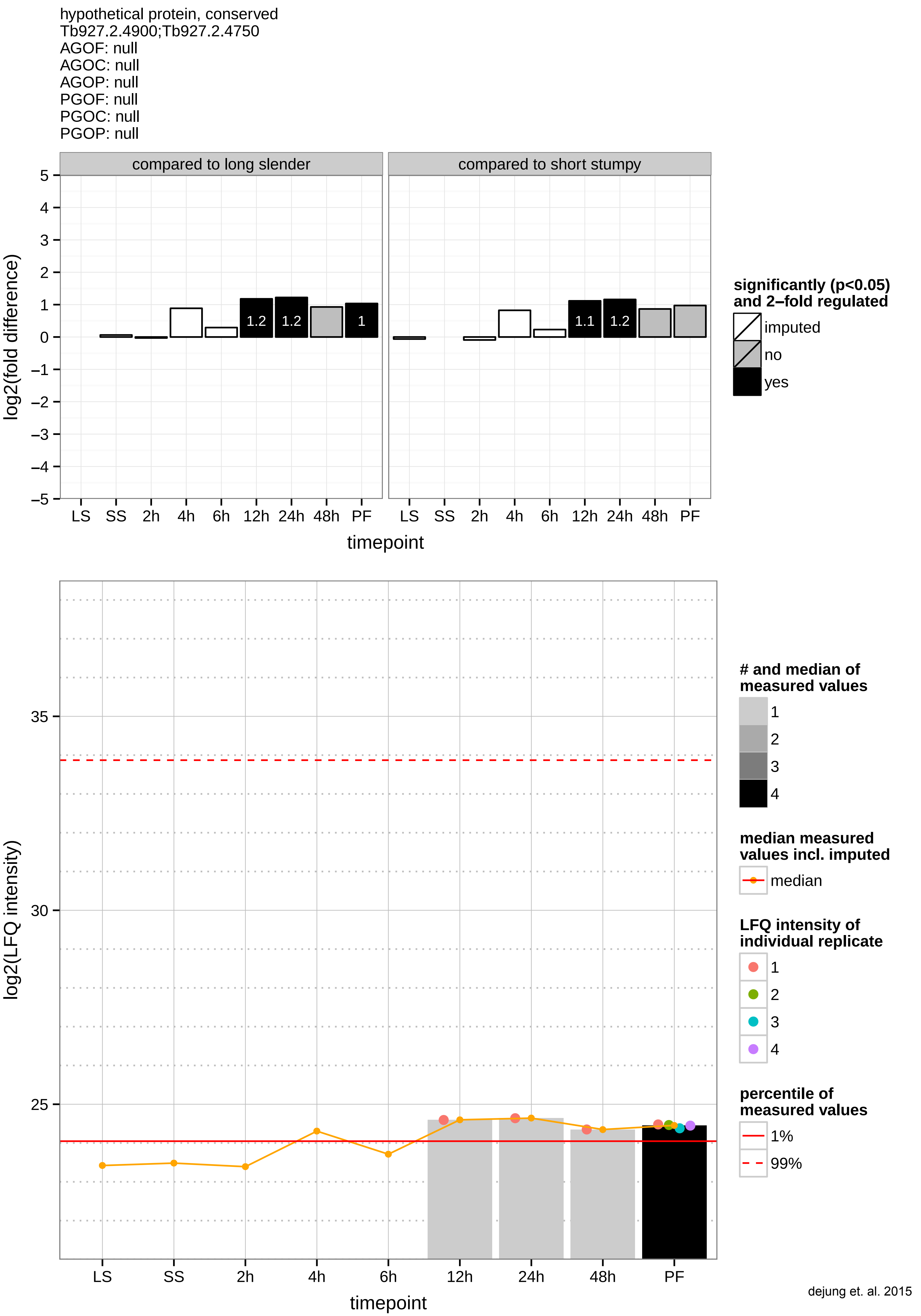
hypothetical protein, conserved
Tb927.2.4900;Tb927.2.4750
AGOF: null
AGOC: null
AGOP: null
PGOF: null
PGOC: null
PGOP: null
compared to long slender
compared to short stumpy
log2(fold difference)
timepoint
LS
SS
2h
4h
6h
12h
24h
48h
PF
1.2
1.2
1
1.1
1.2
significantly (p<0.05) and 2–fold regulated
imputed
no
yes
log2(LFQ intensity)
# and median of measured values
median measured values incl. imputed
median
LFQ intensity of individual replicate
percentile of measured values
1%
99%
dejung et. al. 2015

hypothetical protein, conserved
Tb927.3.1260
AGOF: null
AGOC: null
AGOP: null
PGOF: null
PGOC: null
PGOP: null

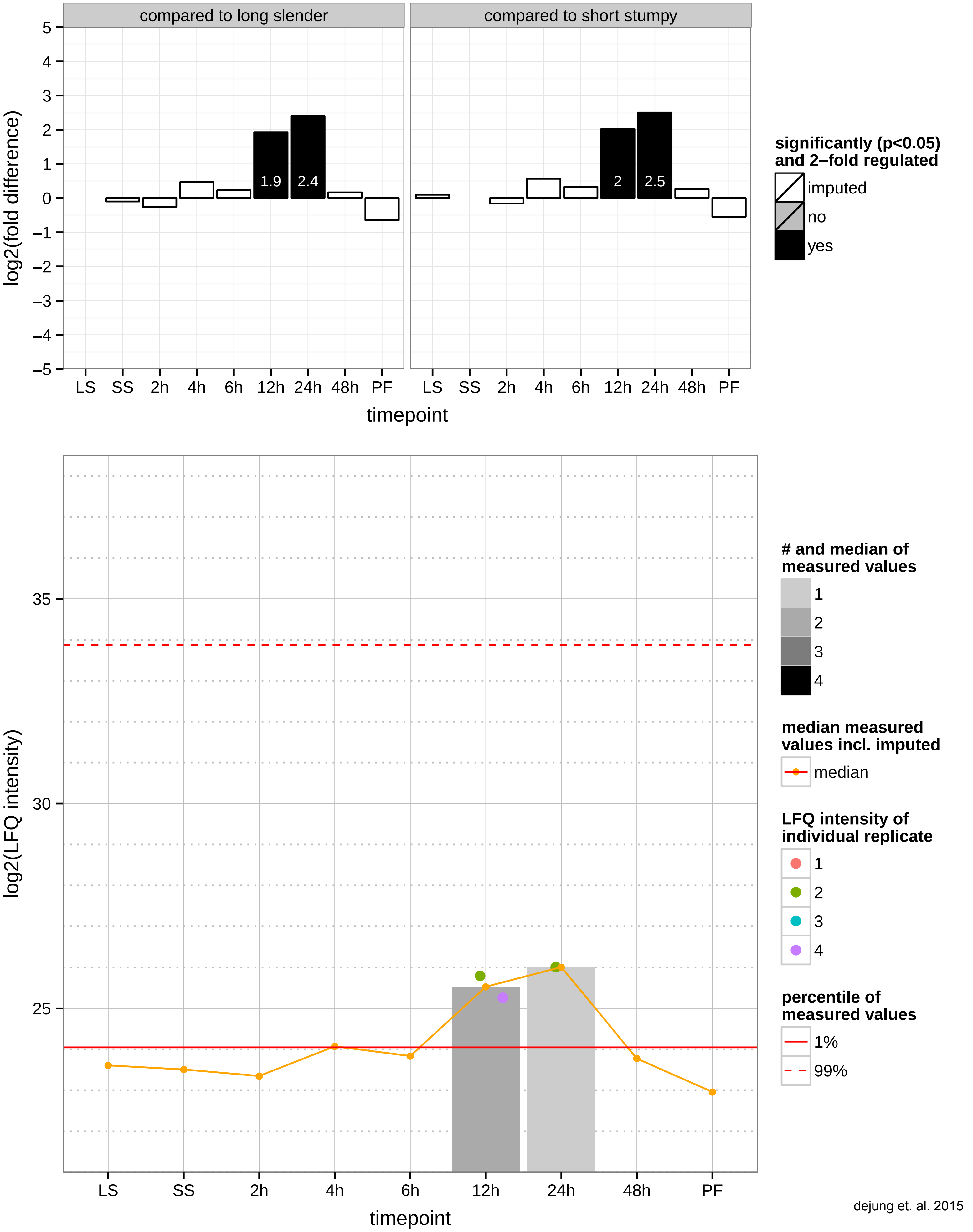

dejung et. al. 2015

hypothetical protein, conserved
Tb927.3.1600
AGOF: null
AGOC: null
AGOP: null
PGOF: null
PGOC: mitochondrial intermembrane space protein transporter complex
PGOP: protein import into mitochondrial inner membrane, protein targeting to mitochondrion

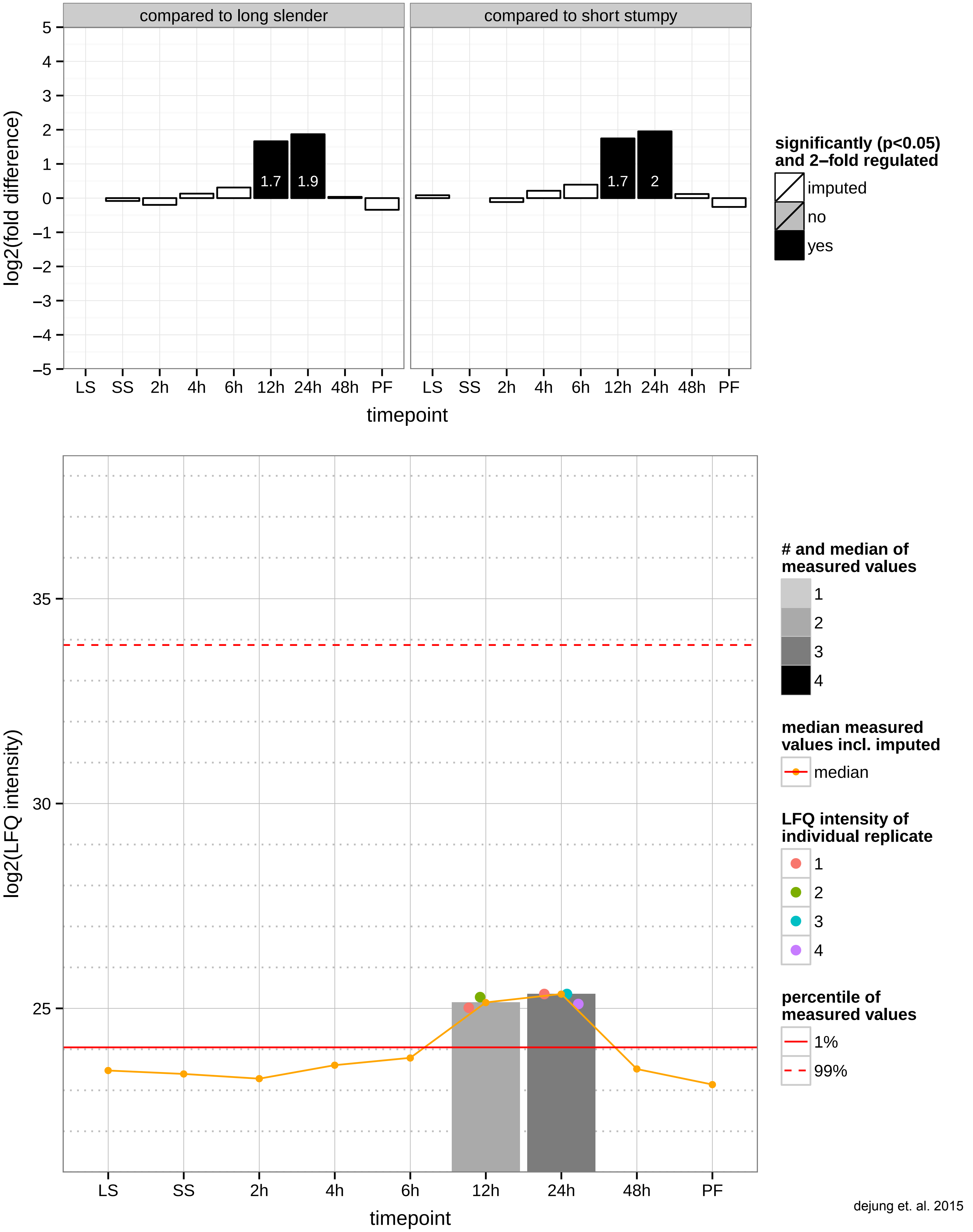

dejung et. al. 2015

hypothetical protein, conserved
Tb927.4.1120
AGOF: null
AGOC: null
AGOP: null
PGOF: null
PGOC: null
PGOP: null

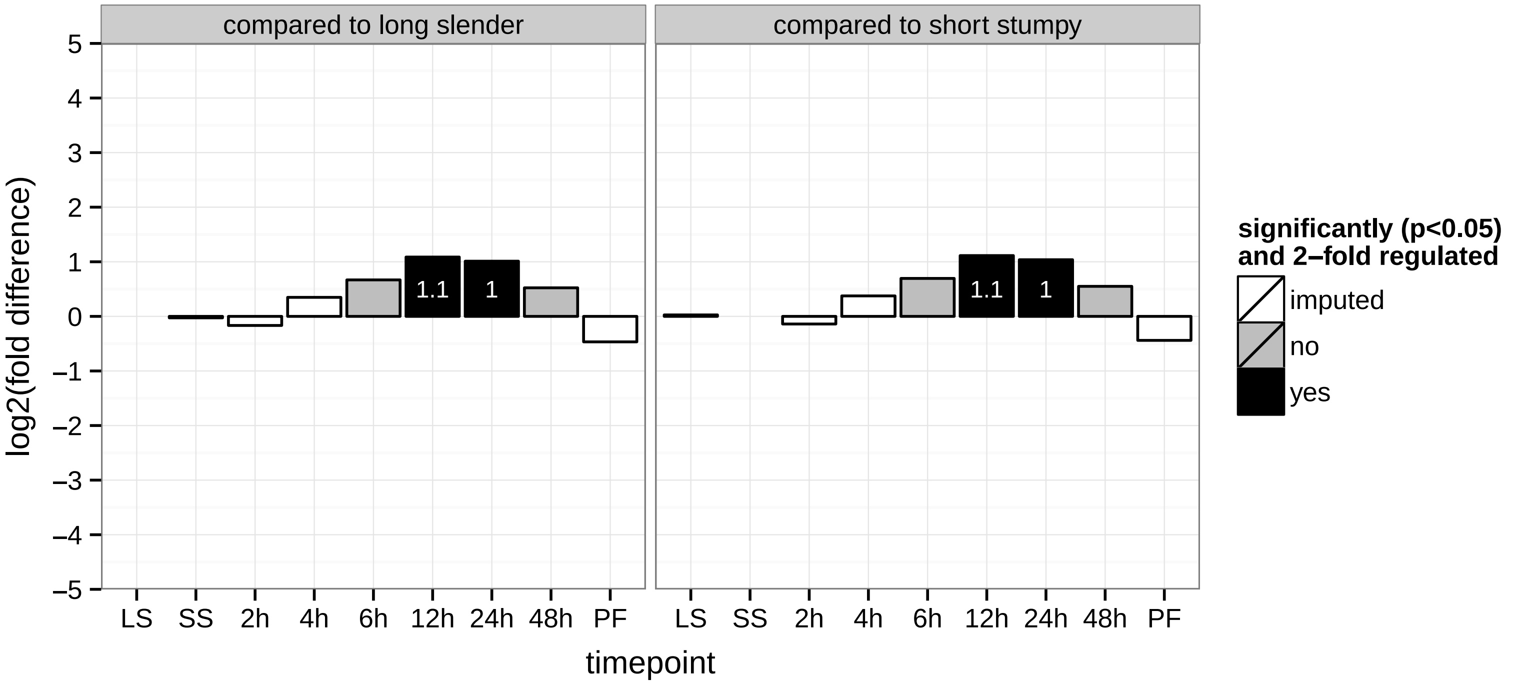

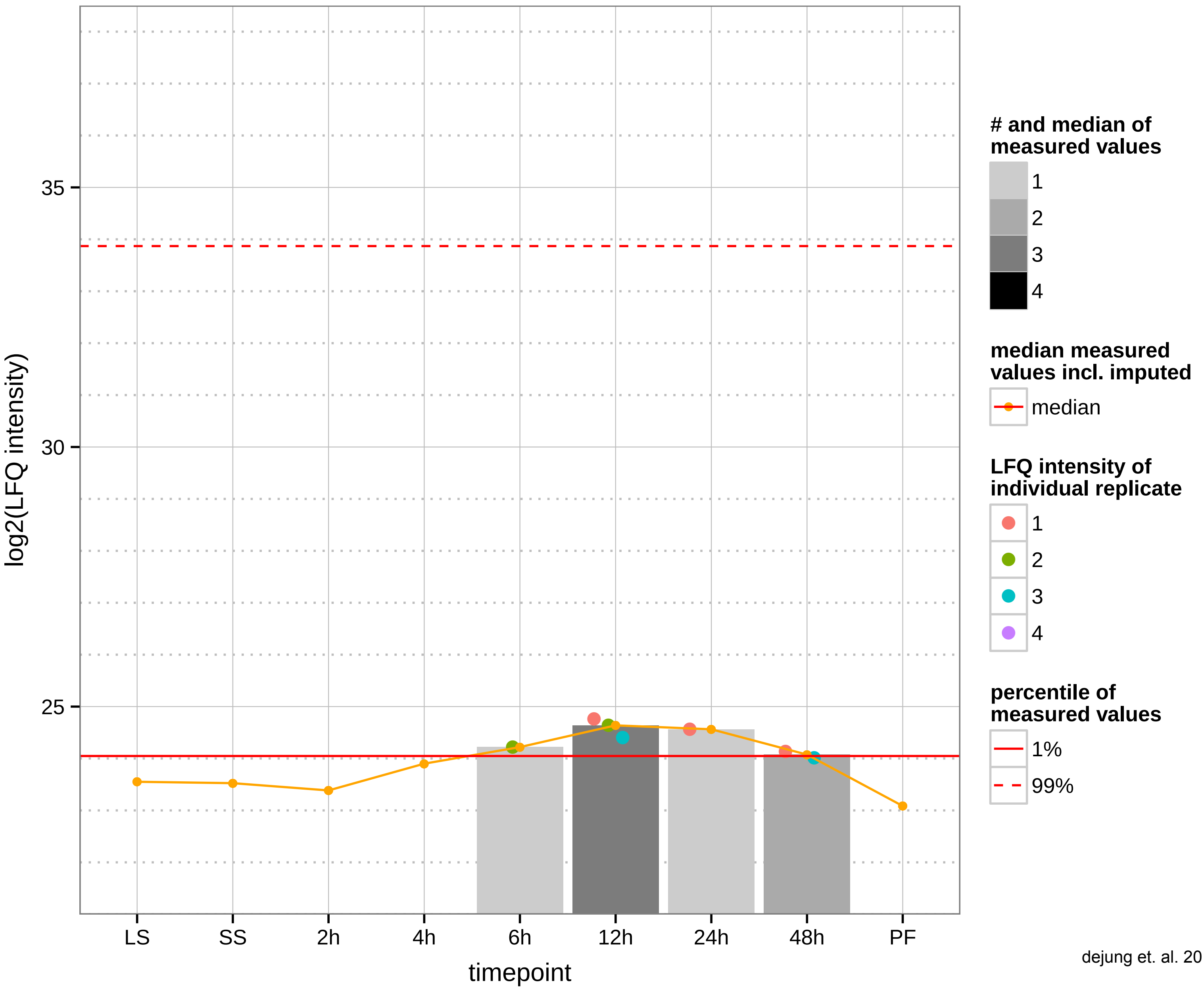

dejung et. al. 2015

ubiquitin, putative
Tb927.4.2540
AGOF: null
AGOC: null
AGOP: null
PGOF: protein binding
PGOC: null
PGOP: null

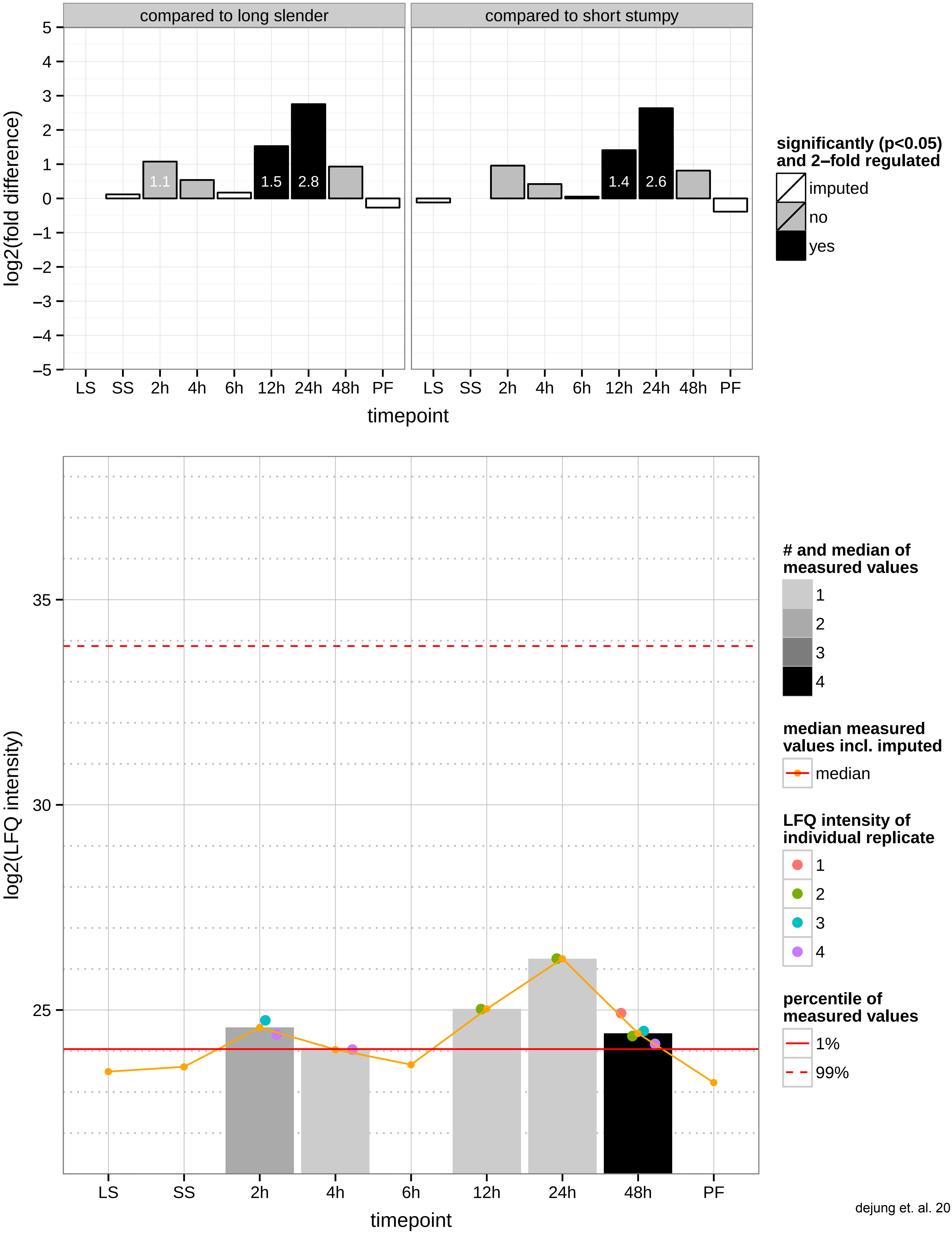

dejung et. al. 2015

hypothetical protein, conserved
Tb927.4.3430
AGOF: null
AGOC: null
AGOP: null
PGOF: null
PGOC: null
PGOP: null

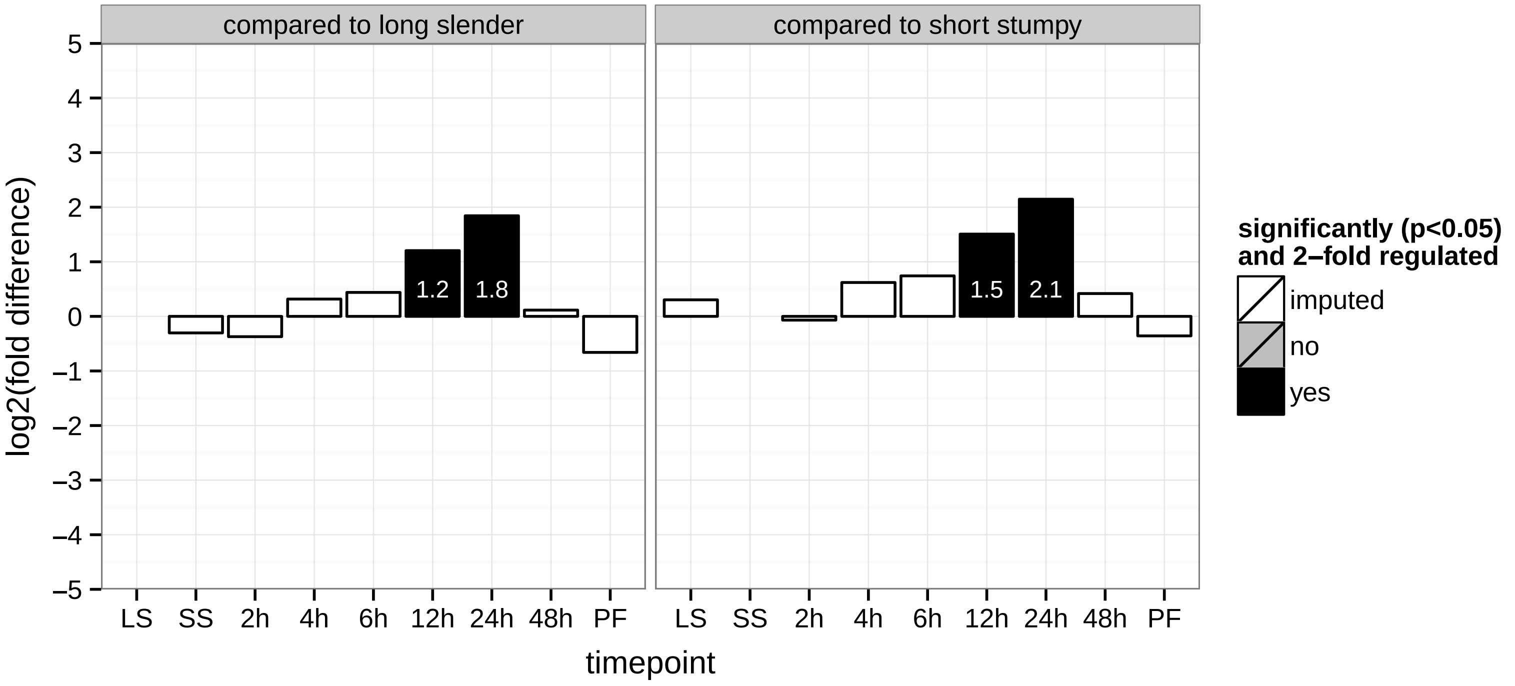

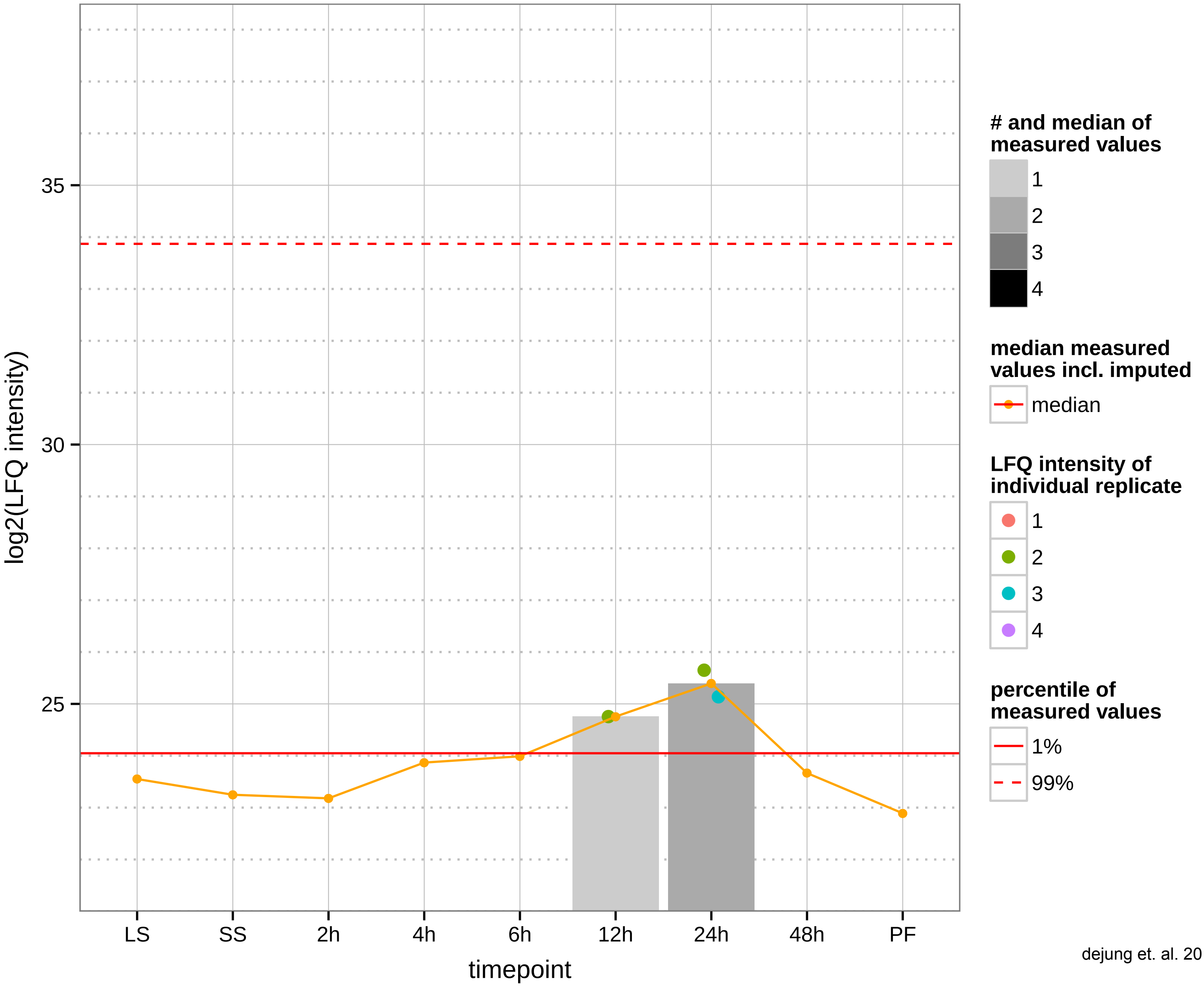

dejung et. al. 2015

phosphoglycerate mutase protein, putative
Tb927.5.3580
AGOF: catalytic activity
AGOC: null
AGOP: metabolic process, purine ribonucleotide biosynthetic process
PGOF: null
PGOC: null
PGOP: null

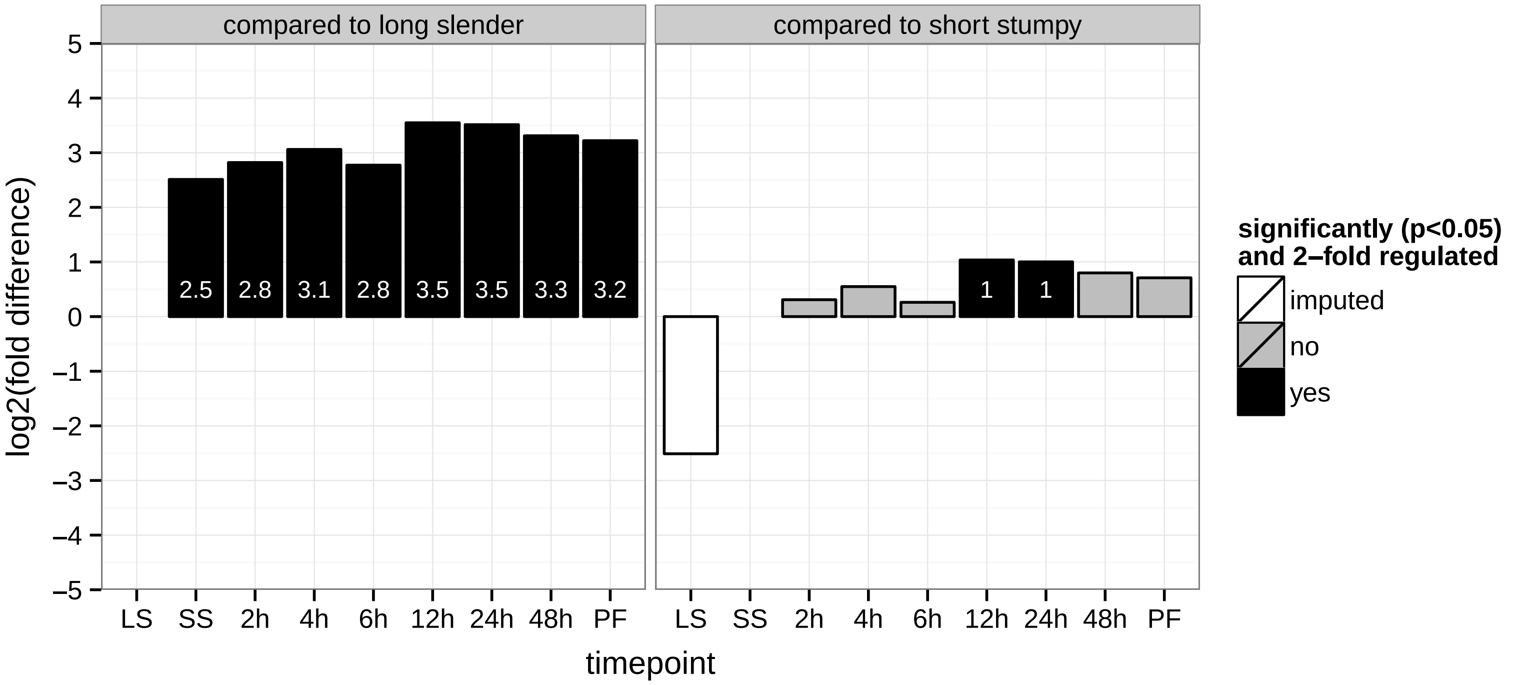

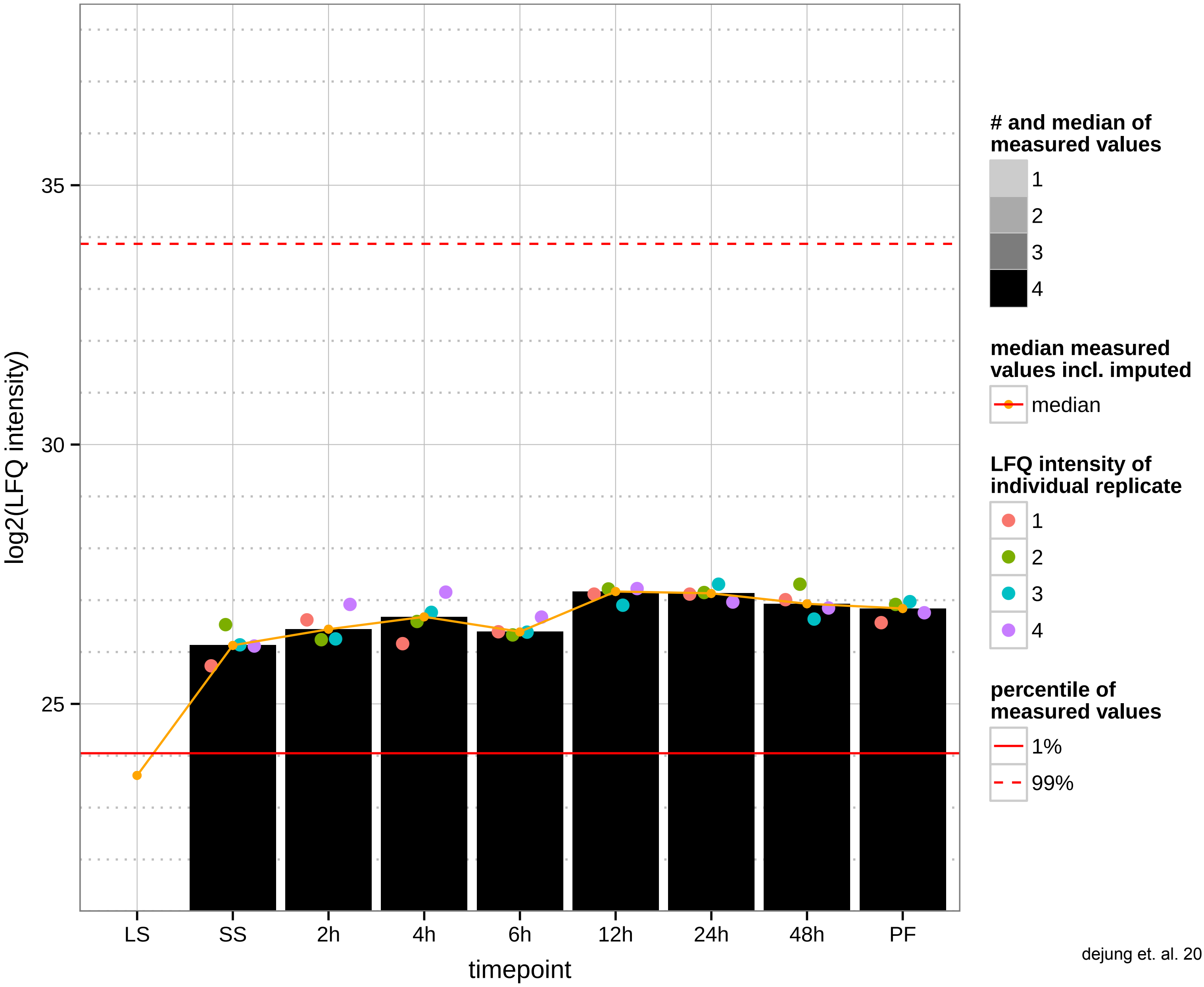

dejung et. al. 2015

hypothetical protein, conserved
Tb927.7.2160
AGOF: null
AGOC: null
AGOP: null
PGOF: null
PGOC: null
PGOP: null

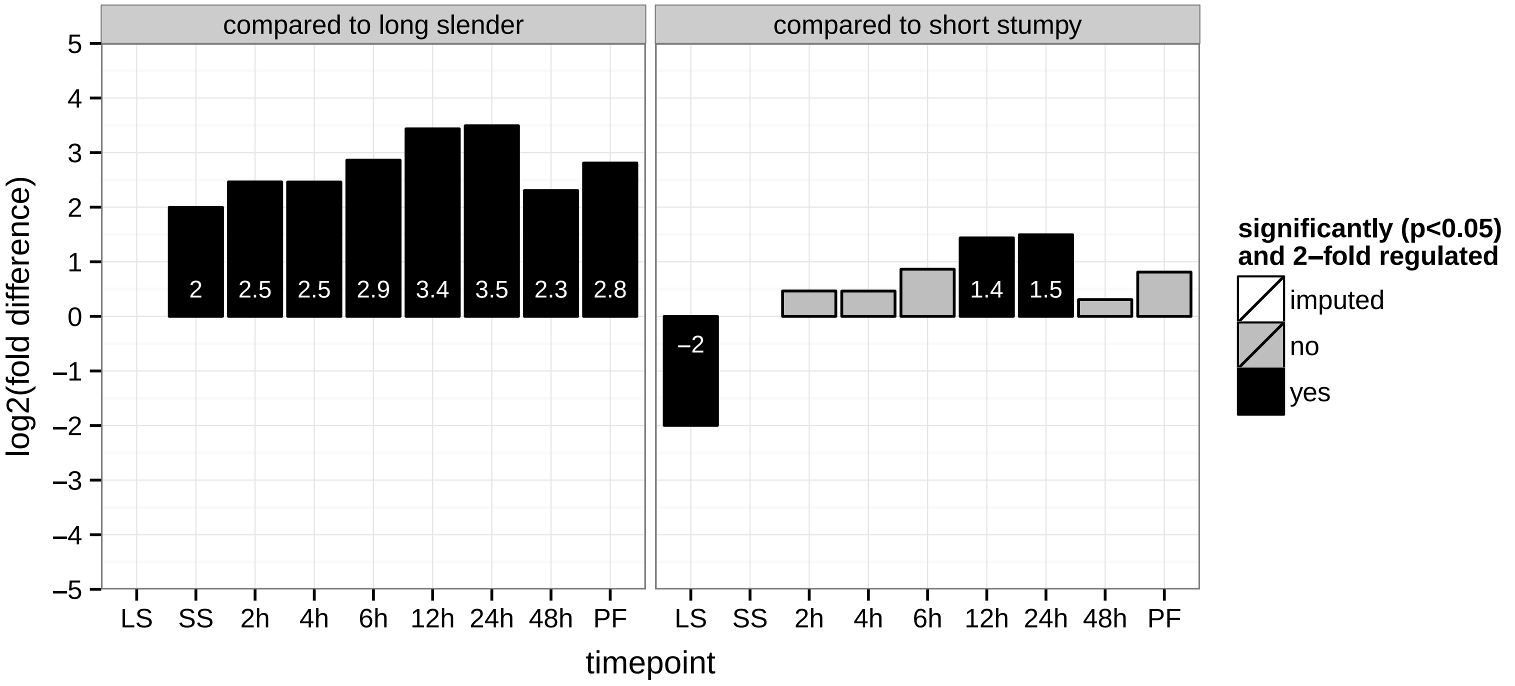

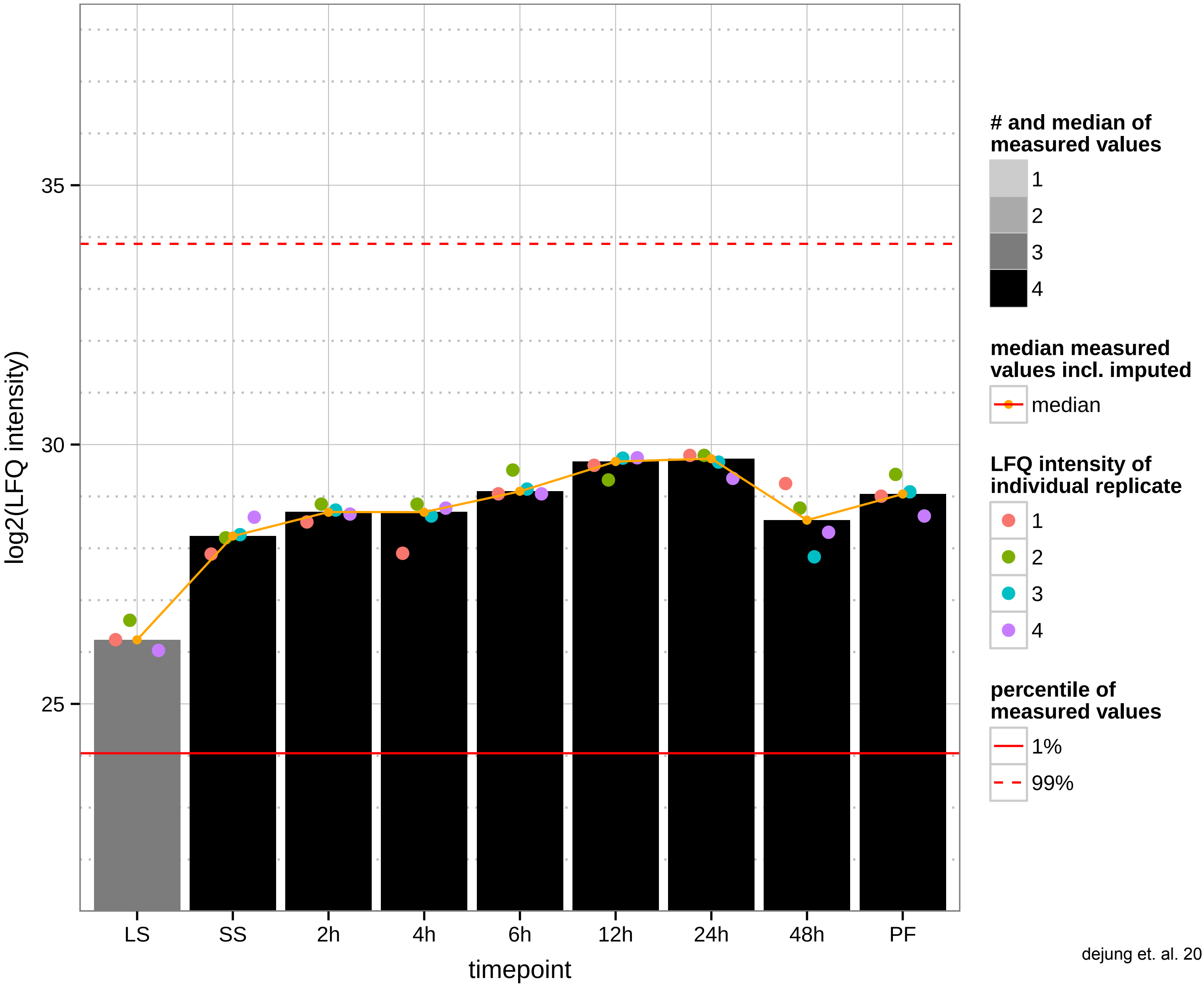

dejung et. al. 2015

hypothetical protein, conserved
Tb927.7.6110;Tb927.7.6170;Tb927.7.6150;Tb927.7.6130
AGOF: null
AGOC: null
AGOP: null
PGOF: null
PGOC: null
PGOP: null

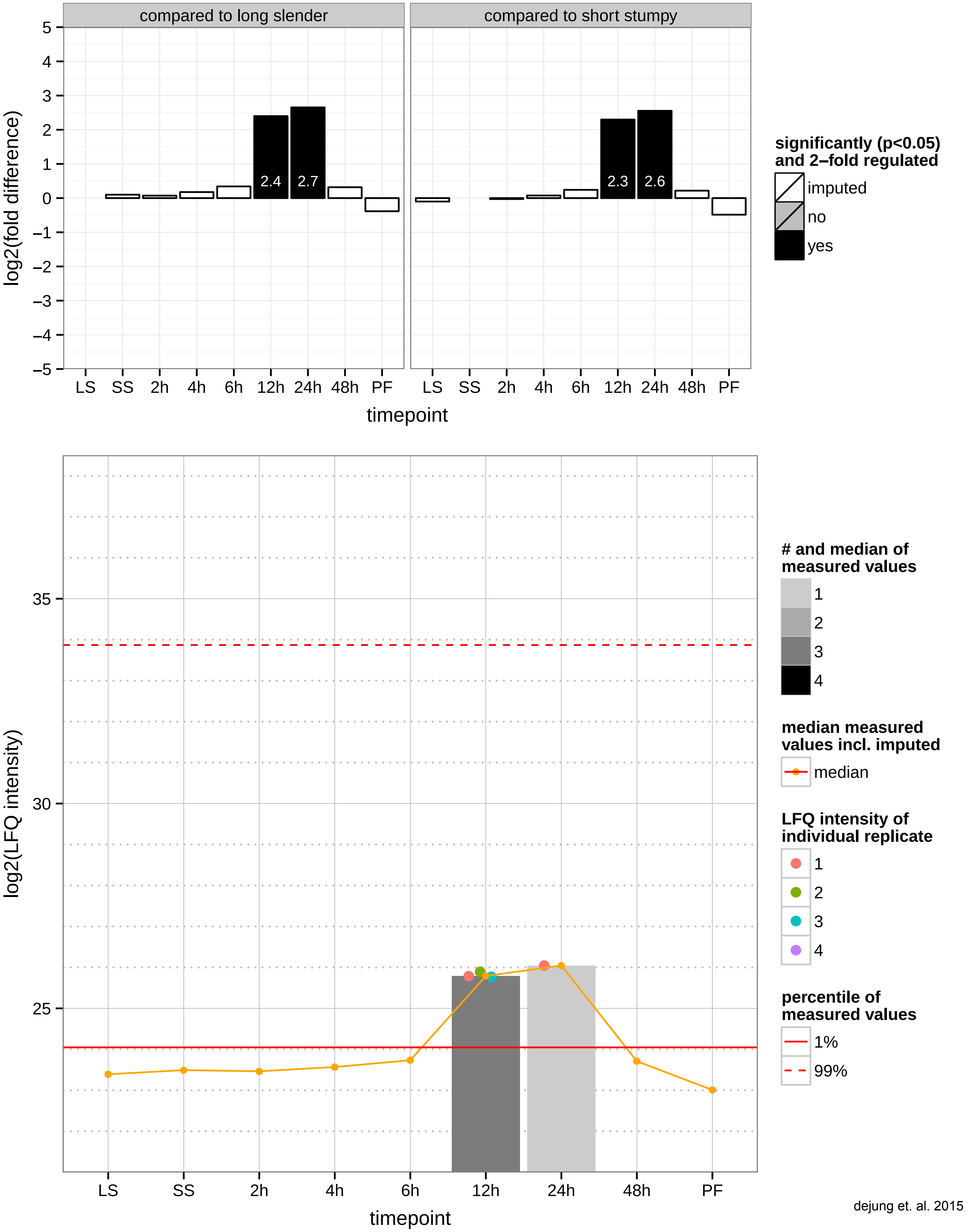

dejung et. al. 2015

hypothetical protein, conserved
Tb927.8.1250
AGOF: null
AGOC: null
AGOP: null
PGOF: null
PGOC: null
PGOP: null

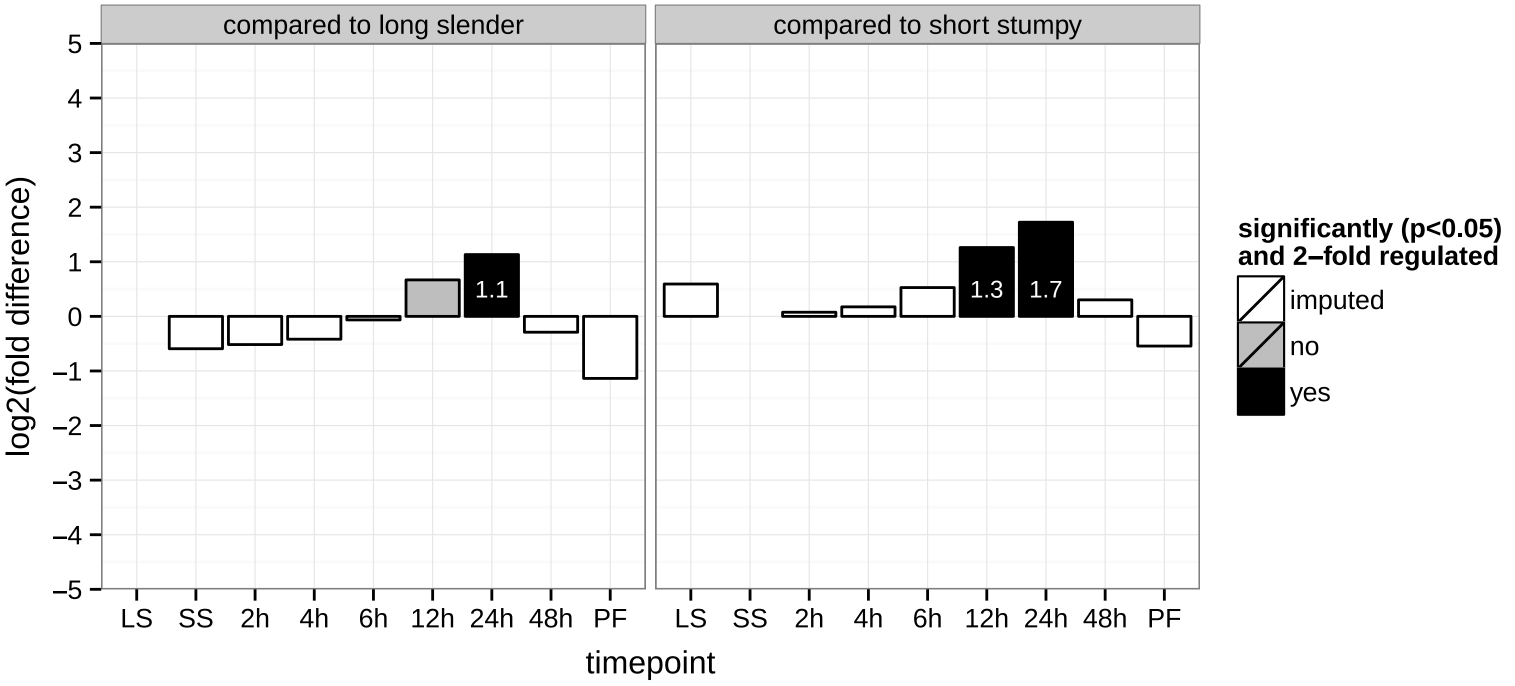

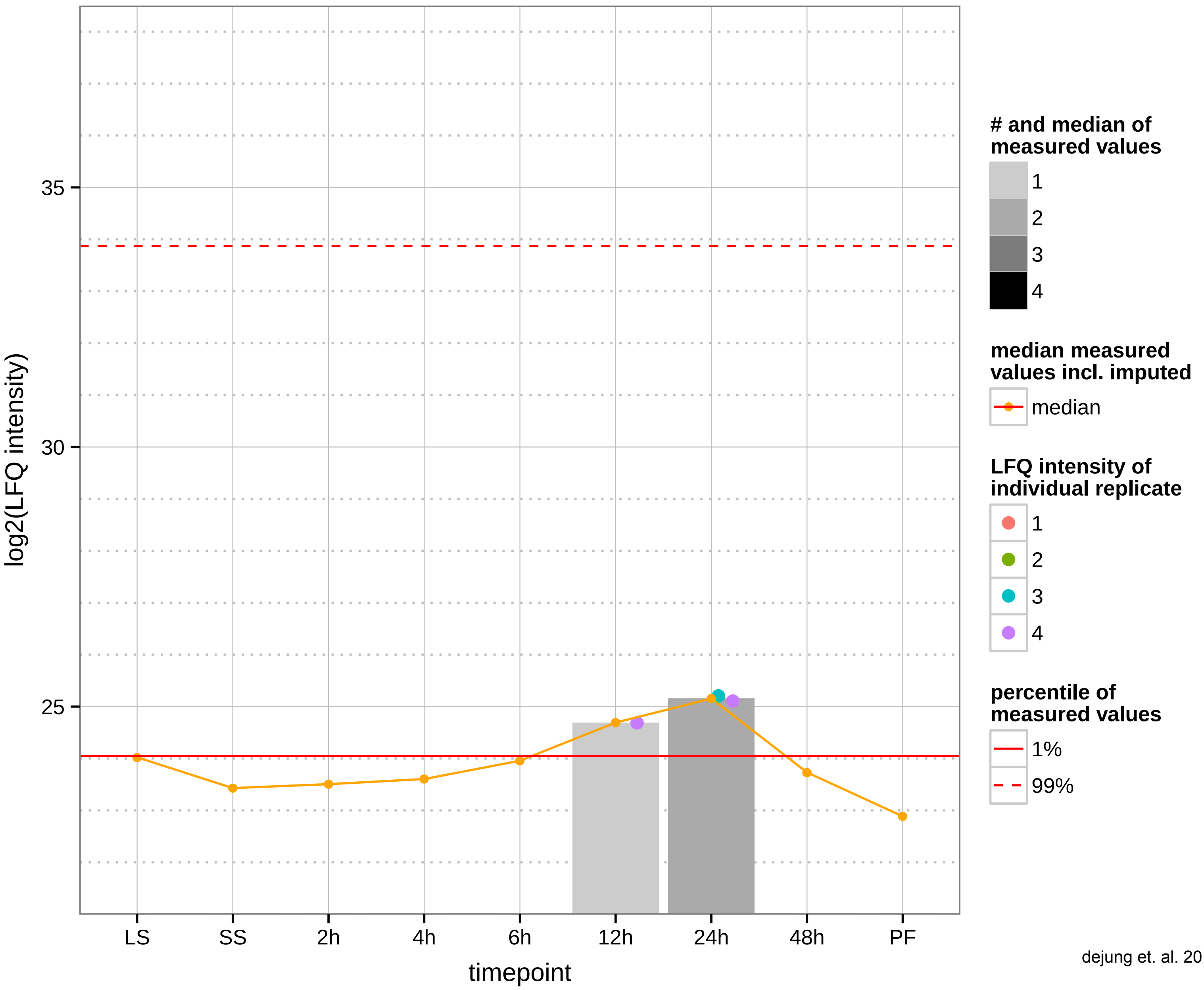

dejung et. al. 2015

metal–ion transporter, putative
Tb927.8.7460;Tb927.4.4960;Tb11.v5.0836
AGOF: null, cation transmembrane transporter activity, metal ion transmembrane transporter activity
AGOC: null, membrane, acidocalcisome, integral to membrane, membrane
AGOP: null, cation transport, response to metal ion
PGOF: cation transmembrane transporter activity
PGOC: integral to membrane
PGOP: cation transport, transmembrane transport

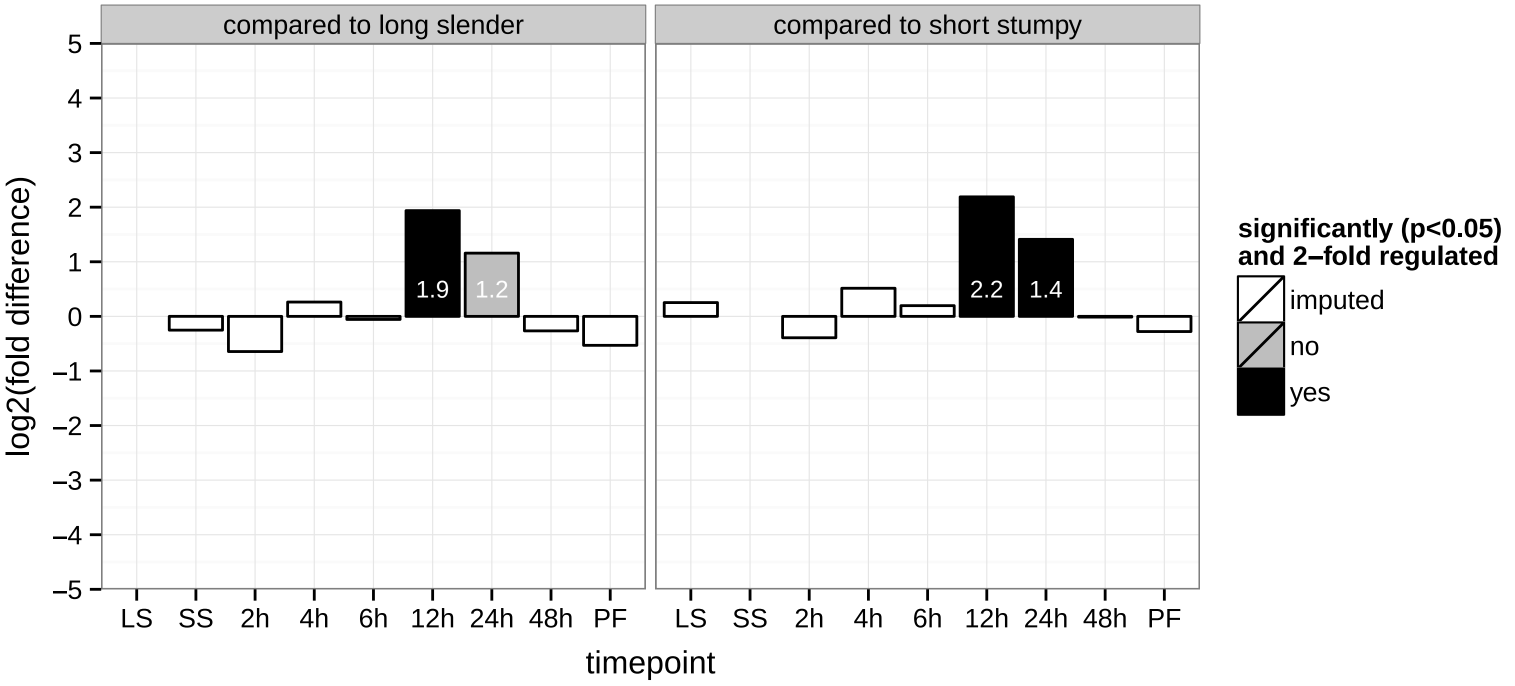

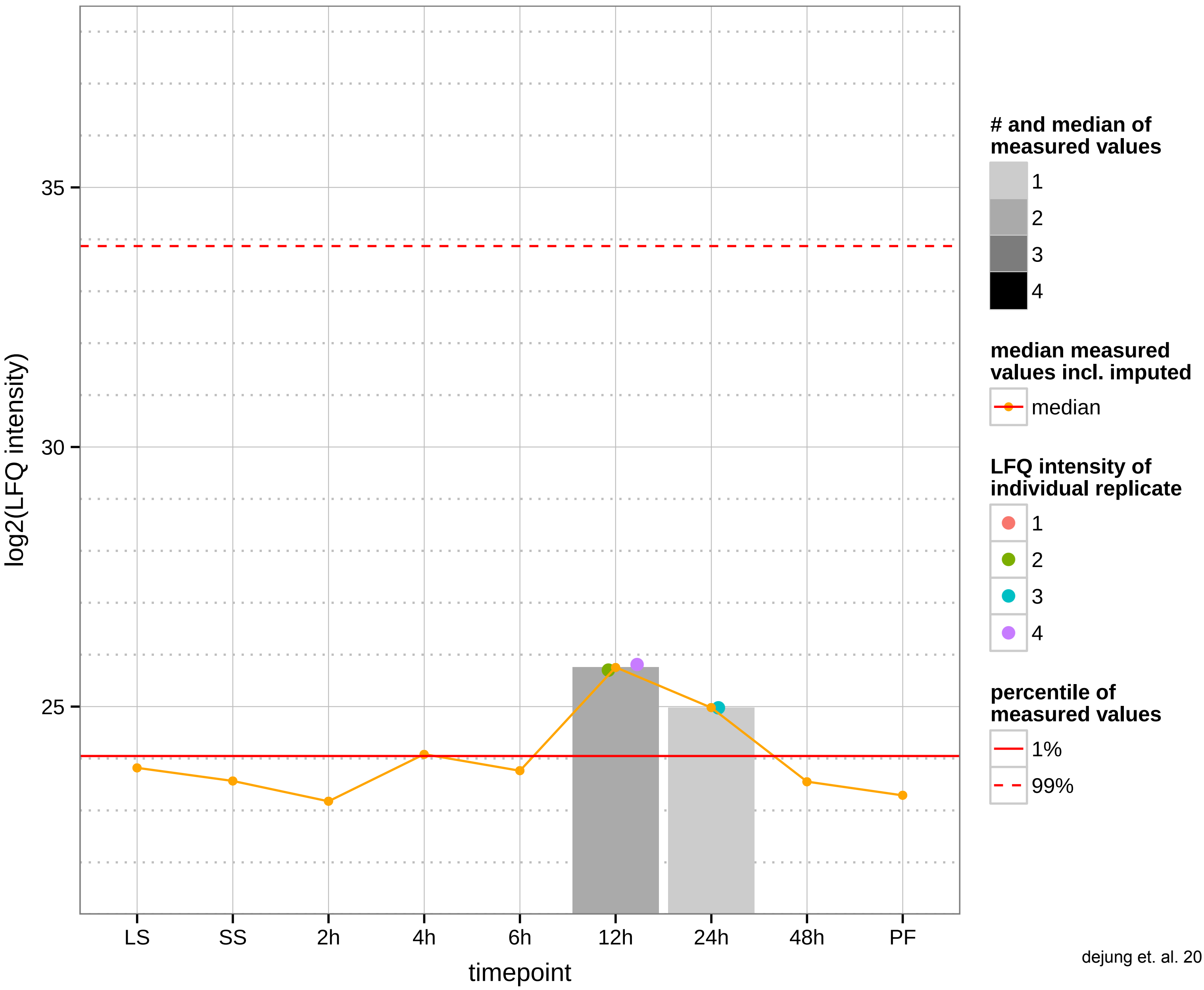

dejung et. al. 2015

hypothetical protein, conserved
Tb927.9.12330
AGOF: null
AGOC: null
AGOP: null
PGOF: null
PGOC: null
PGOP: cell redox homeostasis

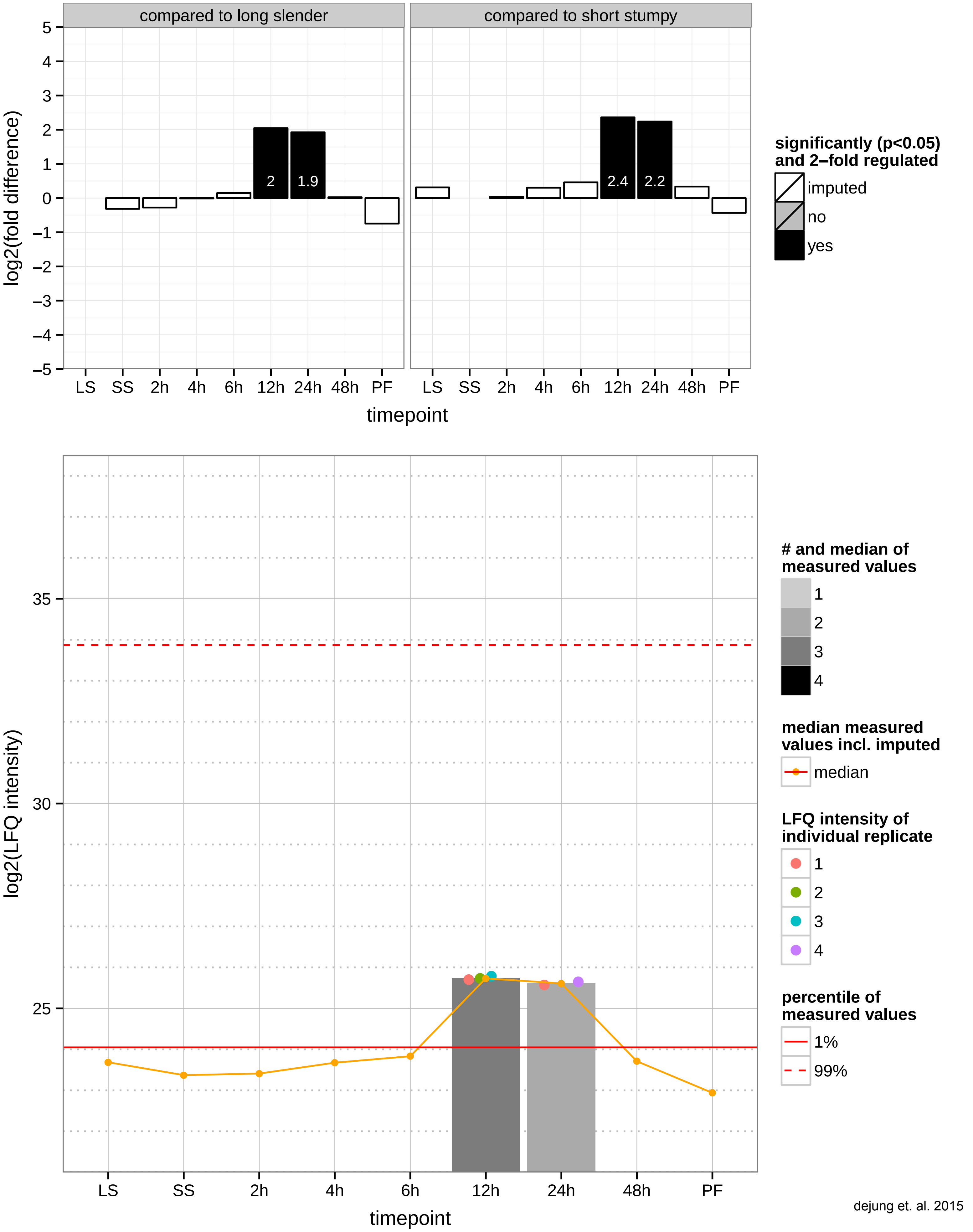

dejung et. al. 2015

hypothetical protein, conserved
Tb927.9.3780
AGOF: null
AGOC: mitochondrion
AGOP: null
PGOF: null
PGOC: null
PGOP: null

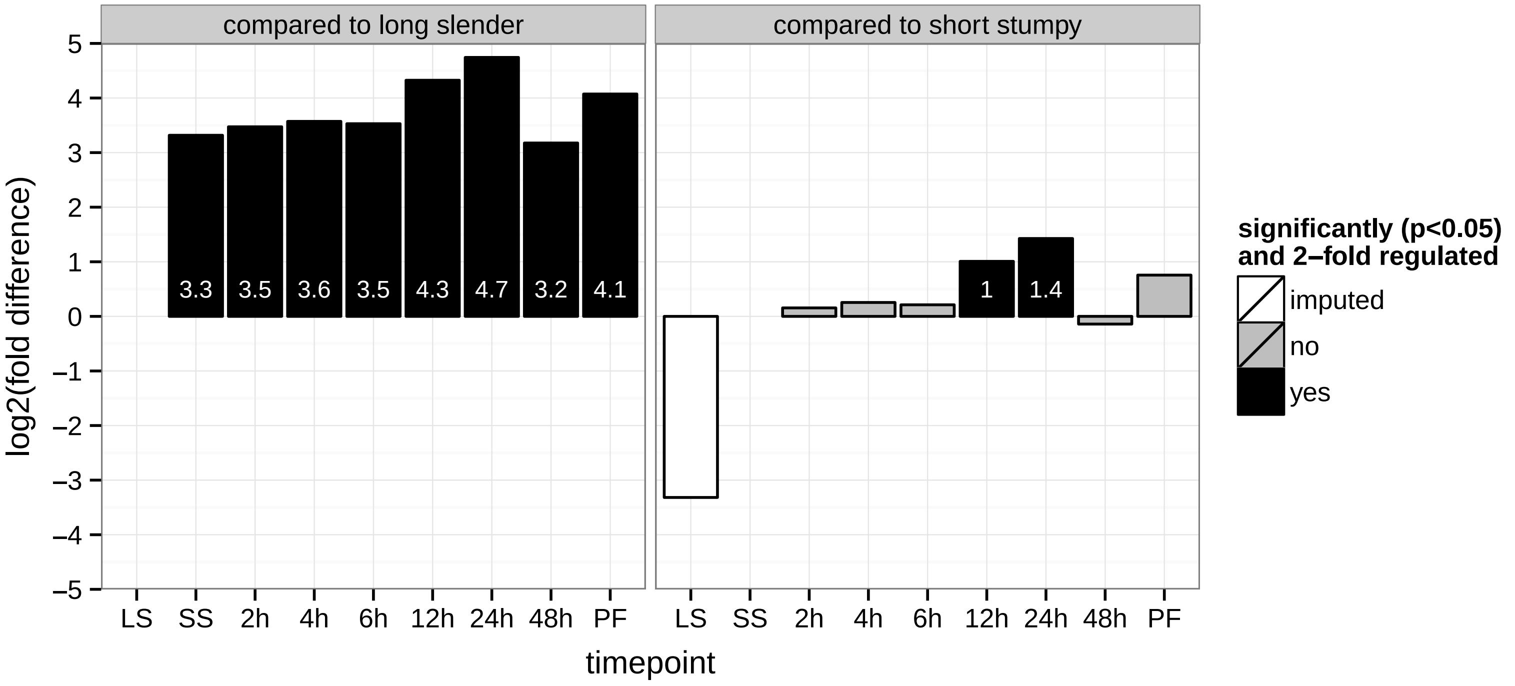

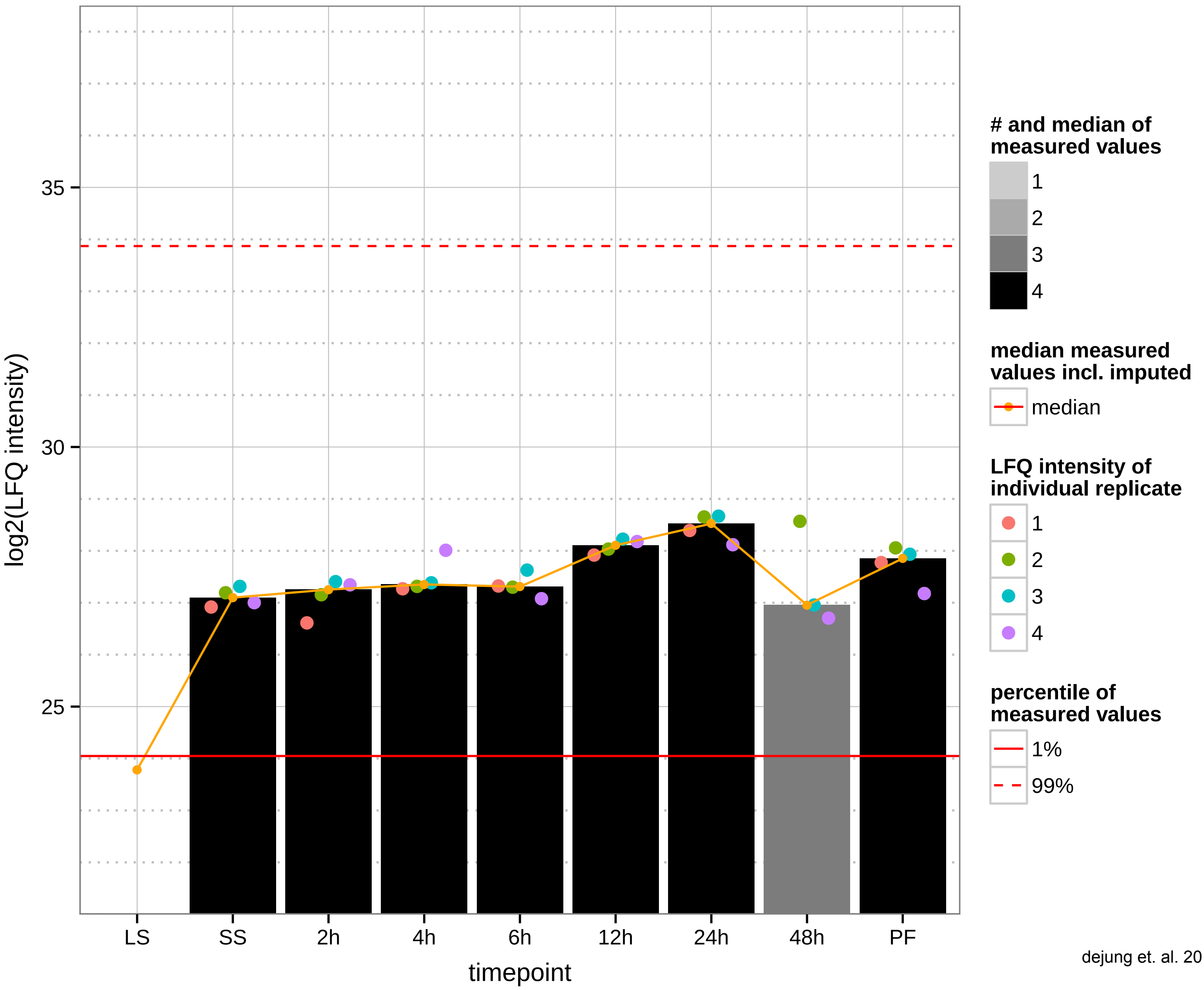

dejung et. al. 2015

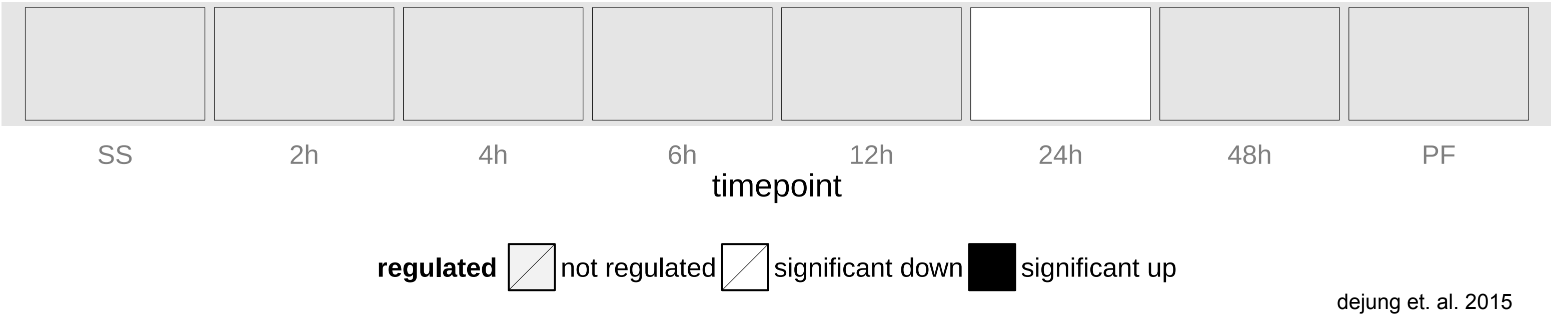
SS
2h
4h
6h
12h
24h
48h
PF
timepoint
regulated
not regulated
significant down
significant up
dejung et. al. 2015

leucine−rich repeat protein (LRRP), putative
Tb927.1.4180
AGOF: null
AGOC: null
AGOP: null
PGOF: null
PGOC: null
PGOP: null

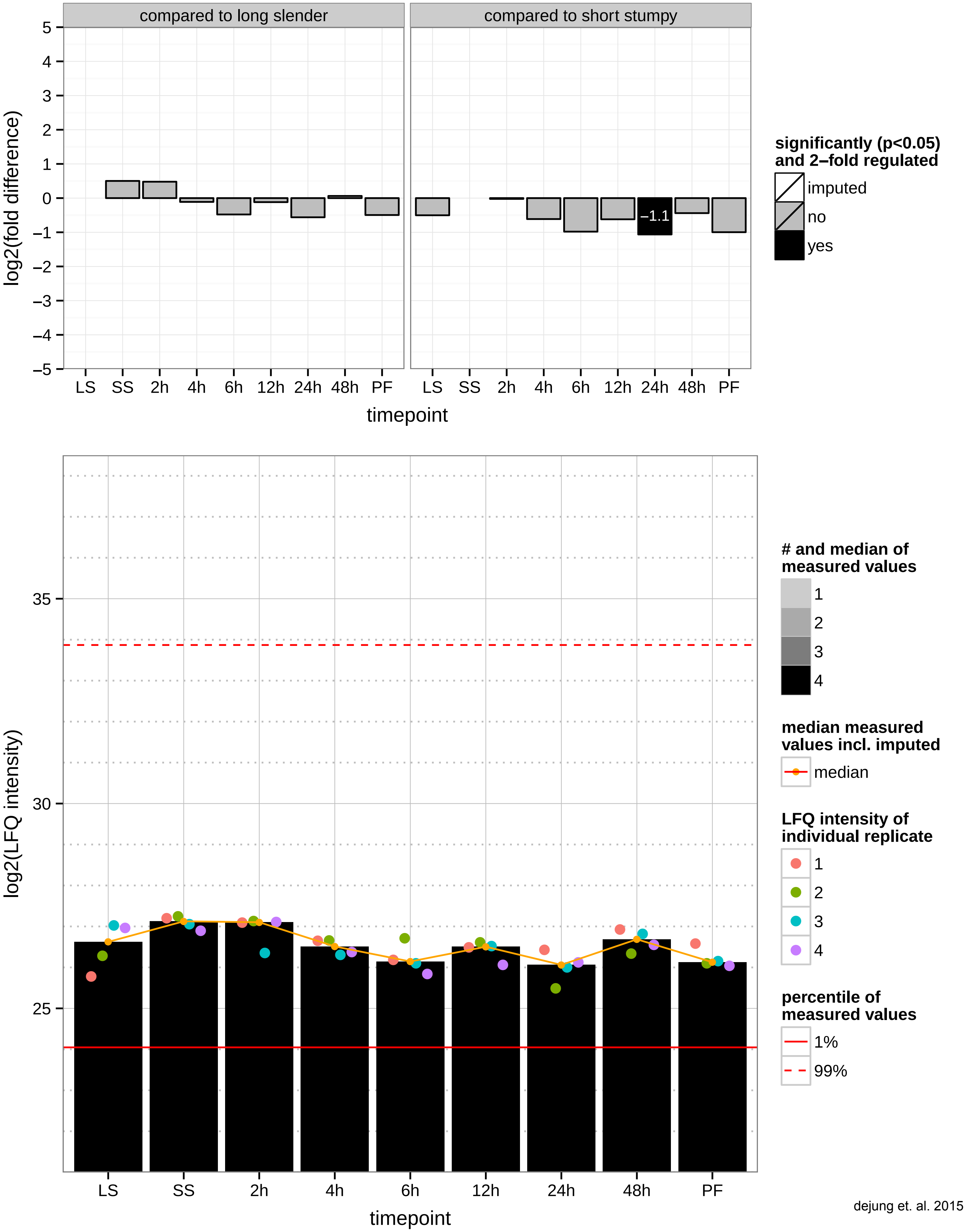

dejung et. al. 2015

GPI transamidase component GAA1 (TbGAA1)
Tb927.10.210
AGOF: GPI–anchor transamidase activity
AGOC: GPI–anchor transamidase complex, anchored to plasma membrane
AGOP: GPI anchor biosynthetic process
PGOF: null
PGOC: GPI–anchor transamidase complex, integral to membrane
PGOP: null

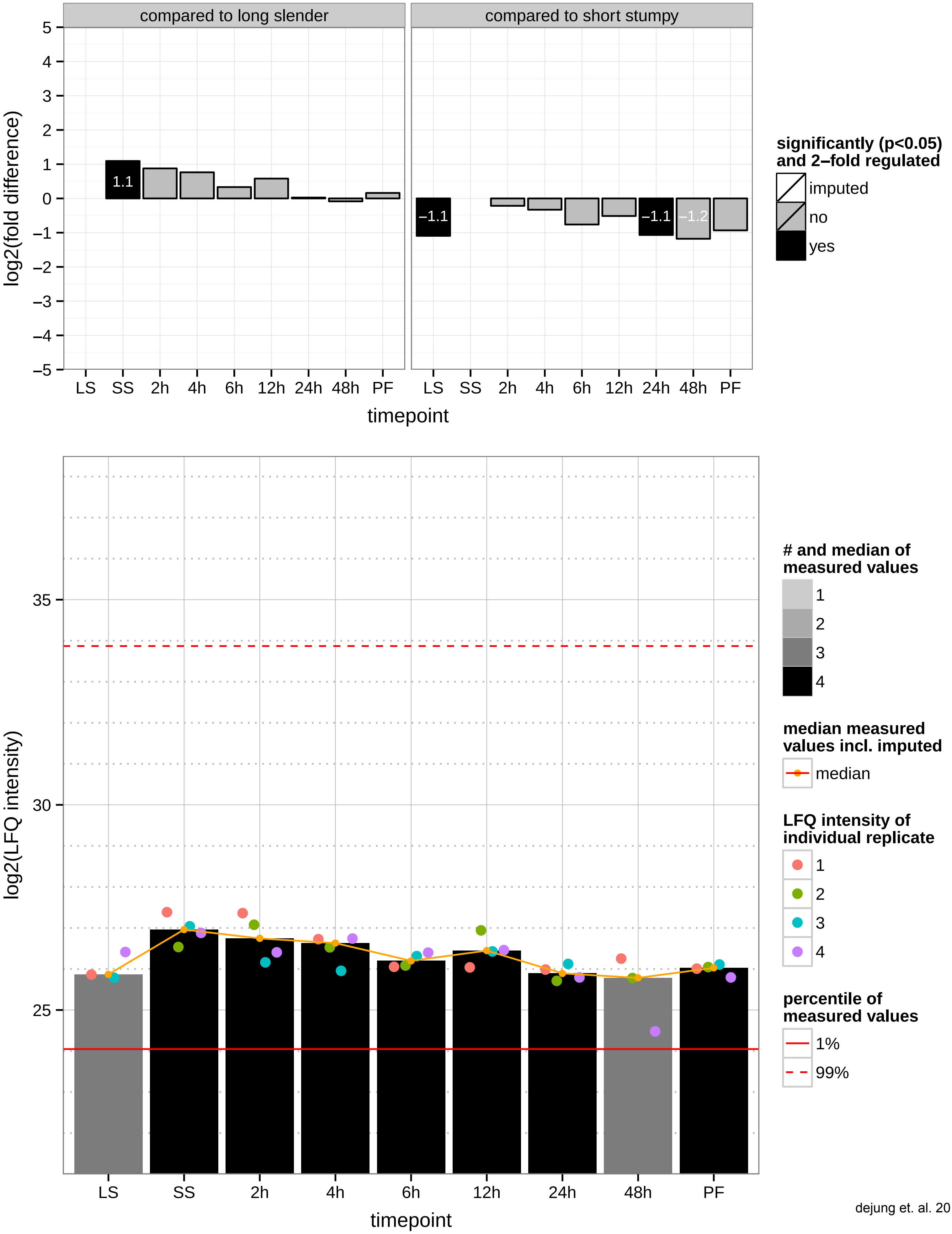

dejung et. al. 2015

hypothetical protein, conserved
Tb927.10.3310
AGOF: null
AGOC: null
AGOP: ciliary or flagellar motility
PGOF: null
PGOC: null
PGOP: null

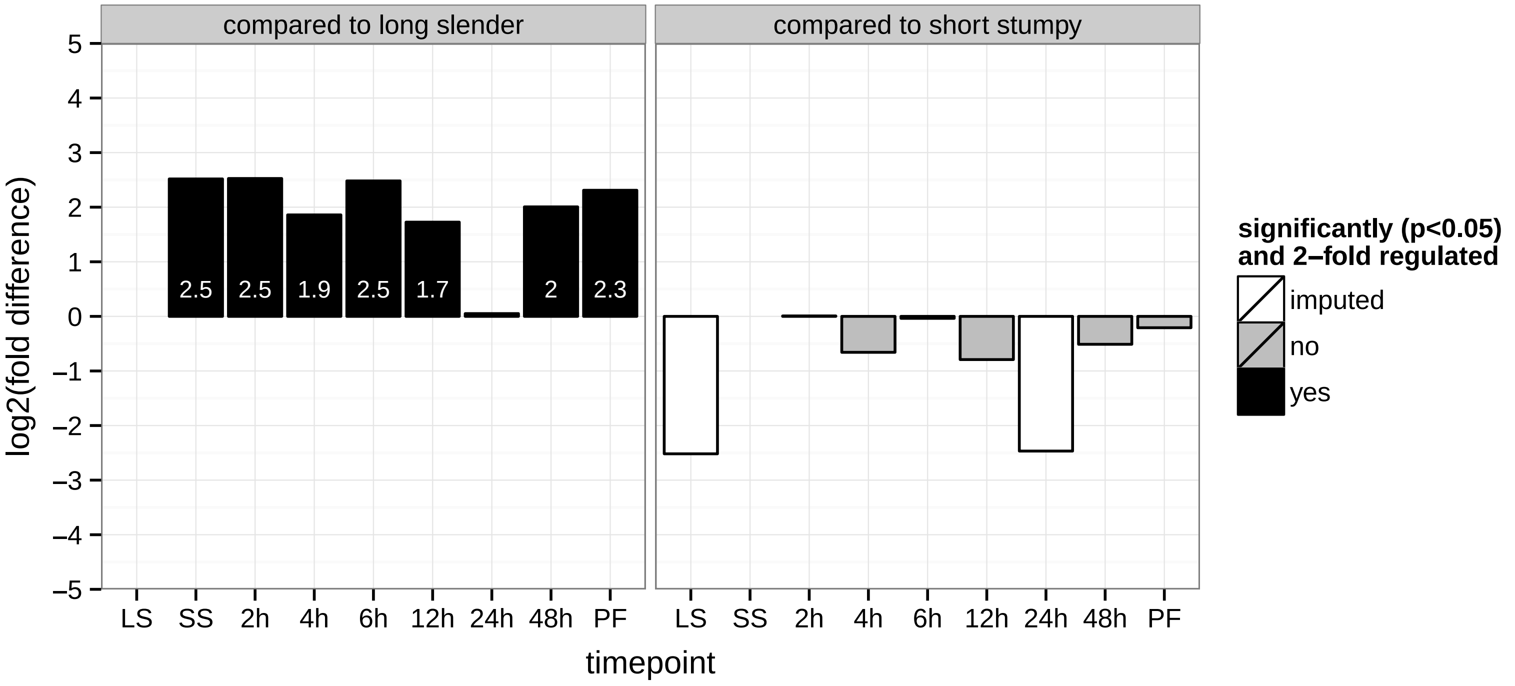

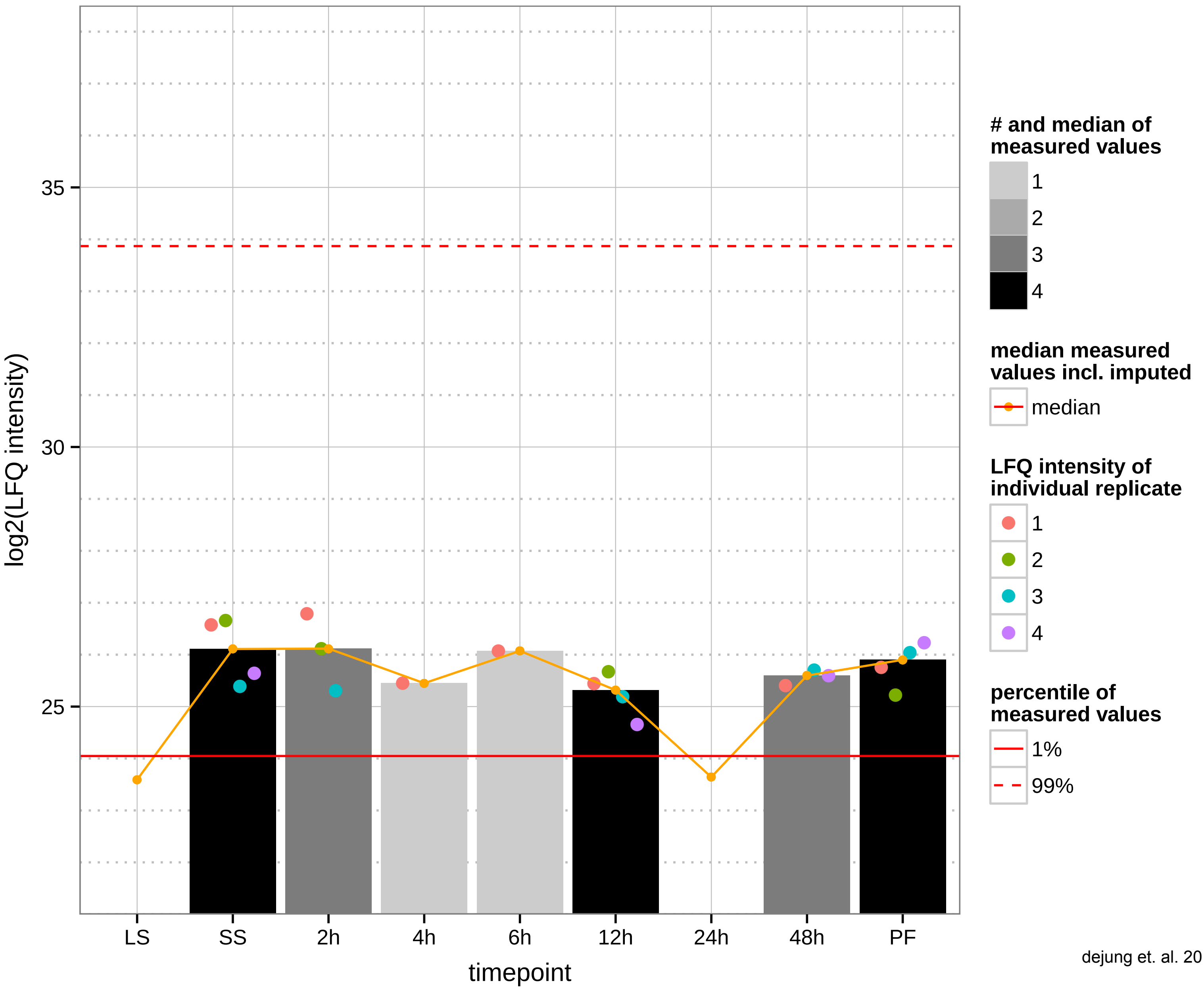

dejung et. al. 2015

paraflagellar rod component, putative (PFC17)
Tb927.11.11210
AGOF: null
AGOC: null
AGOP: null
PGOF: null
PGOC: null
PGOP: null

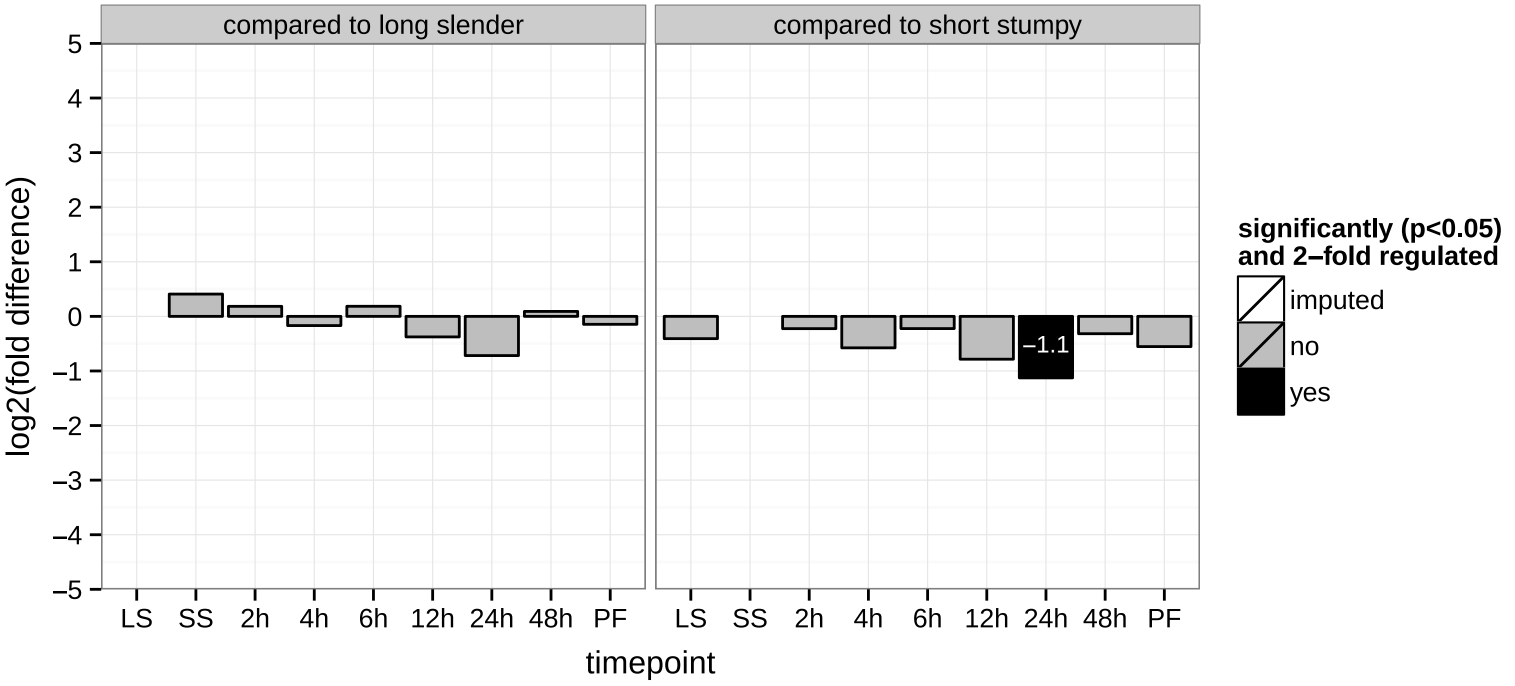

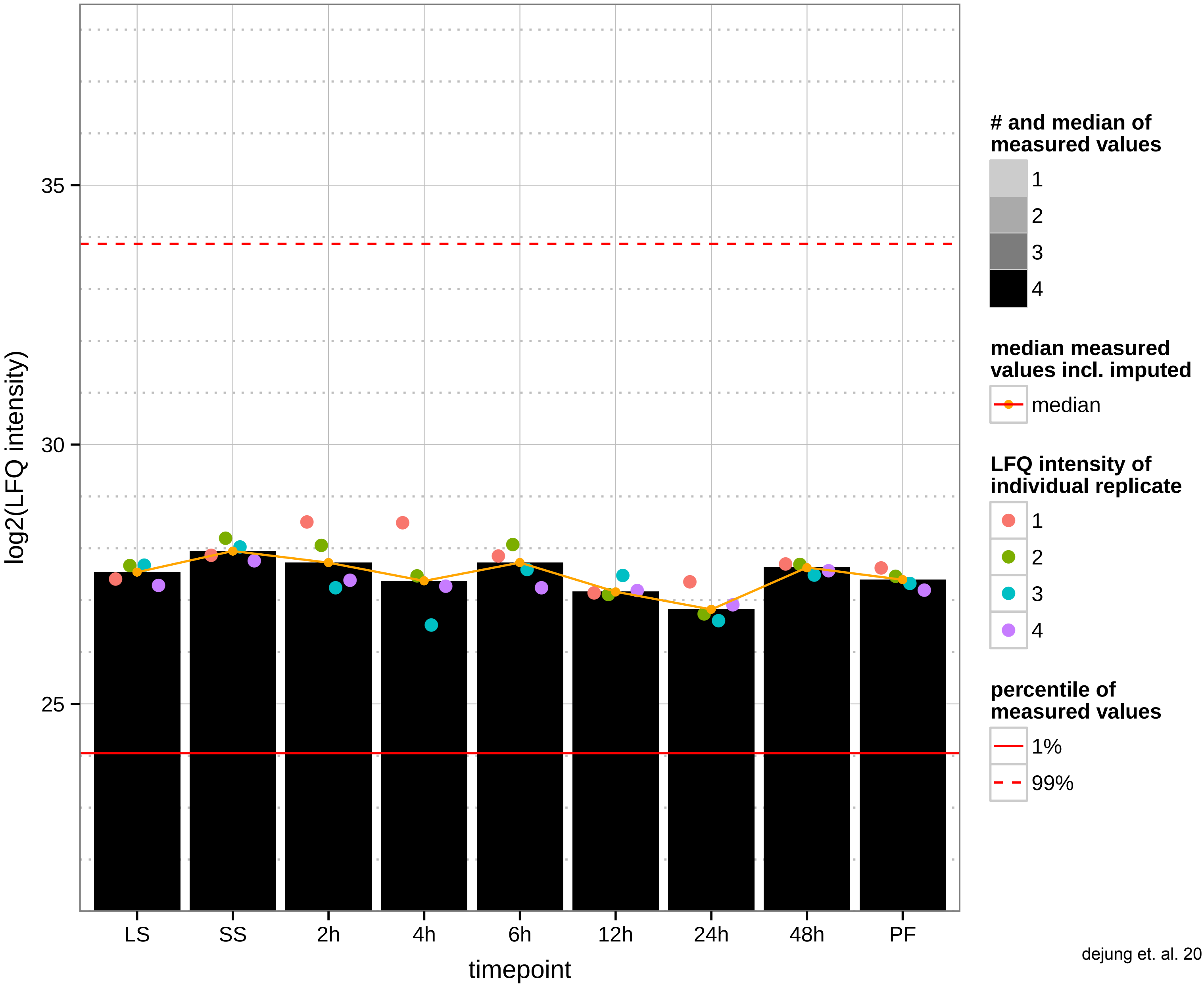

dejung et. al. 2015

hypothetical protein, conserved
Tb927.11.11260
AGOF: null
AGOC: null
AGOP: null
PGOF: null
PGOC: null
PGOP: null

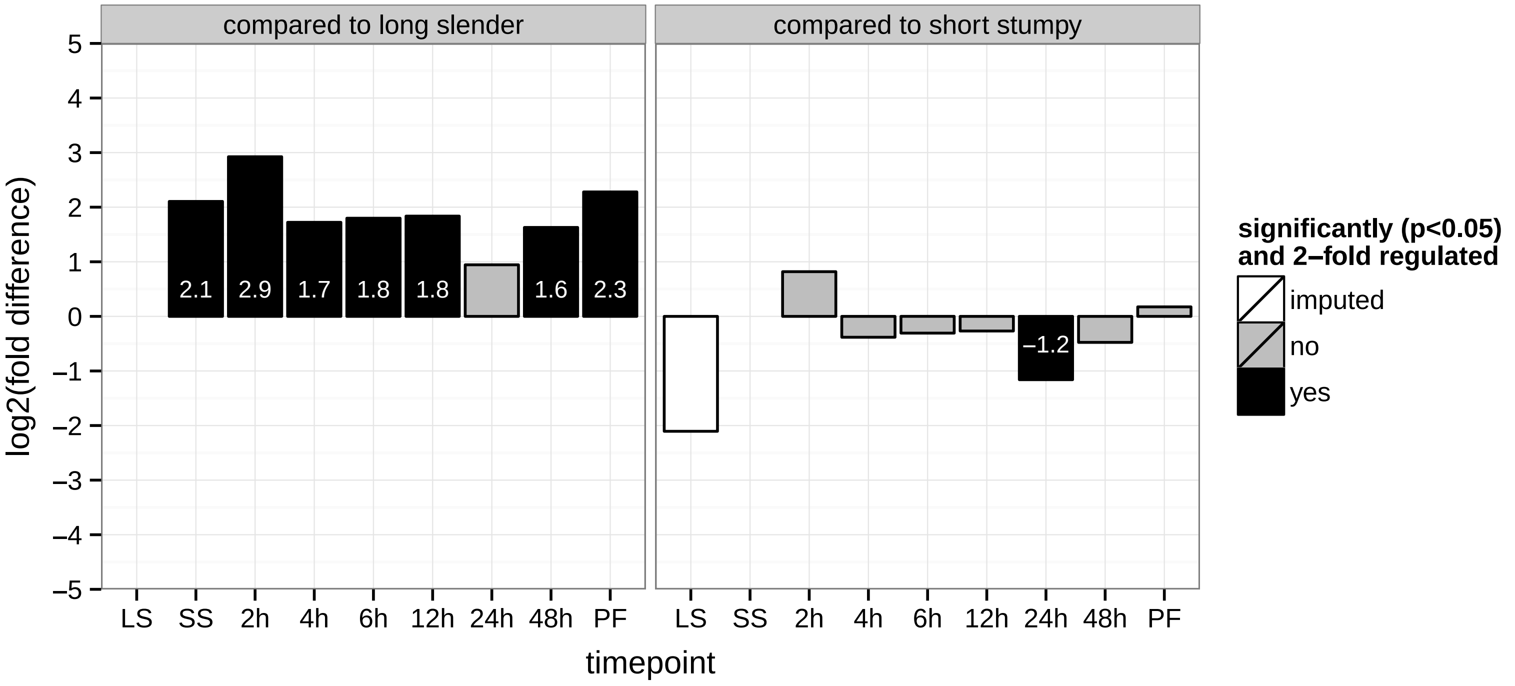

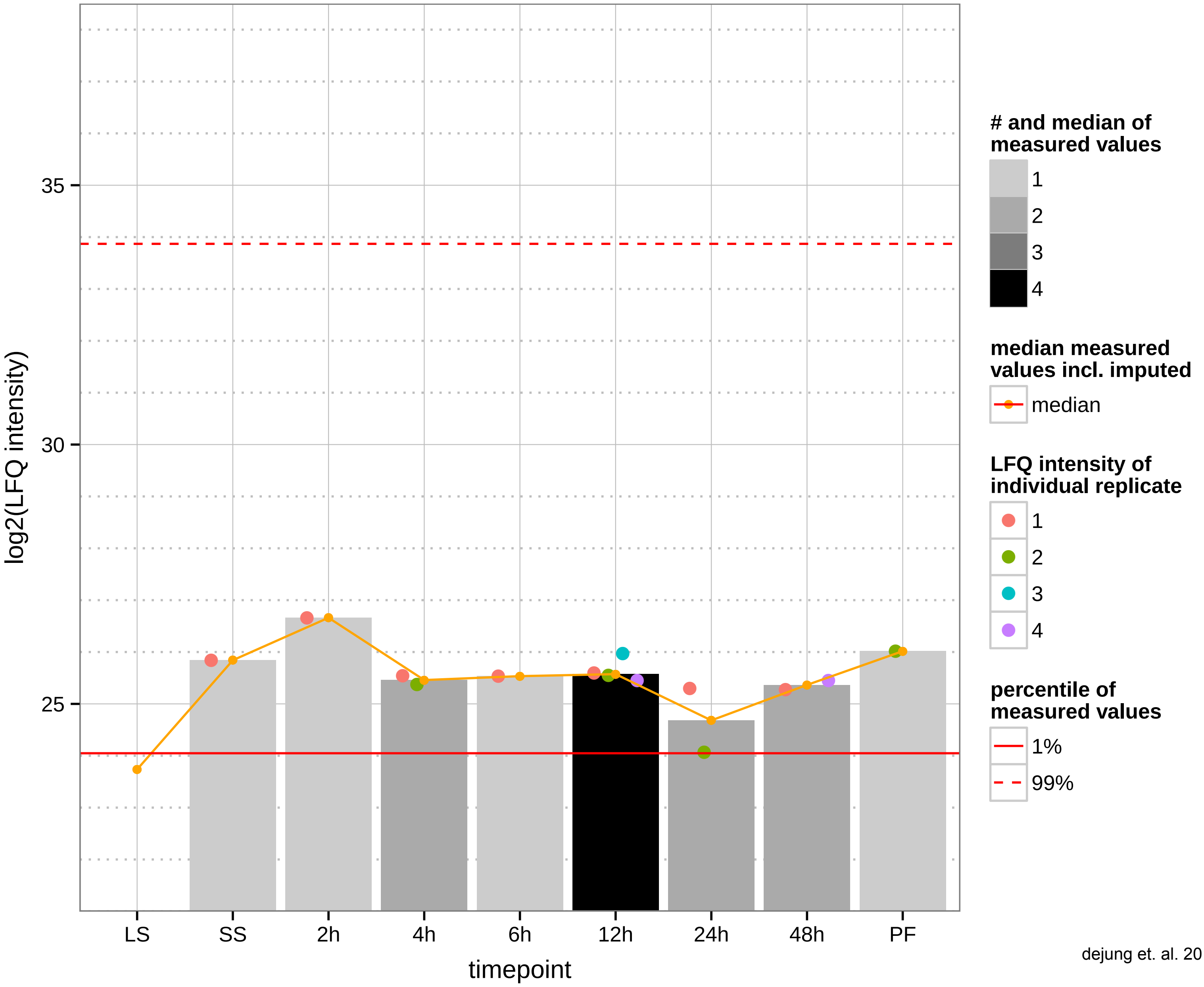

dejung et. al. 2015

hypothetical protein, conserved
Tb927.3.1200
AGOF: null
AGOC: null
AGOP: null
PGOF: null
PGOC: null
PGOP: null

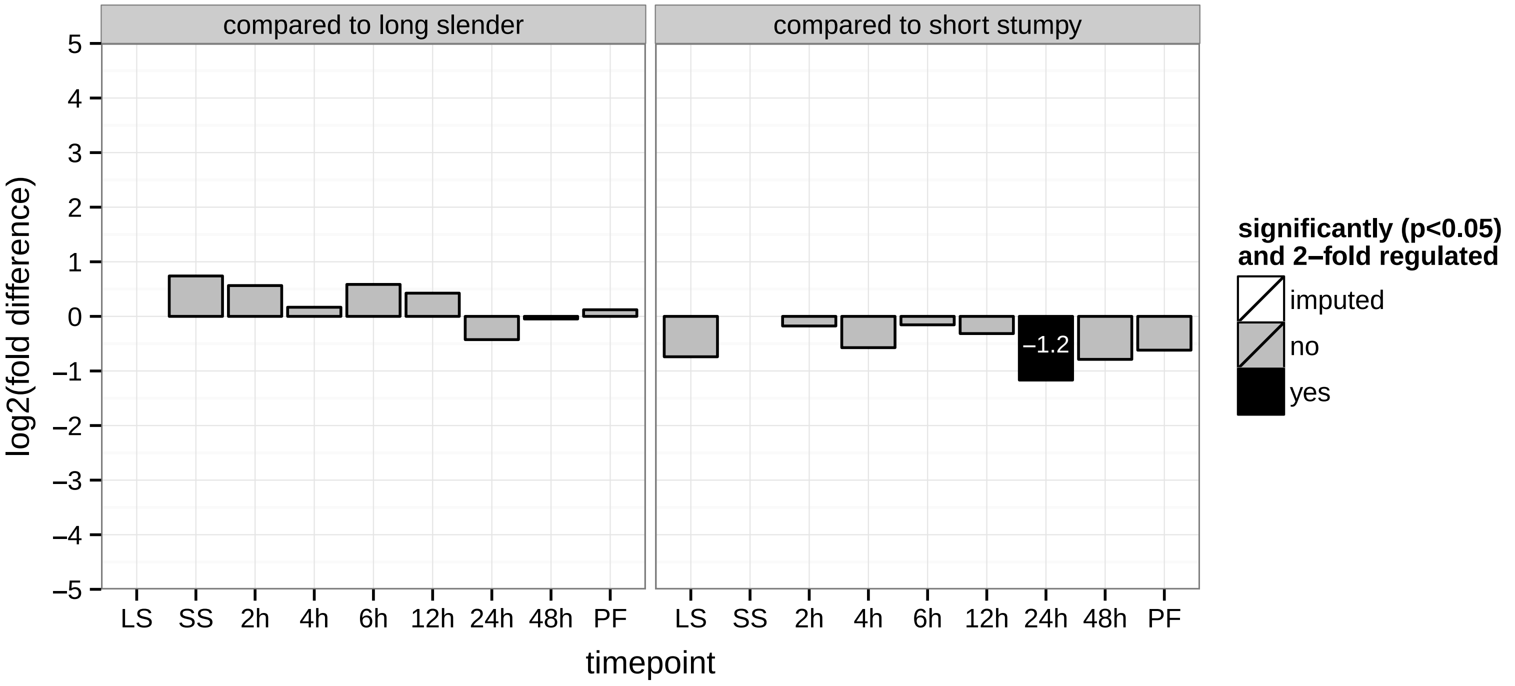

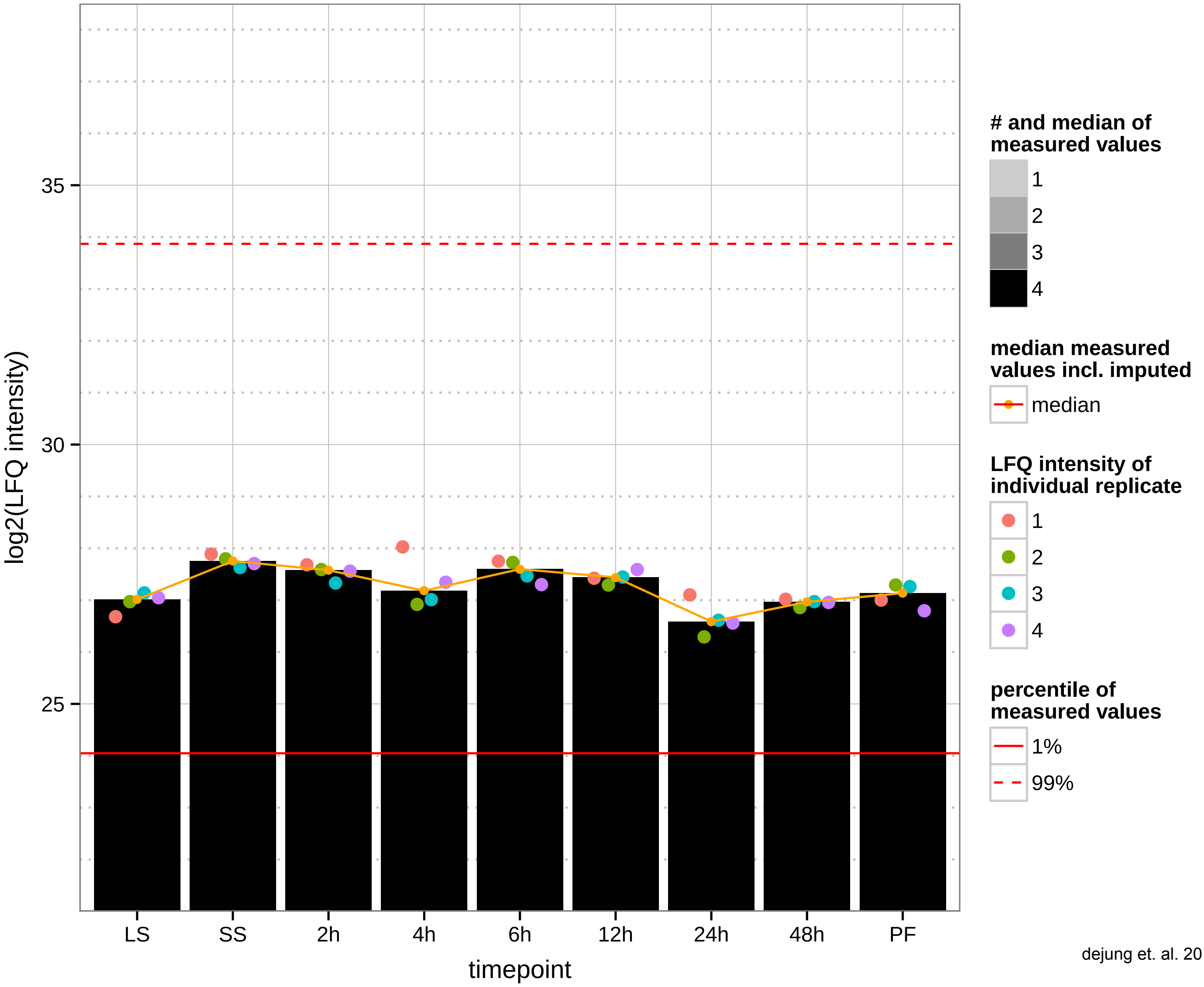

dejung et. al. 2015

hypothetical protein, conserved
Tb927.3.4270
AGOF: null
AGOC: null
AGOP: null
PGOF: null
PGOC: null
PGOP: null

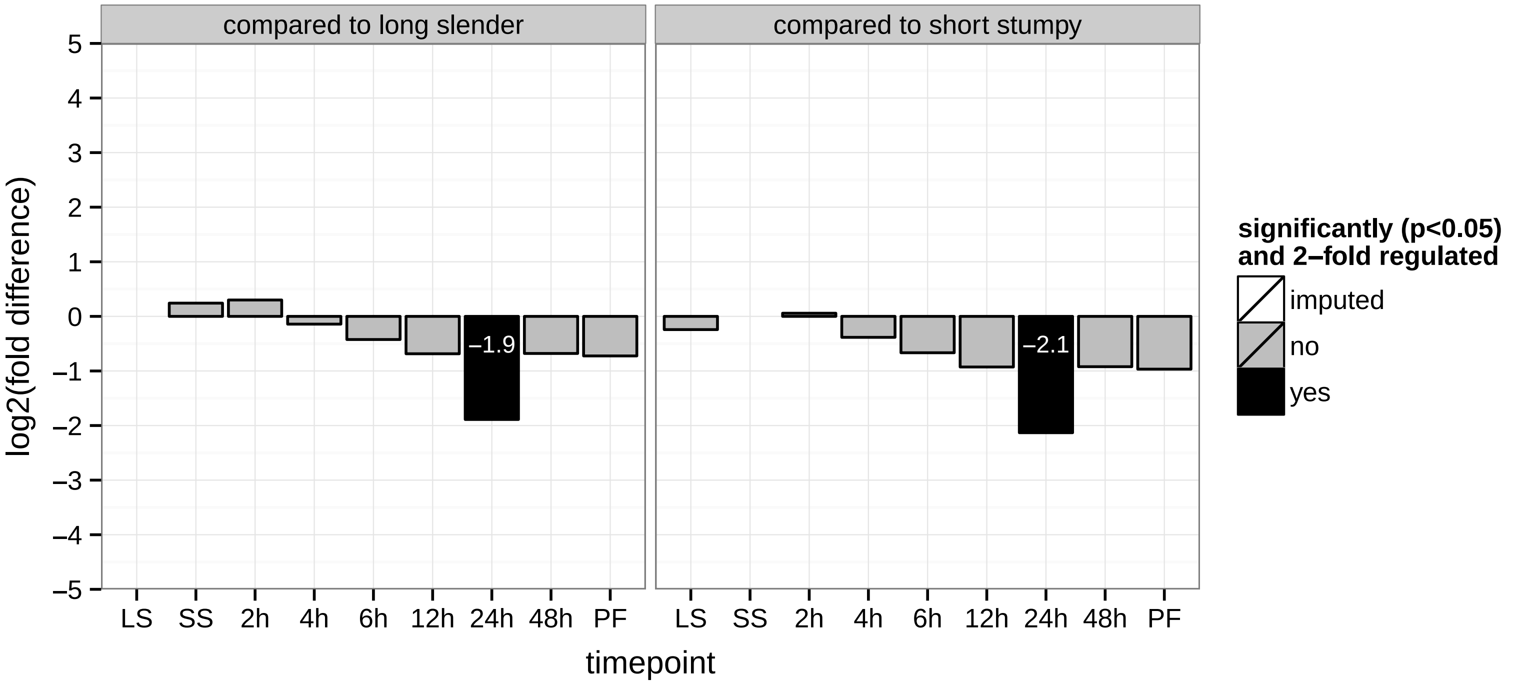

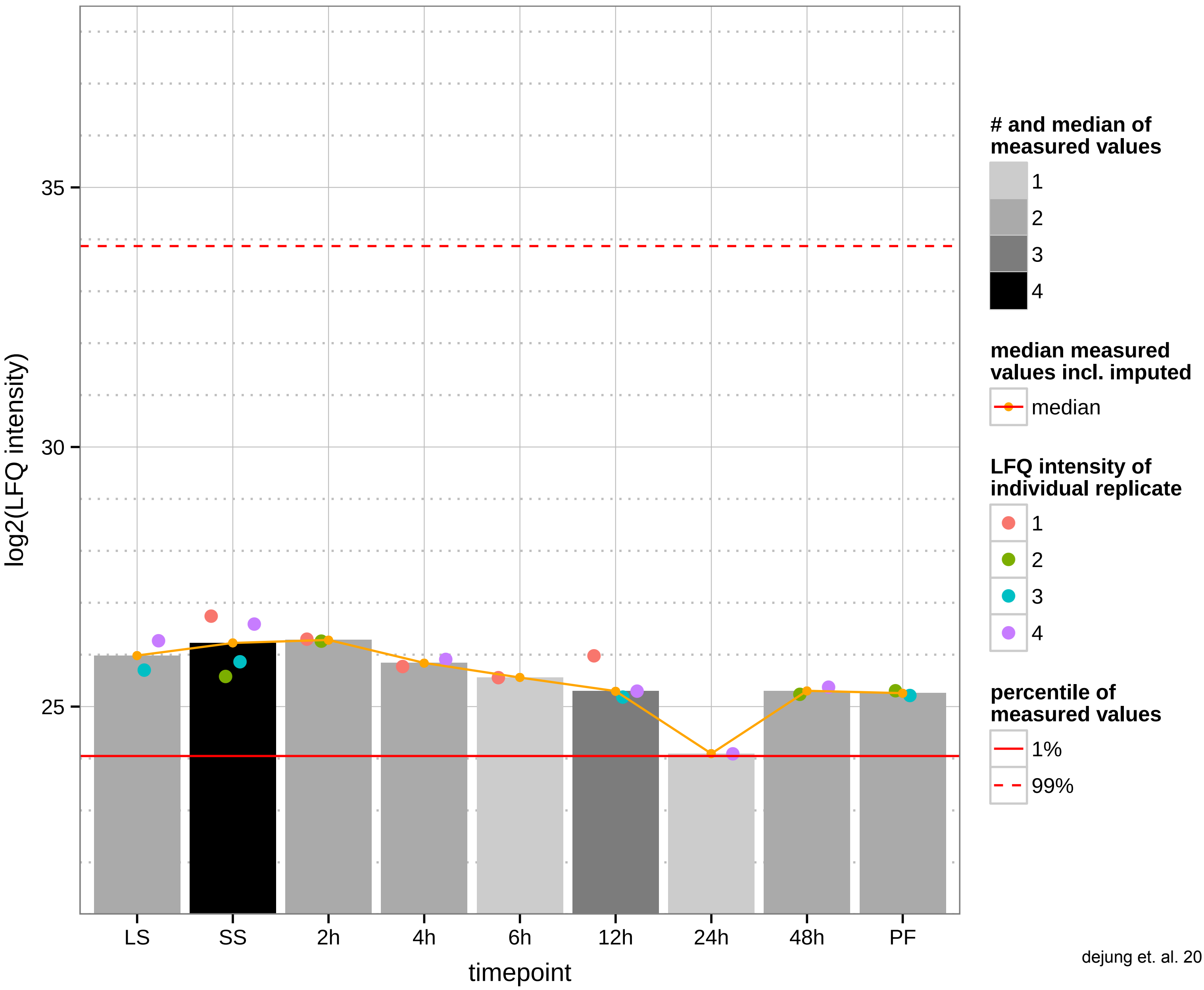

dejung et. al. 2015

kinesin, putative
Tb927.6.2880
AGOF: ATP binding, microtubule motor activity, motor activity
AGOC: microtubule associated complex
AGOP: microtubule–based movement
PGOF: ATP binding, microtubule motor activity
PGOC: null
PGOP: microtubule–based movement

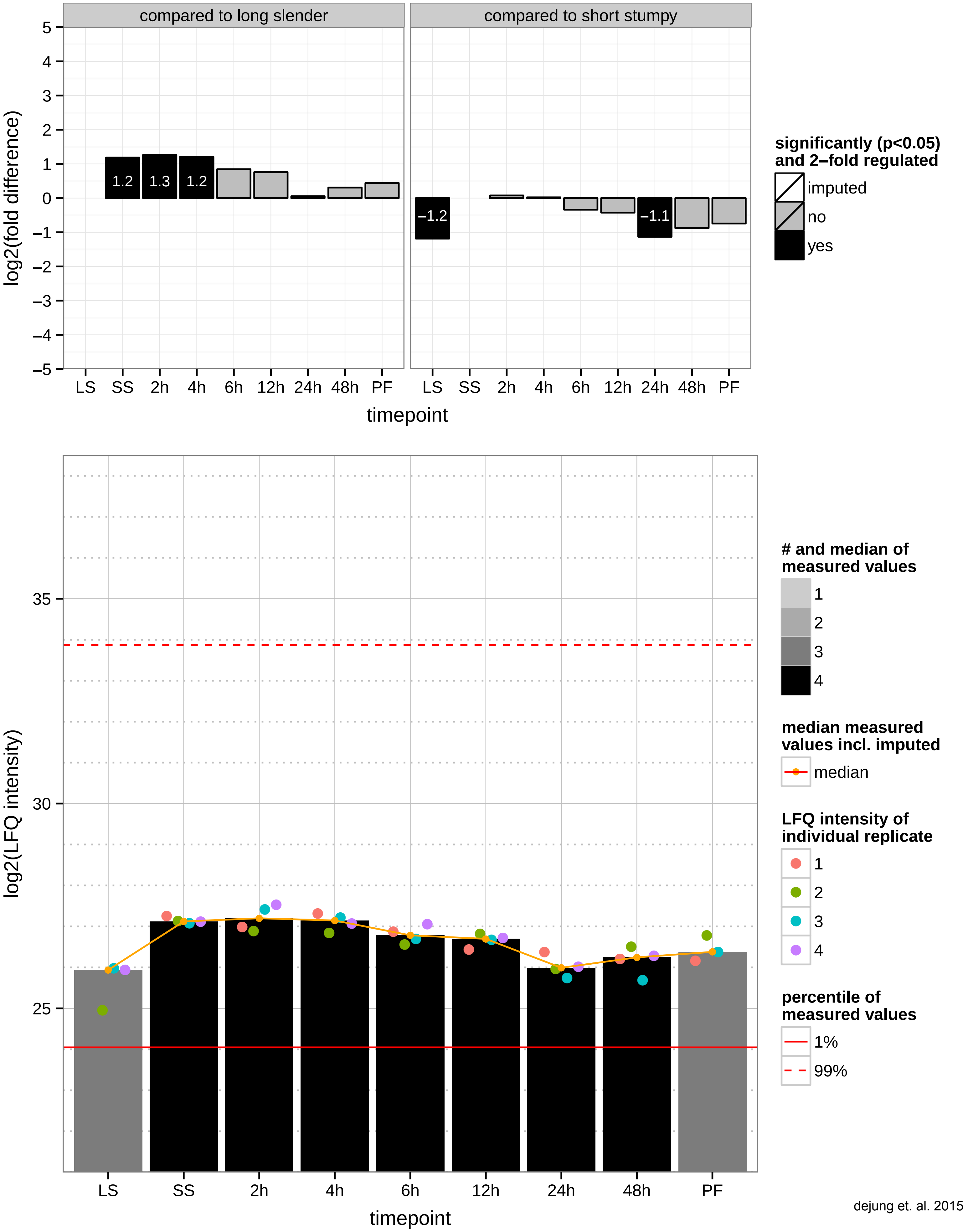

dejung et. al. 2015

RING finger protein, putative
Tb927.6.3780
AGOF: nucleic acid binding, ubiquitin–protein ligase activity, zinc ion binding
AGOC: null
AGOP: histone ubiquitination
PGOF: protein binding, zinc ion binding
PGOC: null
PGOP: null

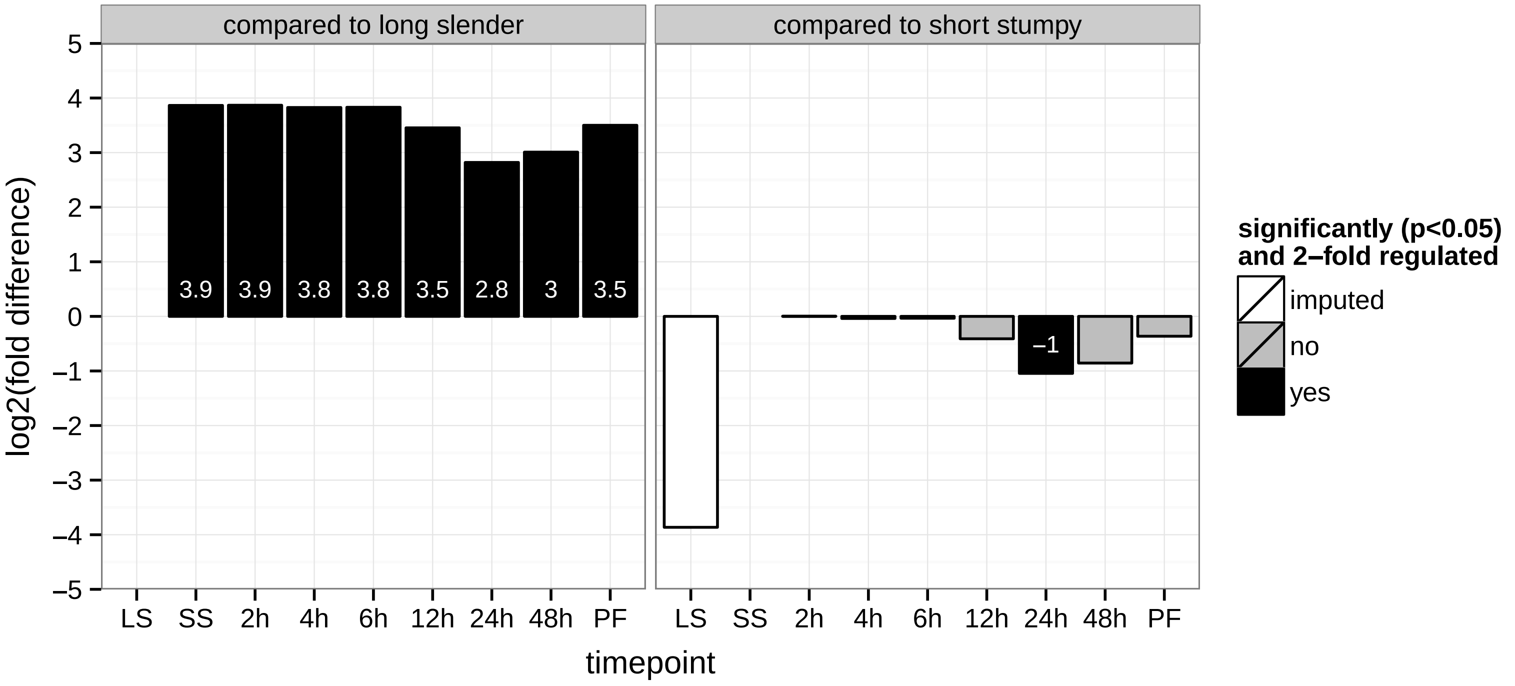

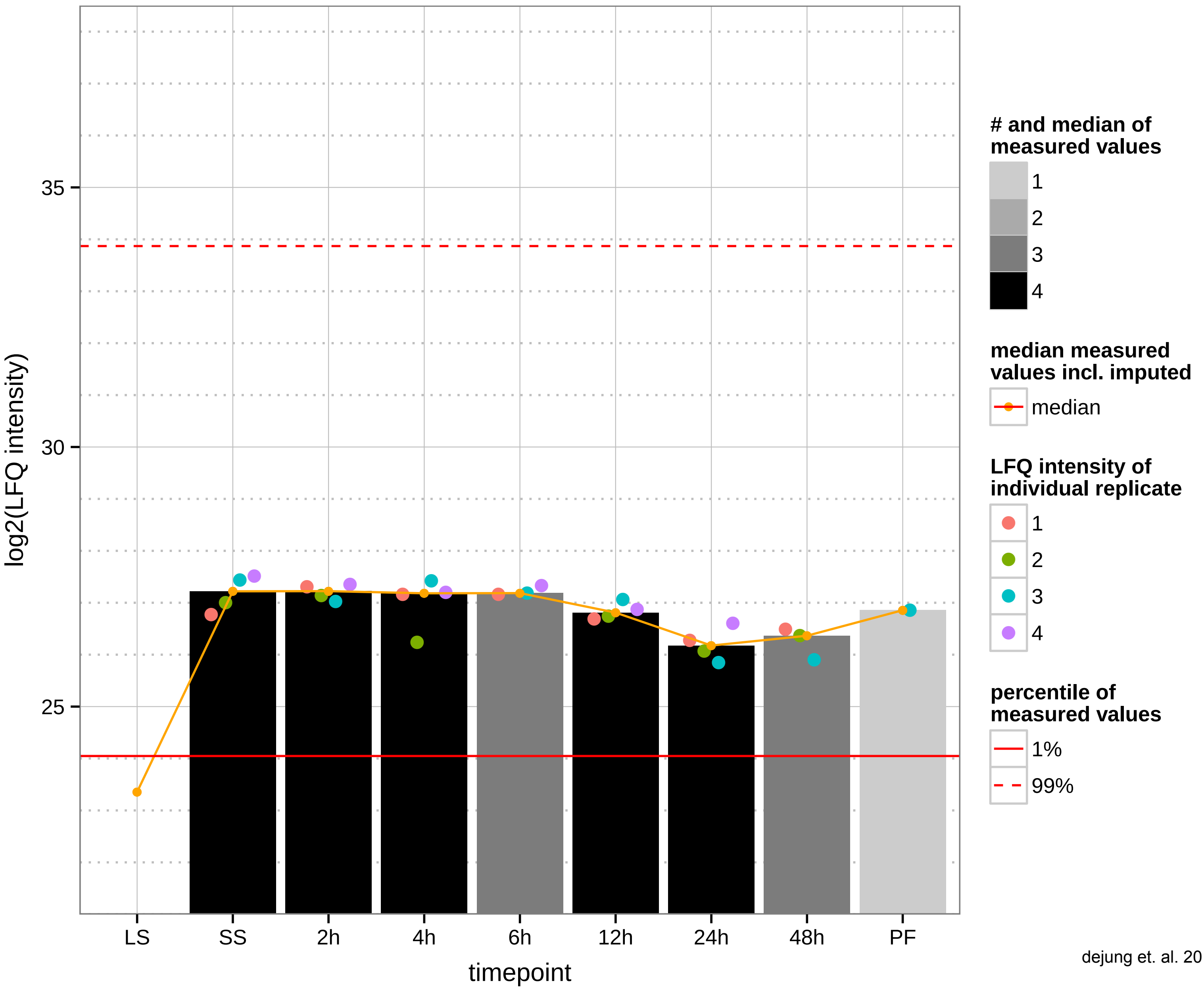

dejung et. al. 2015

hypothetical protein, conserved
Tb927.6.840
AGOF: null
AGOC: null
AGOP: null
PGOF: protein binding
PGOC: null
PGOP: null

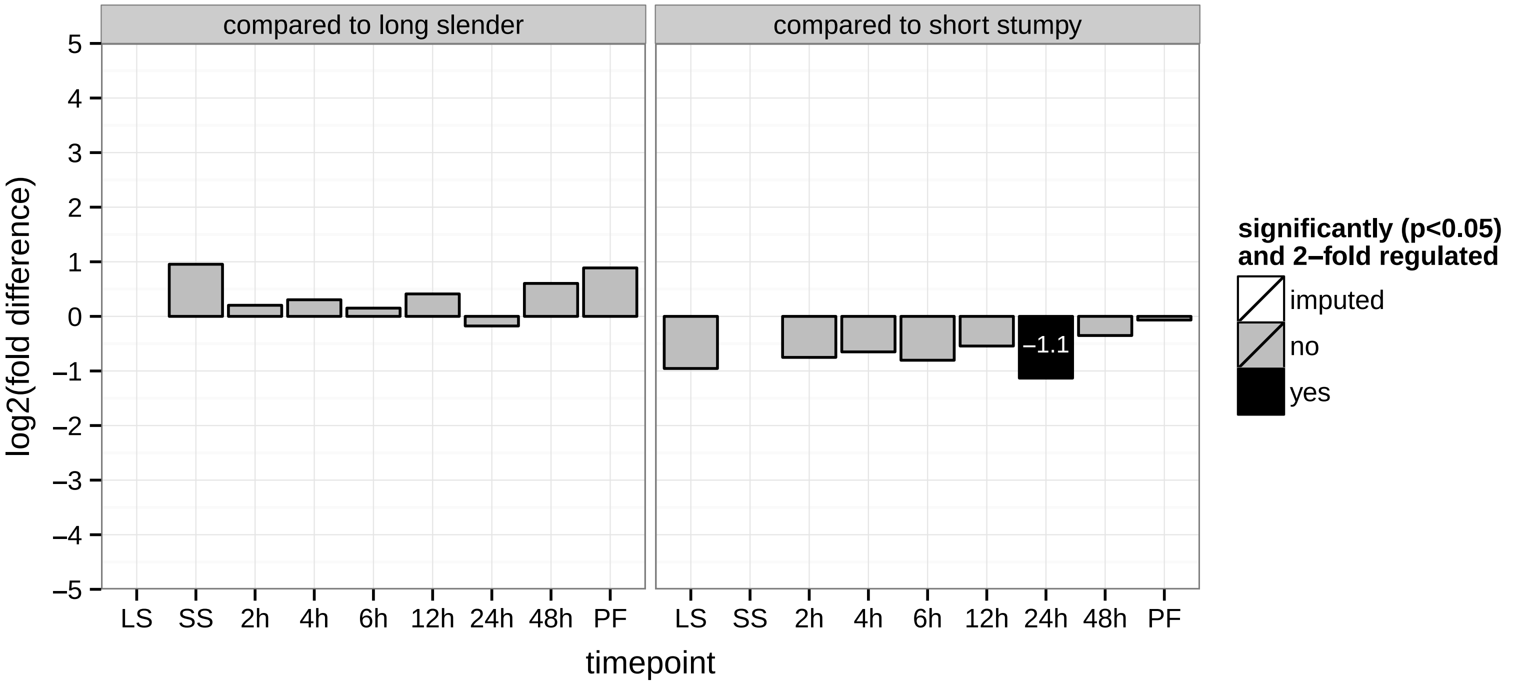

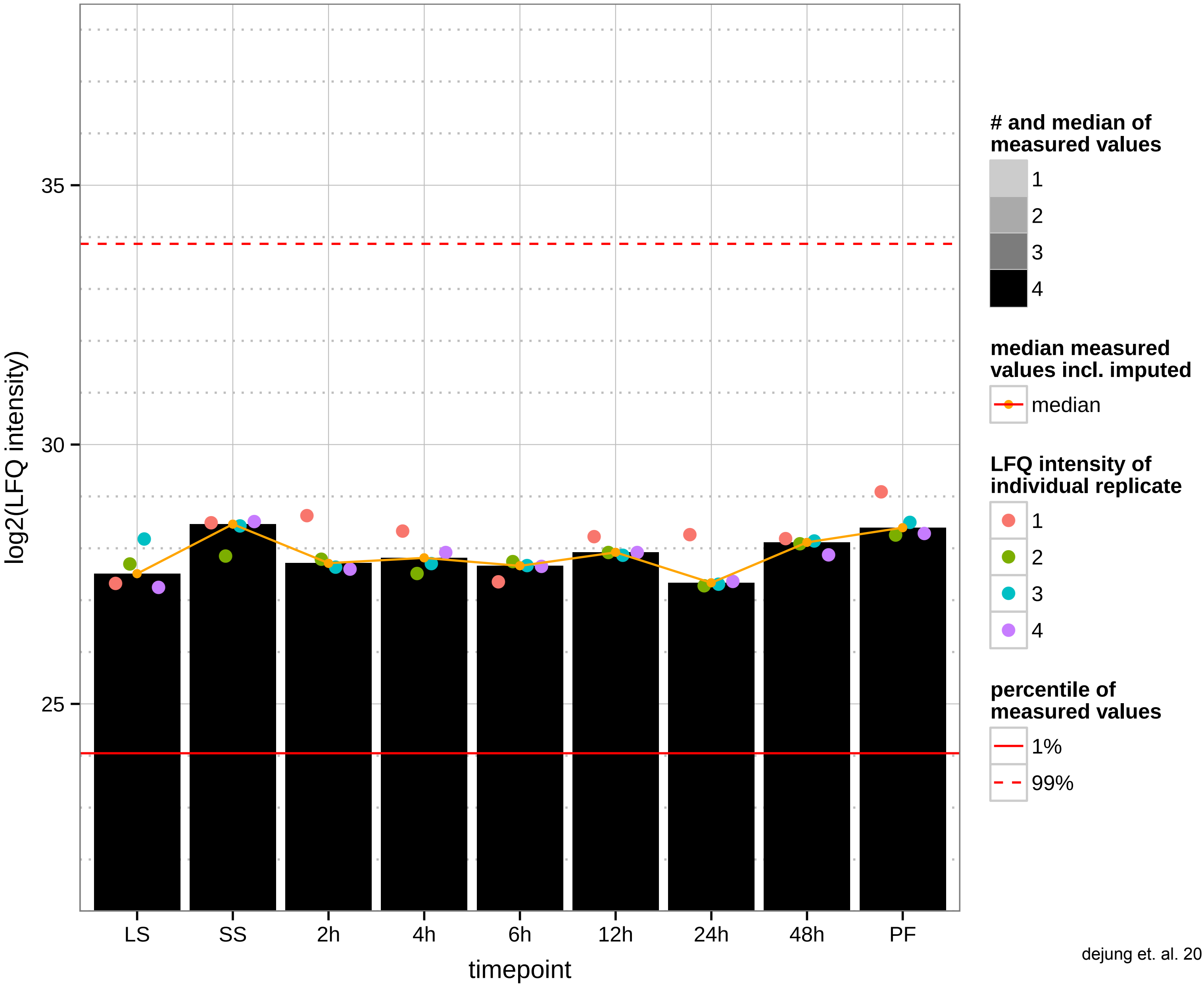

dejung et. al. 2015

hypothetical protein, conserved
Tb927.7.1200
AGOF: null
AGOC: null
AGOP: null
PGOF: null
PGOC: null
PGOP: null

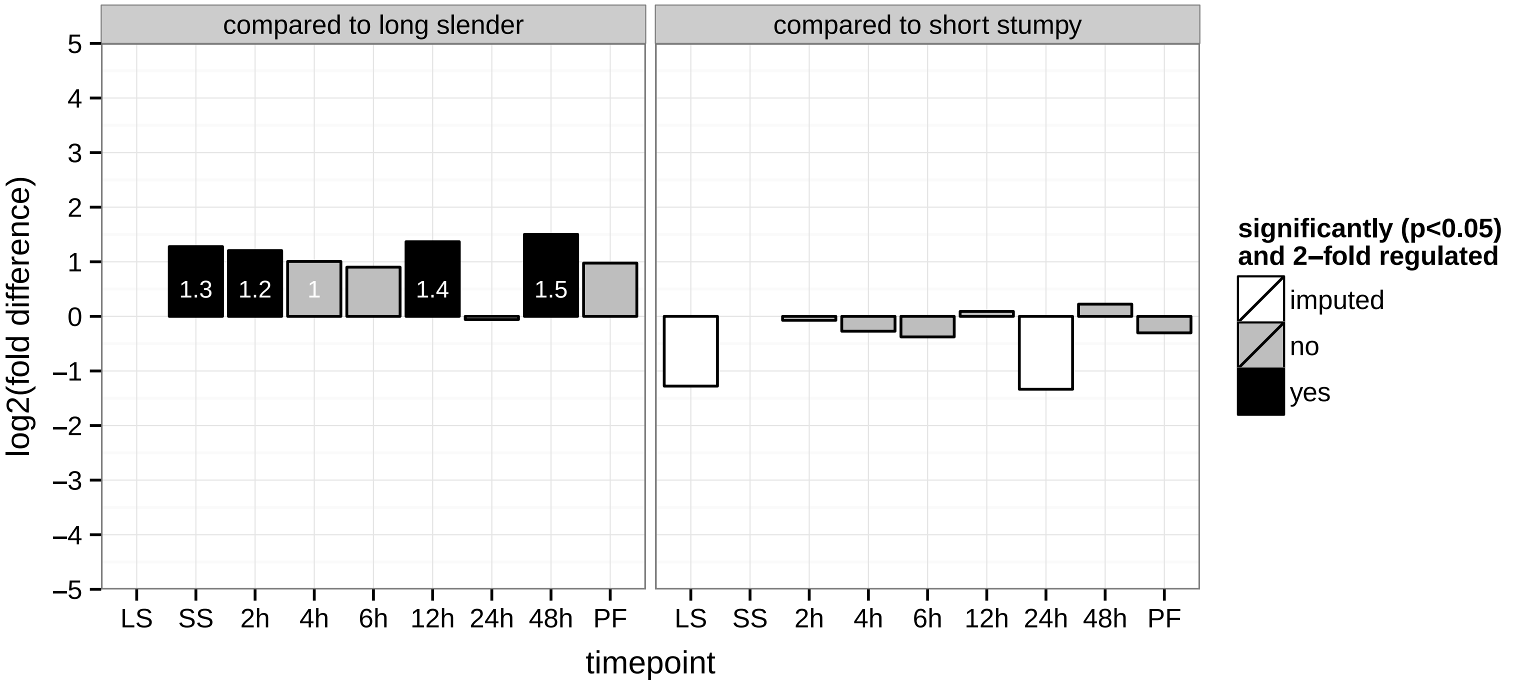

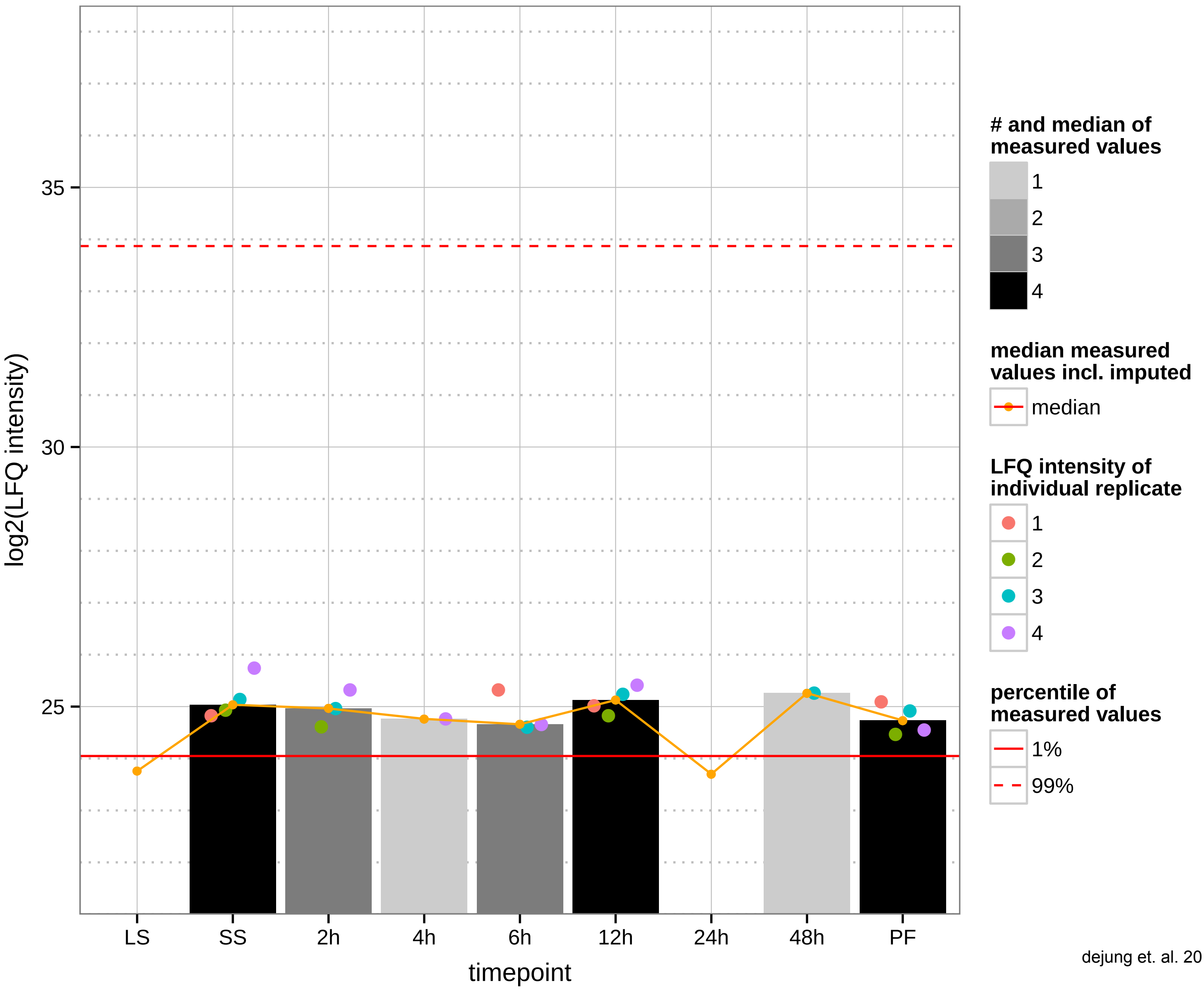

dejung et. al. 2015

hypothetical protein, conserved
Tb927.7.3910
AGOF: null
AGOC: mitochondrion
AGOP: null
PGOF: null
PGOC: null
PGOP: null

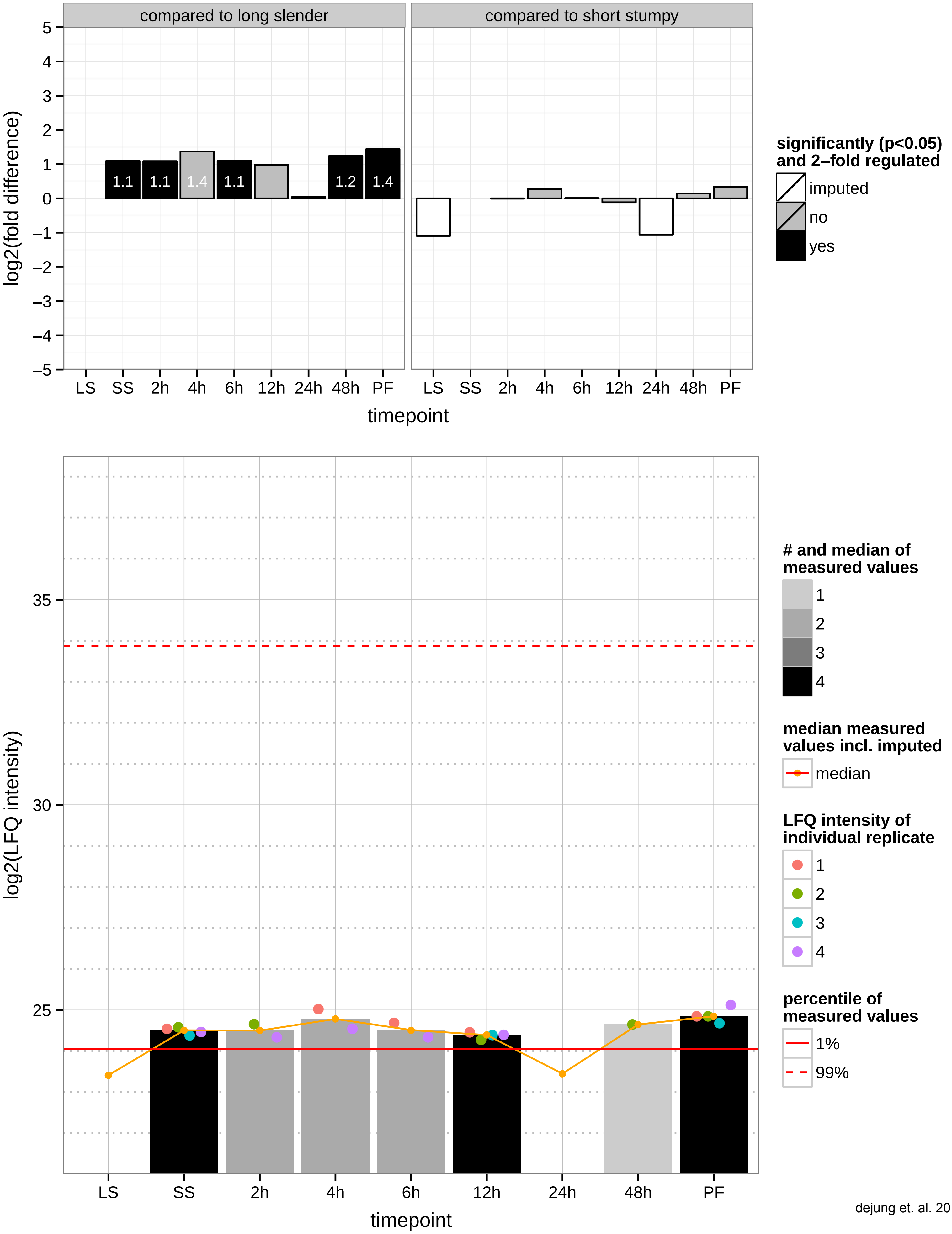

dejung et. al. 2015

NADH–ubiquinone oxidoreductase, mitochondrial, putative
Tb927.7.6350
AGOF: NAD binding, electron carrier activity, oxidoreductase activity
AGOC: mitochondrion
AGOP: null
PGOF: NAD binding, oxidoreductase activity
PGOC: null
PGOP: oxidation–reduction process

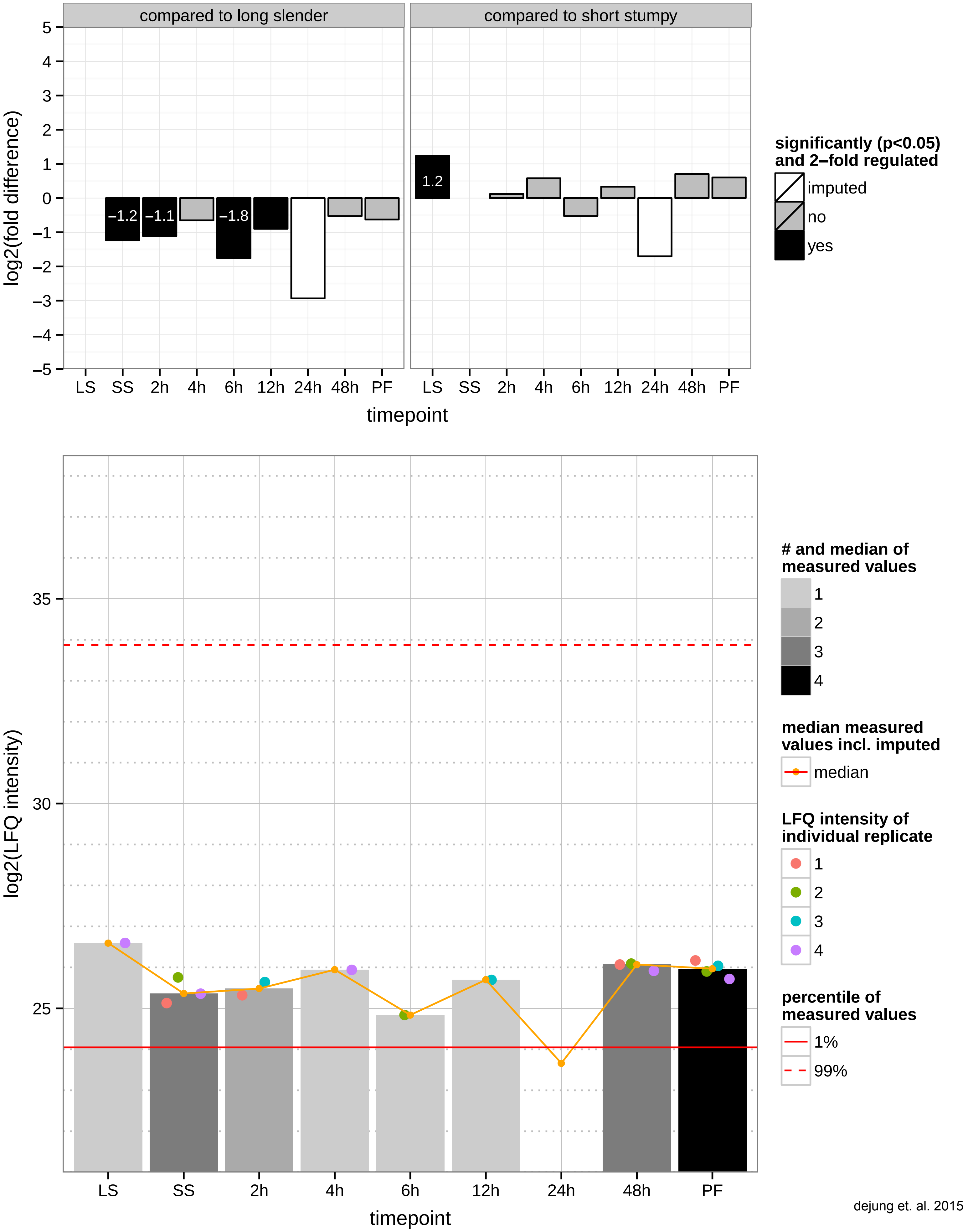

dejung et. al. 2015

actin, putative
Tb927.9.5440
AGOF: structural constituent of cytoskeleton
AGOC: actin cytoskeleton
AGOP: cytoskeleton organization
PGOF: null
PGOC: null
PGOP: null

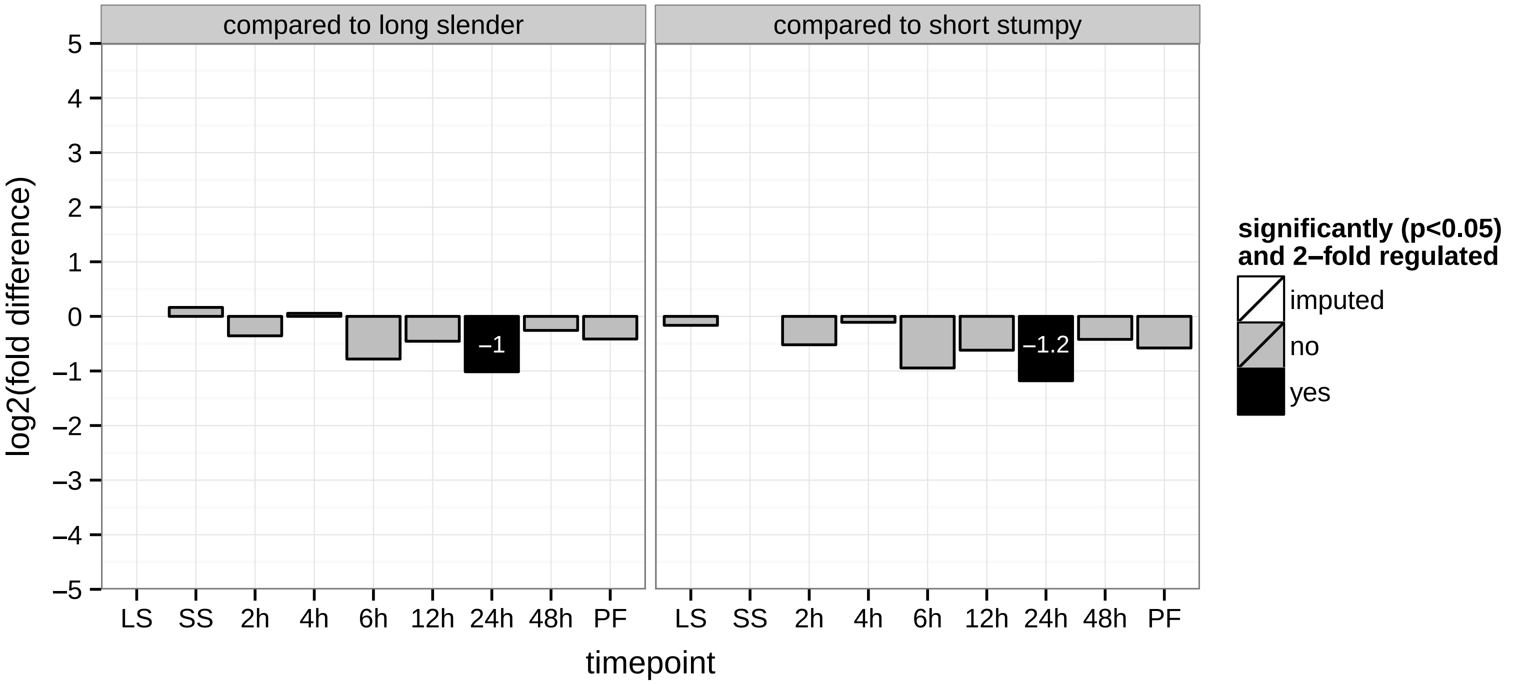

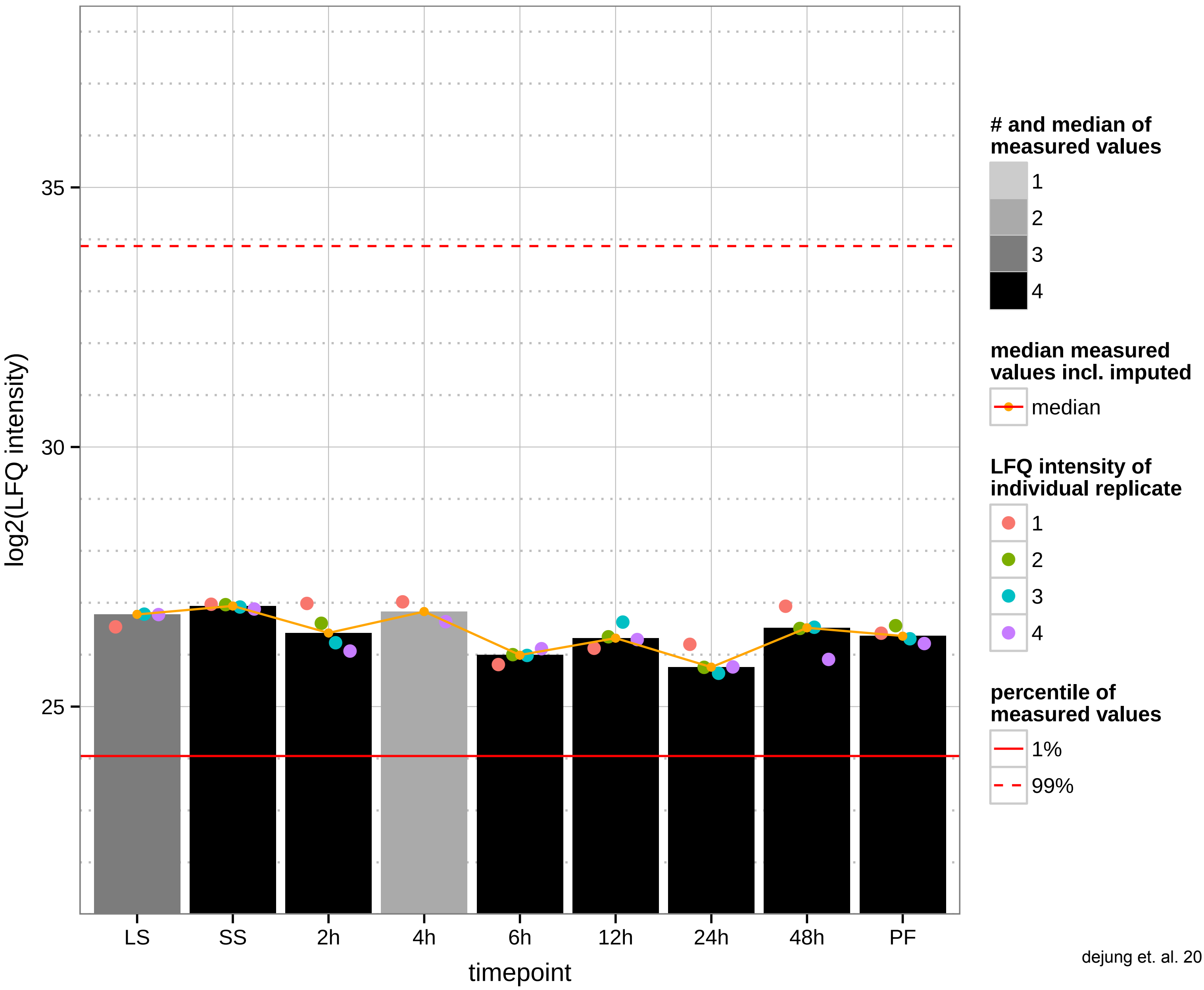

dejung et. al. 2015

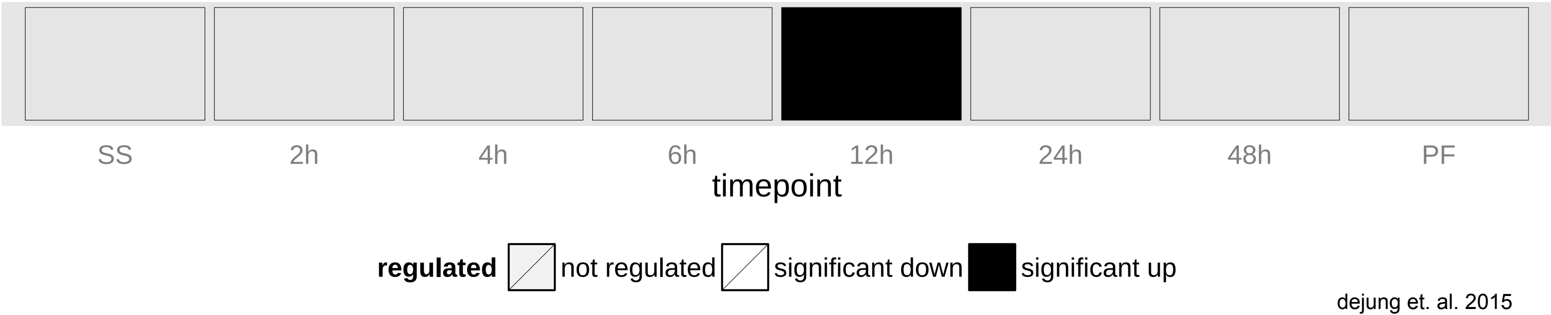
SS
2h
4h
6h
12h
24h
48h
PF
timepoint
regulated
not regulated
significant down
significant up
dejung et. al. 2015

hypothetical PIN domain–containing protein, conserved
Tb927.1.1790
AGOF: RNA binding
AGOC: null
AGOP: null
PGOF: null
PGOC: null
PGOP: null

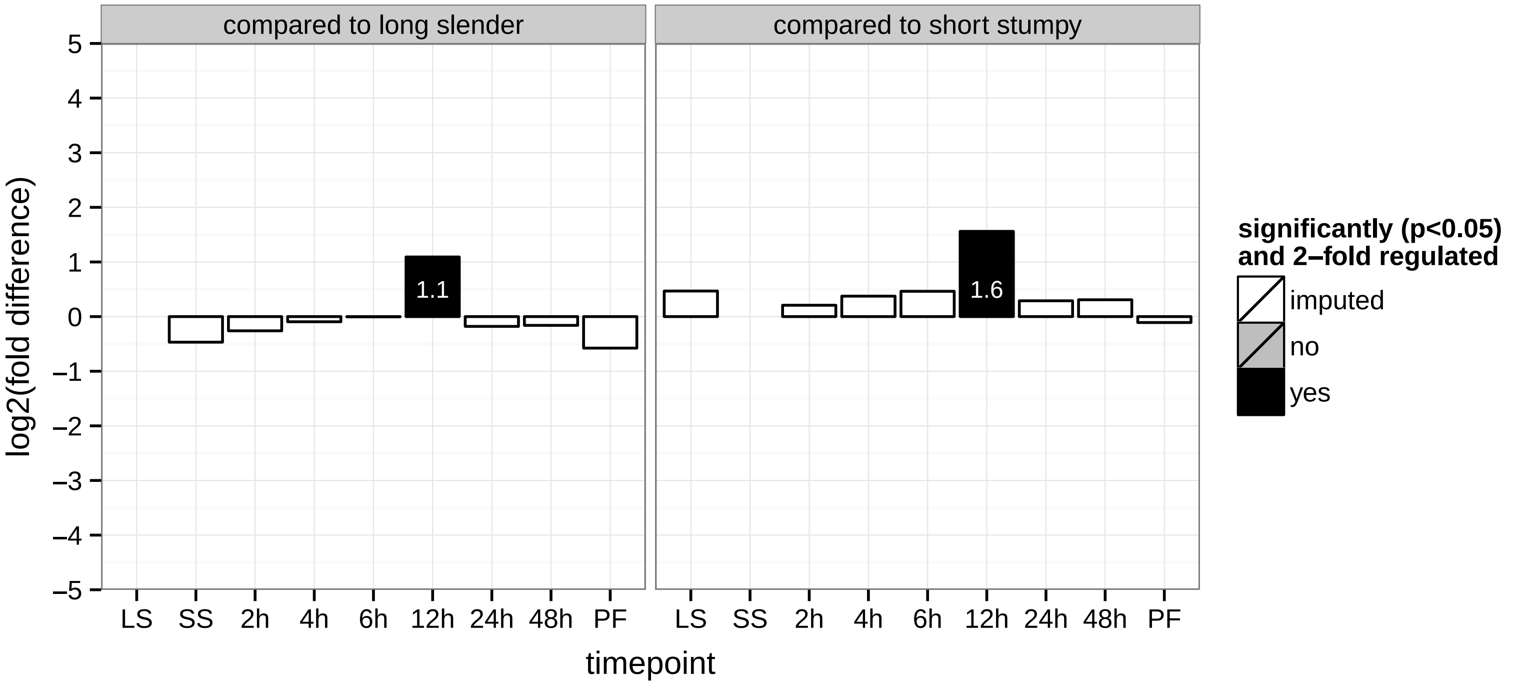

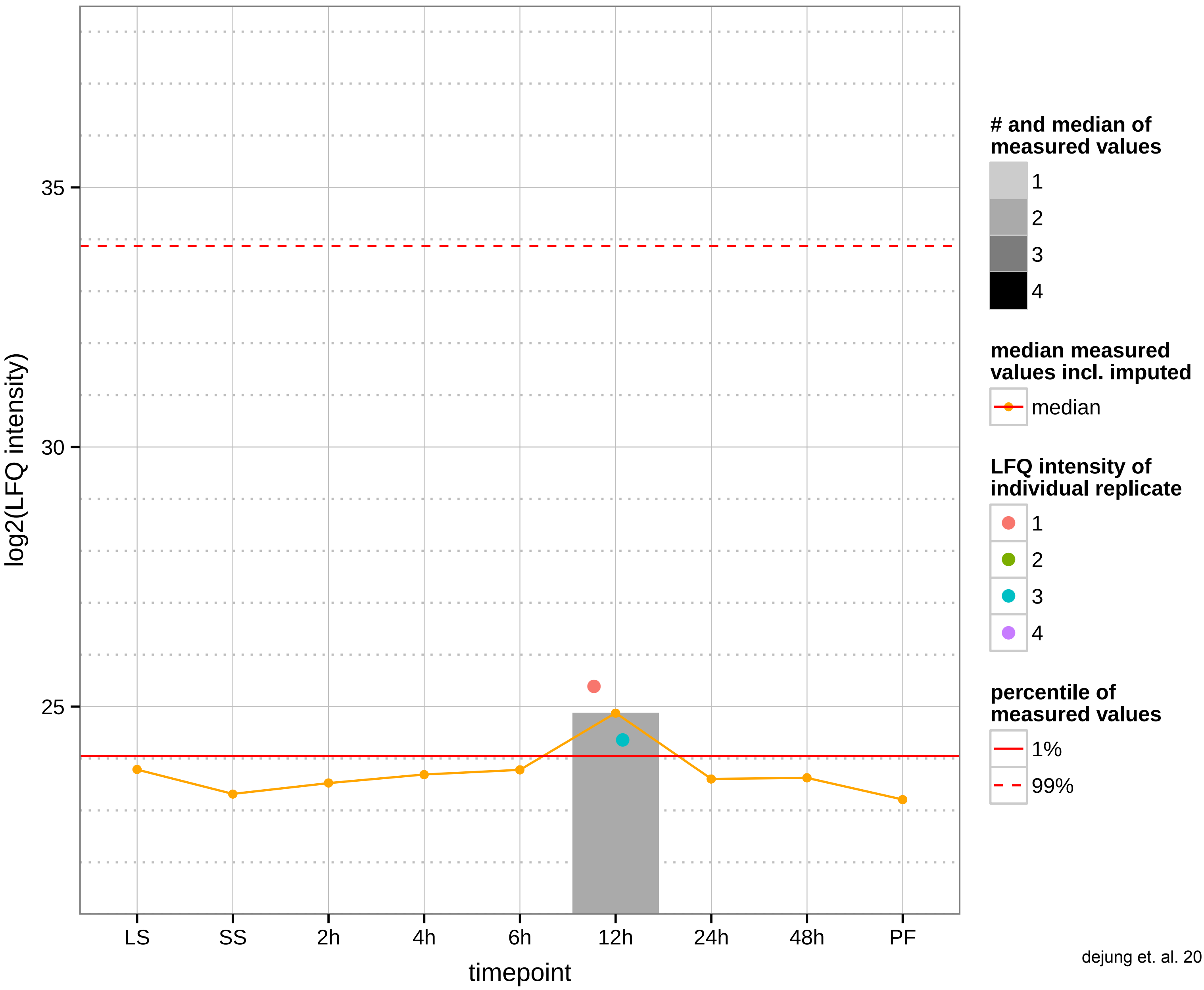

dejung et. al. 2015

hypothetical protein, conserved
Tb927.1.2190
AGOF: null
AGOC: null
AGOP: null
PGOF: protein binding
PGOC: null
PGOP: null

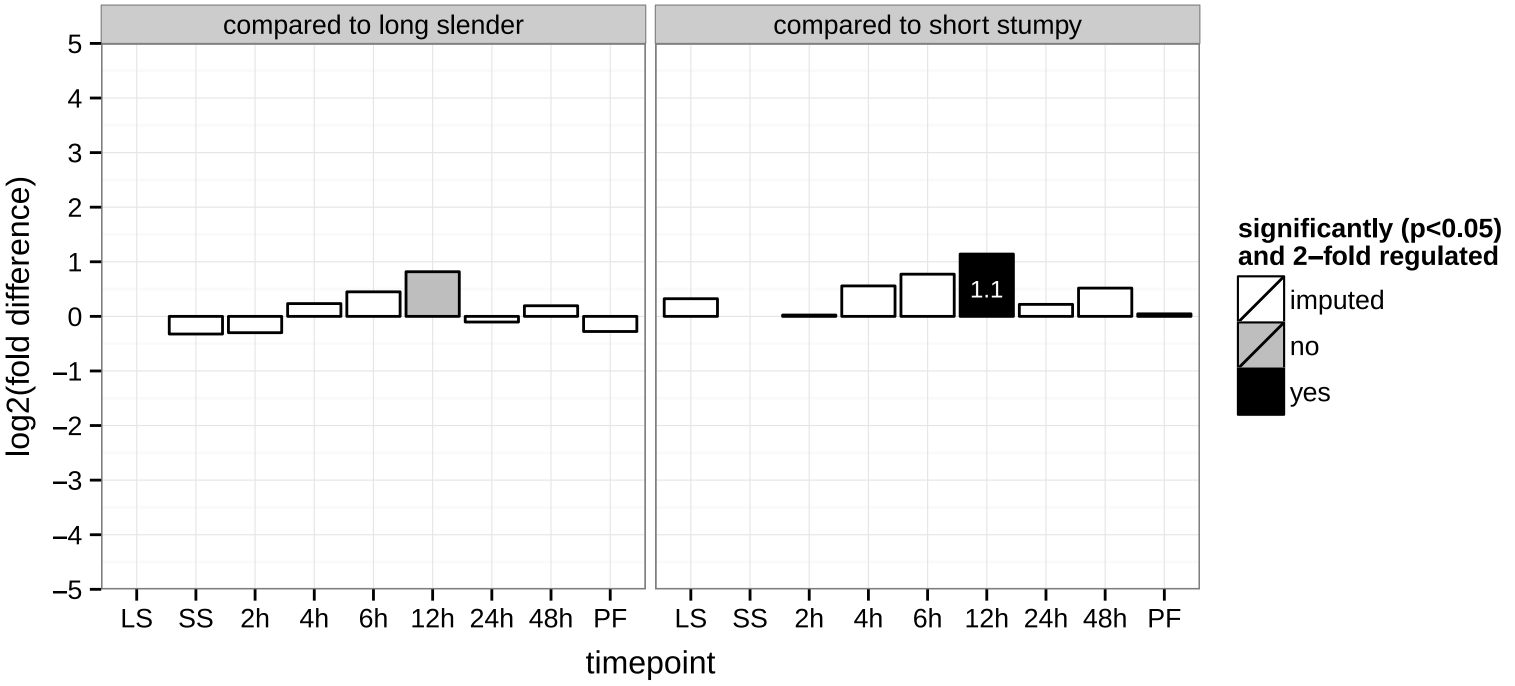

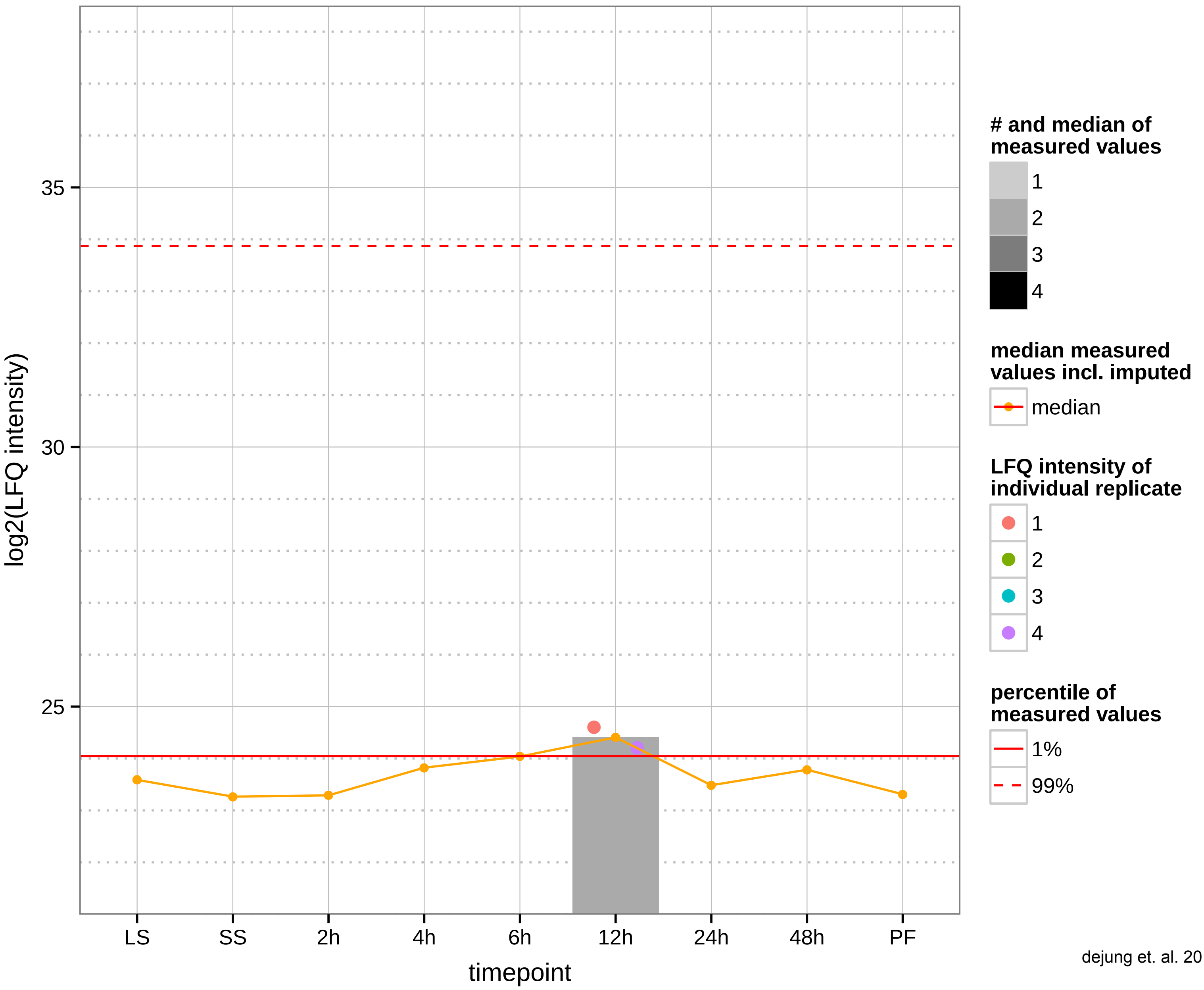

dejung et. al. 2015

Histone–lysine N–methyltransferase, H3 lysine–76 specific, Histone H3–K76 methyltransferase (DOT1B)
Tb927.1.570
AGOF: histone–lysine N–methyltransferase activity
AGOC: null
AGOP: antigenic variation, regulation of cell cycle
PGOF: histone–lysine N–methyltransferase activity
PGOC: null
PGOP: null

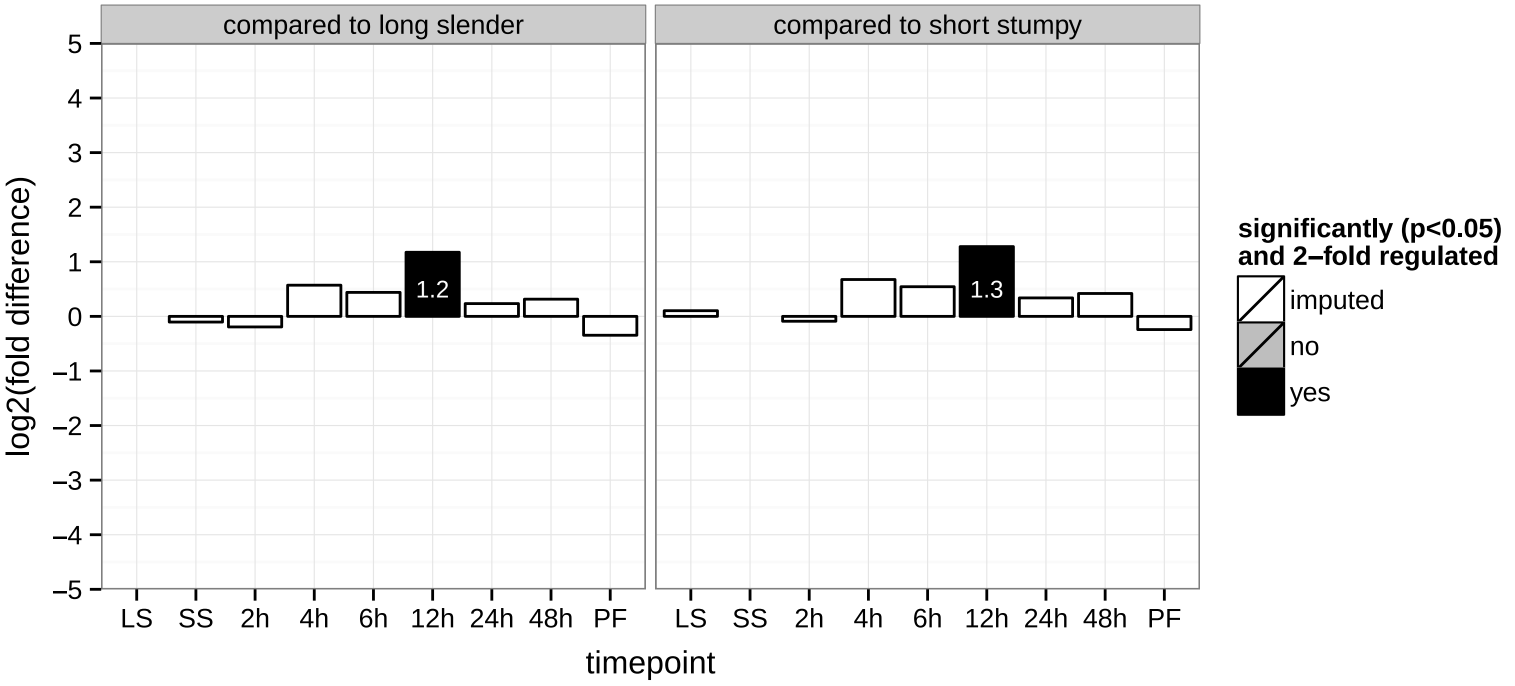

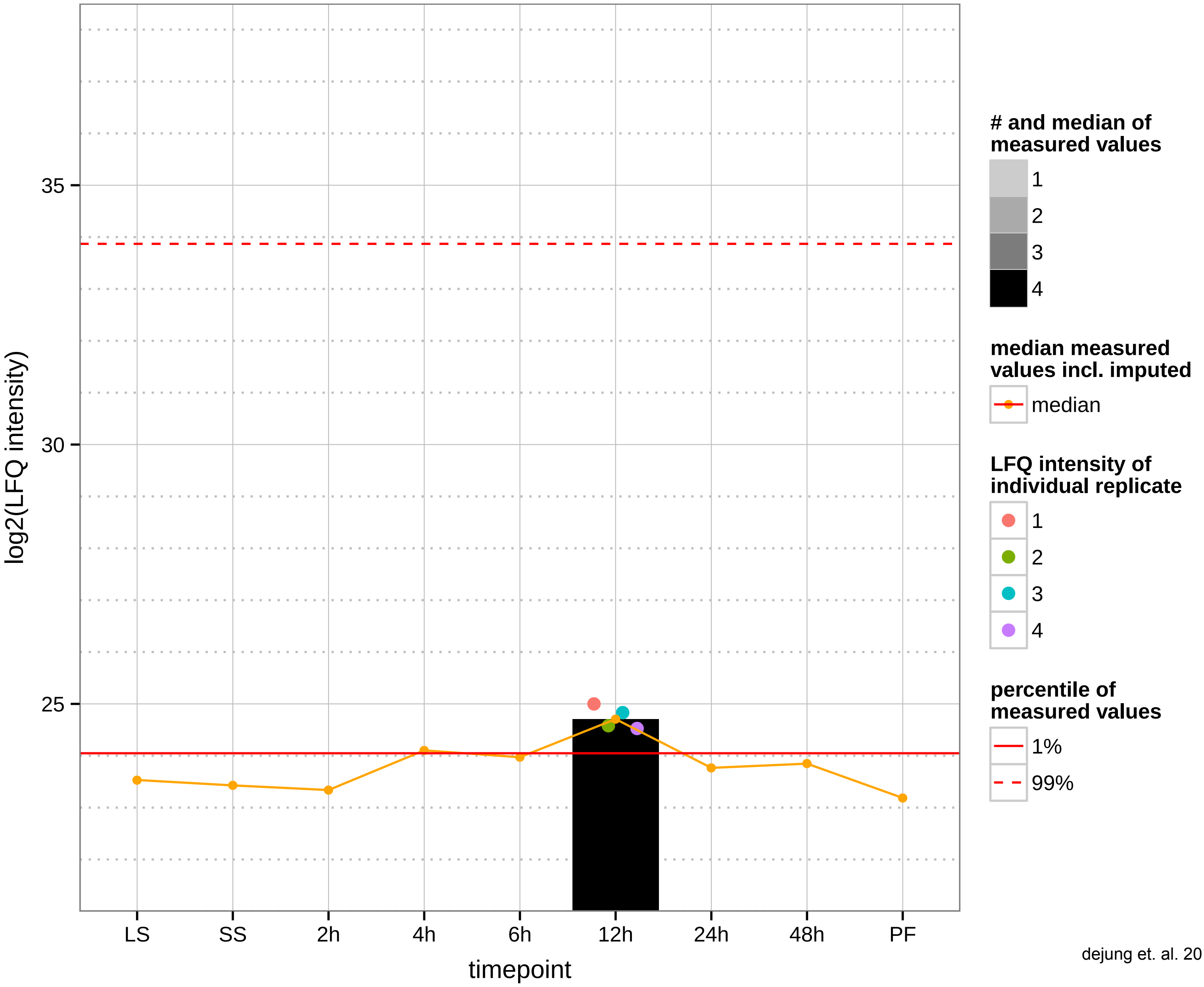

dejung et. al. 2015

hypothetical protein, conserved
Tb927.10.14650
AGOF: null
AGOC: null
AGOP: null
PGOF: null
PGOC: null
PGOP: null

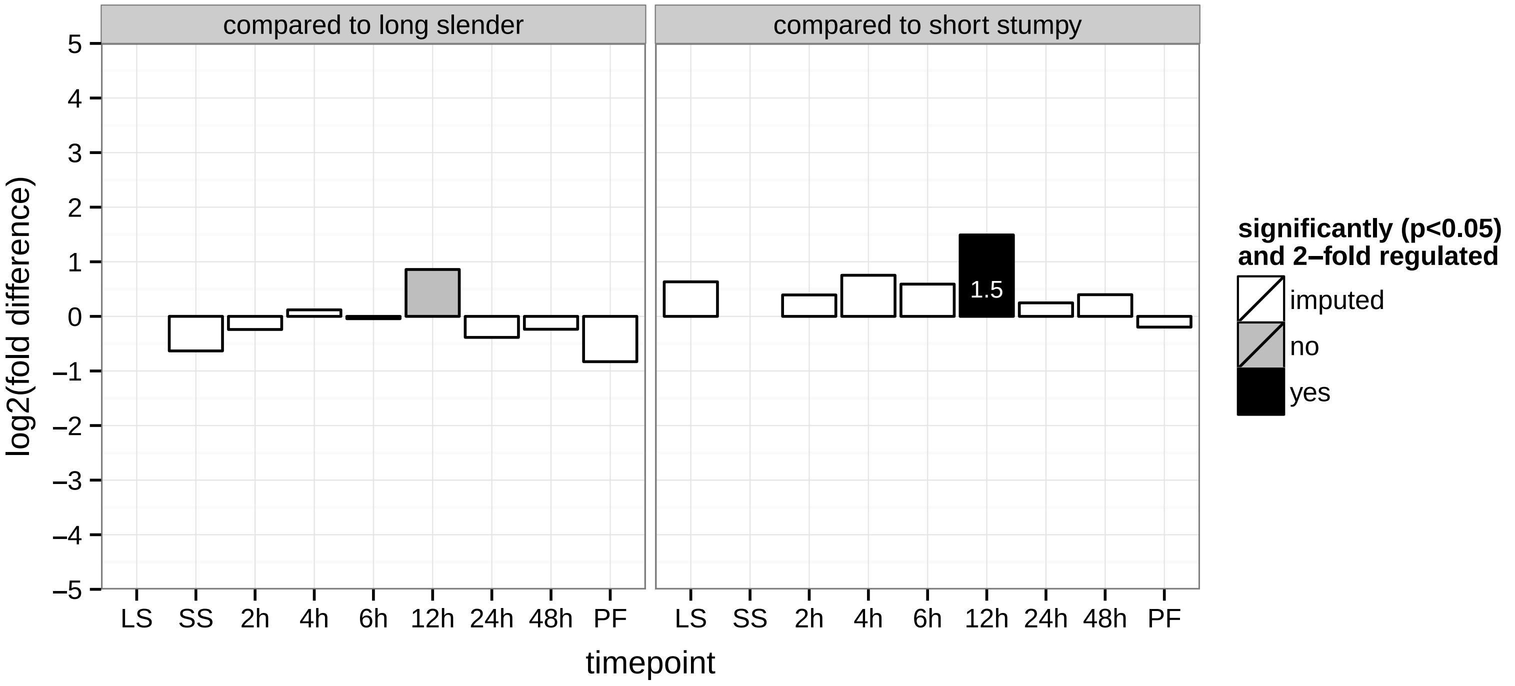

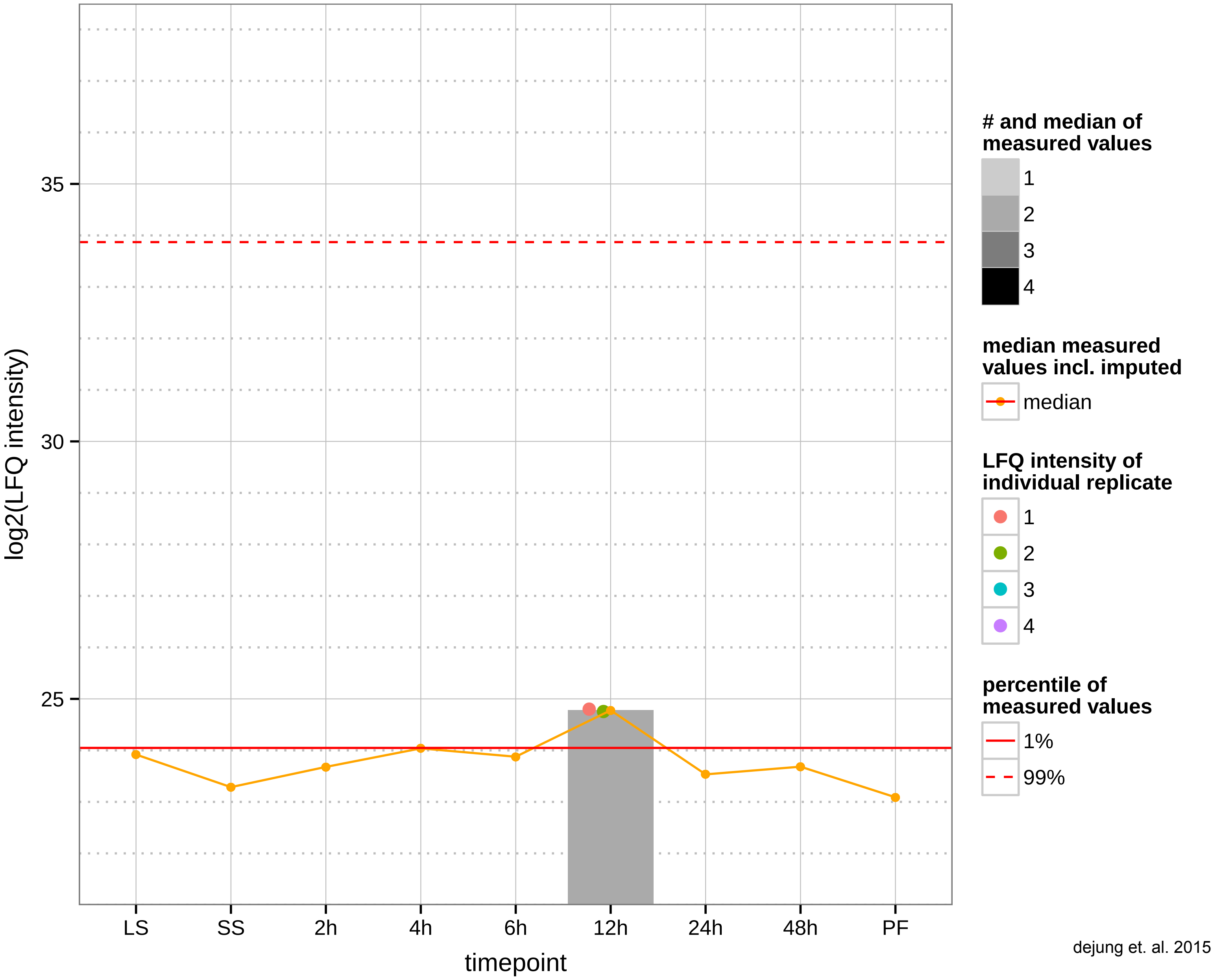

dejung et. al. 2015

calcineurin B subunit, putative
Tb927.10.370
AGOF: calcium ion binding
AGOC: null
AGOP: null
PGOF: calcium ion binding
PGOC: null
PGOP: null

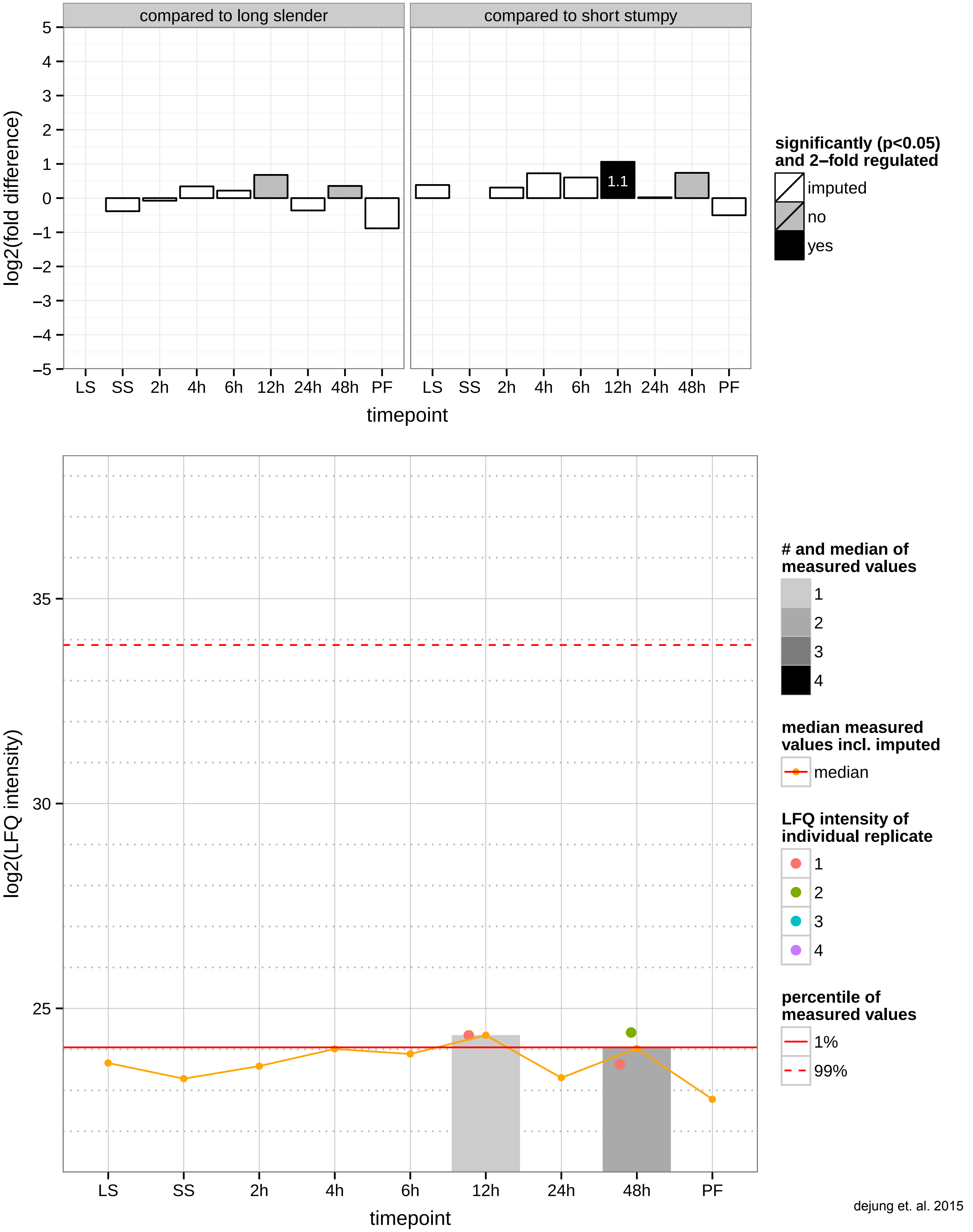

dejung et. al. 2015

hypothetical protein, conserved
Tb927.10.4830
AGOF: null
AGOC: null
AGOP: null
PGOF: null
PGOC: null
PGOP: null

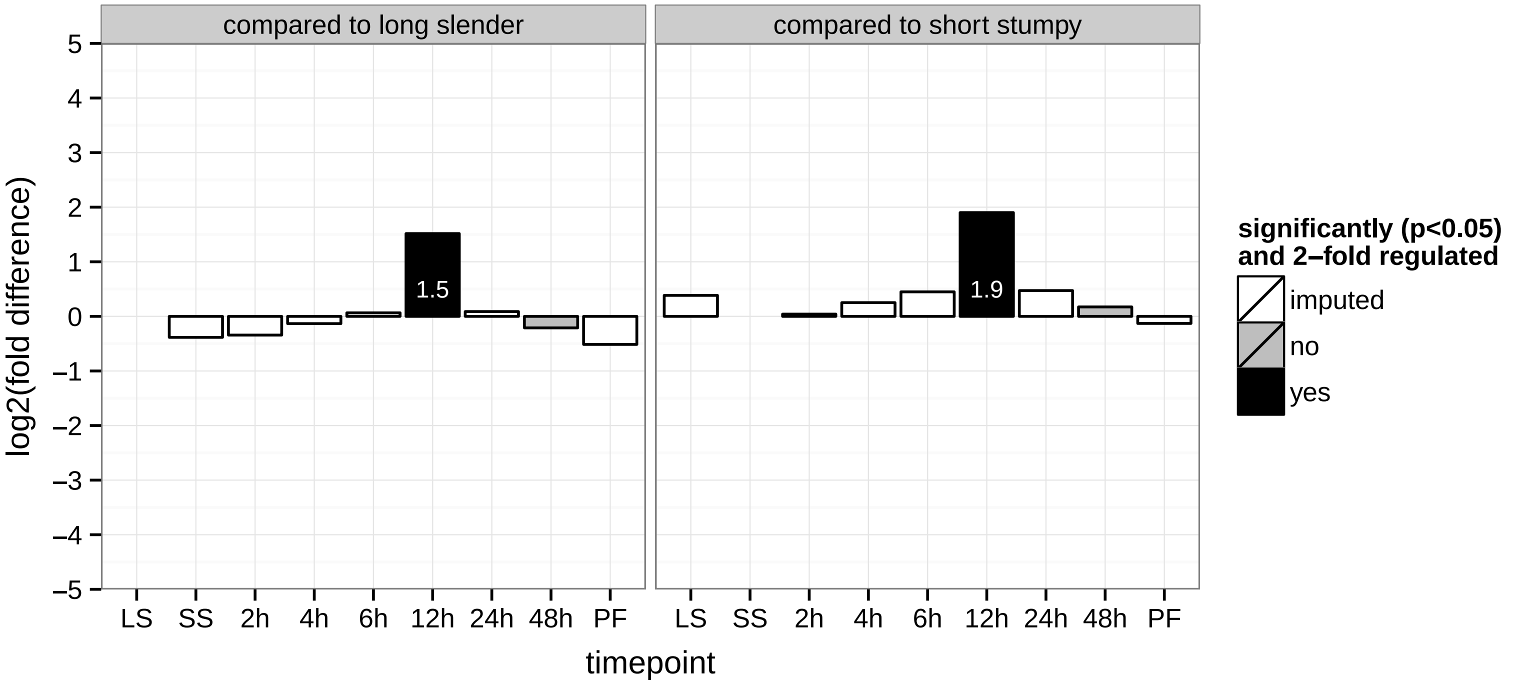

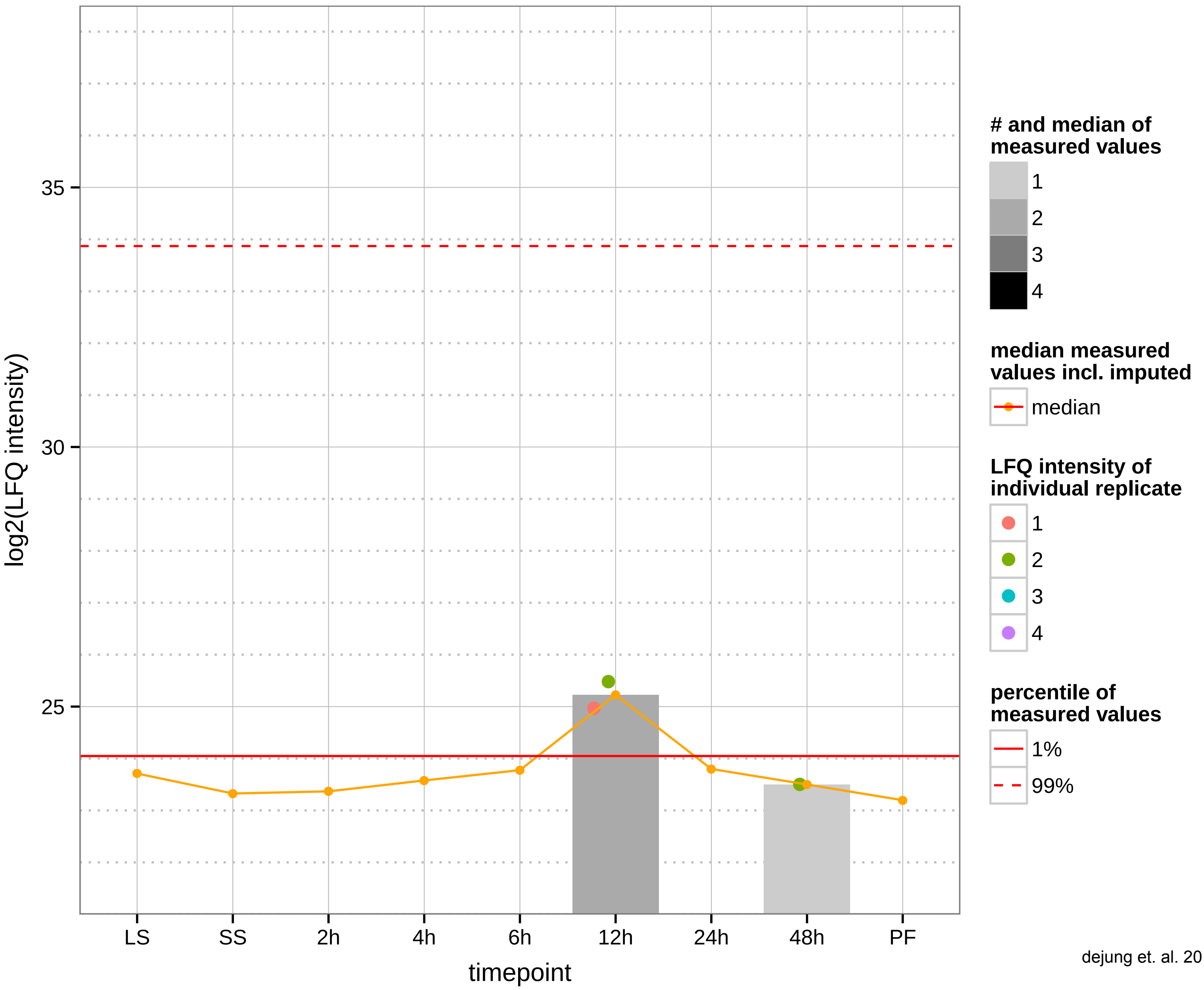

dejung et. al. 2015

amino acid tansporter, putative
Tb927.10.5780
AGOF: null
AGOC: membrane
AGOP: amino acid transport
PGOF: null
PGOC: null
PGOP: null

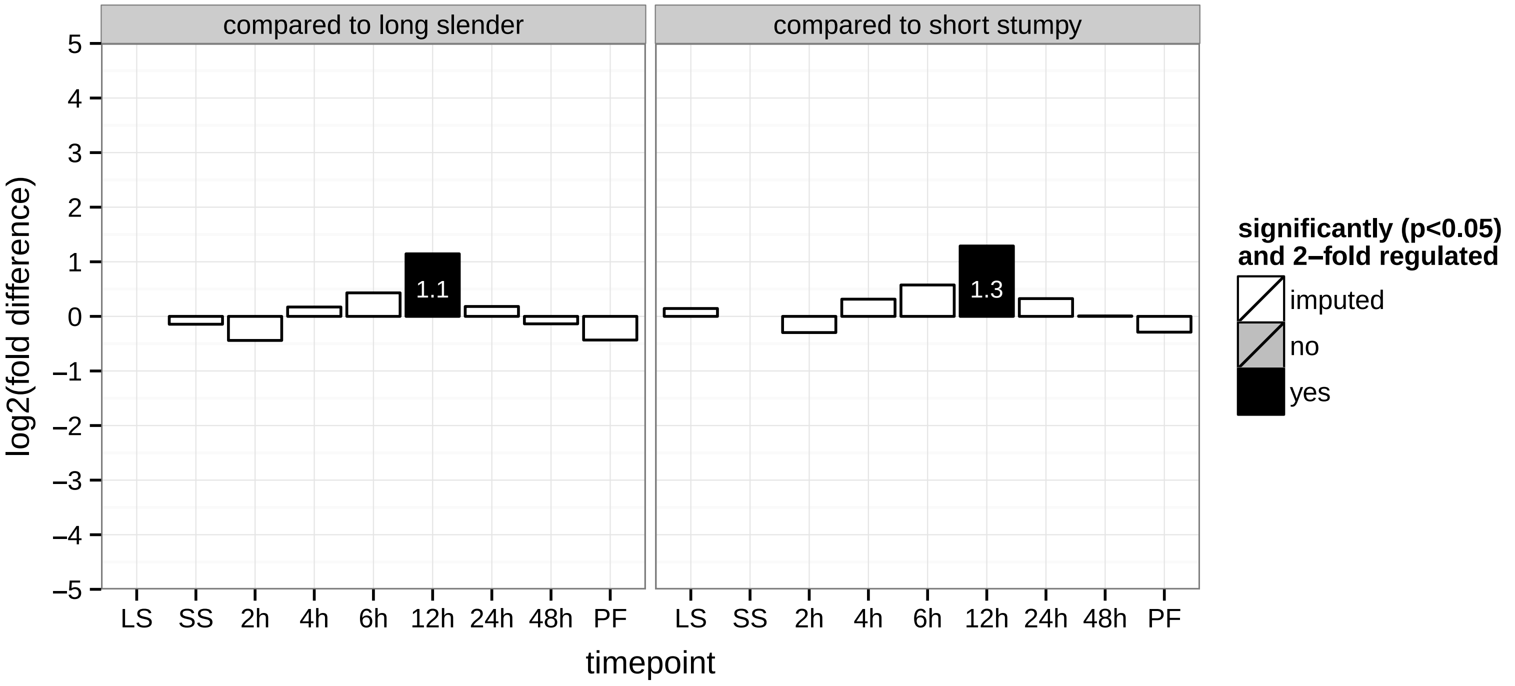

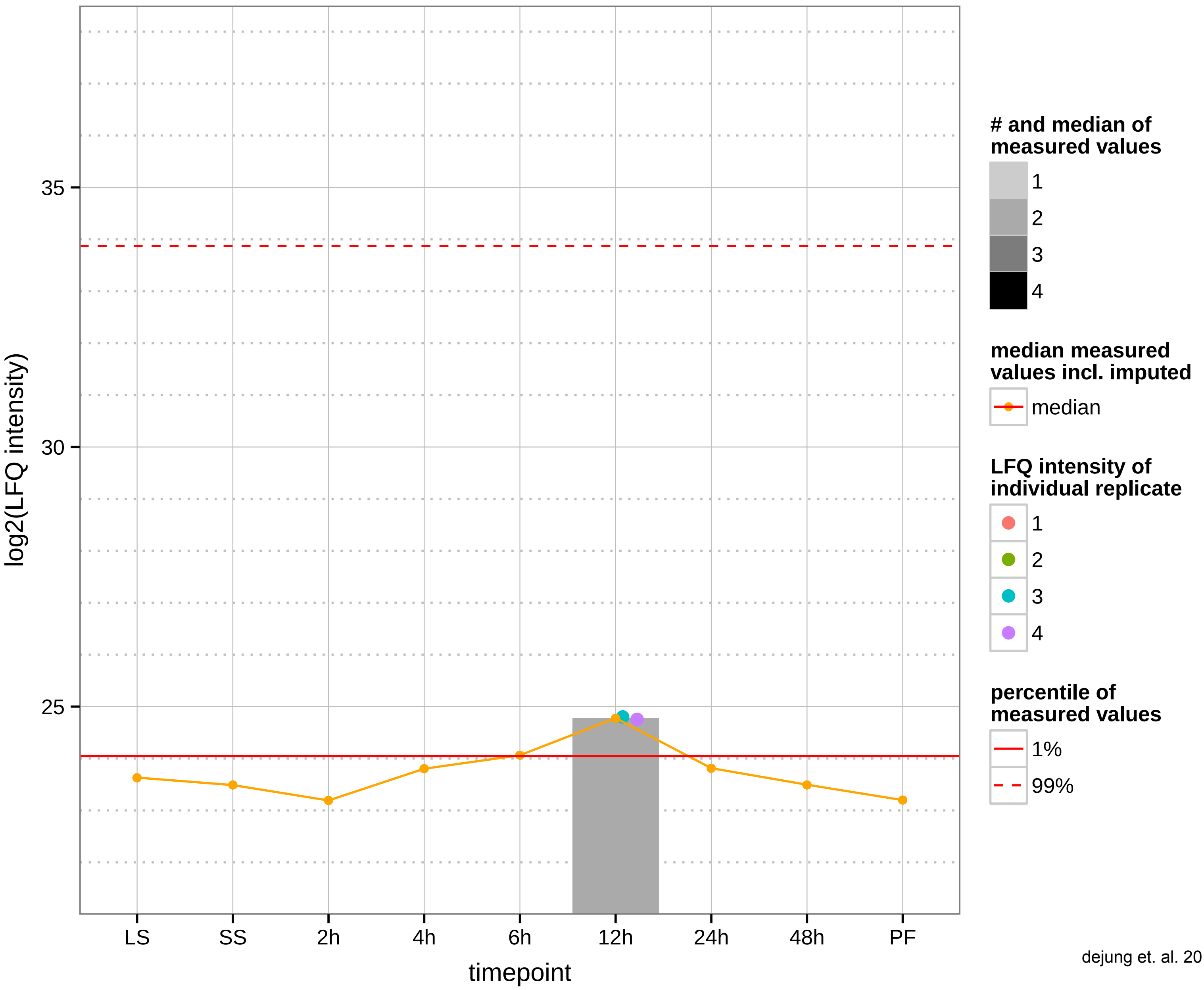

dejung et. al. 2015

hypothetical protein, conserved
Tb927.11.1750
AGOF: null
AGOC: null
AGOP: null
PGOF: null
PGOC: null
PGOP: null

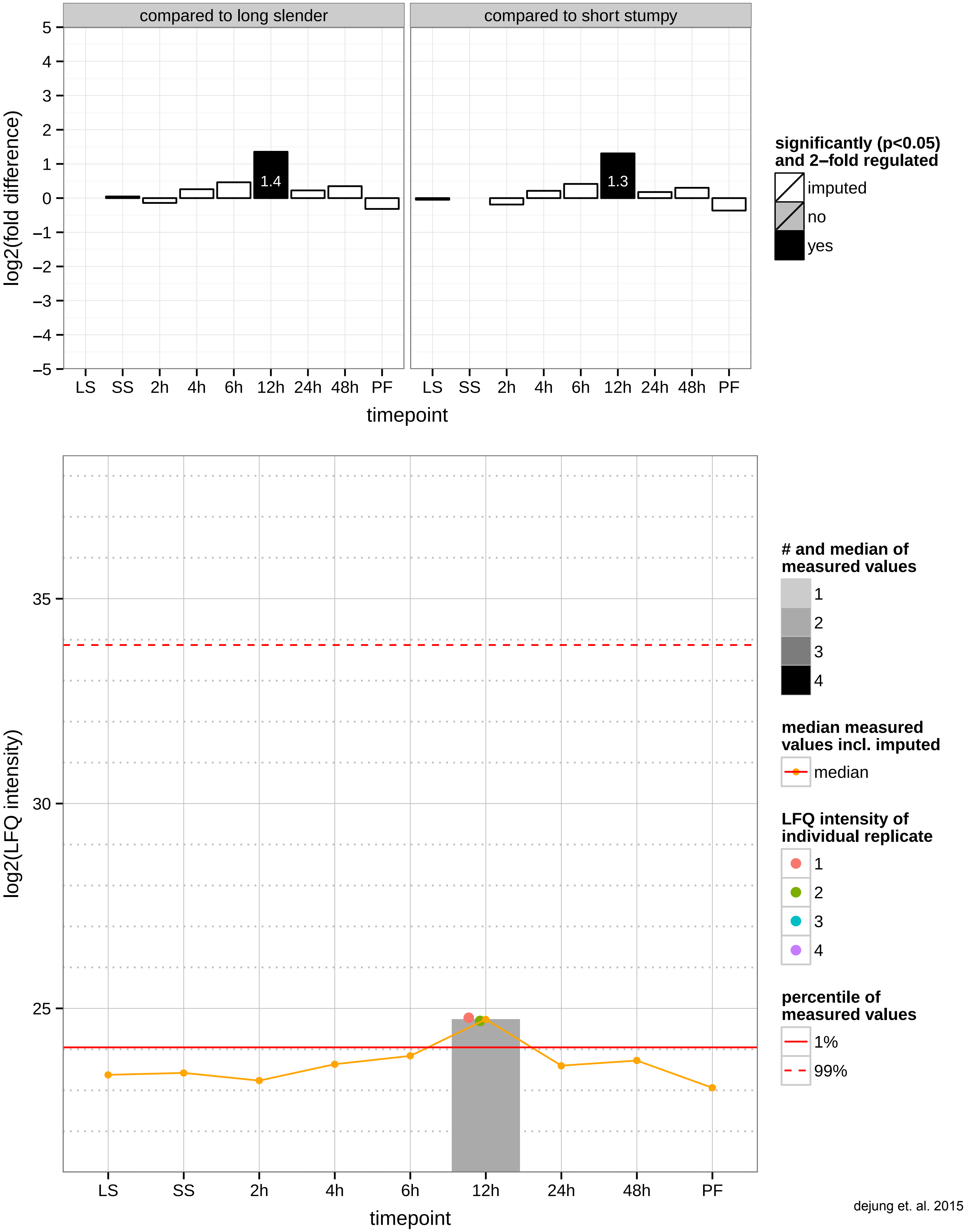

dejung et. al. 2015

hypothetical protein, conserved
Tb927.2.5930
AGOF: null
AGOC: mitochondrion
AGOP: null
PGOF: null
PGOC: null
PGOP: null

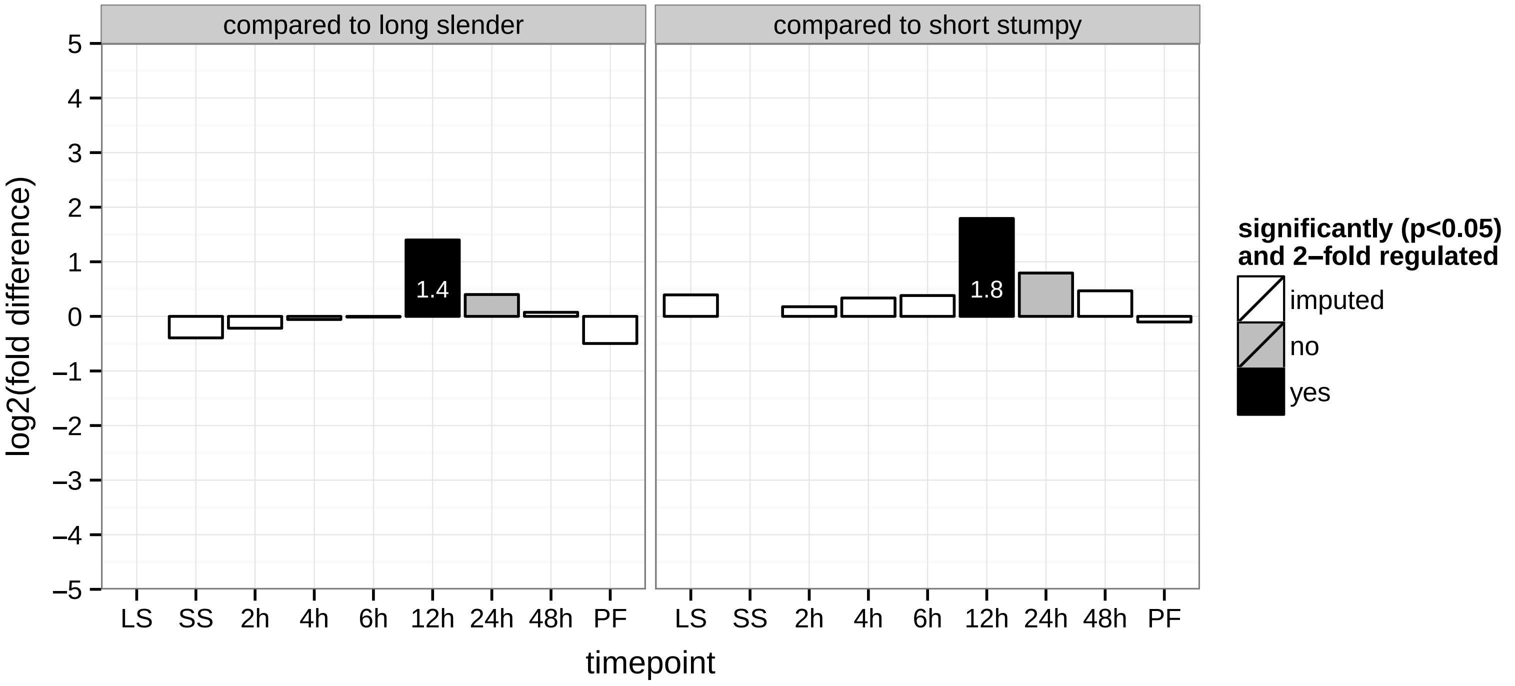

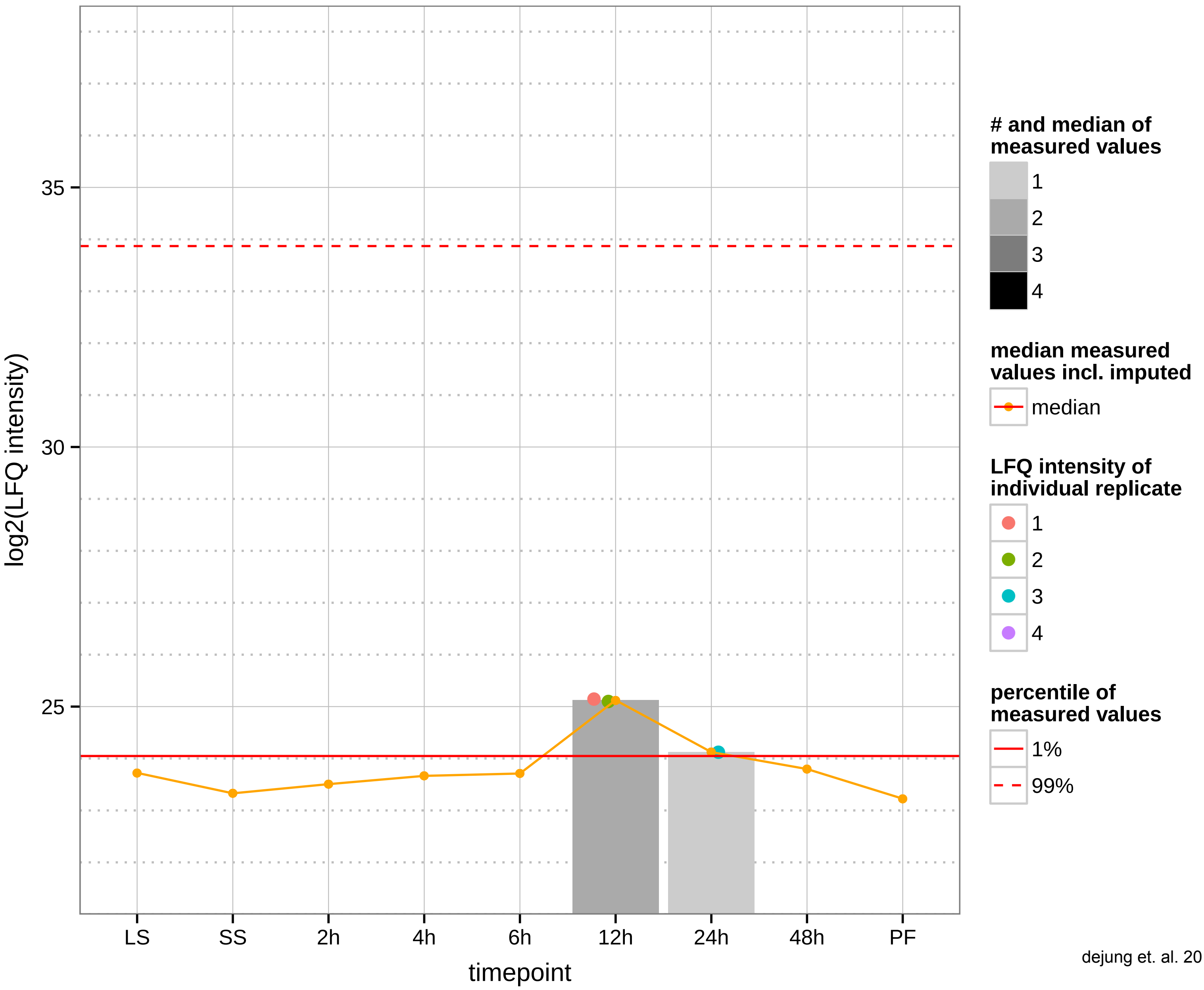

dejung et. al. 2015

metalloprotease, putative
Tb927.5.1870
AGOF: ATP binding, nucleoside−triphosphatase activity
AGOC: null
AGOP: null
PGOF: ATP binding, nucleoside−triphosphatase activity, nucleotide binding
PGOC: null
PGOP: null

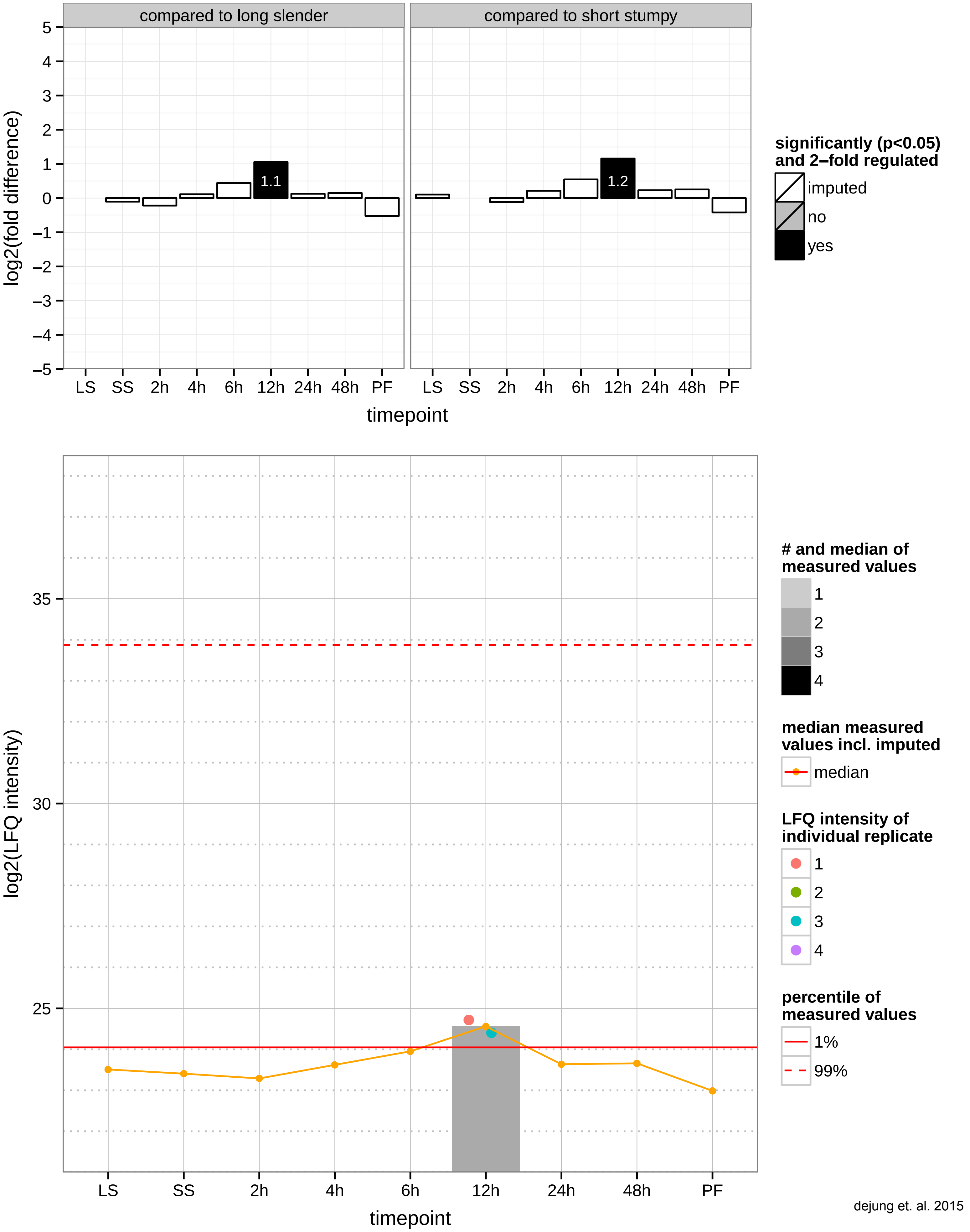

dejung et. al. 2015

hypothetical protein, conserved
Tb927.5.2620
AGOF: null
AGOC: null
AGOP: null
PGOF: null
PGOC: null
PGOP: null

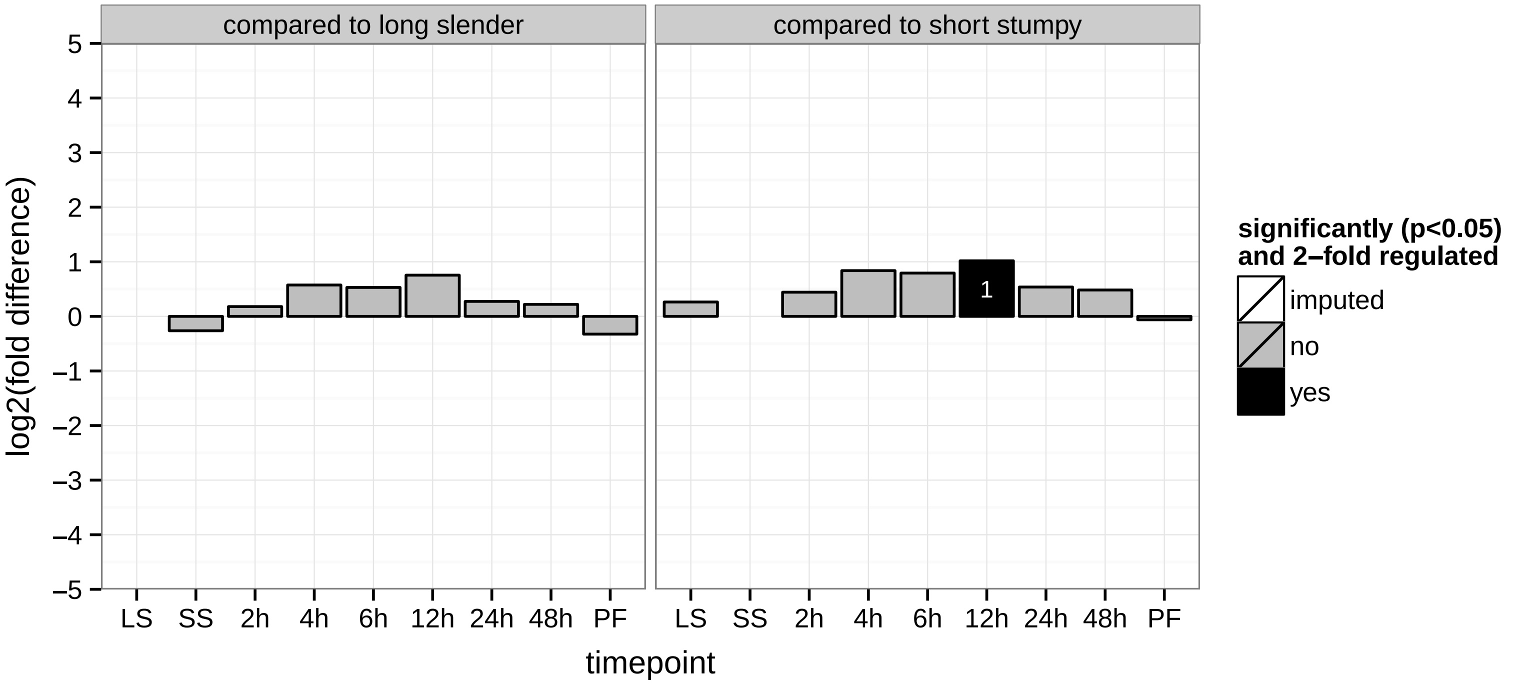

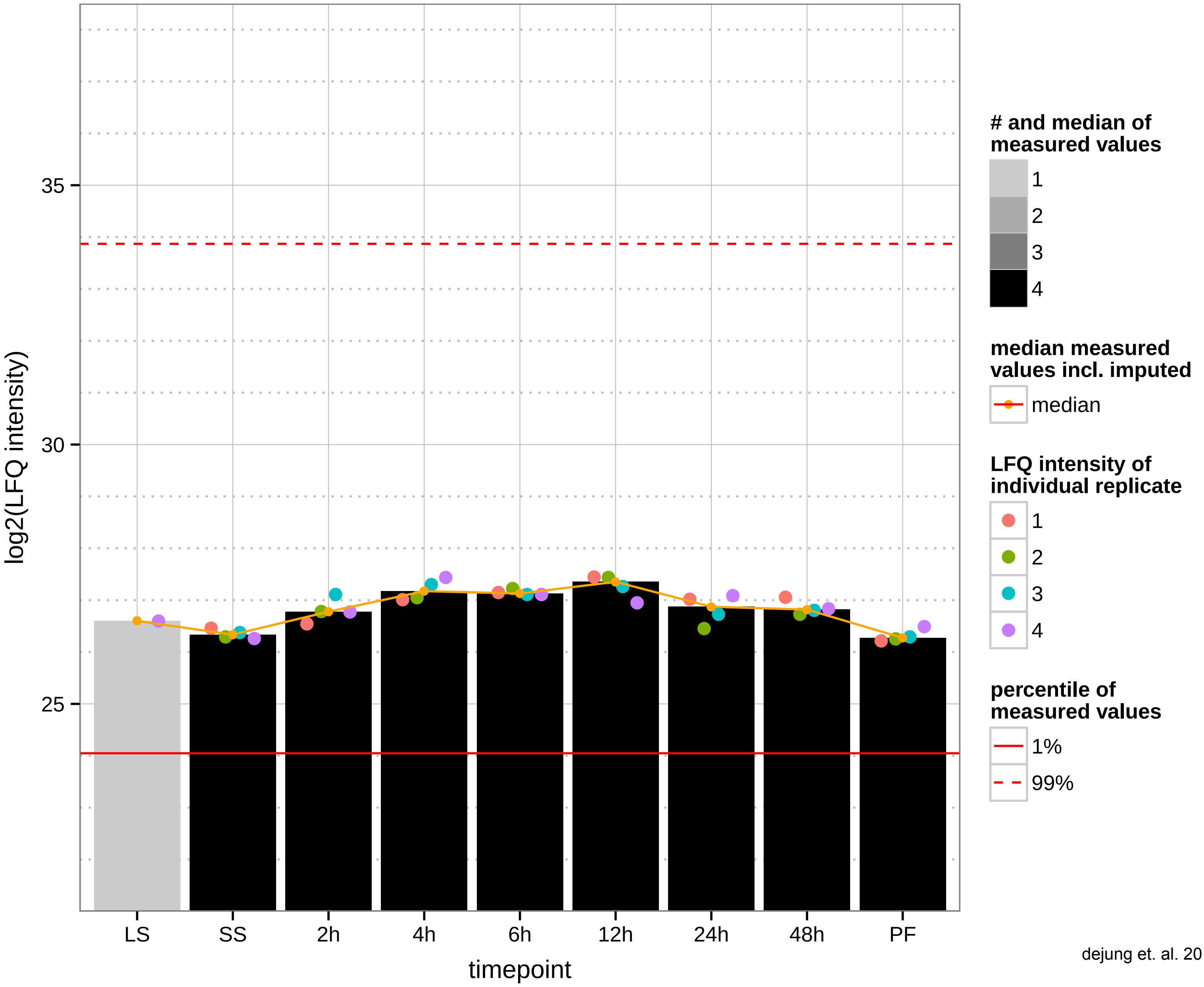

dejung et. al. 2015

hypothetical protein, conserved
Tb927.5.3190
AGOF: null
AGOC: null
AGOP: null
PGOF: null
PGOC: null
PGOP: null

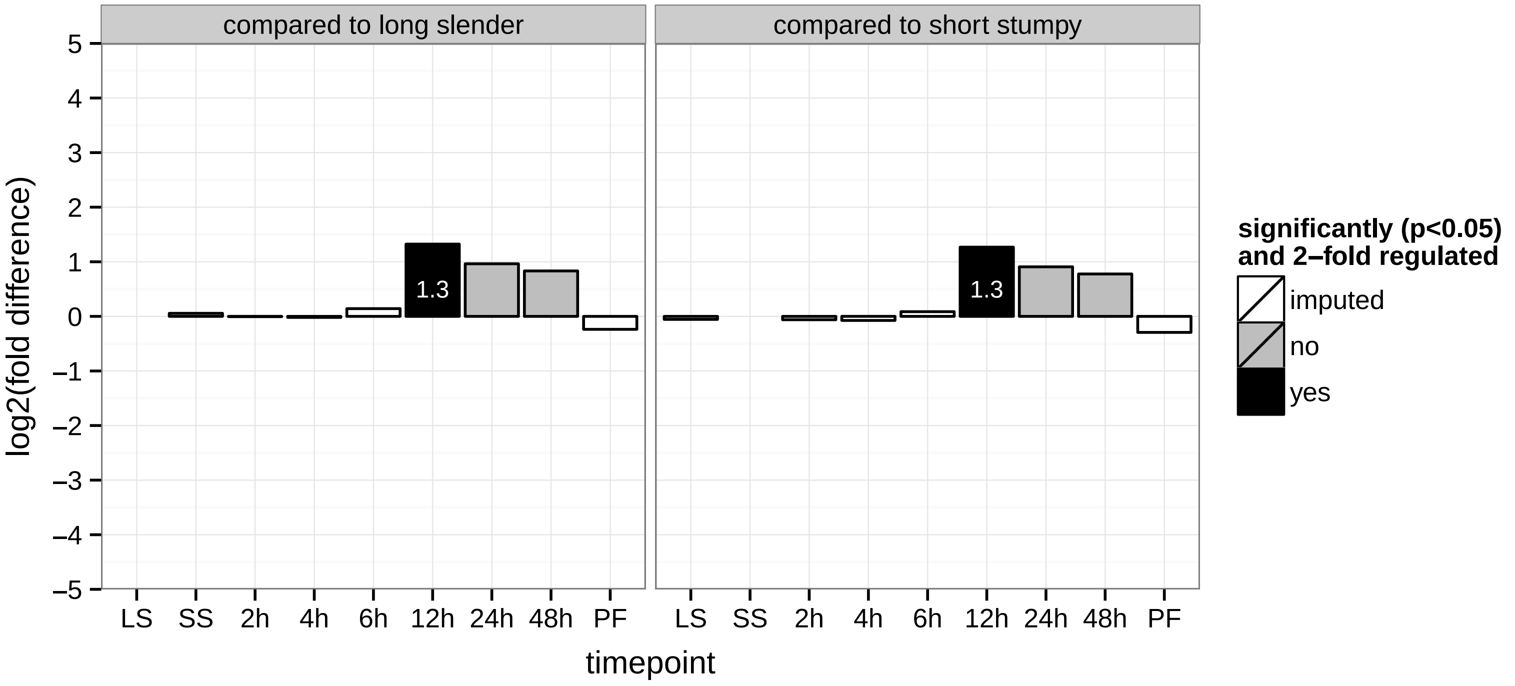

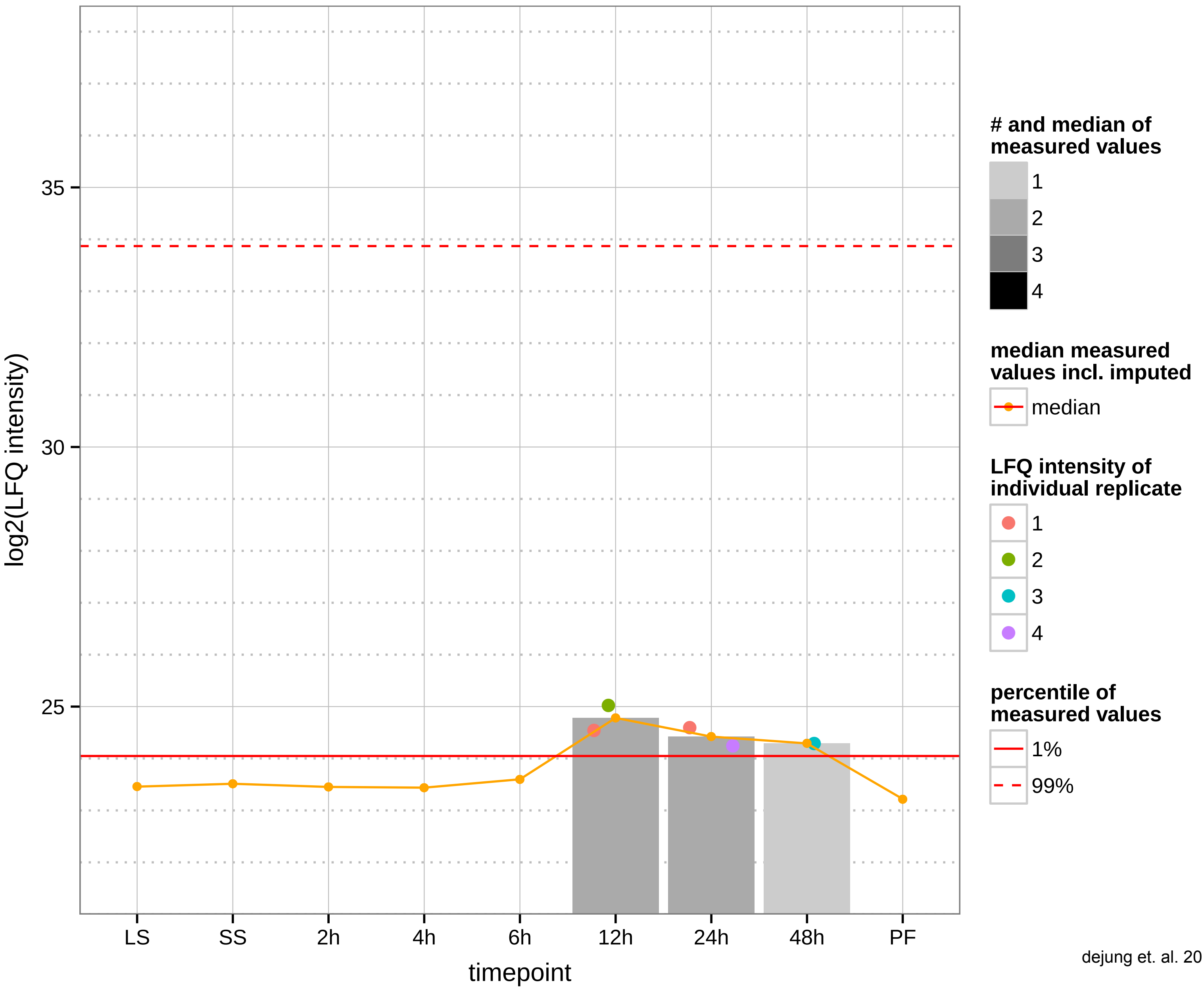

dejung et. al. 2015

hypothetical protein, conserved
Tb927.7.1680
AGOF: null
AGOC: null
AGOP: null
PGOF: null
PGOC: null
PGOP: null

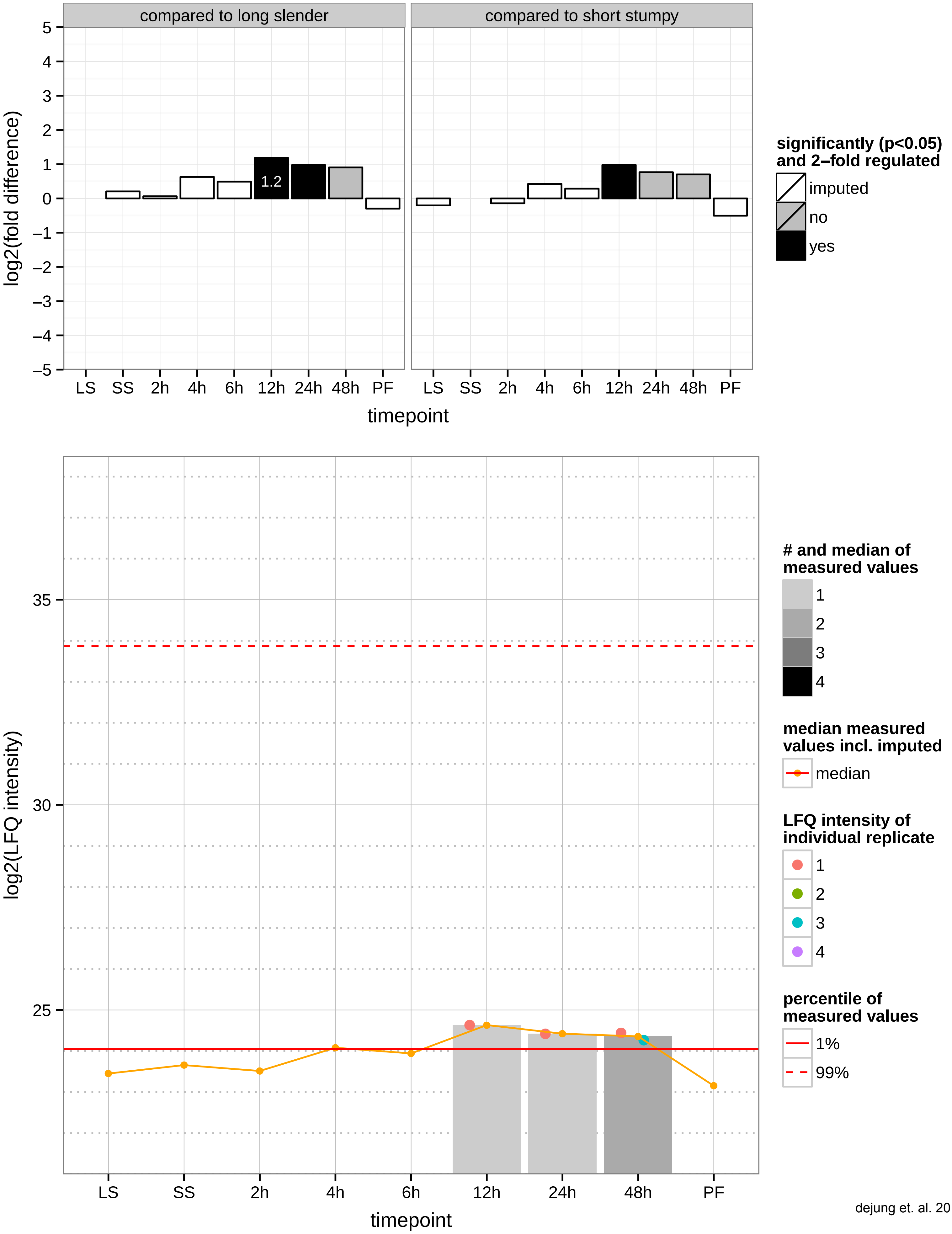

dejung et. al. 2015

hypothetical protein, conserved, unspecified product
Tb927.9.4000;Tb927.9.3930
AGOF: null
AGOC: null
AGOP: null
PGOF: null
PGOC: null
PGOP: null

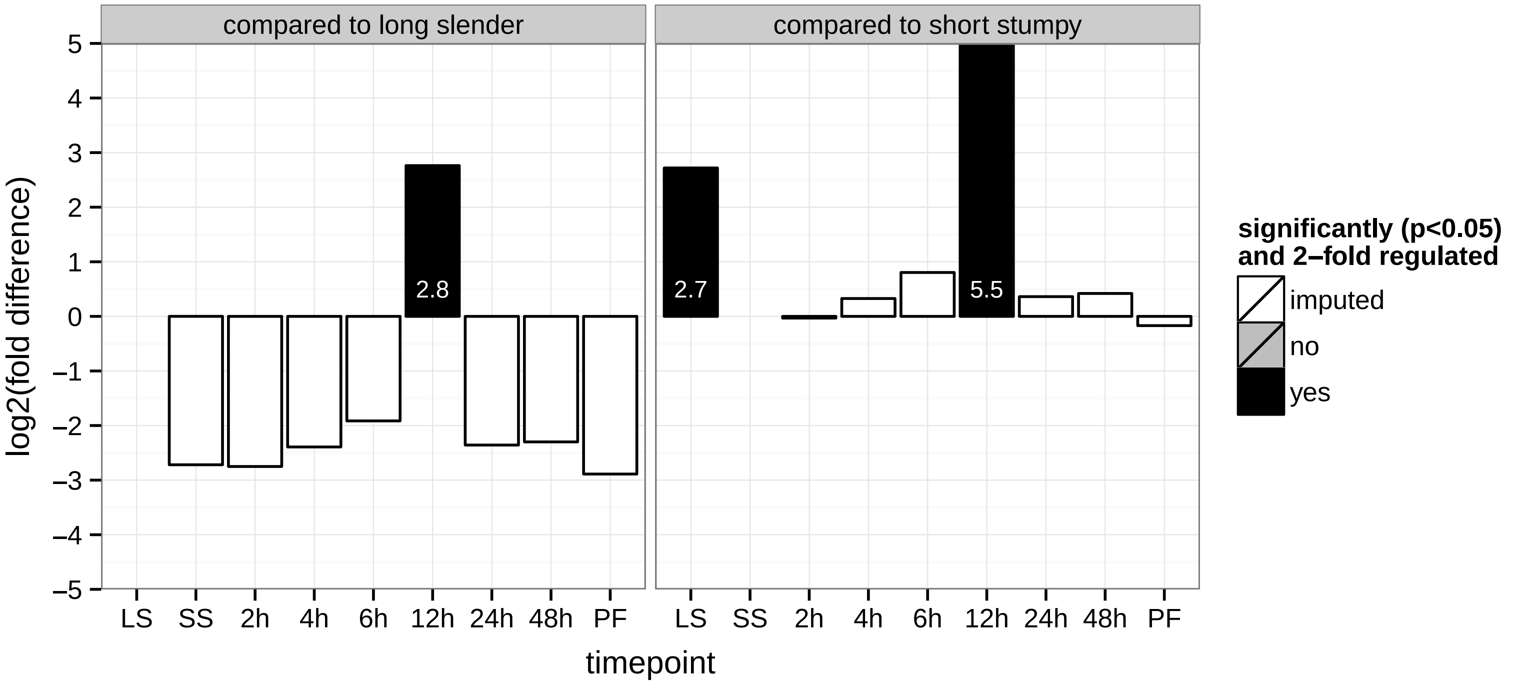

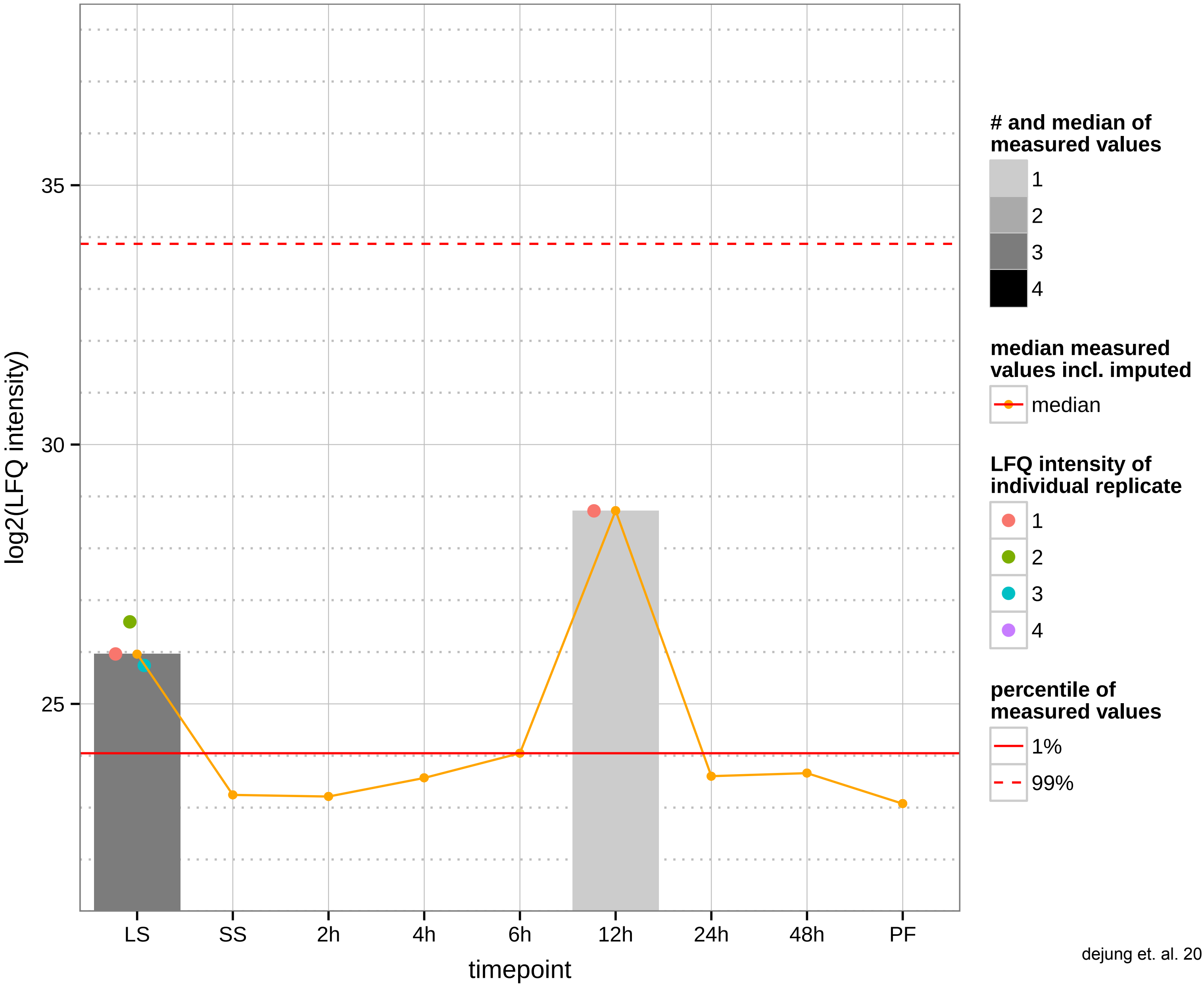

dejung et. al. 2015

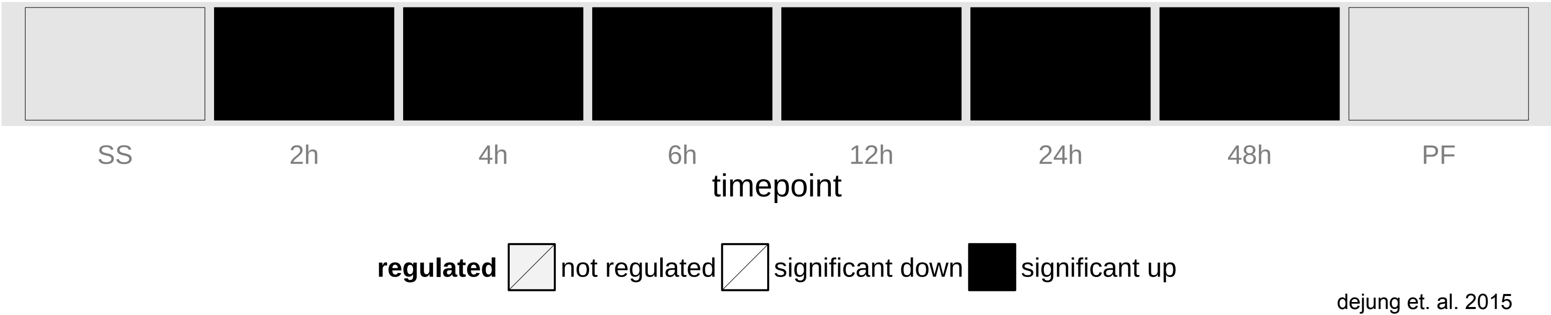

SS
2h
4h
6h
12h
24h
48h
PF
timepoint
regulated
not regulated
significant down
significant up
dejung et. al. 2015

expression site–associated gene (ESAG) protein, putative, expression site–associated gene 5 (ESAG5) protein
Tb927.5.340;Tb11.v5.0282
AGOF: null, lipid binding
AGOC: null
AGOP: null
PGOF: lipid binding
PGOC: null
PGOP: null

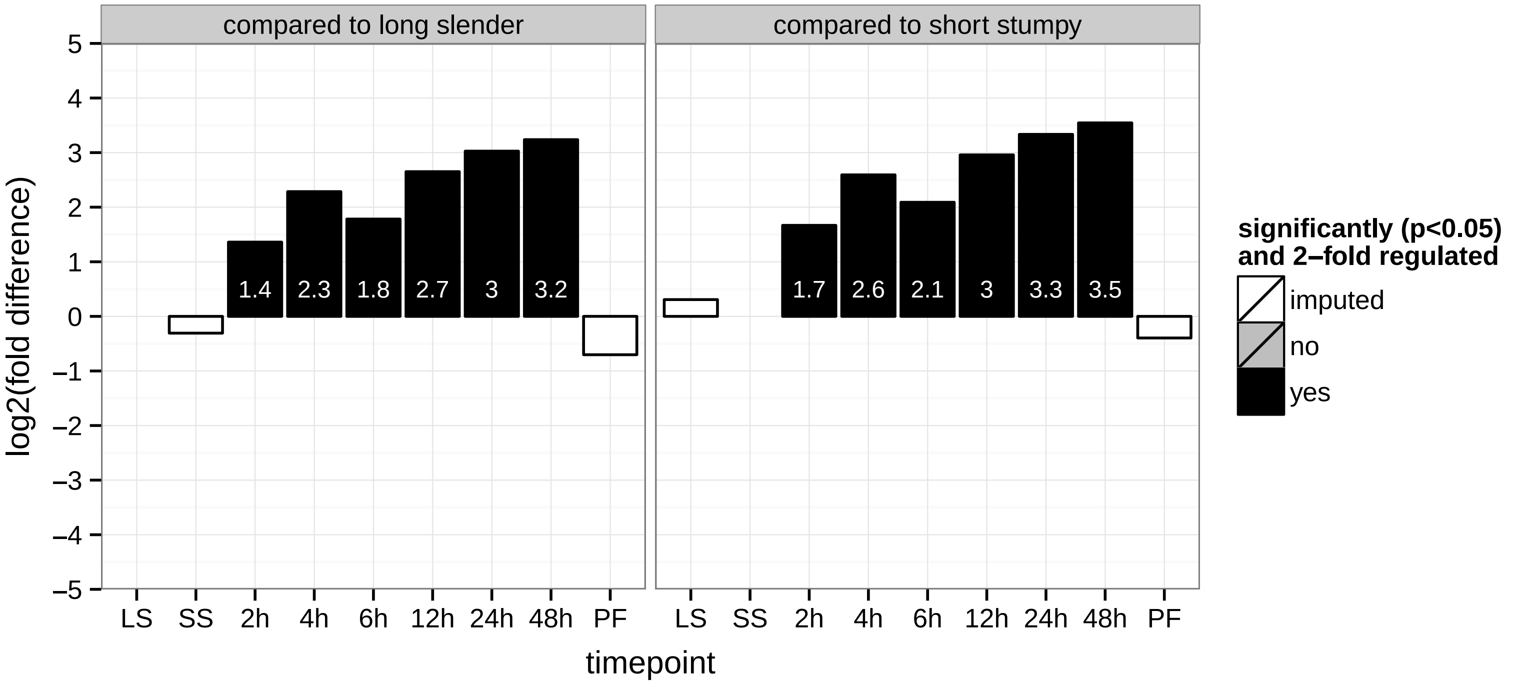

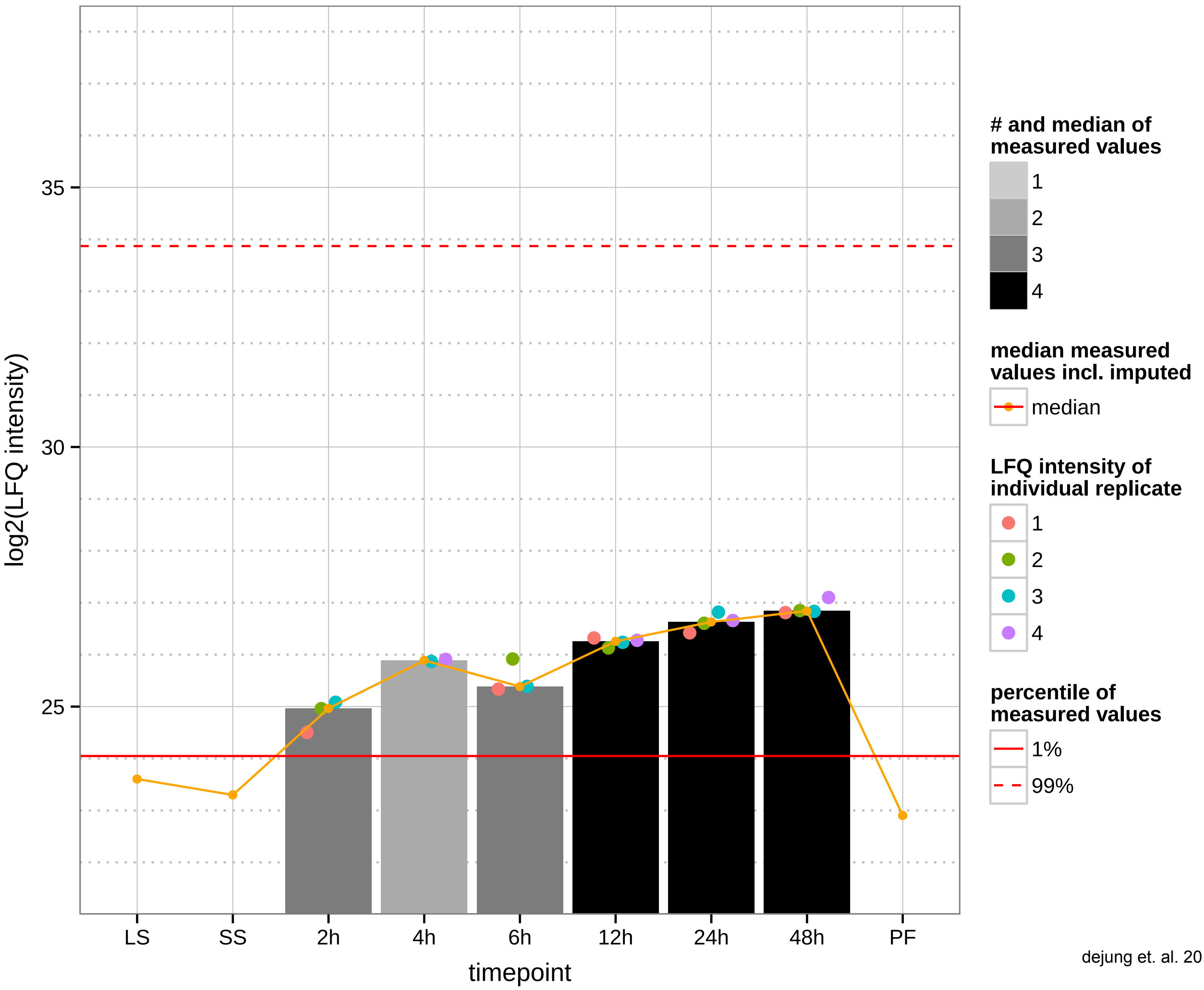

dejung et. al. 2015

hypothetical protein, conserved
Tb927.10.2790
AGOF: null
AGOC: null
AGOP: null
PGOF: protein binding
PGOC: Golgi transport complex
PGOP: intra–Golgi vesicle–mediated transport

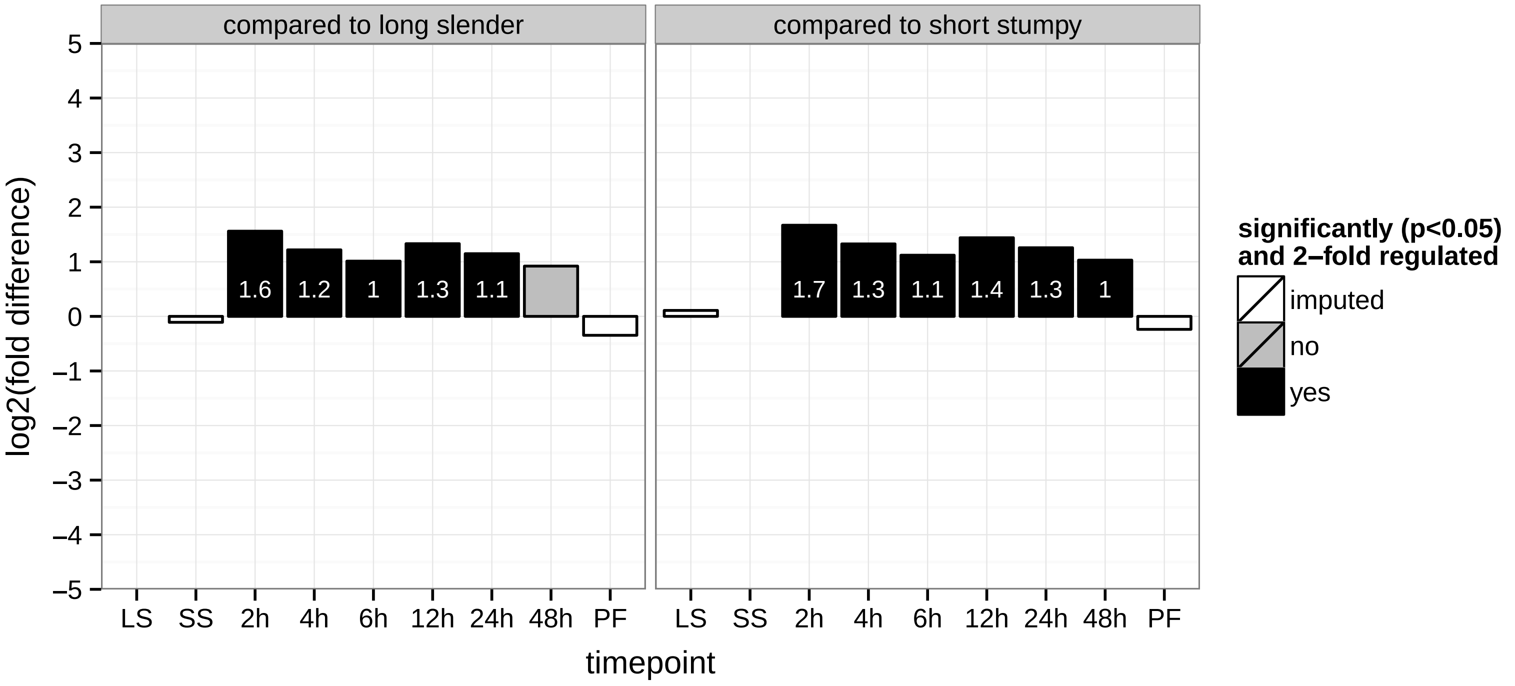

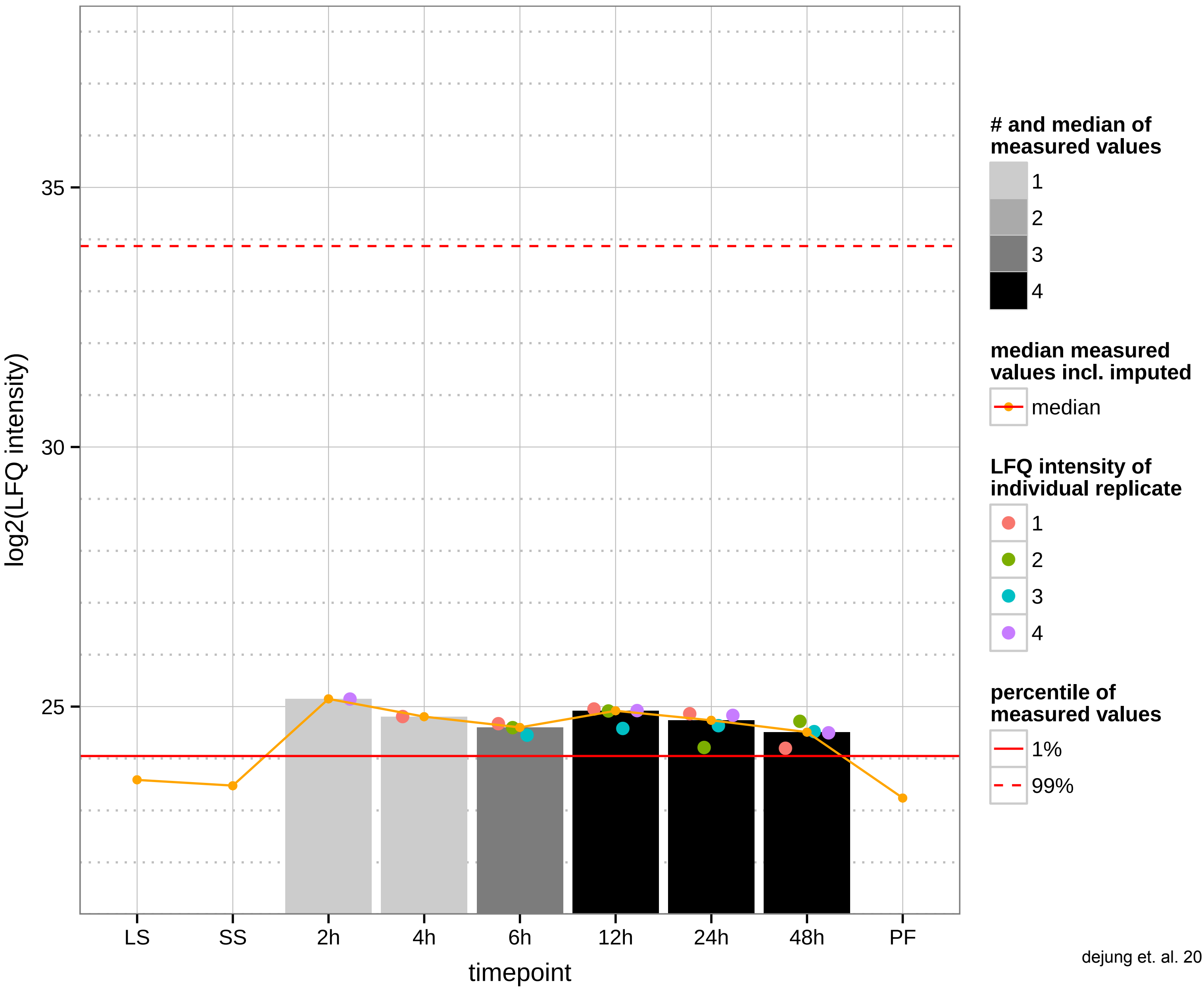

dejung et. al. 2015

small GTPase, putative (RAB23)
Tb927.10.8560
AGOF: GTP binding, GTPase activity
AGOC: intracellular
AGOP: intracellular protein transport, nucleocytoplasmic transport, small GTPase mediated signal transduction
PGOF: GTP binding, GTPase activity
PGOC: intracellular, membrane
PGOP: GTP catabolic process, intracellular protein transport, nucleocytoplasmic transport, protein transport, signal transducti

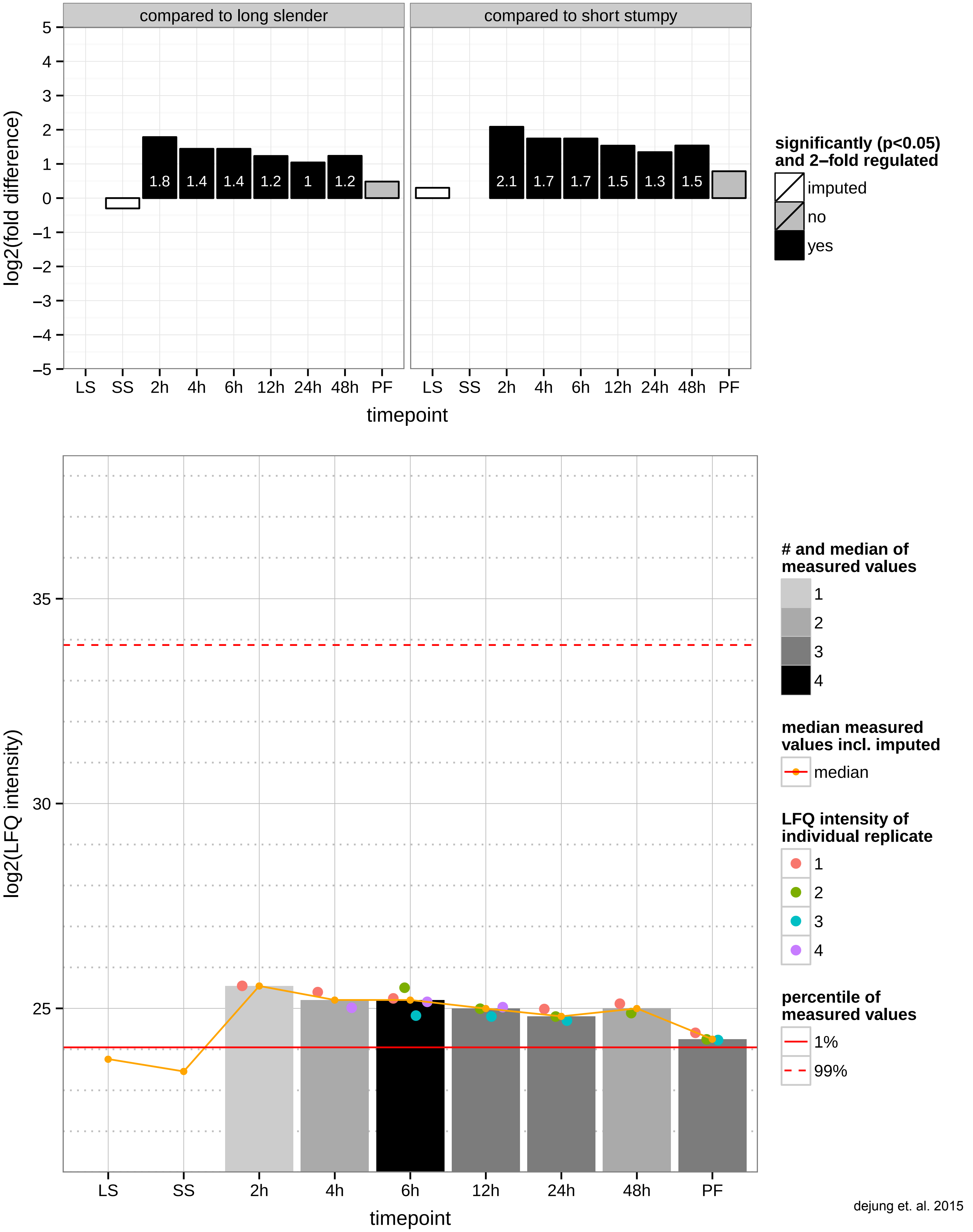

dejung et. al. 2015

expression site–associated gene 4 (ESAG4) protein, putative, receptor–type adenylate cyclase GRESAG 4
Tb927.11.150;Tb11.v5.1015
AGOF: null, adenylate cyclase activity
AGOC: null, integral to membrane
AGOP: null, cyclic nucleotide biosynthetic process, intracellular signal transduction
PGOF: phosphorus–oxygen lyase activity
PGOC: null
PGOP: cyclic nucleotide biosynthetic process, intracellular signal transduction

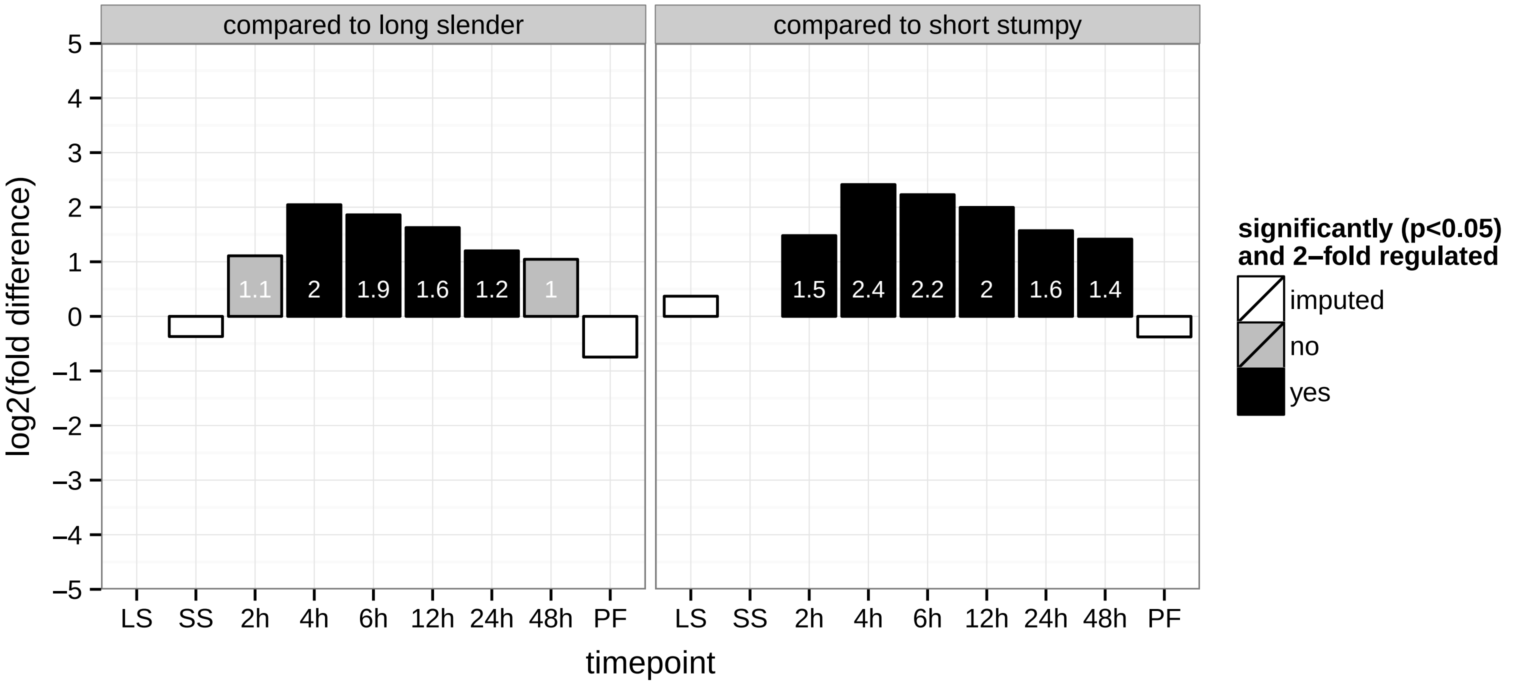

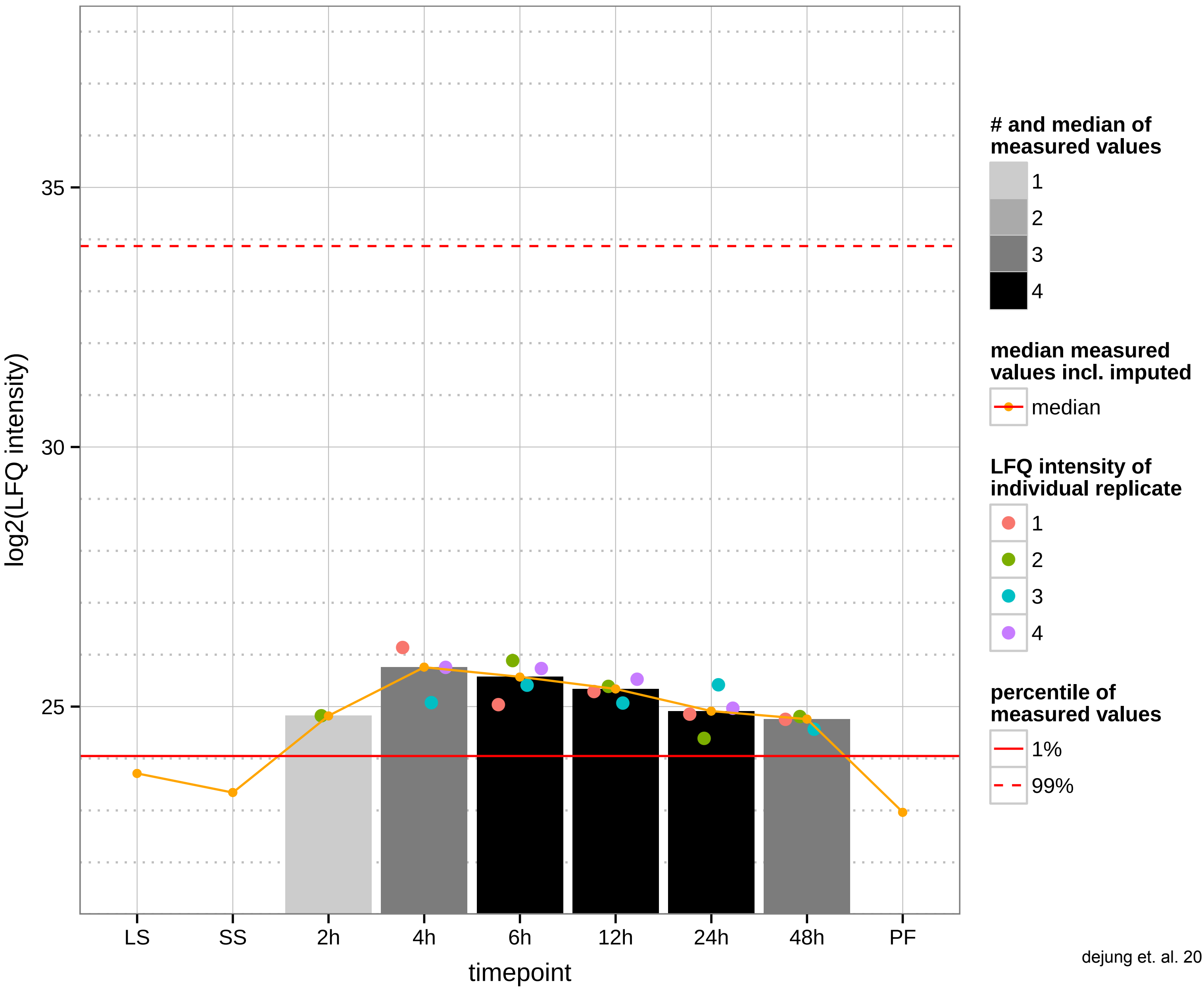

dejung et. al. 2015

hypothetical protein, conserved
Tb927.11.6100
AGOF: null
AGOC: mitochondrion
AGOP: null
PGOF: null
PGOC: null
PGOP: null

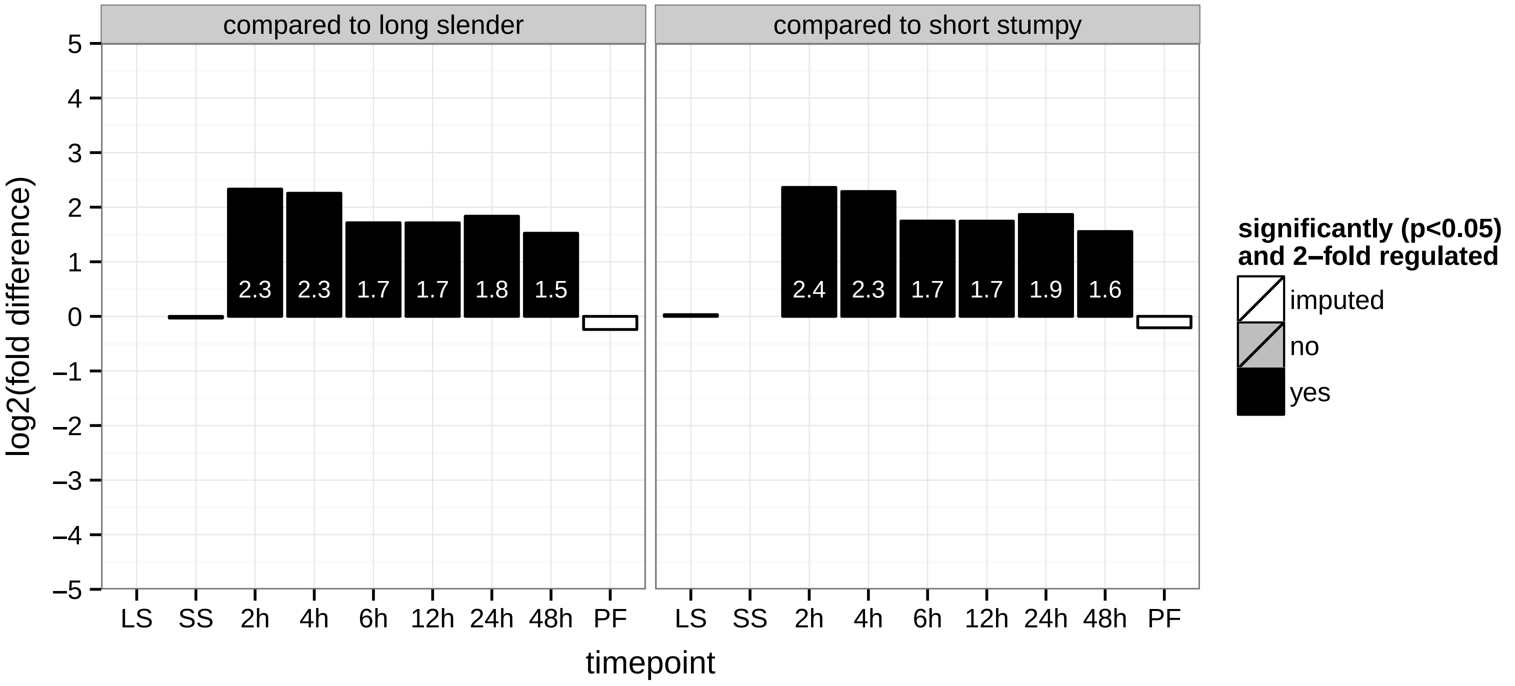

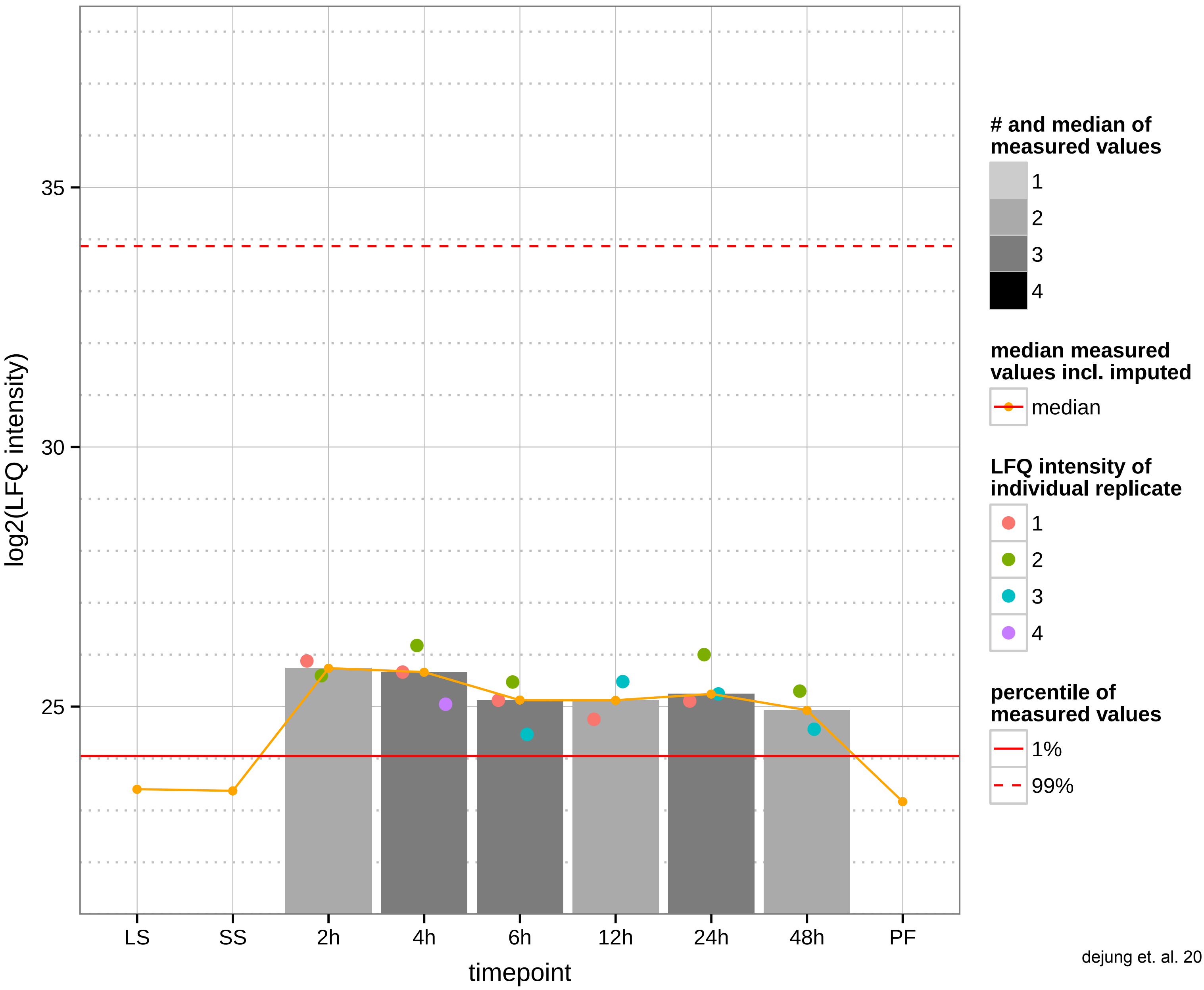

dejung et. al. 2015

hypothetical protein, conserved
Tb927.3.2350
AGOF: null
AGOC: null
AGOP: null
PGOF: null
PGOC: null
PGOP: null

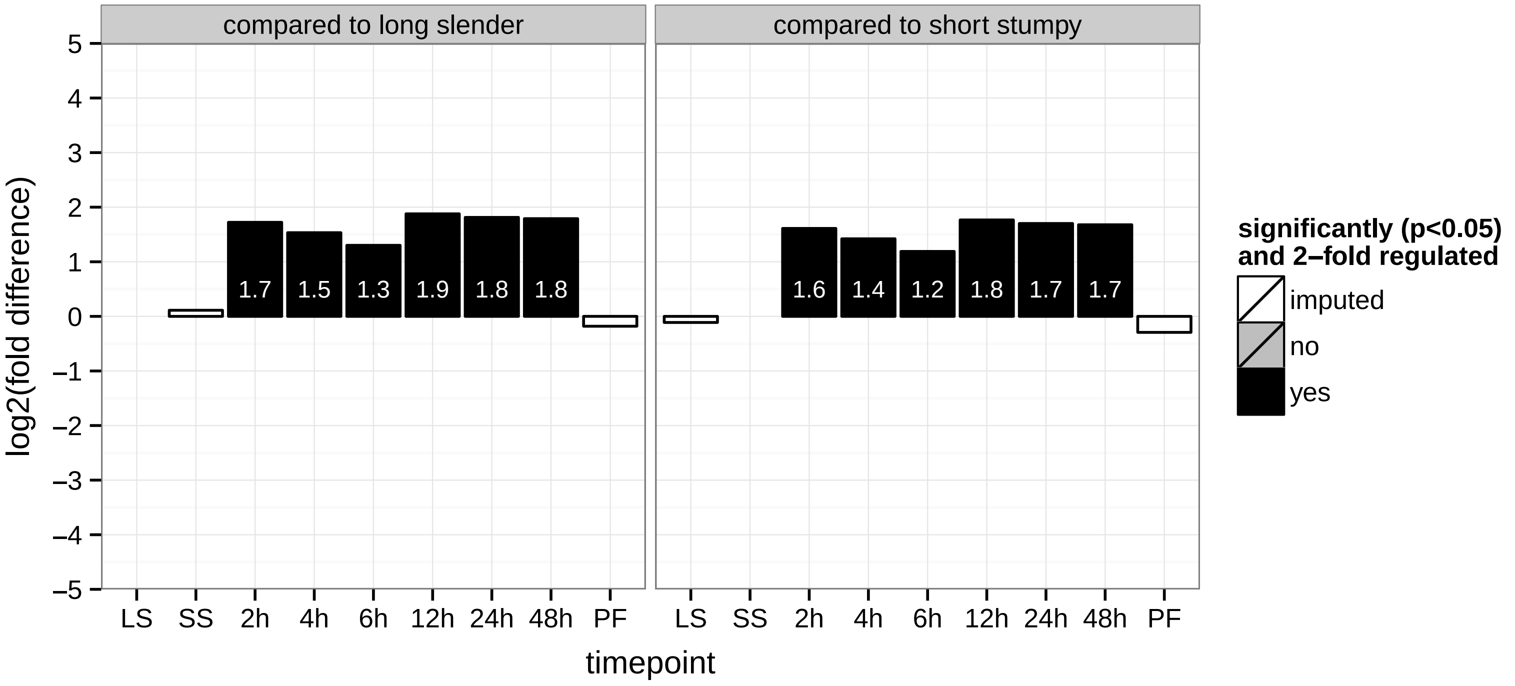

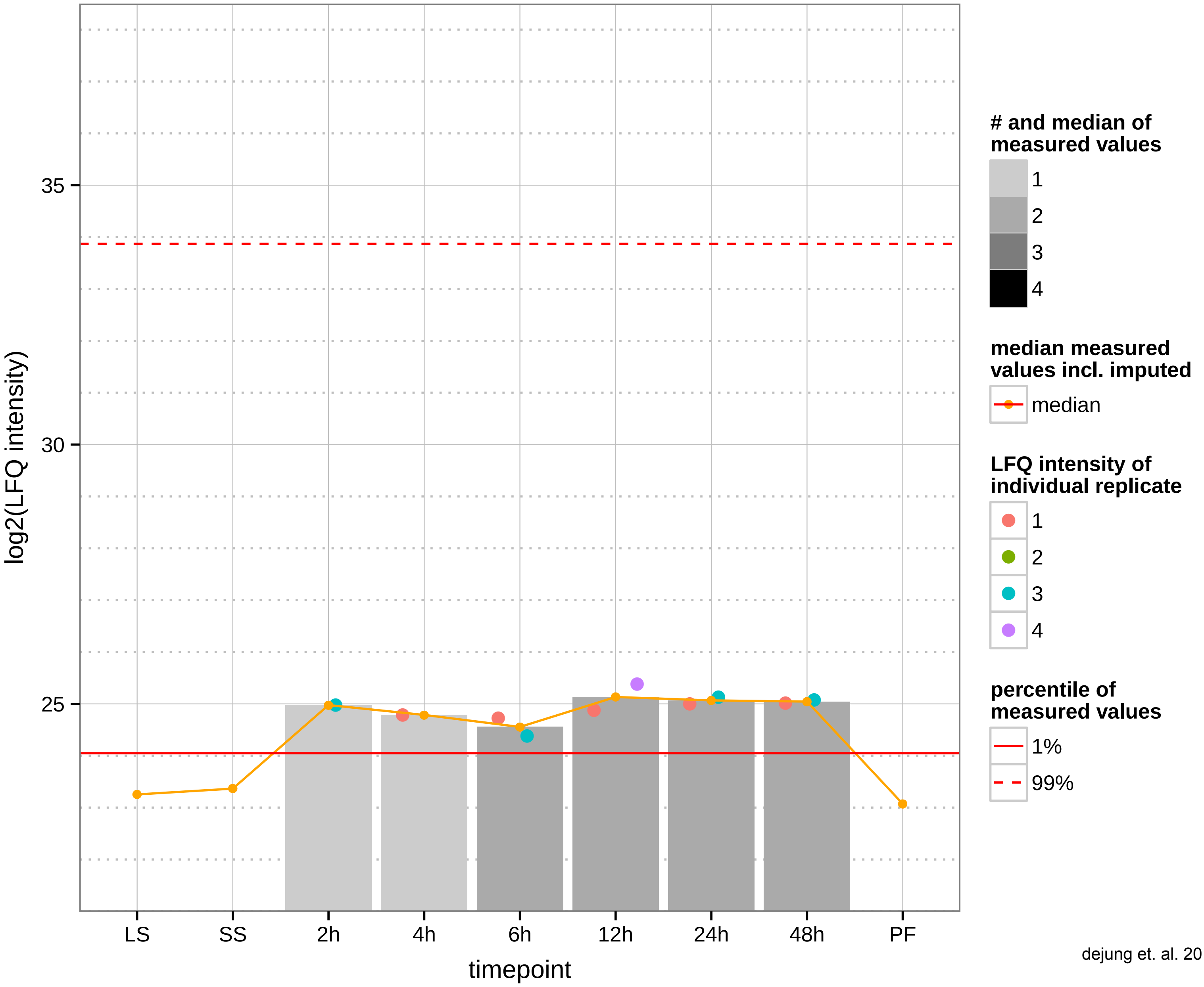

dejung et. al. 2015

hypothetical protein, conserved
Tb927.3.4580
AGOF: null
AGOC: null
AGOP: null
PGOF: null
PGOC: null
PGOP: null

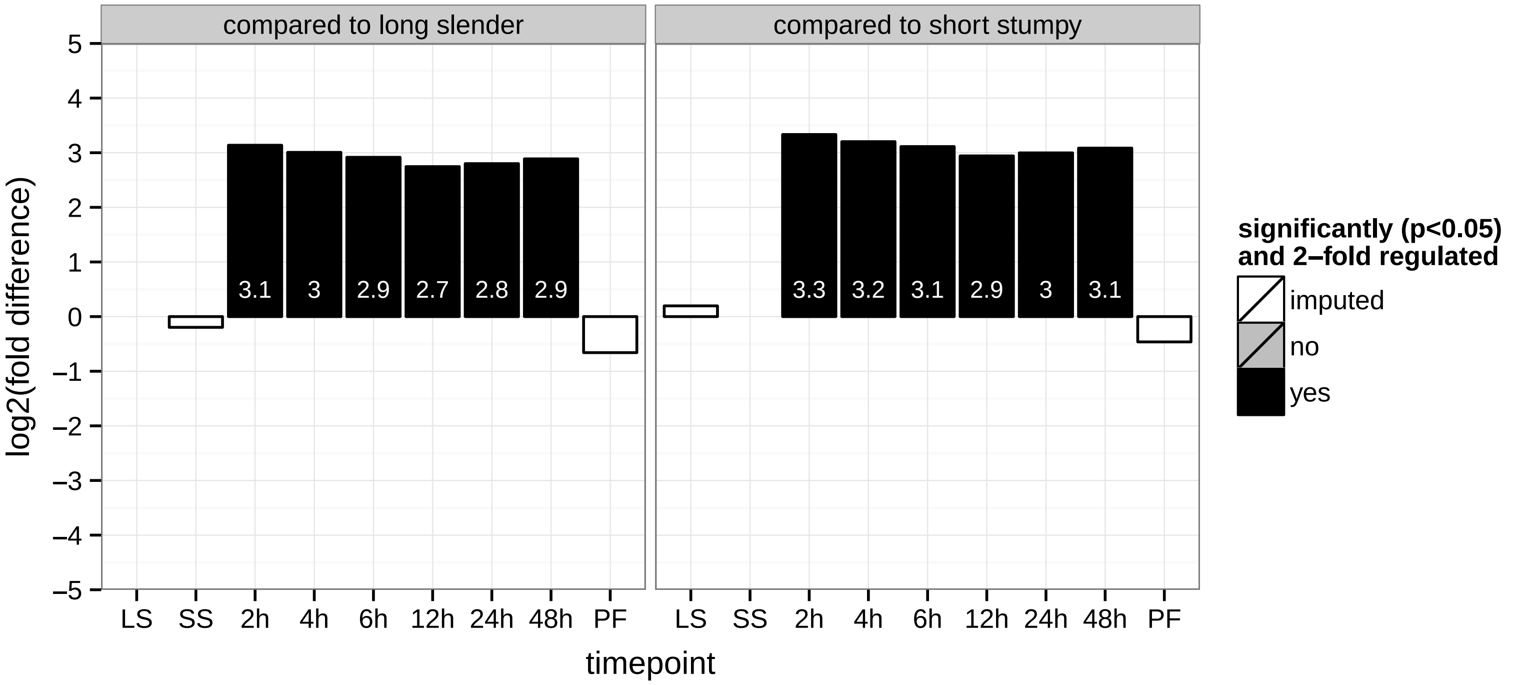

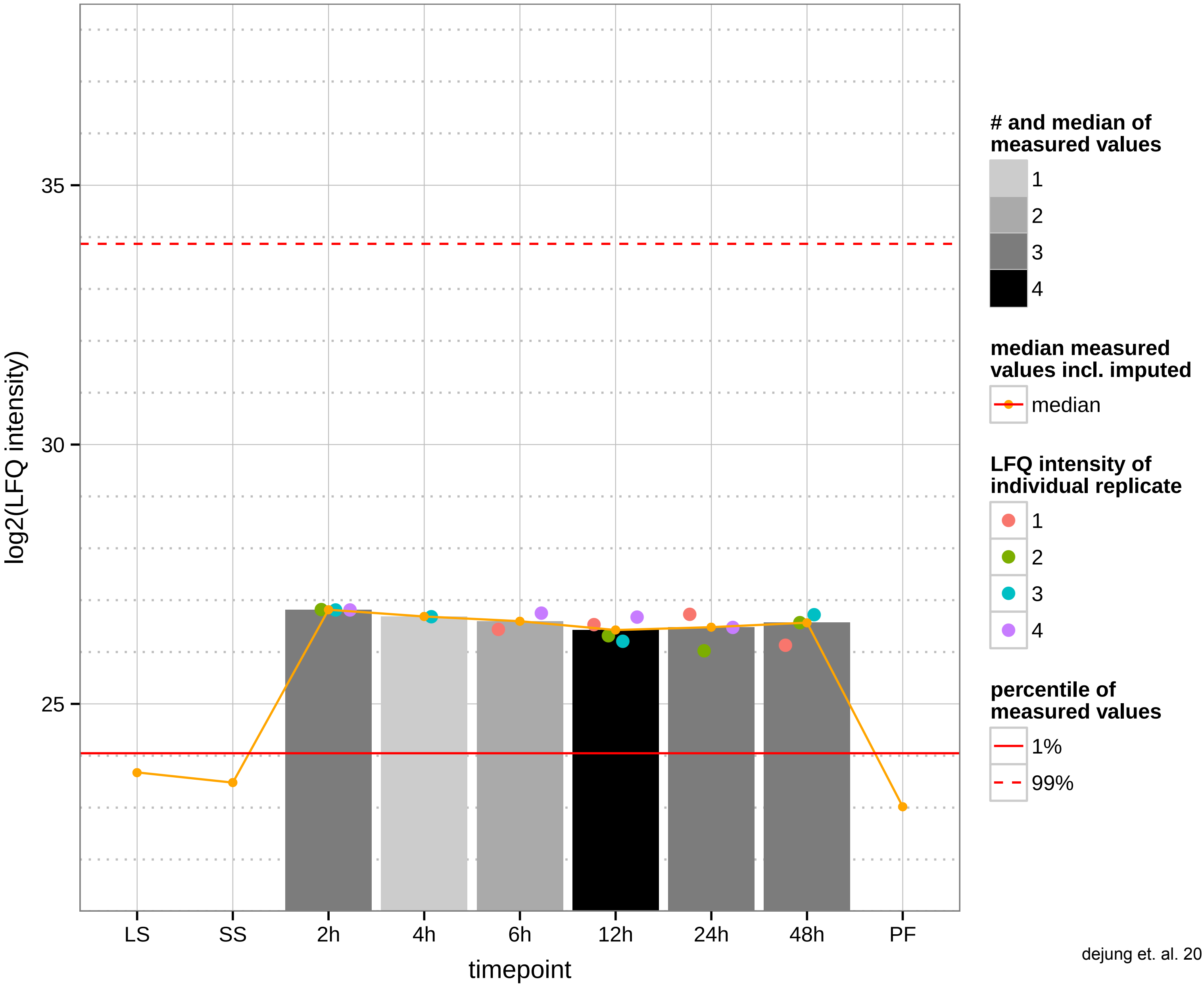

dejung et. al. 2015

protein kinase, putative, eukaryotic translation initiation factor 2−alpha kinase precursor
Tb927.4.2500
AGOF: ATP binding, elongation factor−2 kinase activity, protein serine/threonine kinase activity, protein tyrosine kinase activity
AGOC: integral to membrane
AGOP: protein phosphorylation
PGOF: ATP binding, protein kinase activity, transferase activity, transferring phosphorus−containing groups
PGOC: null
PGOP: protein phosphorylation

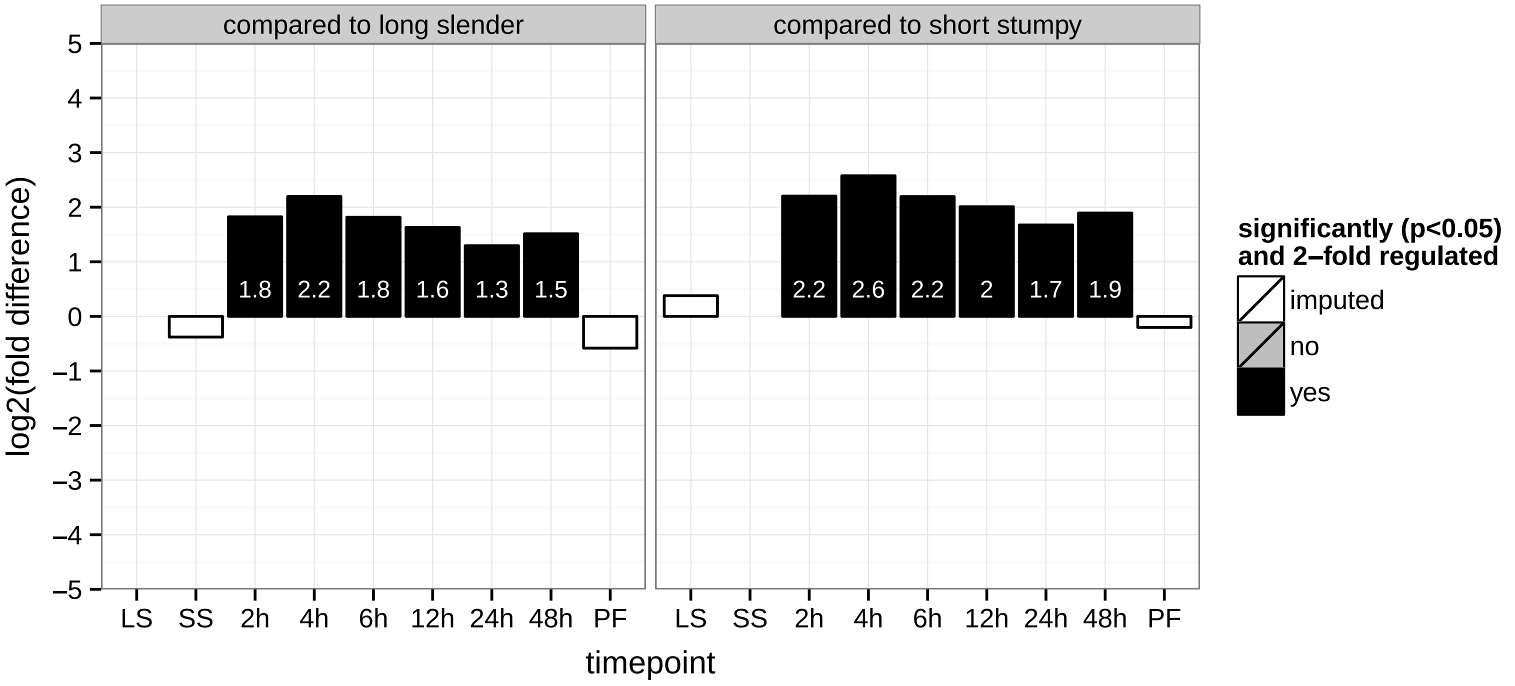

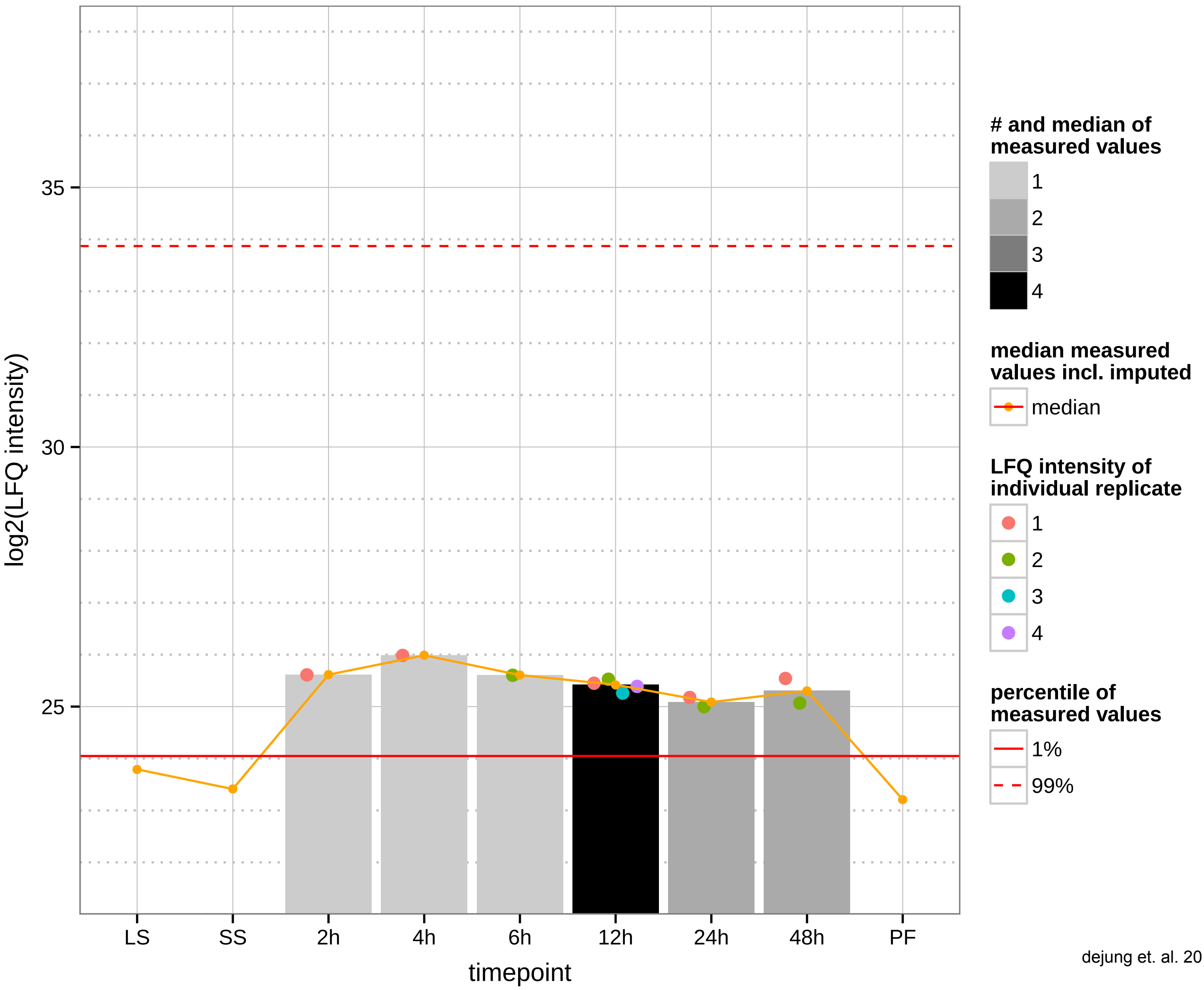

dejung et. al. 2015

hypothetical protein, conserved
Tb927.4.2750
AGOF: null
AGOC: null
AGOP: null
PGOF: null
PGOC: null
PGOP: null

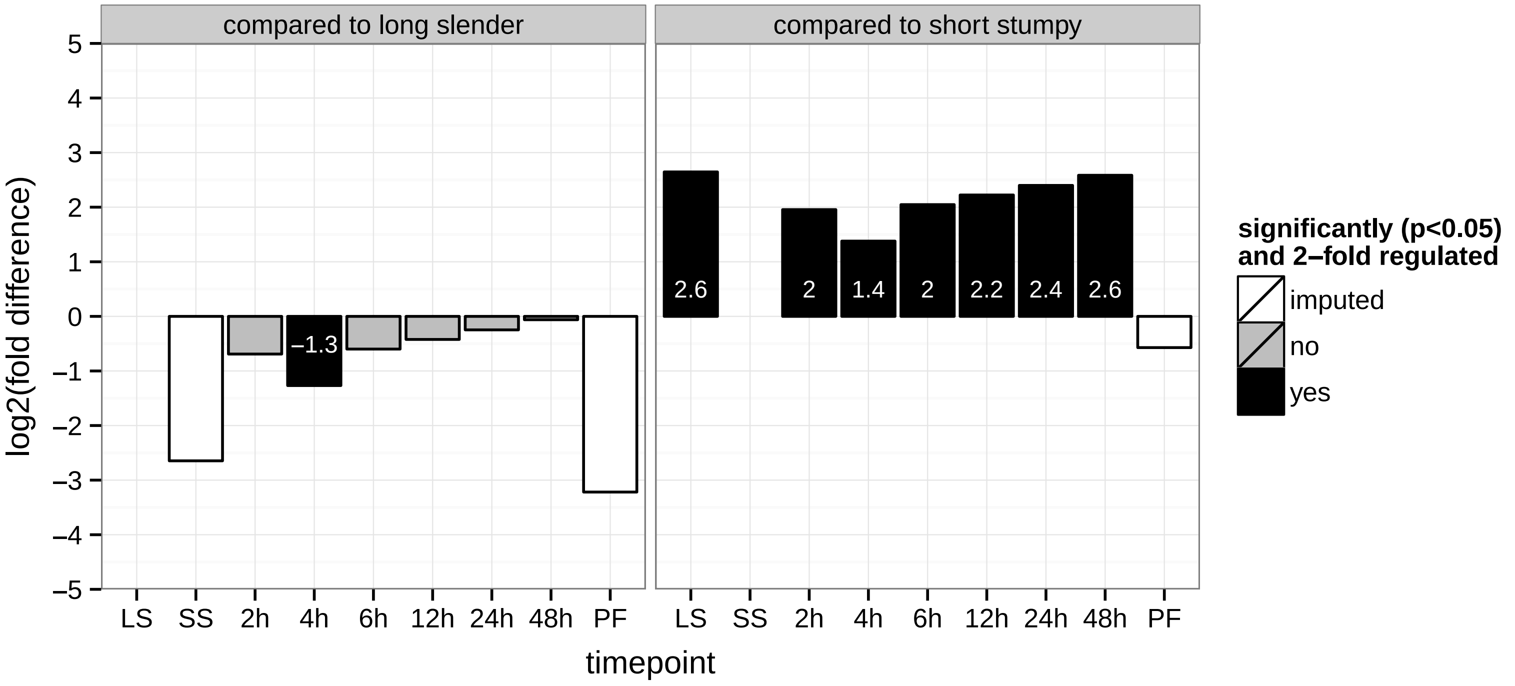

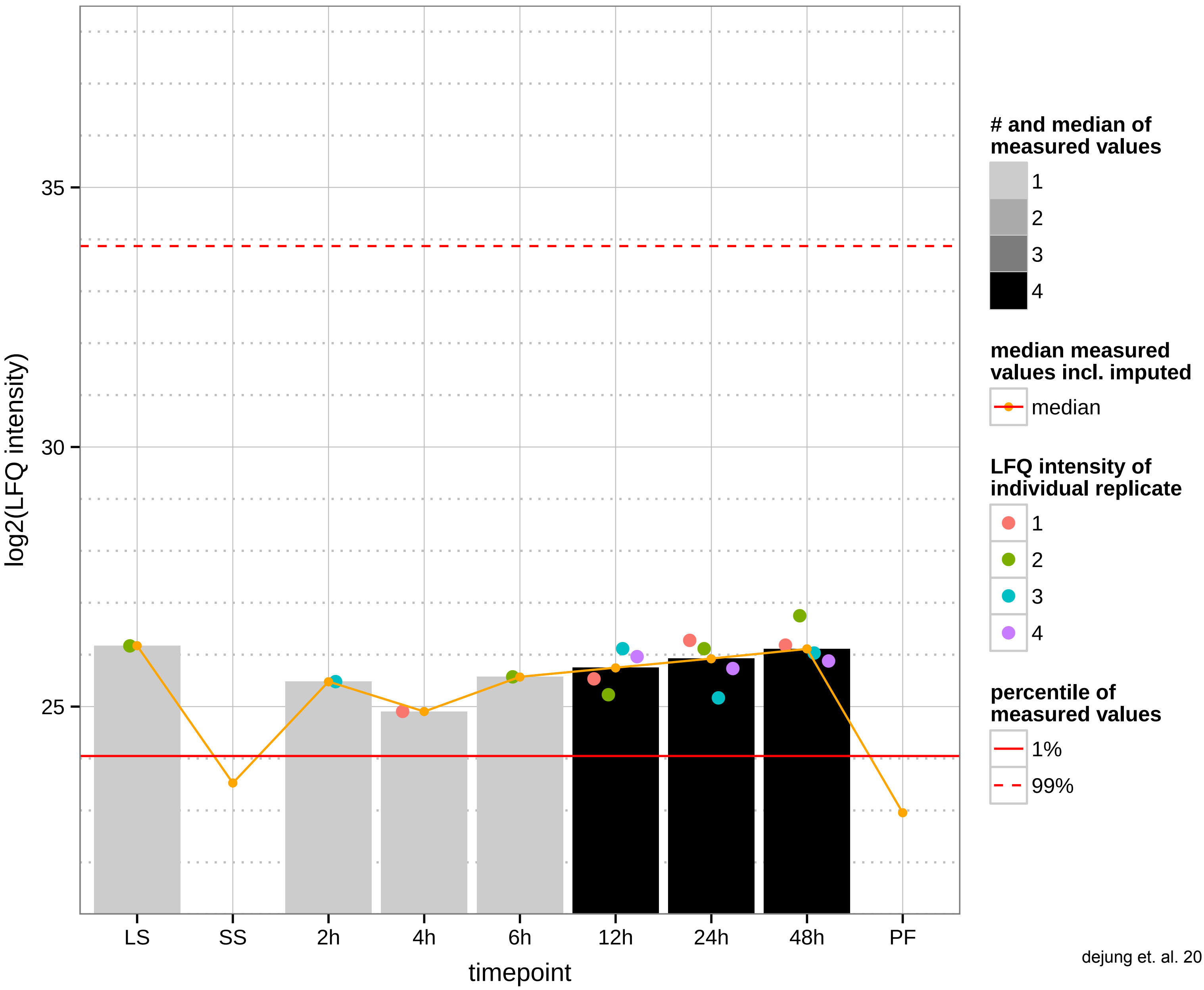

dejung et. al. 2015

amino acid transporter, putative
Tb927.4.3930
AGOF: amine transmembrane transporter activity
AGOC: integral to membrane
AGOP: amino acid transport
PGOF: null
PGOC: null
PGOP: null

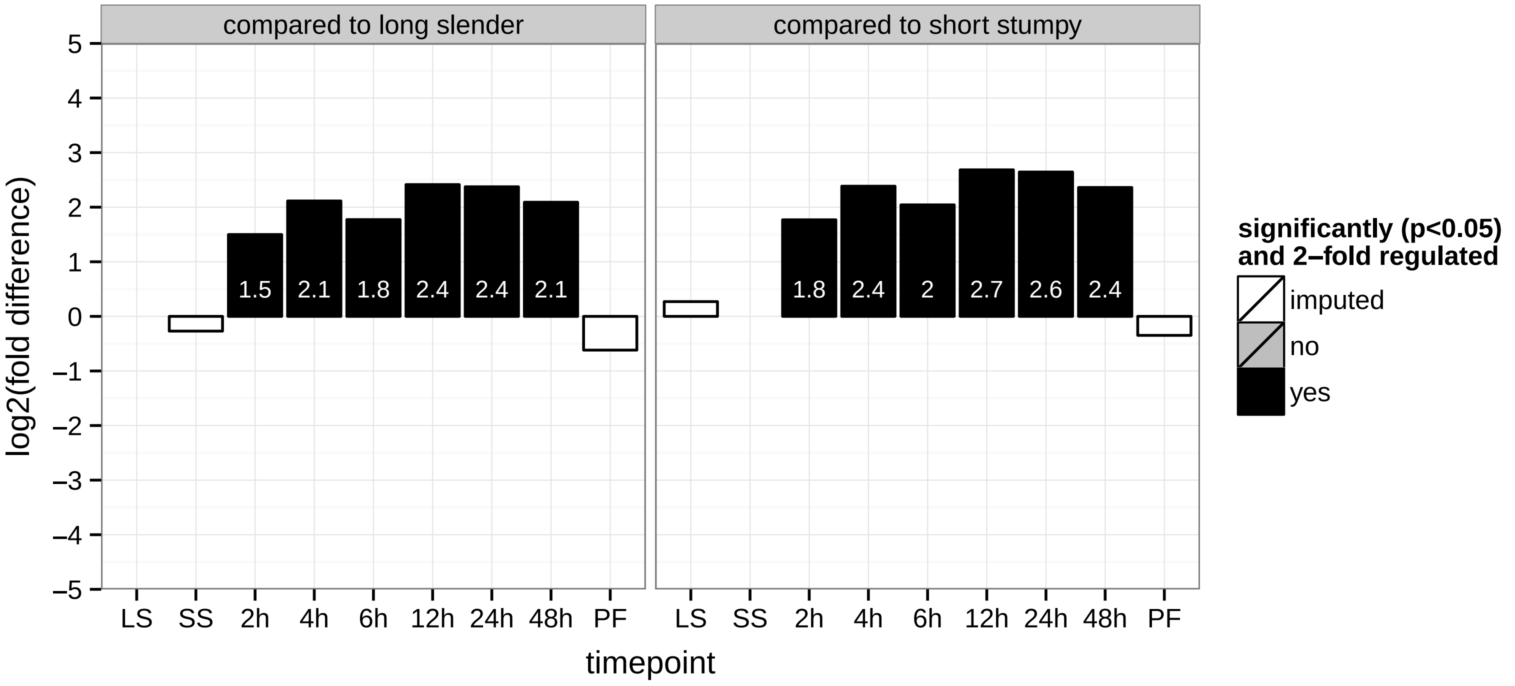

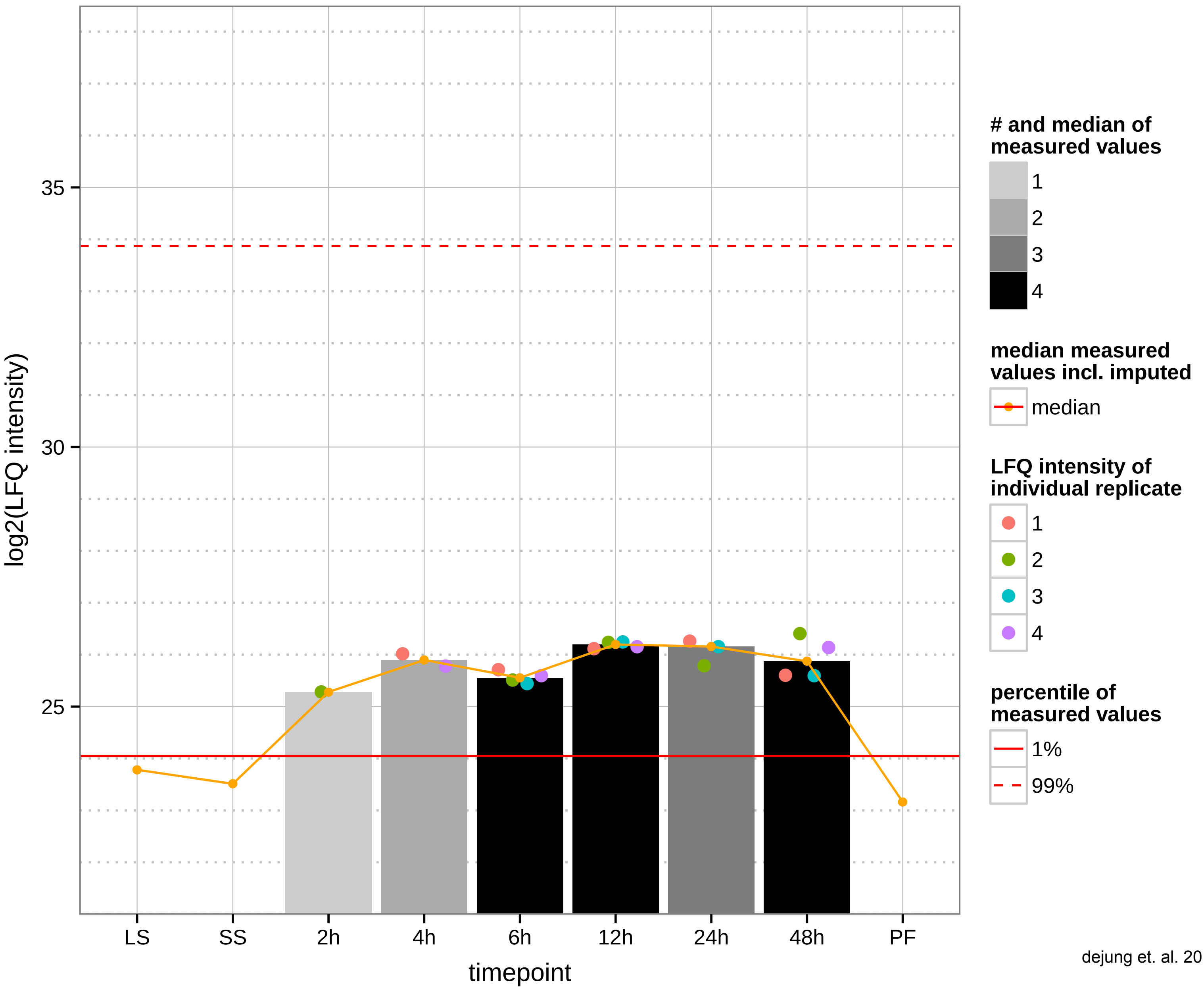

dejung et. al. 2015

centrin, putative, caltractin
Tb927.8.1080
AGOF: calcium ion binding
AGOC: centrosome
AGOP: null
PGOF: calcium ion binding
PGOC: null
PGOP: null

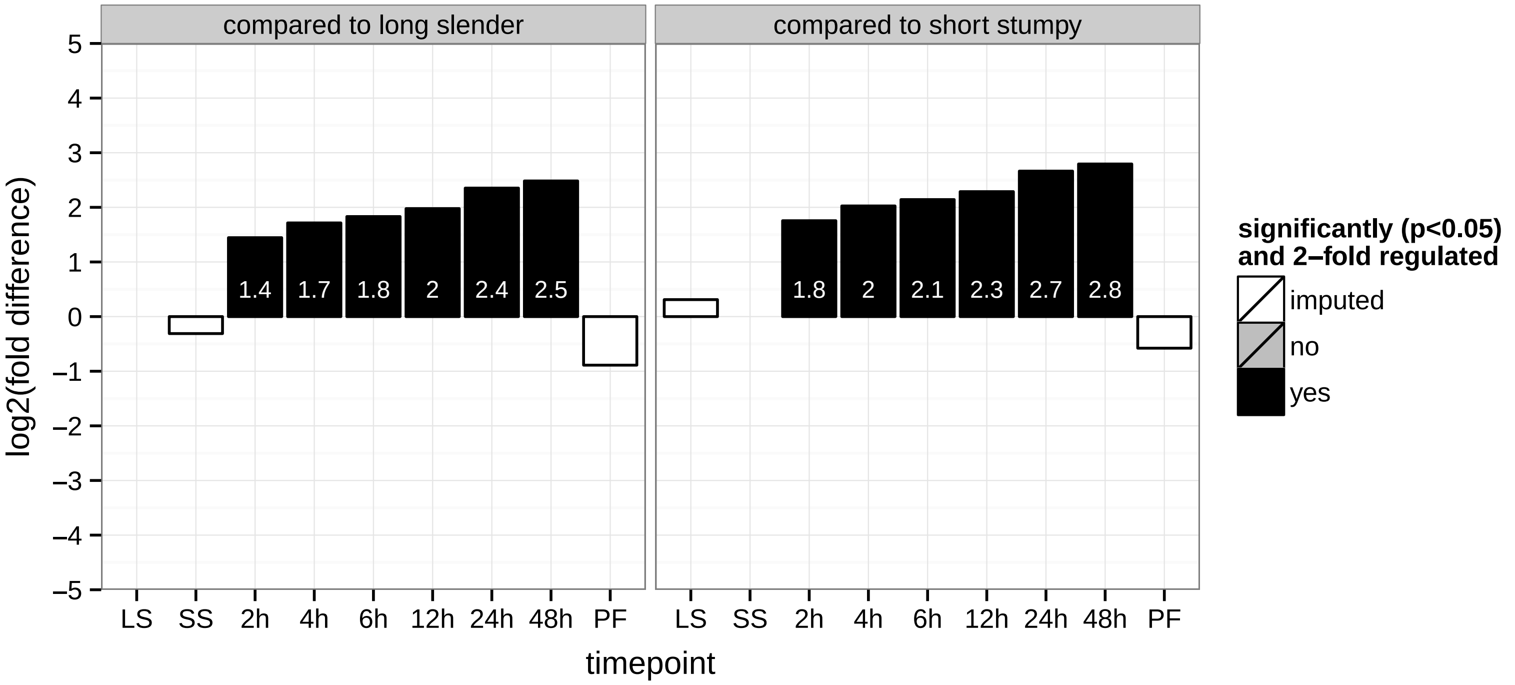

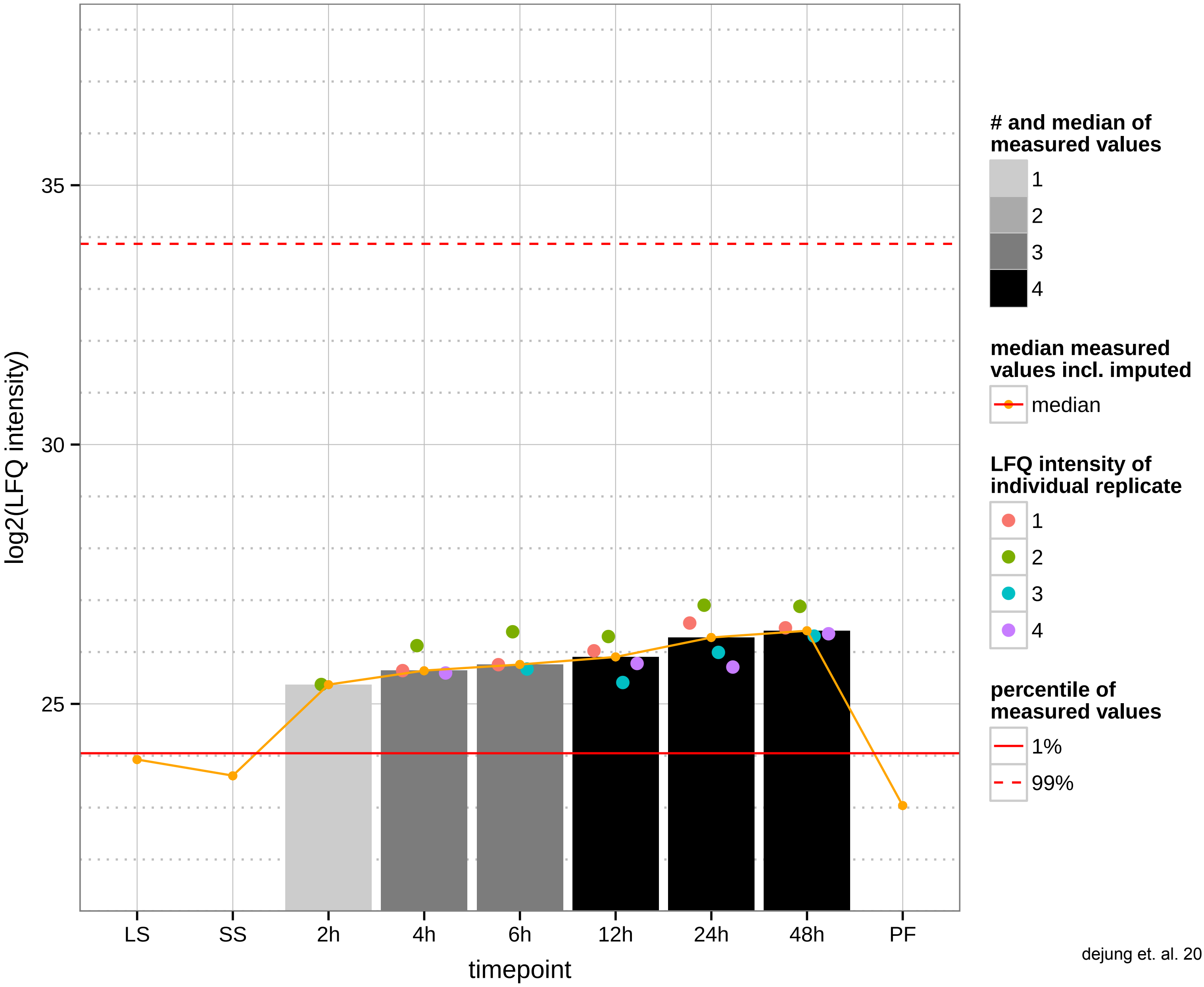

dejung et. al. 2015

hypothetical protein, conserved
Tb927.8.3070
AGOF: null
AGOC: mitochondrion
AGOP: null
PGOF: null
PGOC: null
PGOP: null

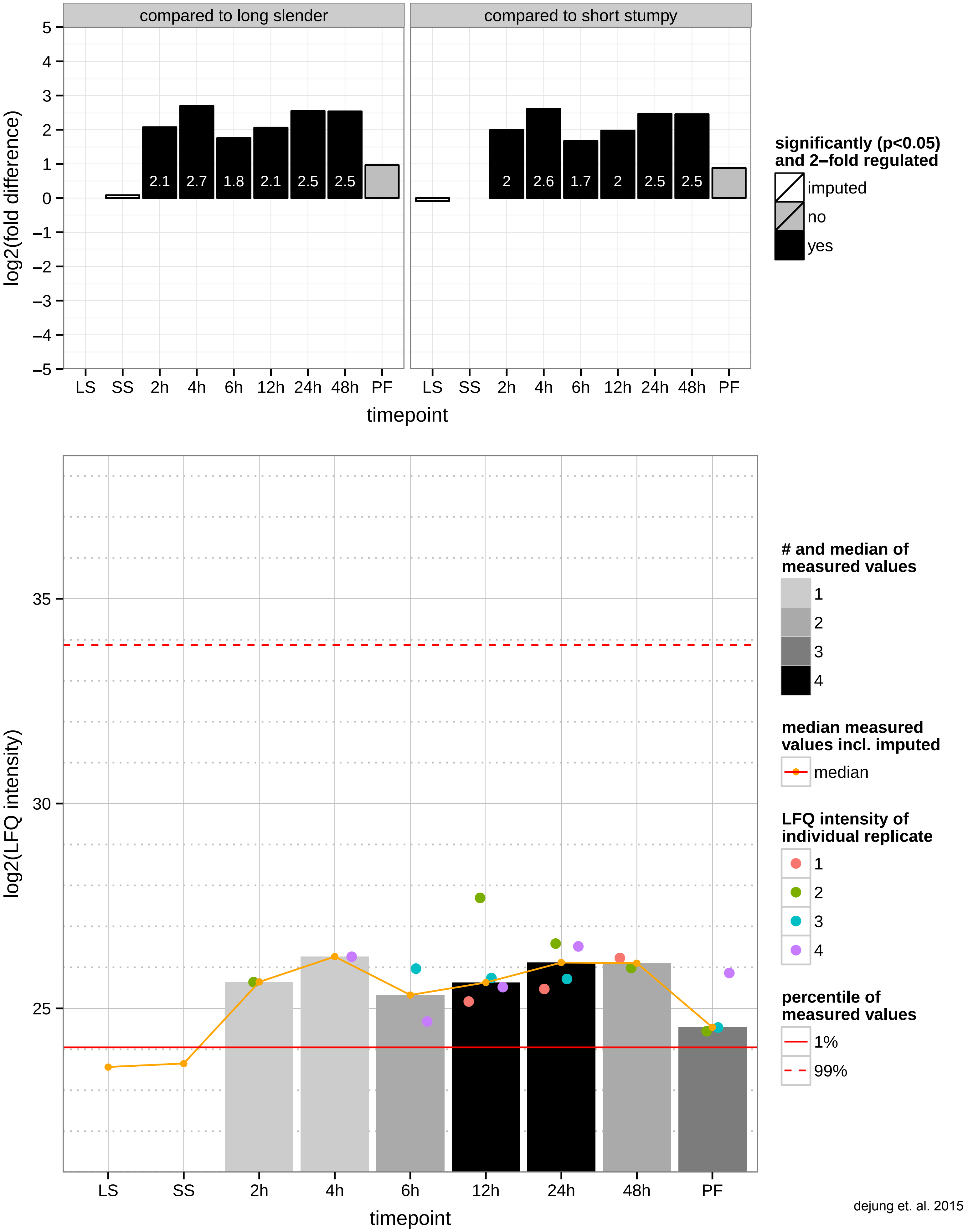

dejung et. al. 2015

cyclophilin type peptidyl–prolyl cis–trans isomerase, putative (PPIase)
Tb927.9.11740
AGOF: peptidyl–prolyl cis–trans isomerase activity
AGOC: null
AGOP: protein folding
PGOF: peptidyl–prolyl cis–trans isomerase activity
PGOC: null
PGOP: protein folding

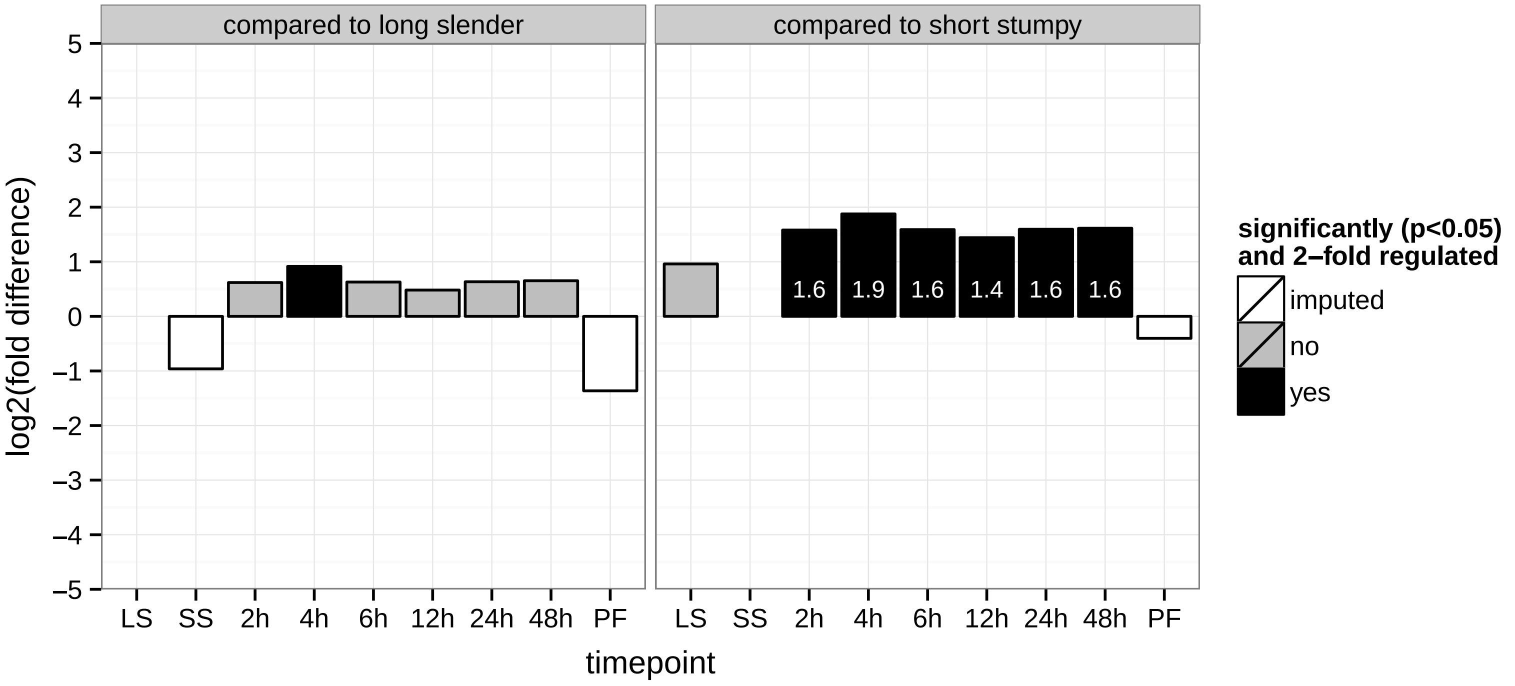

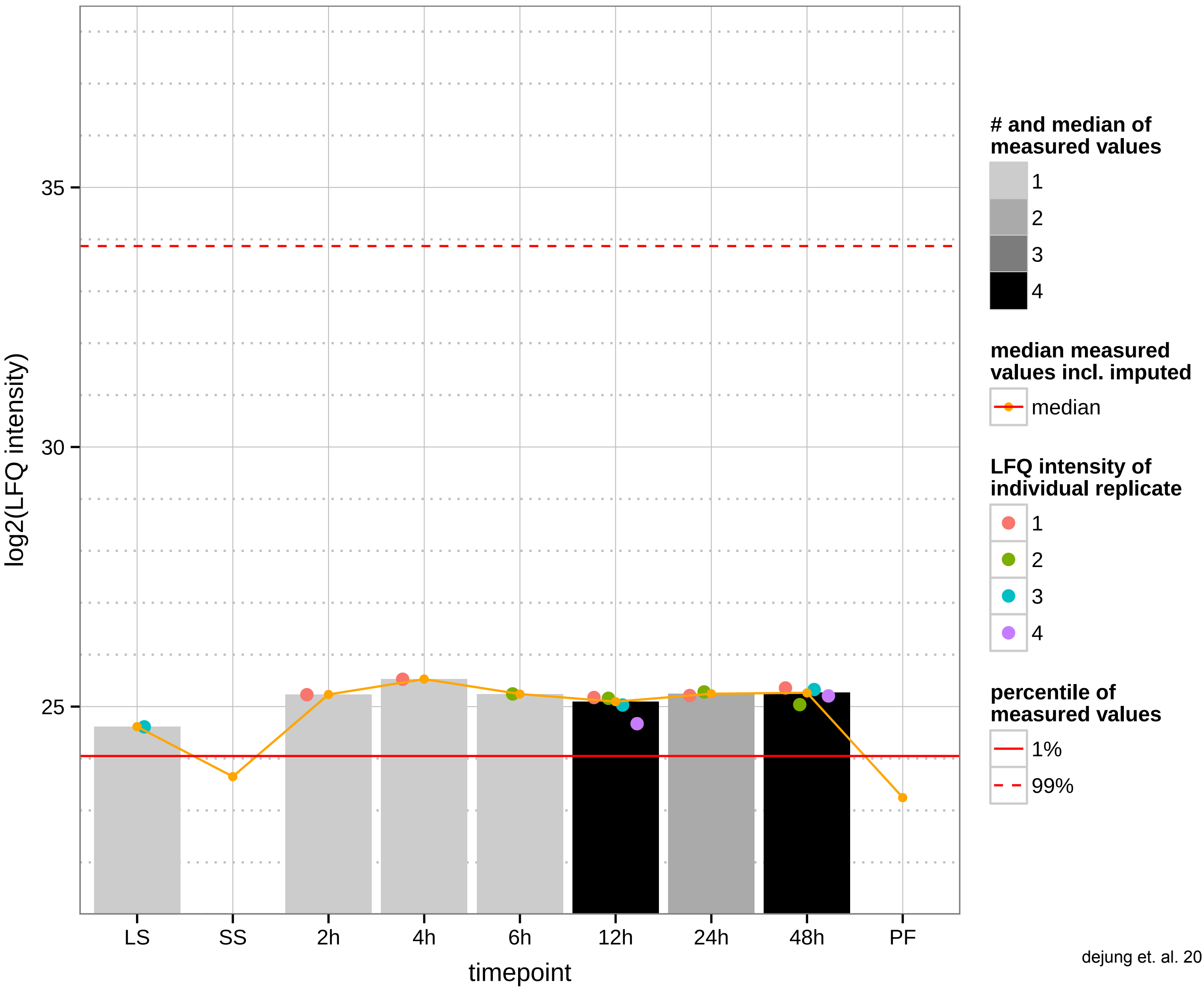

dejung et. al. 2015

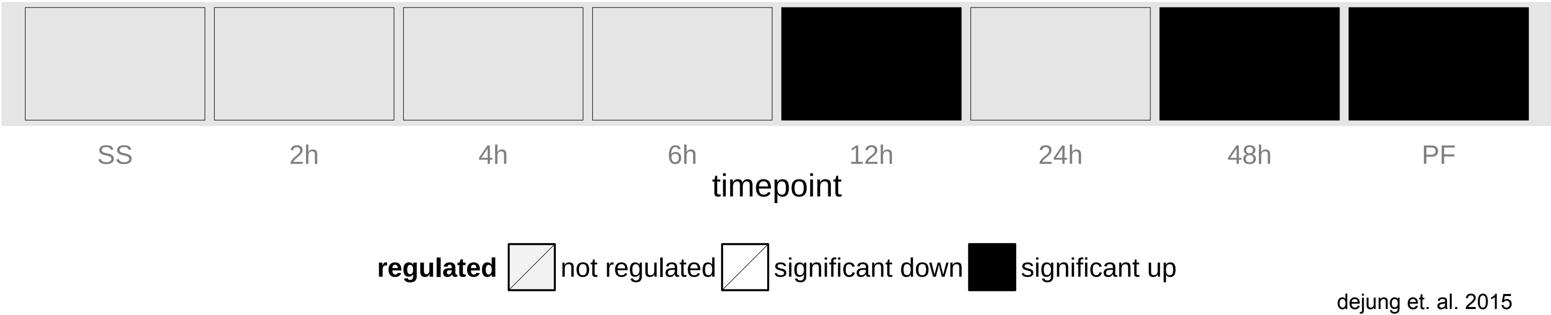
SS
2h
4h
6h
12h
24h
48h
PF
timepoint
regulated
not regulated
significant down
significant up
dejung et. al. 2015

hypothetical protein, conserved
Tb927.10.1200;Tb11.v5.0487
AGOF: null
AGOC: null, cytoplasm
AGOP: null
PGOF: null
PGOC: cytoplasm
PGOP: null

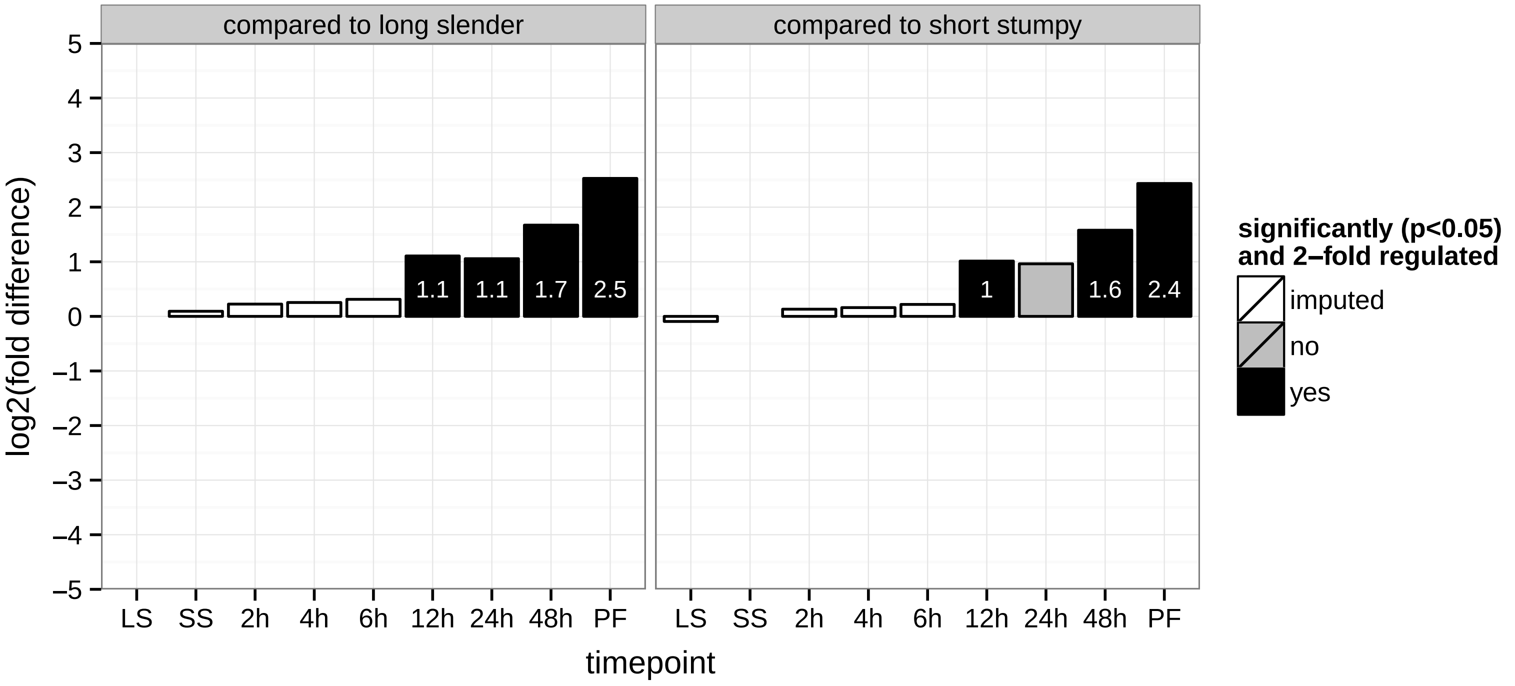

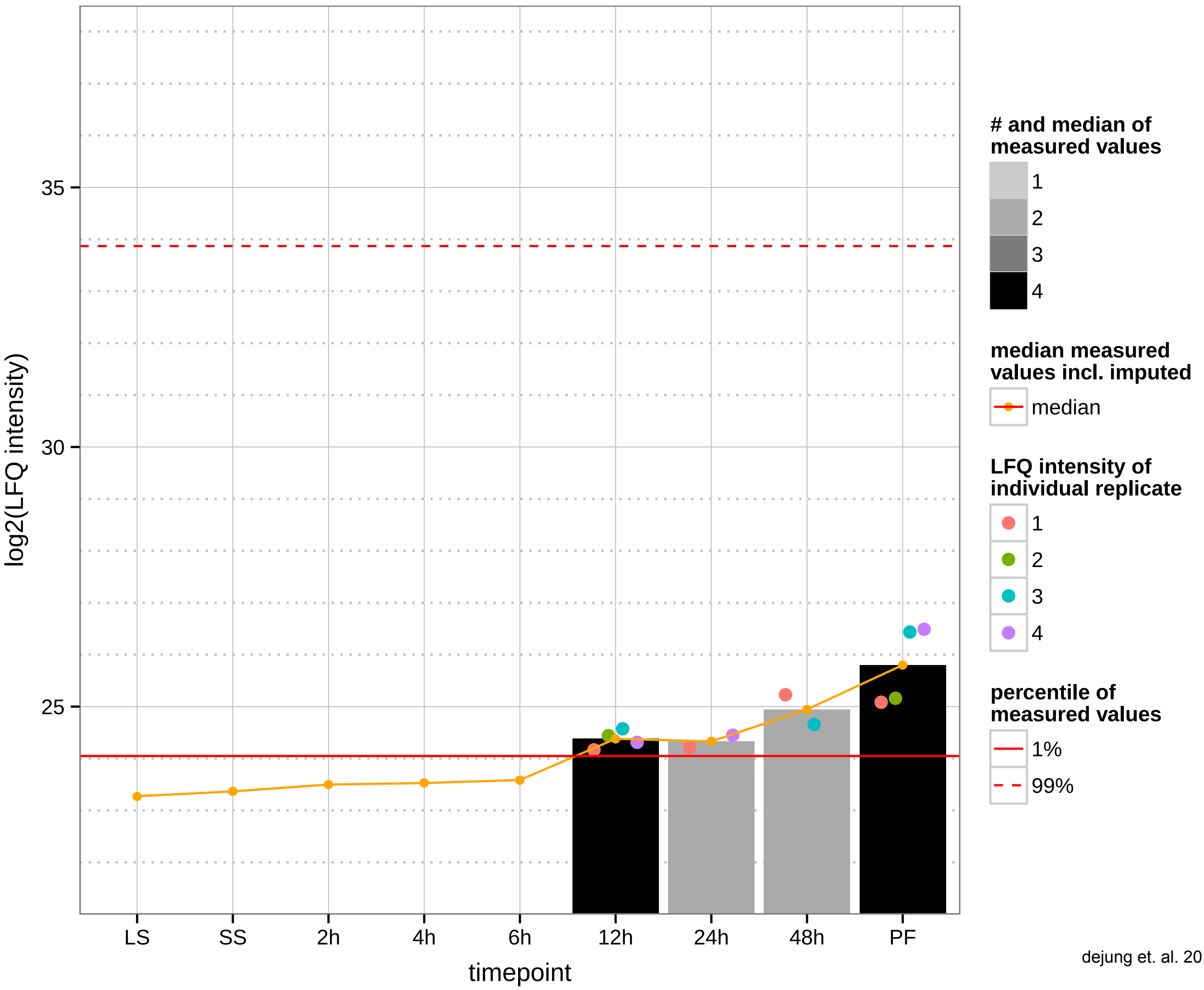

dejung et. al. 2015

ubiquitin hydrolase, putative
Tb11.v5.0553
AGOF: null
AGOC: null
AGOP: null
PGOF: exonuclease activity, nucleic acid binding
PGOC: intracellular
PGOP: null

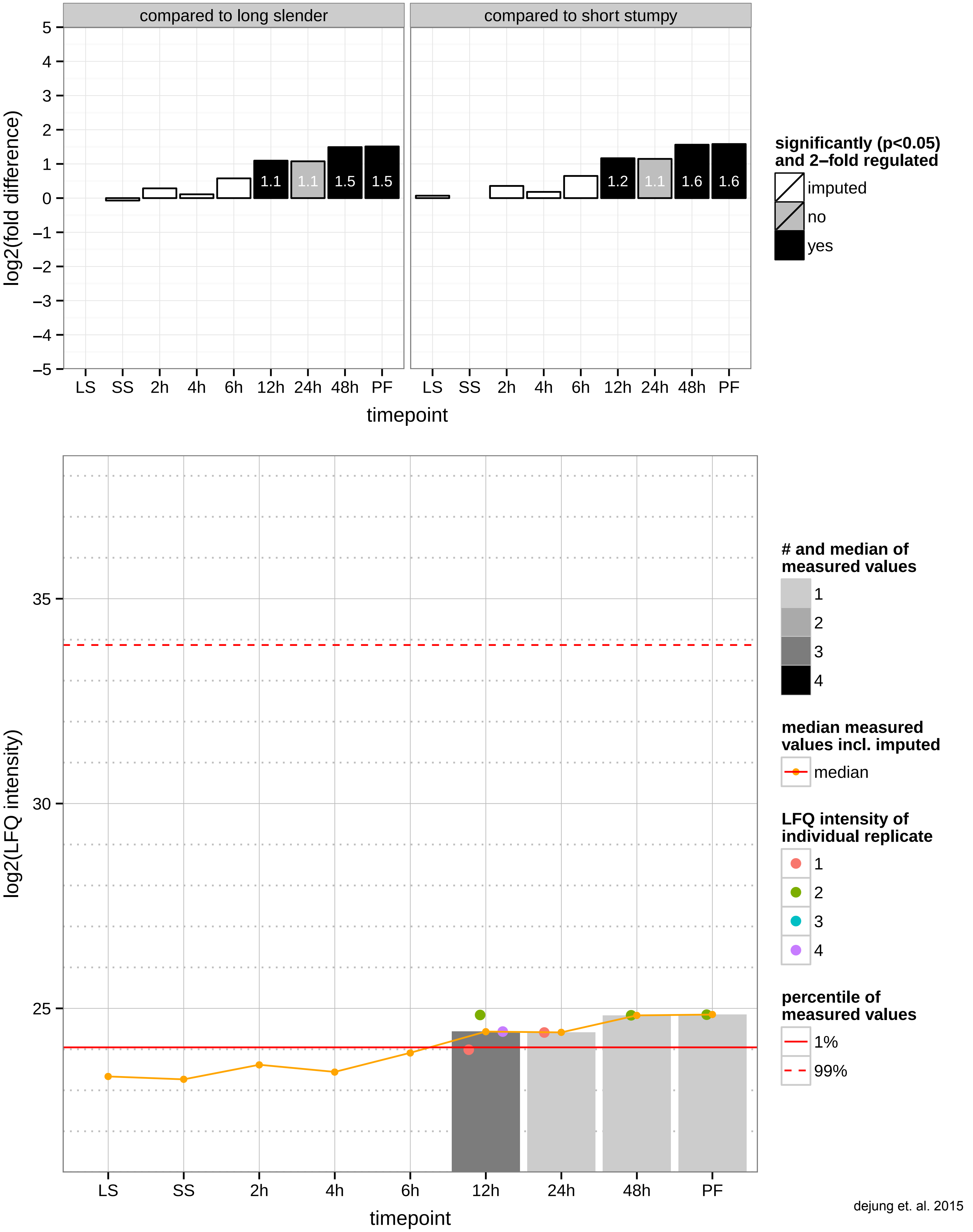

dejung et. al. 2015

hypothetical protein, conserved
Tb927.10.11400
AGOF: null
AGOC: null
AGOP: null
PGOF: protein binding
PGOC: null
PGOP: null

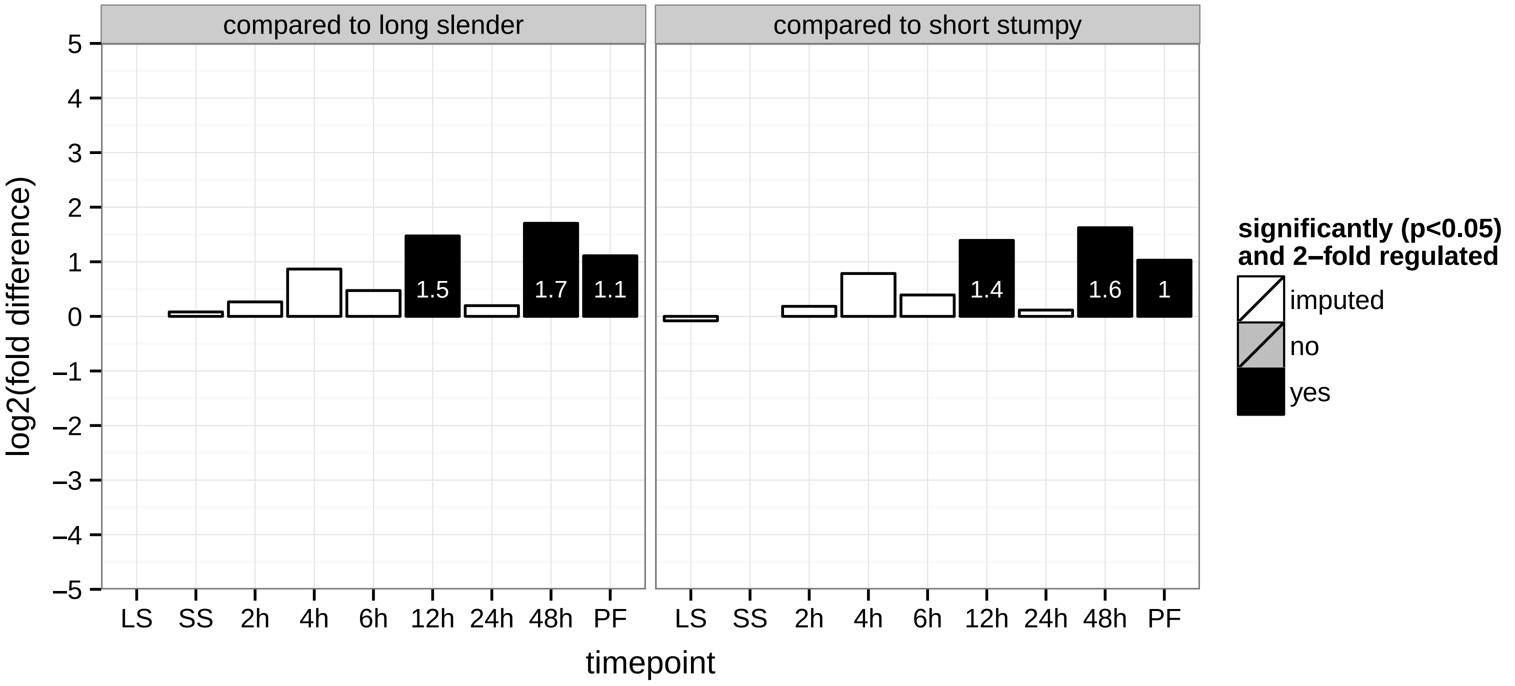

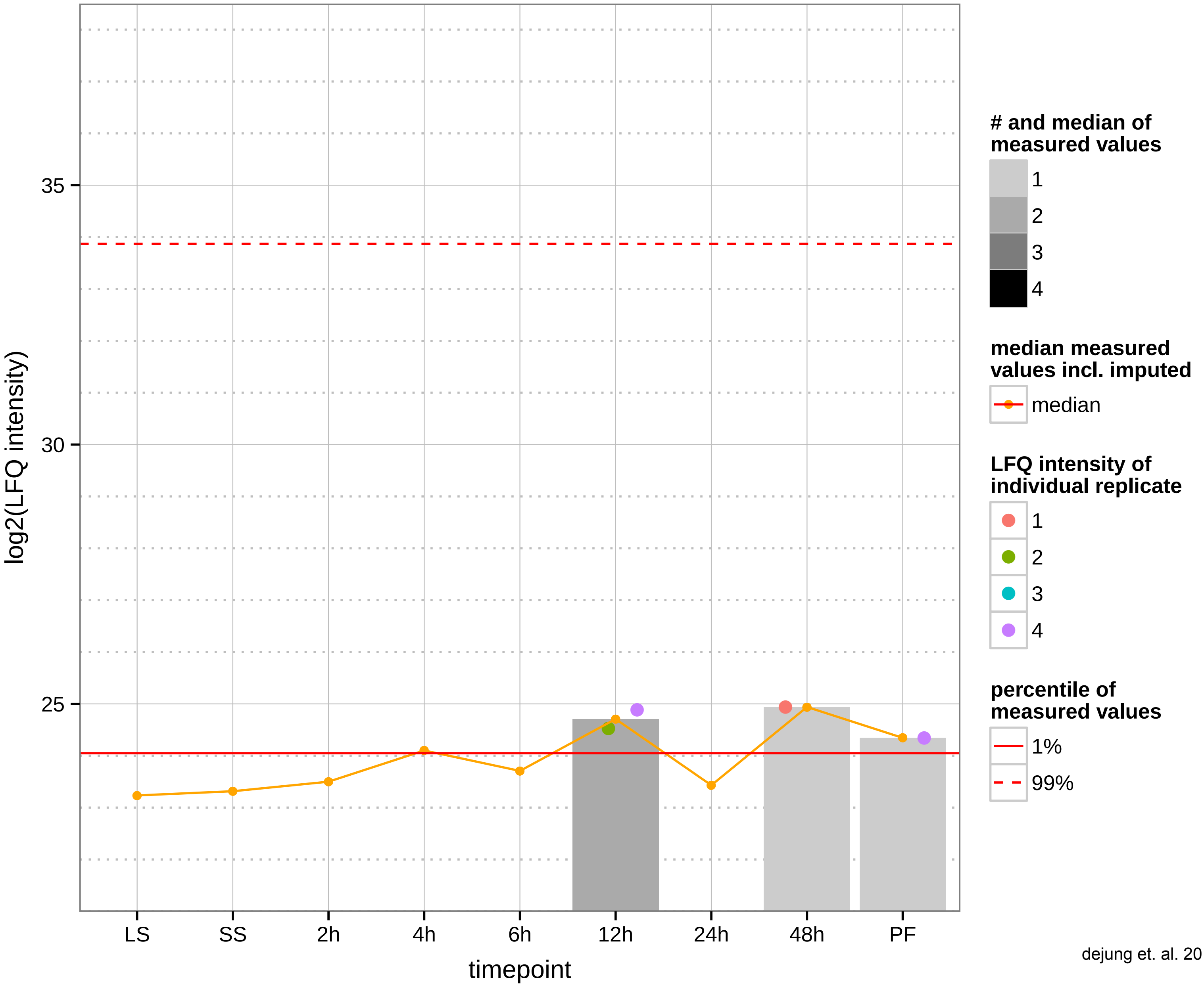

dejung et. al. 2015

U3 small nucleolar ribonucleoprotein protein IMP3, putative
Tb927.11.13930
AGOF: RNA binding, rRNA binding
AGOC: intracellular, nucleus
AGOP: maturation of SSU−rRNA
PGOF: RNA binding
PGOC: null
PGOP: null

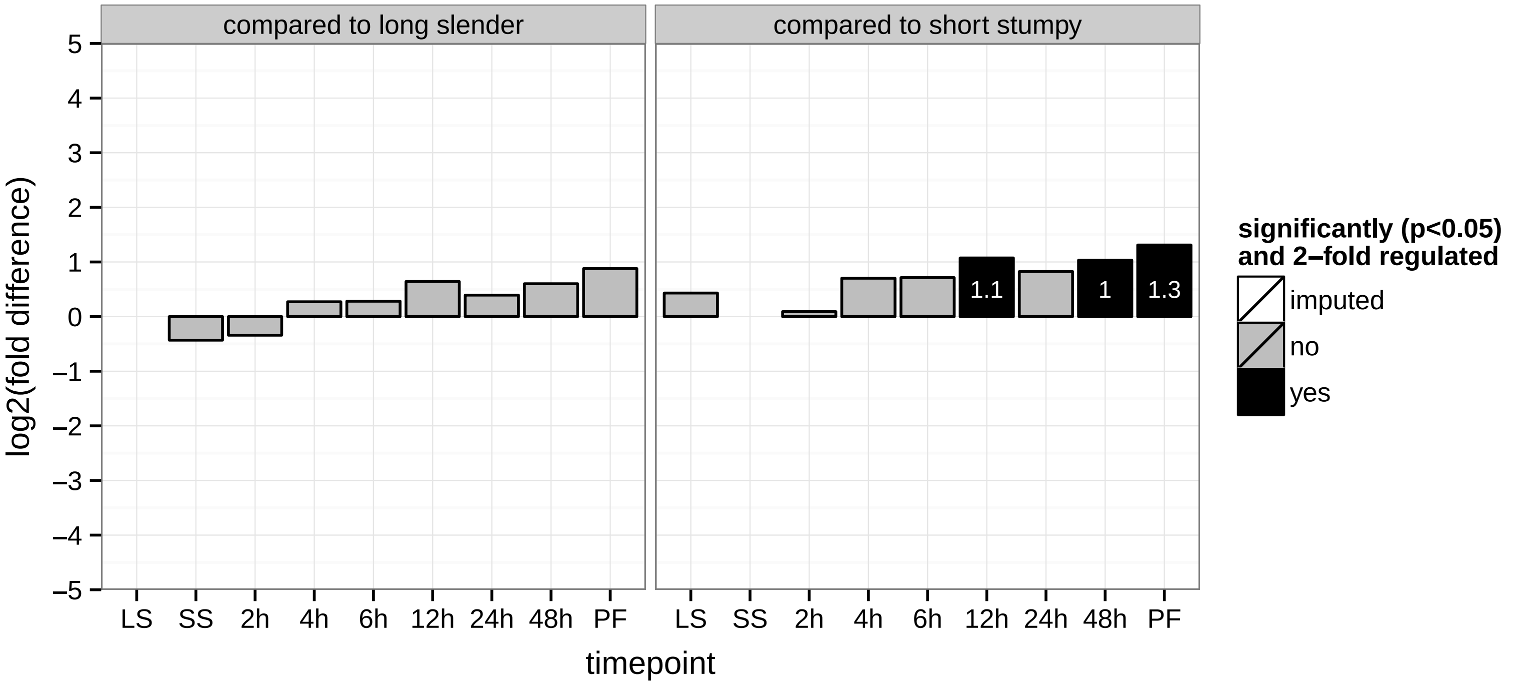

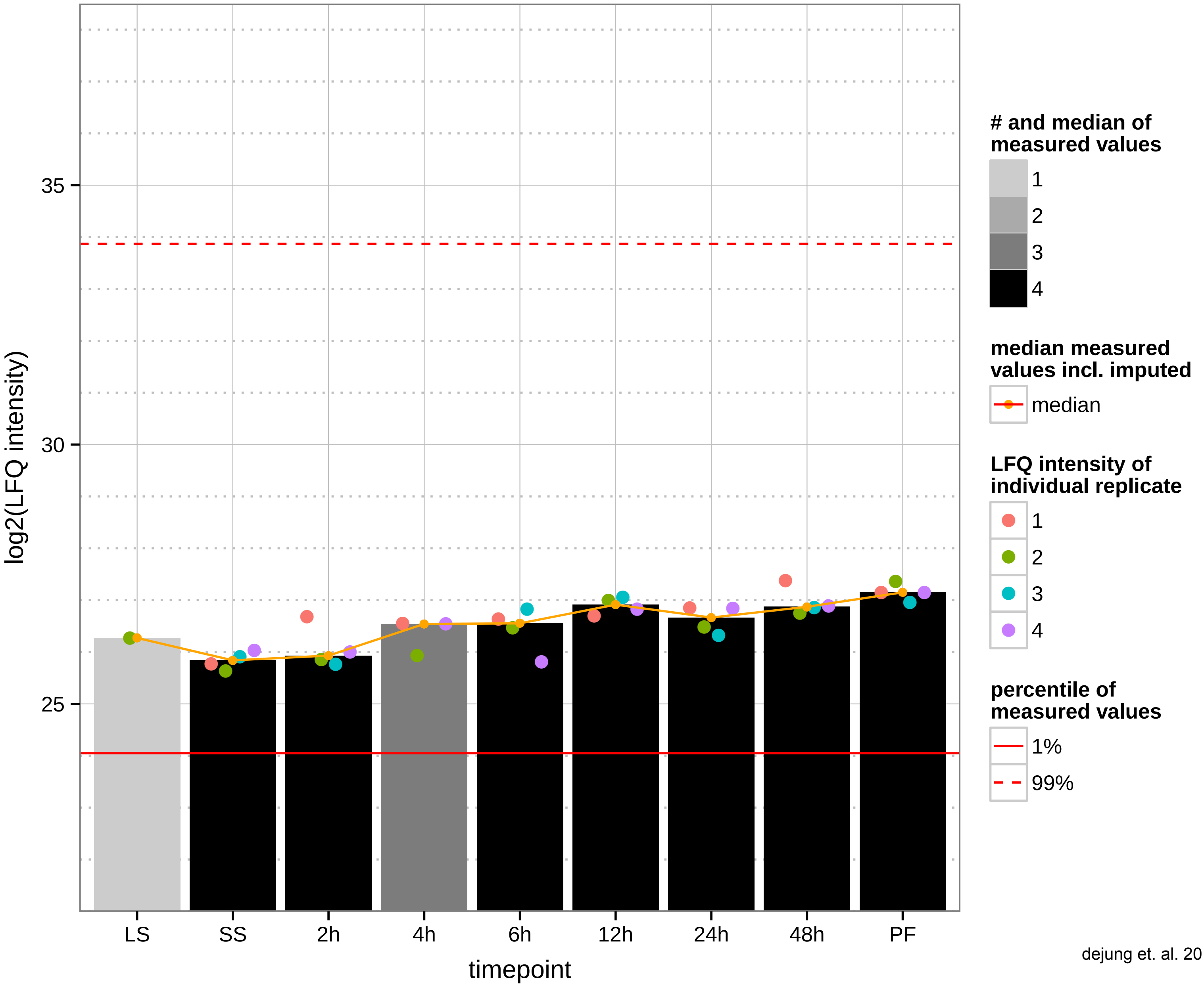

dejung et. al. 2015

paraflagellar rod component, putative (PFC9)
Tb927.11.14880
AGOF: null
AGOC: null
AGOP: null
PGOF: null
PGOC: null
PGOP: null

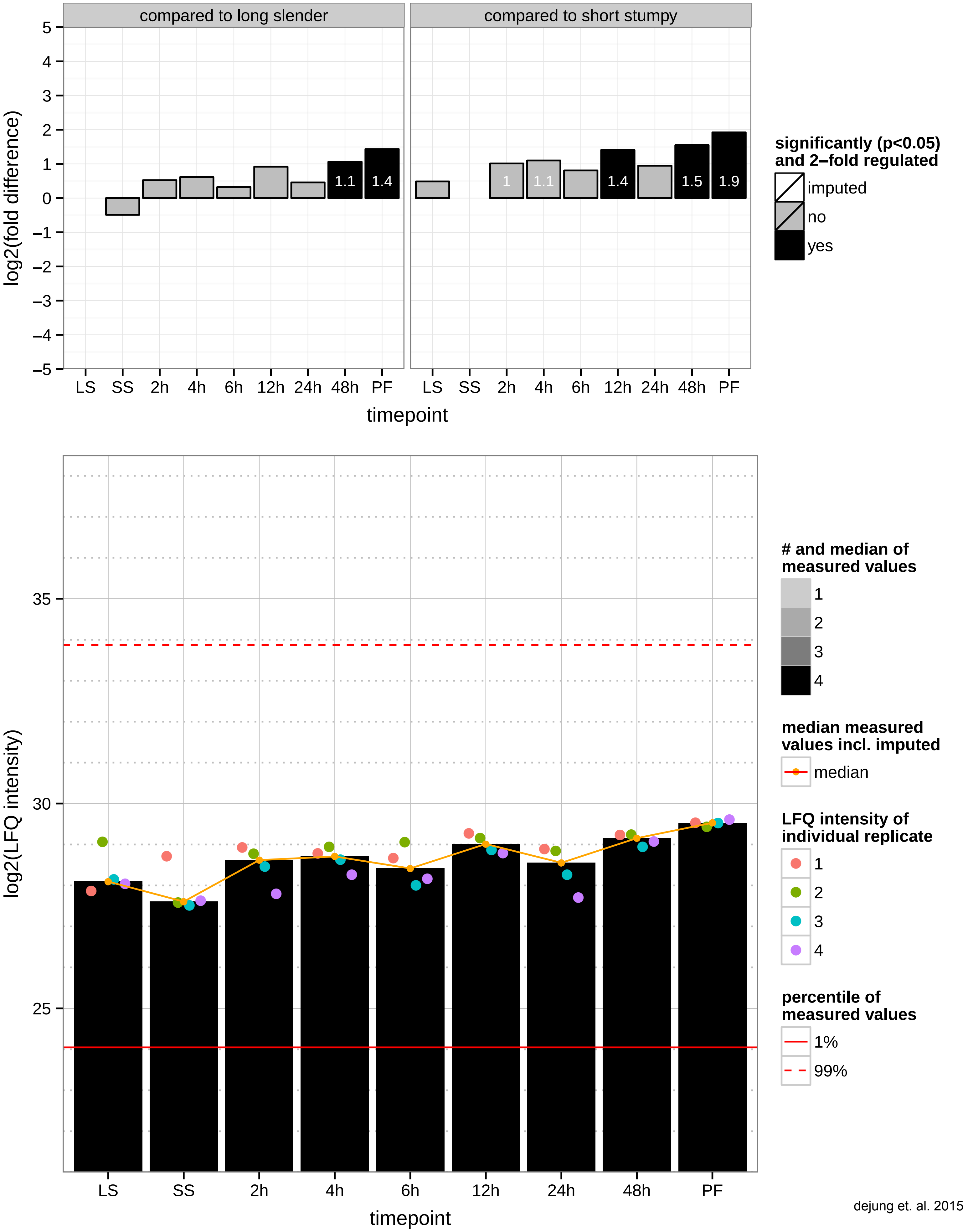

dejung et. al. 2015

hypothetical protein, conserved
Tb927.11.1630
AGOF: null
AGOC: mitochondrion
AGOP: null
PGOF: null
PGOC: null
PGOP: null

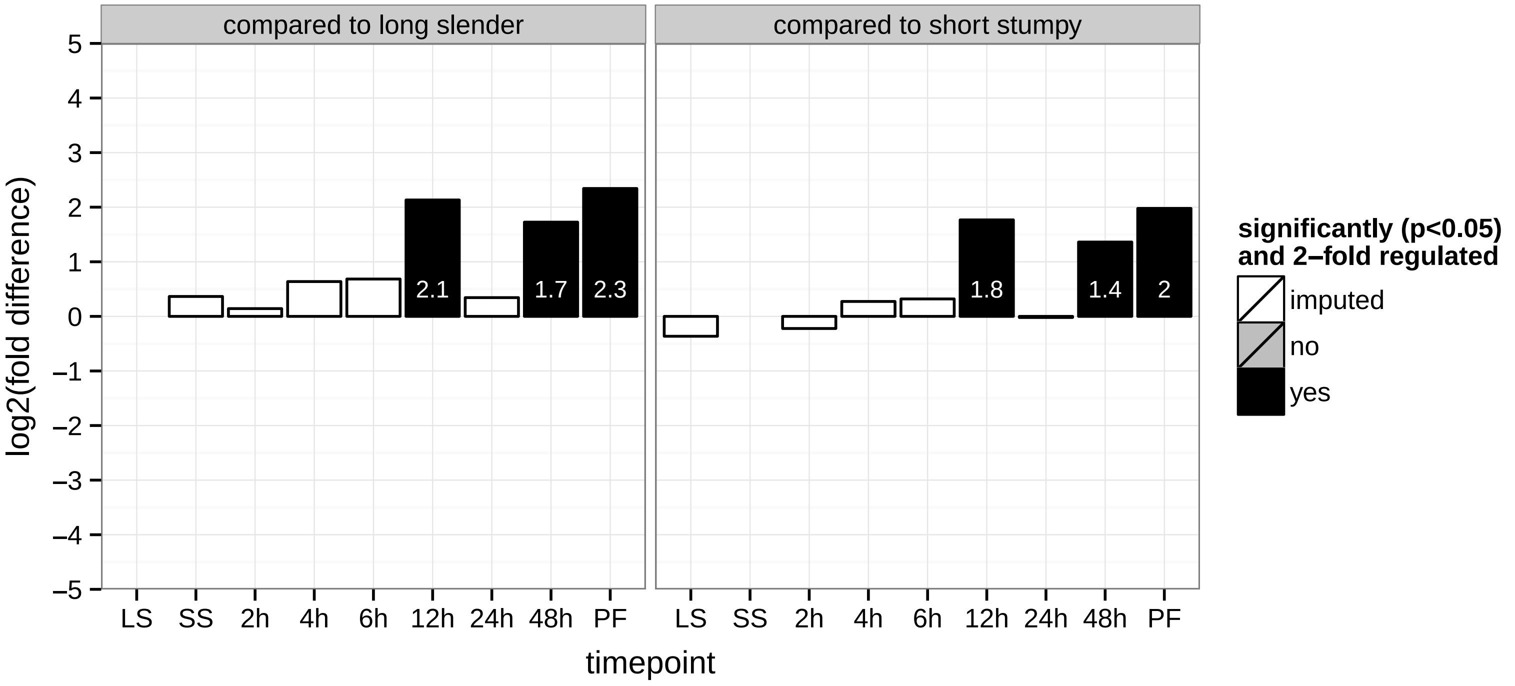

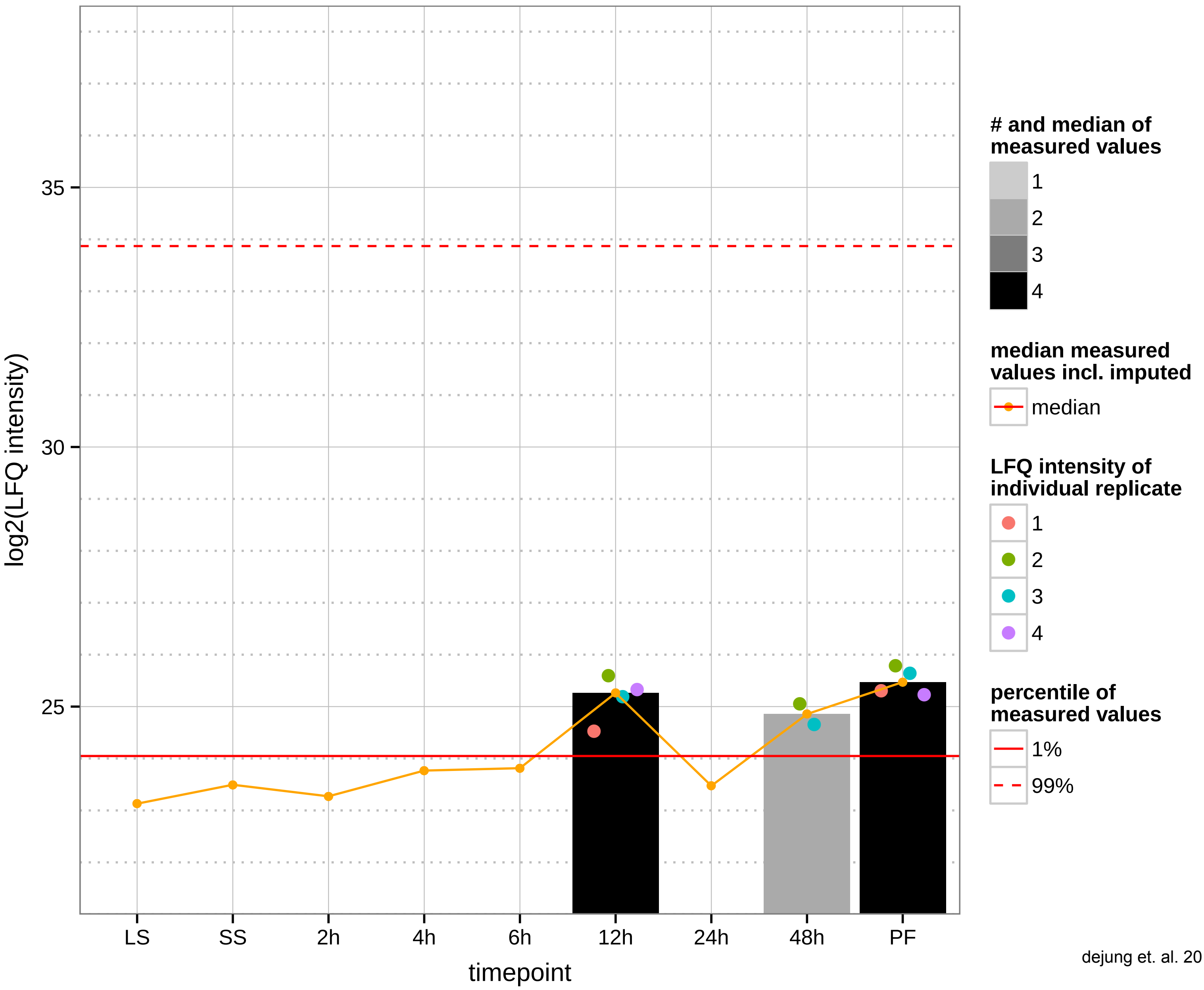

dejung et. al. 2015

hypothetical protein, conserved
Tb927.11.8040
AGOF: null
AGOC: mitochondrion
AGOP: null
PGOF: null
PGOC: null
PGOP: null

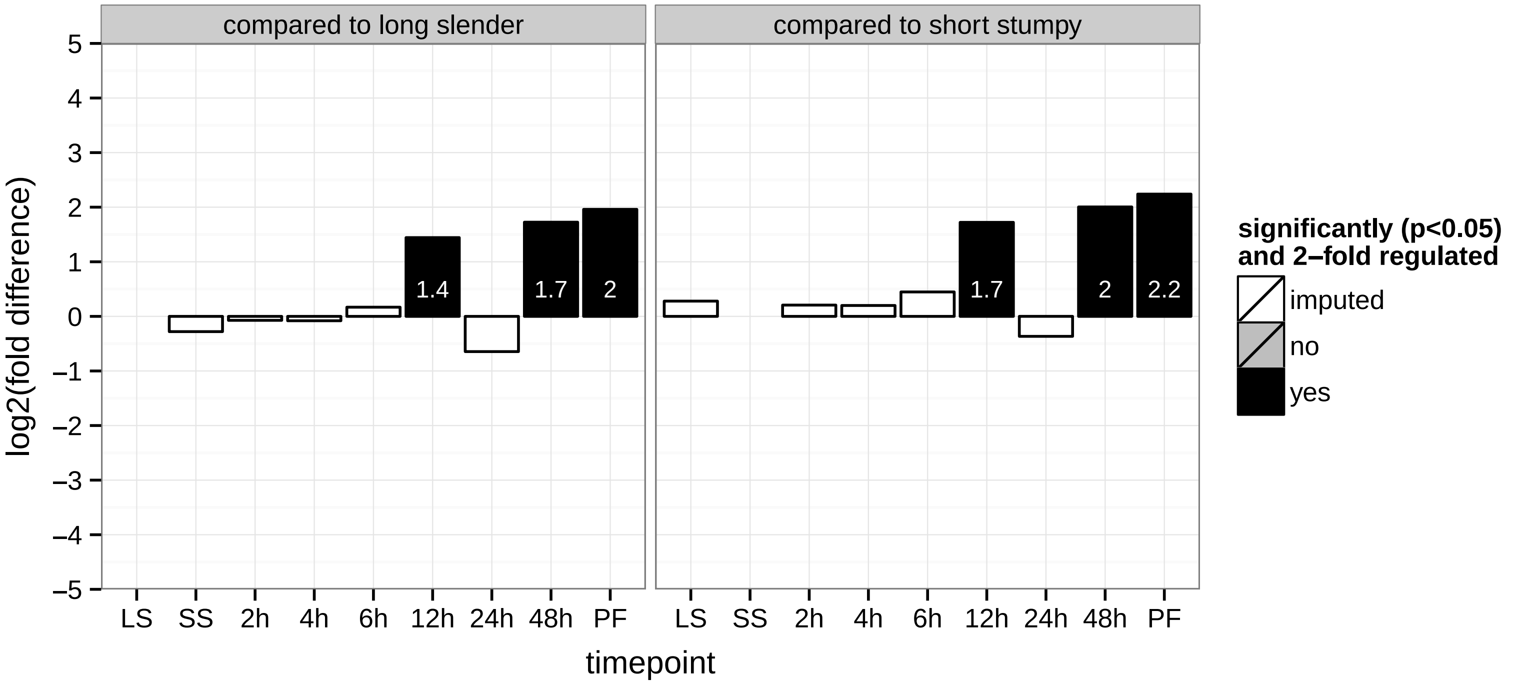

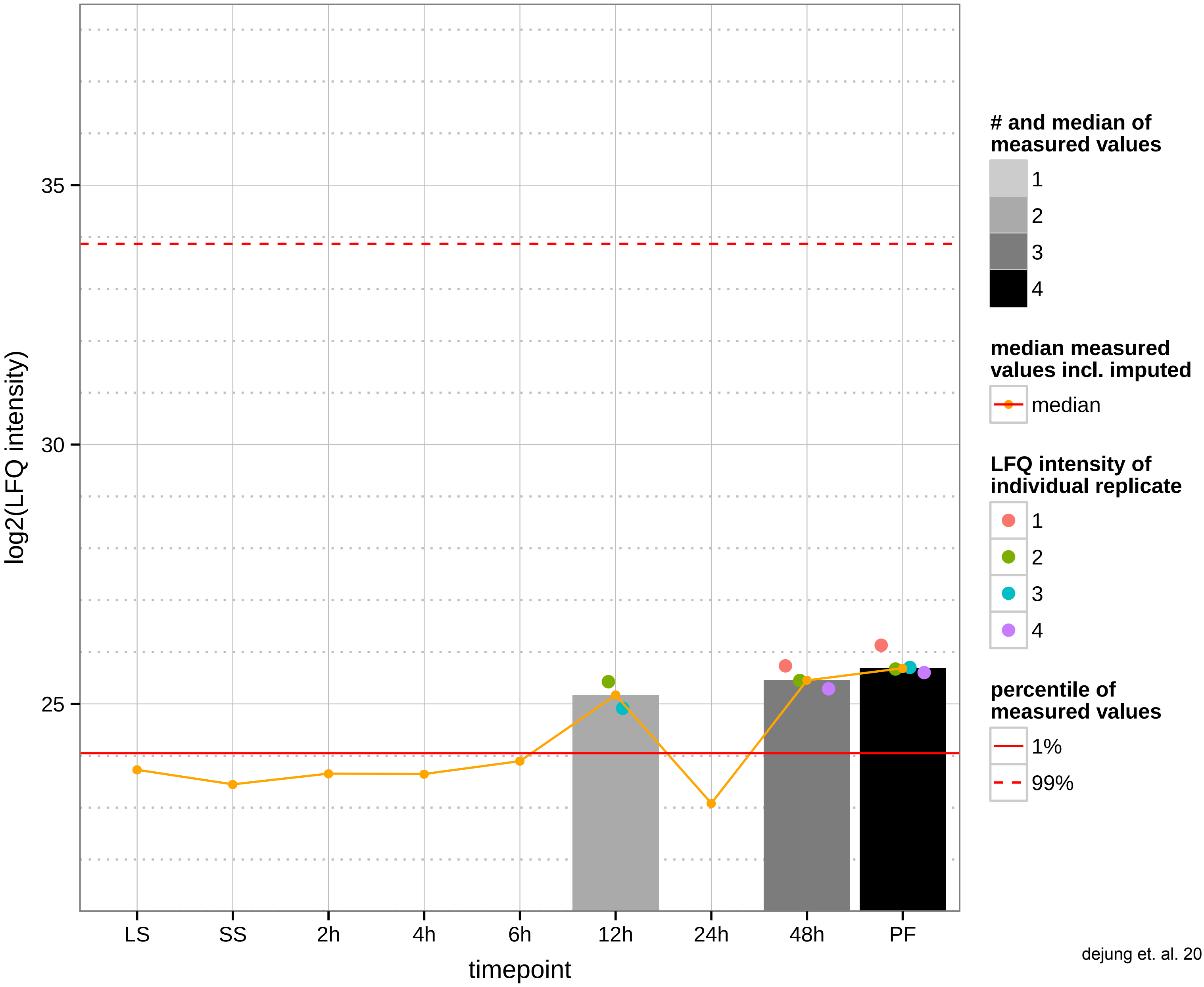

dejung et. al. 2015

hypothetical protein, conserved
Tb927.3.700
AGOF: null
AGOC: null
AGOP: null
PGOF: null
PGOC: null
PGOP: null

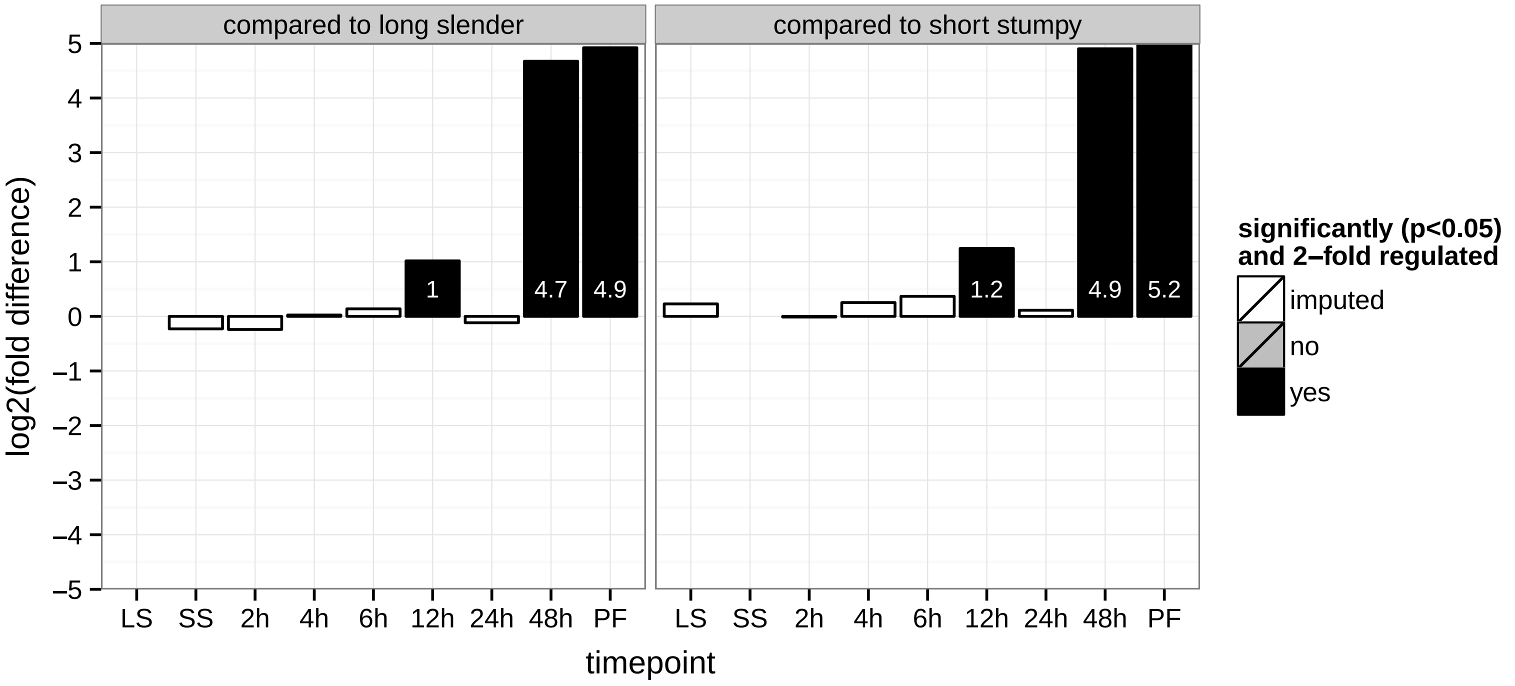

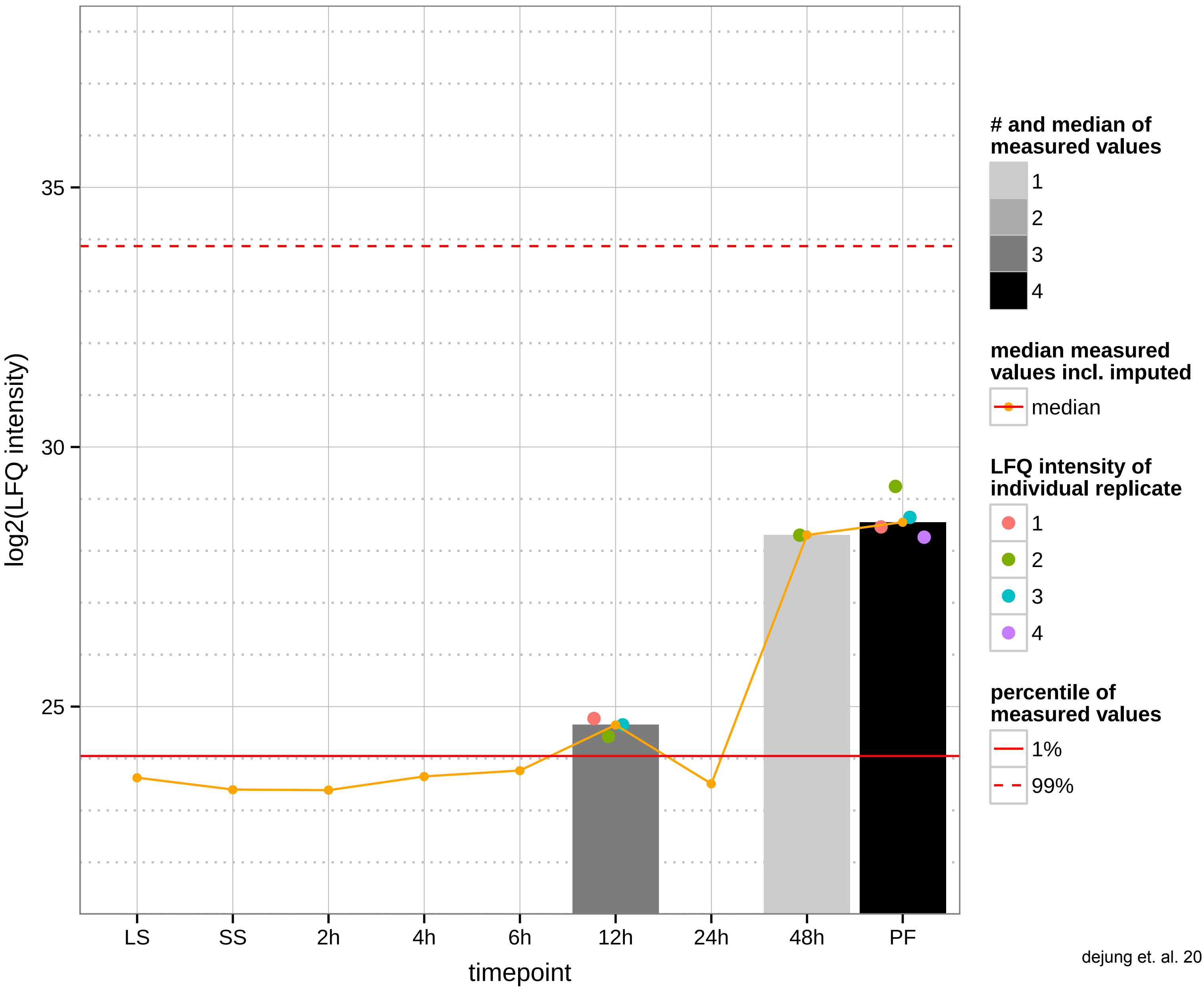

dejung et. al. 2015

hypothetical protein, conserved
Tb927.5.2810
AGOF: null
AGOC: mitochondrion
AGOP: null
PGOF: null
PGOC: null
PGOP: null

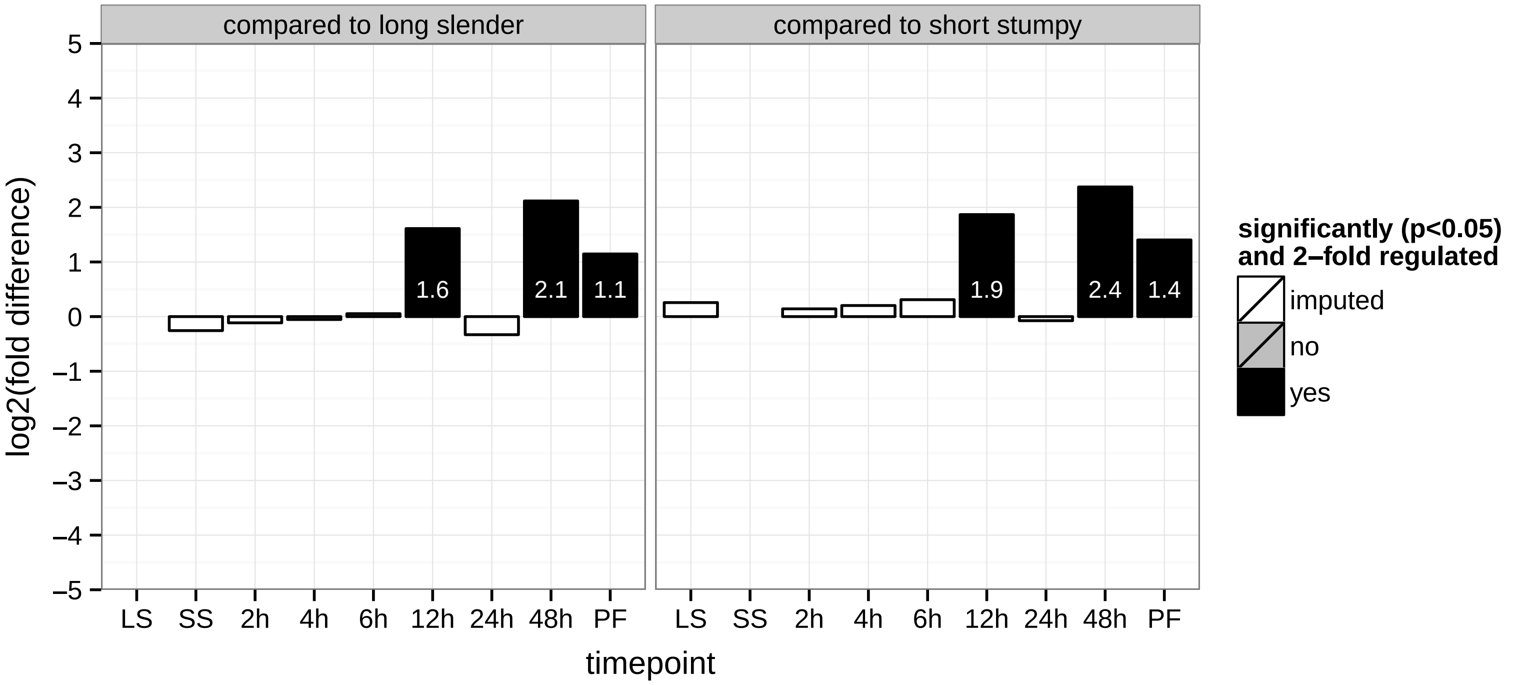

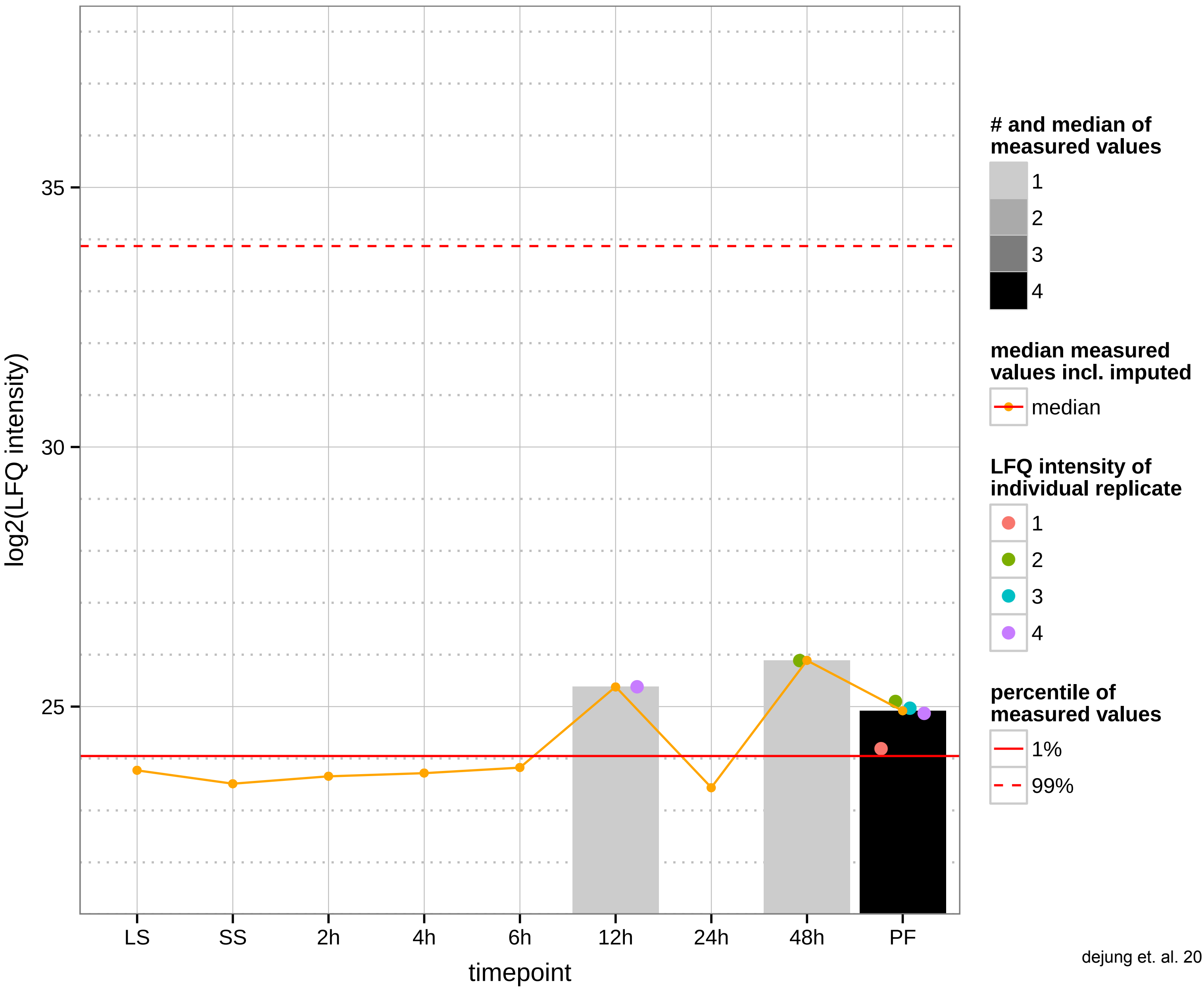

dejung et. al. 2015

hypothetical protein, conserved
Tb927.6.630
AGOF: null
AGOC: null
AGOP: null
PGOF: null
PGOC: null
PGOP: null

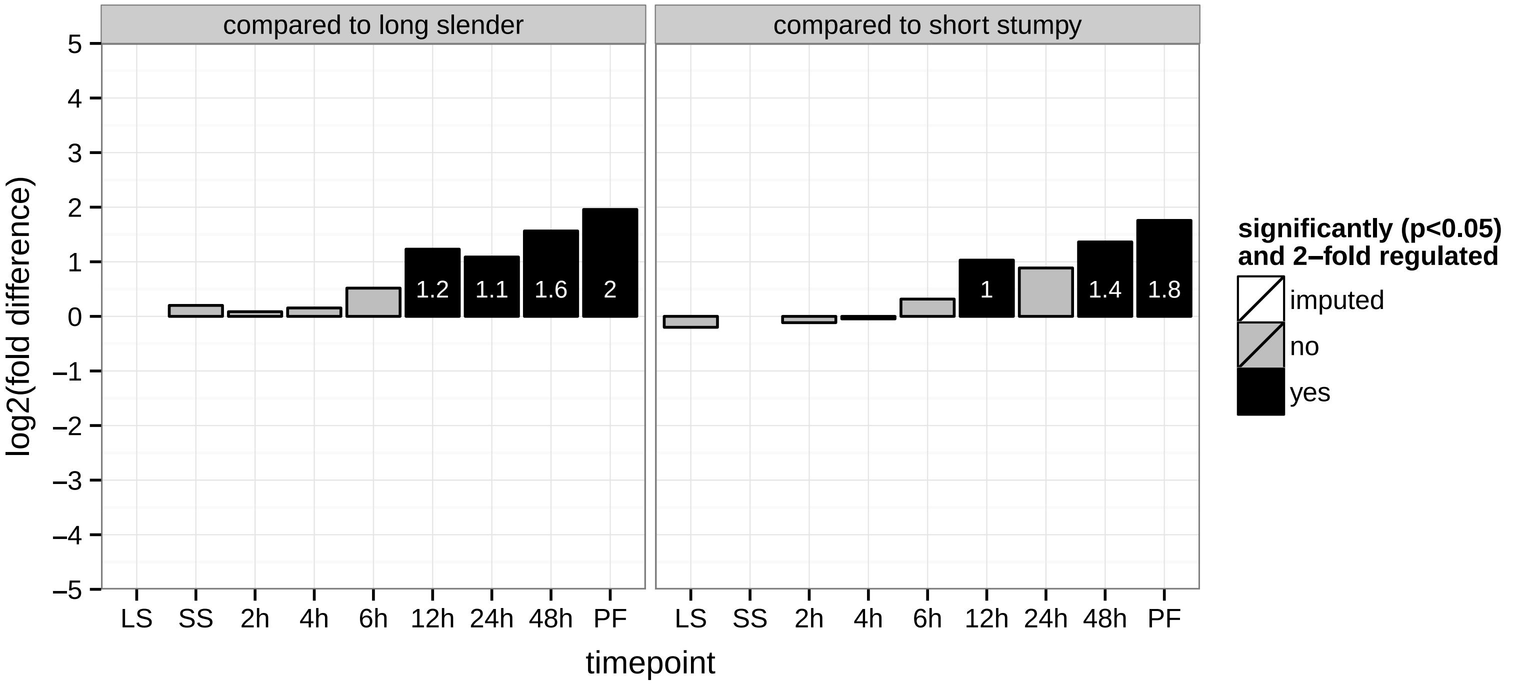

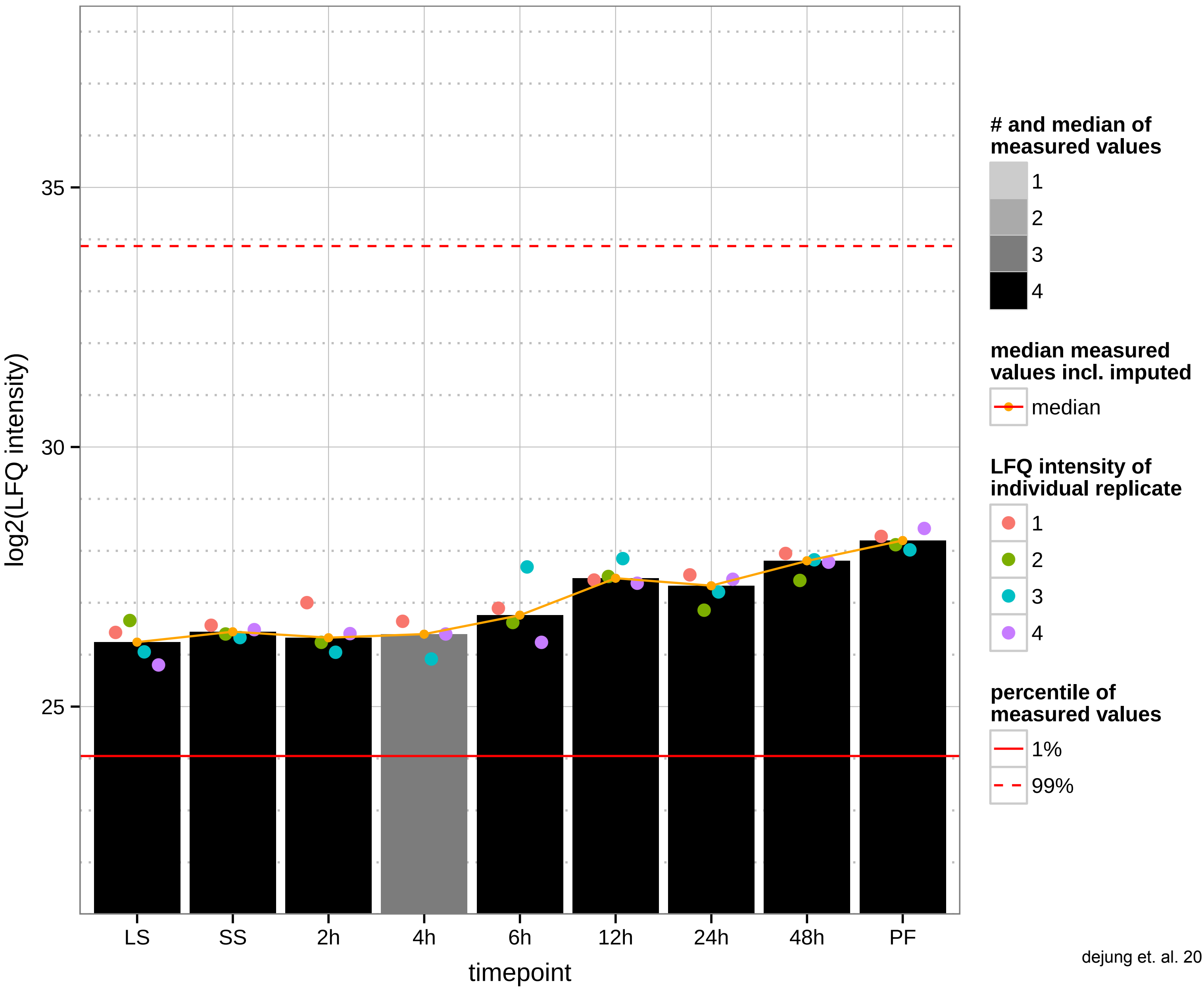

dejung et. al. 2015

clathrin assembly sigma–adaptin protein 3, putative
Tb927.8.6680
AGOF: protein transporter activity
AGOC: AP–3 adaptor complex, clathrin vesicle coat
AGOP: intracellular protein transport, vesicle–mediated transport
PGOF: protein transporter activity
PGOC: null
PGOP: protein transport, transport

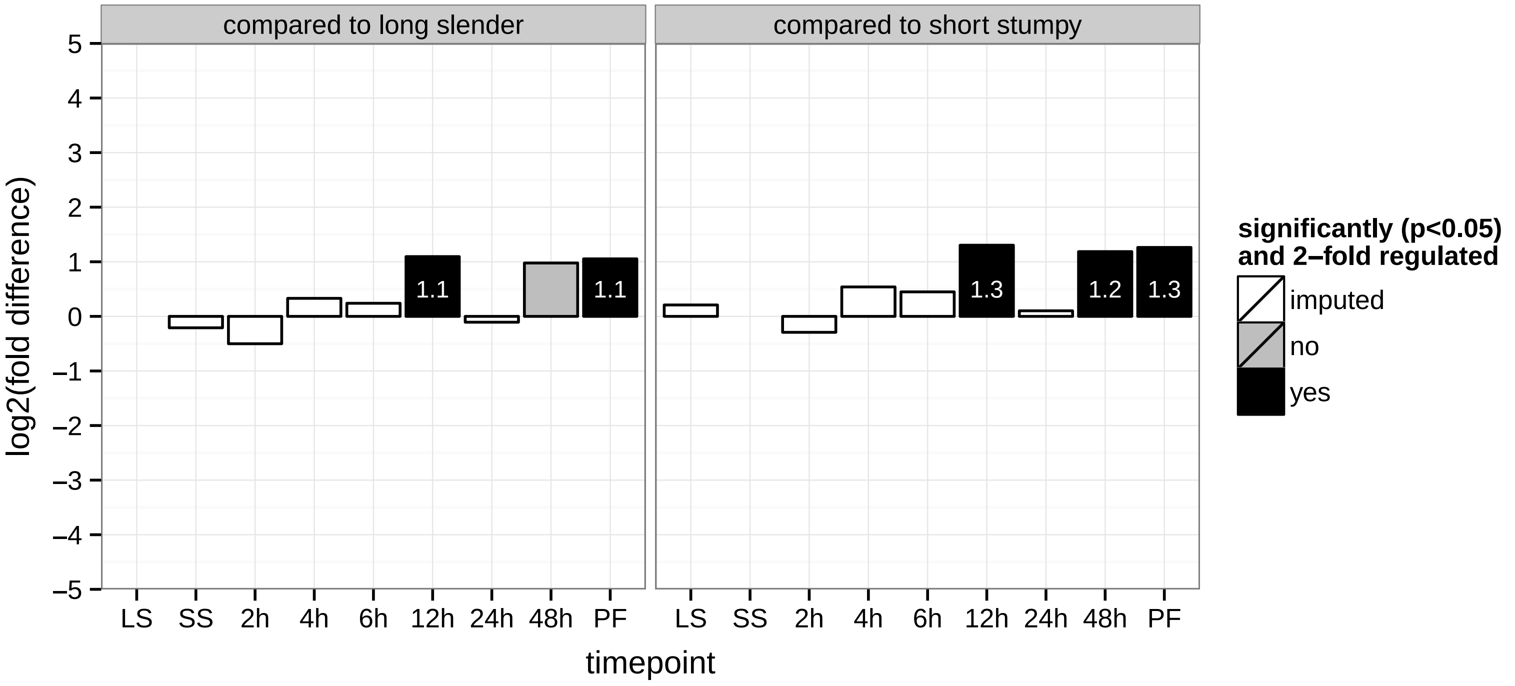

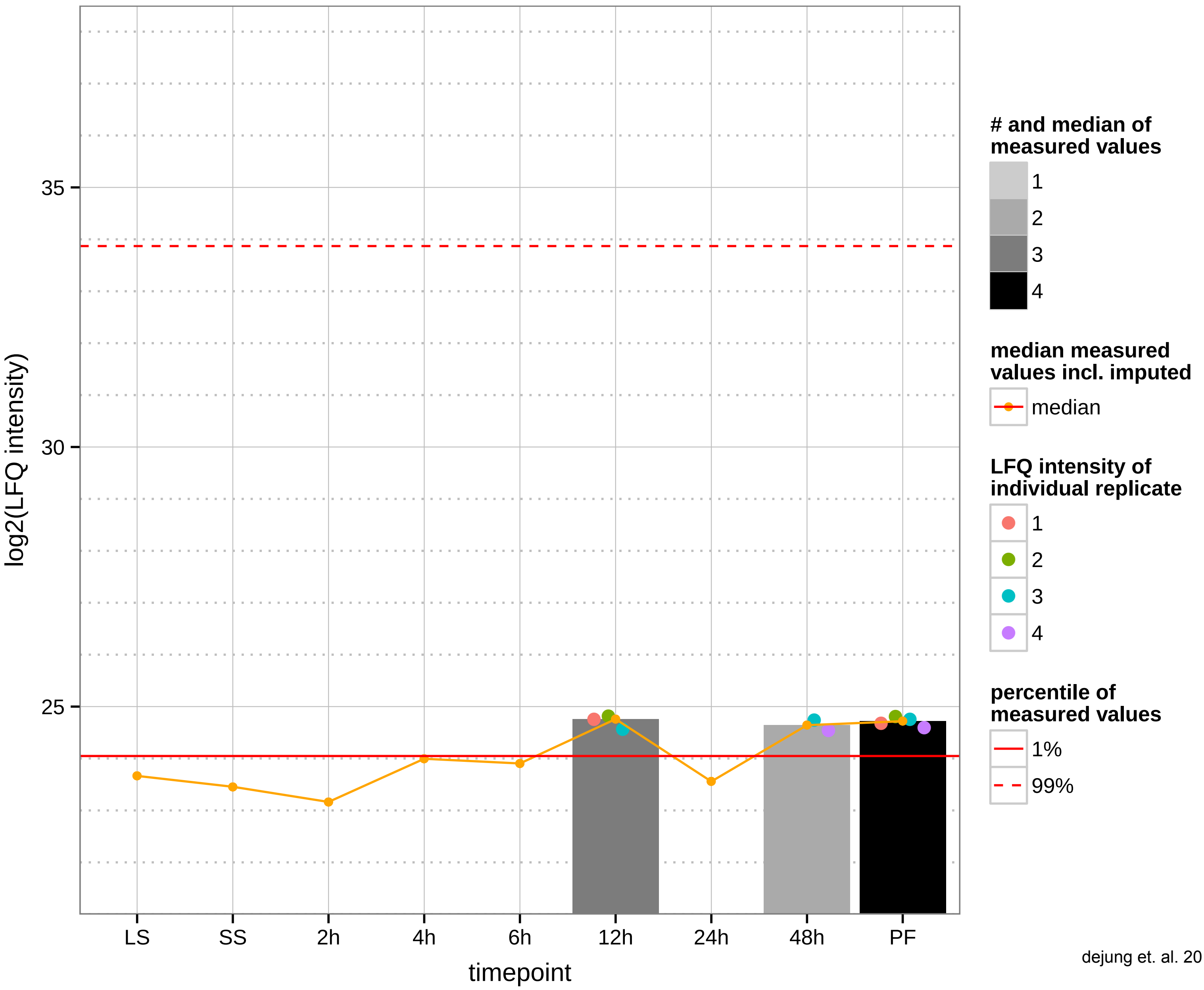

dejung et. al. 2015

hypothetical protein, conserved
Tb927.9.9220
AGOF: antiporter activity, drug transmembrane transporter activity
AGOC: membrane
AGOP: drug transmembrane transport
PGOF: null
PGOC: null
PGOP: null

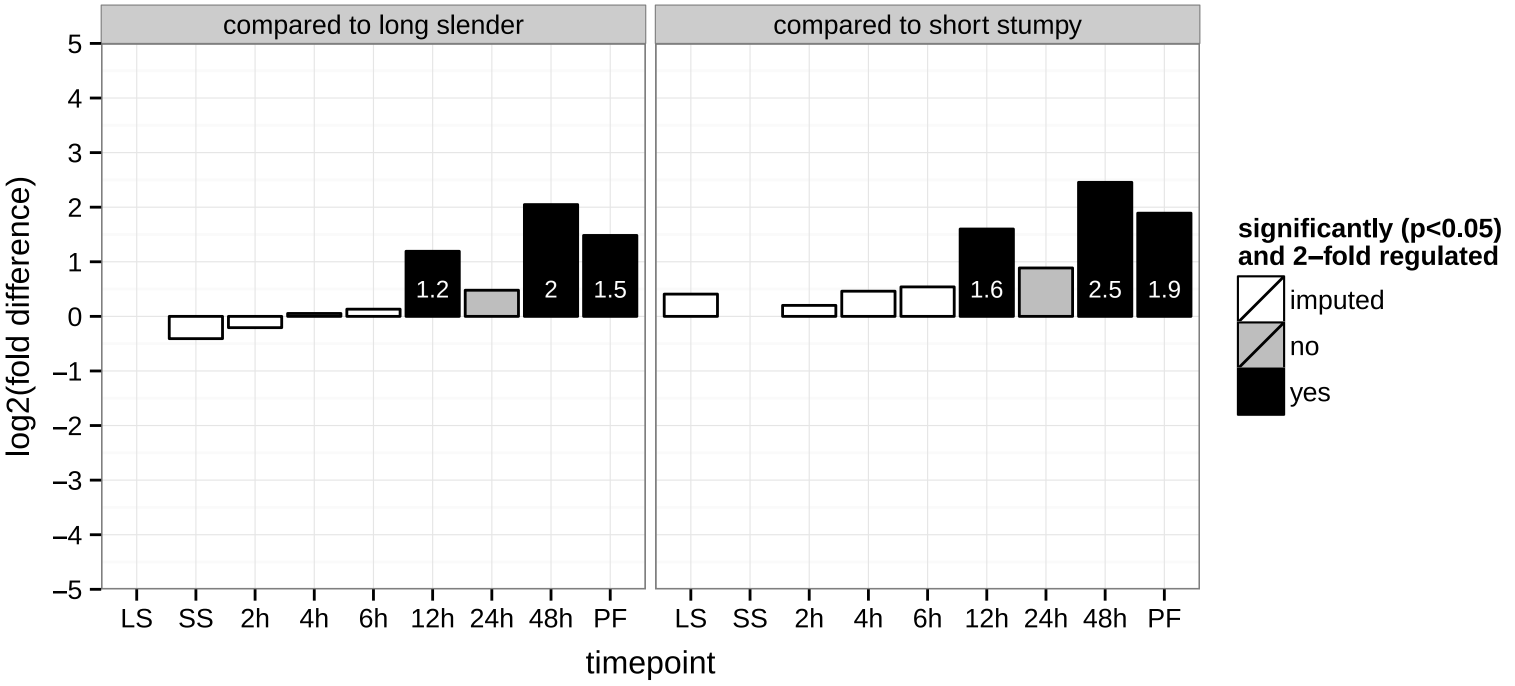

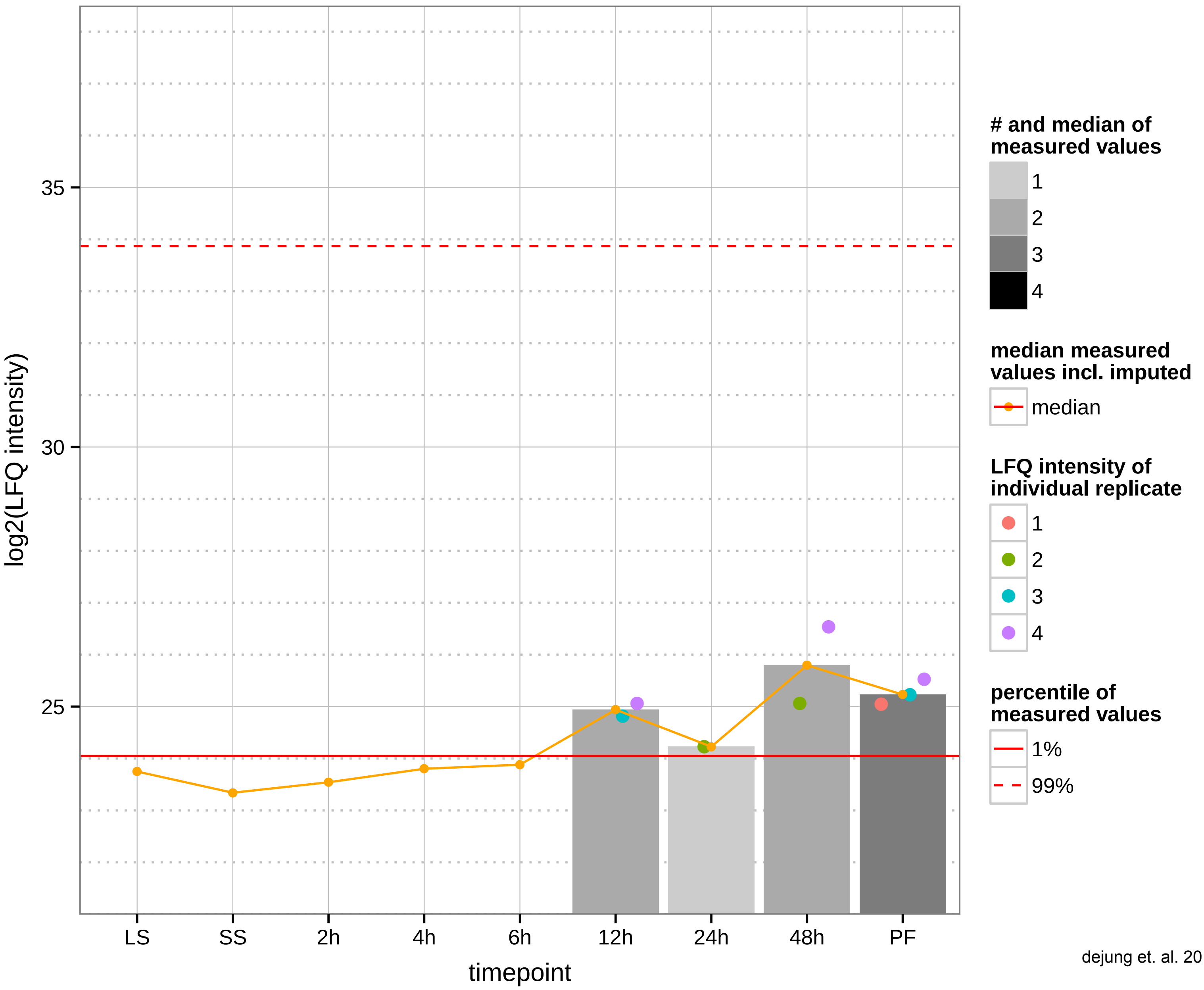

dejung et. al. 2015

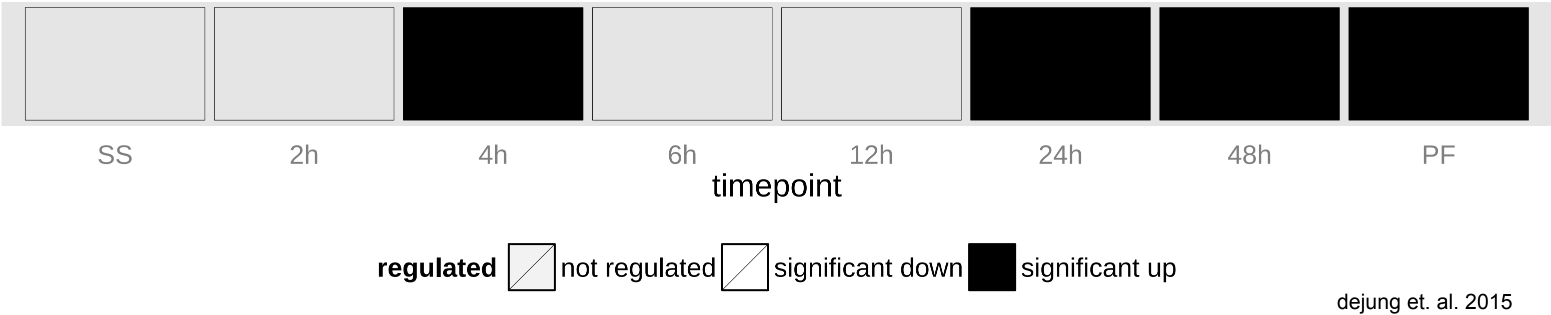
SS
2h
4h
6h
12h
24h
48h
PF
timepoint
regulated
not regulated
significant down
significant up
dejung et. al. 2015

SSU ribosomal protein, mitochondrial (MRPS15)
Tb927.1.1200
AGOF: null
AGOC: mitochondrial small ribosomal subunit, mitochondrion
AGOP: null
PGOF: null
PGOC: null
PGOP: null

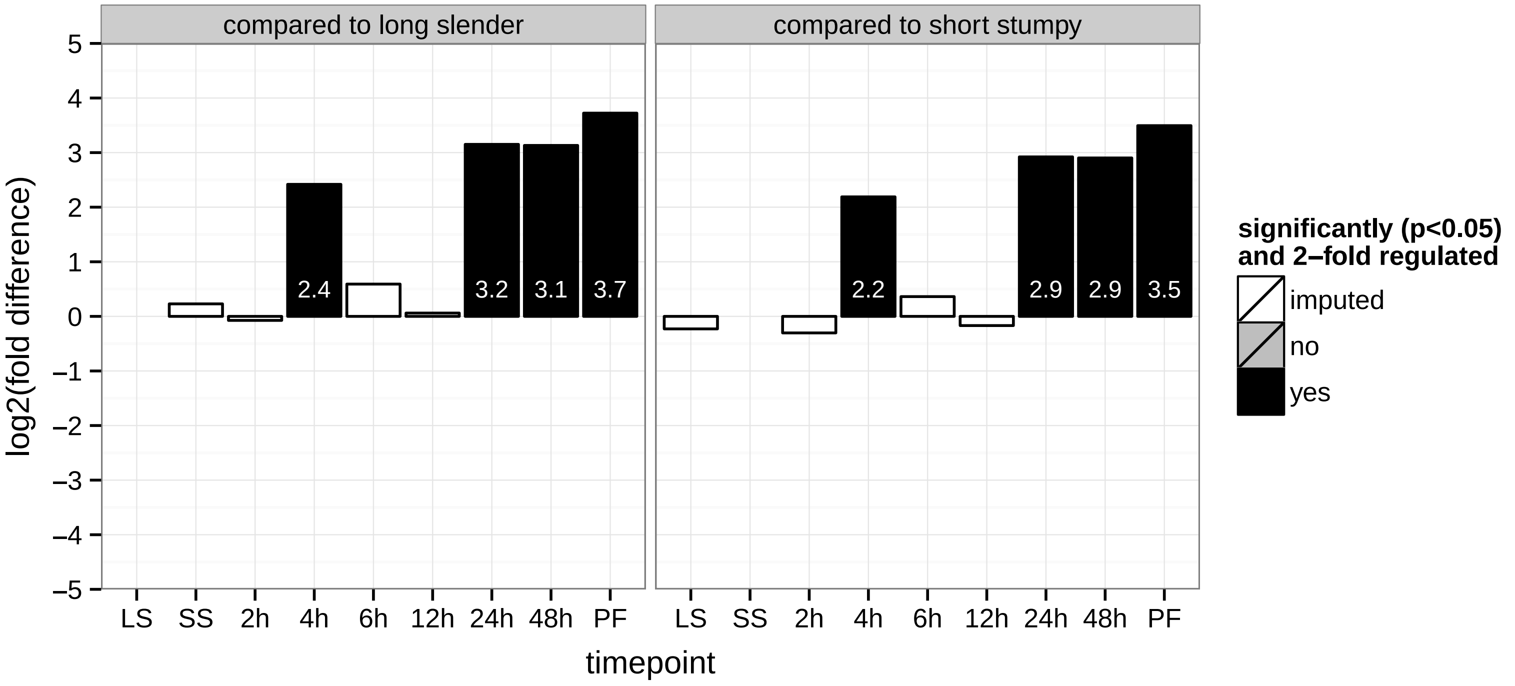

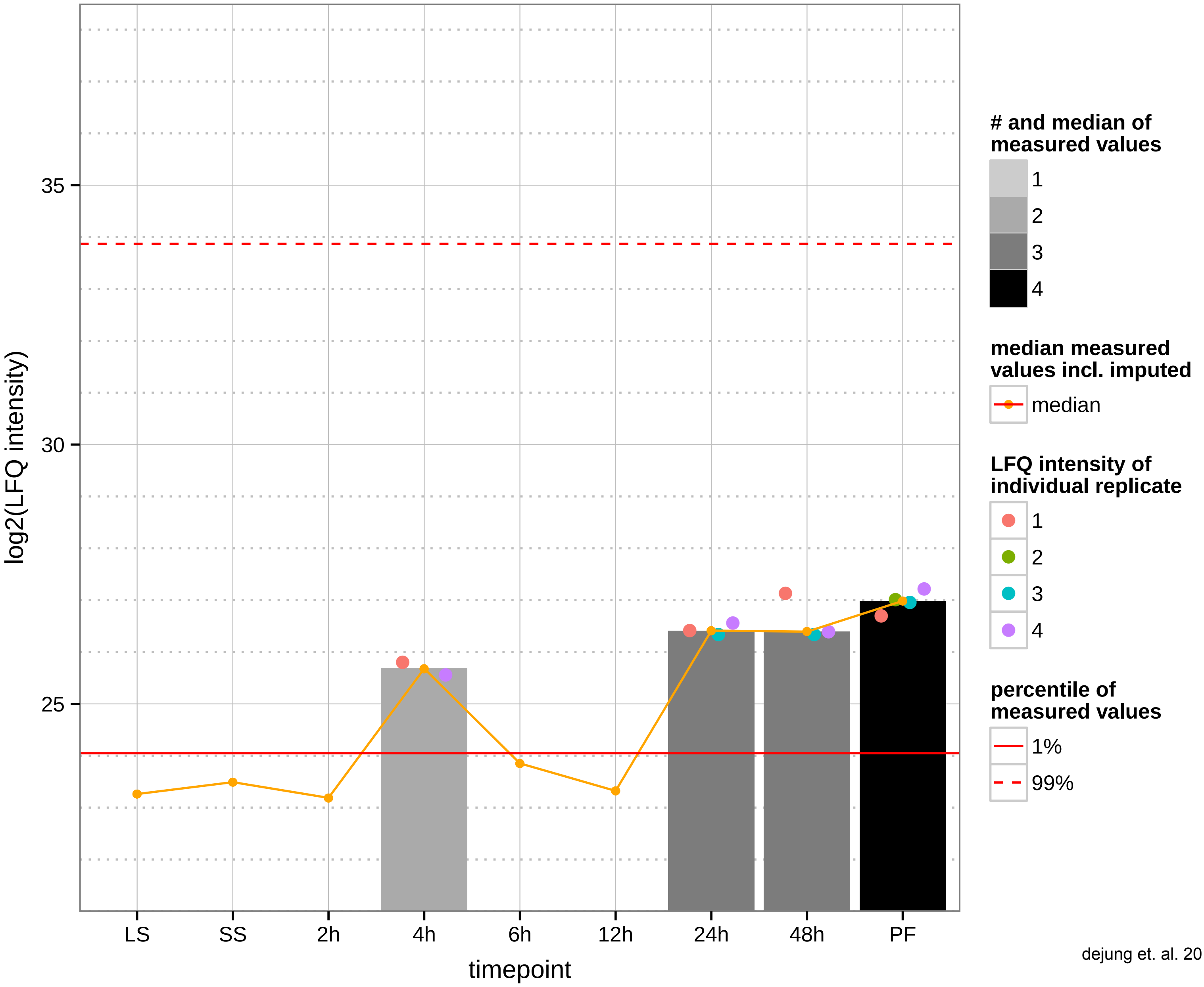

dejung et. al. 2015

glutaredoxin, putative
Tb927.1.1770
AGOF: disulfide oxidoreductase activity, electron carrier activity, protein disulfide isomerase activity, protein disulfide oxidoredu
AGOC: null
AGOP: cell redox homeostasis
PGOF: electron carrier activity, protein disulfide oxidoreductase activity
PGOC: null
PGOP: cell redox homeostasis

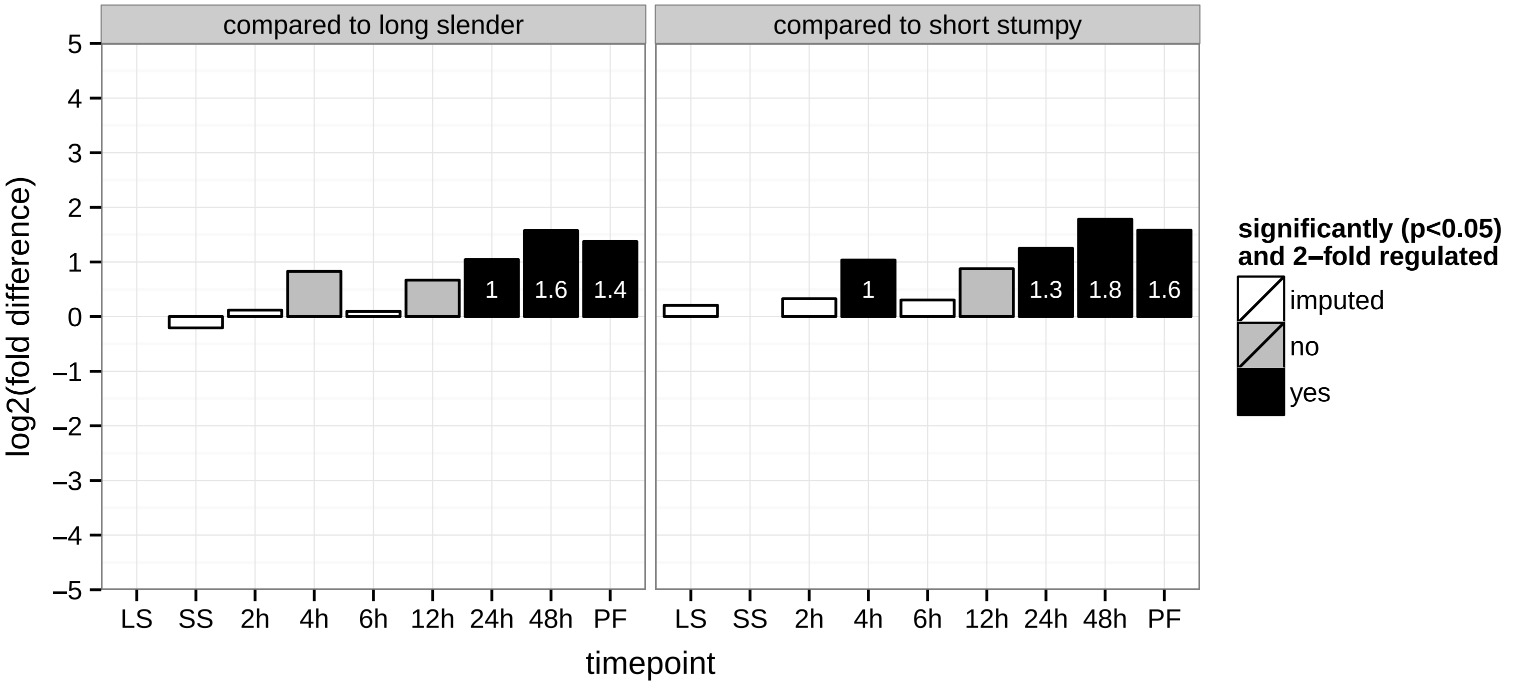

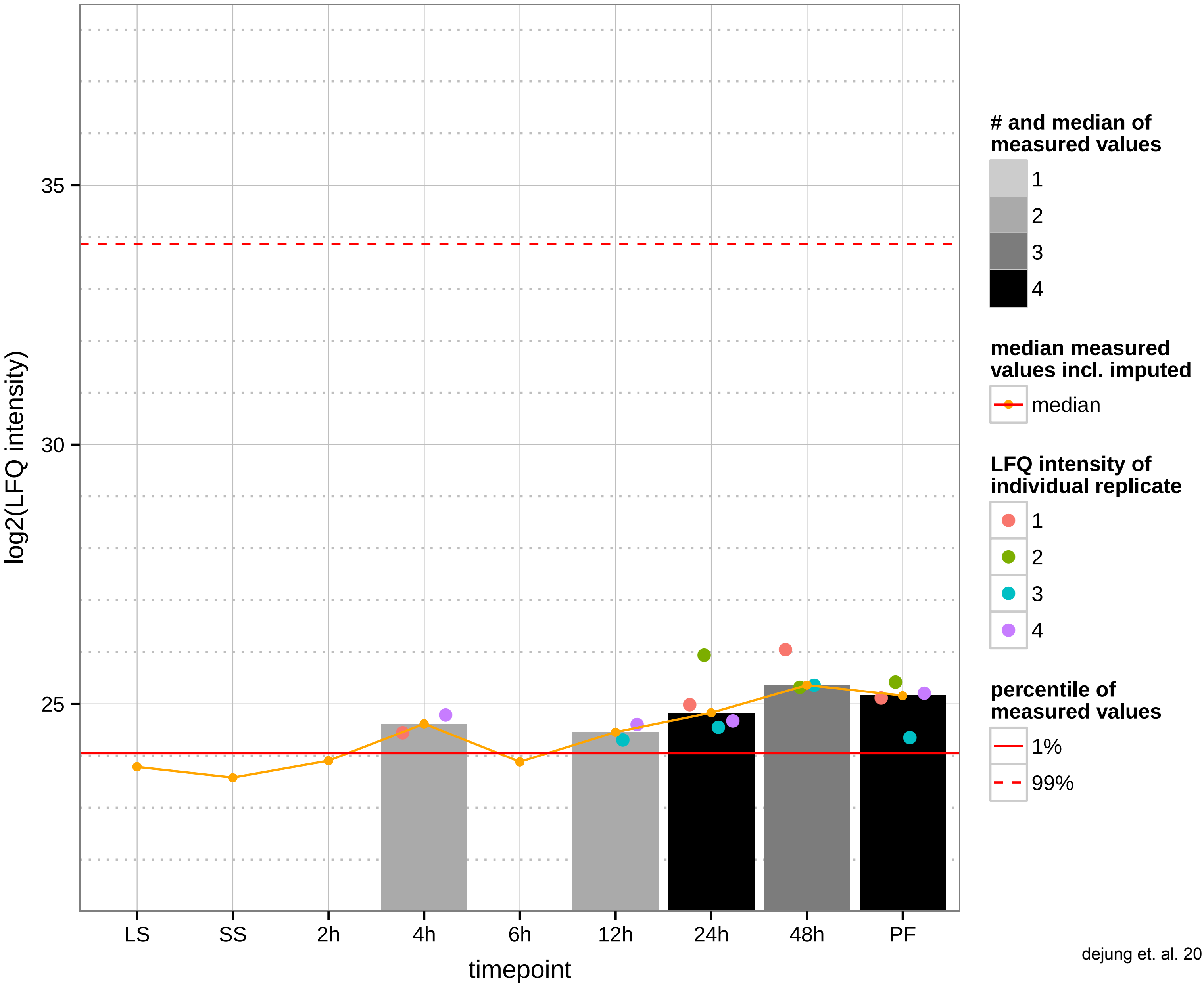

dejung et. al. 2015

hypothetical protein, conserved
Tb927.10.9920
AGOF: null
AGOC: null
AGOP: null
PGOF: protein binding
PGOC: null
PGOP: null

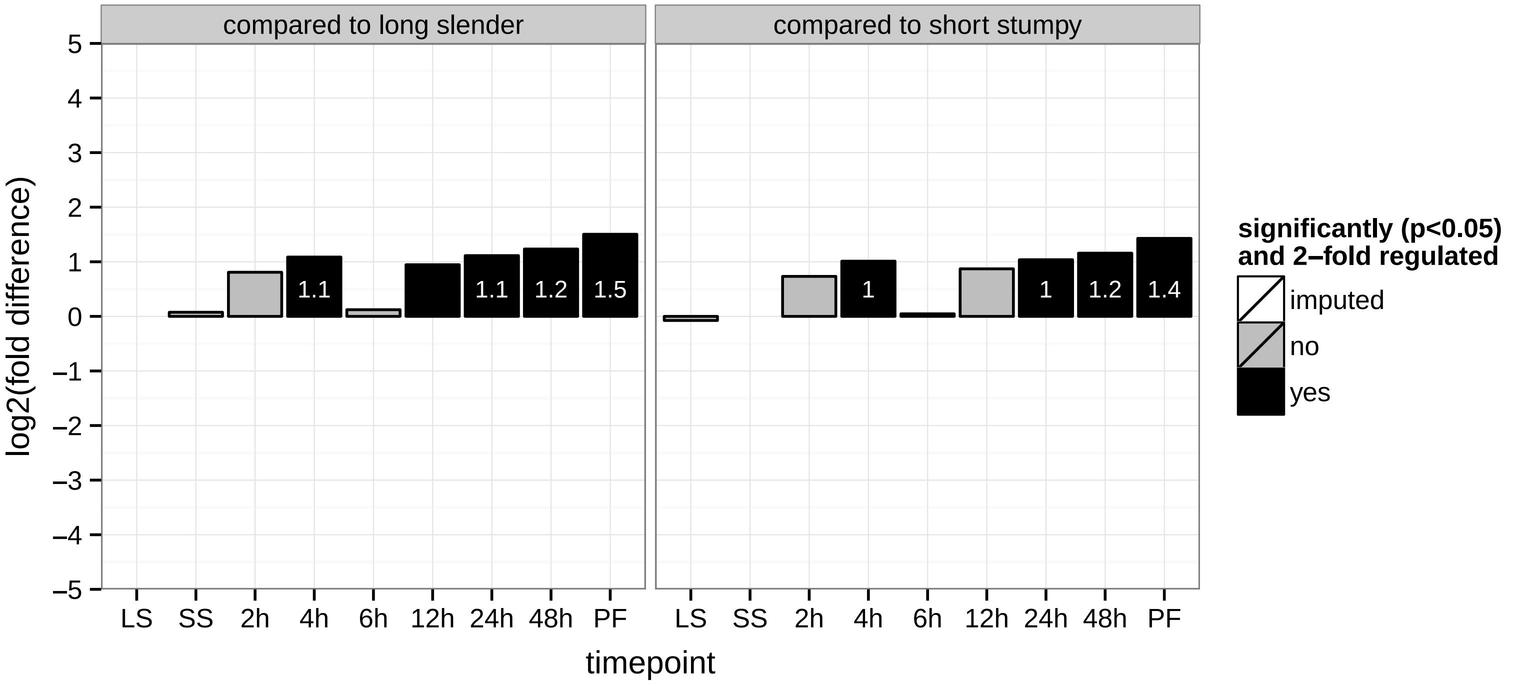

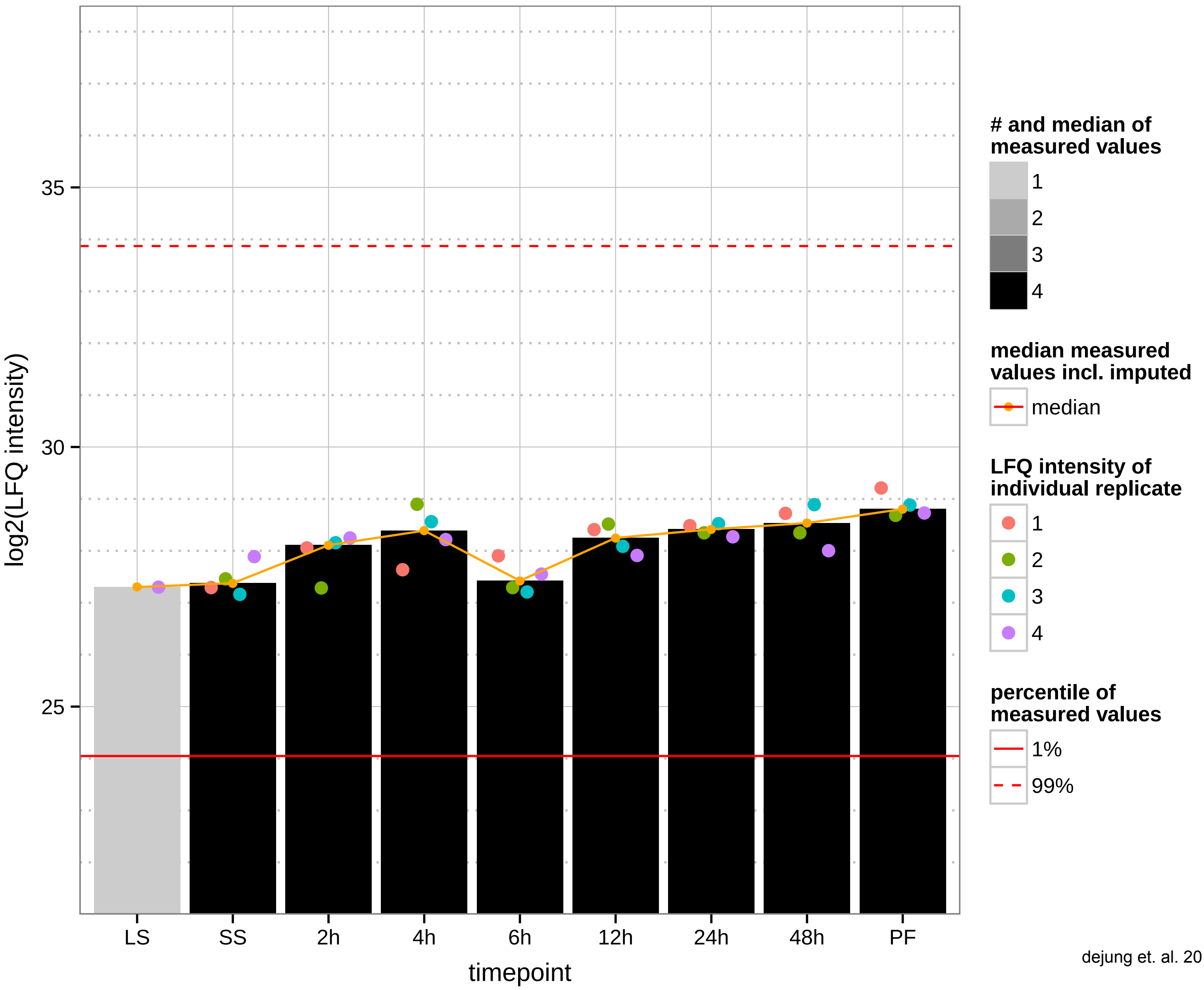

dejung et. al. 2015

hypothetical protein, conserved
Tb927.11.11310
AGOF: null
AGOC: null
AGOP: null
PGOF: null
PGOC: null
PGOP: null

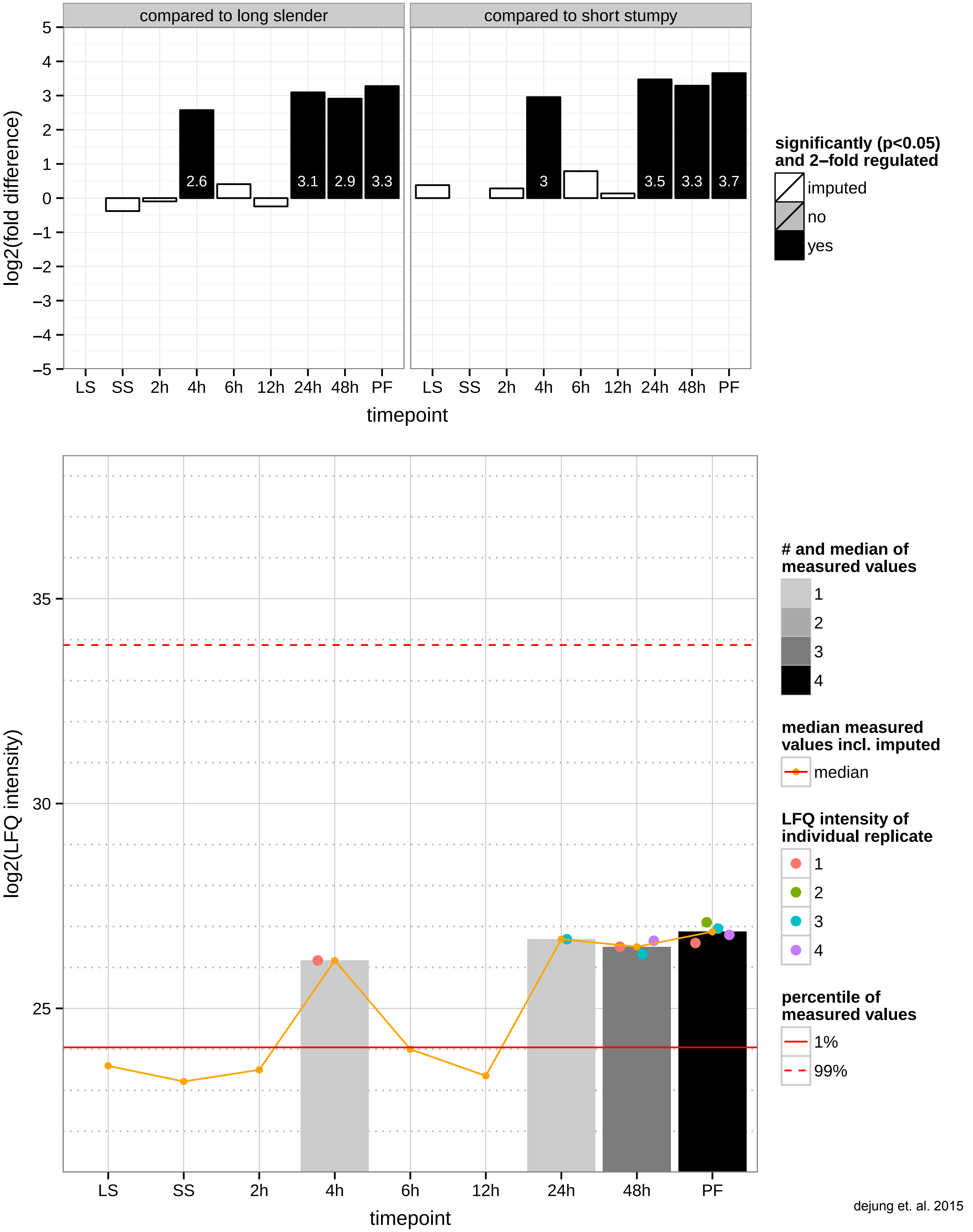

dejung et. al. 2015

hypothetical protein, conserved
Tb927.11.12300
AGOF: null
AGOC: null
AGOP: null
PGOF: null
PGOC: null
PGOP: null

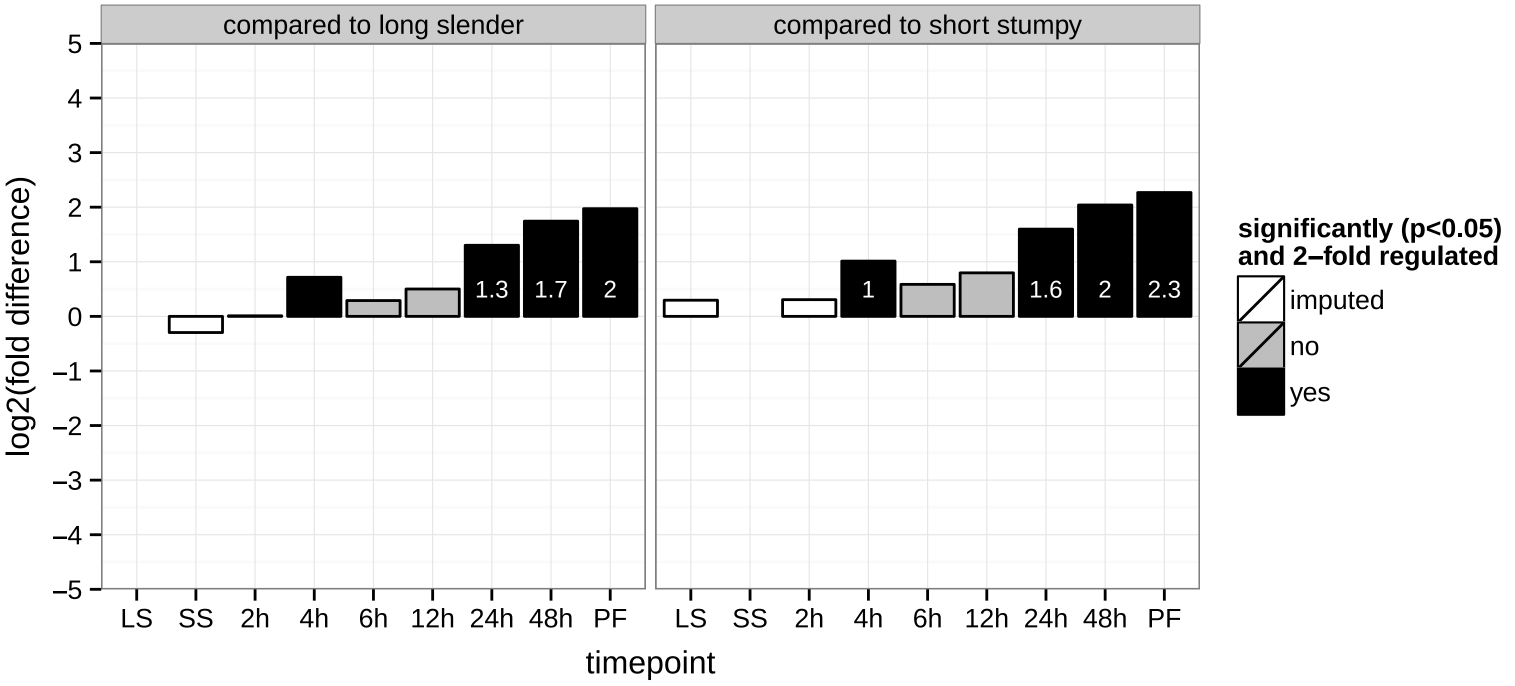

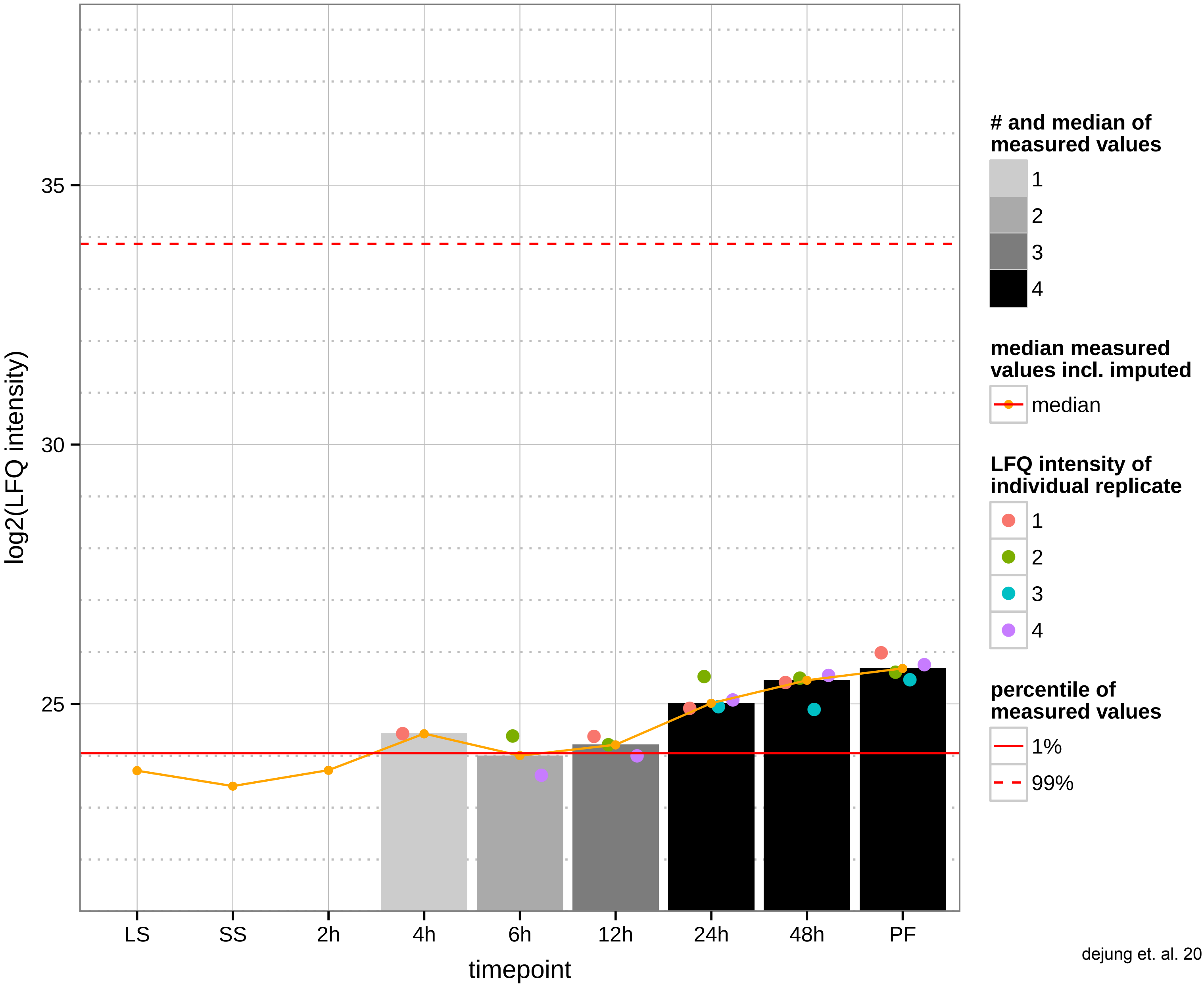

dejung et. al. 2015

leucine−rich repeat protein 1 (LRRP1), putative, leucine−rich repeat protein (LRRP), (fragment), leucine rich repeat protein, le
Tb927.11.1500
AGOF: null
AGOC: null
AGOP: null
PGOF: null, protein binding
PGOC: null
PGOP: null

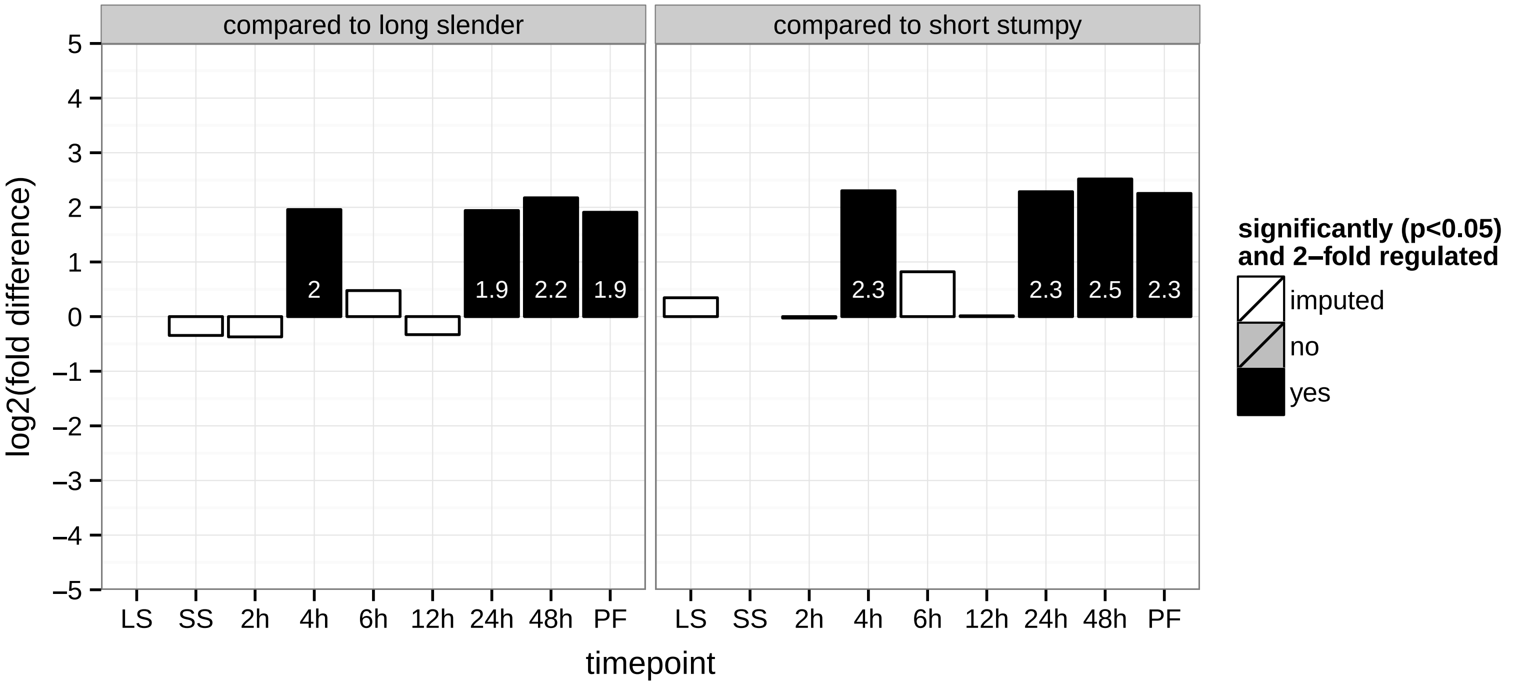

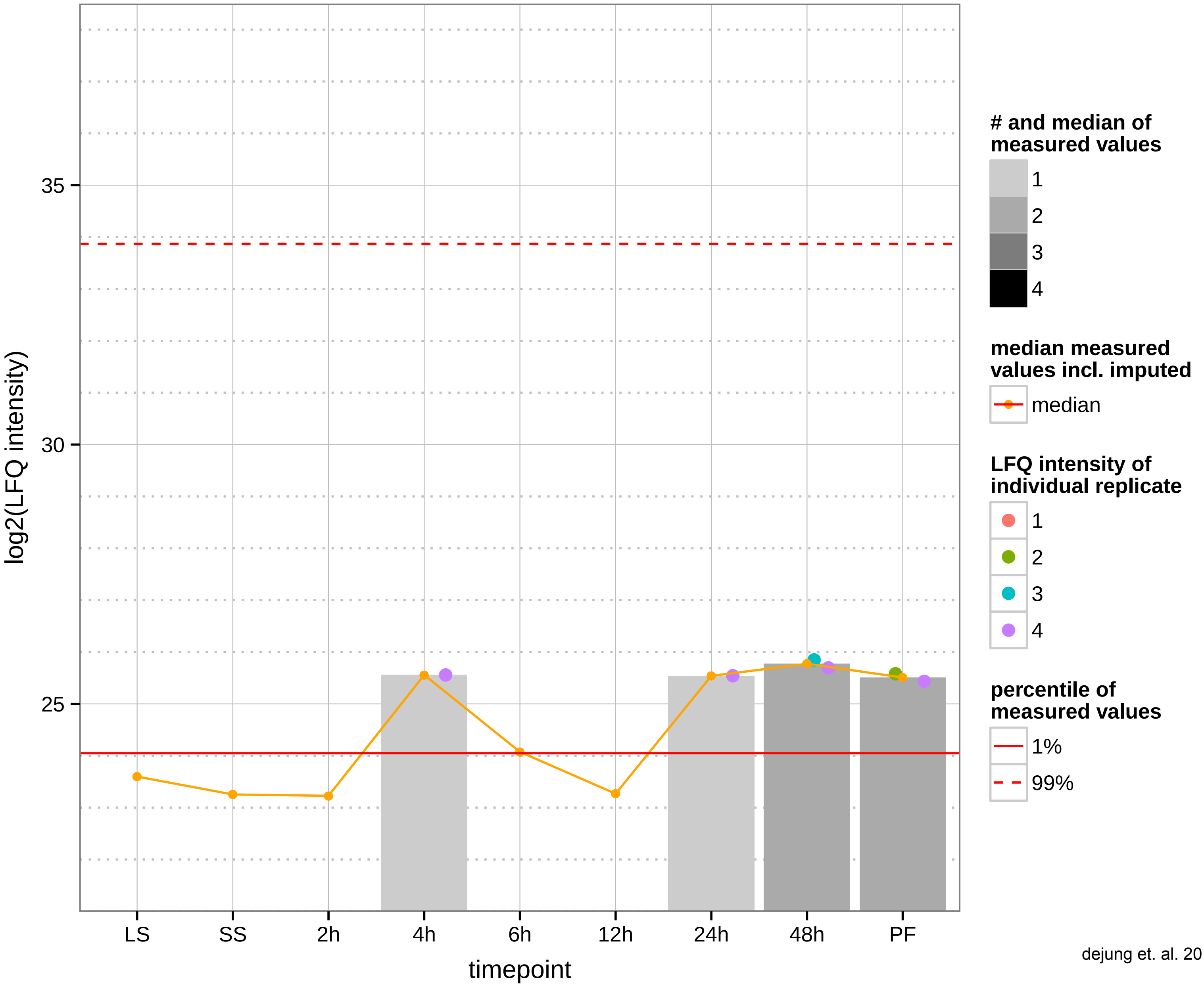

dejung et. al. 2015

RNA–binding protein (RPB28)
Tb927.3.1030
AGOF: nucleic acid binding
AGOC: null
AGOP: null
PGOF: nucleic acid binding
PGOC: null
PGOP: null

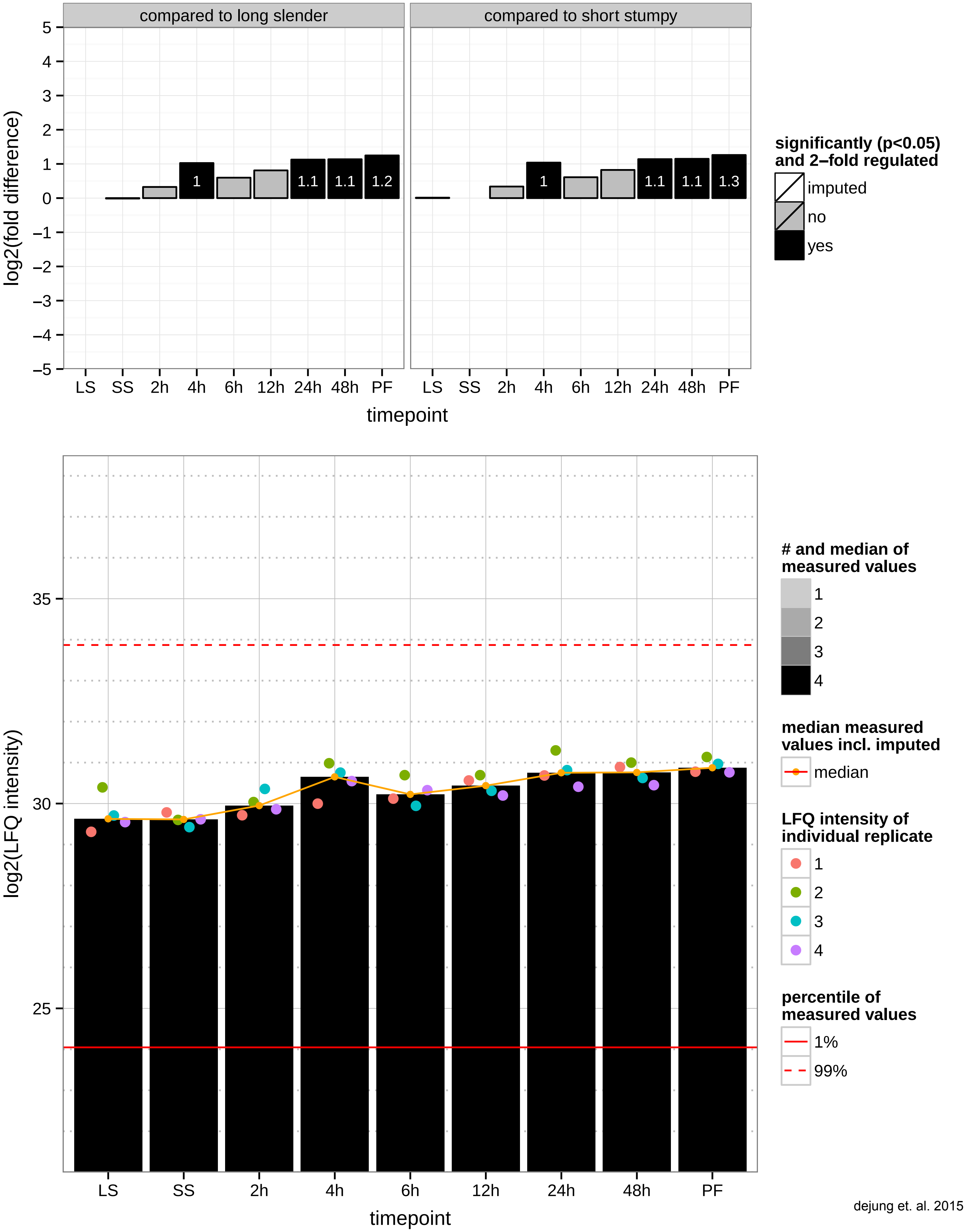

dejung et. al. 2015

hypothetical protein, conserved
Tb927.3.3440
AGOF: null
AGOC: null
AGOP: null
PGOF: null
PGOC: null
PGOP: null

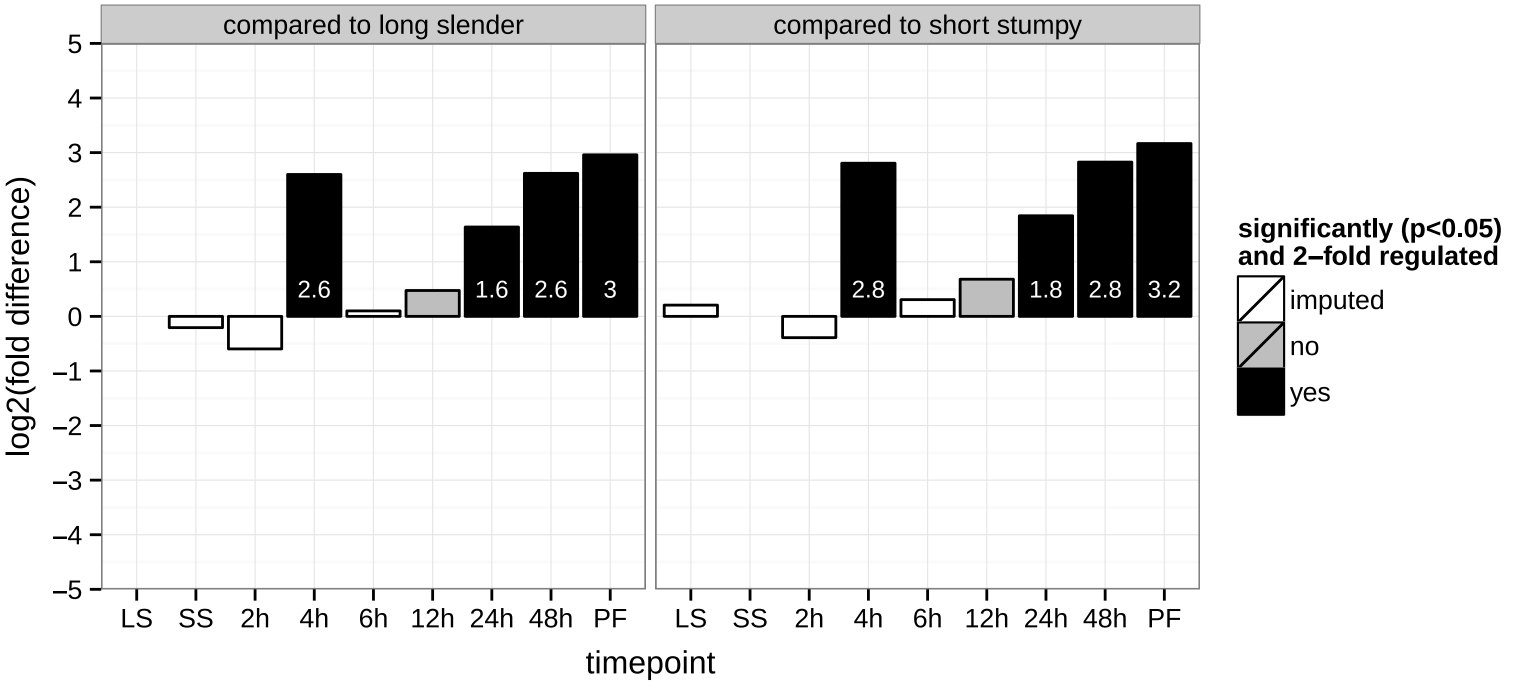

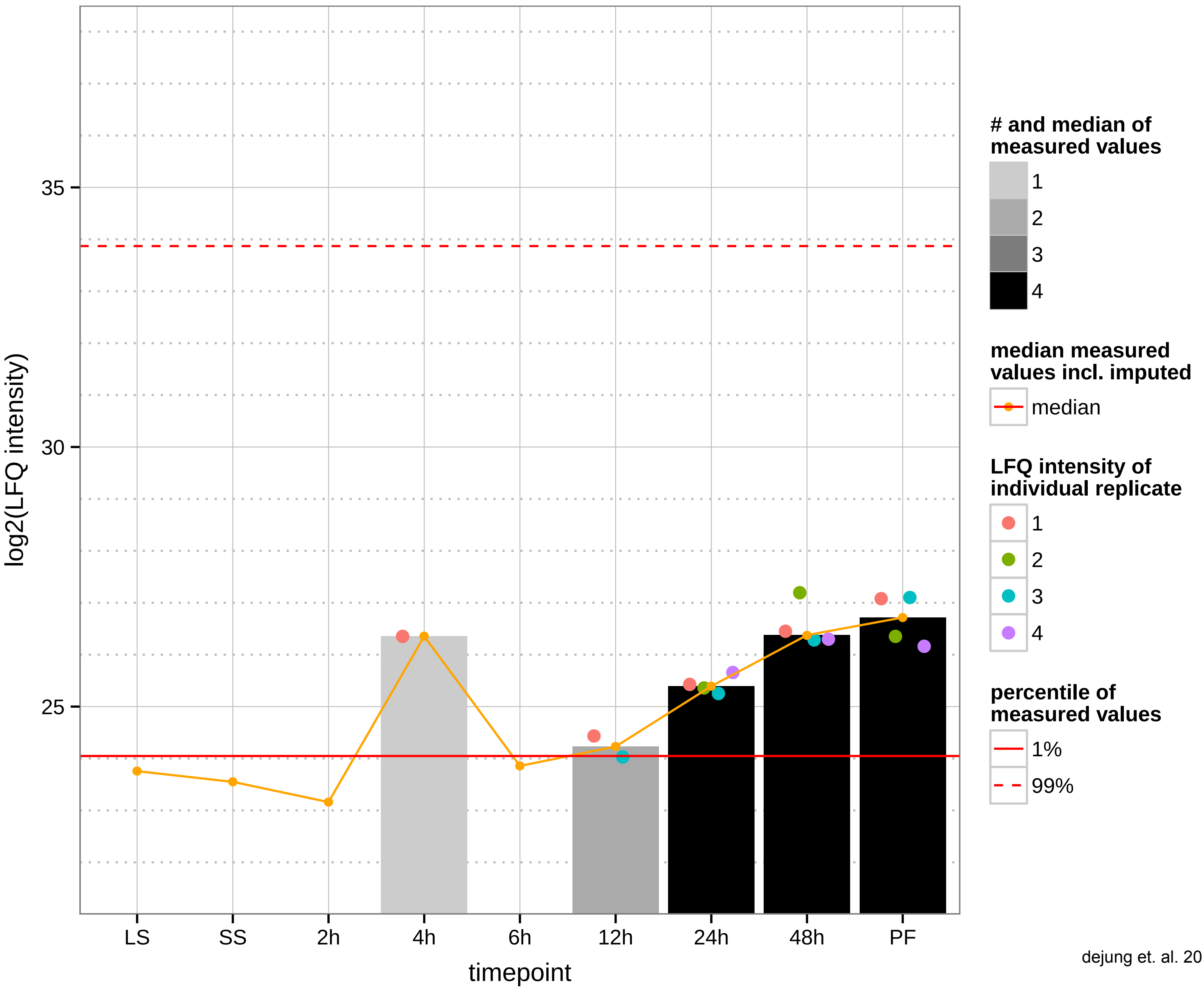

dejung et. al. 2015

RNA–binding protein, putative (EIF3D)
Tb927.4.1930
AGOF: RNA binding
AGOC: null
AGOP: RNA processing
PGOF: nucleic acid binding
PGOC: null
PGOP: null

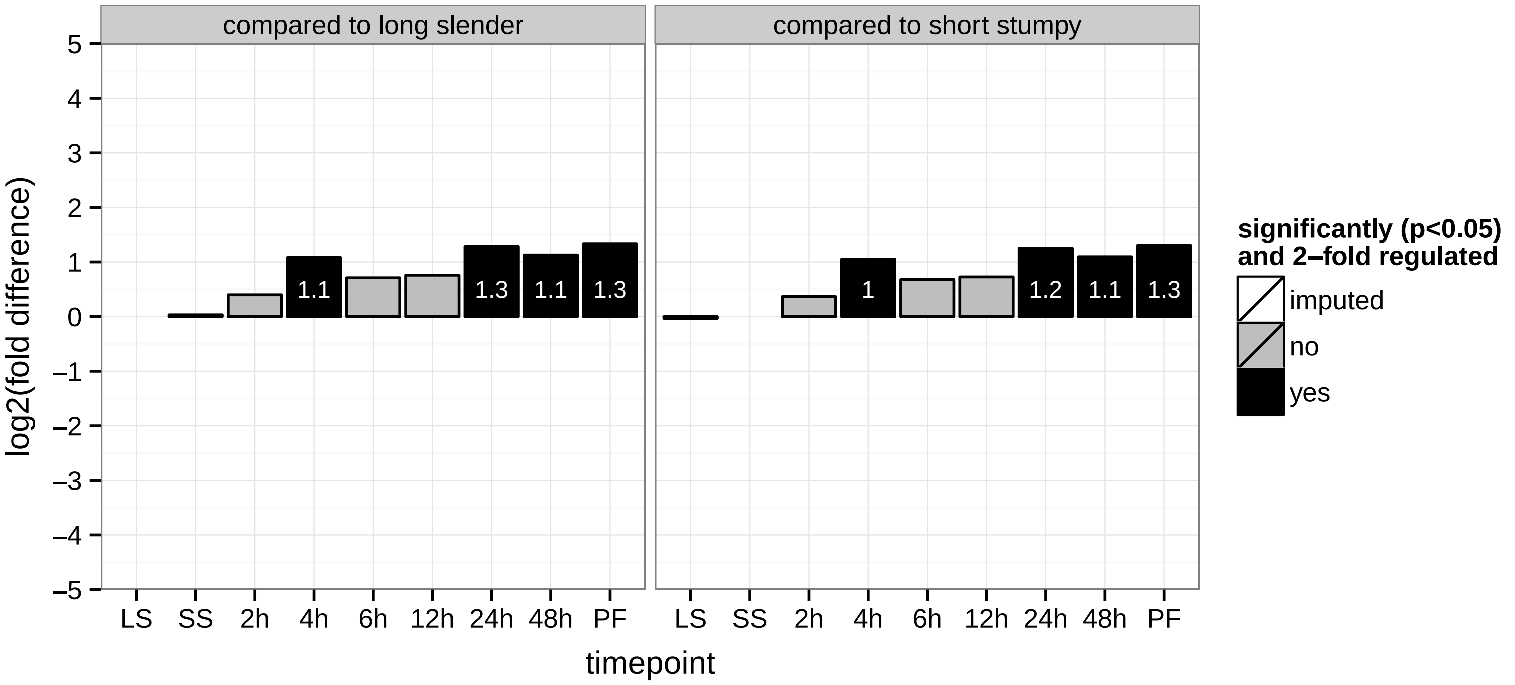

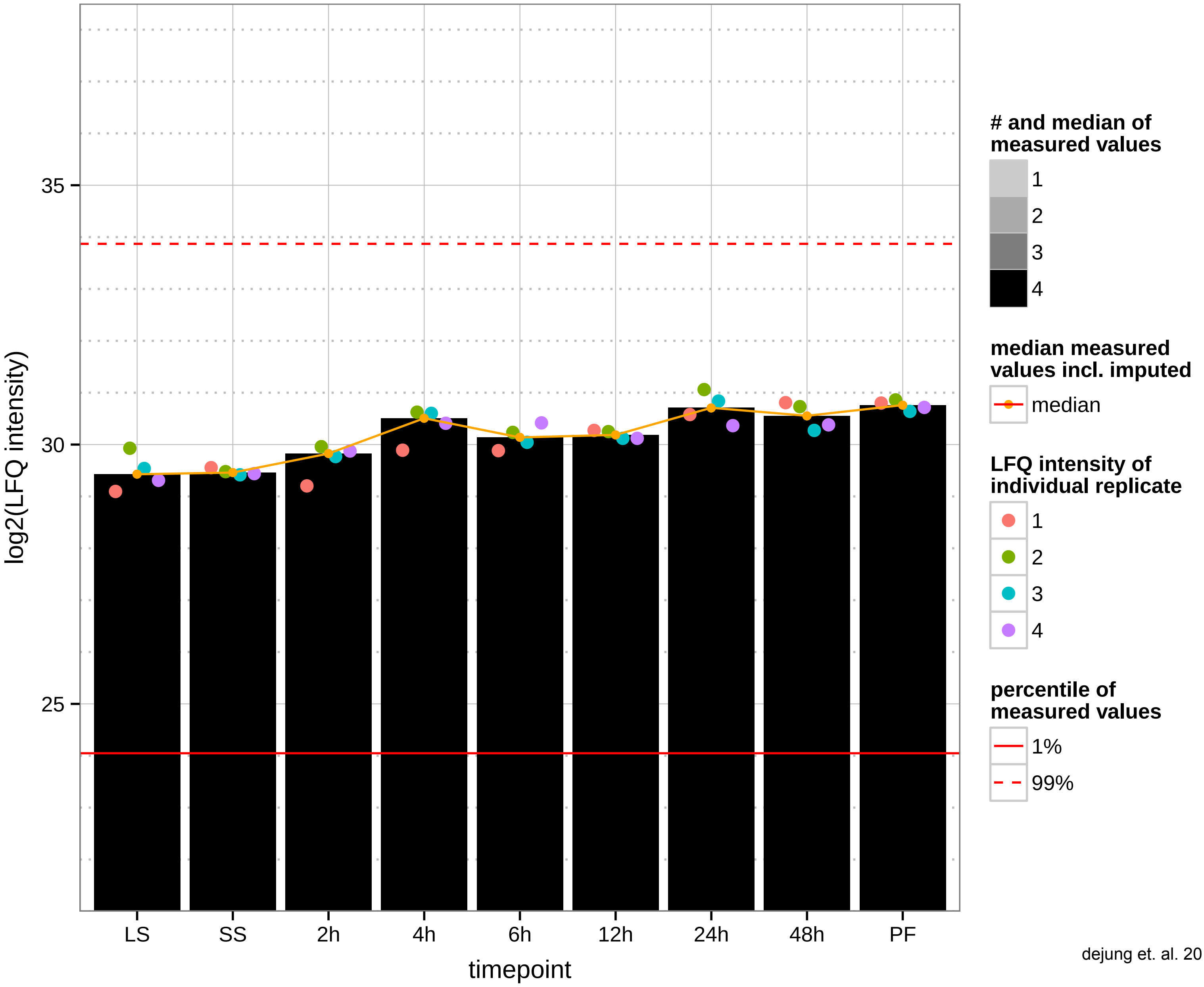

dejung et. al. 2015

ATP synthase, epsilon chain, putative
Tb927.6.4990
AGOF: hydrogen ion transporting ATP synthase activity, rotational mechanism, proton–transporting ATPase activity
AGOC: mitochondrion, proton–transporting ATP synthase complex, catalytic core F(1)
AGOP: ATP synthesis coupled proton transport
PGOF: hydrogen ion transporting ATP synthase activity, rotational mechanism, proton–transporting ATPase activity
PGOC: proton–transporting ATP synthase complex, catalytic core F(1)
PGOP: ATP synthesis coupled proton transport

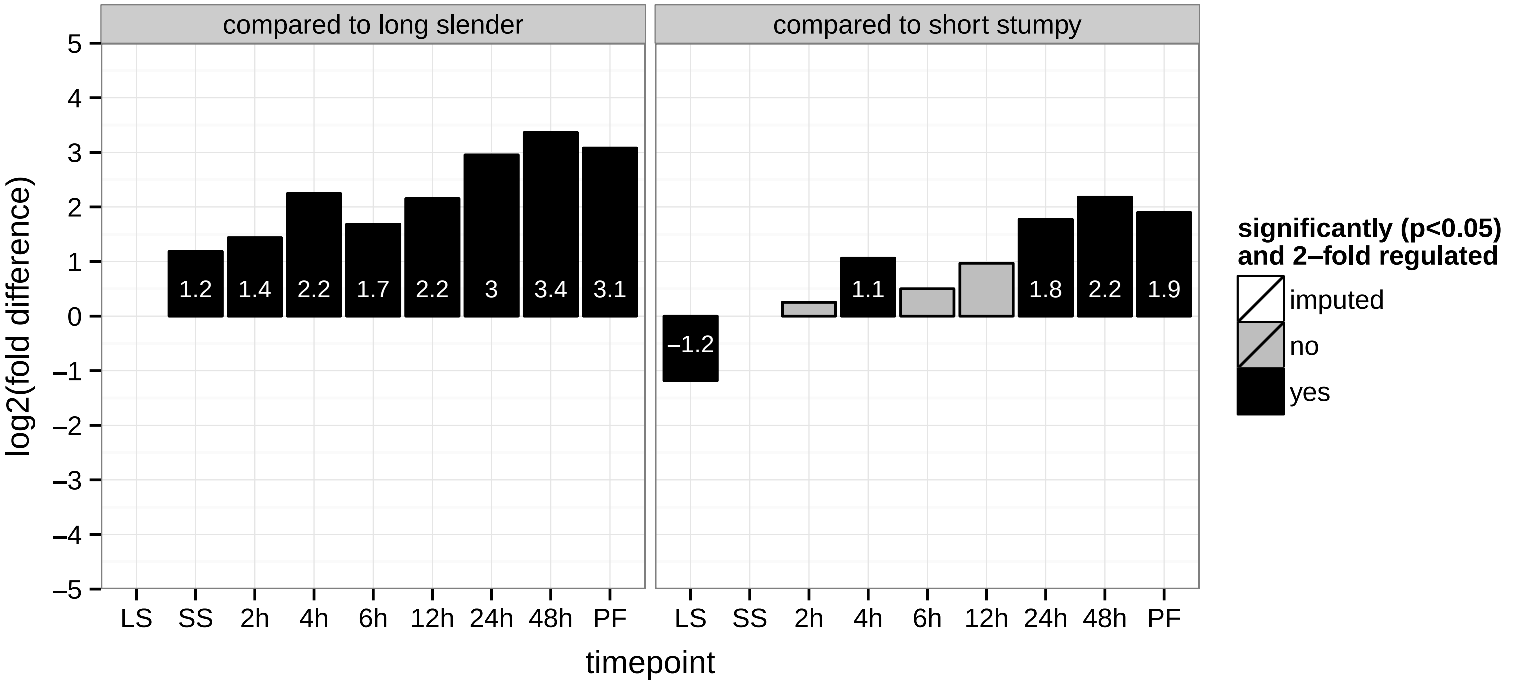

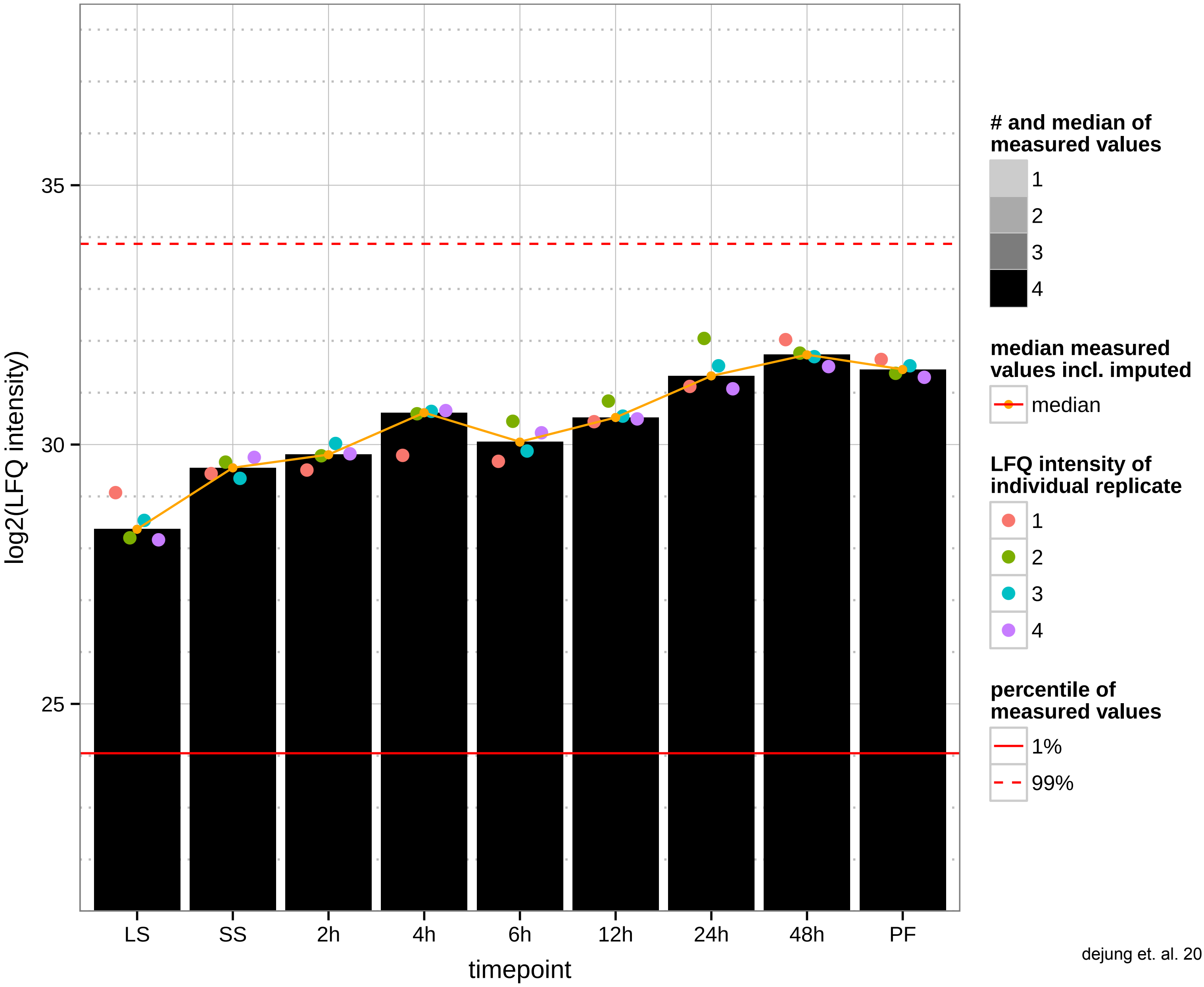

dejung et. al. 2015

hypothetical protein, conserved
Tb927.8.1950
AGOF: null
AGOC: null
AGOP: null
PGOF: null
PGOC: null
PGOP: null

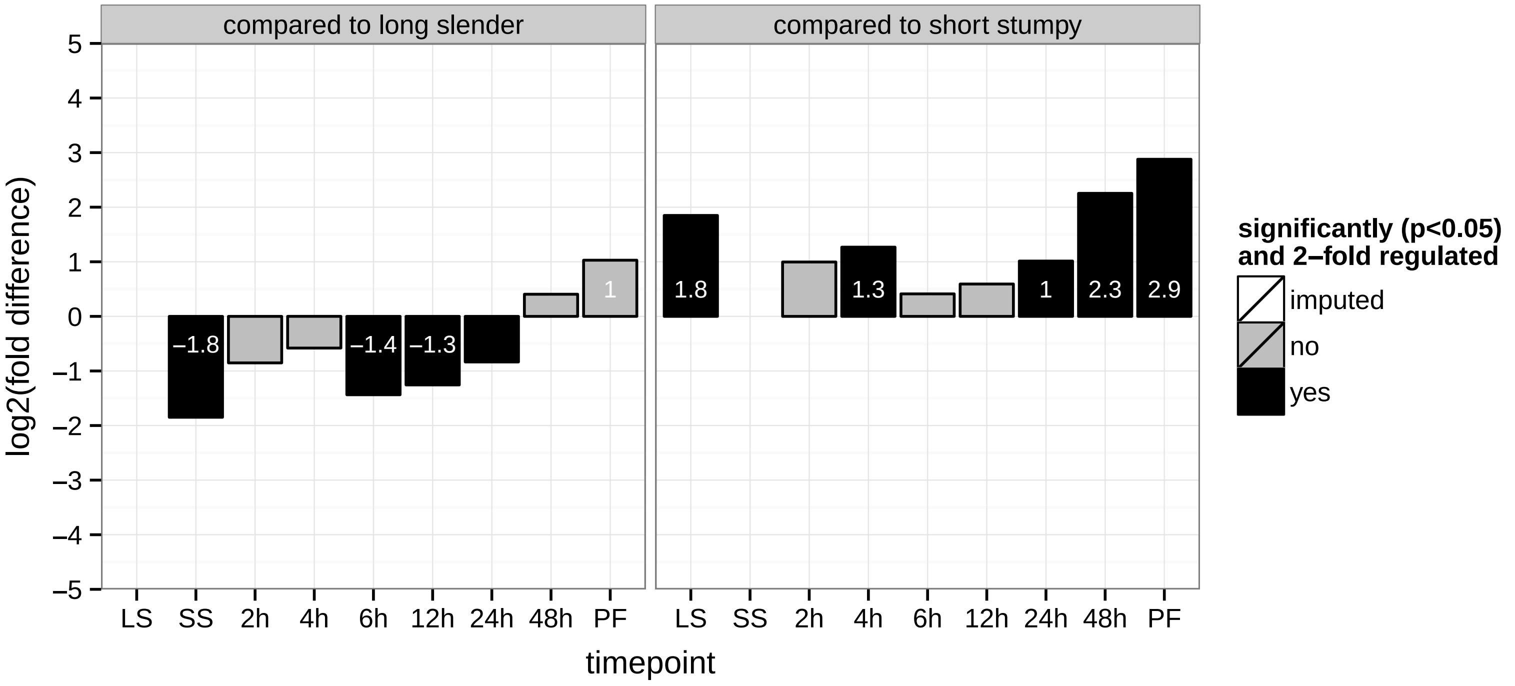

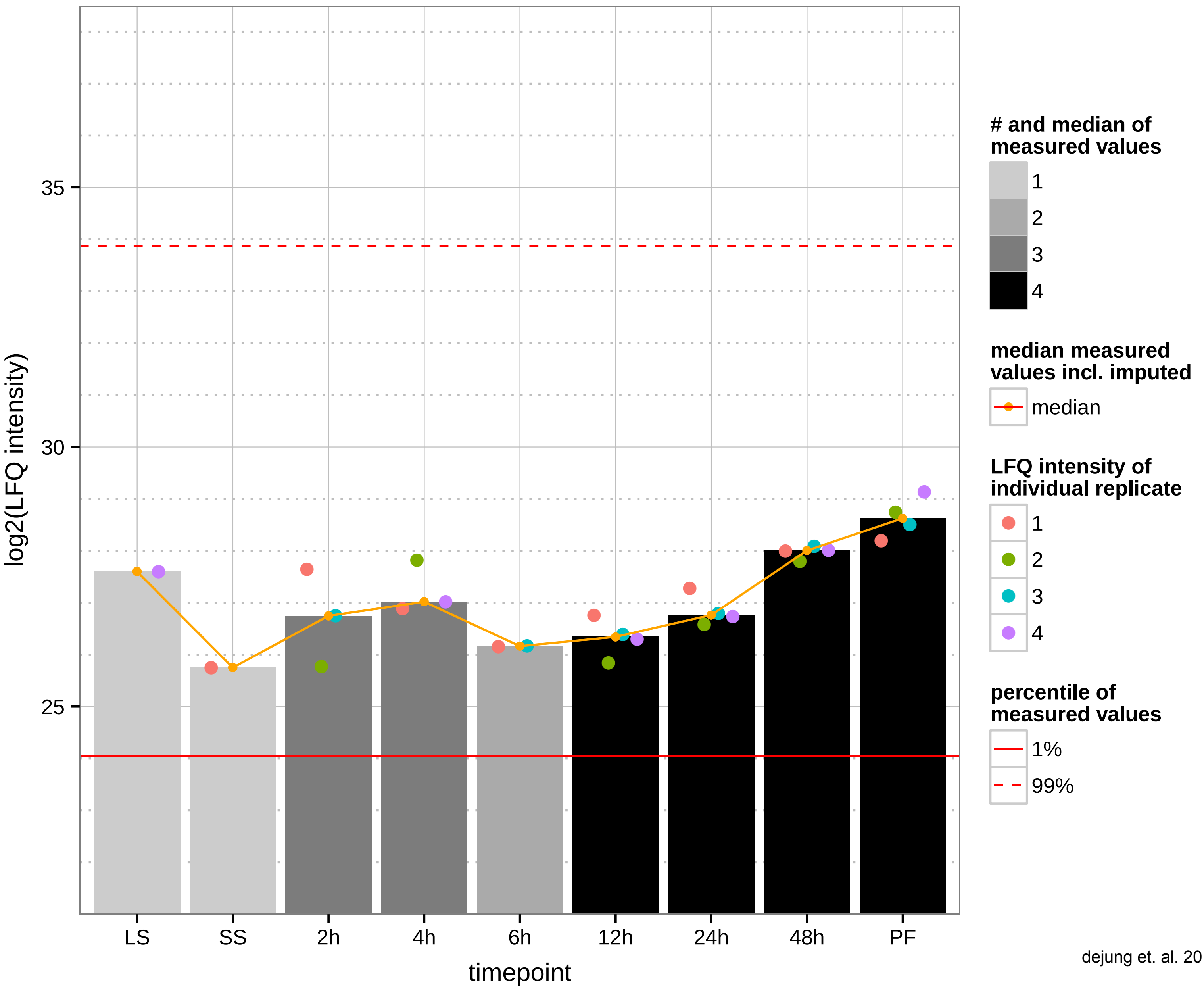

dejung et. al. 2015

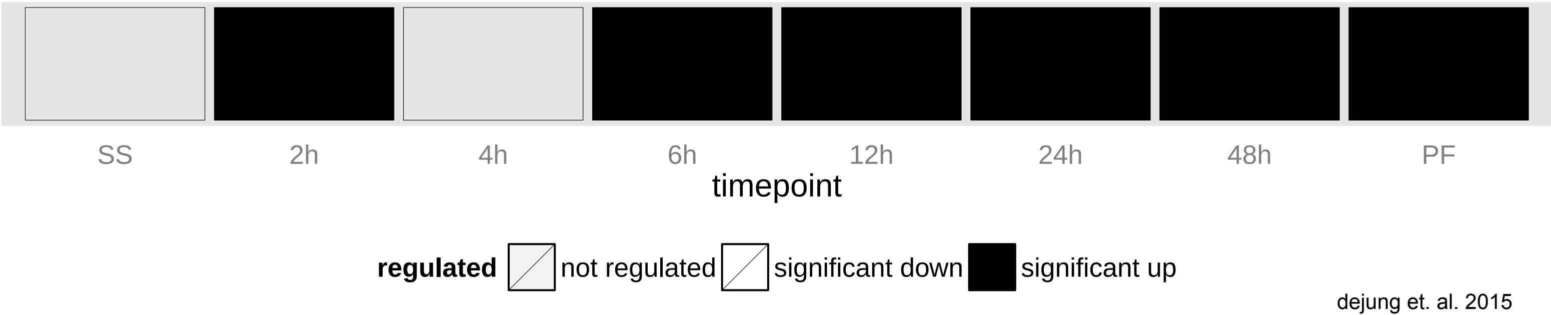
SS
2h
4h
6h
12h
24h
48h
PF
timepoint
regulated
not regulated
significant down
significant up
dejung et. al. 2015

cytochrome oxidase subunit VI (COXVI)
Tb927.10.280
AGOF: cytochrome−c oxidase activity
AGOC: mitochondrion
AGOP: oxidation−reduction process
PGOF: cytochrome−c oxidase activity
PGOC: mitochondrion
PGOP: null

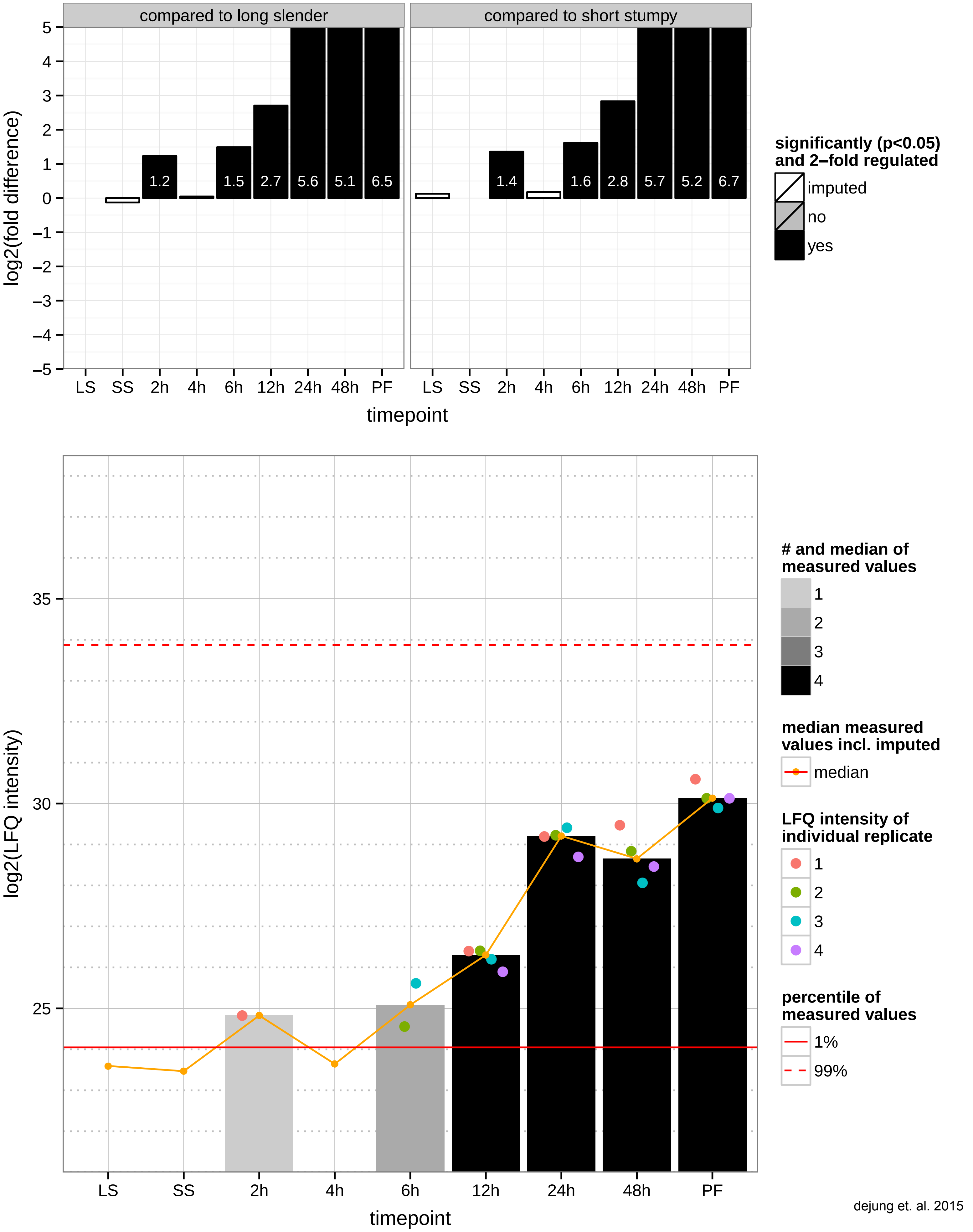

dejung et. al. 2015

peptidyl–prolyl cis–trans isomerase, putative (PPIase)
Tb927.10.4620
AGOF: FK506 binding, peptidyl–prolyl cis–trans isomerase activity
AGOC: null
AGOP: protein folding
PGOF: null
PGOC: null
PGOP: protein folding

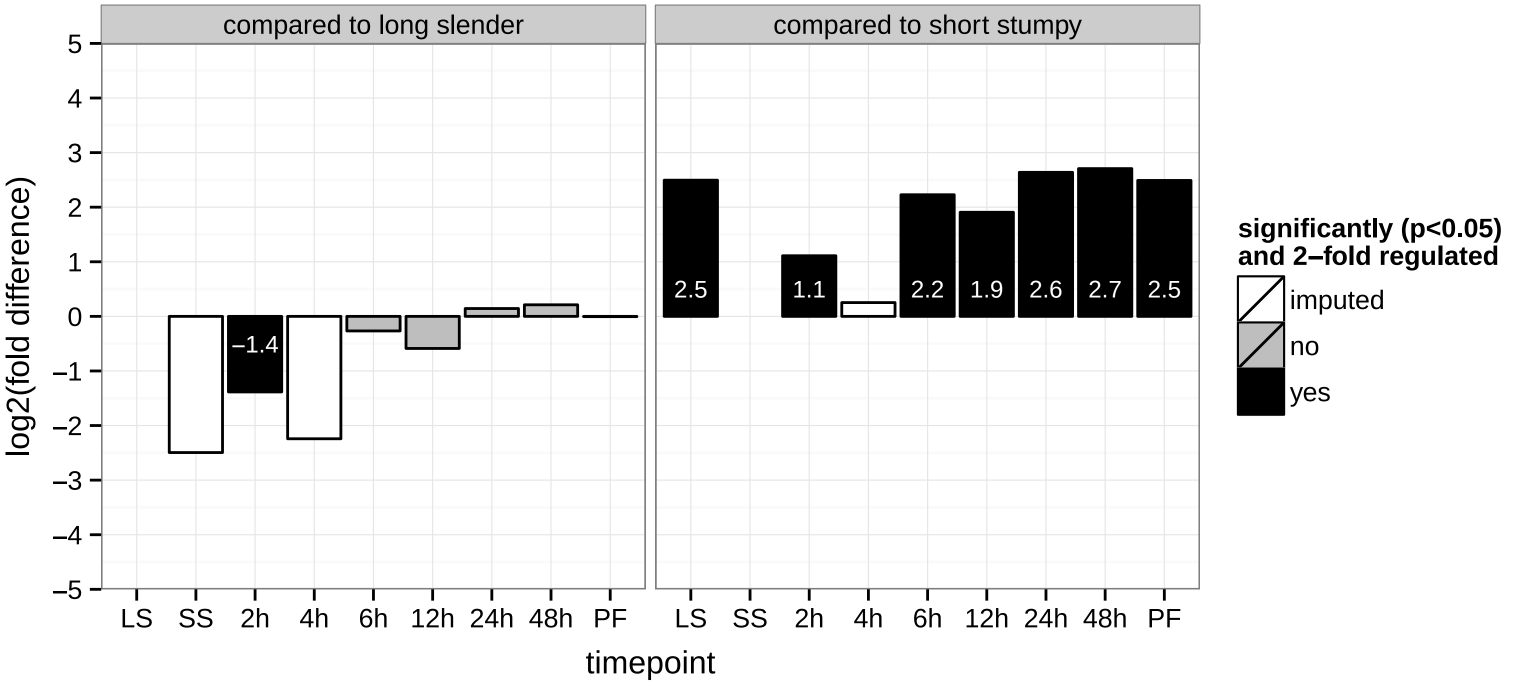

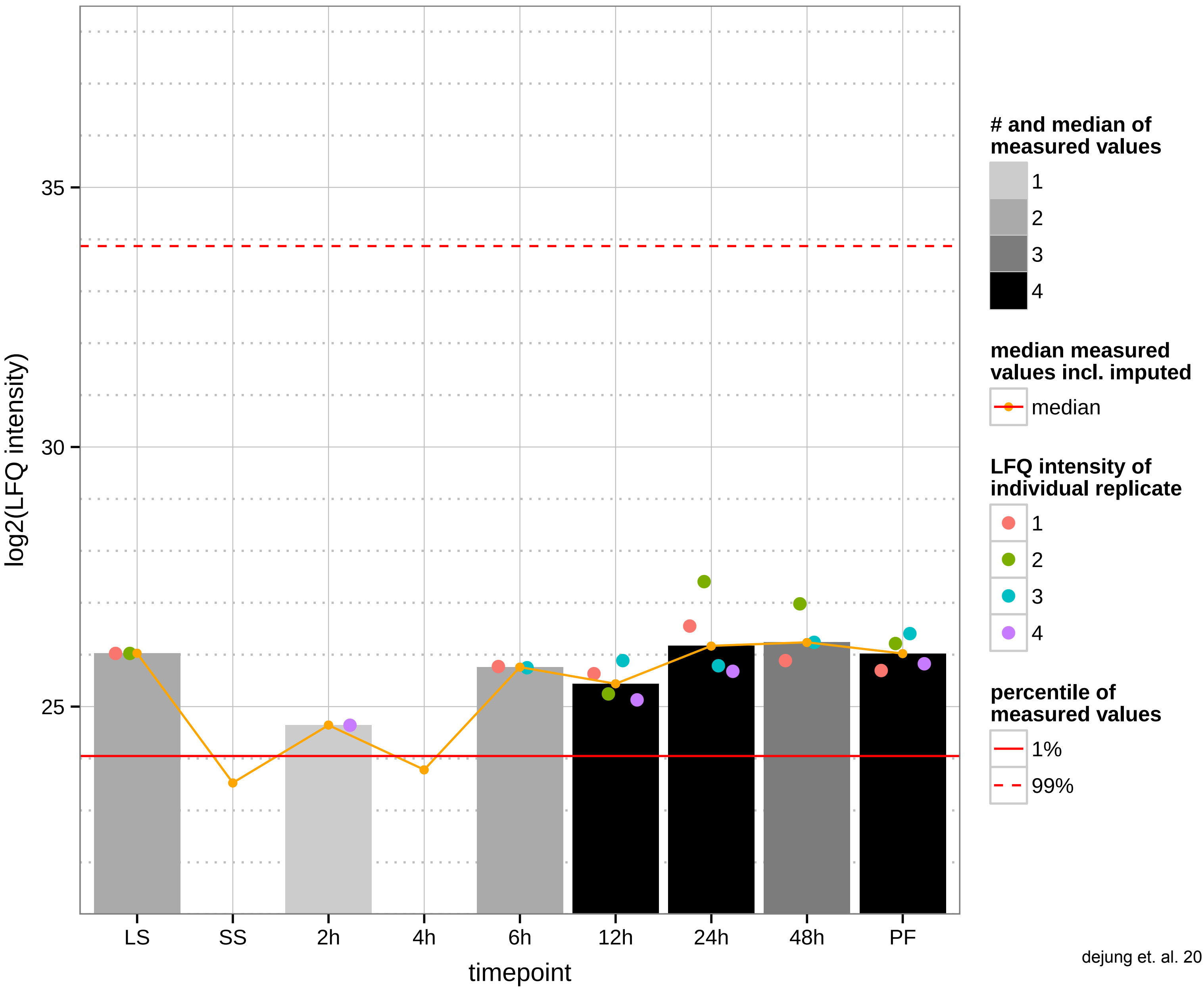

dejung et. al. 2015

hypothetical protein, conserved
Tb927.10.7730
AGOF: null
AGOC: null
AGOP: null
PGOF: null
PGOC: null
PGOP: null

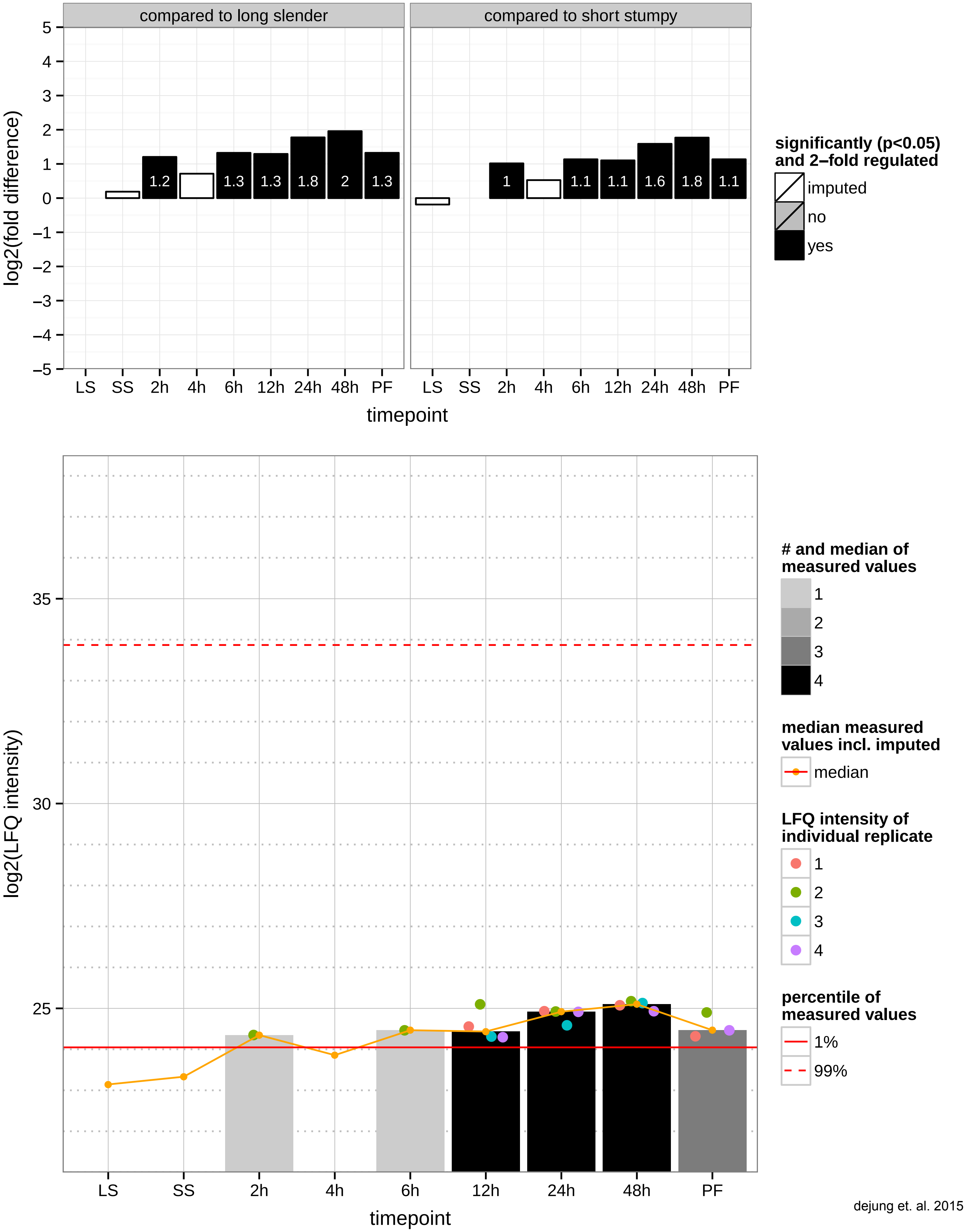

dejung et. al. 2015

hypothetical protein, conserved
Tb927.11.11470
AGOF: null
AGOC: mitochondrion
AGOP: modulation of development of symbiont involved in interaction with host, quorum sensing involved in interaction with ho
PGOF: null
PGOC: null
PGOP: null

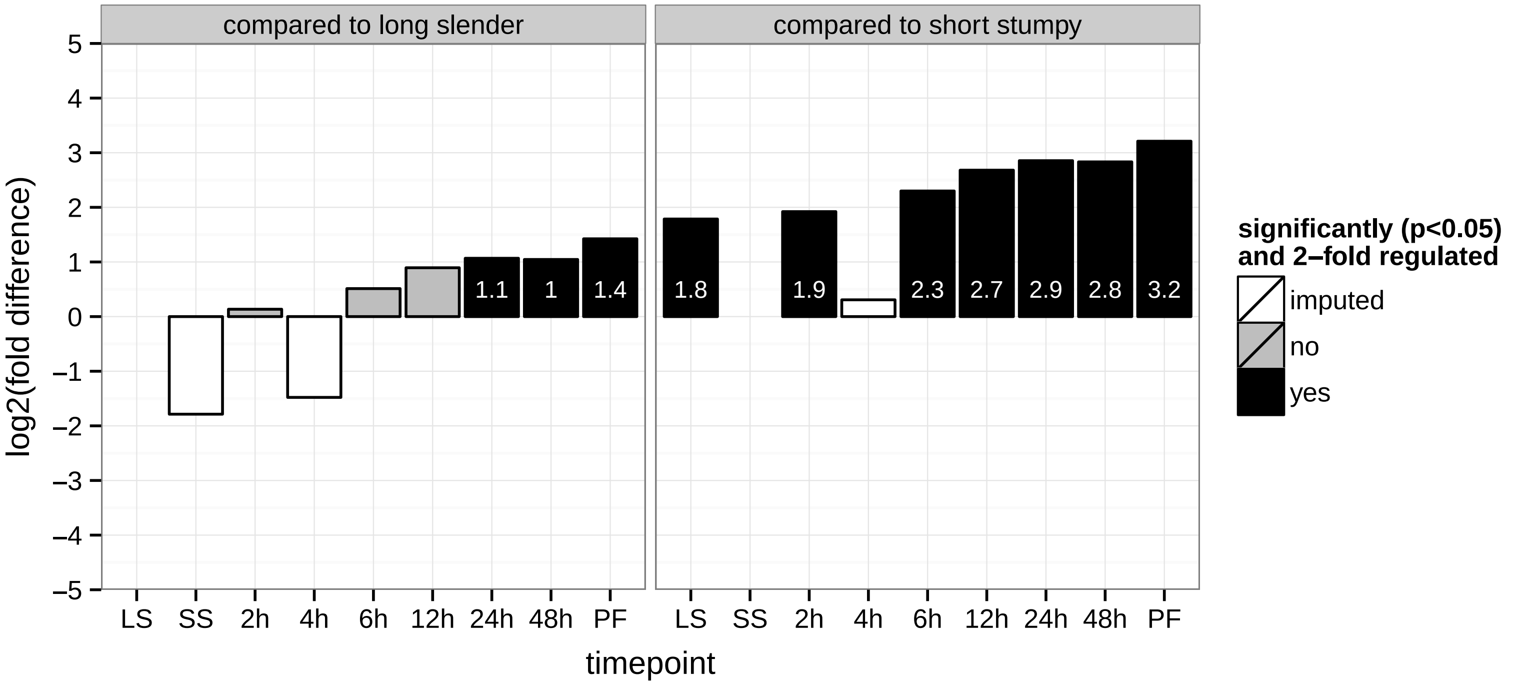

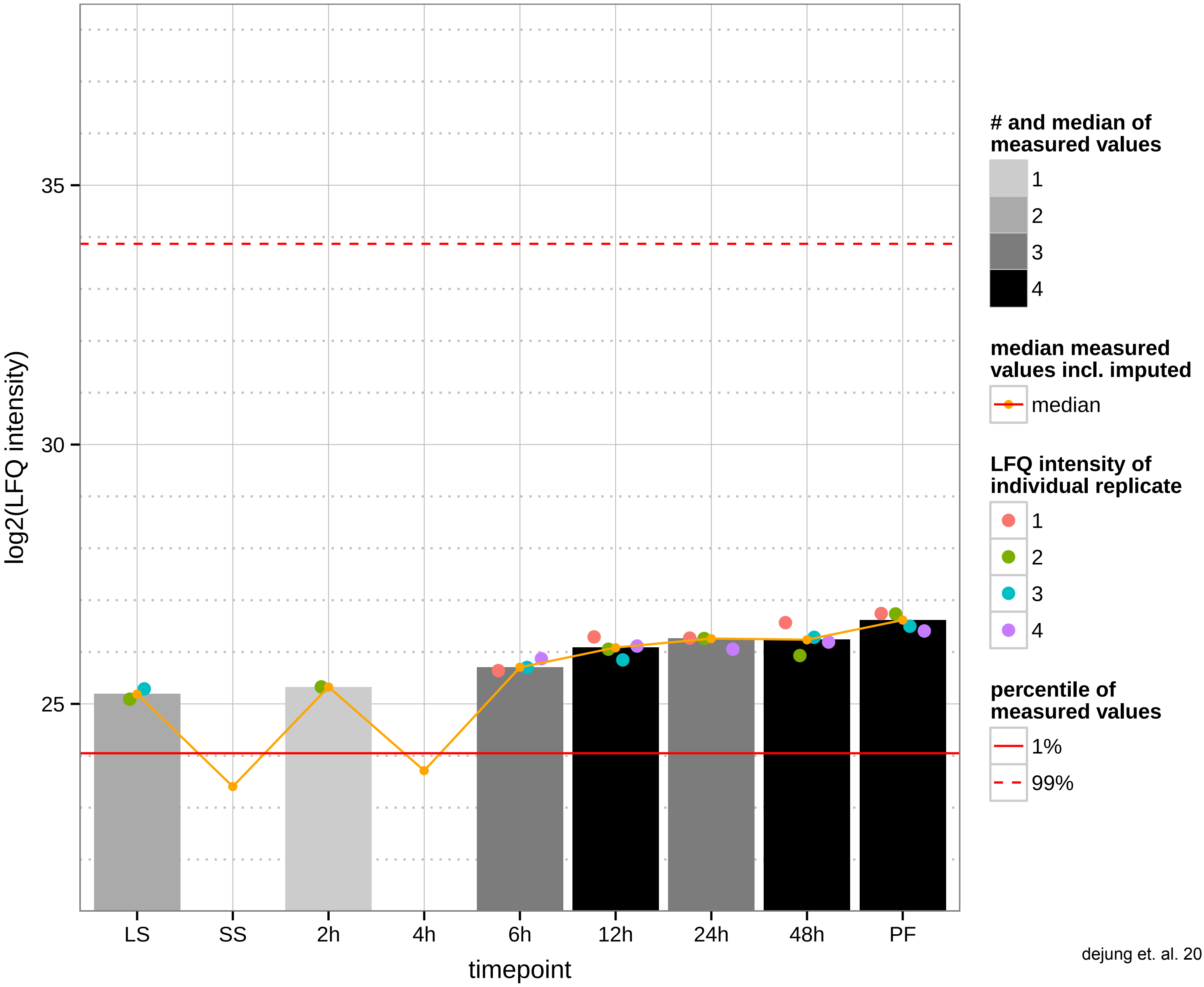

dejung et. al. 2015

NADH–cytochrome b5 reductase, putative (B5R)
Tb927.11.3750
AGOF: cytochrome–b5 reductase activity, electron carrier activity
AGOC: integral to membrane
AGOP: oxidation–reduction process
PGOF: oxidoreductase activity
PGOC: null
PGOP: oxidation–reduction process

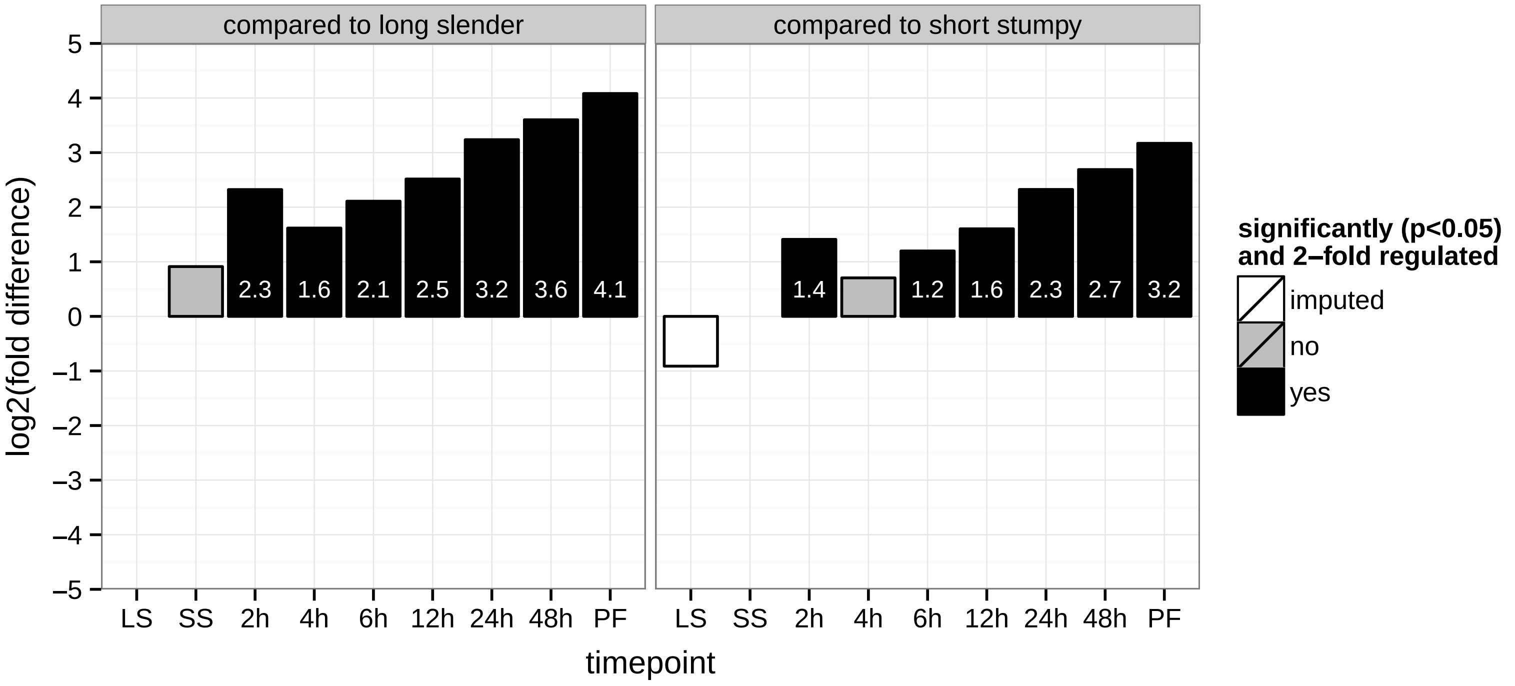

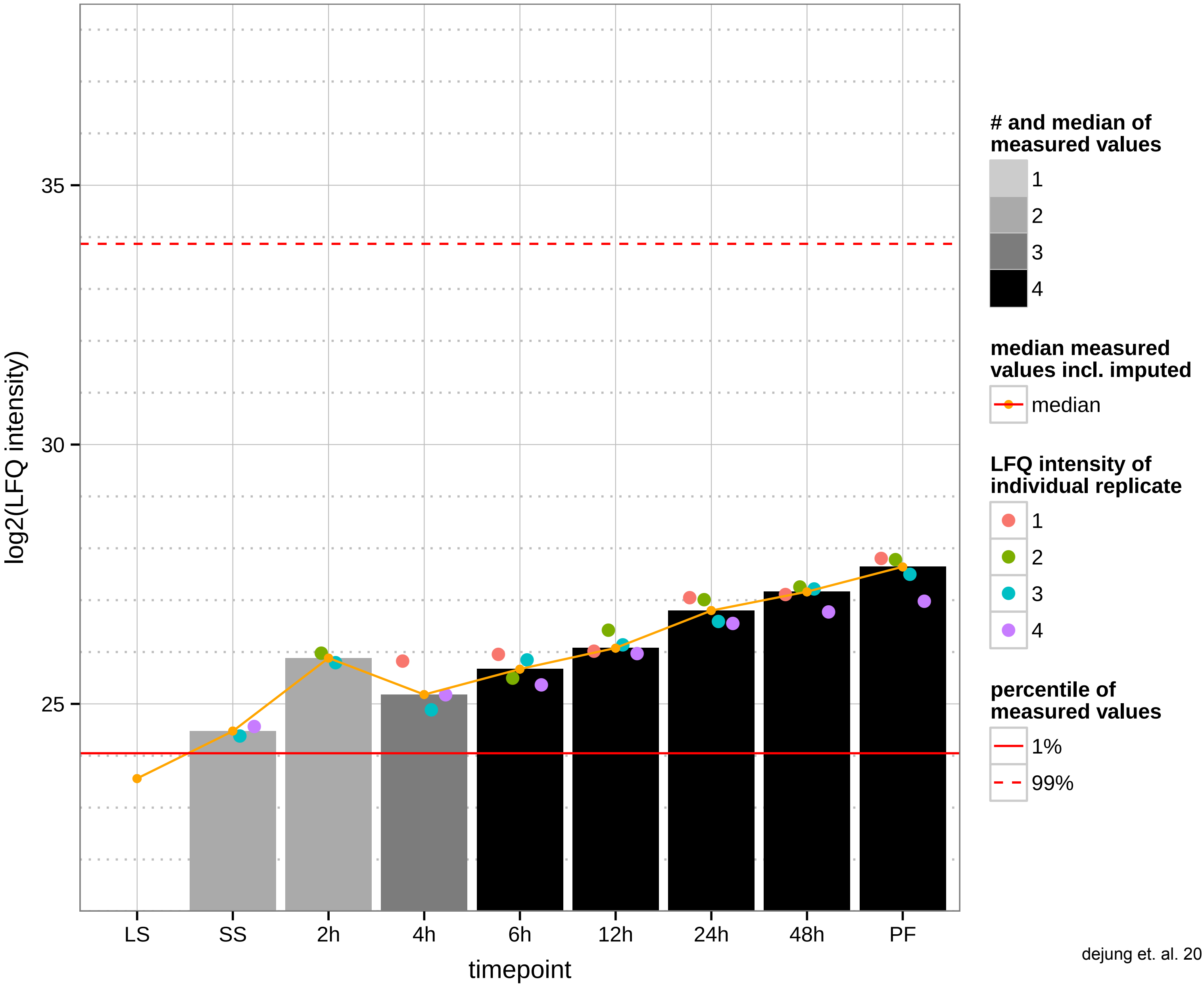

dejung et. al. 2015

mitochondrial DNA–directed RNA polymerase (MTRNAP)
Tb927.11.5780
AGOF: DNA binding, DNA–directed RNA polymerase activity
AGOC: mitochondrion
AGOP: transcription, DNA–dependent
PGOF: DNA binding, DNA–directed RNA polymerase activity
PGOC: null
PGOP: transcription, DNA–dependent

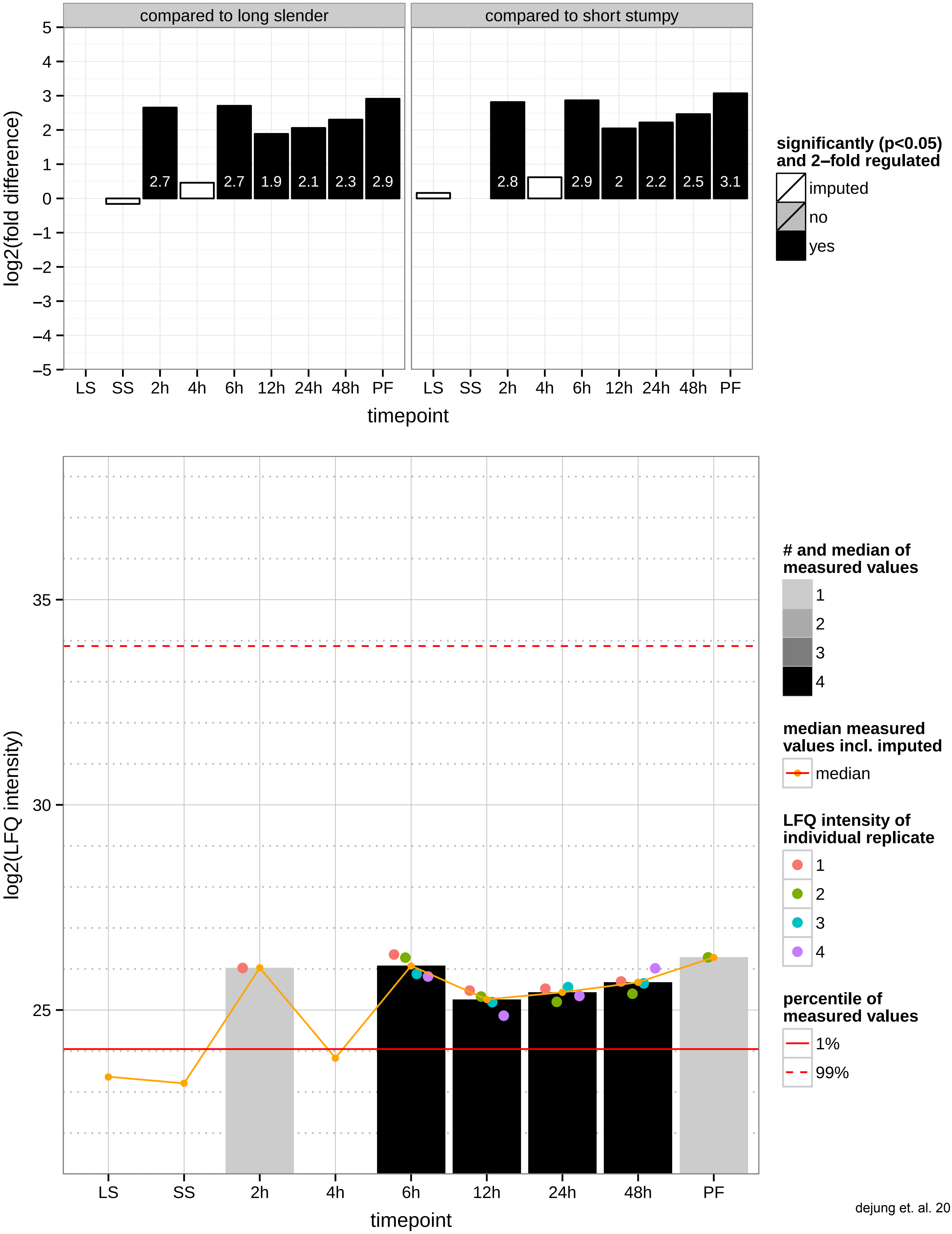

dejung et. al. 2015

hypothetical protein, conserved
Tb927.2.2340
AGOF: glycerophosphodiester phosphodiesterase activity, phospholipase C activity
AGOC: null
AGOP: glycerol metabolic process, intracellular signal transduction, lipid metabolic process
PGOF: glycerophosphodiester phosphodiesterase activity, phospholipase C activity, phosphoric diester hydrolase activity
PGOC: null
PGOP: glycerol metabolic process, intracellular signal transduction, lipid metabolic process

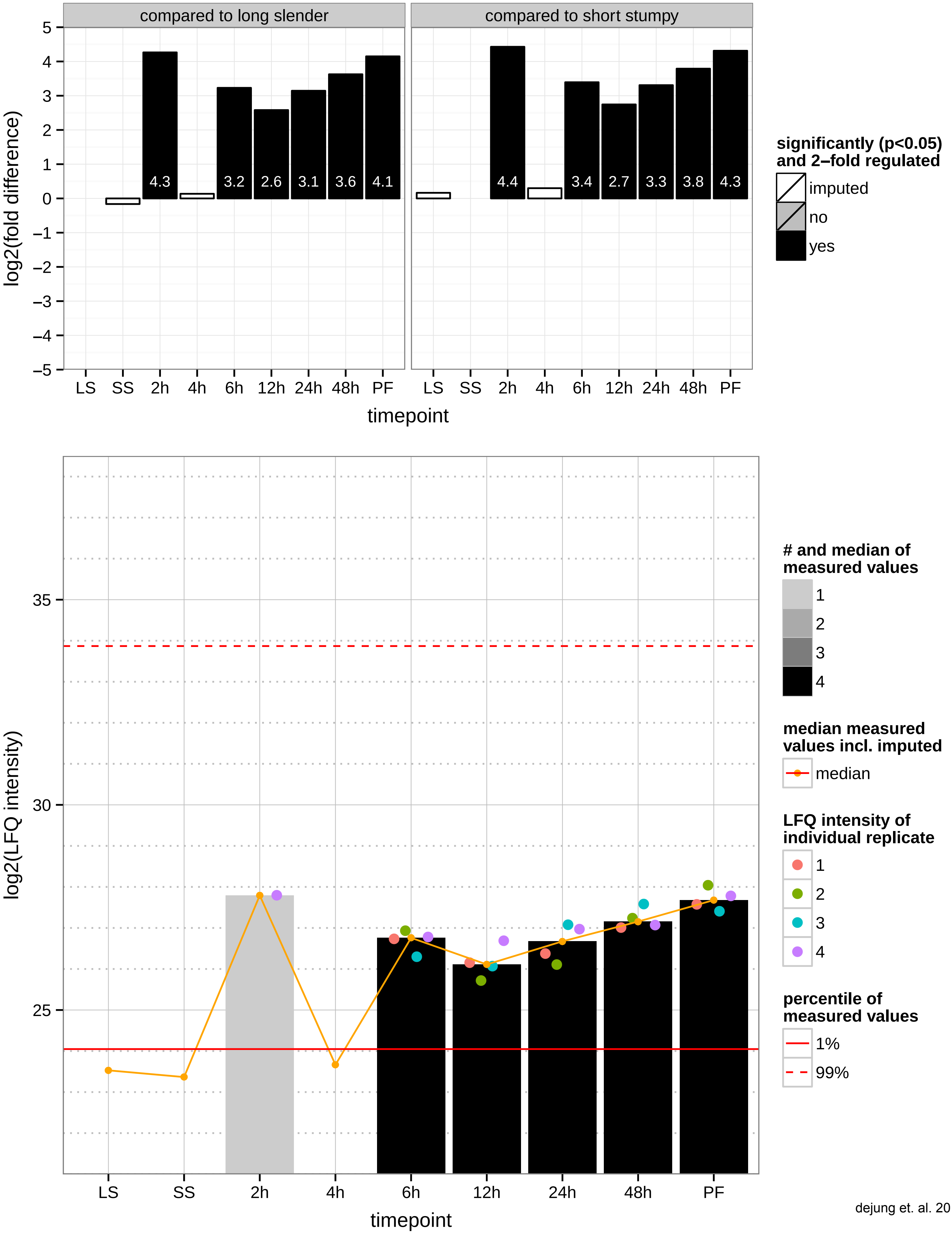

dejung et. al. 2015

hypothetical protein, conserved
Tb927.4.3140
AGOF: null
AGOC: null
AGOP: null
PGOF: null
PGOC: null
PGOP: ribosome biogenesis

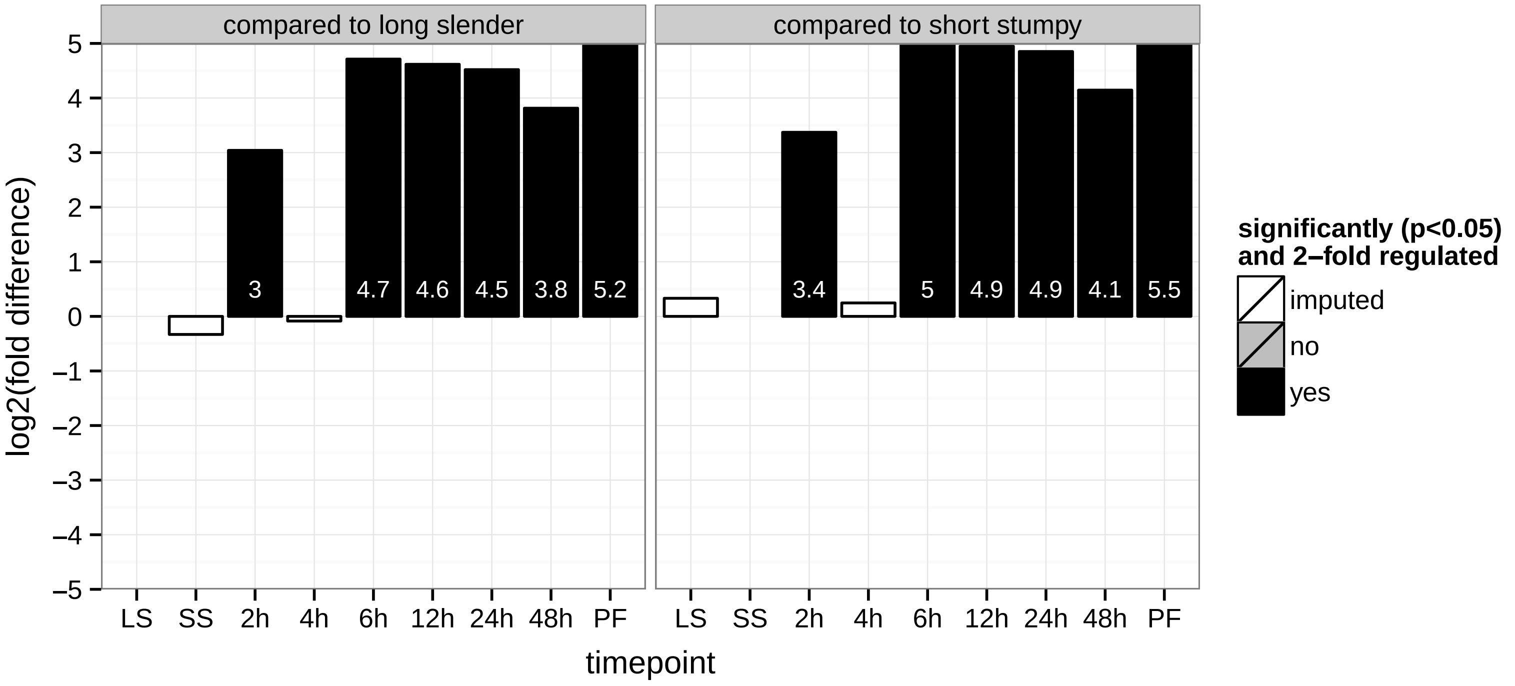

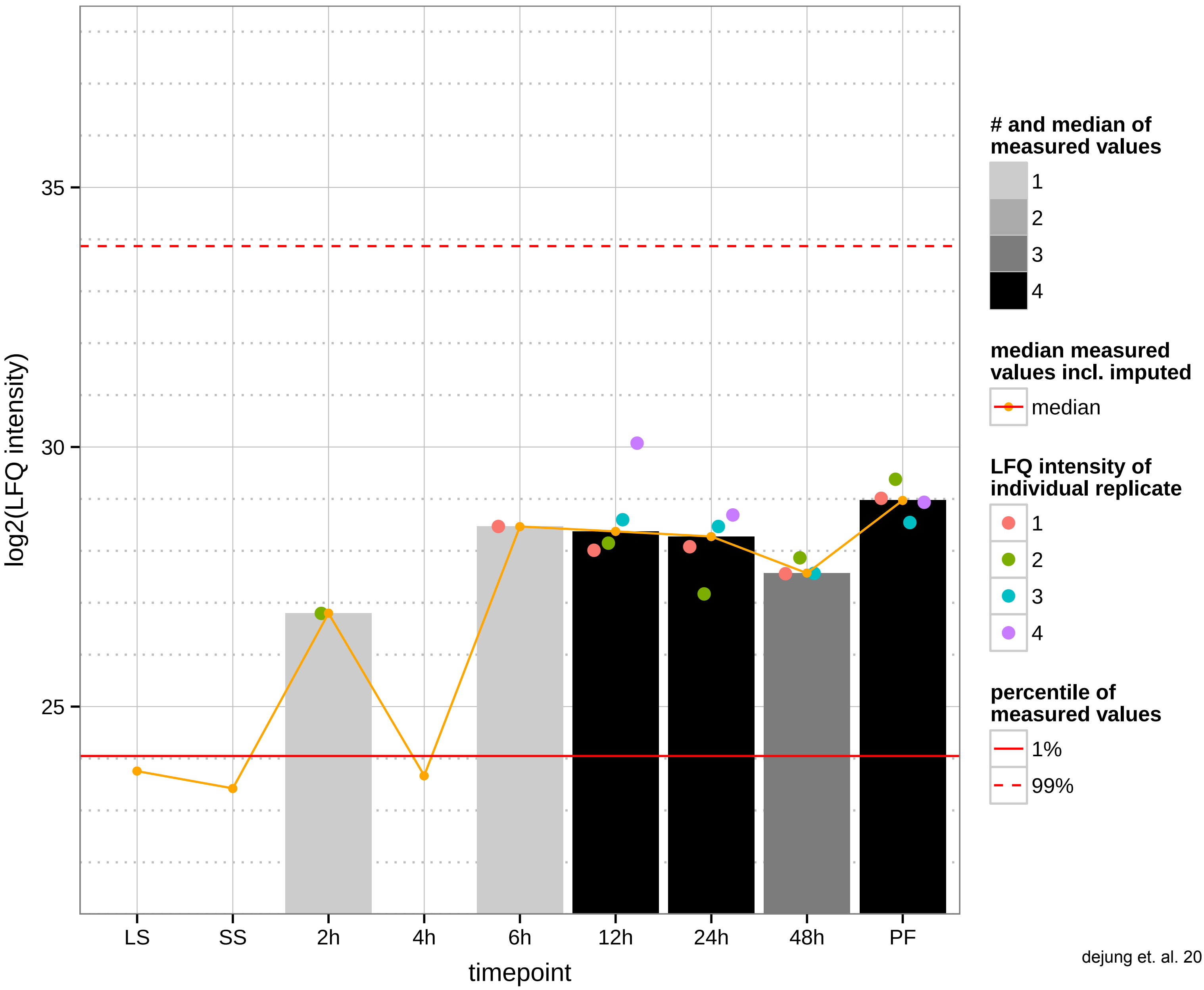

dejung et. al. 2015

hypothetical protein, conserved, inositol 1, 4, 5–trisphosphate receptor (IP3R)
Tb927.8.2770
AGOF: null
AGOC: null, membrane
AGOP: null, ion transport
PGOF: binding, calcium channel activity, calcium channel activity, ion channel activity
PGOC: membrane
PGOP: calcium ion transmembrane transport, ion transport, transmembrane transport

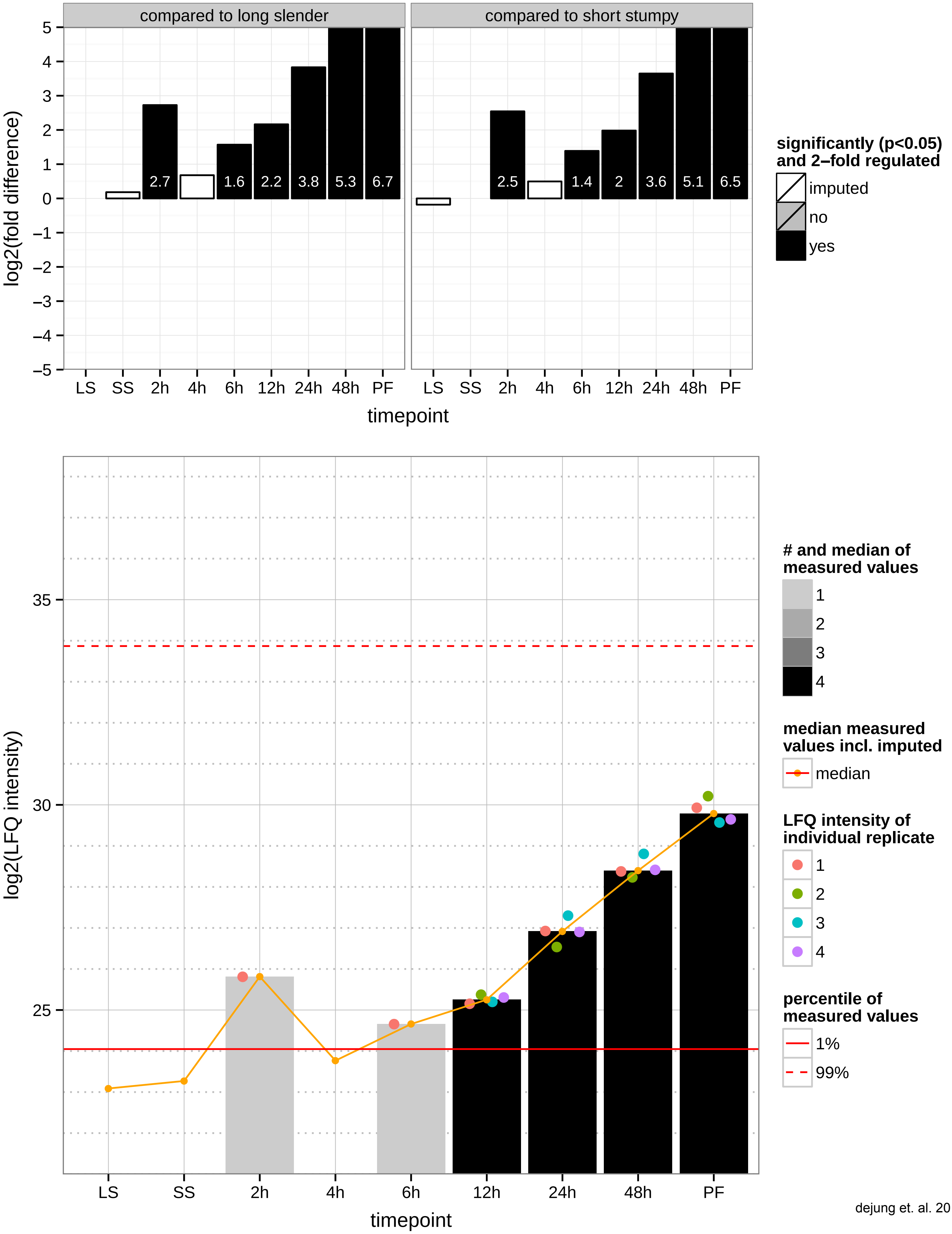

dejung et. al. 2015

small nuclear ribonucleoprotein Sm–F (Sm–F)
Tb927.9.10250
AGOF: null
AGOC: nucleus, small nucleolar ribonucleoprotein complex
AGOP: mRNA processing, nuclear mRNA trans splicing, SL addition
PGOF: null
PGOC: null
PGOP: null

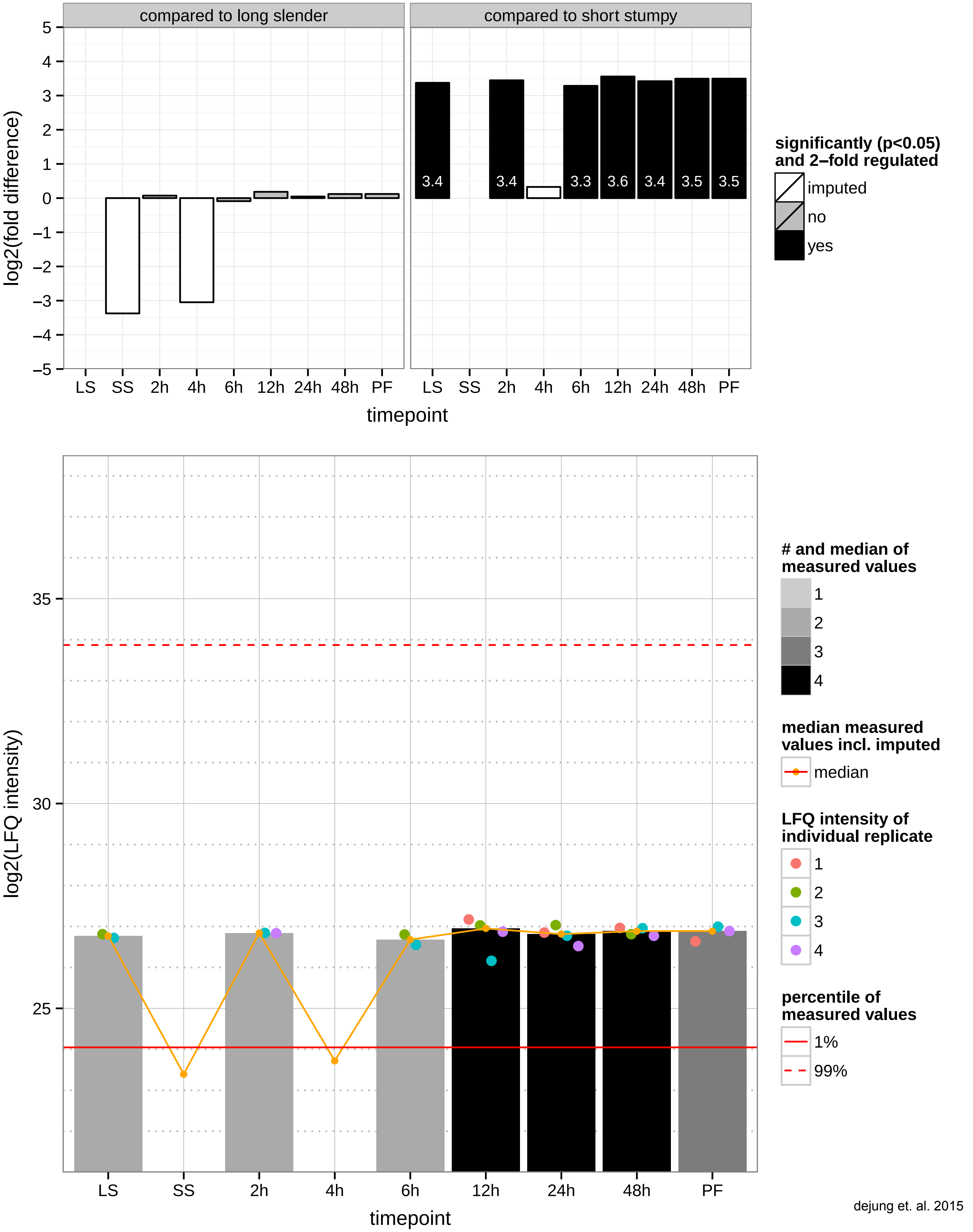

dejung et. al. 2015

hypothetical protein, conserved
Tb927.9.10560
AGOF: null
AGOC: mitochondrion
AGOP: null
PGOF: null
PGOC: null
PGOP: null

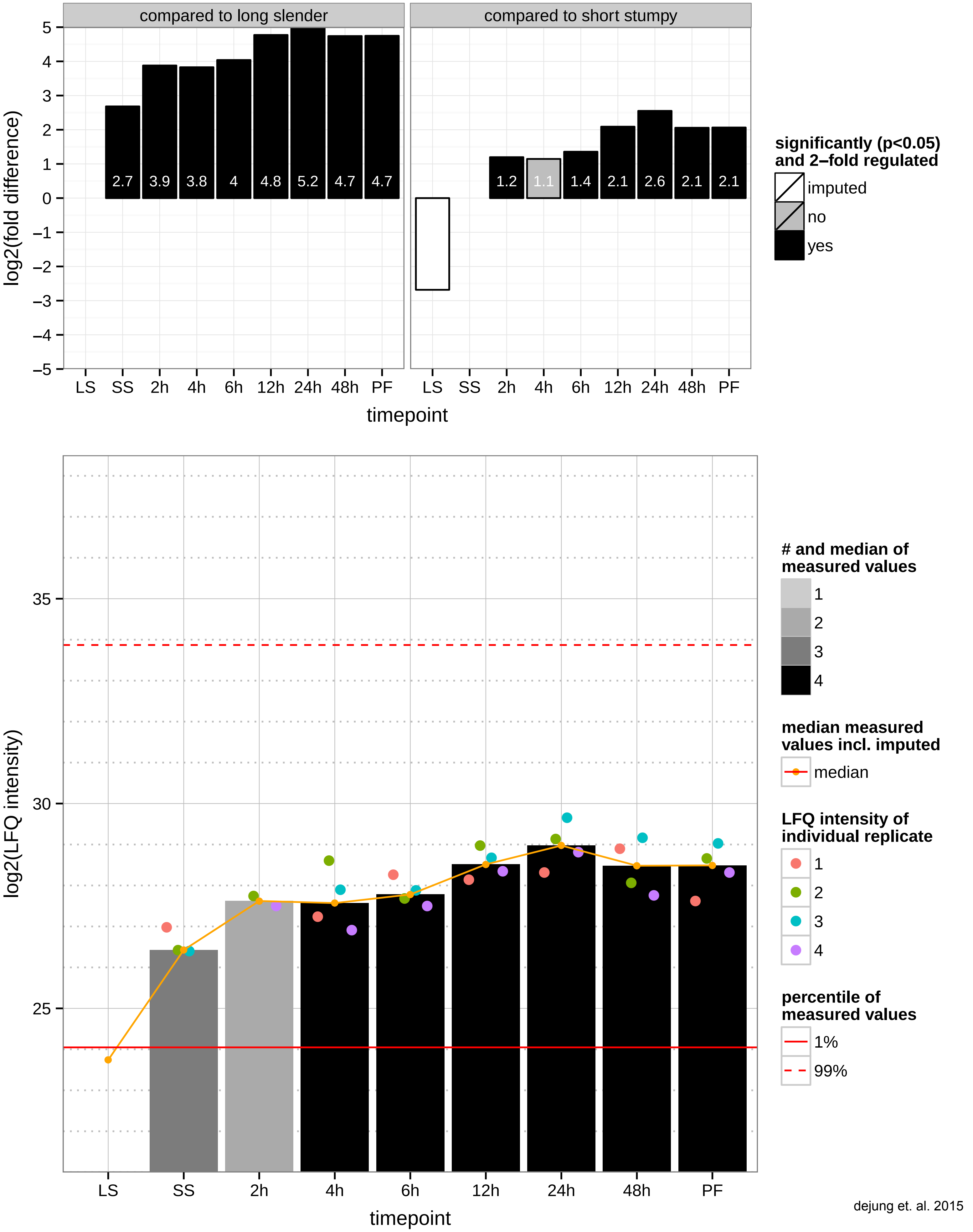

dejung et. al. 2015

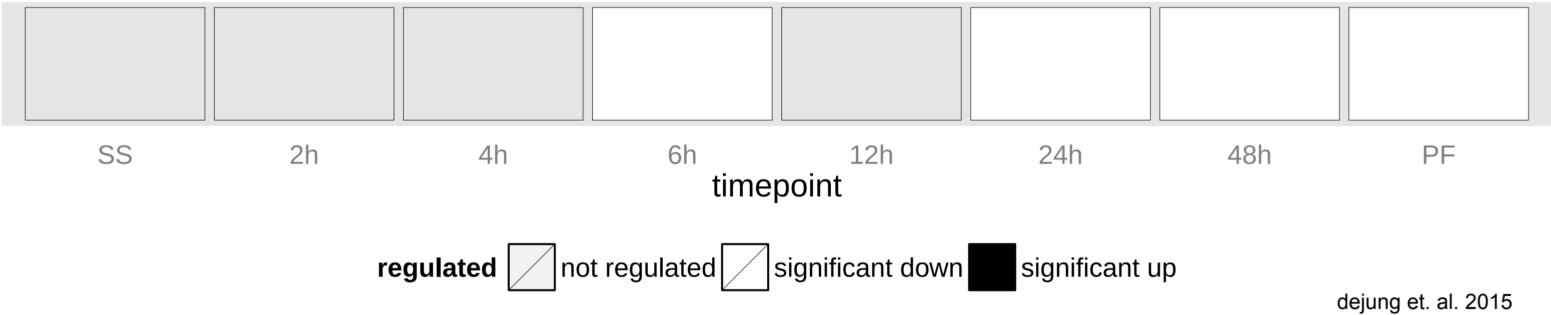
SS
2h
4h
6h
12h
24h
48h
PF
timepoint
regulated
not regulated
significant down
significant up
dejung et. al. 2015

hypothetical protein, conserved
Tb927.5.309b;Tb11.v5.0216
AGOF: null
AGOC: null
AGOP: null
PGOF: null
PGOC: null
PGOP: null

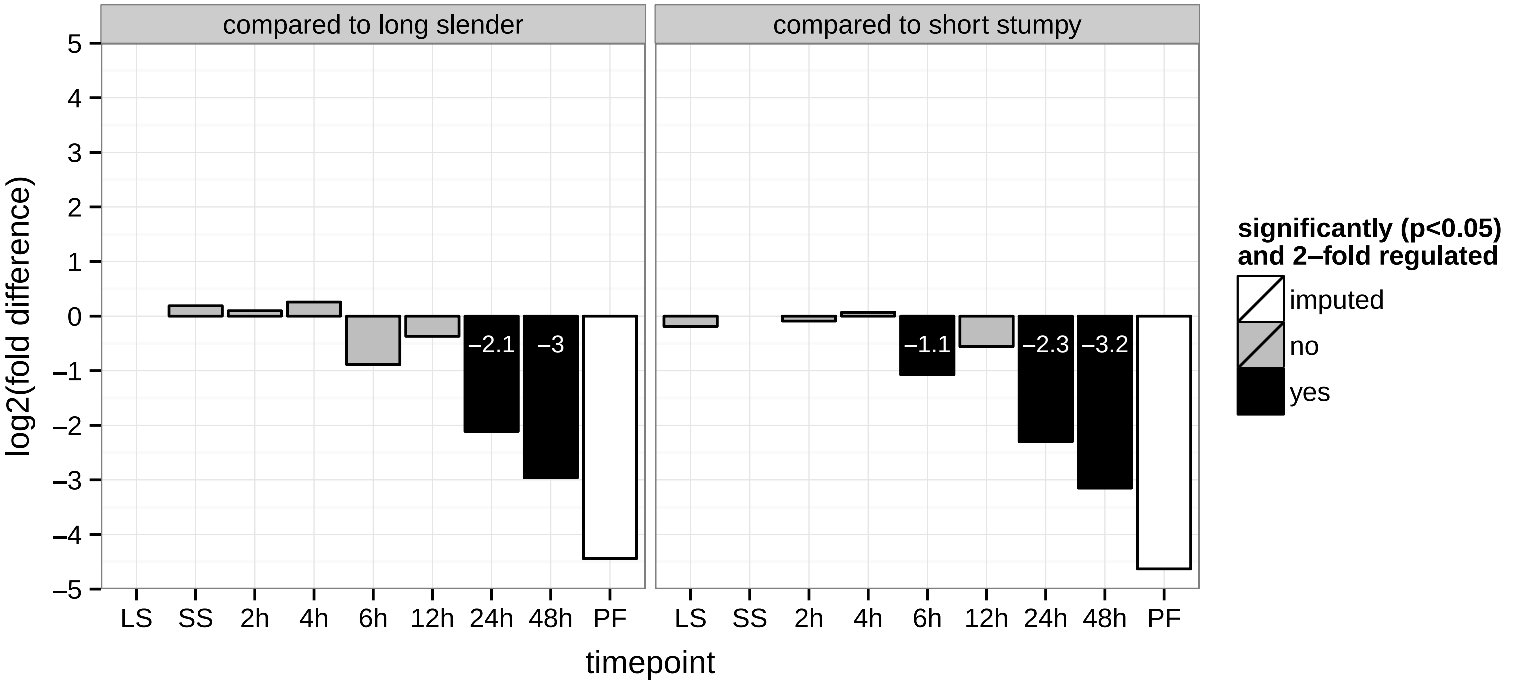

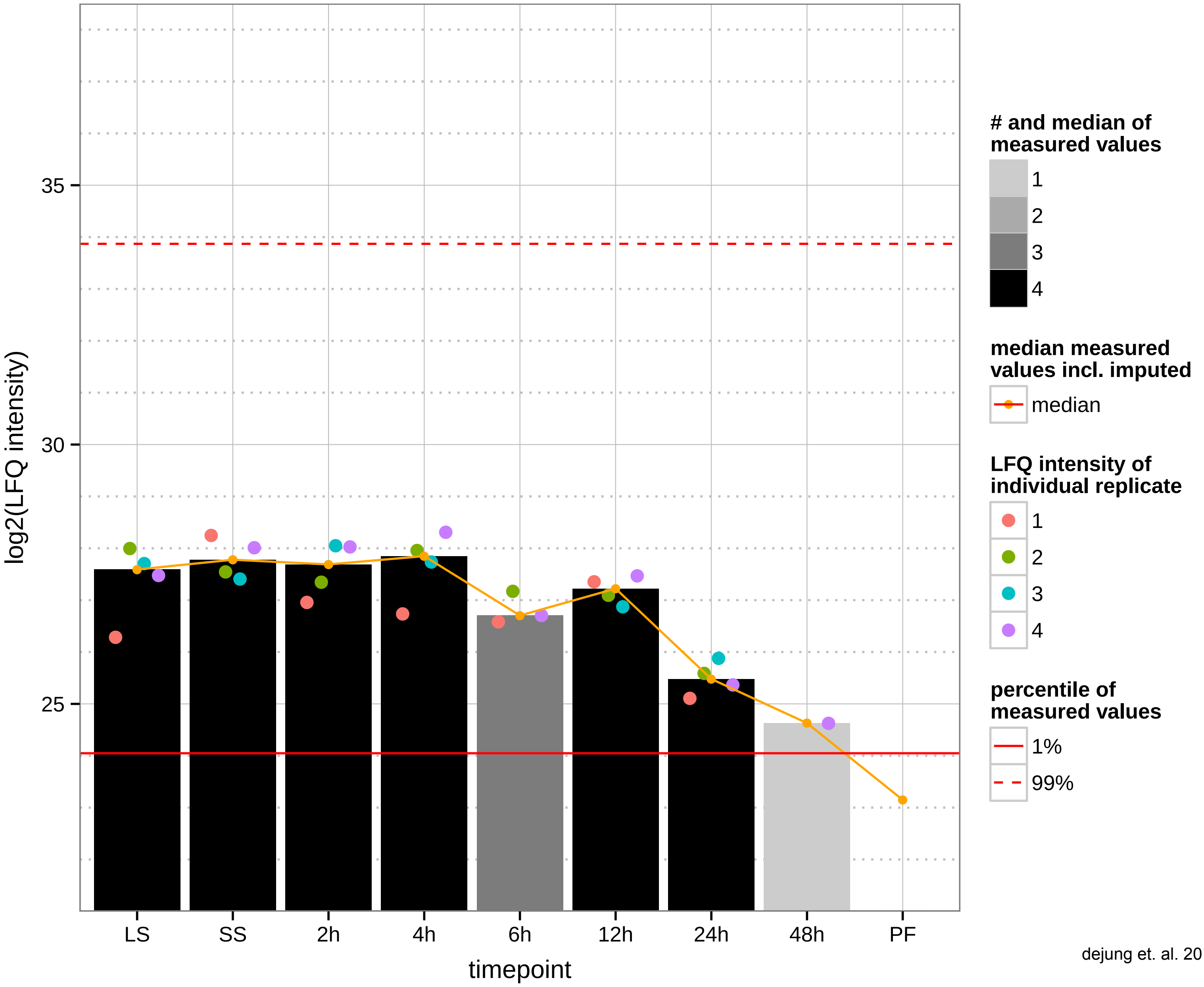

dejung et. al. 2015

hypothetical protein, conserved
Tb927.10.10840
AGOF: null
AGOC: membrane
AGOP: null
PGOF: null
PGOC: membrane
PGOP: null

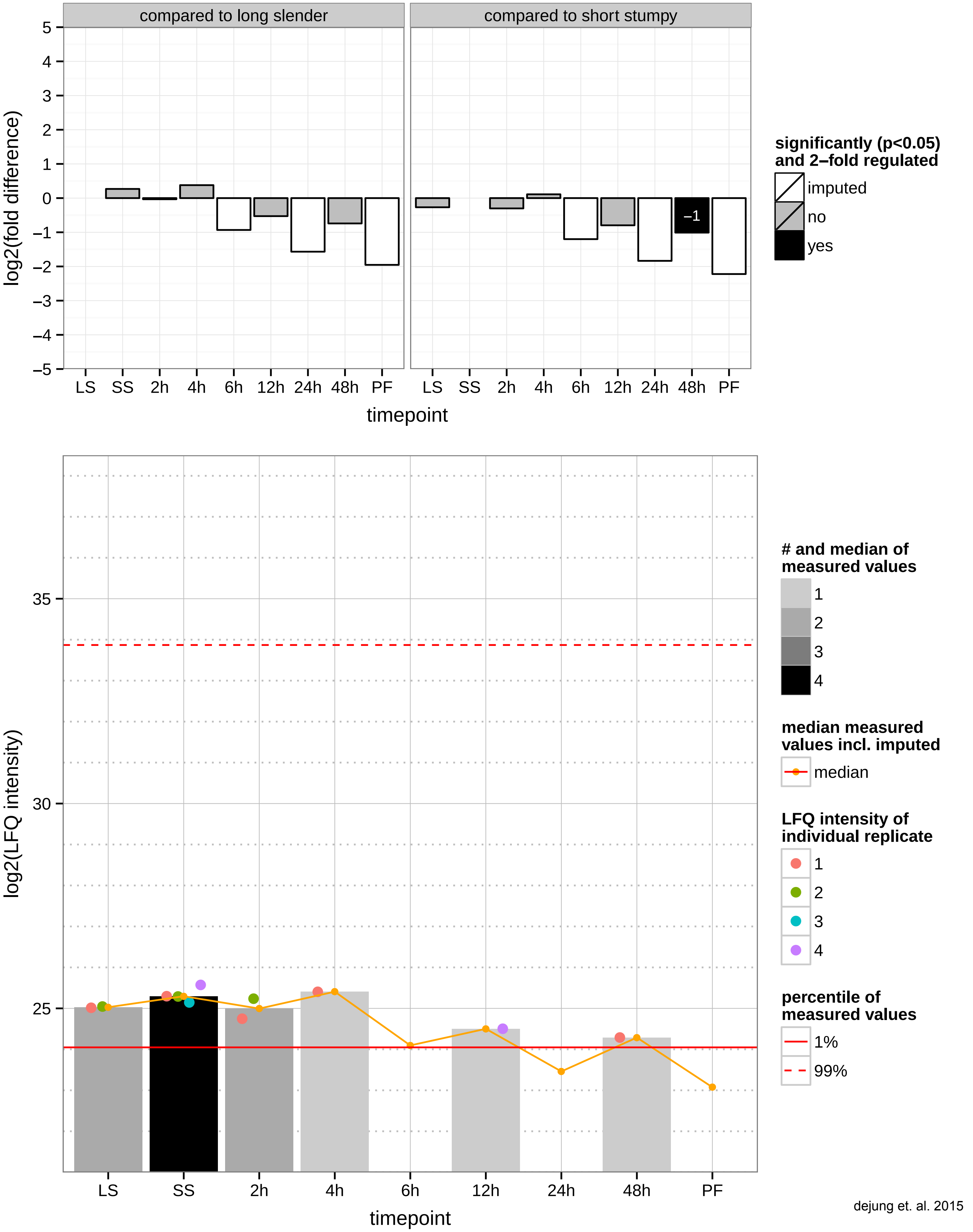

dejung et. al. 2015

hypothetical protein, conserved
Tb927.10.3750
AGOF: null
AGOC: null
AGOP: null
PGOF: null
PGOC: null
PGOP: null

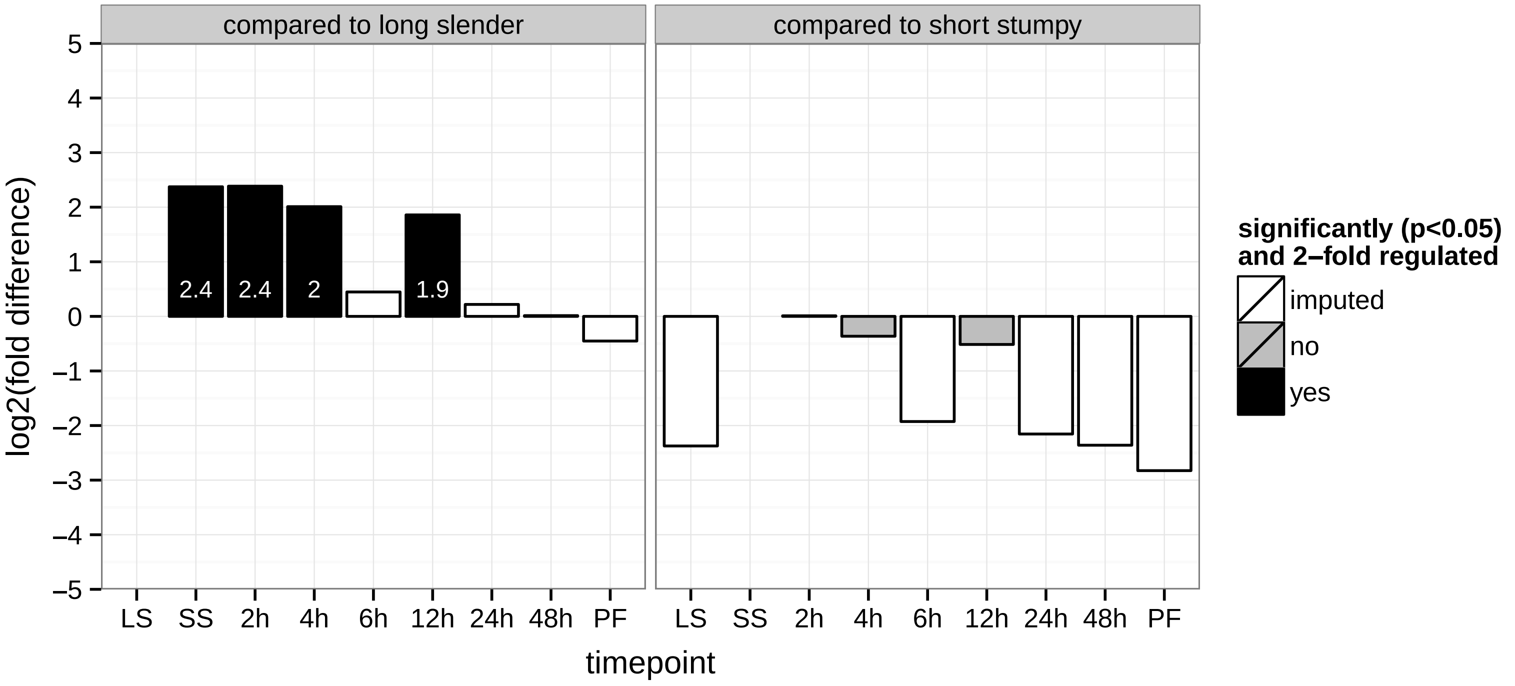

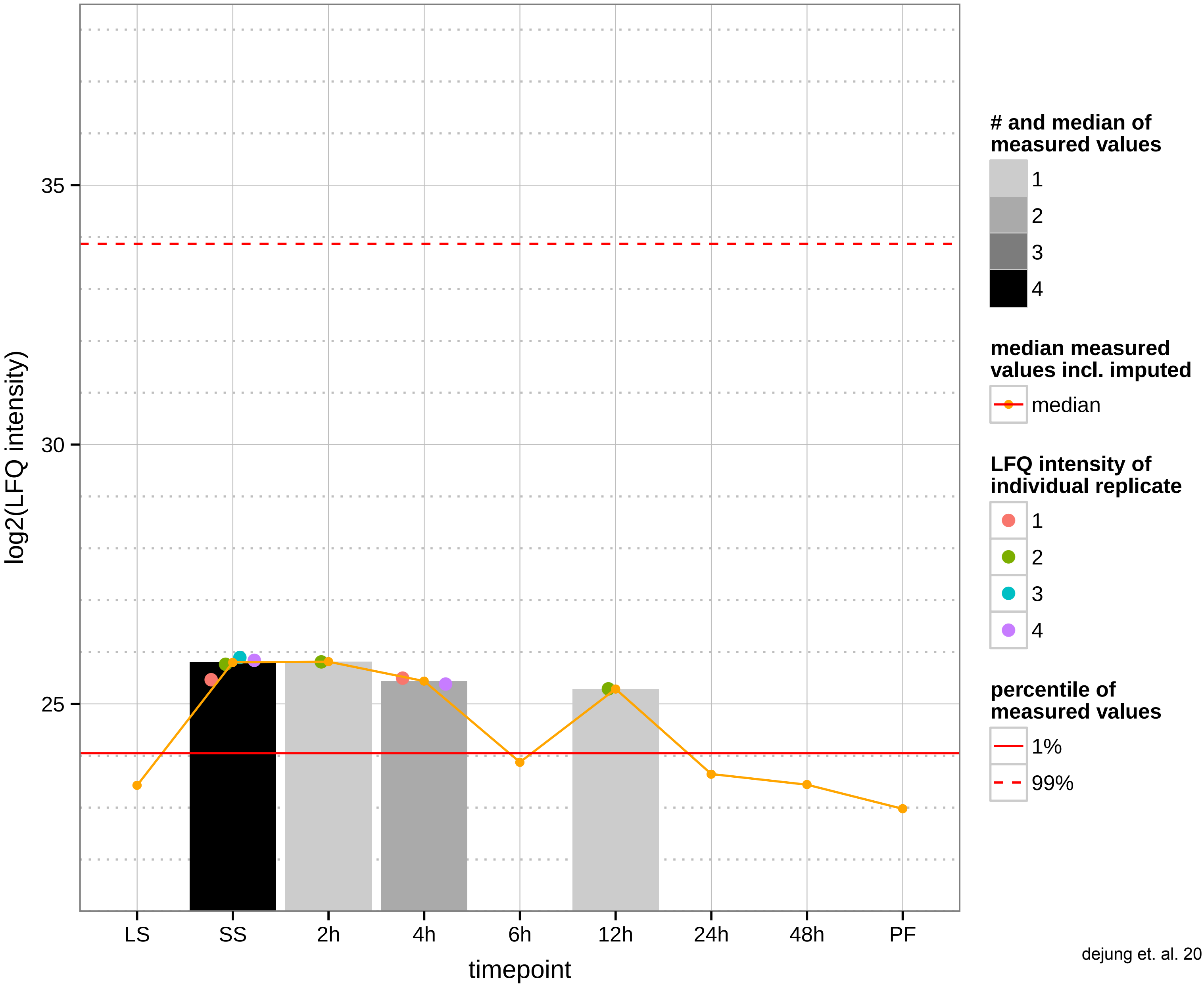

dejung et. al. 2015

ras–like small GTPase, putative (TbRLJ)
Tb927.11.12950
AGOF: GTP binding, GTPase activity
AGOC: intracellular
AGOP: protein transport, small GTPase mediated signal transduction
PGOF: GTP binding, GTPase activity
PGOC: intracellular, membrane
PGOP: GTP catabolic process, protein transport, signal transduction, small GTPase mediated signal transduction

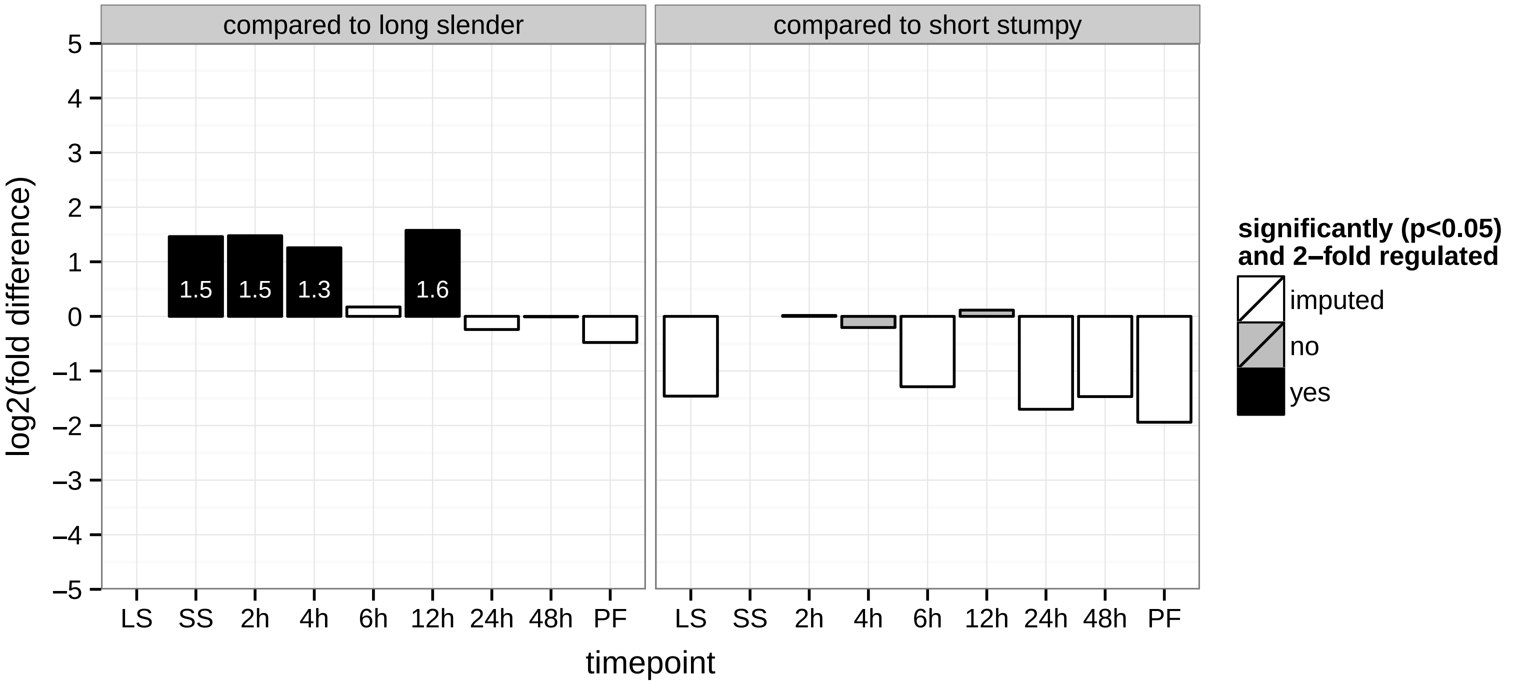

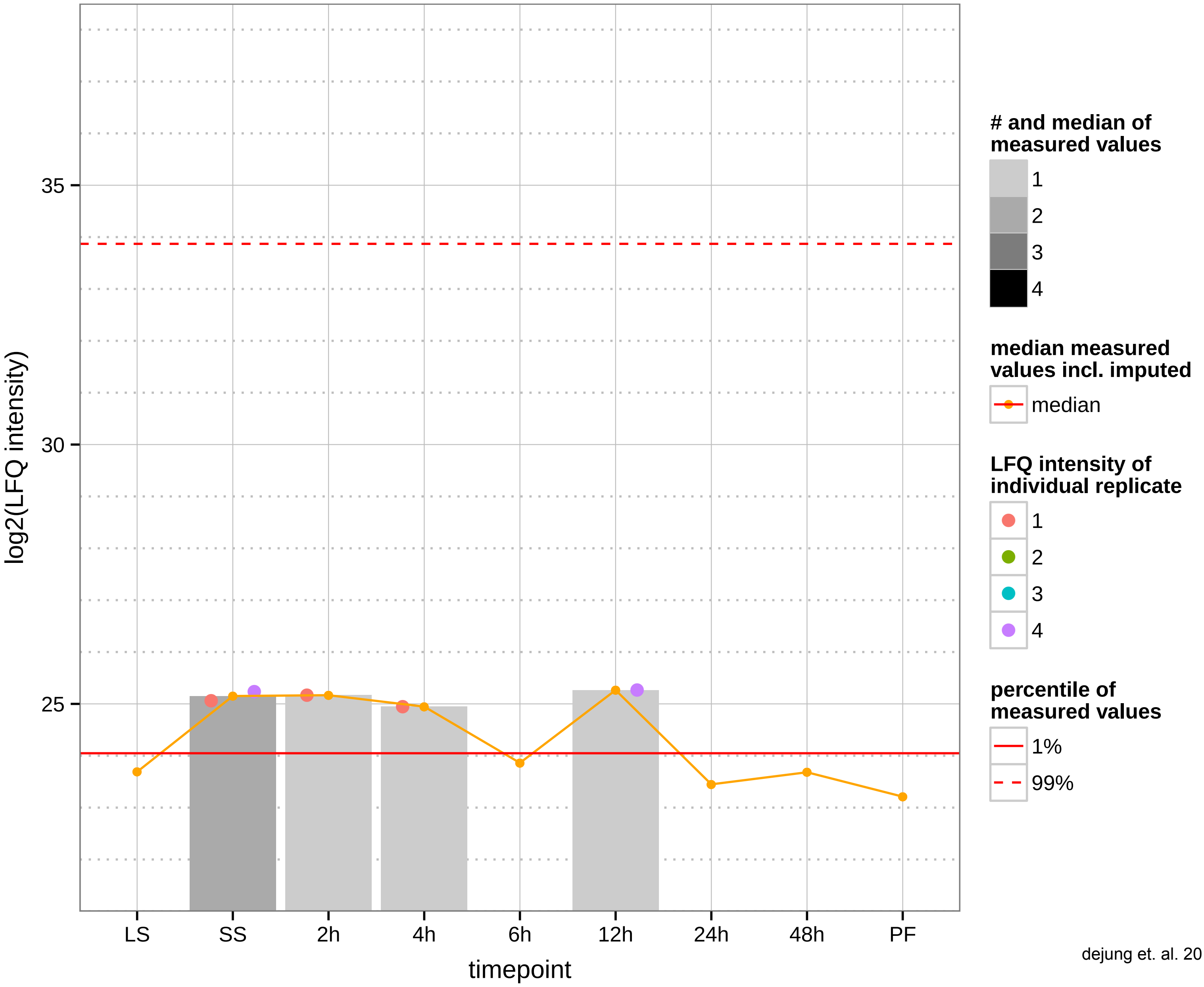

dejung et. al. 2015

glutathionylspermidine synthetase, putative
Tb927.11.8820
AGOF: glutathionylspermidine synthase activity
AGOC: integral to membrane
AGOP: cellular biogenic amine metabolic process
PGOF: null
PGOC: null
PGOP: null

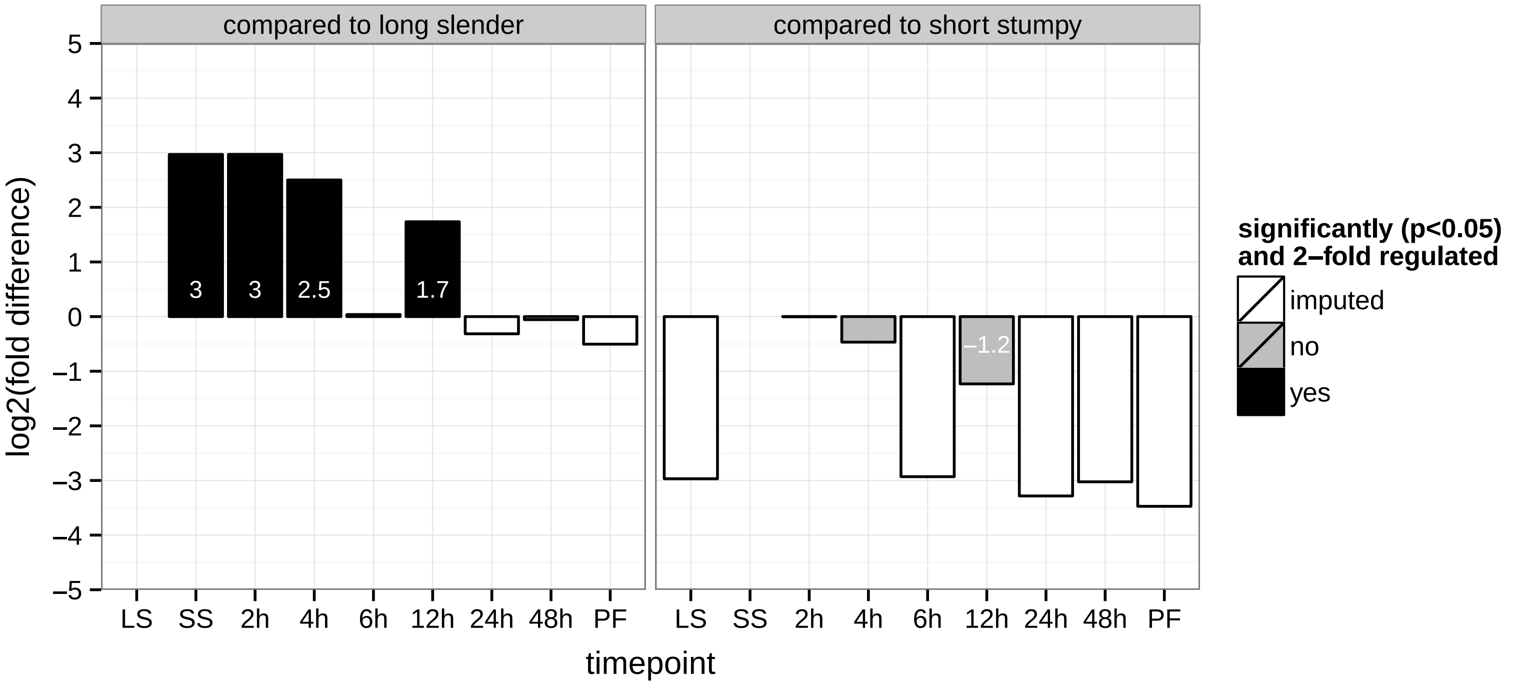

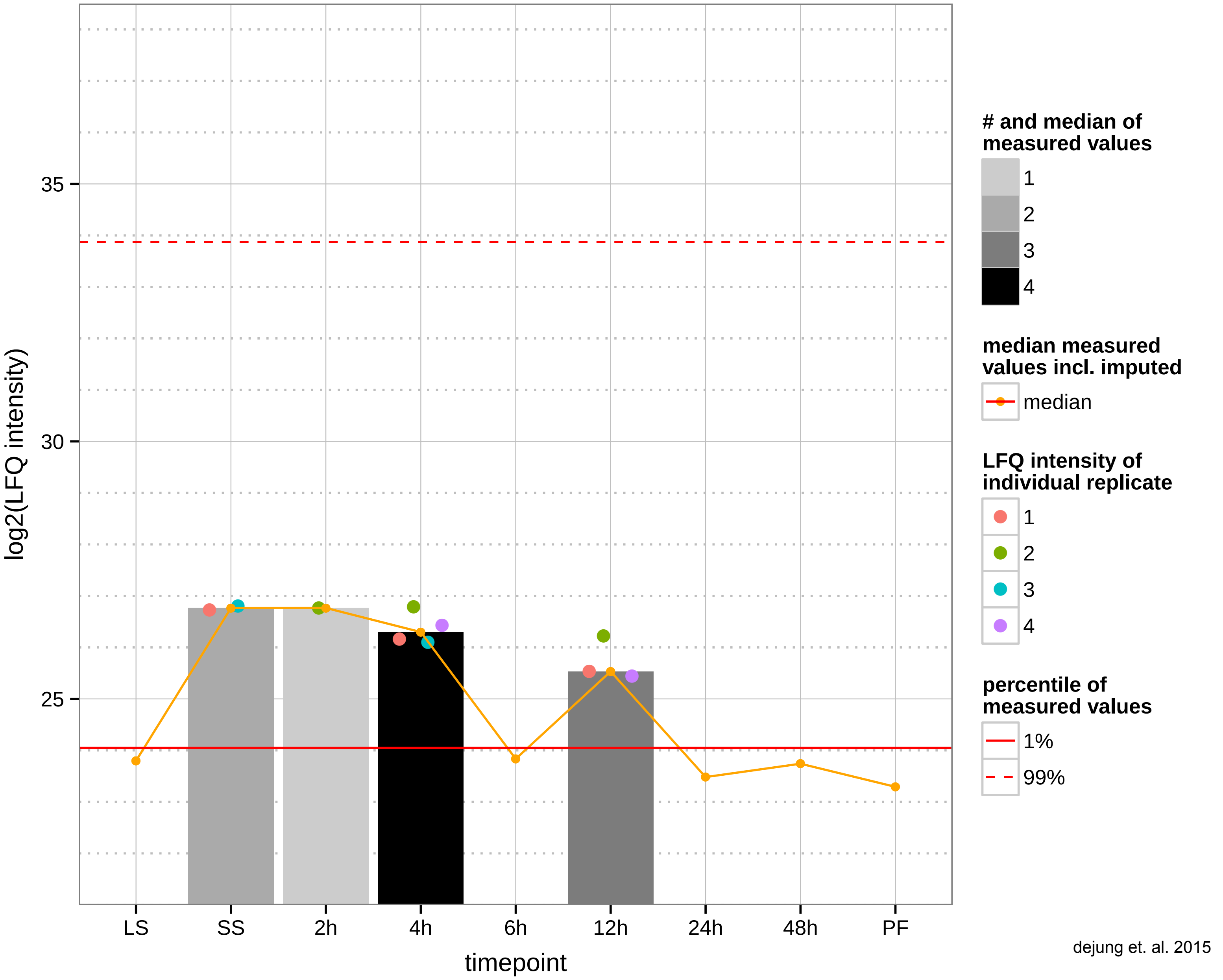

dejung et. al. 2015

acylphosphatase, putative
Tb927.3.2030
AGOF: acylphosphatase activity
AGOC: null
AGOP: null
PGOF: null
PGOC: null
PGOP: null

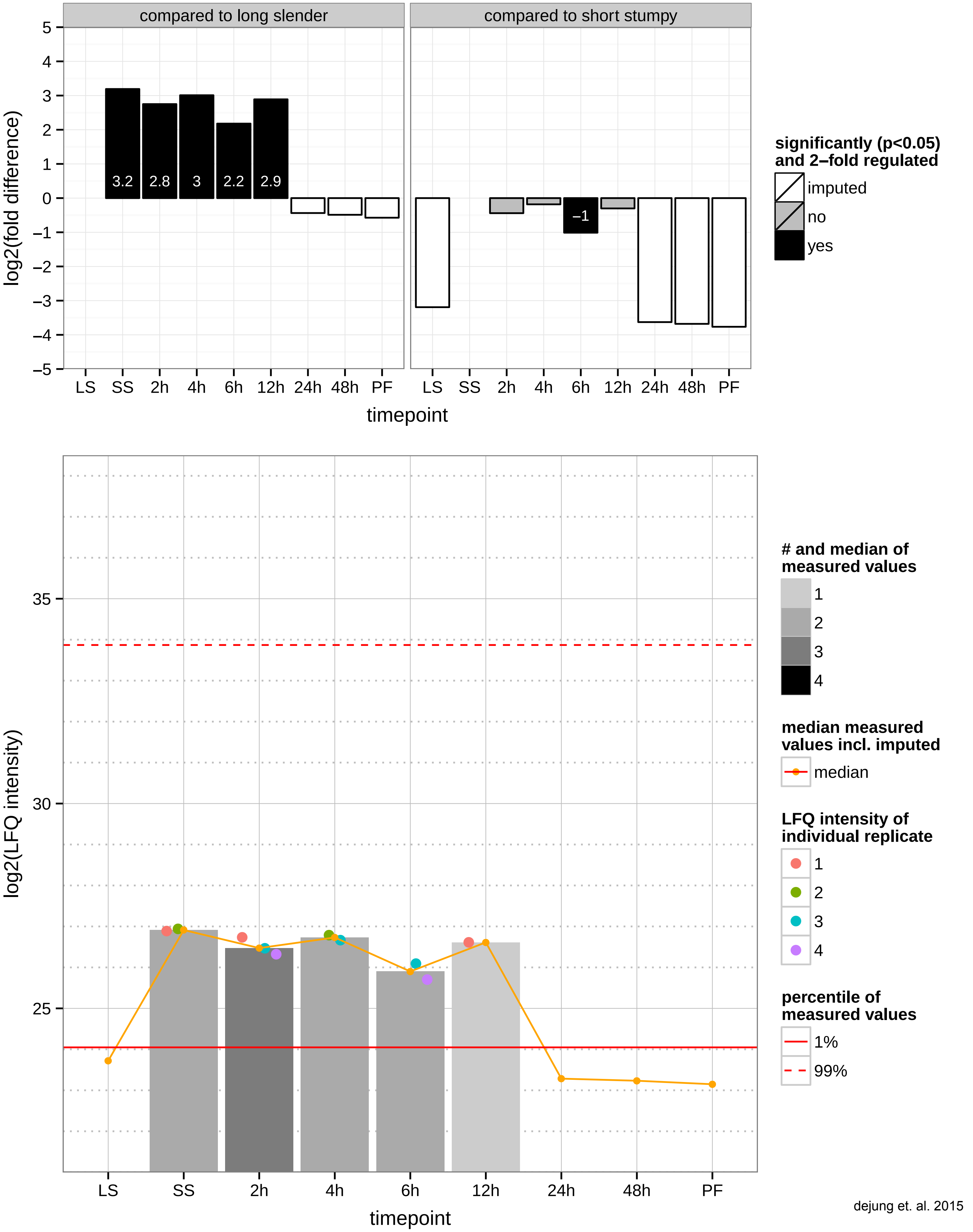

dejung et. al. 2015

DNA–directed RNA polymerase III subunit, putative, DNA–directed rna polymerase II subunit, putative (RPB6), DNA–directed
Tb927.4.3510;Tb927.4.3490
AGOF: DNA binding, DNA–directed RNA polymerase activity
AGOC: null
AGOP: transcription, DNA–dependent
PGOF: DNA binding, DNA–directed RNA polymerase activity
PGOC: null
PGOP: transcription, DNA–dependent

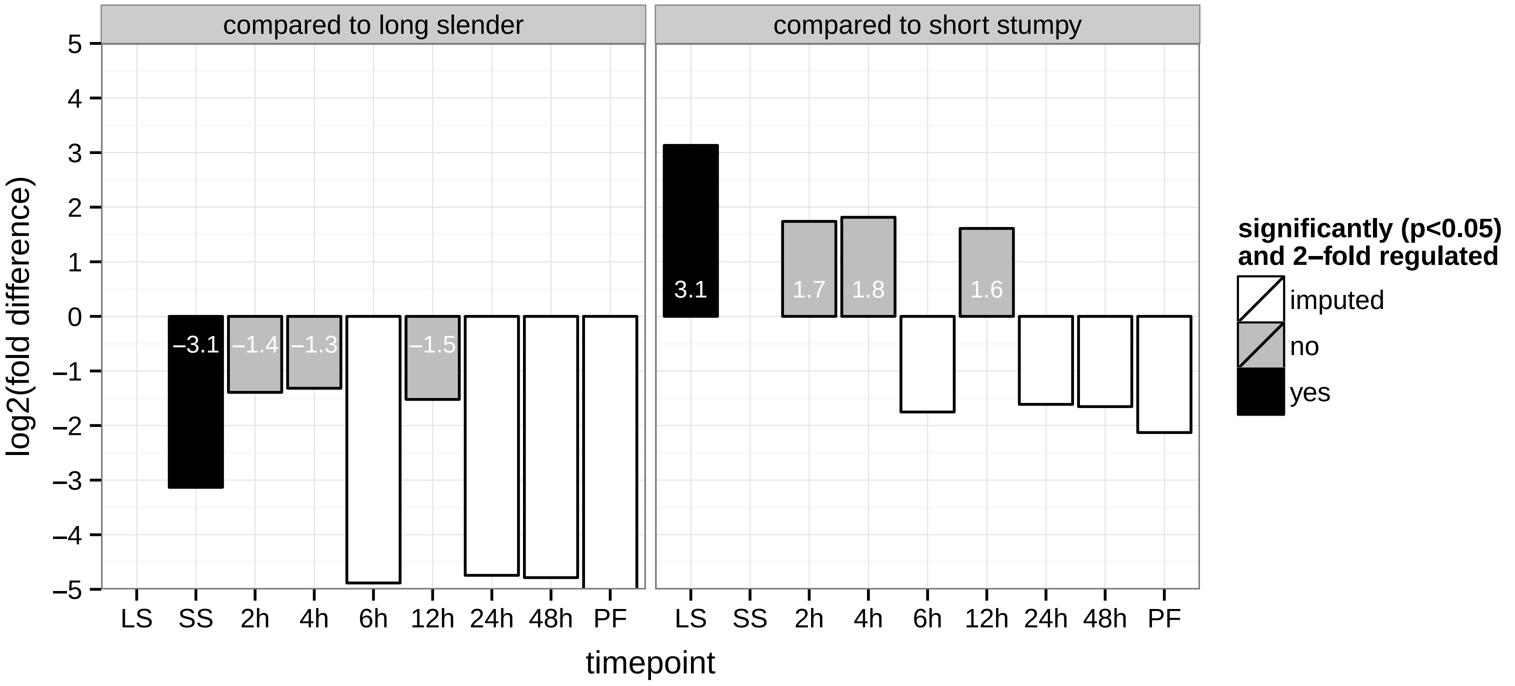

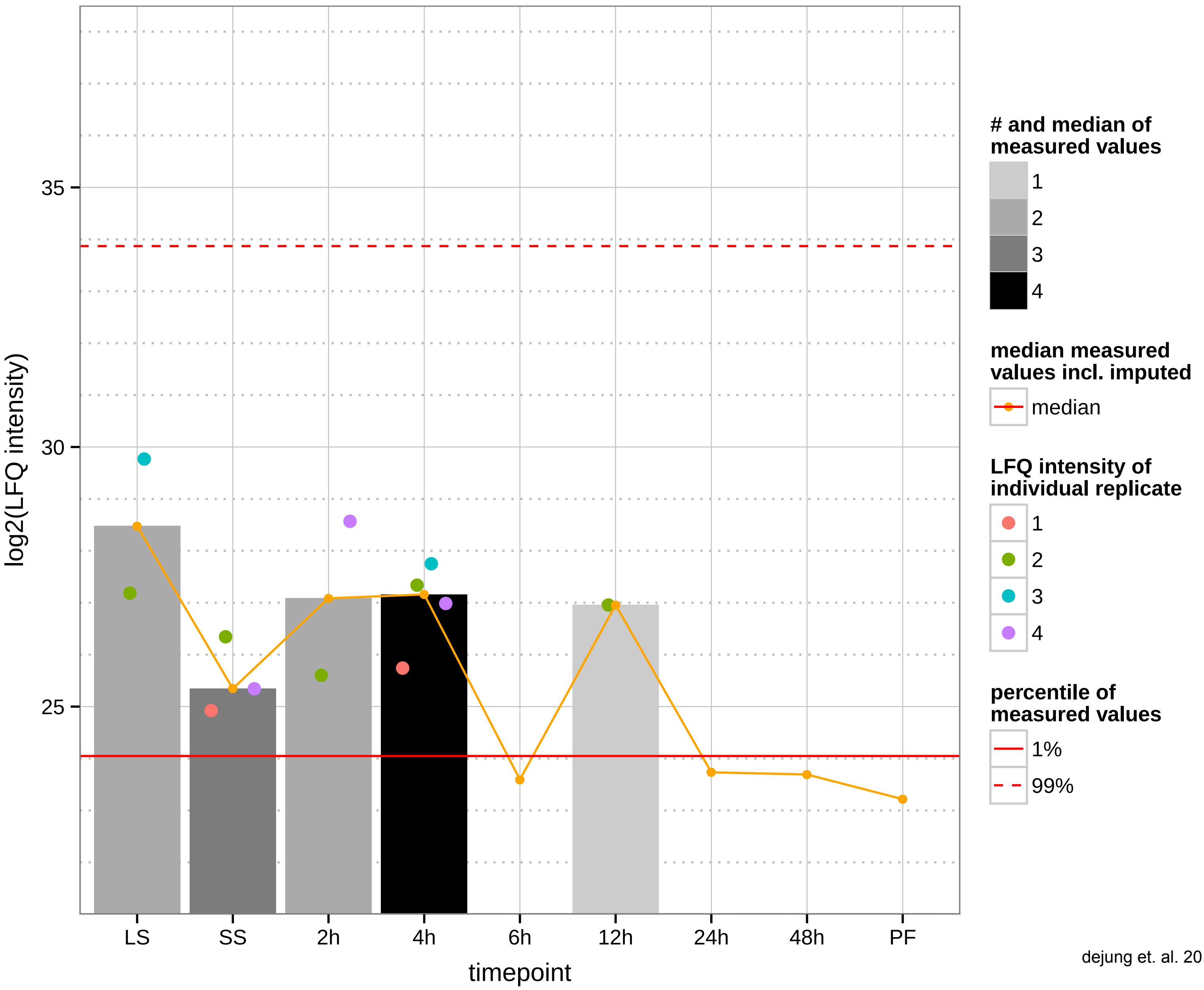

dejung et. al. 2015

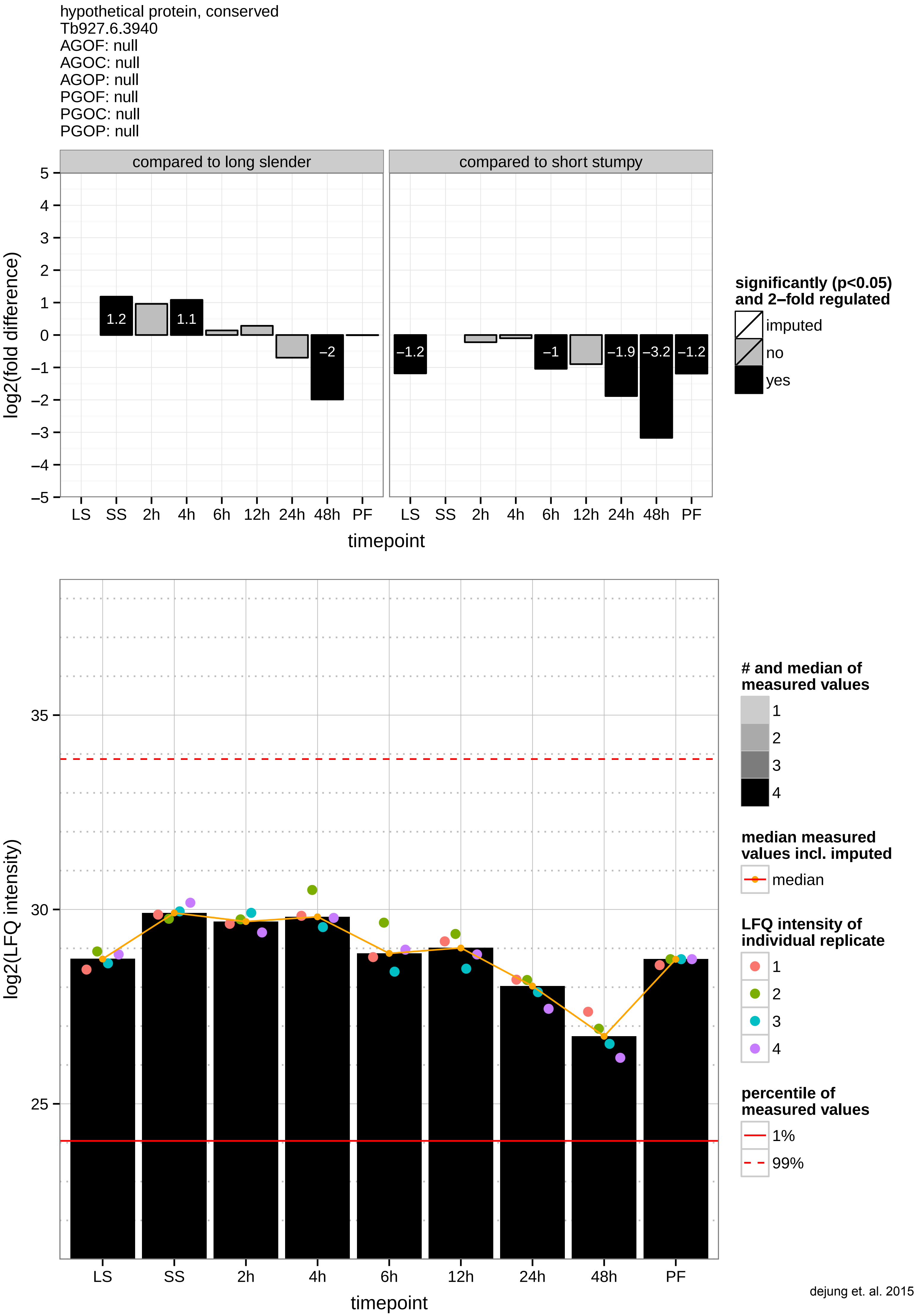
hypothetical protein, conserved
Tb927.6.3940
AGOF: null
AGOC: null
AGOP: null
PGOF: null
PGOC: null
PGOP: null
compared to long slender
compared to short stumpy
log2(fold difference)
1.2
1.1
−2
−1.2
−1
−1.9
−3.2
−1.2
LS SS 2h 4h 6h 12h 24h 48h PF
timepoint
significantly (p<0.05) and 2−fold regulated
imputed
no
yes
log2(LFQ intensity)
# and median of measured values
median measured values incl. imputed
median
LFQ intensity of individual replicate
percentile of measured values
1%
99%
dejung et. al. 2015

diacylglycerol kinase, putative
Tb927.8.5140
AGOF: diacylglycerol kinase activity
AGOC: cytosol, mitochondrion
AGOP: fatty acid oxidation, protein kinase C–activating G–protein coupled receptor signaling pathway
PGOF: NAD+ kinase activity, diacylglycerol kinase activity
PGOC: null
PGOP: metabolic process, protein kinase C–activating G–protein coupled receptor signaling pathway

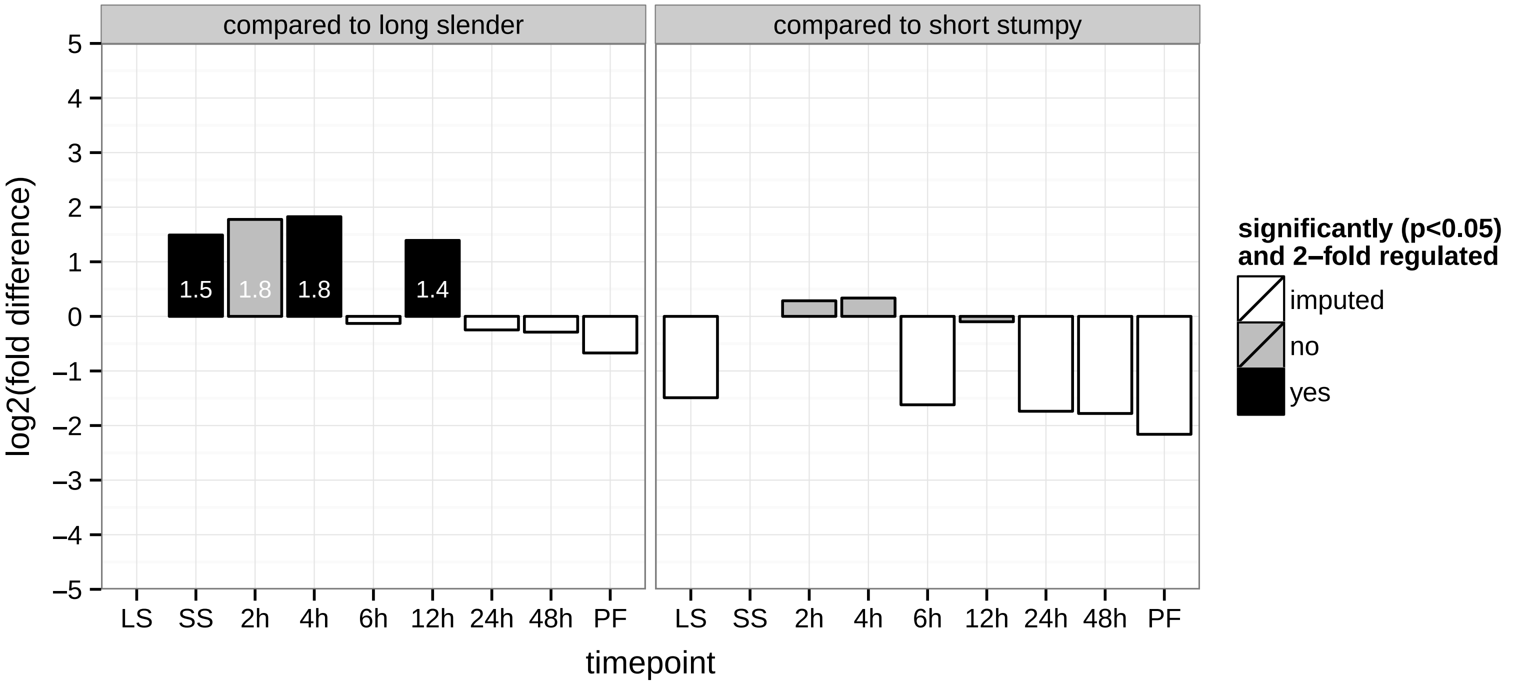

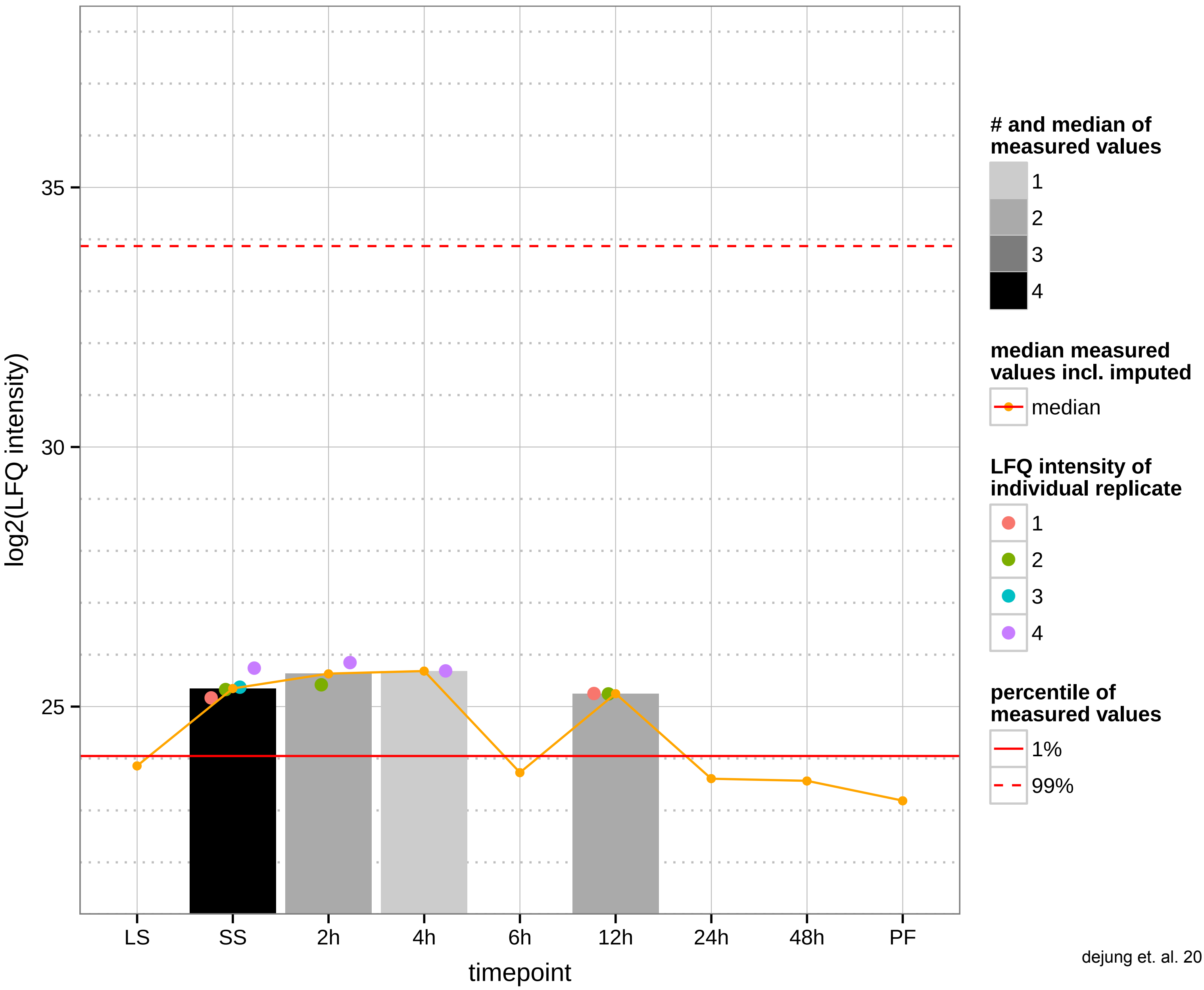

dejung et. al. 2015

hypothetical protein, conserved
Tb927.9.12380
AGOF: ATP binding, catalytic activity
AGOC: null
AGOP: fructose 2, 6−bisphosphate metabolic process
PGOF: null
PGOC: null
PGOP: null

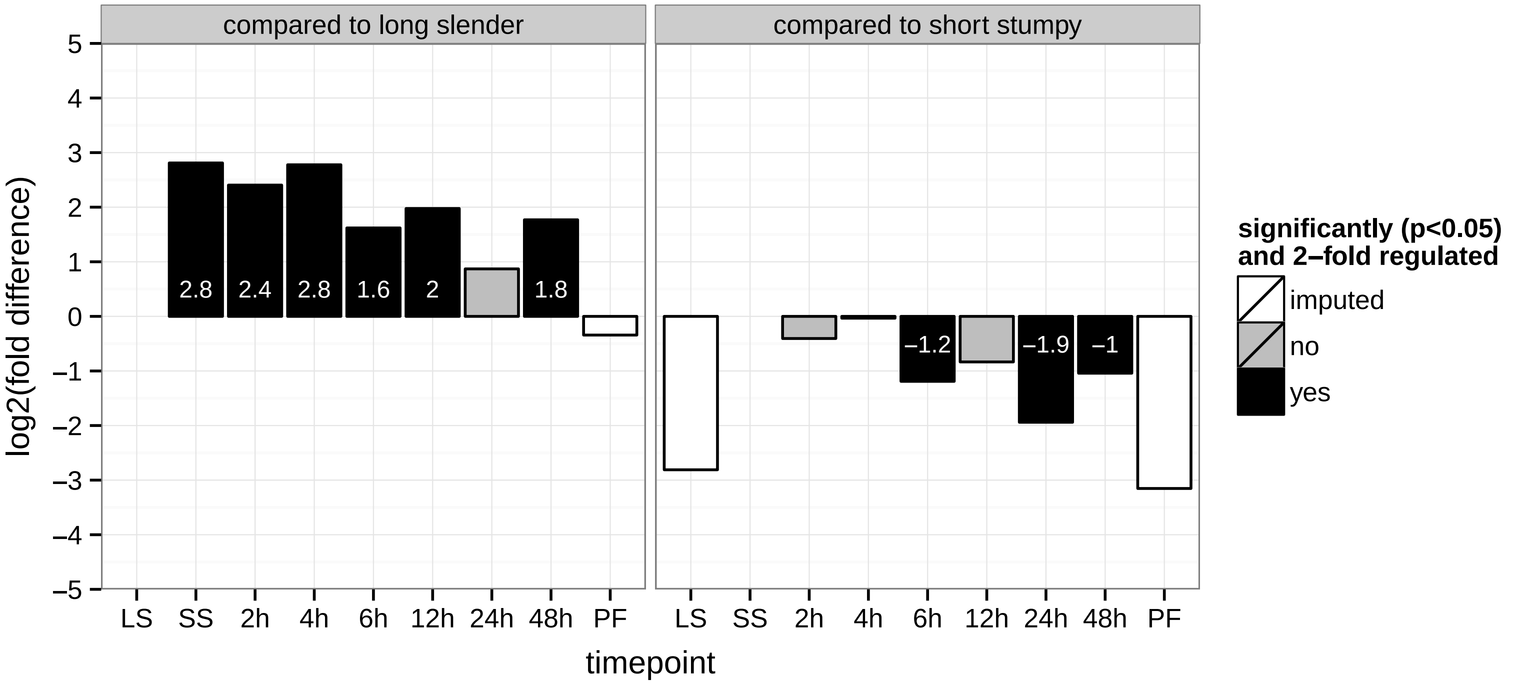

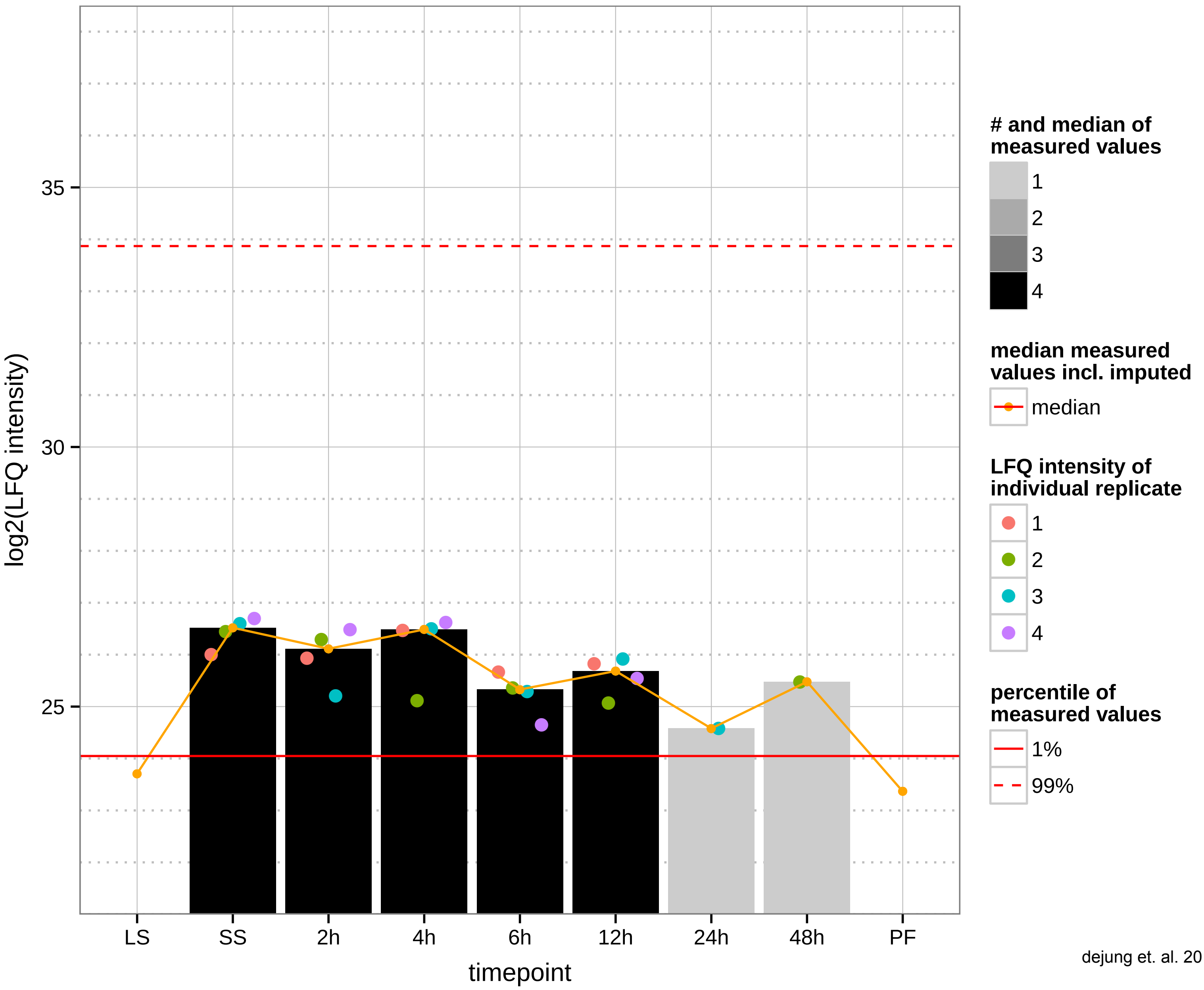

dejung et. al. 2015

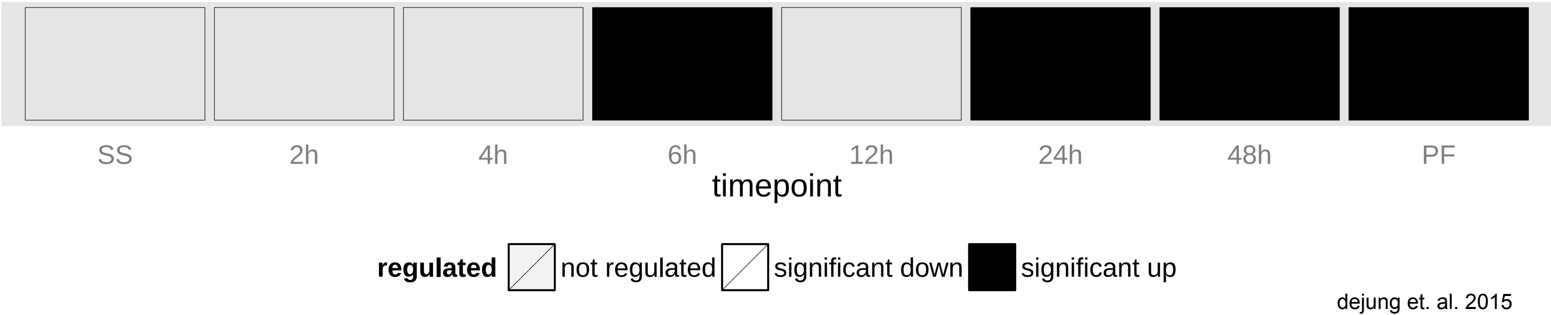
SS
2h
4h
6h
12h
24h
48h
PF
timepoint
regulated
not regulated
significant down
significant up
dejung et. al. 2015

hypothetical protein, conserved
Tb927.10.11720
AGOF: zinc ion binding
AGOC: null
AGOP: null
PGOF: zinc ion binding
PGOC: null
PGOP: null

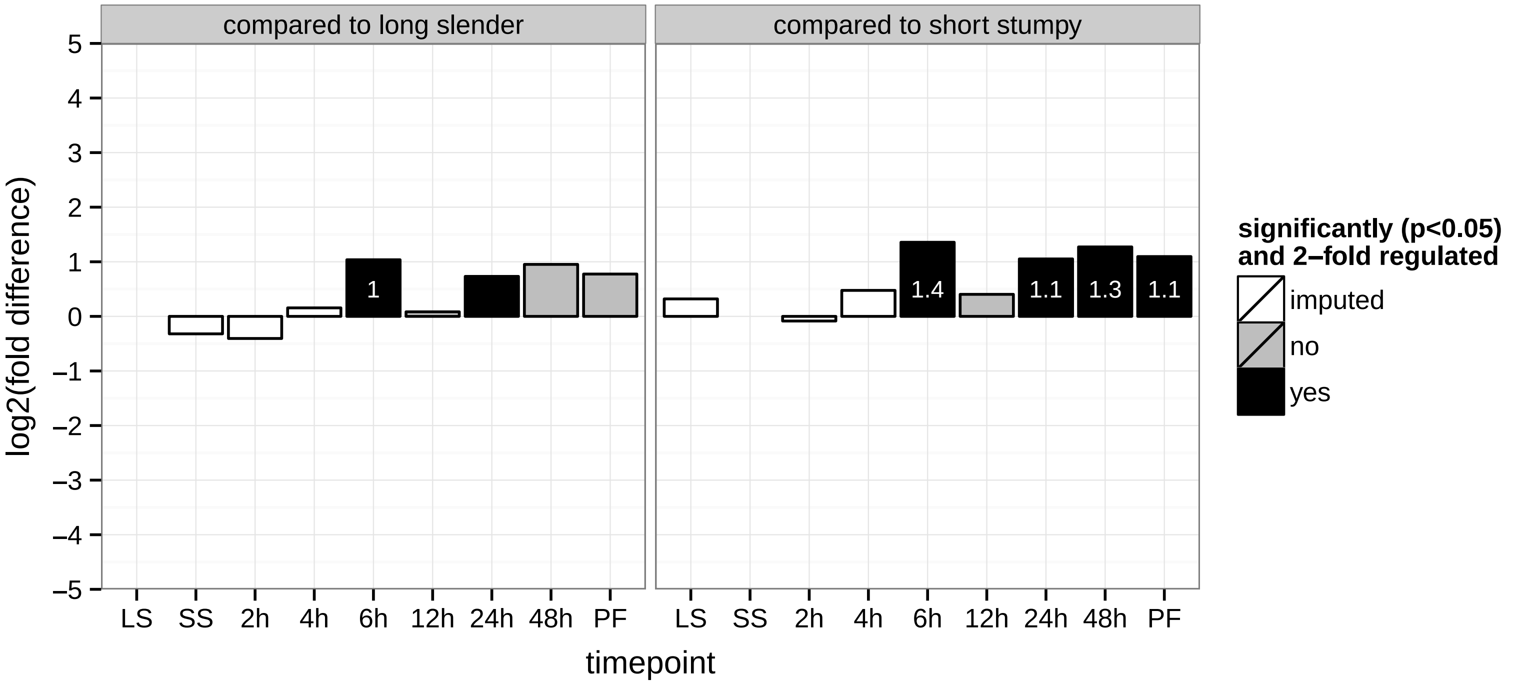

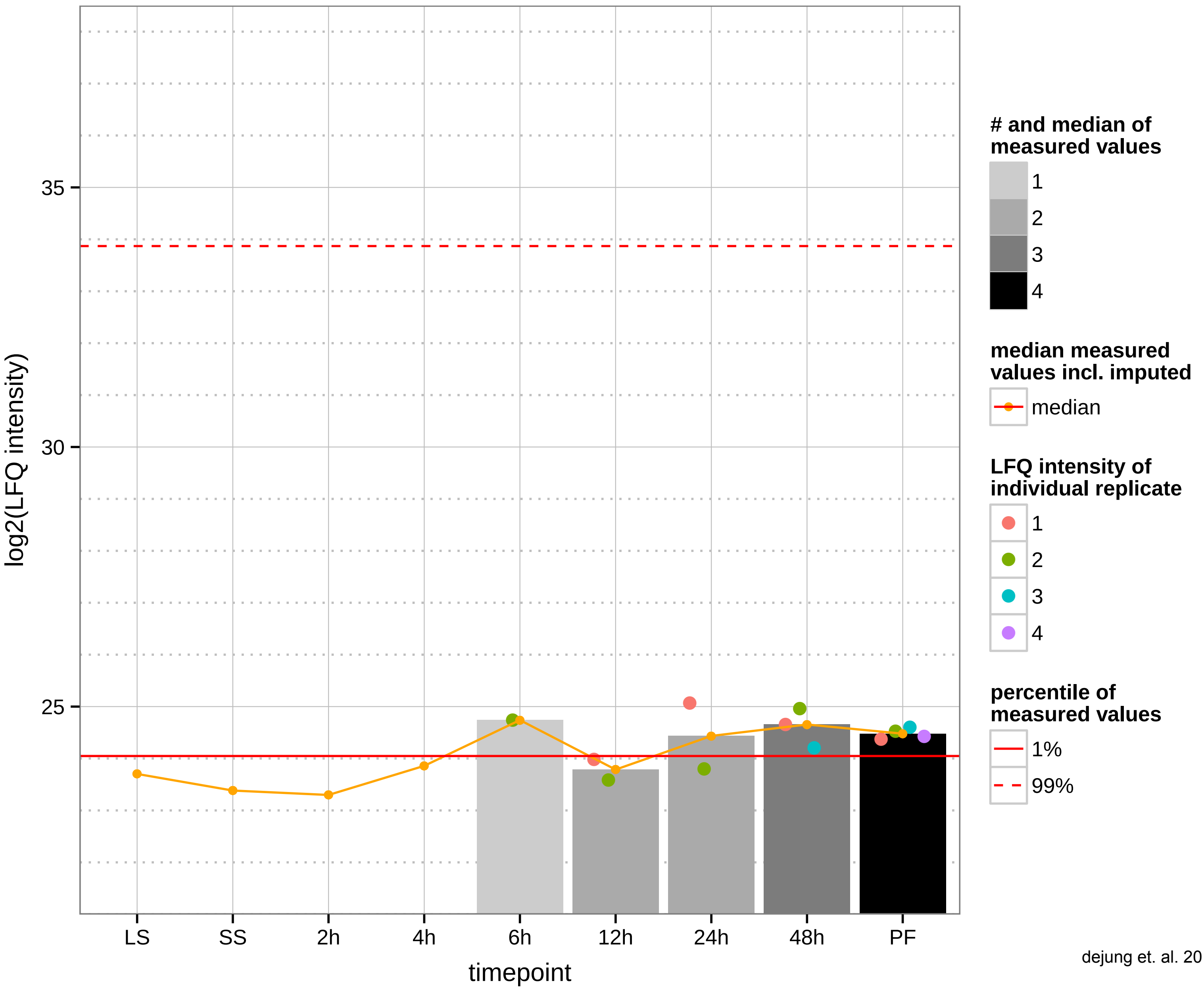

dejung et. al. 2015

hypothetical protein, conserved
Tb927.10.7670
AGOF: null
AGOC: null
AGOP: null
PGOF: null
PGOC: null
PGOP: null

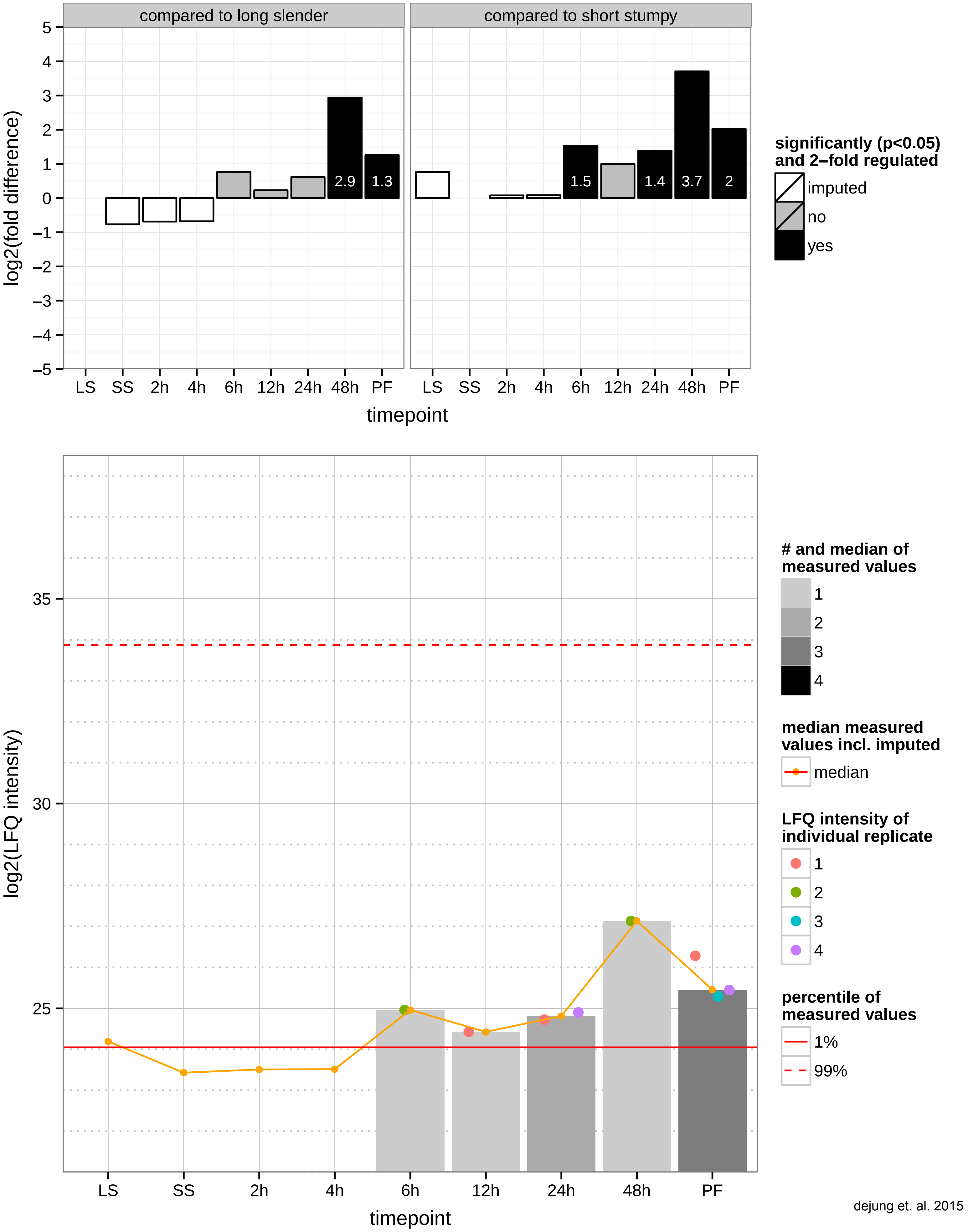

dejung et. al. 2015

hypothetical protein, conserved
Tb927.11.16420
AGOF: null
AGOC: mitochondrion
AGOP: null
PGOF: null
PGOC: null
PGOP: null

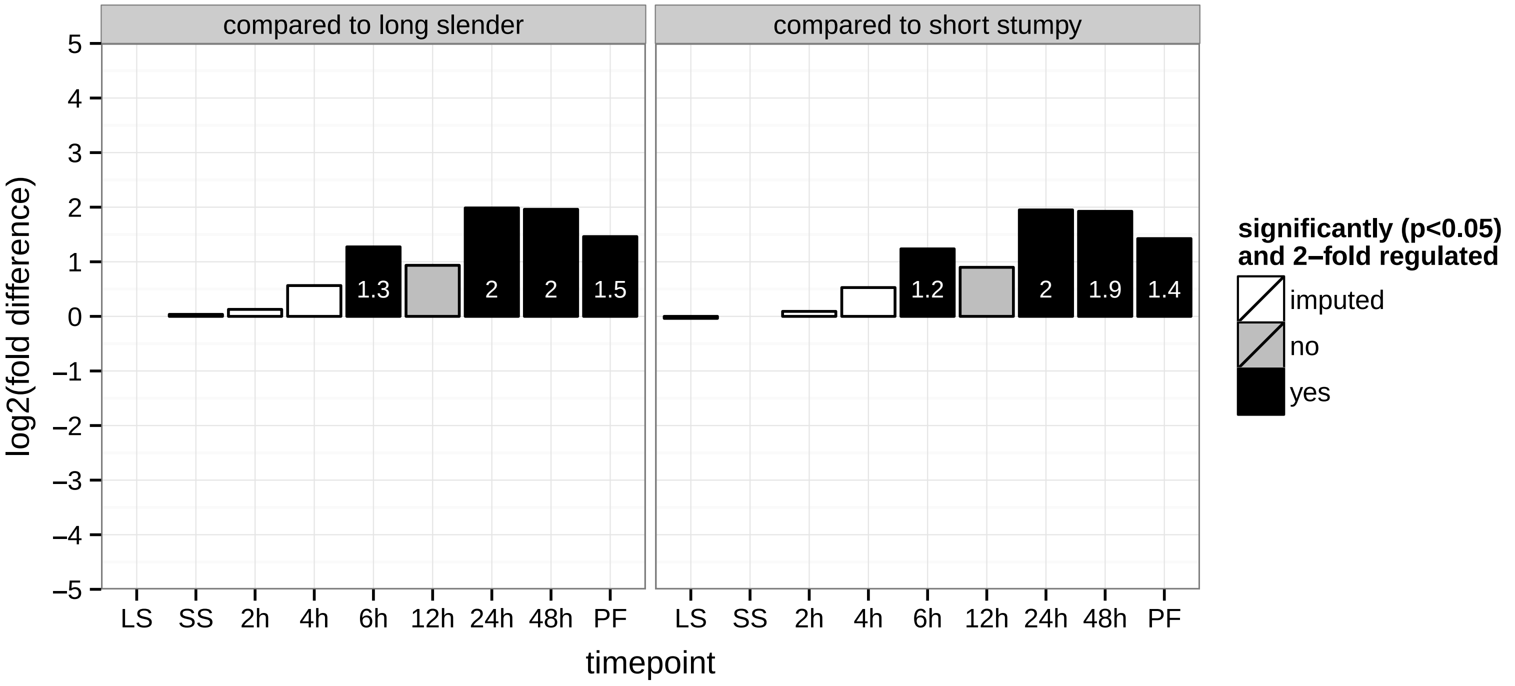

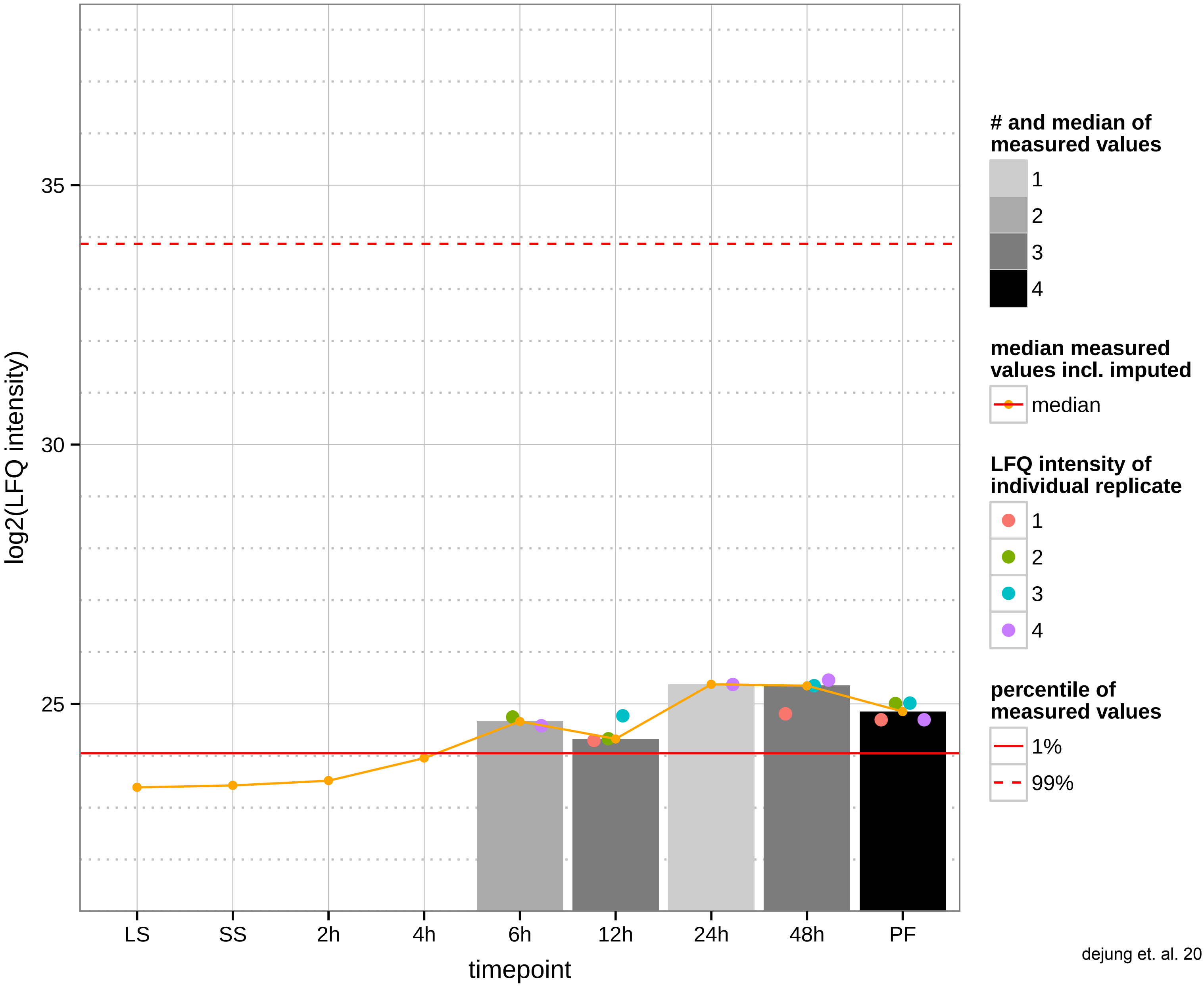

dejung et. al. 2015

aurora B kinase (AUK1)
Tb927.11.8220
AGOF: ATP binding, protein binding, protein serine/threonine kinase activity, protein tyrosine kinase activity
AGOC: chromosome passenger complex, nucleus, spindle
AGOP: chromosome segregation, cytokinesis, histone H3–S10 phosphorylation, protein phosphorylation, regulation of mitosis
PGOF: ATP binding, protein kinase activity, transferase activity, transferring phosphorus–containing groups
PGOC: null
PGOP: protein phosphorylation

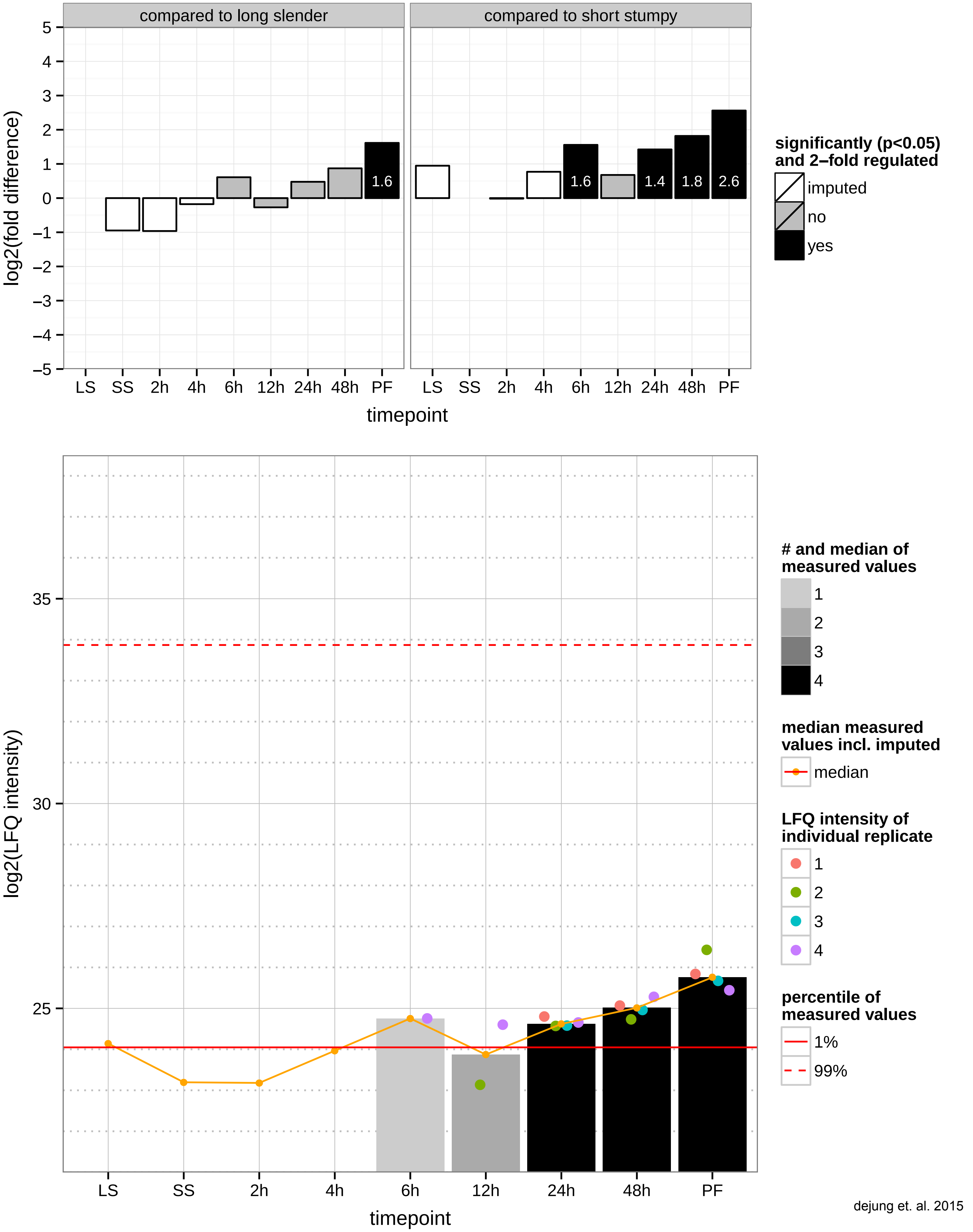

dejung et. al. 2015

translation elongation factor EF−2, putative
Tb927.3.2170
AGOF: GTP binding, GTPase activity
AGOC: null
AGOP: mature ribosome assembly
PGOF: GTP binding, GTPase activity
PGOC: null
PGOP: null

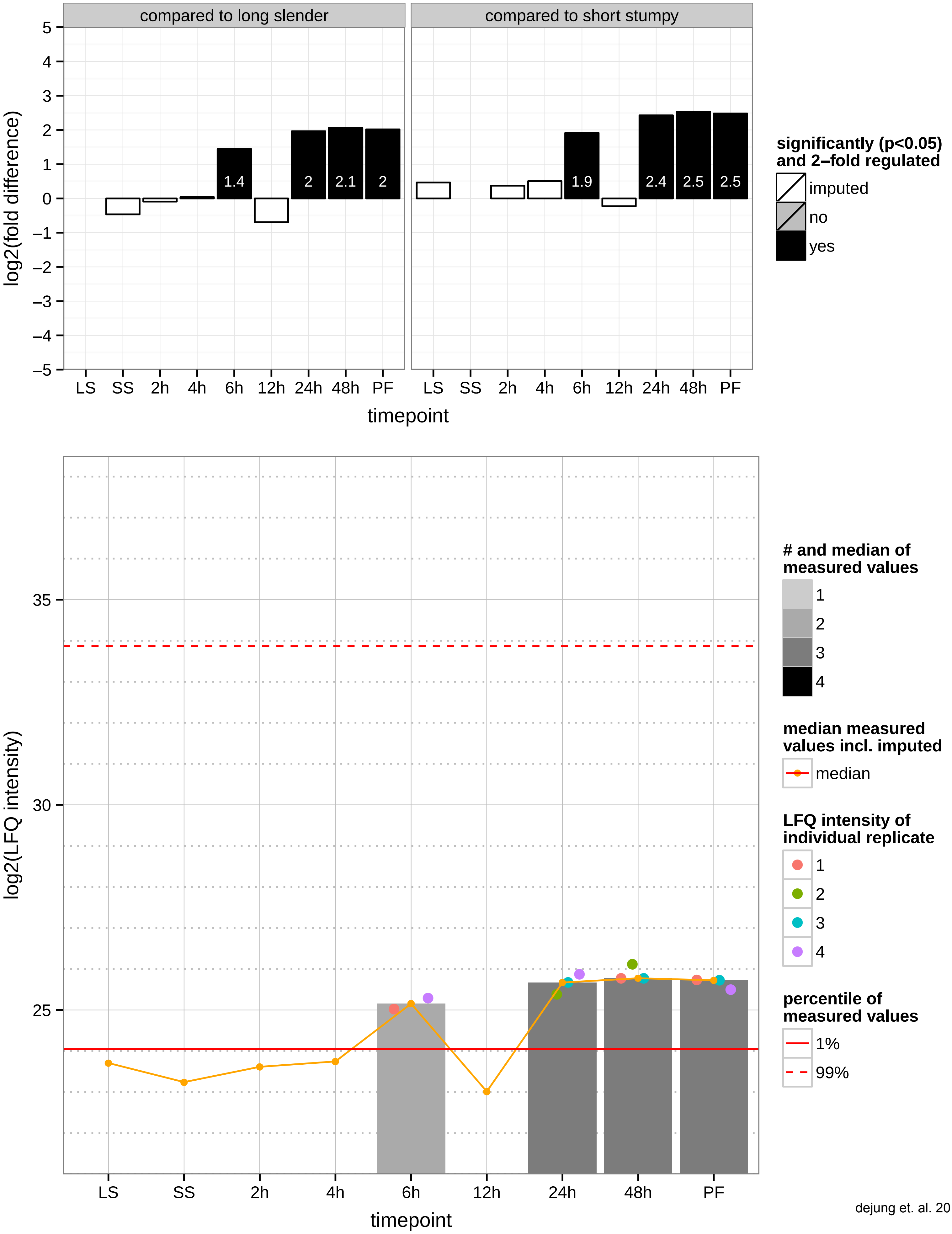

dejung et. al. 2015

hypothetical protein, conserved
Tb927.4.2430
AGOF: null
AGOC: null
AGOP: null
PGOF: null
PGOC: null
PGOP: null

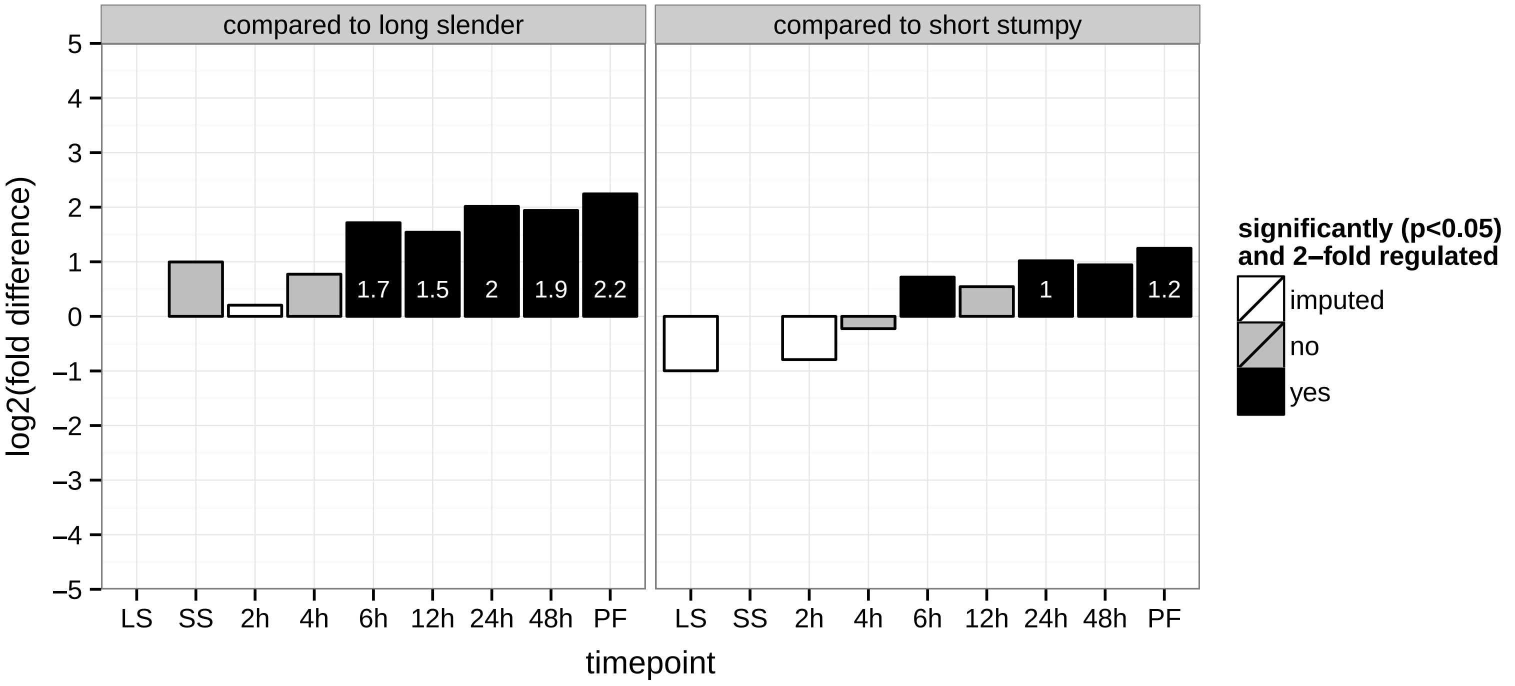

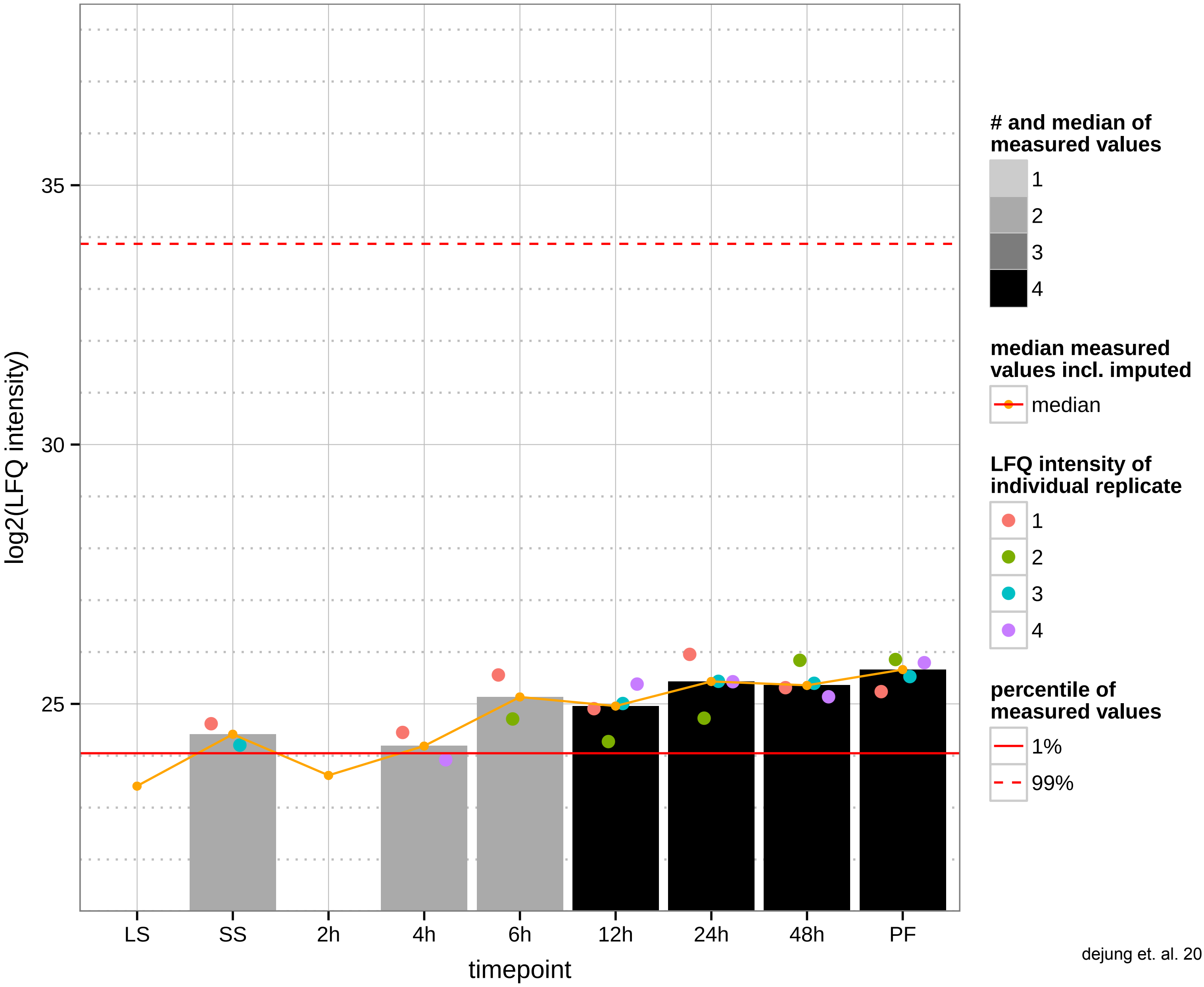

dejung et. al. 2015

hypothetical protein, conserved
Tb927.4.2580
AGOF: null
AGOC: mitochondrion
AGOP: null
PGOF: null
PGOC: null
PGOP: null

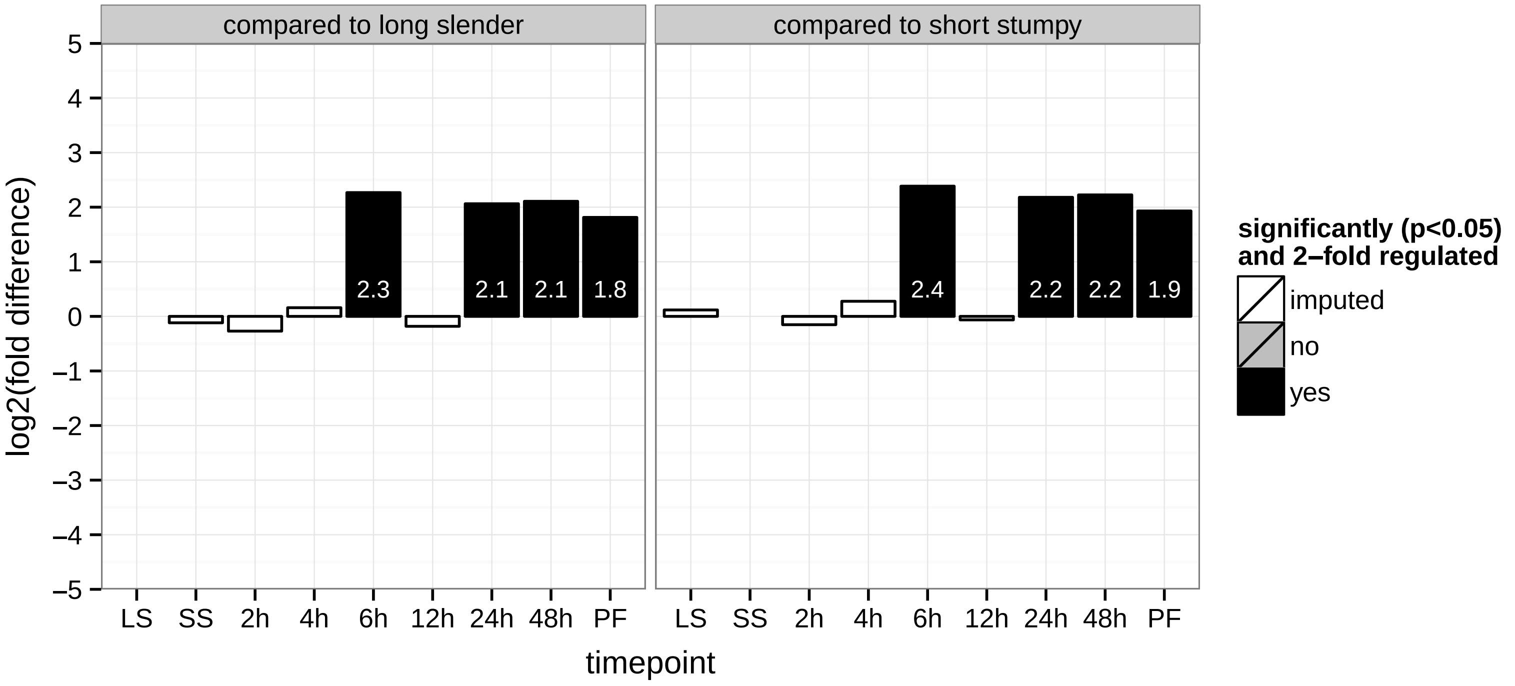

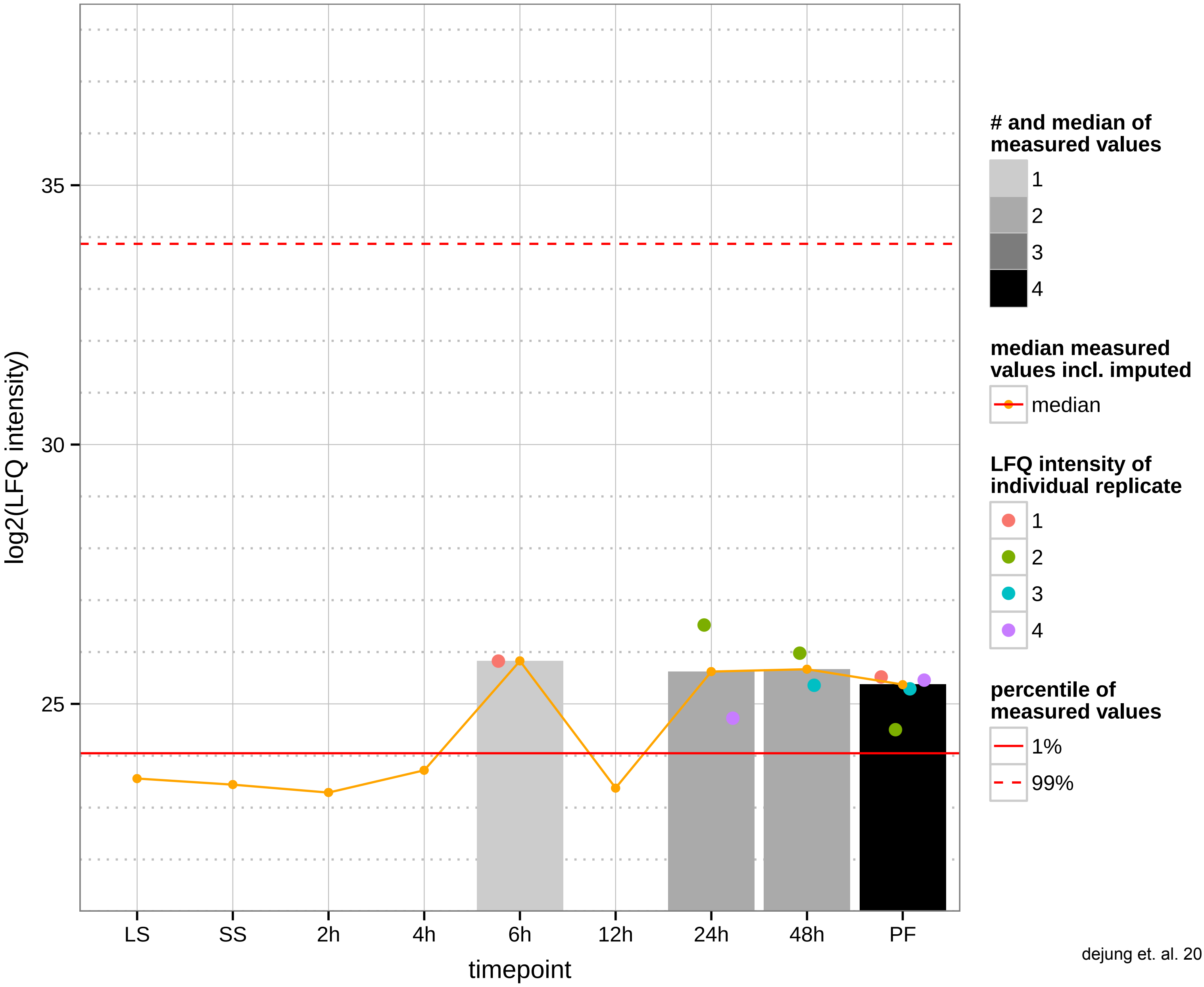

dejung et. al. 2015

DNA primase small subunit, putative
Tb927.7.2310
AGOF: DNA primase activity
AGOC: null
AGOP: DNA replication, synthesis of RNA primer
PGOF: DNA primase activity
PGOC: null
PGOP: DNA replication, synthesis of RNA primer

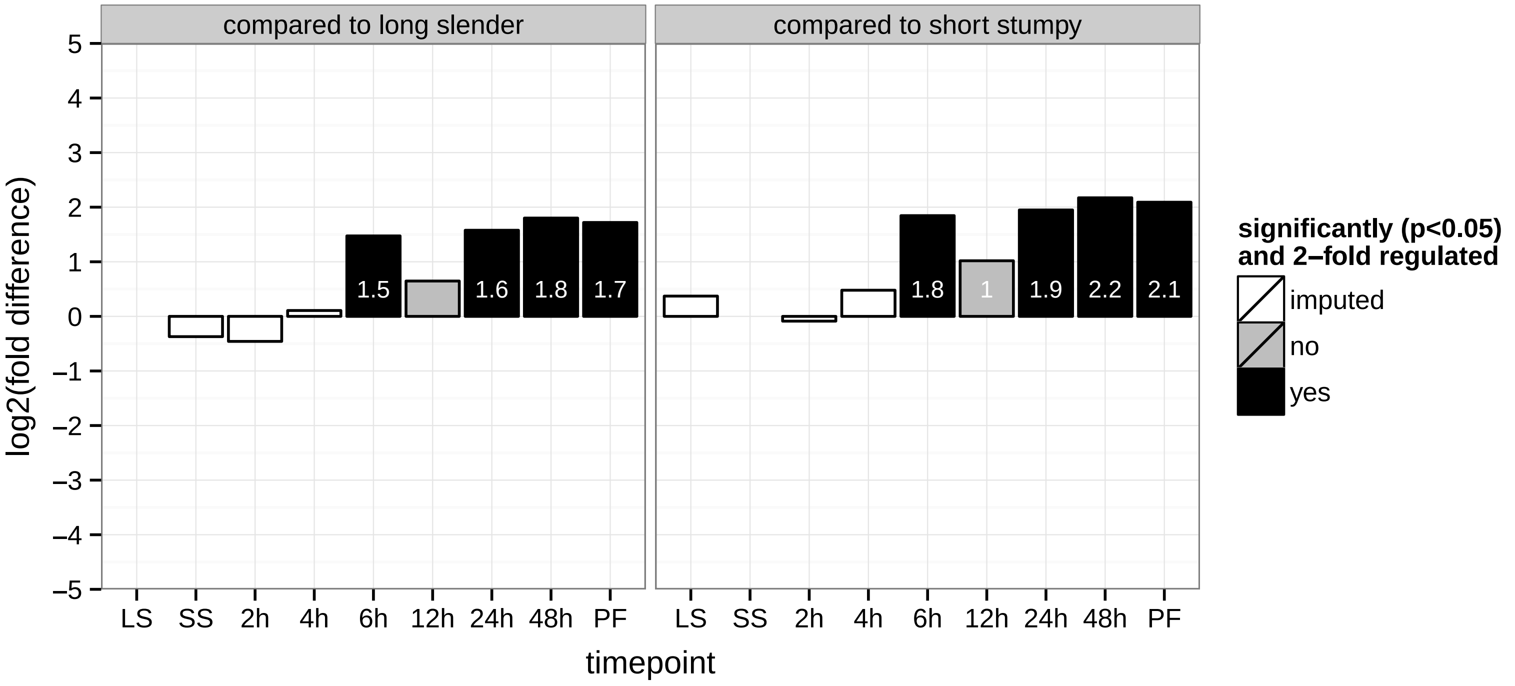

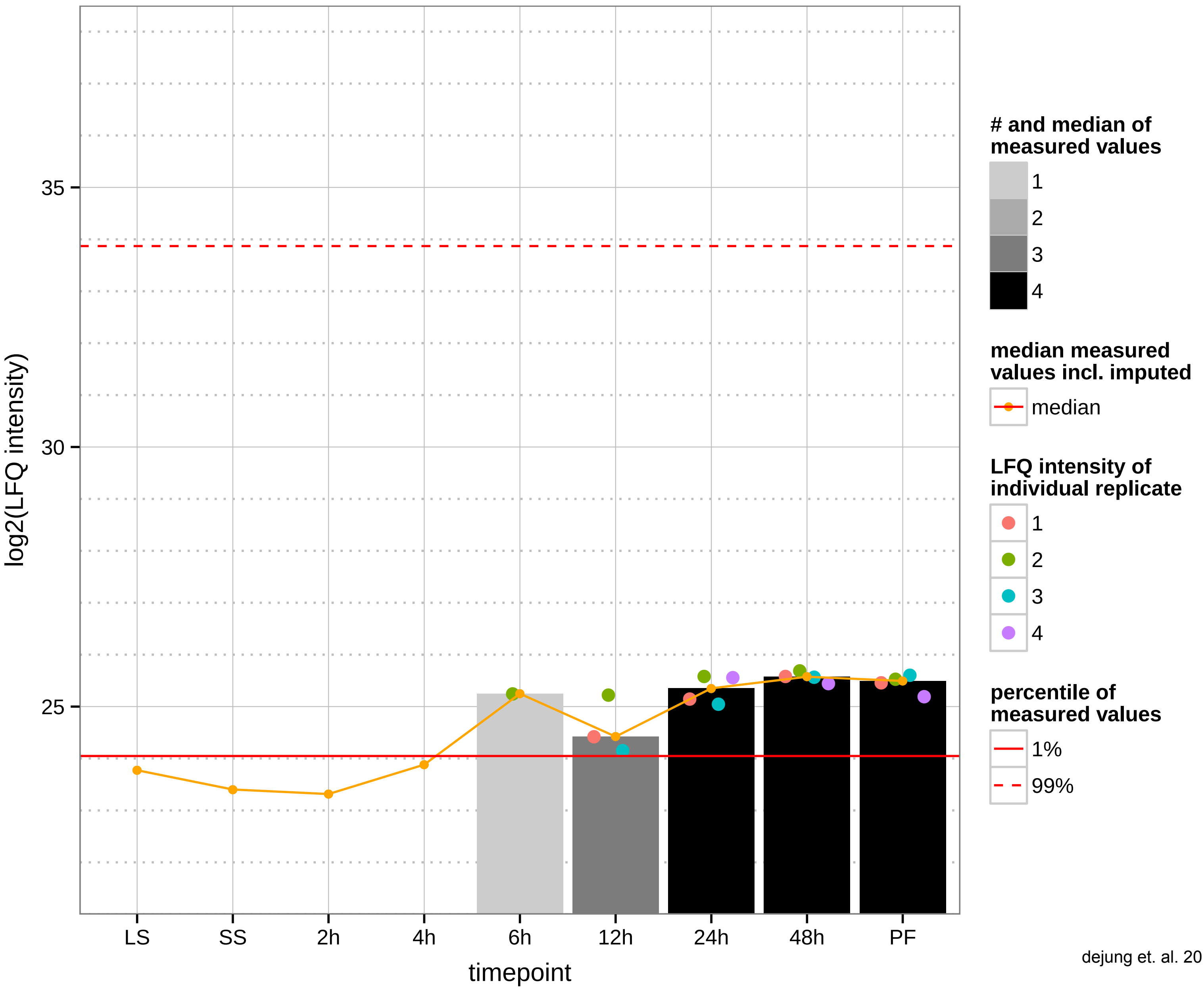

dejung et. al. 2015

hypothetical protein, conserved
Tb927.9.11120
AGOF: null
AGOC: mitochondrion
AGOP: null
PGOF: null
PGOC: null
PGOP: null

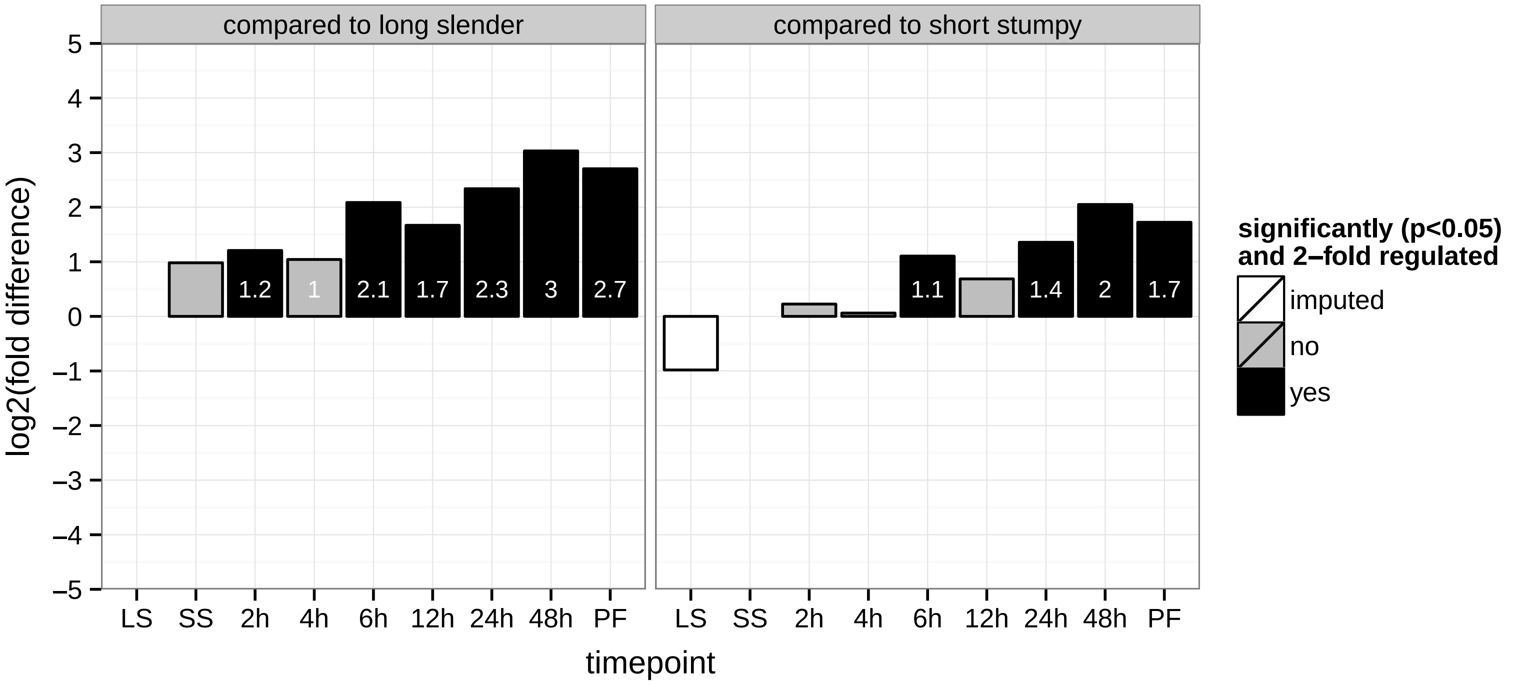

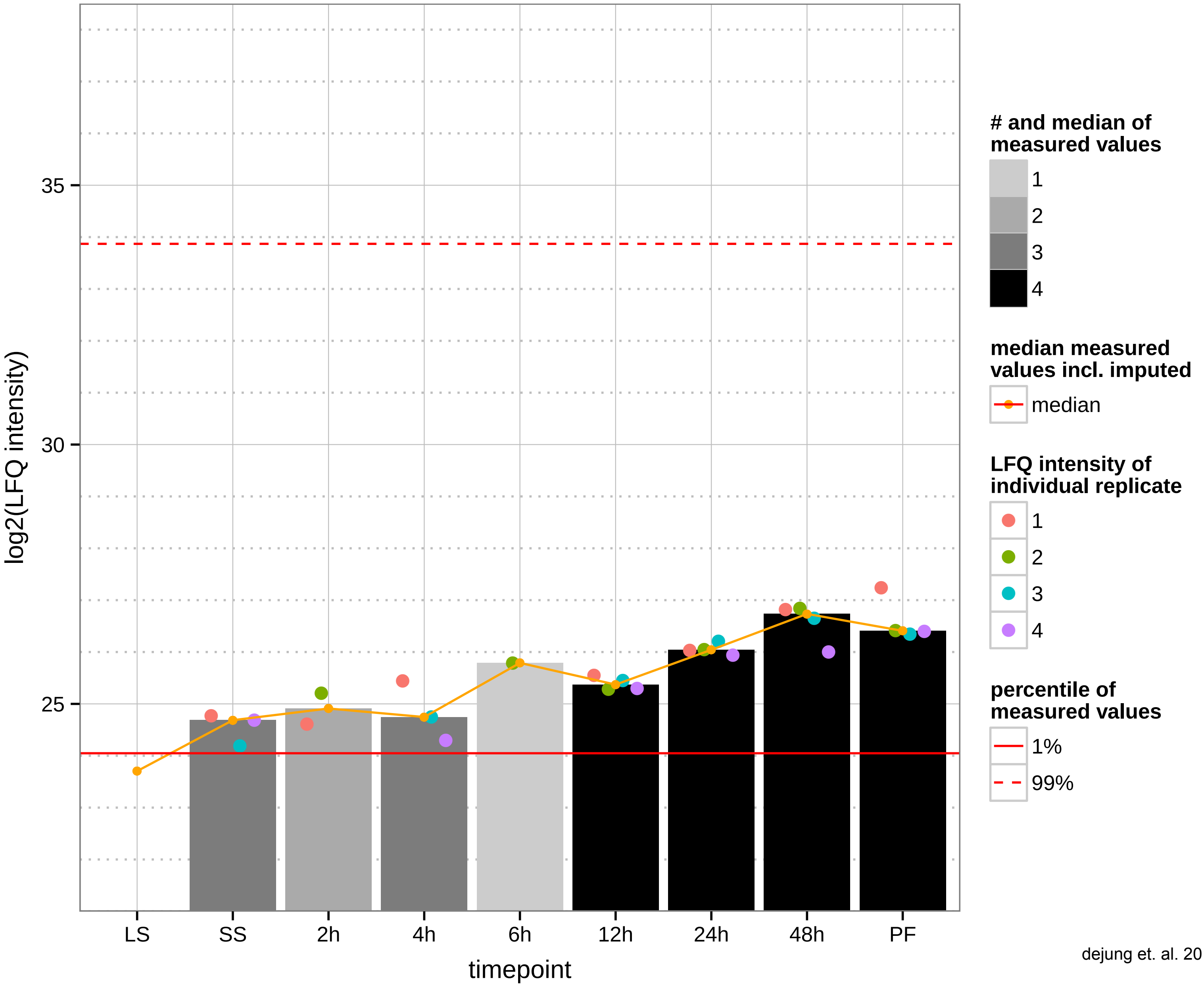

dejung et. al. 2015

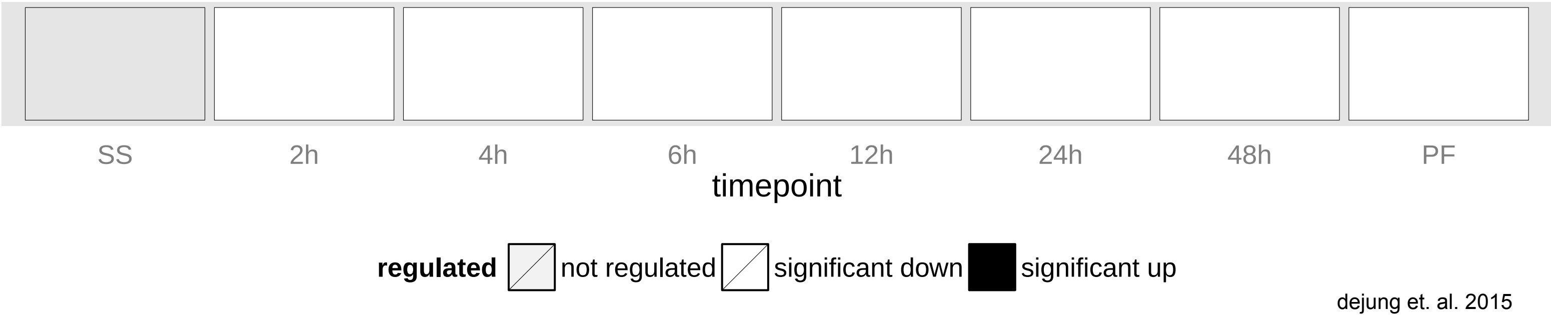
SS
2h
4h
6h
12h
24h
48h
PF
timepoint
regulated
not regulated
significant down
significant up
dejung et. al. 2015

protein kinase, putative
Tb927.1.1530
AGOF: null, ATP binding, protein kinase activity, protein serine/threonine kinase activity, protein tyrosine kinase activity, signal
AGOC: null
AGOP: null, protein phosphorylation, regulation of transcription, DNA–dependent, signal transduction
PGOF: ATP binding, protein kinase activity, transferase activity, transferring phosphorus–containing groups, signal transducer
PGOC: null
PGOP: protein phosphorylation, regulation of transcription, DNA–dependent, signal transduction

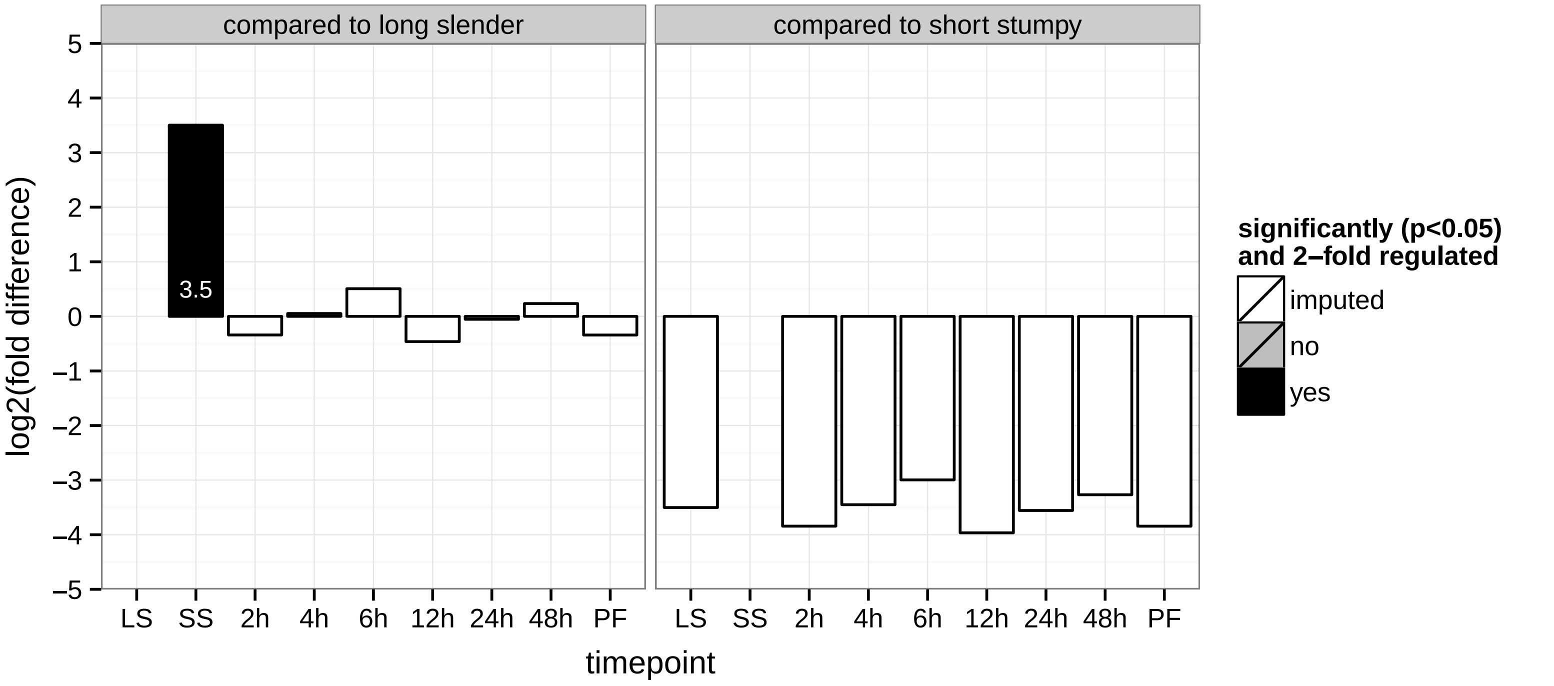

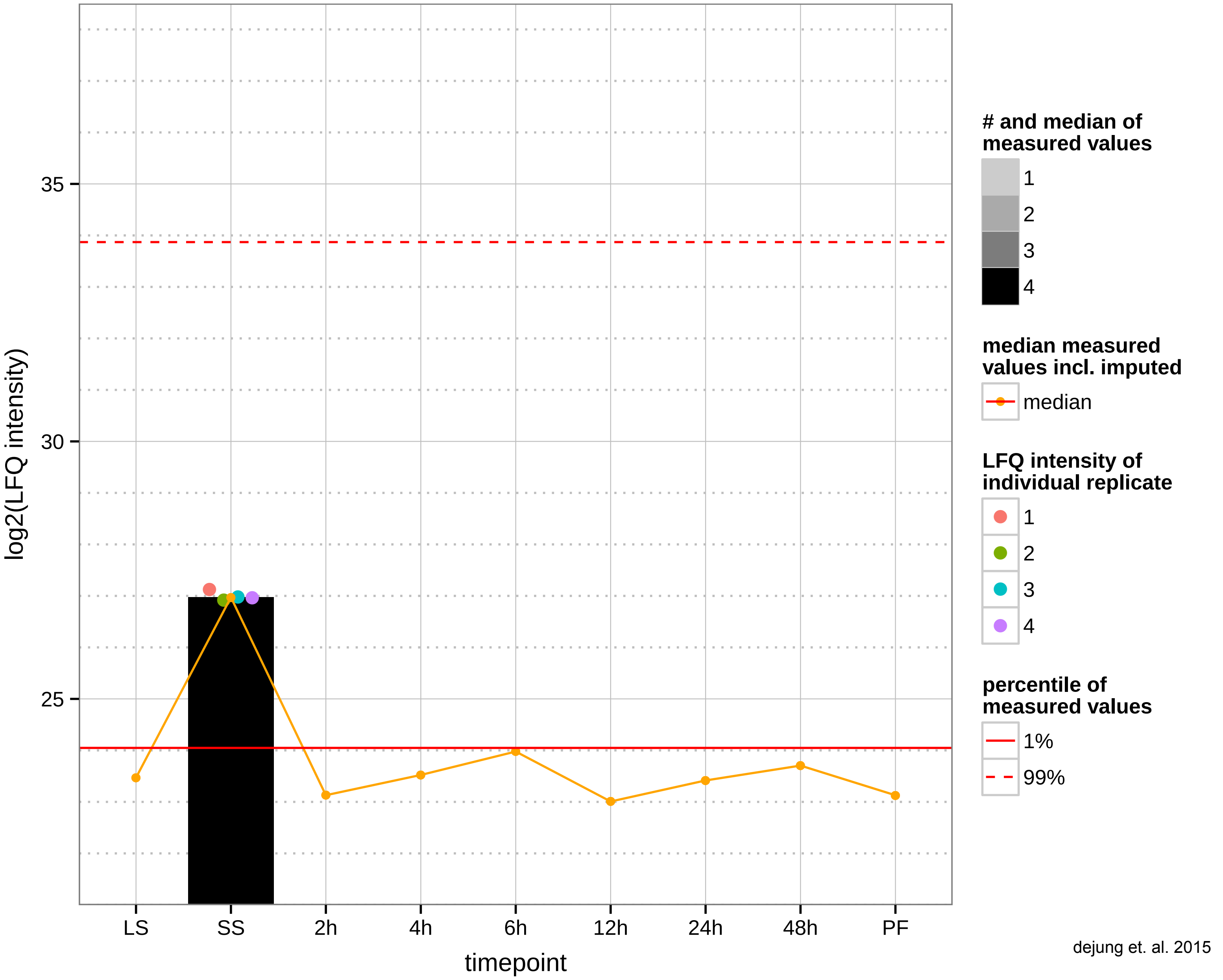

dejung et. al. 2015

cyclin–like F–box protein 2 (CFB2)
Tb927.1.4650
AGOF: null
AGOC: null
AGOP: mitochondrial DNA replication
PGOF: protein binding
PGOC: null
PGOP: null

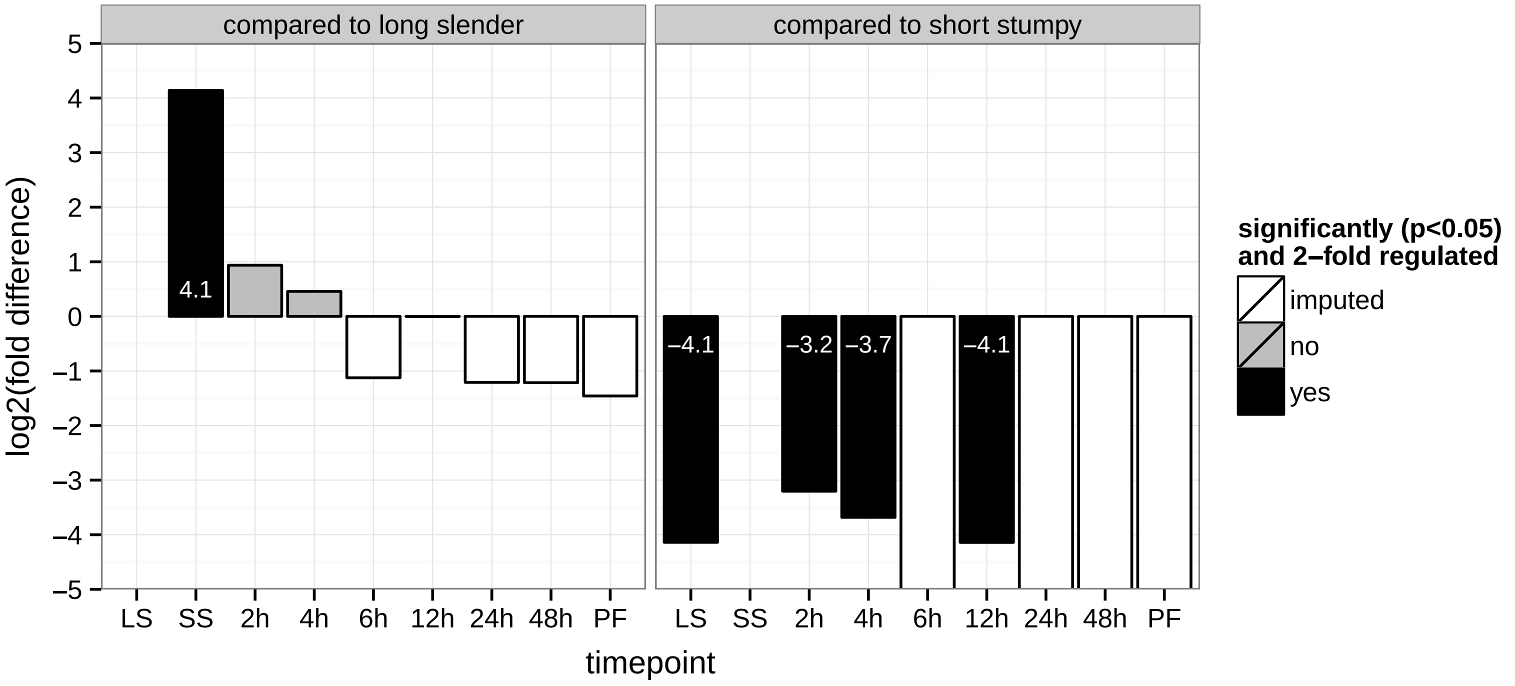

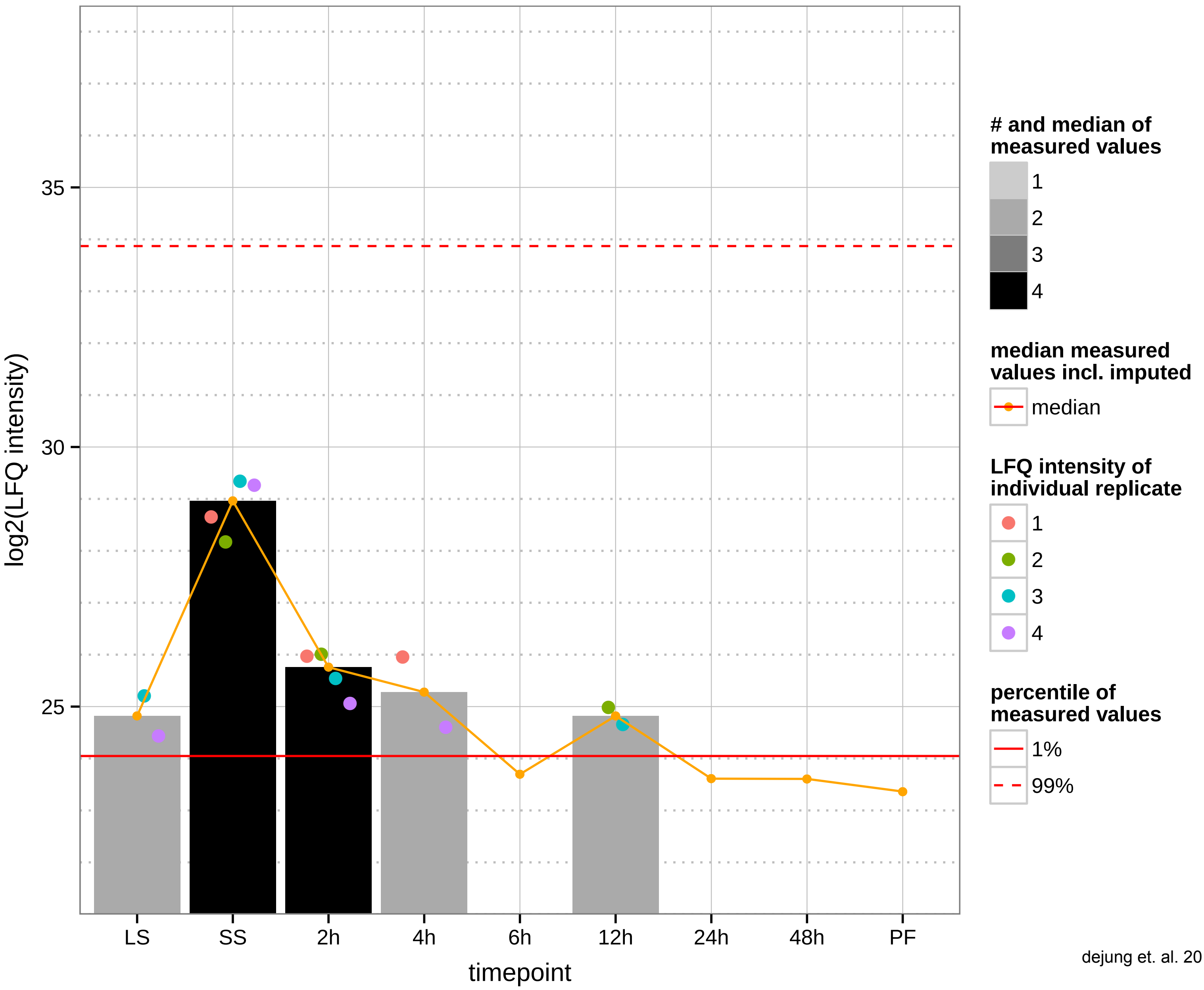

dejung et. al. 2015

MSP–C, putative (MSP–C)
Tb927.10.2410
AGOF: metalloendopeptidase activity, zinc ion binding
AGOC: membrane
AGOP: cell adhesion, proteolysis
PGOF: metalloendopeptidase activity, zinc ion binding
PGOC: membrane
PGOP: cell adhesion, proteolysis

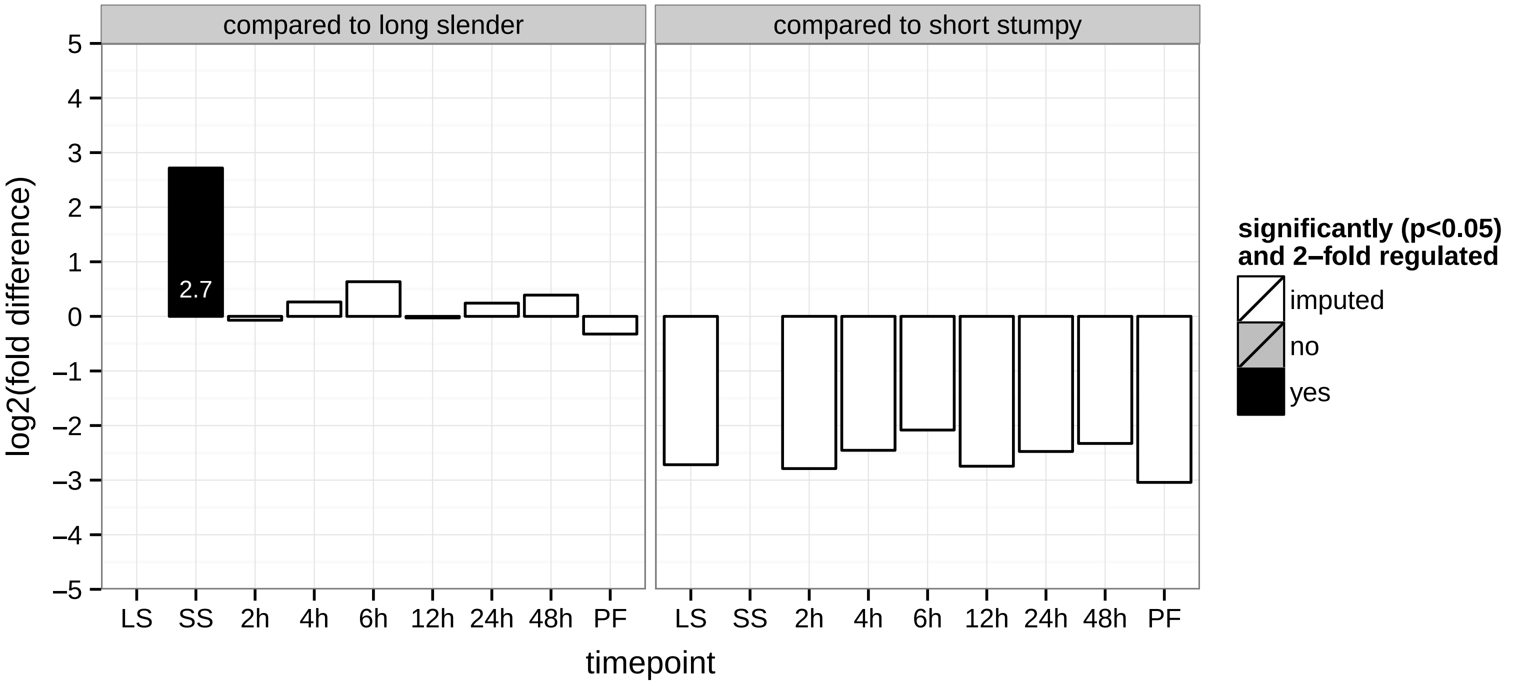

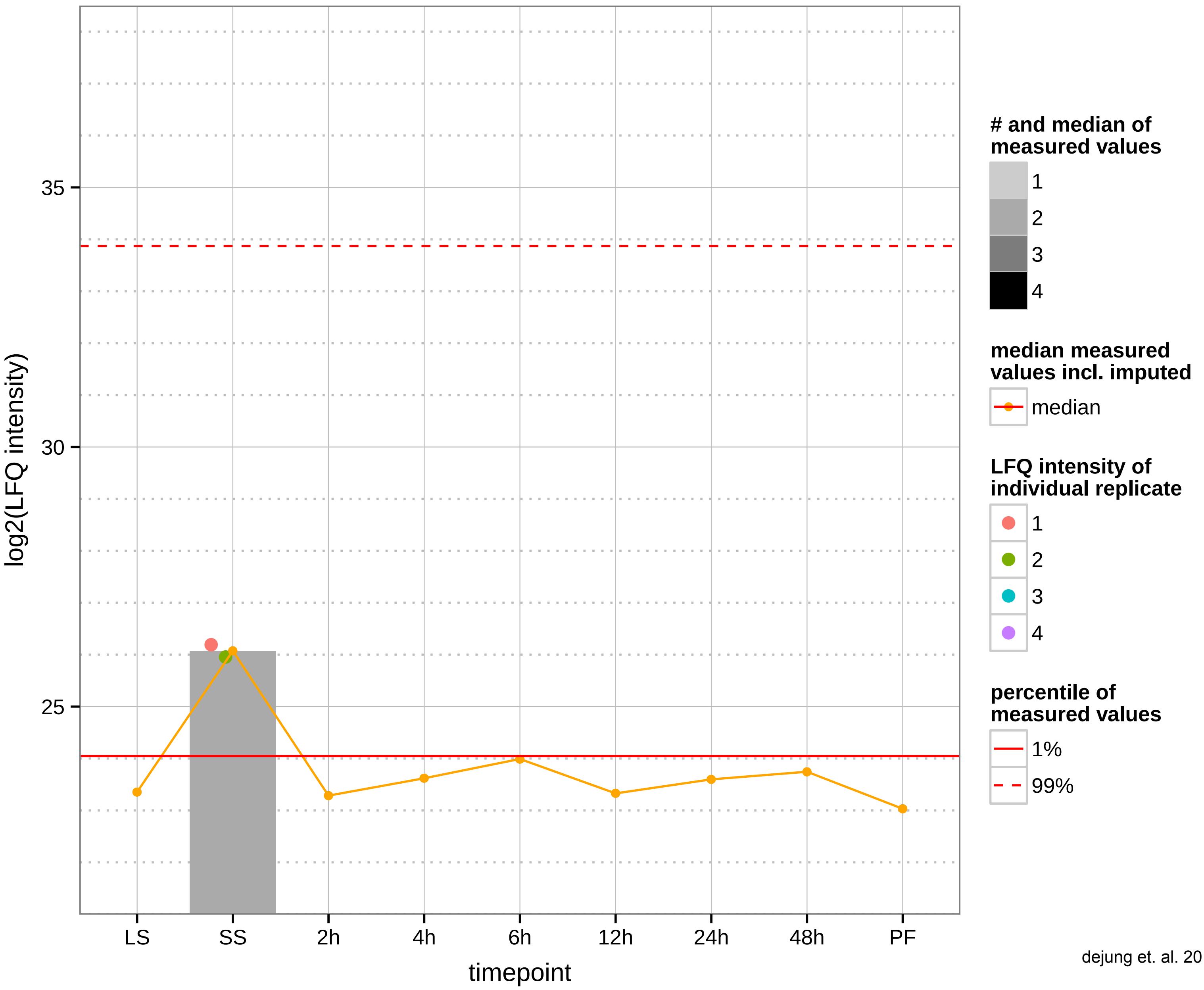

dejung et. al. 2015

hypothetical protein
Tb927.4.1230
AGOF: null
AGOC: null
AGOP: null
PGOF: null
PGOC: null
PGOP: null

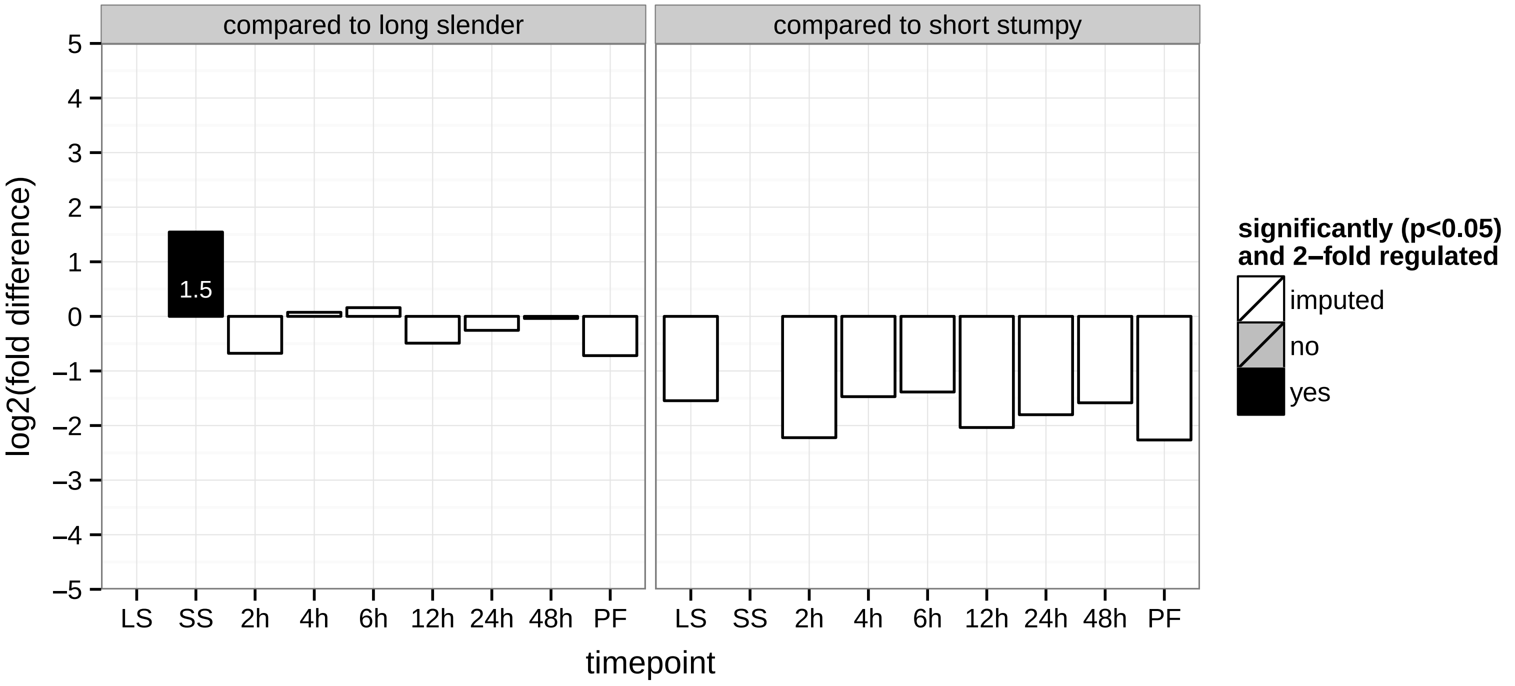

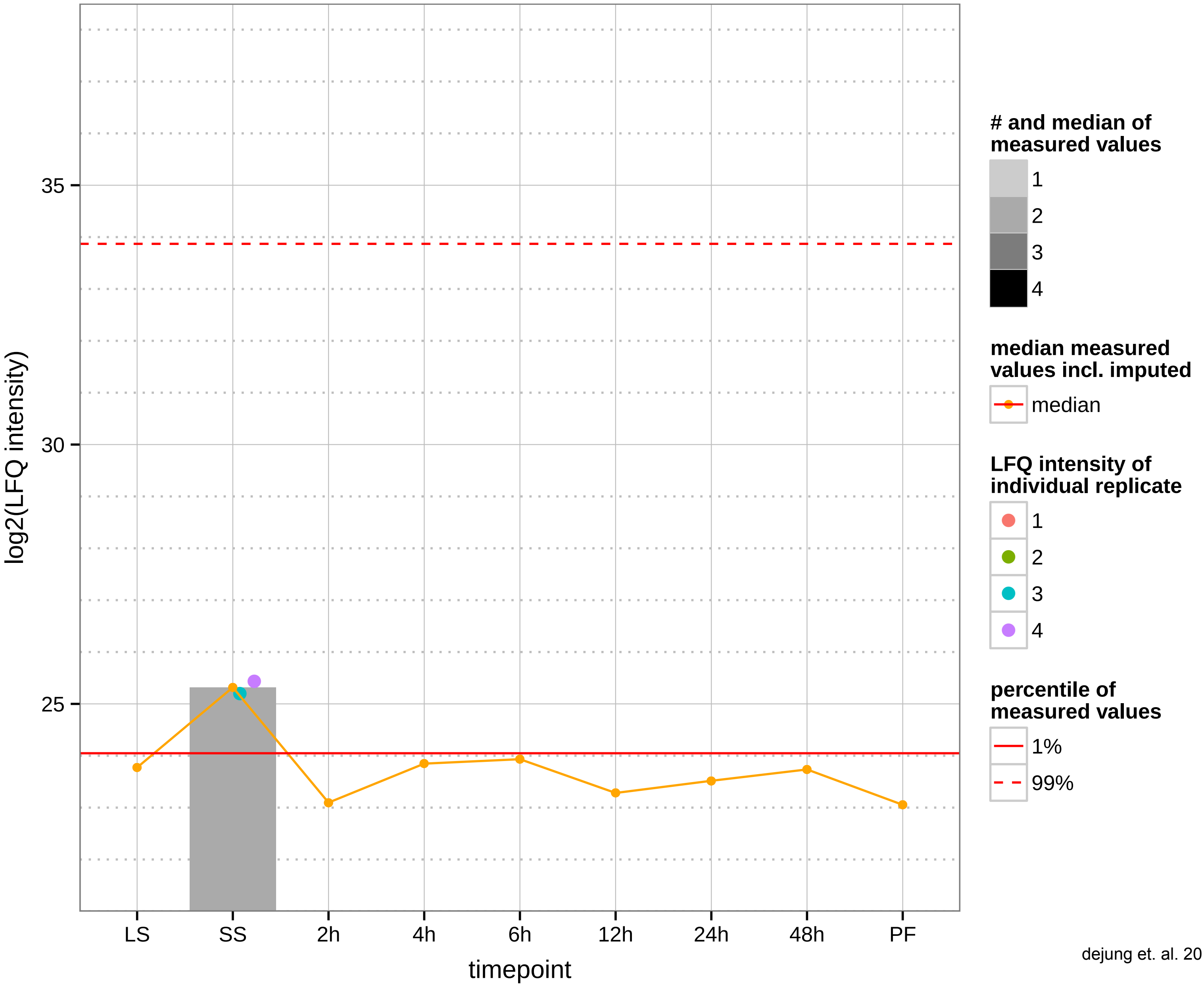

dejung et. al. 2015

serine threonine–protein phosphatase PP1, putative (PP1–5), putative (PP1–4)
Tb927.4.3640;Tb927.4.3630
AGOF: hydrolase activity, protein serine/threonine phosphatase activity
AGOC: null
AGOP: modulation of development of symbiont involved in interaction with host, protein dephosphorylation, quorum sensing i
PGOF: hydrolase activity
PGOC: null
PGOP: null

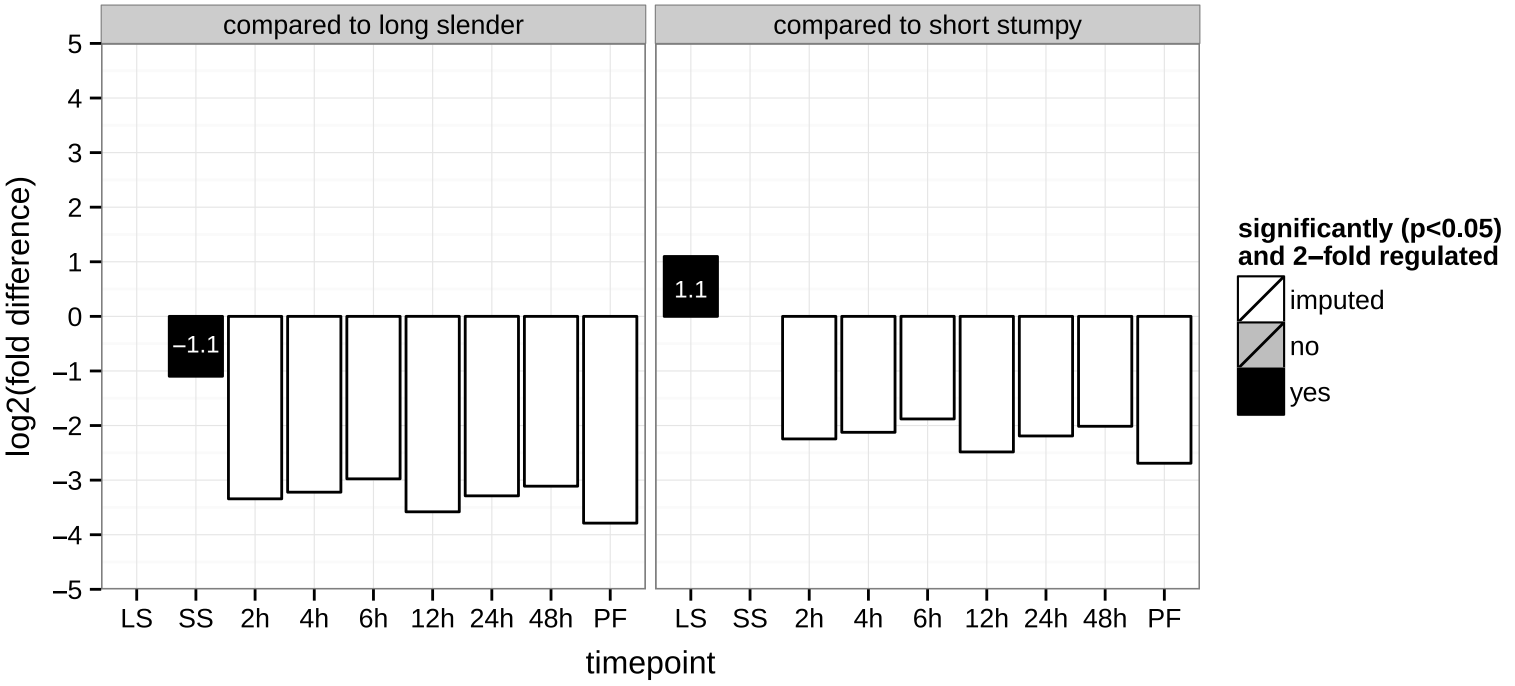

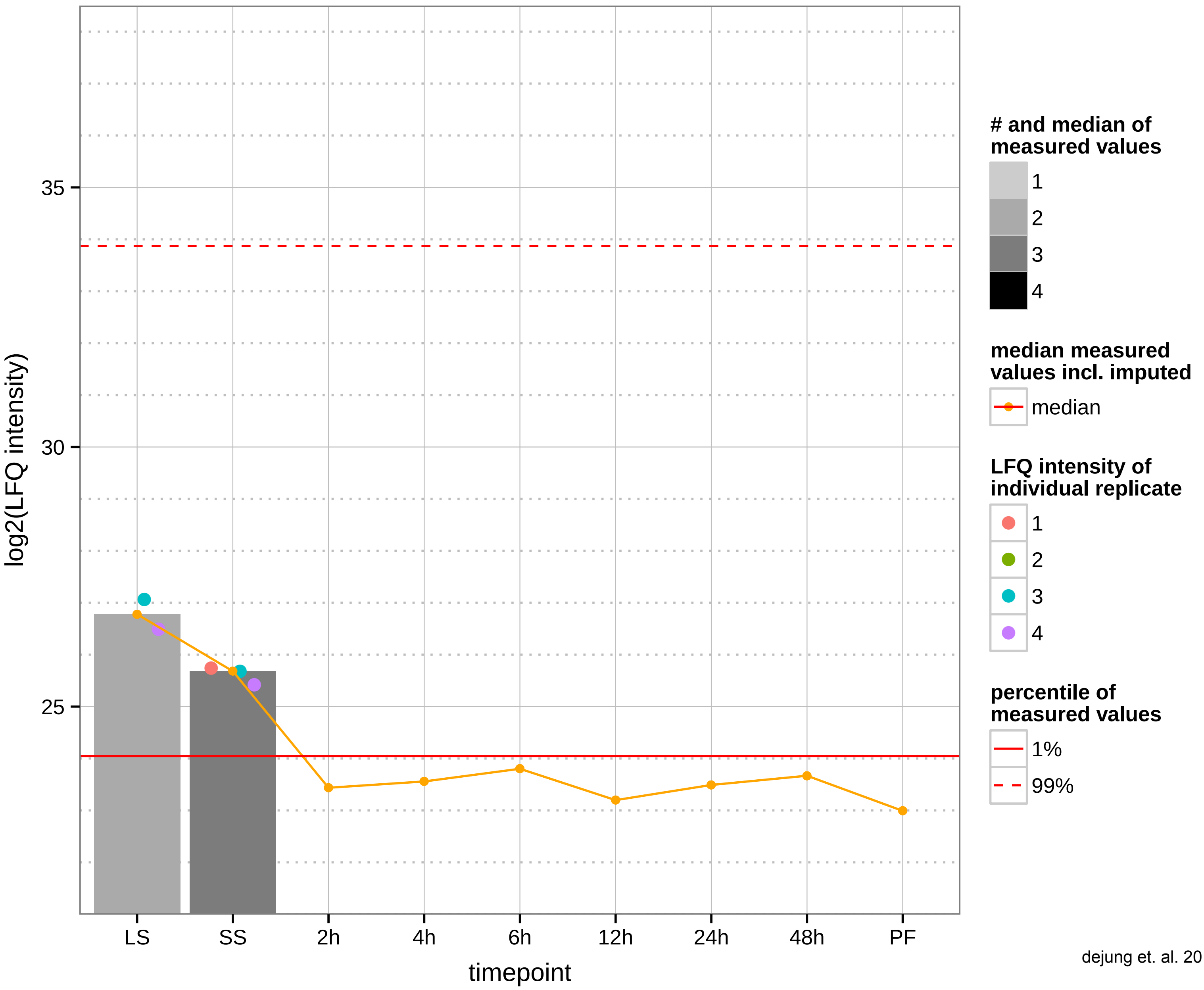

dejung et. al. 2015

phospholipid−translocating P−type ATPase (flippase), putative
Tb927.6.3550
AGOF: ATP binding, ATPase activity, coupled to transmembrane movement of ions, phosphorylative mechanism, magnesium
AGOC: integral to membrane, membrane
AGOP: ATP biosynthetic process, phospholipid transport
PGOF: ATP binding, ATPase activity, coupled to transmembrane movement of ions, phosphorylative mechanism, hydrolase ac
PGOC: integral to membrane, membrane
PGOP: cation transport, phospholipid transport

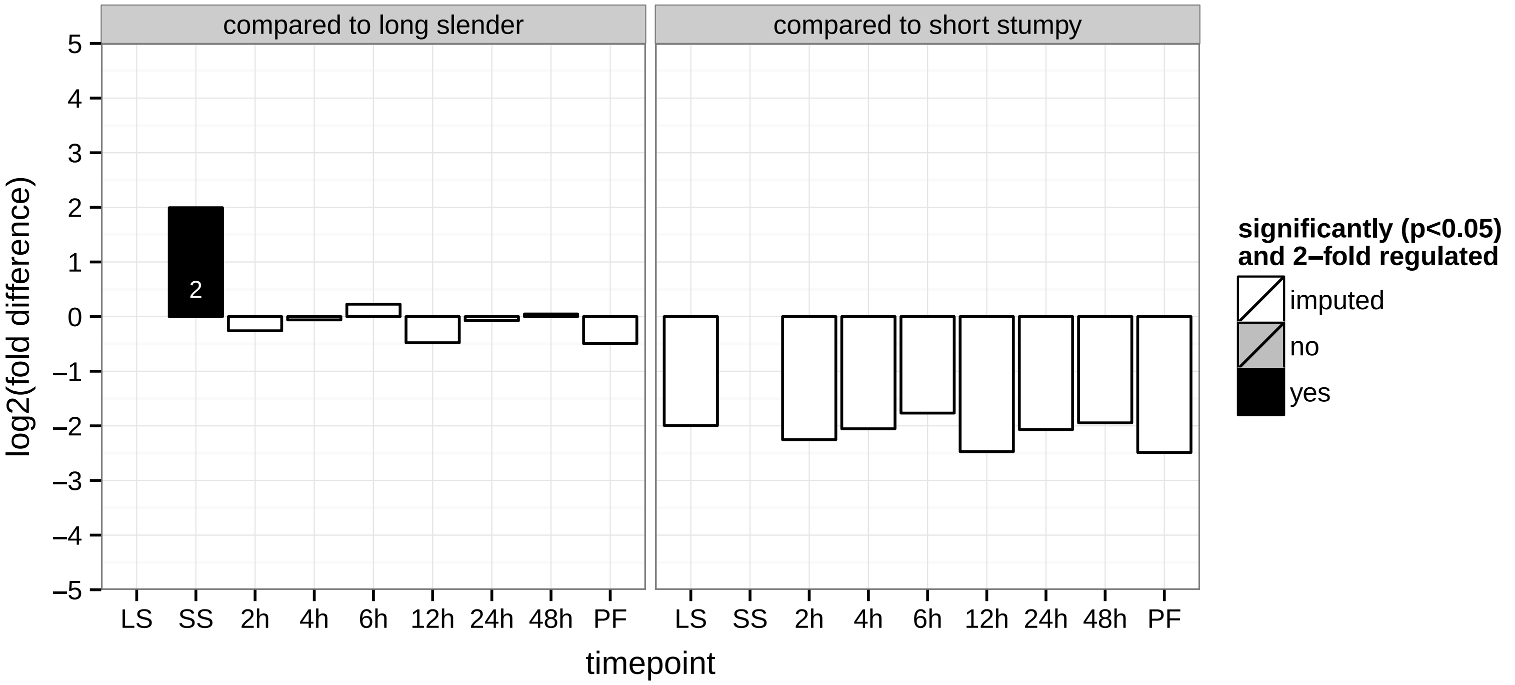

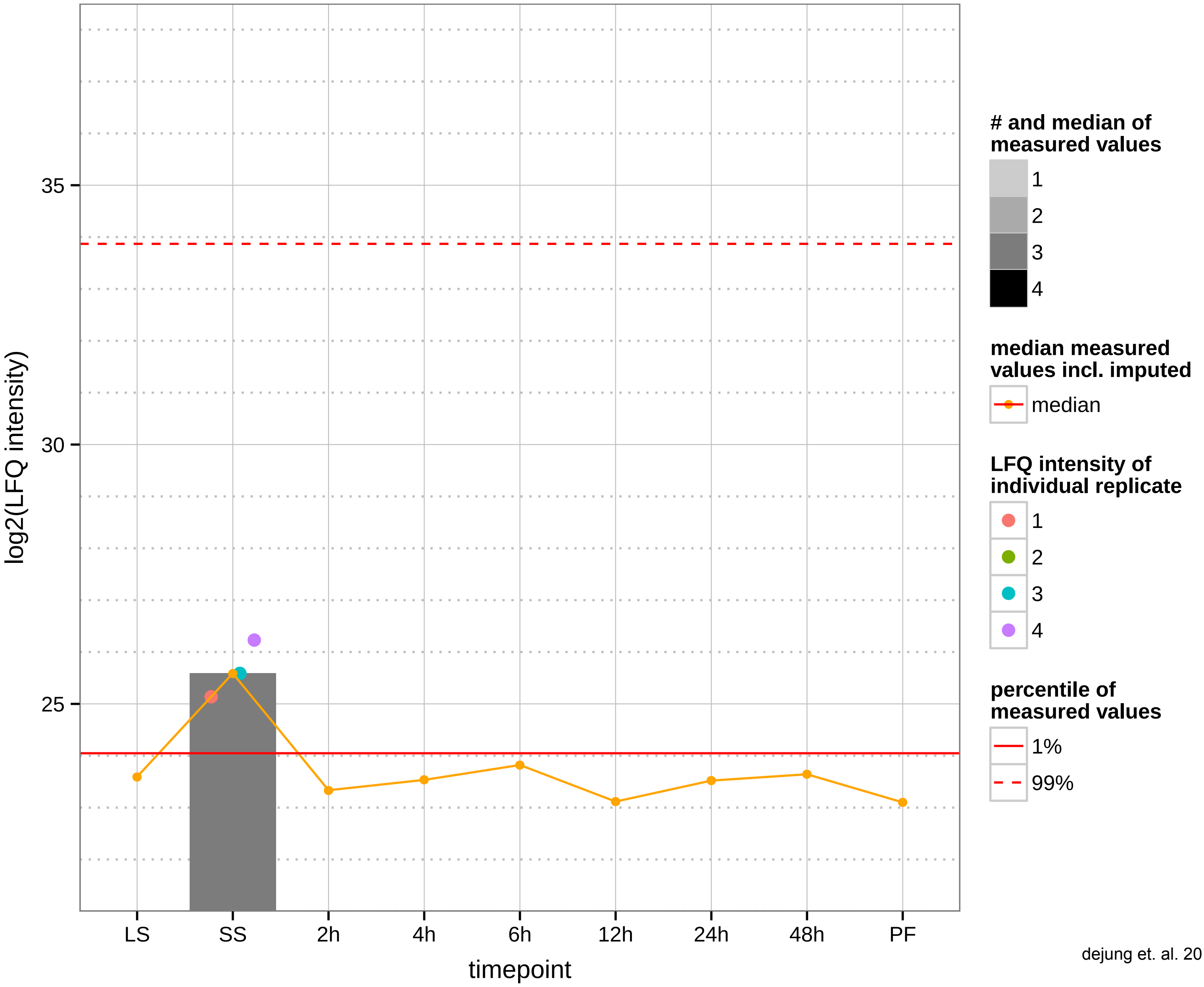

dejung et. al. 2015

hypothetical protein, conserved
Tb927.7.6570
AGOF: null
AGOC: null
AGOP: null
PGOF: null
PGOC: null
PGOP: null

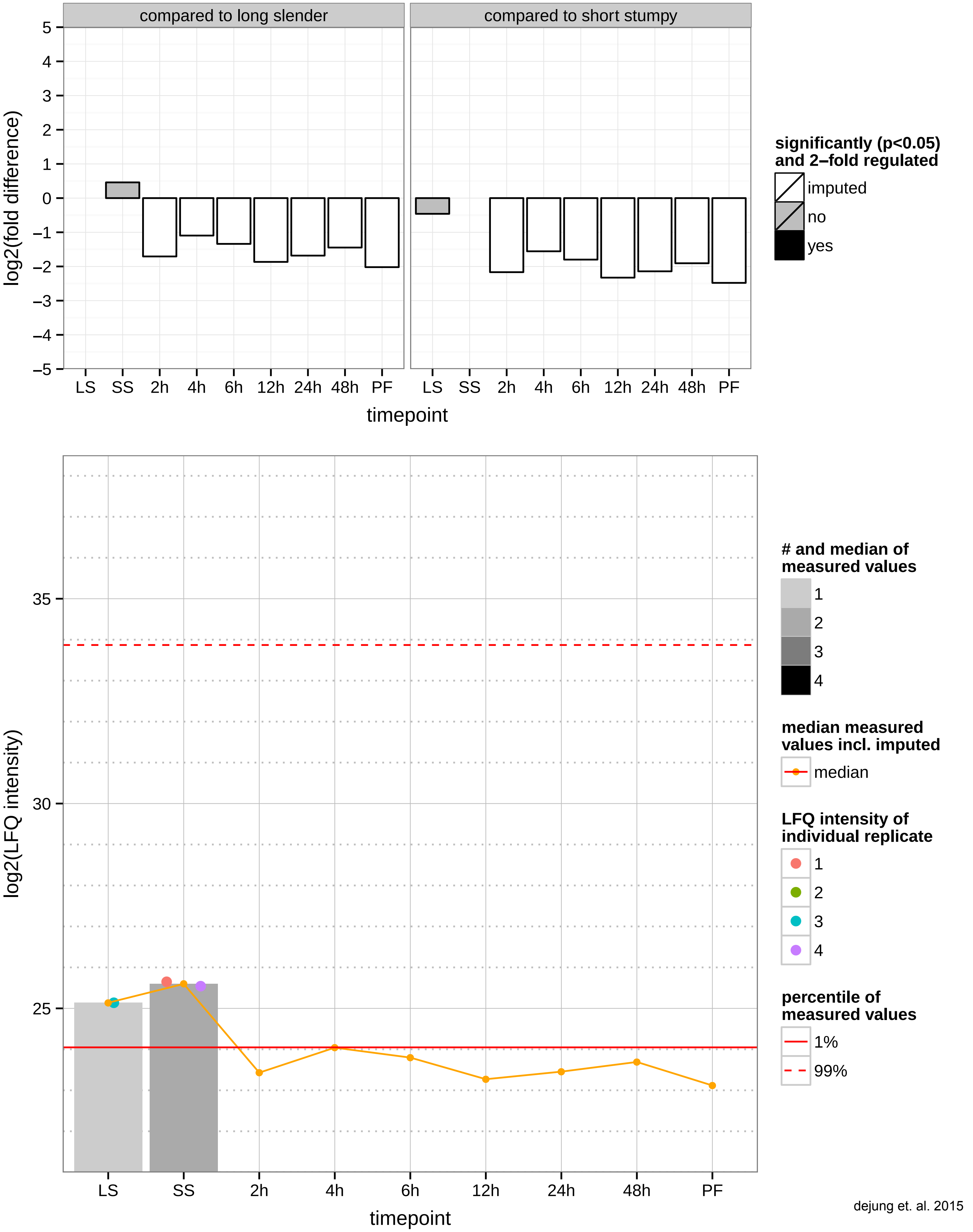

dejung et. al. 2015

hypothetical protein, conserved
Tb927.8.2580
AGOF: null
AGOC: cytoplasm
AGOP: exocytosis, vesicle docking
PGOF: null
PGOC: cytoplasm
PGOP: exocytosis, vesicle docking

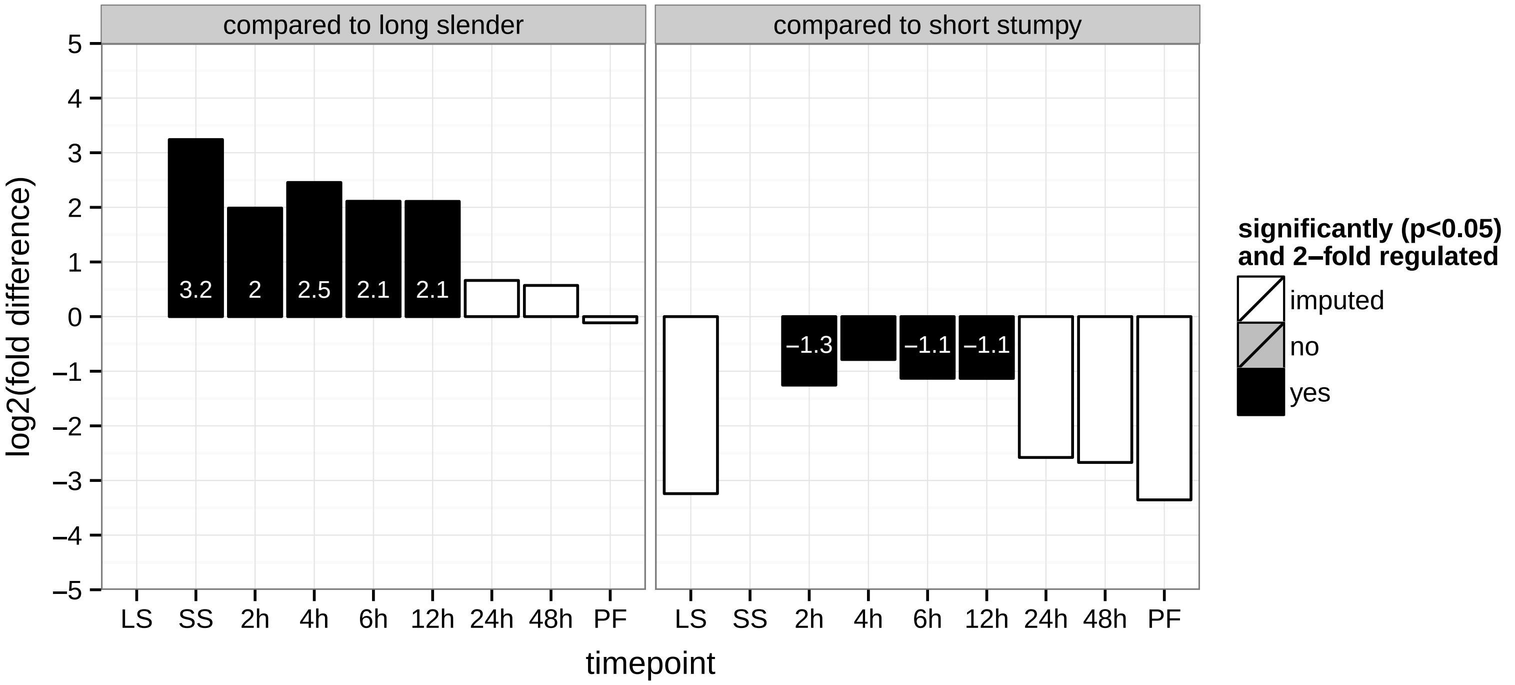

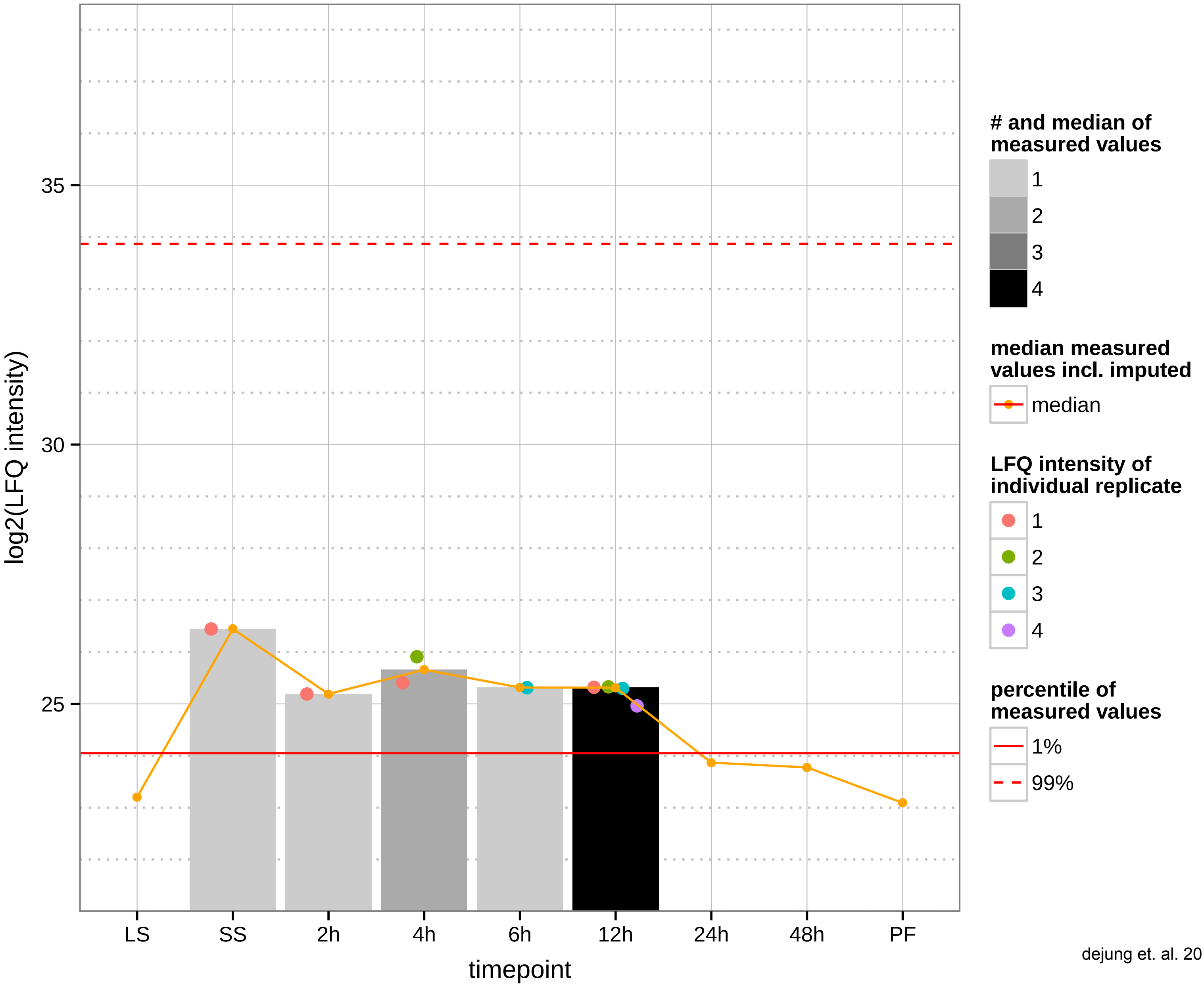

dejung et. al. 2015

hypothetical protein, conserved
Tb927.9.5620
AGOF: null
AGOC: null
AGOP: null
PGOF: protein binding
PGOC: null
PGOP: null

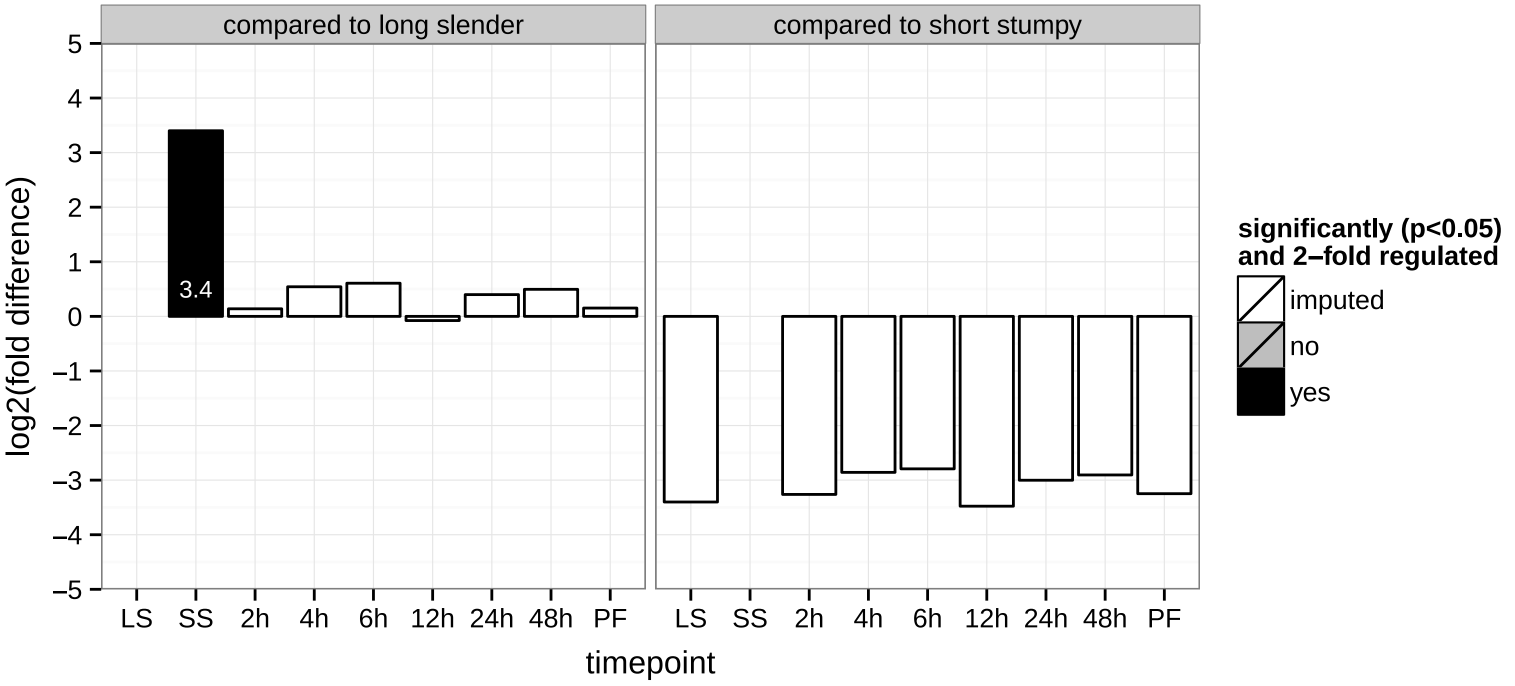

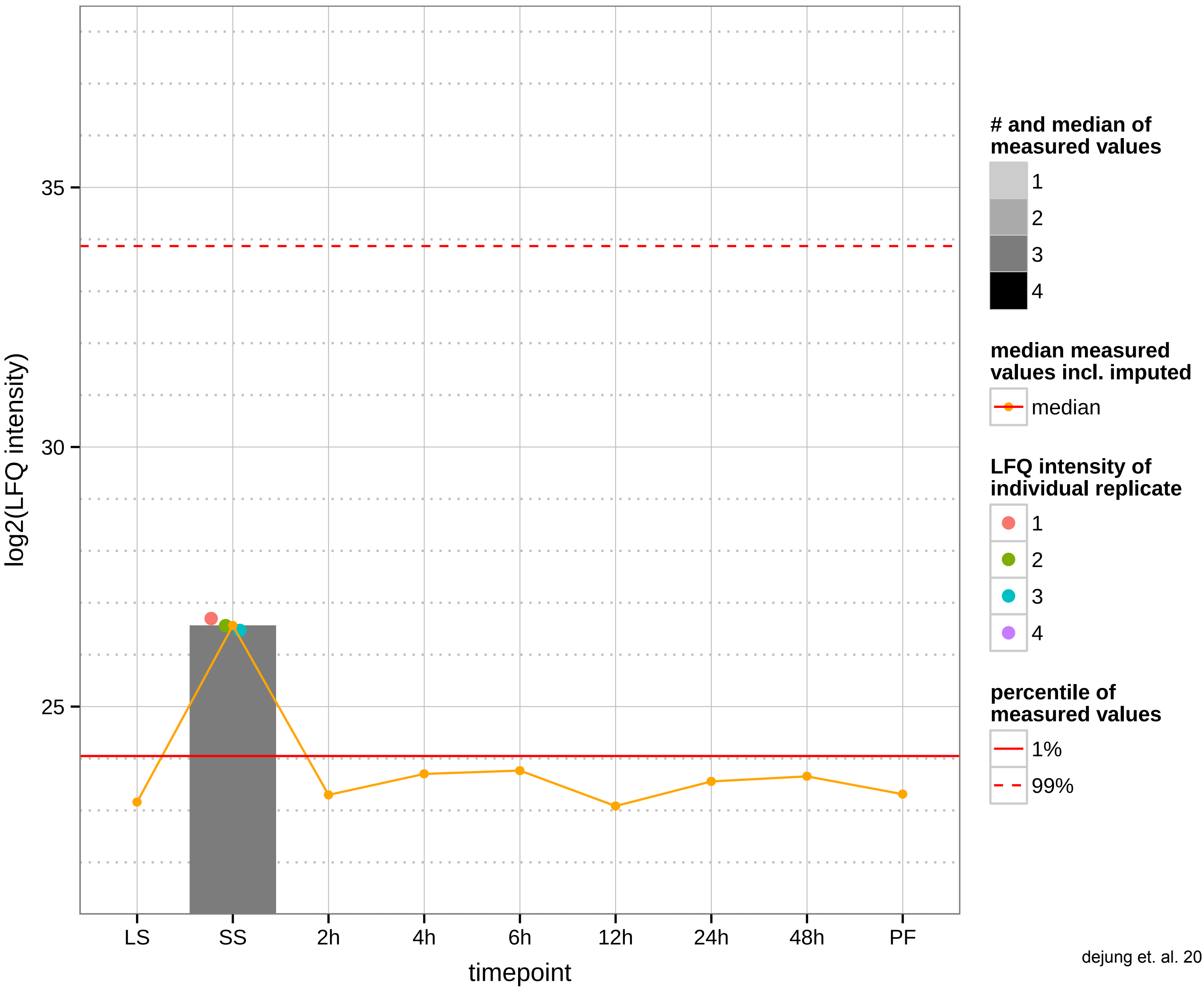

dejung et. al. 2015

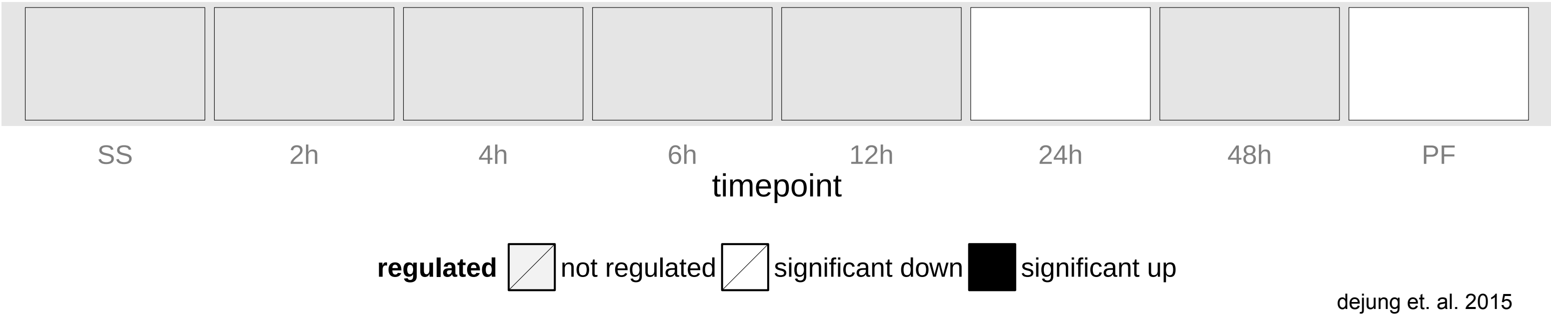
SS
2h
4h
6h
12h
24h
48h
PF
timepoint
regulated
not regulated
significant down
significant up
dejung et. al. 2015

hypothetical protein, conserved
Tb927.10.11600
AGOF: null
AGOC: null
AGOP: null
PGOF: null
PGOC: null
PGOP: null

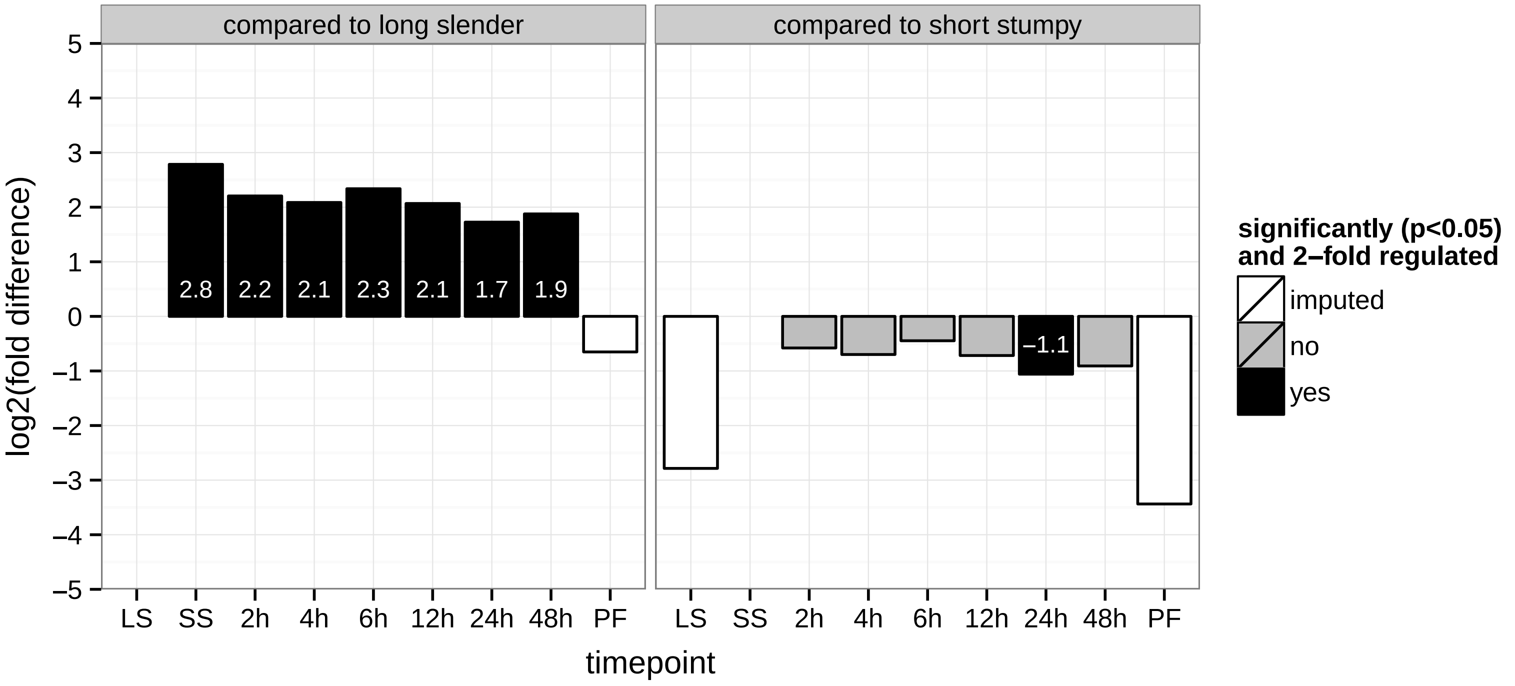

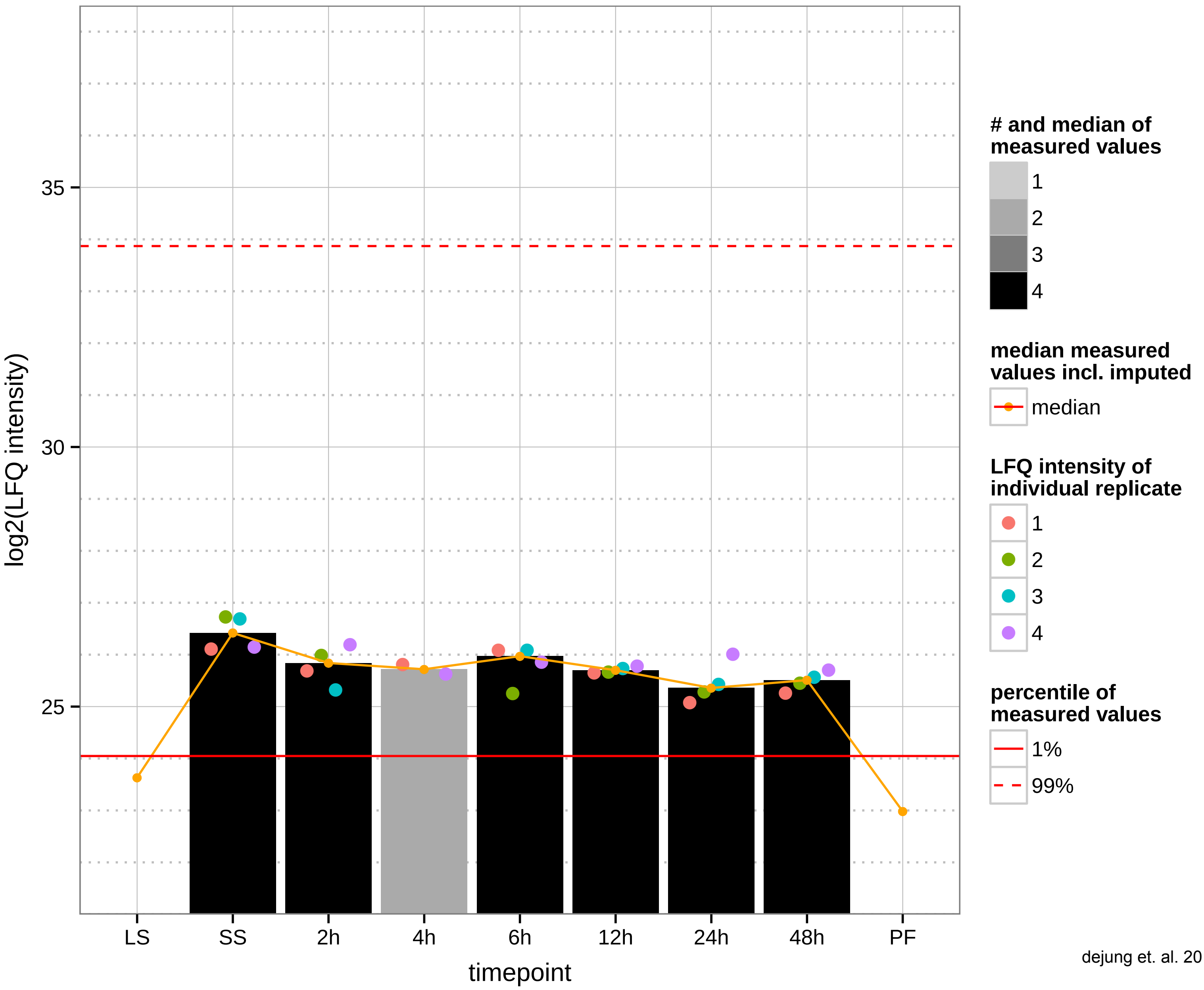

dejung et. al. 2015

procyclin–associated gene 2 (PAG2) protein, putative
Tb927.10.5690
AGOF: null
AGOC: null
AGOP: evasion or tolerance of host immune response
PGOF: null
PGOC: null
PGOP: evasion or tolerance of host immune response

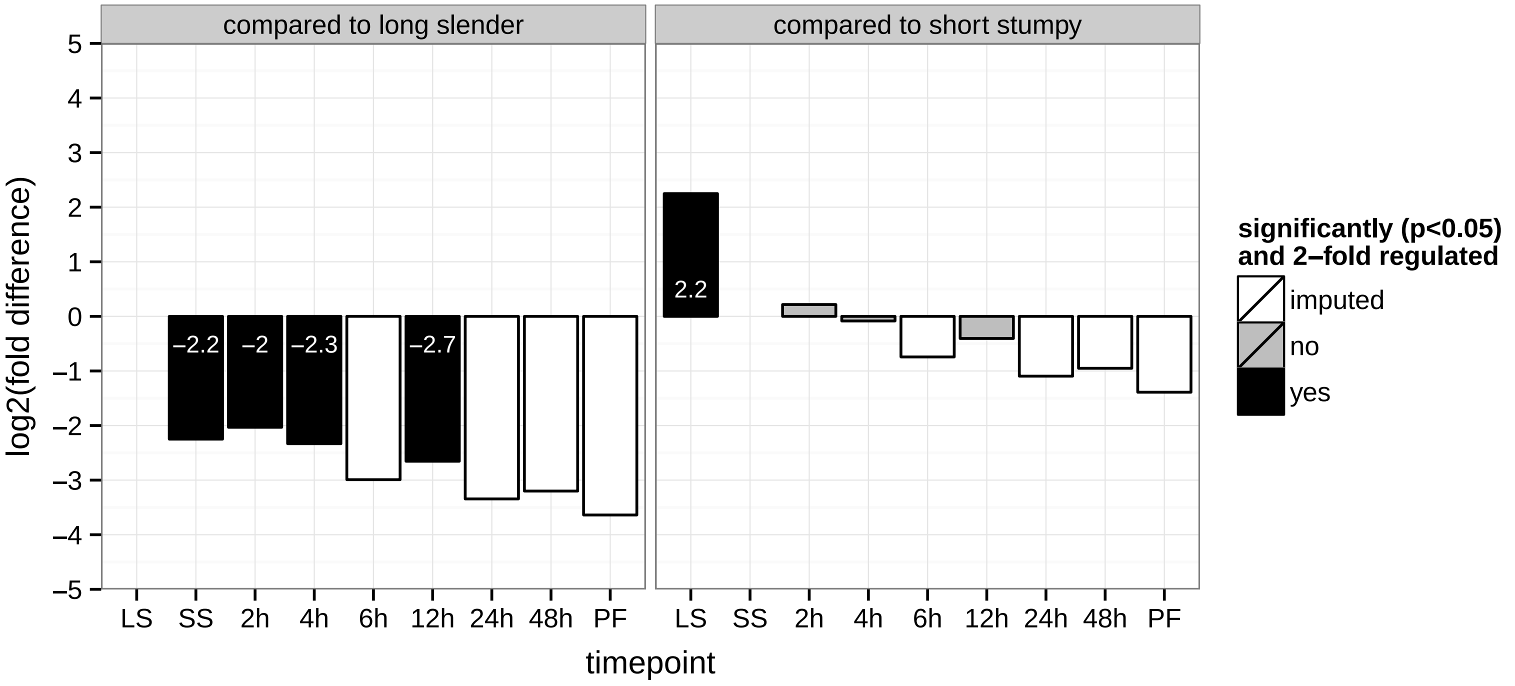

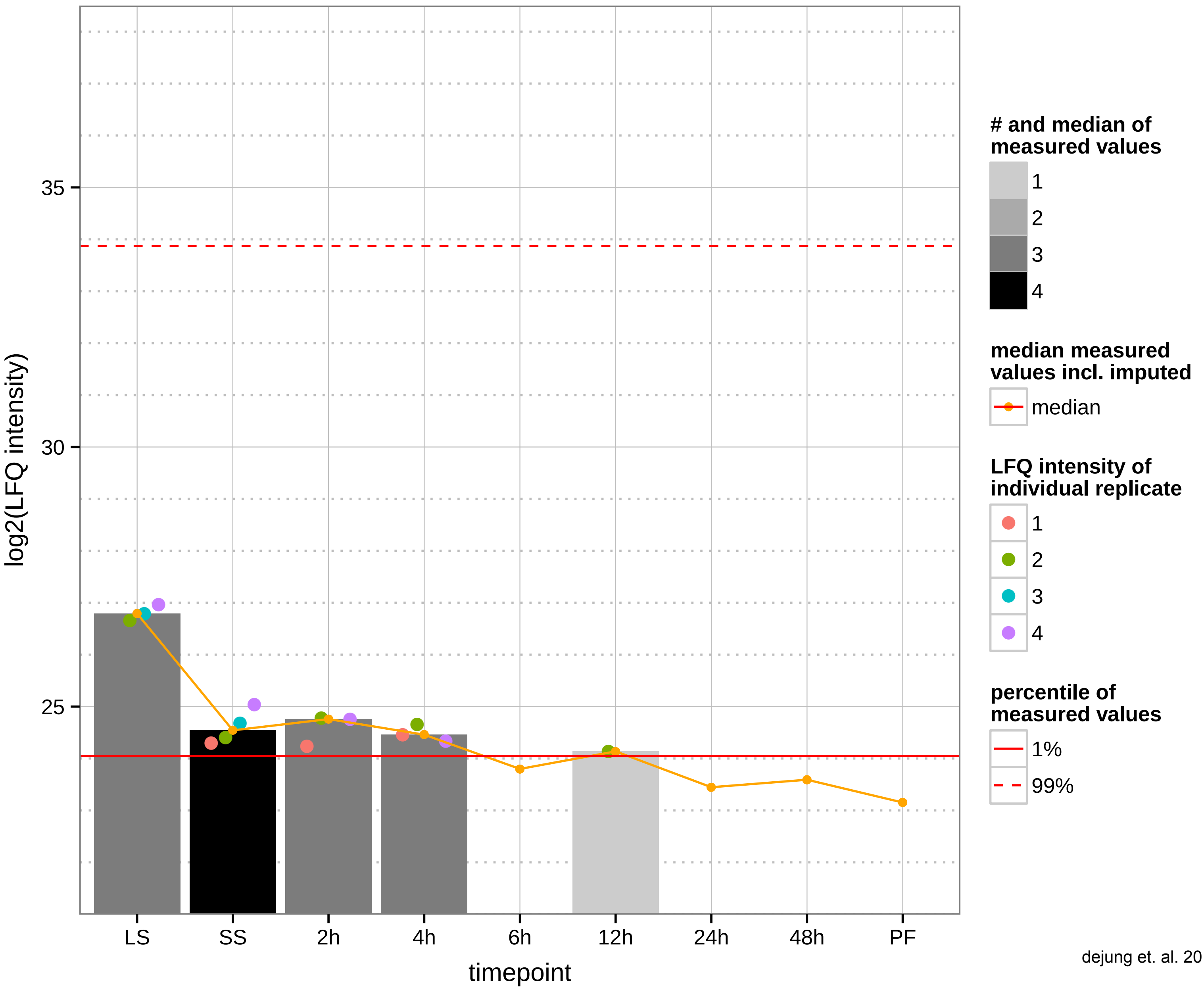

dejung et. al. 2015

cAMP response protein, putative (CARP2a)
Tb927.11.12860.2;Tb927.11.12860.1
AGOF: null
AGOC: null
AGOP: response to drug
PGOF: null
PGOC: null
PGOP: null

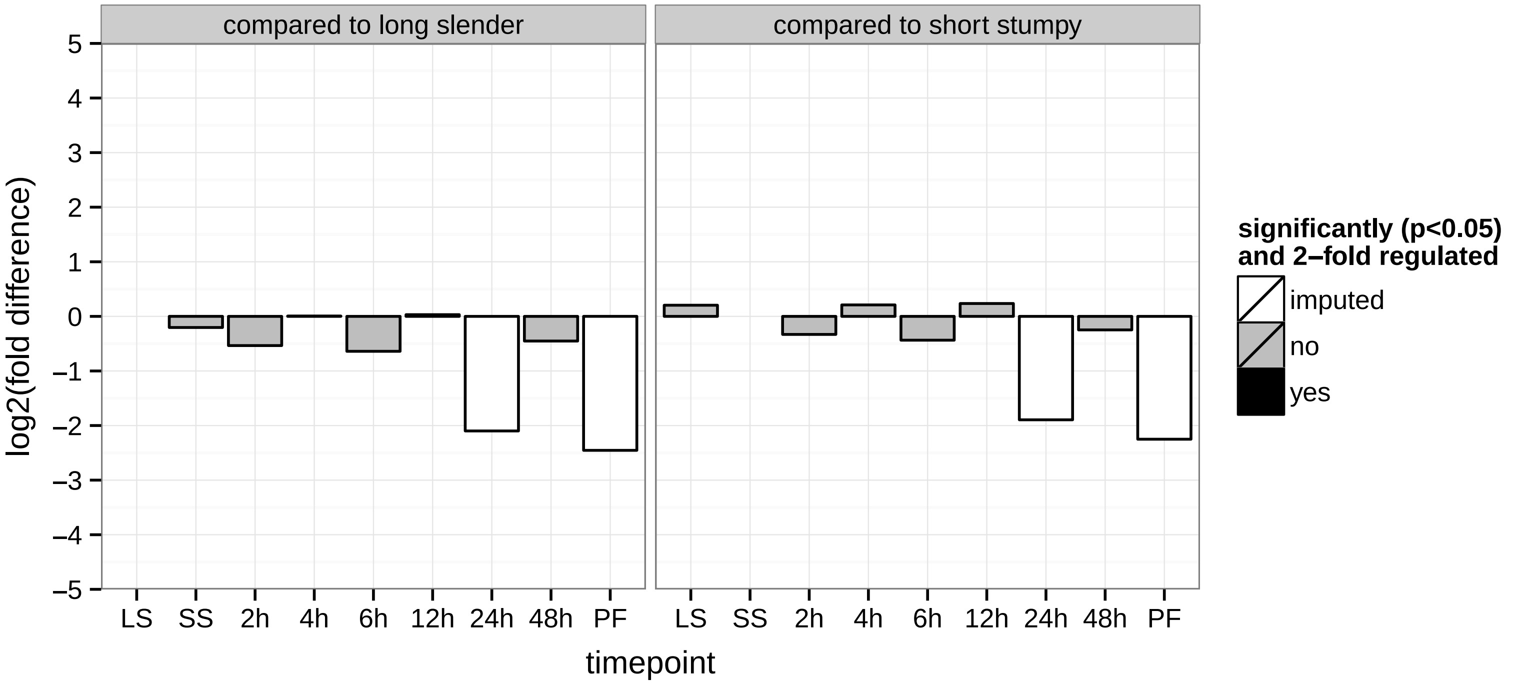

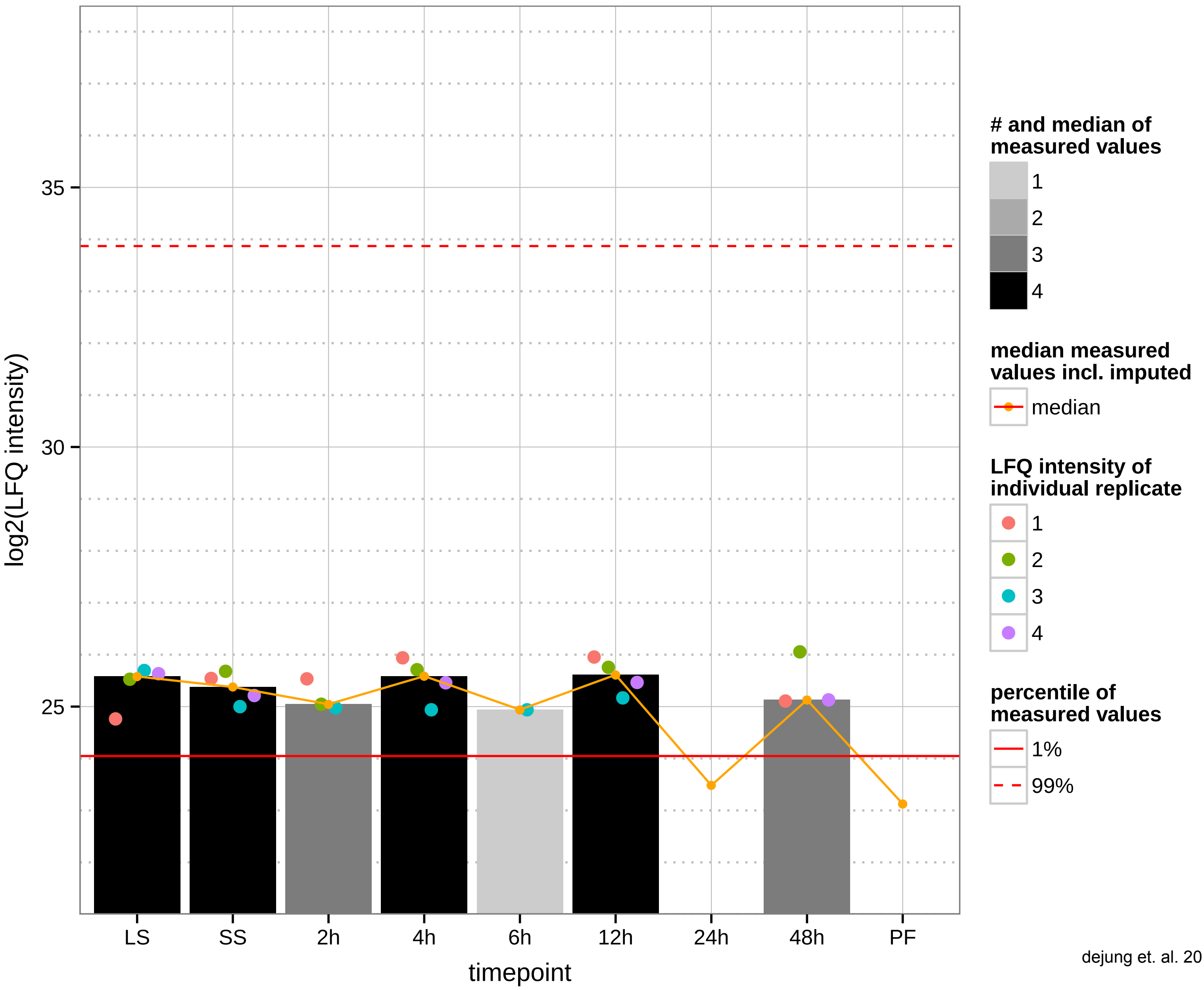

dejung et. al. 2015

hypothetical protein, conserved
Tb927.2.1700
AGOF: null
AGOC: null
AGOP: null
PGOF: protein binding
PGOC: null
PGOP: null

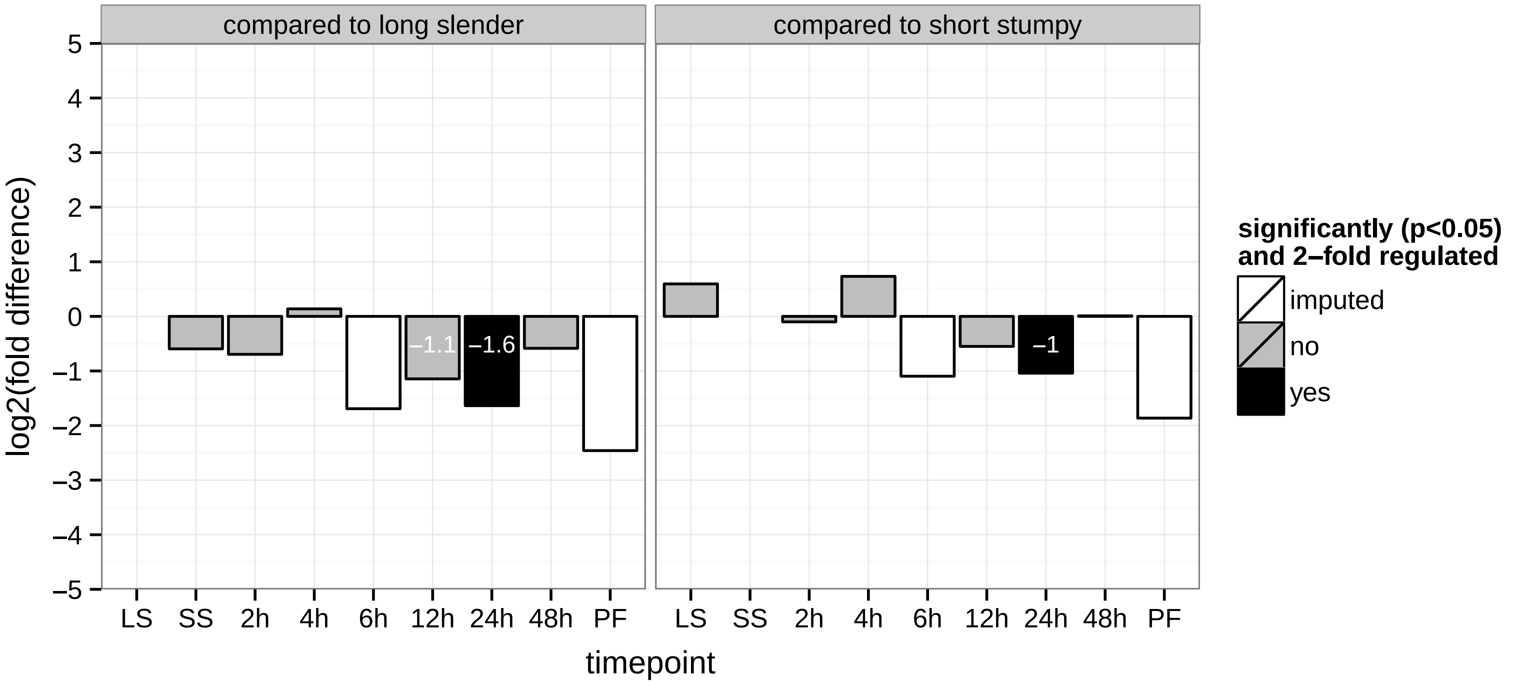

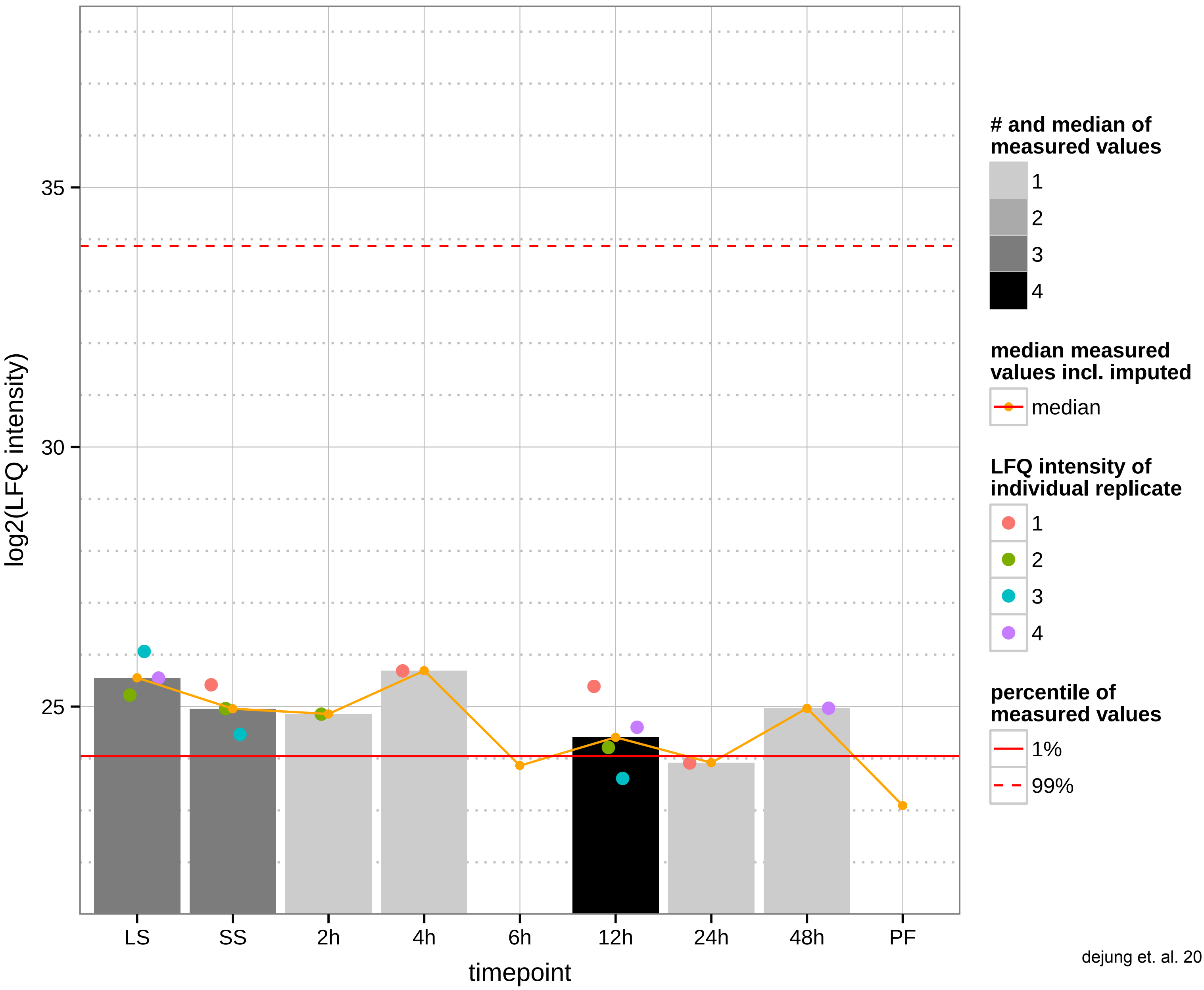

dejung et. al. 2015

hypothetical protein, conserved
Tb927.2.1850
AGOF: null
AGOC: null
AGOP: null
PGOF: protein binding
PGOC: null
PGOP: null

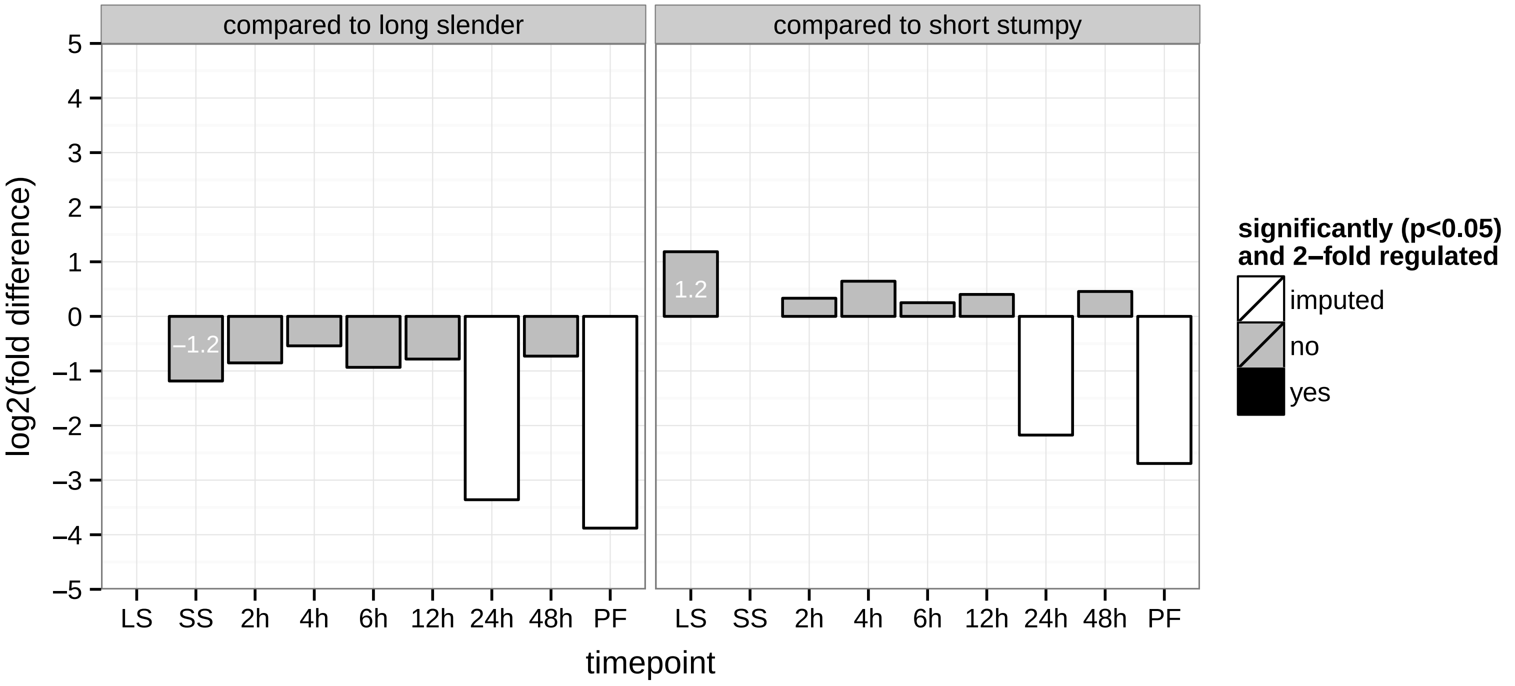

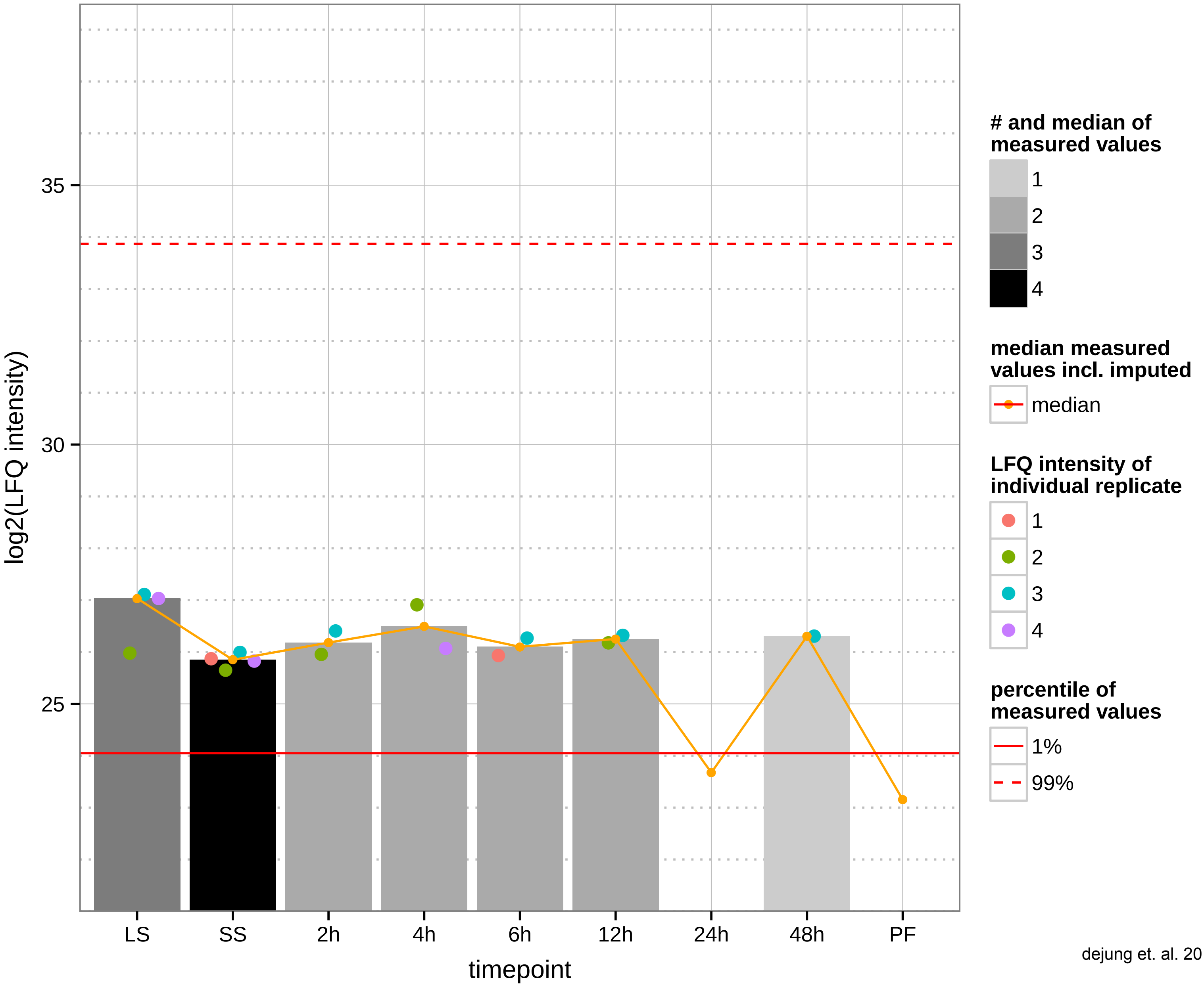

dejung et. al. 2015

UDP–glucose:glycoprotein glucosyltransferase, putative
Tb927.3.4630
AGOF: UDP–glucose:glycoprotein glucosyltransferase activity
AGOC: endoplasmic reticulum
AGOP: protein glycosylation
PGOF: UDP–glucose:glycoprotein glucosyltransferase activity, transferase activity, transferring glycosyl groups
PGOC: null
PGOP: protein glycosylation

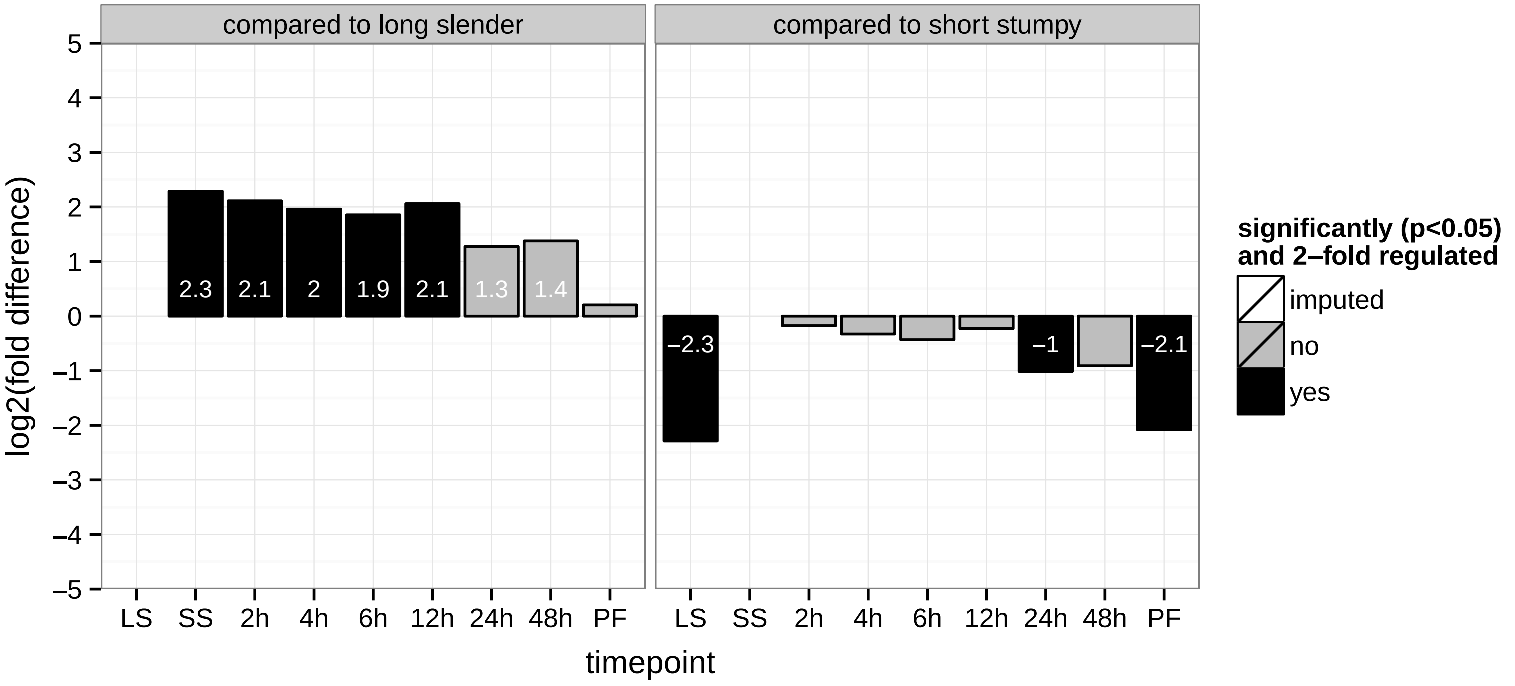

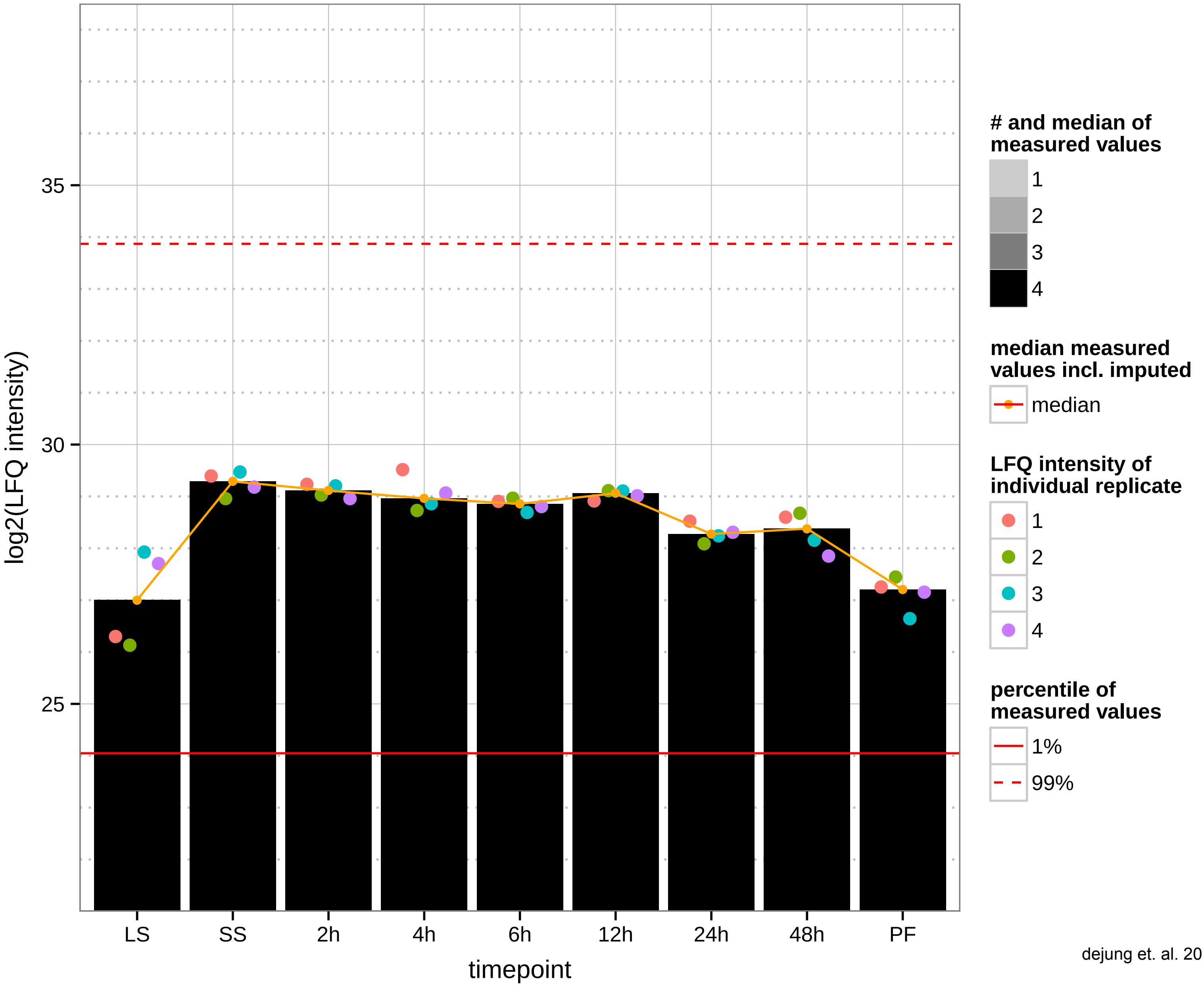

dejung et. al. 2015

hypothetical protein, conserved
Tb927.6.2200
AGOF: null
AGOC: null
AGOP: null
PGOF: null
PGOC: null
PGOP: null

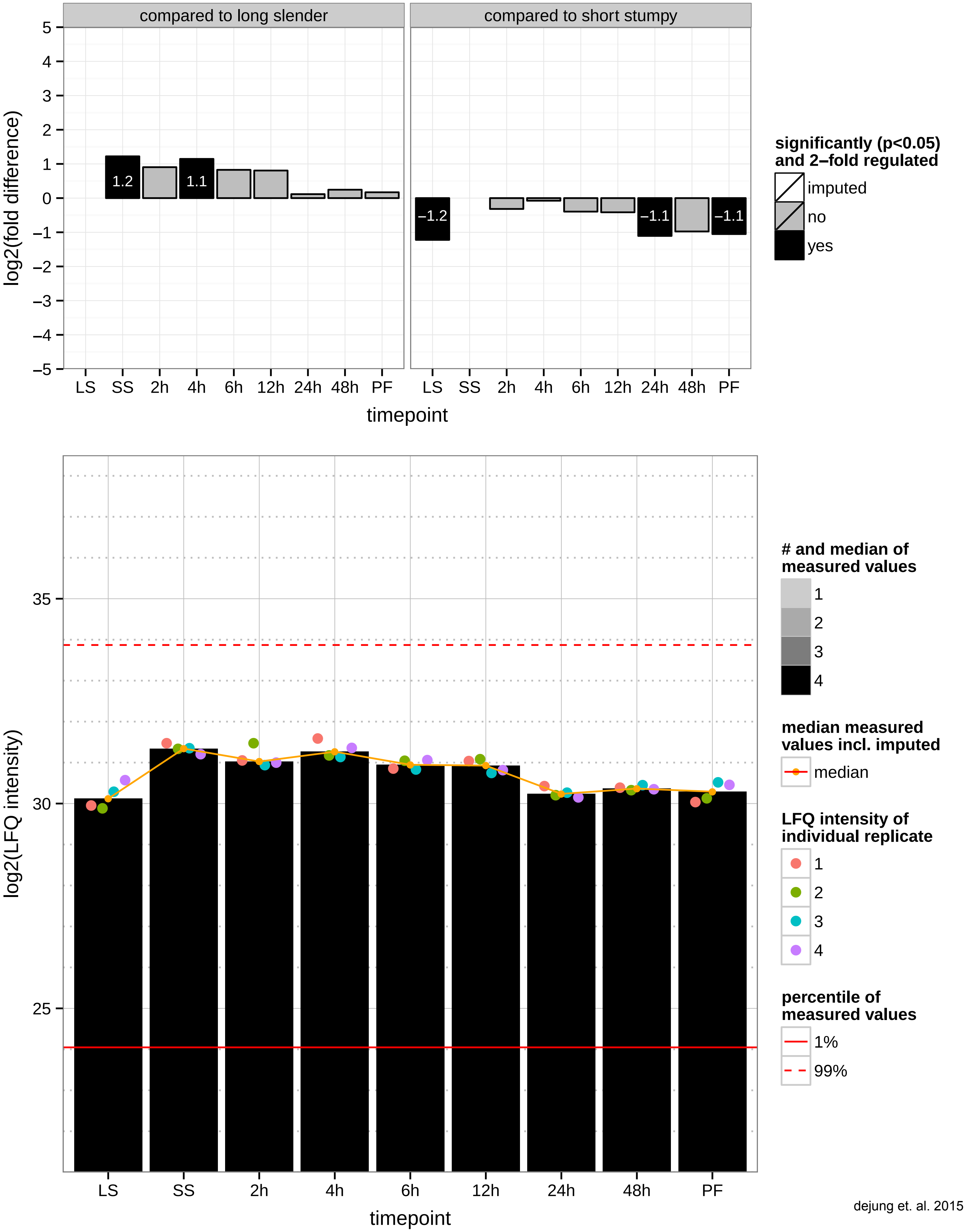

dejung et. al. 2015

hypothetical protein, conserved
Tb927.7.5150
AGOF: null
AGOC: null
AGOP: null
PGOF: null
PGOC: null
PGOP: null

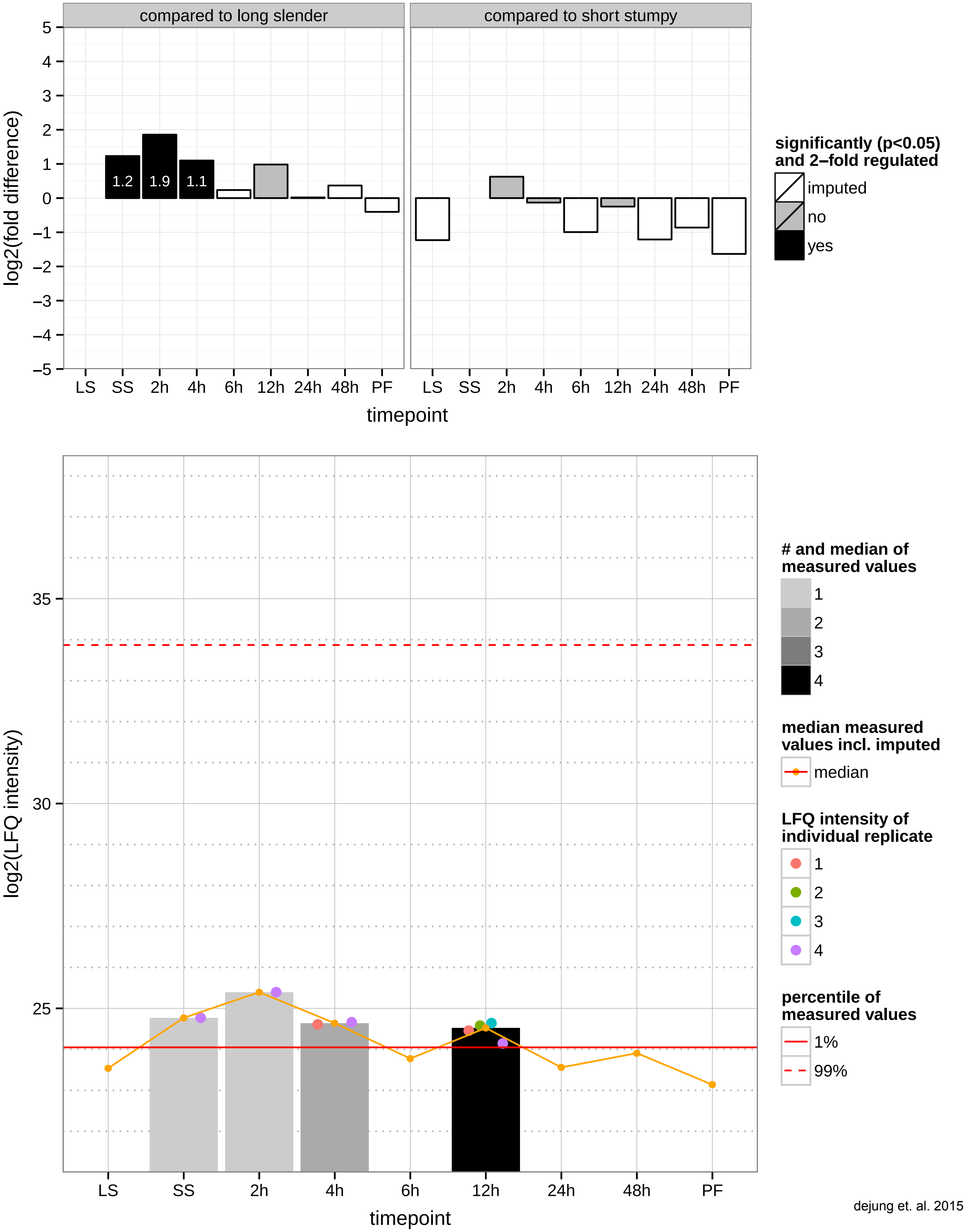

dejung et. al. 2015

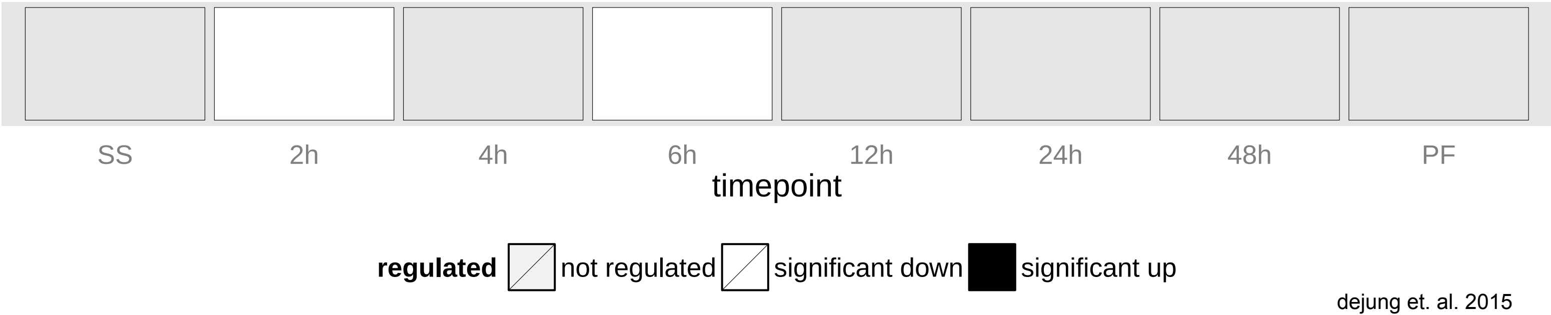

SS
2h
4h
6h
12h
24h
48h
PF
timepoint
regulated
not regulated
significant down
significant up
dejung et. al. 2015

aspartyl–tRNA synthetase, putative
Tb927.10.1260
AGOF: ATP binding, asparagine–tRNA ligase activity, aspartate–tRNA ligase activity, nucleic acid binding
AGOC: cytoplasm, mitochondrion
AGOP: asparaginyl–tRNA aminoacylation, aspartyl–tRNA aminoacylation, translation
PGOF: ATP binding, aminoacyl–tRNA ligase activity, nucleic acid binding, nucleotide binding
PGOC: cytoplasm
PGOP: tRNA aminoacylation for protein translation

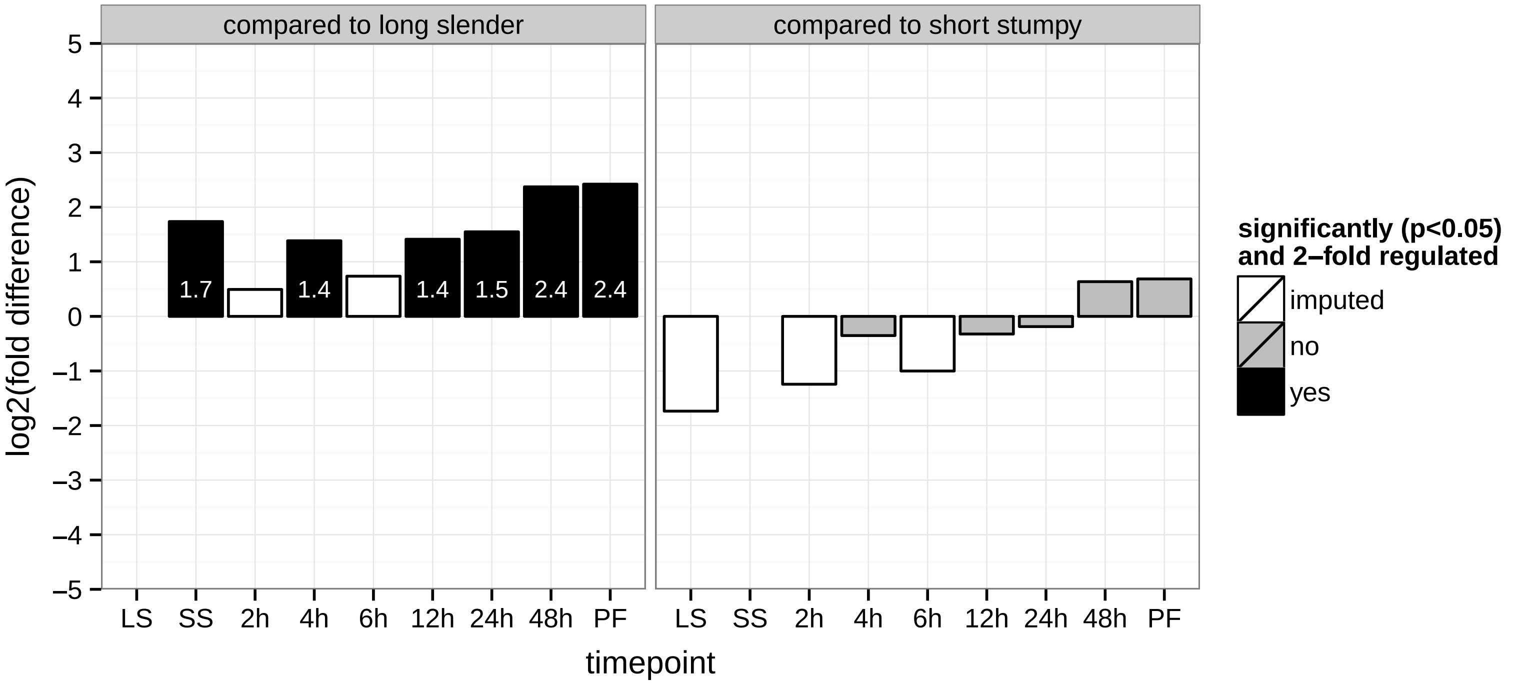

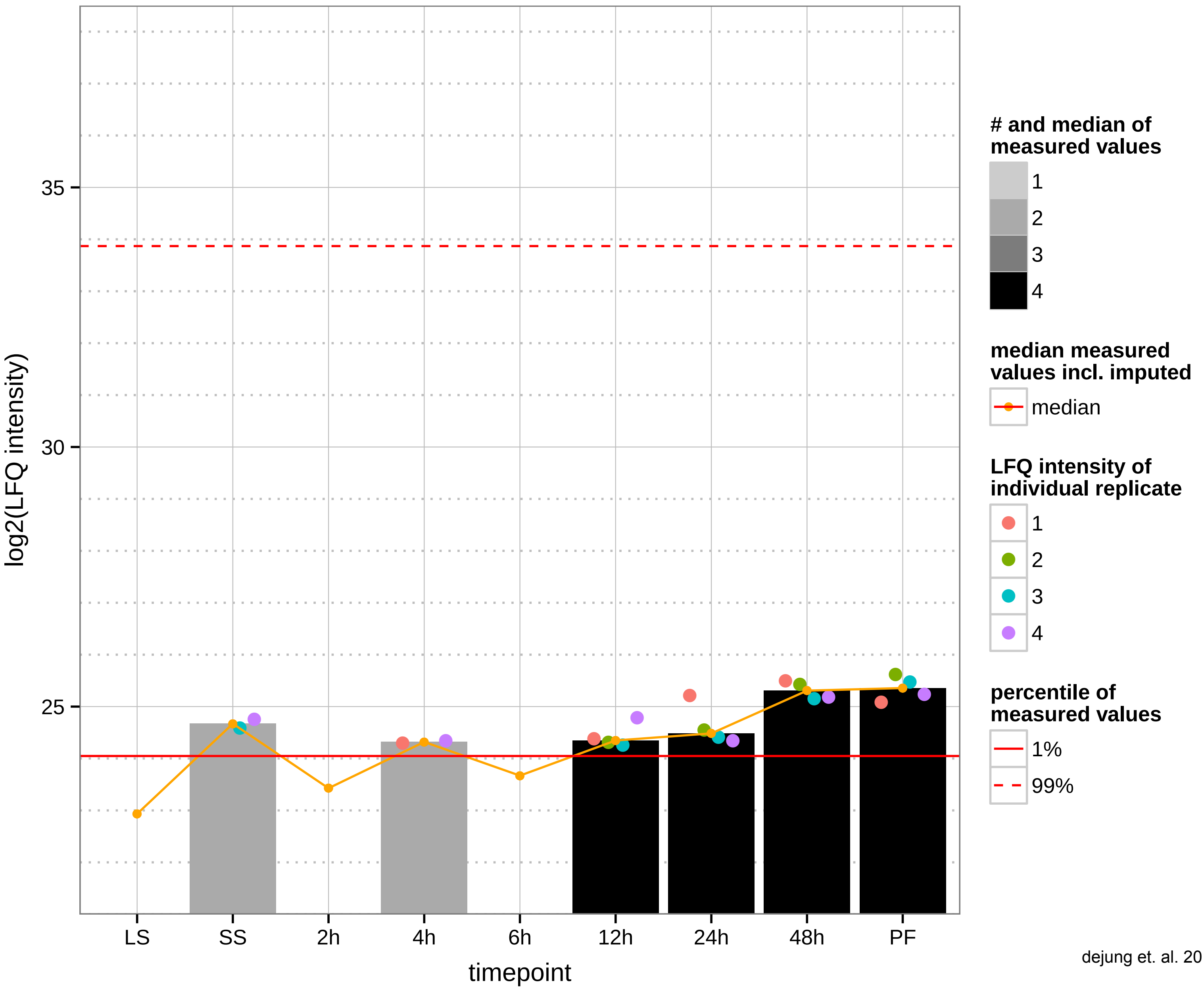

dejung et. al. 2015

hypothetical protein
Tb927.10.8880
AGOF: null
AGOC: null
AGOP: null
PGOF: null
PGOC: null
PGOP: null

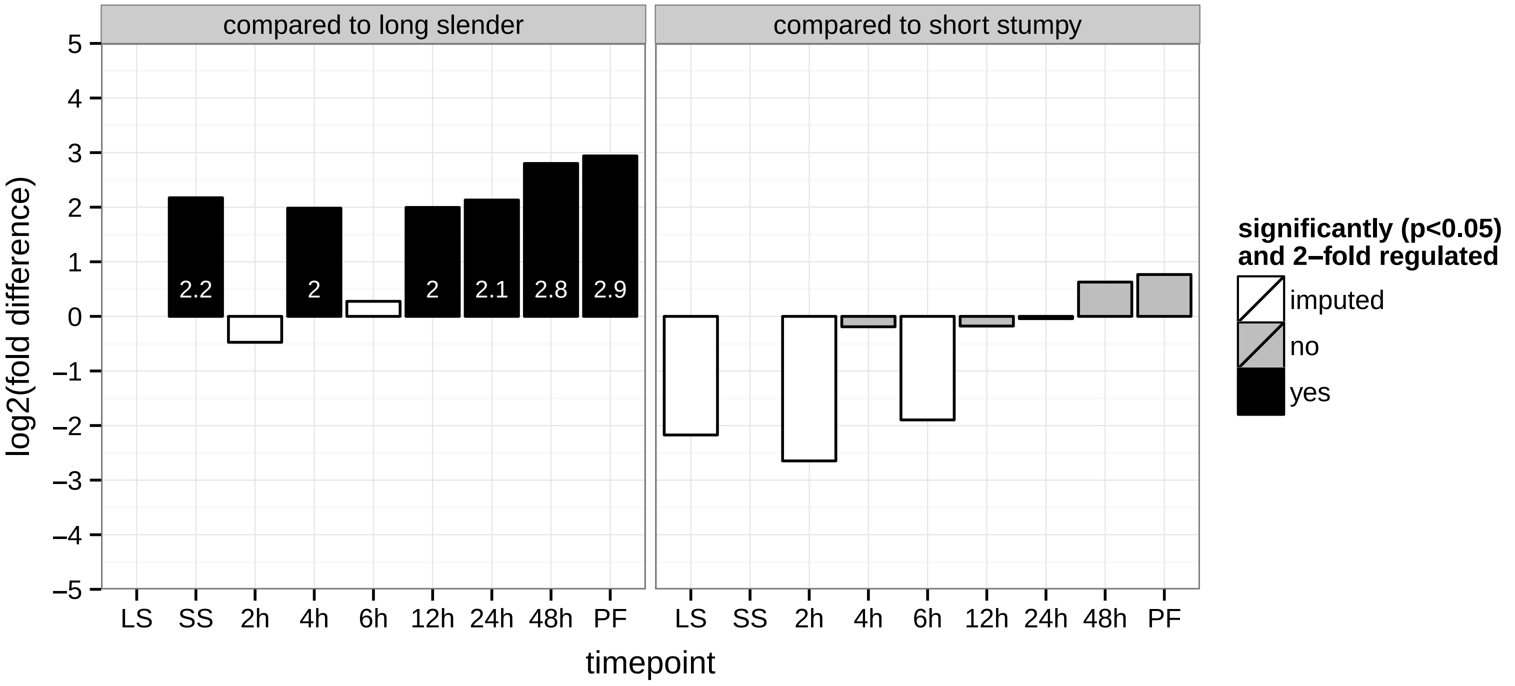

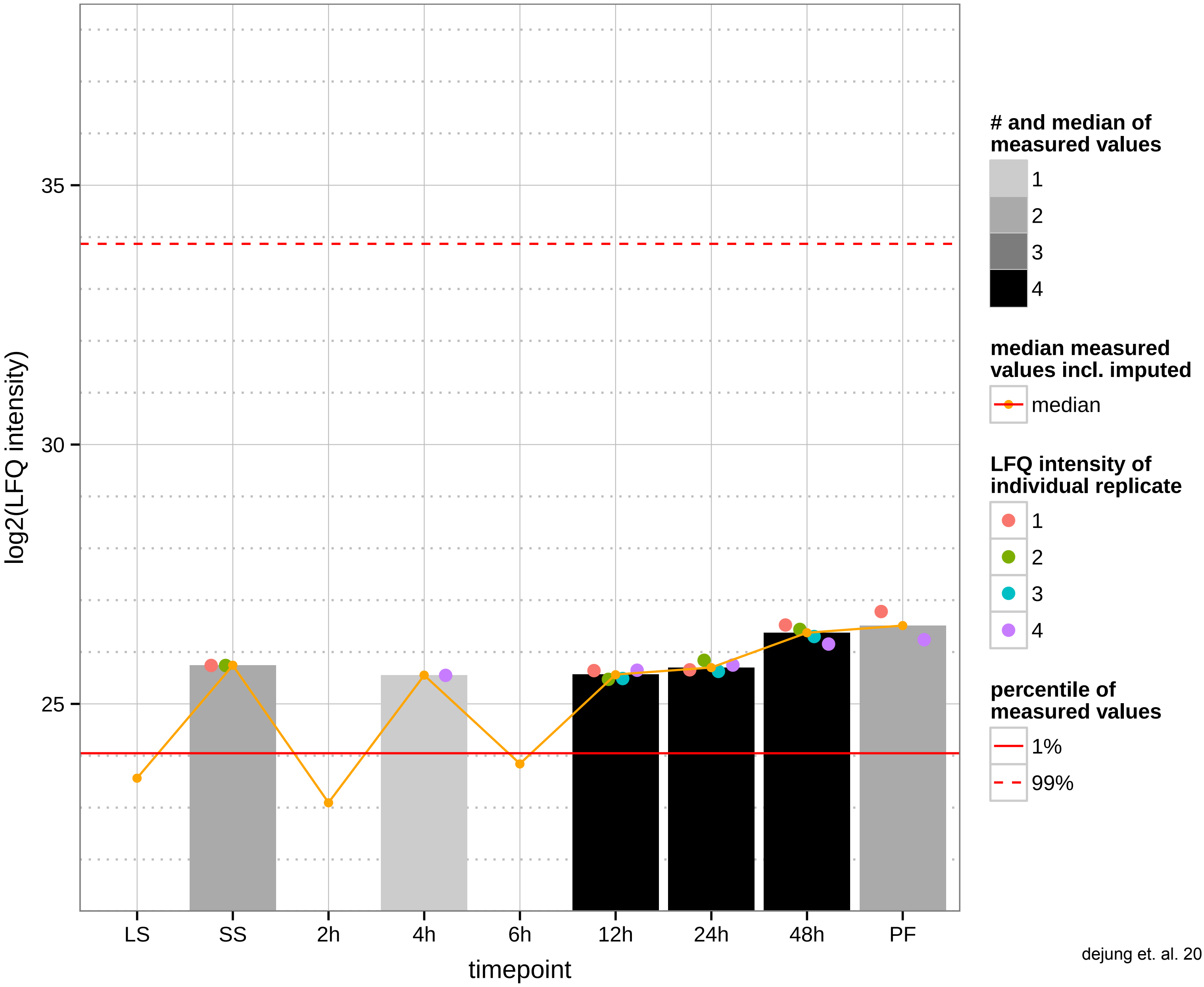

dejung et. al. 2015

RNA–binding protein, putative
Tb927.11.350
AGOF: null
AGOC: eukaryotic translation initiation factor 4F complex
AGOP: null
PGOF: nucleic acid binding
PGOC: null
PGOP: null

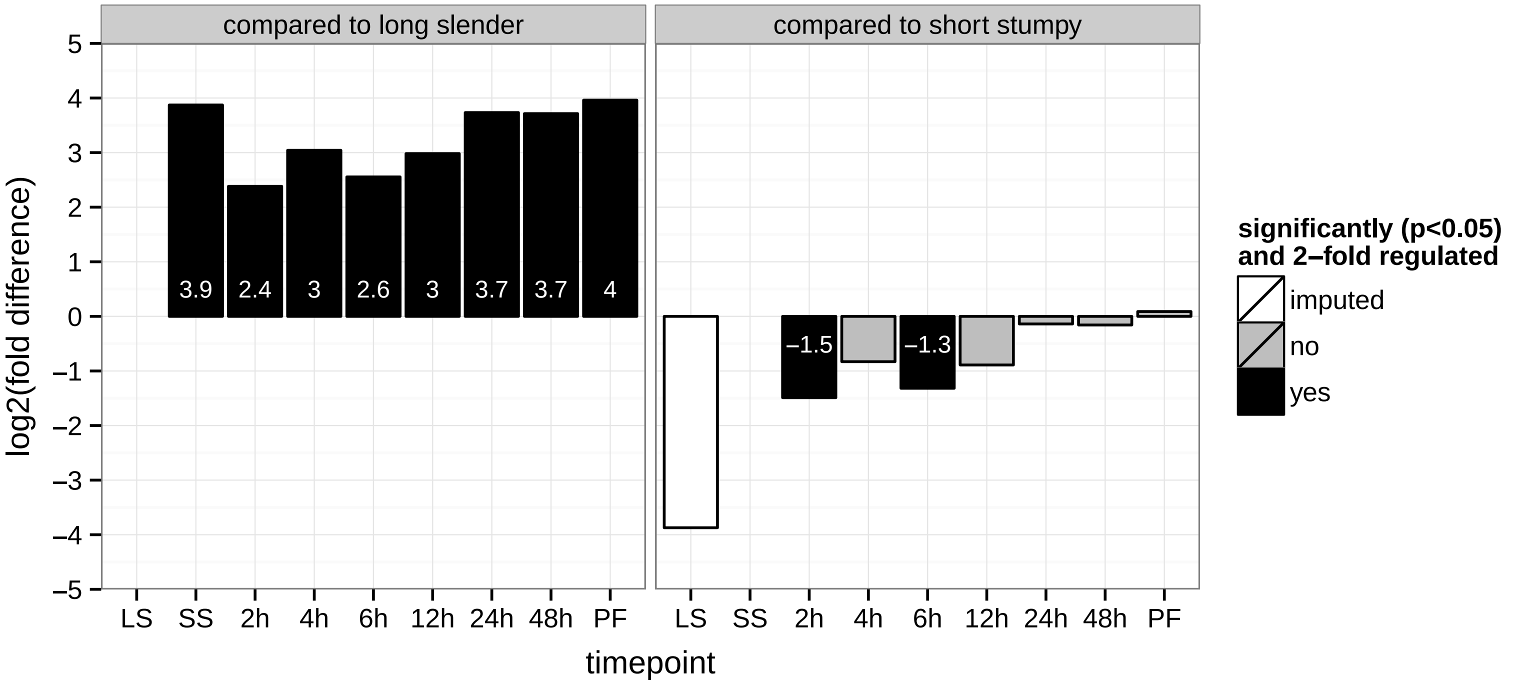

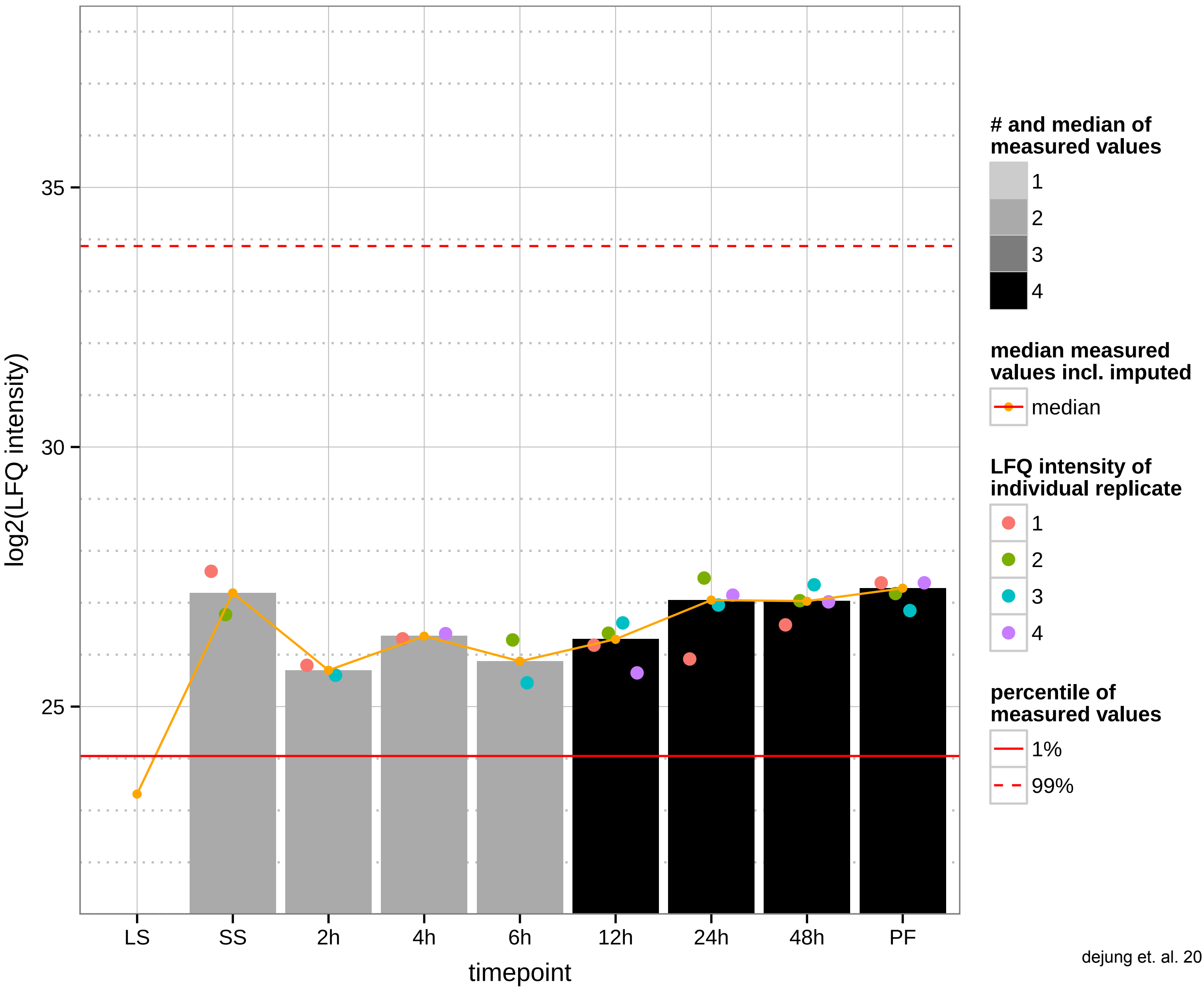

dejung et. al. 2015

ubiquitin carboxyl–terminal hydrolase, putative, cysteine peptidase, Clan CA, family C19
Tb927.11.5270
AGOF: cysteine–type endopeptidase activity, ubiquitin thiolesterase activity
AGOC: null
AGOP: ubiquitin–dependent protein catabolic process
PGOF: ubiquitin thiolesterase activity
PGOC: null
PGOP: ubiquitin–dependent protein catabolic process

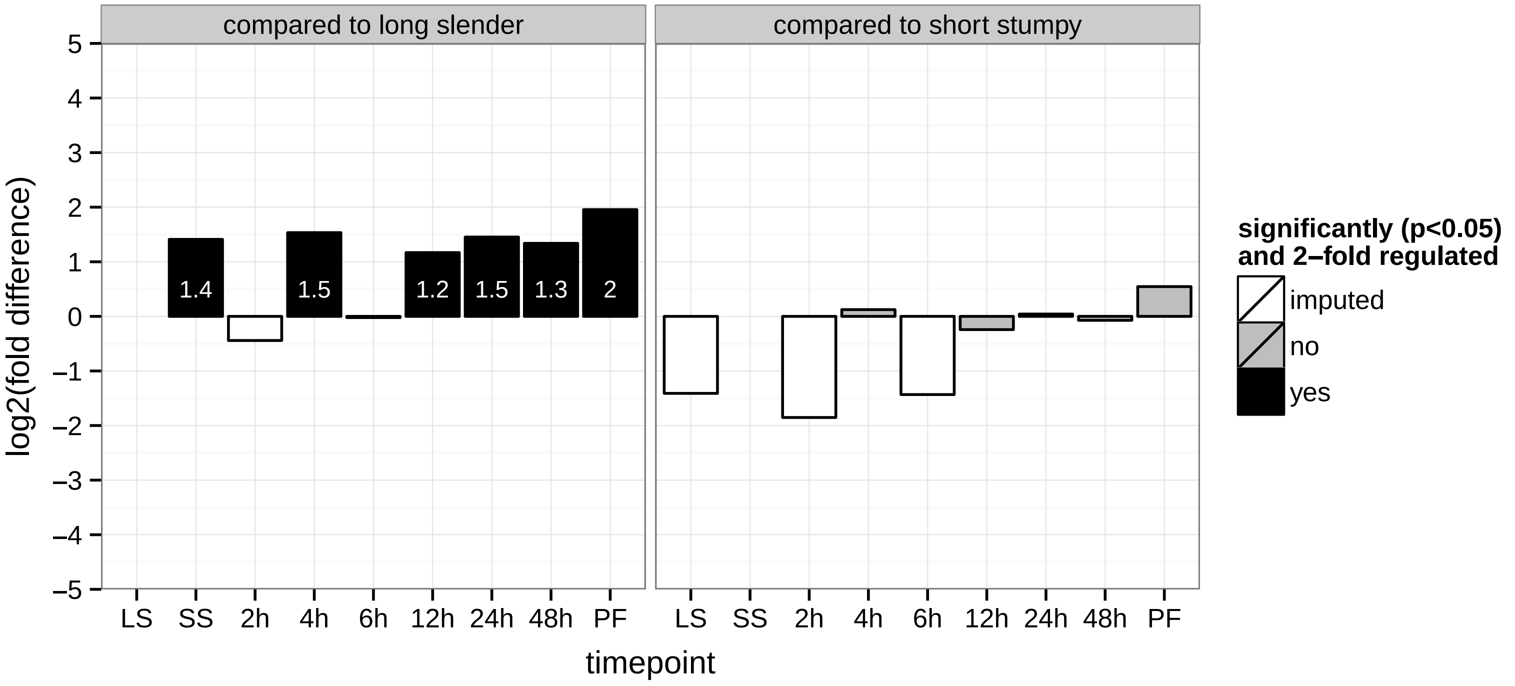

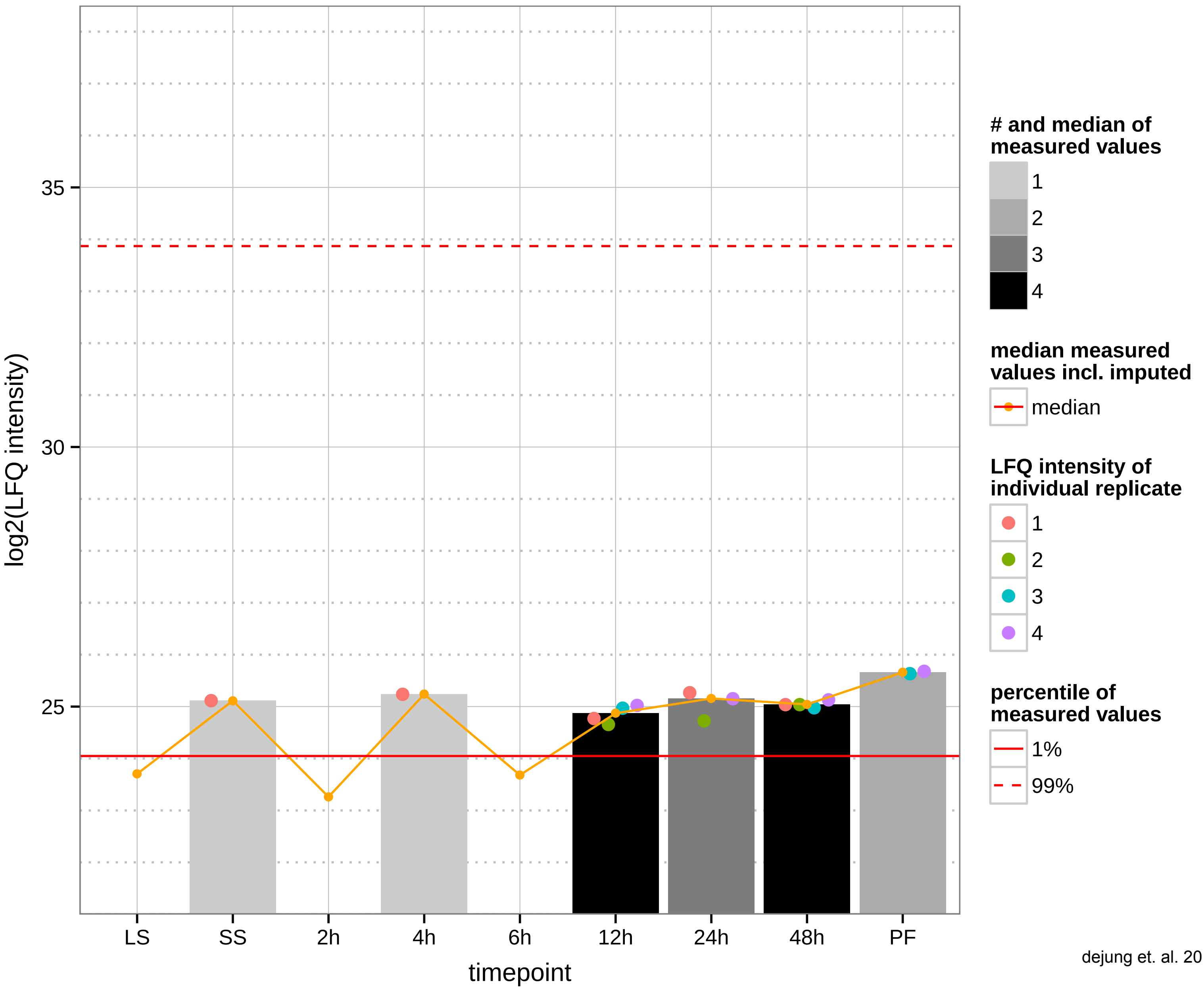

dejung et. al. 2015

ubiquitin–conjugating enzyme E2, putative
Tb927.2.2460
AGOF: ubiquitin–protein ligase activity
AGOC: nucleus
AGOP: cellular protein modification process, post–translational protein modification, regulation of protein metabolic process
PGOF: acid–amino acid ligase activity
PGOC: null
PGOP: null

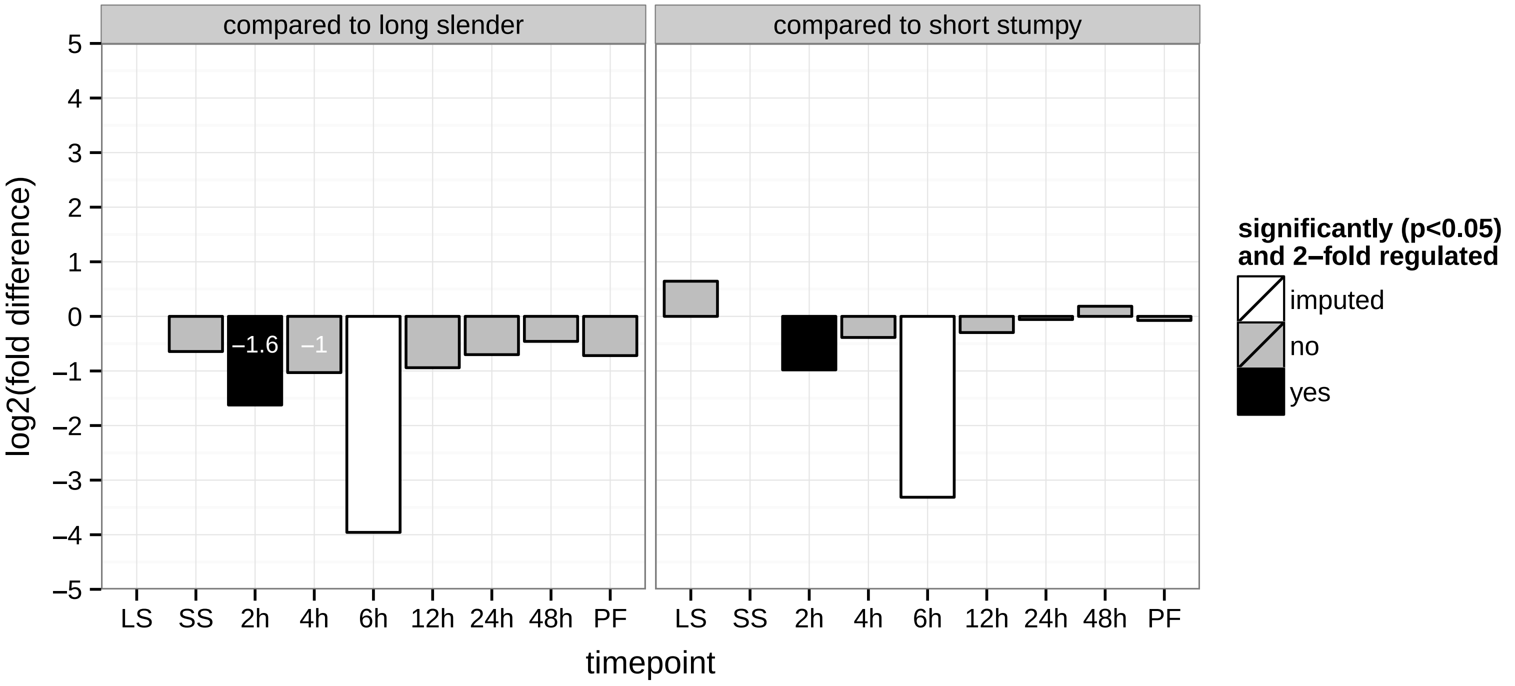

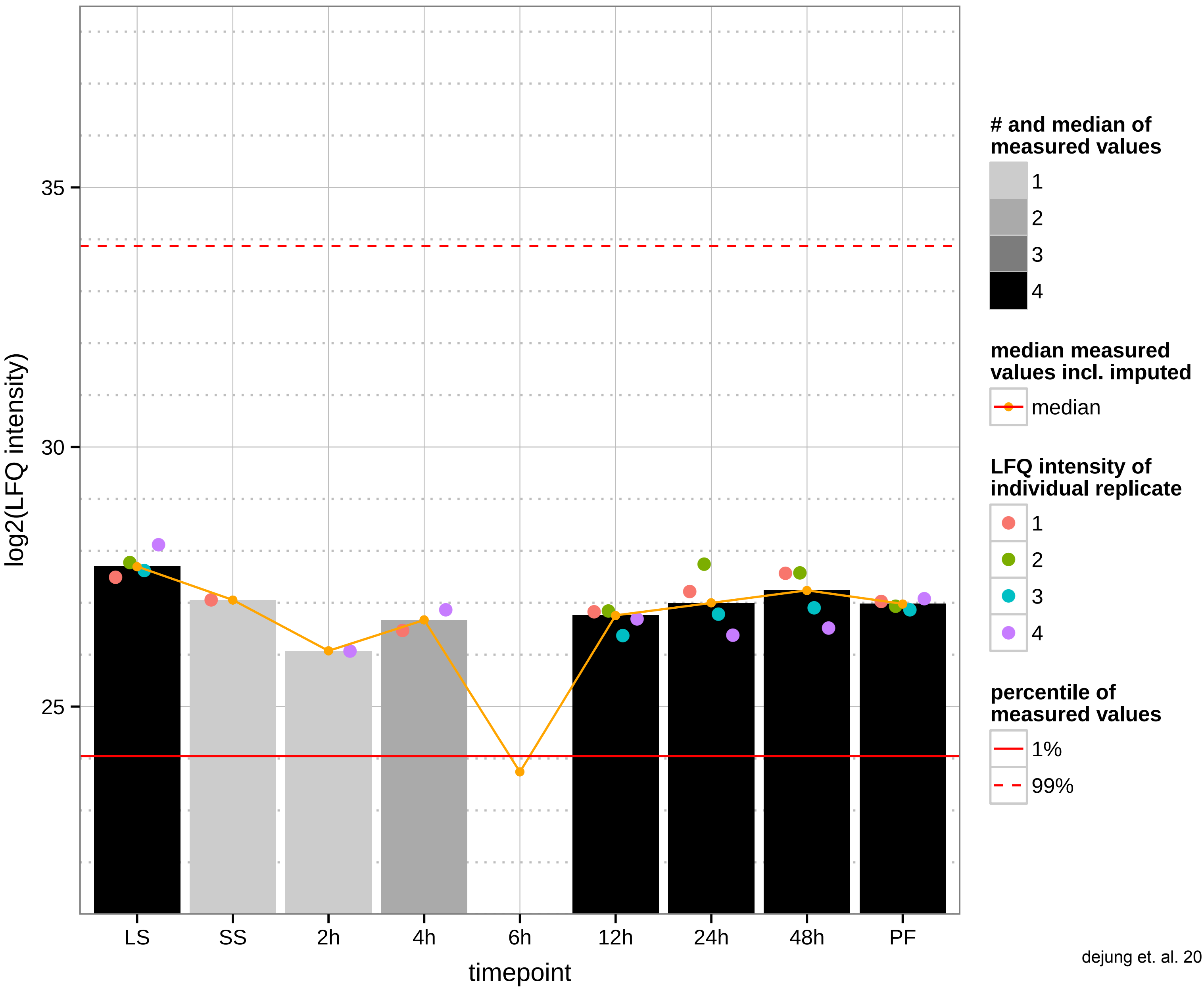

dejung et. al. 2015

50S ribosomal protein L13, putative
Tb927.4.1070
AGOF: structural constituent of ribosome
AGOC: intracellular, mitochondrion, ribosome
AGOP: translation
PGOF: structural constituent of ribosome
PGOC: ribosome
PGOP: translation

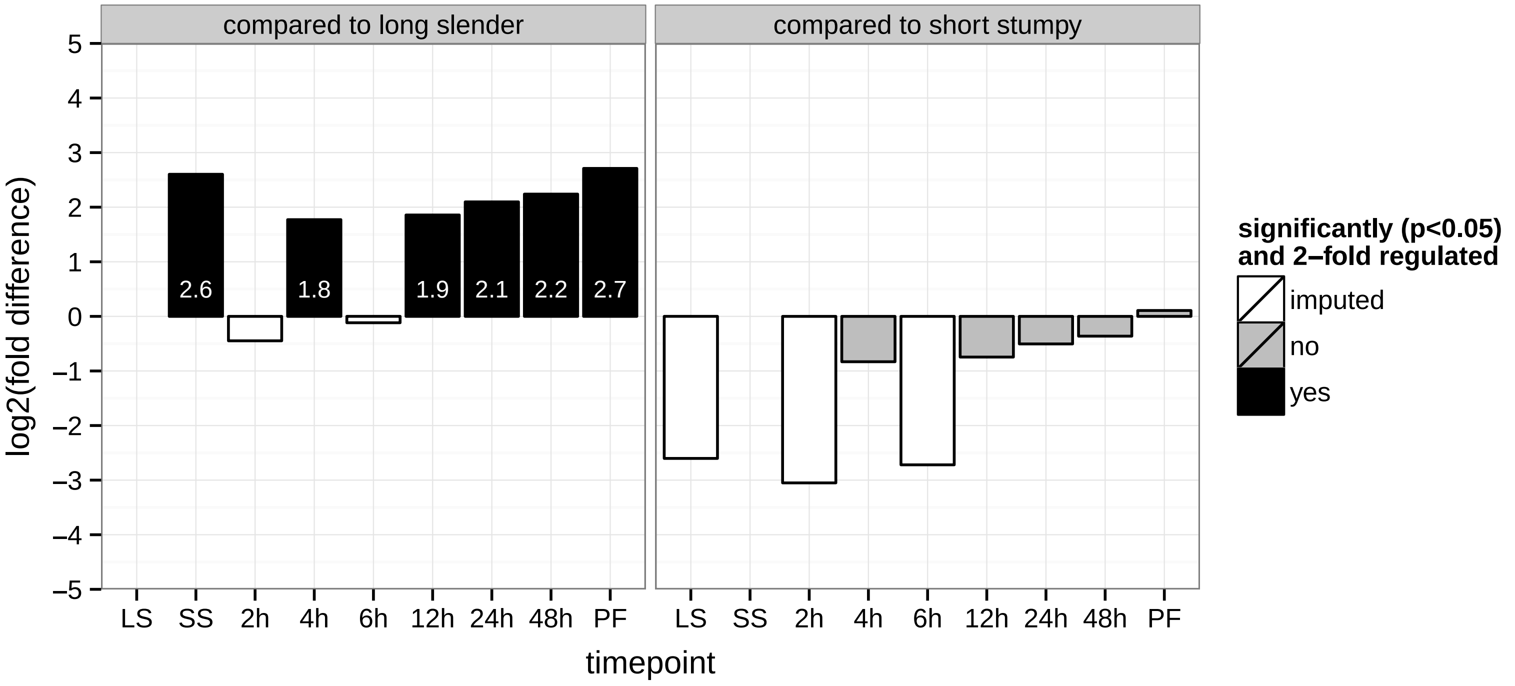

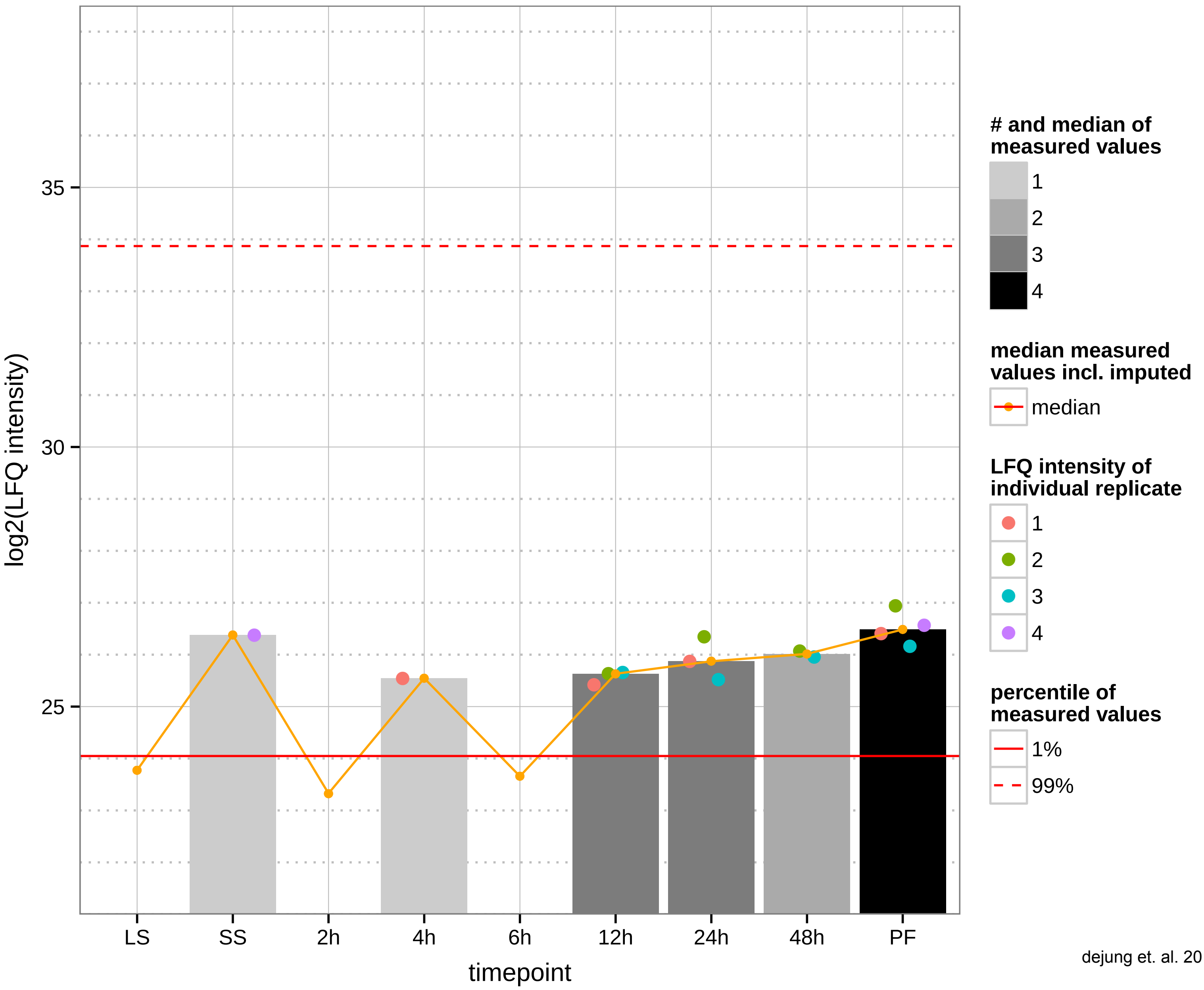

dejung et. al. 2015

Alkylated DNA repair protein (alkB homolog), putative
Tb927.4.460
AGOF: null
AGOC: null
AGOP: null
PGOF: oxidoreductase activity, oxidoreductase activity, acting on paired donors, with incorporation or reduction of molecular o
PGOC: null
PGOP: oxidation−reduction process

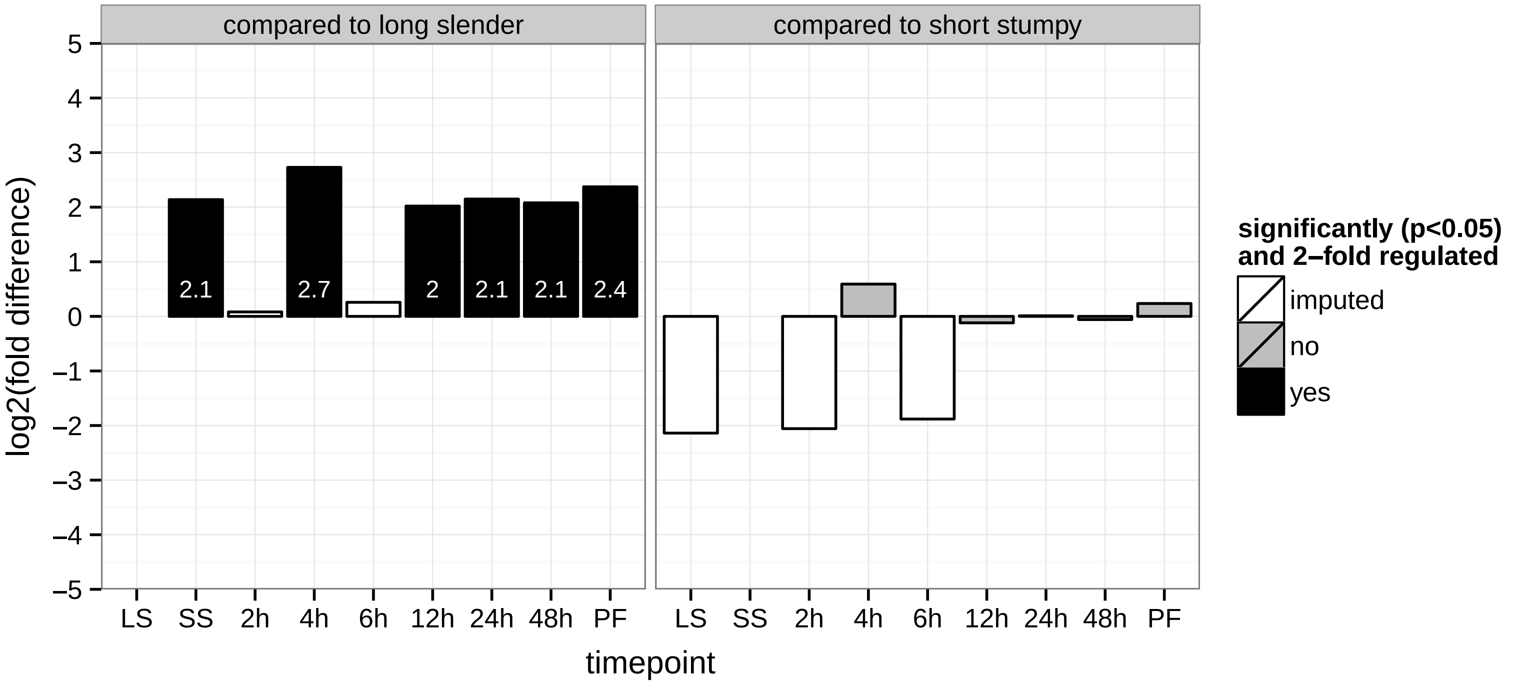

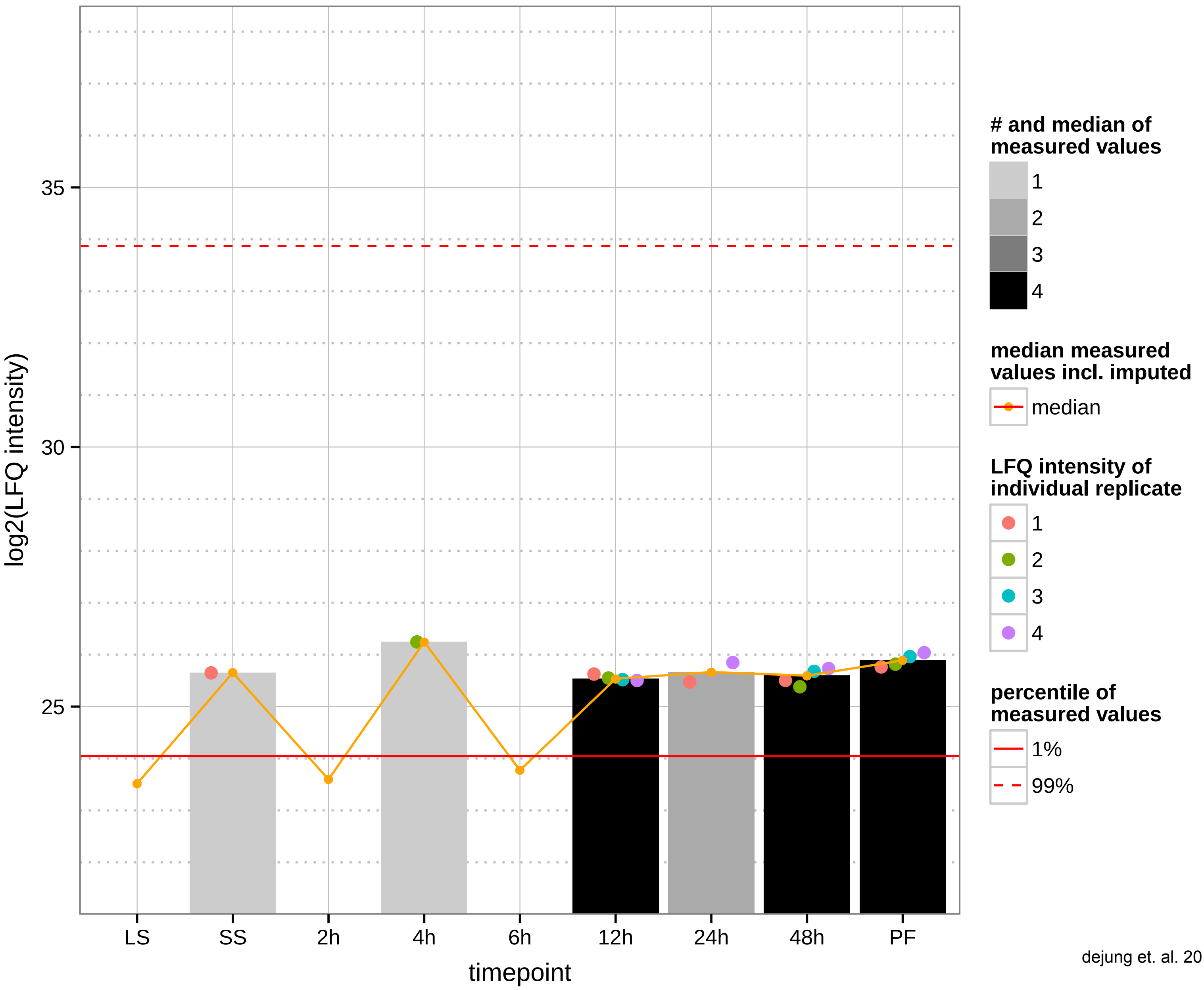

dejung et. al. 2015

hypothetical protein, conserved
Tb927.5.3730
AGOF: null
AGOC: null
AGOP: null
PGOF: null
PGOC: null
PGOP: null

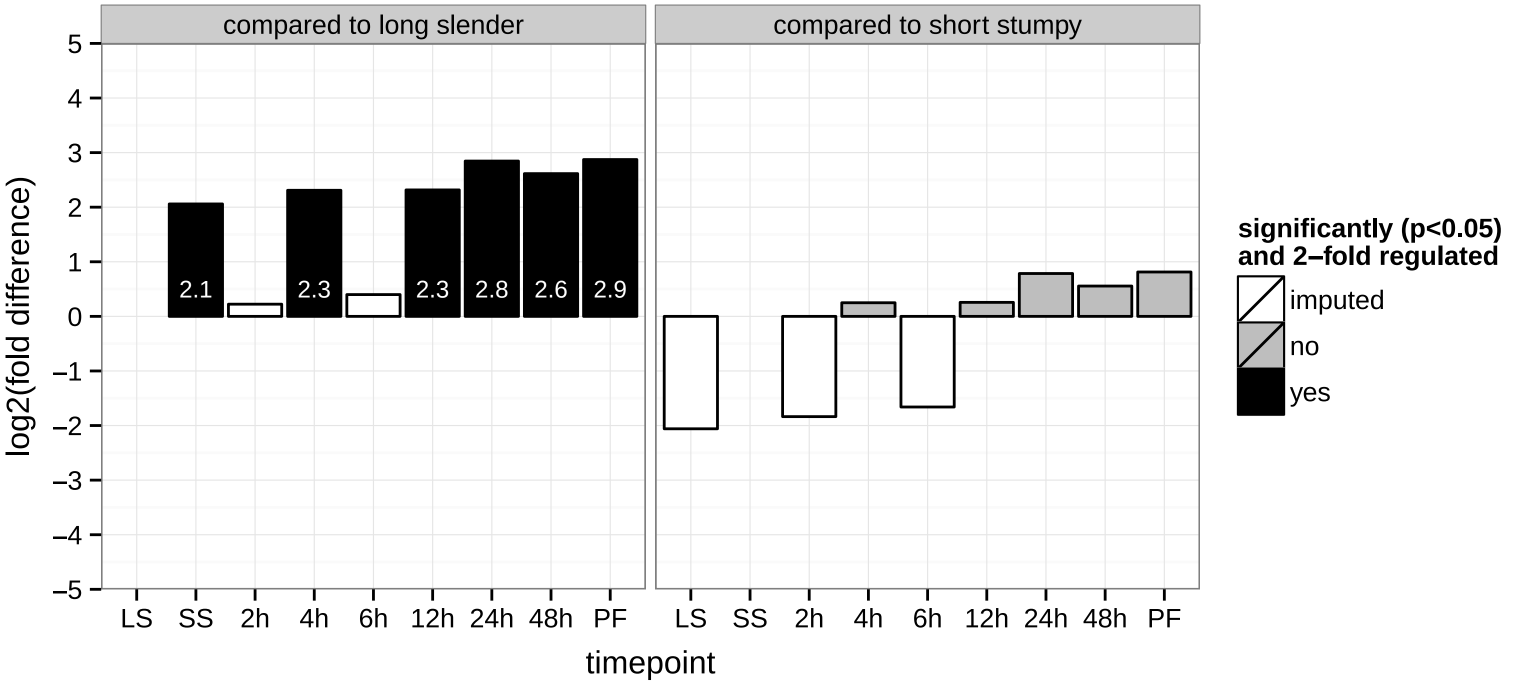

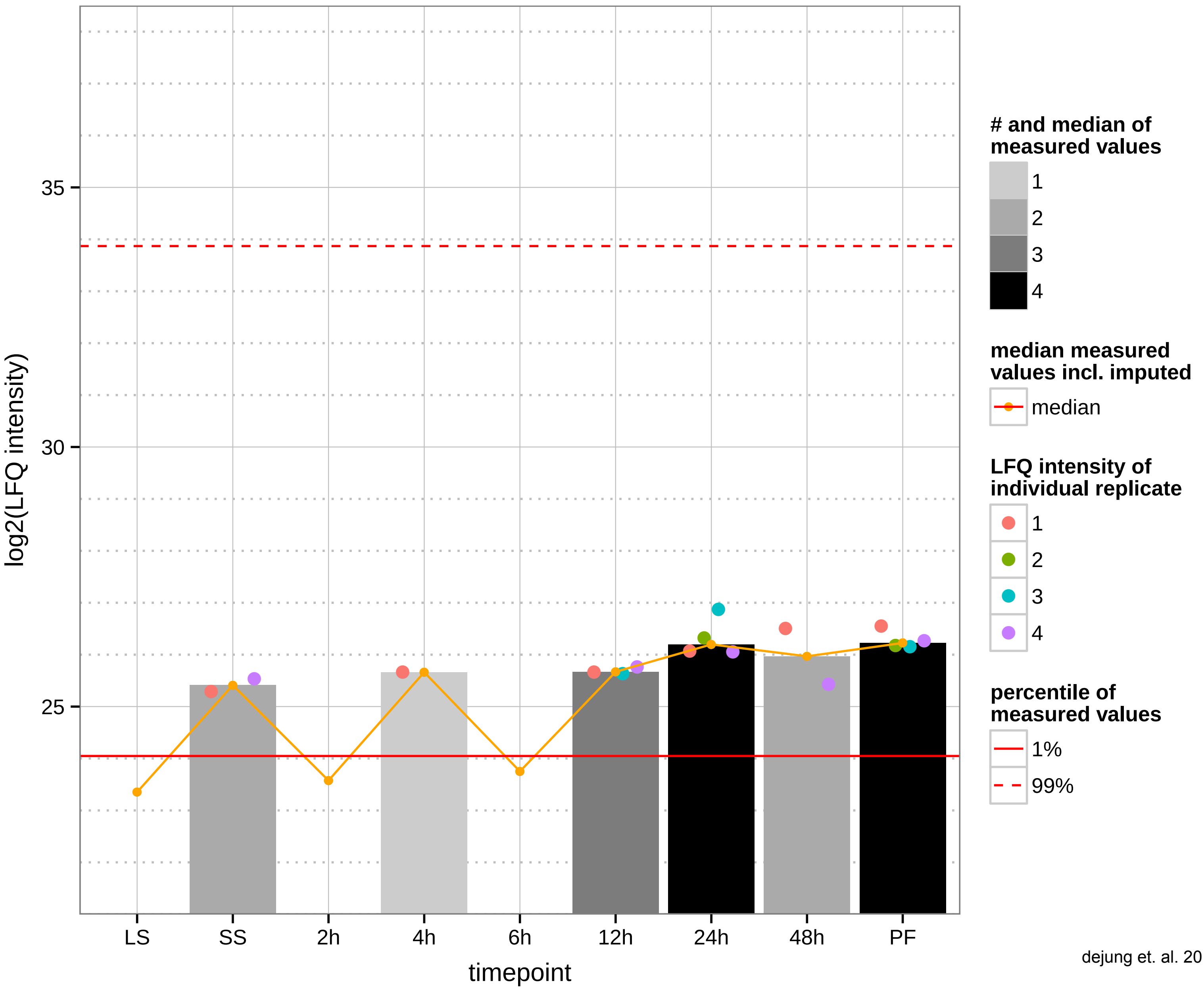

dejung et. al. 2015

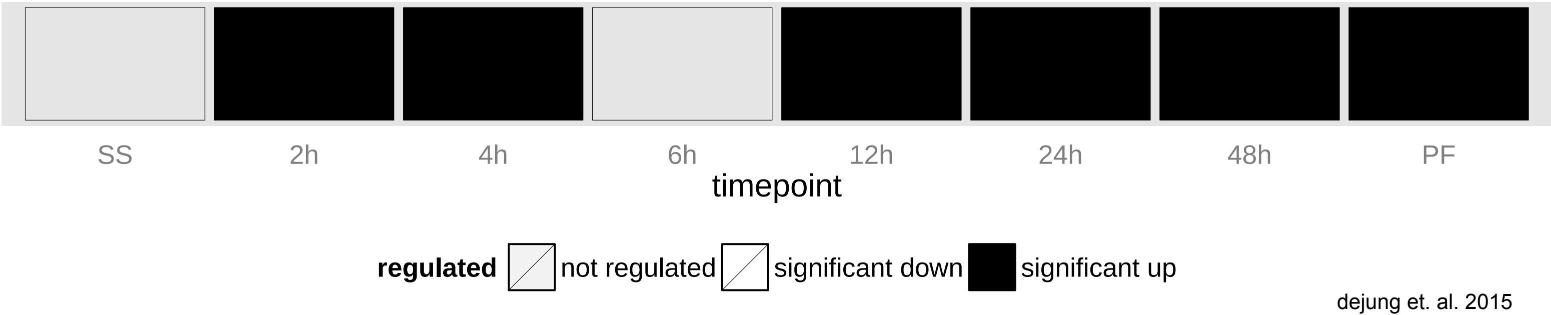
SS
2h
4h
6h
12h
24h
48h
PF
timepoint
regulated
not regulated
significant down
significant up
dejung et. al. 2015

NADH–ubiquinone oxidoreductase complex I subunit, putative, ferredoxin, putative (NDUFS1)
Tb927.10.12540
AGOF: electron carrier activity, glutathione–disulfide reductase activity, iron–sulfur cluster binding
AGOC: mitochondrion
AGOP: oxidation–reduction process
PGOF: electron carrier activity, iron–sulfur cluster binding, oxidoreductase activity
PGOC: null
PGOP: oxidation–reduction process

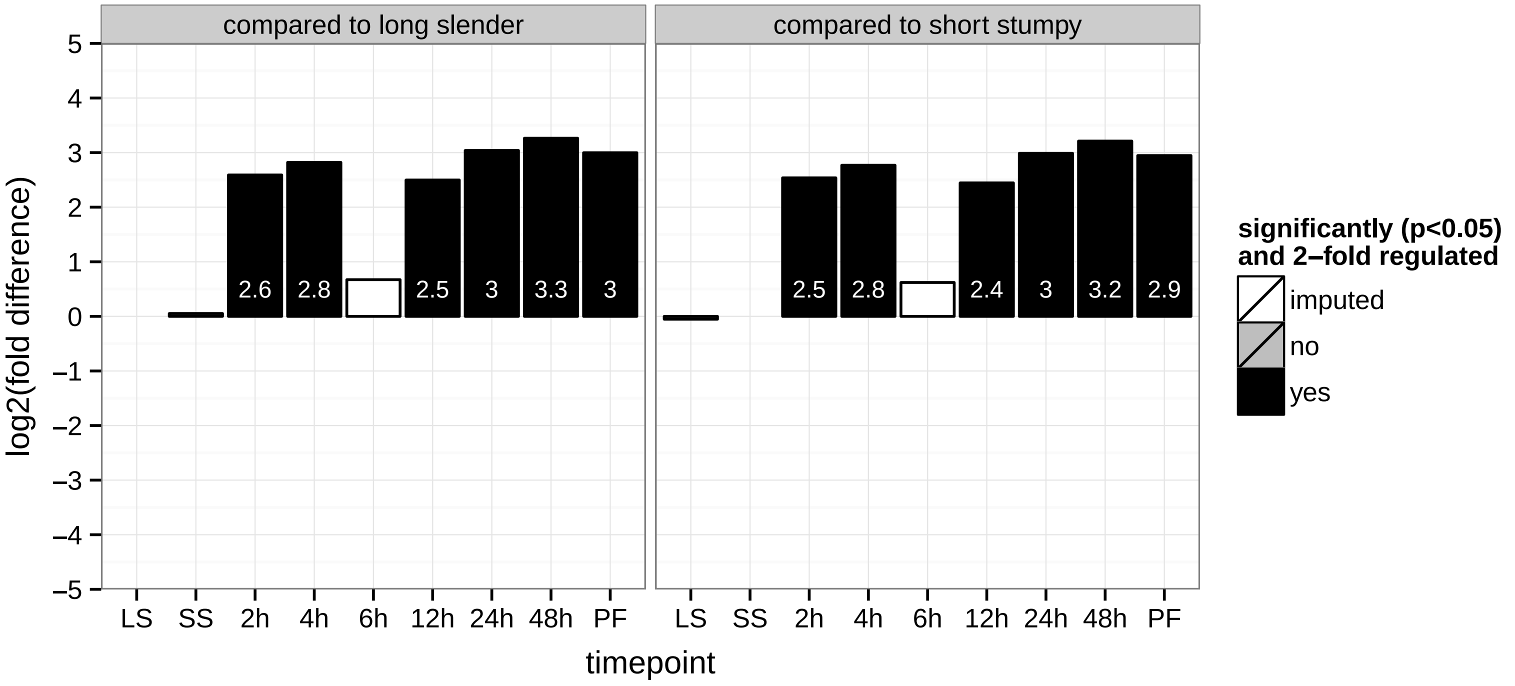

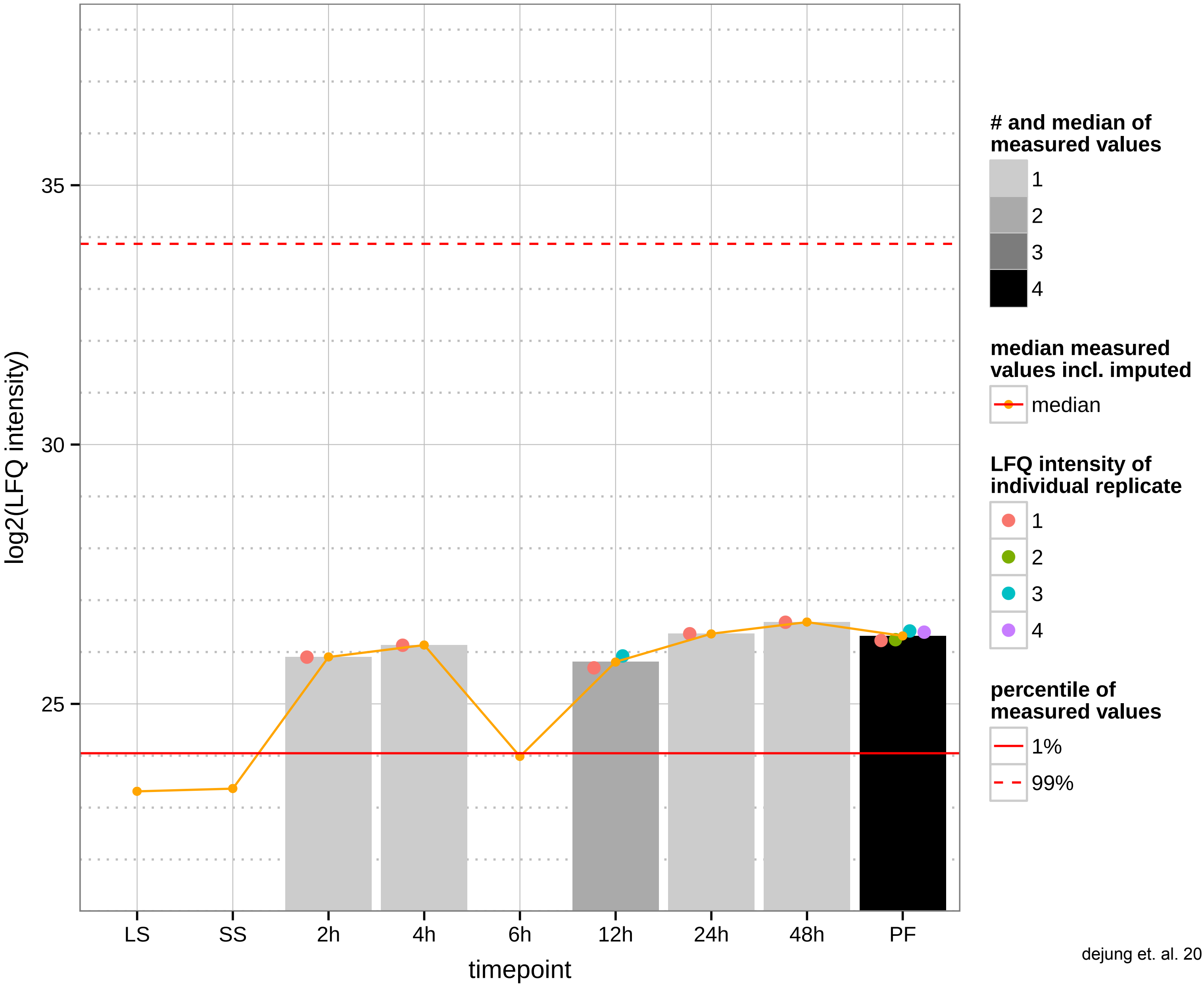

dejung et. al. 2015

survival of motor neuron (SMN)–like protein (TbSMN)
Tb927.11.15000
AGOF: null
AGOC: nucleus
AGOP: null
PGOF: null
PGOC: null
PGOP: null

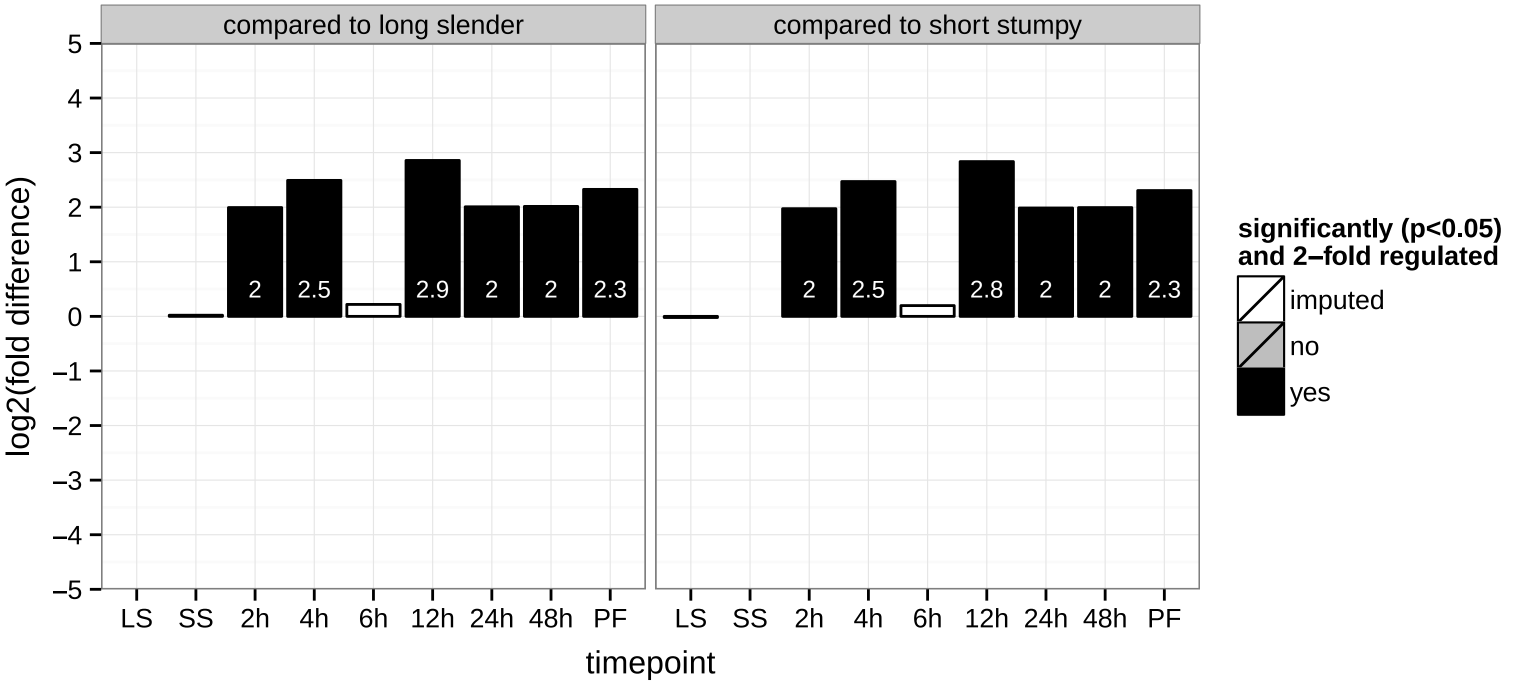

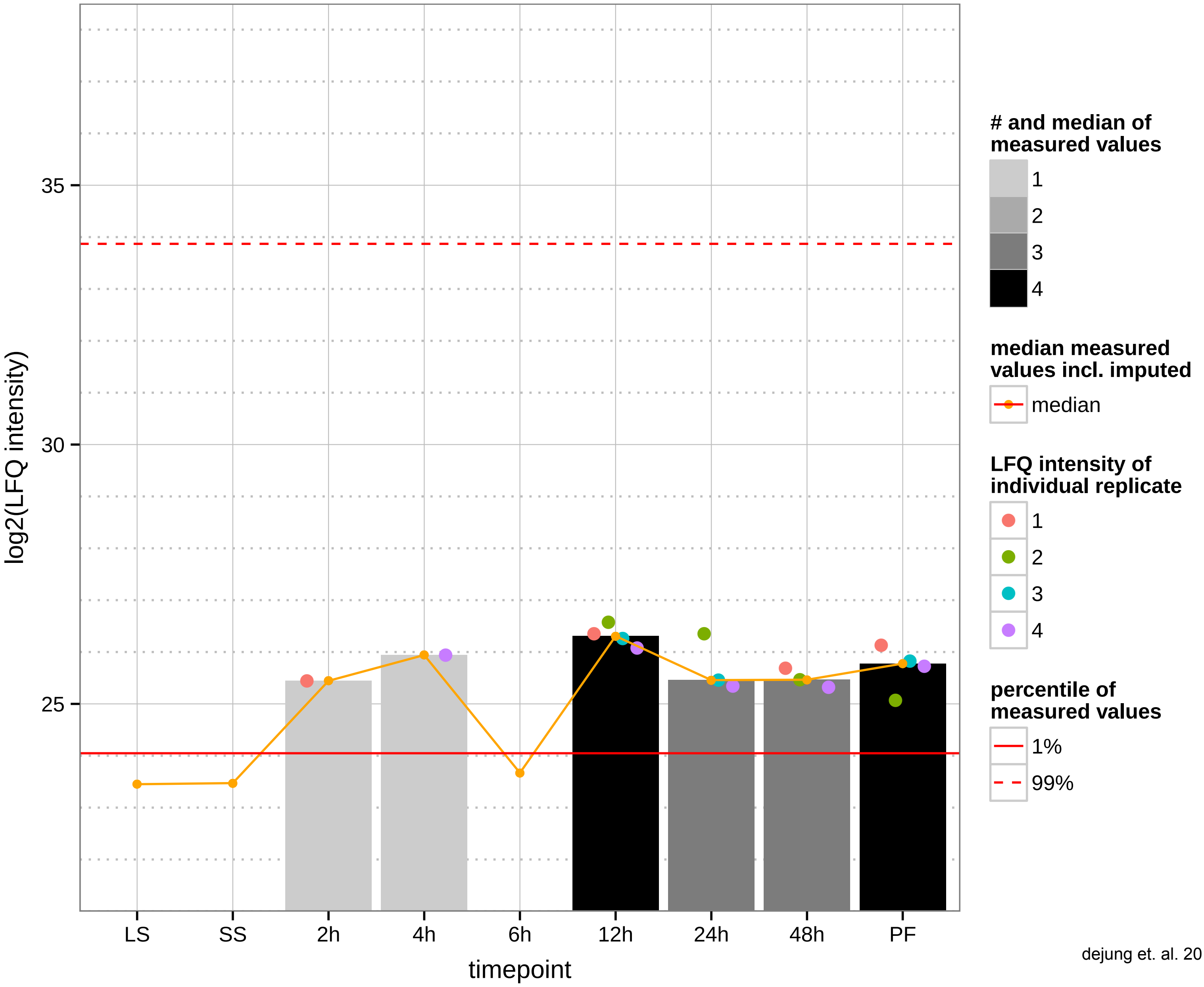

dejung et. al. 2015

kinteoplast poly(A) polymerase complex 1 subunit
Tb927.11.15850
AGOF: RNA binding
AGOC: mitochondrion
AGOP: RNA polyadenylation, mRNA stabilization
PGOF: null
PGOC: null
PGOP: null

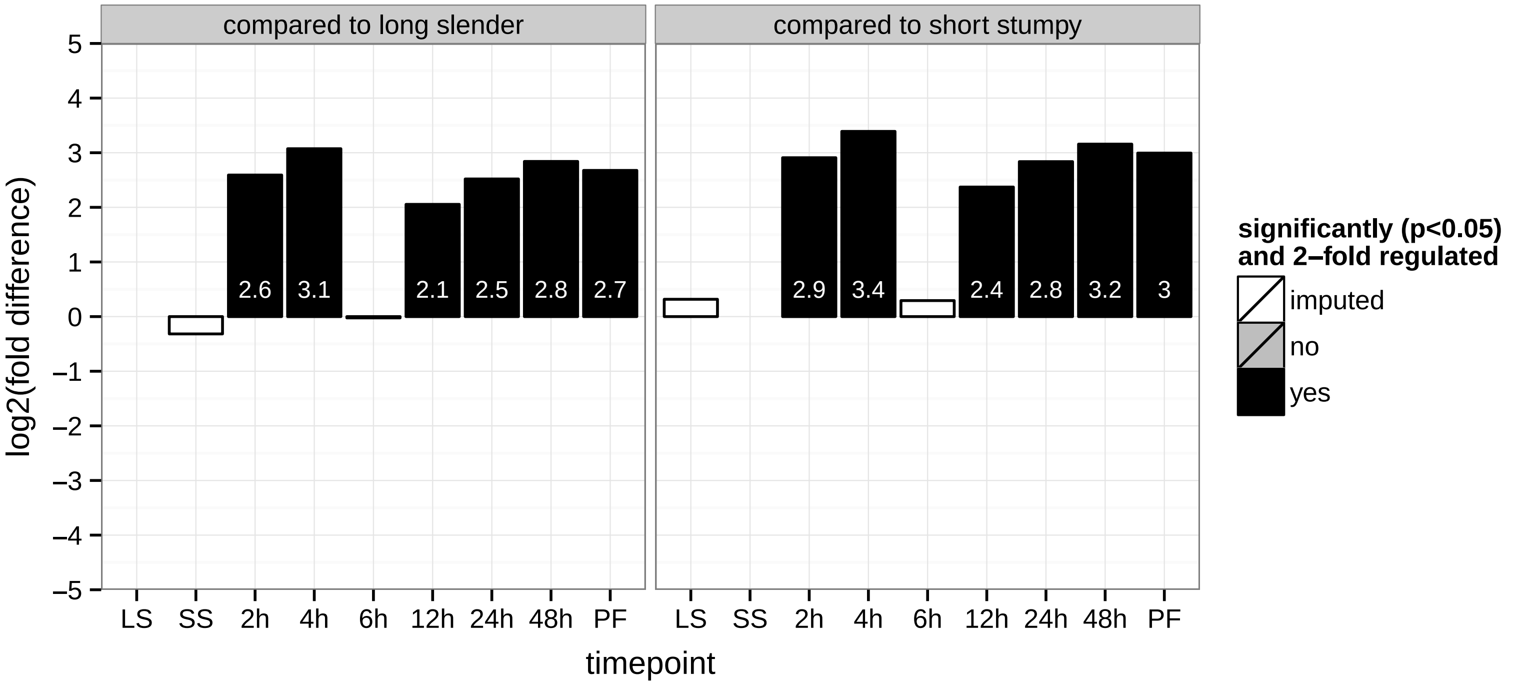

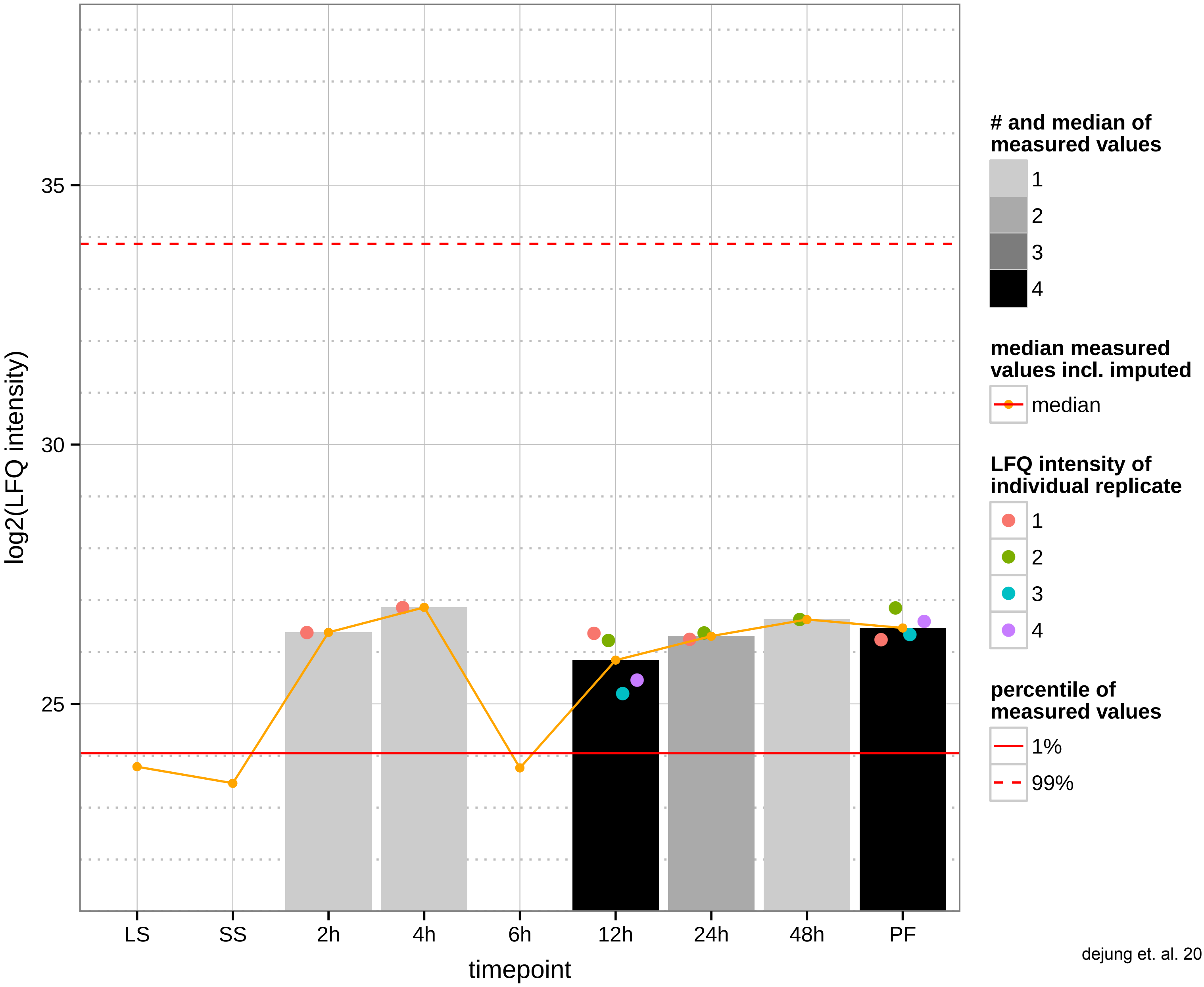

dejung et. al. 2015

hypothetical protein, conserved
Tb927.6.2560
AGOF: null
AGOC: null
AGOP: null
PGOF: null
PGOC: null
PGOP: null

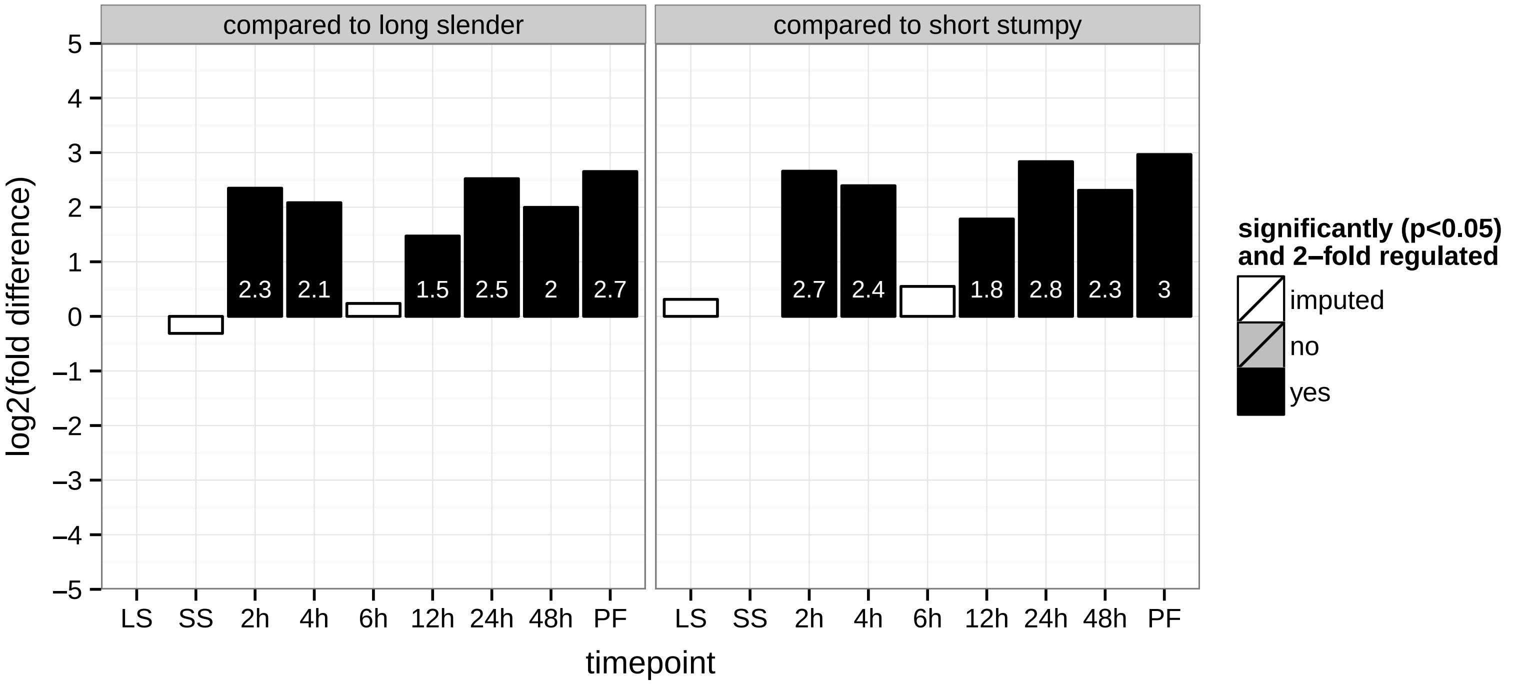

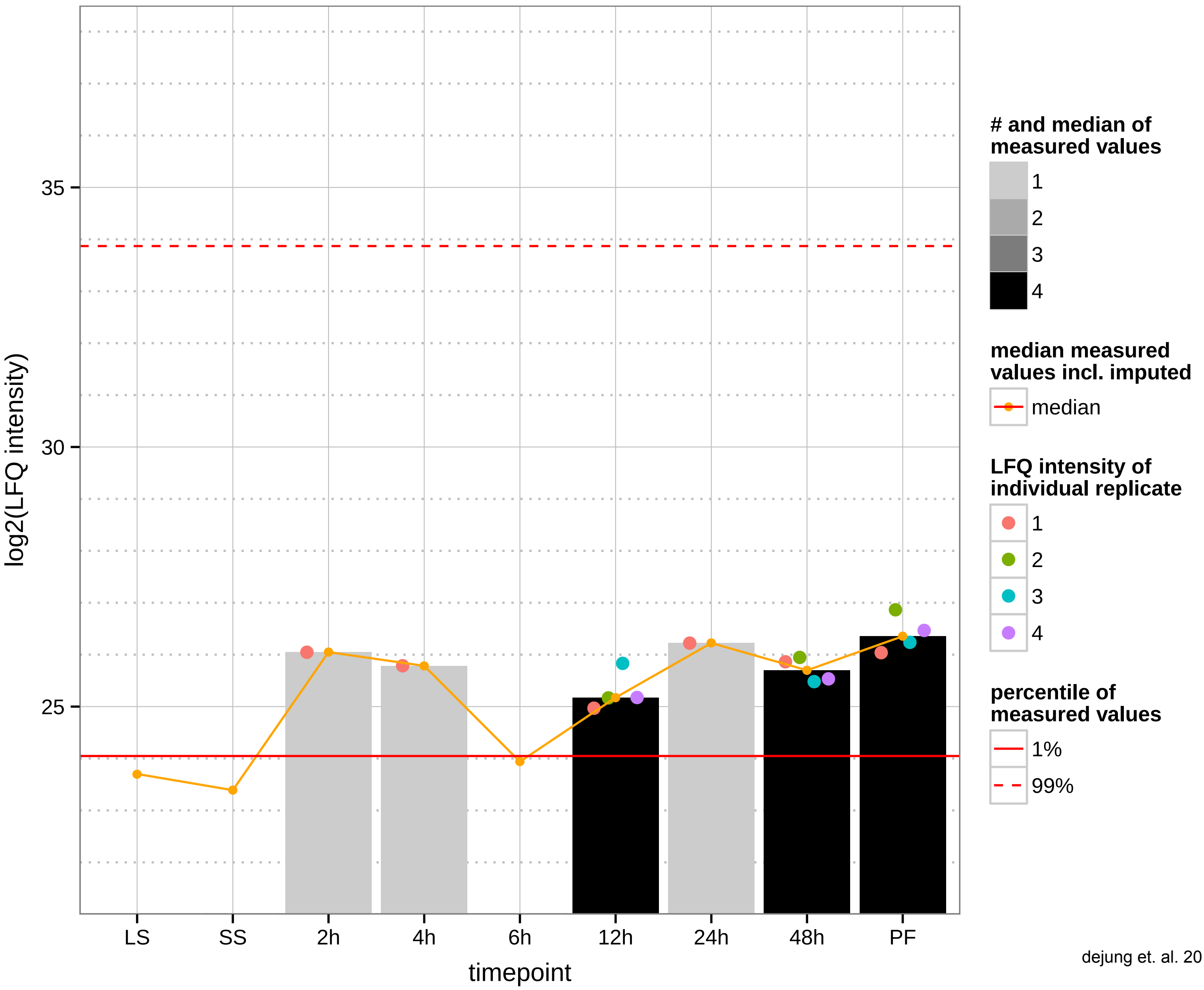

dejung et. al. 2015

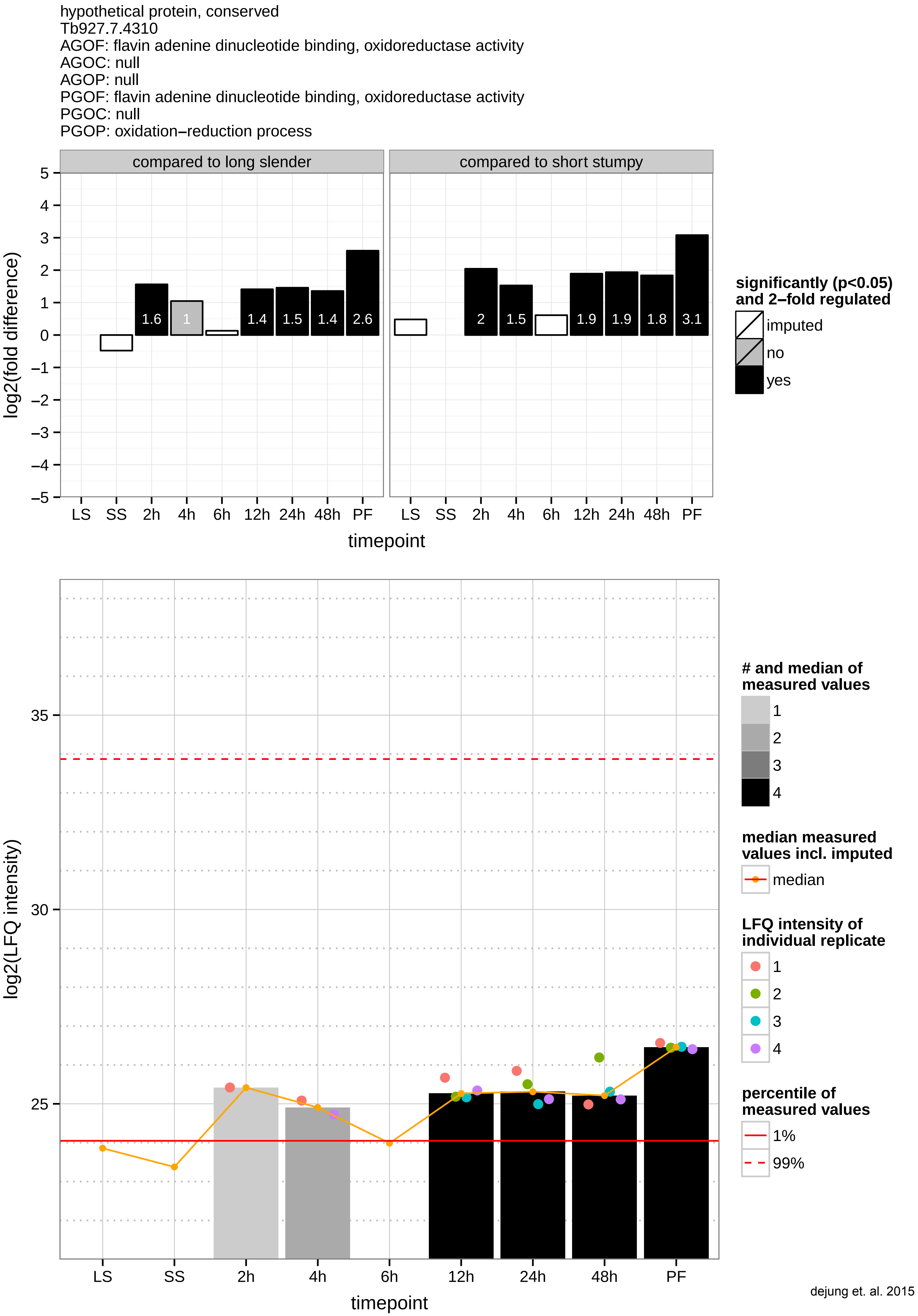
hypothetical protein, conserved
Tb927.7.4310
AGOF: flavin adenine dinucleotide binding, oxidoreductase activity
AGOC: null
AGOP: null
PGOF: flavin adenine dinucleotide binding, oxidoreductase activity
PGOC: null
PGOP: oxidation–reduction process
compared to long slender
compared to short stumpy
log2(fold difference)
timepoint
LS
SS
2h
4h
6h
12h
24h
48h
PF
1.6
1
1.4
1.5
1.4
2.6
2
1.5
1.9
1.9
1.8
3.1
significantly (p<0.05) and 2–fold regulated
imputed
no
yes
log2(LFQ intensity)
# and median of measured values
median measured values incl. imputed
median
LFQ intensity of individual replicate
percentile of measured values
1%
99%
dejung et. al. 2015

WD–repeat containing protein
Tb927.8.3860
AGOF: null
AGOC: null
AGOP: null
PGOF: protein binding
PGOC: null
PGOP: null

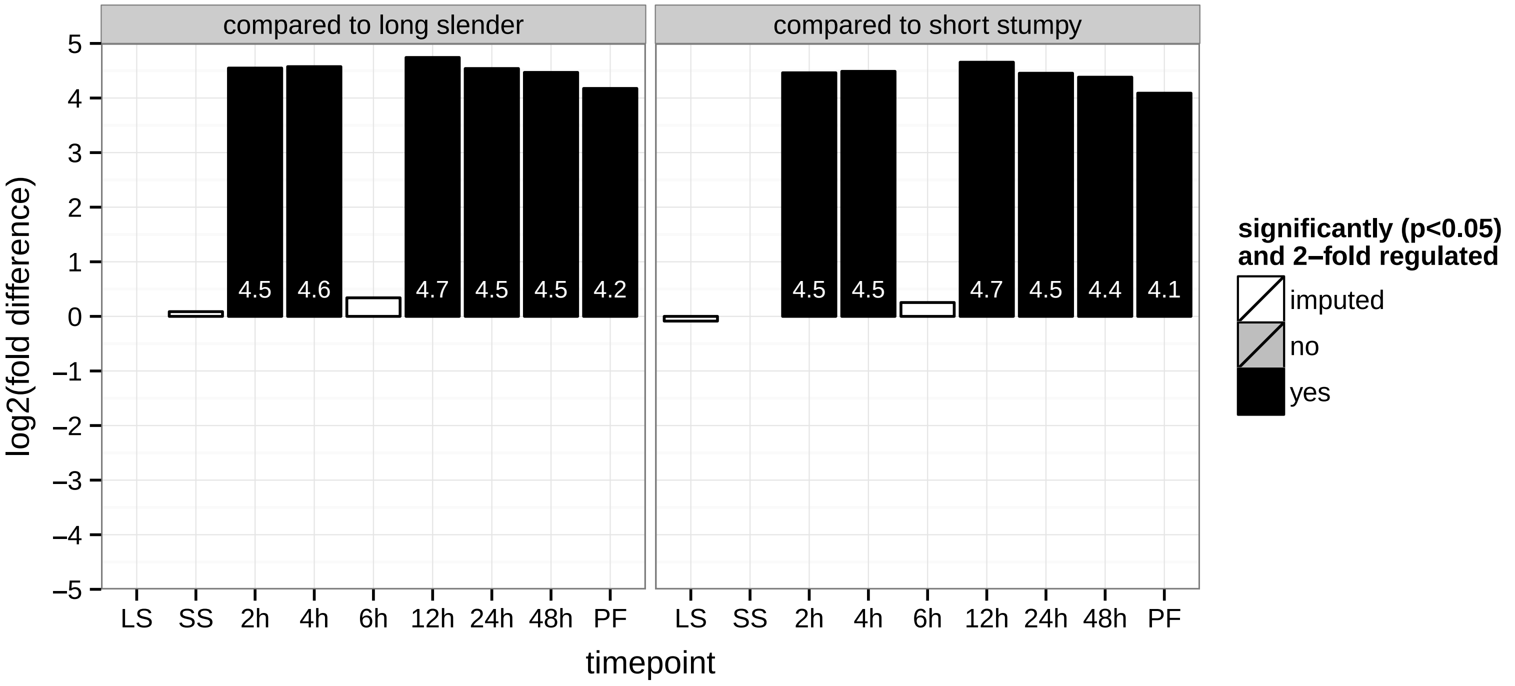

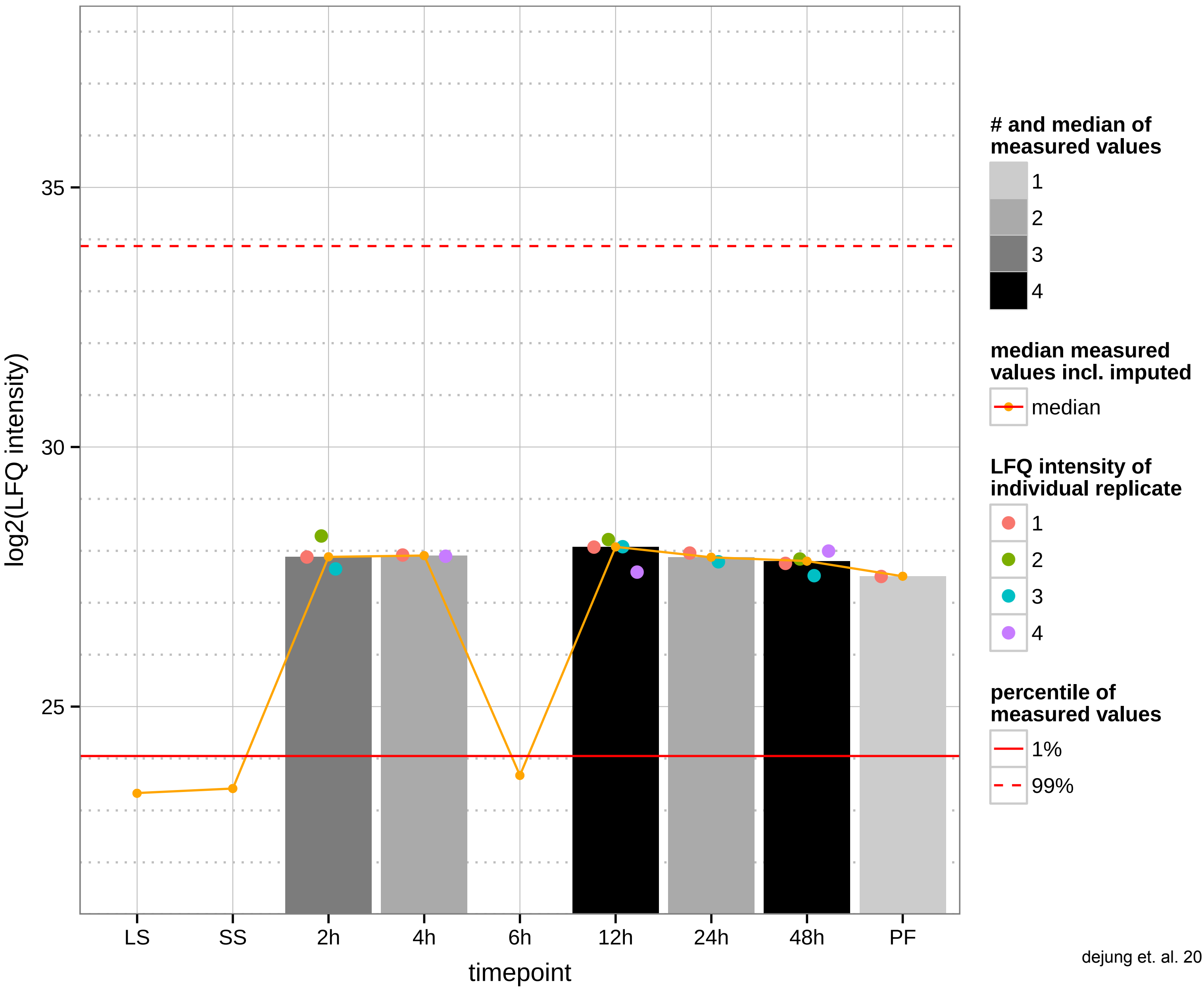

dejung et. al. 2015

predicted zinc finger protein
Tb927.9.11070
AGOF: zinc ion binding
AGOC: null
AGOP: null
PGOF: zinc ion binding
PGOC: null
PGOP: null

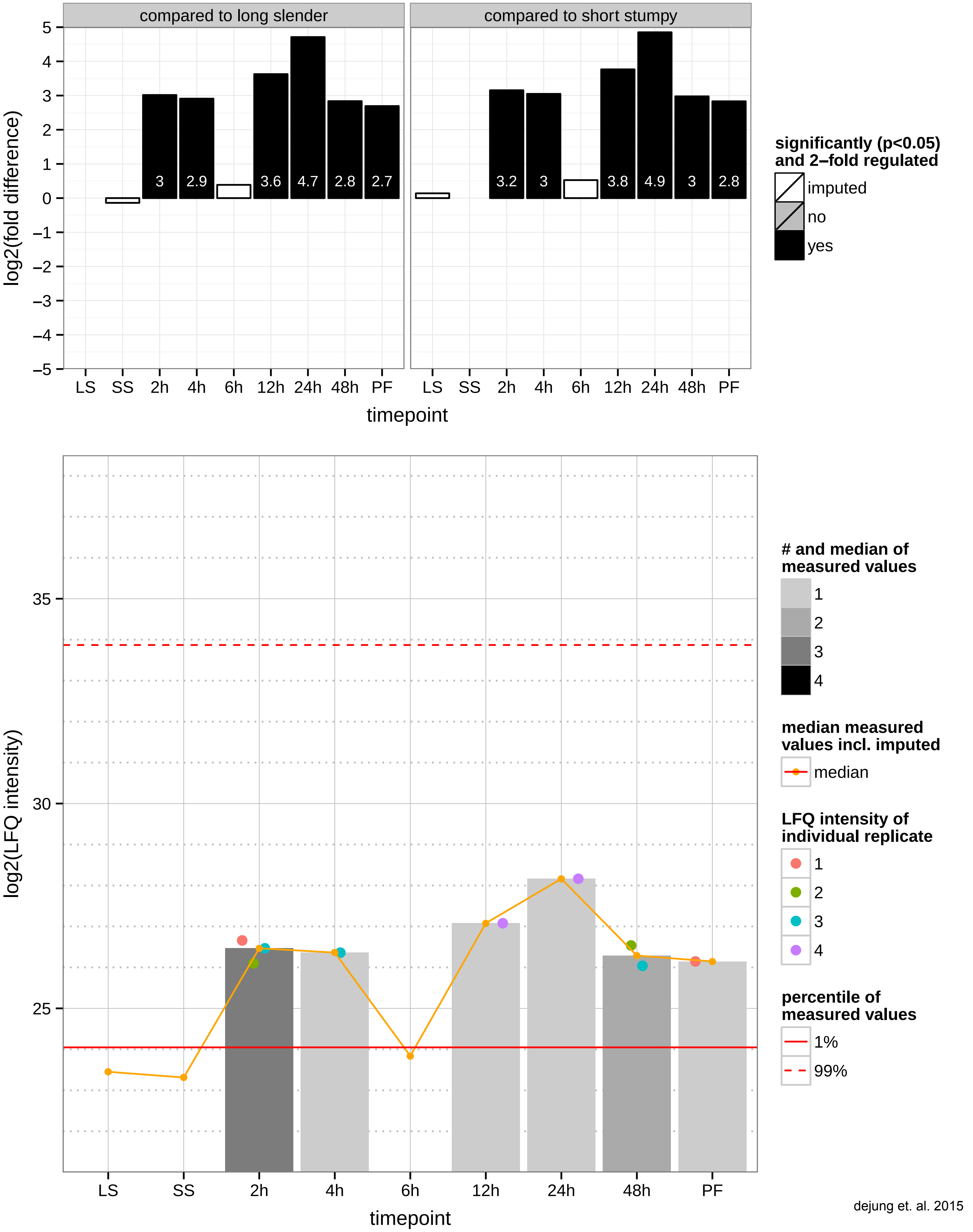

dejung et. al. 2015

unspecified product
Tb927.9.12120
AGOF: null
AGOC: null
AGOP: null
PGOF: null
PGOC: null
PGOP: null

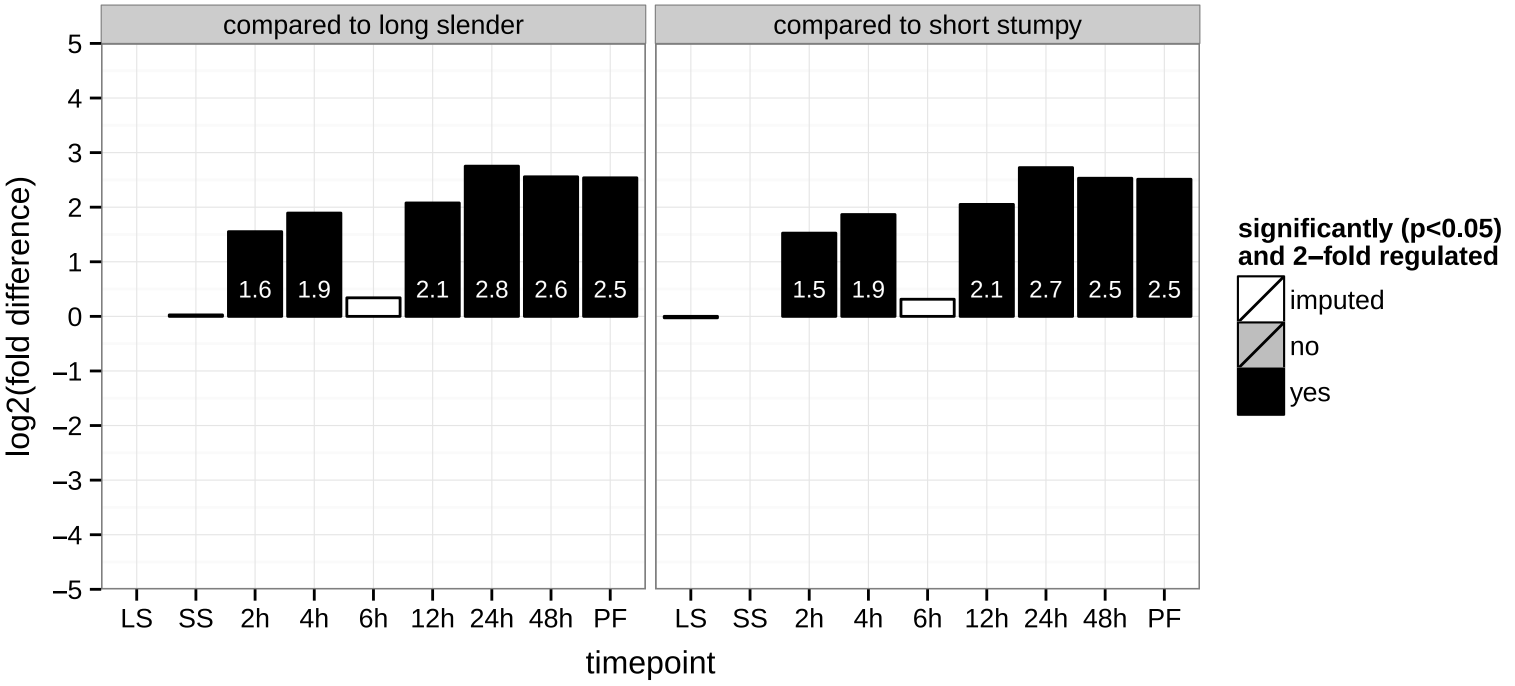

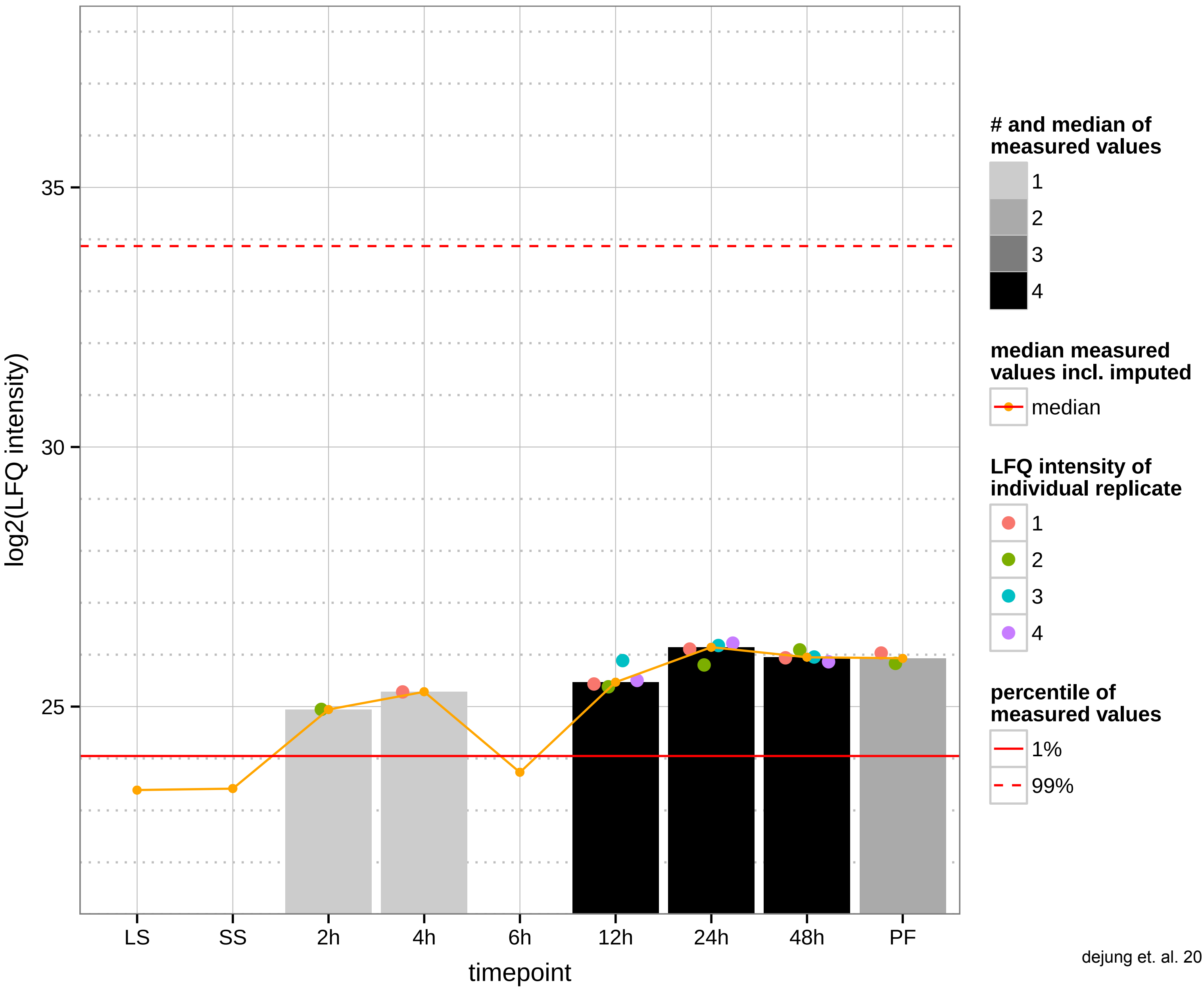

dejung et. al. 2015

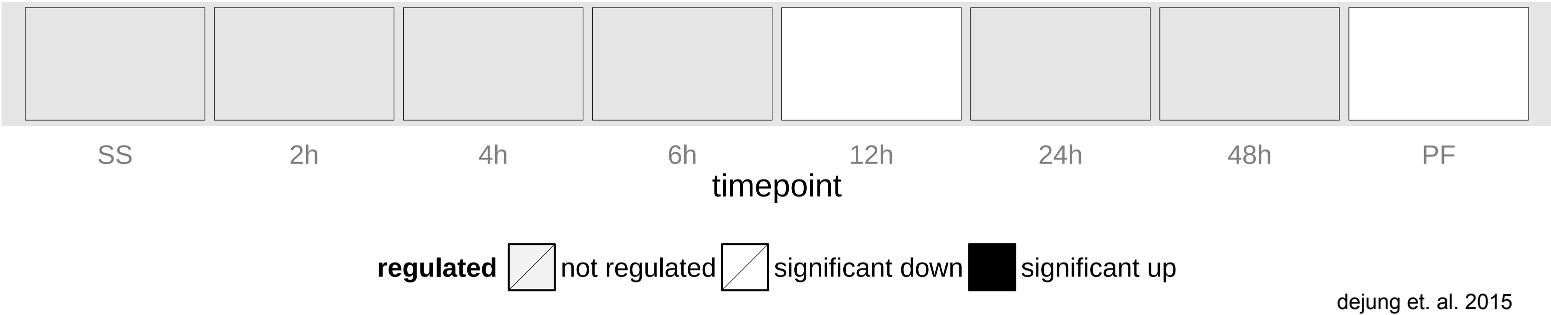
SS
2h
4h
6h
12h
24h
48h
PF
timepoint
regulated
not regulated
significant down
significant up
dejung et. al. 2015

deoxyhypusine synthase, putative
Tb927.10.2750
AGOF: deoxyhypusine synthase activity
AGOC: cytosol
AGOP: peptidyl–lysine modification to hypusine, post–translational protein modification
PGOF: null
PGOC: null
PGOP: peptidyl–lysine modification to hypusine

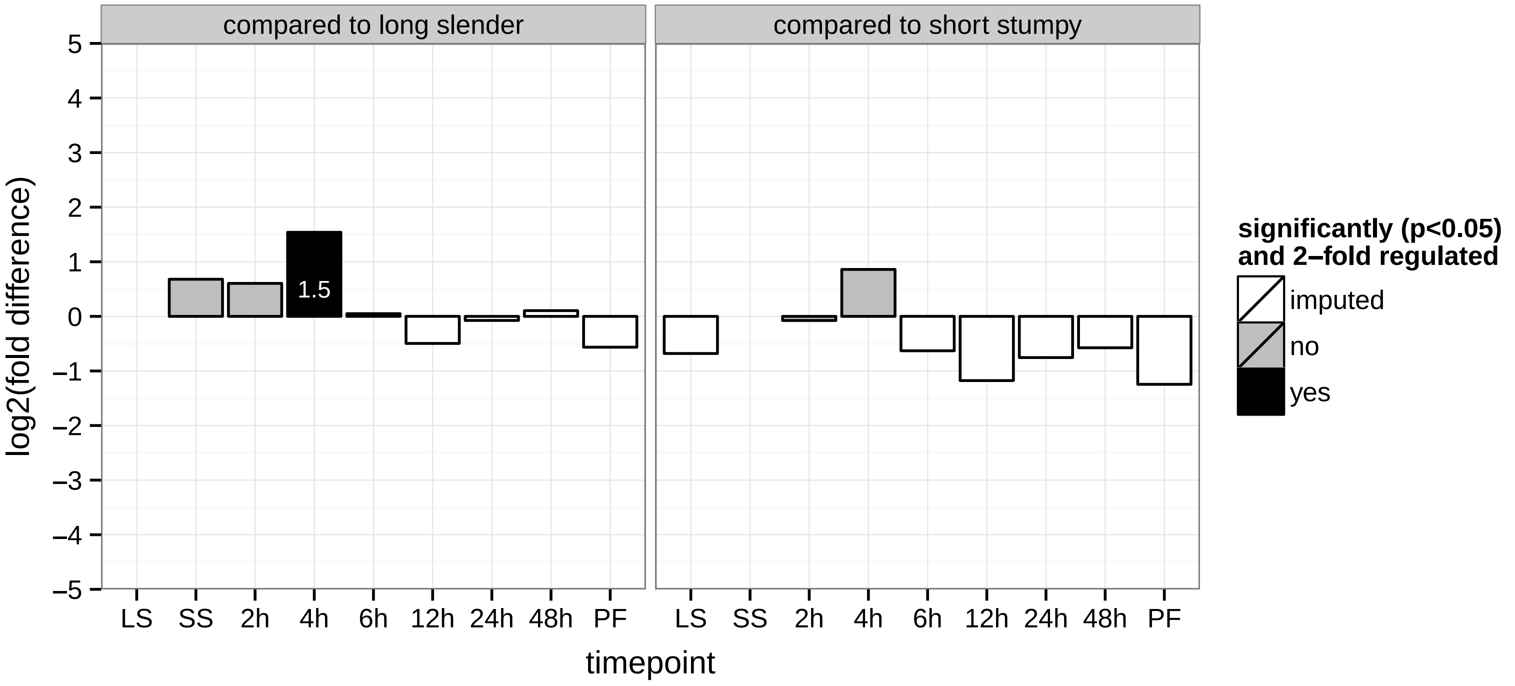

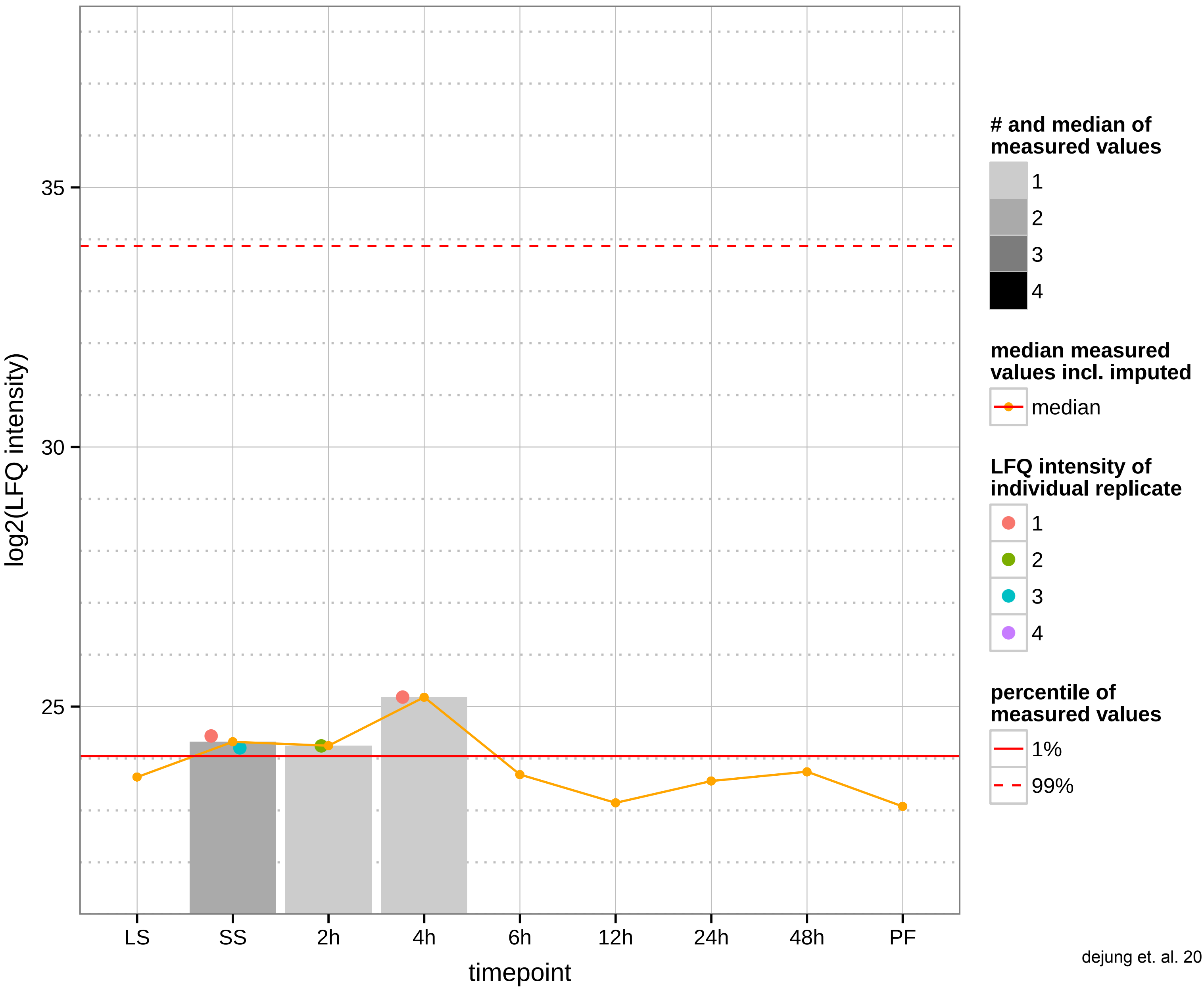

dejung et. al. 2015

hypothetical protein, conserved
Tb927.10.420
AGOF: null
AGOC: null
AGOP: null
PGOF: protein binding
PGOC: null
PGOP: null

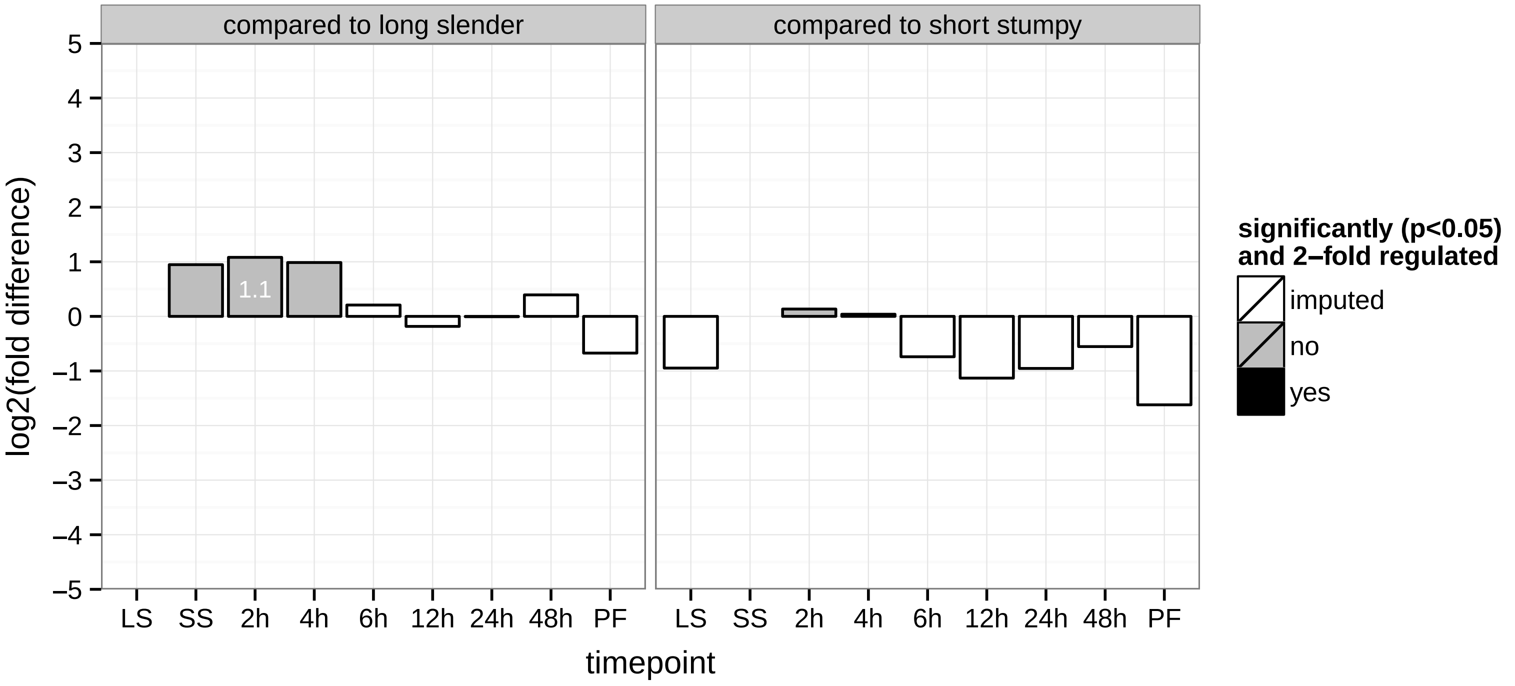

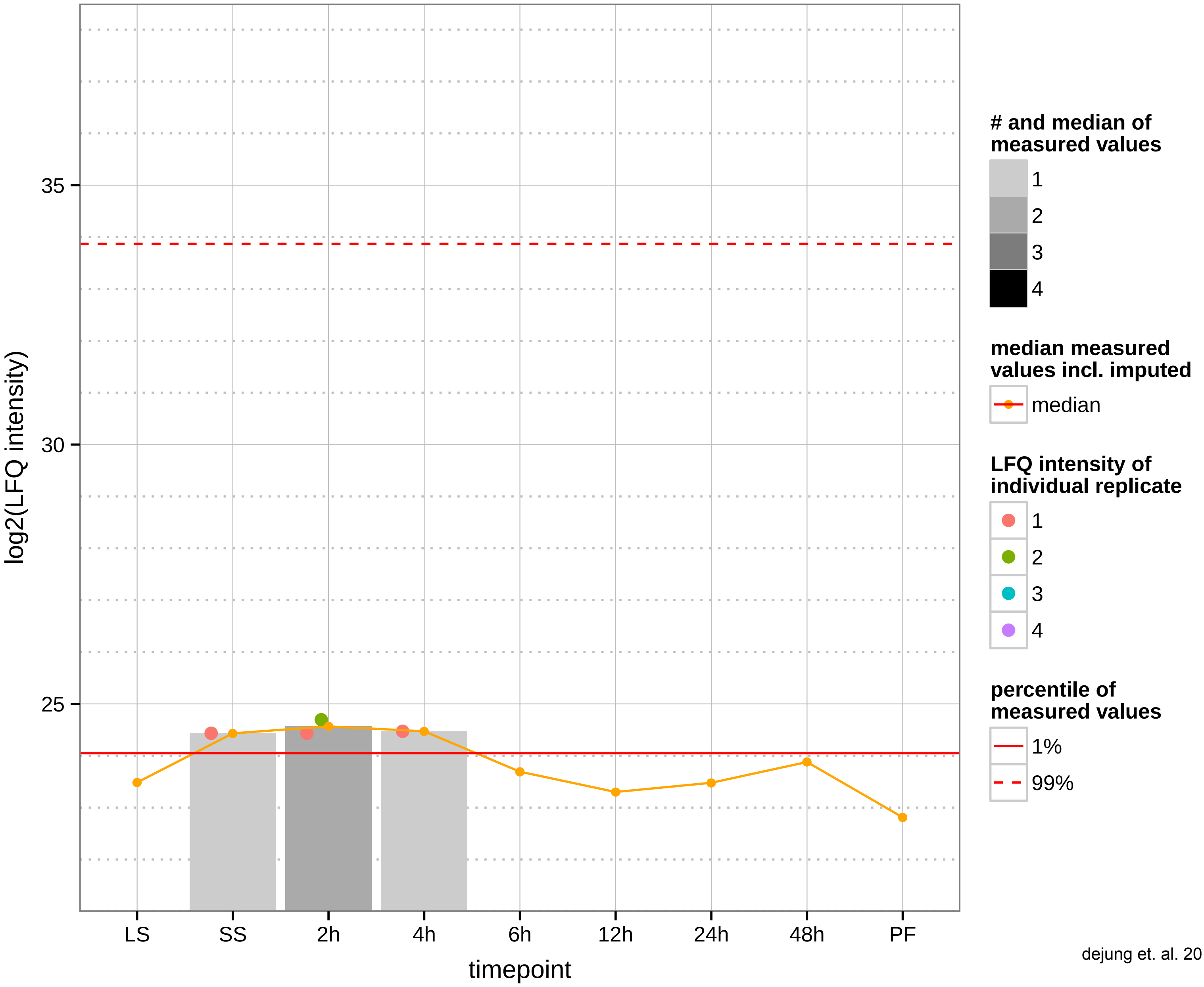

dejung et. al. 2015

hypothetical protein, conserved
Tb927.2.4050
AGOF: zinc ion binding
AGOC: null
AGOP: null
PGOF: zinc ion binding
PGOC: null
PGOP: null

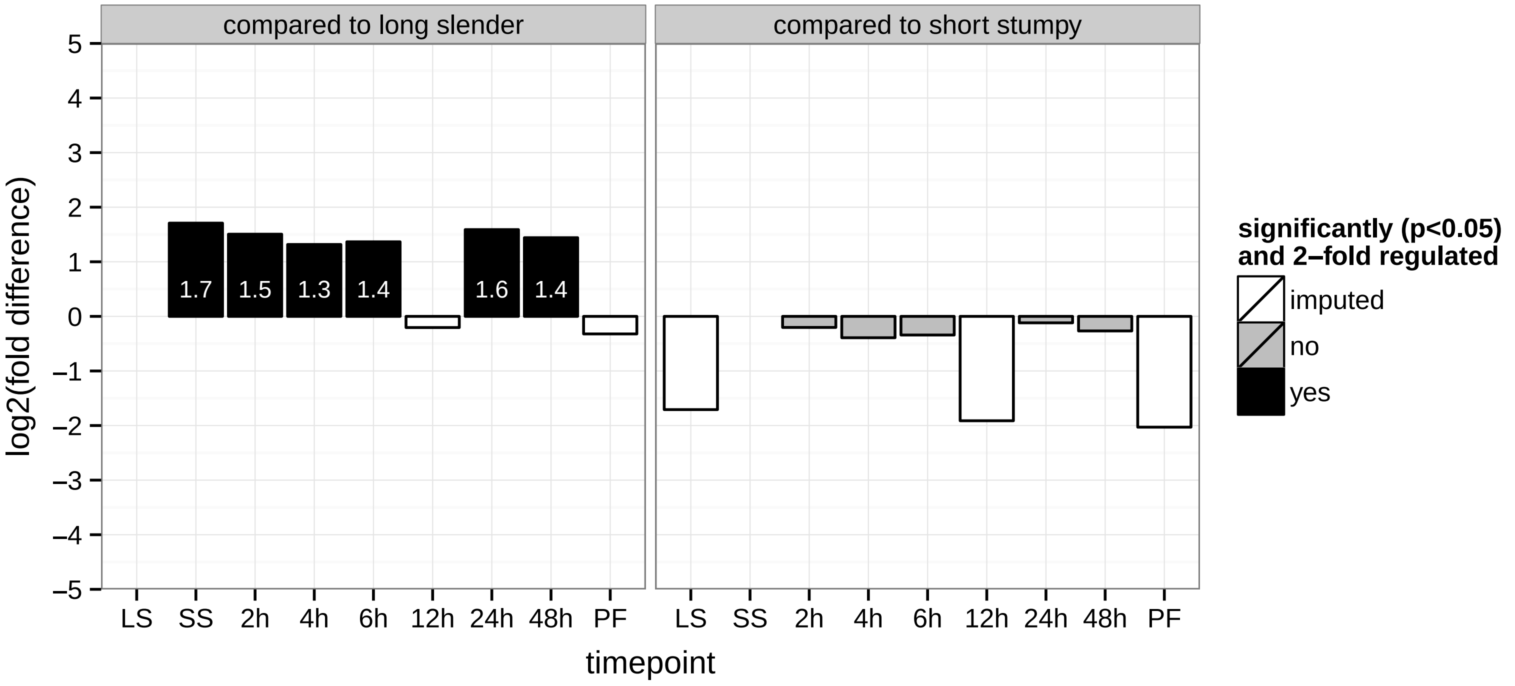

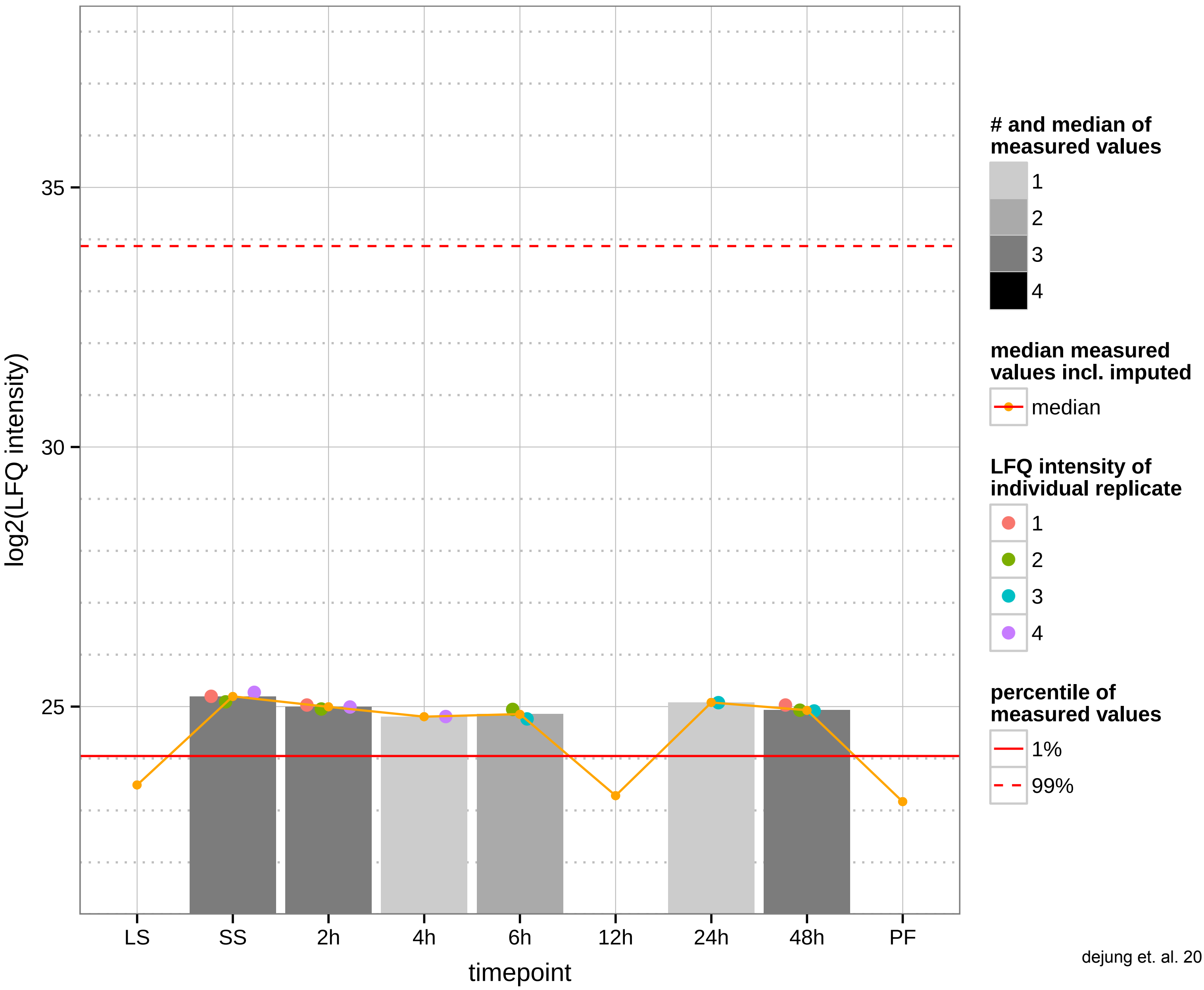

dejung et. al. 2015

protein kinase, putative
Tb927.7.3580
AGOF: ATP binding, protein serine/threonine kinase activity, protein tyrosine kinase activity
AGOC: null
AGOP: protein phosphorylation
PGOF: ATP binding, protein kinase activity, transferase activity, transferring phosphorus–containing groups
PGOC: null
PGOP: protein phosphorylation

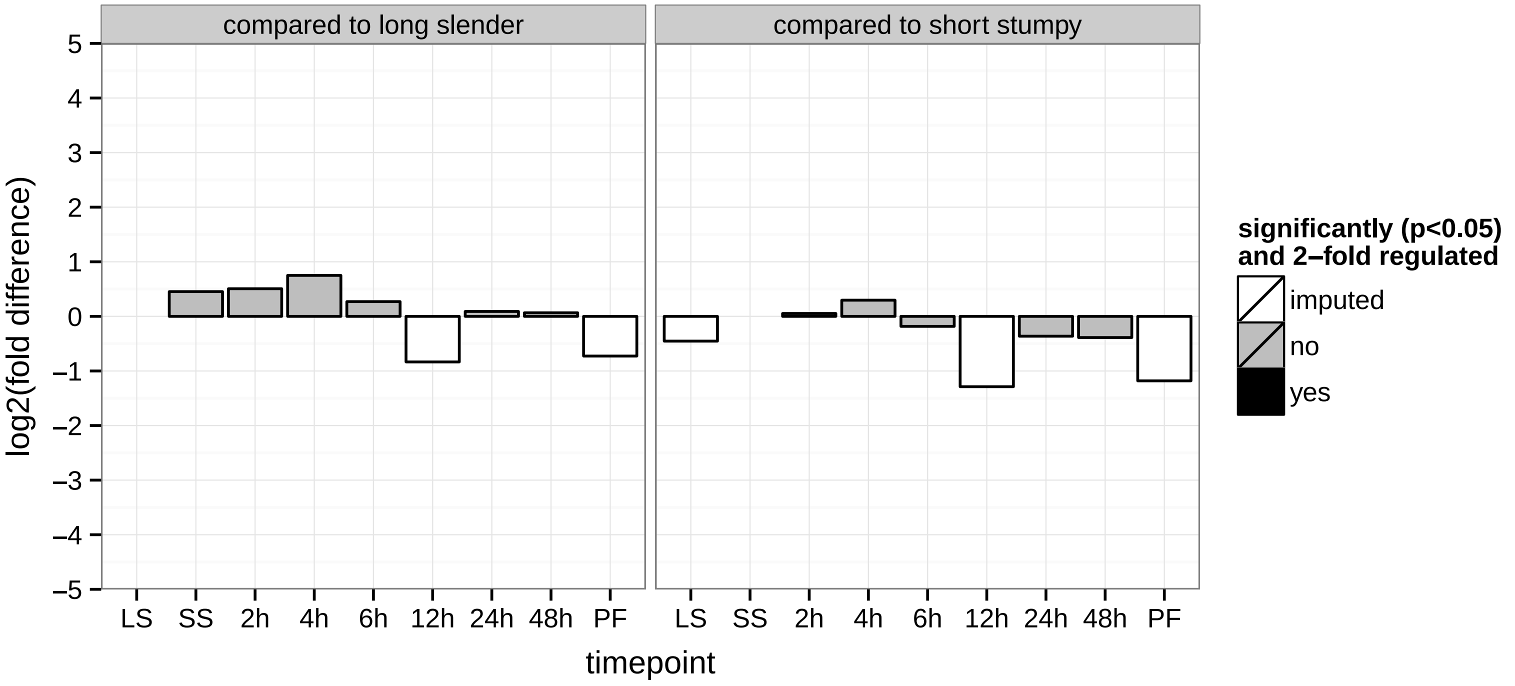

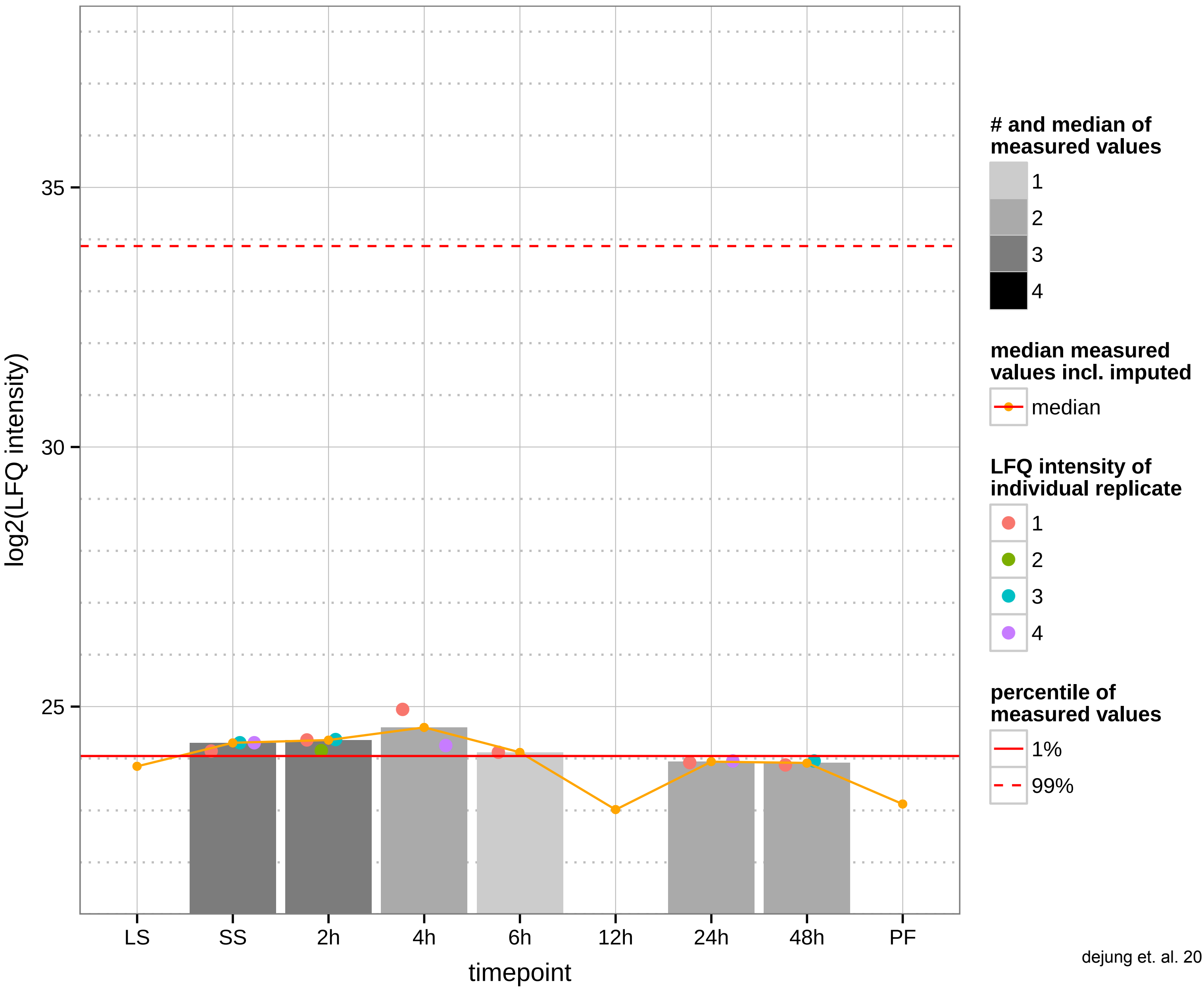

dejung et. al. 2015

inhibitor of cysteine peptidase (ICP)
Tb927.8.6450
AGOF: endopeptidase inhibitor activity
AGOC: null
AGOP: regulation of proteolysis
PGOF: null
PGOC: null
PGOP: null

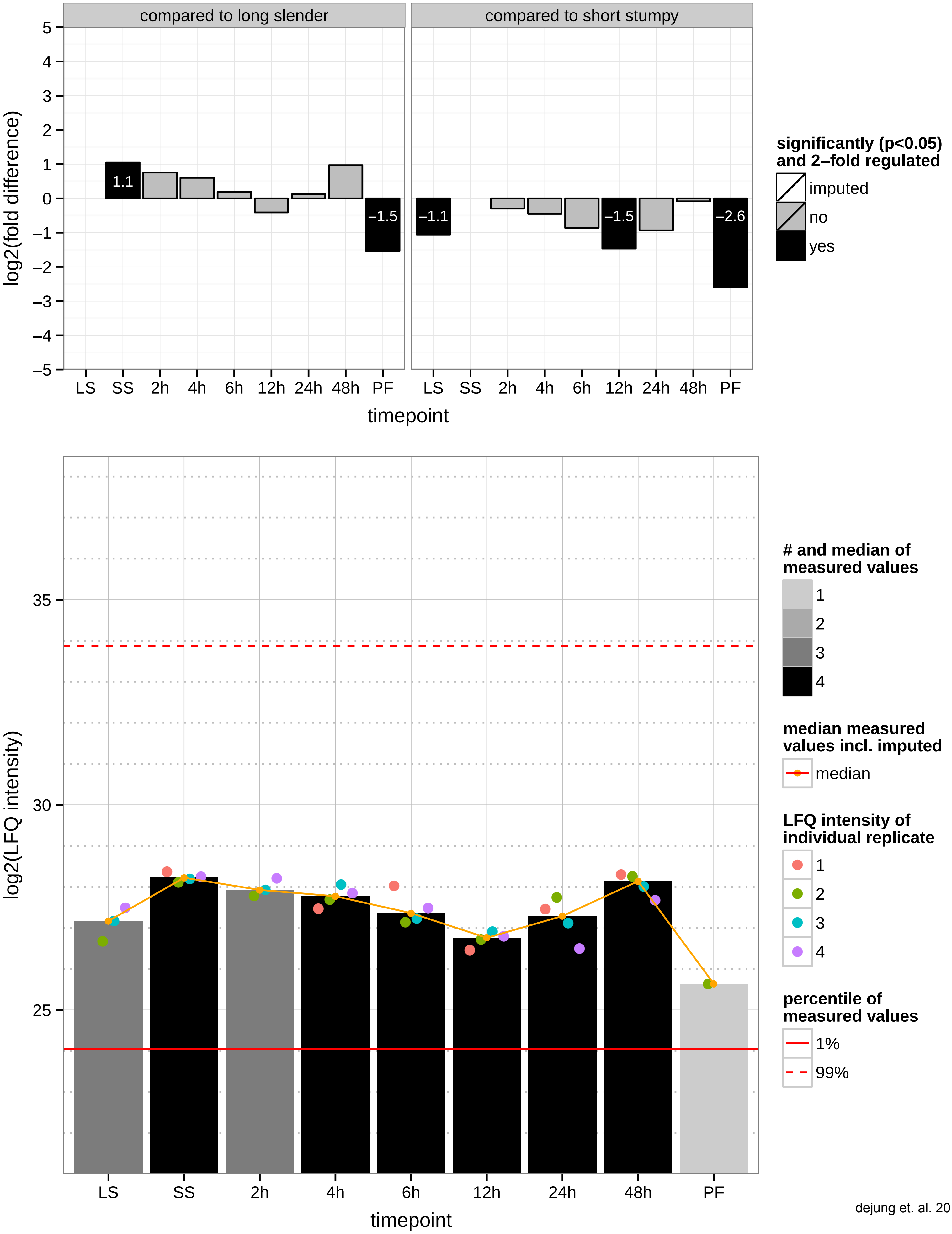

dejung et. al. 2015

tRNA–dihydrouridine synthase 1, putative
Tb927.8.7570
AGOF: flavin adenine dinucleotide binding, tRNA dihydrouridine synthase activity
AGOC: null
AGOP: oxidation–reduction process, tRNA modification
PGOF: flavin adenine dinucleotide binding, tRNA dihydrouridine synthase activity
PGOC: null
PGOP: oxidation–reduction process, tRNA processing

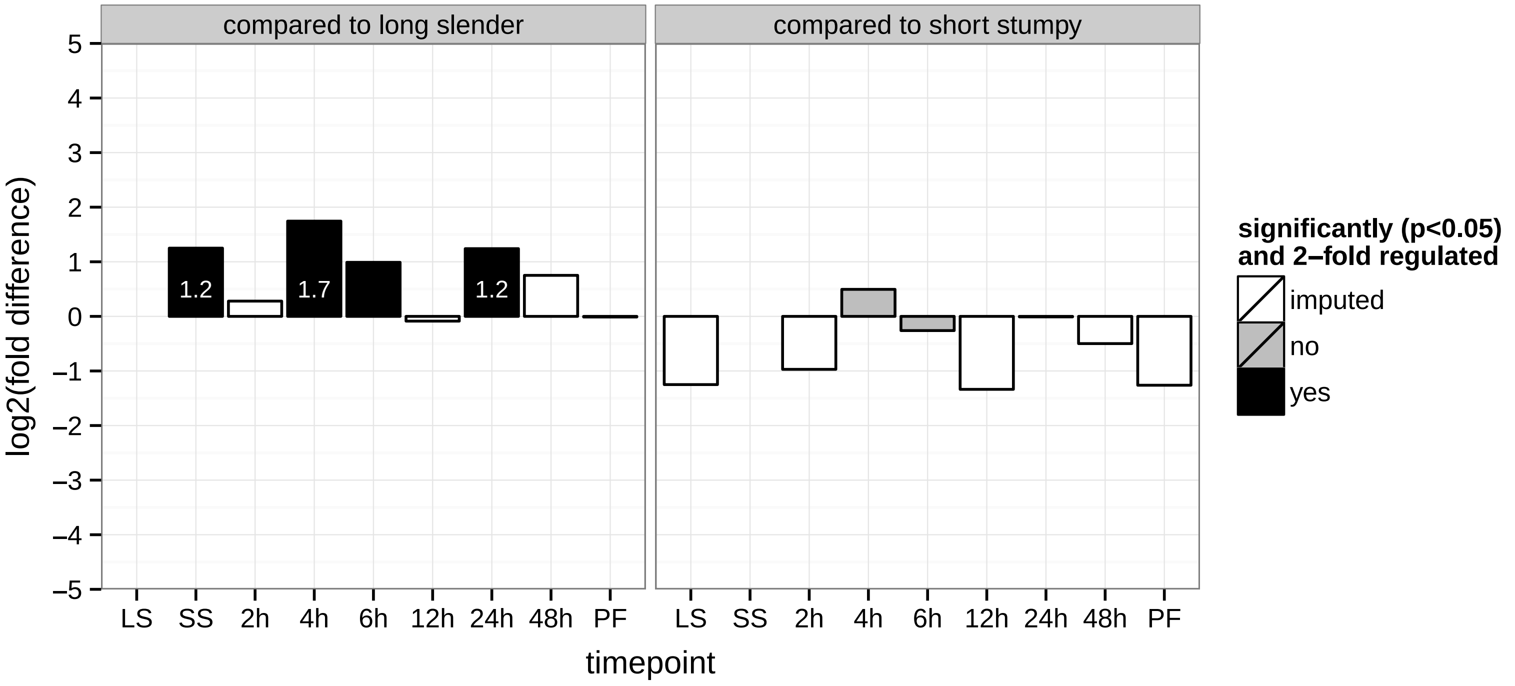

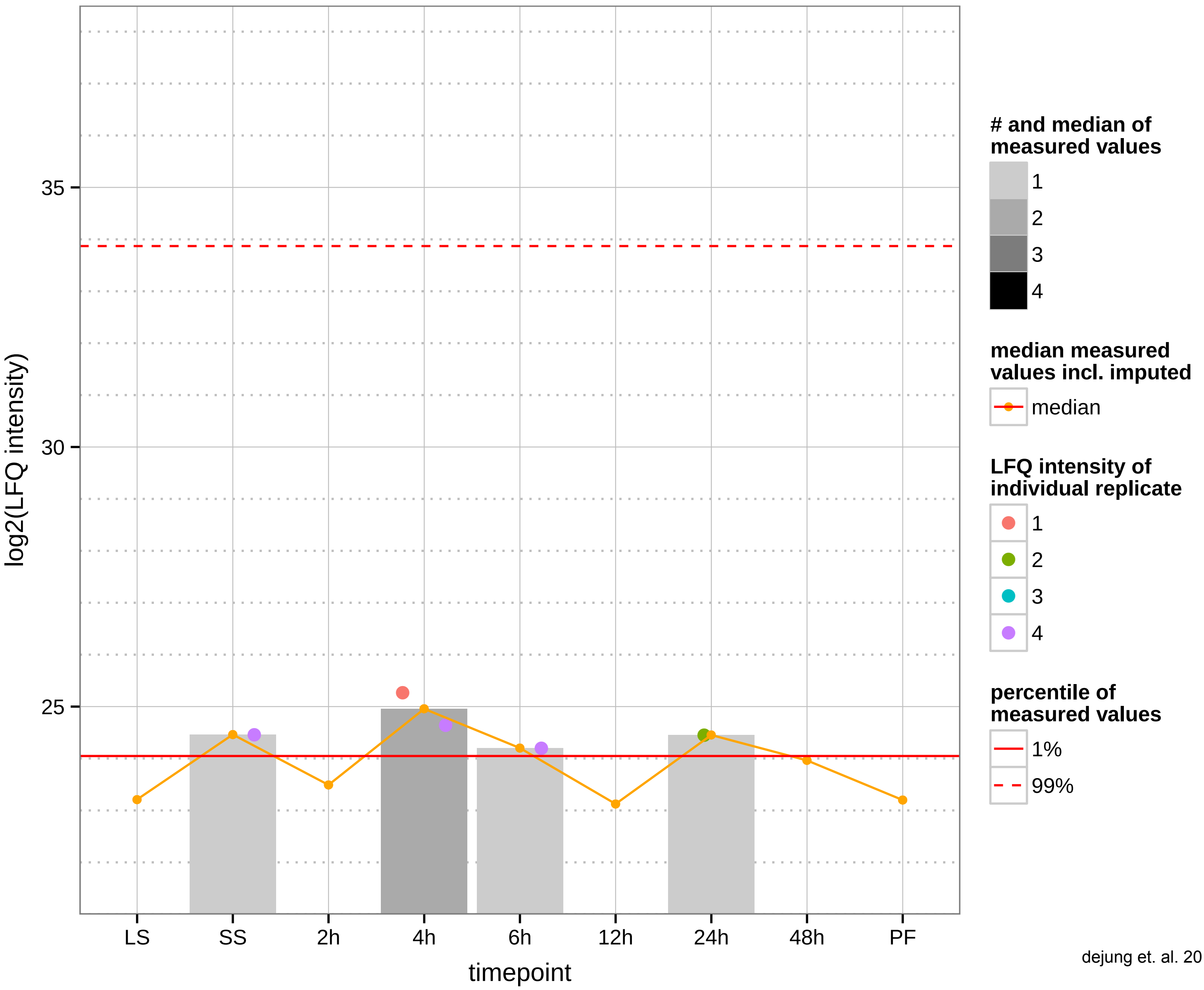

dejung et. al. 2015

ankyrin–repeat protein, putative
Tb927.9.15400
AGOF: null
AGOC: null
AGOP: null
PGOF: protein binding
PGOC: null
PGOP: null

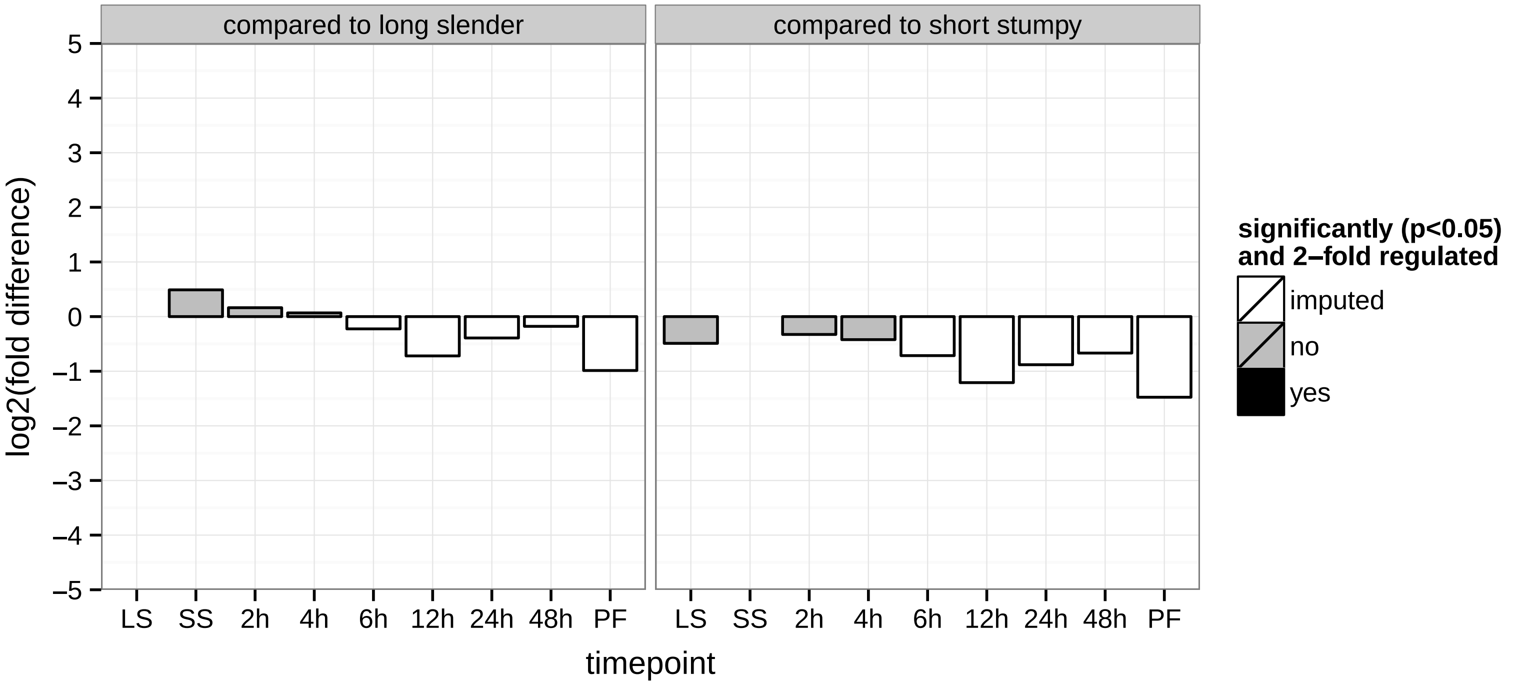

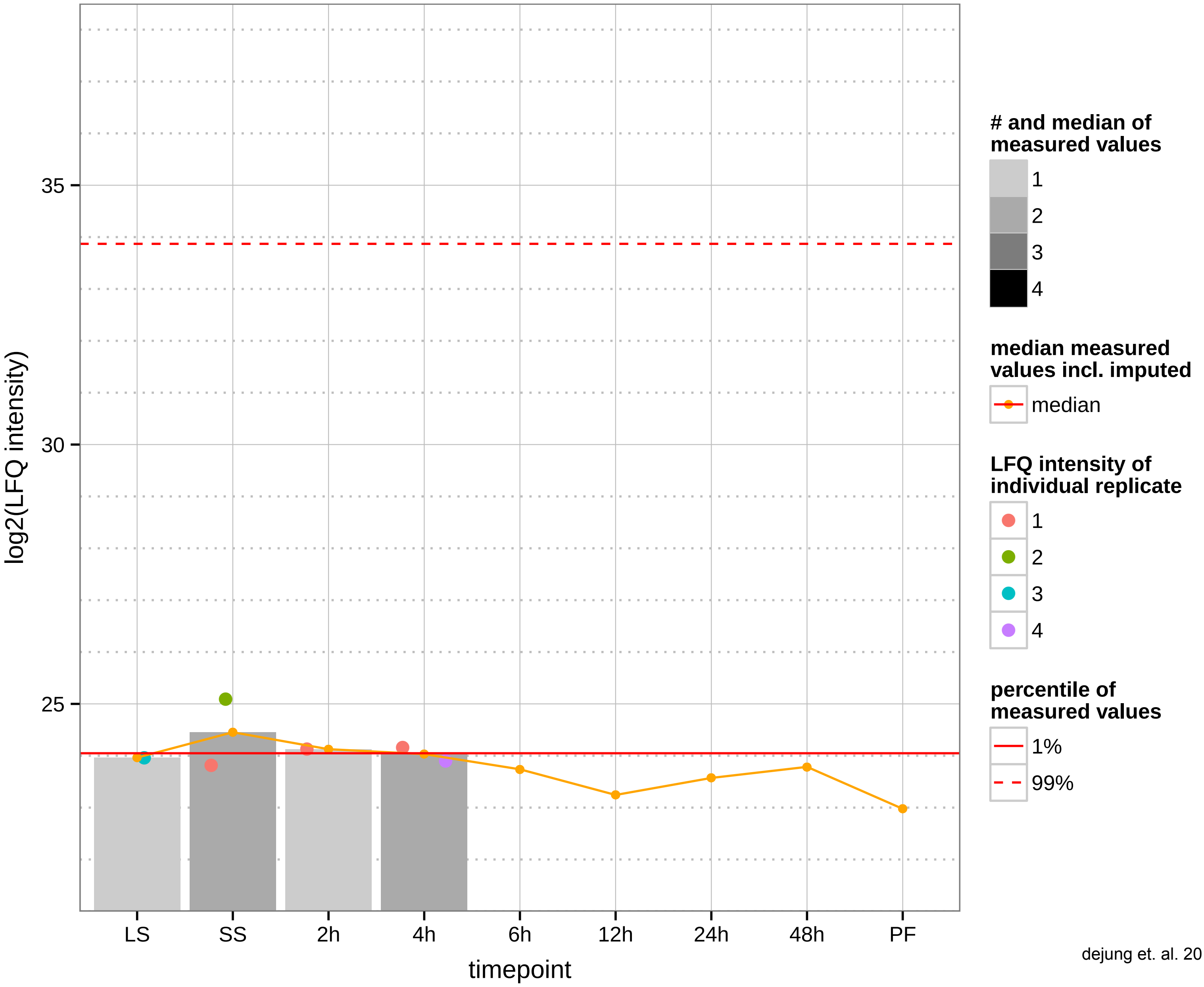

dejung et. al. 2015

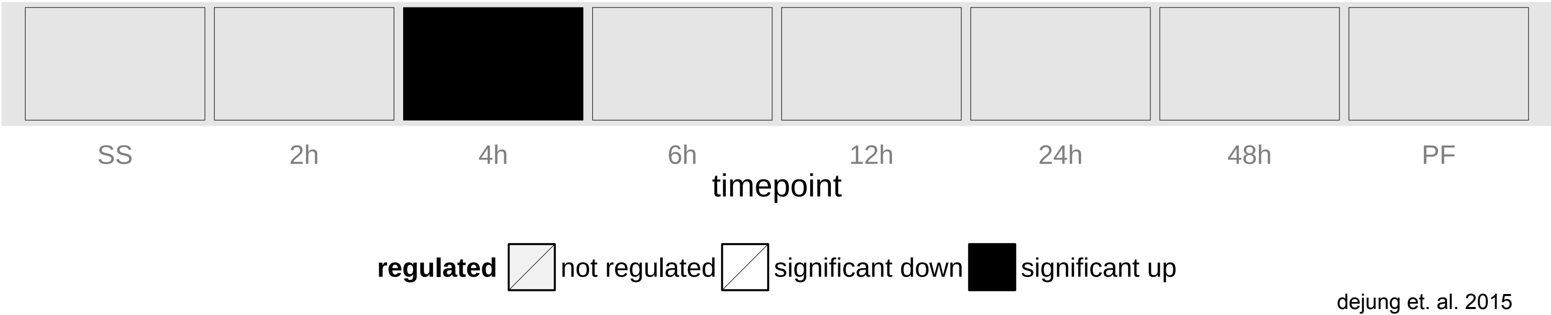
SS
2h
4h
6h
12h
24h
48h
PF
timepoint
regulated
not regulated
significant down
significant up
dejung et. al. 2015

N(2), N(2)–dimethylguanosine tRNA methyltransferase, putative
Tb927.11.3890
AGOF: RNA binding, tRNA (guanine–N2–)–methyltransferase activity
AGOC: null
AGOP: tRNA processing
PGOF: RNA binding, tRNA (guanine–N2–)–methyltransferase activity
PGOC: null
PGOP: tRNA processing

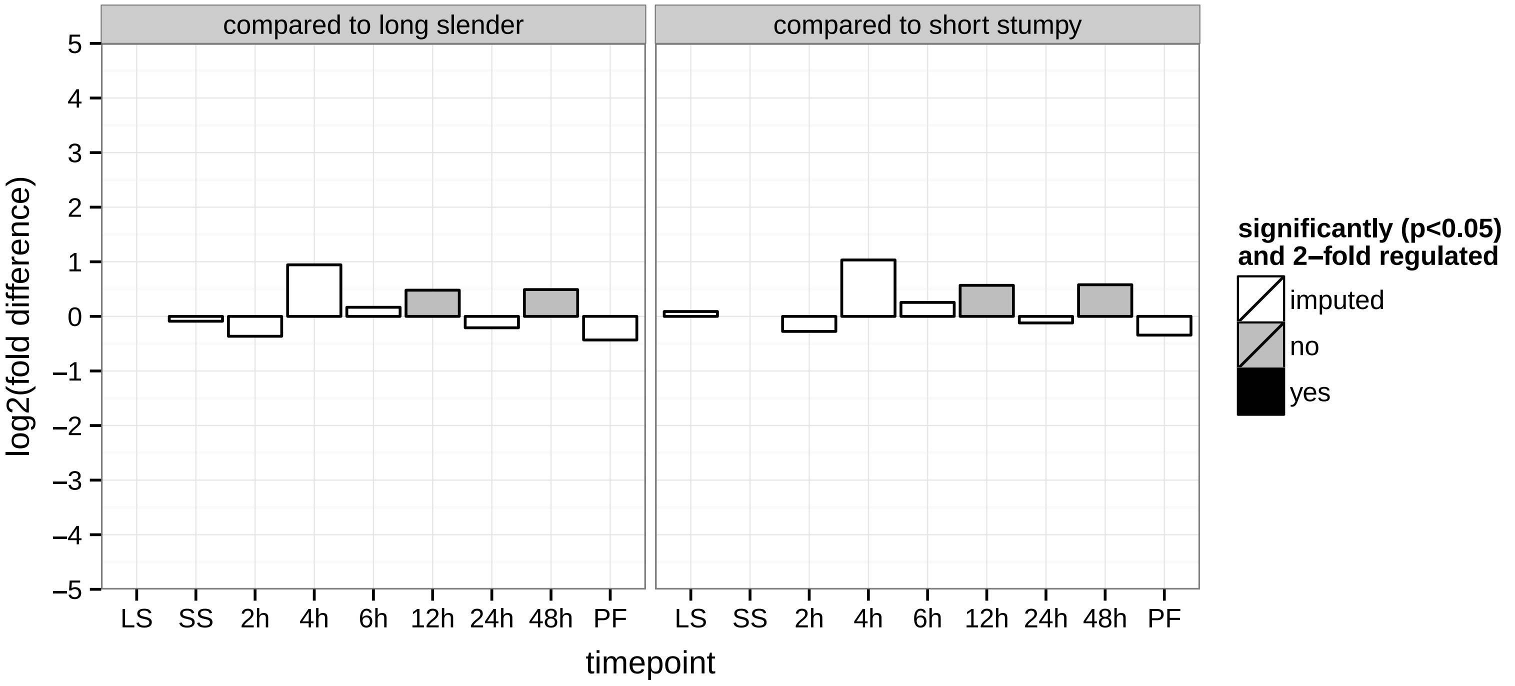

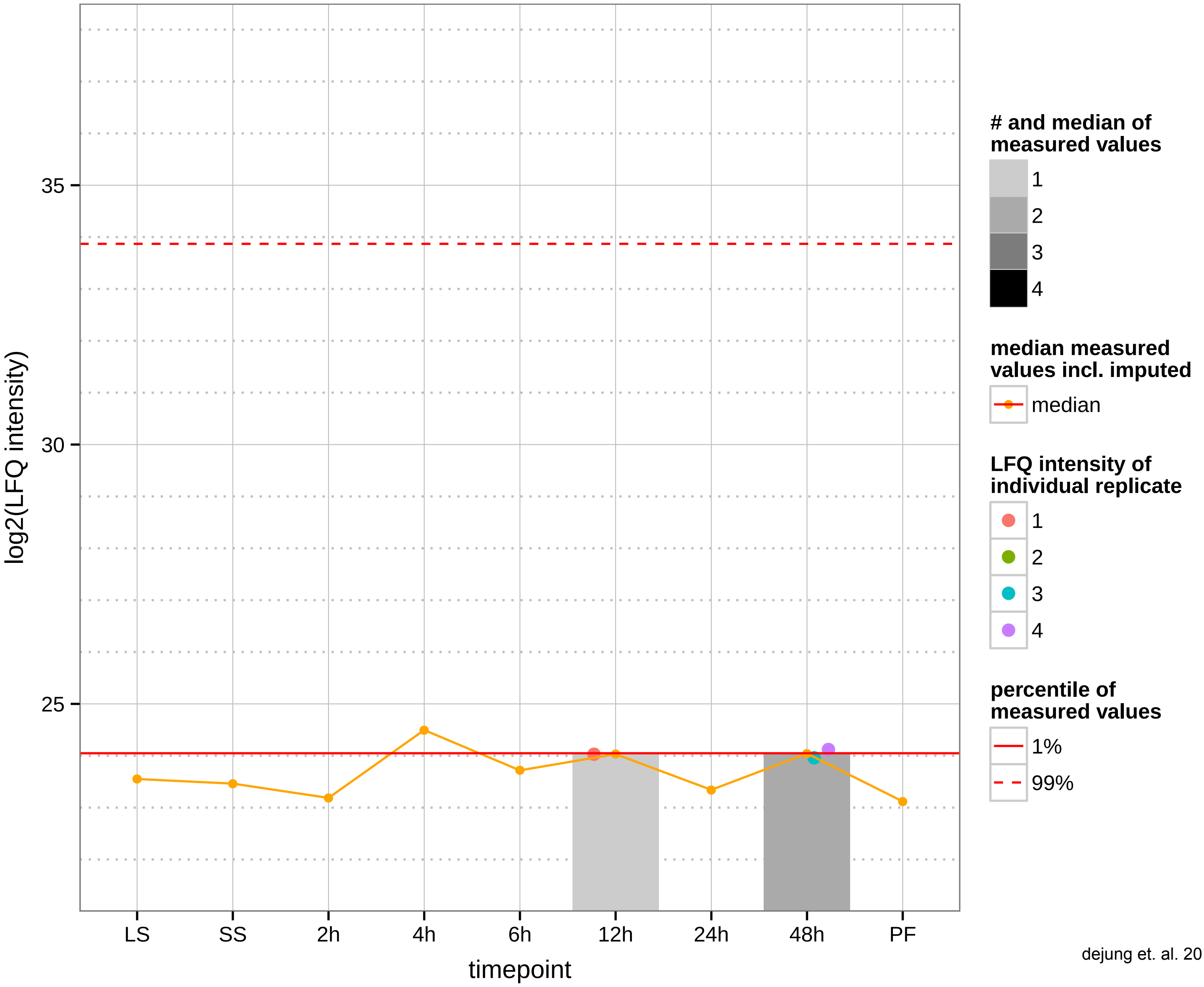

dejung et. al. 2015

hypothetical protein, conserved
Tb927.11.4630
AGOF: null
AGOC: null
AGOP: null
PGOF: null
PGOC: null
PGOP: null

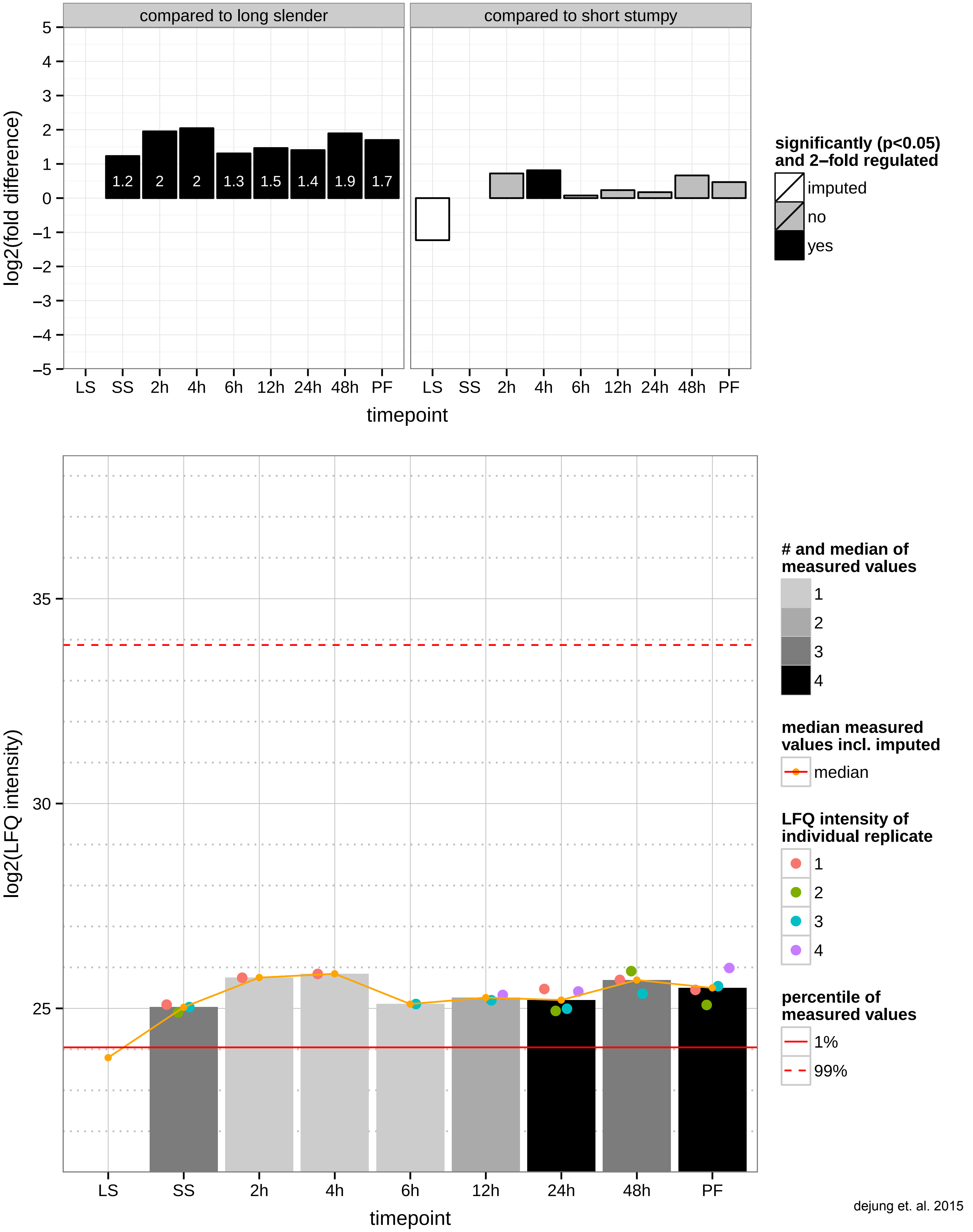

dejung et. al. 2015

hypothetical protein, conserved
Tb927.3.5470
AGOF: null
AGOC: null
AGOP: null
PGOF: protein phosphatase inhibitor activity
PGOC: null
PGOP: regulation of phosphoprotein phosphatase activity, regulation of signal transduction

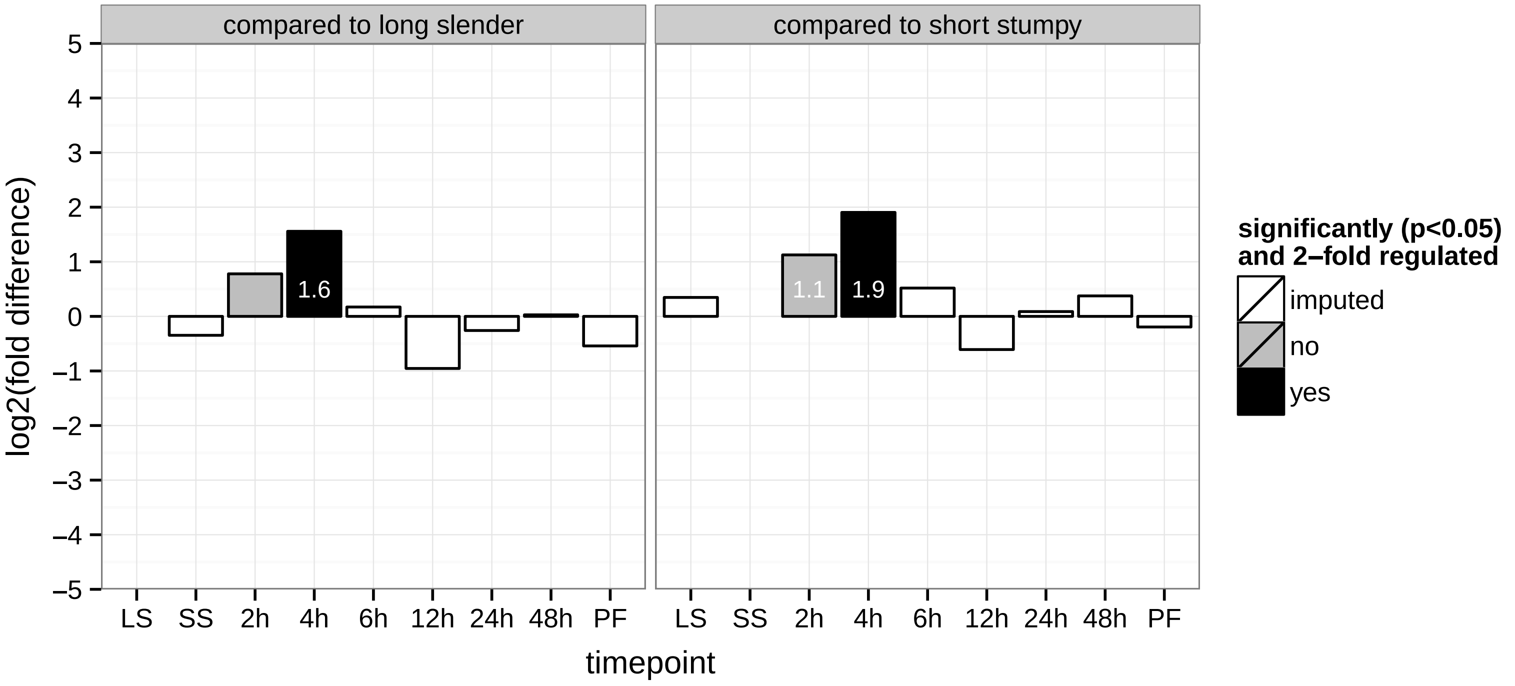

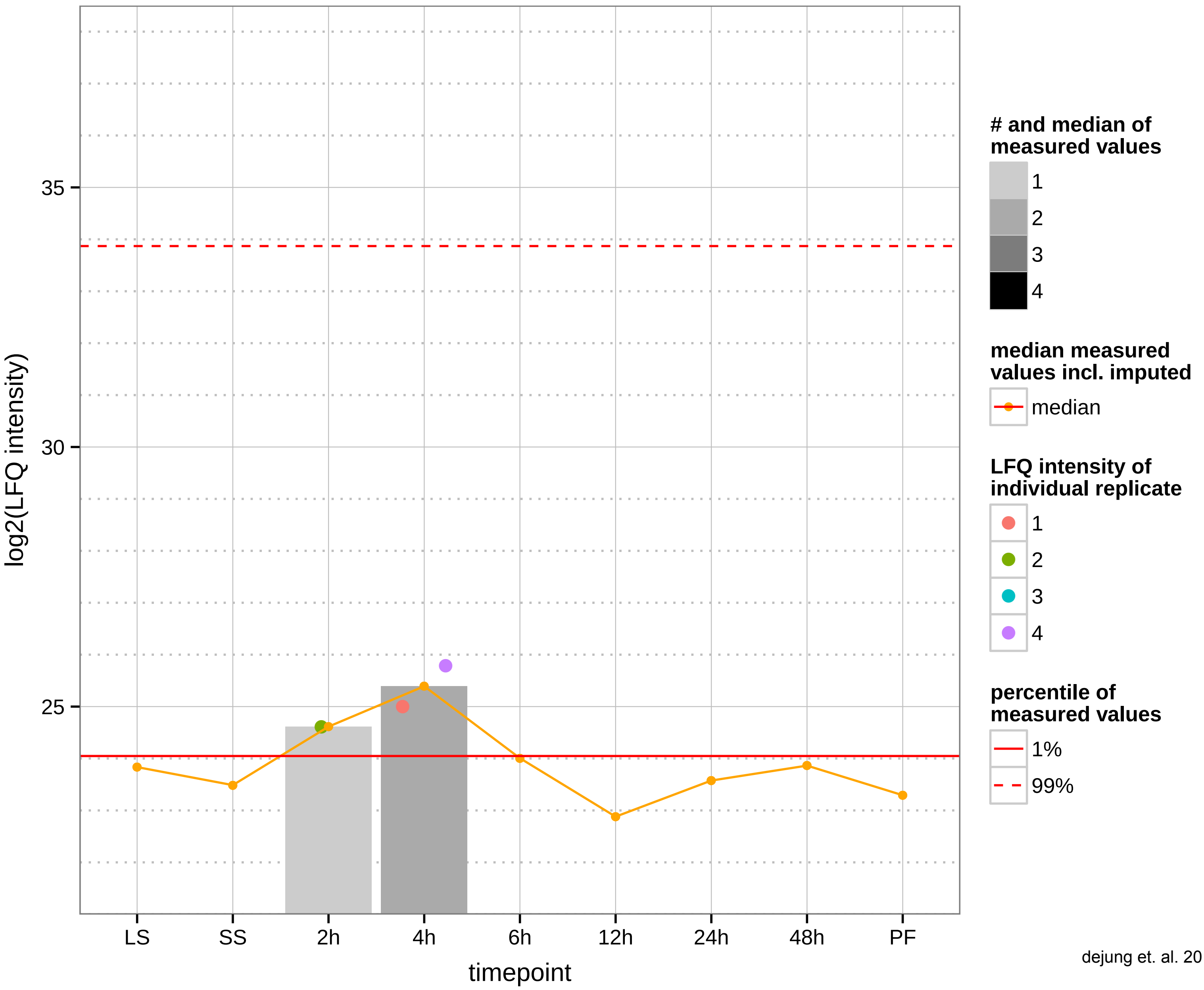

dejung et. al. 2015

hypothetical protein, conserved
Tb927.7.4030
AGOF: null
AGOC: null
AGOP: null
PGOF: null
PGOC: null
PGOP: null

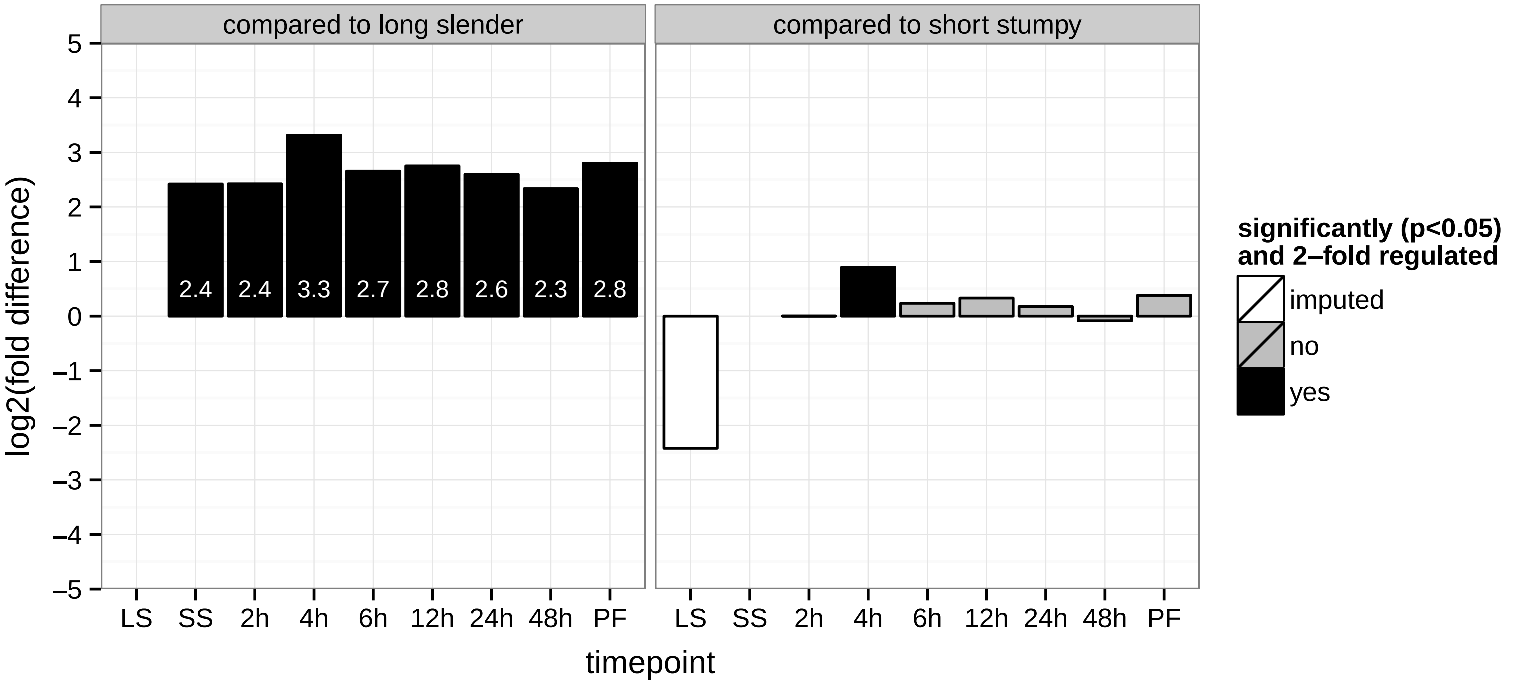

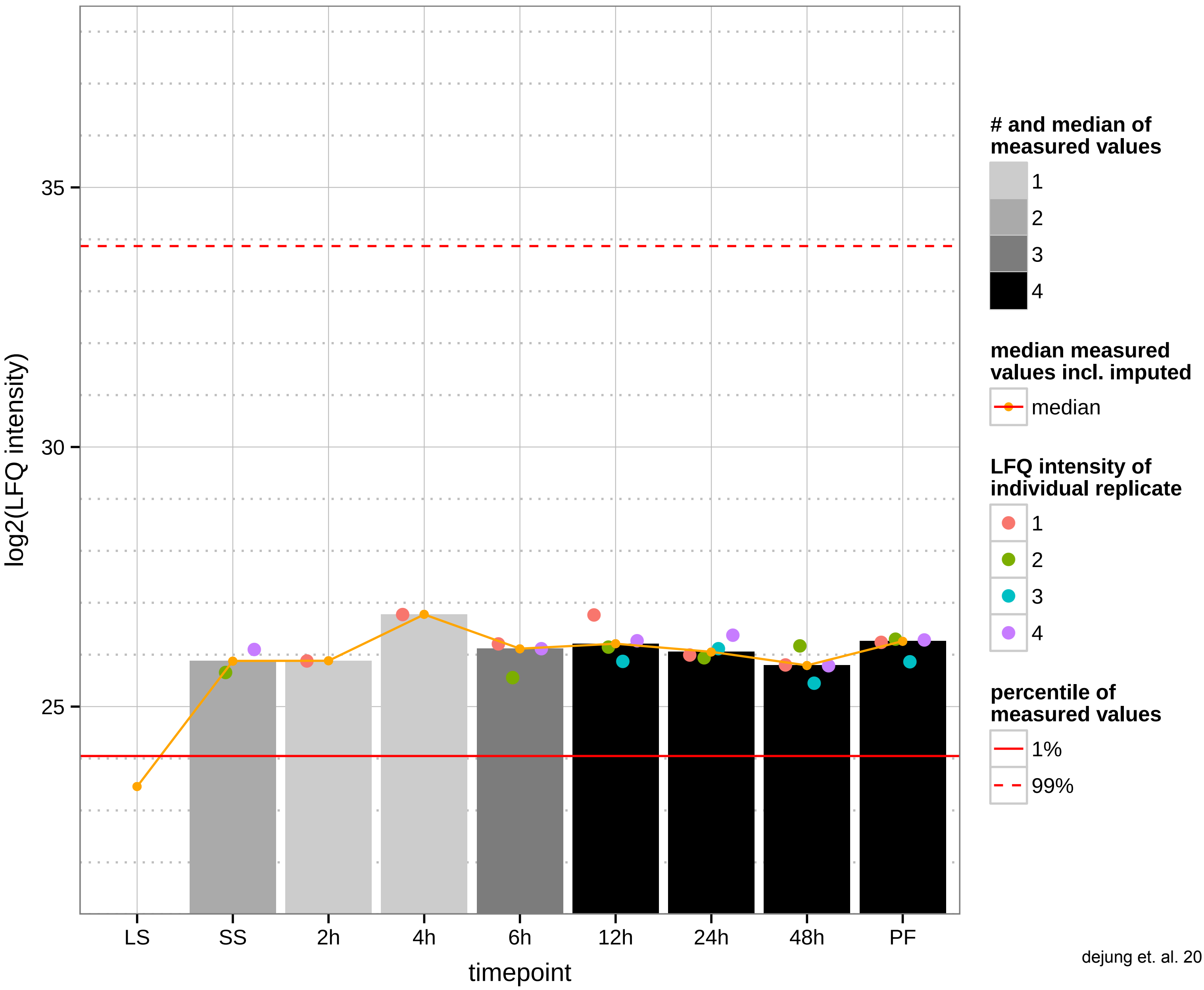

dejung et. al. 2015

hypothetical protein, conserved
Tb927.7.6210
AGOF: null
AGOC: null
AGOP: null
PGOF: null
PGOC: null
PGOP: null

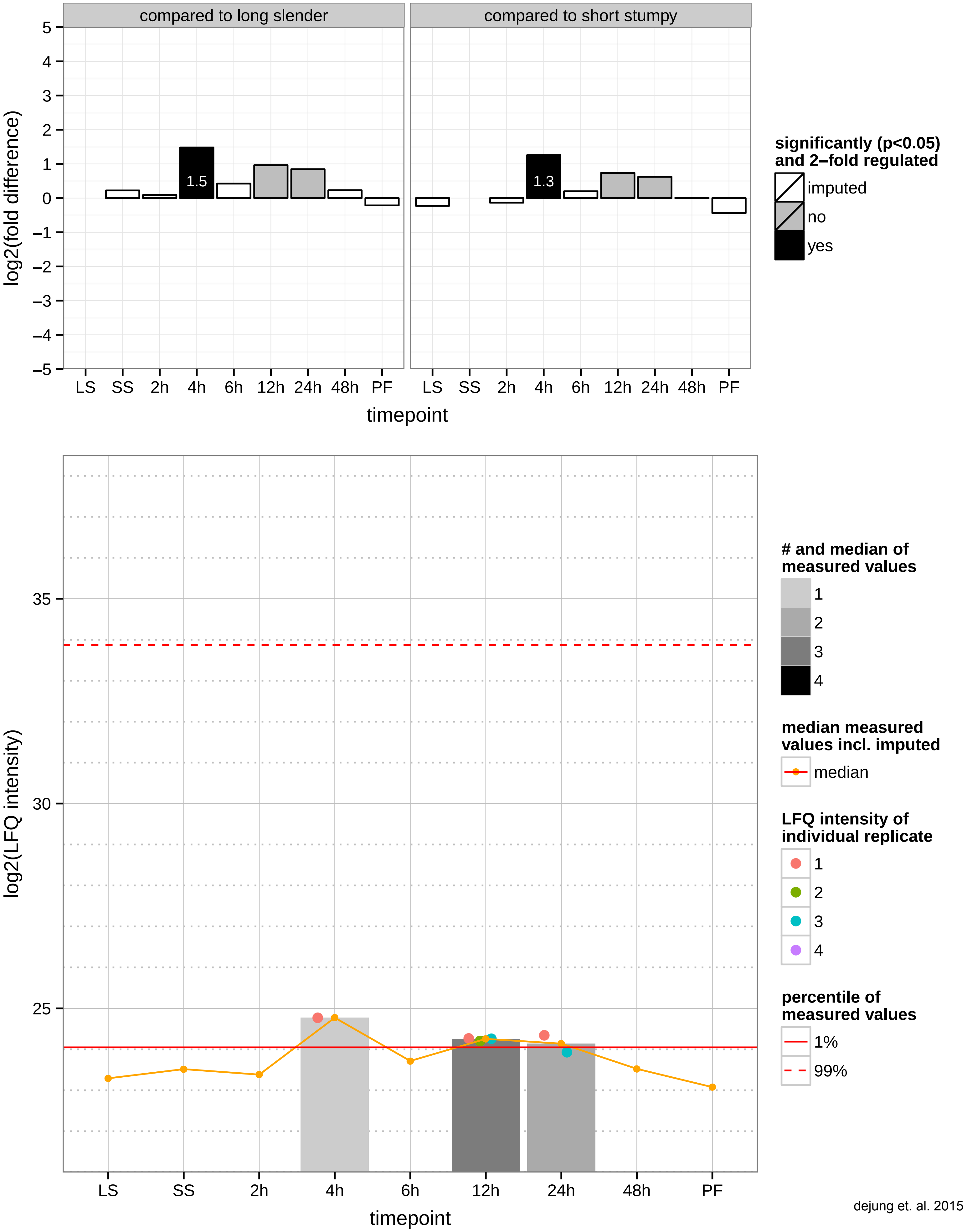

hypothetical protein, conserved
Tb927.8.2840
AGOF: null
AGOC: null
AGOP: null
PGOF: null
PGOC: null
PGOP: null

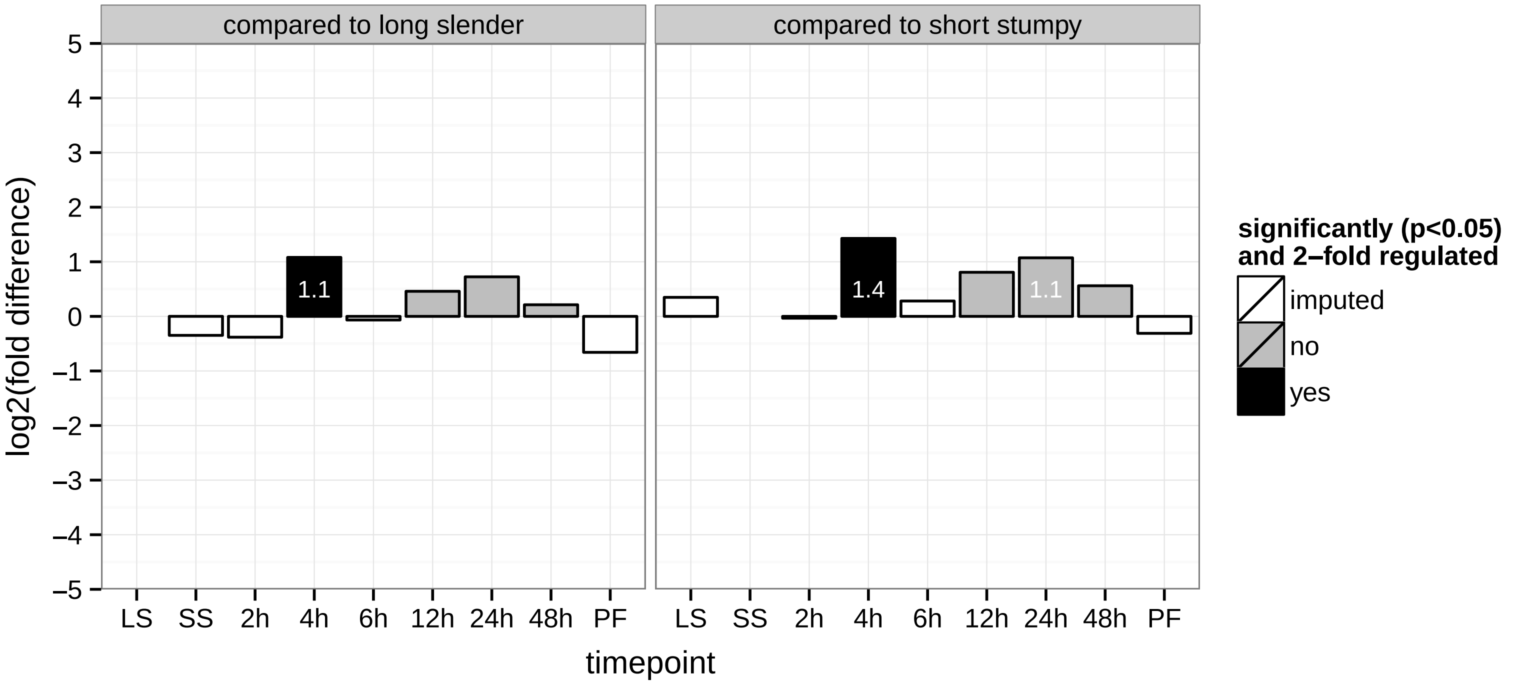

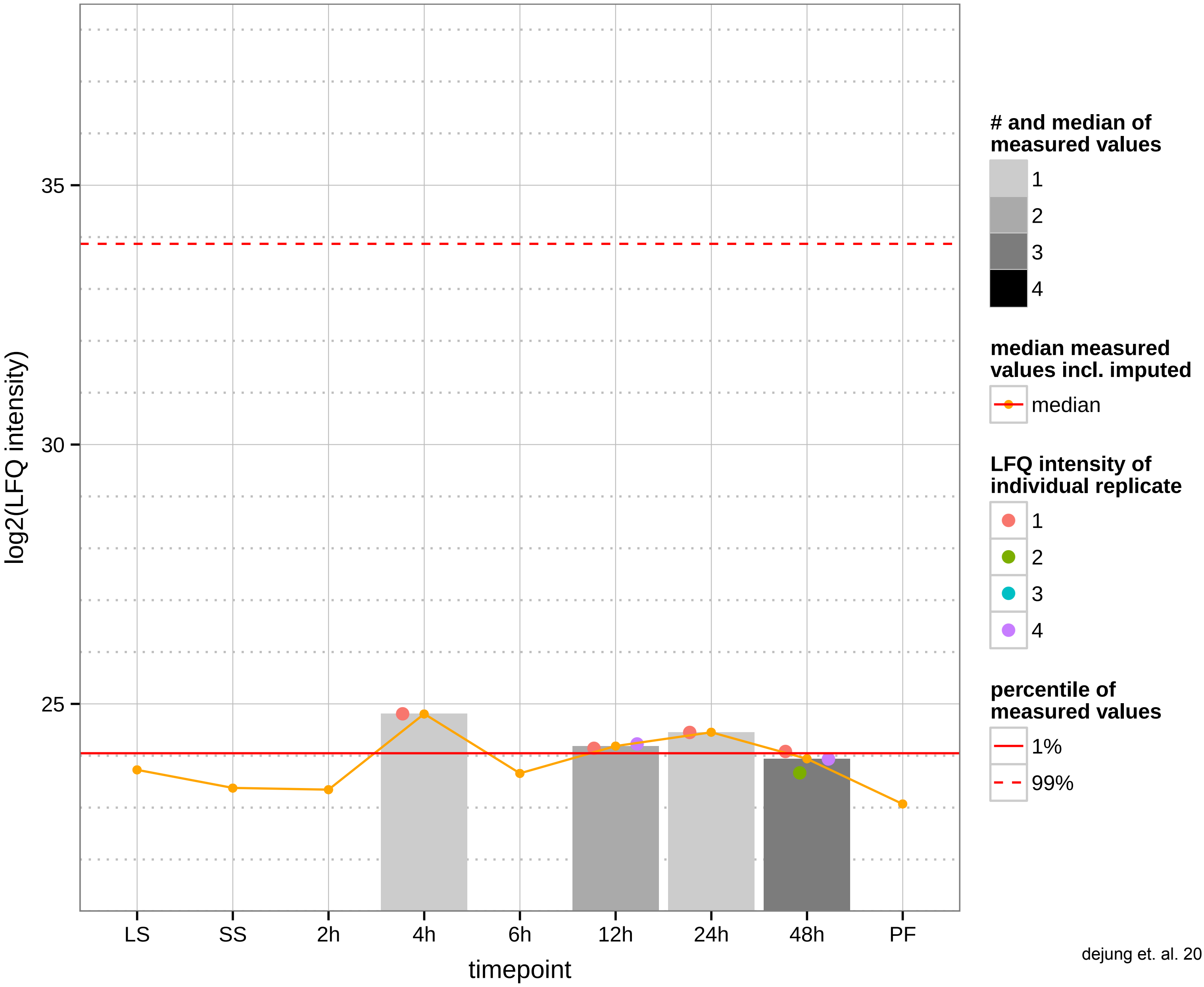

dejung et. al. 2015

hypothetical protein, conserved
Tb927.8.5720
AGOF: null
AGOC: null
AGOP: null
PGOF: transferase activity
PGOC: null
PGOP: null

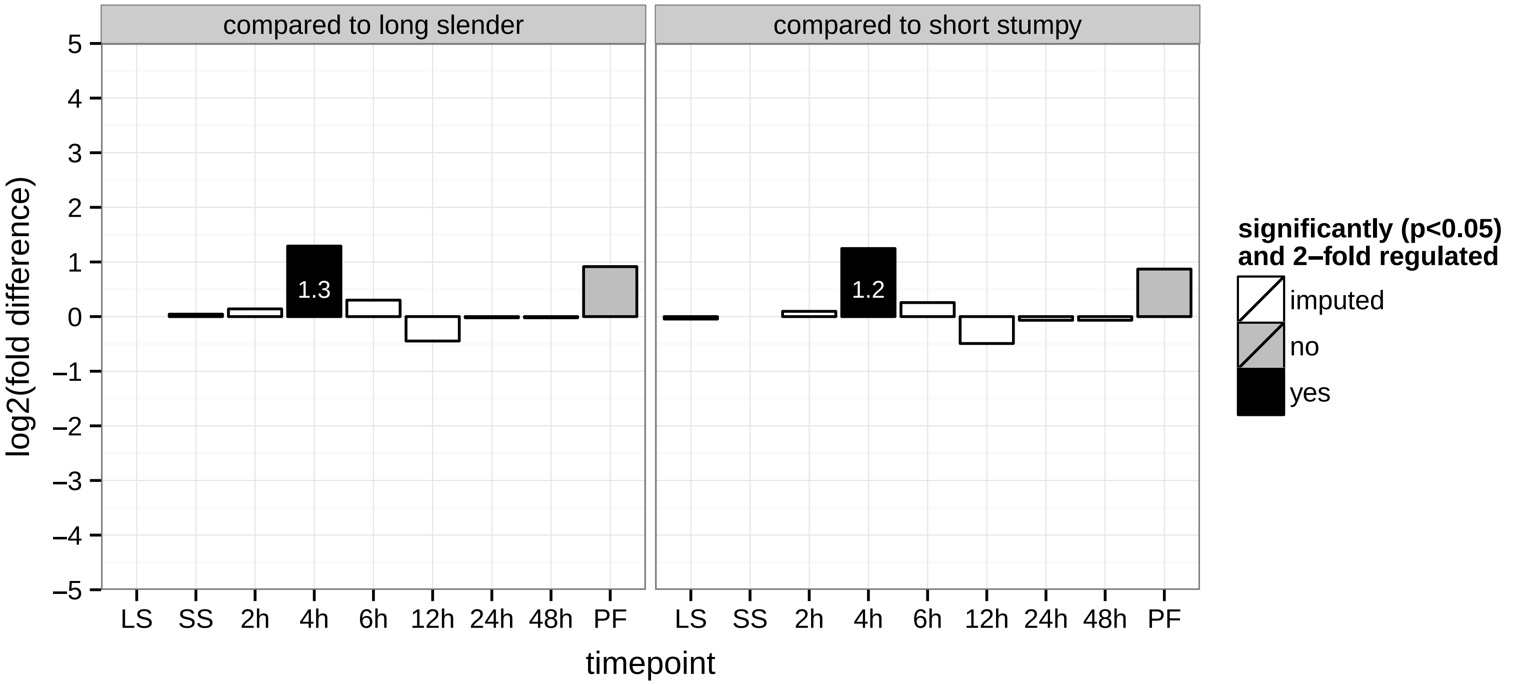

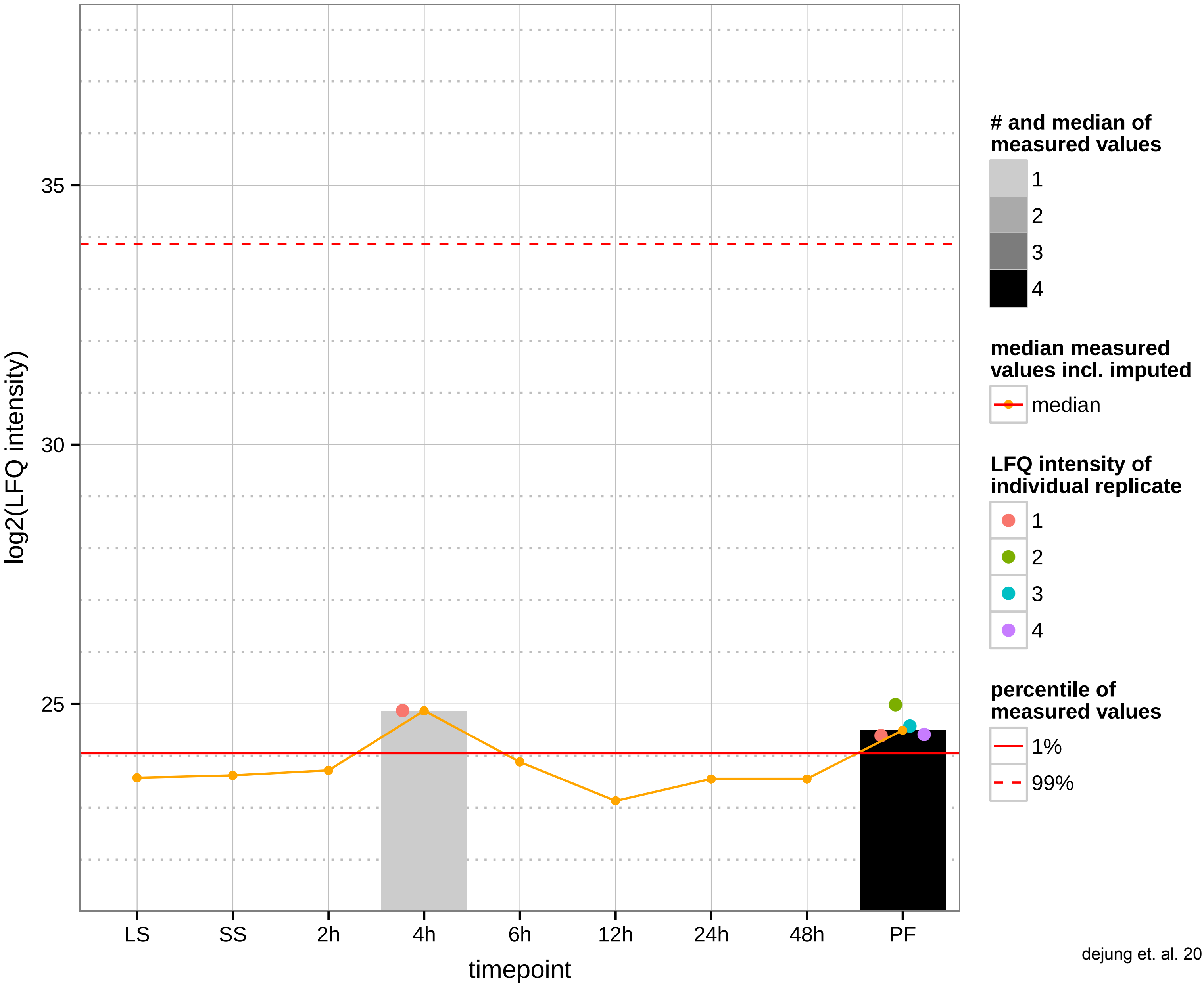

dejung et. al. 2015

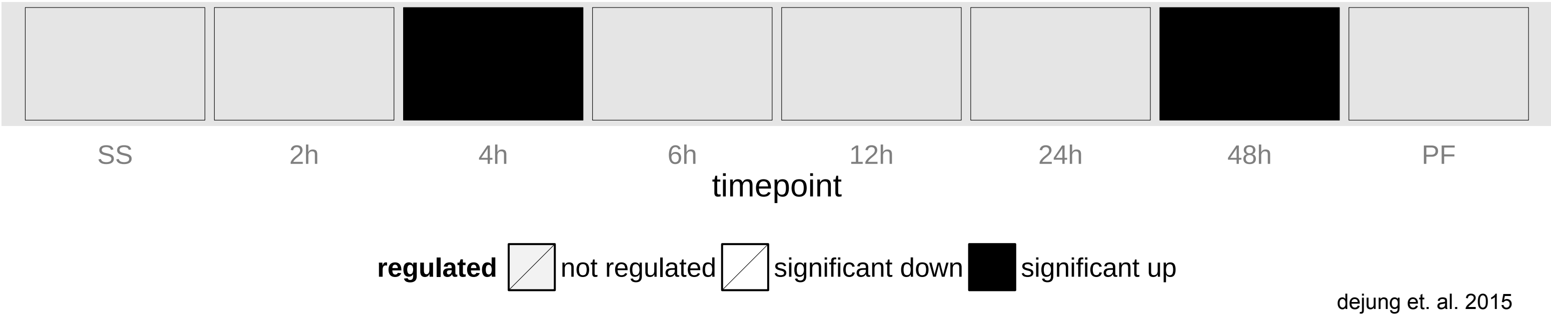
SS
2h
4h
6h
12h
24h
48h
PF
timepoint
regulated
not regulated
significant down
significant up
dejung et. al. 2015

hypothetical protein, conserved
Tb927.1.1390
AGOF: null
AGOC: null
AGOP: null
PGOF: null
PGOC: null
PGOP: null

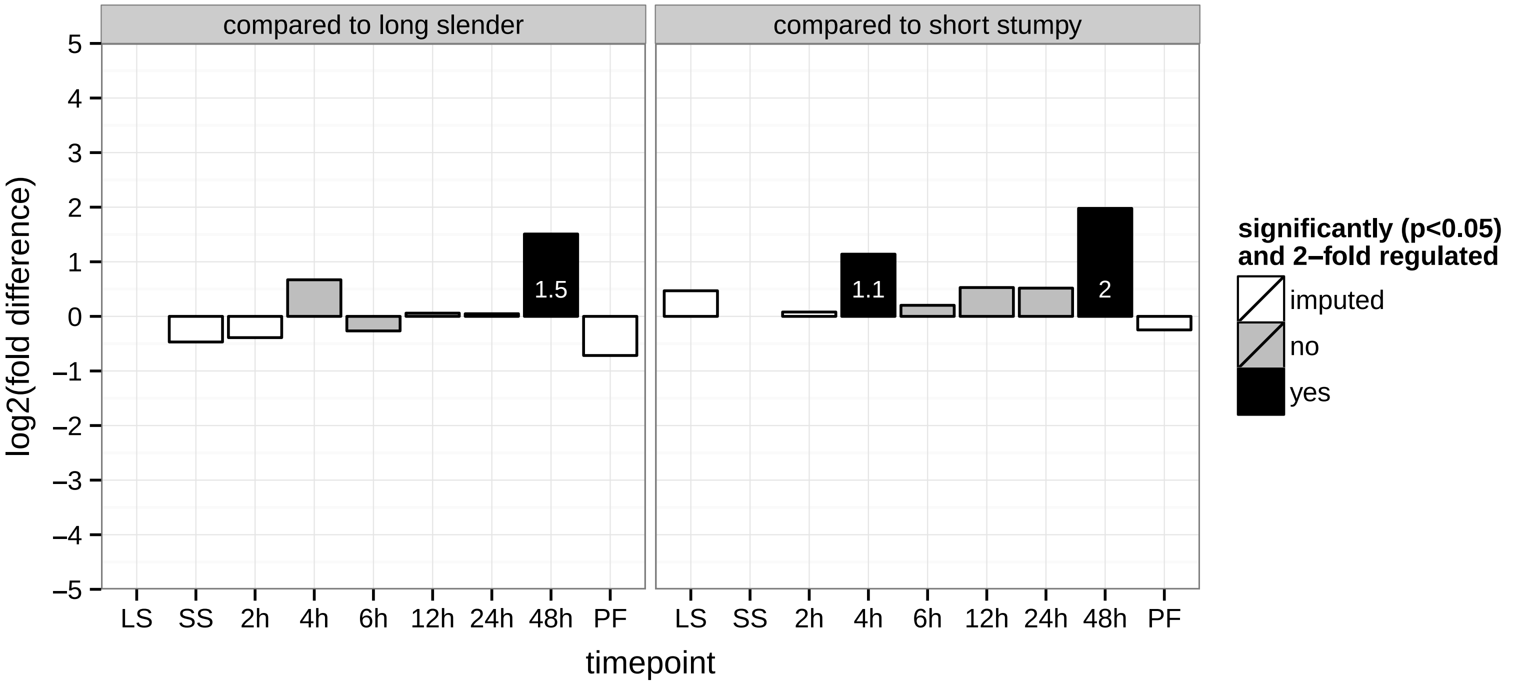

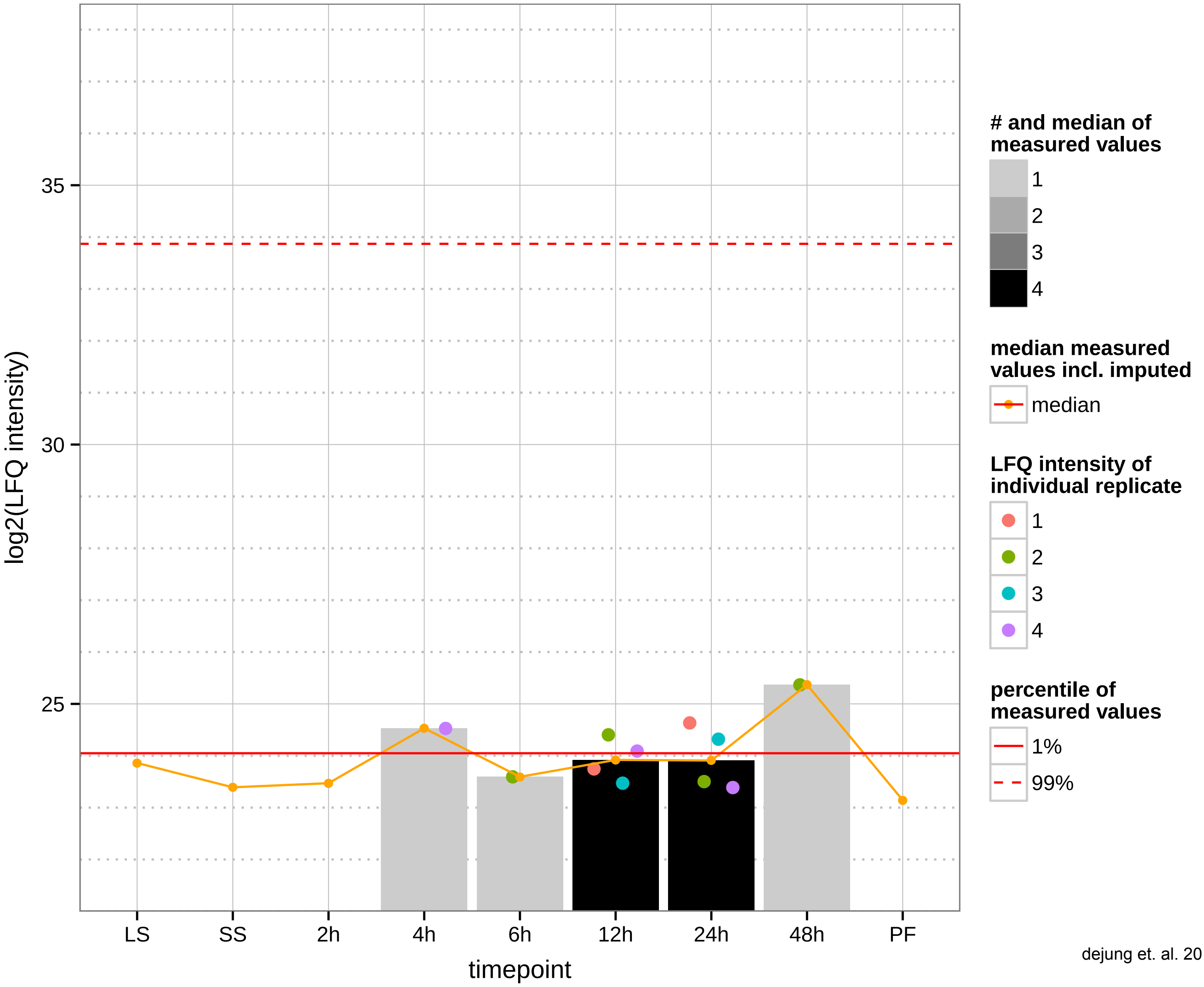

dejung et. al. 2015

hypothetical protein, conserved
Tb927.3.1910
AGOF: null
AGOC: null
AGOP: null
PGOF: null
PGOC: null
PGOP: null

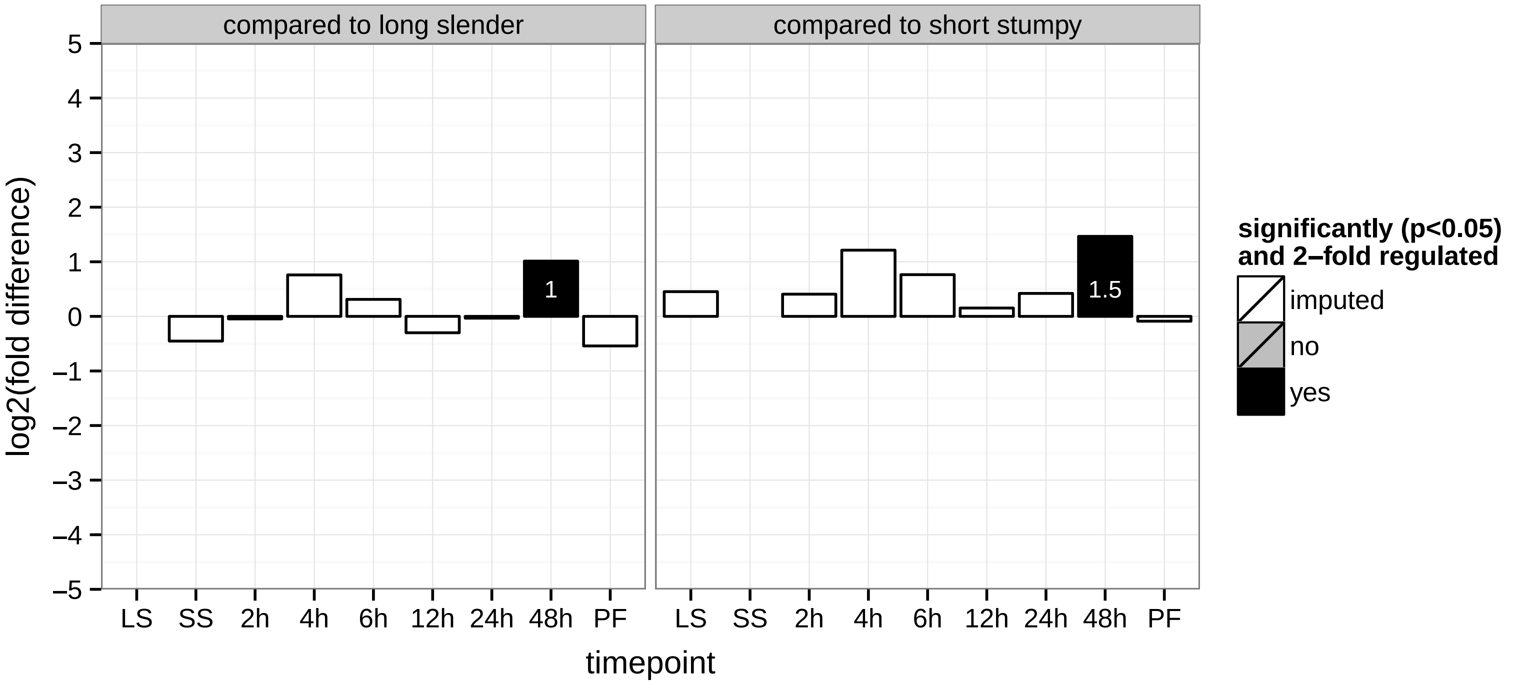

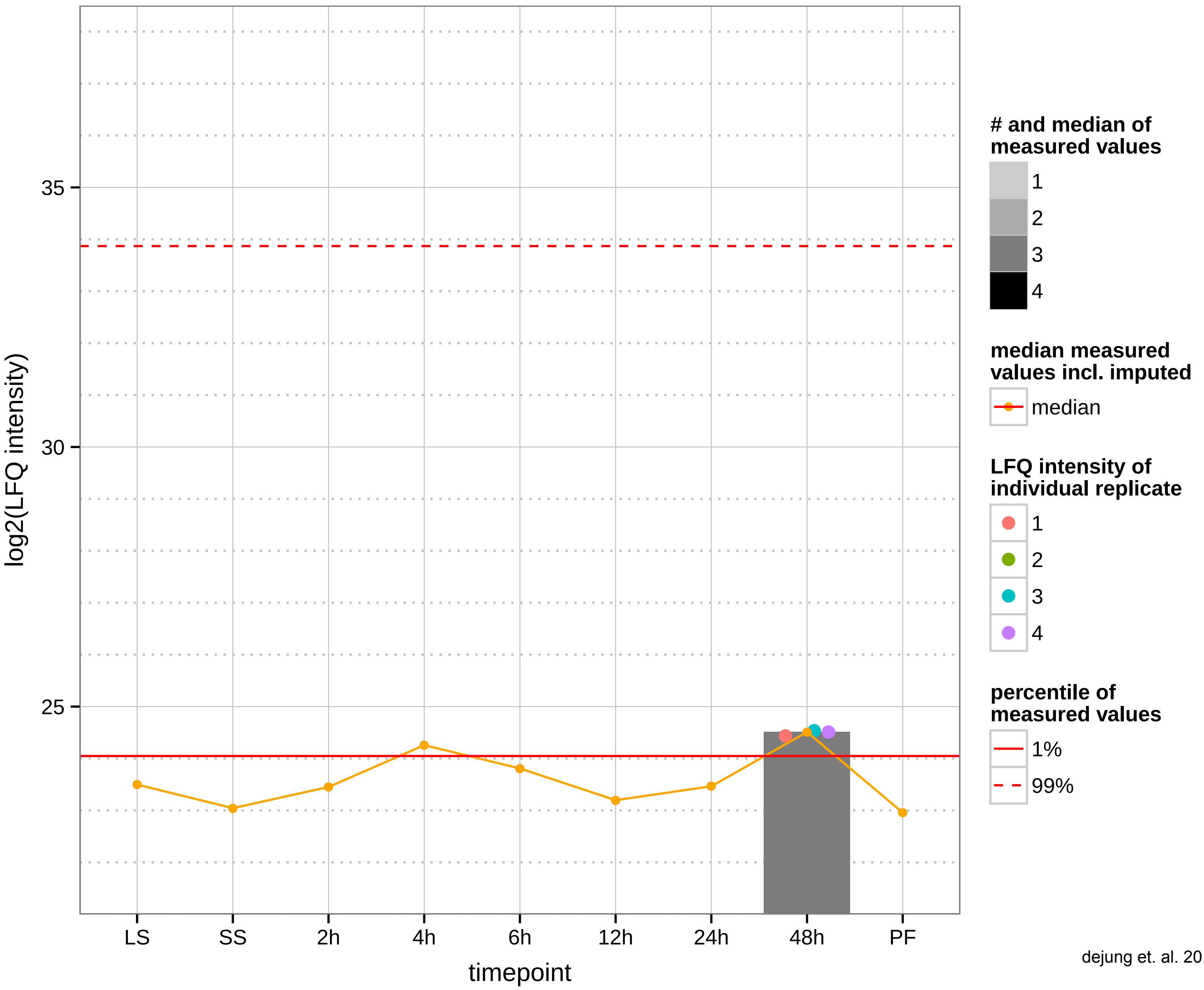

dejung et. al. 2015

hypothetical protein, conserved
Tb927.3.1930
AGOF: null
AGOC: null
AGOP: null
PGOF: protein binding
PGOC: null
PGOP: null

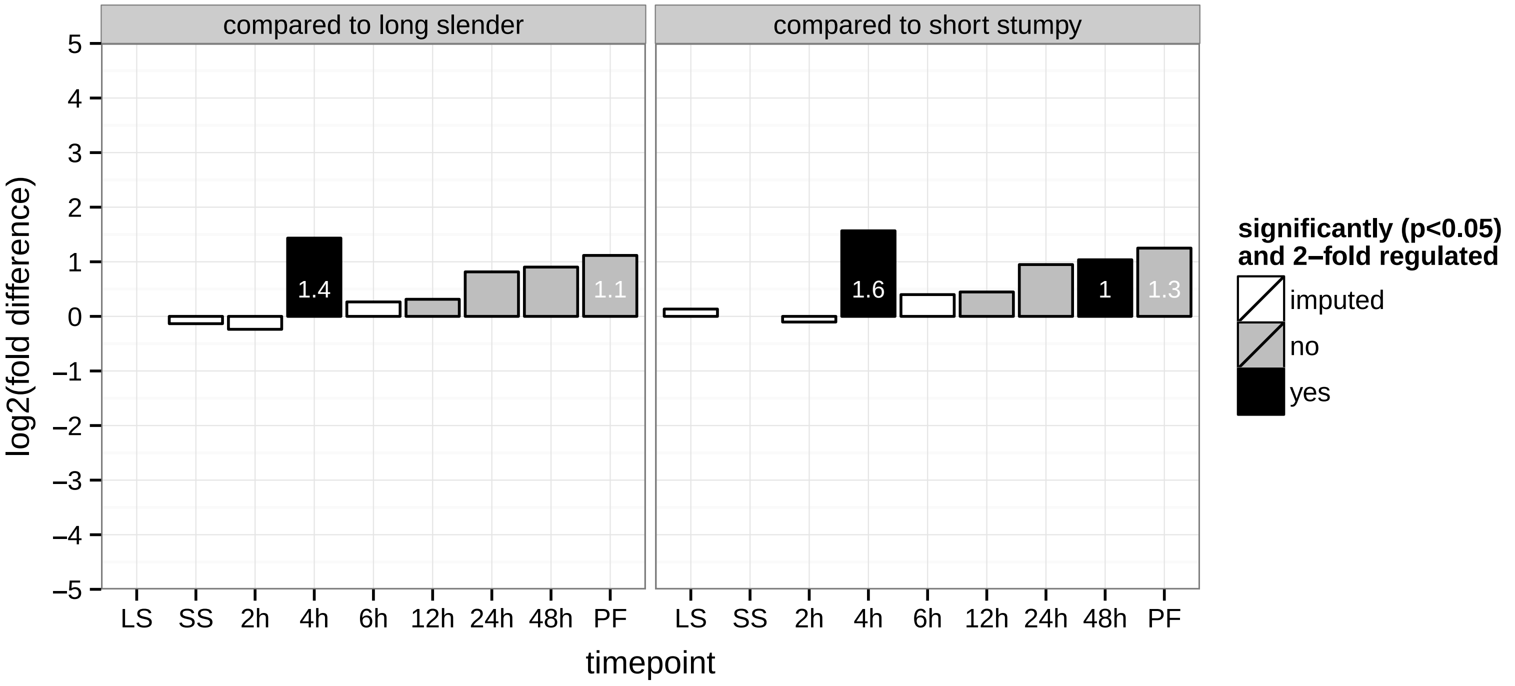

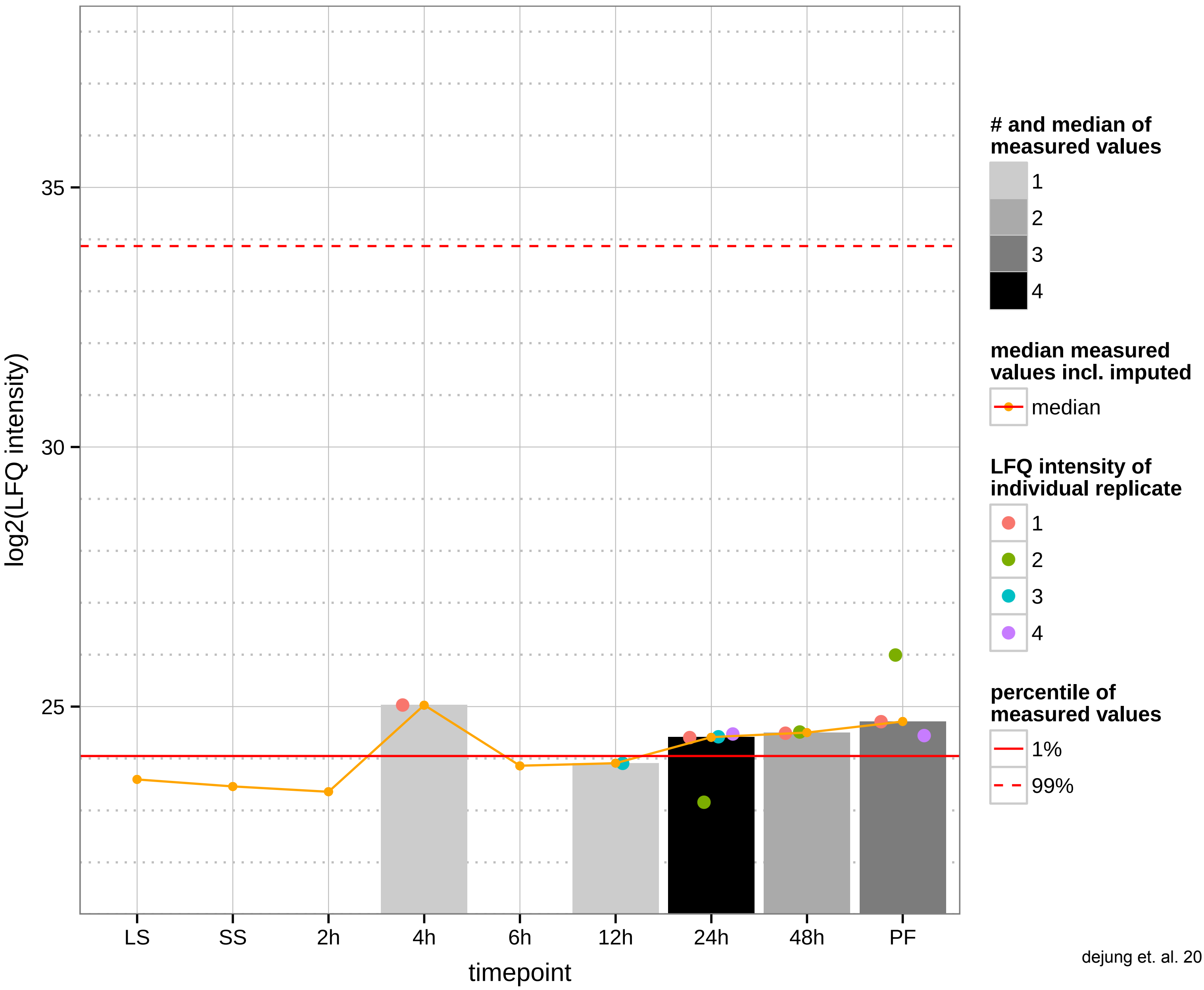

dejung et. al. 2015

tousled–like kinase I (TLK1), tousled–like kinase II (TLK2)
Tb927.4.5180;Tb927.8.7220
AGOF: ATP binding, protein binding, protein serine/threonine kinase activity, protein tyrosine kinase activity
AGOC: nucleus, spindle pole
AGOP: DNA replication, chromosome segregation, histone H3–S10 phosphorylation, protein phosphorylation, spindle assem
PGOF: ATP binding, protein kinase activity, transferase activity, transferring phosphorus–containing groups
PGOC: null
PGOP: protein phosphorylation

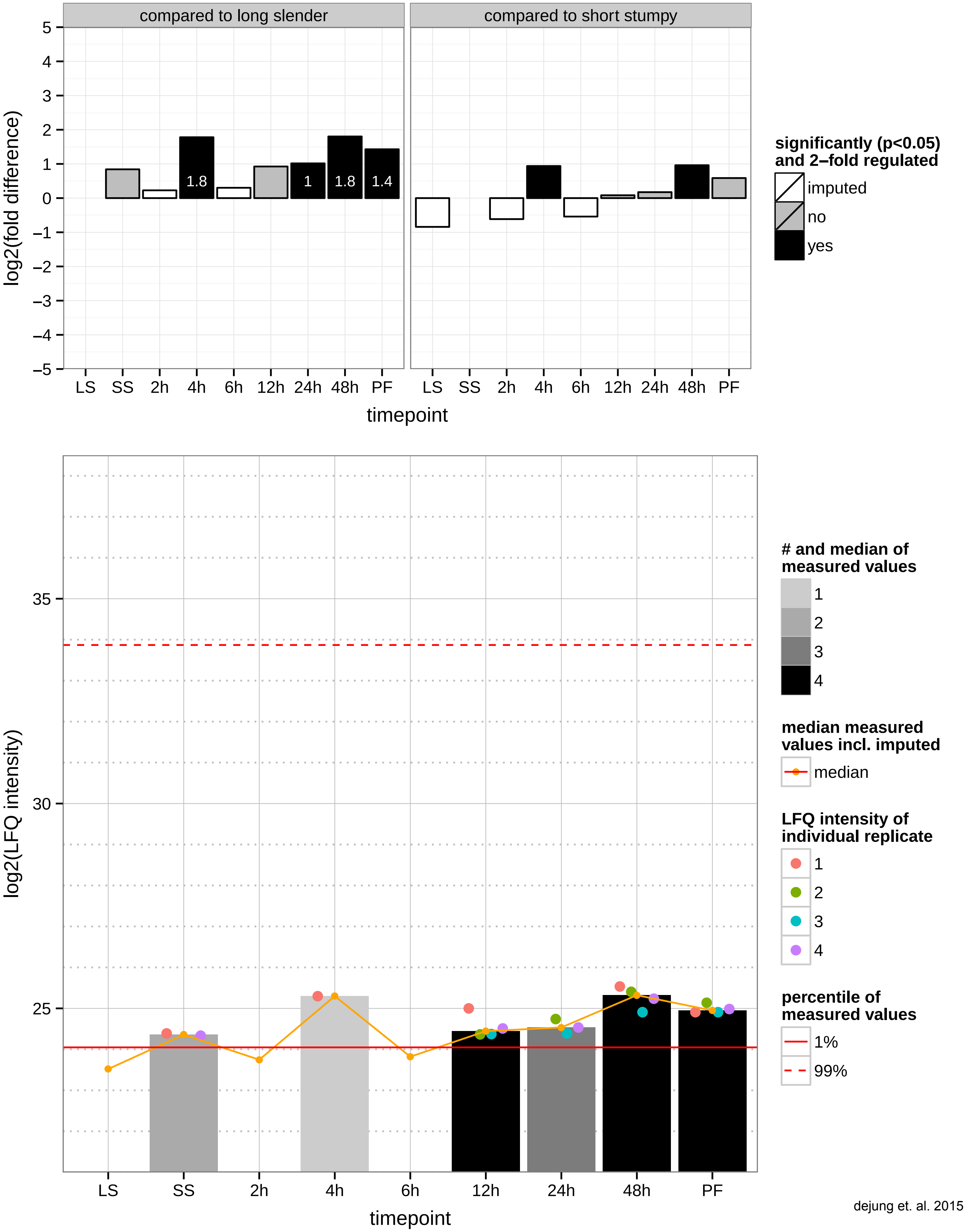

dejung et. al. 2015

RNA–editing complex protein, RNA–editing 3' terminal uridylyl transferase 1, KRET1 (KRET1)
Tb927.7.3950
AGOF: RNA uridylyltransferase activity, nucleotide binding, polynucleotide adenylyltransferase activity
AGOC: mitochondrion
AGOP: null
PGOF: null
PGOC: null
PGOP: null

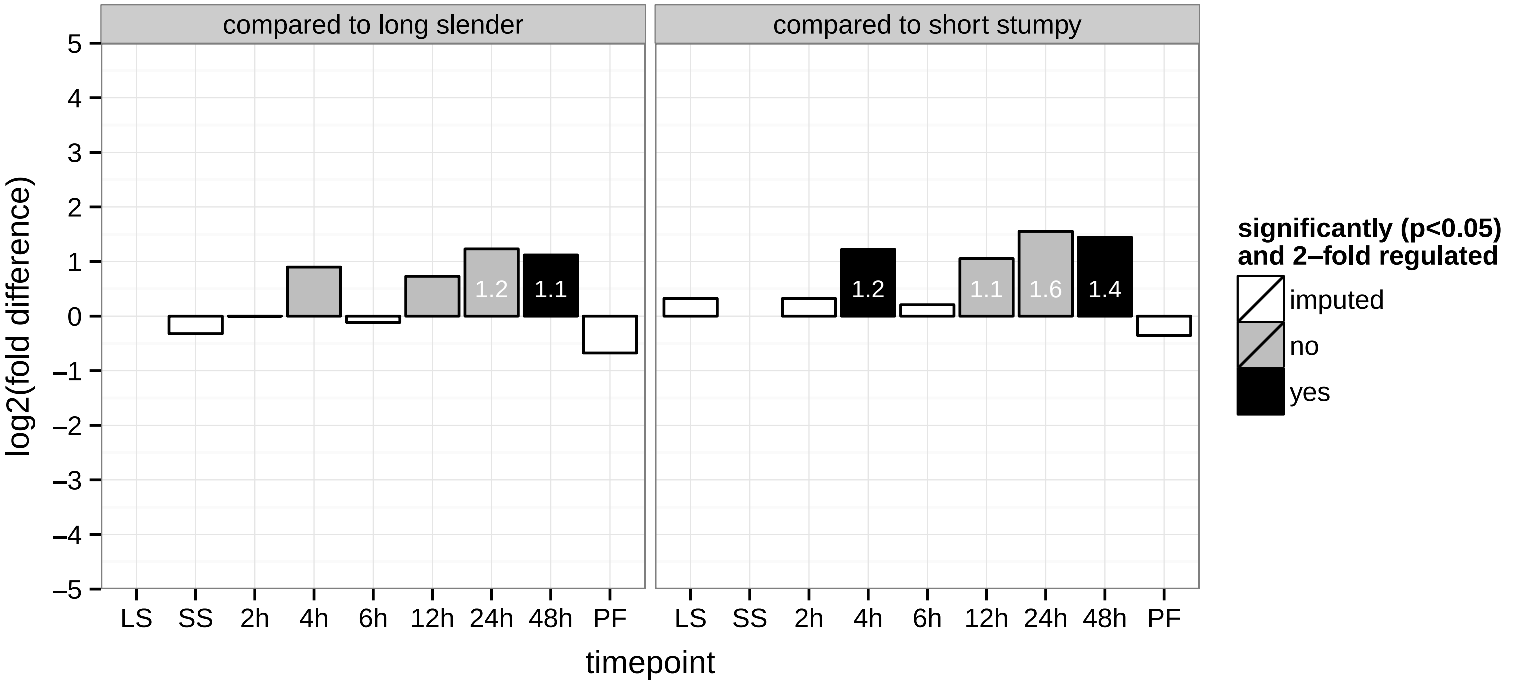

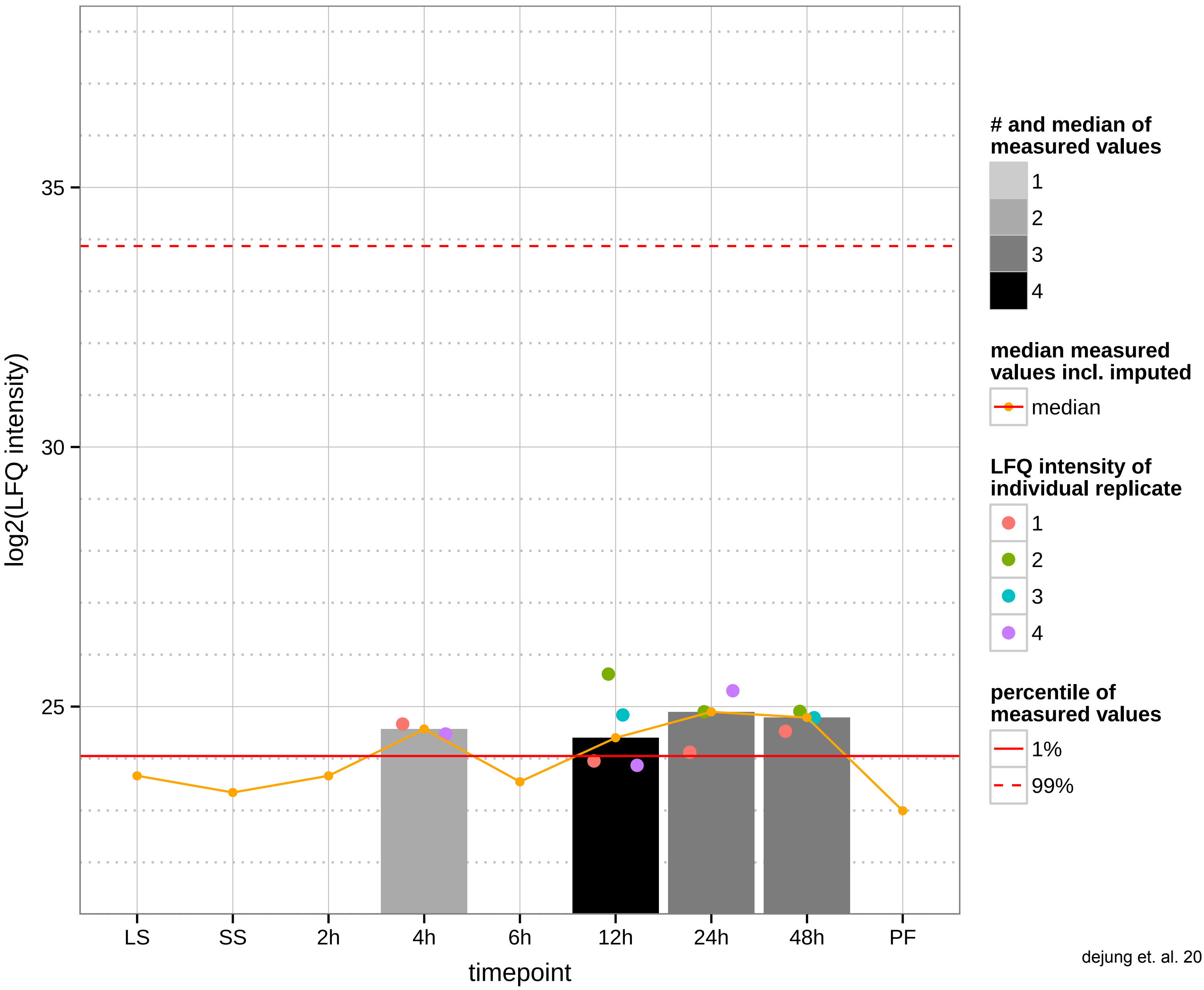

dejung et. al. 2015

hypothetical protein, conserved
Tb927.8.2430
AGOF: null
AGOC: null
AGOP: null
PGOF: null
PGOC: null
PGOP: null

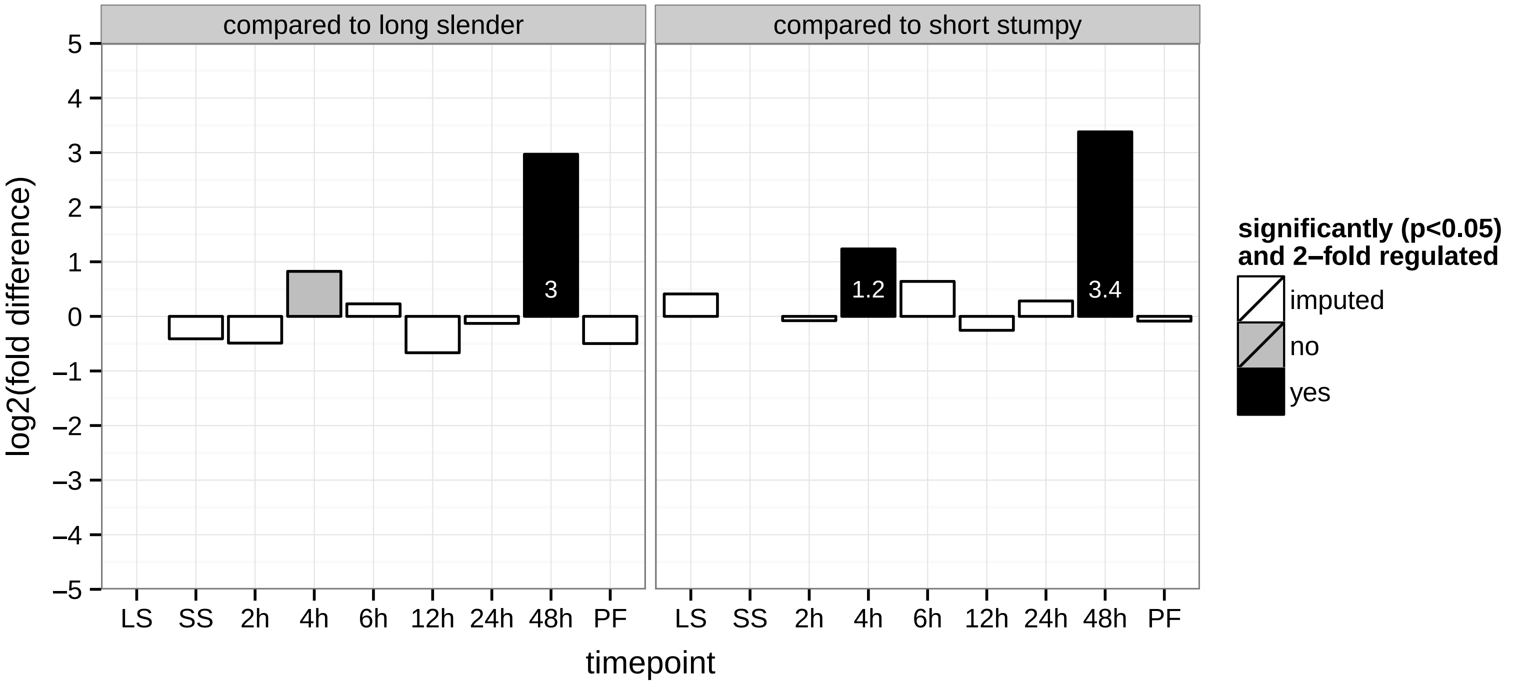

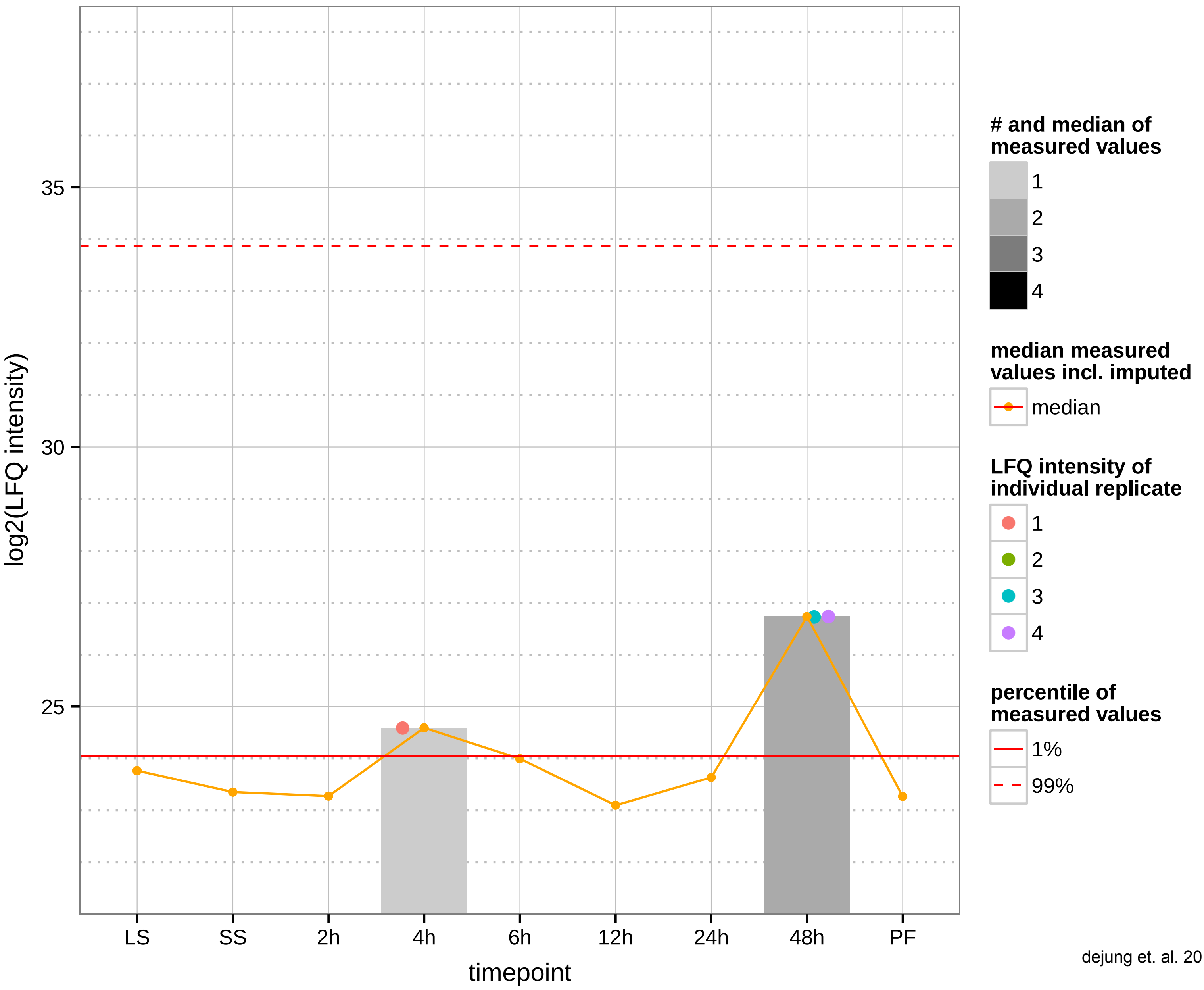

dejung et. al. 2015

nucleosome assembly protein–like protein
Tb927.9.5730
AGOF: histone binding
AGOC: nucleus
AGOP: nucleosome assembly
PGOF: null
PGOC: nucleus
PGOP: nucleosome assembly

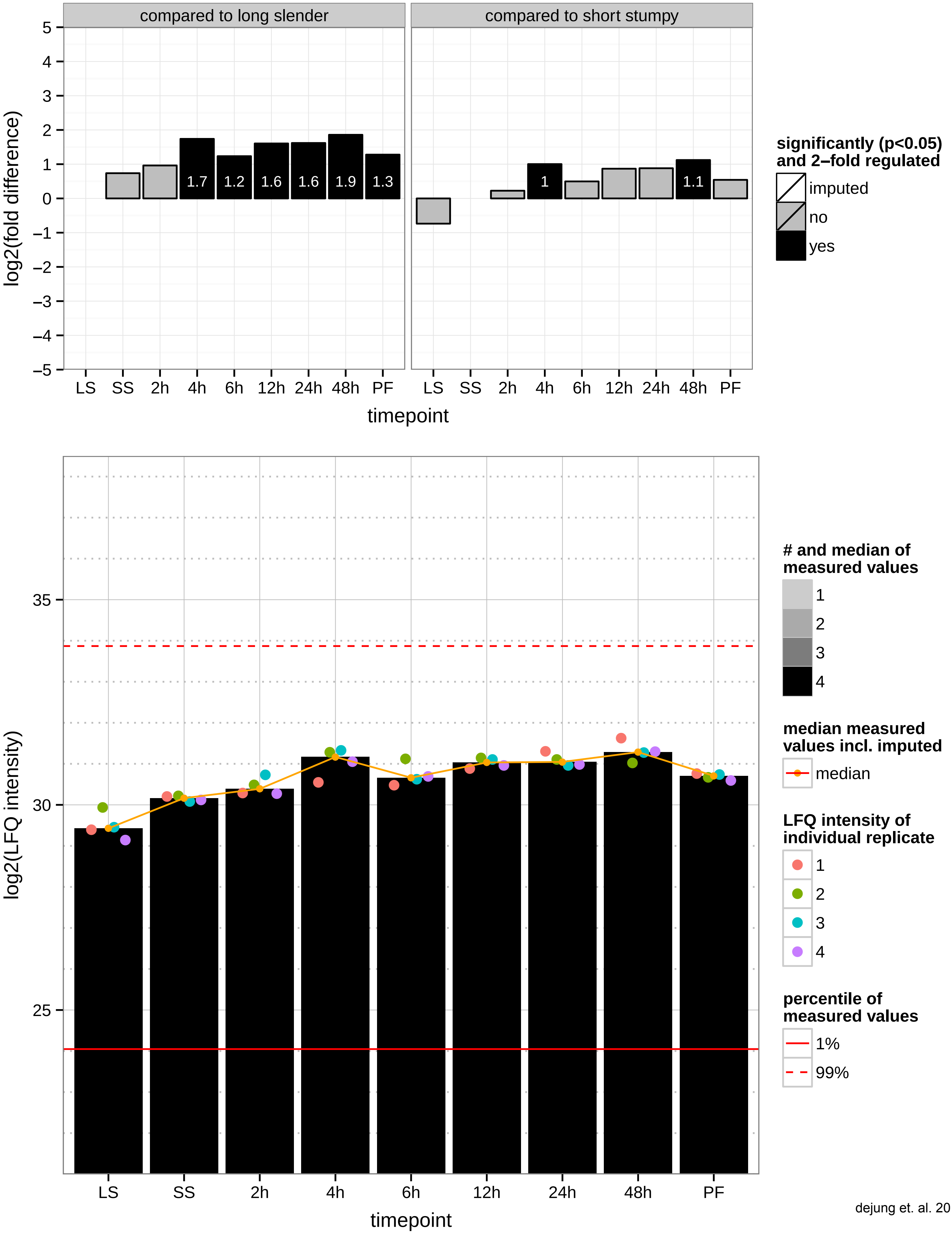

dejung et. al. 2015

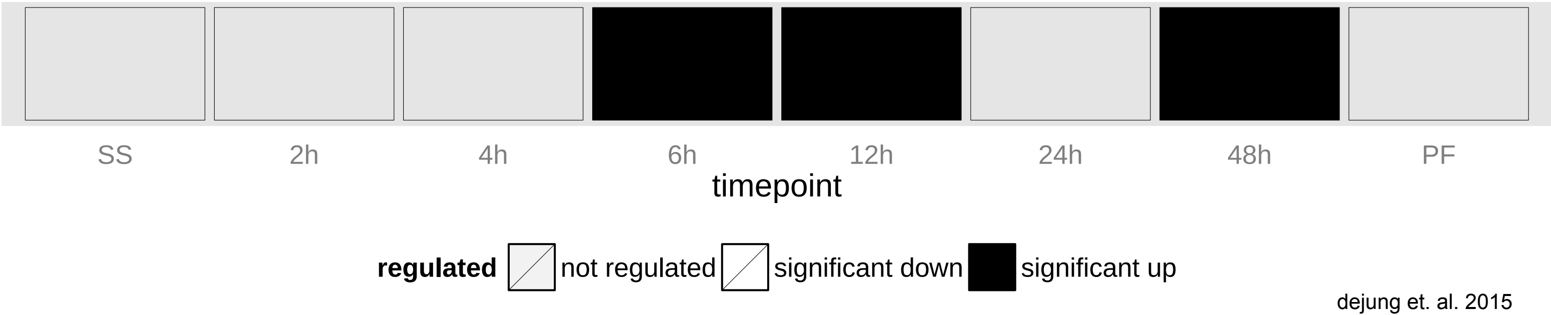
SS
2h
4h
6h
12h
24h
48h
PF
timepoint
regulated
not regulated
significant down
significant up
dejung et. al. 2015

kinetoplastid kinetochore protein 19, protein kinase, putative (kkt19)
Tb927.11.12420
AGOF: ATP binding, protein serine/threonine kinase activity, protein tyrosine kinase activity
AGOC: null
AGOP: protein phosphorylation
PGOF: ATP binding, protein kinase activity, transferase activity, transferring phosphorus–containing groups
PGOC: null
PGOP: protein phosphorylation

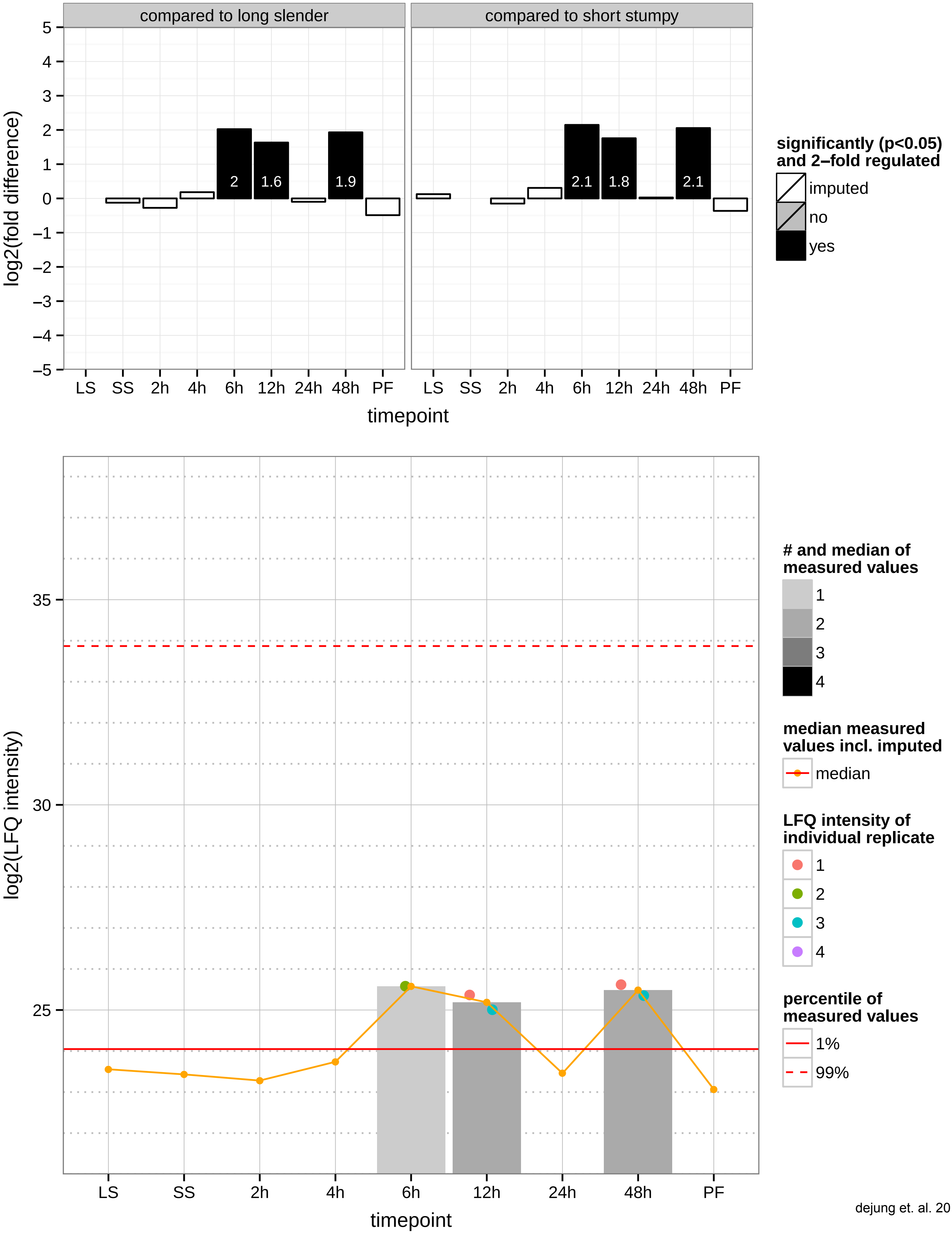

dejung et. al. 2015

hypothetical protein, conserved
Tb927.2.2540
AGOF: null
AGOC: null
AGOP: null
PGOF: null
PGOC: null
PGOP: null

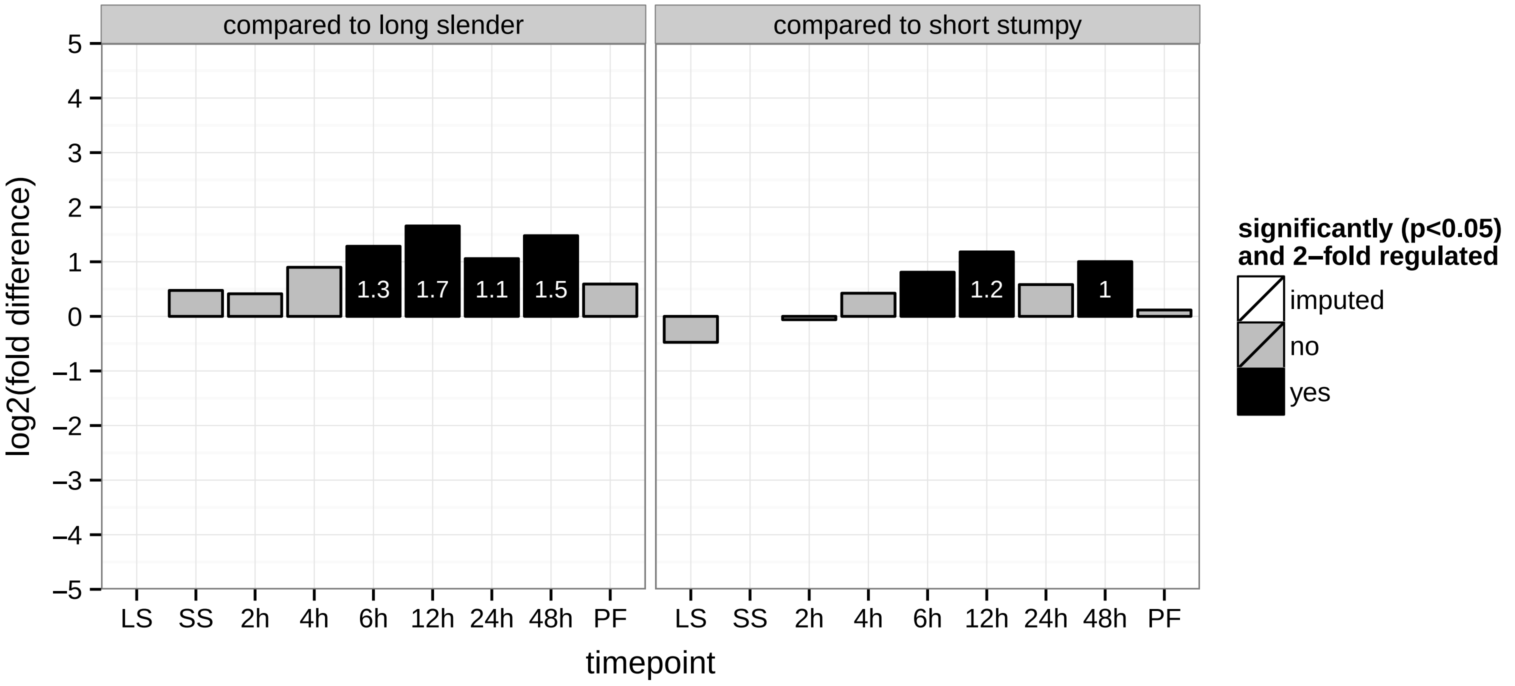

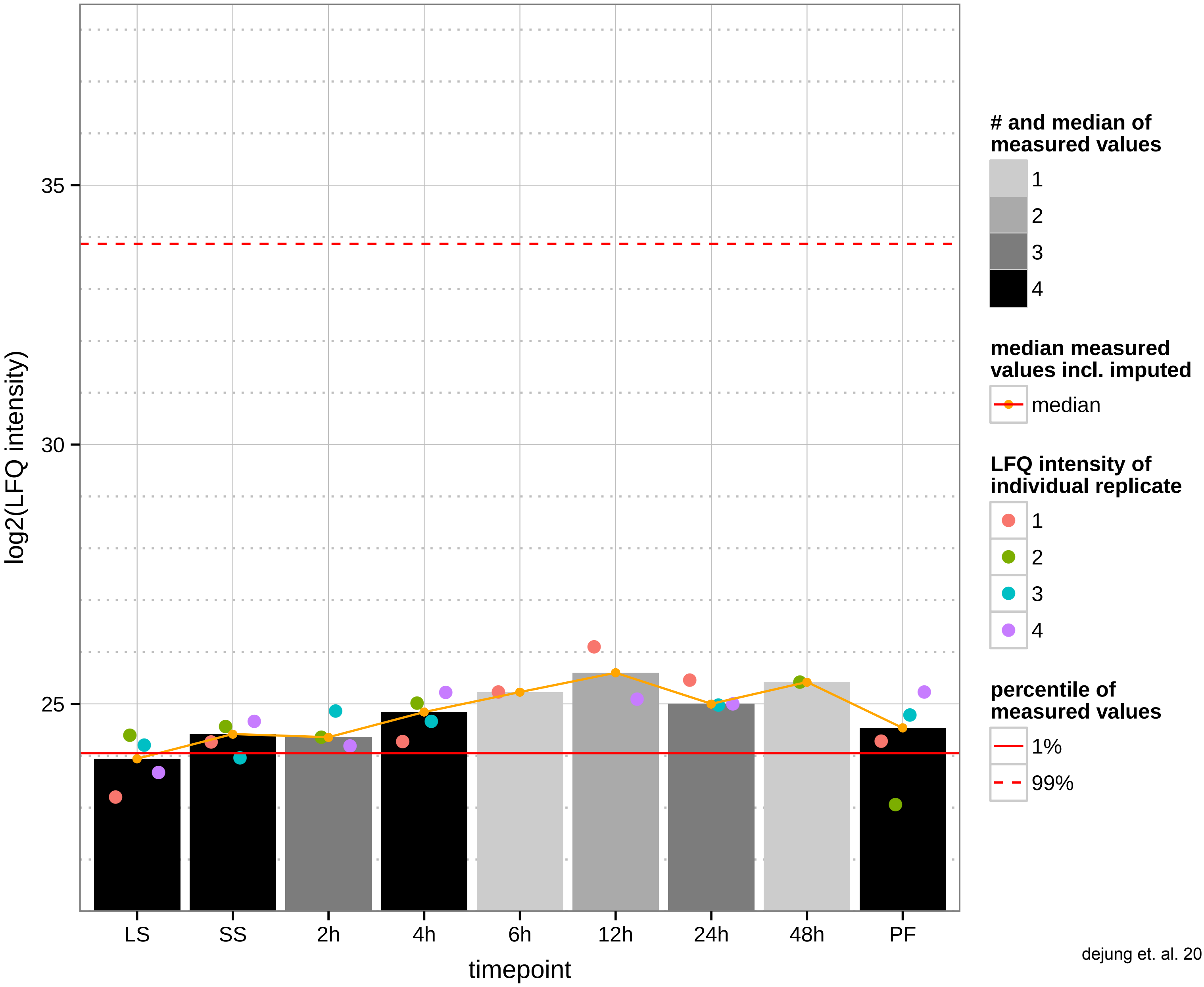

dejung et. al. 2015

hypothetical protein, conserved
Tb927.3.4210
AGOF: null
AGOC: mitochondrion
AGOP: null
PGOF: null
PGOC: null
PGOP: null

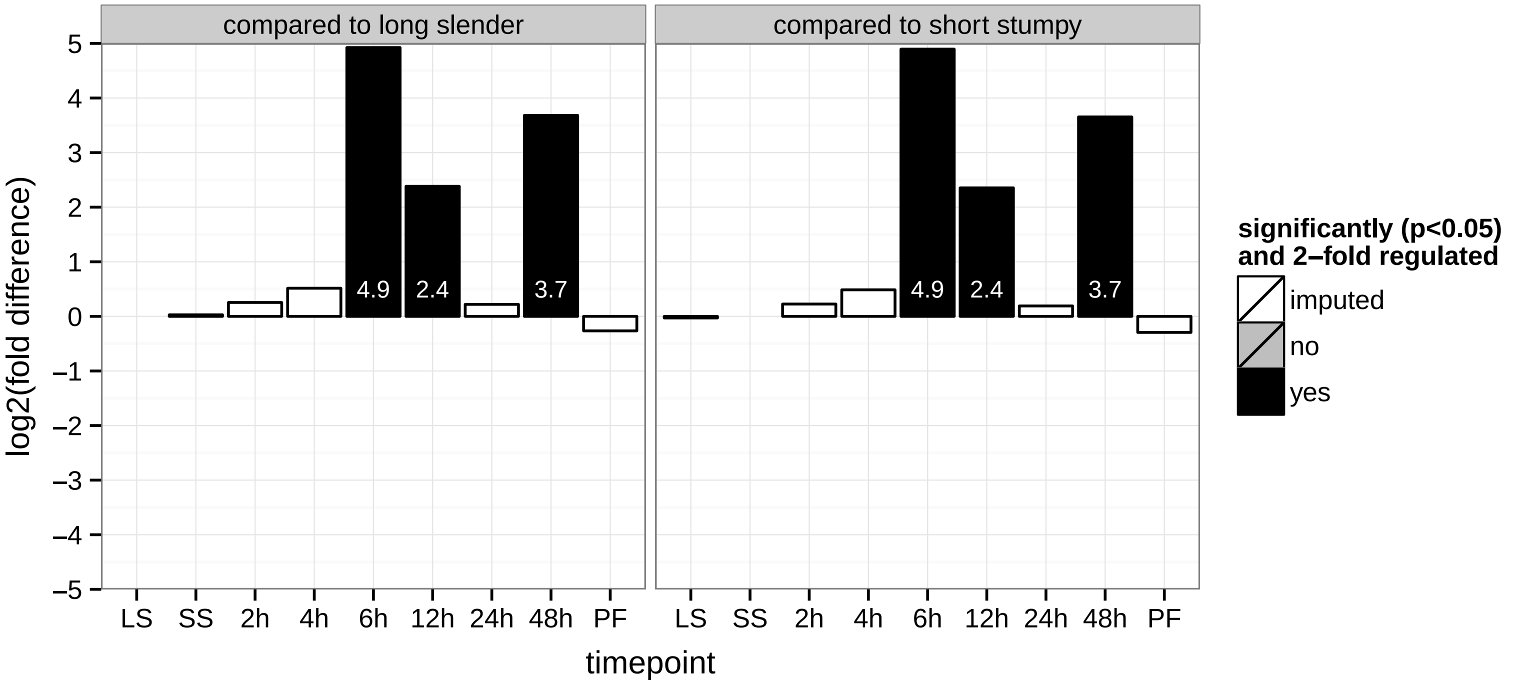

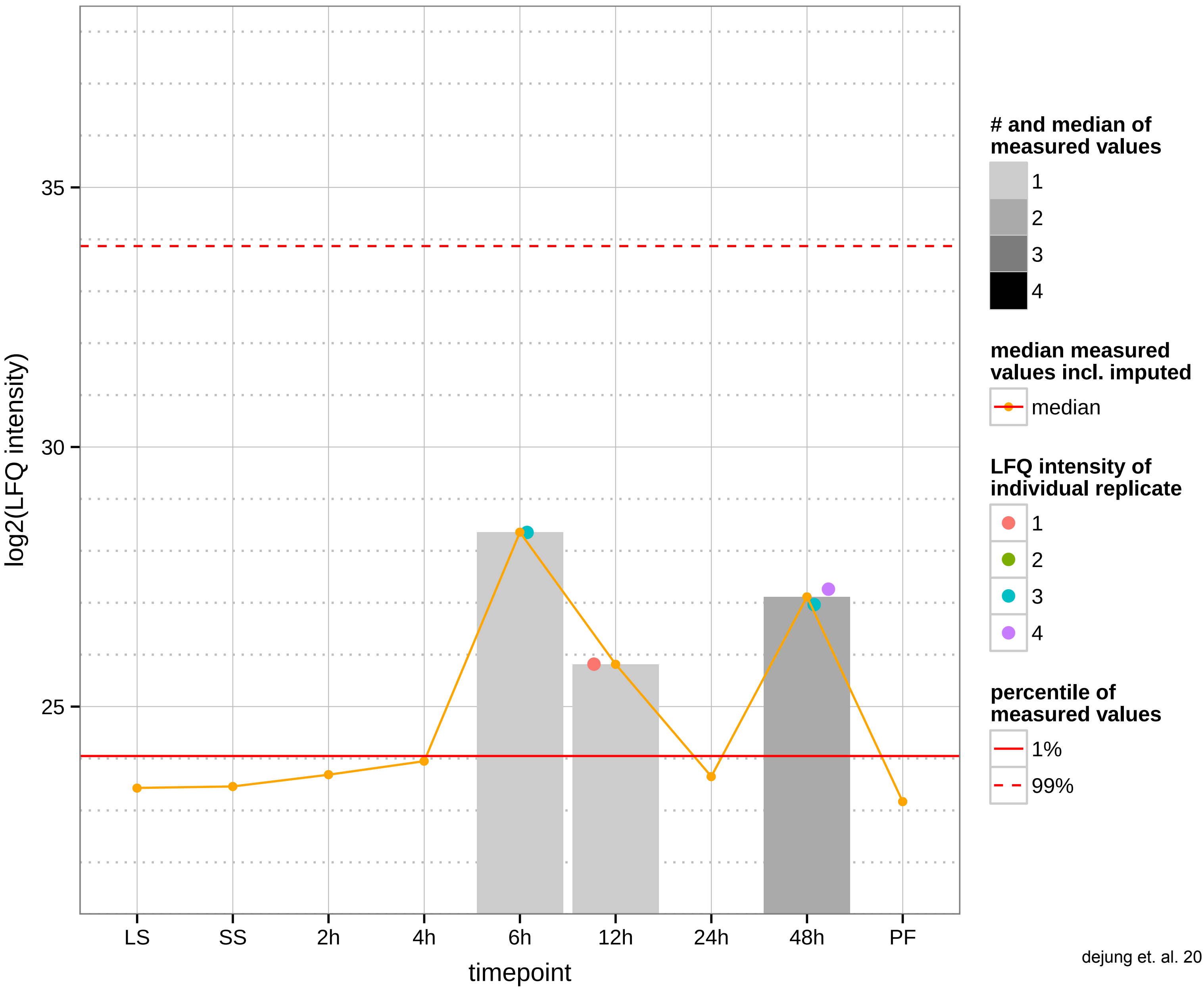

dejung et. al. 2015

hypothetical protein, conserved
Tb927.7.3060
AGOF: null
AGOC: null
AGOP: null
PGOF: null
PGOC: null
PGOP: null

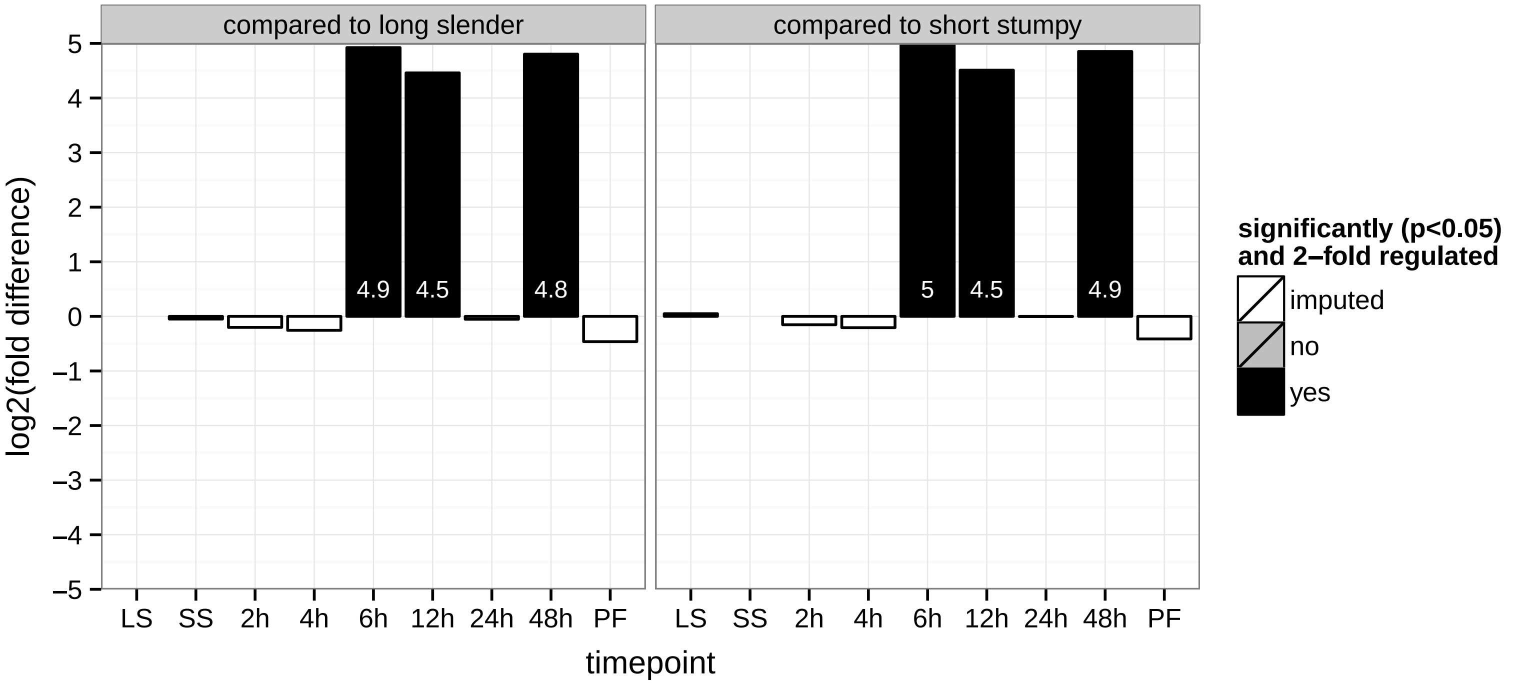

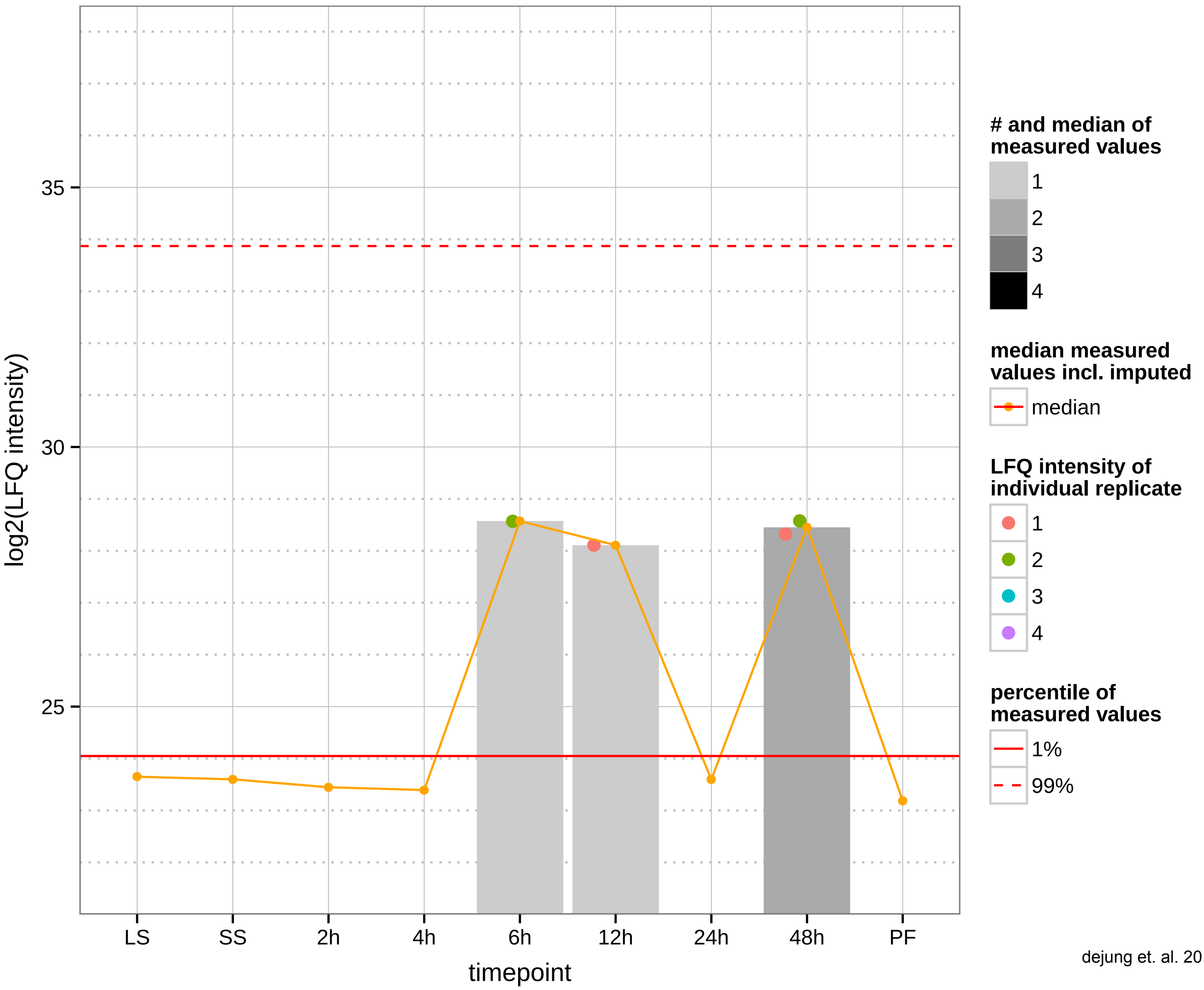

dejung et. al. 2015

hypothetical protein, conserved
Tb927.7.660
AGOF: null
AGOC: null
AGOP: null
PGOF: protein binding
PGOC: null
PGOP: null

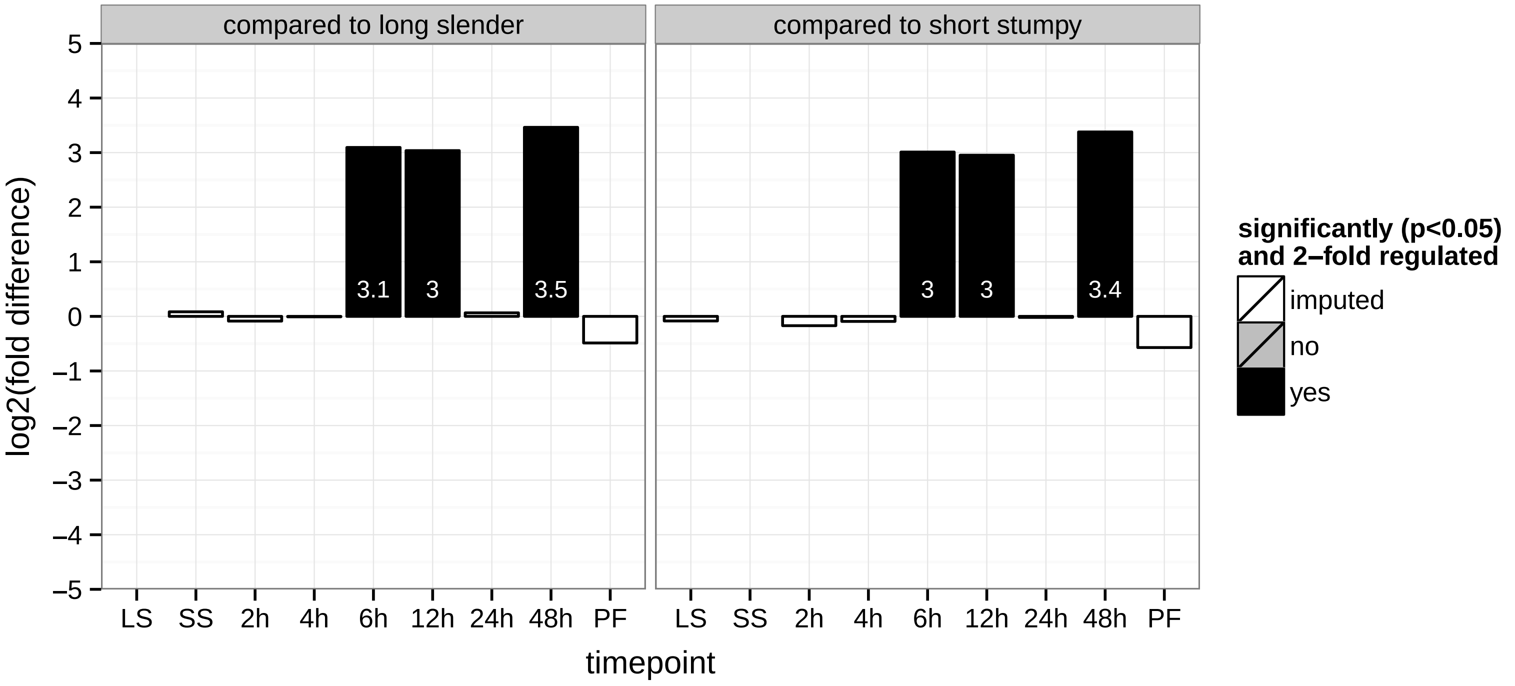

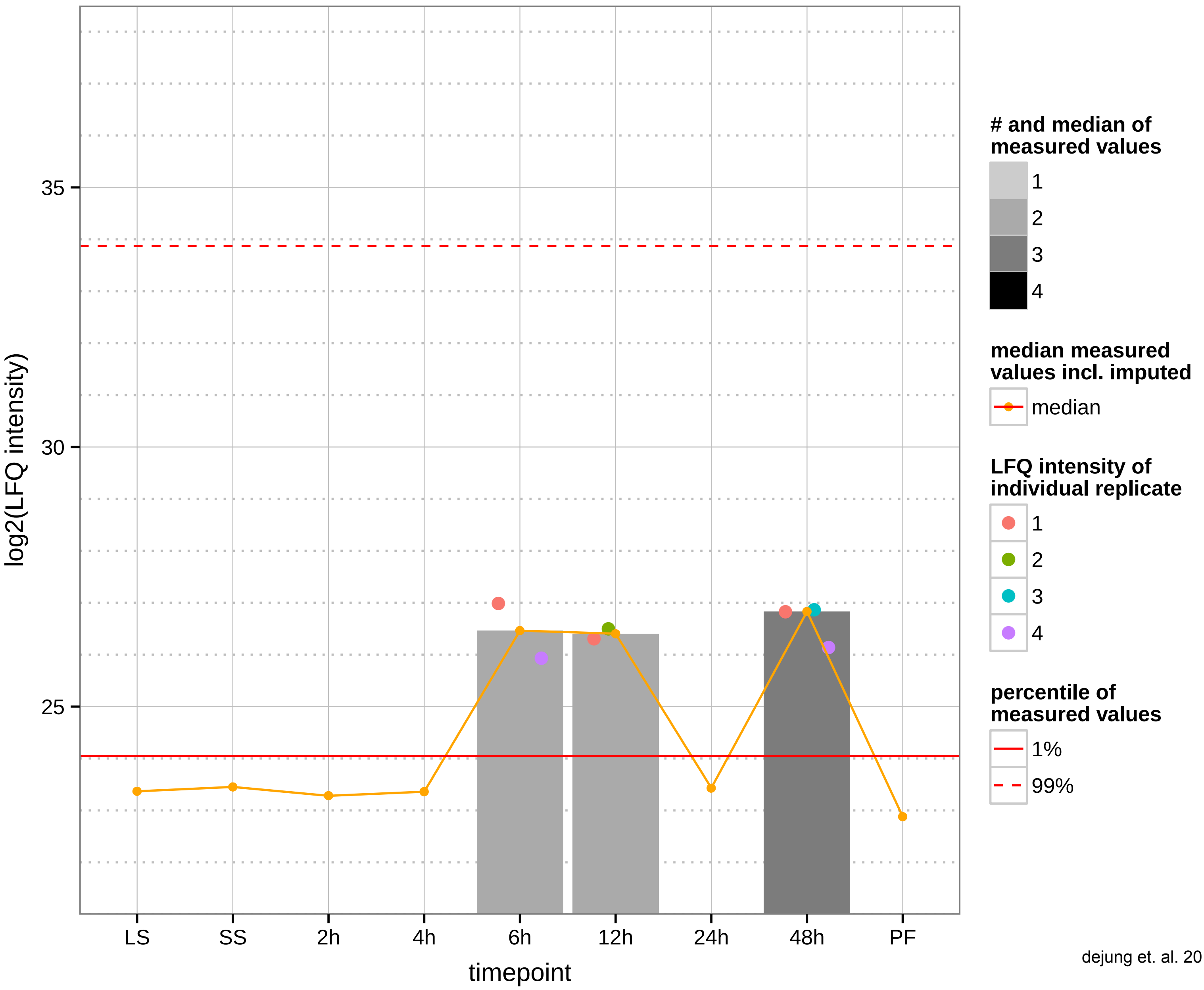

dejung et. al. 2015

hypothetical protein, conserved
Tb927.8.7080
AGOF: null
AGOC: null
AGOP: null
PGOF: null
PGOC: null
PGOP: null

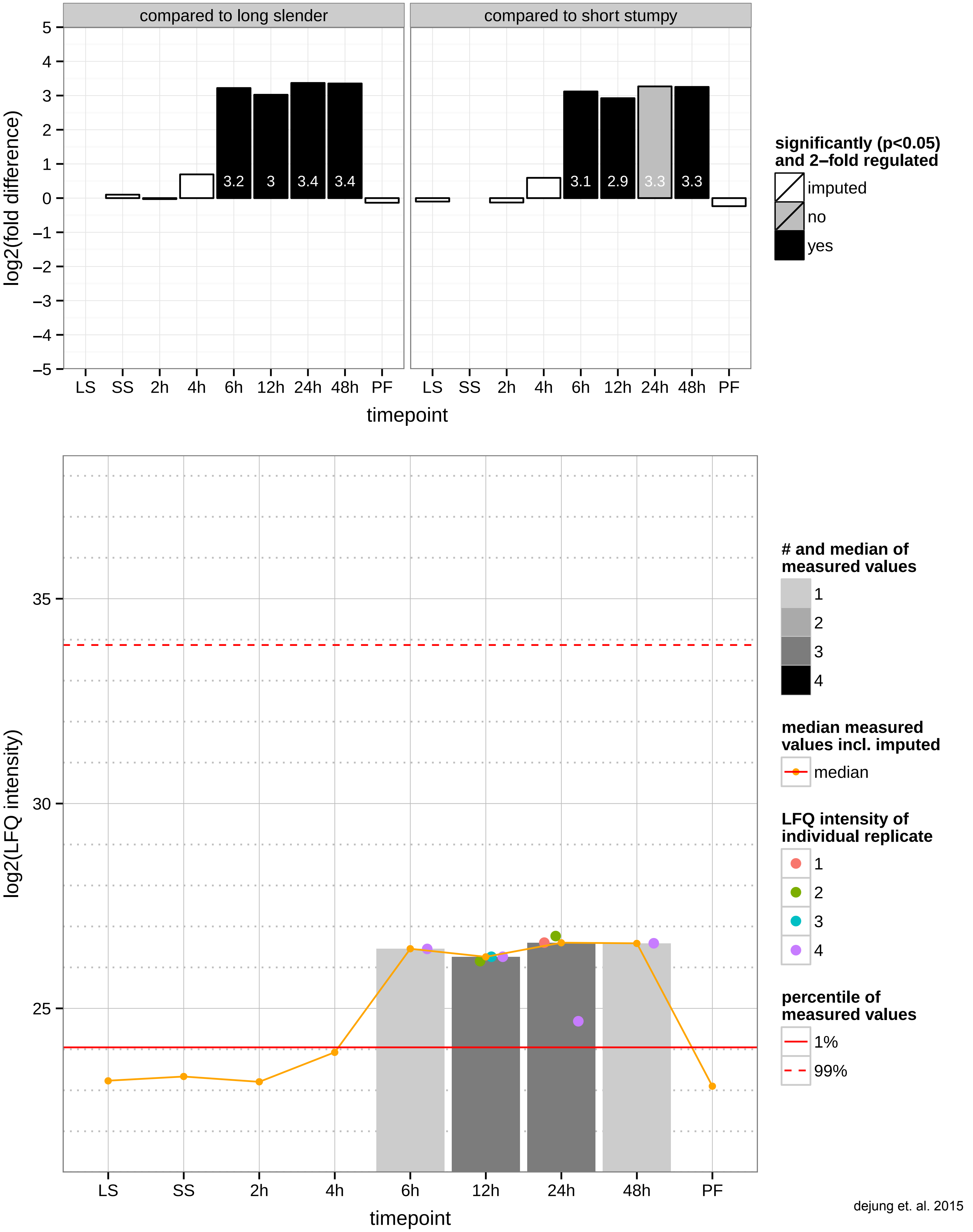

dejung et. al. 2015

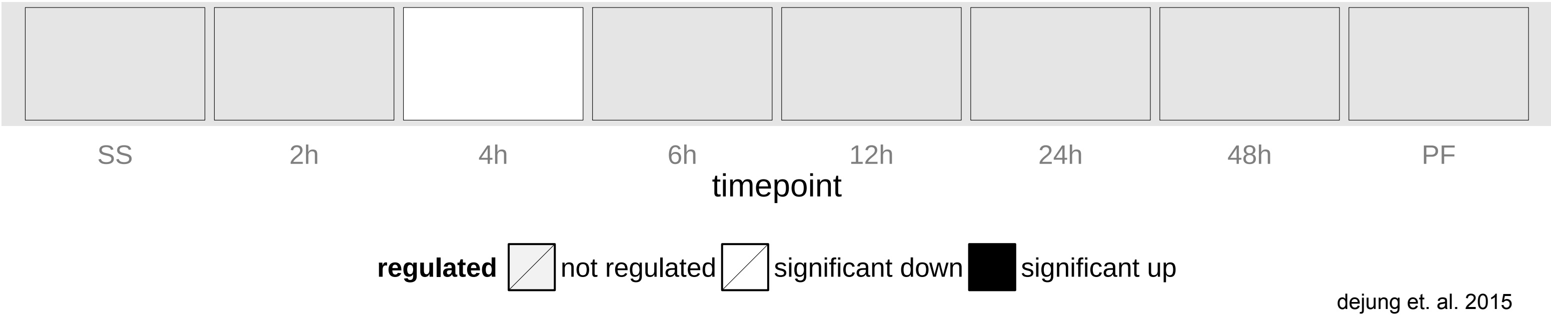
SS
2h
4h
6h
12h
24h
48h
PF
timepoint
regulated
not regulated
significant down
significant up
dejung et. al. 2015

protein kinase A catalytic subunit, putative, protein kinase A catalytic subunit (PKAC3)
Tb927.10.13010;Tb11.v5.0671
AGOF: null, ATP binding, protein serine/threonine kinase activity, protein tyrosine kinase activity
AGOC: null
AGOP: null, protein phosphorylation
PGOF: ATP binding, protein kinase activity, protein serine/threonine kinase activity, transferase activity, transferring phosphoru
PGOC: null
PGOP: protein phosphorylation

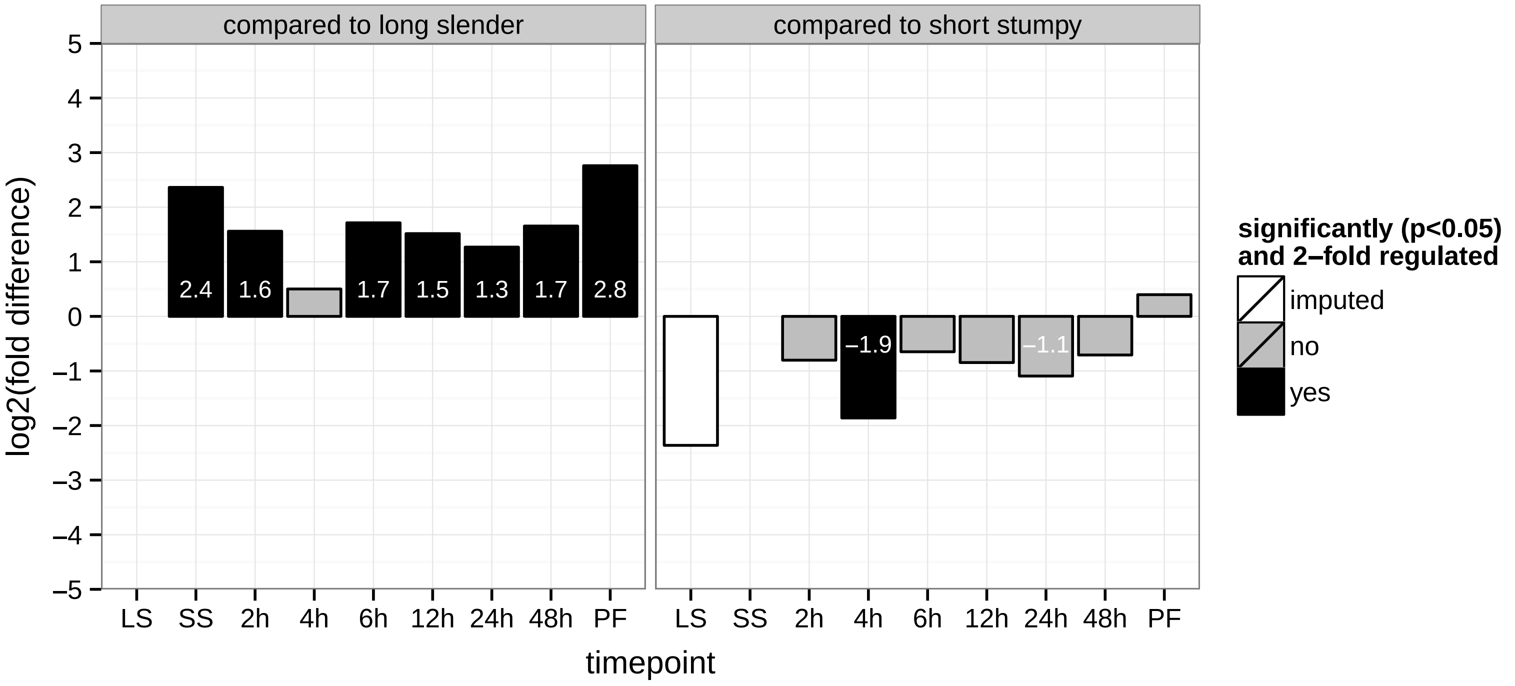

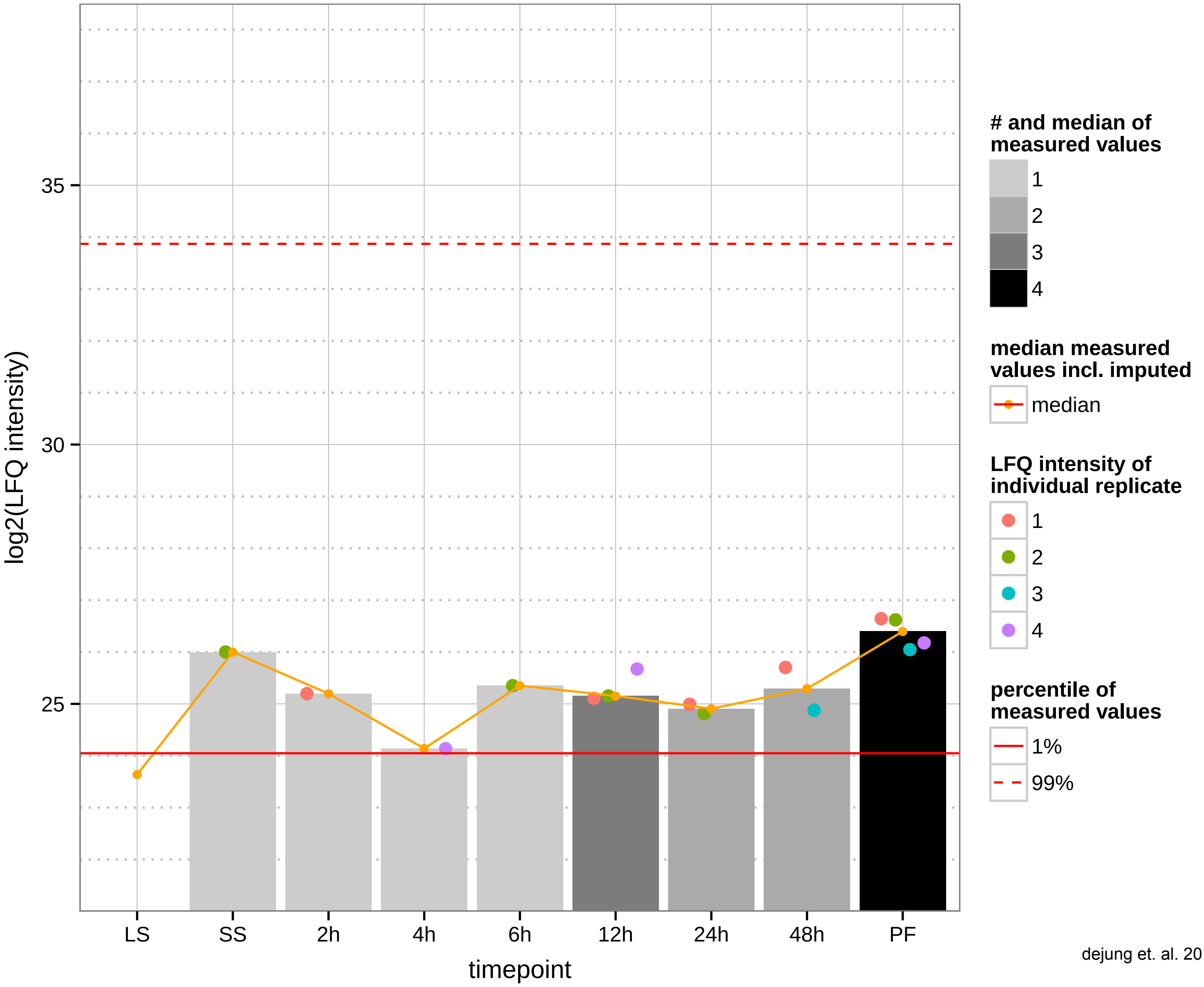

dejung et. al. 2015

Protein C21orf2 homolog, putative
Tb927.10.5130
AGOF: null
AGOC: mitochondrion
AGOP: cilium morphogenesis, cytoskeleton organization, regulation of cell shape
PGOF: null
PGOC: null
PGOP: null

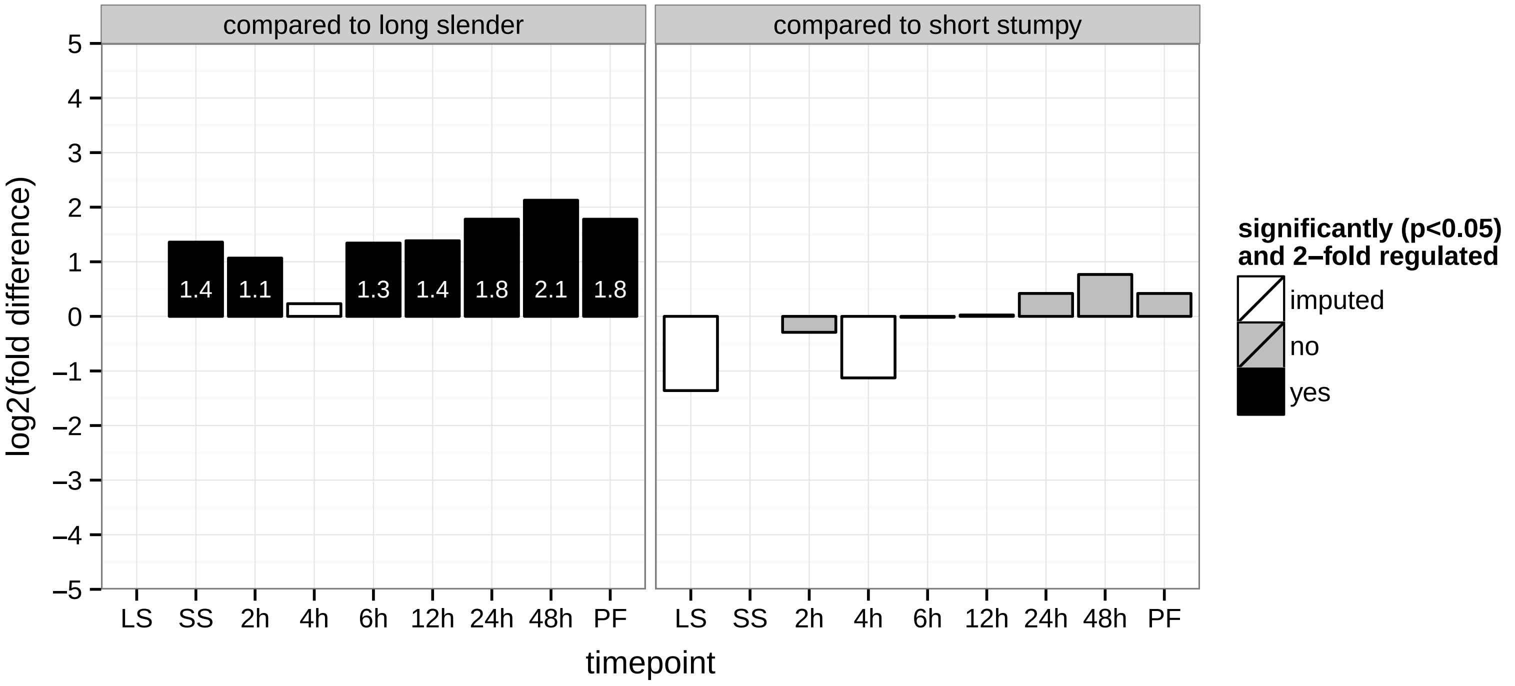

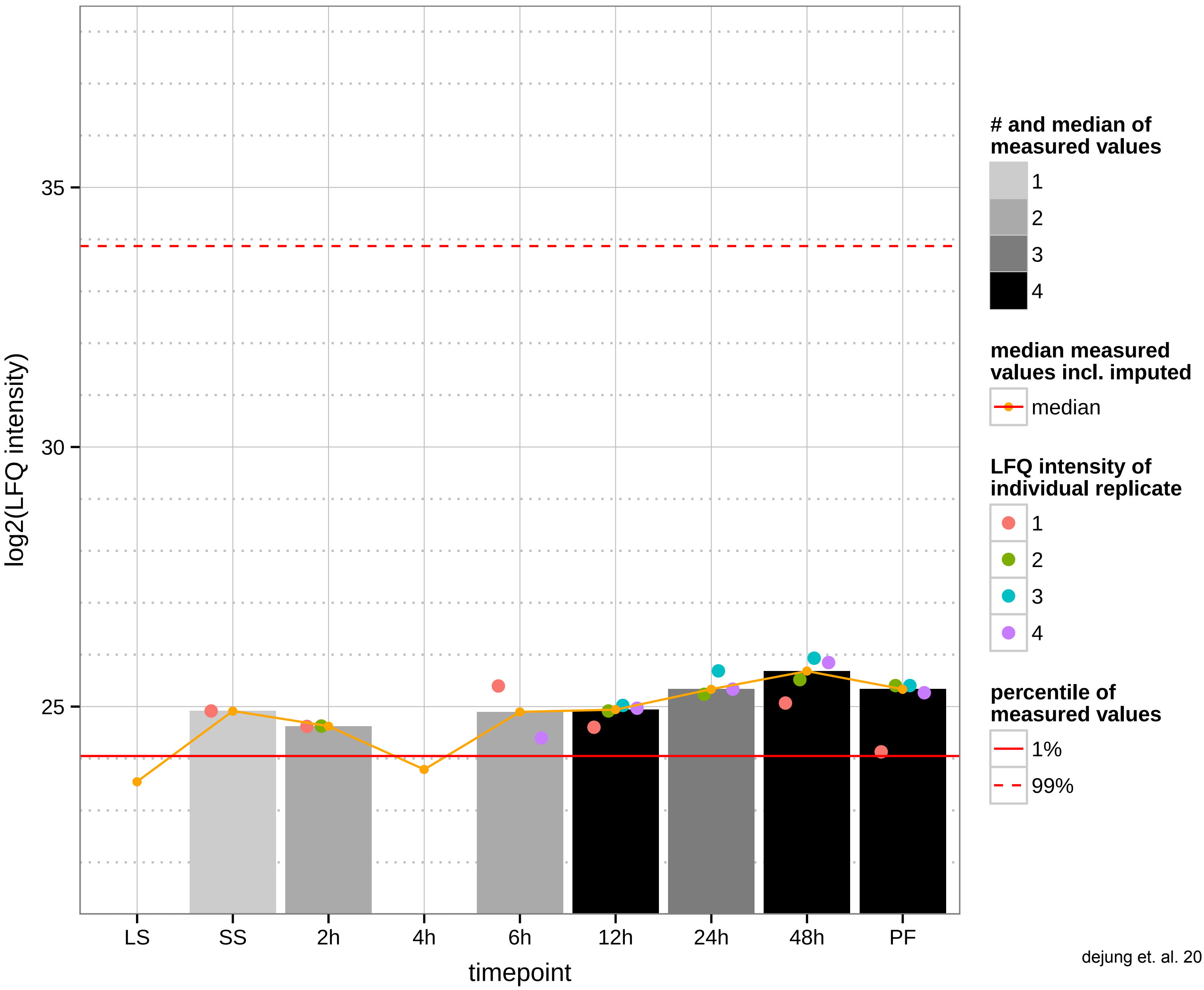

dejung et. al. 2015

hypothetical protein, conserved
Tb927.6.2920
AGOF: null
AGOC: null
AGOP: null
PGOF: null
PGOC: null
PGOP: null

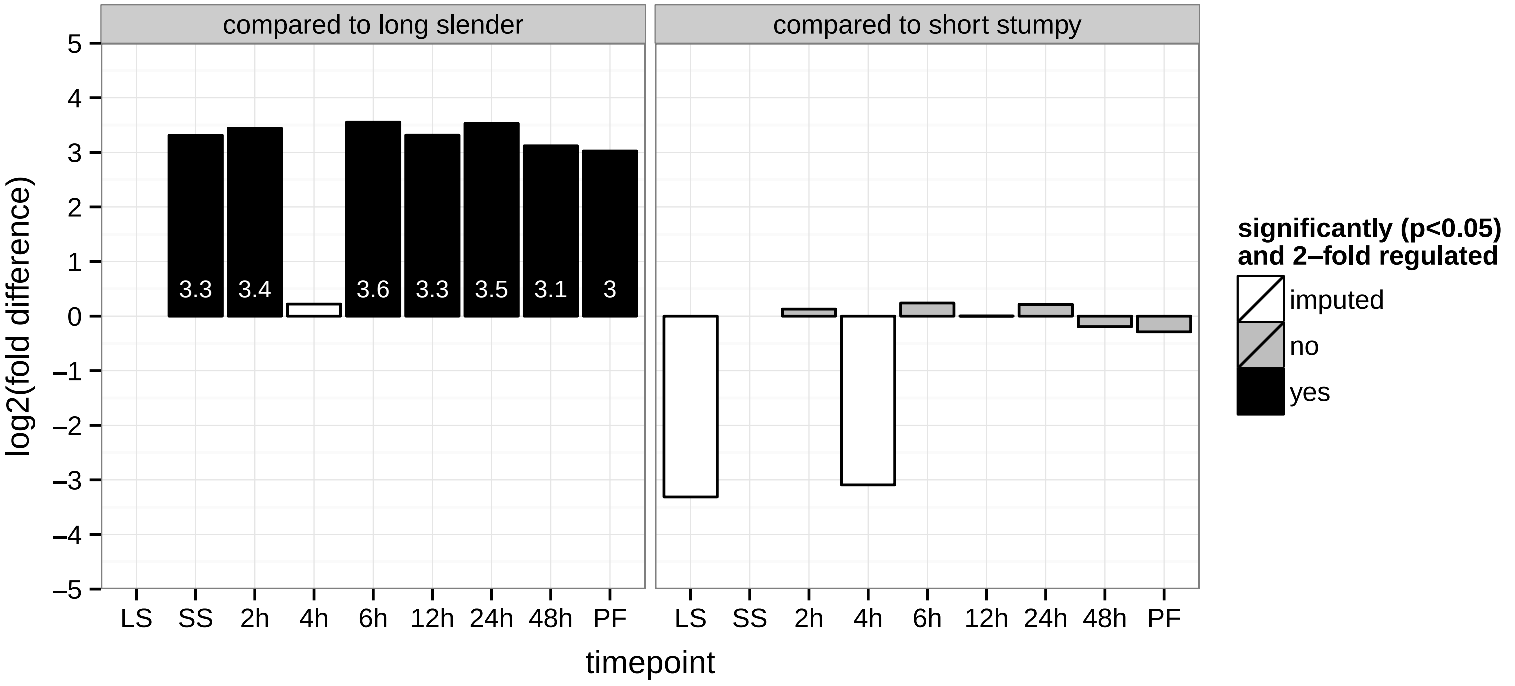

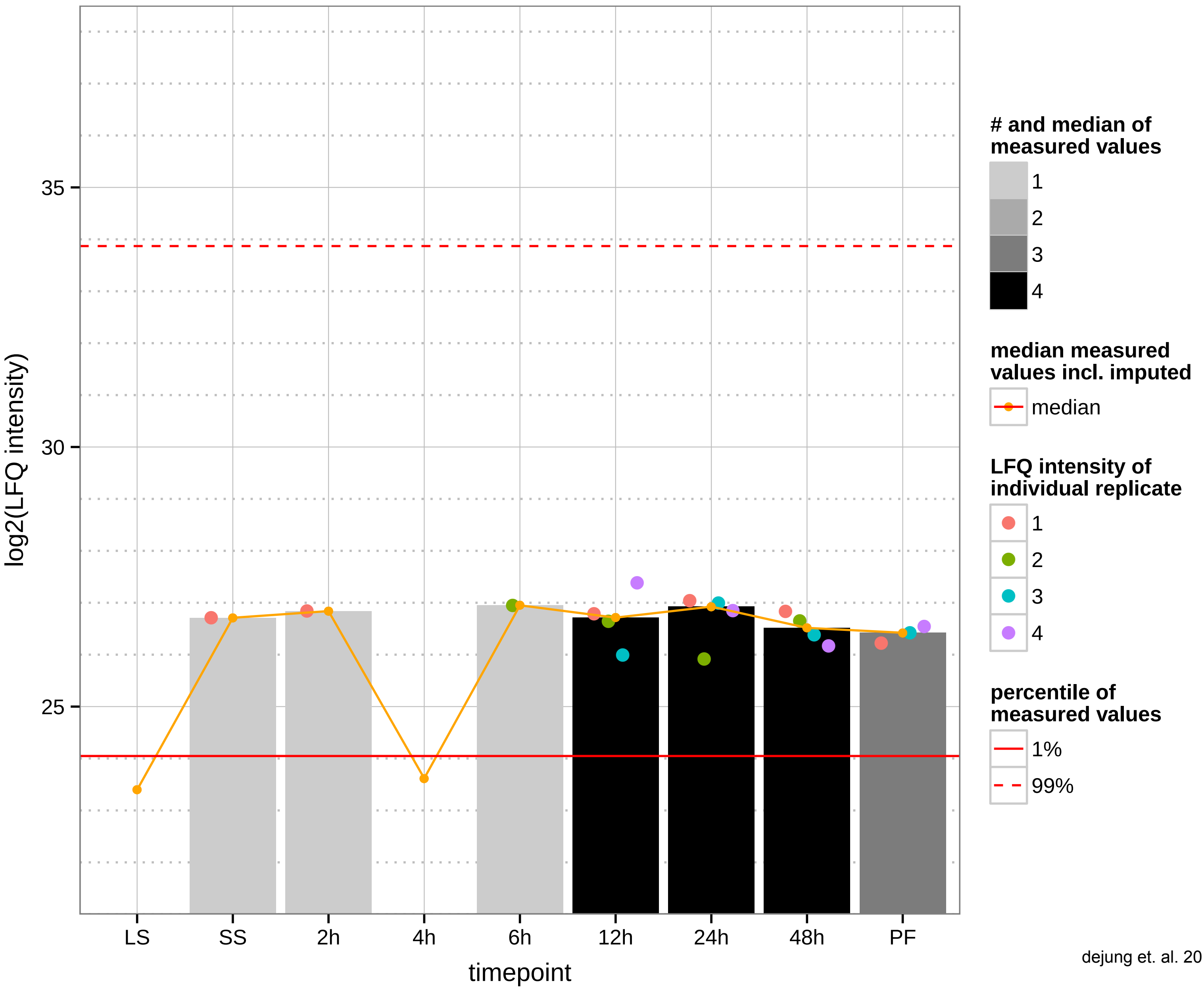

dejung et. al. 2015

hypothetical protein, conserved
Tb927.7.6610
AGOF: null
AGOC: null
AGOP: null
PGOF: protein binding
PGOC: null
PGOP: null

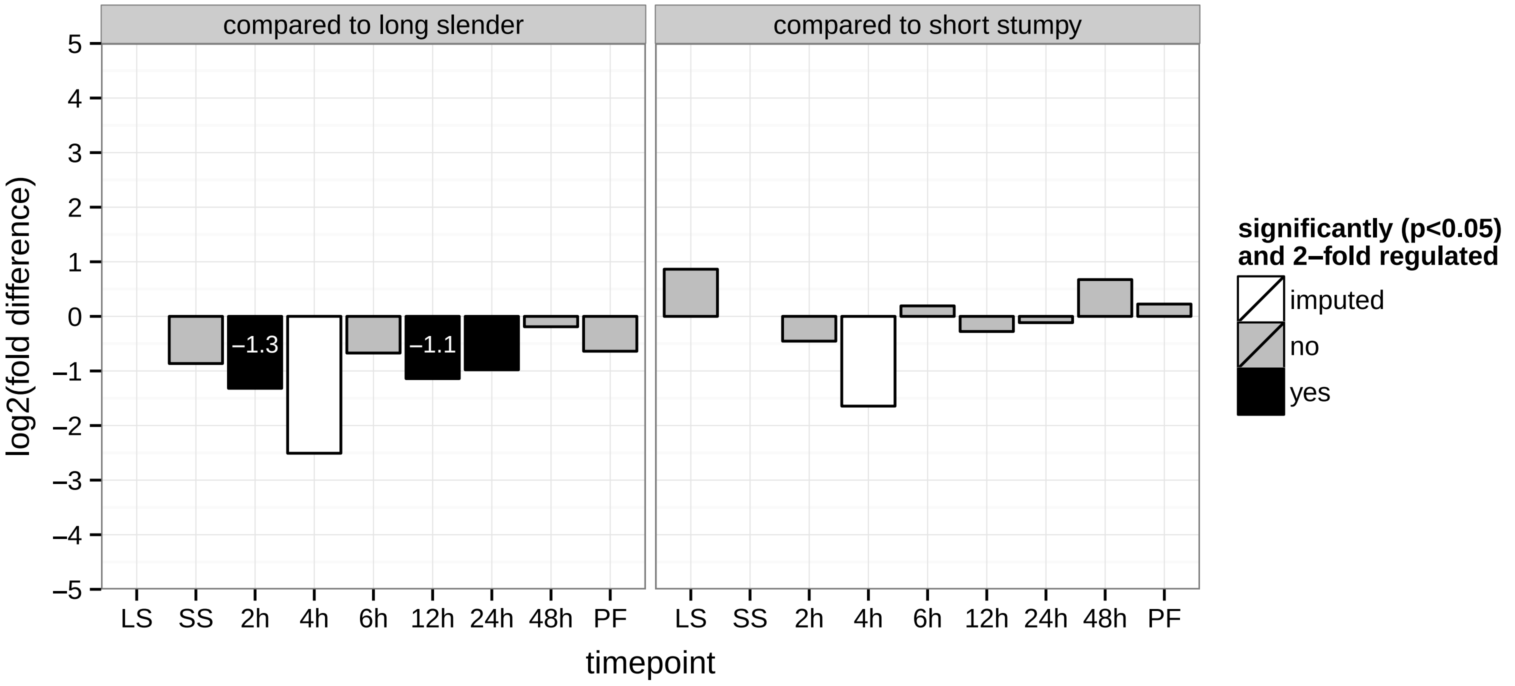

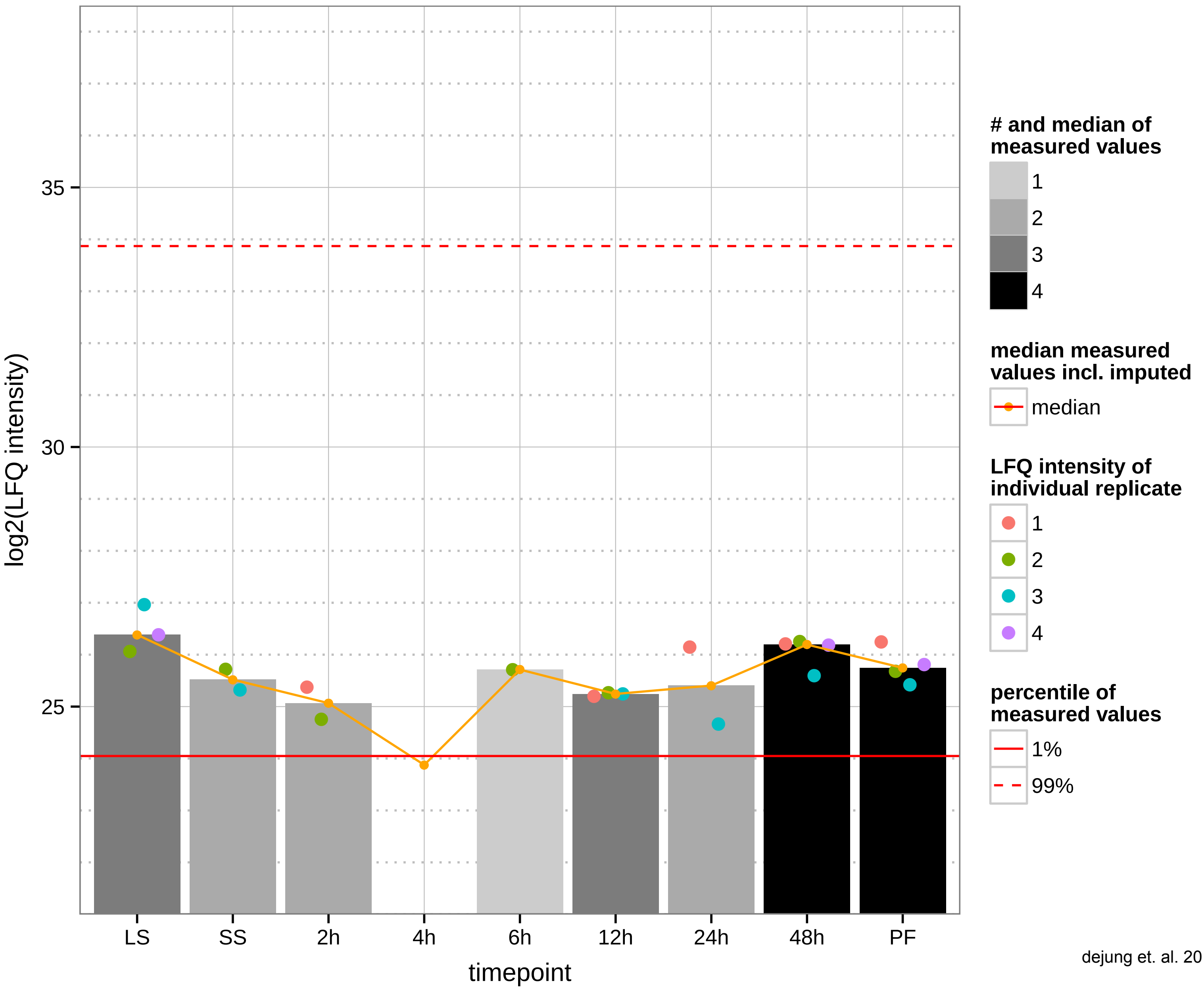

dejung et. al. 2015

hypothetical protein, conserved
Tb927.8.6730
AGOF: null
AGOC: null
AGOP: null
PGOF: null
PGOC: null
PGOP: null

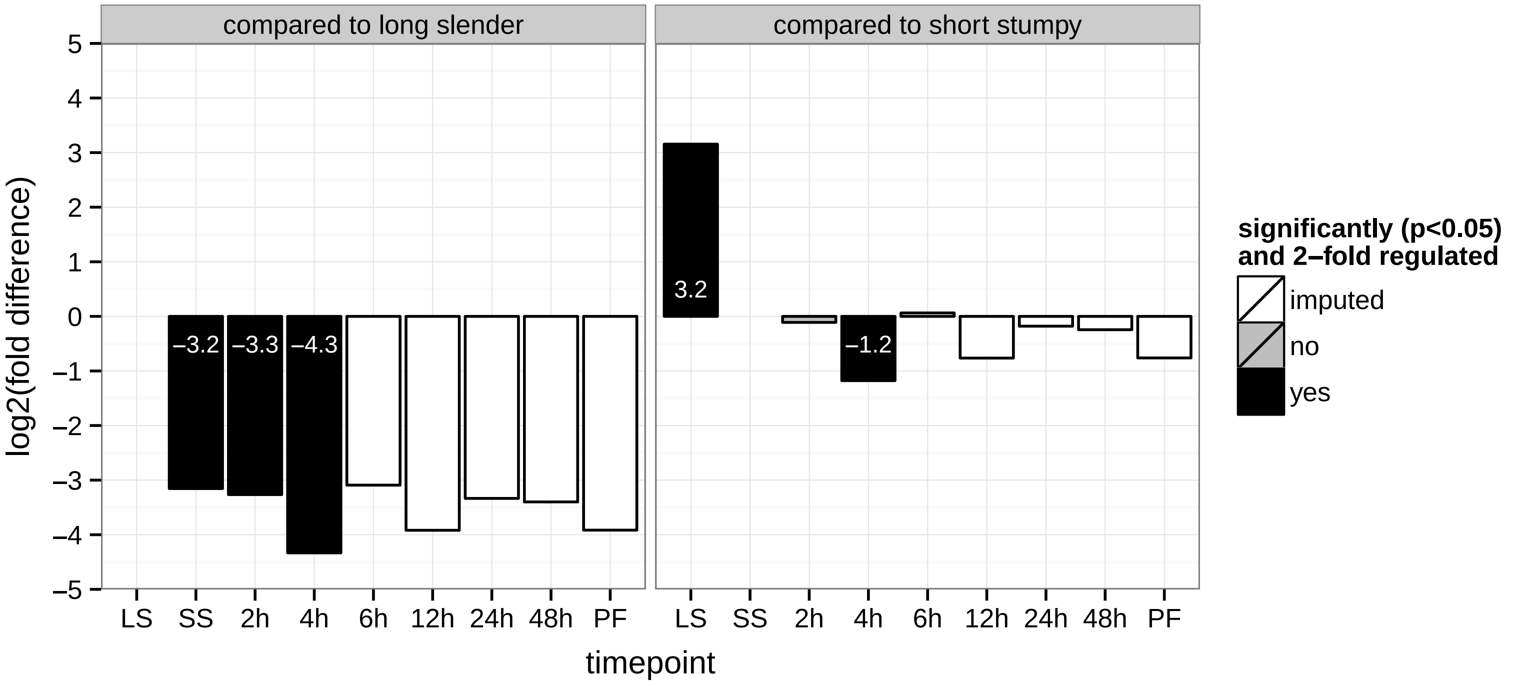

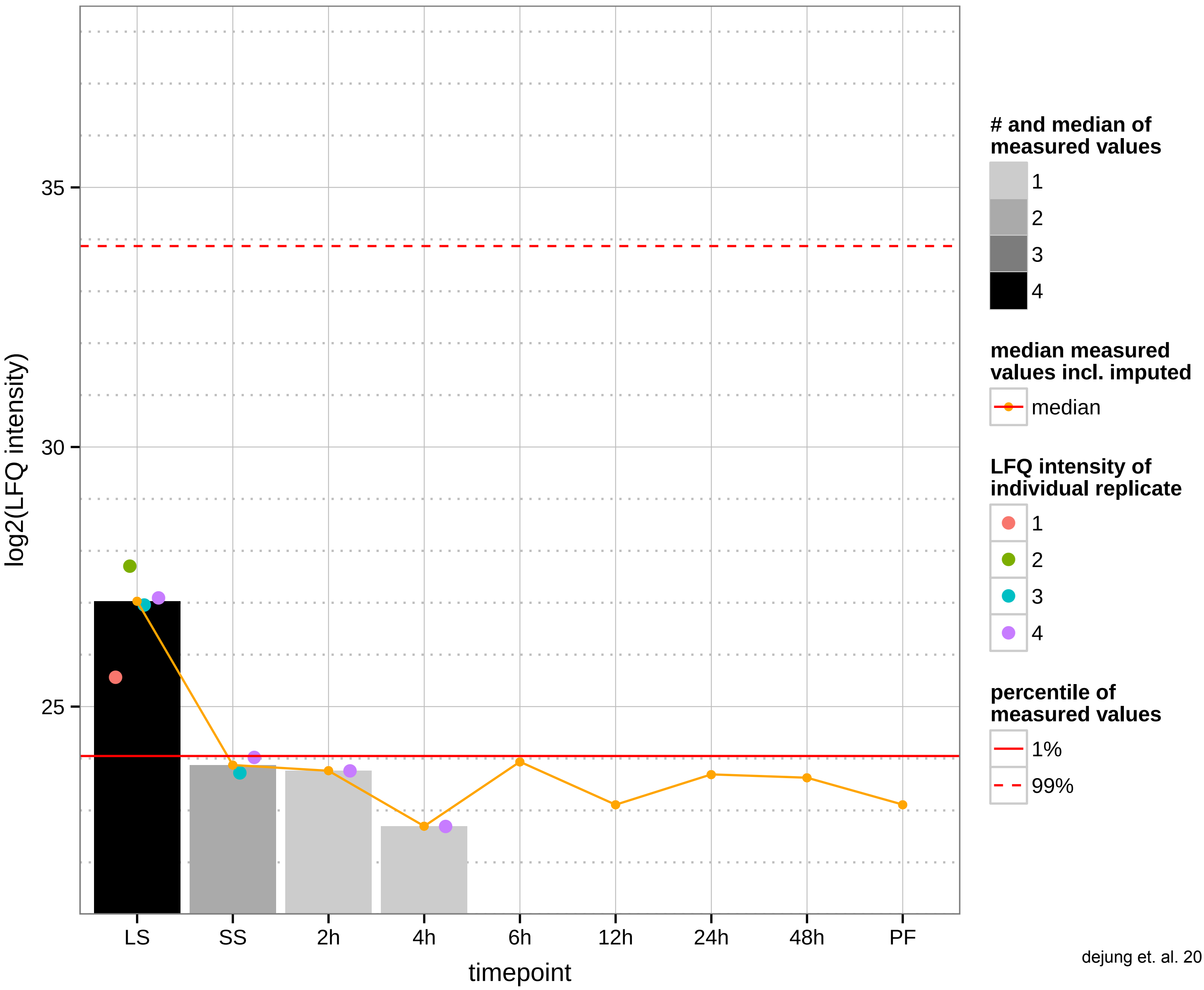

dejung et. al. 2015

hypothetical protein, conserved
Tb927.9.8350
AGOF: null
AGOC: null
AGOP: null
PGOF: protein binding
PGOC: null
PGOP: null

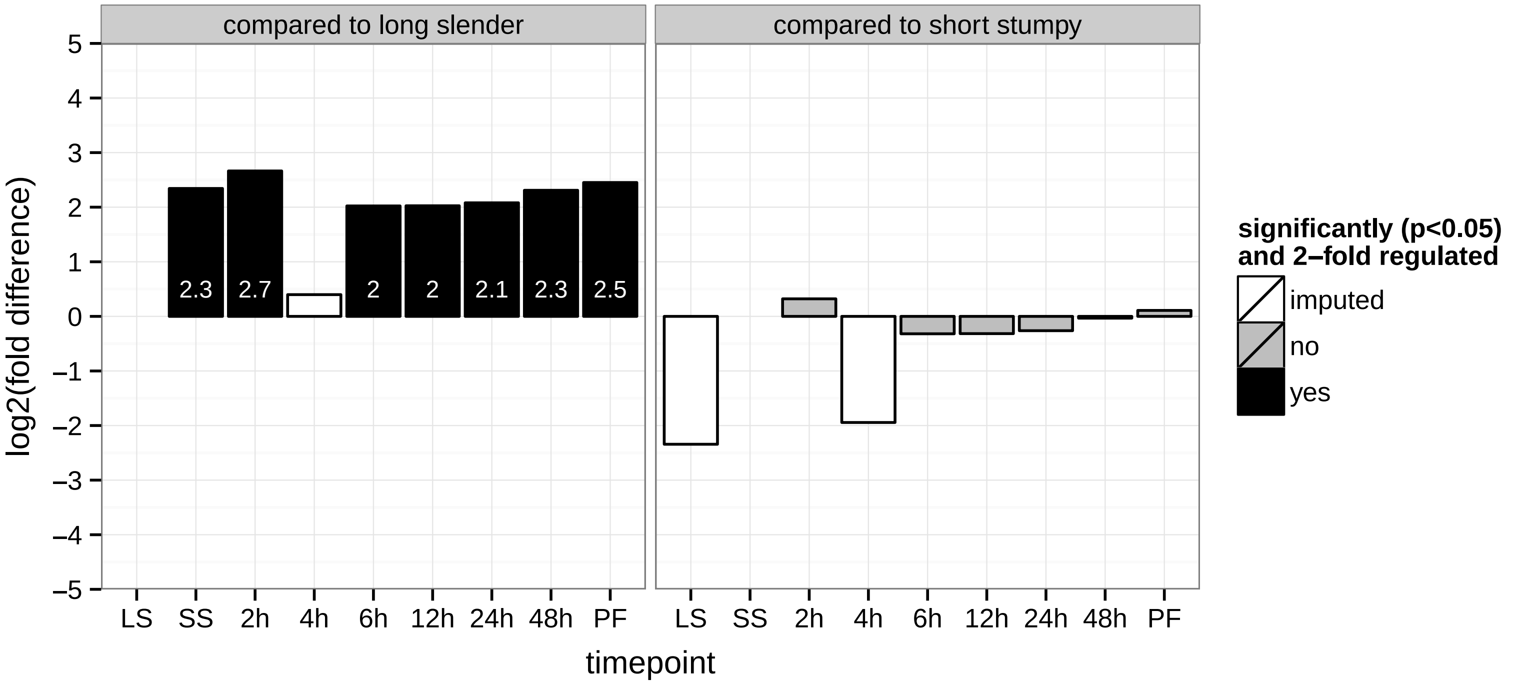

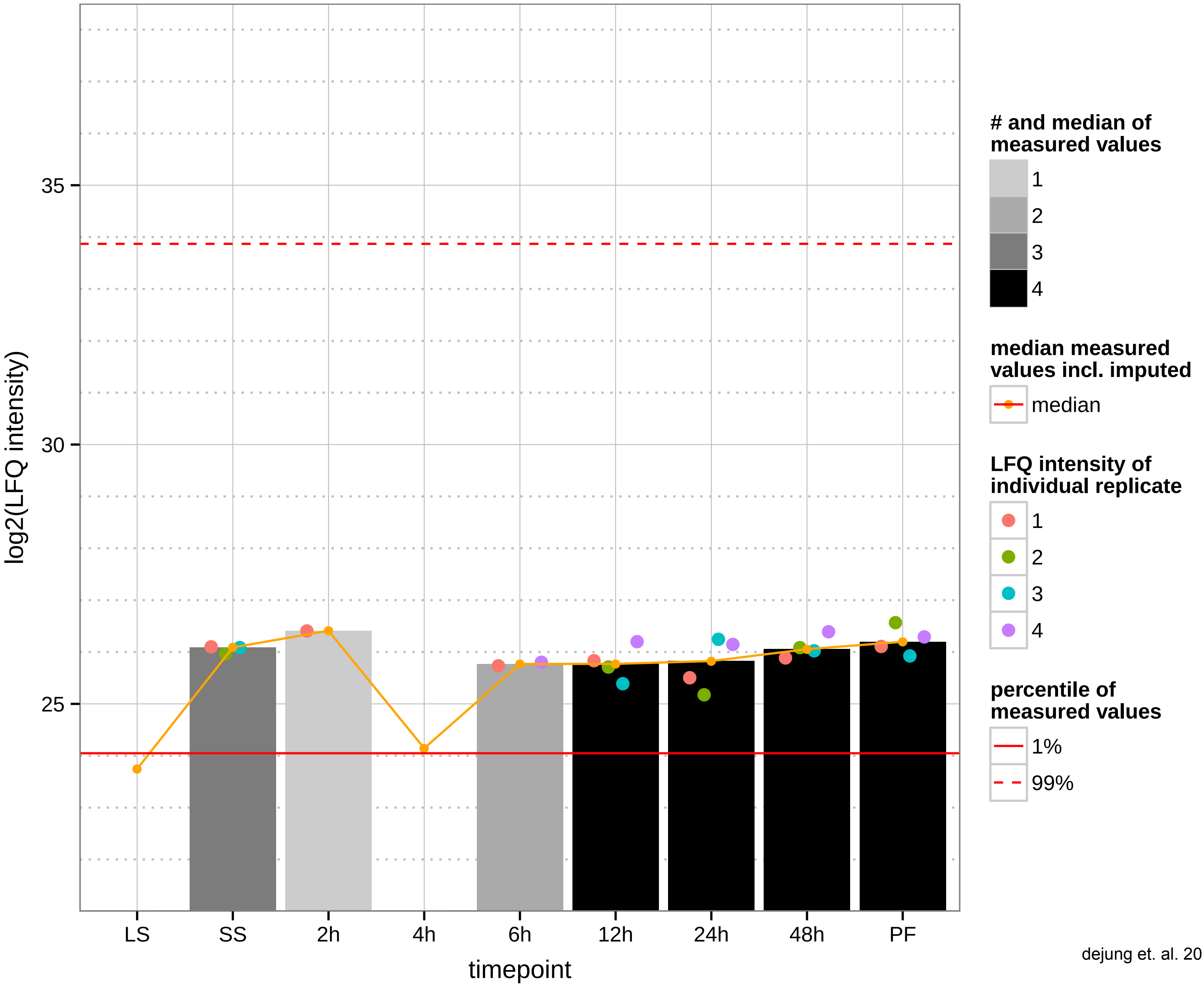

dejung et. al. 2015

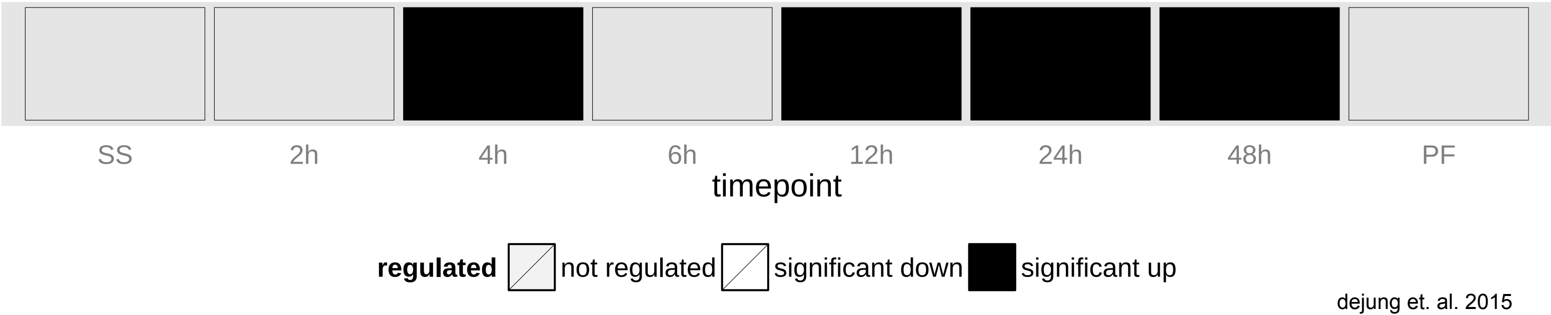
SS
2h
4h
6h
12h
24h
48h
PF
timepoint
regulated
not regulated
significant down
significant up
dejung et. al. 2015

Cell cycle checkpoint protein RAD1–like, putative
Tb927.1.1060
AGOF: exodeoxyribonuclease activity
AGOC: null
AGOP: DNA repair
PGOF: damaged DNA binding
PGOC: nucleus
PGOP: DNA repair

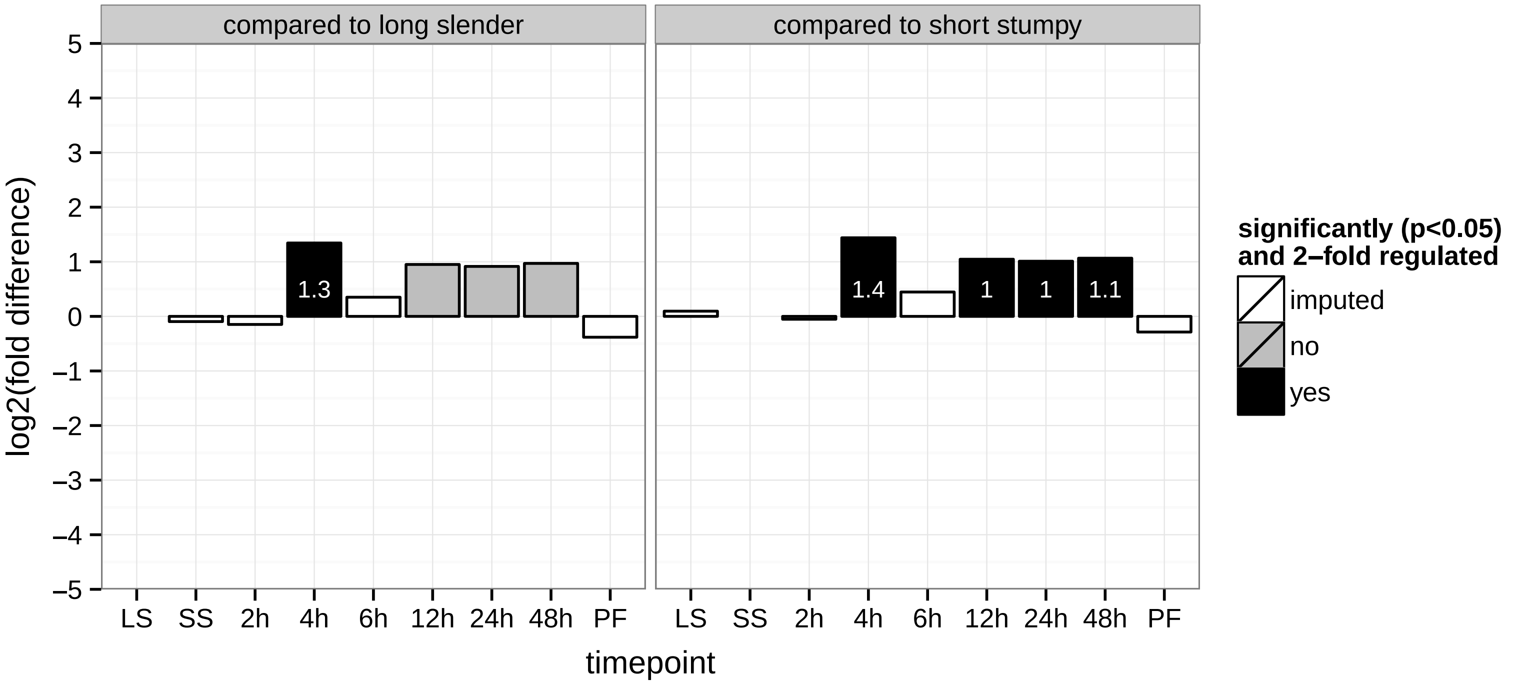

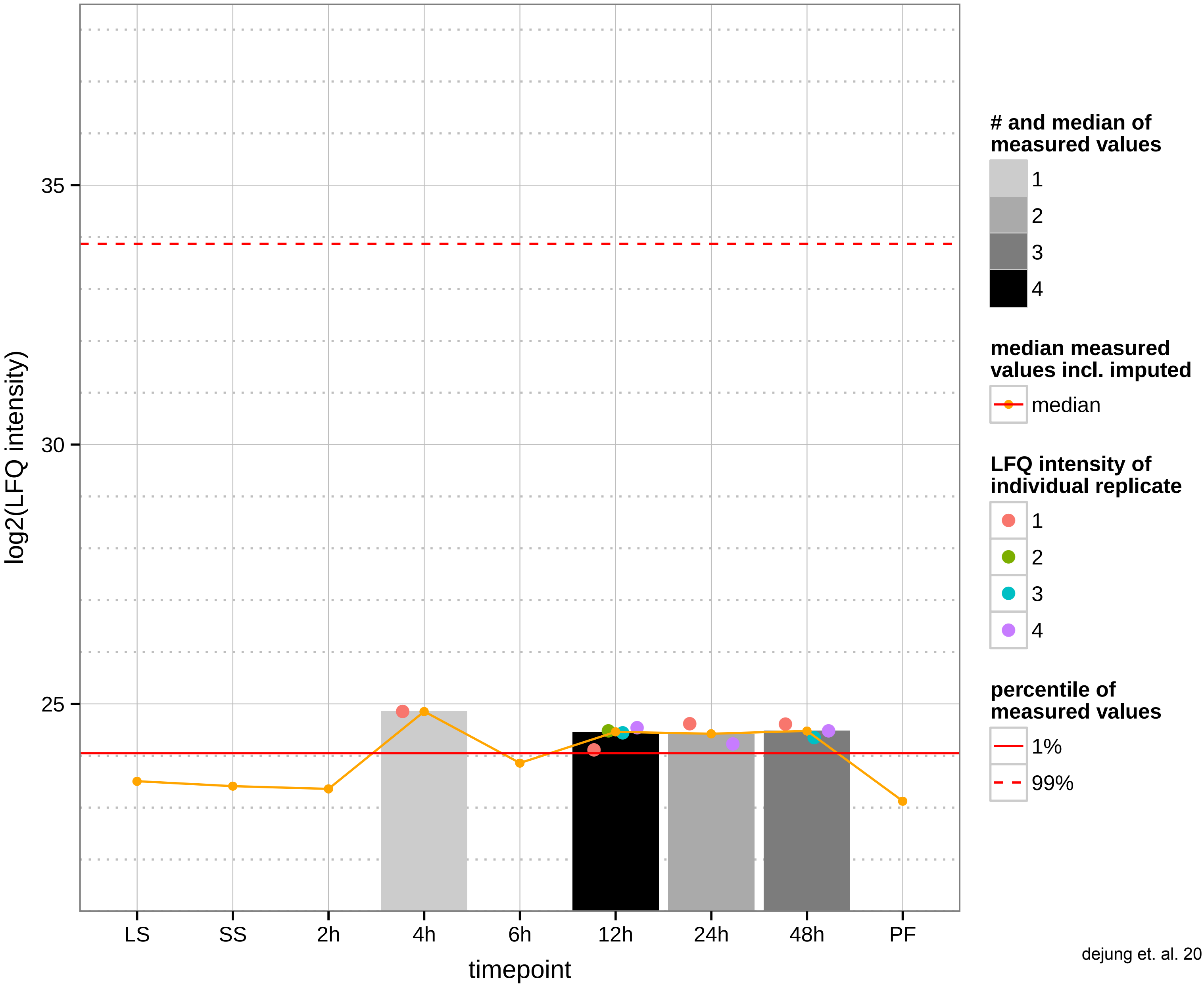

dejung et. al. 2015

hypothetical protein, conserved
Tb927.11.6540
AGOF: null
AGOC: null
AGOP: null
PGOF: null
PGOC: null
PGOP: null

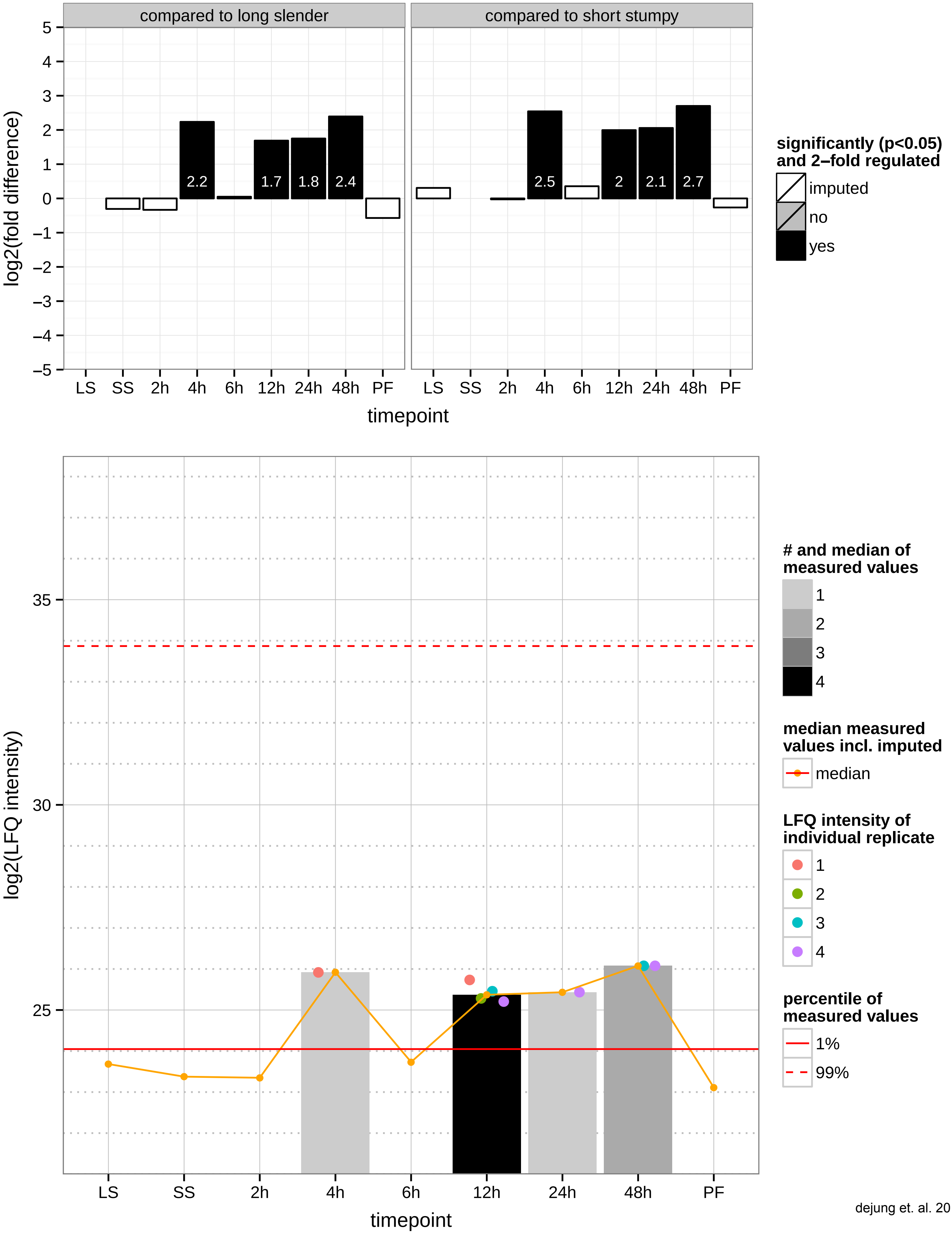

dejung et. al. 2015

small nuclear RNA (snRNA) U1 (TbU1–24K)
Tb927.3.1090
AGOF: null
AGOC: null
AGOP: null
PGOF: null
PGOC: null
PGOP: null

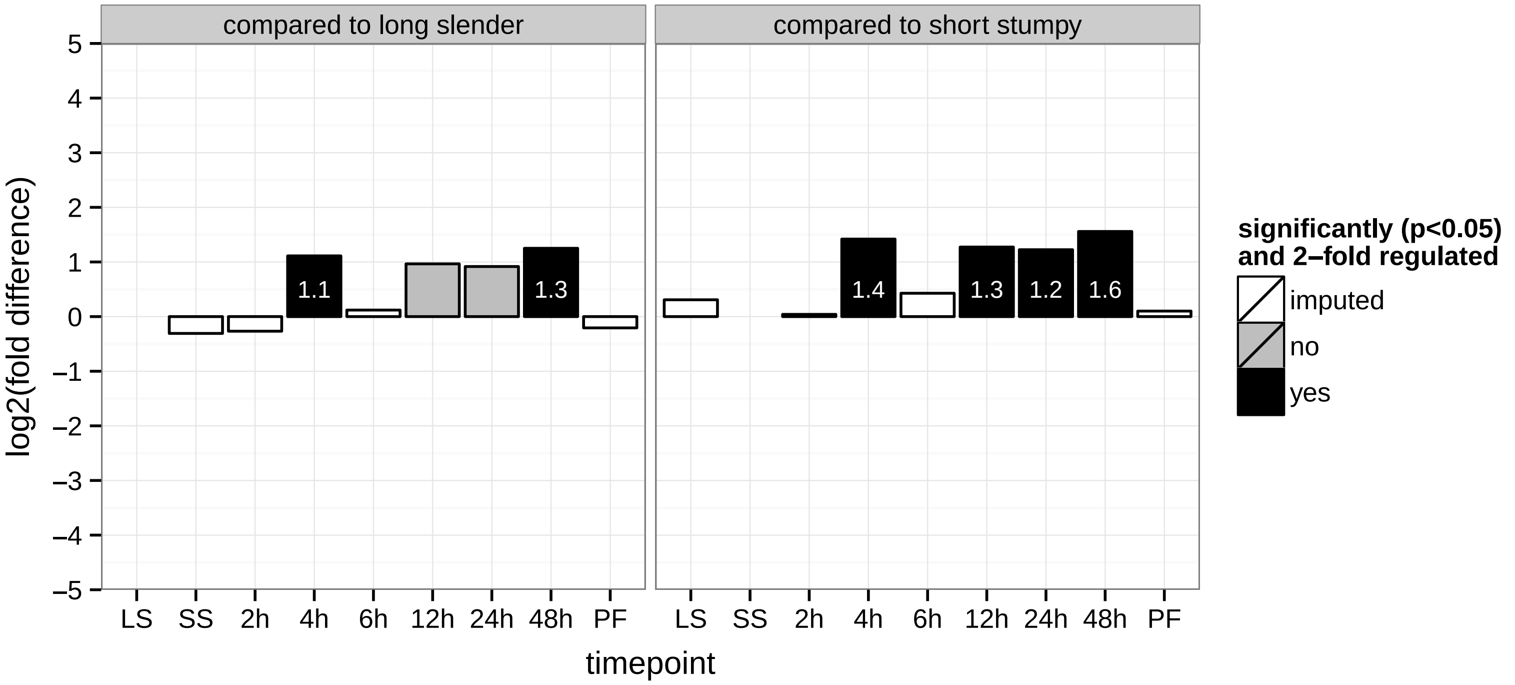

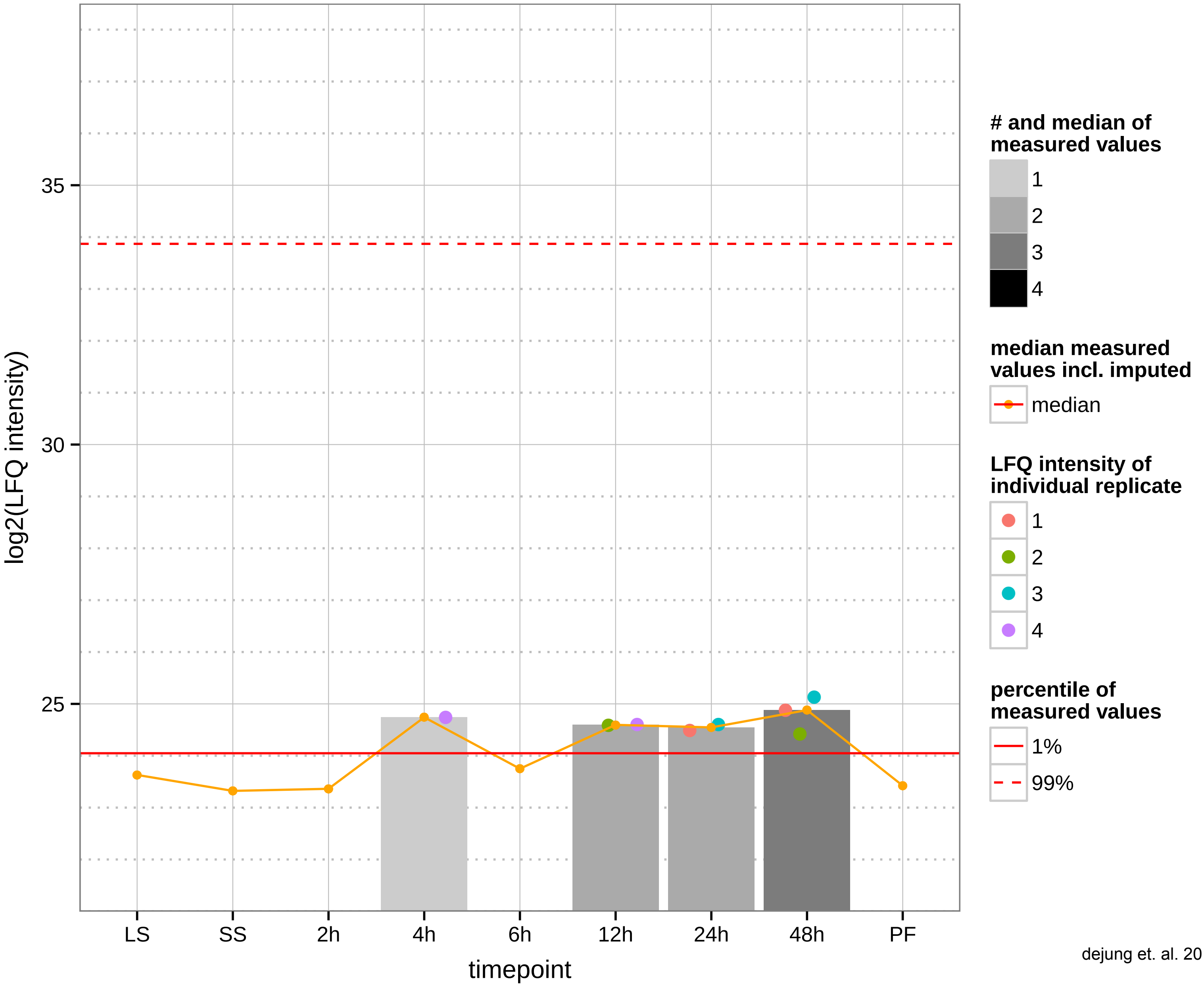

dejung et. al. 2015

hypothetical protein, conserved
Tb927.7.3800
AGOF: null
AGOC: mitochondrion
AGOP: null
PGOF: null
PGOC: null
PGOP: null

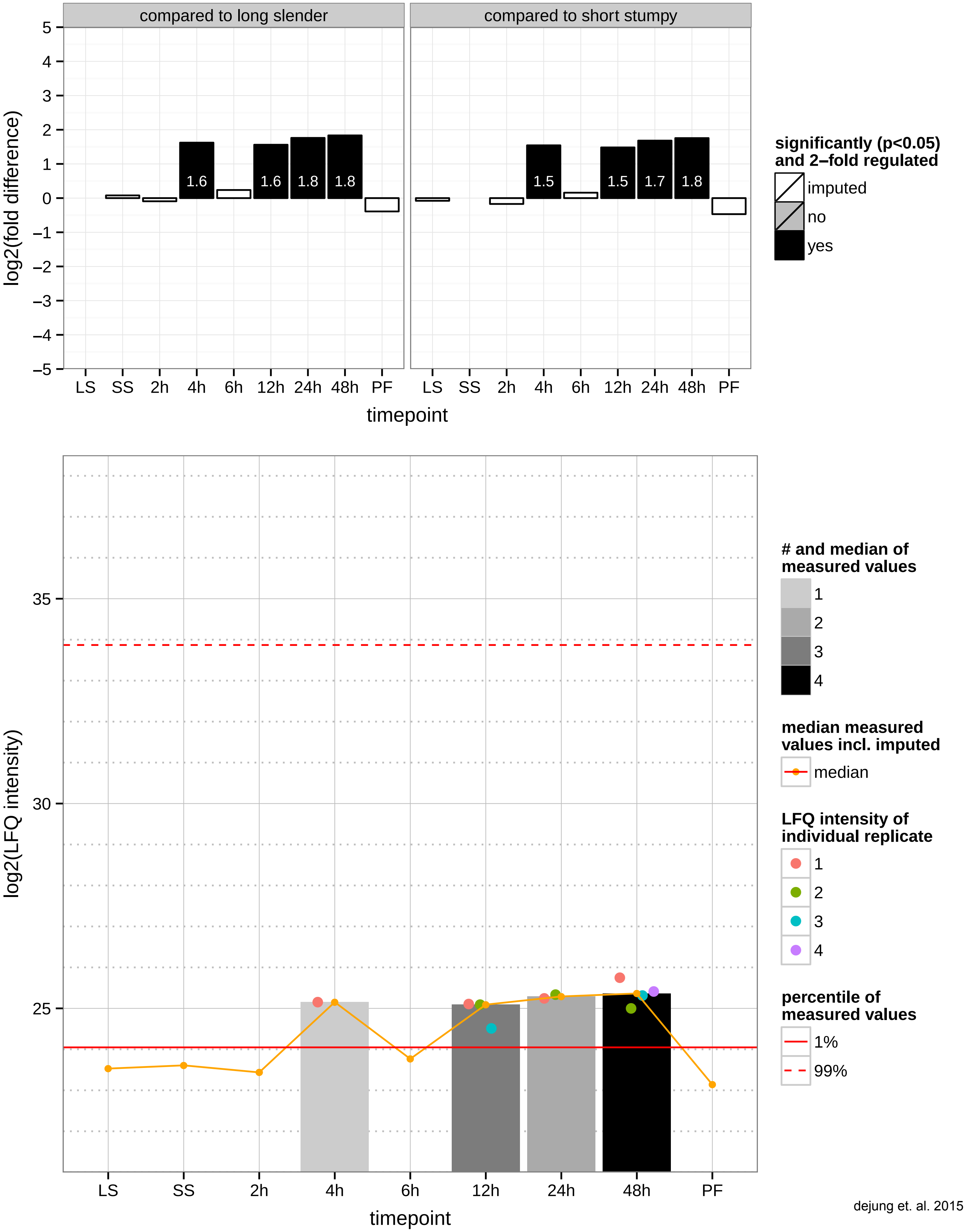

dejung et. al. 2015

chaperone protein DNAj, putative
Tb927.7.6660
AGOF: heat shock protein binding, unfolded protein binding
AGOC: integral to membrane, mitochondrion
AGOP: protein folding
PGOF: heat shock protein binding
PGOC: null
PGOP: null

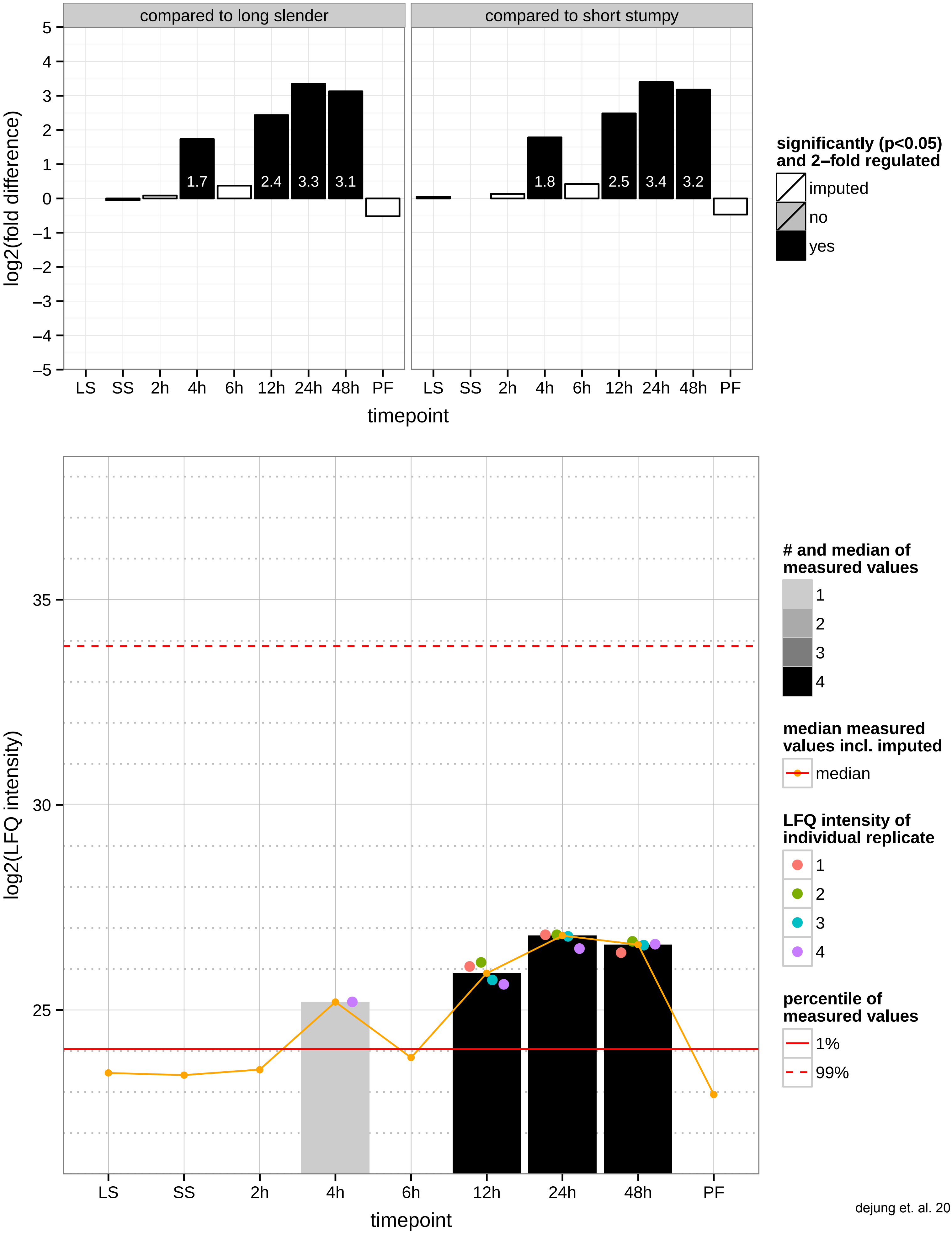

dejung et. al. 2015

hypothetical protein, conserved
Tb927.7.7010
AGOF: null
AGOC: mitochondrion
AGOP: null
PGOF: null
PGOC: null
PGOP: null

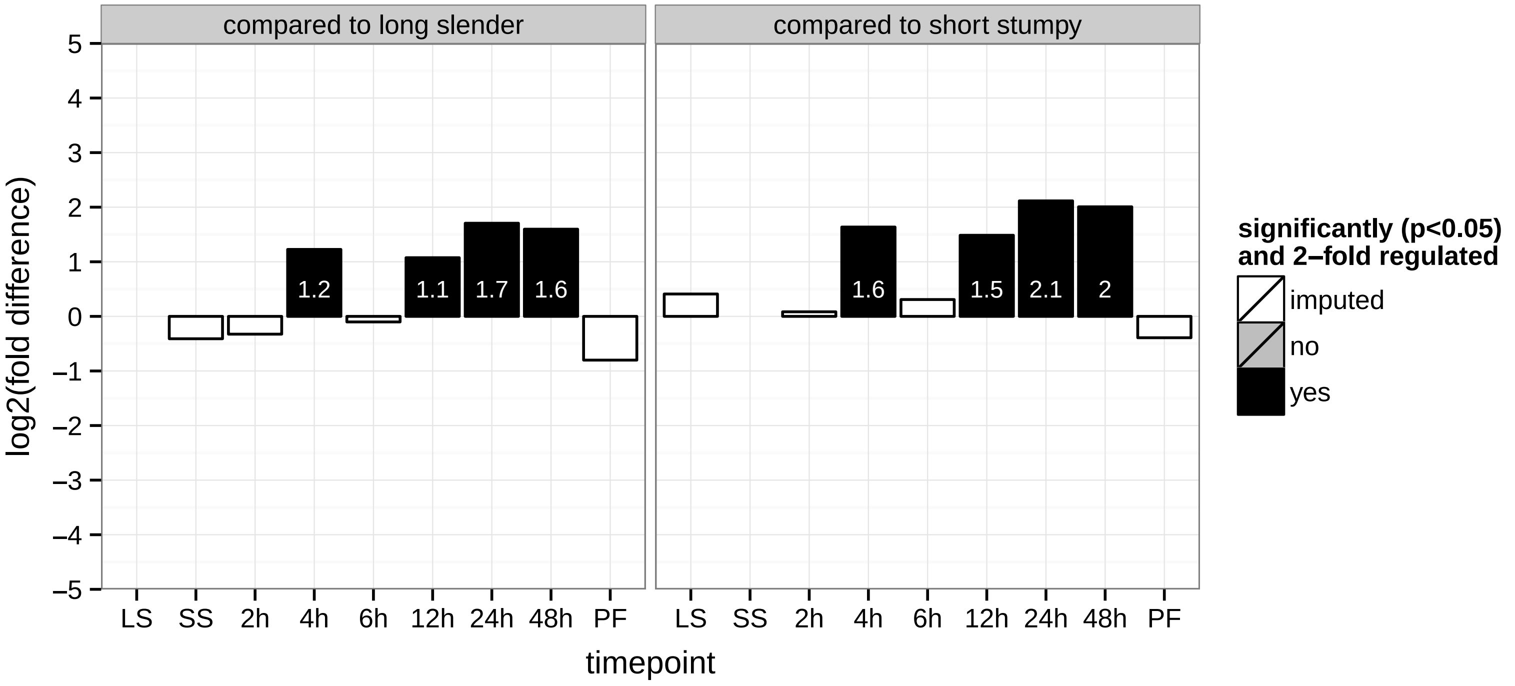

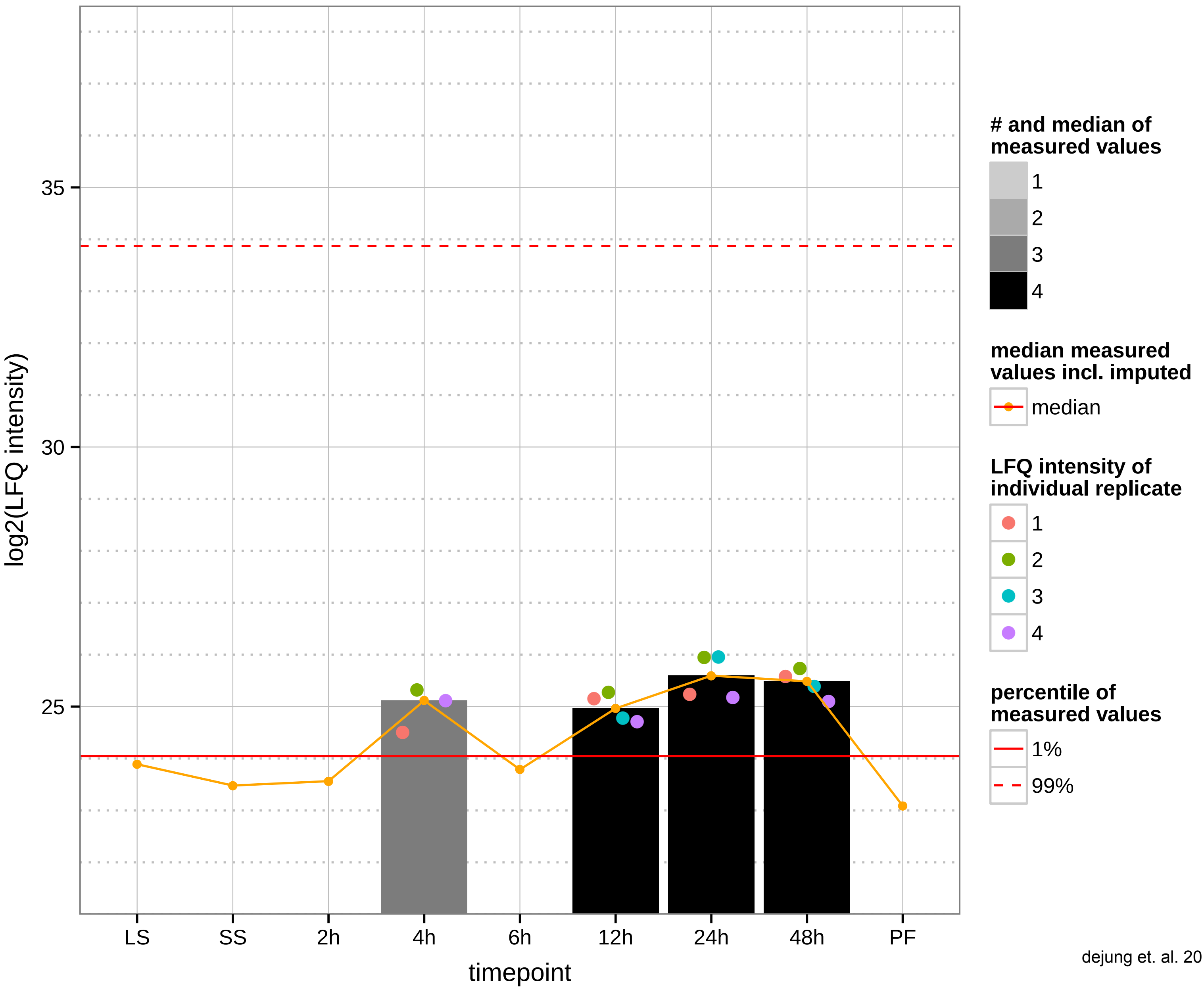

dejung et. al. 2015

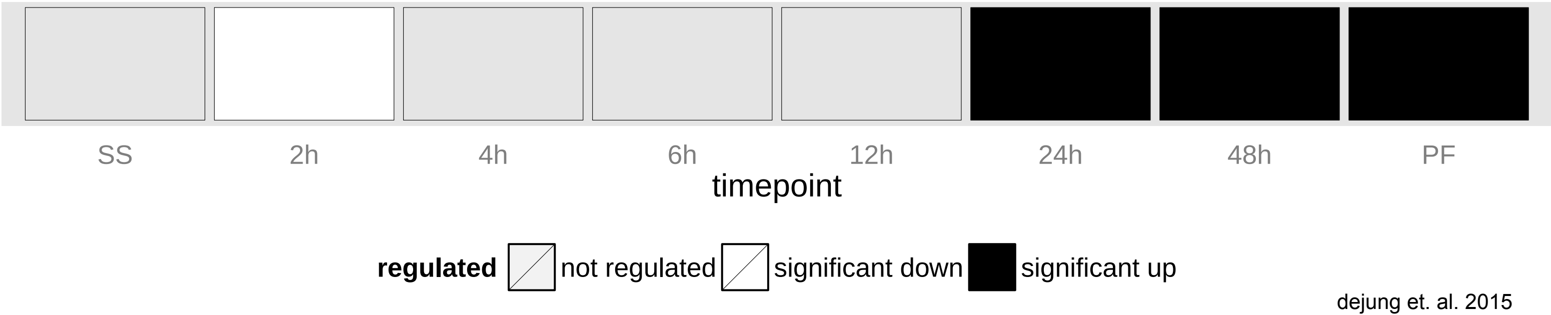

SS
2h
4h
6h
12h
24h
48h
PF
timepoint
regulated
not regulated
significant down
significant up
dejung et. al. 2015

DNA mismatch repair protein, putative, DNA mismatch repair protein MSH2, MutS protein homolog 2, putative (MSH2)
Tb927.10.11020
AGOF: null, ATP binding, damaged DNA binding, mismatched DNA binding
AGOC: null, MutSalpha complex, MutSbeta complex
AGOP: null, DNA metabolic process, mismatch repair
PGOF: ATP binding, mismatched DNA binding
PGOC: null, MutSalpha complex, MutSbeta complex
PGOP: mismatch repair

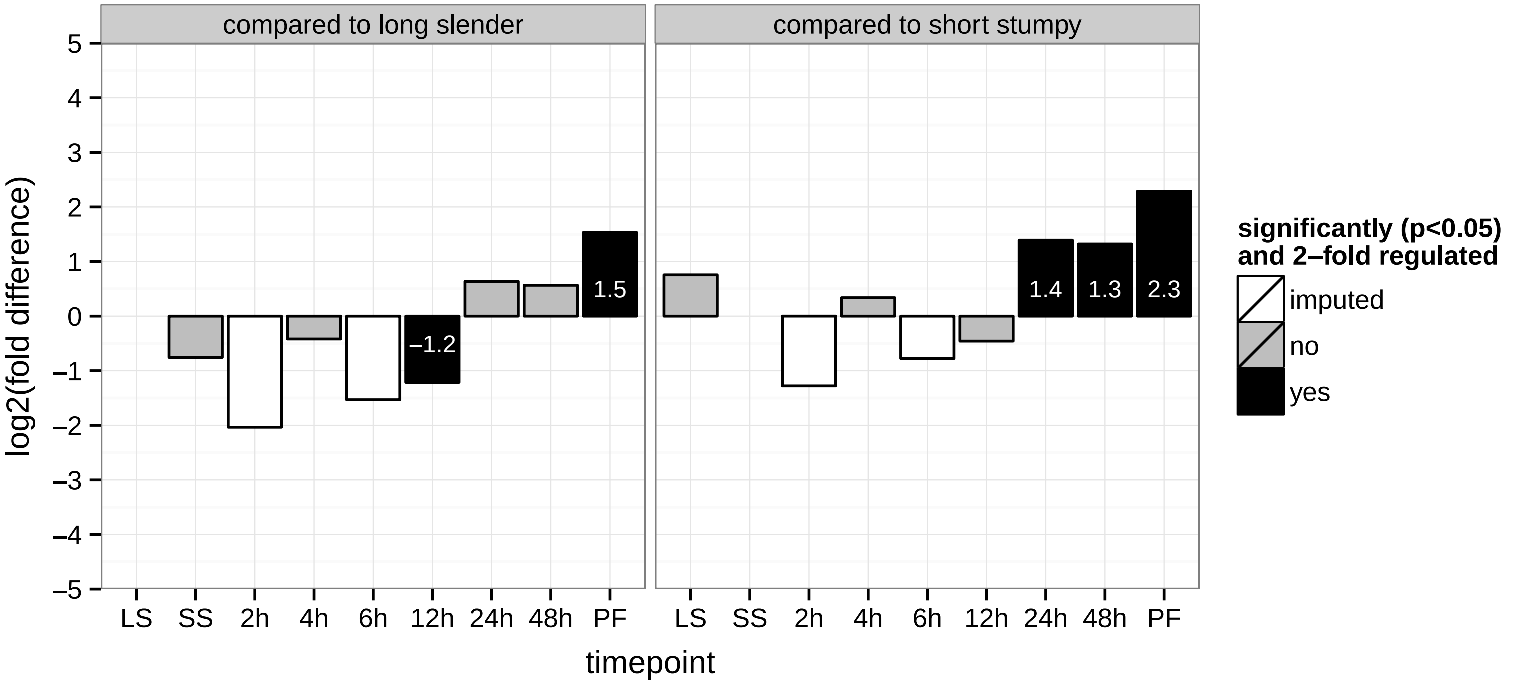

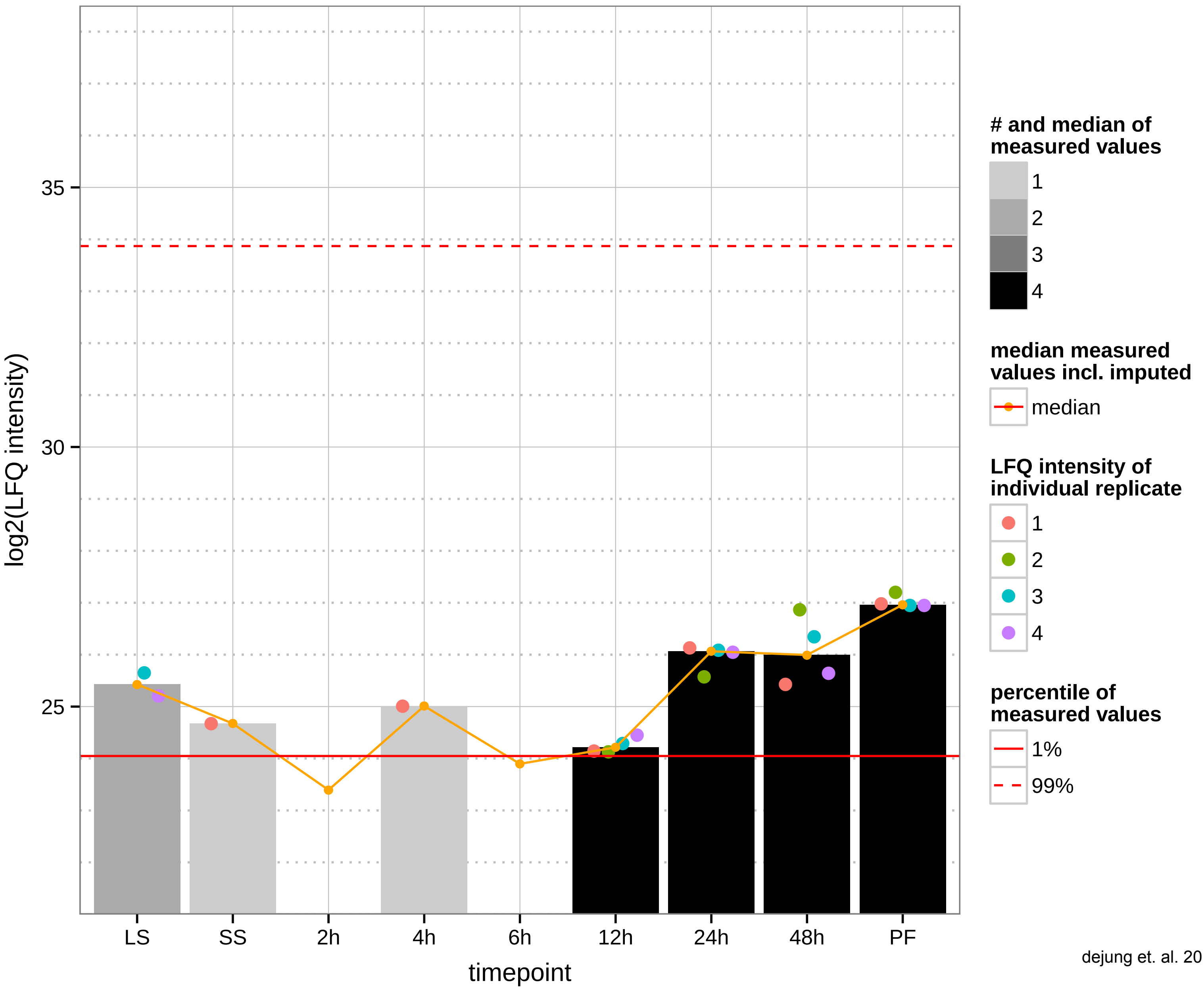

dejung et. al. 2015

hypothetical protein, conserved
Tb927.10.11380
AGOF: null
AGOC: null
AGOP: null
PGOF: protein binding
PGOC: null
PGOP: null

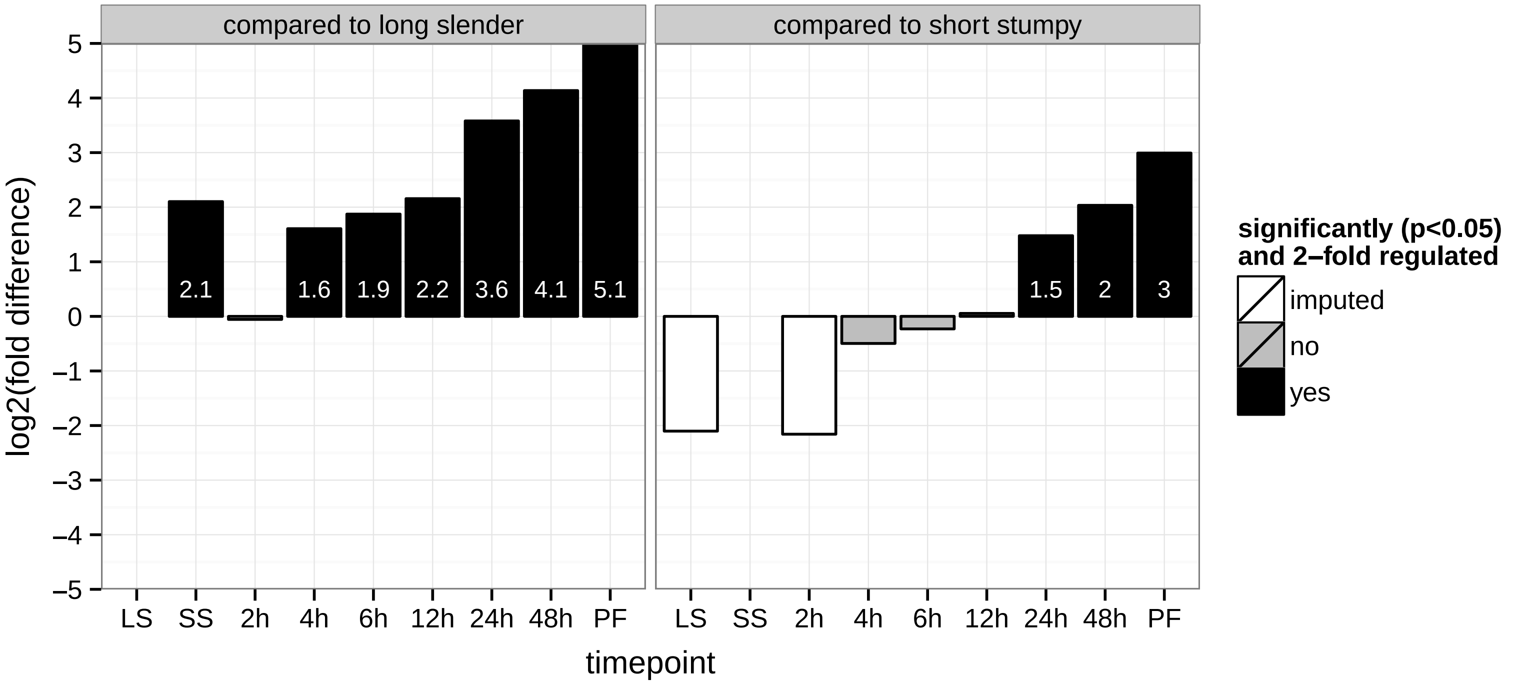

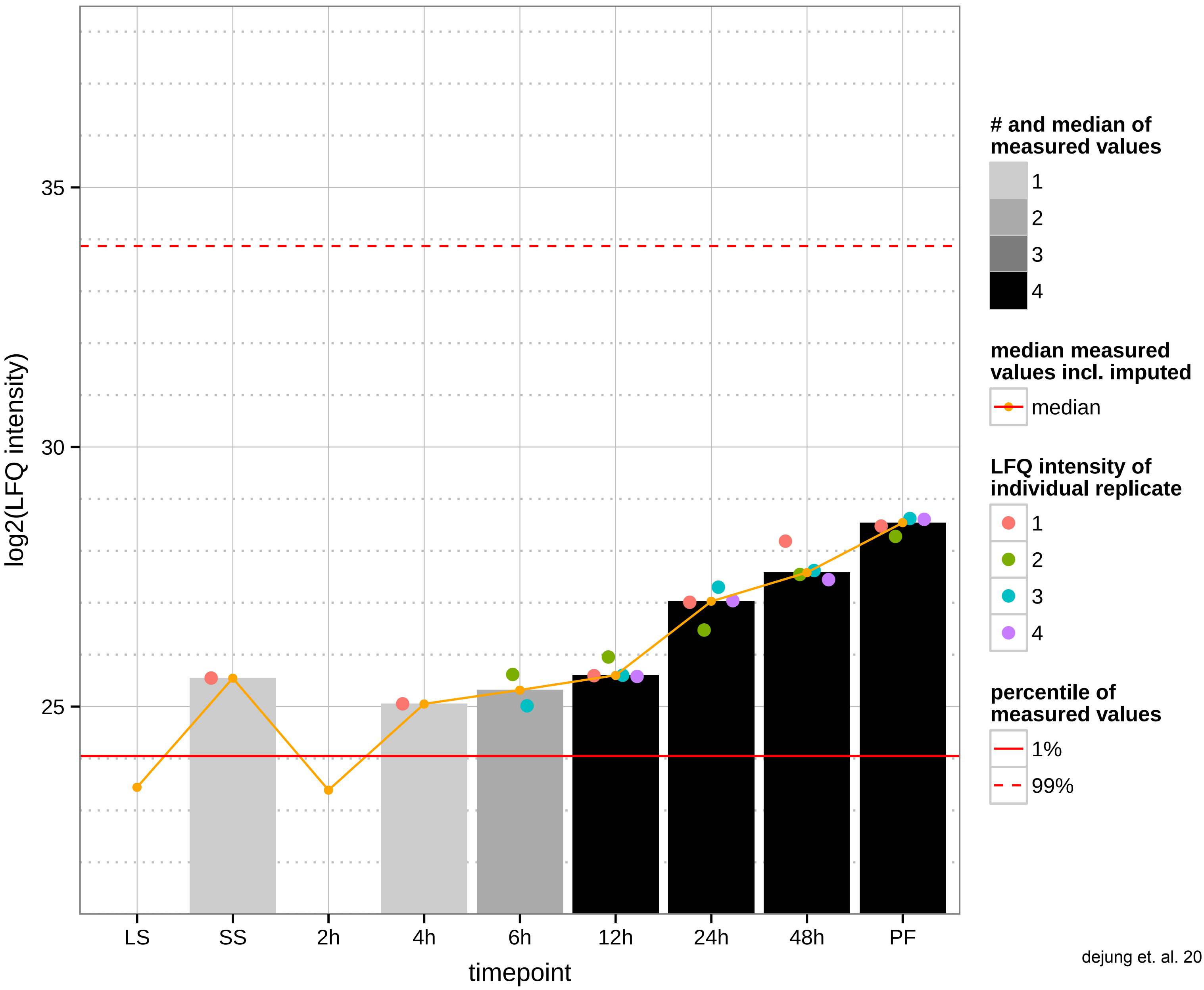

dejung et. al. 2015

ribosomal protein L11, putative
Tb927.2.4890;Tb927.2.4740
AGOF: structural constituent of ribosome
AGOC: intracellular, mitochondrion, ribosome
AGOP: translation
PGOF: structural constituent of ribosome
PGOC: ribosome
PGOP: translation

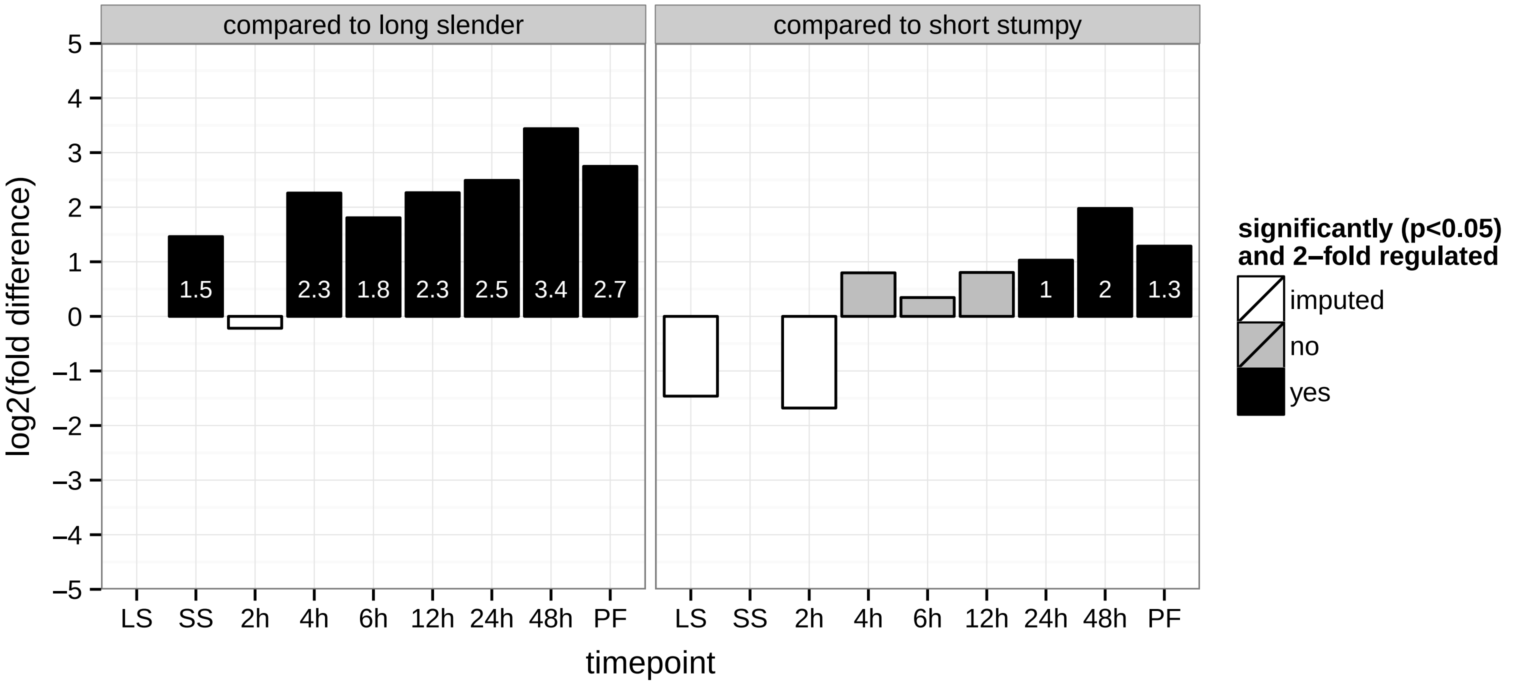

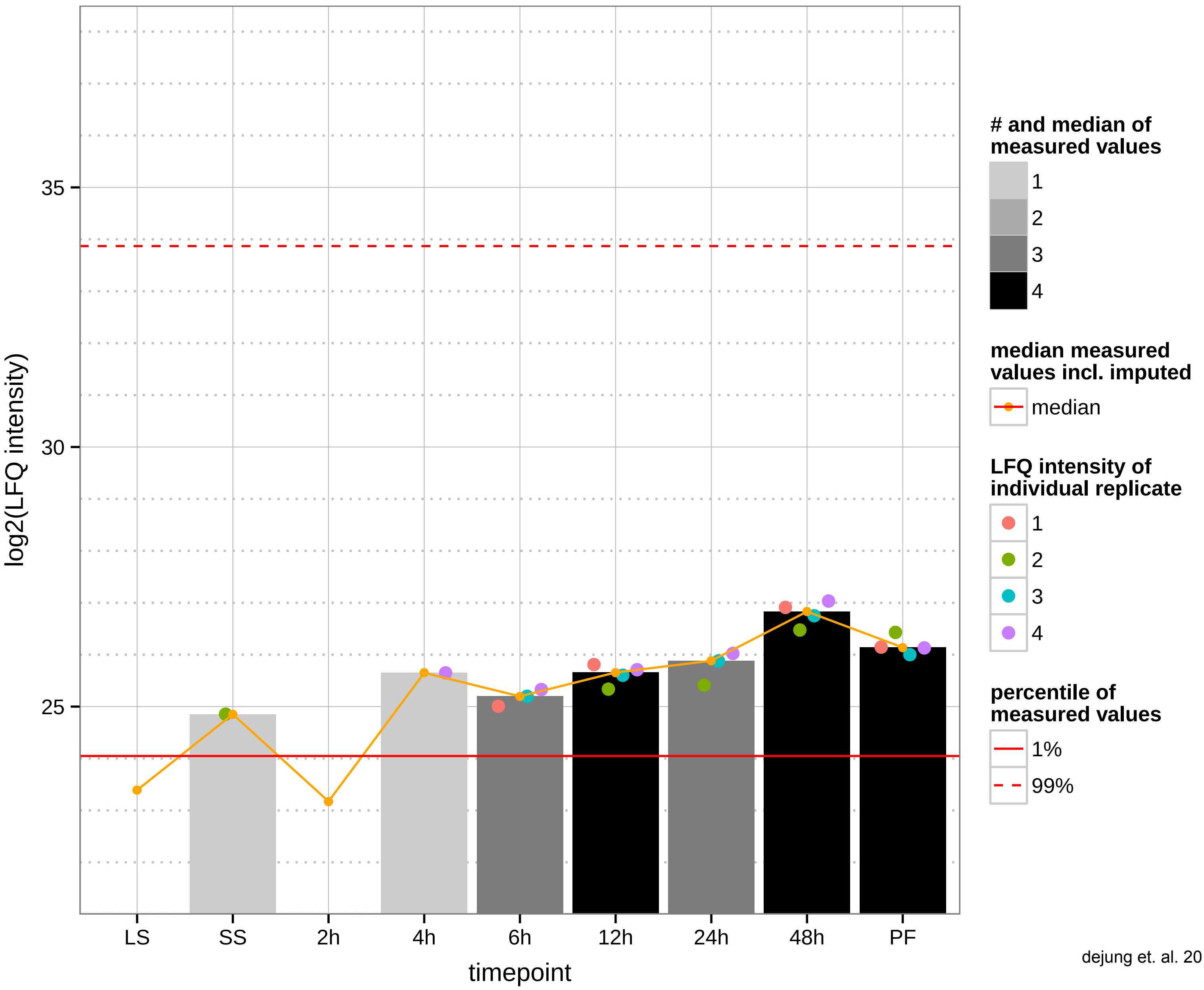

dejung et. al. 2015

hypothetical protein, conserved
Tb927.5.3410
AGOF: null
AGOC: mitochondrion
AGOP: null
PGOF: null
PGOC: null
PGOP: null

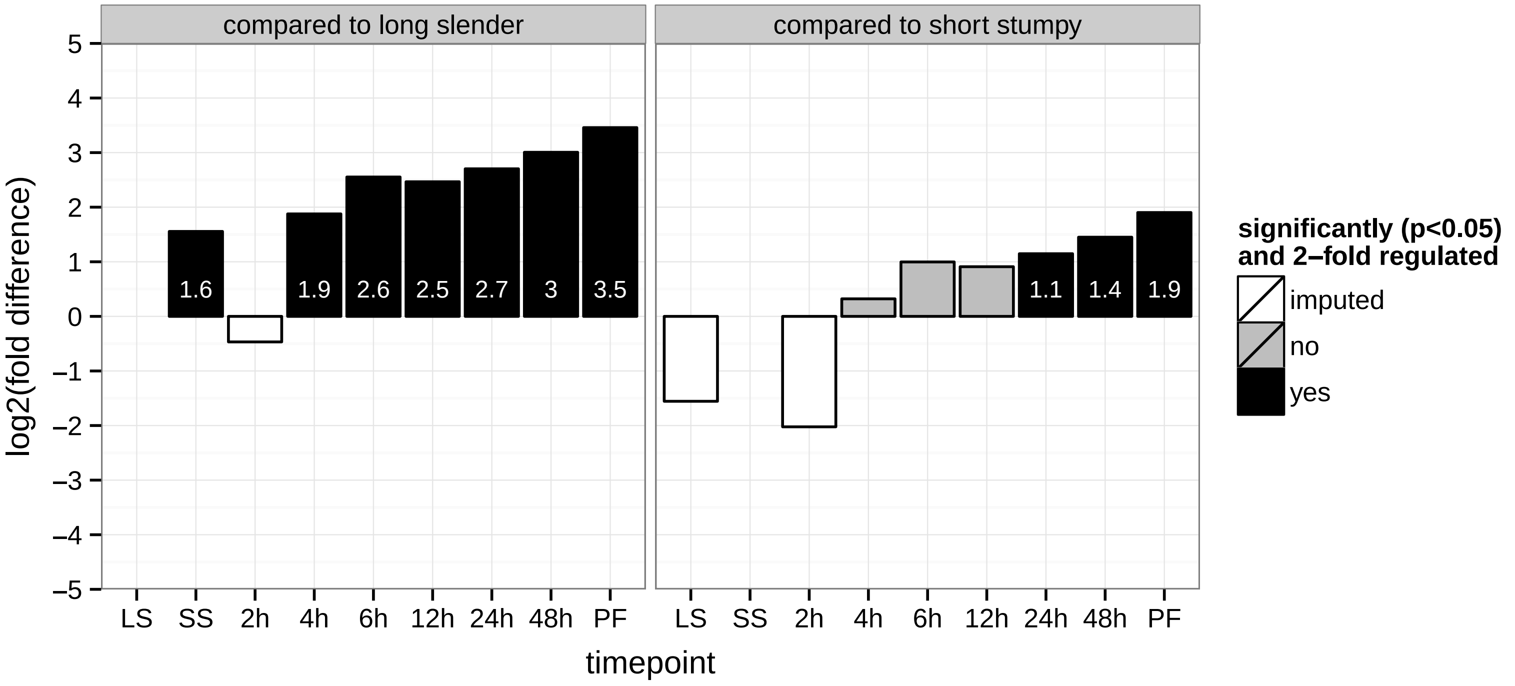

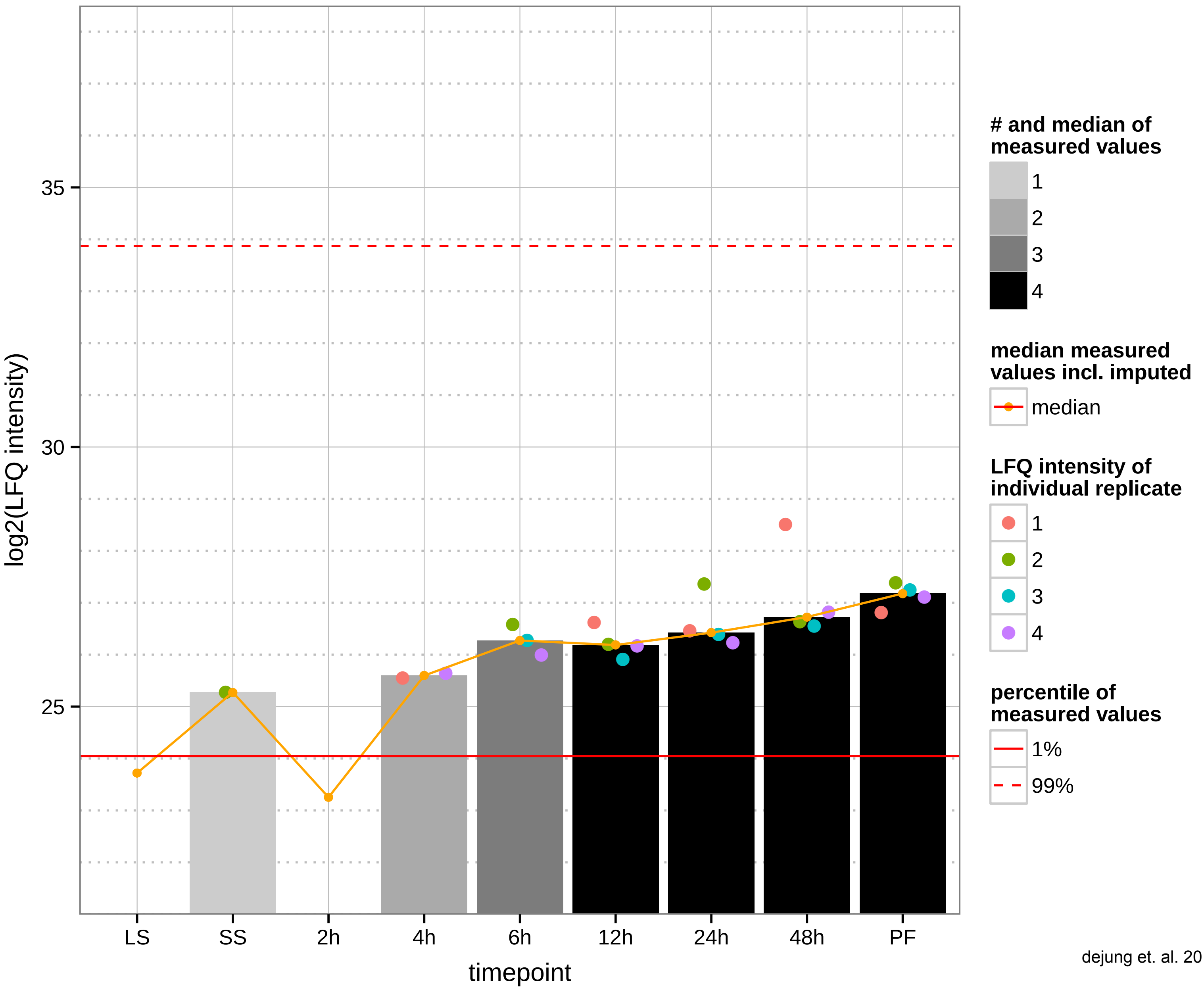

dejung et. al. 2015

hypothetical protein, conserved
Tb927.6.4130
AGOF: null
AGOC: mitochondrion
AGOP: null
PGOF: null
PGOC: null
PGOP: null

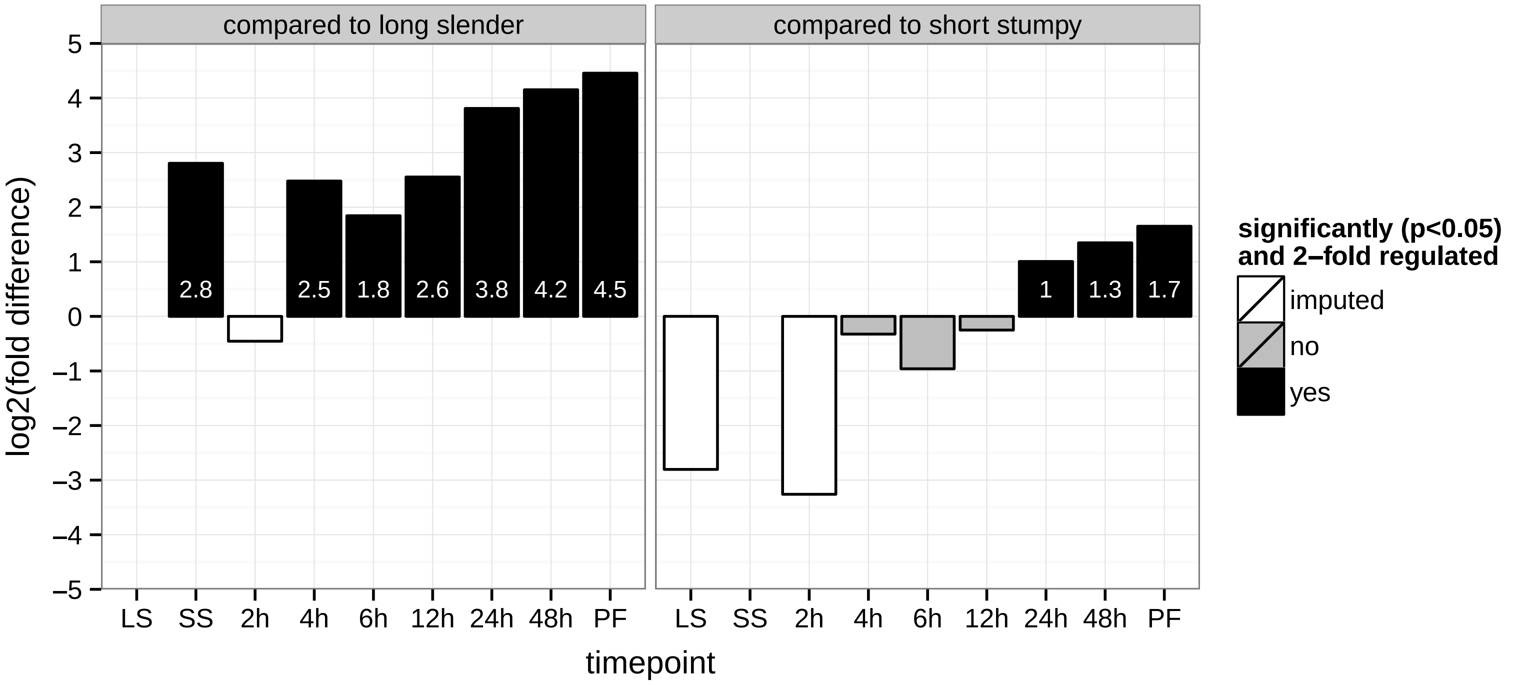

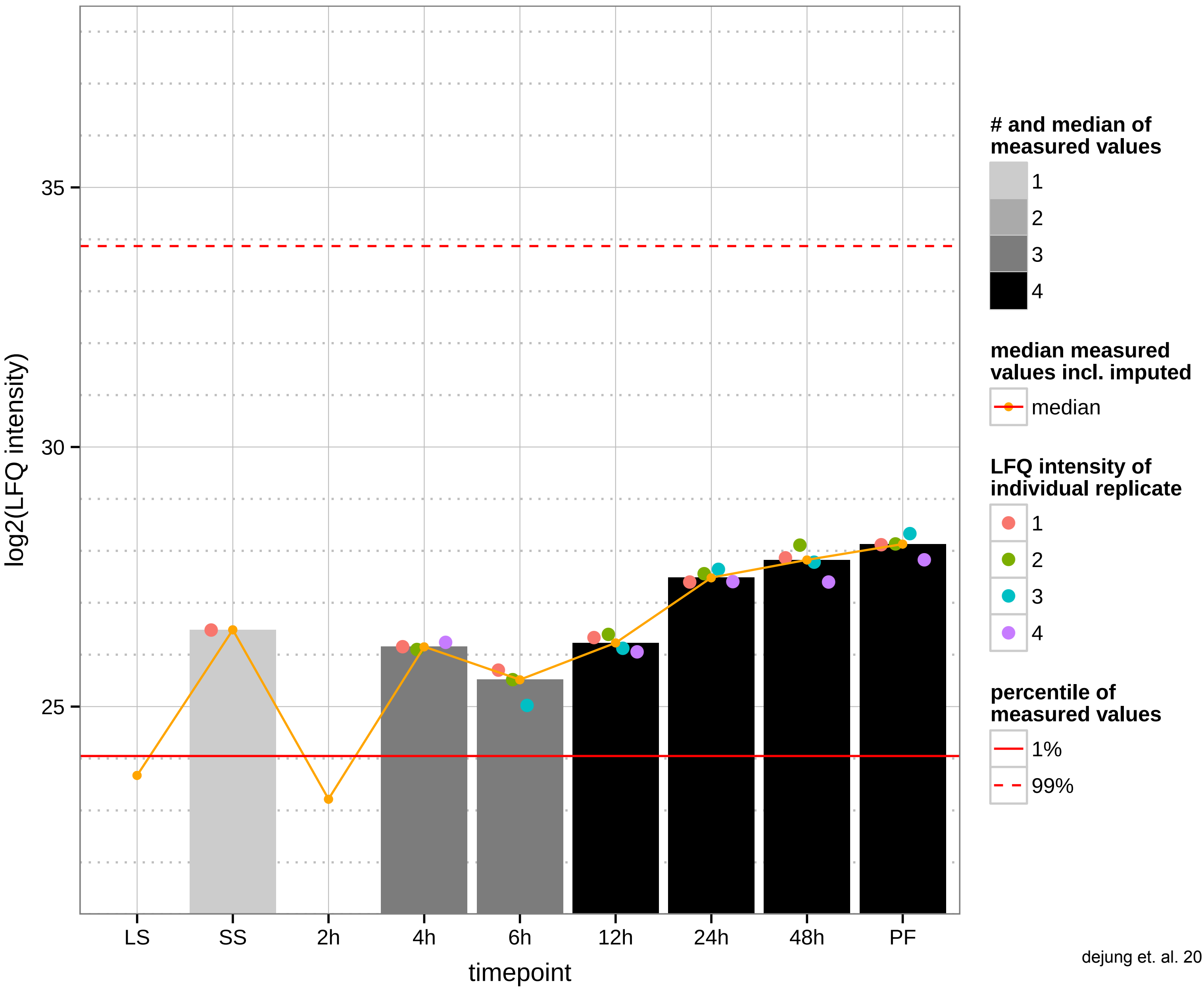

dejung et. al. 2015

hypothetical protein, conserved
Tb927.8.3300
AGOF: null
AGOC: mitochondrion
AGOP: null
PGOF: null
PGOC: null
PGOP: null

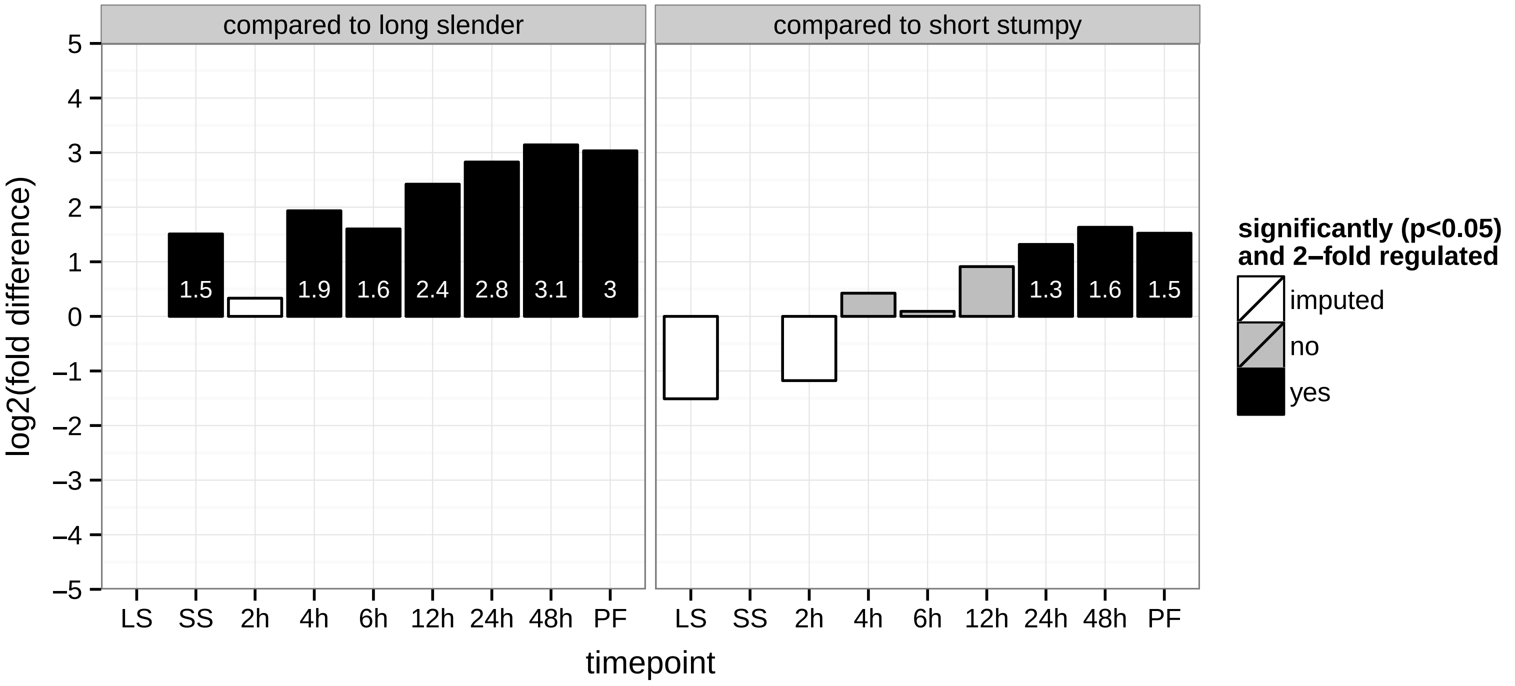

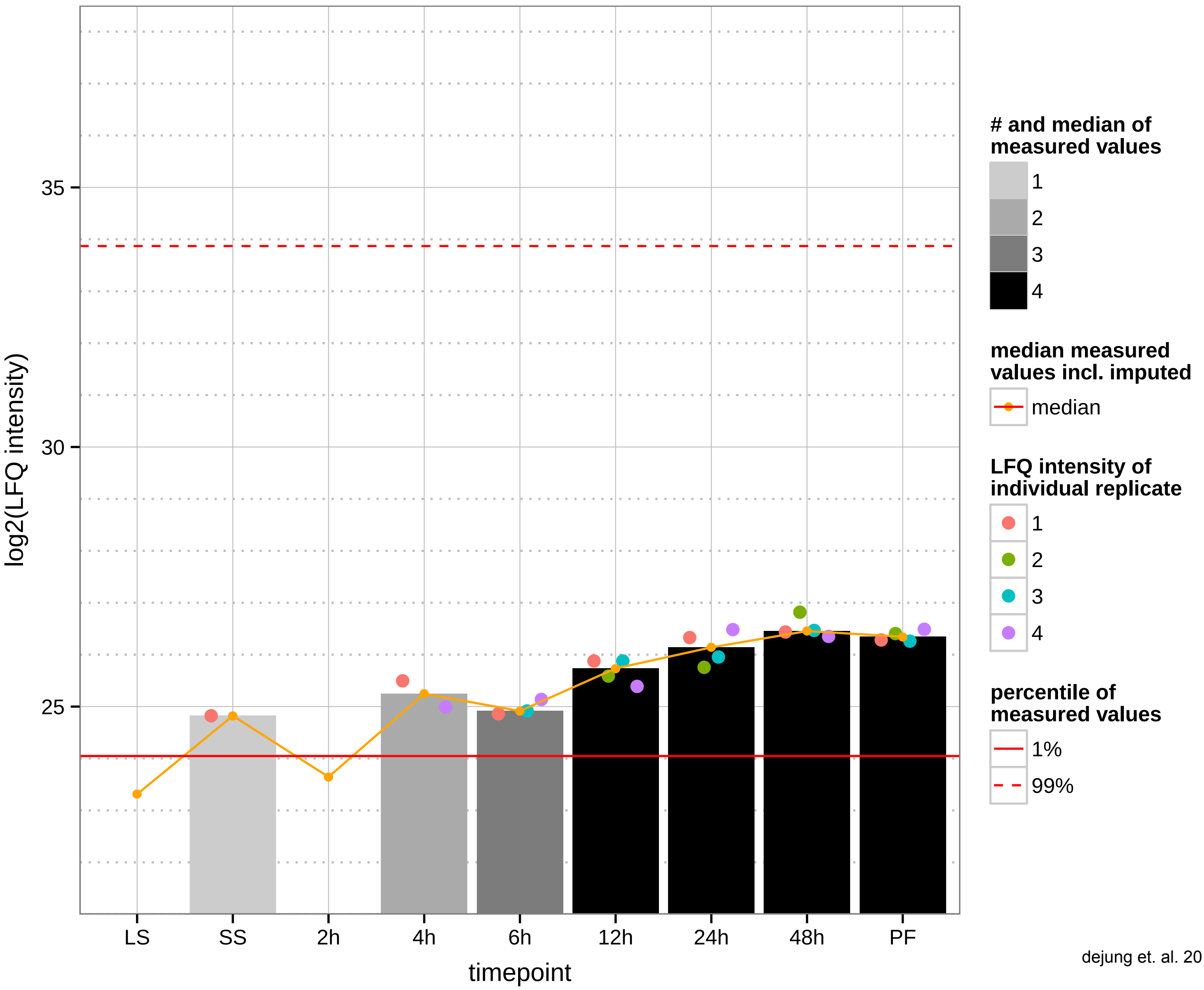

dejung et. al. 2015

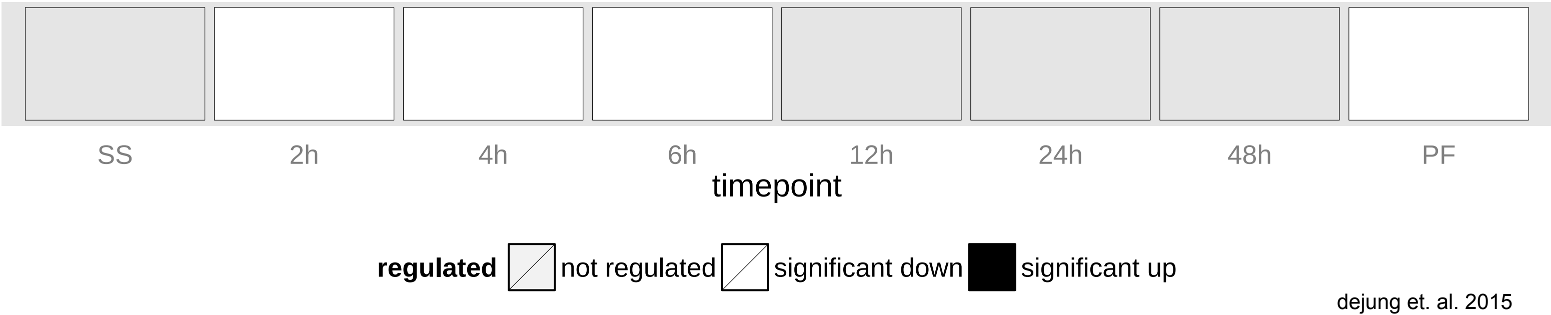
SS
2h
4h
6h
12h
24h
48h
PF
timepoint
regulated
not regulated
significant down
significant up
dejung et. al. 2015

ATP−dependent DEAH−box RNA helicase, putative
Tb927.4.3020;Tb11.v5.0965
AGOF: null, ATP binding, double−stranded RNA binding, helicase activity
AGOC: null, intracellular
AGOP: null
PGOF: helicase activity, ATP binding, ATP−dependent helicase activity, double−stranded RNA binding, helicase activity, nucle
PGOC: null, intracellular
PGOP: null

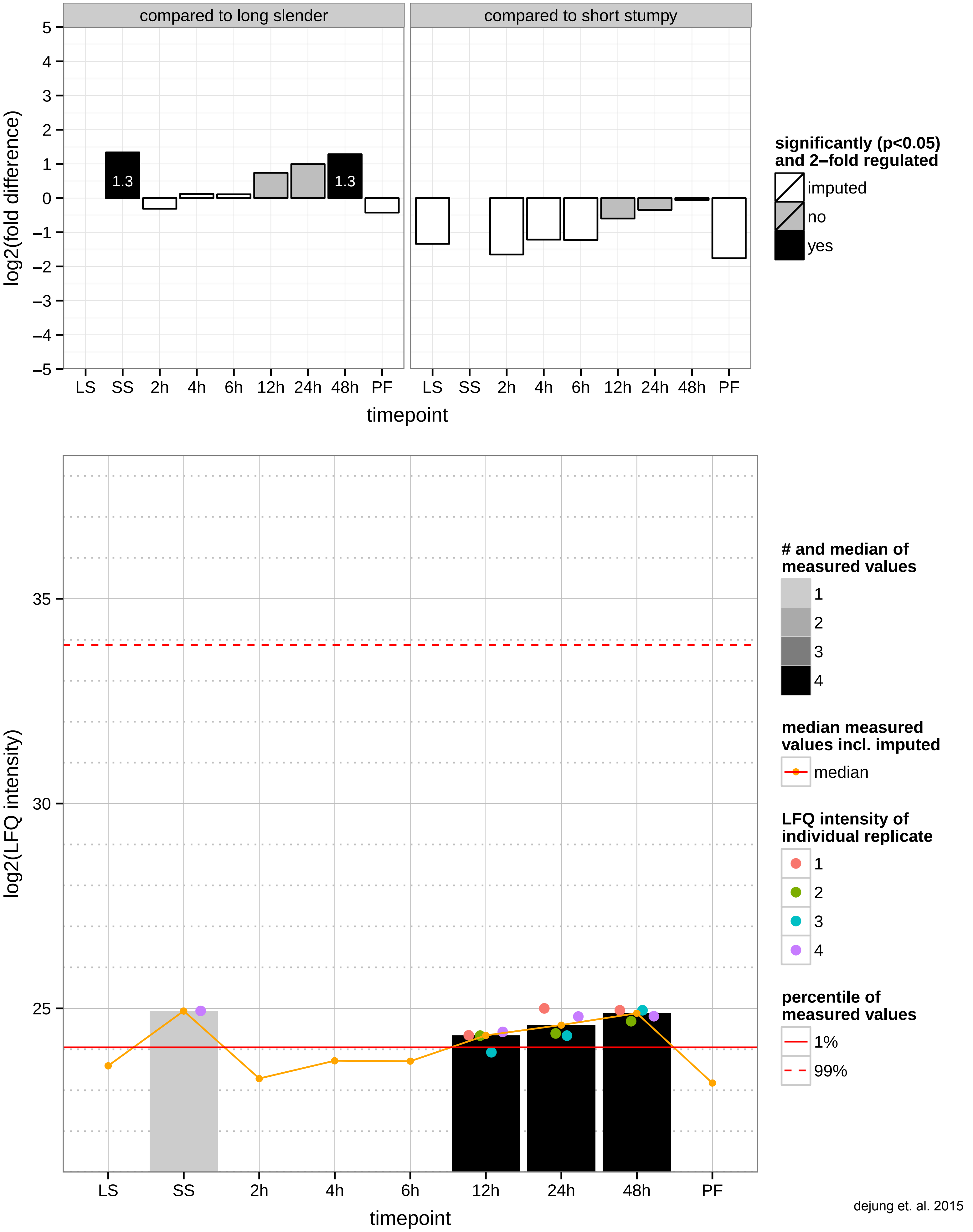

dejung et. al. 2015

coatomer subunit zeta, putative
Tb927.10.4270
AGOF: null
AGOC: membrane coat
AGOP: intracellular protein transport, vesicle−mediated transport
PGOF: null
PGOC: null
PGOP: transport

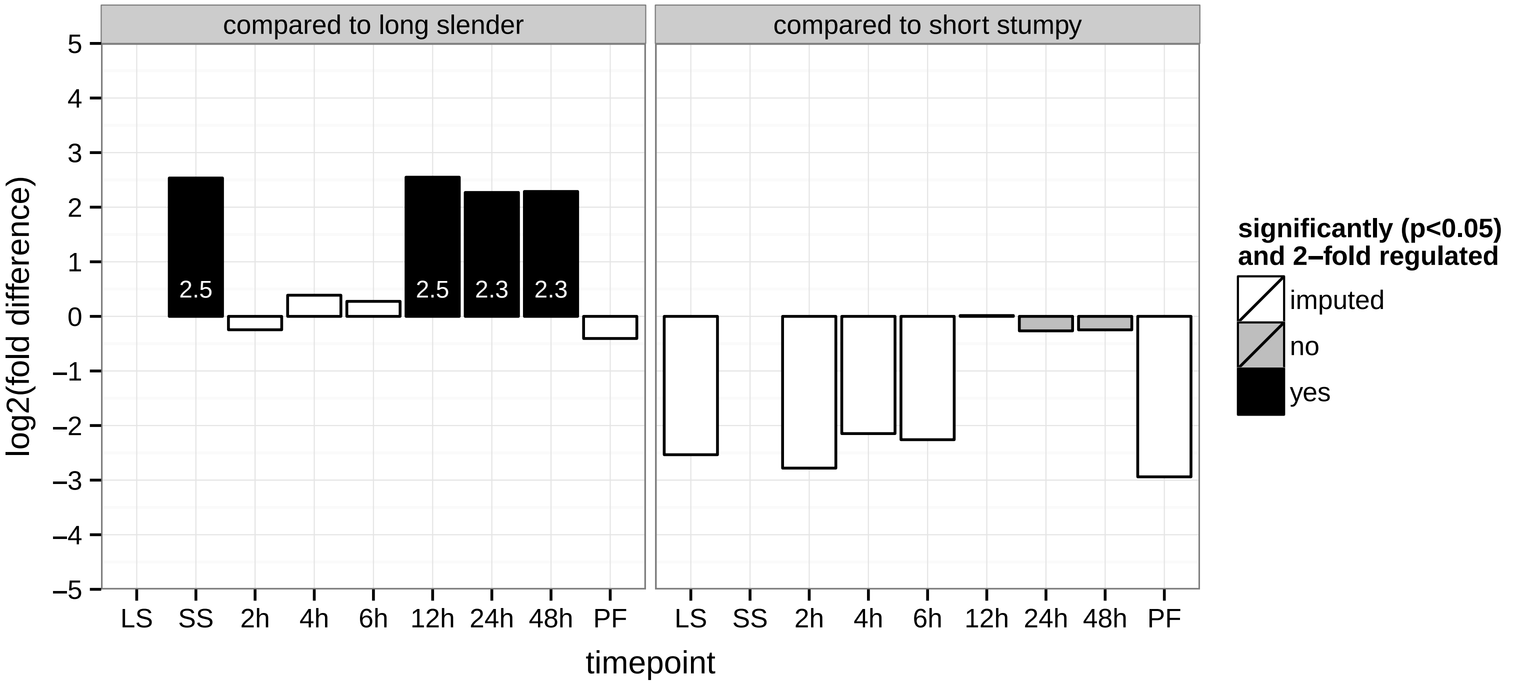

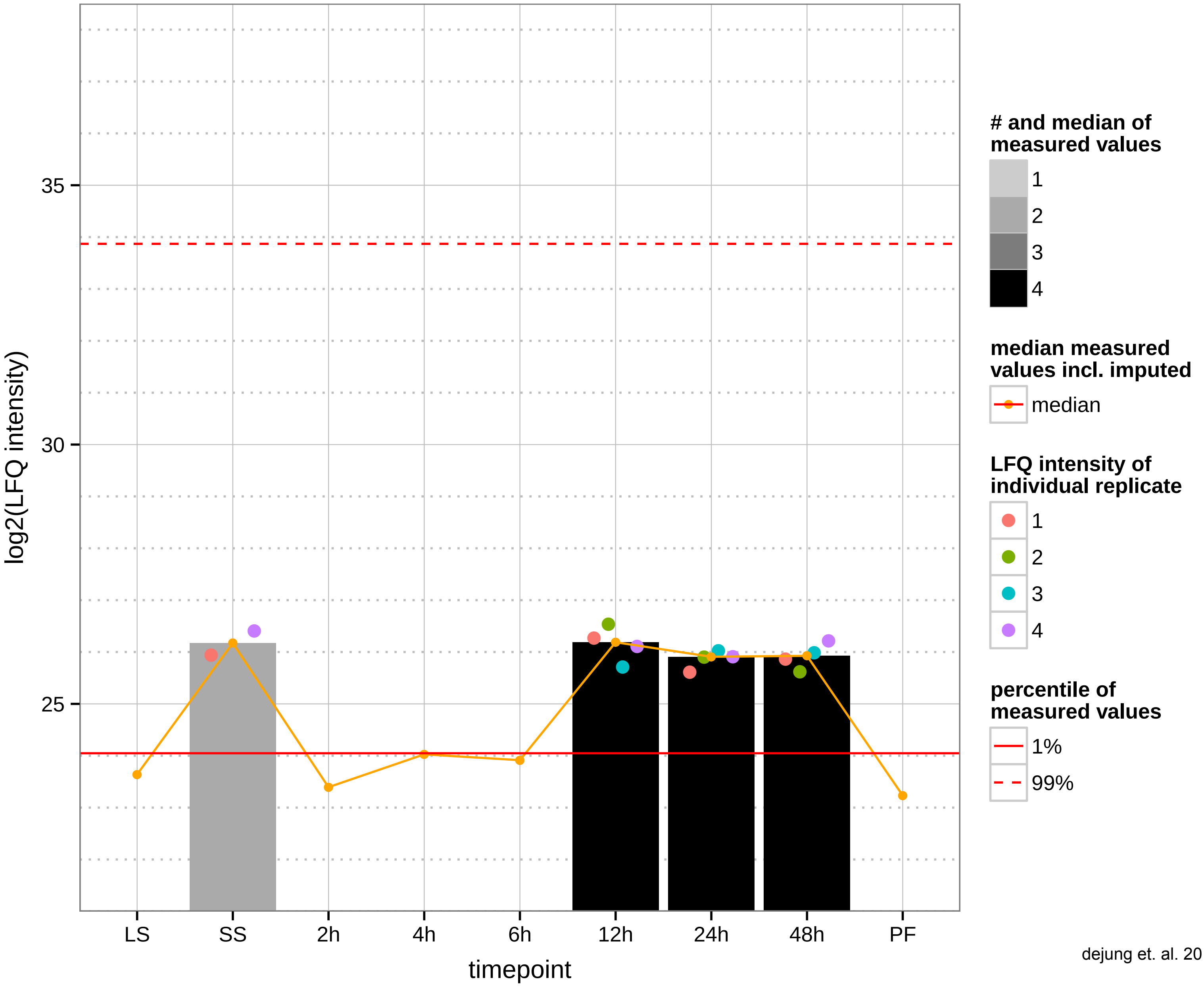

dejung et. al. 2015

intergrin alpha chain protein, putative, FG−GAP repeat protein
Tb927.3.3640
AGOF: null
AGOC: null
AGOP: null
PGOF: protein binding
PGOC: null
PGOP: null

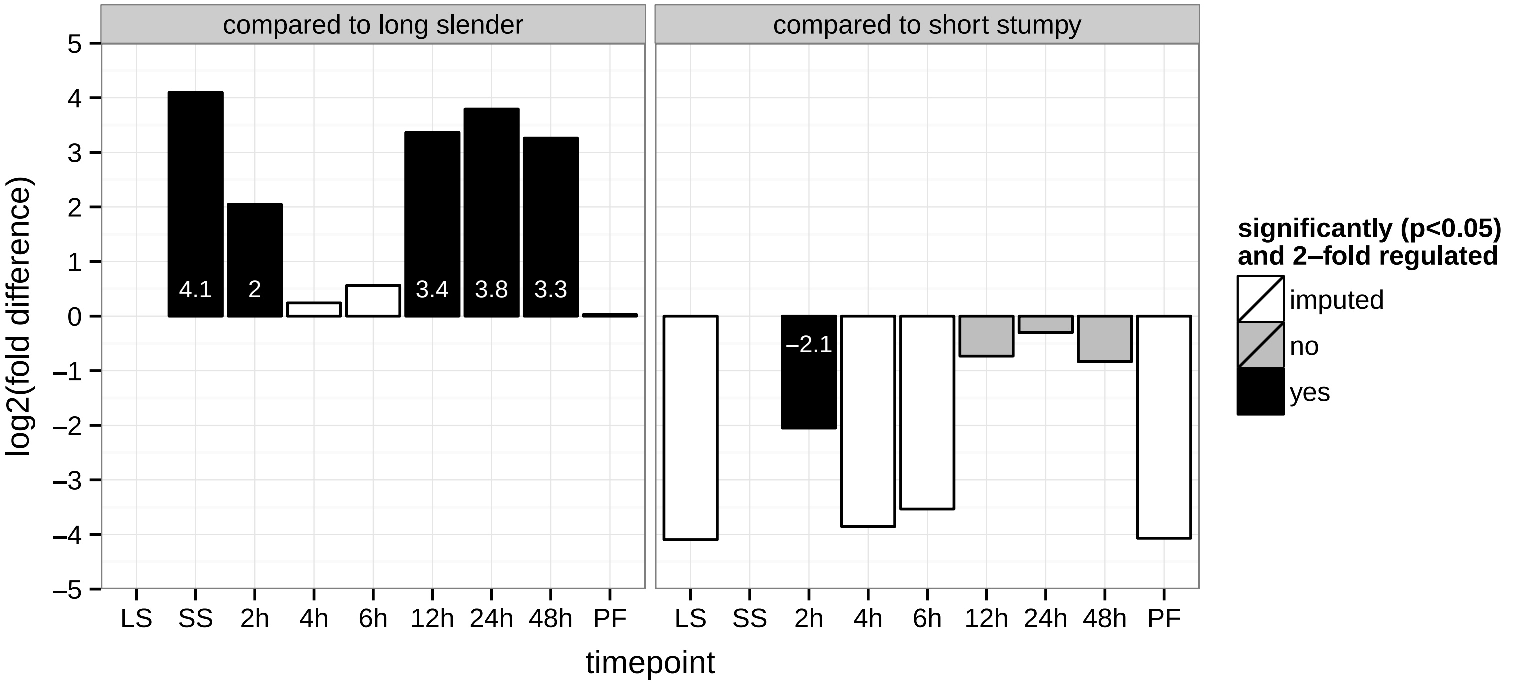

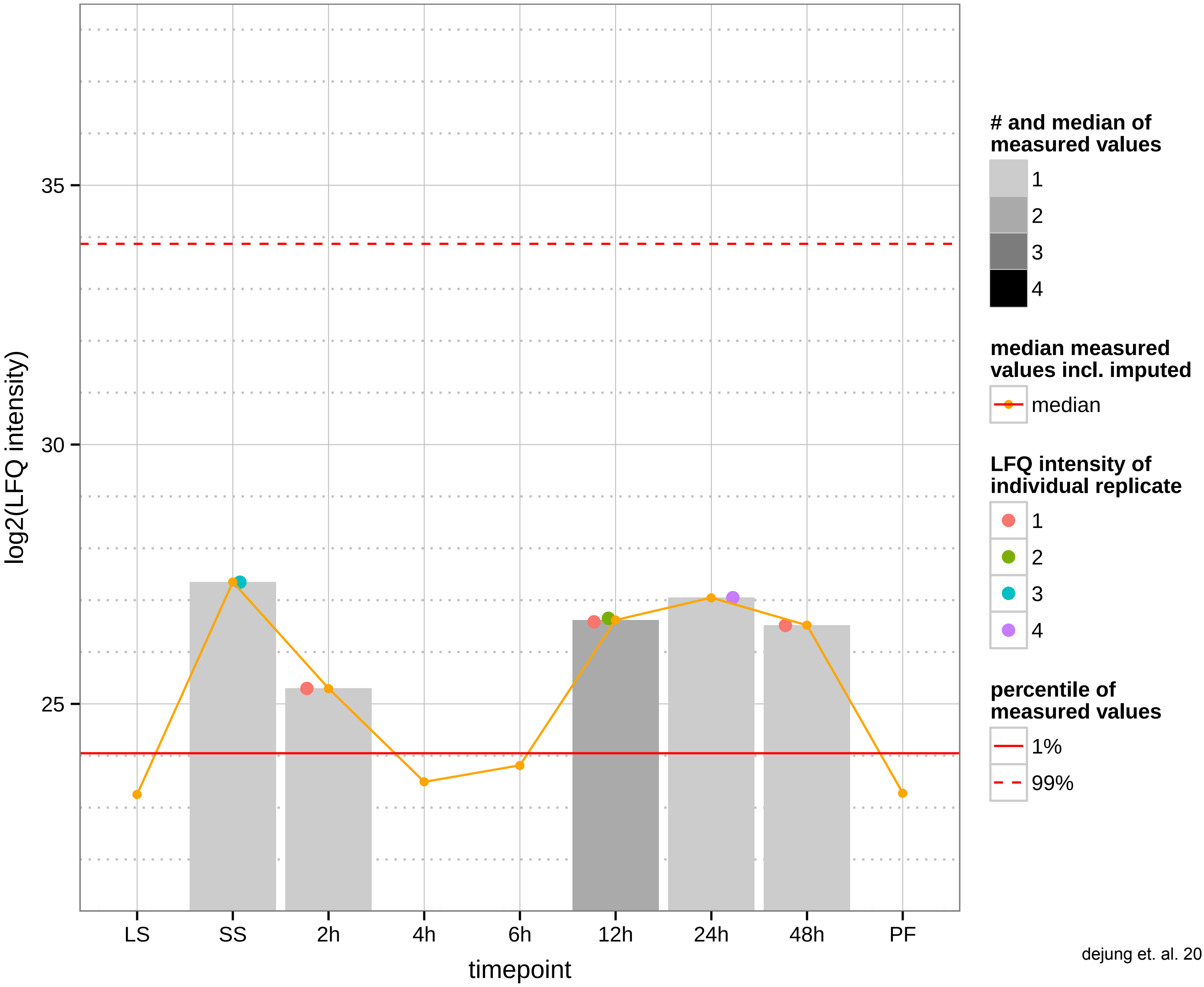

dejung et. al. 2015

glycosyltransferase family 28 protein, putative
Tb927.6.1960
AGOF: carbohydrate binding, transferase activity, transferring hexosyl groups
AGOC: null
AGOP: carbohydrate metabolic process, lipid glycosylation
PGOF: carbohydrate binding, transferase activity, transferring hexosyl groups
PGOC: null
PGOP: carbohydrate metabolic process, lipid glycosylation

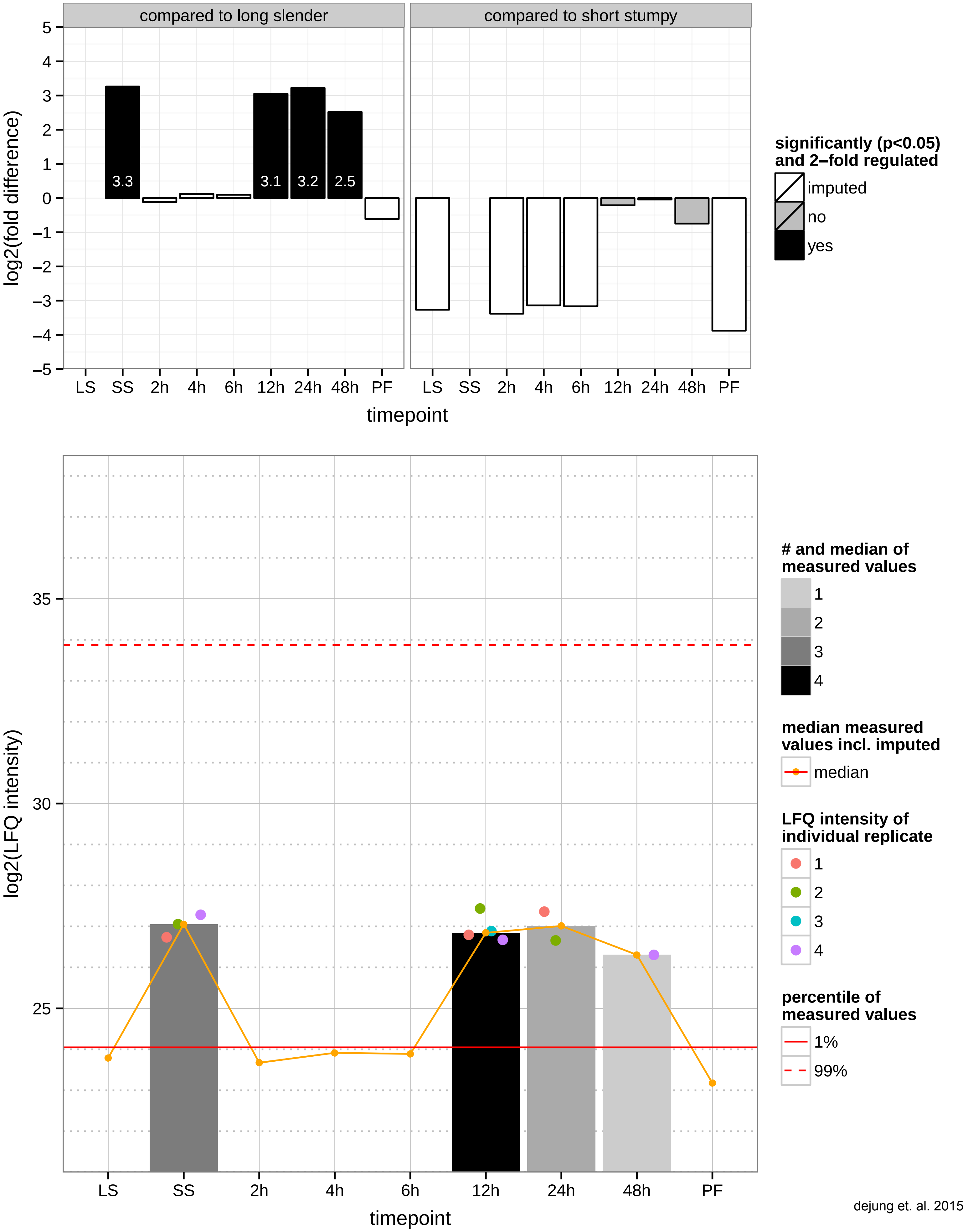

dejung et. al. 2015

intergrin alpha chain protein, putative, FG−GAP repeat protein
Tb927.6.2020
AGOF: null
AGOC: null
AGOP: null
PGOF: protein binding
PGOC: null
PGOP: null

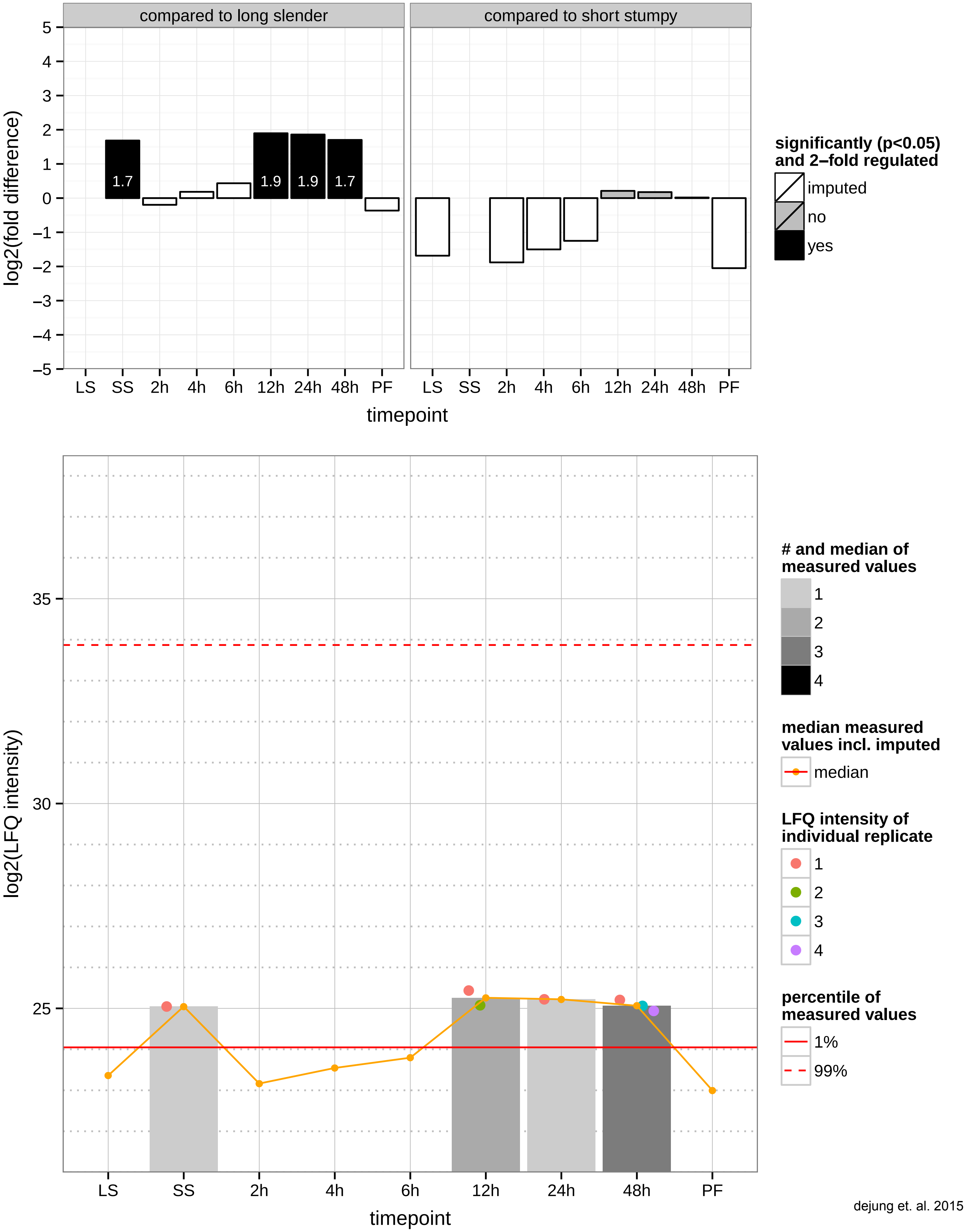

dejung et. al. 2015

hypothetical protein, conserved
Tb927.8.2040
AGOF: null
AGOC: null
AGOP: null
PGOF: null
PGOC: null
PGOP: null

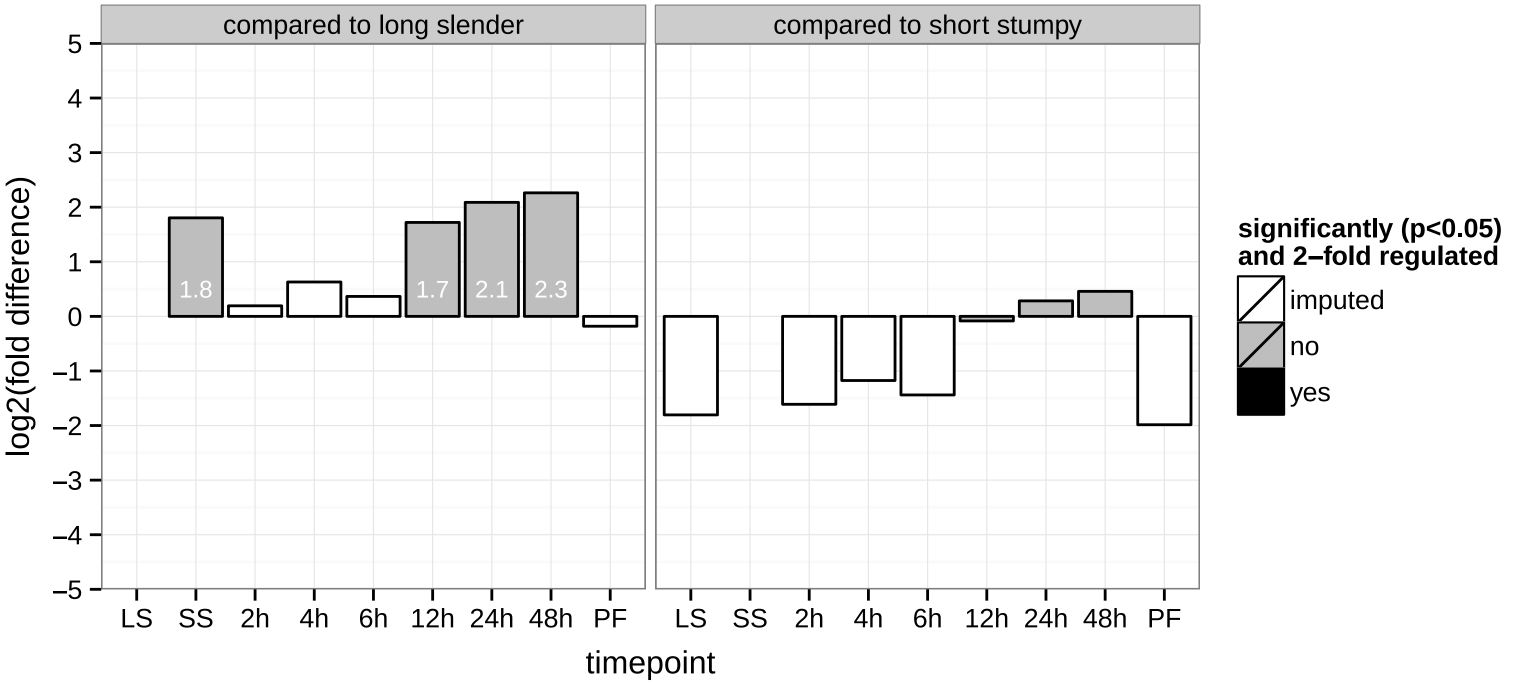

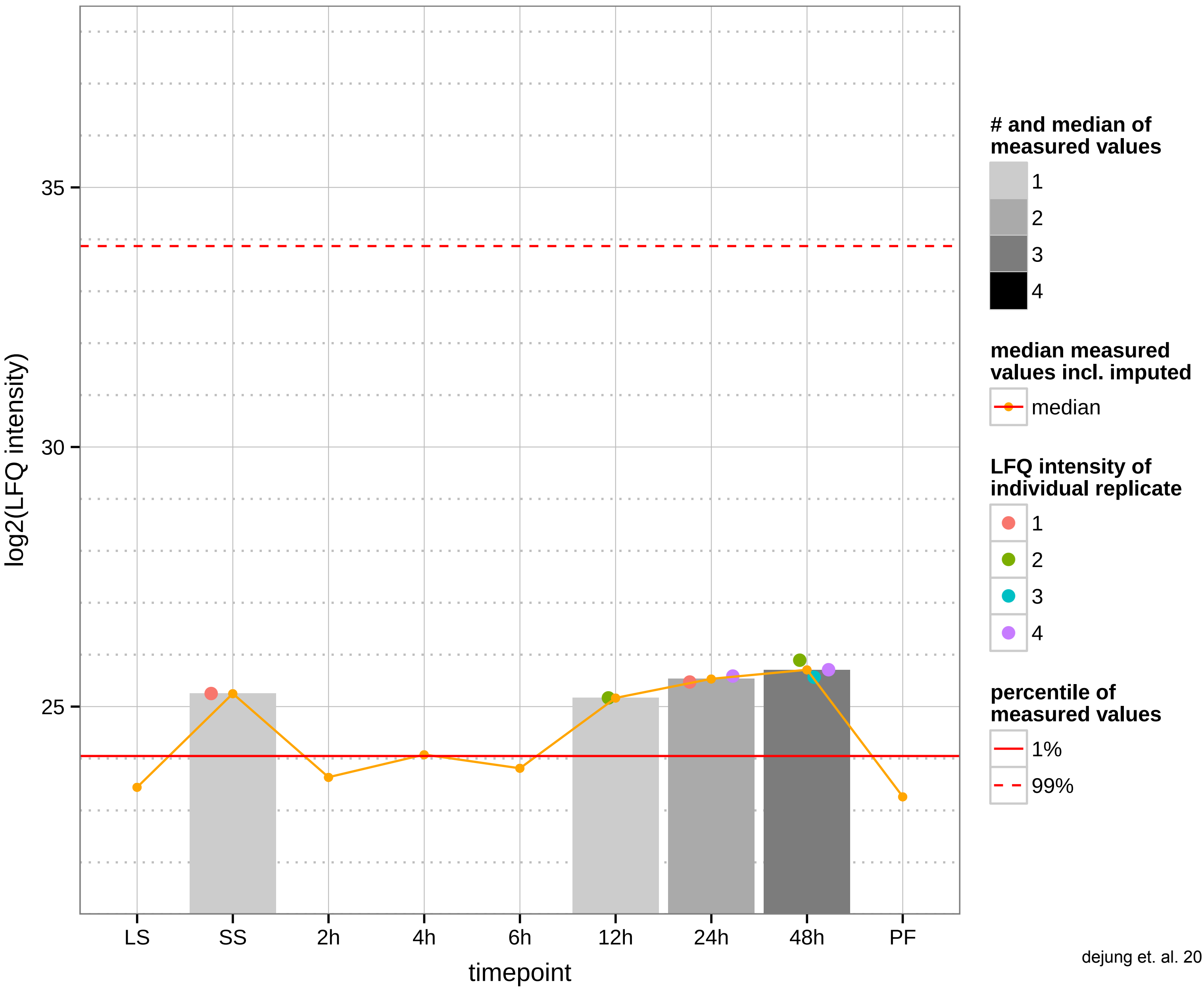

dejung et. al. 2015

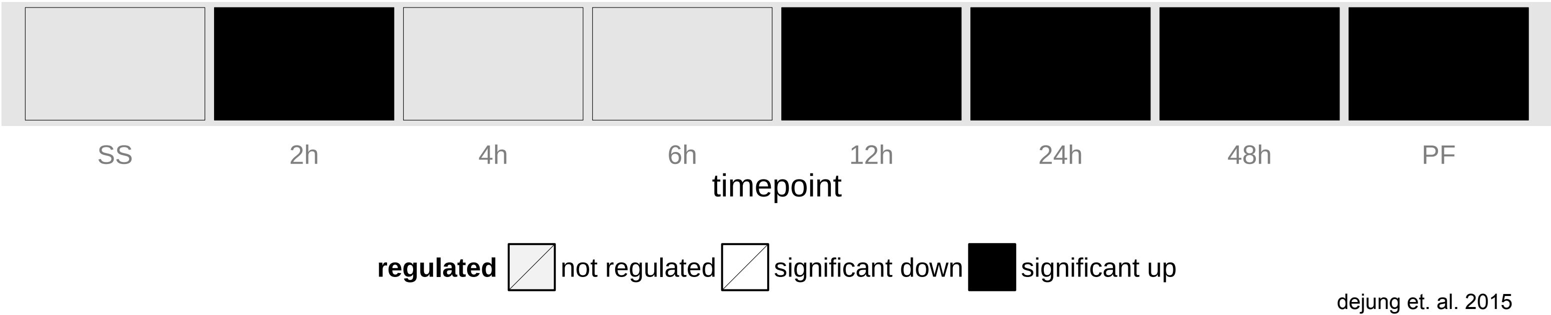
SS
2h
4h
6h
12h
24h
48h
PF
timepoint
regulated
not regulated
significant down
significant up
dejung et. al. 2015

beta–D–hydroxybutyrate dehydrogenase
Tb927.10.11930
AGOF: 3–hydroxybutyrate dehydrogenase activity
AGOC: mitochondrion
AGOP: ketone body biosynthetic process
PGOF: 3–hydroxybutyrate dehydrogenase activity, oxidoreductase activity
PGOC: null
PGOP: metabolic process, oxidation–reduction process

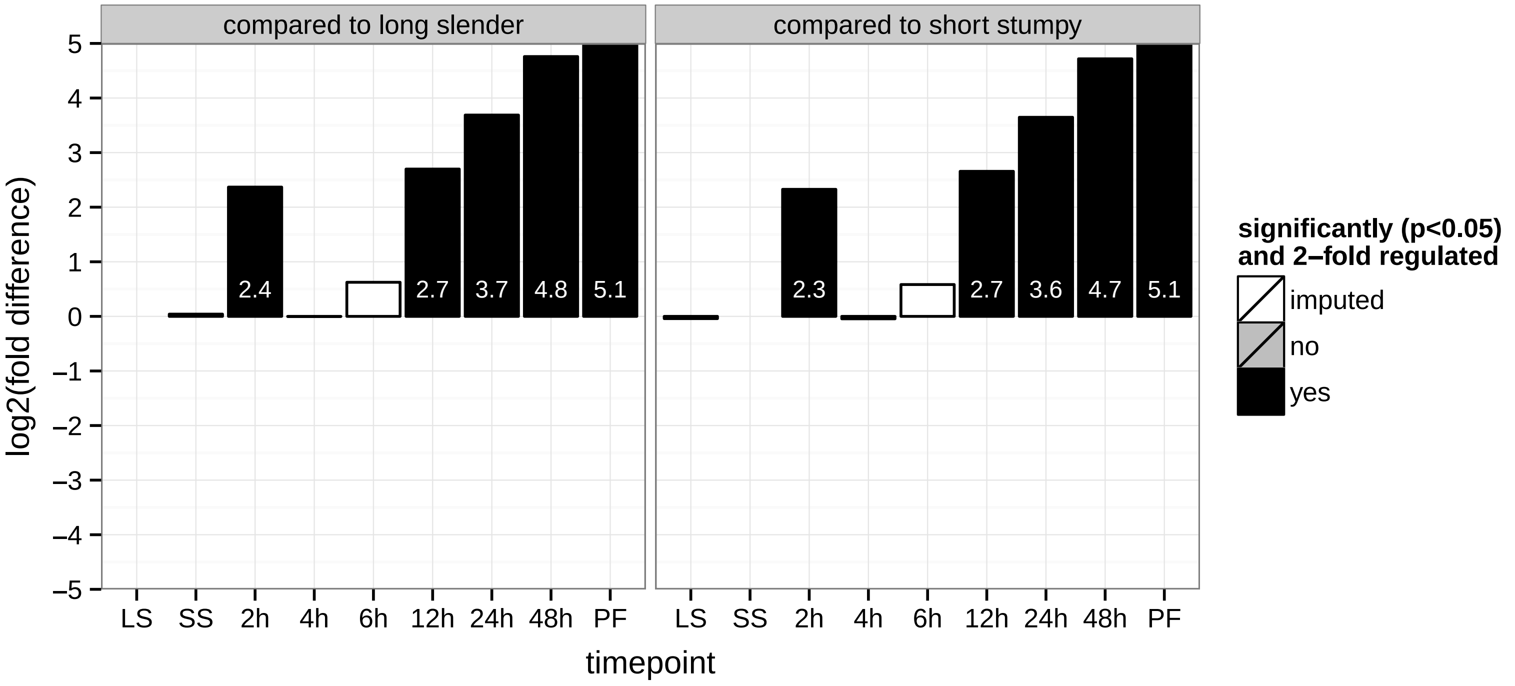

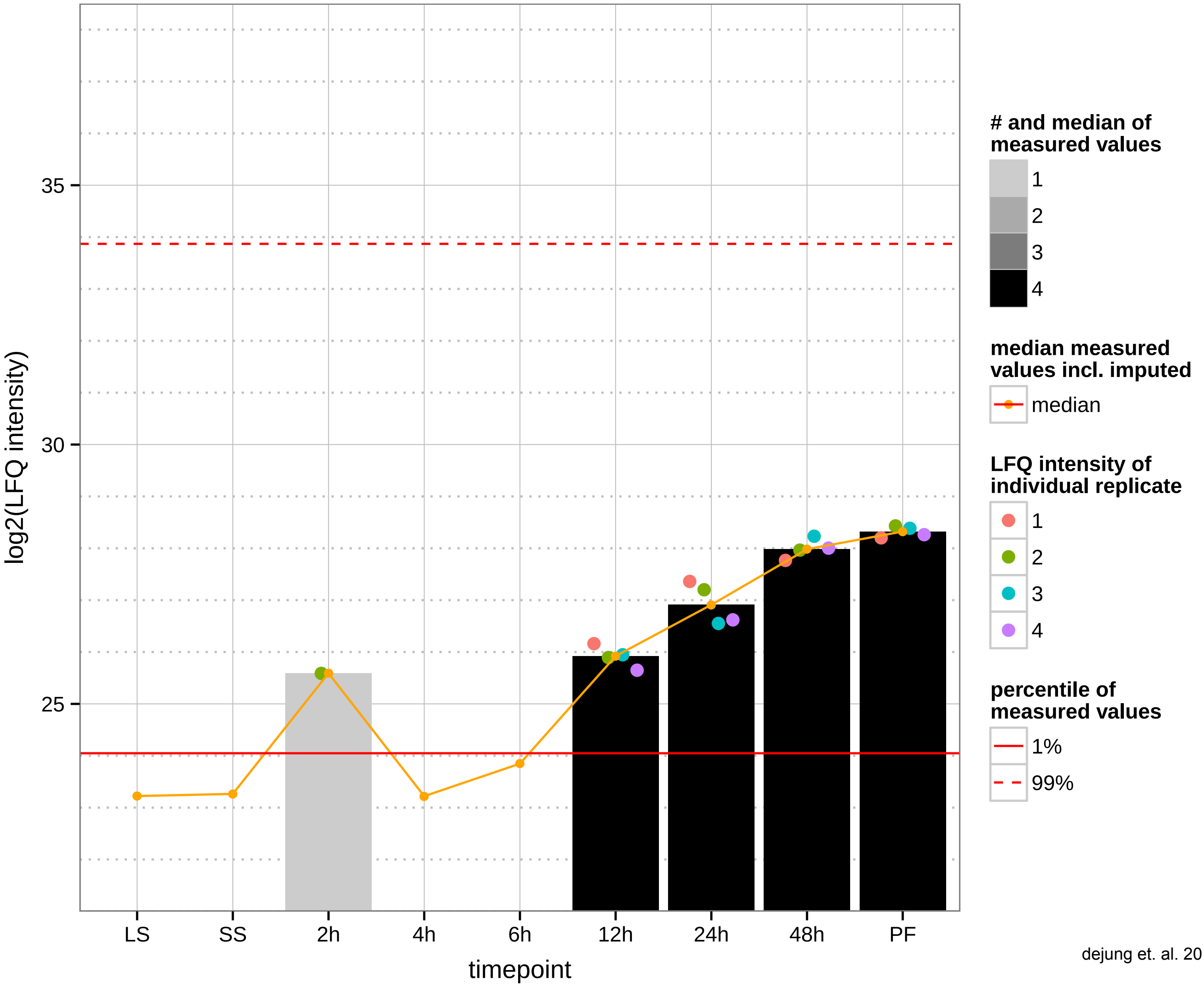

dejung et. al. 2015

hypothetical protein, conserved
Tb927.11.14750
AGOF: null
AGOC: null
AGOP: null
PGOF: null
PGOC: null
PGOP: null

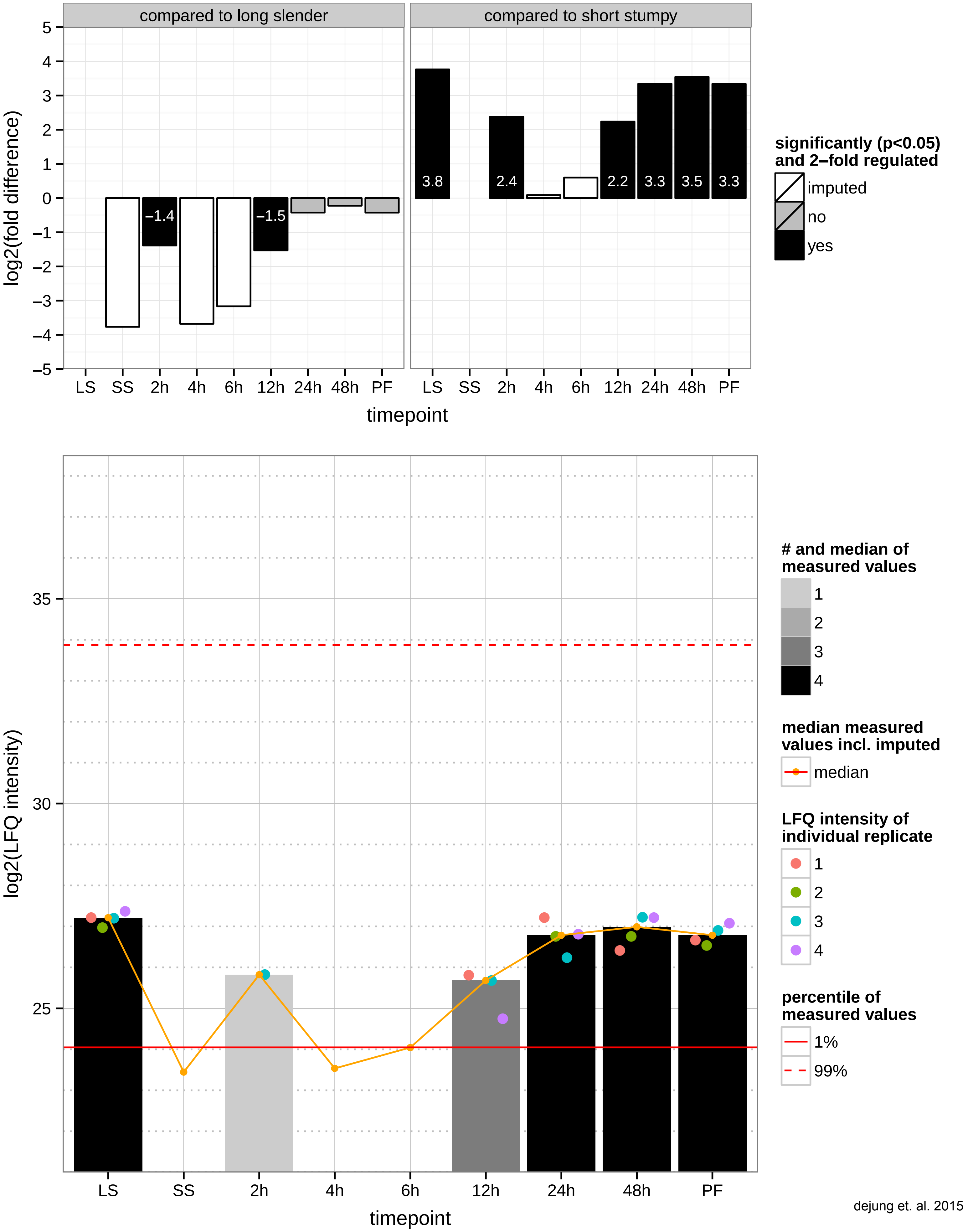

dejung et. al. 2015

hypothetical protein, conserved
Tb927.5.530
AGOF: null
AGOC: mitochondrion
AGOP: null
PGOF: null
PGOC: null
PGOP: null

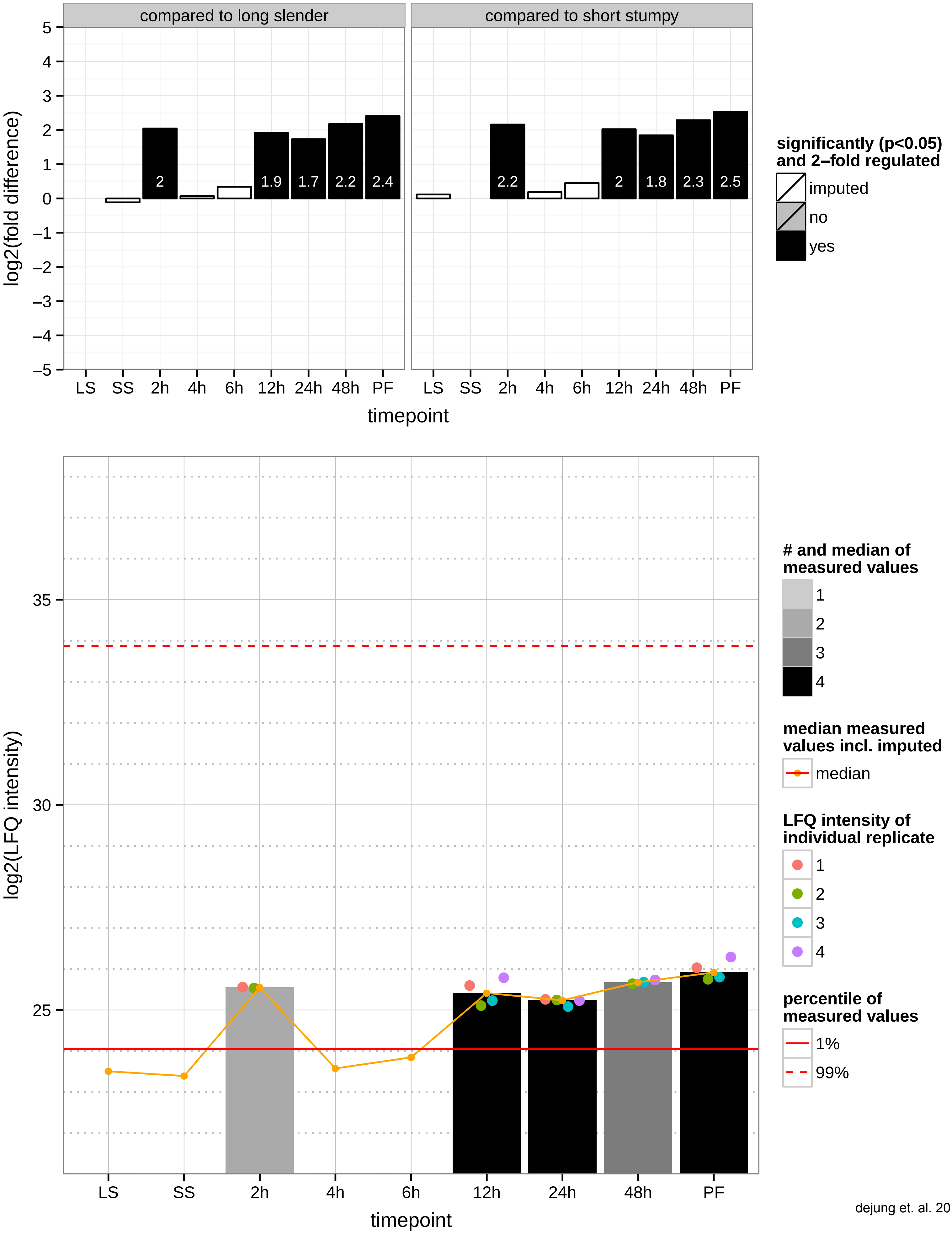

dejung et. al. 2015

ribosomal RNA adenine dimethylase family protein, conserved
Tb927.6.1610
AGOF: rRNA (adenine−N6, N6−)−dimethyltransferase activity
AGOC: null
AGOP: rRNA modification
PGOF: rRNA (adenine) methyltransferase activity, rRNA (adenine−N6, N6−)−dimethyltransferase activity, rRNA methyltransfer
PGOC: null
PGOP: rRNA modification, rRNA processing

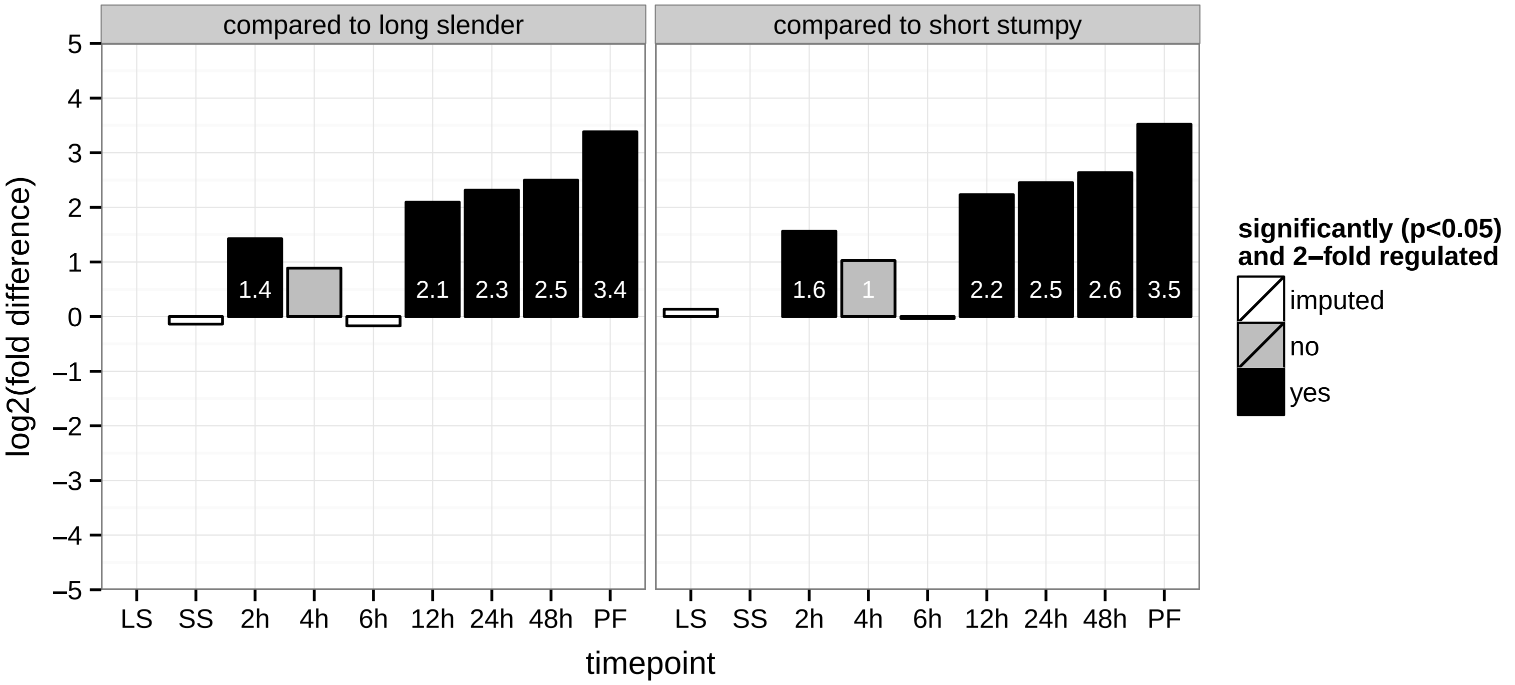

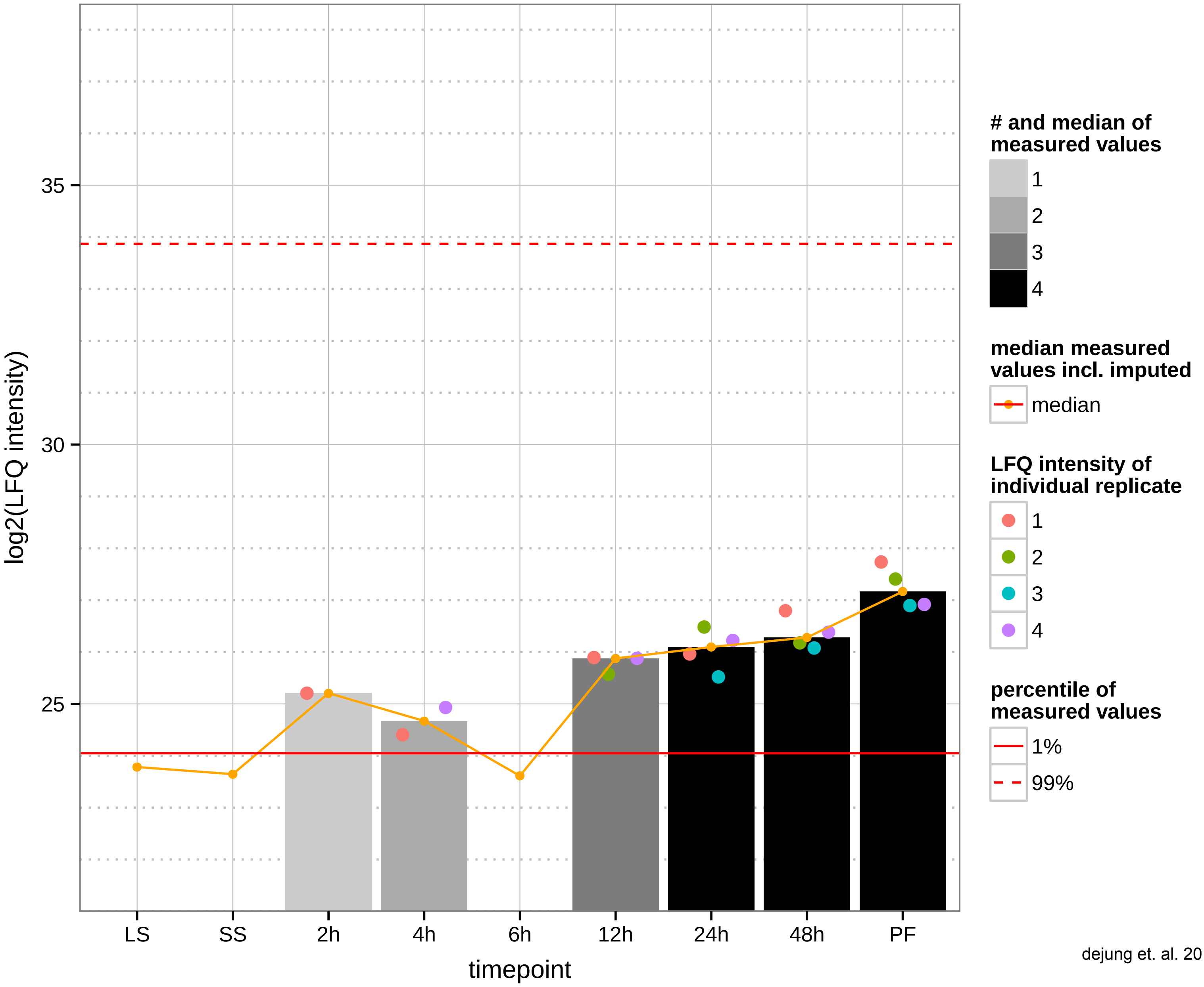

dejung et. al. 2015

hypothetical protein, conserved
Tb927.7.4710
AGOF: null
AGOC: mitochondrion
AGOP: null
PGOF: hydrolase activity
PGOC: null
PGOP: null

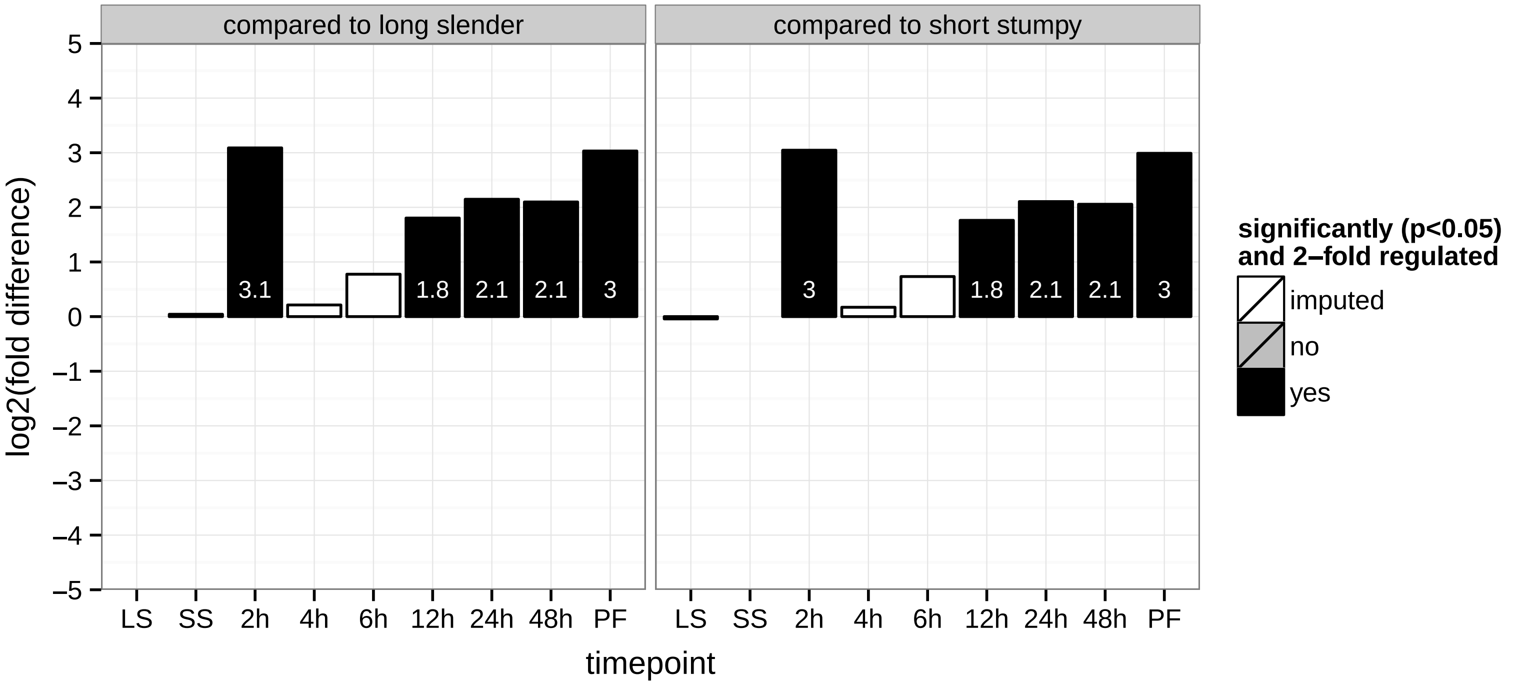

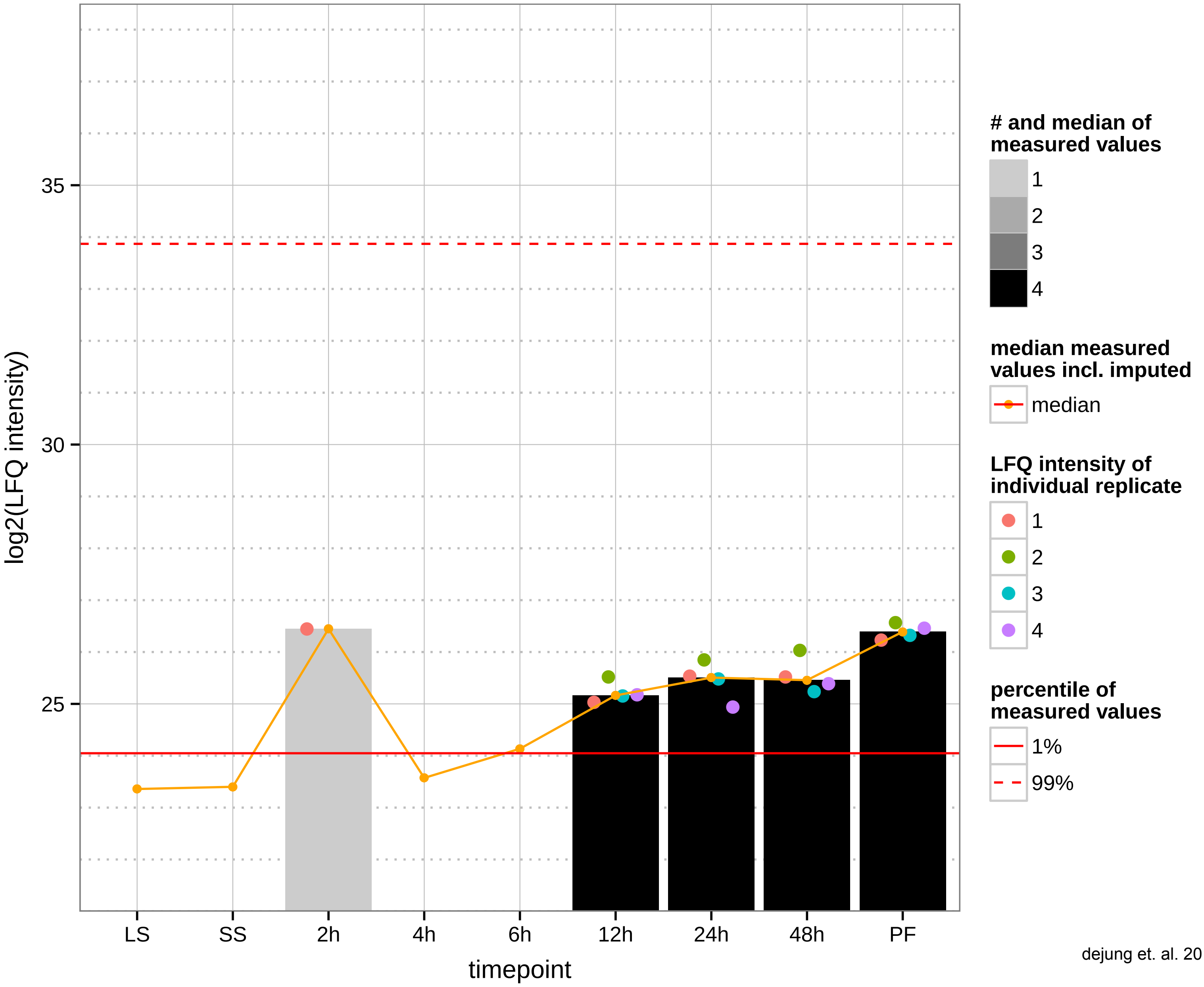

dejung et. al. 2015

KREPA3, RNA−editing complex protein MP42 (KREPA3)
Tb927.8.620
AGOF: 3'−5'−exoribonuclease activity, endoribonuclease activity, producing 5'−phosphomonoesters, zinc ion binding
AGOC: intracellular, mitochondrial mRNA editing complex, mitochondrion
AGOP: mRNA modification, response to metal ion
PGOF: zinc ion binding
PGOC: intracellular
PGOP: null

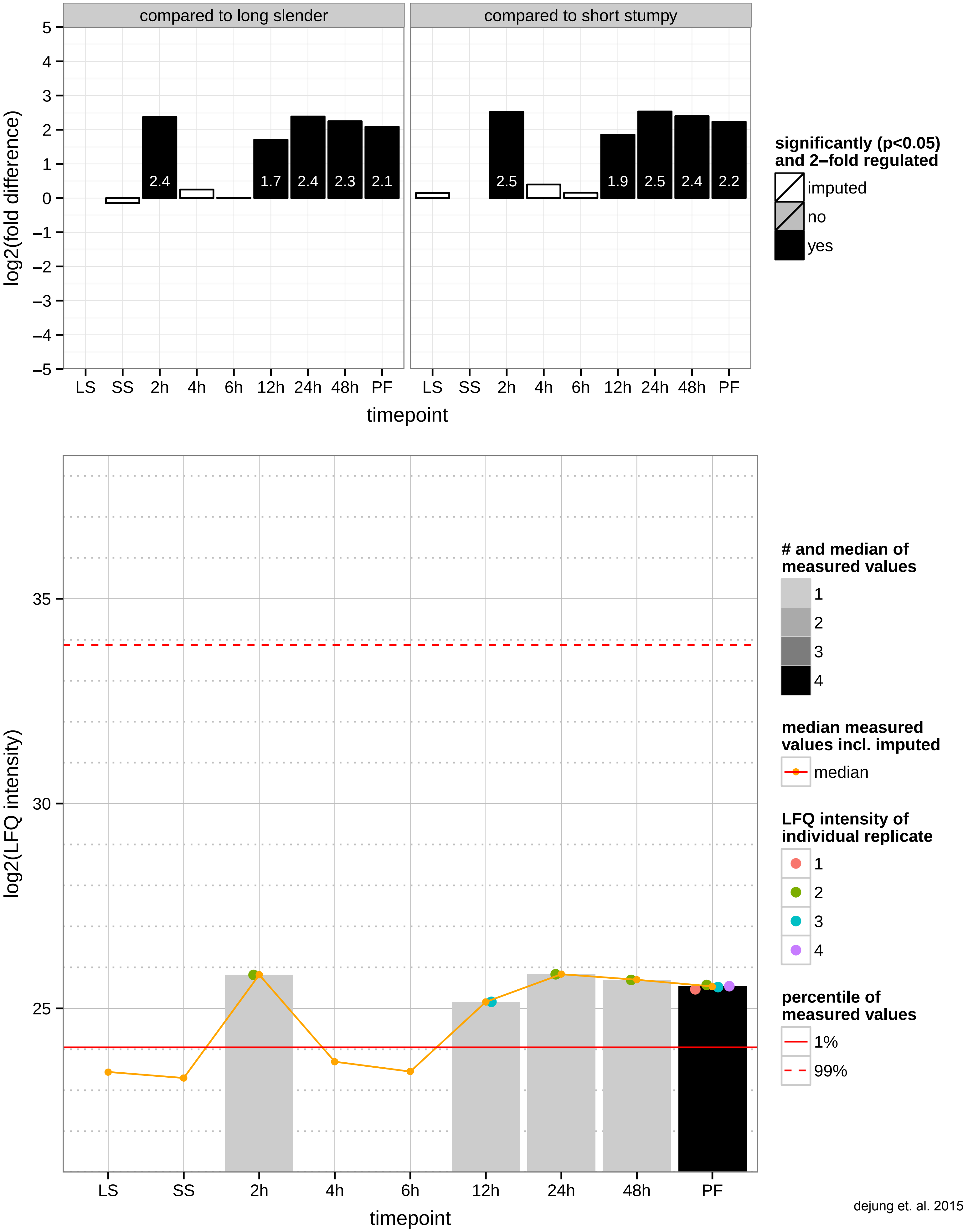

dejung et. al. 2015

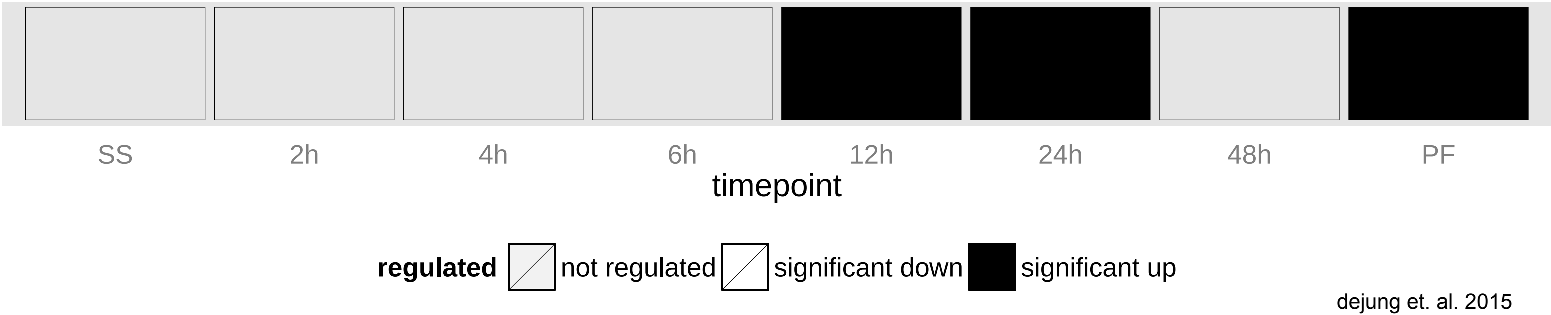
SS
2h
4h
6h
12h
24h
48h
PF
timepoint
regulated
not regulated
significant down
significant up
dejung et. al. 2015

septum formation protein MAF homologue, putative
Tb927.1.3280
AGOF: null
AGOC: cytoplasm
AGOP: null
PGOF: null
PGOC: cytoplasm
PGOP: null

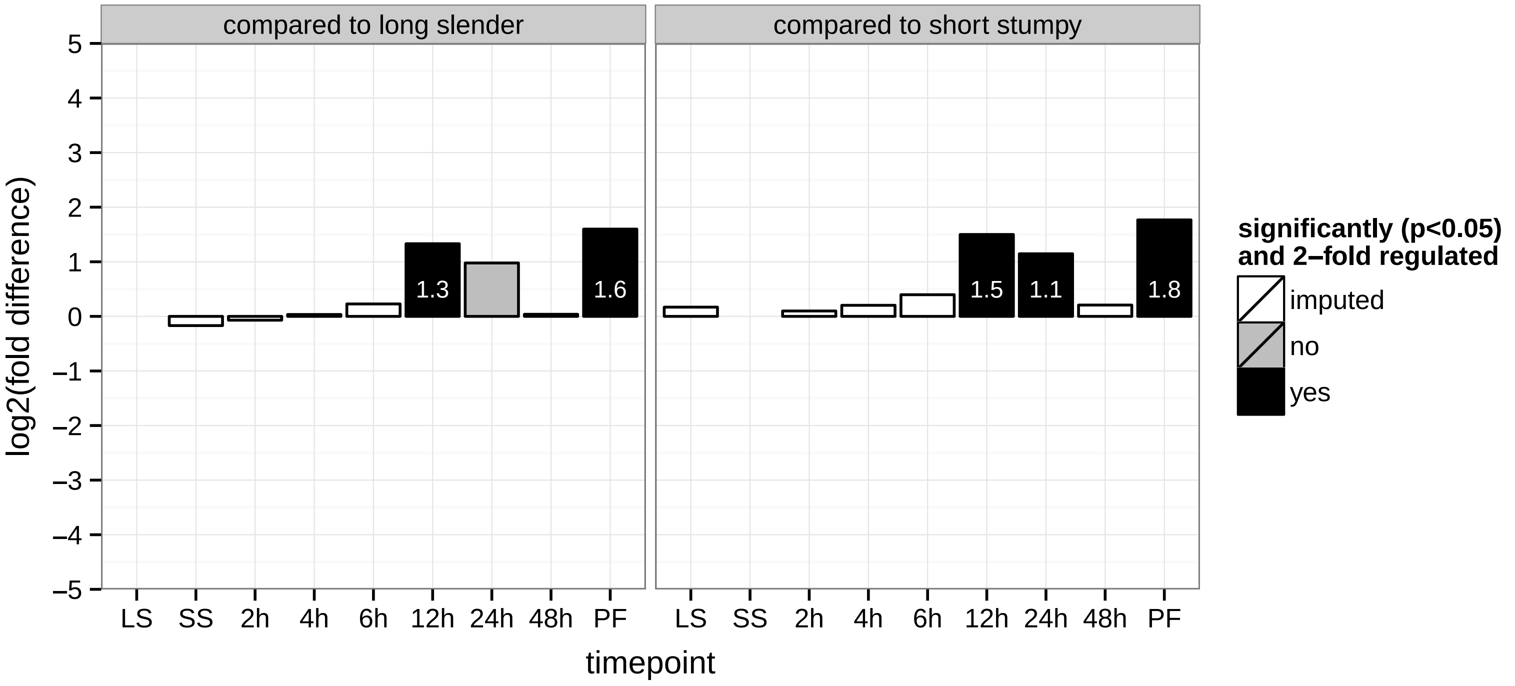

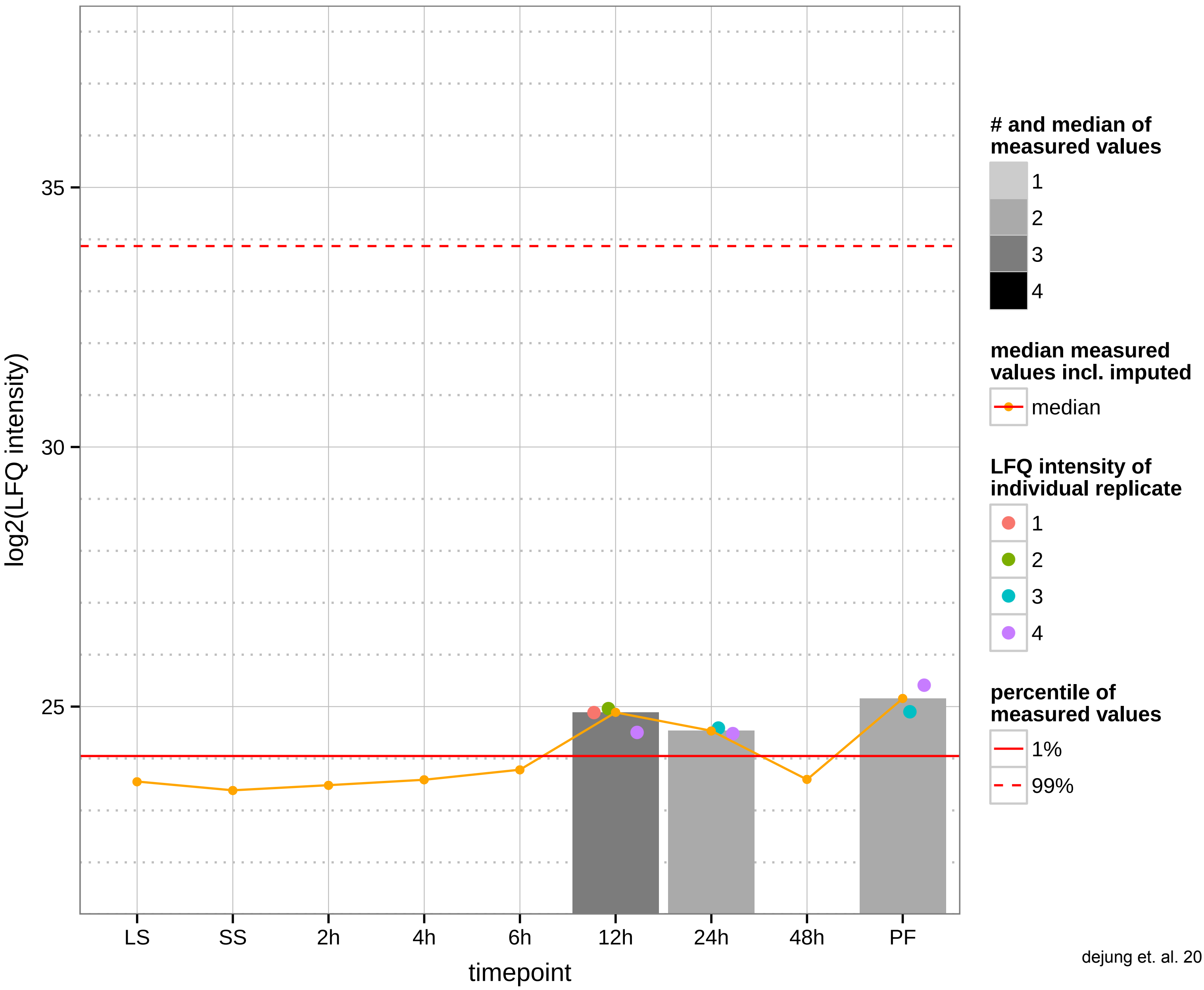

dejung et. al. 2015

cullin, putative
Tb927.10.7490
AGOF: ubiquitin protein ligase binding
AGOC: cullin–RING ubiquitin ligase complex
AGOP: cell cycle, ubiquitin–dependent protein catabolic process
PGOF: ubiquitin protein ligase binding
PGOC: cullin–RING ubiquitin ligase complex
PGOP: ubiquitin–dependent protein catabolic process

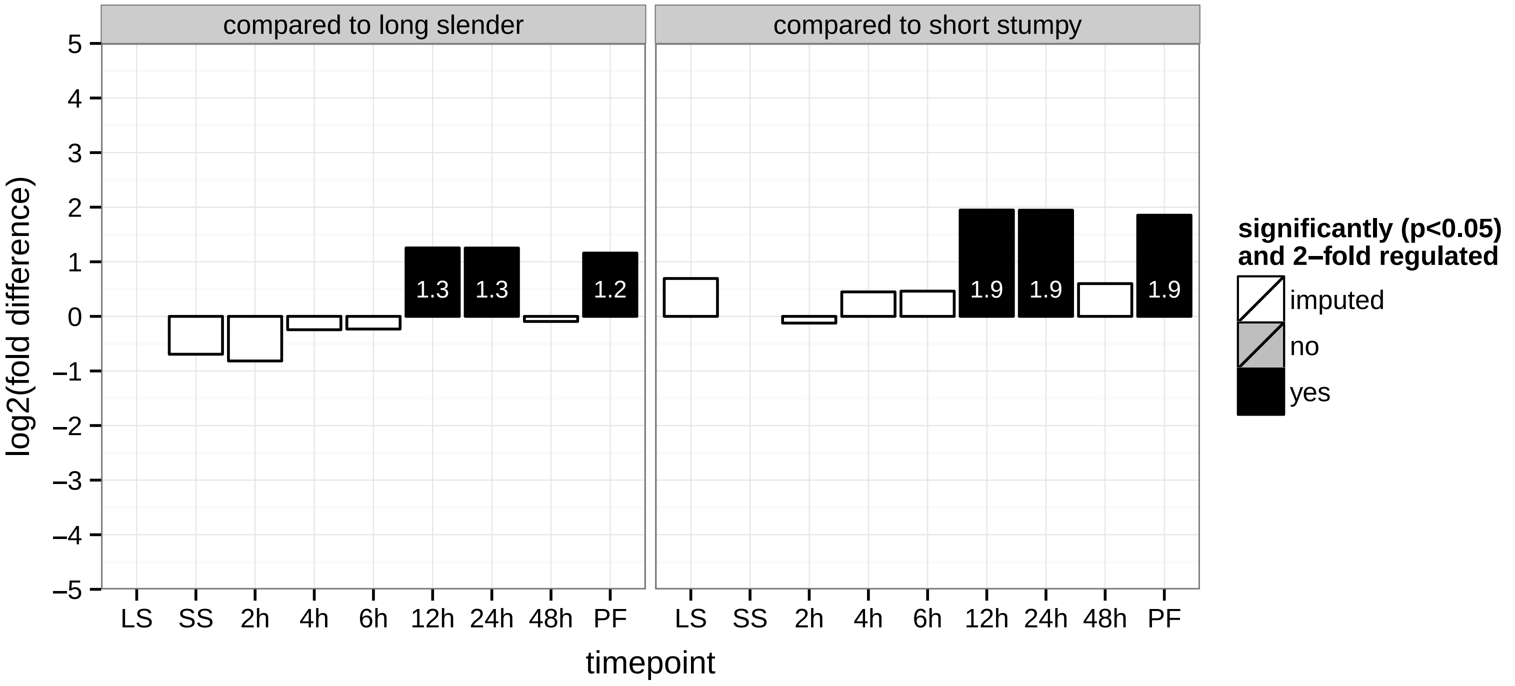

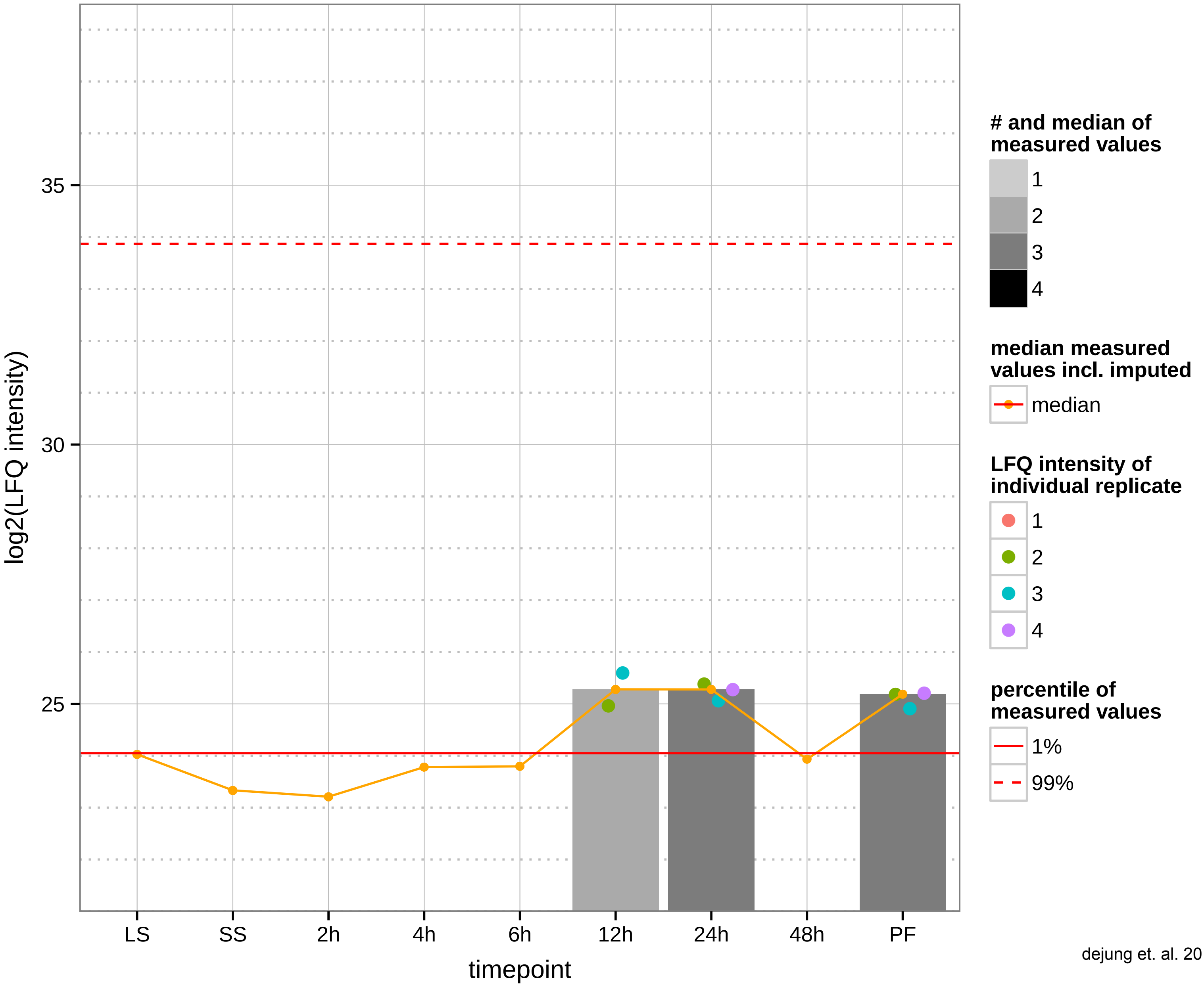

dejung et. al. 2015

hypothetical protein, conserved
Tb927.11.15080
AGOF: null
AGOC: membrane
AGOP: null
PGOF: null
PGOC: membrane
PGOP: null

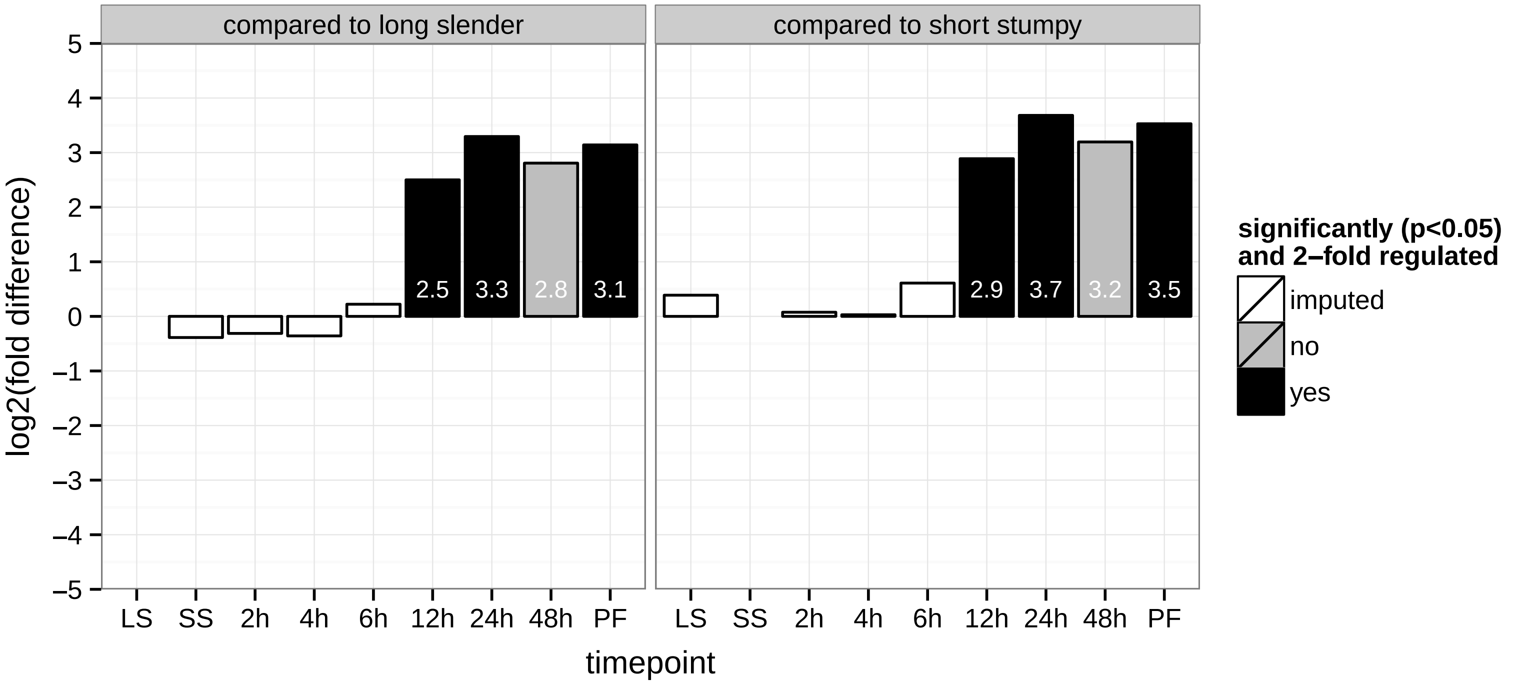

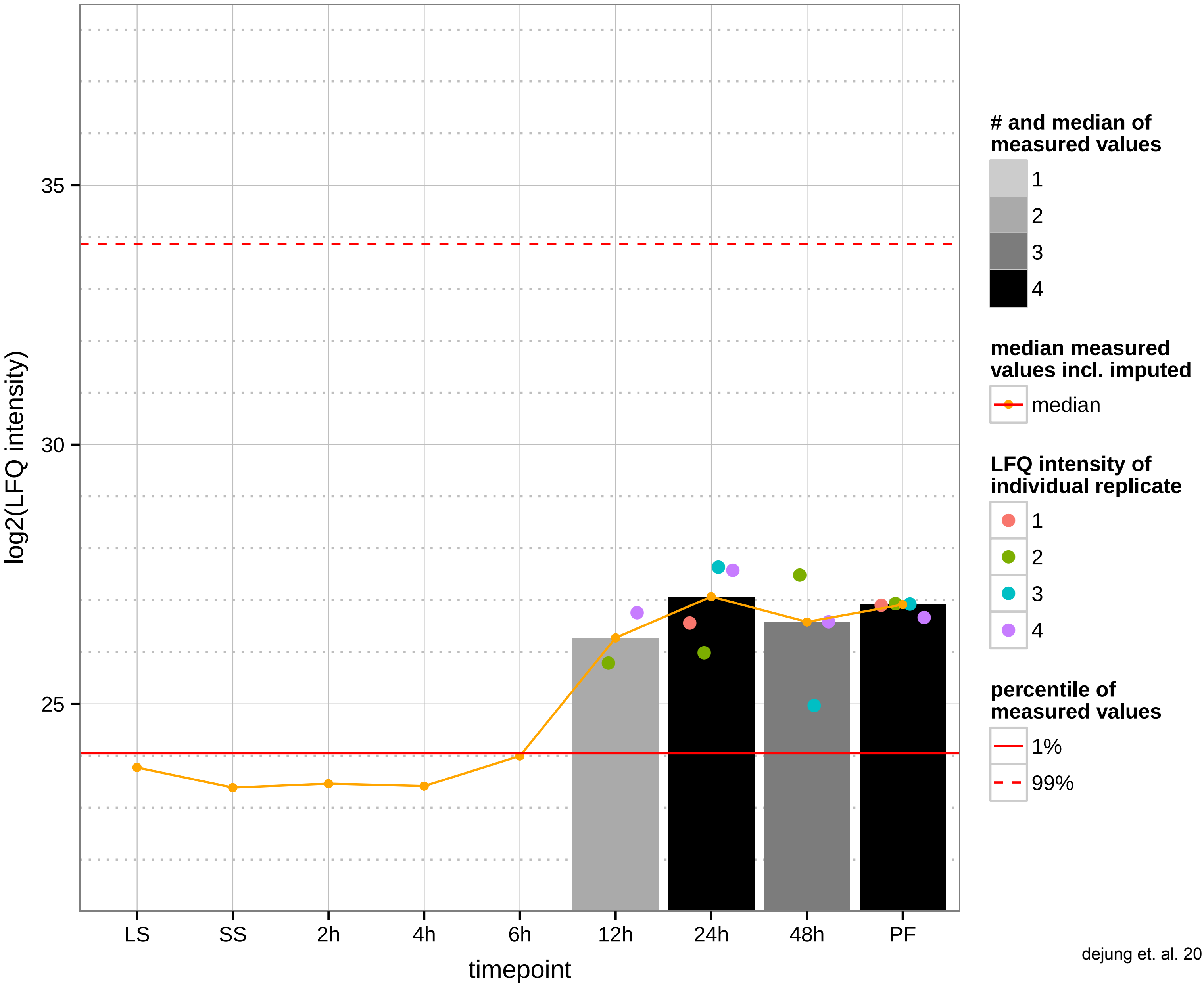

dejung et. al. 2015

hypothetical protein, conserved
Tb927.8.4690
AGOF: null
AGOC: null
AGOP: null
PGOF: null
PGOC: null
PGOP: null

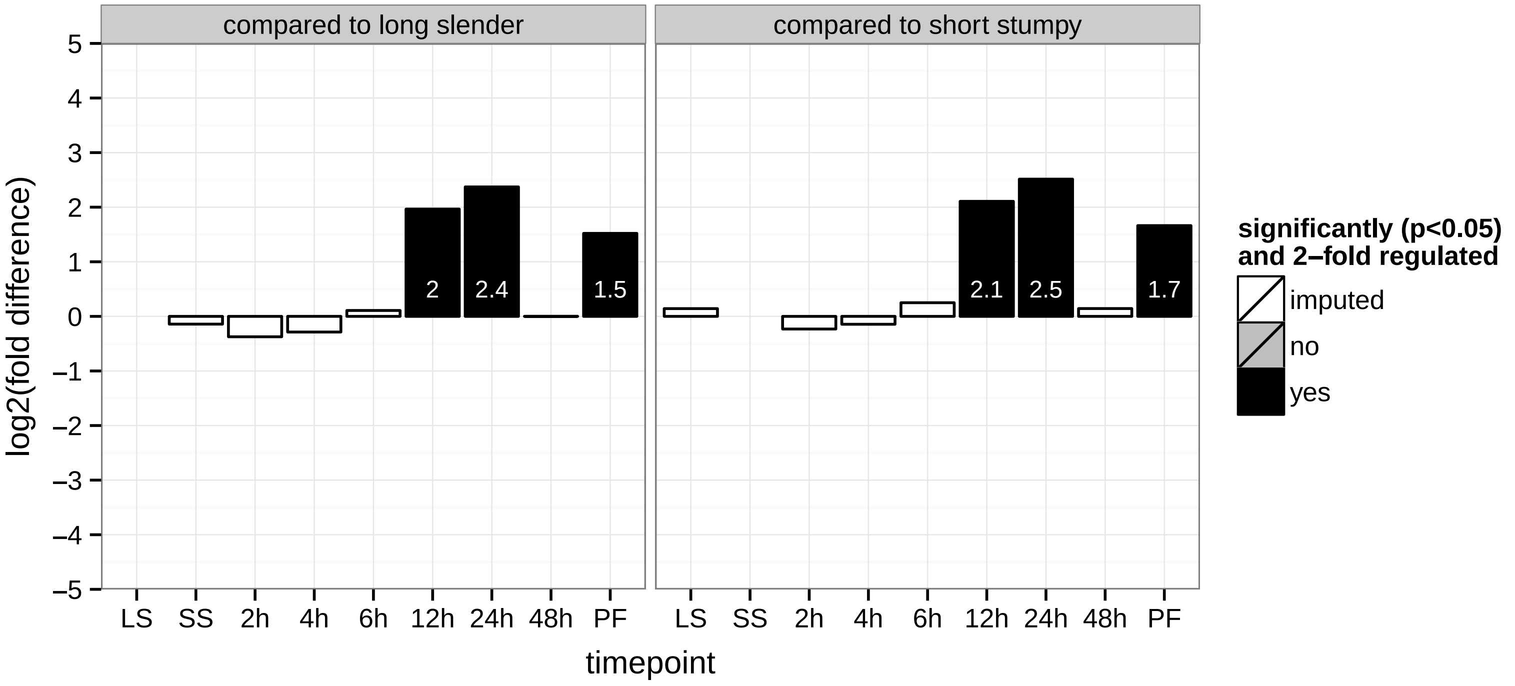

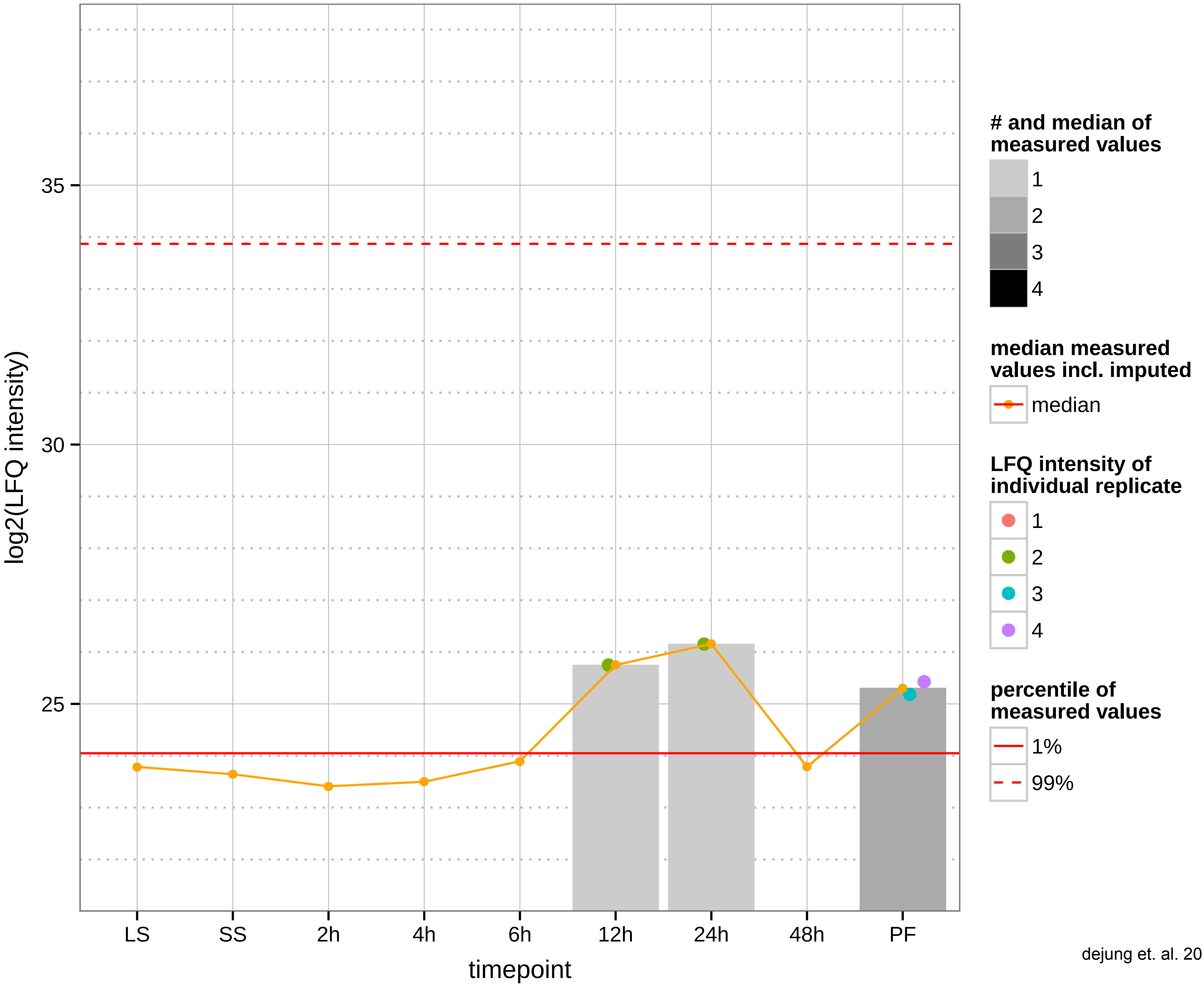

dejung et. al. 2015

flavoprotein monooxygenase, putative
Tb927.9.6710
AGOF: monooxygenase activity
AGOC: mitochondrion
AGOP: cellular aromatic compound metabolic process
PGOF: null
PGOC: null
PGOP: null

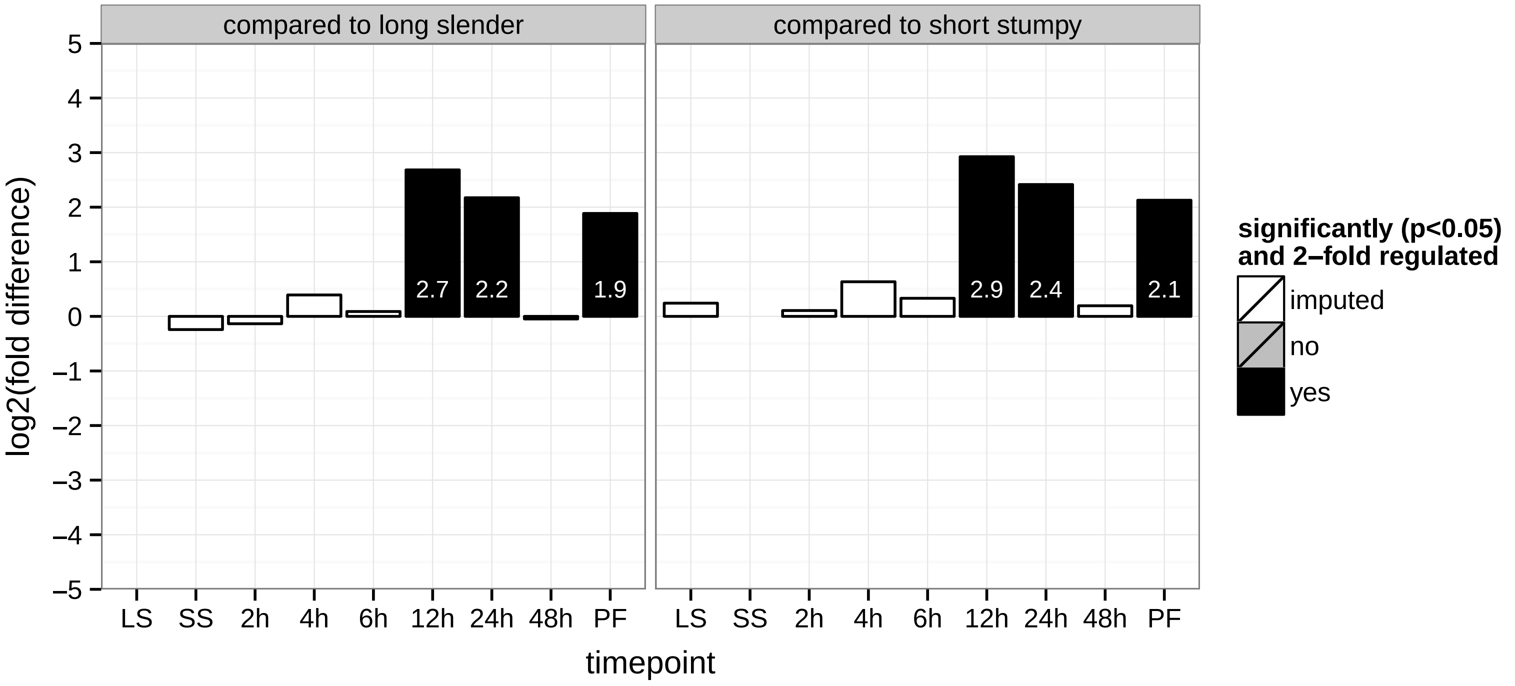

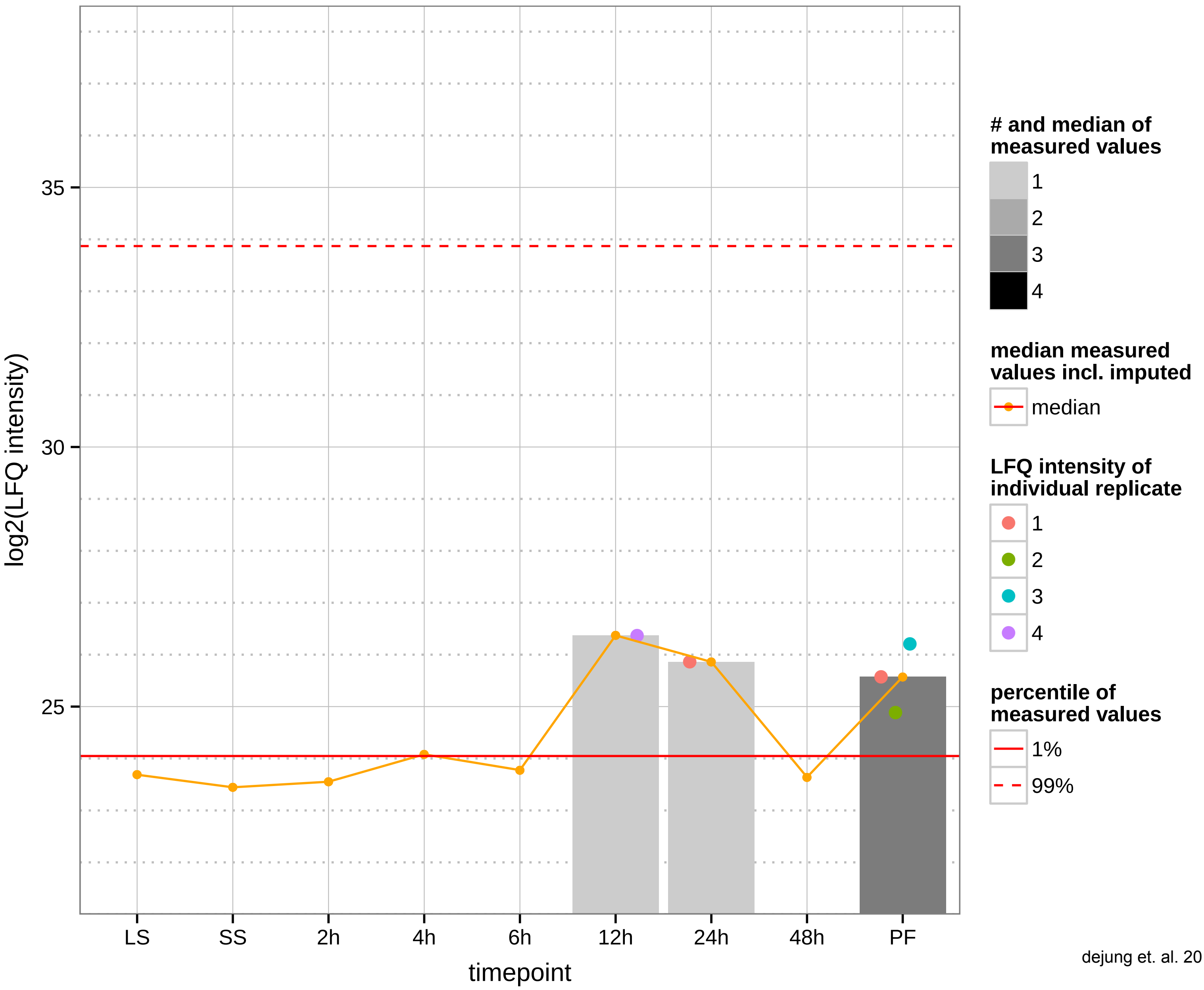

dejung et. al. 2015

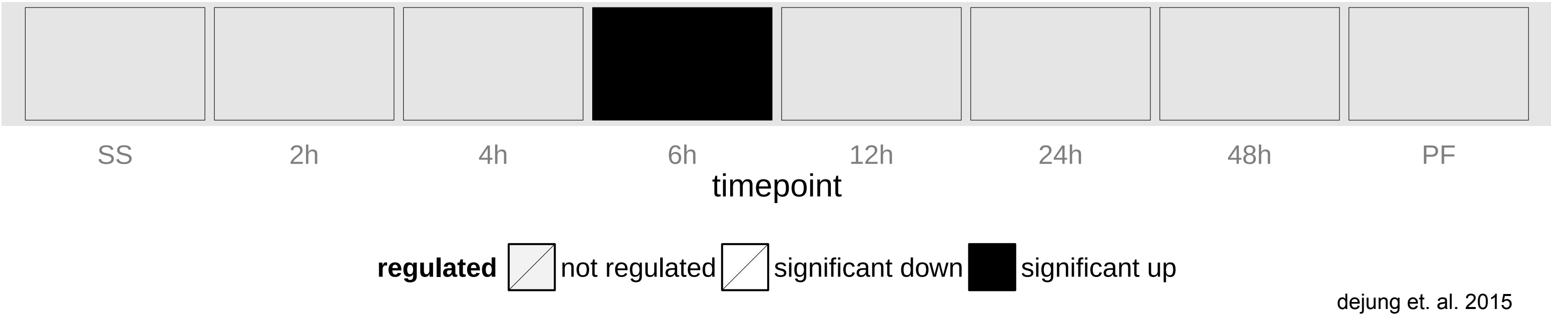
SS
2h
4h
6h
12h
24h
48h
PF
timepoint
regulated
not regulated
significant down
significant up
dejung et. al. 2015

protein kinase, putative
Tb927.3.3290;Tb11.v5.0474
AGOF: null, ATP binding, protein kinase activity, protein serine/threonine kinase activity, protein tyrosine kinase activity
AGOC: null
AGOP: null, protein phosphorylation
PGOF: ATP binding, protein kinase activity, transferase activity, transferring phosphorus–containing groups
PGOC: null
PGOP: protein phosphorylation

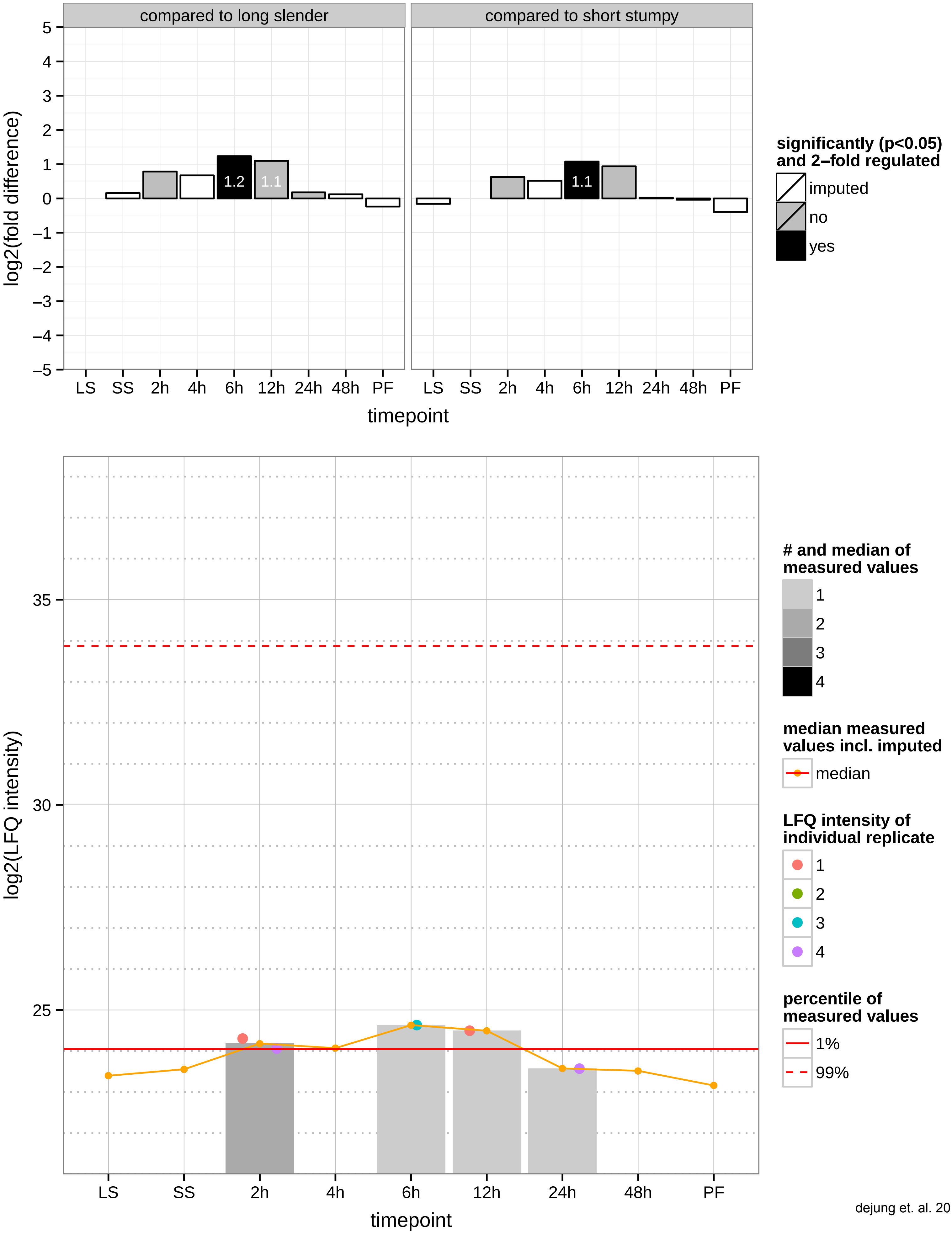

dejung et. al. 2015

histone deacetylase 4, histone deacetylase–like protein HDO4 (HDAC4)
Tb927.5.2900
AGOF: histone deacetylase activity
AGOC: cytoplasm, nucleus
AGOP: cell cycle, histone deacetylation
PGOF: null
PGOC: null
PGOP: null

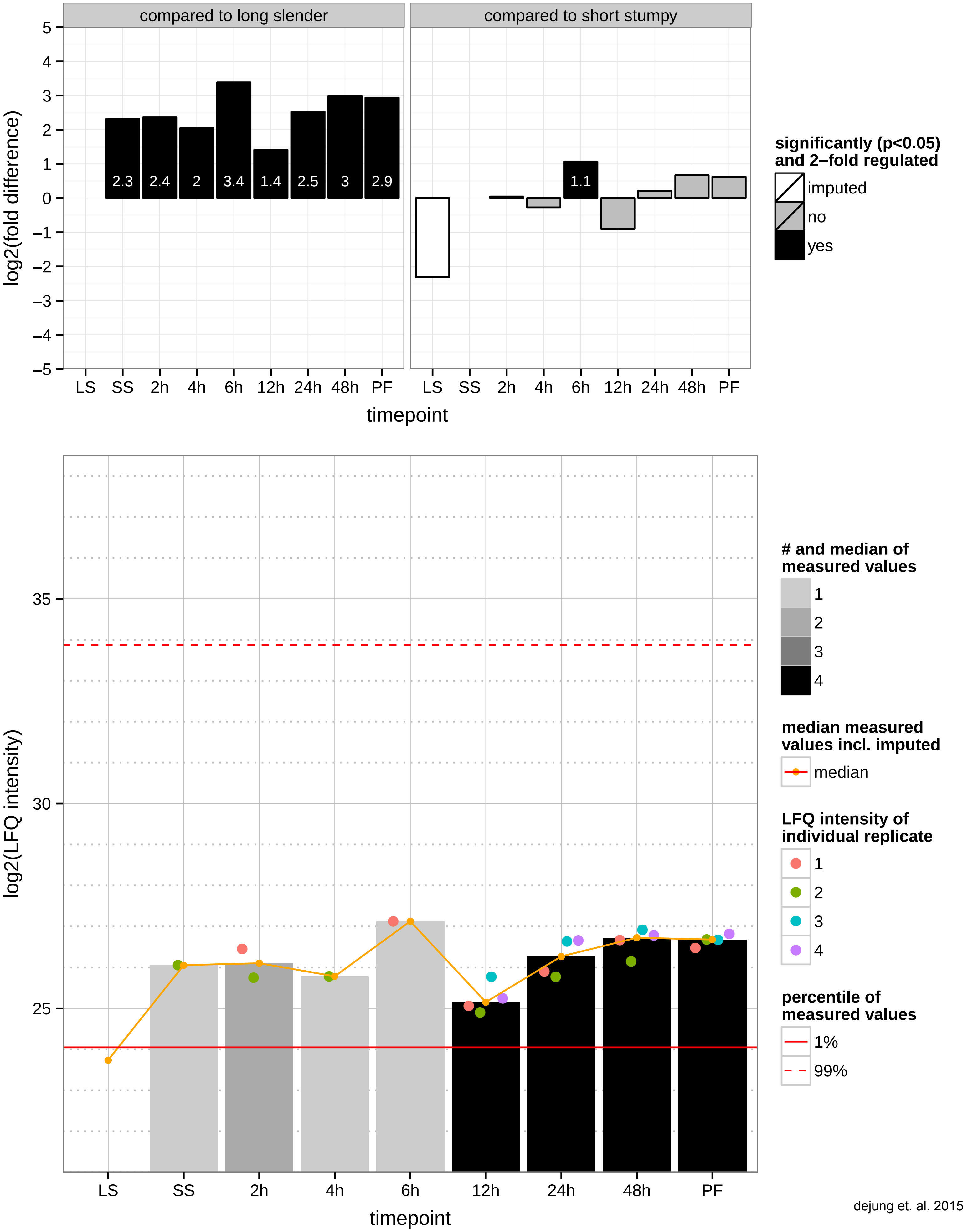

dejung et. al. 2015

ubiquitin hydrolase, pseudogene, putative, cysteine peptidase, Clan CA, family C19, frameshift, frameshift (PAN2)
Tb927.6.1670
AGOF: ubiquitin thiolesterase activity
AGOC: null
AGOP: ubiquitin–dependent protein catabolic process
PGOF: protein binding
PGOC: null
PGOP: null

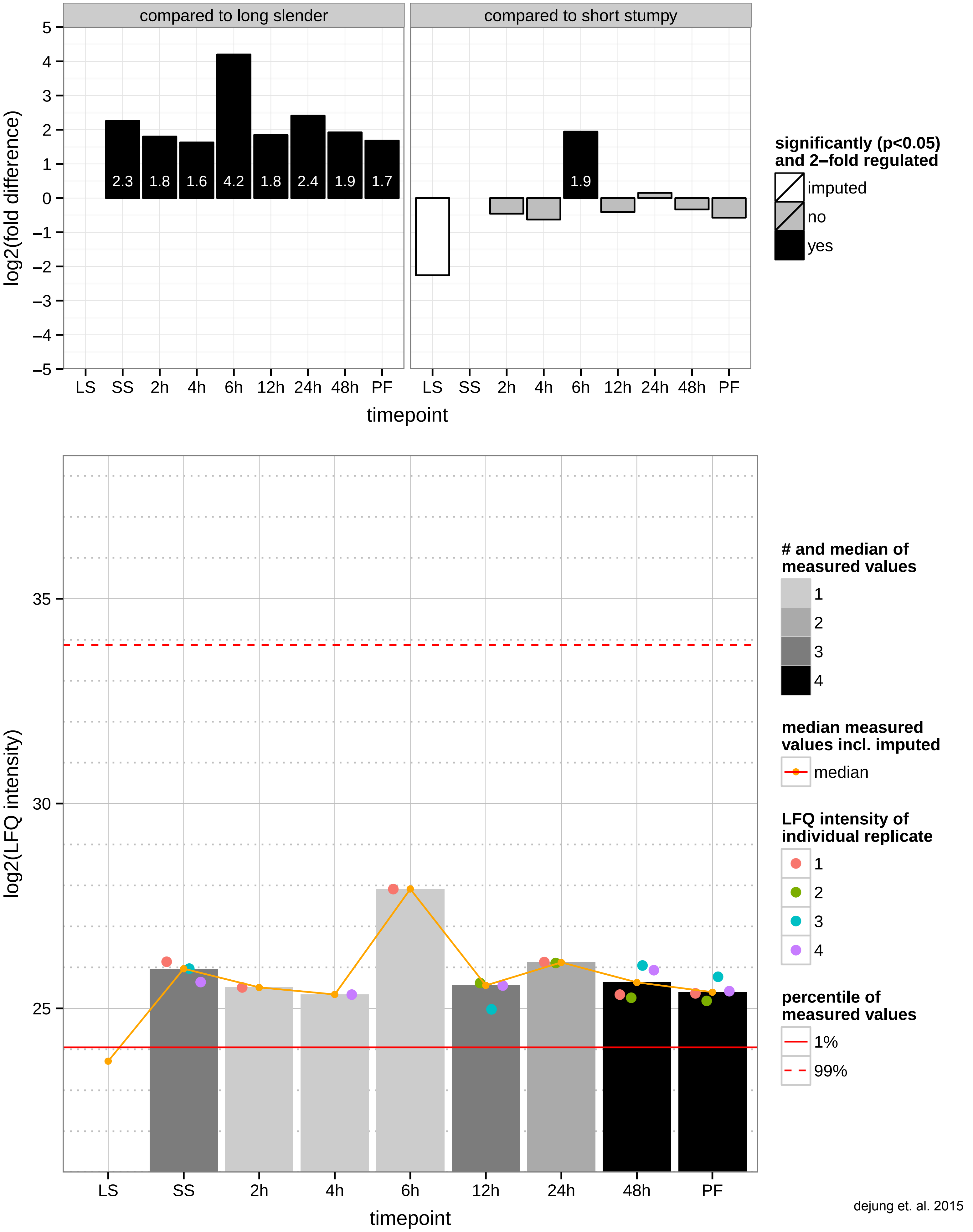

dejung et. al. 2015

ABC transporter, putative
Tb927.6.2810
AGOF: ATP binding, ATPase activity
AGOC: integral to membrane
AGOP: transport
PGOF: ATP binding, ATPase activity, nucleoside−triphosphatase activity, nucleotide binding
PGOC: null
PGOP: null

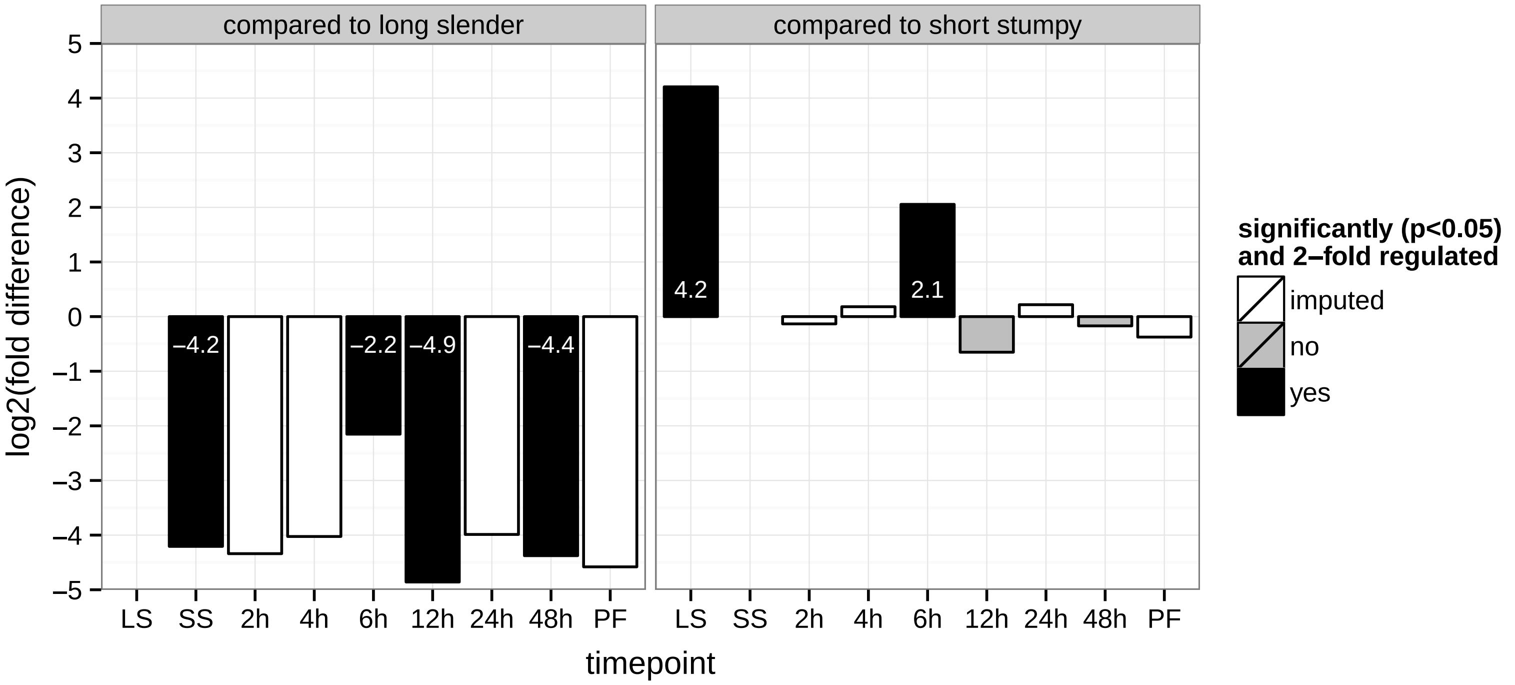

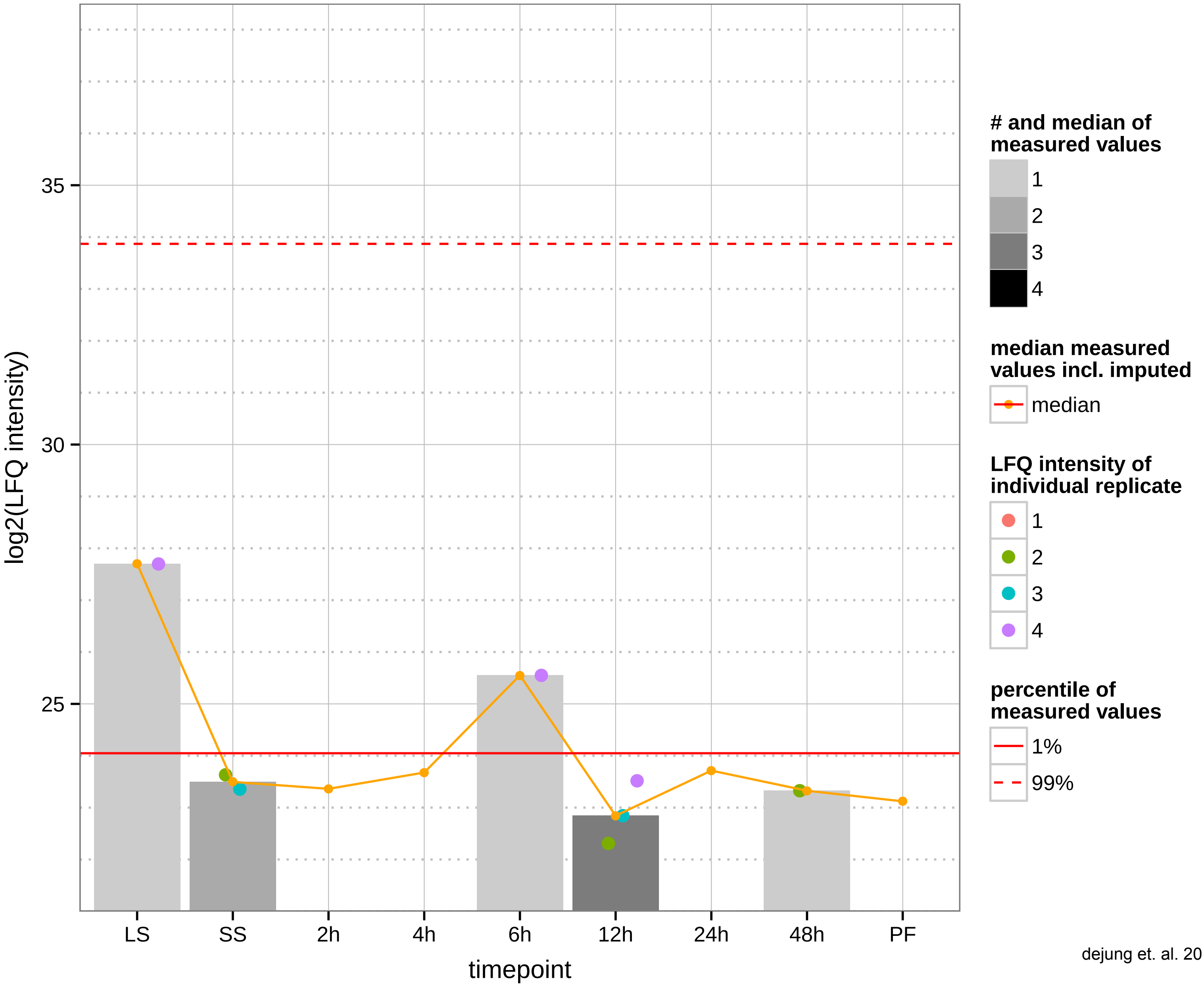

dejung et. al. 2015

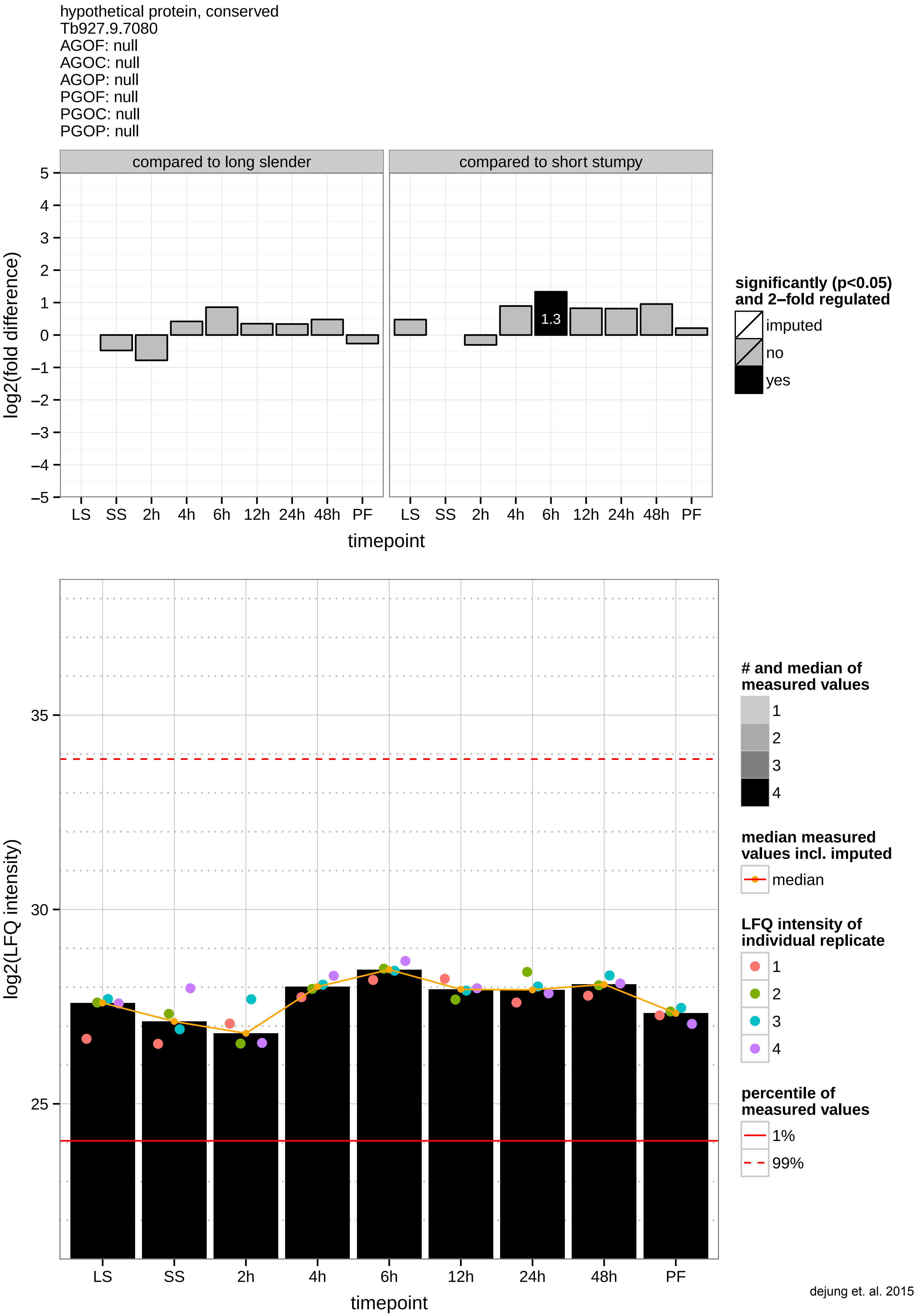
hypothetical protein, conserved
Tb927.9.7080
AGOF: null
AGOC: null
AGOP: null
PGOF: null
PGOC: null
PGOP: null
compared to long slender
compared to short stumpy
log2(fold difference)
1.3
LS SS 2h 4h 6h 12h 24h 48h PF
timepoint
significantly (p<0.05) and 2−fold regulated
imputed
no
yes
log2(LFQ intensity)
35
30
25
and median of measured values
1
2
3
4
median measured values incl. imputed
median
LFQ intensity of individual replicate
1
2
3
4
percentile of measured values
1%
99%
dejung et. al. 2015

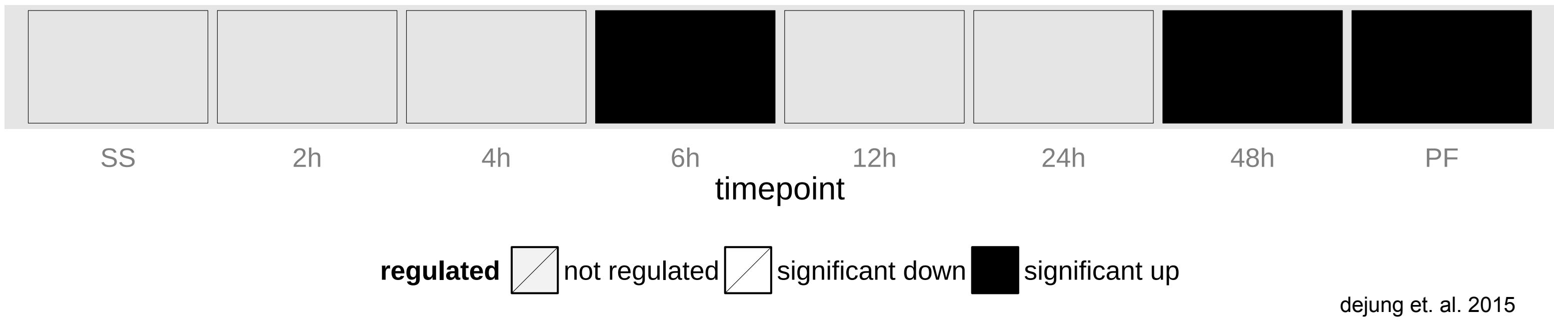
SS
2h
4h
6h
12h
24h
48h
PF
timepoint
regulated
not regulated
significant down
significant up
dejung et. al. 2015

DNA replication licensing factor MCM4, putative (MCM4)
Tb927.11.12250
AGOF: ATP binding, DNA binding
AGOC: PCNA complex, nucleus
AGOP: DNA replication, DNA–dependent DNA replication initiation
PGOF: ATP binding, DNA binding
PGOC: null
PGOP: DNA replication

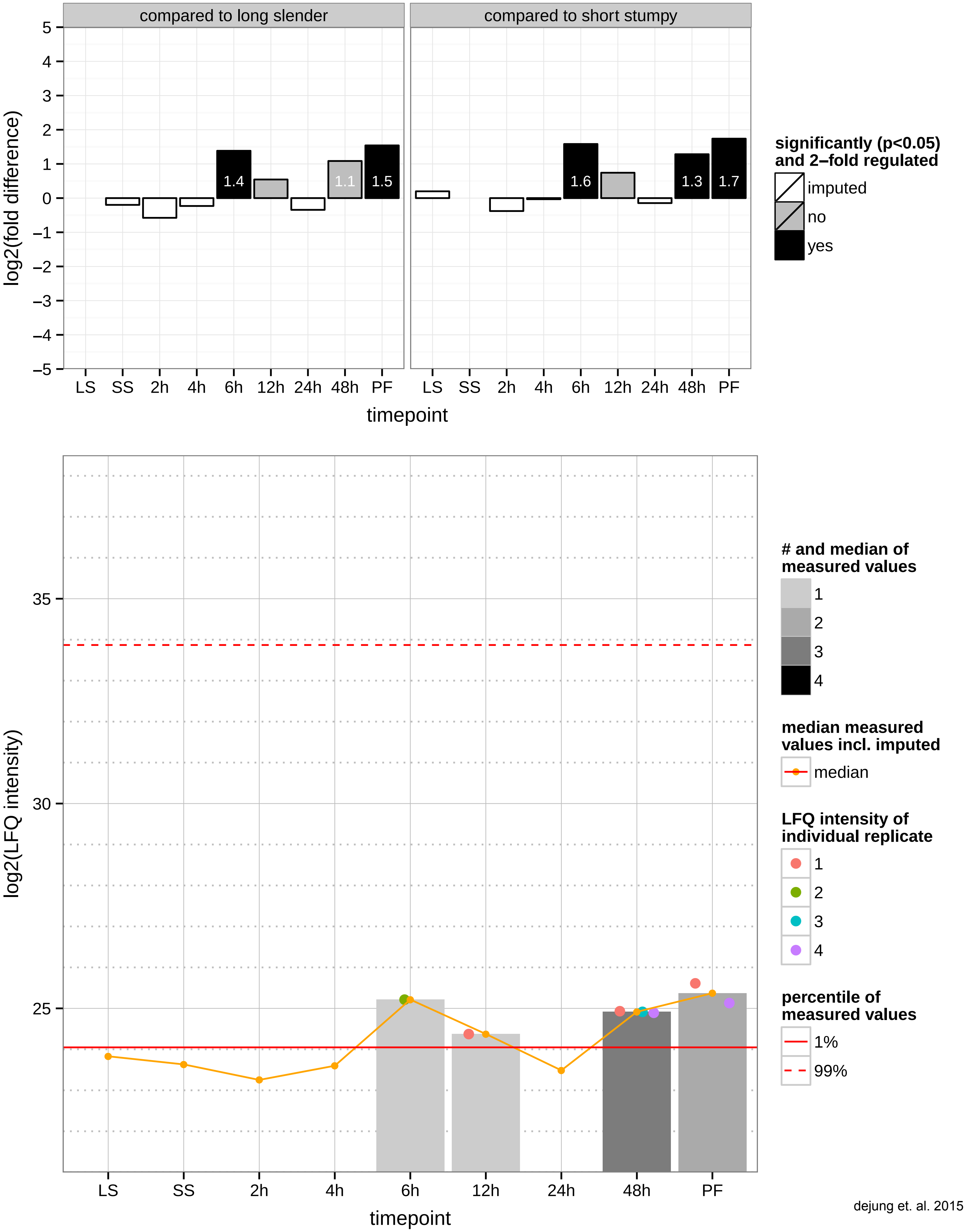

dejung et. al. 2015

hypothetical protein, conserved
Tb927.11.5820
AGOF: null
AGOC: mitochondrion
AGOP: null
PGOF: null
PGOC: null
PGOP: null

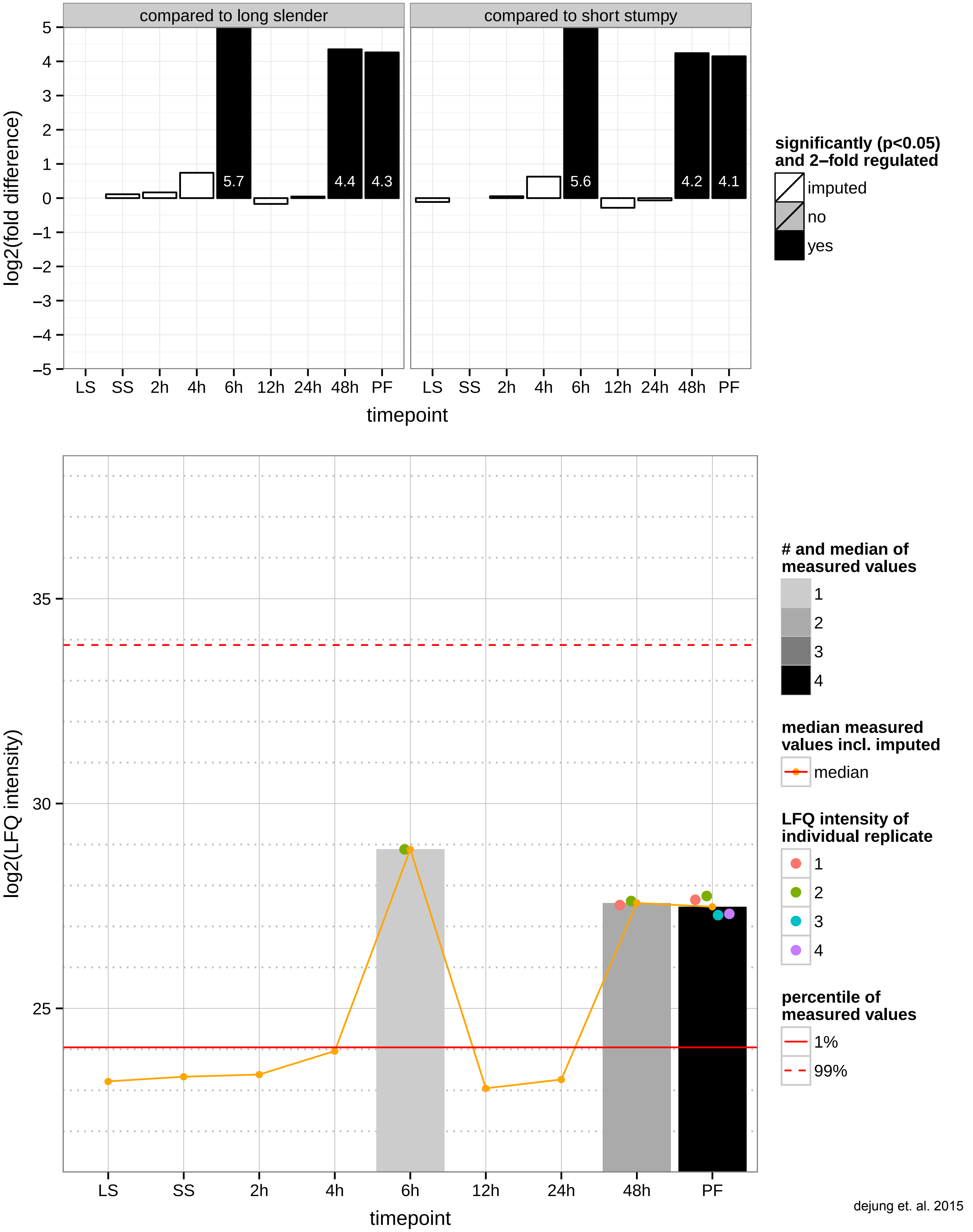

dejung et. al. 2015

hypothetical protein, conserved
Tb927.4.4300
AGOF: null
AGOC: mitochondrion
AGOP: null
PGOF: protein binding
PGOC: null
PGOP: null

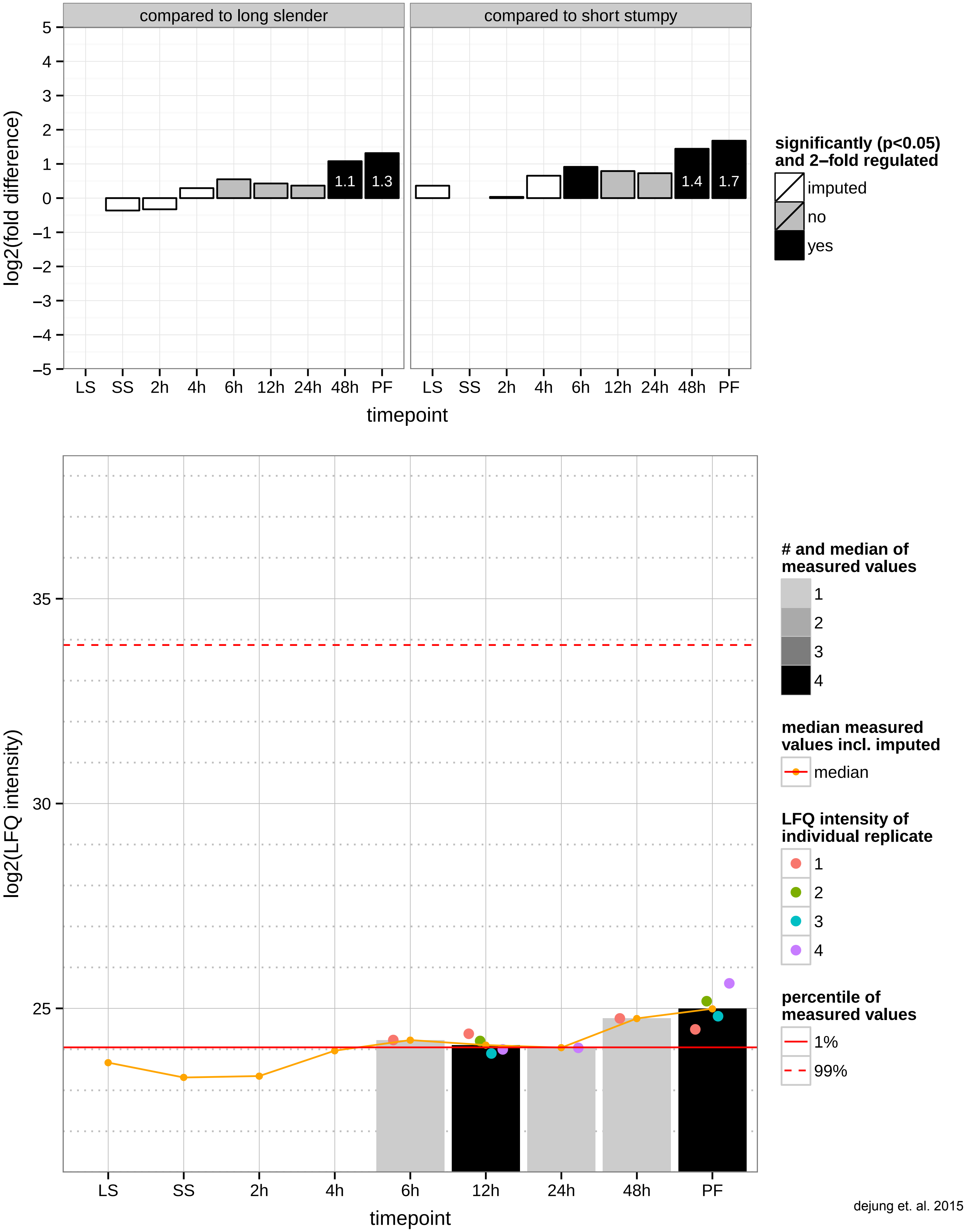

dejung et. al. 2015

disulfide isomerase, putative
Tb927.5.1020
AGOF: null
AGOC: null
AGOP: cell redox homeostasis
PGOF: null
PGOC: null
PGOP: cell redox homeostasis

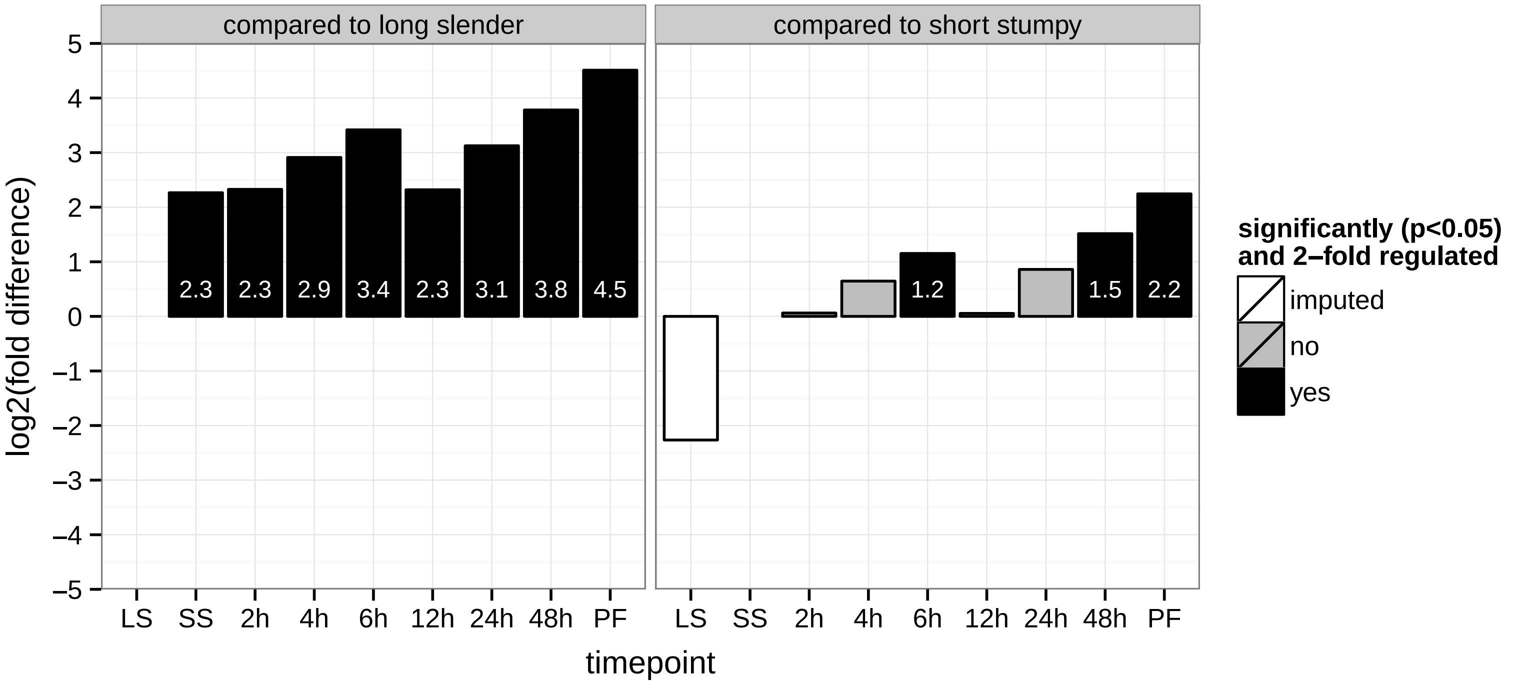

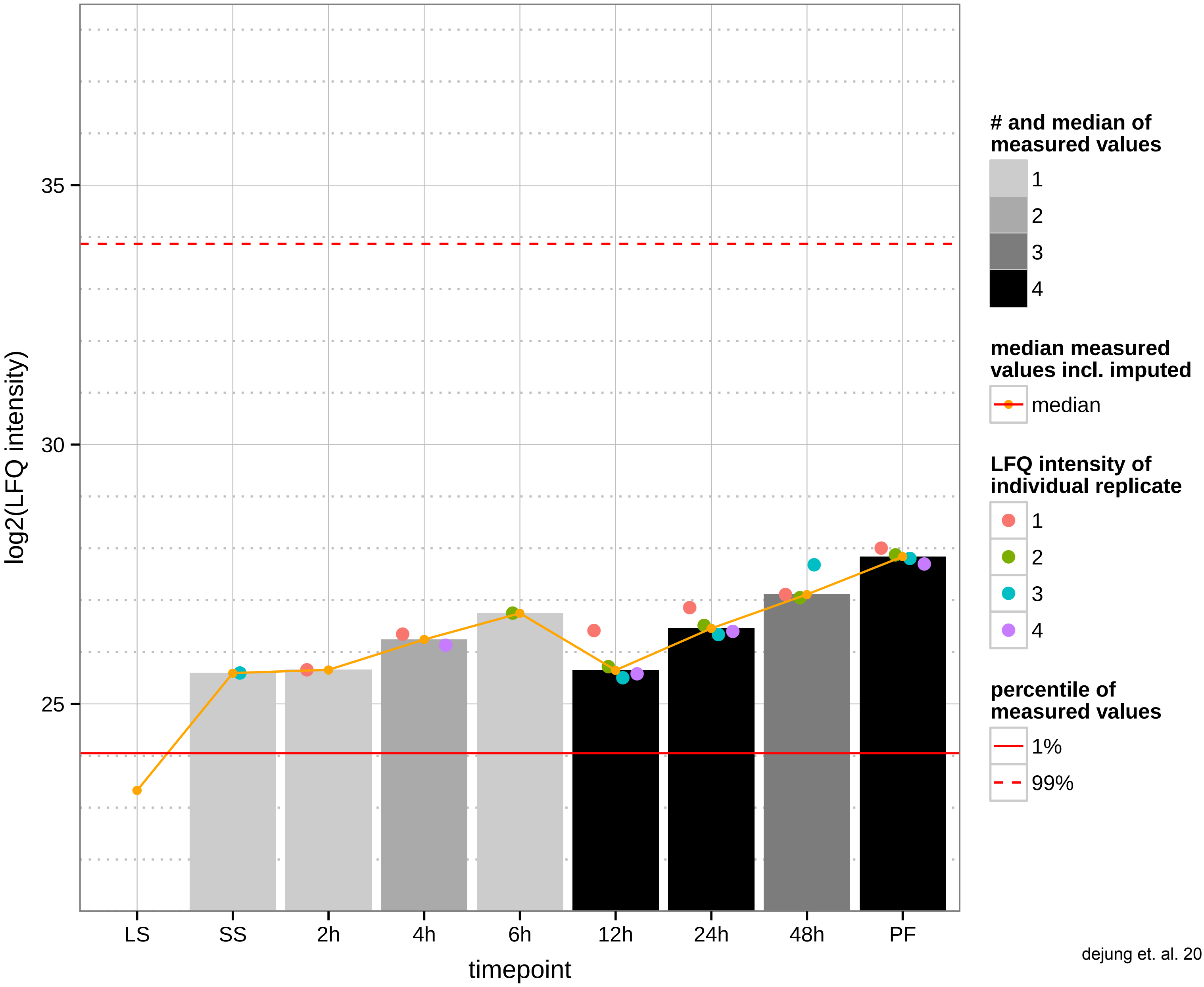

dejung et. al. 2015

ubiquitin−conjugating enzyme E2, putative, ubiquitin carrier protein, ubiquitin−protein ligase
Tb927.7.2540
AGOF: ubiquitin−protein ligase activity
AGOC: null
AGOP: cellular protein modification process, post−translational protein modification, regulation of protein metabolic process
PGOF: acid−amino acid ligase activity
PGOC: null
PGOP: null

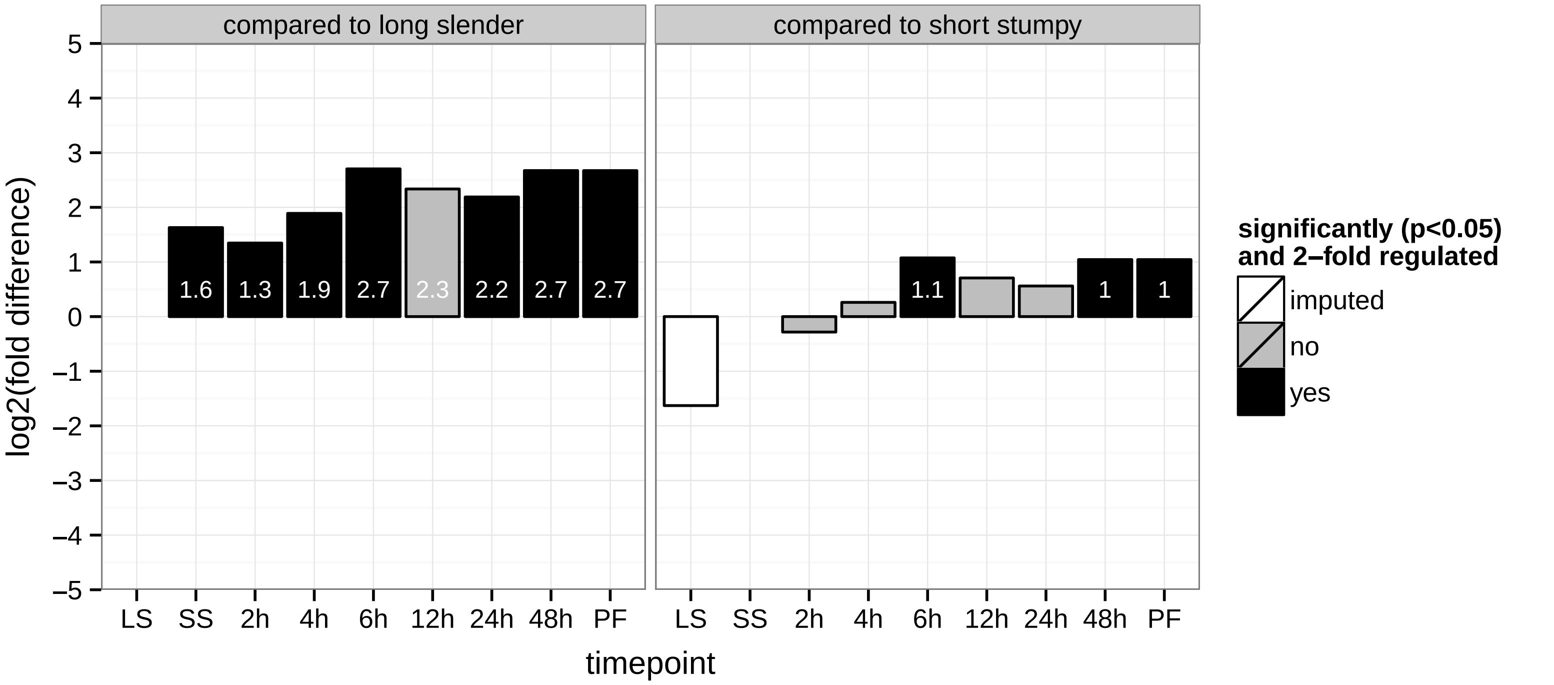

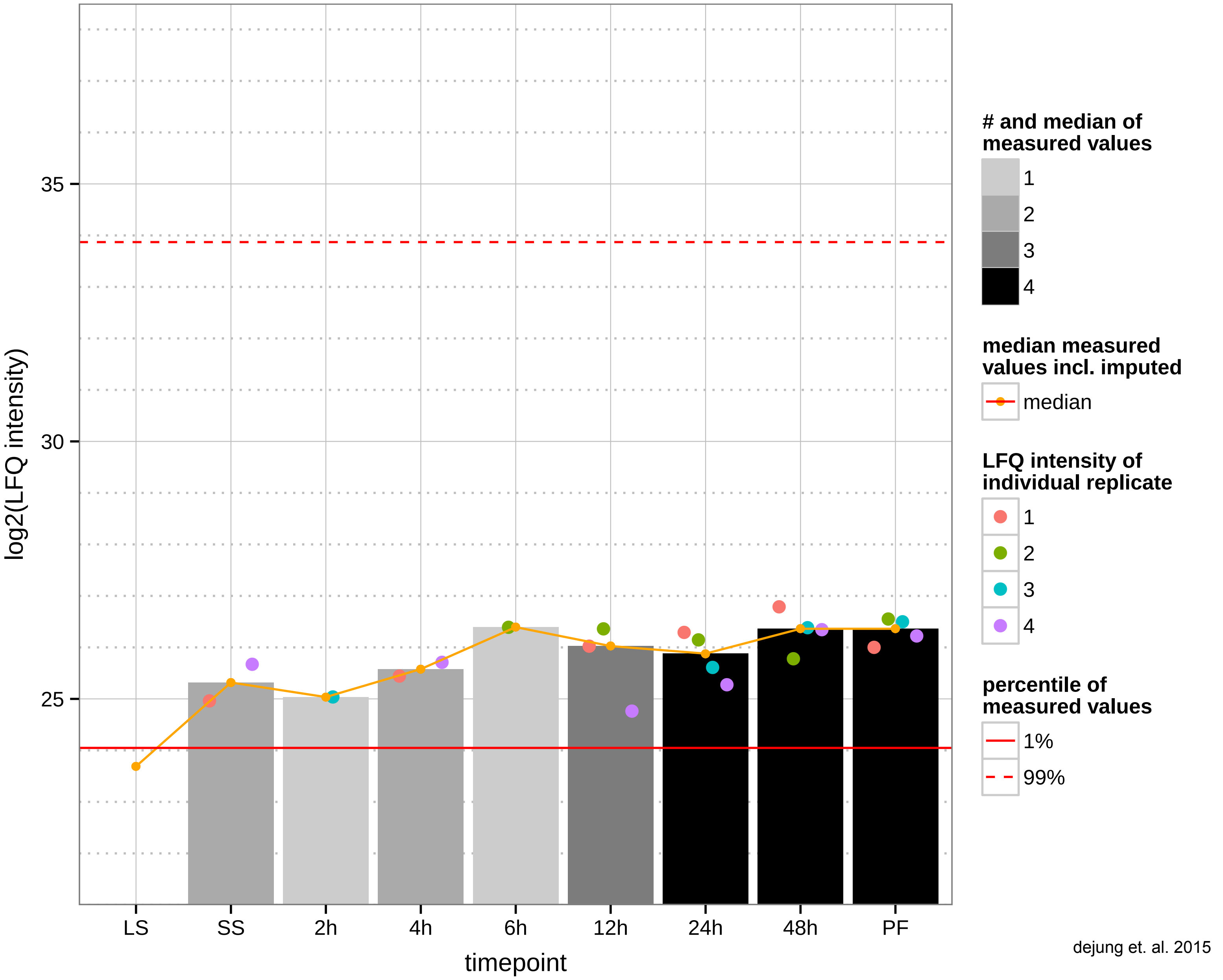

dejung et. al. 2015

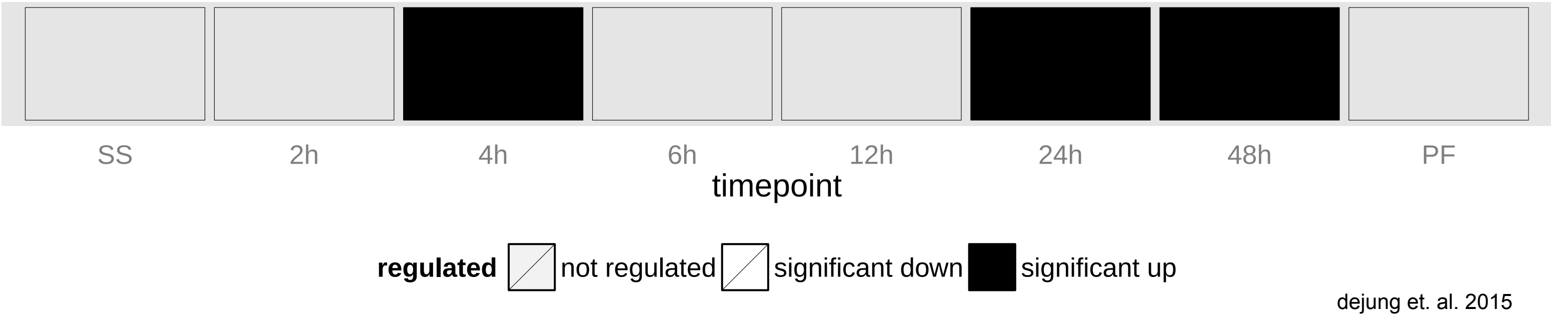
SS
2h
4h
6h
12h
24h
48h
PF
timepoint
regulated
not regulated
significant down
significant up
dejung et. al. 2015

hypothetical protein, conserved
Tb927.11.10950
AGOF: heat shock protein binding
AGOC: null
AGOP: null
PGOF: heat shock protein binding
PGOC: null
PGOP: null

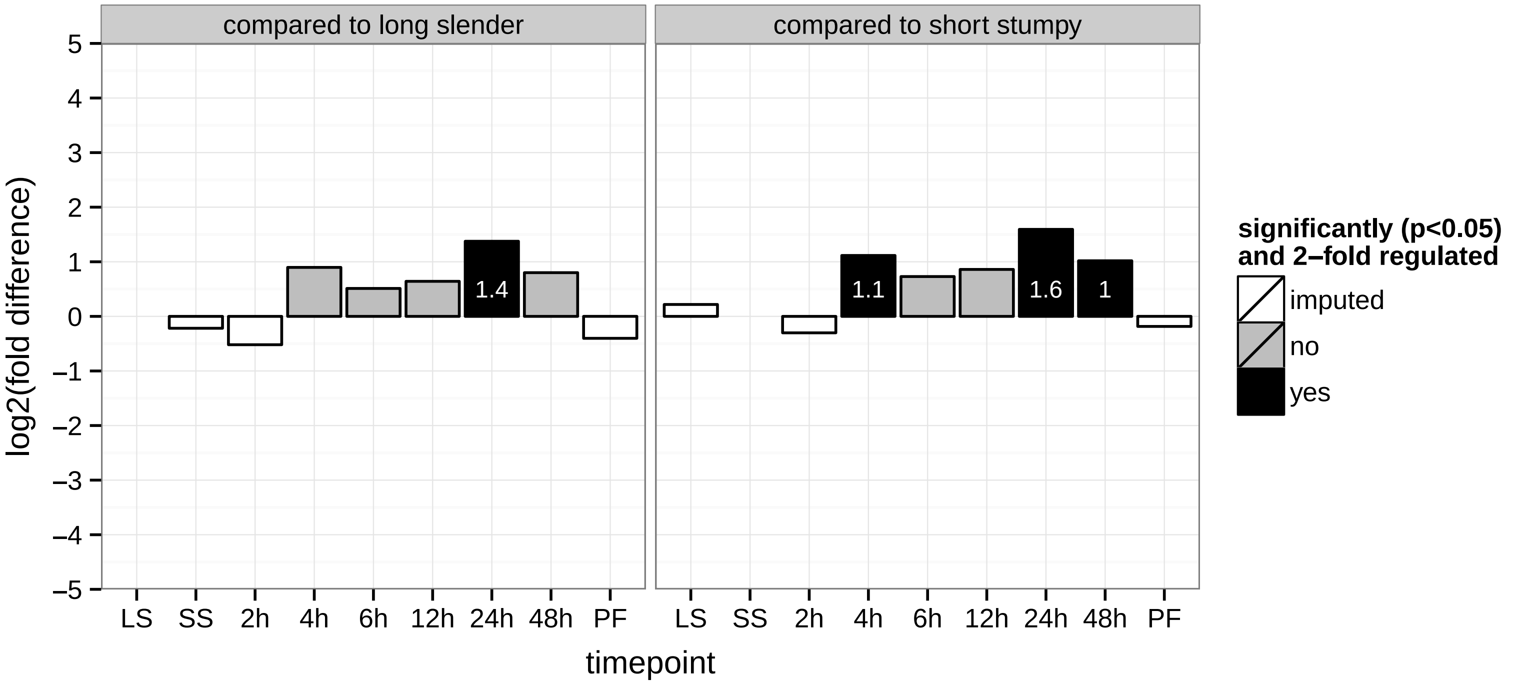

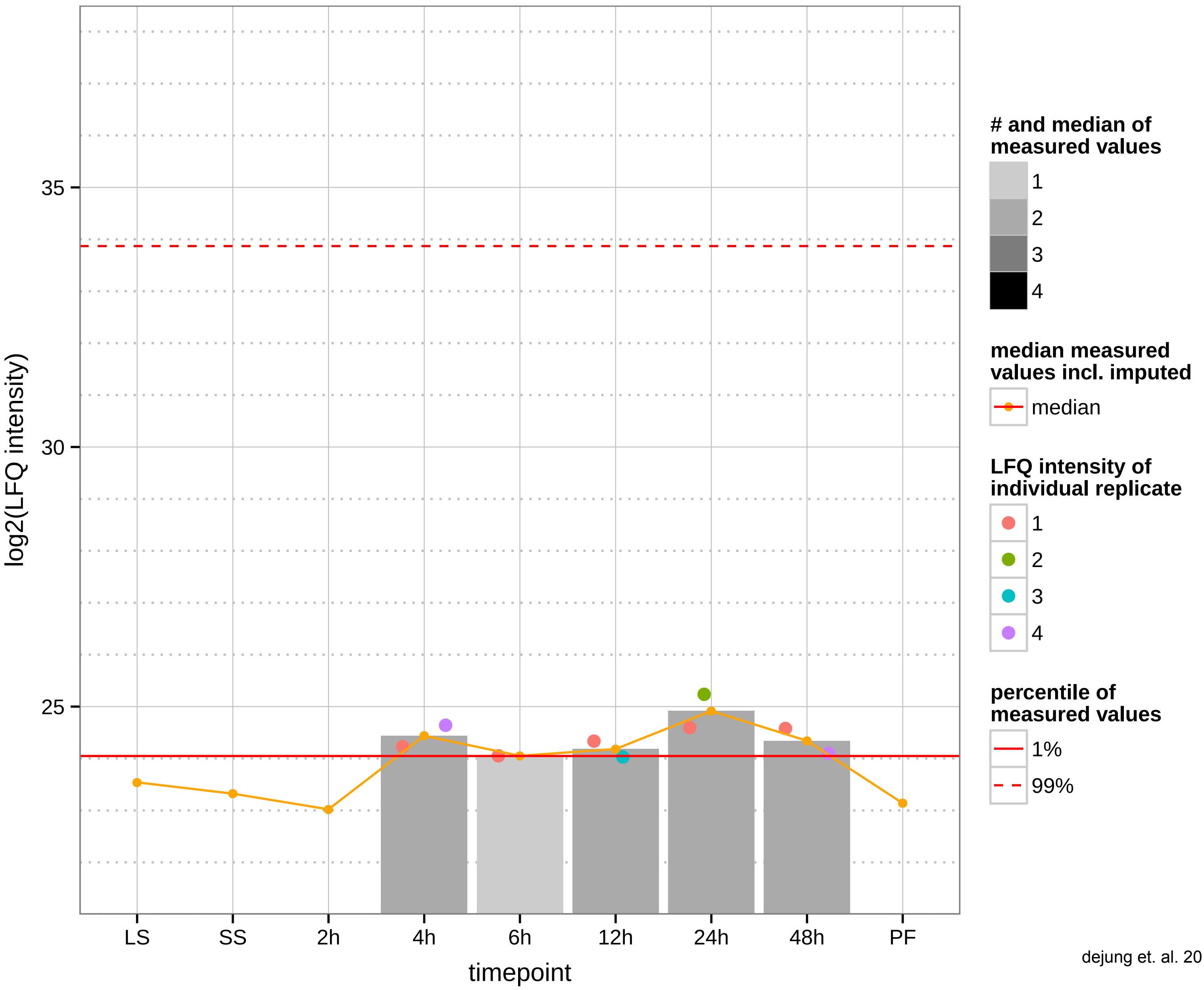

dejung et. al. 2015

nascent polypeptide associated complex alpha subunit, putative
Tb927.11.9700
AGOF: sequence−specific DNA binding transcription factor activity
AGOC: nascent polypeptide−associated complex
AGOP: positive regulation of growth rate
PGOF: null
PGOC: null
PGOP: null

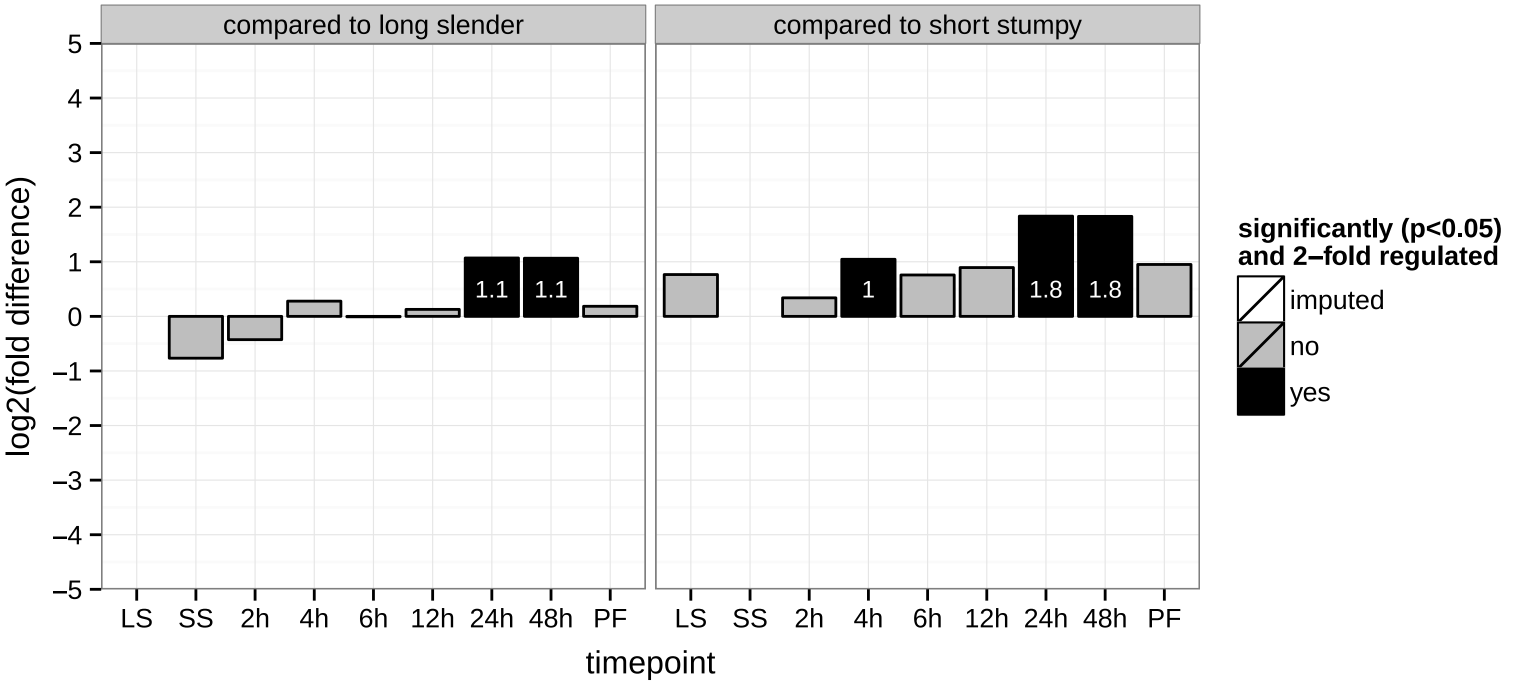

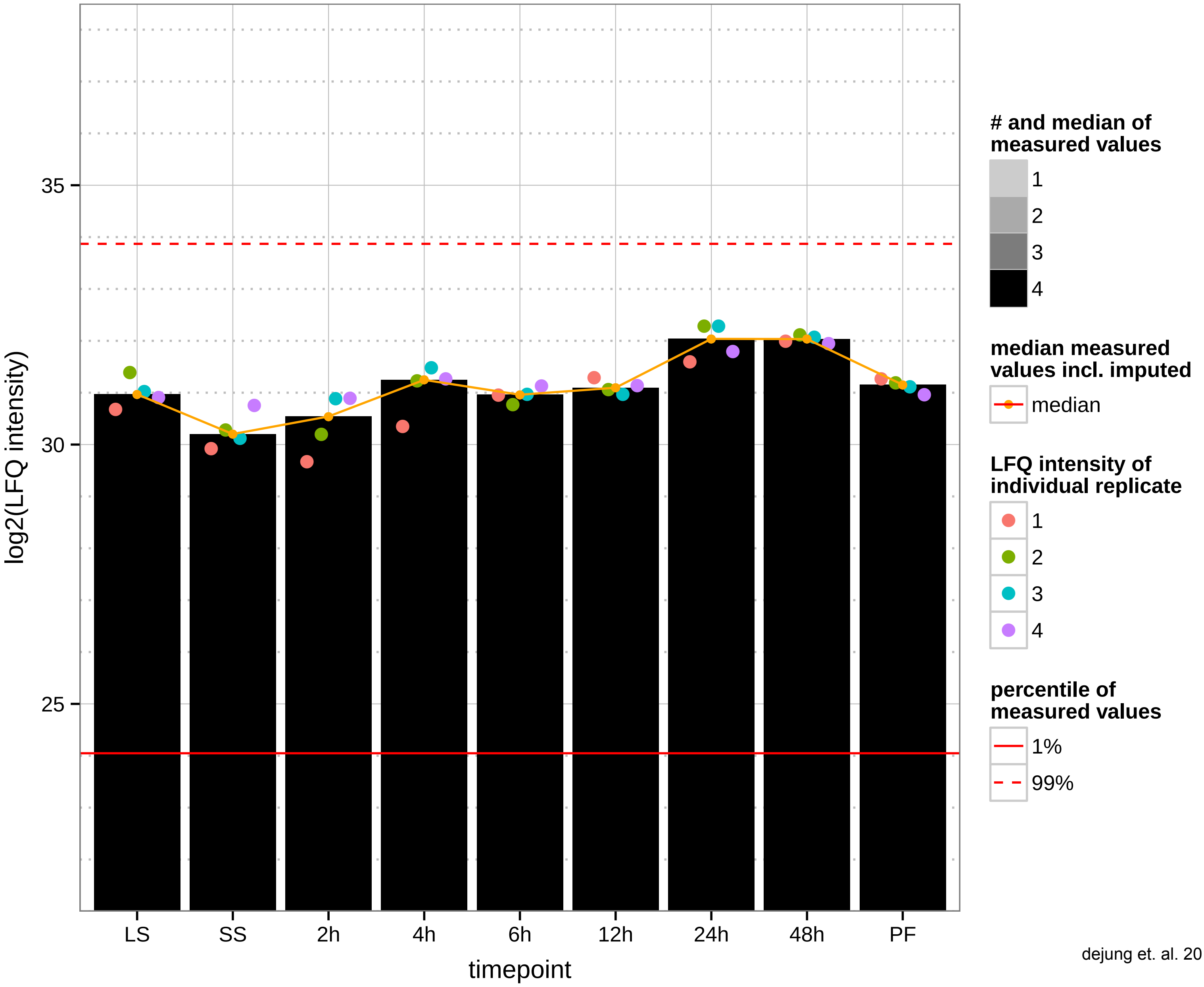

dejung et. al. 2015

hypothetical protein, conserved
Tb927.11.9910
AGOF: null
AGOC: null
AGOP: null
PGOF: null
PGOC: null
PGOP: null

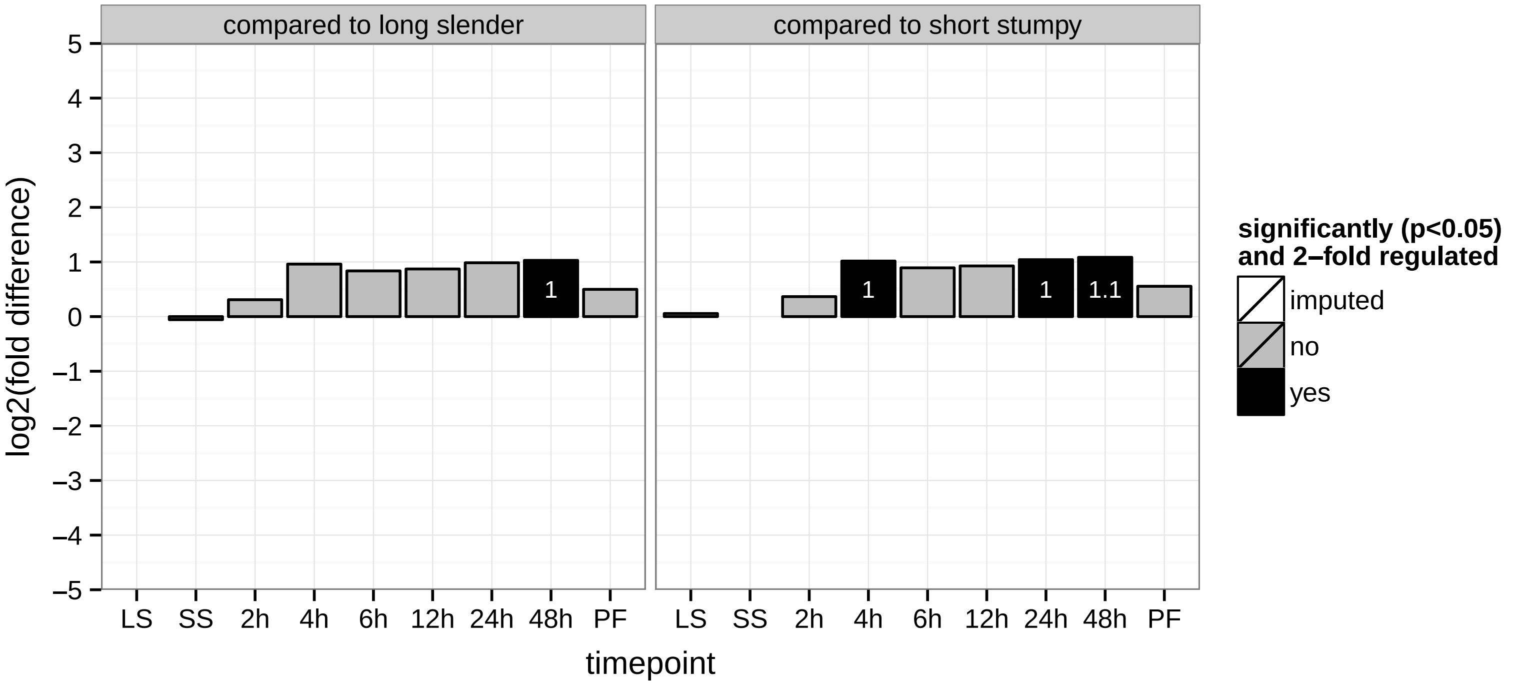

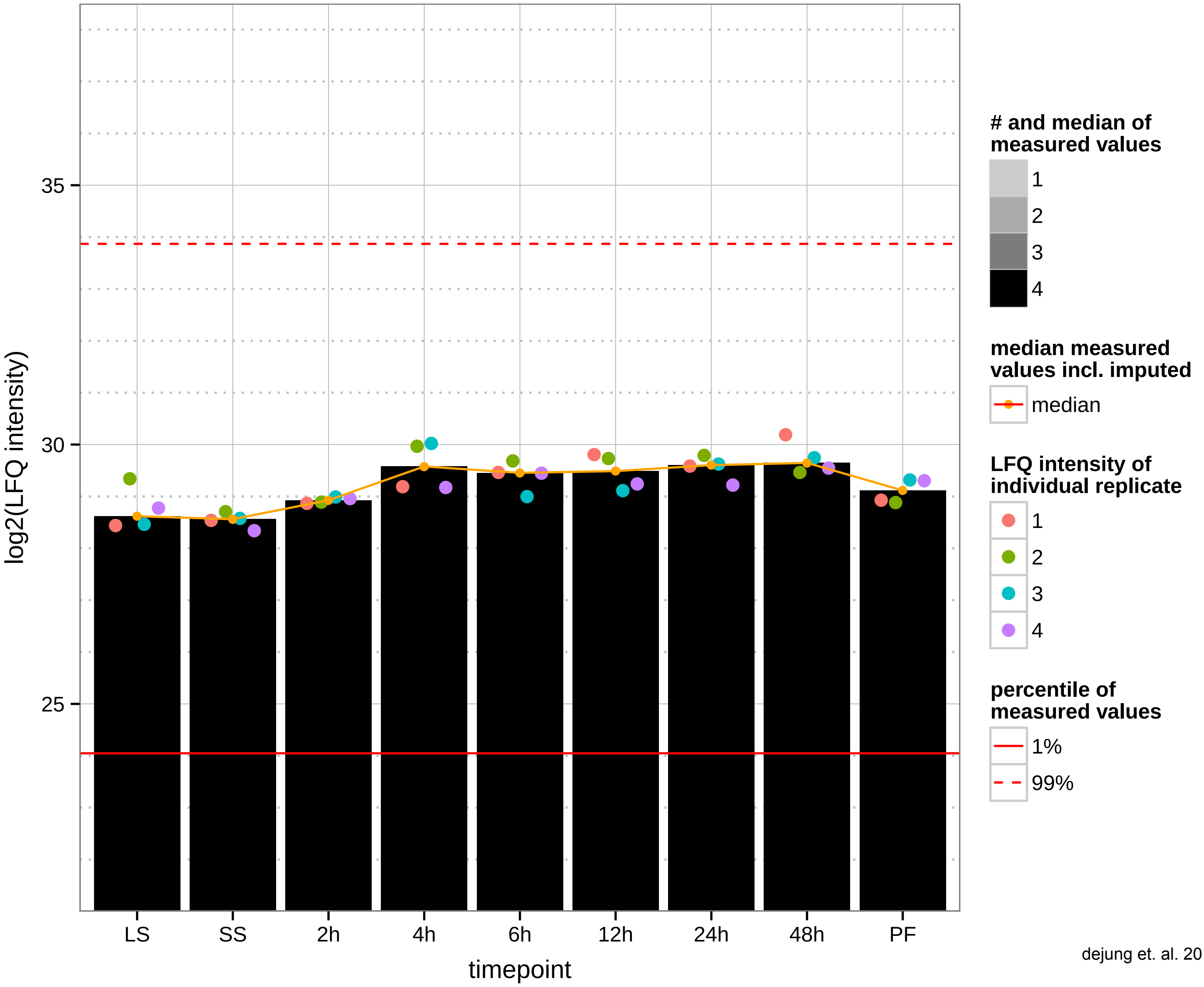

dejung et. al. 2015

hypothetical protein, conserved
Tb927.4.3070
AGOF: null
AGOC: mitochondrion
AGOP: null
PGOF: null
PGOC: null
PGOP: null

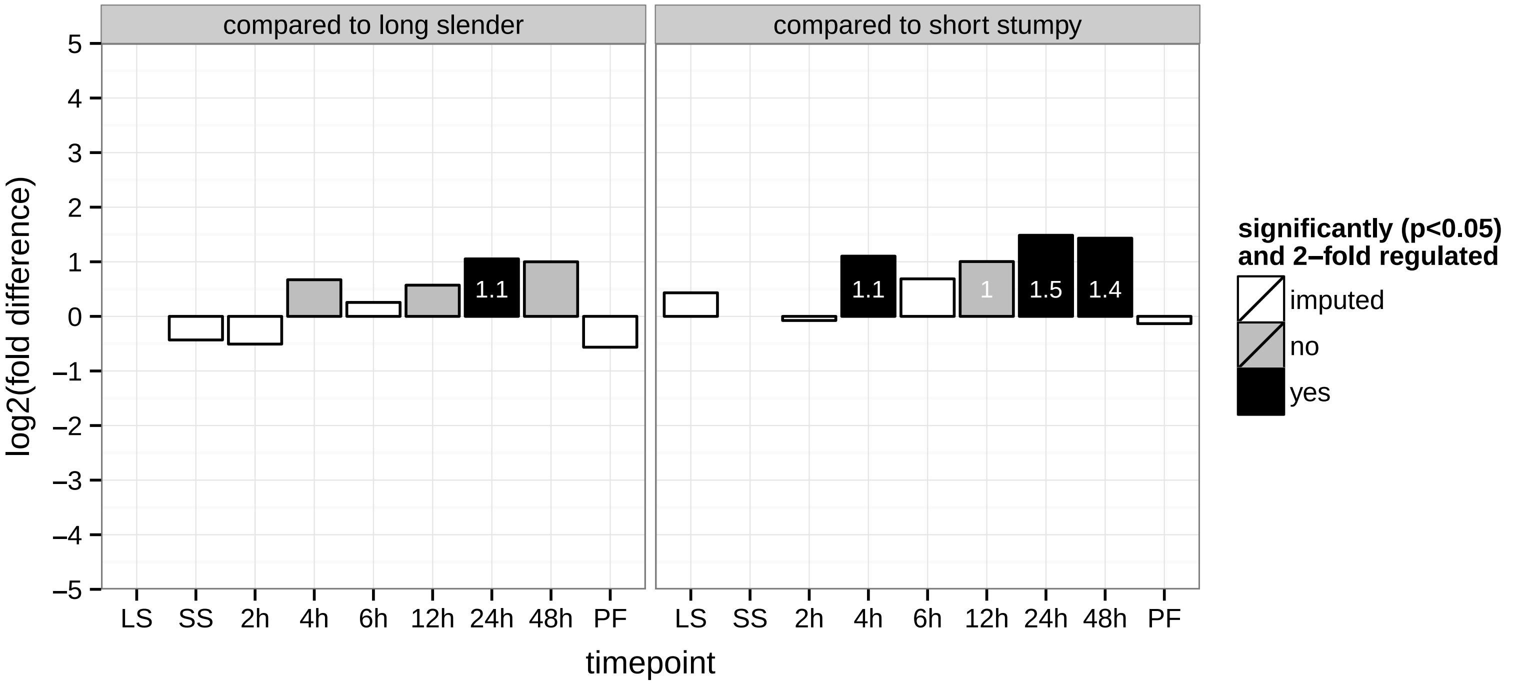

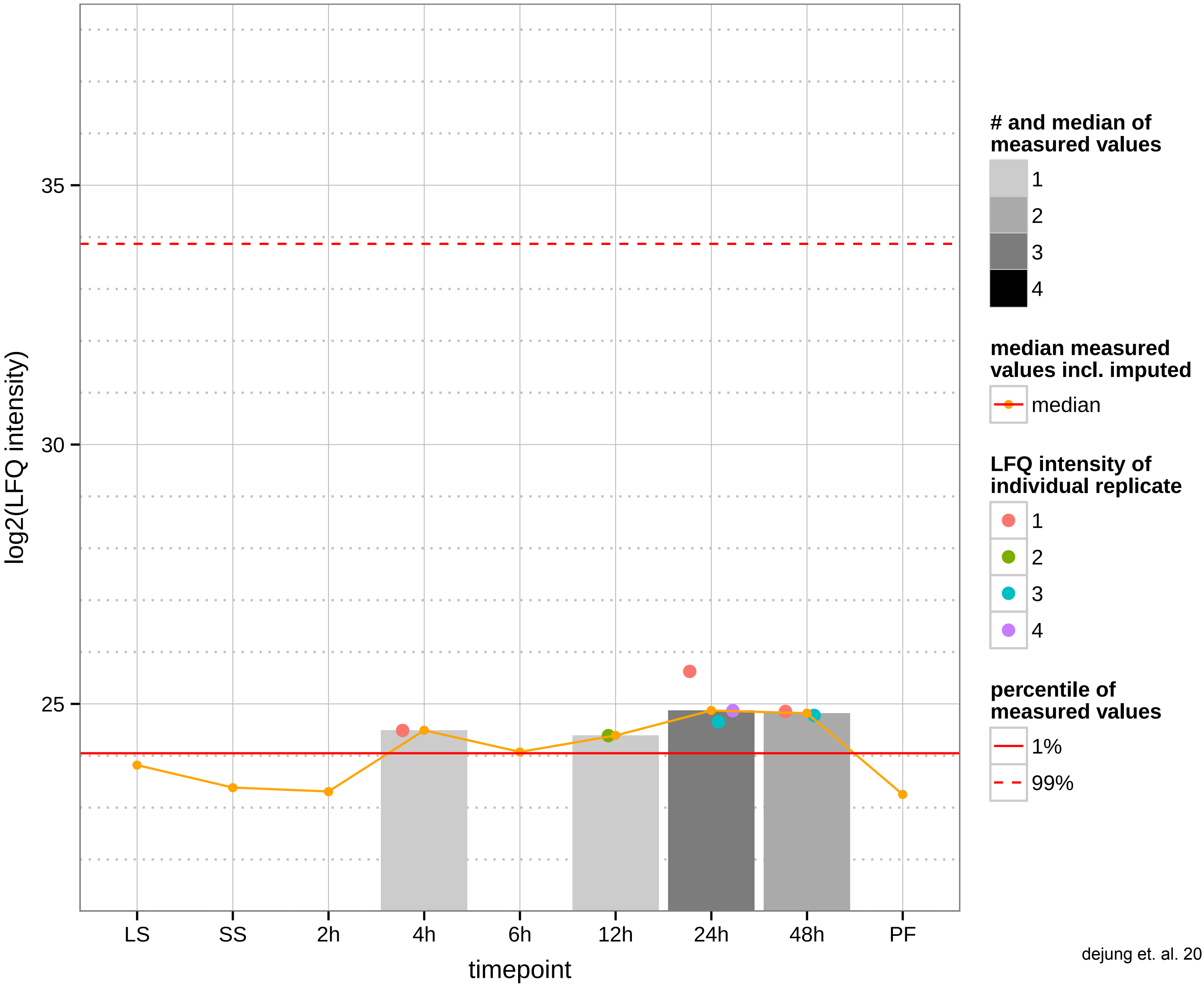

dejung et. al. 2015

hypothetical protein, conserved
Tb927.7.2200
AGOF: null
AGOC: mitochondrial intermembrane space protein transporter complex, mitochondrion
AGOP: protein import into mitochondrial inner membrane
PGOF: null
PGOC: mitochondrial intermembrane space protein transporter complex
PGOP: protein import into mitochondrial inner membrane, protein targeting to mitochondrion

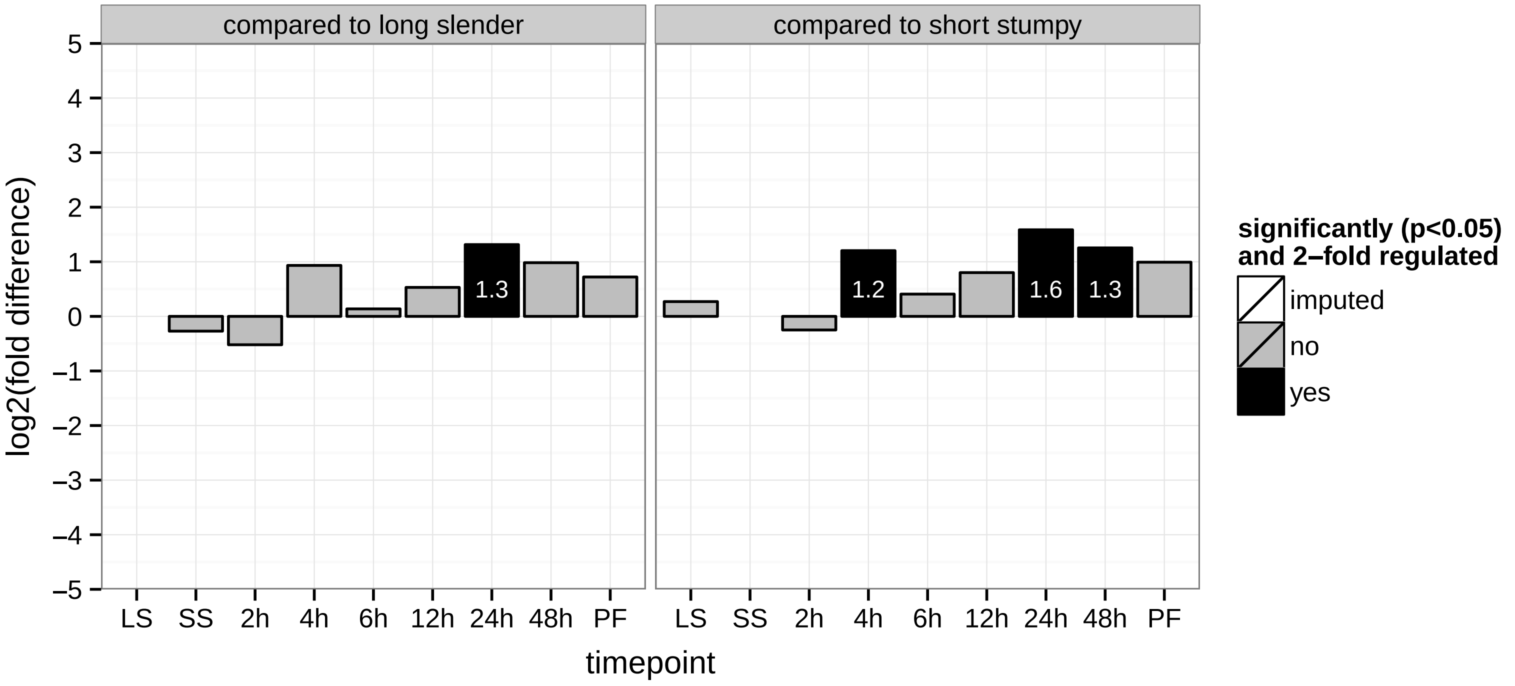

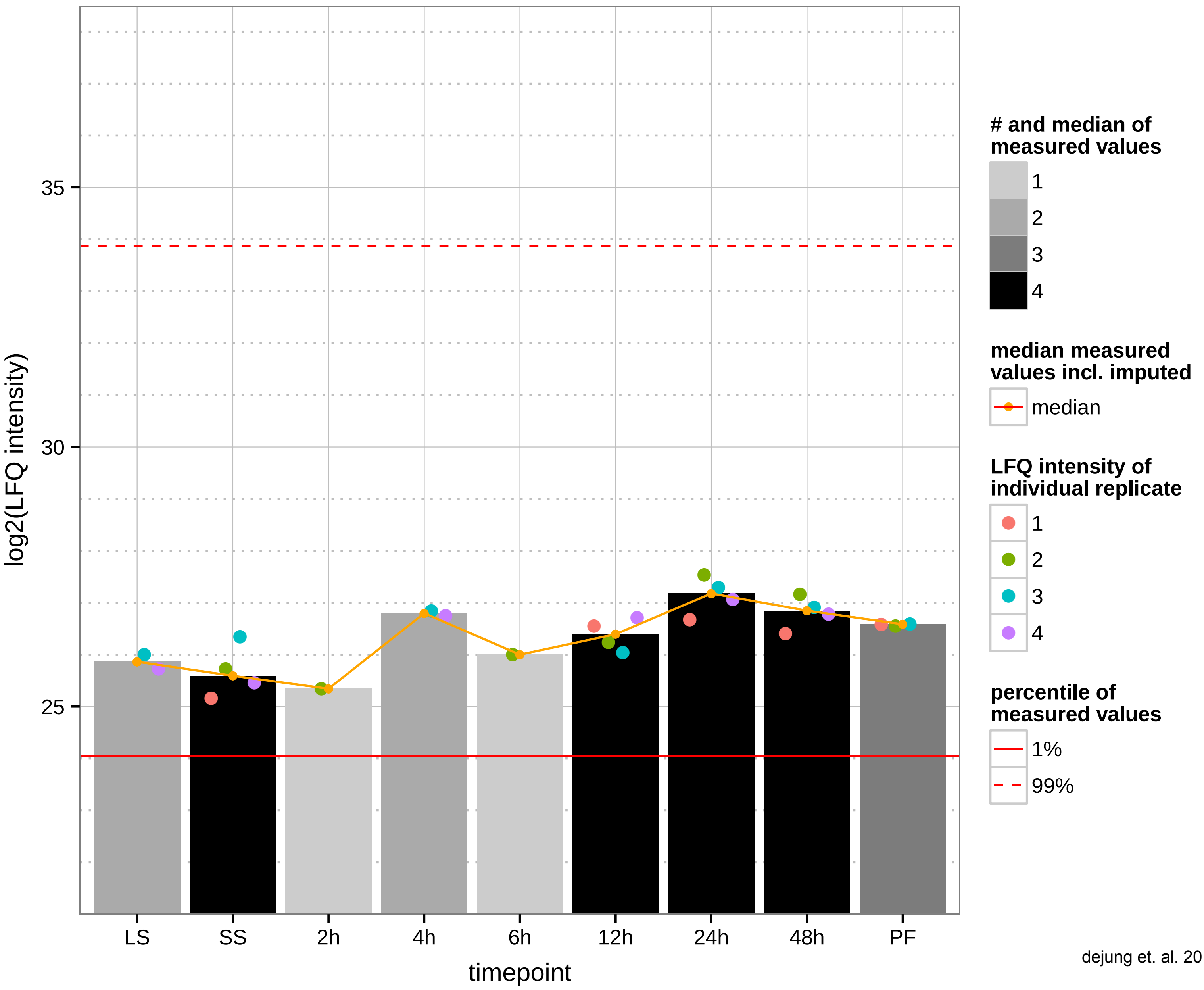

dejung et. al. 2015

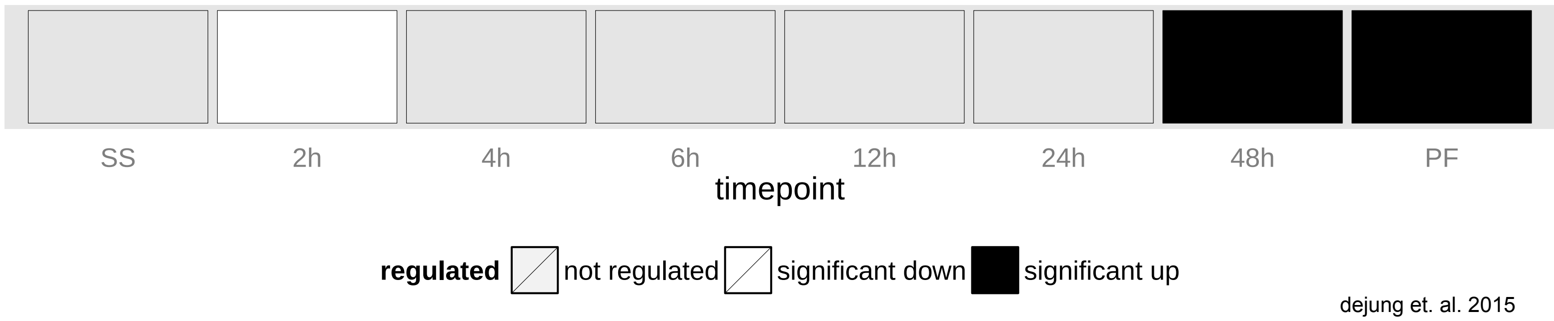
SS
2h
4h
6h
12h
24h
48h
PF
timepoint
regulated
not regulated
significant down
significant up
dejung et. al. 2015

hypothetical protein, conserved
Tb927.10.6300;Tb11.v5.0276
AGOF: null, structural constituent of ribosome
AGOC: null, intracellular, mitochondrion, ribosome
AGOP: null, translation
PGOF: structural constituent of ribosome
PGOC: intracellular, ribosome
PGOP: translation

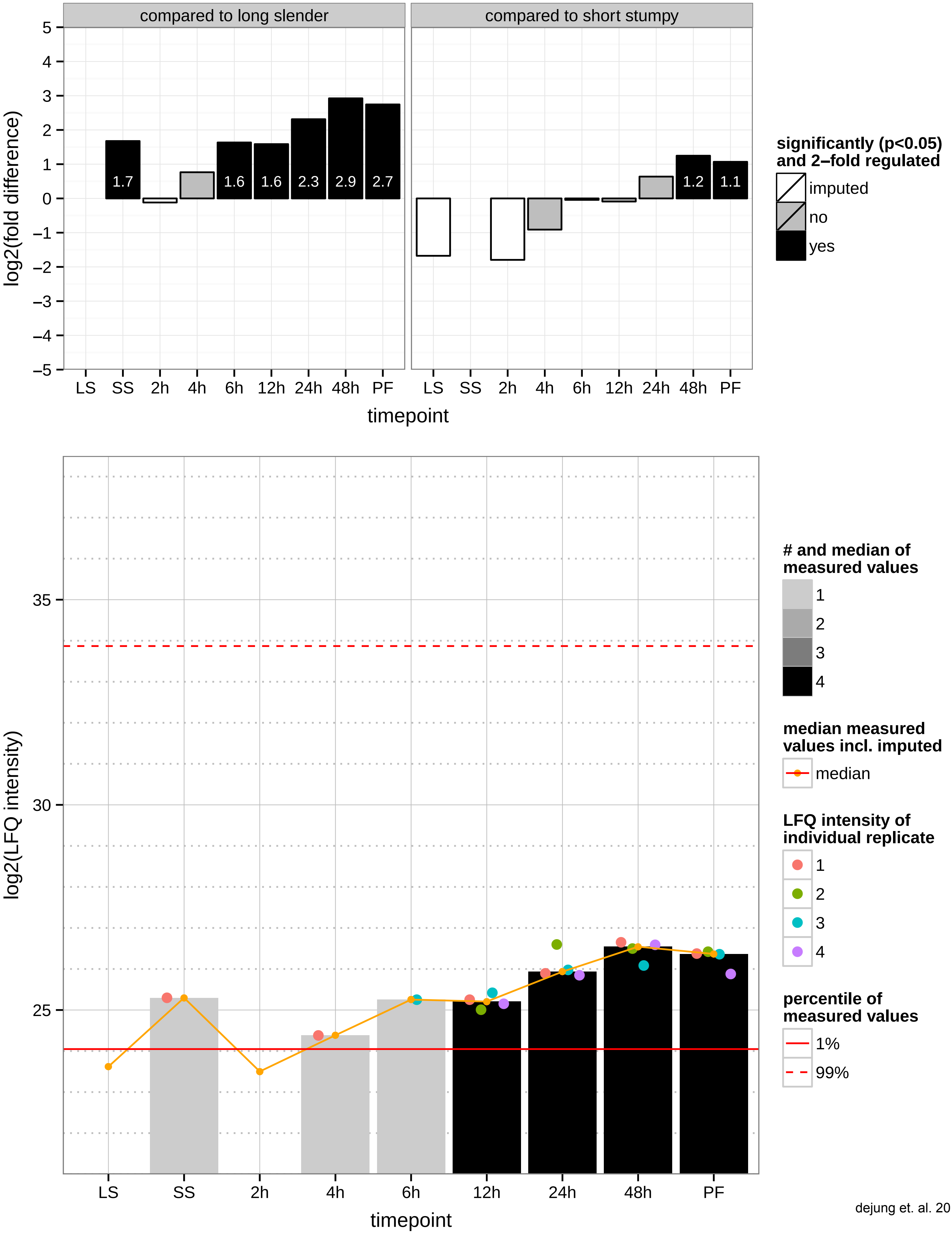

dejung et. al. 2015

hypothetical protein, conserved
Tb927.4.4340
AGOF: null
AGOC: null
AGOP: null
PGOF: null
PGOC: null
PGOP: null

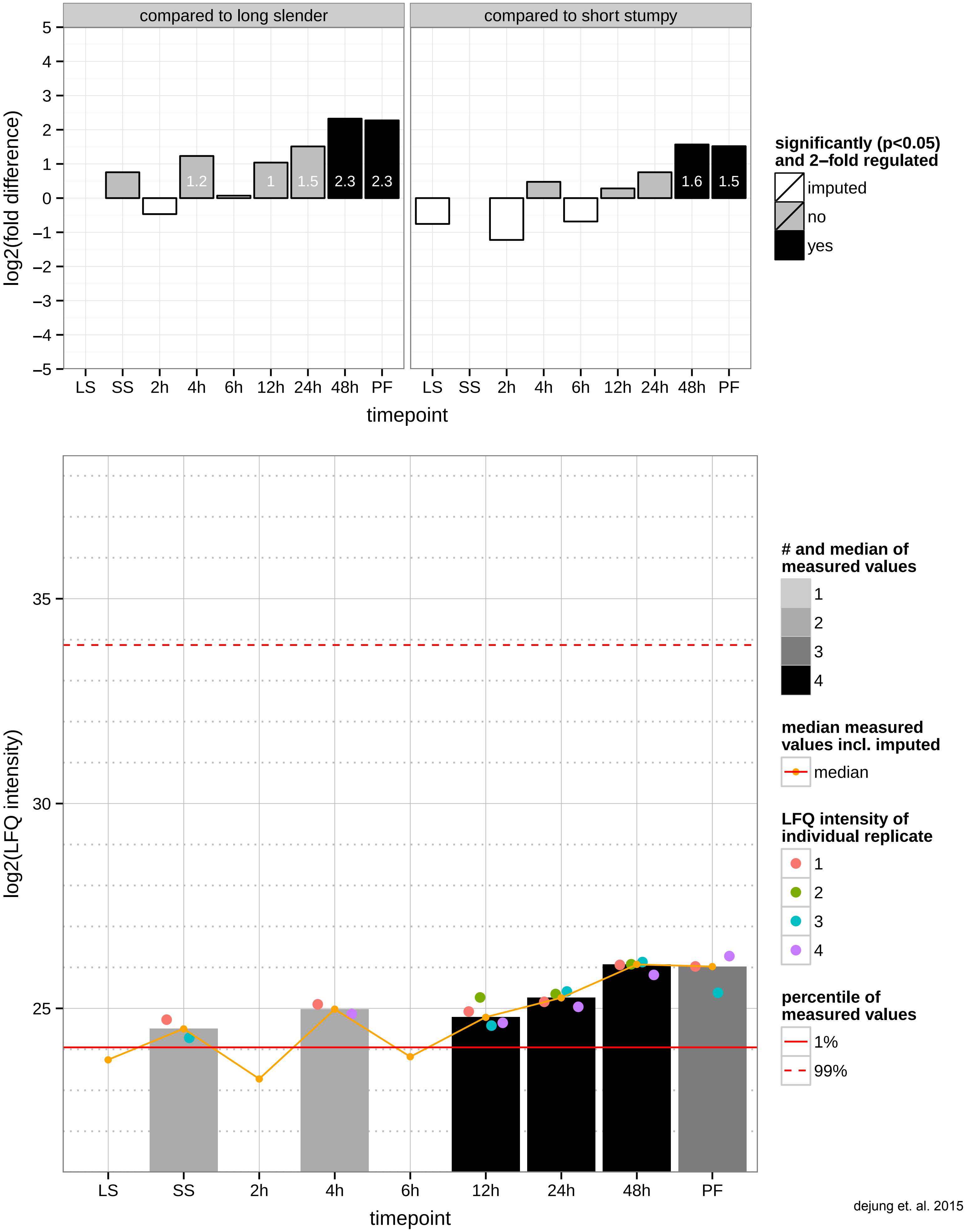

hypothetical protein, conserved, BAC from homologous region on chr5
Tb927.5.4360;Tb05.5K5.20
AGOF: null
AGOC: null
AGOP: null
PGOF: null
PGOC: null
PGOP: null

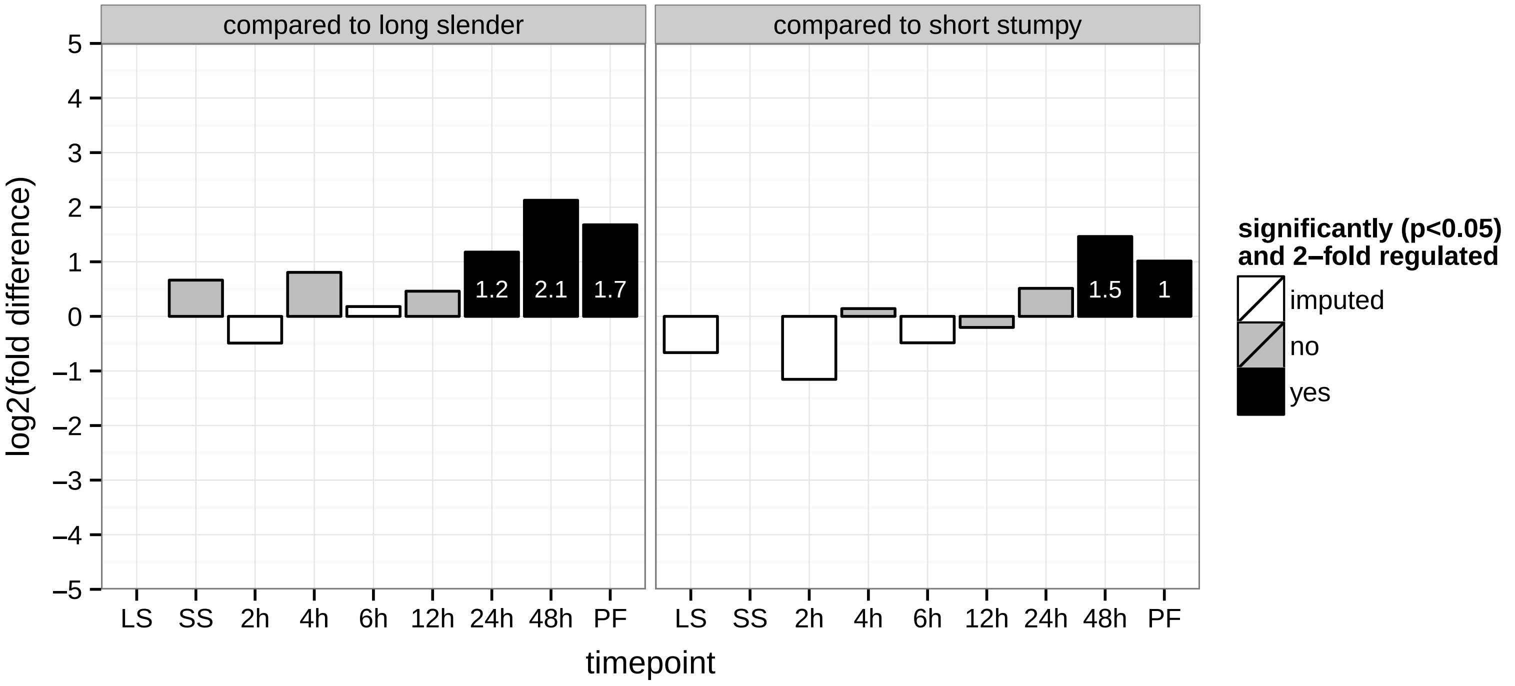

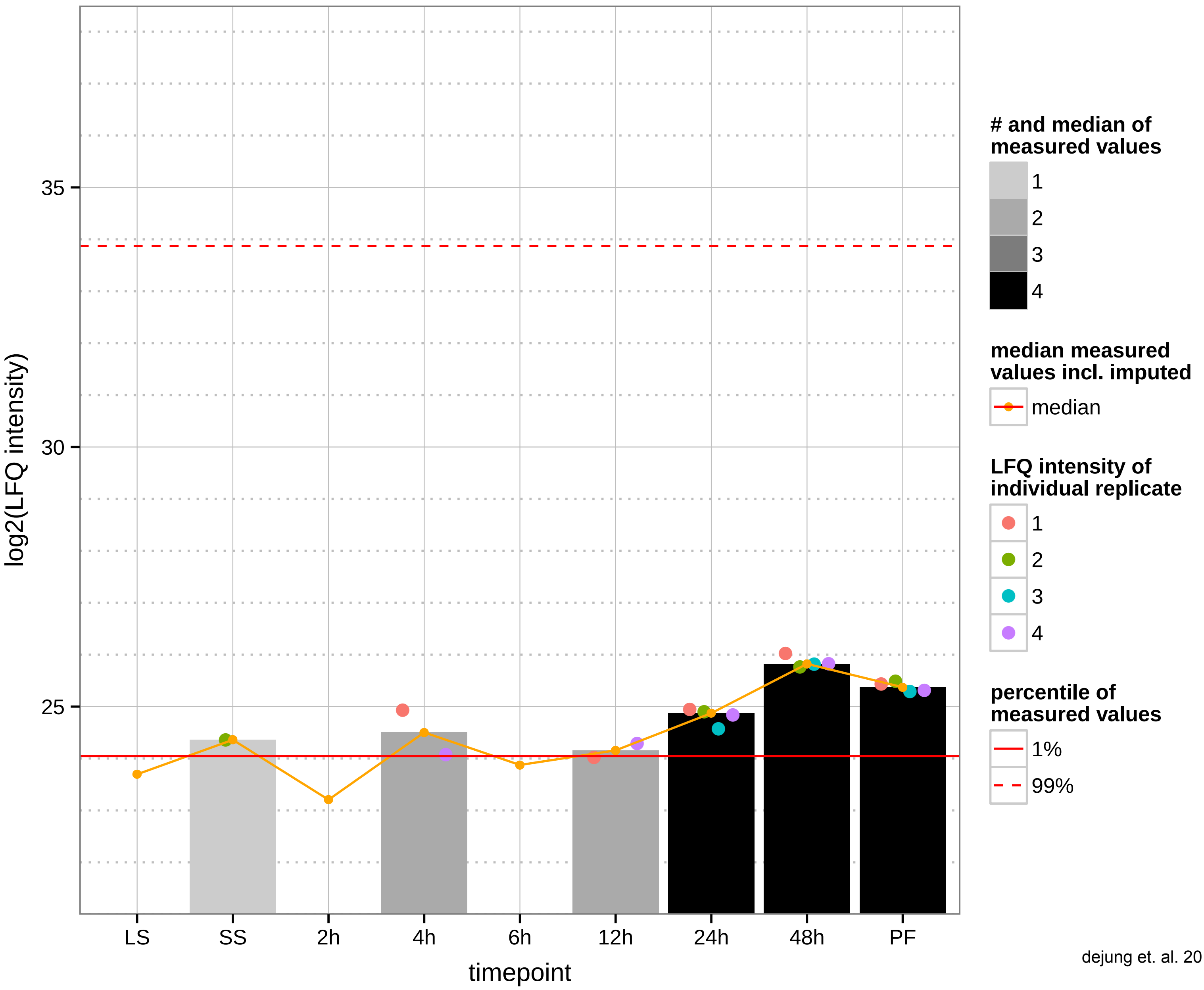

dejung et. al. 2015

hypothetical protein, conserved
Tb927.8.6770
AGOF: null
AGOC: null
AGOP: null
PGOF: protein binding
PGOC: null
PGOP: null

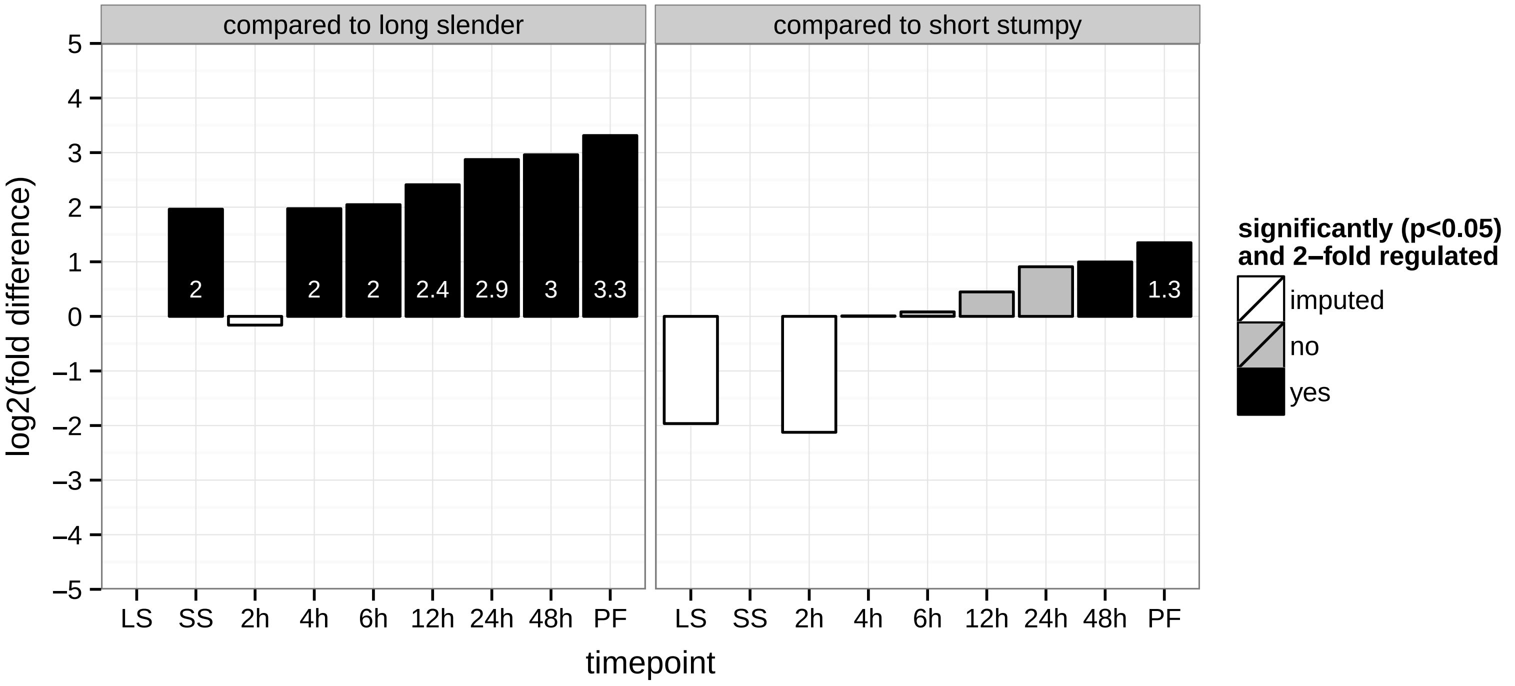

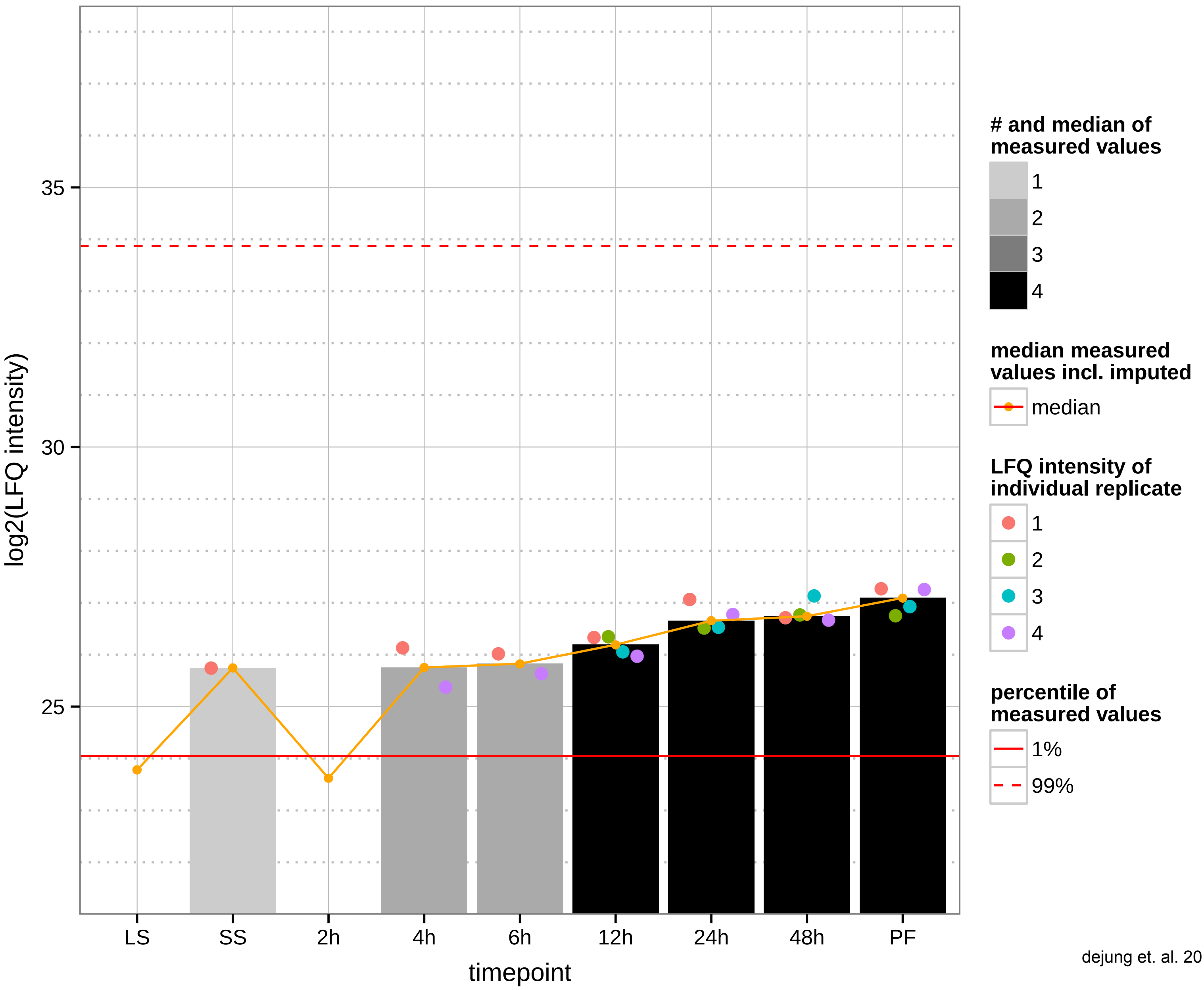

dejung et. al. 2015

trans−sialidase, putative, neuraminidase
Tb927.8.7340
AGOF: exo−alpha−sialidase activity
AGOC: null
AGOP: pathogenesis
PGOF: null
PGOC: null
PGOP: null

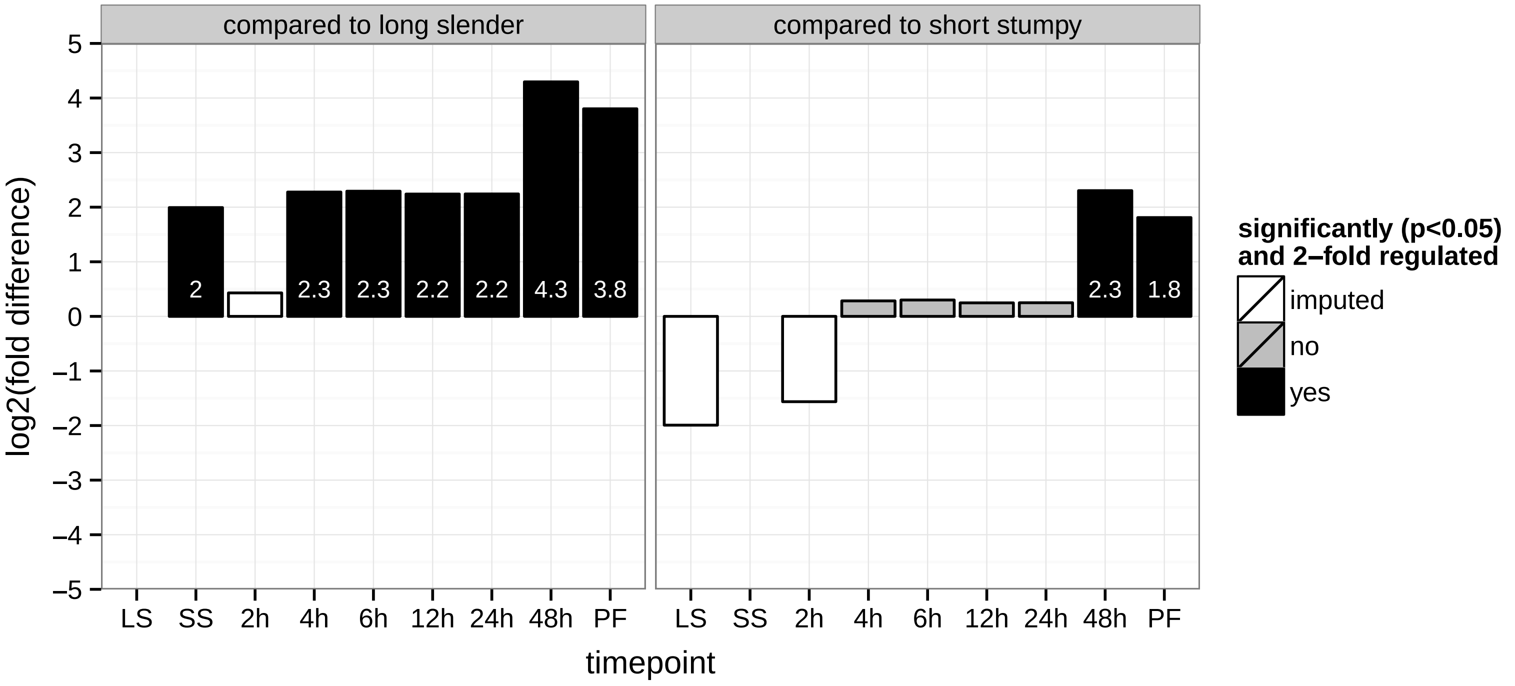

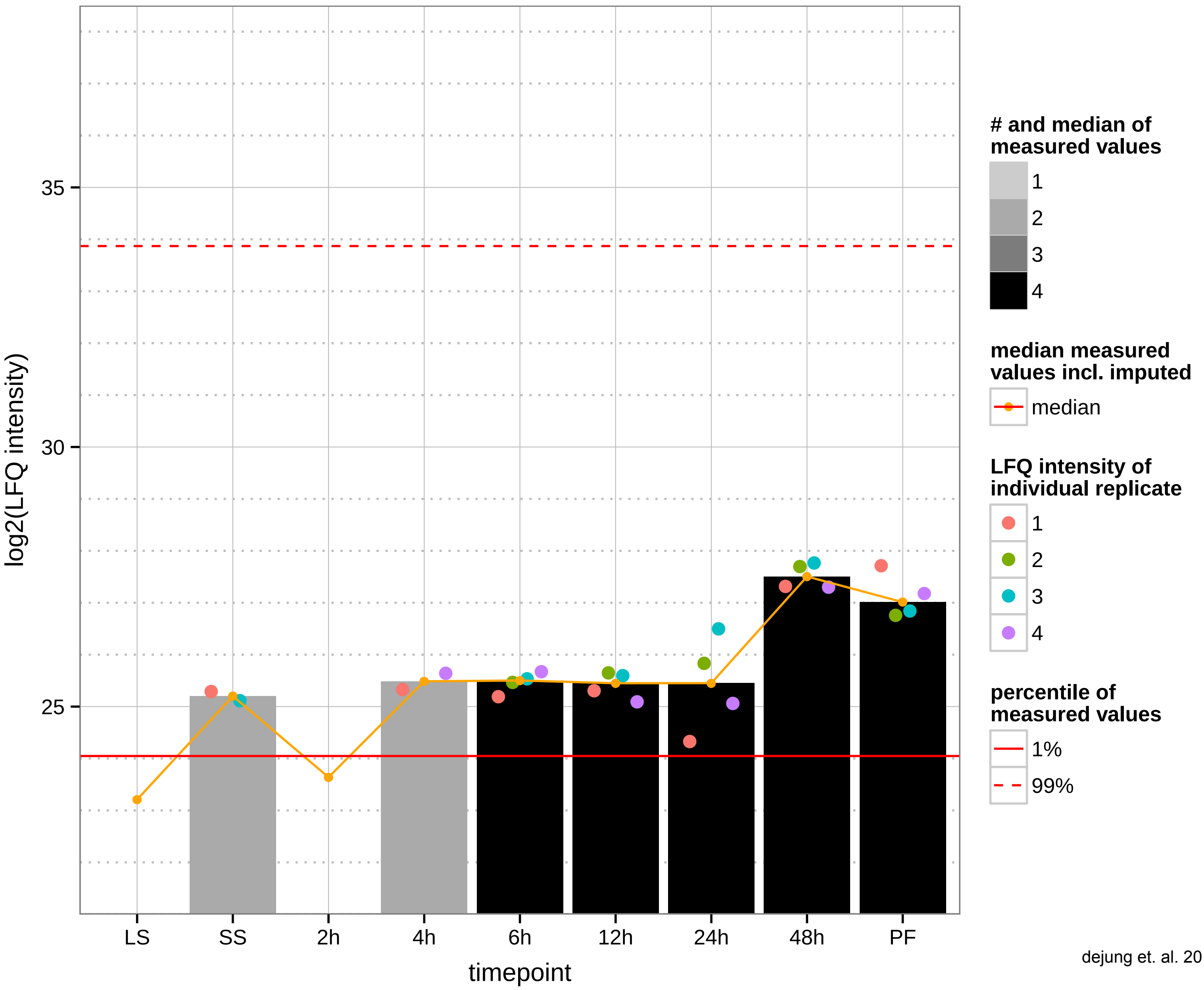

dejung et. al. 2015

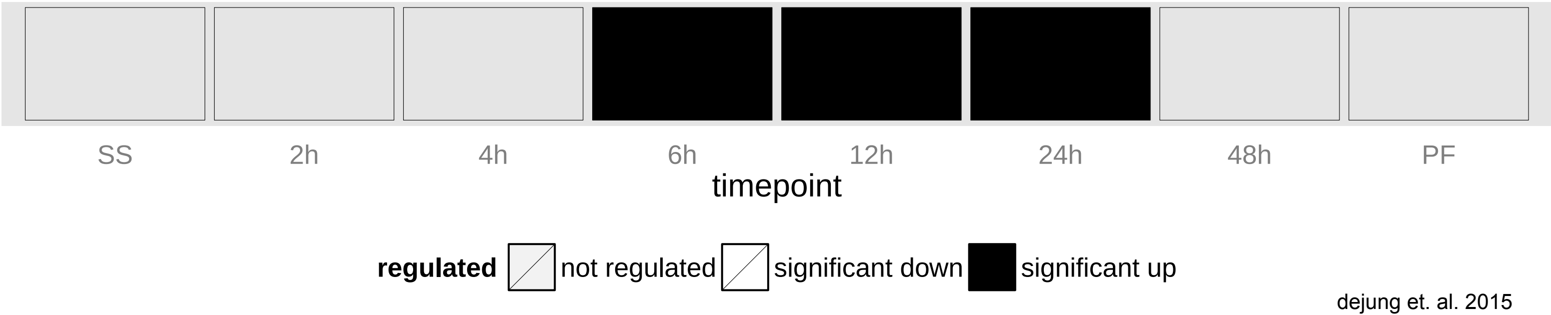
SS
2h
4h
6h
12h
24h
48h
PF
timepoint
regulated
not regulated
significant down
significant up
dejung et. al. 2015

GPI inositol deacylase precursor (GPIdeAc)
Tb927.10.4780
AGOF: phosphatidylinositol deacylase activity
AGOC: membrane
AGOP: GPI anchor biosynthetic process
PGOF: null
PGOC: null
PGOP: null

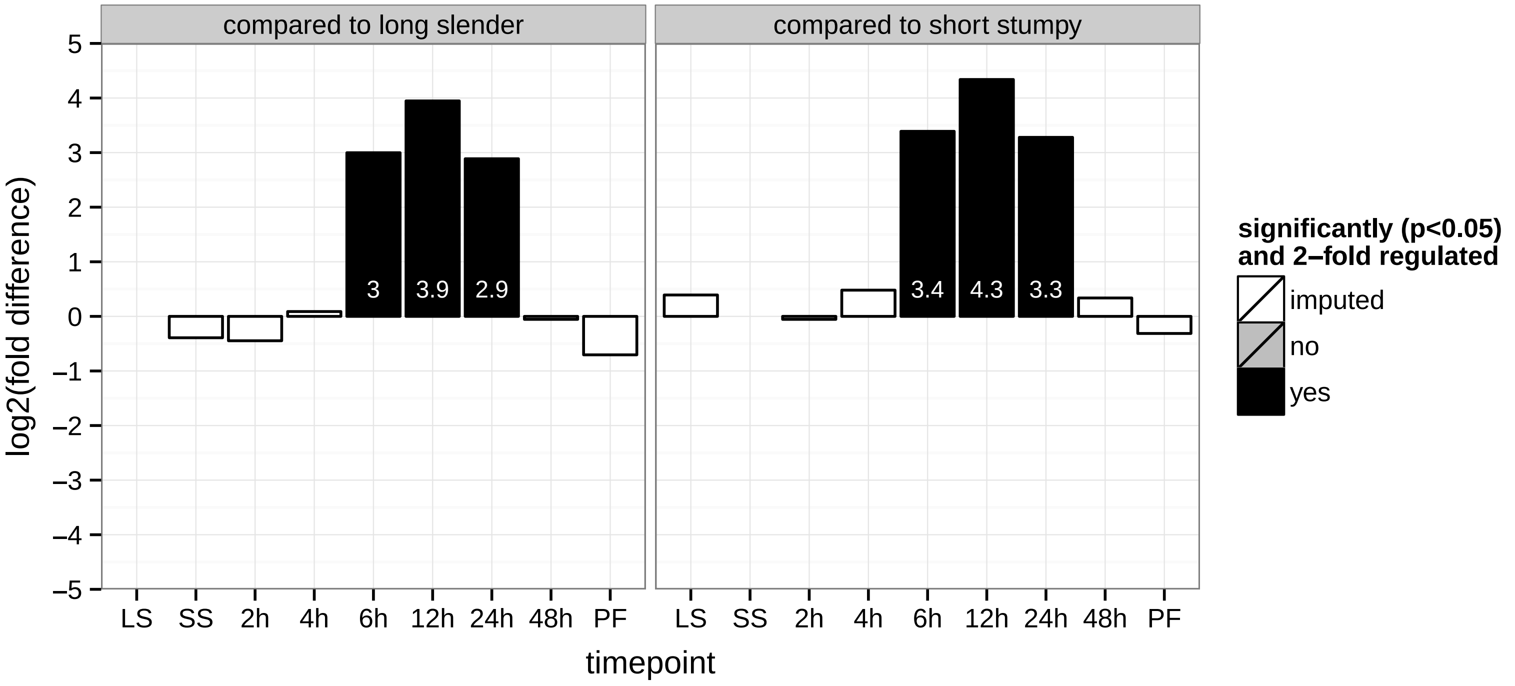

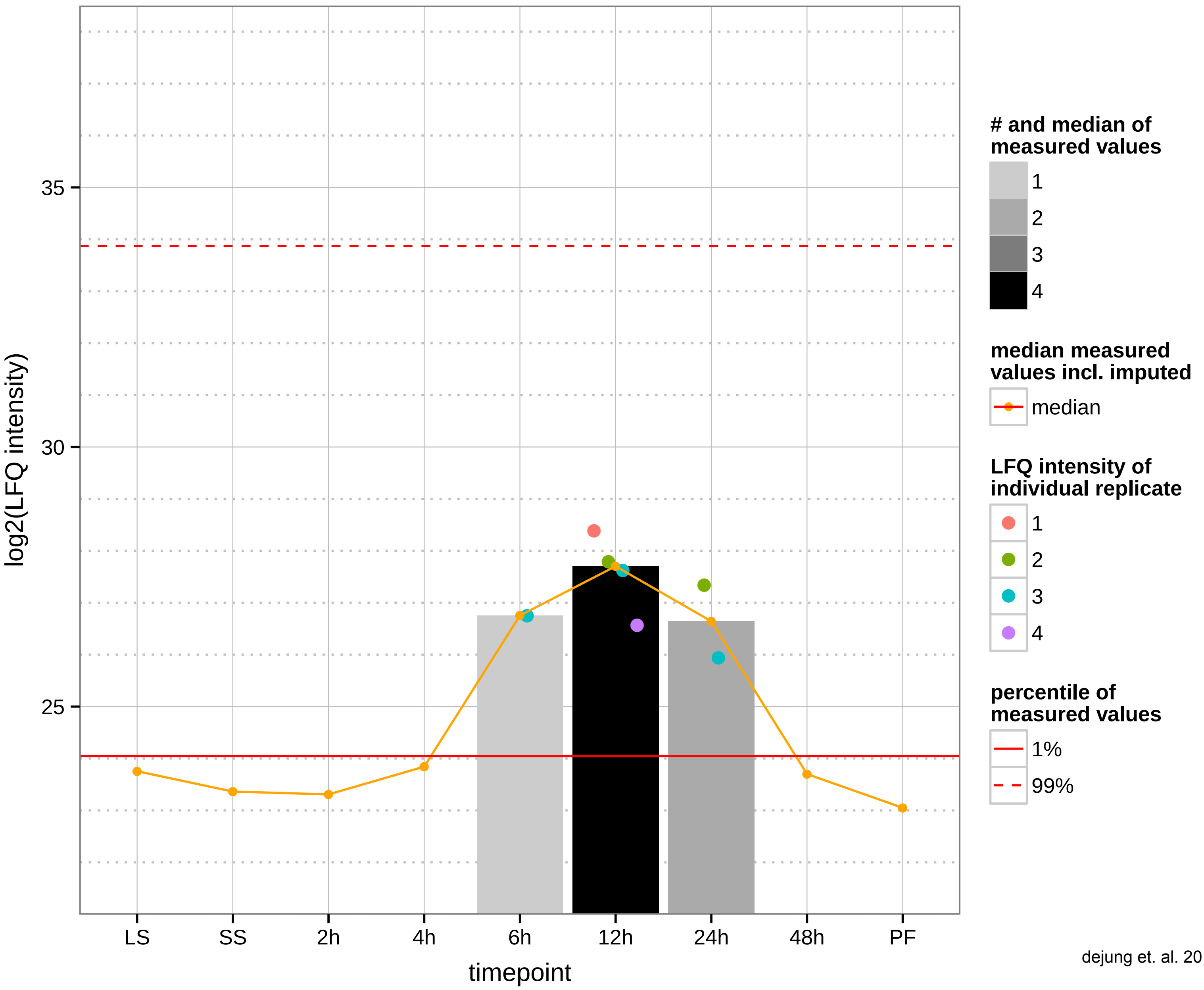

dejung et. al. 2015

hypothetical protein, conserved
Tb927.10.6730
AGOF: null
AGOC: null
AGOP: null
PGOF: null
PGOC: null
PGOP: null

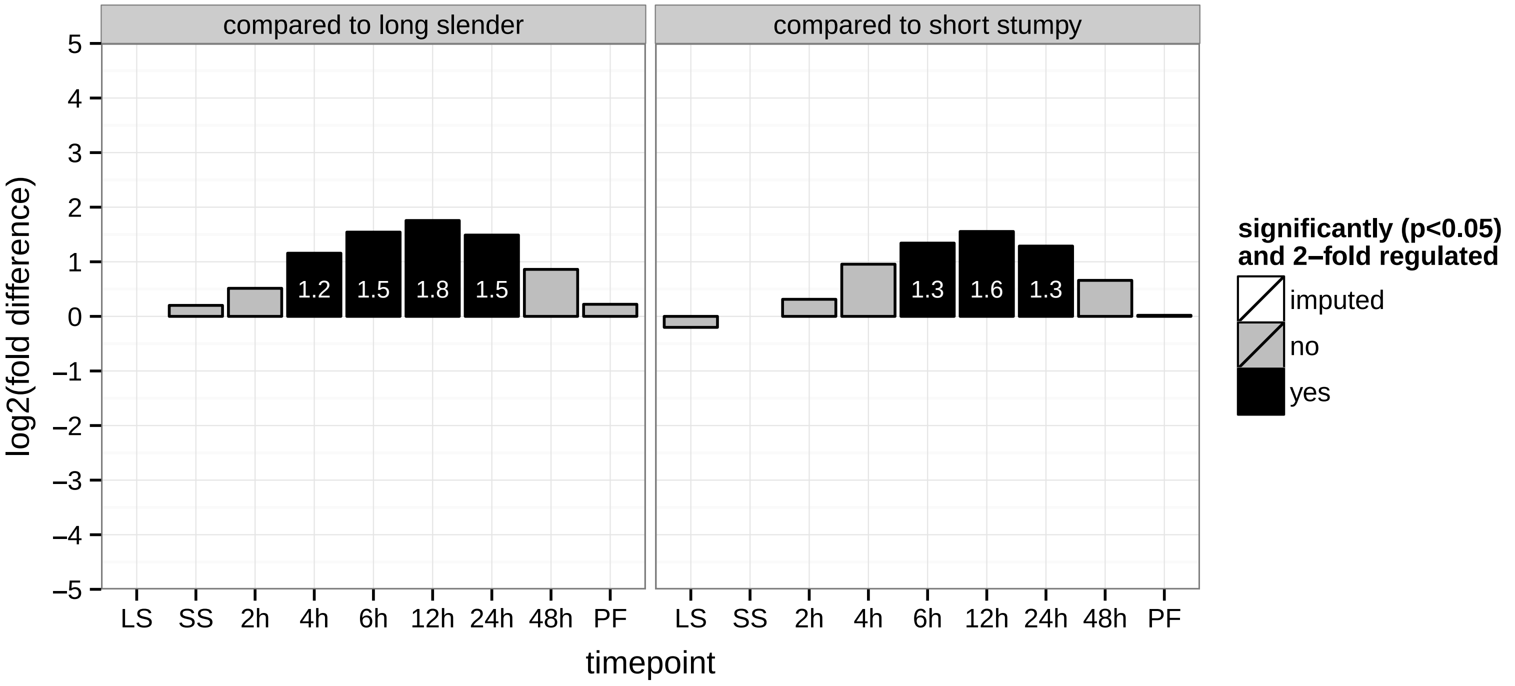

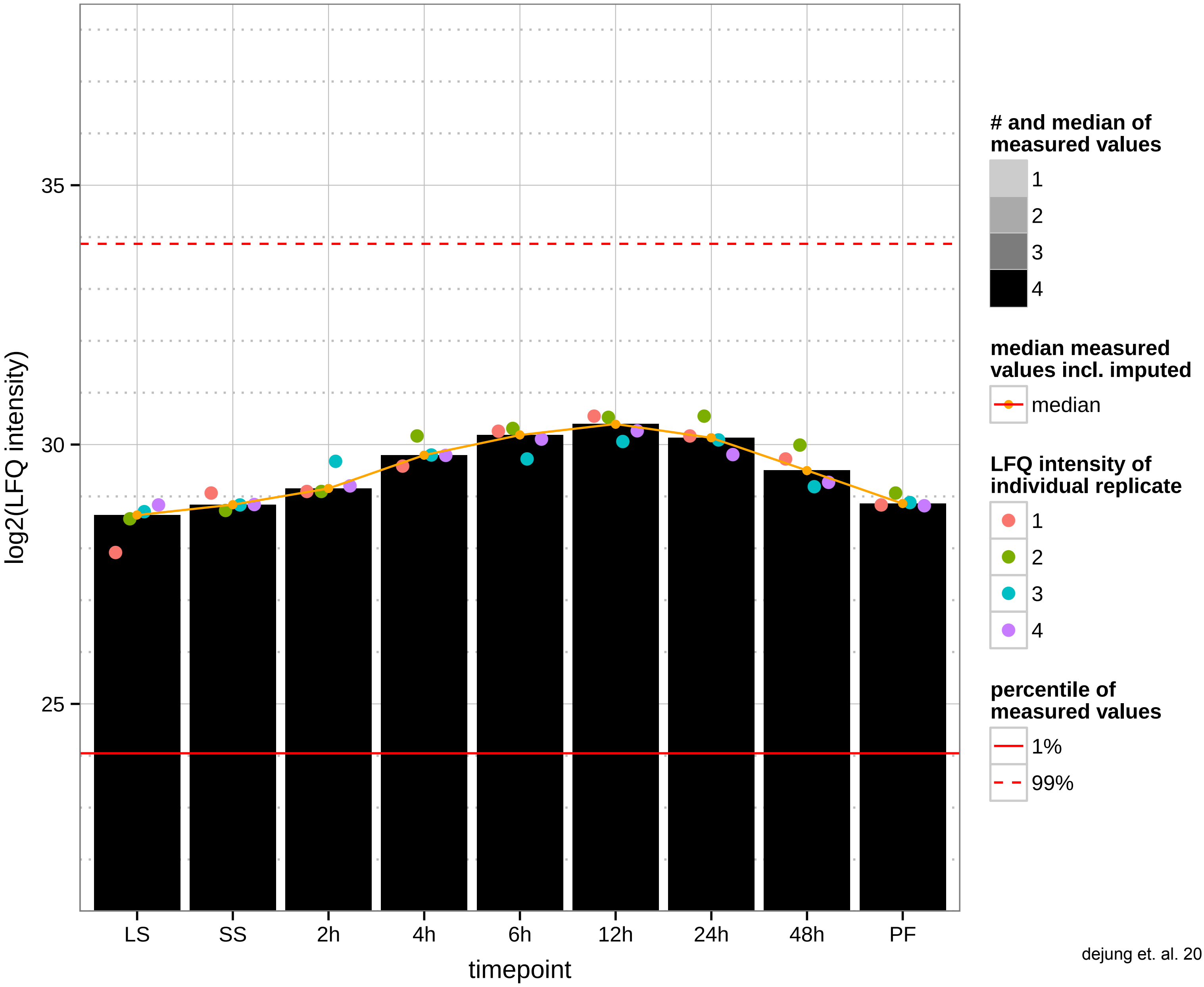

dejung et. al. 2015

hypothetical protein, conserved
Tb927.7.4360
AGOF: null
AGOC: null
AGOP: null
PGOF: null
PGOC: null
PGOP: null

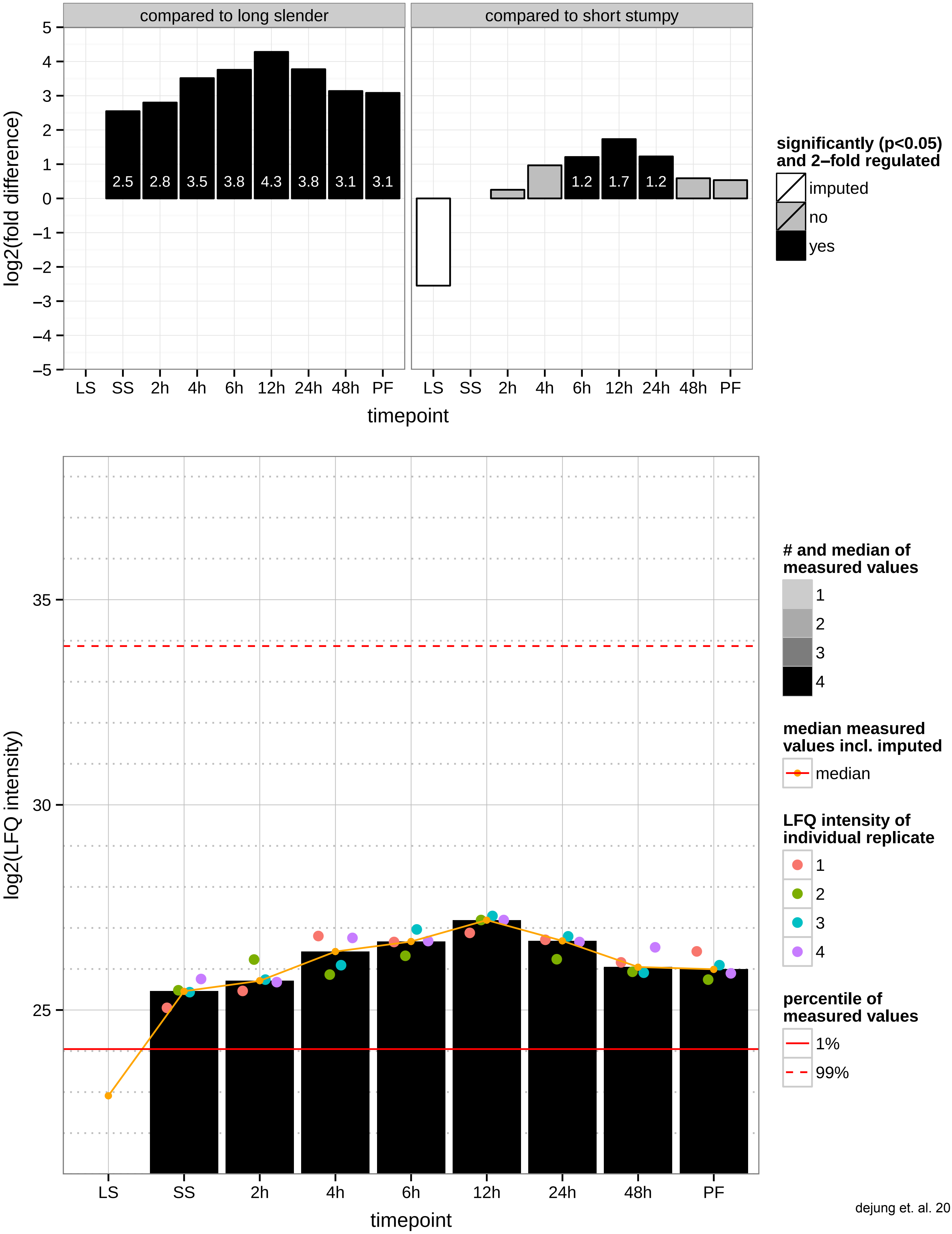

dejung et. al. 2015

hypothetical protein
Tb927.8.6630
AGOF: null
AGOC: null
AGOP: null
PGOF: null
PGOC: null
PGOP: null

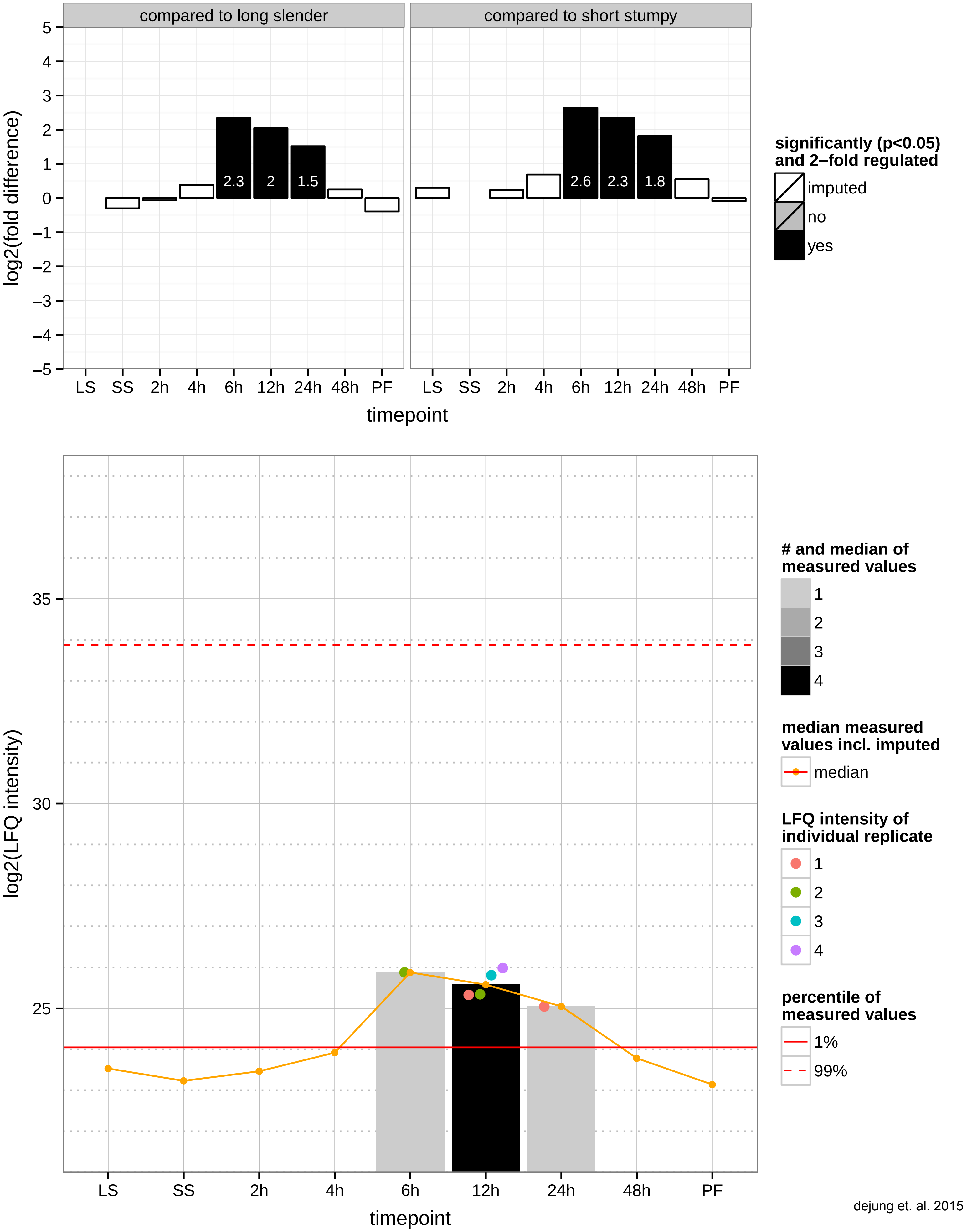

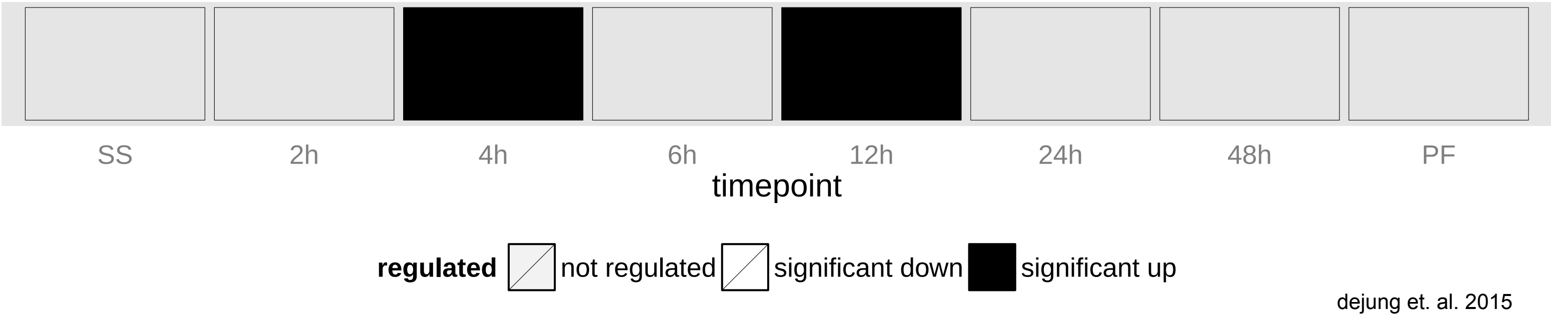
SS
2h
4h
6h
12h
24h
48h
PF
timepoint
regulated
not regulated
significant down
significant up
dejung et. al. 2015

hypothetical protein, conserved
Tb927.11.1380
AGOF: null
AGOC: null
AGOP: null
PGOF: null
PGOC: null
PGOP: null

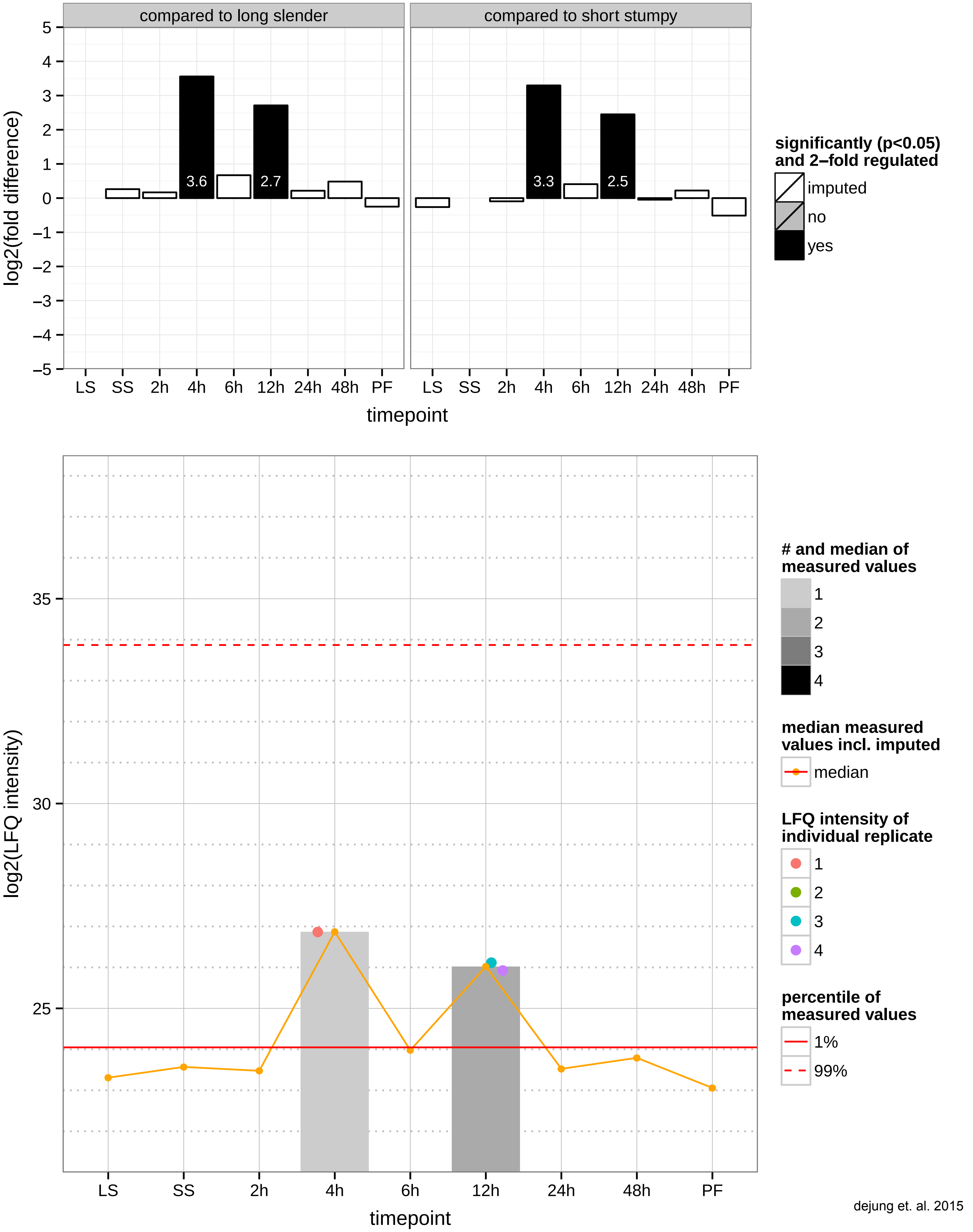

dejung et. al. 2015

hypothetical protein, conserved
Tb927.11.1610
AGOF: null
AGOC: null
AGOP: null
PGOF: null
PGOC: null
PGOP: null

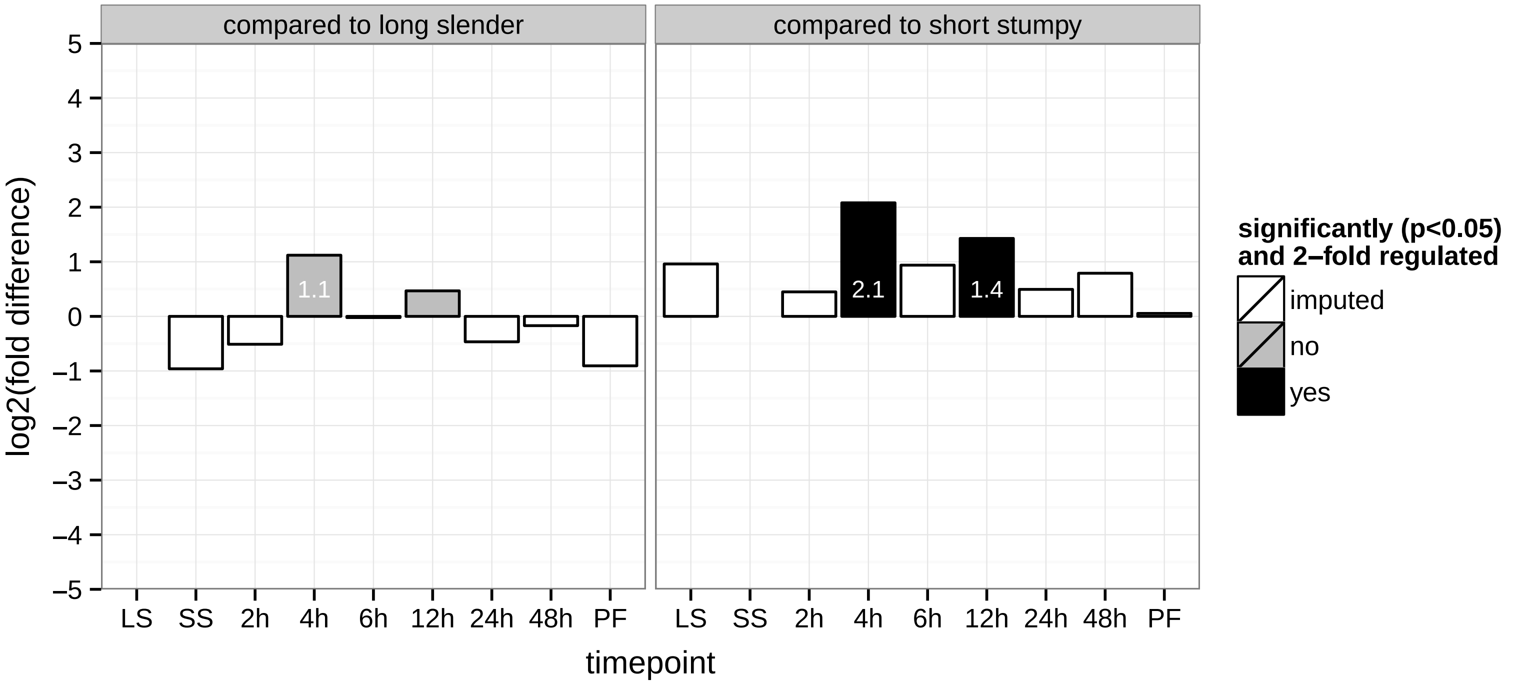

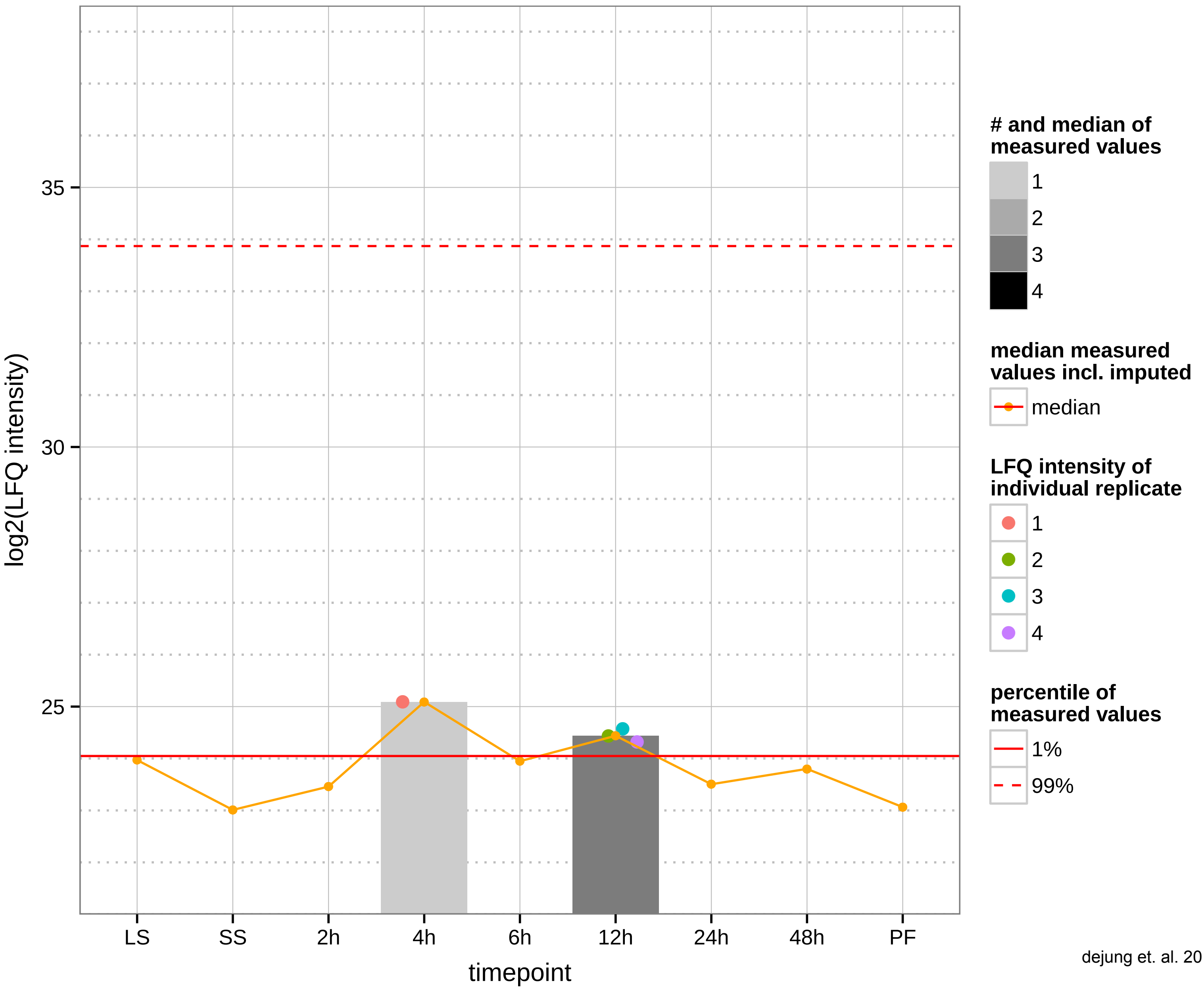

dejung et. al. 2015

carnitine O−palmitoyltransferase II, putative
Tb927.7.2250
AGOF: transferase activity, transferring acyl groups
AGOC: null
AGOP: null
PGOF: transferase activity, transferring acyl groups
PGOC: null
PGOP: null

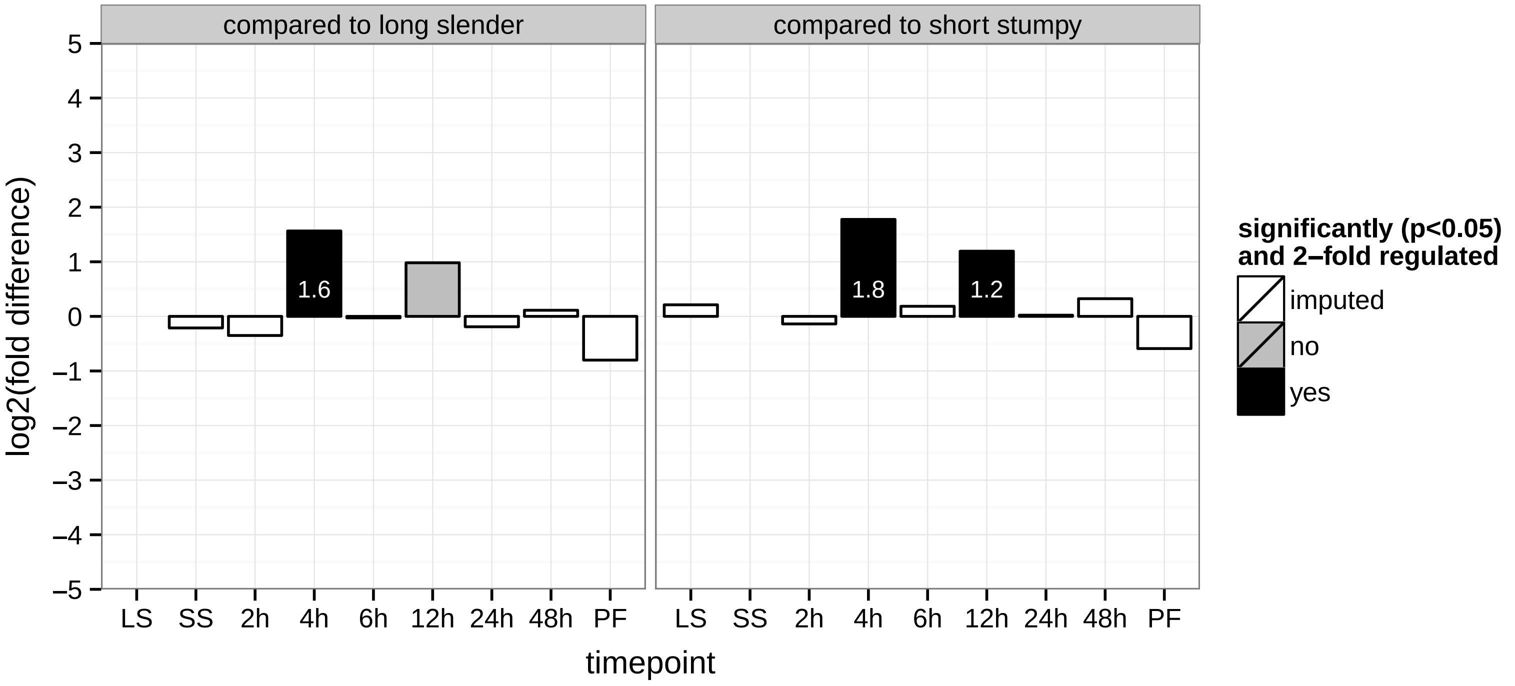

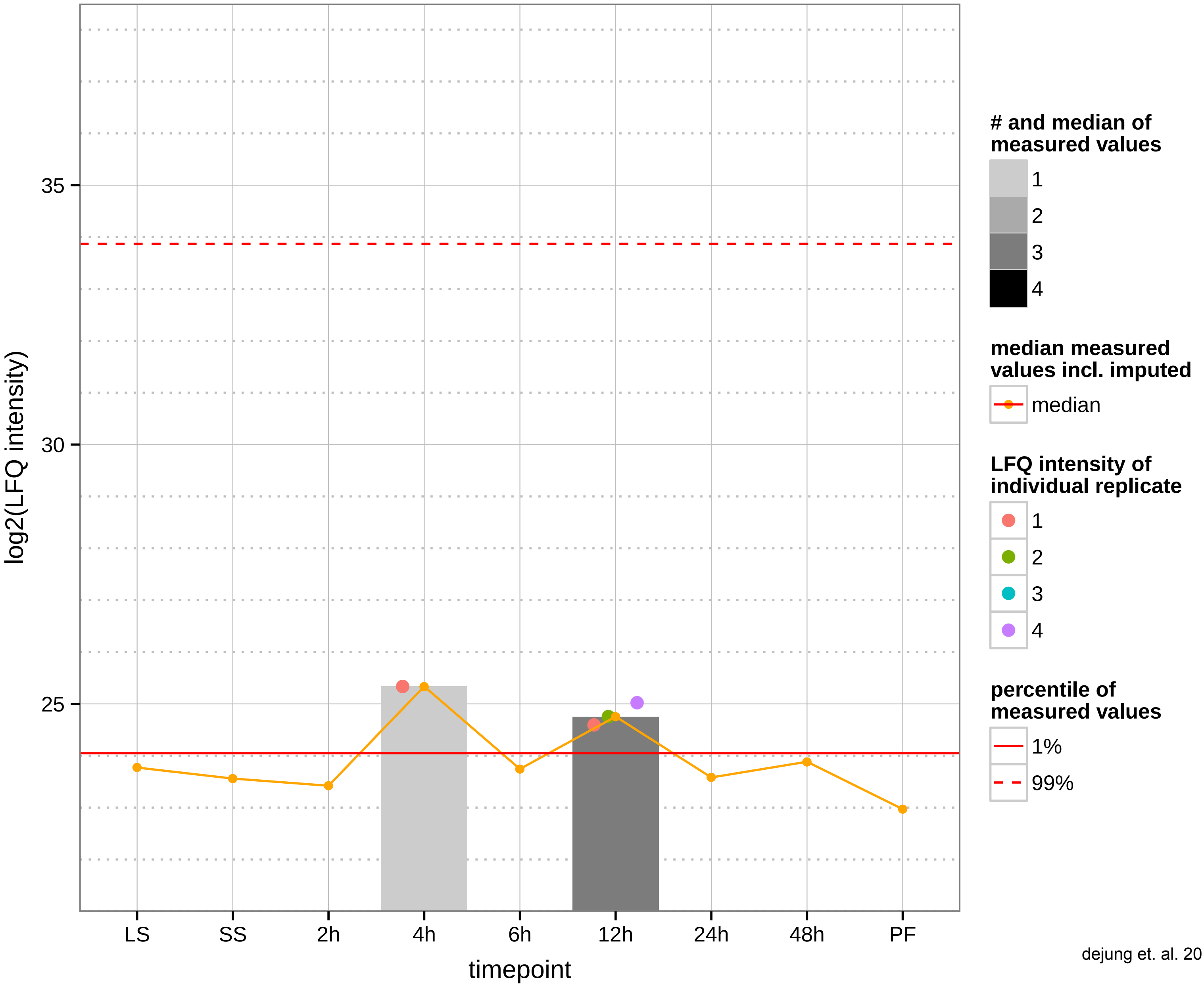

dejung et. al. 2015

CDP−diacylglycerol−−inositol 3−phosphatidyltransferase, putative, phosphatidyltransferase
Tb927.9.1610
AGOF: CDP−diacylglycerol−inositol 3−phosphatidyltransferase activity, phosphatidylinositol transporter activity
AGOC: Golgi apparatus, endoplasmic reticulum, membrane
AGOP: phospholipid biosynthetic process
PGOF: phosphotransferase activity, for other substituted phosphate groups
PGOC: membrane
PGOP: phospholipid biosynthetic process

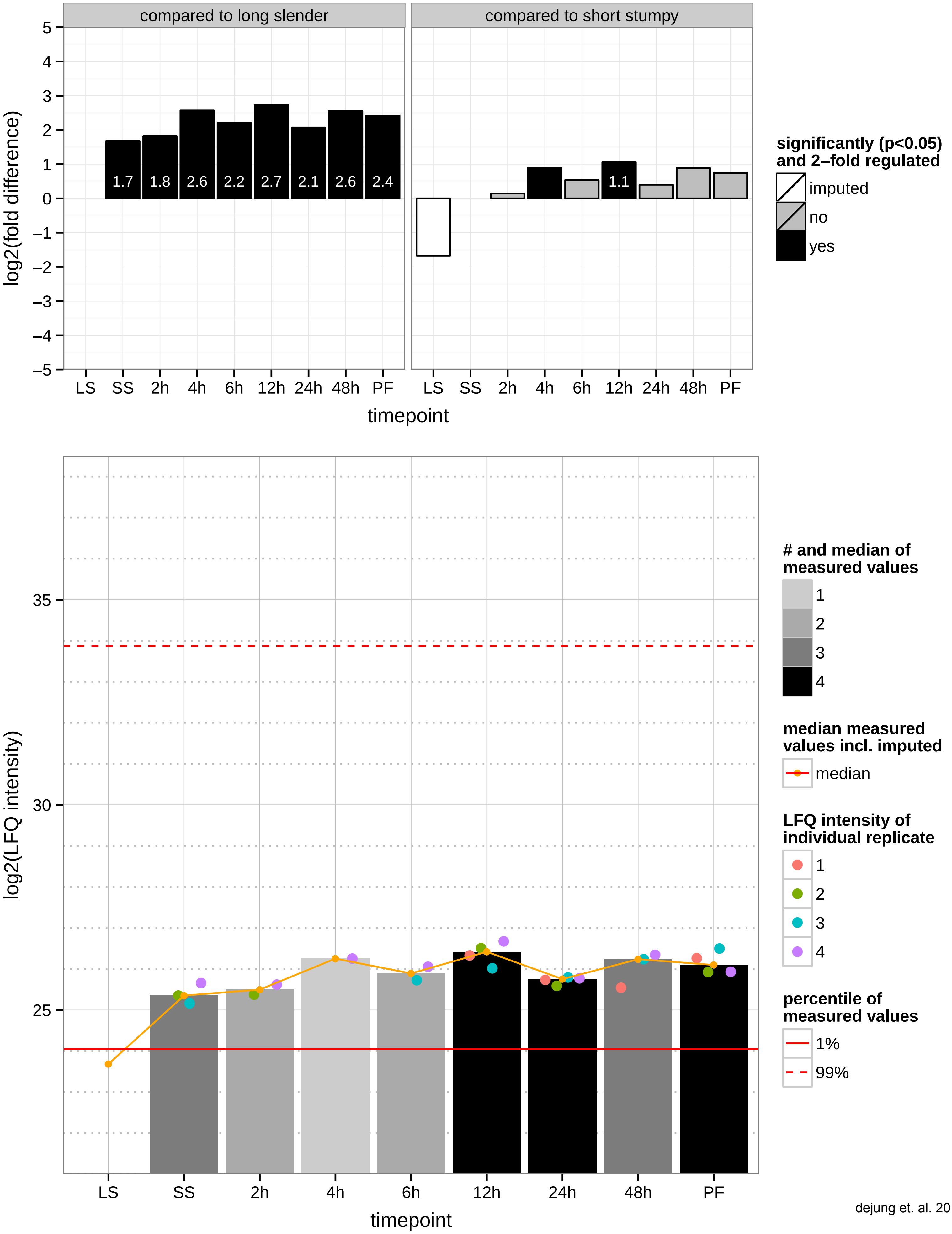

dejung et. al. 2015

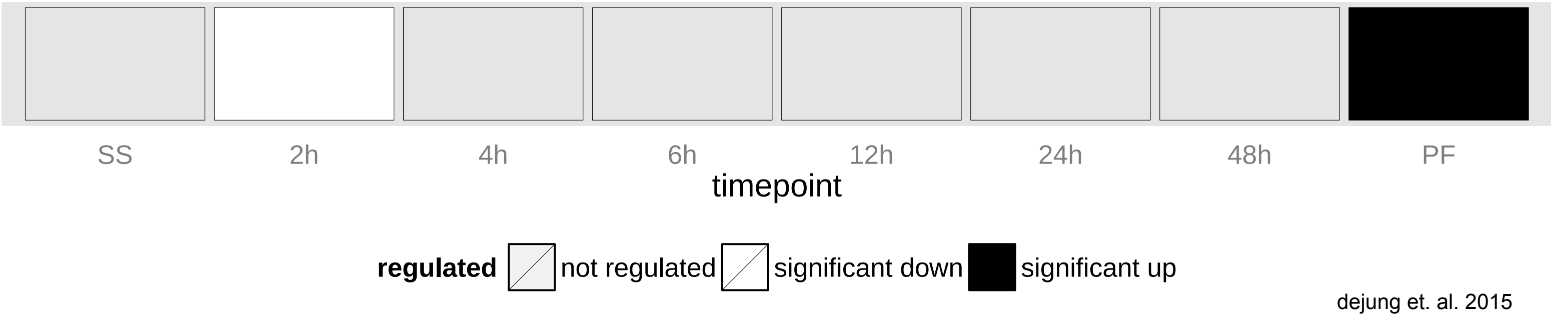
SS
2h
4h
6h
12h
24h
48h
PF
timepoint
regulated
not regulated
significant down
significant up
dejung et. al. 2015

hypothetical protein, conserved
Tb927.10.1660
AGOF: null
AGOC: mitochondrion
AGOP: null
PGOF: null
PGOC: null
PGOP: null

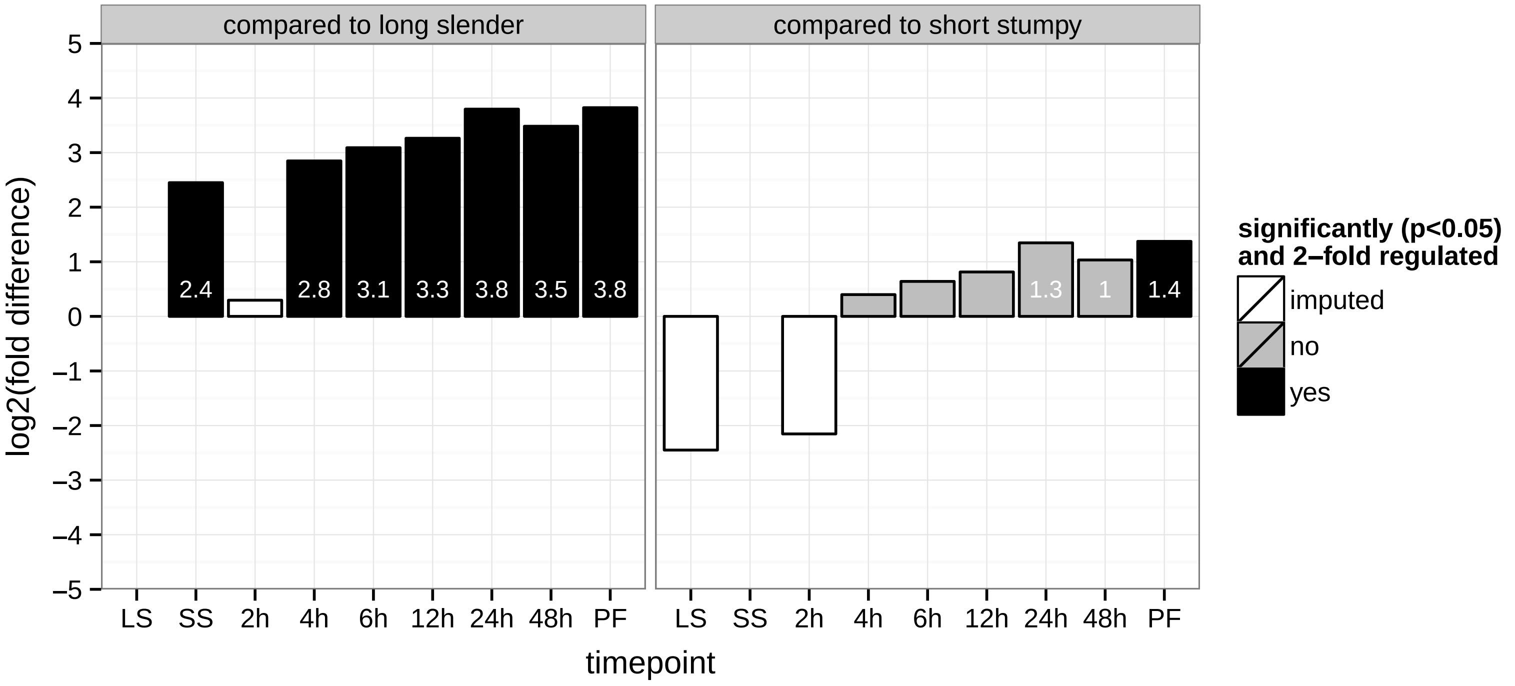

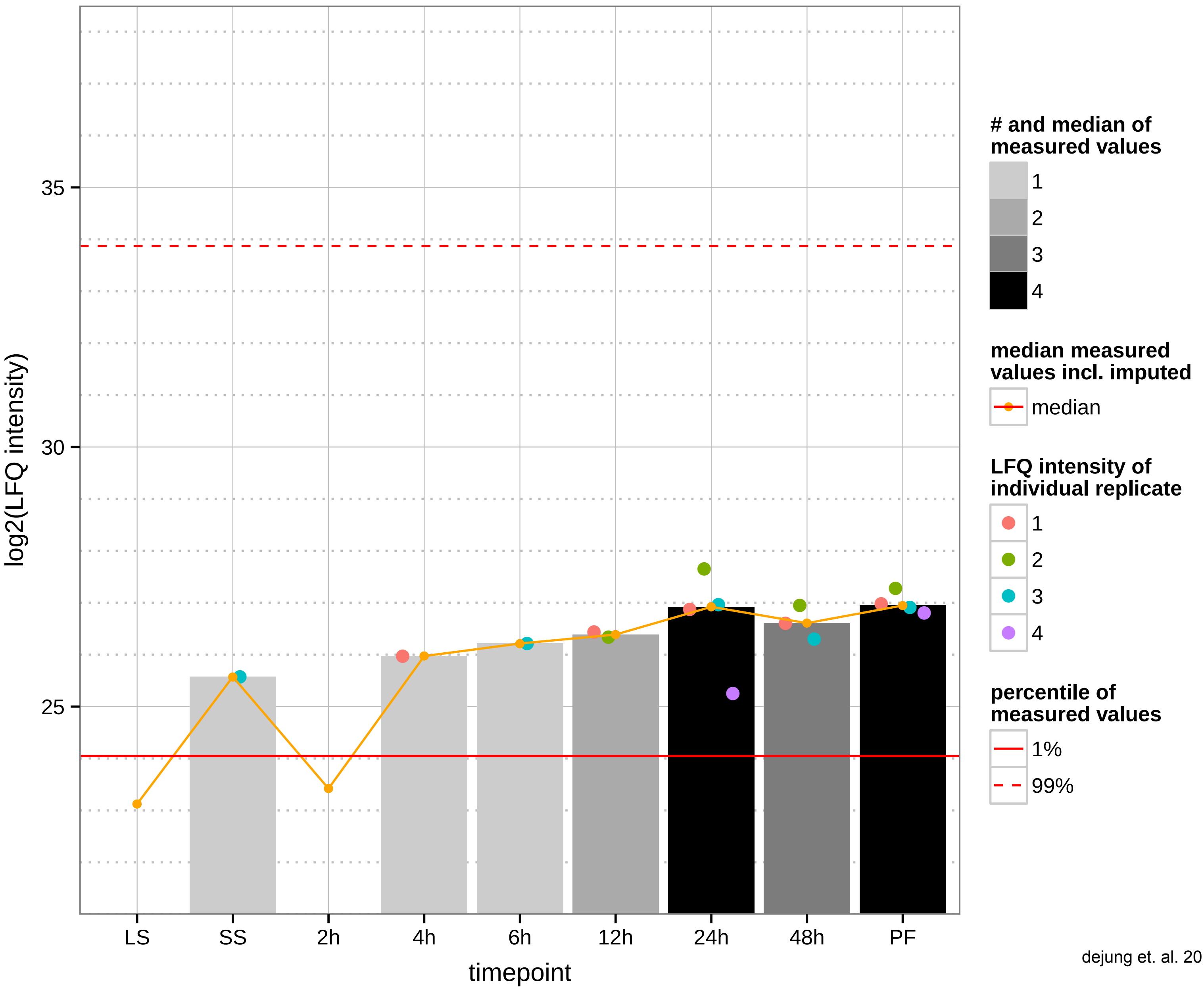

dejung et. al. 2015

kinetoplastid–specific dual specificity phosphatase, putative
Tb927.2.4280
AGOF: protein tyrosine/serine/threonine phosphatase activity
AGOC: null
AGOP: protein dephosphorylation
PGOF: phosphatase activity, protein tyrosine/serine/threonine phosphatase activity
PGOC: null
PGOP: dephosphorylation, protein dephosphorylation

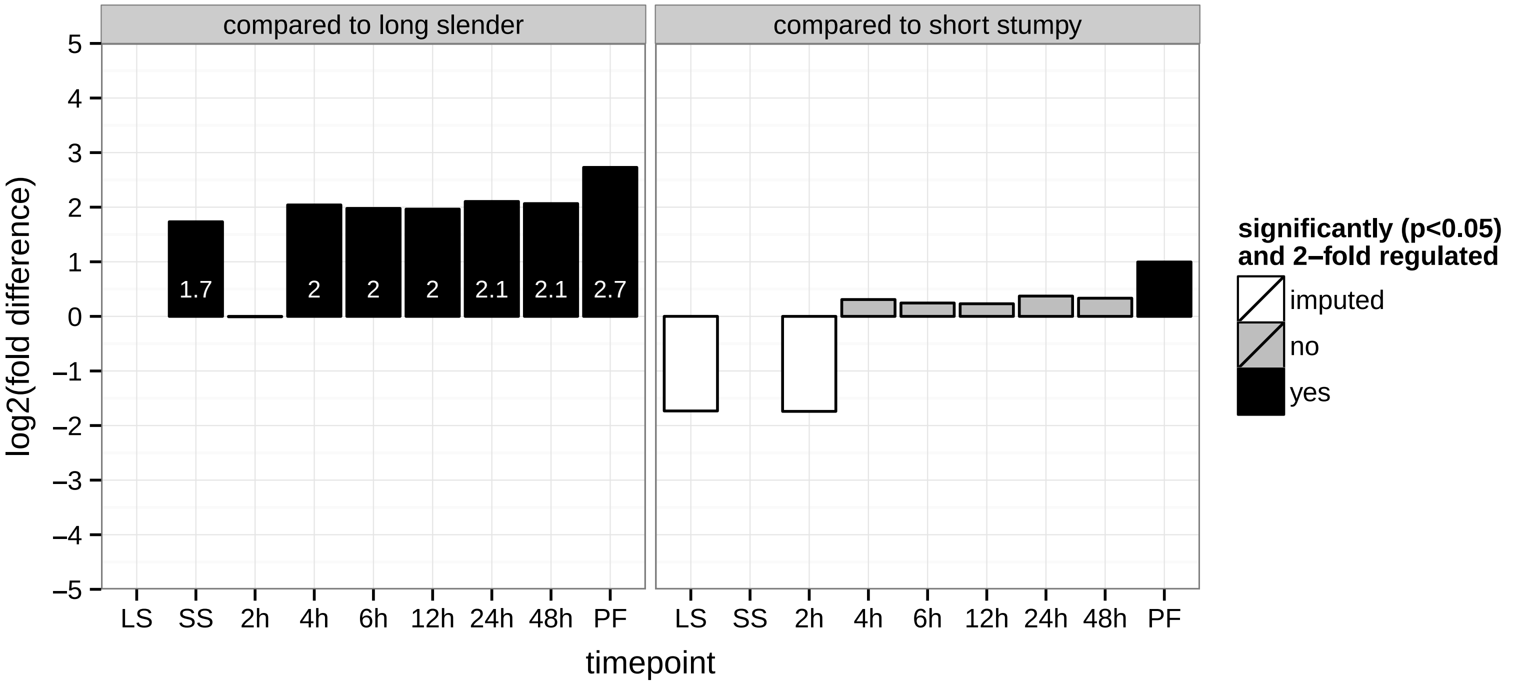

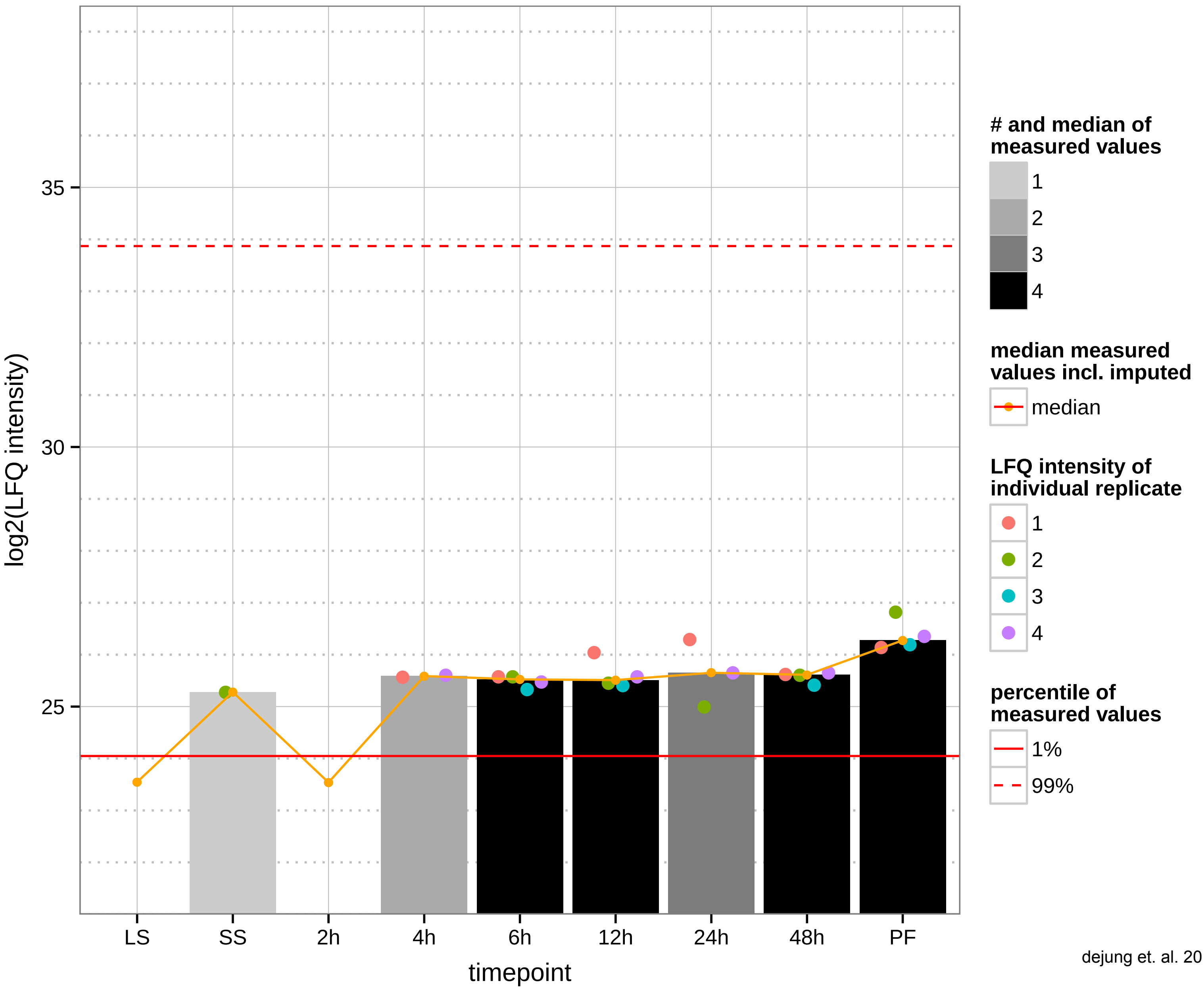

dejung et. al. 2015

ribosomal protein L3 mitochondrial, putative
Tb927.3.5610
AGOF: structural constituent of ribosome
AGOC: intracellular, mitochondrion, ribosome
AGOP: translation
PGOF: structural constituent of ribosome
PGOC: intracellular, ribosome
PGOP: translation

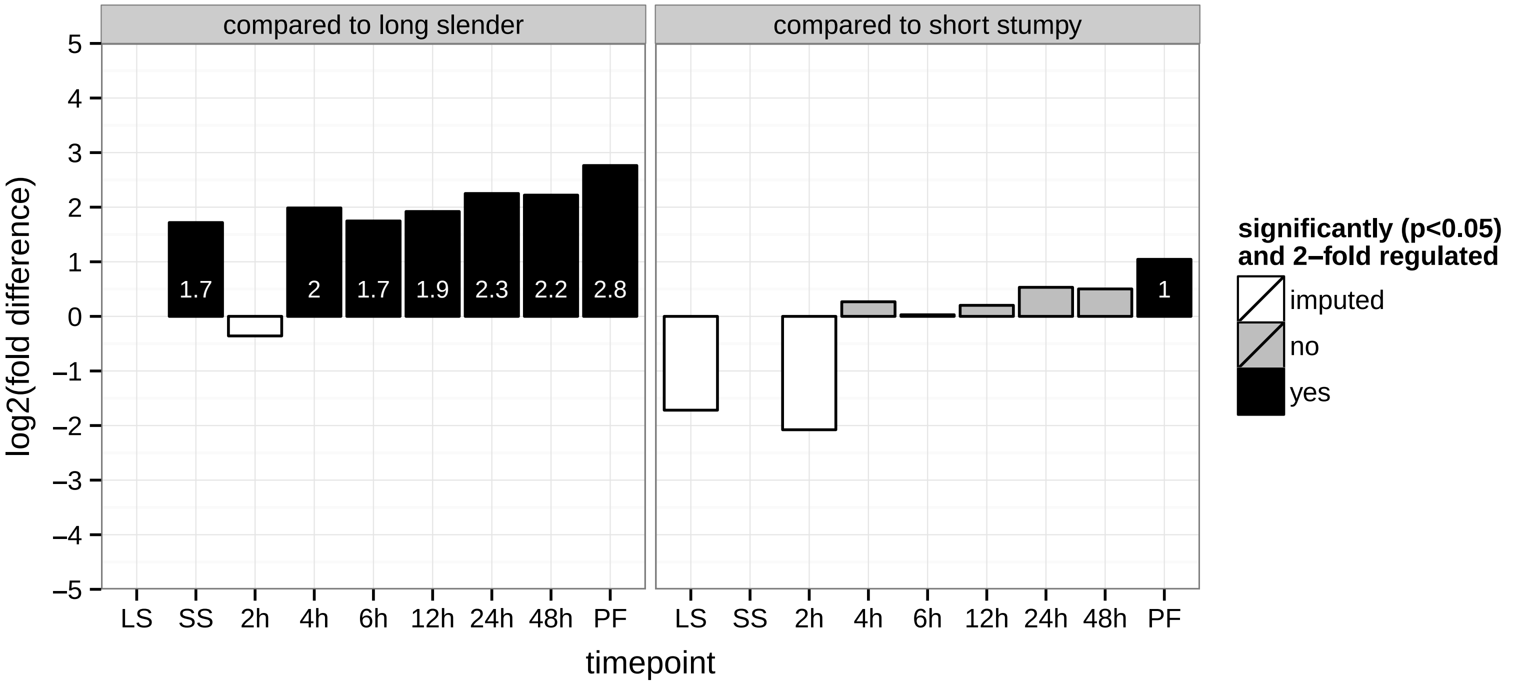

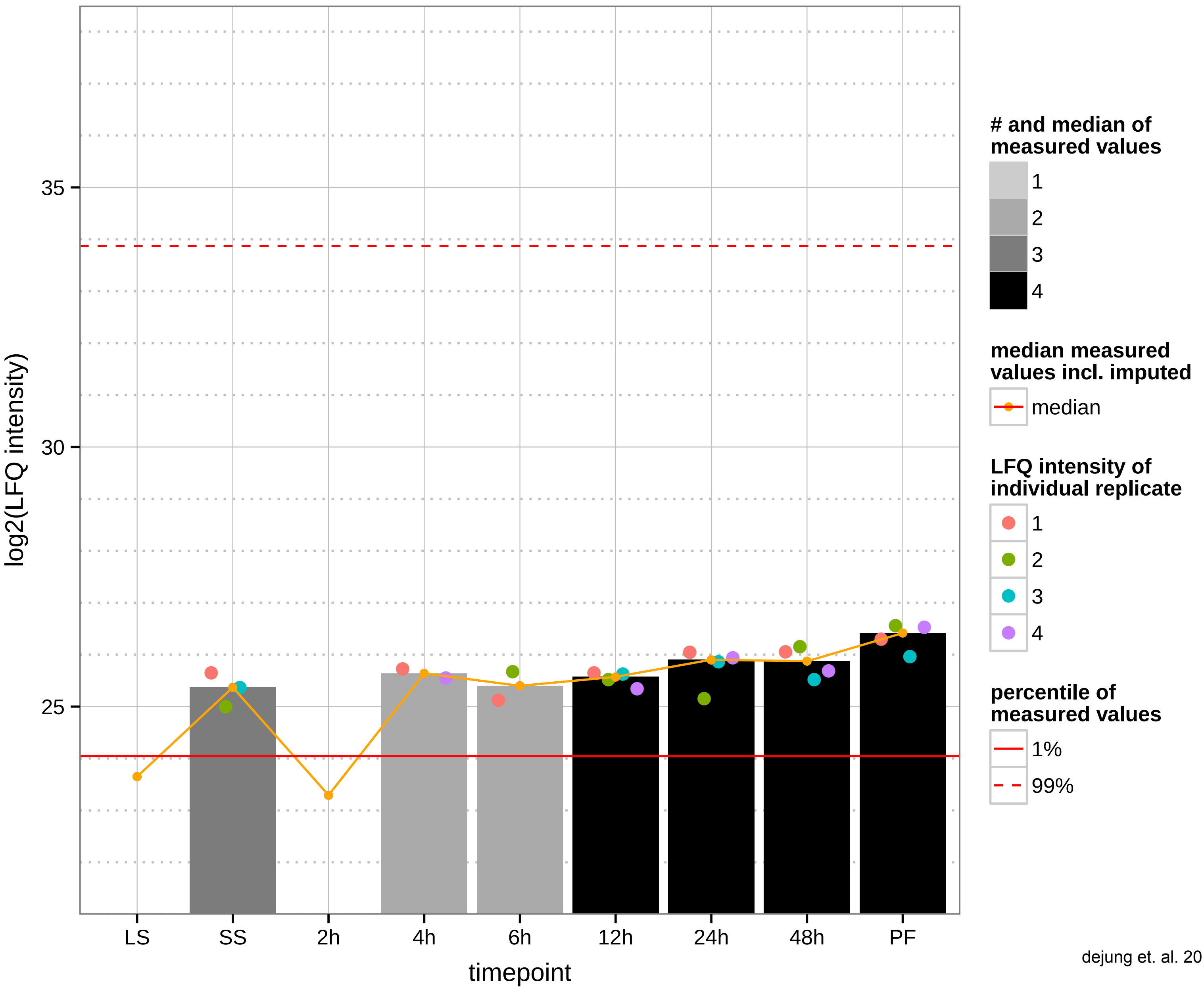

dejung et. al. 2015

hypothetical protein, conserved
Tb927.4.1900
AGOF: methyltransferase activity
AGOC: null
AGOP: metabolic process
PGOF: null
PGOC: null
PGOP: null

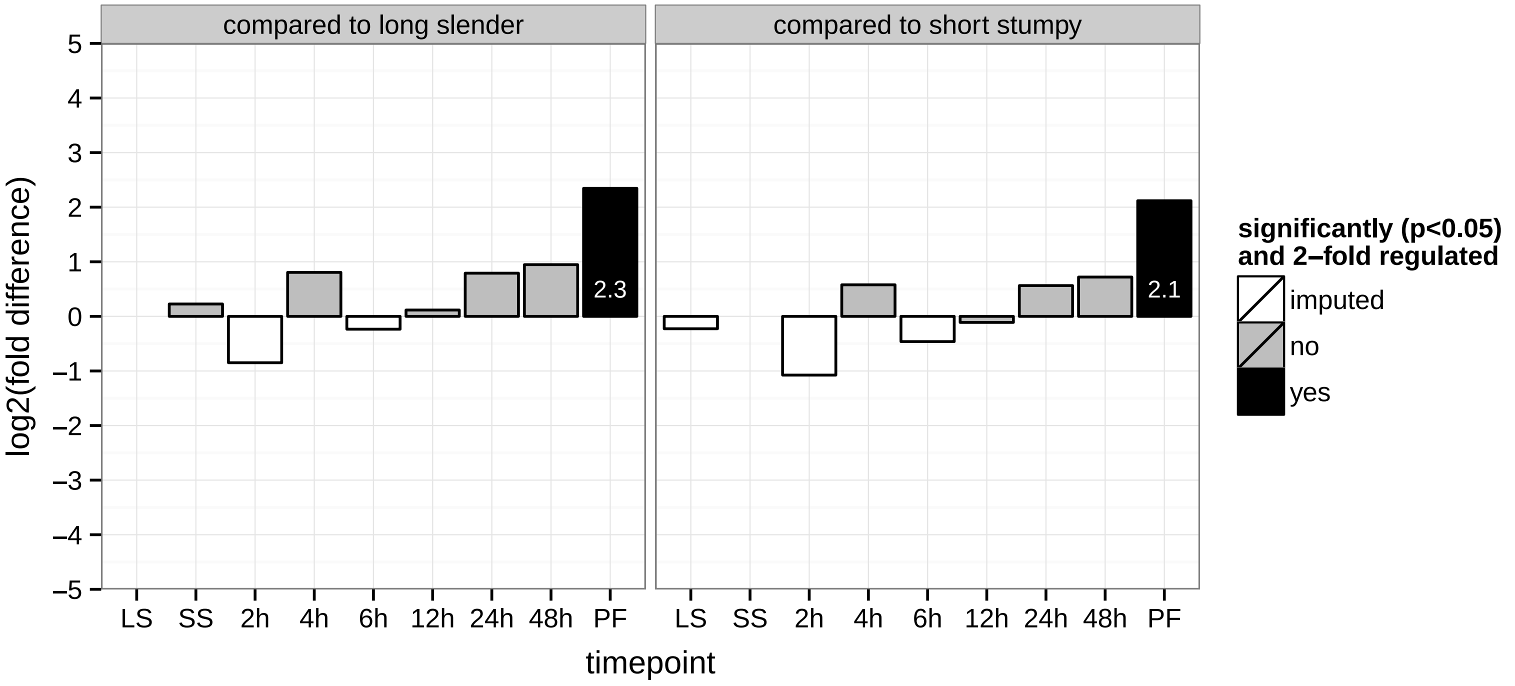

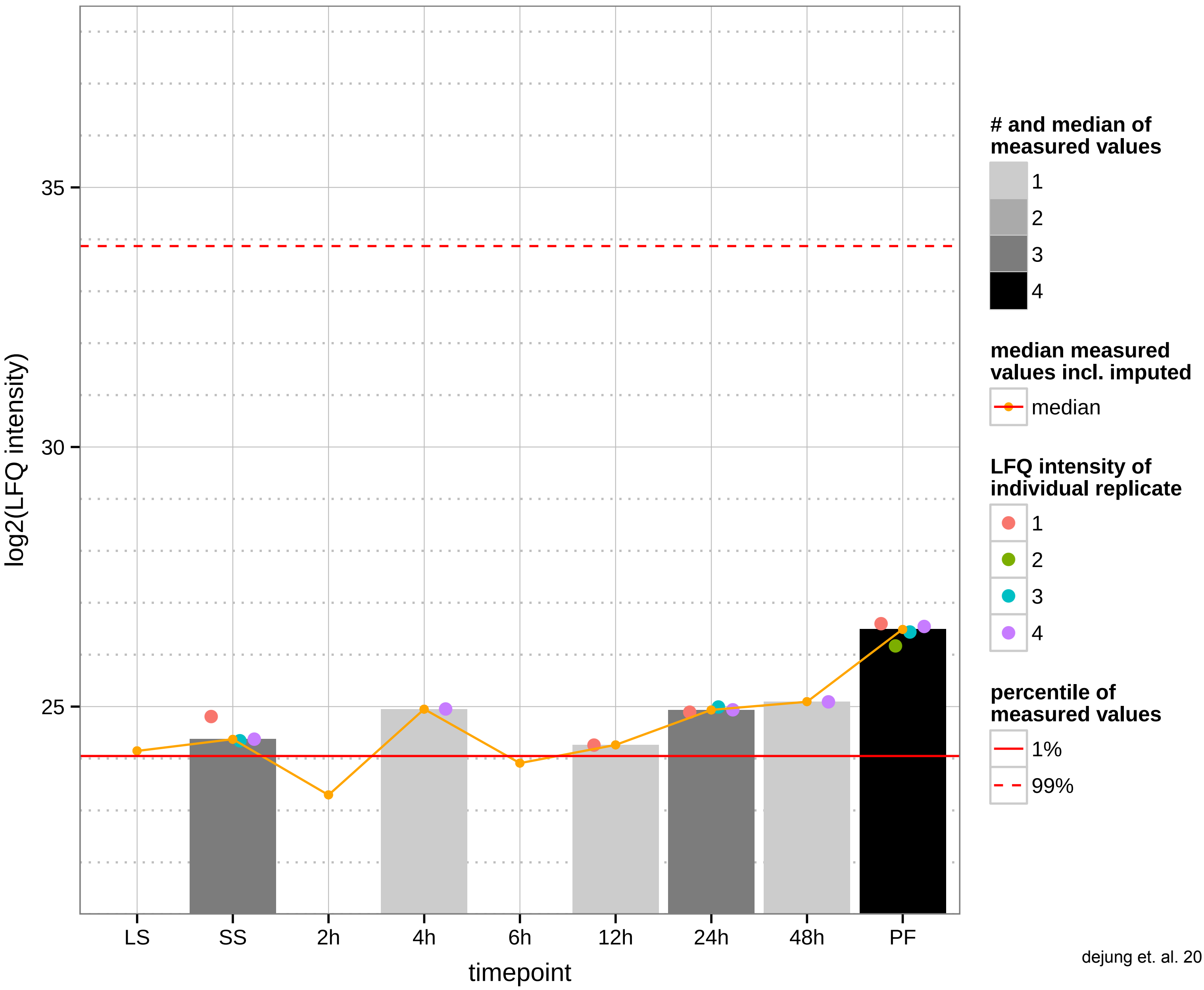

dejung et. al. 2015

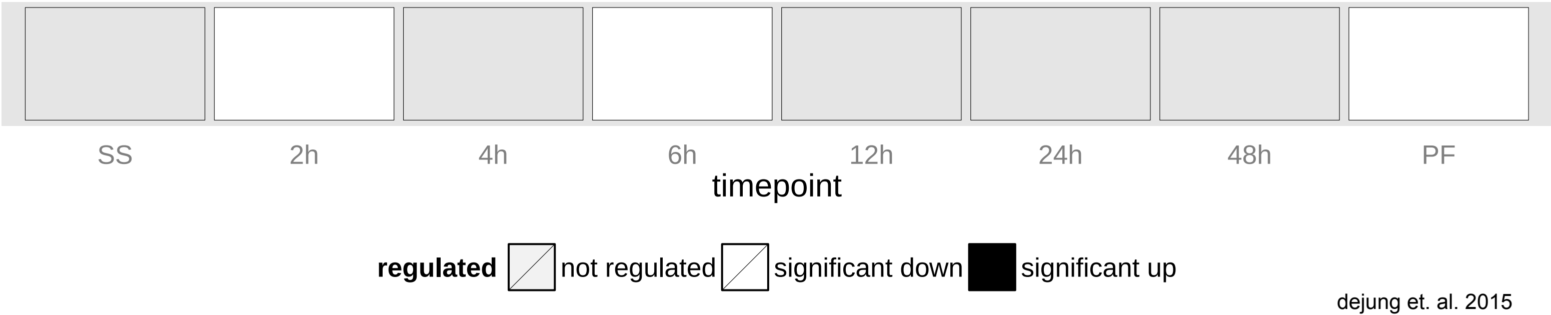
SS
2h
4h
6h
12h
24h
48h
PF
timepoint
regulated
not regulated
significant down
significant up
dejung et. al. 2015

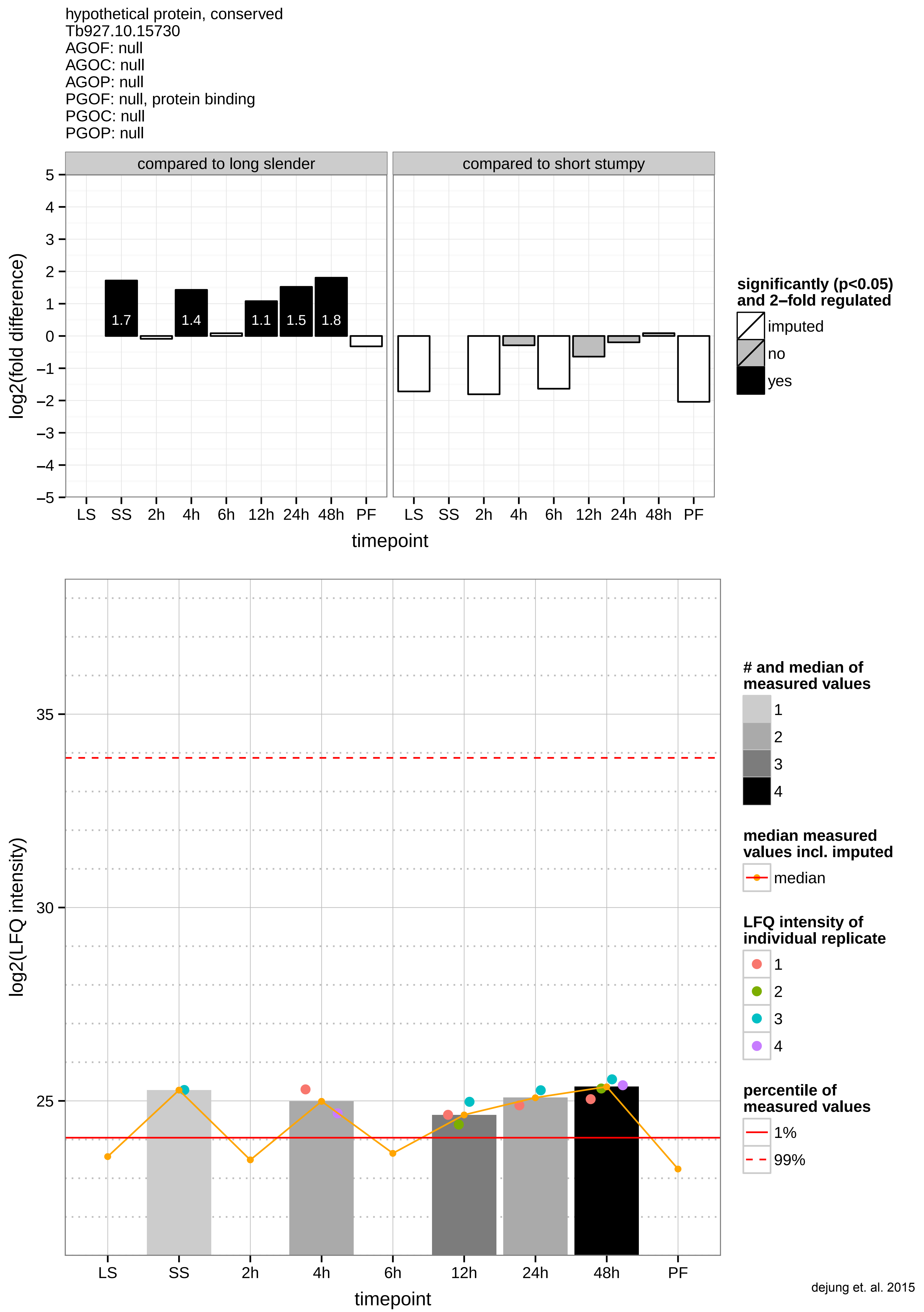

hypothetical protein, conserved
Tb927.10.15730
AGOF: null
AGOC: null
AGOP: null
PGOF: null, protein binding
PGOC: null
PGOP: null
compared to long slender
compared to short stumpy
log2(fold difference)
1.7
1.4
1.1
1.5
1.8
LS
SS
2h
4h
6h
12h
24h
48h
PF
timepoint
significantly (p<0.05) and 2–fold regulated
imputed
no
yes
log2(LFQ intensity)
# and median of measured values
1
2
3
4
median measured values incl. imputed
median
LFQ intensity of individual replicate
percentile of measured values
1%
99%
dejung et. al. 2015

protein phosphatase 2A, putative
Tb927.11.11940
AGOF: hydrolase activity
AGOC: null
AGOP: null
PGOF: hydrolase activity
PGOC: null
PGOP: null

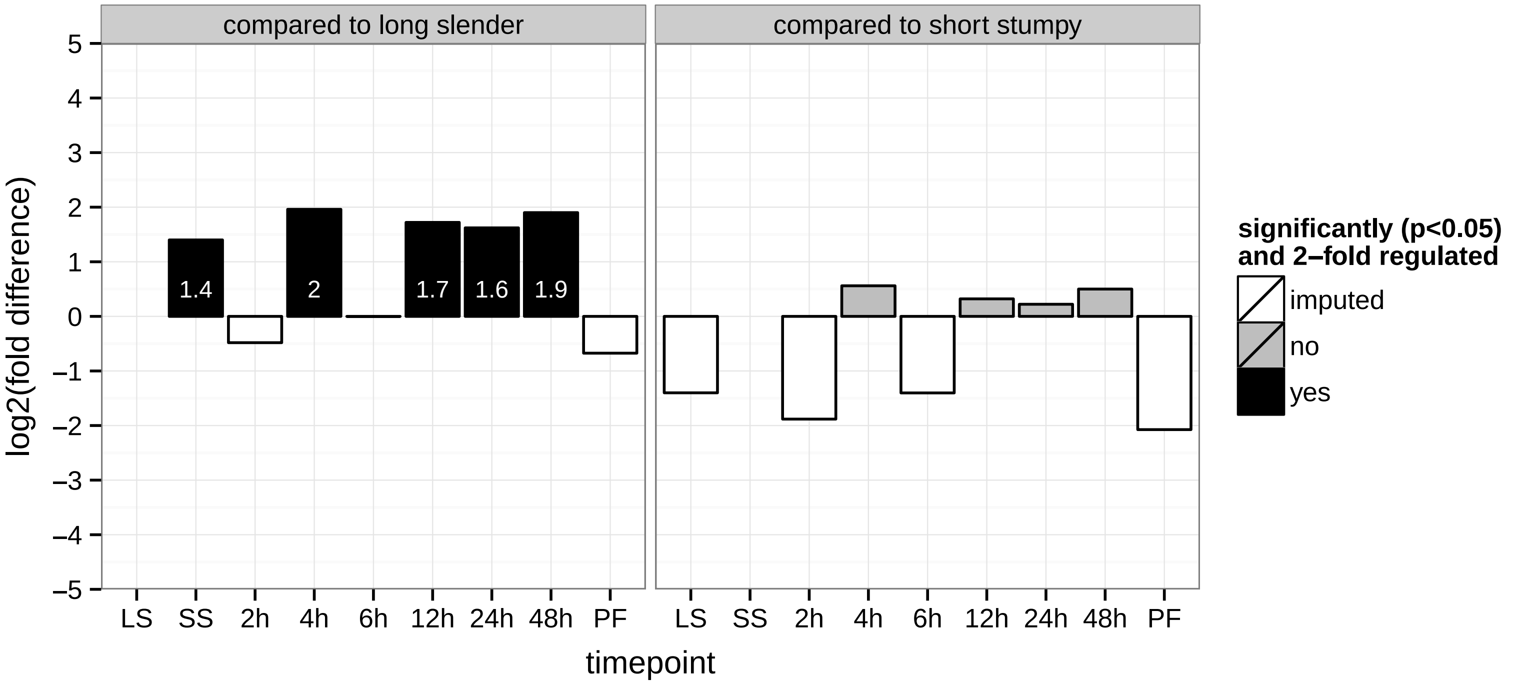

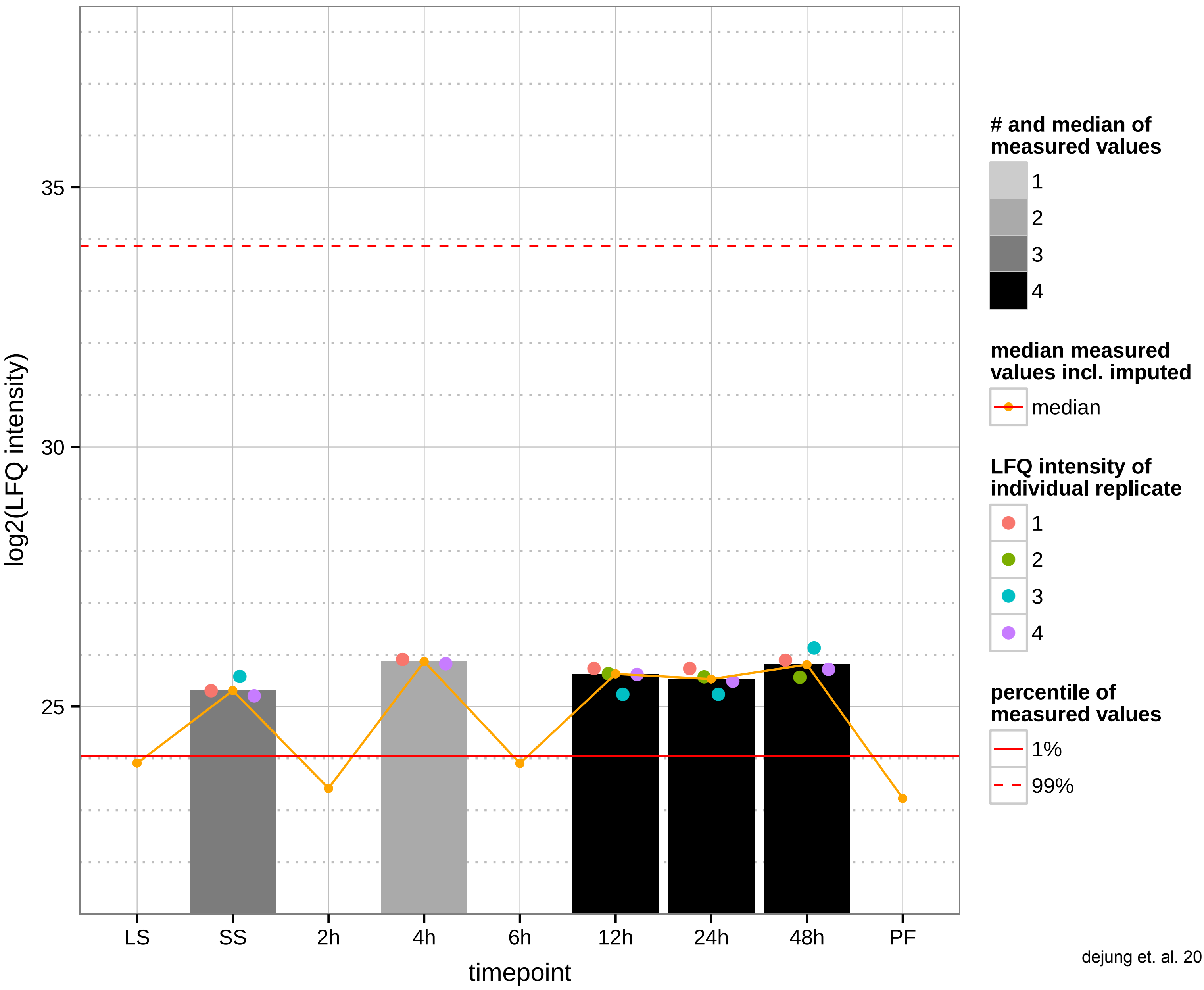

dejung et. al. 2015

ATP−dependent DEAD/H RNA helicase, putative
Tb927.11.15460
AGOF: ATP binding, ATP−dependent helicase activity, helicase activity, nucleic acid binding
AGOC: null
AGOP: nucleobase−containing compound metabolic process
PGOF: ATP binding, ATP−dependent helicase activity, helicase activity, nucleic acid binding
PGOC: null
PGOP: null

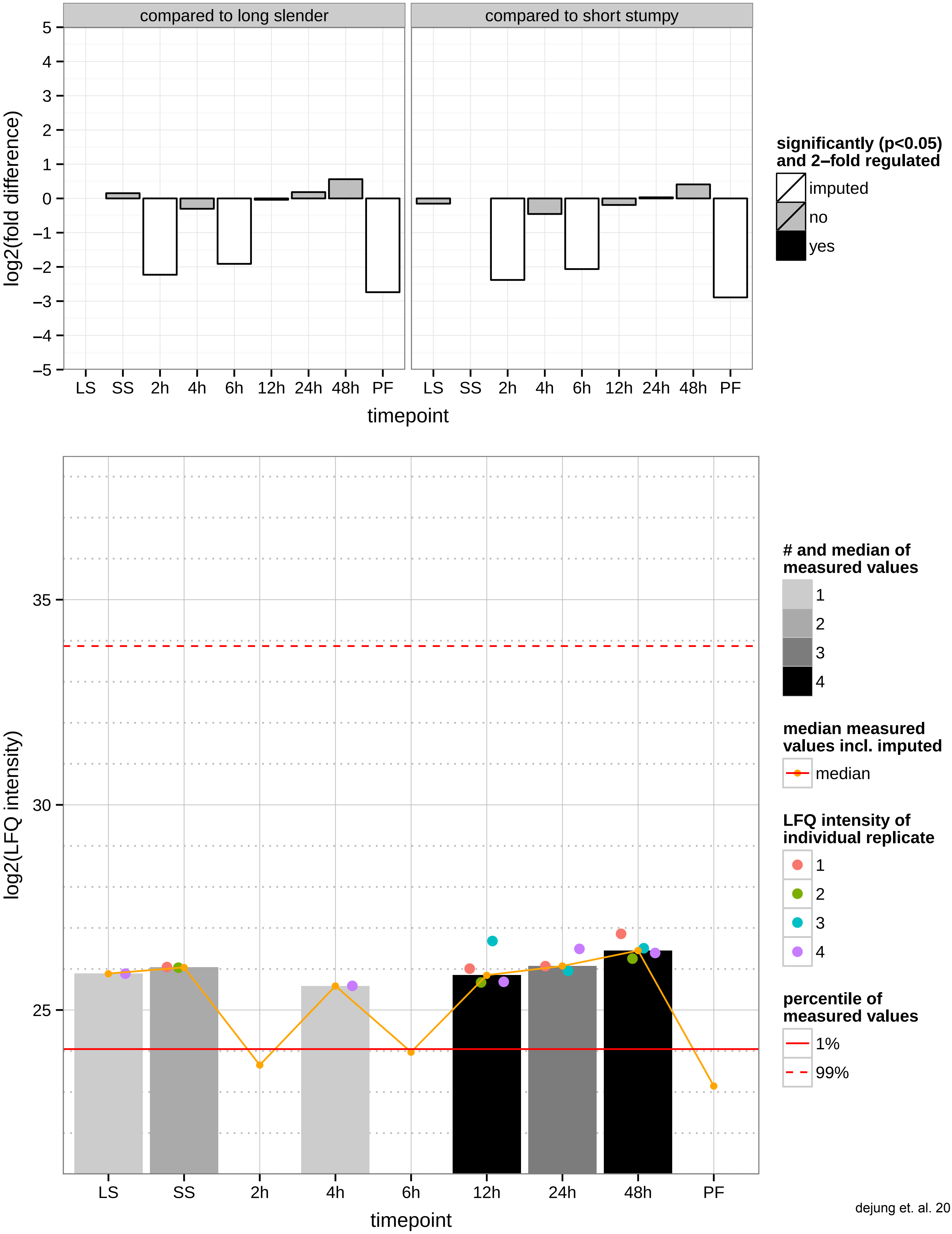

dejung et. al. 2015

unspecified product
Tb927.9.11280
AGOF: null
AGOC: null
AGOP: null
PGOF: structural constituent of ribosome
PGOC: intracellular, ribosome
PGOP: translation

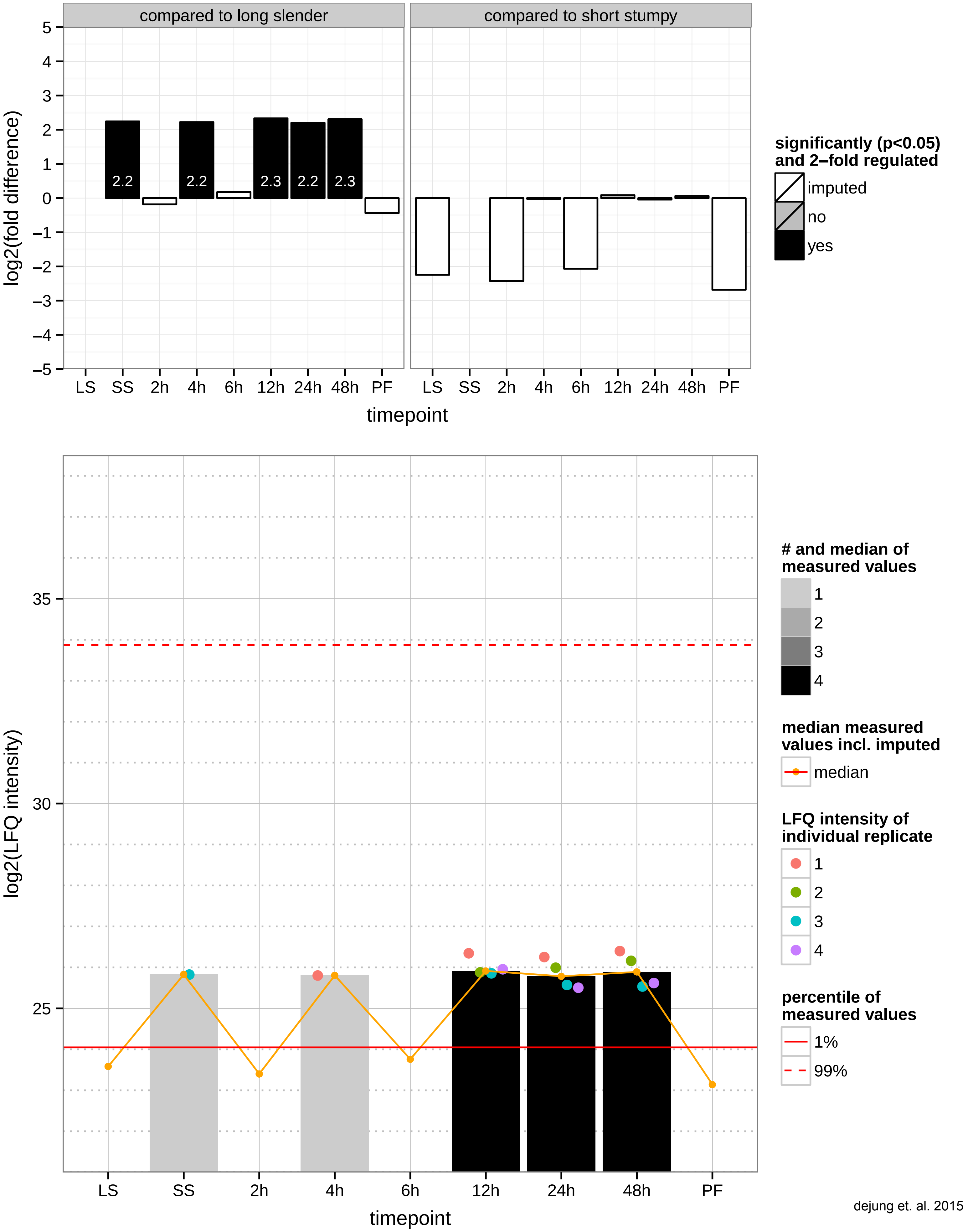

dejung et. al. 2015

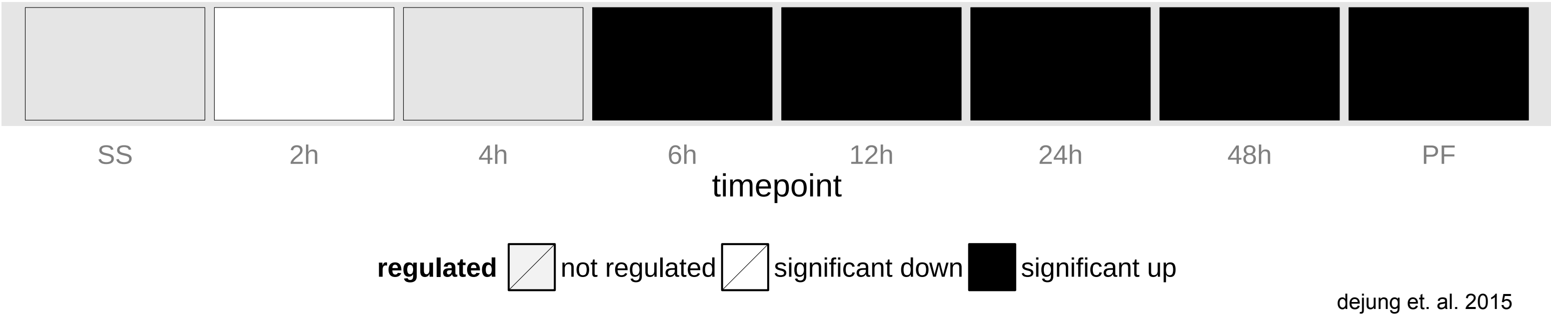
SS
2h
4h
6h
12h
24h
48h
PF
timepoint
regulated
not regulated
significant down
significant up
dejung et. al. 2015

hypothetical protein, conserved
Tb927.11.240
AGOF: null
AGOC: mitochondrion
AGOP: null
PGOF: null
PGOC: null
PGOP: null

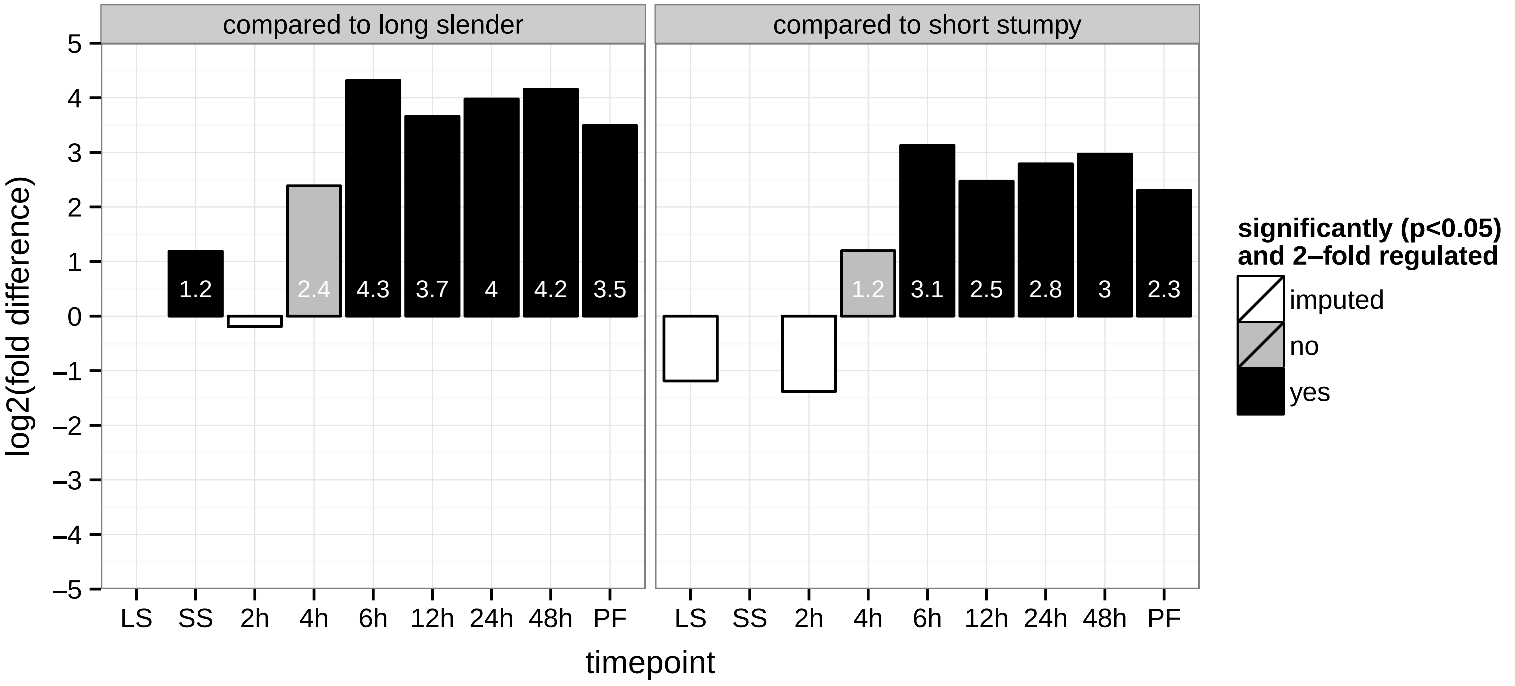

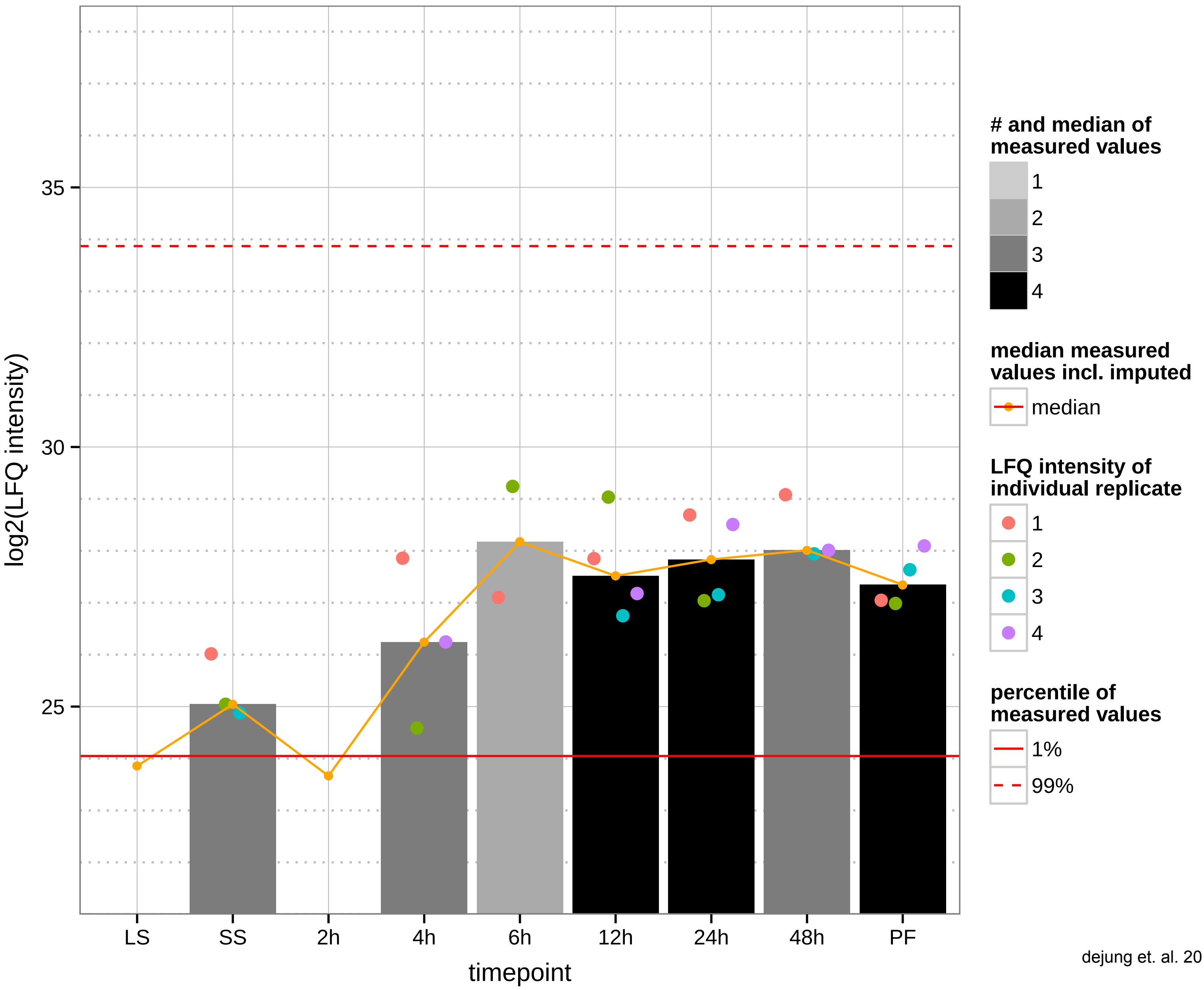

dejung et. al. 2015

hypothetical protein, conserved
Tb927.4.3370
AGOF: null
AGOC: null
AGOP: null
PGOF: binding
PGOC: null
PGOP: null

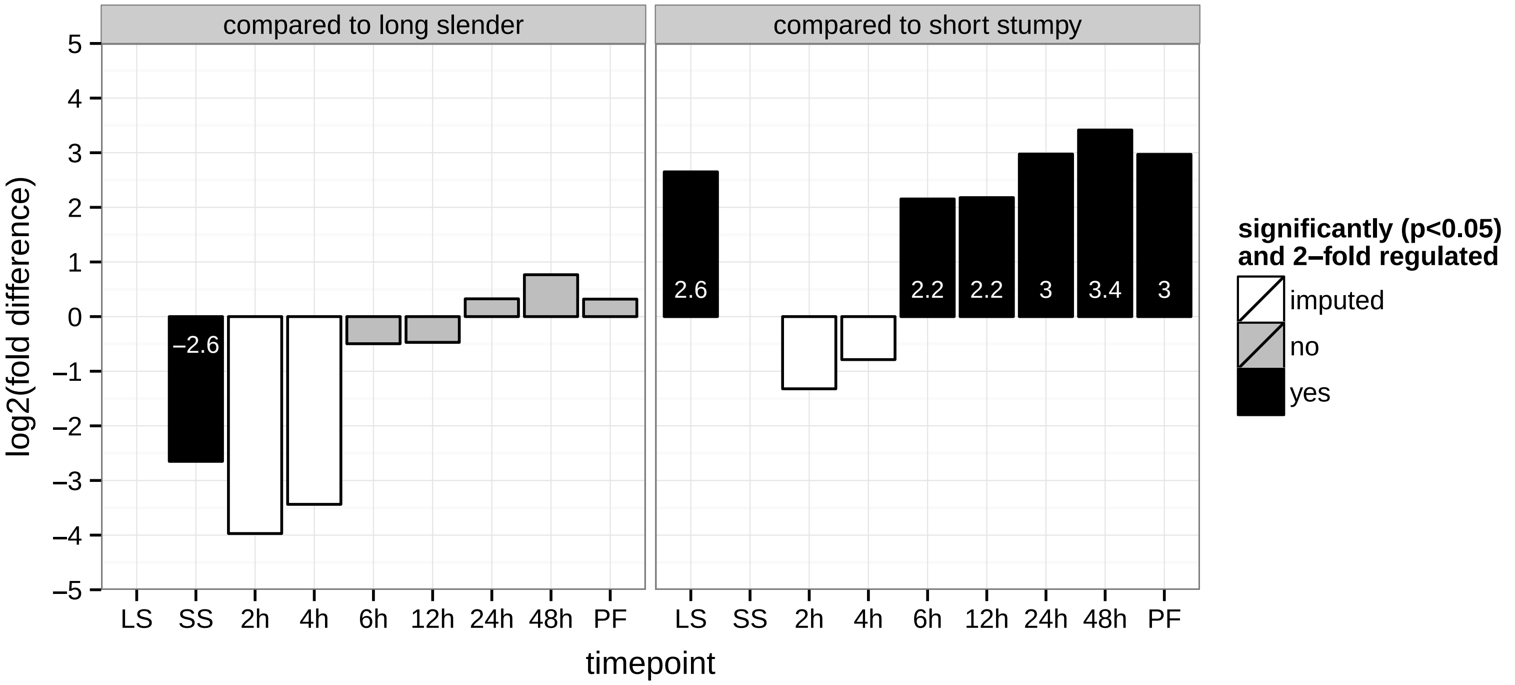

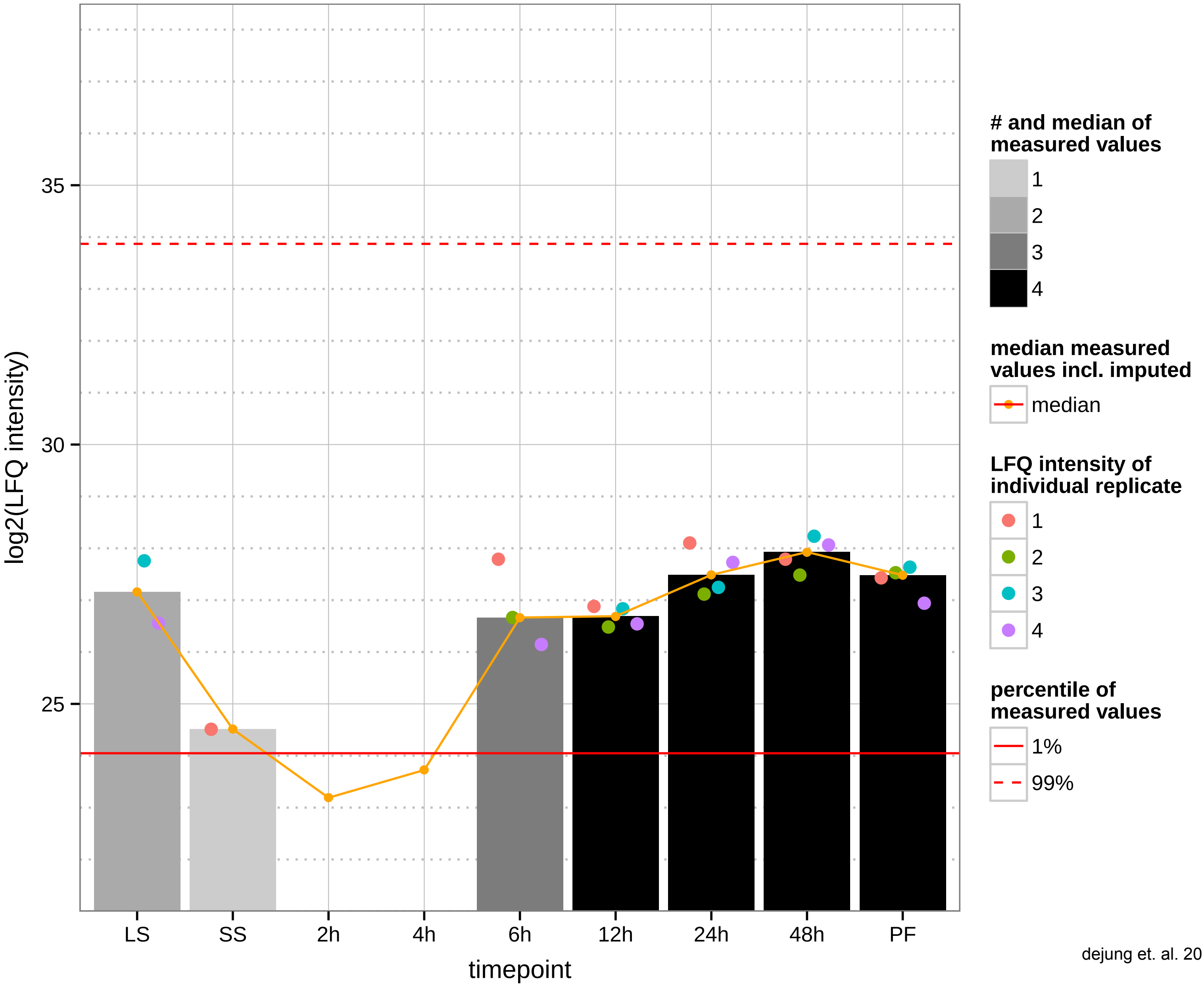

dejung et. al. 2015

mitochondrial processing peptidase, beta subunit, putative, metallo−peptidase, Clan ME, Family M16
Tb927.5.1060
AGOF: catalytic activity, metalloendopeptidase activity, zinc ion binding
AGOC: mitochondrion
AGOP: proteolysis
PGOF: catalytic activity, metal ion binding, metalloendopeptidase activity, zinc ion binding
PGOC: null
PGOP: proteolysis

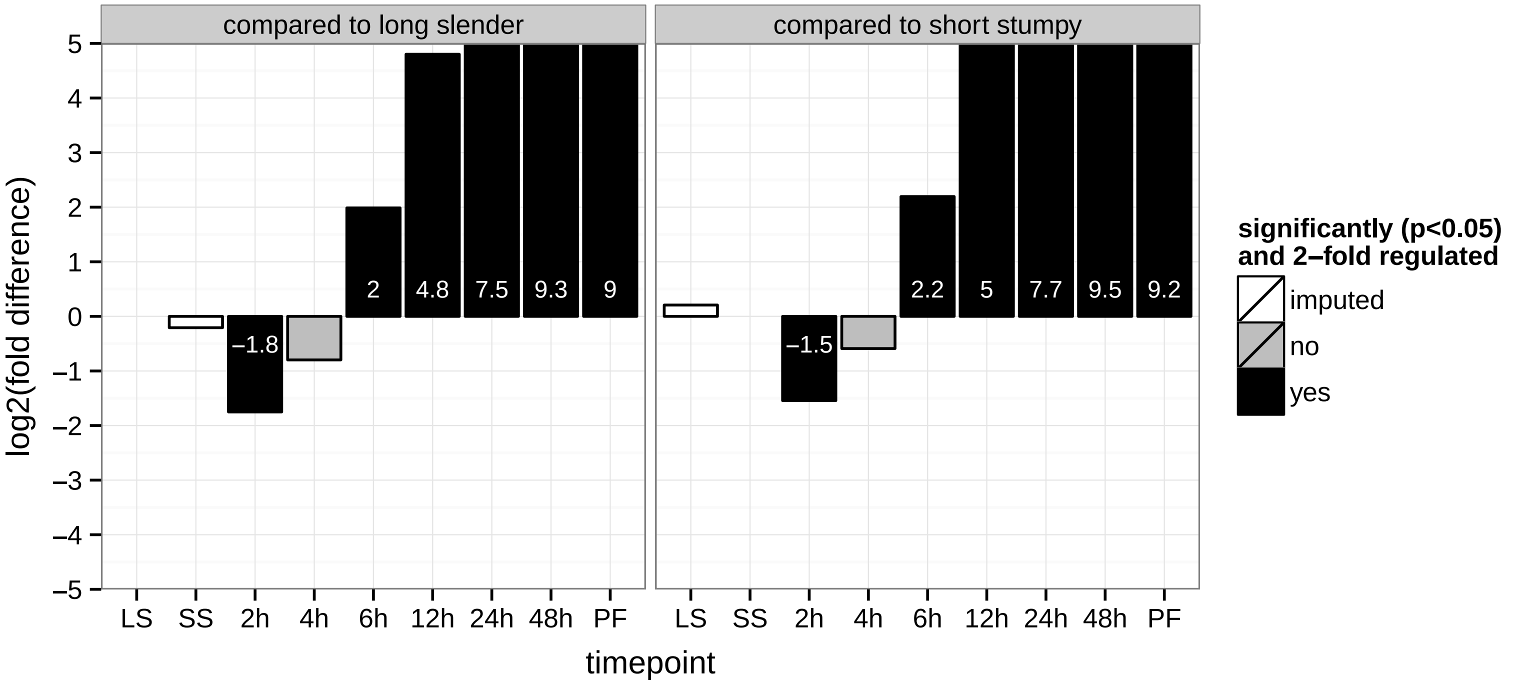

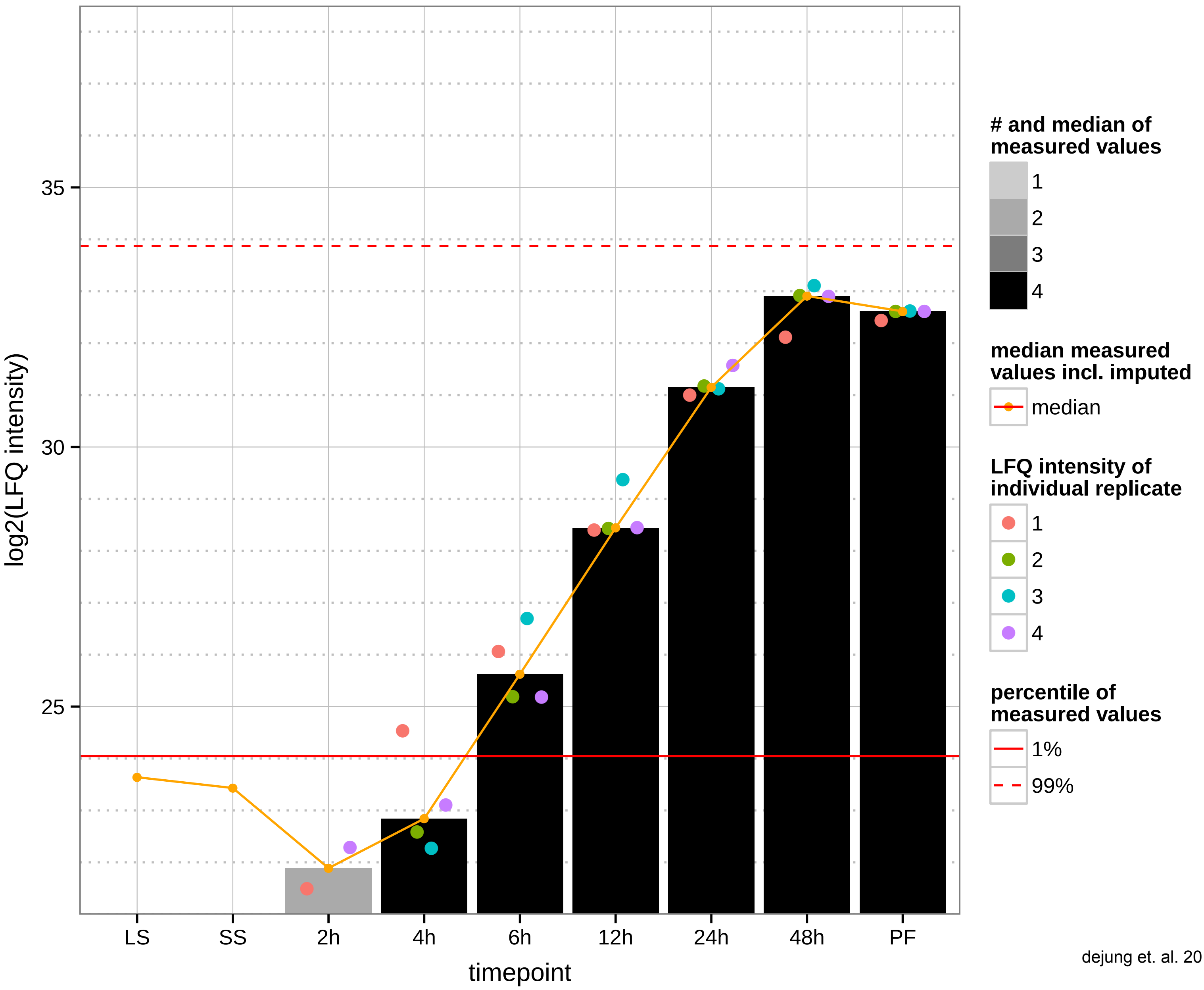

dejung et. al. 2015

hypothetical protein, conserved
Tb927.8.5200
AGOF: null
AGOC: mitochondrion
AGOP: null
PGOF: null
PGOC: null
PGOP: null

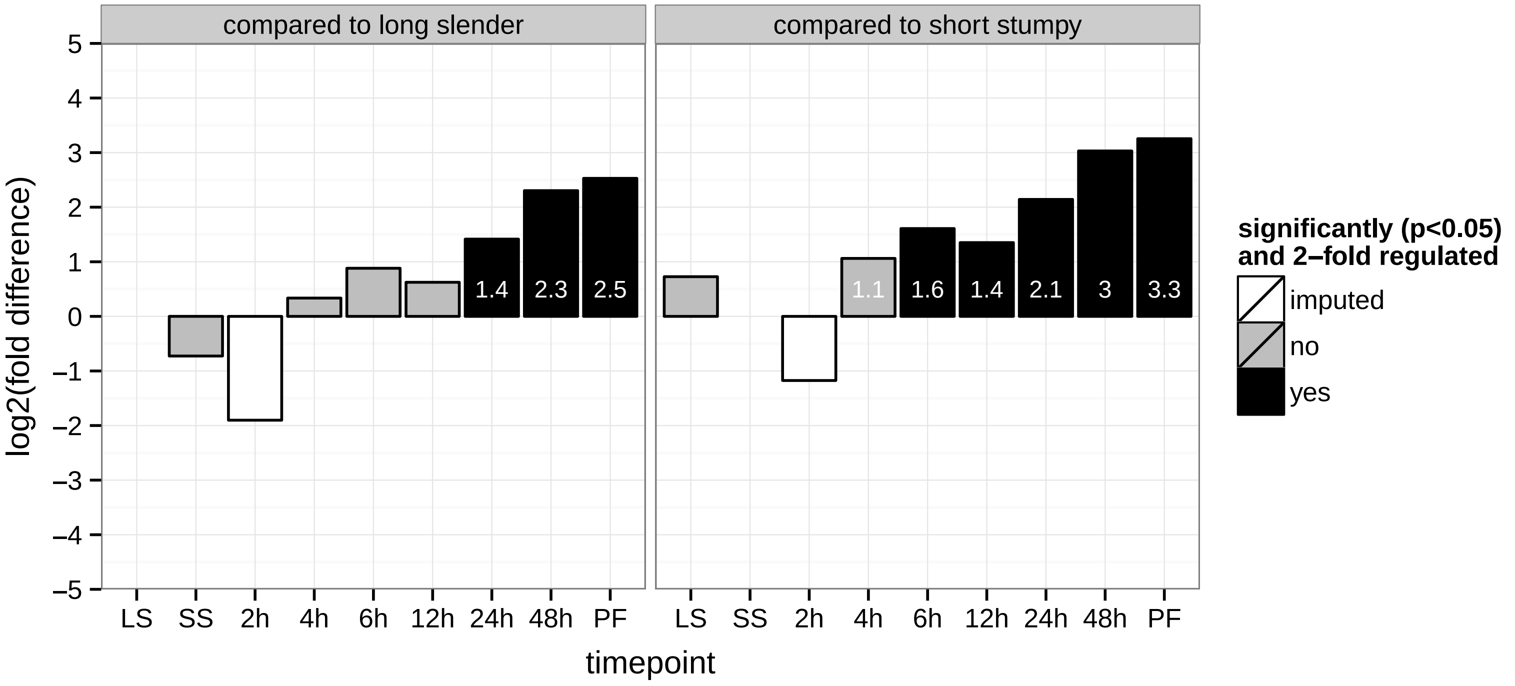

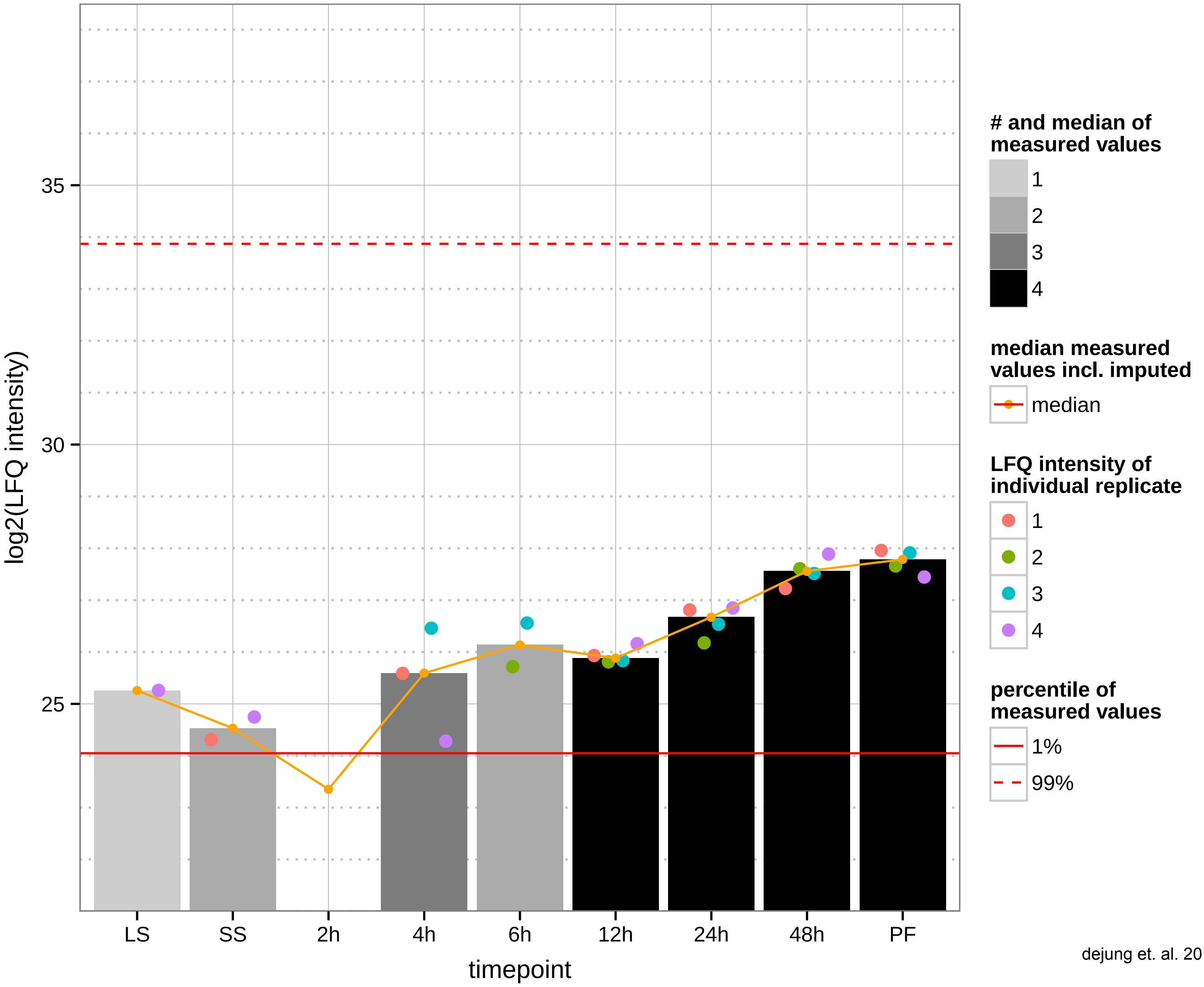

dejung et. al. 2015

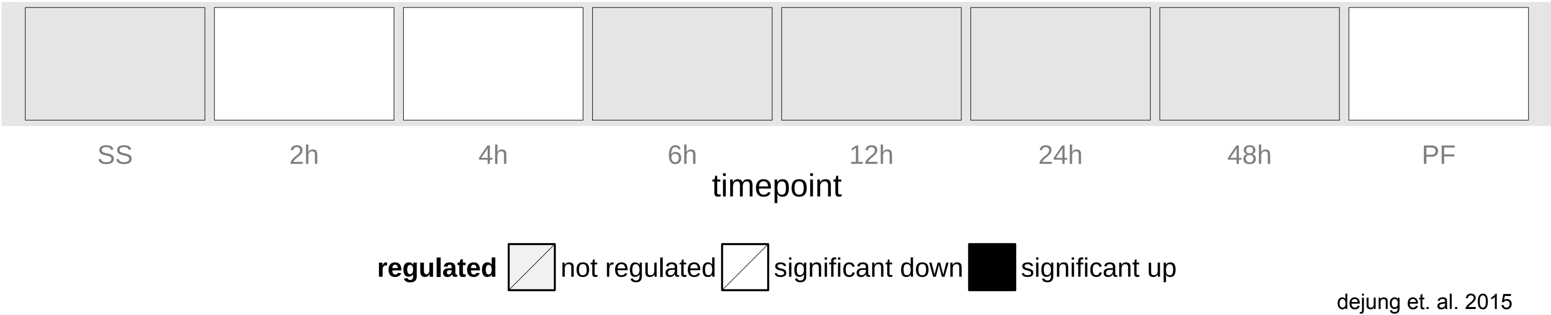
SS
2h
4h
6h
12h
24h
48h
PF
timepoint
regulated
not regulated
significant down
significant up
dejung et. al. 2015

hypothetical protein, conserved
Tb927.10.14760
AGOF: structural molecule activity
AGOC: clathrin coat of coated pit, clathrin coat of trans–Golgi network vesicle
AGOP: intracellular protein transport, vesicle–mediated transport
PGOF: structural molecule activity
PGOC: clathrin coat of coated pit, clathrin coat of trans–Golgi network vesicle
PGOP: intracellular protein transport, vesicle–mediated transport

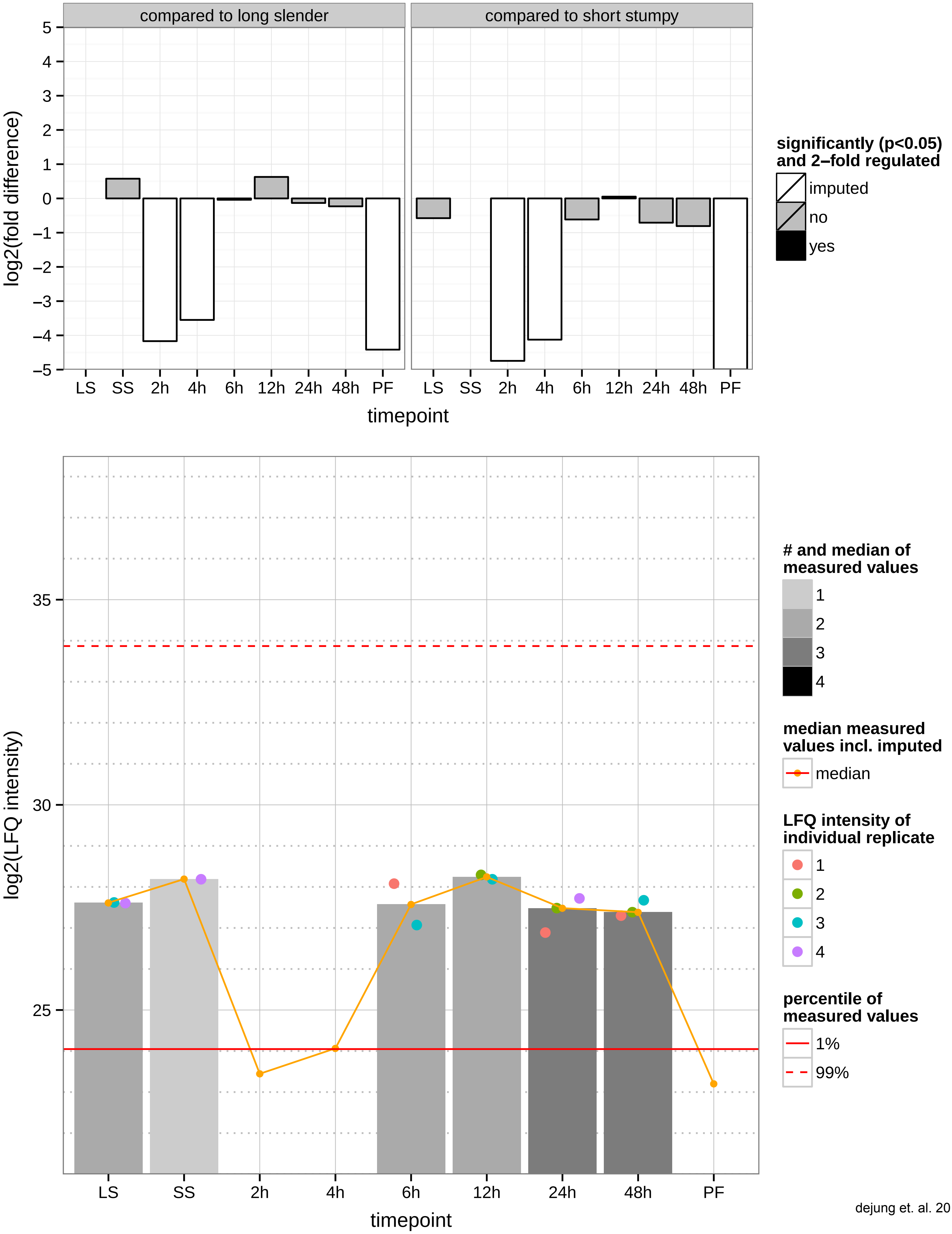

dejung et. al. 2015

hypothetical protein, conserved
Tb927.10.2130
AGOF: null
AGOC: null
AGOP: null
PGOF: null
PGOC: null
PGOP: null

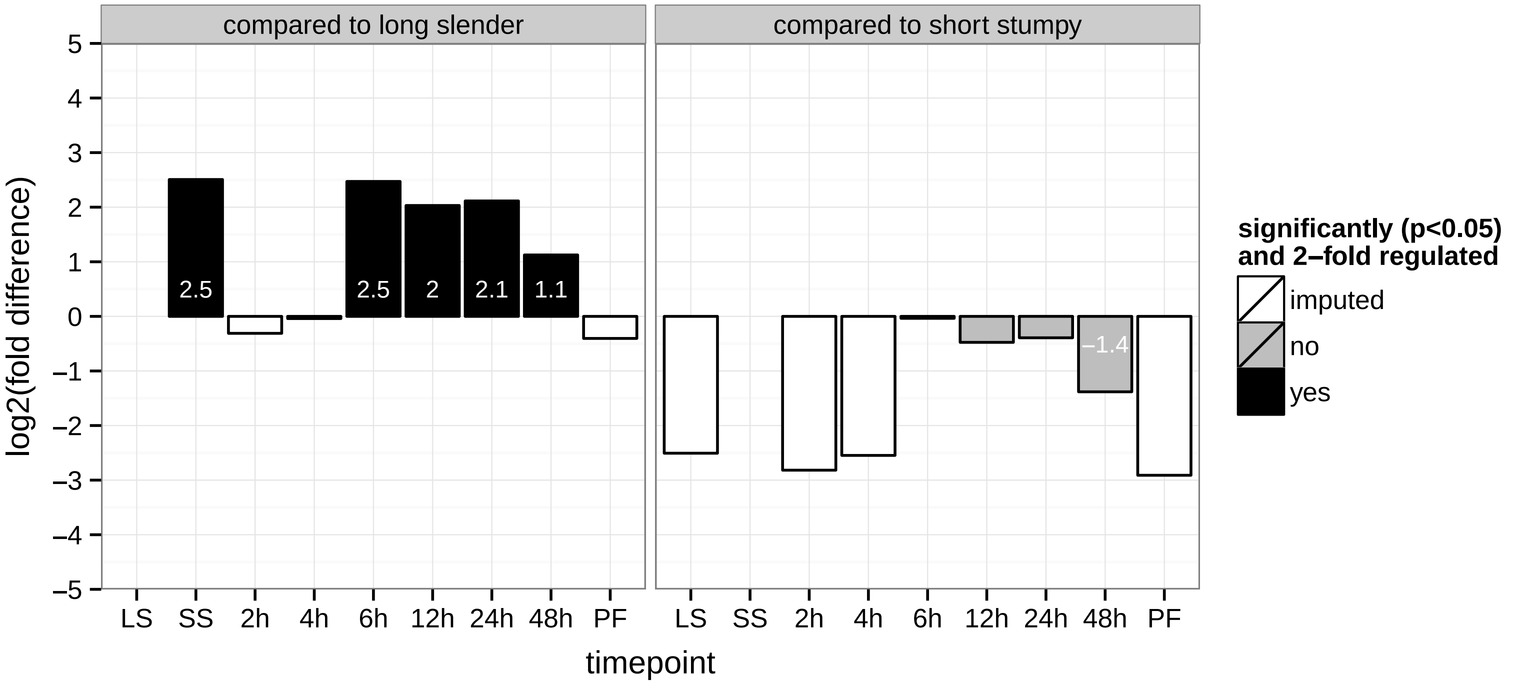

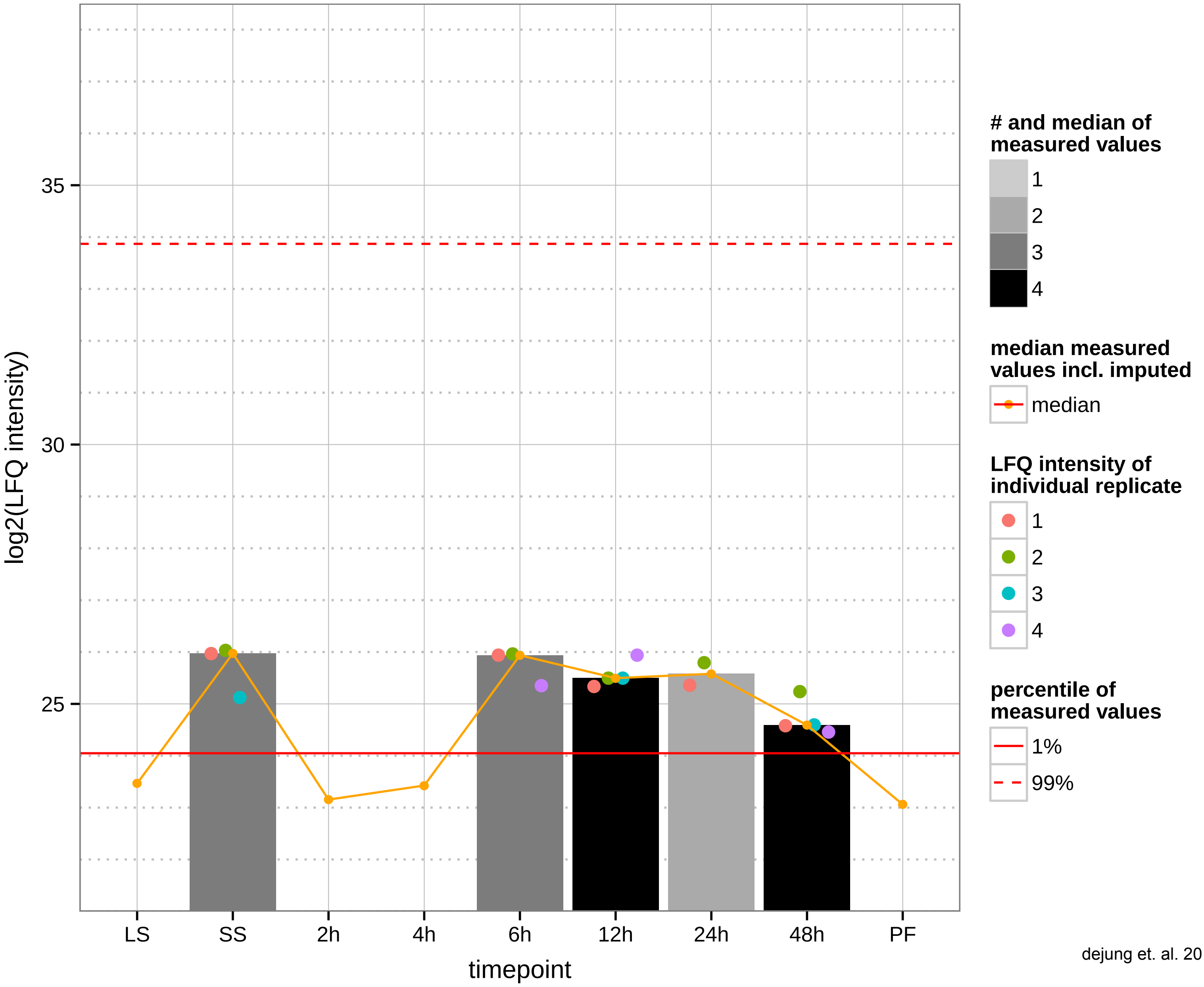

dejung et. al. 2015

TOR–like 1 (TOR–like 1)
Tb927.4.800
AGOF: phosphotransferase activity, alcohol group as acceptor
AGOC: cytosol
AGOP: cellular response to glucose starvation, regulation of response to osmotic stress
PGOF: binding, phosphotransferase activity, alcohol group as acceptor, protein binding, transferase activity, transferring phosp
PGOC: null
PGOP: null

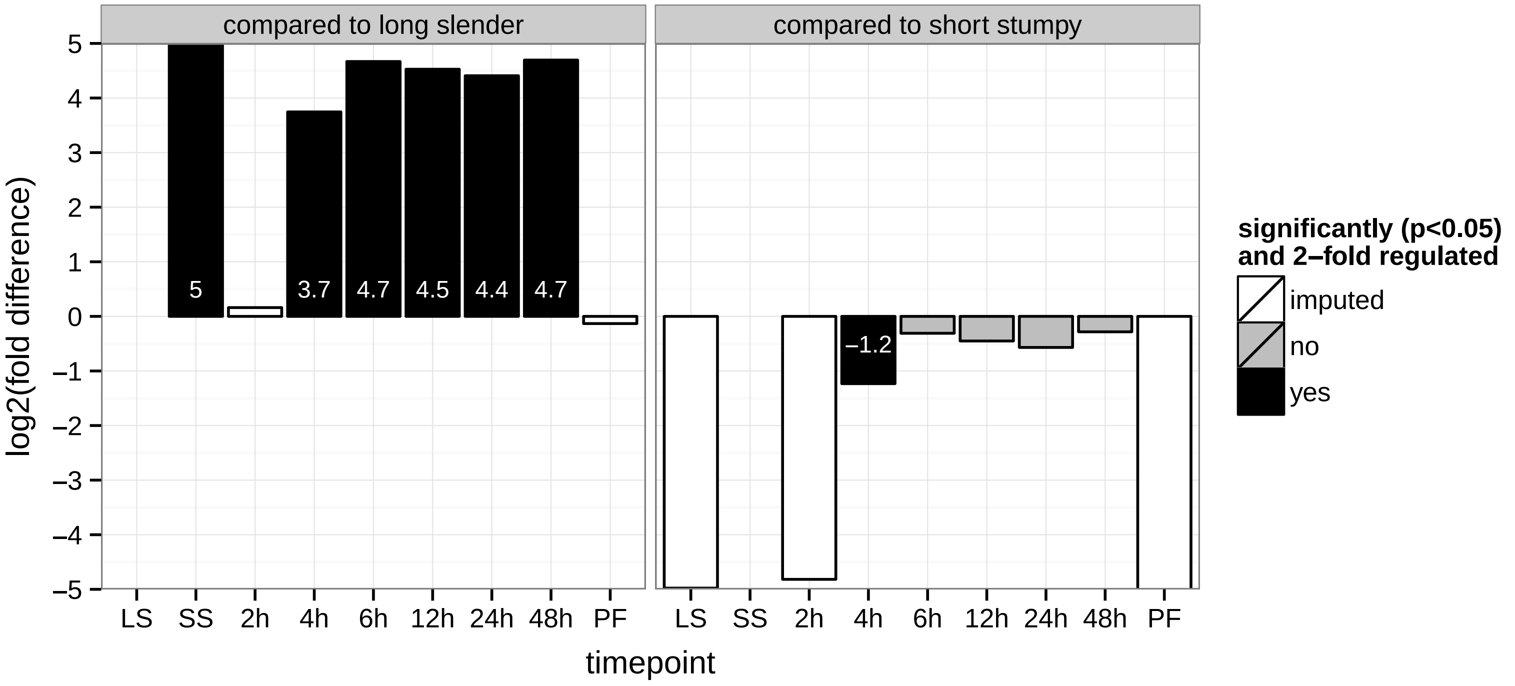

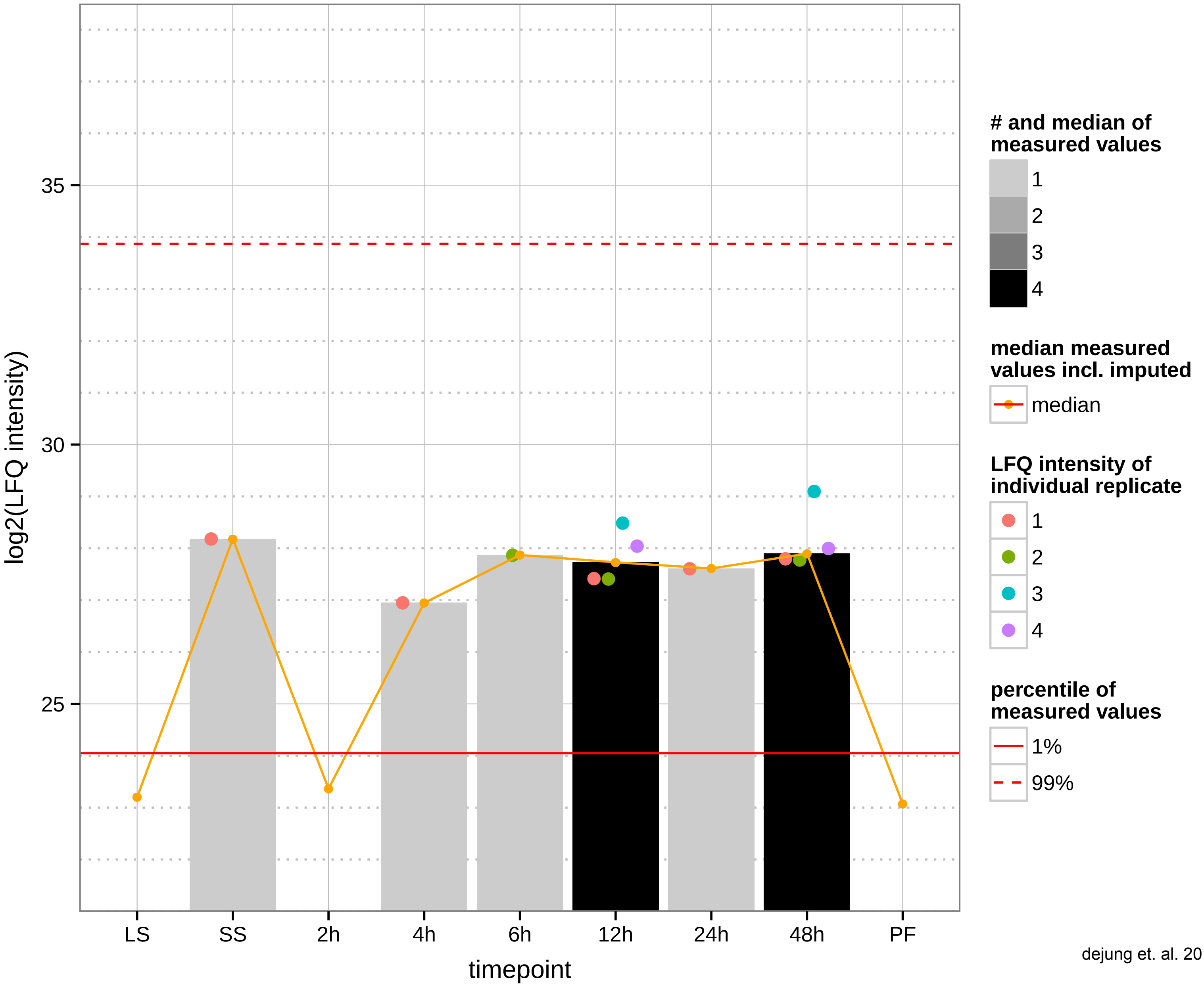

dejung et. al. 2015

hypothetical protein, conserved
Tb927.9.12780
AGOF: protein C−terminal carboxyl O−methyltransferase activity
AGOC: null
AGOP: cellular protein modification process
PGOF: methyltransferase activity, protein binding
PGOC: null
PGOP: null

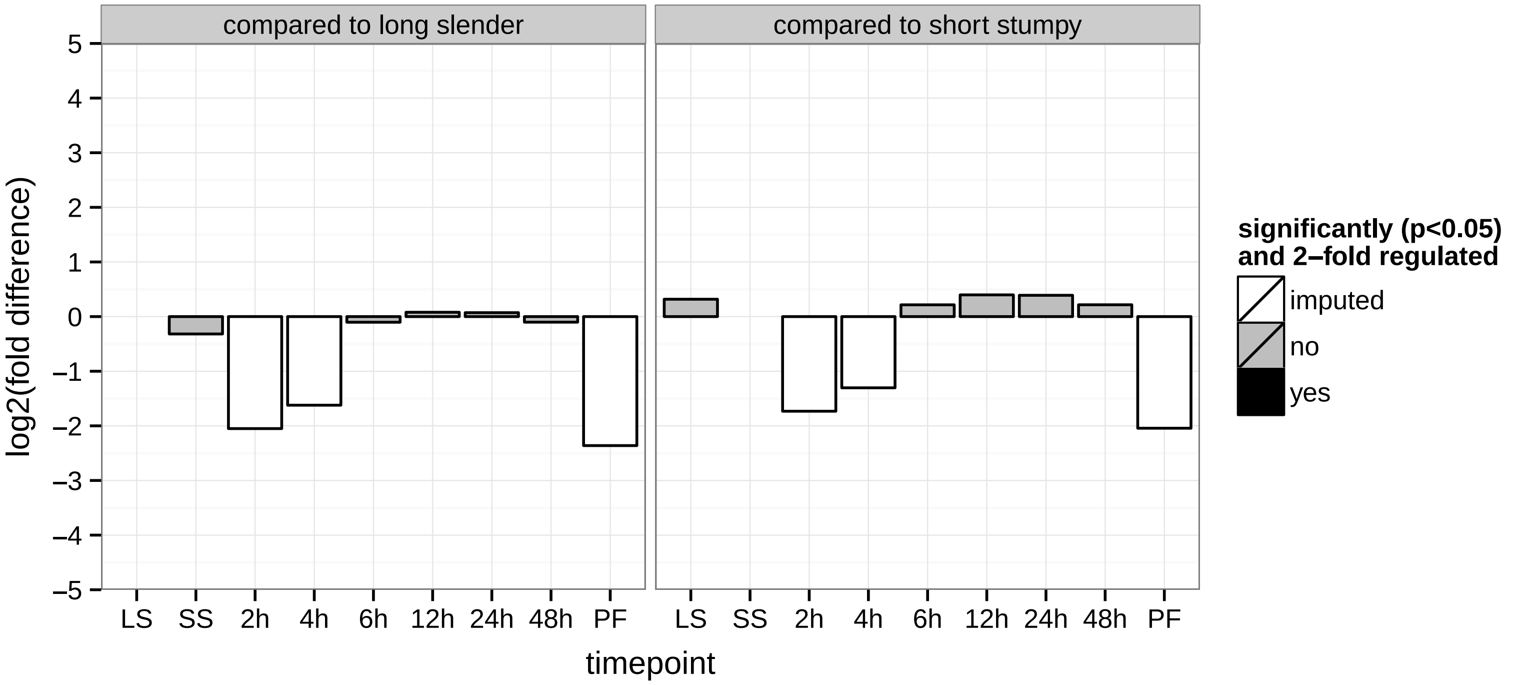

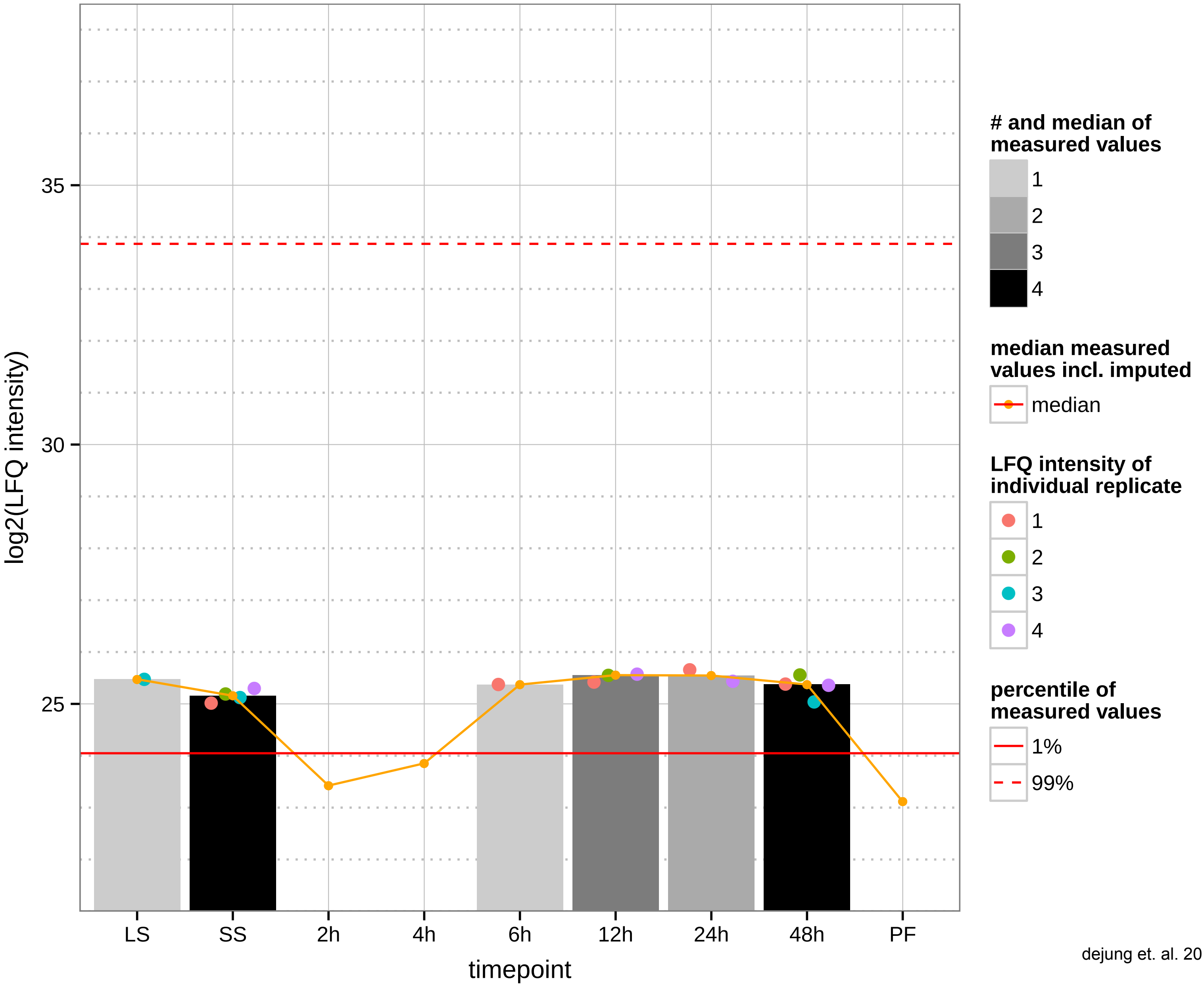

dejung et. al. 2015

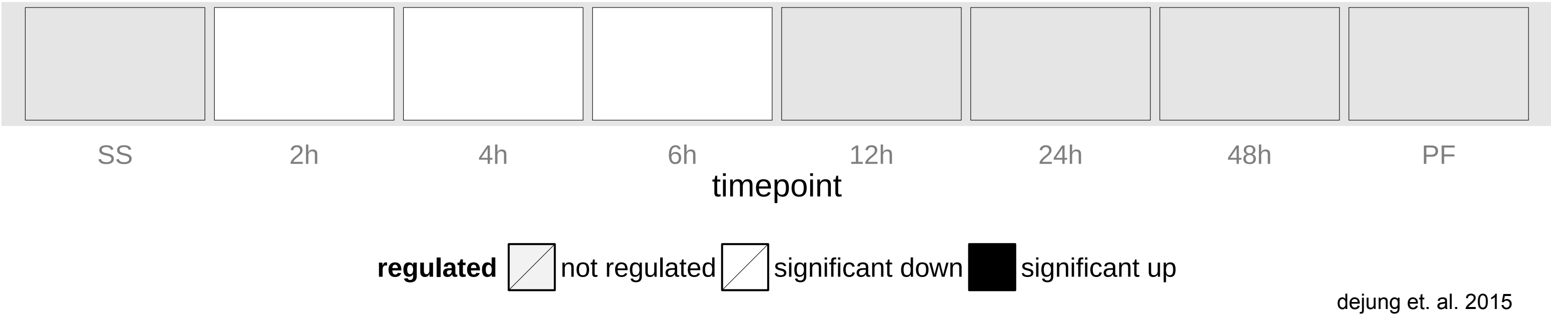
SS
2h
4h
6h
12h
24h
48h
PF
timepoint
regulated
not regulated
significant down
significant up
dejung et. al. 2015

hypothetical protein, conserved
Tb927.3.3680
AGOF: null
AGOC: mitochondrion
AGOP: null
PGOF: null
PGOC: null
PGOP: null

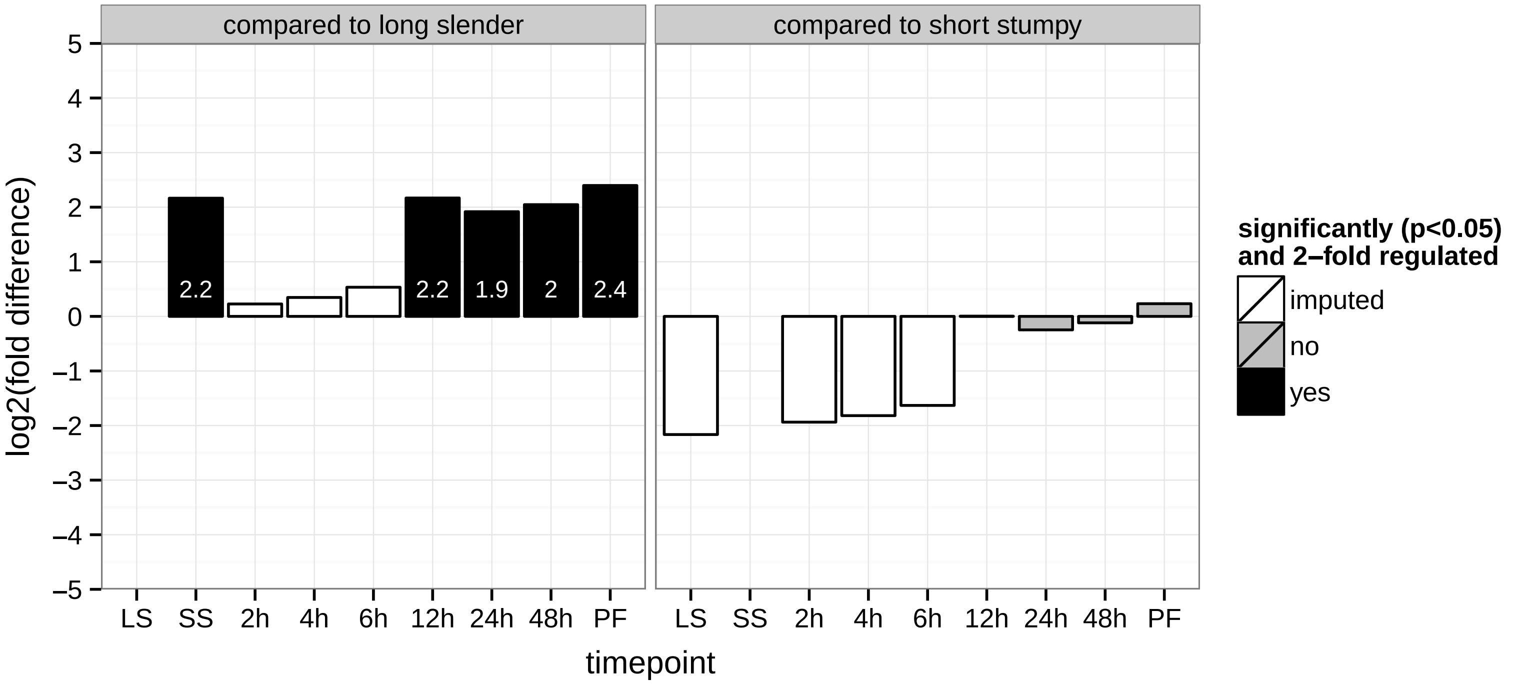

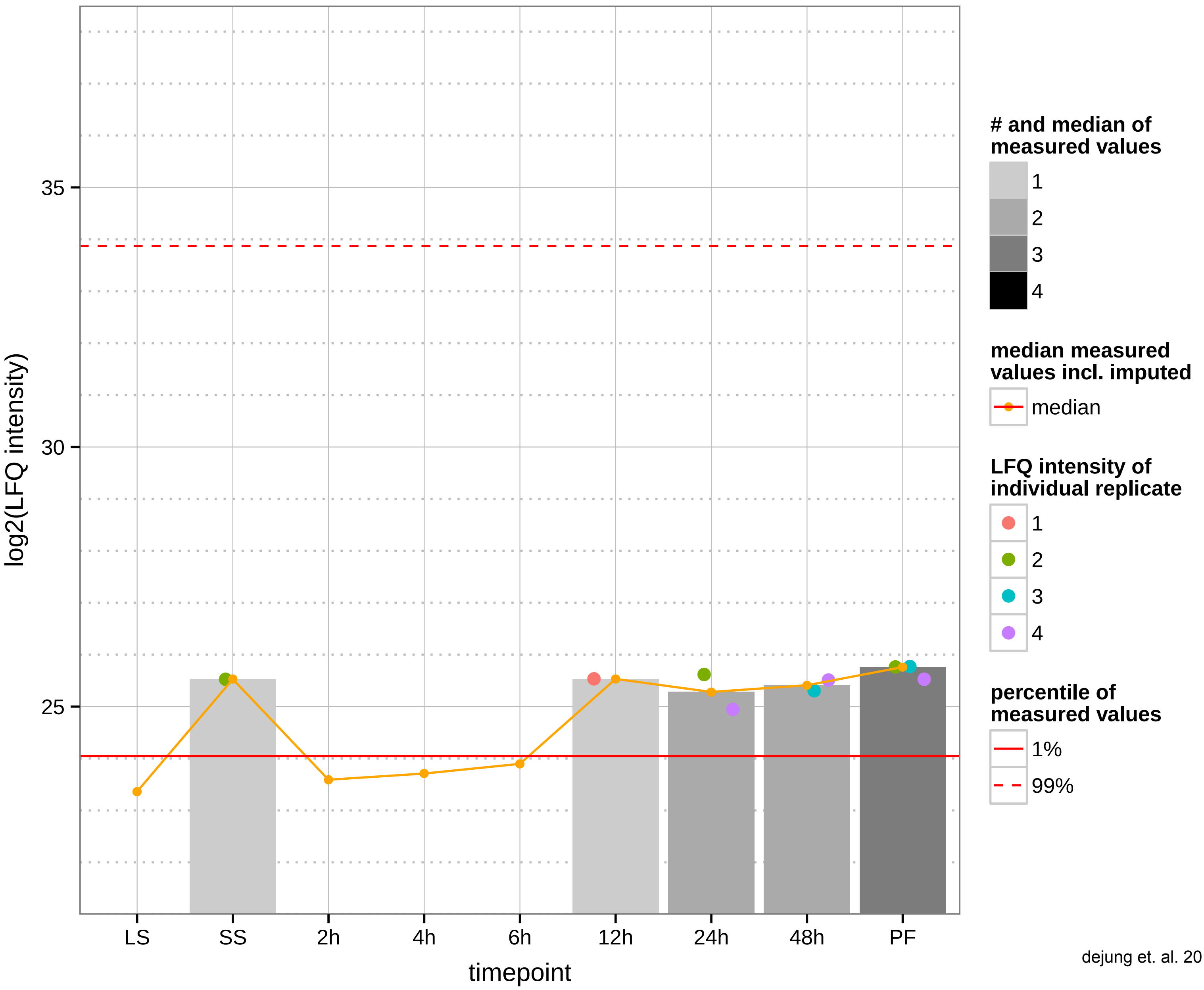

dejung et. al. 2015

hypothetical protein, conserved
Tb927.7.2760
AGOF: structural constituent of ribosome
AGOC: intracellular, mitochondrion, ribosome
AGOP: translation
PGOF: structural constituent of ribosome
PGOC: intracellular, ribosome
PGOP: translation

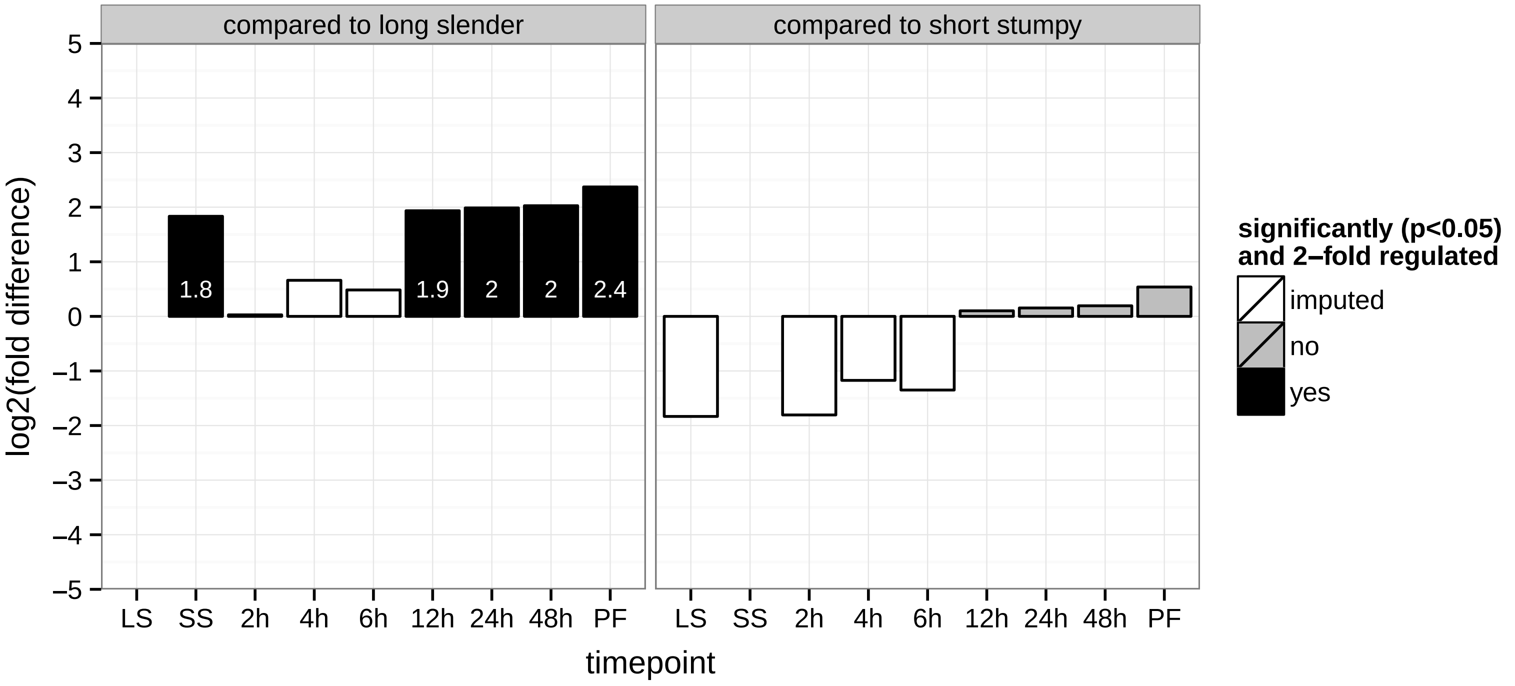

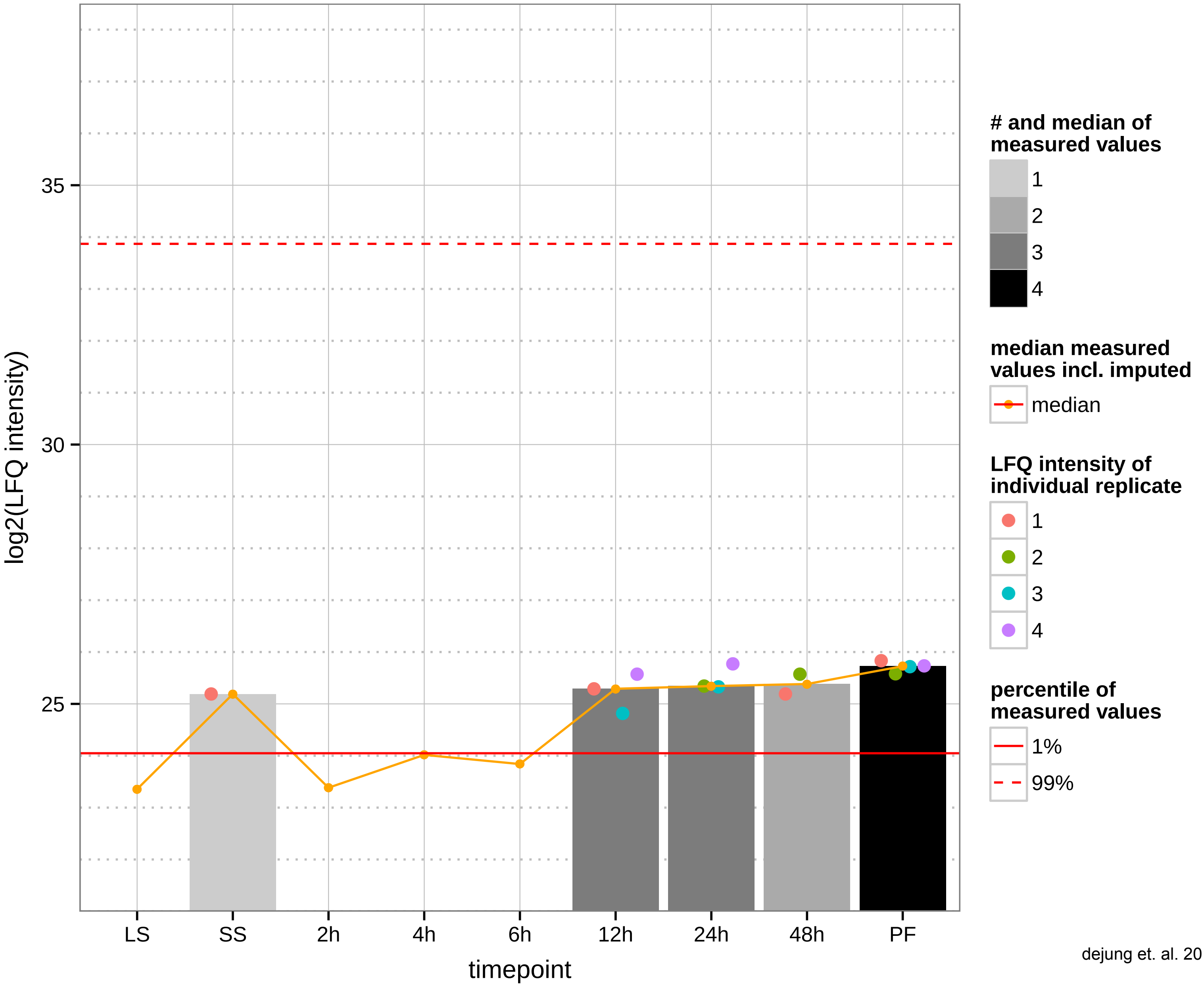

dejung et. al. 2015

hypothetical protein, conserved
Tb927.8.6480
AGOF: aminomethyltransferase activity
AGOC: cytoplasm, extracellular region
AGOP: glycine decarboxylation via glycine cleavage system
PGOF: aminomethyltransferase activity
PGOC: cytoplasm
PGOP: glycine catabolic process

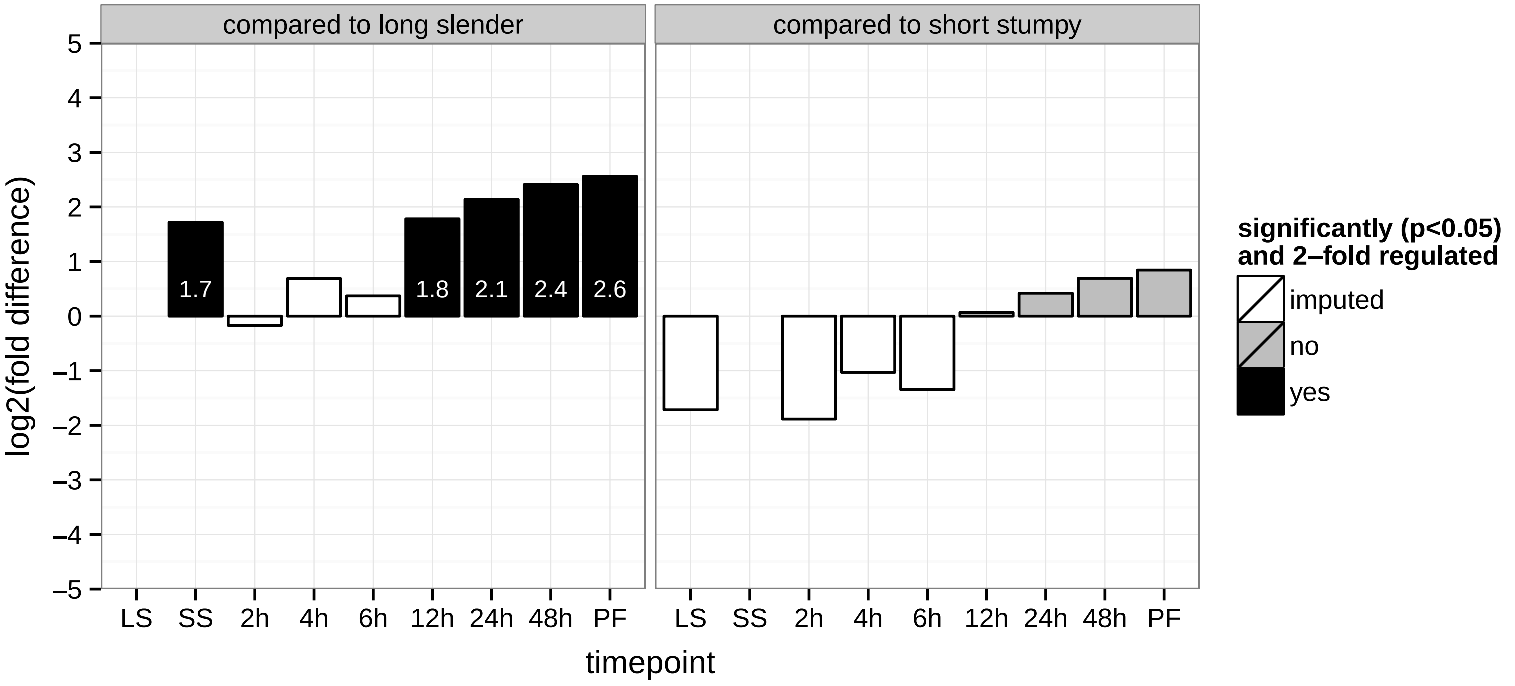

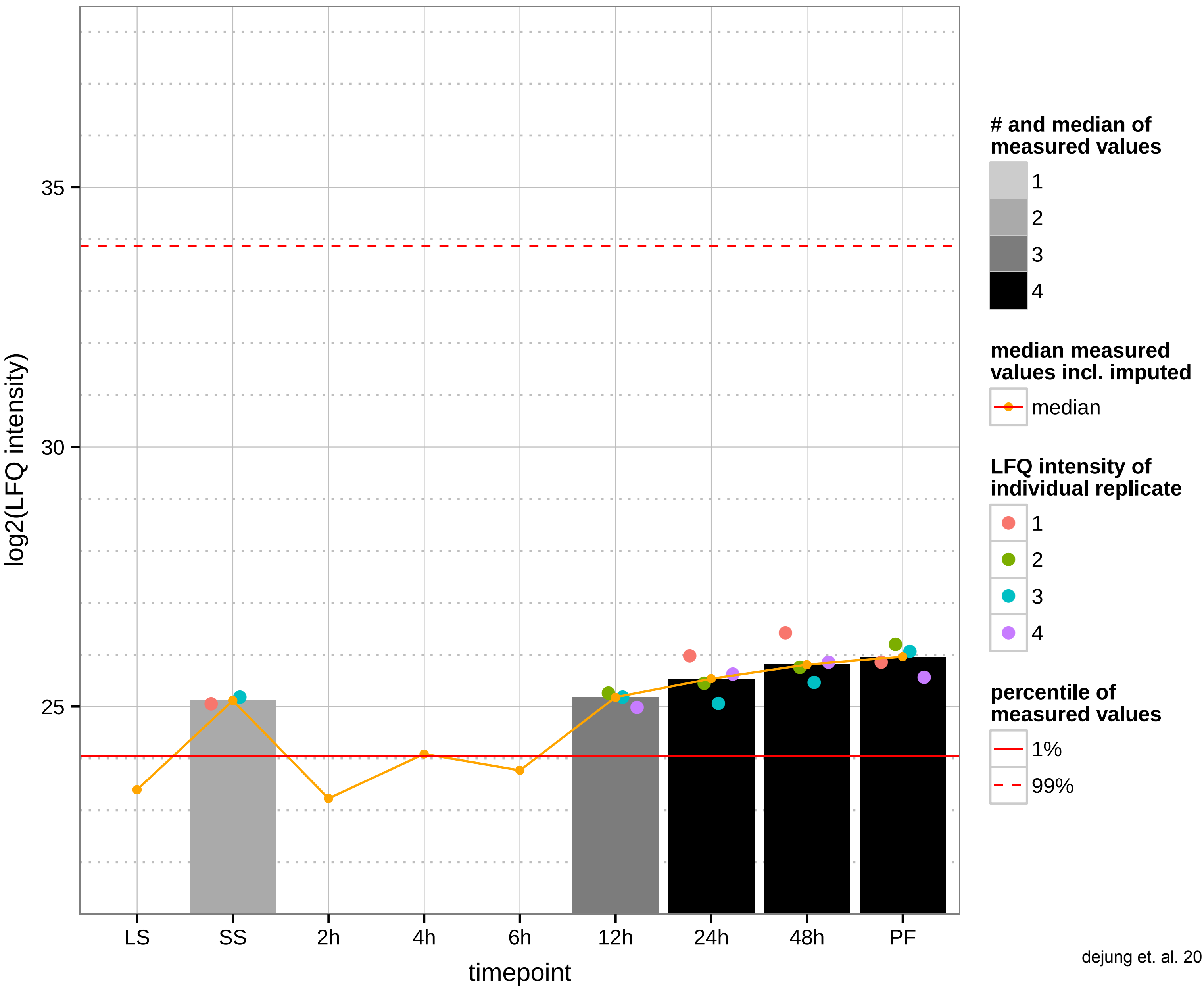

dejung et. al. 2015

hypothetical protein, conserved
Tb927.9.3560
AGOF: null
AGOC: null
AGOP: null
PGOF: null
PGOC: null
PGOP: null

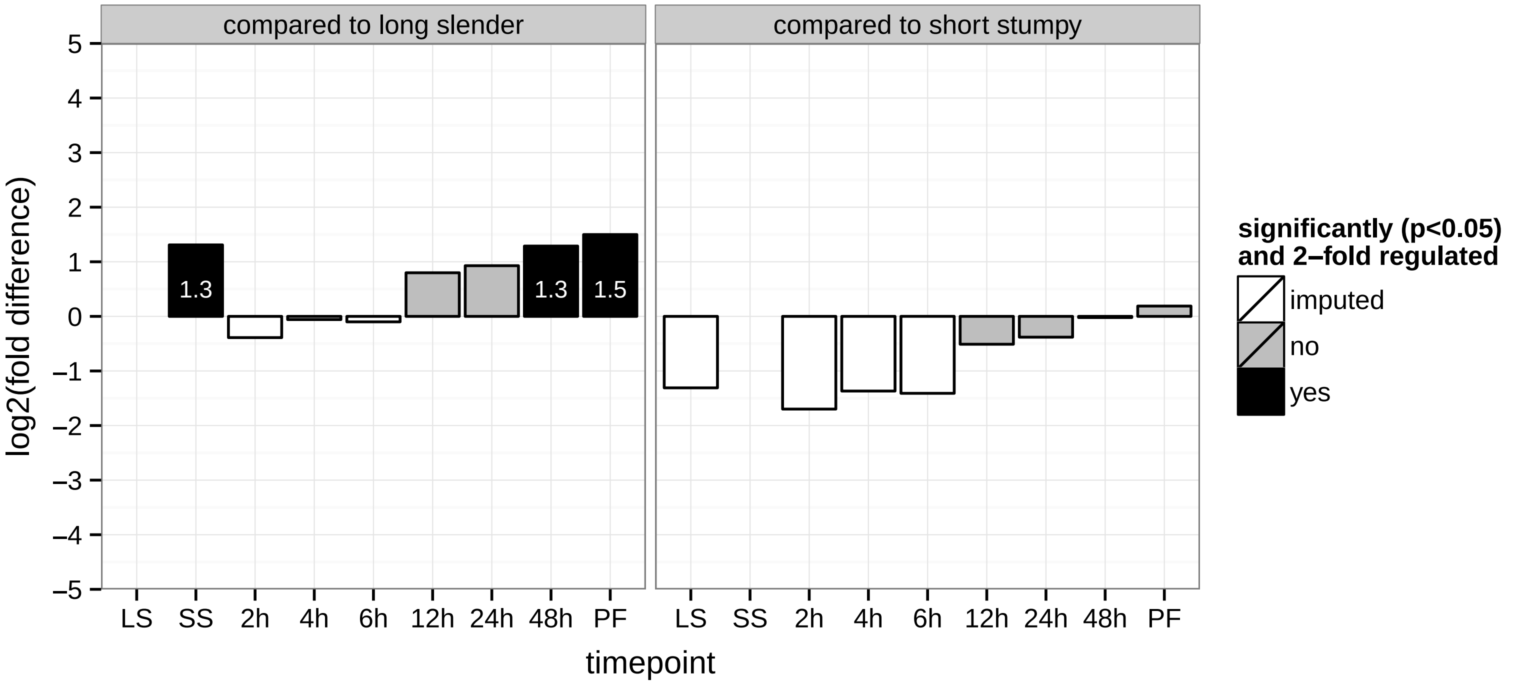

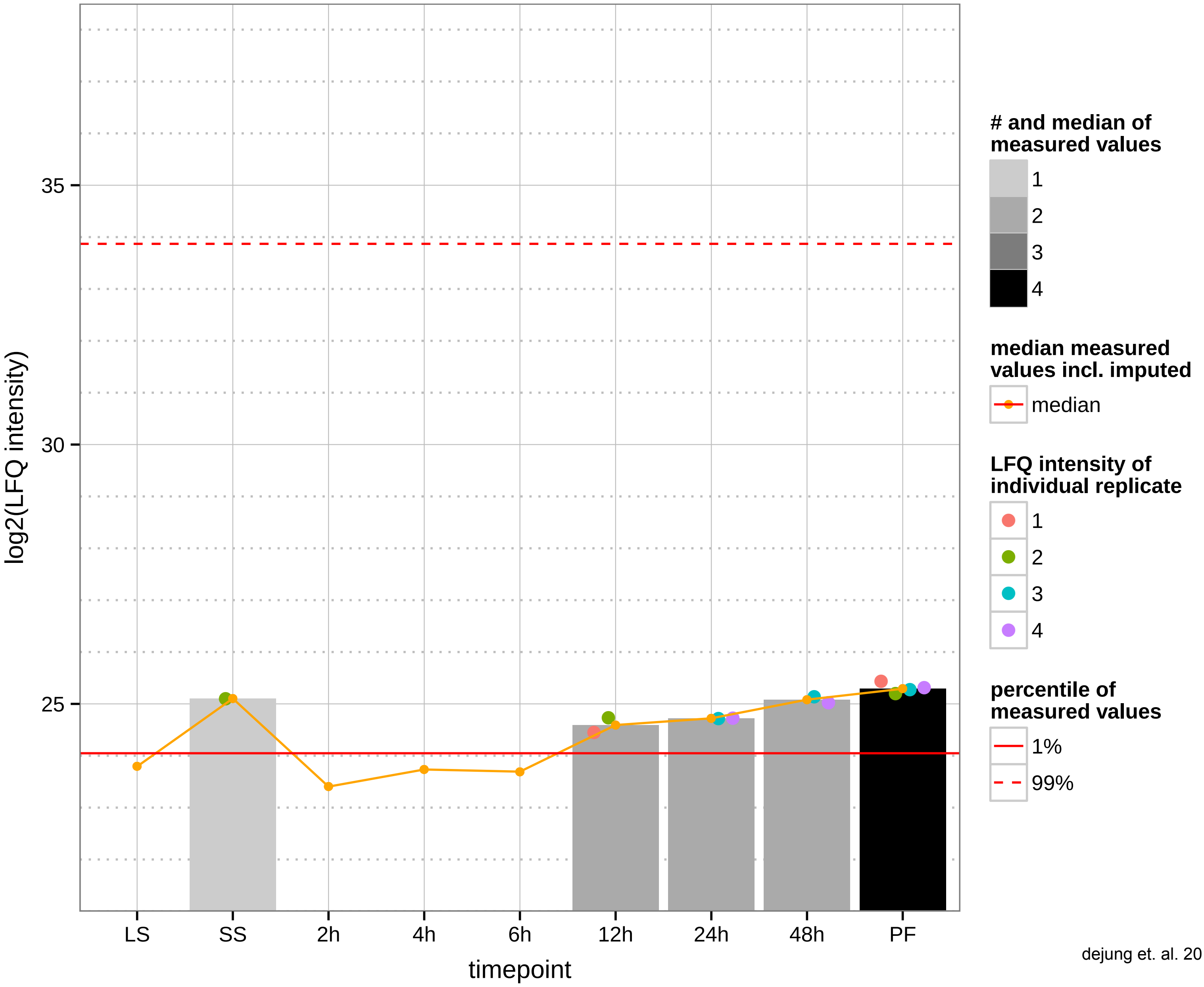

dejung et. al. 2015

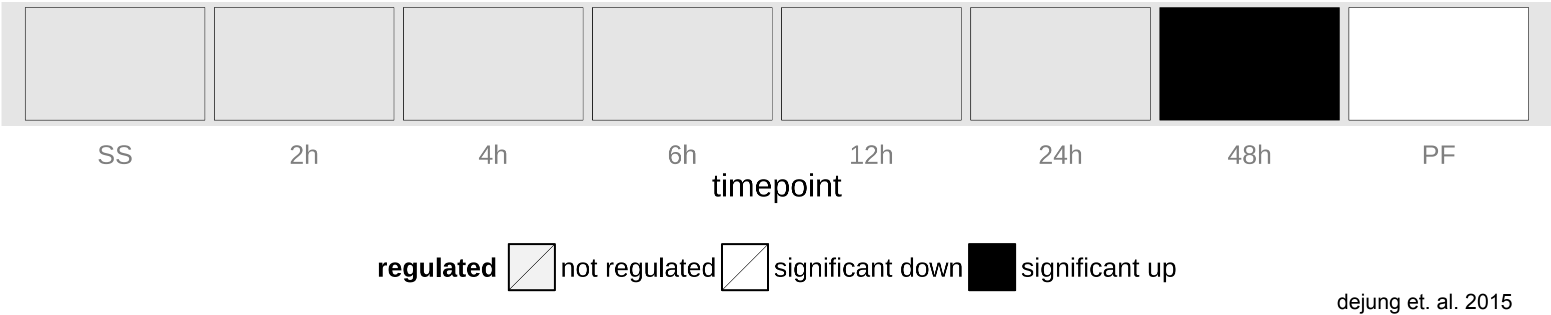

SS
2h
4h
6h
12h
24h
48h
PF
timepoint
regulated
not regulated
significant down
significant up
dejung et. al. 2015

retrotransposon hot spot (RHS) protein, putative
Tb11.v5.0326
AGOF: null
AGOC: null
AGOP: null
PGOF: null
PGOC: null
PGOP: null

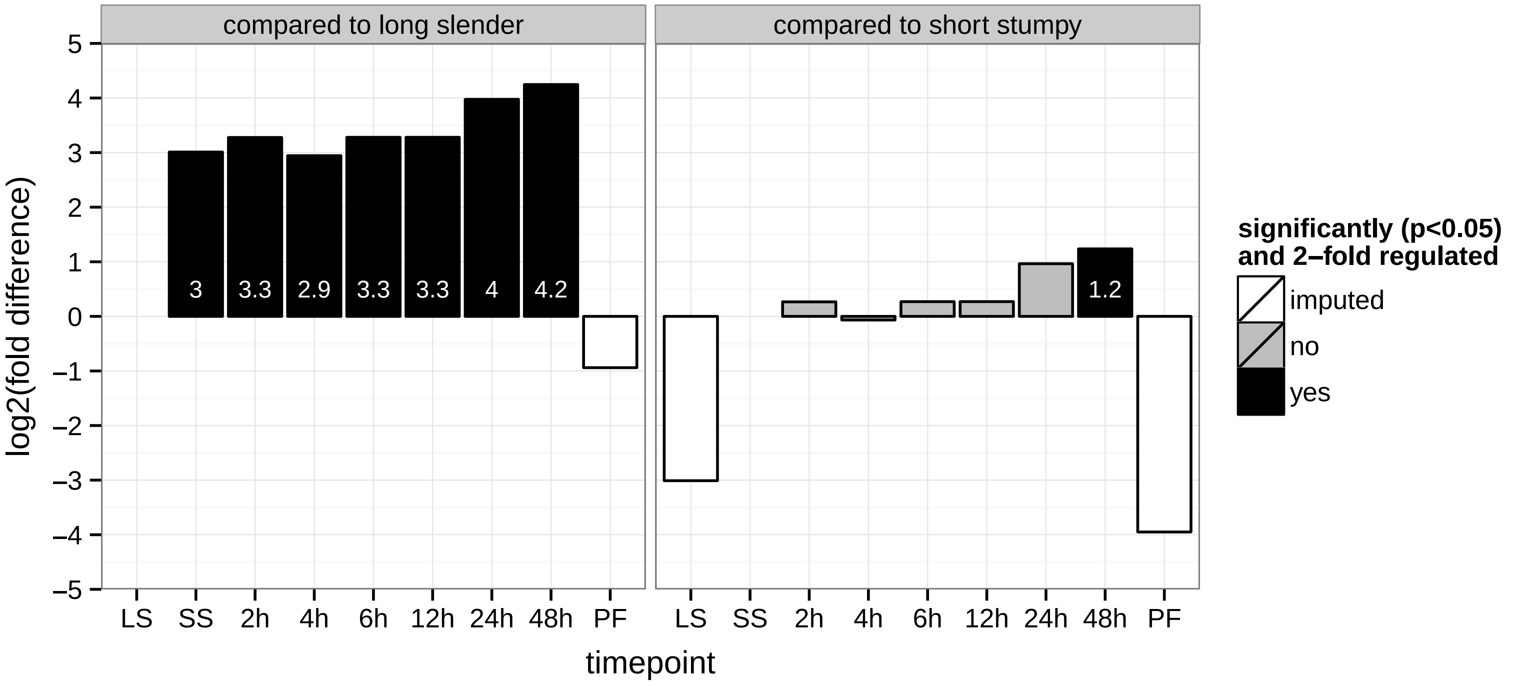

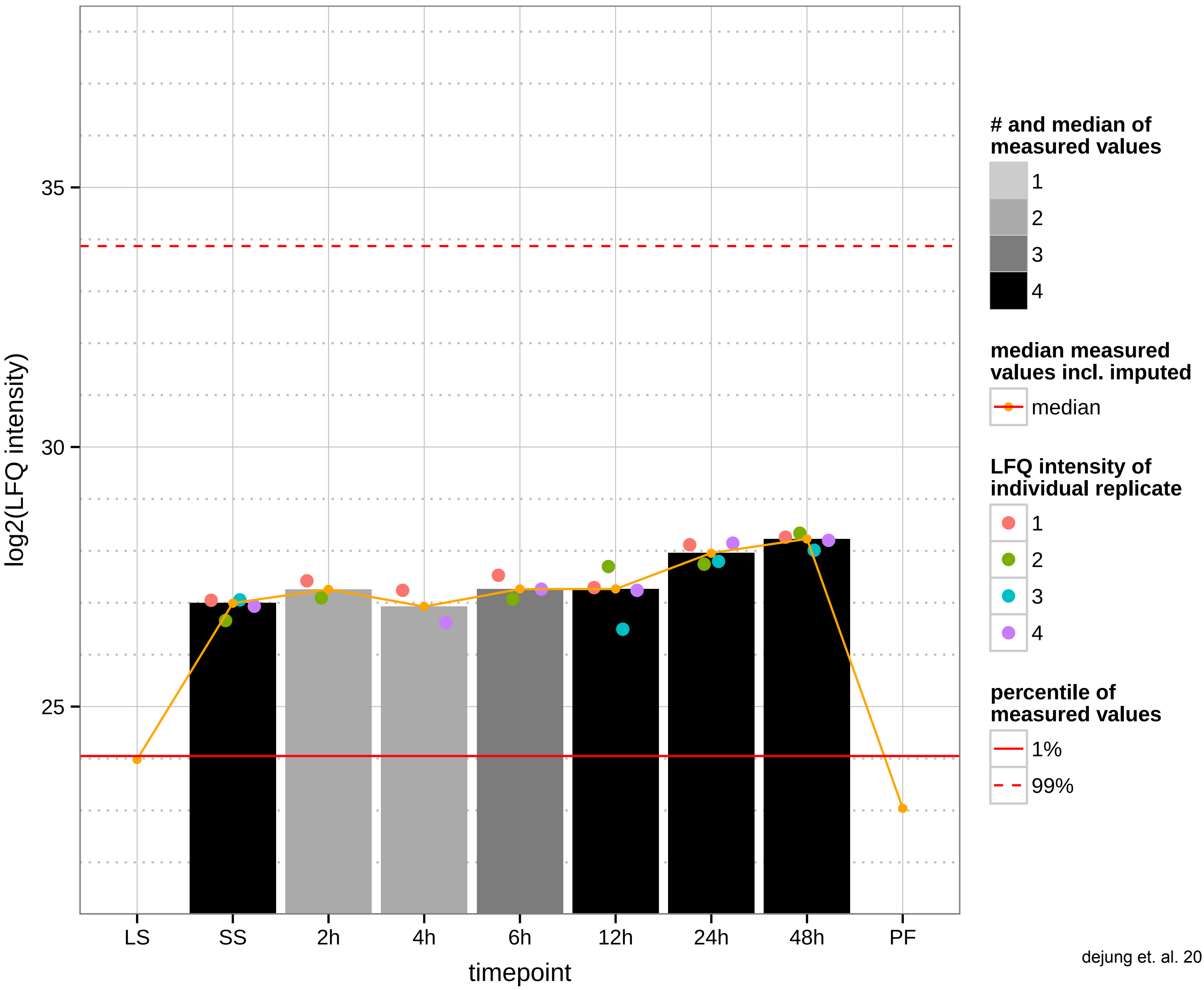

dejung et. al. 2015

hypothetical protein, conserved
Tb927.10.8850
AGOF: RNA binding, catalytic activity
AGOC: null
AGOP: null
PGOF: catalytic activity
PGOC: null
PGOP: null

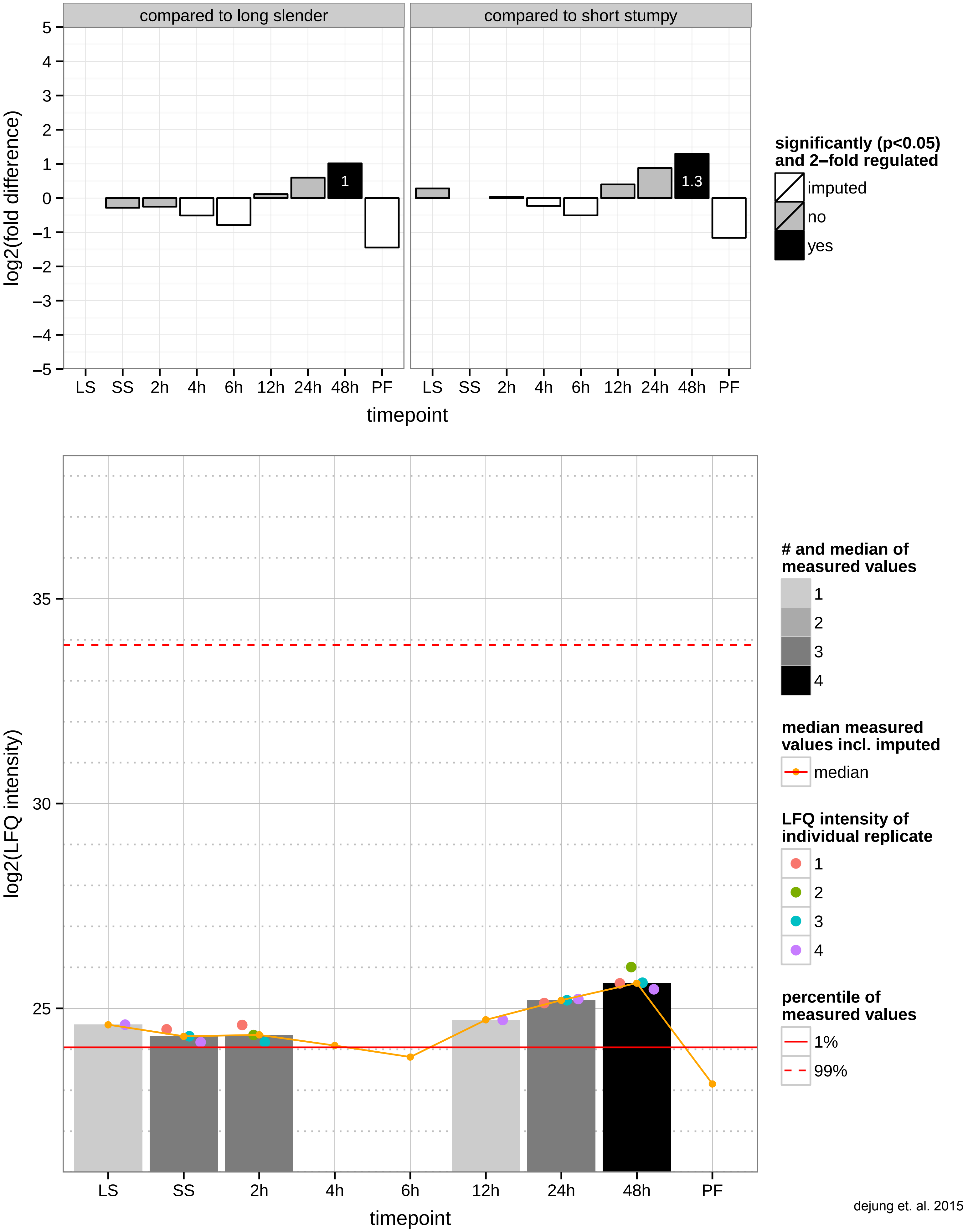

dejung et. al. 2015

hypothetical protein, conserved
Tb927.7.2330
AGOF: null
AGOC: null
AGOP: null
PGOF: null
PGOC: null
PGOP: null

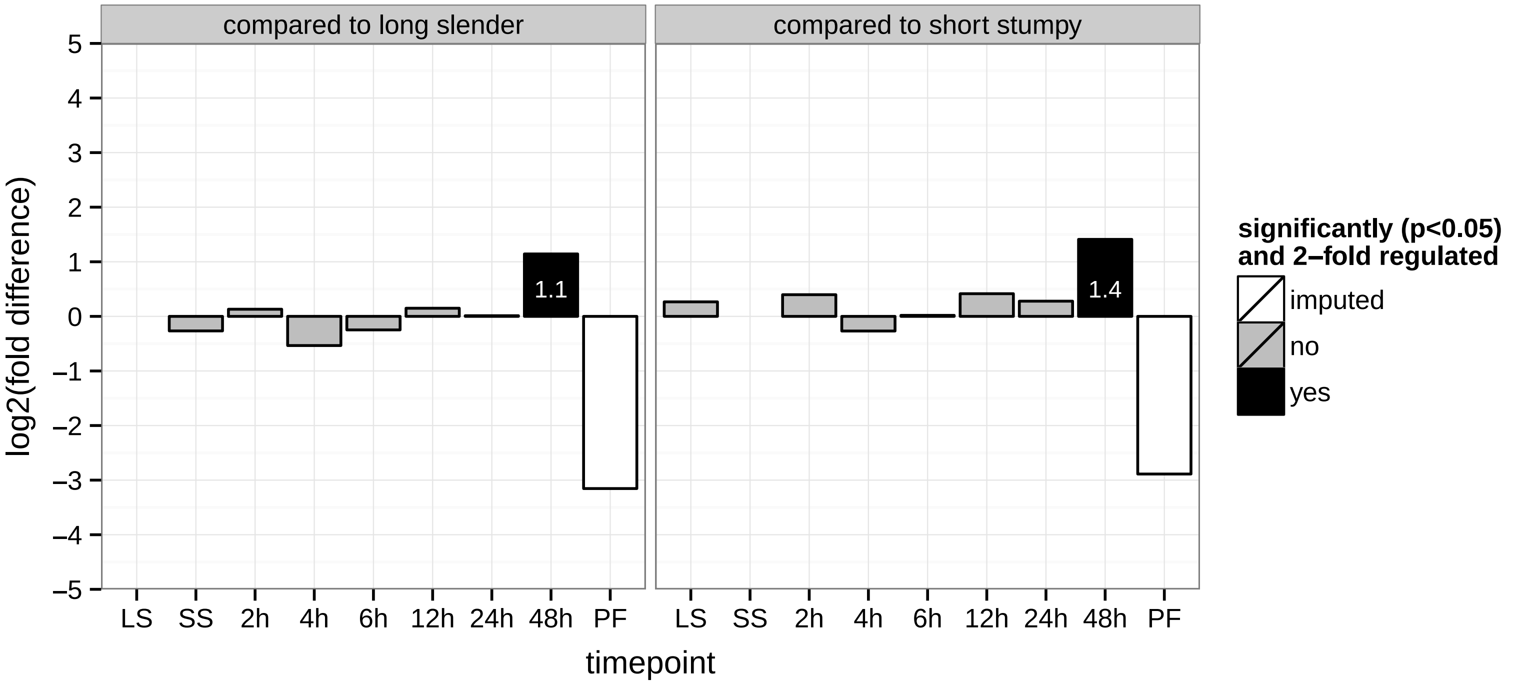

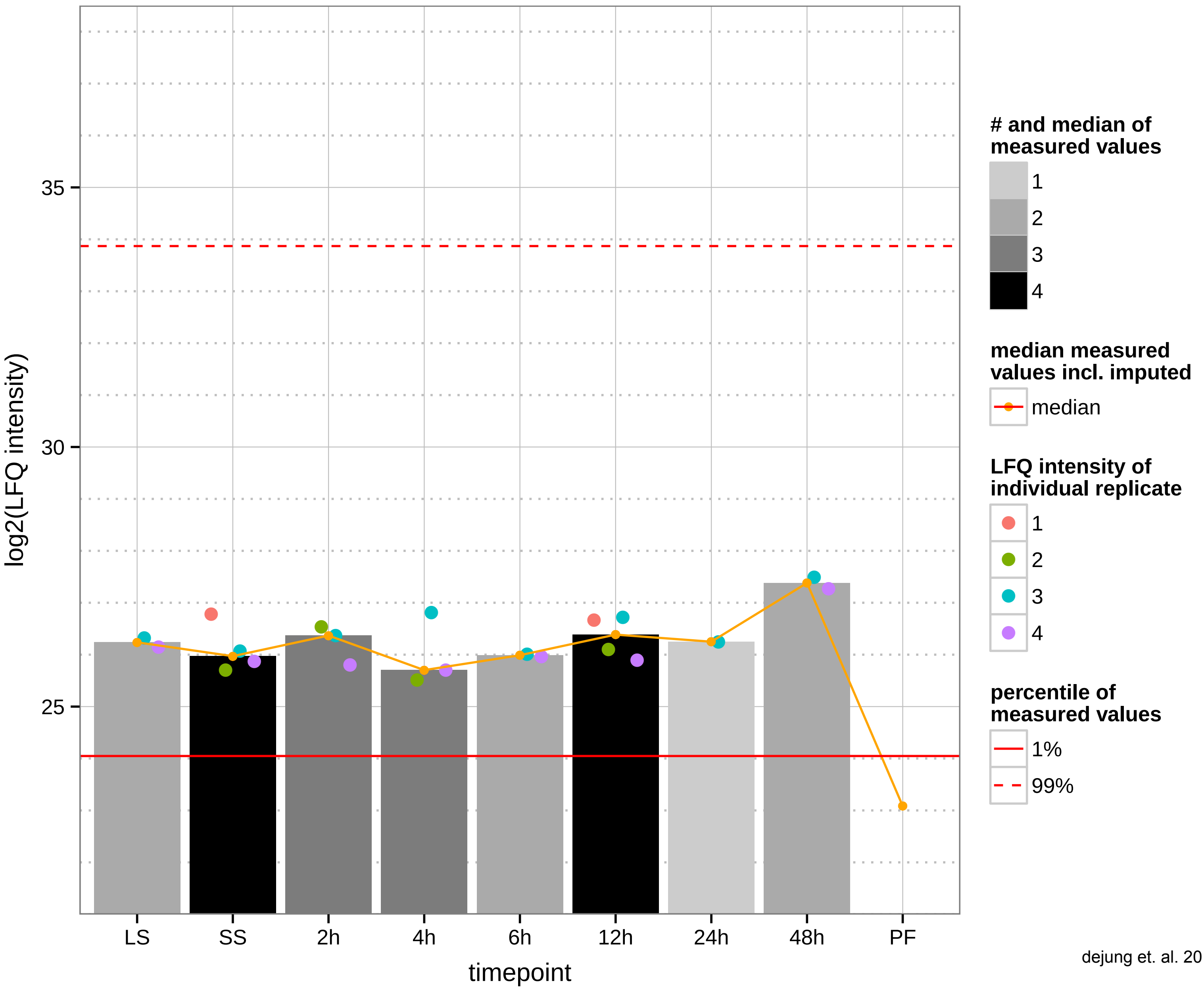

dejung et. al. 2015

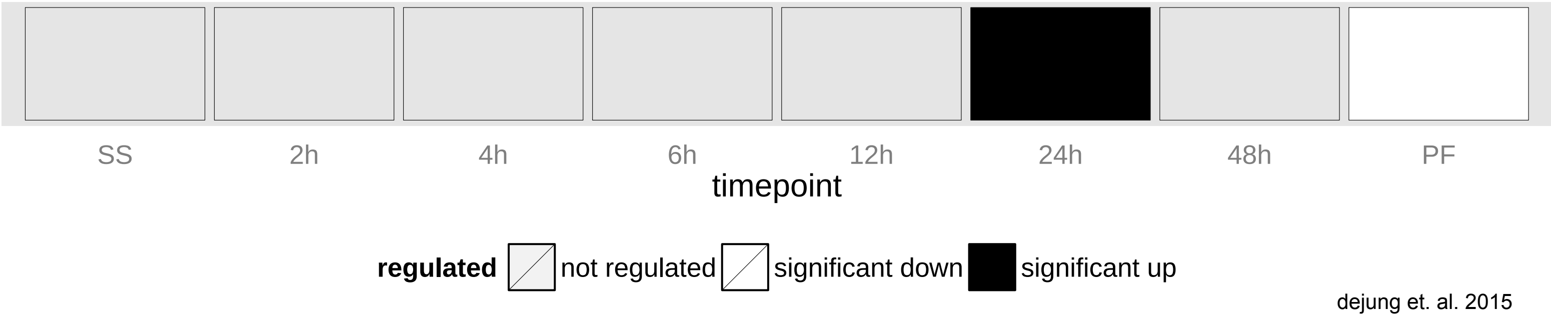

SS
2h
4h
6h
12h
24h
48h
PF
timepoint
regulated
not regulated
significant down
significant up
dejung et. al. 2015

hypothetical protein, conserved
Tb927.10.2580
AGOF: null
AGOC: null
AGOP: null
PGOF: null
PGOC: null
PGOP: null

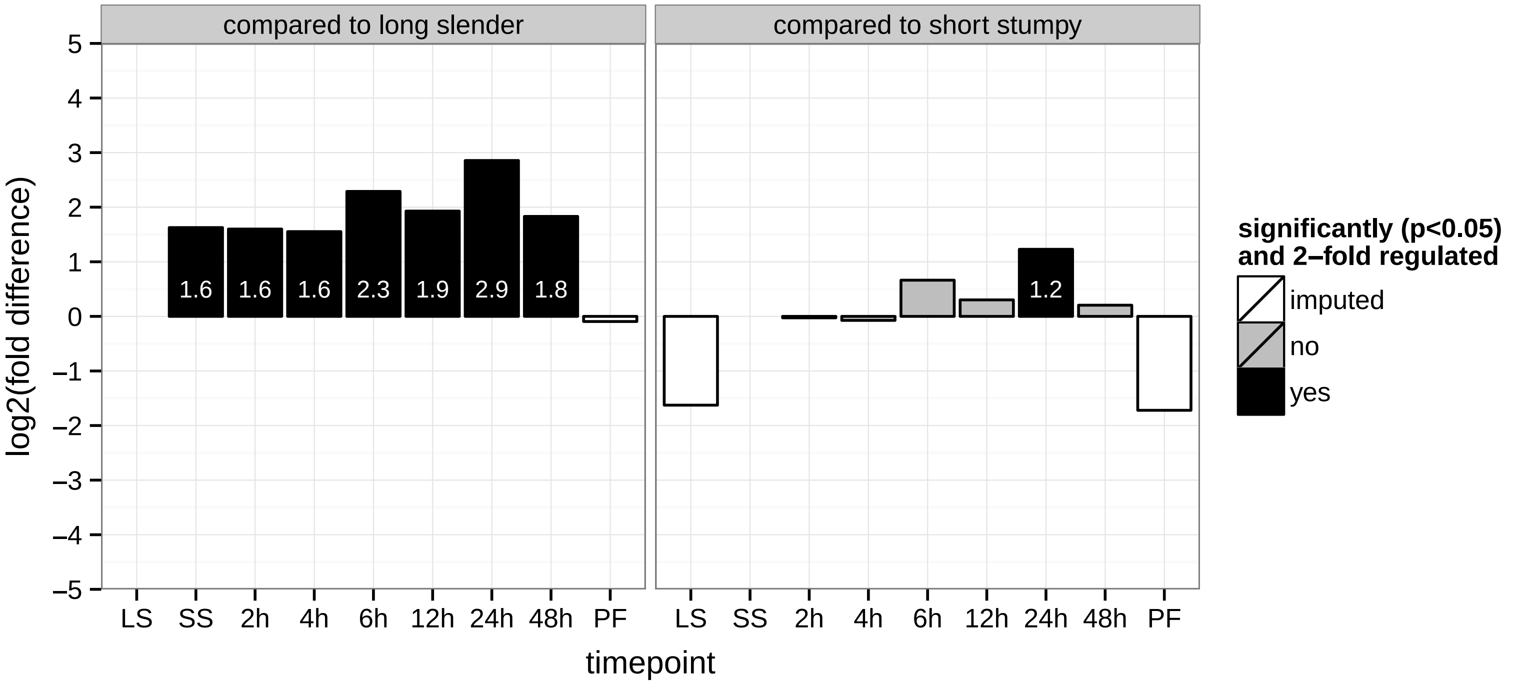

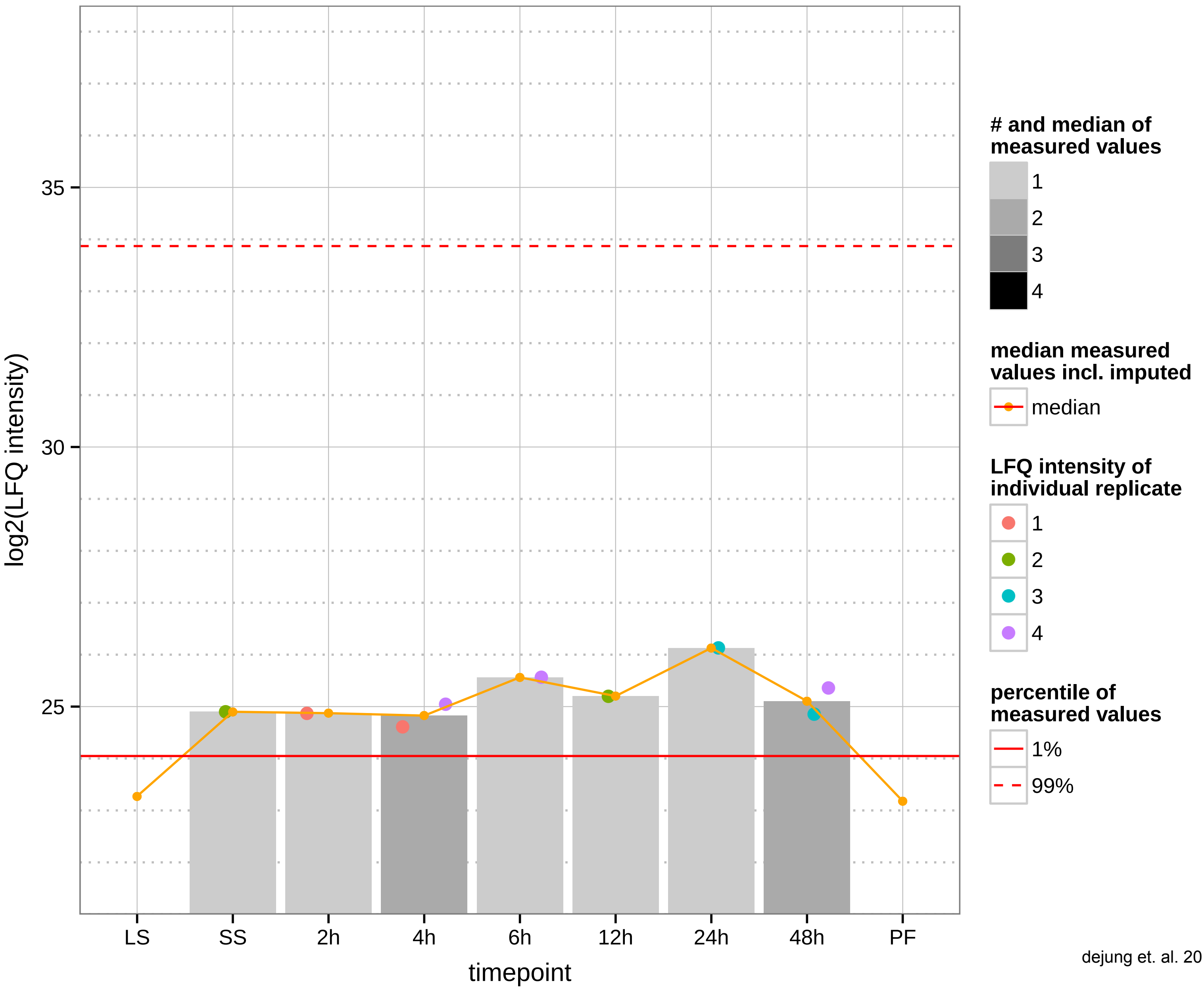

dejung et. al. 2015

hypothetical protein, conserved
Tb927.11.10170
AGOF: null
AGOC: mitochondrion
AGOP: null
PGOF: null
PGOC: null
PGOP: null

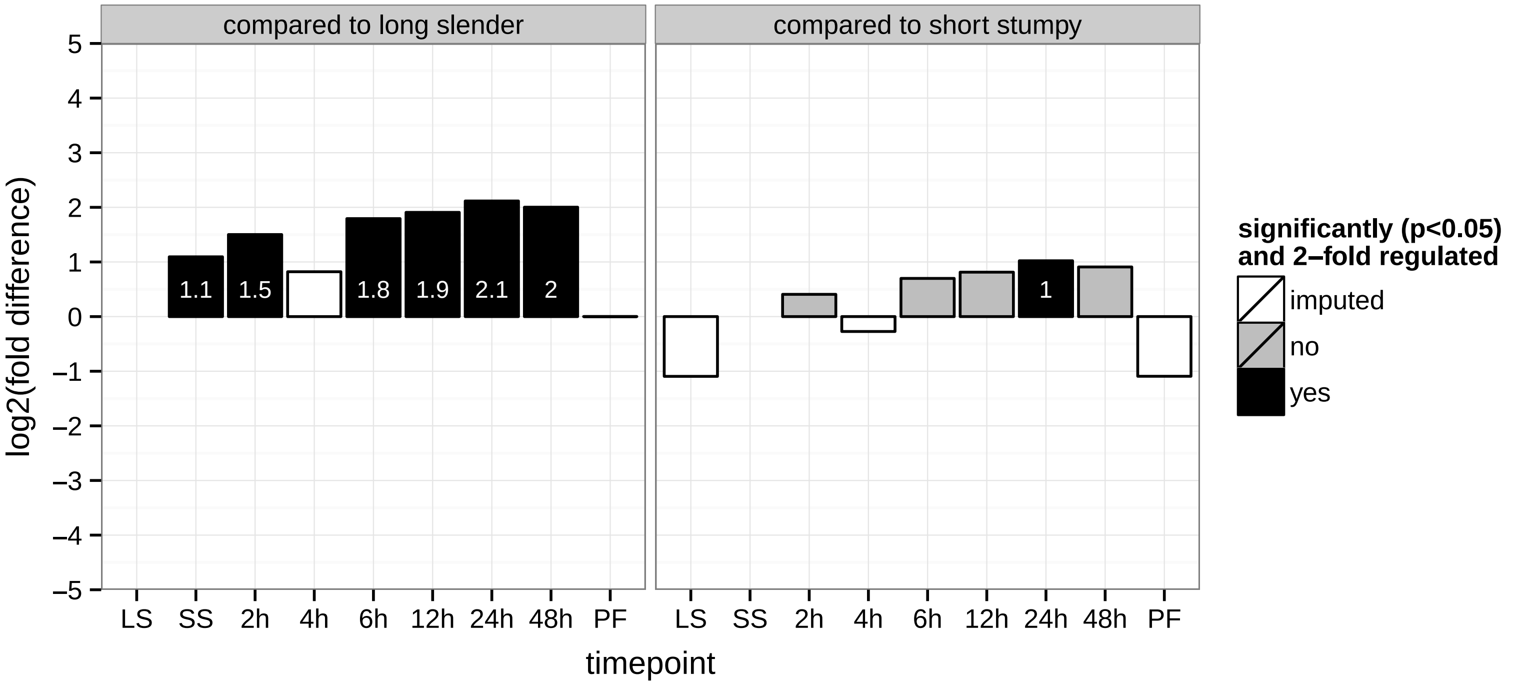

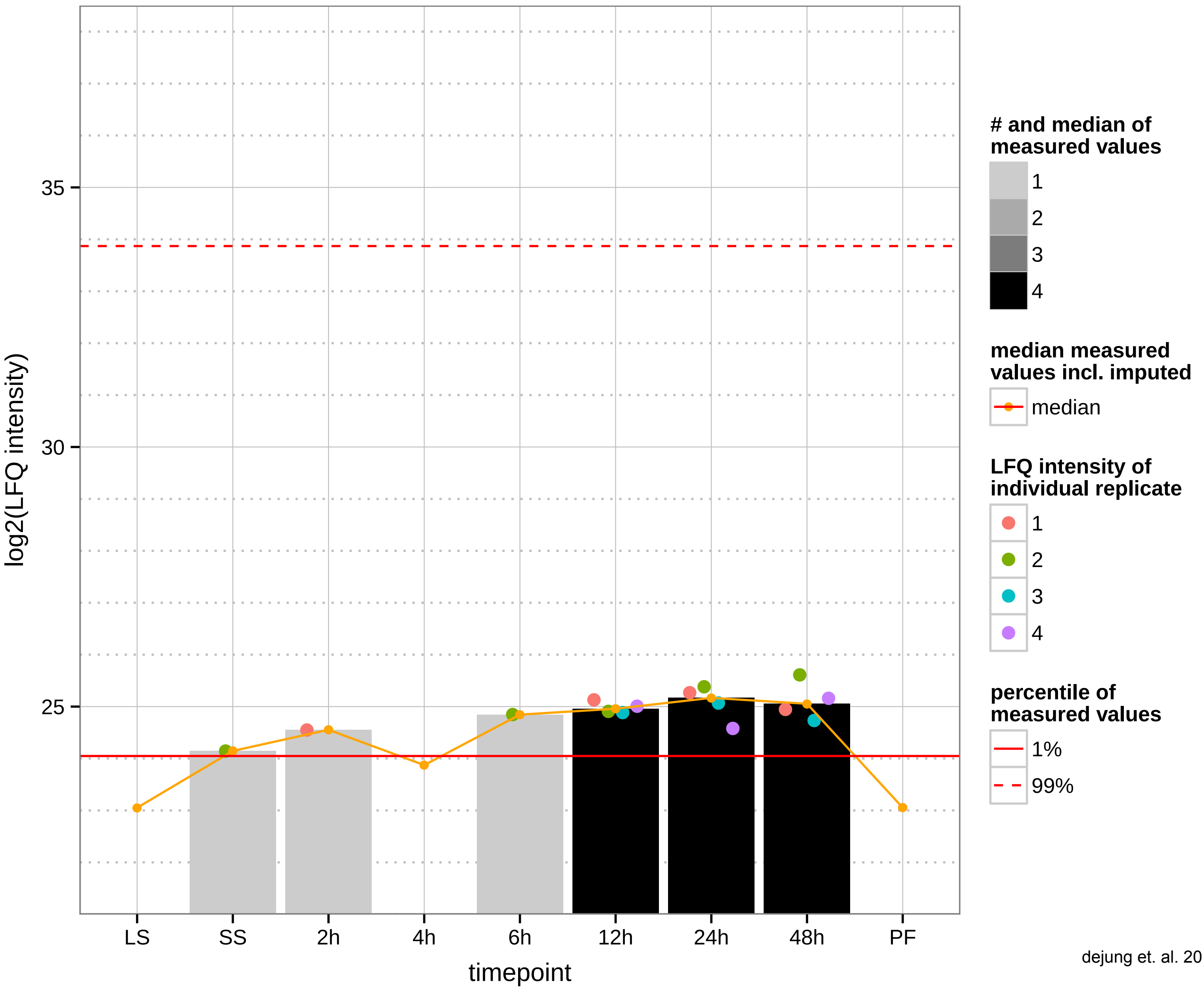

dejung et. al. 2015

conserved protein
Tb927.6.1860
AGOF: null
AGOC: mitochondrion
AGOP: translation
PGOF: null
PGOC: null
PGOP: null

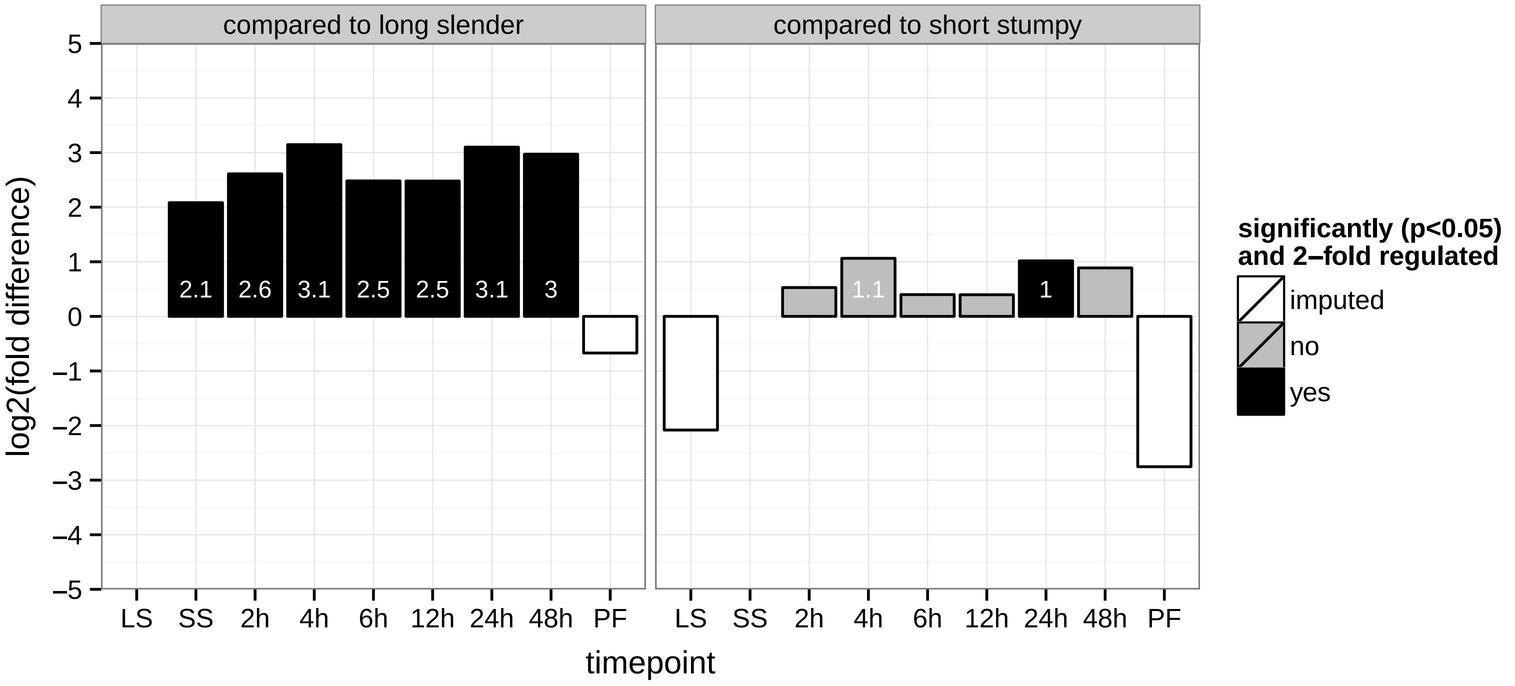

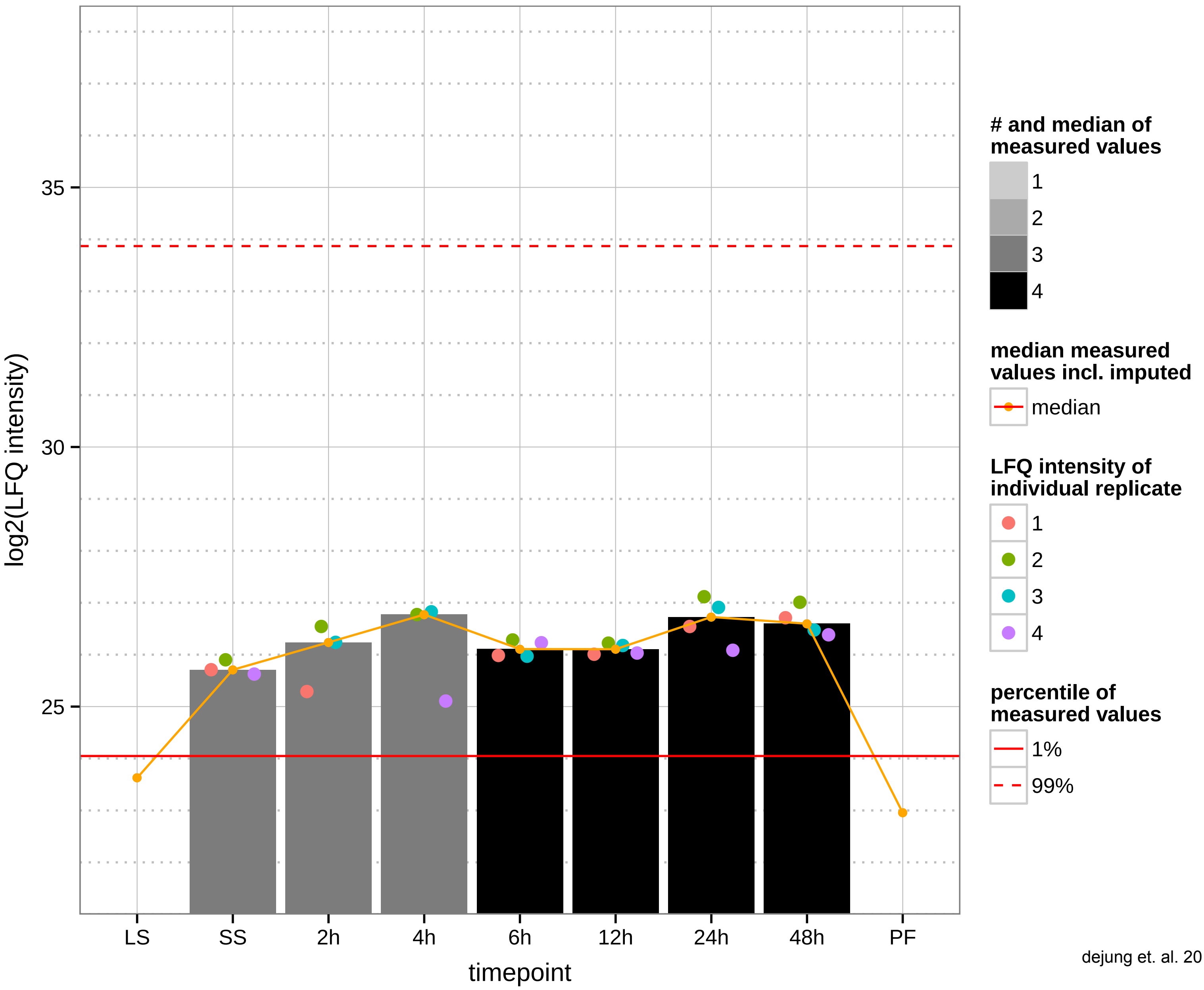

dejung et. al. 2015

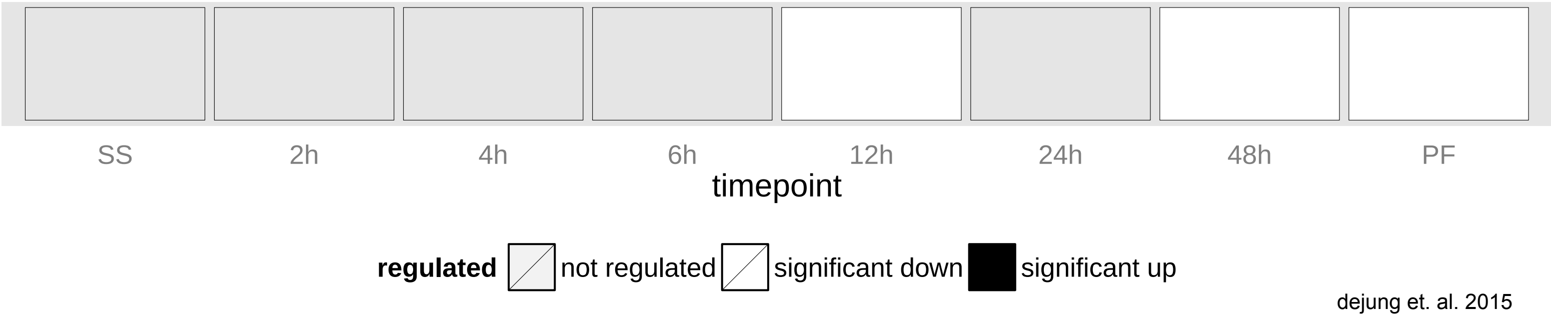
SS
2h
4h
6h
12h
24h
48h
PF
timepoint
regulated
not regulated
significant down
significant up
dejung et. al. 2015

hypothetical protein, conserved
Tb927.2.4410
AGOF: null
AGOC: integral to membrane
AGOP: null
PGOF: null
PGOC: integral to membrane
PGOP: null

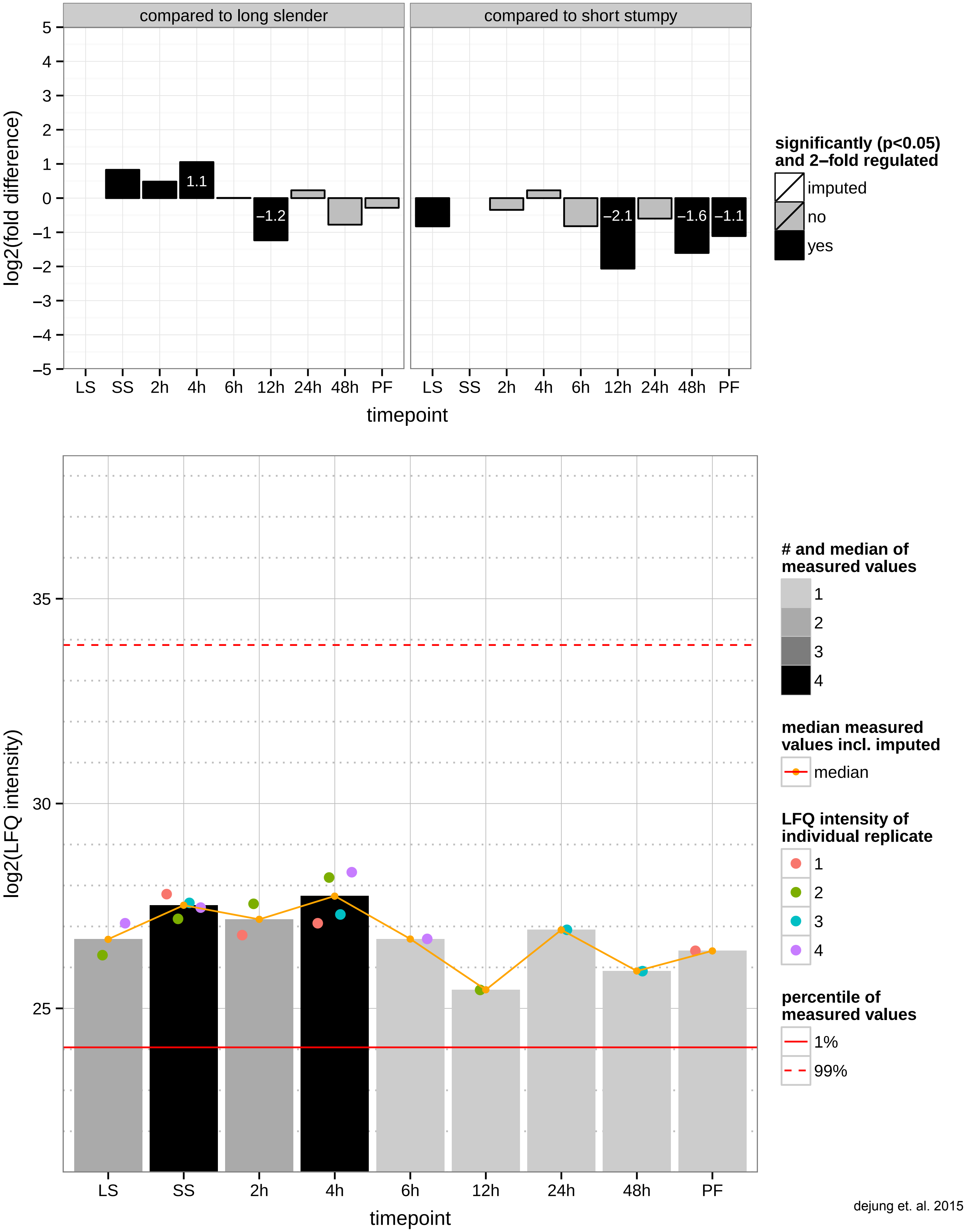

dejung et. al. 2015

p22 protein precursor, putative
Tb927.7.3470
AGOF: null
AGOC: mitochondrial matrix, mitochondrion
AGOP: null
PGOF: null
PGOC: mitochondrial matrix
PGOP: null

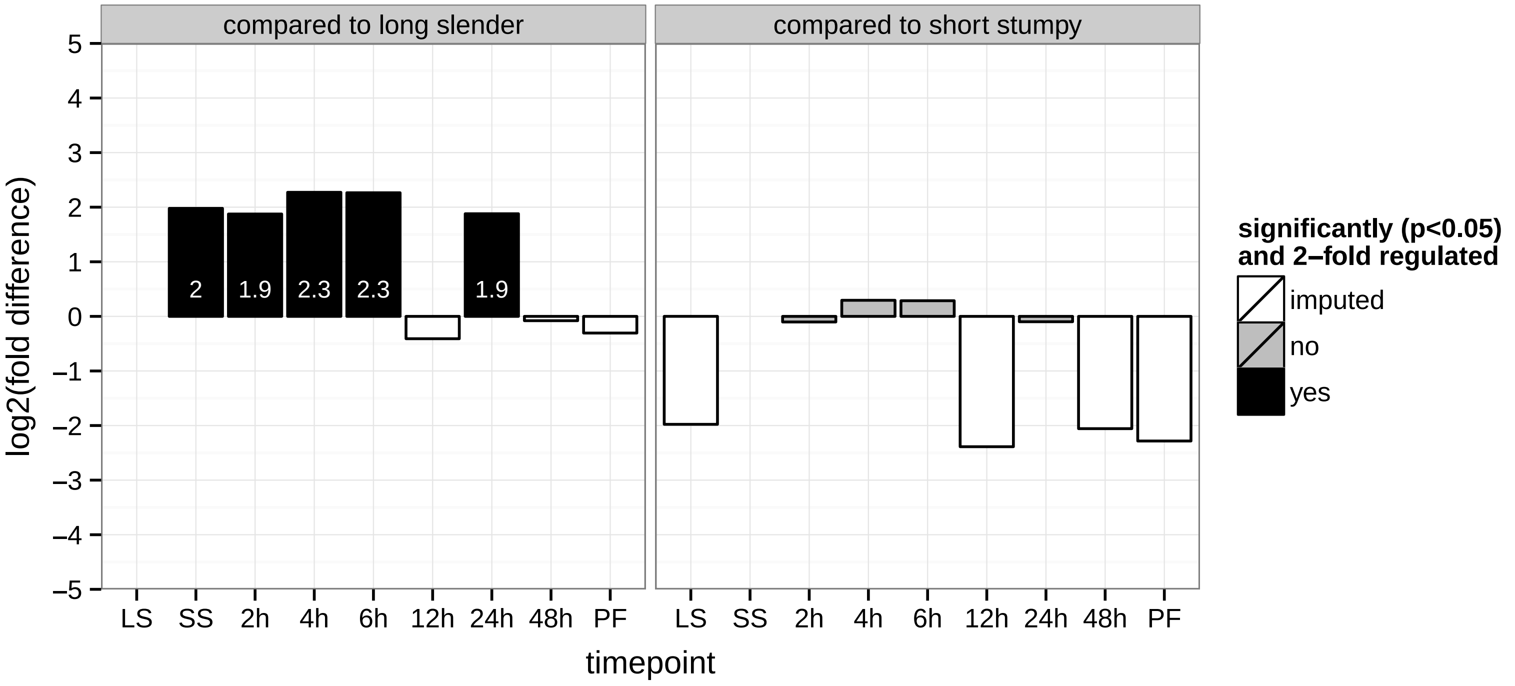

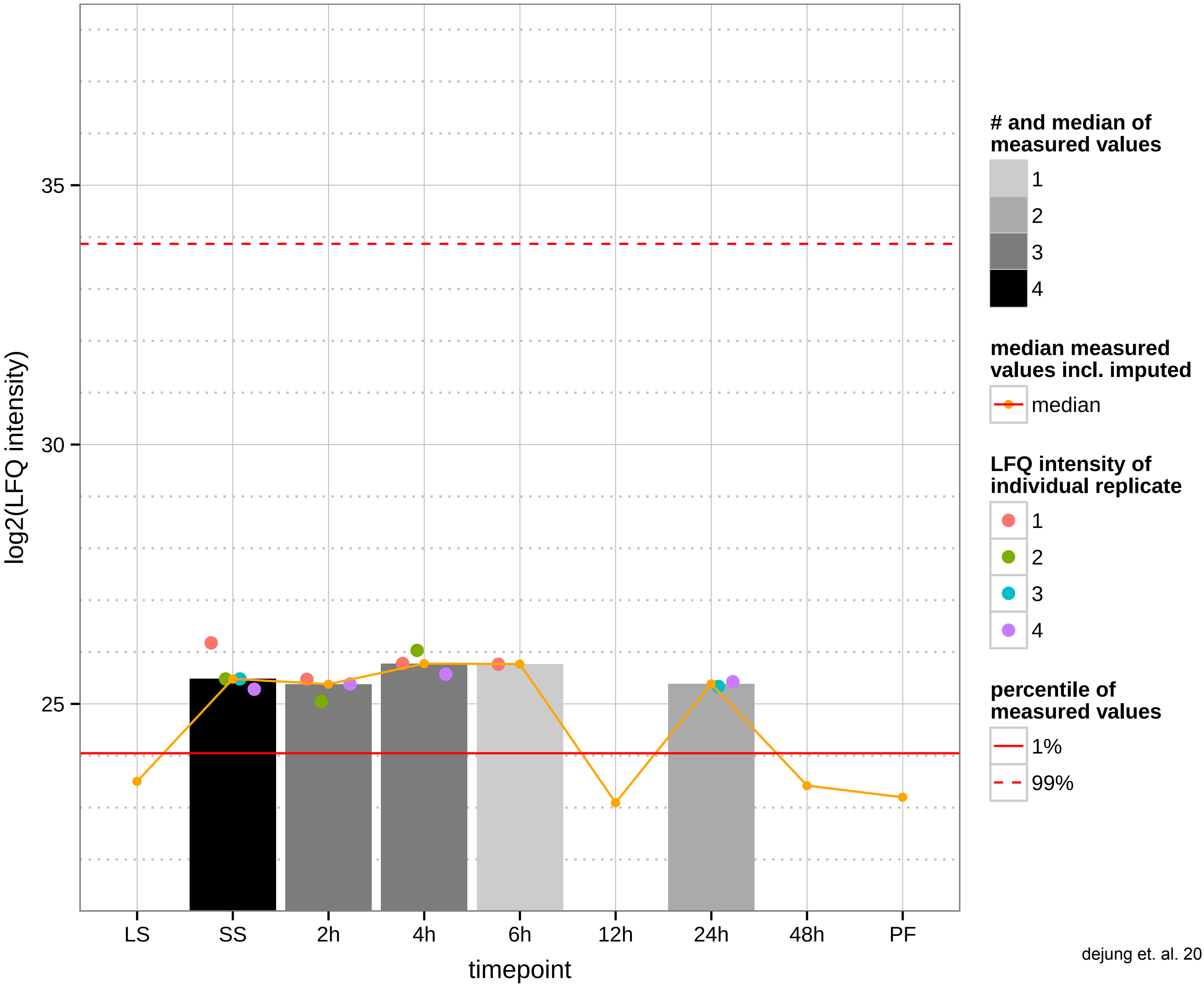

dejung et. al. 2015

hypothetical protein, conserved
Tb927.8.7760
AGOF: null
AGOC: null
AGOP: null
PGOF: null
PGOC: null
PGOP: null

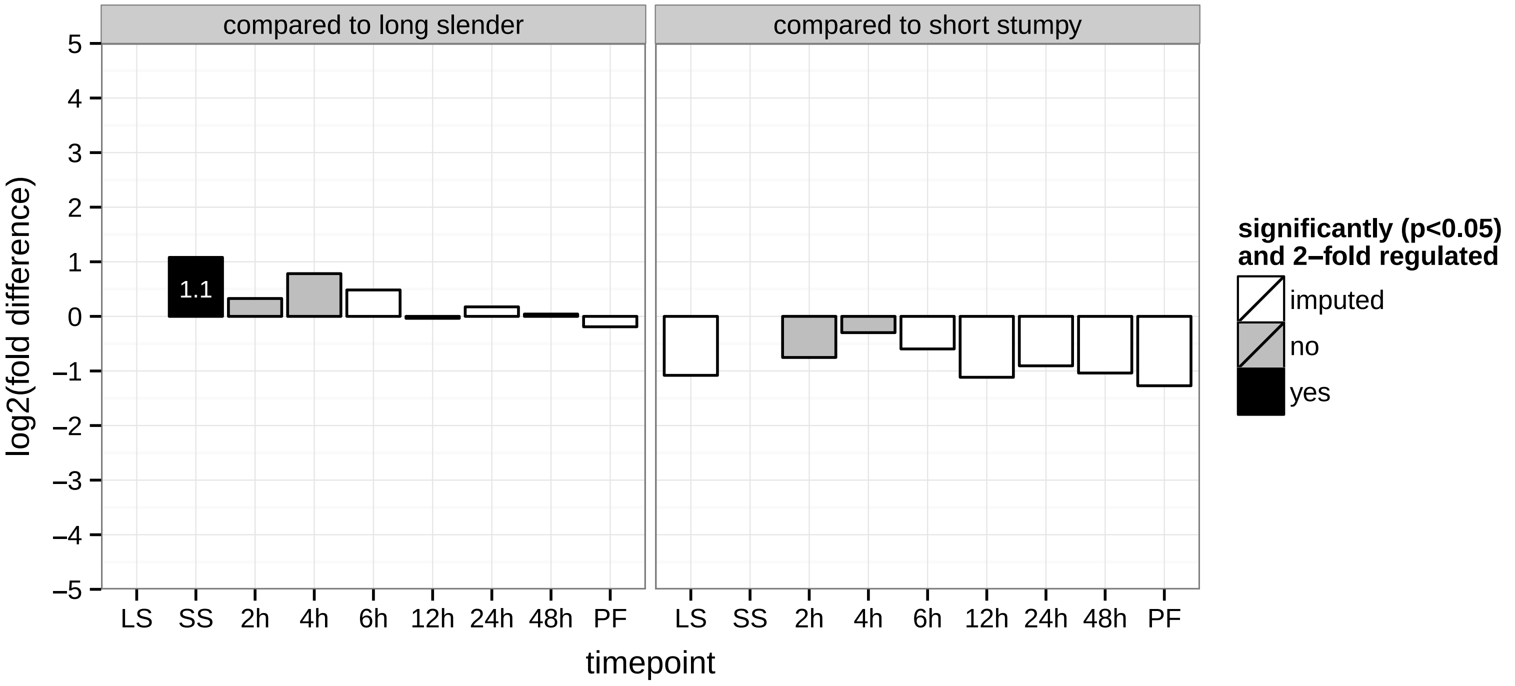

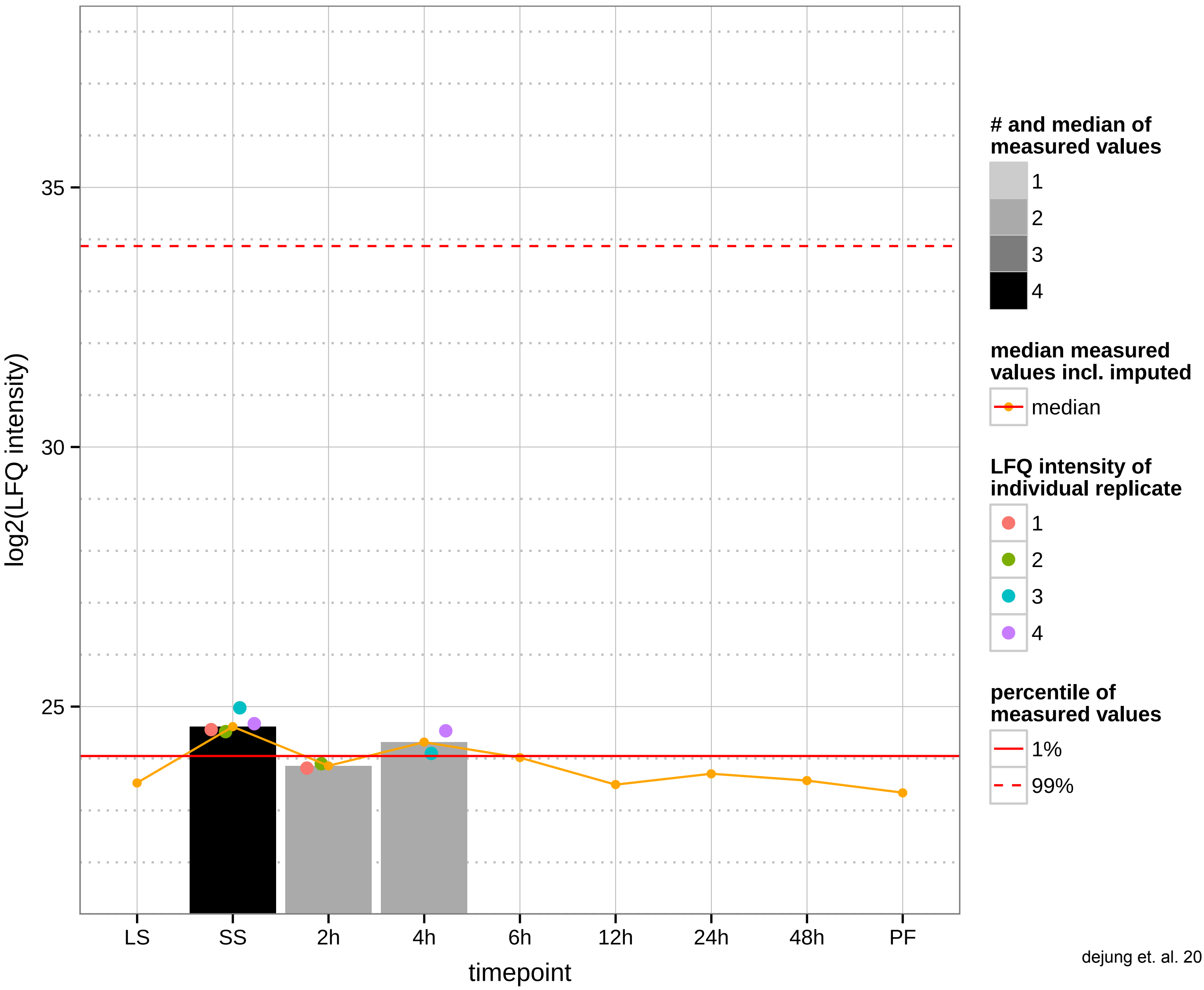

dejung et. al. 2015

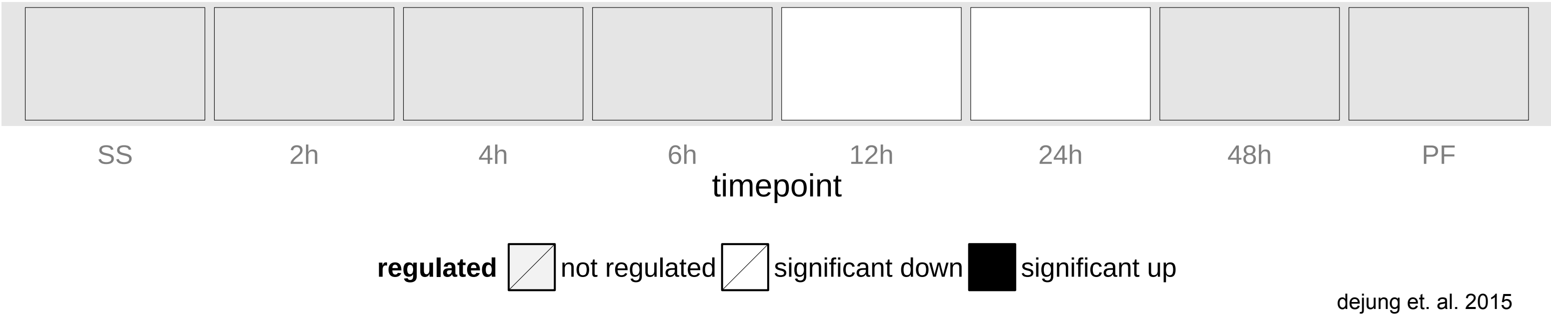
SS
2h
4h
6h
12h
24h
48h
PF
timepoint
regulated
not regulated
significant down
significant up
dejung et. al. 2015

Dynein light chain Tctex−type, putative
Tb927.10.10320
AGOF: microtubule motor activity
AGOC: microtubule associated complex
AGOP: microtubule−based process
PGOF: null
PGOC: null
PGOP: null

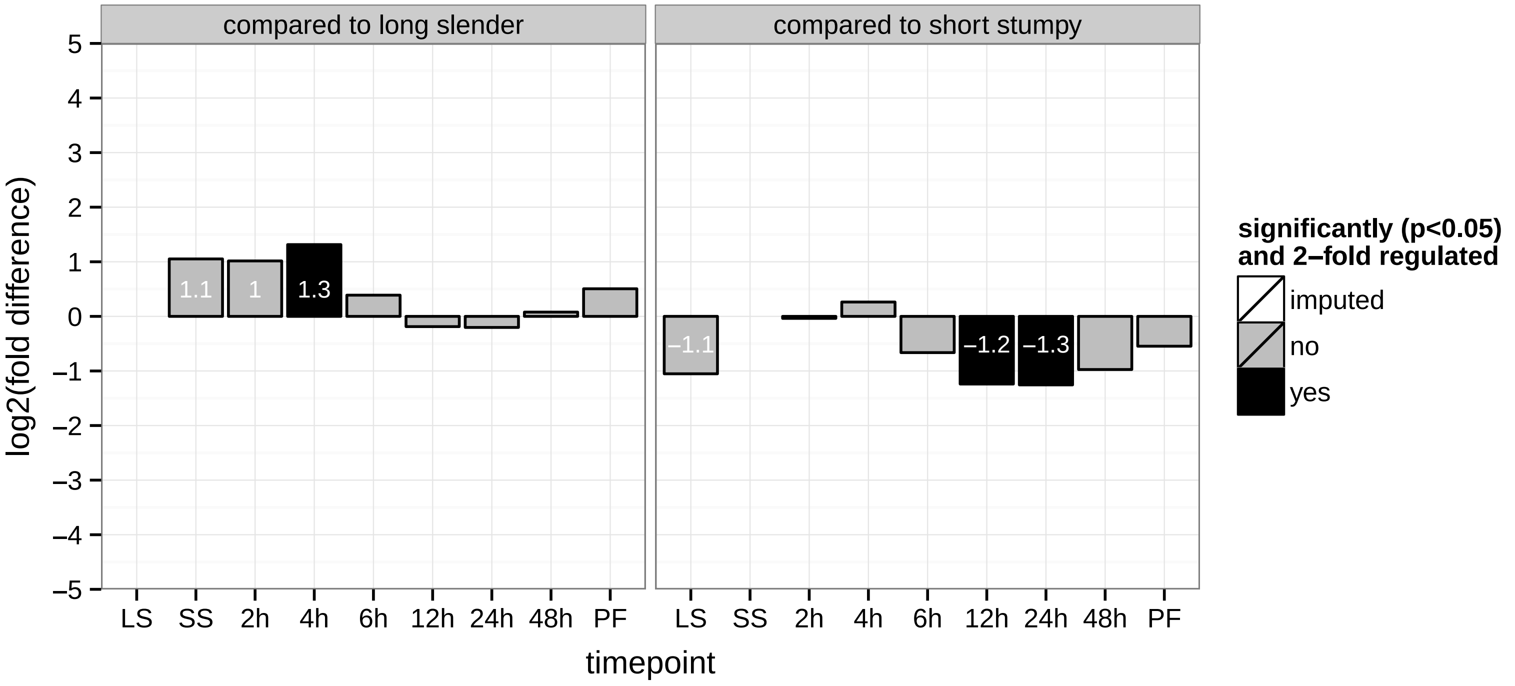

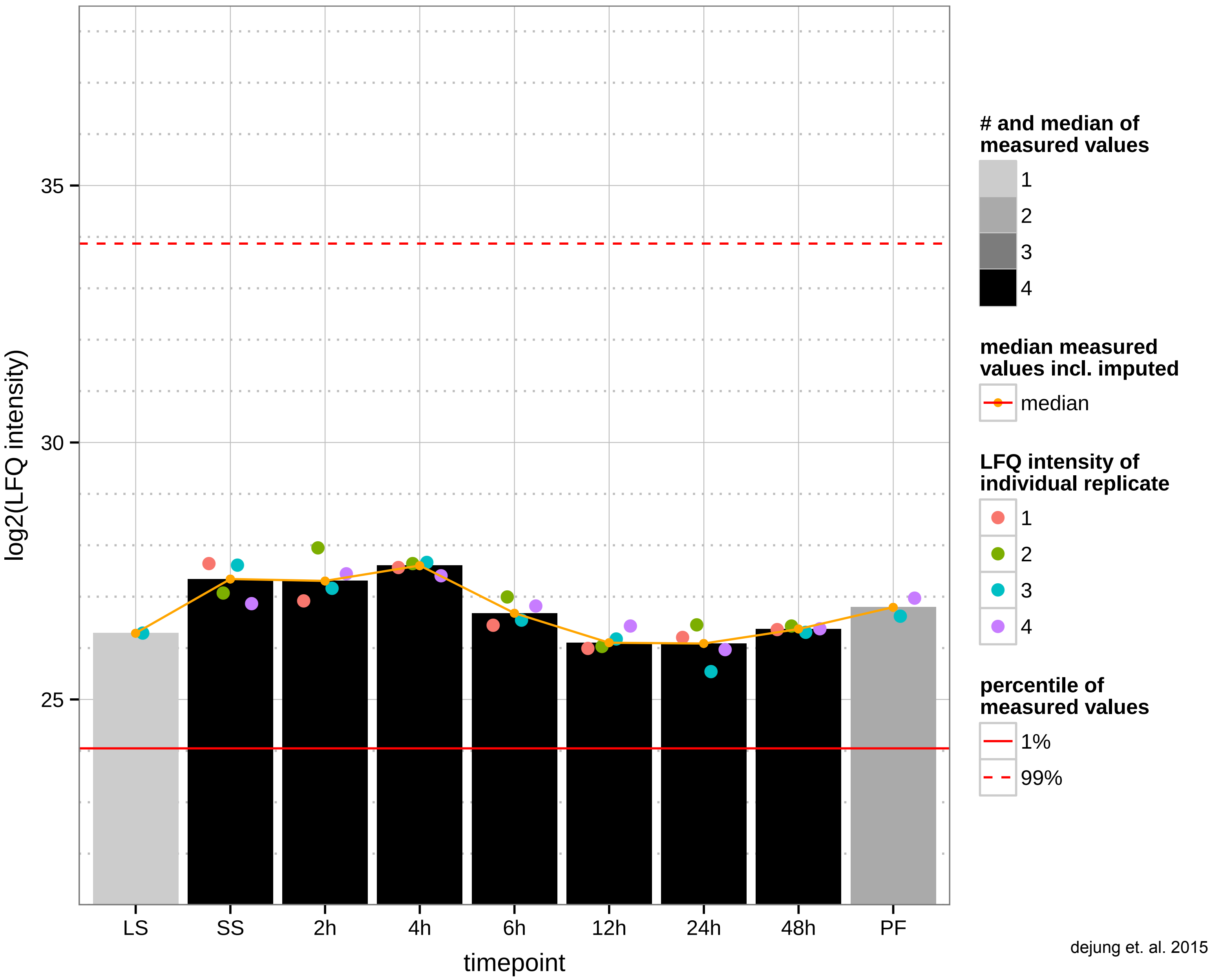

dejung et. al. 2015

RNA–binding protein, putative (RBP39)
Tb927.11.15350
AGOF: RNA binding
AGOC: null
AGOP: null
PGOF: nucleic acid binding
PGOC: null
PGOP: null

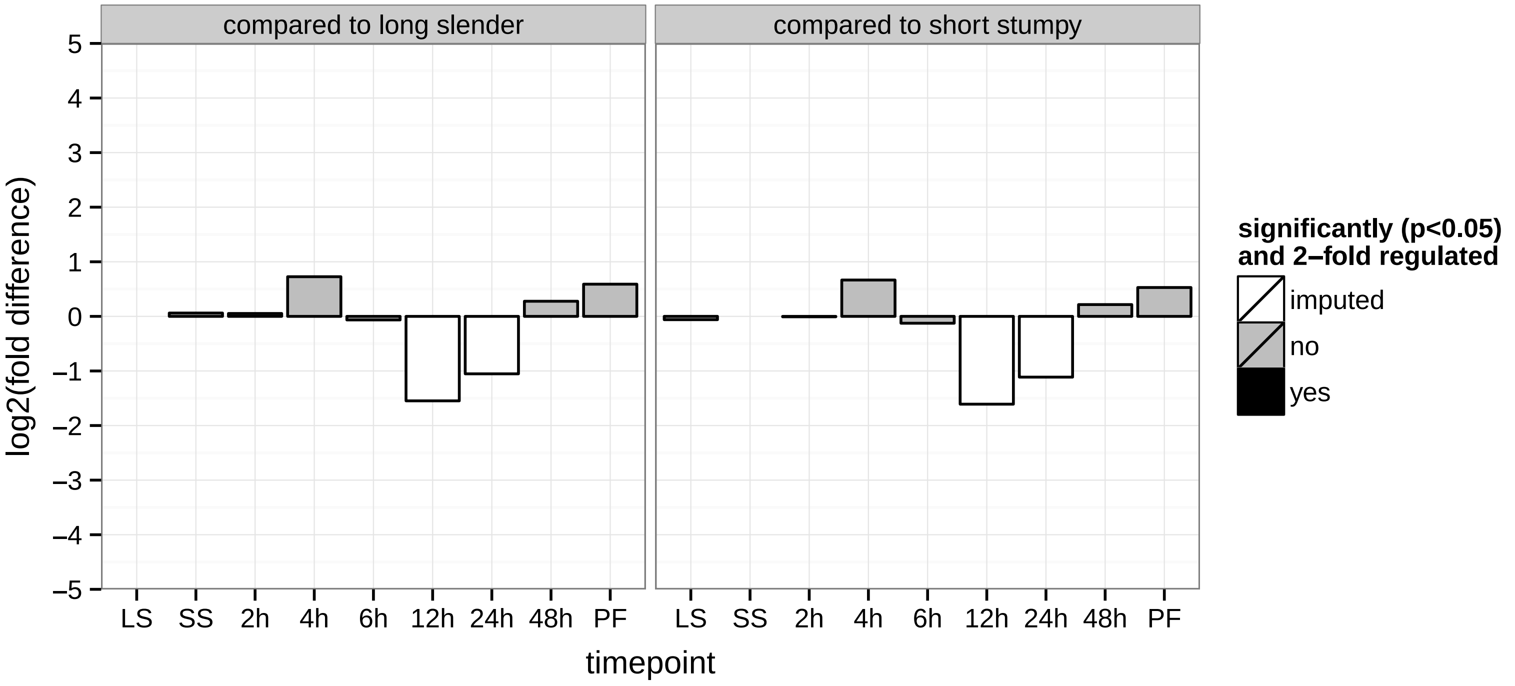

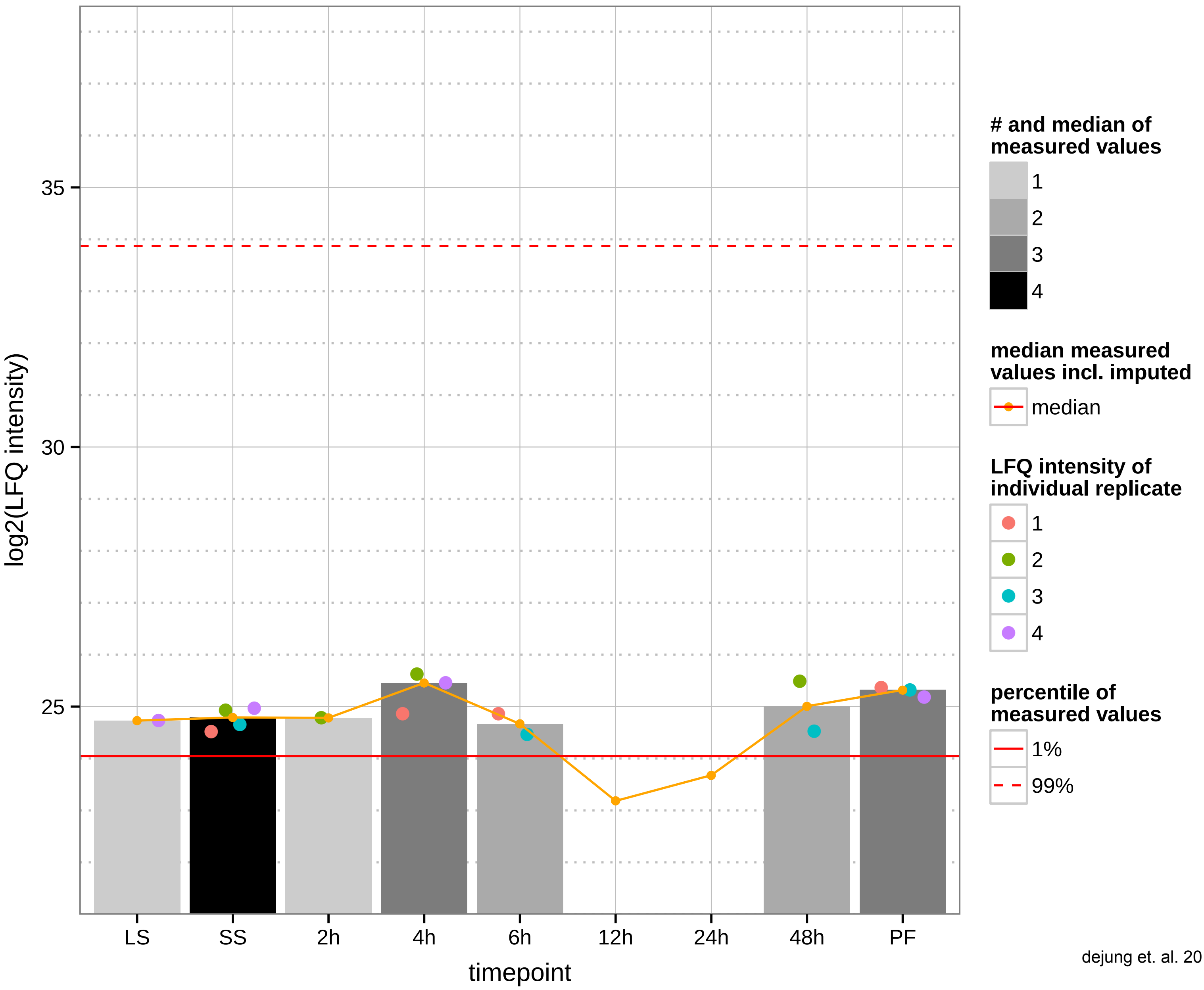

dejung et. al. 2015

ADP−ribosylation factor GTPase activating protein, putative
Tb927.9.15490
AGOF: ARF GTPase activator activity, DNA binding, zinc ion binding
AGOC: nucleus
AGOP: regulation of ARF GTPase activity
PGOF: ARF GTPase activator activity, zinc ion binding
PGOC: null
PGOP: regulation of ARF GTPase activity

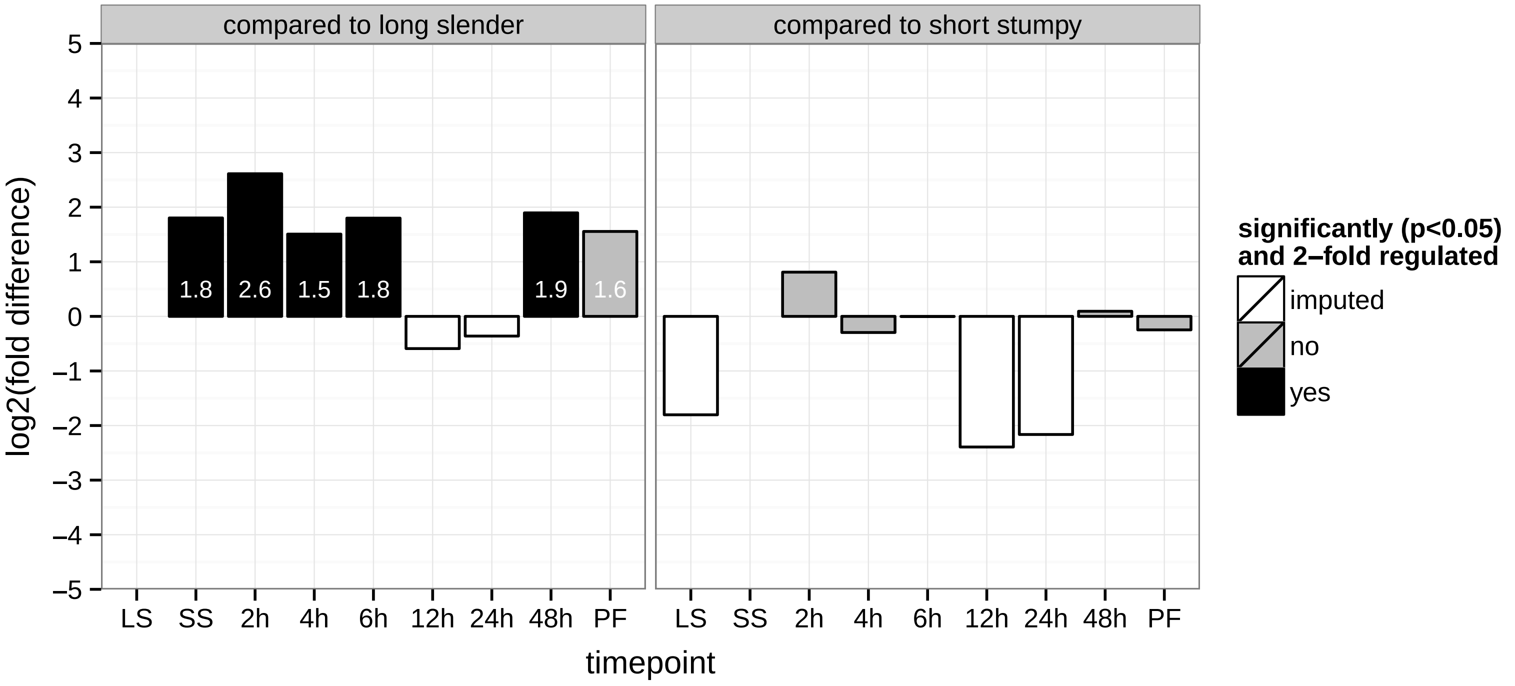

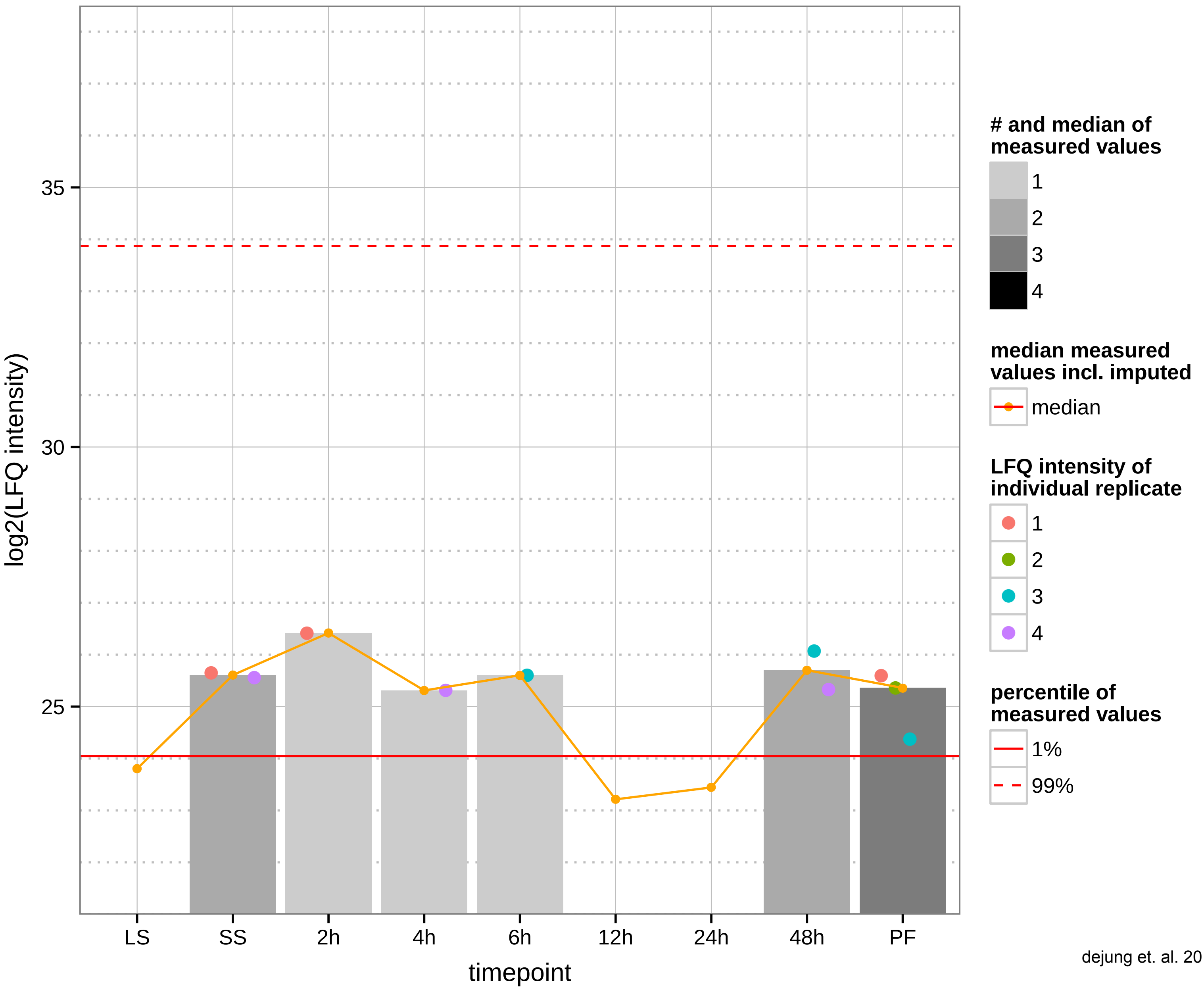

dejung et. al. 2015

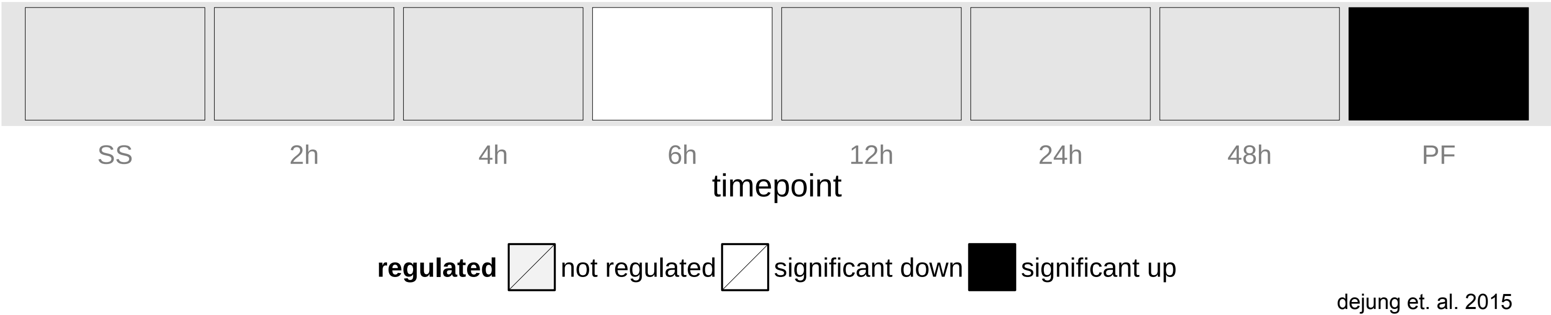
SS
2h
4h
6h
12h
24h
48h
PF
timepoint
regulated
not regulated
significant down
significant up
dejung et. al. 2015

dihydrolipoamide dehydrogenase, putative (GCVL−1)
Tb927.3.4390
AGOF: dihydrolipoyl dehydrogenase activity, flavin adenine dinucleotide binding
AGOC: cytoplasm
AGOP: cell redox homeostasis
PGOF: flavin adenine dinucleotide binding, oxidoreductase activity
PGOC: cytoplasm
PGOP: cell redox homeostasis, oxidation−reduction process

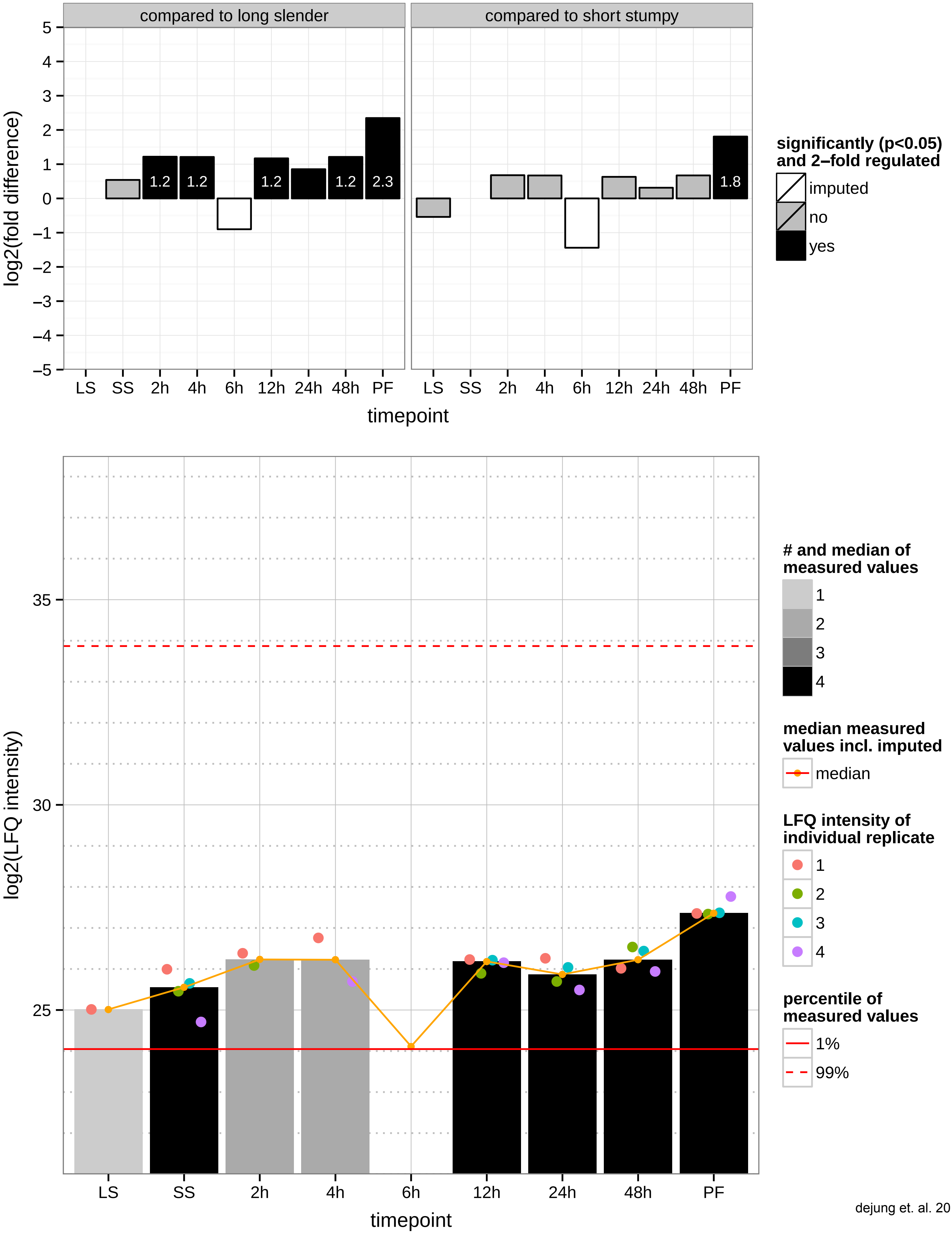

dejung et. al. 2015

hypothetical protein, conserved
Tb927.4.2360
AGOF: null
AGOC: mitochondrion
AGOP: null
PGOF: null
PGOC: null
PGOP: null

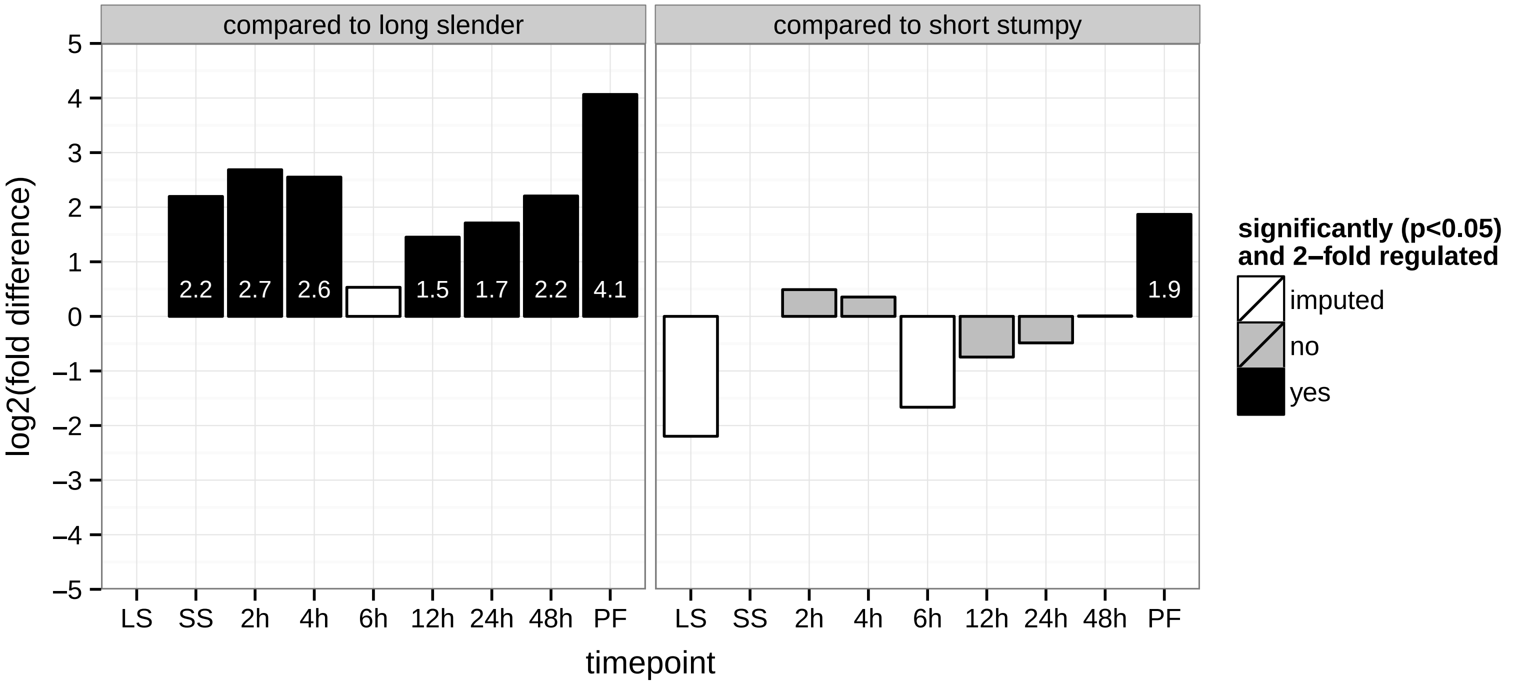

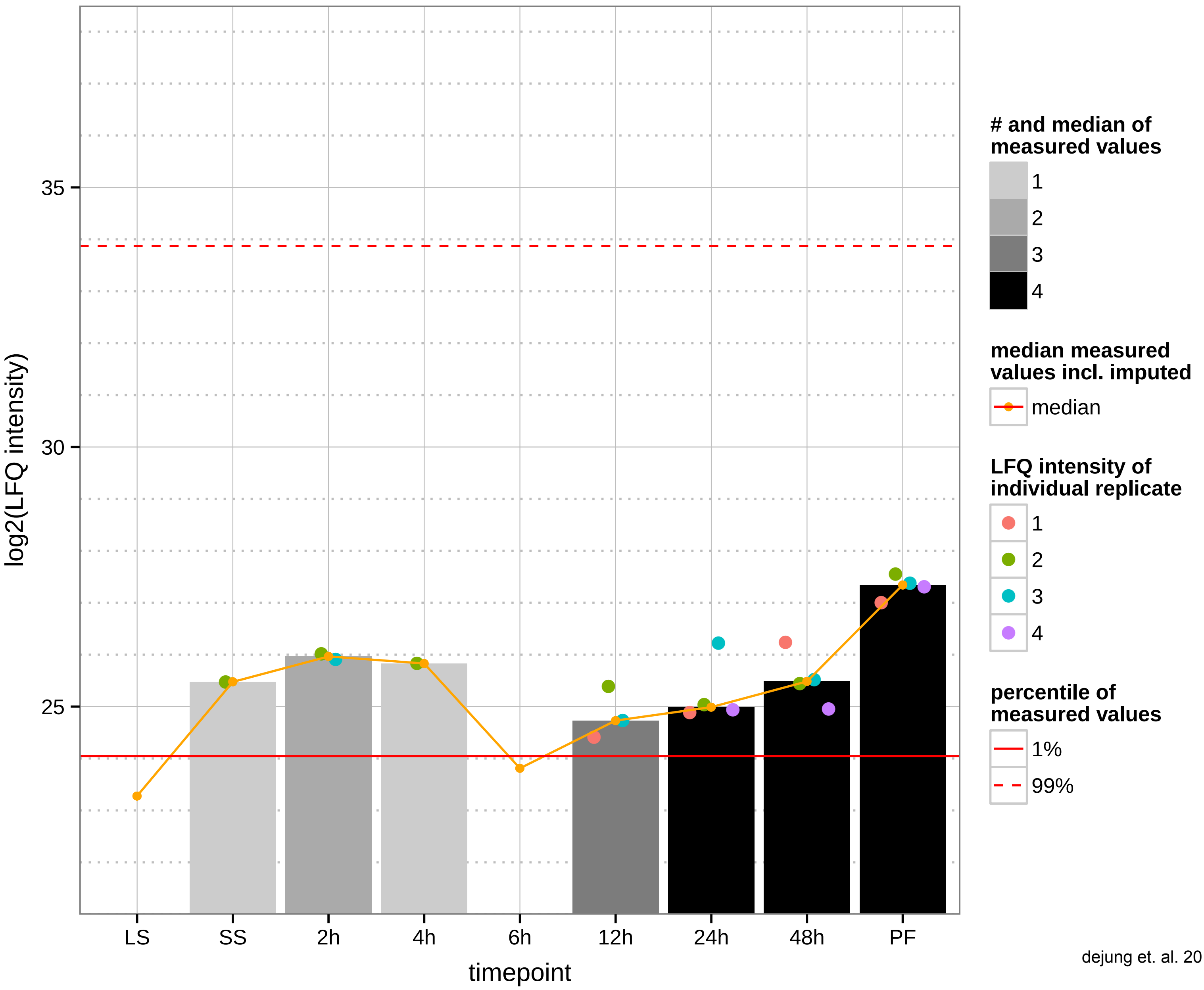

dejung et. al. 2015

hypothetical protein, conserved
Tb927.8.3210
AGOF: null
AGOC: null
AGOP: null
PGOF: null
PGOC: null
PGOP: null

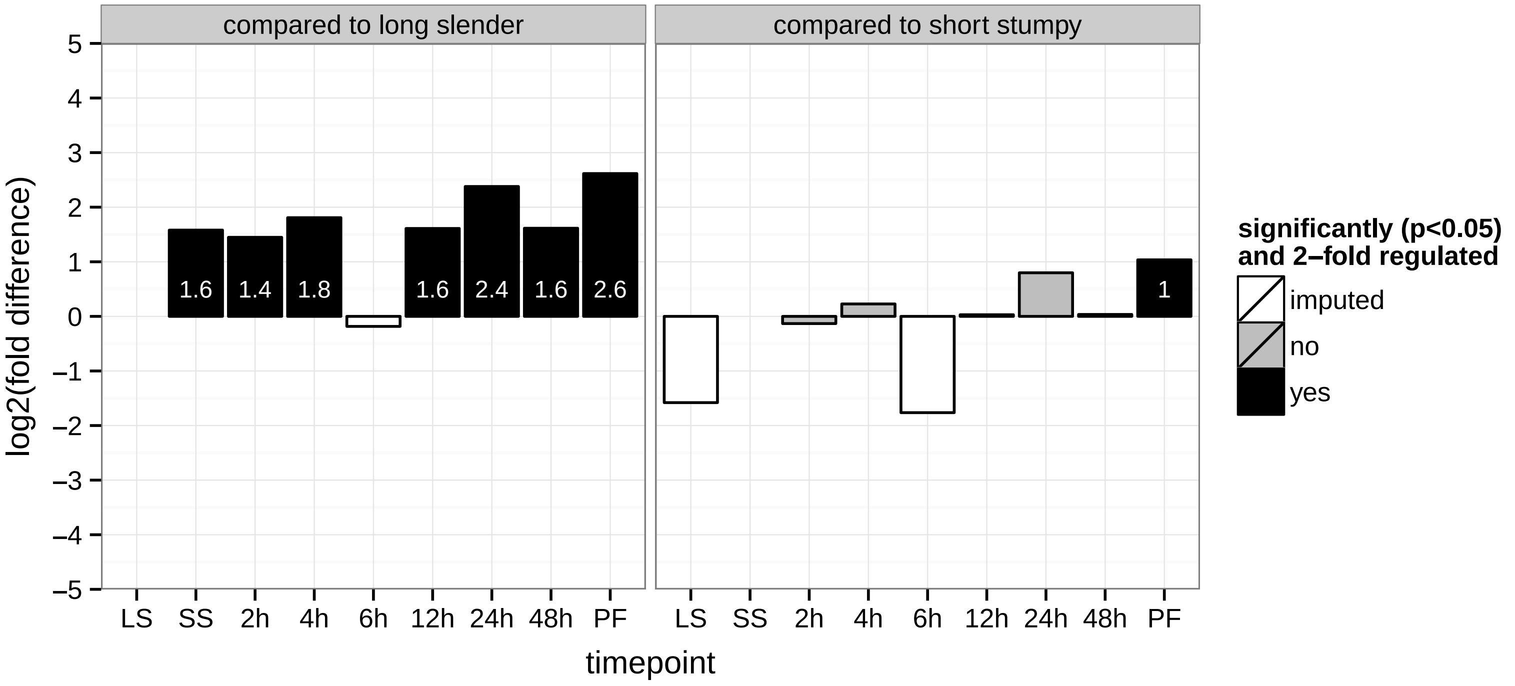

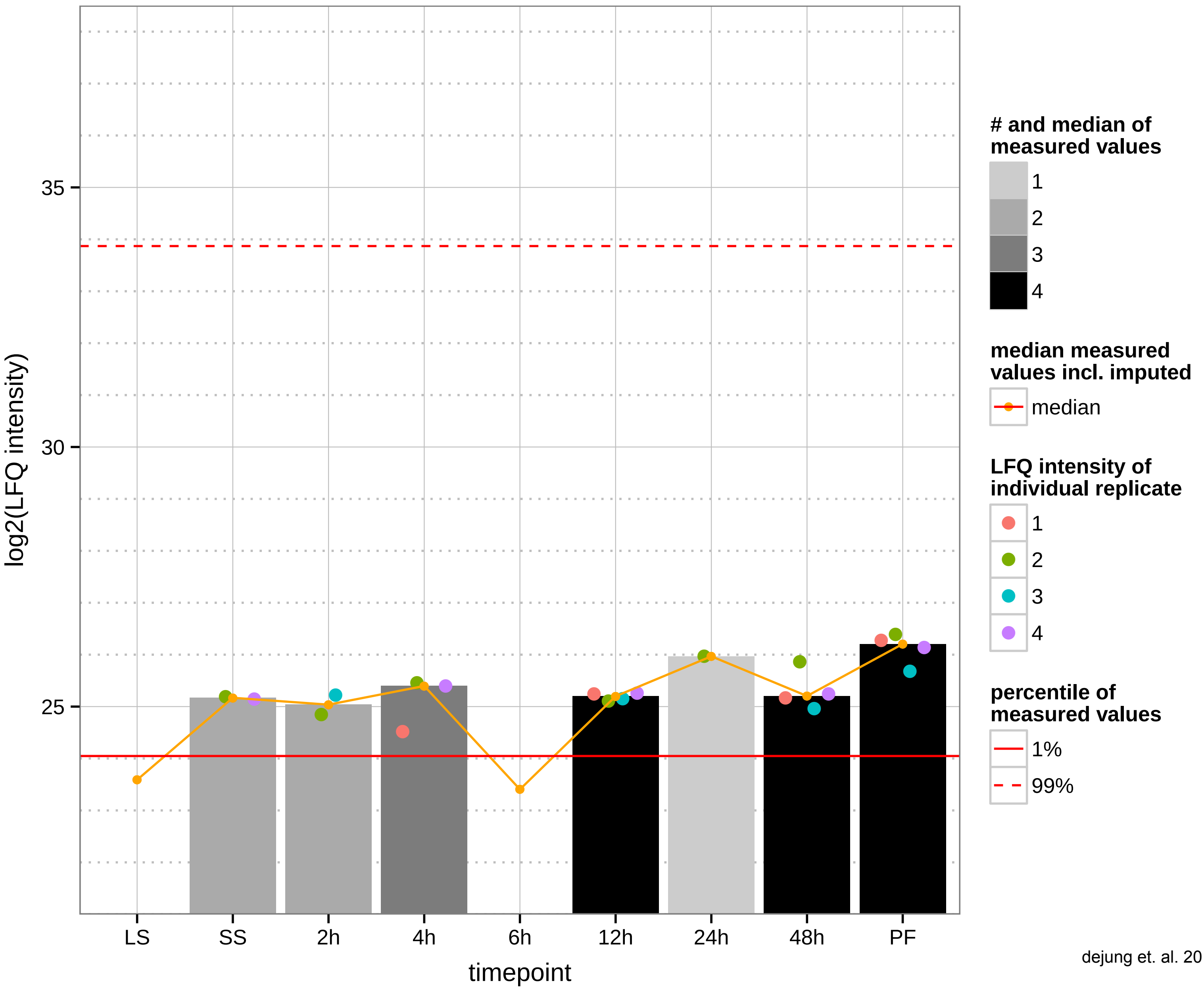

dejung et. al. 2015

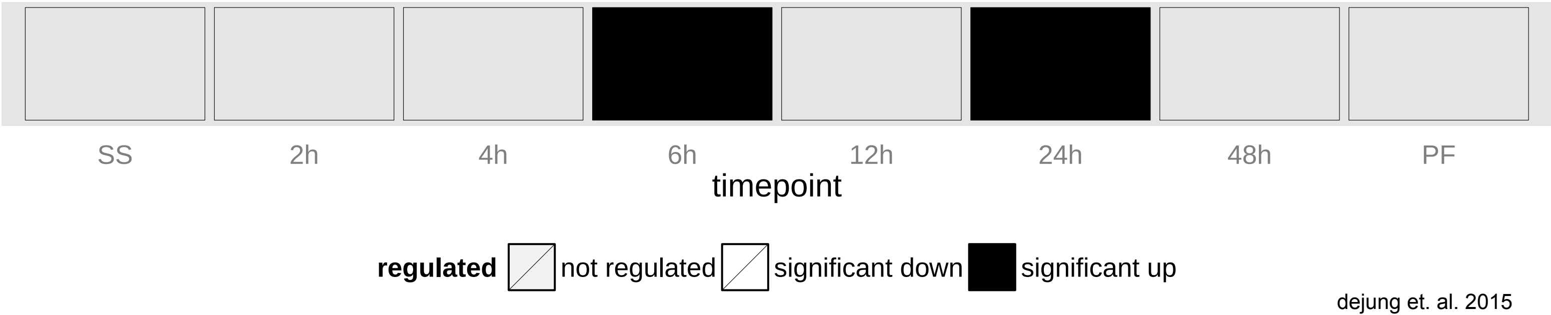
SS
2h
4h
6h
12h
24h
48h
PF
timepoint
regulated
not regulated
significant down
significant up
dejung et. al. 2015

mitochondrial carrier protein (MCP14)
Tb927.10.13120
AGOF: null
AGOC: membrane, mitochondrial part
AGOP: transport
PGOF: null
PGOC: null
PGOP: null

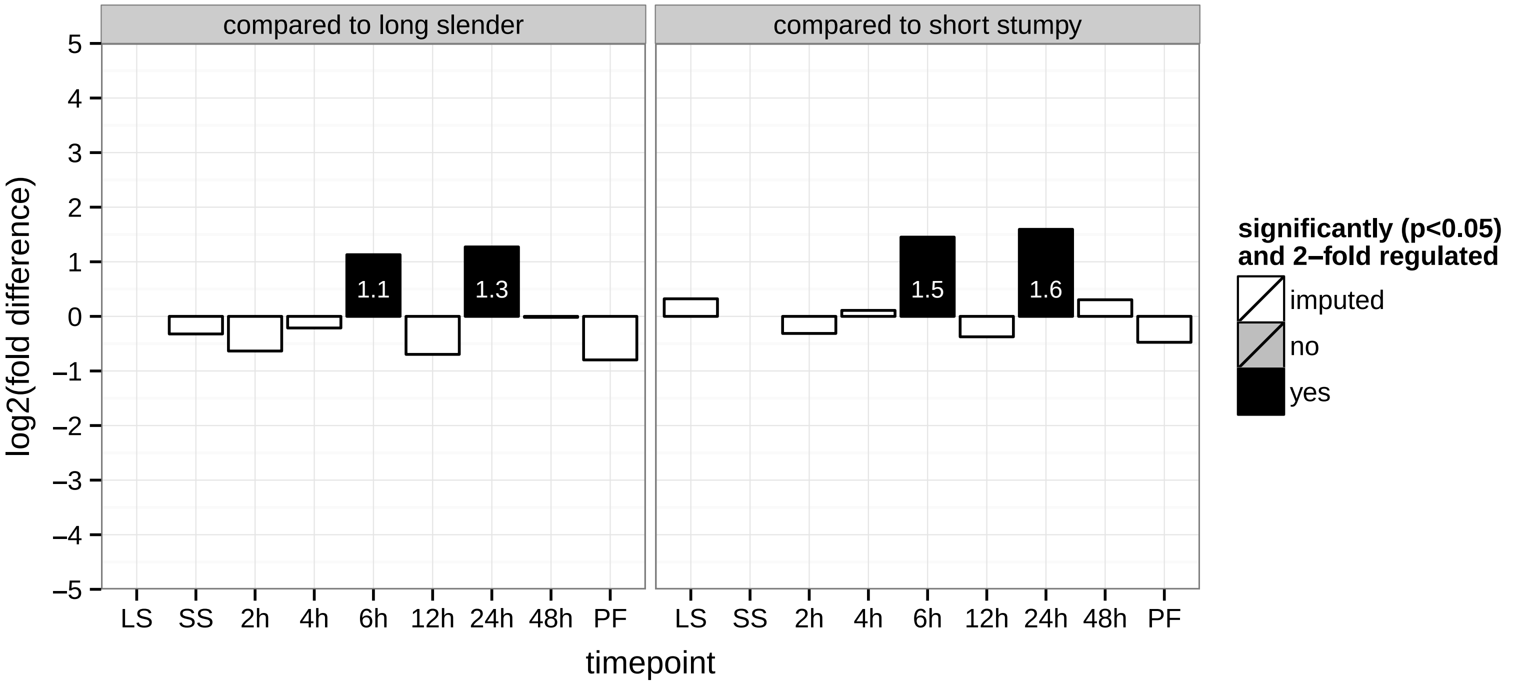

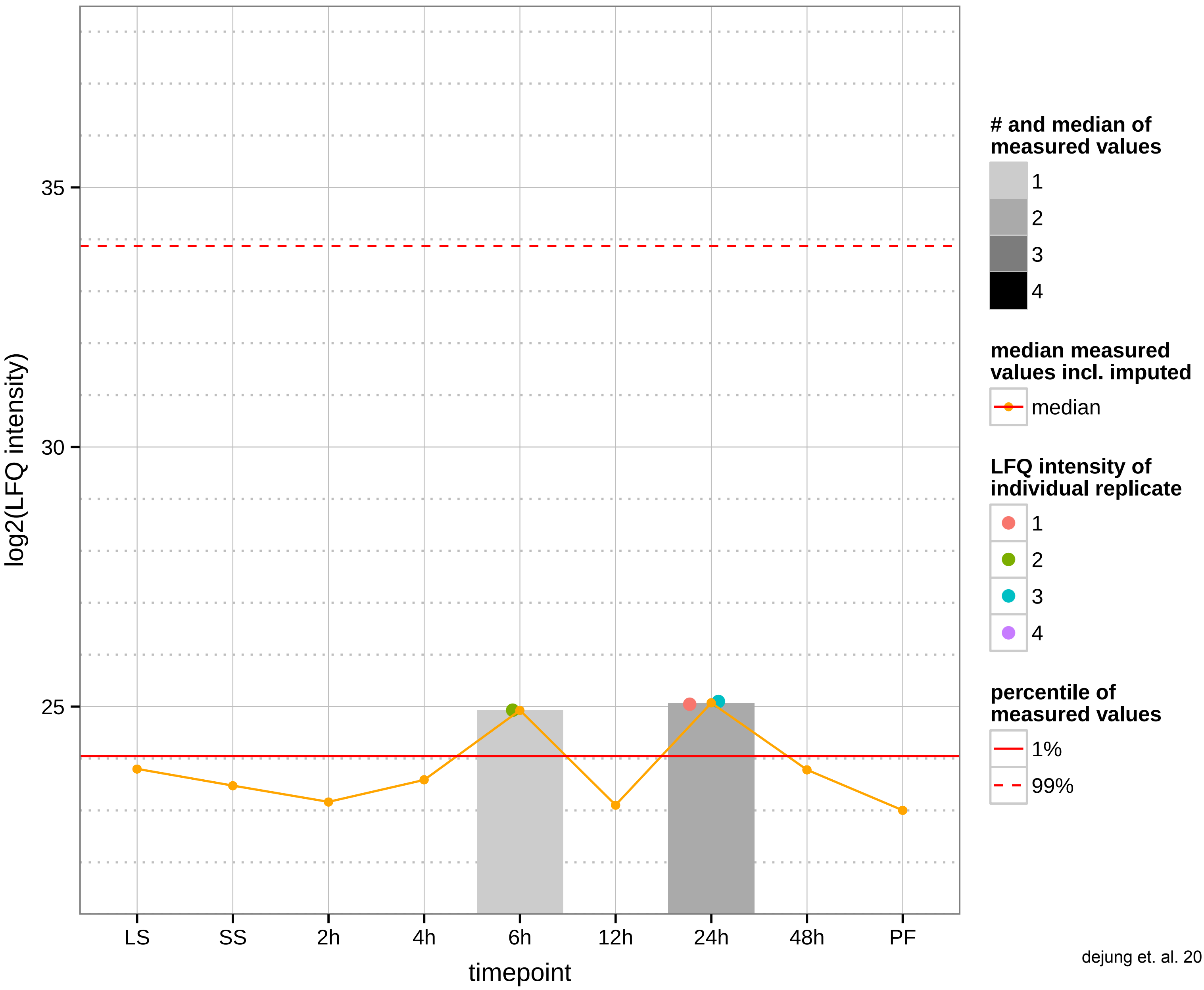

dejung et. al. 2015

hypothetical protein, conserved
Tb927.10.2510;Tb11.v5.0623
AGOF: null
AGOC: null
AGOP: null
PGOF: null
PGOC: null
PGOP: null

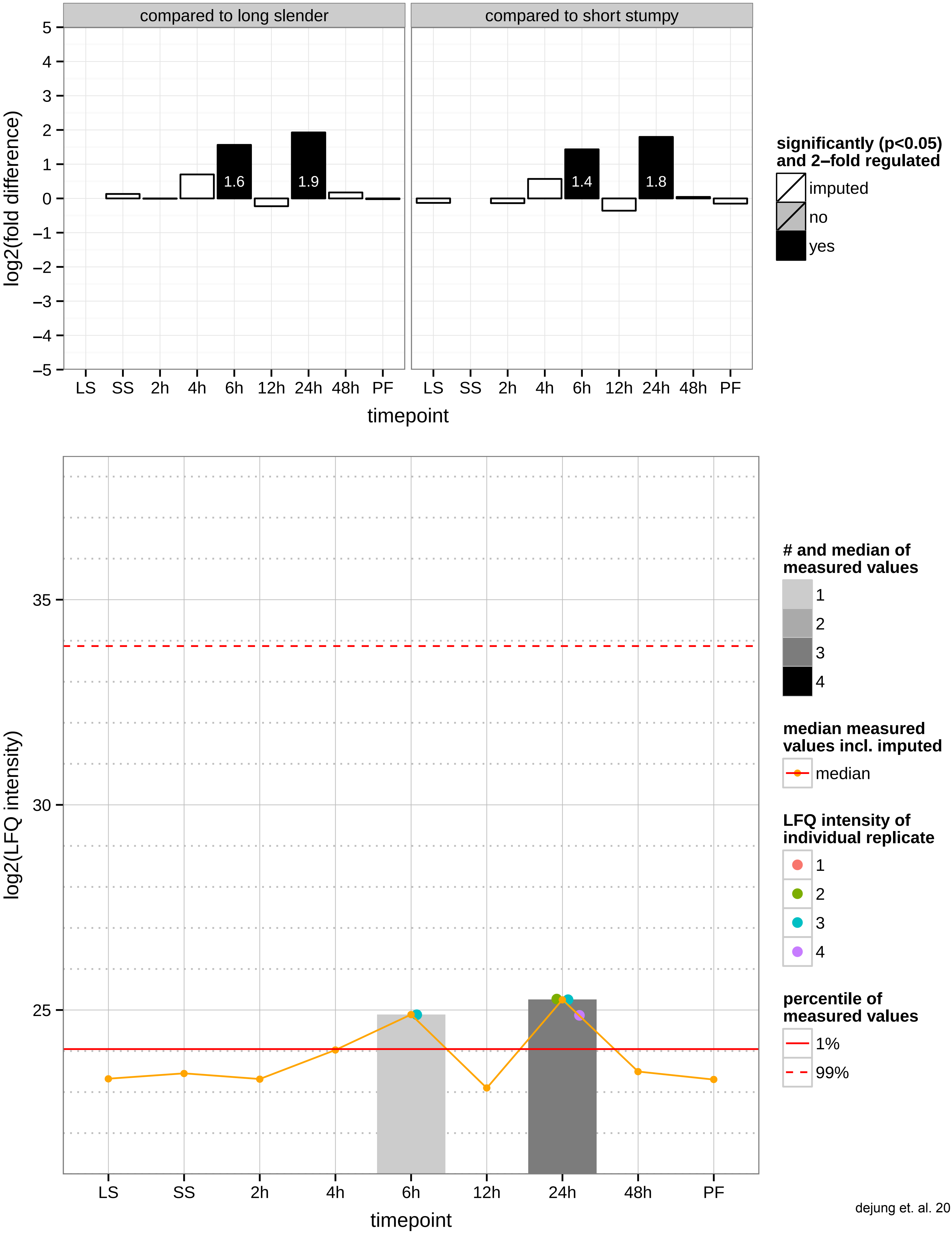

dejung et. al. 2015

centrin, putative
Tb927.10.8710
AGOF: calcium ion binding
AGOC: null
AGOP: null
PGOF: calcium ion binding
PGOC: null
PGOP: null

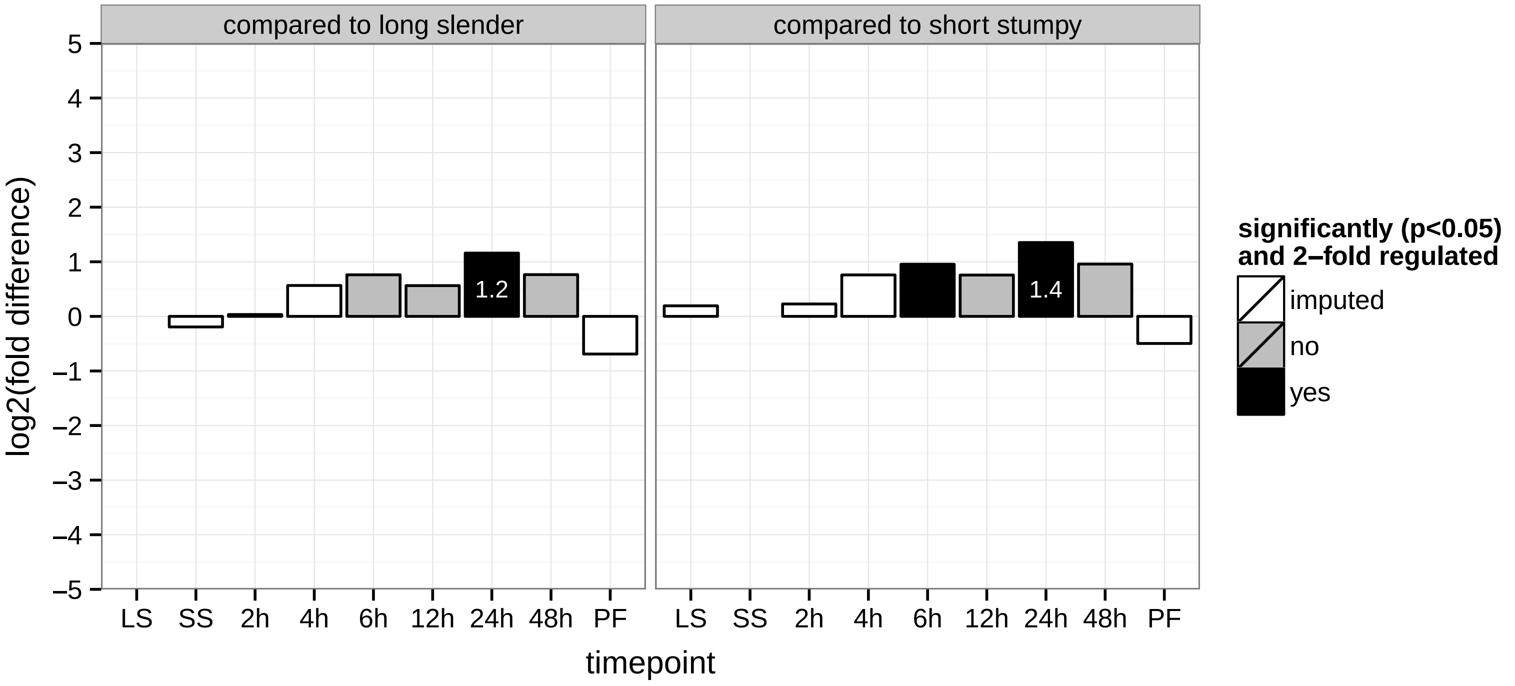

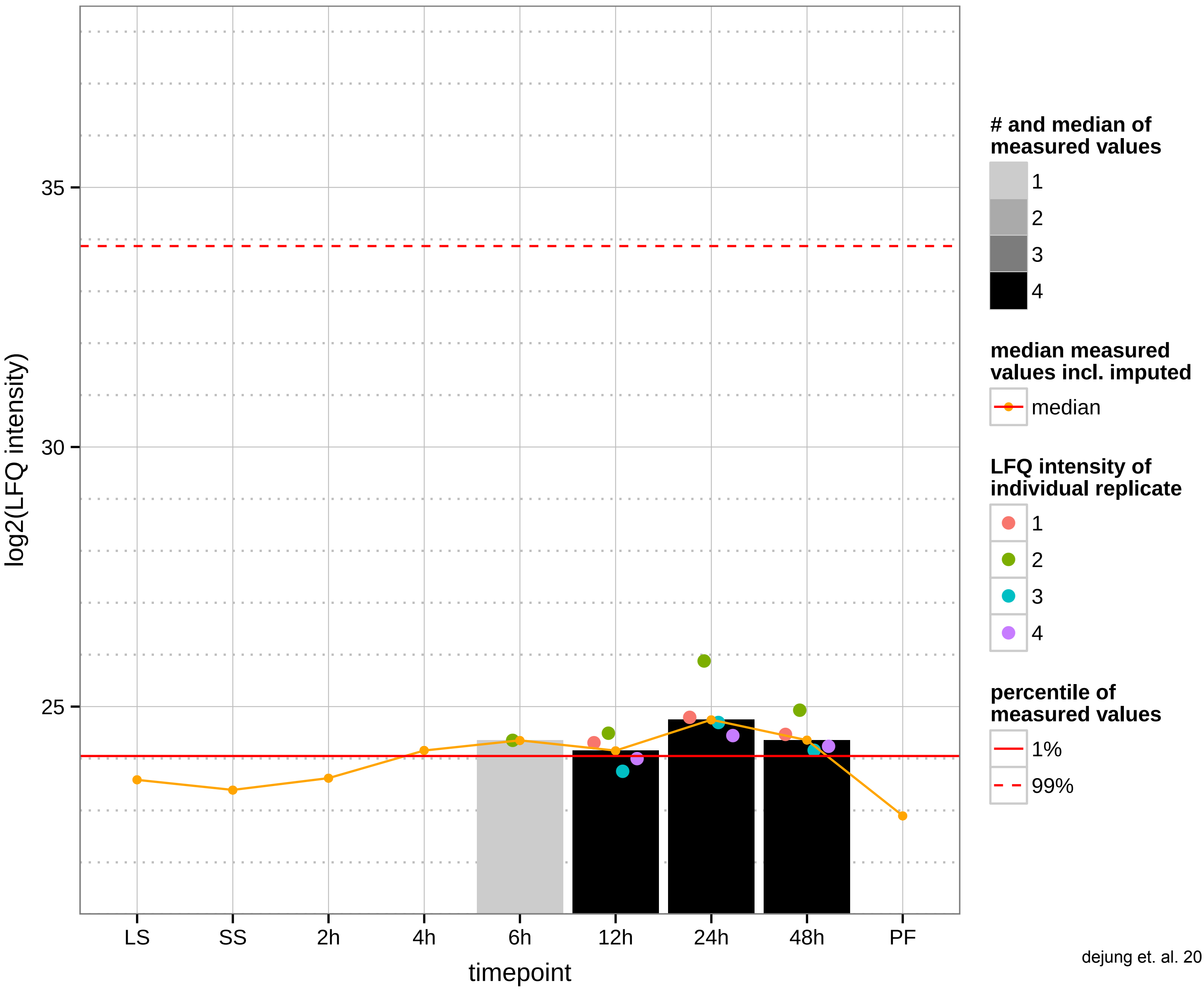

dejung et. al. 2015

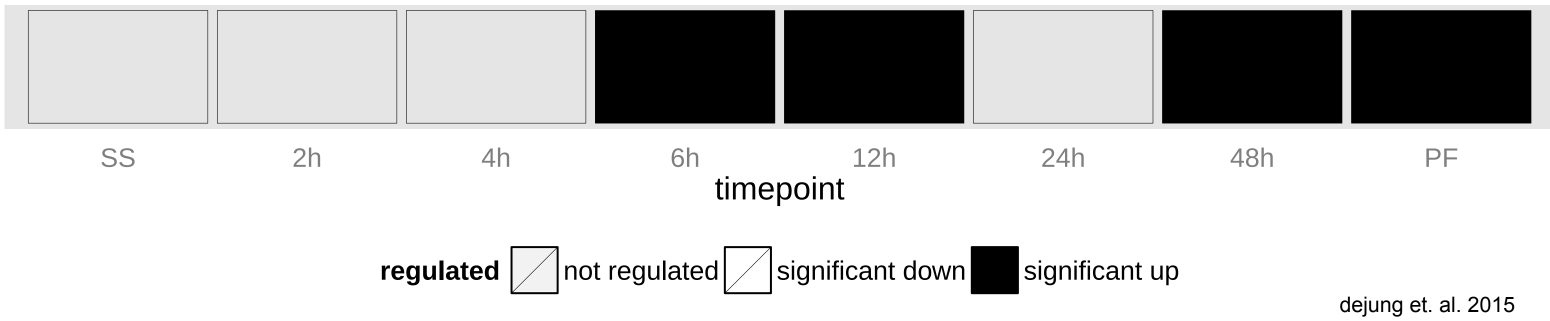

SS
2h
4h
6h
12h
24h
48h
PF
timepoint
regulated
not regulated
significant down
significant up
dejung et. al. 2015

transmembrane protein, putative
Tb927.1.860
AGOF: null
AGOC: null
AGOP: null
PGOF: null
PGOC: null
PGOP: null

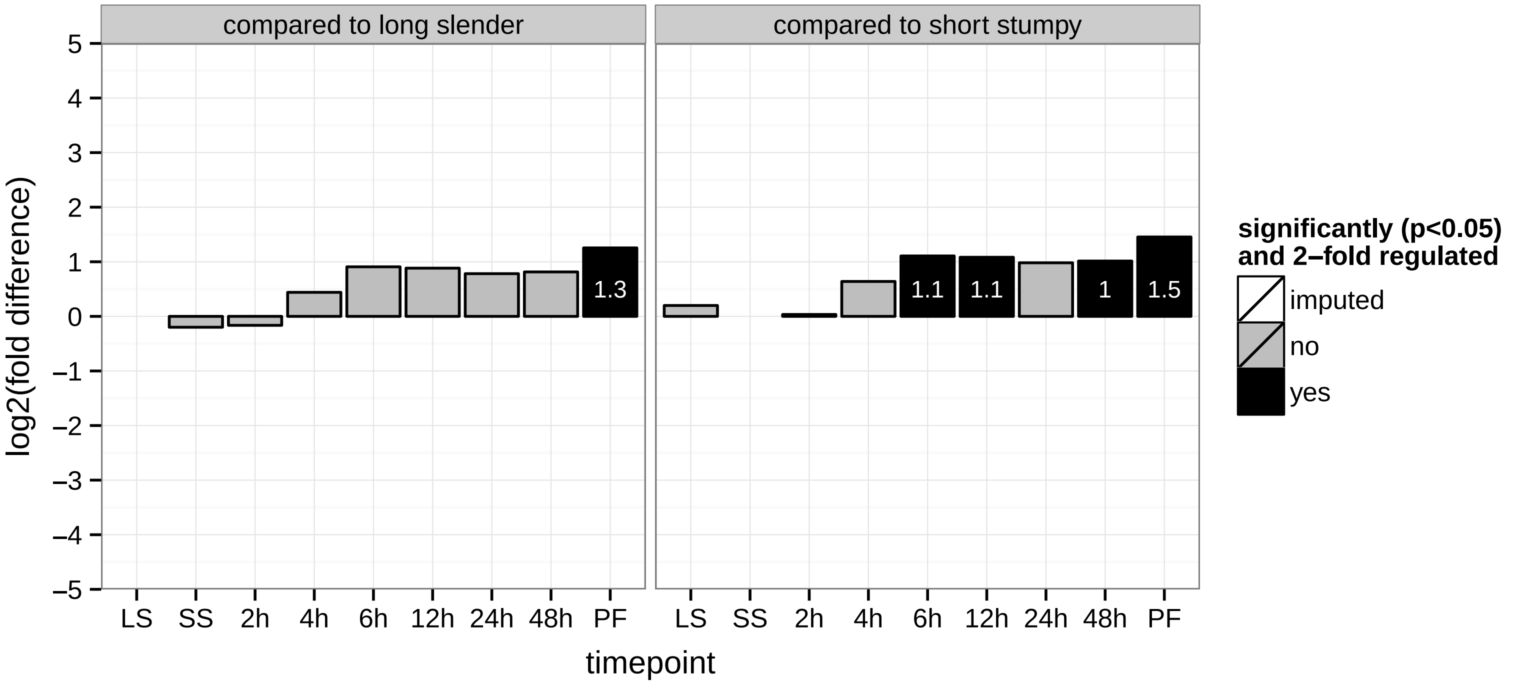

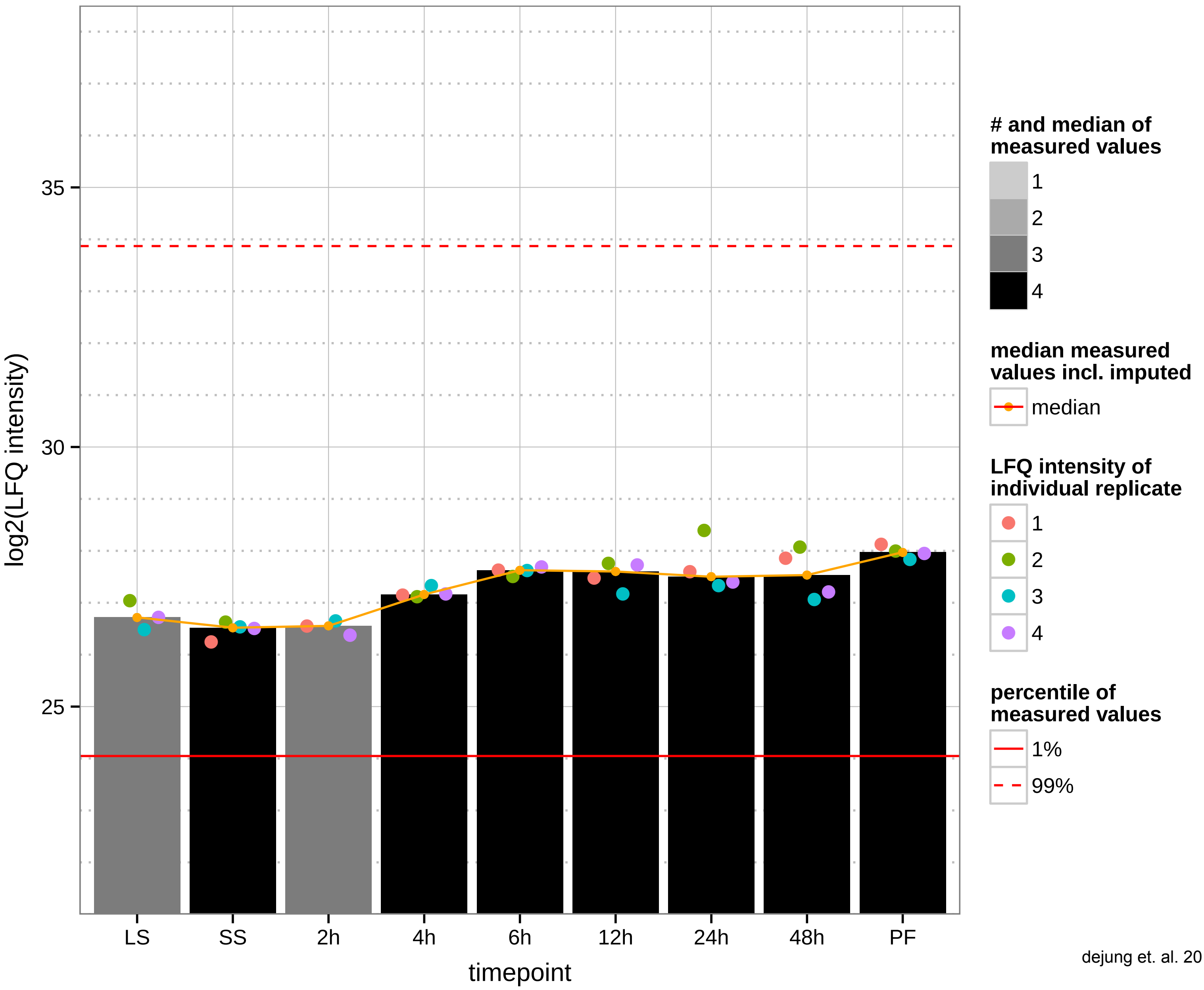

dejung et. al. 2015

hypothetical protein, conserved
Tb927.10.13550
AGOF: null
AGOC: null
AGOP: null
PGOF: null
PGOC: null
PGOP: null

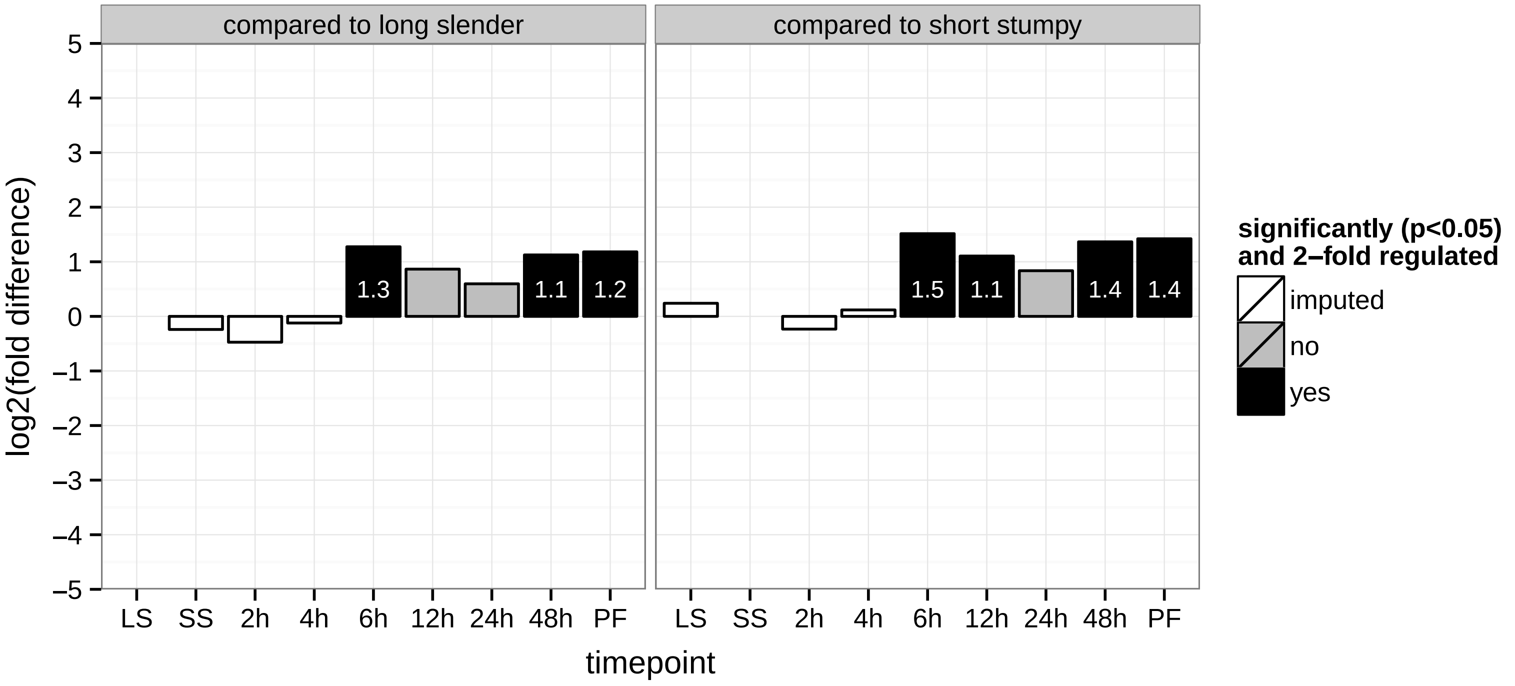

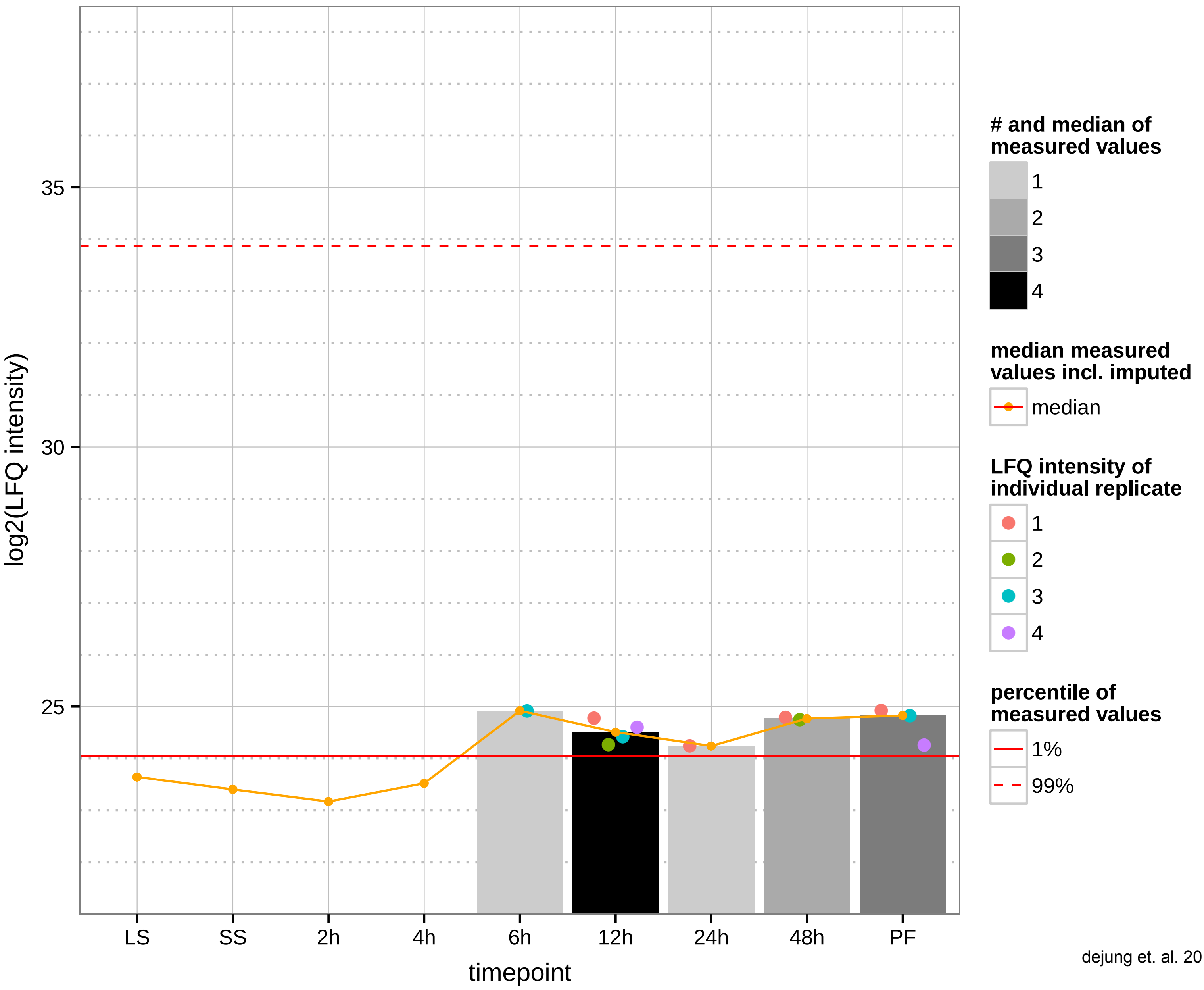

dejung et. al. 2015

zinc–finger protein, conserved
Tb927.6.3540
AGOF: zinc ion binding
AGOC: intracellular
AGOP: null
PGOF: zinc ion binding
PGOC: intracellular
PGOP: null

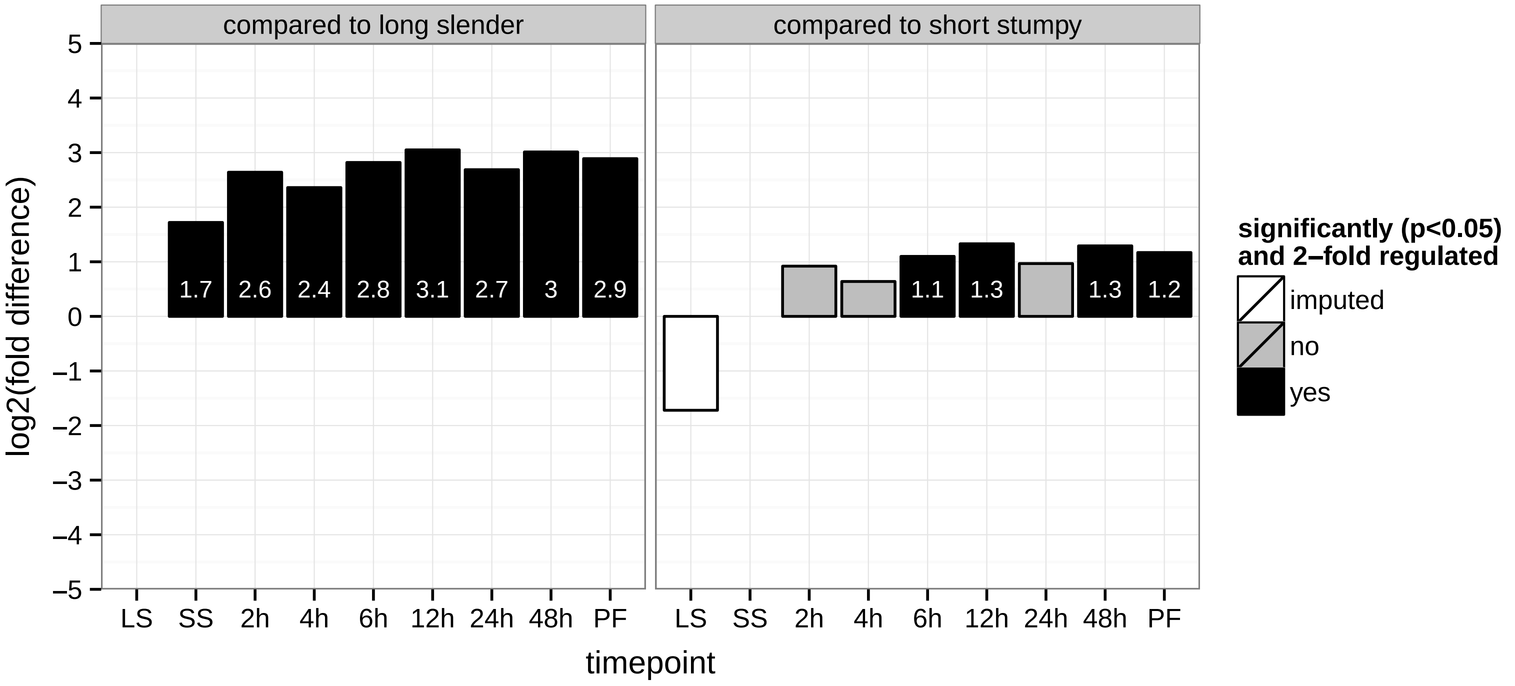

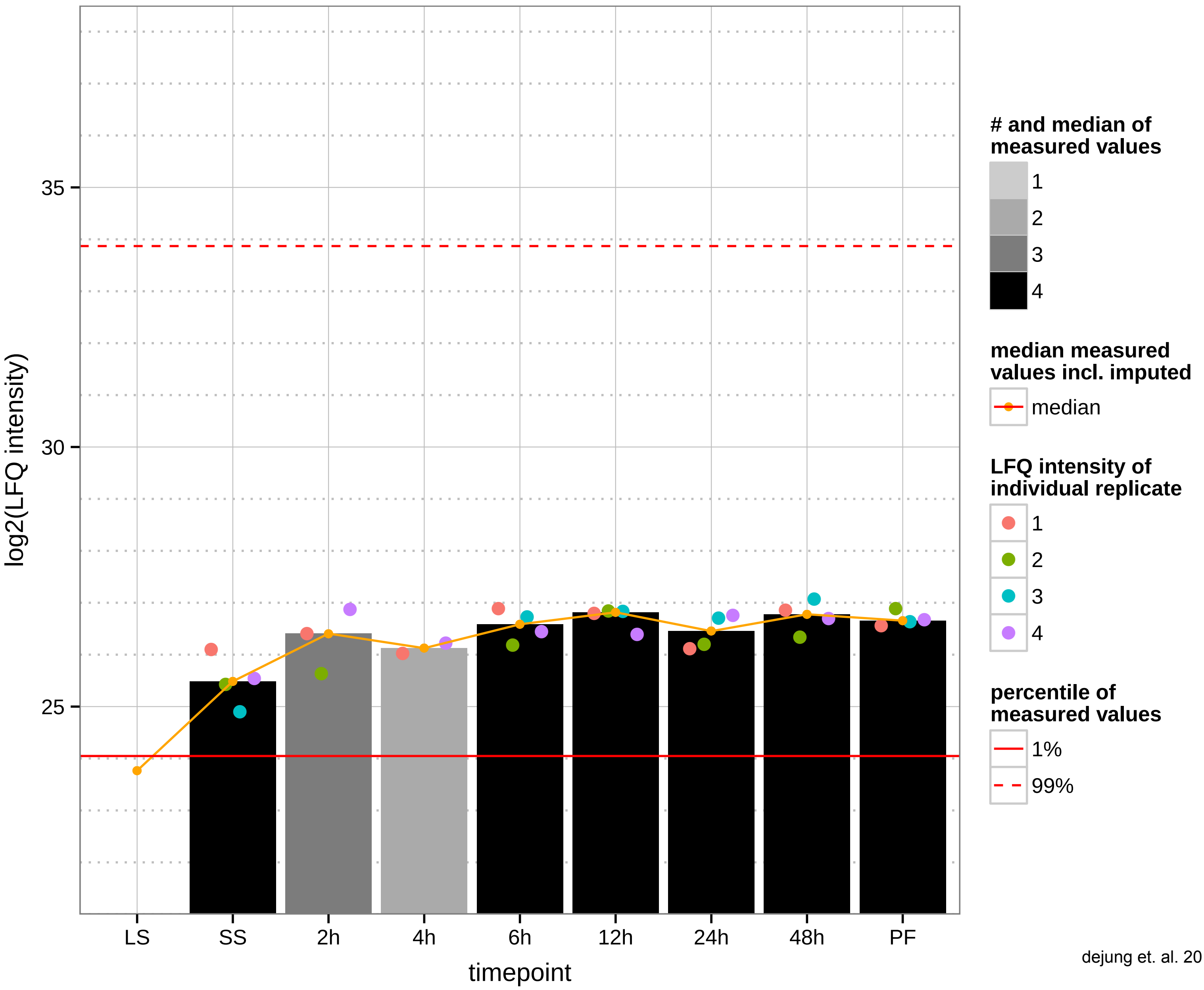

dejung et. al. 2015

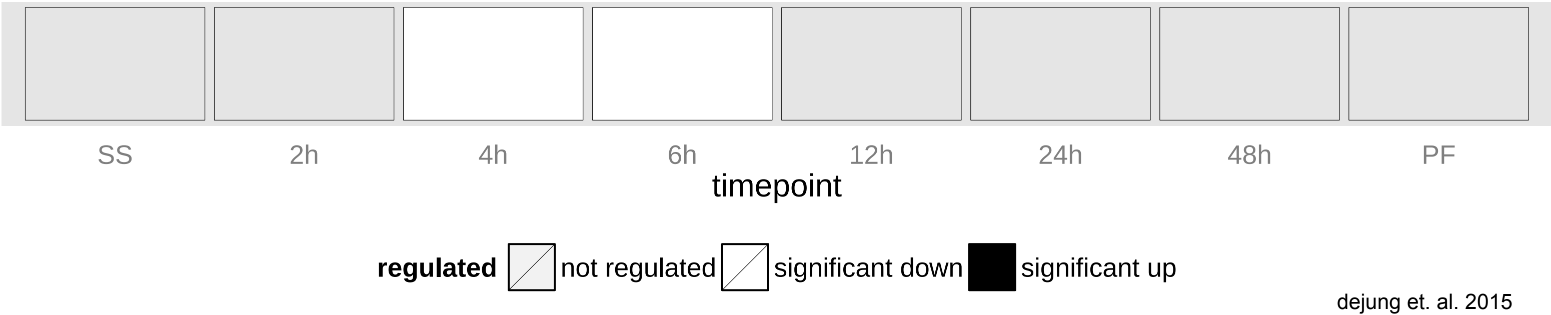
SS
2h
4h
6h
12h
24h
48h
PF
timepoint
regulated
not regulated
significant down
significant up
dejung et. al. 2015

mitochondrial RNA binding complex 1 subunit (MRB6070)
Tb927.2.6070
AGOF: RNA binding, zinc ion binding
AGOC: intracellular, mitochondrion
AGOP: cytidine to uridine editing, mRNA stabilization
PGOF: zinc ion binding
PGOC: intracellular
PGOP: null

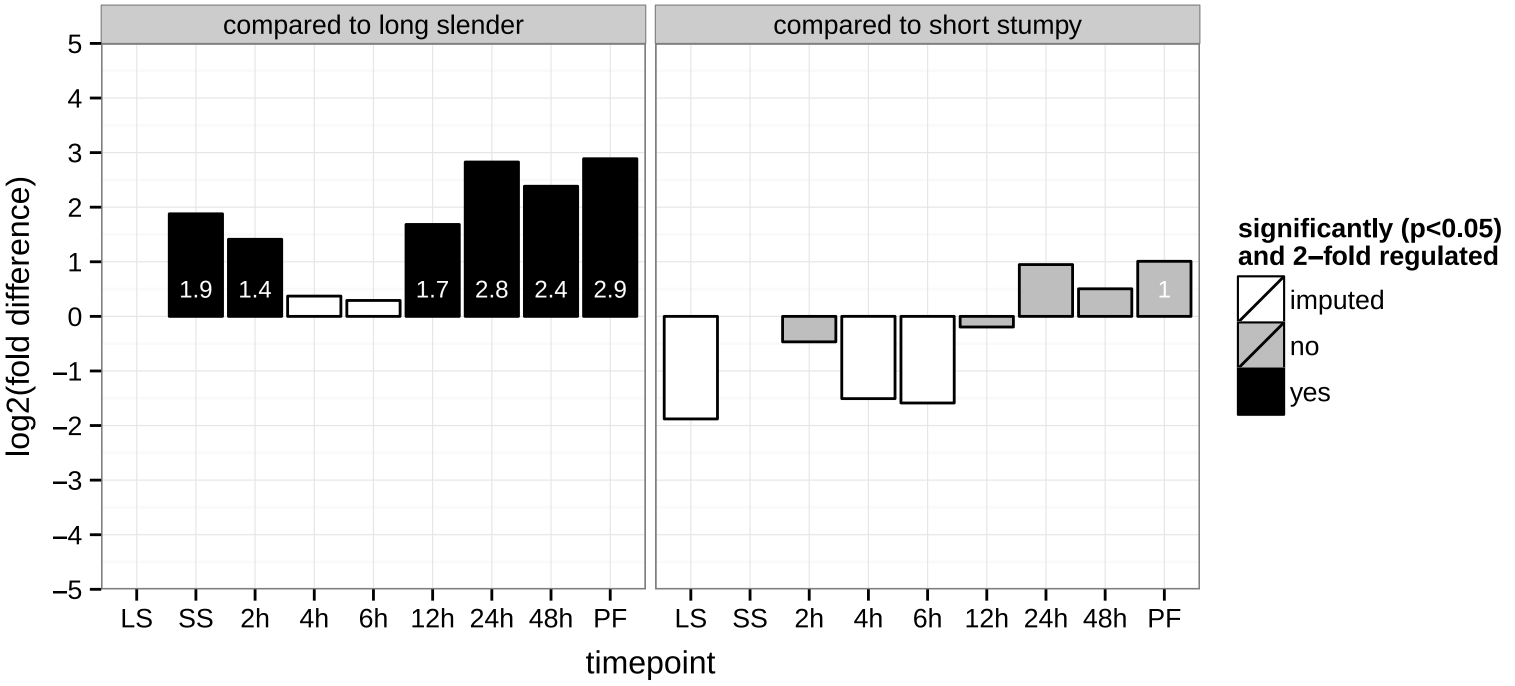

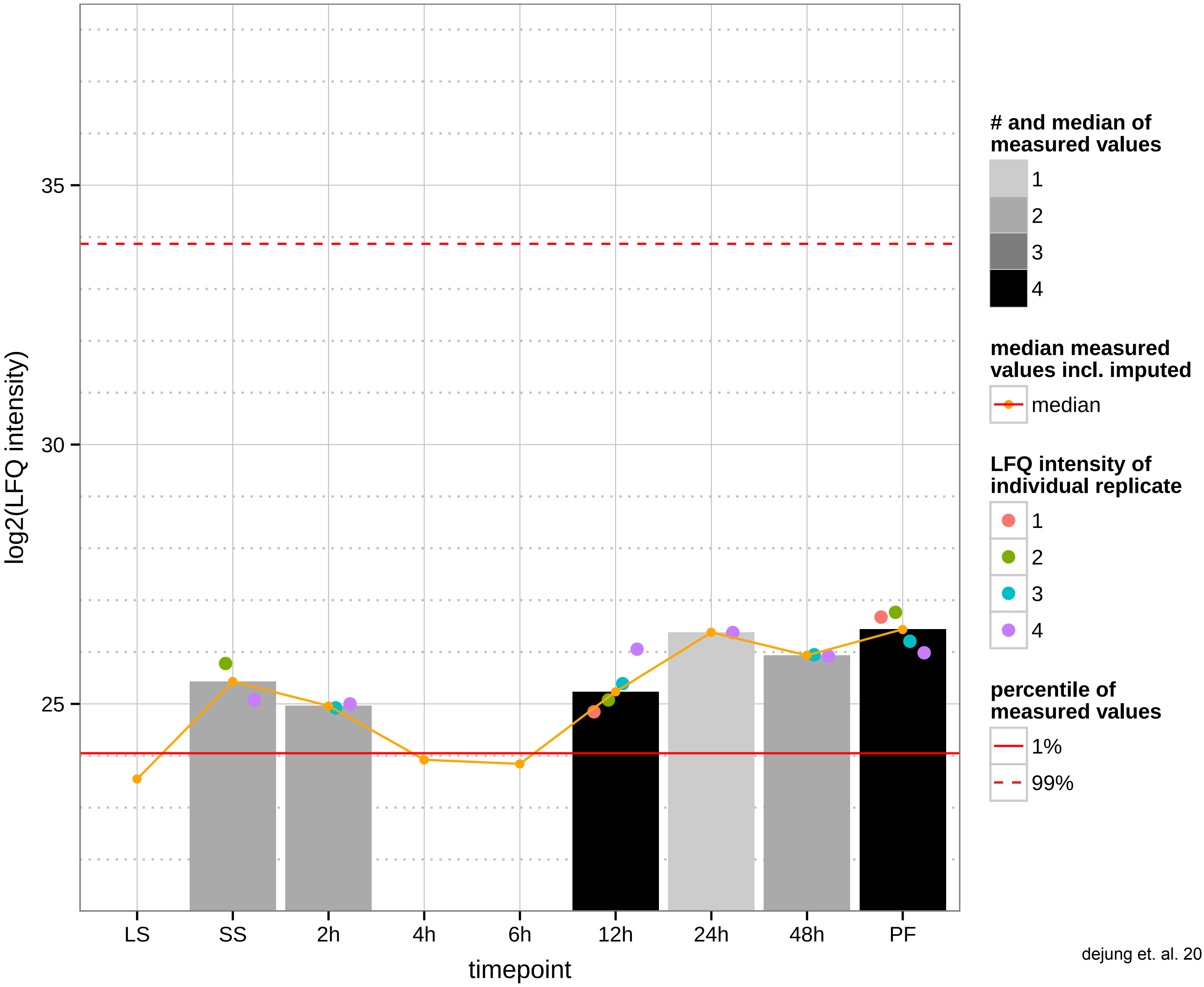

dejung et. al. 2015

hypothetical protein, conserved
Tb927.3.5240
AGOF: null
AGOC: mitochondrion
AGOP: null
PGOF: null
PGOC: null
PGOP: null

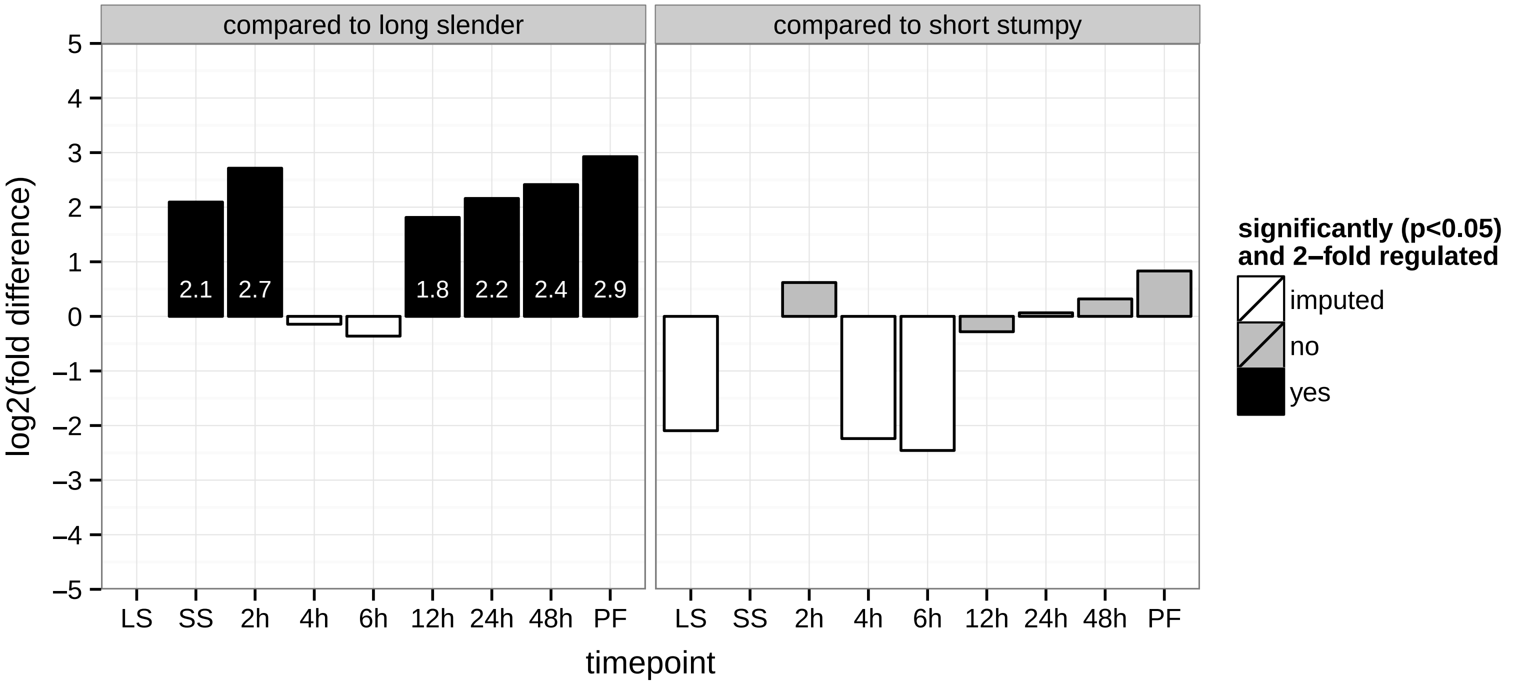

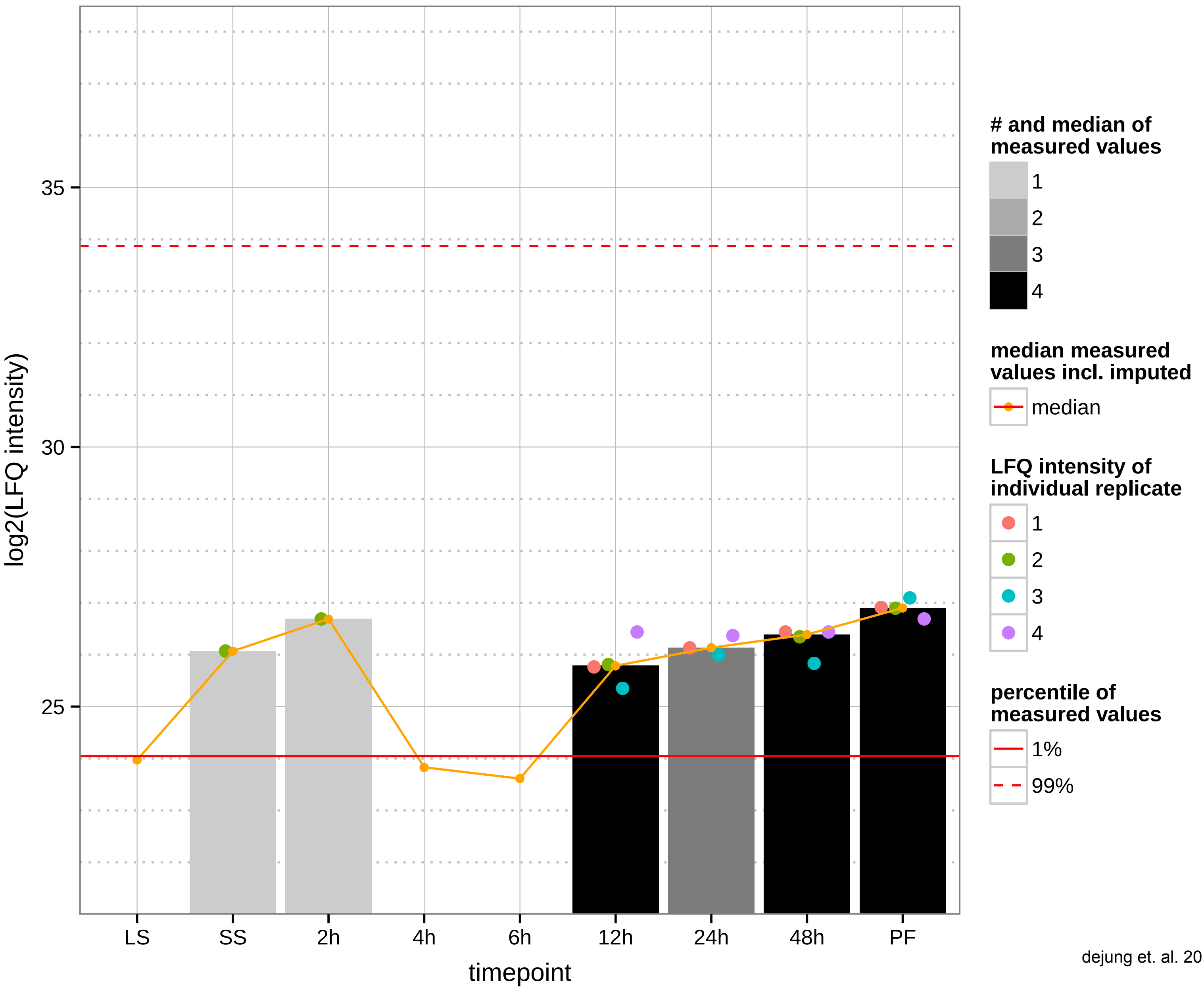

dejung et. al. 2015

hypothetical protein, conserved
Tb927.4.730
AGOF: null
AGOC: null
AGOP: null
PGOF: null
PGOC: null
PGOP: null

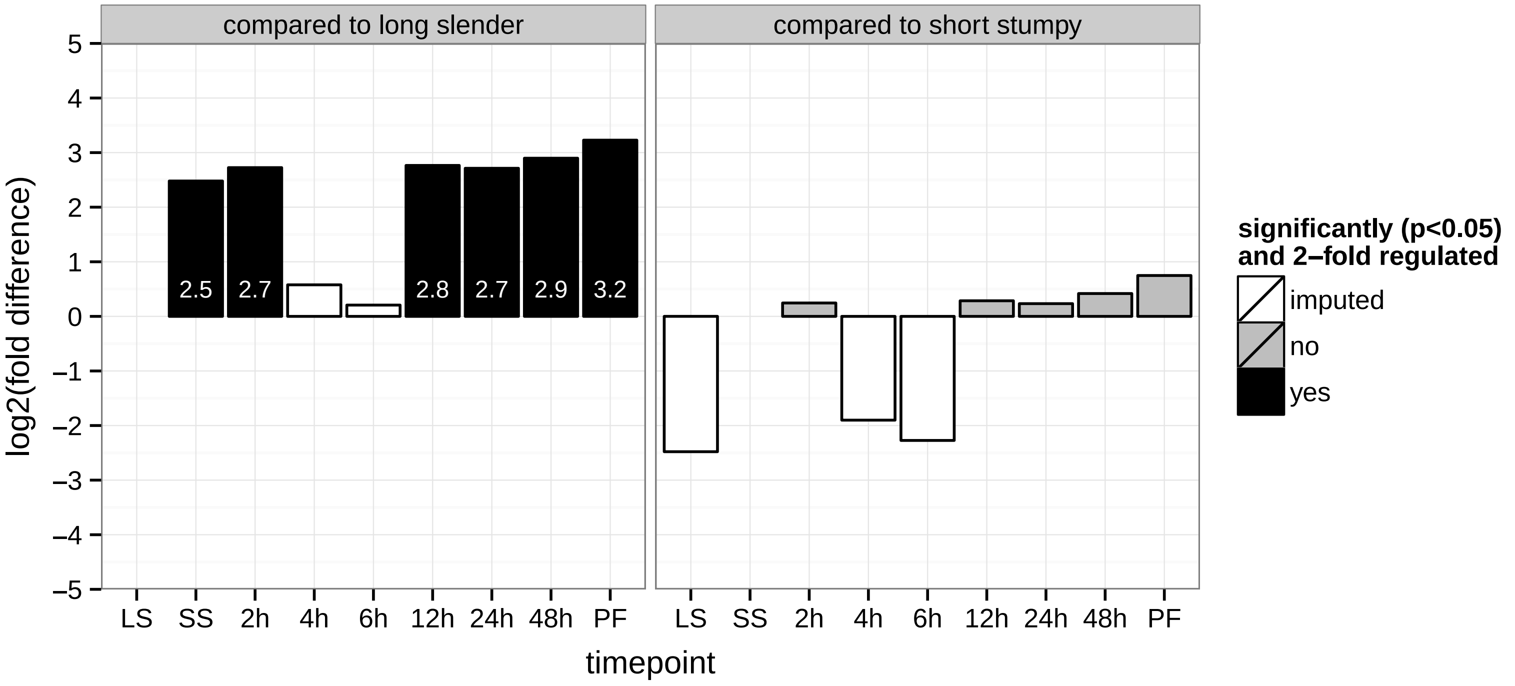

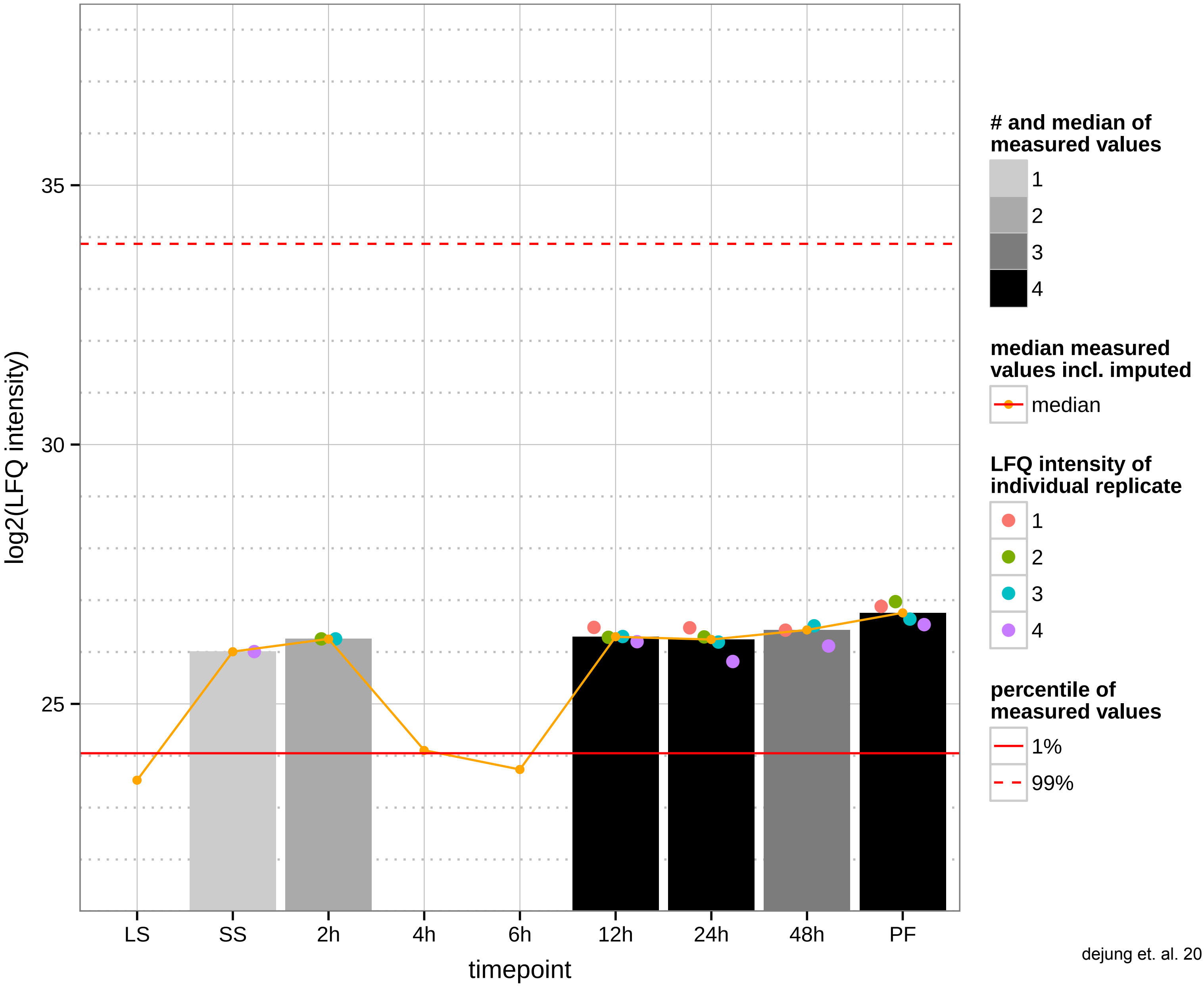

dejung et. al. 2015

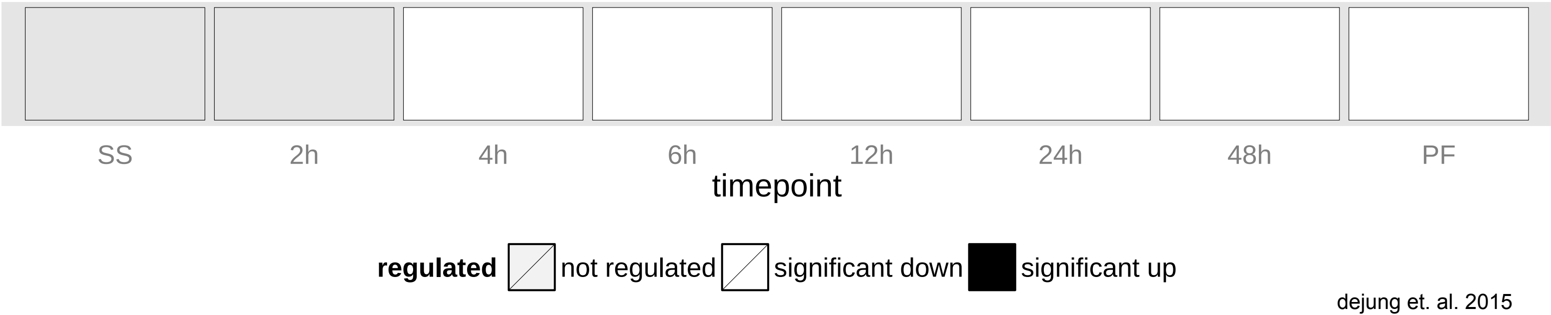
SS
2h
4h
6h
12h
24h
48h
PF
timepoint
regulated
not regulated
significant down
significant up
dejung et. al. 2015

75 kDa invariant surface glycoprotein, putative
Tb927.5.390;Tb11.v5.0228
AGOF: null
AGOC: null, plasma membrane
AGOP: null
PGOF: null
PGOC: null
PGOP: null

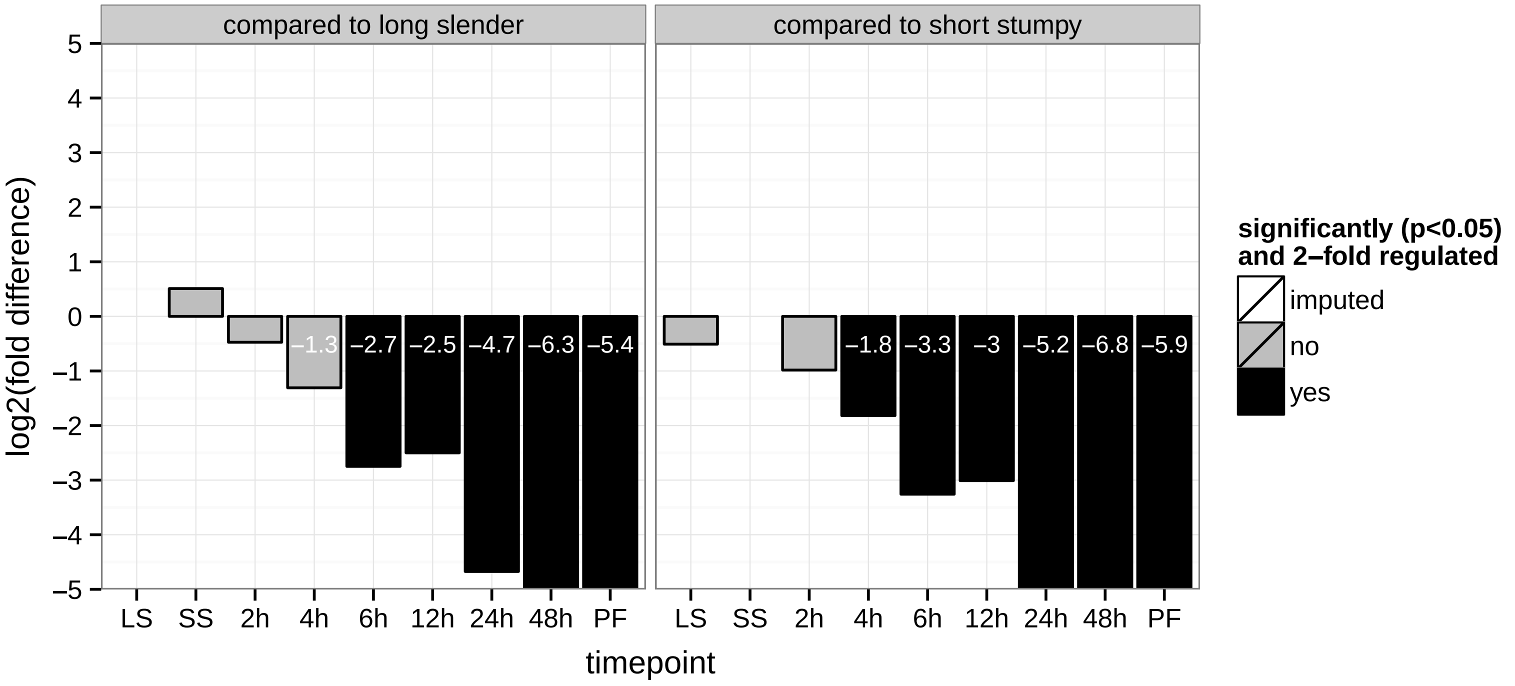

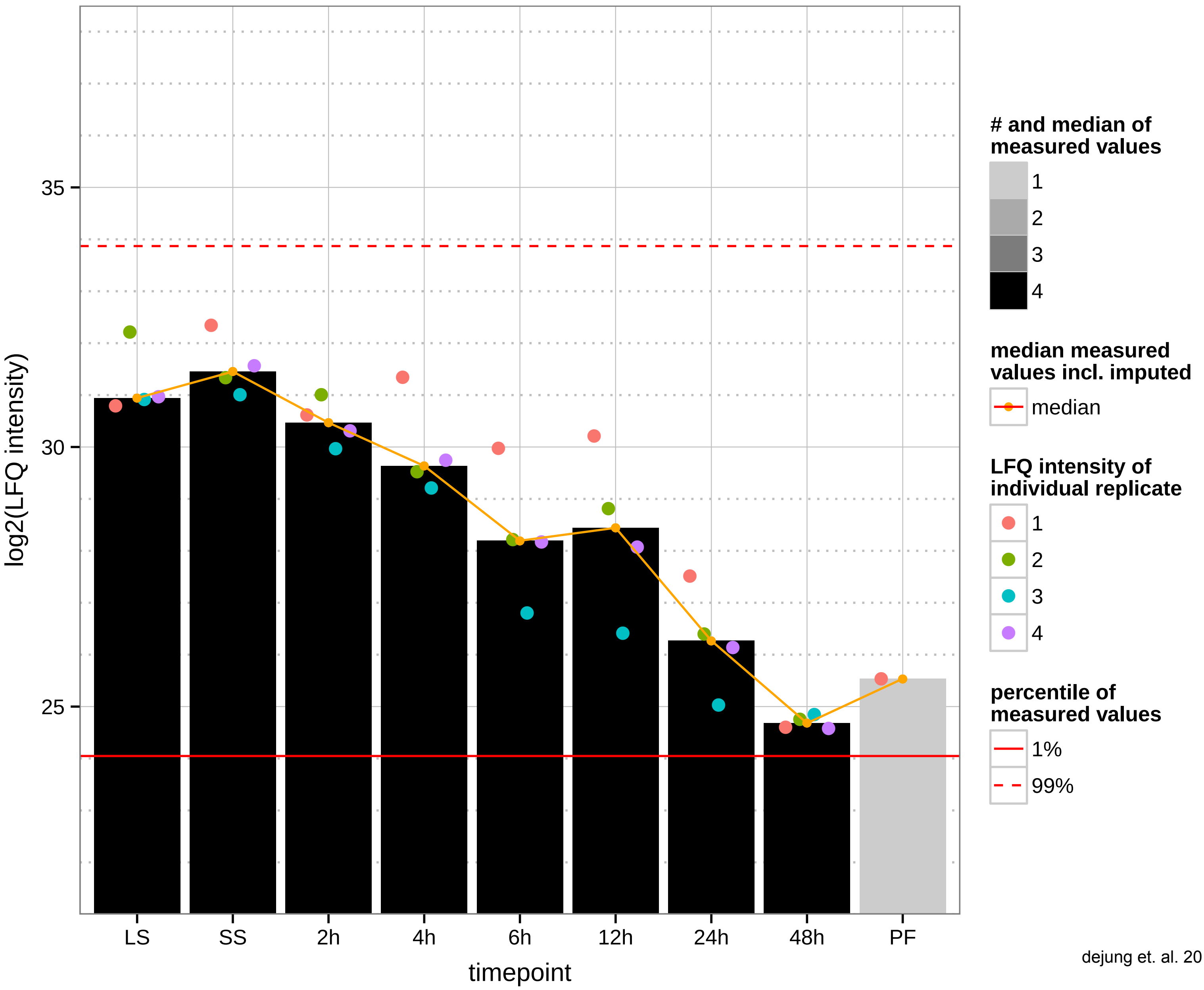

dejung et. al. 2015

hypothetical protein, conserved
Tb927.10.2080
AGOF: null
AGOC: null
AGOP: null
PGOF: null
PGOC: null
PGOP: null

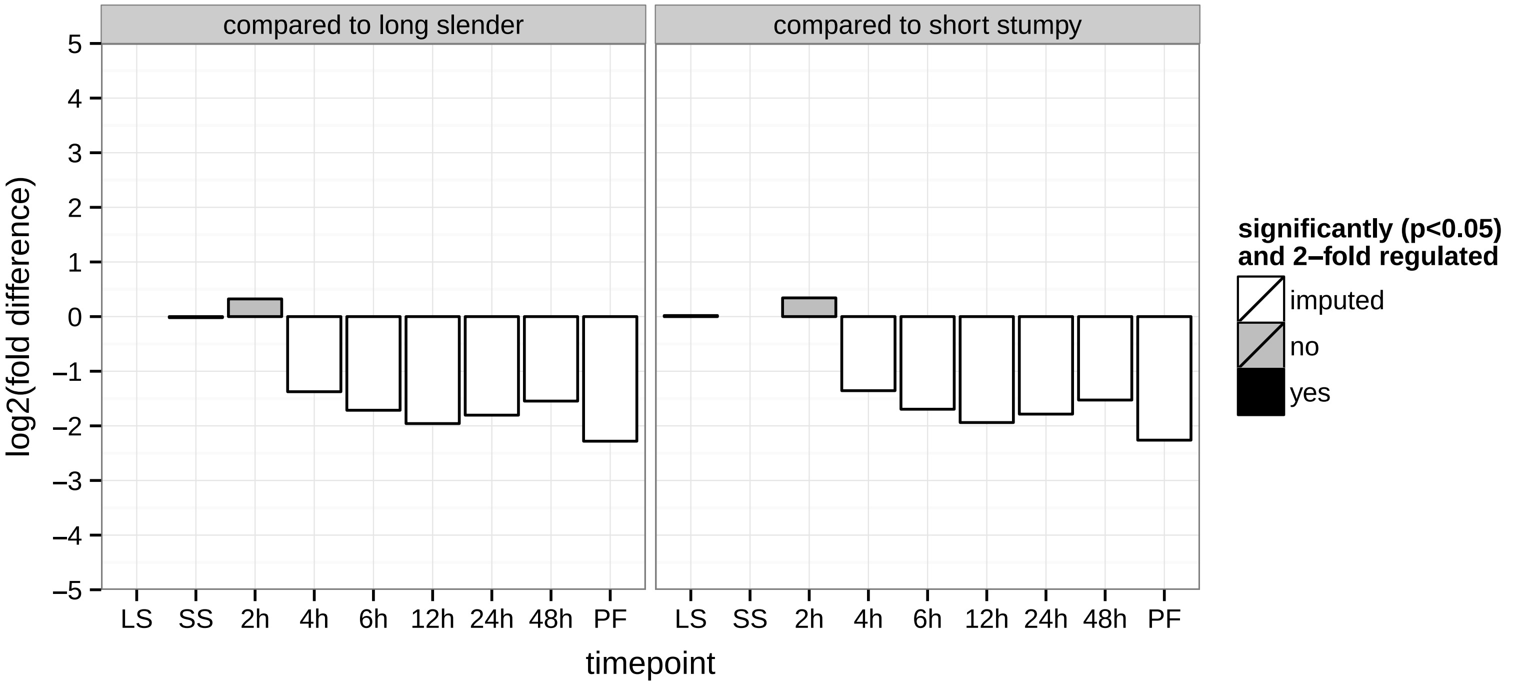

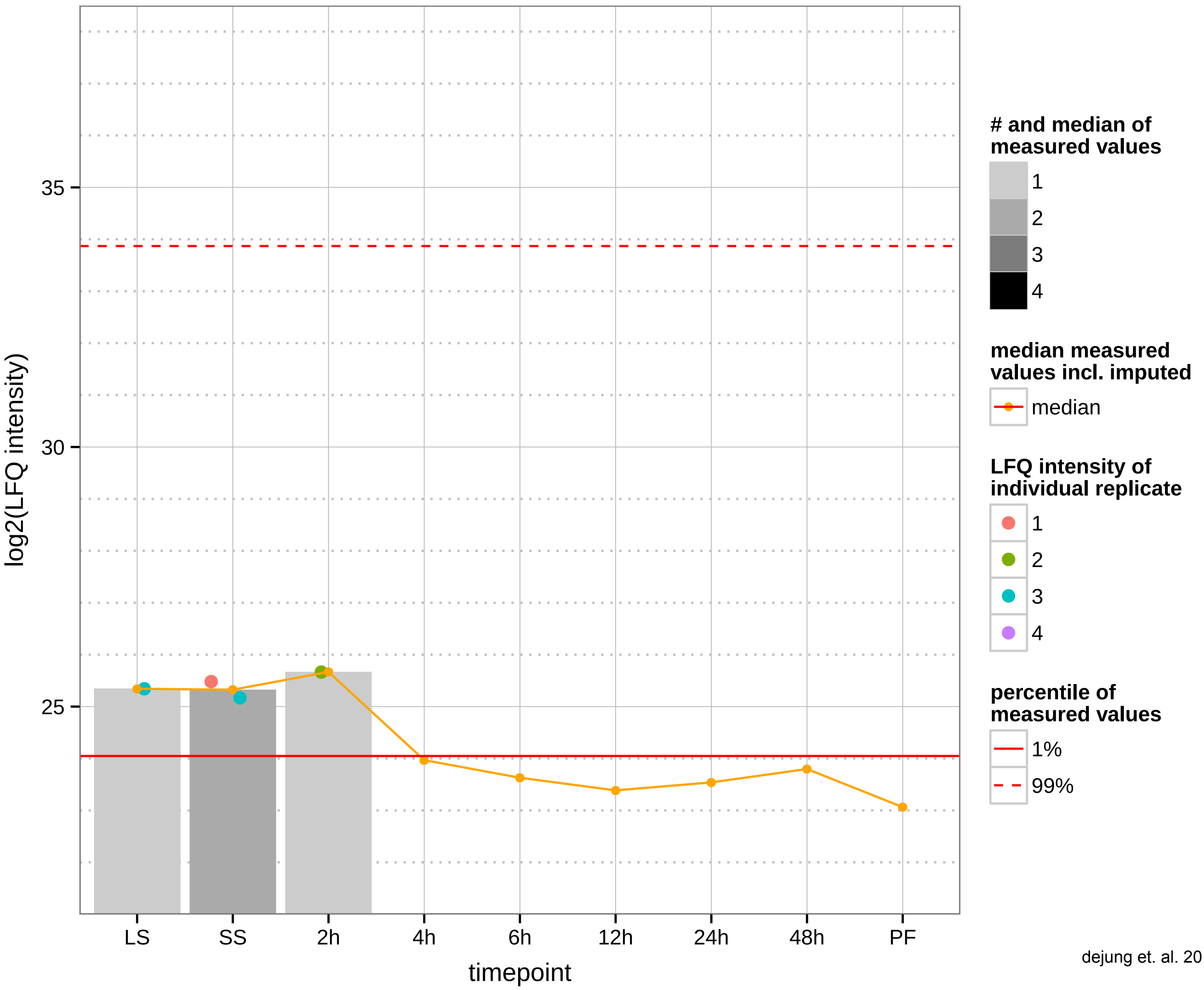

dejung et. al. 2015

UDP−GlcNAc:alpha3−D−mannoside beta−1, 2−N−acetylglucosaminyltransferase I, N−Acetylglucosaminyltransferase I (GnTI)
Tb927.3.5660
AGOF: UDP−glycosyltransferase activity, alpha−1, 3−mannosylglycoprotein 2−beta−N−acetylglucosaminyltransferase activity
AGOC: Golgi apparatus, integral to membrane
AGOP: GPI anchor biosynthetic process, N−glycan processing, cell proliferation, pathogenesis
PGOF: null
PGOC: null
PGOP: null

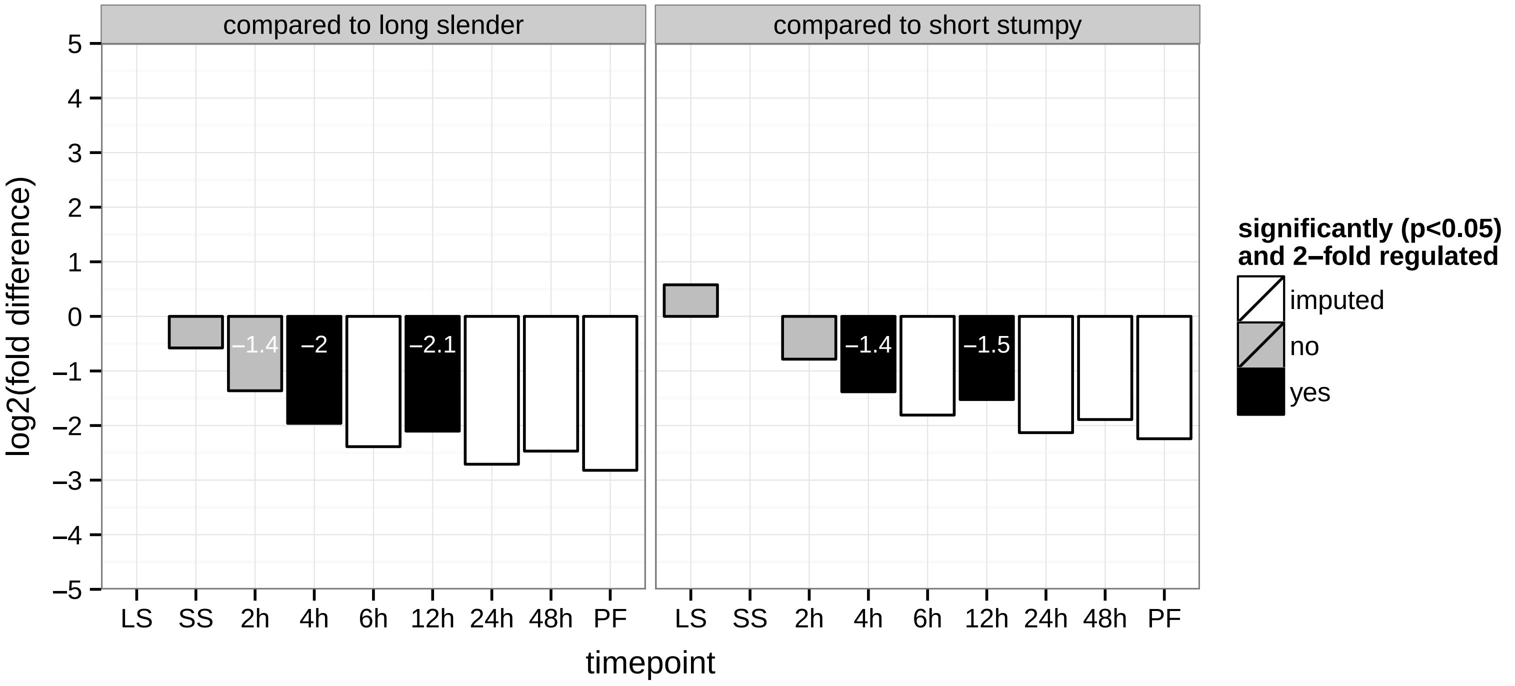

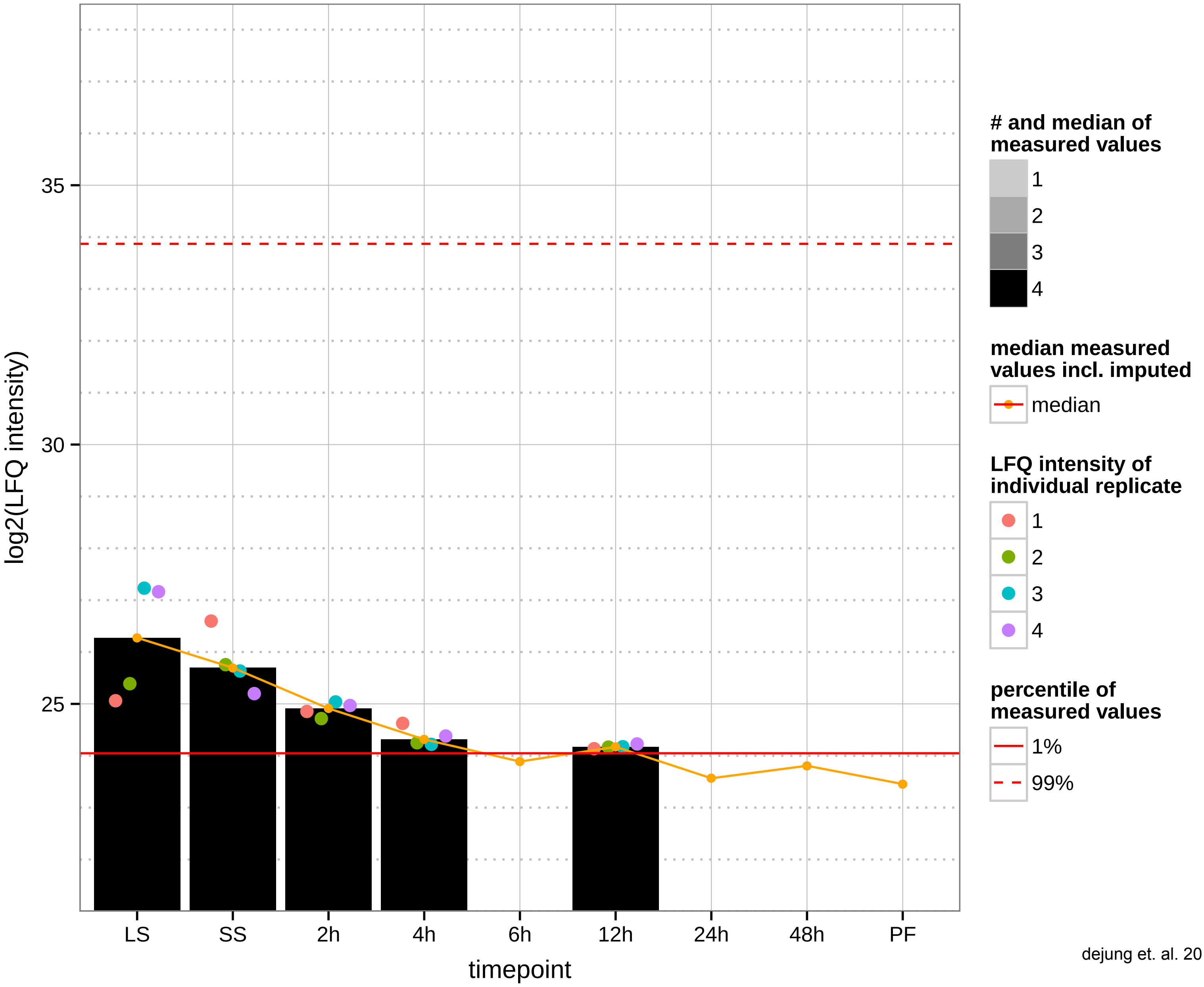

dejung et. al. 2015

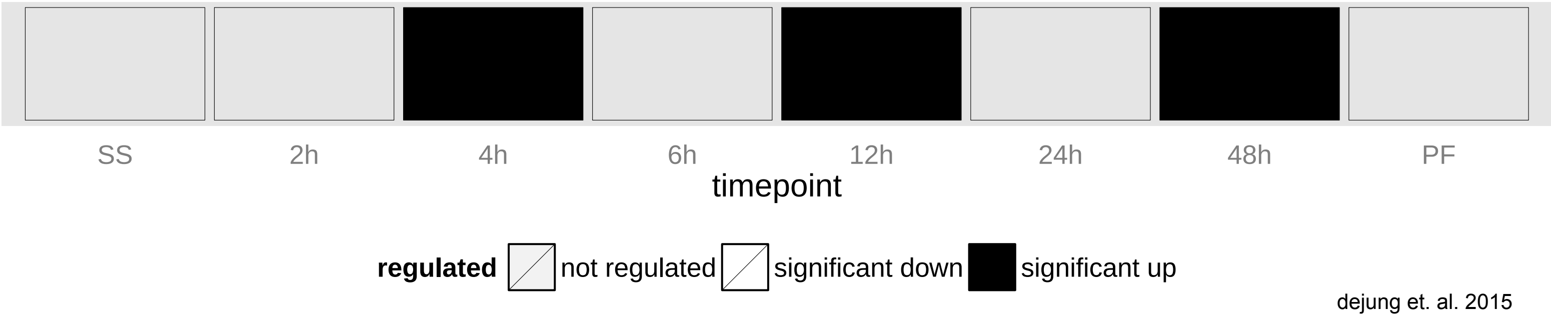
SS
2h
4h
6h
12h
24h
48h
PF
timepoint
regulated
not regulated
significant down
significant up
dejung et. al. 2015

hypothetical protein, conserved
Tb927.11.9630
AGOF: null
AGOC: null
AGOP: protein transport, vesicle–mediated transport
PGOF: null
PGOC: null
PGOP: protein transport

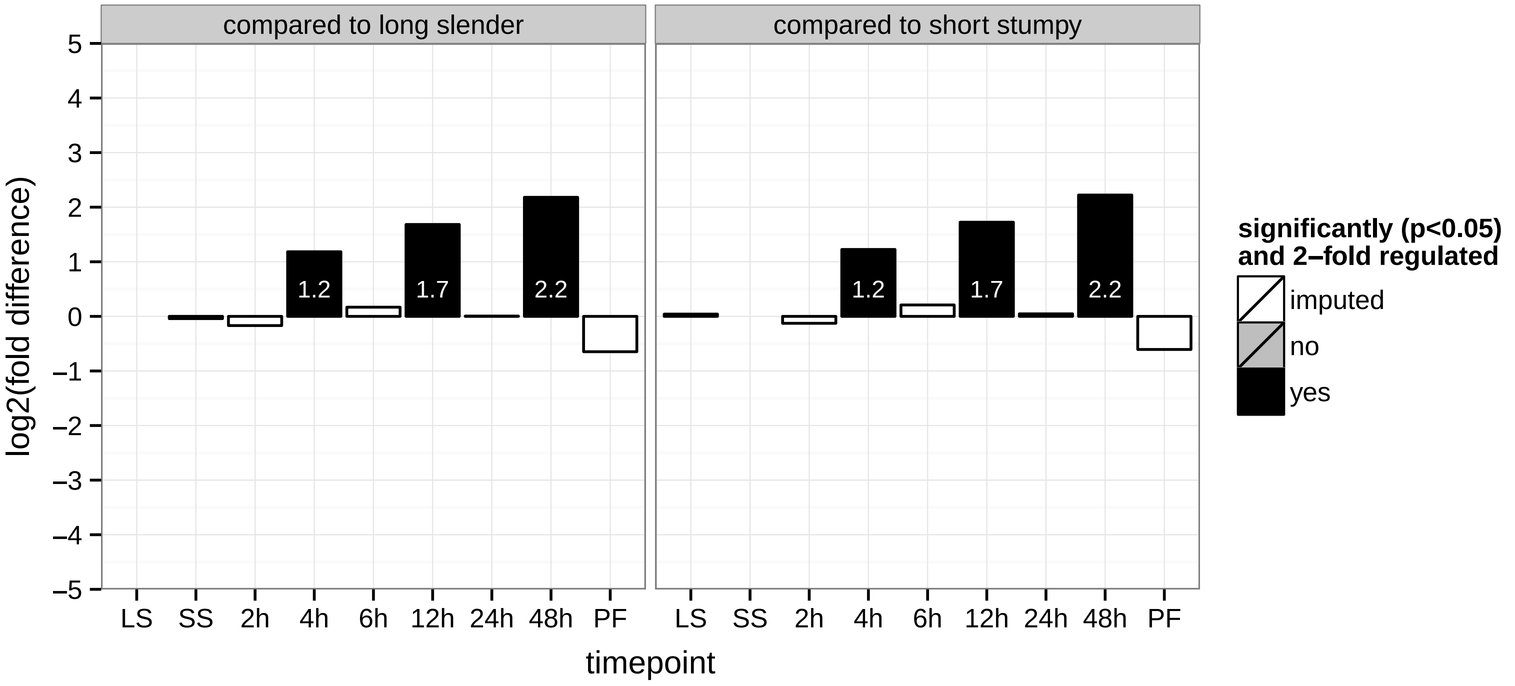

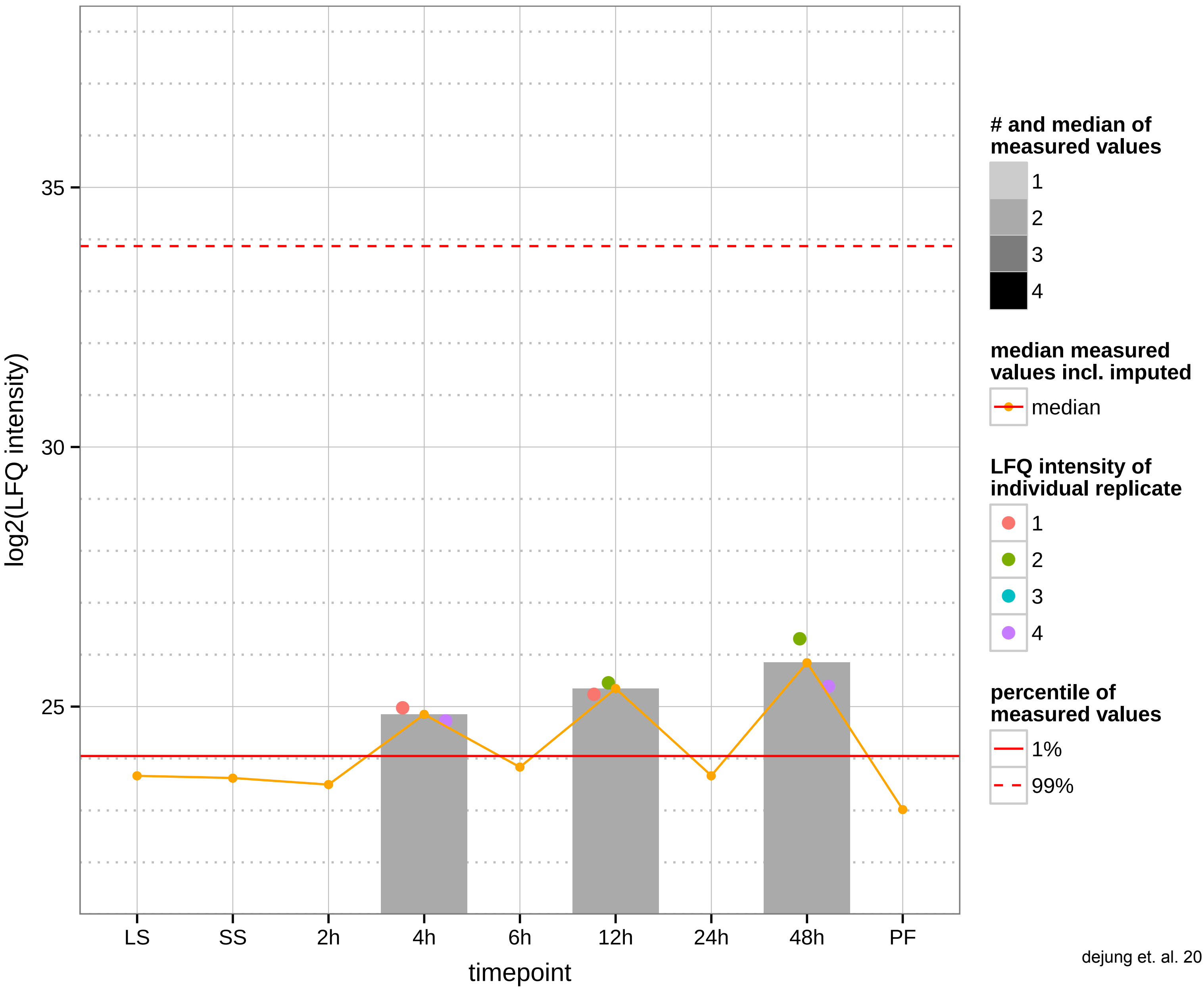

dejung et. al. 2015

peroxisome assembly protein, putative
Tb927.3.2410
AGOF: zinc ion binding
AGOC: peroxisomal membrane
AGOP: peroxisome organization
PGOF: protein binding, zinc ion binding
PGOC: null
PGOP: null

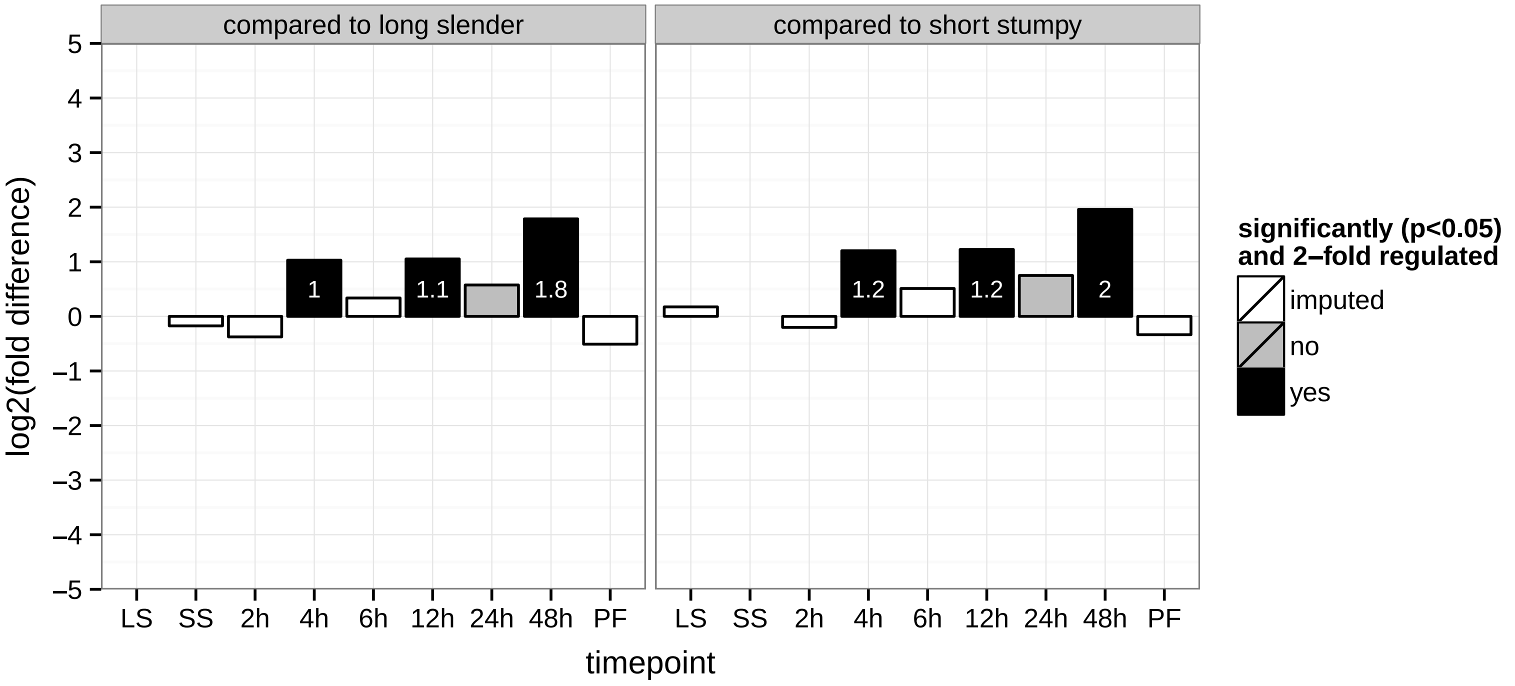

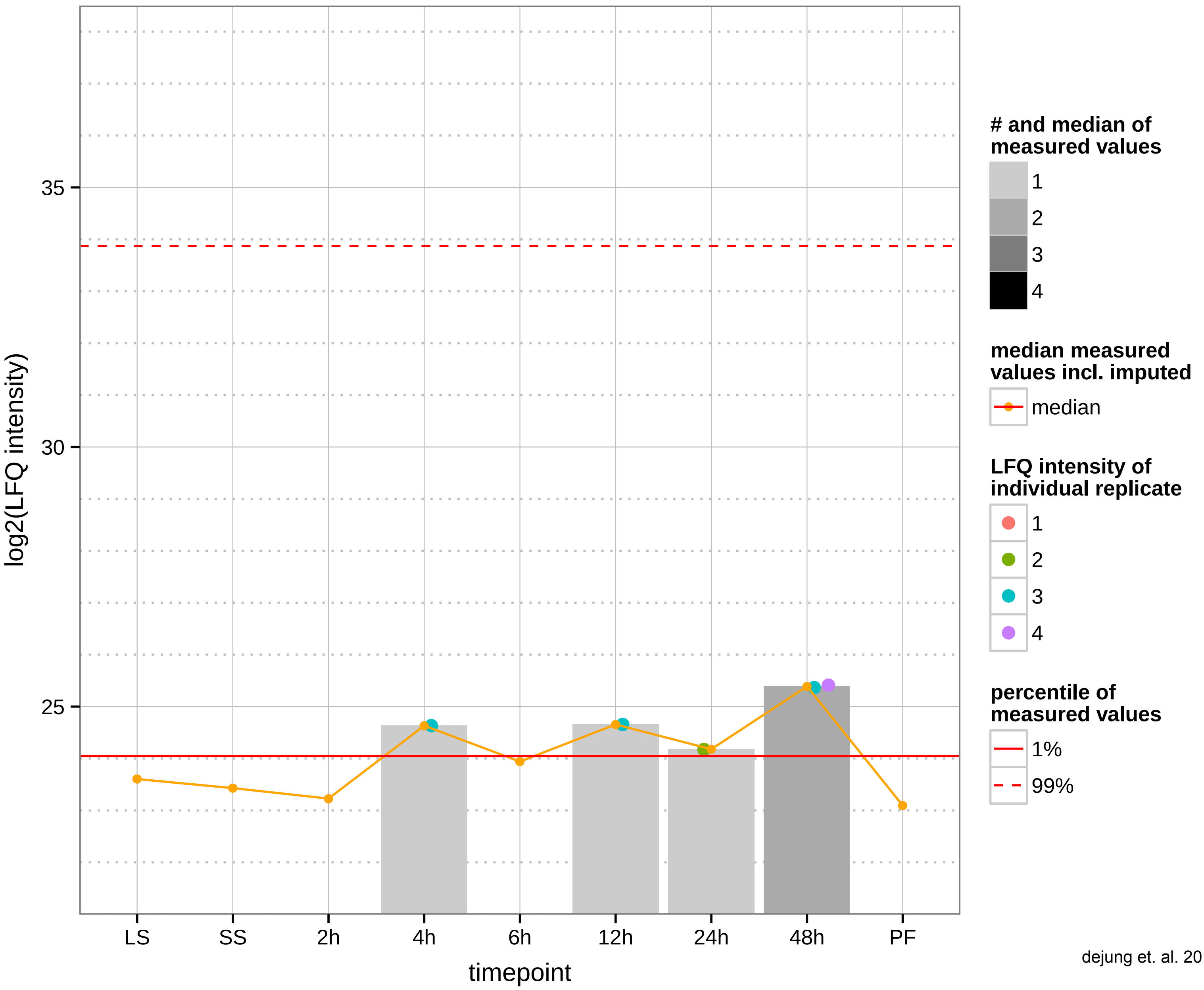

dejung et. al. 2015

hypothetical protein, conserved
Tb927.9.11660
AGOF: null
AGOC: mitochondrion
AGOP: null
PGOF: NADH dehydrogenase (ubiquinone) activity, NADH dehydrogenase activity
PGOC: mitochondrion
PGOP: null

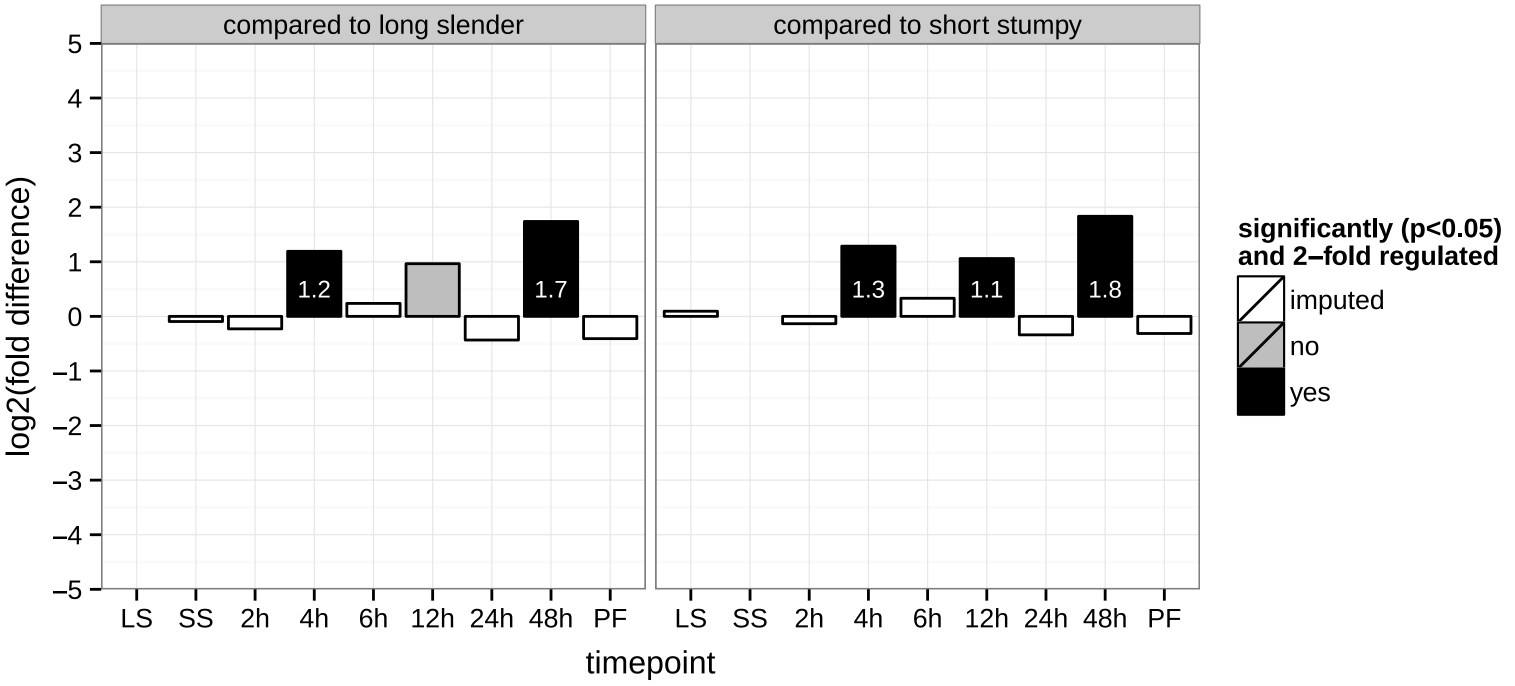

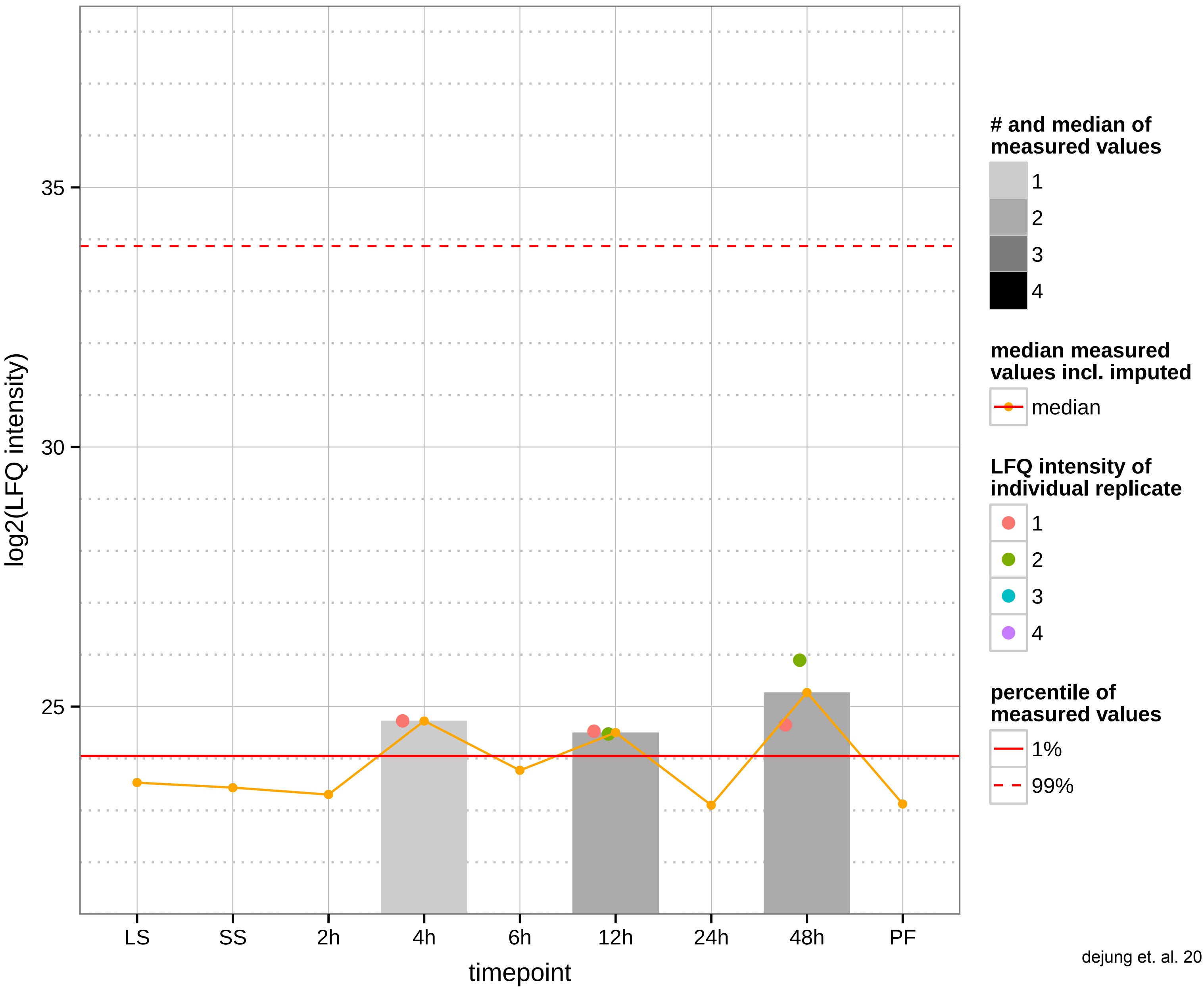

dejung et. al. 2015

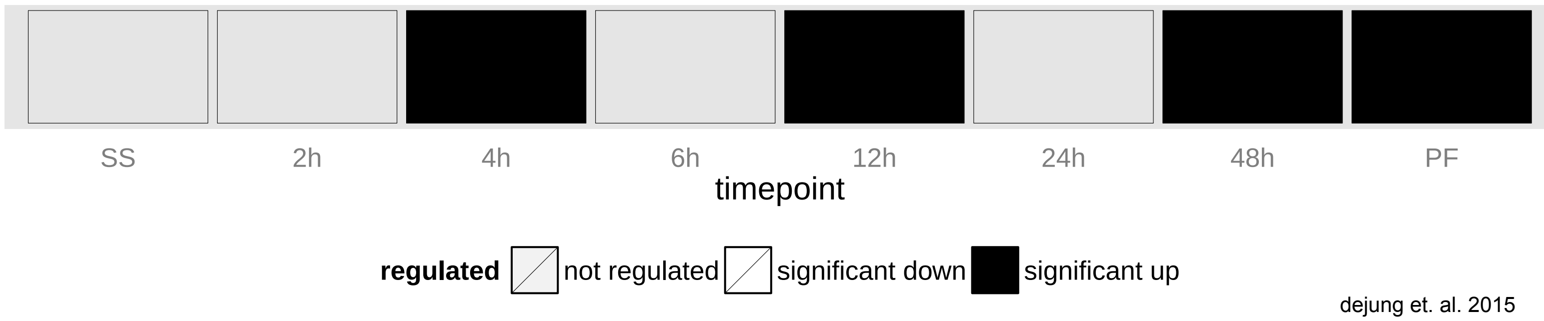
SS
2h
4h
6h
12h
24h
48h
PF
timepoint
regulated
not regulated
significant down
significant up
dejung et. al. 2015

hypothetical protein, conserved
Tb927.10.1980
AGOF: null
AGOC: null
AGOP: null
PGOF: null
PGOC: null
PGOP: null

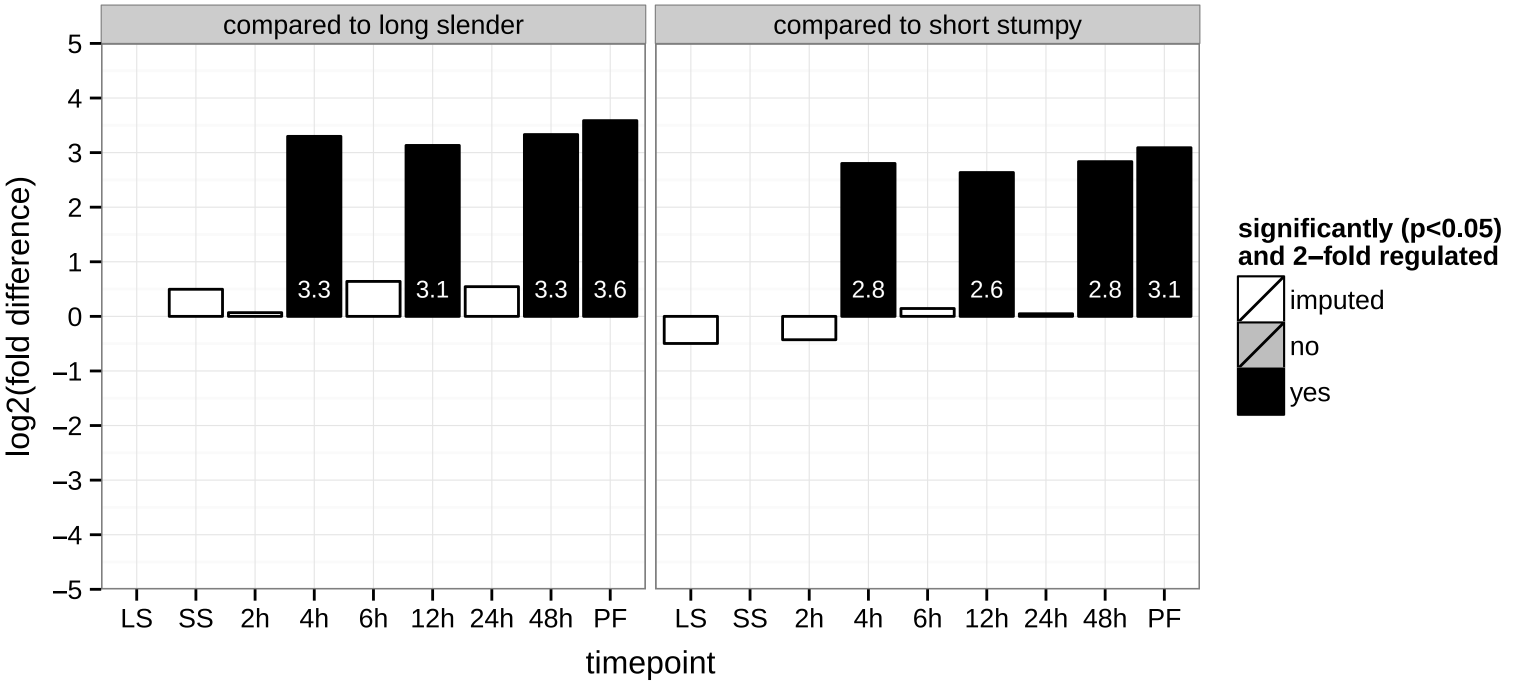

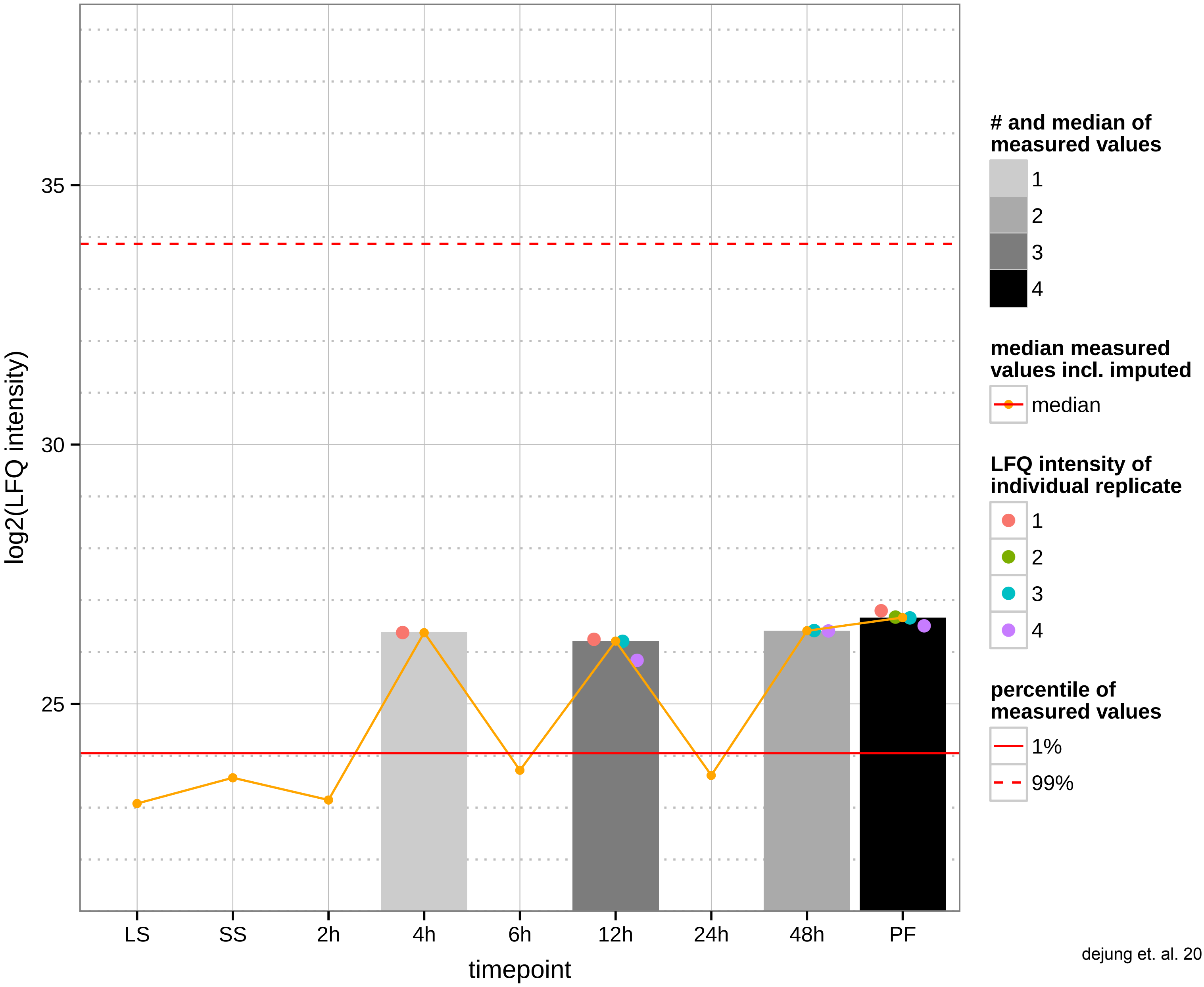

dejung et. al. 2015

hypothetical protein, conserved
Tb927.11.1200
AGOF: null
AGOC: null
AGOP: null
PGOF: protein binding
PGOC: null
PGOP: null

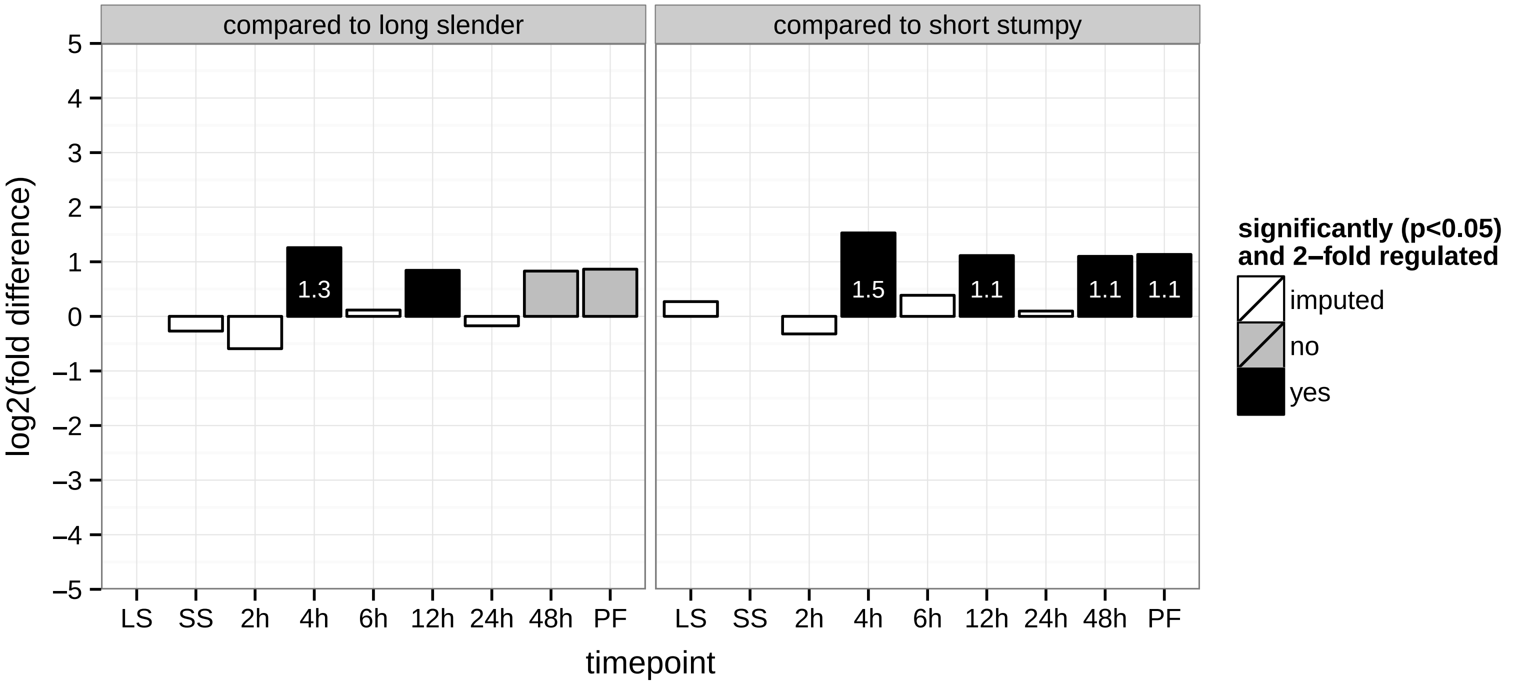

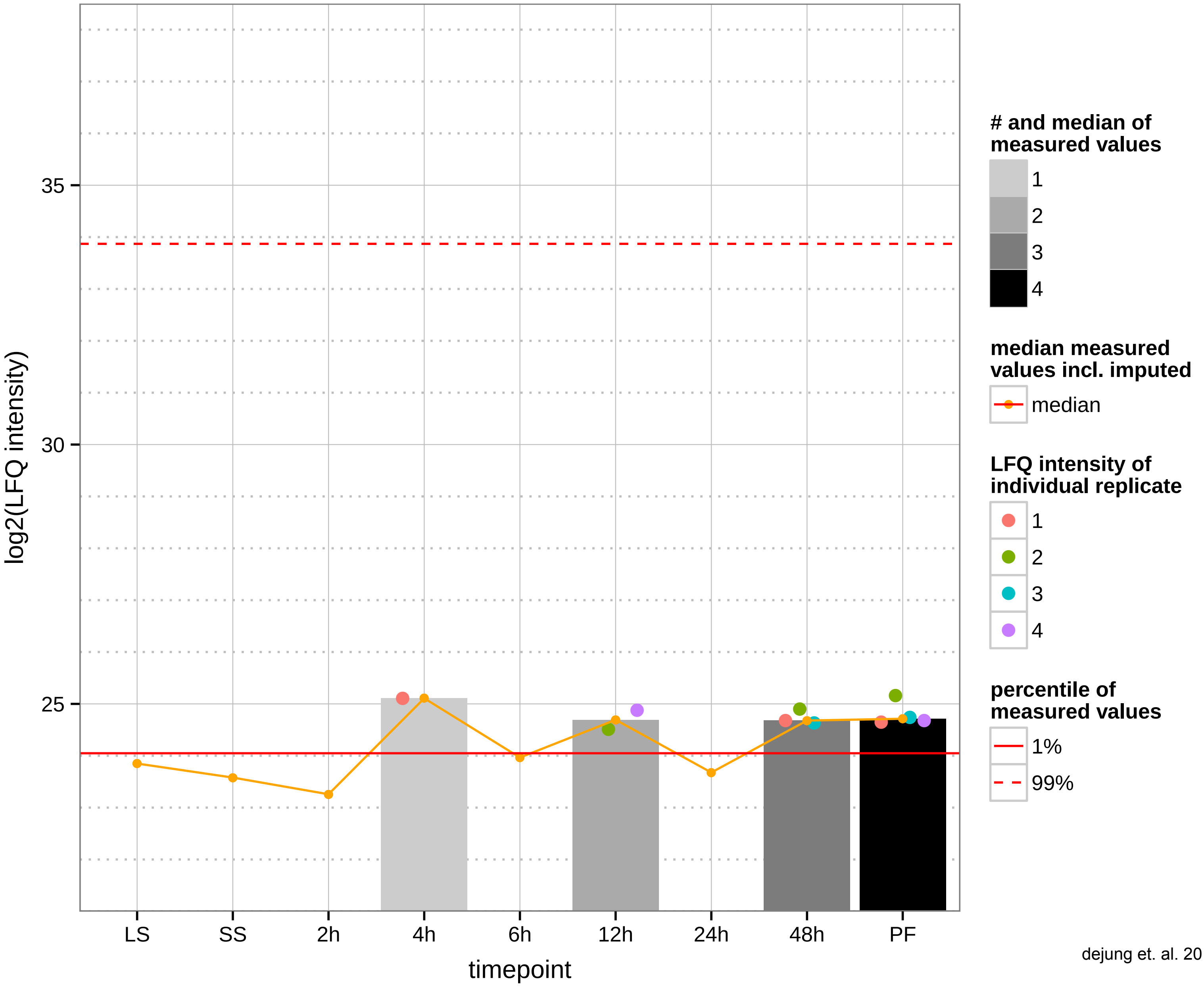

dejung et. al. 2015

hypothetical protein, conserved
Tb927.3.2260
AGOF: null
AGOC: mitochondrion
AGOP: null
PGOF: protein binding
PGOC: null
PGOP: null

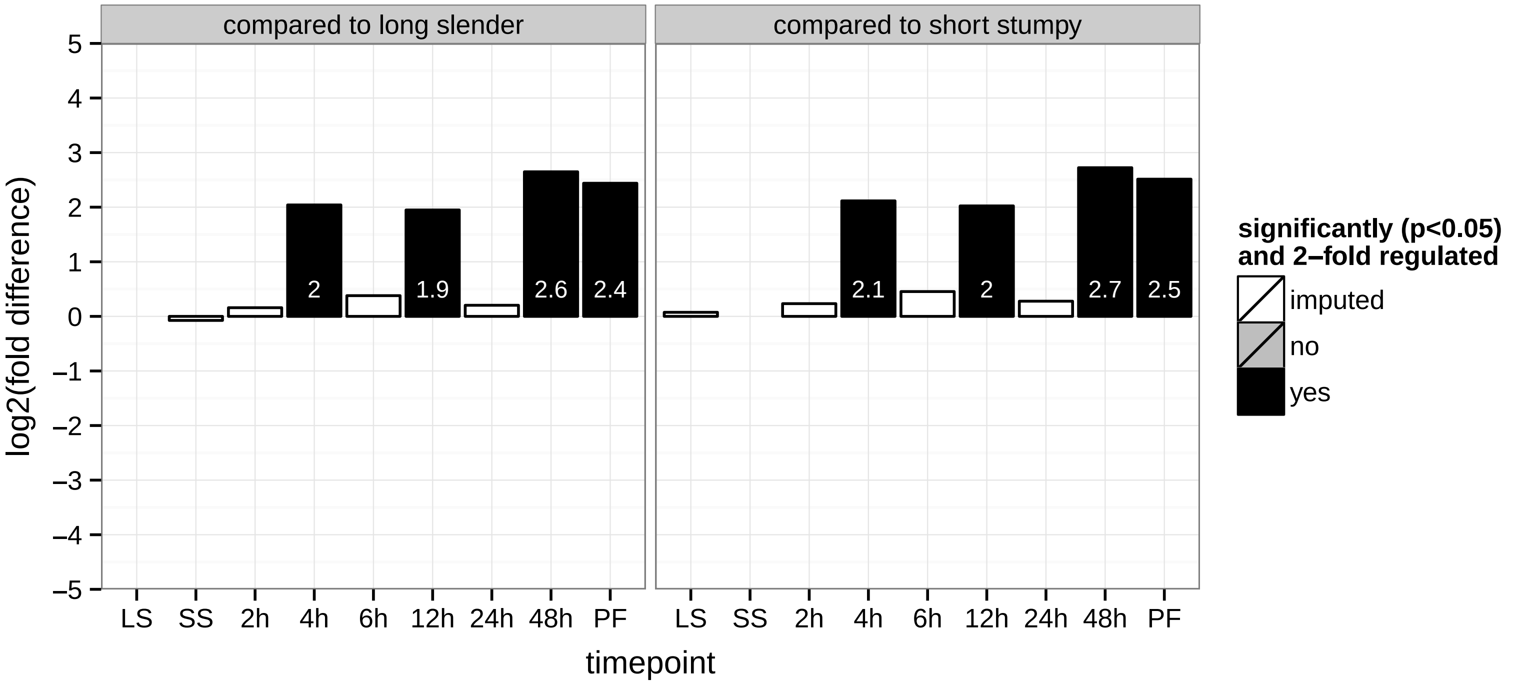

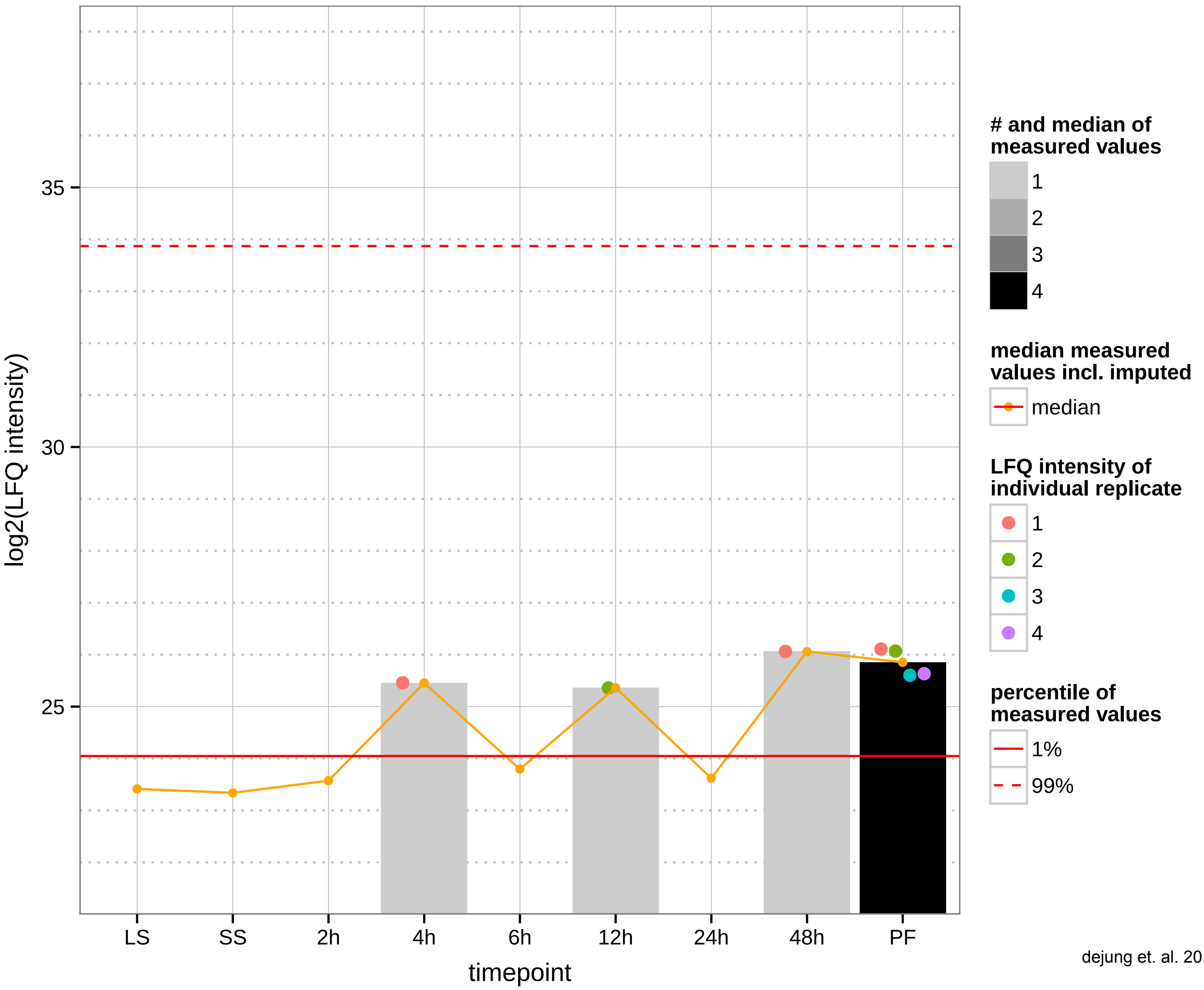

dejung et. al. 2015

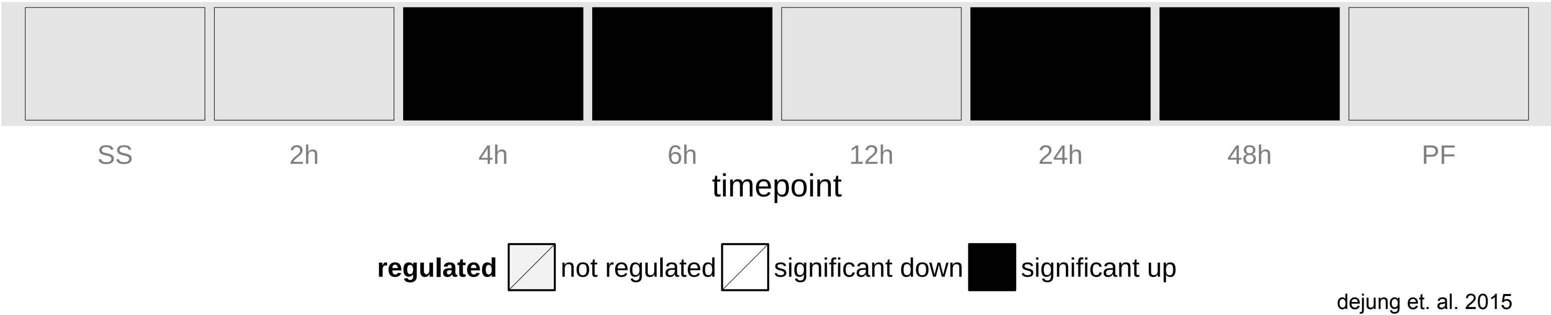

SS
2h
4h
6h
12h
24h
48h
PF
timepoint
regulated
not regulated
significant down
significant up
dejung et. al. 2015

hypothetical protein, conserved
Tb927.11.11050
AGOF: null
AGOC: null
AGOP: null
PGOF: null
PGOC: null
PGOP: null

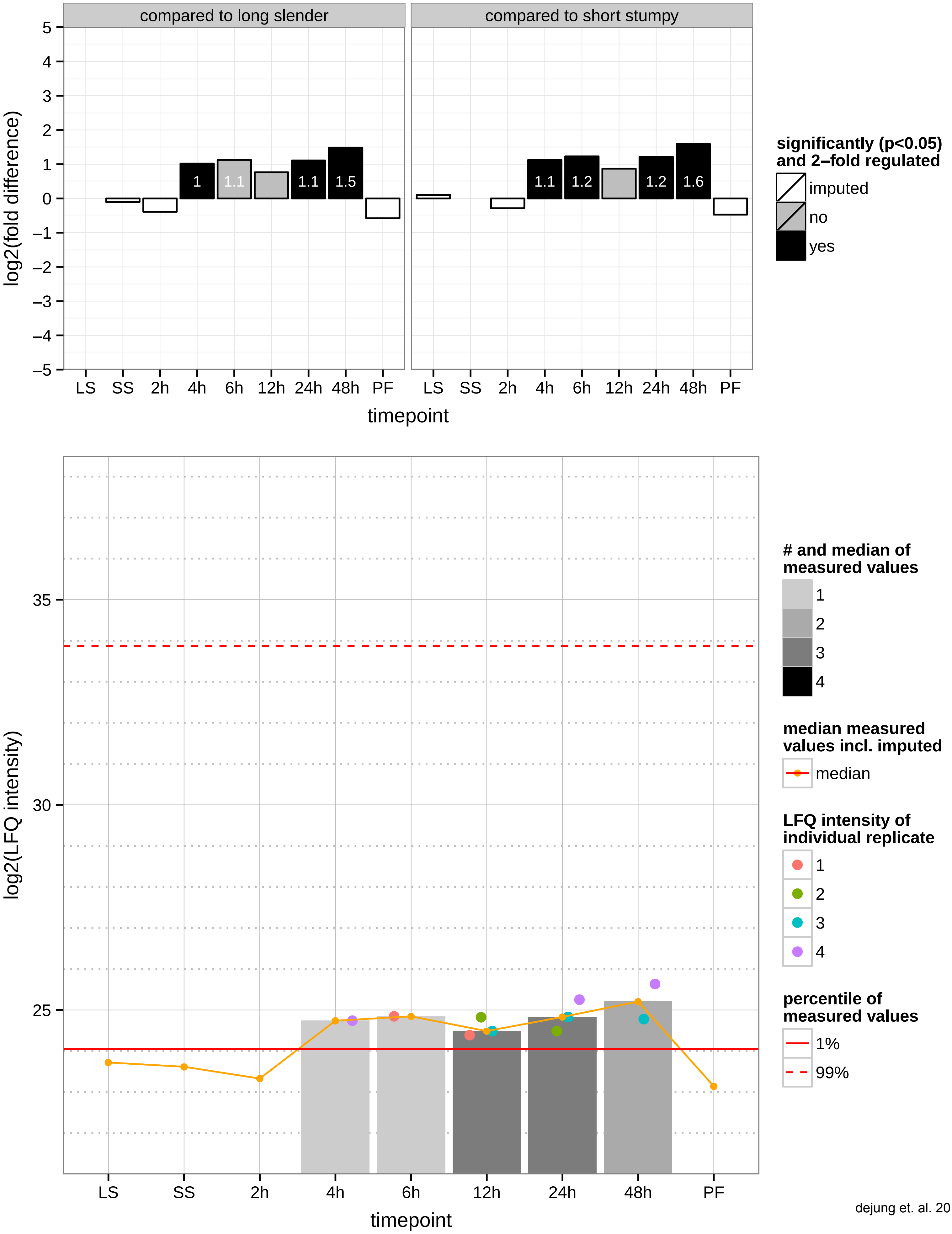

dejung et. al. 2015

hypothetical protein, conserved
Tb927.11.14990
AGOF: null
AGOC: null
AGOP: null
PGOF: null
PGOC: null
PGOP: null

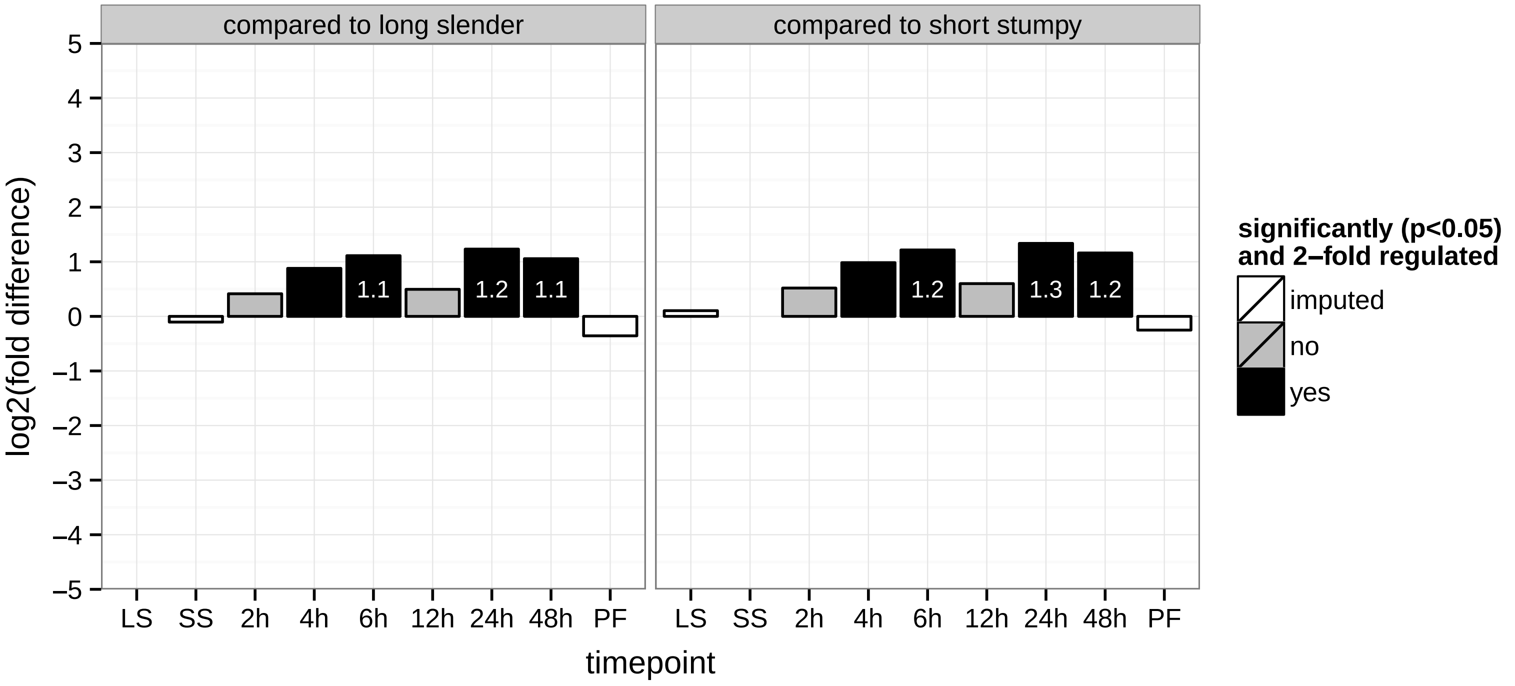

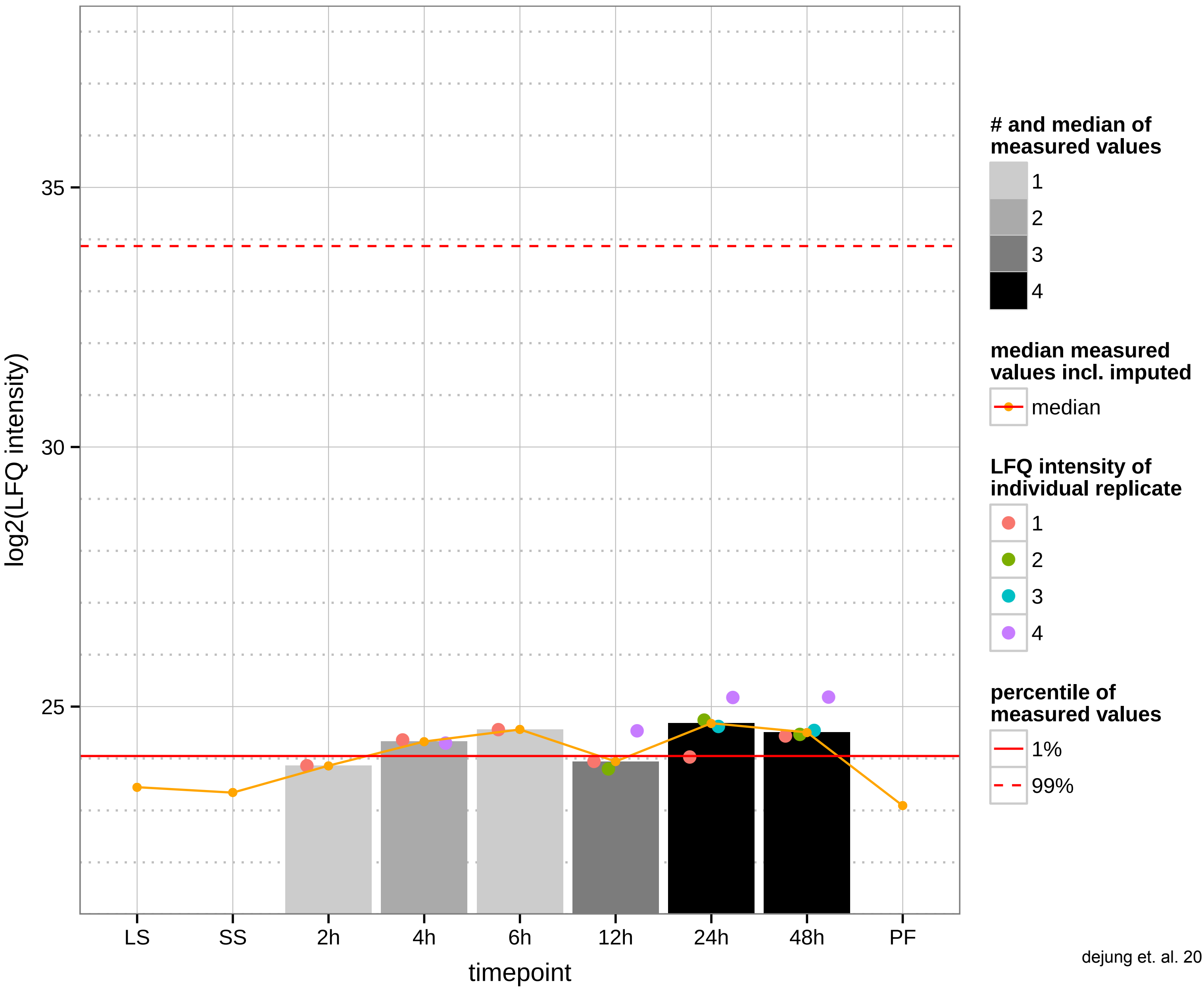

dejung et. al. 2015

L–Lysine transport protein, putative
Tb927.11.15860;Tb927.11.15840
AGOF: L–lysine transmembrane transporter activity, amine transmembrane transporter activity
AGOC: flagellar pocket, integral to membrane, kinetoplast, nucleus, plasma membrane
AGOP: lysine transport, amino acid transport, lysine transport
PGOF: null
PGOC: null
PGOP: null

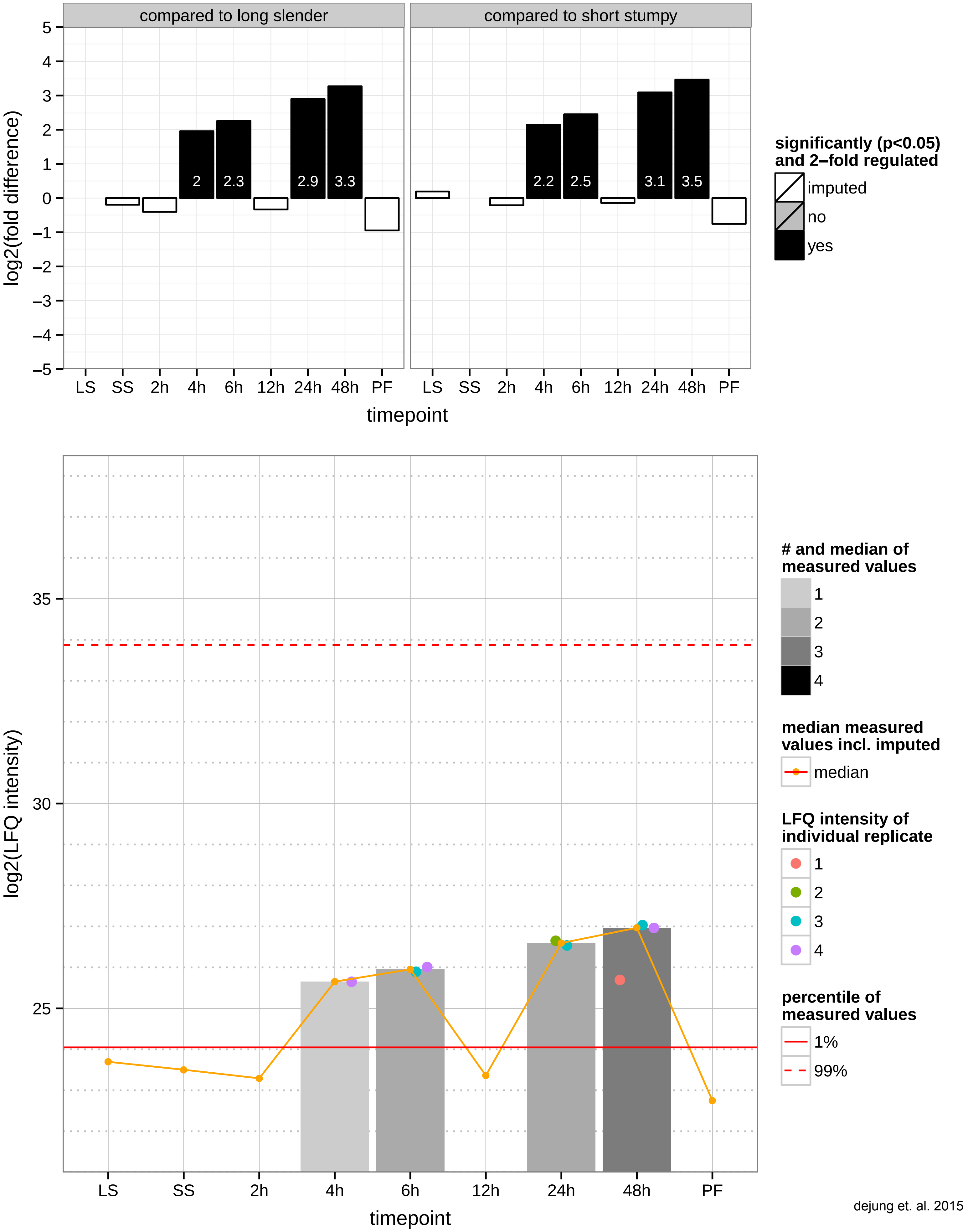

dejung et. al. 2015

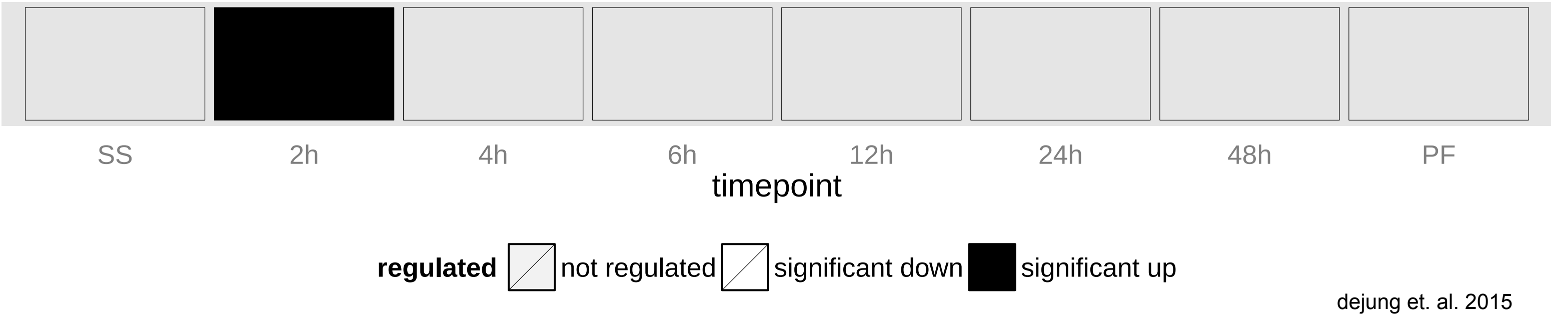

SS
2h
4h
6h
12h
24h
48h
PF
timepoint
regulated
not regulated
significant down
significant up
dejung et. al. 2015

hypothetical protein, conserved
Tb927.11.9290
AGOF: ATP binding, protein serine/threonine kinase activity, protein tyrosine kinase activity
AGOC: null
AGOP: protein phosphorylation
PGOF: ATP binding, protein kinase activity, transferase activity, transferring phosphorus–containing groups
PGOC: null
PGOP: protein phosphorylation

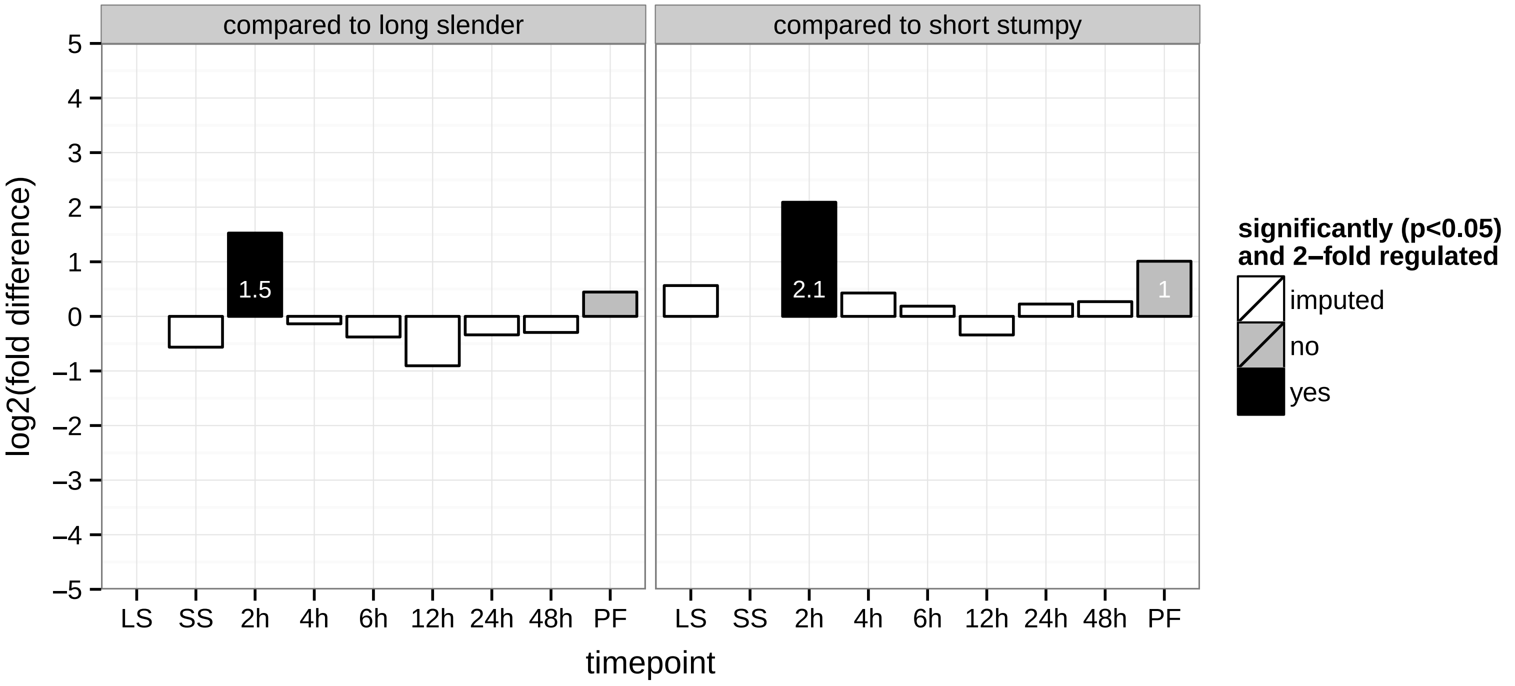

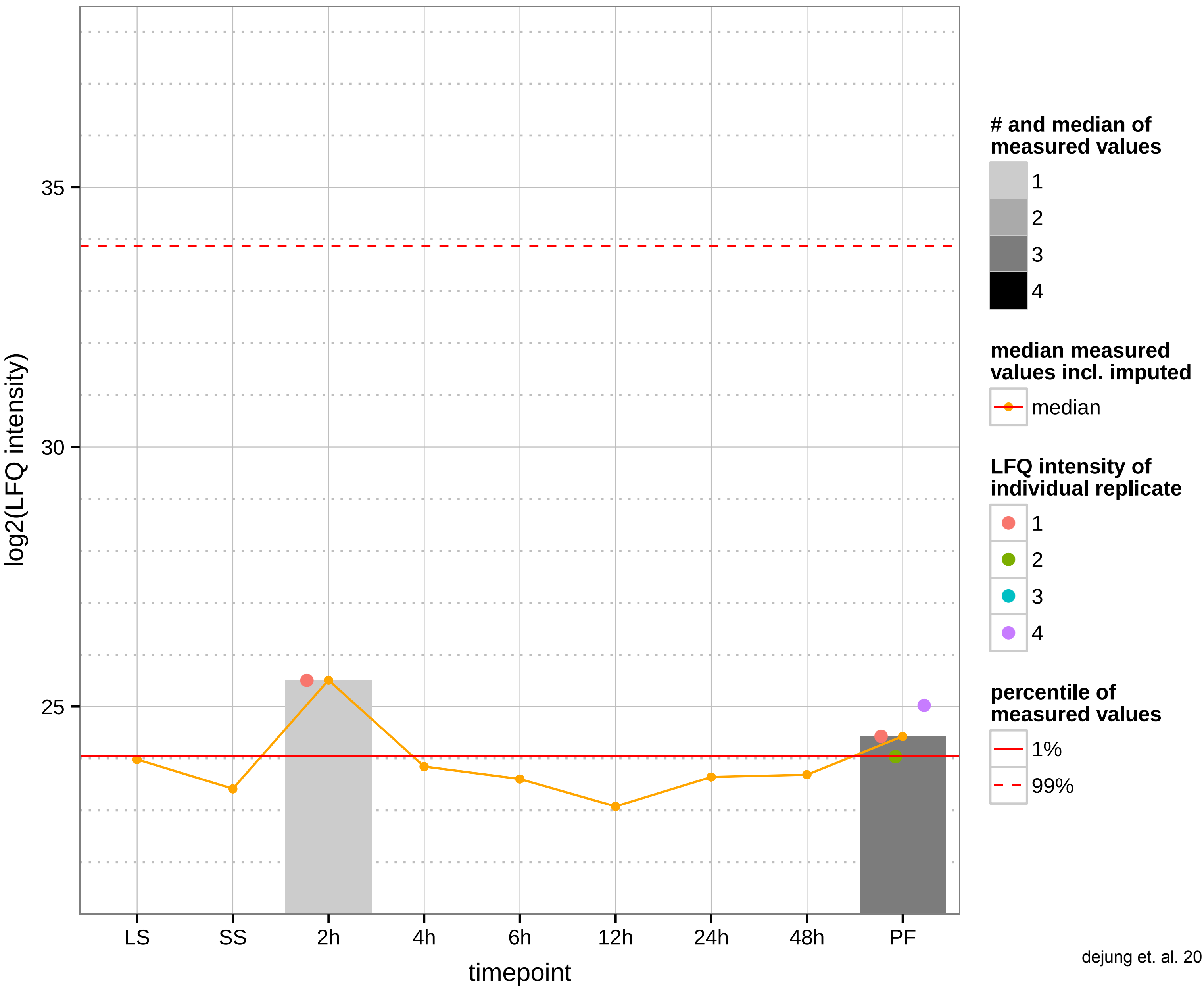

dejung et. al. 2015

hypothetical protein, conserved
Tb927.5.2150
AGOF: null
AGOC: mitochondrion
AGOP: null
PGOF: null
PGOC: null
PGOP: null

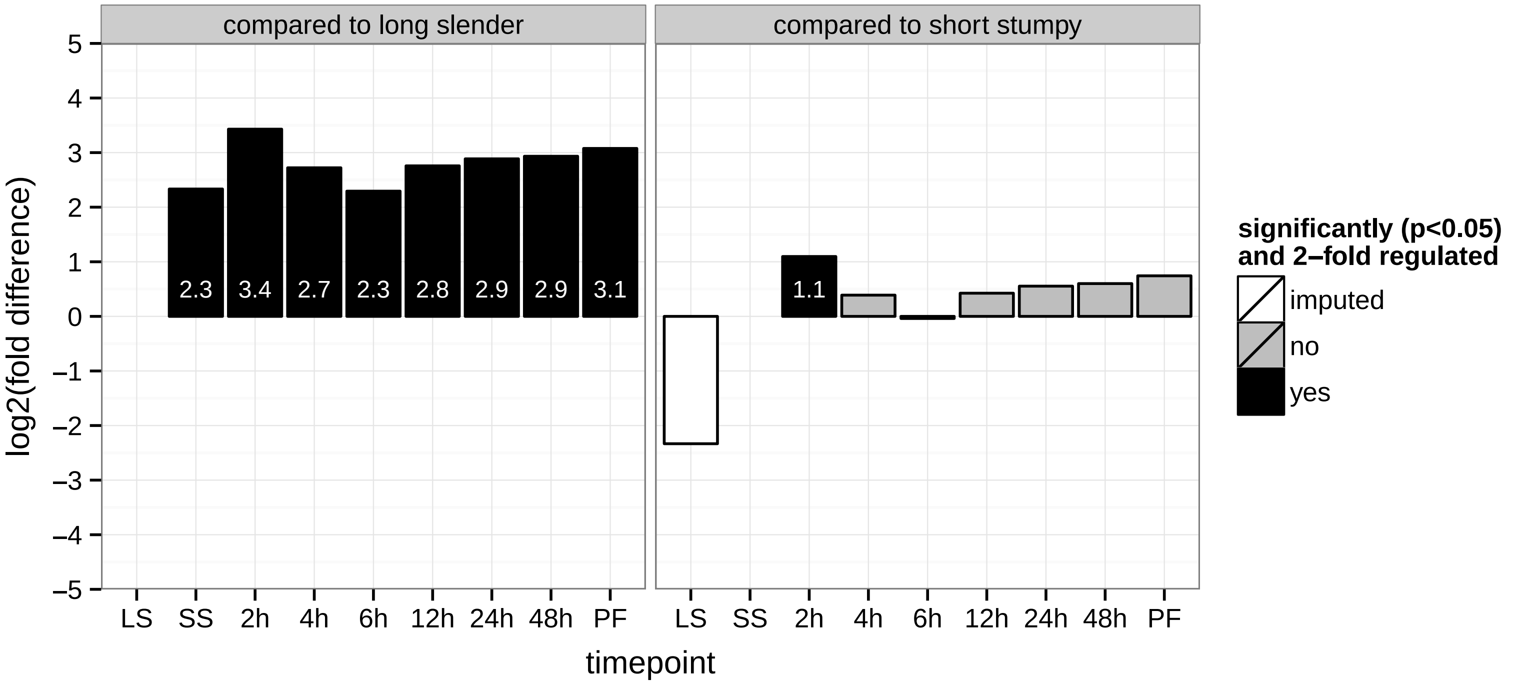

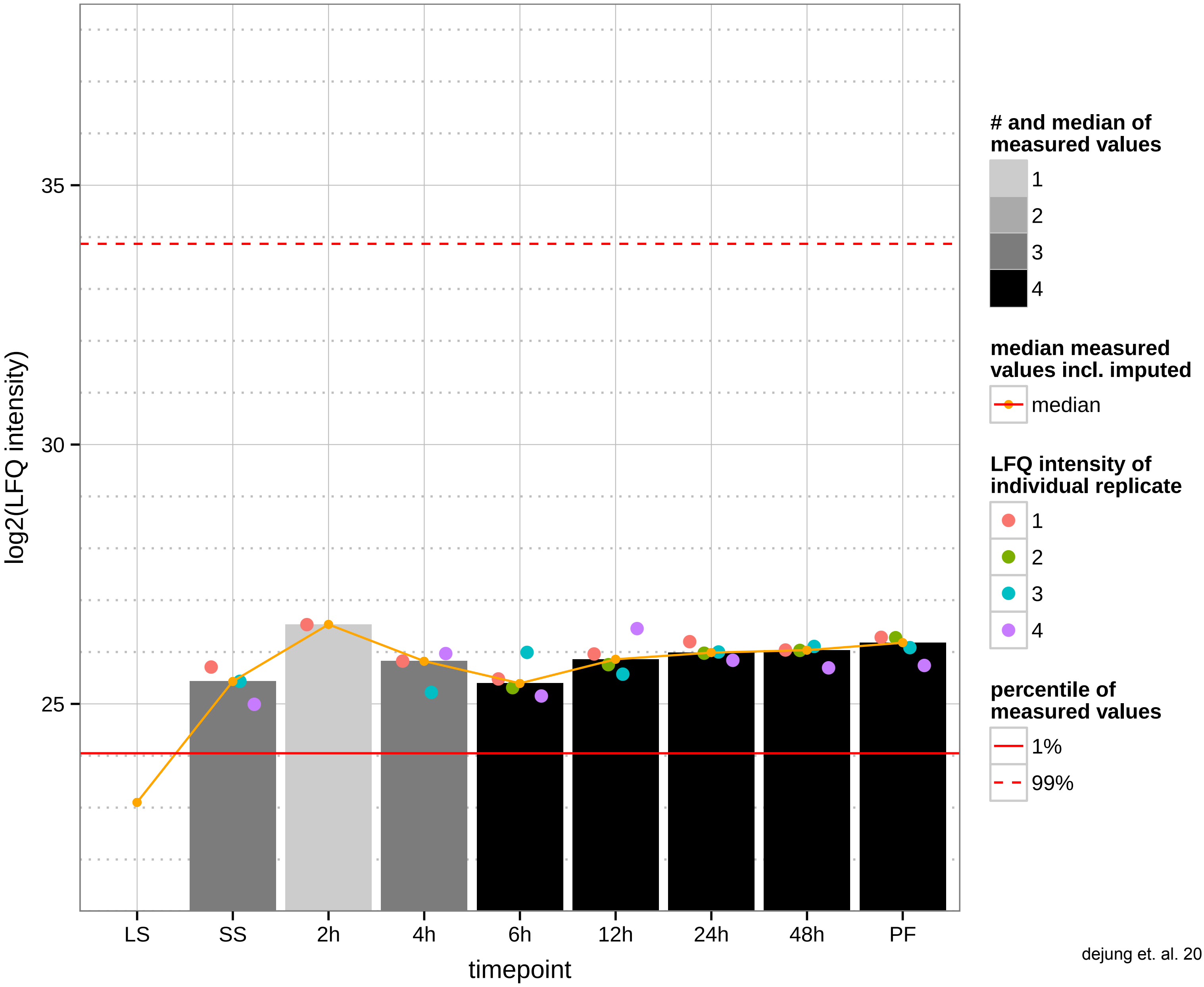

dejung et. al. 2015

hypothetical protein, conserved
Tb927.9.13470
AGOF: null
AGOC: null
AGOP: null
PGOF: protein binding
PGOC: null
PGOP: null

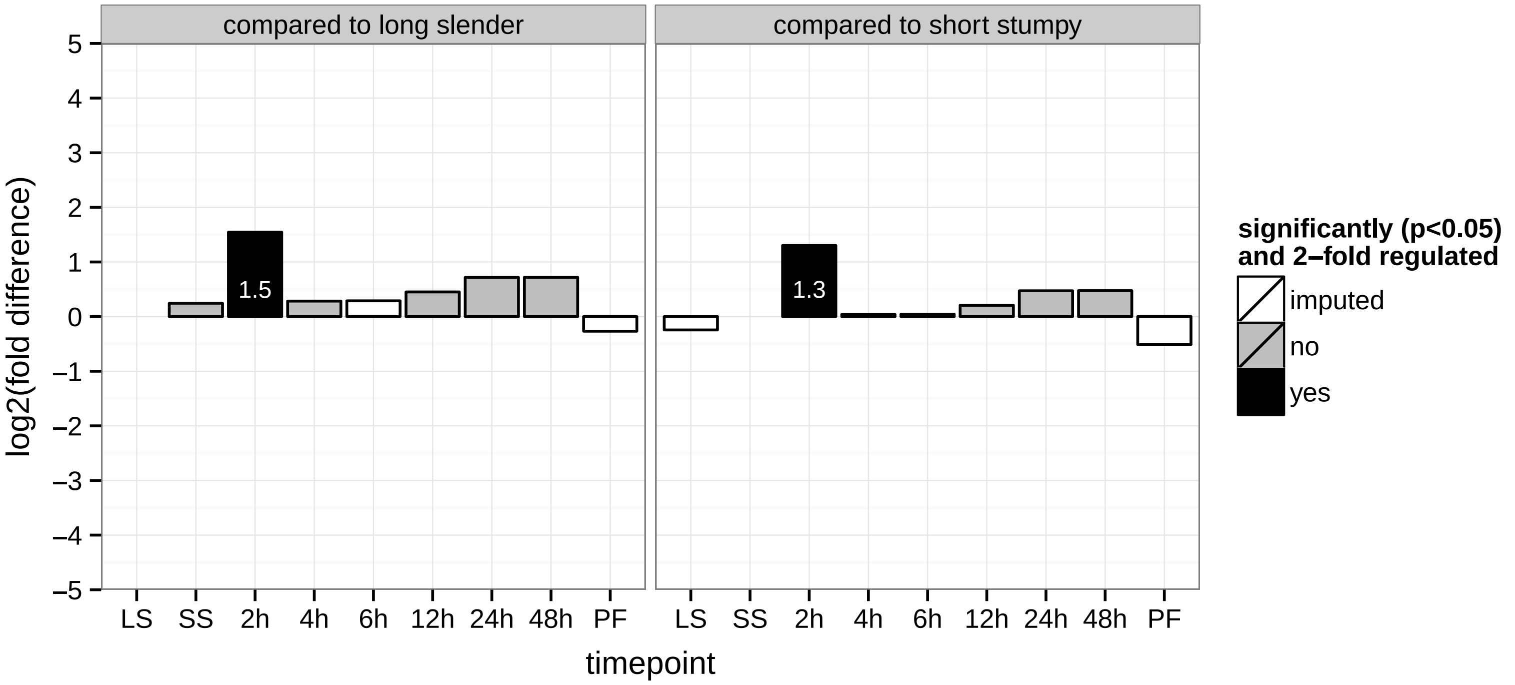

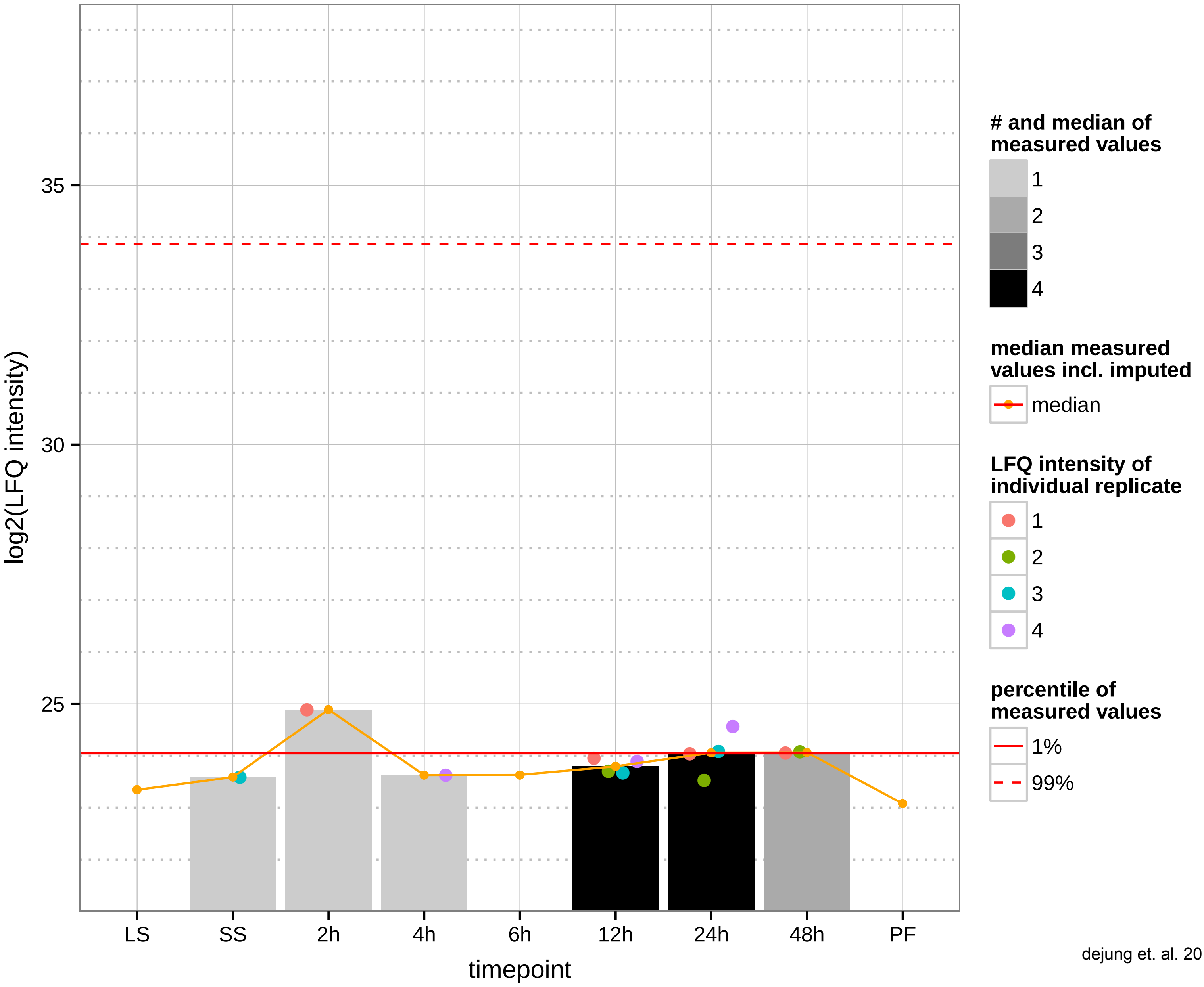

dejung et. al. 2015

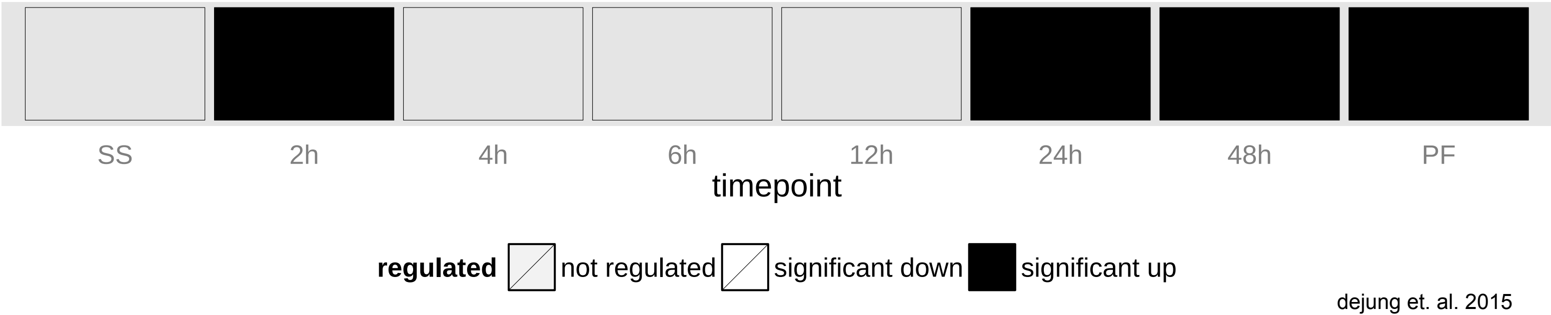
SS
2h
4h
6h
12h
24h
48h
PF
timepoint
regulated
not regulated
significant down
significant up
dejung et. al. 2015

acetyltransferase, putative
Tb927.5.2280
AGOF: transferase activity, transferring acyl groups
AGOC: null
AGOP: metabolic process
PGOF: transferase activity, transferring acyl groups
PGOC: null
PGOP: metabolic process

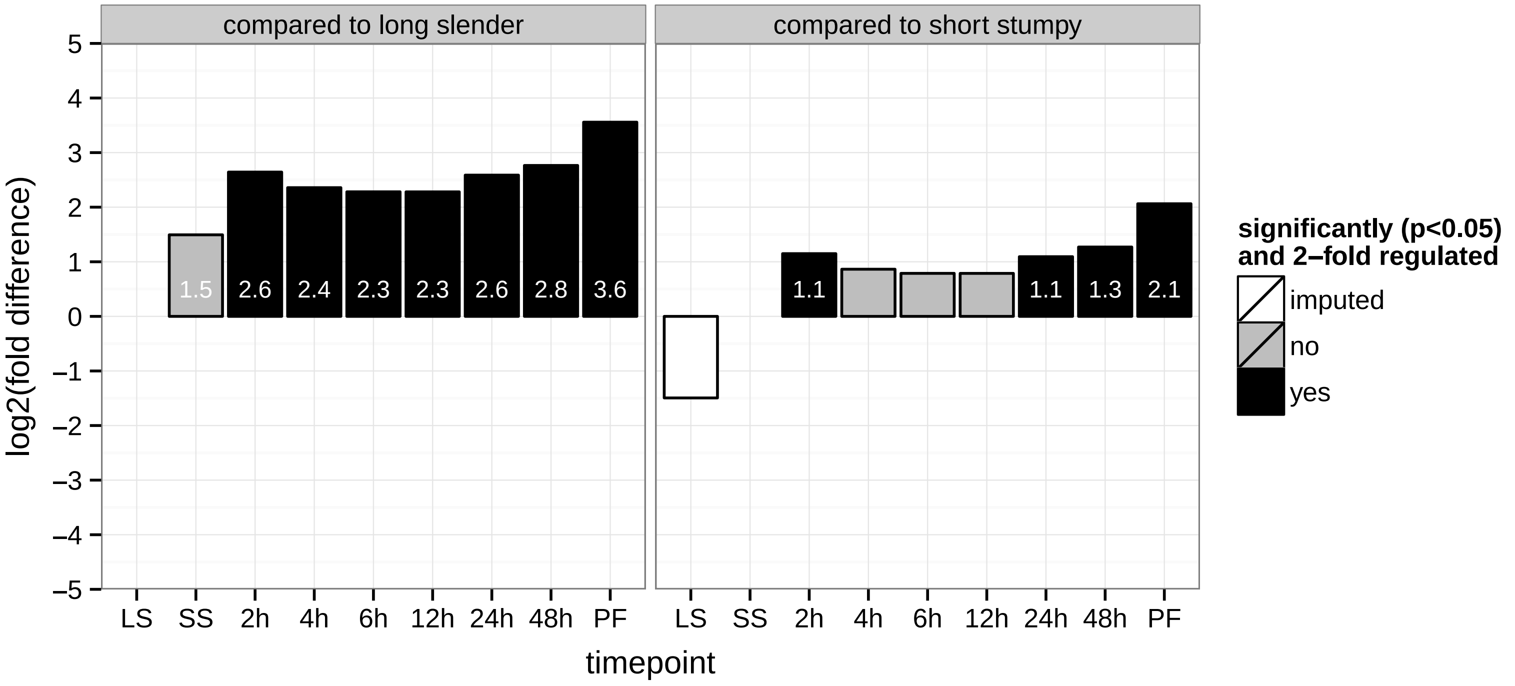

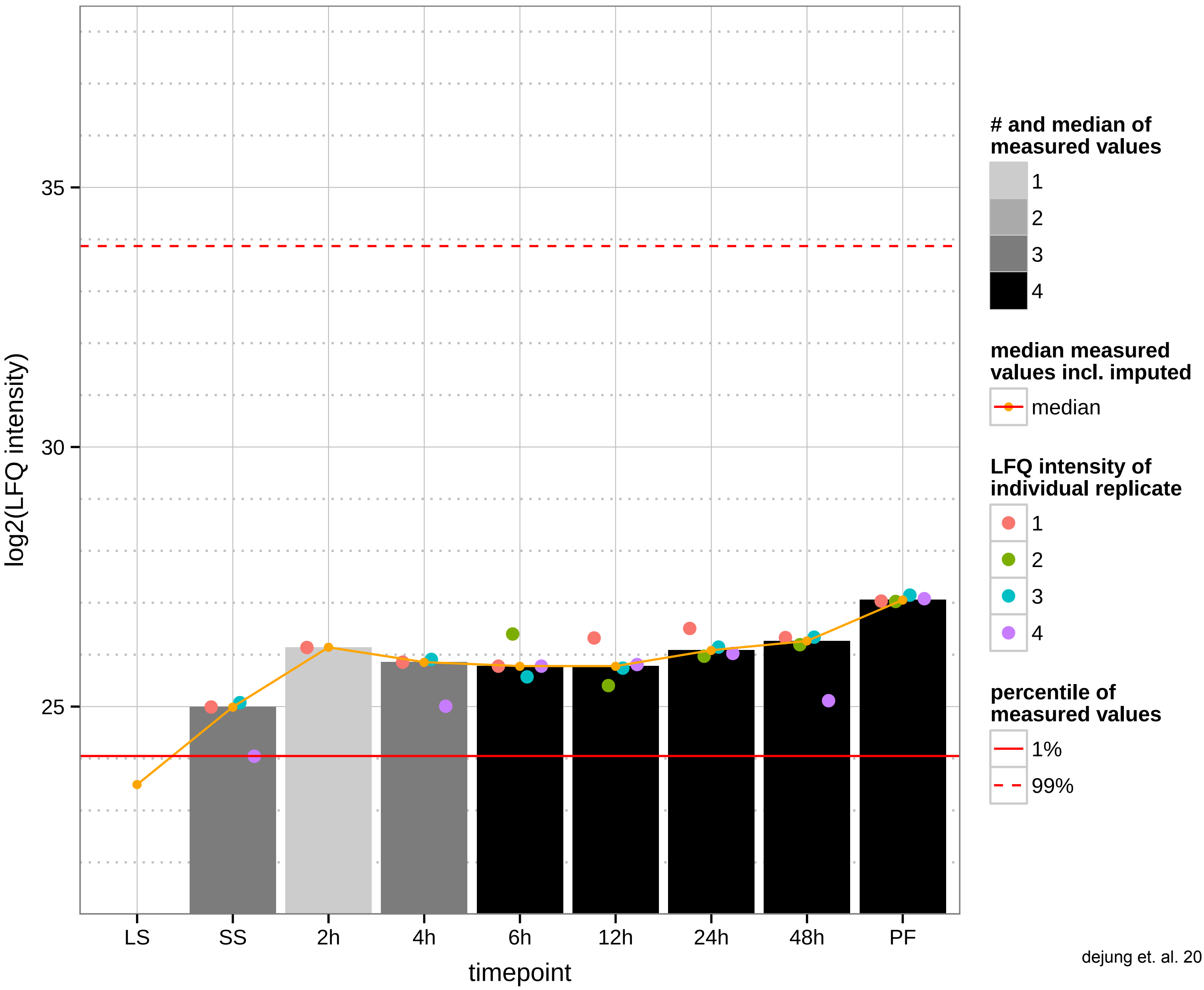

dejung et. al. 2015

hypothetical protein, conserved
Tb927.5.2520
AGOF: null
AGOC: null
AGOP: null
PGOF: protein binding
PGOC: null
PGOP: null

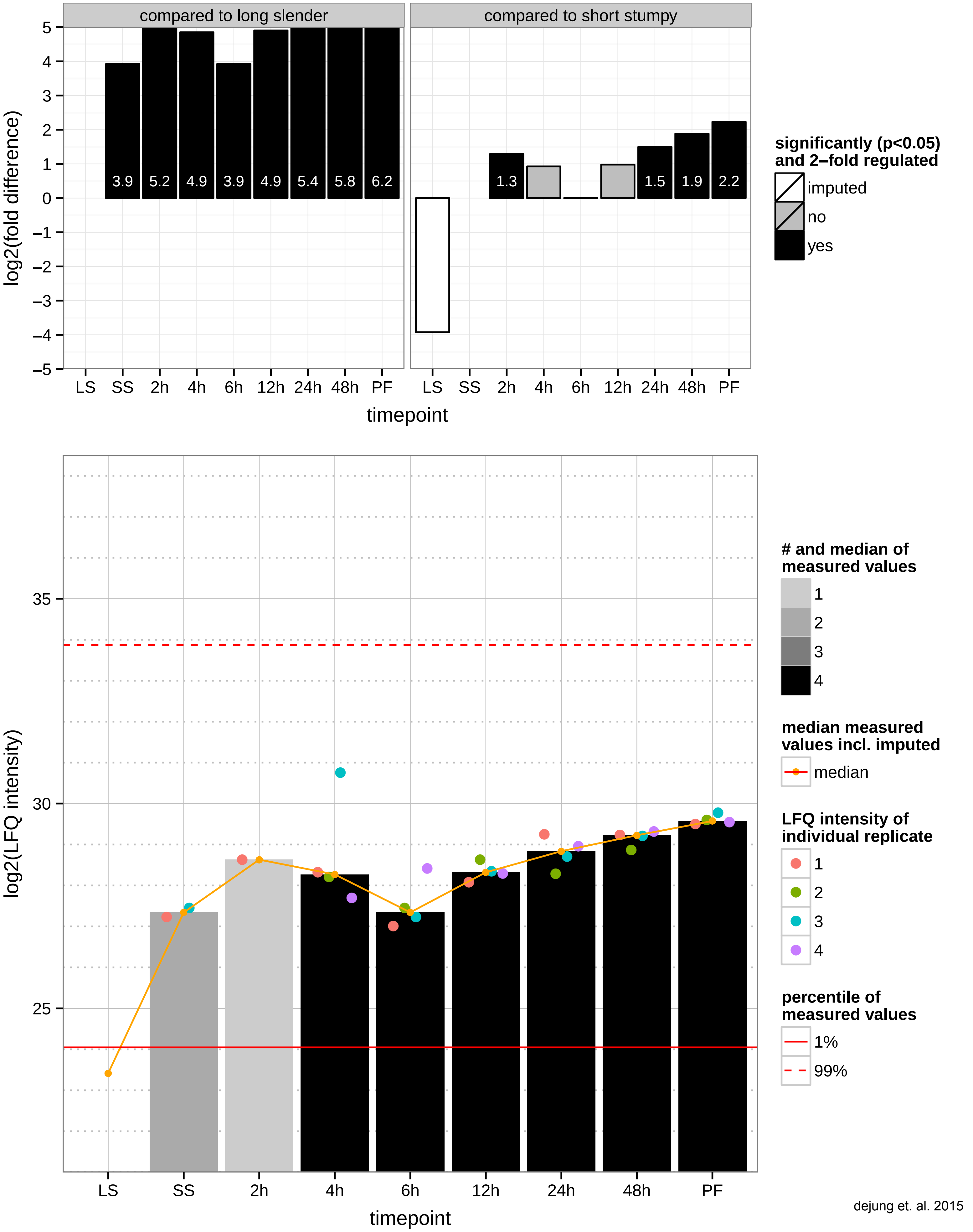

dejung et. al. 2015

thiosulfate sulfurtransferase, mitochondrial, putative
Tb927.6.4930
AGOF: null
AGOC: mitochondrial small ribosomal subunit, mitochondrion
AGOP: null
PGOF: null
PGOC: null
PGOP: null

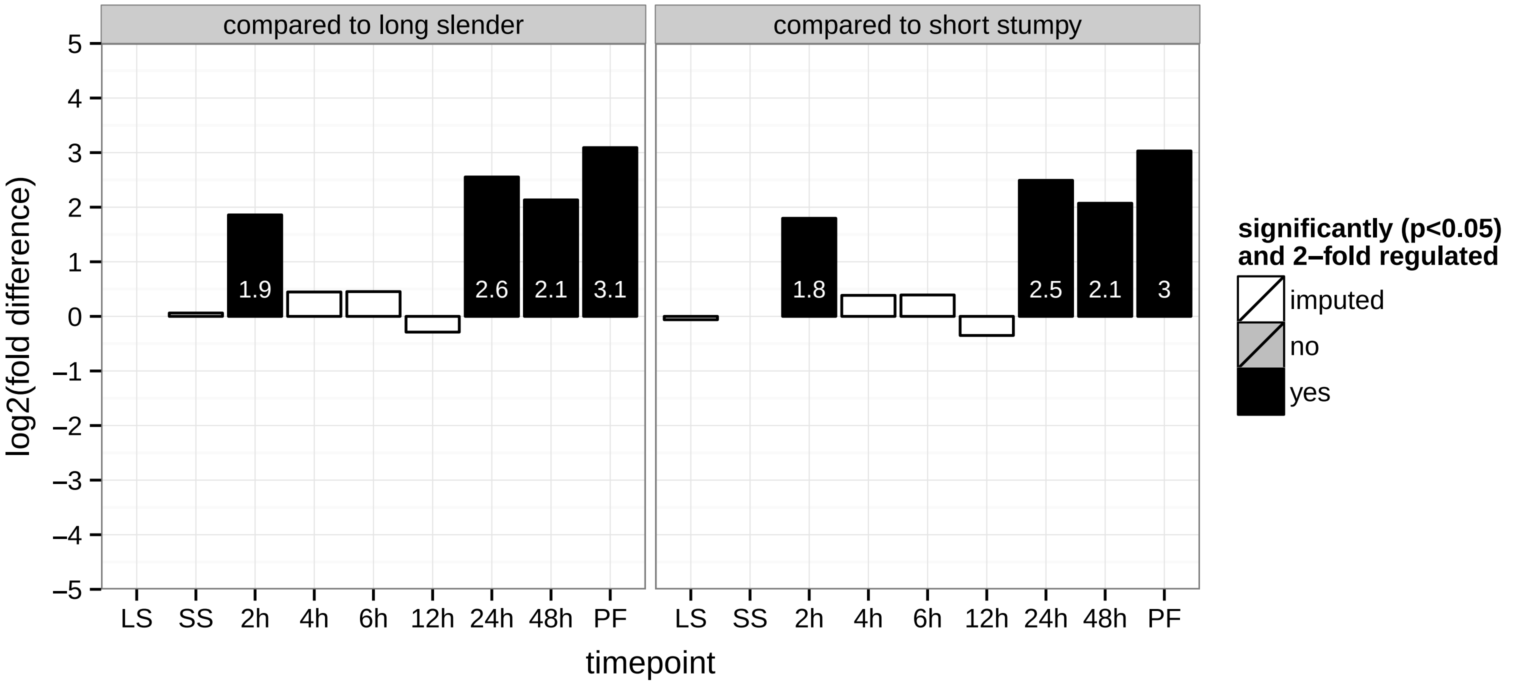

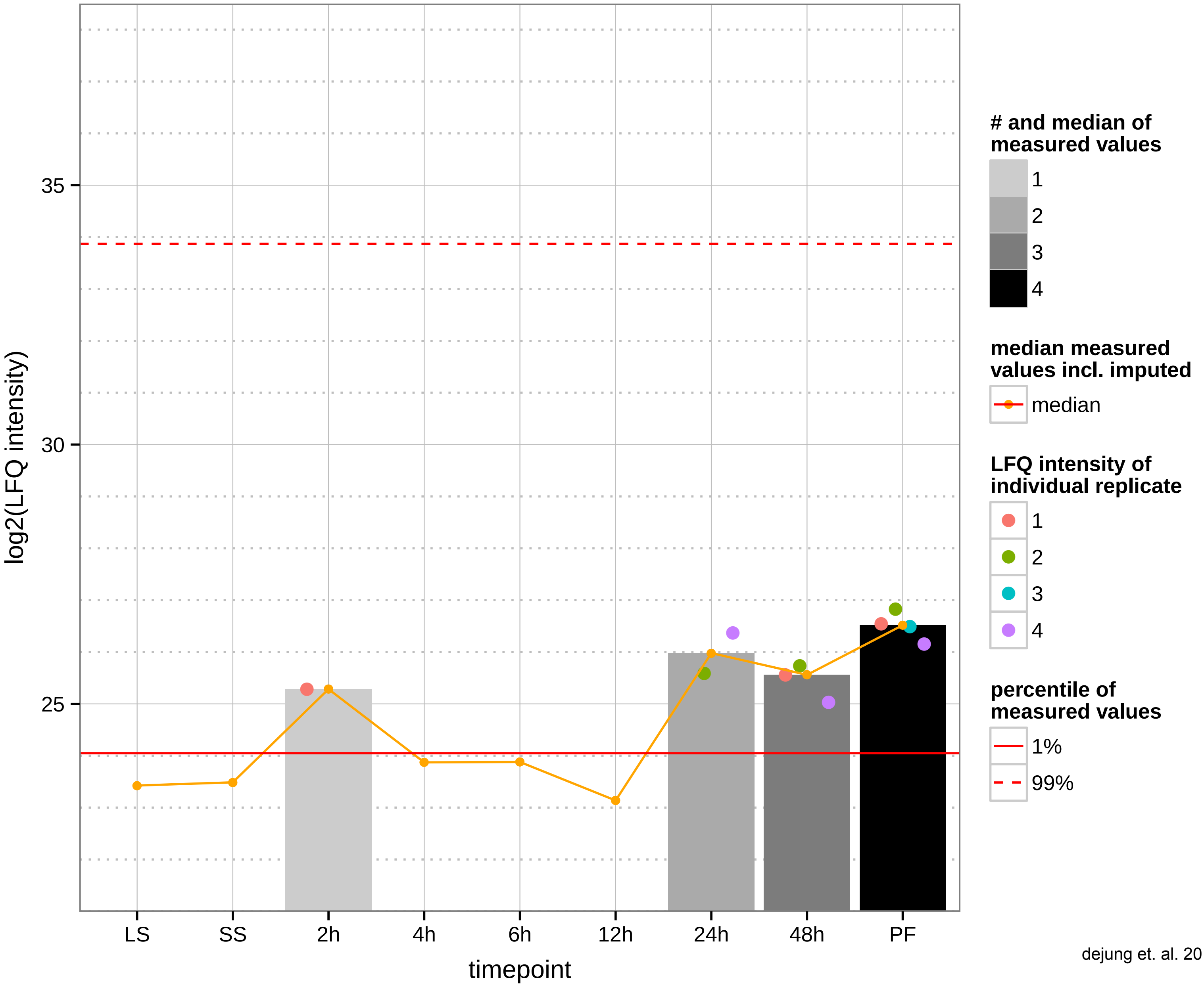

dejung et. al. 2015

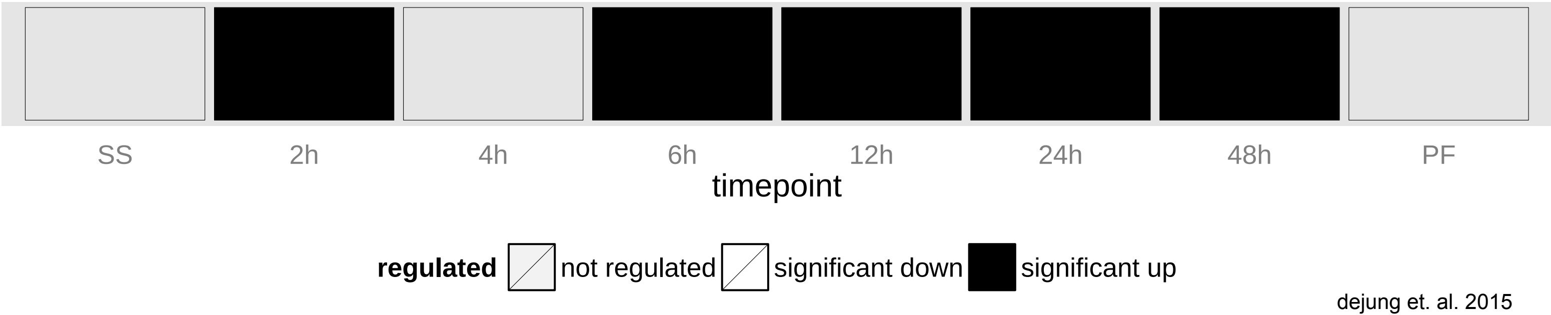
SS
2h
4h
6h
12h
24h
48h
PF
timepoint
regulated
not regulated
significant down
significant up
dejung et. al. 2015

RNA–binding protein, putative (RBP15)
Tb927.10.7470
AGOF: RNA binding
AGOC: null
AGOP: RNA processing
PGOF: nucleic acid binding
PGOC: null
PGOP: null

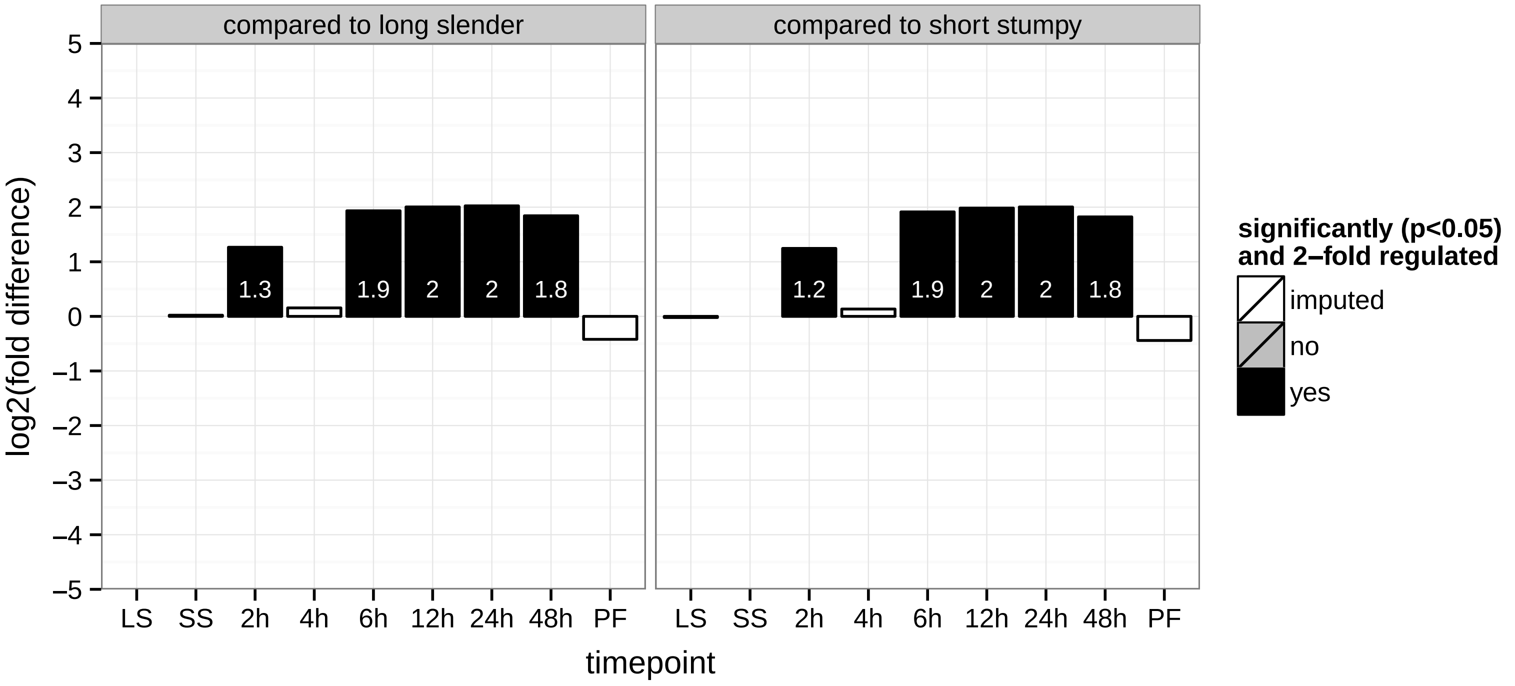

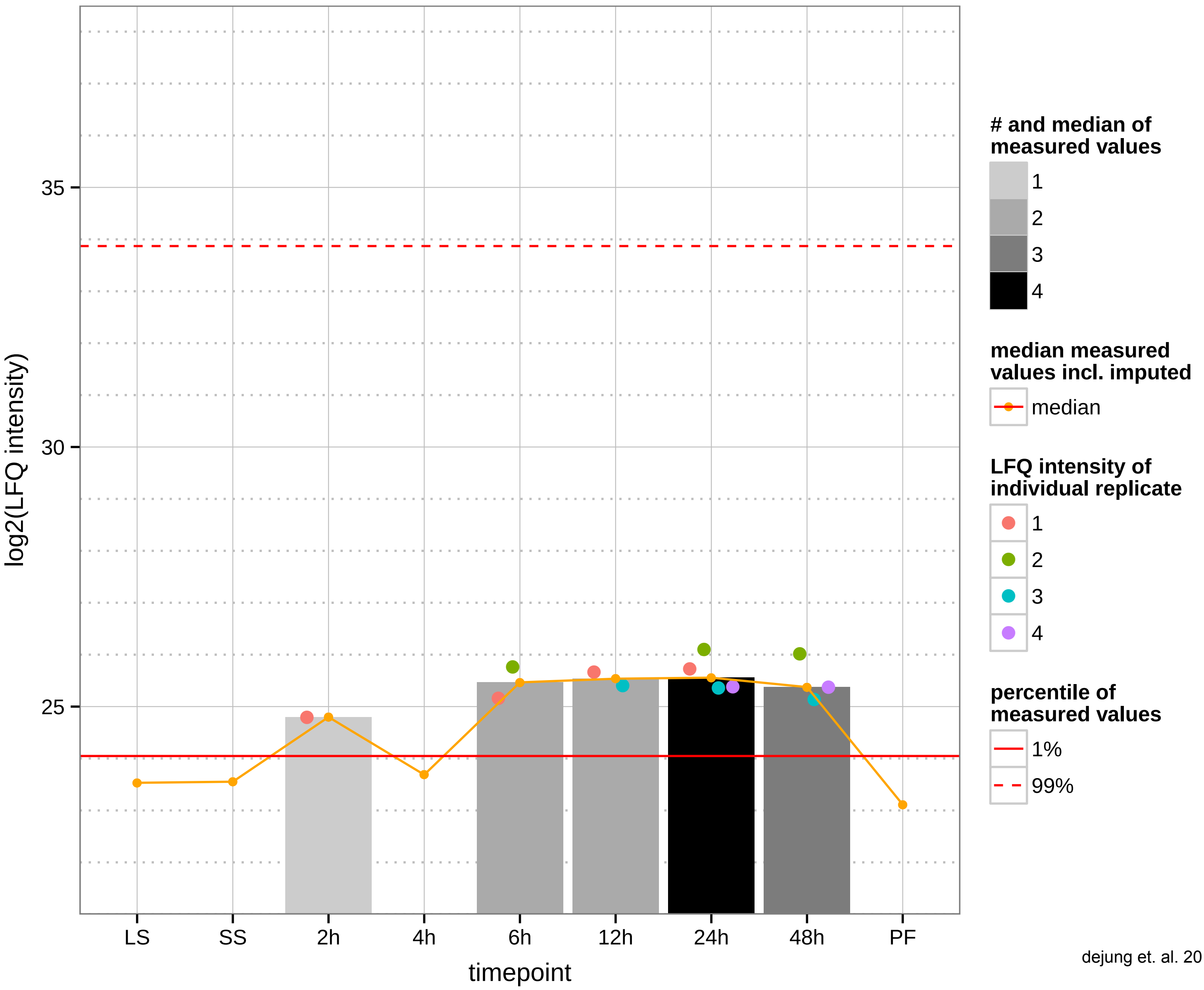

dejung et. al. 2015

hypothetical protein, conserved
Tb927.3.1560
AGOF: null
AGOC: null
AGOP: null
PGOF: null
PGOC: null
PGOP: null

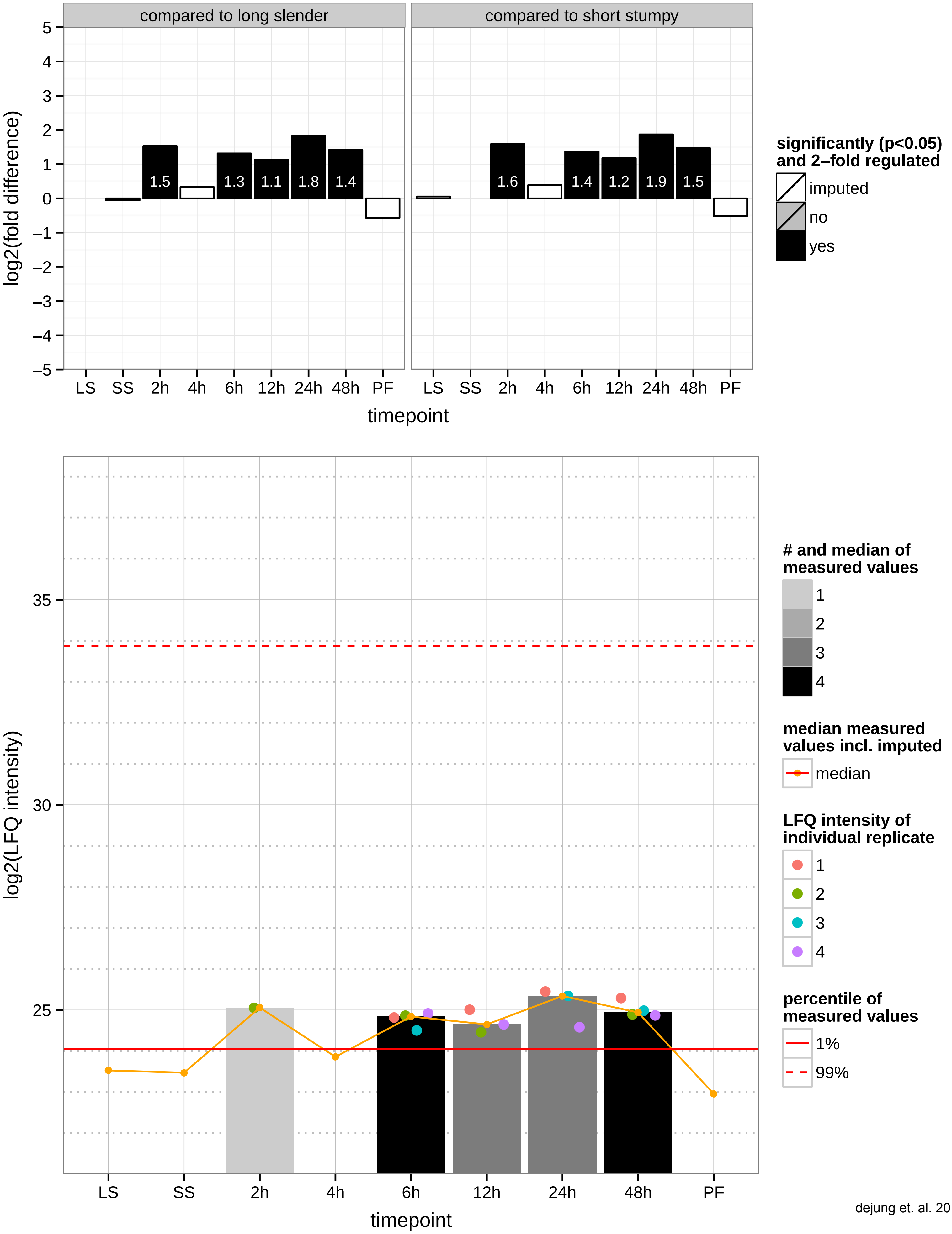

dejung et. al. 2015

hypothetical protein, conserved
Tb927.7.3270
AGOF: null
AGOC: mitochondrion
AGOP: null
PGOF: null
PGOC: null
PGOP: null

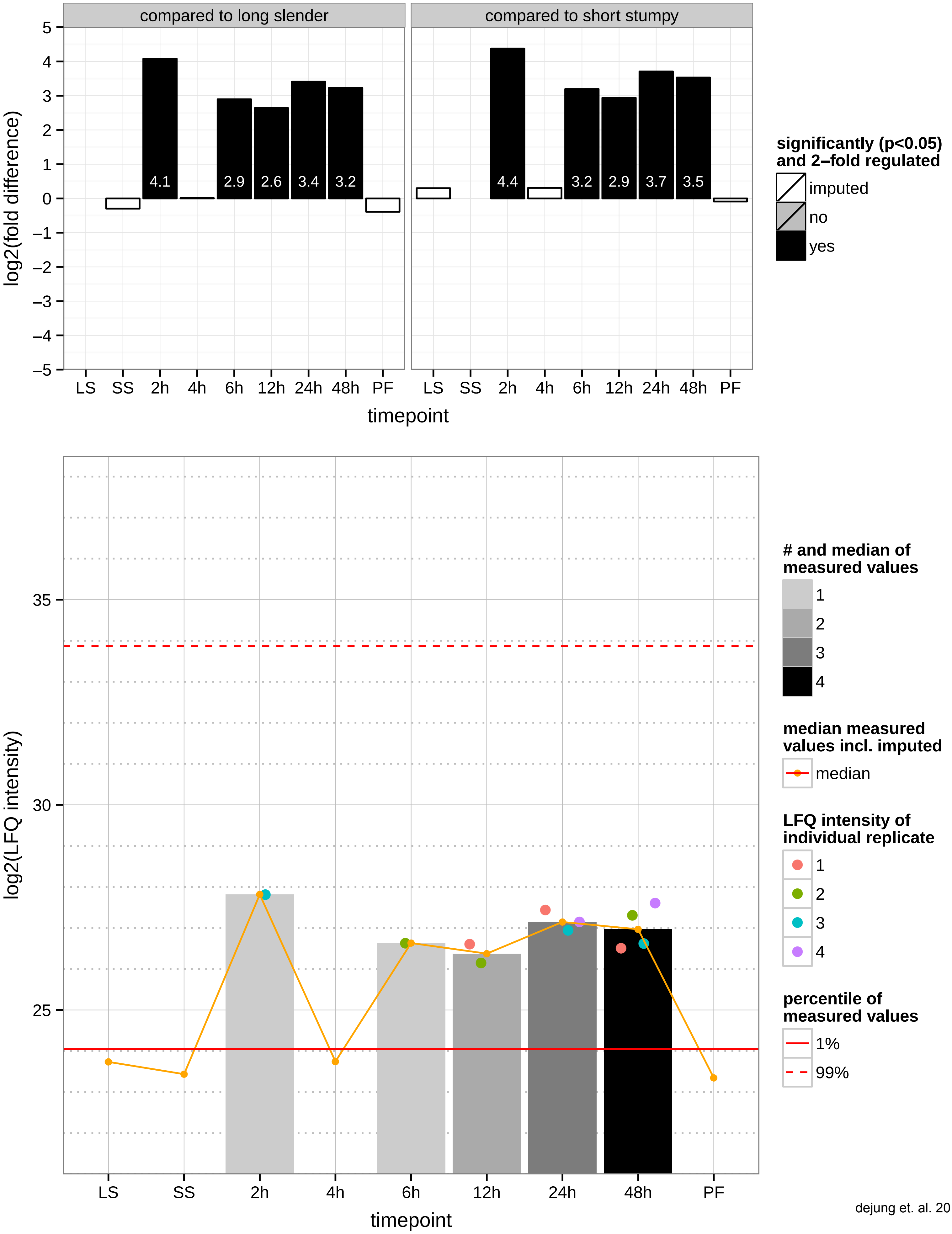

dejung et. al. 2015

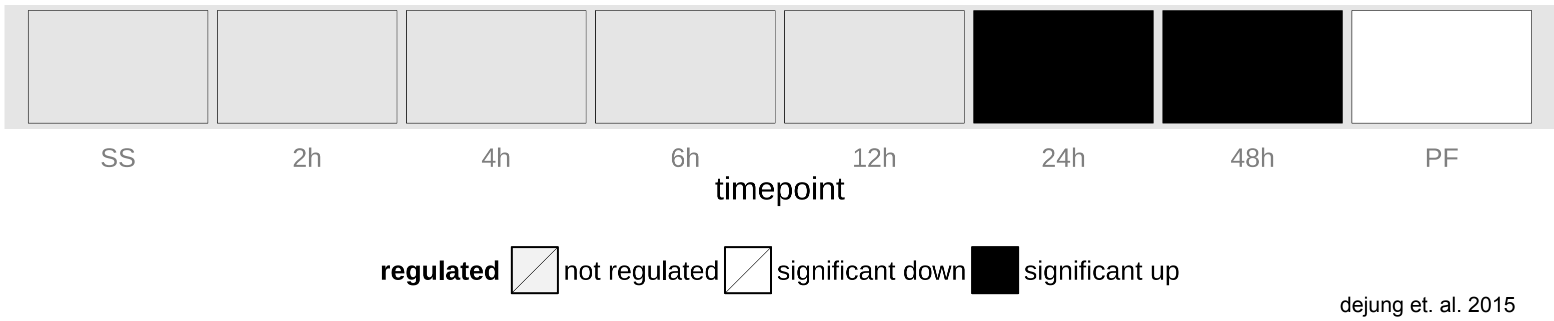
SS
2h
4h
6h
12h
24h
48h
PF
timepoint
regulated
not regulated
significant down
significant up
dejung et. al. 2015

hypothetical protein, conserved
Tb927.10.15830
AGOF: null
AGOC: mitochondrion
AGOP: null
PGOF: null
PGOC: null
PGOP: null

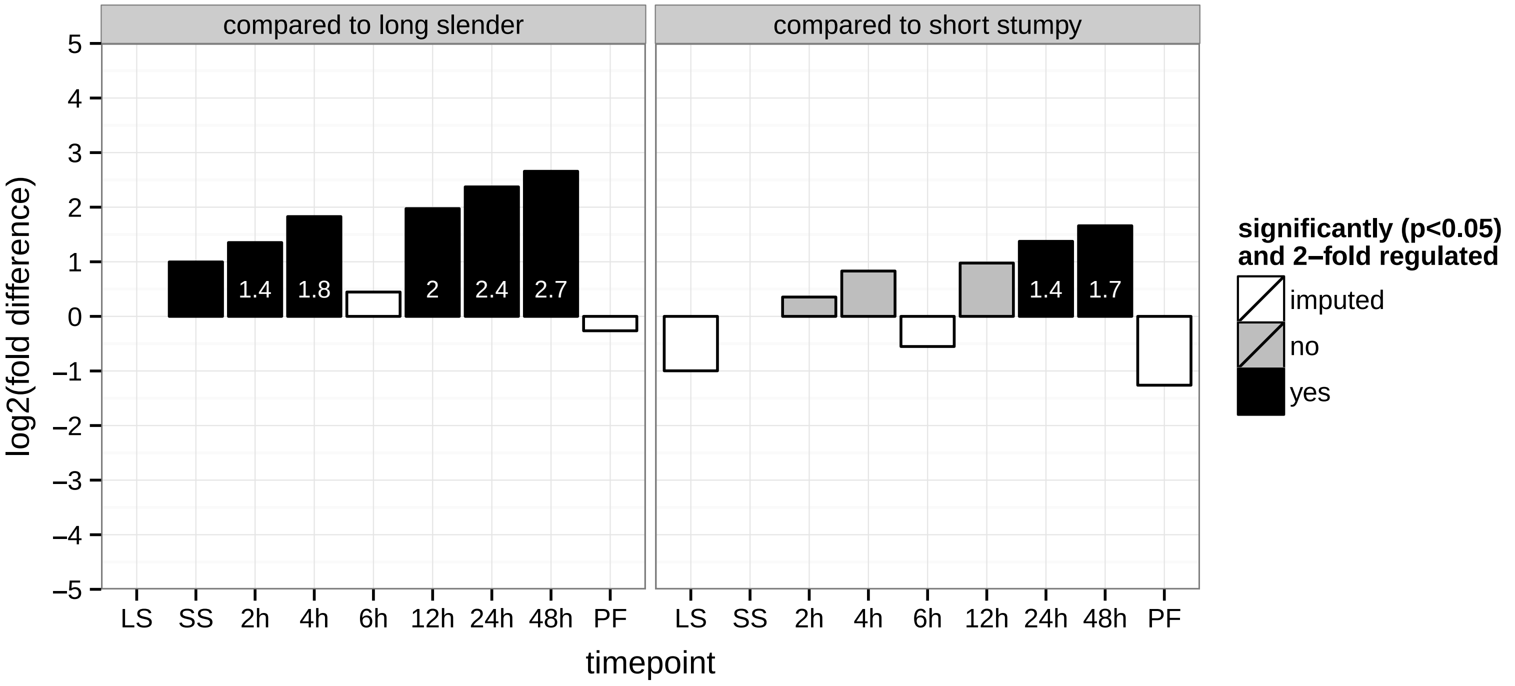

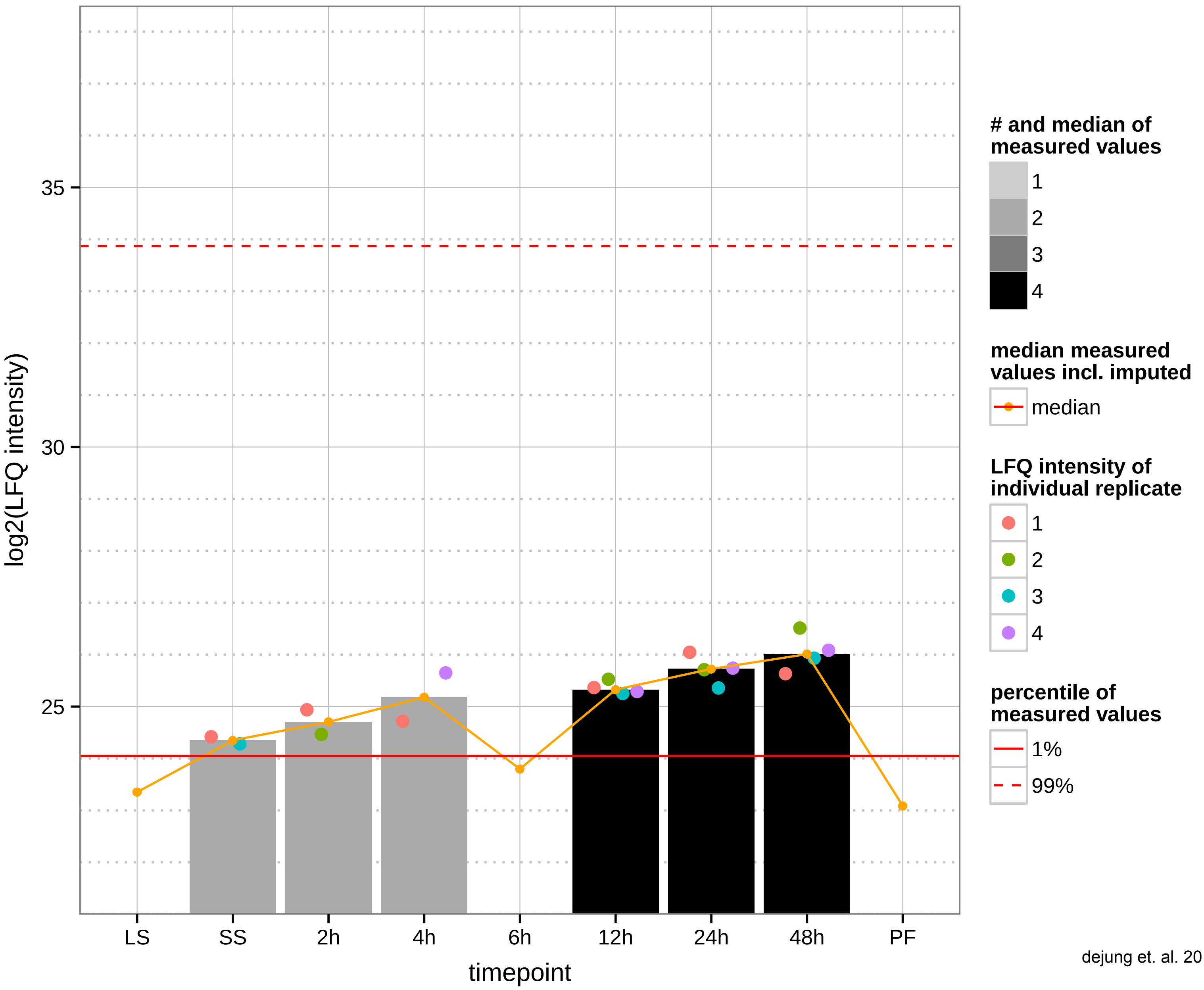

dejung et. al. 2015

hypothetical protein, conserved
Tb927.5.1510
AGOF: null
AGOC: mitochondrion
AGOP: null
PGOF: null
PGOC: null
PGOP: null

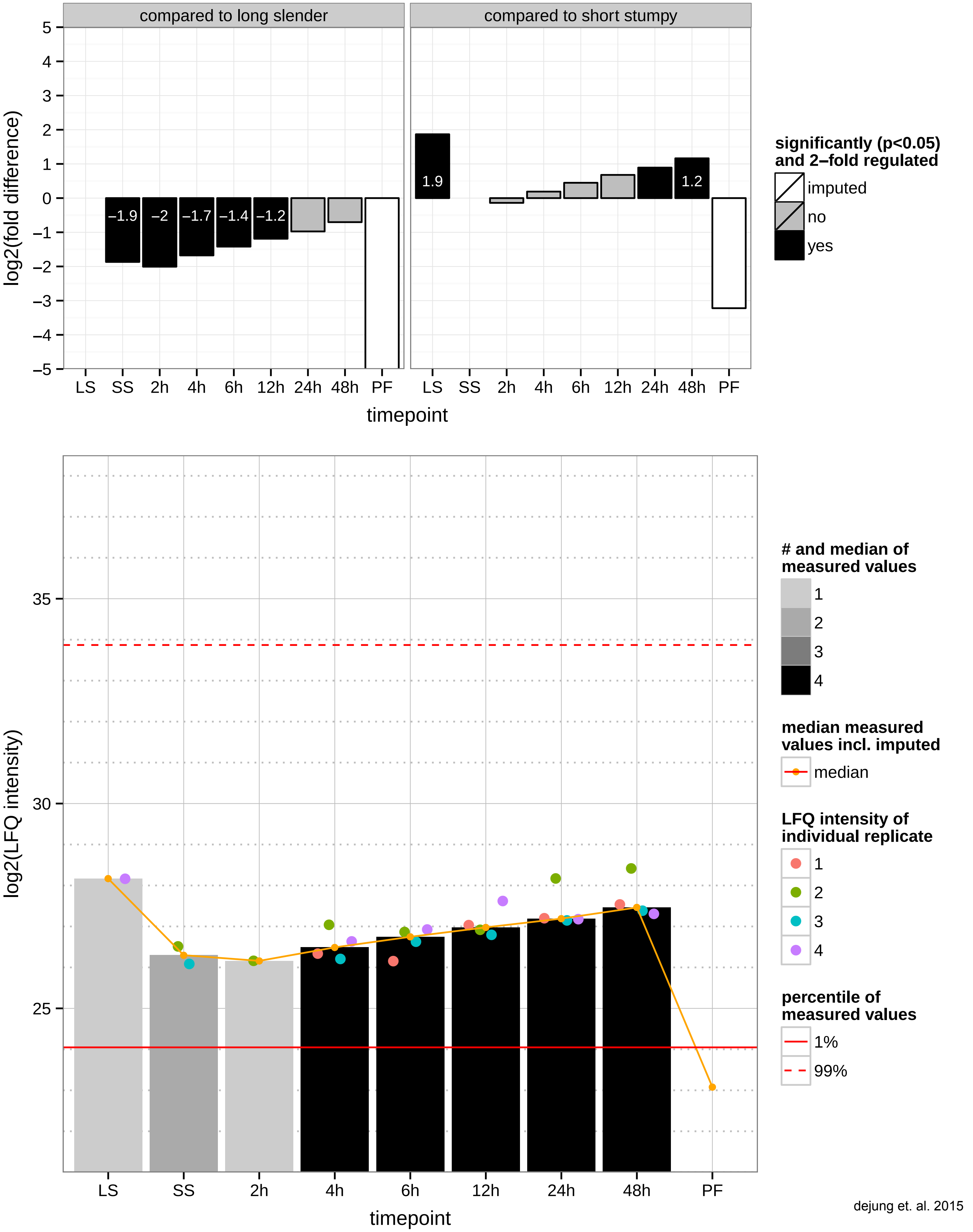

dejung et. al. 2015

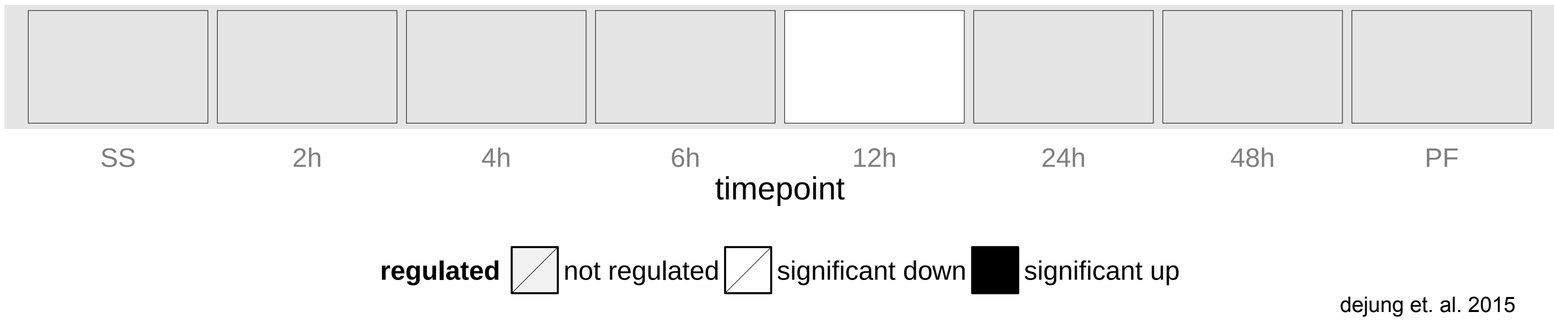
SS
2h
4h
6h
12h
24h
48h
PF
timepoint
regulated
not regulated
significant down
significant up
dejung et. al. 2015

hypothetical protein, conserved
Tb927.10.12130
AGOF: null
AGOC: null
AGOP: null
PGOF: protein binding
PGOC: null
PGOP: null

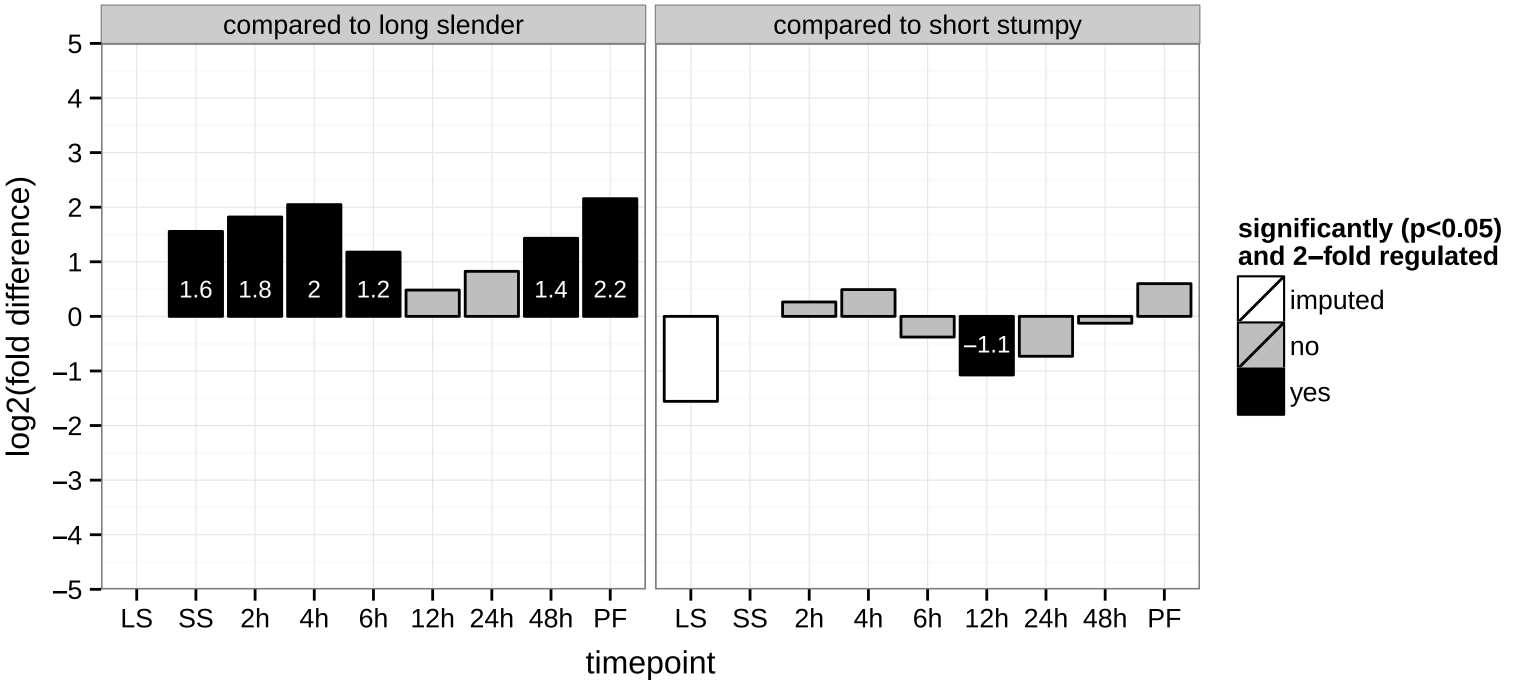

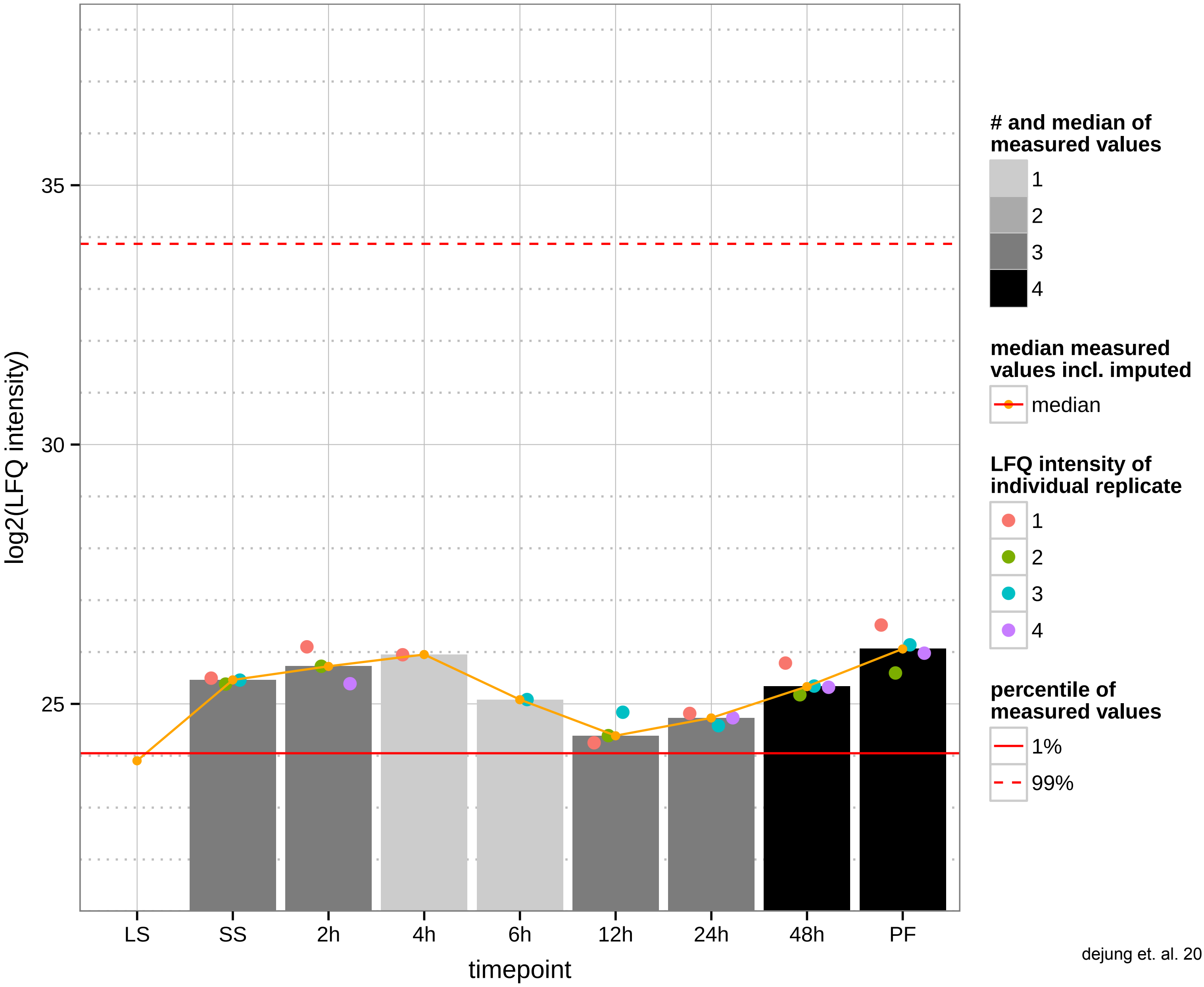

dejung et. al. 2015

thymidylate kinase, putative
Tb927.8.3510;Tb927.8.3910;Tb11.v5.0738
AGOF: null, ATP binding, thymidylate kinase activity
AGOC: null
AGOP: null, TTP biosynthetic process, dTDP biosynthetic process, pyrimidine–containing compound salvage
PGOF: null
PGOC: null
PGOP: null

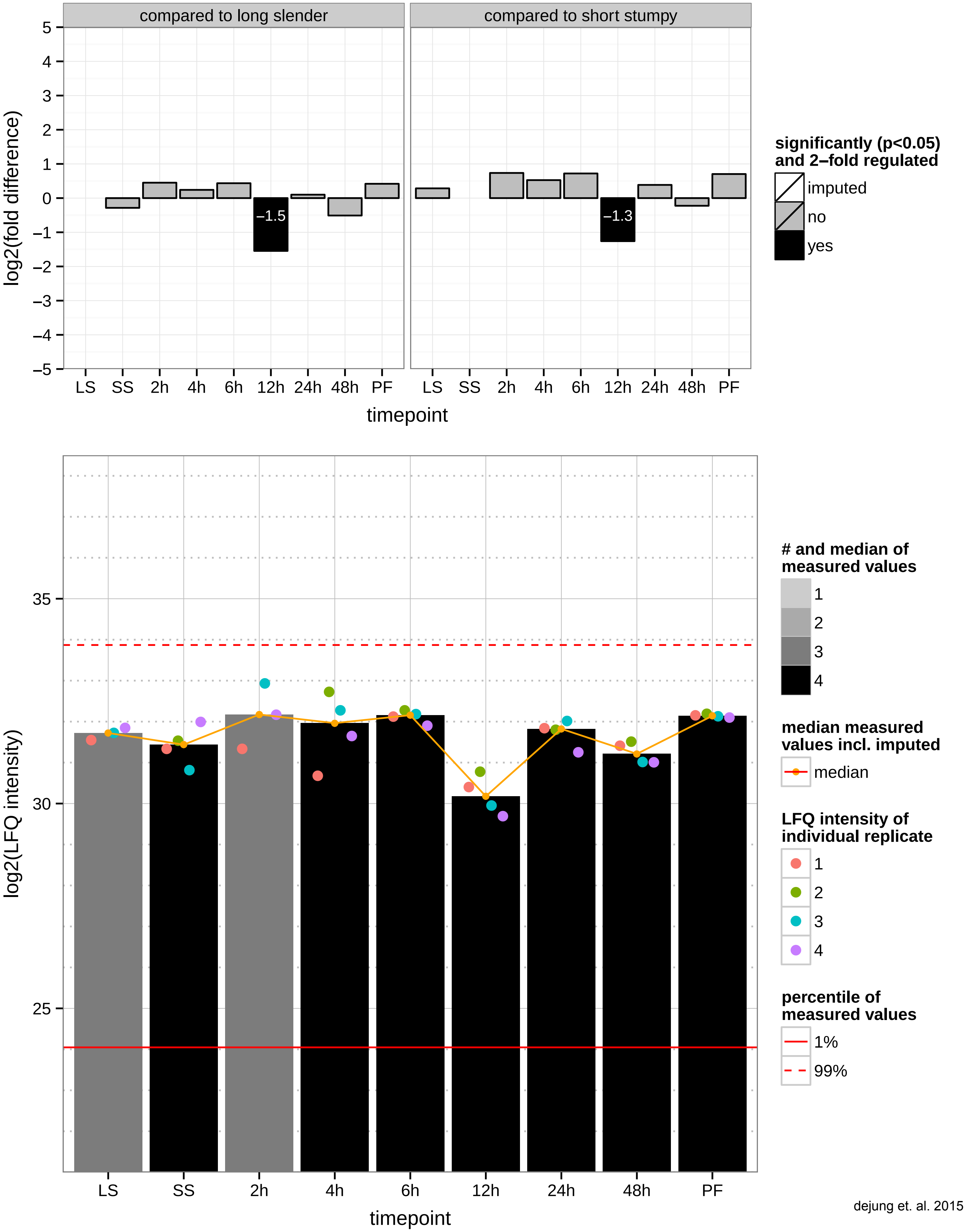

dejung et. al. 2015

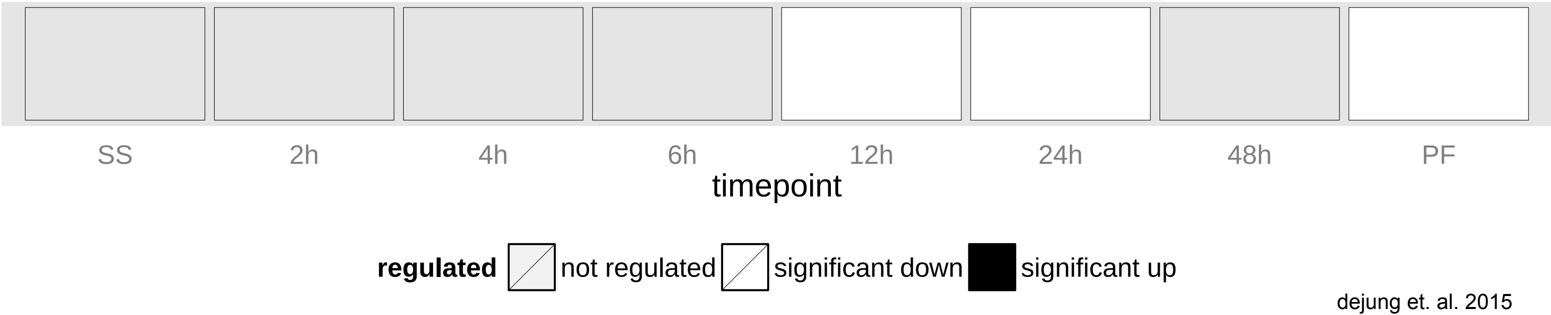
SS
2h
4h
6h
12h
24h
48h
PF
timepoint
regulated
not regulated
significant down
significant up
dejung et. al. 2015

mitochondrial carrier protein (MCP20)
Tb927.10.14280
AGOF: null
AGOC: membrane, mitochondrion
AGOP: transport
PGOF: null
PGOC: null
PGOP: null

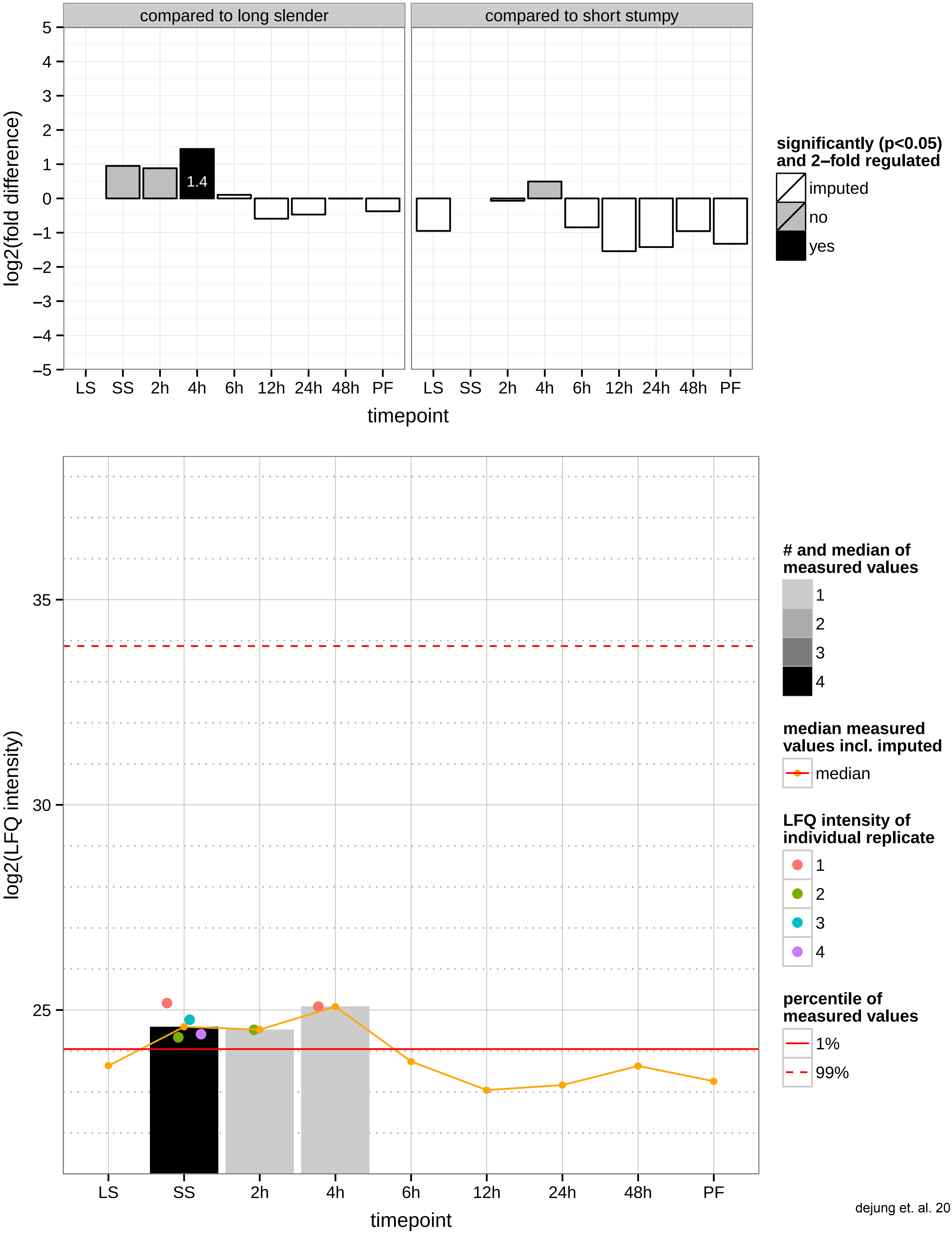

dejung et. al. 2015

hypothetical protein, conserved
Tb927.11.5770
AGOF: null
AGOC: null
AGOP: null
PGOF: protein binding
PGOC: null
PGOP: null

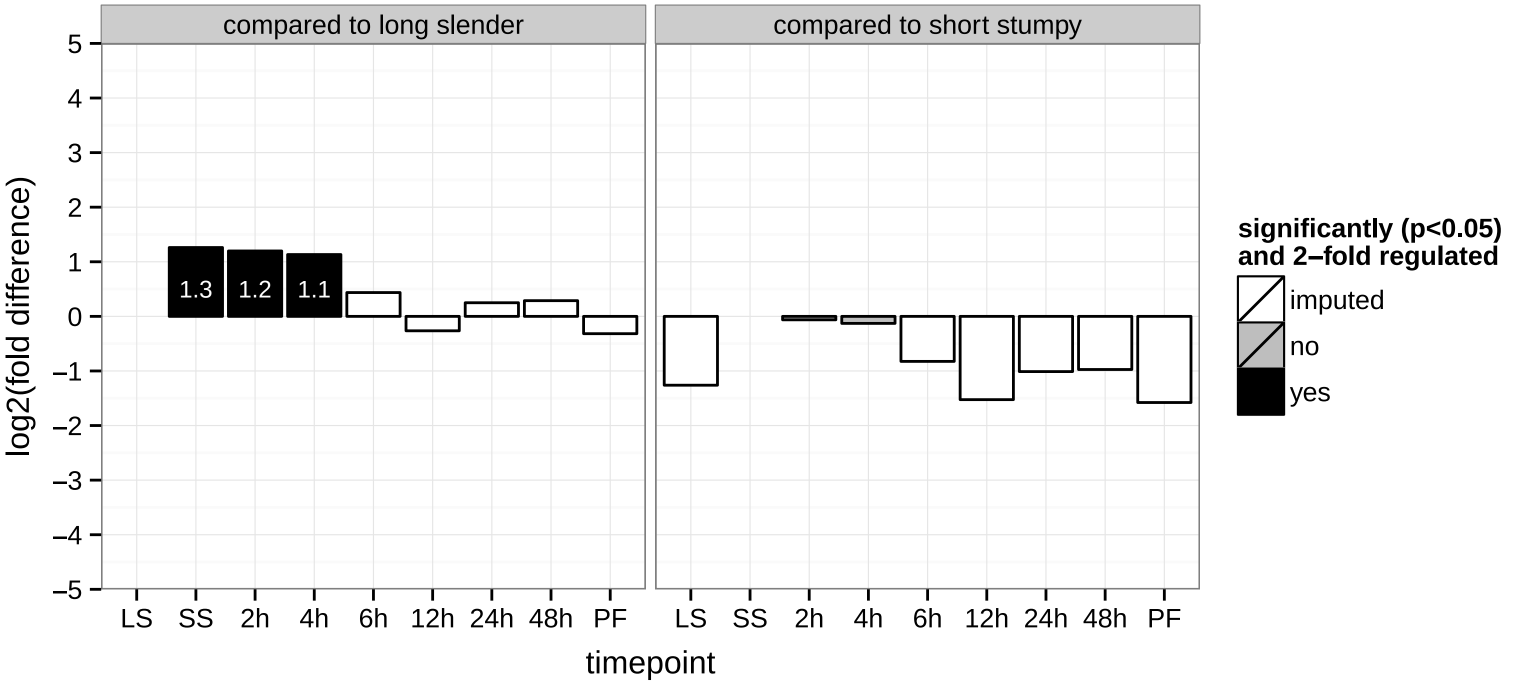

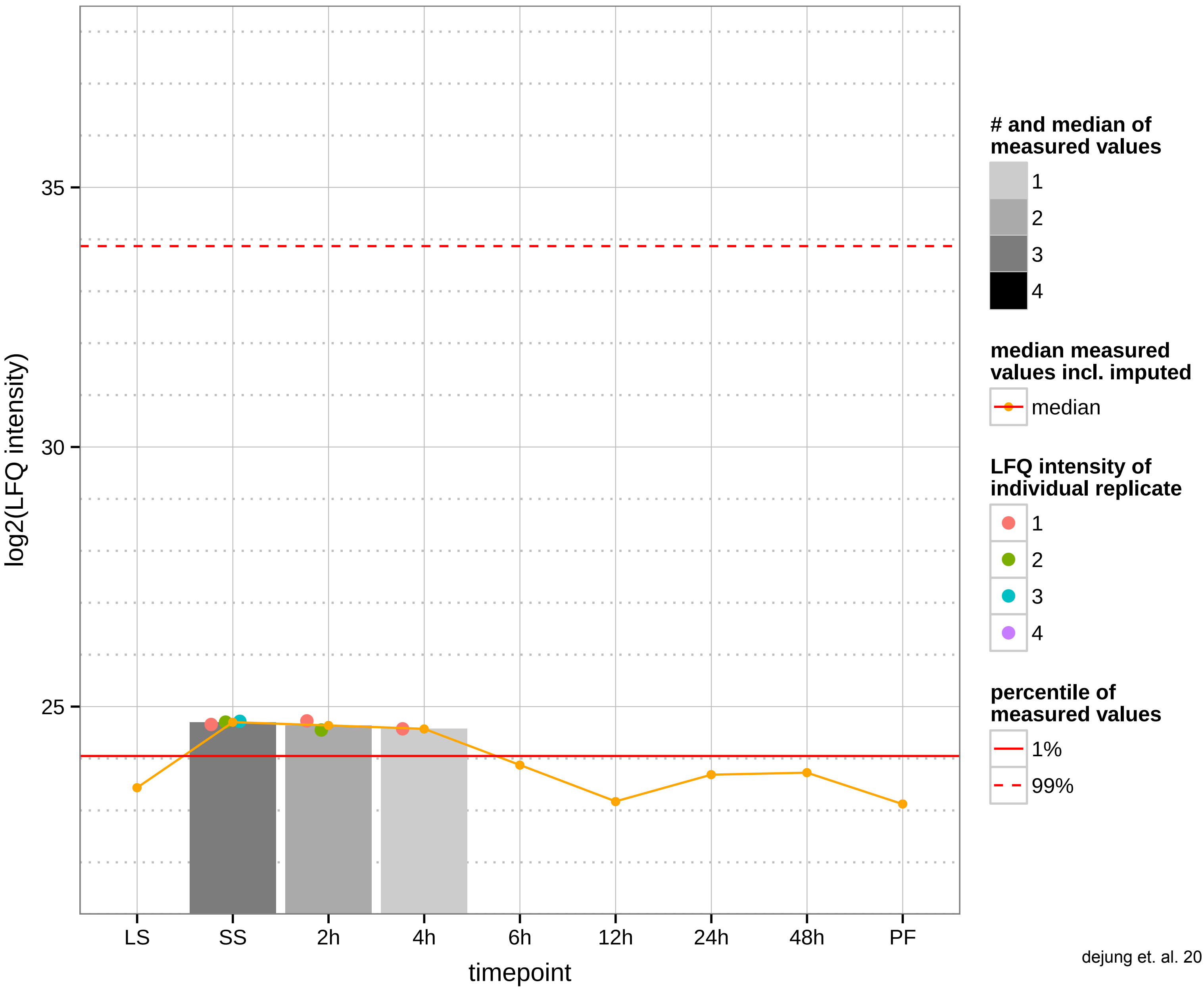

dejung et. al. 2015

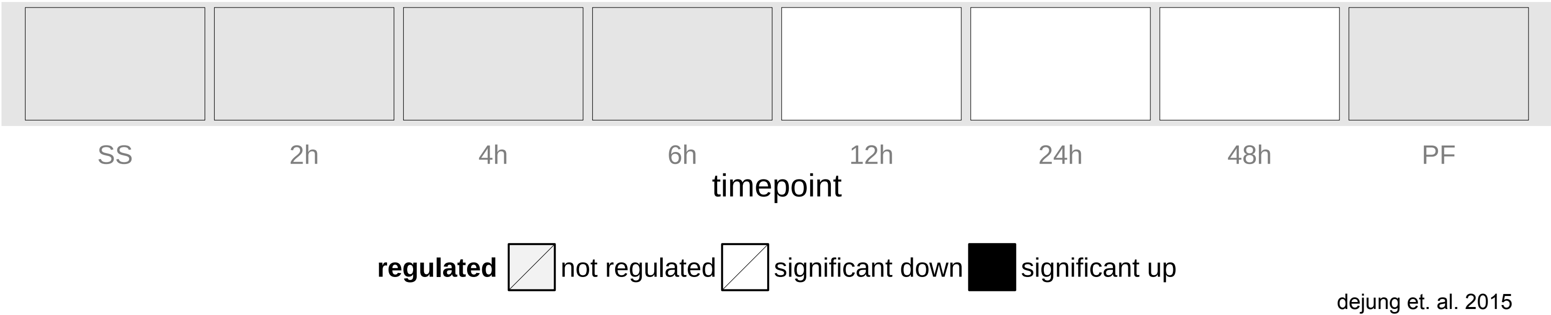
SS
2h
4h
6h
12h
24h
48h
PF
timepoint
regulated
not regulated
significant down
significant up
dejung et. al. 2015

vacuolar protein sorting–associated protein 45, putative (VPS45)
Tb927.10.6780
AGOF: SNARE binding
AGOC: null
AGOP: vesicle docking involved in exocytosis, vesicle–mediated transport
PGOF: null
PGOC: null
PGOP: vesicle docking involved in exocytosis, vesicle–mediated transport

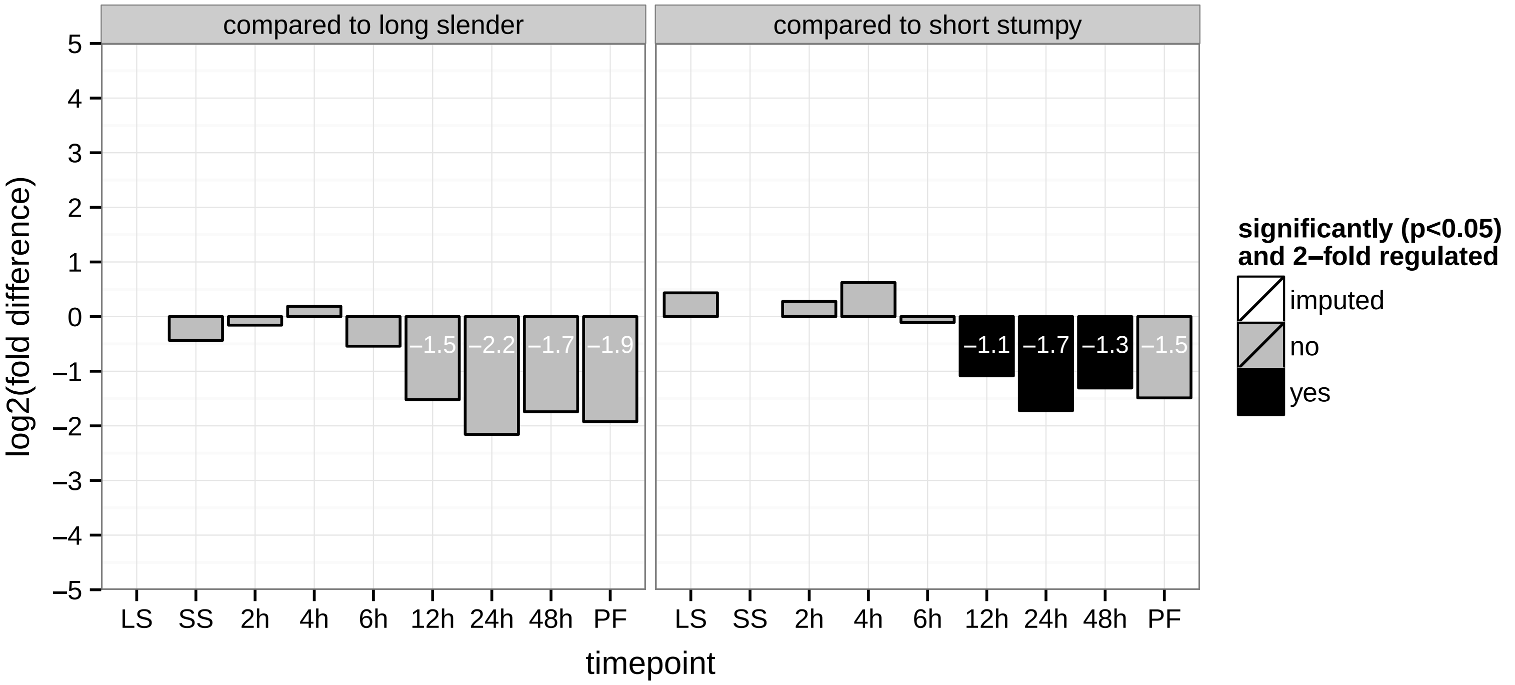

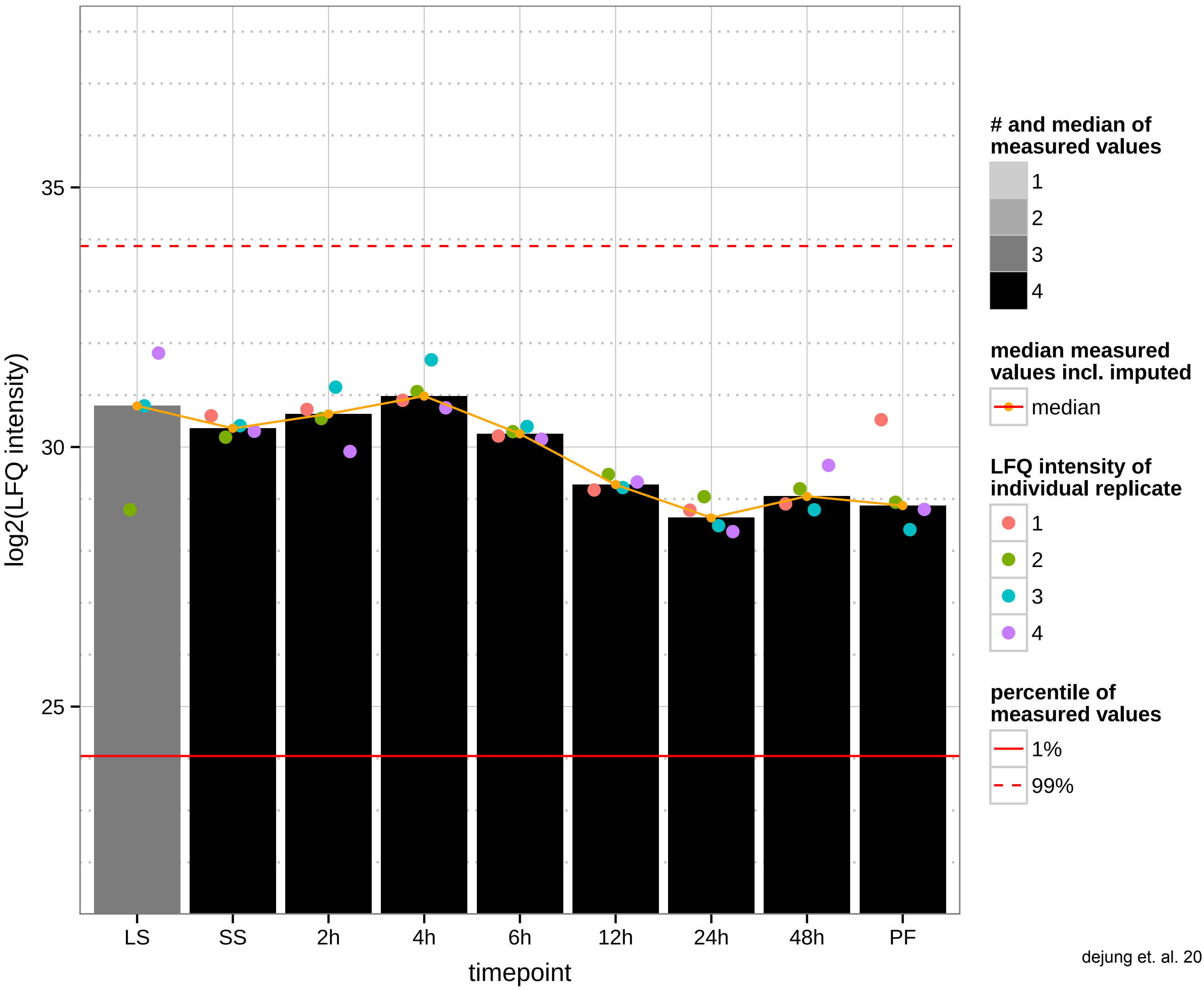

dejung et. al. 2015

engulfment and cell motility domain 2, putative
Tb927.9.2670
AGOF: null
AGOC: cytoskeleton
AGOP: phagocytosis
PGOF: null
PGOC: cytoskeleton
PGOP: phagocytosis

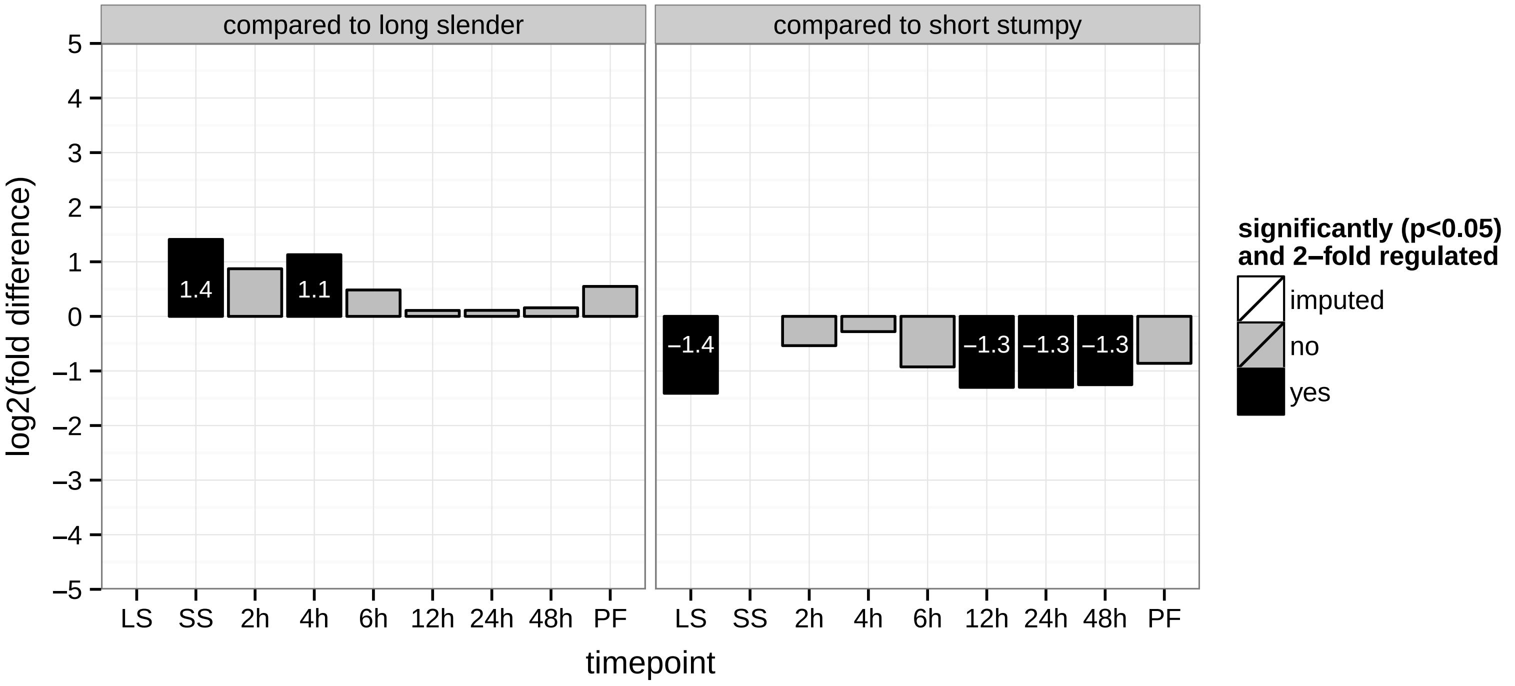

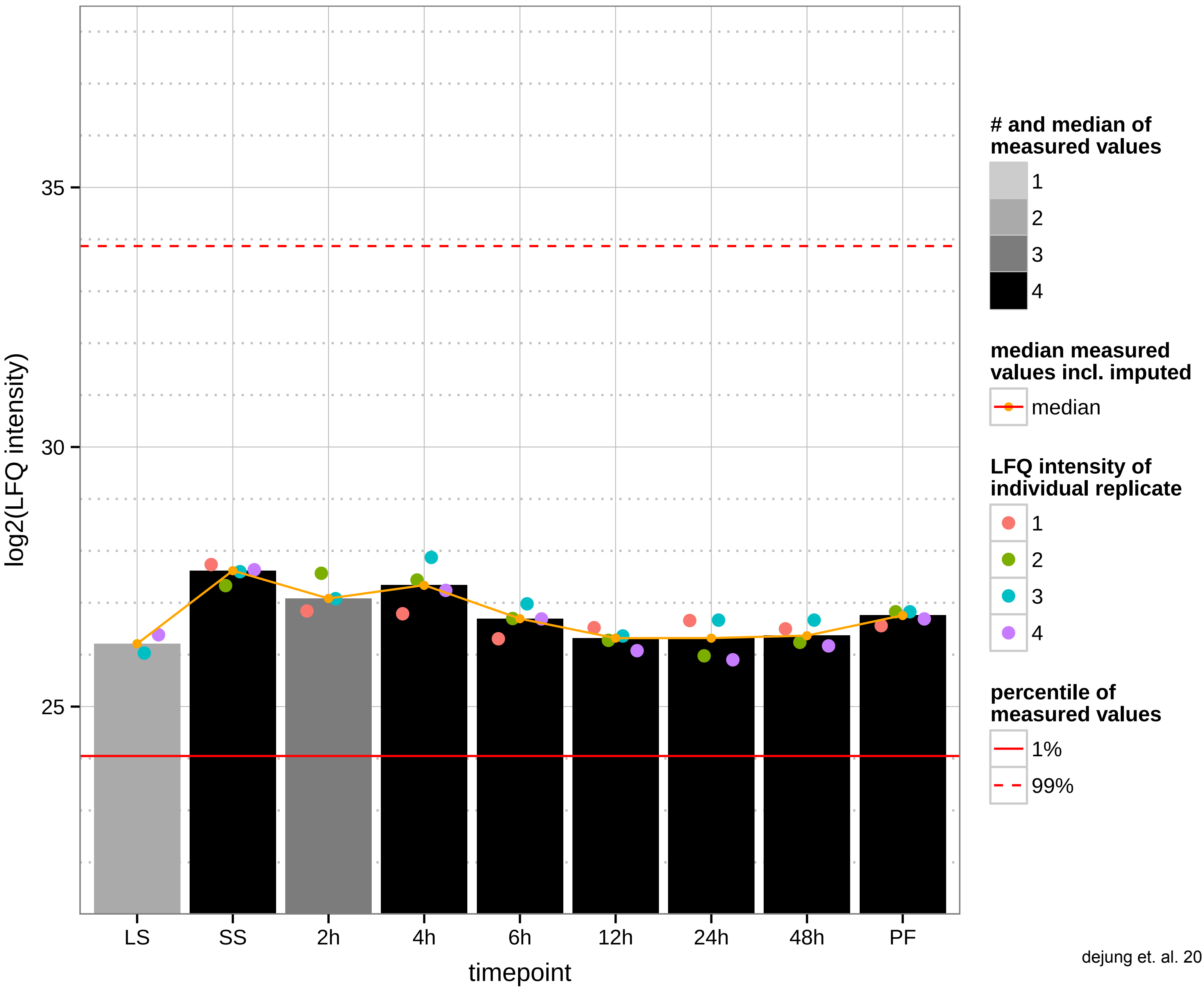

dejung et. al. 2015

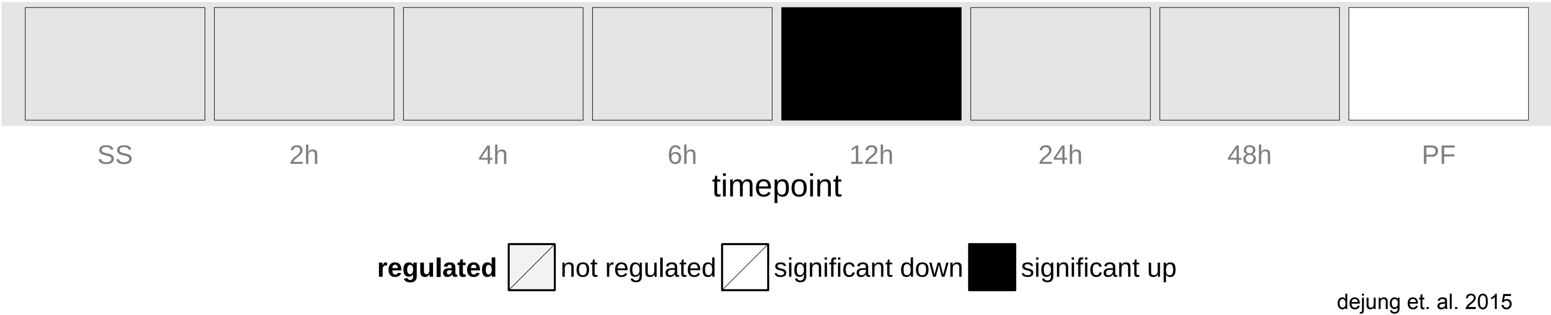
SS
2h
4h
6h
12h
24h
48h
PF
timepoint
regulated
not regulated
significant down
significant up
dejung et. al. 2015

Ribosomal RNA–processing protein 8, putative (RRP8)
Tb927.1.1120
AGOF: methyltransferase activity
AGOC: null
AGOP: null
PGOF: methyltransferase activity
PGOC: null
PGOP: null

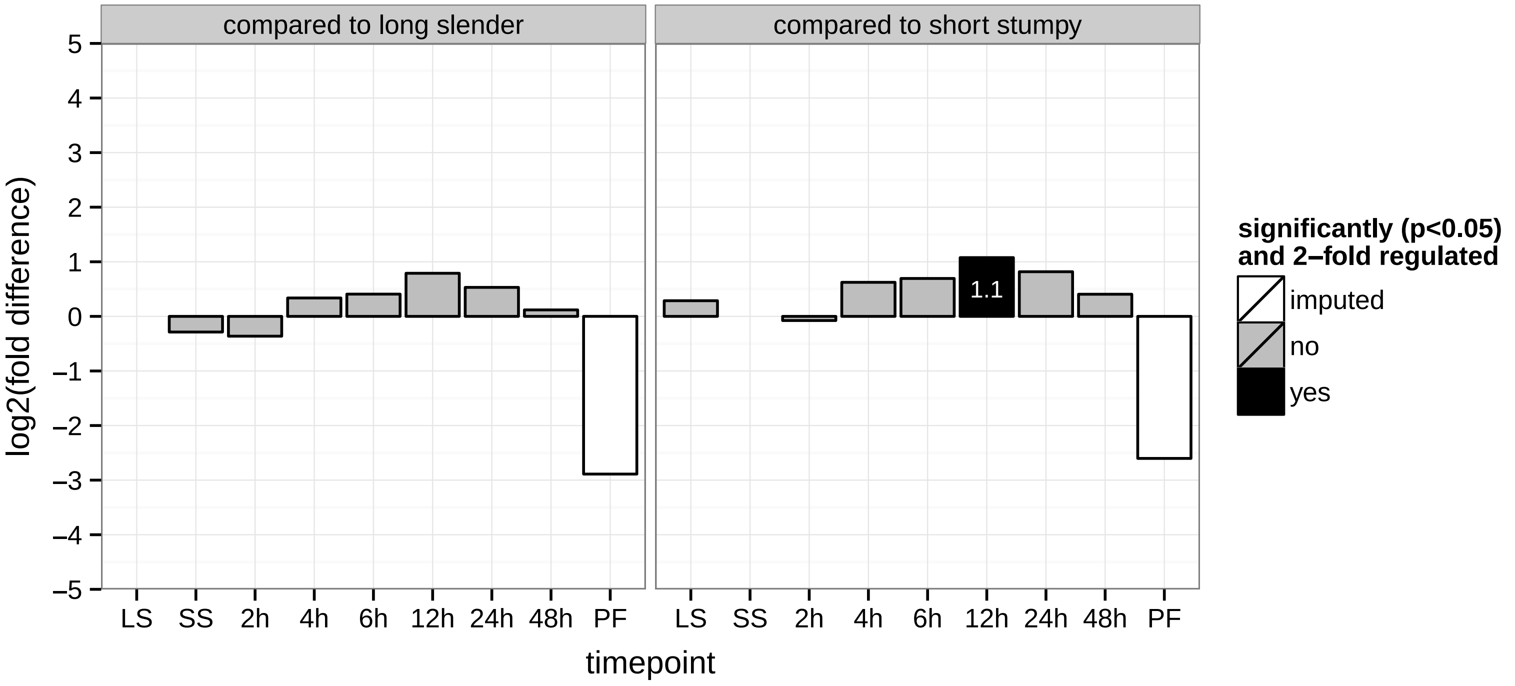

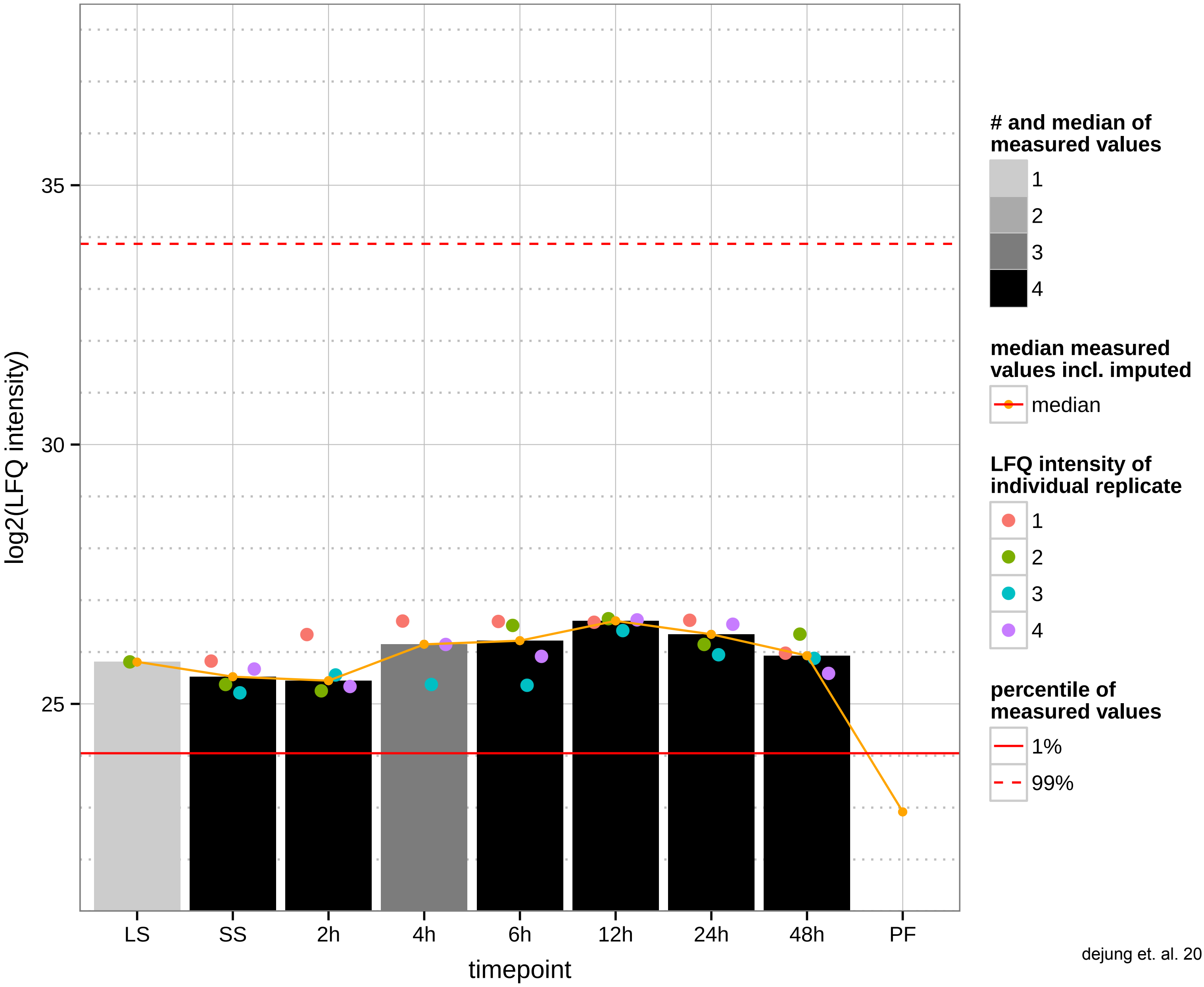

dejung et. al. 2015

IgE–dependent histamine–releasing factor, putative
Tb927.8.6760
AGOF: null
AGOC: cytoplasm
AGOP: null
PGOF: null
PGOC: null
PGOP: null

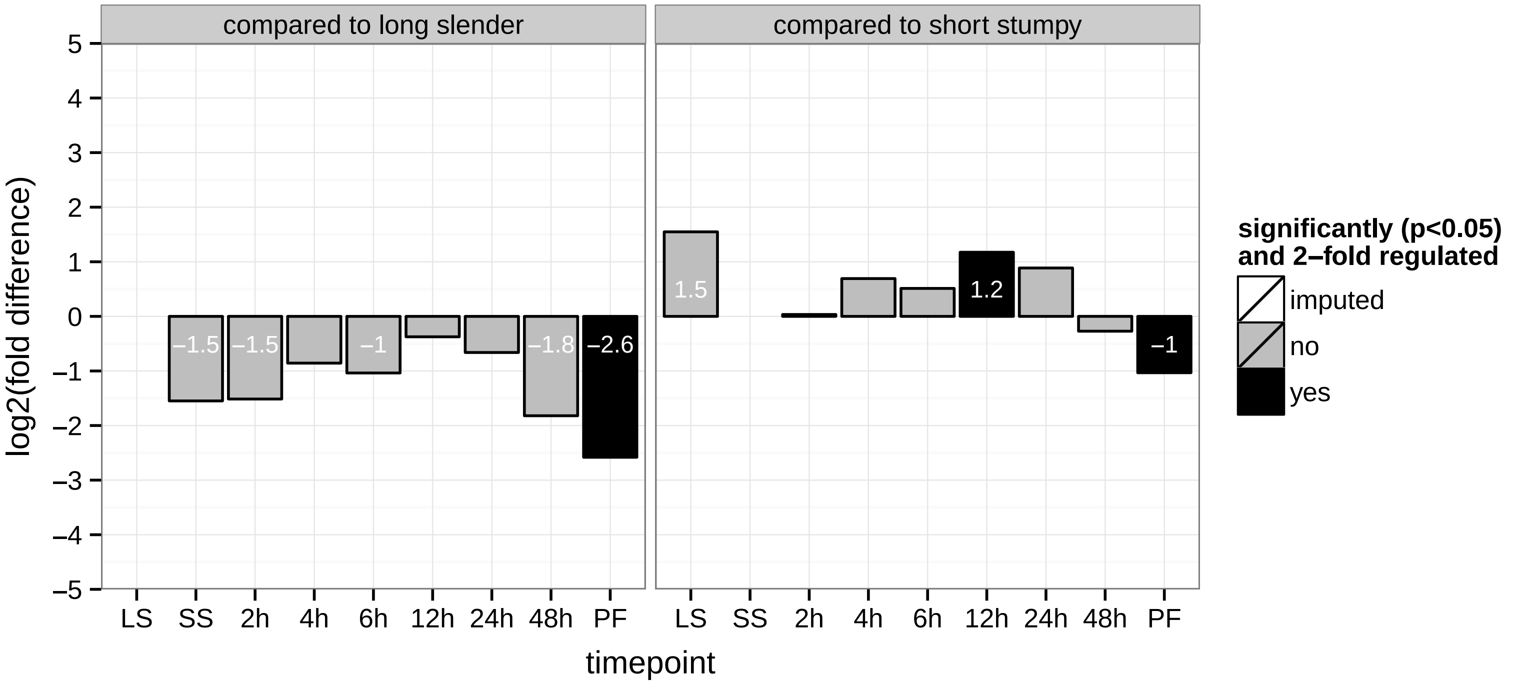

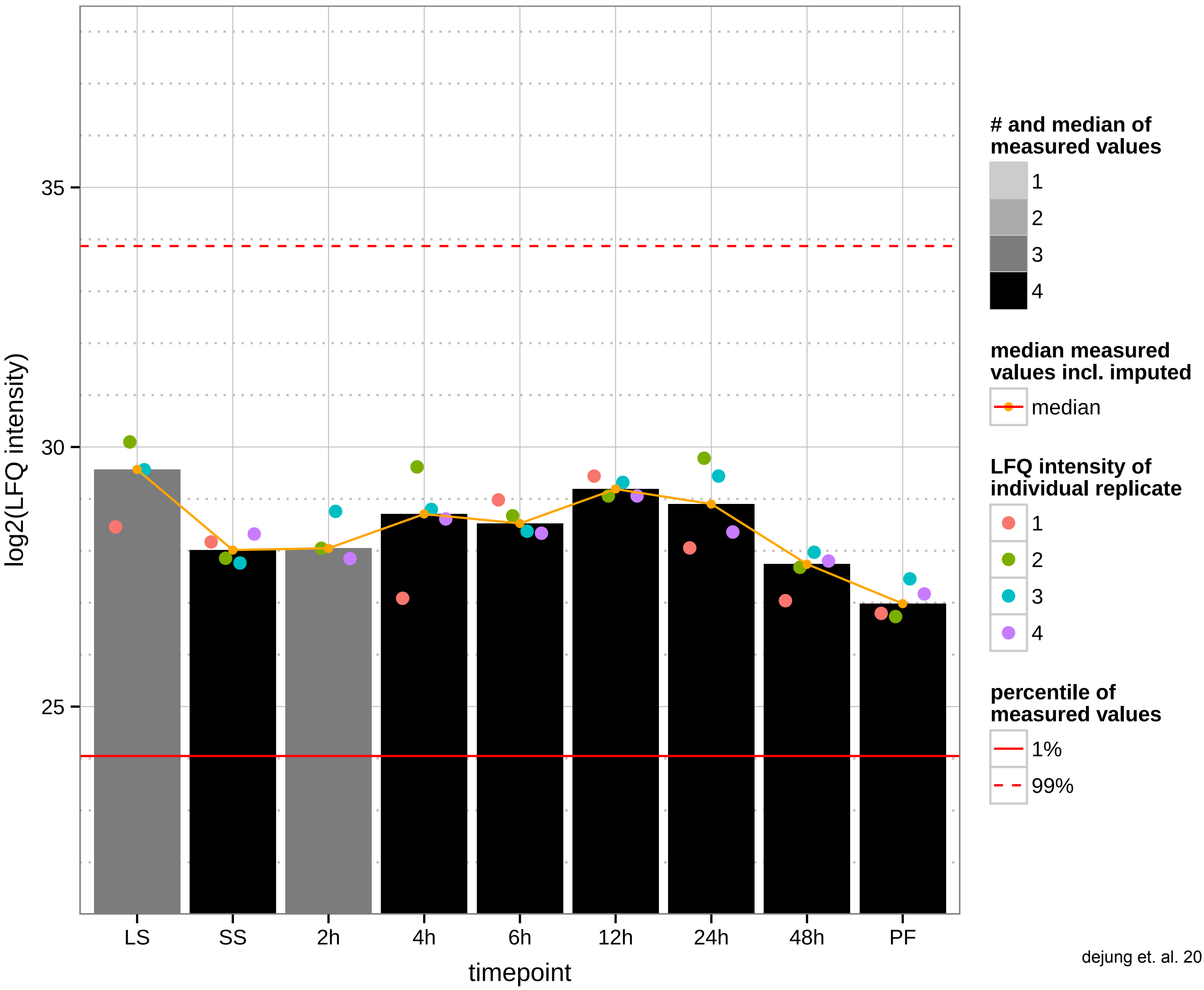

dejung et. al. 2015

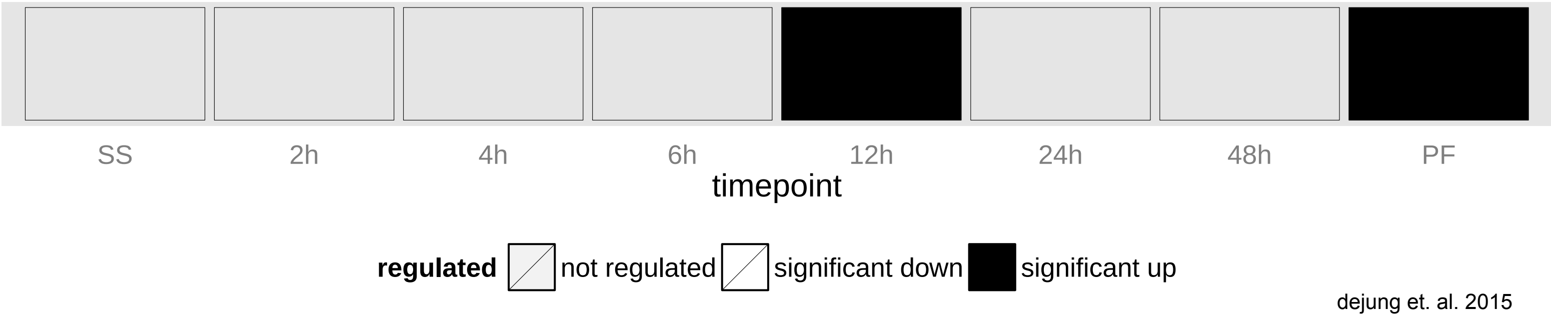
SS
2h
4h
6h
12h
24h
48h
PF
timepoint
regulated
not regulated
significant down
significant up
dejung et. al. 2015

chaperone protein DNAj, putative
Tb11.v5.0778;Tb927.7.680
AGOF: null, heat shock protein binding, unfolded protein binding
AGOC: null
AGOP: null, protein folding
PGOF: heat shock protein binding
PGOC: null
PGOP: null

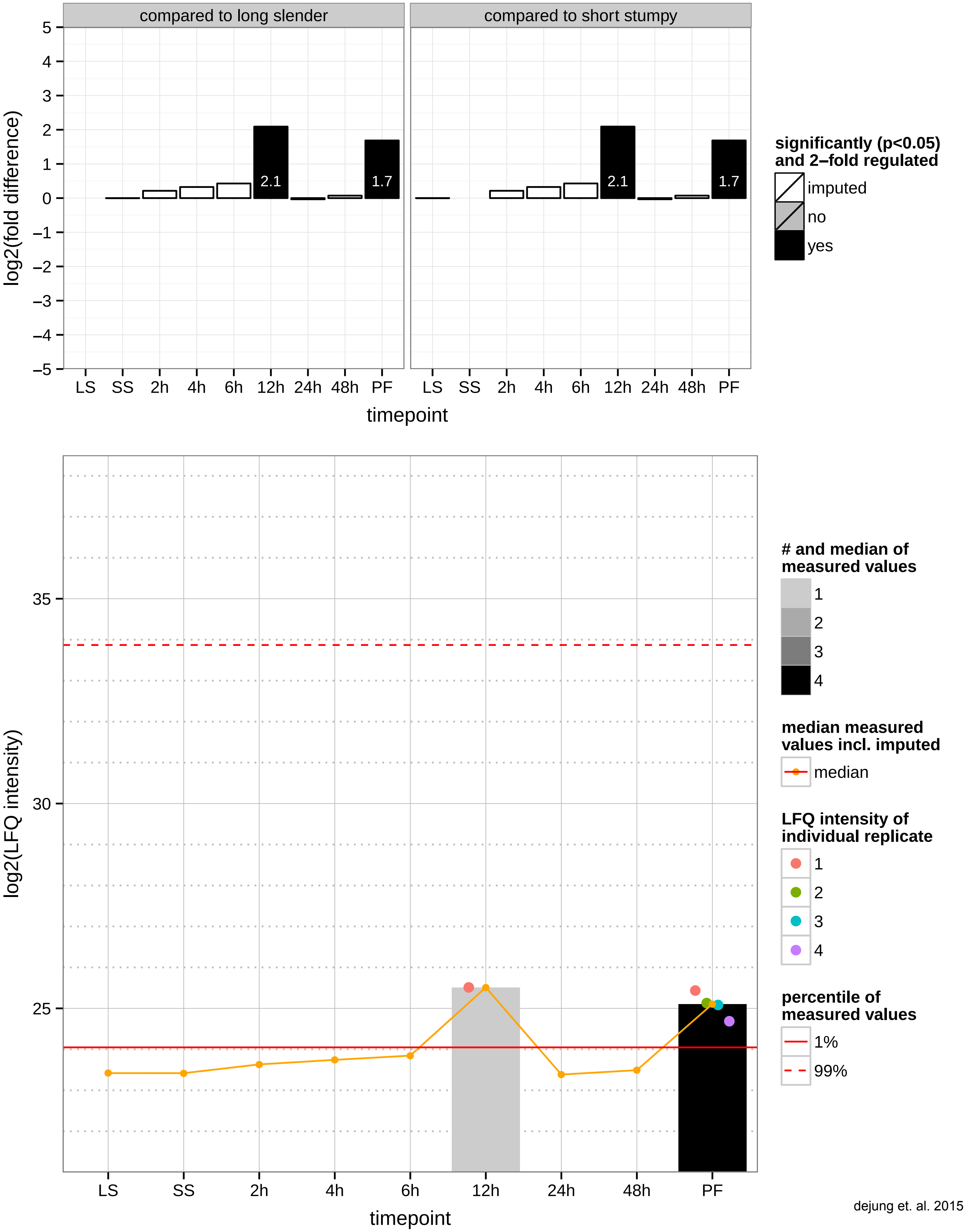

dejung et. al. 2015

hypothetical protein, conserved
Tb927.11.13660
AGOF: null
AGOC: null
AGOP: null
PGOF: null
PGOC: null
PGOP: null

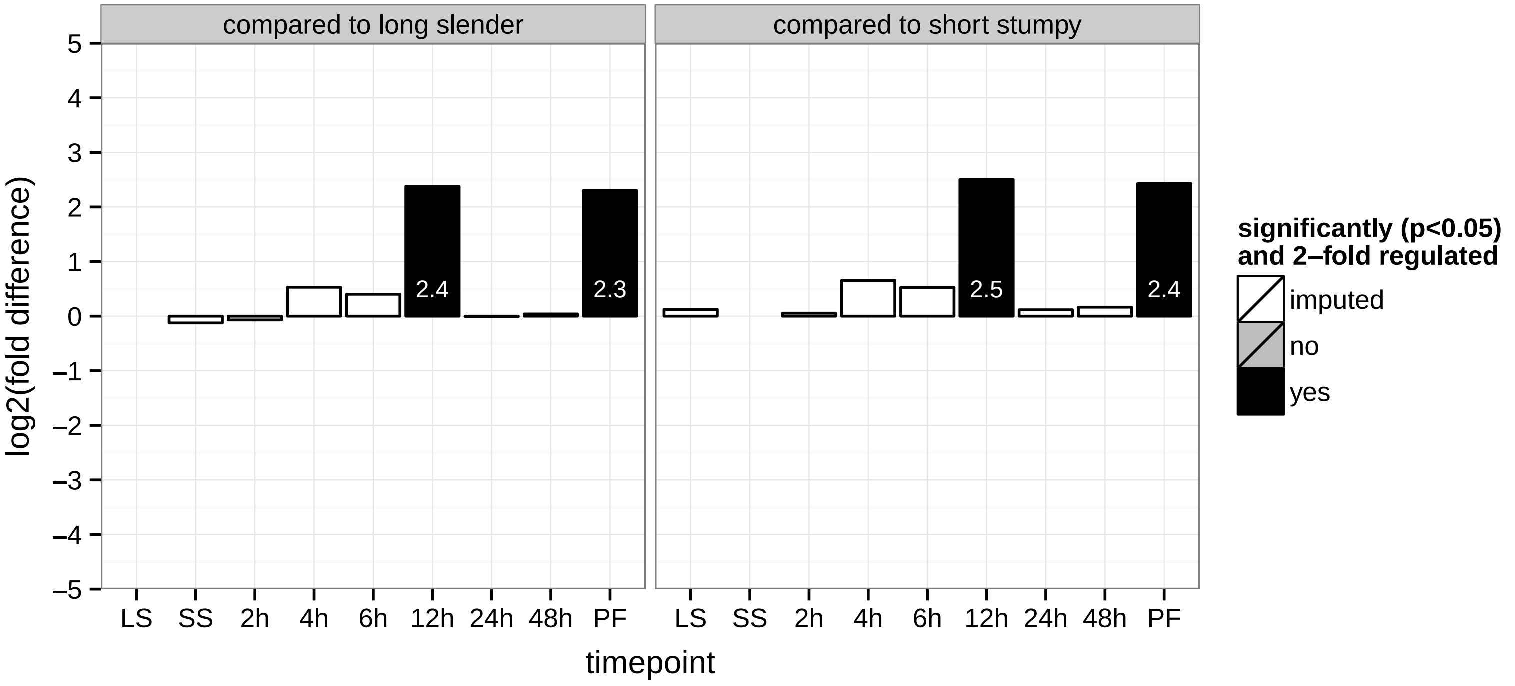

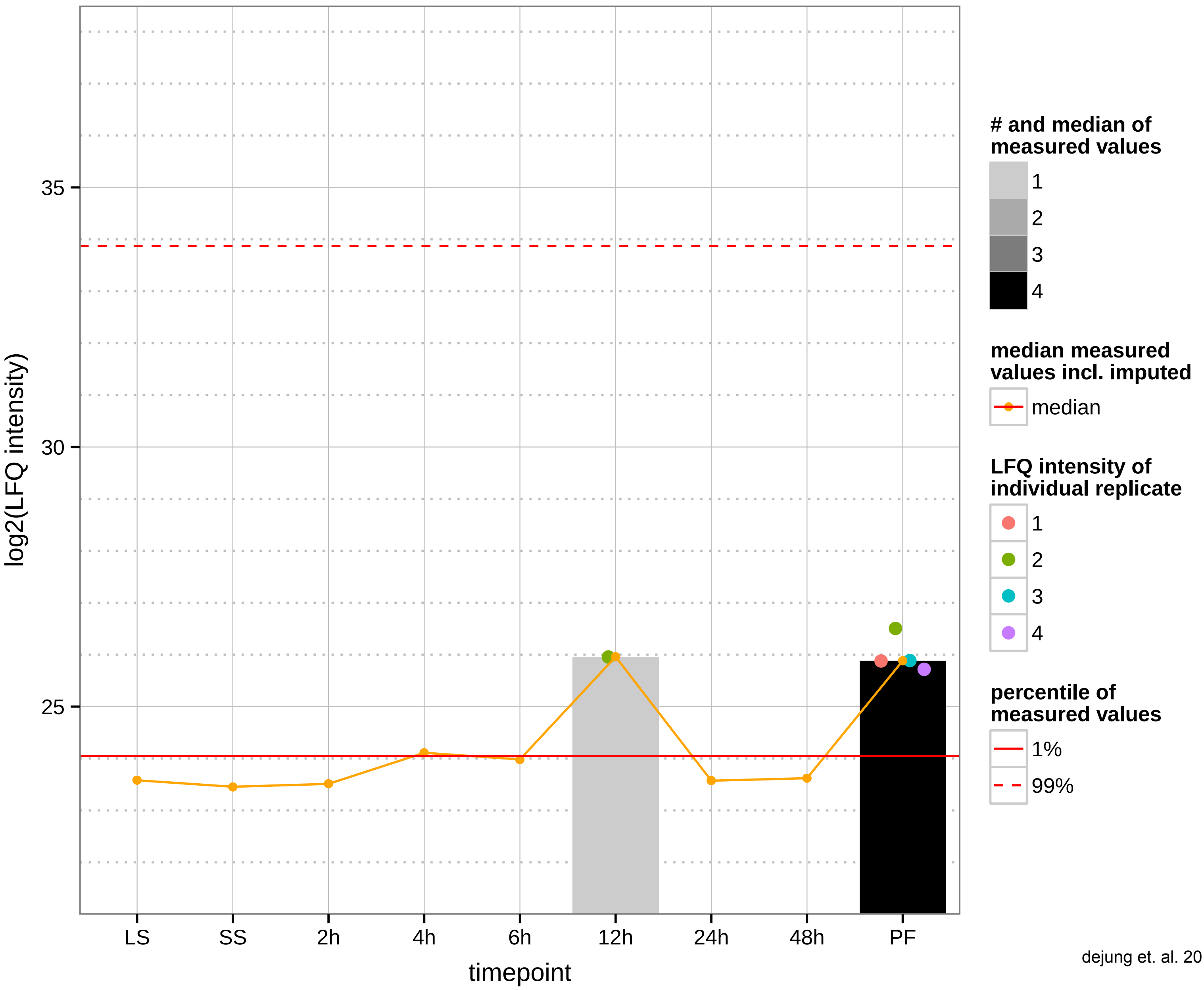

dejung et. al. 2015

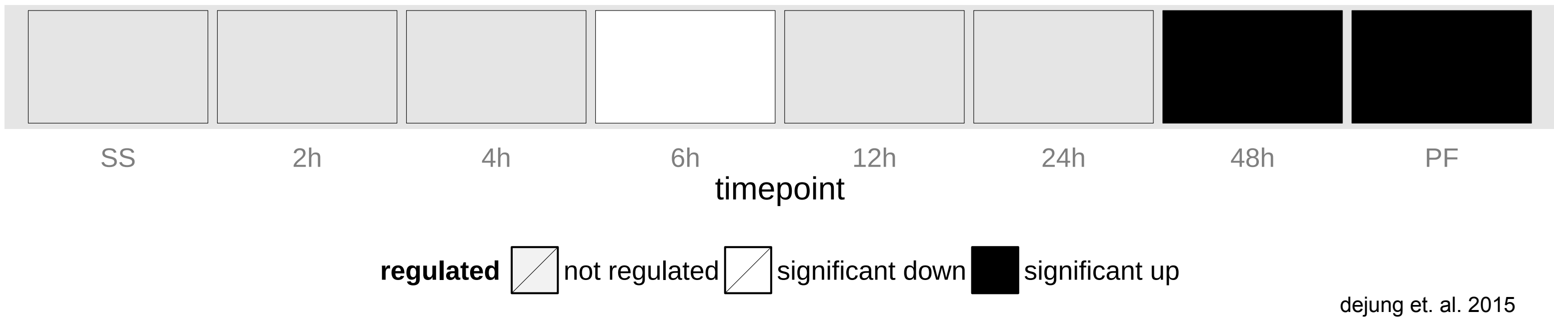
SS
2h
4h
6h
12h
24h
48h
PF
timepoint
regulated
not regulated
significant down
significant up
dejung et. al. 2015

conserved protein, unknown function
Tb927.1.1420
AGOF: null
AGOC: null
AGOP: null
PGOF: null
PGOC: null
PGOP: null

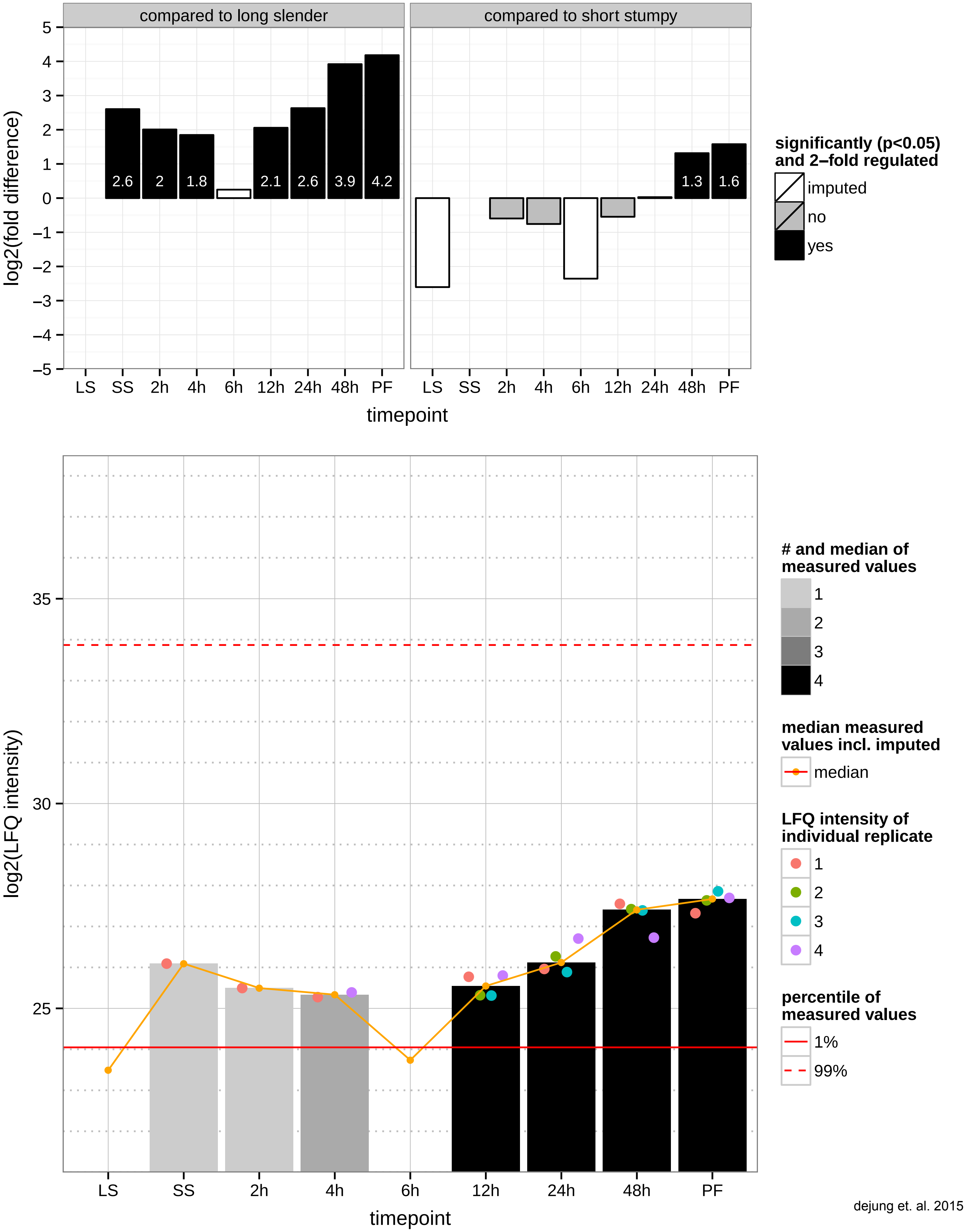

dejung et. al. 2015

fatty acid desaturase, putative, oleate desaturase
Tb927.2.3080
AGOF: oxidoreductase activity
AGOC: null
AGOP: fatty acid biosynthetic process
PGOF: null
PGOC: null
PGOP: lipid metabolic process

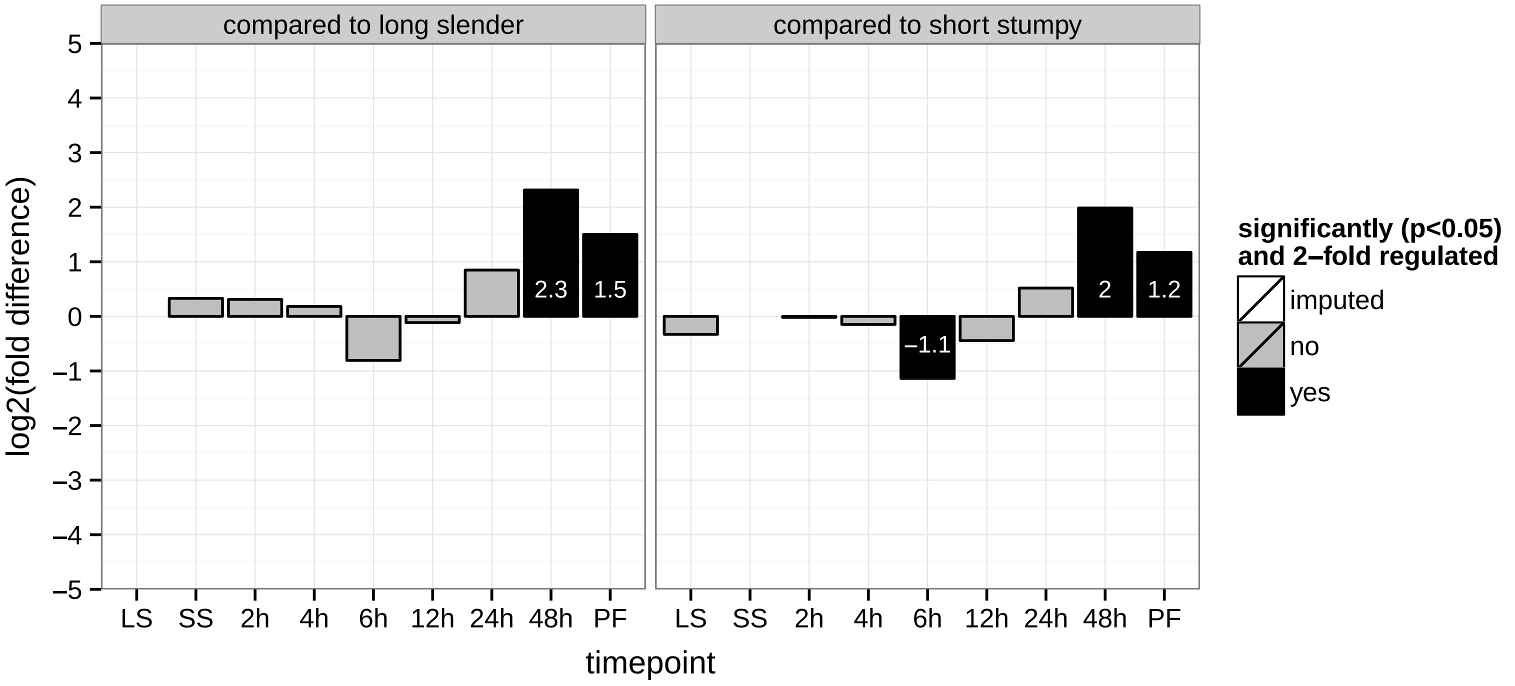

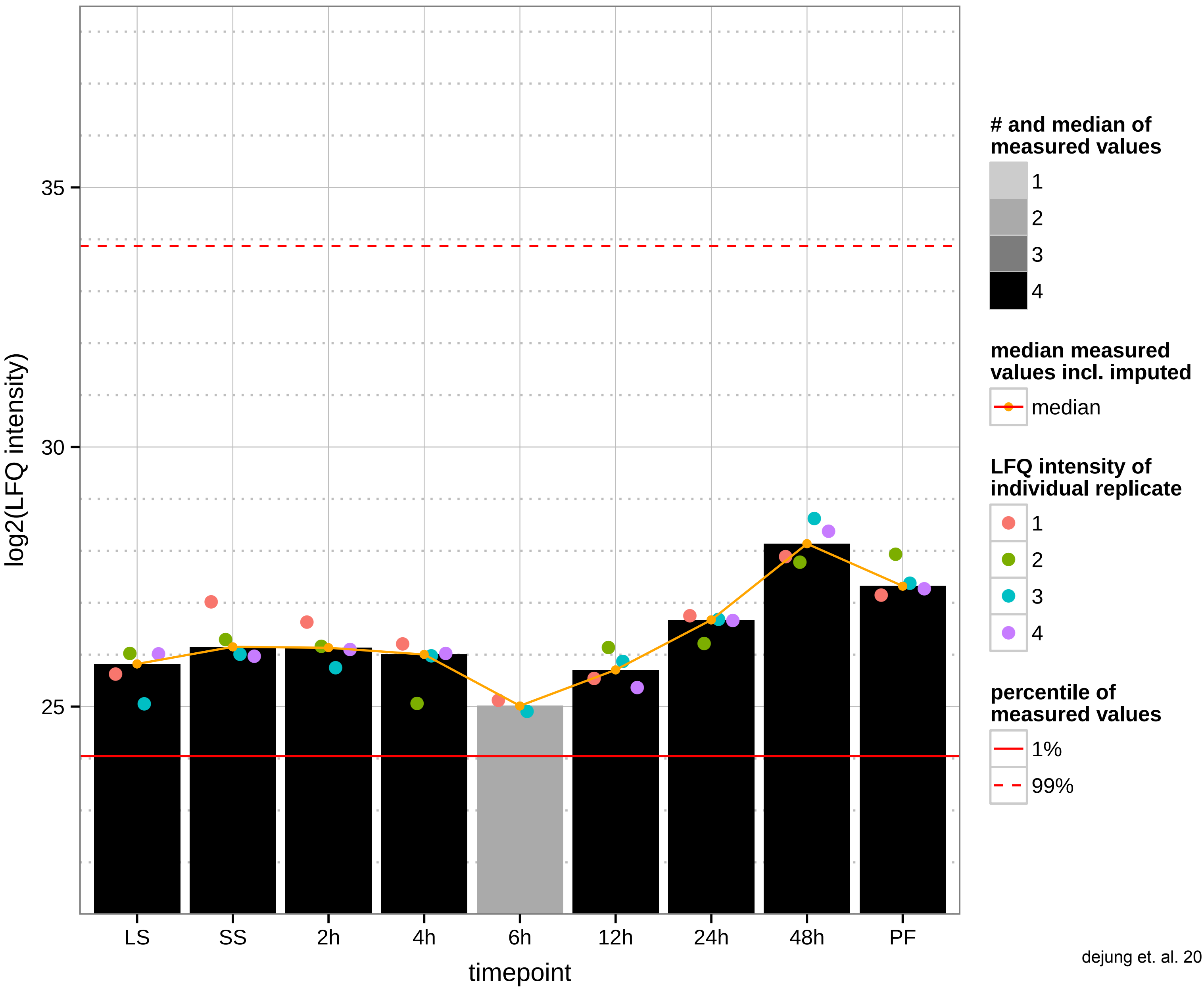

dejung et. al. 2015

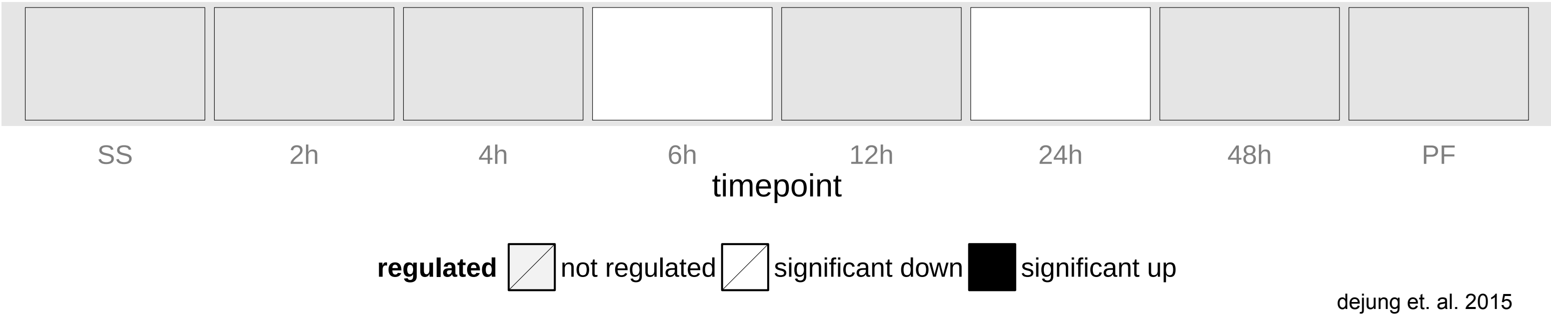
SS
2h
4h
6h
12h
24h
48h
PF
timepoint
regulated
not regulated
significant down
significant up
dejung et. al. 2015

short–chain dehydrogenase, putative
Tb927.10.6870
AGOF: oxidoreductase activity
AGOC: null
AGOP: metabolic process
PGOF: oxidoreductase activity
PGOC: null
PGOP: metabolic process

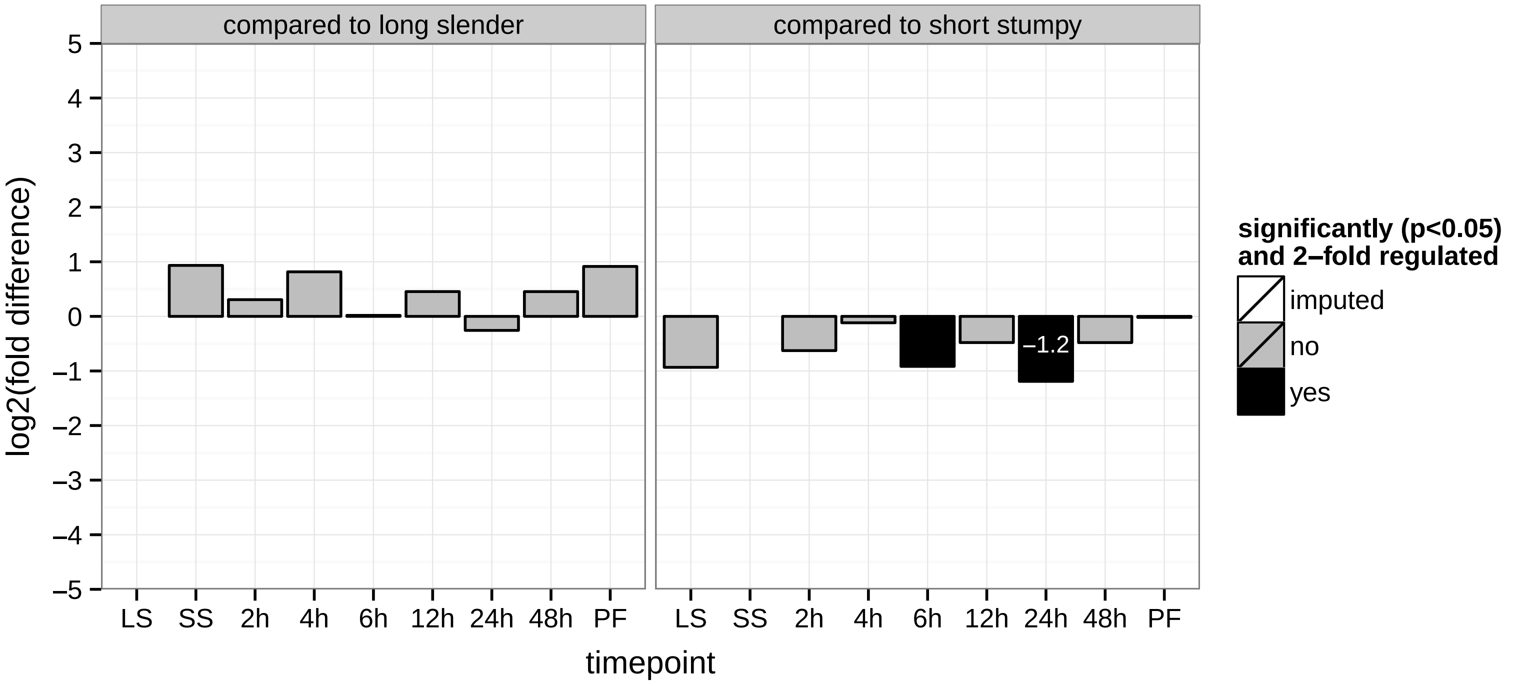

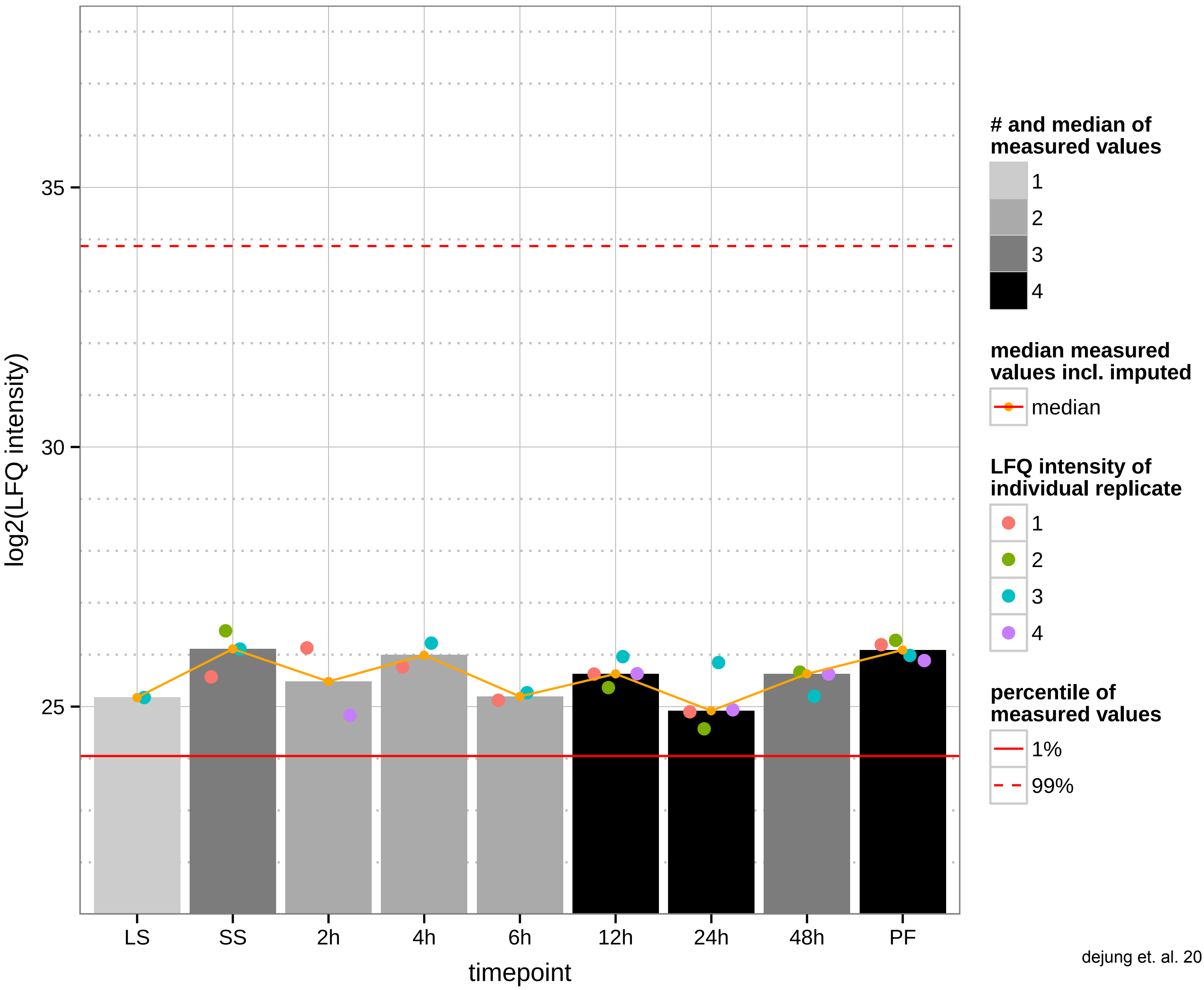

dejung et. al. 2015

enolase, putative
Tb927.11.16410
AGOF: phosphopyruvate hydratase activity
AGOC: phosphopyruvate hydratase complex
AGOP: glycolysis
PGOF: magnesium ion binding, phosphopyruvate hydratase activity
PGOC: phosphopyruvate hydratase complex
PGOP: glycolysis

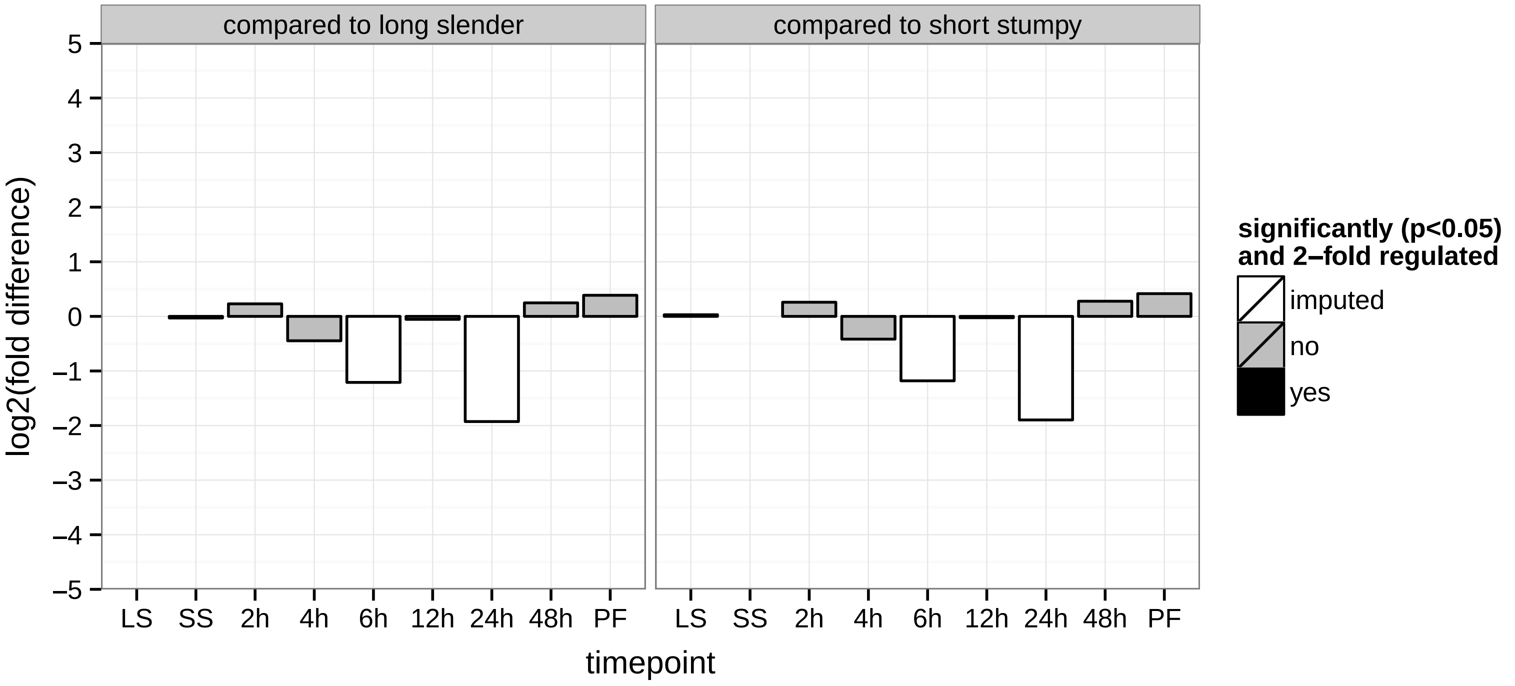

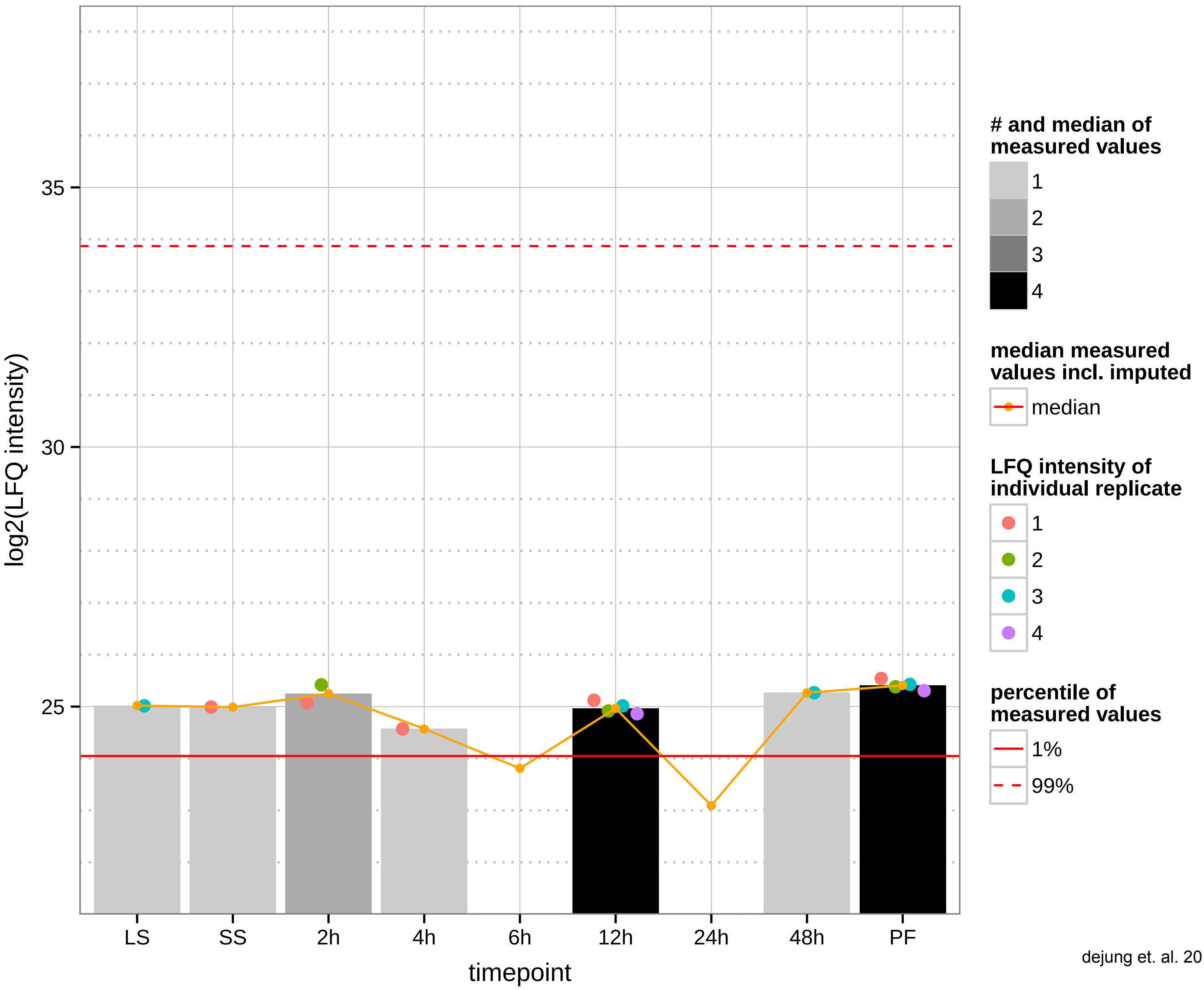

dejung et. al. 2015

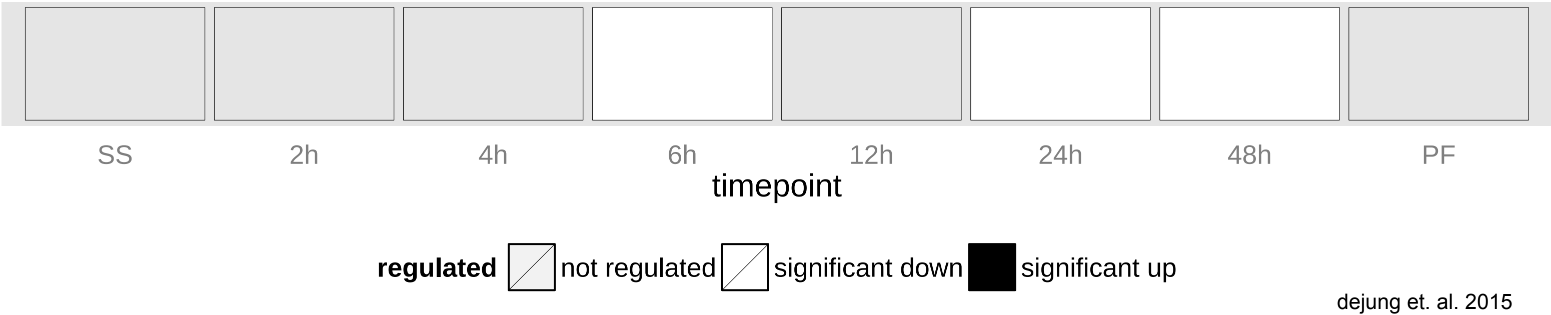
SS
2h
4h
6h
12h
24h
48h
PF
timepoint
regulated
not regulated
significant down
significant up
dejung et. al. 2015

vacuolar ATP synthase, putative
Tb927.5.550
AGOF: hydrogen ion transmembrane transporter activity
AGOC: proton−transporting V−type ATPase, V0 domain
AGOP: ATP synthesis coupled proton transport
PGOF: hydrogen ion transmembrane transporter activity
PGOC: proton−transporting V−type ATPase, V0 domain, proton−transporting two−sector ATPase complex, proton−transportin
PGOP: ATP hydrolysis coupled proton transport

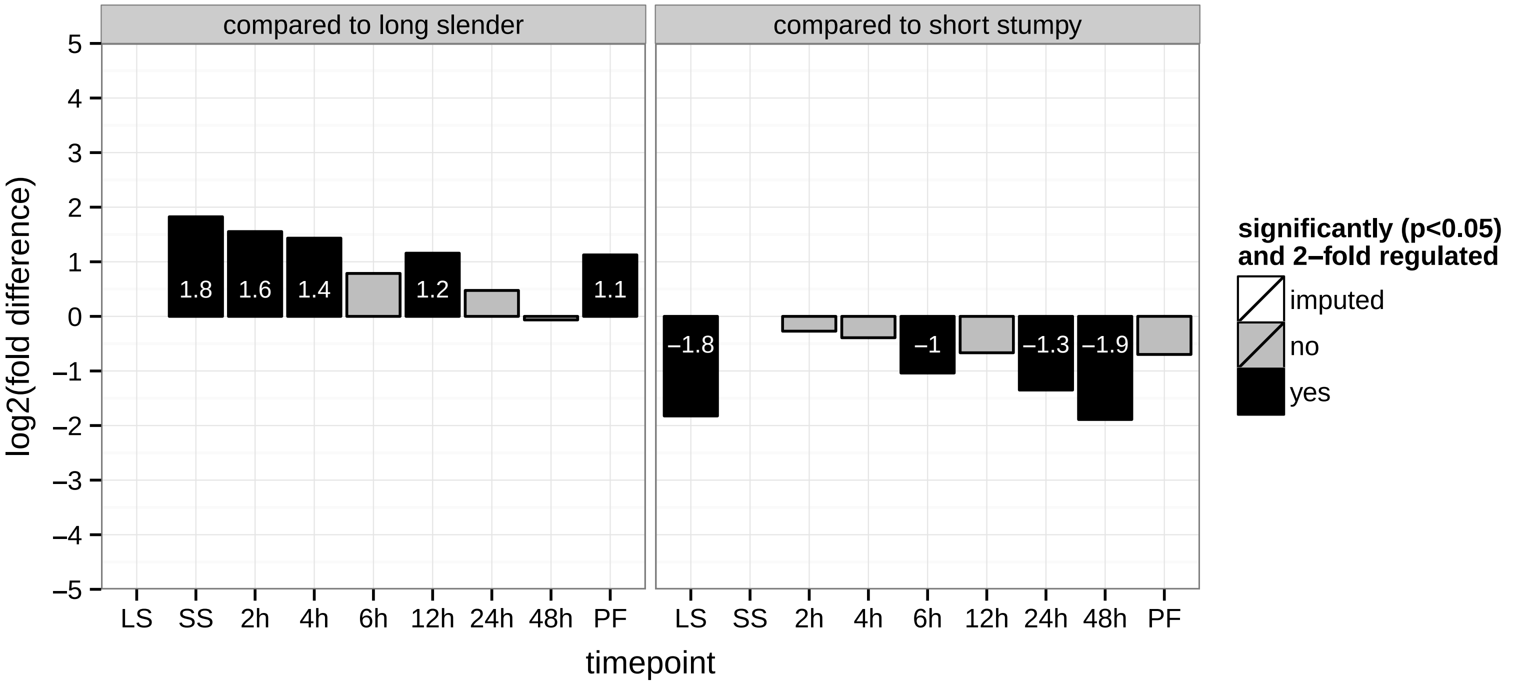

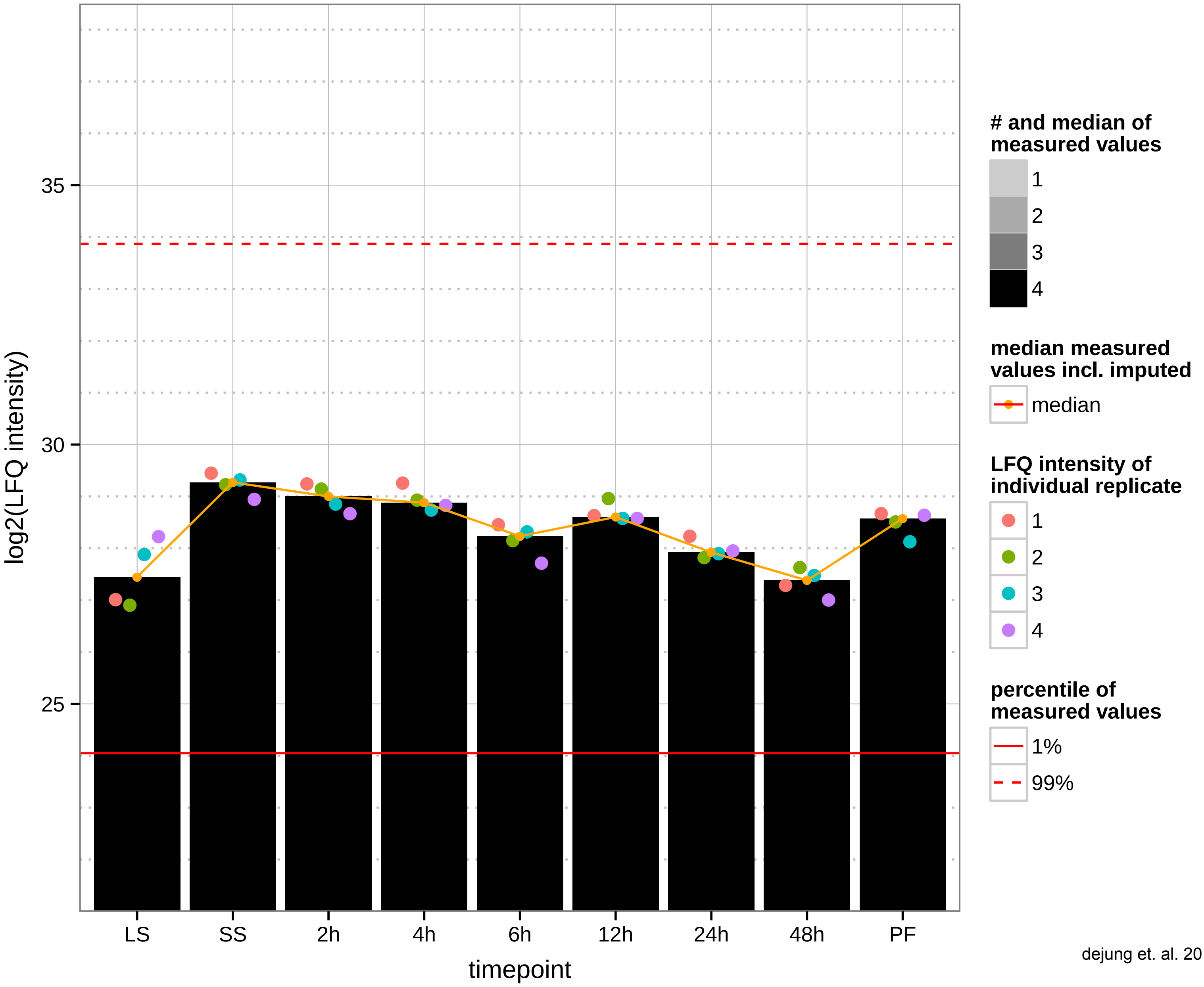

dejung et. al. 2015

hypothetical protein, conserved
Tb927.8.7200
AGOF: zinc ion binding
AGOC: null
AGOP: null
PGOF: binding, protein binding, zinc ion binding
PGOC: null
PGOP: intracellular protein transport, vesicle–mediated transport

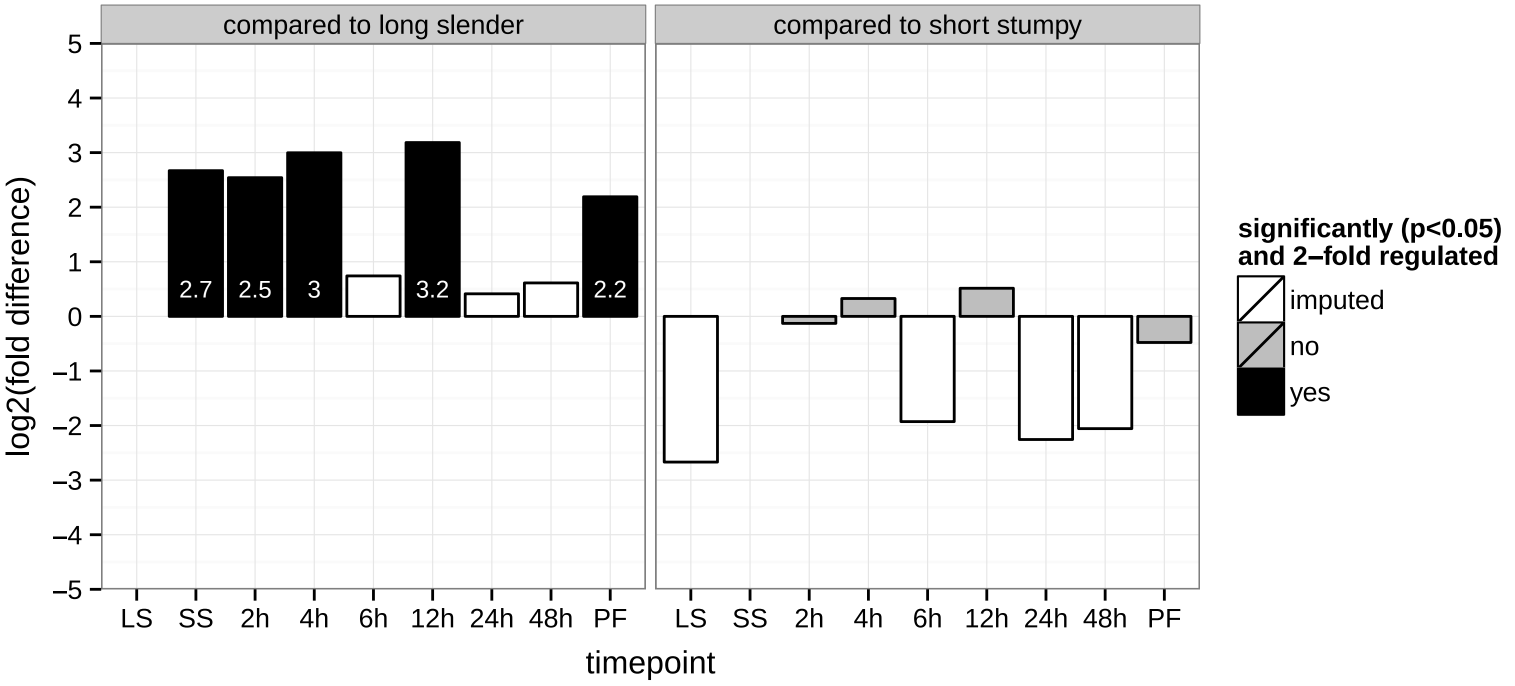

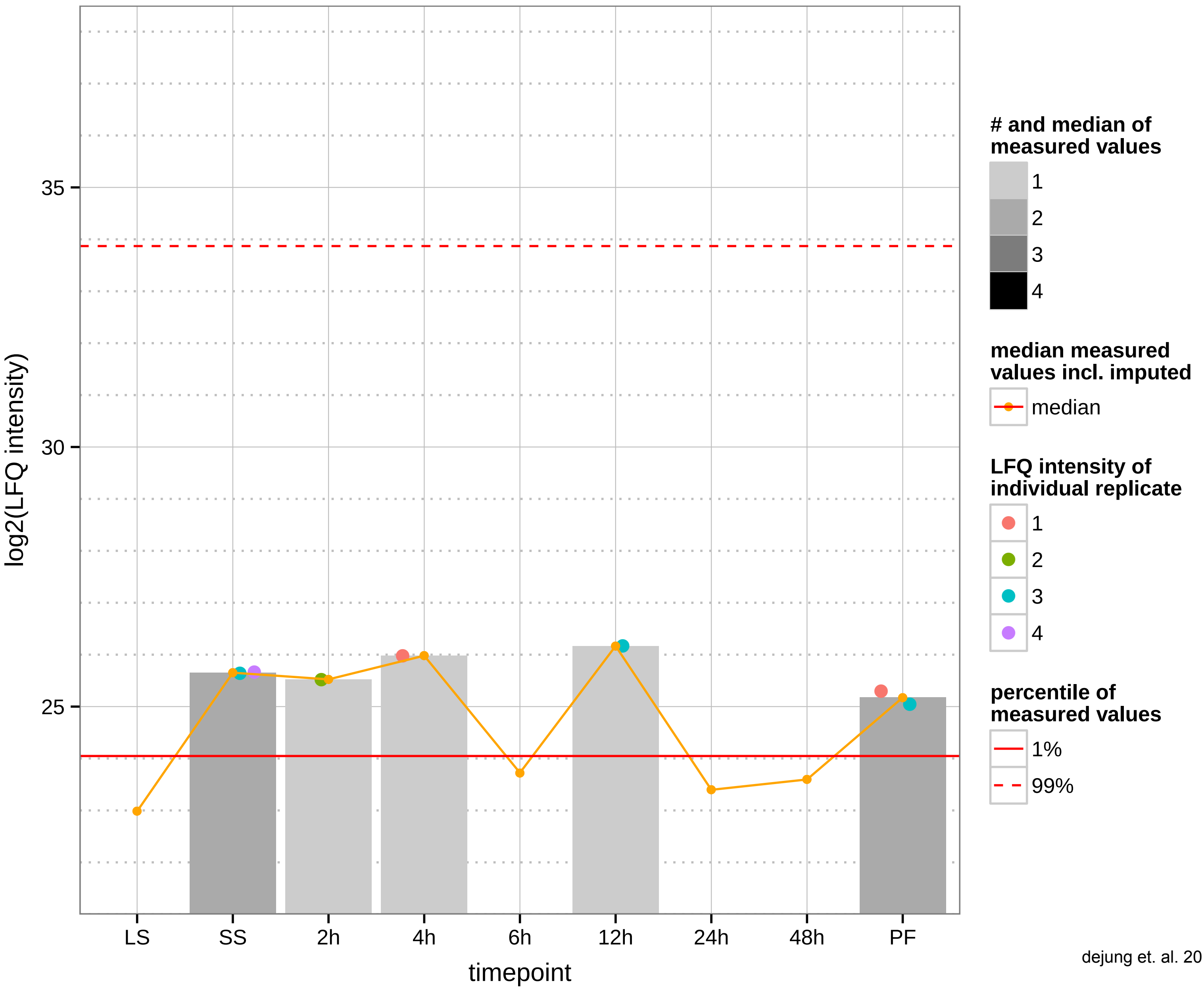

dejung et. al. 2015

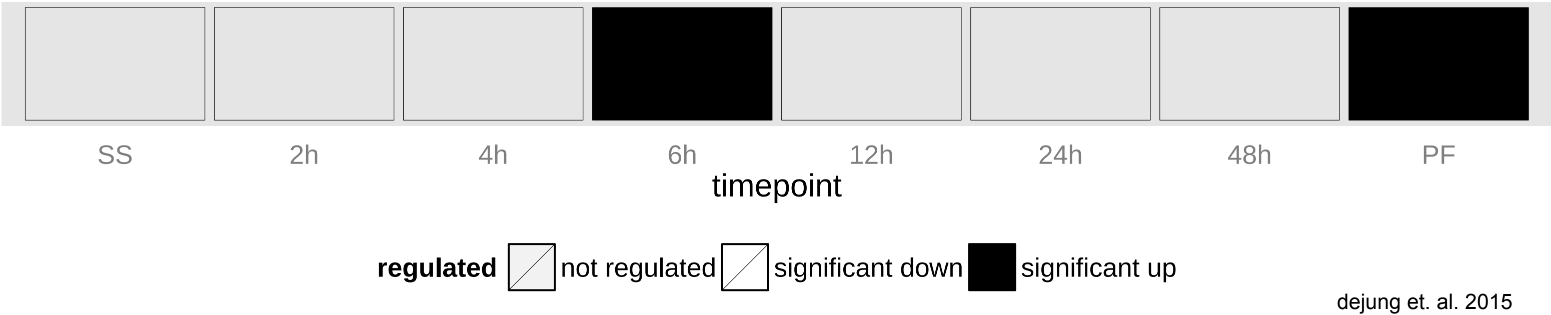
SS
2h
4h
6h
12h
24h
48h
PF
timepoint
regulated
not regulated
significant down
significant up
dejung et. al. 2015

glycosyltransferase, putative, dolichyl−P−Man:GDP−ManGlcNAc2−PP−dolichyl beta−1, 4−mannosyltransferase
Tb927.10.13210;Tb11.v5.0694
AGOF: null, dolichyl−phosphate beta−D−mannosyltransferase activity
AGOC: null, endoplasmic reticulum membrane
AGOP: null, protein mannosylation
PGOF: null
PGOC: null
PGOP: null

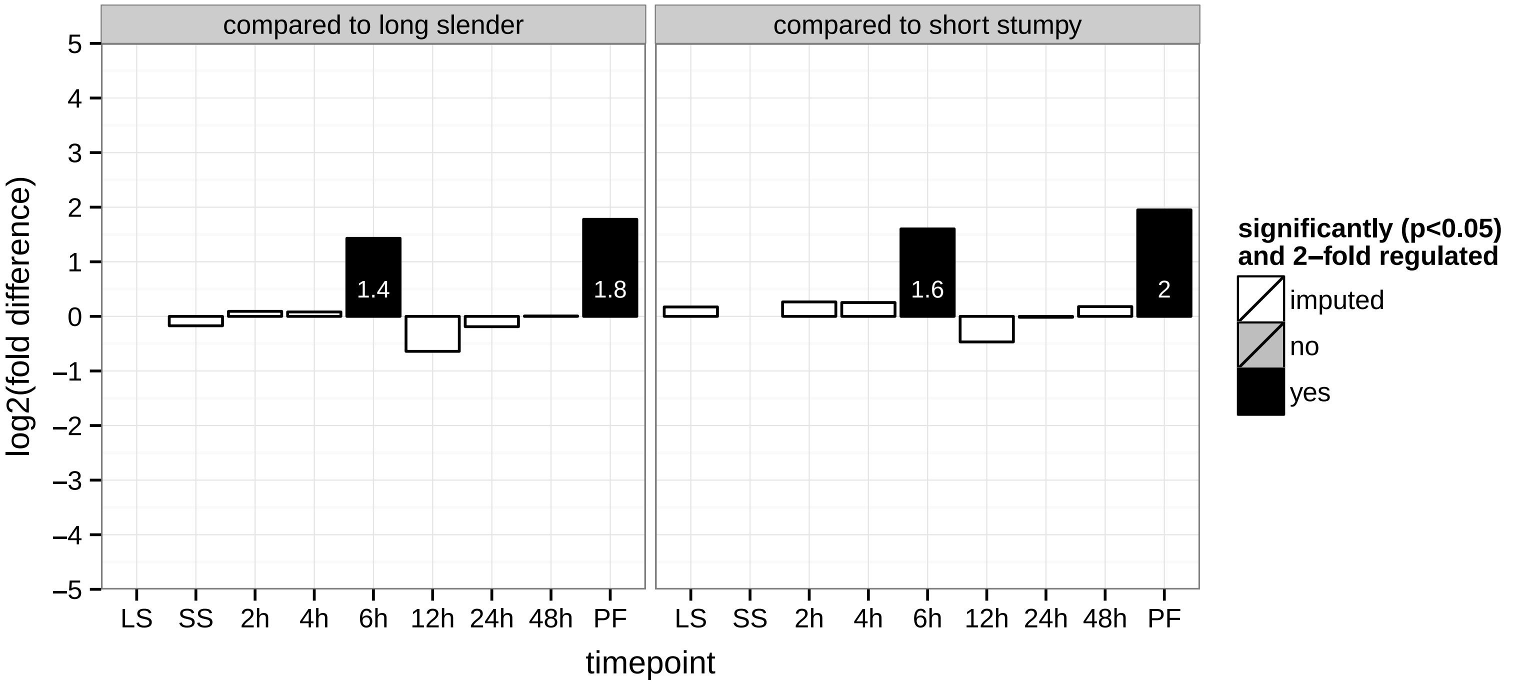

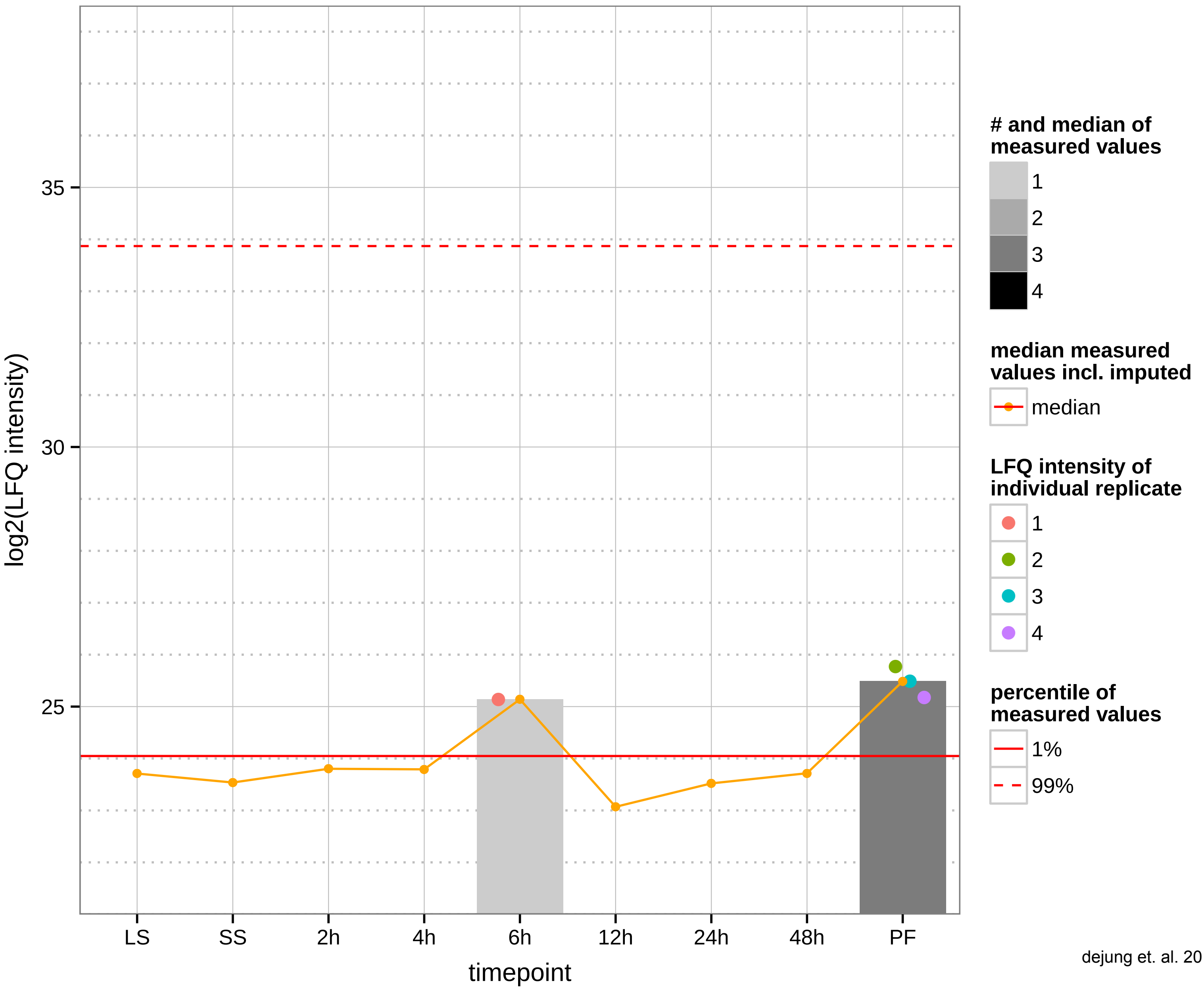

dejung et. al. 2015

hypothetical protein, conserved
Tb927.11.7180
AGOF: null
AGOC: null
AGOP: null
PGOF: null
PGOC: null
PGOP: null

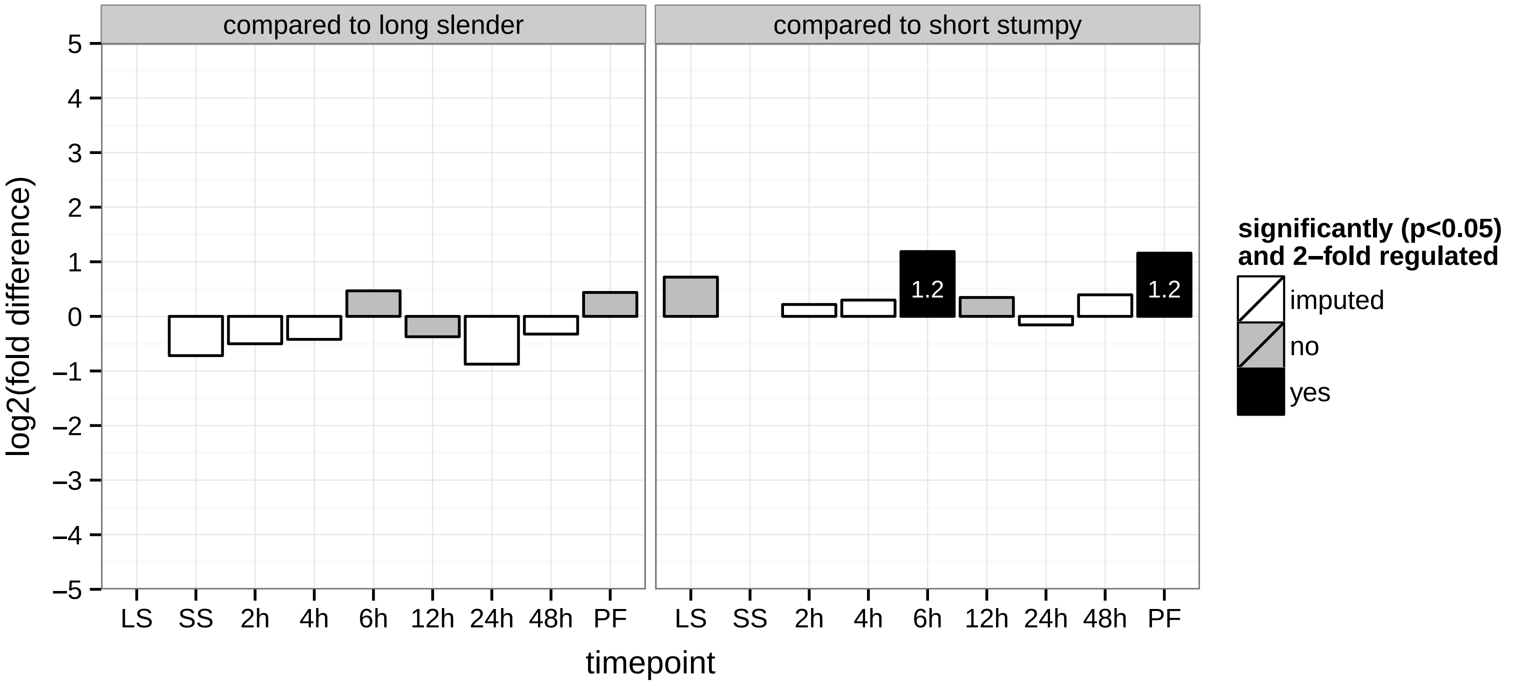

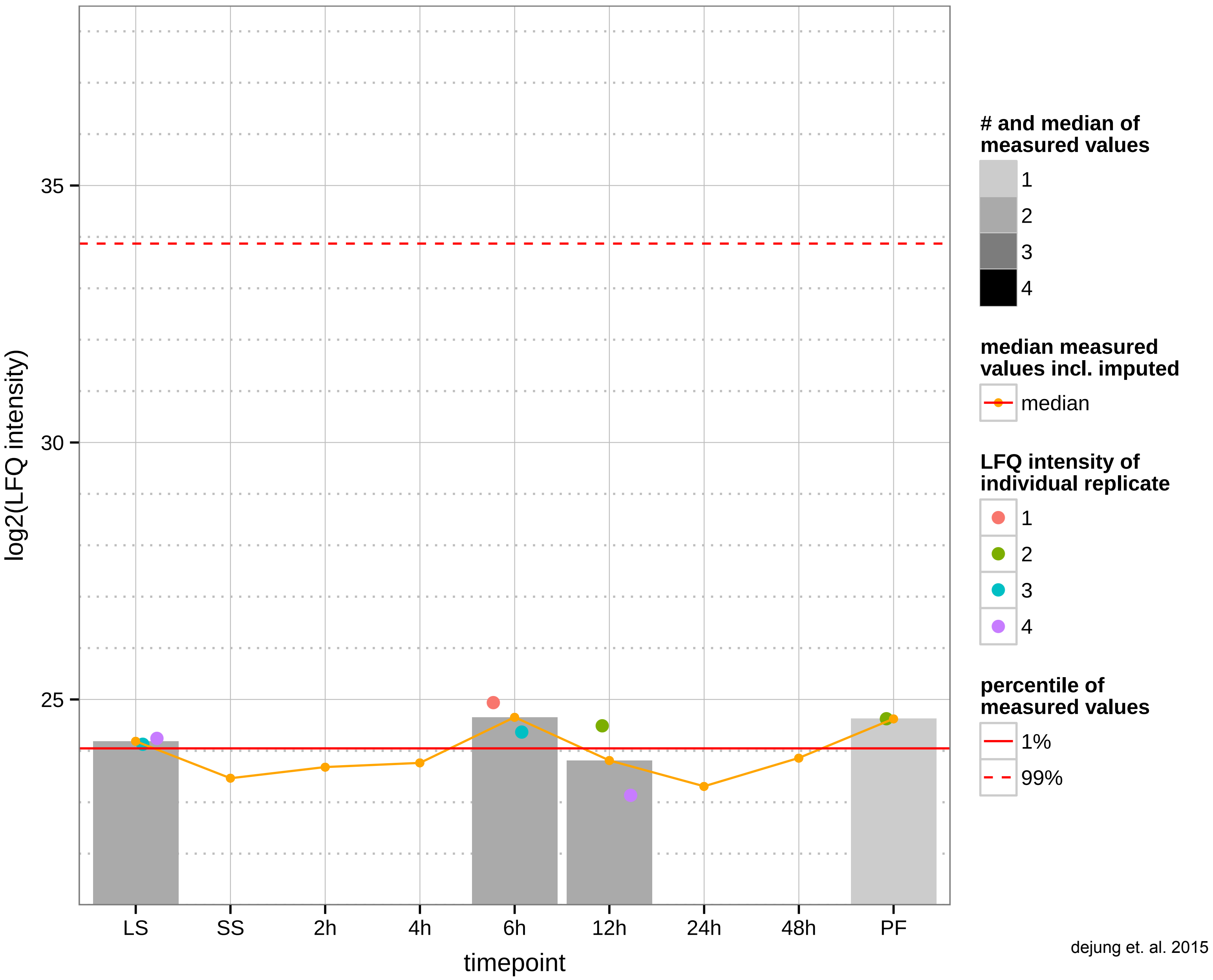

dejung et. al. 2015

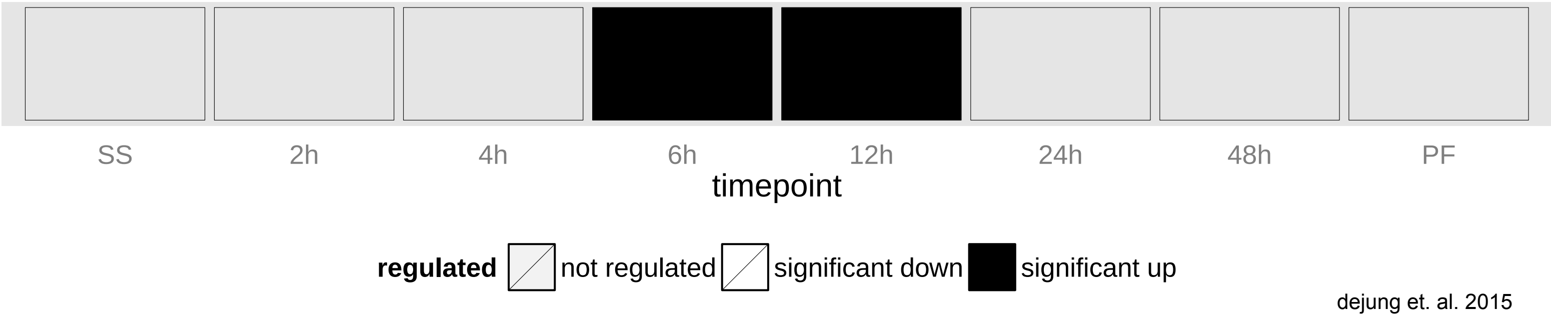

SS
2h
4h
6h
12h
24h
48h
PF
timepoint
regulated
not regulated
significant down
significant up
dejung et. al. 2015

hypothetical protein, conserved
Tb927.3.4510
AGOF: null
AGOC: null
AGOP: null
PGOF: null
PGOC: null
PGOP: null

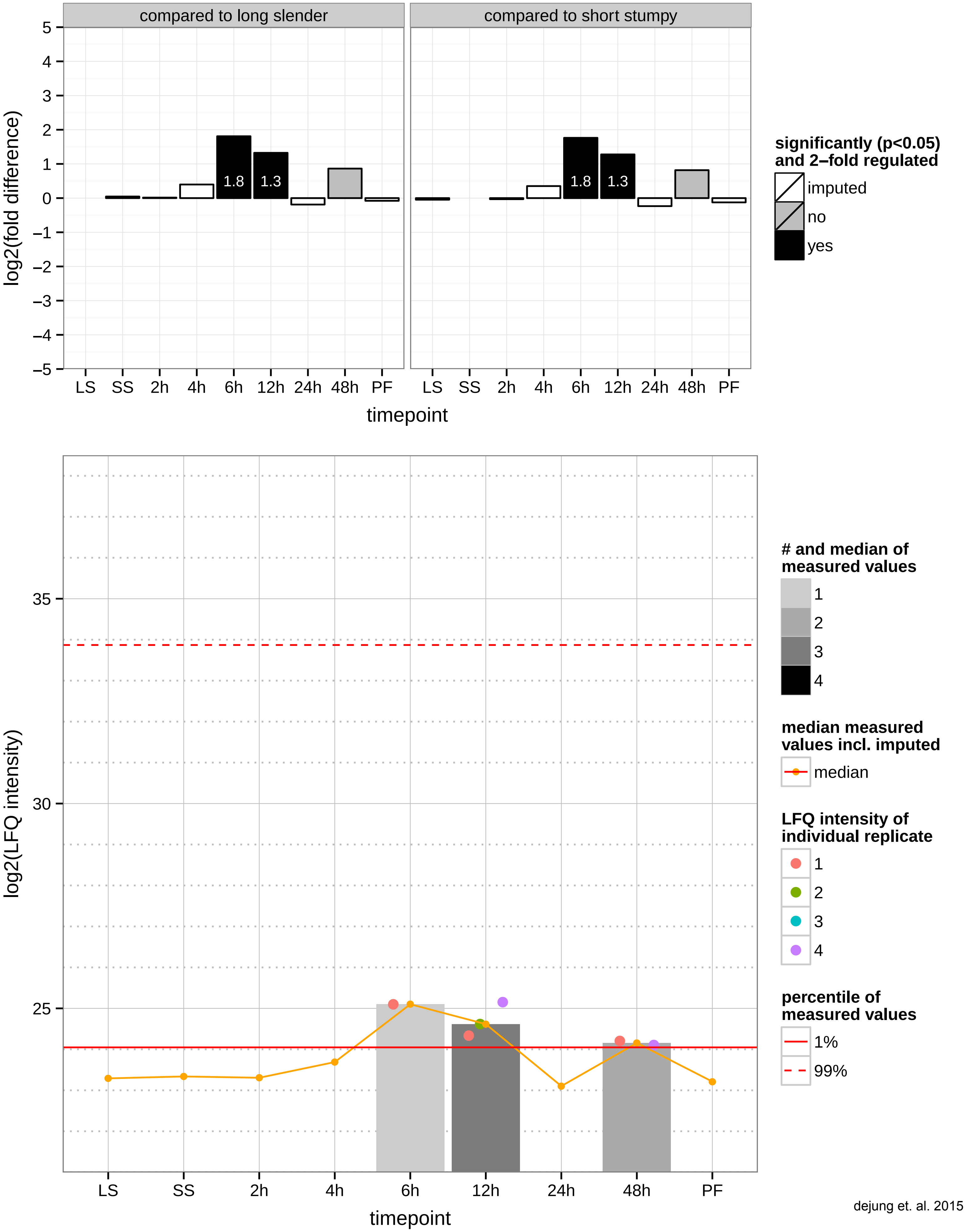

dejung et. al. 2015

hypothetical protein, conserved
Tb927.9.10140
AGOF: null
AGOC: null
AGOP: null
PGOF: null
PGOC: null
PGOP: null

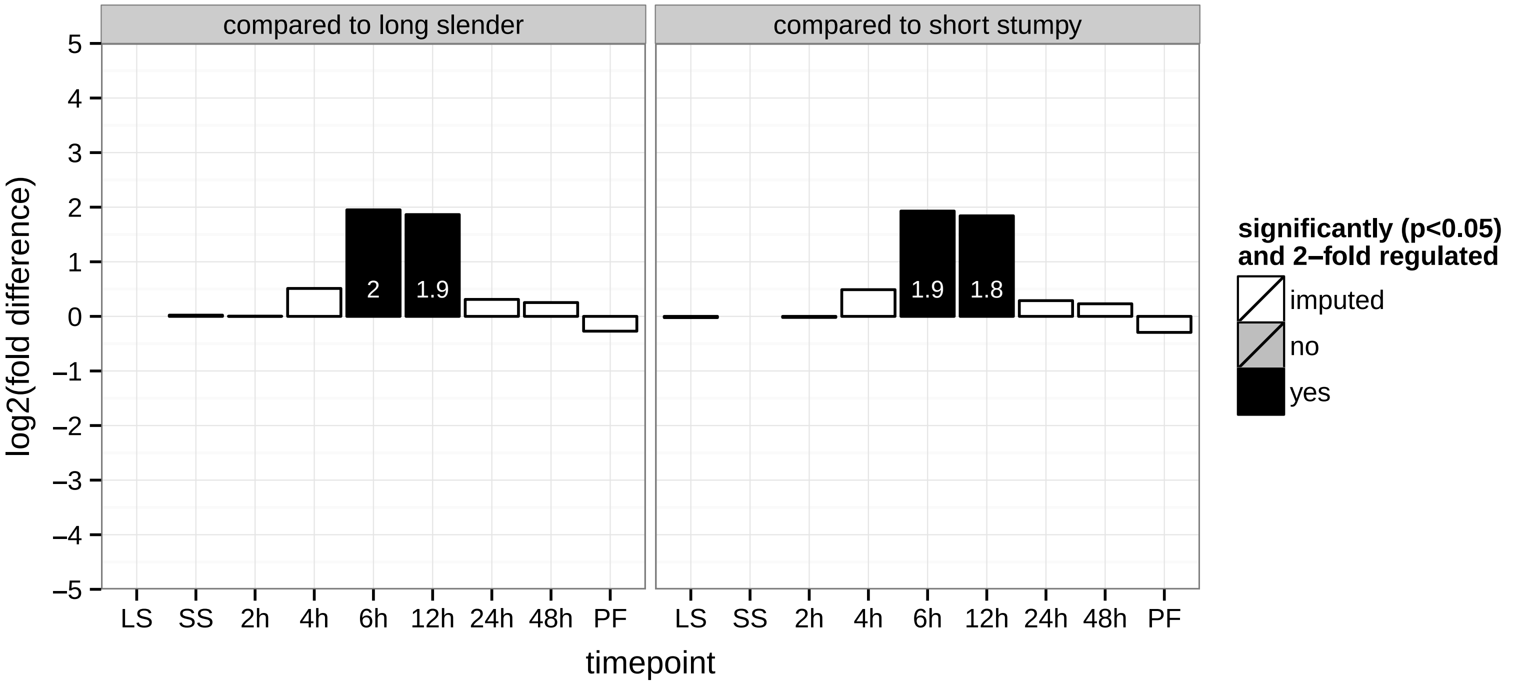

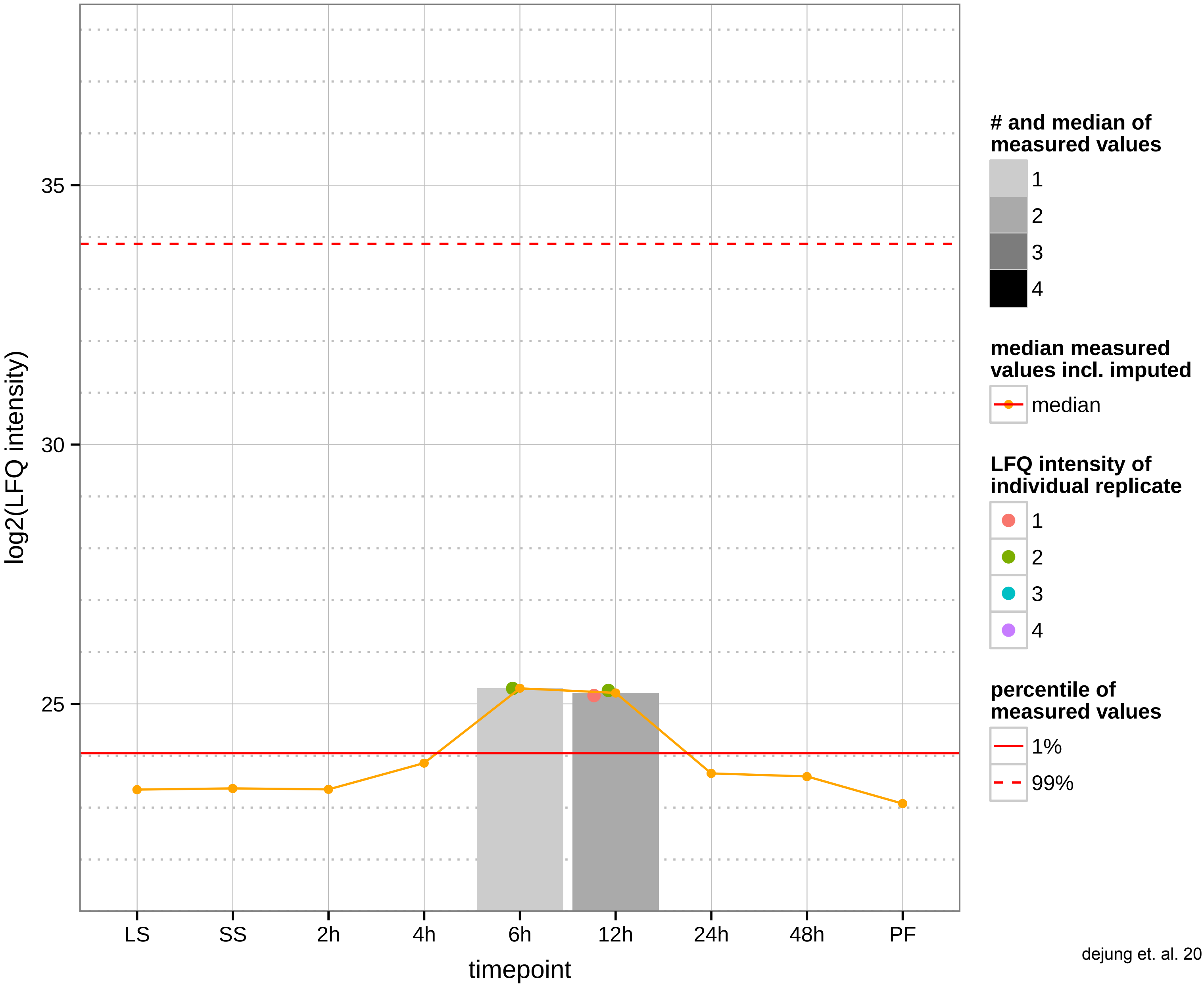

dejung et. al. 2015

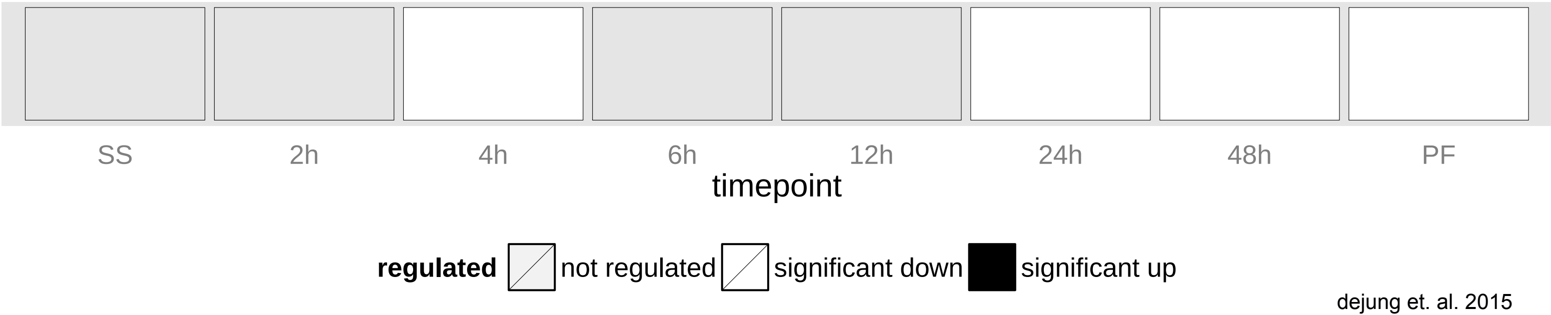
SS
2h
4h
6h
12h
24h
48h
PF
timepoint
regulated
not regulated
significant down
significant up
dejung et. al. 2015

leucine−rich repeat−containing protein
Tb927.1.1020
AGOF: null
AGOC: null
AGOP: null
PGOF: null
PGOC: null
PGOP: null

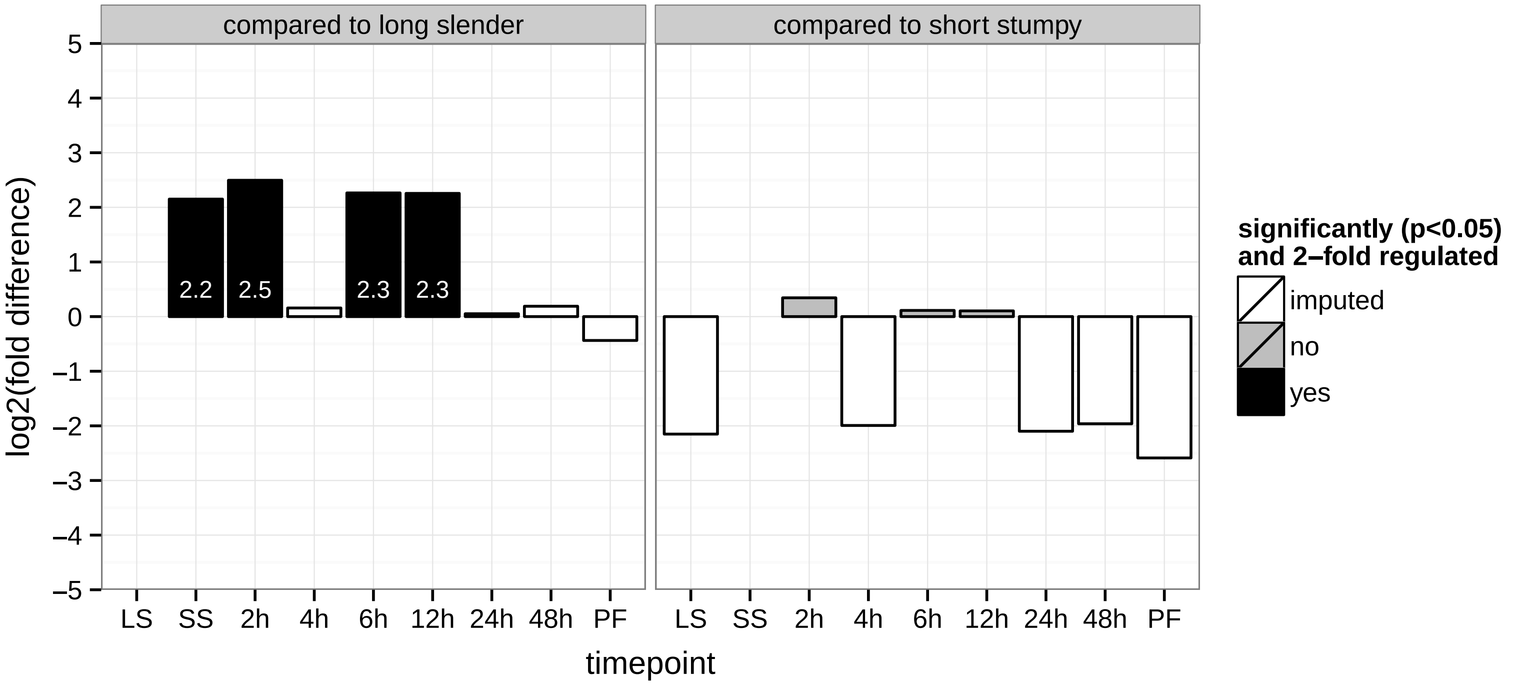

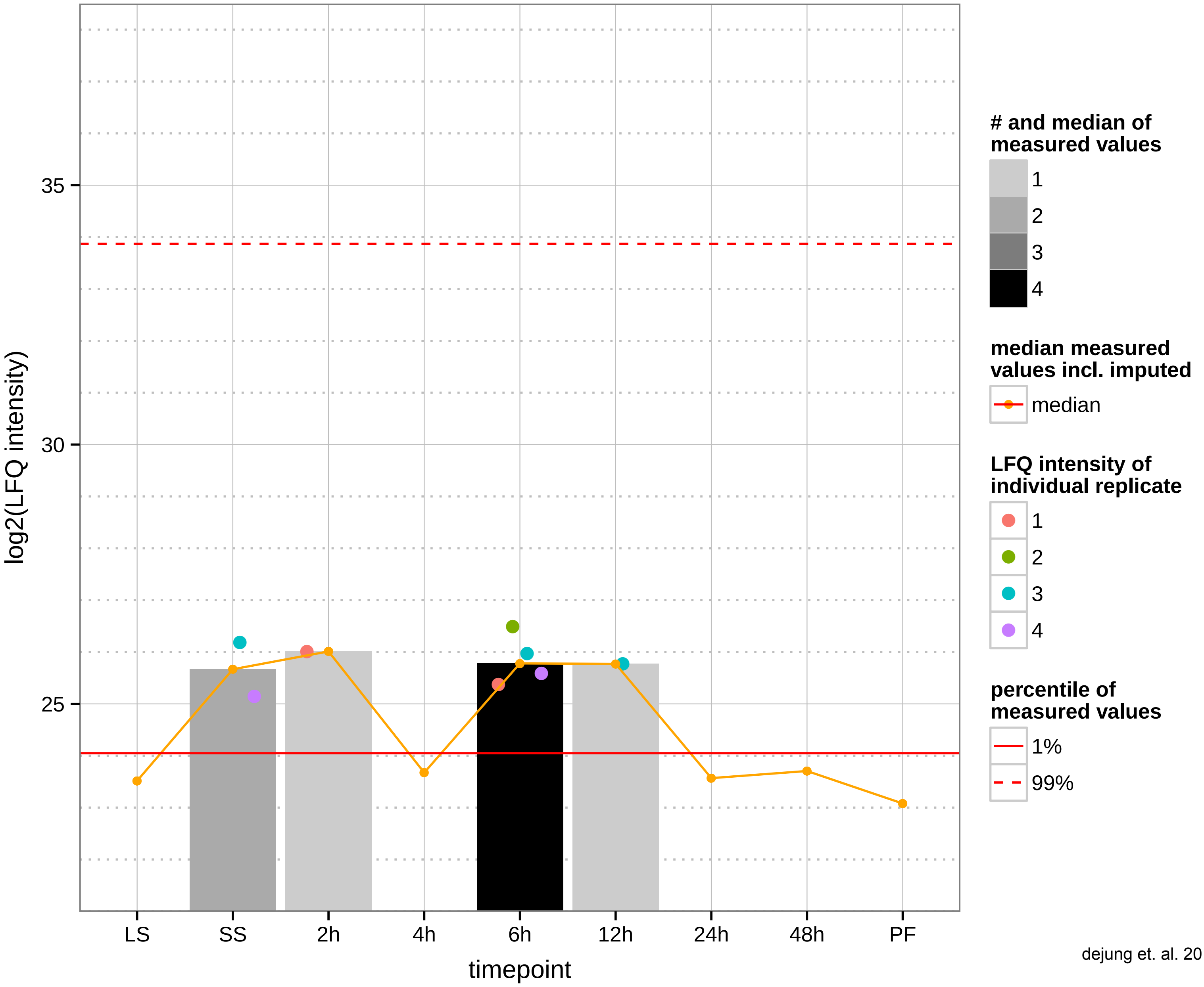

dejung et. al. 2015

hypothetical protein, conserved
Tb927.8.6020
AGOF: nucleoside–triphosphatase activity, nucleotide binding
AGOC: null
AGOP: null
PGOF: nucleoside–triphosphatase activity, nucleotide binding
PGOC: null
PGOP: null

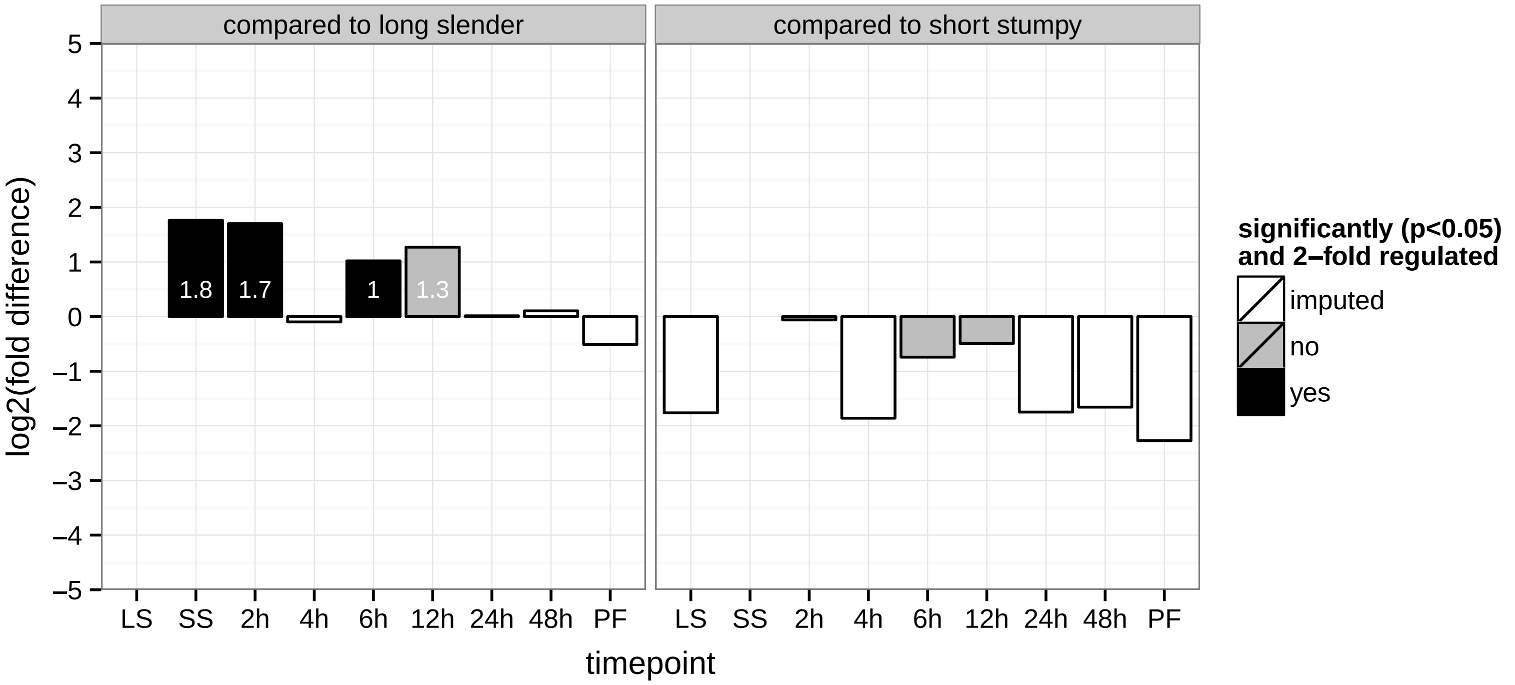

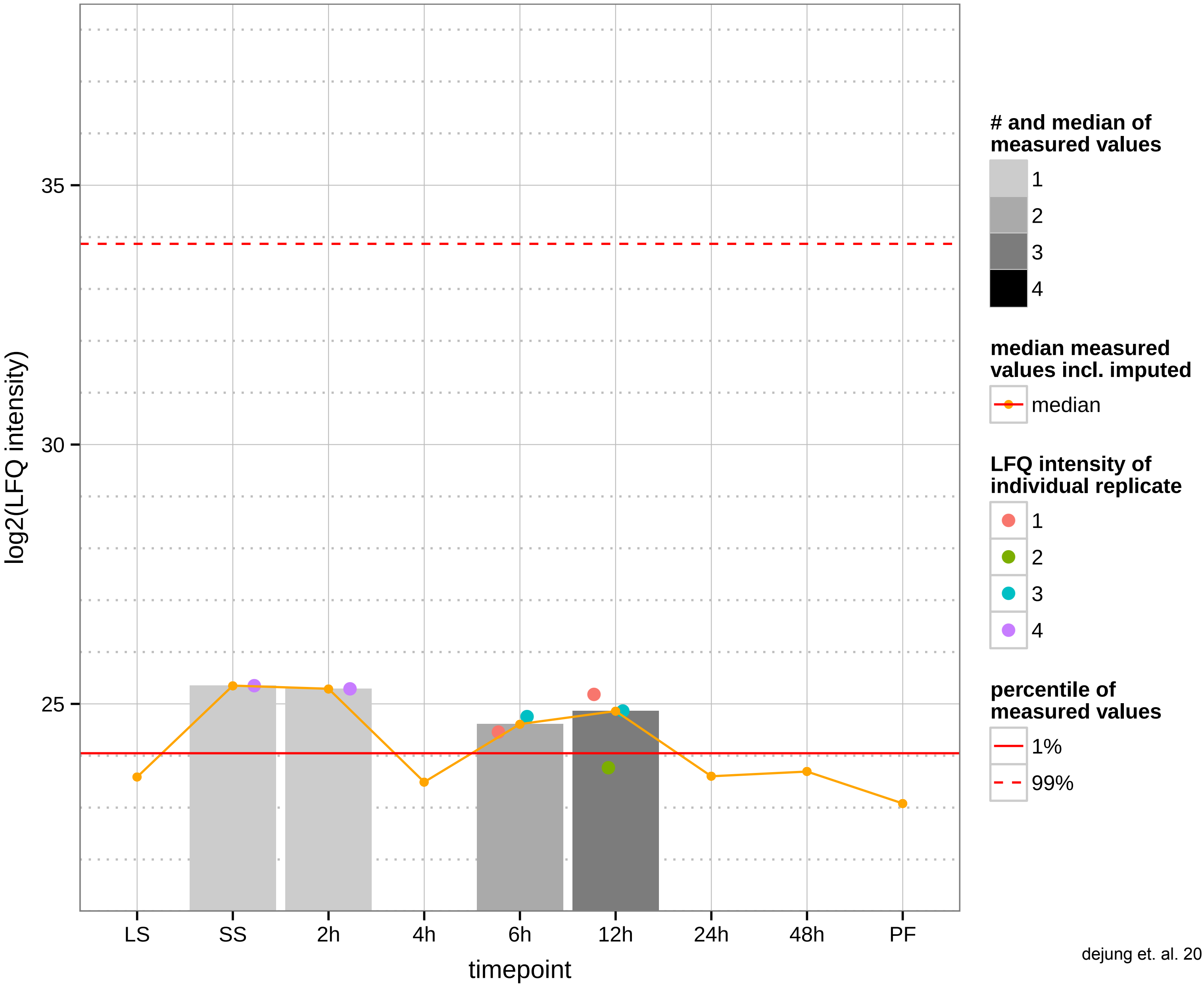

dejung et. al. 2015

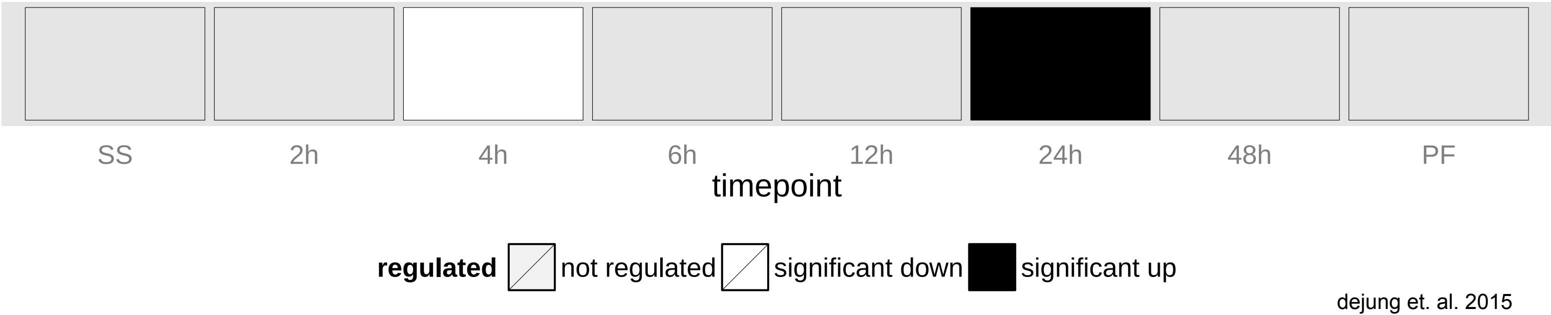
SS
2h
4h
6h
12h
24h
48h
PF
timepoint
regulated
not regulated
significant down
significant up
dejung et. al. 2015

retrotransposon hot spot protein 5 (RHS5), putative
Tb927.1.420
AGOF: null
AGOC: nucleus
AGOP: null
PGOF: null
PGOC: null
PGOP: null

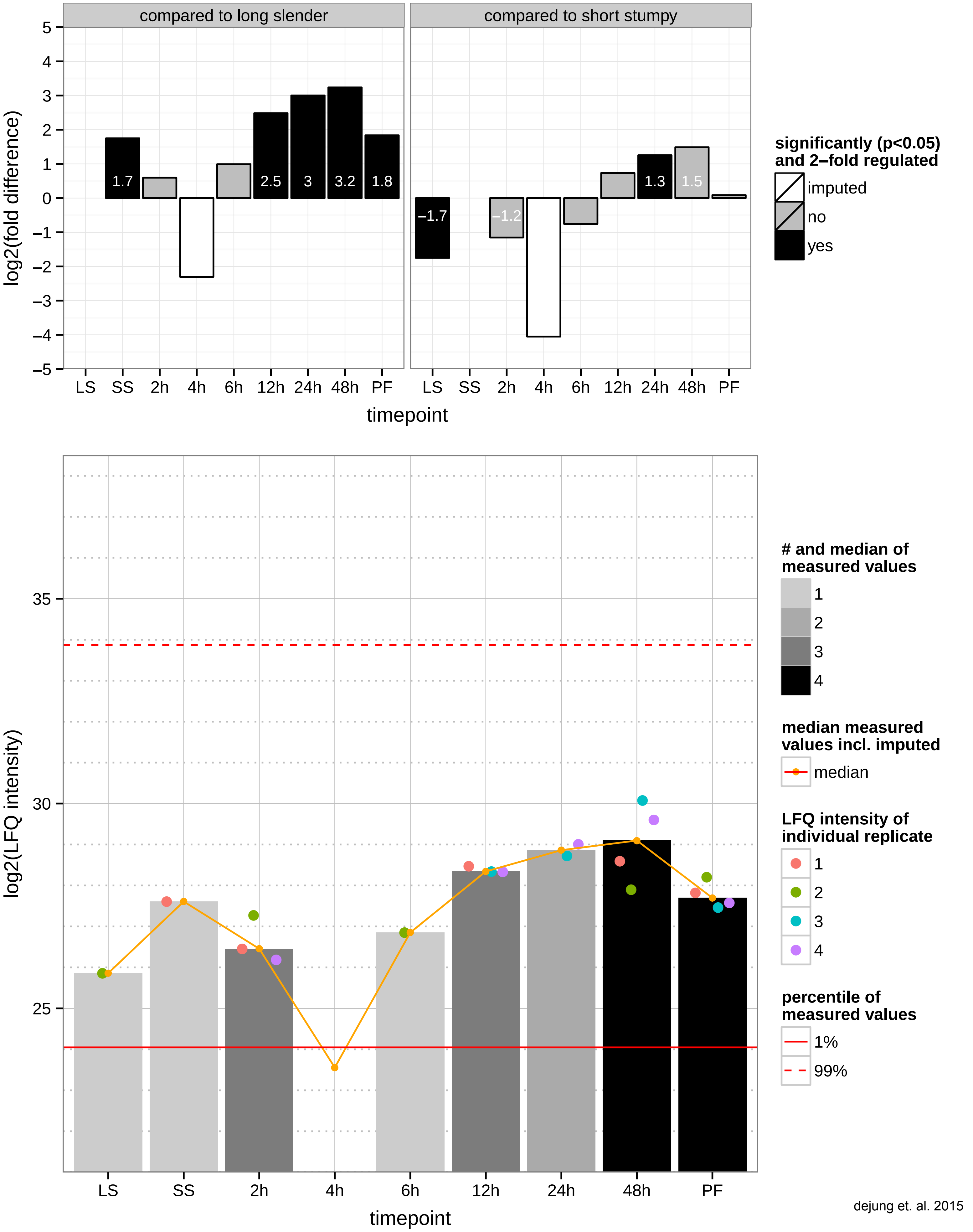

dejung et. al. 2015

hypothetical protein, conserved
Tb927.4.3540
AGOF: null
AGOC: null
AGOP: null
PGOF: null
PGOC: null
PGOP: null

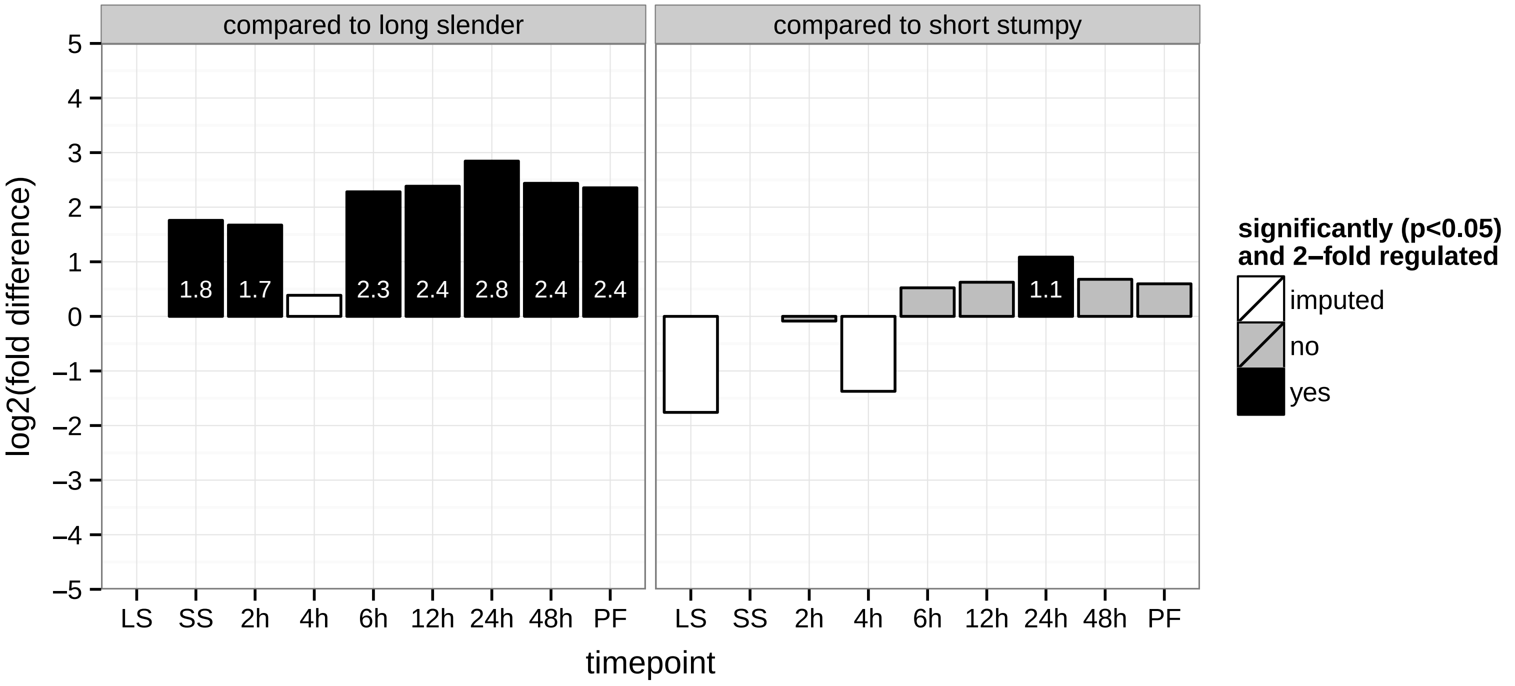

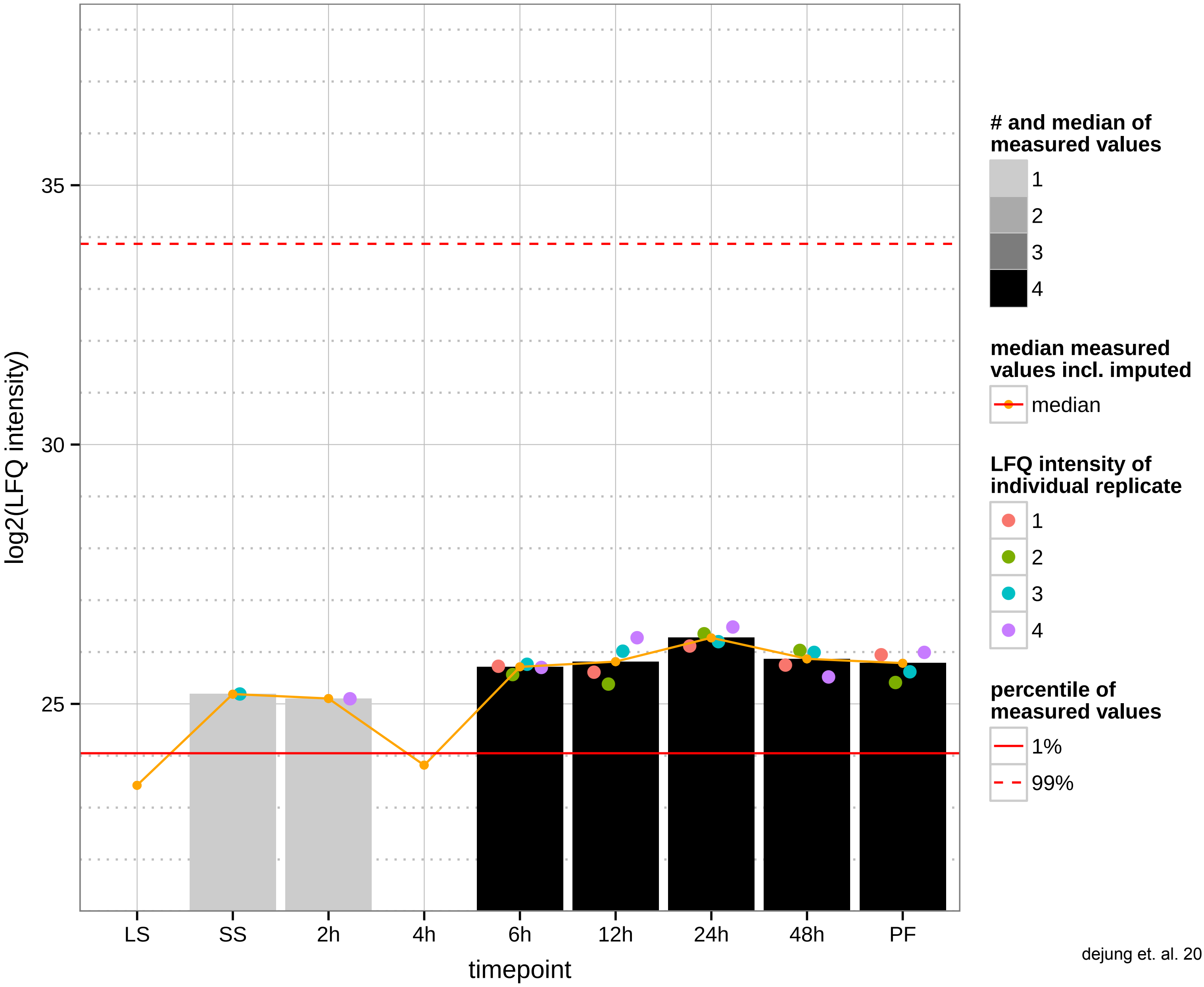

dejung et. al. 2015

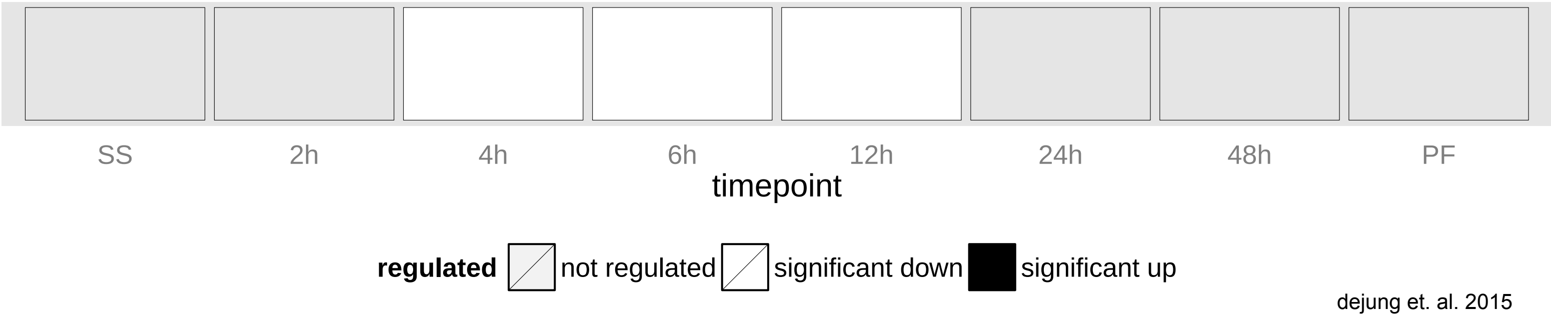

SS
2h
4h
6h
12h
24h
48h
PF
timepoint
regulated
not regulated
significant down
significant up
dejung et. al. 2015

NADH–ubiquinone oxidoreductase, mitochondrial, putative, putative (NUBM)
Tb927.5.450;Tb11.v5.0222
AGOF: null, 4 iron, 4 sulfur cluster binding, FMN binding, NAD binding, oxidoreductase activity, acting on NADH or NADPH
AGOC: null, mitochondrion, respiratory chain complex I
AGOP: null, oxidation–reduction process
PGOF: 4 iron, 4 sulfur cluster binding, FMN binding, NAD binding, oxidoreductase activity, acting on NADH or NADPH
PGOC: null
PGOP: oxidation–reduction process

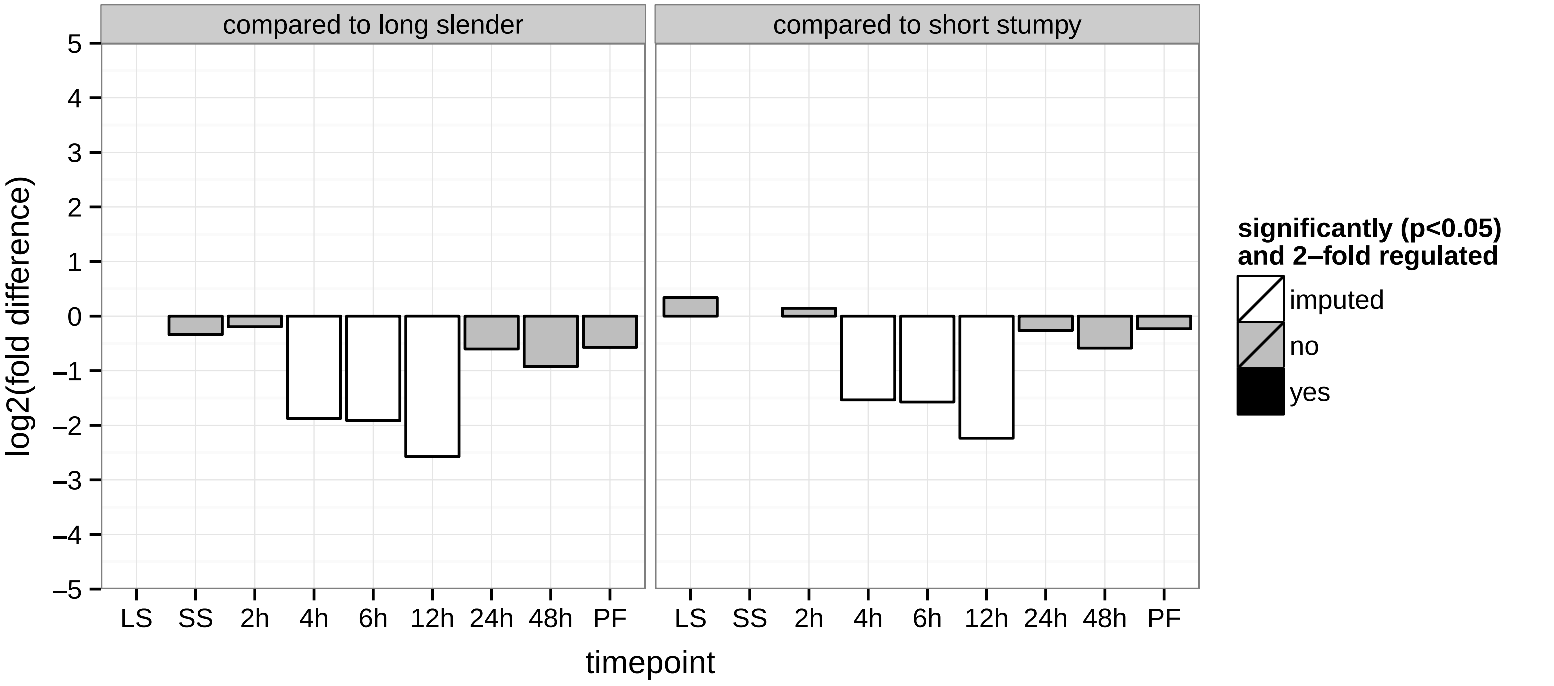

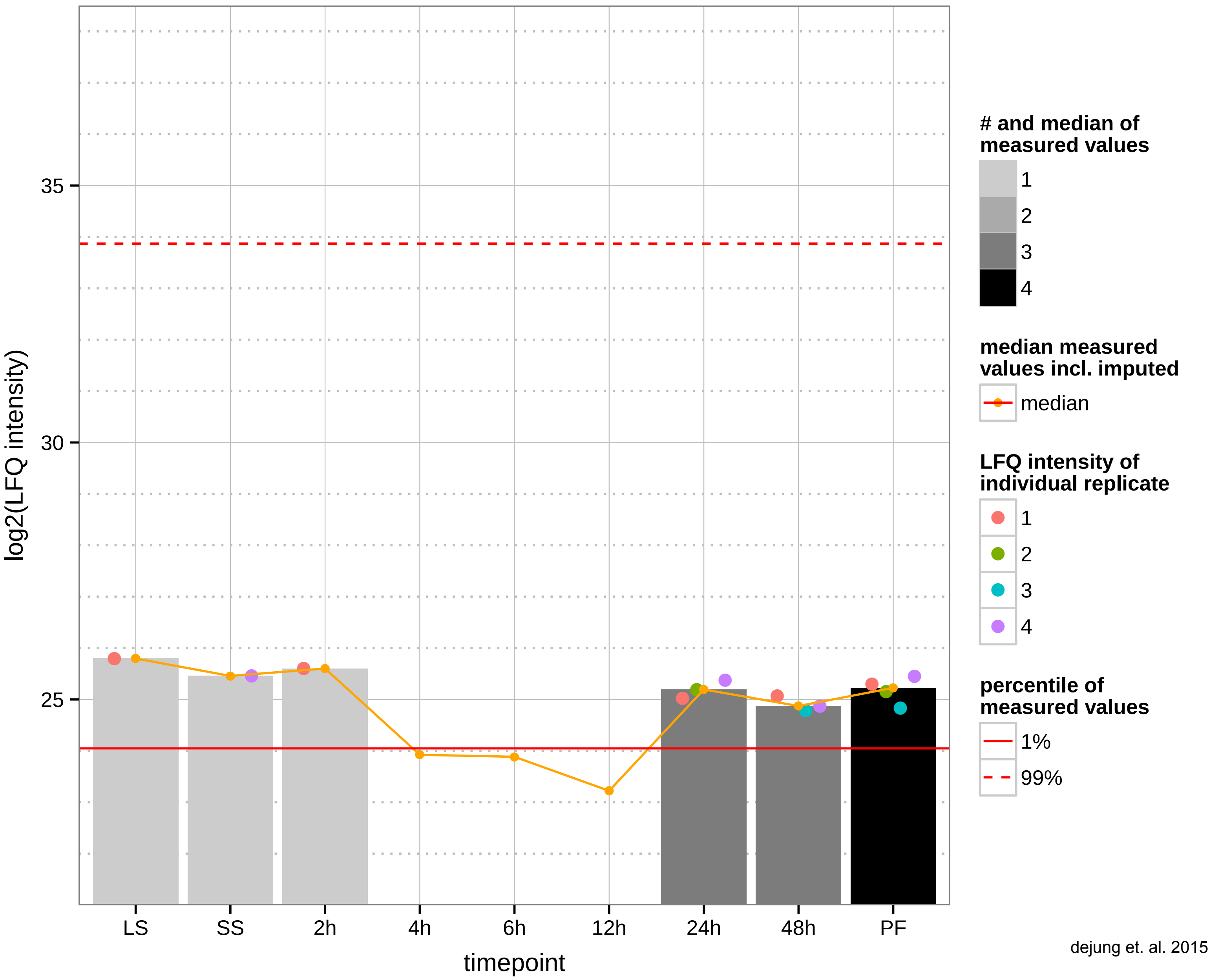

dejung et. al. 2015

hypothetical protein, conserved
Tb927.10.3810
AGOF: null
AGOC: null
AGOP: null
PGOF: null
PGOC: null
PGOP: null

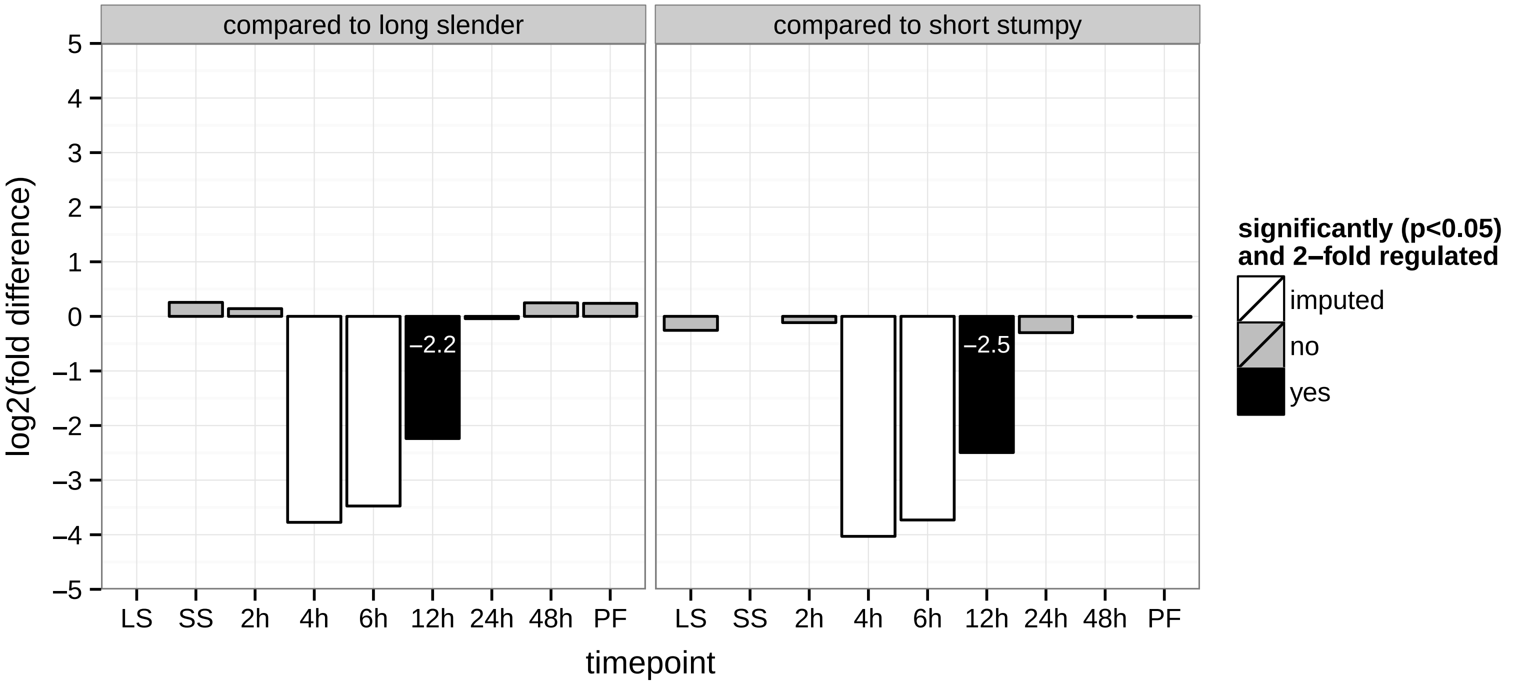

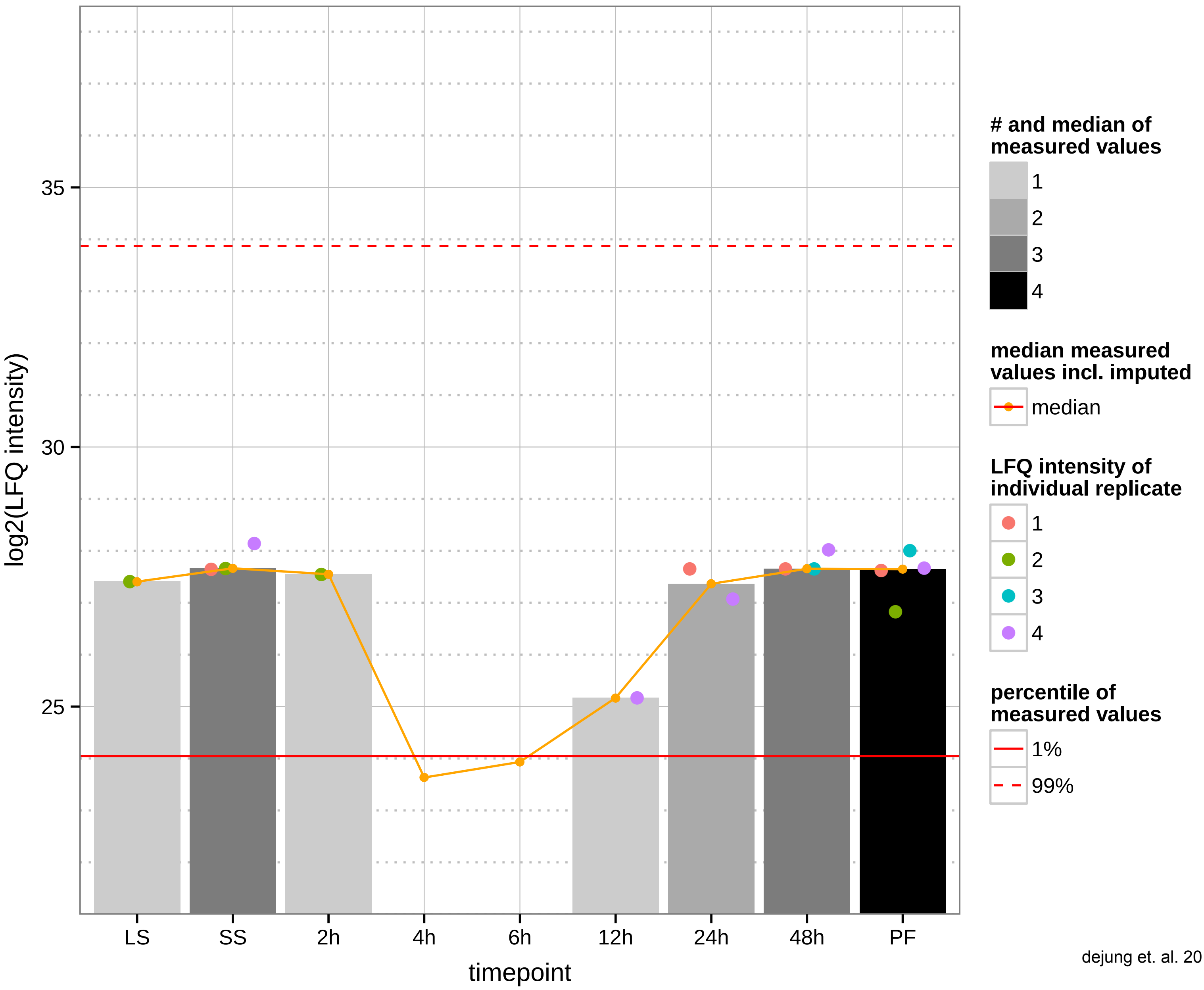

dejung et. al. 2015

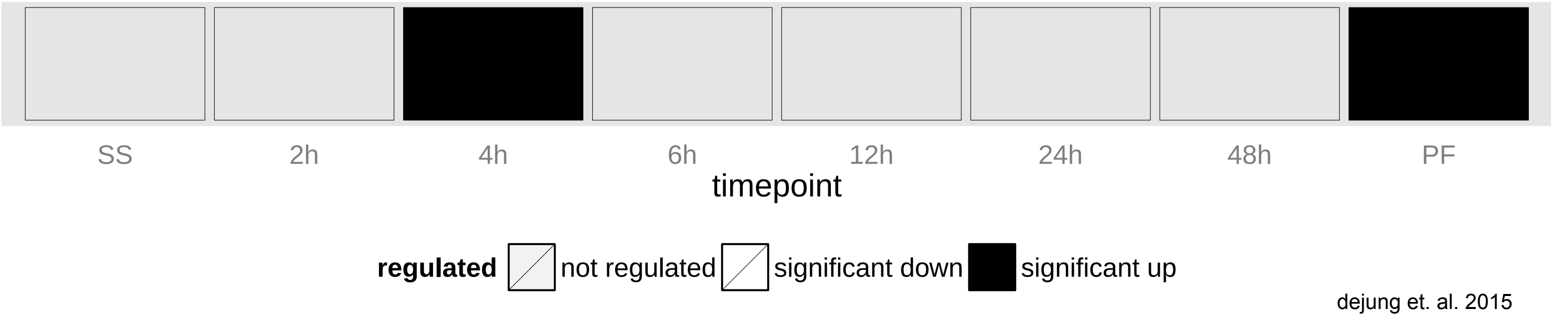
SS
2h
4h
6h
12h
24h
48h
PF
timepoint
regulated
not regulated
significant down
significant up
dejung et. al. 2015

hypothetical protein, conserved
Tb927.11.2220
AGOF: null
AGOC: null
AGOP: null
PGOF: null
PGOC: null
PGOP: null

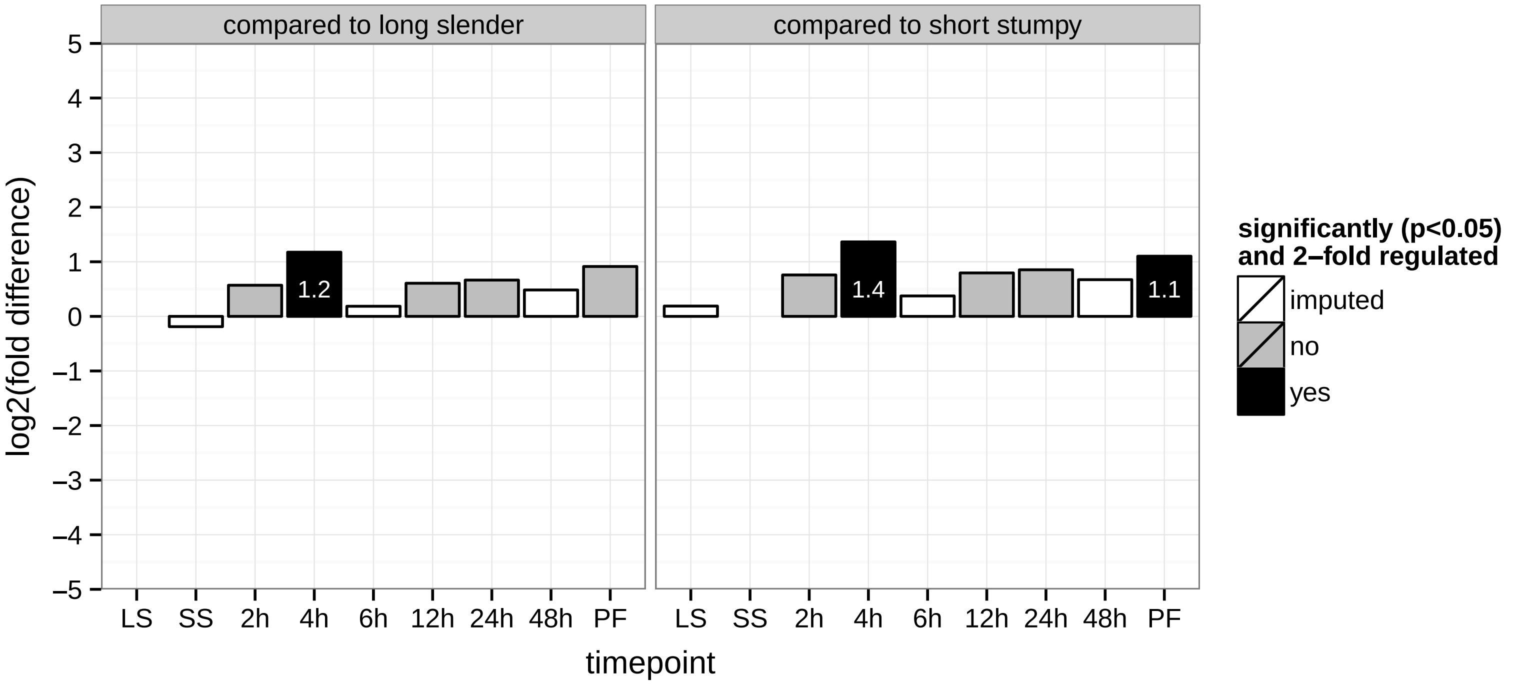

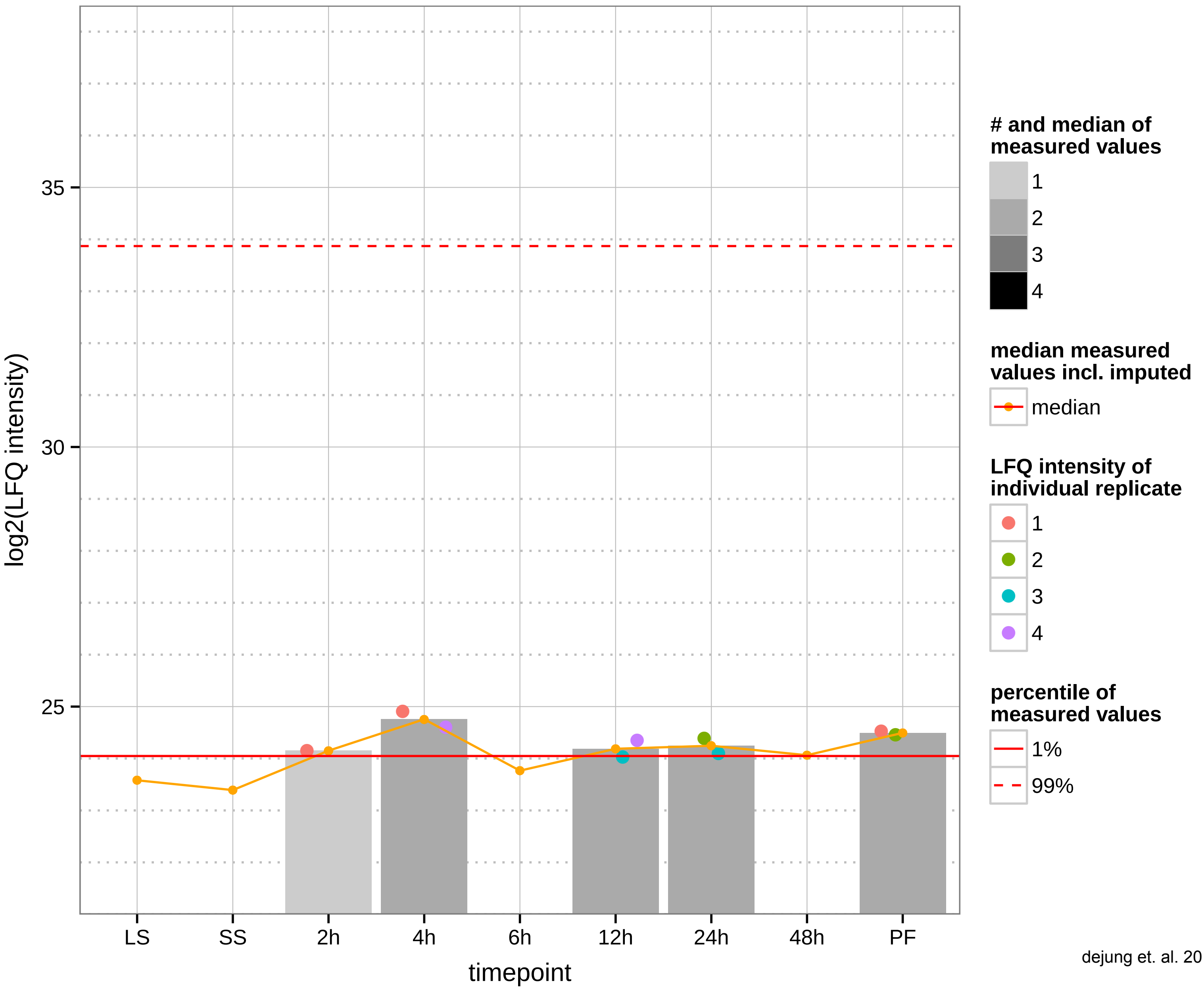

dejung et. al. 2015

retrotransposon hot spot protein 1 (RHS1), putative
Tb927.6.160
AGOF: null
AGOC: null
AGOP: null
PGOF: null
PGOC: null
PGOP: null

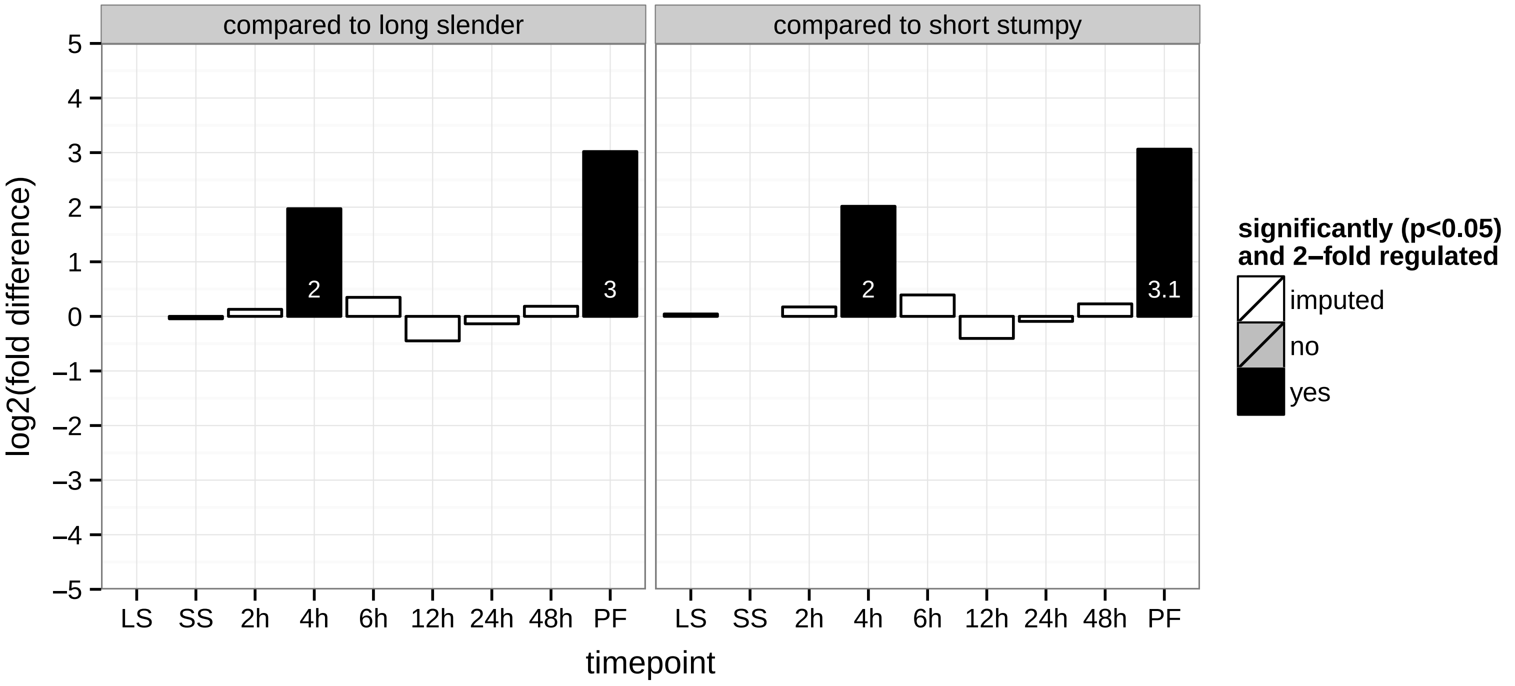

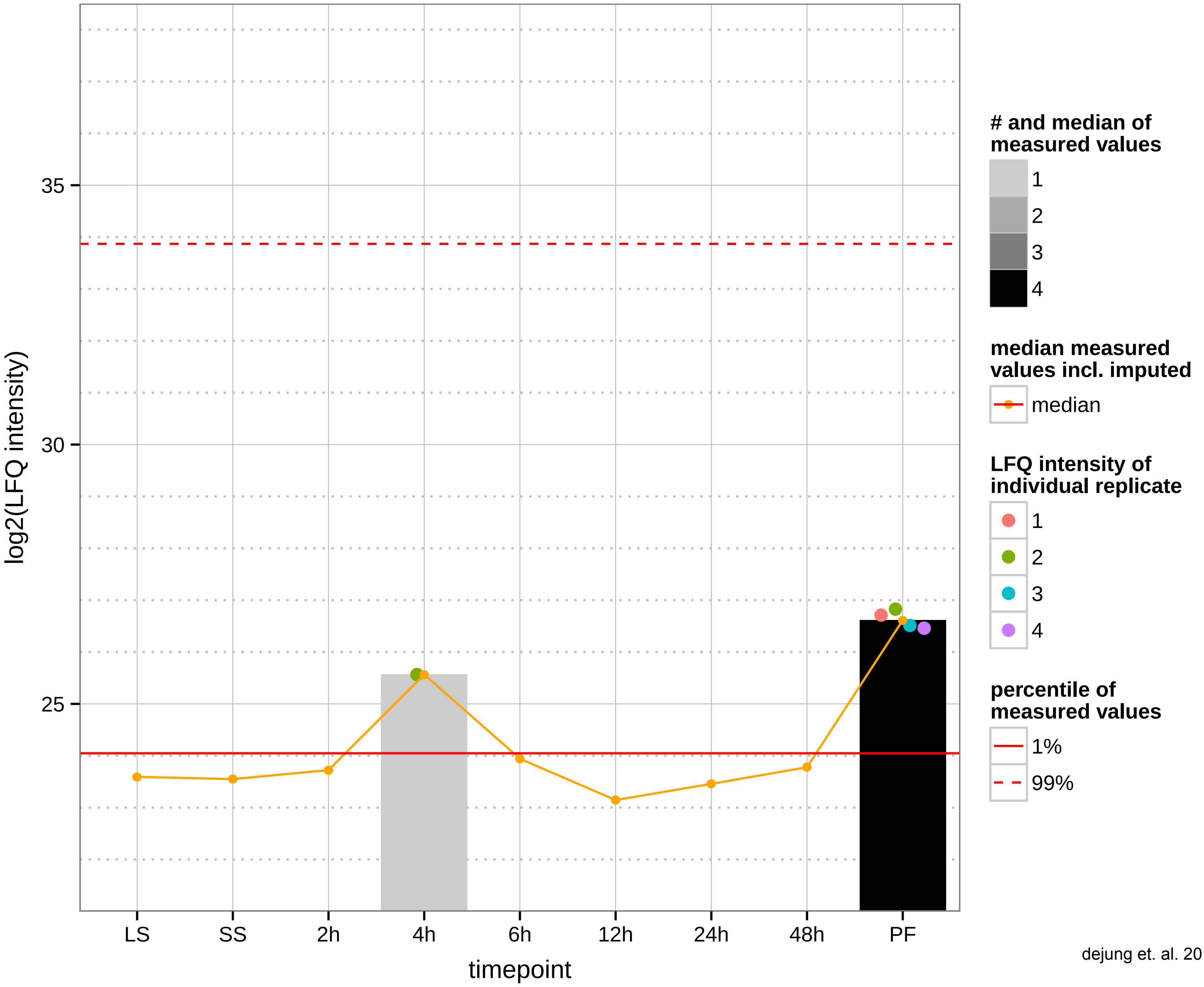

dejung et. al. 2015

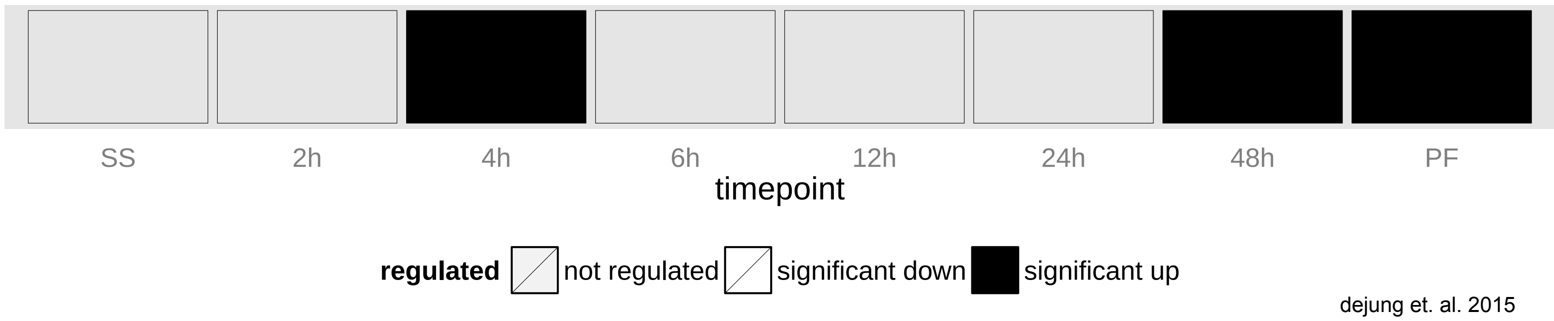
SS
2h
4h
6h
12h
24h
48h
PF
timepoint
regulated
not regulated
significant down
significant up
dejung et. al. 2015

tubulin tyrosine ligase protein, putative
Tb927.10.13870
AGOF: tubulin−tyrosine ligase activity
AGOC: null
AGOP: cellular protein modification process
PGOF: tubulin−tyrosine ligase activity
PGOC: null
PGOP: cellular protein modification process

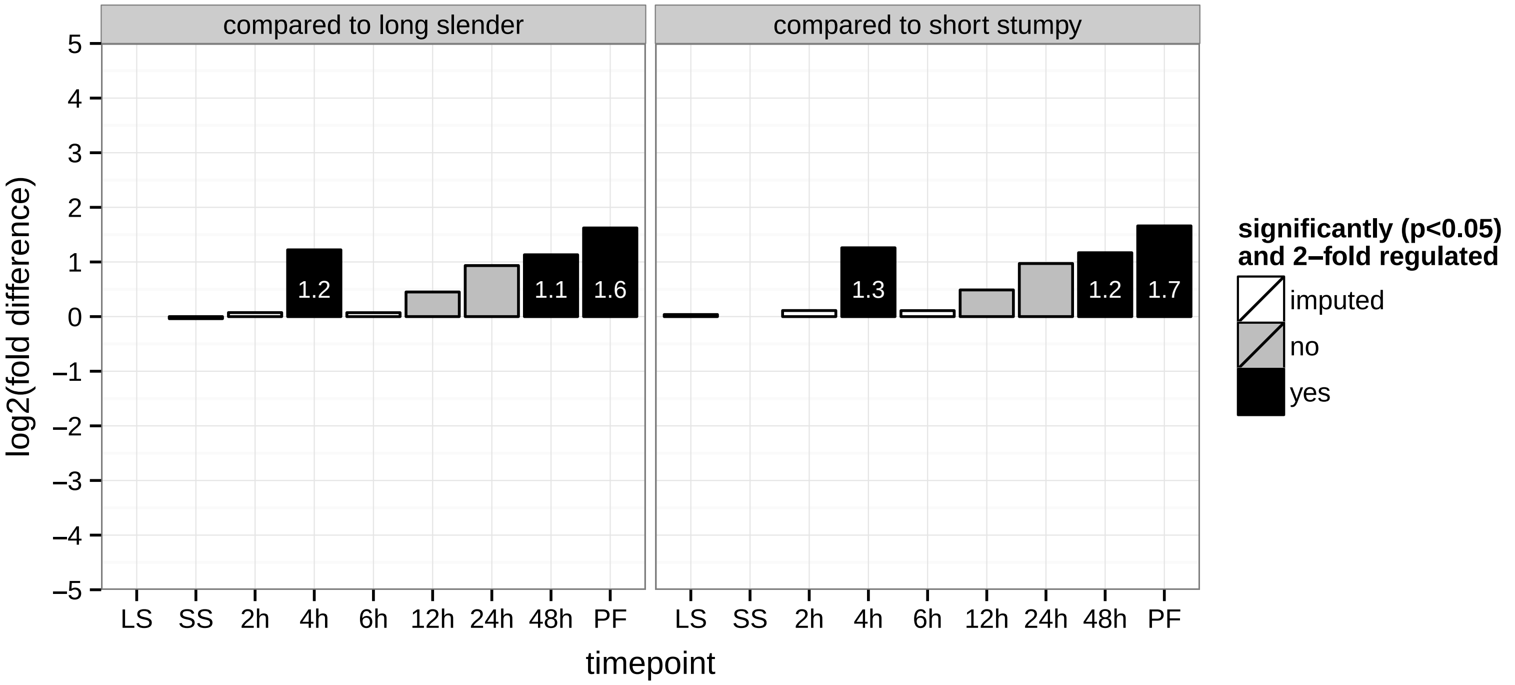

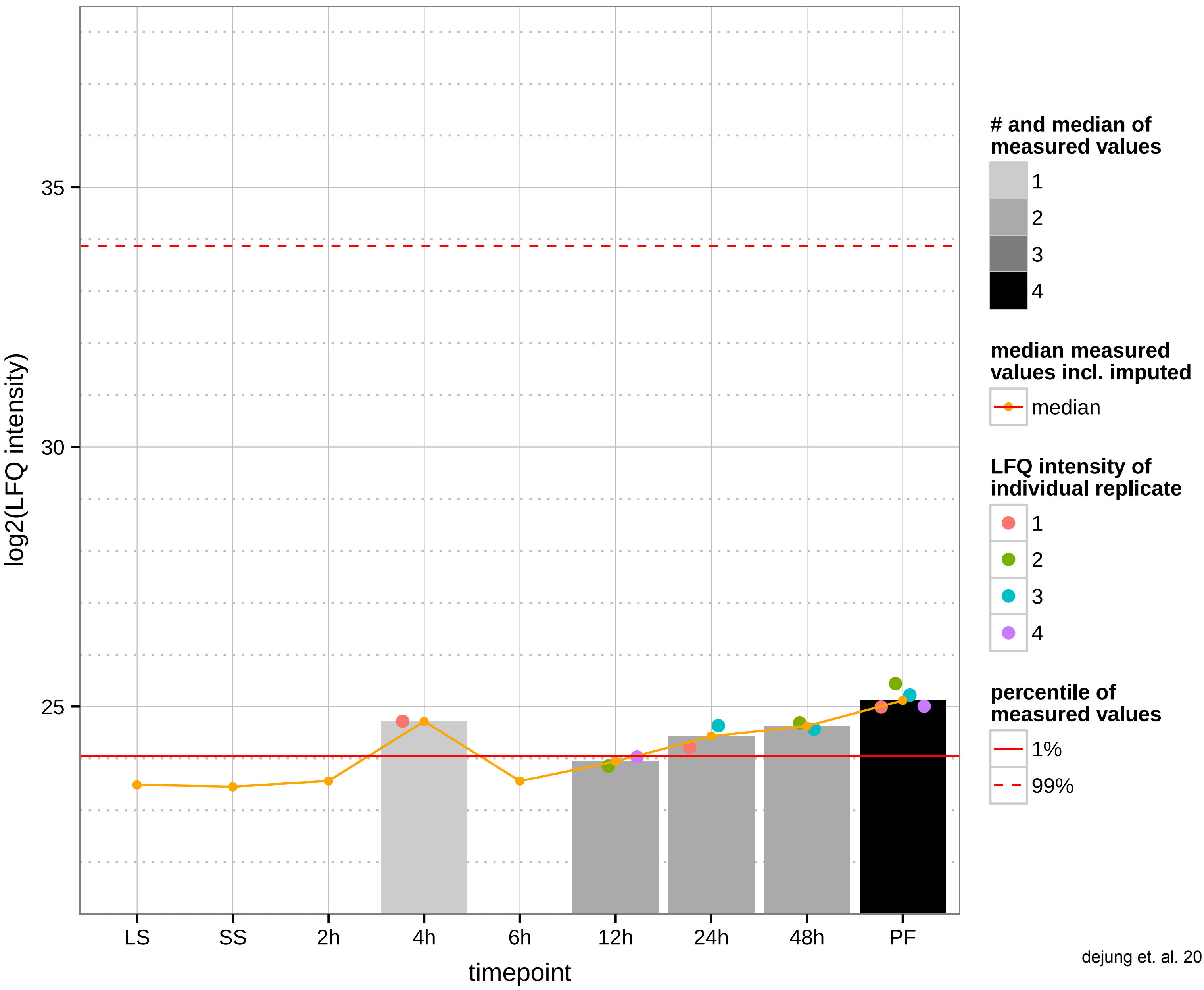

dejung et. al. 2015

hydrolase, alpha/beta fold family, putative
Tb927.5.2370
AGOF: null
AGOC: null
AGOP: null
PGOF: null
PGOC: null
PGOP: null

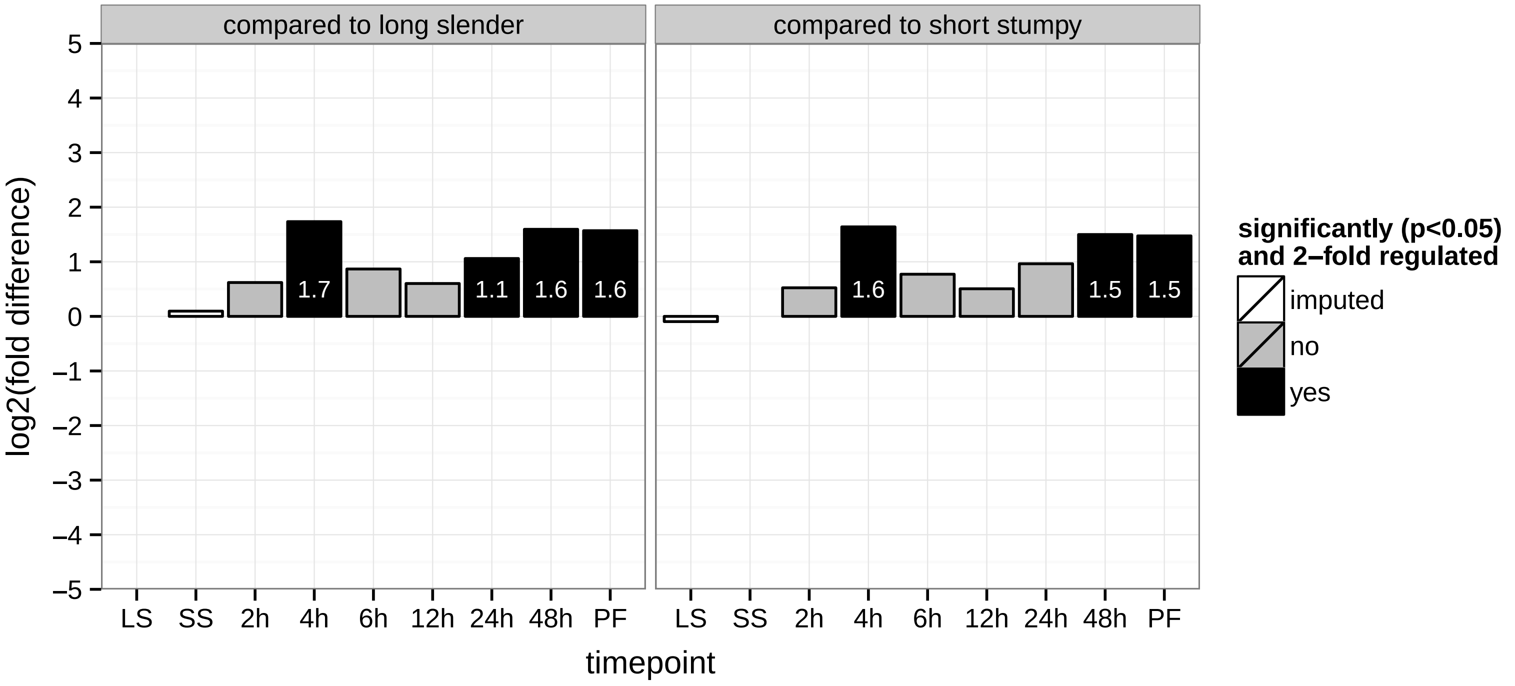

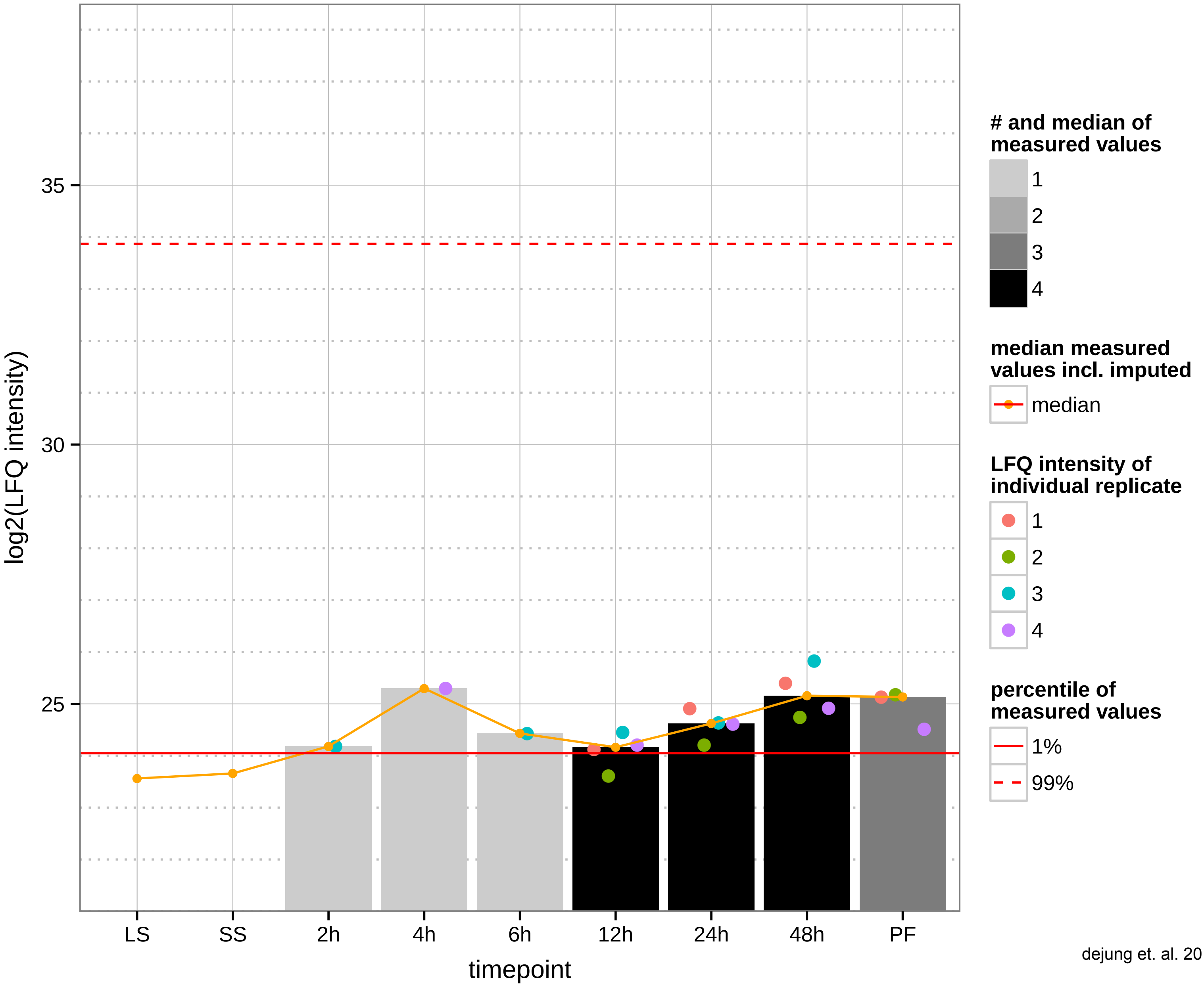

dejung et. al. 2015

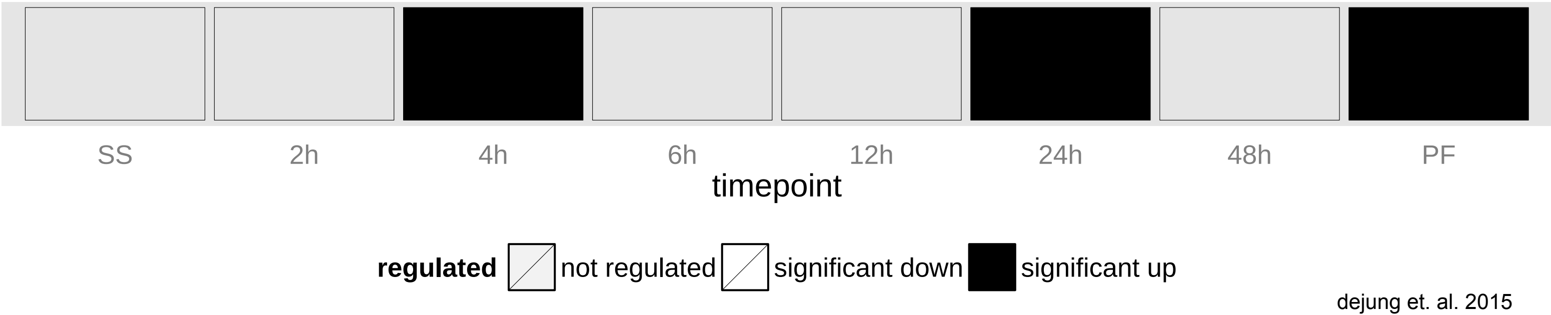
SS
2h
4h
6h
12h
24h
48h
PF
timepoint
regulated
not regulated
significant down
significant up
dejung et. al. 2015

hypothetical protein, conserved
Tb927.10.4550
AGOF: null
AGOC: mitochondrion
AGOP: null
PGOF: null
PGOC: null
PGOP: null

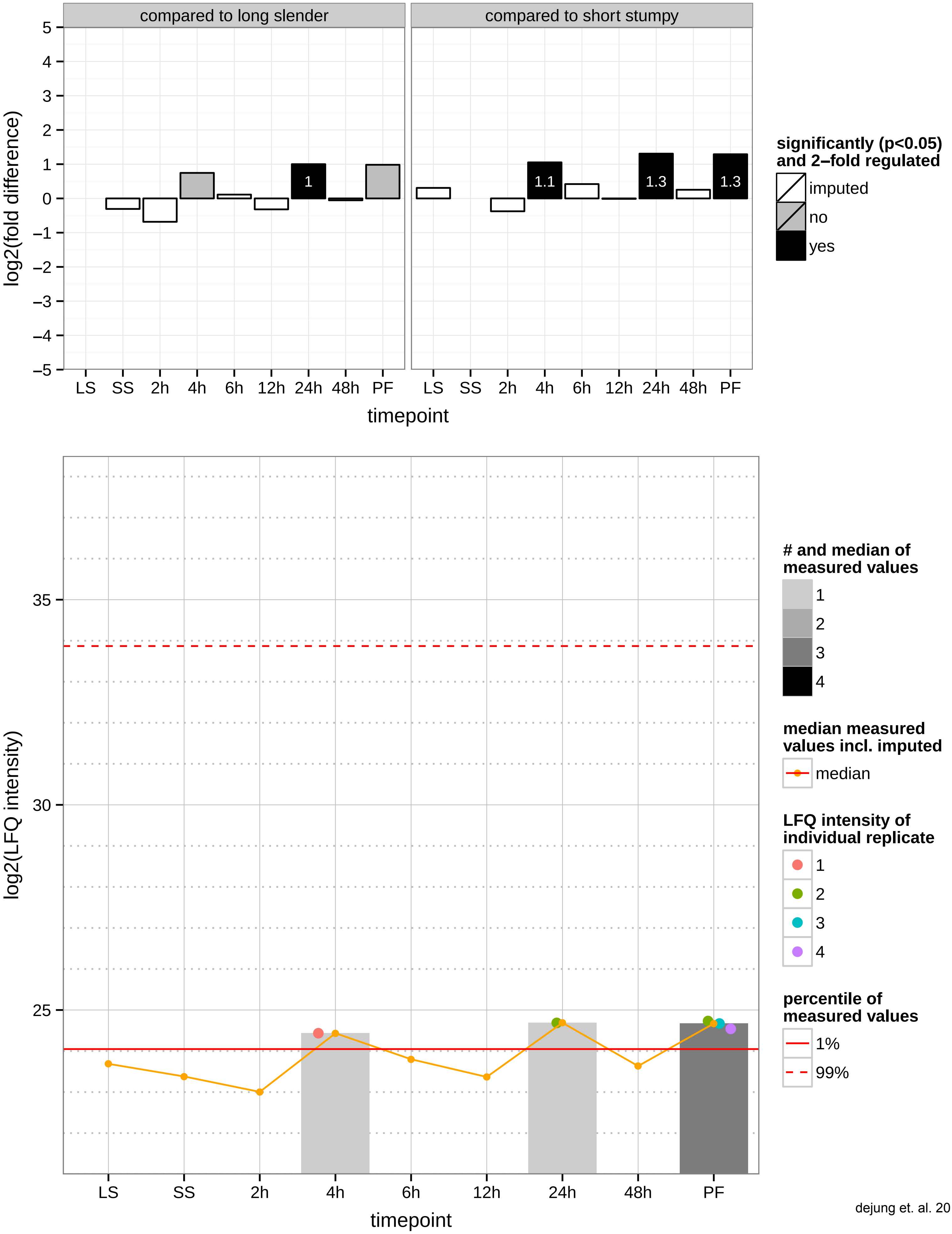

dejung et. al. 2015

hypothetical protein, conserved
Tb927.11.9580.1;Tb927.11.9580.2
AGOF: null
AGOC: null
AGOP: null
PGOF: null
PGOC: null
PGOP: null

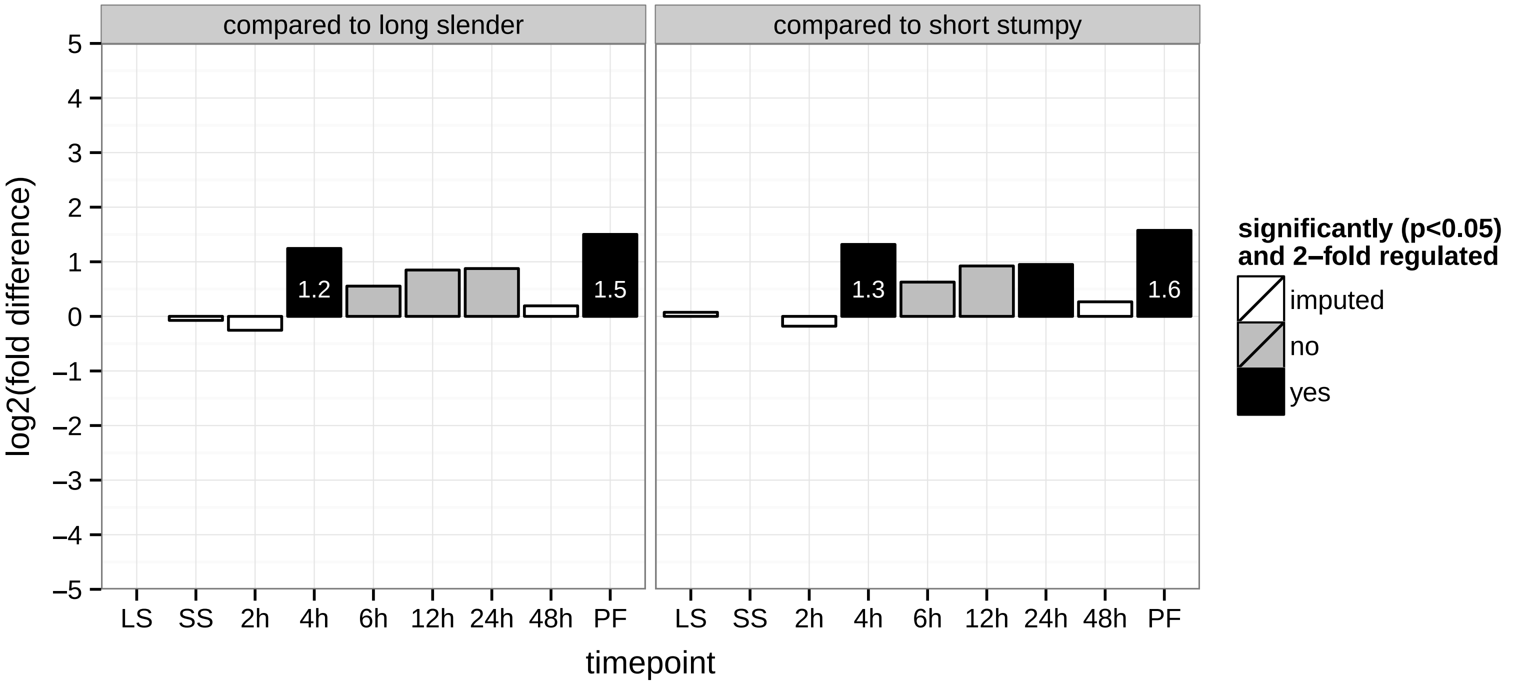

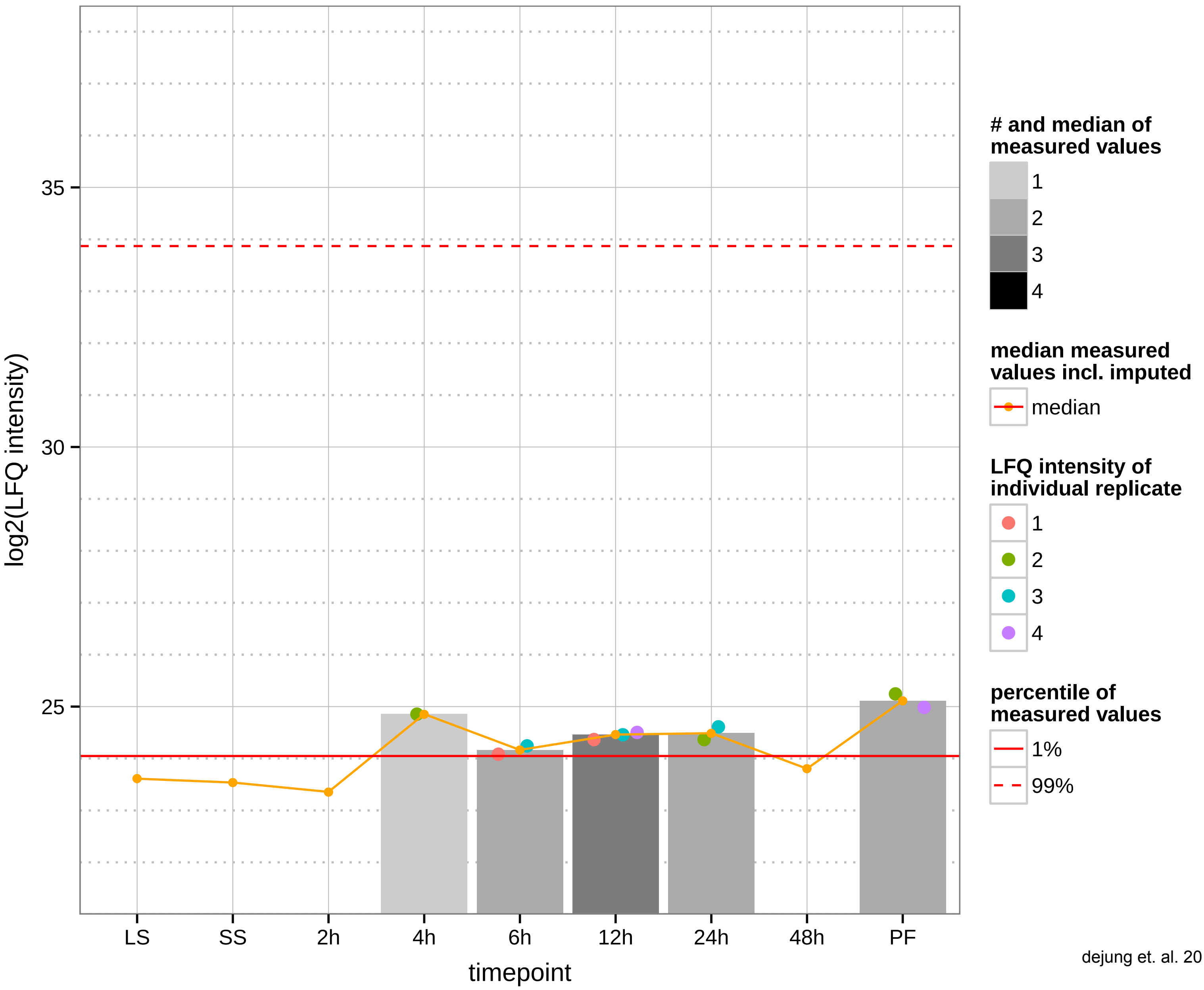

dejung et. al. 2015

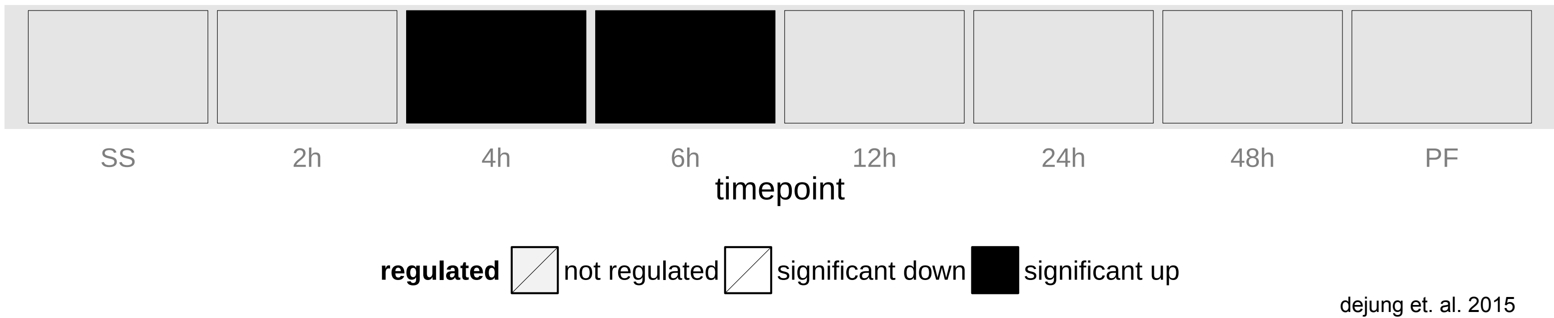
SS
2h
4h
6h
12h
24h
48h
PF
timepoint
regulated
not regulated
significant down
significant up
dejung et. al. 2015

ATP synthase subunit, putative
Tb927.1.3820
AGOF: hydrogen ion transporting ATP synthase activity, rotational mechanism, proton−transporting ATPase activity
AGOC: proton−transporting V−type ATPase, V1 domain
AGOP: ATP hydrolysis coupled proton transport
PGOF: hydrogen ion transporting ATP synthase activity, rotational mechanism, proton−transporting ATPase activity
PGOC: proton−transporting V−type ATPase, V1 domain, proton−transporting two−sector ATPase complex, catalytic domain
PGOP: ATP hydrolysis coupled proton transport

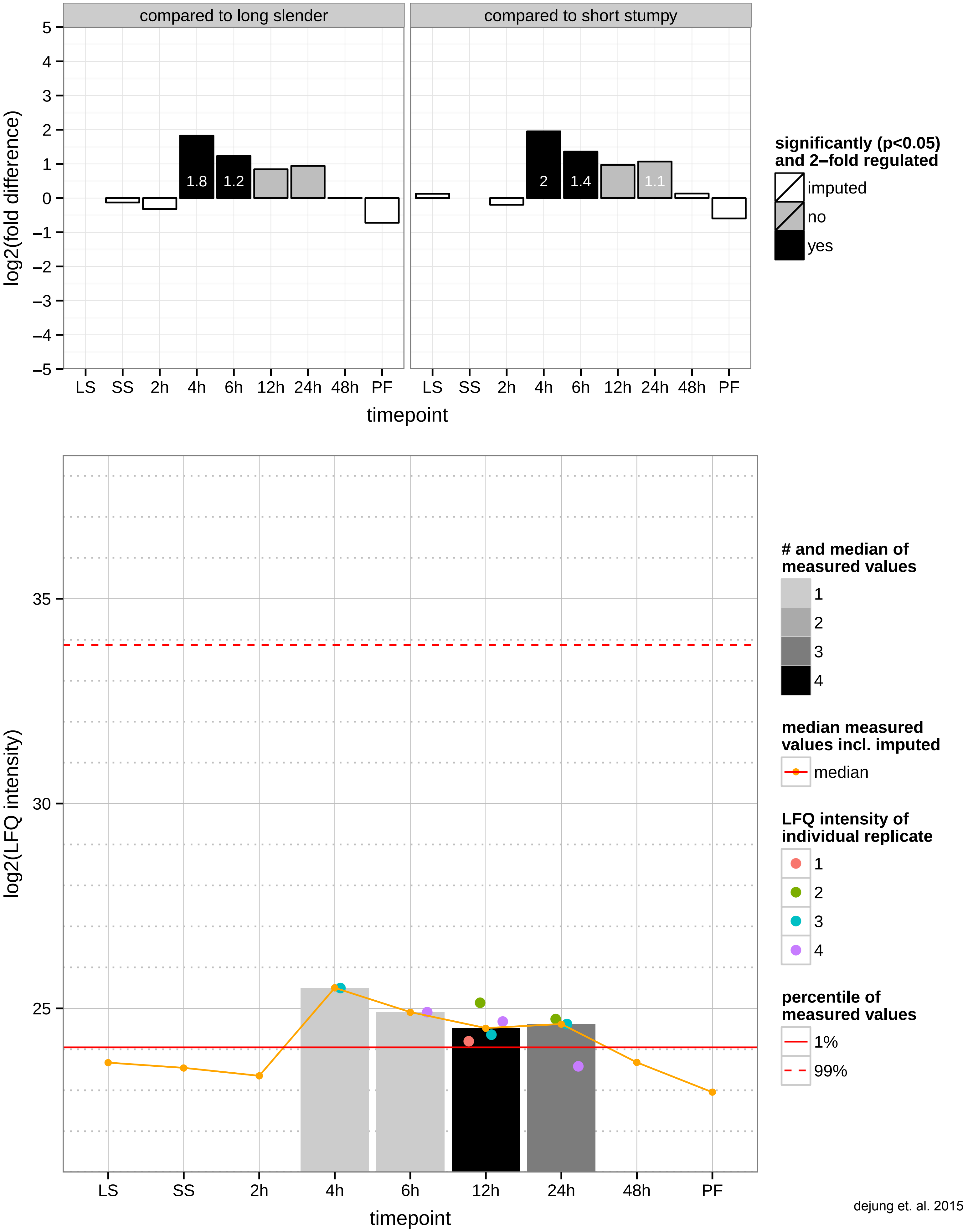

dejung et. al. 2015

hypothetical protein, conserved
Tb927.9.8050
AGOF: null
AGOC: null
AGOP: null
PGOF: null
PGOC: null
PGOP: null

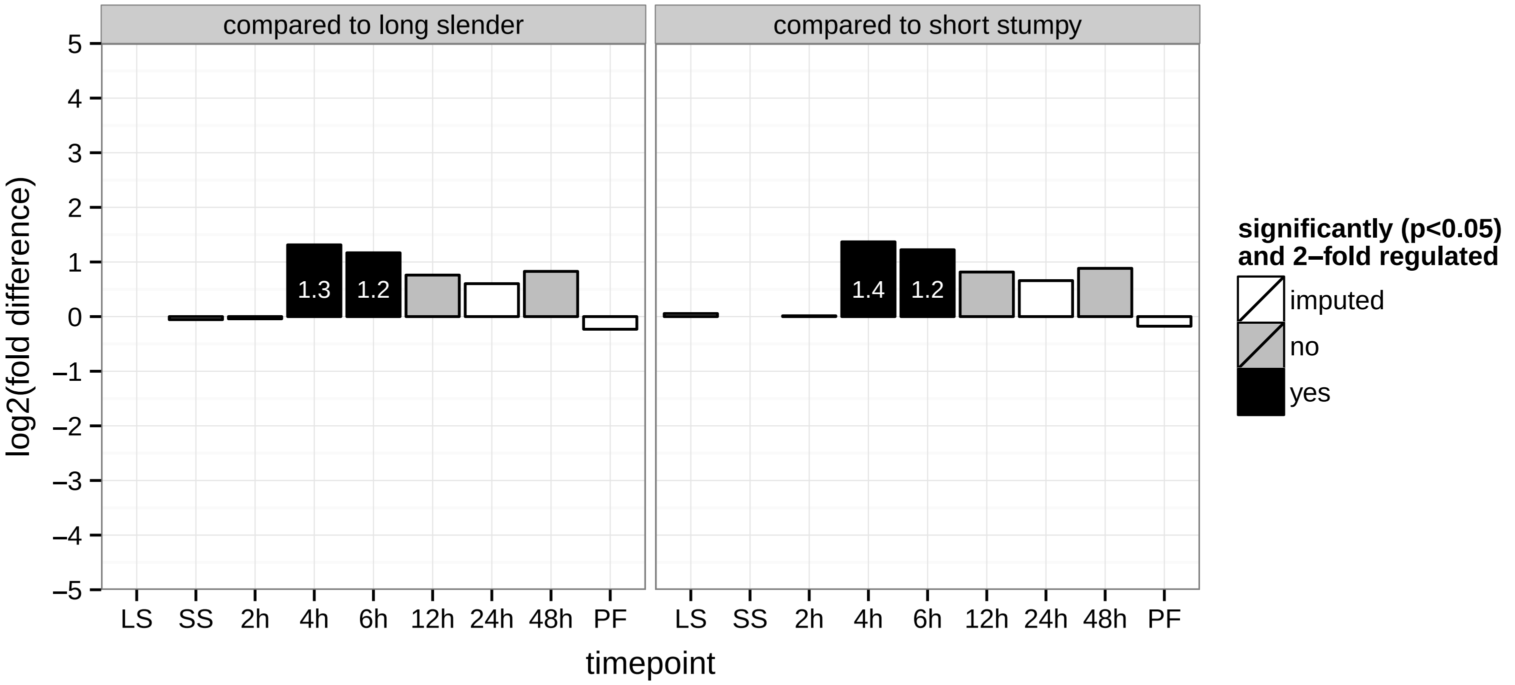

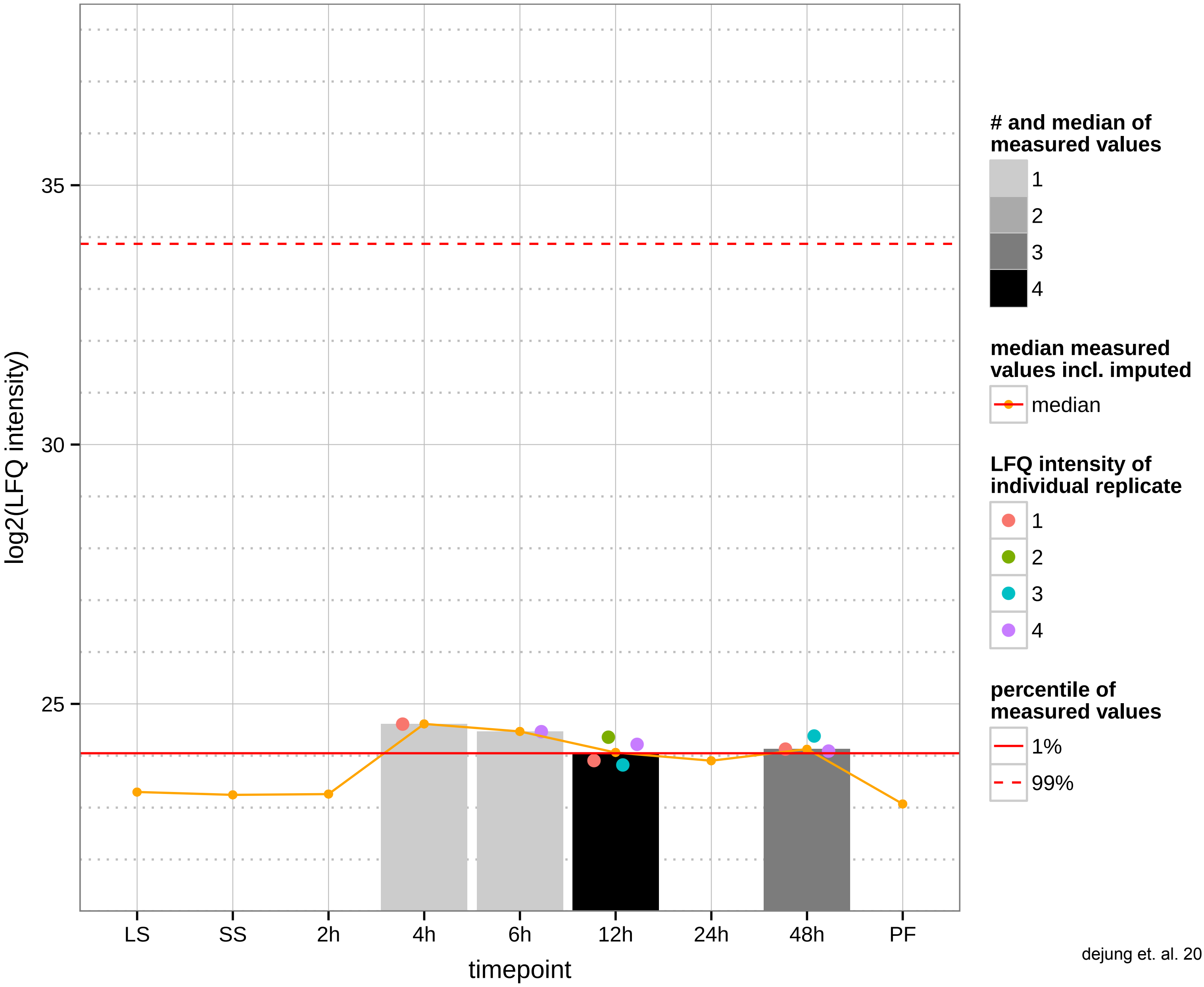

dejung et. al. 2015

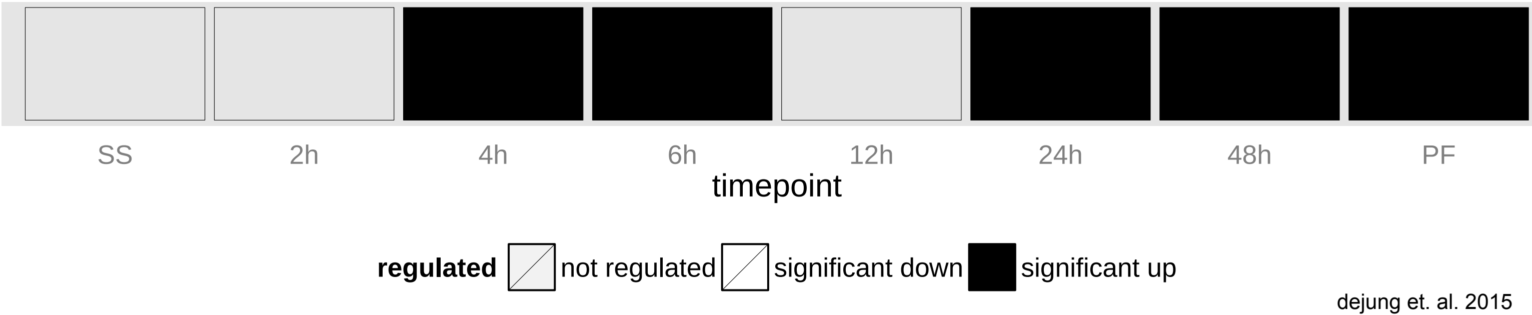
SS
2h
4h
6h
12h
24h
48h
PF
timepoint
regulated
not regulated
significant down
significant up
dejung et. al. 2015

hypothetical protein, conserved
Tb927.11.16080
AGOF: null
AGOC: Golgi apparatus, endoplasmic reticulum
AGOP: intracellular protein transport
PGOF: null
PGOC: null
PGOP: null

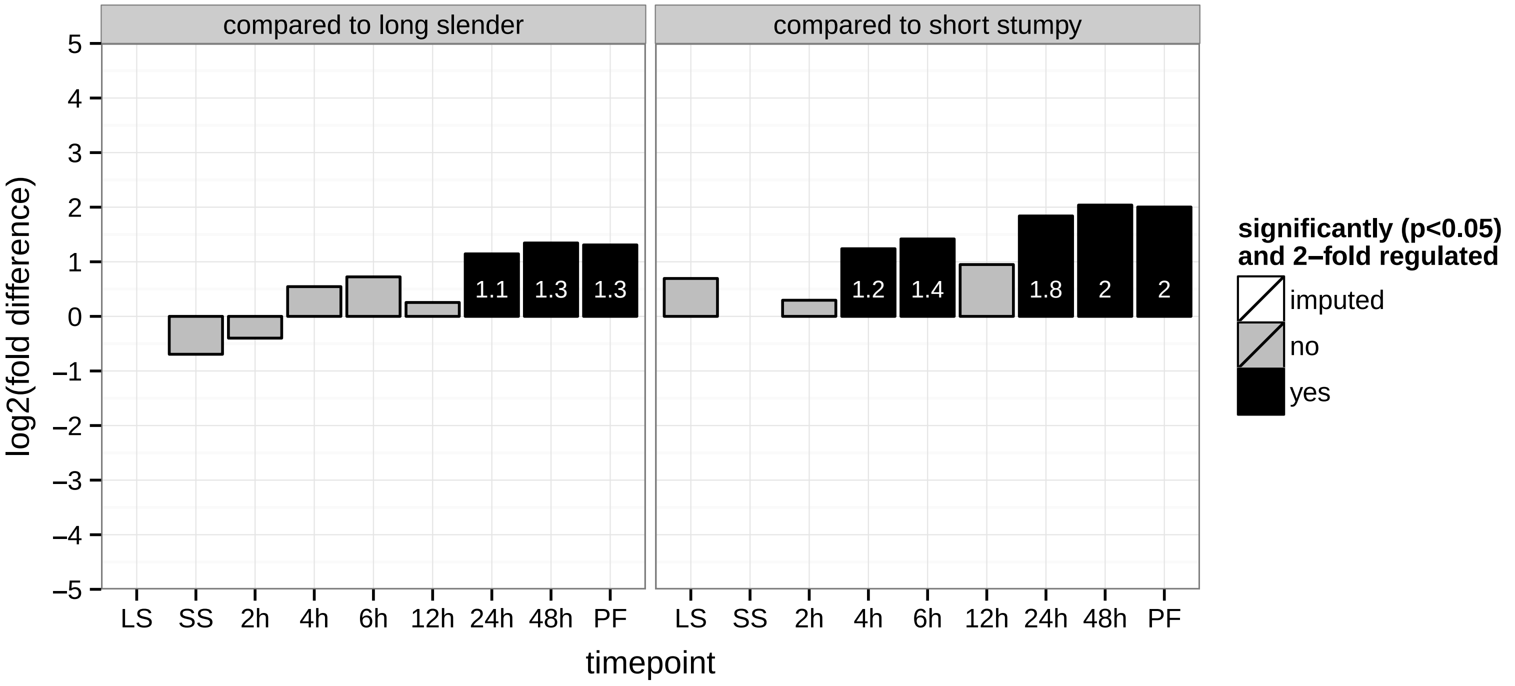

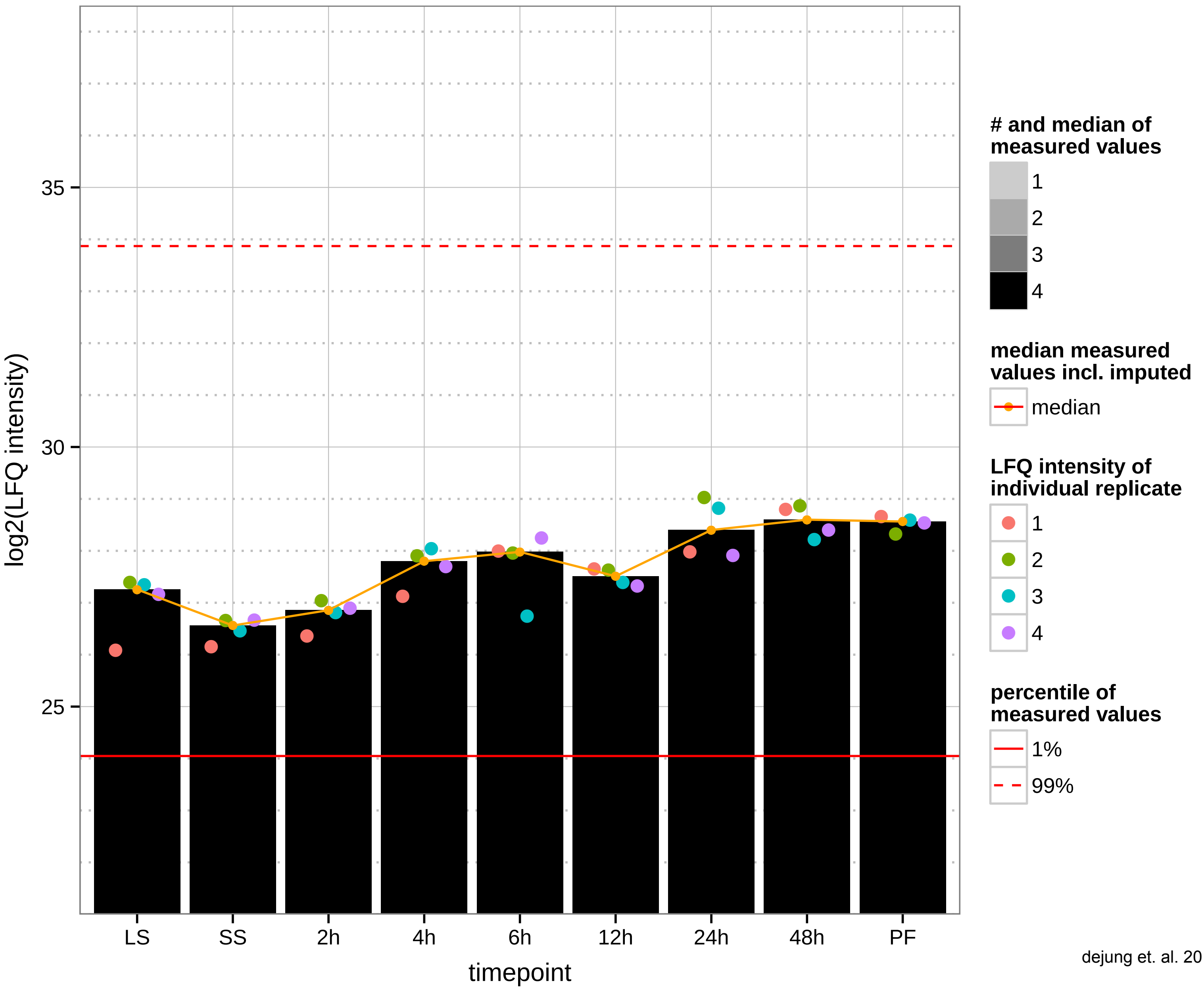

dejung et. al. 2015

ATP−dependent chaperone, putative, mitochondrial chaperone BCS1
Tb927.8.3580
AGOF: ATP binding, nucleoside−triphosphatase activity
AGOC: mitochondrion
AGOP: aerobic respiration
PGOF: ATP binding, nucleoside−triphosphatase activity, nucleotide binding
PGOC: null
PGOP: null

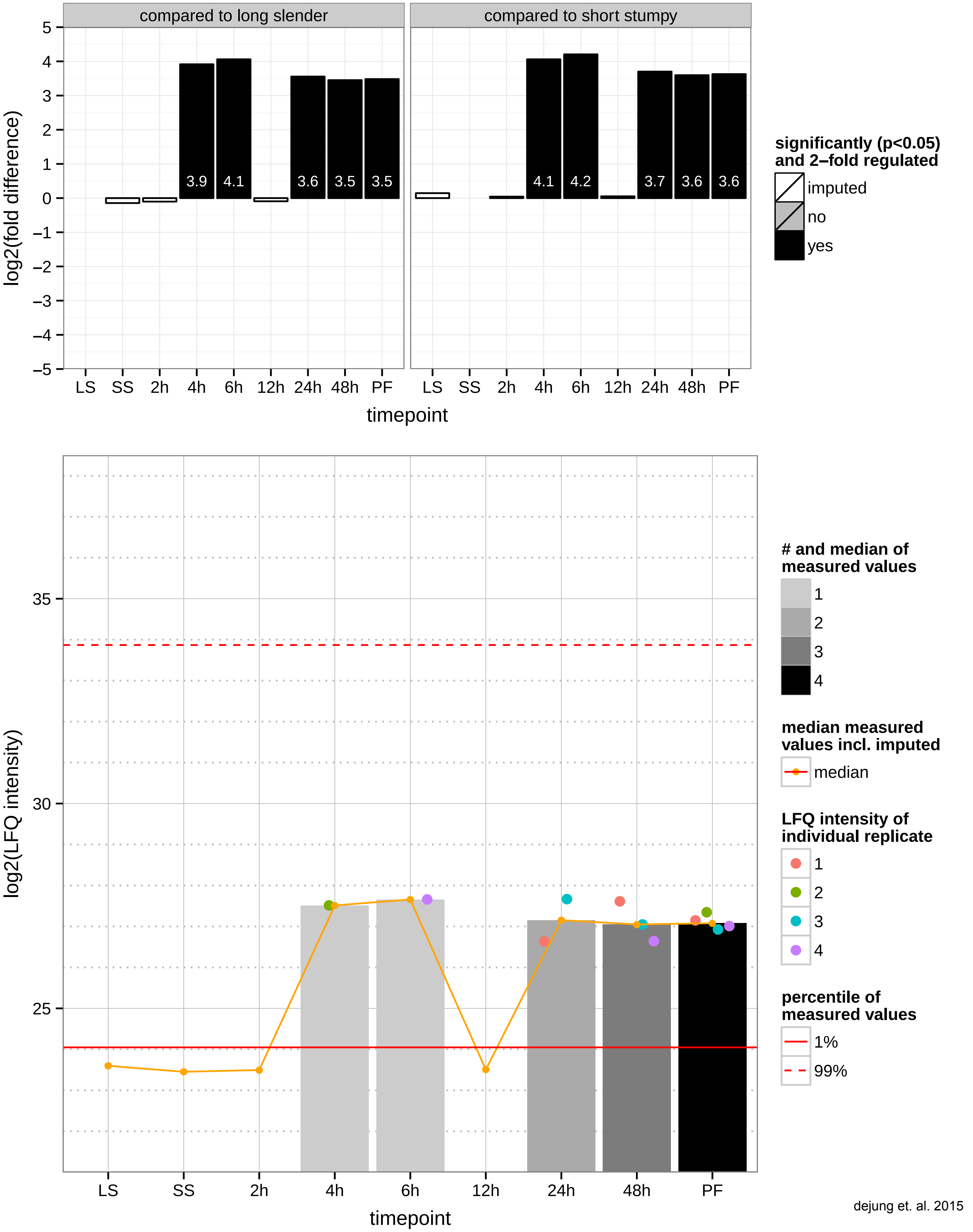

dejung et. al. 2015

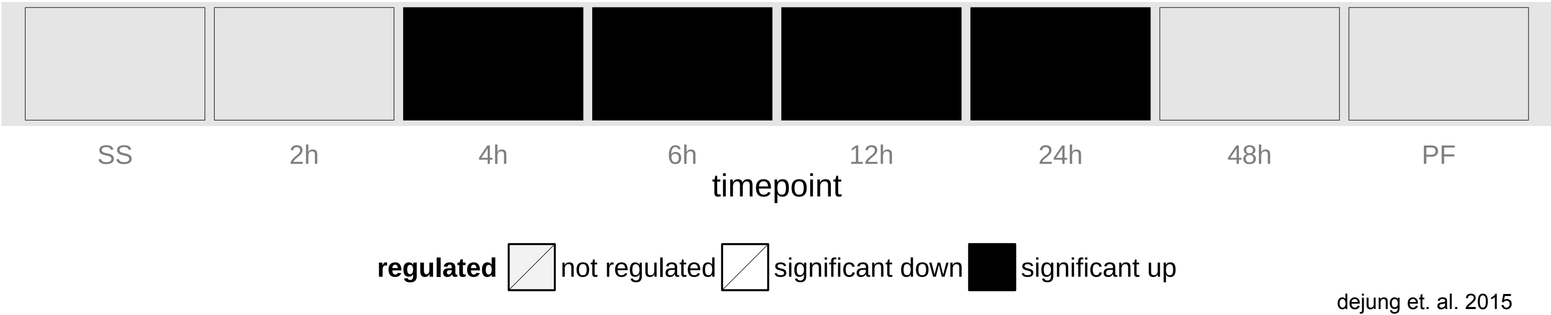
SS
2h
4h
6h
12h
24h
48h
PF
timepoint
regulated
not regulated
significant down
significant up
dejung et. al. 2015

hypothetical protein, conserved
Tb927.10.7290
AGOF: null
AGOC: null
AGOP: null
PGOF: glutathione gamma–glutamylcysteinyltransferase activity, metal ion binding
PGOC: null
PGOP: phytochelatin biosynthetic process, response to metal ion

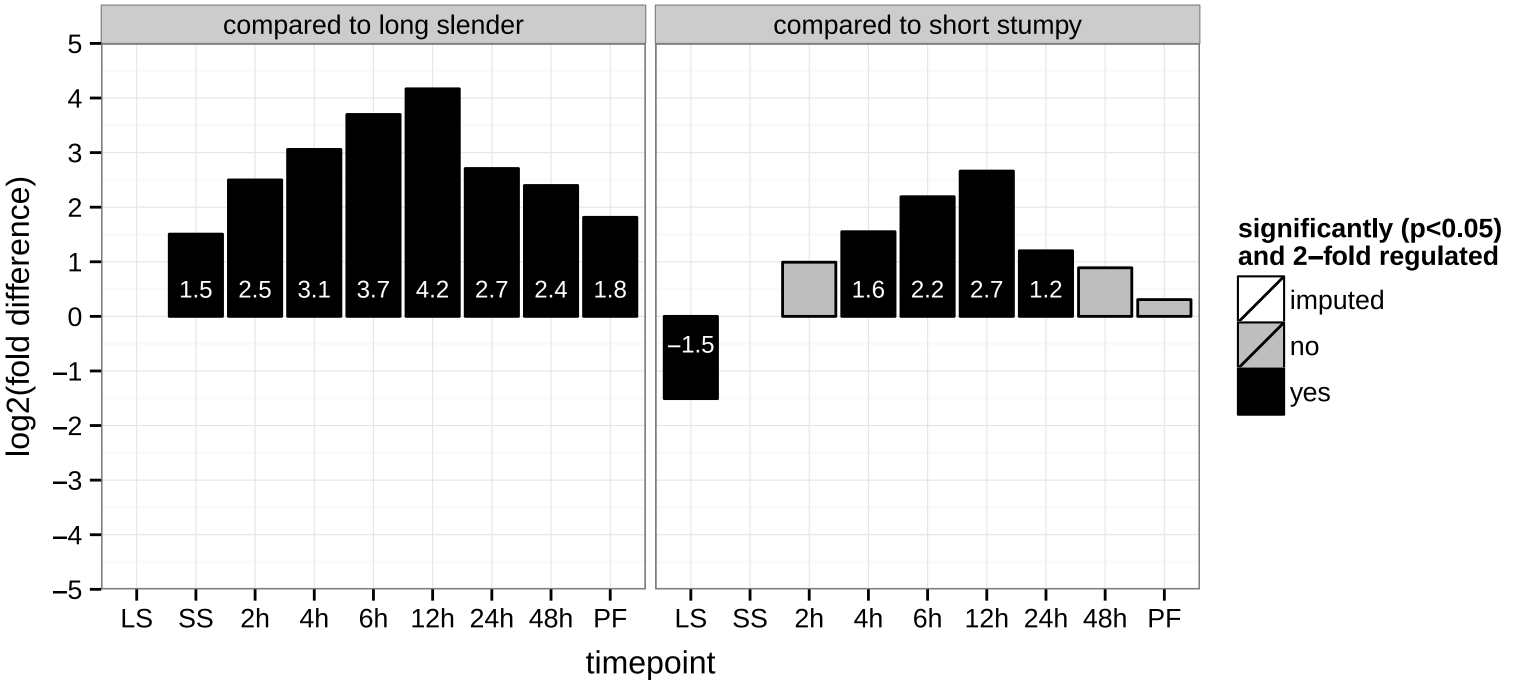

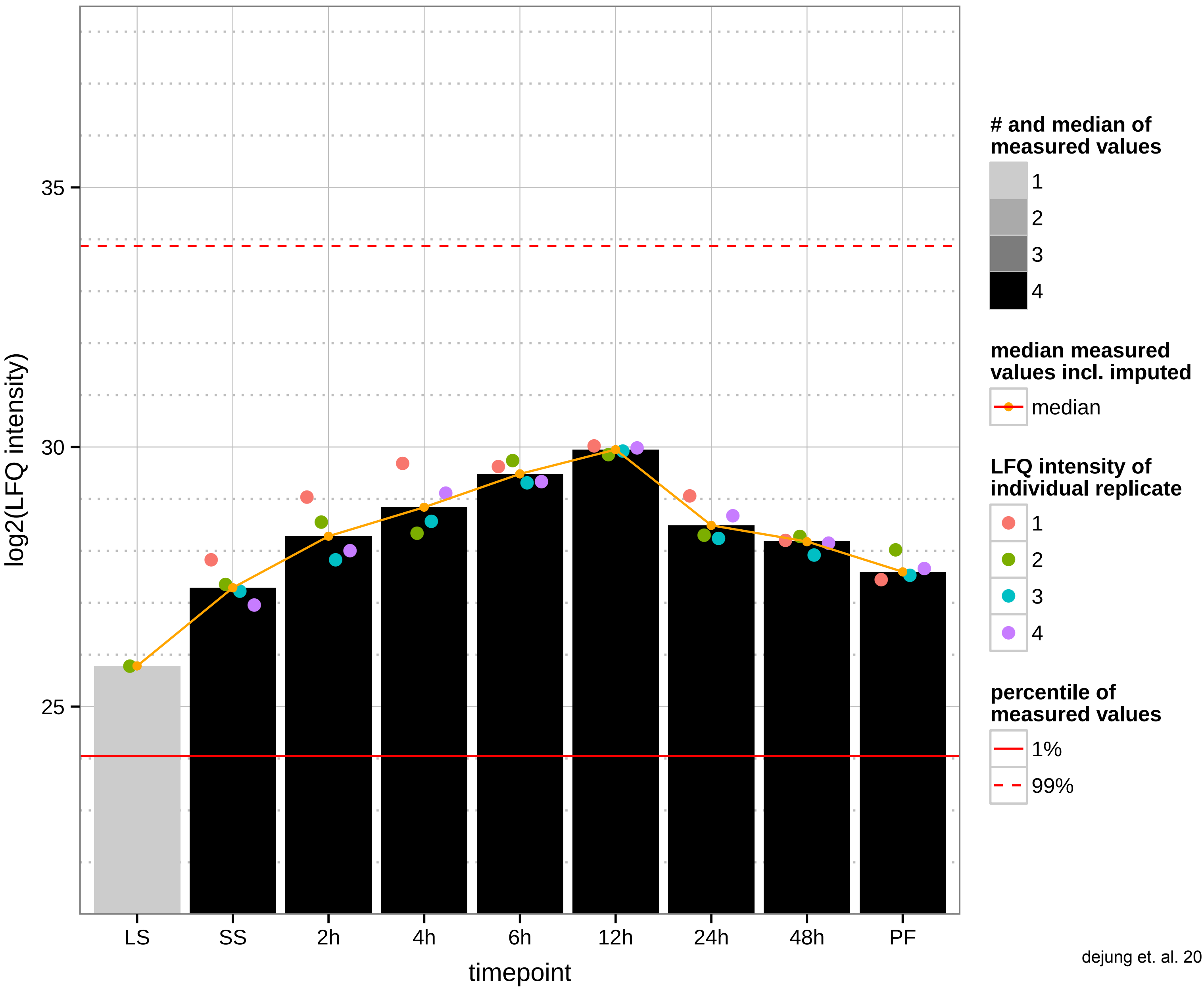

dejung et. al. 2015

hypothetical protein, conserved
Tb927.4.4540
AGOF: null
AGOC: null
AGOP: null
PGOF: null
PGOC: null
PGOP: null

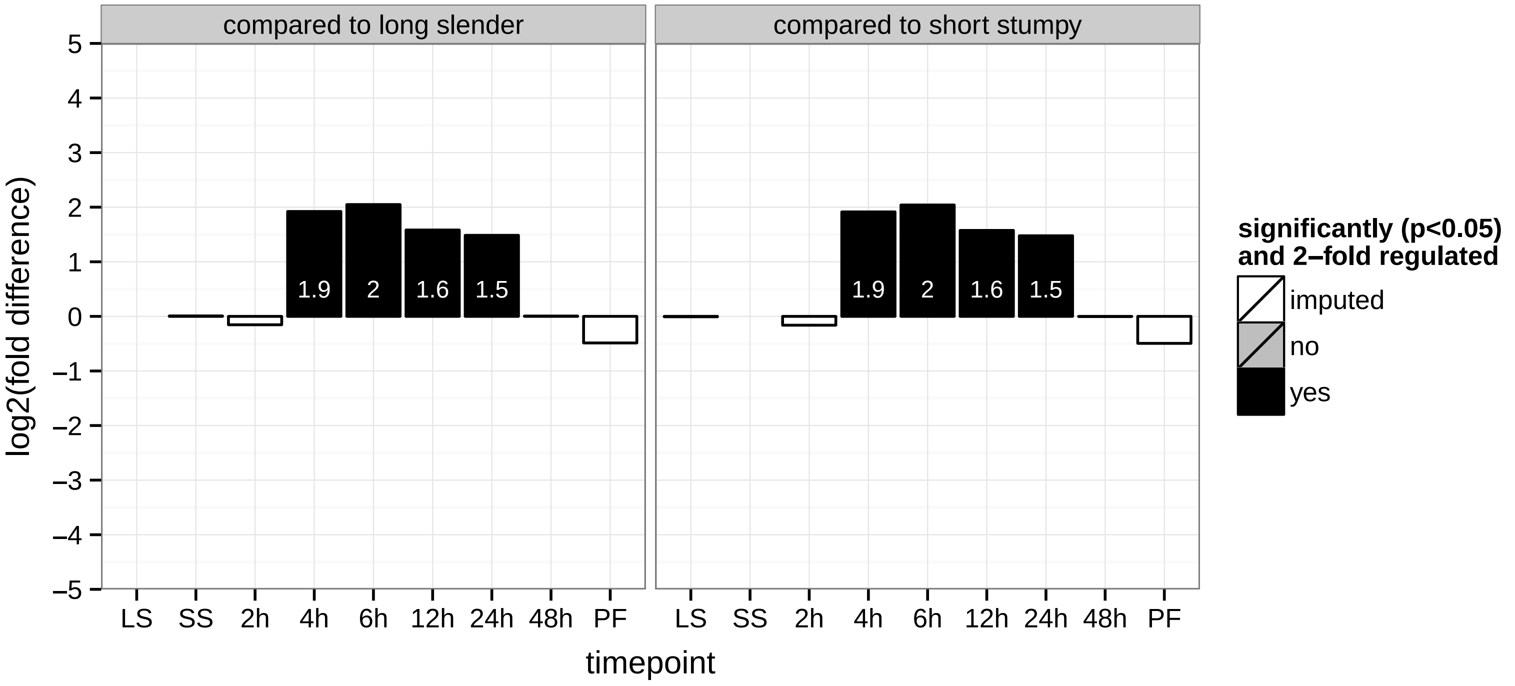

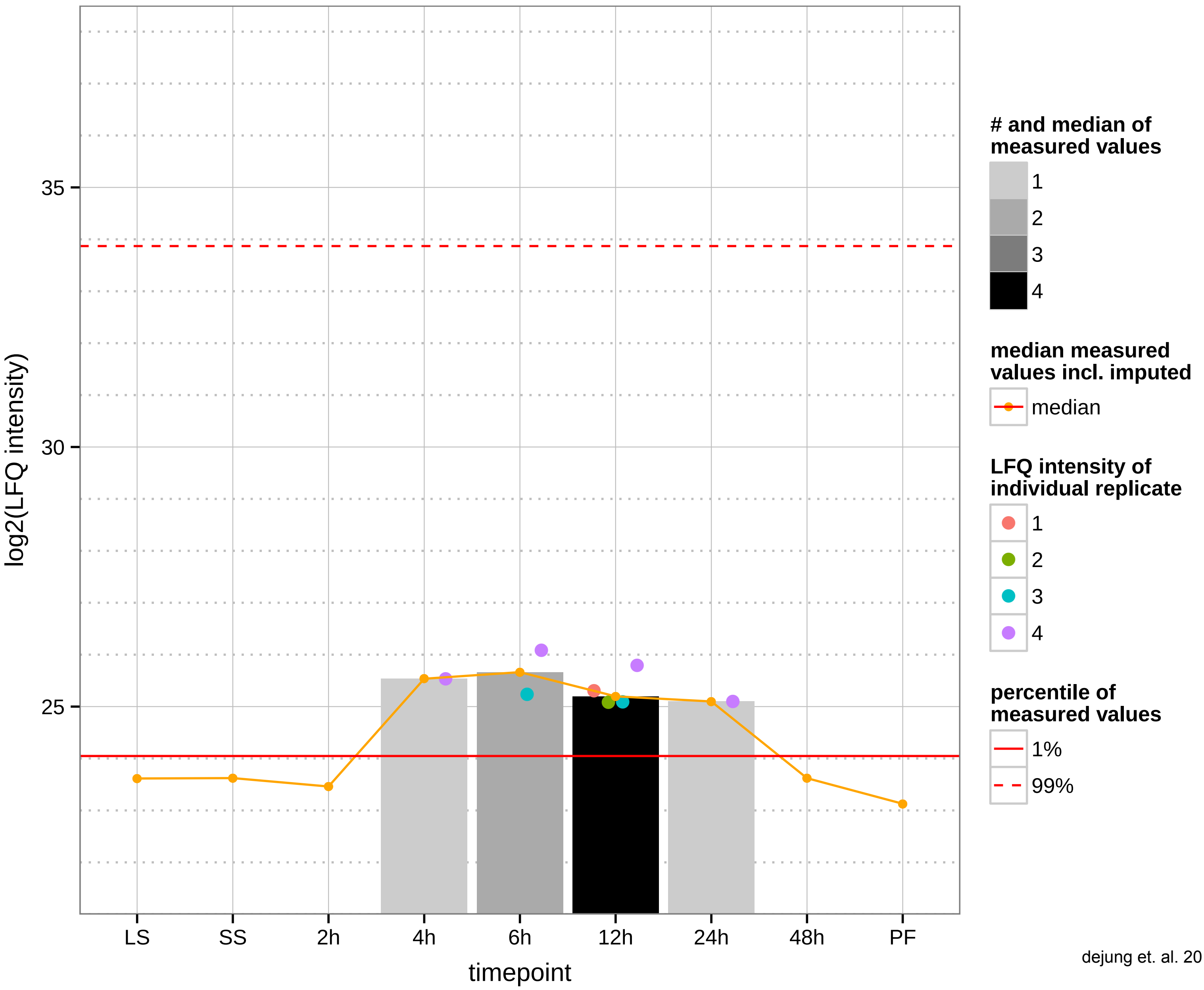

dejung et. al. 2015

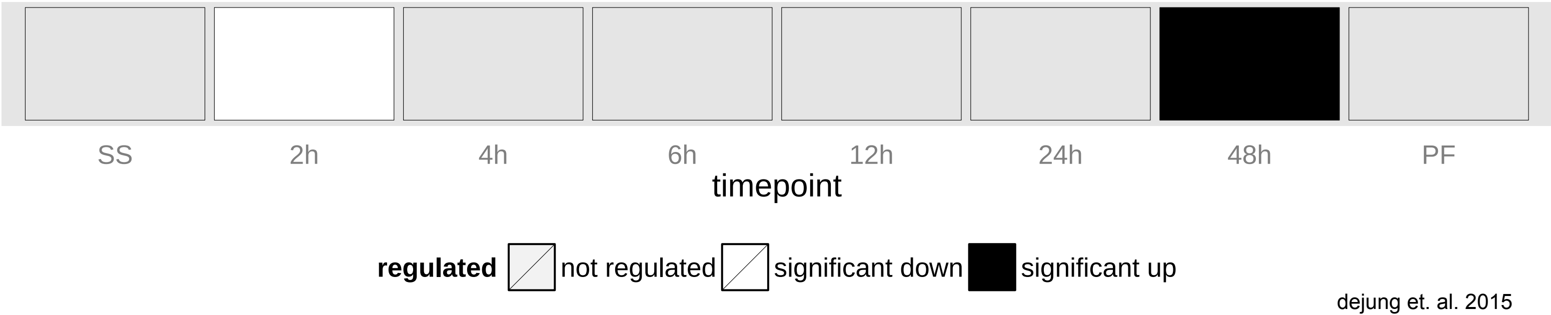
SS
2h
4h
6h
12h
24h
48h
PF
timepoint
regulated
not regulated
significant down
significant up
dejung et. al. 2015

hypothetical protein, conserved
Tb927.7.7030
AGOF: zinc ion binding
AGOC: null
AGOP: null
PGOF: null
PGOC: null
PGOP: null

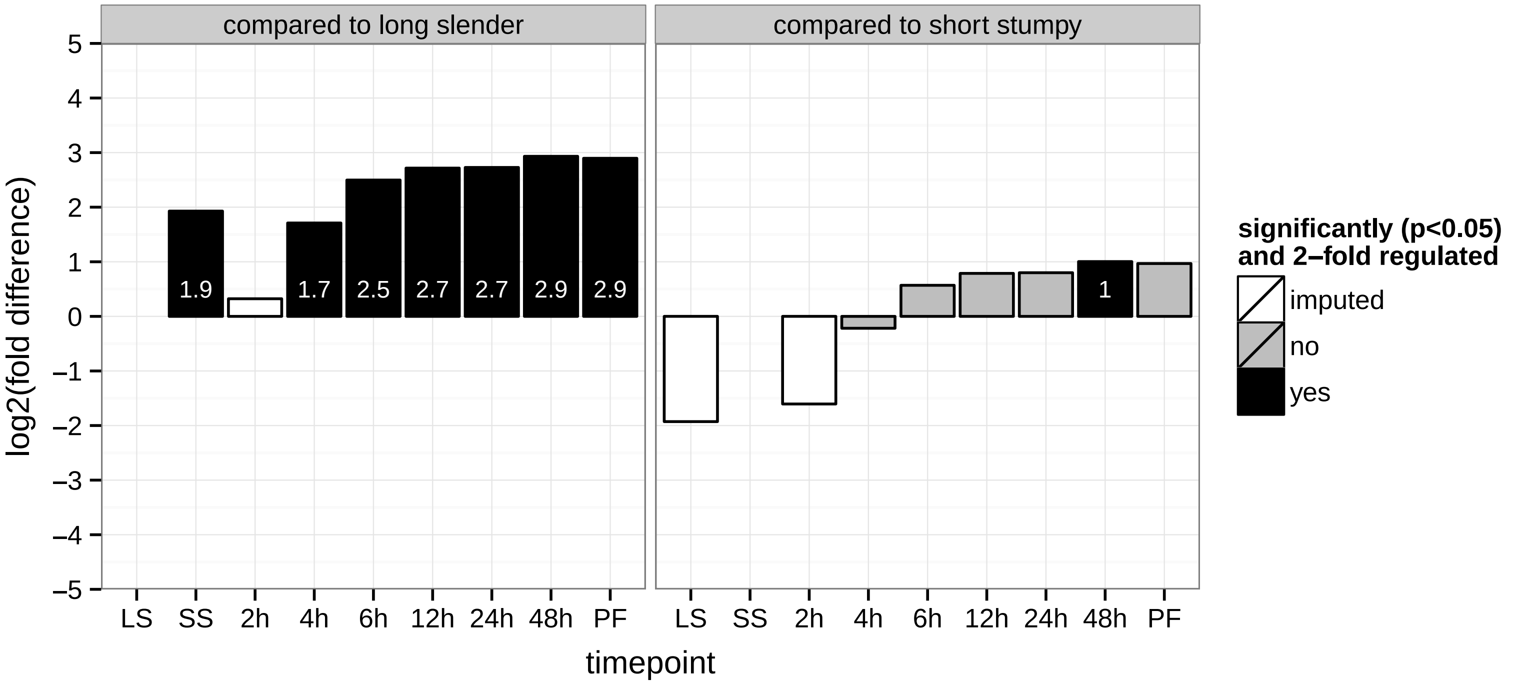

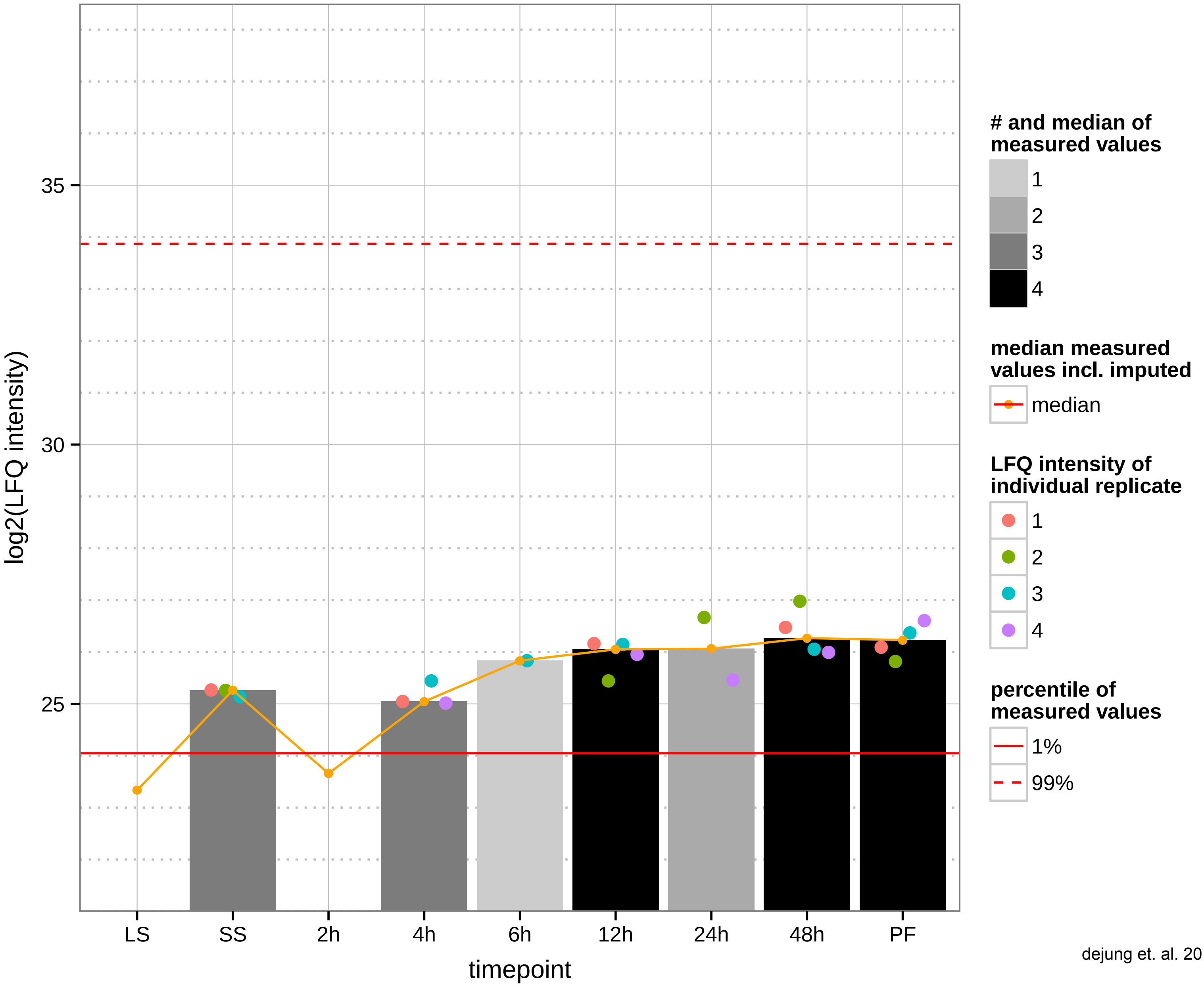

dejung et. al. 2015

general transcription factor IIB (tf2b)
Tb927.9.5710
AGOF: null
AGOC: null
AGOP: transcription from RNA polymerase II promoter, transcription, DNA−dependent
PGOF: null
PGOC: null
PGOP: null

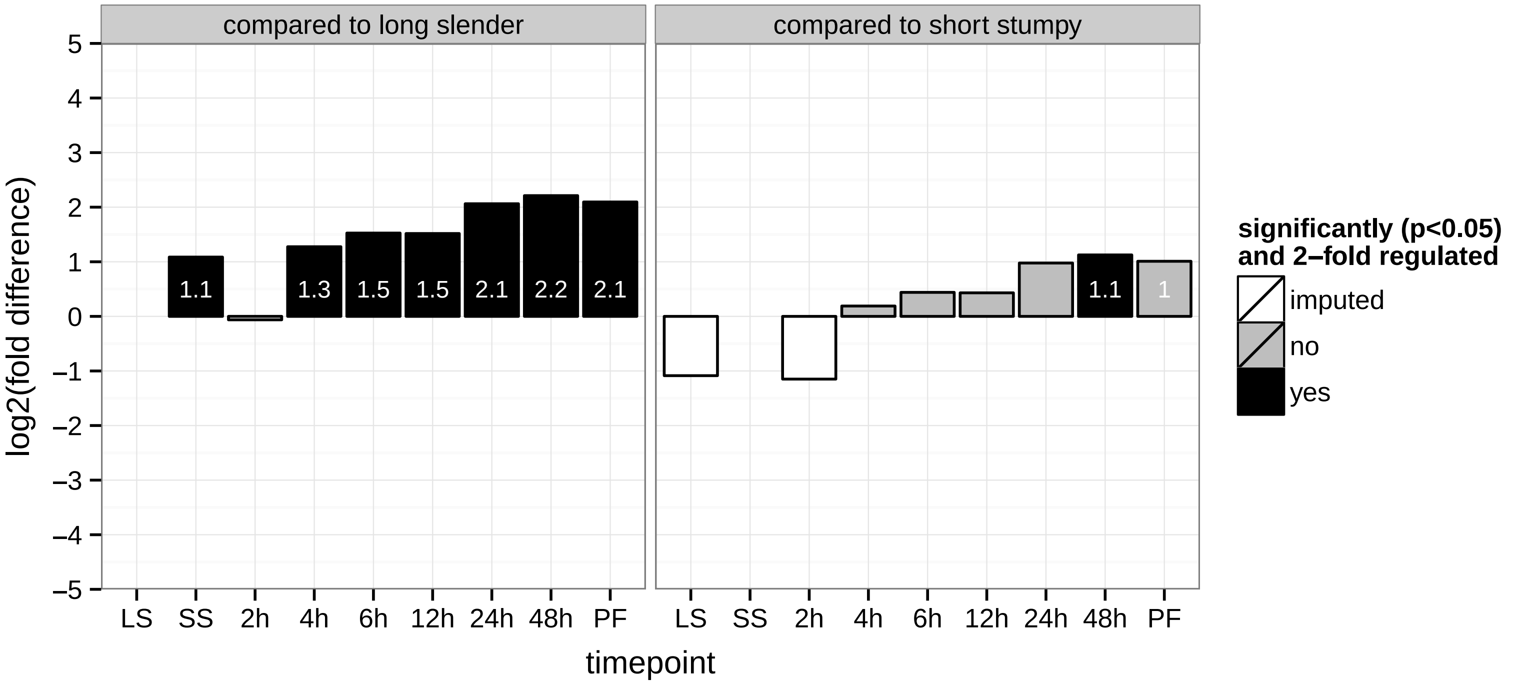

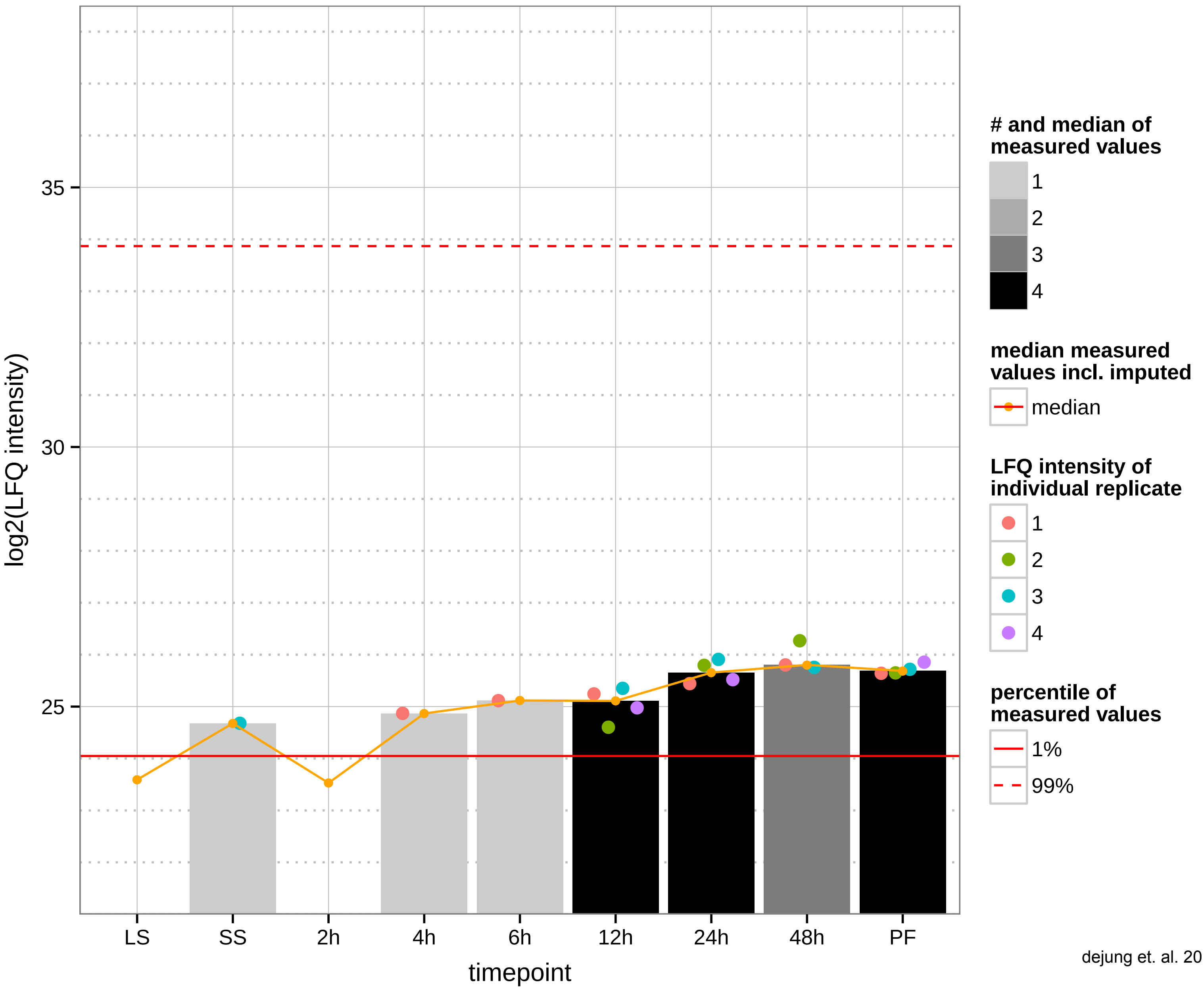

dejung et. al. 2015

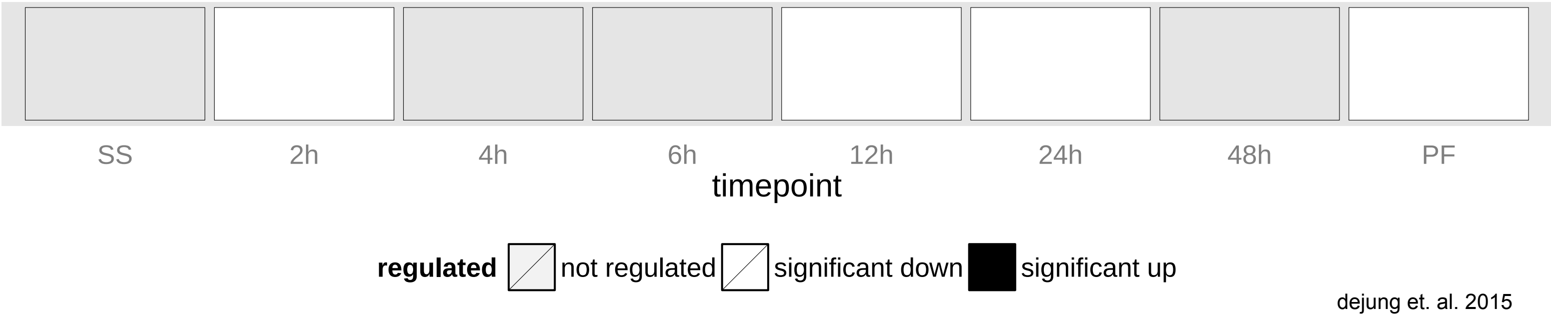

SS
2h
4h
6h
12h
24h
48h
PF
timepoint
regulated
not regulated
significant down
significant up
dejung et. al. 2015

hypothetical protein, conserved
Tb927.10.5000
AGOF: null
AGOC: null
AGOP: null
PGOF: null
PGOC: null
PGOP: null

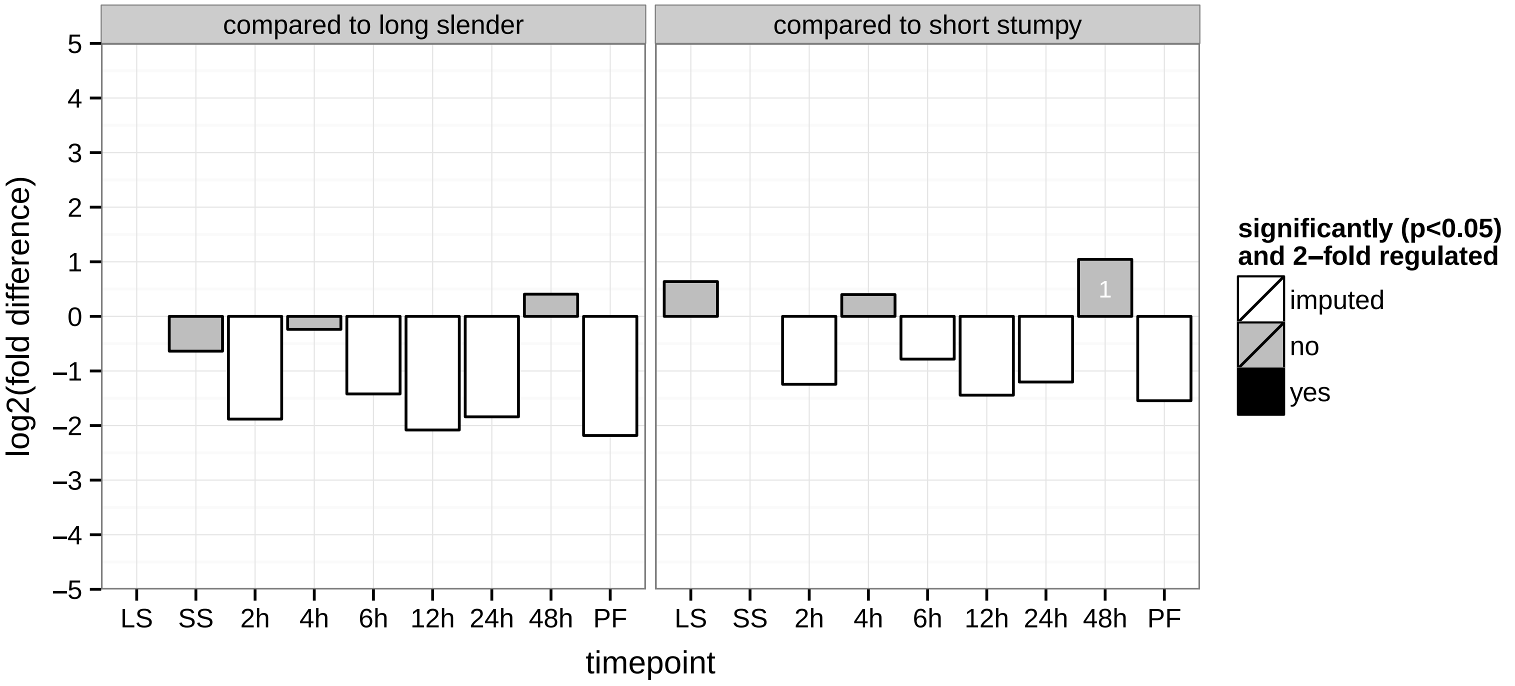

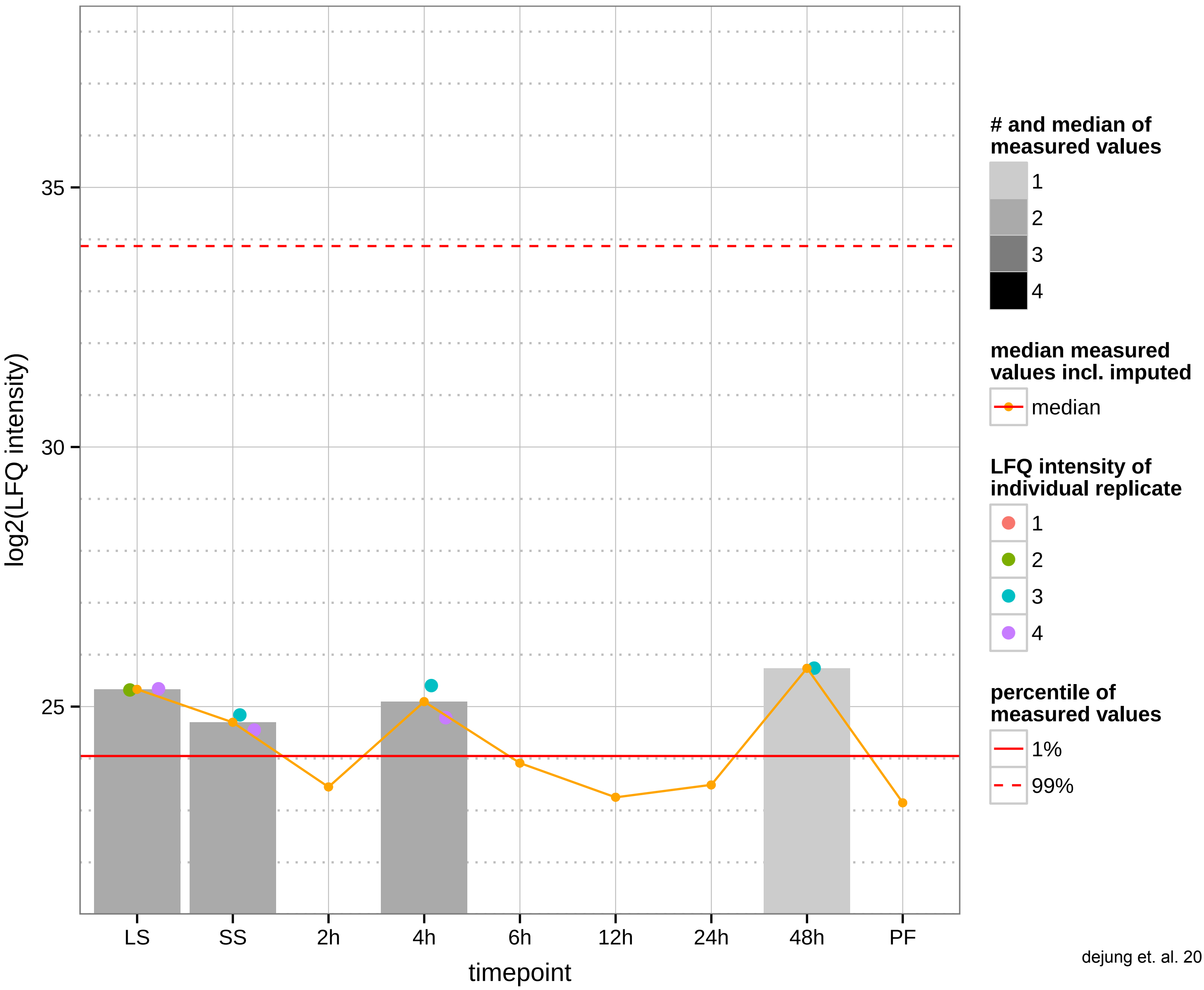

dejung et. al. 2015

hypothetical protein, conserved
Tb927.11.9950
AGOF: null
AGOC: null
AGOP: null
PGOF: null
PGOC: null
PGOP: null

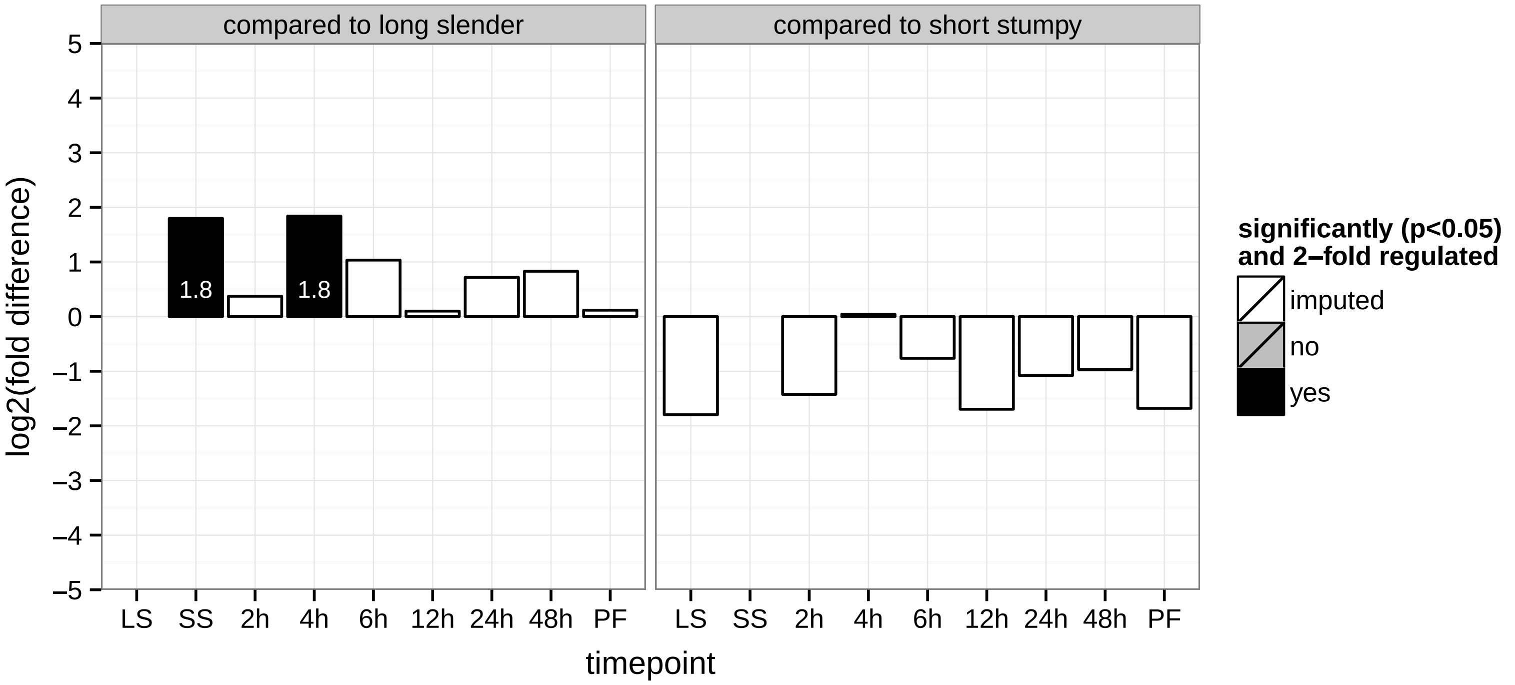

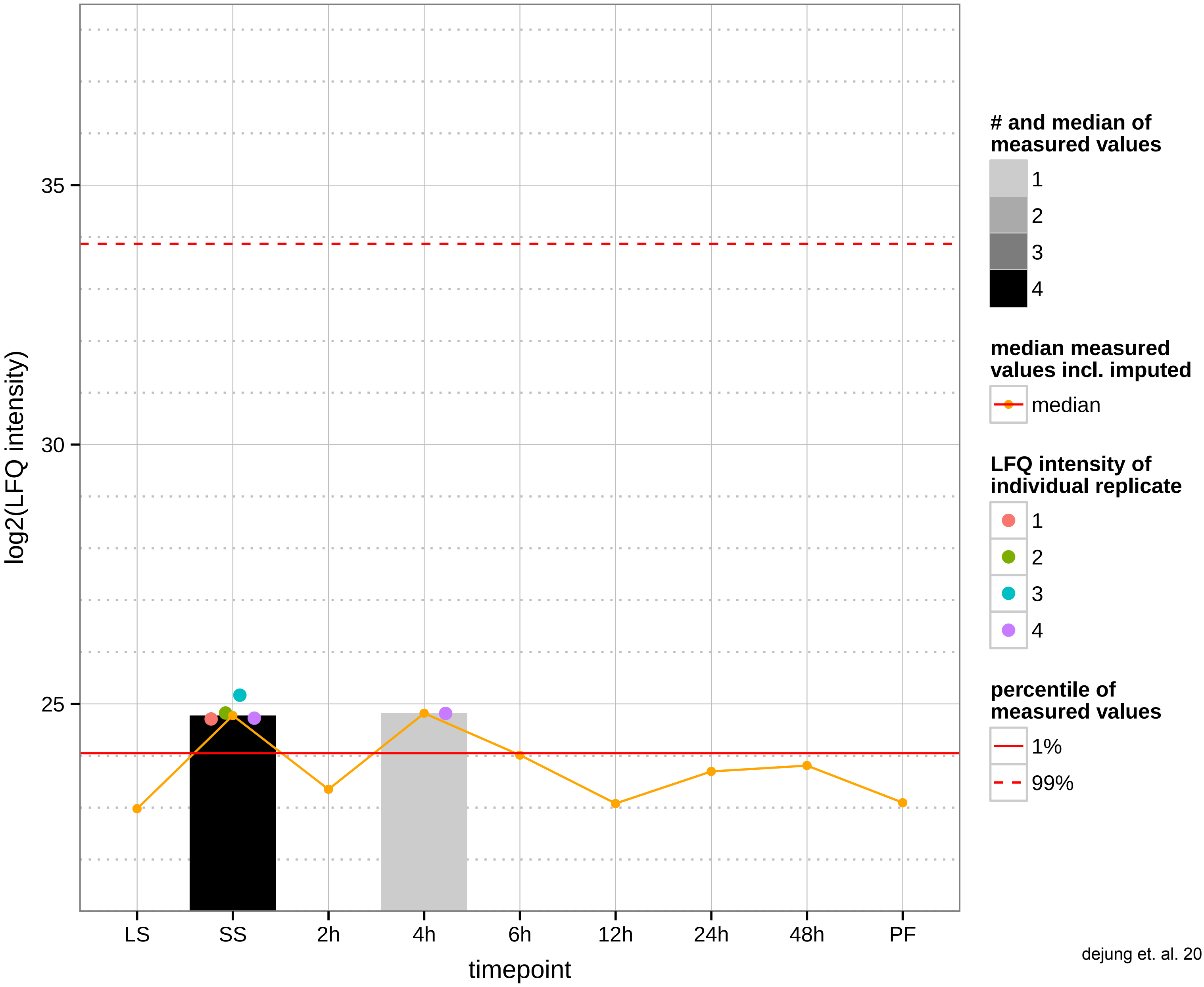

dejung et. al. 2015

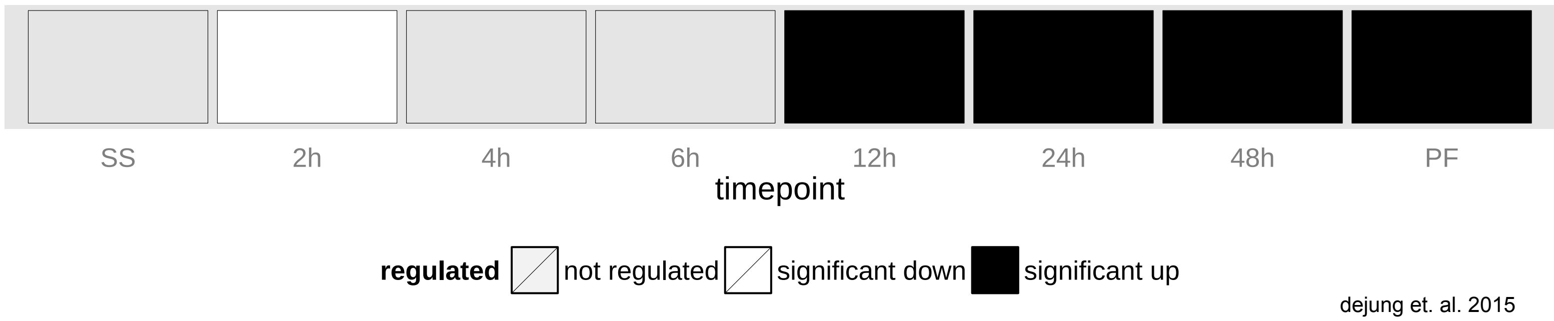

SS
2h
4h
6h
12h
24h
48h
PF
timepoint
regulated
not regulated
significant down
significant up
dejung et. al. 2015

hypothetical protein, conserved
Tb927.11.3860
AGOF: null
AGOC: mitochondrion
AGOP: null
PGOF: null
PGOC: null
PGOP: null

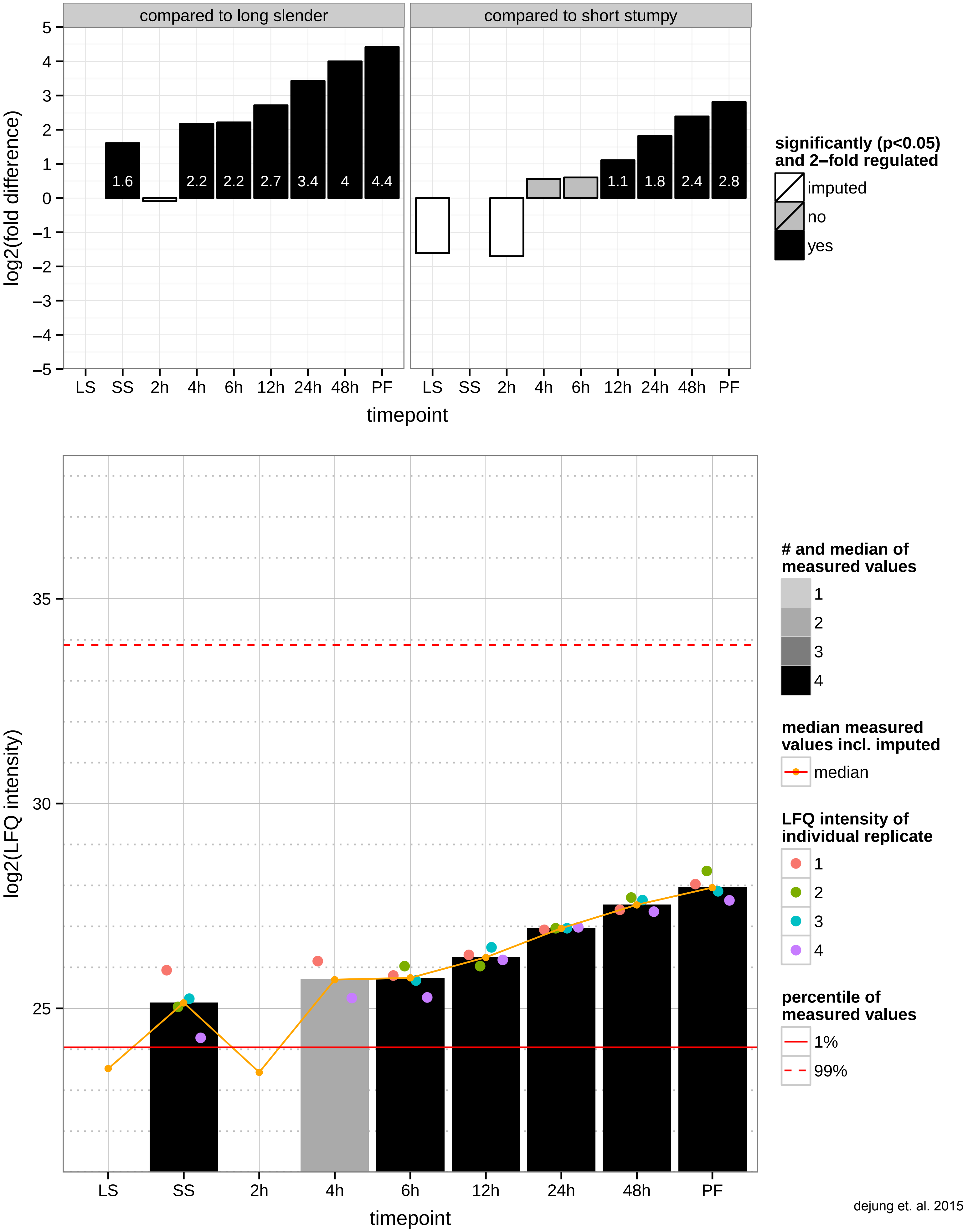

dejung et. al. 2015

cytochrome P450, putative
Tb927.3.680
AGOF: electron carrier activity, heme binding, iron ion binding, monooxygenase activity
AGOC: null
AGOP: null
PGOF: electron carrier activity, heme binding, iron ion binding, oxidoreductase activity, acting on paired donors, with incorpora
PGOC: null
PGOP: oxidation–reduction process

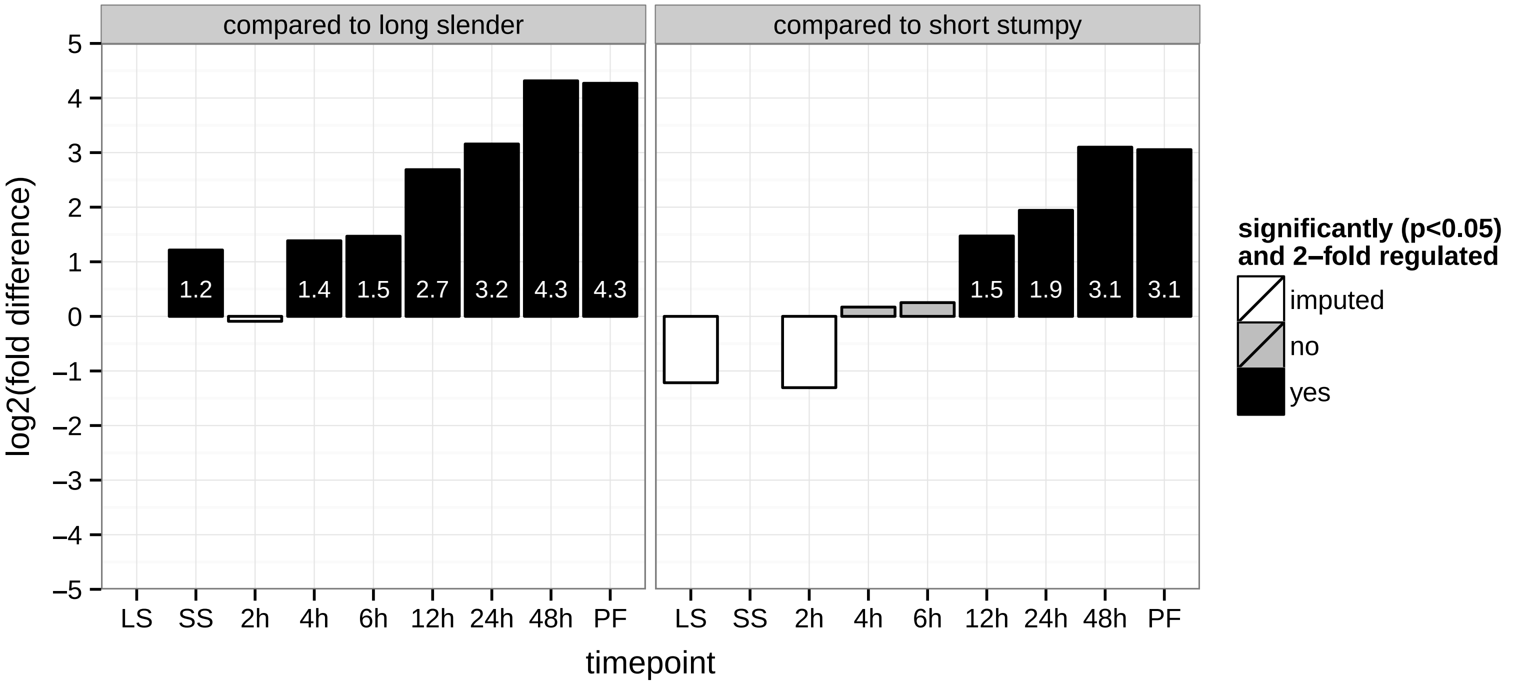

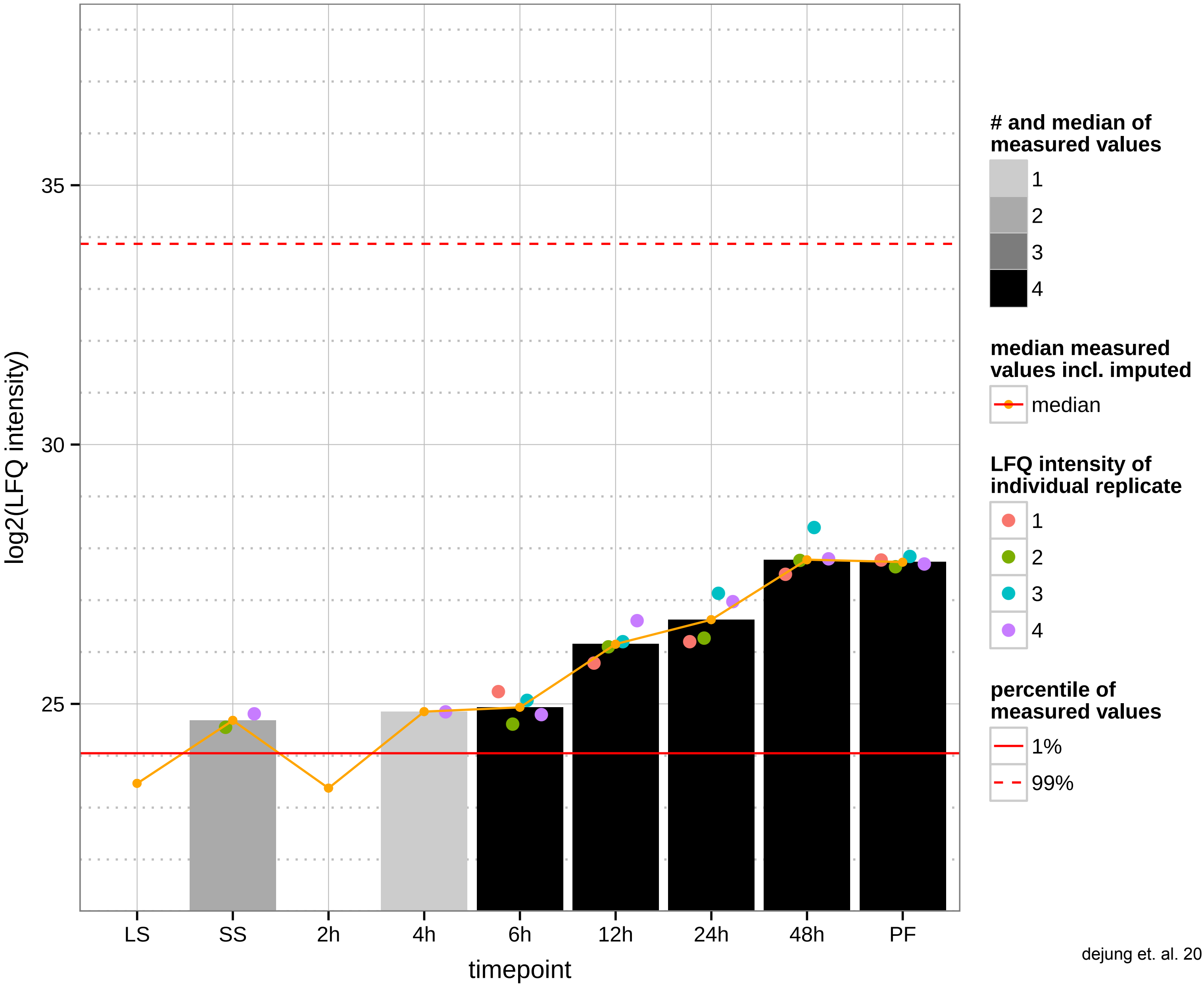

dejung et. al. 2015

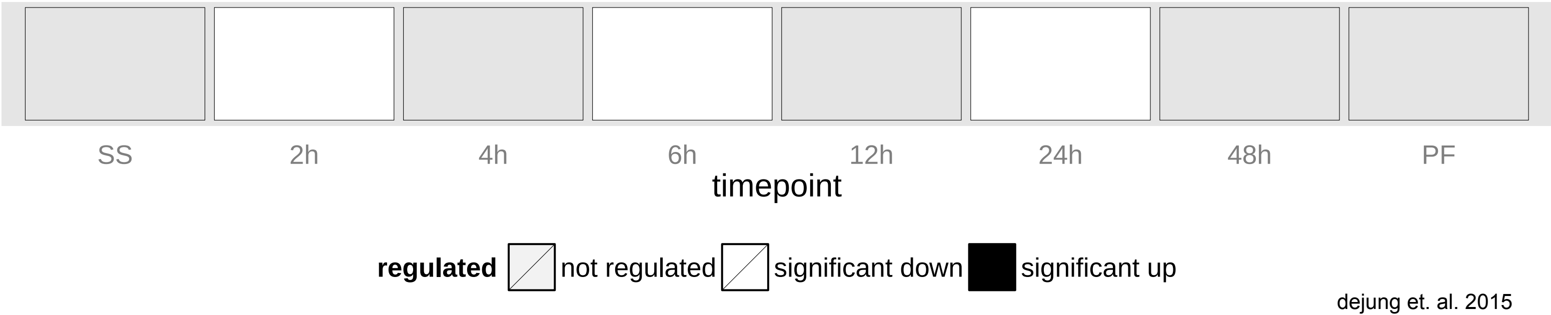
SS
2h
4h
6h
12h
24h
48h
PF
timepoint
regulated
not regulated
significant down
significant up
dejung et. al. 2015

hypothetical protein, conserved
Tb927.11.12720;Tb11.v5.0433
AGOF: null
AGOC: null
AGOP: null
PGOF: null
PGOC: null
PGOP: null

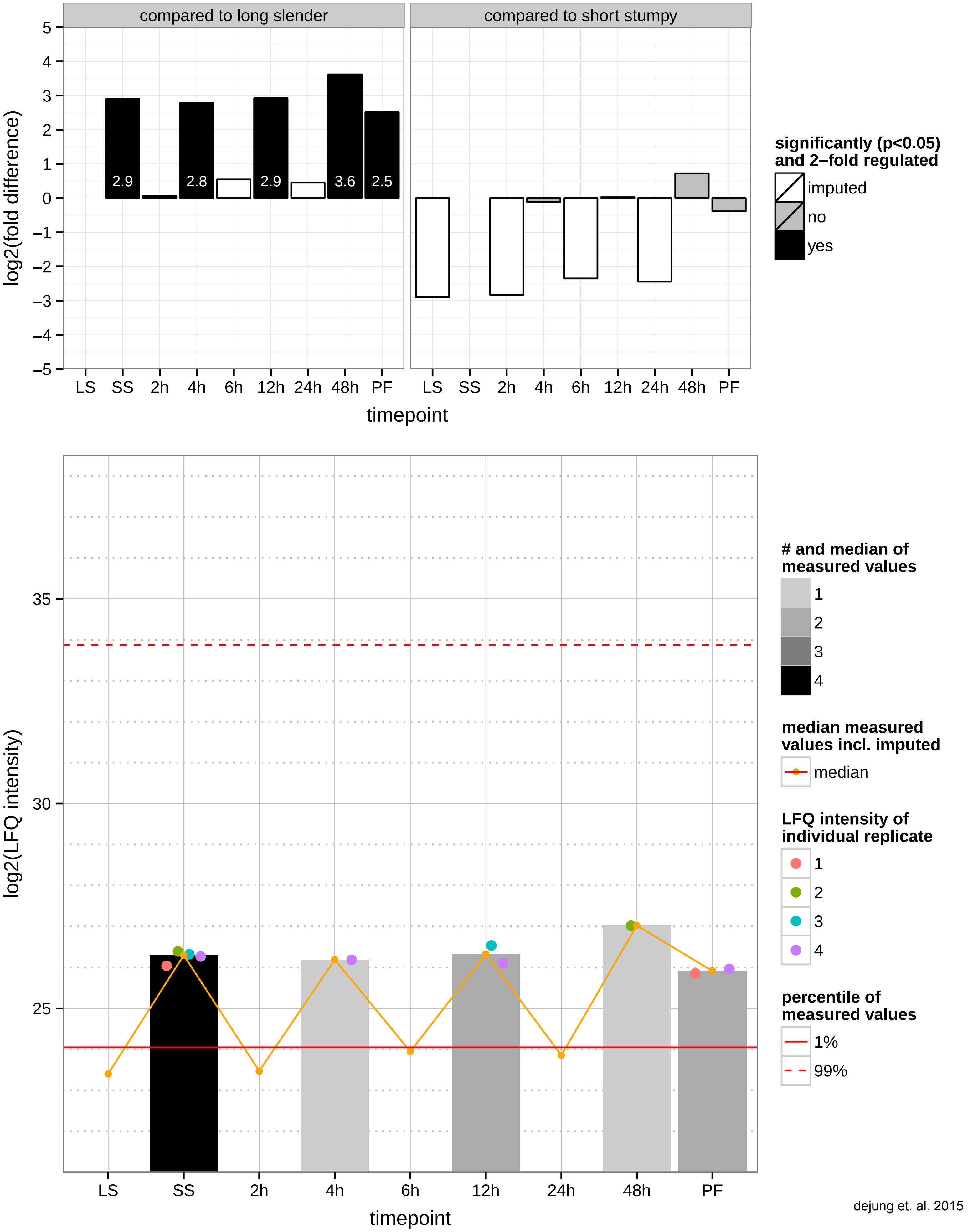

dejung et. al. 2015

hypothetical protein, conserved
Tb927.4.2920
AGOF: null
AGOC: null
AGOP: null
PGOF: null
PGOC: null
PGOP: null

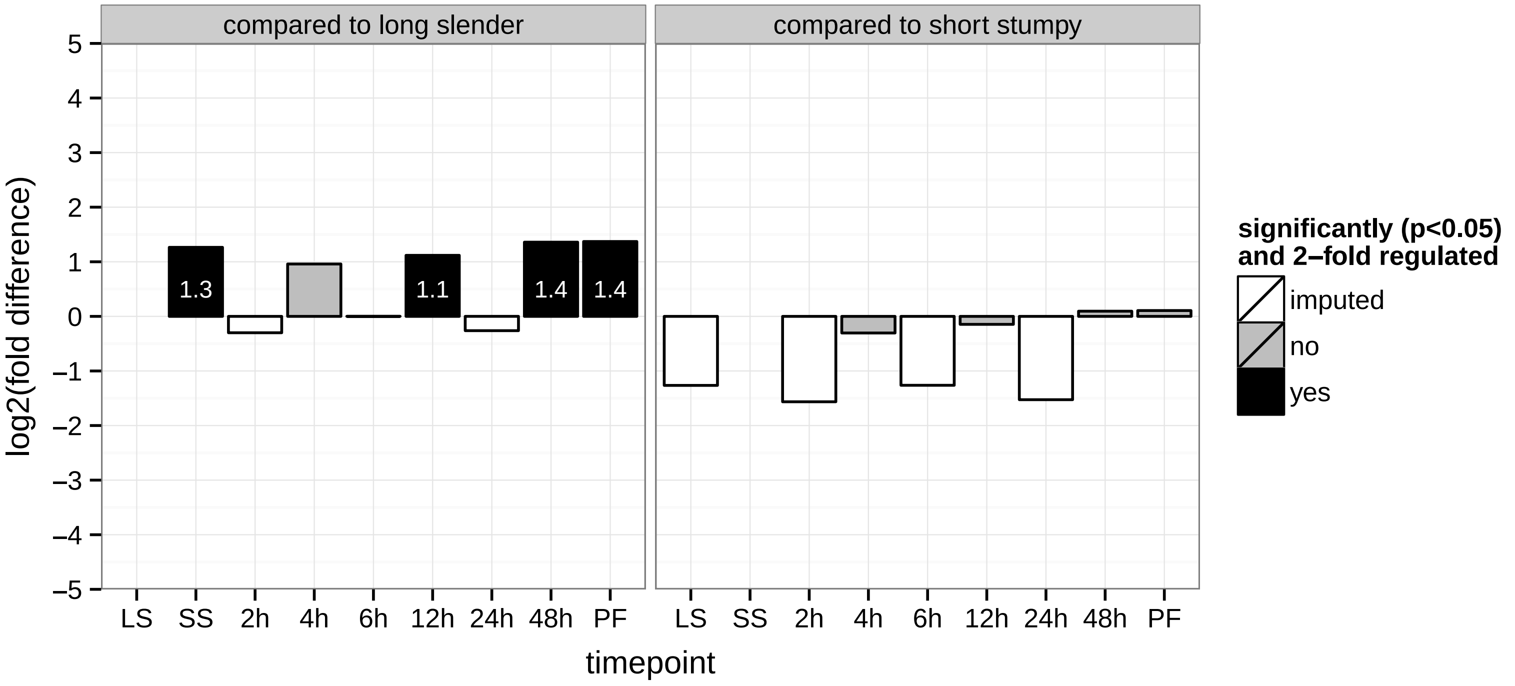

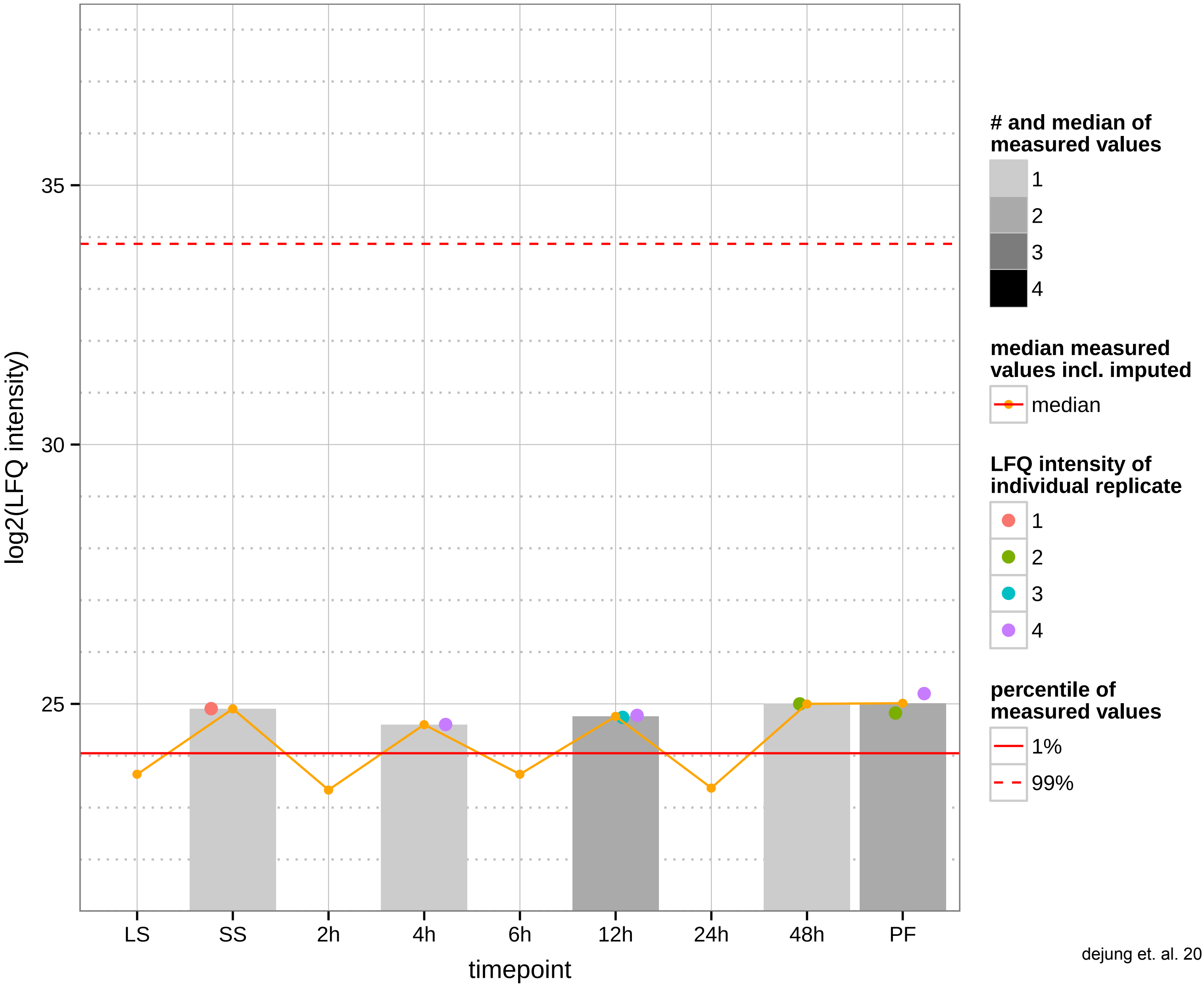

dejung et. al. 2015

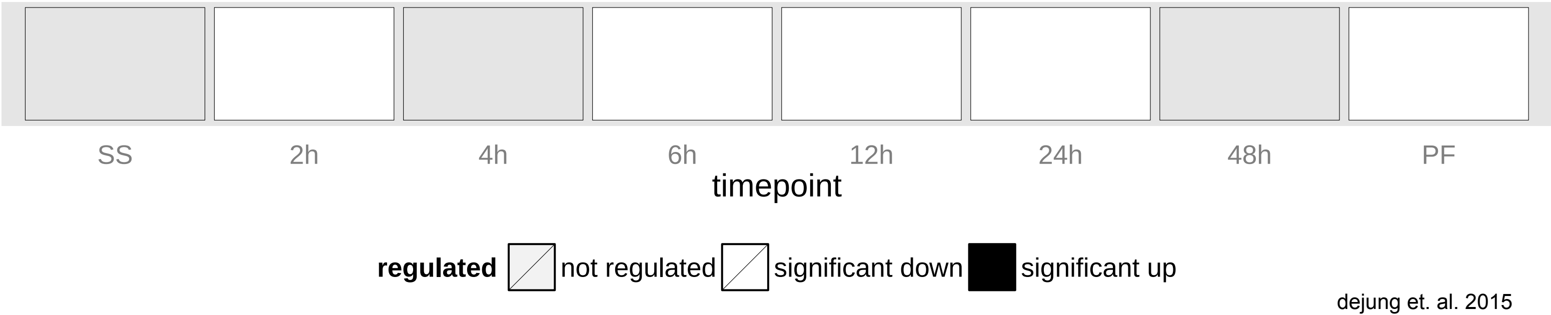
SS
2h
4h
6h
12h
24h
48h
PF
timepoint
regulated
not regulated
significant down
significant up
dejung et. al. 2015

hypothetical protein, conserved
Tb927.3.5190
AGOF: null
AGOC: null
AGOP: null
PGOF: null
PGOC: null
PGOP: null

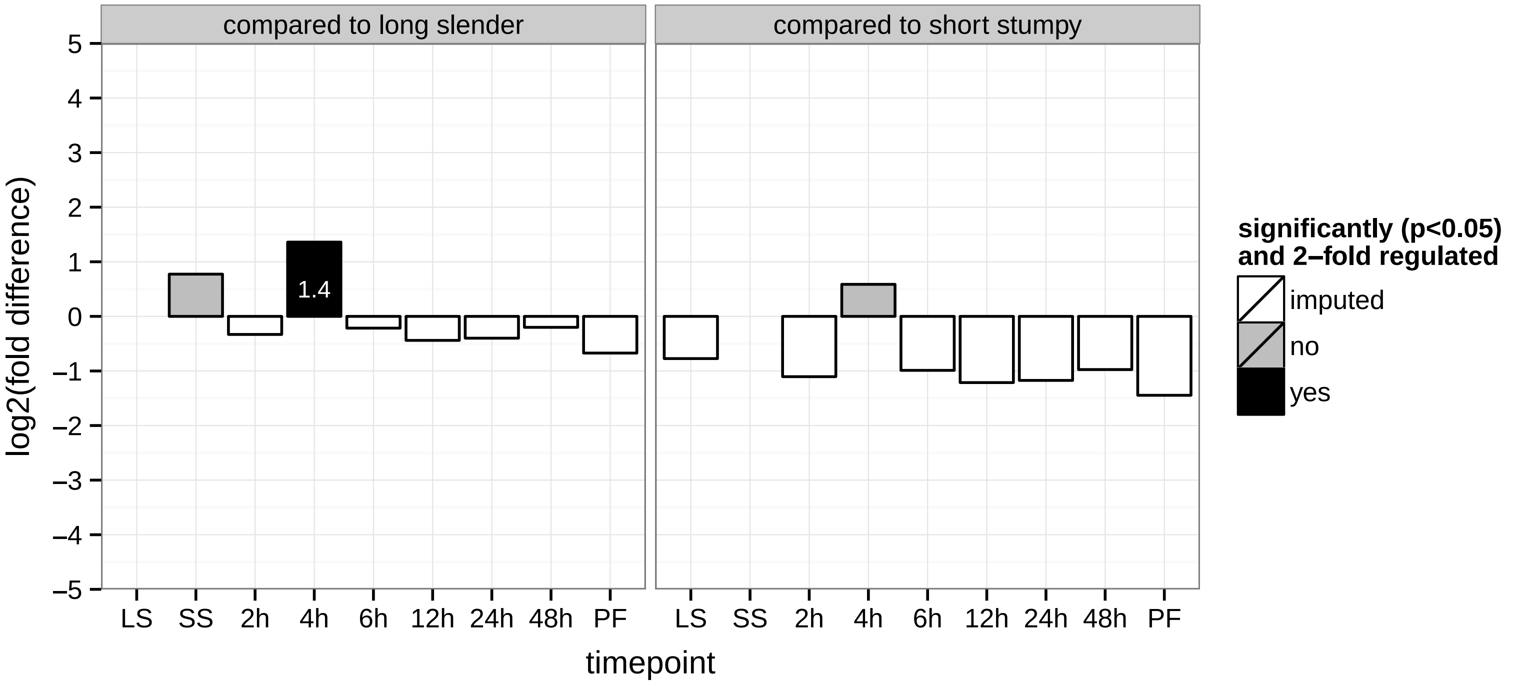

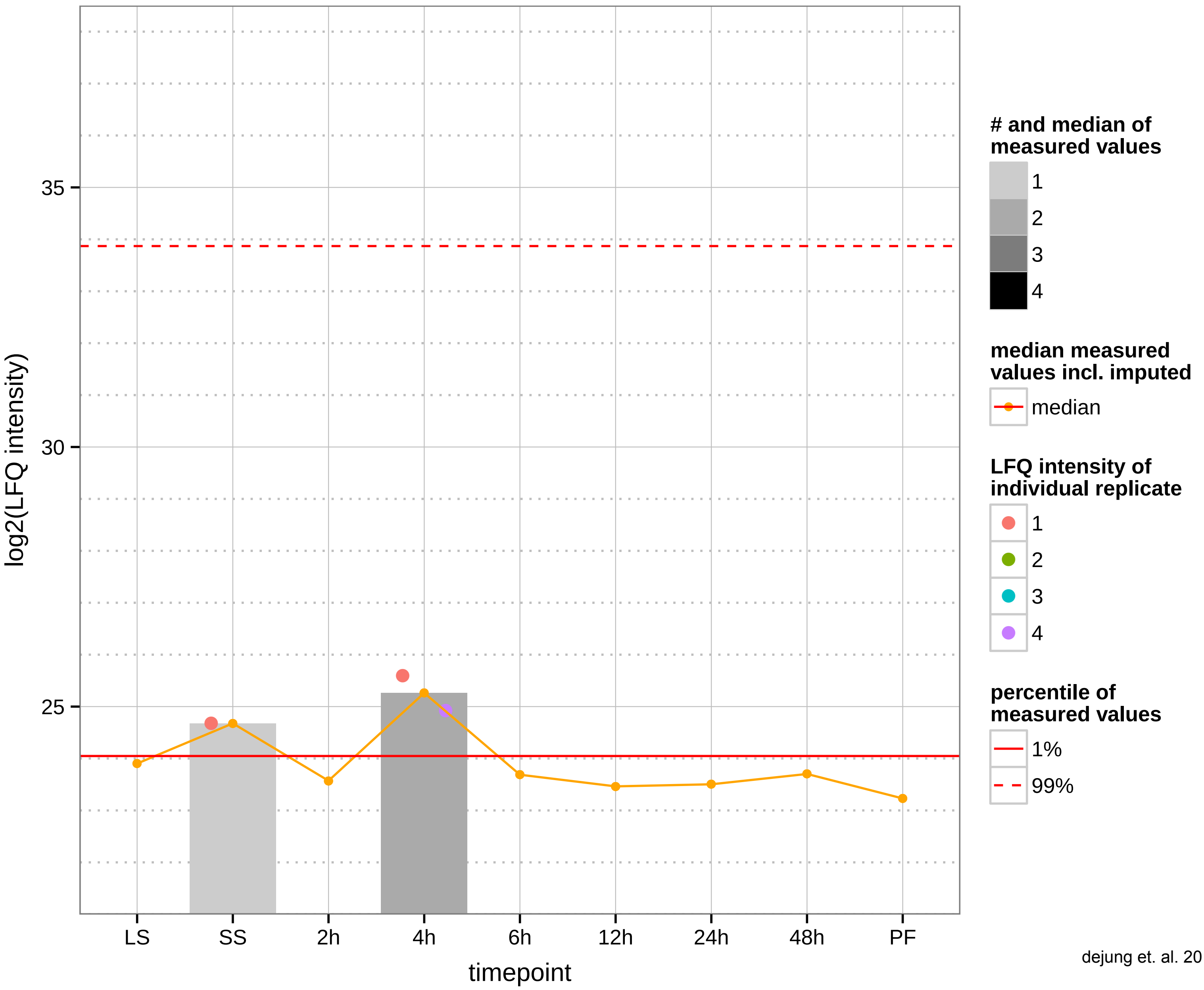

dejung et. al. 2015

hypothetical protein, conserved
Tb927.8.3930
AGOF: null
AGOC: null
AGOP: null
PGOF: null
PGOC: null
PGOP: null

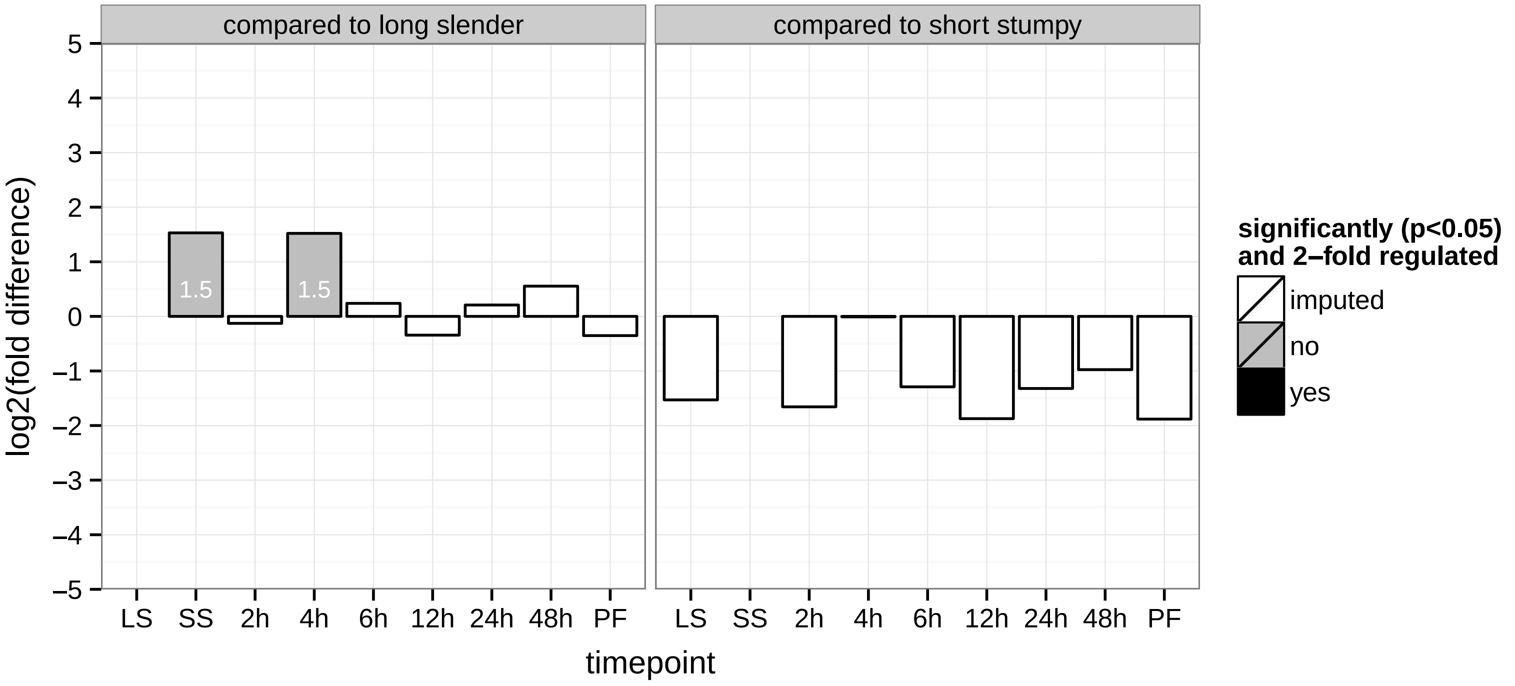

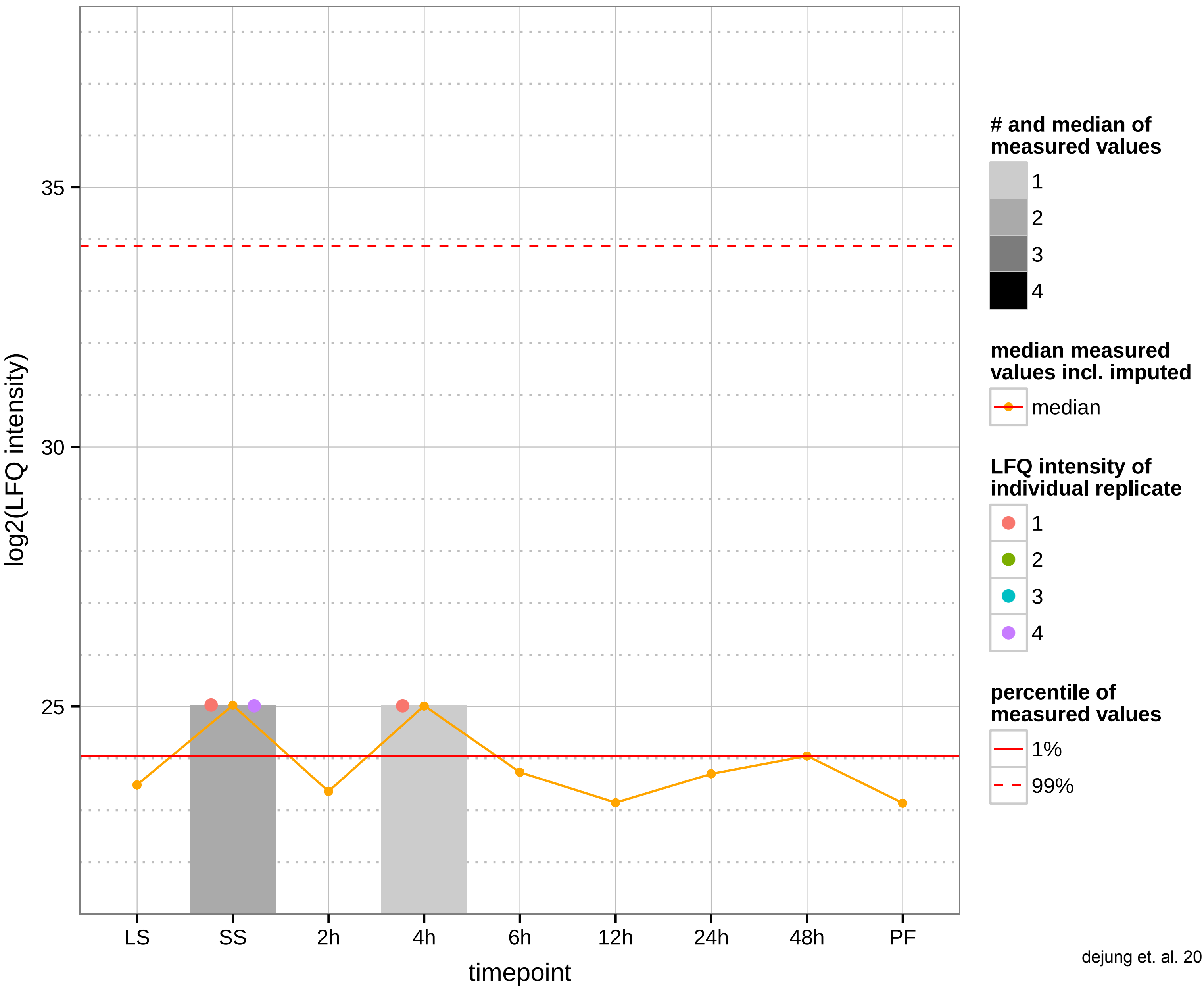

dejung et. al. 2015

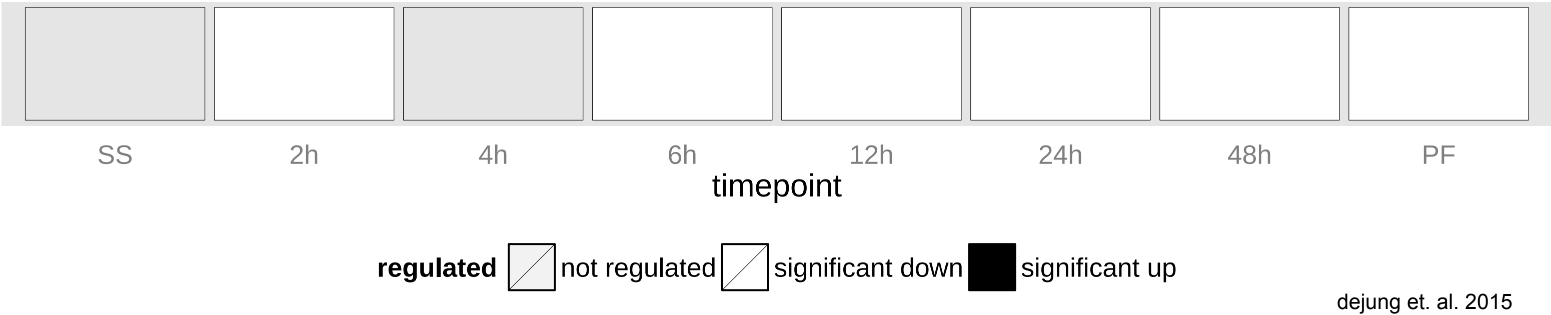
SS
2h
4h
6h
12h
24h
48h
PF
timepoint
regulated
not regulated
significant down
significant up
dejung et. al. 2015

conserved protein, unknown function
Tb927.1.1500
AGOF: null
AGOC: null
AGOP: null
PGOF: null
PGOC: null
PGOP: null

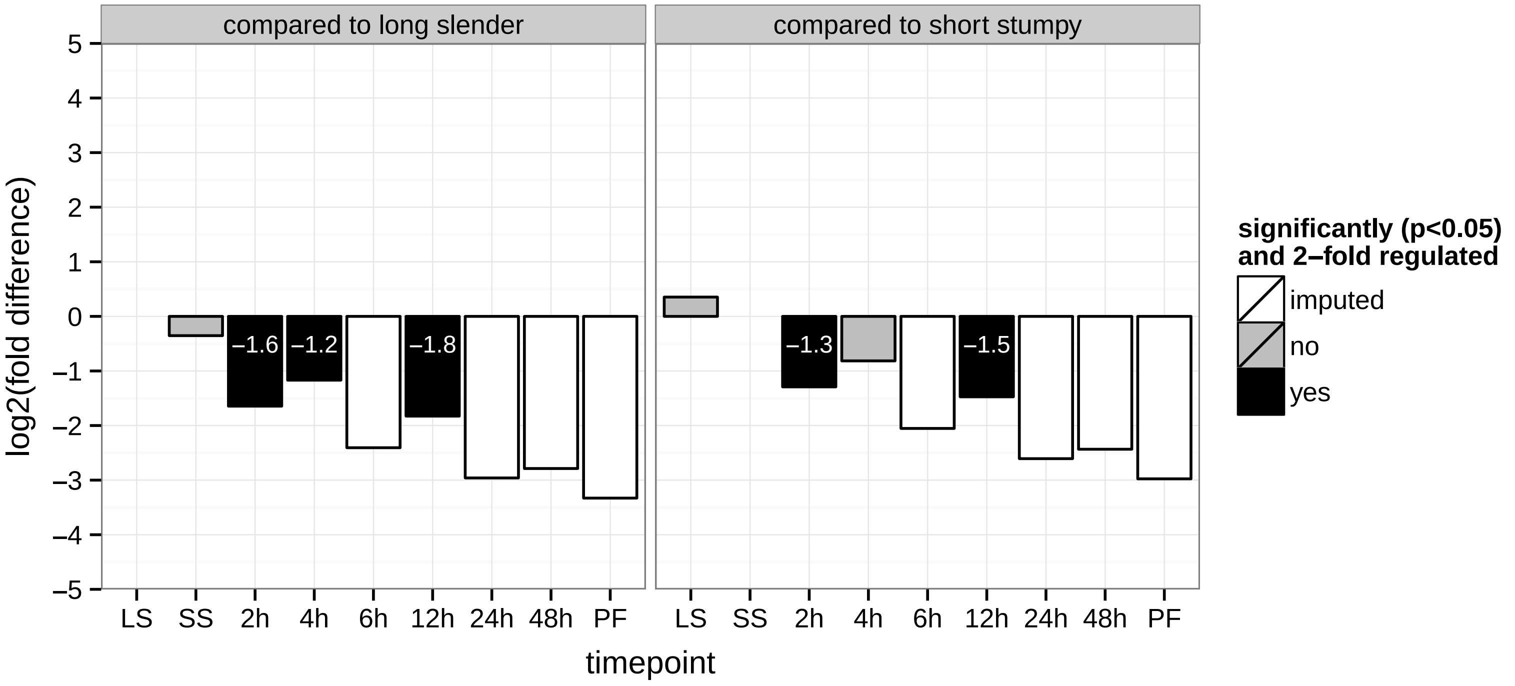

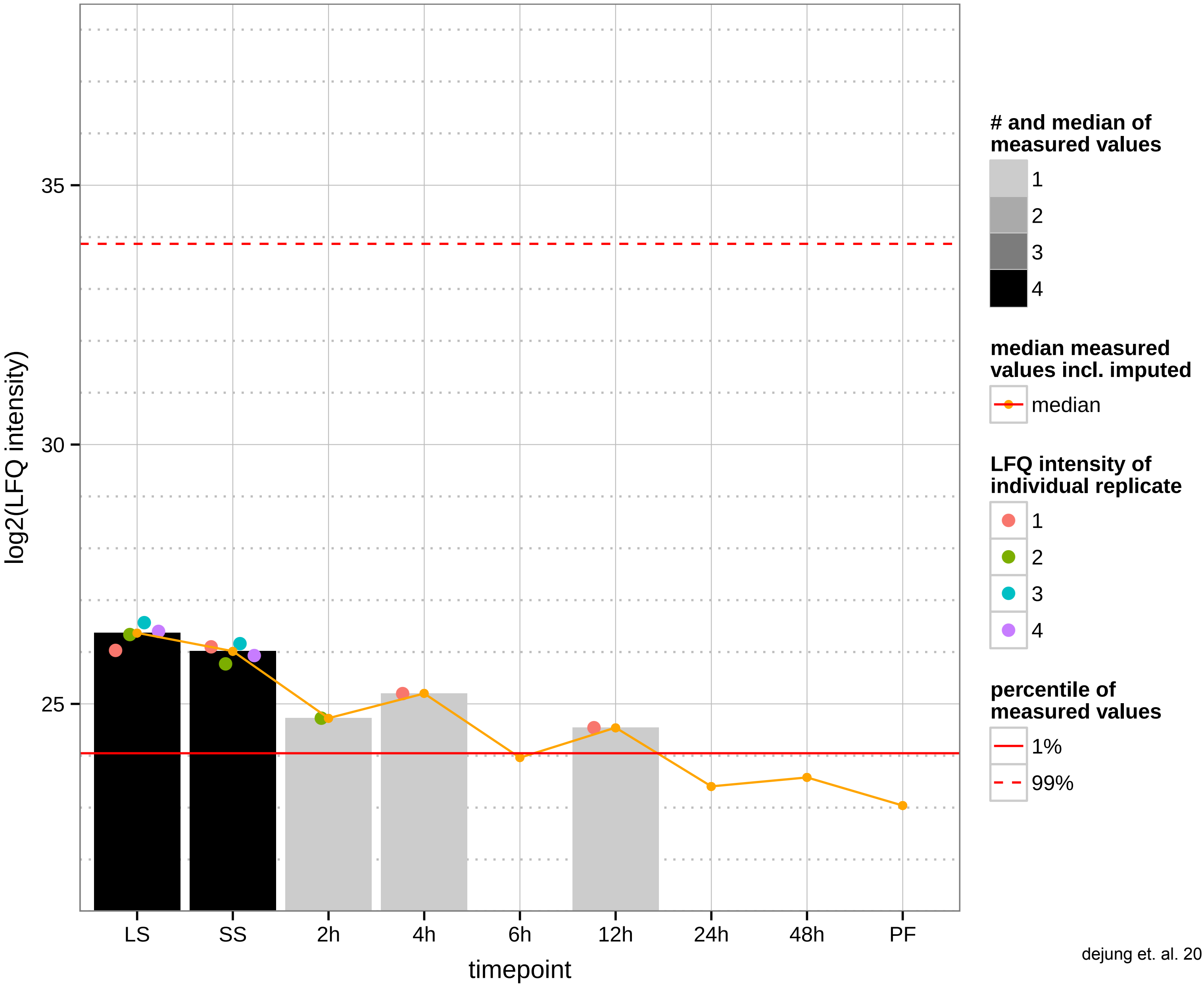

dejung et. al. 2015

hypothetical PIN domain−containing protein
Tb927.1.1820
AGOF: RNA binding
AGOC: null
AGOP: null
PGOF: null
PGOC: null
PGOP: null

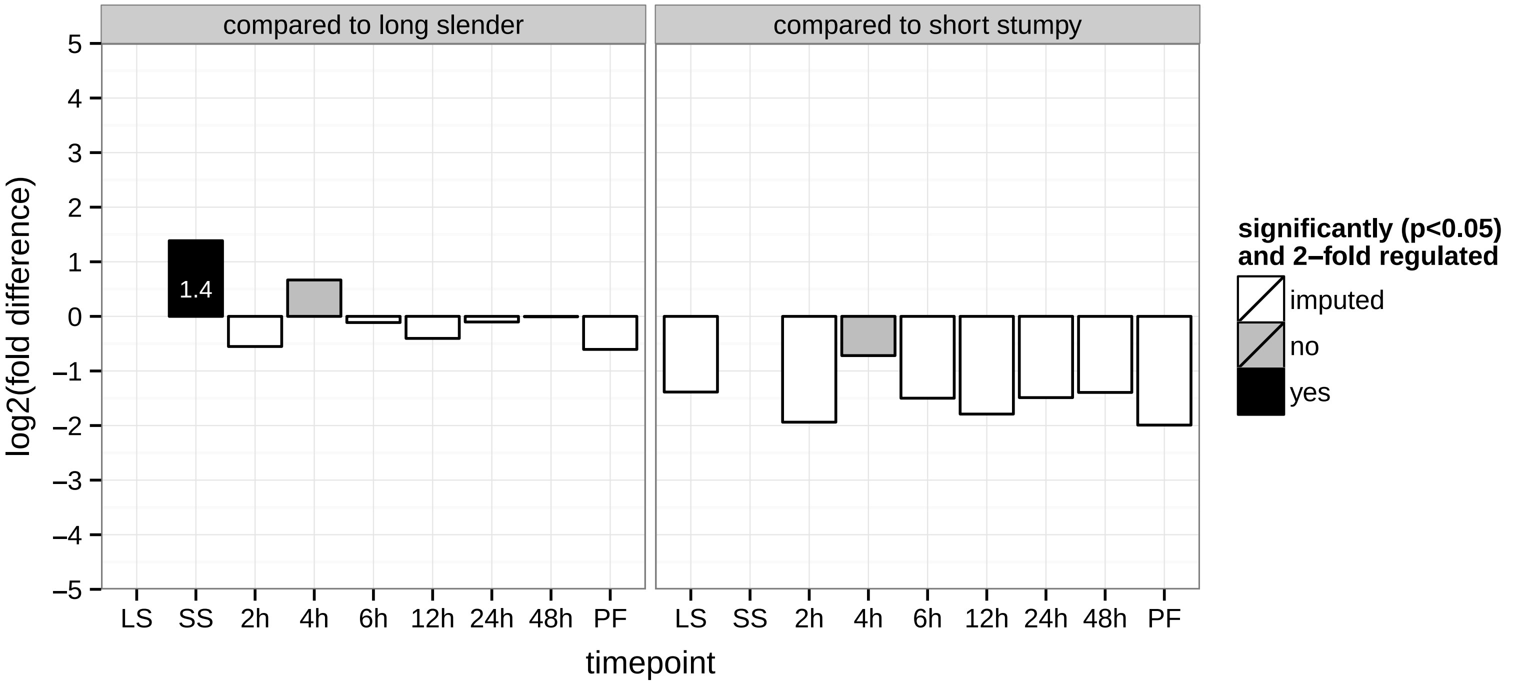

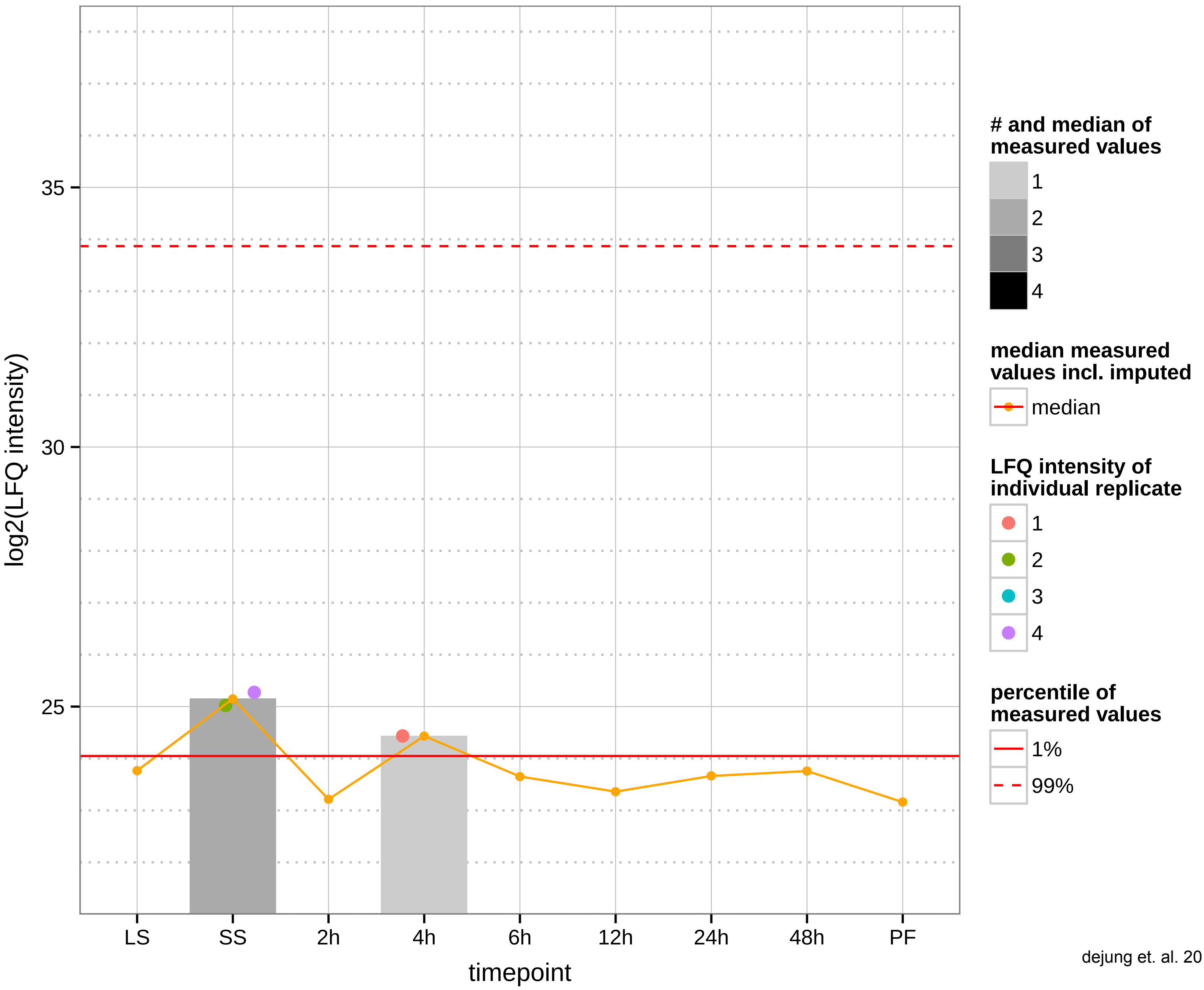

dejung et. al. 2015

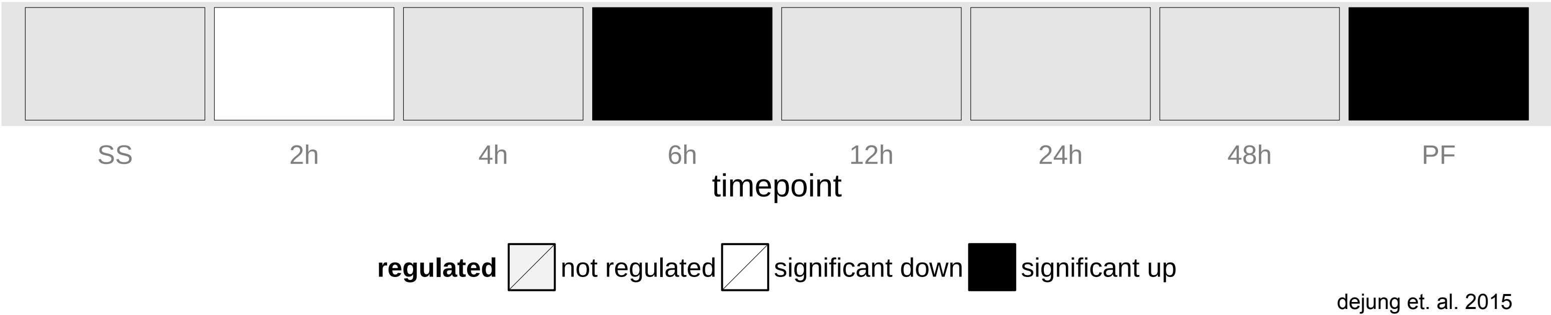
SS
2h
4h
6h
12h
24h
48h
PF
timepoint
regulated
not regulated
significant down
significant up
dejung et. al. 2015

zinc finger protein family member, putative (FIP1)
Tb927.5.4320
AGOF: RNA binding, zinc ion binding
AGOC: null
AGOP: null
PGOF: nucleic acid binding, zinc ion binding
PGOC: null
PGOP: null

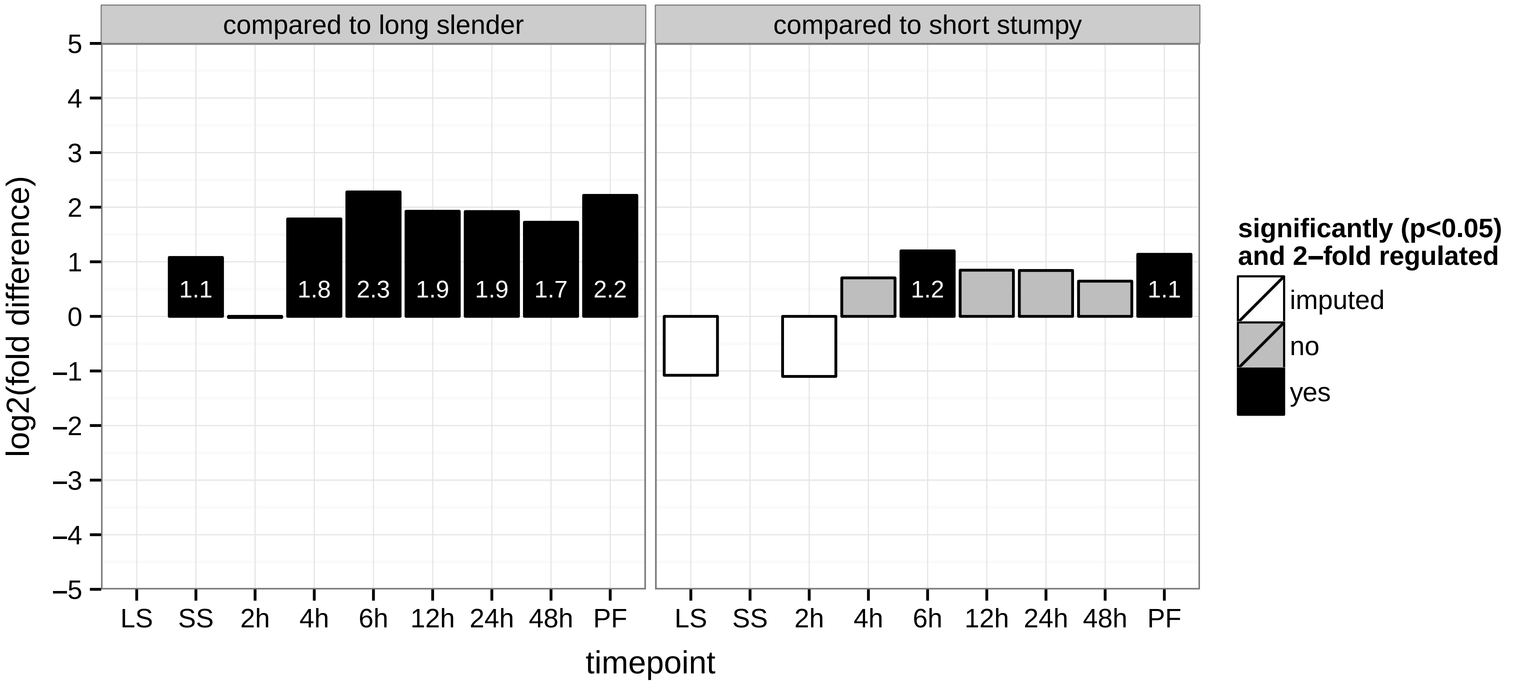

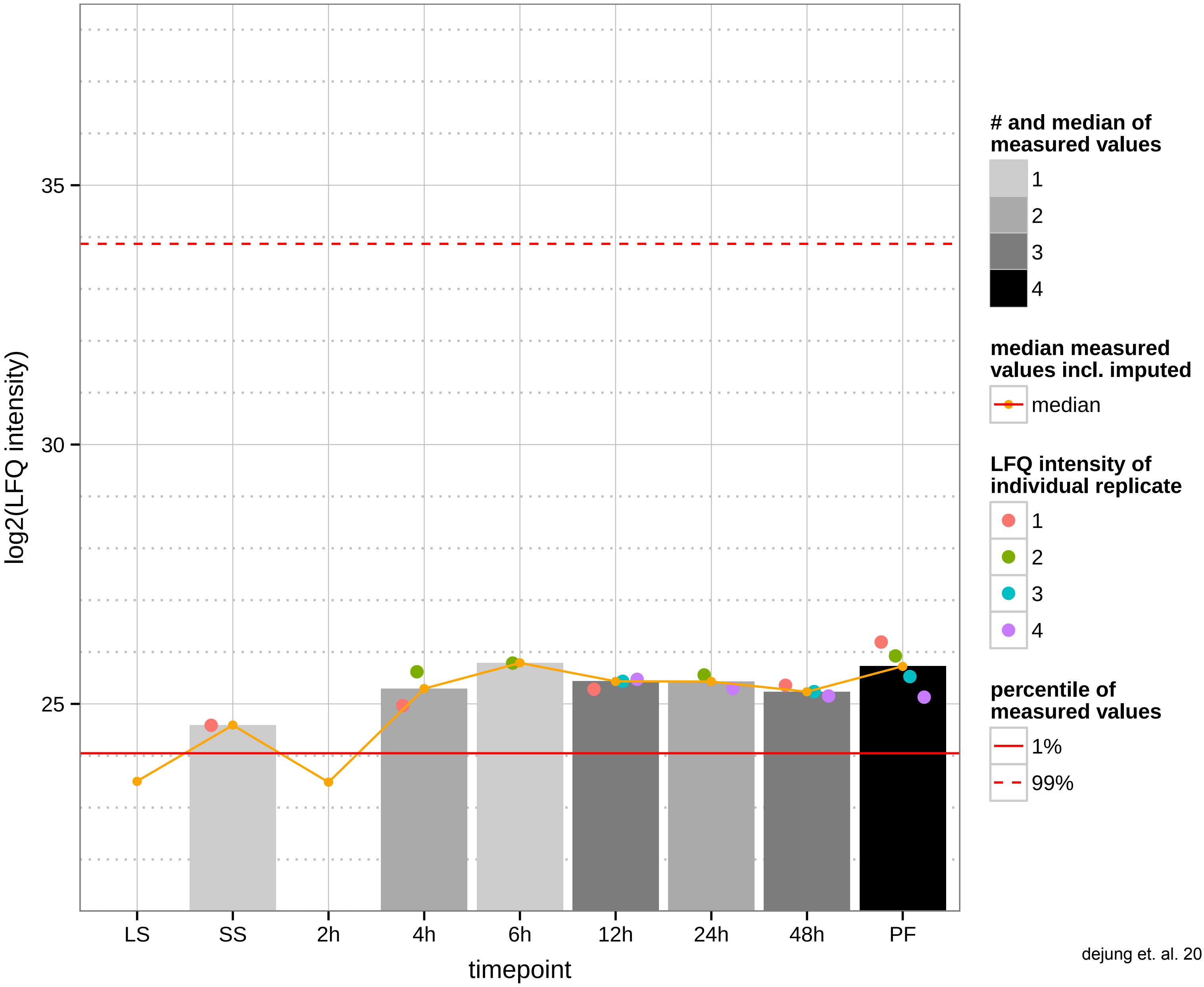

dejung et. al. 2015

hypothetical protein, conserved
Tb927.8.3960
AGOF: null
AGOC: mitochondrion
AGOP: null
PGOF: null
PGOC: null
PGOP: null

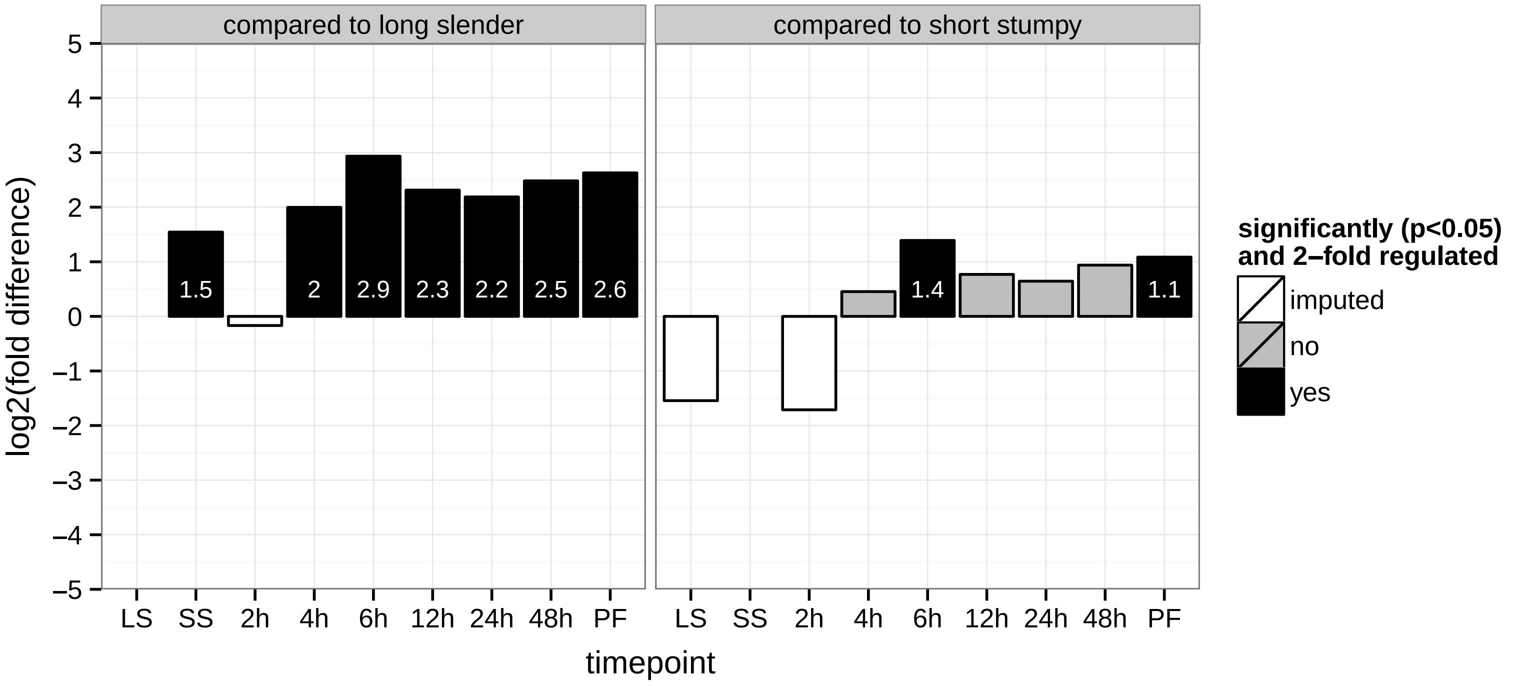

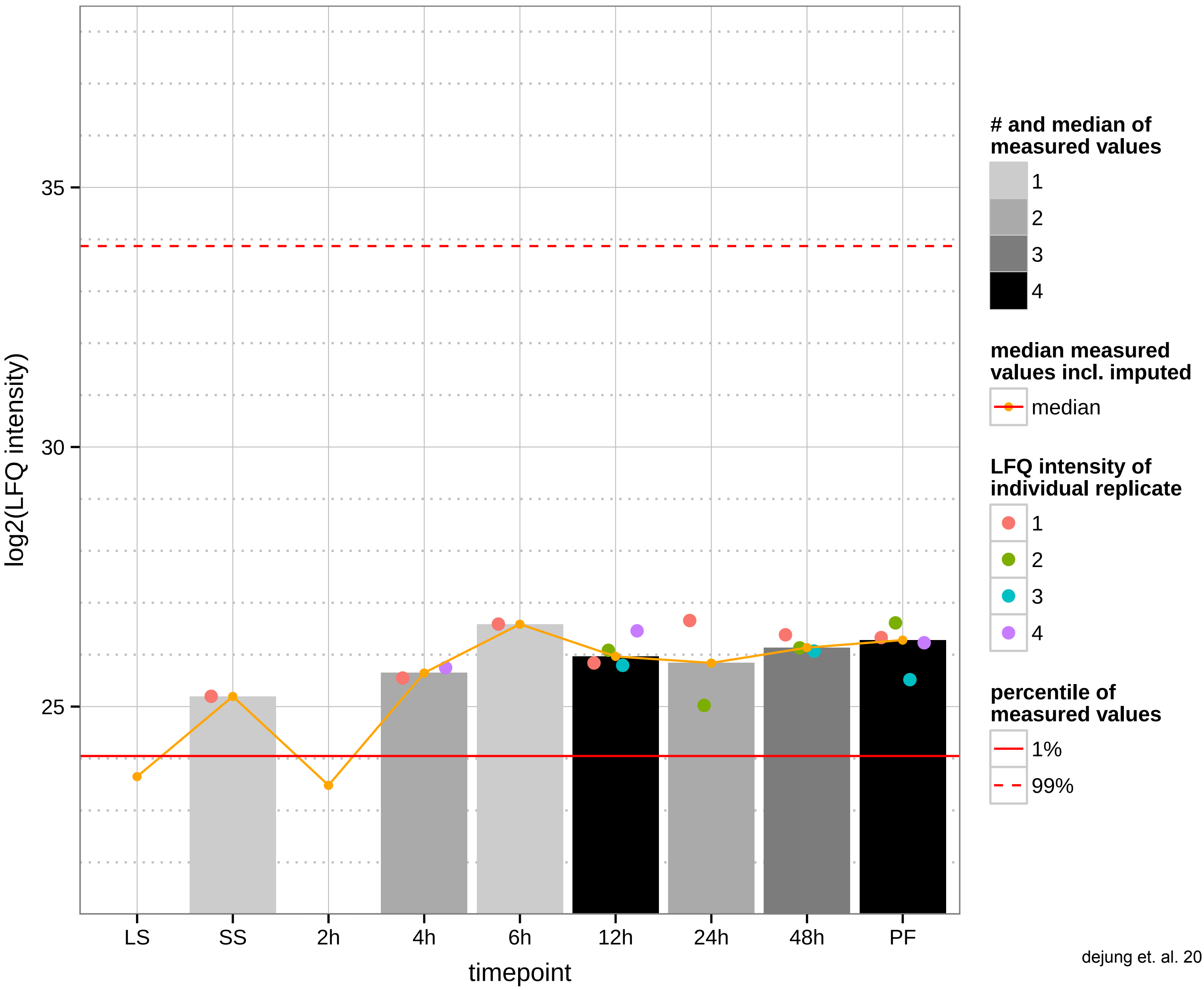

dejung et. al. 2015

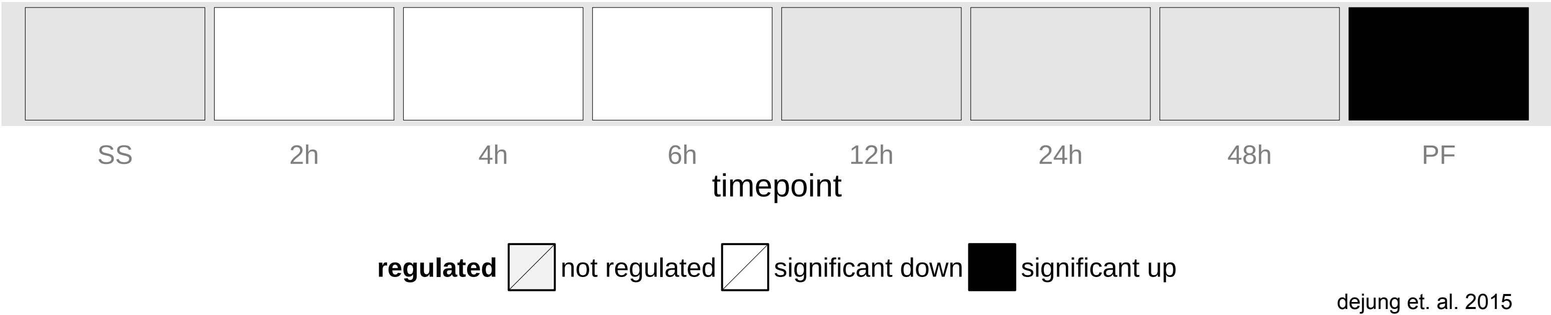
SS
2h
4h
6h
12h
24h
48h
PF
timepoint
regulated
not regulated
significant down
significant up
dejung et. al. 2015

hypothetical protein, conserved
Tb927.5.2540;Tb11.v5.0574
AGOF: null
AGOC: null
AGOP: null
PGOF: null
PGOC: null
PGOP: null

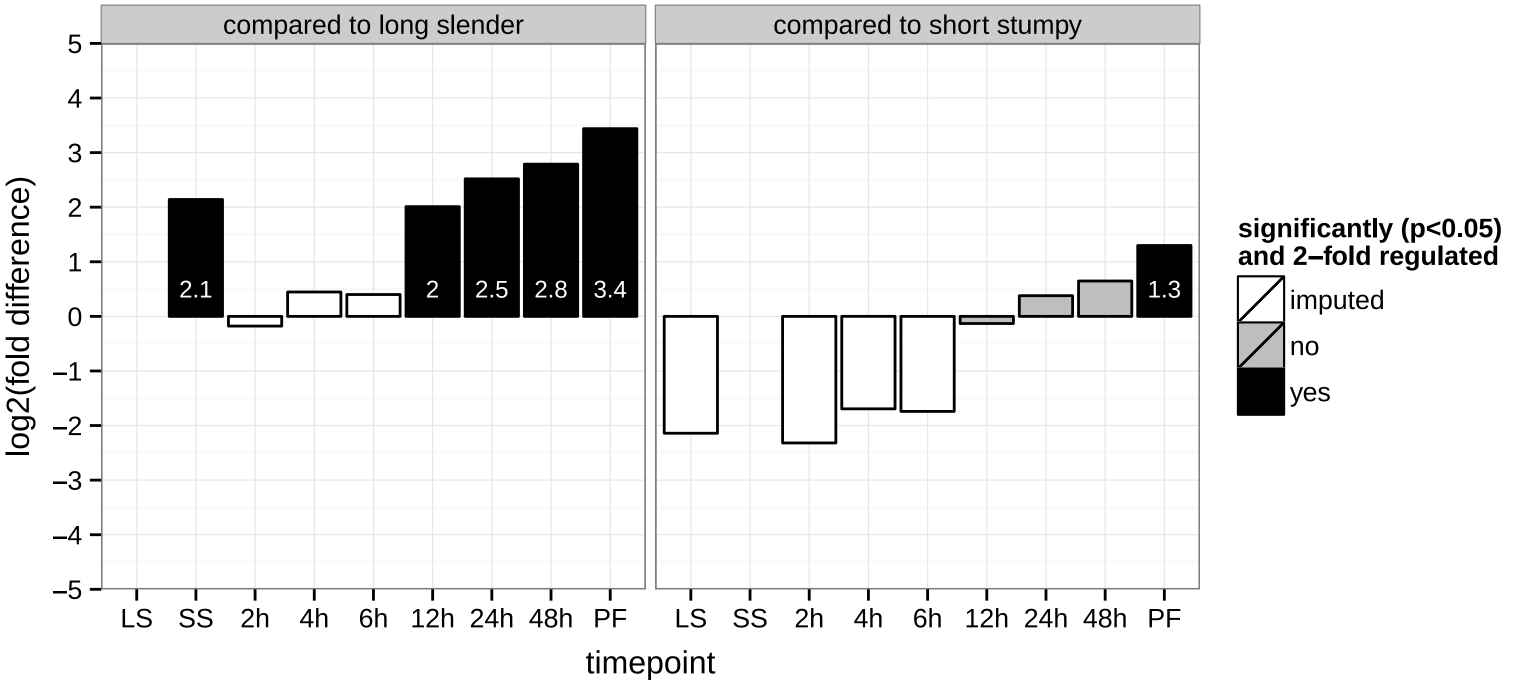

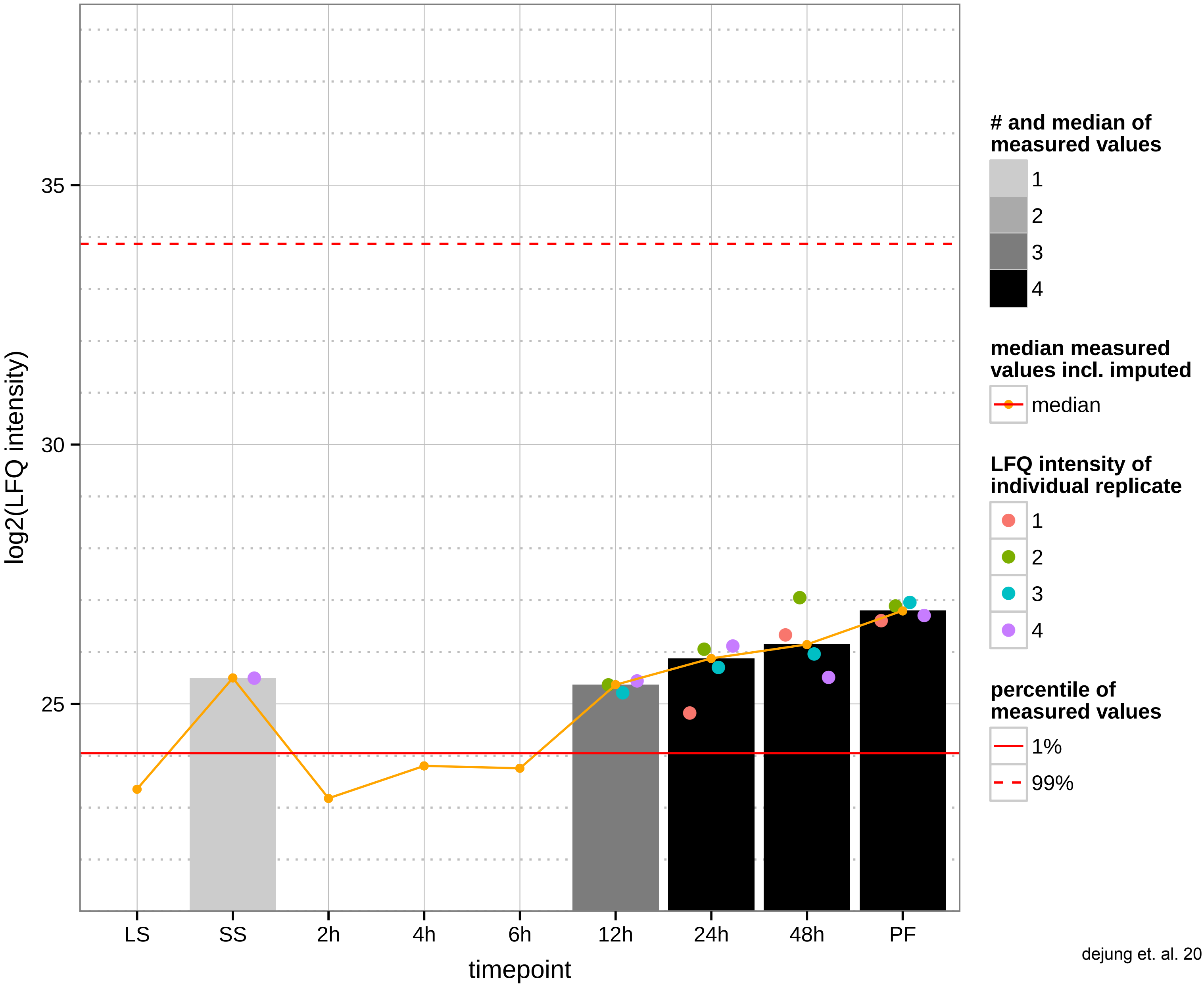

dejung et. al. 2015

hypothetical protein, conserved
Tb927.1.3170
AGOF: null
AGOC: null
AGOP: null
PGOF: null
PGOC: null
PGOP: null

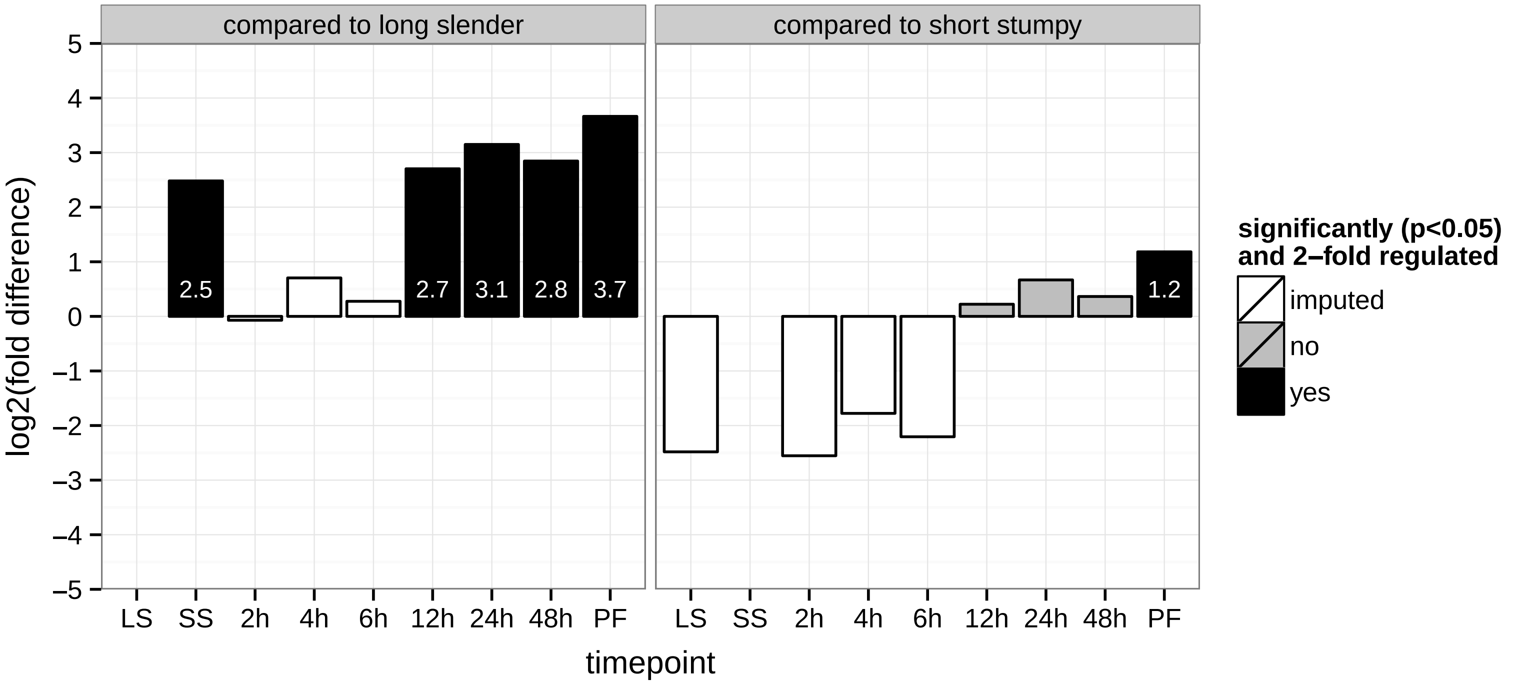

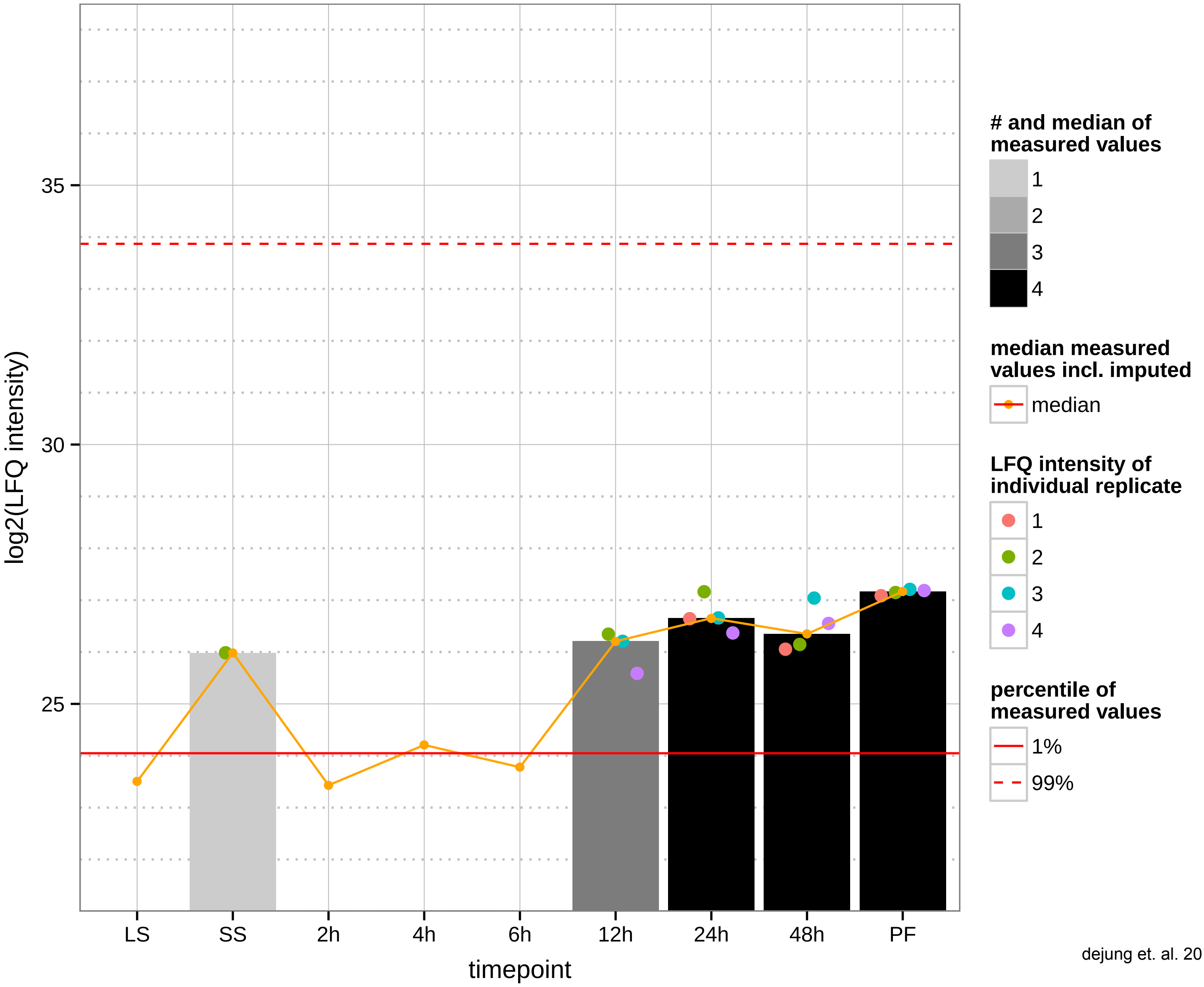

dejung et. al. 2015

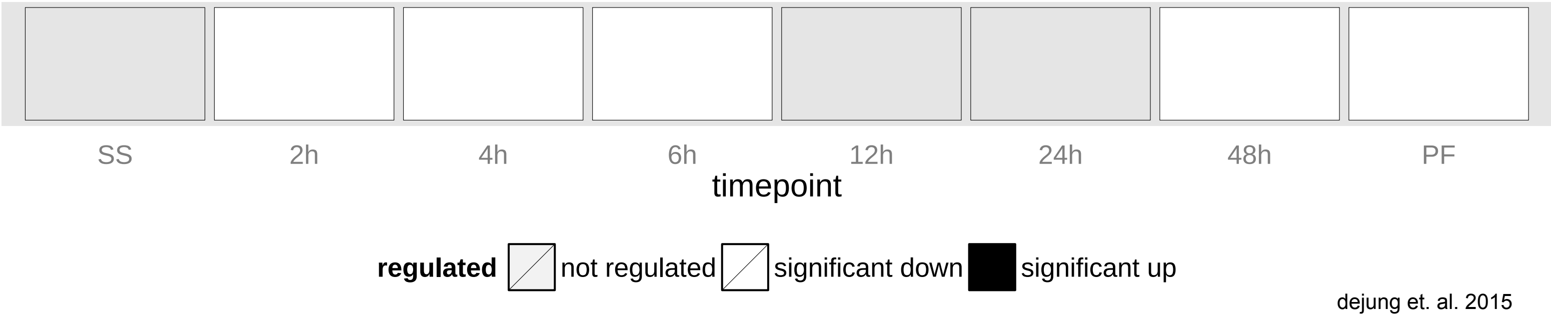
SS
2h
4h
6h
12h
24h
48h
PF
timepoint
regulated
not regulated
significant down
significant up
dejung et. al. 2015

hypothetical protein, conserved
Tb927.10.1740
AGOF: null
AGOC: null
AGOP: null
PGOF: null
PGOC: null
PGOP: null

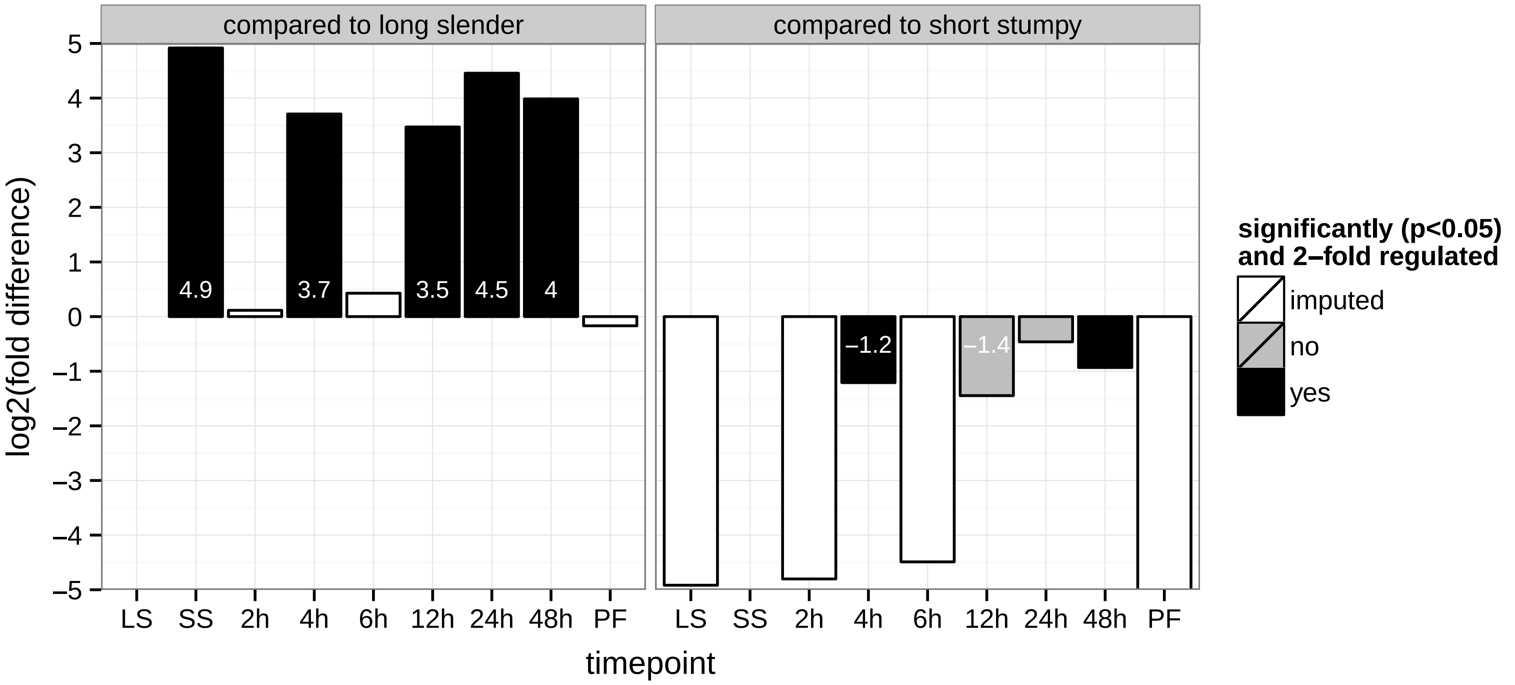

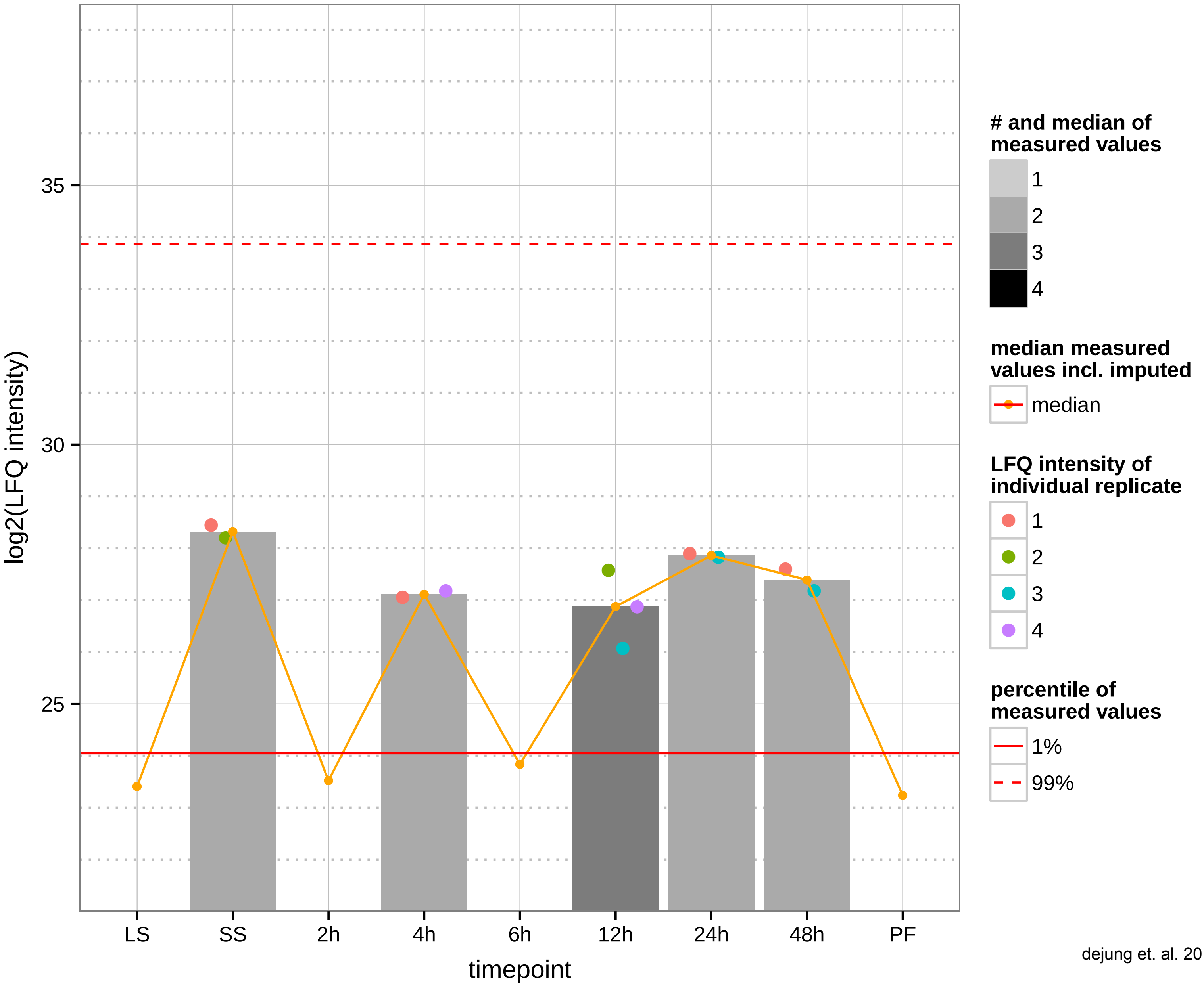

dejung et. al. 2015

hypothetical protein, conserved
Tb927.10.8380
AGOF: null
AGOC: null
AGOP: null
PGOF: null
PGOC: null
PGOP: null

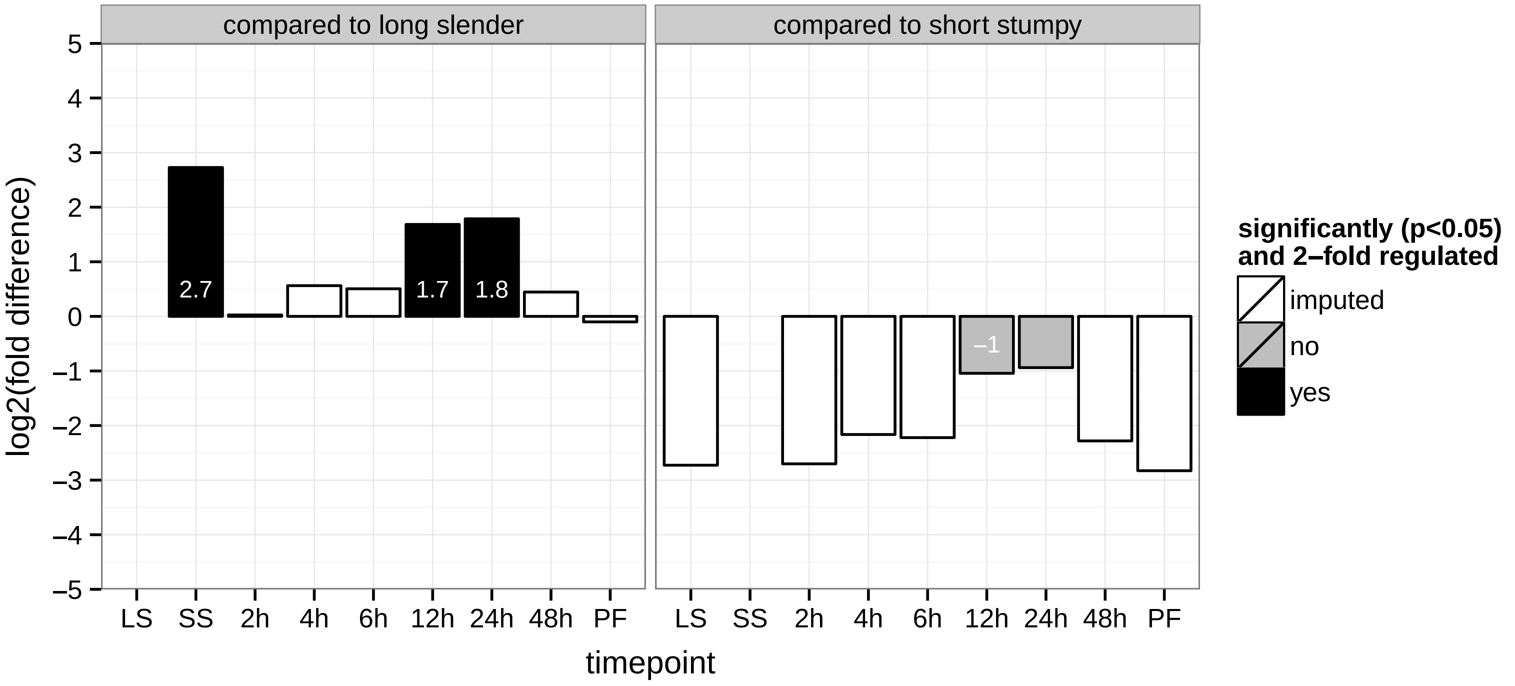

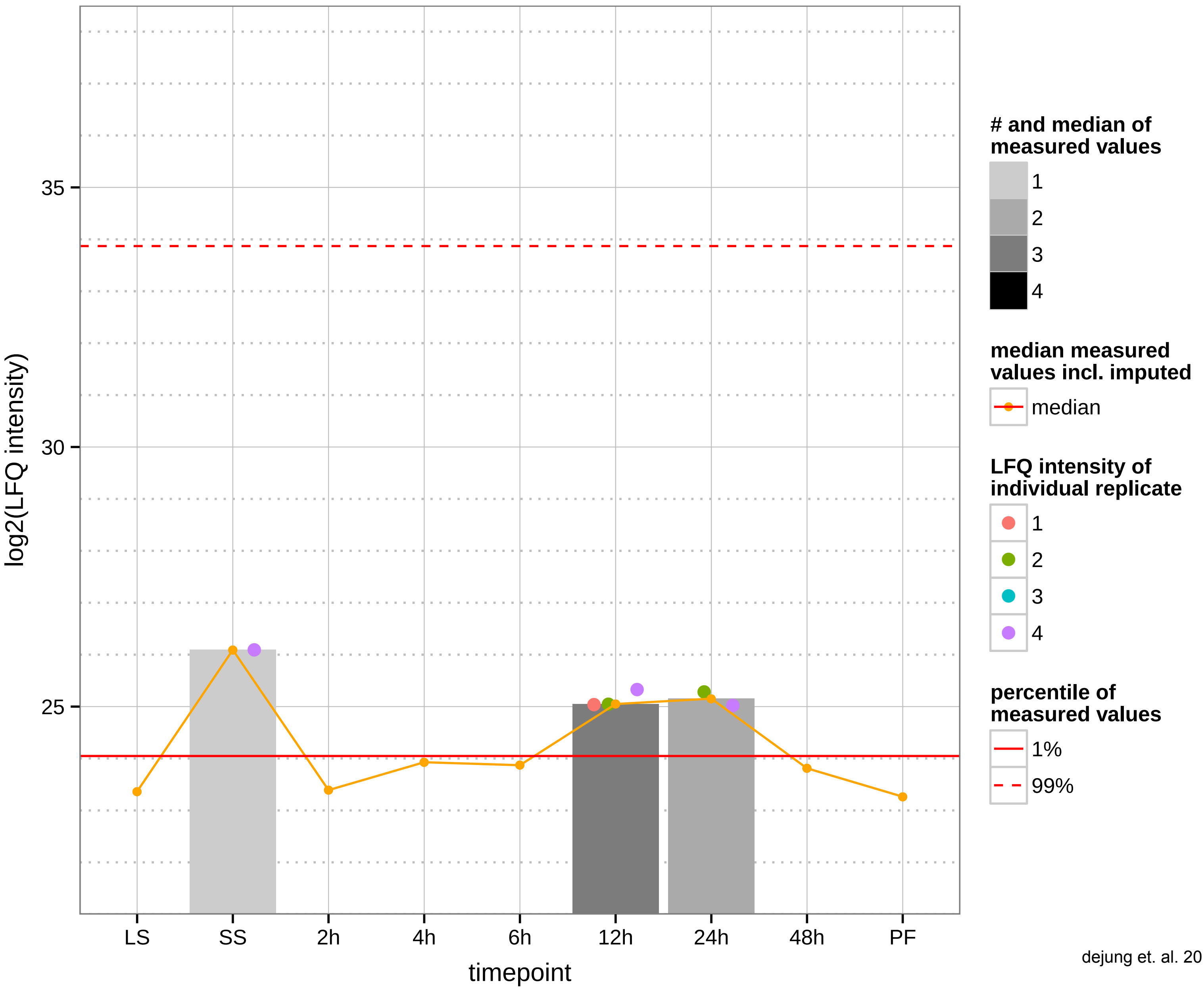

dejung et. al. 2015

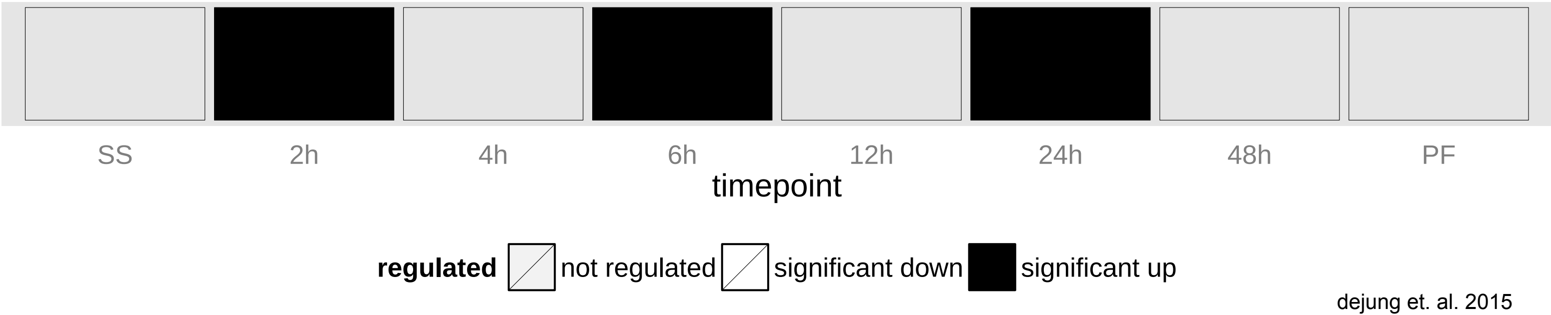
SS
2h
4h
6h
12h
24h
48h
PF
timepoint
regulated
not regulated
significant down
significant up
dejung et. al. 2015

hypothetical protein, conserved
Tb927.10.4070
AGOF: null
AGOC: null
AGOP: null
PGOF: null
PGOC: null
PGOP: null

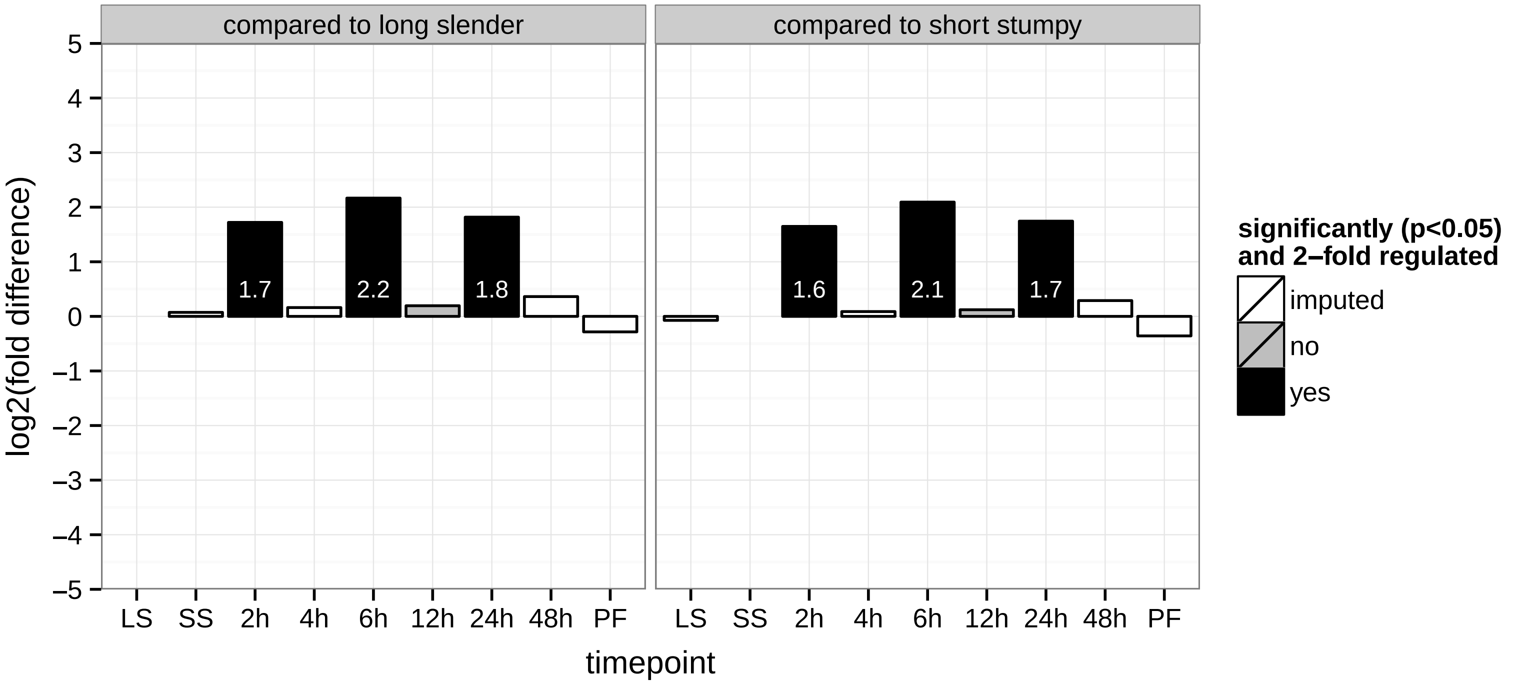

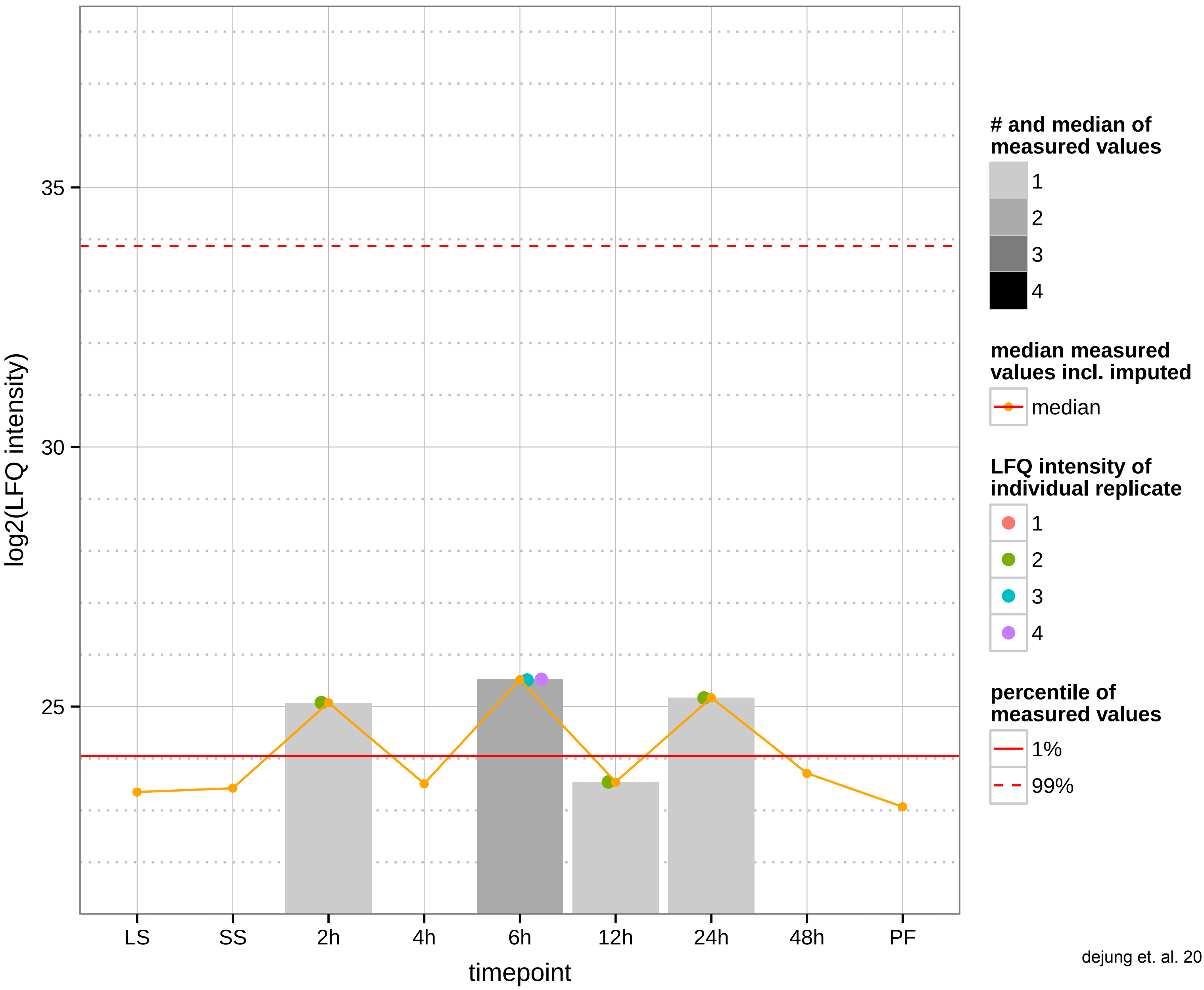

dejung et. al. 2015

transcription elongation factor s–II, putative (TFIIS1)
Tb927.11.4960
AGOF: DNA binding, sequence–specific DNA binding transcription factor activity, zinc ion binding
AGOC: null
AGOP: DNA–dependent transcription, elongation, regulation of transcription, DNA–dependent, transcription
PGOF: nucleic acid binding, zinc ion binding
PGOC: null
PGOP: transcription, DNA–dependent

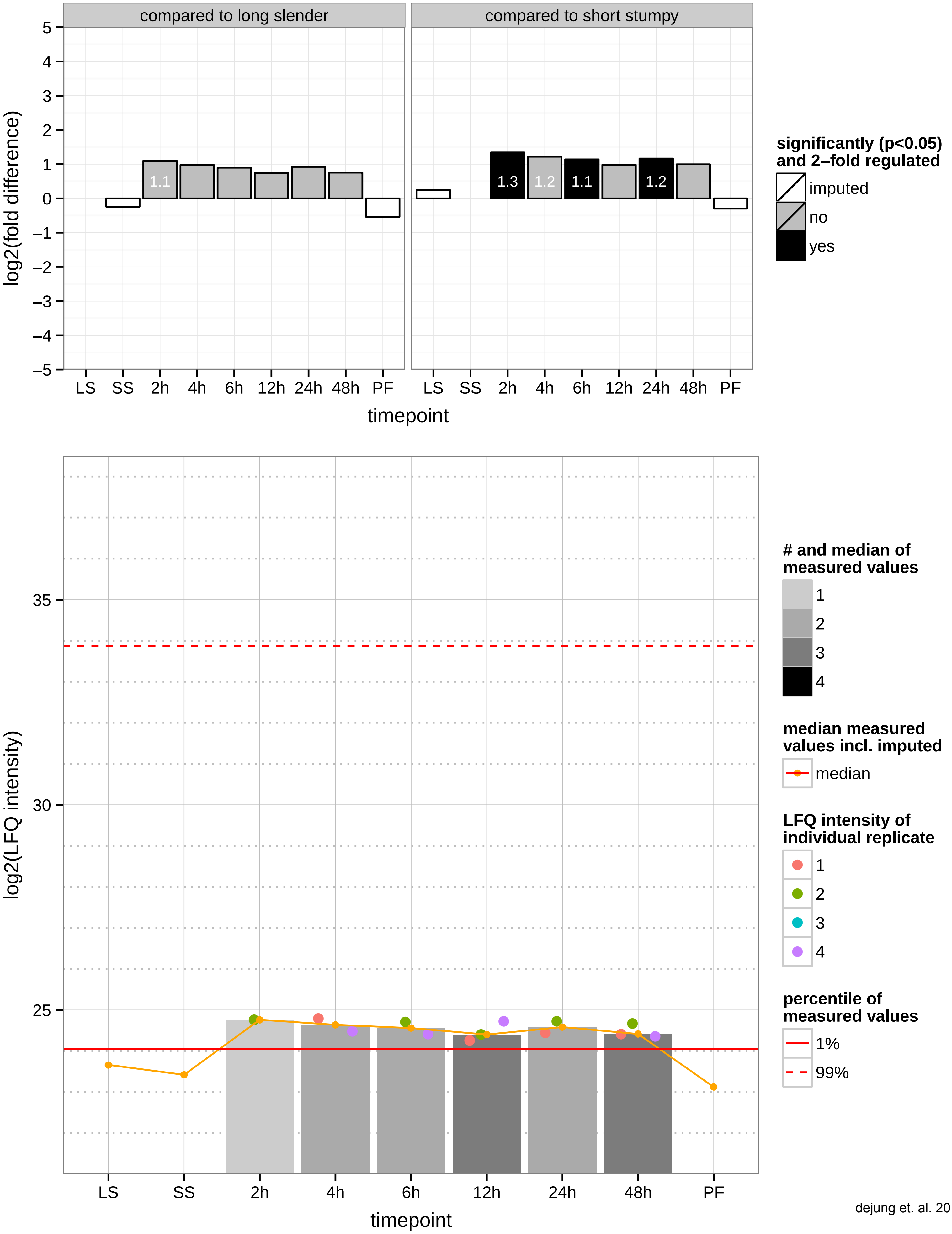

dejung et. al. 2015

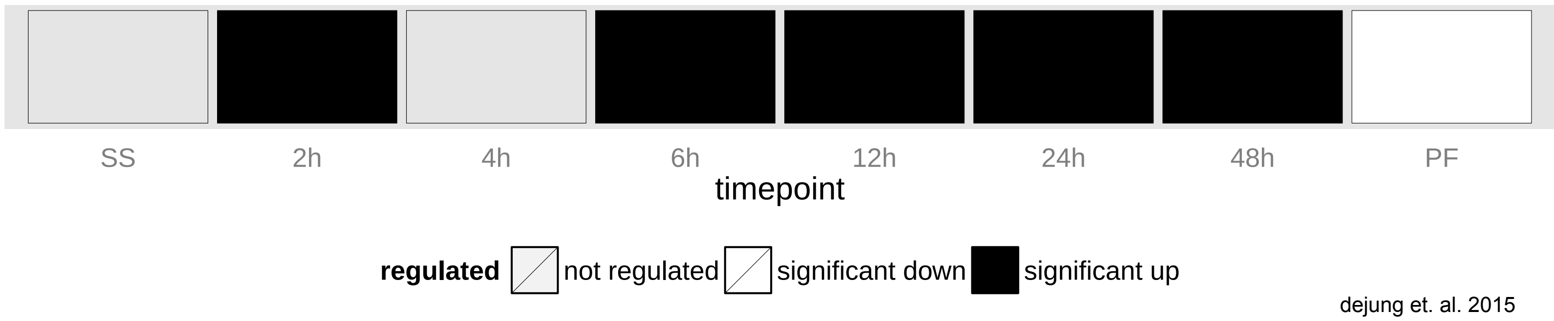

SS
2h
4h
6h
12h
24h
48h
PF
timepoint
regulated
not regulated
significant down
significant up
dejung et. al. 2015

calpain–like protein fragment, putative
Tb927.1.2230
AGOF: null
AGOC: null
AGOP: null
PGOF: null
PGOC: null
PGOP: null

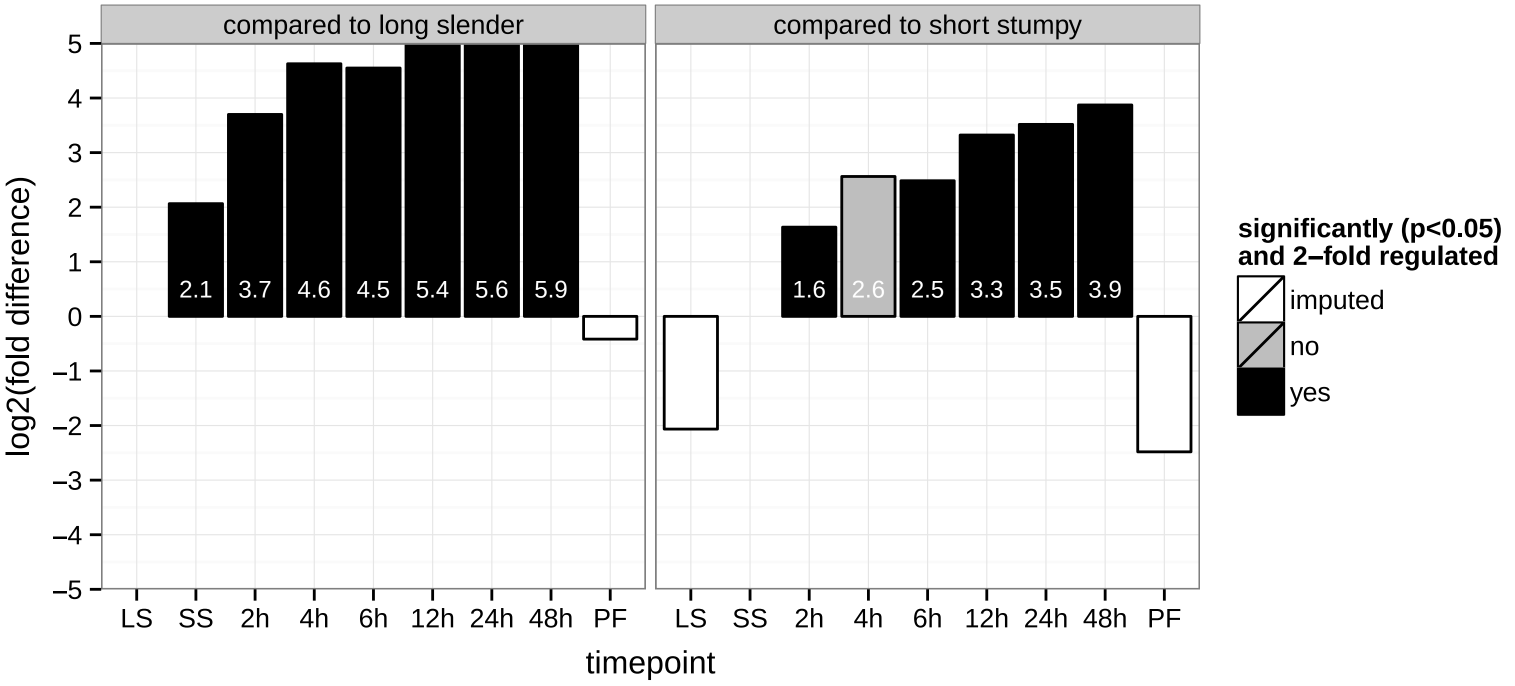

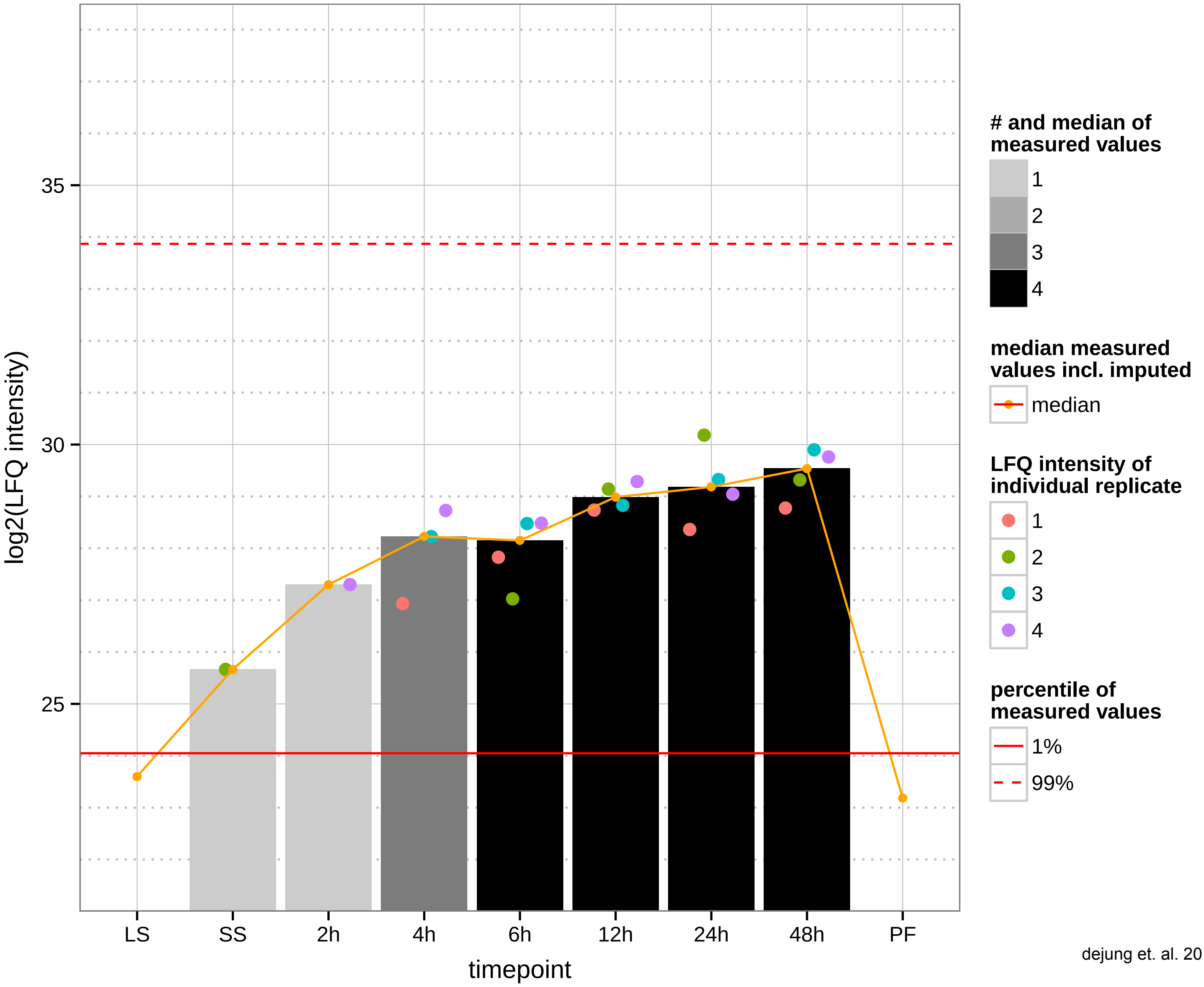

dejung et. al. 2015

dynein heavy chain, cytosolic, putative, Cytoplasmic dynein 1 heavy chain (DYNC1H1)
Tb927.7.3160
AGOF: null, ATP binding, ATPase activity, microtubule motor activity
AGOC: null, dynein complex
AGOP: null, chromosome segregation, microtubule–based movement, minus–end–directed vesicle transport along microtubul
PGOF: null, ATP binding, ATPase activity, microtubule motor activity, nucleoside–triphosphatase activity, nucleotide binding
PGOC: null, dynein complex
PGOP: null, microtubule–based movement

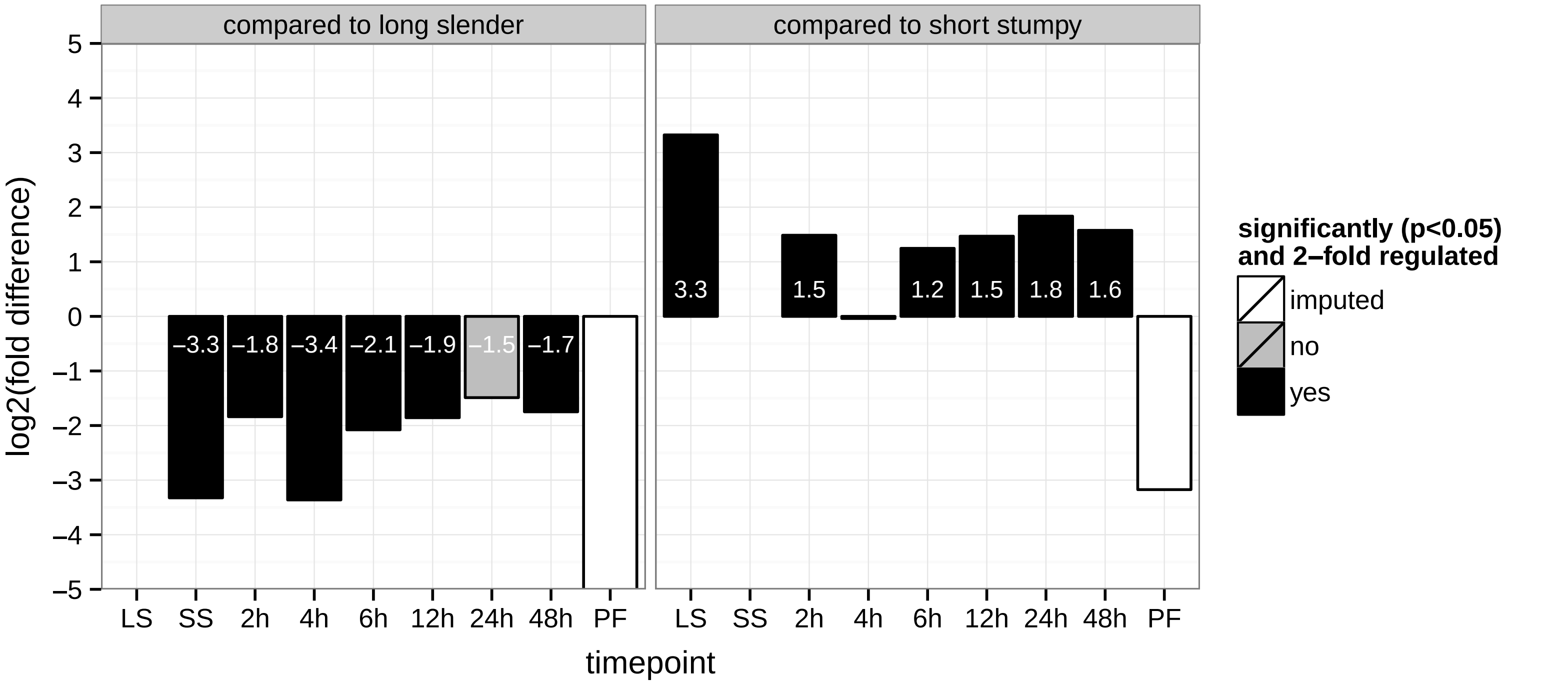

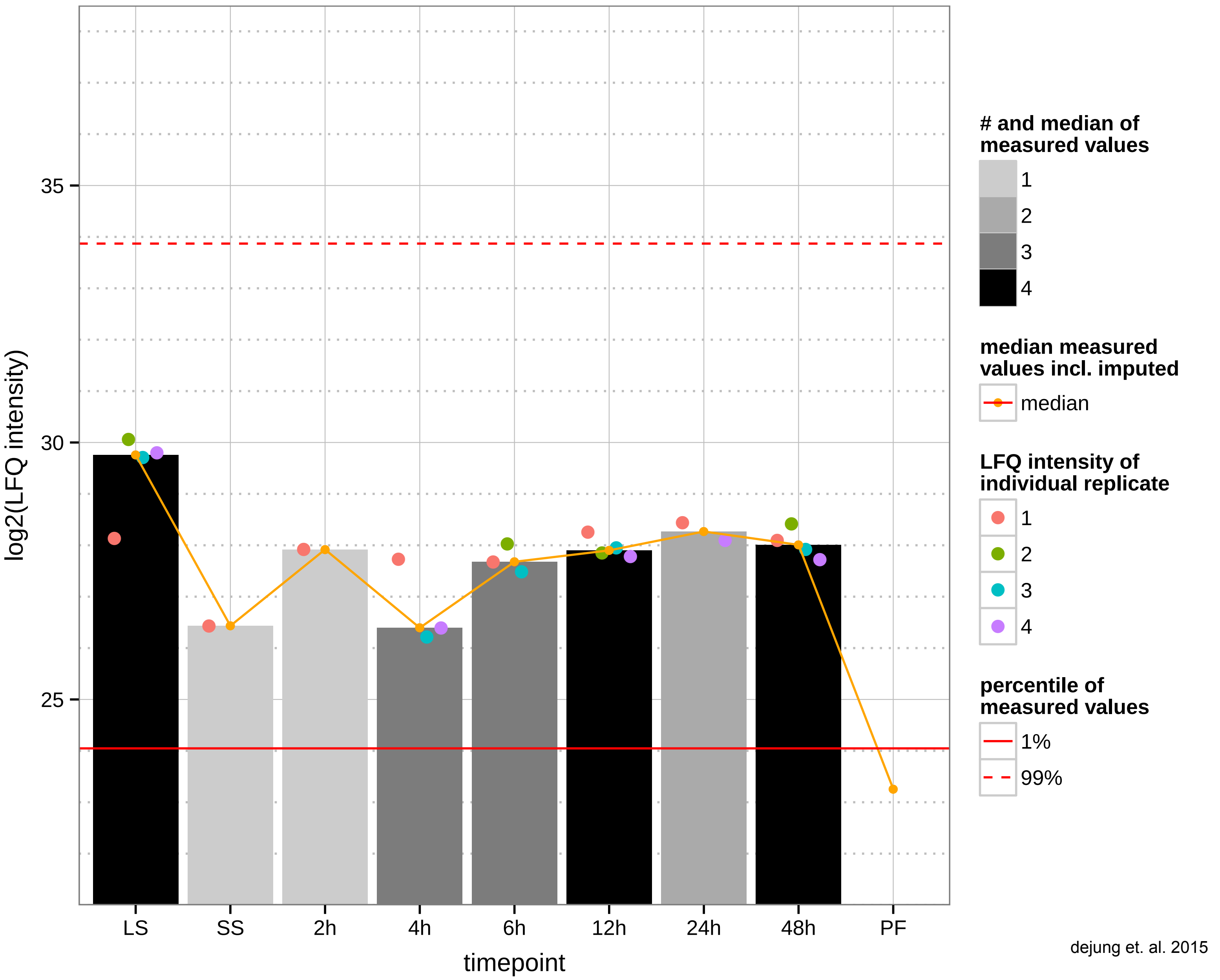

dejung et. al. 2015

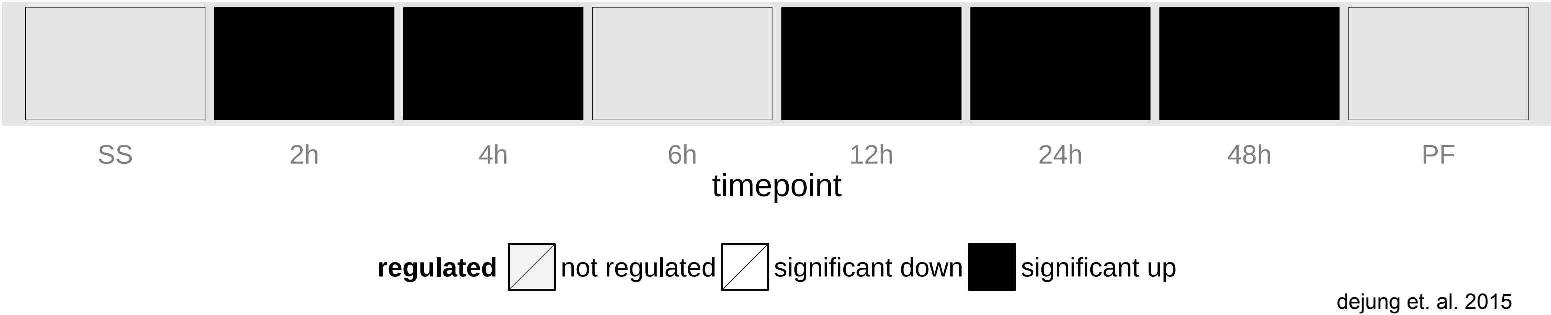
SS
2h
4h
6h
12h
24h
48h
PF
timepoint
regulated
not regulated
significant down
significant up
dejung et. al. 2015

iron–sulfur cluster assembly protein, putative
Tb927.10.12000
AGOF: iron–sulfur cluster binding
AGOC: cytoplasm, mitochondrion
AGOP: tRNA thio–modification
PGOF: null
PGOC: null
PGOP: null

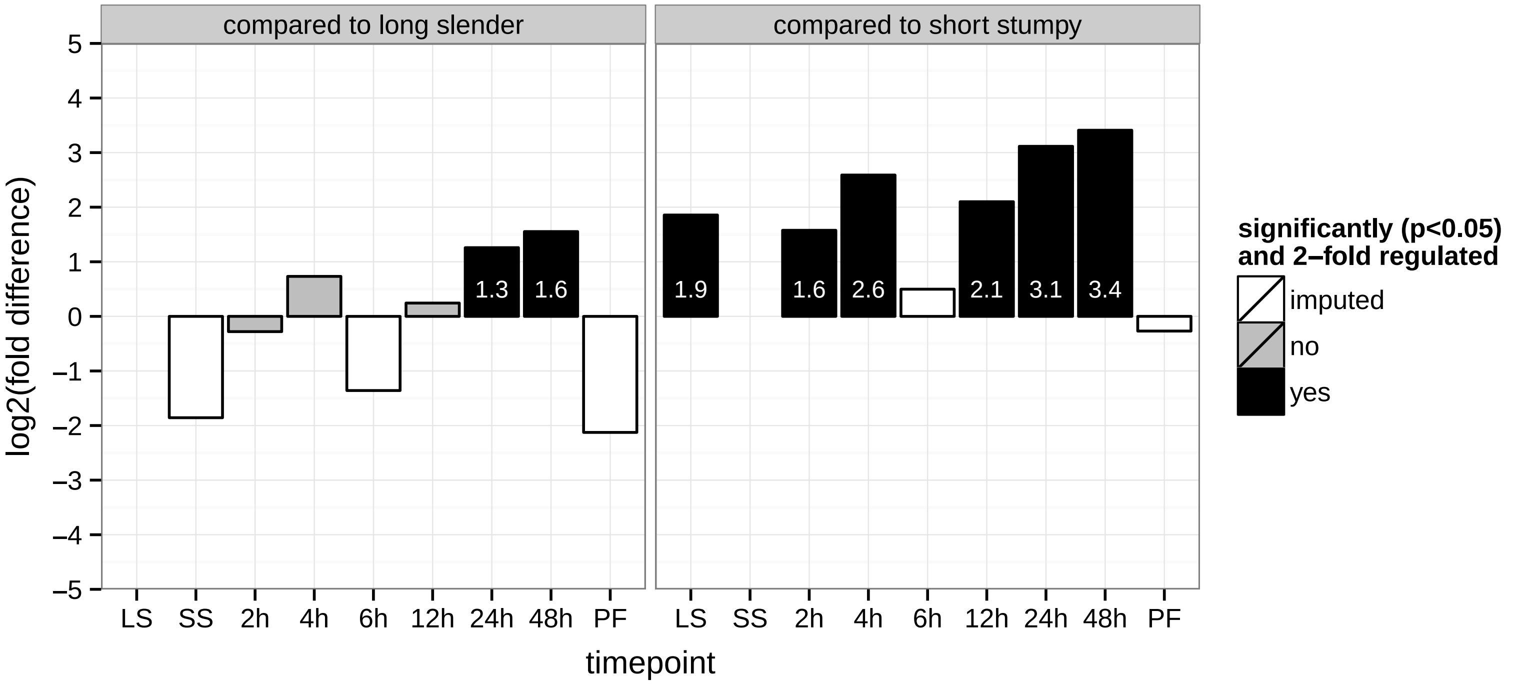

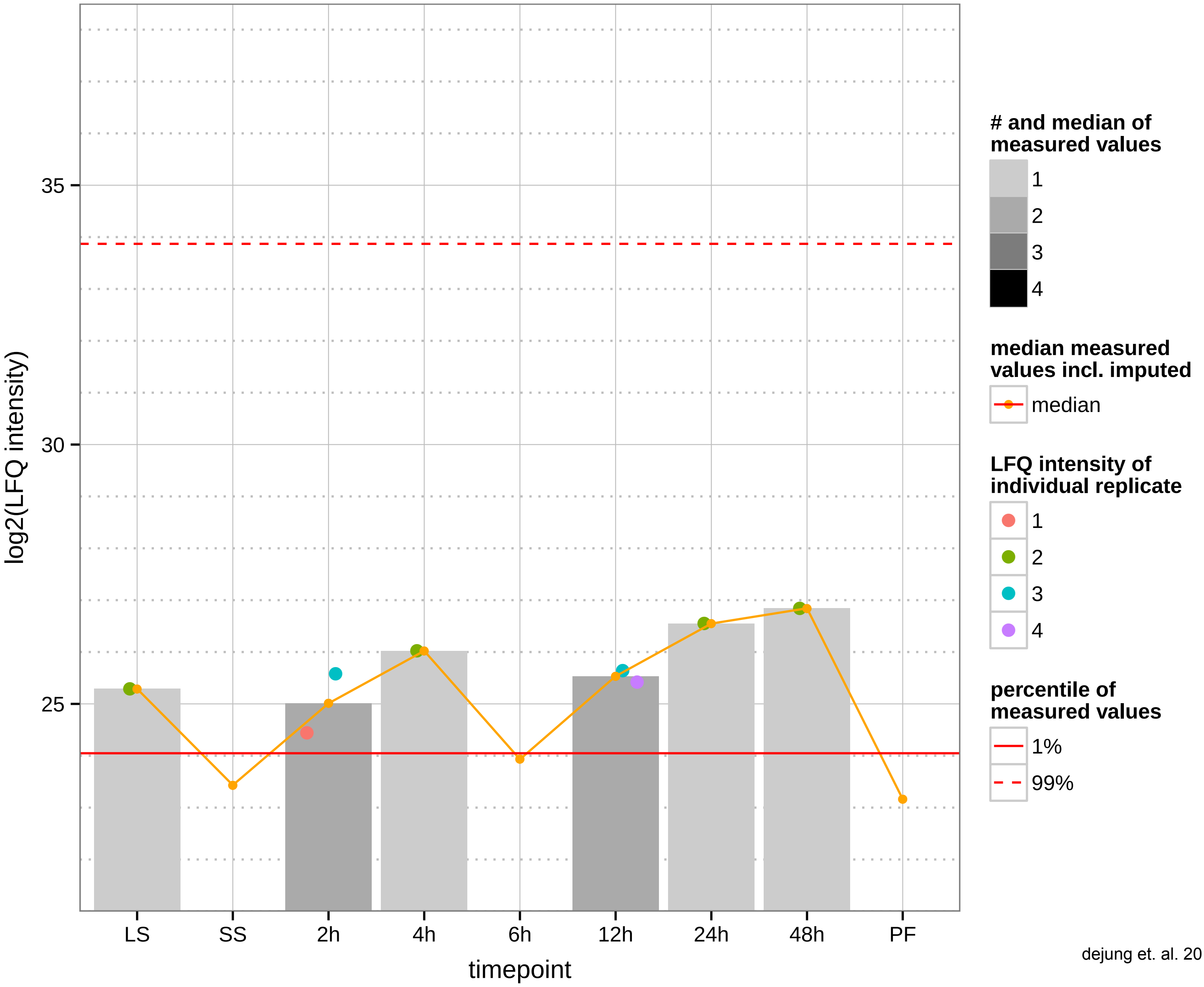

dejung et. al. 2015

DNA–directed RNA polymerases II subunit, putative (RPB4)
Tb927.3.5270
AGOF: DNA–directed RNA polymerase activity, protein binding
AGOC: null
AGOP: transcription, DNA–dependent
PGOF: DNA–directed RNA polymerase activity, catalytic activity, nucleotide binding
PGOC: null
PGOP: cellular metabolic process, transcription, DNA–dependent

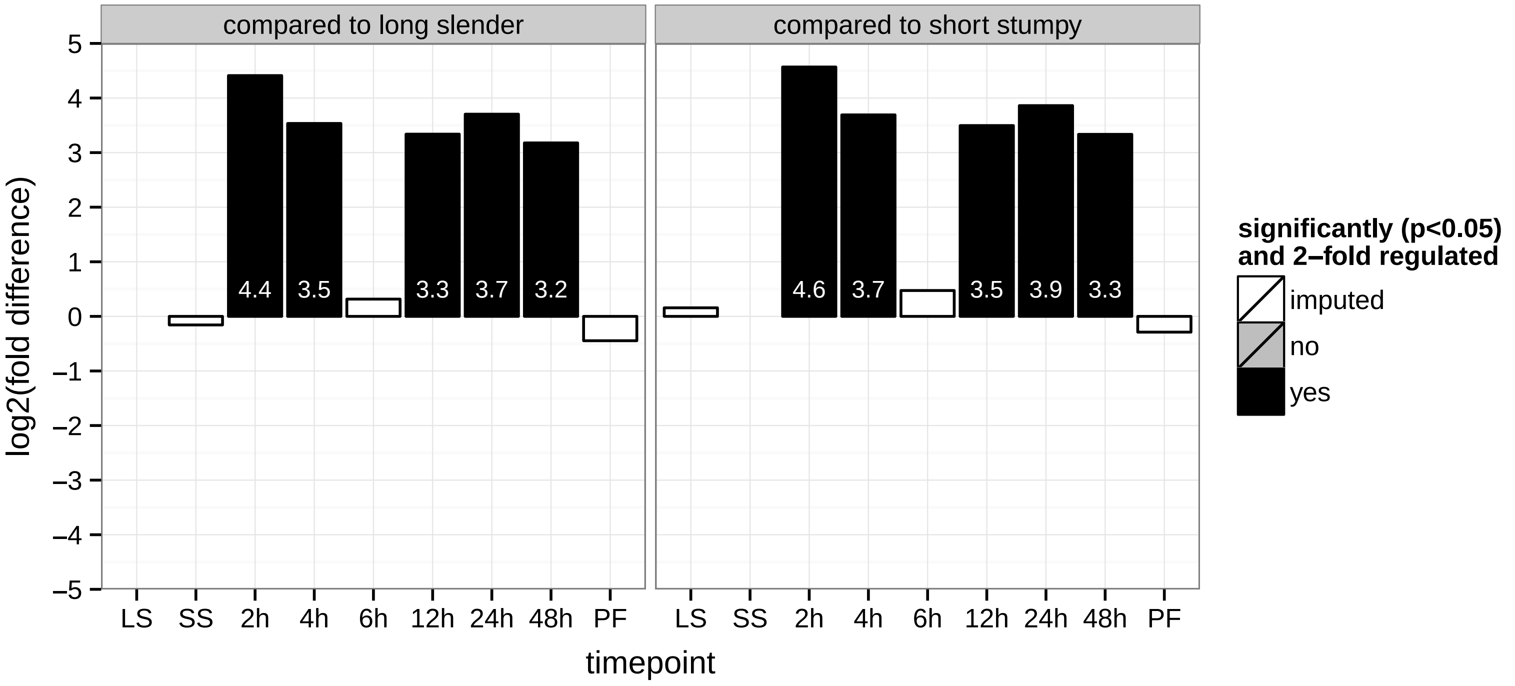

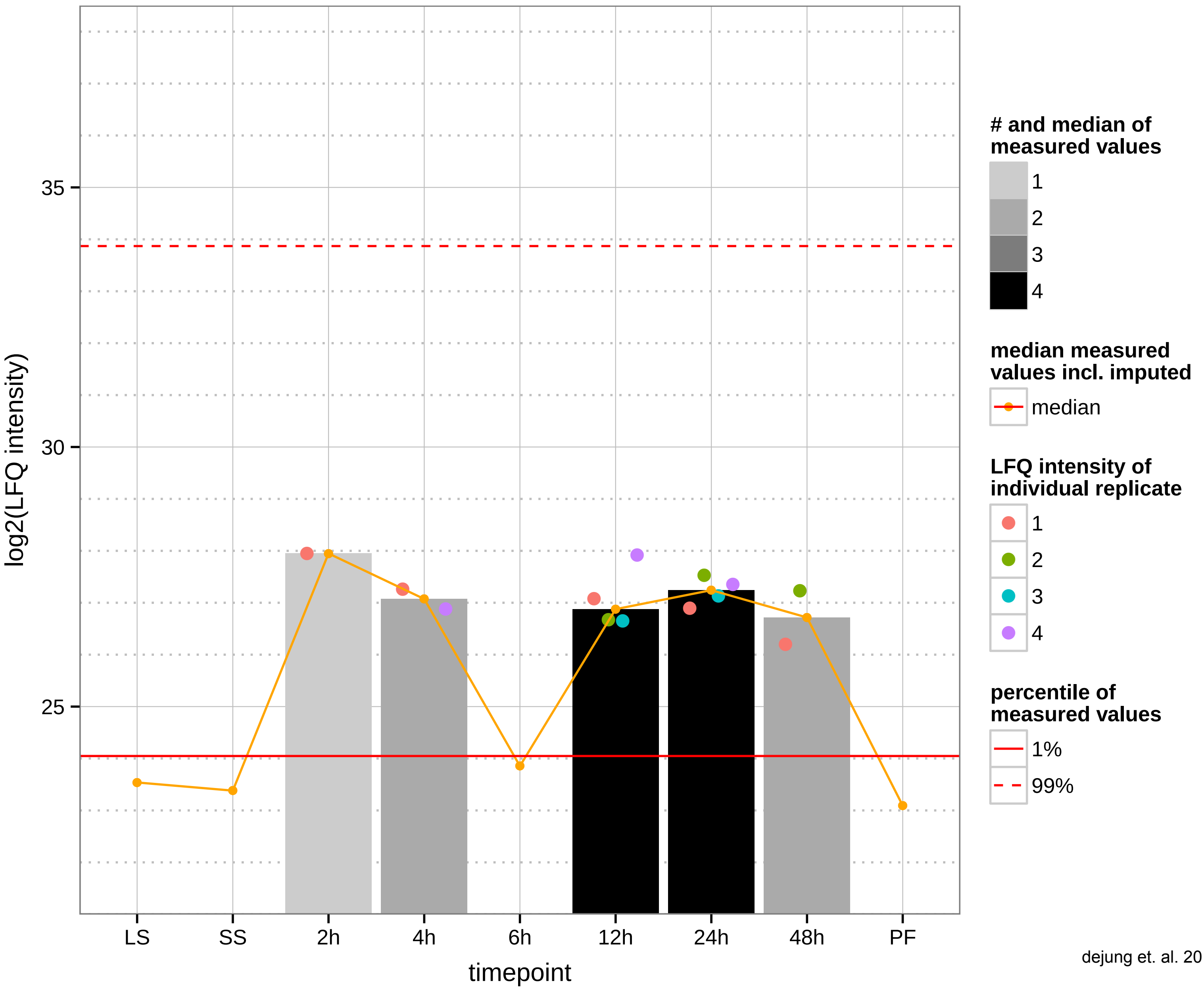

dejung et. al. 2015

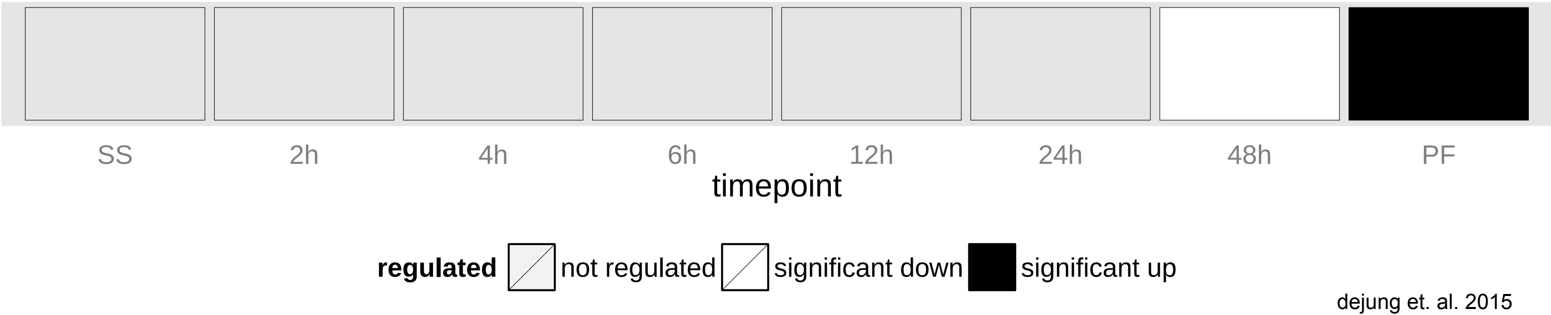
SS
2h
4h
6h
12h
24h
48h
PF
timepoint
regulated
not regulated
significant down
significant up
dejung et. al. 2015

hypothetical protein, conserved
Tb927.11.10690
AGOF: null
AGOC: endoplasmic reticulum
AGOP: null
PGOF: null
PGOC: null
PGOP: null

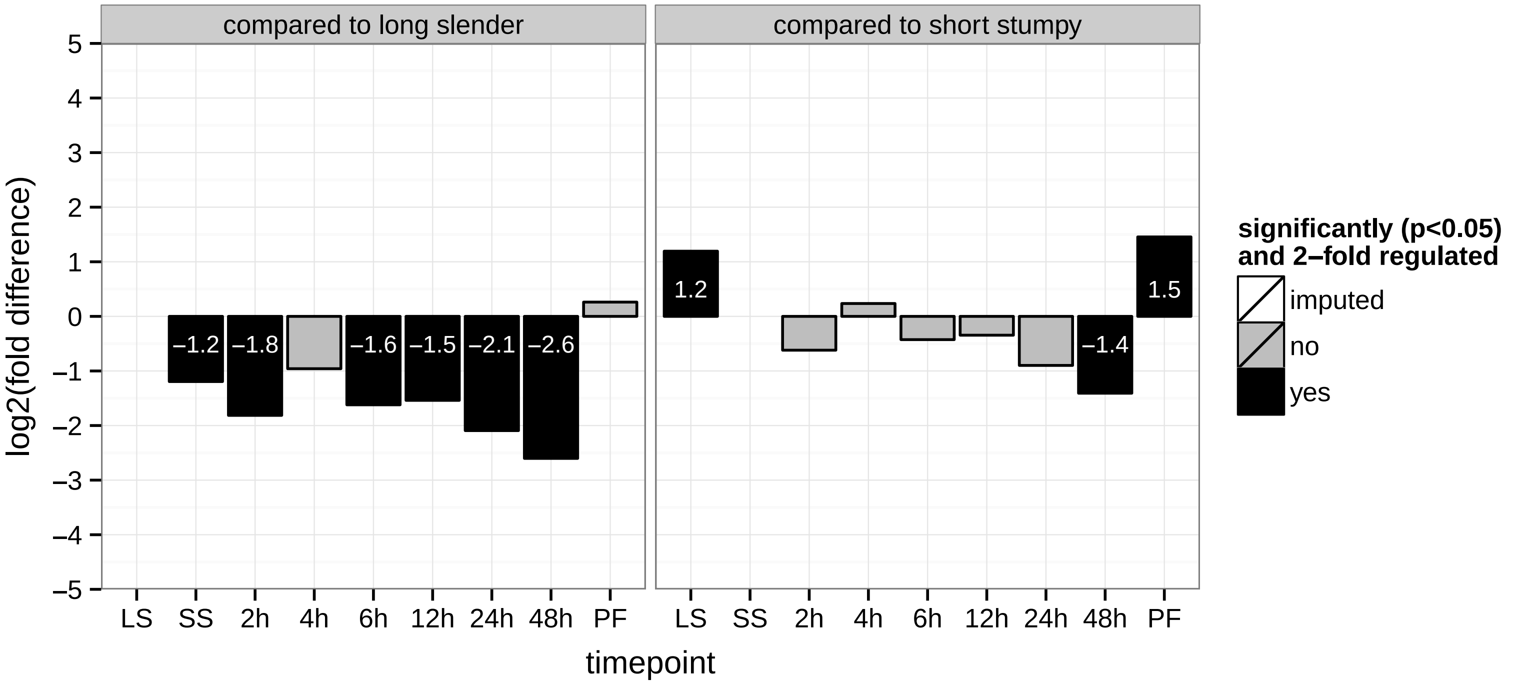

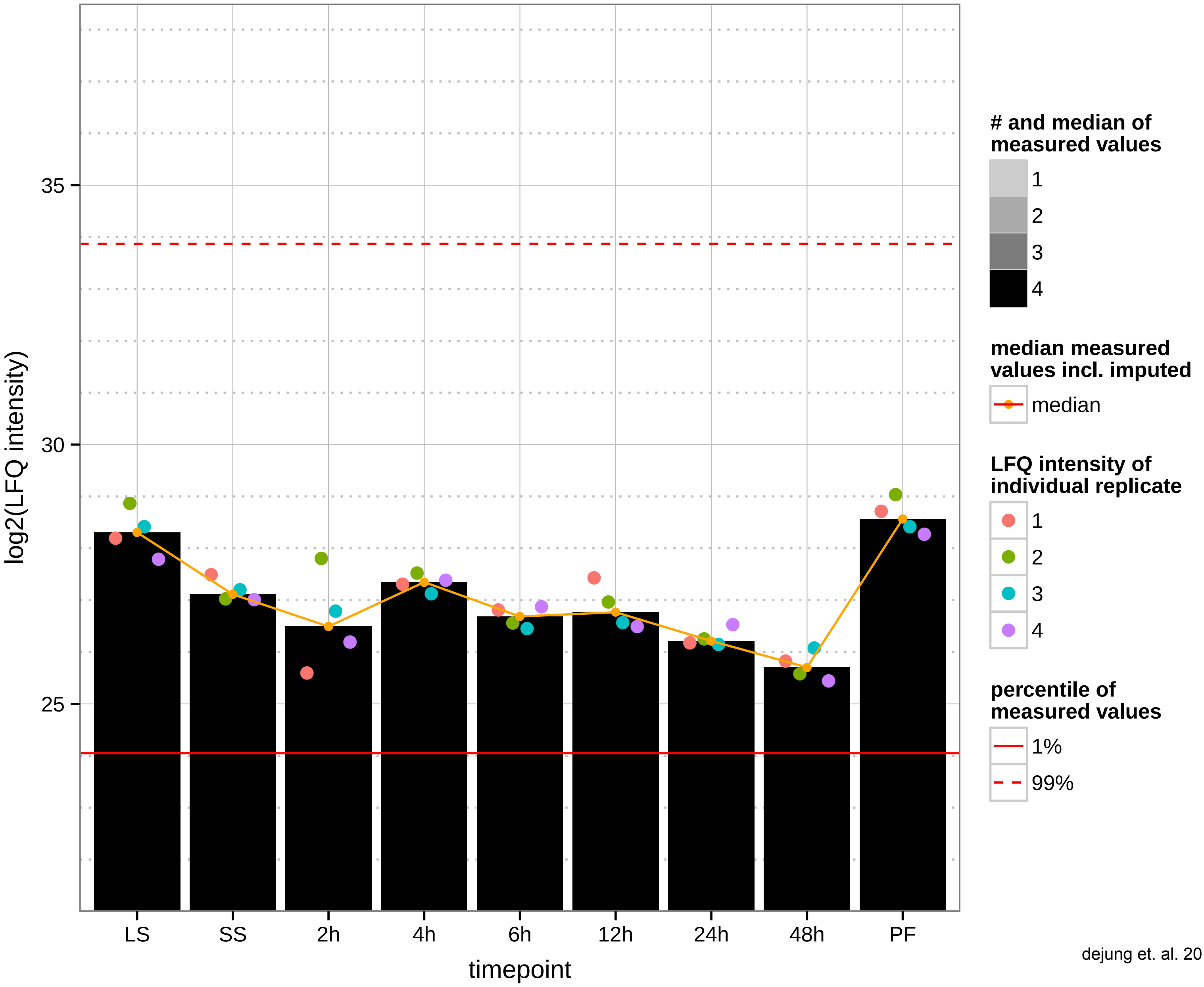

dejung et. al. 2015

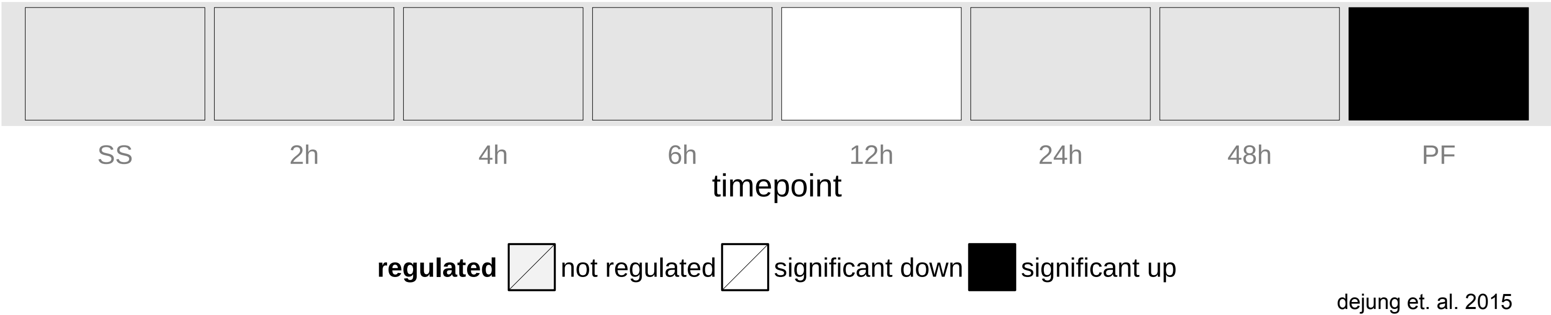

SS
2h
4h
6h
12h
24h
48h
PF
timepoint
regulated
not regulated
significant down
significant up
dejung et. al. 2015

hypothetical protein, conserved
Tb927.11.14700
AGOF: null
AGOC: mitochondrion
AGOP: null
PGOF: null
PGOC: null
PGOP: null

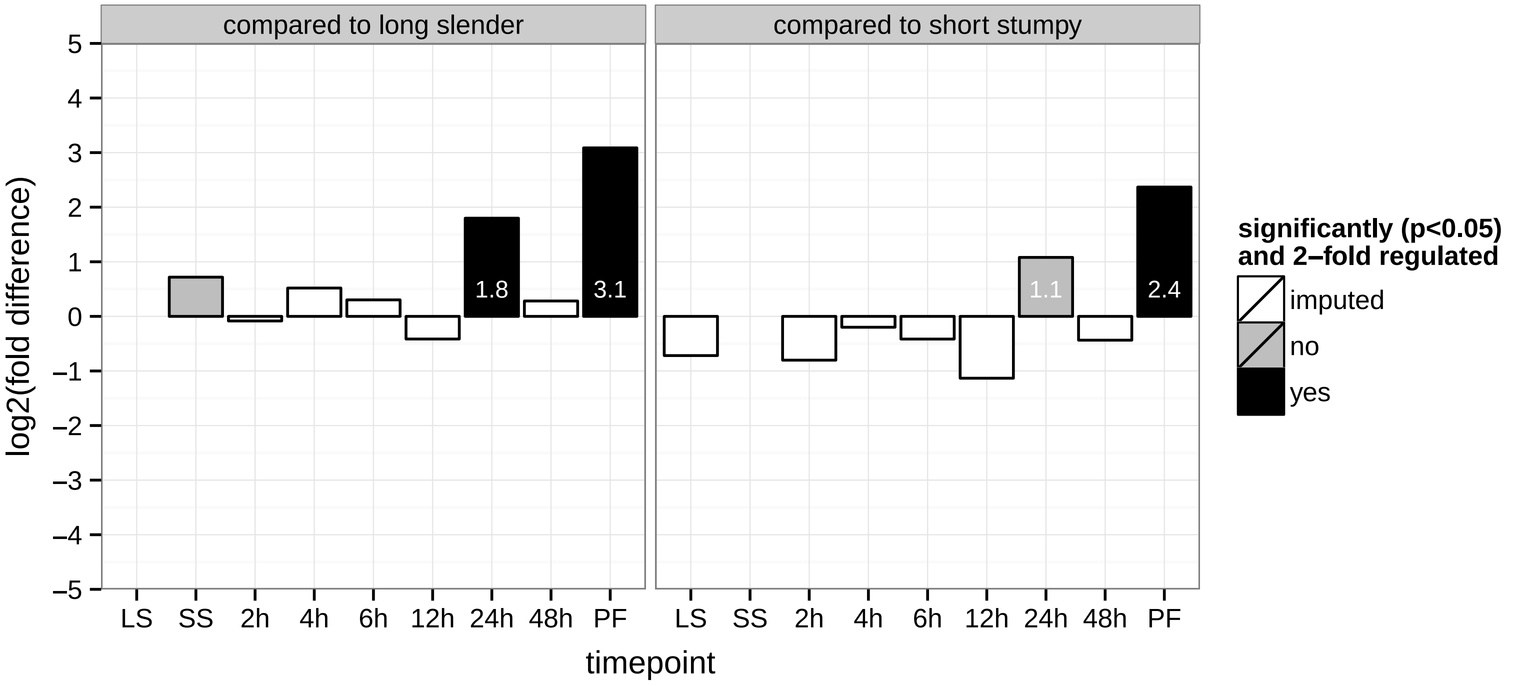

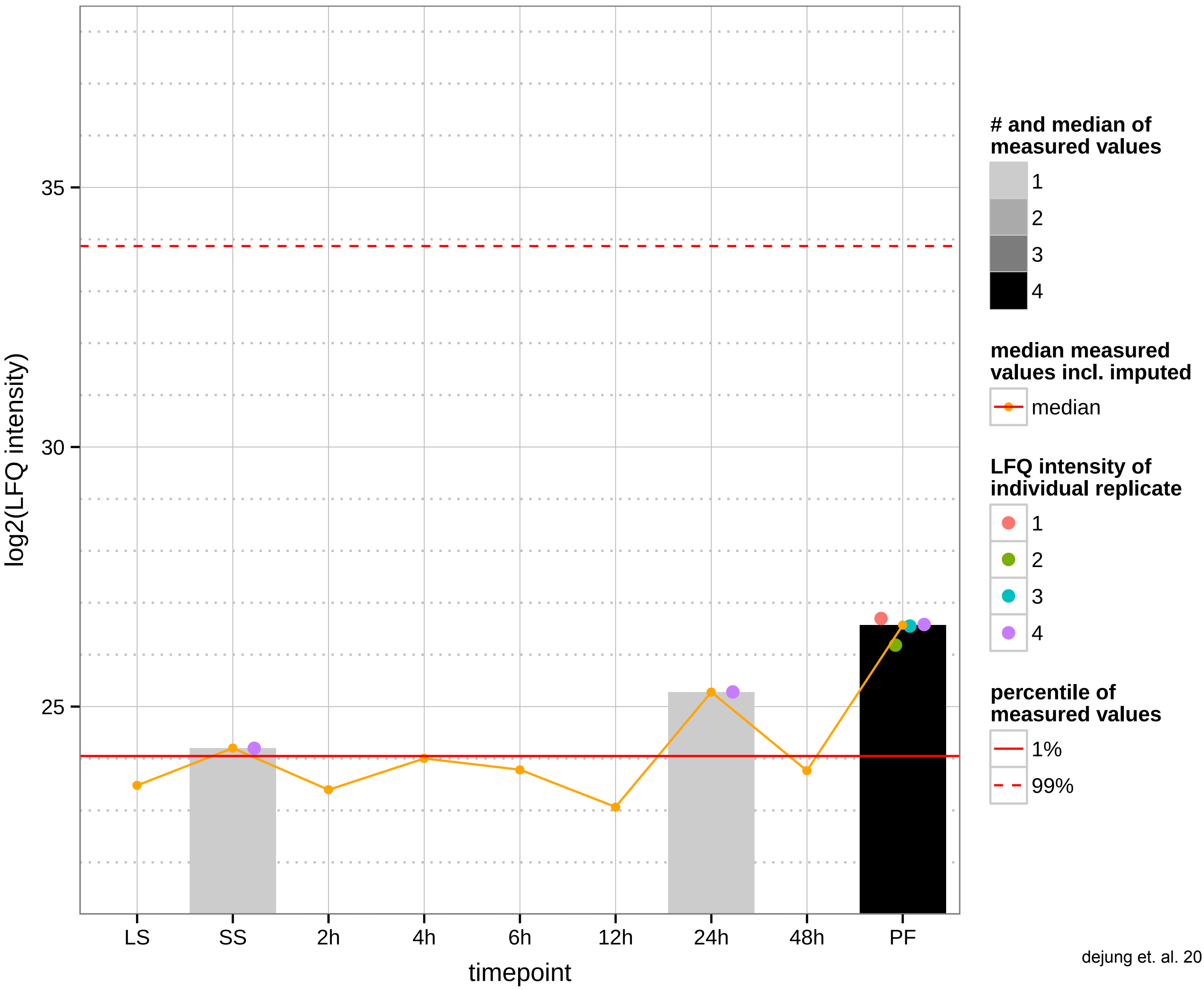

dejung et. al. 2015

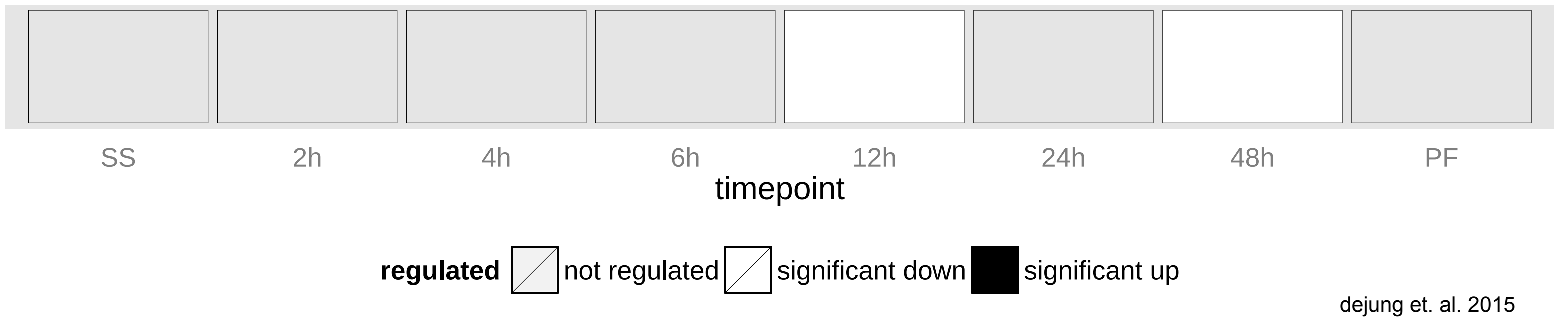
SS
2h
4h
6h
12h
24h
48h
PF
timepoint
regulated
not regulated
significant down
significant up
dejung et. al. 2015

ATP−binding protein, putative
Tb927.11.360
AGOF: null
AGOC: null
AGOP: null
PGOF: null
PGOC: null
PGOP: null

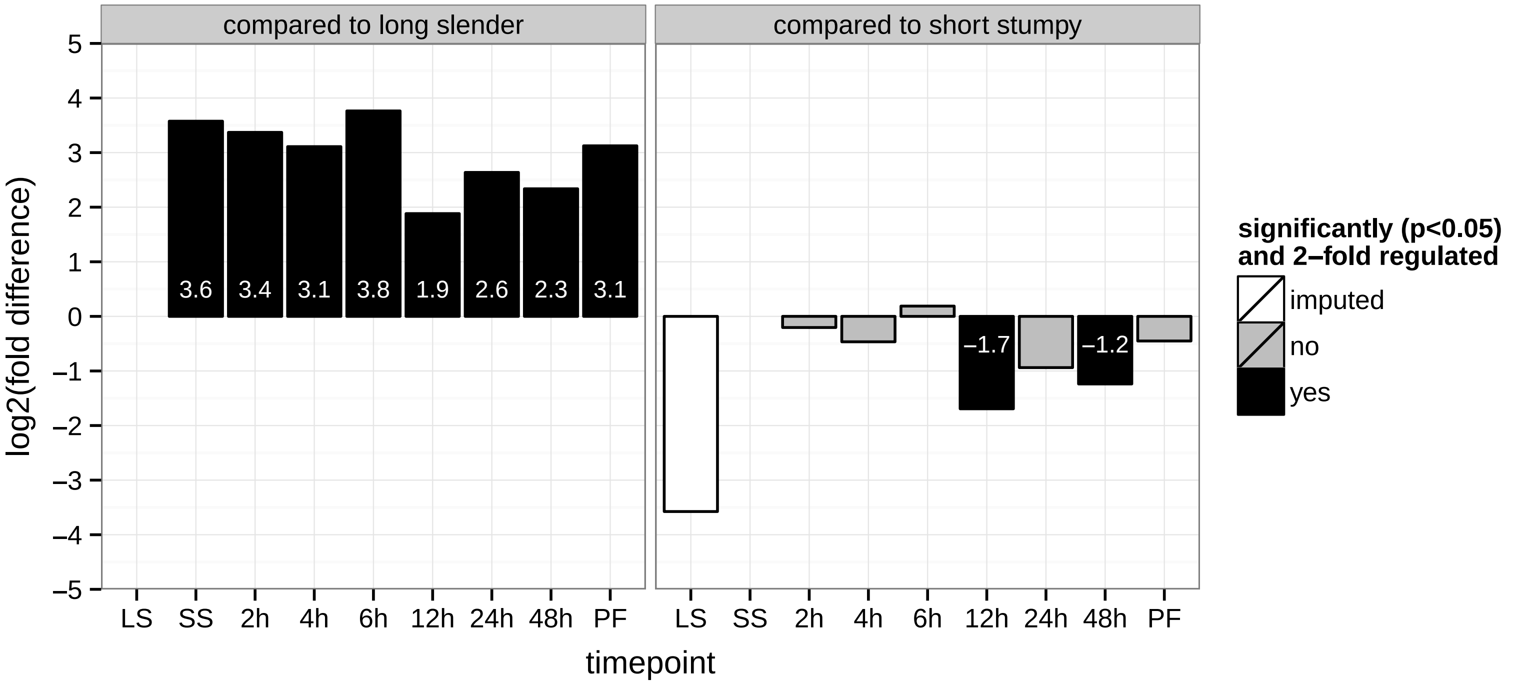

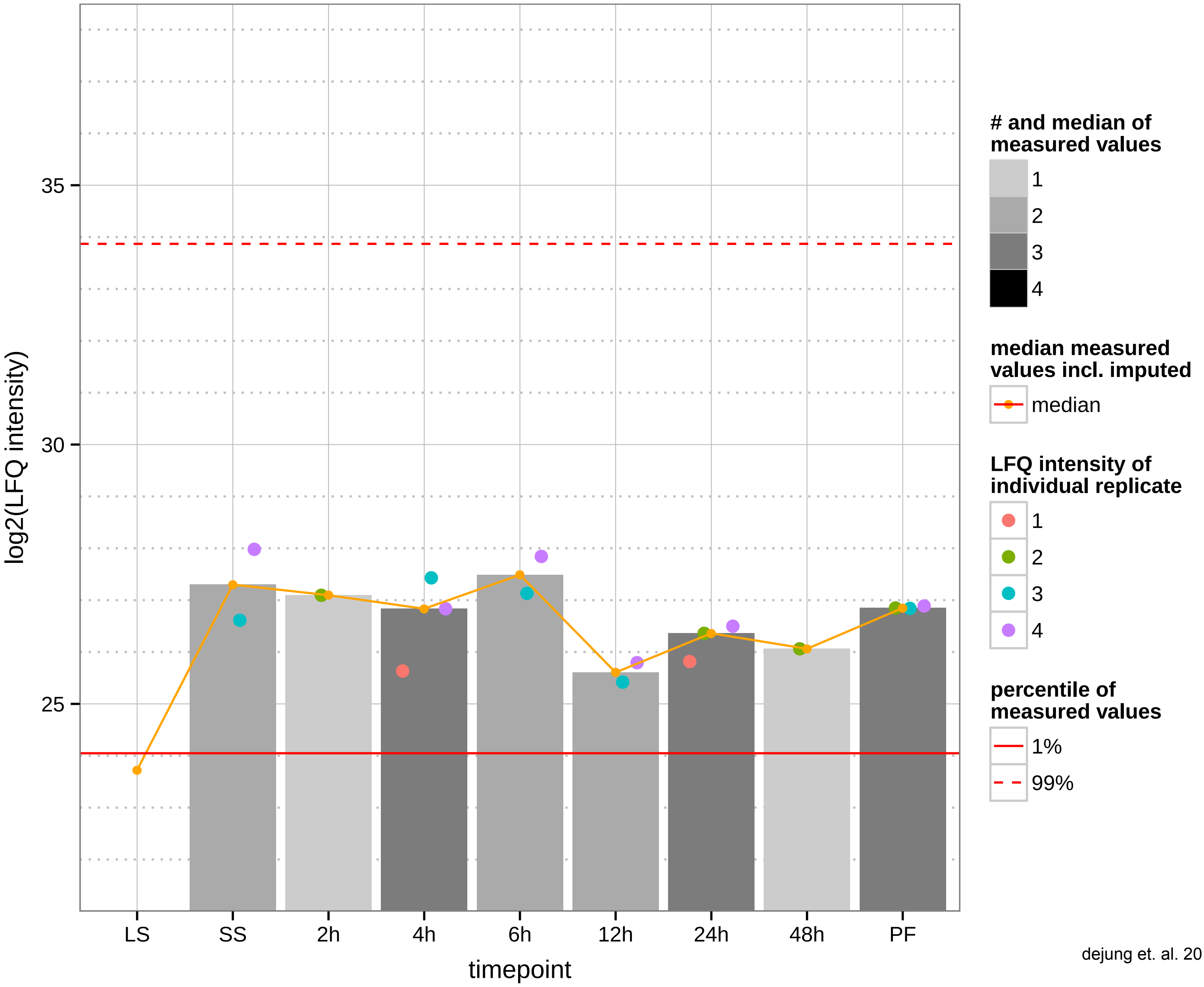

dejung et. al. 2015

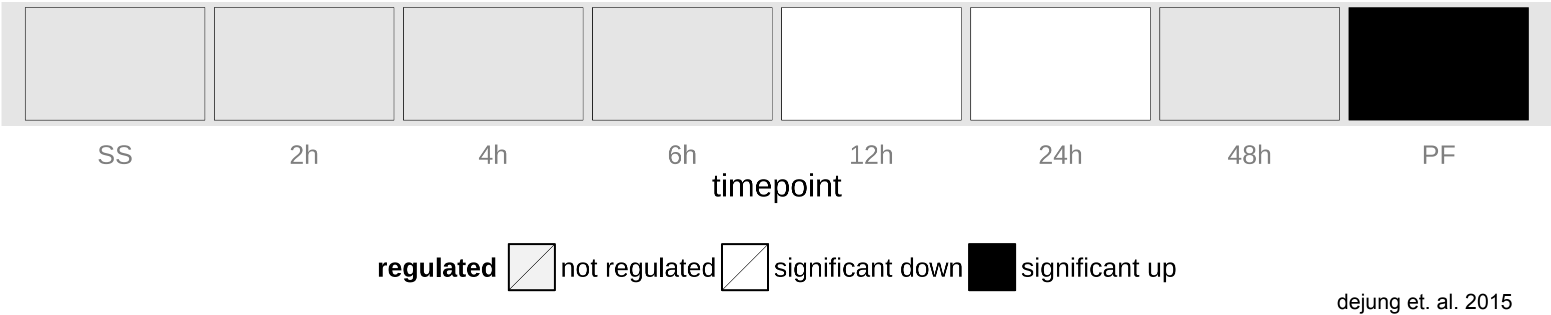
SS
2h
4h
6h
12h
24h
48h
PF
timepoint
regulated
not regulated
significant down
significant up
dejung et. al. 2015

hypothetical protein, conserved
Tb927.7.3860
AGOF: null
AGOC: null
AGOP: null
PGOF: null
PGOC: null
PGOP: null

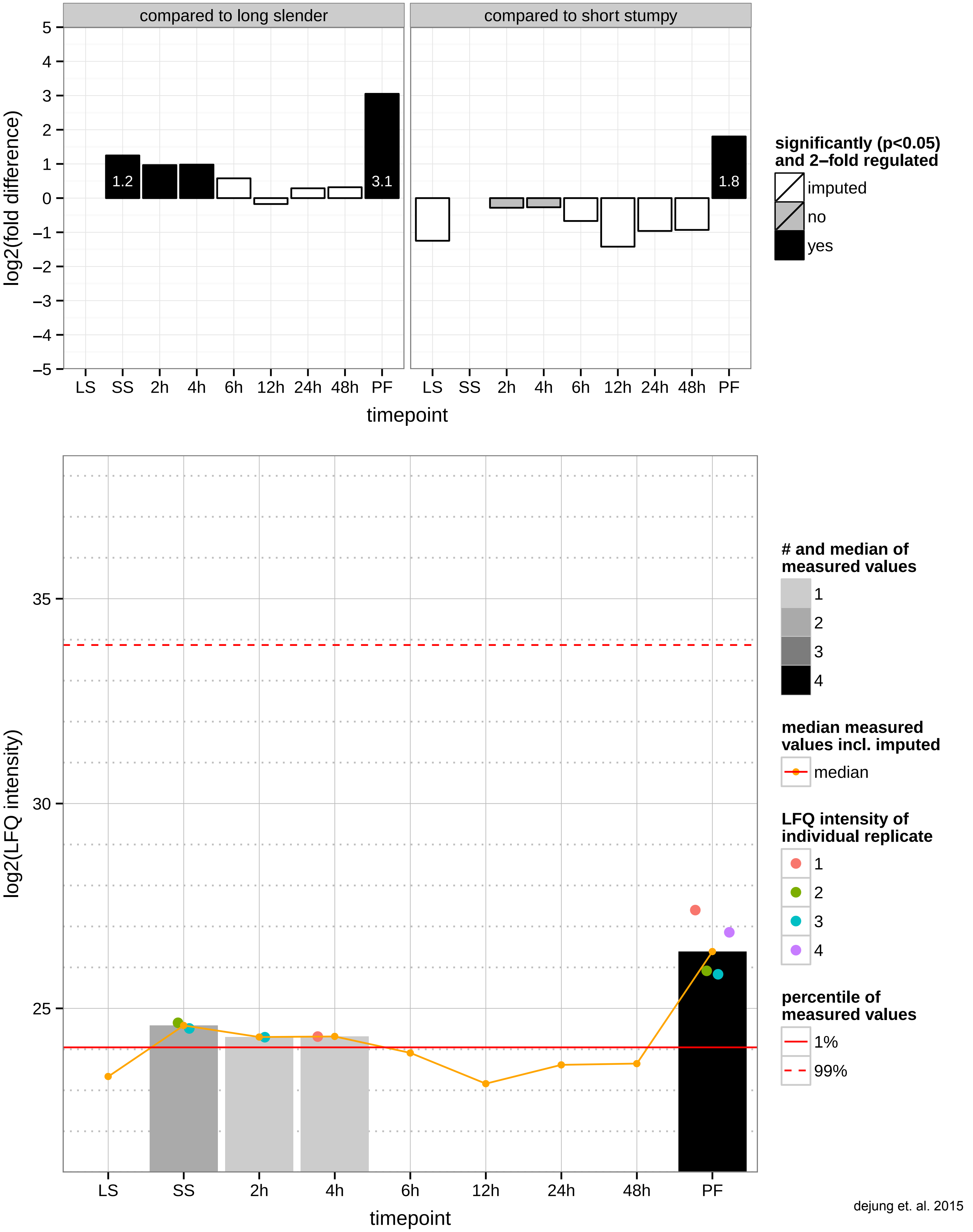

dejung et. al. 2015

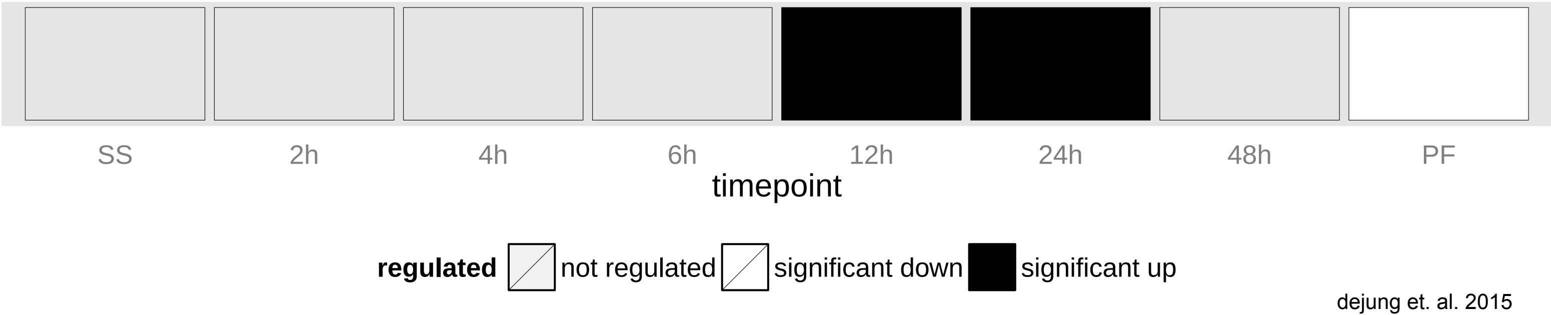
SS
2h
4h
6h
12h
24h
48h
PF
timepoint
regulated
not regulated
significant down
significant up
dejung et. al. 2015

hypothetical protein, conserved
Tb927.6.2220
AGOF: null
AGOC: null
AGOP: null
PGOF: null
PGOC: null
PGOP: null

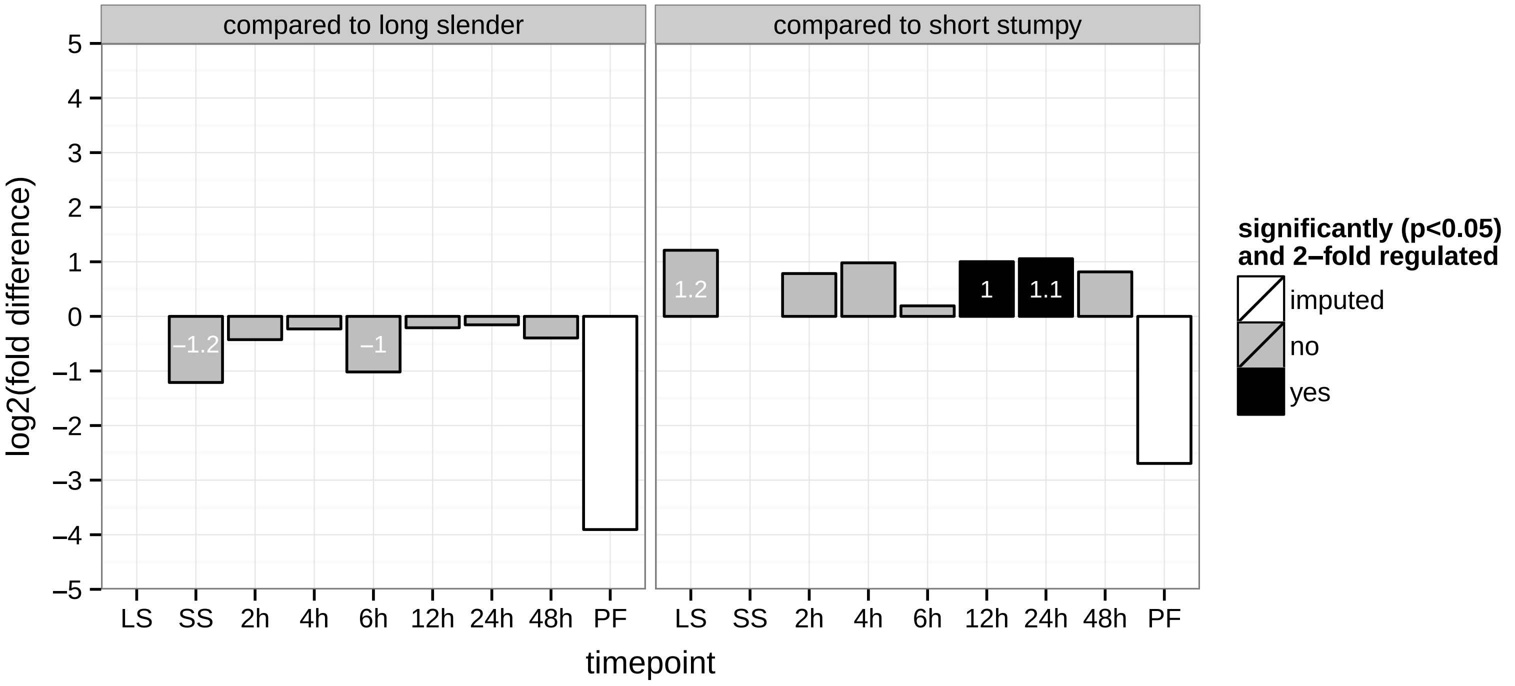

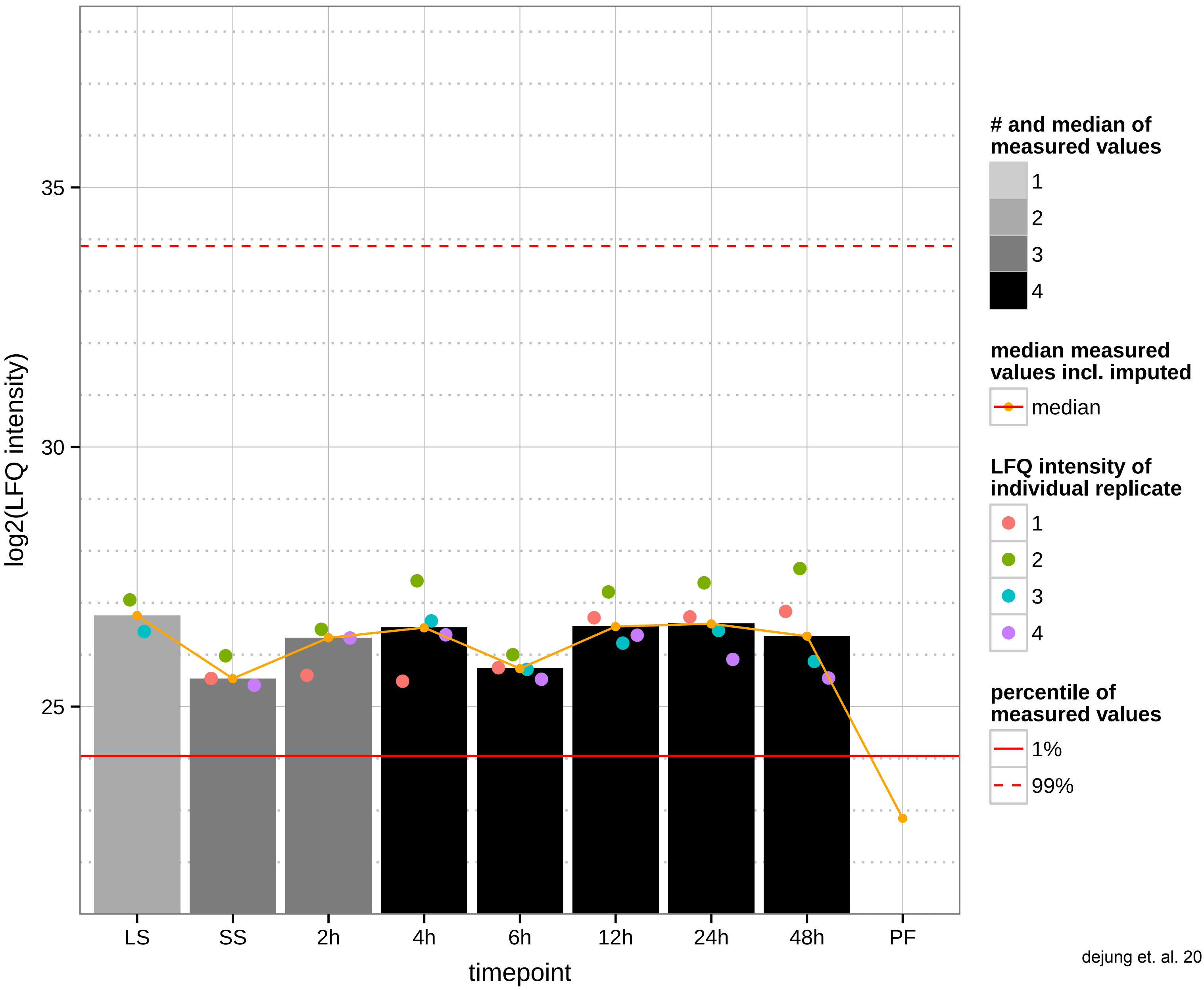

dejung et. al. 2015

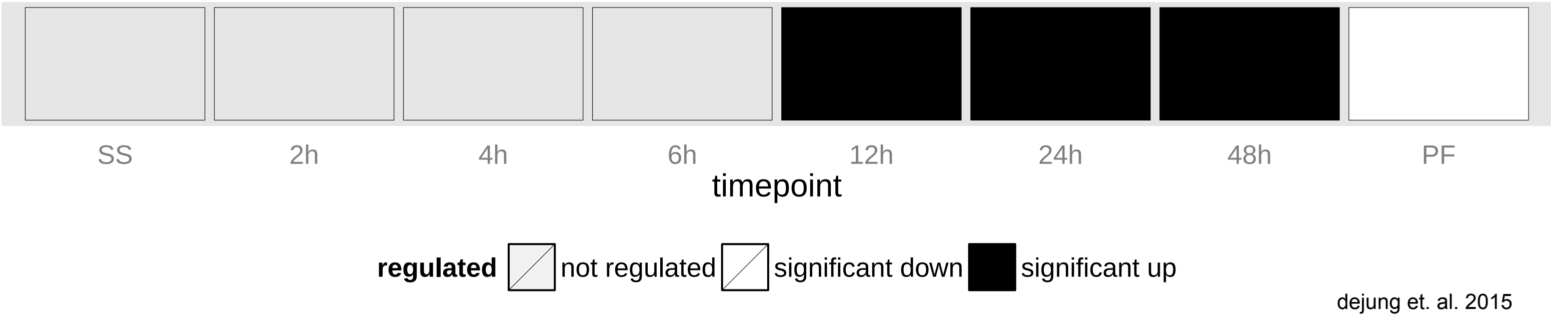

SS
2h
4h
6h
12h
24h
48h
PF
timepoint
regulated
not regulated
significant down
significant up
dejung et. al. 2015

hypothetical protein, conserved
Tb927.11.12840
AGOF: null
AGOC: null
AGOP: null
PGOF: null
PGOC: null
PGOP: null

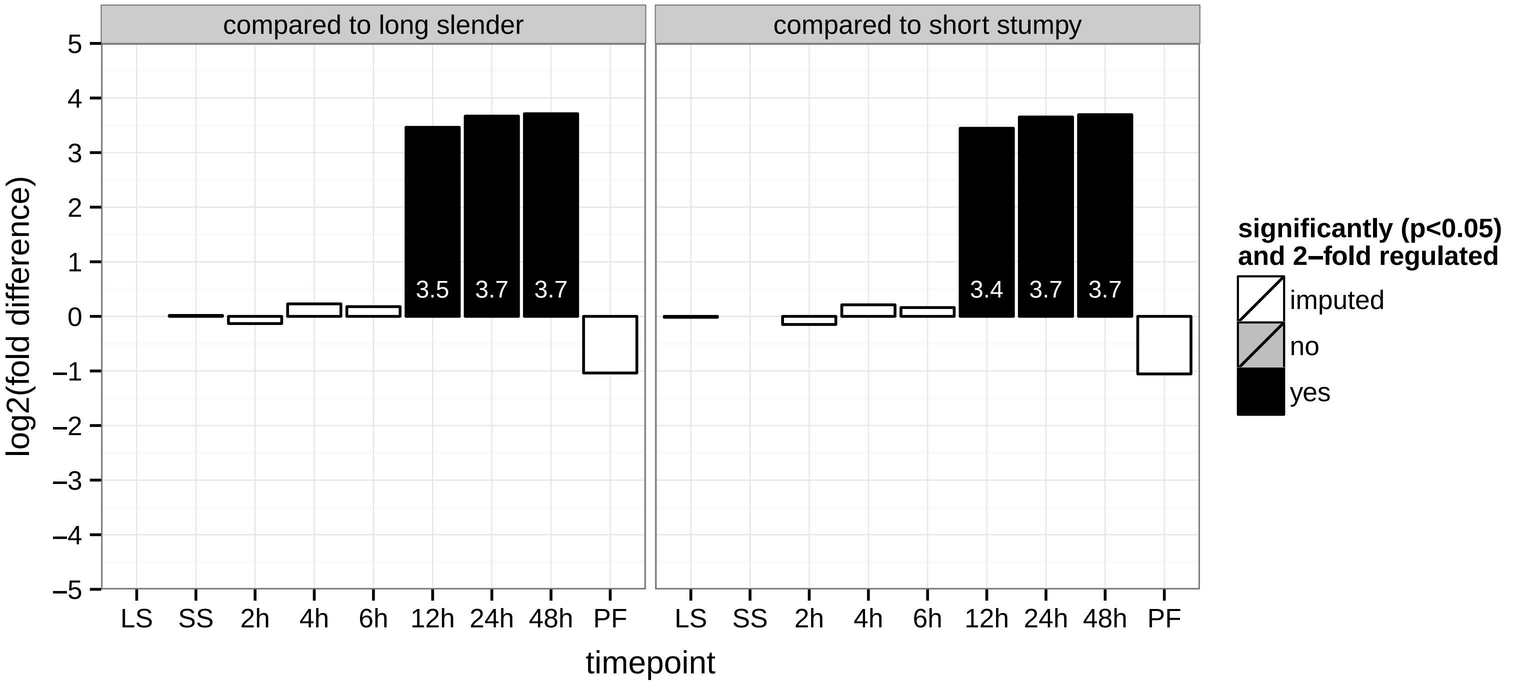

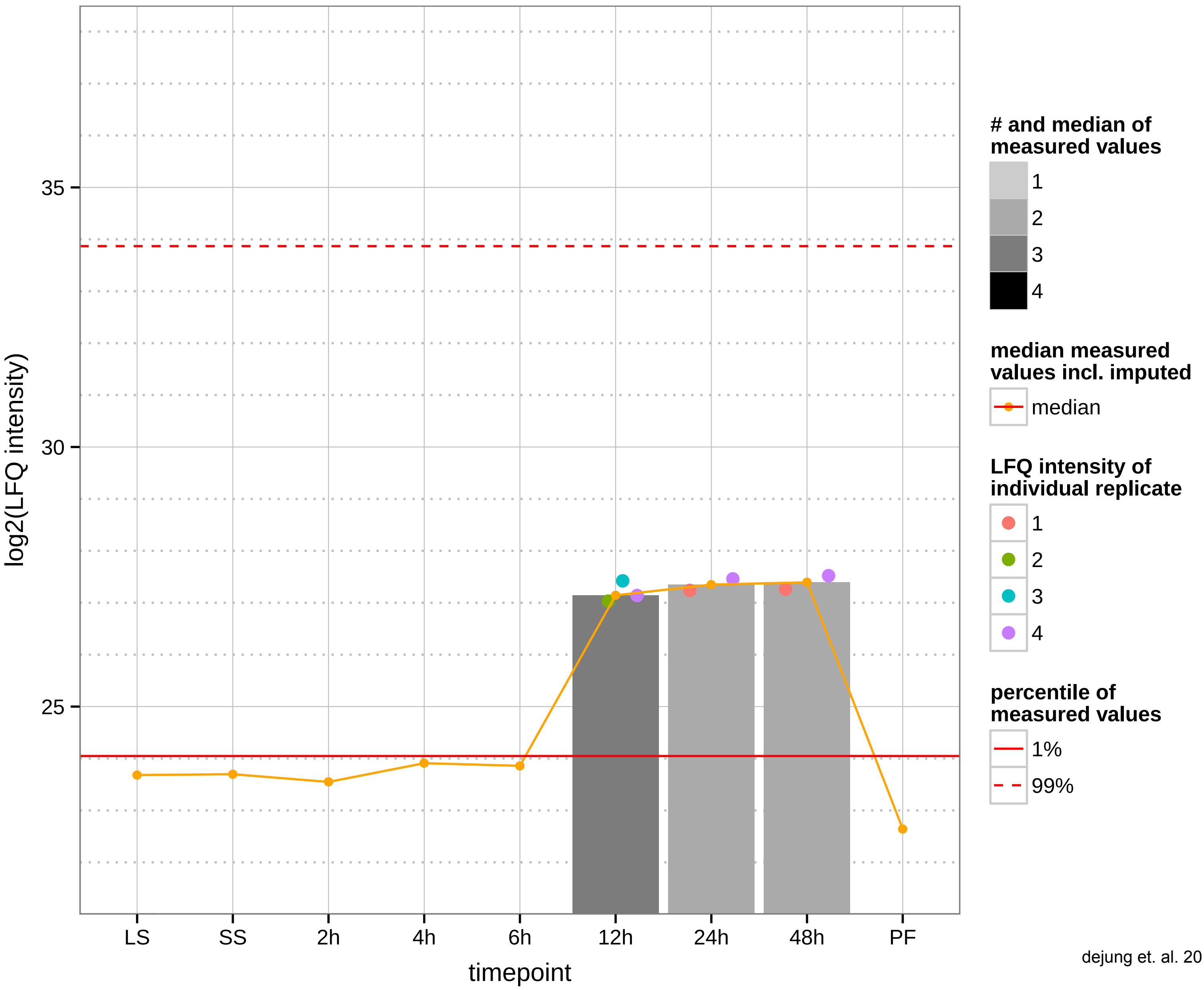

dejung et. al. 2015

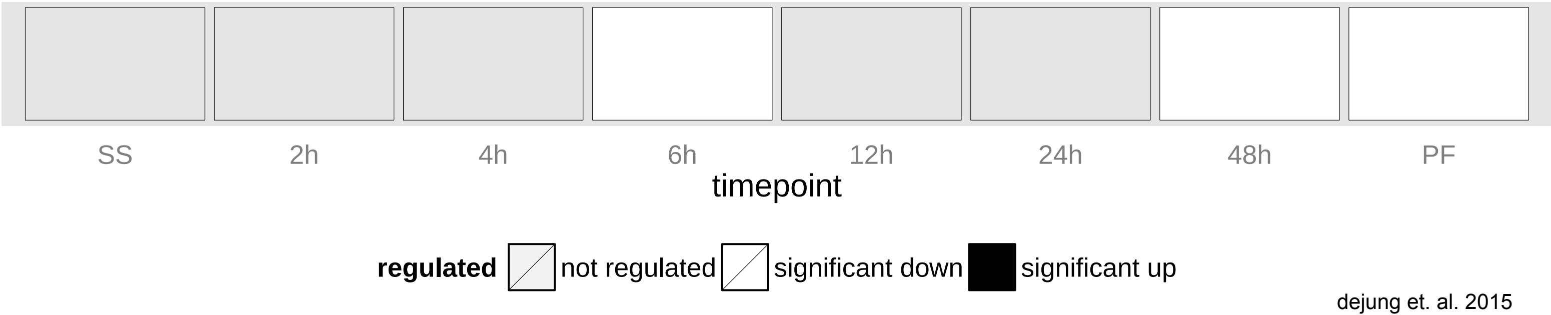
SS
2h
4h
6h
12h
24h
48h
PF
timepoint
regulated
not regulated
significant down
significant up
dejung et. al. 2015

hypothetical protein, conserved
Tb927.9.10060
AGOF: null
AGOC: null
AGOP: null
PGOF: null
PGOC: null
PGOP: null

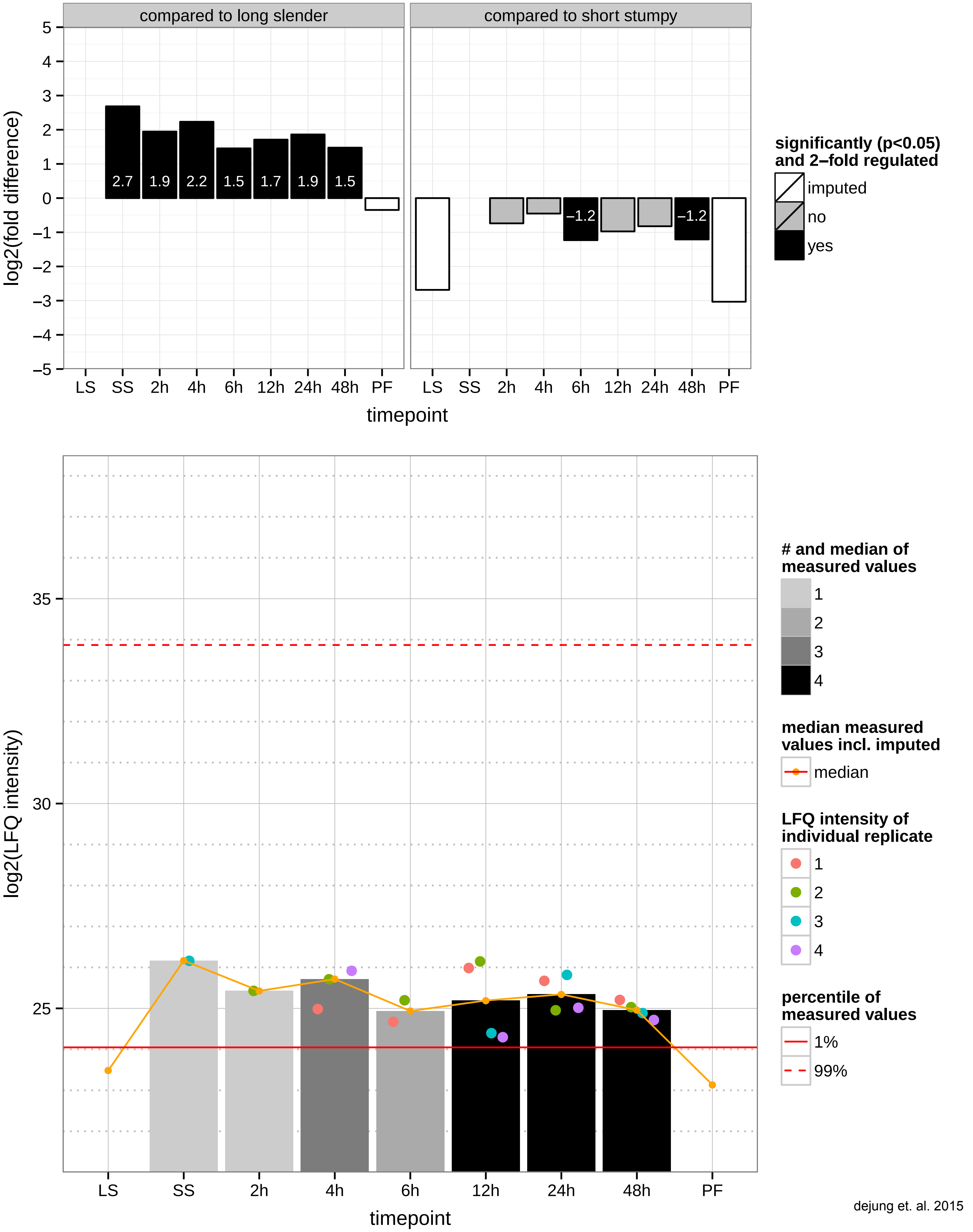

dejung et. al. 2015

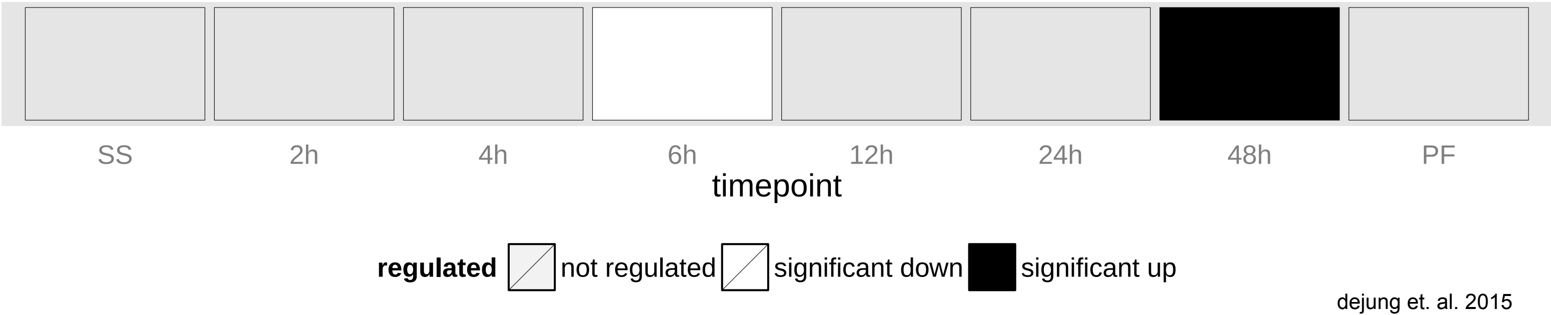
SS
2h
4h
6h
12h
24h
48h
PF
timepoint
regulated
not regulated
significant down
significant up
dejung et. al. 2015

phosphoglycerate kinase (PGKB)
Tb927.1.710
AGOF: phosphoglycerate kinase activity
AGOC: cytoplasm
AGOP: glycolysis
PGOF: phosphoglycerate kinase activity
PGOC: null
PGOP: glycolysis

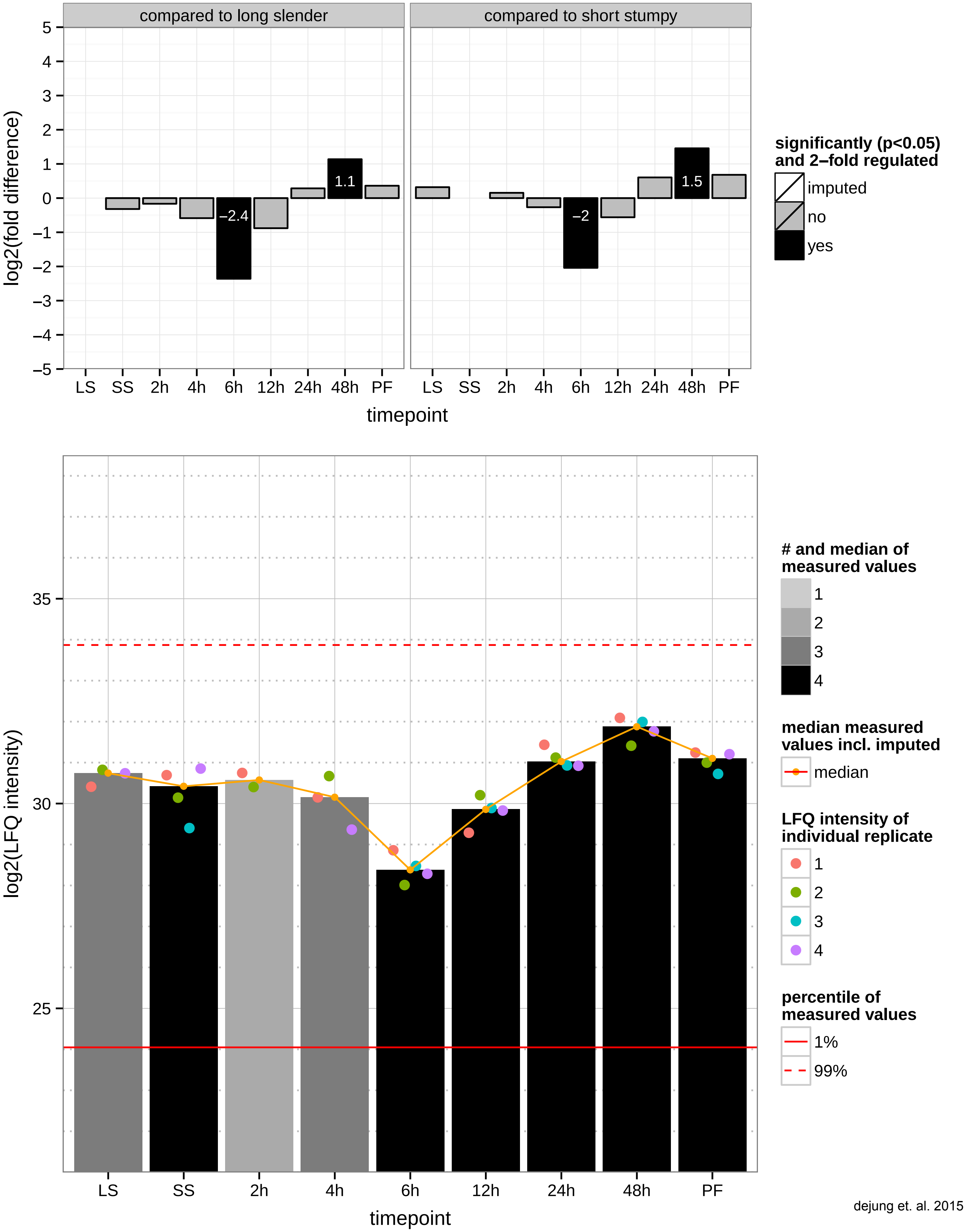

dejung et. al. 2015

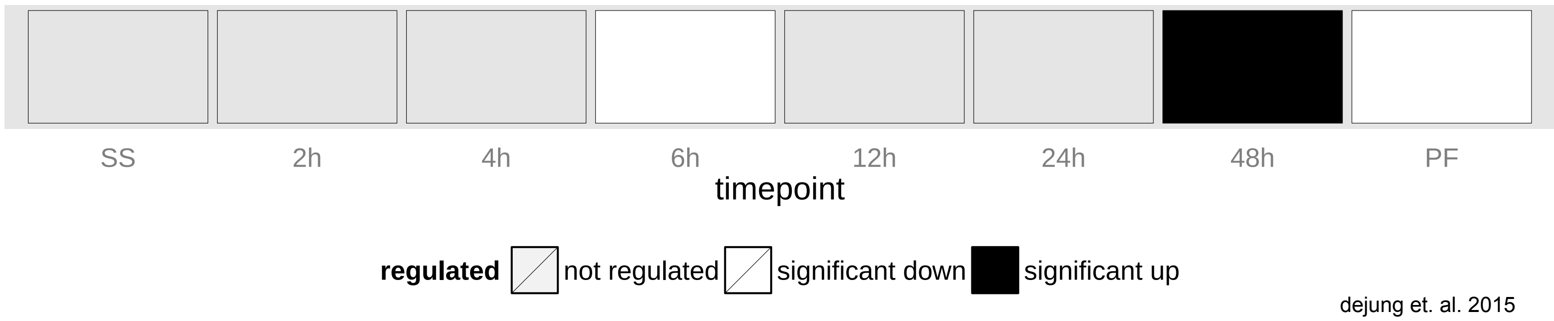
SS
2h
4h
6h
12h
24h
48h
PF
timepoint
regulated
not regulated
significant down
significant up
dejung et. al. 2015

chrX additional, unordered contigs, hypothetical protein, conserved
Tb927.10.1160;Tb11.0550
AGOF: null
AGOC: null, mitochondrion
AGOP: null
PGOF: null
PGOC: null
PGOP: null

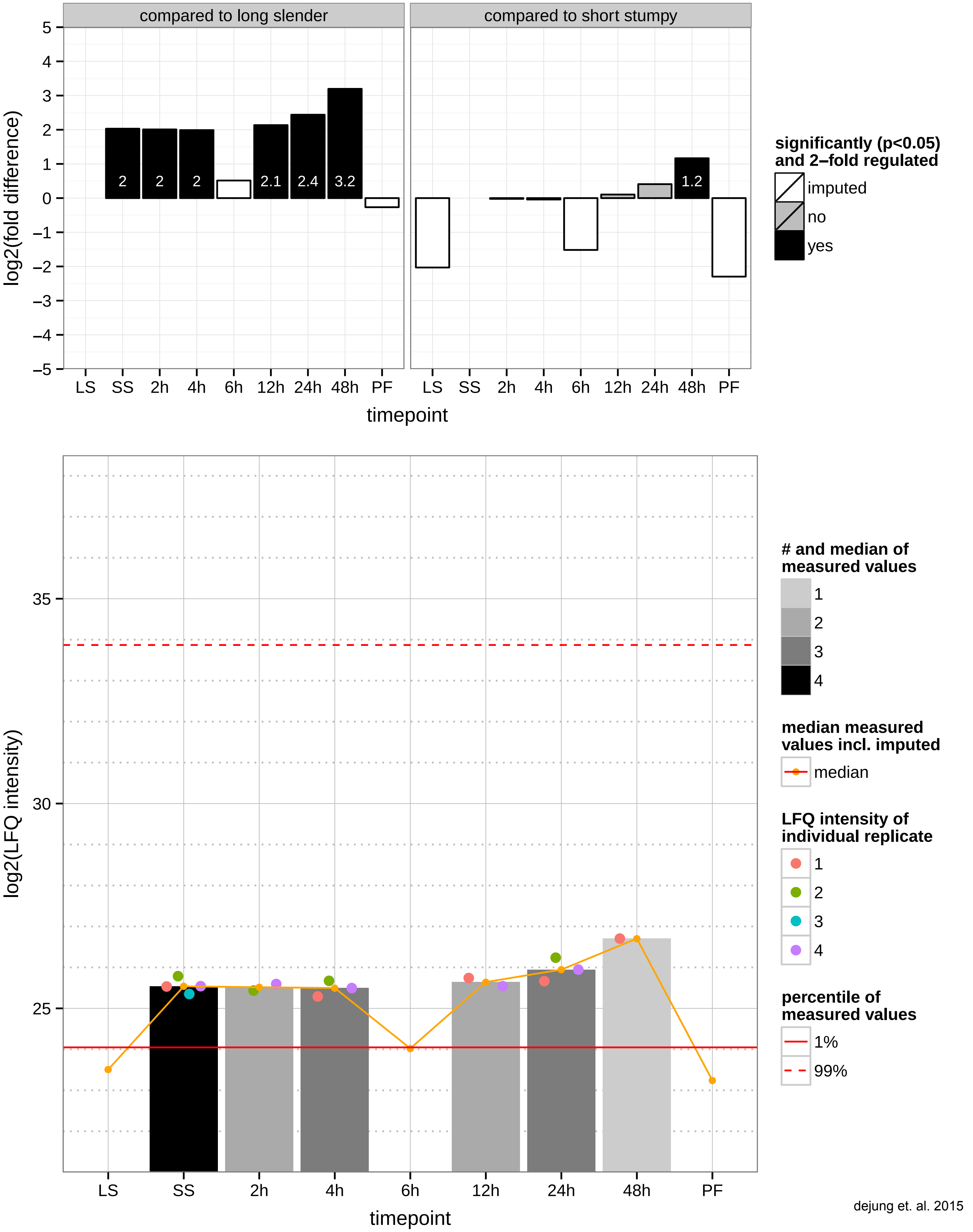

dejung et. al. 2015

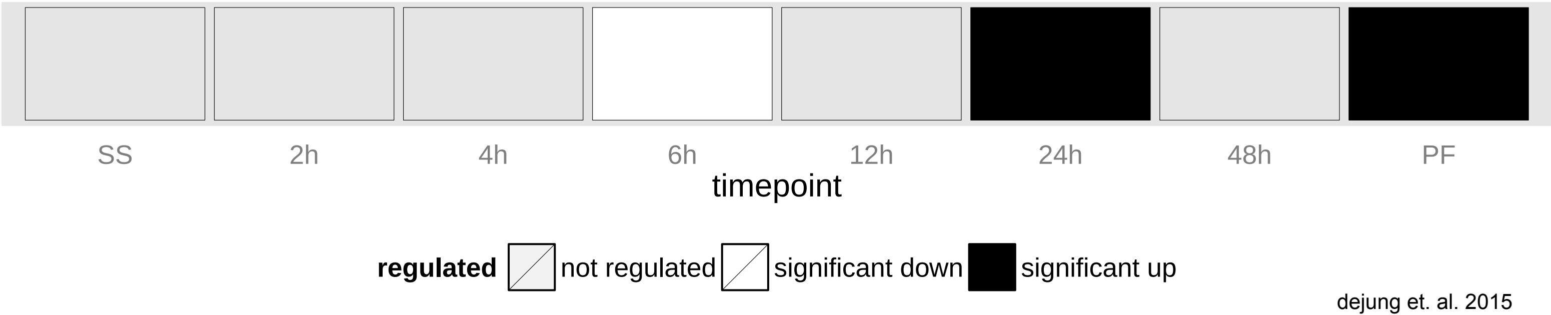
SS
2h
4h
6h
12h
24h
48h
PF
timepoint
regulated
not regulated
significant down
significant up
dejung et. al. 2015

hypothetical protein, conserved
Tb927.3.820
AGOF: null
AGOC: mitochondrion
AGOP: null
PGOF: null
PGOC: null
PGOP: null

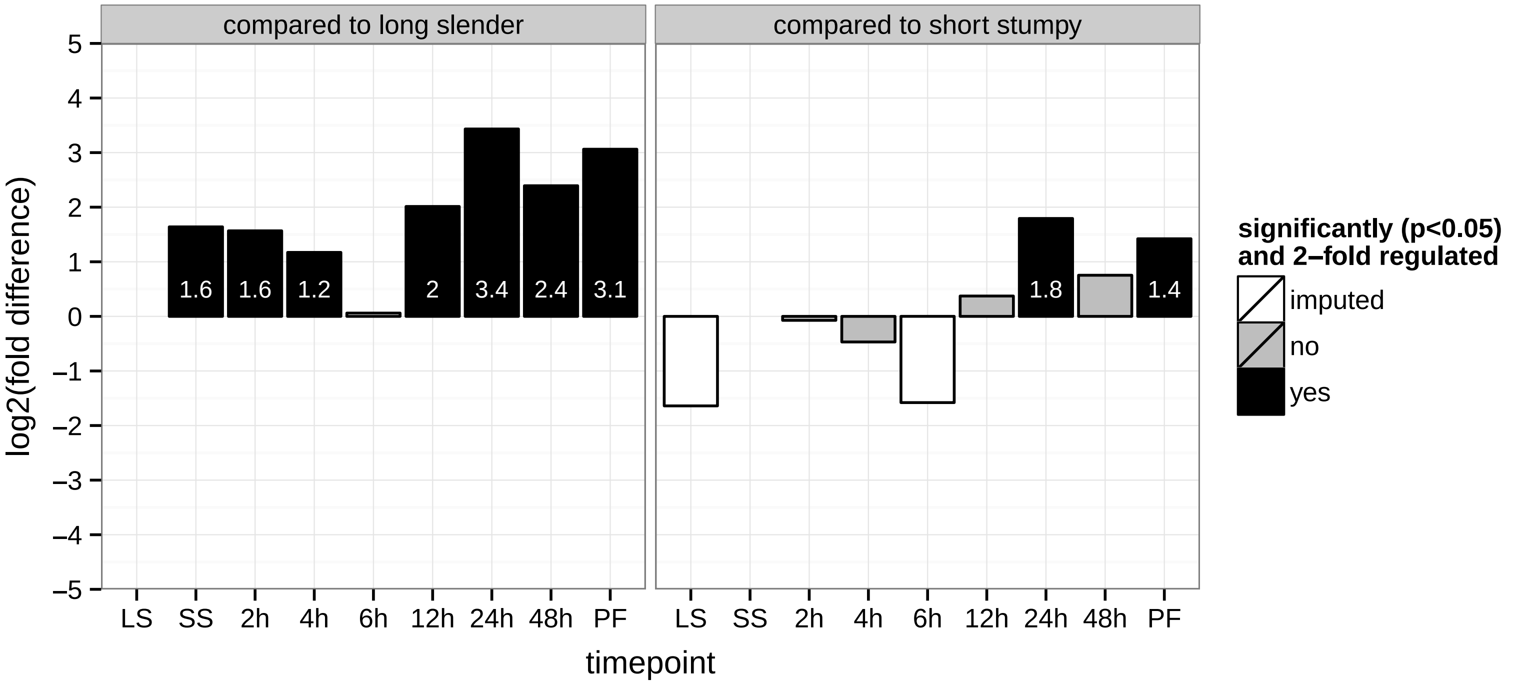

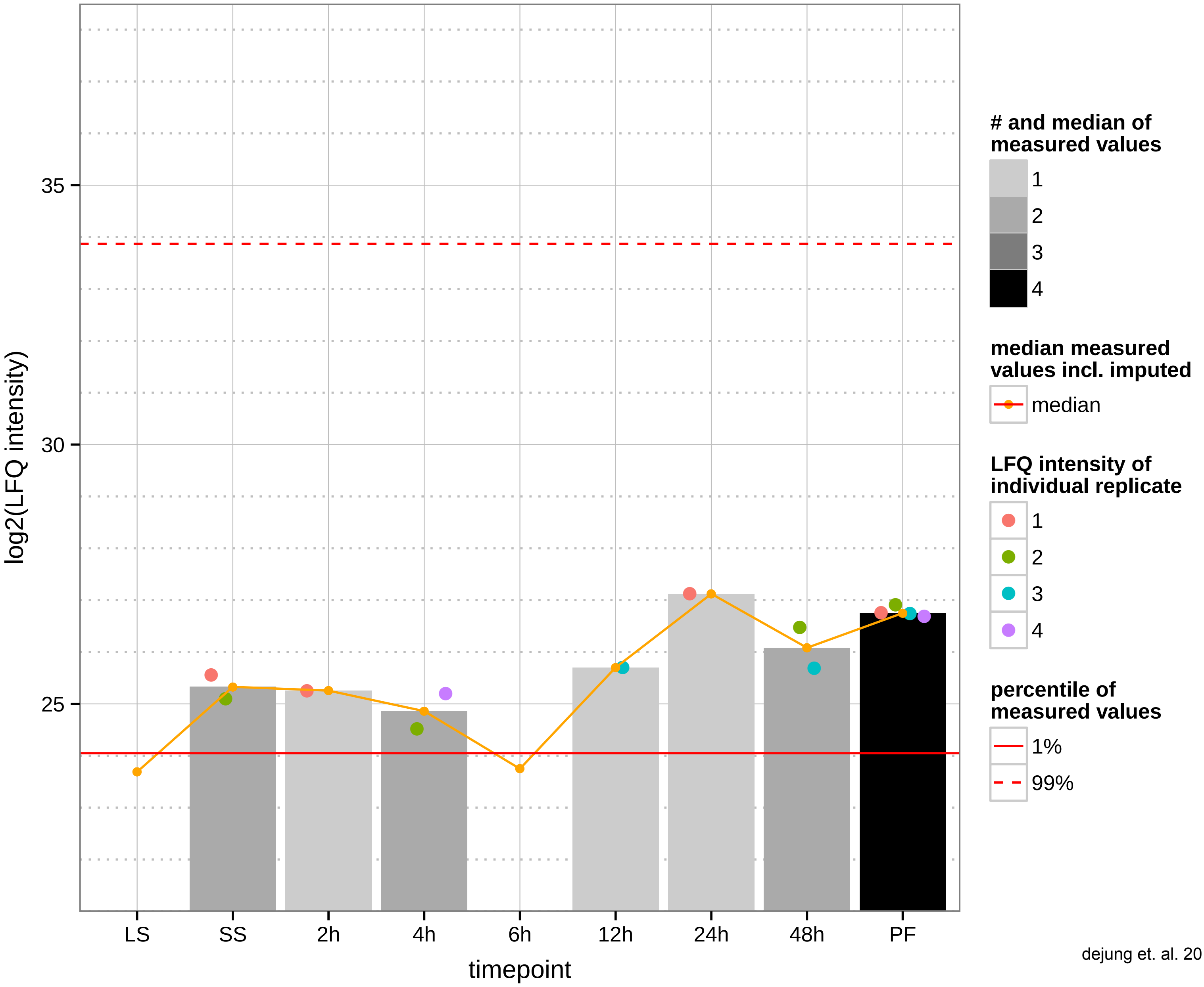

dejung et. al. 2015

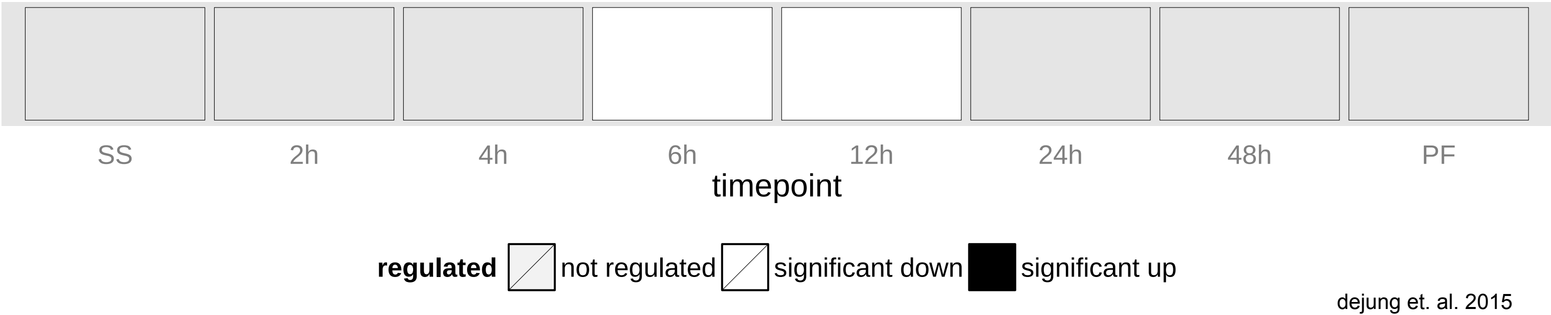
SS
2h
4h
6h
12h
24h
48h
PF
timepoint
regulated
not regulated
significant down
significant up
dejung et. al. 2015

class I transcription factor A, subunit 6 (CITFA−6)
Tb927.5.970
AGOF: null
AGOC: null
AGOP: null
PGOF: null
PGOC: null
PGOP: null

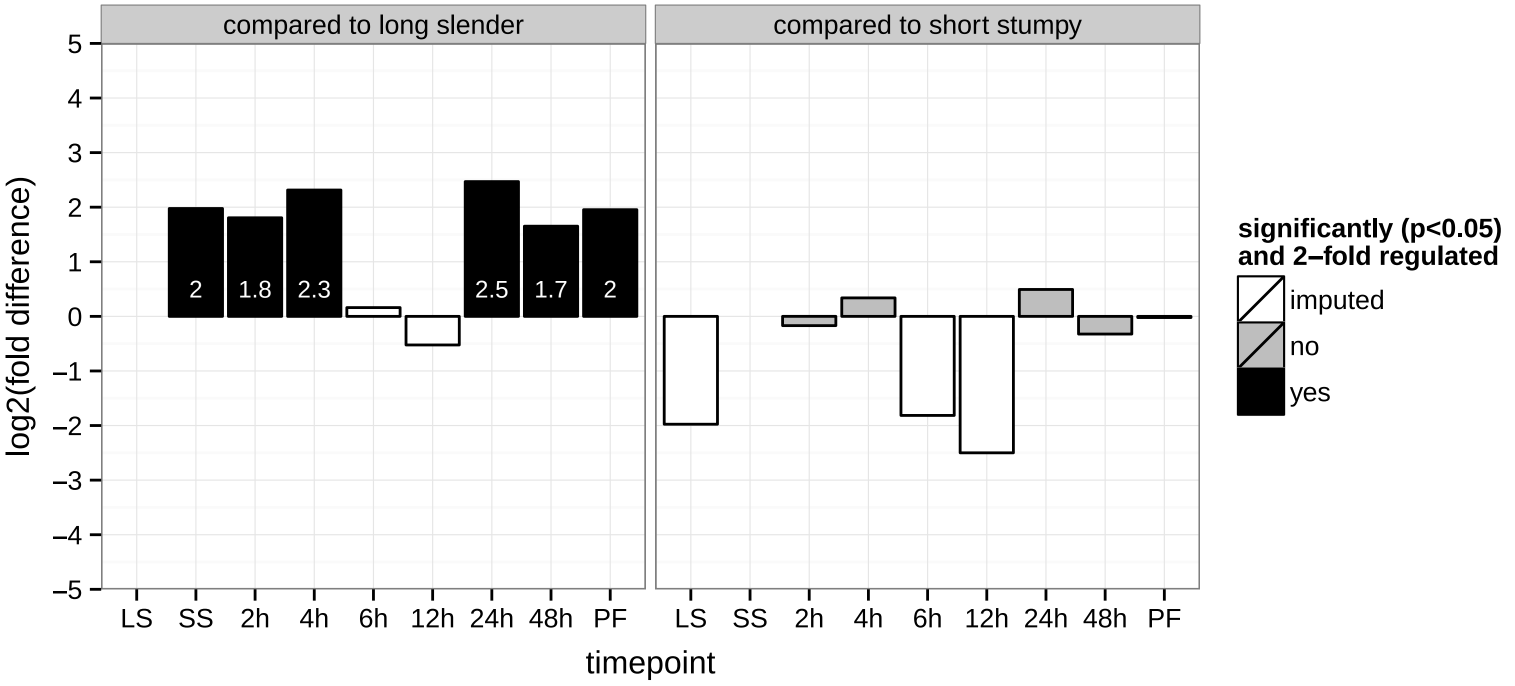

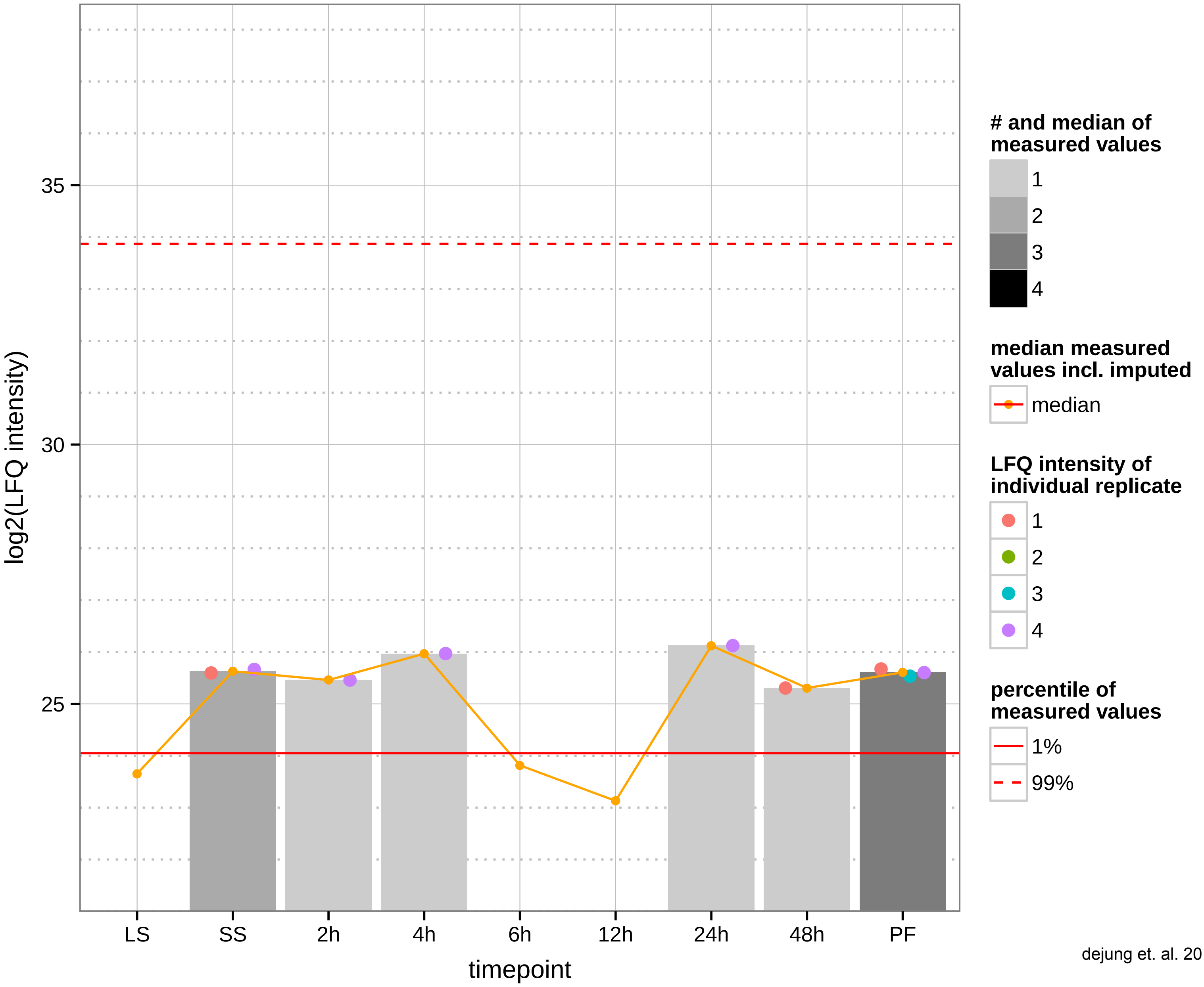

dejung et. al. 2015

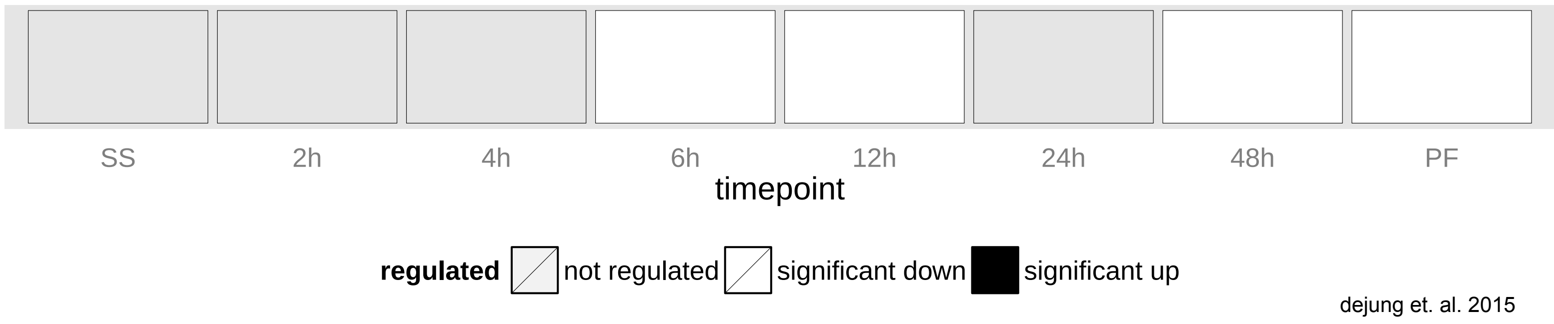
SS
2h
4h
6h
12h
24h
48h
PF
timepoint
regulated
not regulated
significant down
significant up
dejung et. al. 2015

ATG8/AUT7/APG8/PAZ2, putative, ubiquitin–like protein (ATG8A), ubiquitin–like protein (ATG8B)
Tb927.7.5910;Tb927.7.5900
AGOF: null
AGOC: null
AGOP: null
PGOF: null
PGOC: null
PGOP: null

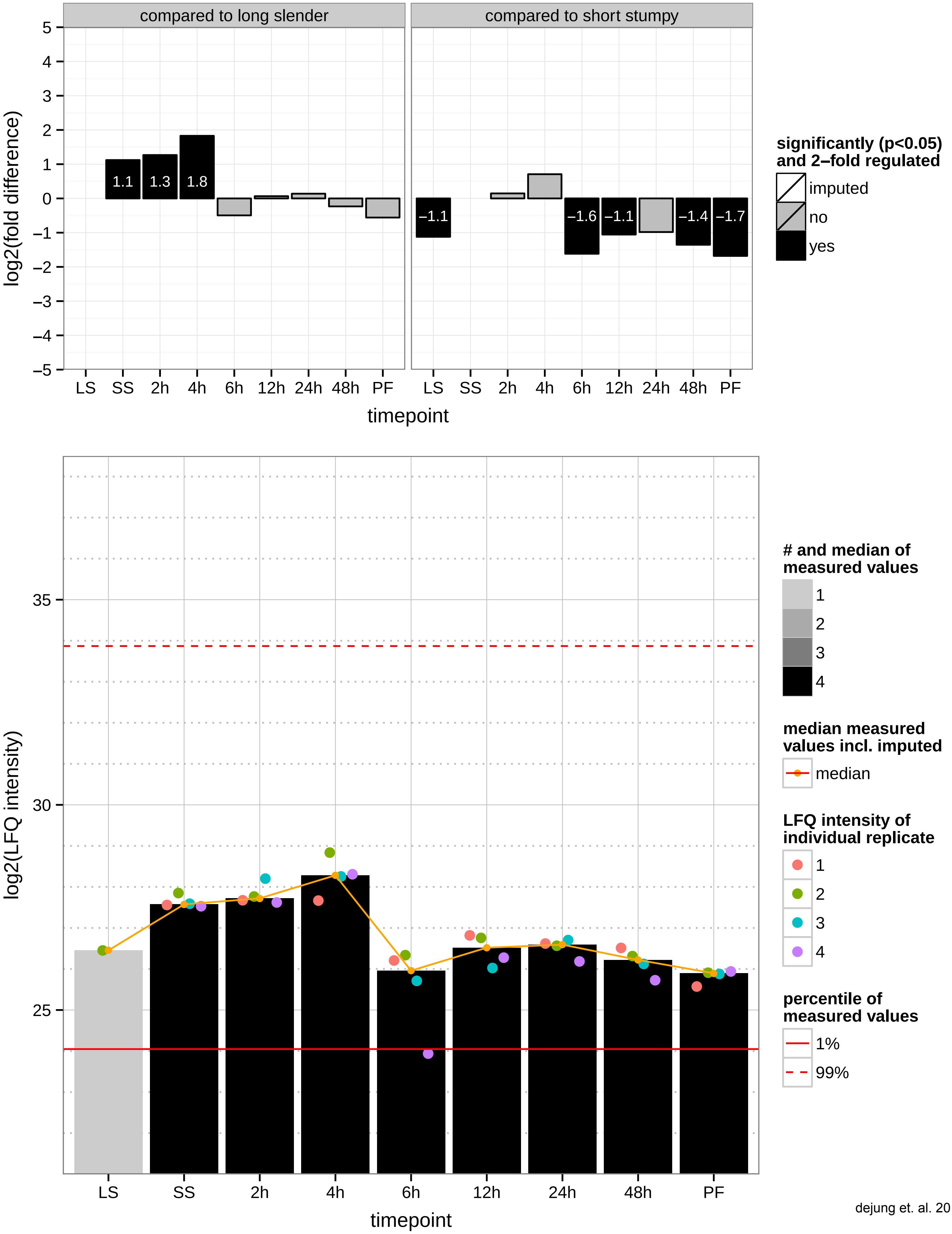

dejung et. al. 2015

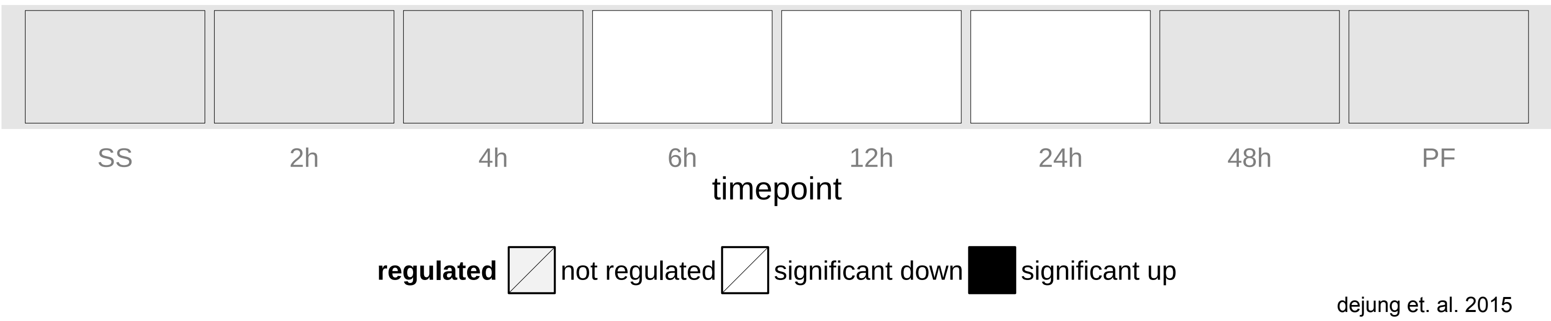

SS
2h
4h
6h
12h
24h
48h
PF
timepoint
regulated
not regulated
significant down
significant up
dejung et. al. 2015

hypothetical protein, conserved
Tb927.8.4910
AGOF: null
AGOC: null
AGOP: null
PGOF: null
PGOC: null
PGOP: null

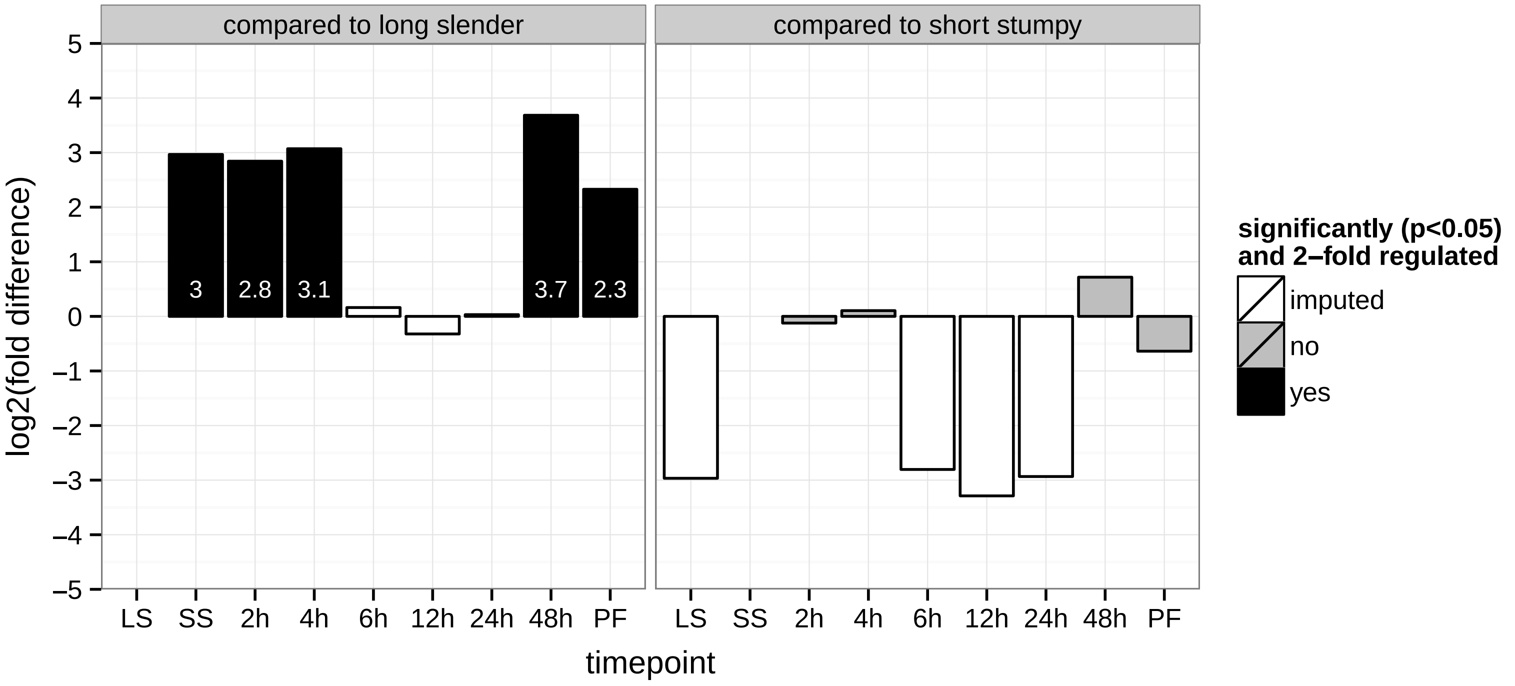

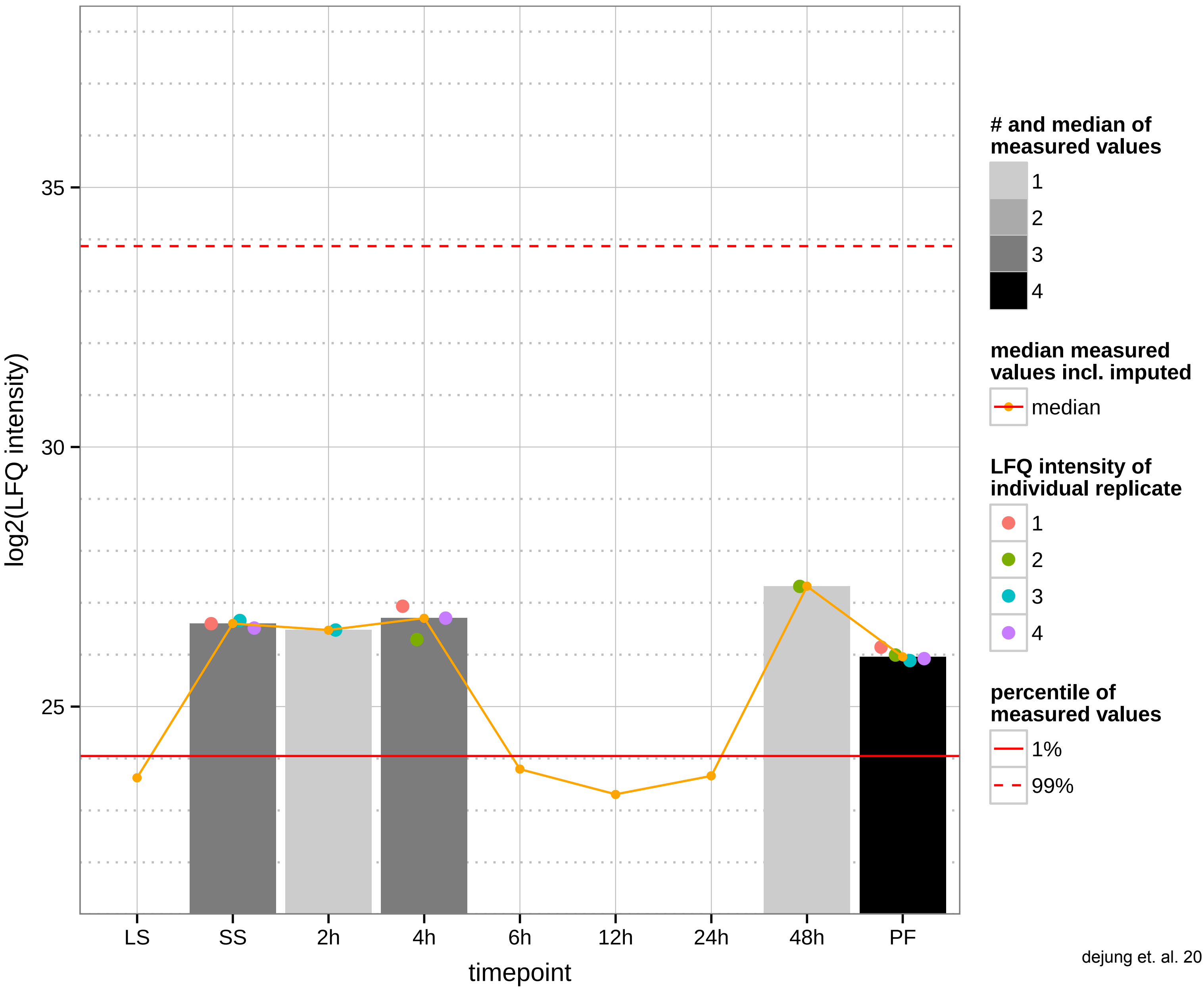

dejung et. al. 2015

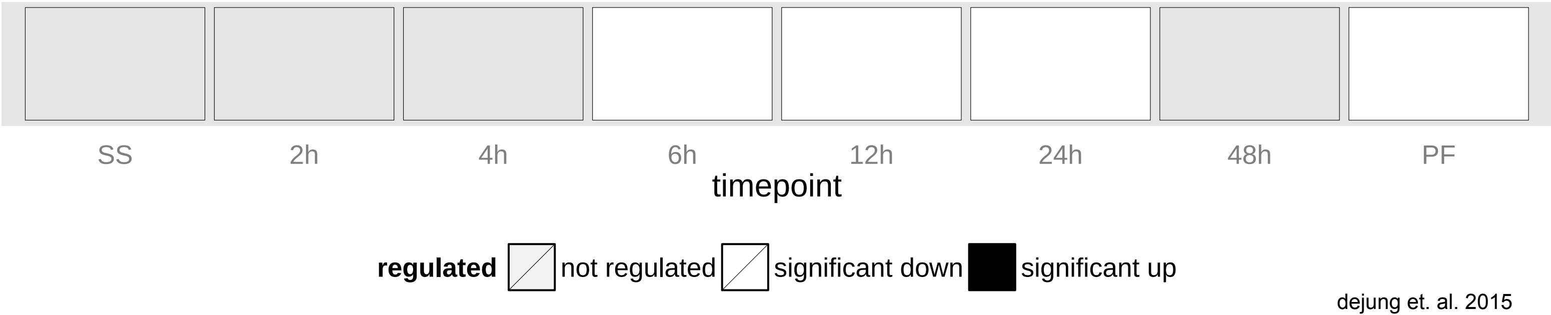
SS
2h
4h
6h
12h
24h
48h
PF
timepoint
regulated
not regulated
significant down
significant up
dejung et. al. 2015

hypothetical protein, conserved
Tb927.11.4550
AGOF: null
AGOC: null
AGOP: null
PGOF: null
PGOC: null
PGOP: null

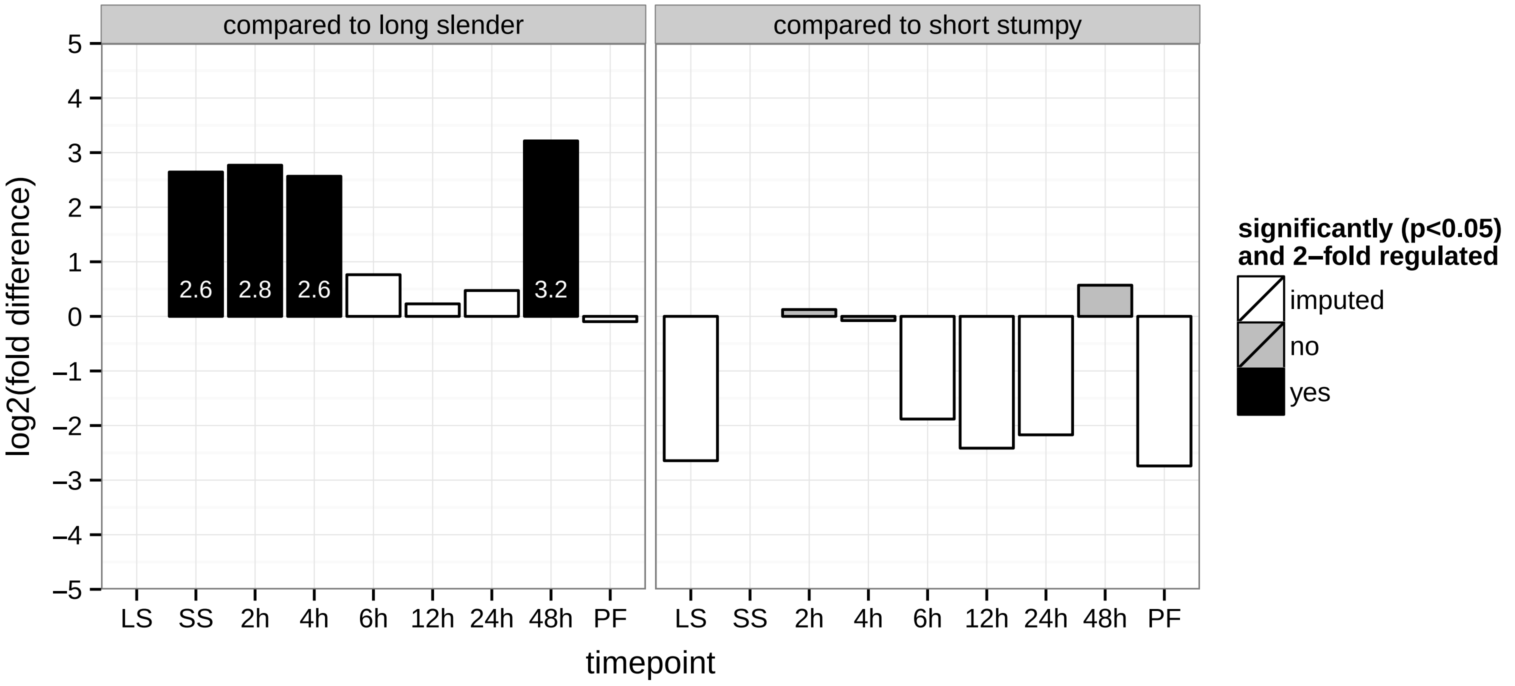

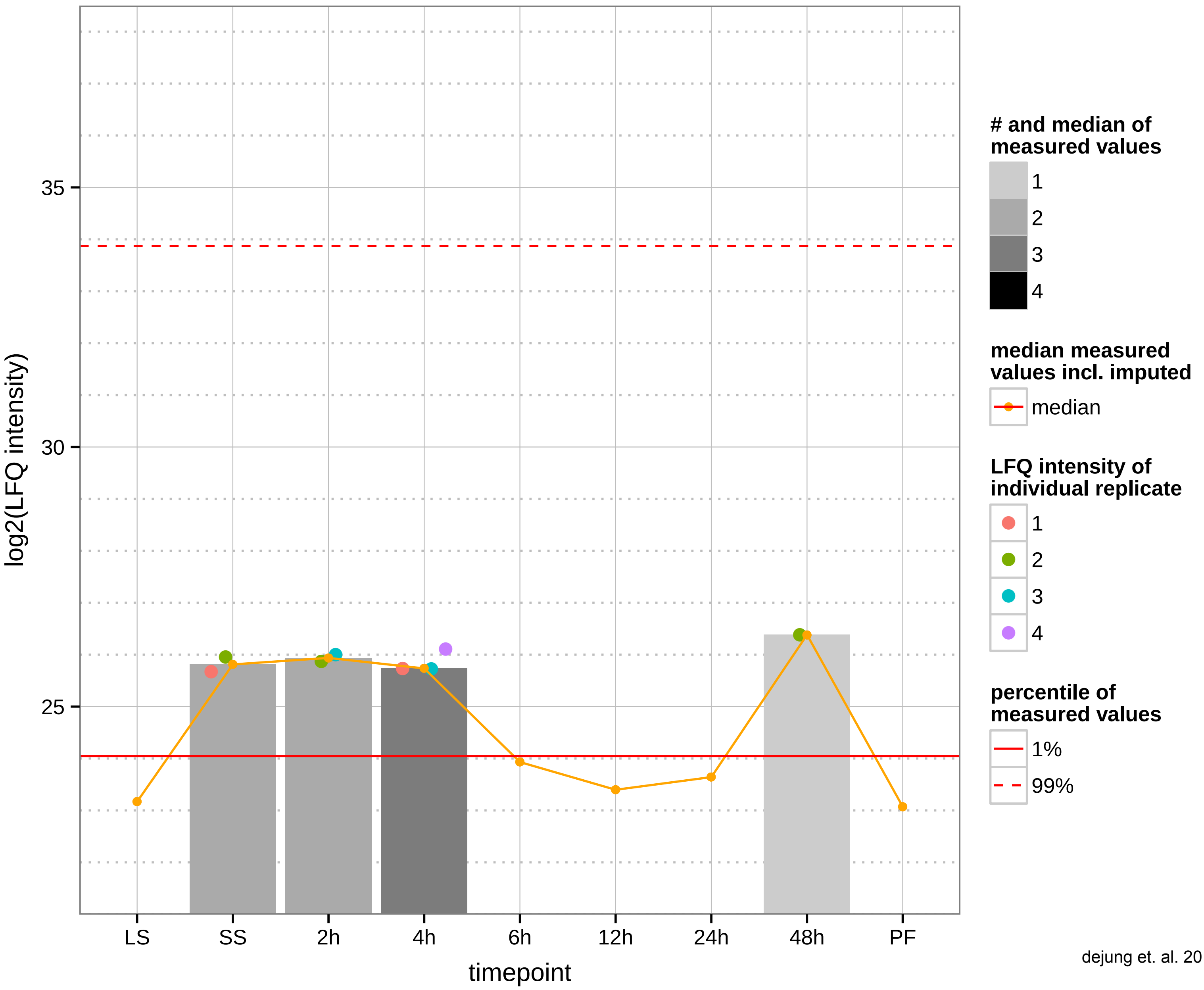

dejung et. al. 2015

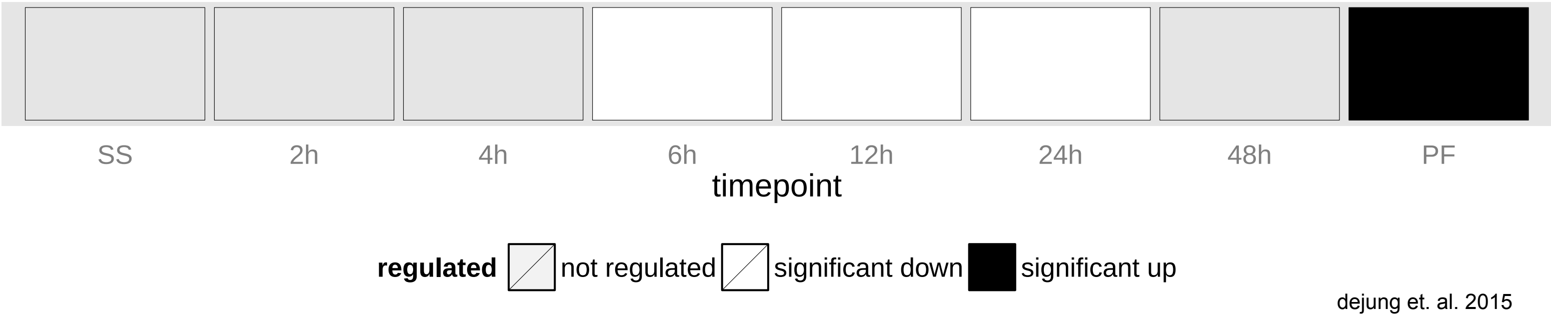
SS
2h
4h
6h
12h
24h
48h
PF
timepoint
regulated
not regulated
significant down
significant up
dejung et. al. 2015

endosomal integral membrane protein, putative
Tb927.8.1940
AGOF: null
AGOC: integral to membrane
AGOP: null
PGOF: null
PGOC: integral to membrane
PGOP: null

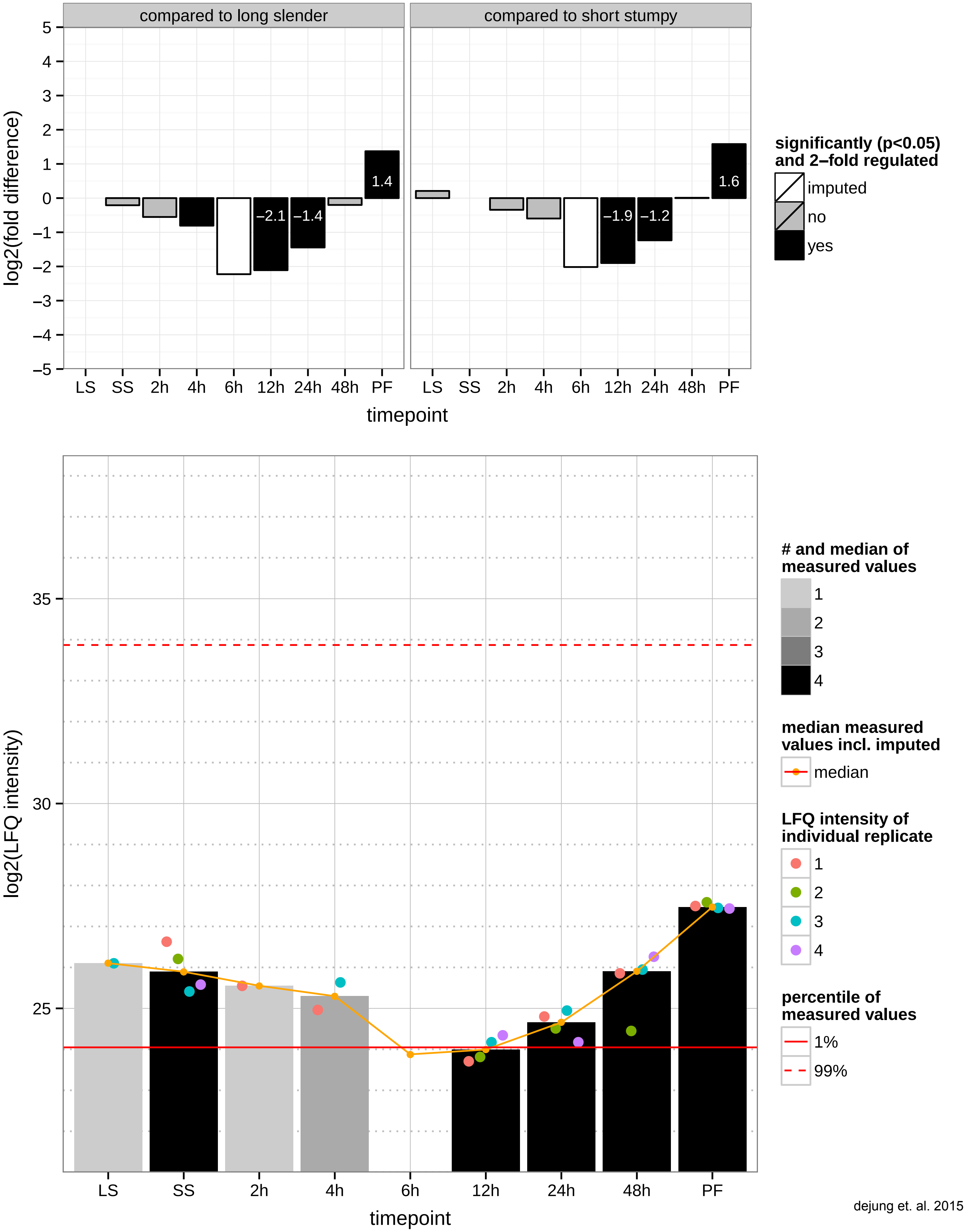

dejung et. al. 2015

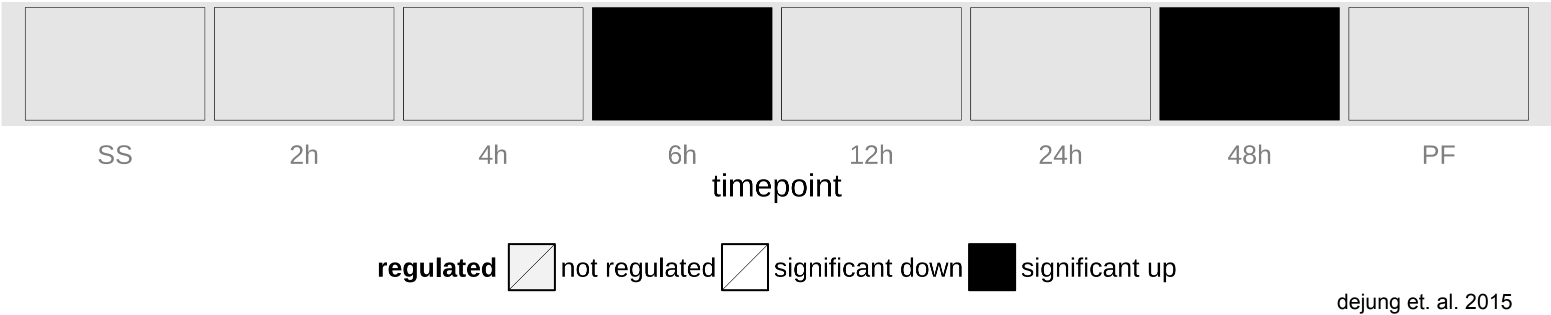
SS
2h
4h
6h
12h
24h
48h
PF
timepoint
regulated
not regulated
significant down
significant up
dejung et. al. 2015

expression site–associated gene (ESAG, pseudogene), putative, expression site–associated gene 4 (ESAG4), degenerate
Tb927.2.1260
AGOF: phosphorus–oxygen lyase activity
AGOC: null
AGOP: cyclic nucleotide biosynthetic process, intracellular signal transduction
PGOF: phosphorus–oxygen lyase activity
PGOC: null
PGOP: cyclic nucleotide biosynthetic process, intracellular signal transduction

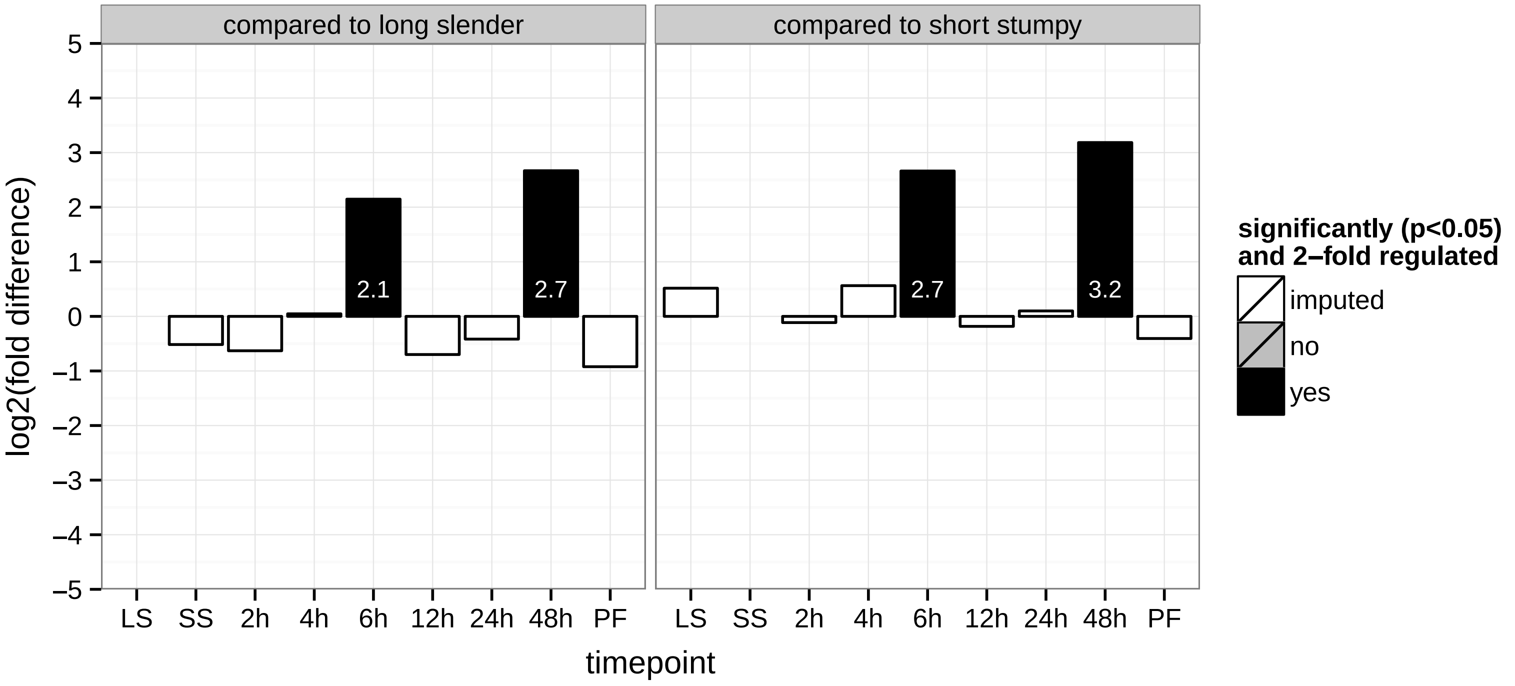

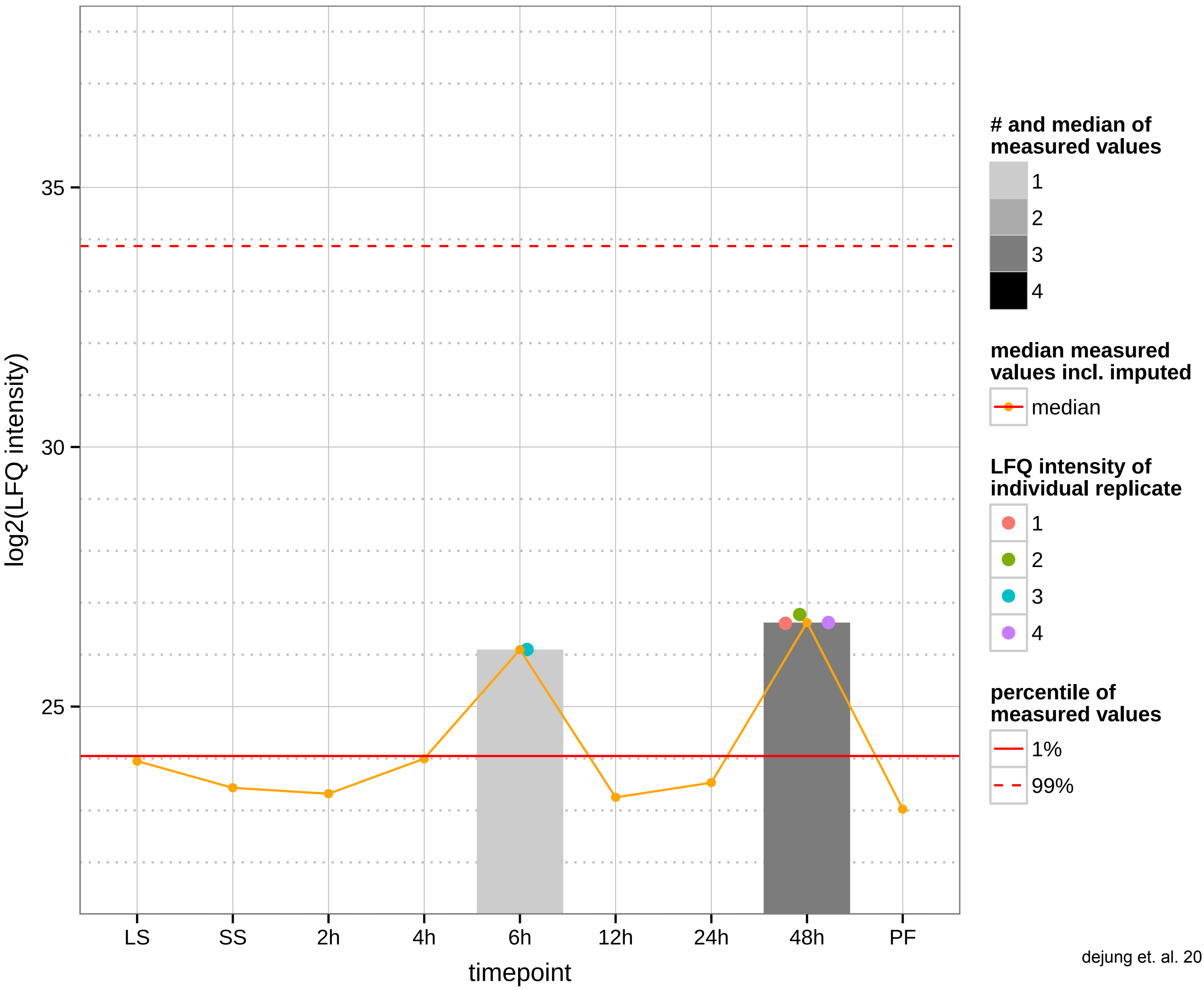

dejung et. al. 2015

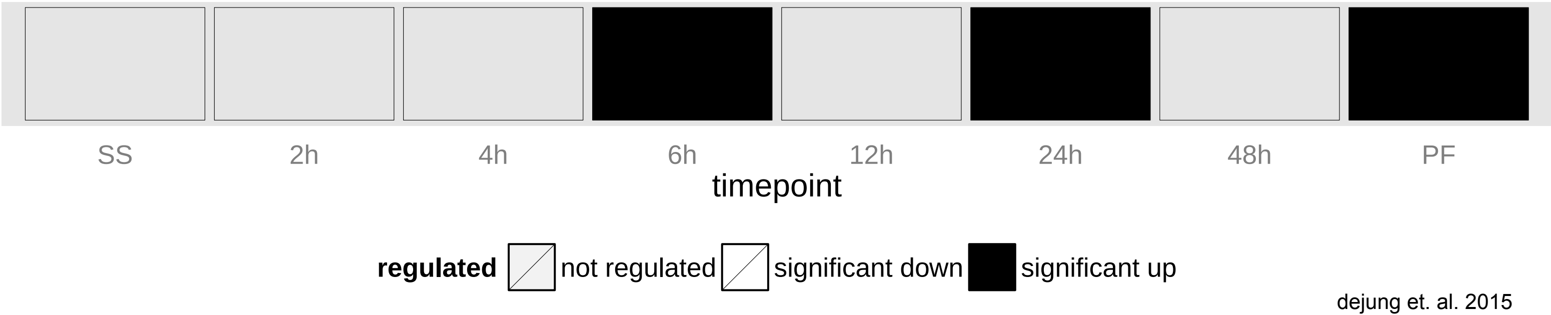
SS
2h
4h
6h
12h
24h
48h
PF
timepoint
regulated
not regulated
significant down
significant up
dejung et. al. 2015

COP9 signalosome complex subunit 5, putative, JAB−MPN domain protein
Tb927.8.5530
AGOF: null
AGOC: signalosome
AGOP: null
PGOF: protein binding
PGOC: null
PGOP: null

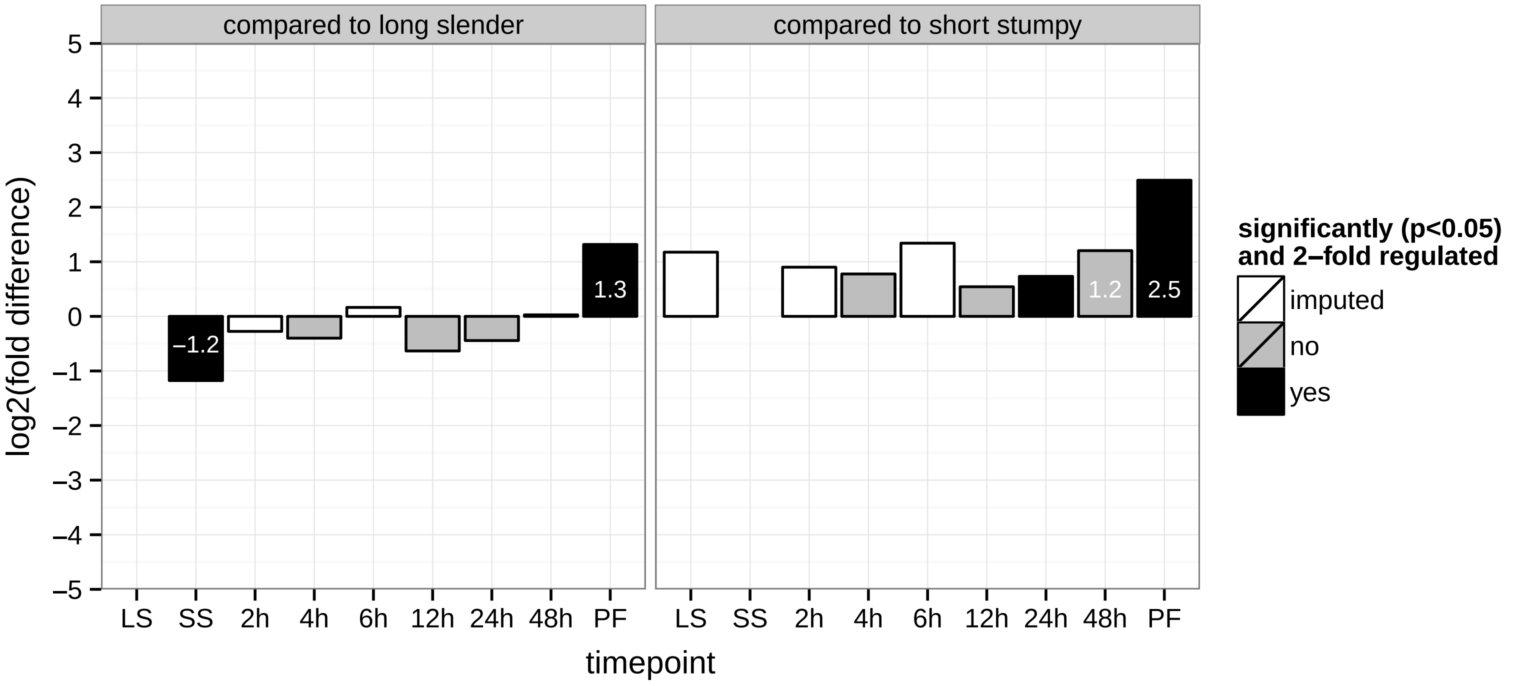

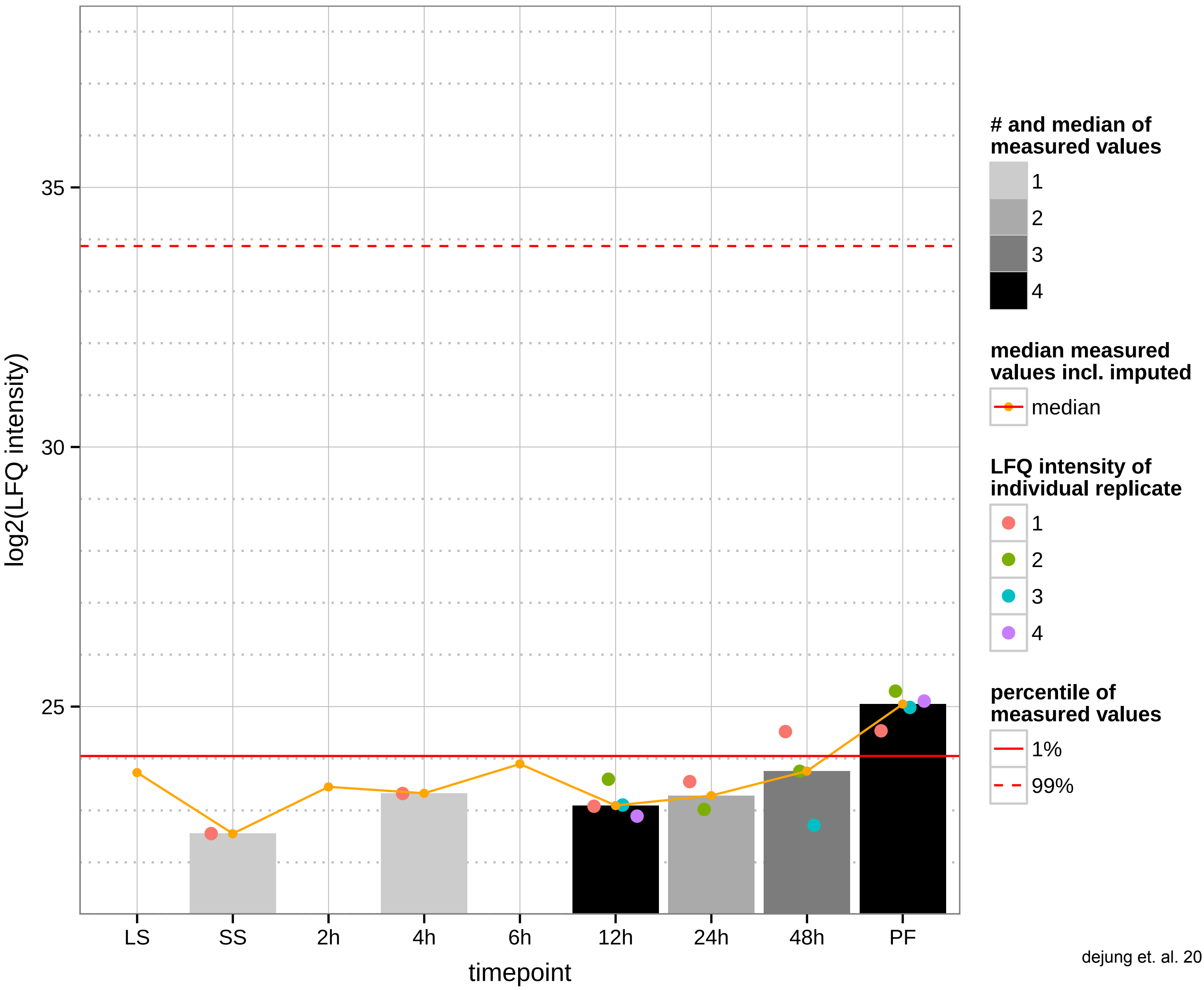

dejung et. al. 2015

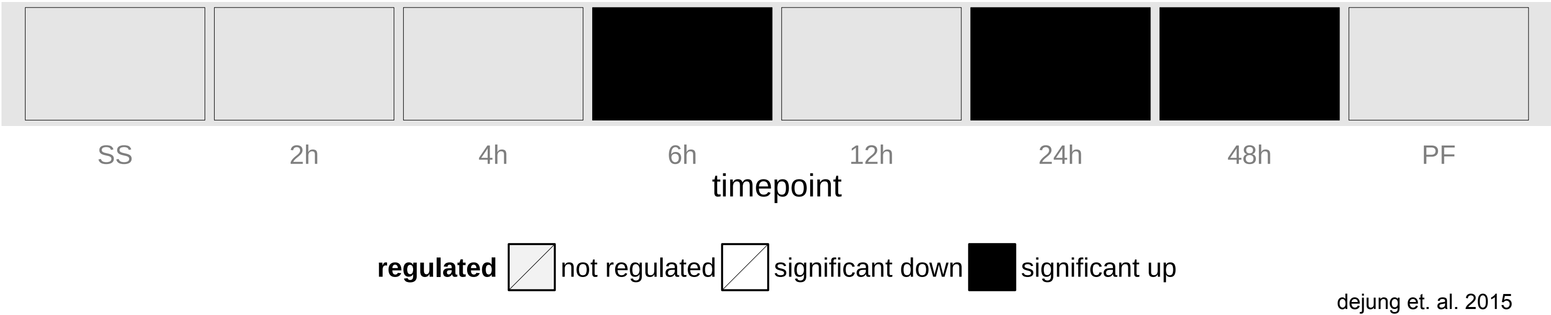
SS
2h
4h
6h
12h
24h
48h
PF
timepoint
regulated
not regulated
significant down
significant up
dejung et. al. 2015

hypothetical protein, conserved
Tb927.9.11940
AGOF: null
AGOC: mitochondrion
AGOP: null
PGOF: null
PGOC: null
PGOP: null

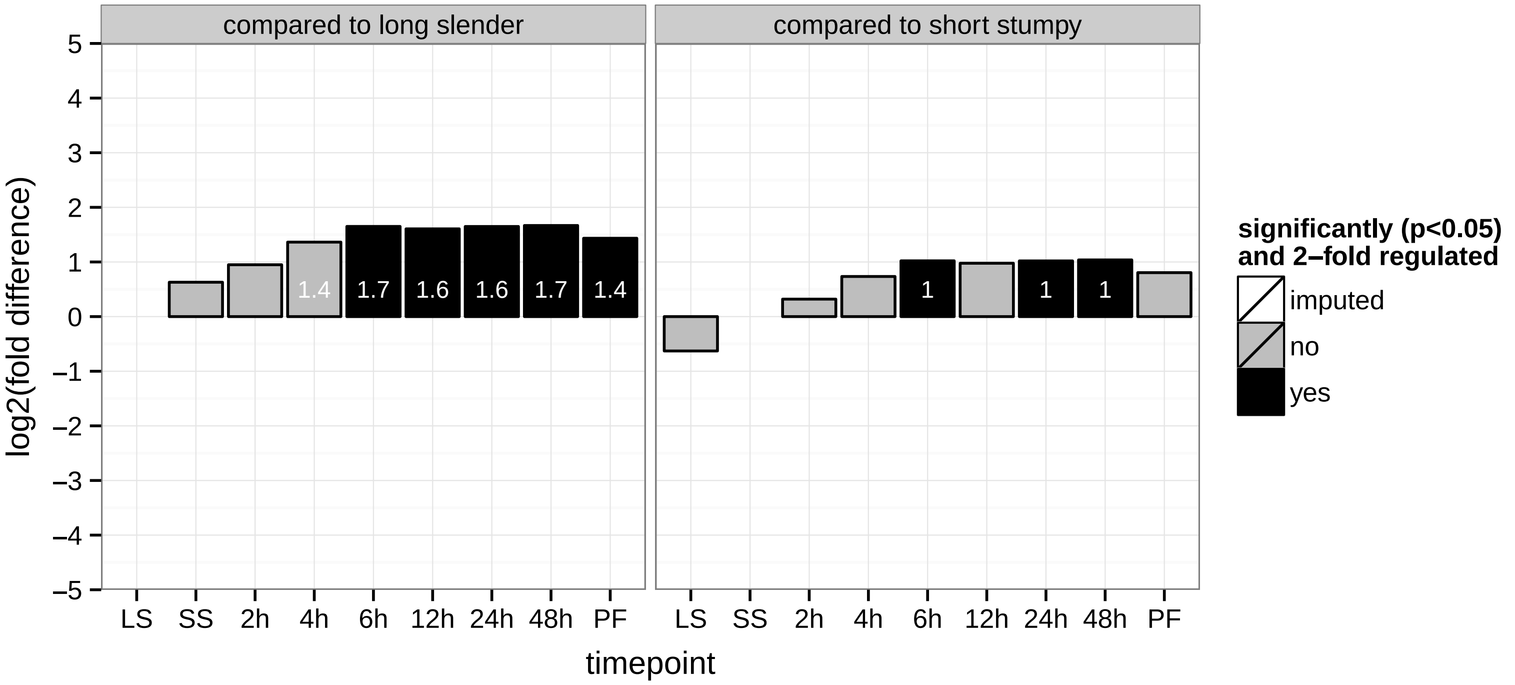

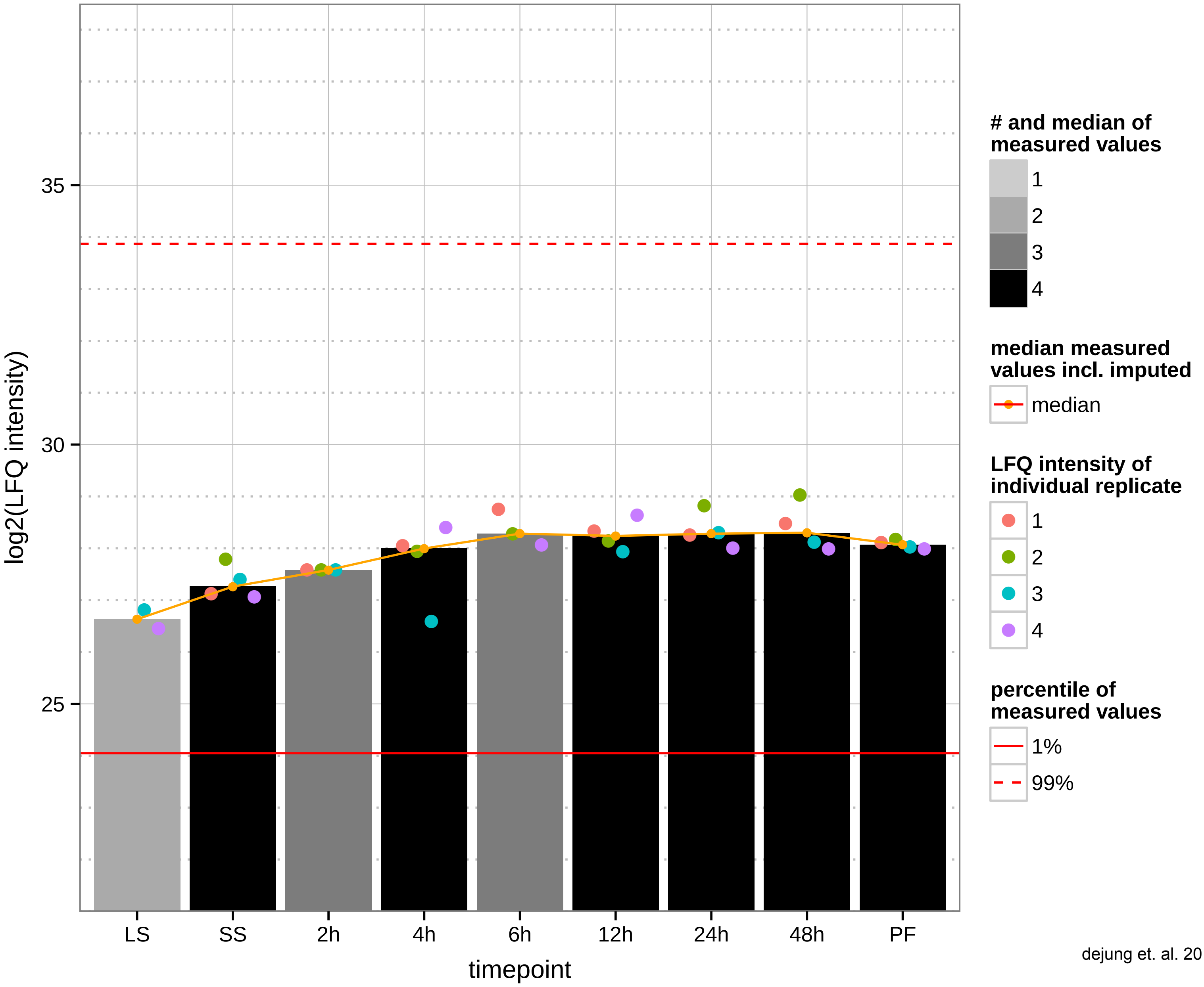

dejung et. al. 2015

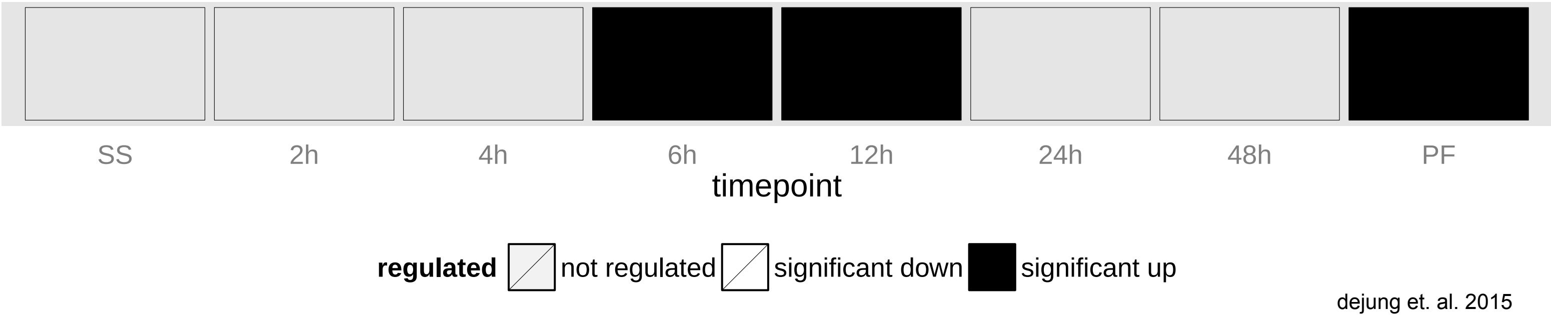
SS
2h
4h
6h
12h
24h
48h
PF
timepoint
regulated
not regulated
significant down
significant up
dejung et. al. 2015

hypothetical protein, conserved
Tb927.8.3810
AGOF: null
AGOC: mitochondrion
AGOP: null
PGOF: null
PGOC: null
PGOP: null

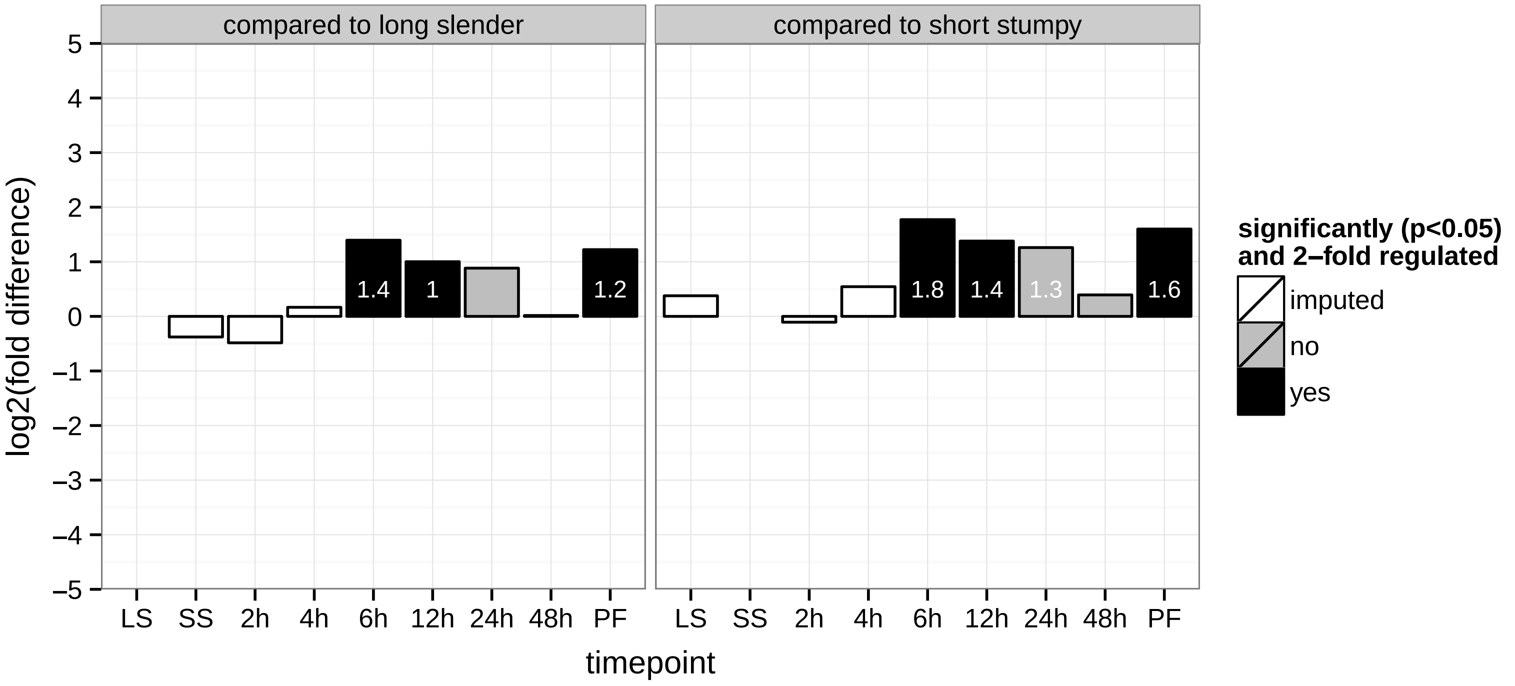

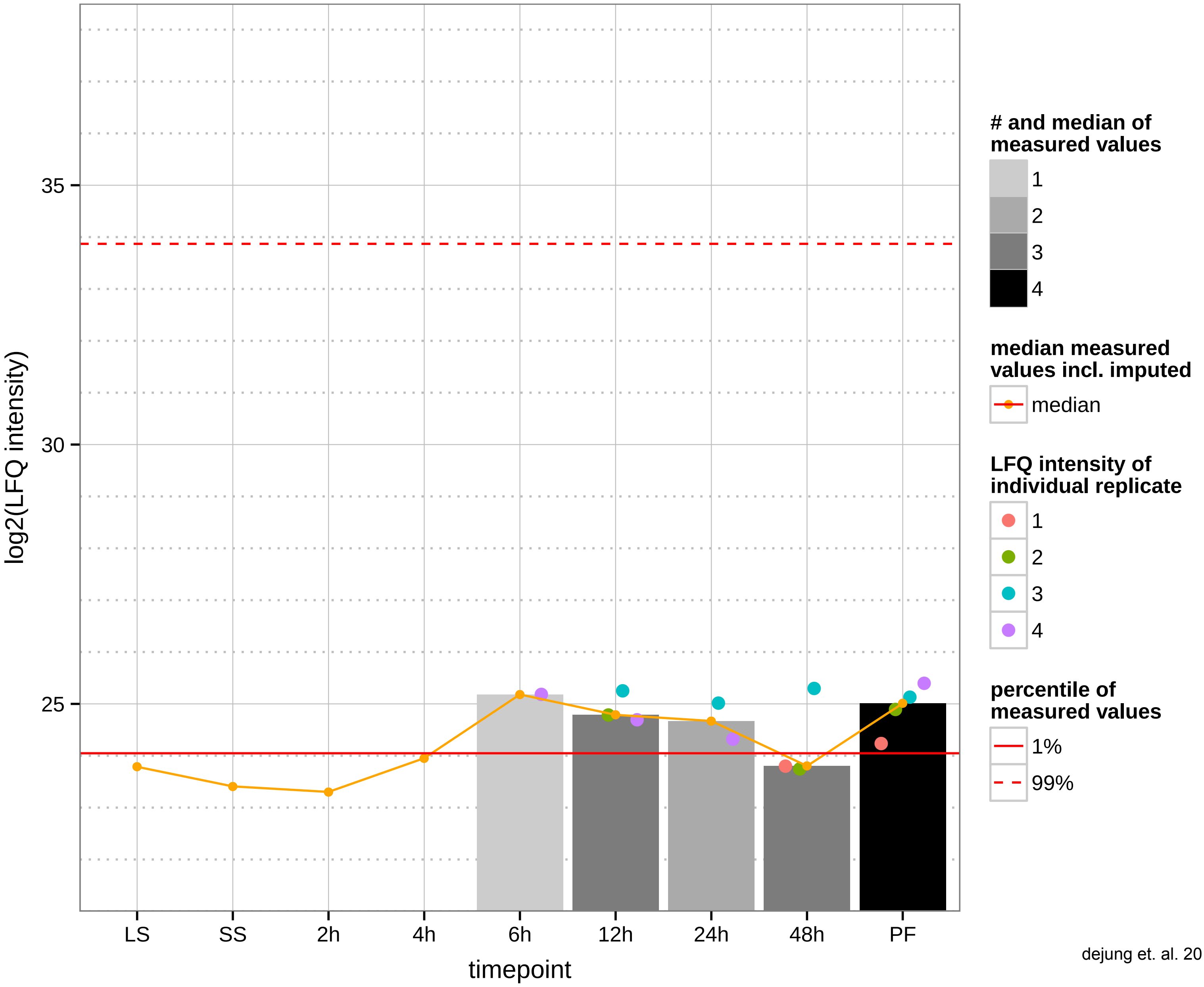

dejung et. al. 2015

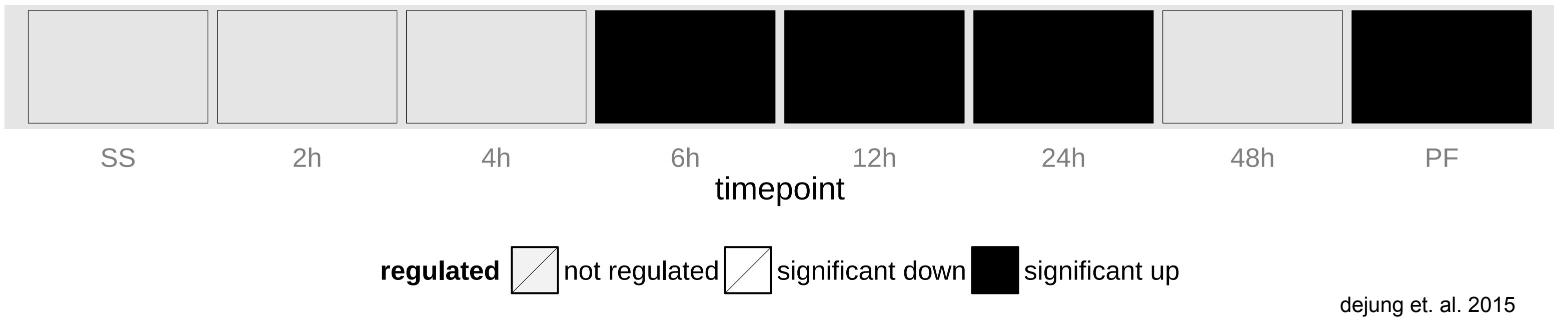
SS
2h
4h
6h
12h
24h
48h
PF
timepoint
regulated
not regulated
significant down
significant up
dejung et. al. 2015

beta–ketoacyl–ACP reductase 2 (KAR2)
Tb927.8.6420
AGOF: 3–oxoacyl–[acyl–carrier–protein] reductase (NADPH) activity
AGOC: mitochondrion
AGOP: fatty acid elongation
PGOF: oxidoreductase activity
PGOC: null
PGOP: metabolic process

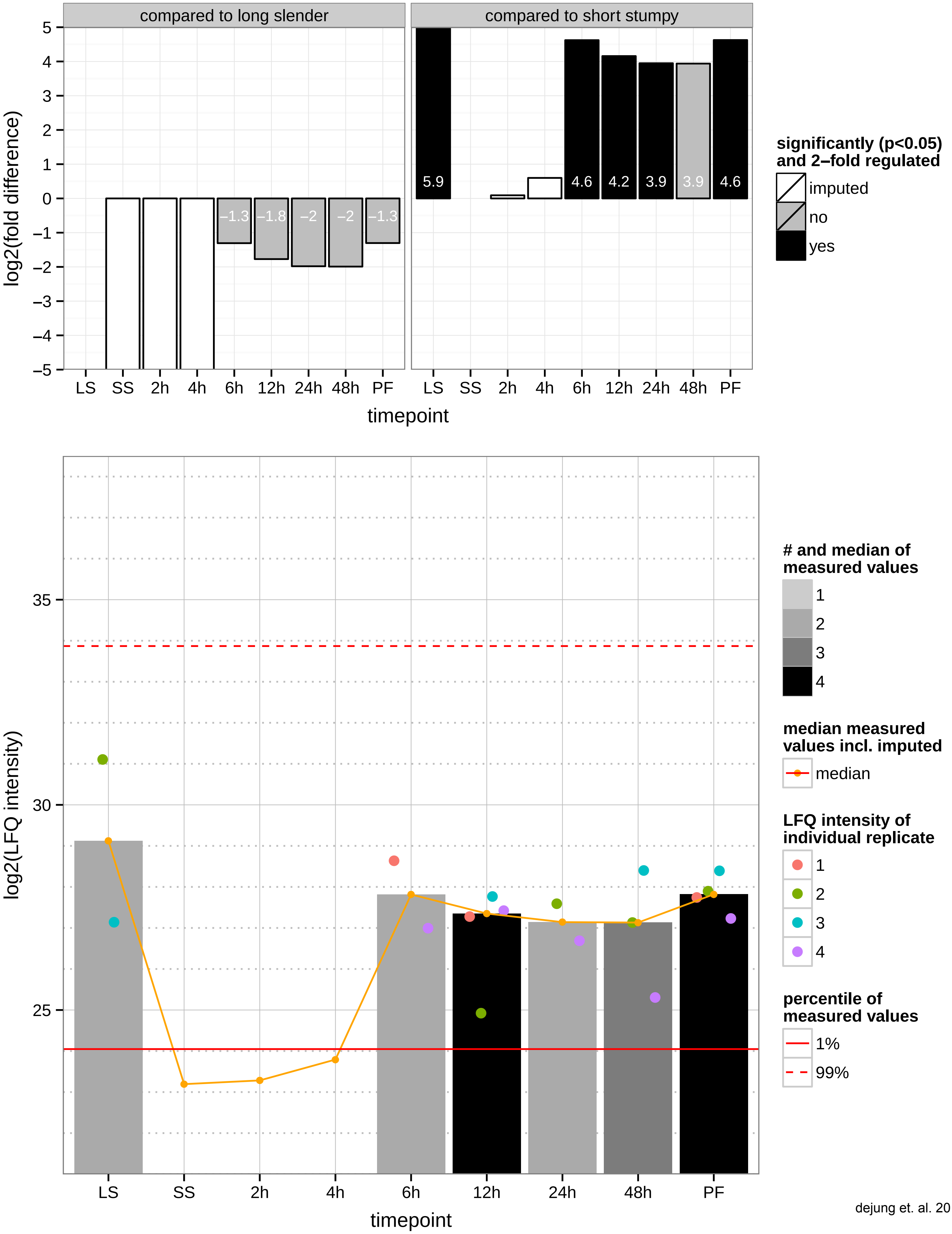

dejung et. al. 2015

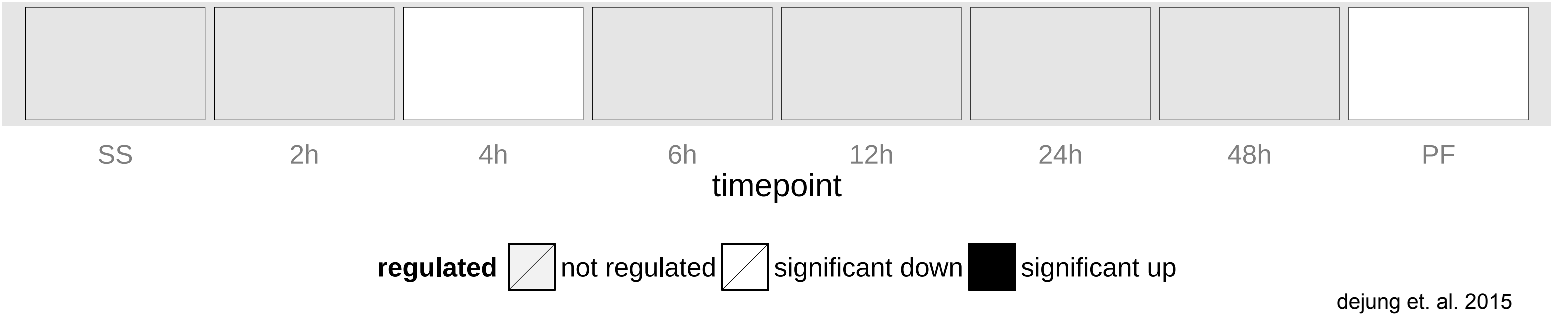
SS
2h
4h
6h
12h
24h
48h
PF
timepoint
regulated
not regulated
significant down
significant up
dejung et. al. 2015

hypothetical protein, conserved
Tb927.9.2940
AGOF: null
AGOC: null
AGOP: null
PGOF: null
PGOC: null
PGOP: null

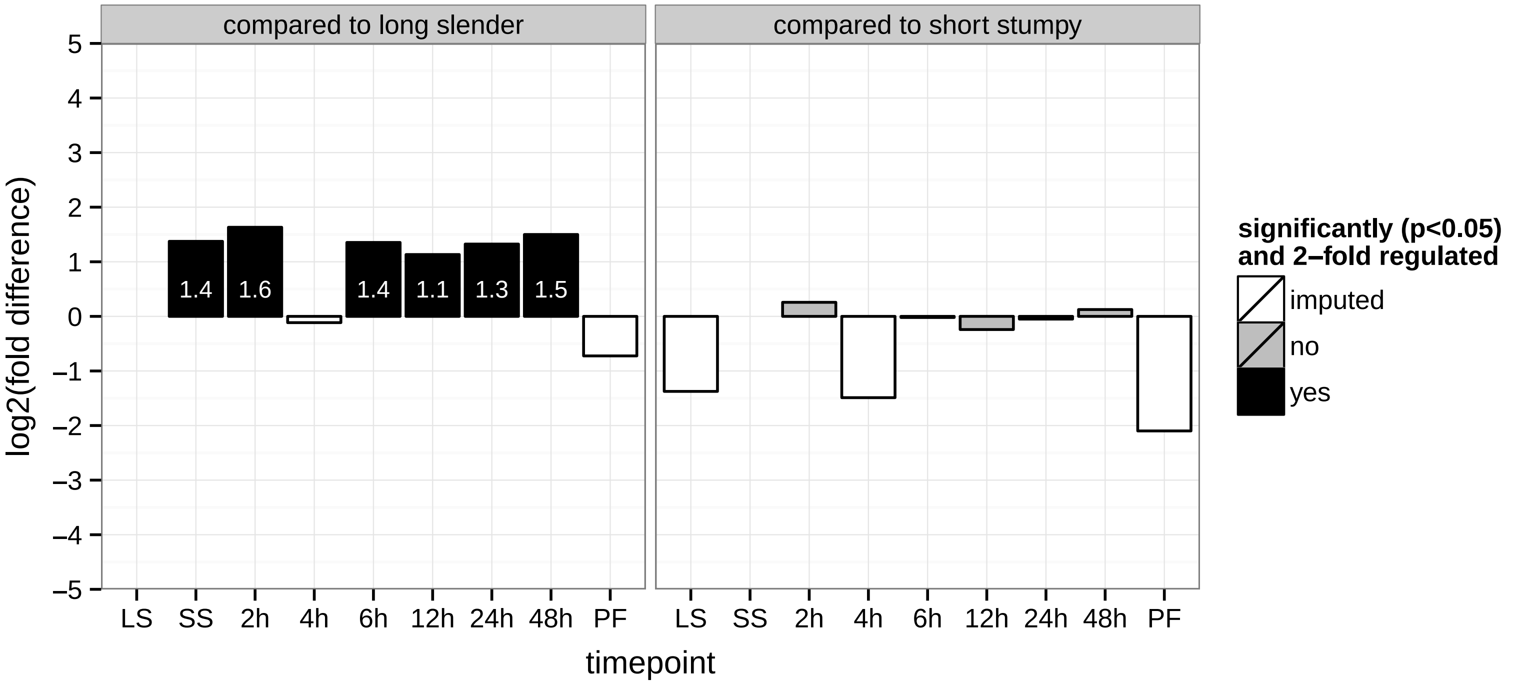

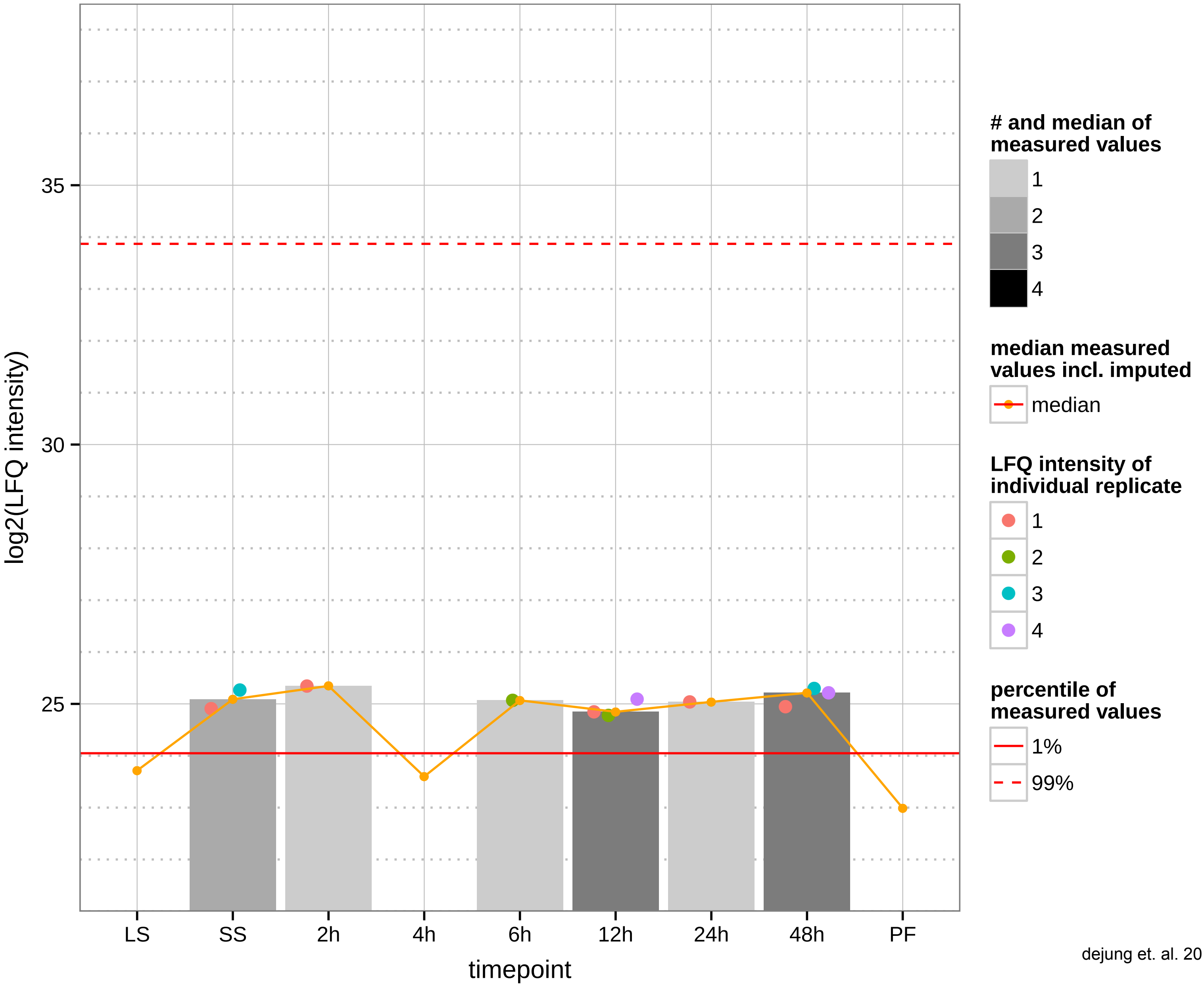

dejung et. al. 2015

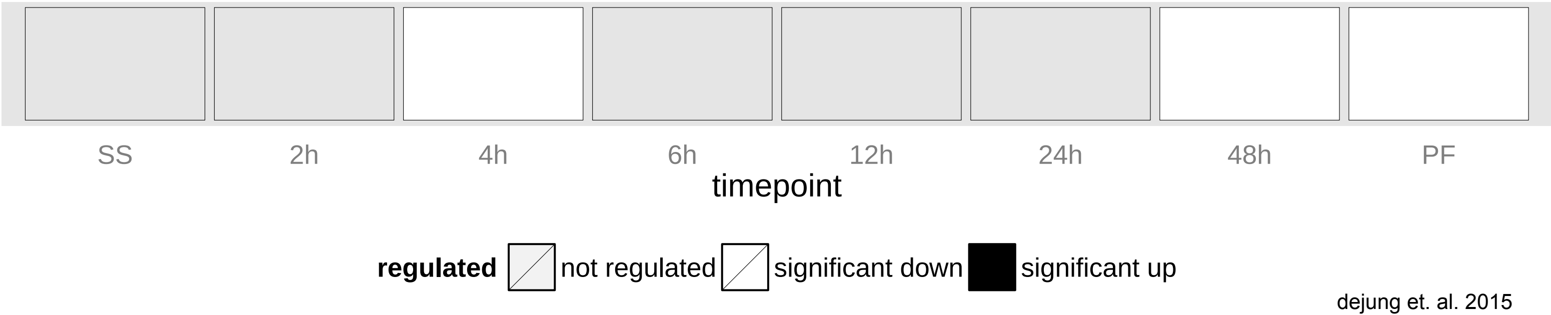
SS
2h
4h
6h
12h
24h
48h
PF
timepoint
regulated
not regulated
significant down
significant up
dejung et. al. 2015

cdc2–related kinase, putative, cell division control protein, putative (CRK10)
Tb927.3.4670
AGOF: ATP binding, protein kinase activity, protein serine/threonine kinase activity, protein tyrosine kinase activity
AGOC: null
AGOP: cell cycle, protein phosphorylation
PGOF: ATP binding, protein kinase activity, transferase activity, transferring phosphorus–containing groups
PGOC: null
PGOP: protein phosphorylation

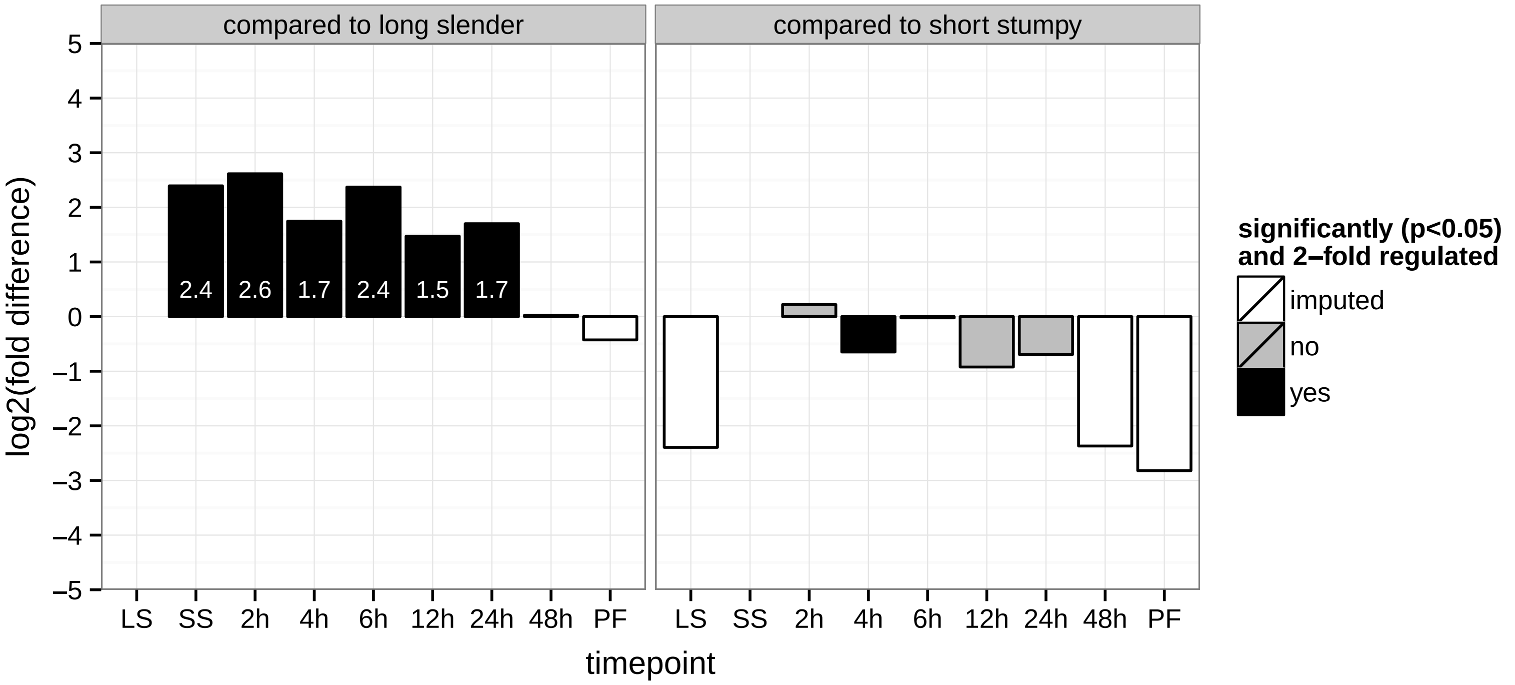

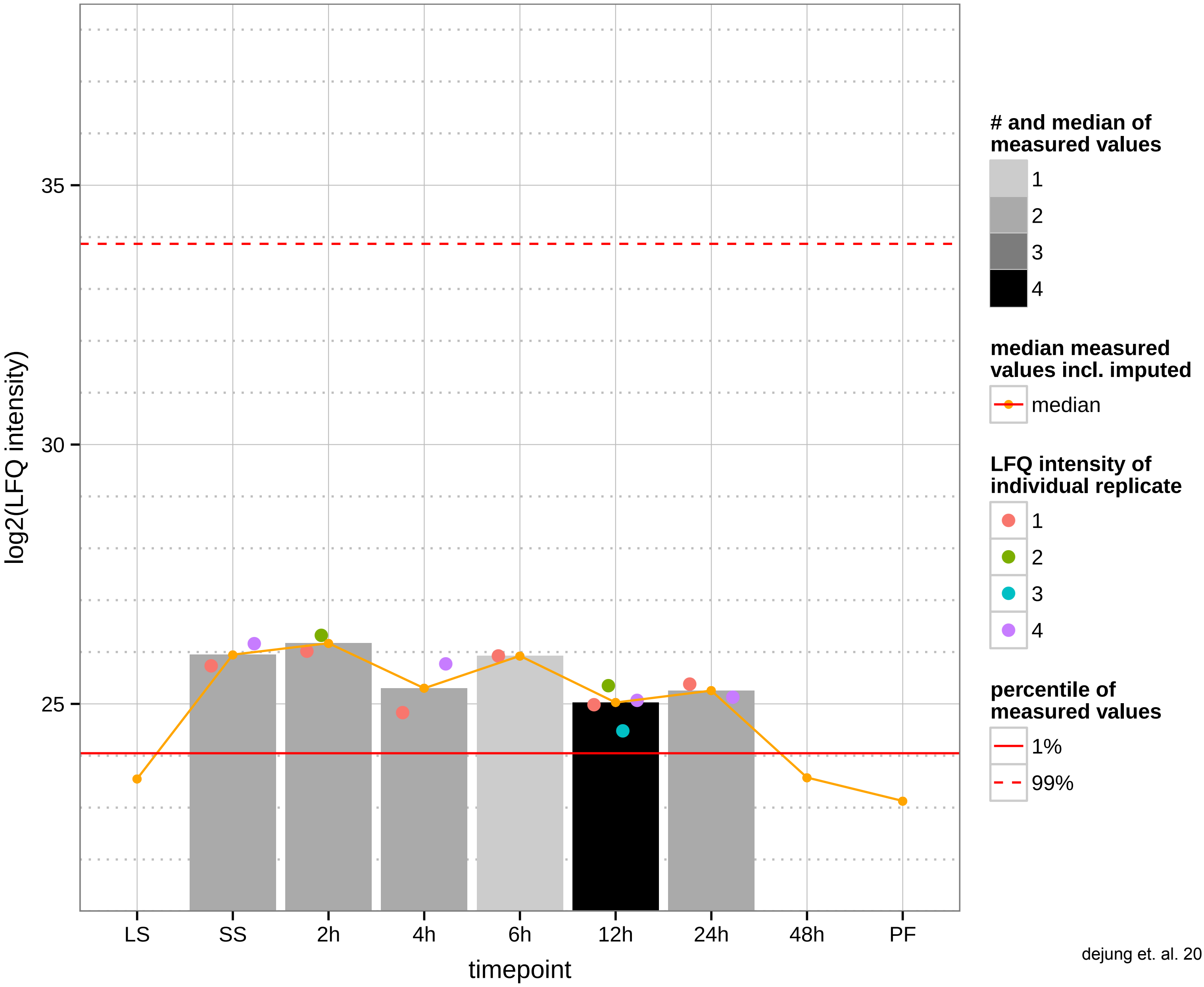

dejung et. al. 2015

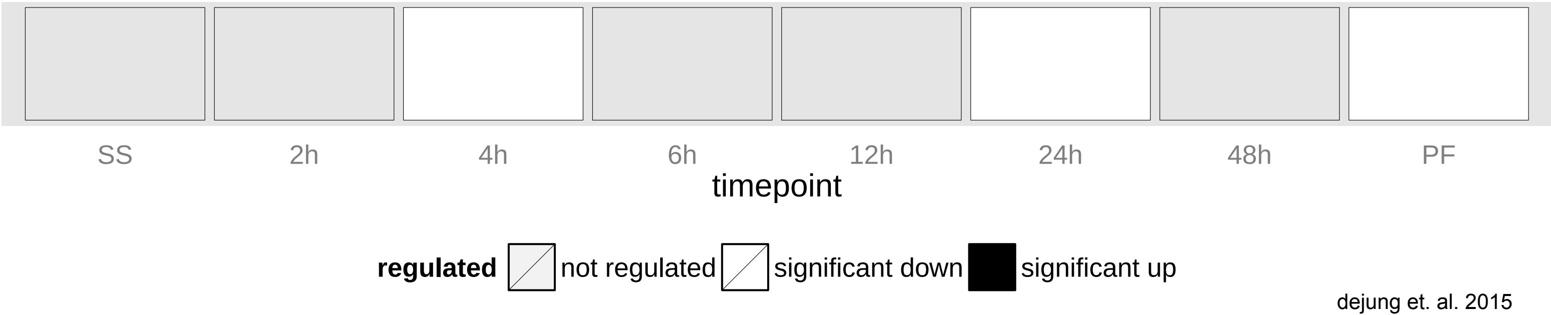
SS
2h
4h
6h
12h
24h
48h
PF
timepoint
regulated
not regulated
significant down
significant up
dejung et. al. 2015

hypothetical protein, conserved
Tb927.10.14310
AGOF: null
AGOC: null
AGOP: null
PGOF: null
PGOC: null
PGOP: null

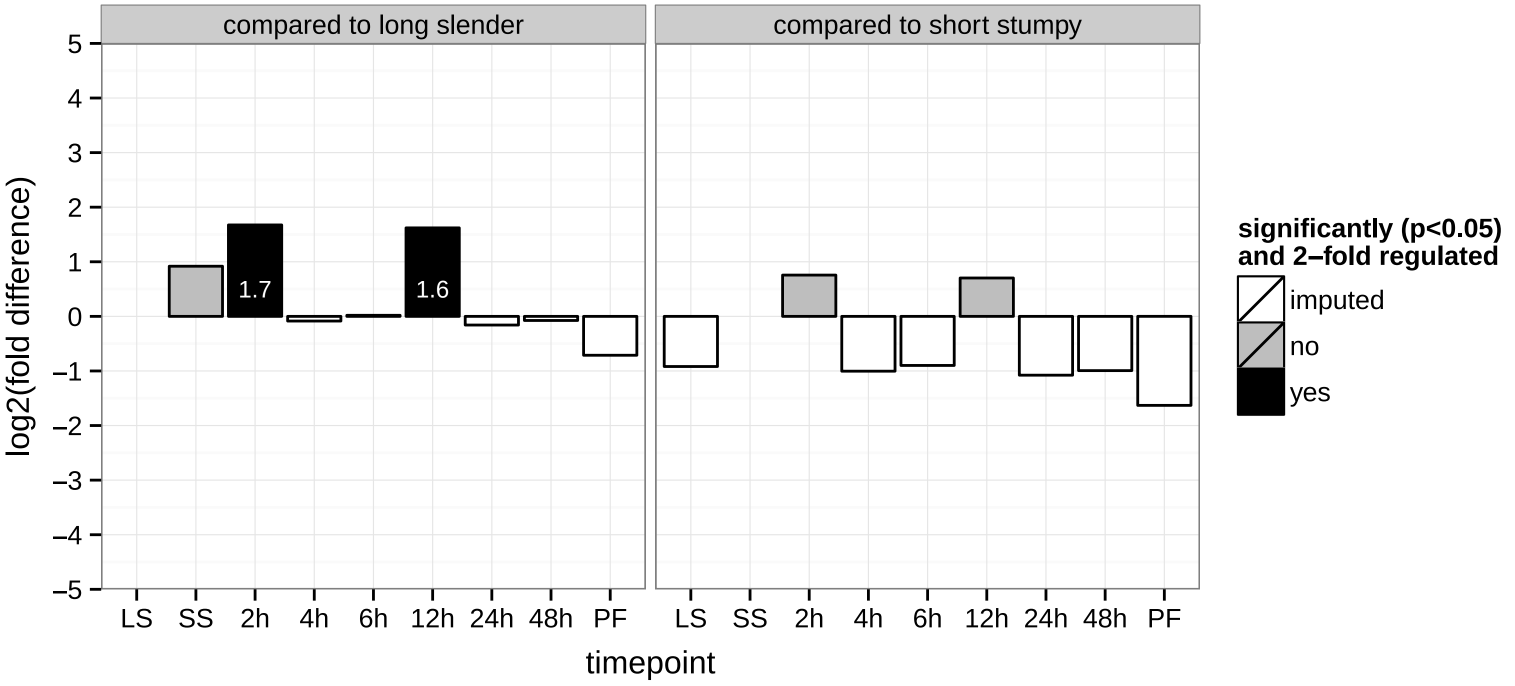

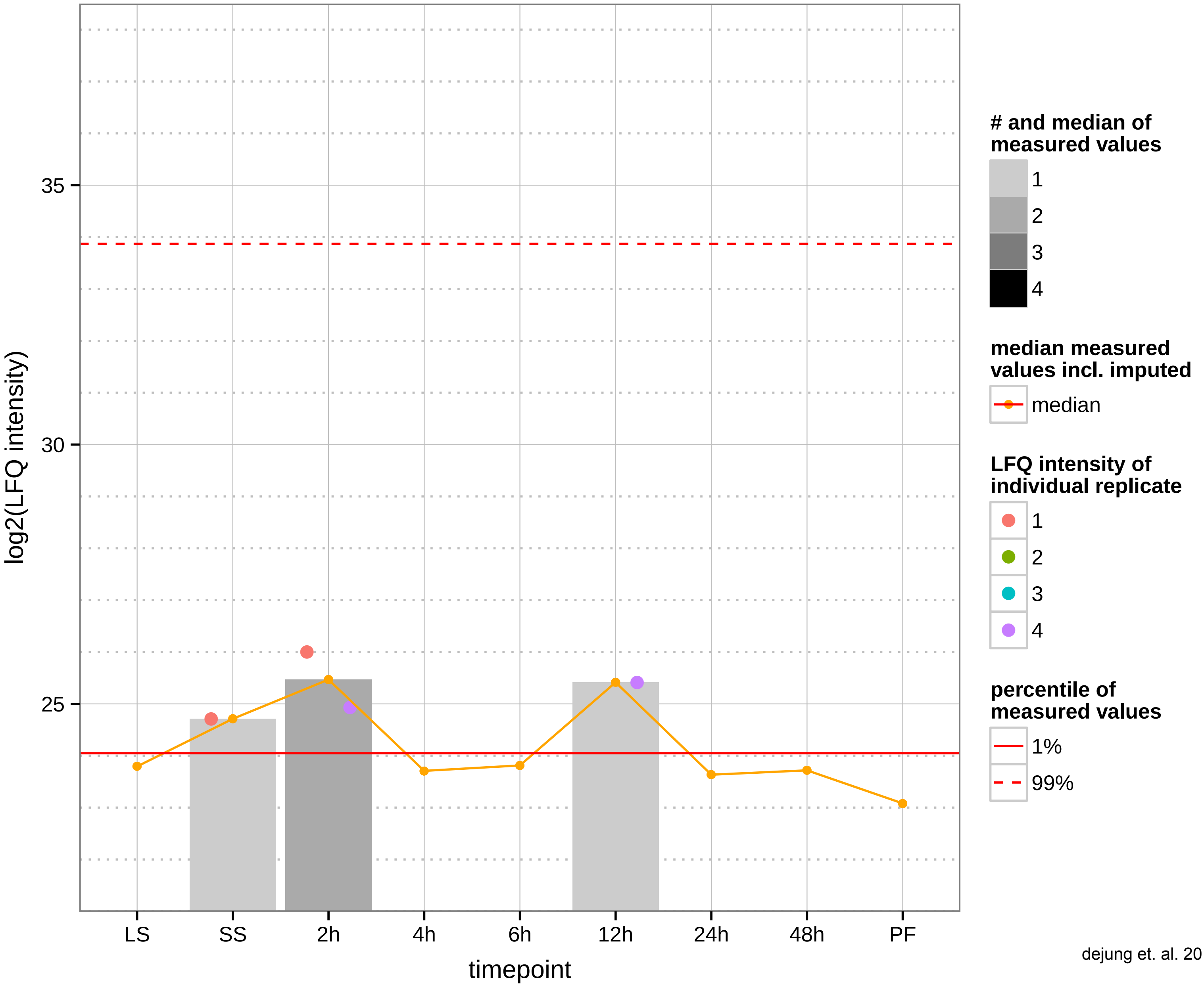

dejung et. al. 2015

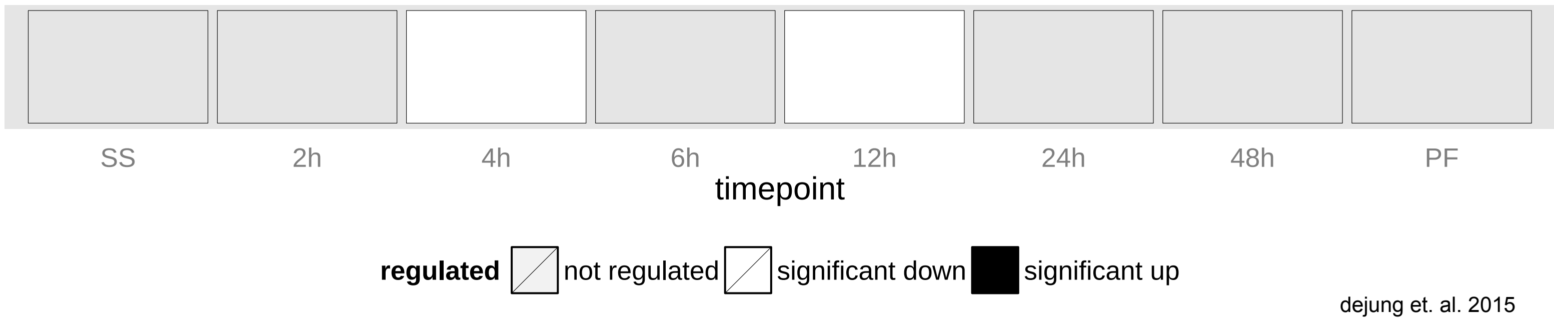
SS
2h
4h
6h
12h
24h
48h
PF
timepoint
regulated
not regulated
significant down
significant up
dejung et. al. 2015

hypothetical protein, conserved
Tb927.9.15260
AGOF: null
AGOC: null
AGOP: null
PGOF: protein binding
PGOC: null
PGOP: null

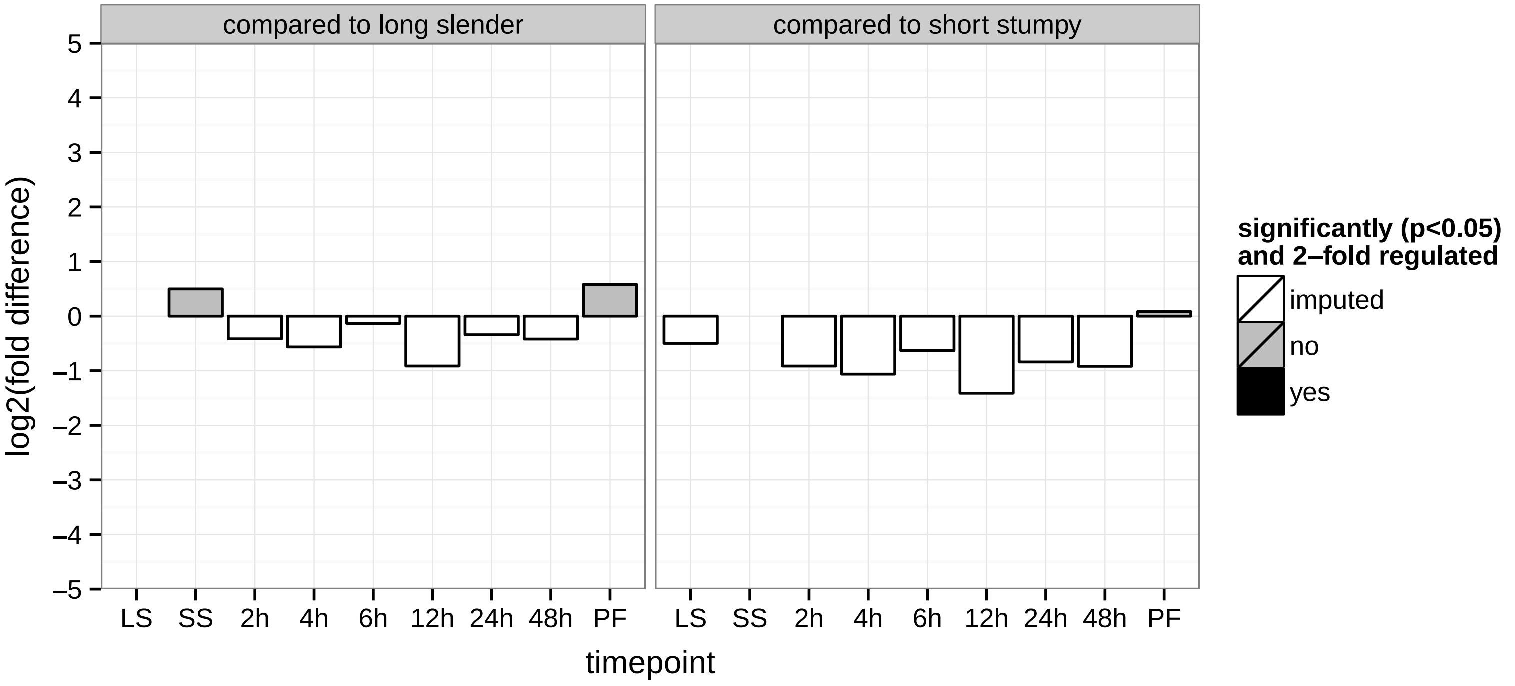

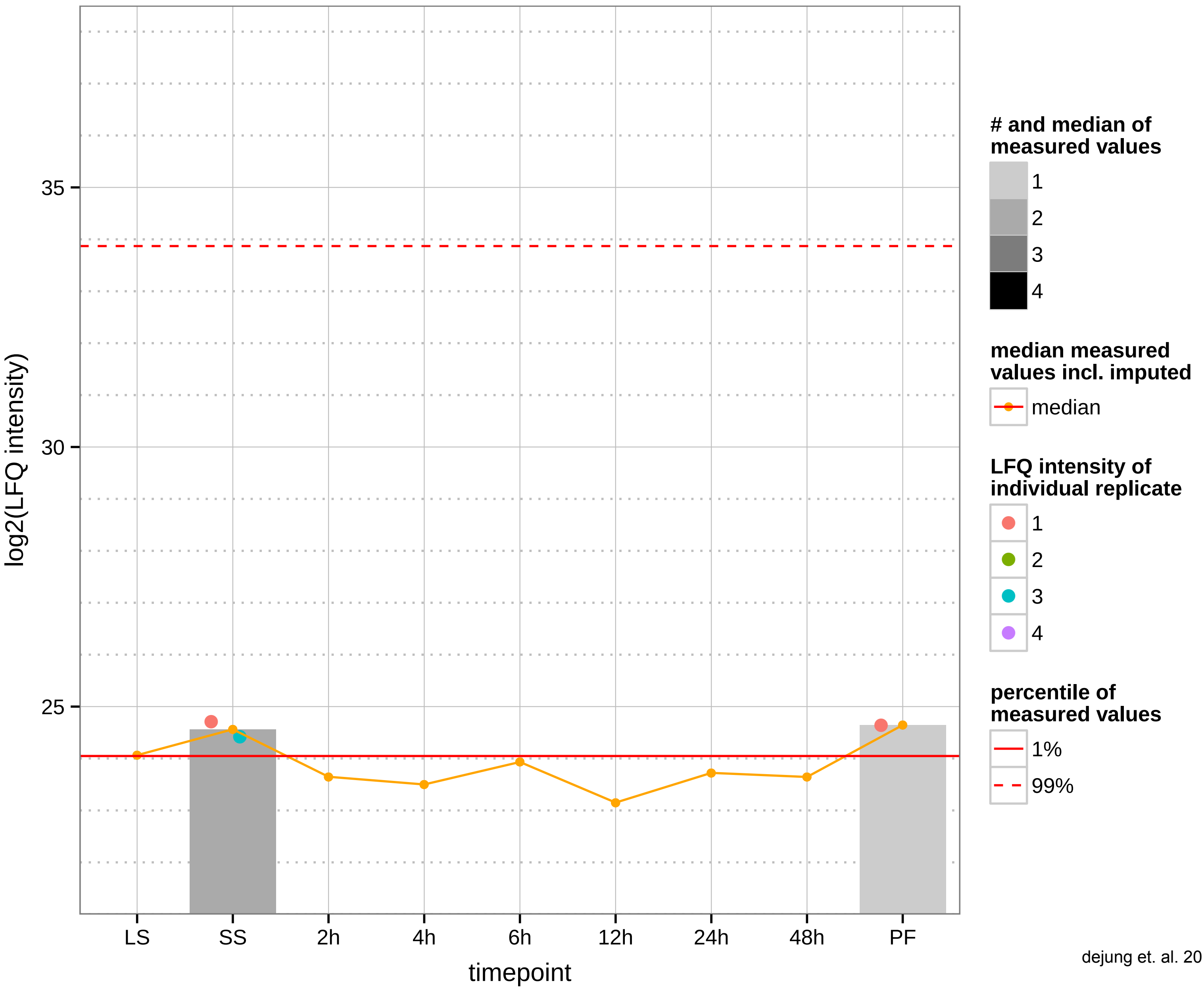

dejung et. al. 2015

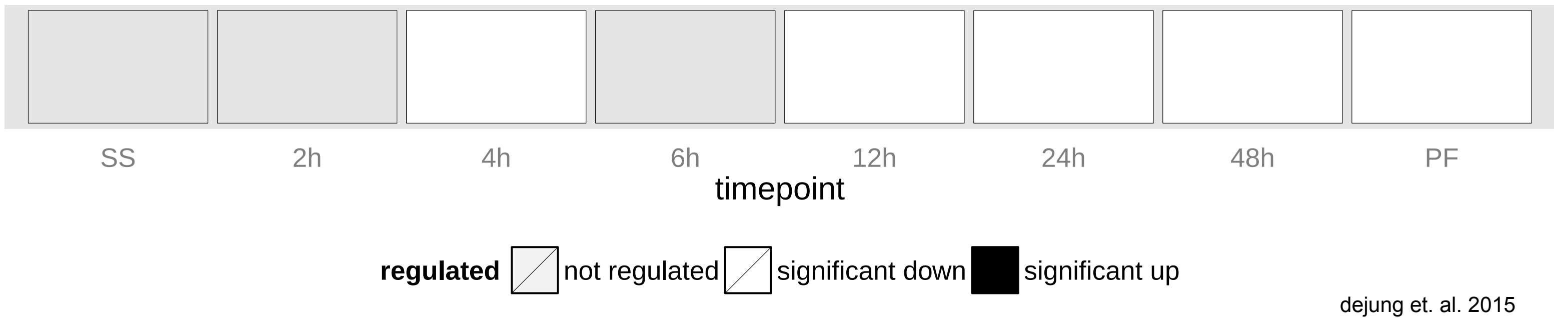
SS
2h
4h
6h
12h
24h
48h
PF
timepoint
regulated
not regulated
significant down
significant up
dejung et. al. 2015

ubiquitin–protein ligase, putative (upl3)
Tb927.9.13360
AGOF: ubiquitin–protein ligase activity
AGOC: intracellular
AGOP: cellular protein modification process
PGOF: acid–amino acid ligase activity
PGOC: intracellular
PGOP: cellular protein modification process

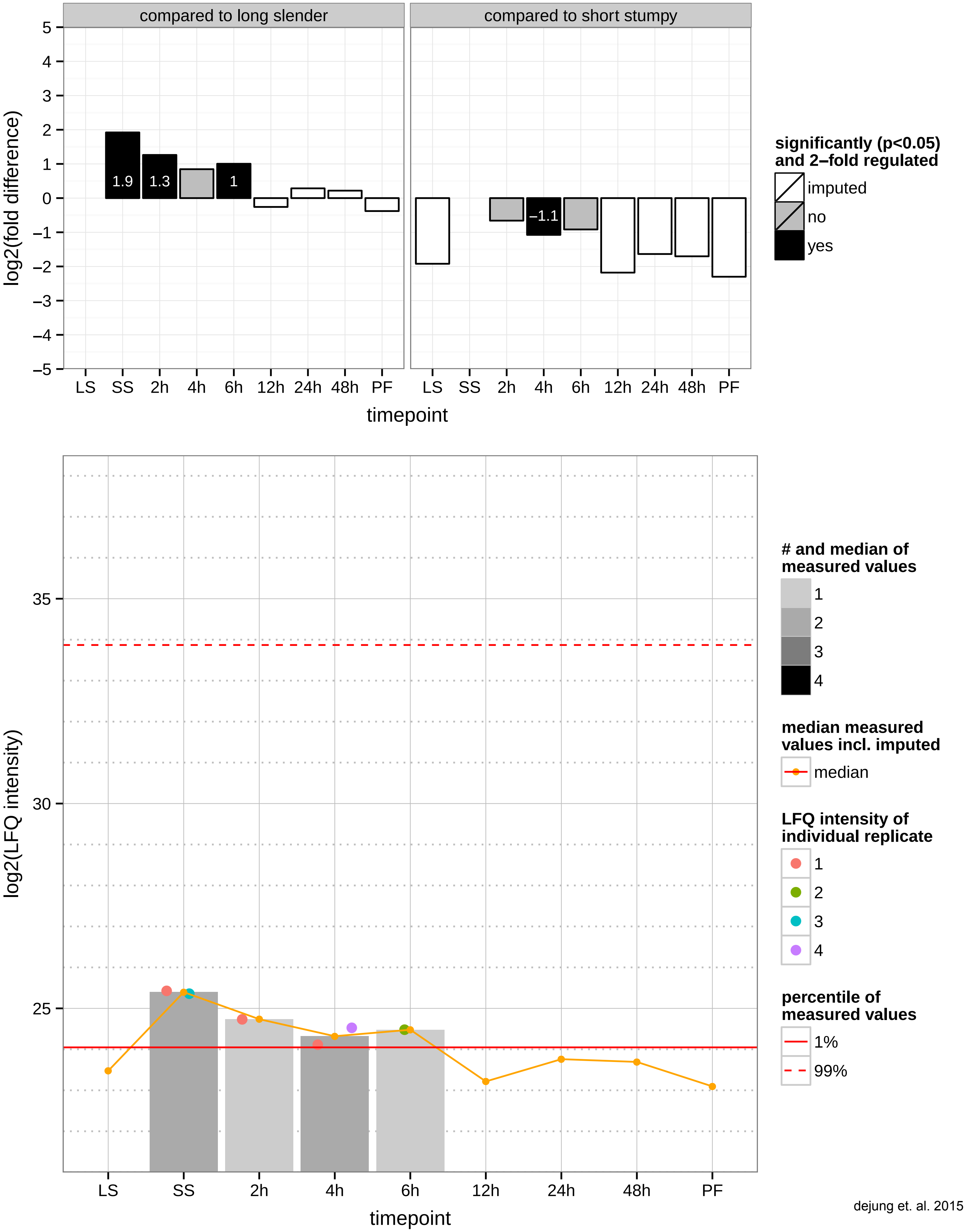

dejung et. al. 2015

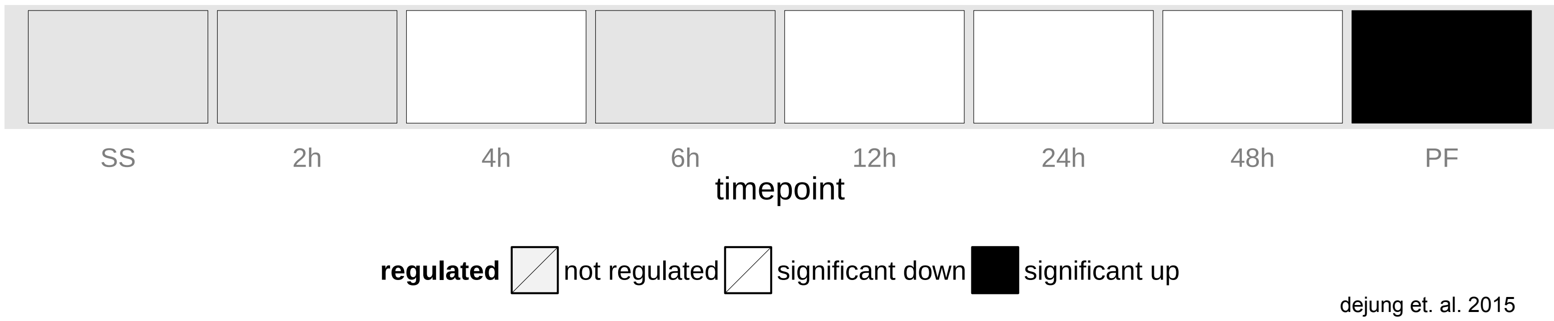
SS
2h
4h
6h
12h
24h
48h
PF
timepoint
regulated
not regulated
significant down
significant up
dejung et. al. 2015

amino acid transporter 8, putative, amino acid transporter 10, putative (AATP10), amino acid transporter 7, putative (AATP7),
Tb927.4.4860;Tb11.v5.0540;Tb927.4.4840;Tb927.4.4820
AGOF: null, amine transmembrane transporter activity
AGOC: null, membrane
AGOP: null, amino acid transport
PGOF: null
PGOC: null
PGOP: null

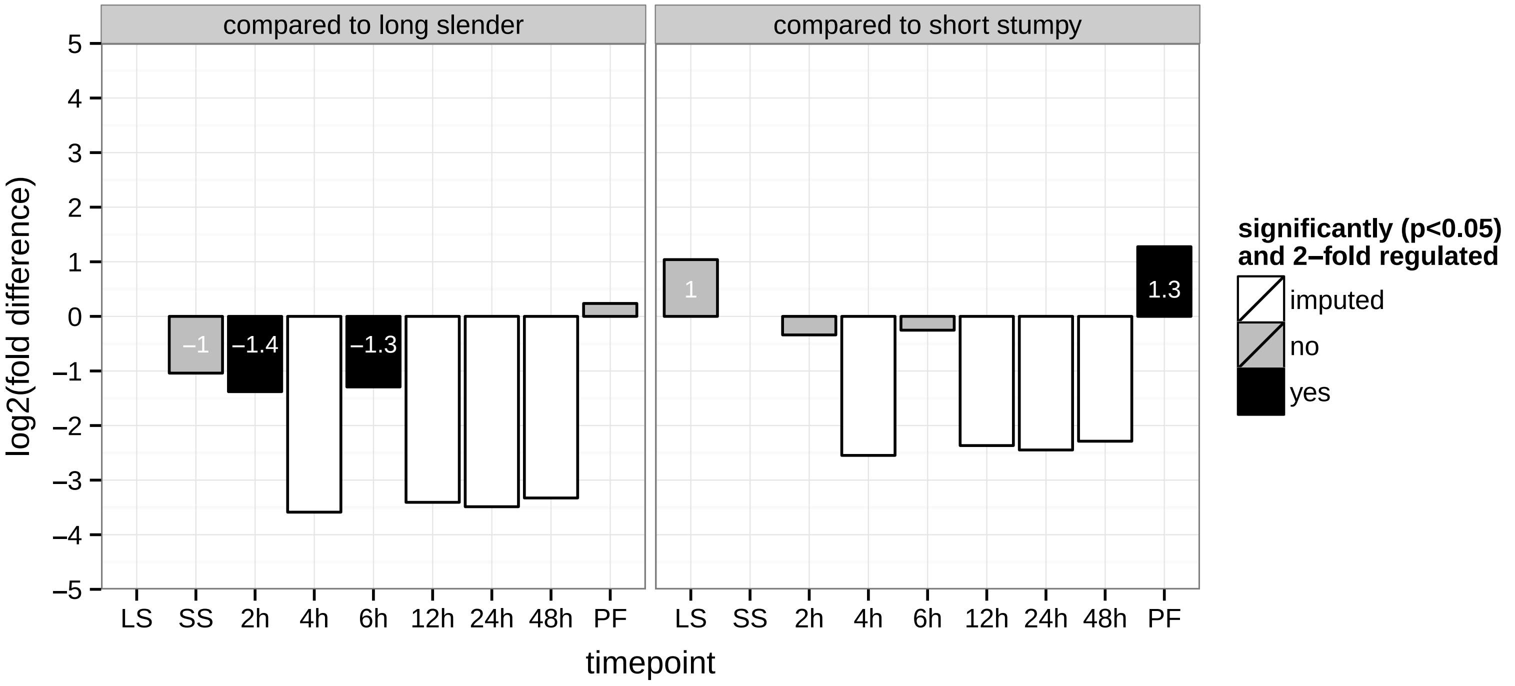

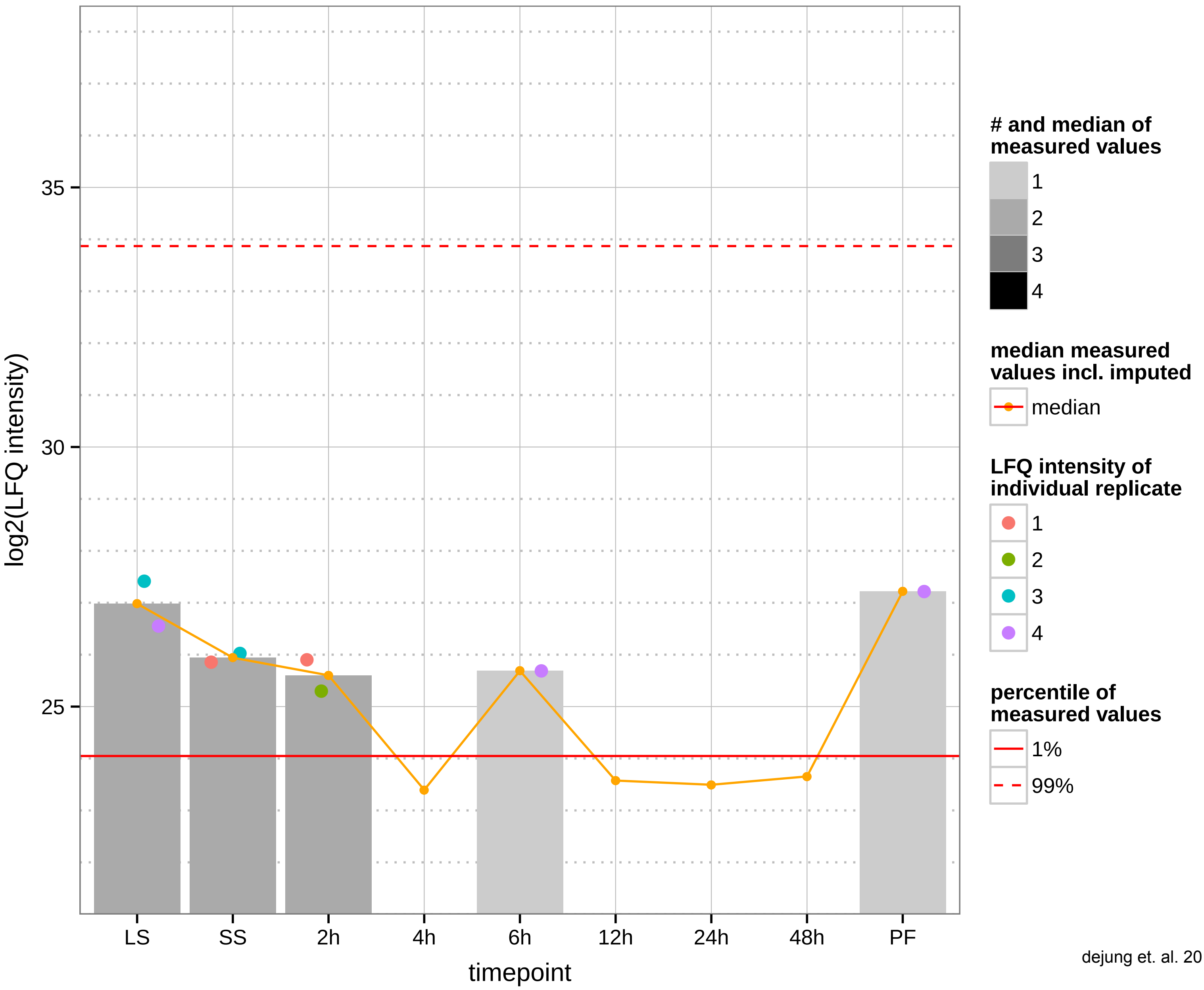

dejung et. al. 2015

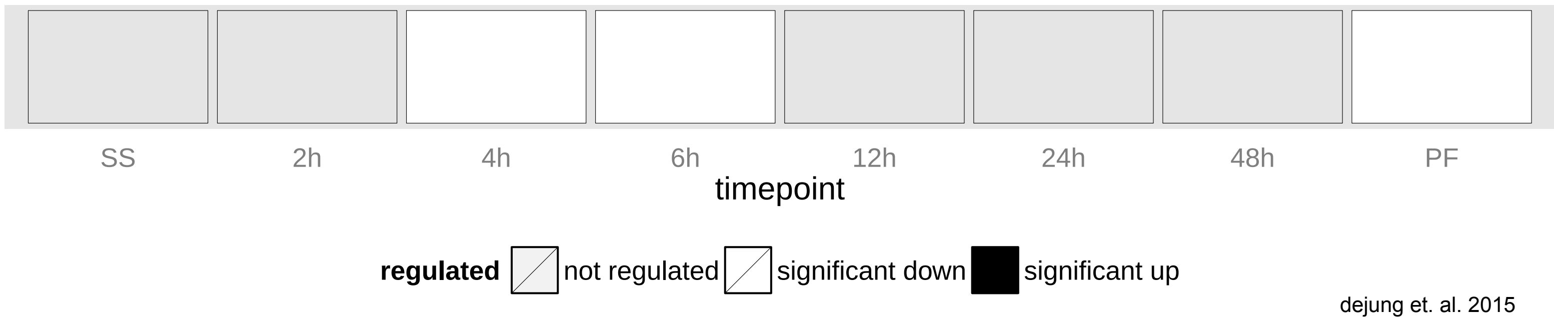
SS
2h
4h
6h
12h
24h
48h
PF
timepoint
regulated
not regulated
significant down
significant up
dejung et. al. 2015

hypothetical protein, conserved
Tb927.11.10120
AGOF: null
AGOC: null
AGOP: null
PGOF: null
PGOC: null
PGOP: null

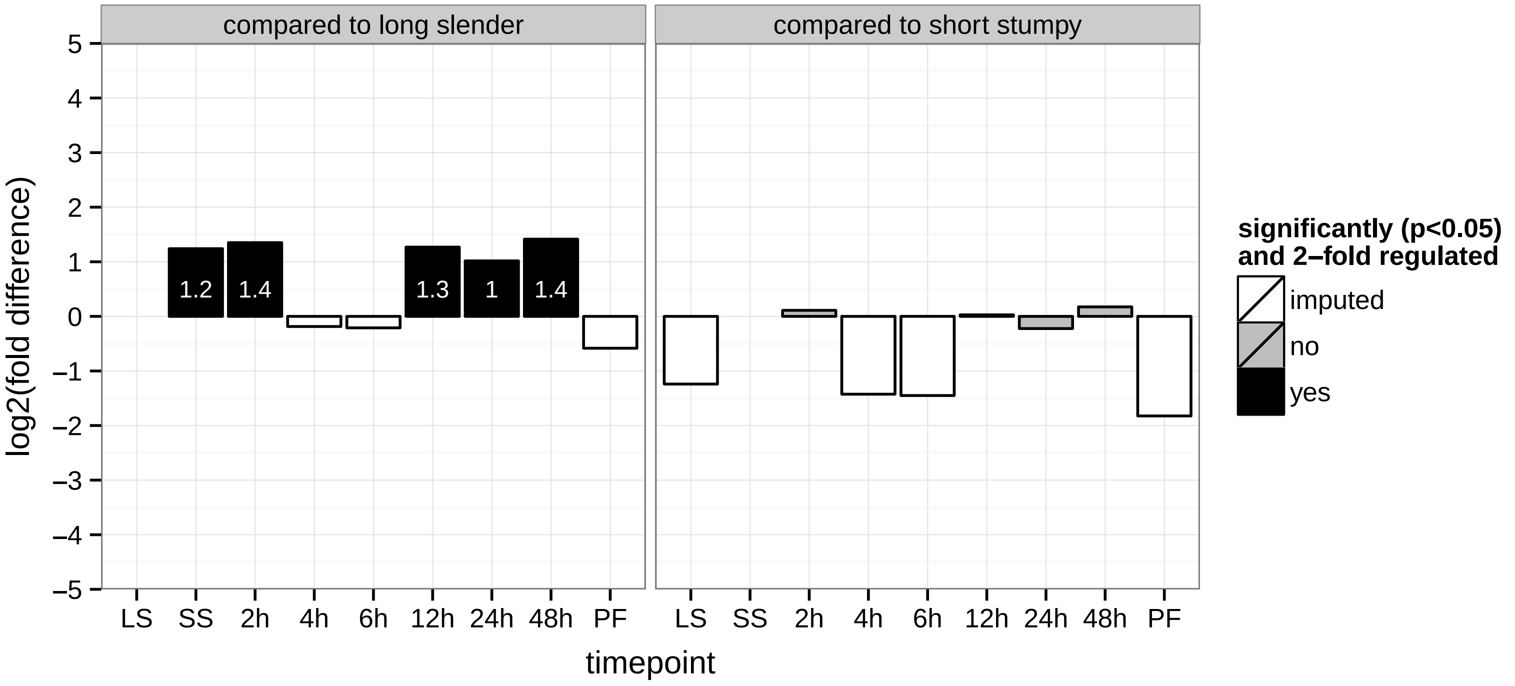

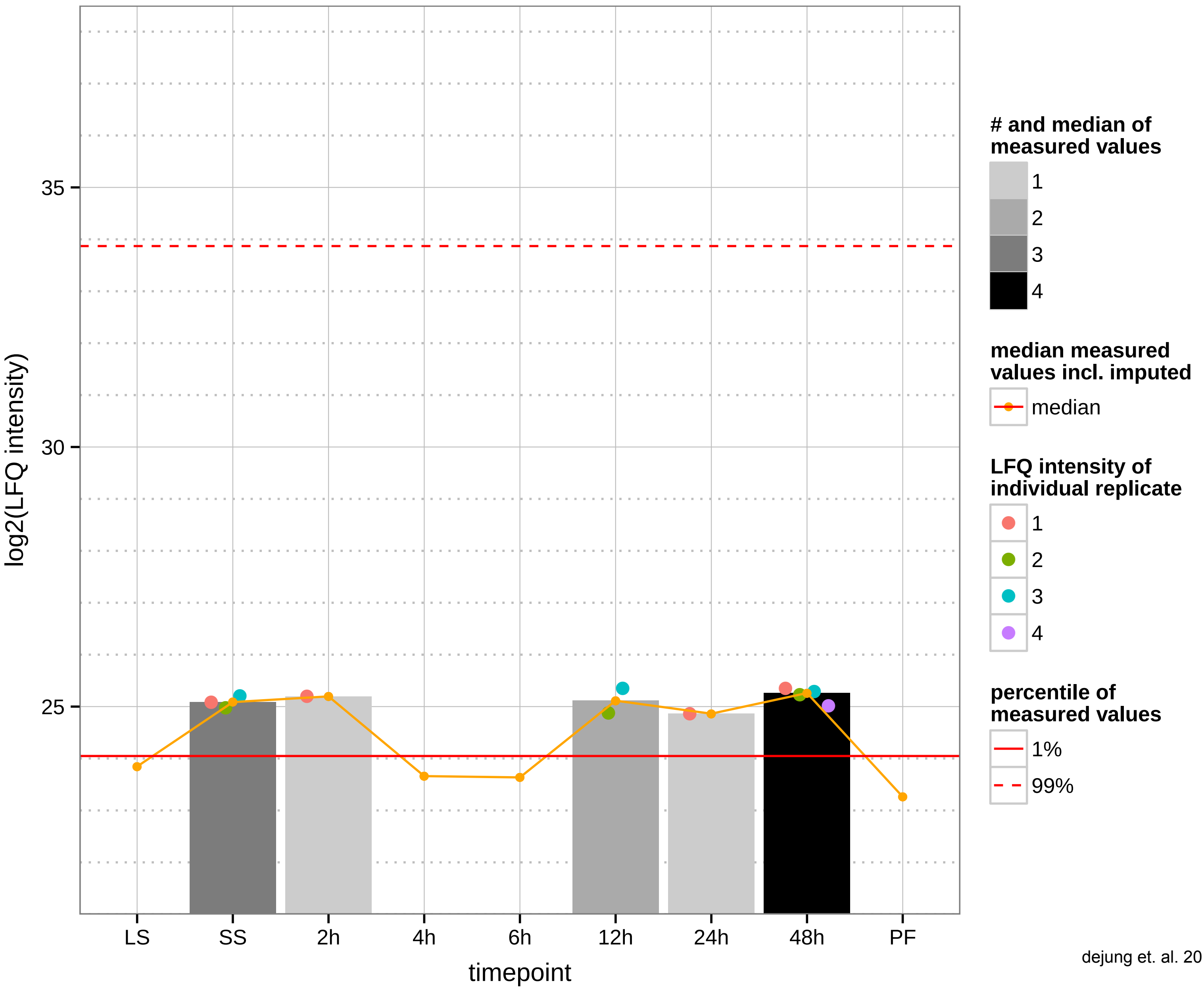

dejung et. al. 2015

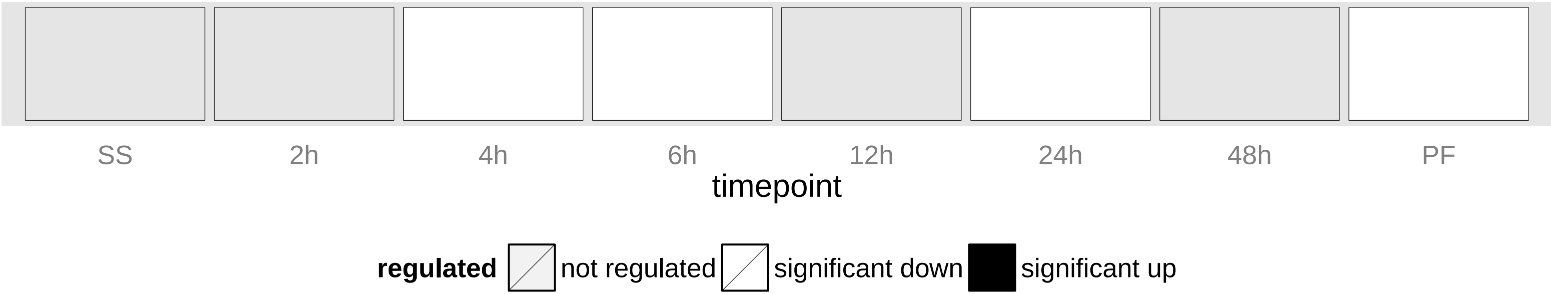

dejung et. al. 2015

hypothetical protein, conserved
Tb927.7.2520
AGOF: null
AGOC: null
AGOP: null
PGOF: null
PGOC: null
PGOP: null

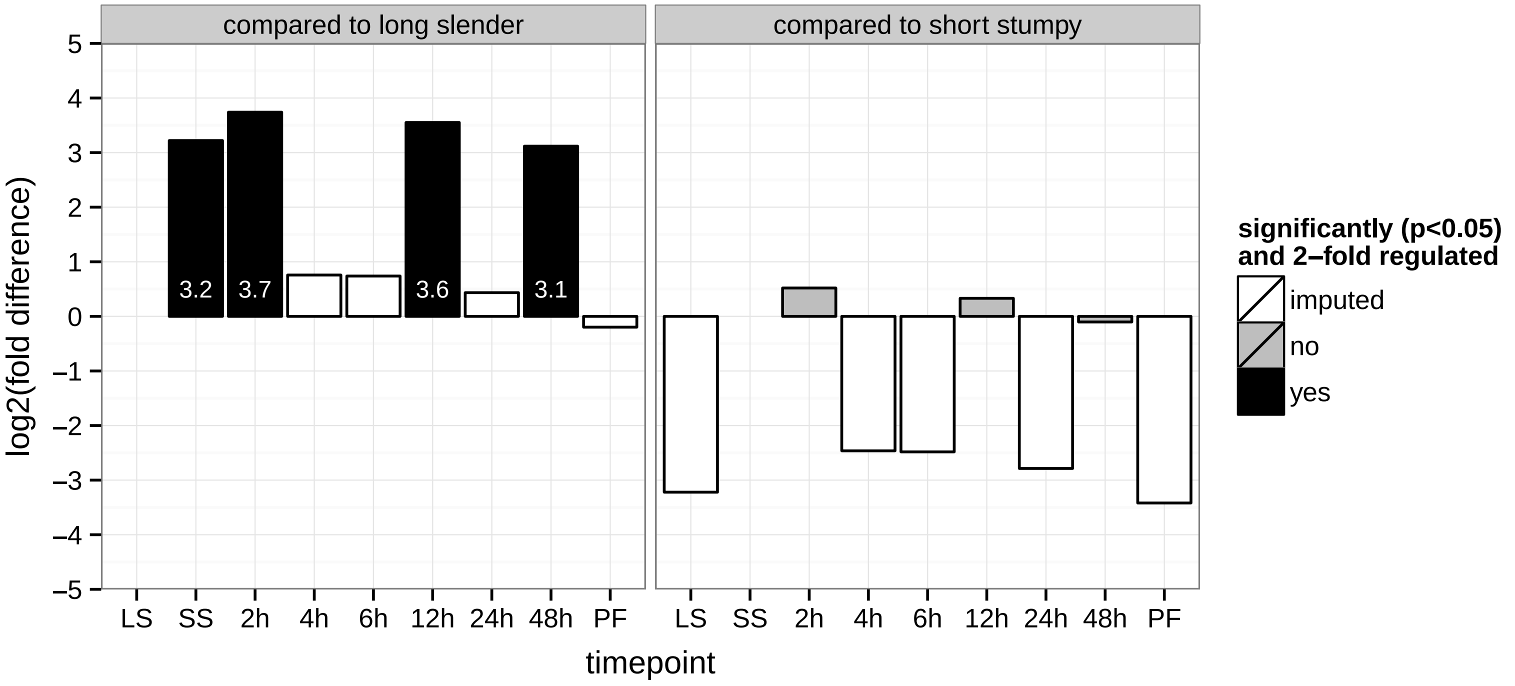

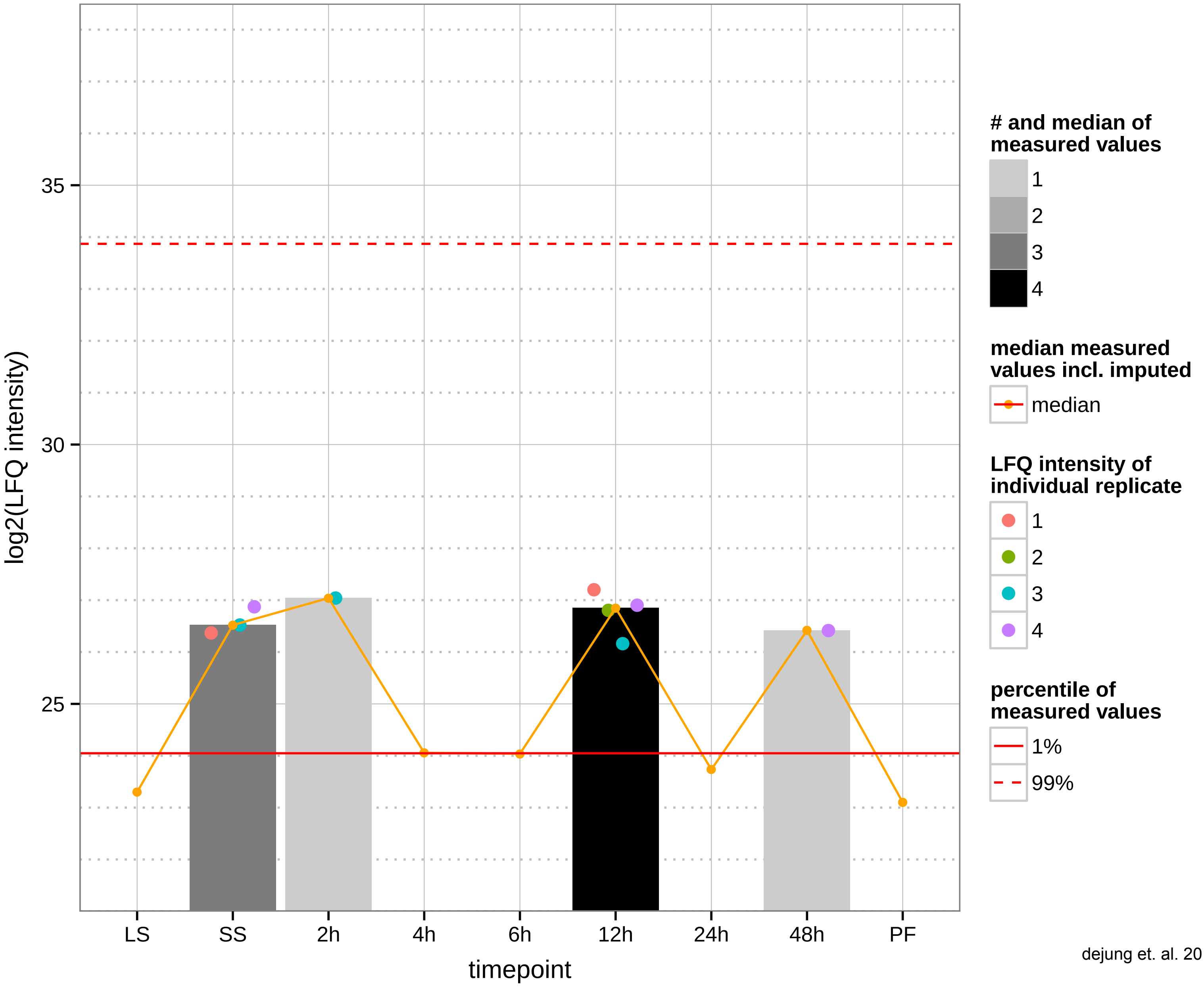

dejung et. al. 2015

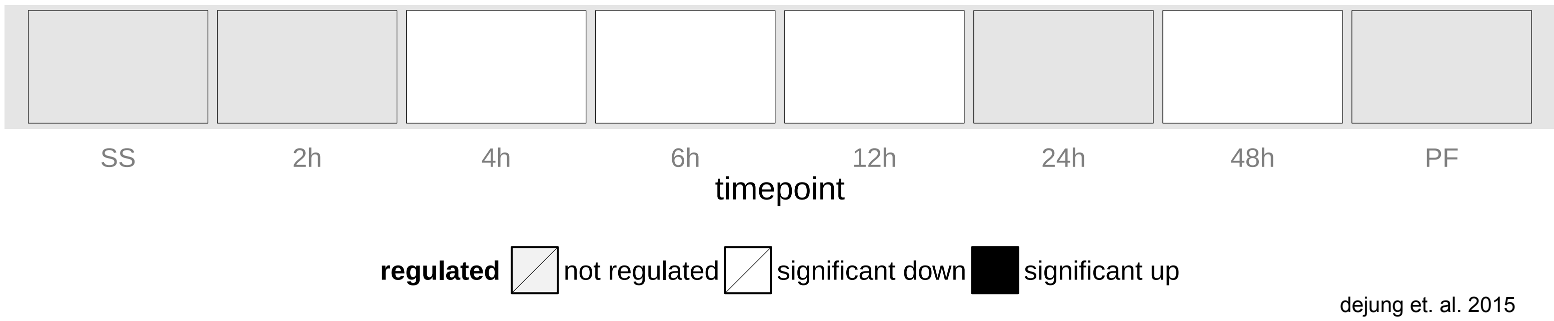
SS
2h
4h
6h
12h
24h
48h
PF
timepoint
regulated
not regulated
significant down
significant up
dejung et. al. 2015

Nuclear Dbf2–related kinase, PK53 (PK53)
Tb927.7.5770
AGOF: ATP binding, protein kinase activity, protein serine/threonine kinase activity, protein tyrosine kinase activity
AGOC: cytosol
AGOP: cell proliferation, cytokinesis, protein phosphorylation, regulation of cell cycle
PGOF: ATP binding, protein kinase activity, protein serine/threonine kinase activity, transferase activity, transferring phosphoru
PGOC: null
PGOP: protein phosphorylation

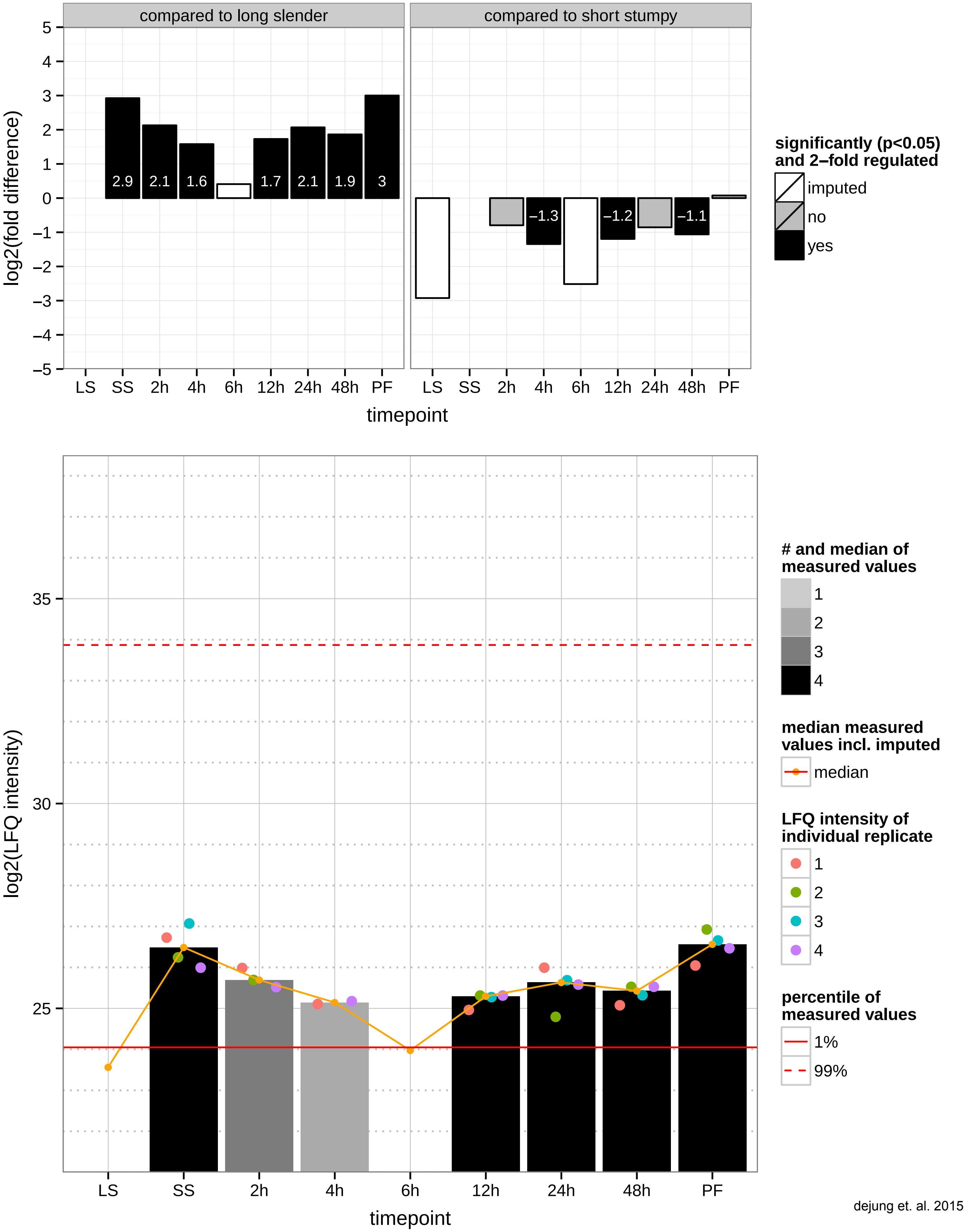

dejung et. al. 2015

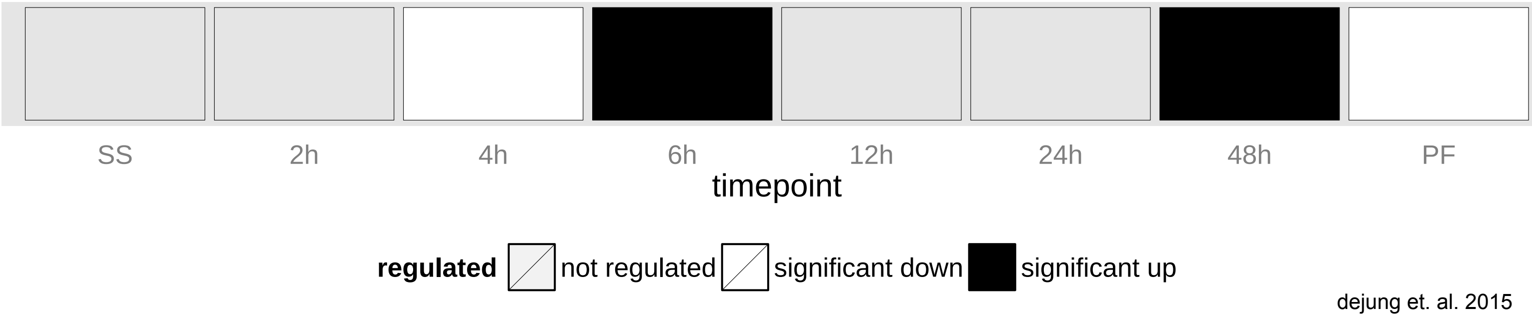
SS
2h
4h
6h
12h
24h
48h
PF
timepoint
regulated
not regulated
significant down
significant up
dejung et. al. 2015

hypothetical protein, conserved
Tb927.11.11170
AGOF: null
AGOC: null
AGOP: null
PGOF: null
PGOC: null
PGOP: null

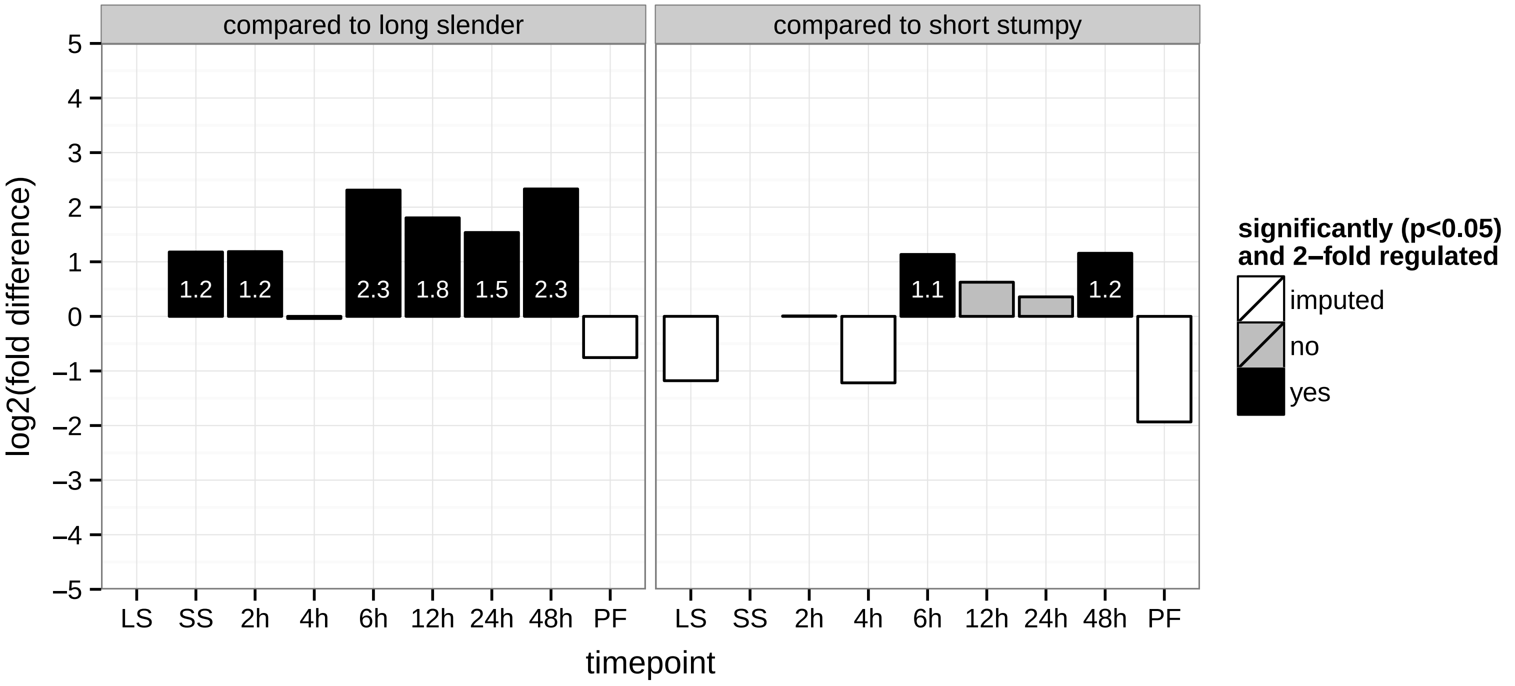

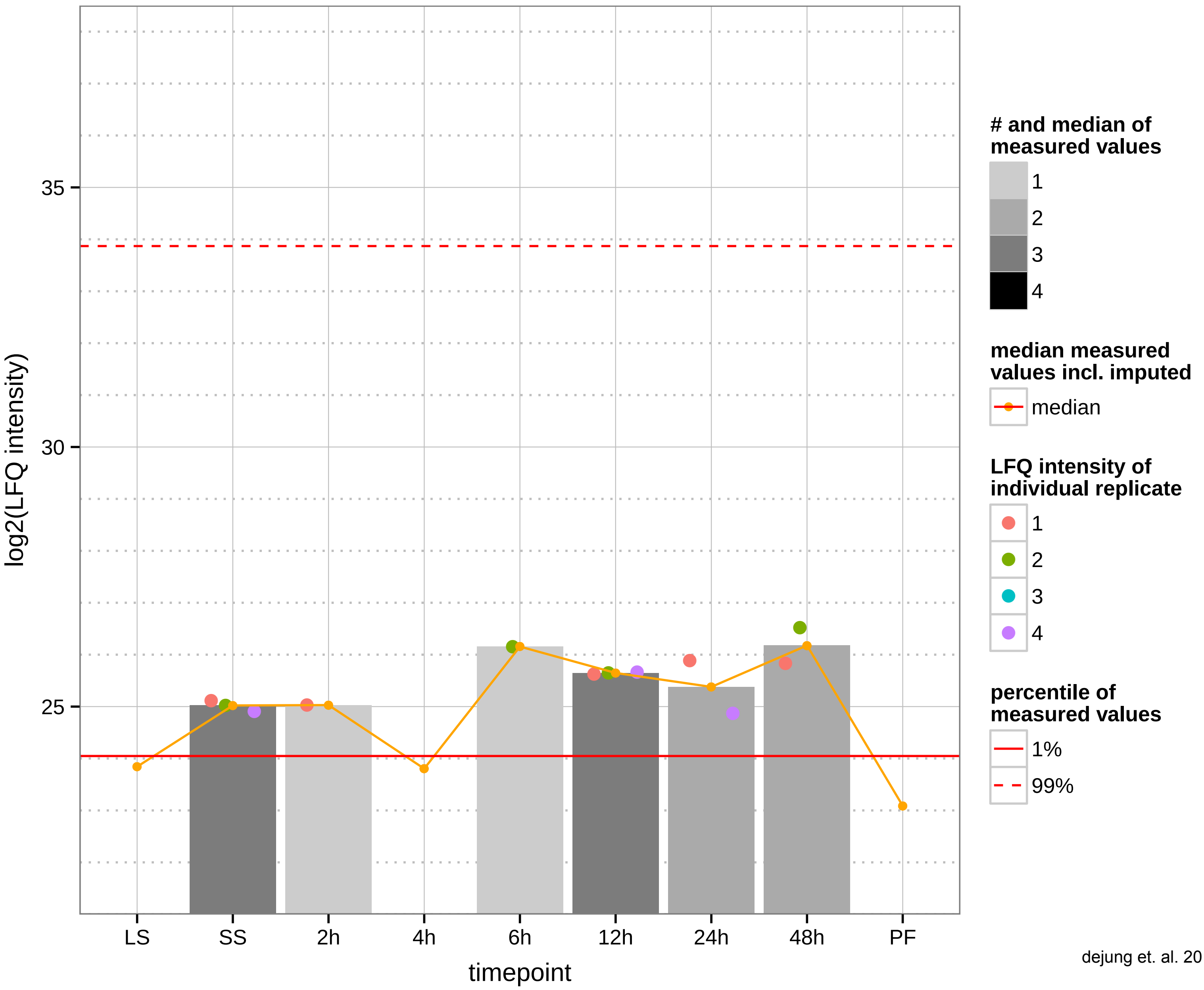

dejung et. al. 2015

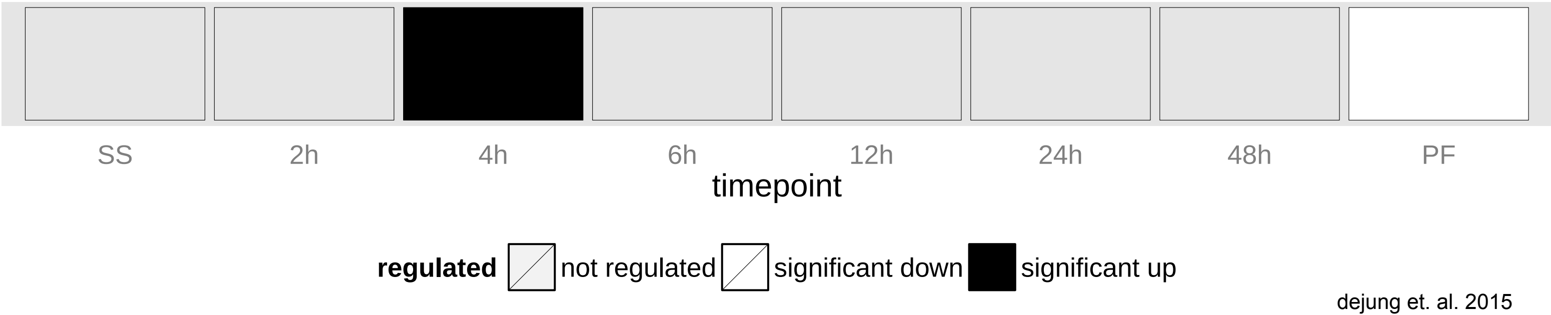
SS
2h
4h
6h
12h
24h
48h
PF
timepoint
regulated
not regulated
significant down
significant up
dejung et. al. 2015

SKP1–like protein
Tb927.10.11610
AGOF: null
AGOC: null
AGOP: ubiquitin–dependent protein catabolic process
PGOF: null
PGOC: null
PGOP: ubiquitin–dependent protein catabolic process

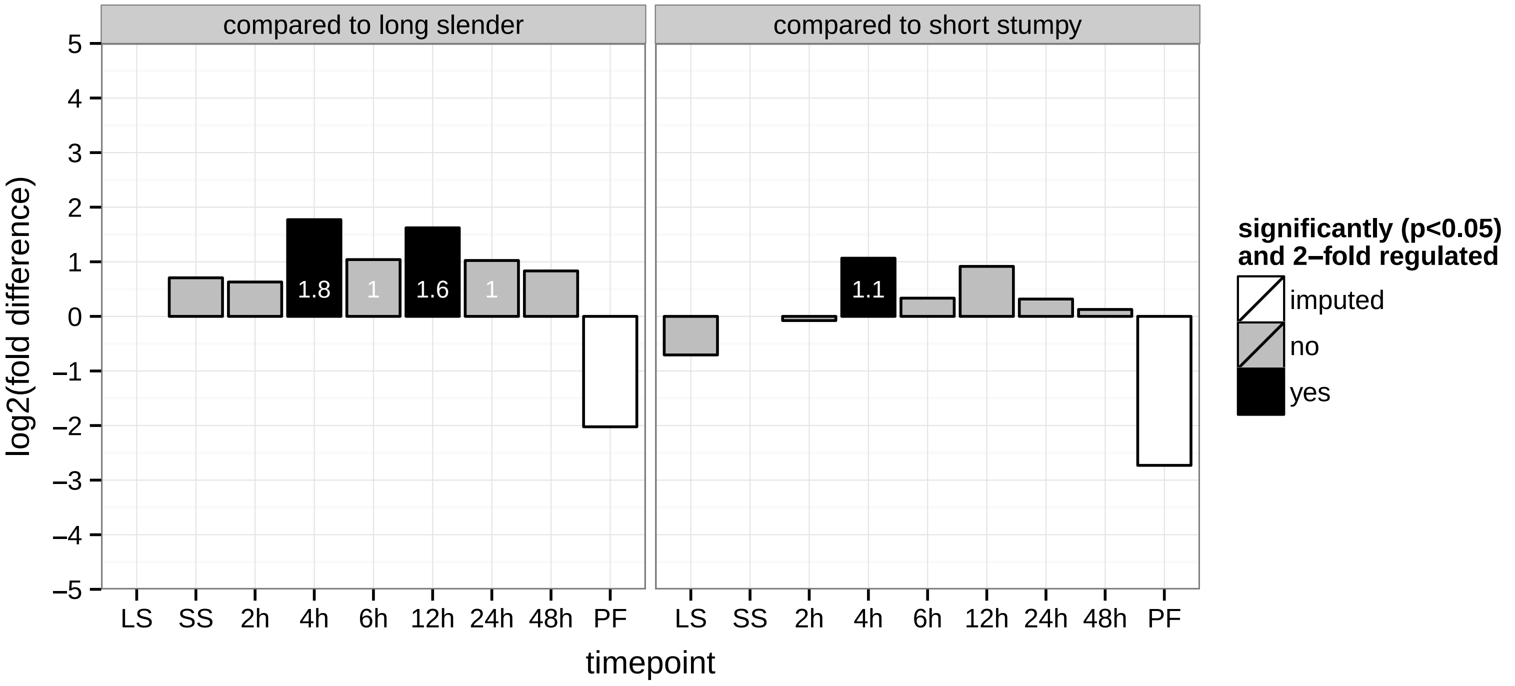

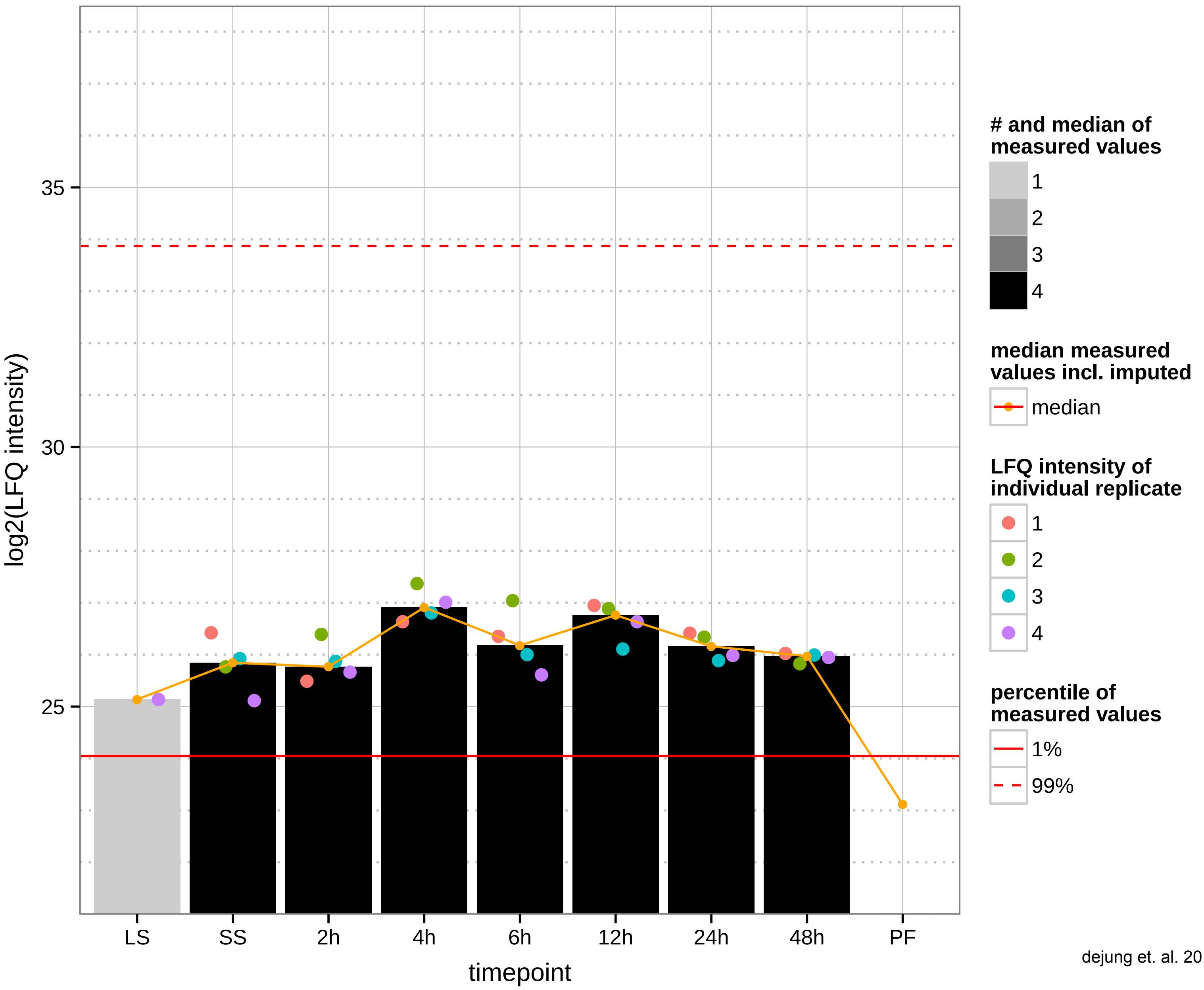

dejung et. al. 2015

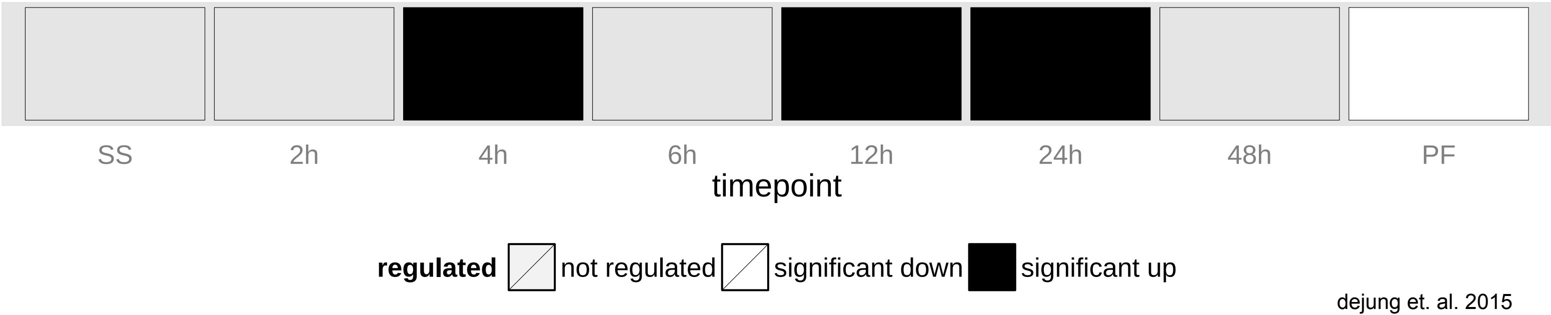
SS
2h
4h
6h
12h
24h
48h
PF
timepoint
regulated
not regulated
significant down
significant up
dejung et. al. 2015

prefoldin subunit, putative
Tb927.5.580
AGOF: unfolded protein binding
AGOC: prefoldin complex
AGOP: protein folding
PGOF: unfolded protein binding
PGOC: prefoldin complex
PGOP: protein folding

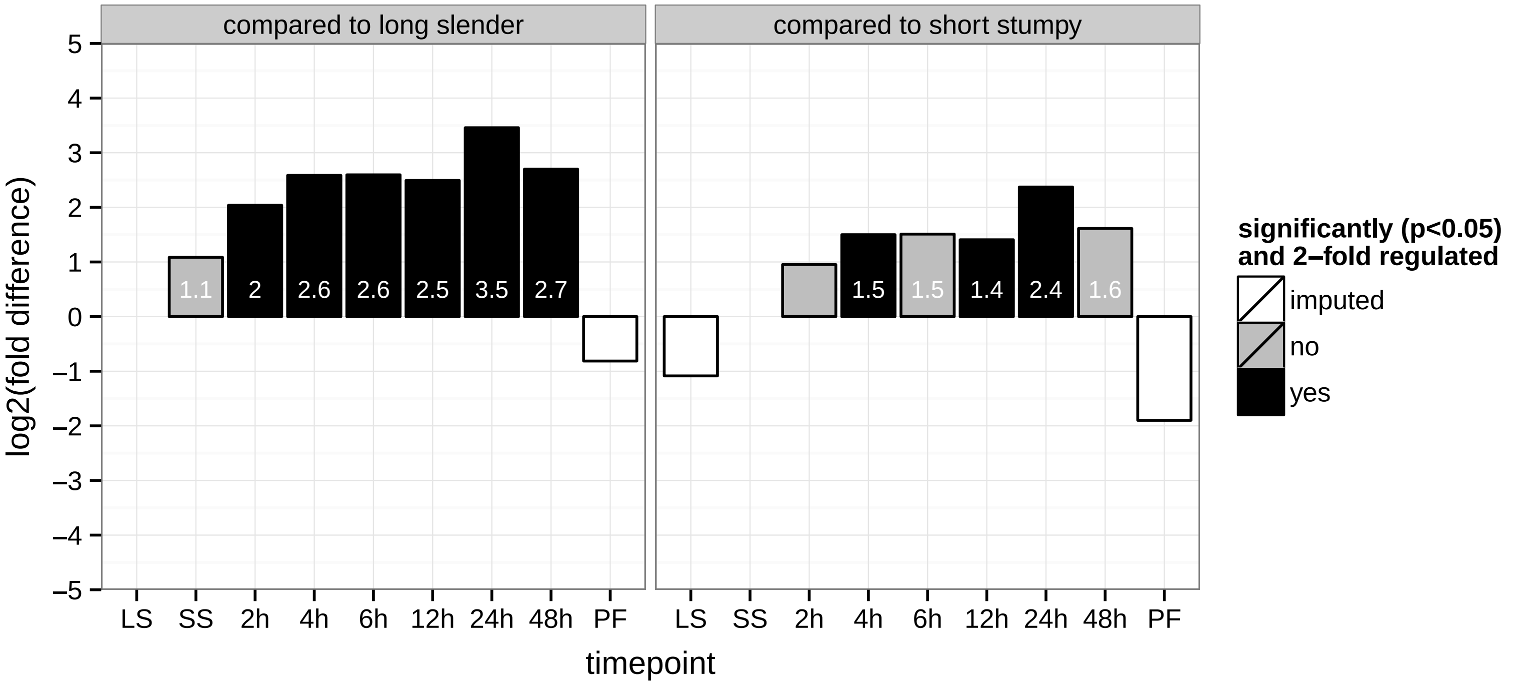

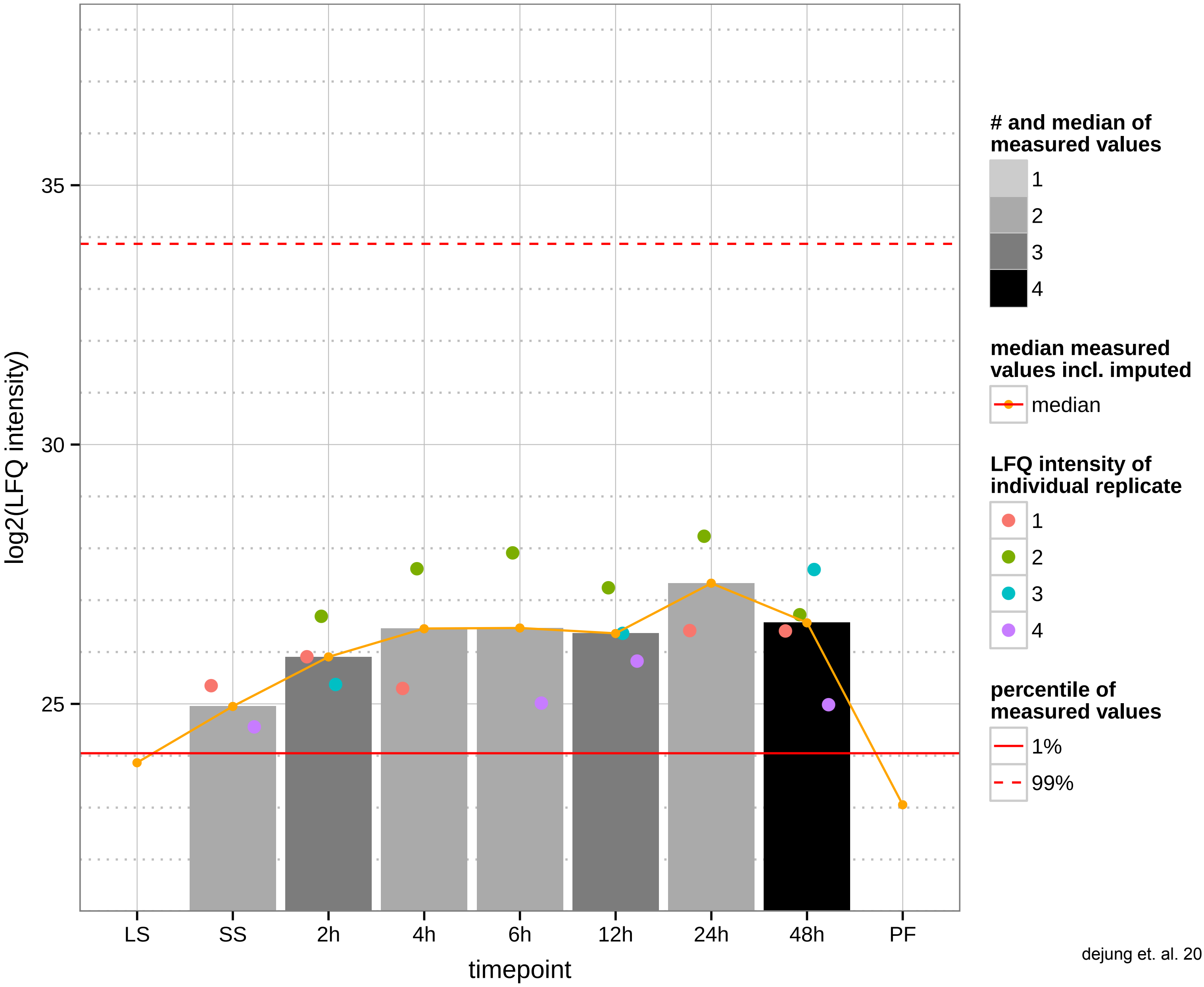

dejung et. al. 2015

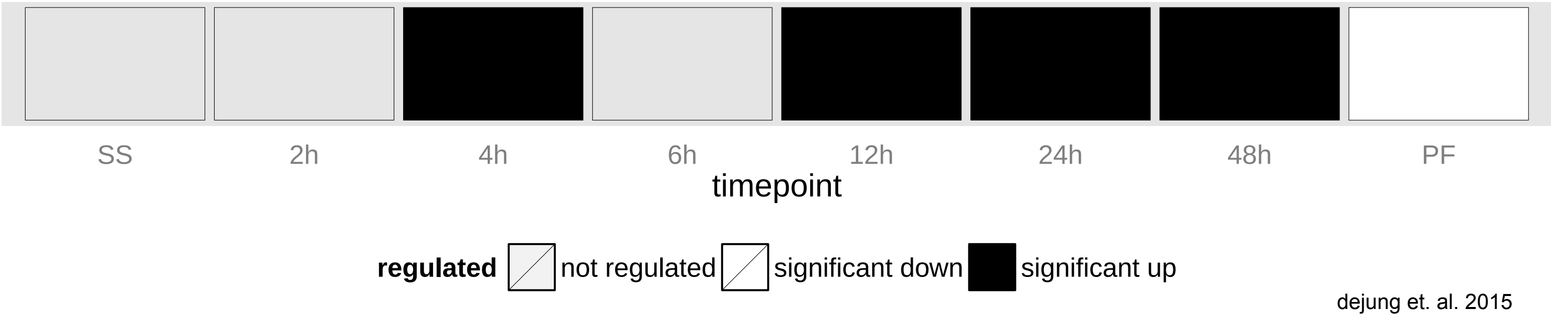

SS
2h
4h
6h
12h
24h
48h
PF
timepoint
regulated
not regulated
significant down
significant up
dejung et. al. 2015

hypothetical protein, conserved
Tb927.2.4445
AGOF: null
AGOC: null
AGOP: null
PGOF: null
PGOC: null
PGOP: null

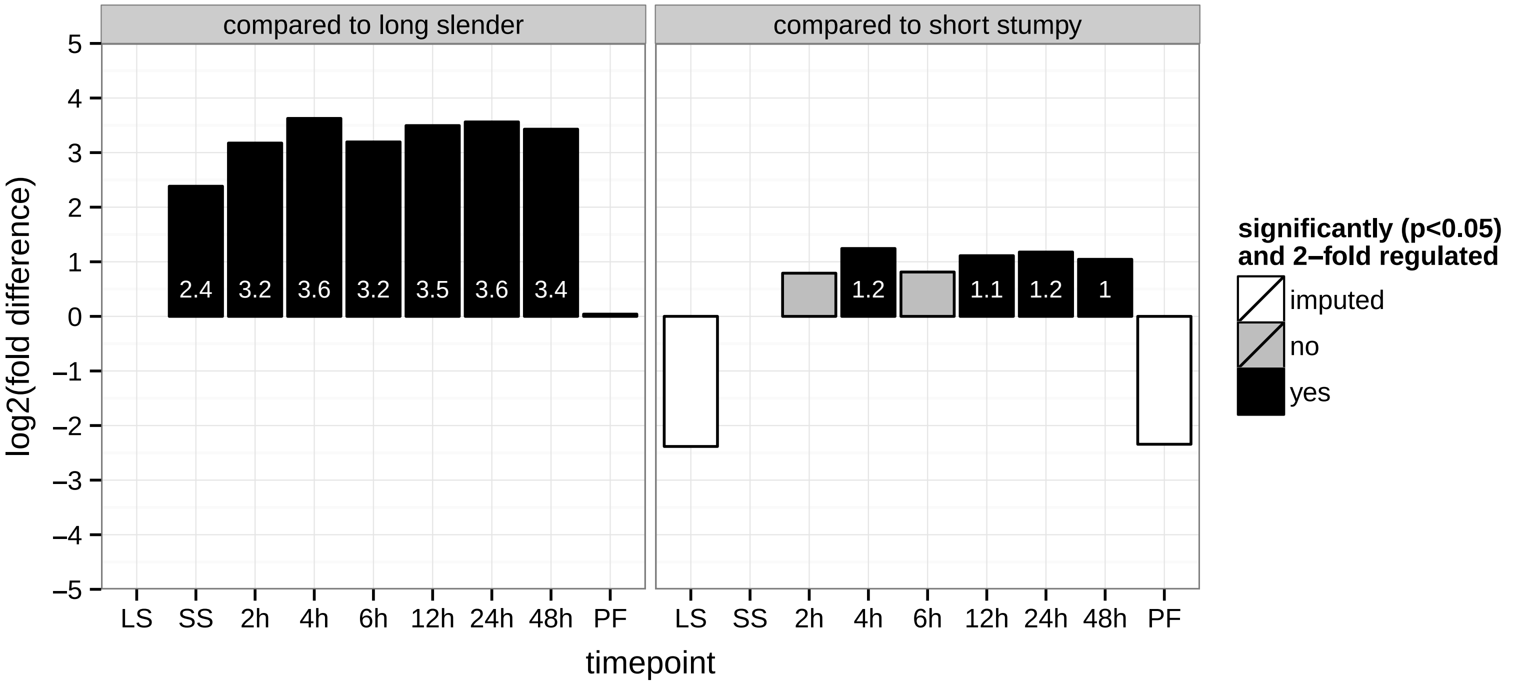

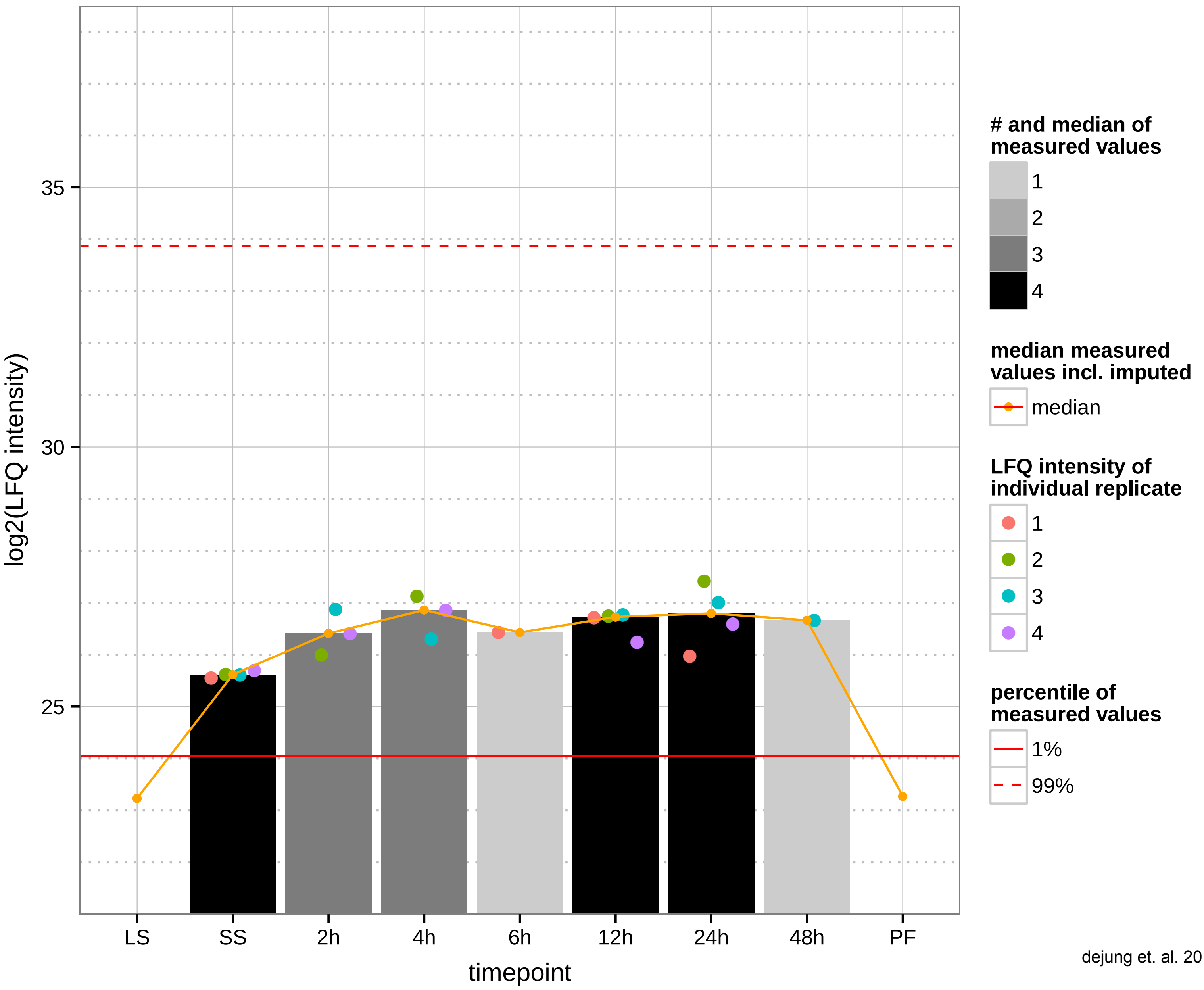

dejung et. al. 2015

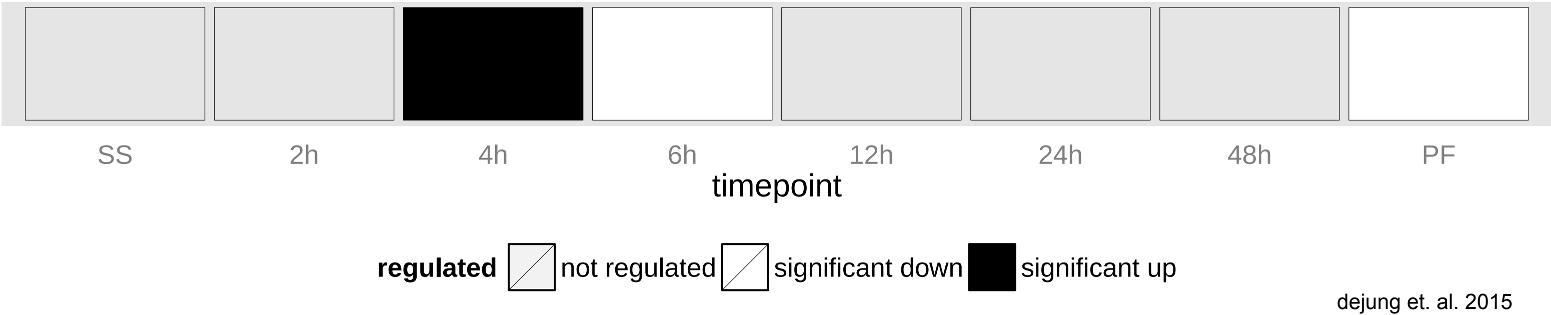
SS
2h
4h
6h
12h
24h
48h
PF
timepoint
regulated
not regulated
significant down
significant up
dejung et. al. 2015

hypothetical protein, conserved
Tb927.5.3950
AGOF: L−ascorbic acid binding, iron ion binding, oxidoreductase activity, acting on paired donors, with incorporation or reduct
AGOC: null
AGOP: oxidation−reduction process
PGOF: L−ascorbic acid binding, iron ion binding, oxidoreductase activity, acting on paired donors, with incorporation or reduct
PGOC: null
PGOP: oxidation−reduction process

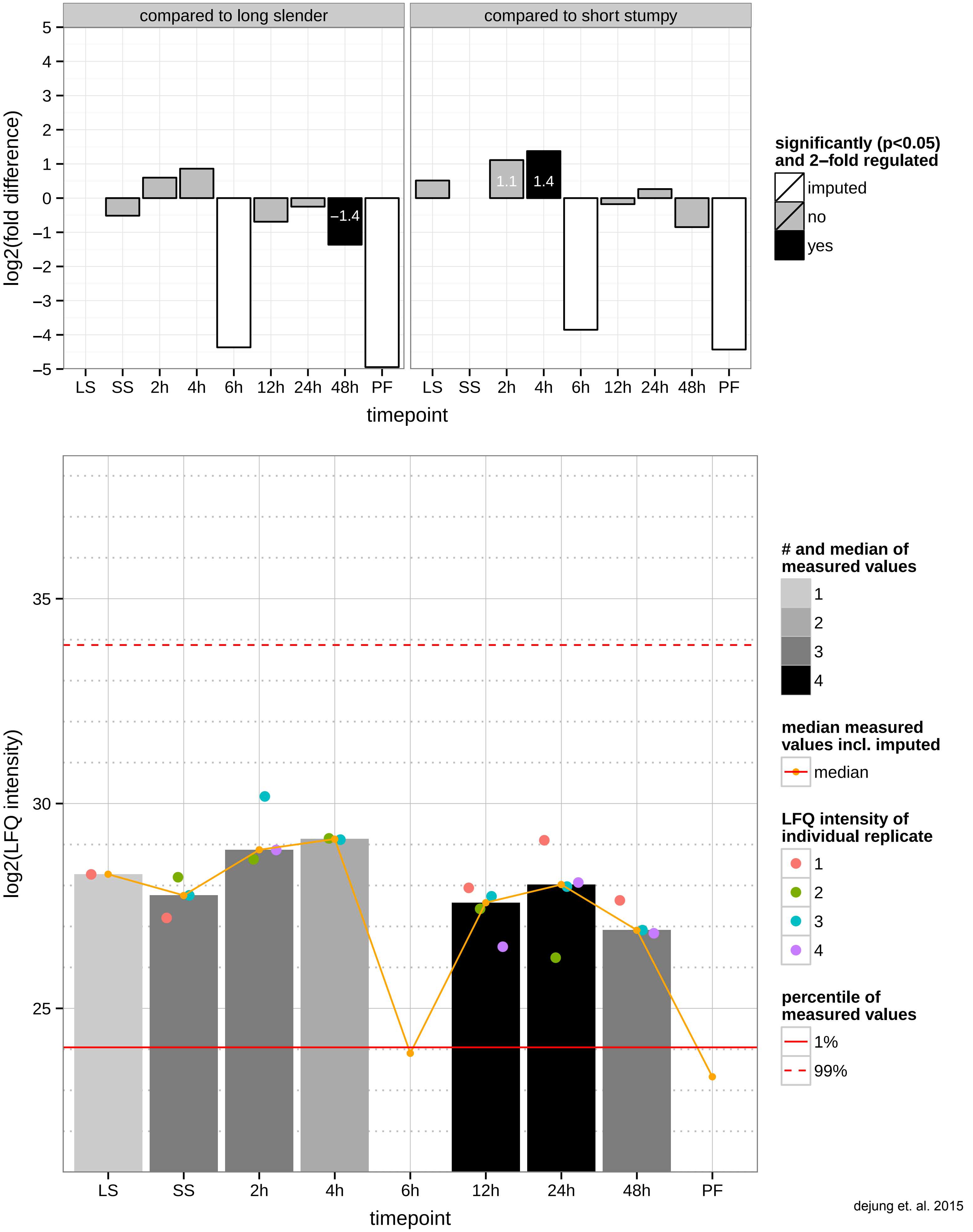

dejung et. al. 2015

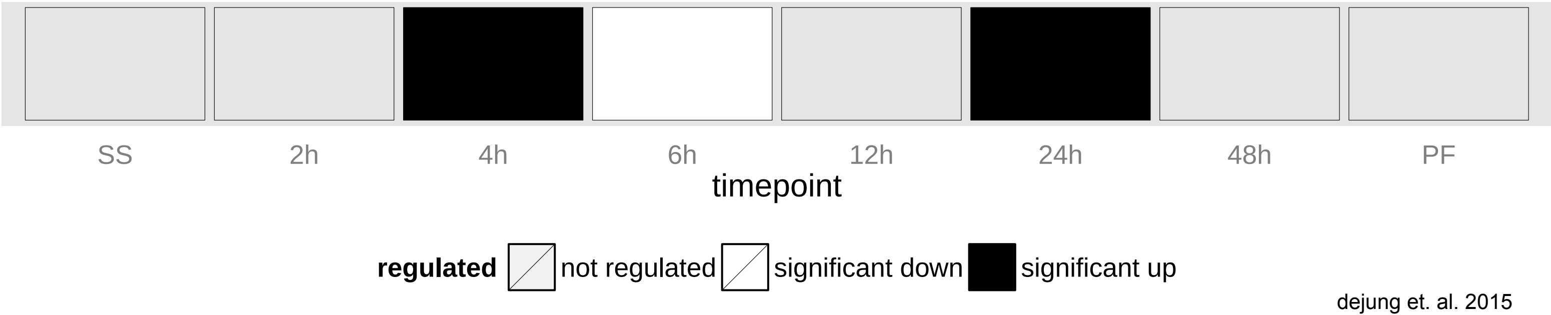

SS
2h
4h
6h
12h
24h
48h
PF
timepoint
regulated
not regulated
significant down
significant up
dejung et. al. 2015

hypothetical protein, conserved
Tb927.10.2340
AGOF: null
AGOC: null
AGOP: null
PGOF: protein binding
PGOC: null
PGOP: null

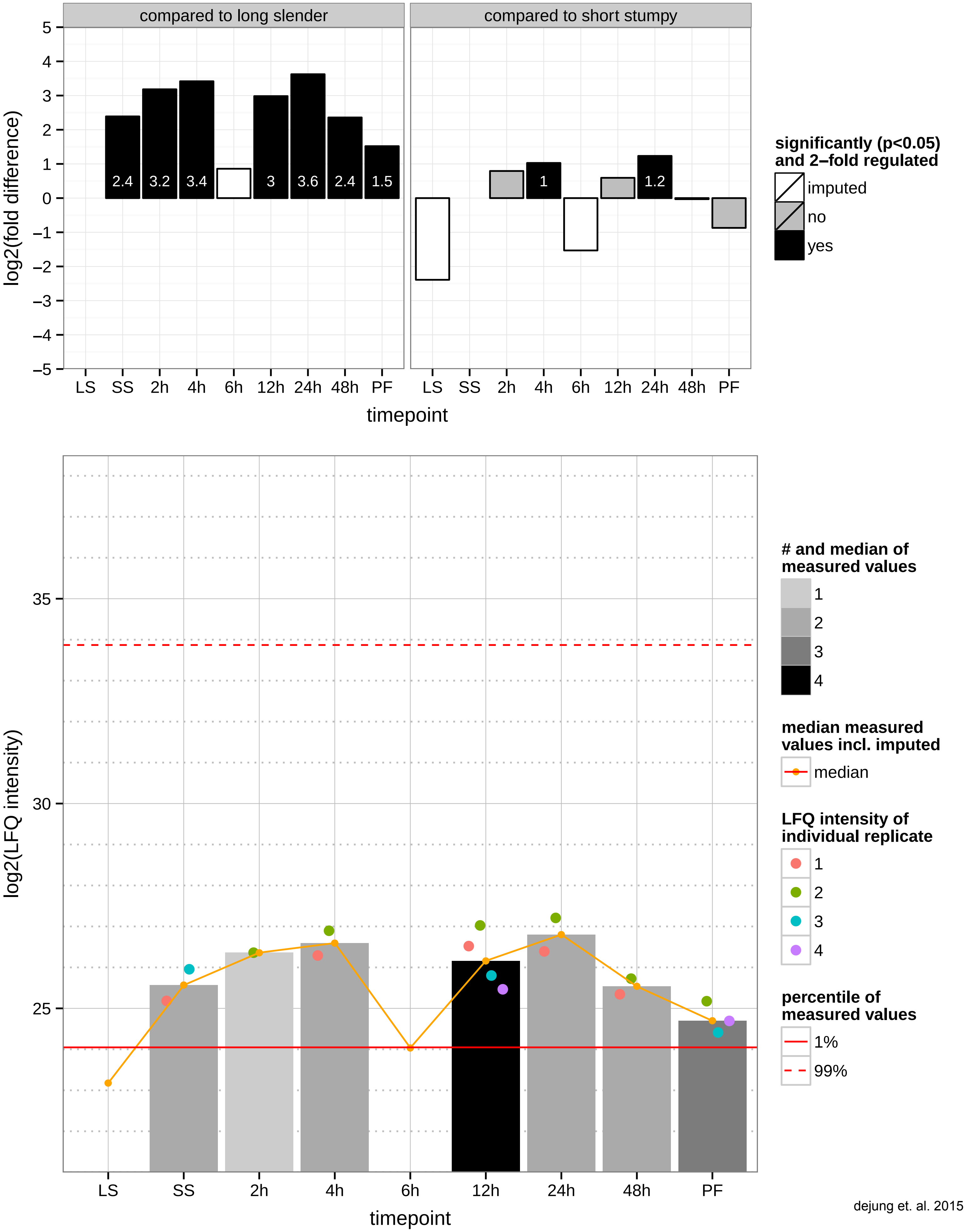

dejung et. al. 2015

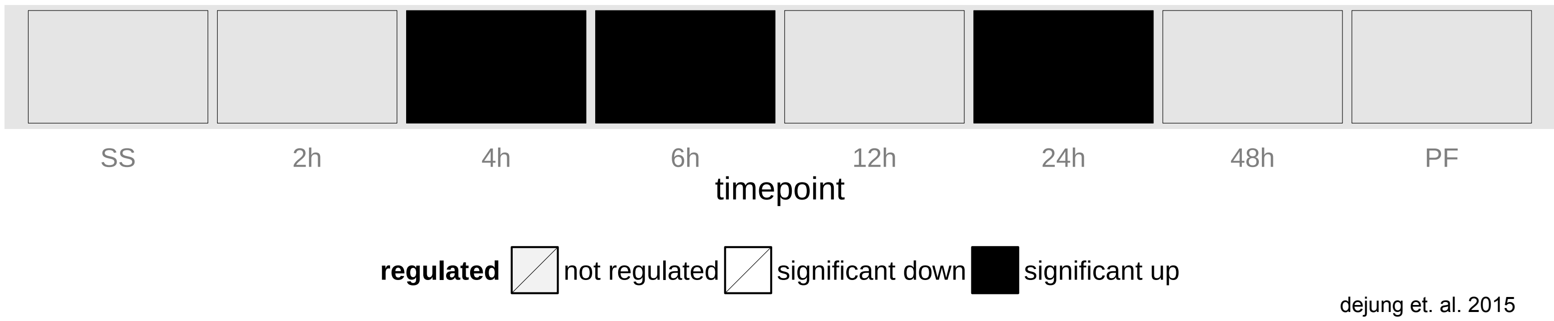
SS
2h
4h
6h
12h
24h
48h
PF
timepoint
regulated
not regulated
significant down
significant up
dejung et. al. 2015

hypothetical protein, conserved
Tb927.9.8290
AGOF: structural constituent of ribosome
AGOC: mitochondrion, ribosome
AGOP: translation
PGOF: null
PGOC: null
PGOP: null

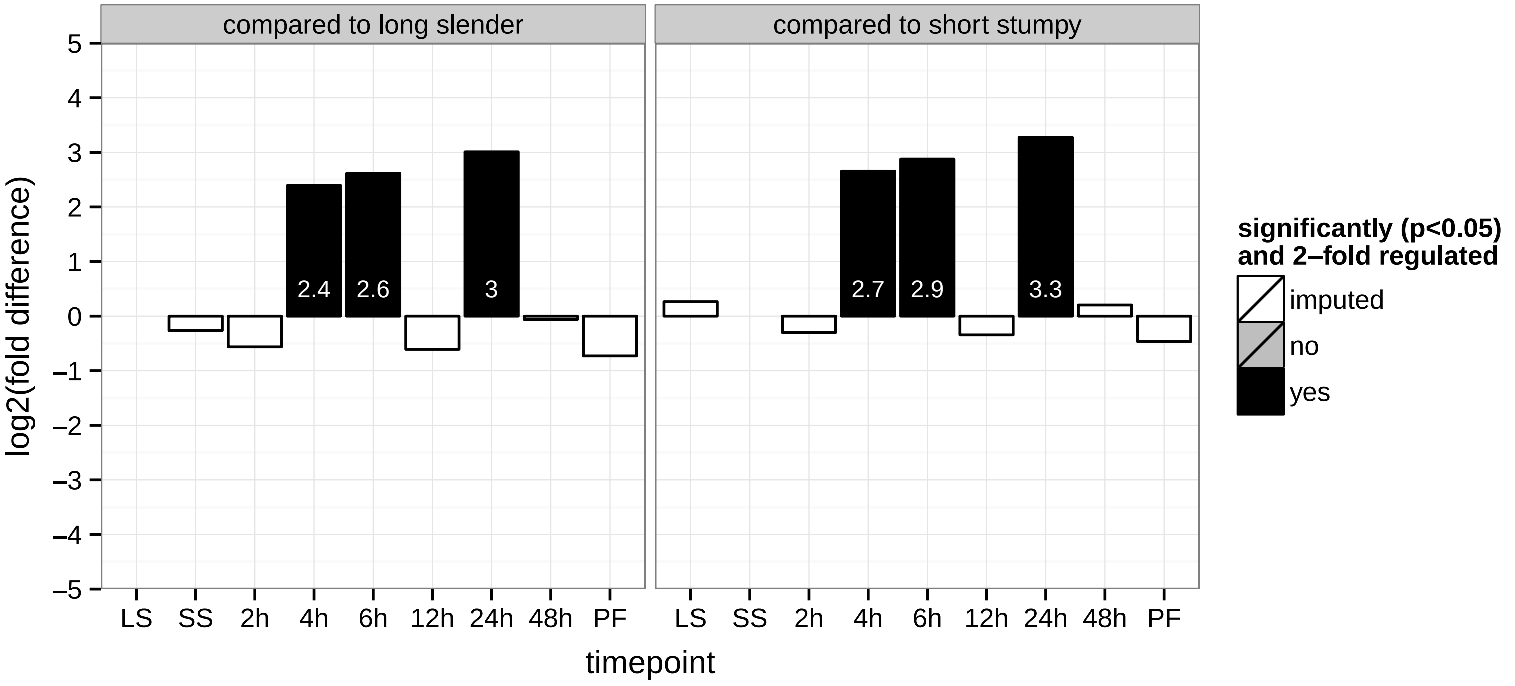

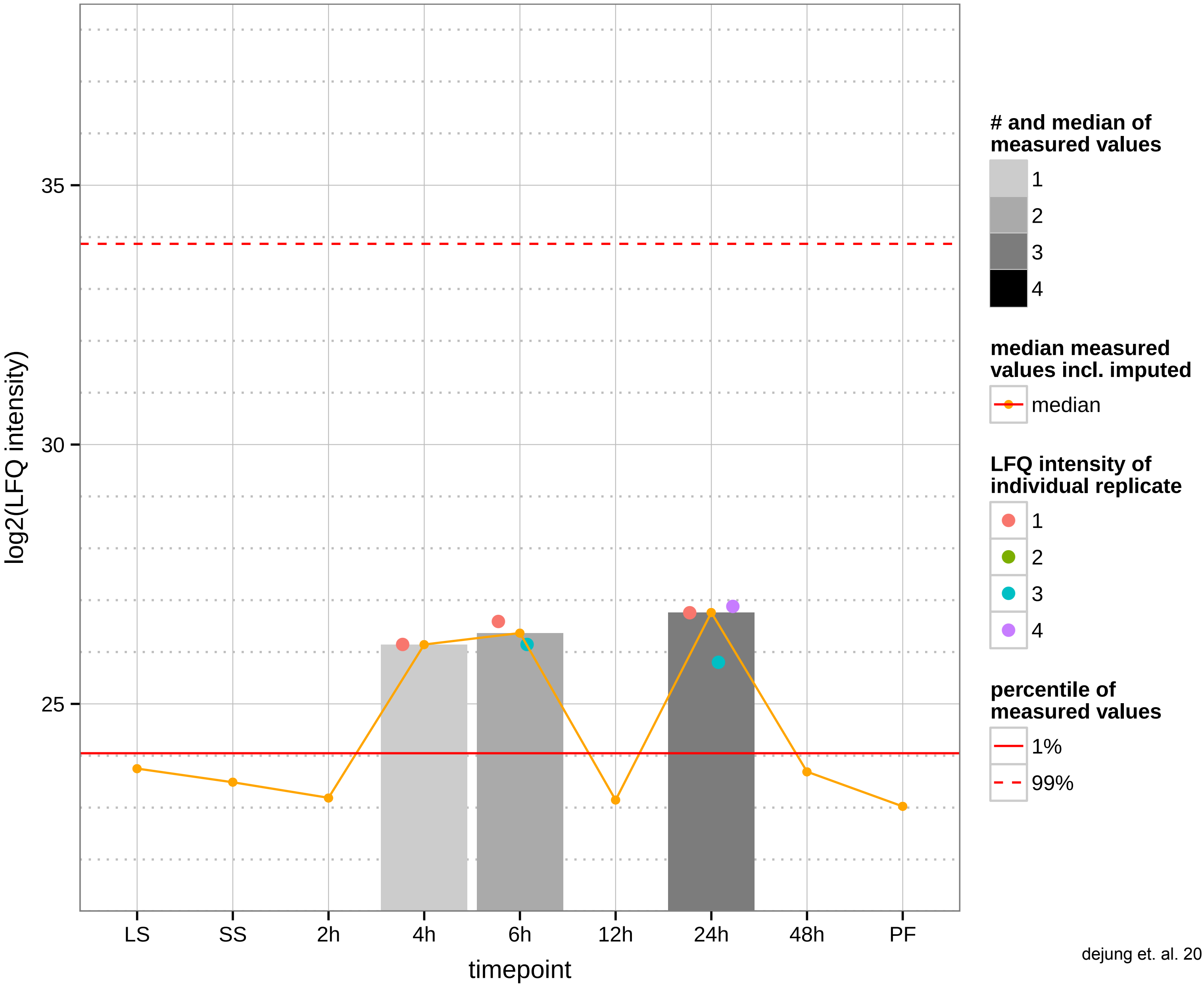

dejung et. al. 2015

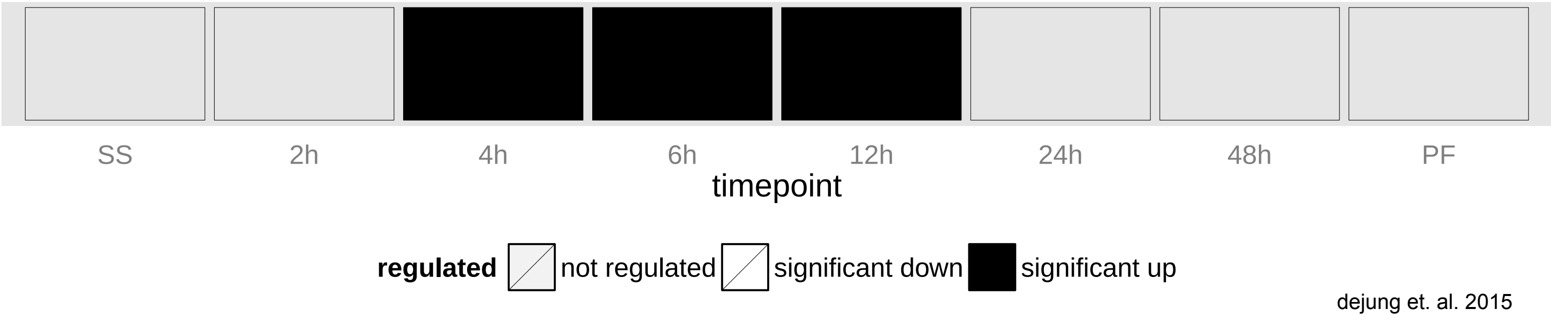
SS
2h
4h
6h
12h
24h
48h
PF
timepoint
regulated
not regulated
significant down
significant up
dejung et. al. 2015

ubiquitin−conjugating enzyme E2, putative, ubiquitin carrier protein, ubiquitin−protein ligase
Tb927.4.2710
AGOF: ubiquitin−protein ligase activity
AGOC: null
AGOP: cellular protein modification process, post−translational protein modification, regulation of protein metabolic process
PGOF: acid−amino acid ligase activity
PGOC: null
PGOP: null

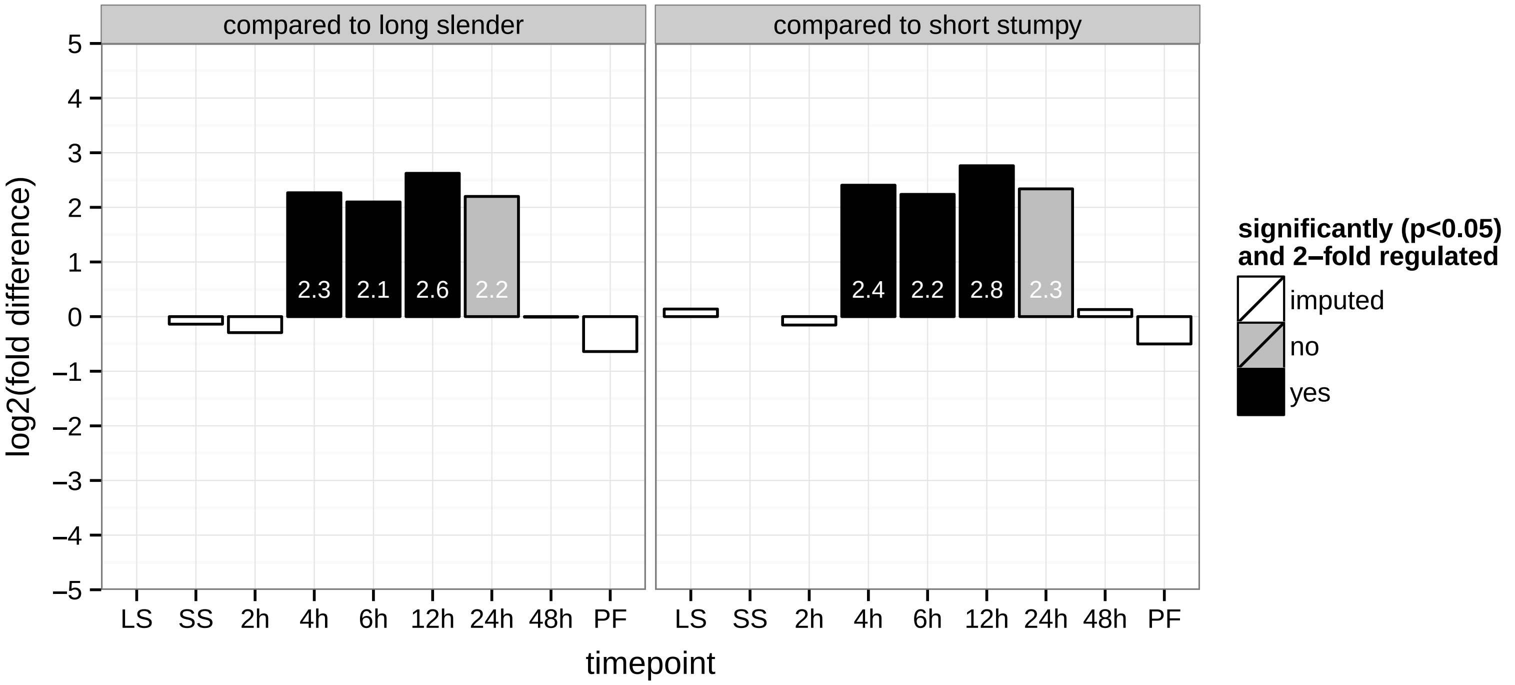

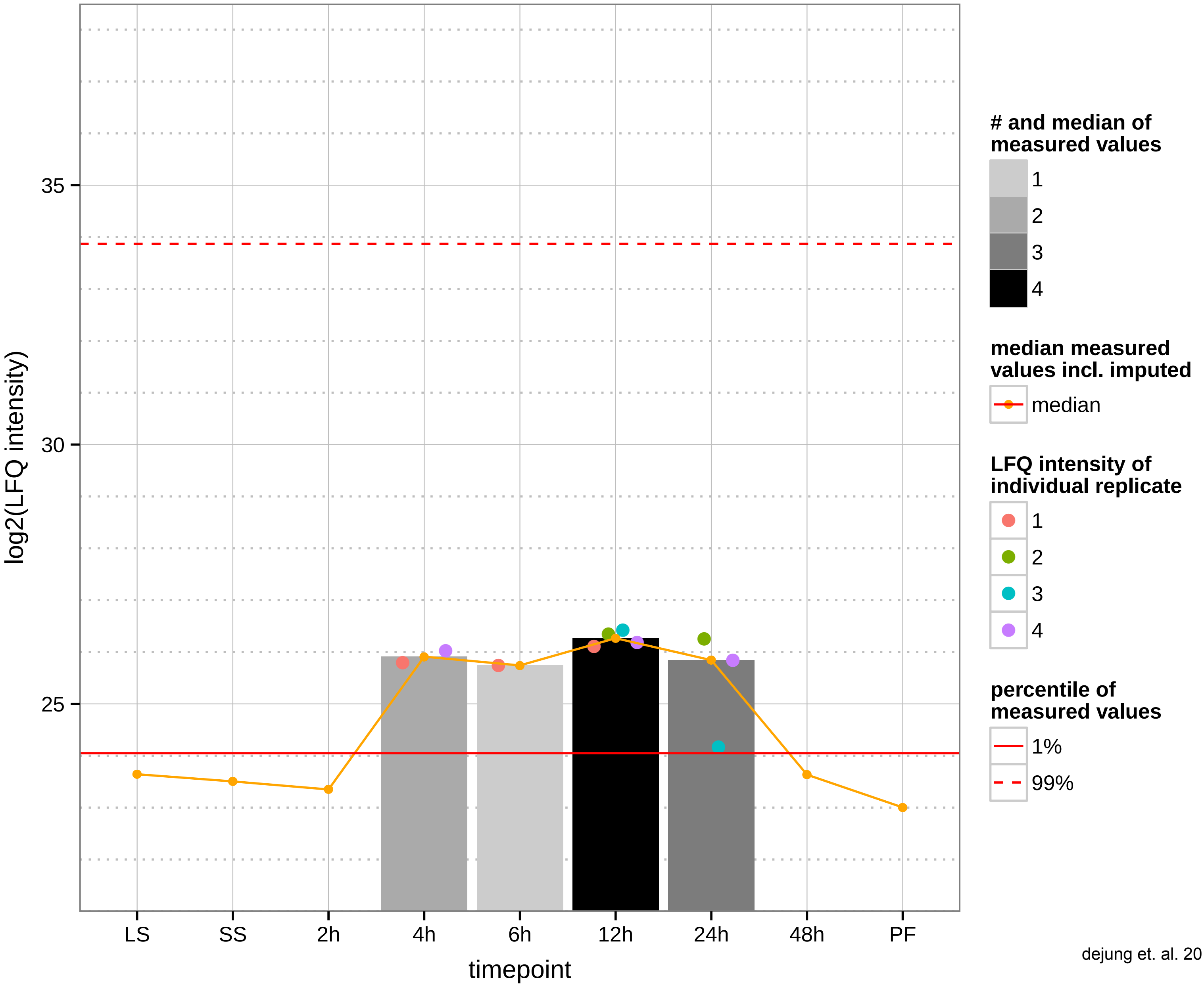

dejung et. al. 2015

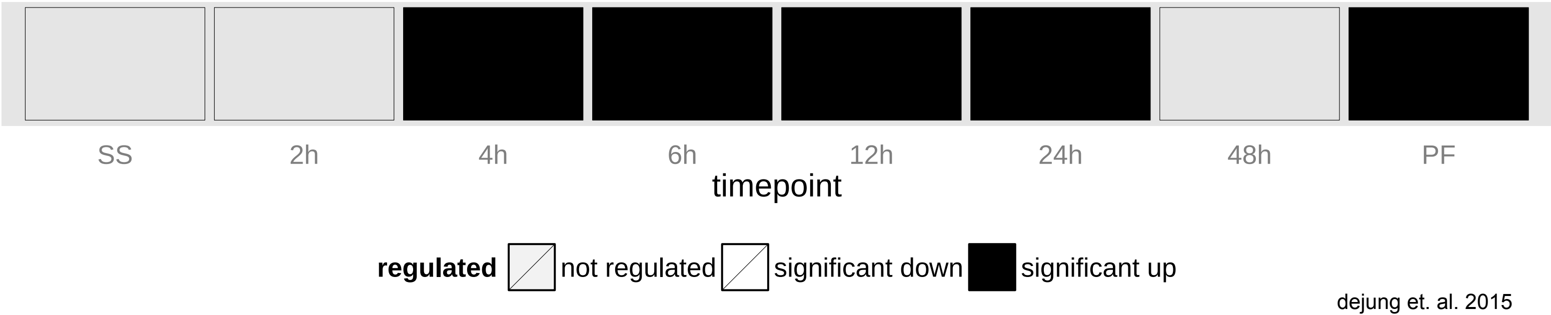
SS
2h
4h
6h
12h
24h
48h
PF
timepoint
regulated
not regulated
significant down
significant up
dejung et. al. 2015

chaperone protein DNAj, putative
Tb927.9.8160
AGOF: heat shock protein binding, unfolded protein binding
AGOC: integral to membrane
AGOP: protein folding
PGOF: heat shock protein binding
PGOC: null
PGOP: null

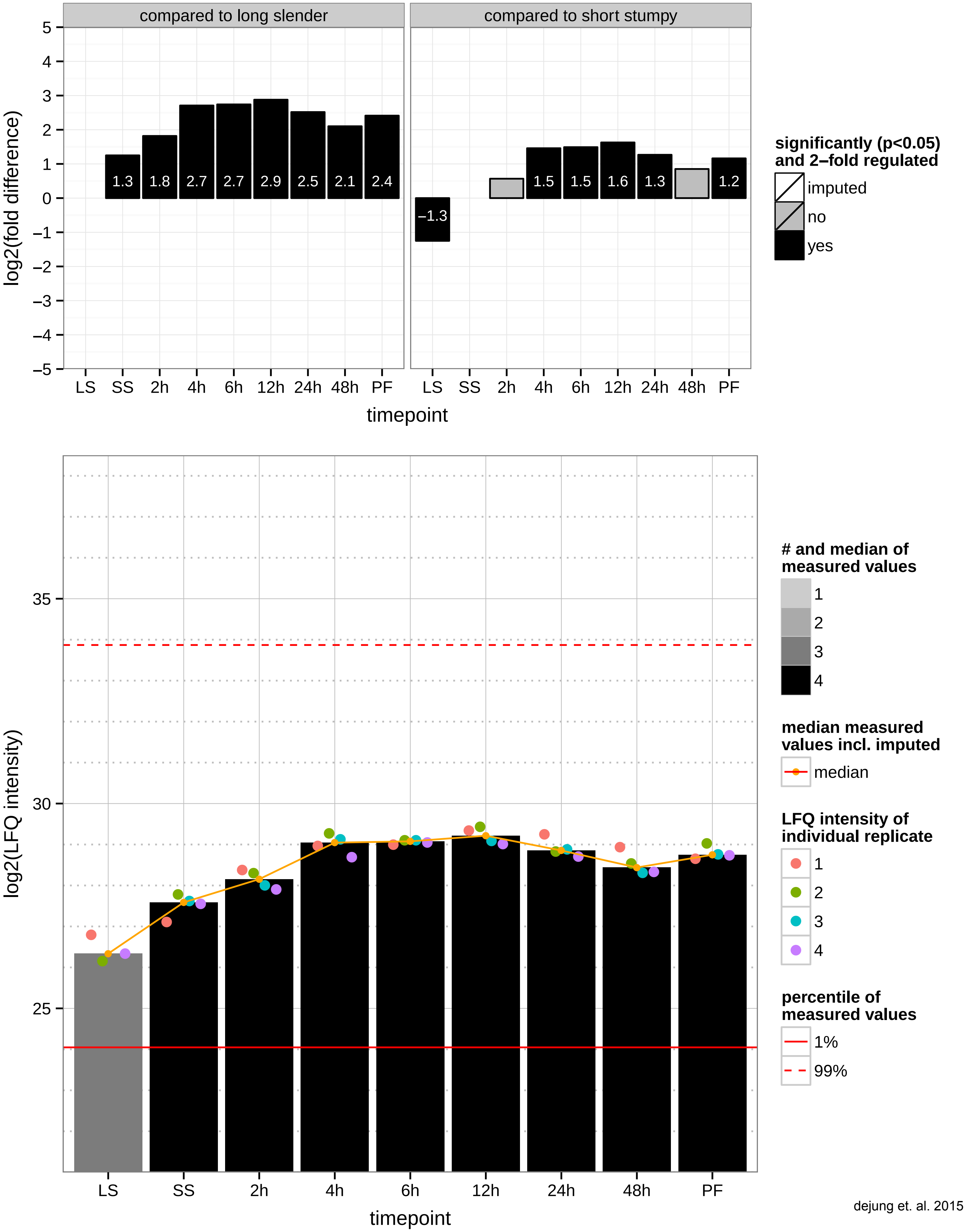

dejung et. al. 2015

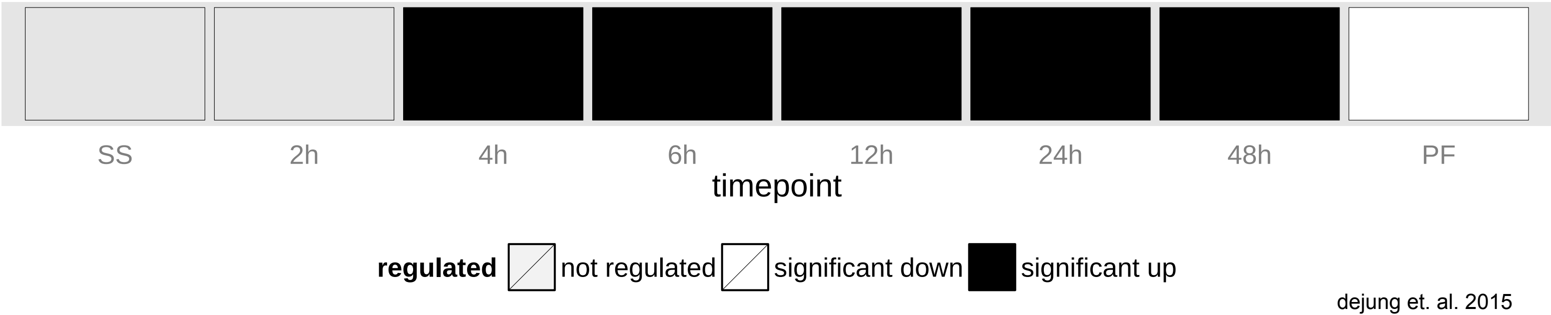
SS
2h
4h
6h
12h
24h
48h
PF
timepoint
regulated
not regulated
significant down
significant up
dejung et. al. 2015

hypothetical protein
Tb927.5.4020
AGOF: null
AGOC: null
AGOP: null
PGOF: null
PGOC: null
PGOP: null

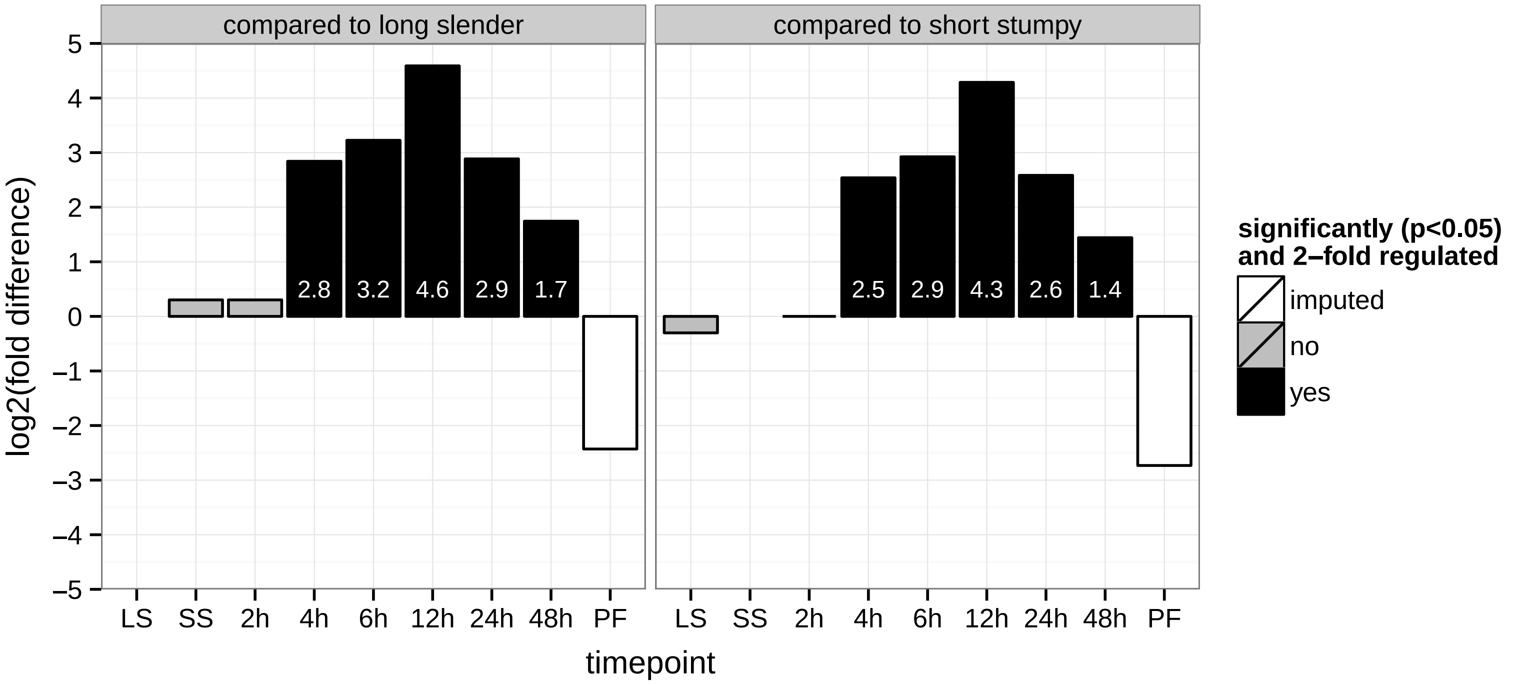

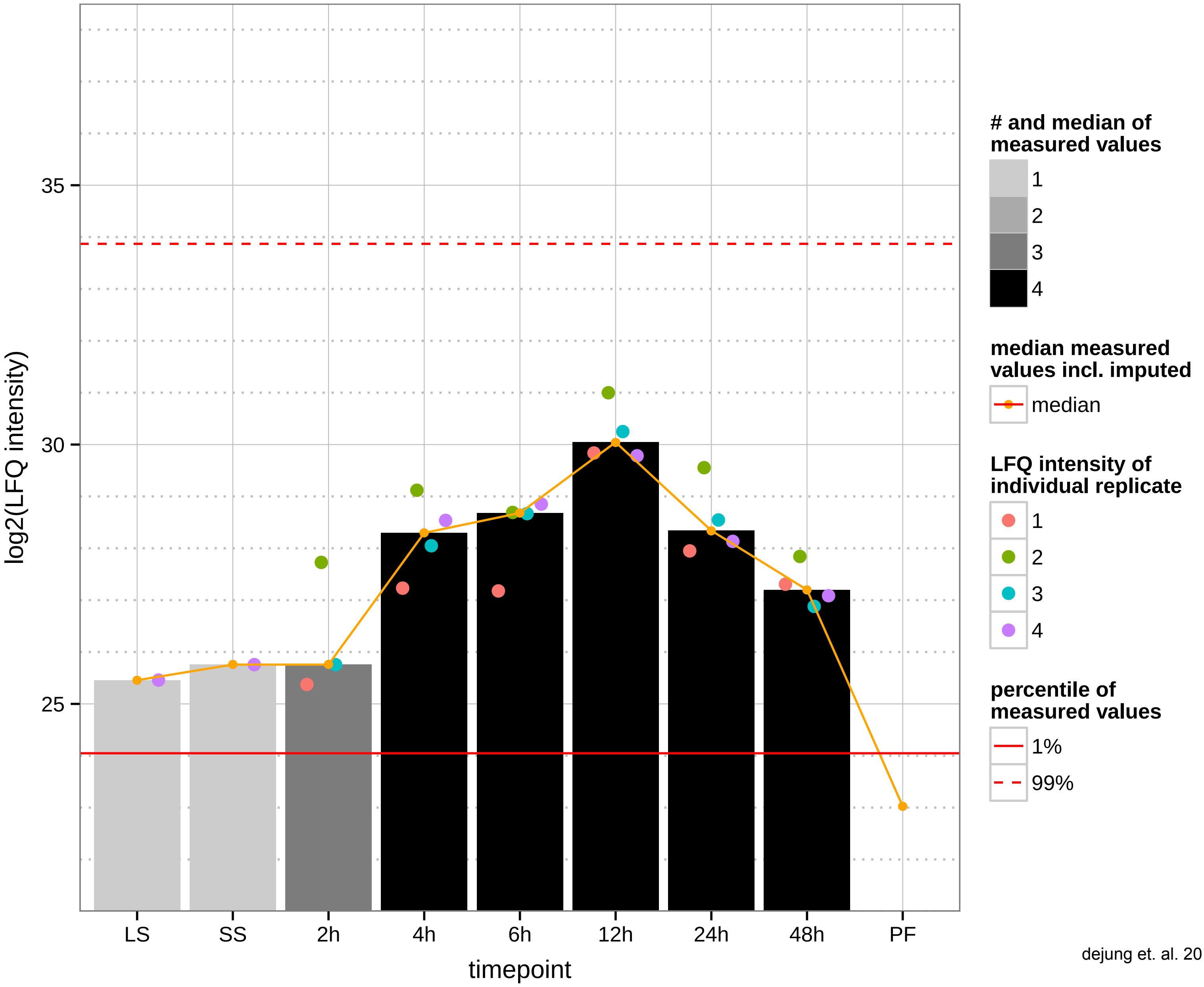

dejung et. al. 2015

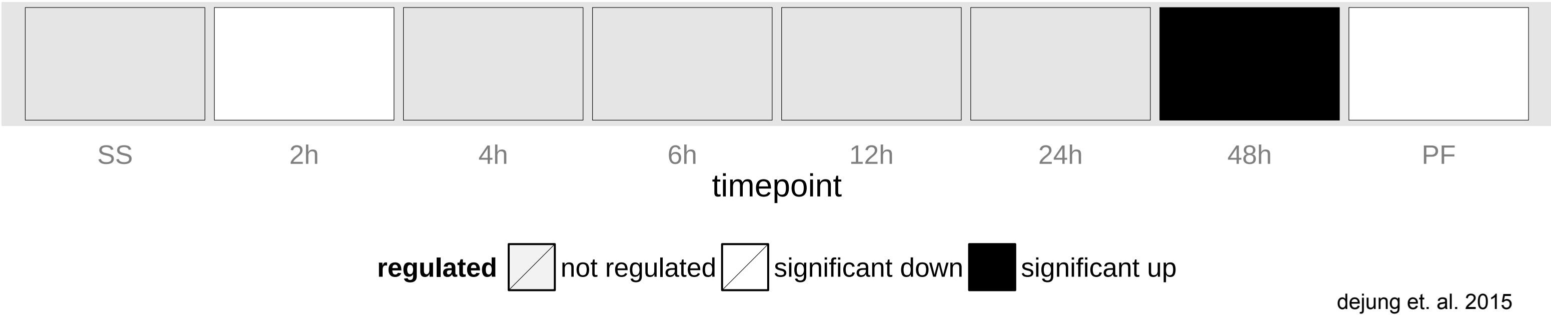
SS
2h
4h
6h
12h
24h
48h
PF
timepoint
regulated
not regulated
significant down
significant up
dejung et. al. 2015

hypothetical protein, conserved
Tb927.8.3980
AGOF: null
AGOC: null
AGOP: null
PGOF: null
PGOC: null
PGOP: null

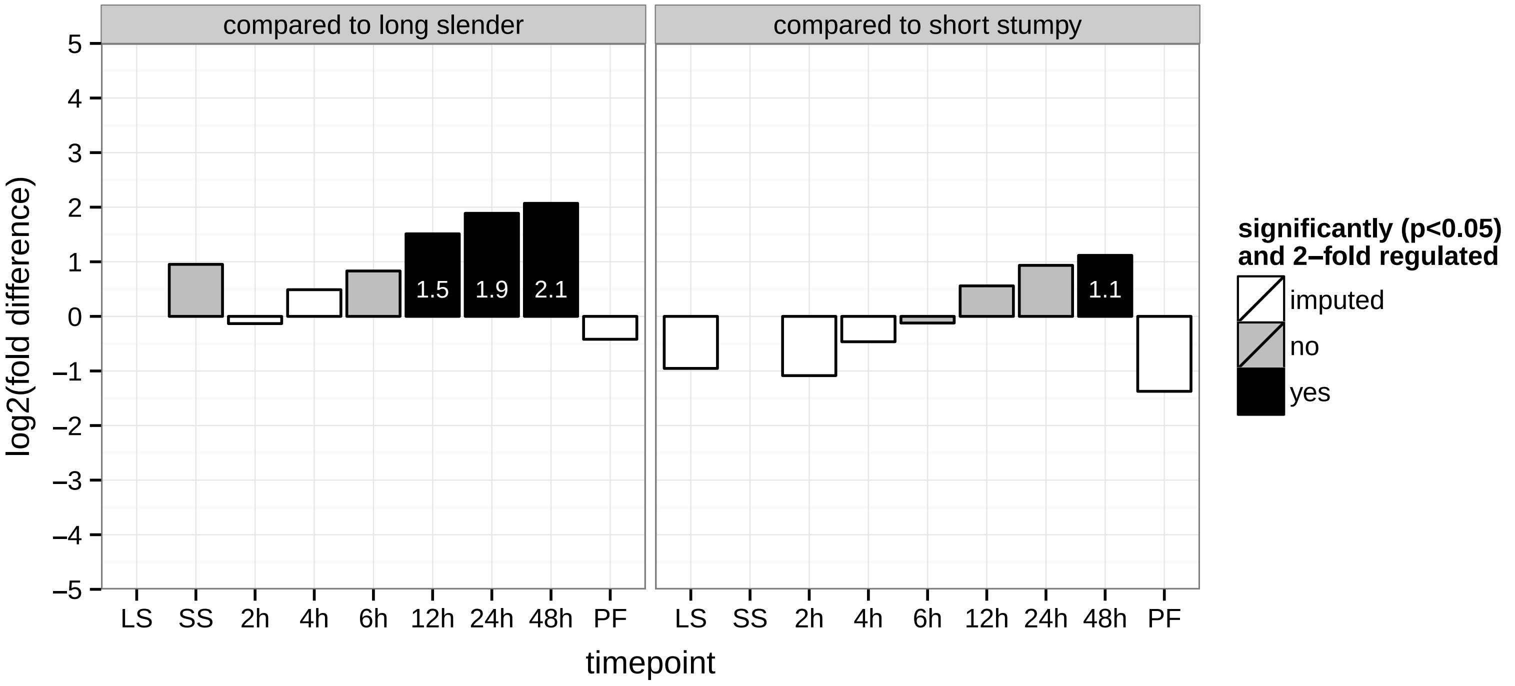

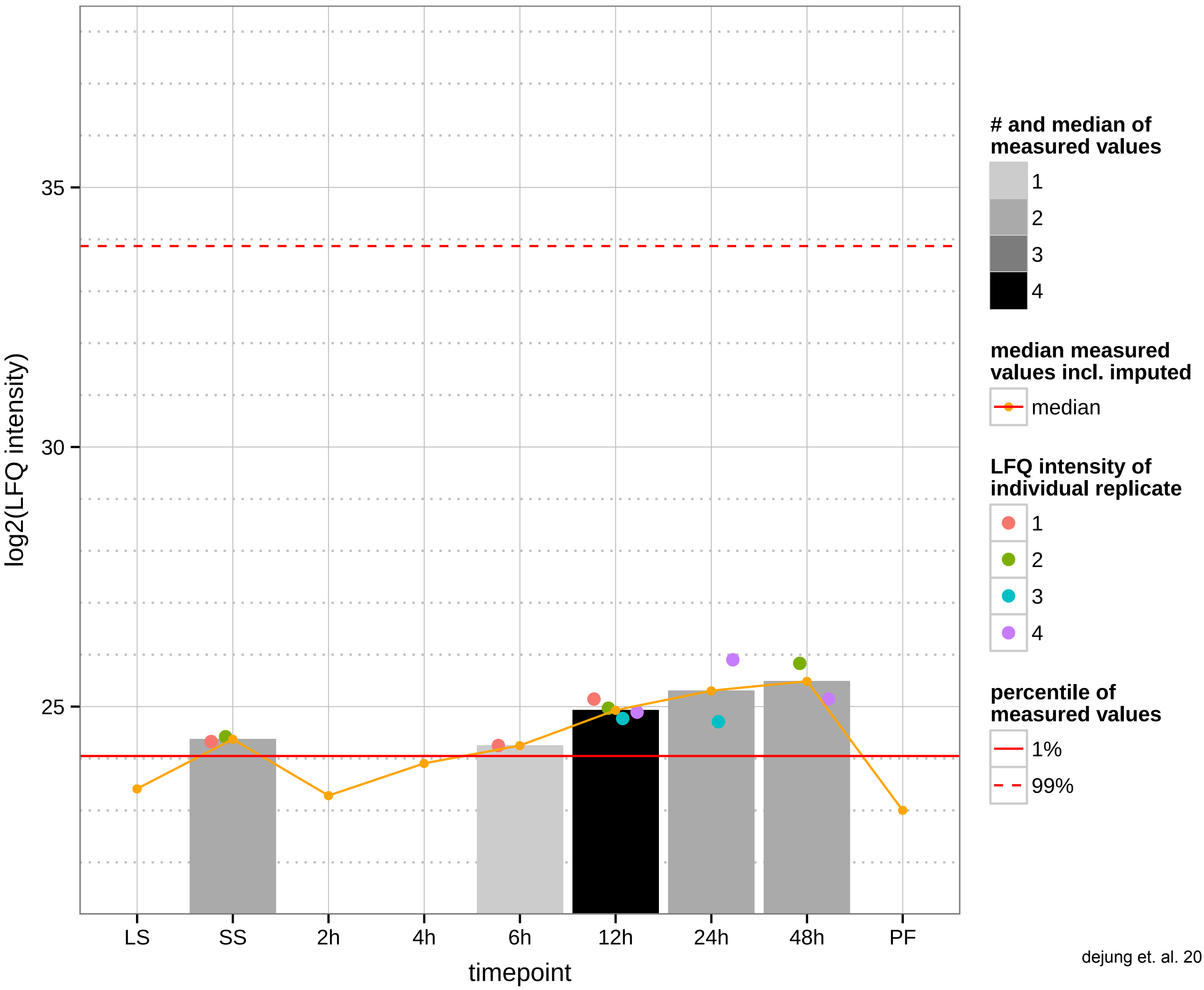

dejung et. al. 2015

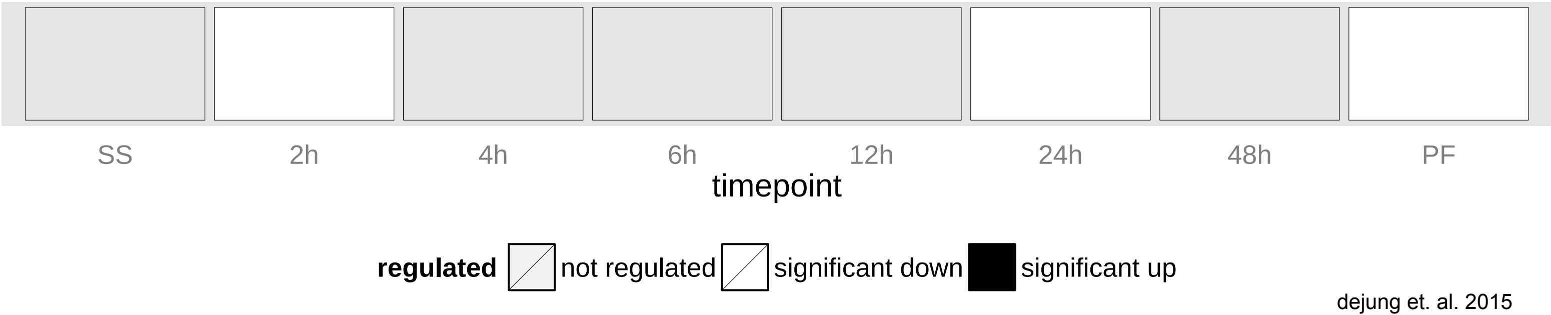
SS
2h
4h
6h
12h
24h
48h
PF
timepoint
regulated
not regulated
significant down
significant up
dejung et. al. 2015

hypothetical protein, conserved, class I transcription factor A, subunit 5a (CITFA−5a)
Tb927.8.4080;Tb927.8.4030;Tb11.v5.0861
AGOF: null
AGOC: null
AGOP: null
PGOF: null
PGOC: null
PGOP: null

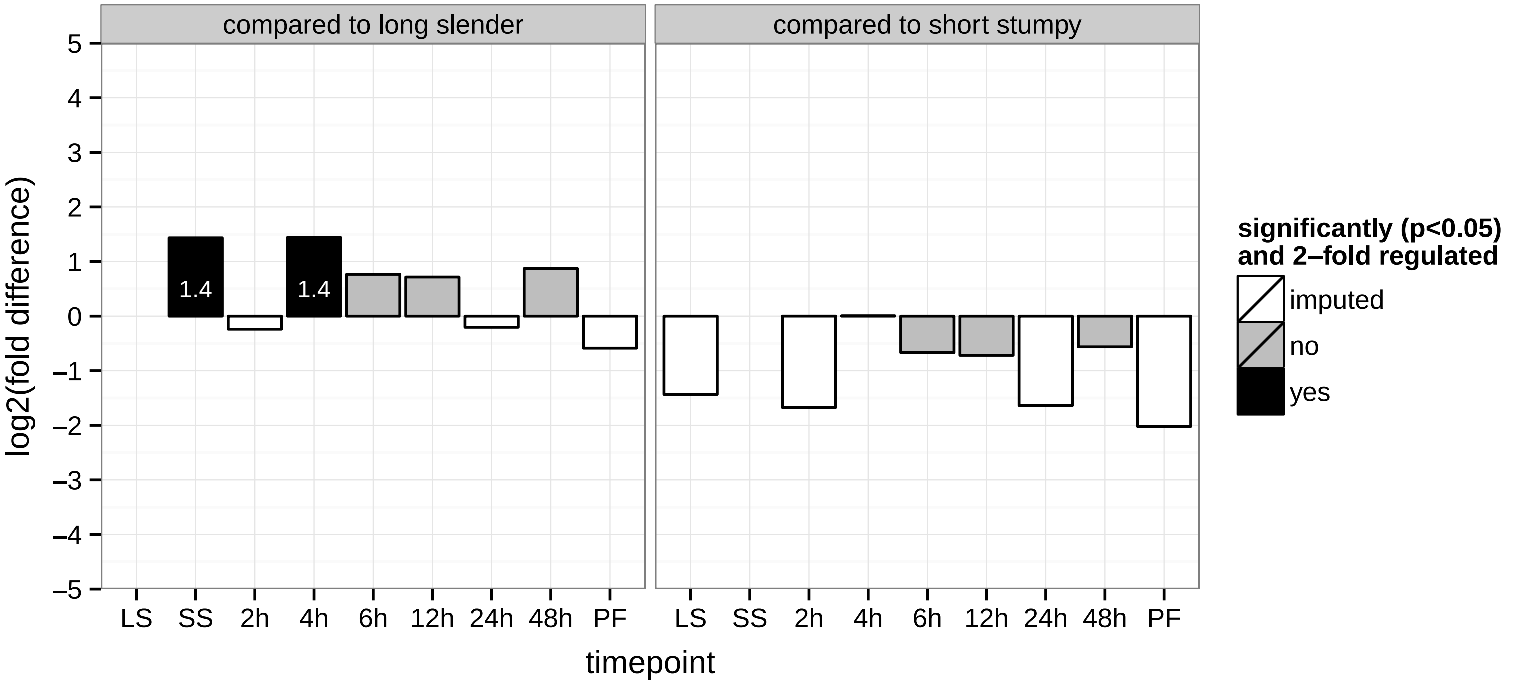

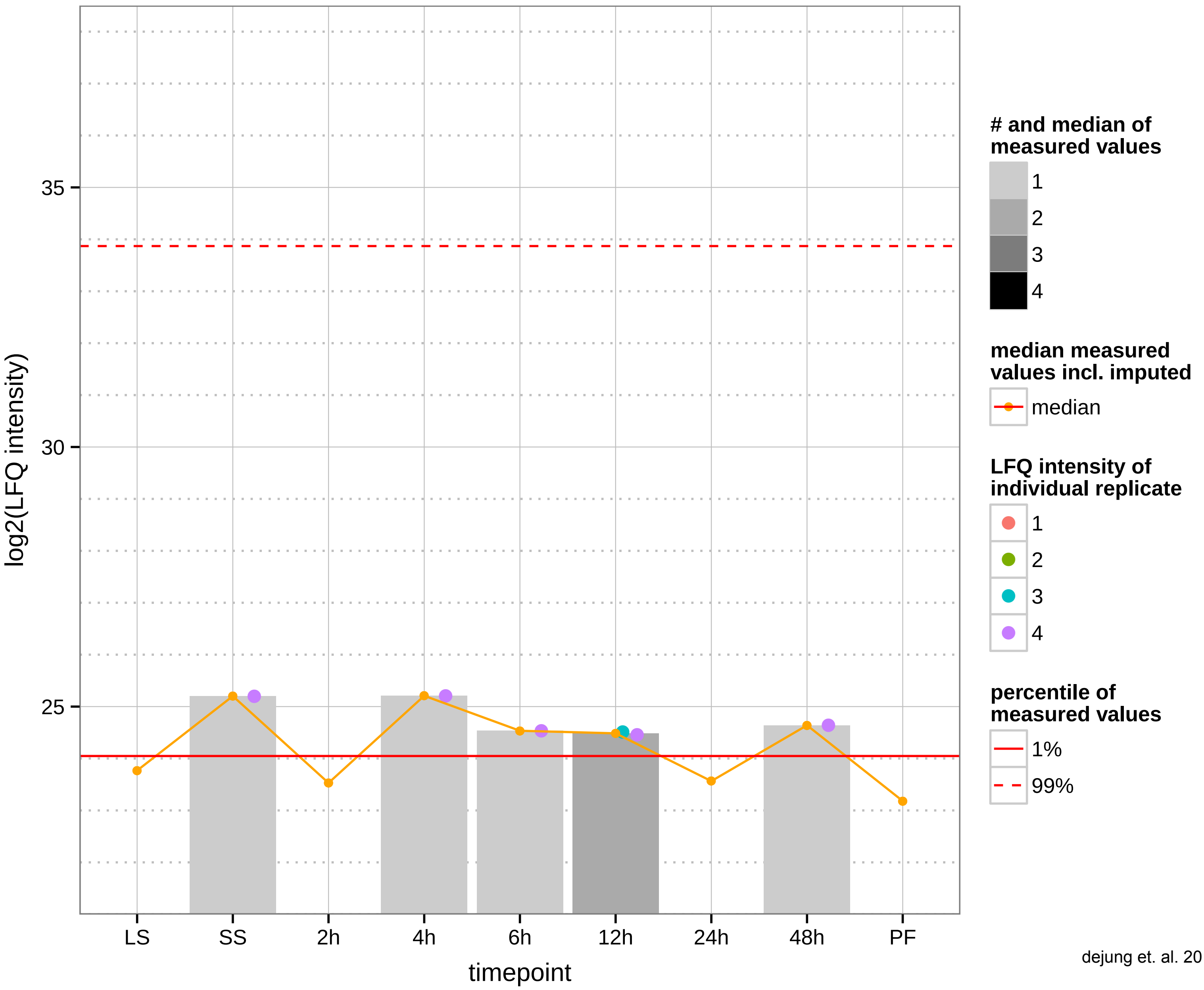

dejung et. al. 2015

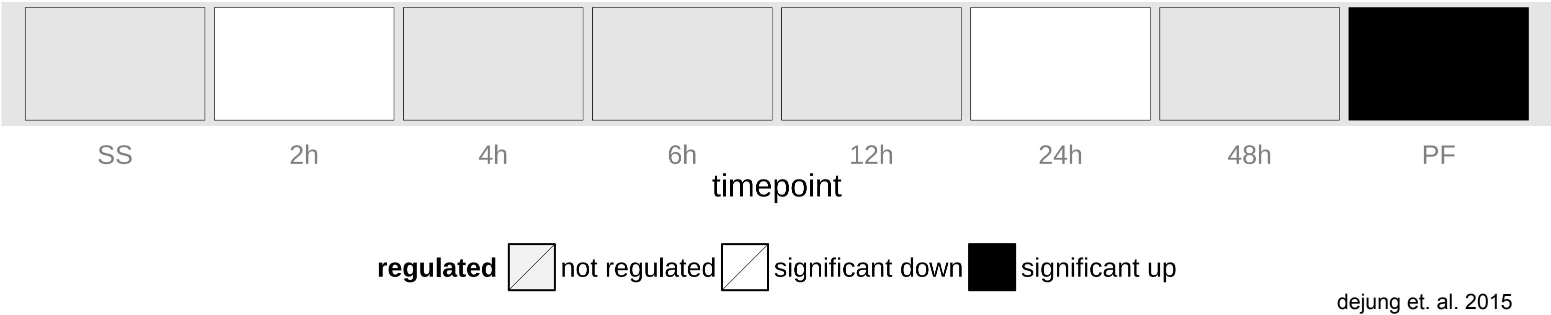
SS
2h
4h
6h
12h
24h
48h
PF
timepoint
regulated
not regulated
significant down
significant up
dejung et. al. 2015

hypothetical protein, conserved
Tb927.10.2200
AGOF: null
AGOC: null
AGOP: null
PGOF: null
PGOC: null
PGOP: null

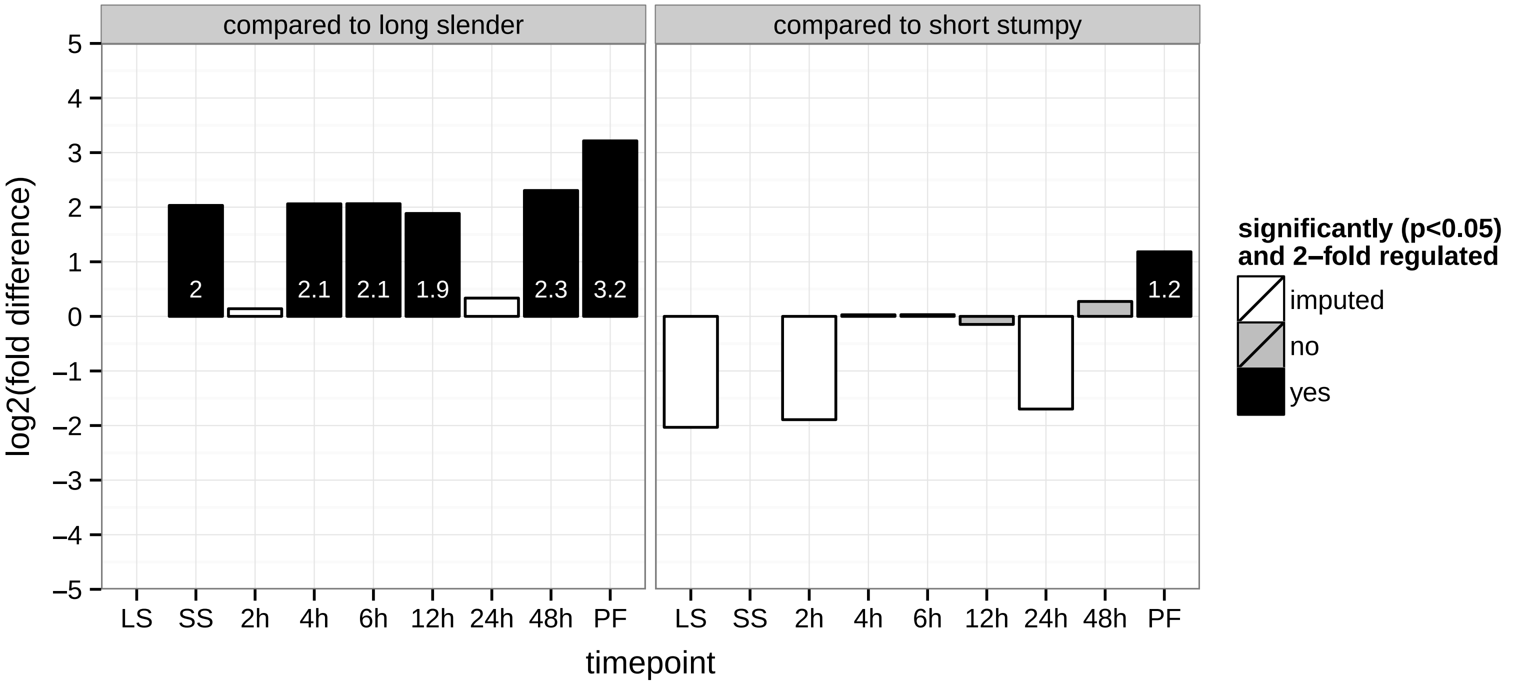

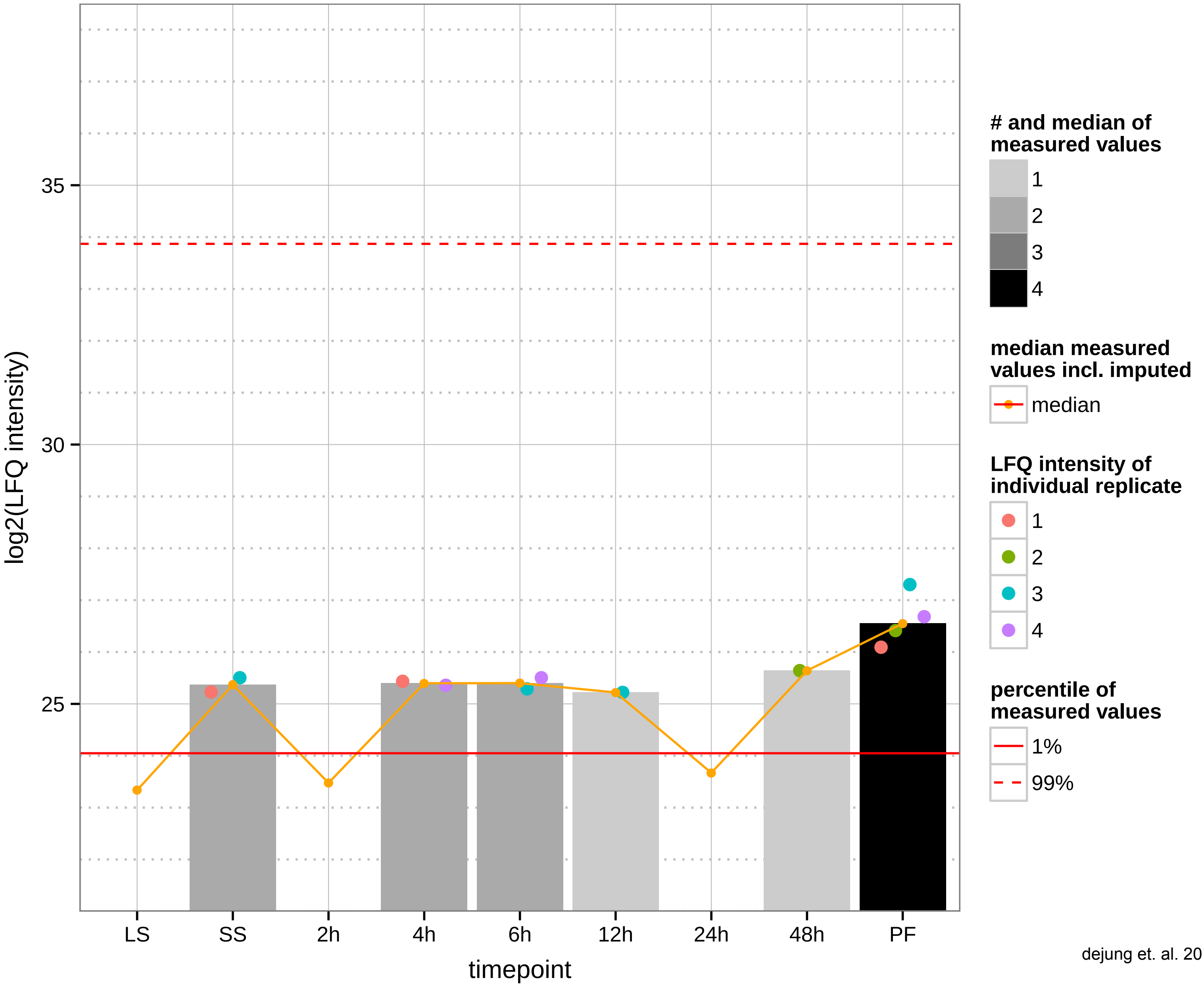

dejung et. al. 2015

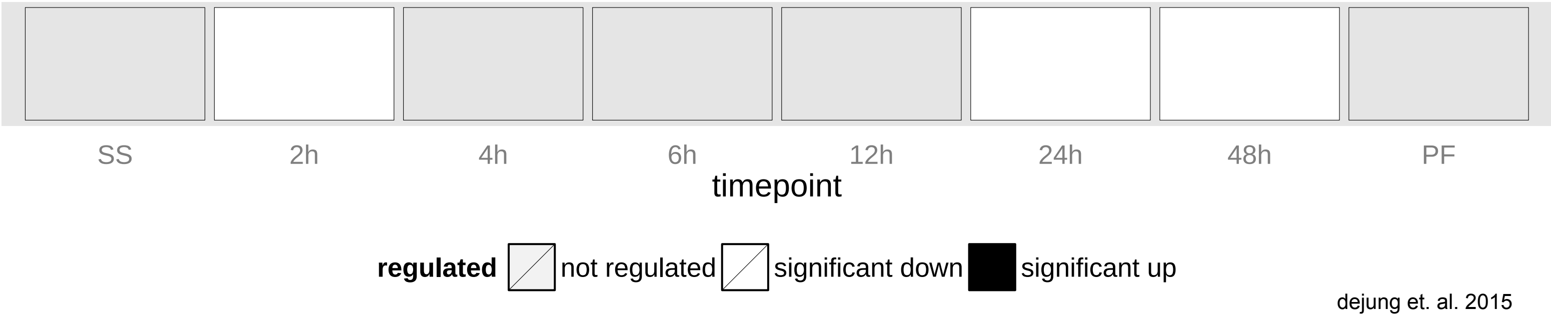
SS
2h
4h
6h
12h
24h
48h
PF
timepoint
regulated
not regulated
significant down
significant up
dejung et. al. 2015

trypanothione/tryparedoxin dependent peroxidase 1, cytosolic, glutathione peroxidase–like protein 1 (TDPX1)
Tb927.7.1120
AGOF: glutathione peroxidase activity
AGOC: null
AGOP: response to oxidative stress
PGOF: glutathione peroxidase activity
PGOC: null
PGOP: oxidation–reduction process, response to oxidative stress

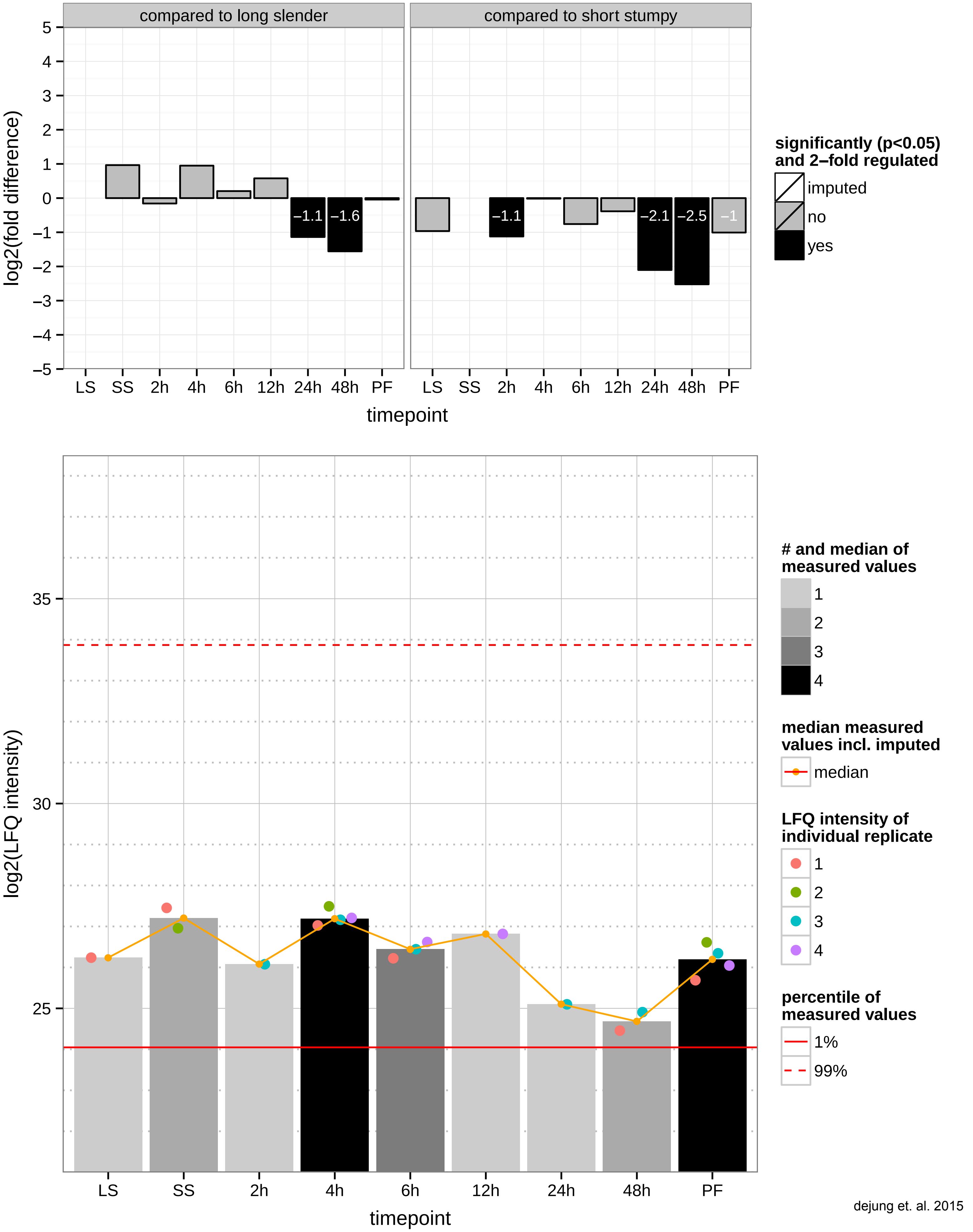

dejung et. al. 2015

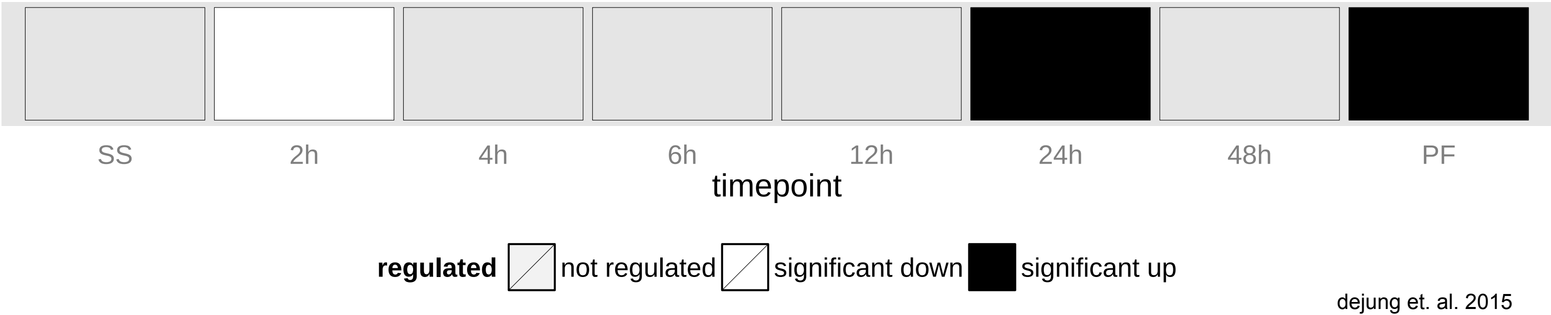
SS
2h
4h
6h
12h
24h
48h
PF
timepoint
regulated
not regulated
significant down
significant up
dejung et. al. 2015

hypothetical protein, conserved
Tb927.8.2570
AGOF: null
AGOC: null
AGOP: null
PGOF: null
PGOC: null
PGOP: null

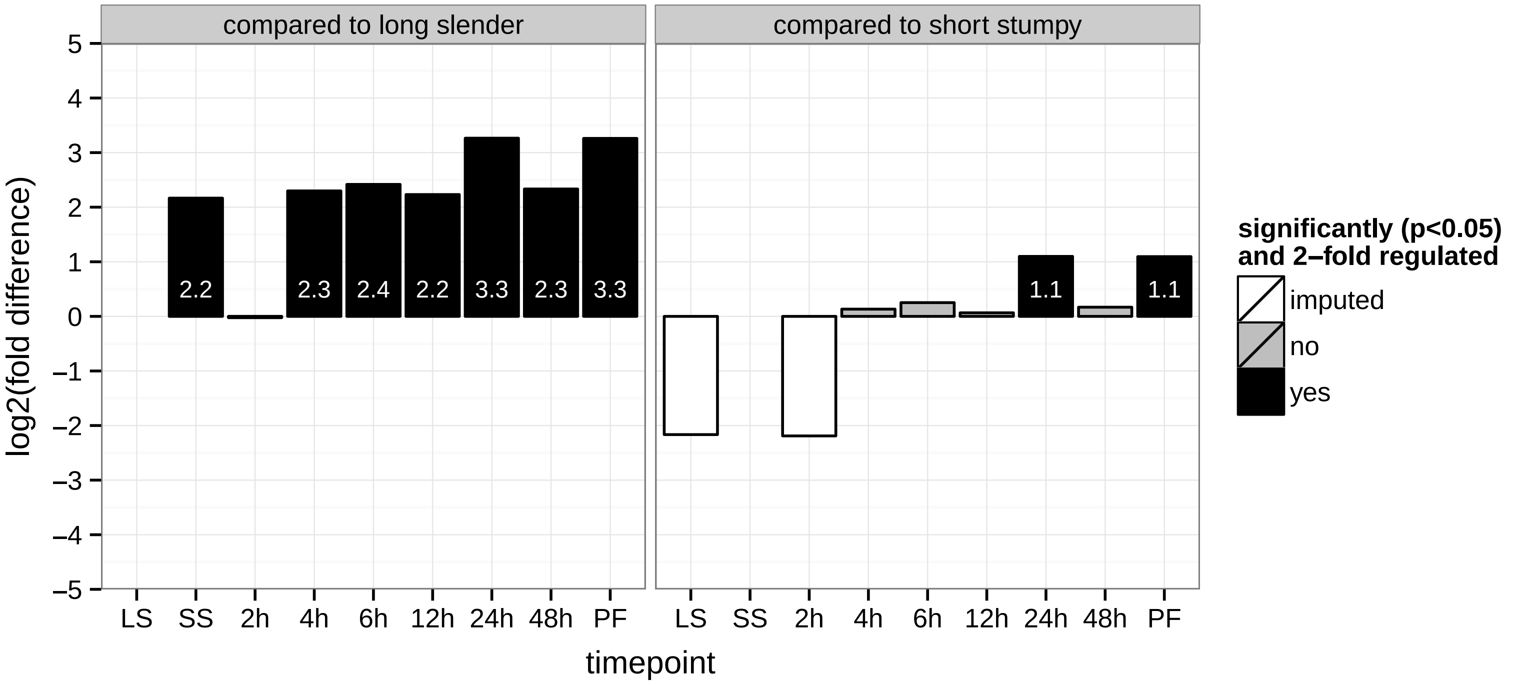

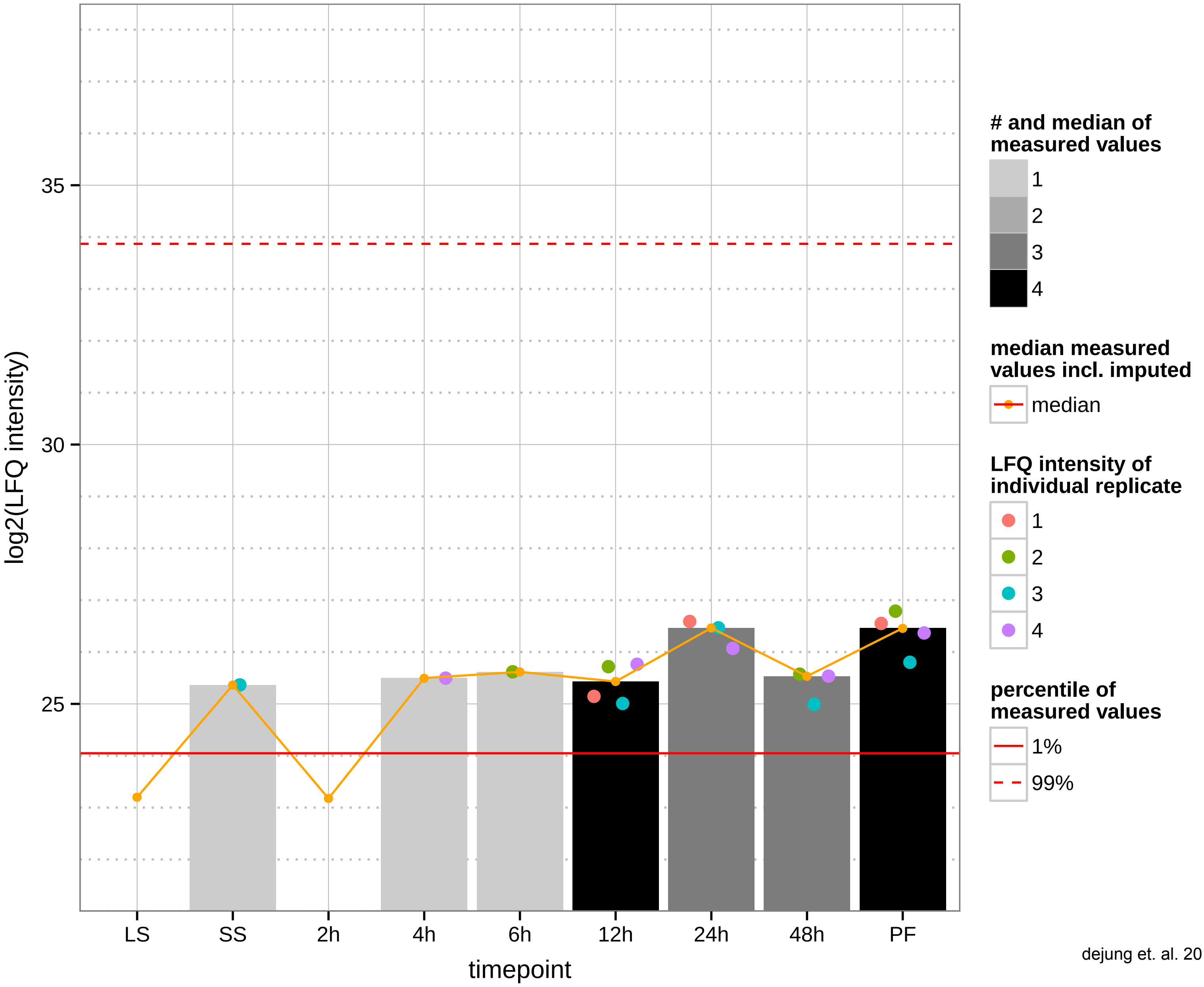

dejung et. al. 2015

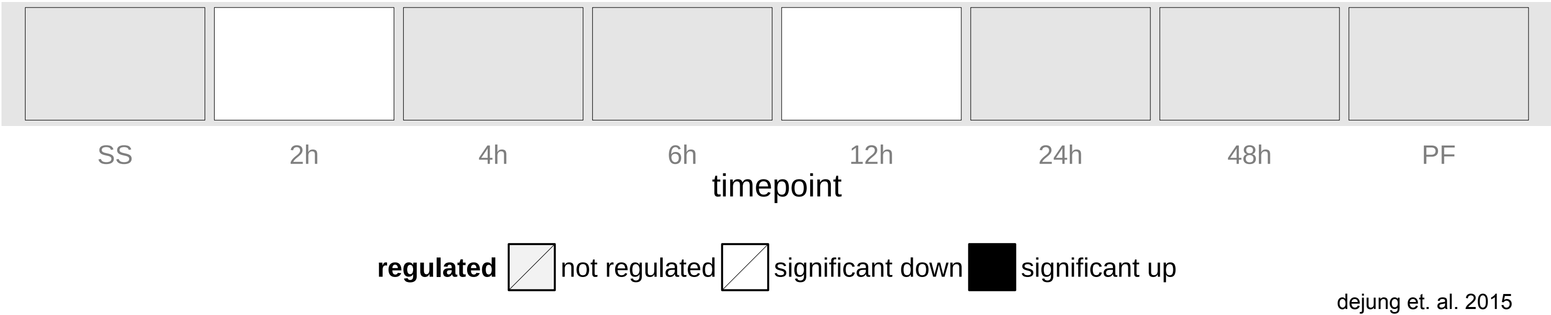

SS
2h
4h
6h
12h
24h
48h
PF
timepoint
regulated
not regulated
significant down
significant up
dejung et. al. 2015

hypothetical protein, conserved
Tb927.9.6010
AGOF: null
AGOC: null
AGOP: null
PGOF: null
PGOC: null
PGOP: null

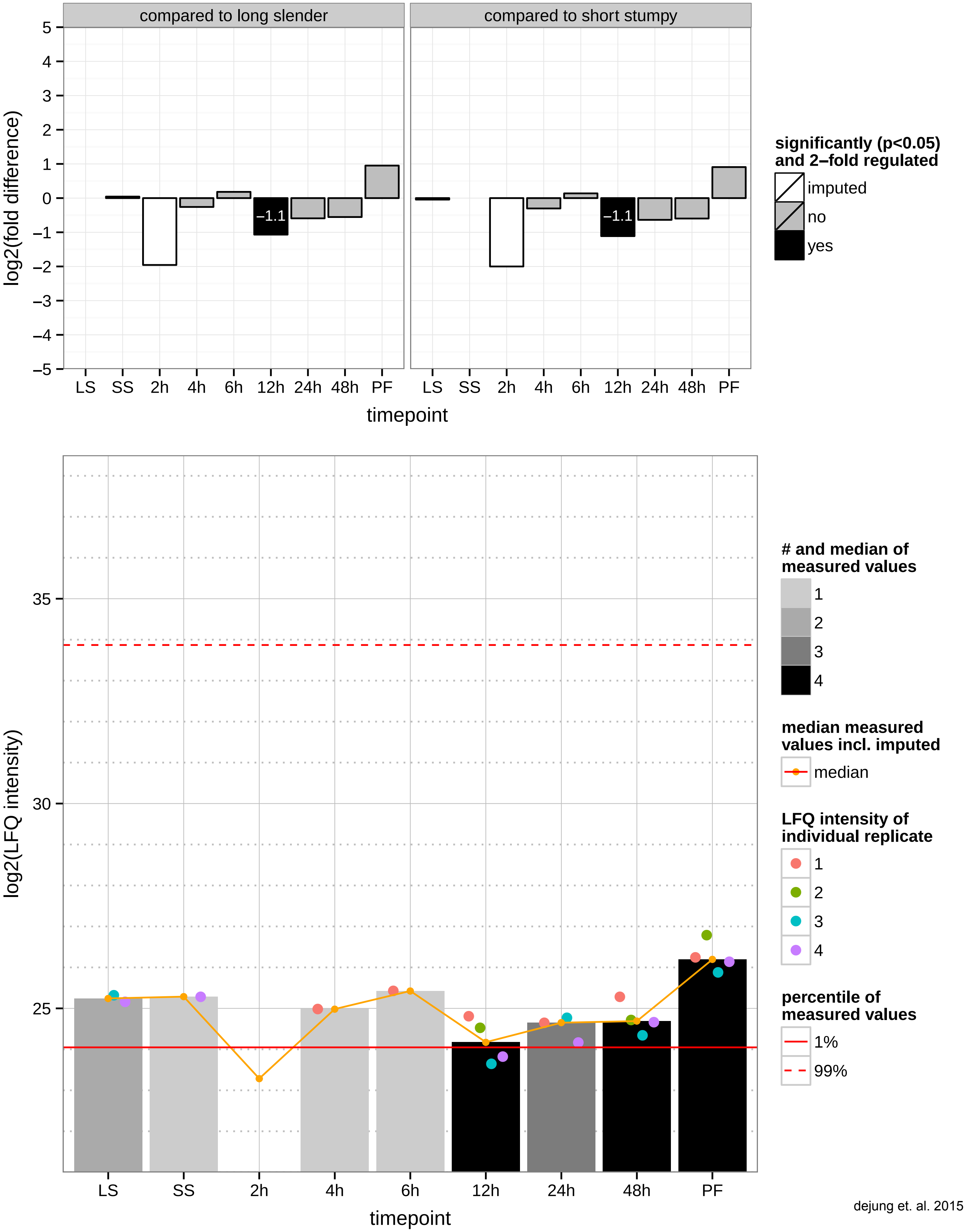

dejung et. al. 2015

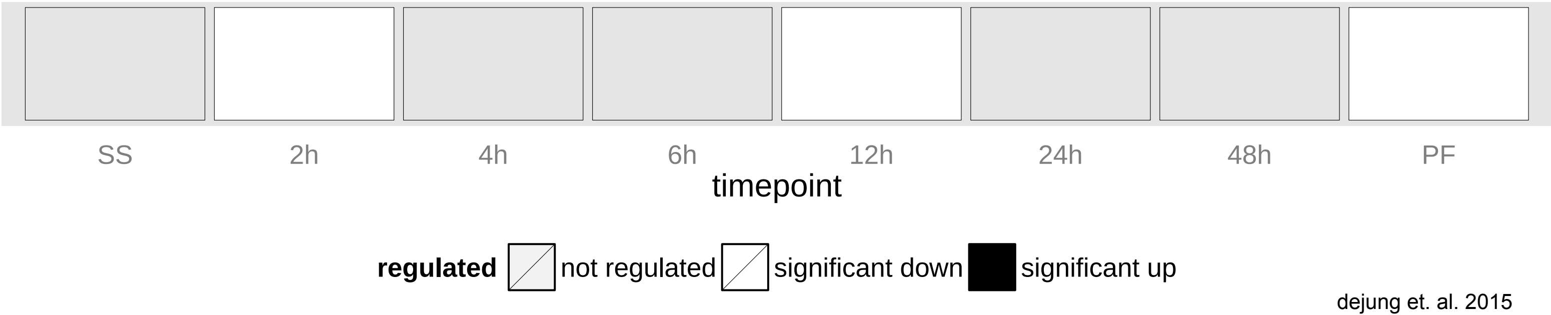
SS
2h
4h
6h
12h
24h
48h
PF
timepoint
regulated
not regulated
significant down
significant up
dejung et. al. 2015

5−methyltetrahydropteroyltriglutamate−homocysteine S−methyltransferase, putative, cobalamin−independent methionine synt
Tb927.8.2610
AGOF: 5−methyltetrahydropteroyltriglutamate−homocysteine S−methyltransferase activity, zinc ion binding
AGOC: null
AGOP: methionine biosynthetic process
PGOF: 5−methyltetrahydropteroyltriglutamate−homocysteine S−methyltransferase activity, zinc ion binding
PGOC: null
PGOP: cellular amino acid biosynthetic process, methionine biosynthetic process

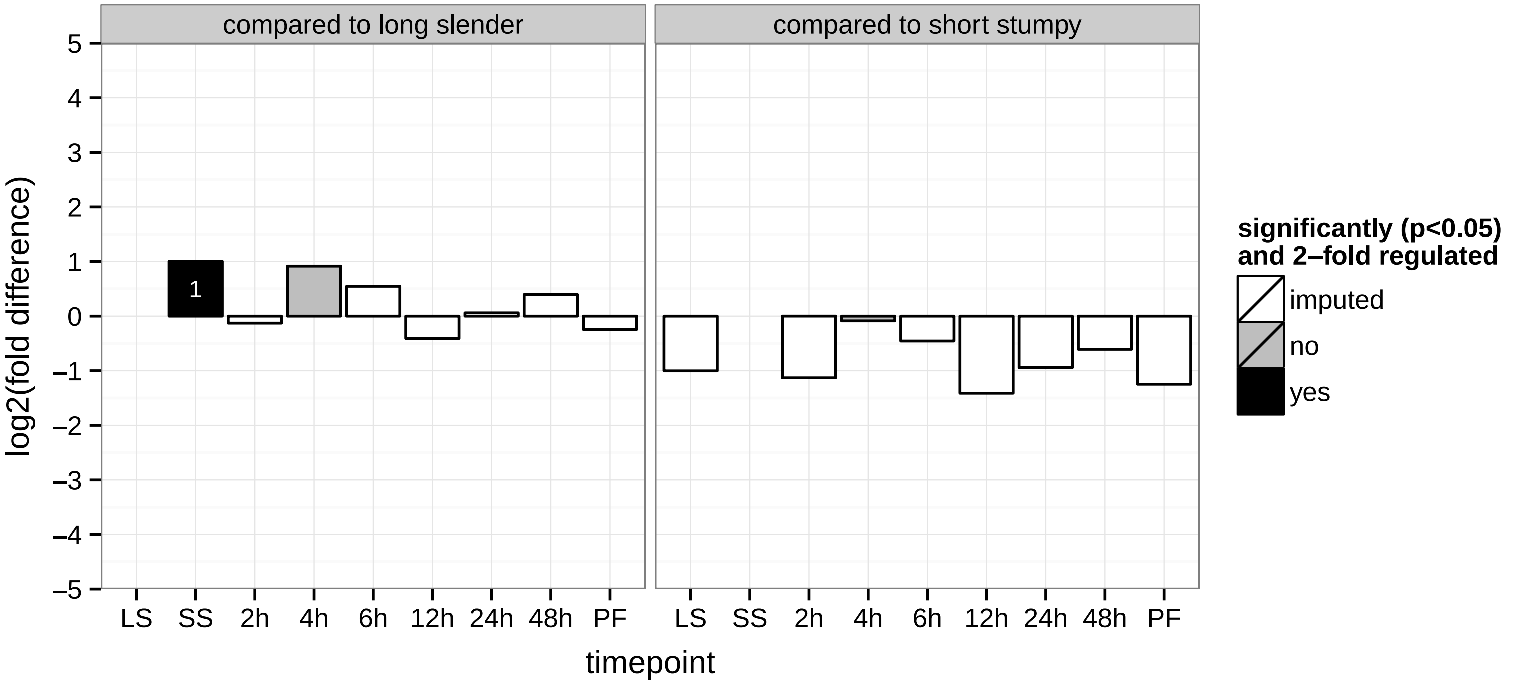

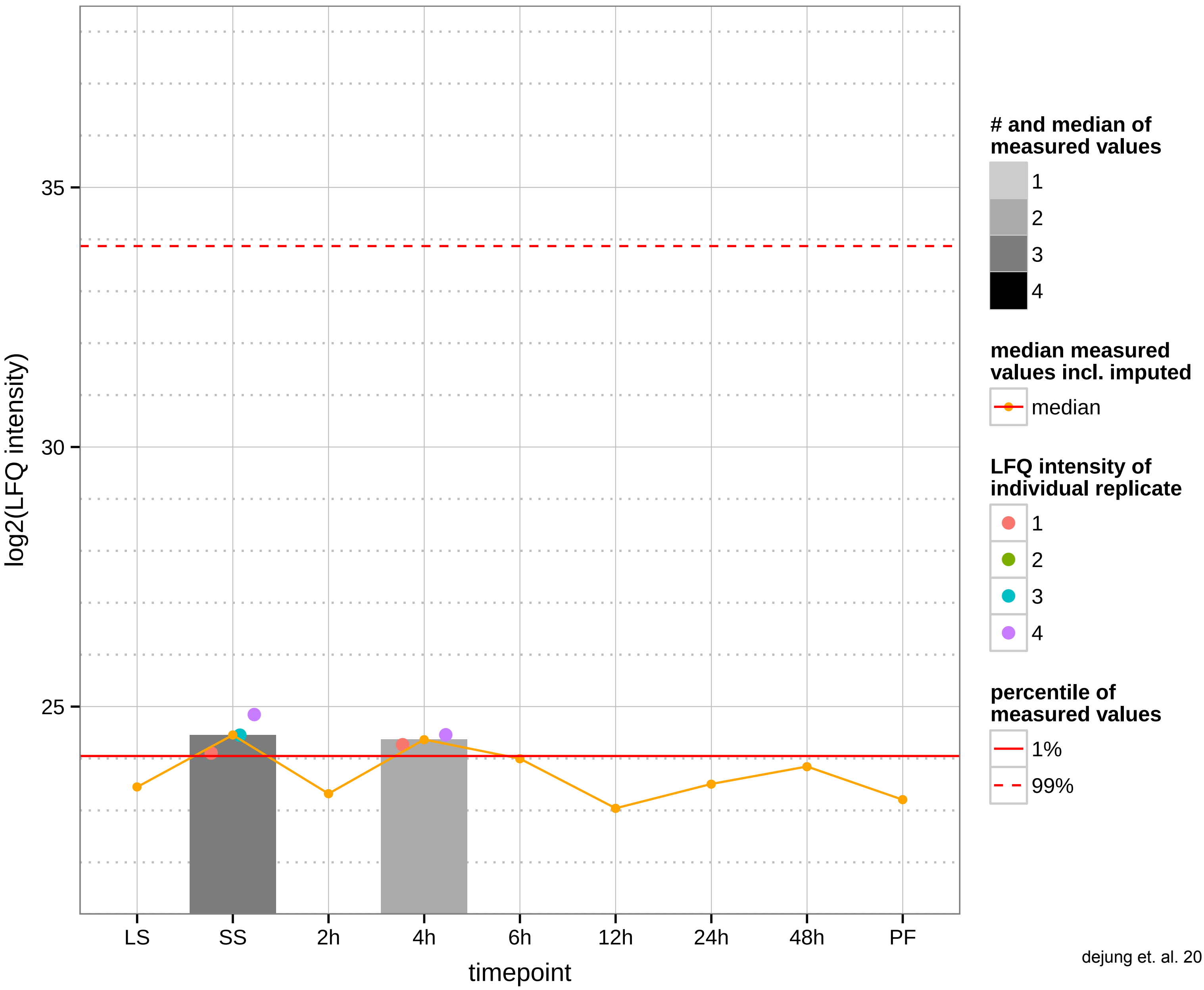

dejung et. al. 2015

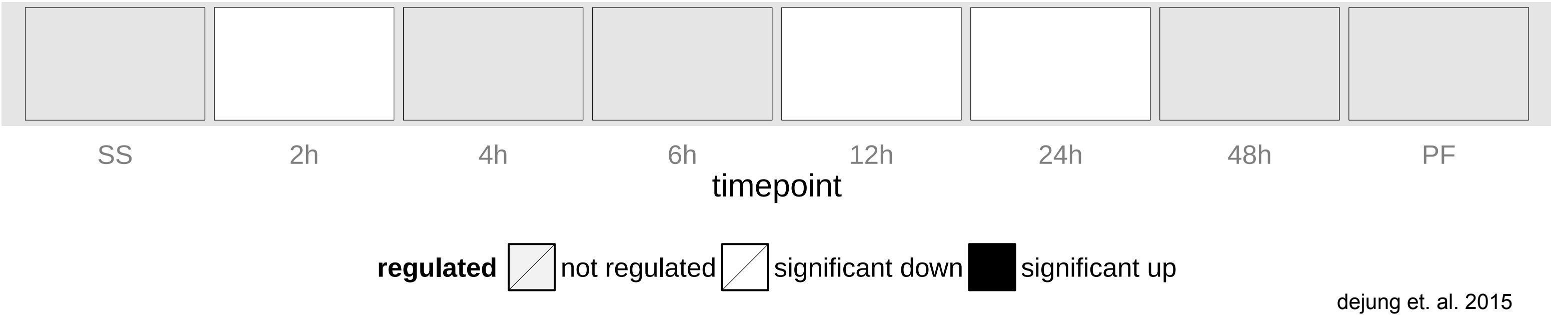
SS
2h
4h
6h
12h
24h
48h
PF
timepoint
regulated
not regulated
significant down
significant up
dejung et. al. 2015

kinetoplastid kinetochore protein 4 (kkt4)
Tb927.8.3680
AGOF: null
AGOC: intracellular, kinetochore
AGOP: chromosome segregation
PGOF: null
PGOC: null
PGOP: null

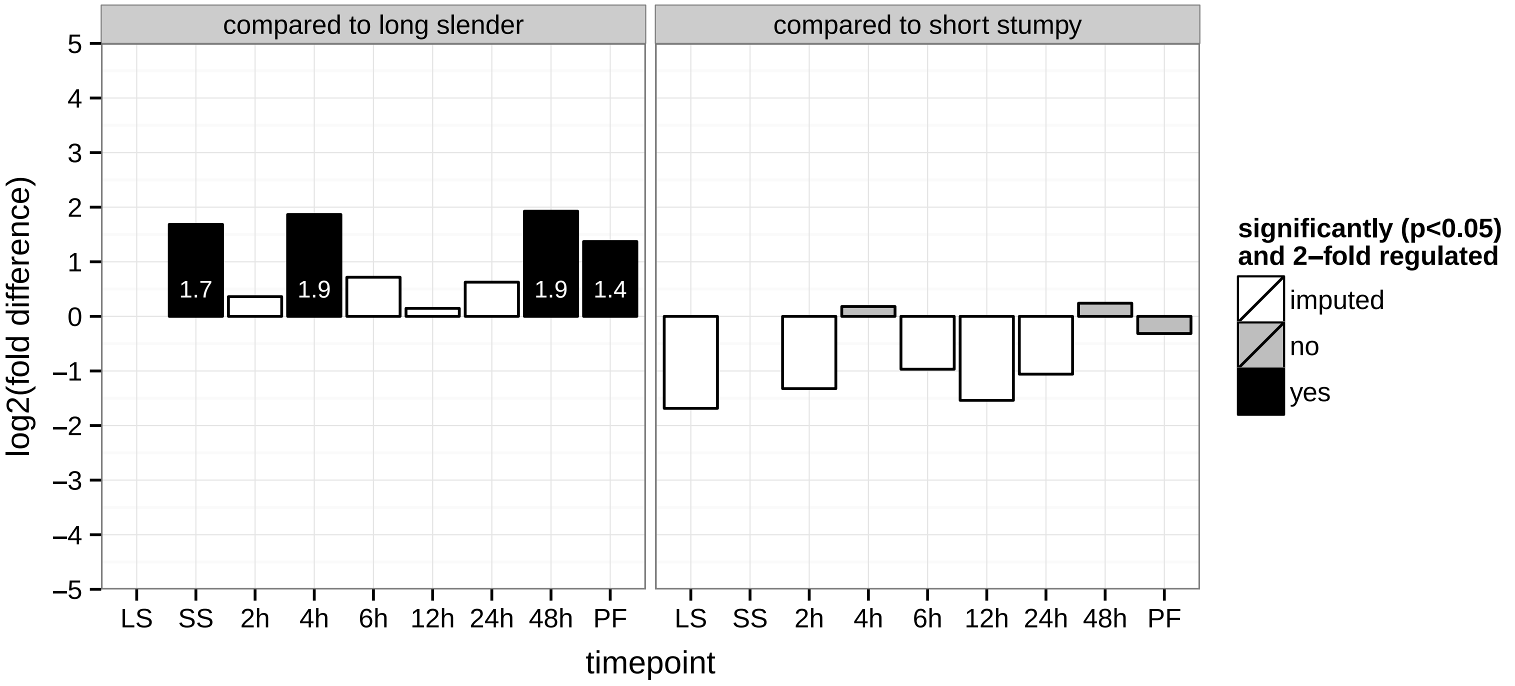

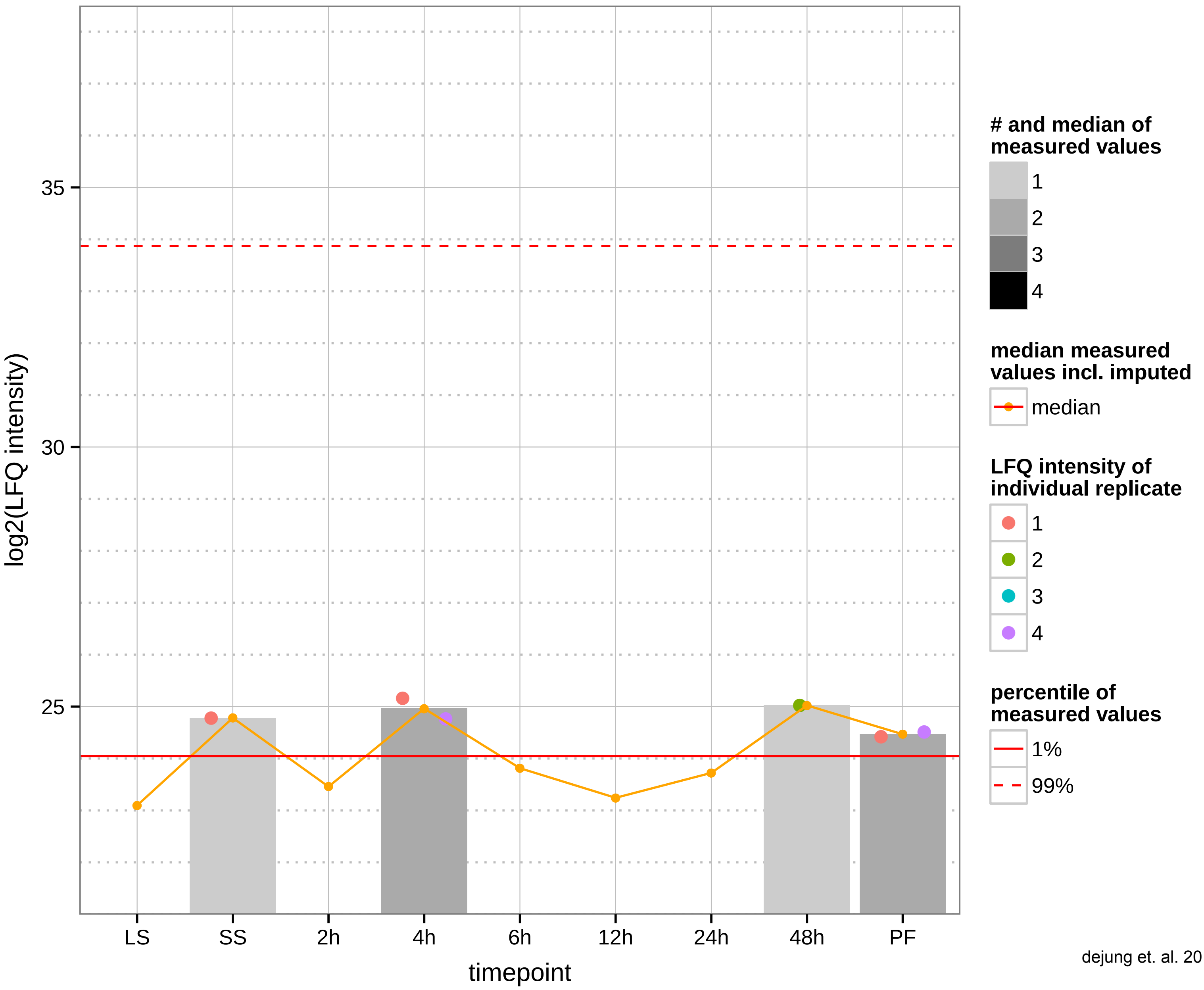

dejung et. al. 2015

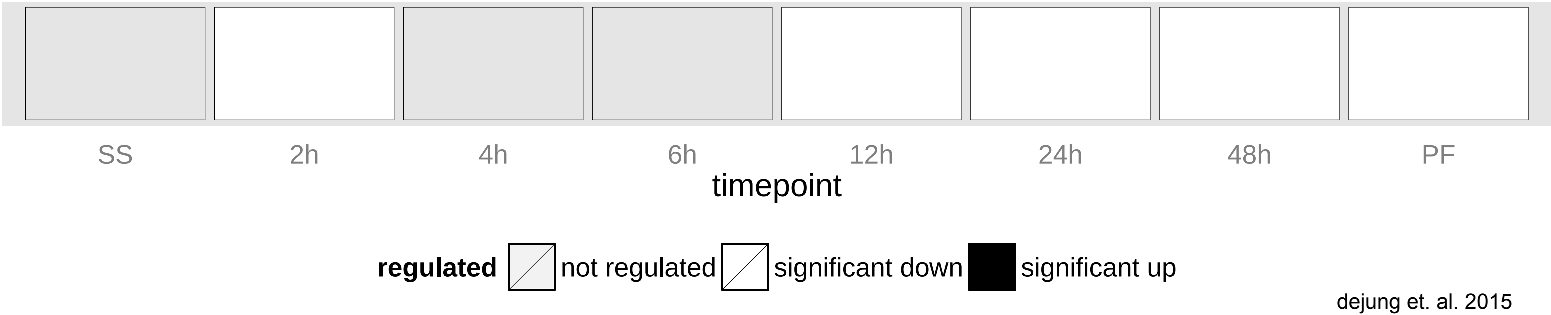
SS
2h
4h
6h
12h
24h
48h
PF
timepoint
regulated
not regulated
significant down
significant up
dejung et. al. 2015

hypothetical protein, conserved
Tb927.10.2760
AGOF: ATP binding, nucleoside−triphosphatase activity
AGOC: null
AGOP: null
PGOF: ATP binding, nucleoside−triphosphatase activity, nucleotide binding
PGOC: null
PGOP: null

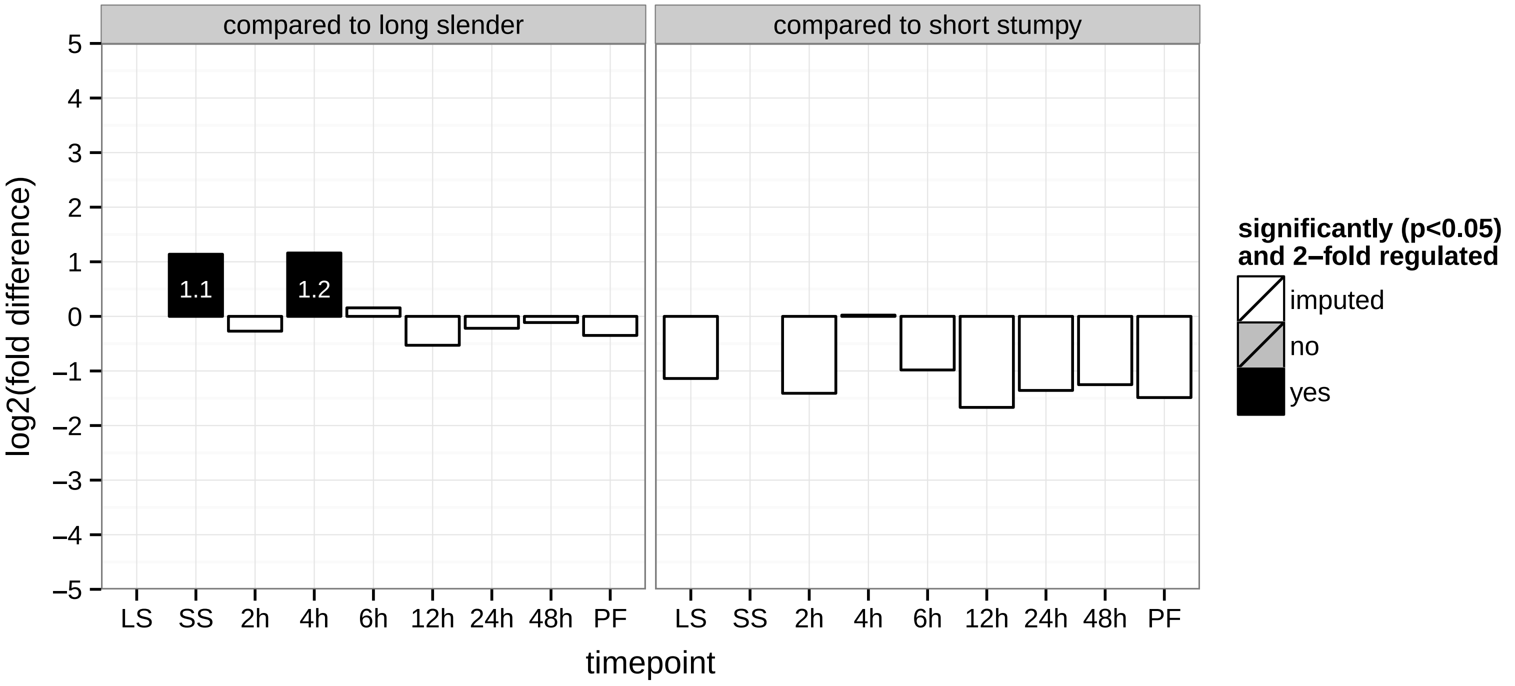

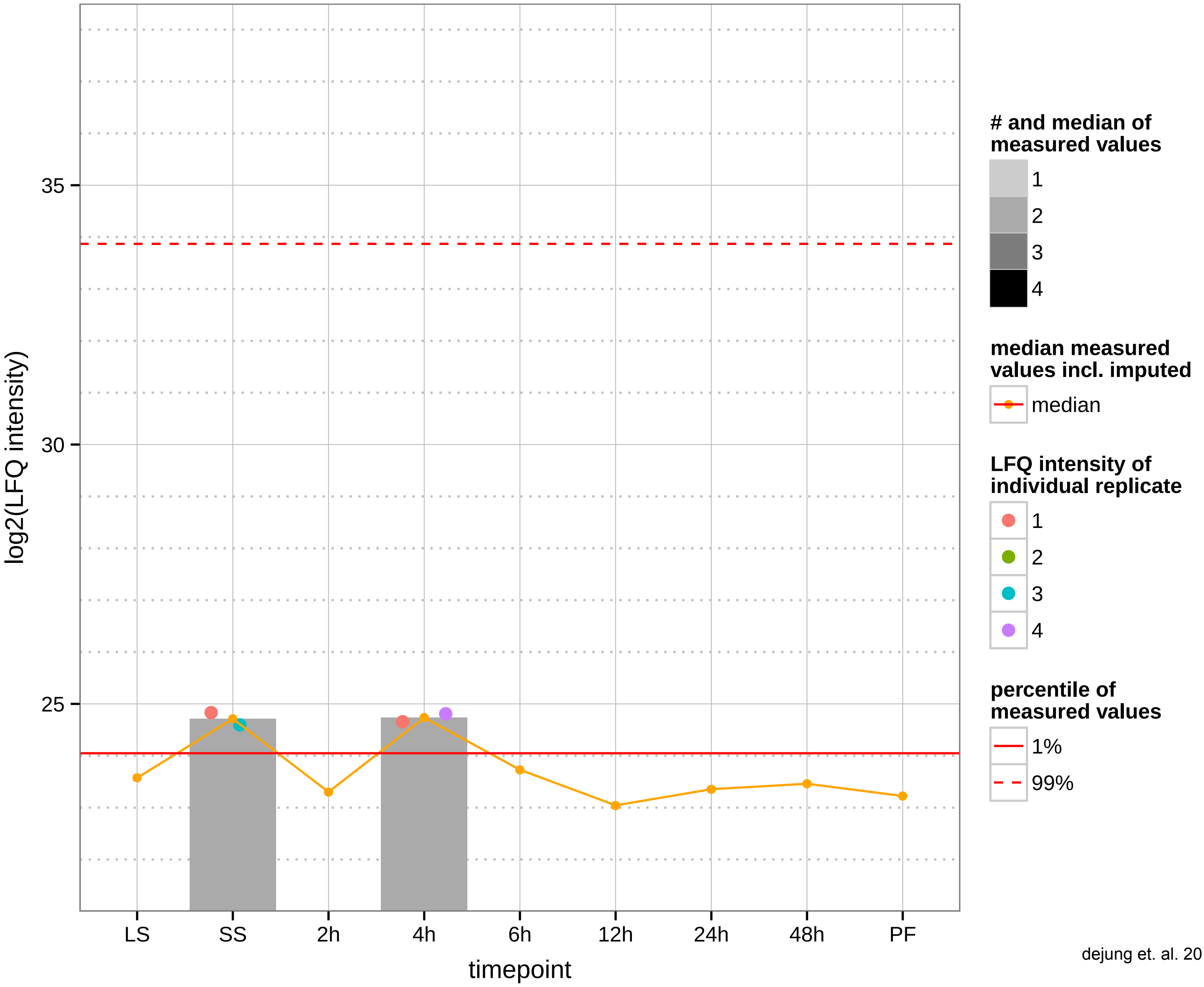

dejung et. al. 2015

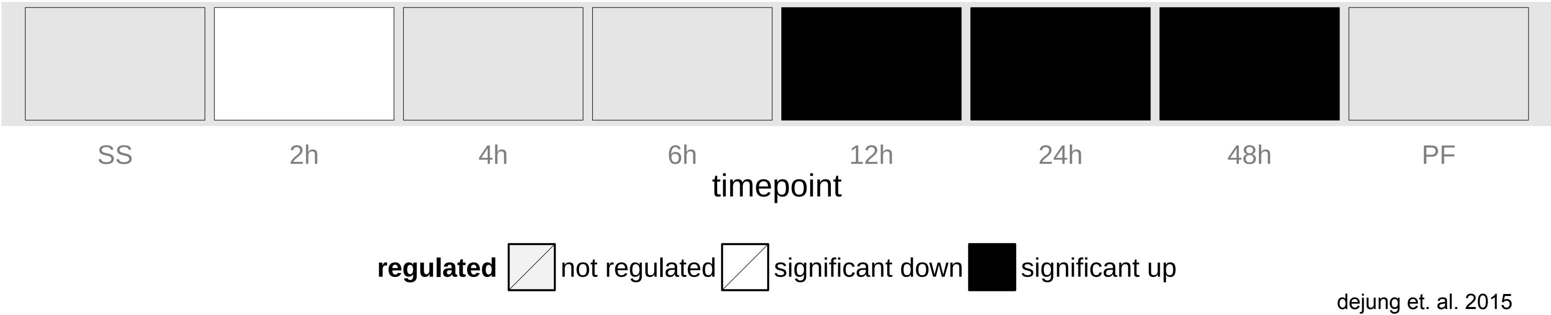
SS
2h
4h
6h
12h
24h
48h
PF
timepoint
regulated
not regulated
significant down
significant up
dejung et. al. 2015

hypothetical protein, conserved
Tb927.11.2630
AGOF: null
AGOC: null
AGOP: null
PGOF: null
PGOC: cytoplasm
PGOP: peptidyl–diphthamide biosynthetic process from peptidyl–histidine

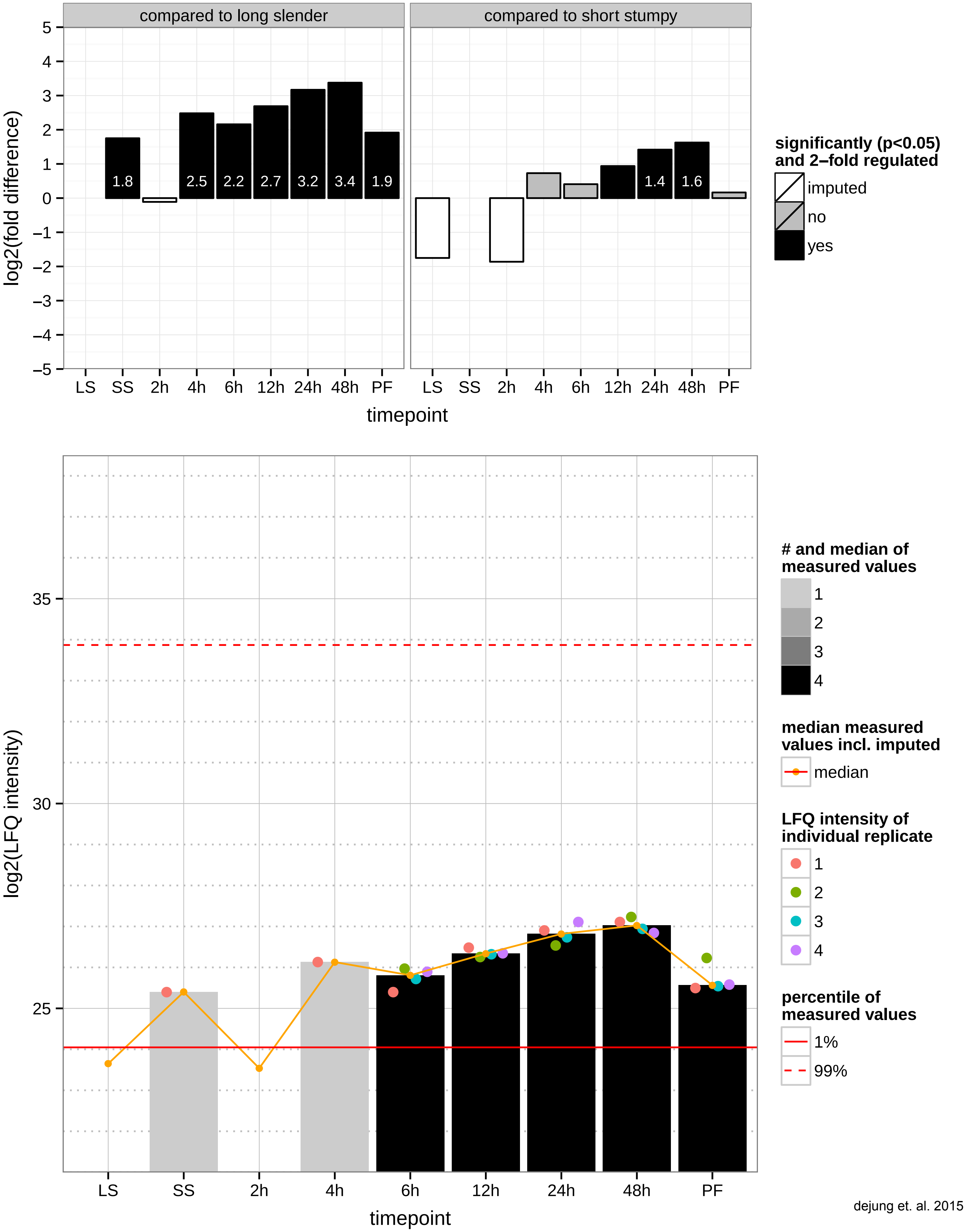

dejung et. al. 2015

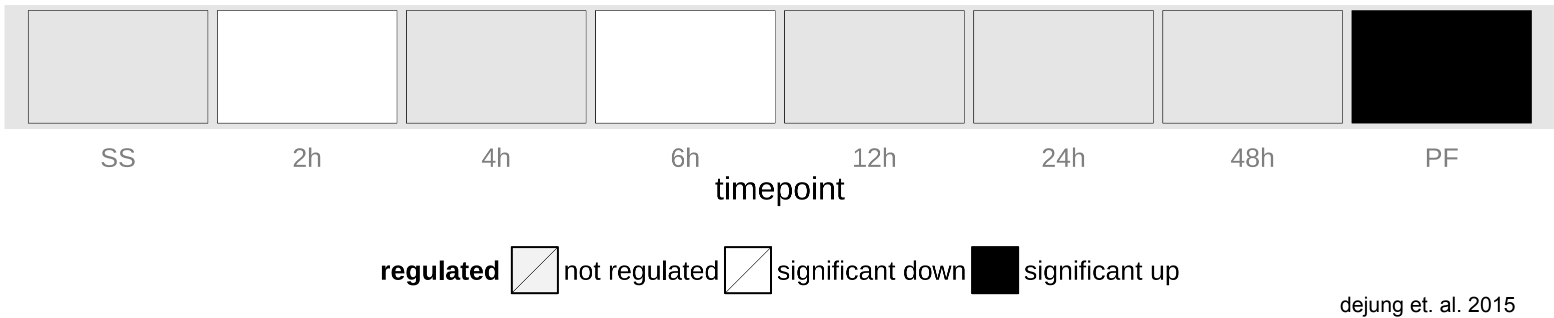
SS
2h
4h
6h
12h
24h
48h
PF
timepoint
regulated
not regulated
significant down
significant up
dejung et. al. 2015

retrotransposon hot spot (RHS) protein, putative, retrotransposon hot spot protein 5 (RHS5)
Tb11.v5.0567;Tb927.6.140
AGOF: null
AGOC: null
AGOP: null
PGOF: null
PGOC: null
PGOP: null

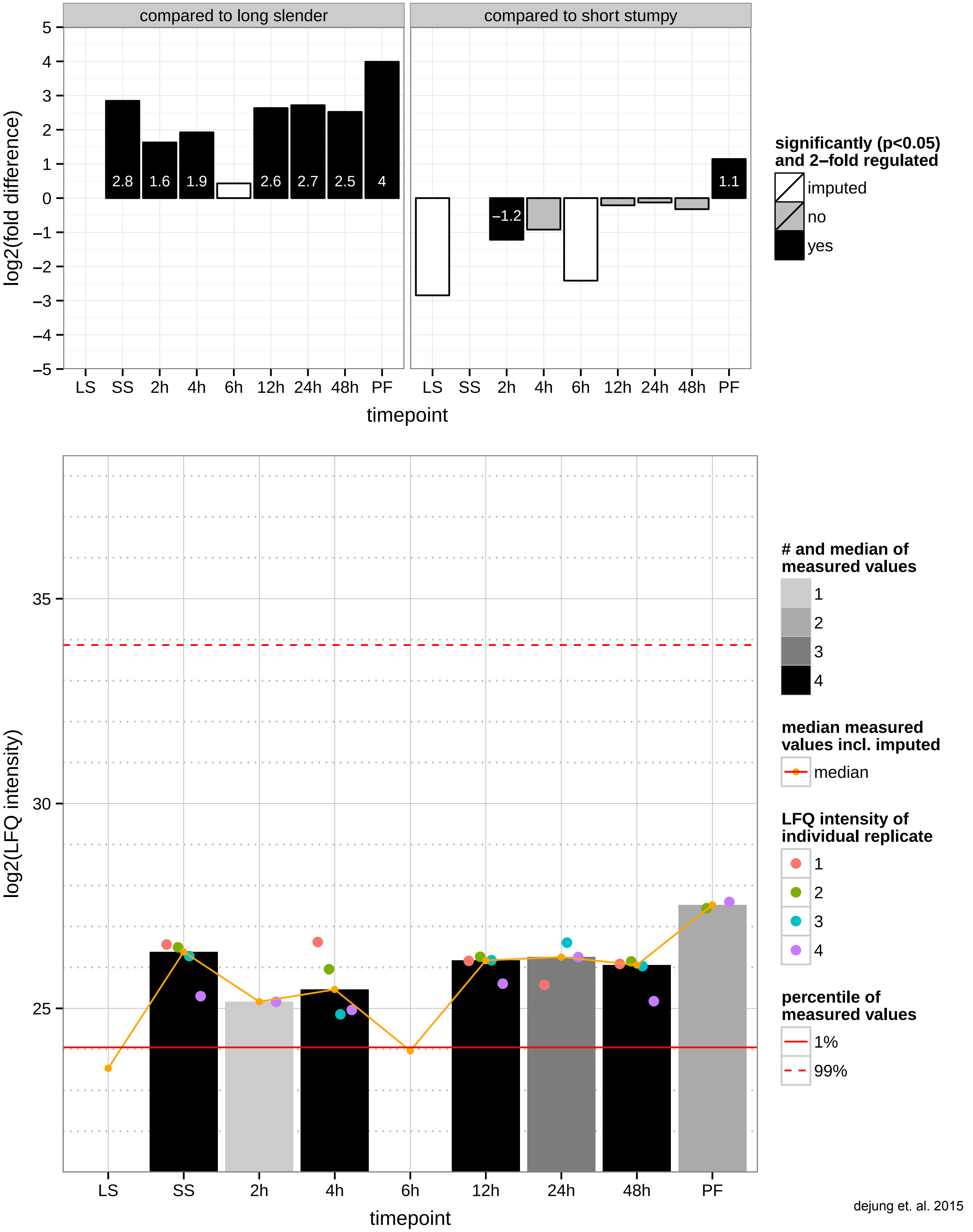

dejung et. al. 2015

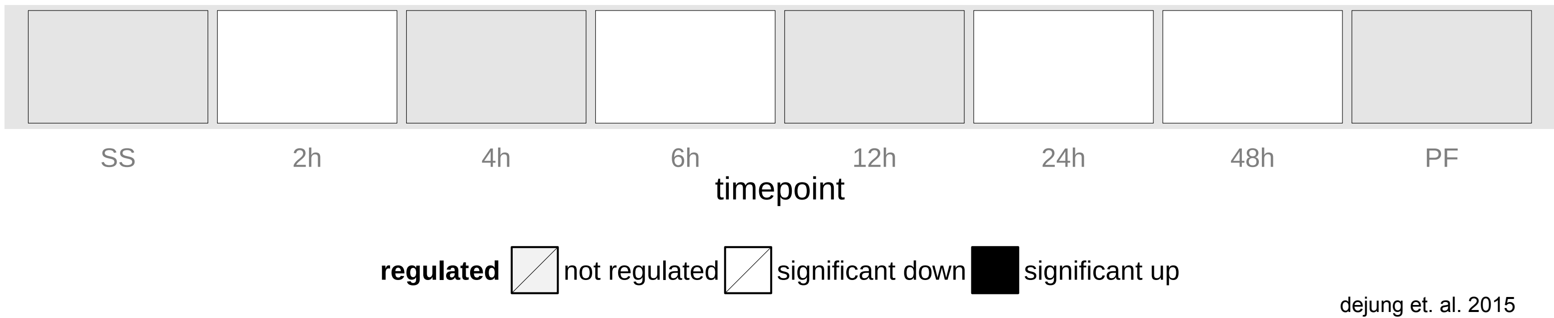
SS
2h
4h
6h
12h
24h
48h
PF
timepoint
regulated
not regulated
significant down
significant up
dejung et. al. 2015

hypothetical protein, conserved
Tb927.11.12500
AGOF: null
AGOC: null
AGOP: null
PGOF: null
PGOC: null
PGOP: null

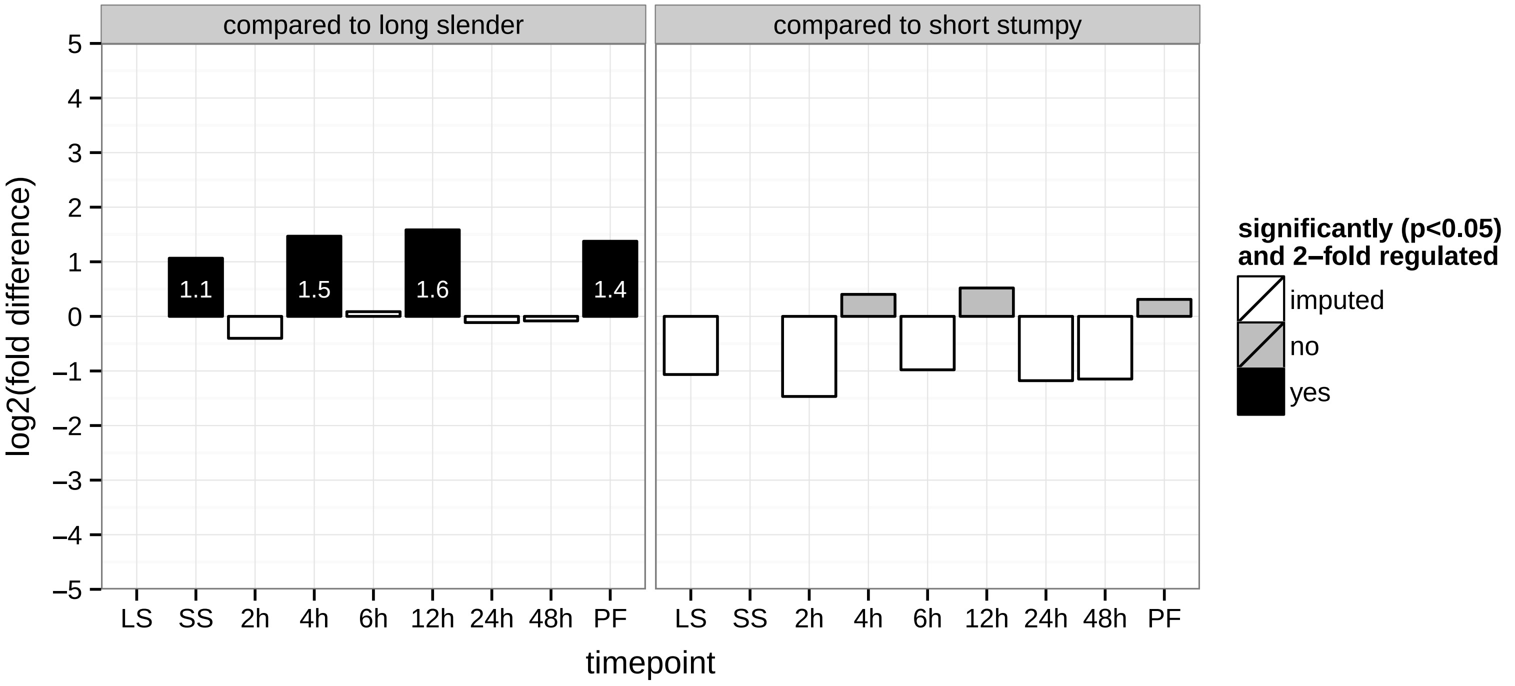

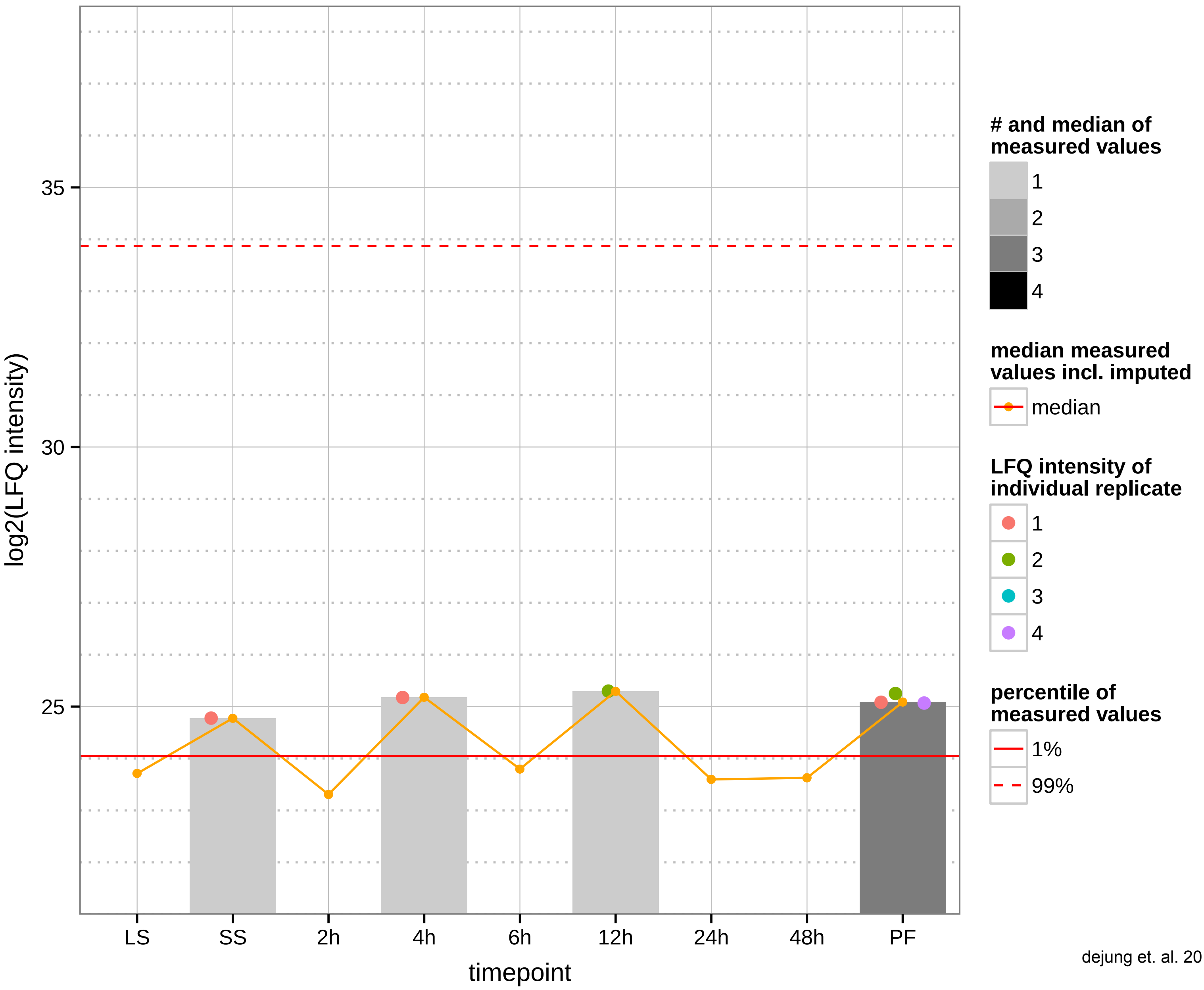

dejung et. al. 2015

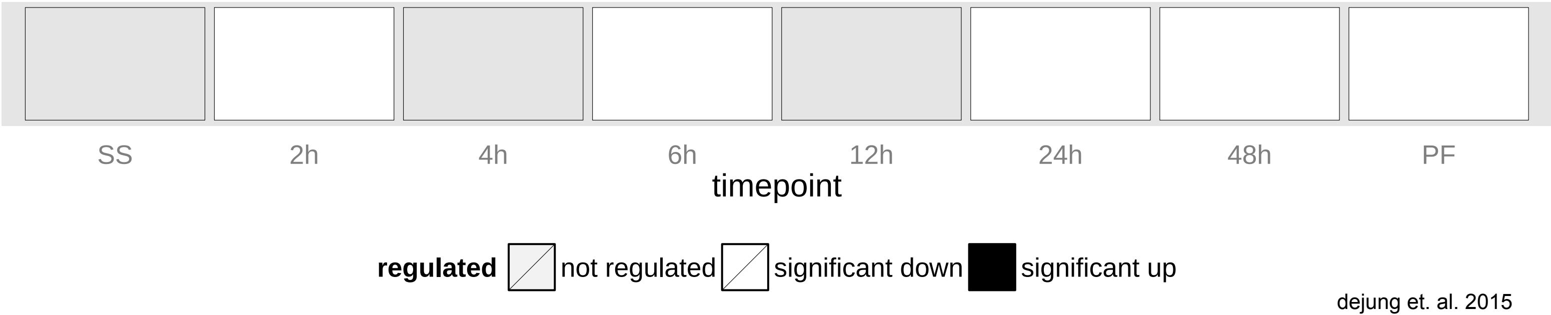
SS
2h
4h
6h
12h
24h
48h
PF
timepoint
regulated
not regulated
significant down
significant up
dejung et. al. 2015

hypothetical protein, chrIX additional, unordered contigs
Tb927.9.15850;Tb09.v4.0110;Tb09.v4.0014
AGOF: null
AGOC: null
AGOP: null
PGOF: null
PGOC: null
PGOP: null

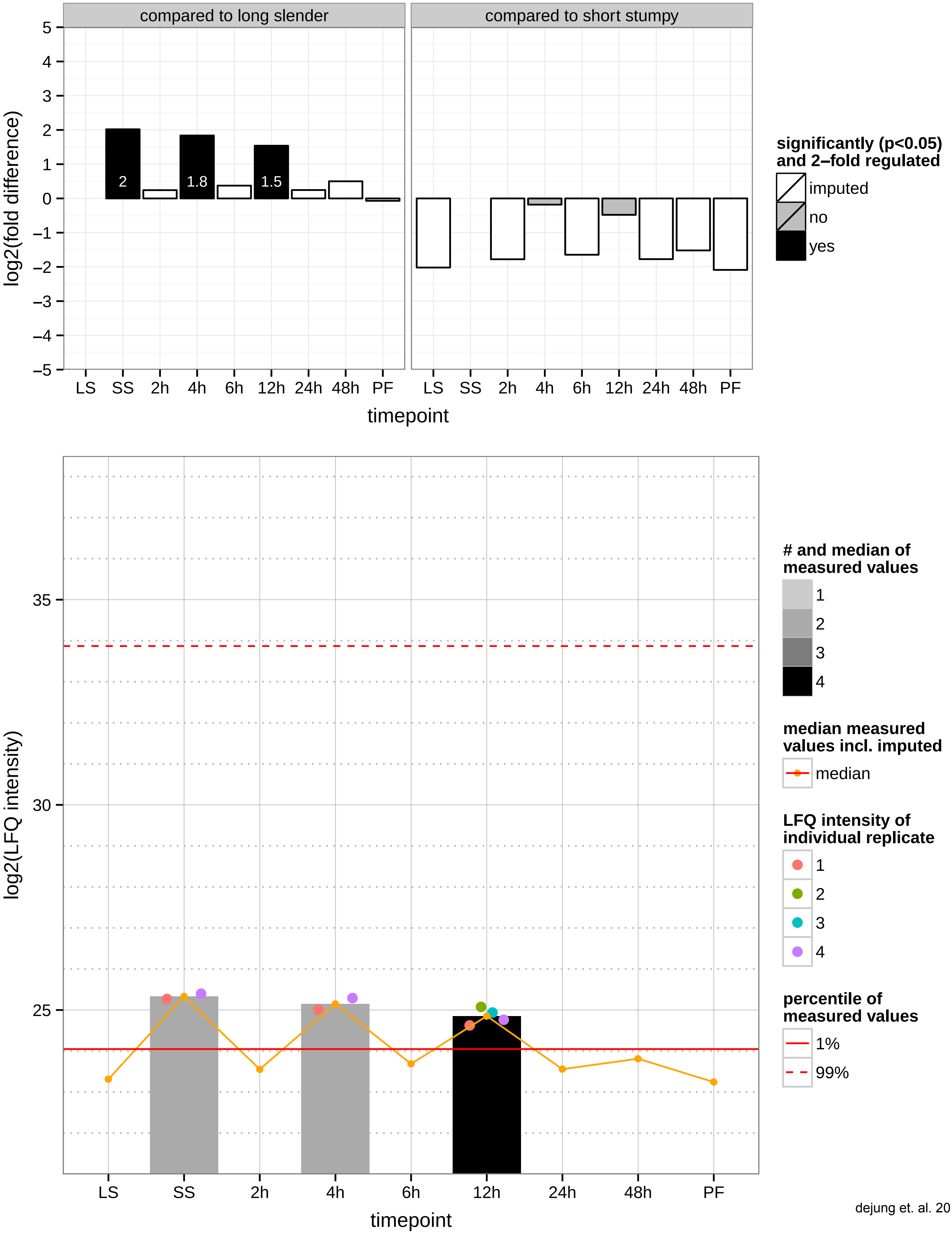

dejung et. al. 2015

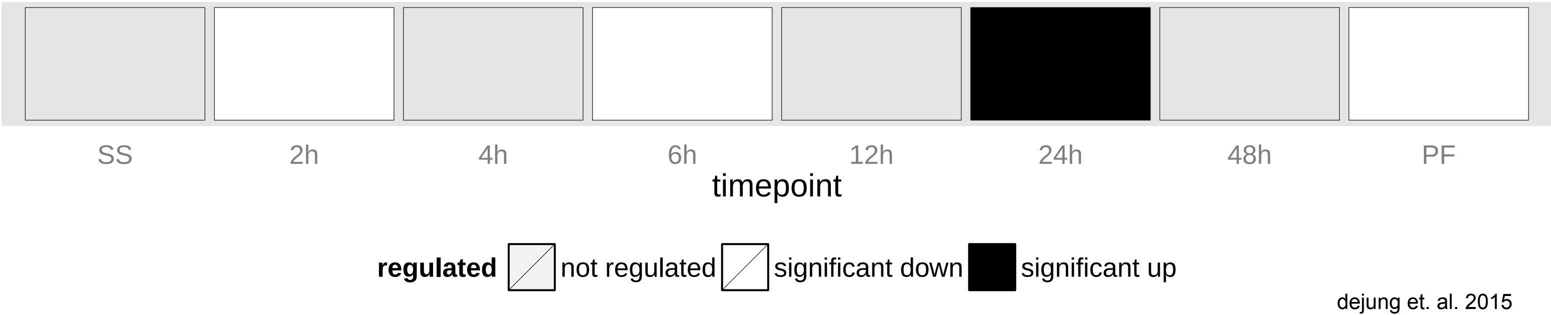
SS
2h
4h
6h
12h
24h
48h
PF
timepoint
regulated
not regulated
significant down
significant up
dejung et. al. 2015

hypothetical protein, conserved
Tb927.11.13570
AGOF: null
AGOC: null
AGOP: null
PGOF: null
PGOC: null
PGOP: null

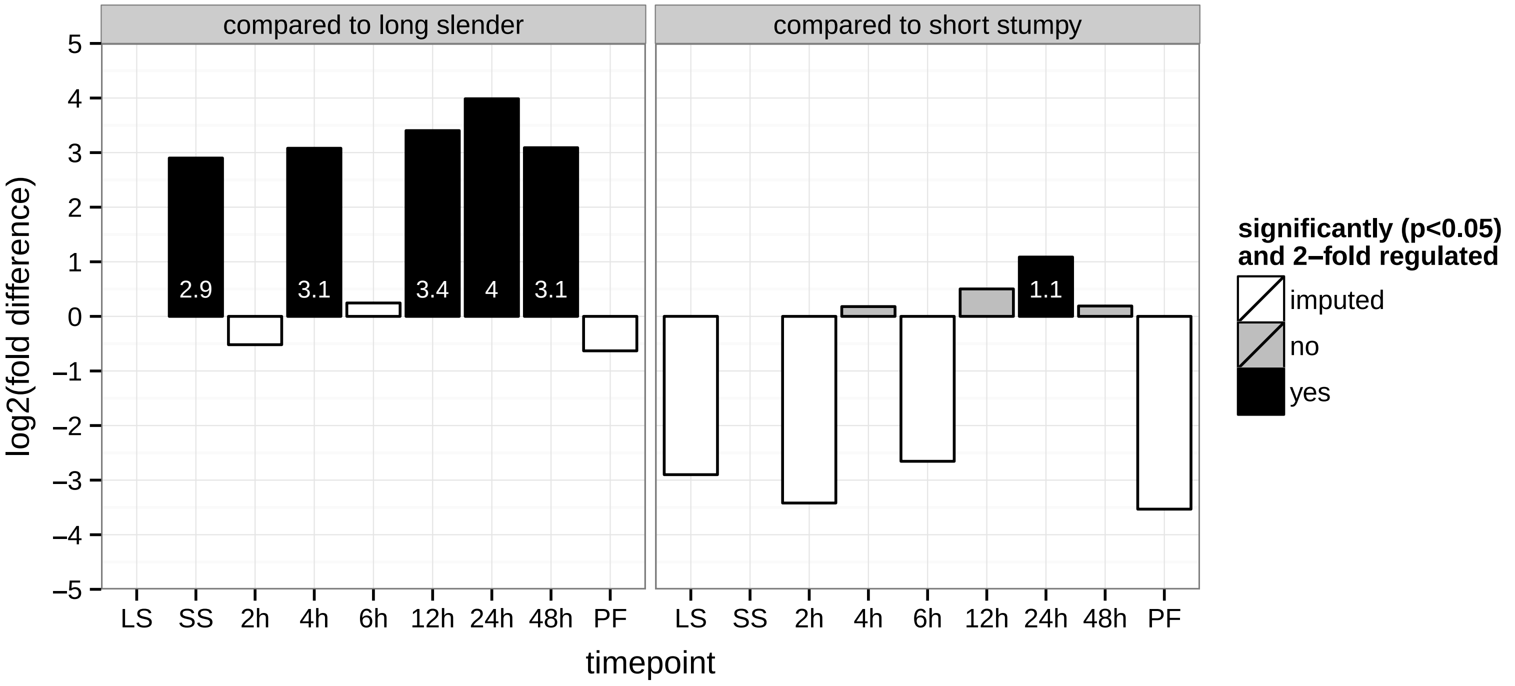

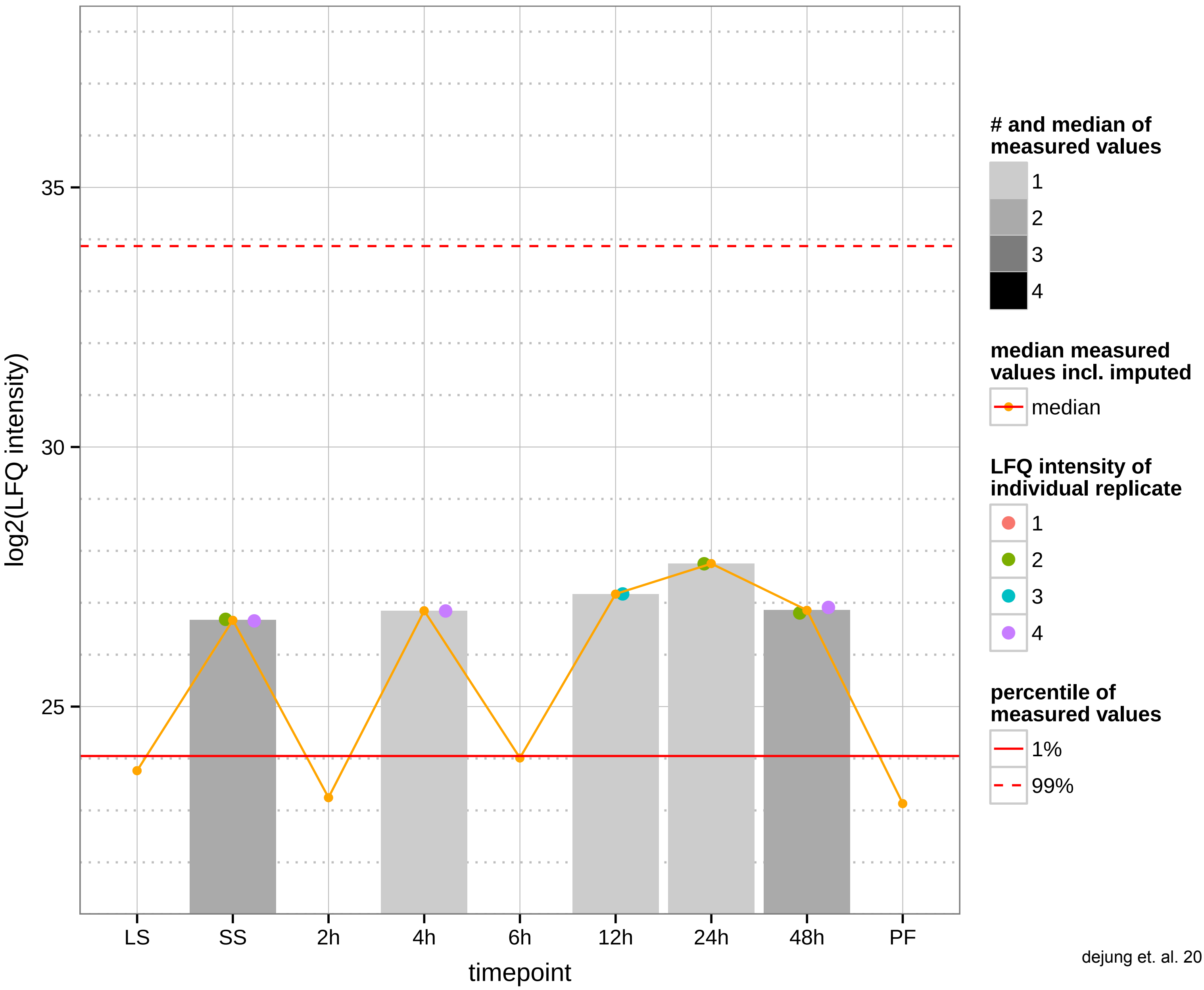

dejung et. al. 2015

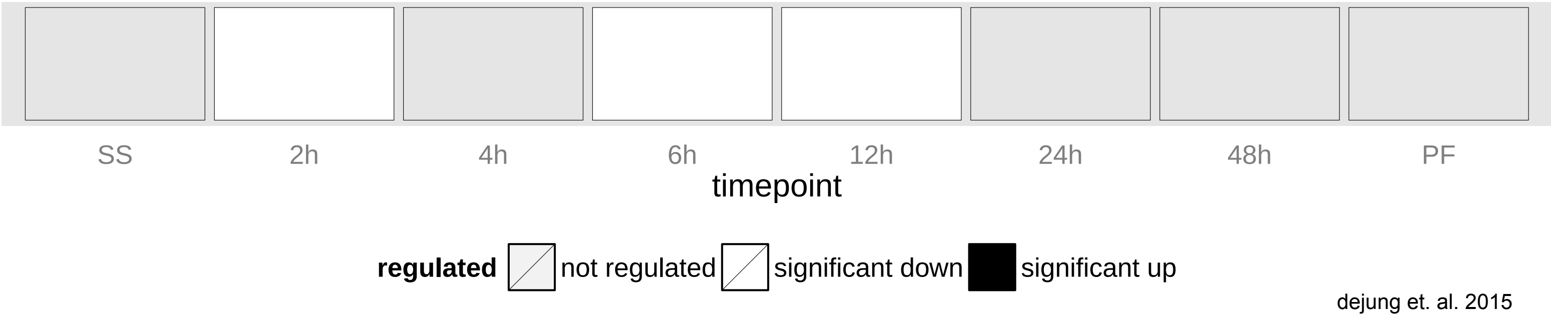
SS
2h
4h
6h
12h
24h
48h
PF
timepoint
regulated
not regulated
significant down
significant up
dejung et. al. 2015

hypothetical protein, conserved
Tb927.11.2010
AGOF: null
AGOC: mitochondrion
AGOP: null
PGOF: null
PGOC: null
PGOP: null

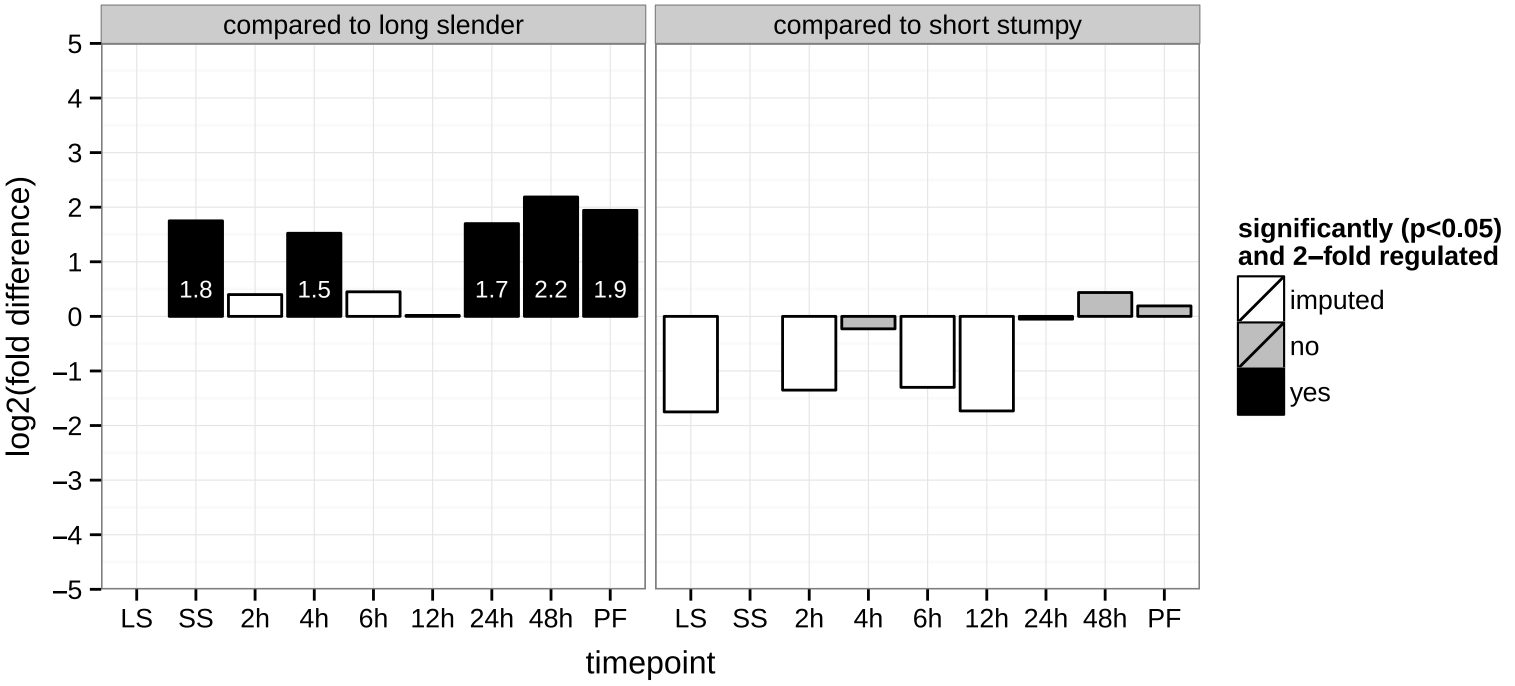

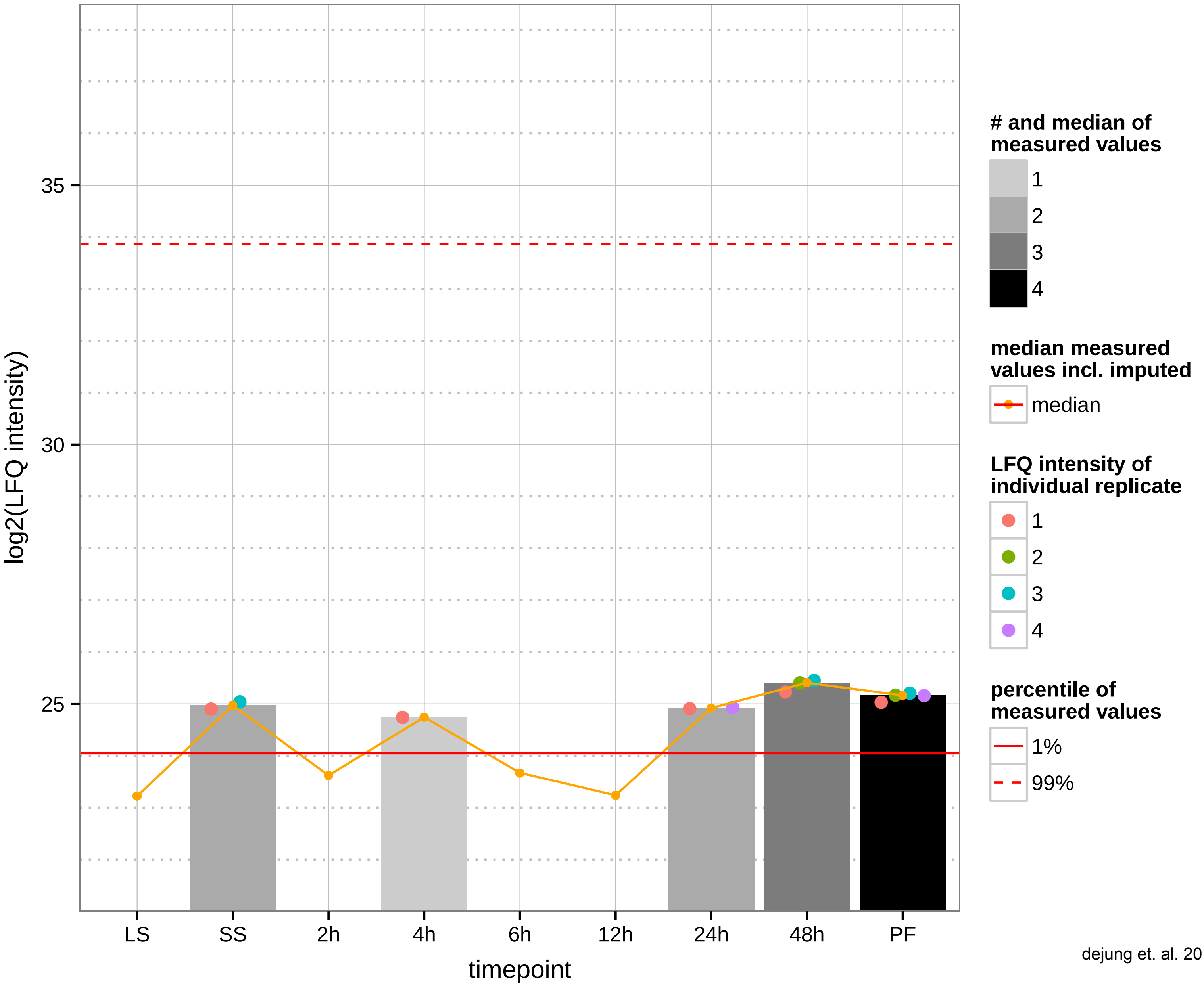

dejung et. al. 2015

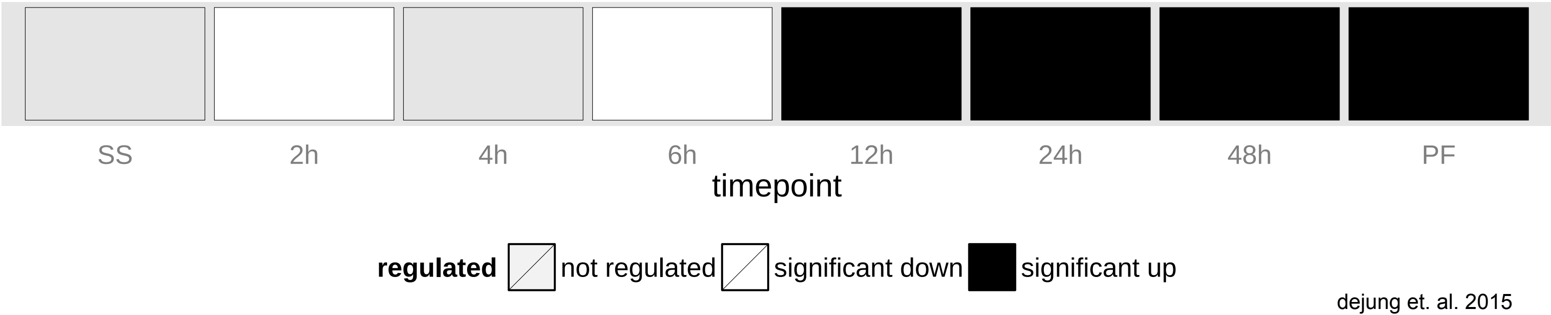
SS
2h
4h
6h
12h
24h
48h
PF
timepoint
regulated
not regulated
significant down
significant up
dejung et. al. 2015

cyclin 1, serine peptidase family S51, peptidase E, putative
Tb927.11.12610
AGOF: null
AGOC: null
AGOP: null
PGOF: serine–type peptidase activity
PGOC: null
PGOP: proteolysis

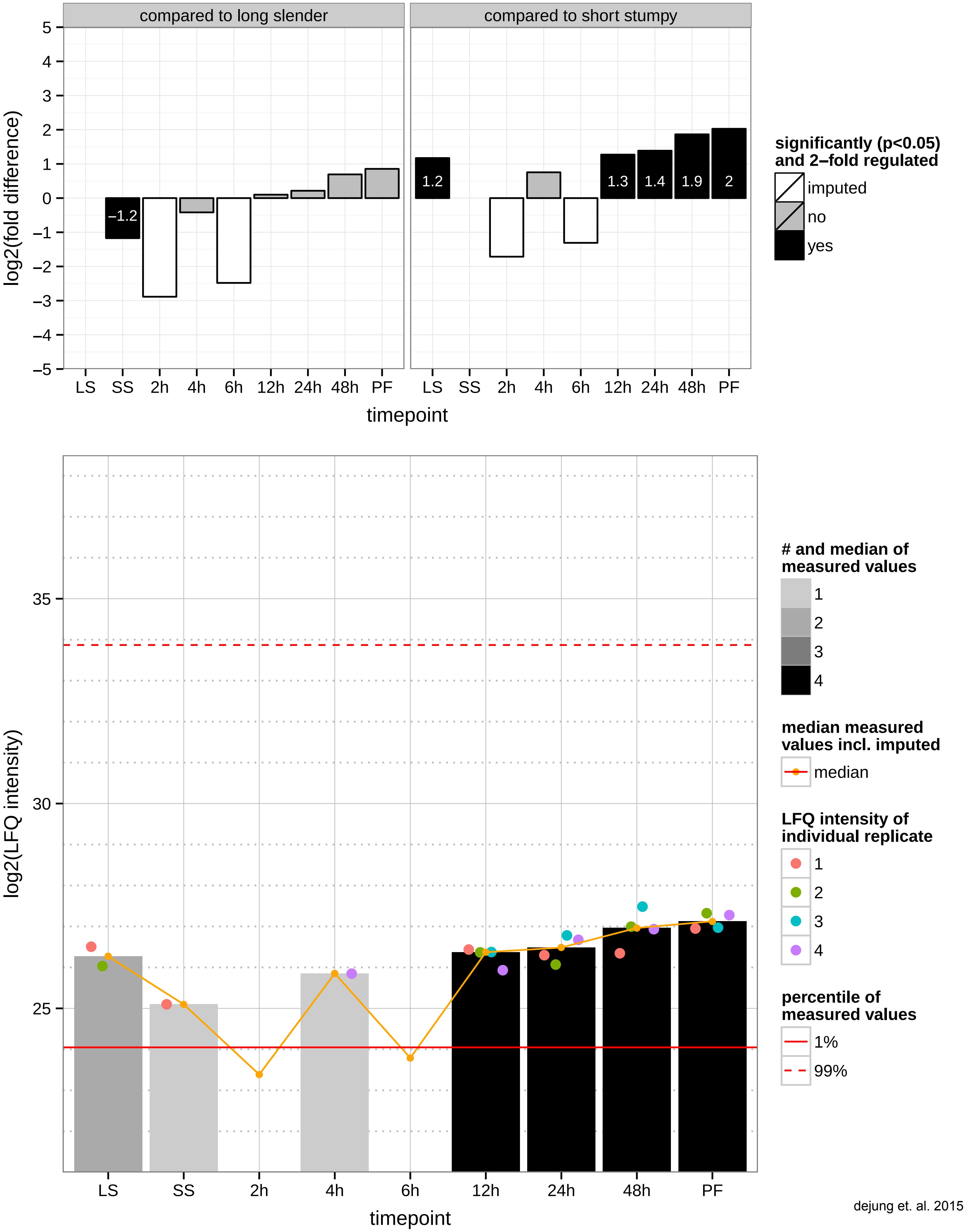

dejung et. al. 2015

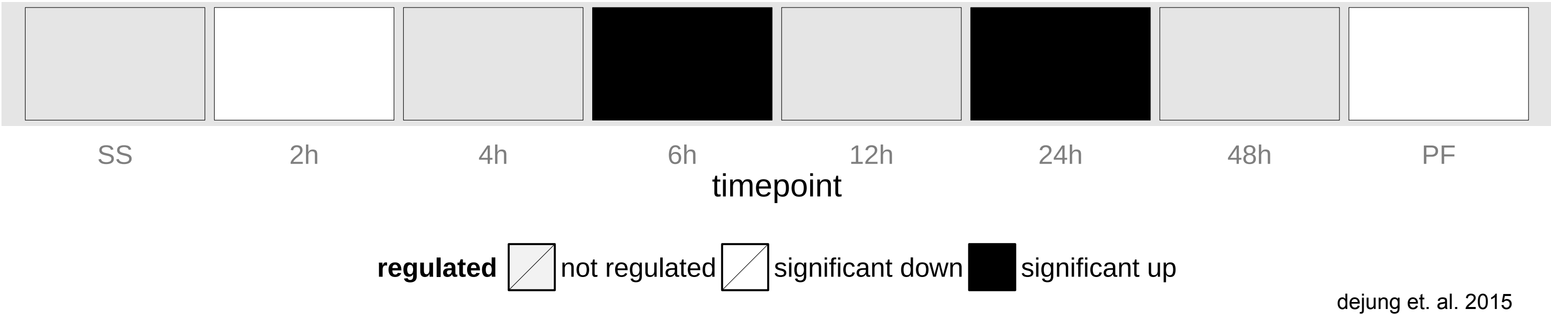
SS
2h
4h
6h
12h
24h
48h
PF
timepoint
regulated
not regulated
significant down
significant up
dejung et. al. 2015

small GTP–binding protein Rab7, putative (Trab7)
Tb927.8.4620
AGOF: GTP binding, GTPase activity
AGOC: intracellular
AGOP: intracellular protein transport, nucleocytoplasmic transport, small GTPase mediated signal transduction
PGOF: GTP binding, GTPase activity
PGOC: intracellular, membrane
PGOP: GTP catabolic process, intracellular protein transport, nucleocytoplasmic transport, protein transport, signal transducti

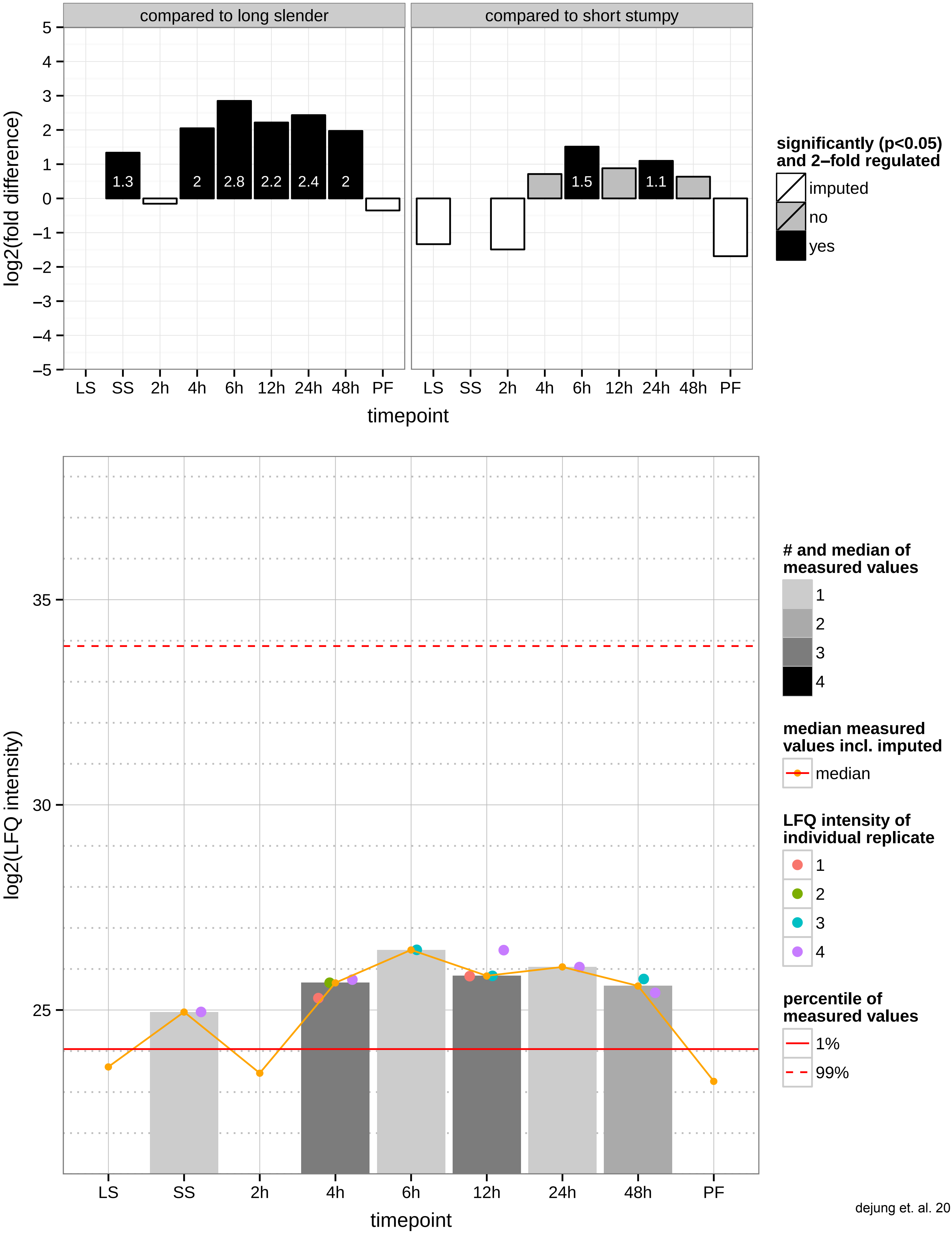

dejung et. al. 2015

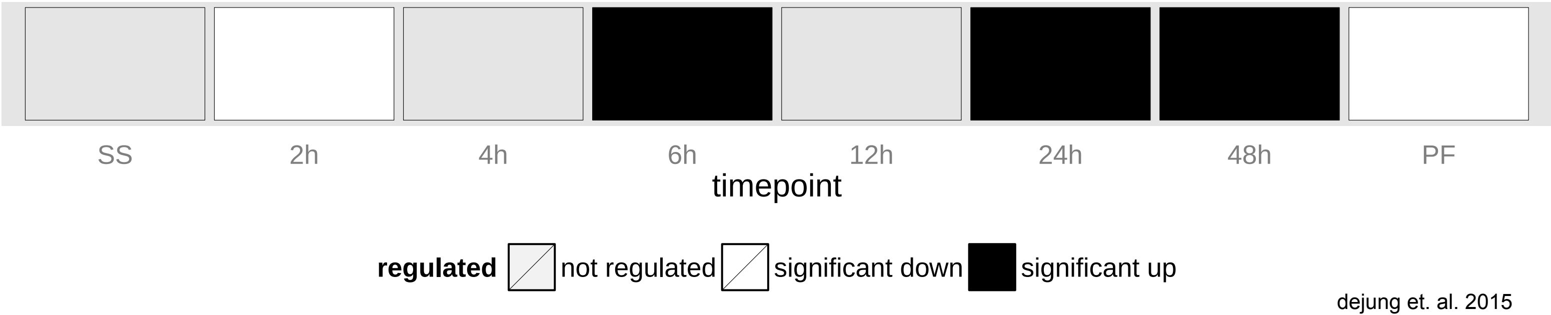
SS
2h
4h
6h
12h
24h
48h
PF
timepoint
regulated
not regulated
significant down
significant up
dejung et. al. 2015

hypothetical protein, conserved
Tb927.10.13980
AGOF: voltage−gated potassium channel activity
AGOC: membrane, voltage−gated potassium channel complex
AGOP: potassium ion transport
PGOF: protein binding
PGOC: null
PGOP: protein homooligomerization

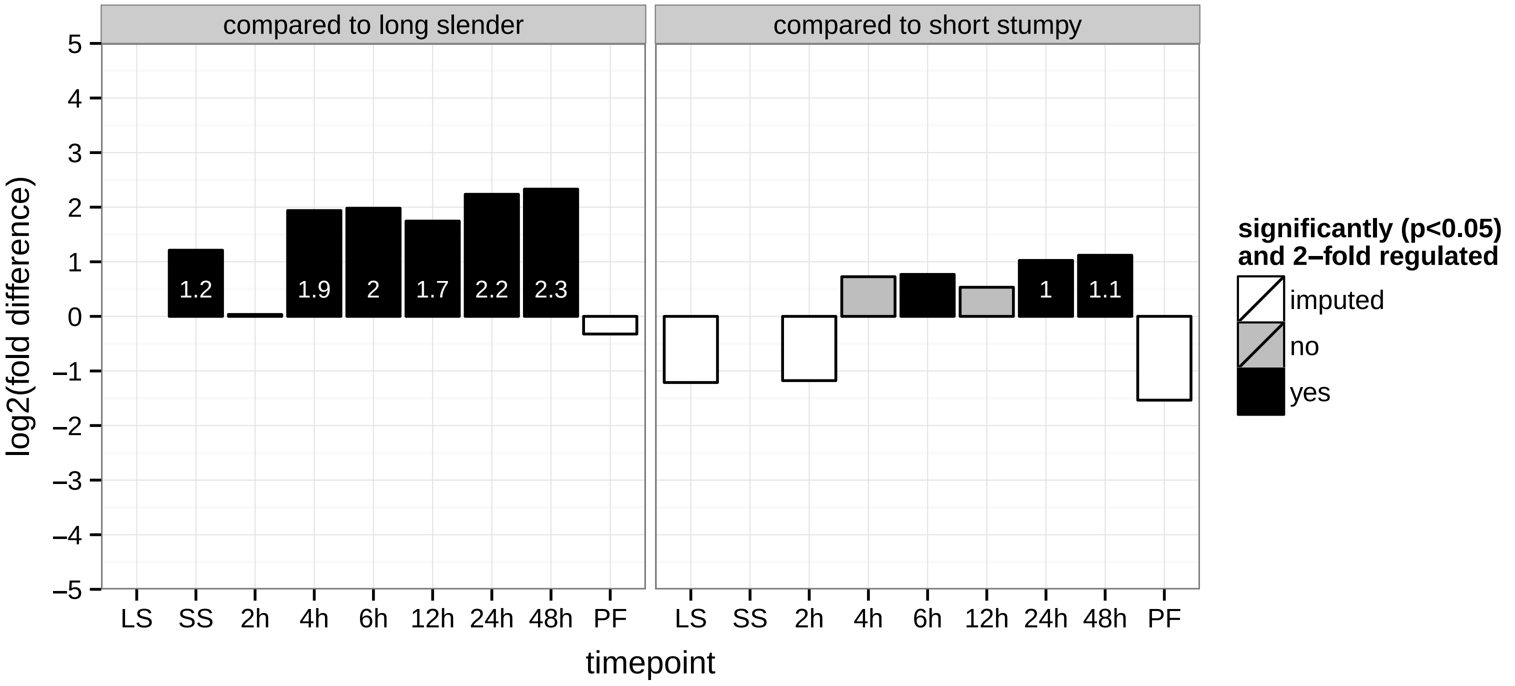

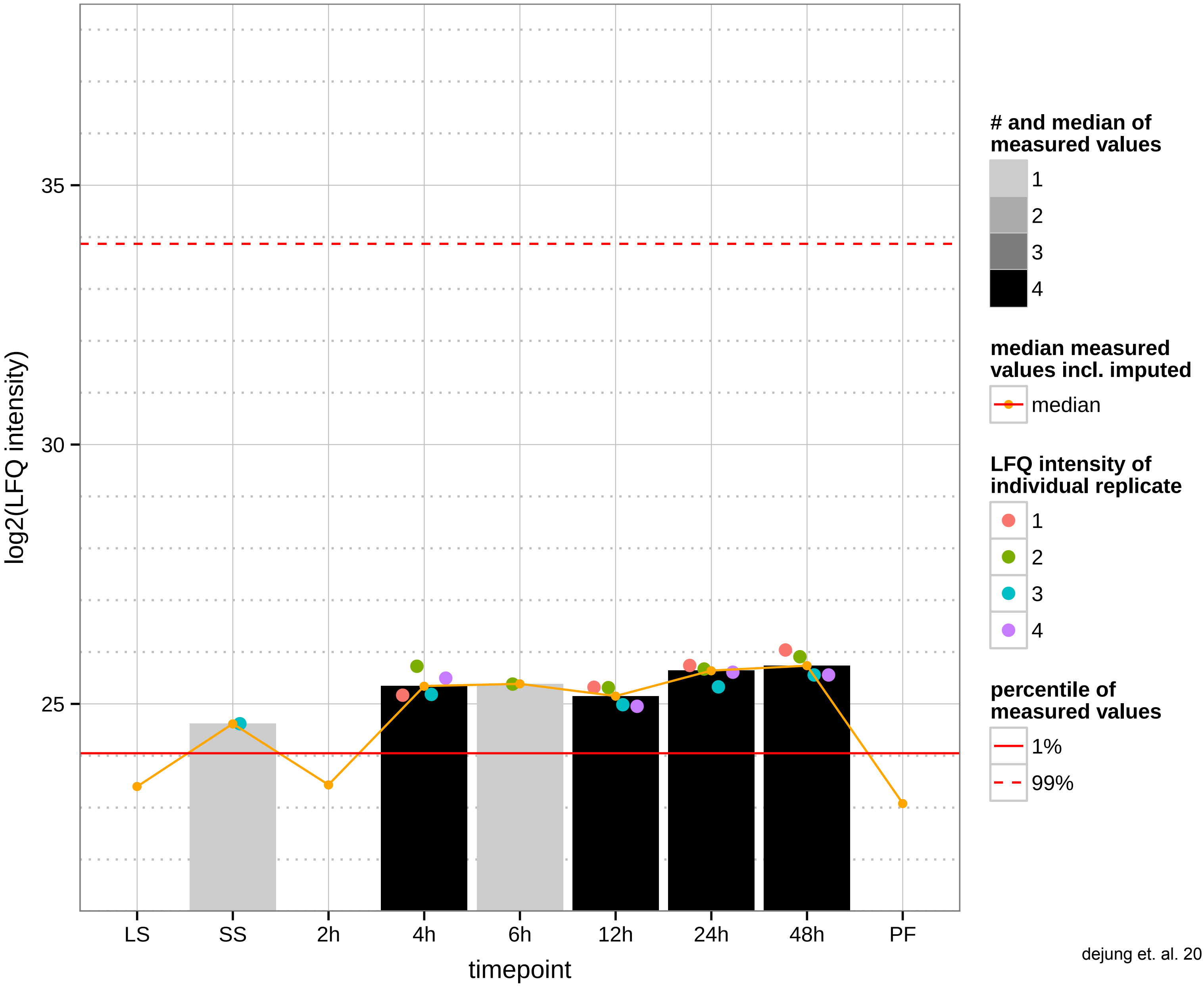

dejung et. al. 2015

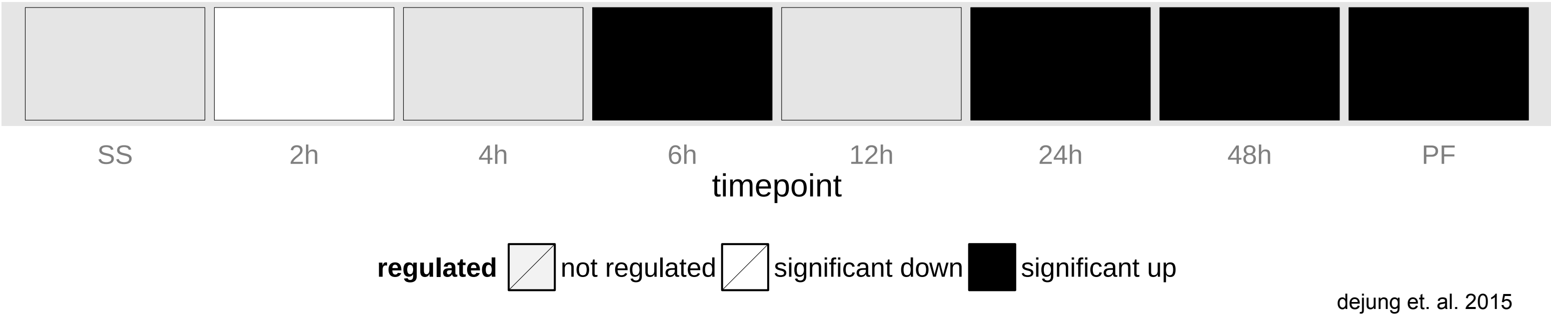
SS
2h
4h
6h
12h
24h
48h
PF
timepoint
regulated
not regulated
significant down
significant up
dejung et. al. 2015

60S ribosomal protein–like, putative
Tb927.7.4550;Tb11.v5.0268
AGOF: null, structural constituent of ribosome
AGOC: null, intracellular, ribosome
AGOP: null, translation
PGOF: structural constituent of ribosome
PGOC: intracellular, ribosome
PGOP: translation

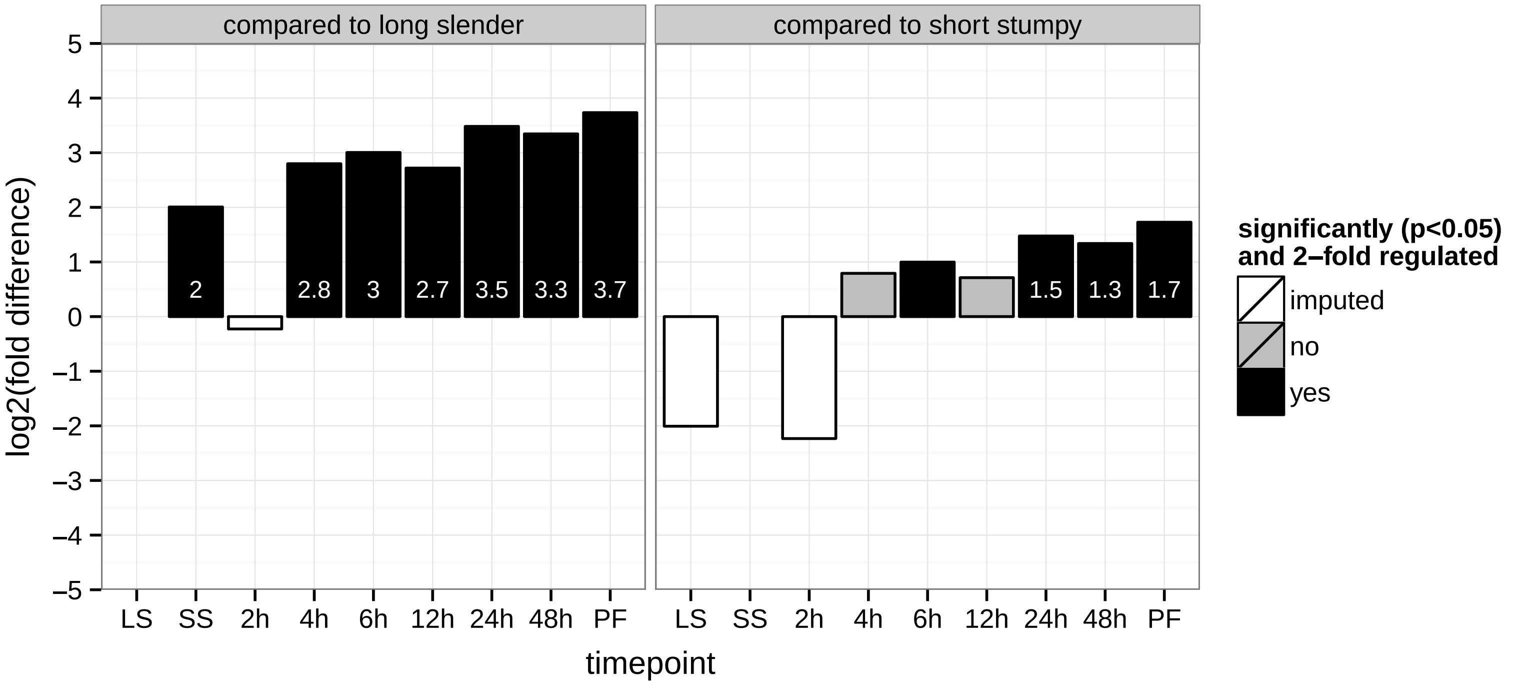

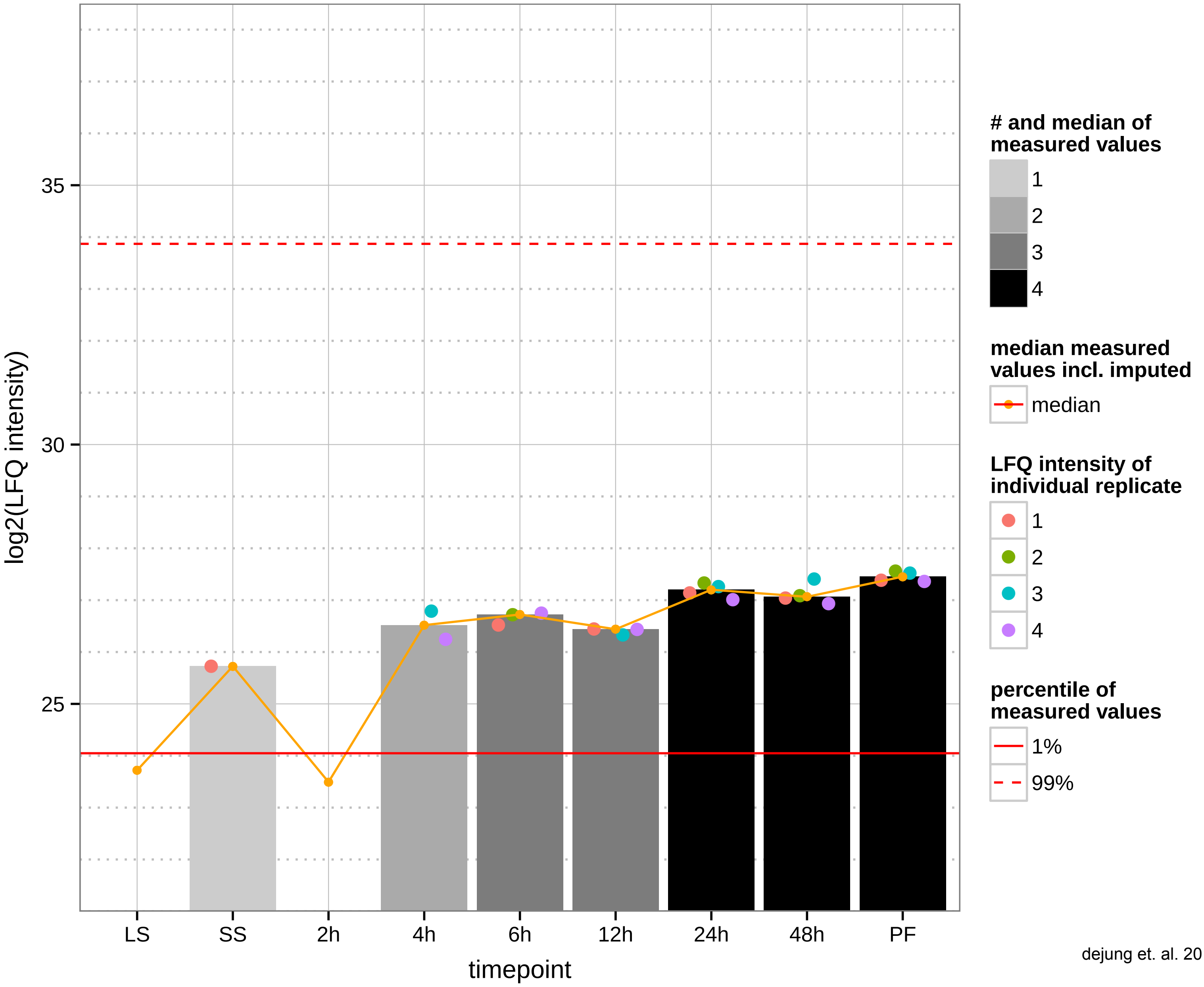

dejung et. al. 2015

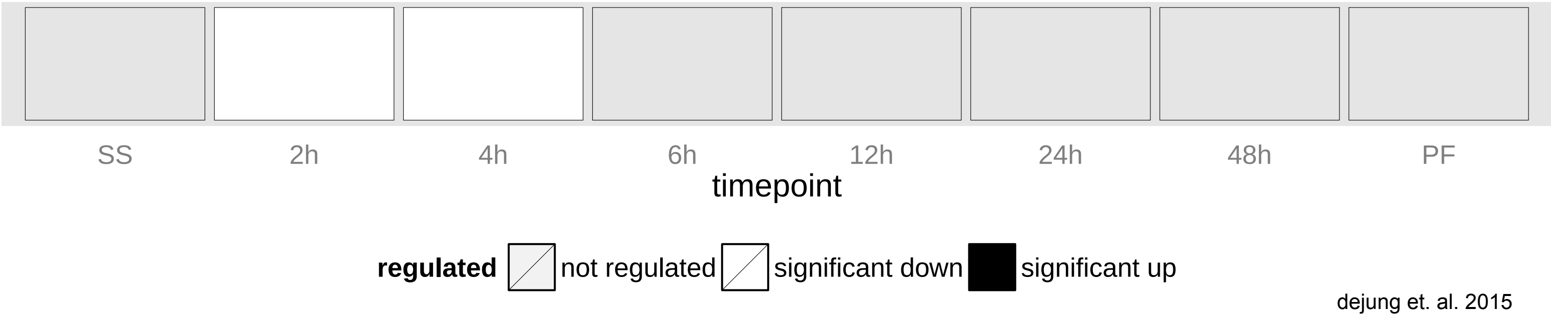
SS
2h
4h
6h
12h
24h
48h
PF
timepoint
regulated
not regulated
significant down
significant up
dejung et. al. 2015

hypothetical protein, conserved
Tb927.8.6800
AGOF: null
AGOC: mitochondrion
AGOP: null
PGOF: null
PGOC: null
PGOP: null

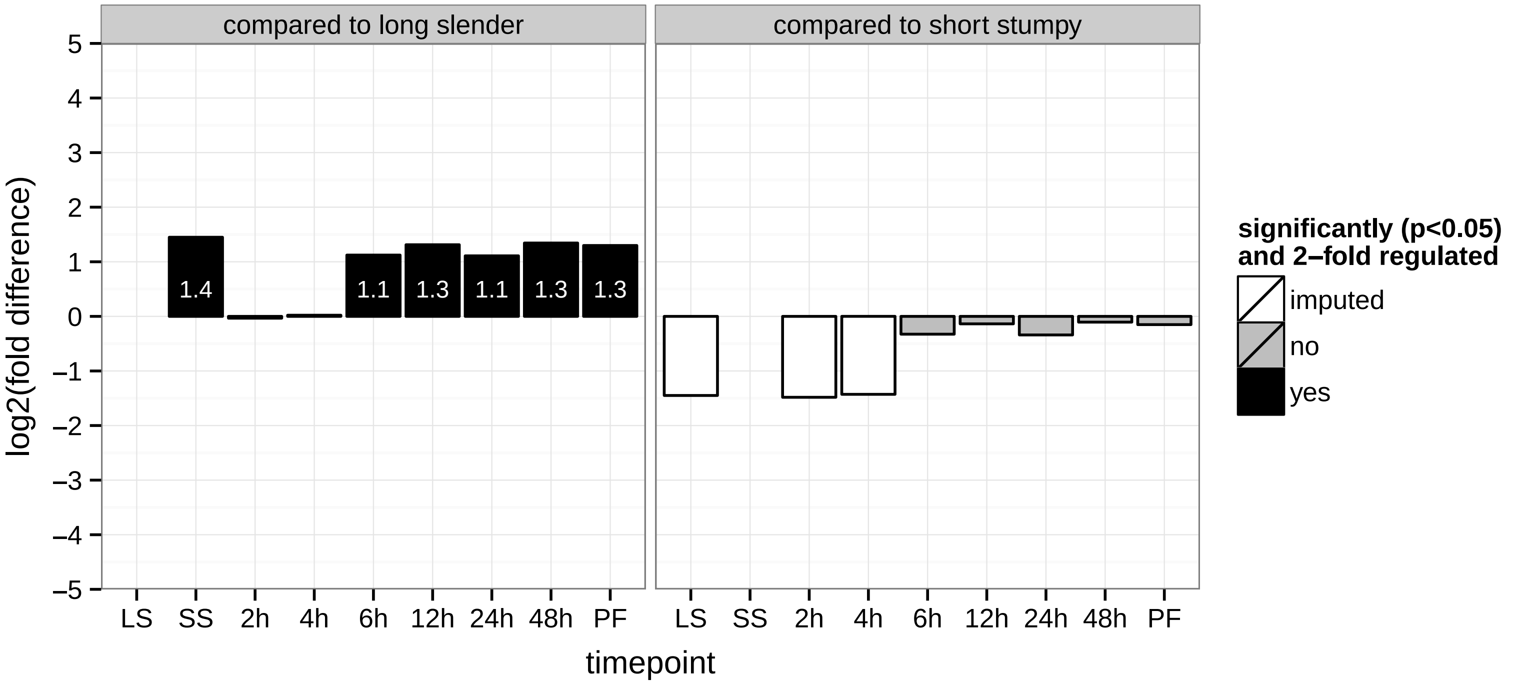

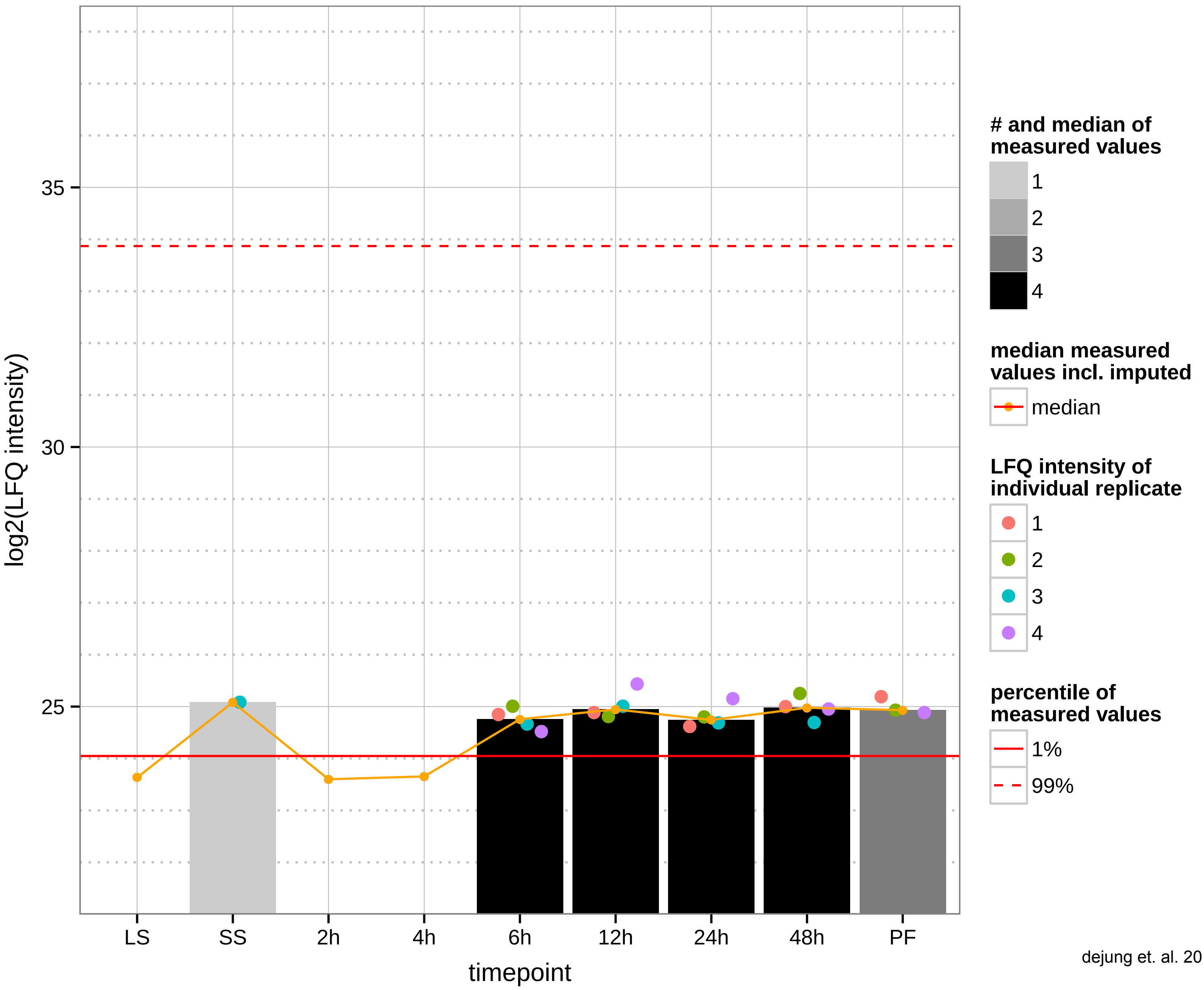

dejung et. al. 2015

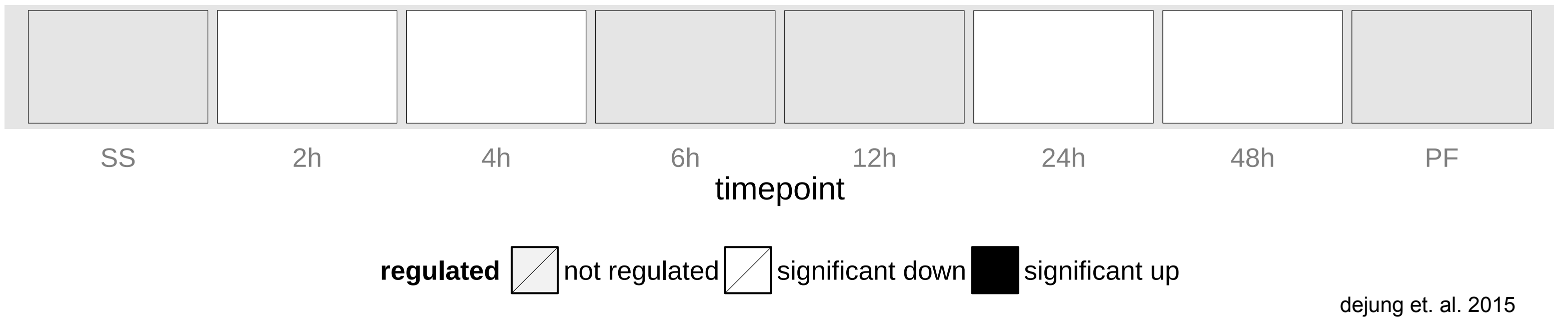
SS
2h
4h
6h
12h
24h
48h
PF
timepoint
regulated
not regulated
significant down
significant up
dejung et. al. 2015

GPI alpha–mannosyltransferase III, GPI10 (GPI10)
Tb927.10.5560
AGOF: mannosyltransferase activity
AGOC: integral to membrane, intrinsic to endoplasmic reticulum membrane
AGOP: GPI anchor biosynthetic process, growth
PGOF: transferase activity, transferring glycosyl groups
PGOC: intrinsic to endoplasmic reticulum membrane
PGOP: GPI anchor biosynthetic process

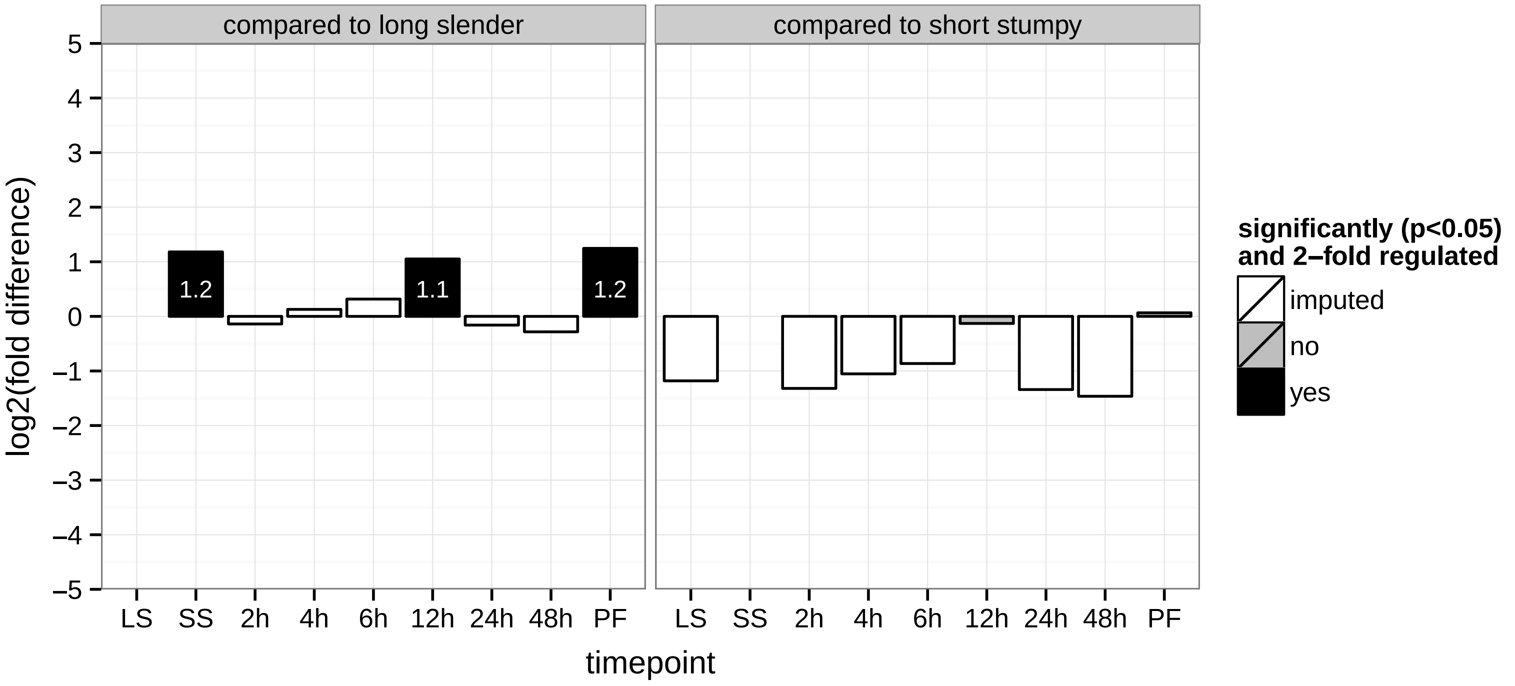

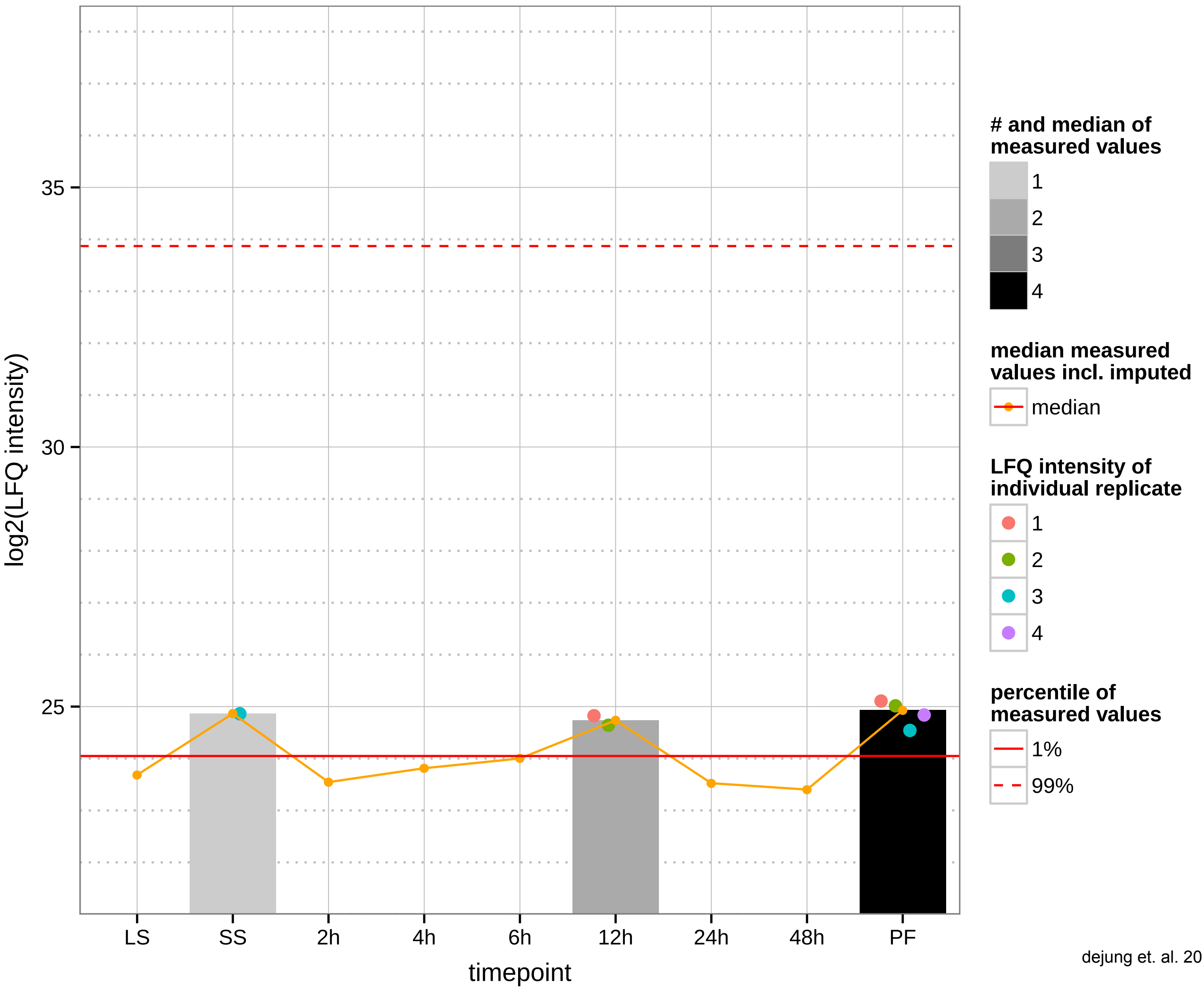

dejung et. al. 2015

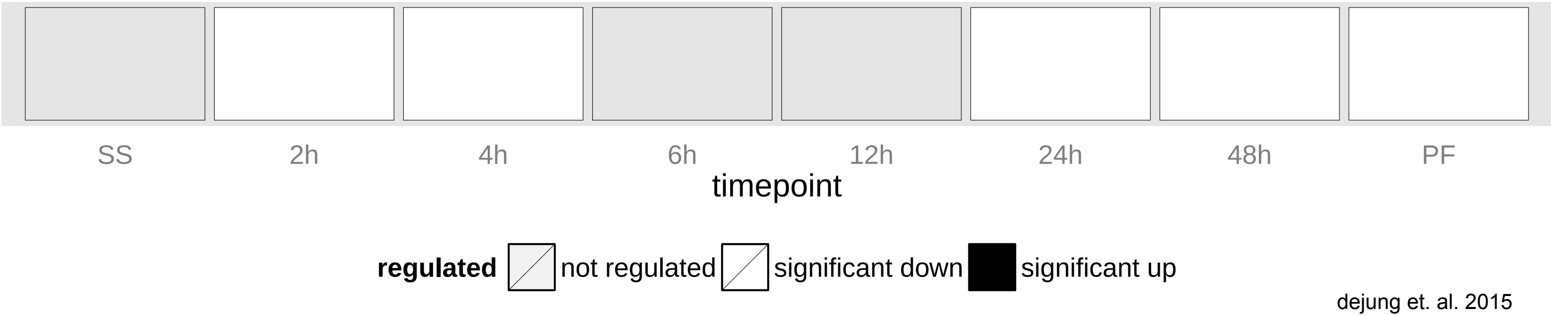
SS
2h
4h
6h
12h
24h
48h
PF
timepoint
regulated
not regulated
significant down
significant up
dejung et. al. 2015

variant surface glycoprotein, fragment, hypothetical protein, conserved, chrX additional, unordered contigs, predicted SAP dor
Tb927.10.4440;Tb10.v4.0034
AGOF: null, nucleic acid binding
AGOC: null
AGOP: null
PGOF: null, nucleic acid binding
PGOC: null
PGOP: evasion or tolerance of host immune response, null

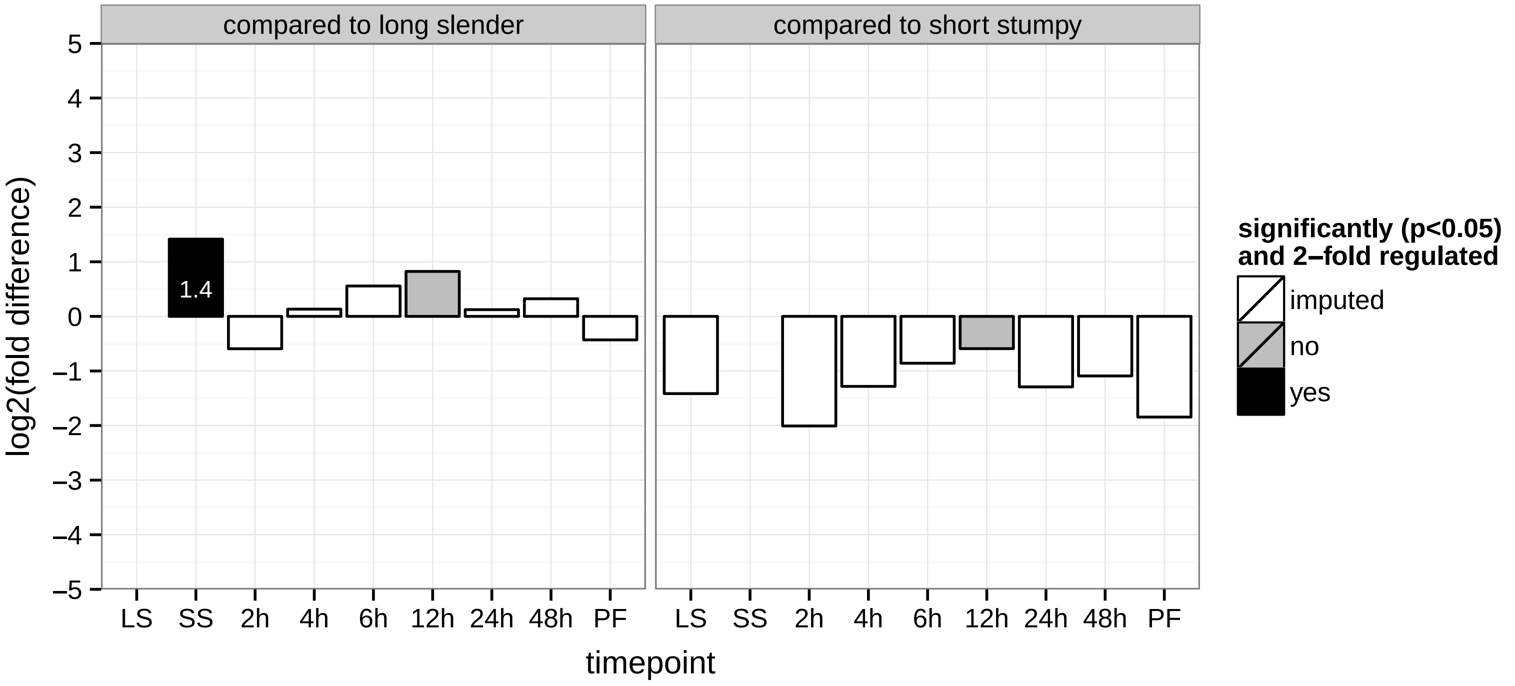

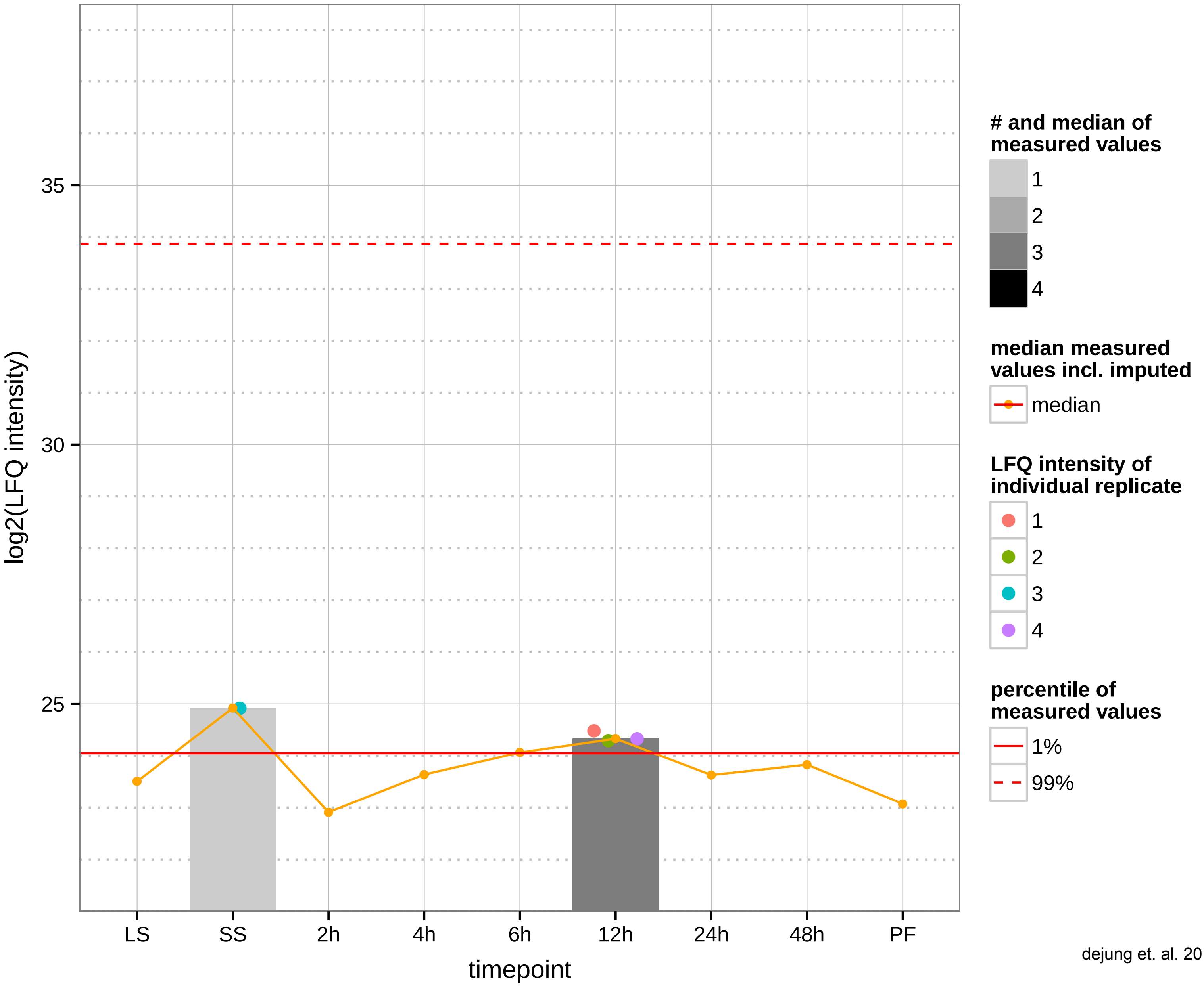

dejung et. al. 2015

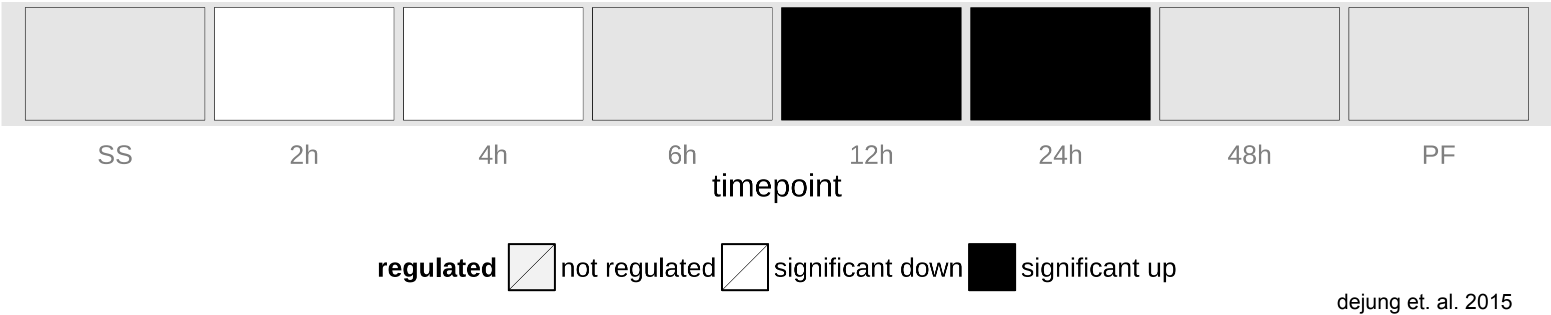
SS
2h
4h
6h
12h
24h
48h
PF
timepoint
regulated
not regulated
significant down
significant up
dejung et. al. 2015

hypothetical protein, conserved
Tb927.10.15810
AGOF: null
AGOC: null
AGOP: null
PGOF: null
PGOC: null
PGOP: null

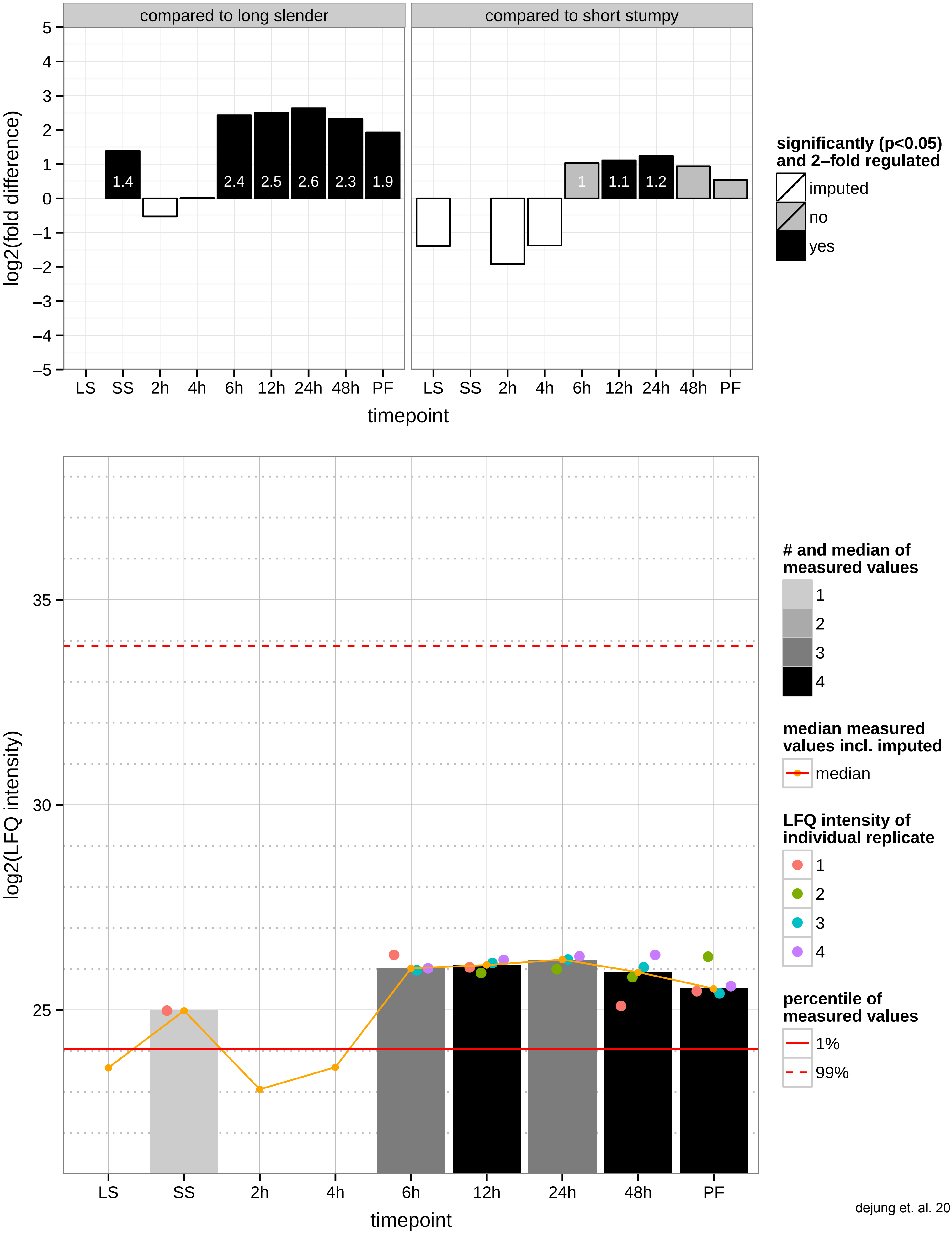

dejung et. al. 2015

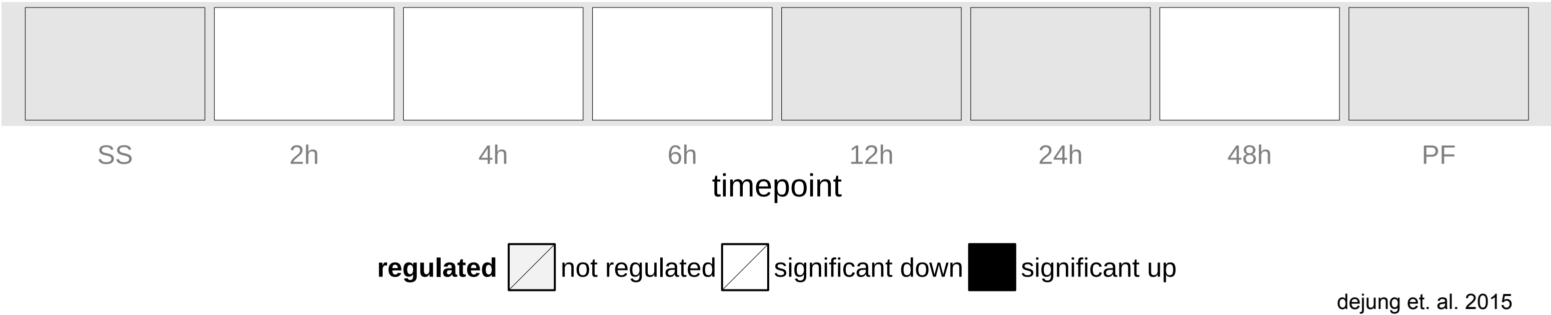
SS
2h
4h
6h
12h
24h
48h
PF
timepoint
regulated
not regulated
significant down
significant up
dejung et. al. 2015

hypothetical protein, conserved
Tb927.11.14230
AGOF: null
AGOC: null
AGOP: null
PGOF: null
PGOC: null
PGOP: null

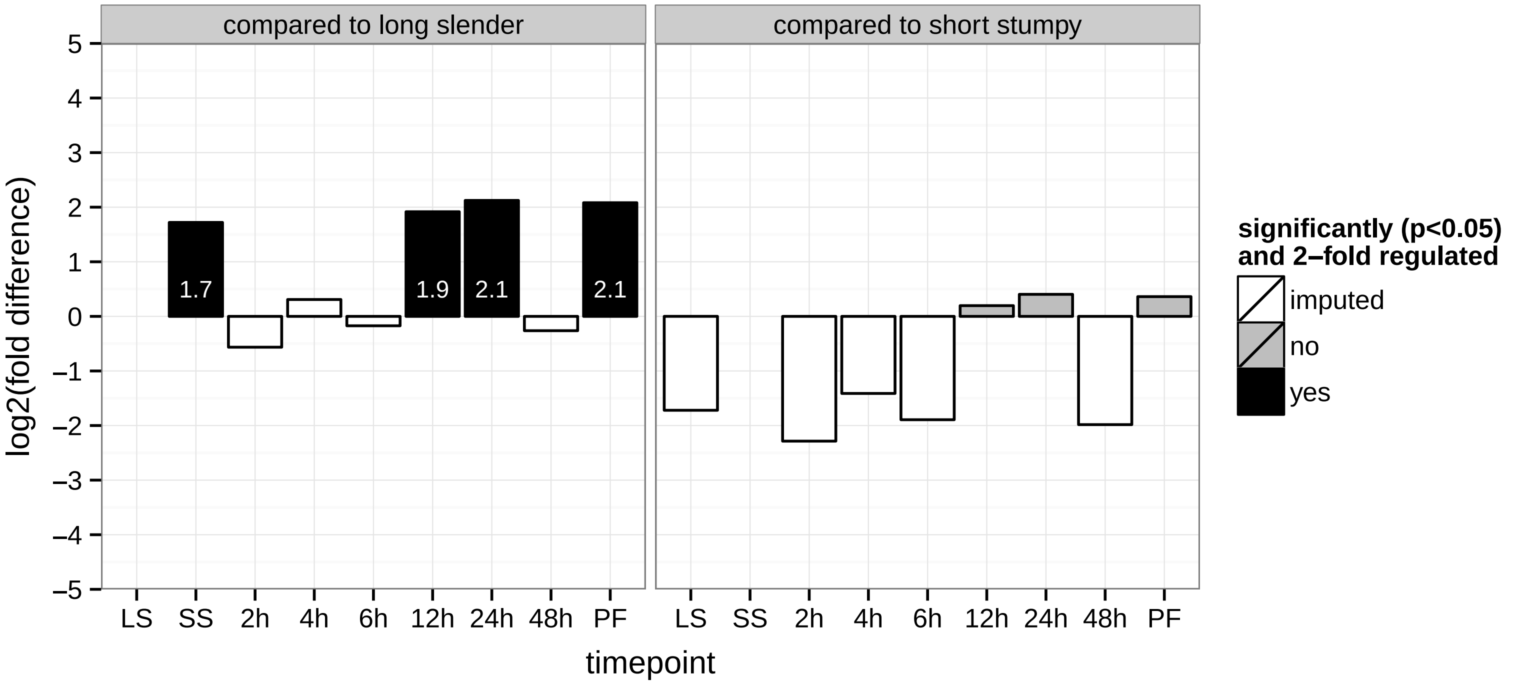

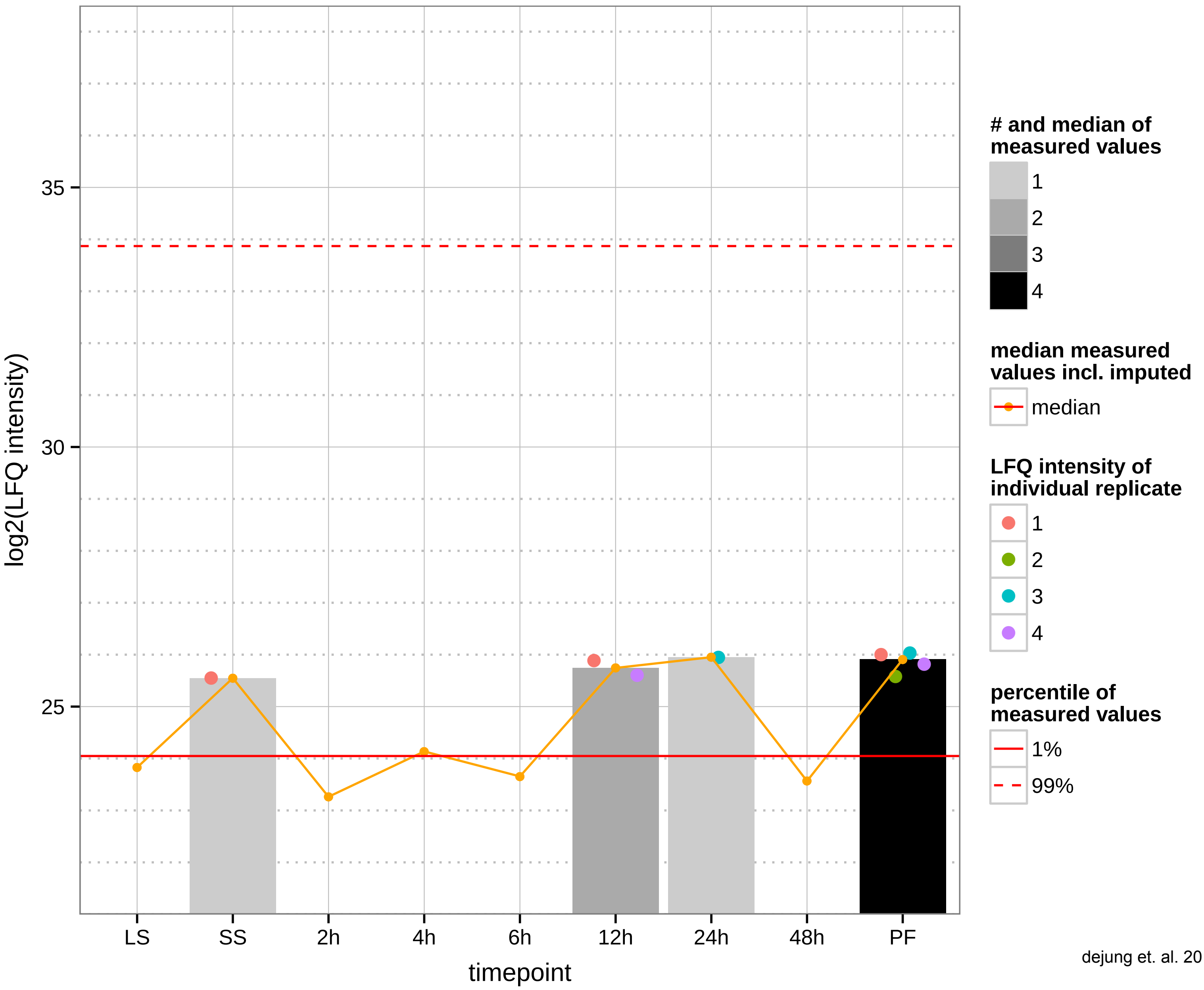

dejung et. al. 2015

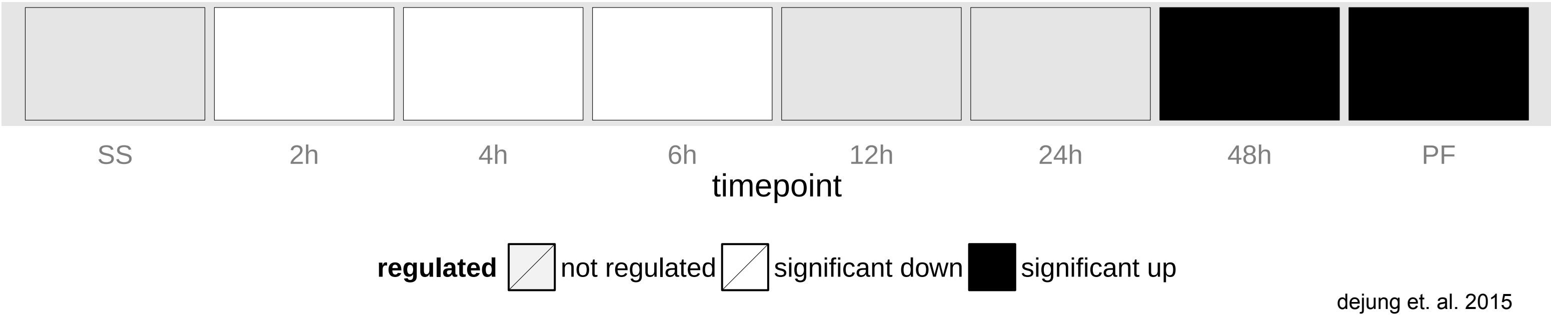
SS
2h
4h
6h
12h
24h
48h
PF
timepoint
regulated
not regulated
significant down
significant up
dejung et. al. 2015

hypothetical protein, conserved
Tb927.5.1780
AGOF: null
AGOC: null
AGOP: null
PGOF: null
PGOC: null
PGOP: null

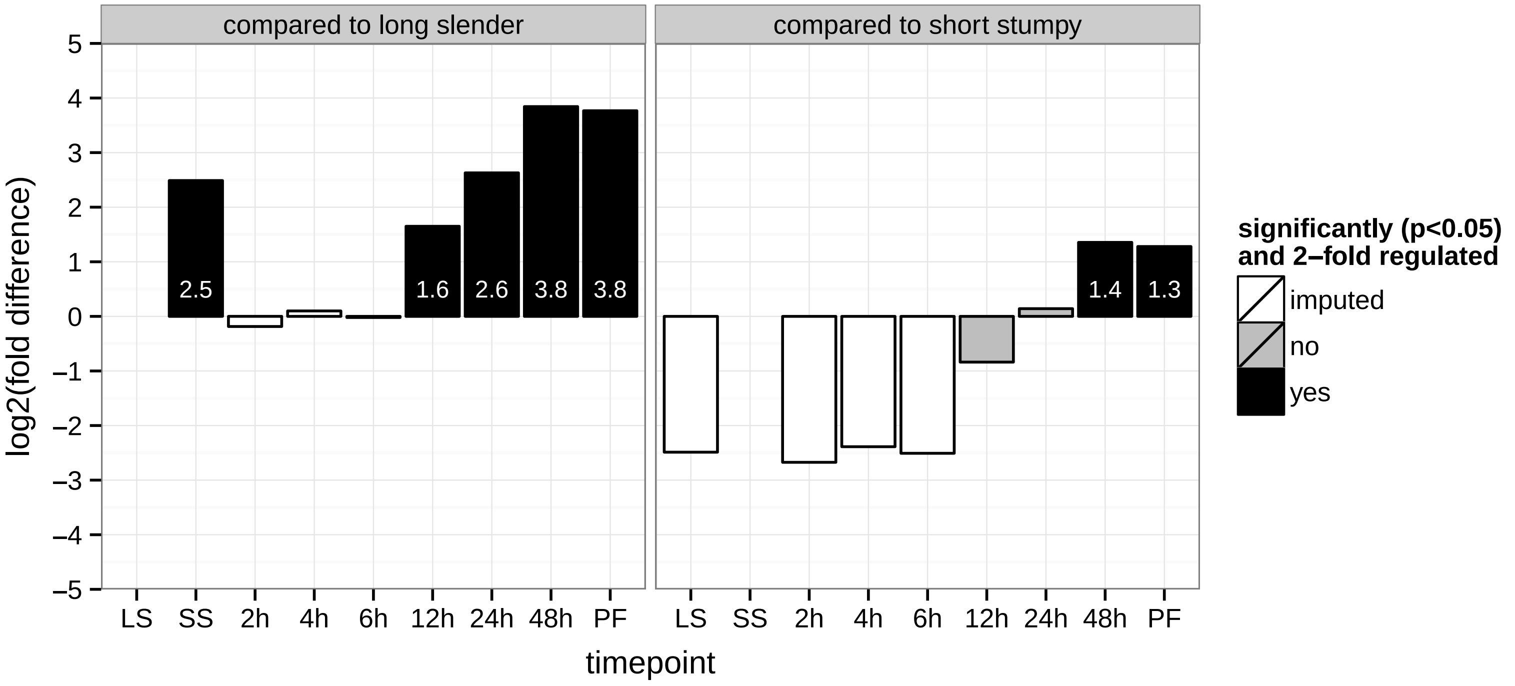

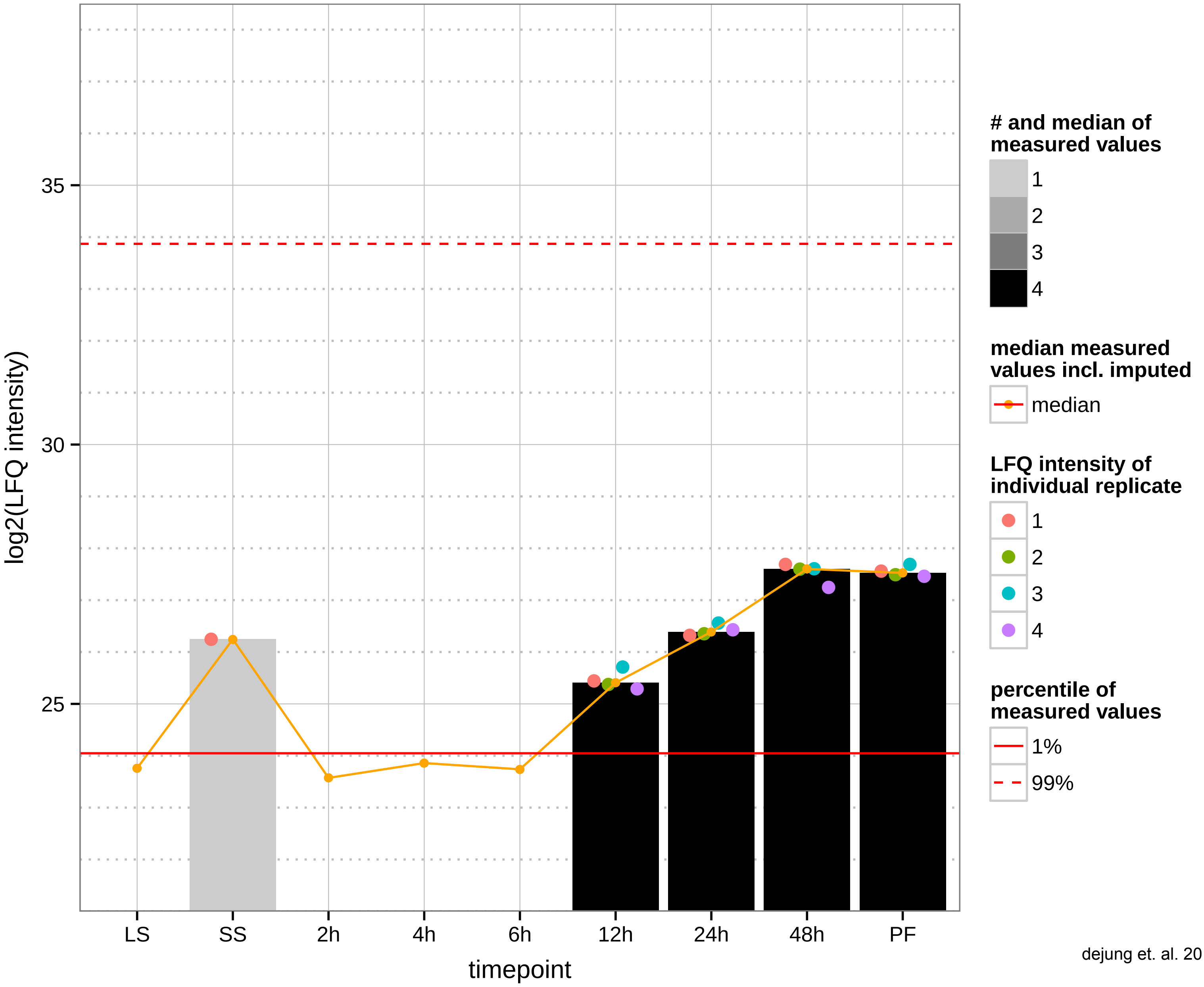

dejung et. al. 2015

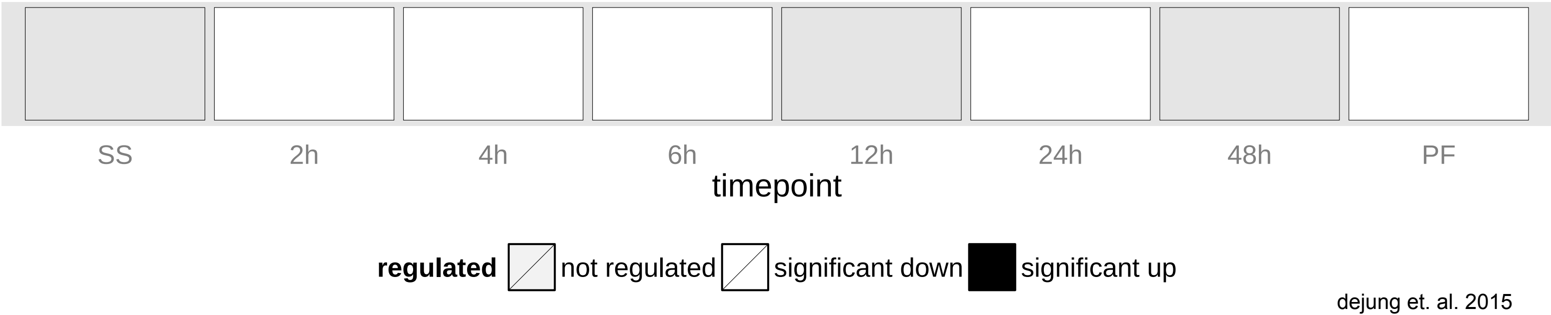
SS
2h
4h
6h
12h
24h
48h
PF
timepoint
regulated
not regulated
significant down
significant up
dejung et. al. 2015

hypothetical protein, conserved
Tb927.2.2530
AGOF: null
AGOC: null
AGOP: null
PGOF: null
PGOC: null
PGOP: null

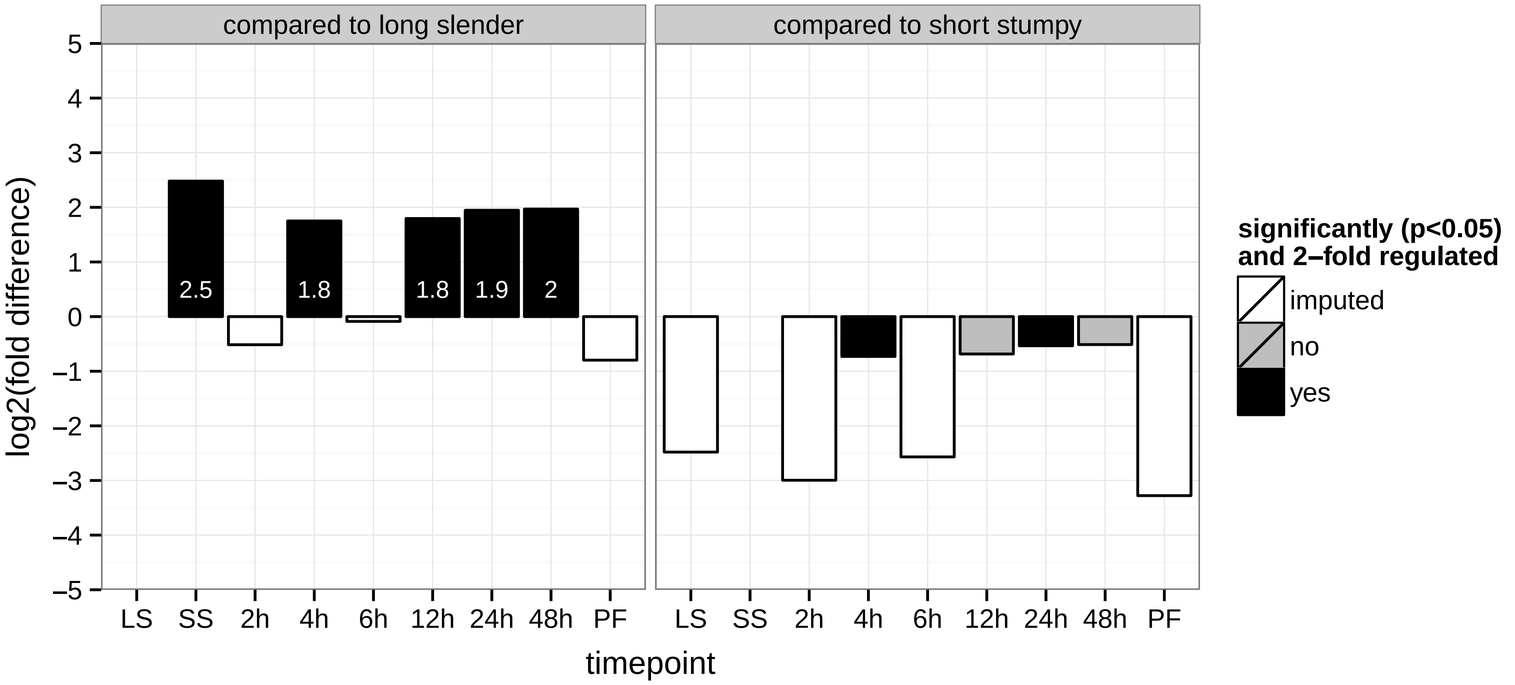

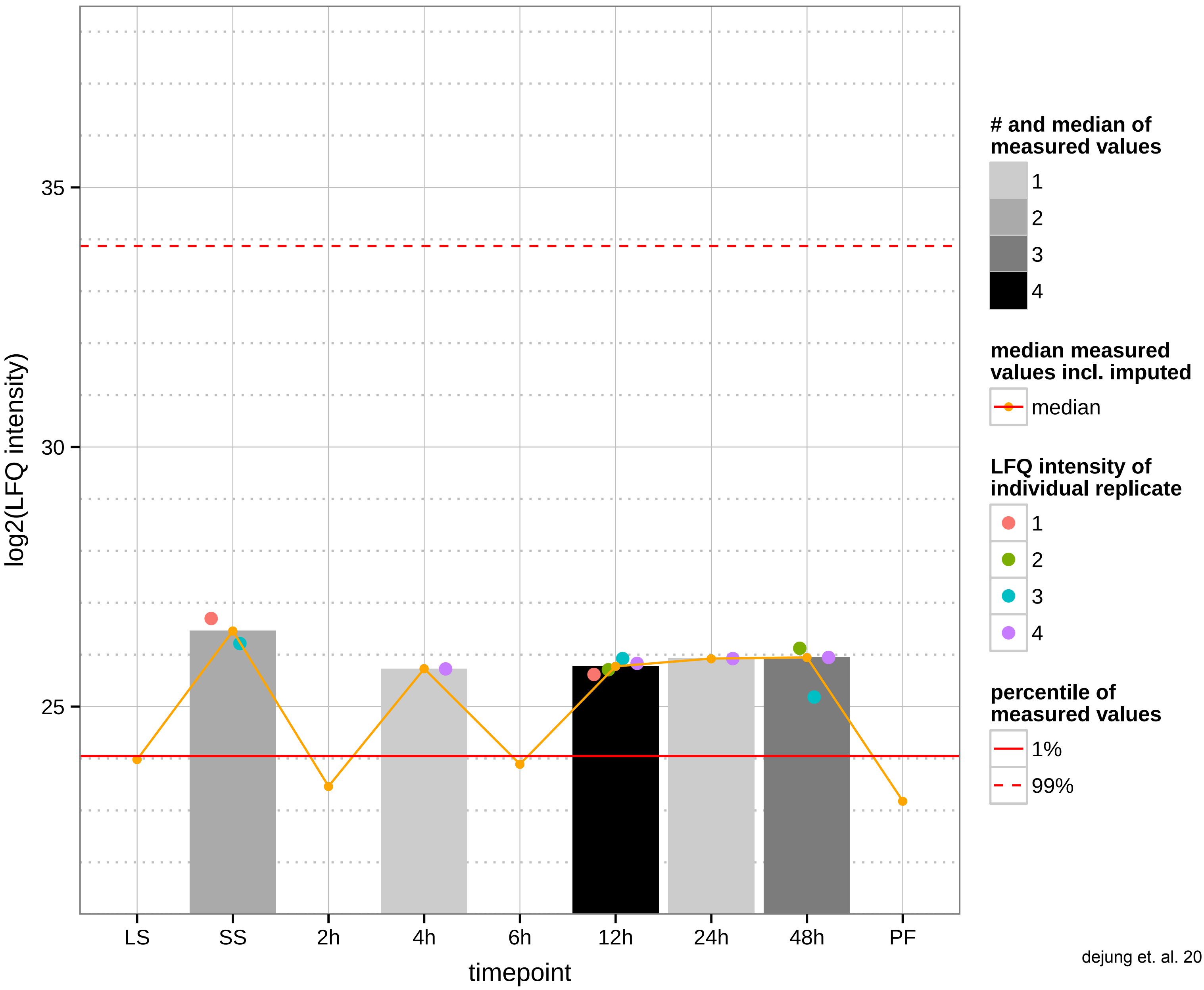

dejung et. al. 2015

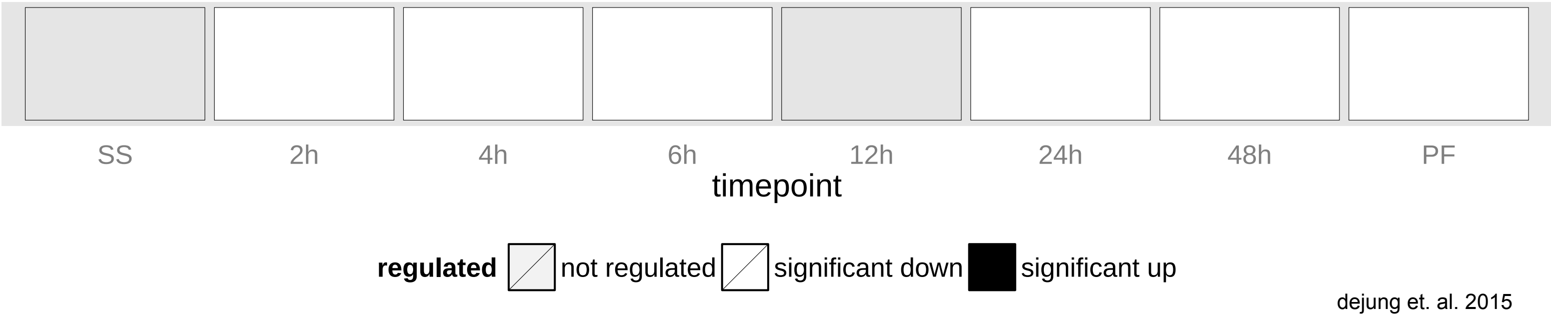
SS
2h
4h
6h
12h
24h
48h
PF
timepoint
regulated
not regulated
significant down
significant up
dejung et. al. 2015

AAA ATPase, putative
Tb927.11.6350
AGOF: ATP binding, ATPase activity
AGOC: null
AGOP: protein metabolic process
PGOF: ATP binding, nucleoside−triphosphatase activity, nucleotide binding
PGOC: null
PGOP: null

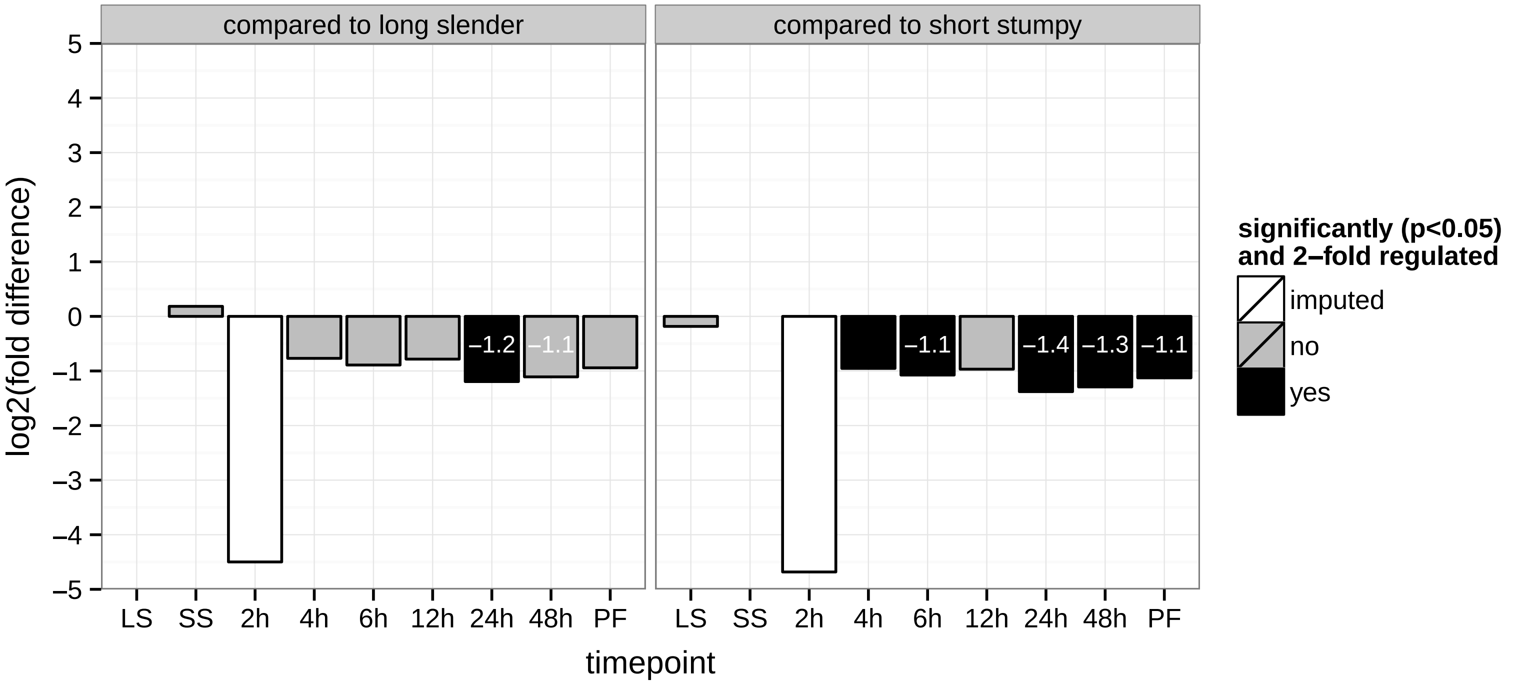

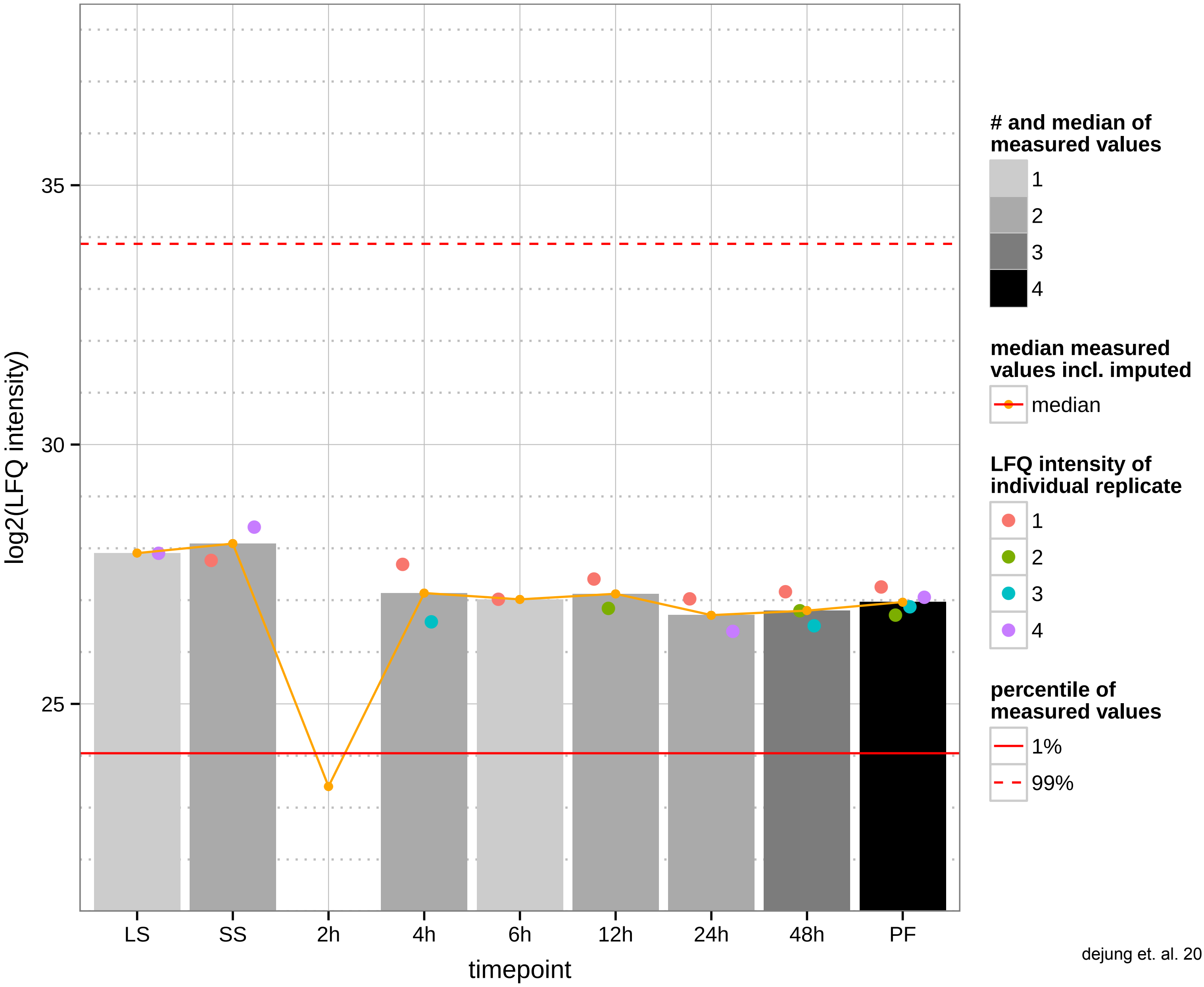

dejung et. al. 2015

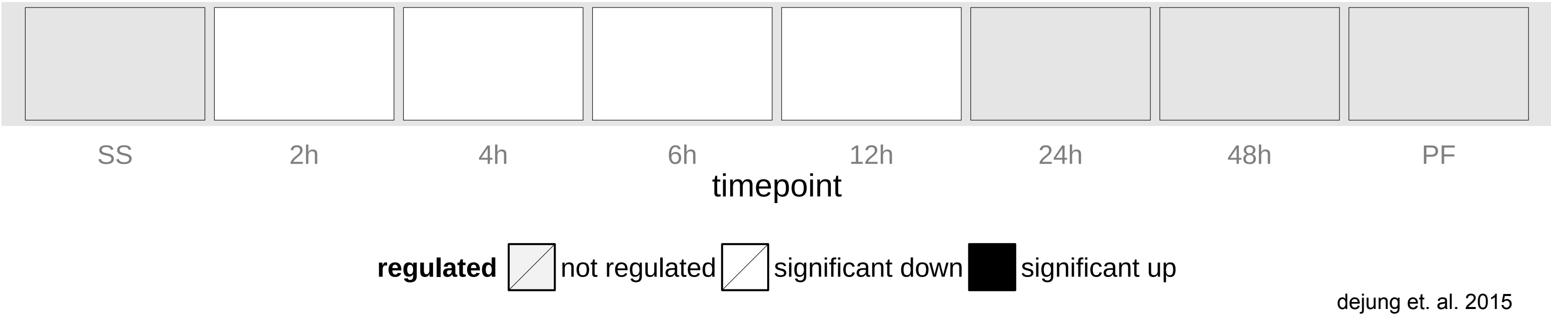
SS
2h
4h
6h
12h
24h
48h
PF
timepoint
regulated
not regulated
significant down
significant up
dejung et. al. 2015

amino acid transporter, putative (AATP11), putative
Tb927.4.4730;Tb927.8.7740
AGOF: amine transmembrane transporter activity
AGOC: null
AGOP: amino acid transport
PGOF: null
PGOC: null
PGOP: null

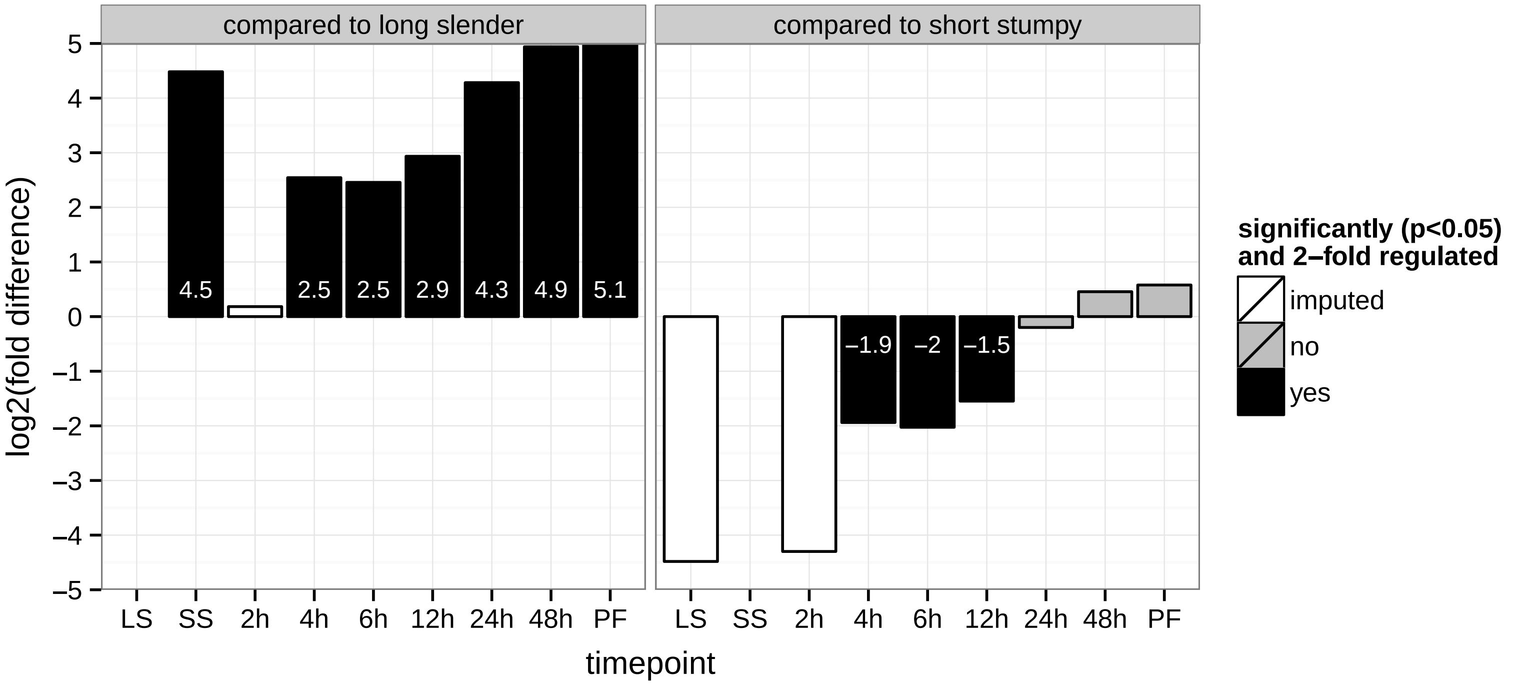

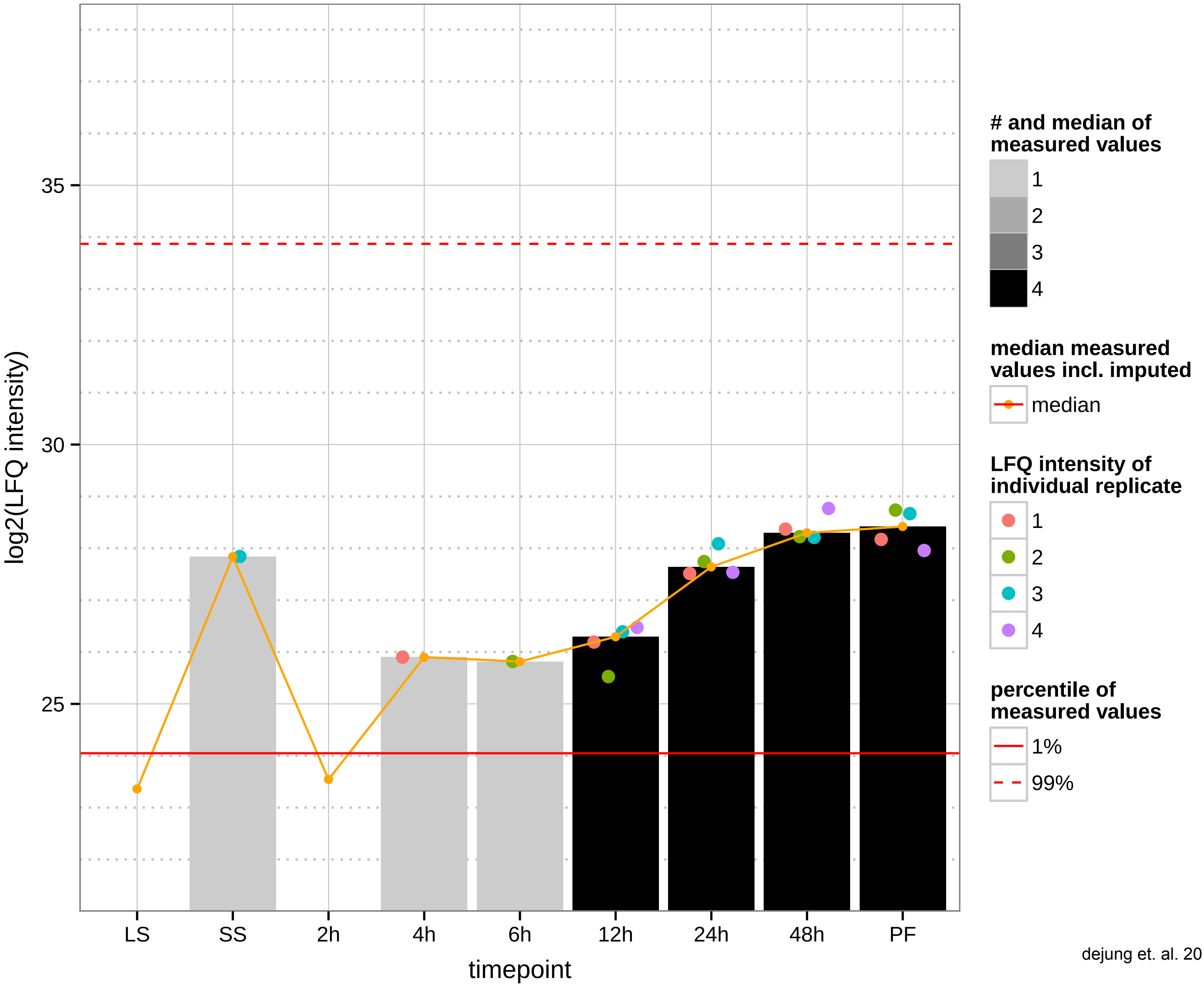

dejung et. al. 2015

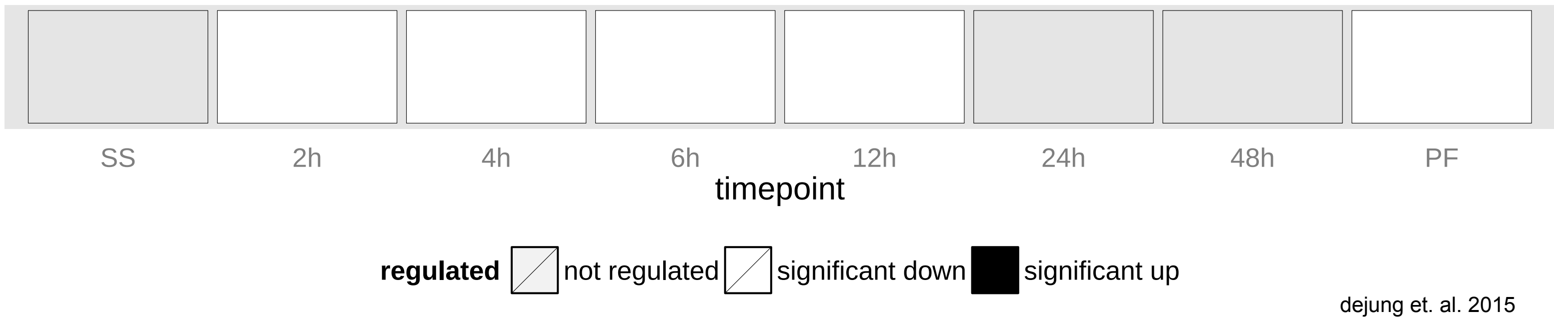
SS
2h
4h
6h
12h
24h
48h
PF
timepoint
regulated
not regulated
significant down
significant up
dejung et. al. 2015

mitochondrial RNA binding protein (MRB4160)
Tb927.4.4160
AGOF: RNA binding
AGOC: mitochondrion
AGOP: RNA metabolic process
PGOF: null
PGOC: null
PGOP: null

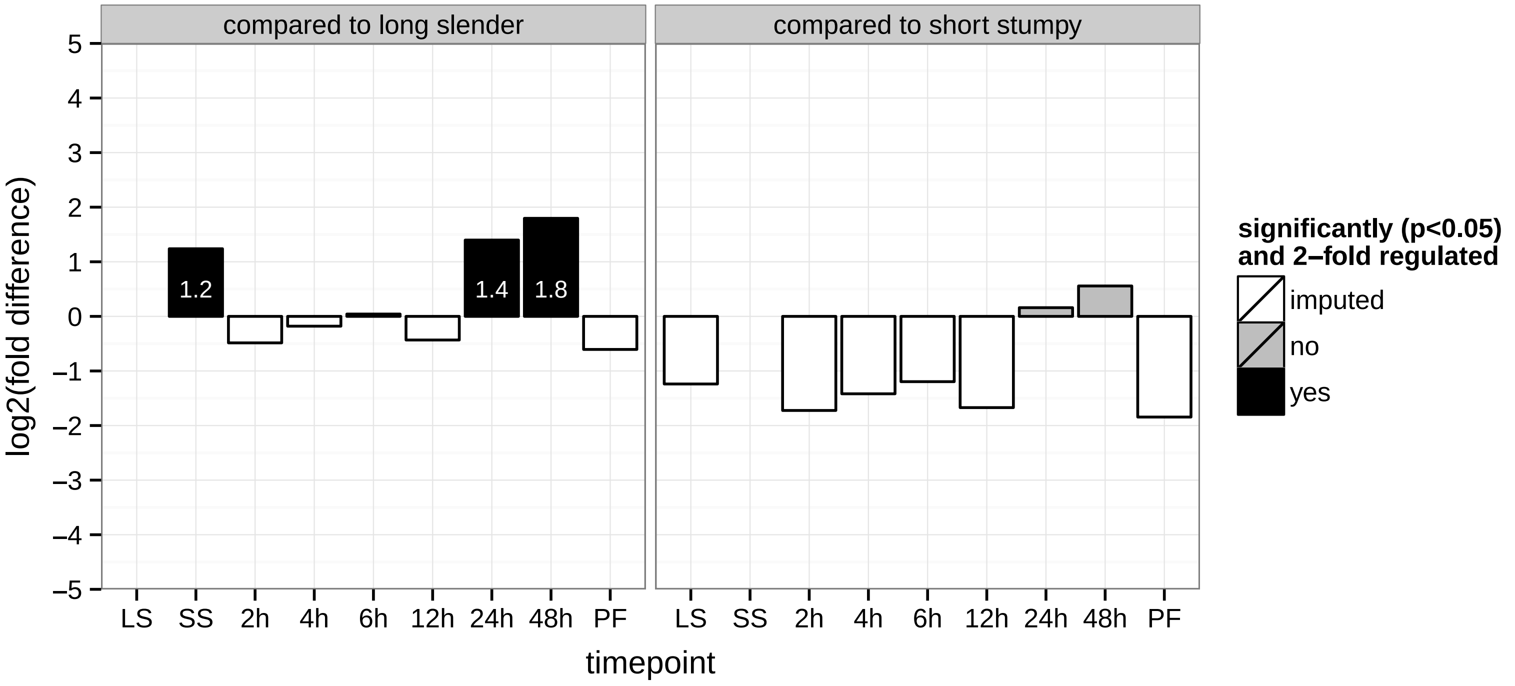

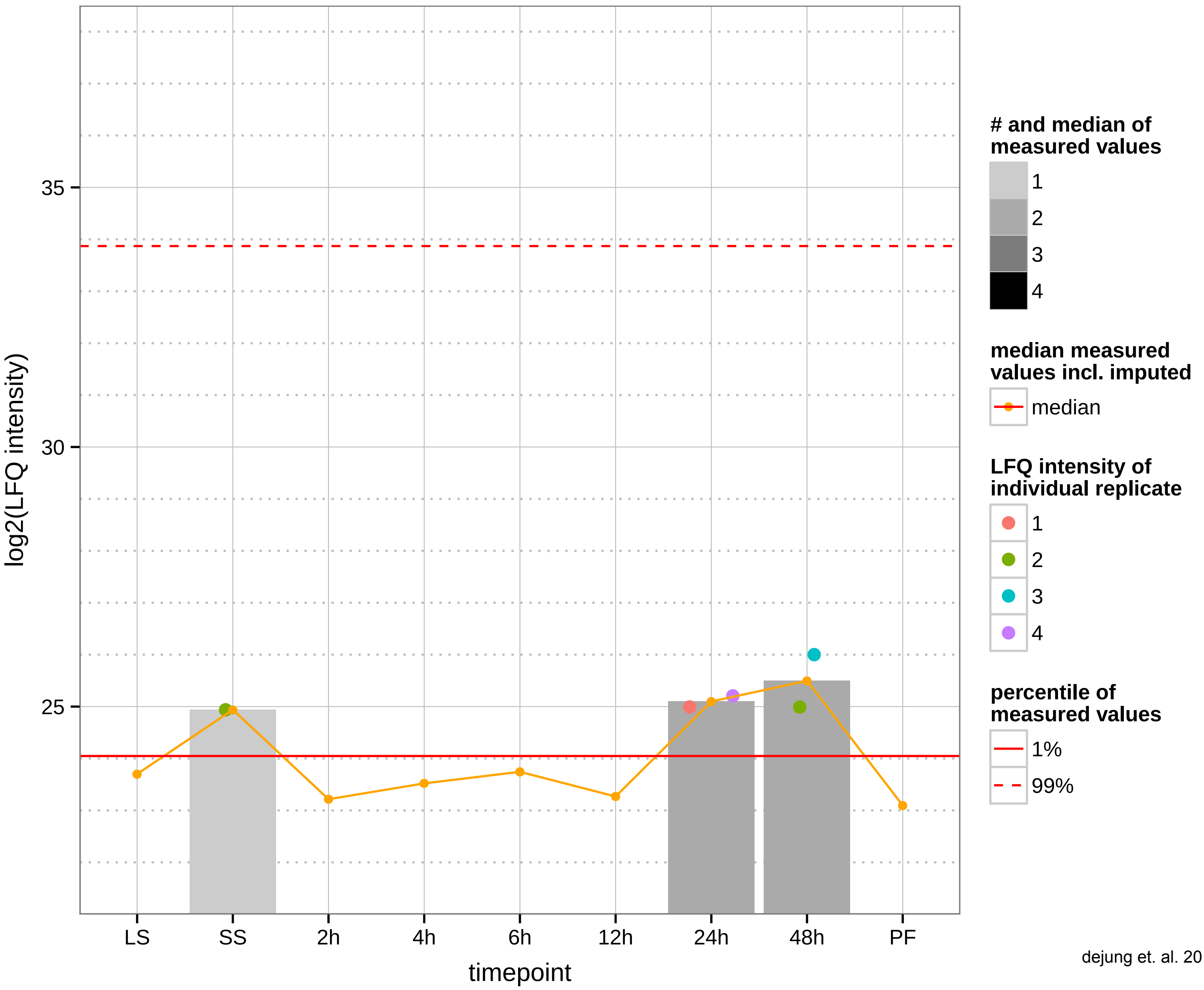

dejung et. al. 2015

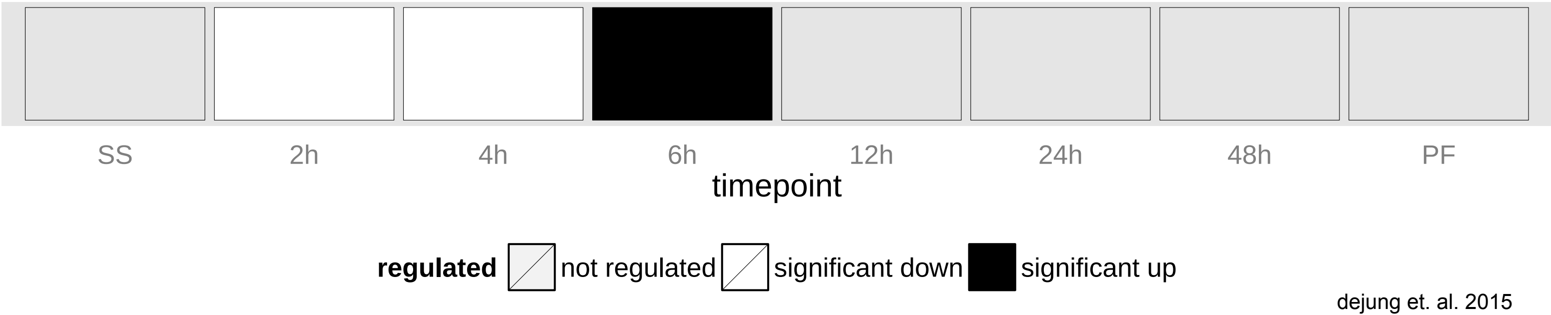
SS
2h
4h
6h
12h
24h
48h
PF
timepoint
regulated
not regulated
significant down
significant up
dejung et. al. 2015

TPR–repeat–containing chaperone protein DNAJ, putative
Tb927.4.2220
AGOF: heat shock protein binding
AGOC: null
AGOP: null
PGOF: heat shock protein binding, protein binding
PGOC: null
PGOP: null

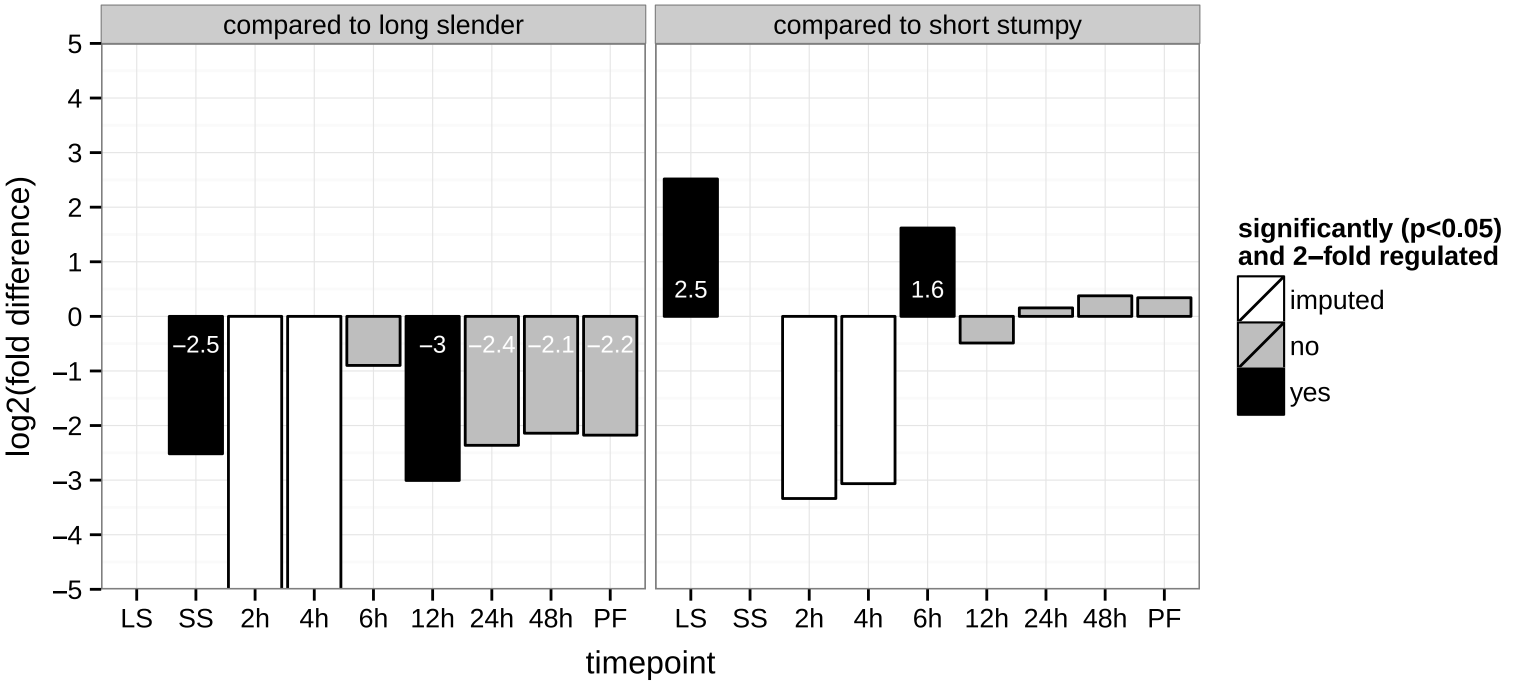

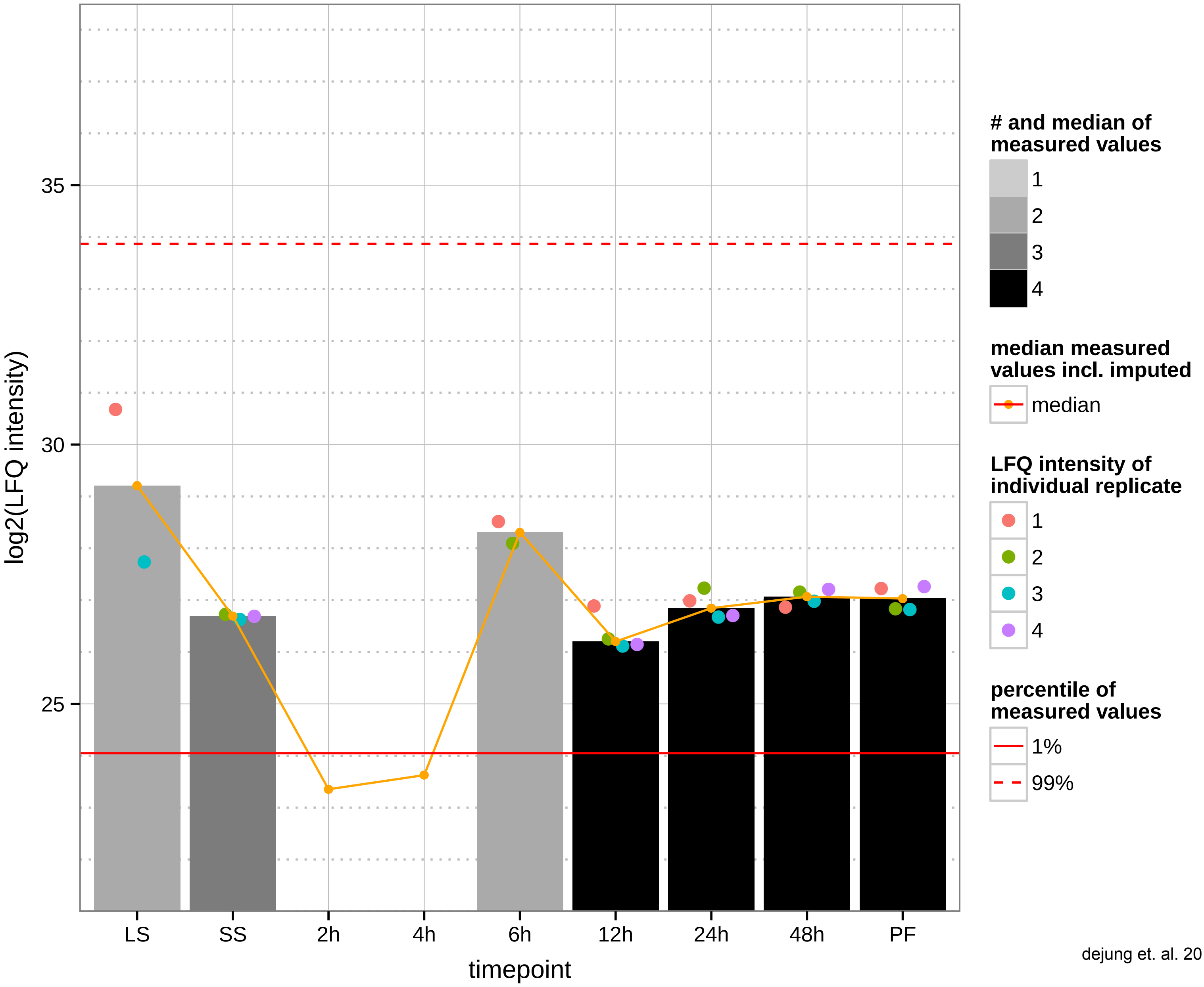

dejung et. al. 2015

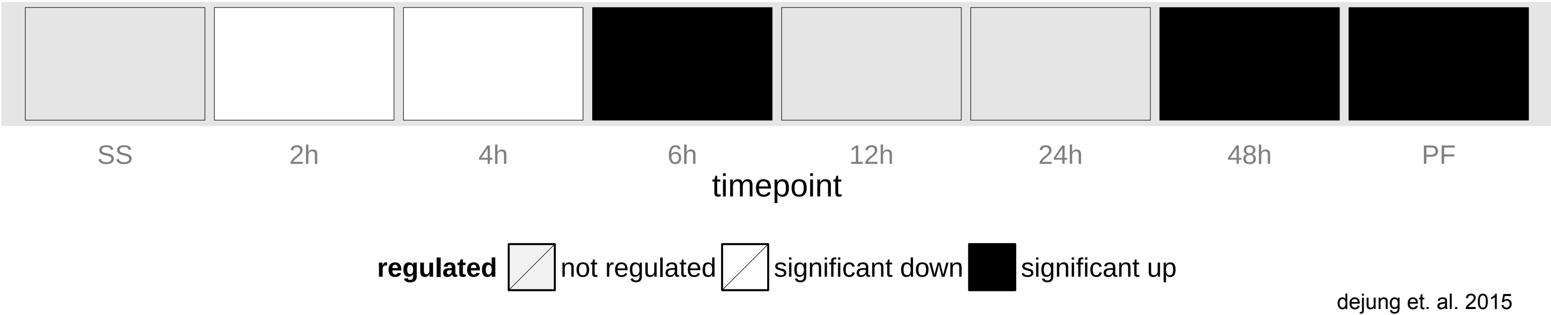
SS
2h
4h
6h
12h
24h
48h
PF
timepoint
regulated
not regulated
significant down
significant up
dejung et. al. 2015

metacaspase 5, putative (MCA5)
Tb927.9.14220
AGOF: cysteine−type endopeptidase activity
AGOC: null
AGOP: apoptotic process, cellular response to hydrogen peroxide, induction of apoptosis by oxidative stress, protein autoproc
PGOF: cysteine−type endopeptidase activity
PGOC: null
PGOP: proteolysis

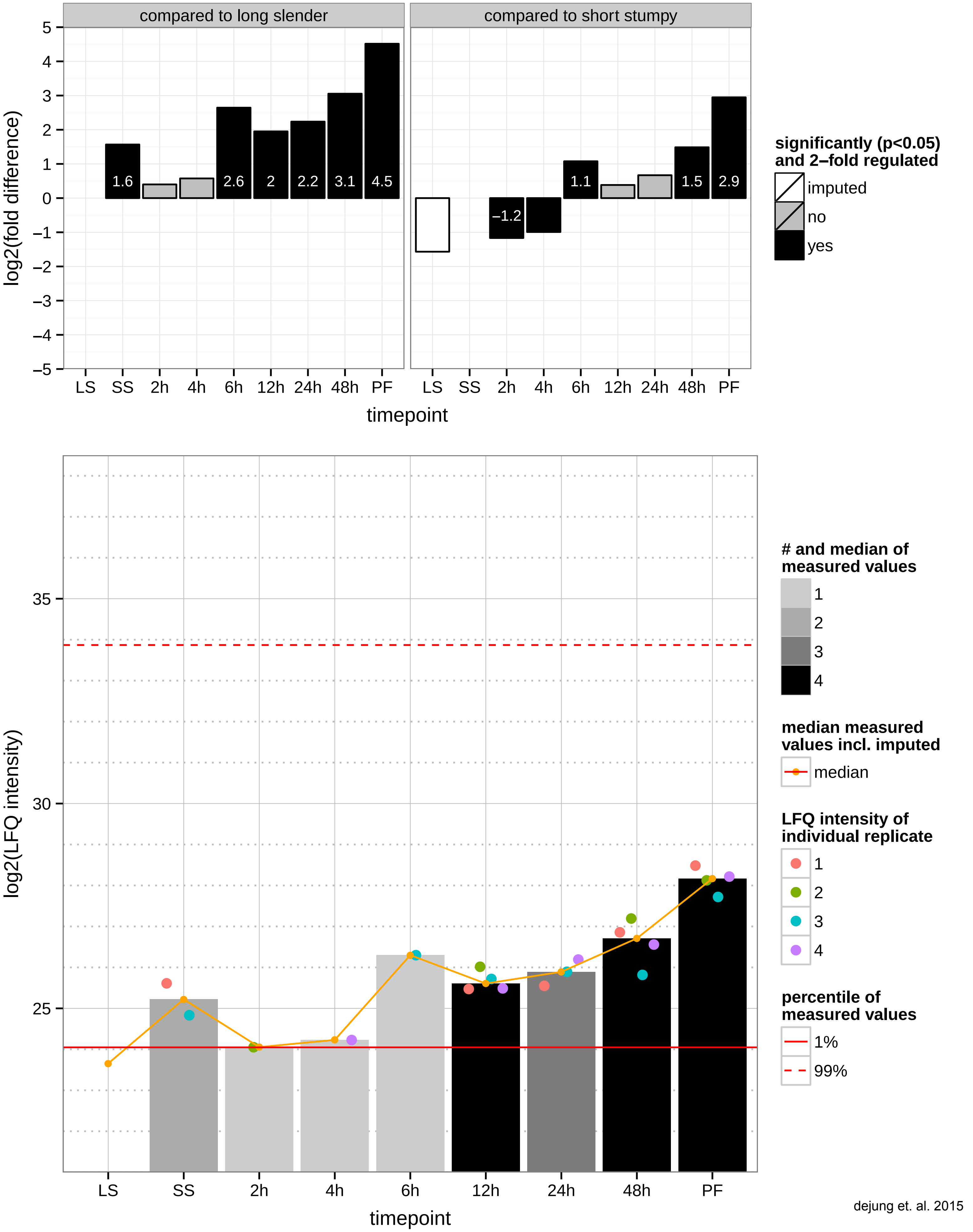

dejung et. al. 2015

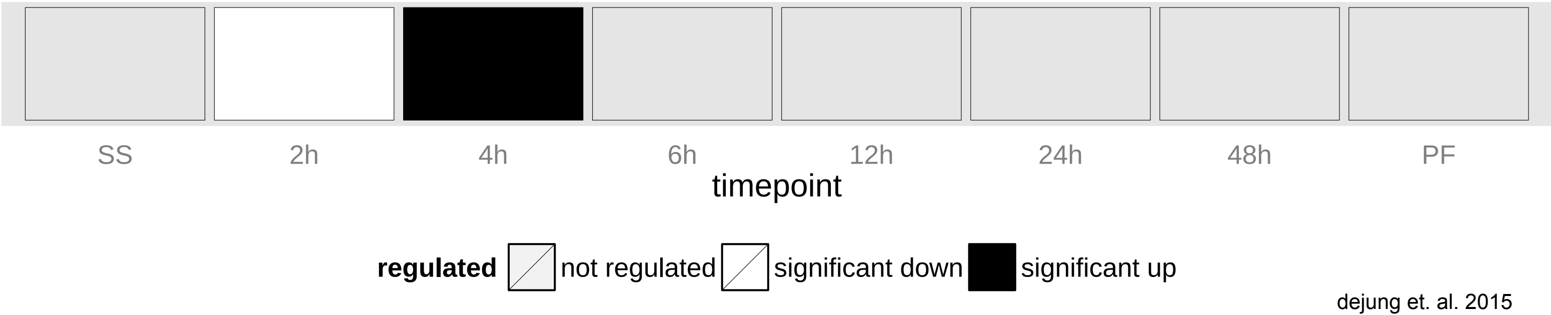
SS
2h
4h
6h
12h
24h
48h
PF
timepoint
regulated
not regulated
significant down
significant up
dejung et. al. 2015

Golgi reassembly stacking protein (GRASP homologue), putative
Tb927.11.2660
AGOF: null
AGOC: Golgi stack
AGOP: Golgi organization
PGOF: protein binding
PGOC: null
PGOP: null

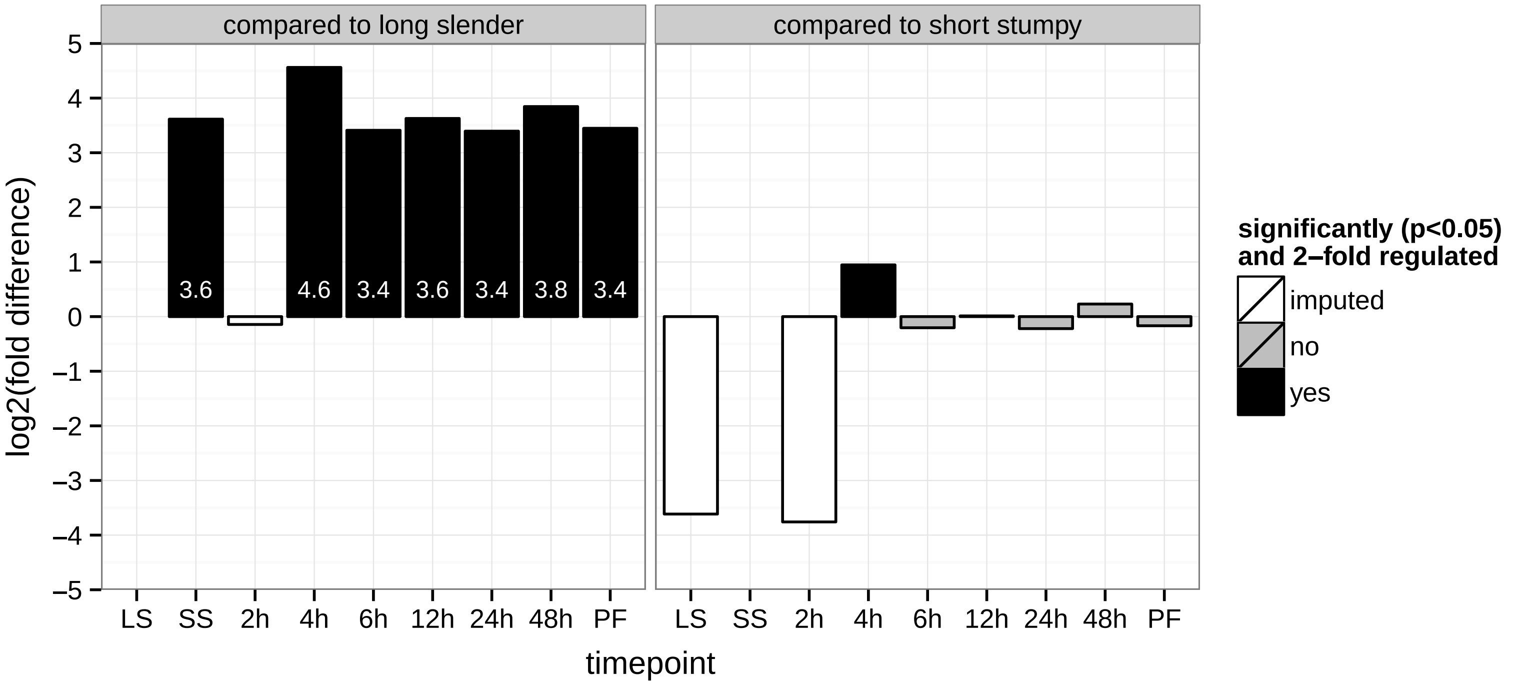

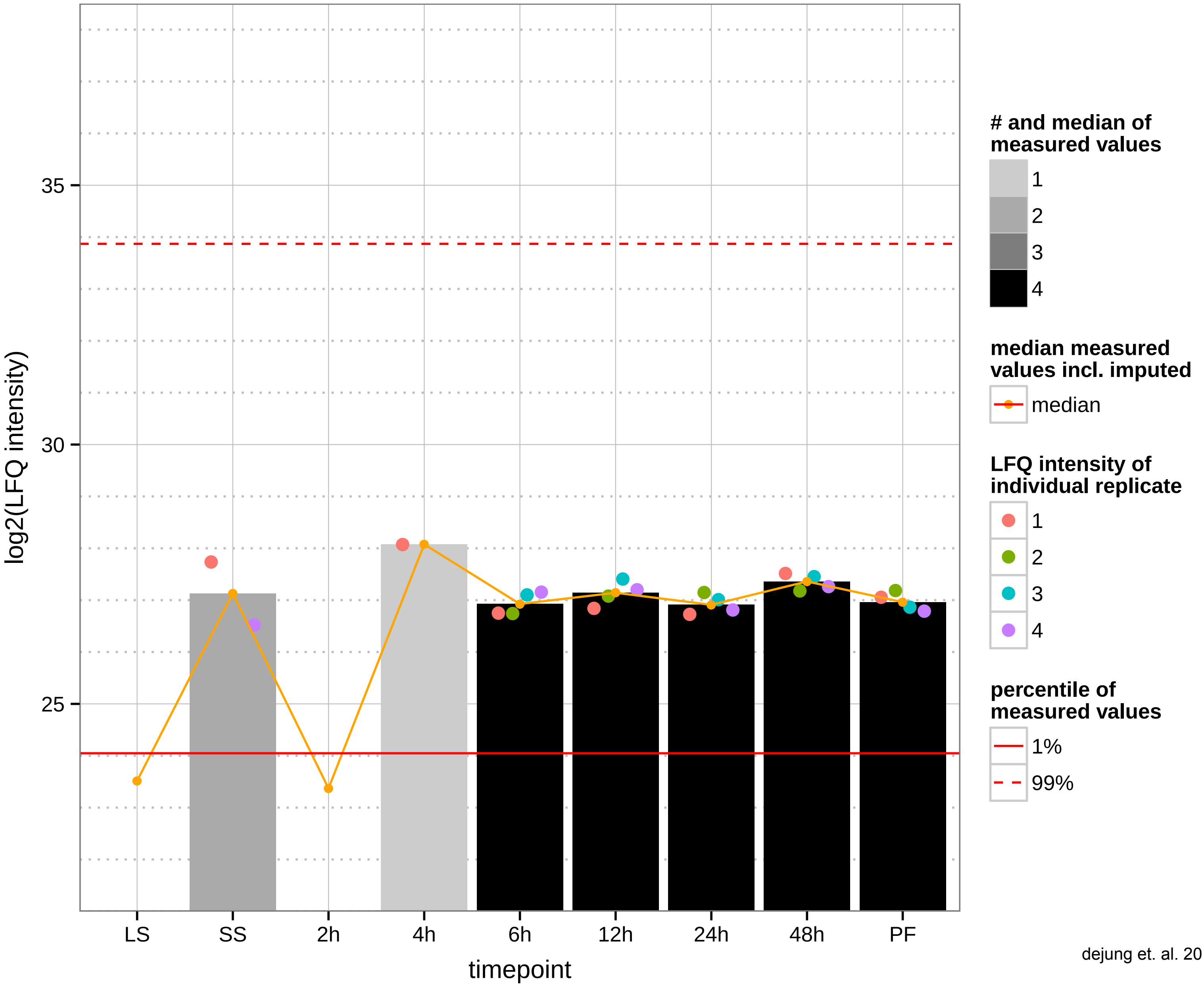

dejung et. al. 2015

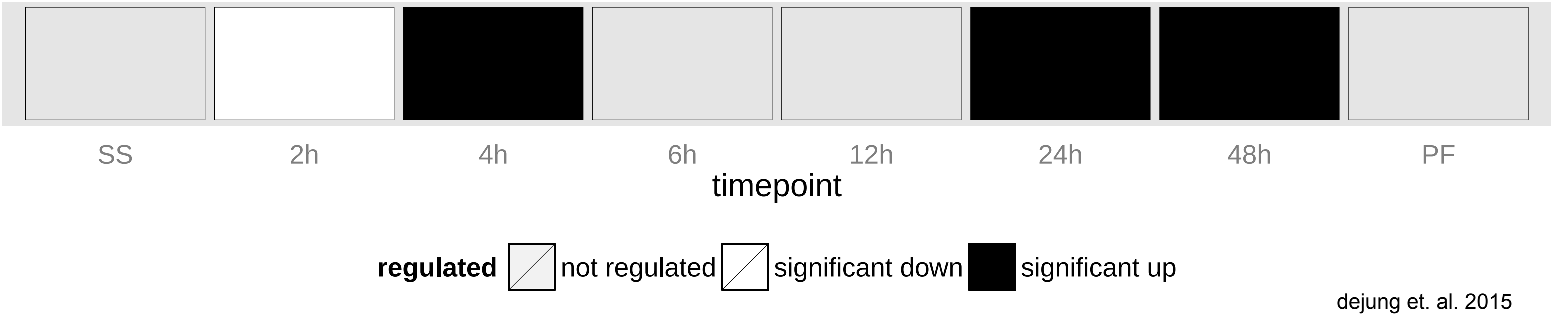
SS
2h
4h
6h
12h
24h
48h
PF
timepoint
regulated
not regulated
significant down
significant up
dejung et. al. 2015

hypothetical protein, conserved
Tb927.3.3110
AGOF: null
AGOC: null
AGOP: null
PGOF: binding
PGOC: null
PGOP: null

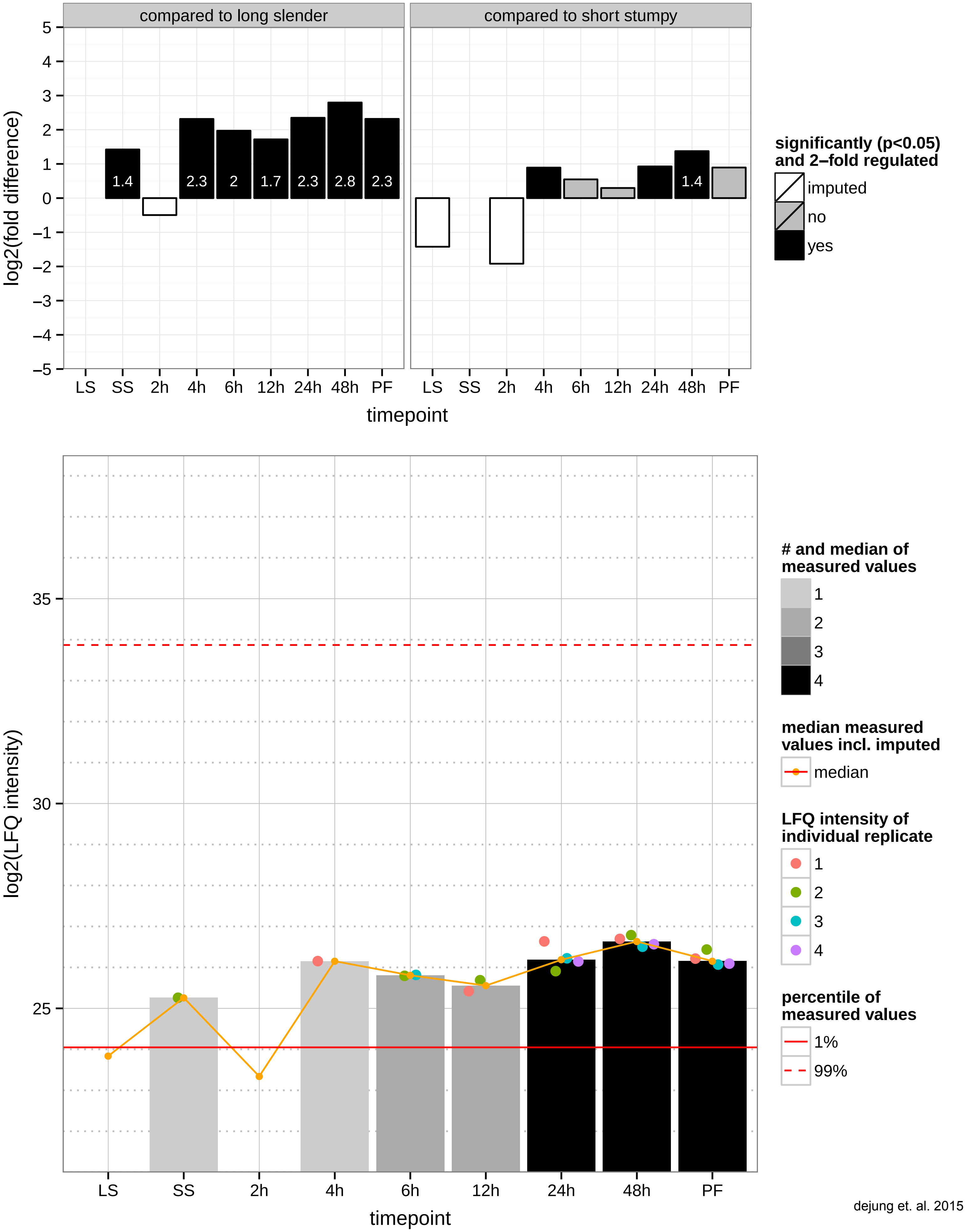

dejung et. al. 2015

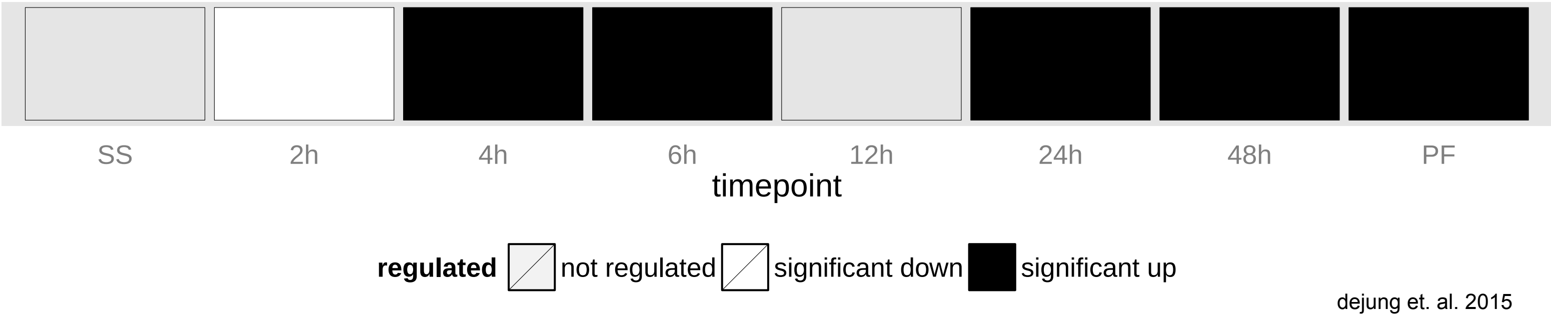
SS
2h
4h
6h
12h
24h
48h
PF
timepoint
regulated
not regulated
significant down
significant up
dejung et. al. 2015

hypothetical protein, conserved
Tb927.4.4370
AGOF: null
AGOC: null
AGOP: null
PGOF: null
PGOC: null
PGOP: null

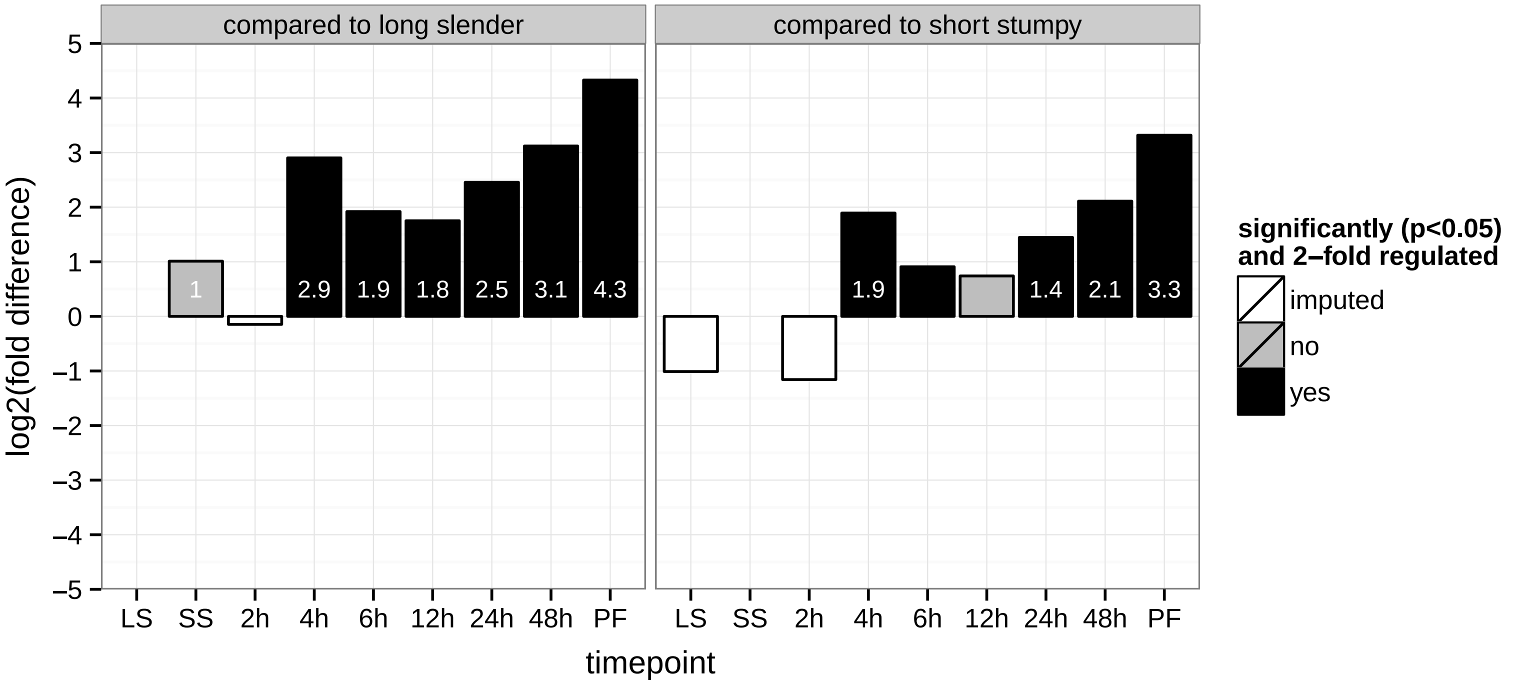

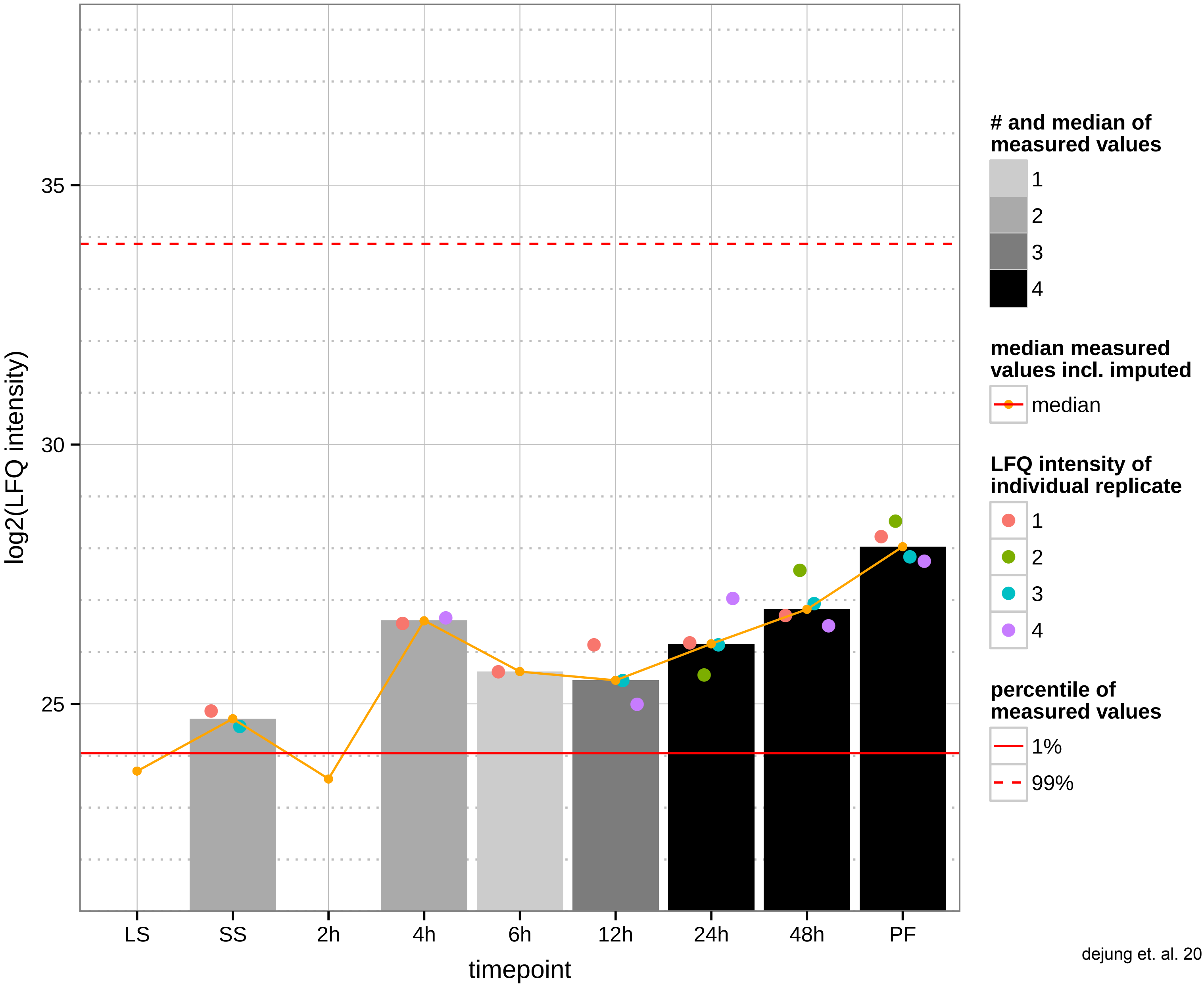

dejung et. al. 2015

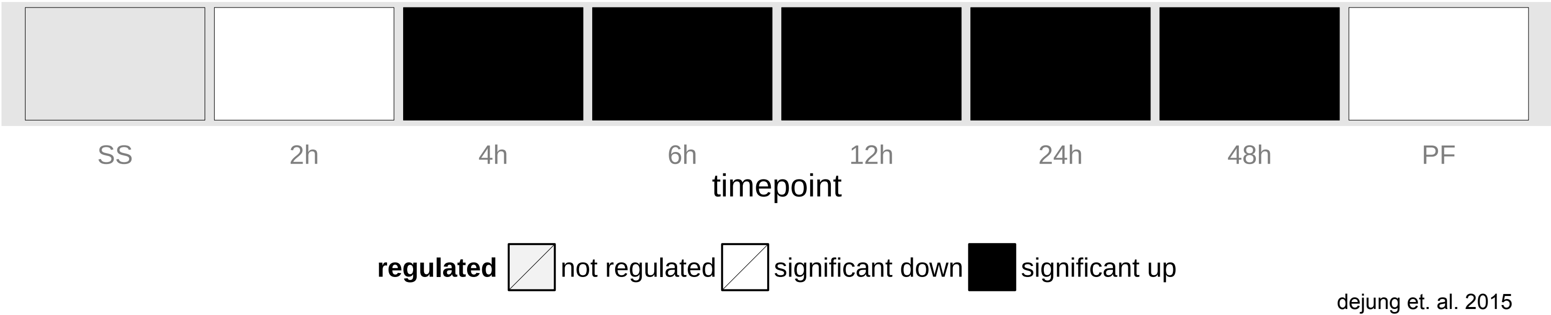
SS
2h
4h
6h
12h
24h
48h
PF
timepoint
regulated
not regulated
significant down
significant up
dejung et. al. 2015

TFIIF–stimulated CTD phosphatase, putative
Tb927.9.6100
AGOF: null
AGOC: mitochondrion
AGOP: null
PGOF: protein binding
PGOC: null
PGOP: null

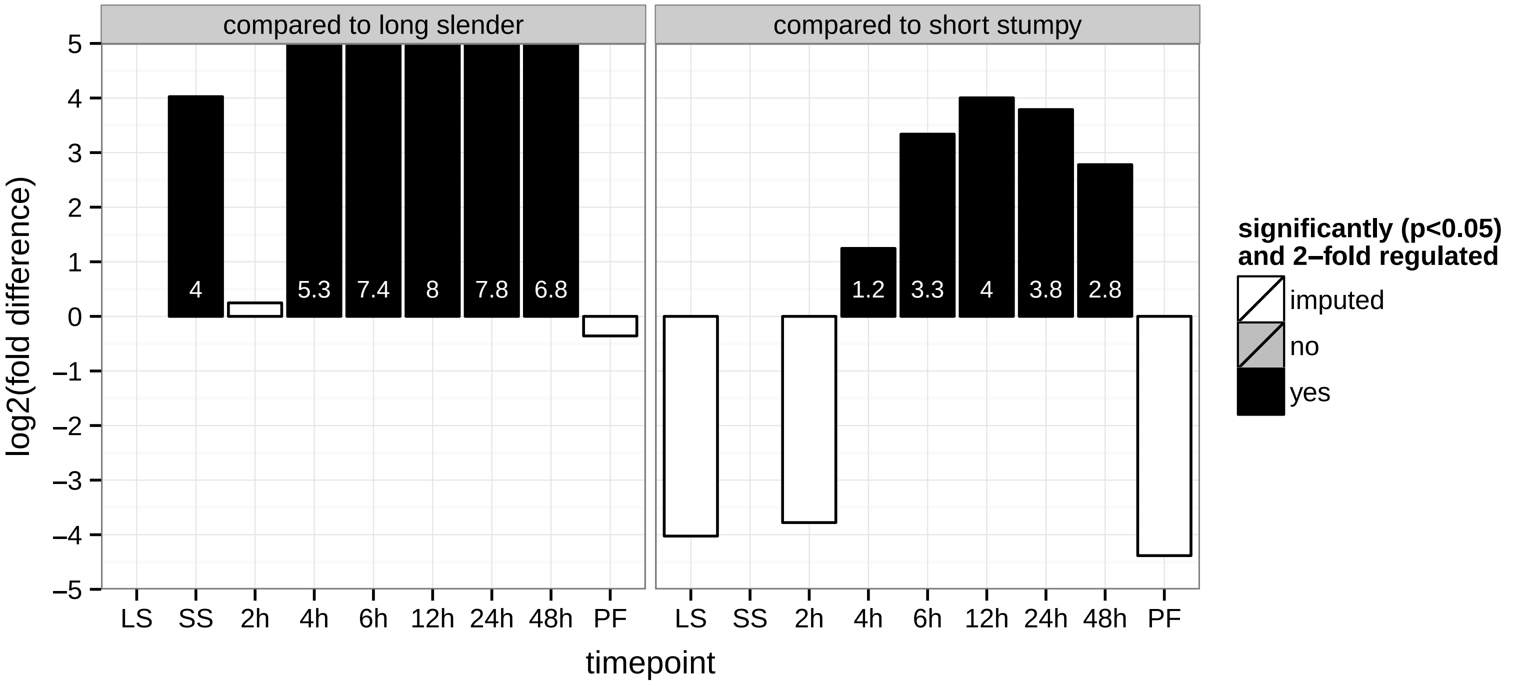

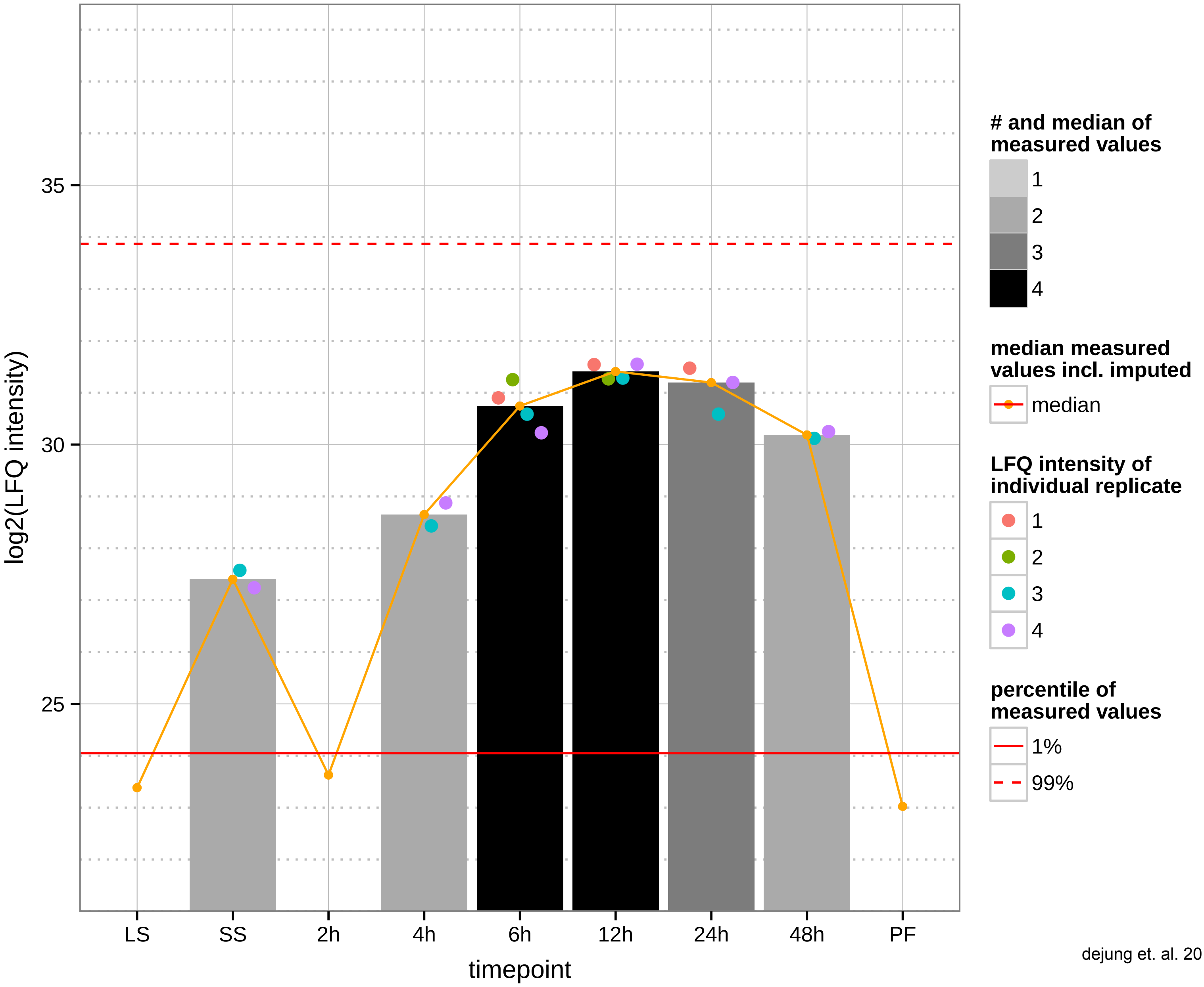

dejung et. al. 2015

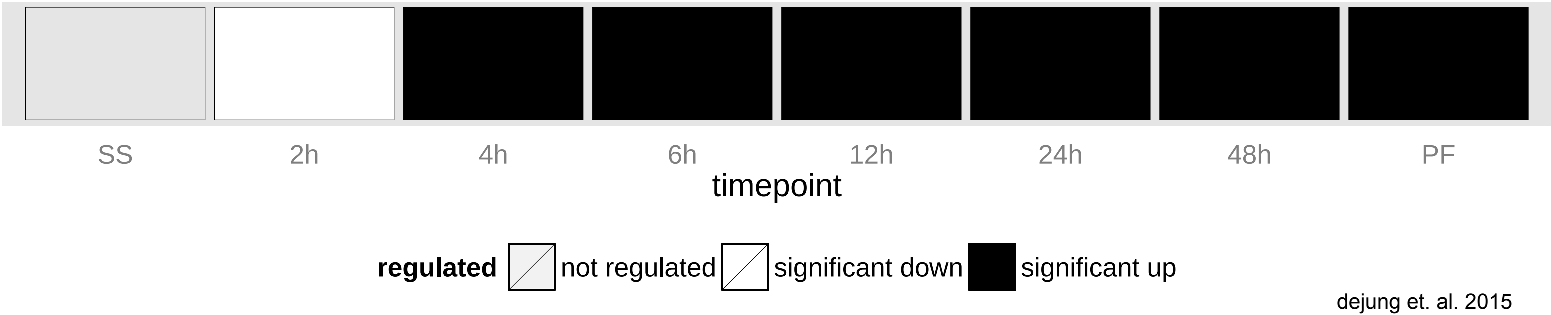
SS
2h
4h
6h
12h
24h
48h
PF
timepoint
regulated
not regulated
significant down
significant up
dejung et. al. 2015

gamma−glutamylcysteine synthetase (GCS)
Tb927.10.12370
AGOF: glutamate−cysteine ligase activity
AGOC: null
AGOP: glutathione biosynthetic process, response to reactive oxygen species, trypanothione biosynthetic process
PGOF: glutamate−cysteine ligase activity
PGOC: null
PGOP: glutathione biosynthetic process

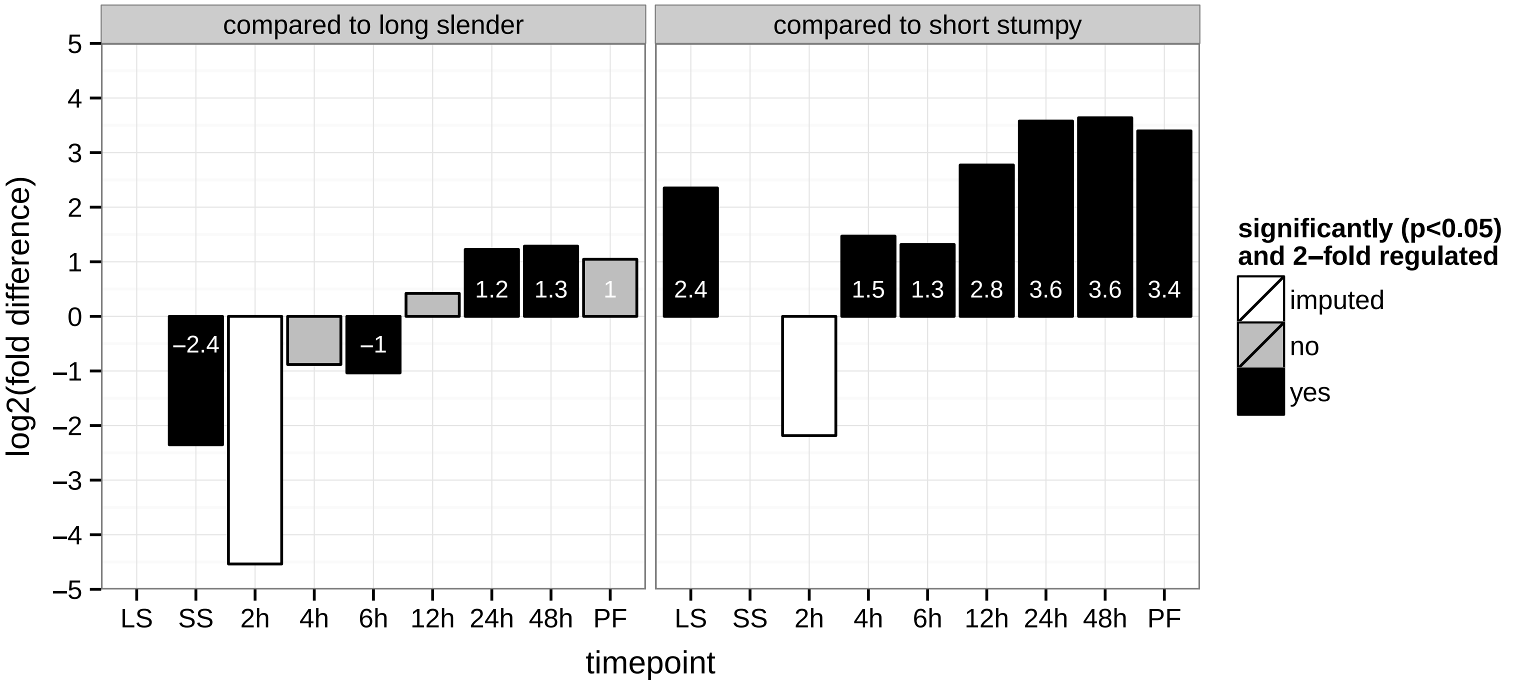

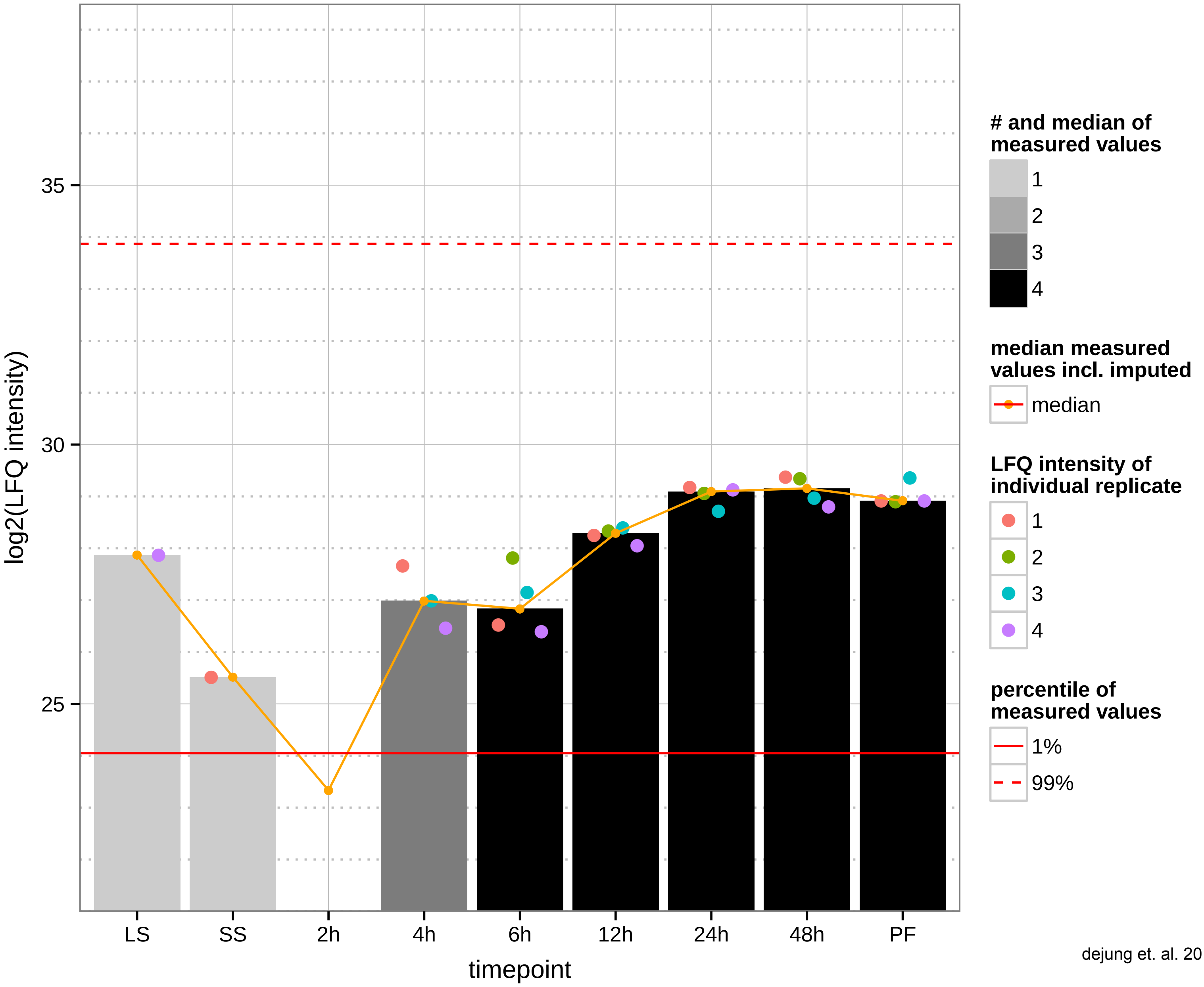

dejung et. al. 2015

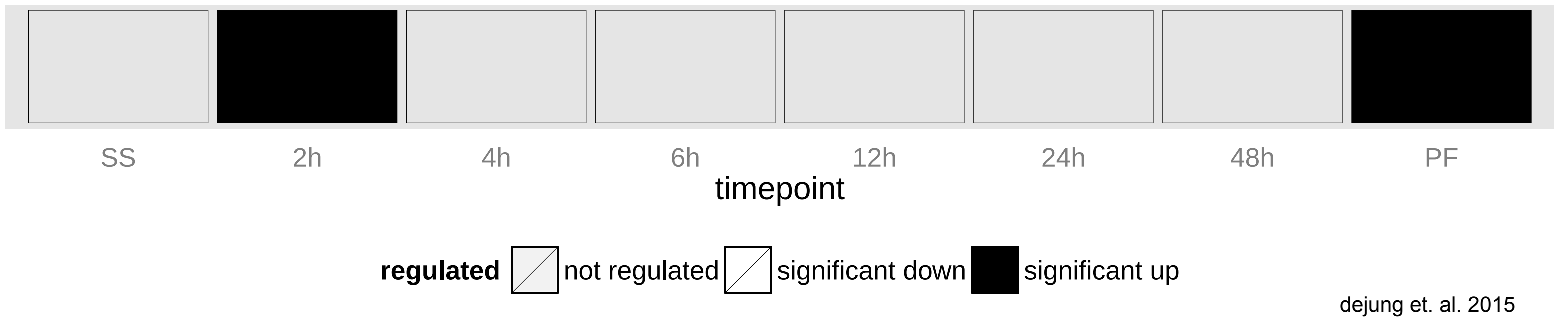
SS
2h
4h
6h
12h
24h
48h
PF
timepoint
regulated
not regulated
significant down
significant up
dejung et. al. 2015

hypothetical protein, conserved
Tb927.3.3500
AGOF: null
AGOC: null
AGOP: null
PGOF: null
PGOC: null
PGOP: null

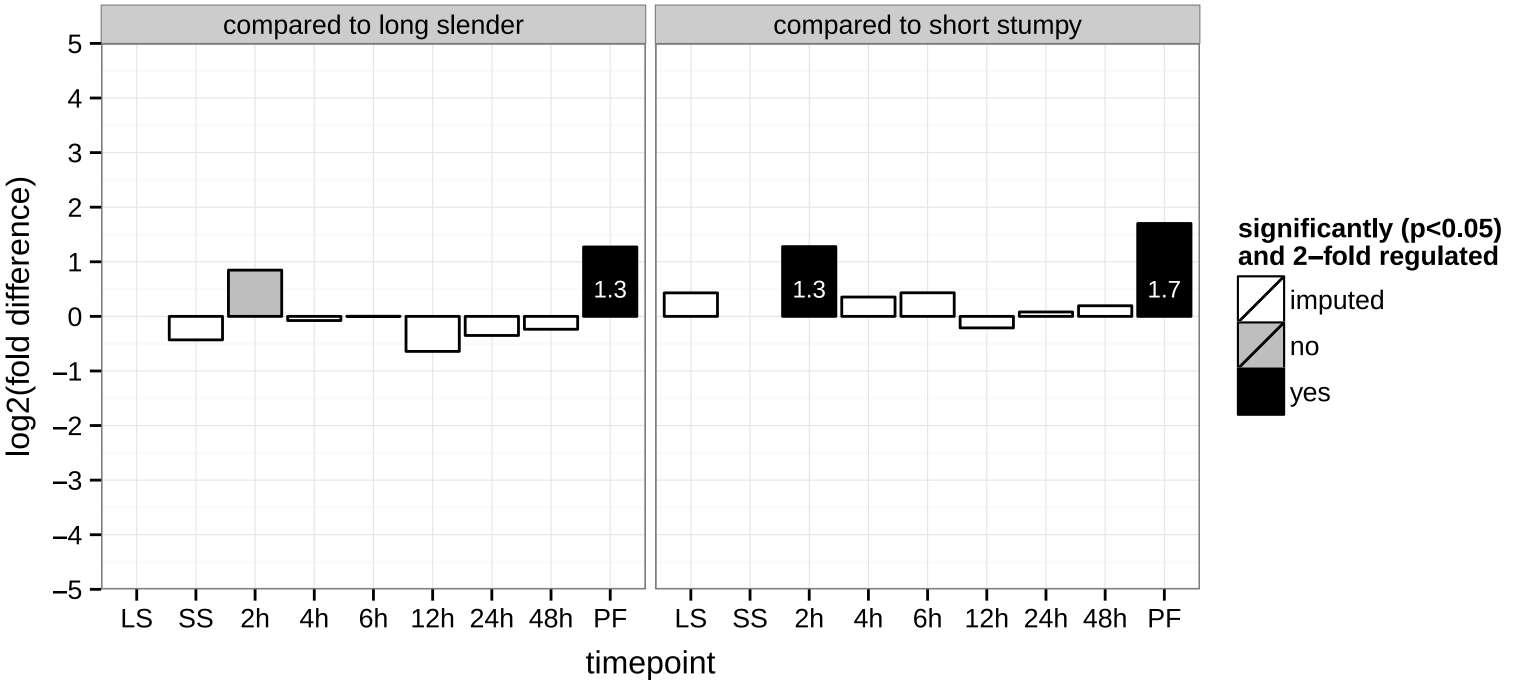

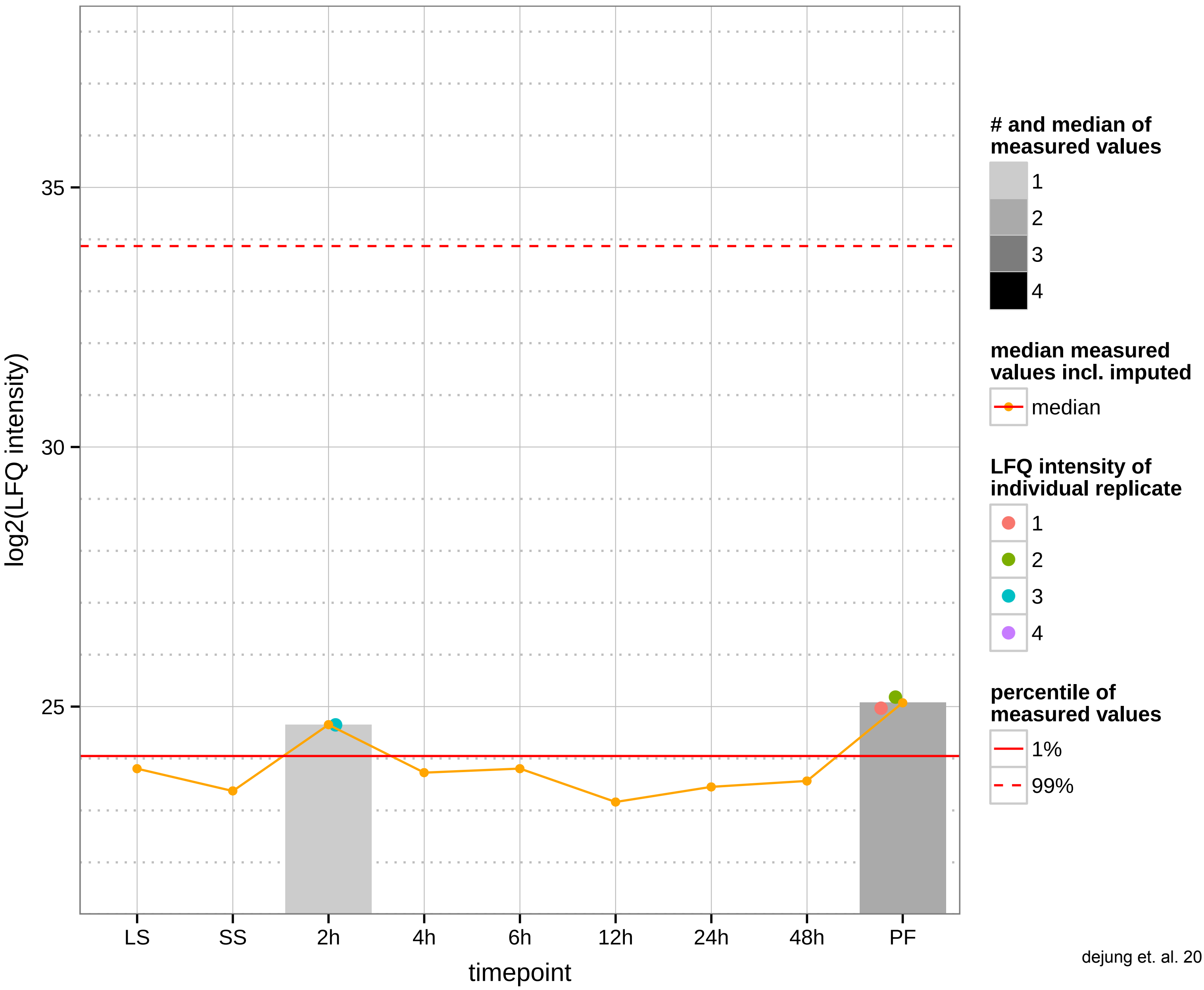

dejung et. al. 2015

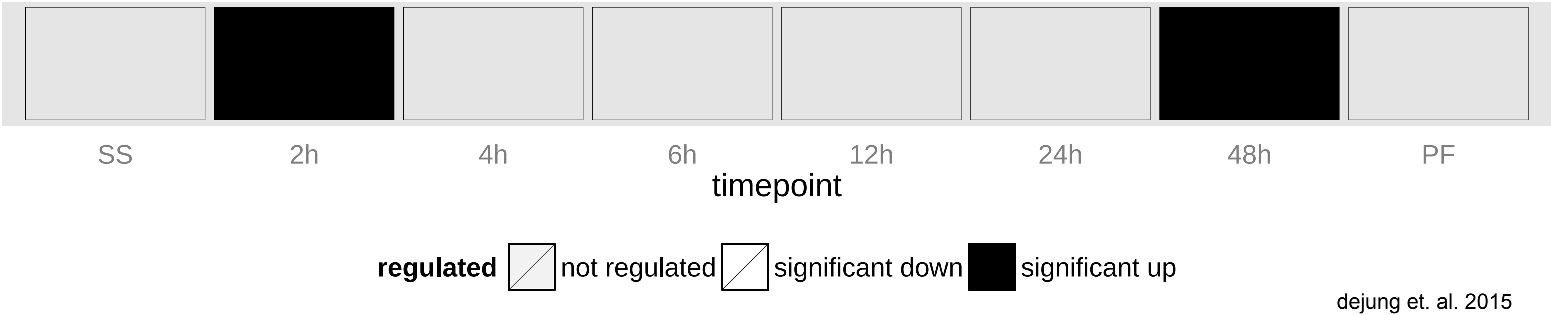

SS
2h
4h
6h
12h
24h
48h
PF
timepoint
regulated
not regulated
significant down
significant up
dejung et. al. 2015

hypothetical protein, conserved
Tb927.8.5170
AGOF: null
AGOC: null
AGOP: null
PGOF: null
PGOC: null
PGOP: null

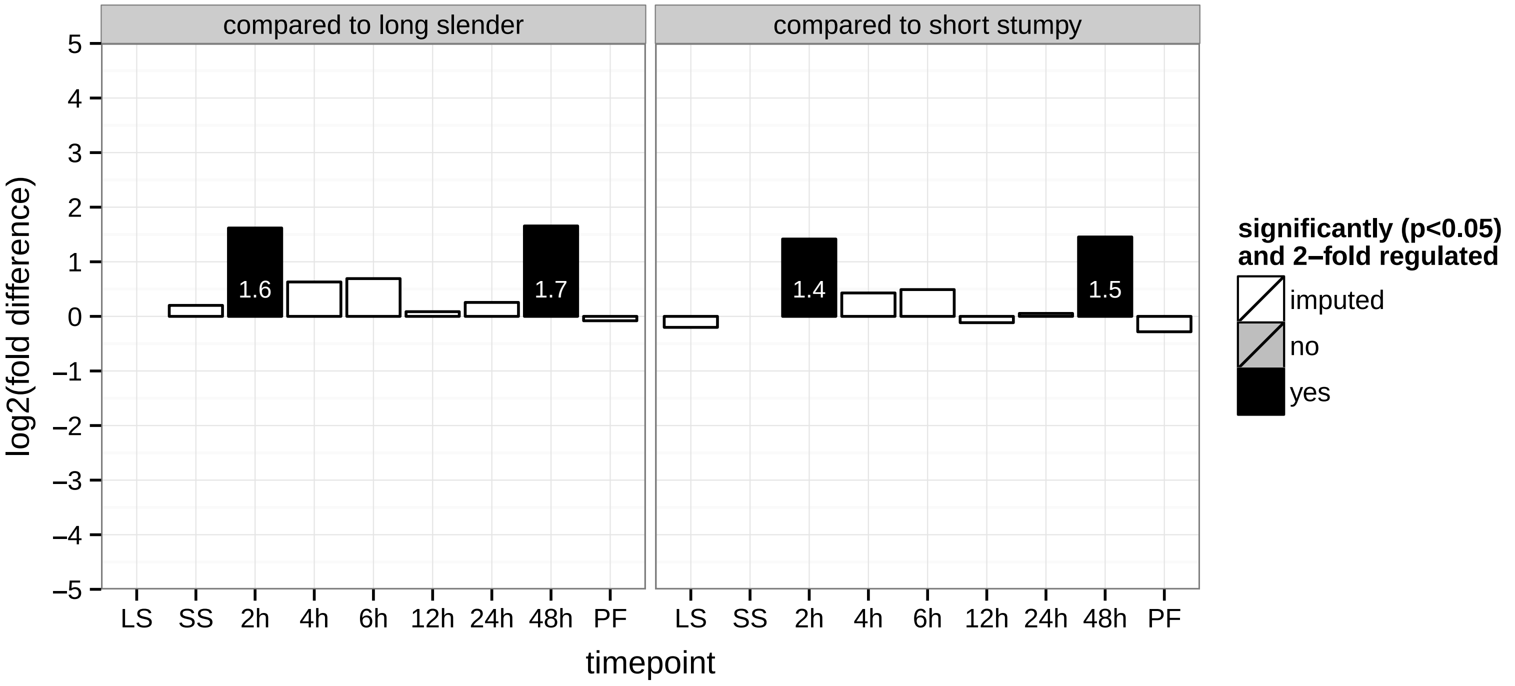

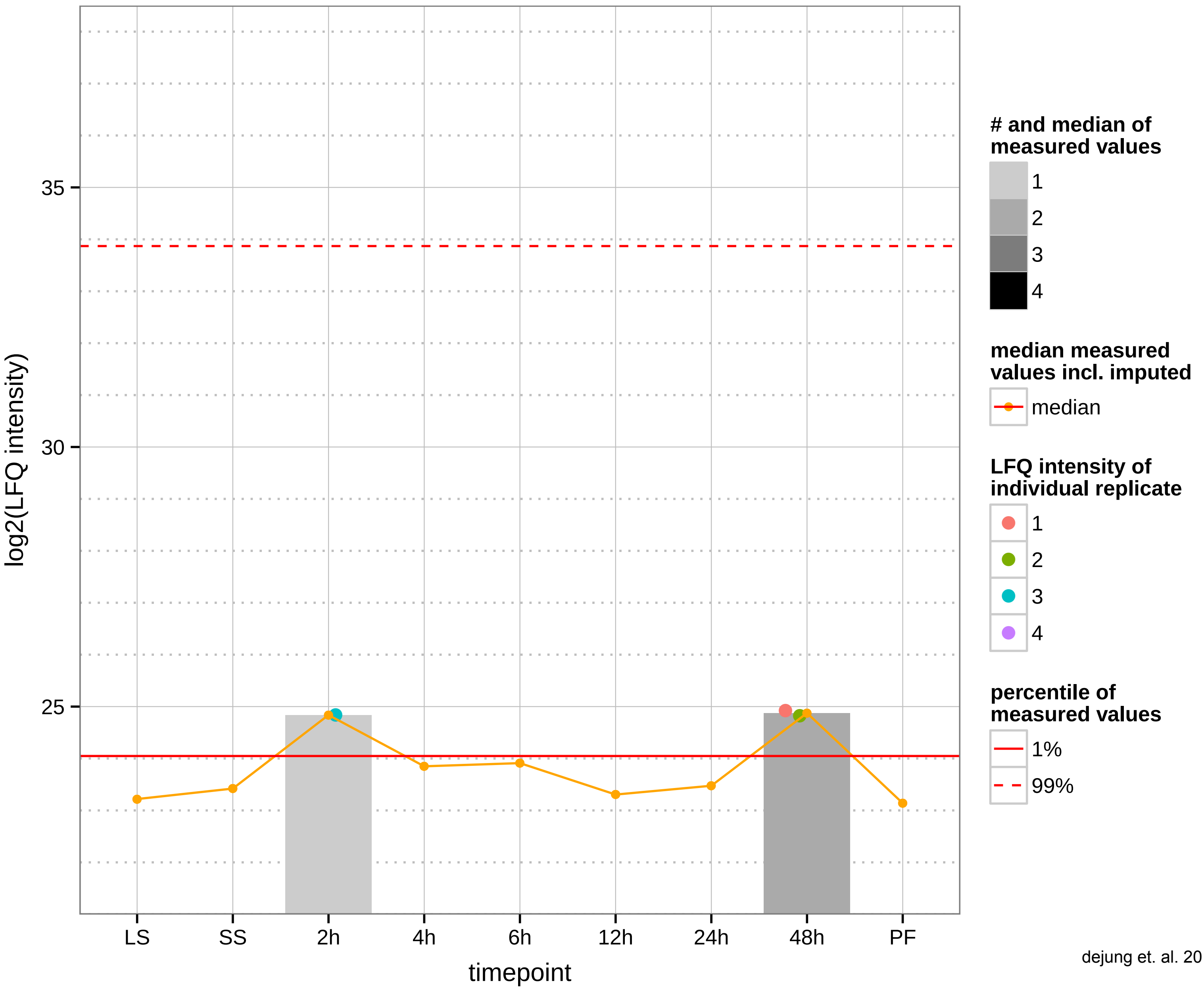

dejung et. al. 2015

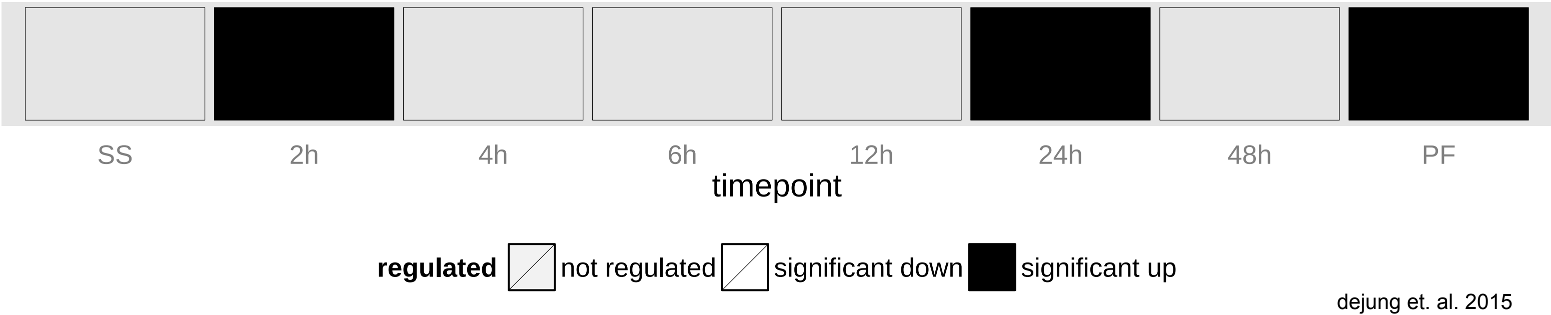
SS
2h
4h
6h
12h
24h
48h
PF
timepoint
regulated
not regulated
significant down
significant up
dejung et. al. 2015

cyclosome subunit 1, putative
Tb927.6.3280
AGOF: null
AGOC: null
AGOP: null
PGOF: null
PGOC: null
PGOP: null

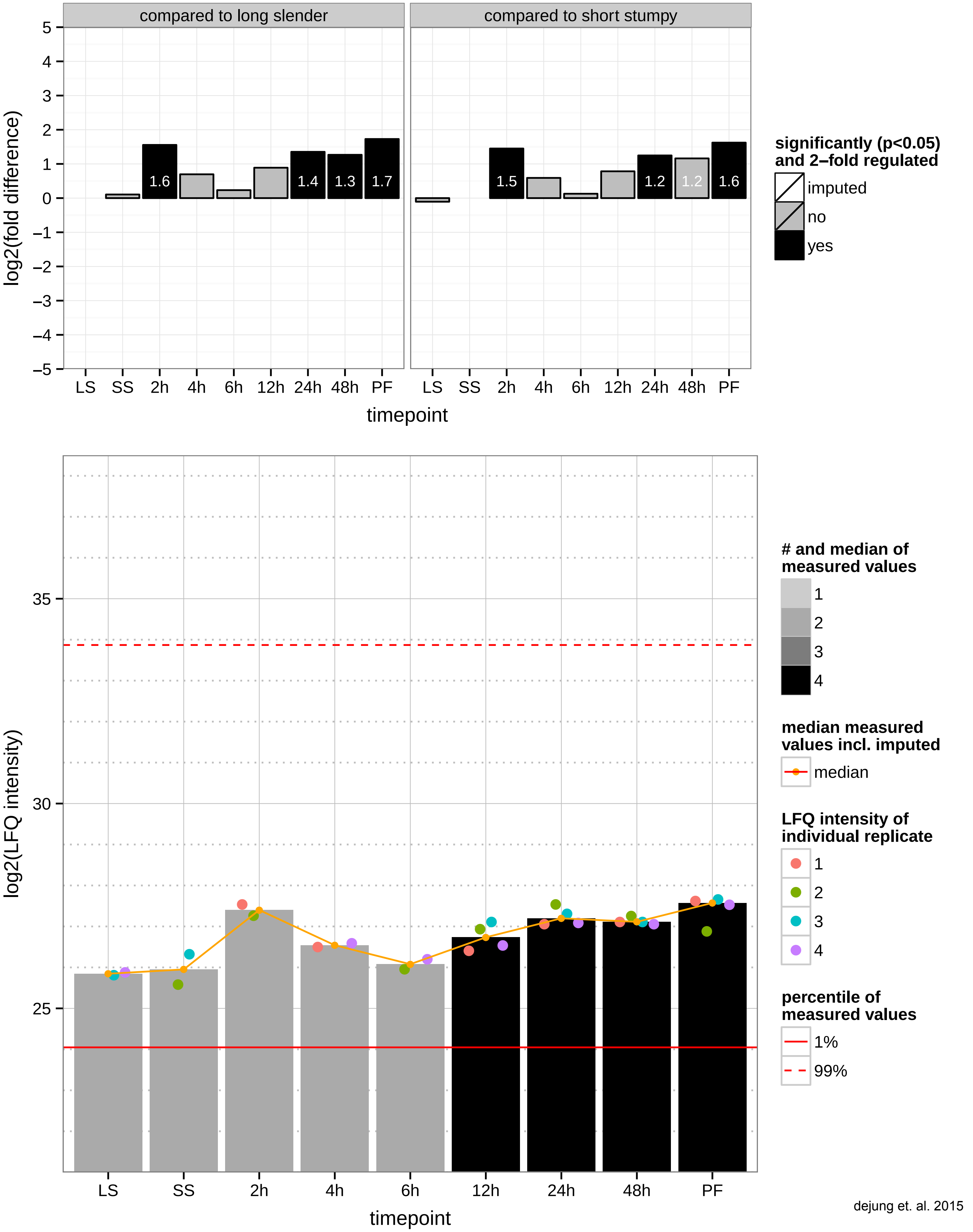

dejung et. al. 2015

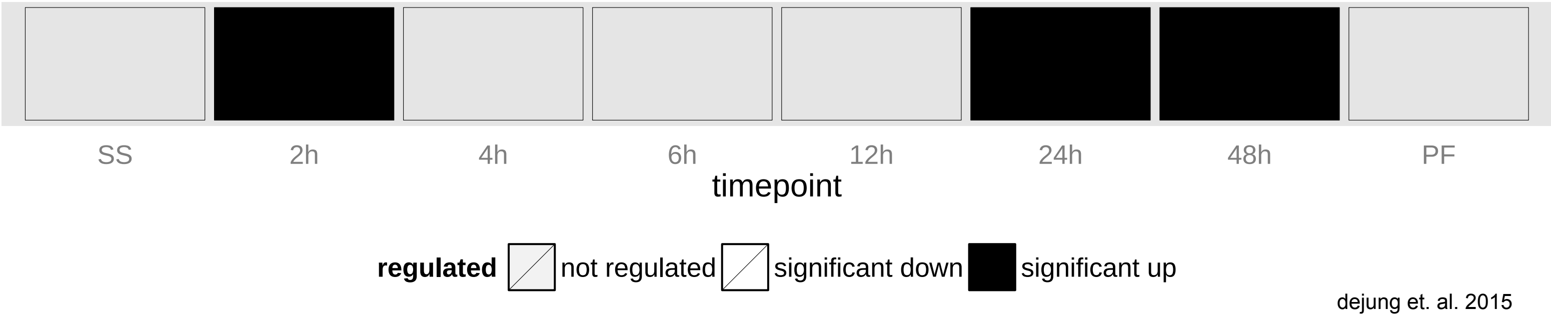
SS
2h
4h
6h
12h
24h
48h
PF
timepoint
regulated
not regulated
significant down
significant up
dejung et. al. 2015

peptide methionine sulfoxide reductase, putative, peptide Met(O) reductase, methionine−S−sulfoxide reductase
Tb927.8.550
AGOF: peptide−methionine−(S)−S−oxide reductase activity
AGOC: null
AGOP: cellular protein modification process, oxidation−reduction process
PGOF: oxidoreductase activity, acting on a sulfur group of donors, disulfide as acceptor
PGOC: null
PGOP: oxidation−reduction process, protein metabolic process

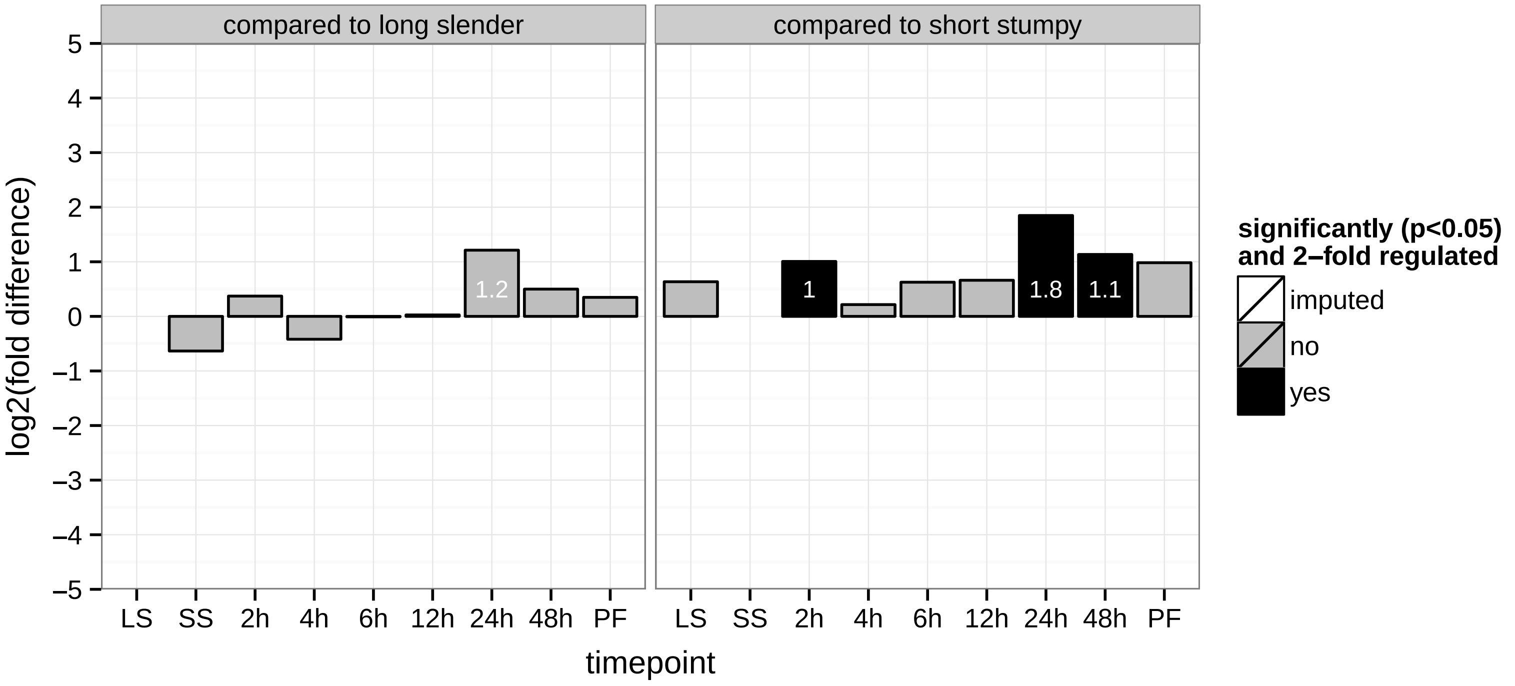

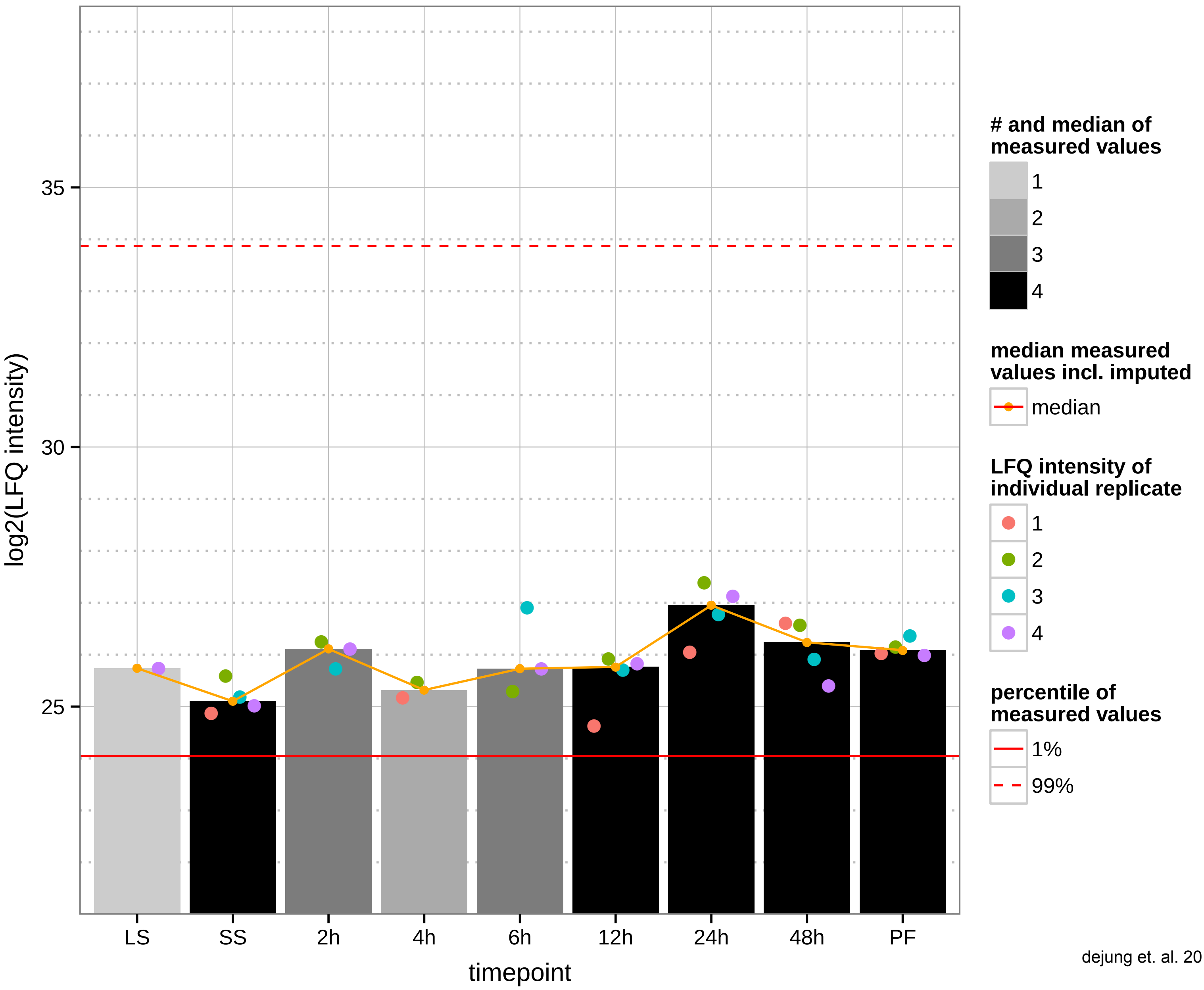

dejung et. al. 2015

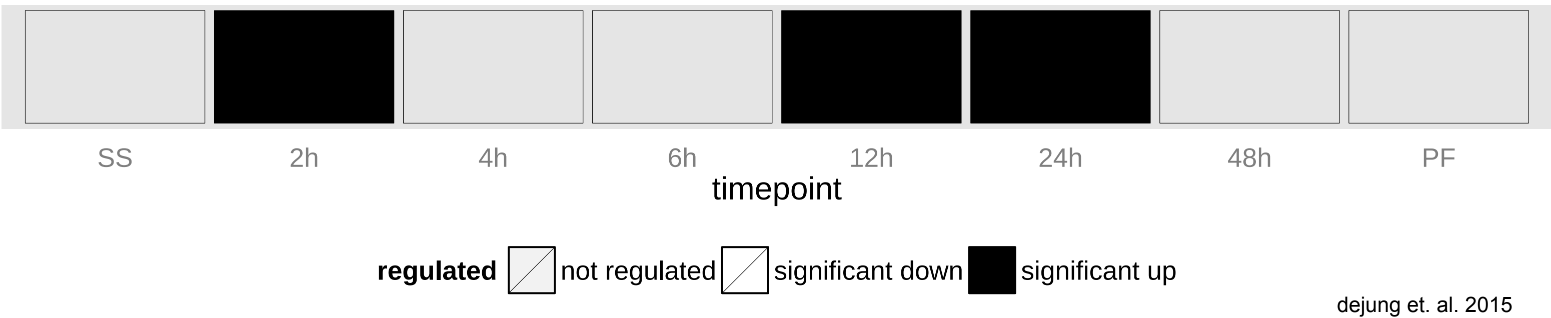
SS
2h
4h
6h
12h
24h
48h
PF
timepoint
regulated
not regulated
significant down
significant up
dejung et. al. 2015

kinesin, putative
Tb927.10.12490
AGOF: ATP binding, microtubule motor activity, motor activity
AGOC: microtubule associated complex
AGOP: microtubule−based movement
PGOF: ATP binding, microtubule motor activity
PGOC: null
PGOP: microtubule−based movement

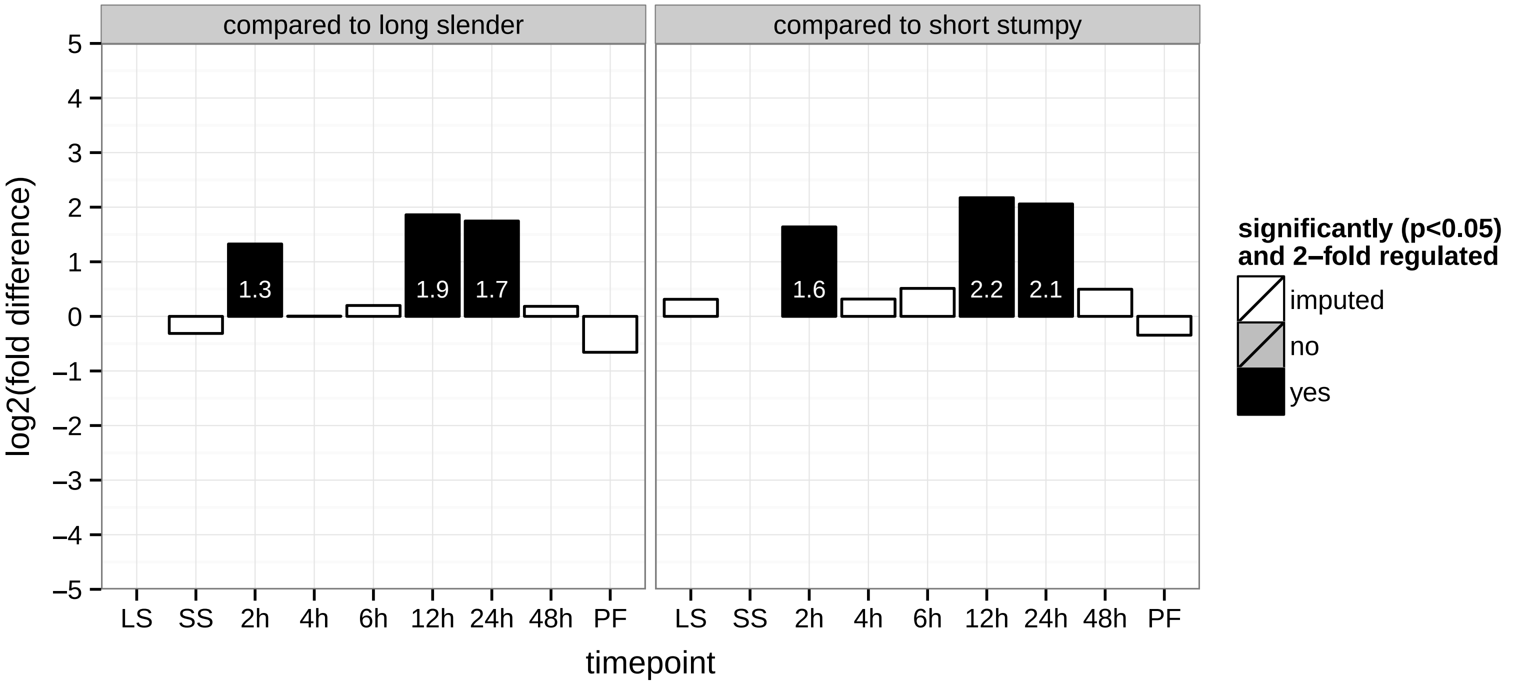

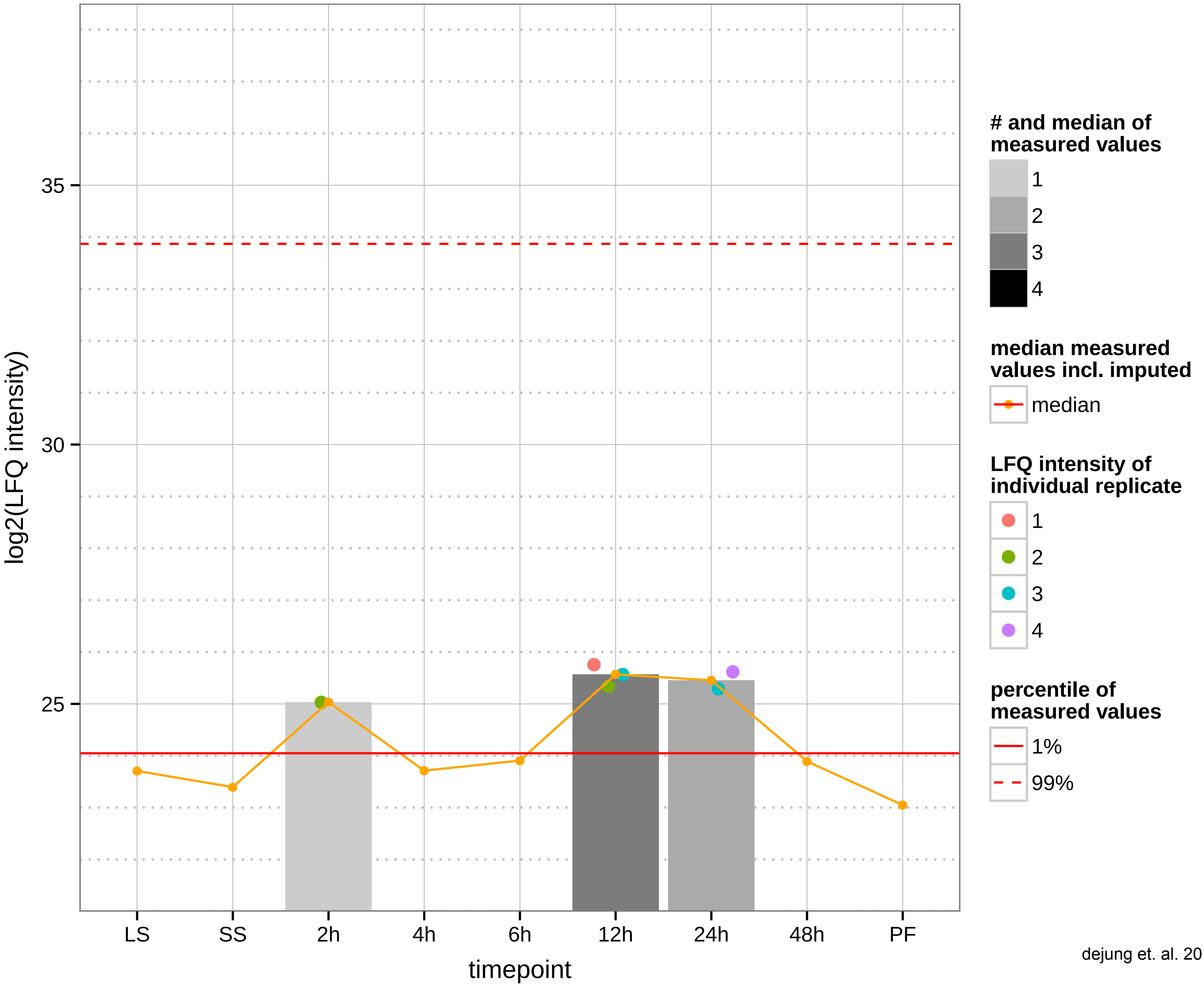

dejung et. al. 2015

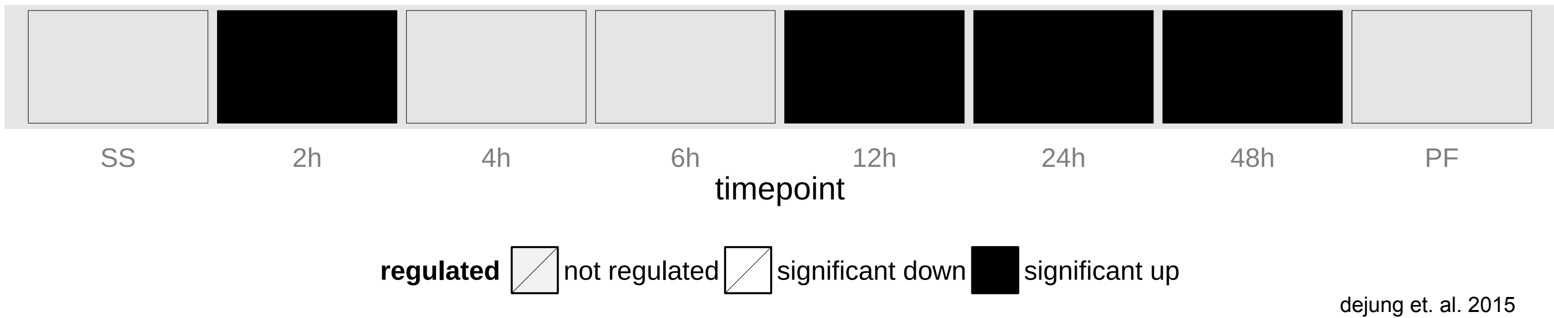
SS
2h
4h
6h
12h
24h
48h
PF
timepoint
regulated
not regulated
significant down
significant up
dejung et. al. 2015

hypothetical protein, conserved
Tb927.5.510
AGOF: null
AGOC: mitochondrial membrane, mitochondrion
AGOP: null
PGOF: null
PGOC: null
PGOP: null

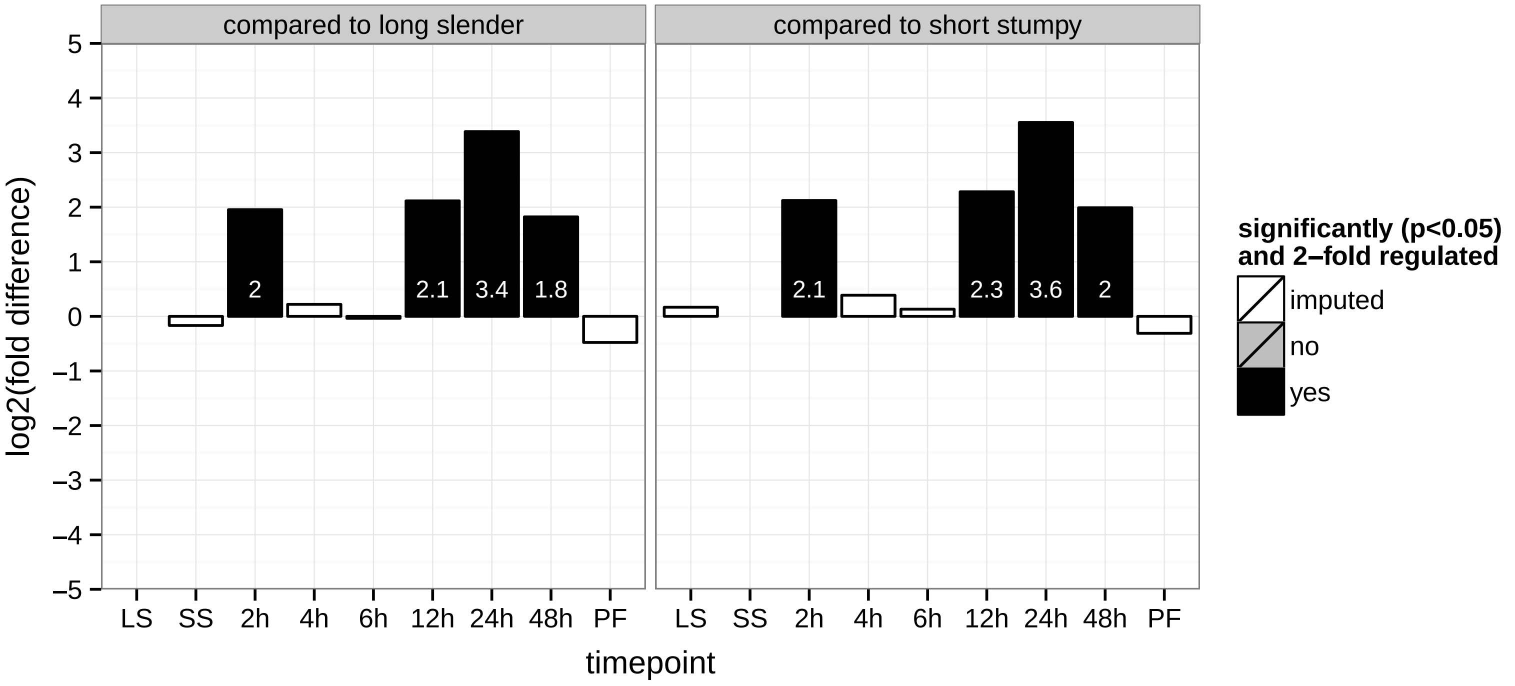

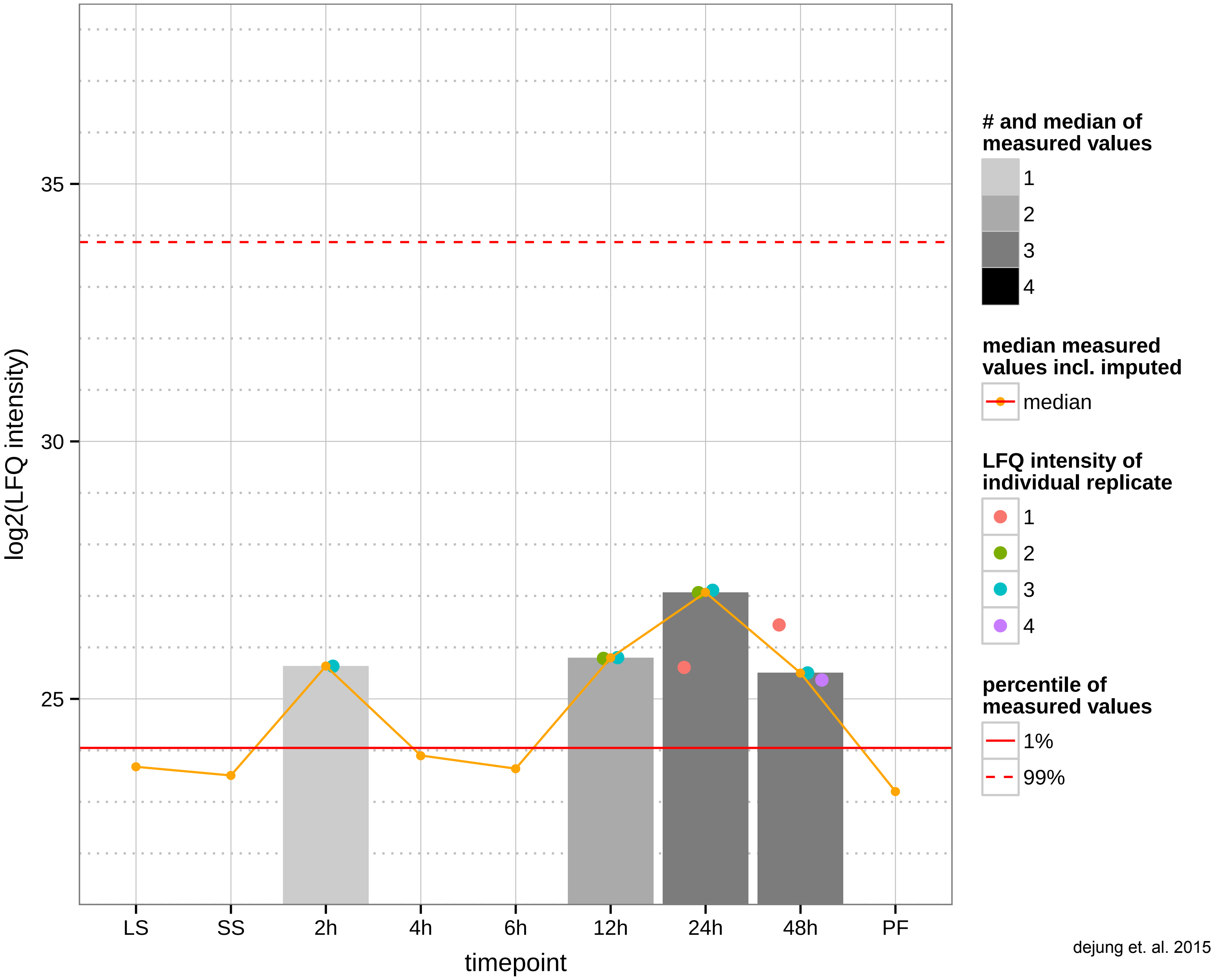

dejung et. al. 2015

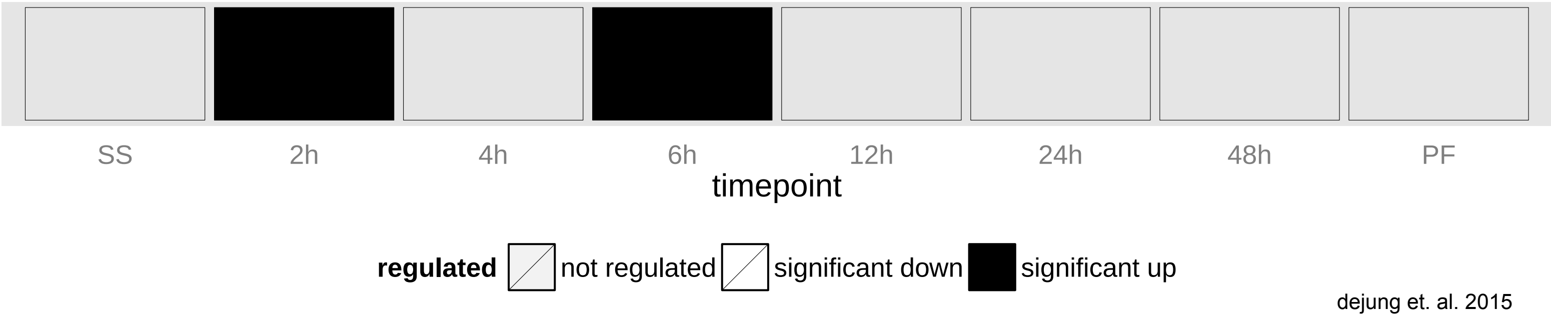
SS
2h
4h
6h
12h
24h
48h
PF
timepoint
regulated
not regulated
significant down
significant up
dejung et. al. 2015

65 kDa invariant surface glycoprotein
Tb927.2.3310;Tb927.2.3300;Tb927.2.3290;Tb927.2.3280
AGOF: null
AGOC: plasma membrane
AGOP: plasma membrane organization
PGOF: null
PGOC: null
PGOP: null

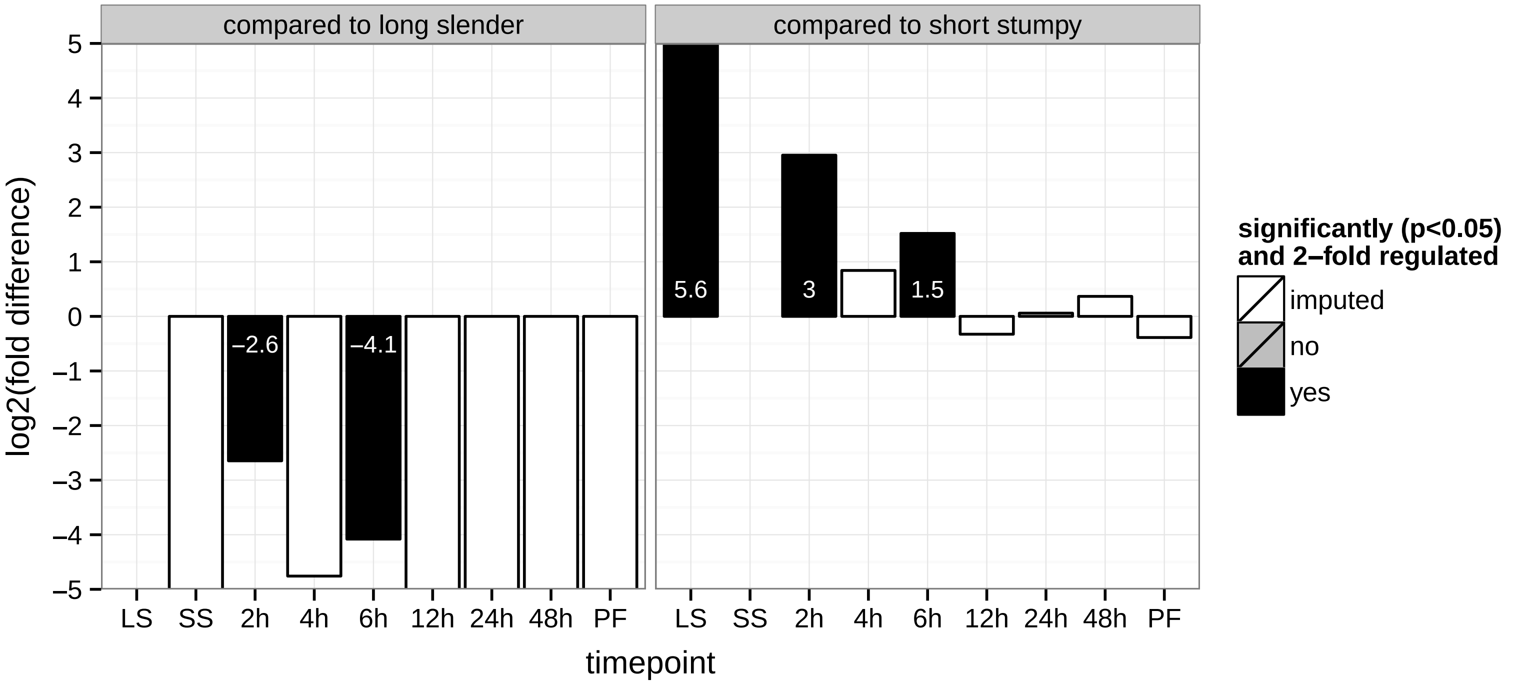

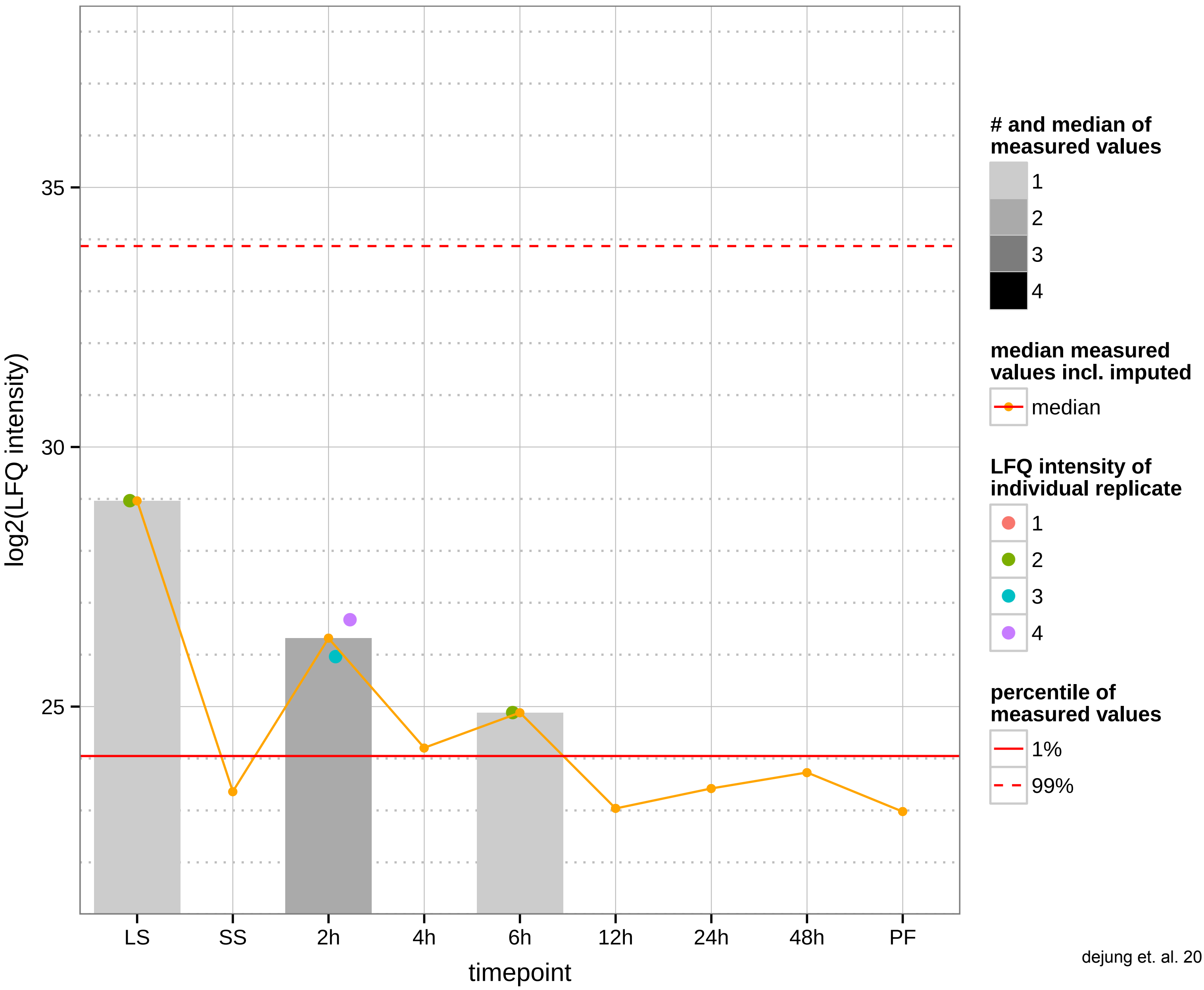

dejung et. al. 2015

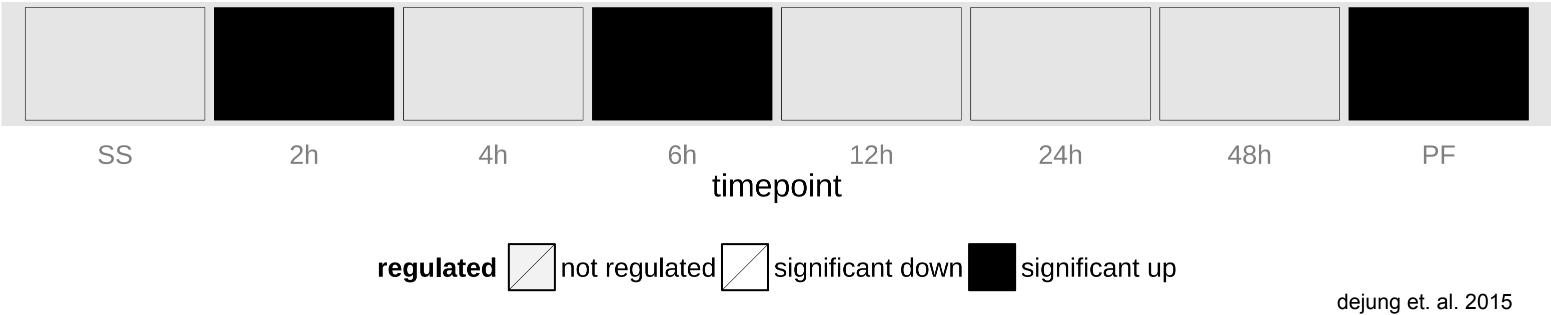

SS
2h
4h
6h
12h
24h
48h
PF
timepoint
regulated
not regulated
significant down
significant up
dejung et. al. 2015

hypothetical protein, conserved
Tb927.8.5280
AGOF: null
AGOC: mitochondrion
AGOP: null
PGOF: null
PGOC: null
PGOP: null

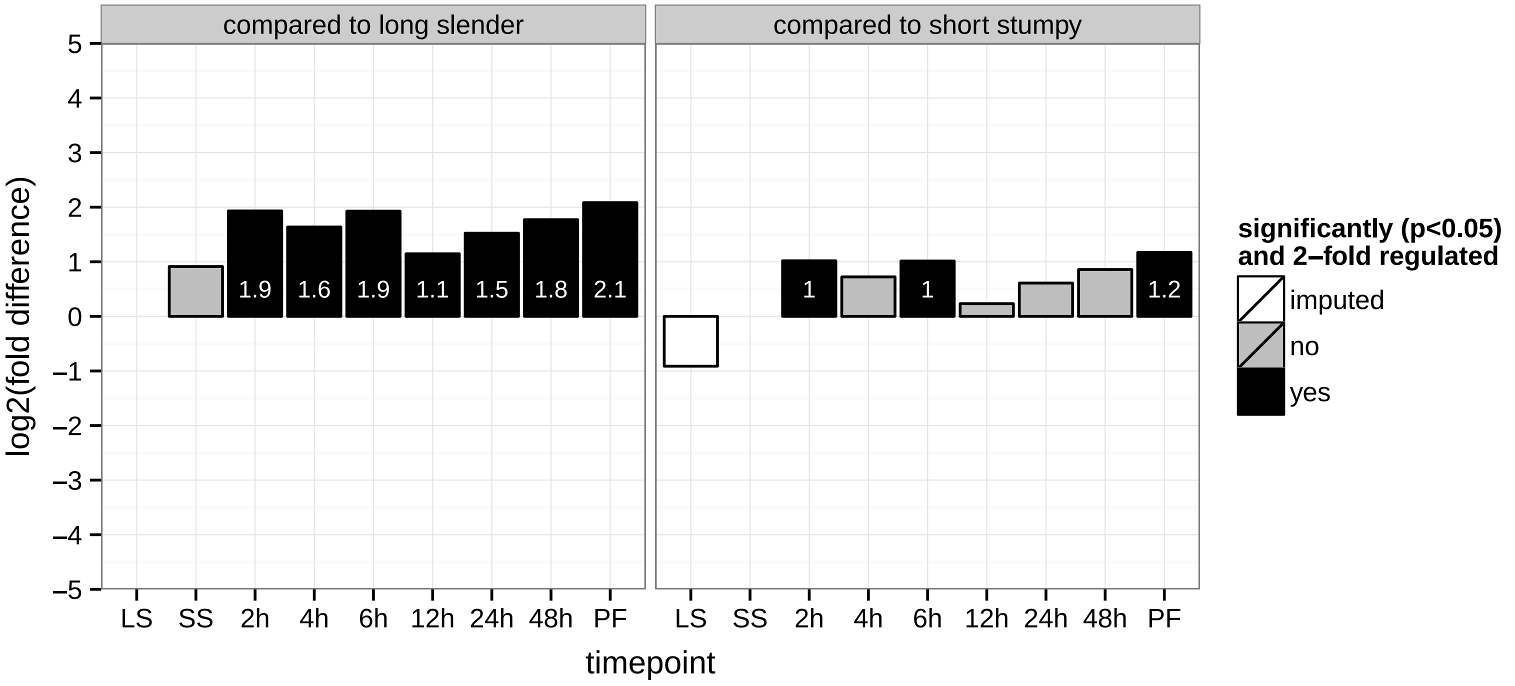

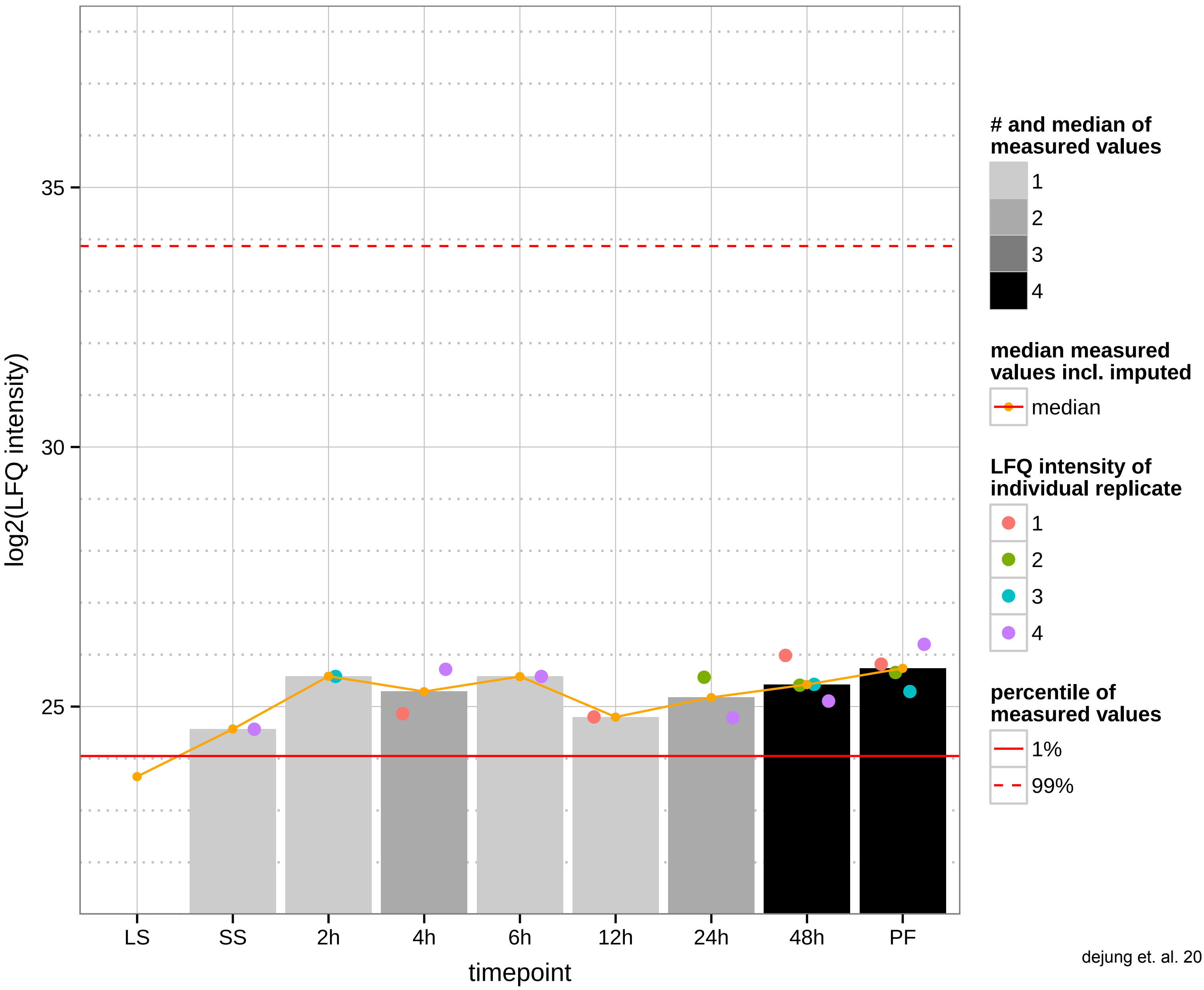

dejung et. al. 2015

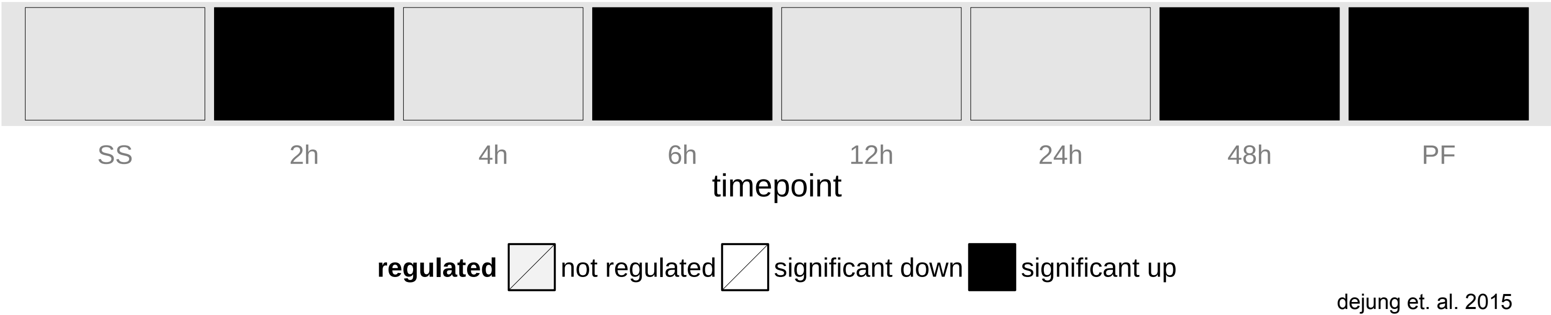
SS
2h
4h
6h
12h
24h
48h
PF
timepoint
regulated
not regulated
significant down
significant up
dejung et. al. 2015

RNA Interference Factor 5 (RIF5)
Tb927.10.10730
AGOF: null
AGOC: null
AGOP: null
PGOF: null
PGOC: null
PGOP: null

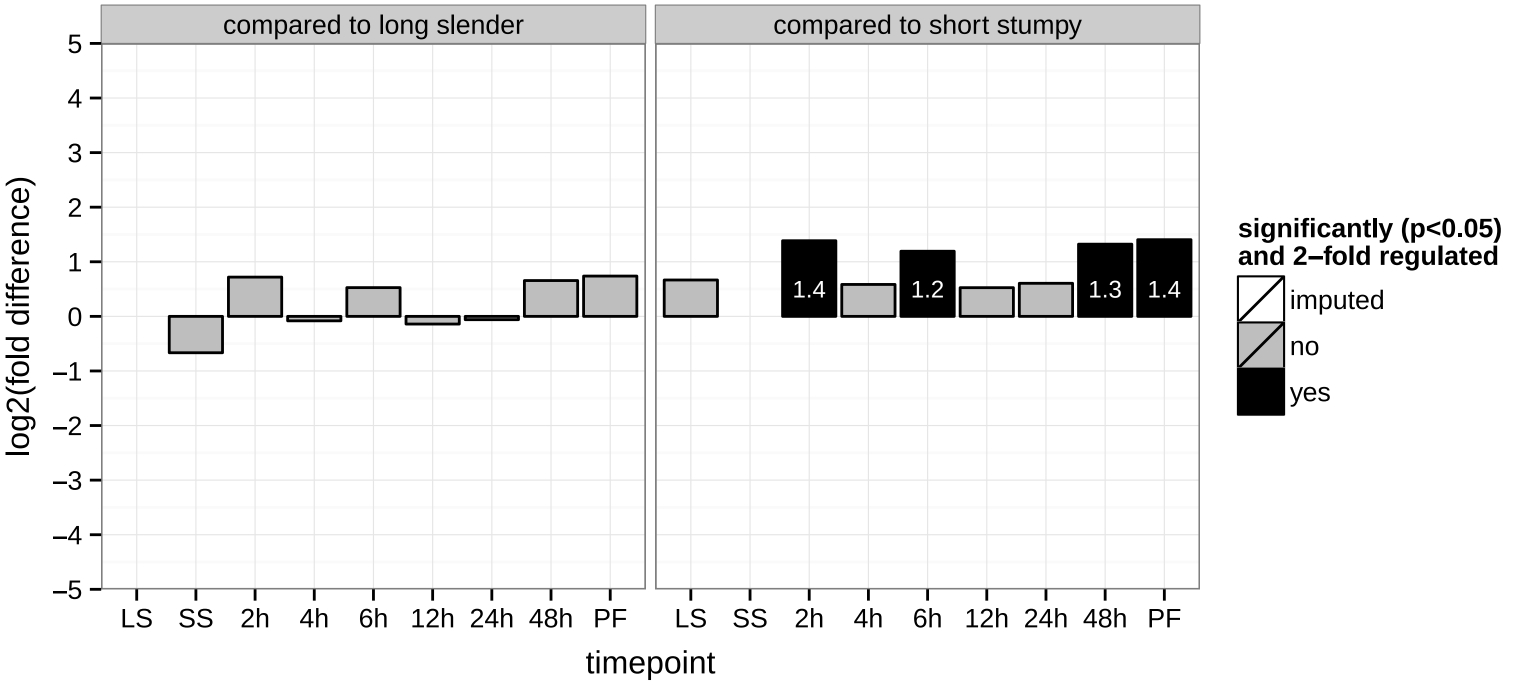

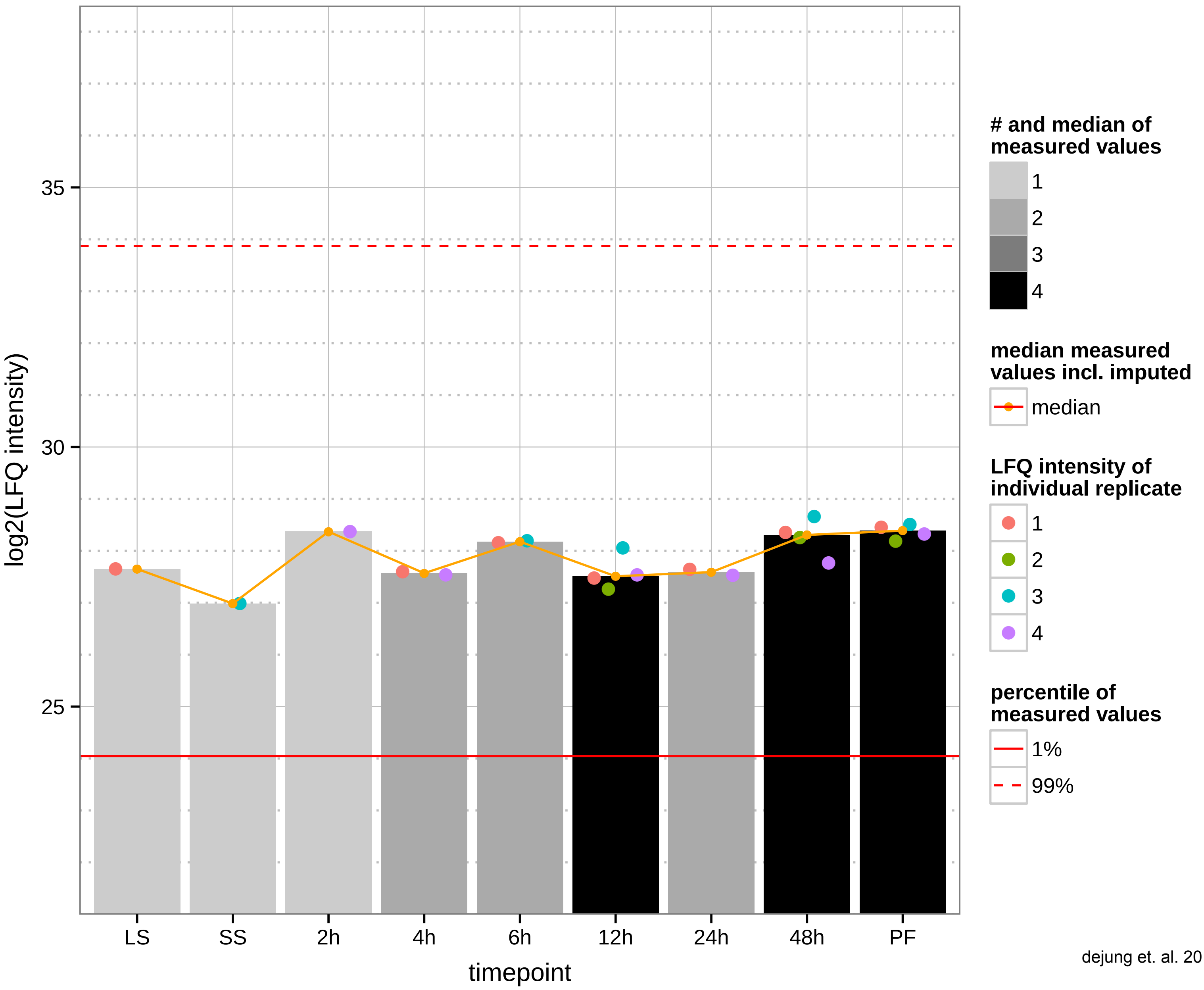

dejung et. al. 2015

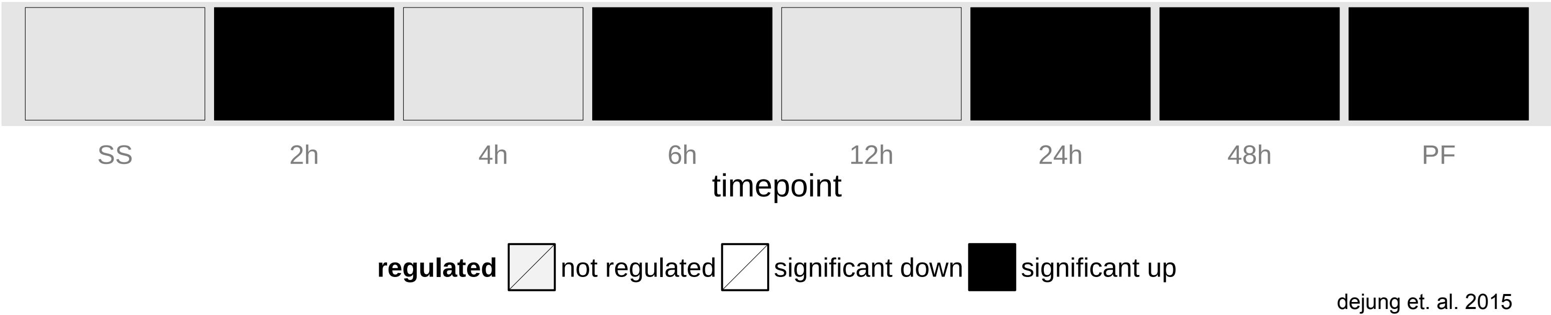
SS
2h
4h
6h
12h
24h
48h
PF
timepoint
regulated
not regulated
significant down
significant up
dejung et. al. 2015

protein kinase, putative
Tb927.3.690
AGOF: ATP binding, protein serine/threonine kinase activity, protein tyrosine kinase activity
AGOC: null
AGOP: cytokinesis after mitosis, protein phosphorylation
PGOF: ATP binding, protein kinase activity, transferase activity, transferring phosphorus–containing groups
PGOC: null
PGOP: protein phosphorylation

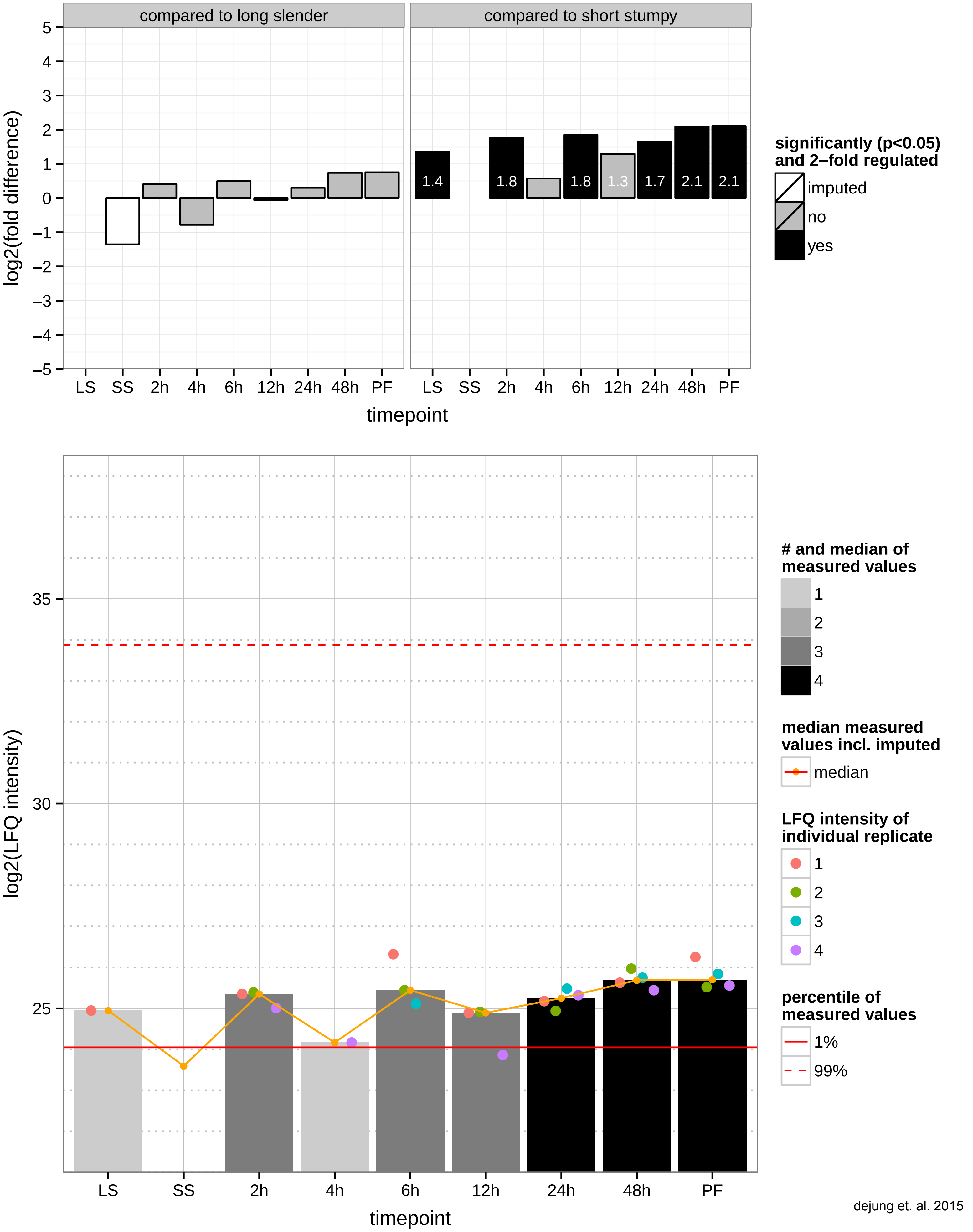

dejung et. al. 2015

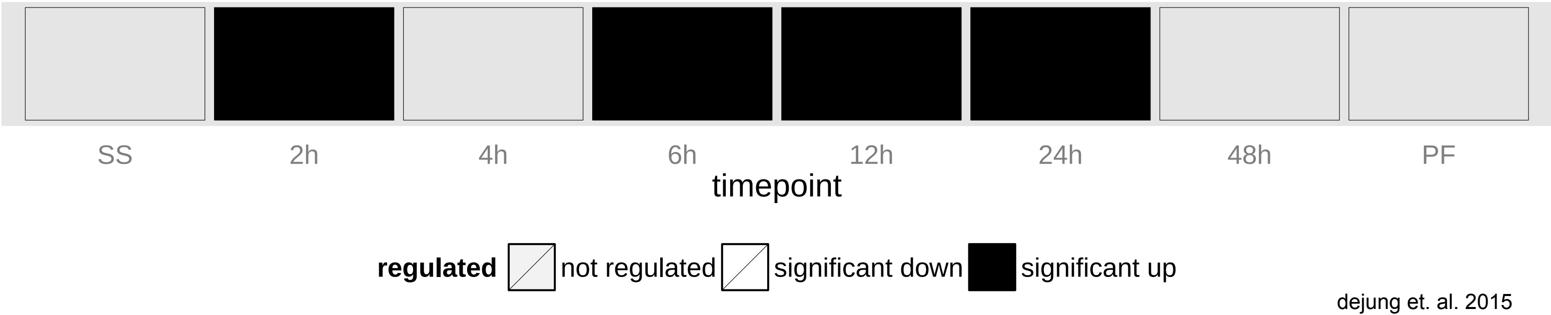
SS
2h
4h
6h
12h
24h
48h
PF
timepoint
regulated
not regulated
significant down
significant up
dejung et. al. 2015

kinetoplastid−specific phospho−protein phosphatase, putative
Tb927.4.1870
AGOF: protein serine/threonine phosphatase activity
AGOC: null
AGOP: protein dephosphorylation
PGOF: hydrolase activity
PGOC: null
PGOP: null

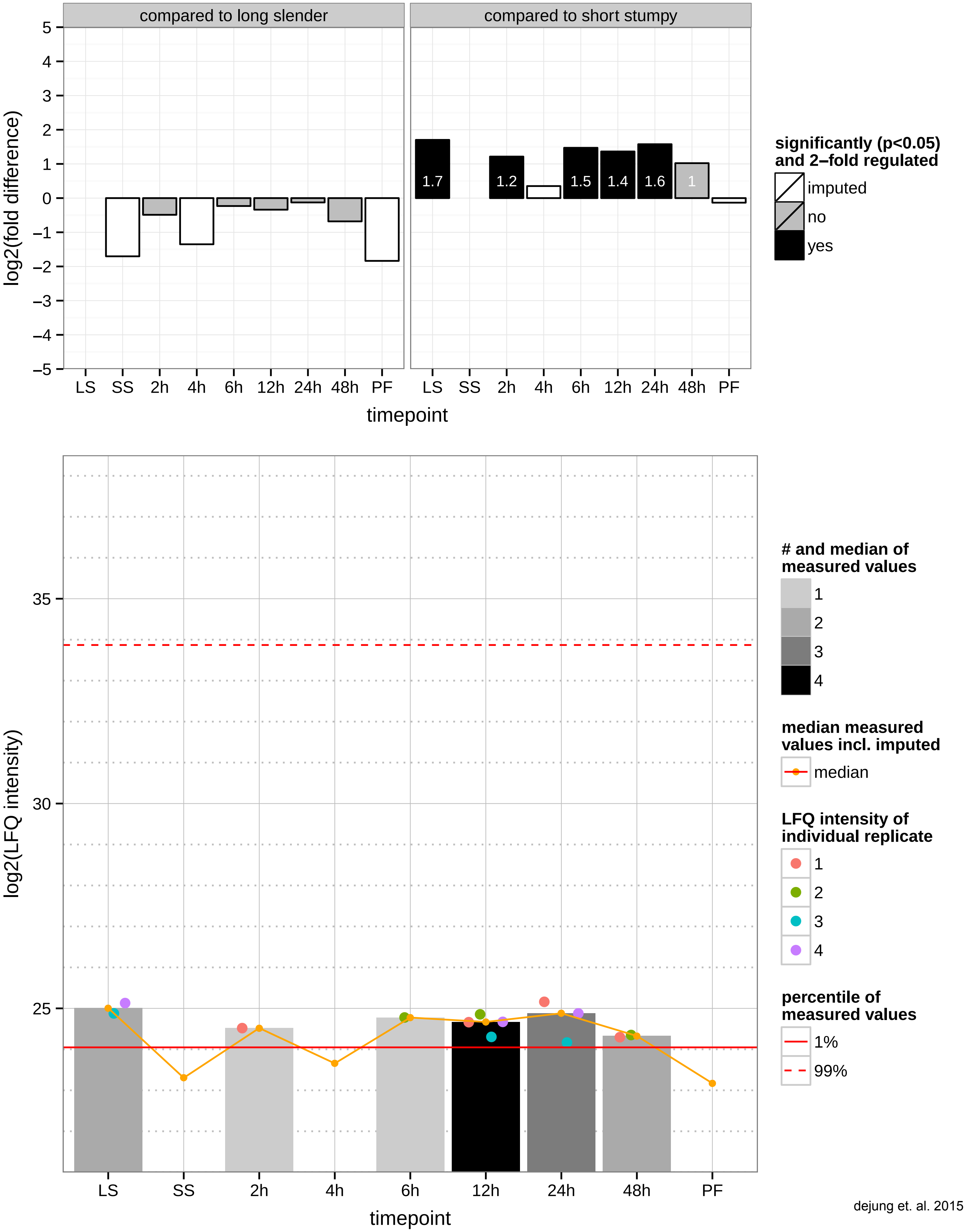

dejung et. al. 2015

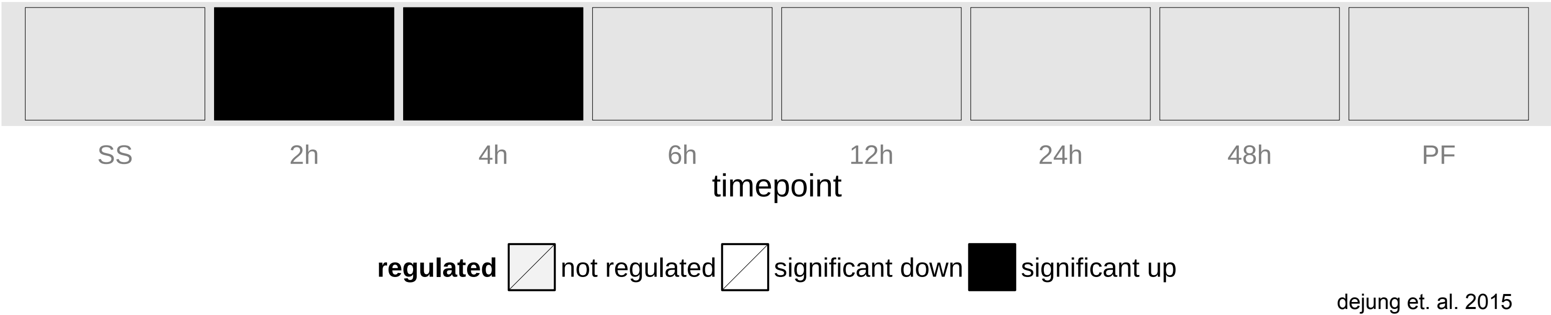
SS
2h
4h
6h
12h
24h
48h
PF
timepoint
regulated
not regulated
significant down
significant up
dejung et. al. 2015

double−strand−break repair protein rad21 homolog, putative
Tb927.7.6900
AGOF: null
AGOC: nuclear chromosome
AGOP: null
PGOF: protein binding
PGOC: nuclear chromosome
PGOP: null

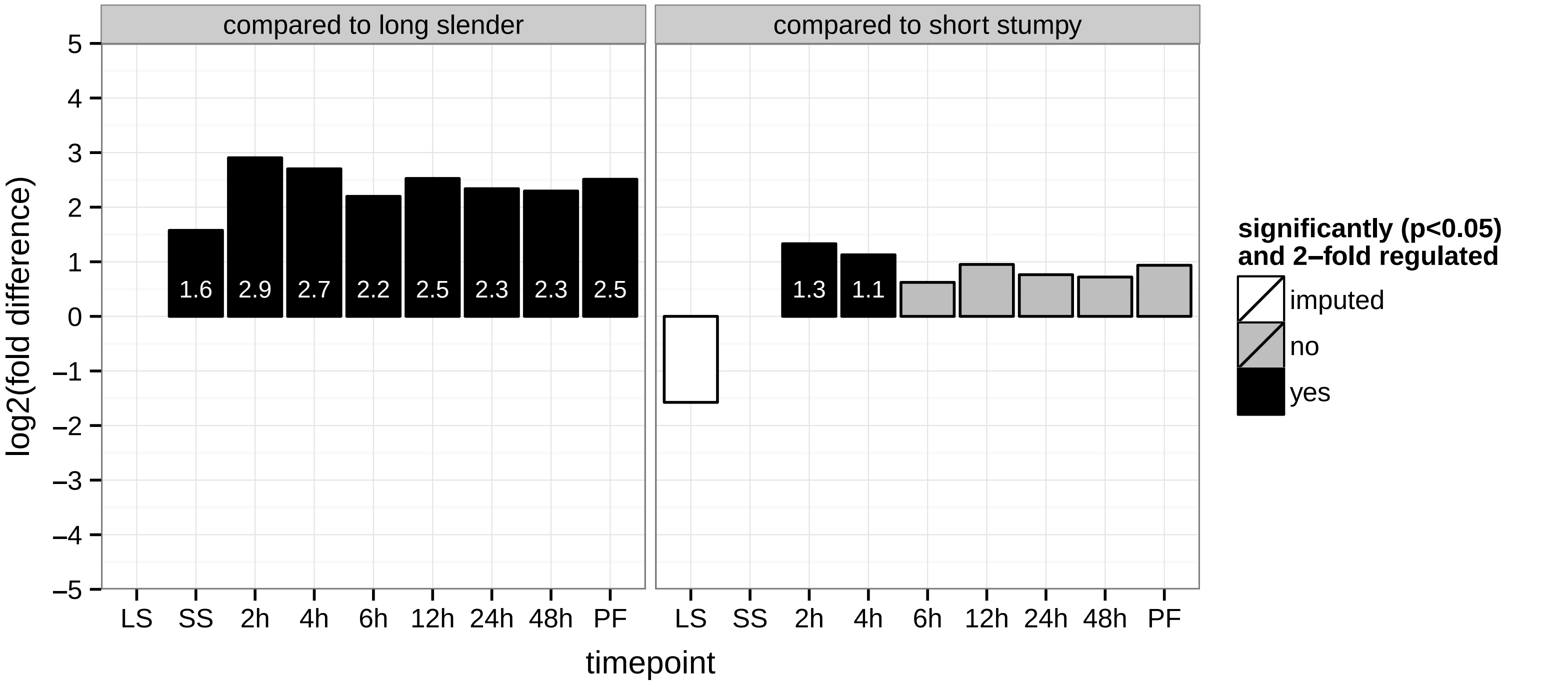

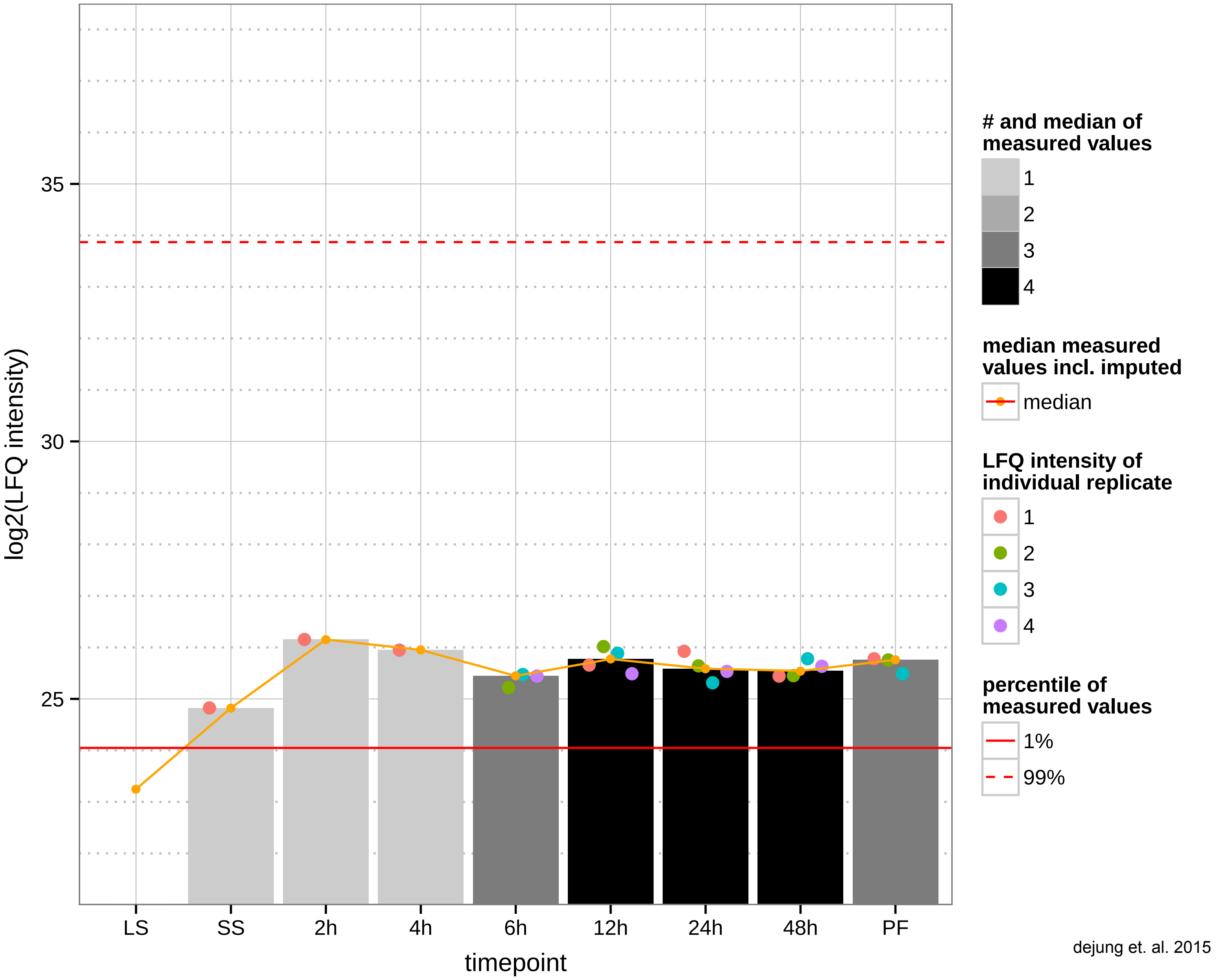

dejung et. al. 2015

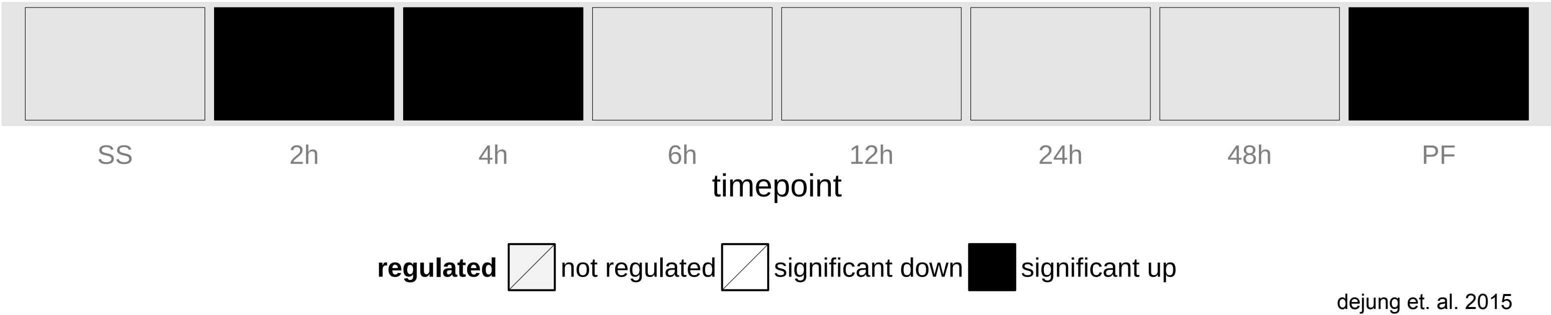
SS
2h
4h
6h
12h
24h
48h
PF
timepoint
regulated
not regulated
significant down
significant up
dejung et. al. 2015

hypothetical protein, conserved
Tb927.8.3890;Tb927.8.3900;Tb927.8.3880
AGOF: null, structural constituent of cell wall
AGOC: integral to membrane
AGOP: null, plant–type cell wall organization
PGOF: null
PGOC: null
PGOP: null

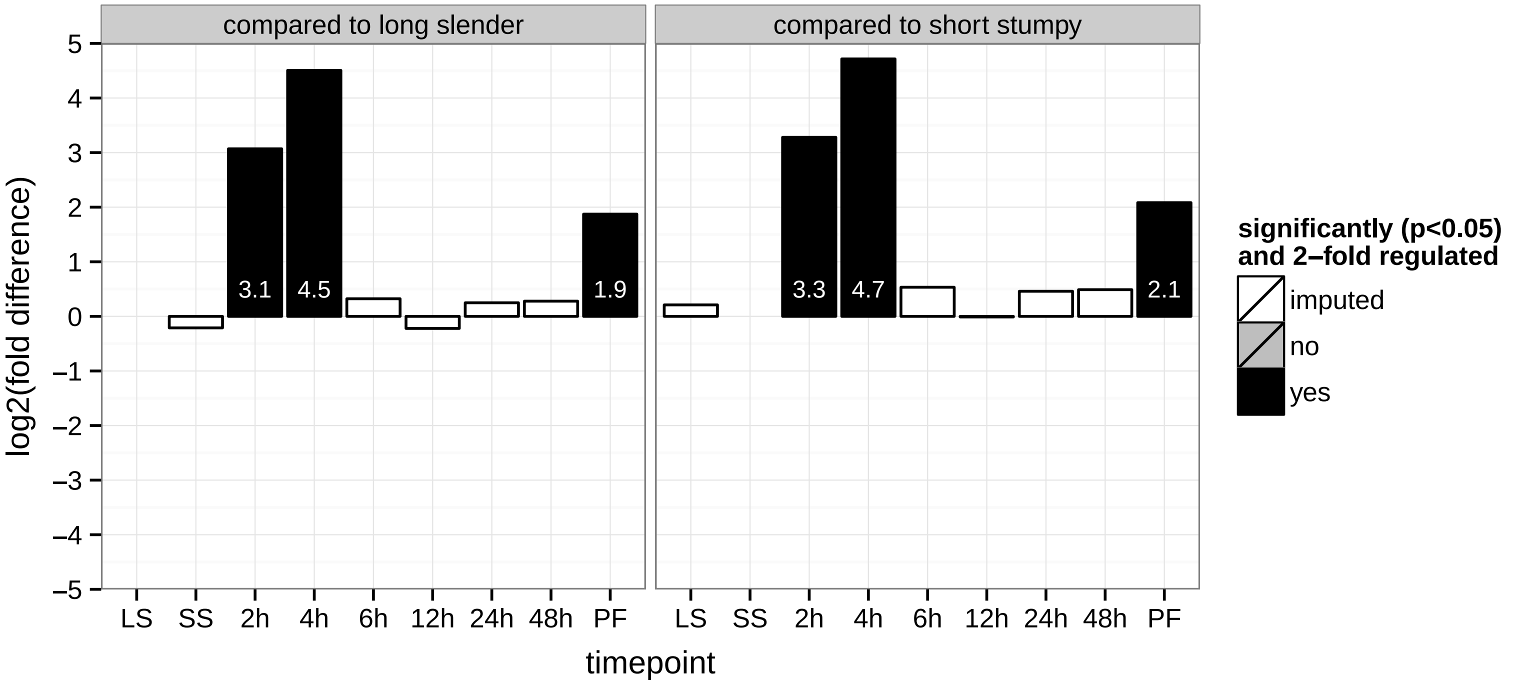

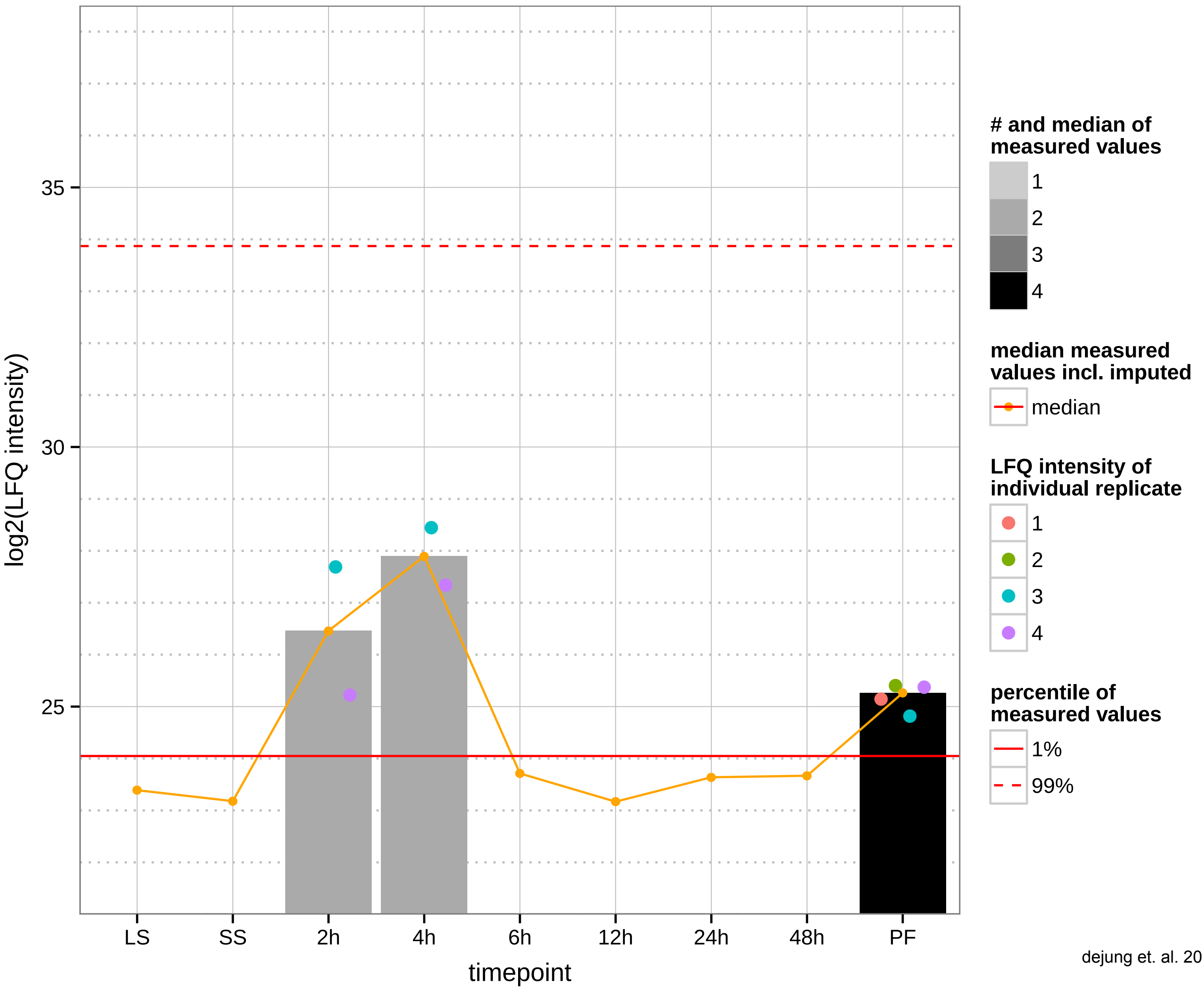

dejung et. al. 2015

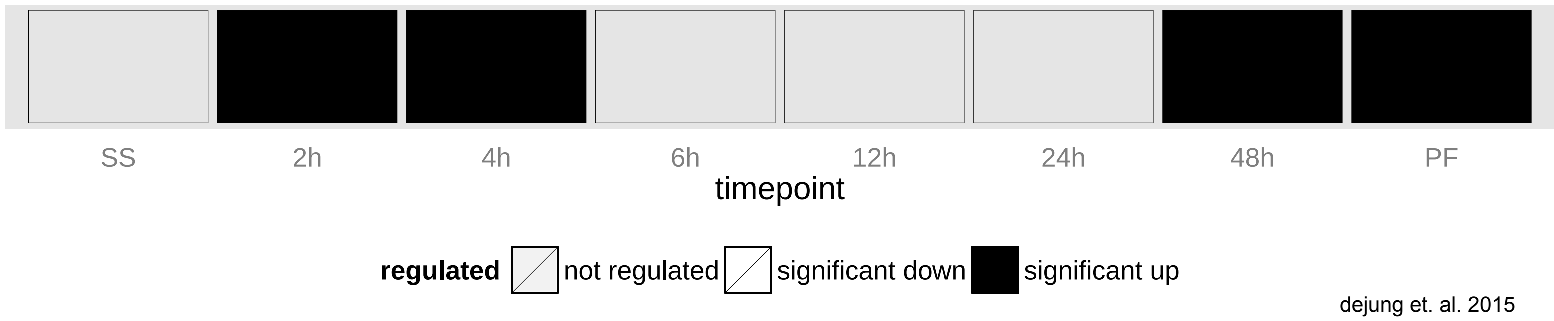
SS
2h
4h
6h
12h
24h
48h
PF
timepoint
regulated
not regulated
significant down
significant up
dejung et. al. 2015

60S ribosomal protein L39, putative
Tb927.8.5260
AGOF: structural constituent of ribosome
AGOC: intracellular, ribosome
AGOP: translation
PGOF: structural constituent of ribosome
PGOC: intracellular, ribosome
PGOP: translation

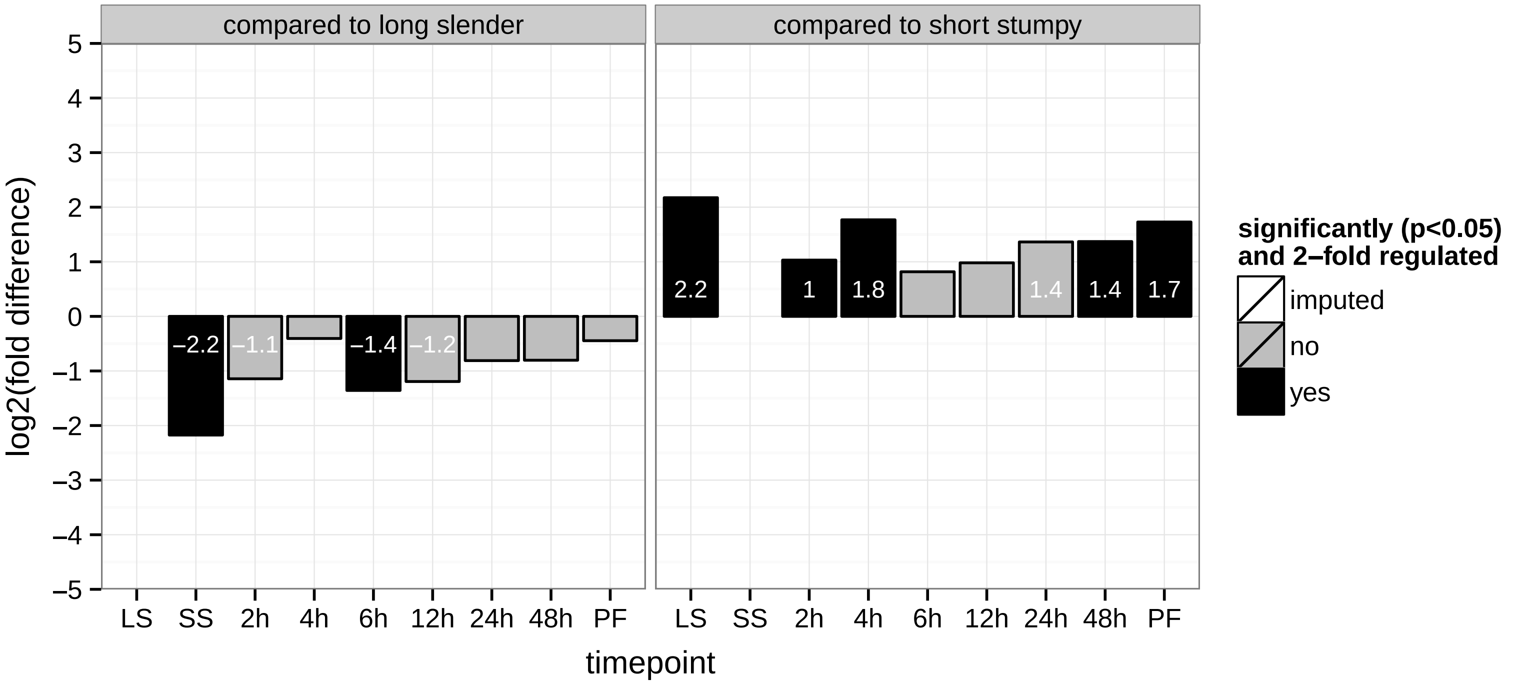

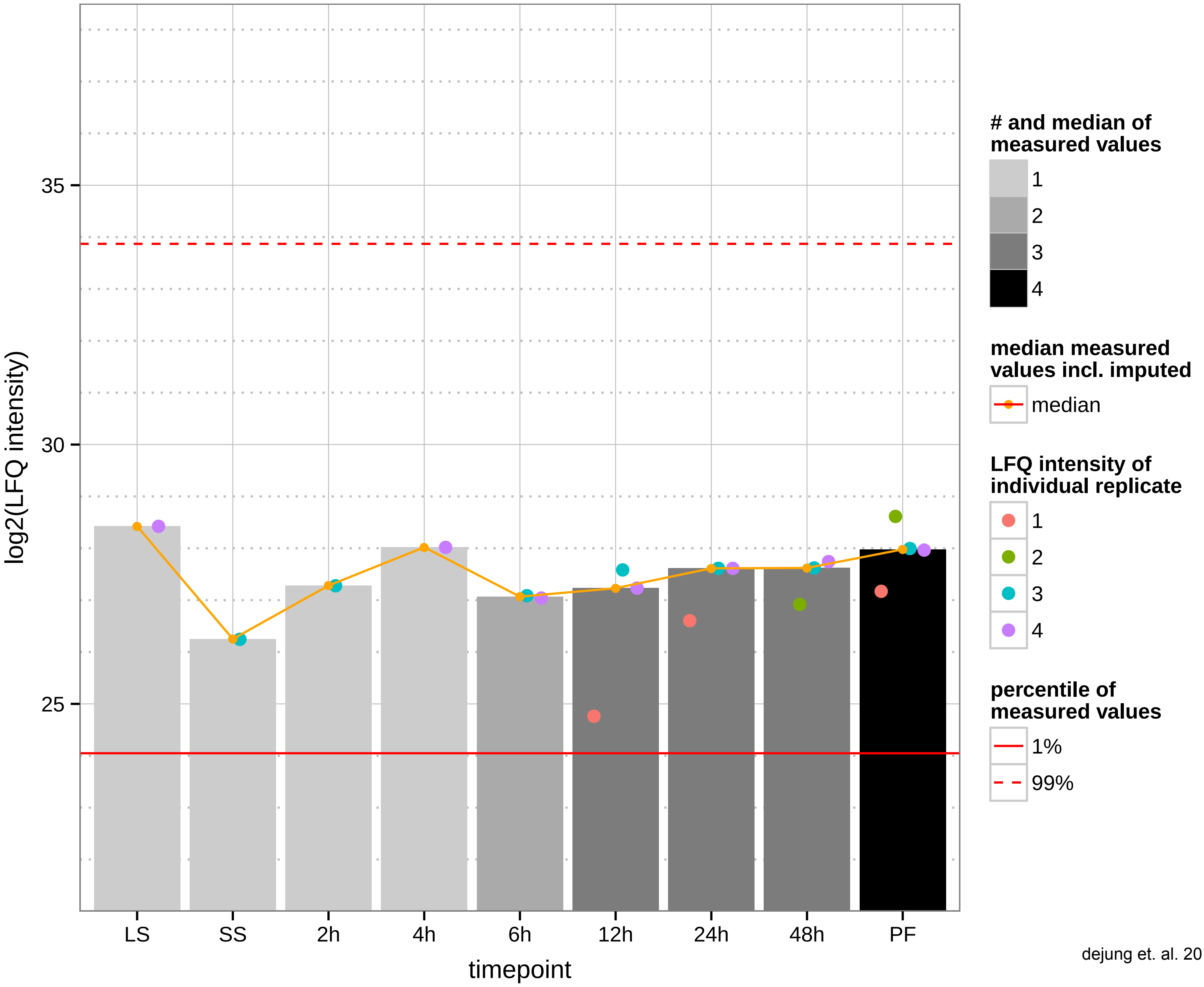

dejung et. al. 2015

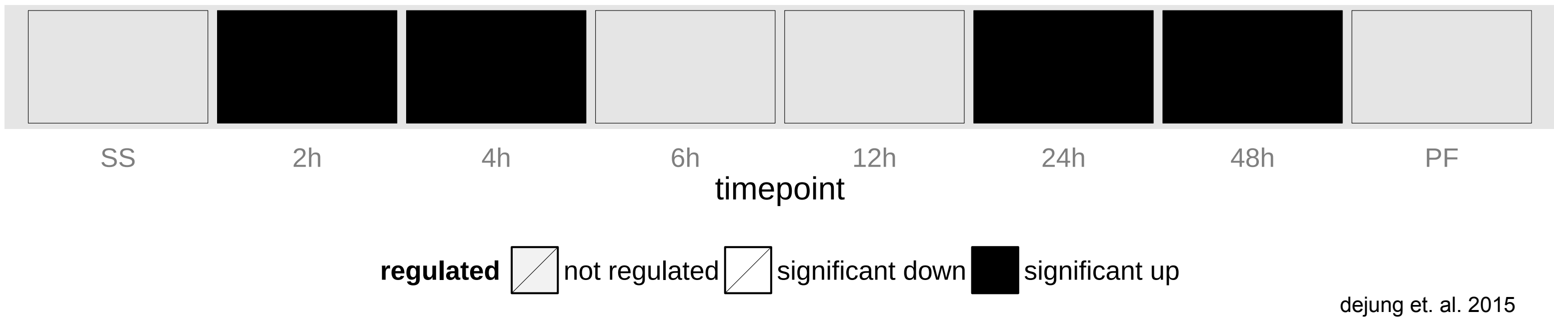
SS
2h
4h
6h
12h
24h
48h
PF
timepoint
regulated
not regulated
significant down
significant up
dejung et. al. 2015

ribulose−5−phosphate 3−epimerase, putative
Tb927.10.12210
AGOF: ribulose−phosphate 3−epimerase activity
AGOC: null
AGOP: pentose−phosphate shunt
PGOF: catalytic activity, racemase and epimerase activity, acting on carbohydrates and derivatives, ribulose−phosphate 3−epi
PGOC: null
PGOP: carbohydrate metabolic process, metabolic process, pentose−phosphate shunt

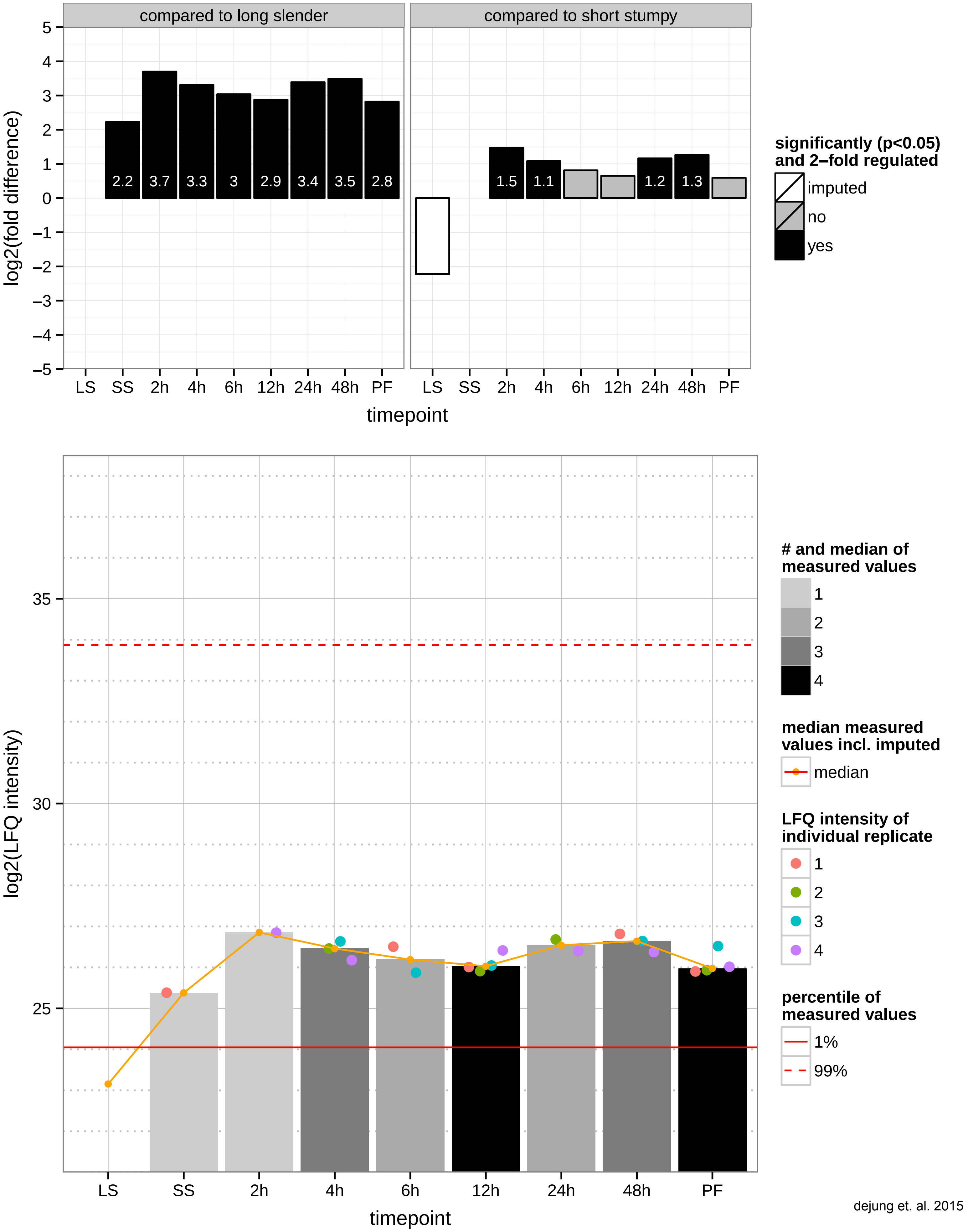

dejung et. al. 2015

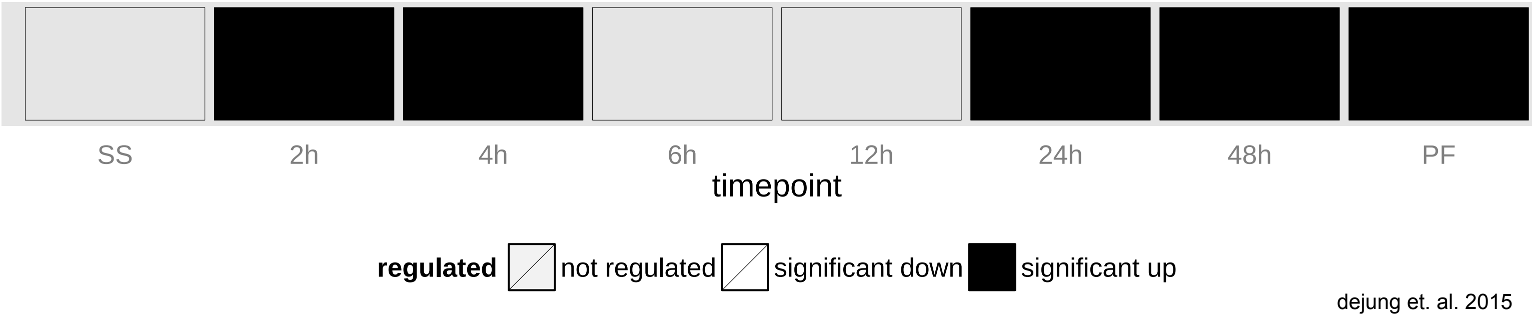
SS
2h
4h
6h
12h
24h
48h
PF
timepoint
regulated
not regulated
significant down
significant up
dejung et. al. 2015

hypothetical protein, conserved
Tb927.10.11520
AGOF: null
AGOC: mitochondrion
AGOP: null
PGOF: null
PGOC: null
PGOP: null

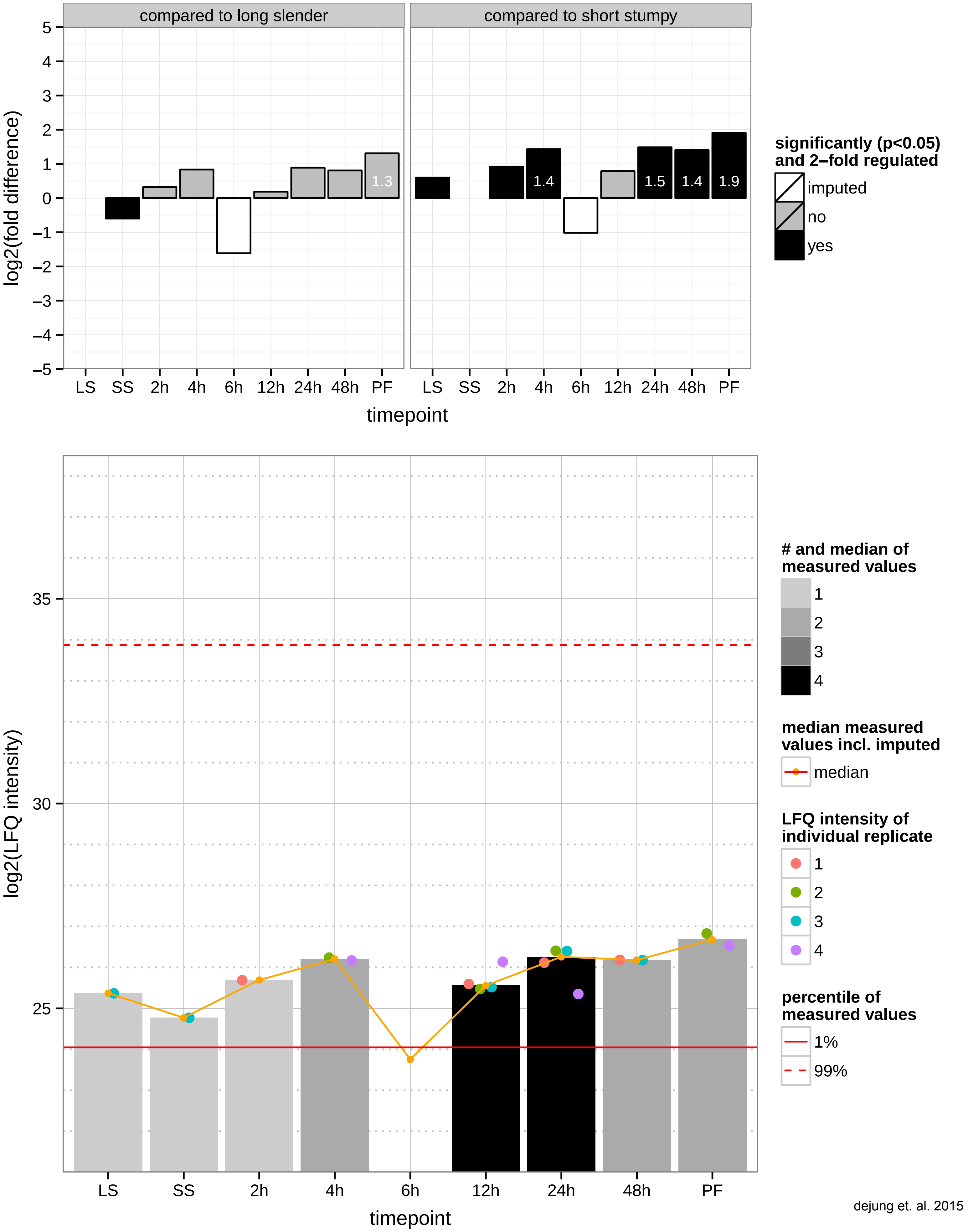

dejung et. al. 2015

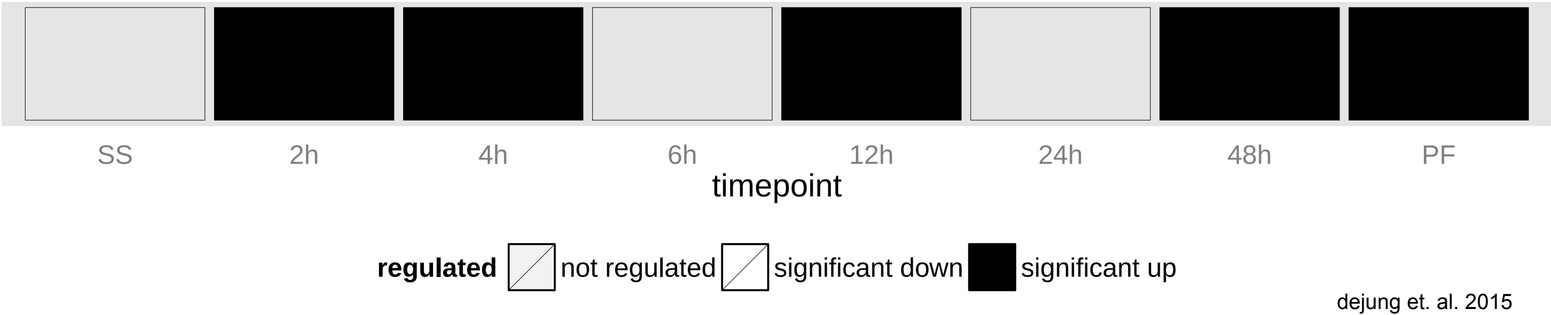
SS
2h
4h
6h
12h
24h
48h
PF
timepoint
regulated
not regulated
significant down
significant up
dejung et. al. 2015

hypothetical protein, conserved
Tb927.5.4120
AGOF: null
AGOC: mitochondrion
AGOP: null
PGOF: null
PGOC: null
PGOP: null

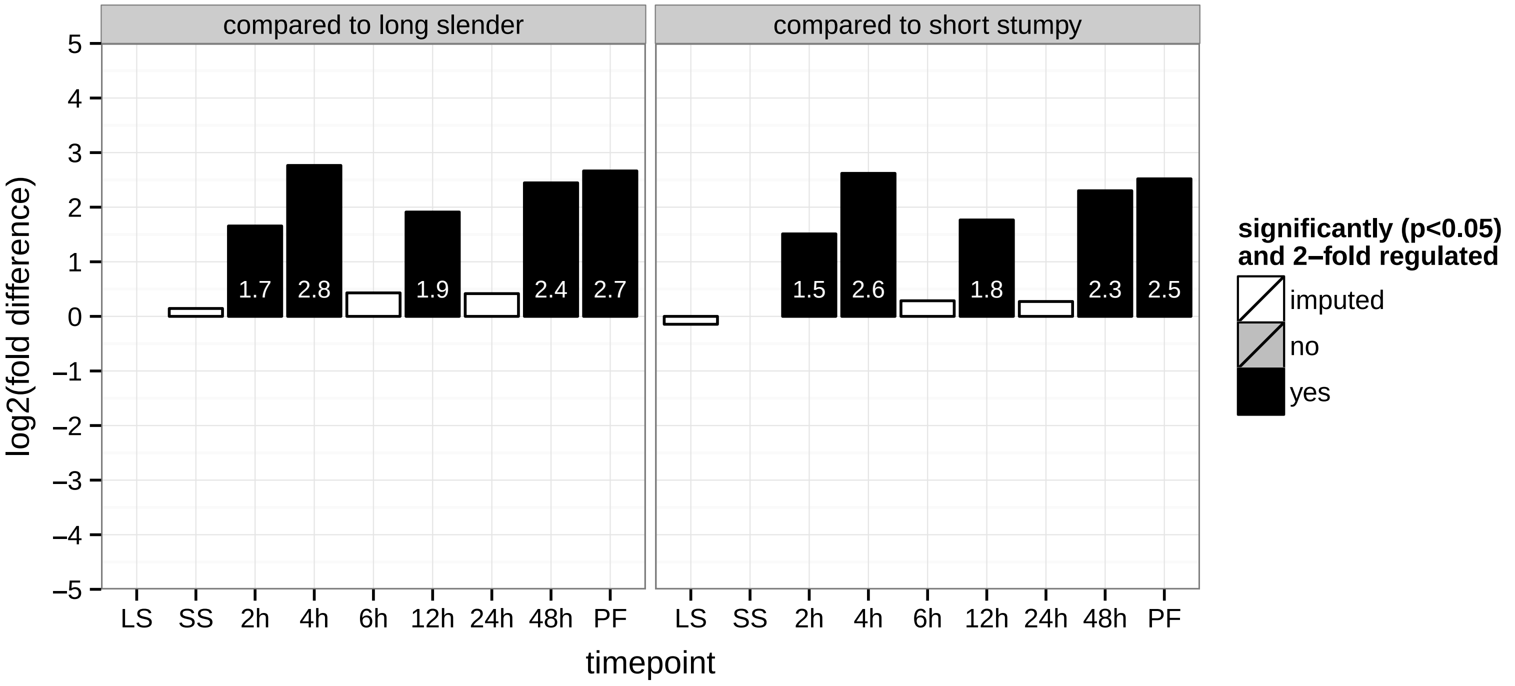

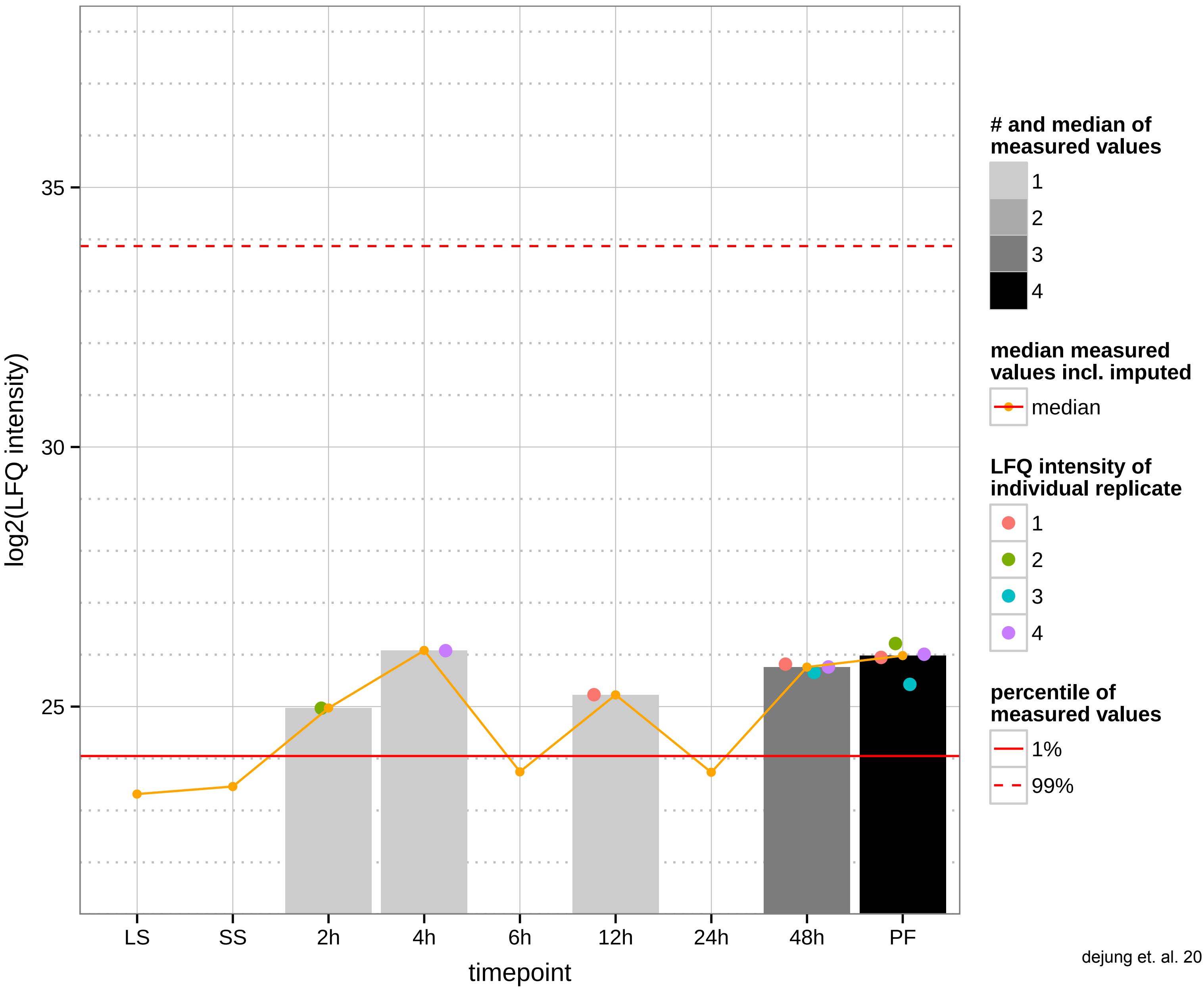

dejung et. al. 2015

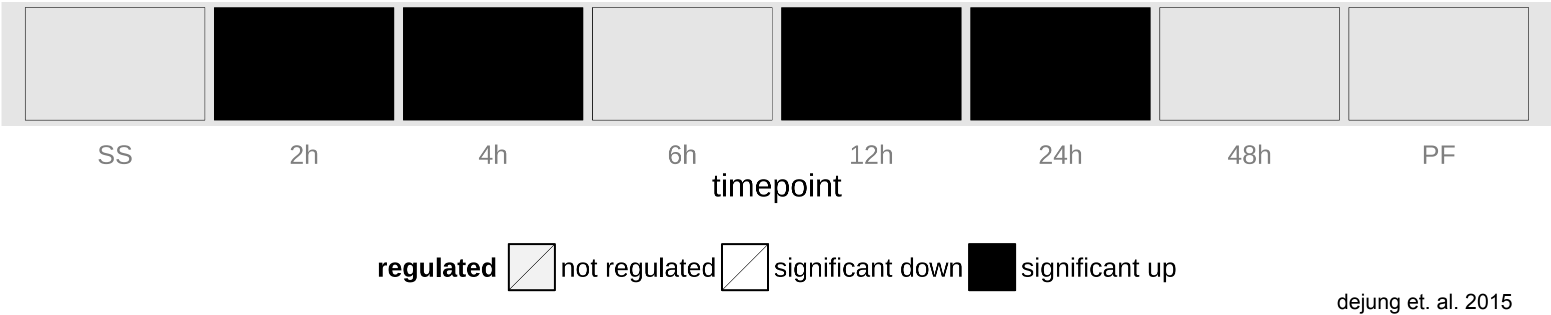
SS
2h
4h
6h
12h
24h
48h
PF
timepoint
regulated
not regulated
significant down
significant up
dejung et. al. 2015

hypothetical protein, conserved
Tb927.11.15210
AGOF: null
AGOC: null
AGOP: null
PGOF: null
PGOC: null
PGOP: null

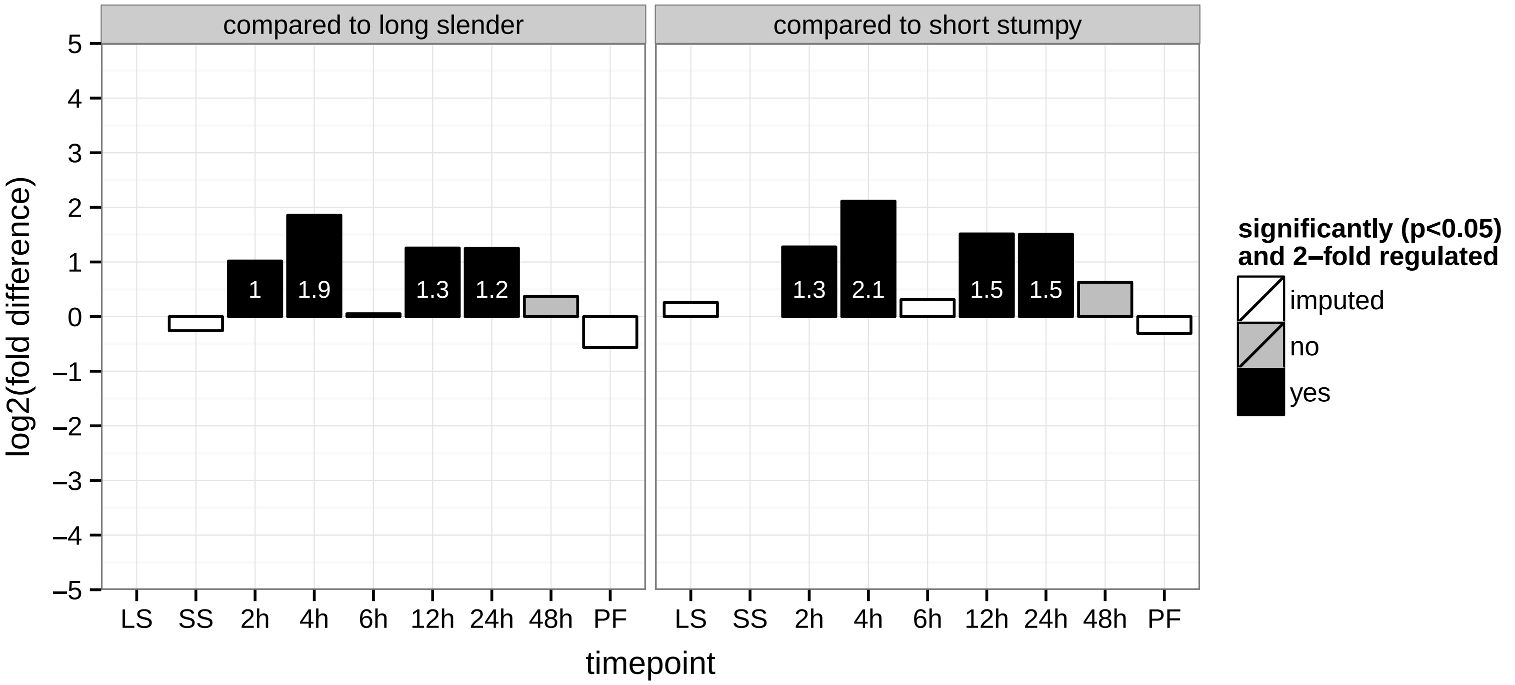

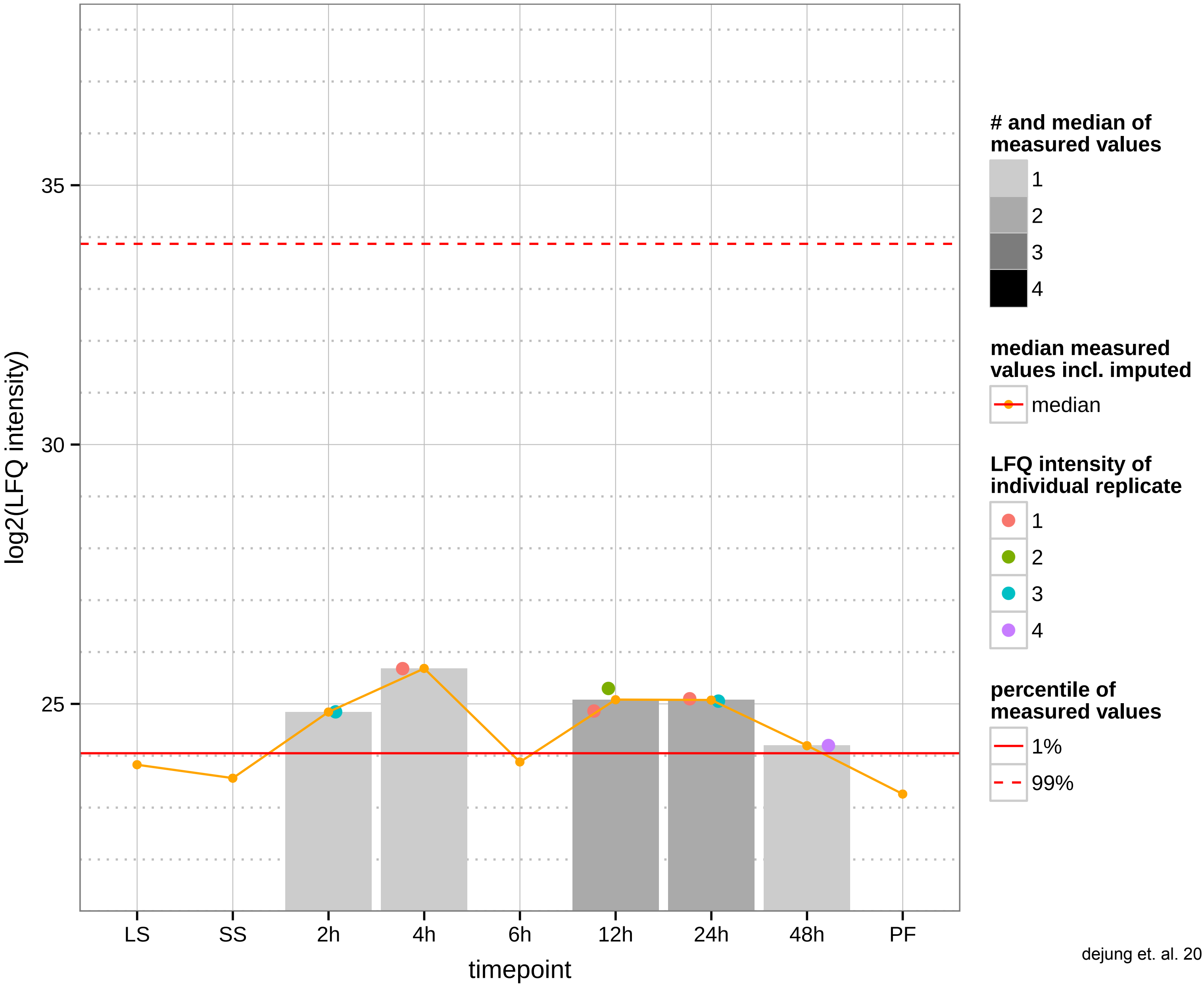

dejung et. al. 2015

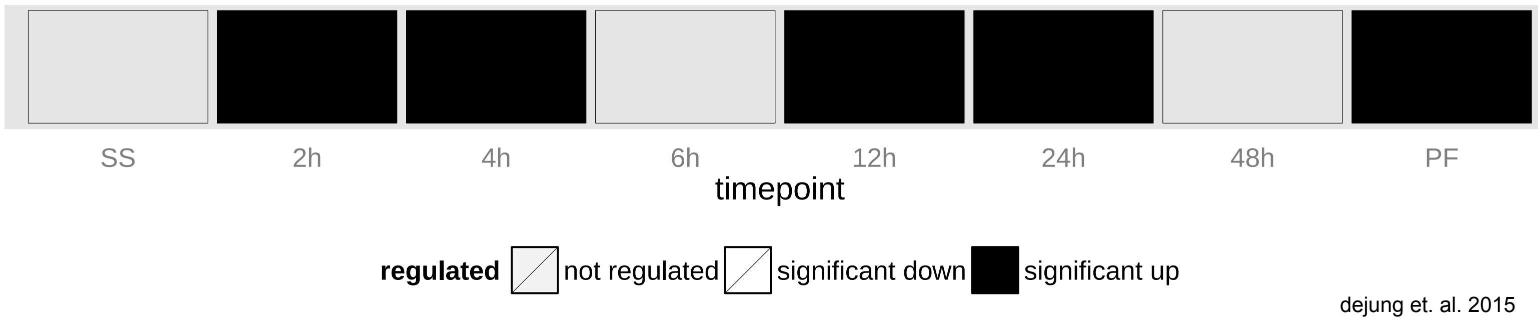
SS
2h
4h
6h
12h
24h
48h
PF
timepoint
regulated
not regulated
significant down
significant up
dejung et. al. 2015

hypothetical protein, conserved
Tb927.10.11890
AGOF: null
AGOC: mitochondrion
AGOP: null
PGOF: null
PGOC: null
PGOP: null

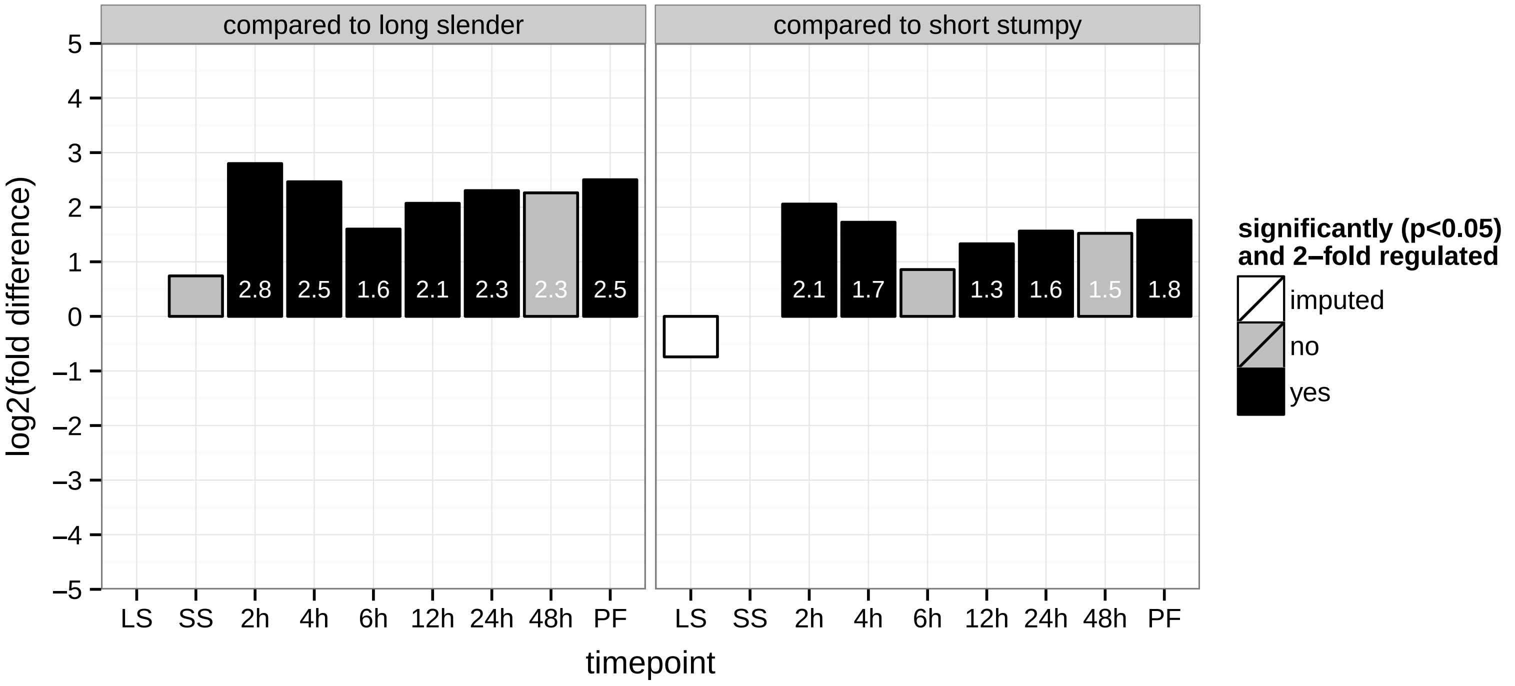

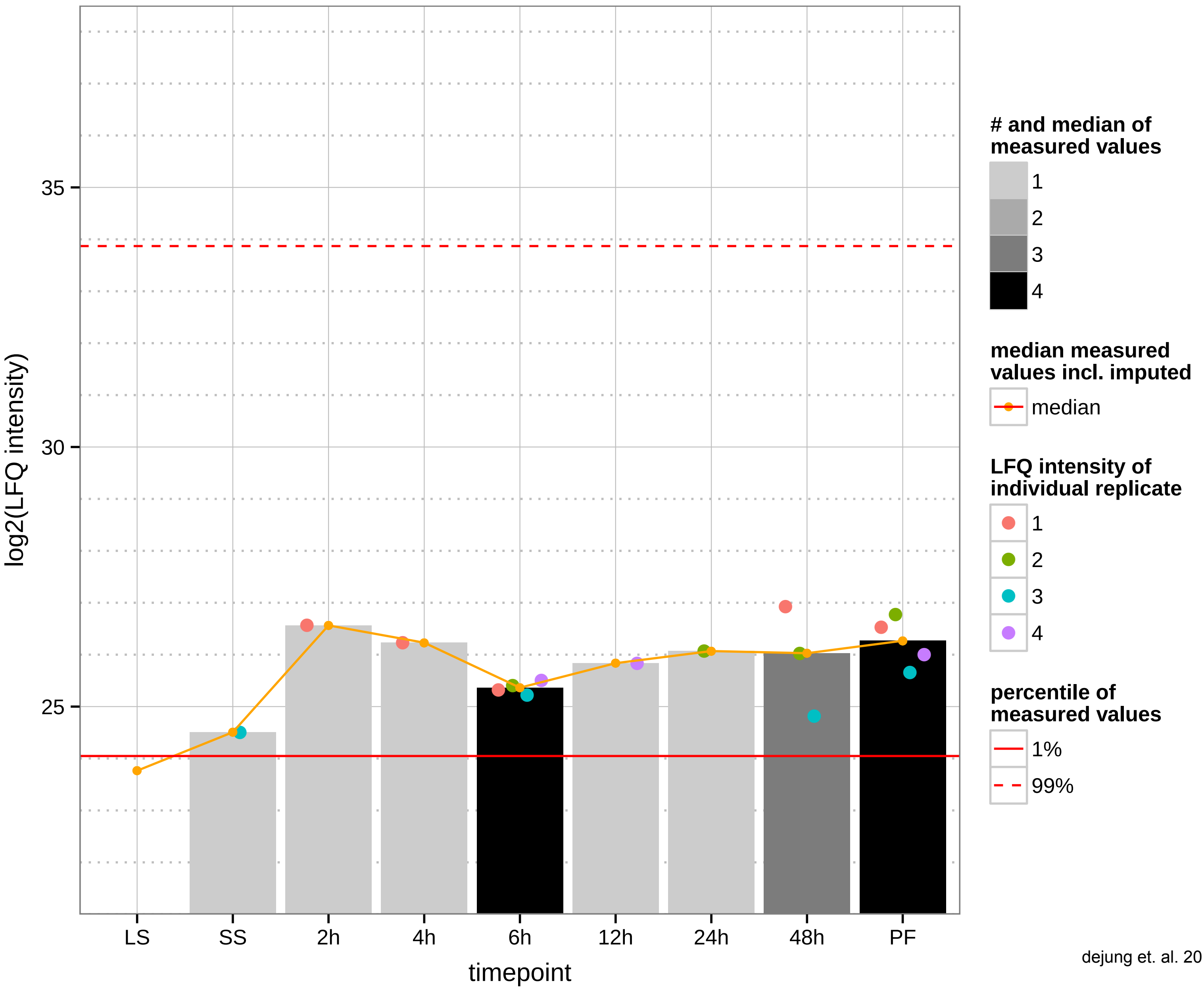

dejung et. al. 2015

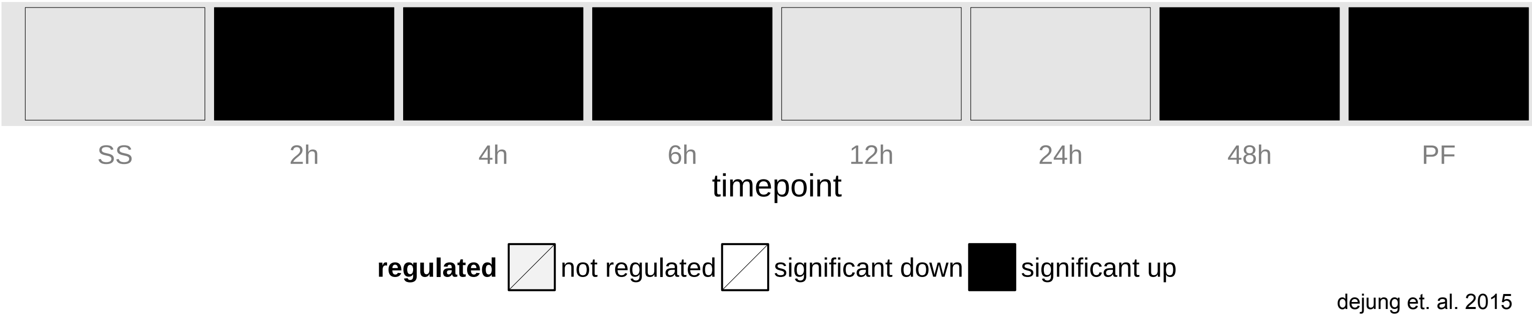
SS
2h
4h
6h
12h
24h
48h
PF
timepoint
regulated
not regulated
significant down
significant up
dejung et. al. 2015

hypothetical protein, conserved
Tb927.4.5160
AGOF: null
AGOC: null
AGOP: null
PGOF: null
PGOC: null
PGOP: null

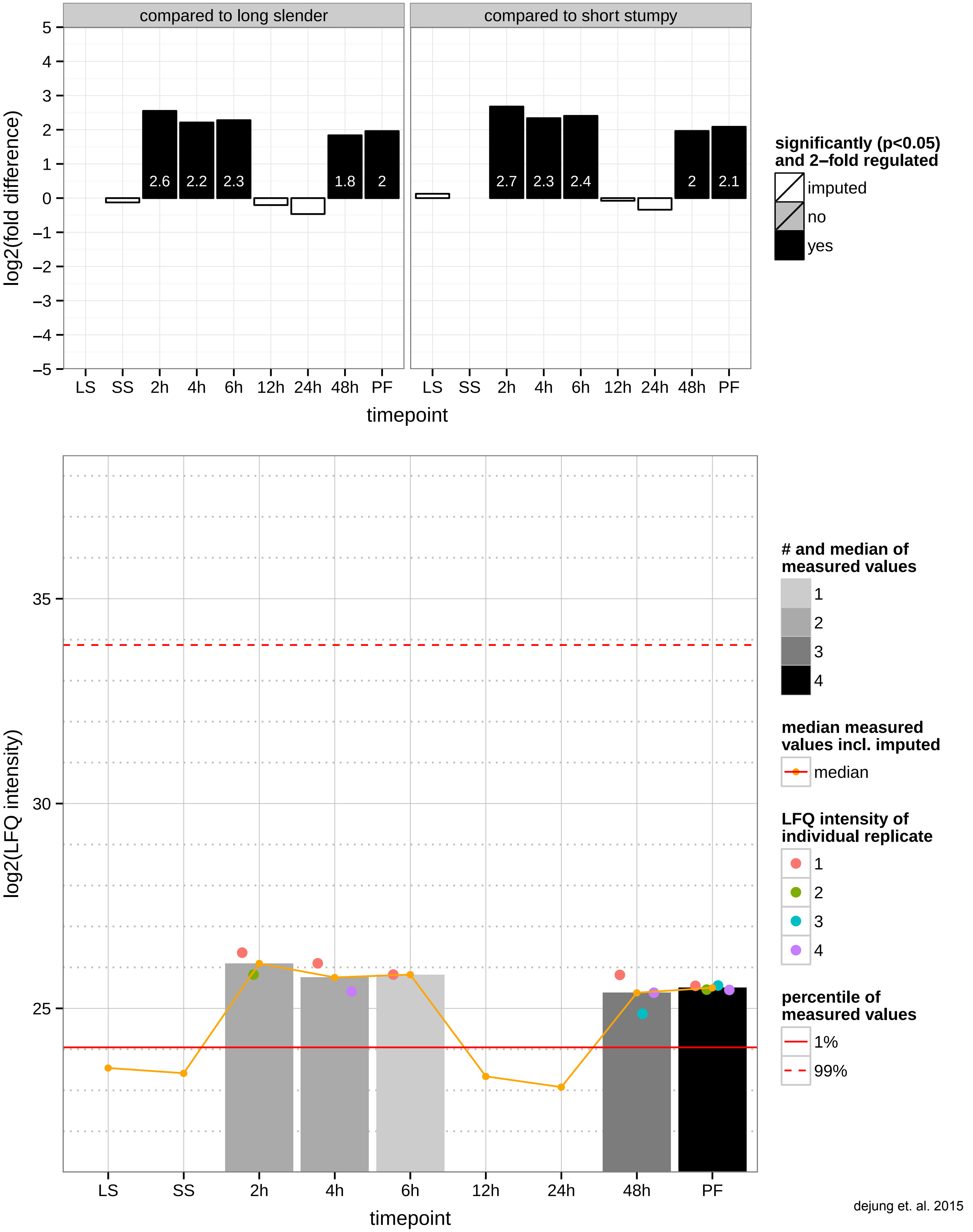

dejung et. al. 2015

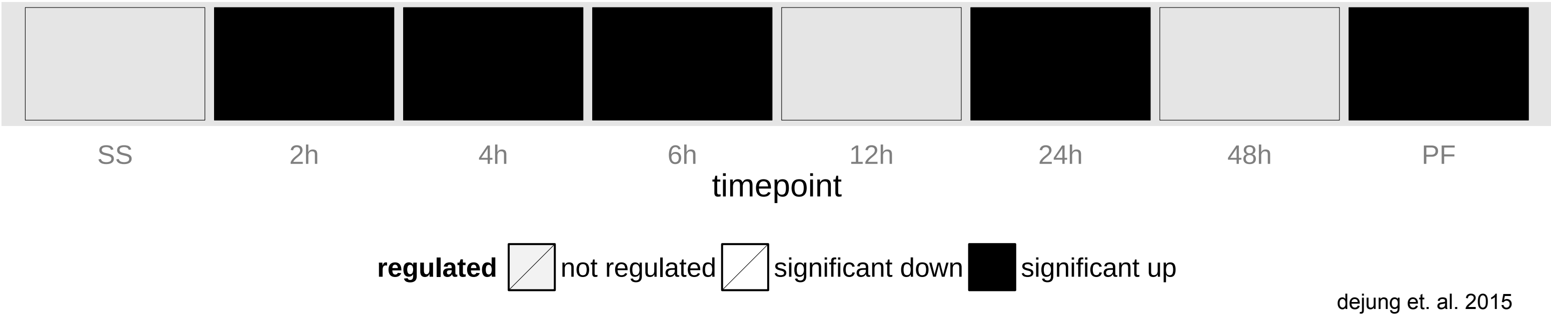
SS
2h
4h
6h
12h
24h
48h
PF
timepoint
regulated
not regulated
significant down
significant up
dejung et. al. 2015

hypothetical protein, conserved
Tb927.9.2260
AGOF: null
AGOC: null
AGOP: null
PGOF: null
PGOC: null
PGOP: null

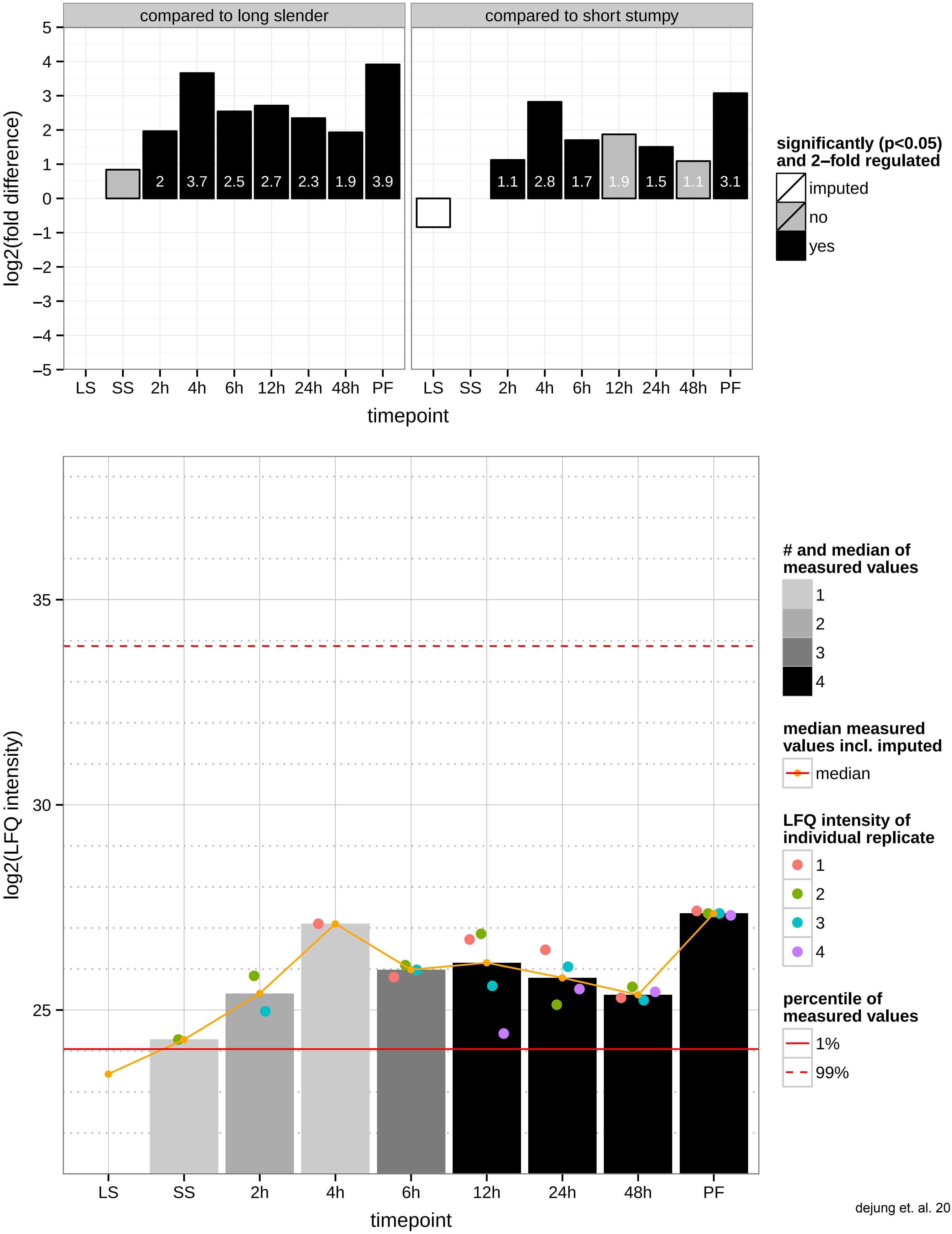

dejung et. al. 2015

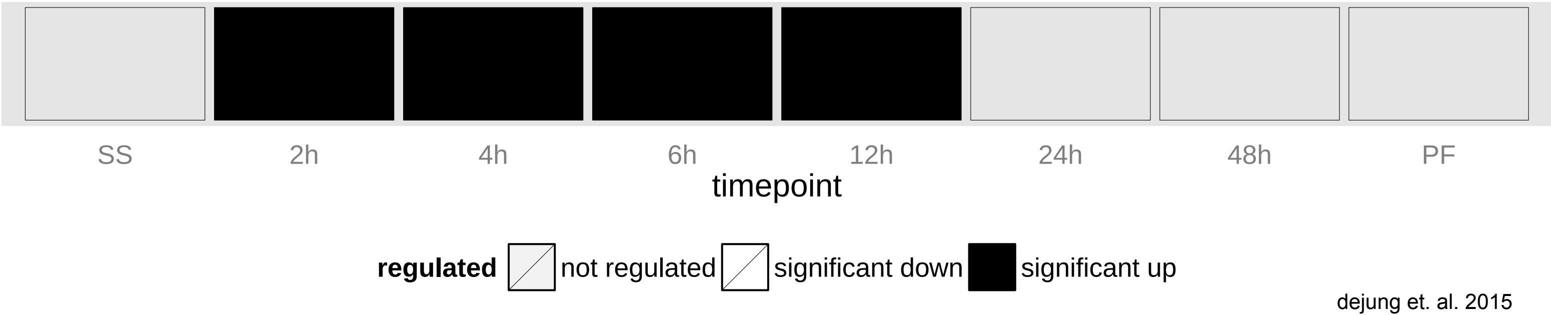
SS
2h
4h
6h
12h
24h
48h
PF
timepoint
regulated
not regulated
significant down
significant up
dejung et. al. 2015

hypothetical protein, conserved
Tb927.10.11630
AGOF: null
AGOC: null
AGOP: null
PGOF: null
PGOC: null
PGOP: null

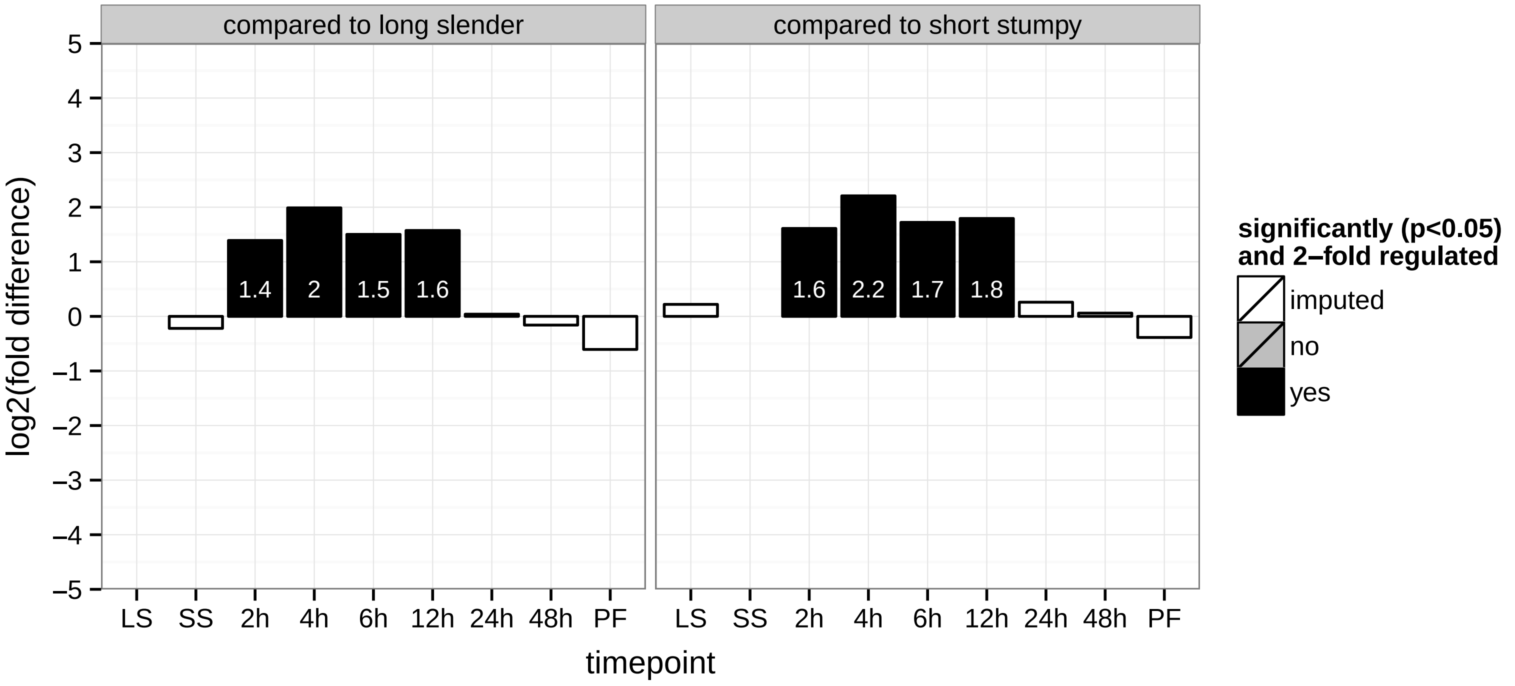

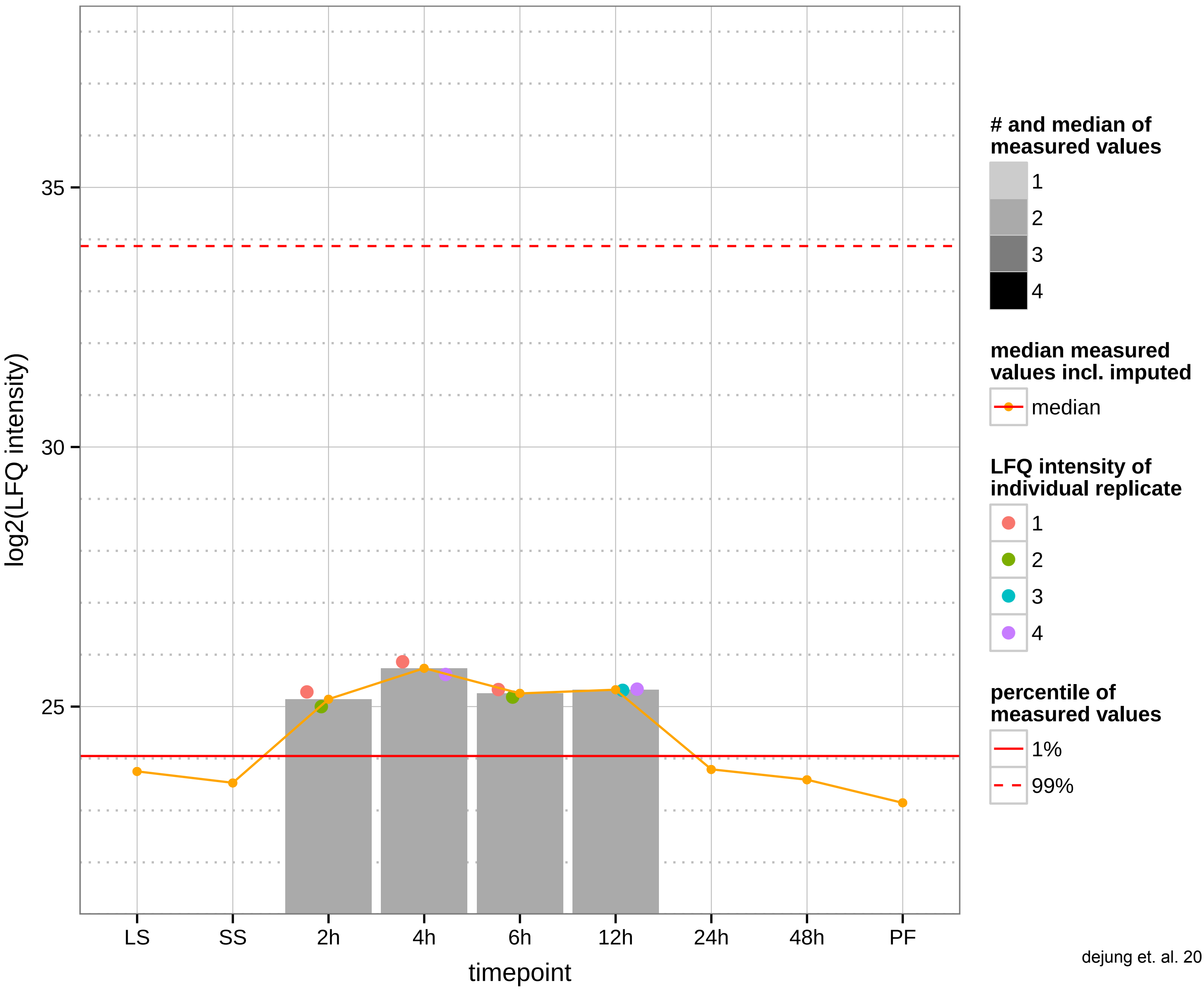

dejung et. al. 2015

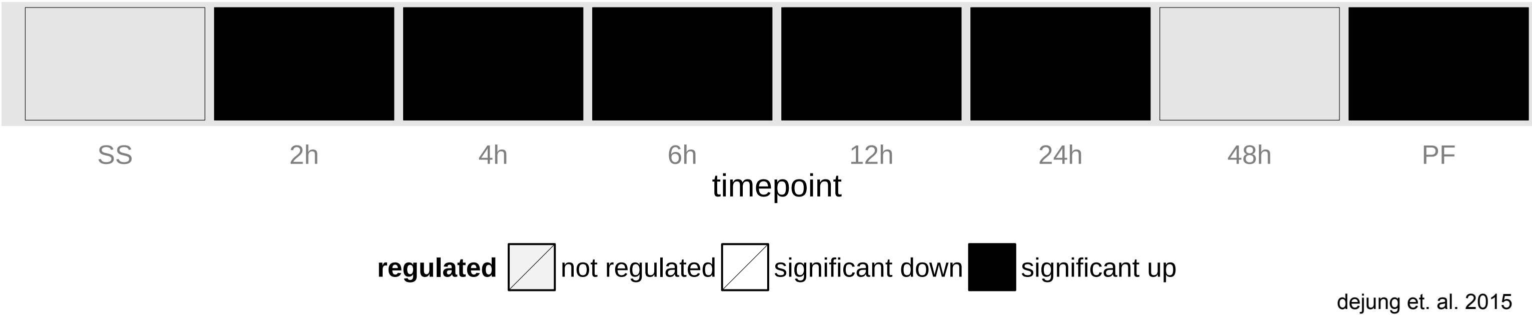
SS
2h
4h
6h
12h
24h
48h
PF
timepoint
regulated
not regulated
significant down
significant up
dejung et. al. 2015

hypothetical protein, conserved
Tb927.11.11300
AGOF: null
AGOC: null
AGOP: null
PGOF: null
PGOC: null
PGOP: null

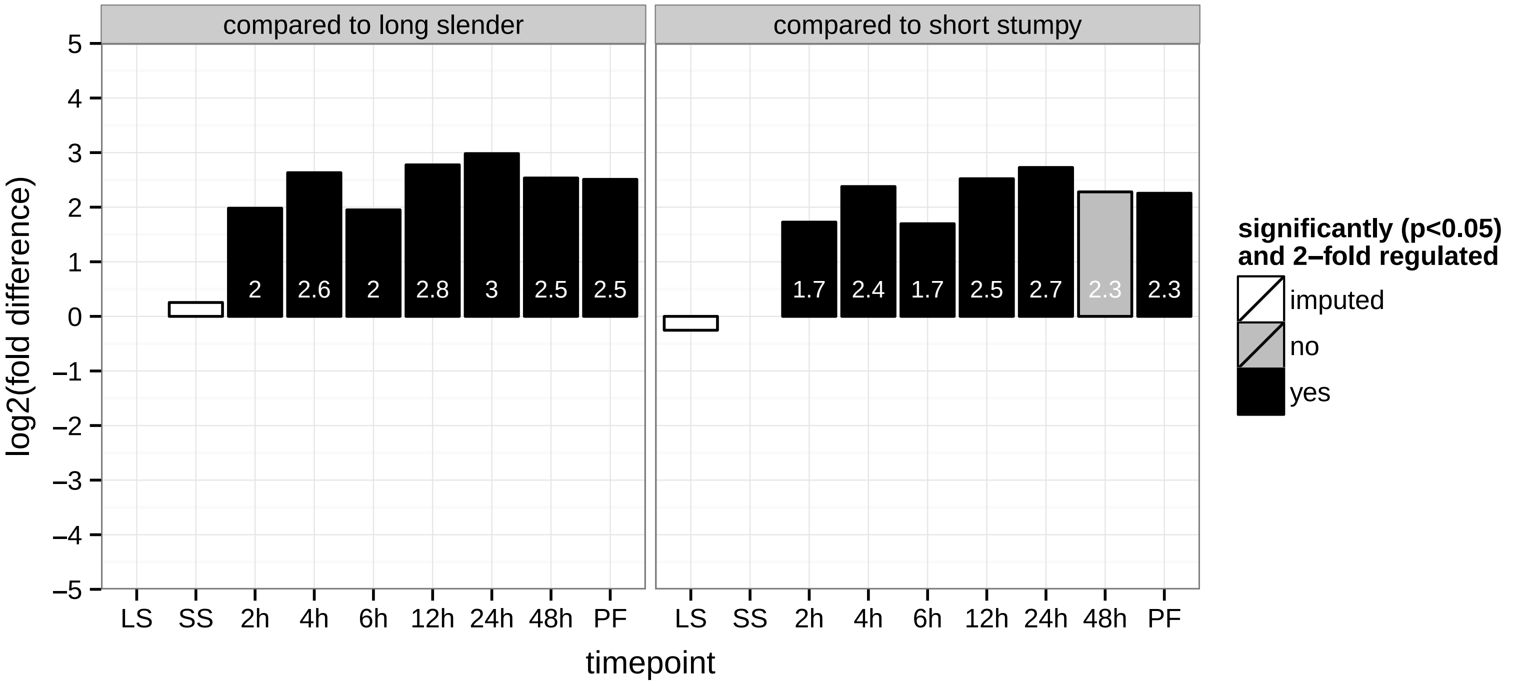

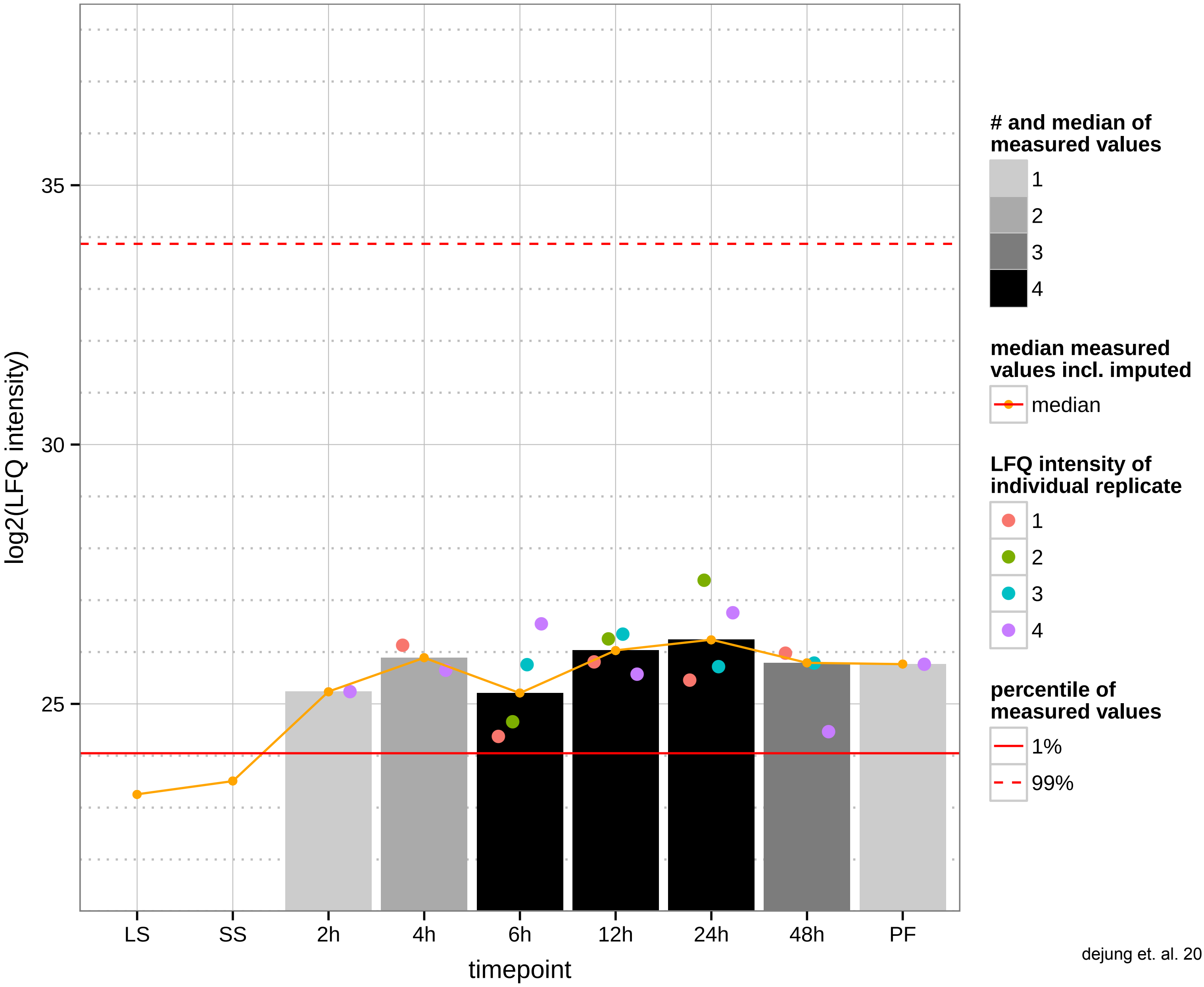

dejung et. al. 2015
